# Insights into the mechanism(s) of digestion of crystalline cellulose by plant class C GH9 endoglucanases

**DOI:** 10.1007/s00894-019-4133-1

**Published:** 2019-07-23

**Authors:** Siddhartha Kundu

**Affiliations:** Department of Biochemistry, Army College of Medical Sciences, Brar Square, Delhi Cantt., New Delhi, 110010 India

**Keywords:** Active-site geometry, Carbohydrate binding module, Class C GH9 endoglucanases, Crystalline cellulose, Glycoside hydrolase, Homology modelling, Interaction surface

## Abstract

Biofuels such as *γ*-valerolactone, bioethanol, and biodiesel are derived from potentially fermentable cellulose and vegetable oils. Plant class C GH9 endoglucanases are CBM49-encompassing hydrolases that cleave the *β* (1 → 4) glycosidic linkage of contiguous *D*-glucopyranose residues of crystalline cellulose. Here, I analyse 3D-homology models of characterised and putative class C enzymes to glean insights into the contribution of the GH9, linker, and CBM49 to the mechanism(s) of crystalline cellulose digestion. Crystalline cellulose may be accommodated in a surface groove which is imperfectly bounded by the GH9_CBM49, GH9_linker, and linker_CBM49 surfaces and thence digested in a solvent accessible subsurface cavity. The physical dimensions and distortions thereof, of the groove, are mediated in part by the bulky side chains of aromatic amino acids that comprise it and may also result in a strained geometry of the bound cellulose polymer. These data along with an almost complete absence of measurable cavities, along with poorly conserved, hydrophobic, and heterogeneous amino acid composition, increased atomic motion of the CBM49_linker junction, and docking experiements with ligands of lower degrees of polymerization suggests a modulatory rather than direct role for CBM49 in catalysis. Crystalline cellulose is the de facto substrate for CBM-containing plant and non-plant GH9 enzymes, a finding supported by exceptional sequence- and structural-homology. However, despite the implied similarity in general acid-base catalysis of crystalline cellulose, this study also highlights qualitative differences in substrate binding and glycosidic bond cleavage amongst class C members. Results presented may aid the development of novel plant-based GH9 endoglucanases that could extract and utilise potential fermentable carbohydrates from biomass.

**Electronic supplementary material:**

The online version of this article (10.1007/s00894-019-4133-1) contains supplementary material, which is available to authorized users.

**Graphical Abstract:**
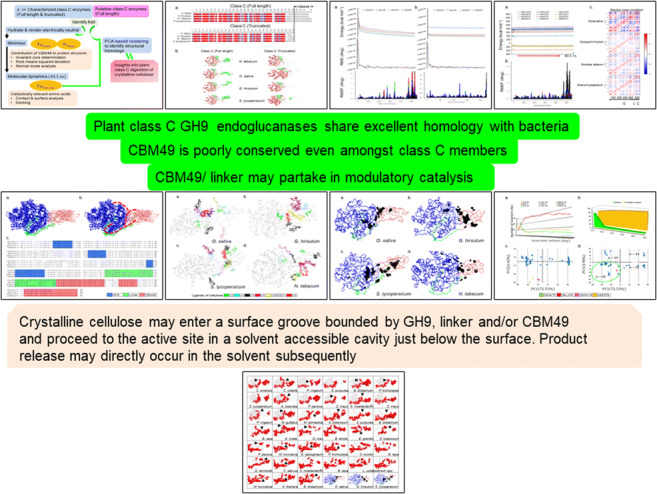
Crystalline cellulose digestion by plant class C GH9 endoglucanases - an in silico assessment of function.

## Introduction

The microfibrillar structure of cellulose is constituted and strengthened by islands of hydrogen-bonded inter-glucan chains. These microcrystalline regions (*I*_*α*_, *I*_*β*_) render cellulose chemically inert and recalcitrant to most physical stressors, an attribute that is desirable to land plants (xylem, phloem), sporulating bacteria and fungi, and quorum sensing by microbial biofilms [1–8]. Most organisms (bacteria, fungi, protists) possess enzymes (oxidoreductases, EC 1.x.y.z; transferases, EC 2.x.y.z; hydrolases, EC 3.x.y.z) that can cleave cellulose into physiologically relevant oligo- and mono-saccharides (RXNs 1–3) [2, 9–16].

RXN 1 $$ \left\{{C}_2,{C}_{2+}\right\}+\left\{{e}^{-};2{e}^{-}\right\}\rightleftharpoons \left\{{C}_2=O,{C}_{2+}=O\right\} $$

RXN 2 $$ {C}_n+{H}_iP{O}_4\leftrightharpoons C- OP{O}_3+{C}_{n-1} $$

RXN 3 $$ {C}_n+{kH}_2O\leftrightharpoons +\left({m}_1\right)\left({C}_{n-i}\right)+{kH}_2O\leftrightharpoons \left({m}_2\right)\left({C}_{2-5}\right)+{kH}_2O\leftrightharpoons \left({m}_n\right)\left({C}_1\right) $$

$$ {\displaystyle \begin{array}{ccc}{C}_n& := & \mathrm{Glucan}\\ {}C& := & D\left(\alpha \right)-\mathrm{glucopyranose}\ \mathrm{phosphate}\\ {}i& \in & \left\{1,2,3\right\}\\ {}{C}_2& := & \mathrm{Cellulose}\ \mathrm{with}\ \mathrm{degree}\ \mathrm{of}\ \mathrm{polymerization}\ \left( DP=2\right)\\ {}{C}_{2+}& := & \mathrm{Cellulose}\ \mathrm{with}\ \mathrm{degree}\ \mathrm{of}\ \mathrm{polymerization}\ \left( DP>2\right)\\ {}{C}_2=O& := & \mathrm{Lactone}\ \mathrm{form}\ \mathrm{of}\ {C}_2\\ {}{C}_{2+}=O& := & \mathrm{Lactone}\ \mathrm{form}\ \mathrm{of}\ {C}_{2+}\\ {}{C}_{n-i}& := & \mathrm{Shorter}\ \mathrm{chain}\ \mathrm{glucans}\\ {}{C}_{2-5}& := & \mathrm{Oligosacchrides}\ \left( DP\in \left\{2,3,4,5\right\}\right)\ \mathrm{of}\ \beta (D)-\mathrm{glucopyranose}\\ {}{C}_1& := & \mathrm{Monosaccharide}\ \mathrm{of}\ \beta (D)-\mathrm{glucopyranose}\\ {}{m}_j& := & \mathrm{Stoichiometry}\ \mathrm{of}\ \mathrm{short}\ \mathrm{chain}\ \mathrm{glucans}\ \left({m}_1<{m}_2<{m}_3<\dots ..{m}_n\right)\end{array}} $$

Glycoside hydrolase 9 (GH9) endoglucanases (*EC* 3.2.1.4) hydrolytically cleave the *β* (1 → 4)-glycoside linkage between contiguous (*D*)-glucopyranose residues and accomplish this with the aid of one or more carbohydrate binding modules (CBMs). Detailed phylogenetics analysis and molecular dating has shown that GH9 (≅480 *AA*) is very well conserved amongst taxa and has been so for ≈3000 Mya [8, 17]. The presence of active site residues in GH9 further imply that catalysis of crystalline cellulose proceeds by a relatively unchanged generic acid-base mechanism and may deploy aspartic (*D*) and/or glutamic (*E*) acids as alternating proton donors/acceptors. The arrangement of these, i.e. {*EE*, *DD*, *DE*, *ED*}, may then dictate the position of the −OH at the hemiacetal/acetal carbon (anomeric carbon; {*C*1, *C*2}) of the oligosaccharide products thereby retaining or inverting the configuration of the parent compound [18].

Carbohydrate-binding modules (CBMs) or carbohydrate-binding domains (CBDs) form distinct subsequences in eukaryotes (plants, CBM49; yeast, CBM54), protists (*Dictyostelium discoideum*, CBM8), fungi (CBM1), and bacteria (CBMs 2-4) [8, 17, 18]. Most CBMs are separated by linkers (<100 *AA*) from the GH domain(s) and vary in length (≈40 − 200 *AA*), number, position (N-, C-termini, central), substrate affinity, and contribution to catalysis [8, 17–41]. For example, GH9 endoglucanases from vascular land plants possess a unique subpopulation of CBM49-encompassing crystalline cellulose-digesting enzymes (class C) in addition to the amorphous cellulose cleaving subsets (classes A and B) [17, 18, 42–44]. The presence of one or more CBMs may also extend the range of substrates of GH9 enzymes to include complex heteropolymeric moieties (chitin, CBM5, 12, 14, 18, 33; polygalactouronic acid, CBM32; lipopolysaccharide/lipoteichoic acid, CBM39) [8, 17, 19, 35–41]. The precise mechanism(s) by which CBM-mediated catalysis proceeds is(are) debatable with several plausible explanations for the observed kinetic data [20–34]. Most CBMs possess non-contiguous aromatic amino acids (tryptophan/phenylalanine/tyrosine) interspersed with amino acids with shorter side chains. These could result in concomitant and non-uniform interactions with the glycosidic linkage(s) and consecutive cycles of stretching and relaxation. This mechanism favours the introduction of strain with consequent weakening of the glycosidic linkage [33, 34, 45–47]. Alternatively, there are reports that polar amino acids (serine/threonine/cysteine) could form complexes with calcium (CBM35, 36, 60) which, even in the absence of an overt CBM may mediate cleavage [48–50].

Extant structures of non-plant GH9 enzymes suggest that crystalline cellulose may be digested in subtle fully enclosed tunnels (processive), or in larger, open solvent accessible grooves/clefts (non-processive), although a mixed mode is likely to prevail in most enzymes [51–60]. The binding site(s) are labelled as plus (substrate, entrance) and minus (product, exit) sites with hydrolytic cleavage occurring between the +1 and −1 sites [51–57]. The length of the tunnel itself (≈50 *Ang*) is consistent amongst other GH9 enzymes and consists of about ten subsites (−7 *to* + 2), where amino acids make contact with the glucan chain [51–57]. Further insights into the mechanistic contributions of GH9, linker, and/or the CBMs may be gleaned from the X-ray structures of enzymes in complex with simple (*DP* < 9; *DP* = {2, 3, 5}) or complex (*DP* = 10; −*SH*) oligosaccharides [58–60]. For example, GH9 and CBM3 are distinct spatial entities (Cel9G, *Clostridium cellulolyticum*; CelE4, *Thermomonospora fusca*) with an interaction surface that comprises a network of hydrogen-bonded residues [59, 60]. However, in the absence of an active enzyme substrate (ES) complex (*DP* ≥ 6), the manner in which polymeric crystalline cellulose is processed by GH9 enzymes is not known [59]. Interestingly, the authors also report an inter-dependence or quasi-allostericity of the GH9 and CMBs in binding crystalline cellulose, a substrate-binding groove that is lined with polar and aromatic acid residues, and the possibility of a polyfunctional CelE4 with exo- and endo-glucanase activities [59, 60]. Crystalline cellulose is the cognate substrate for GH9 endoglucanases in non-plant taxa such as bacteria, archaea, fungi, protists, and arthropods, and may predate plant GH9 enzymes by several millions of years [8]. This, when combined with the similarity between the GH9 domains, suggests that the active site architecture of plant class C enzymes and subsequent reaction chemistry may be similar [8, 51, 52]. Whilst, the data generated vide supra is able to offer insights into the origin and evolution of plant class C enzymes, mechanistic details of the same are fundamental to comprehending the precise manner in which catalysis of crystalline cellulose may proceed. Here, I analyse homology models of putative and characterised plant class C sequences, i.e. with a single wel-defined CBM49 subsequence, to classify and infer the contribution(s) of the GH9, CBM49, and linker to the catalysis of crystalline cellulose.

## Methods

### Model generation, geometry optimization, equilibration, and MD of class C enzymes

**Fig. 1:**
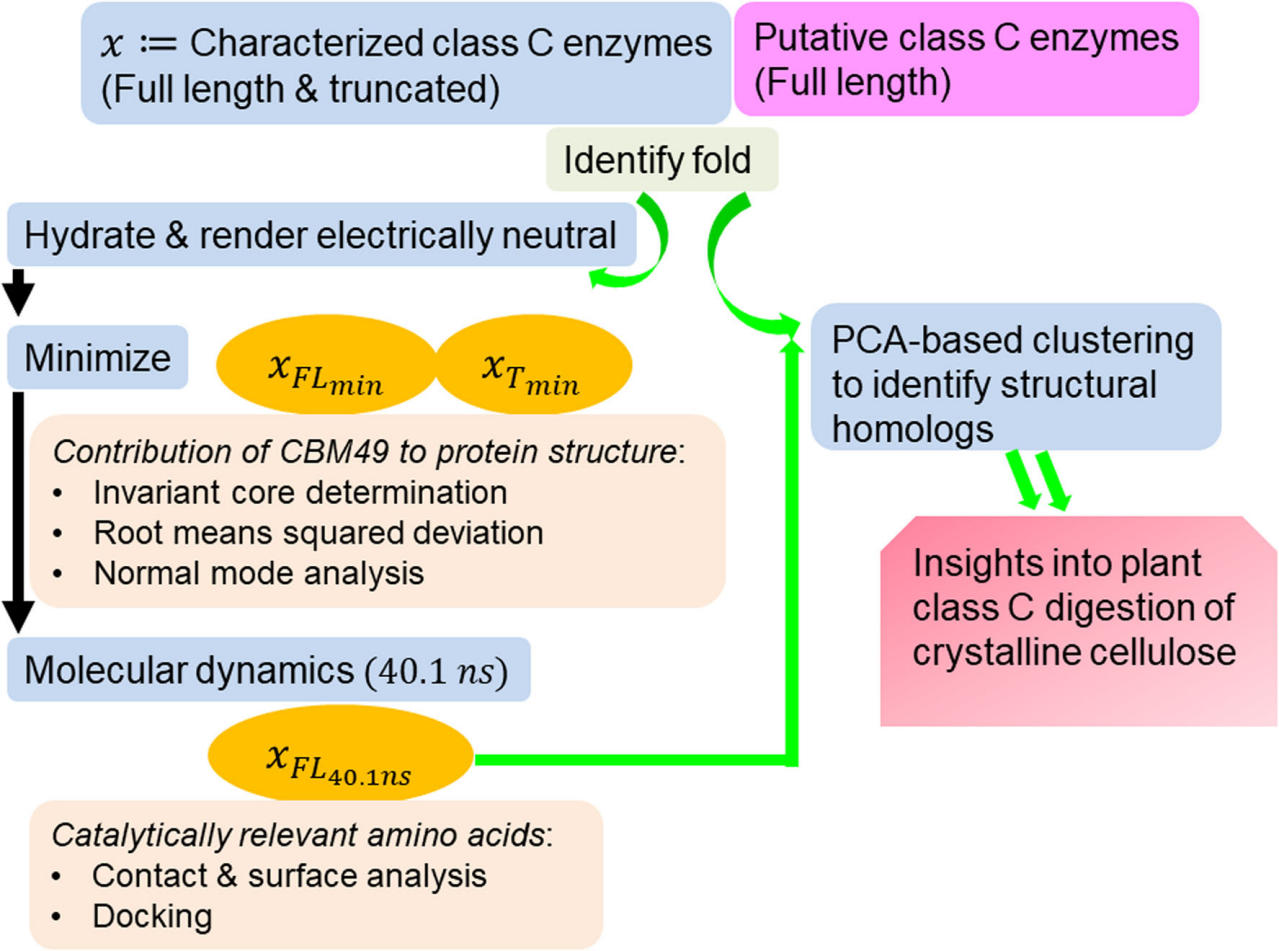
Schema for biophysical characterization of class C GH9 enzymes. Generic protocol to assess contribution of GH9, CBM49, and the linker to catalysis of crystalline cellulose by plant class C enzymes. These steps consisted of fold identification, 3D protein and ligand geometry optimization, invariant core determination and normal mode analysis, surface analysis, cavity and groove delineation, and docking. Folds of characterised (full length, truncated) class C enzymes and putative class C sequences were initially identified. 3D models of class C enzymes with the top scoring templates (non-plant) were used for all further analysis; energy minimization (*E*_min_) of the 3D models was used to compare the effects of truncation on the structural integrity of the protein. Equilibrium structures (40.1 *ns*) were used subsequently to delineate the active site architecture of plant class C GH9 endoglucanases as well as conduct detailed docking studies with cellulose based ligands. Abbreviations—GH, glycoside hydrolase; CBM, carbohydrate binding module; Phyre2, protein homology/analogy recognition engine

A generic protocol to assess the contribution(s) of GH9, linker, and CBM49 has been outlined (Fig. 1). Laboratory-characterised full length (*FL*) and truncated (*T*) class C sequences (*x*) from *Oryza sativa* (*Q*5*NAT*0), *Gossypium hirsutum* (*Q*8*LJP*6), *Nicotiana tabacum* (*Q*93*WY*9), *Solanum lycopersicum* (*Q*9*ZSP*9), i.e. *x*(*FL*) = *x*_*FL*_ = *GH*9 ∪ *L* ∪ *CBM*49; *x*(*T*) = *x*_*T*_ = *GH*9 ∪ *L*; *x* ∈ {*Q*5*NAT*0, *Q*8*LJP*6, *Q*93*WY*9, *Q*9*ZSP*9}, along with full-length putative class C sequences (*n* = 92) identified in previous work were submitted to Phyre2 (www.sbg.bio.ic.ac.uk/phyre2) [8, 18, 61]. The templates were graded in terms of the root mean squared deviation (*rmsd*) of their *Cα*-backbones from the predicted model, presence of an extant homologous structure (confidence), proportion of the sequence modelled (coverage), and sequence identity.

The LeAP module of AMBERTOOLS v17.0 was used to explicitly add water molecules (TIP3P) to the 3D models of characterised class C enzymes (*n* = 4; *x*_*FL*_, *x*_*T*_) and render the modelled structures electrically neutral ({*Na*^+^, *Cl*^−^} ≥ 1) (Fig. 1) [62]. The models were optimised by minimizing their computed energies in a bi-phasic (*n*_min1_ = *n*_min2_ = 5000) implementation of the steepest descent algorithm with (100 Kcal mol^−^*Ang*^2^) and without positional restraints for the amino acids (Fig. 1, Table 1). The minimised models $\left({x}_{FL_{\mathrm{min}}},{x}_{T_{\mathrm{min}}}\right)$ were utilised for comparative analyses to ascertain the significance and relevance of CBM49 to the structural integrity of the protein. Full length minimised structures were perturbed (Temp : 0.0K → 300.0K; constant volume; 20 *ps*) with low energy (10.0 Kcal mol^−1^*Ang*^2^) positional restraints for the amino acids, which was followed by an unrestrained (Temp = 300.0K; constant pressure; 100 *ps*) and a production grade run (40.1 *ns*) MD run with NAMD v2.13 (nanoscale molecular dynamics) and VMD v1.9.3 (visual molecular dynamics; configuration files) (Fig. 1, Table 1) [63, 64]. These models, i.e. ${x}_{FL_{40.1 ns}};x\in \left\{Q5 NAT0,Q8 LJP6,Q93 WY9,Q9 ZSP9\right\}$, were used to infer active site architecture, perform docking experiments, and identifying structural homologues of selected characterised class C enzymes (Fig. 1, Table 1).

**Table 1:** Parameters for minimizing, equilibrating, and simulating 3D structures of characterised class C GH9 endoglucanases

		Min_1	Min_2	Equil_1	Equil_2	40.1 ns
Algorithm		SD	SD	Shake	Shake	
imin		1	1	0	0	0
irest		0	0	0	1	–
maxcyc		5000	5000	–	–	–
ncyc		10000	10000	–	–	–
ntc		–	–	2.0	2.0	–
ntf		–	–	2.0	2.0	–
ntr		1	0	1	0	–
Force (Kcal mol^−1^) Ang^2^)		100.0	–	10.0	–	–
ntt		–	–	3.0	3.0	–
tempi (K)		–	–	0	300.0	300.0
temp0 (K)		–	–	300.0	300.0	300.0
igb		0	0	0	0	–
cut (Ang)		12.0	12.0	10.0	10.0	10.0
nstlim				10000	50000	20,050,000
dt				0.002	0.002	0.002
ntb		–	–	1	2	–
ntp		–	–	0.0	1.0	–
pres0		–	–	–	1.0	
taup		–	–	–	2.0	
ntpr		100	100	1000	10000	10000
ntwx		–	–	1000	10000	10000
ntwr		–	–	5000	10000	10000
Dielectric		–	–	–	–	1.0
Simulation space partitioning	Switching	–	–	–	–	On
	Switchdist (Ang)					9
	Pairlistdist (Ang)					12
	exclude					Scaled 1–4
	1–4scaling					1.0
firsttimestep	Timestep	–	–	–	–	1
	Stepspercycle					20
	nonbondedFreq					2
	fullElectFrequency					4
Langevin dynamics	Langevin	–	–	–	–	On
	LangevinDamping					1.0
	LangevinTemp					300
	LangevinHydrogen					No
	LangevinPiston					On
	LangevinPistonTarget					1.01325
	LangevinPistonPeriod					2000 fs
	LangevinPistonDecay					1000 fs
	LangevinPistonTemp					300
	useFlexibleCell					No
	useGroupPressure					No
	fixedAtomsForces					Off
Cell basis vectors	CellBasisVector1	–	–	–	–	x-coord,0,0
	CellBasisVector2					0,y-coord,0
	CellBasisVector3					0,0,z-coord
	wrapAll	–	–	–	–	No
	dcdUnitCell					Yes

### Invariant core analysis of characterised and putative class C enzymes

The invariant core is a measure of inferring structural variation from the *xyz* coordinates of aligned atoms of amino acids at specific site(s) and was utilised to assess the conservation of GH9, linker, and CBM49. This was accomplished by generating multiple sequence alignments (MSA) with a standalone version of multiple sequence alignment by computing log-expectation (MUSCLE; http://drive5.com/muscle) in association with the R-package Bio3D (http://thegrantlab.org/bio3d) and with scripts developed in house (Fig. 1) [65–67]. The volume of the invariant core was then iteratively computed and is defined as the least volume (*V* < 1.0 *Ang*^3^) from all volumes of arbitrary ellipsoids (*V* ≥ 1.00 *Ang*^3^). Here, an ellipsoid comprises the variance of eigenvalues along its three principle axes of the atomic *xyz* coordinates of amino acid(s) at every aligned position of the combined and ungapped MSA, whilst its volume represents the structural variation at the given position(s) [67–70]. Although Alanine is not the most hydrophobic amino acid (*kdH*_*Ala*_ < *kdH*_*Met*_ < *kdH*_*Cys*_ < *kdH*_*Phe*_ < *kdH*_*Leu*_ < *kdH*_*Val*_ < *kdH*_*Ile*_; *kdH* ≔ Kyte Doolittle Hydrophobicity index), its non-bulky and unbranched side chain renders it an excellent index of invariance of a given structure. Since truncating the proteins might be expected to dramatically alter the behaviour of the GH9 of the 3D models, a corrected subset (*O. sativa*, #*AA* = 456; *N. tabacum*, #*AA* = 466; *G. hirsutum*, #*AA* = 464; *S. lycopersicum*, #*AA* = 476) that comprised matched residues of full length proteins was used $\left(x\left({cFL}_{\mathrm{min}}\right)={x}_{cFL_{\mathrm{min}}}\right)$, i.e.

1 $$ {x}_{cF{L}_{\mathrm{min}}}={x}_{F{L}_{\mathrm{min}}}-\left({x}_{F{L}_{\mathrm{min}}}-{x}_{T_{\mathrm{min}}}\right) $$

for comparative analyses $\left({x}_{cFL_{\mathrm{min}}}\  vs\ {x}_{T_{\mathrm{min}}}\right)$ where *x* ∈ {*Q*5*NAT*0, *Q*8*LJP*6, *Q*93*WY*9, *Q*9*ZSP*9}. Since, the number of characterised class C enzymes was small (*n* = 4), a larger MSA, which included 3D models of putative class C enzymes (*n* = 92) was generated. The eigenvalues of the lowest invariant core (0 < *V*(*Ang*) ≤ 1.0) were then investigated with principal component analysis (PCA), which in turn was used to cluster and identify structural homologues of characterised class C enzymes. The aligned models were thence utilised to infer plausible active-site architecture(s) of plant class C enzymes.

### Structural analysis of 3D models of plant class C GH9 enzymes

Low frequency (*ω*) and non-trivial normal modes (NM) (*ω*(*NM*) > 0, *NM* > 6; *ω* ∈ *ℝ*, *NM* ∈ *ℕ*) of the superposed 3D models as well as individual protein sequences of the minimised $\left( NM\left({x}_{FL_{\mathrm{min}}}\right)\right.={NM}_{x_{FL_{\mathrm{min}}}}, NM\left({x}_{T_{\mathrm{min}}}\right)={NM}_{x_{T_{\mathrm{min}}}}, NM\left({x}_{cFL_{\mathrm{min}}}\right)={NM}_{x_{c{FL}_{\mathrm{min}}}}\Big)$ and 40.1 *ns* MD trajectories $\left( NM\left({x}_{FL_{40.1 ns}}\right)={NM}_{x_{FL_{40.1 ns}}}\right)$ was done [67, 71, 72]. Each normal mode investigated was an eigenvector and was computed from the combined oscillatory motion of the *Cα*-atoms under a generic force field and possessed a characteristic eigenvalue (Fig. 1). As discussed vide supra, the corrected subset (*x*_*cFL*_) of each protein was used for comparative analyses $\left({x}_{cFL_{\mathrm{min}}}\  vs\ {x}_{T_{\mathrm{min}}}\right)$ where *x* ∈ {*Q*5*NAT*0, *Q*8*LJP*6, *Q*93*WY*9, *Q*9*ZSP*9}. A modified rmsf-based score $\left(\mathrm{rmsf}\left({x}_{cFL_{\mathrm{min}}}\right)={\mathrm{rmsf}}_{x_{c{FL}_{\mathrm{min}}}},\mathrm{rmsf}\left({x}_{T_{\mathrm{min}}}\right)={\mathrm{rmsf}}_{x_{T_{\mathrm{min}}}}\right)$ was formulated as under:

2 $$ \Delta  {\mathrm{rmsf}}_{x_{c{FL}_{\mathrm{min}}}}=\max \left({\mathrm{rmsf}}_{x_{c{FL}_{\mathrm{min}}}}\right)-\min \left({\mathrm{rmsf}}_{x_{c{FL}_{\mathrm{min}}}}\right) $$

3 $$ \Delta  {\mathrm{rmsf}}_{x_{T_{\mathrm{min}}}}=\max \left({\mathrm{rmsf}}_{x_{T_{\mathrm{min}}}}\right)-\min \left({\mathrm{rmsf}}_{x_{T_{\mathrm{min}}}}\right) $$

These, in tandem with the standard deviation $\left({\sigma}_{\mathrm{rmsf}}\left({x}_{cFL_{\mathrm{min}}},{x}_{T_{\mathrm{min}}}\right)\right)$, were used to assess and compare the influence of atomic motion on the structural organization of characterised class C proteins. The presence of correlated displacements of residues for each full length protein after the MD run $\left({x}_{FL_{40.1 ns}}\right)$ was also examined by the dynamic cross correlation map (DCCM), i.e. the covariance matrix of the root mean square fluctuations $\left(\mathrm{rmsf}\left({x}_{FL_{40.1 ns}}\right)={\mathrm{rmsf}}_{x_{FL_{40.1 ns}}}\right)$ of every *Cα* atom of each class C protein $\left(\operatorname{cov}\left({x}_{F{L}_{40.1 ns}},{x}_{F{L}_{40.1 ns}}\right)\right)\forall$*x* ∈ {*Q*5*NAT*0, *Q*8*LJP*6, *Q*93*WY*9, *Q*9*ZSP*9} (Fig. 1). These investigations were complemented by computing the surfaces, cavities, and, grooves present in the GH9, linker, and CBM49 regions or at their interfaces using the SPDBV (Swiss protein data bank viewer) suite of programs (https://spdbv.vital-it.ch) (Fig. 1) [73]. A cylinder of minimum area and volume was used to model and thence approximate the dimensions (radius ≔ *r*, height ≔ *h*, length ≔ *l*; *r*, *h*, *l* ∈ *ℝ*_+_) of the predicted substrate binding and cleaving groove(s) necessary to accommodate and digest crystalline. These formulas were derived and are as under:

4 $$ {A}_o\cong \varnothing +{A}_c=(2)\left(\pi \right)(r)\left(r+h\right) $$

5 $$ {V}_o\cong \beta +{V}_c=\left(\pi \right)\left({r}^2\right)(h) $$

Differentiating *w*, *r*, *t*, *h* and solving for *r* and *h* results in the formulae

6 $$ r=\sqrt{A_o/(4)\left(\pi \right)} $$

7 $$ h=(4)\left({V}_o\right)/{A}_o $$

8 $$ l={A}_0/r $$

$$ {\displaystyle \begin{array}{ccc}{A}_o& := & \mathrm{Computed}\ \mathrm{area}\ \mathrm{of}\ \mathrm{wide}\ \mathrm{groove}\ \left({Ang}^2\right)\\ {}{V}_o& := & \mathrm{Computed}\ \mathrm{volume}\ \mathrm{of}\ \mathrm{wide}\ \mathrm{groove}\ \left({Ang}^3\right)\\ {}r& := & \mathrm{Radius}\ \mathrm{of}\ \mathrm{approximating}\ \mathrm{cylinder}\\ {}h& := & \mathrm{Height}\ \mathrm{of}\ \mathrm{approximating}\ \mathrm{cylinder}\\ {}l& := & \mathrm{Length}\ \mathrm{of}\ \mathrm{groove}\ (Ang)\\ {}{A}_c& := & \mathrm{Computed}\ \mathrm{area}\ \mathrm{of}\ \mathrm{approximating}\ \mathrm{cylinder}\ \left({Ang}^2\right)\\ {}{V}_c& := & \mathrm{Computed}\ \mathrm{volume}\ \mathrm{of}\ \mathrm{approximating}\ \mathrm{cylinder}\ \left({Ang}^3\right)\\ {}\varnothing & := & \mathrm{Constant}\ \mathrm{of}\ \mathrm{approximation}\ (Area)\\ {}\beta & := & \mathrm{Constant}\ \mathrm{of}\ \mathrm{approximation}\ (Volume)\end{array}} $$

The difference data, i.e. ∅ = |*A*_*o*_ − *A*_*c*_|; *β* = |*V*_*o*_ − *V*_*c*_|, was then used to quantify and characterise this approximation.

### Ligand preparation and utilization

The degree of polymerization (*DP*) was utilised to shortlist potential candidates of cellulose oligomers (2 ≤ *DP* ≤ 8) and their stereoisomers, from the ZINC12 and PubChem databases (http://www.ncbi.nlm.nih.gov/pubchem;http://zinc.docking.org) [74, 75]. Briefly, for 2 ≤ *DP* ≤ 4 (*n* = 3) and for 5 ≤ *DP* ≤ 8 (*n* = 1) were utilised (*n* = 13 = 3 ∗ (3) + 4) for this analysis (Fig. 1, Table 2). The ligands were downloaded in the isomeric SMILES format and built with ChemSketch installed locally. Geometry isomerization was initially performed with Chemsketch itself, followed by a further 500 − 2000 cycles of optimization with the steepest descent and the Broyden–Fletcher–Goldfarb–Shanno (BFGS) algorithms [76]. These were implemented with a local installation of Arguslab using the universal force field (UFF) parameter of the molecular mechanics component (http://www.arguslab.com/arguslab.com) [77]. Additional relevant parameters for this step were the cutoff for non-bonded interactions (8.0 *Ang*) and data updates after every 20 steps. The optimization converged for all the ligands tested with a net energy of < − 8 Kcal mol^−^*Ang*^2^. The *xyz* coordinates along with other relevant information was encoded as a pdb file and uploaded to the DockingServer (https://www.dockingserver.com/web) [78]. The geometry of all the ligands (*n* = 13) uploaded were finally optimised using the semi-empirical (PM6) method of partial charge addition, the Merck molecular force field (MMFF94), with all rotatable bonds delineated and non-polar hydrogen atoms merged [79, 80].

**Table 2:** Ligands utilised in docking experiments

Formula	Database	ID	LID	MW	Isomeric SMILES
C_12_H_22_O_11_	ZINC12	53683219	C21	342.297	C([C@H]1[C@@H]([C@H]([C@@H]([C@@H](O1)O[C@@H]2[C@@H](O[C@H]([C@@H]([C@H]2O)O)O)CO)O)O)O)O
		3978744	C22	342.297	C([C@@H]1[C@H]([C@@H]([C@H]([C@@H](O1)O[C@@H]2[C@H](O[C@@H]([C@@H]([C@H]2O)O)O)CO)O)O)O)O
		4097113	C23	342.297	C([C@@H]1[C@H]([C@@H]([C@H]([C@@H](O1)O[C@@H]2[C@H](O[C@H]([C@@H]([C@H]2O)O)O)CO)O)O)O)O
C_18_H_32_O_16_	ZINC12	53683224	C31	504.438	C([C@@H]1[C@H]([C@@H]([C@H]([C@@H](O1)O[C@@H]2[C@@H](O[C@H]([C@@H]([C@H]2O)O)O[C@@H]3[C@@H](O[C@H]([C@@H]([C@H]3O)O)O)CO)CO)O)O)O)O
		8216112	C32	504.438	C([C@@H]1[C@H]([C@@H]([C@H]([C@@H](O1)O[C@@H]2[C@H](O[C@H]([C@@H]([C@H]2O)O)O[C@@H]3[C@H](O[C@@H]([C@@H]([C@H]3O)O)O)CO)CO)O)O)O)O
		8220386	C33	504.438	C([C@@H]1[C@H]([C@@H]([C@H]([C@@H](O1)O[C@@H]2[C@H](O[C@H]([C@@H]([C@H]2O)O)O[C@@H]3[C@H](O[C@H]([C@@H]([C@H]3O)O)O)CO)CO)O)O)O)O
C_24_H_42_O_22_	ZINC12	96006066	C41	682.578	C([C@H]1[C@@H]([C@H]([C@@H]([C@@H](O1)O[C@@H]2[C@@H](O[C@H]([C@@H]([C@H]2O)O)O[C@@H]3[C@@H](O[C@H]([C@@H]([C@H]3O)O)O[C@@H]4[C@@H](OC([C@@H]([C@H]4O)O)(O)O)CO)CO)CO)O)O)O)O
		85603797	C42	666.579	C([C@@H]1[C@H]([C@@H]([C@H]([C@@H](O1)O[C@@H]2[C@H](O[C@H]([C@@H]([C@H]2O)O)O[C@@H]3[C@H](O[C@H]([C@@H]([C@H]3O)O)O[C@@H]4[C@H](O[C@H]([C@@H]([C@H]4O)O)O)CO)CO)CO)O)O)O)O
		87528241	C43	666.579	C([C@@H]1[C@H]([C@@H]([C@H]([C@@H](O1)O[C@@H]2[C@H](O[C@H]([C@@H]([C@H]2O)O)O[C@@H]3[C@H](O[C@H]([C@@H]([C@H]3O)O)O[C@@H]4[C@H](O[C@@H]([C@@H]([C@H]4O)O)O)CO)CO)CO)O)O)O)O
C_30_H_52_O_26_	ZINC12	52940142	C5	828.7183	C([C@@H]1[C@H]([C@@H]([C@H]([C@@H](O1)O[C@@H]2[C@H](O[C@H]([C@@H]([C@H]2O)O)O[C@@H]3[C@H](O[C@H]([C@@H]([C@H]3O)O)O[C@@H]4[C@H](O[C@H]([C@@H]([C@H]4O)O)O[C@H]([C@@H](CO)O)[C@@H]([C@H](C=O)O)O)CO)CO)CO)O)O)O)O
C_36_H_62_O_31_	PUBCHEM	74539963	C6	990.85888	C([C@@H]1[C@H]([C@@H]([C@H]([C@@H](O1)O[C@@H]2[C@H](O[C@H]([C@@H]([C@H]2O)O)O[C@@H]3[C@H](O[C@H]([C@@H]([C@H]3O)O)O[C@@H]4[C@H](O[C@H]([C@@H]([C@H]4O)O)O[C@@H]5[C@H](O[C@H]([C@@H]([C@H]5O)O)O[C@@H]6[C@H](OC([C@@H]([C@H]6O)O)O)CO)CO)CO)CO)CO)O)O)O)O
C_42_H_72_O_36_	PUBCHEM	440947	C7	1152.99948	C([C@@H]1[C@H]([C@@H]([C@H]([C@@H](O1)O[C@@H]2[C@H](O[C@H]([C@@H]([C@H]2O)O)O[C@@H]3[C@H](O[C@H]([C@@H]([C@H]3O)O)O[C@@H]4[C@H](O[C@H]([C@@H]([C@H]4O)O)O[C@@H]5[C@H](O[C@H]([C@@H]([C@H]5O)O)O[C@@H]6[C@H](O[C@H]([C@@H]([C@H]6O)O)O[C@@H]7[C@H](O[C@H]([C@@H]([C@H]7O)O)O)CO)CO)CO)CO)CO)CO)O)O)O)O
C_48_H_80_O_40_	PUBCHEM	5287407	C8	1297.1248	C([C@@H]1[C@@H]2[C@@H]([C@H]([C@H](O1)O[C@@H]3[C@H](O[C@@H]([C@@H]([C@H]3O)O)O[C@@H]4[C@H](O[C@@H]([C@@H]([C@H]4O)O)O[C@@H]5[C@H](O[C@@H]([C@@H]([C@H]5O)O)O[C@@H]6[C@H](O[C@@H]([C@@H]([C@H]6O)O)O[C@@H]7[C@H](O[C@@H]([C@@H]([C@H]7O)O)O[C@@H]8[C@H](O[C@@H]([C@@H]([C@H]8O)O)O[C@@H]9[C@H](O[C@H](O2)[C@@H]([C@H]9O)O)CO)CO)CO)CO)CO)CO)CO)O)O)O

*ZINC*, zinc is not commercial; *LID*, ligand identity; *MW*, molecular weight

### Docking experiments of characterised plant class C GH9 endoglucanases

3D models of characterised plant class C GH9 endoglucanases $\left({x}_{FL_{40.1 ns}};x\in \left\{Q5 NAT0,Q8 LJP6,Q93 WY9,Q9 ZSP9\right\}\right)$ were uploaded to the DockingServer (https://www.dockingserver.com/web) [78]. The server with the aid of AutoDock, added the necessary hydrogens, atomic charges, and utilised a grid of 100 × 100 × 100 points with a spacing of 0.375 *Ang* [81]. The final positions of the coordinates on this grid were modified to include the previously delineated interaction surfaces of GH9, linker, and CBM49, for all the proteins. Computation of the non-covalent bonds (van der Waals, electrostatics) was accomplished using the parameter set from AutoDock. Docking was performed using the Lamarckian genetic algorithm and a local search method after the initial position, orientation, and torsion angles of the ligand molecules were set randomly [81, 82]. Data for a single experiment was derived from 100 different runs (*∆*translation = 0.2 *Ang*; *∆*torsion = *∆*quaternion = 5). These were set to terminate after a previously set limit of energy evaluations (E_evals =2500000, population =150). The contribution of these residues to the catalysis of crystalline cellulose was inferred from the free energy $\left(x\left({\Delta  G}_y\right)={x}_{{\Delta  G}_y}\right)$ and constant of inhibition $\left(x\left({Ki}_y\right)={x}_{Ki_y}\right)$ ∀*x* ∈ {*Q*5*NAT*0, *Q*8*LJP*6, *Q*93*WY*9, *Q*9*ZSP*9}; *y* ∈ {*C*21, *C*22, *C*23, *C*31, *C*32, *C*33, *C*41, *C*5, *C*6, *C*7, *C*8}.

## Results

### Data organization and arrangement

**Fig. 2:**
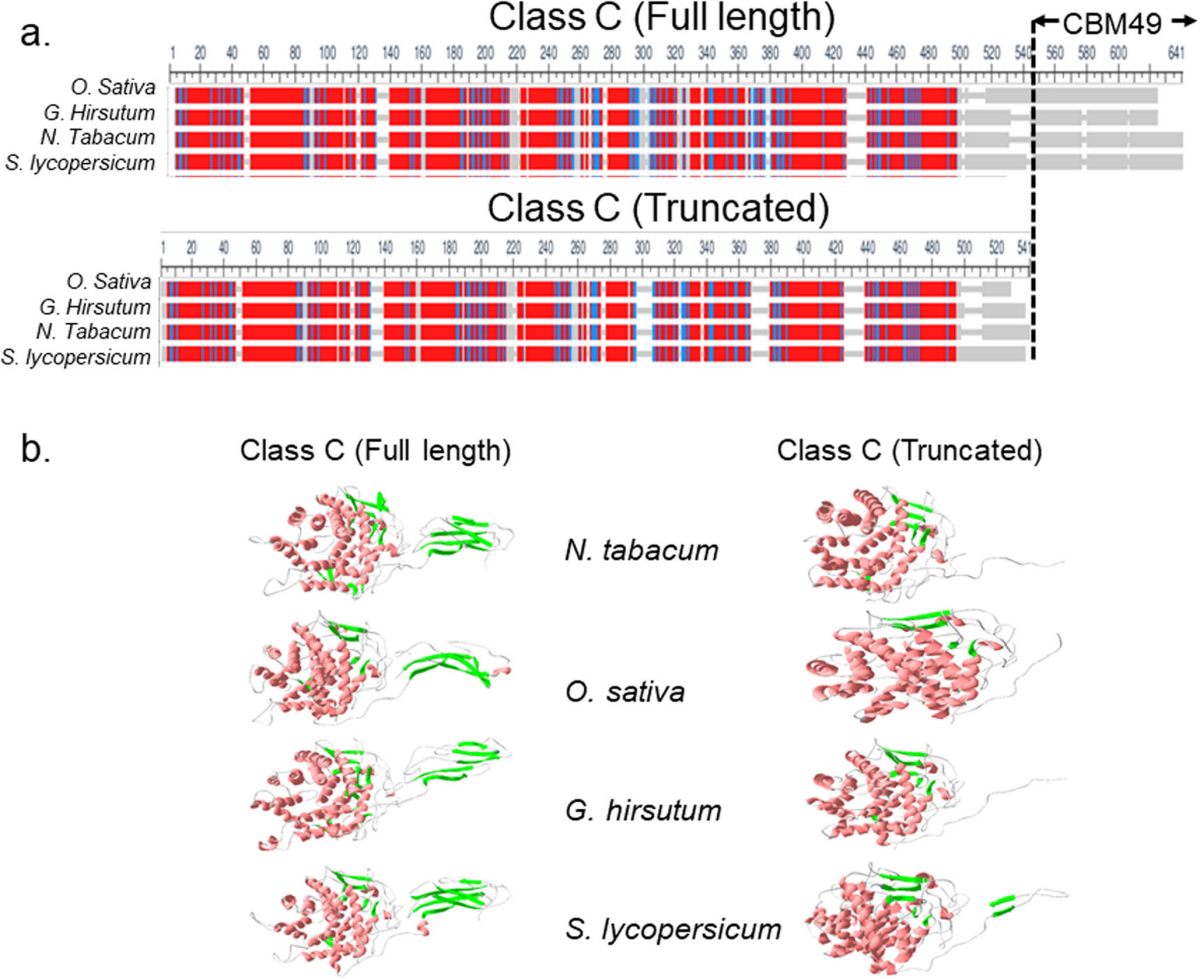
3D models of full length and truncated plant class C endoglucanases. **a** Full length (*GH*9 ∪ *L* ∪ *CBM*49) and truncated (*GH*9 ∪ *L*) sequences of characterised (*n* = 4; *Oryza sativa*; *Gossypium hirsutum*; *Solanum lycopersicum*; *Nicotiana tabaccum*) plant class C GH9 endonucleases along with full-length sequences of putative class C enzymes (*n* = 92) were submitted to Phyre2; **b** The 3D models that represented the best approximation to the template X-ray structures *Thermomonospora fusca* (PDB: 1JS4; *UID* : *Q*8*LJP*6) and *Clostridium cellulolyticum* (PDB: 1GA2; *UIDs* : *Q*5*NAT*0, *Q*9*ZSP*9, *Q*93*WY*9) were used for all further investigations. The parameters used to evaluate these were sequence identity, presence of an homologous structure (confidence), and the percentage of the protein that could be modelled (coverage). Abbreviations—GH9, glycoside hydrolase; L, linker sequence; CBM49, carbohydrate binding module; MUSCLE, multiple sequence comparison by log-expectation; PDB, protein data bank; Phyre2, protein homology/analogy recognition engine; *UID* : *Q*5*NAT*0, *O. sativa*; *UID* : *Q*8*LJP*6, *G. hirsutum*; *UID* : *Q*93*WY*9, *N. tabacum*; *UID* : *Q*9*ZSP*9, *S. lycopersicum*

**Fig. 3:**
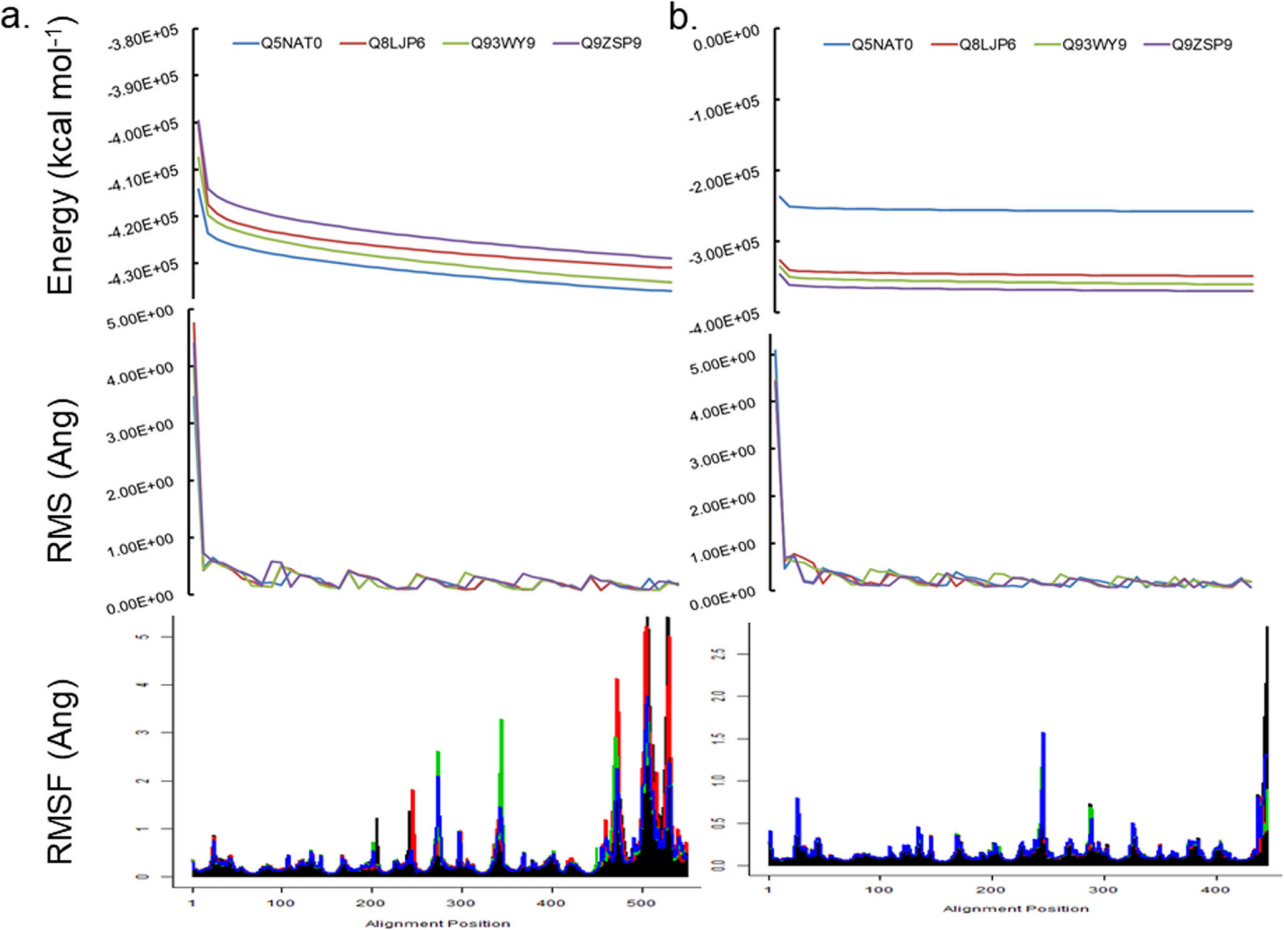
Comparative analyses of full length and truncated 3D models of class C GH9 endoglucanases. **a**, **b** Energy minimization (*E*_*min*_ < 0.0) of 3D models of full length and truncated $\left({x}_{FL_{min}},{x}_{T_{min}};x\in \left\{Q5 NAT0,Q8 LJP6,Q93 WY9,Q9 ZSP9\right\}\right)$ characterised class C GH9 endoglucanases was carried out and monitored by the root mean squared deviation of the intermediate structures. The absence of significant variation of the total (ETOT) energy for the models studied suggested that these were stable and could be examined further. **c** Normal mode analysis of these minimised models suggested that the carboxy-terminal end of the linker region and the CBM49 regions experienced increased oscillatory motion, an observation which is mitigated when these were truncated. The frequencies for *O. sativa* $\Big(Q5 NAT{0}_{T_{\mathrm{min}}}\gg Q5 NAT{0}_{FL_{\mathrm{min}}}$) and was more pronounced for the lower non-trivial modes as opposed to the proteins from *S. lycopersicum* and *N. tabacum* $\left({x}_{T_{\mathrm{min}}}<{x}_{FL_{\mathrm{min}}};x\in \left\{Q8 LJP6,Q93 WY9,Q9 ZSP9\right\}\right)$. Abbreviations—GH, glycoside hydrolase; CBM, carbohydrate binding module; FL, full length; T, truncated; *UID* : *Q*5*NAT*0, *O. sativa*; *UID* : *Q*8*LJP*6, *G. hirsutum*; *UID* : *Q*93*WY*9, *N. tabacum*; *UID* : *Q*9*ZSP*9, *S. lycopersicum*

**Fig. 4:**
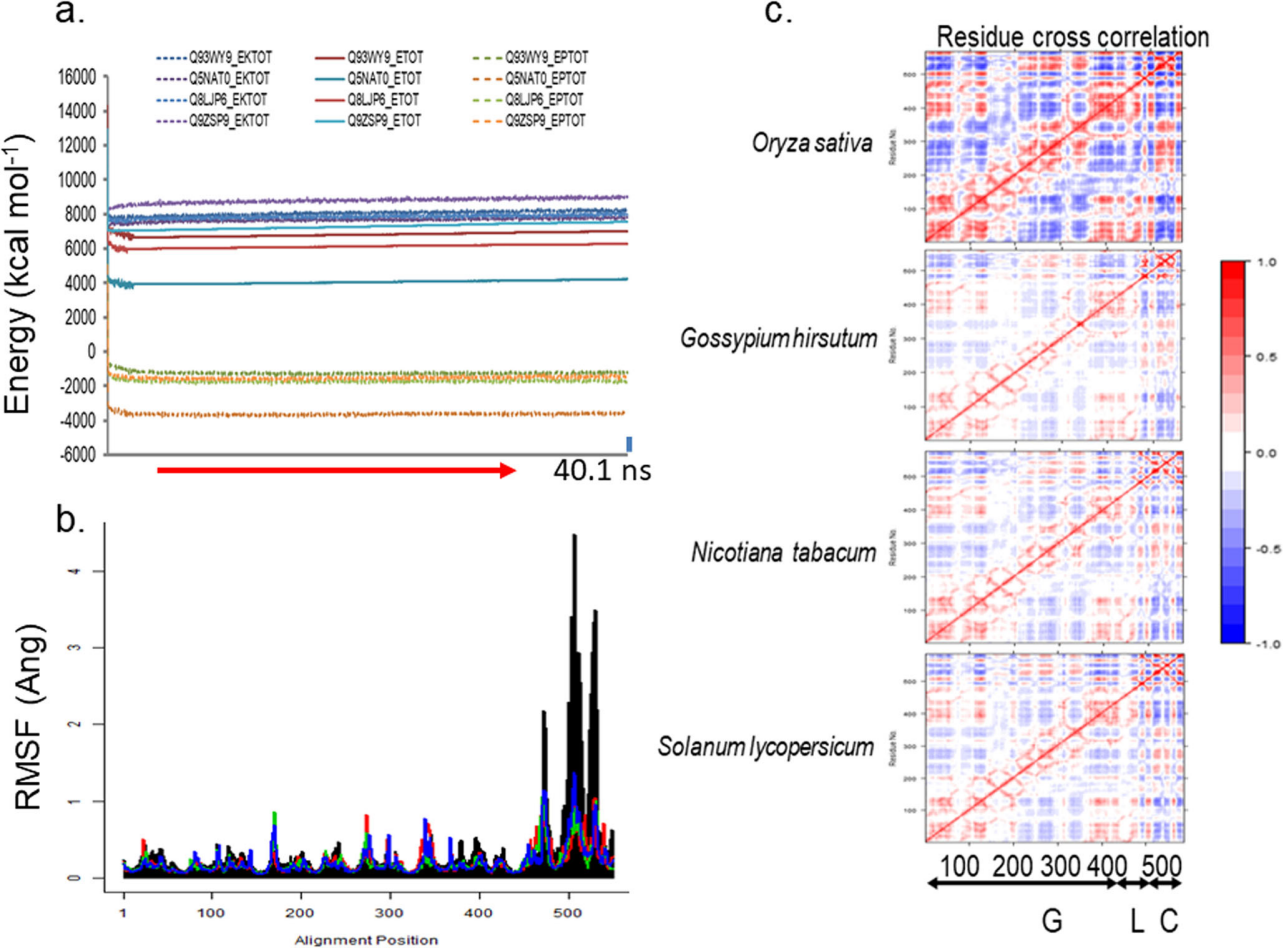
Structural analyses of 3D models of class C GH9 endoglucanases. **a**, **b** 3D models of full length characterised class C GH9 endoglucanases, at equilibrium, were monitored by the root mean squared deviation of the intermediate structures, and the absence of significant variation of the kinetic (EKTOT), potential (EPTOT), and thence the total (ETOT) energies. **c** Normal mode analysis and root means square fluctuations (*rmsf*) suggested that the carboxy-terminal end of the linker region and/or CBM49 regions are flexible and may contribute to an adaptable active site geometry and **d** dynamic cross-correlation map of residues of characterised class C GH9 enzymes. The coavariance matrix of the *rmsf* values of each residue per protein was computed. The dynamic cross correlation map ($\mathit{\operatorname{cov}}\left({rmsf}_{x_{FL_{40.1 ns}}},{rmsf}_{x_{FL_{40.1 ns}}}\right)$ ∀*x* ∈ {*Q*5*NAT*0, *Q*8*LJP*6, *Q*93*WY*9, *Q*9*ZSP*9}) was examined for areas of positive correlation (red, *r* → 1.00) across all modes of vibrational motion. The off-diagonal data suggests that like the bacterial templates, class C enzymes may also possess different non-contiguous segments (*G*, *L*, *C*) whose atomic displacements might be correlated. Evaluating the positive *r*−coefficients suggests the existence of multiple interaction surfaces between these. Abbreviations—*C*, carbohydrate binding module 49; cov, covariance matrix; *G*, glycoside hydrolase 9; *L*, linker; *r*, correlation coefficient; GH, glycoside hydrolase; CBM, carbohydrate binding module; FL, full length; *UID* : *Q*5*NAT*0, *O. sativa*; *UID* : *Q*8*LJP*6, *G. hirsutum*; *UID* : *Q*93*WY*9, *N. tabacum*; *UID* : *Q*9*ZSP*9, *S. lycopersicum*

**Fig. 5:**
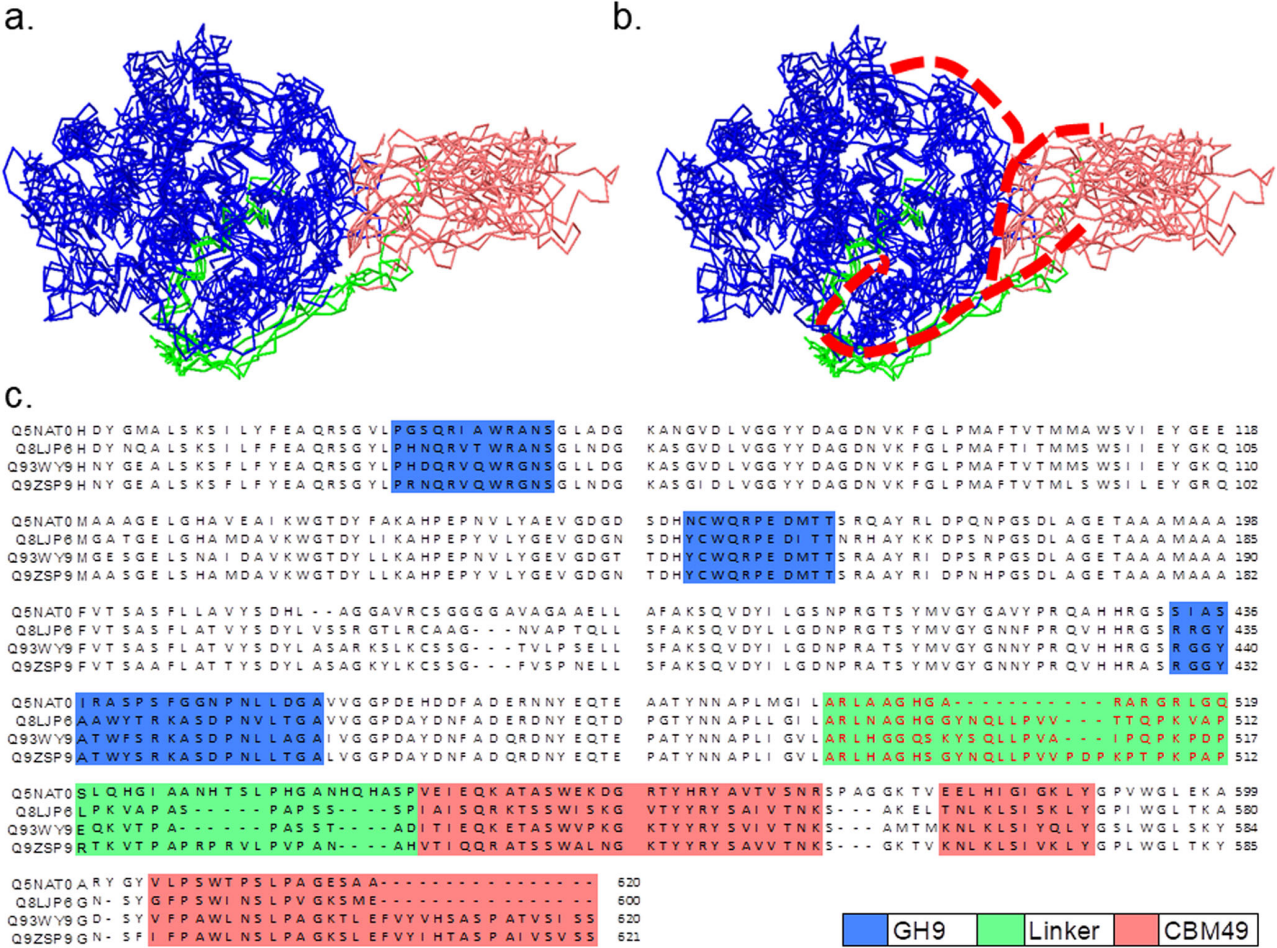
Characterizing non-contiguous interacting residues of class C enzymes. The NMA- and DCCM -data of full length characterised class C enzymes $\left({x}_{FL_{40.1 ns}};x\in \left\{Q5 NAT0,Q8 LJP6,Q93 WY9,Q9 ZSP9\right\}\right)$ were analysed for the presence of interacting residues of GH9, linker, and CBM49. The MSA of these suggests that GH9 and CBM49 are comprised of three potential regions of interactions (*G*1, *G*2, *G*3; *C*1, *C*2, *C*3), by which they interact with each other as well as the intervening linker. These may be summarised as ${IS}_x^{GC},{IS}_x^{GL},{IS}_x^{CL}$ and exceptionally ${IS}_x^{GLC}$. Here *x* ∈ {*Q*5*NAT*0, *Q*8*LJP*6, *Q*93*WY*9, *Q*9*ZSP*9}. These residues were additionally, submitted for docking with ligands of cellulose to ascertain their functional relevance. Abbreviations—CBM49≡C, carbohydrate binding module; DCCM, dynamic cross-correlation map; FL, full length; GH9≡G, glycoside hydrolase 9; IS, interaction surface; L, linker; MSA, multiple sequence alignment; NMA, normal mode analysis

**Fig. 6:**
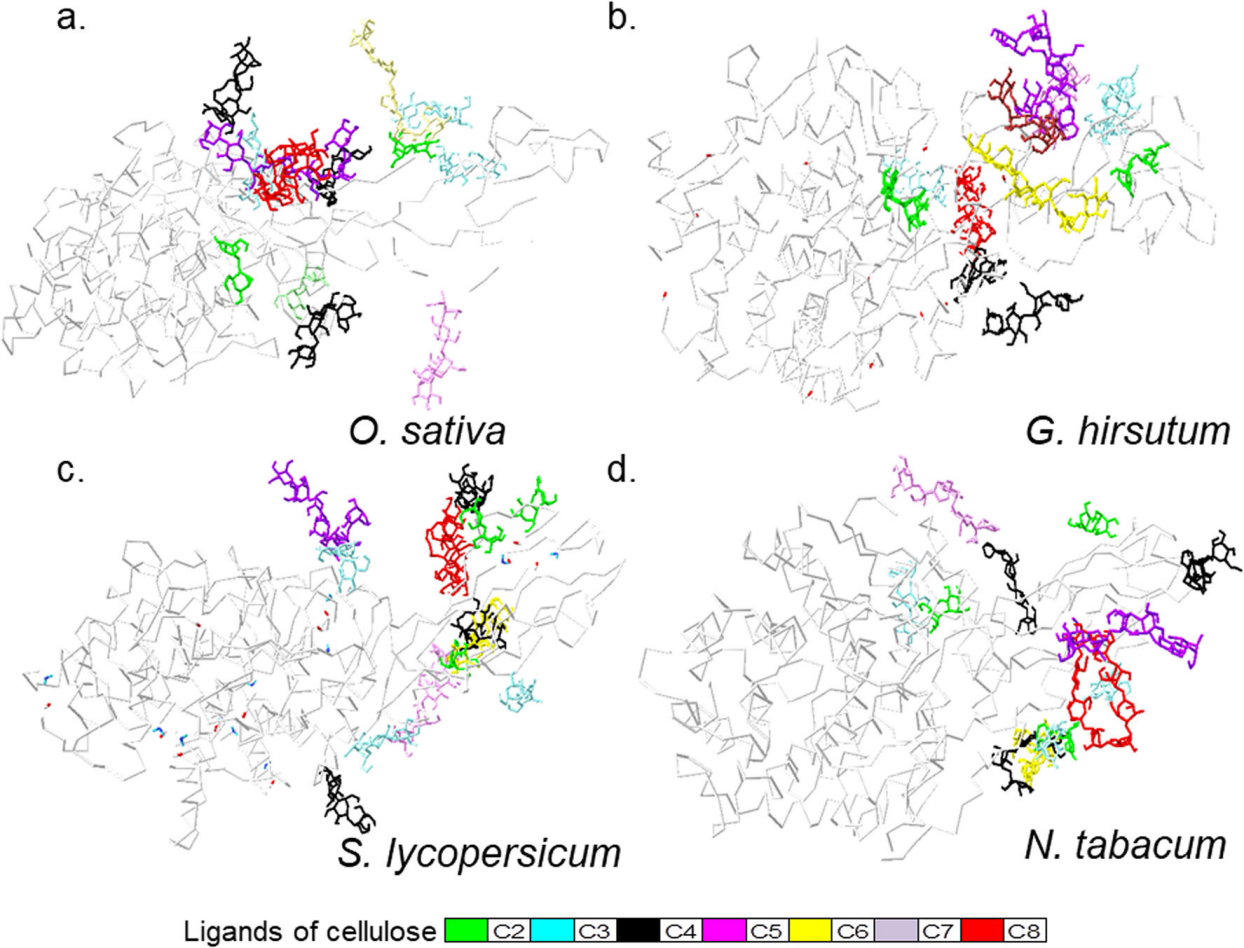
Docking experiments to determine energetically favourable amino acids of characterised plant class C GH9 endoglucanases. Optimised ligands of cellulose (2 ≤ *DP* ≤ 8) were docked with 3D models of class C GH9 endoglucanases $\left({x}_{FL_{40.1 ns}};x\in \left\{Q5 NAT0,Q8 LJP6,Q93 WY9,Q9 ZSP9\right\}\right)$ using the potentially interacting surface residues of GH9, CBM49, and linker regions as potential contacts. The results were the top ranked, i.e. $\min \left({x}_{\Delta  {G}_y}\right);x\in \left\{Q5 NAT0,Q8 LJP6,Q93 WY9,Q9 ZSP9\right\},y\in \left\{C21,C22,C23,C31,C32,C33,C41,C42,C43,C5,C6,C7,C8\right\}$ of all considered runs. Here, despite *C*8 being the largest ligand the free energy of binding was the least $\left(\min \left({x}_{\Delta  {G}_y}\right)=\min \left({x}_{\Delta  {G}_{C8}}\right)\le -7.36\  kcal\ {mol}^{-1}\right)$. In contrast, the results for *C*7 were the exact opposite $\left(\max \left({x}_{\Delta  {G}_y}\right)=\min \left({x}_{\Delta  {G}_{C7}}\right)\ge 2.97\ \mathrm{kcal}\ {\mathrm{mol}}^{-1}\right)$. These data while applicable to smaller ligands are unlikely to extend to the full length cellulose polymer. Here, the interactions are expected to interact uniformly with all the groove binding residues to accomplish substrate modification and catalysis. Abbreviations—CBM49, carbohydrate binding module; *DP*, degree of polymerization; GH9, glycoside hydrolase; *UID* : *Q*5*NAT*0, *O. sativa*; *UID* : *Q*8*LJP*6, *G. hirsutum*; *UID* : *Q*93*WY*9, *N. tabacum*; *UID* : *Q*9*ZSP*9, *S. lycopersicum*

**Fig. 7:**
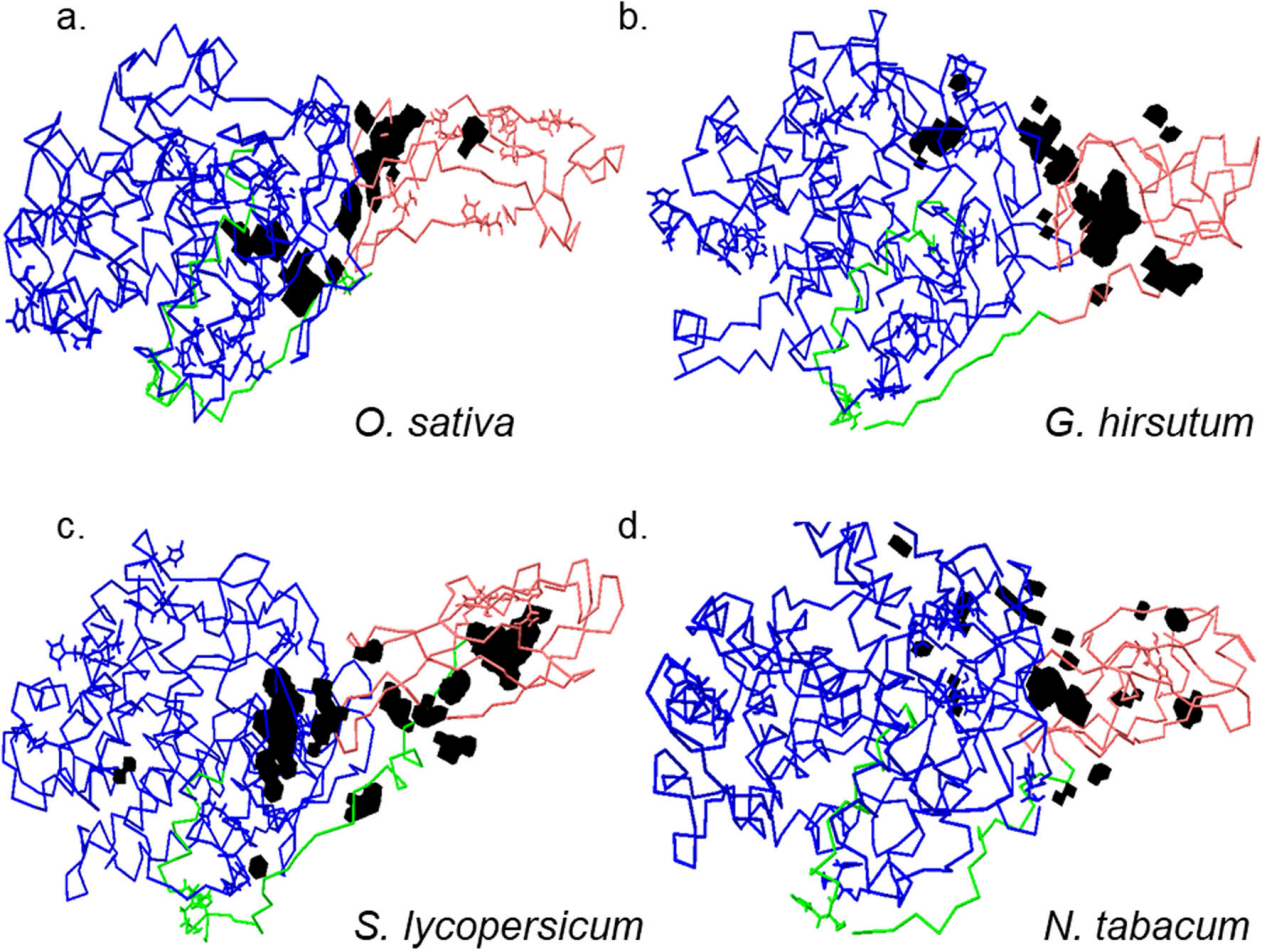
Putative architecture of active site of class C GH9 endoglucanases. A combination of analytic tools were used to establish the putative active site of characterised class C enzymes. These included the amino acids that comprised the interaction surface $\left({AA}_x^{IS}\right)$, resulted in energetically favourable interactions with ligands of cellulose $\left({AA}_x^{Dock}\subset {AA}_x^{IS}\right)$, and those that formed part of numerous cavities and grooves along the surface $\left({AA}_x^{CvG}\right)$ *x* ∈ {*Q*5*NAT*0, *Q*8*LJP*6, *Q*93*WY*9, *Q*9*ZSP*9}. The combined list, *i.e*, $\left({AA}_x=\left({AA}_x^{Dock}\cap {AA}_x^{CvG}\right)\subset {AA}_x^{IS}\right)$, was completely devoid of the aspartic (*D*), glutamic (*E*) acids, cysteine (*C*), and histidine (*H*), amino acids with known propensity for catalysis. The absence of a single continuous groove/cavity and the distribution of amino acids suggests a dual/discontinuous mode, wherein the +1 and −1 sites, are present in a subsurface cavity, while crystalline cellulose itself may interact and be modified by residues at the surface before entering the catalytic site. Despite these variations the probable length (*l* ≥ 100 − 200 *Ang*) of the relevant cavities and grooves suggest a well-adapted mechanism for the intact cellulose polymer. Colour codes for GH9 (blue), linker (green), and CBM49 (red), and relevant cavity and grooves (black). Abbreviations—AA, amino acids; CvG, cavities and grooves; Dock; docking experiment; *r*, *h*, *l*, radius, height, and height of groove-approximating cylinder; GH9, glycoside hydrolase 9; IS, interaction surface

**Fig. 8:**
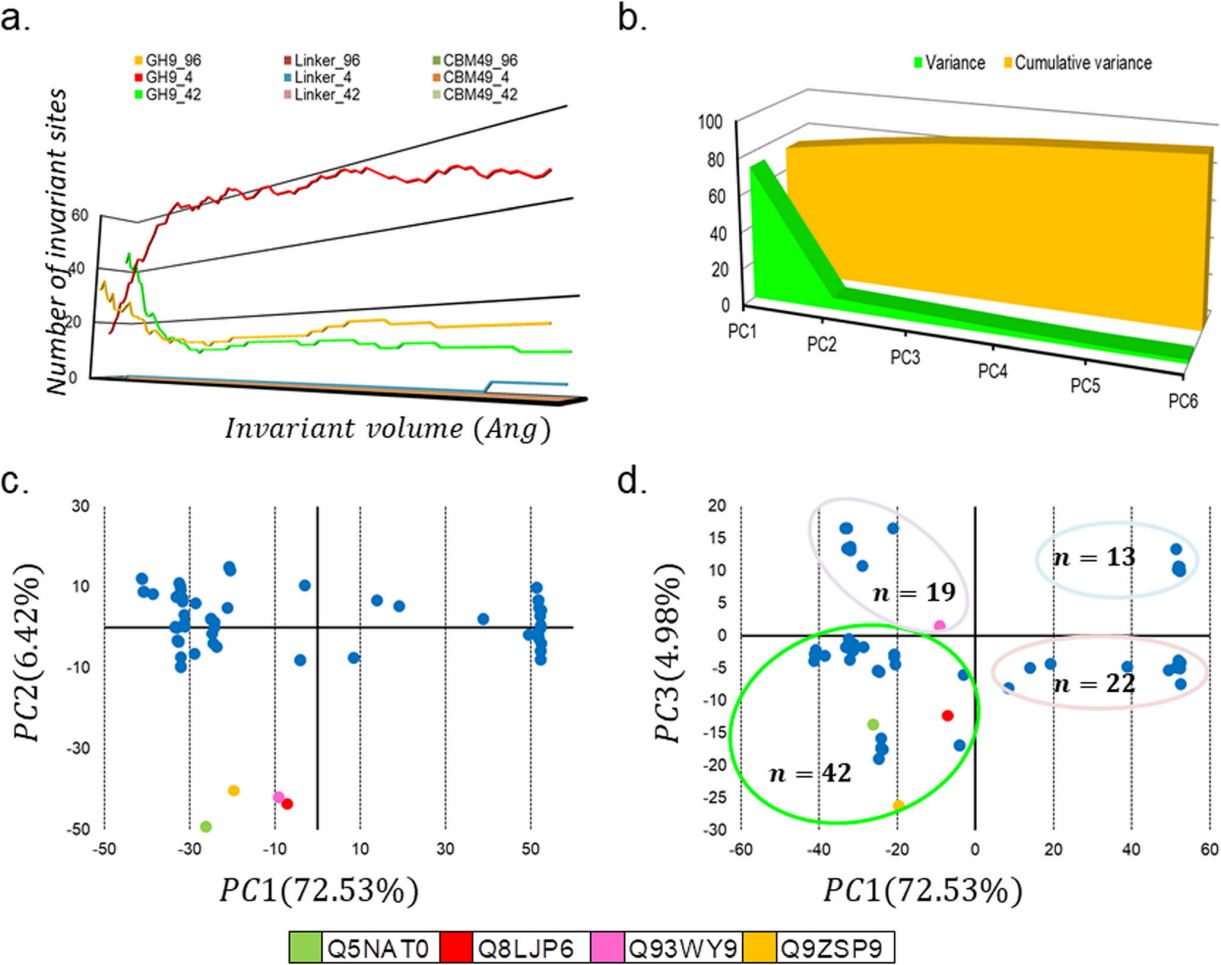
PCA-based inferential clustering of plant class C enzymes. **a** The contribution of the GH9, linker, and CBM49 was assessed as a function of the volume (0 < *V*(*Ang*^3^) ≤ 100) of the invariant core computed and the number of sites that participate from each subsegment. The data suggests that while GH9 is universally conserved, CBM49 is not, even amongst class C members. **b** Principal component analysis of putative and characterised class C GH9 endoglucanases (*n* = 96) was also done to assess the variation in coordinates across the 3D models of class C enzymes Here, **c** *PC*1*vs PC*2 and **d** *PC*1*vsPC*3 were considered. Despite, the higher contribution of *PC*2 to the variance (≅6.42%) as compared with *PC*3 (≅5%), there was greater resolution of the sequences and a greater number of sequences (*n* = 39 *vs n* = 22) with the latter. Further, three characterised members (*O. sativa*, *G. hirsutum*, *S. lycopersicum*) also clustered into this quadrant (−, −) as opposed to only *N. tabacum* (−, +)*.* These data suggest that the *x* −  & *z* − *axes* (*PC*1, *PC*3) might represent the principal axes of class C enzymes. Abbreviations—PC1–6, principal components 1–6; GH9, glycoside hydroxyl 9; CBM49, carbohydrate binding module; V, invariant core volume

**Fig. 9:**
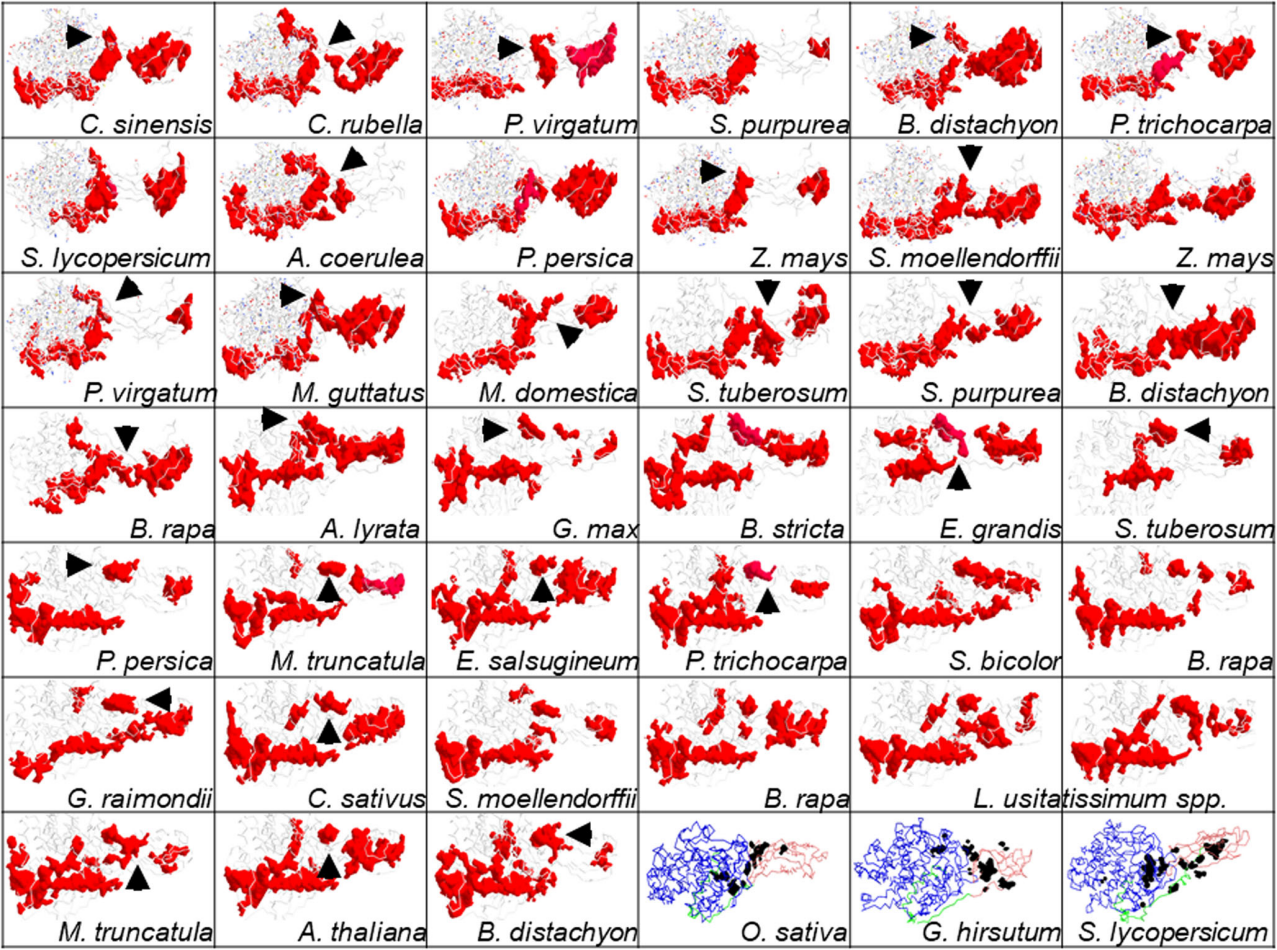
Mechanistic insights into digestion of crystalline cellulose by plant class C endoglucanases. The data presented suggest that CBM49 along with the linker is poorly conserved and exhibits considerable heterogeneity, even amongst plant class C enzymes. Since, the effects of similar CBMs on catalysis are well characterised at least in non-plant taxa, any model would have to consider modulation by CBM49 of the catalytic residues which are present on GH9. This would imply that while catalysis may occur in a solvent accessible subsurface cavity, the surface groove(s) leading to it must involve CBM49 and the linker. The multi-modal approach adopted here (interactions surface definition and amino acid enumeration, docking, cavity and surface analysis) suggests that the extended side chains of aromatic amino acid effect could interact and thereby render crystalline cellulose amenable to subsequent cleavage. Residues such as proline and stabilizing electrostatic interations involving arginine, lysine, asparagine, glutamine, serine, and threonine, along with several smaller hydrophobic residues along the interaction surfaces of the linker and CBM49, heightened oscillatory motion, could result in physical alteration of the groove itself, whilst concomitantly influencing the reactions that cleave crystalline cellulose. Additionally, the selection of substrates/polymer may also be determined by these residues. In support of these analyses 3D models of several homologues were analysed. Clearly, a large and extended groove formed by GH9, linker, and CBM49 and could lead to the catalytic site is observed with *C. sinensis*, *C. rubella*, *A. coerulea*, *P. persica*, and *M. domestica*. Although, segment spanning and overlapping grooves (*B. distachyon*, *M. truncatula*) are also present, it is unlikely that these may contribute to catalysis. However, the clear presence of large disjoint grooves along the interaction surfaces, along with the complete absence of catalytically competent residues corroborates a dual mode of interaction/modification and catalysis by plant class C enzymes. Abbreviations—CBM49, carbohydrate binding module; GH9, glycoside hydrolase

A pipeline comprising each step and the relevant data generated are presented as under the following steps:

- Step 0: Parameters were defined for protocols to minimise, equilibriate, and preliminarily characterise 3D models of plant class C GH9 endoglucanases and ligands of cellulose (Fig. 1, Tables 1 and 2)
- Step 1: The 3D fold of sequences of characterised (full length, truncated) and putative plant class C GH9 endoglucanases was determined (Figs. 1 and 2, Table 3; Supplementary Text 1).
- Step 2: The 3D models of characterised class C enzymes were minimised and used to assess contributions of the linker and CBM49 to the structural integrity of protein (potential energy calculations, rms deviation, normal mode analysis, root mean square fluctuations) (Fig. 3, Table 4; Supplementary Texts 2–5).
- Step 3: The minimised full length 3D models of characterised class C enzymes were perturb, equilibriate (300K; 120*ps*), and simulated with a molecular dynamics run (300K; 40.1 *ns*) (Fig. 4, Supplementary Text 6).
- Step 4: The MD simulated characterised class C plant GH9 endoglucanases were analysed (invariant core analysis, surface contact analysis, cavity and groove delineation, normal mode analysis, docking) to garner insights into the architecture and composition of putative active sites (Figs. 5, 6, and 7, Tables 5, 6, 7, and 8; Supplementary Texts 7–9).
- Step 5: Structural homologues of selected characterised and putative class C enzymes were identified with a PCA-based clustering schema and analysed to derive insignts into the mechanism(s) of digesting crystalline cellulose by plant class C GH9 endoglucanases (Figs. 8 and 9, Table 9; Supplementary Text 10).

**Table 3:** Fold identification by homology modelling of plant GH9 endoglucanases

UID	PDBID	Organism	R(Ang)	Cv(%)	SI(%)	Co(%)	Range	Ref.
Q8LJP6	1JS4	*T. fusca*	2.0	92	37	100	26–600	60
Q5NAT0	1GA2	*C. cellulolyticum*	1.7	90	33	100	39–620	59
Q9ZSP9			1.7	89	32	100	23–621	
Q93WV9			1.7	94	33	100	31–620	

*UID*, uniprot identity; *PDBID*, protein data bank identity; *R*, root mean squared deviation; *Cv*, coverage; *SI*, sequence identity; *Co*, confidence; *Ref*, reference

**Table 4:** Frequencies of non-trivial low frequency modes of 3D models of characterised and minimised class C enzymes

*x*	Q5NAT0	Q8LJP6	Q93WY9	Q9ZSP9
*x*_*FL*min_				
NM	1704	1680	1728	1755
7	0.003	0.003	0.003	0.003
8	0.003	0.004	0.004	0.004
9	0.005	0.006	0.006	0.006
10	0.009	0.010	0.009	0.009
11	0.011	0.011	0.011	0.012
12	0.012	0.013	0.012	0.013
13	0.013	0.013	0.014	0.014
14	0.013	0.015	0.015	0.015
15	0.015	0.015	0.016	0.016
16	0.016	0.017	0.017	0.016
17	0.016	0.017	0.017	0.018
18	0.018	0.018	0.019	0.019
*x*_*T*min_				
NM	1368	1392	1398	1428
7	0.012	0.001	0.001	0.002
8	0.015	0.001	0.001	0.002
9	0.016	0.001	0.001	0.004
10	0.017	0.004	0.004	0.007
11	0.018	0.004	0.005	0.009
12	0.021	0.006	0.006	0.011
13	0.022	0.007	0.007	0.015
14	0.022	0.009	0.009	0.015
15	0.023	0.009	0.010	0.016
16	0.023	0.011	0.011	0.017
17	0.024	0.014	0.014	0.018
18	0.025	0.015	0.014	0.020

*GH9*, glycoside hydrolase 9; *M*, normal modes; *x*_*FL*min_, full length minimised 3D model of class C sequence; *x*_*T*min_, truncated minimised 3D model of class C sequence; *Q5NAT0*, *Oryza sativa*; *Q8LJP6*, *Gossypium hirsutum*; *Q93WY9*, *Nicotiana tabacum*; *Q9ZSP9*, *Solanum lycopersicum*

**Table 5:** Dimensions of putative crystalline cellulose binding cleft of full length characterised class C enzymes after 40.1 ns MD-run

Sequence ID	Organism	*A*_*o*_	*V*_*o*_	*r*	*h*	*l*	*A*_*c*_	*V*_*c*_	*∆A* = ∅	*∆V* = *β*
Q5NAT0	*O. sativa*	905	616	8.49	2.72	106.62	597.64	616.00	307.36	0.00
Q8LJP6	*G. hirsutum*	1272	705	10.06	2.22	126.40	776.11	705.00	495.89	0.00
Q93WY9	*N. tabacum*	778	537	7.87	2.76	98.85	525.46	537.00	252.54	0.00
Q9ZSP9	*S. lycopersicum*	1352	858	10.38	2.54	130.31	841.40	858.00	510.60	0.00

MD, molecular dynamics simulations; *A*_*o*_, observed area of wide groove (*Ang*^2^); *V*_*o*_, observed volume of wide groove (*Ang*^3^); *r*, radius of approximating cylinder (*Ang*); *h*, height of approximating cylinder (*Ang*); *l*, length of wide groove (*Ang*); *A*_*c*_, computed area of approximating cylinder (*Ang*^2^); *V*_*c*_, computed volume of approximating cylinder (*Ang*^3^); *∆A*, area differential ∣A_o_ − A_c_∣

**Table 6:** Computed data for cleaned and prepared ligands

	pKa	pKb	logP = logD	logK	TC	HAC	HDO	RBC	PSA	R(G)	molpol	MMFF94	MOPAC
C21	11.58	− 2.9	− 5.38	− 17.44	0	11	8	4	189.53	20.11	28.97	177.5862	− 469.9
C22					0.003							205.8188	− 464.852
C23					0.001							178.4911	− 468.472
C31	11.55	− 3.49	− 7.49	− 50.179	0	16	11	7	268.68	29.45	42.78	285.7057	− 663.263
C32					0.005							274.8125	− 674.849
C33					0.001							275.0043	− 674.058
C41	10.56	− 3.52	− 9.78	− 103.062	0	22	15	10	368.06	39.36	57.32	318.1659	− 979.702
C42	11.53		− 9.61	− 99.559	0.002	21	14		347.83	38.8	56.6	359.4289	− 951.688
C43					0.003							400.5905	− 930.327
C5	11.7	− 3.52	− 12.47	− 165.71	− 0.001	26	17	17	434.82	48.13	70.51	472.1967	− 1087.63
C6	11.51	− 3.52	− 13.84	− 248.049	0.002	31	20	16	506.13	57.49	84.23	567.8972	− 1298.93
C7	11.49	− 3.73	− 15.96	− 347.241	0.003	36	23	19	585.28	66.84	98.04	694.9641	− 1490.82
C8	11.95	− 3.74	− 16.93	− 444.031	0.001	40	24	8	633.2	74.77	110.52	813.1349	− 1659.38

*pKa*, log acid dissociation constant; *HDO*, hydrogen bond donor; *pKb*, log base dissociation constant; *RBC*, rotatable bond count; *LogP*, log partition-coefficient; *PSA*, polar surface area; *LogD*, log distribution coefficient; *R(G)*, Randic index; *LogK*, log binding constant; *MMFF94*, Merck molecular force field (kcal mol^−1^); *HAC*, hydrogen bond acceptor; *MOPAC*, molecular orbital package (kcal mol^−1^); *TC*, total charge

**Table 7:** Docking calculations to assess contribution of ligand interacting amino acids in full length class C enzyme after 40.1 ns MD-run

Ligand	Sequence	*∆G*_*B*_	Ki	NC	E	T	F	IS
C21	*N. tabacum*	− 4.02	1.13 mM	− 3.31	− 0.18	− 3.49	2%	445.487
	*G. hirsutum*	− 4.68	3.69 uM	− 4.24	− 0.12	− 4.36	1%	532.405
	*O. sativa*	− 4.01	1.15 mM	− 3.20	− 0.07	− 3.27	1%	411.143
	*S. lycopersicum*	− 3.72	1.87 mM	− 3.03	− 0.09	− 3.13	1%	403.271
C22	*N. tabacum*	− 3.35	3.52 mM	− 3.10	− 0.13	− 3.23	1%	371.54
	*G. hirsutum*	− 3.27	4.04 mM	− 3.44	− 0.11	− 3.56	1%	391.544
	*O. sativa*	− 3.64	2.13 mM	− 3.97	− 0.29	− 4.26	1%	561.366
	*S. lycopersicum*	− 3.82	1.59 mM	− 3.75	− 0.35	− 4.10	1%	471.261
C23	*N. tabacum*	− 3.72	1.88 mM	− 2.95	− 0.13	− 3.09	1%	321.025
	*G. hirsutum*	− 3.64	2.14 mM	− 3.60	− 0.06	− 3.65	1%	531.849
	*O. sativa*	− 3.37	3.40 mM	− 2.74	− 0.01	− 2.76	2%	364.286
	*S. lycopersicum*	− 3.62	2.21 mM	− 2.70	+ 0.02	− 2.68	1%	404.028
C31	*N. tabacum*	− 4.98	224.64 uM	− 3.80	− 0.04	− 3.84	2%	515.486
	*G. hirsutum*	− 4.72	346.9 uM	− 2.77	+ 0.06	− 2.71	1%	439.364
	*O. sativa*	− 4.44	556.51 uM	− 2.23	− 0.01	− 2.25	1%	353.431
	*S. lycopersicum*	− 4.19	841.75 uM	− 2.96	− 0.25	− 3.20	1%	431.316
C32	*N. tabacum*	− 5.13	172.83 uM	− 3.57	− 0.09	− 3.66	1%	537.325
	*G. hirsutum*	− 5.15	166.98 uM	− 2.77	− 0.05	− 2.82	1%	417.736
	*O. sativa*	− 4.67	377.39 uM	− 3.06	− 0.01	− 3.07	1%	472.404
	*S. lycopersicum*	− 4.38	619.66 uM	− 2.15	− 0.02	− 2.17	1%	508.931
C33	*N. tabacum*	− 5.16	165.33 uM	− 3.51	− 0.02	− 3.53	1%	454.372
	*G. hirsutum*	− 4.81	296.47 uM	− 2.80	− 0.03	− 2.83	1%	472.293
	*O. sativa*	− 4.50	505.89 uM	− 2.47	− 0.10	− 2.57	1%	422.942
	*S. lycopersicum*	− 4.59	431.31 uM	− 4.01	− 0.03	− 4.04	1%	565.025
C41	*N. tabacum*	− 4.78	315.42 uM	− 2.26	+ 0.02	− 2.24	1%	324.88
	*G. hirsutum*	− 5.16	165.42 uM	− 2.90	− 0.03	− 2.93	1%	472.96
	*O. sativa*	− 5.45	100.64 uM	− 1.78	− 0.10	− 1.87	1%	252.991
	*S. lycopersicum*	− 5.35	119.05 uM	− 1.35	− 0.02	− 1.37	1%	247.16
C42	*N. tabacum*	− 4.34	658.16 uM	− 3.62	− 0.25	− 3.87	1%	569.419
	*G. hirsutum*	− 3.90	1.37 mM	− 2.13	− 0.00	− 2.13	1%	497.506
	*O. sativa*	− 4.58	437.91 uM	− 2.54	− 0.20	− 2.74	1%	402.801
	*S. lycopersicum*	− 4.22	806.11 uM	− 1.59	− 0.05	− 1.64	1%	358.607
C43	*N. tabacum*	− 4.56	451.74 uM	− 1.61	− 0.06	− 1.67	1%	376.196
	*G. hirsutum*	− 5.06	200.60 uM	− 3.55	− 0.14	− 3.69	1%	605.308
	*O. sativa*	− 5.00	217.10 uM	− 2.12	− 0.01	− 2.14	1%	355.299
	*S. lycopersicum*	− 4.54	469.07 uM	− 1.38	− 0.01	− 1.39	1%	382.023
C5	*N. tabacum*	− 5.14	171.69 uM	− 2.15	− 0.12	− 2.27	1%	529.463
	*G. hirsutum*	− 4.56	457.13 uM	− 0.37	+ 0.14	− 0.23	1%	257.428
	*O. sativa*	− 5.40	111.01 uM	− 0.83	− 0.11	− 0.94	1%	221.706
	*S. lycopersicum*	− 6.22	27.42 uM	− 2.31	− 0.14	− 2.45	1%	439.826
C6	*N. tabacum*	− 5.40	109.29 uM	− 2.54	− 0.02	− 2.56	1%	438.28
	*G. hirsutum*	− 4.54	470.52 uM	− 2.44	− 0.10	− − 2.54	1%	386.471
	*O. sativa*	− 4.19	850.37 uM	− 1.57	− 0.02	− 1.59	1%	337.564
	*S. lycopersicum*	− 4.74	337.11 uM	− 2.08	− 0.09	− 2.17	1%	458.349
C7	*N. tabacum*	− 2.97	6.63 mM	− 0.72	− 0.05	− 0.77	1%	158.073
	*G. hirsutum*	− 2.64	11.66 mM	− 1.78	− 0.05	− 1.83	1%	437.98
	*O. sativa*	− 2.43	16.50 mM	− 1.95	− 0.09	− 2.04	1%	534.30
	*S. lycopersicum*	− 2.64	11.63 mM	− 2.26	+ 0.12	− 2.14	1%	424.185
C8	*N. tabacum*	− 7.60	2.70 uM	− 2.01	− 0.09	− 2.10	1%	324.657
	*G. hirsutum*	− 7.36	4.00 uM	− 1.73	− 0.05	− 1.79	1%	334.515
	*O. sativa*	− 7.40	3.79 uM	− 2.34	− 0.04	− 2.38	1%	338.932
	*S. lycopersicum*	− 7.40	3.75 uM	− 2.56	− 0.10	− 2.66	1%	450.992

*MD*, molecular dynamics simulations; *∆G*_*B*_, estimated free energy of binding; *Ki*, inhibition constant; *NC*, non-covalent energy (van Der Waals, Hydrogen bond, desolvation); E, electrostatic energy; *T*, total energy; *F*, frequency; *IS*, interaction surface (*Ang*^2^)

**Table 8:** Distribution and composition of amino acids that may interact with cellulose-based ligands (2 ≤ DP ≤ 8)

Sequence ID	Organism	Amino acids (AA)
Q5NAT0	*O. sativa*	L60, Q64, A67, A75, K78, A79, S172, V417, Y423, R425, S442, F443, G459, P461, L463, D465, S520, L521, Q522, L532, W554, Y561, R563, Y590, V593, V605, P607, W609
Q8LJP6	*G. hirsutum*	L47, Q51, T54, A57, N62, D80, V82, F84, T157, N159, T223, V224, Q226, Y227, Y228, R414, A436, W437, Y469, Y496, Q498, L499, L500, V503, T504, L512, P513, K514, A516, I538, V542, T543, Y544, I550, N561, K563, L569, Y570, S587, W588, I589, S597, M598
Q93WY9	*N. tabacum*	L67, Y142, T149, T150, Y153, W155, T162, Y233, Y440, T442, W443, F444, P451, V508, A509, I510, P511, P513, K514, V521, T522, P525, Q536, K547, T548, Y550, M464, L567, K568, L569, Y572, K583, Y584
Q9ZSP9	*S. lycopersicum*	Y43, N47, R49, N55, L58, K62, S106, D139, N141, T142, Y145, W435, S437, D451, P502, P504, T507, K509, A511, P512, K515, P520, R521, P522, R523, V524, L525, P526, T534, L545, T549, Y550, Y551, R552, Y553, L575, P578, L579, F591, L595, N596, V607, V619

${AA}_x^{Dock}$, amino acids that are energetically favourable (Black+Red); ${AA}_x^{CvG}$, amino acids that form part of cavities and grooves (Red); *DP*, ligands of cellulose with varying degrees of polymerization

**Table 9:** Major groove dimensions of putative plant class C enzymes (*n* = 39)

Sequence ID	Organism	*r*_*GH*9_	*h*_*GH*9_	*l*_*GH*9_	*r*_*CBM*49_	*h*_*CBM*49_	*l*_*CBM*49_
orange1.1g043219m	*Citrus sinensis*	13.60	3.50	170.78	11.29	4.40	141.76
ppa022524m	*Prunus persica*	11.65	3.71	146.38	13.29	4.06	166.87
SapurV1A.0237s0330.1.p	*Salix purpurea*	13.68	3.74	171.84	6.09	3.73	76.50
Carubv10003874m	*Capsella rubella*	13.50	3.28	169.59	10.11	3.90	126.99
Pavir.Eb00189.1.p	*Panicum virgatum*	13.55	3.53	170.15	8.76	4.06	110.04
Bradi5g026010.1.p	*Bradipodium distachyon*	9.79	3.64	123.02	14.46	4.08	181.58
Brara.E01714.1.p	*Brassica rapa*	8.06	4.00	101.18	10.23	4.28	128.52
Pavir.Ea00142.1.p	*Panicum virgatum*	12.12	3.66	152.23	6.31	2.70	79.25
GRMZM2G143747_P01	*Zea mays*	15.45	3.32	194.11	9.71	4.61	121.95
SapurV1A.0035s0560.1.p	*Salix purpurea*	14.64	3.47	183.88	8.48	3.78	106.56
Bradi2g07150.1.p	*Bradipodium distachyon*	13.45	3.94	168.96	–	–	–
PGSC0003DMP400021750	*Solanum tuberosum*	14.02	3.50	176.10	9.77	2.94	122.77
Migut.D01909.1.p	*Mimulus guttatus*	10.15	3.26	127.54	11.60	4.19	145.69
234652	*Selaginella moellendorffii*	9.30	4.07	116.79	10.93	4.09	137.30
MDP0000131267	*Malus domestica*	12.20	3.81	153.26	8.13	4.22	102.16
Potri.001G092200.1	*Populus trichocarpa*	12.44	3.79	156.30	9.47	3.20	118.92
GRMZM2G453565_P01	*Zea mays*	12.86	3.71	161.55	7.27	4.68	91.25
Solyc02g014220.2.1	*Solanum lycopersicum*	8.73	3.05	109.64	9.66	4.43	121.38
Aquca_037_00141.1	*Aquilegia coerulea*	12.83	3.96	161.16	6.80	2.67	85.35
Glyma.05G216400.1.p	*Glycine max*	13.34	3.68	167.55	5.91	3.24	74.26
489943	*Arabidopsis lyrata*	12.62	3.74	158.53	14.59	3.44	183.19
Bostr.25463s0223.1.p	*Boechera stricta*	13.46	3.67	169.11	12.65	3.65	158.85
Eucgr.J00862.1	*Eucalyptus grandis*	9.11	4.41	114.46	12.83	3.54	161.13
ppa002939m	*Prunus persica*	14.83	3.79	186.22	8.16	3.85	102.47
PGSC0003DMP400034548	*Solanum tuberosum*	8.54	3.40	107.32	8.66	3.21	108.72
Thhalv10028514m	*Eutrema salsugineum*	13.92	3.71	174.81	13.35	3.97	167.66
Medtr4g074960.1	*Medicago truncatula*	9.77	3.75	122.77	8.91	4.48	111.90
Gorai.005G210200.1	*Gossypium raimondii*	11.43	3.57	143.52	10.27	3.78	128.96
Potri.003G139600.1	*Populus trichocarpa*	13.60	3.89	170.81	7.71	3.66	96.86
Sobic.003G015700.1.p	*Sorghum bicolor*	13.31	3.57	167.13	10.85	3.60	136.29
Brara.I01325.1.p	*Brassica rapa*	13.90	3.84	174.63	6.05	3.10	75.93
Cucsa.107370.1	*Cucumis sativus*	14.22	4.11	178.58	12.41	4.04	155.86
99802	*Selaginella moellendorffii*	9.71	4.13	122.00	8.00	4.70	100.49
Brara.C02656.1.p	*Brassica rapa*	13.86	3.68	174.09	12.82	3.95	160.97
Lus10032377	*Linum usitatissimum*	12.70	3.99	159.48	7.76	3.98	97.44
Lus10003888	*Linum usitatissimum*	12.45	4.17	156.38	6.27	2.83	78.77
Medtr8g099410.1	*Medicago truncatula*	13.71	3.81	172.20	7.92	3.04	99.49
AT4G11050.1	*Arabidopsis thaliana*	13.53	3.84	169.89	12.86	4.03	161.55
Bradi2g32270.1.p	*Bradipodium distachyon*	14.21	3.61	178.47	5.64	2.68	70.88

*r*, radius of approximating cylinder (*Ang*); *h*, height of approximating cylinder (*Ang*); *l*, length of wide groove (*Ang*); *GH9*, glycoside hydrolase 9; *CBM49*, carbohydrate binding module

### Homology modelling and assessment of characterised class C GH9 endoglucanases

An intersequence pairwise alignment suggests that despite a high degree of identity (≈75 − 83%) between the class C enzymes of *S. lycopersicum*, *G. hirsutum*, and *N. tabacum*, the preferred template for *G. hirsutum* was from *T. fusca* (*PDBID* : 1*JS*4). Conversely, the sequence identity for *O. sativa* was marginally lower (≈62 % *identity*), yet shared the same top ranked template, i.e. *C. cellulolyticum* (*PDBID* : 1*GA*2), with *S. lycopersicum* and *N. tabacum* (Table 3; Supplementary Text 1). However, the average sequence identity with the templates (≈32 − 40%) was similar for all class C enzymes investigated (Table 3). The superposed ungapped MSA of the truncated (*x*_*T*_) class C proteins additionally resulted in the exclusion of the linker, i.e. *CBM*49 ≡ *CBM*49 ∪ *L*, from the MSA, i.e. *x*_*T*_ = *GH*9 − *CBM*49 = *GH*9 − (*CBM*49 ∪ *L*); *x* ∈ {*Q*5*NAT*0, *Q*8*LJP*6, *Q*93*WY*9, *Q*9*ZSP*9}, (Fig. 2). The results (rmsd (template, *x*) < 2 *Ang*) suggest that the catalytic machinery for digesting crystalline may be conserved in plants and other non-plant taxa most notably bacteria (Table 3; Supplementary Text 1) [8, 17, 20–34, 59, 60]. The models also indicate that in addition to GH9, CBM49 and the linker (coverage = 89 − 94%) may partake in digesting crystalline cellulose (Table 3) [8, 17, 59, 60]. Since, solvent addition was explicit, minimization of energy (*E*_min_) was carried out exclusively by the steepest descent algorithm (*ncyc* > *maxcyc*) for the full length $\left({E}_{\mathrm{min}}\left(Q5 NAT{0}_{FL_{\mathrm{min}}}\right)\cong -4.36\ast {10}^5\ \mathrm{kcal}\ {\mathrm{mol}}^{-1};{E}_{\mathrm{min}}\left(Q93 WY{9}_{FL_{\mathrm{min}}}\right)\cong -4.34\ast {10}^5\ \mathrm{kcal}\ {\mathrm{mol}}^{-1};{E}_{\mathrm{min}}\left(Q8 LJP{6}_{FL_{\mathrm{min}}}\right)\cong -4.31\ast {10}^5\ \mathrm{kcal}\ {\mathrm{mol}}^{-1};{E}_{\mathrm{min}}\left(Q9 ZSP{9}_{FL_{\mathrm{min}}}\right)\cong -4.29\ast {10}^5\mathrm{kcal}\ {\mathrm{mol}}^{-1}\right)$ and truncated $\left({E}_{\mathrm{min}}\left(Q5 NAT{0}_{T_{\mathrm{min}}}\right)\cong -2.58\ast {10}^5\ \mathrm{kcal}\ {\mathrm{mol}}^{-1};{E}_{\mathrm{min}}\left(Q93 WY{9}_{T_{\mathrm{min}}}\right)\cong -3.61\ast {10}^5\ \mathrm{kcal}\ {\mathrm{mol}}^{-1};{E}_{\mathrm{min}}\left(Q8 LJP{6}_{T_{\mathrm{min}}}\right)\cong -3.49\ast {10}^5\ \mathrm{kcal}\ {\mathrm{mol}}^{-1};{E}_{\mathrm{min}}\left(Q9 ZSP{9}_{T_{\mathrm{min}}}\right)\cong -3.70\ast {10}^5\ \mathrm{kcal}\ {\mathrm{mol}}^{-1}\right)$ models (Fig. 3, Tables 1 and 3; Supplementary Text 3). Interestingly, whilst, the data $\left(\mathrm{Rank}\left({E}_{\mathrm{min}}\left({x}_{FL_{\mathrm{min}}}\right)\right)=\mathrm{Rank}\left({E}_{\mathrm{min}}\left({x}_{T_{\mathrm{min}}}\right)\right)=\left\{2,3\right\};x=\left\{Q93 WY9,Q8 LJP6\right\}\right)$ were consistent for *N. tabacum* and *G. hirsutum*, there was a complete reversal of the same for *O. sativa* and *S. lycopersicum* $\left(\mathrm{Rank}\ \left({E}_{\mathrm{min}}\left(Q5 NAT{0}_{FL_{\mathrm{min}}},Q9 ZSP{9}_{T_{\mathrm{min}}}\right)\right)\propto 1/\mathrm{Rank}\left({E}_{\mathrm{min}}\left(Q5 NAT{0}_{T_{\mathrm{min}}},Q9 ZSP{9}_{FL_{\mathrm{min}}}\right)\right)\right)$ (Fig. 3; Supplementary Text 3). These data suggest that full length class C enzymes may adopt a stable conformation earlier than their truncated counterparts. Interestingly, the rms deviations of the minimised full length class C enzymes from *O. sativa* $\left({E}_{\mathrm{min}}\left(Q5 NAT{0}_{FL_{\mathrm{min}}}\right)/{E}_{\mathrm{min}}\left(Q5 NAT{0}_{T_{\mathrm{min}}}\right)\cong 2.31\right)$, *G. hirsutum* $\left({E}_{\mathrm{min}}\left(Q8 LJP{6}_{FL_{\mathrm{min}}}\right)/{E}_{\mathrm{min}}\left(Q8 LJP{6}_{T_{\mathrm{min}}}\right)\cong 1.05\right)$, and *S. lycopersicum* $\left({E}_{\mathrm{min}}\left(Q9 ZSP{9}_{FL_{\mathrm{min}}}\right)/{E}_{\mathrm{min}}\left(Q9 ZSP{9}_{T_{\mathrm{min}}}\right)\cong 2.84\right)$ were higher as compared with the truncated forms while the reverse was observed for *N. tabacum* $\left({E}_{\mathrm{min}}\left(Q93 WY{9}_{FL_{\mathrm{min}}}\right)/{E}_{\mathrm{min}}\left(Q93 WY{9}_{T_{\mathrm{min}}}\right)\cong 0.89\right)$ (Fig. 3; Supplementary Text 3).

### Assessing the contribution of CBM49 to the structural integrity of class C enzymes

The core data for the 3D models of all full length characterised plant class C enzymes suggests that while GH9 is well conserved (#*Cα*_0.0 < *V* ≤ 100.0_(*GH*9) > 0), CBM49 is not (#*Cα*_0.0 < *V* ≤ 100.0_(*CBM*49) = 0). The N- and C-terminal regions of the linker does, however, exhibit partial conservation (#*Cα*_8.0 < *V* ≤ 100.0_(*Linker*) = {1, 3}), a trend which is unlikely to be sustained for larger datasets (Supplementary Text 2). Low frequency non-trivial modes, i.e. ${NM}_{{x_{FL}}_{\mathrm{min}}}={NM}_{{x_T}_{\mathrm{min}}}=7-18$, were also assessed to garner additional information about the possible role(s) of CBM49 and the linker in influencing structure of the GH9 (Table 4; Supplementary Texts 4 and 5). With the exception of *O. sativa*, the frequencies of these modes for all other full length $\left(\Delta  \omega \left({NM}_{{x_{FL}}_{\mathrm{min}}}\right)\right)$ class C members (*G. hirsutum*, *N. tabacum*, *S. lycopersicum*) were ≈2 − 3 fold higher than those for their truncated forms $\left(\Delta  \omega \left({NM}_{{x_{FL}}_{\mathrm{min}}}\right)\right.>\left(\Delta  \omega \left({NM}_{{x_T}_{\mathrm{min}}}\right)\right)$ (Table 4; Supplementary Texts 4 and 5). The frequency of these for the truncated models $\left(\Delta  \omega \left({NM}_{{x_T}_{\mathrm{min}}}\right)\right)$ of *O. sativa* in general was ≈2 − 5 fold higher for all modes examined or as in *S. lycopersicum* for the higher frequency modes $\left(\Delta  \omega \left({NM}_{{x_{FL}}_{\mathrm{min}}}\right)\right.<\left(\Delta  \omega \left({NM}_{{x_T}_{\mathrm{min}}}\right)\right)$ (Table 4; Supplementary Texts 4 and 5). The atomic fluctuation data for *G. hirsutum* $\left(\Delta  {\mathrm{rmsf}}_{{x_{cFL}}_{\mathrm{min}}}\cong 1.74,\sigma \left({\mathrm{rmsf}}_{{x_{cFL}}_{\mathrm{min}}}\right)\cong 0.21;\Delta  {\mathrm{rmsf}}_{{x_T}_{\mathrm{min}}}\cong 758.00,\sigma \left({\mathrm{rmsf}}_{{x_T}_{\mathrm{min}}}\right)\cong 41.12\right)$, *N. tabacum* $\left(\Delta  {\mathrm{rmsf}}_{{x_{cFL}}_{\mathrm{min}}}\cong 3.28,\sigma \left({\mathrm{rmsf}}_{{x_{cFL}}_{\mathrm{min}}}\right)\cong 0.29;\Delta  {\mathrm{rmsf}}_{{x_T}_{\mathrm{min}}}\cong 673.61,\sigma \left({\mathrm{rmsf}}_{{x_T}_{\mathrm{min}}}\right)\cong 37.56\right)$, and *S. lycopersicum* $\left(\Delta  {\mathrm{rmsf}}_{{x_{cFL}}_{\mathrm{min}}}\cong 2.1,\sigma \left({\mathrm{rmsf}}_{{x_{cFL}}_{\mathrm{min}}}\right)\cong 0.22;\Delta  {\mathrm{rmsf}}_{{x_T}_{\mathrm{min}}}\cong 46.29,\sigma \left({\mathrm{rmsf}}_{{x_T}_{\mathrm{min}}}\right)\cong 4.30\right)$, exhibited greater variance as compared with the full length proteins, i.e. $\sigma \left({\mathrm{rmsf}}_{{x_T}_{\mathrm{min}}}\right)>>\sigma \left({\mathrm{rmsf}}_{{x_{cFL}}_{\mathrm{min}}}\right)$ (Fig. 3; Supplementary Texts 4 and 5). Interestingly, the corresponding data for *O. sativa* only differed marginally $\left(\Delta  {\mathrm{rmsf}}_{{x_{cFL}}_{\mathrm{min}}}\cong 2.49,\sigma \left({\mathrm{rmsf}}_{{x_{cFL}}_{\mathrm{min}}}\right)\cong 0.26;\Delta  {\mathrm{rmsf}}_{{x_T}_{\mathrm{min}}}\cong 2.77,\sigma \left({\mathrm{rmsf}}_{{x_T}_{\mathrm{min}}}\right)\cong 0.22\right)$ (Fig. 3; Supplementary Texts 4 and 5). The baseline rmsf values were remarkably consistent for all the proteins $\left(\min \left(\Delta  {\mathrm{rmsf}}_{{x_{cFL}}_{\mathrm{min}}}\right)\cong \min \left(\Delta  {\mathrm{rmsf}}_{{x_T}_{\mathrm{min}}}\right)\cong 0.07\right)$ examined, although for *O. sativa* there was a tangible difference, i.e. $\min \left(\Delta  {\mathrm{rmsf}}_{{x_{cFL}}_{\mathrm{min}}}\right)\cong 0.07,\min \left(\Delta  {\mathrm{rmsf}}_{{x_T}_{\mathrm{min}}}\right)\cong 0.05$ (Table 4; Supplementary Texts 4 and 5). A position-specific analysis of this data clearly demonstrates that this heightened oscillatory motion involves the residues of the linker and CBM49 (Fig. 3, Table 4; Supplementary Texts 4 and 5). These data when combined suggests that CBM49 and the linker, despite being poorly conserved even amongst class C members, may deploy corrective hypermobility to rapidly restore equilibrial status secondary to perturbation events such as that observed for substrate binding and subsequent catalysis by enzymes.

### Delineating the active site architecture of characterised plant class C enzymes

An multi-modal approach (surface contact analysis, docking, cavity and groove delineation) was adopted to ascertain the residues and their relevance to crystalline cellulose digestion by plant class C enzymes.

#### Analysing the DCCM to assess and characterise intra-protein residue interactions

The NMA and DCCM data of mature-folded (40.1 *ns*) class C enzymes suggest that several residues that comprise the non-contiguous segments between the GH9, linker, and CBM49 exhibit positively correlated atomic displacements (*r* ≅ 1.00) (Fig. 4, Supplementary Texts 6–9). These data imply that plant class C enzymes, like their bacterial counterparts may also possess well-defined interaction surface(s) $\left( IS=\left\{{IS}_x^{GC},{IS}_x^{CL},{IS}_x^{GL}\right\}\right)$ between GH9, linker, and CBM49 (Fig. 5) [59, 60]. The surface area of interacting residues was variable and ranged from 375 − 517 *Ang*^2^ (CBM49_linker$\equiv {IS}_x^{CL}$), 283 − 481 *Ang*^2^ (GH9_linker$\equiv {IS}_x^{GL}$), and 96−208 *Ang*^2^ (GH9_CBM49$\equiv {IS}_x^{GC}$) where *x* ∈ {*Q*5*NAT*0, *Q*8*LJP*6, *Q*93*WY*9, *Q*9*ZSP*9}. The surfaces themselves may be further decomposed into non-contiguous subsegments, i.e. *G* = *G*1 ∪ *G*2 ∪ *G*3 and *C* = *C*1 ∪ *C*2 ∪ *C*3. Thus, ${IS}_x^{GC}=G2\cup G3\cup C2\cup C3$, ${IS}_x^{GL}=G1\cup G2\cup G3\cup L$, and ${IS}_x^{CL}=C1\cup C2\cup C3\cup L$ (Fig. 5). In general, while the contact surface formed between GH9 and CBM49 was the least, the same for CBM49 and the linker was maximal $\left({IS}_x^{GC}<{IS}_x^{GL}<{IS}_x^{CL},x\in \left\{Q5 NAT0,Q8 LJP6,Q93 WY9,Q9 ZSP9\right\}\right)$. The only exception was for the class C enzyme from *O. sativa* $\left({IS}_{Q5 NAT0}^{CL}>{IS}_{Q5 NAT0}^{GL}\right)$ which can be explained by a large interaction surface spanning GH9, CBM49, and the linker $\left({IS}_{Q5 NAT0}^{GLC}=G1\cup G2\cup G3\cup C1\cup L\right)$, i.e. ${IS}_{Q5 NAT0}^{GL}\equiv {IS}_{Q5 NAT0}^{GL C}$. The bonds between the residues that comprised these protein-protein interaction surfaces $\left({AA}_x^{IS}\right.;x\in \left\{Q5 NAT0,Q8 LJP6,Q93 WY9,Q9 ZSP9\right\}\Big)$were non-covalent (hydrophobic, hydrogen, van der Waals) for *N. tabacum*, *G. hirsutum*, and *S. lycopersicum*. Here, too, the contact surface for the class C enzyme from *O. sativa* was exceptional and included the possibility of a covalent and oxygen-sensitive (−*SS*−) linkage between *C*124 and *M*5/*M*132 (Fig. 5).

#### Docking data suggests qualitative differences between individual class C enzymes

The binding energy of the ligands was lower for the higher molecular weight ligands $\left({\mathrm{x}}_{\Delta  {G}_{C8}}<{\mathrm{x}}_{\Delta  {G}_{C5}}<{\mathrm{x}}_{\Delta  {G}_{C6}}\le {\mathrm{x}}_{\Delta  {G}_{C4}}\le {\mathrm{x}}_{\Delta  {G}_{C3}}<{\mathrm{x}}_{\Delta  {G}_{C2}}<{\mathrm{x}}_{\Delta  {G}_{C7}}\right)$ with *C*8 possessing the lowest $\Big({\mathrm{x}}_{\Delta  {G}_{C8}}\cong -7.44\ \mathrm{kcal}\ {\mathrm{mol}}^{-1}=\min \left({\mathrm{x}}_{\Delta  {G}_y}\right)$, while interestingly, the free energy of binding for *C*7 $\left({\mathrm{x}}_{\Delta  {G}_{C7}}\cong -2.67\ \mathrm{kcal}\ {\mathrm{mol}}^{-1}=\max \left({\mathrm{x}}_{\Delta  {G}_y}\right)\right)$ for all the class C enzymes investigated. These data were also supported by the corresponding *Ki* values, i.e. ${\mathrm{x}}_{Ki_{C8}}\cong 3.56\ \upmu \mathrm{M}=\min \left({\mathrm{x}}_{Ki_y}\right)$ and ${\mathrm{x}}_{Ki_{C7}}\cong 7.58\ \mathrm{mM}=\max \left({\mathrm{x}}_{Ki_y}\right)$ *x* ∈ {*Q*5*NAT*0, *Q*8*LJP*6, *Q*93*WY*9, *Q*9*ZSP*9}; *y* ∈ {*C*21, *C*22, *C*23, *C*31, *C*32, *C*33, *C*41, *C*5, *C*6, *C*7, *C*8}) (Figs. 5 and 6, Tables 5 and 6). The distribution of specific amino acids identified by docking $\left({AA}_x^{\mathrm{Dock}}\subset {AA}_x^{IS}\right.;x\in \left\{Q5 NAT0,Q8 LJP6,Q93 WY9,Q9 ZSP9\right\}\Big)$ suggests a preponderance of residues with small hydrophobic, aromatic, and basic side chains along with serine and threonine. Exceptionally, the catalytic amino acids aspartic (*D*) and glutamic (*E*) acids were almost (*D*465, *O*. *sativa*; *D*139, *D*451, *S*. *lycopersicum*) completely excluded from these calculations as were other amino acids with known proclivity to partake in catalysis, i.e. cysteine (*C*) and histidine (*H*) (Table 7).

#### Delineating the cavities and grooves for crystalline cellulose catalysis and modification by plant class C GH9 endoglucanases

Since, solvent accessibility is a pre-requisite for hydrolytic catalysis of the glycosidic linkage by GH9 endoglucanases, the presence of amino acids identified previously by docking was examined in cavities and grooves of the 3D models of full length characterised class C enzymes. The distribution of these for *O. sativa* (*GH*9 = 27, *L* = 0, *CBM*49 = 1, *LC* = 4, *GC* = 1), *G. hirsutum* (*GH*9 = 21, *L* = 0, *CBM*49 = 0, *LC* = 4, *GC* = 1), *N. tabacum* (*GH*9 = 20, *L* = 0, *CBM*49 = 0, *LC* = 4, *GC* = 0), and *S. lycopersicum* (*GH*9 = 23, *L* = 1, *CBM*49 = 2, *LC* = 1, *GC* = 0) that CBM49/linker may function to modulate catalysis by substrate modification rather participate directly (Figs. 5 and 6, Tables 5, 6, and 7). The amino acids that comprise these were enumerated $\left({AA}_x^{CvG}\right.;x\in \left\{Q5 NAT0,Q8 LJP6,Q93 WY9,Q9 ZSP9\right\}\Big)$ and analysed (Table 7). The amino acid distribution when combined, ${AA}_x=\left({AA}_x^{\mathrm{Dock}}\cap {AA}_x^{CvG}\right)\subset {AA}_x^{IS};x\in \left\{O. sativa,G. hirsutum,N. tabacum,S. lycopersicum\right\}$, was utilised to compute the dimensions (length ≅ 100 − 130 *Ang*, radius ≅ 8.0 − 10.4 *Ang*, height ≅ 2.2 − 2.8 *Ang*) of a probable architecture for the active site(s) of plant class C GH9 endoglucanases (Fig. 7, Tables 7 and 8). Whilst, the volume of the approximating cylinder perfectly matched the observed data (|*V*_*o*_ − *V*_*c*_| = *β* ≅ 0) for all class C enzymes, the differences in the surface areas (∅ ≅ 250 − 510 *Ang*^2^, mean ≅ 679 ± 137.66) could imply an intrinsic heterogeneity in the composition of amino acids viz. their side chains that comprise these grooves (Fig. 7, Tables 7 and 8).

### Principal component-based clustering to identify potential class C homologues

The variance between the *xyz* coordinates of each ungapped aligned position (*n* = 363) was computed and summarised as eigenvalues (*n* = 1089). A scatter plot of the principal components (*PC*1 ≈ 73 %  ≡ *x* axis; *PC*3 ≈ 5 %  ≡ *z* axis) resulted in class C enzymes (*n* = 96) being clustered into 4 distinct groups (*x*, *z* = {(−, −), (−, +), (+, −), (+, +)}). Since most of the characterised members (*n* = 3; *O. sativa*, *S. lycopersicum*, *G. hirsutum*) belonged to a single cluster, these, and associated putative class C members (*n* = 39; *Arabidopsis* spp., *B. stricta*, *B. distachyon*, *B. rapa*, *C. rubella*, *C. sinensis*, *E. grandis*, *E. salsugineum*, *G. max*, *G. raimondii*, *L. usitatissimum*, *M. domestica*, *M. truncatula*, *M. guttatus*, *P. virgatum*, *P. trichocarpa*, *P. persica*, *S. purpurea*, *S. moellendorffii*, *S. lycopersicum*, *S. tuberosum*, *Z. mays*) (Sequence identity ≈ 3 − 49%) could be utilised to draw meaningful inferences about the generic active site and mechanism(s) deployed by plant class C enzymes to digest crystalline cellulose (Fig. 8; Supplementary Table 1 and Supplementary Text 10). Interestingly, members (*n* = 22) of the quadrant (+, −) included the bryophyte *P. patens* spp. and *O. sativa* spp. as compared with sequences (*n* = 39) present (−, −) which included the tracheophyte *S. moellendorffii* spp. (Fig. 8; Supplementary Table 1 and Supplementary Text 10). The presence of these ancestral class C members, i.e. tracheophytes, further strengthened the rationale of selecting this group since it represents organisms that may have evolved over 400 million years ago and therefore any mechanism postulated to digest crystalline cellulose would also likely have remained unchanged for that duration [8]. The quadrants (−, +) whose members (*n* = 19) included the characterised class C enzyme from *N. tabacum*, and (+, +) with *n* = 13 members possessed a similar distribution of plant members as with group 1 (−, −) (Fig. 8; Supplementary Table 1 and Supplementary Text 10).

## Discussion

### Contribution of the GH9, linker, and CBM49 to the architecture of the active site plant class C enzymes

Plant class C enzymes share considerable structural homology with gram-positive and -negative bacterial GH9 members (Tables 1, 2, and 3; Supplementary Texts 1 and 2). Although these results for GH9 are not entirely unexpected, data from this study also supports the involvement of the linker and CBM49 in the catalysis of crystalline cellulose by plant class C enzymes (Table 3; Supplementary Texts 1 and 2) [8, 17, 20–34, 59, 60]. The inclusion of the N- and C-terminal linker, albeit at higher volumes (*V* ∈ (8.0,100.0]) and the complete exclusion of CBM49 even amongst this small subset of class C enzymes suggest poor conservation of these segments (Figs. 5 and 8a; Supplementary Text 2) [8, 81]. These data raise the possibility that the linker and CBM49 may have an indirect or modulatory role in catalysing glycosidic cleavage and may partake in substrate selection/modification rather than direct catalysis (Figs. 5 and 8a; Supplementary Text 2)).

The digestion of crystalline cellulose, in non-plant taxa may occur in a continuous groove that spans the GH9, linker, and the associated CBMs [51–60]. Plant class C enzymes may also do so in a surface groove that is initially bounded by the GH9_linker $\left({IS}_x^{GL}\right)$ at the posterior basolateral surface and continues laterally being bounded in turn by the GH9_linker $\left({IS}_x^{GL}\right)$, CBM49_linker $\left({IS}_x^{CL}\right)$, and GH9_CBM49 $\left({IS}_x^{GC}\right)$ surfaces where *x* ∈ {*Q*5*NAT*0, *Q*8*LJP*6, *Q*93*WY*9, *Q*9*ZSP*9}, finally terminating anteriorly in a solvent accessible cavity that might constitute the principal active site (Fig. 5). Physically, although the *IS*-bounded grooves appear discontinuous at the surface, a thorough analysis suggests the presence of several subsurface cavities that could maintain contiuity (Figs. 5, 6, and 7). Further, an almost complete absence of measurable cavities in CBM49/linker could also ensure that the substrate-facing surface through which crystalline cellulose traverses was chemically inert. The model precludes the existence of disparate active sites whilst, concomitantly asserts a preparatory/modulatory effect by CBM49/linker which may then be followed by the hydrolytic cleavage of the glycosidic bond at the active site (Fig. 7). The rmsf and DCCM data in concert with the invariant core volumes further suggests that the *IS* that bounds the linker and CBM49 $\left({IS}_x^{CL};x\in \left\{Q5 NAT0,Q8 LJP6,Q93 WY9,Q9 ZSP9\right\}\right)$ surface may exhibit heightened low frequency motion, a factor that could confer upon class C enzymes the propensity to accommodate varying lengths of crystalline cellulose (4^7^, Tables 6, 7, and 8; Supplementary Text 2, 6–9).

### Molecular dissection of a putative active site of class C enzymes

Any plausible model of the active site architecture of plant class C enzymes would have to explain as well as include extant empirical data. 3D models of full length minimised and characterised members from *O. sativa*, *G. hirsutum*, *N. tabacum*, and *S. lycopersicum* were simulated in vacuo for 40.1 *ns* and thence examined for amino acids that may contribute to substrate binding and/or catalysis. The combined list of functionally relevant amino acids, i.e. ${AA}_x=\left({AA}_x^{\mathrm{Dock}}\cap {AA}_x^{CvG}\right)\subset {AA}_x^{IS};x\in \left\{O. sativa,G. hirsutum,N. tabacum,S. lycopersicum\right\}$, were enumerated and utilised for these analyses (Table 7). The paucity/absence of residues that support generic acid-base mediated cleavage of the *β* (1 → 4) glycosidic bond for crystalline cellulose as well as known active site amino acids $\left(\left\{E,C,H\right\}\notin {AA}_x^{\mathrm{Dock}}\right)$, despite being present contiguously with those that are $\left(\left\{D,E,C,H,P,R,K,N,Q,L,I,V,A,M,W,F,Y,G,S,T\right\}\in {AA}_x^{IS}\cup {AA}_x^{CvG}\right)$ suggests that catalysis might occur in a superficial cavity just below the surface of the protein (Fig. 5, Table 7). However, the preponderance of energetically favourable aromatic amino acids along the interaction surfaces and various grooves (*AAA* = {*W*, *F*, *Y*} ≈ 15 − 41%; {*W*, *F*, *Y*} ∈ *AA*_*x*_) when taken in tandem with previously conducted mutagenesis experiments on the CBMs suggest that cellulose may physically interact with these residues on the surface prior to entering the cavity for catalysis (Figs. 5, 6, and 7, Tables 6, 7, and 8). The formation of this purported groove may be supported/strengthened by the uniform presence of proline (*P* ≈ 3.7 − 25%), as well as stabilizing electrostatic interactions involving arginine (*R*), lysine (*K*), asparagine (*N*), glutamine (*Q*), serine (*S*), and threonine (*T*) ([*RKNQ*] ≈ 12 − 25%; [*ST*] ≈ 10 − 18.5%), while remaining chemically inert throughout its length with several amino acids with shorter hydrophobic side chains lining the groove, i.e. leucine (*L*), isoleucine (*I*), valine (*V*), methionine (*M*), and exceptionally alanine (*A*) (*HSC* ≡ [*LIVAM*] ≈ 25 − 37%) (Table 7).

### Mechanistic insights into crystalline cellulose digestion by plant class C enzymes

The aforementioned discussion notwithstanding the small sample size could preclude meaningful inference of the mechanism(s) of crystalline cellulose digestion by plant class C GH9 endoglucanases. This was offset by examining 3D models of putative structural homologues of selected class C members (*n* = 39) (Fig. 8a; Supplementary Table 1 and Supplementary Text 2 and 10). Data from these suggest that the largest uninterrupted grooves that span GH9 (*l*_*GH*9_ ≅ 101 − 194 *Ang*) and CBM49 (*l*_*CBM*49_ ≅ 71 − 183 *Ang*) are disjoint and distinct, the only exceptions being the sequences from *L. usitatissimum* spp. and *B. distachyon* spp. (Fig. 9, Table 9). Further support for the mechanism(s) purported for digesting crystalline cellulose plant class C enzymes may be gleaned by examining the 3D models for *IS*-bounded surface grooves $\left({IS}_x^{GL},{IS}_x^{CL},{IS}_x^{GC}\right)$ in *A. coerulea*, *C. sinensis*, *C. rubella*, *S. purpurea*, *P. persica* spp., *M. domestica*, *P. virgatum* spp., *A. lyrata*, *B. rapa* spp., *Z. mays* spp., and *B. distachyon* spp. (Fig. 9, Table 9). Interestingly, the groove located at the interaction surface and bounded by GH9, linker, and CBM49 concomitantly $\left({IS}_x^{GLC}\right)$ as in *L usitatissimum* spp., *P. trichocarpa*, *M. truncatula* spp., *G. max*, and *B. distachyon* spp. may exert significant influence on crystalline cellulose in comparison with the distally located and smaller CBM49-bounded grooves (*l*_*CBM*49_ ≤ 100 *Ang*) (Fig. 9). This data further complements the hypothesis that plant class C GH9 endoglucanases may possess a dual mode (processive, non-processive) of action wherein crystalline cellulose is initially acted upon and thereby modified by the indenting side chains of aromatic amino in a quasi-continuous surface groove at the interface(s) of GH9, linker, and CBM49, which is inert and stable. Once modified (induced strain on the glycosidic linkage), crystalline cellulose is driven towards a solvent accessible subsurface cavity. Here, the GH9 conserved catalytic residues of aspartic (*D*) and/or glutamic (*E*) acids utilise an acid-base catalytic mechanism to cleave the *β* (1 → 4) linkage between glucopyranose units. These may then be acted upon by exoglucanases to release oligosaccharides (*C*2 − *C*4). This mechanism not only corroborates extant kinetic data such as CBM-mediated modulatory catalysis, but also offers a molecular explanation for substrate promiscuity observed for this group of enzymes, whilst conforming to available structural data from non-plant taxa (Figs. 4, 5, 6, 7, 8, and 9, Tables 4, 5, 6, 7, 8, and 9; Supplementary Tables 1 and Supplementary Texts 2–10) [51–60].

### Evolutionary significance for CBM49-mediated digestion of crystalline cellulose

The ability to cleave crystalline cellulose by plant class C members is dependent on the presence of CBM49 and may have evolved directly from non-plant taxa (≈500 Mya) [8, 17, 20–34, 59, 60]. An additional premise explored previously was that plant class C enzymes may not just predate but, could potentially diverge into classes A and B after CBM49 was excised during processing of the mature mRNA transcript [8, 18, 46, 83–85]. A mechanistic understanding of these processes is clearly desirable with much of the aforementioned generated data involving kinetic parameters, mRNA expression levels, and sequence information. The present study highlights variations in the CBM49/linker even amongst class C enzymes, provides insights into the architecture, position, plasticity, and composition of the *IS*-enclosed surface grooves, delineates the position and composition of a contiguous subsurface cavity for catalytic cleavage of the glycosidic linkage, enumerates functionally relevant amino acids that participate in substrate selection/modification, and offers a mechanistic explanation of CBM49-mediated reaction chemistry (Figs. 1, 2, 3, 4, 5, 6, 7, 8, and 9; Tables 1, 2, 3, 4, 5, 6, 7, 8, and 9; Supplementary Table 1 and Supplementary Texts 1–10). Additionally, a definitive body of literature indicates that hyperflexible regions may be intrinsically disordered and therefore have short *t*_1/2_ [63, 86, 87]. This would imply that proteins with the CBM49_linker may be evolutionarily at a disadvantage than those without. Alternatively, these might be encoded by nucleotides with a tendency to form higher order substructures in mRNA such as stem loops, bulges, and bends. These in turn could delay or irreversibly interrupt the ribosomal apparatus and prevent effective translation of the mRNA, and thereby contribute to decreased expression of class C enzymes. Since CBM49 is central to the ability of plant class C enzymes to digest crystalline cellulose, it would follow this loss could lead to a decrease in class C enzymes or conversely an increase in classes A and B [8].

## Conclusions

A detailed biophysical analysis of homology models of characterised and putative class C endoglucanases was carried out to assess the contribution(s) of the GH9, linker, and CBM49 to catalysis/modification of crystalline cellulose. The work presented in this manuscript corroborates the notion that the linker and CBM49 may complement generic acid-base catalysis by aspartic/glutamic residues of GH9, and may do so in a multitude of ways. These include an influence on the structural organization of the protein, participation in critical intra-protein interactions, facilitate formation of inert and structurally plastic surface grooves, and render crystalline cellulose amenable to hydrolytic cleavage. Despite being entirely computational, the findings presented here offer profound insights into not just the active site geometry of plant class C GH9 endoglucanases, but also offer valuable clues into their evolutionary divergence. Whilst, most these findings await experimental valiation the analyses conducted suggests that plant-based conversion of biomass is feasible and may constitute a viable alternative to bacterial-, fungal-, and algal-based protocols.

## Electronic supplementary material

ESM 1 (PDF 20 kb)

ESM 2 (PDF 1857 kb)

ESM 3 (PDF 2382 kb)

ESM 4 (PDF 276 kb)

ESM 5 (PDF 208 kb)

ESM 6 (PDF 215 kb)

ESM 7 (PDF 282 kb)

ESM 8 (PDF 2217 kb)

ESM 9 (PDF 123 kb)

ESM 10 (PDF 155 kb)

ESM 11 (PDF 129 kb)

## Abbreviations

AA: Amino acids
CBM: Carbohydrate binding module
DP: Degree of polymerization
EC: Enzyme commission
FL: Full length
GH: Glycoside hydrolase
IS: Interaction surface
L: Linker
NMA: Normal mode analysis
T: Truncated
