# Correction to: Insights into the mechanism(s) of digestion of crystalline cellulose by plant class C GH9 endoglucanases

**DOI:** 10.1007/s00894-020-04465-7

**Published:** 2020-07-27

**Authors:** Siddhartha Kundu

**Affiliations:** Department of Biochemistry, Army College of Medical Sciences, Brar Square, Delhi Cantt, New Delhi, 110010 India

**Correction to: Journal of Molecular Modeling (2019) 25: 240**

10.1007/s00894-019-4133-1

The article Insights into the mechanism(s) of digestion of crystalline cellulose by plant class C GH9 endoglucanases, written by Siddhartha Kundu, was originally published Online First without Open Access. After publication in volume 25, issue 8, article number 240 the author decided to opt for Open Choice and to make the article an Open Access publication. Therefore, the copyright of the article has been changed to © The Author(s) 2020 and the article is forthwith distributed under the terms of the Creative Commons Attribution 4.0 International License (http://creativecommons.org/licenses/by/4.0/), which permits use, duplication, adaptation, distribution and reproduction in any medium or format, as long as you give appropriate credit to the original author(s) and the source, provide a link to the Creative Commons license, and indicate if changes were made.

