# Supplementary material for: Insights into the mechanism(s) of digestion of crystalline cellulose by plant class C GH9 endoglucanases
Source: J Mol Model. 2019 Jul 23;25(8):240. doi: 10.1007/s00894-019-4133-1 (PMC7385011; doi:10.1007/s00894-019-4133-1)
Supplement: Supplementary file 1 — (PDF 20 kb) [file 894_2019_4133_MOESM1_ESM.pdf]

Supplementary Table 1: Principal components of characterized and putative class C GH9 endoglucanases (n=96)

	Sequence ID	Organism	Principal components			
			PC1	PC3	PC2	
1	Q5NAT0	*Oryza sativa*	-26.175	-13.655	-49.195	
2	Q8LJP6	*Gossypium hirsutum*	-7.1372	-12.302	-43.569	
3	Q9ZSP9	*Solanum lycopersicum*	-19.659	-26.247	-40.287	
4	orange1.1g043219m	*Citrus sinensis*	-24.106	-15.803	0.06297	
5	ppa022524m	*Prunus persica*	-24.135	-17.287	1.31338	
6	SapurV1A.0237s0330.1.p	*Salix purpurea*	-40.74	-2.866	8.82545	
7	Carubv10003874m	*Capsella rubella*	-40.74	-2.866	8.82545	
8	Pavir.Ea00142.1.p	*Panicum virgatum*	-24.485	-5.5735	-3.9026	
9	Bradi2g07150.1.p	*Bradipodium distachyon*	-3.0182	-6.0425	10.4498	
10	Brara.E01714.1.p	*Brassica rapa*	-31.214	-1.7171	3.1937	
11	Pavir.Eb00189.1.p	*Panicum virgatum*	-25.013	-5.4123	2.20198	
12	GRMZM2G453565_P01	*Zea mays*	-31.698	-2.084	7.12213	
13	SapurV1A.0035s0560.1.p	*Salix purpurea*	-25.013	-5.4123	2.20198	
14	Bradi5g26010.1.p	*Bradipodium distachyon*	-23.608	-17.579	-4.774	
15	PGSC0003DMP400021750	*Solanum tuberosum*	-31.698	-2.084	7.12213	
16	Migut.D01909.1.p	*Mimulus guttatus*	-24.136	-17.417	1.3305	
17	234652	*Selaginella moellendorffii*	-31.698	-2.084	7.12213	
18	MDP0000131267	*Malus domestica*	-41.208	-3.8202	12.1763	
19	Potri.001G092200.1	*Populus trichocarpa*	-38.546	-3.047	8.28083	
20	GRMZM2G143747_P01	*Zea mays*	-31.698	-2.084	7.12213	
21	Solyc02g014220.2.1	*Solanum lycopersicum*	-31.15	-1.2201	0.31682	n=42
22	Aquca_037_00141.1	*Aquilegia coerulea*	-4.0553	-16.919	-8.072	
23	Glyma.05G216400.1.p	*Glycine max*	-25.013	-5.4123	2.20198	
24	489943	*Arabidopsis lyrata*	-20.422	-4.4213	14.1454	
25	Bostr.25463s0223.1.p	*Boechera stricta*	-20.714	-3.2022	15.0352	
26	Eucgr.J00862.1	*Eucalyptus grandis*	-24.715	-18.956	-1.4259	
27	ppa002939m	*Prunus persica*	-32.386	-0.5027	10.9578	
28	PGSC0003DMP400034548	*Solanum tuberosum*	-31.678	-1.0589	6.42137	
29	Thhalv10028514m	*Eutrema salsugineum*	-20.704	-2.9152	14.82	
30	Medtr4g074960.1	*Medicago truncatula*	-28.611	-1.7237	5.96221	
31	Gorai.005G210200.1	*Gossypium raimondii*	-31.17	-2.2451	1.01758	
32	Potri.003G139600.1	*Populus trichocarpa*	-40.74	-2.866	8.82545	
33	Sobic.003G015700.1.p	*Sorghum bicolor*	-33.132	-1.7338	7.59862	
34	Brara.I01325.1.p	*Brassica rapa*	-25.013	-5.4123	2.20198	
35	Cucsa.107370.1	*Cucumis sativus*	-32.09	-3.6424	9.27177	
36	99802	*Selaginella moellendorffii*	-40.795	-2.1137	8.90289	
37	Brara.C02656.1.p	*Brassica rapa*	-20.704	-2.9152	14.82	
38	Lus10032377	*Linum usitatissimum*	-4.0553	-16.919	-8.072	
39	Lus10003888	*Linum usitatissimum*	-4.0553	-16.919	-8.072	
40	Medtr8g099410.1	*Medicago truncatula*	-41.145	-2.7908	11.9865	
41	AT4G11050.1	*Arabidopsis thaliana*	-20.704	-2.9152	14.82	
42	Bradi2g32270.1.p	*Bradipodium distachyon*	-32.103	-2.0088	10.2831	
1	Q93WY9	*Nicotiana tabacum*	-9.1093	1.52235	-41.947	
2	474038	*Arabidopsis lyrata*	-32.505	13.459	-3.4165	
3	Pavir.Ca00497.1.p	*Panicum virgatum*	-32.005	13.4669	-9.7136	
4	MDP0000276676	*Malus domestica*	-32.505	13.459	-3.4165	
5	Cucsa.303850.1	*Cucumis sativus*	-28.903	10.8242	-6.4089	
6	cassava4.1_003698m	*Manihot esculenta*	-33.307	16.6434	0.17342	
7	Sobic.009G079900.1.p	*Sorghum bicolor*	-32.022	13.7921	-7.3446	
8	GSVIVT01019523001	*Vitis vinifera*	-32.505	13.459	-3.4165	
9	Carubv10012624m	*Capsella rubella*	-32.505	13.459	-3.4165	
10	evm.TU.contig_27379.2	*Carica papaya*	-33.307	16.6434	0.17342	n=19
11	Bostr.26675s0278.1.p	*Boechera stricta*	-31.979	13.234	-9.5598	
12	Gorai.008G254000.1	*Gossypium raimondii*	-21.125	16.6217	4.85636	

13	orange1.1g006835m	*Citrus sinensis*	-32.902	16.5897	-3.1781	
14	Lus10001833	*Linum usitatissimum*	-32.902	16.5897	-3.1781	
15	Thhalv10011318m	*Eutrema salsugineum*	-31.979	13.234	-9.5598	
16	Gorai.003G080800.1	*Gossypium raimondii*	-32.911	13.5127	-0.065	
17	Ciclev10019301m	*Citrus clementena*	-31.979	13.234	-9.5598	
18	Lus10017402	*Linum usitatissimum*	-32.505	13.459	-3.4165	
19	AT1G48930.1	*Arabidopsis thaliana*	-32.505	13.459	-3.4165	
1	Eucgr.E00858.1	*Eucalyptus grandis*	51.815	-4.8982	6.72738	
2	Phvul.002G297600.1	*Phaseolus vulgaris*	52.2996	-4.5314	2.79895	
3	Phpat.013G084100.1.p	*Physcomitrella patens*	52.3427	-5.0594	0.62283	
4	Phpat.006G094200.1.p	*Physcomitrella patens*	52.5324	-4.1475	4.2756	
5	Bostr.29223s0050.1.p	*Boechera stricta*	51.4097	-4.8231	9.88839	
6	LOC_Os05g12150.1	*Oryza sativa*	51.815	-4.8982	6.72738	
7	Si000642m	*Setaria italica*	19.2078	-4.3037	5.30126	
8	LOC_Os01g12030.1	*Oryza sativa*	38.8555	-4.7061	2.1592	
9	GRMZM2G343144_P02	*Zea mays*	51.815	-4.8982	6.72738	
10	Eucgr.D01733.1	*Eucalyptus grandis*	51.815	-4.8982	6.72738	
11	mrna32087.1-v1.0-hybrid	*Fragaria vesca*	51.815	-4.8982	6.72738	n=22
12	AT1G64390.1	*Arabidopsis thaliana*	51.815	-4.8982	6.72738	
13	Gorai.004G133700.1	*Gossypium raimondii*	51.815	-4.8982	6.72738	
14	Thhalv10023353m	*Eutrema salsugineum*	51.815	-4.8982	6.72738	
15	Phpat.026G000800.1.p	*Physcomitrella patens*	52.4941	-7.4573	-3.8268	
16	Phvul.011G030300.1	*Phaseolus vulgaris*	8.50492	-8.0315	-7.4505	
17	mrna11058.1-v1.0-hybrid	*Fragaria vesca*	52.3632	-4.0344	-0.0779	
18	LOC_Os04g57860.1	*Oryza sativa*	13.9433	-4.8868	6.69178	
19	109529	*Selaginella moellendorffii*	49.547	-5.3287	-1.7579	
20	Potri.007G071200.1	*Populus trichocarpa*	51.815	-4.8982	6.72738	
21	Aquca_013_00114.1	*Aquilegia coerulea*	51.9229	-3.7792	4.87625	
22	Pavir.Cb01721.1.p	*Panicum virgatum*	51.4097	-4.8231	9.88839	
1	29675.m000388	*Ricinus communis*	52.3642	10.0282	-7.8938	
2	Carubv10019981m	*Capsella rubella*	52.3213	10.5863	-5.6785	
3	Lus10010201	*Linum usitatissimum*	51.8379	10.2532	-1.7504	
4	Si021476m	*Setaria italica*	51.8379	10.2532	-1.7504	
5	Cagra.1255s0001.1.p	*Capsella grandiflora*	52.3213	10.5863	-5.6785	
6	Sobic.006G265100.1.p	*Sorghum bicolor*	51.4415	13.3839	-1.512	
7	Aquca_002_01464.1	*Aquilegia coerulea*	51.8379	10.2532	-1.7504	n=13
8	MDP0000826600	*Malus domestica*	52.3213	10.5863	-5.6785	
9	Si021474m	*Setaria italica*	52.0024	10.7011	-2.7862	
10	GSVIVT01012043001	*Vitis vinifera*	52.3213	10.5863	-5.6785	
11	Ciclev10033912m	*Citrus clementena*	52.3642	10.0282	-7.8938	
12	Cucsa.128330.1	*Cucumis sativus*	52.3642	10.0282	-7.8938	
13	Pavir.Ga00244.1.p	*Panicum virgatum*	51.8379	10.2532	-1.7504	
