# Supplementary material for: Insights into the mechanism(s) of digestion of crystalline cellulose by plant class C GH9 endoglucanases
Source: J Mol Model. 2019 Jul 23;25(8):240. doi: 10.1007/s00894-019-4133-1 (PMC7385011; doi:10.1007/s00894-019-4133-1)
Supplement: Supplementary file 2 — (PDF 1857 kb) [file 894_2019_4133_MOESM2_ESM.pdf]

Phyre2

Email	siddhartha_kundu@yahoo.co.in
Description	Q8LJP6___
Date	Thu Feb 1 11:12:10 GMT 2018
Unique Job ID	b166f14fde6eb22a

Detailed template information

#	Template	Alignment Coverage	3D Model	Confidence	% i.d.	Template Information
1	c1js4B_	Alignment	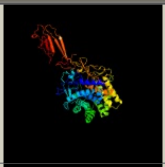	100.0	37	**PDB header:**glycosyl hydrolase<br>**Chain:** B: **PDB Molecule:**endo/exocellulase e4;<br>**PDBTitle:** endo/exocellulase:cellobiose from thermomonospora
2	c1ga2A_	Alignment	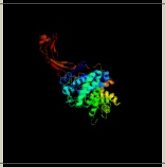	100.0	34	**PDB header:**hydrolase<br>**Chain:** A: **PDB Molecule:**endoglucanase 9g;<br>**PDBTitle:** the crystal structure of endoglucanase 9g from clostridium2 cellulolyticum complexed with cellobiose
3	c5gxyA_	Alignment	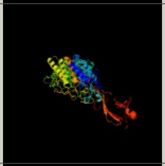	100.0	32	**PDB header:**hydrolase<br>**Chain:** A: **PDB Molecule:**glucanase;<br>**PDBTitle:** crystal structure of endoglucanase celq from clostridium thermocellum2 complexed with cellobiose and tris
4	d1g87a1	Alignment	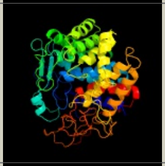	100.0	37	**Fold:**alpha/alpha toroid<br>**Superfamily:**Six-hairpin glycosidases<br>**Family:**Cellulases catalytic domain
5	d1tf4a1	Alignment	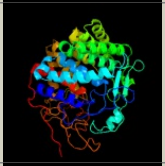	100.0	42	**Fold:**alpha/alpha toroid<br>**Superfamily:**Six-hairpin glycosidases<br>**Family:**Cellulases catalytic domain
6	c2yikA_	Alignment	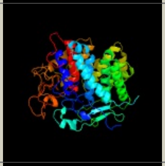	100.0	31	**PDB header:**hydrolase<br>**Chain:** A: **PDB Molecule:**endoglucanase;<br>**PDBTitle:** catalytic domain of clostridium thermocellum celt
7	c2xfgA_	Alignment	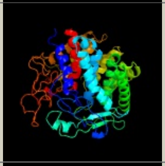	100.0	40	**PDB header:**hydrolase/sugar binding protein<br>**Chain:** A: **PDB Molecule:**endoglucanase 1;<br>**PDBTitle:** reassembly and co-crystallization of a family 9 processive2 endoglucanase from separately expressed gh9 and cbm3c3 modules
8	c3wc3A_	Alignment	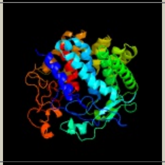	100.0	43	**PDB header:**hydrolase<br>**Chain:** A: **PDB Molecule:**endo-1, 4-beta-glucanase;<br>**PDBTitle:** crystal structure of endo-1,4-beta-glucanase from eisenia fetida
9	d1ia6a_	Alignment	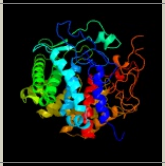	100.0	36	**Fold:**alpha/alpha toroid<br>**Superfamily:**Six-hairpin glycosidases<br>**Family:**Cellulases catalytic domain
10	d1ks8a_	Alignment	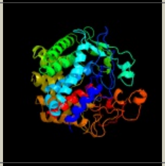	100.0	48	**Fold:**alpha/alpha toroid<br>**Superfamily:**Six-hairpin glycosidases<br>**Family:**Cellulases catalytic domain
11	d1ut9a1	Alignment	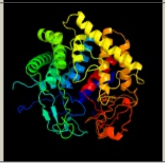	100.0	19	**Fold:**alpha/alpha toroid<br>**Superfamily:**Six-hairpin glycosidases<br>**Family:**Cellulases catalytic domain

12	c1clcA_	Alignment	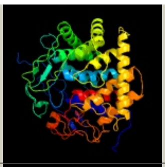	100.0	19	**PDB header:**glycosyl hydrolase<br>**Chain:** A: **PDB Molecule:**endoglucanase celd; ec: 3.2.1.4;<br>**PDBTitle:** three-dimensional structure of endoglucanase d at 1.92 angstroms resolution
13	d1clca1	Alignment	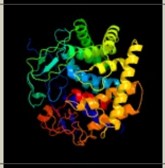	100.0	20	**Fold:**alpha/alpha toroid<br>**Superfamily:**Six-hairpin glycosidases<br>**Family:**Cellulases catalytic domain
14	c1ut9A_	Alignment	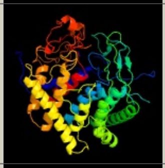	100.0	20	**PDB header:**hydrolase<br>**Chain:** A: **PDB Molecule:**cellulose 1,4-beta-cellobiosidase;<br>**PDBTitle:** structural basis for the exocellulase activity of the2 cellobiohydrolase cbha from c. thermocellum
15	c3gzkA_	Alignment	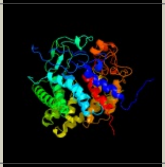	100.0	20	**PDB header:**hydrolase<br>**Chain:** A: **PDB Molecule:**cellulase;<br>**PDBTitle:** structure of a. acidocaldarius cellulase cela
16	c3x17B_	Alignment	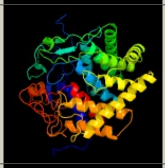	100.0	21	**PDB header:**hydrolase<br>**Chain:** B: **PDB Molecule:**endoglucanase;<br>**PDBTitle:** crystal structure of metagenome-derived glycoside hydrolase family 92 endoglucanase
17	c5dgrB_	Alignment	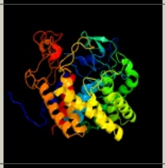	100.0	15	**PDB header:**hydrolase<br>**Chain:** B: **PDB Molecule:**putative endoglucanase-related protein;<br>**PDBTitle:** crystal structure of gh9 exo-beta-d-glucosaminidase pbpra0520,2 glucosamine complex
18	c3h7lC_	Alignment	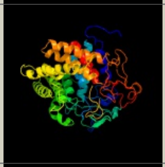	100.0	17	**PDB header:**hydrolase<br>**Chain:** C: **PDB Molecule:**endoglucanase;<br>**PDBTitle:** crystal structure of endoglucanase-related protein from vibrio2 parahaemolyticus
19	c2xfgB_	Alignment	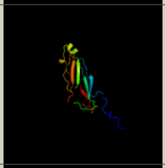	99.6	13	**PDB header:**hydrolase/sugar binding protein<br>**Chain:** B: **PDB Molecule:**endoglucanase 1;<br>**PDBTitle:** reassembly and co-crystallization of a family 9 processive2 endoglucanase from separately expressed gh9 and cbm3c3 modules
20	d1tf4a2	Alignment	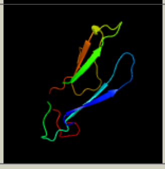	99.2	14	**Fold:**Common fold of diphtheria toxin/transcription factors/cytochrome f<br>**Superfamily:**Carbohydrate-binding domain<br>**Family:**Cellulose-binding domain family III
21	d1g87a2	Alignment	not modelled	99.1	17	**Fold:**Common fold of diphtheria toxin/transcription factors/cytochrome f<br>**Superfamily:**Carbohydrate-binding domain<br>**Family:**Cellulose-binding domain family III
22	c4v1sA_	Alignment	not modelled	98.9	10	**PDB header:**hydrolase<br>**Chain:** A: **PDB Molecule:**alpha-1,6-mannanase;<br>**PDBTitle:** structure of the gh76 alpha-mannanase bt2949 from2 bacteroides thetaiotaomicron
23	c4z4lA_	Alignment	not modelled	98.4	11	**PDB header:**isomerase<br>**Chain:** A: **PDB Molecule:**cellobiose 2-epimerase;<br>**PDBTitle:** crystal structure of cellobiose 2-epimerase from caldicellulosiruptor2 saccharolyticus dsm 8903
24	c4c1sA_	Alignment	not modelled	98.4	15	**PDB header:**hydrolase<br>**Chain:** A: **PDB Molecule:**glycoside hydrolase family 76 mannosidase;<br>**PDBTitle:** glycoside hydrolase family 76 (mannosidase) bt3792 from2 bacteroides thetaiotaomicron vpi-5482
25	c3wkgA_	Alignment	not modelled	98.4	12	**PDB header:**isomerase<br>**Chain:** A: **PDB Molecule:**cellobiose 2-epimerase;<br>**PDBTitle:** crystal structure of cellobiose 2-epimerase in complex with2 glucosylmannose
26	c3vw5B_	Alignment	not modelled	98.3	17	**PDB header:**isomerase<br>**Chain:** B: **PDB Molecule:**cellobiose 2-epimerase;<br>**PDBTitle:** crystal structure of sugar epimerase from ruminal bacterium
27	c3k7xA_	Alignment	not modelled	98.2	14	**PDB header:**structural genomics, unknown function<br>**Chain:** A: **PDB Molecule:**lin0763 protein;<br>**PDBTitle:** crystal structure of the lin0763 protein from listeria innocua.2 northeast structural genomics consortium target lkr23.
28	c4qk0C_	Alignment	not modelled	98.0	13	**PDB header:**hydrolase<br>**Chain:** C: **PDB Molecule:**gh127 beta-l-arabinofuranoside;<br>**PDBTitle:** crystal structure of ara127n-se, a gh127 beta-l-

						arabinofuranosidase2 from geobacillus stearothermophilus t6
29	c2wo4A_	Alignment	not modelled	97.8	12	**PDB header:**hydrolase<br>**Chain:** A: **PDB Molecule:**glycoside hydrolase, family 9;<br>**PDBTitle:** 3b' carbohydrate-binding module from the cel9v glycoside2 hydrolase from clostridium thermocellum, in-house data
30	c5mqoA_	Alignment	not modelled	97.8	13	**PDB header:**hydrolase<br>**Chain:** A: **PDB Molecule:**non-reducing end beta-l-arabinofuranosidase;<br>**PDBTitle:** glycoside hydrolase bt_1003
31	d1nbca_	Alignment	not modelled	97.7	20	**Fold:**Common fold of diphtheria toxin/transcription factors/cytochrome f<br>**Superfamily:**Carbohydrate-binding domain<br>**Family:**Cellulose-binding domain family III
32	c4bojC_	Alignment	not modelled	97.6	17	**PDB header:**hydrolase<br>**Chain:** C: **PDB Molecule:**alpha-1,6-mannanase;<br>**PDBTitle:** crystal structure of bacillus circulans tn-31 aman6 in2 complex with mannobiose
33	c4mu9B_	Alignment	not modelled	97.6	11	**PDB header:**hydrolase<br>**Chain:** B: **PDB Molecule:**glycoside hydrolase family 73;<br>**PDBTitle:** crystal structure of a putative glycosylhydrolase (bt_3782) from2 bacteroides thetaiotaomicron vpi-5482 at 1.89 a resolution
34	c4b9pA_	Alignment	not modelled	97.5	14	**PDB header:**carbohydrate-binding protein<br>**Chain:** A: **PDB Molecule:**type 3a cellulose-binding domain protein;<br>**PDBTitle:** biomass sensoring module from putative rsgi2 protein of2 clostridium thermocellum resemble family 3 carbohydrate-3 binding module of cellulosome
35	c3gt5A_	Alignment	not modelled	97.5	15	**PDB header:**isomerase<br>**Chain:** A: **PDB Molecule:**n-acetylglucosamine 2-epimerase;<br>**PDBTitle:** crystal structure of an n-acetylglucosamine 2-epimerase family protein2 from xylella fastidiosa
36	d1exha_	Alignment	not modelled	97.5	14	**Fold:**Common fold of diphtheria toxin/transcription factors/cytochrome f<br>**Superfamily:**Carbohydrate-binding domain<br>**Family:**Cellulose-binding domain family II
37	d1g43a_	Alignment	not modelled	97.4	16	**Fold:**Common fold of diphtheria toxin/transcription factors/cytochrome f<br>**Superfamily:**Carbohydrate-binding domain<br>**Family:**Cellulose-binding domain family III
38	c3wkxA_	Alignment	not modelled	97.4	16	**PDB header:**hydrolase<br>**Chain:** A: **PDB Molecule:**non-reducing end beta-l-arabinofuranosidase;<br>**PDBTitle:** crystal structure of gh127 beta-l-arabinofuranosidase hypba1 from2 bifidobacterium longum arabinose complex form
39	c2xbtA_	Alignment	not modelled	97.3	13	**PDB header:**sugar binding protein<br>**Chain:** A: **PDB Molecule:**cellulosomal scaffoldin;<br>**PDBTitle:** structure of a scaffoldin carbohydrate-binding module family 3b from2 the cellulosome of bacteroides cellulosolvens: structural diversity3 and implications for carbohydrate binding
40	c4b97A_	Alignment	not modelled	97.2	13	**PDB header:**sugar binding protein<br>**Chain:** A: **PDB Molecule:**cellulose binding domain-containing protein;<br>**PDBTitle:** biomass sensing modules from putative rsgi-like proteins2 of clostridium thermocellum resemble family 3 carbohydrate-3 binding module of cellulosome
41	c2rttA_	Alignment	not modelled	97.2	14	**PDB header:**hydrolase<br>**Chain:** A: **PDB Molecule:**chic;<br>**PDBTitle:** solution structure of the chitin-binding domain of chi18ac from2 streptomyces coelicolor
42	c2l8aA_	Alignment	not modelled	97.2	14	**PDB header:**hydrolase<br>**Chain:** A: **PDB Molecule:**endoglucanase;<br>**PDBTitle:** structure of a novel cbm3 lacking the calcium-binding site
43	c3zqwA_	Alignment	not modelled	97.1	13	**PDB header:**carbohydrate-binding protein<br>**Chain:** A: **PDB Molecule:**cellulosomal scaffoldin;<br>**PDBTitle:** structure of cbm3b of major scaffoldin subunit scaa from2 acetivibrio cellulolyticus
44	c4b96A_	Alignment	not modelled	96.7	13	**PDB header:**sugar binding protein<br>**Chain:** A: **PDB Molecule:**cellulose binding domain-containing protein;<br>**PDBTitle:** family 3b carbohydrate-binding module from the biomass2 sensoring system of clostridium clariflavum
45	c2ylkD_	Alignment	not modelled	96.6	9	**PDB header:**hydrolase<br>**Chain:** D: **PDB Molecule:**cellulose 1,4-beta-cellobiosidase;<br>**PDBTitle:** carbohydrate-binding module cbm3b from the cellulosomal2 cellobiohydrolase 9a from clostridium thermocellum
46	c2gz6B_	Alignment	not modelled	96.6	9	**PDB header:**isomerase<br>**Chain:** B: **PDB Molecule:**n-acetyl-d-glucosamine 2-epimerase;<br>**PDBTitle:** crystal structure of anabaena sp. ch1 n-acetyl-d-glucosamine 2-2 epimerase at 2.0 a
47	c3ndyG_	Alignment	not modelled	96.6	8	**PDB header:**hydrolase<br>**Chain:** G: **PDB Molecule:**endoglucanase d;<br>**PDBTitle:** the structure of the catalytic and carbohydrate binding domain of2 endoglucanase d from clostridium cellulovorans
48	c4b9cA_	Alignment	not modelled	96.6	9	**PDB header:**carbohydrate-binding protein<br>**Chain:** A: **PDB Molecule:**type 3a cellulose-binding domain protein;<br>**PDBTitle:** biomass sensoring modules from putative rsgi-like proteins2 of clostridium thermocellum resemble family 3 carbohydrate-3 binding module of cellulosome
49	c4xuvB_	Alignment	not modelled	96.5	12	**PDB header:**hydrolase<br>**Chain:** B: **PDB Molecule:**glycoside hydrolase family 105 protein;<br>**PDBTitle:** crystal structure of a glycoside hydrolase family 105 (gh105) enzyme2 from thielavia terrestris
50	c4wu0B_	Alignment	not modelled	96.3	12	**PDB header:**hydrolase<br>**Chain:** B: **PDB Molecule:**similar to yter (bacilus subtilis);<br>**PDBTitle:** structural analysis of c. acetobutylicum atcc 824 glycoside hydrolase2 from family 105
51	c3wiwA_	Alignment	not modelled	96.2	9	**PDB header:**hydrolase<br>**Chain:** A: **PDB Molecule:**glycosyl hydrolase family 88;<br>**PDBTitle:** crystal structure of unsaturated glucuronyl hydrolase specific for2 heparin
52	d2afaa1	Alignment	not modelled	96.2	10	**Fold:**alpha/alpha toroid<br>**Superfamily:**Six-hairpin glycosidases<br>**Family:**N-acylglucosamine (NAG) epimerase

53	c5no8A_	Alignment	not modelled	95.6	15	**PDB header:**lyase **Chain:** A: **PDB Molecule:**baccell_00875; **PDBTitle:** polysaccharide lyase baccell_00875
54	c2zzrA_	Alignment	not modelled	95.0	16	**PDB header:**hydrolase **Chain:** A: **PDB Molecule:**unsaturated glucuronyl hydrolase; **PDBTitle:** crystal structure of unsaturated glucuronyl hydrolase from2 streptcoccus agalactiae
55	c5noaA_	Alignment	not modelled	94.5	8	**PDB header:**hydrolase **Chain:** A: **PDB Molecule:**family 88 glycosyl hydrolase; **PDBTitle:** polysaccharide lyase baccell_00875
56	c2cwrA_	Alignment	not modelled	94.2	7	**PDB header:**hydrolase **Chain:** A: **PDB Molecule:**chitinase; **PDBTitle:** crystal structure of chitin biding domain of chitinase from2 pyrococcus furiosus
57	c5dhdA_	Alignment	not modelled	94.2	13	**PDB header:**hydrolase **Chain:** A: **PDB Molecule:**chitinase; **PDBTitle:** crystal structure of chbd2 from thermococcus kodakarensis kod1
58	d1nc5a_	Alignment	not modelled	93.8	7	**Fold:**alpha/alpha toroid **Superfamily:**Six-hairpin glycosidases **Family:**Hypothetical protein YteR
59	d1gxma_	Alignment	not modelled	93.8	18	**Fold:**alpha/alpha toroid **Superfamily:**Family 10 polysaccharide lyase **Family:**Family 10 polysaccharide lyase
60	d1fp3a_	Alignment	not modelled	93.7	19	**Fold:**alpha/alpha toroid **Superfamily:**Six-hairpin glycosidases **Family:**N-acylglucosamine (NAG) epimerase
61	c3e6uA_	Alignment	not modelled	93.7	22	**PDB header:**signaling protein **Chain:** A: **PDB Molecule:**lanc-like protein 1; **PDBTitle:** crystal structure of human lancl1
62	c3k11A_	Alignment	not modelled	92.2	11	**PDB header:**hydrolase **Chain:** A: **PDB Molecule:**putative glycosyl hydrolase; **PDBTitle:** crystal structure of putative glycosyl hydrolase (np_813087.1) from2 bacteroides thetaiotaomicron vpi-5482 at 1.80 a resolution
63	c4q88B_	Alignment	not modelled	88.2	7	**PDB header:**structural genomics, unknown function **Chain:** B: **PDB Molecule:**uncharacterized protein; **PDBTitle:** glycosyl hydrolase family 88 from bacteroides vulgatus
64	c3pmmA_	Alignment	not modelled	86.1	13	**PDB header:**hydrolase **Chain:** A: **PDB Molecule:**putative cytoplasmic protein; **PDBTitle:** the crystal structure of a possible member of gh105 family from2 klebsiella pneumoniae subsp. pneumoniae mgh 78578
65	d2d5ja1	Alignment	not modelled	85.9	12	**Fold:**alpha/alpha toroid **Superfamily:**Six-hairpin glycosidases **Family:**Glycosyl Hydrolase Family 88
66	c4ce7B_	Alignment	not modelled	85.5	11	**PDB header:**hydrolase **Chain:** B: **PDB Molecule:**unsaturated 3s-rhamnoglycuronyl hydrolase; **PDBTitle:** crystal structure of a novel unsaturated beta-glucuronyl2 hydrolase enzyme, belonging to family gh105, involved in3 ulvan degradation
67	c5a29A_	Alignment	not modelled	84.4	24	**PDB header:**lyase **Chain:** A: **PDB Molecule:**exopolygalacturonate lyase; **PDBTitle:** family 2 pectate lyase from vibrio vulnificus
68	d2ri9a1	Alignment	not modelled	81.0	10	**Fold:**alpha/alpha toroid **Superfamily:**Seven-hairpin glycosidases **Family:**Class I alpha-1;2-mannosidase, catalytic domain
69	c1krfA_	Alignment	not modelled	79.9	10	**PDB header:**hydrolase **Chain:** A: **PDB Molecule:**mannosyl-oligosaccharide alpha-1,2-mannosidase; **PDBTitle:** structure of p. citrinum alpha 1,2-mannosidase reveals the basis for2 differences in specificity of the er and golgi class i enzymes
70	c3icgD_	Alignment	not modelled	77.4	11	**PDB header:**hydrolase **Chain:** D: **PDB Molecule:**endoglucanase d; **PDBTitle:** crystal structure of the catalytic and carbohydrate binding domain of2 endoglucanase d from clostridium cellulovorans
71	d1qaza_	Alignment	not modelled	74.5	12	**Fold:**alpha/alpha toroid **Superfamily:**Chondroitin AC/alginate lyase **Family:**Alginate lyase A1-III
72	d1hcua_	Alignment	not modelled	73.5	10	**Fold:**alpha/alpha toroid **Superfamily:**Seven-hairpin glycosidases **Family:**Class I alpha-1;2-mannosidase, catalytic domain
73	d1nxca_	Alignment	not modelled	73.0	5	**Fold:**alpha/alpha toroid **Superfamily:**Seven-hairpin glycosidases **Family:**Class I alpha-1;2-mannosidase, catalytic domain
74	c1x9dA_	Alignment	not modelled	72.6	13	**PDB header:**hydrolase **Chain:** A: **PDB Molecule:**endoplasmic reticulum mannosyl-oligosaccharide 1,2-alpha- **PDBTitle:** crystal structure of human class i alpha-1,2-mannosidase in complex2 with thio-disaccharide substrate analogue
75	d1x9da1	Alignment	not modelled	72.6	13	**Fold:**alpha/alpha toroid **Superfamily:**Seven-hairpin glycosidases **Family:**Class I alpha-1;2-mannosidase, catalytic domain
76	c4ayqA_	Alignment	not modelled	66.6	18	**PDB header:**hydrolase **Chain:** A: **PDB Molecule:**mannosyl-oligosaccharide 1,2-alpha-mannosidase; **PDBTitle:** structure of the gh47 processing alpha-1,2-mannosidase from2 caulobacter strain k31 in complex with mannoimidazole
77	c2v8kA_	Alignment	not modelled	65.5	21	**PDB header:**lyase **Chain:** A: **PDB Molecule:**pectate lyase; **PDBTitle:** structure of a family 2 pectate lyase in complex with2 trigalacturonic acid

78	d1r76a_	Alignment	not modelled	63.5	14	**Fold:**alpha/alpha toroid<br>**Superfamily:**Family 10 polysaccharide lyase<br>**Family:**Family 10 polysaccharide lyase
79	d1dl2a_	Alignment	not modelled	57.7	15	**Fold:**alpha/alpha toroid<br>**Superfamily:**Seven-hairpin glycosidases<br>**Family:**Class I alpha-1;2-mannosidase, catalytic domain
80	c1g6iA_	Alignment	not modelled	57.5	15	**PDB header:**hydrolase<br>**Chain:** A: **PDB Molecule:**class i alpha-1,2-mannosidase;<br>**PDBTitle:** crystal structure of the yeast alpha-1,2-mannosidase with bound 1-2 deoxymannojirimycin at 1.59 a resolution
81	c2kmgA_	Alignment	not modelled	55.7	20	**PDB header:**gene regulation<br>**Chain:** A: **PDB Molecule:**klca;<br>**PDBTitle:** the structure of the klca and ardb proteins show a novel2 fold and antirestriction activity against type i dna3 restriction systems in vivo but not in vitro
82	c2fuqA_	Alignment	not modelled	55.0	5	**PDB header:**sugar binding protein<br>**Chain:** A: **PDB Molecule:**heparinase ii protein;<br>**PDBTitle:** crystal structure of heparinase ii
83	c3nfvA_	Alignment	not modelled	53.3	11	**PDB header:**lyase<br>**Chain:** A: **PDB Molecule:**alginate lyase;<br>**PDBTitle:** crystal structure of an alginate lyase (bacova_01668) from bacteroides2 ovatus at 1.95 a resolution
84	c5bp8A_	Alignment	not modelled	47.8	12	**PDB header:**isomerase<br>**Chain:** A: **PDB Molecule:**ent-copalyl diphosphate synthase;<br>**PDBTitle:** ent-copalyl diphosphate synthase from streptomyces platensis
85	c4ok2A_	Alignment	not modelled	41.1	14	**PDB header:**lyase<br>**Chain:** A: **PDB Molecule:**putative alginate lyase;<br>**PDBTitle:** crystal structure of alg17c mutant y258a
86	d1e5ba_	Alignment	not modelled	34.6	13	**Fold:**Common fold of diphtheria toxin/transcription factors/cytochrome f<br>**Superfamily:**Carbohydrate-binding domain<br>**Family:**Cellulose-binding domain family II
87	c4fnvA_	Alignment	not modelled	26.8	9	**PDB header:**lyase<br>**Chain:** A: **PDB Molecule:**heparinase iii protein, heparitin sulfate lyase;<br>**PDBTitle:** crystal structure of heparinase iii
88	c4mcjC_	Alignment	not modelled	24.2	24	**PDB header:**transferase<br>**Chain:** C: **PDB Molecule:**uncharacterized protein;<br>**PDBTitle:** crystal structure of a putative nucleoside deoxyribosyltransferase2 (bdi_0649) from parabacteroides distasonis atcc 8503 at 2.40 a3 resolution
89	d1f53a_	Alignment	not modelled	19.3	70	**Fold:**gamma-Crystallin-like<br>**Superfamily:**gamma-Crystallin-like<br>**Family:**Killer toxin-like protein SKLP
90	c4qawA_	Alignment	not modelled	17.8	24	**PDB header:**hydrolase<br>**Chain:** A: **PDB Molecule:**xyn30d;<br>**PDBTitle:** structure of modular xyn30d from paenibacillus barcinonensis
91	c3adyA_	Alignment	not modelled	17.5	14	**PDB header:**proton transport<br>**Chain:** A: **PDB Molecule:**dotd;<br>**PDBTitle:** crystal structure of dotd from legionella
92	c4zlgA_	Alignment	not modelled	16.0	21	**PDB header:**transferase<br>**Chain:** A: **PDB Molecule:**putative b-glycan phosphorylase;<br>**PDBTitle:** cellobionic acid phosphorylase - gluconic acid complex
93	c4ozwA_	Alignment	not modelled	15.8	12	**PDB header:**lyase<br>**Chain:** A: **PDB Molecule:**alginate lyase;<br>**PDBTitle:** crystal structure of the periplasmic alginate lyase algl h202a mutant
94	c5eurC_	Alignment	not modelled	13.5	56	**PDB header:**unknown function<br>**Chain:** C: **PDB Molecule:**uncharacterized protein;<br>**PDBTitle:** hypothetical protein sf216 from shigella flexneri 5a m90t
95	c3w5mA_	Alignment	not modelled	13.3	16	**PDB header:**hydrolase<br>**Chain:** A: **PDB Molecule:**putative rhamnosidase;<br>**PDBTitle:** crystal structure of streptomyces avermitilis alpha-l-rhamnosidase
96	c3zr6A_	Alignment	not modelled	13.0	8	**PDB header:**hydrolase<br>**Chain:** A: **PDB Molecule:**galactocerebrosidase;<br>**PDBTitle:** structure of galactocerebrosidase from mouse in complex with2 galactose
97	c3a0oB_	Alignment	not modelled	12.6	8	**PDB header:**lyase<br>**Chain:** B: **PDB Molecule:**oligo alginate lyase;<br>**PDBTitle:** crystal structure of alginate lyase from agrobacterium tumefaciens c58
98	c1ayeA_	Alignment	not modelled	12.2	16	**PDB header:**serine protease<br>**Chain:** A: **PDB Molecule:**procarboxypeptidase a2;<br>**PDBTitle:** human procarboxypeptidase a2
99	d1iyna_	Alignment	not modelled	10.2	16	**Fold:**Heme-dependent peroxidases<br>**Superfamily:**Heme-dependent peroxidases<br>**Family:**CCP-like

Phyre2

Email	siddhartha_kundu@yahoo.co.in
Description	Q5NAT0___
Date	Thu Feb 1 11:15:32 GMT 2018
Unique Job ID	19edadbfa51e60b5

Detailed template information

#	Template	Alignment Coverage	3D Model	Confidence	% i.d.	Template Information
1	c1ga2A_	Alignment	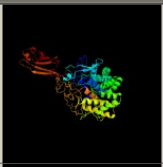	100.0	33	**PDB header:**hydrolase **Chain:** A: **PDB Molecule:**endoglucanase 9g; **PDBTitle:** the crystal structure of endoglucanase 9g from clostridium2 cellulolyticum complexed with cellobiose
2	c1js4B_	Alignment	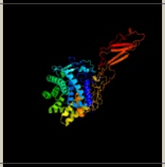	100.0	35	**PDB header:**glycosyl hydrolase **Chain:** B: **PDB Molecule:**endo/exocellulase e4; **PDBTitle:** endo/exocellulase:cellobiose from thermomonospora
3	c5gxyA_	Alignment	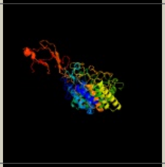	100.0	31	**PDB header:**hydrolase **Chain:** A: **PDB Molecule:**glucanase; **PDBTitle:** crystal structure of endoglucanase celq from clostridium thermocellum2 complexed with cellobiose and tris
4	d1g87a1	Alignment	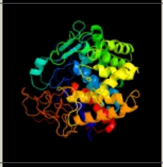	100.0	37	**Fold:**alpha/alpha toroid **Superfamily:**Six-hairpin glycosidases **Family:**Cellulases catalytic domain
5	c2yikA_	Alignment	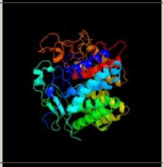	100.0	31	**PDB header:**hydrolase **Chain:** A: **PDB Molecule:**endoglucanase; **PDBTitle:** catalytic domain of clostridium thermocellum celt
6	d1tf4a1	Alignment	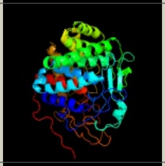	100.0	41	**Fold:**alpha/alpha toroid **Superfamily:**Six-hairpin glycosidases **Family:**Cellulases catalytic domain
7	c2xfgA_	Alignment	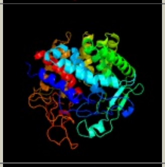	100.0	40	**PDB header:**hydrolase/sugar binding protein **Chain:** A: **PDB Molecule:**endoglucanase 1; **PDBTitle:** reassembly and co-crystallization of a family 9 processive2 endoglucanase from separately expressed gh9 and cbm3c3 modules
8	c3wc3A_	Alignment	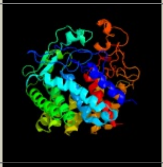	100.0	42	**PDB header:**hydrolase **Chain:** A: **PDB Molecule:**endo-1, 4-beta-glucanase; **PDBTitle:** crystal structure of endo-1,4-beta-glucanase from eisenia fetida
9	d1ia6a_	Alignment	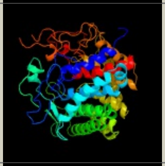	100.0	35	**Fold:**alpha/alpha toroid **Superfamily:**Six-hairpin glycosidases **Family:**Cellulases catalytic domain
10	d1ks8a_	Alignment	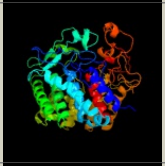	100.0	45	**Fold:**alpha/alpha toroid **Superfamily:**Six-hairpin glycosidases **Family:**Cellulases catalytic domain
11	d1ut9a1	Alignment	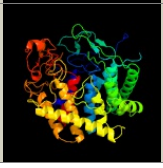	100.0	19	**Fold:**alpha/alpha toroid **Superfamily:**Six-hairpin glycosidases **Family:**Cellulases catalytic domain

12	d1clca1	Alignment	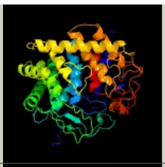	100.0	21	**Fold:**alpha/alpha toroid<br>**Superfamily:**Six-hairpin glycosidases<br>**Family:**Cellulases catalytic domain
13	c1clcA_	Alignment	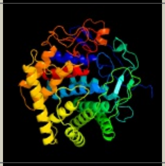	100.0	22	**PDB header:**glycosyl hydrolase<br>**Chain:** A: **PDB Molecule:**endoglucanase celd; ec: 3.2.1.4;<br>**PDBTitle:** three-dimensional structure of endoglucanase d at 1.92 angstroms resolution
14	c3gzkA_	Alignment	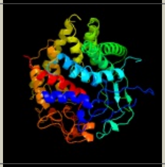	100.0	21	**PDB header:**hydrolase<br>**Chain:** A: **PDB Molecule:**cellulase;<br>**PDBTitle:** structure of a. acidocaldarius cellulase cela
15	c1ut9A_	Alignment	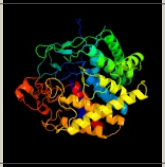	100.0	20	**PDB header:**hydrolase<br>**Chain:** A: **PDB Molecule:**cellulose 1,4-beta-cellobiosidase;<br>**PDBTitle:** structural basis for the exocellulase activity of the2 cellobiohydrolase cbha from c. thermocellum
16	c3x17B_	Alignment	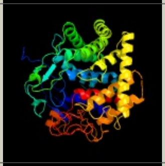	100.0	22	**PDB header:**hydrolase<br>**Chain:** B: **PDB Molecule:**endoglucanase;<br>**PDBTitle:** crystal structure of metagenome-derived glycoside hydrolase family 92 endoglucanase
17	c5dgrB_	Alignment	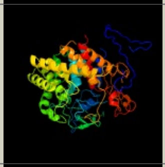	100.0	17	**PDB header:**hydrolase<br>**Chain:** B: **PDB Molecule:**putative endoglucanase-related protein;<br>**PDBTitle:** crystal structure of gh9 exo-beta-d-glucosaminidase pbpra0520,2 glucosamine complex
18	c3h7lC_	Alignment	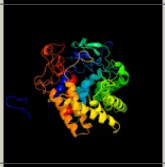	100.0	18	**PDB header:**hydrolase<br>**Chain:** C: **PDB Molecule:**endoglucanase;<br>**PDBTitle:** crystal structure of endoglucanase-related protein from vibrio2 parahaemolyticus
19	c2xfgB_	Alignment	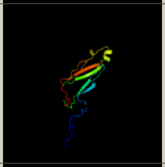	99.6	11	**PDB header:**hydrolase/sugar binding protein<br>**Chain:** B: **PDB Molecule:**endoglucanase 1;<br>**PDBTitle:** reassembly and co-crystallization of a family 9 processive2 endoglucanase from separately expressed gh9 and cbm3c3 modules
20	d1g87a2	Alignment	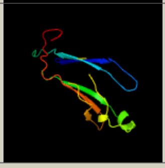	99.1	12	**Fold:**Common fold of diphtheria toxin/transcription factors/cytochrome f<br>**Superfamily:**Carbohydrate-binding domain<br>**Family:**Cellulose-binding domain family III
21	d1tf4a2	Alignment	not modelled	99.1	8	**Fold:**Common fold of diphtheria toxin/transcription factors/cytochrome f<br>**Superfamily:**Carbohydrate-binding domain<br>**Family:**Cellulose-binding domain family III
22	c4v1sA_	Alignment	not modelled	98.8	9	**PDB header:**hydrolase<br>**Chain:** A: **PDB Molecule:**alpha-1,6-mannanase;<br>**PDBTitle:** structure of the gh76 alpha-mannanase bt2949 from2 bacteroides thetaiotaomicron
23	c3wkgA_	Alignment	not modelled	98.7	15	**PDB header:**isomerase<br>**Chain:** A: **PDB Molecule:**cellobiose 2-epimerase;<br>**PDBTitle:** crystal structure of cellobiose 2-epimerase in complex with2 glucosylmannose
24	c4c1sA_	Alignment	not modelled	98.7	11	**PDB header:**hydrolase<br>**Chain:** A: **PDB Molecule:**glycoside hydrolase family 76 mannosidase;<br>**PDBTitle:** glycoside hydrolase family 76 (mannosidase) bt3792 from2 bacteroides thetaiotaomicron vpi-5482
25	c3vw5B_	Alignment	not modelled	98.5	14	**PDB header:**isomerase<br>**Chain:** B: **PDB Molecule:**cellobiose 2-epimerase;<br>**PDBTitle:** crystal structure of sugar epimerase from ruminal bacterium
26	c4qk0C_	Alignment	not modelled	98.5	15	**PDB header:**hydrolase<br>**Chain:** C: **PDB Molecule:**gh127 beta-l-arabinofuranoside;<br>**PDBTitle:** crystal structure of ara127n-se, a gh127 beta-l-arabinofuranosidase2 from geobacillus stearothermophilus t6
27	c3wkxA_	Alignment	not modelled	98.4	21	**PDB header:**hydrolase<br>**Chain:** A: **PDB Molecule:**non-reducing end beta-l-arabinofuranosidase;<br>**PDBTitle:** crystal structure of gh127 beta-l-arabinofuranosidase hypba1 from2 bifidobacterium longum arabinose complex form
28	c4z4lA_	Alignment	not modelled	98.3	13	**PDB header:**isomerase<br>**Chain:** A: **PDB Molecule:**cellobiose 2-epimerase;

28	c4z4iA_	Alignment	not modelled	[illegible]	[illegible]	**PDBTitle:** crystal structure of cellobiose 2-epimerase from caldicellulosiruptor2 saccharolyticus dsm 8903
29	c3k7xA_	Alignment	not modelled	98.3	11	**PDB header:**structural genomics, unknown function<br>**Chain:** A: **PDB Molecule:**lin0763 protein;<br>**PDBTitle:** crystal structure of the lin0763 protein from listeria innocua.2 northeast structural genomics consortium target lkr23.
30	c2wo4A_	Alignment	not modelled	98.2	16	**PDB header:**hydrolase<br>**Chain:** A: **PDB Molecule:**glycoside hydrolase, family 9;<br>**PDBTitle:** 3b' carbohydrate-binding module from the cel9v glycoside2 hydrolase from clostridium thermocellum, in-house data
31	c4b9pA_	Alignment	not modelled	98.2	17	**PDB header:**carbohydrate-binding protein<br>**Chain:** A: **PDB Molecule:**type 3a cellulose-binding domain protein;<br>**PDBTitle:** biomass sensoring module from putative rsgi2 protein of2 clostridium thermocellum resemble family 3 carbohydrate-3 binding module of cellulosome
32	c4bojC_	Alignment	not modelled	98.0	13	**PDB header:**hydrolase<br>**Chain:** C: **PDB Molecule:**alpha-1,6-mannanase;<br>**PDBTitle:** crystal structure of bacillus circulans tn-31 aman6 in2 complex with mannobiose
33	d1nbca_	Alignment	not modelled	98.0	15	**Fold:**Common fold of diphtheria toxin/transcription factors/cytochrome f<br>**Superfamily:**Carbohydrate-binding domain<br>**Family:**Cellulose-binding domain family III
34	c5mqoA_	Alignment	not modelled	98.0	12	**PDB header:**hydrolase<br>**Chain:** A: **PDB Molecule:**non-reducing end beta-l-arabinofuranosidase;<br>**PDBTitle:** glycoside hydrolase bt_1003
35	c2l8aA_	Alignment	not modelled	97.9	17	**PDB header:**hydrolase<br>**Chain:** A: **PDB Molecule:**endoglucanase;<br>**PDBTitle:** structure of a novel cbm3 lacking the calcium-binding site
36	d1g43a_	Alignment	not modelled	97.8	17	**Fold:**Common fold of diphtheria toxin/transcription factors/cytochrome f<br>**Superfamily:**Carbohydrate-binding domain<br>**Family:**Cellulose-binding domain family III
37	c3gt5A_	Alignment	not modelled	97.8	15	**PDB header:**isomerase<br>**Chain:** A: **PDB Molecule:**n-acetylglucosamine 2-epimerase;<br>**PDBTitle:** crystal structure of an n-acetylglucosamine 2-epimerase family protein2 from xylella fastidiosa
38	c3zqwA_	Alignment	not modelled	97.7	15	**PDB header:**carbohydrate-binding protein<br>**Chain:** A: **PDB Molecule:**cellulosomal scaffoldin;<br>**PDBTitle:** structure of cbm3b of major scaffoldin subunit scaa from2 acetivibrio cellulolyticus
39	c4b97A_	Alignment	not modelled	97.6	20	**PDB header:**sugar binding protein<br>**Chain:** A: **PDB Molecule:**cellulose binding domain-containing protein;<br>**PDBTitle:** biomass sensing modules from putative rsgi-like proteins2 of clostridium thermocellum resemble family 3 carbohydrate-3 binding module of cellulosome
40	c4mu9B_	Alignment	not modelled	97.5	10	**PDB header:**hydrolase<br>**Chain:** B: **PDB Molecule:**glycoside hydrolase family 73;<br>**PDBTitle:** crystal structure of a putative glycosylhydrolase (bt_3782) from2 bacteroides thetaiotaomicron vpi-5482 at 1.89 a resolution
41	c2xbtA_	Alignment	not modelled	97.5	14	**PDB header:**sugar binding protein<br>**Chain:** A: **PDB Molecule:**cellulosomal scaffoldin;<br>**PDBTitle:** structure of a scaffoldin carbohydrate-binding module family 3b from2 the cellulosome of bacteroides cellulosolvens: structural diversity3 and implications for carbohydrate binding
42	c4b9cA_	Alignment	not modelled	97.3	10	**PDB header:**carbohydrate-binding protein<br>**Chain:** A: **PDB Molecule:**type 3a cellulose-binding domain protein;<br>**PDBTitle:** biomass sensoring modules from putative rsgi-like proteins2 of clostridium thermocellum resemble family 3 carbohydrate-3 binding module of cellulosome
43	d1nc5a_	Alignment	not modelled	97.2	15	**Fold:**alpha/alpha toroid<br>**Superfamily:**Six-hairpin glycosidases<br>**Family:**Hypothetical protein YteR
44	c2ylkD_	Alignment	not modelled	97.1	17	**PDB header:**hydrolase<br>**Chain:** D: **PDB Molecule:**cellulose 1,4-beta-cellobiosidase;<br>**PDBTitle:** carbohydrate-binding module cbm3b from the cellulosomal2 cellobiohydrolase 9a from clostridium thermocellum
45	c4b96A_	Alignment	not modelled	97.1	14	**PDB header:**sugar binding protein<br>**Chain:** A: **PDB Molecule:**cellulose binding domain-containing protein;<br>**PDBTitle:** family 3b carbohydrate-binding module from the biomass2 sensoring system of clostridium clariflavum
46	c4wu0B_	Alignment	not modelled	96.9	20	**PDB header:**hydrolase<br>**Chain:** B: **PDB Molecule:**similar to yter (bacilus subtilis);<br>**PDBTitle:** structural analysis of c. acetobutylicum atcc 824 glycoside hydrolase2 from family 105
47	c3k11A_	Alignment	not modelled	96.7	20	**PDB header:**hydrolase<br>**Chain:** A: **PDB Molecule:**putative glycosyl hydrolase;<br>**PDBTitle:** crystal structure of putative glycosyl hydrolase (np_813087.1) from2 bacteroides thetaiotaomicron vpi-5482 at 1.80 a resolution
48	c4xuvB_	Alignment	not modelled	96.7	13	**PDB header:**hydrolase<br>**Chain:** B: **PDB Molecule:**glycoside hydrolase family 105 protein;<br>**PDBTitle:** crystal structure of a glycoside hydrolase family 105 (gh105) enzyme2 from thielavia terrestris
49	c2gz6B_	Alignment	not modelled	96.6	12	**PDB header:**isomerase<br>**Chain:** B: **PDB Molecule:**n-acetyl-d-glucosamine 2-epimerase;<br>**PDBTitle:** crystal structure of anabaena sp. ch1 n-acetyl-d-glucosamine 2-2 epimerase at 2.0 a
50	c5no8A_	Alignment	not modelled	96.6	15	**PDB header:**lyase<br>**Chain:** A: **PDB Molecule:**baccell_00875;<br>**PDBTitle:** polysaccharide lyase baccell_00875
51	c3pmmA_	Alignment	not modelled	96.0	15	**PDB header:**hydrolase<br>**Chain:** A: **PDB Molecule:**putative cytoplasmic protein;<br>**PDBTitle:** the crystal structure of a possible member of gh105 family from2 klebsiella pneumoniae subsp. pneumoniae mgh 78578
52	d2afaa1	Alignment	not modelled	95.5	15	**Fold:**alpha/alpha toroid<br>**Superfamily:**Six-hairpin glycosidases<br>**Family:**N-acylglucosamine (NAG) epimerase

53	c4ce7B_	Alignment	not modelled	94.3	11	**PDB header:**hydrolase<br>**Chain:** B: **PDB Molecule:**unsaturated 3s-rhamnoglycuronyl hydrolase;<br>**PDBTitle:** crystal structure of a novel unsaturated beta-glucuronyl2 hydrolase enzyme, belonging to family gh105, involved in3 ulvan degradation
54	d2d5ja1	Alignment	not modelled	93.6	13	**Fold:**alpha/alpha toroid<br>**Superfamily:**Six-hairpin glycosidases<br>**Family:**Glycosyl Hydrolase Family 88
55	d1fp3a_	Alignment	not modelled	93.5	17	**Fold:**alpha/alpha toroid<br>**Superfamily:**Six-hairpin glycosidases<br>**Family:**N-acylglucosamine (NAG) epimerase
56	c2zzrA_	Alignment	not modelled	93.5	14	**PDB header:**hydrolase<br>**Chain:** A: **PDB Molecule:**unsaturated glucuronyl hydrolase;<br>**PDBTitle:** crystal structure of unsaturated glucuronyl hydrolase from2 streptcoccus agalactiae
57	c3e6uA_	Alignment	not modelled	91.1	17	**PDB header:**signaling protein<br>**Chain:** A: **PDB Molecule:**lanc-like protein 1;<br>**PDBTitle:** crystal structure of human lancl1
58	c5noaA_	Alignment	not modelled	87.6	12	**PDB header:**hydrolase<br>**Chain:** A: **PDB Molecule:**family 88 glycosyl hydrolase;<br>**PDBTitle:** polysaccharide lyase baccell_00875
59	c3wiwA_	Alignment	not modelled	87.6	12	**PDB header:**hydrolase<br>**Chain:** A: **PDB Molecule:**glycosyl hydrolase family 88;<br>**PDBTitle:** crystal structure of unsaturated glucuronyl hydrolase specific for2 heparin
60	c4q88B_	Alignment	not modelled	86.6	9	**PDB header:**structural genomics, unknown function<br>**Chain:** B: **PDB Molecule:**uncharacterized protein;<br>**PDBTitle:** glycosyl hydrolase family 88 from bacteroides vulgatus
61	c1w6kA_	Alignment	not modelled	86.4	10	**PDB header:**isomerase<br>**Chain:** A: **PDB Molecule:**lanosterol synthase;<br>**PDBTitle:** structure of human osc in complex with lanosterol
62	c3ndyG_	Alignment	not modelled	86.0	19	**PDB header:**hydrolase<br>**Chain:** G: **PDB Molecule:**endoglucanase d;<br>**PDBTitle:** the structure of the catalytic and carbohydrate binding domain of2 endoglucanase d from clostridium cellulovorans
63	c4pw2A_	Alignment	not modelled	85.1	18	**PDB header:**isomerase<br>**Chain:** A: **PDB Molecule:**d-glucuronyl c5 epimerase b;<br>**PDBTitle:** crystal structure of d-glucuronyl c5 epimerase
64	d1gxma_	Alignment	not modelled	84.4	20	**Fold:**alpha/alpha toroid<br>**Superfamily:**Family 10 polysaccharide lyase<br>**Family:**Family 10 polysaccharide lyase
65	c2rttA_	Alignment	not modelled	84.0	18	**PDB header:**hydrolase<br>**Chain:** A: **PDB Molecule:**chic;<br>**PDBTitle:** solution structure of the chitin-binding domain of chi18ac from2 streptomyces coelicolor
66	d1exha_	Alignment	not modelled	84.0	21	**Fold:**Common fold of diphtheria toxin/transcription factors/cytochrome f<br>**Superfamily:**Carbohydrate-binding domain<br>**Family:**Cellulose-binding domain family II
67	c5a29A_	Alignment	not modelled	78.3	32	**PDB header:**lyase<br>**Chain:** A: **PDB Molecule:**exopolygalacturonate lyase;<br>**PDBTitle:** family 2 pectate lyase from vibrio vulnificus
68	c2kmgA_	Alignment	not modelled	72.2	17	**PDB header:**gene regulation<br>**Chain:** A: **PDB Molecule:**klca;<br>**PDBTitle:** the structure of the klca and ardb proteins show a novel2 fold and antirestriction activity against type i dna3 restriction systems in vivo but not in vitro
69	d1qaza_	Alignment	not modelled	67.5	14	**Fold:**alpha/alpha toroid<br>**Superfamily:**Chondroitin AC/alginate lyase<br>**Family:**Alginate lyase A1-III
70	d1r76a_	Alignment	not modelled	65.8	12	**Fold:**alpha/alpha toroid<br>**Superfamily:**Family 10 polysaccharide lyase<br>**Family:**Family 10 polysaccharide lyase
71	c4ayqA_	Alignment	not modelled	56.5	24	**PDB header:**hydrolase<br>**Chain:** A: **PDB Molecule:**mannosyl-oligosaccharide 1,2-alpha-mannosidase;<br>**PDBTitle:** structure of the gh47 processing alpha-1,2-mannosidase from2 caulobacter strain k31 in complex with mannoimidazole
72	c1krfA_	Alignment	not modelled	54.8	18	**PDB header:**hydrolase<br>**Chain:** A: **PDB Molecule:**mannosyl-oligosaccharide alpha-1,2-mannosidase;<br>**PDBTitle:** structure of p. citrinum alpha 1,2-mannosidase reveals the basis for2 differences in specificity of the er and golgi class i enzymes
73	d2ri9a1	Alignment	not modelled	54.8	18	**Fold:**alpha/alpha toroid<br>**Superfamily:**Seven-hairpin glycosidases<br>**Family:**Class I alpha-1;2-mannosidase, catalytic domain
74	d1hcua_	Alignment	not modelled	45.5	21	**Fold:**alpha/alpha toroid<br>**Superfamily:**Seven-hairpin glycosidases<br>**Family:**Class I alpha-1;2-mannosidase, catalytic domain
75	c2v8kA_	Alignment	not modelled	40.5	18	**PDB header:**lyase<br>**Chain:** A: **PDB Molecule:**pectate lyase;<br>**PDBTitle:** structure of a family 2 pectate lyase in complex with2 trigalacturonic acid
76	d1x9da1	Alignment	not modelled	38.1	24	**Fold:**alpha/alpha toroid<br>**Superfamily:**Seven-hairpin glycosidases<br>**Family:**Class I alpha-1;2-mannosidase, catalytic domain
77	c1x9dA_	Alignment	not modelled	38.1	24	**PDB header:**hydrolase<br>**Chain:** A: **PDB Molecule:**endoplasmic reticulum mannosyl-oligosaccharide 1,2-alpha-<br>**PDBTitle:** crystal structure of human class i alpha-1,2-mannosidase in complex2 with thio-disaccharide substrate analogue

78	c3t33A_	Alignment	not modelled	36.7	13	**PDB header:**signaling protein receptor<br>**Chain:** A: **PDB Molecule:**g protein coupled receptor;<br>**PDBTitle:** crystal structure of arabidopsis gcr2
79	d1dl2a_	Alignment	not modelled	35.8	24	**Fold:**alpha/alpha toroid<br>**Superfamily:**Seven-hairpin glycosidases<br>**Family:**Class I alpha-1;2-mannosidase, catalytic domain
80	d1nxca_	Alignment	not modelled	35.0	17	**Fold:**alpha/alpha toroid<br>**Superfamily:**Seven-hairpin glycosidases<br>**Family:**Class I alpha-1;2-mannosidase, catalytic domain
81	c1g6iA_	Alignment	not modelled	33.1	24	**PDB header:**hydrolase<br>**Chain:** A: **PDB Molecule:**class i alpha-1,2-mannosidase;<br>**PDBTitle:** crystal structure of the yeast alpha-1,2-mannosidase with bound 1-2 deoxymannojirimycin at 1.59 a resolution
82	c4ok2A_	Alignment	not modelled	29.5	18	**PDB header:**lyase<br>**Chain:** A: **PDB Molecule:**putative alginate lyase;<br>**PDBTitle:** crystal structure of alg17c mutant y258a
83	d1tzaa_	Alignment	not modelled	25.5	23	**Fold:**Immunoglobulin-like beta-sandwich<br>**Superfamily:**ApaG-like<br>**Family:**ApaG-like
84	c4ozwA_	Alignment	not modelled	23.8	15	**PDB header:**lyase<br>**Chain:** A: **PDB Molecule:**alginate lyase;<br>**PDBTitle:** crystal structure of the periplasmic alginate lyase algl h202a mutant
85	c2fuqA_	Alignment	not modelled	23.2	8	**PDB header:**sugar binding protein<br>**Chain:** A: **PDB Molecule:**heparinase ii protein;<br>**PDBTitle:** crystal structure of heparinase ii
86	c3nfvA_	Alignment	not modelled	21.5	17	**PDB header:**lyase<br>**Chain:** A: **PDB Molecule:**alginate lyase;<br>**PDBTitle:** crystal structure of an alginate lyase (bacova_01668) from bacteroides2 ovatus at 1.95 a resolution
87	d1llaa3	Alignment	not modelled	21.4	22	**Fold:**Immunoglobulin-like beta-sandwich<br>**Superfamily:**E set domains<br>**Family:**Arthropod hemocyanin, C-terminal domain
88	c5hdwA_	Alignment	not modelled	19.1	13	**PDB header:**protein binding<br>**Chain:** A: **PDB Molecule:**f-box only protein 3;<br>**PDBTitle:** apag domain of fbxo3
89	d1xvsa_	Alignment	not modelled	19.1	18	**Fold:**Immunoglobulin-like beta-sandwich<br>**Superfamily:**ApaG-like<br>**Family:**ApaG-like
90	c3a0oB_	Alignment	not modelled	19.0	12	**PDB header:**lyase<br>**Chain:** B: **PDB Molecule:**oligo alginate lyase;<br>**PDBTitle:** crystal structure of alginate lyase from agrobacterium tumefaciens c58
91	d1f53a_	Alignment	not modelled	18.3	64	**Fold:**gamma-Crystallin-like<br>**Superfamily:**gamma-Crystallin-like<br>**Family:**Killer toxin-like protein SKLP
92	c5bp8A_	Alignment	not modelled	17.7	14	**PDB header:**isomerase<br>**Chain:** A: **PDB Molecule:**ent-copalyl diphosphate synthase;<br>**PDBTitle:** ent-copalyl diphosphate synthase from streptomyces platensis
93	c2f1eA_	Alignment	not modelled	16.9	25	**PDB header:**structural genomics, unknown function<br>**Chain:** A: **PDB Molecule:**protein apag;<br>**PDBTitle:** solution structure of apag protein
94	c2laaA_	Alignment	not modelled	16.7	15	**PDB header:**hydrolase<br>**Chain:** A: **PDB Molecule:**beta/alpha-amylase;<br>**PDBTitle:** solution strucuture of the cbm25-1 of beta/alpha-amylase from2 paenibacillus polymyxa
95	c1ug9A_	Alignment	not modelled	15.5	20	**PDB header:**hydrolase<br>**Chain:** A: **PDB Molecule:**glucodextranase;<br>**PDBTitle:** crystal structure of glucodextranase from arthrobacter globiformis i42
96	d1w8oa1	Alignment	not modelled	14.3	20	**Fold:**Immunoglobulin-like beta-sandwich<br>**Superfamily:**E set domains<br>**Family:**E-set domains of sugar-utilizing enzymes
97	c5eurC_	Alignment	not modelled	13.4	56	**PDB header:**unknown function<br>**Chain:** C: **PDB Molecule:**uncharacterized protein;<br>**PDBTitle:** hypothetical protein sf216 from shigella flexneri 5a m90t
98	d2g0da1	Alignment	not modelled	13.4	12	**Fold:**alpha/alpha toroid<br>**Superfamily:**LanC-like<br>**Family:**LanC-like
99	c3q7aB_	Alignment	not modelled	12.6	17	**PDB header:**transferase/transferase inhibitor<br>**Chain:** B: **PDB Molecule:**farnesyltransferase beta subunit;<br>**PDBTitle:** cryptococcus neoformans protein farnesyltransferase in complex with2 fpp and l-778,123

Phyre2

Email	siddhartha_kundu@yahoo.co.in
Description	Q93WY9___
Date	Sat Aug 15 22:30:43 BST 2015
Unique Job ID	5130cfbdfa612b38

Detailed template information

#	Template	Alignment Coverage	3D Model	Confidence	% i.d.	Template Information
1	c1ga2A_	Alignment	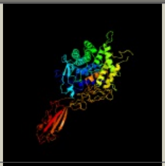	100.0	33	**PDB header:**hydrolase **Chain:** A: **PDB Molecule:**endoglucanase 9g; **PDBTitle:** the crystal structure of endoglucanase 9g from clostridium2 cellulolyticum complexed with cellobiose
2	c1js4B_	Alignment	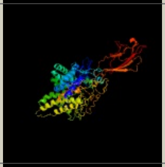	100.0	39	**PDB header:**glycosyl hydrolase **Chain:** B: **PDB Molecule:**endo/exocellulase e4; **PDBTitle:** endo/exocellulase:cellobiose from thermomonospora
3	d1g87a1	Alignment	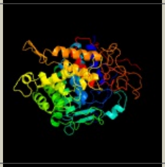	100.0	38	**Fold:**alpha/alpha toroid **Superfamily:**Six-hairpin glycosidases **Family:**Cellulases catalytic domain
4	c2yikA_	Alignment	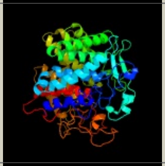	100.0	32	**PDB header:**hydrolase **Chain:** A: **PDB Molecule:**endoglucanase; **PDBTitle:** catalytic domain of clostridium thermocellum celt
5	d1tf4a1	Alignment	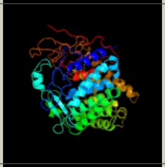	100.0	44	**Fold:**alpha/alpha toroid **Superfamily:**Six-hairpin glycosidases **Family:**Cellulases catalytic domain
6	c3wc3A_	Alignment	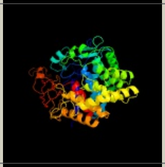	100.0	44	**PDB header:**hydrolase **Chain:** A: **PDB Molecule:**endo-1, 4-beta-glucanase; **PDBTitle:** crystal structure of endo-1,4-beta-glucanase from eisenia fetida
7	c2xfgA_	Alignment	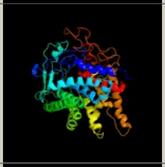	100.0	41	**PDB header:**hydrolase/sugar binding protein **Chain:** A: **PDB Molecule:**endoglucanase 1; **PDBTitle:** reassembly and co-crystallization of a family 9 processive2 endoglucanase from separately expressed gh9 and cbm3c3 modules
8	d1ks8a_	Alignment	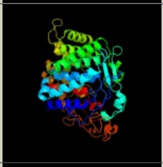	100.0	47	**Fold:**alpha/alpha toroid **Superfamily:**Six-hairpin glycosidases **Family:**Cellulases catalytic domain
9	d1ia6a_	Alignment	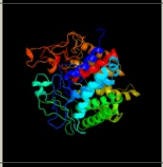	100.0	35	**Fold:**alpha/alpha toroid **Superfamily:**Six-hairpin glycosidases **Family:**Cellulases catalytic domain
10	d1ut9a1	Alignment	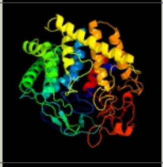	100.0	21	**Fold:**alpha/alpha toroid **Superfamily:**Six-hairpin glycosidases **Family:**Cellulases catalytic domain
11	c3gzkA_	Alignment	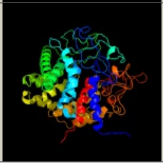	100.0	21	**PDB header:**hydrolase **Chain:** A: **PDB Molecule:**cellulase; **PDBTitle:** structure of a. acidocaldarius cellulase cela

12	c1clcA_	Alignment	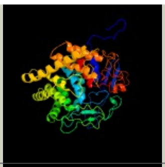	100.0	21	**PDB header:**glycosyl hydrolase<br>**Chain:** A: **PDB Molecule:**endoglucanase celd; ec: 3.2.1.4;<br>**PDBTitle:** three-dimensional structure of endoglucanase d at 1.92 angstroms resolution
13	c1ut9A_	Alignment	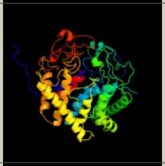	100.0	22	**PDB header:**hydrolase<br>**Chain:** A: **PDB Molecule:**cellulose 1,4-beta-cellobiosidase;<br>**PDBTitle:** structural basis for the exocellulase activity of the2 cellobiohydrolase cbha from c. thermocellum
14	d1clca1	Alignment	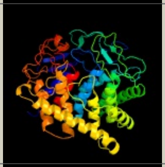	100.0	21	**Fold:**alpha/alpha toroid<br>**Superfamily:**Six-hairpin glycosidases<br>**Family:**Cellulases catalytic domain
15	c3x17B_	Alignment	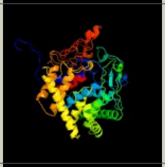	100.0	23	**PDB header:**hydrolase<br>**Chain:** B: **PDB Molecule:**endoglucanase;<br>**PDBTitle:** crystal structure of metagenome-derived glycoside hydrolase family 92 endoglucanase
16	c3h7lC_	Alignment	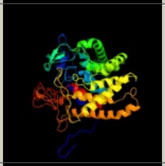	100.0	17	**PDB header:**hydrolase<br>**Chain:** C: **PDB Molecule:**endoglucanase;<br>**PDBTitle:** crystal structure of endoglucanase-related protein from vibrio2 parahaemolyticus
17	c2xfgB_	Alignment	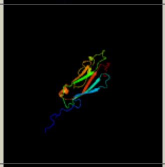	99.8	20	**PDB header:**hydrolase/sugar binding protein<br>**Chain:** B: **PDB Molecule:**endoglucanase 1;<br>**PDBTitle:** reassembly and co-crystallization of a family 9 processive2 endoglucanase from separately expressed gh9 and cbm3c3 modules
18	d1tf4a2	Alignment	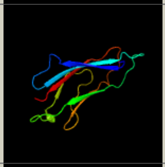	99.4	16	**Fold:**Common fold of diphtheria toxin/transcription factors/cytochrome f<br>**Superfamily:**Carbohydrate-binding domain<br>**Family:**Cellulose-binding domain family III
19	d1g87a2	Alignment	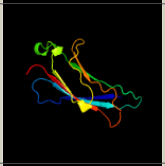	99.4	12	**Fold:**Common fold of diphtheria toxin/transcription factors/cytochrome f<br>**Superfamily:**Carbohydrate-binding domain<br>**Family:**Cellulose-binding domain family III
20	c4b9pA_	Alignment	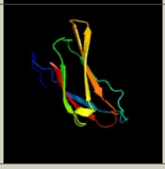	98.8	17	**PDB header:**carbohydrate-binding protein<br>**Chain:** A: **PDB Molecule:**type 3a cellulose-binding domain protein;<br>**PDBTitle:** biomass sensoring module from putative rsgi2 protein of2 clostridium thermocellum resemble family 3 carbohydrate-3 binding module of cellulosome
21	c4v1sA_	Alignment	not modelled	98.7	9	**PDB header:**hydrolase<br>**Chain:** A: **PDB Molecule:**alpha-1,6-mannanase;<br>**PDBTitle:** structure of the gh76 alpha-mannanase bt2949 from2 bacteroides thetaiotaomicron
22	d1g43a_	Alignment	not modelled	98.7	17	**Fold:**Common fold of diphtheria toxin/transcription factors/cytochrome f<br>**Superfamily:**Carbohydrate-binding domain<br>**Family:**Cellulose-binding domain family III
23	d1nbca_	Alignment	not modelled	98.7	12	**Fold:**Common fold of diphtheria toxin/transcription factors/cytochrome f<br>**Superfamily:**Carbohydrate-binding domain<br>**Family:**Cellulose-binding domain family III
24	c2wo4A_	Alignment	not modelled	98.7	15	**PDB header:**hydrolase<br>**Chain:** A: **PDB Molecule:**glycoside hydrolase, family 9;<br>**PDBTitle:** 3b' carbohydrate-binding module from the cel9v glycoside2 hydrolase from clostridium thermocellum, in-house data
25	c2l8aA_	Alignment	not modelled	98.6	13	**PDB header:**hydrolase<br>**Chain:** A: **PDB Molecule:**endoglucanase;<br>**PDBTitle:** structure of a novel cbm3 lacking the calcium-binding site
26	c4b97A_	Alignment	not modelled	98.6	14	**PDB header:**sugar binding protein<br>**Chain:** A: **PDB Molecule:**cellulose binding domain-containing protein;<br>**PDBTitle:** biomass sensing modules from putative rsgi-like proteins2 of clostridium thermocellum resemble family 3 carbohydrate-3 binding module of cellulosome
27	c4c1sA_	Alignment	not modelled	98.6	14	**PDB header:**hydrolase<br>**Chain:** A: **PDB Molecule:**glycoside hydrolase family 76 mannosidase;<br>**PDBTitle:** glycoside hydrolase family 76 (mannosidase) bt3792 from2 bacteroides thetaiotaomicron vpi-5482
[illegible]	[illegible]	[illegible]	[illegible]	[illegible]	[illegible]	**PDB header:**carbohydrate-binding protein<br>[illegible]

28	c3zqwA_	Alignment	not modelled	98.5	14	**Chain:** A: **PDB Molecule:**cellulosomal scaffoldin;<br>**PDBTitle:** structure of cbm3b of major scaffoldin subunit scaa from2 acetivibrio cellulolyticus
29	c4qk0C_	Alignment	not modelled	98.5	14	**PDB header:**hydrolase<br>**Chain:** C: **PDB Molecule:**gh127 beta-l-arabinofuranoside;<br>**PDBTitle:** crystal structure of ara127n-se, a gh127 beta-l-arabinofuranosidase2 from geobacillus stearothermophilus t6
30	c3vw5B_	Alignment	not modelled	98.5	19	**PDB header:**isomerase<br>**Chain:** B: **PDB Molecule:**cellobiose 2-epimerase;<br>**PDBTitle:** crystal structure of sugar epimerase from ruminal bacterium
31	c3wkgA_	Alignment	not modelled	98.4	12	**PDB header:**isomerase<br>**Chain:** A: **PDB Molecule:**cellobiose 2-epimerase;<br>**PDBTitle:** crystal structure of cellobiose 2-epimerase in complex with2 glucosylmannose
32	c2xbtA_	Alignment	not modelled	98.4	13	**PDB header:**sugar binding protein<br>**Chain:** A: **PDB Molecule:**cellulosomal scaffoldin;<br>**PDBTitle:** structure of a scaffoldin carbohydrate-binding module family 3b from2 the cellulosome of bacteroides cellulosolvens: structural diversity3 and implications for carbohydrate binding
33	c3k7xA_	Alignment	not modelled	98.2	12	**PDB header:**structural genomics, unknown function<br>**Chain:** A: **PDB Molecule:**lin0763 protein;<br>**PDBTitle:** crystal structure of the lin0763 protein from listeria2 innocua. northeast structural genomics consortium target3 lkr23.
34	c4b96A_	Alignment	not modelled	98.2	11	**PDB header:**sugar binding protein<br>**Chain:** A: **PDB Molecule:**cellulose binding domain-containing protein;<br>**PDBTitle:** family 3b carbohydrate-binding module from the biomass2 sensoring system of clostridium clariflavum
35	c3wkxA_	Alignment	not modelled	98.2	16	**PDB header:**hydrolase<br>**Chain:** A: **PDB Molecule:**non-reducing end beta-l-arabinofuranosidase;<br>**PDBTitle:** crystal structure of gh127 beta-l-arabinofuranosidase hypba1 from2 bifidobacterium longum arabinose complex form
36	c4b9cA_	Alignment	not modelled	98.1	10	**PDB header:**carbohydrate-binding protein<br>**Chain:** A: **PDB Molecule:**type 3a cellulose-binding domain protein;<br>**PDBTitle:** biomass sensoring modules from putative rsgi-like proteins2 of clostridium thermocellum resemble family 3 carbohydrate-3 binding module of cellulosome
37	c4bojC_	Alignment	not modelled	98.0	15	**PDB header:**hydrolase<br>**Chain:** C: **PDB Molecule:**alpha-1,6-mannanase;<br>**PDBTitle:** crystal structure of bacillus circulans tn-31 aman6 in2 complex with mannobiose
38	c4mu9B_	Alignment	not modelled	98.0	14	**PDB header:**hydrolase<br>**Chain:** B: **PDB Molecule:**glycoside hydrolase family 73;<br>**PDBTitle:** crystal structure of a putative glycosylhydrolase (bt_3782) from2 bacteroides thetaiotaomicron vpi-5482 at 1.89 a resolution
39	c2ylkD_	Alignment	not modelled	98.0	12	**PDB header:**hydrolase<br>**Chain:** D: **PDB Molecule:**cellulose 1,4-beta-cellobiosidase;<br>**PDBTitle:** carbohydrate-binding module cbm3b from the cellulosomal2 cellobiohydrolase 9a from clostridium thermocellum
40	c3gt5A_	Alignment	not modelled	97.6	17	**PDB header:**isomerase<br>**Chain:** A: **PDB Molecule:**n-acetylglucosamine 2-epimerase;<br>**PDBTitle:** crystal structure of an n-acetylglucosamine 2-epimerase family protein2 from xylella fastidiosa
41	c4xuvB_	Alignment	not modelled	97.4	11	**PDB header:**hydrolase<br>**Chain:** B: **PDB Molecule:**glycoside hydrolase family 105 protein;<br>**PDBTitle:** crystal structure of a glycoside hydrolase family 105 (gh105) enzyme2 from thielavia terrestris
42	d2d5ja1	Alignment	not modelled	97.1	10	**Fold:**alpha/alpha toroid<br>**Superfamily:**Six-hairpin glycosidases<br>**Family:**Glycosyl Hydrolase Family 88
43	d1nc5a_	Alignment	not modelled	97.1	16	**Fold:**alpha/alpha toroid<br>**Superfamily:**Six-hairpin glycosidases<br>**Family:**Hypothetical protein YteR
44	c4wu0B_	Alignment	not modelled	96.7	13	**PDB header:**hydrolase<br>**Chain:** B: **PDB Molecule:**similar to yter (bacilus subtilis);<br>**PDBTitle:** structural analysis of c. acetobutylicum atcc 824 glycoside hydrolase2 from family 105
45	c2gz6B_	Alignment	not modelled	96.6	10	**PDB header:**isomerase<br>**Chain:** B: **PDB Molecule:**n-acetyl-d-glucosamine 2-epimerase;<br>**PDBTitle:** crystal structure of anabaena sp. ch1 n-acetyl-d-glucosamine 2-2 epimerase at 2.0 a
46	c3wiwA_	Alignment	not modelled	96.5	12	**PDB header:**hydrolase<br>**Chain:** A: **PDB Molecule:**glycosyl hydrolase family 88;<br>**PDBTitle:** crystal structure of unsaturated glucuronyl hydrolase specific for2 heparin
47	d2afaa1	Alignment	not modelled	96.3	12	**Fold:**alpha/alpha toroid<br>**Superfamily:**Six-hairpin glycosidases<br>**Family:**N-acylglucosamine (NAG) epimerase
48	c3pmmA_	Alignment	not modelled	95.9	10	**PDB header:**hydrolase<br>**Chain:** A: **PDB Molecule:**putative cytoplasmic protein;<br>**PDBTitle:** the crystal structure of a possible member of gh105 family from2 klebsiella pneumoniae subsp. pneumoniae mgh 78578
49	c4ce7B_	Alignment	not modelled	94.7	10	**PDB header:**hydrolase<br>**Chain:** B: **PDB Molecule:**unsaturated 3s-rhamnoglycuronyl hydrolase;<br>**PDBTitle:** crystal structure of a novel unsaturated beta-glucuronyl2 hydrolase enzyme, belonging to family gh105, involved in3 ulvan degradation
50	c2zzrA_	Alignment	not modelled	94.6	14	**PDB header:**hydrolase<br>**Chain:** A: **PDB Molecule:**unsaturated glucuronyl hydrolase;<br>**PDBTitle:** crystal structure of unsaturated glucuronyl hydrolase from2 streptcoccus agalactiae
51	c3k11A_	Alignment	not modelled	92.4	17	**PDB header:**hydrolase<br>**Chain:** A: **PDB Molecule:**putative glycosyl hydrolase;<br>**PDBTitle:** crystal structure of putative glycosyl hydrolase (np_813087.1) from2 bacteroides thetaiotaomicron vpi-5482 at 1.80 a resolution
52	c3e6uA_	Alignment	not modelled	89.1	17	**PDB header:**signaling protein<br>**Chain:** A: **PDB Molecule:**lanc-like protein 1;

						**PDBTitle:** crystal structure of human lancl1
53	d1fp3a_	Alignment	not modelled	88.7	15	**Fold:**alpha/alpha toroid<br>**Superfamily:**Six-hairpin glycosidases<br>**Family:**N-acylglucosamine (NAG) epimerase
54	c1w6kA_	Alignment	not modelled	85.8	15	**PDB header:**isomerase<br>**Chain:** A: **PDB Molecule:**lanosterol synthase;<br>**PDBTitle:** structure of human osc in complex with lanosterol
55	c4q88B_	Alignment	not modelled	85.4	10	**PDB header:**structural genomics, unknown function<br>**Chain:** B: **PDB Molecule:**uncharacterized protein;<br>**PDBTitle:** glycosyl hydrolase family 88 from bacteroides vulgatus
56	c4pw2A_	Alignment	not modelled	84.5	18	**PDB header:**isomerase<br>**Chain:** A: **PDB Molecule:**d-glucuronyl c5 epimerase b;<br>**PDBTitle:** crystal structure of d-glucuronyl c5 epimerase
57	d1gxma_	Alignment	not modelled	80.3	16	**Fold:**alpha/alpha toroid<br>**Superfamily:**Family 10 polysaccharide lyase<br>**Family:**Family 10 polysaccharide lyase
58	d1r76a_	Alignment	not modelled	77.4	11	**Fold:**alpha/alpha toroid<br>**Superfamily:**Family 10 polysaccharide lyase<br>**Family:**Family 10 polysaccharide lyase
59	c5a29A_	Alignment	not modelled	77.4	29	**PDB header:**lyase<br>**Chain:** A: **PDB Molecule:**exopolygalacturonate lyase;<br>**PDBTitle:** family 2 pectate lyase from vibrio vulnificus
60	c3t33A_	Alignment	not modelled	77.0	15	**PDB header:**signaling protein receptor<br>**Chain:** A: **PDB Molecule:**g protein coupled receptor;<br>**PDBTitle:** crystal structure of arabidopsis gcr2
61	c3ndyG_	Alignment	not modelled	64.7	18	**PDB header:**hydrolase<br>**Chain:** G: **PDB Molecule:**endoglucanase d;<br>**PDBTitle:** the structure of the catalytic and carbohydrate binding domain of2 endoglucanase d from clostridium cellulovorans
62	c2kmgA_	Alignment	not modelled	63.9	22	**PDB header:**gene regulation<br>**Chain:** A: **PDB Molecule:**klca;<br>**PDBTitle:** the structure of the klca and ardb proteins show a novel2 fold and antirestriction activity against type i dna3 restriction systems in vivo but not in vitro
63	d1exha_	Alignment	not modelled	62.5	20	**Fold:**Common fold of diphtheria toxin/transcription factors/cytochrome f<br>**Superfamily:**Carbohydrate-binding domain<br>**Family:**Cellulose-binding domain family II
64	c4ayqA_	Alignment	not modelled	60.4	10	**PDB header:**hydrolase<br>**Chain:** A: **PDB Molecule:**mannosyl-oligosaccharide 1,2-alpha-mannosidase;<br>**PDBTitle:** structure of the gh47 processing alpha-1,2-mannosidase from2 caulobacter strain k31 in complex with mannoimidazole
65	d1nxca_	Alignment	not modelled	59.2	19	**Fold:**alpha/alpha toroid<br>**Superfamily:**Seven-hairpin glycosidases<br>**Family:**Class I alpha-1;2-mannosidase, catalytic domain
66	d1dl2a_	Alignment	not modelled	58.9	19	**Fold:**alpha/alpha toroid<br>**Superfamily:**Seven-hairpin glycosidases<br>**Family:**Class I alpha-1;2-mannosidase, catalytic domain
67	d1x9da1	Alignment	not modelled	58.1	19	**Fold:**alpha/alpha toroid<br>**Superfamily:**Seven-hairpin glycosidases<br>**Family:**Class I alpha-1;2-mannosidase, catalytic domain
68	c1x9dA_	Alignment	not modelled	58.1	19	**PDB header:**hydrolase<br>**Chain:** A: **PDB Molecule:**endoplasmic reticulum mannosyl-oligosaccharide 1,<br>**PDBTitle:** crystal structure of human class i alpha-1,2-mannosidase in2 complex with thio-disaccharide substrate analogue
69	c1g6iA_	Alignment	not modelled	56.7	19	**PDB header:**hydrolase<br>**Chain:** A: **PDB Molecule:**class i alpha-1,2-mannosidase;<br>**PDBTitle:** crystal structure of the yeast alpha-1,2-mannosidase with bound 1-2 deoxymannojirimycin at 1.59 a resolution
70	c4ok2A_	Alignment	not modelled	53.1	13	**PDB header:**lyase<br>**Chain:** A: **PDB Molecule:**putative alginate lyase;<br>**PDBTitle:** crystal structure of alg17c mutant y258a
71	c2fuqA_	Alignment	not modelled	47.1	5	**PDB header:**sugar binding protein<br>**Chain:** A: **PDB Molecule:**heparinase ii protein;<br>**PDBTitle:** crystal structure of heparinase ii
72	c2v8kA_	Alignment	not modelled	35.0	21	**PDB header:**lyase<br>**Chain:** A: **PDB Molecule:**pectate lyase;<br>**PDBTitle:** structure of a family 2 pectate lyase in complex with2 trigalacturonic acid
73	c2ee2A_	Alignment	not modelled	34.5	14	**PDB header:**signaling protein<br>**Chain:** A: **PDB Molecule:**contactin-1;<br>**PDBTitle:** solution structures of the fn3 domain of human contactin 1
74	c3cihA_	Alignment	not modelled	31.7	11	**PDB header:**hydrolase<br>**Chain:** A: **PDB Molecule:**putative alpha-rhamnosidase;<br>**PDBTitle:** crystal structure of a putative alpha-rhamnosidase from2 bacteroides thetaiotaomicron
75	d1qaza_	Alignment	not modelled	31.6	13	**Fold:**alpha/alpha toroid<br>**Superfamily:**Chondroitin AC/alginate lyase<br>**Family:**Alginate lyase A1-III
76	d2dn7a1	Alignment	not modelled	30.8	14	**Fold:**Immunoglobulin-like beta-sandwich<br>**Superfamily:**Fibronectin type III<br>**Family:**Fibronectin type III
77	d1q38a_	Alignment	not modelled	23.7	10	**Fold:**Immunoglobulin-like beta-sandwich<br>**Superfamily:**Fibronectin type III<br>**Family:**Fibronectin type III
78	c2rttA_	Alignment	not modelled	19.9	22	**PDB header:**hydrolase<br>**Chain:** A: **PDB Molecule:**chic;<br>**PDBTitle:** solution structure of the chitin-binding domain of chi18ac

						from2 streptomyces coelicolor
79	c1o79A_	Alignment	not modelled	19.3	12	**PDB header:**isomerase<br>**Chain:** A: **PDB Molecule:**squalene--hopene cyclase;<br>**PDBTitle:** structures of human oxidosqualene cyclase inhibitors bound2 to a homolgous enzyme
80	c2dbjA_	Alignment	not modelled	19.2	11	**PDB header:**transferase, signaling protein<br>**Chain:** A: **PDB Molecule:**proto-oncogene tyrosine-protein kinase mer<br>**PDBTitle:** solution structures of the fn3 domain of human proto-2 oncogene tyrosine-protein kinase mer precursor
81	c4mcjC_	Alignment	not modelled	17.1	27	**PDB header:**transferase<br>**Chain:** C: **PDB Molecule:**uncharacterized protein;<br>**PDBTitle:** crystal structure of a putative nucleoside deoxyribosyltransferase2 (bdi_0649) from parabacteroides distasonis atcc 8503 at 2.40 a3 resolution
82	c2kbgA_	Alignment	not modelled	17.1	14	**PDB header:**cell adhesion<br>**Chain:** A: **PDB Molecule:**neural cell adhesion molecule 2;<br>**PDBTitle:** solution structure of the second fibronectin type-iii module2 of ncam2
83	d1f53a_	Alignment	not modelled	17.0	64	**Fold:**gamma-Crystallin-like<br>**Superfamily:**gamma-Crystallin-like<br>**Family:**Killer toxin-like protein SKLP
84	d1vzsa_	Alignment	not modelled	16.3	18	**Fold:**Mitochondrial ATP synthase coupling factor 6<br>**Superfamily:**Mitochondrial ATP synthase coupling factor 6<br>**Family:**Mitochondrial ATP synthase coupling factor 6
85	c5bp8A_	Alignment	not modelled	15.6	16	**PDB header:**isomerase<br>**Chain:** A: **PDB Molecule:**ent-copalyl diphosphate synthase;<br>**PDBTitle:** ent-copalyl diphosphate synthase from streptomyces platensis
86	c3a0oB_	Alignment	not modelled	15.2	11	**PDB header:**lyase<br>**Chain:** B: **PDB Molecule:**oligo alginate lyase;<br>**PDBTitle:** crystal structure of alginate lyase from agrobacterium tumefaciens c58
87	c3teuA_	Alignment	not modelled	13.0	14	**PDB header:**de novo protein<br>**Chain:** A: **PDB Molecule:**fibcon;<br>**PDBTitle:** crystal structure of fibcon
88	d3csba1	Alignment	not modelled	12.1	11	**Fold:**Immunoglobulin-like beta-sandwich<br>**Superfamily:**Fibronectin type III<br>**Family:**Fibronectin type III
89	d1g4ma2	Alignment	not modelled	11.8	18	**Fold:**Immunoglobulin-like beta-sandwich<br>**Superfamily:**E set domains<br>**Family:**Arrestin/Vps26-like
90	d2g0da1	Alignment	not modelled	11.7	13	**Fold:**alpha/alpha toroid<br>**Superfamily:**LanC-like<br>**Family:**LanC-like
91	c4lsdF_	Alignment	not modelled	11.5	11	**PDB header:**hormone<br>**Chain:** F: **PDB Molecule:**fibronectin type iii domain-containing protein 5;<br>**PDBTitle:** myokine structure
92	c3q7aB_	Alignment	not modelled	10.7	13	**PDB header:**transferase/transferase inhibitor<br>**Chain:** B: **PDB Molecule:**farnesyltransferase beta subunit;<br>**PDBTitle:** cryptococcus neoformans protein farnesyltransferase in complex with2 fpp and l-778,123
93	d2crza1	Alignment	not modelled	10.7	10	**Fold:**Immunoglobulin-like beta-sandwich<br>**Superfamily:**Fibronectin type III<br>**Family:**Fibronectin type III
94	d1qg3a1	Alignment	not modelled	10.5	16	**Fold:**Immunoglobulin-like beta-sandwich<br>**Superfamily:**Fibronectin type III<br>**Family:**Fibronectin type III
95	d1pjwa_	Alignment	not modelled	10.5	33	**Fold:**Immunoglobulin-like beta-sandwich<br>**Superfamily:**E set domains<br>**Family:**Class II viral fusion proteins C-terminal domain
96	d2clyc1	Alignment	not modelled	10.3	17	**Fold:**Mitochondrial ATP synthase coupling factor 6<br>**Superfamily:**Mitochondrial ATP synthase coupling factor 6<br>**Family:**Mitochondrial ATP synthase coupling factor 6
97	d1fnha1	Alignment	not modelled	10.1	20	**Fold:**Immunoglobulin-like beta-sandwich<br>**Superfamily:**Fibronectin type III<br>**Family:**Fibronectin type III
98	c4ozwA_	Alignment	not modelled	10.0	13	**PDB header:**lyase<br>**Chain:** A: **PDB Molecule:**alginate lyase;<br>**PDBTitle:** crystal structure of the periplasmic alginate lyase algl h202a mutant
99	c3nfvA_	Alignment	not modelled	9.7	15	**PDB header:**lyase<br>**Chain:** A: **PDB Molecule:**alginate lyase;<br>**PDBTitle:** crystal structure of an alginate lyase (bacova_01668) from bacteroides2 ovatus at 1.95 a resolution

Phyre2

Email	siddhartha_kundu@yahoo.co.in
Description	Q9ZSP9___
Date	Sat Aug 15 22:35:40 BST 2015
Unique Job ID	f8bd735ab827e260

Detailed template information

#	Template	Alignment Coverage	3D Model	Confidence	% i.d.	Template Information
1	c1ga2A_	Alignment	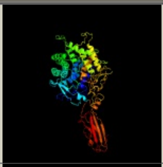	100.0	32	**PDB header:**hydrolase<br>**Chain:** A: **PDB Molecule:**endoglucanase 9g;<br>**PDBTitle:** the crystal structure of endoglucanase 9g from clostridium2 cellulolyticum complexed with cellobiose
2	c1js4B_	Alignment	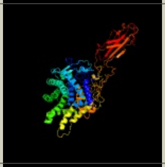	100.0	38	**PDB header:**glycosyl hydrolase<br>**Chain:** B: **PDB Molecule:**endo/exocellulase e4;<br>**PDBTitle:** endo/exocellulase:cellobiose from thermomonospora
3	d1g87a1	Alignment	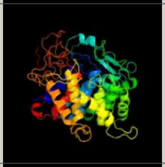	100.0	37	**Fold:**alpha/alpha toroid<br>**Superfamily:**Six-hairpin glycosidases<br>**Family:**Cellulases catalytic domain
4	c2yikA_	Alignment	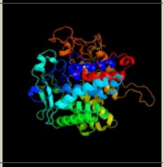	100.0	32	**PDB header:**hydrolase<br>**Chain:** A: **PDB Molecule:**endoglucanase;<br>**PDBTitle:** catalytic domain of clostridium thermocellum celt
5	d1tf4a1	Alignment	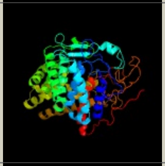	100.0	43	**Fold:**alpha/alpha toroid<br>**Superfamily:**Six-hairpin glycosidases<br>**Family:**Cellulases catalytic domain
6	c2xfgA_	Alignment	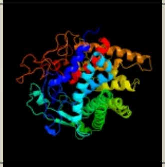	100.0	41	**PDB header:**hydrolase/sugar binding protein<br>**Chain:** A: **PDB Molecule:**endoglucanase 1;<br>**PDBTitle:** reassembly and co-crystallization of a family 9 processive2 endoglucanase from separately expressed gh9 and cbm3c3 modules
7	c3wc3A_	Alignment	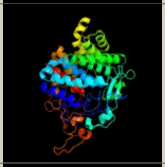	100.0	45	**PDB header:**hydrolase<br>**Chain:** A: **PDB Molecule:**endo-1, 4-beta-glucanase;<br>**PDBTitle:** crystal structure of endo-1,4-beta-glucanase from eisenia fetida
8	d1ia6a_	Alignment	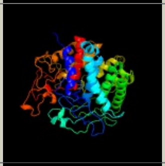	100.0	35	**Fold:**alpha/alpha toroid<br>**Superfamily:**Six-hairpin glycosidases<br>**Family:**Cellulases catalytic domain
9	d1ks8a_	Alignment	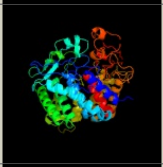	100.0	47	**Fold:**alpha/alpha toroid<br>**Superfamily:**Six-hairpin glycosidases<br>**Family:**Cellulases catalytic domain
10	d1ut9a1	Alignment	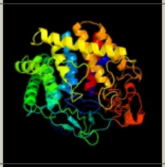	100.0	20	**Fold:**alpha/alpha toroid<br>**Superfamily:**Six-hairpin glycosidases<br>**Family:**Cellulases catalytic domain
11	c3gzkA_	Alignment	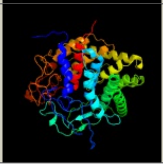	100.0	21	**PDB header:**hydrolase<br>**Chain:** A: **PDB Molecule:**cellulase;<br>**PDBTitle:** structure of a. acidocaldarius cellulase cela

12	c1ut9A_	Alignment	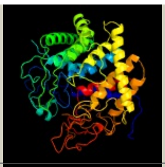	100.0	21	**PDB header:**hydrolase<br>**Chain:** A: **PDB Molecule:**cellulose 1,4-beta-cellobiosidase;<br>**PDBTitle:** structural basis for the exocellulase activity of the2 cellobiohydrolase cbha from c. thermocellum
13	d1clca1	Alignment	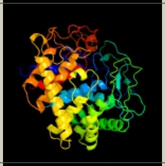	100.0	21	**Fold:**alpha/alpha toroid<br>**Superfamily:**Six-hairpin glycosidases<br>**Family:**Cellulases catalytic domain
14	c1clcA_	Alignment	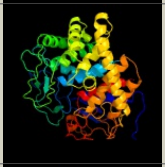	100.0	21	**PDB header:**glycosyl hydrolase<br>**Chain:** A: **PDB Molecule:**endoglucanase celd; ec: 3.2.1.4;<br>**PDBTitle:** three-dimensional structure of endoglucanase d at 1.92 angstroms resolution
15	c3x17B_	Alignment	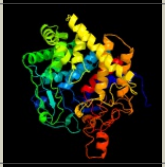	100.0	23	**PDB header:**hydrolase<br>**Chain:** B: **PDB Molecule:**endoglucanase;<br>**PDBTitle:** crystal structure of metagenome-derived glycoside hydrolase family 92 endoglucanase
16	c3h7lC_	Alignment	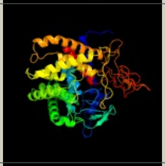	100.0	18	**PDB header:**hydrolase<br>**Chain:** C: **PDB Molecule:**endoglucanase;<br>**PDBTitle:** crystal structure of endoglucanase-related protein from vibrio2 parahaemolyticus
17	c2xfgB_	Alignment	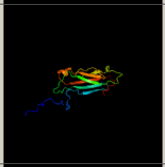	99.8	17	**PDB header:**hydrolase/sugar binding protein<br>**Chain:** B: **PDB Molecule:**endoglucanase 1;<br>**PDBTitle:** reassembly and co-crystallization of a family 9 processive2 endoglucanase from separately expressed gh9 and cbm3c3 modules
18	d1tf4a2	Alignment	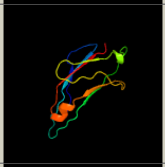	99.5	14	**Fold:**Common fold of diphtheria toxin/transcription factors/cytochrome f<br>**Superfamily:**Carbohydrate-binding domain<br>**Family:**Cellulose-binding domain family III
19	d1g87a2	Alignment	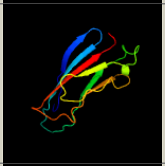	99.5	11	**Fold:**Common fold of diphtheria toxin/transcription factors/cytochrome f<br>**Superfamily:**Carbohydrate-binding domain<br>**Family:**Cellulose-binding domain family III
20	c4b9pA_	Alignment	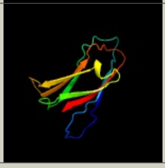	98.9	14	**PDB header:**carbohydrate-binding protein<br>**Chain:** A: **PDB Molecule:**type 3a cellulose-binding domain protein;<br>**PDBTitle:** biomass sensoring module from putative rsgi2 protein of2 clostridium thermocellum resemble family 3 carbohydrate-3 binding module of cellulosome
21	d1nbca_	Alignment	not modelled	98.9	12	**Fold:**Common fold of diphtheria toxin/transcription factors/cytochrome f<br>**Superfamily:**Carbohydrate-binding domain<br>**Family:**Cellulose-binding domain family III
22	d1g43a_	Alignment	not modelled	98.8	17	**Fold:**Common fold of diphtheria toxin/transcription factors/cytochrome f<br>**Superfamily:**Carbohydrate-binding domain<br>**Family:**Cellulose-binding domain family III
23	c2wo4A_	Alignment	not modelled	98.8	14	**PDB header:**hydrolase<br>**Chain:** A: **PDB Molecule:**glycoside hydrolase, family 9;<br>**PDBTitle:** 3b' carbohydrate-binding module from the cel9v glycoside2 hydrolase from clostridium thermocellum, in-house data
24	c2l8aA_	Alignment	not modelled	98.8	11	**PDB header:**hydrolase<br>**Chain:** A: **PDB Molecule:**endoglucanase;<br>**PDBTitle:** structure of a novel cbm3 lacking the calcium-binding site
25	c4v1sA_	Alignment	not modelled	98.7	9	**PDB header:**hydrolase<br>**Chain:** A: **PDB Molecule:**alpha-1,6-mannanase;<br>**PDBTitle:** structure of the gh76 alpha-mannanase bt2949 from2 bacteroides thetaiotaomicron
26	c4b97A_	Alignment	not modelled	98.7	11	**PDB header:**sugar binding protein<br>**Chain:** A: **PDB Molecule:**cellulose binding domain-containing protein;<br>**PDBTitle:** biomass sensing modules from putative rsgi-like proteins2 of clostridium thermocellum resemble family 3 carbohydrate-3 binding module of cellulosome
27	c3zqwA_	Alignment	not modelled	98.7	14	**PDB header:**carbohydrate-binding protein<br>**Chain:** A: **PDB Molecule:**cellulosomal scaffoldin;<br>**PDBTitle:** structure of cbm3b of major scaffoldin subunit scaa from2 acetivibrio cellulolyticus
						**PDB header:**sugar binding protein<br>**Chain:** A: **PDB Molecule:**cellulosomal scaffoldin;

28	c2xbtA_	Alignment	not modelled	98.5	11	[illegible] **PDBTitle:** structure of a scaffoldin carbohydrate-binding module family 3b from2 the cellulosome of bacteroides cellulosolvens: structural diversity3 and implications for carbohydrate binding
29	c4qk0C_	Alignment	not modelled	98.3	16	**PDB header:**hydrolase **Chain:** C: **PDB Molecule:**gh127 beta-l-arabinofuranoside; **PDBTitle:** crystal structure of ara127n-se, a gh127 beta-l-arabinofuranosidase2 from geobacillus stearothermophilus t6
30	c4b96A_	Alignment	not modelled	98.3	11	**PDB header:**sugar binding protein **Chain:** A: **PDB Molecule:**cellulose binding domain-containing protein; **PDBTitle:** family 3b carbohydrate-binding module from the biomass2 sensoring system of clostridium clariflavum
31	c4c1sA_	Alignment	not modelled	98.3	13	**PDB header:**hydrolase **Chain:** A: **PDB Molecule:**glycoside hydrolase family 76 mannosidase; **PDBTitle:** glycoside hydrolase family 76 (mannosidase) bt3792 from2 bacteroides thetaiotaomicron vpi-5482
32	c3wkgA_	Alignment	not modelled	98.2	14	**PDB header:**isomerase **Chain:** A: **PDB Molecule:**cellobiose 2-epimerase; **PDBTitle:** crystal structure of cellobiose 2-epimerase in complex with2 glucosylmannose
33	c2ylkD_	Alignment	not modelled	98.2	8	**PDB header:**hydrolase **Chain:** D: **PDB Molecule:**cellulose 1,4-beta-cellobiosidase; **PDBTitle:** carbohydrate-binding module cbm3b from the cellulosomal2 cellobiohydrolase 9a from clostridium thermocellum
34	c3vw5B_	Alignment	not modelled	98.2	19	**PDB header:**isomerase **Chain:** B: **PDB Molecule:**cellobiose 2-epimerase; **PDBTitle:** crystal structure of sugar epimerase from ruminal bacterium
35	c3wkxA_	Alignment	not modelled	98.2	18	**PDB header:**hydrolase **Chain:** A: **PDB Molecule:**non-reducing end beta-l-arabinofuranosidase; **PDBTitle:** crystal structure of gh127 beta-l-arabinofuranosidase hypba1 from2 bifidobacterium longum arabinose complex form
36	c4b9cA_	Alignment	not modelled	98.2	8	**PDB header:**carbohydrate-binding protein **Chain:** A: **PDB Molecule:**type 3a cellulose-binding domain protein; **PDBTitle:** biomass sensoring modules from putative rsgi-like proteins2 of clostridium thermocellum resemble family 3 carbohydrate-3 binding module of cellulosome
37	c3k7xA_	Alignment	not modelled	98.1	15	**PDB header:**structural genomics, unknown function **Chain:** A: **PDB Molecule:**lin0763 protein; **PDBTitle:** crystal structure of the lin0763 protein from listeria2 innocua. northeast structural genomics consortium target3 lkr23.
38	c4bojC_	Alignment	not modelled	97.9	17	**PDB header:**hydrolase **Chain:** C: **PDB Molecule:**alpha-1,6-mannanase; **PDBTitle:** crystal structure of bacillus circulans tn-31 aman6 in2 complex with mannobiose
39	c3gt5A_	Alignment	not modelled	97.4	15	**PDB header:**isomerase **Chain:** A: **PDB Molecule:**n-acetylglucosamine 2-epimerase; **PDBTitle:** crystal structure of an n-acetylglucosamine 2-epimerase family protein2 from xylella fastidiosa
40	c4mu9B_	Alignment	not modelled	97.3	17	**PDB header:**hydrolase **Chain:** B: **PDB Molecule:**glycoside hydrolase family 73; **PDBTitle:** crystal structure of a putative glycosylhydrolase (bt_3782) from2 bacteroides thetaiotaomicron vpi-5482 at 1.89 a resolution
41	d1nc5a_	Alignment	not modelled	96.8	14	**Fold:**alpha/alpha toroid **Superfamily:**Six-hairpin glycosidases **Family:**Hypothetical protein YteR
42	c4xuvB_	Alignment	not modelled	96.8	11	**PDB header:**hydrolase **Chain:** B: **PDB Molecule:**glycoside hydrolase family 105 protein; **PDBTitle:** crystal structure of a glycoside hydrolase family 105 (gh105) enzyme2 from thielavia terrestris
43	c4wu0B_	Alignment	not modelled	96.4	16	**PDB header:**hydrolase **Chain:** B: **PDB Molecule:**similar to yter (bacilus subtilis); **PDBTitle:** structural analysis of c. acetobutylicum atcc 824 glycoside hydrolase2 from family 105
44	d2afaa1	Alignment	not modelled	96.4	10	**Fold:**alpha/alpha toroid **Superfamily:**Six-hairpin glycosidases **Family:**N-acylglucosamine (NAG) epimerase
45	c3pmmA_	Alignment	not modelled	95.6	9	**PDB header:**hydrolase **Chain:** A: **PDB Molecule:**putative cytoplasmic protein; **PDBTitle:** the crystal structure of a possible member of gh105 family from2 klebsiella pneumoniae subsp. pneumoniae mgh 78578
46	c4ce7B_	Alignment	not modelled	95.6	15	**PDB header:**hydrolase **Chain:** B: **PDB Molecule:**unsaturated 3s-rhamnoglycuronyl hydrolase; **PDBTitle:** crystal structure of a novel unsaturated beta-glucuronyl2 hydrolase enzyme, belonging to family gh105, involved in3 ulvan degradation
47	c3k11A_	Alignment	not modelled	95.1	14	**PDB header:**hydrolase **Chain:** A: **PDB Molecule:**putative glycosyl hydrolase; **PDBTitle:** crystal structure of putative glycosyl hydrolase (np_813087.1) from2 bacteroides thetaiotaomicron vpi-5482 at 1.80 a resolution
48	c2gz6B_	Alignment	not modelled	92.7	10	**PDB header:**isomerase **Chain:** B: **PDB Molecule:**n-acetyl-d-glucosamine 2-epimerase; **PDBTitle:** crystal structure of anabaena sp. ch1 n-acetyl-d-glucosamine 2-2 epimerase at 2.0 a
49	d1exha_	Alignment	not modelled	92.6	23	**Fold:**Common fold of diphtheria toxin/transcription factors/cytochrome f **Superfamily:**Carbohydrate-binding domain **Family:**Cellulose-binding domain family II
50	c3ndyG_	Alignment	not modelled	92.4	16	**PDB header:**hydrolase **Chain:** G: **PDB Molecule:**endoglucanase d; **PDBTitle:** the structure of the catalytic and carbohydrate binding domain of2 endoglucanase d from clostridium cellulovorans
51	c2zzrA_	Alignment	not modelled	92.1	17	**PDB header:**hydrolase **Chain:** A: **PDB Molecule:**unsaturated glucuronyl hydrolase; **PDBTitle:** crystal structure of unsaturated glucuronyl hydrolase from2 streptcoccus agalactiae
						**PDB header:**hydrolase

52	c3wiwA_	Alignment	not modelled	90.2	13	**Chain:** A: **PDB Molecule:**glycosyl hydrolase family 88; **PDBTitle:** crystal structure of unsaturated glucuronyl hydrolase specific for2 heparin
53	d1fp3a_	Alignment	not modelled	89.5	16	**Fold:**alpha/alpha toroid **Superfamily:**Six-hairpin glycosidases **Family:**N-acylglucosamine (NAG) epimerase
54	c4q88B_	Alignment	not modelled	89.0	8	**PDB header:**structural genomics, unknown function **Chain:** B: **PDB Molecule:**uncharacterized protein; **PDBTitle:** glycosyl hydrolase family 88 from bacteroides vulgatus
55	d2d5ja1	Alignment	not modelled	87.9	14	**Fold:**alpha/alpha toroid **Superfamily:**Six-hairpin glycosidases **Family:**Glycosyl Hydrolase Family 88
56	c2rttA_	Alignment	not modelled	87.2	22	**PDB header:**hydrolase **Chain:** A: **PDB Molecule:**chic; **PDBTitle:** solution structure of the chitin-binding domain of chi18ac from2 streptomyces coelicolor
57	c1w6kA_	Alignment	not modelled	86.7	10	**PDB header:**isomerase **Chain:** A: **PDB Molecule:**lanosterol synthase; **PDBTitle:** structure of human osc in complex with lanosterol
58	d1gxma_	Alignment	not modelled	85.7	26	**Fold:**alpha/alpha toroid **Superfamily:**Family 10 polysaccharide lyase **Family:**Family 10 polysaccharide lyase
59	c3e6uA_	Alignment	not modelled	79.3	18	**PDB header:**signaling protein **Chain:** A: **PDB Molecule:**lanc-like protein 1; **PDBTitle:** crystal structure of human lancl1
60	c5a29A_	Alignment	not modelled	79.2	29	**PDB header:**lyase **Chain:** A: **PDB Molecule:**exopolygalacturonate lyase; **PDBTitle:** family 2 pectate lyase from vibrio vulnificus
61	c4pw2A_	Alignment	not modelled	71.4	19	**PDB header:**isomerase **Chain:** A: **PDB Molecule:**d-glucuronyl c5 epimerase b; **PDBTitle:** crystal structure of d-glucuronyl c5 epimerase
62	c3cihA_	Alignment	not modelled	60.1	11	**PDB header:**hydrolase **Chain:** A: **PDB Molecule:**putative alpha-rhamnosidase; **PDBTitle:** crystal structure of a putative alpha-rhamnosidase from2 bacteroides thetaiotaomicron
63	d1hcua_	Alignment	not modelled	55.8	14	**Fold:**alpha/alpha toroid **Superfamily:**Seven-hairpin glycosidases **Family:**Class I alpha-1;2-mannosidase, catalytic domain
64	c2v8kA_	Alignment	not modelled	50.6	20	**PDB header:**lyase **Chain:** A: **PDB Molecule:**pectate lyase; **PDBTitle:** structure of a family 2 pectate lyase in complex with2 trigalacturonic acid
65	d1qaza_	Alignment	not modelled	47.9	12	**Fold:**alpha/alpha toroid **Superfamily:**Chondroitin AC/alginate lyase **Family:**Alginate lyase A1-III
66	c2kmgA_	Alignment	not modelled	45.1	22	**PDB header:**gene regulation **Chain:** A: **PDB Molecule:**klca; **PDBTitle:** the structure of the klca and ardb proteins show a novel2 fold and antirestriction activity against type i dna3 restriction systems in vivo but not in vitro
67	d1dl2a_	Alignment	not modelled	43.8	14	**Fold:**alpha/alpha toroid **Superfamily:**Seven-hairpin glycosidases **Family:**Class I alpha-1;2-mannosidase, catalytic domain
68	c4ayqA_	Alignment	not modelled	41.7	10	**PDB header:**hydrolase **Chain:** A: **PDB Molecule:**mannosyl-oligosaccharide 1,2-alpha-mannosidase; **PDBTitle:** structure of the gh47 processing alpha-1,2-mannosidase from2 caulobacter strain k31 in complex with mannoimidazole
69	c1g6iA_	Alignment	not modelled	40.8	14	**PDB header:**hydrolase **Chain:** A: **PDB Molecule:**class i alpha-1,2-mannosidase; **PDBTitle:** crystal structure of the yeast alpha-1,2-mannosidase with bound 1-2 deoxymannojirimycin at 1.59 a resolution
70	c4zwjA_	Alignment	not modelled	34.9	17	**PDB header:**signaling protein **Chain:** A: **PDB Molecule:**chimera protein of human rhodopsin, mouse s-arrestin, and **PDBTitle:** crystal structure of rhodopsin bound to arrestin by femtosecond x-ray2 laser
71	c2fuqA_	Alignment	not modelled	34.4	5	**PDB header:**sugar binding protein **Chain:** A: **PDB Molecule:**heparinase ii protein; **PDBTitle:** crystal structure of heparinase ii
72	c1krfA_	Alignment	not modelled	32.7	11	**PDB header:**hydrolase **Chain:** A: **PDB Molecule:**mannosyl-oligosaccharide alpha-1,2-mannosidase; **PDBTitle:** structure of p. citrinum alpha 1,2-mannosidase reveals the basis for2 differences in specificity of the er and golgi class i enzymes
73	d2ri9a1	Alignment	not modelled	32.7	11	**Fold:**alpha/alpha toroid **Superfamily:**Seven-hairpin glycosidases **Family:**Class I alpha-1;2-mannosidase, catalytic domain
74	d1nxca_	Alignment	not modelled	31.3	10	**Fold:**alpha/alpha toroid **Superfamily:**Seven-hairpin glycosidases **Family:**Class I alpha-1;2-mannosidase, catalytic domain
75	c4ok2A_	Alignment	not modelled	30.1	17	**PDB header:**lyase **Chain:** A: **PDB Molecule:**putative alginate lyase; **PDBTitle:** crystal structure of alg17c mutant y258a
76	c3nfvA_	Alignment	not modelled	27.6	13	**PDB header:**lyase **Chain:** A: **PDB Molecule:**alginate lyase; **PDBTitle:** crystal structure of an alginate lyase (bacova_01668) from bacteroides2 ovatus at 1.95 a resolution
77	c4zwjC_	Alignment	not modelled	27.3	19	**PDB header:**signaling protein **Chain:** C: **PDB Molecule:**chimera protein of human rhodopsin, mouse s-arrestin, and **PDBTitle:** crystal structure of rhodopsin bound to arrestin by

						femtosecond x-ray2 laser
78	c2okxB_	Alignment	not modelled	20.4	12	**PDB header:**hydrolase<br>**Chain:** B: **PDB Molecule:**rhamnosidase b;<br>**PDBTitle:** crystal structure of gh78 family rhamnosidase of bacillus sp. gl1 at2 1.9 a
79	c4quwA_	Alignment	not modelled	17.4	27	**PDB header:**lyase<br>**Chain:** A: **PDB Molecule:**aldehyde decarbonylase;<br>**PDBTitle:** crystal structure of the apo form of cyanobacterial aldehyde-2 deformylating oxygenase
80	c3a0oB_	Alignment	not modelled	17.1	7	**PDB header:**lyase<br>**Chain:** B: **PDB Molecule:**oligo alginate lyase;<br>**PDBTitle:** crystal structure of alginate lyase from agrobacterium tumefaciens c58
81	d1f53a_	Alignment	not modelled	15.3	70	**Fold:**gamma-Crystallin-like<br>**Superfamily:**gamma-Crystallin-like<br>**Family:**Killer toxin-like protein SKLP
82	c3adyA_	Alignment	not modelled	14.5	14	**PDB header:**proton transport<br>**Chain:** A: **PDB Molecule:**dotd;<br>**PDBTitle:** crystal structure of dotd from legionella
83	d1x9da1	Alignment	not modelled	13.6	7	**Fold:**alpha/alpha toroid<br>**Superfamily:**Seven-hairpin glycosidases<br>**Family:**Class I alpha-1;2-mannosidase, catalytic domain
84	c1x9dA_	Alignment	not modelled	13.6	7	**PDB header:**hydrolase<br>**Chain:** A: **PDB Molecule:**endoplasmic reticulum mannosyl-oligosaccharide 1,<br>**PDBTitle:** crystal structure of human class i alpha-1,2-mannosidase in2 complex with thio-disaccharide substrate analogue
85	c3t33A_	Alignment	not modelled	13.0	13	**PDB header:**signaling protein receptor<br>**Chain:** A: **PDB Molecule:**g protein coupled receptor;<br>**PDBTitle:** crystal structure of arabidopsis gcr2
86	d1pjwa_	Alignment	not modelled	12.2	38	**Fold:**Immunoglobulin-like beta-sandwich<br>**Superfamily:**E set domains<br>**Family:**Class II viral fusion proteins C-terminal domain
87	d1g4ma2	Alignment	not modelled	11.4	17	**Fold:**Immunoglobulin-like beta-sandwich<br>**Superfamily:**E set domains<br>**Family:**Arrestin/Vps26-like
88	c4ozwA_	Alignment	not modelled	11.4	13	**PDB header:**lyase<br>**Chain:** A: **PDB Molecule:**alginate lyase;<br>**PDBTitle:** crystal structure of the periplasmic alginate lyase algl h202a mutant
89	c3vhxF_	Alignment	not modelled	10.8	40	**PDB header:**cell cycle/signaling protein<br>**Chain:** F: **PDB Molecule:**kinesin-like protein kif23;<br>**PDBTitle:** the crystal structure of arf6-mklp1 (mitotic kinesin-like protein 1)2 complex
90	c4fnvA_	Alignment	not modelled	10.7	10	**PDB header:**lyase<br>**Chain:** A: **PDB Molecule:**heparinase iii protein, heparitin sulfate lyase;<br>**PDBTitle:** crystal structure of heparinase iii
91	c5bp8A_	Alignment	not modelled	10.4	12	**PDB header:**isomerase<br>**Chain:** A: **PDB Molecule:**ent-copalyl diphosphate synthase;<br>**PDBTitle:** ent-copalyl diphosphate synthase from streptomyces platensis
92	d2oc5a1	Alignment	not modelled	10.1	19	**Fold:**Ferritin-like<br>**Superfamily:**Ferritin-like<br>**Family:**PMT1231-like
93	c2c3wB_	Alignment	not modelled	9.6	16	**PDB header:**sugar-binding protein<br>**Chain:** B: **PDB Molecule:**alpha-amylase g-6;<br>**PDBTitle:** structure of cbm25 from bacillus halodurans amylase in2 complex with maltotetraose
94	c4hfkC_	Alignment	not modelled	8.6	44	**PDB header:**hydrolase<br>**Chain:** C: **PDB Molecule:**putative cytoplasmic protein;<br>**PDBTitle:** crystal structure of the type vi effector-immunity complex tae4-tai42 from enterobacter cloacae
95	c4zlgA_	Alignment	not modelled	7.8	17	**PDB header:**transferase<br>**Chain:** A: **PDB Molecule:**putative b-glycan phosphorylase;<br>**PDBTitle:** cellobionic acid phosphorylase - gluconic acid complex
96	c3egpA_	Alignment	not modelled	7.4	29	**PDB header:**viral protein<br>**Chain:** A: **PDB Molecule:**envelope protein;<br>**PDBTitle:** crystal structure analysis of dengue-1 envelope protein2 domain iii
97	c2jqmA_	Alignment	not modelled	7.3	33	**PDB header:**transferase<br>**Chain:** A: **PDB Molecule:**envelope protein e;<br>**PDBTitle:** yellow fever envelope protein domain iii nmr structure2 (s288-k398)
98	c4xhcB_	Alignment	not modelled	7.2	16	**PDB header:**hydrolase<br>**Chain:** B: **PDB Molecule:**alpha-l-rhamnosidase;<br>**PDBTitle:** rhamnosidase from klebsiella oxytoca with rhamnose bound
99	c3c67B_	Alignment	not modelled	6.9	11	**PDB header:**hydrolase<br>**Chain:** B: **PDB Molecule:**uncharacterized protein ygjk;<br>**PDBTitle:** escherichia coli k12 ygjk in a complexed with glucose
