# Supplementary material for: Insights into the mechanism(s) of digestion of crystalline cellulose by plant class C GH9 endoglucanases
Source: J Mol Model. 2019 Jul 23;25(8):240. doi: 10.1007/s00894-019-4133-1 (PMC7385011; doi:10.1007/s00894-019-4133-1)
Supplement: Supplementary file 4 — (PDF 276 kb) [file 894_2019_4133_MOESM4_ESM.pdf]

Supplementary Text 2

CORE DATA FOR CHARACTERIZED CLASS C ENZYMES (n=4)

```
core size 548 of 549  vol = 11549.34
core size 547 of 549  vol = 11146
core size 546 of 549  vol = 10800.37
core size 545 of 549  vol = 10520.18
core size 544 of 549  vol = 10225.85
core size 543 of 549  vol = 9952.053
core size 542 of 549  vol = 9725.465
core size 541 of 549  vol = 9458.279
core size 540 of 549  vol = 9238.865
core size 539 of 549  vol = 9085.986
core size 538 of 549  vol = 8936.245
core size 537 of 549  vol = 8779.894
core size 536 of 549  vol = 8600.741
core size 535 of 549  vol = 8465.594
core size 534 of 549  vol = 8320.988
core size 533 of 549  vol = 8200.51
core size 532 of 549  vol = 8069.776
core size 531 of 549  vol = 7918.135
core size 530 of 549  vol = 7777.273
core size 529 of 549  vol = 7616.265
core size 528 of 549  vol = 7452.964
core size 527 of 549  vol = 7304.85
core size 526 of 549  vol = 7165.322
core size 525 of 549  vol = 7002.892
core size 524 of 549  vol = 6868.519
core size 523 of 549  vol = 6758.486
core size 522 of 549  vol = 6588.348
core size 521 of 549  vol = 6457.909
core size 520 of 549  vol = 6339.408
core size 519 of 549  vol = 6193.373
core size 518 of 549  vol = 6072.35
core size 517 of 549  vol = 5985.027
core size 516 of 549  vol = 5889.505
core size 515 of 549  vol = 5748.135
core size 514 of 549  vol = 5626.627
core size 513 of 549  vol = 5489.616
core size 512 of 549  vol = 5384.707
core size 511 of 549  vol = 5282.662
core size 510 of 549  vol = 5211.892
core size 509 of 549  vol = 5138.043
core size 508 of 549  vol = 5052.283
core size 507 of 549  vol = 4932.191
core size 506 of 549  vol = 4826.217
core size 505 of 549  vol = 4717.266
core size 504 of 549  vol = 4615.957
core size 503 of 549  vol = 4499.585
core size 502 of 549  vol = 4407.499
core size 501 of 549  vol = 4336.905
```

core size 500 of 549 vol = 4259.404
core size 499 of 549 vol = 4176.257
core size 498 of 549 vol = 4068.747
core size 497 of 549 vol = 3941.515
core size 496 of 549 vol = 3876.428
core size 495 of 549 vol = 3768.892
core size 494 of 549 vol = 3702.426
core size 493 of 549 vol = 3600.703
core size 492 of 549 vol = 3522.113
core size 491 of 549 vol = 3430.101
core size 490 of 549 vol = 3343.208
core size 489 of 549 vol = 3254.875
core size 488 of 549 vol = 3172.272
core size 487 of 549 vol = 3101.88
core size 486 of 549 vol = 3009.919
core size 485 of 549 vol = 2942.776
core size 484 of 549 vol = 2881.93
core size 483 of 549 vol = 2809.671
core size 482 of 549 vol = 2741.45
core size 481 of 549 vol = 2680.1
core size 480 of 549 vol = 2618.965
core size 479 of 549 vol = 2556.74
core size 478 of 549 vol = 2494.844
core size 477 of 549 vol = 2452.548
core size 476 of 549 vol = 2406.872
core size 475 of 549 vol = 2356.446
core size 474 of 549 vol = 2295.36
core size 473 of 549 vol = 2246.961
core size 472 of 549 vol = 2202.74
core size 471 of 549 vol = 2151.995
core size 470 of 549 vol = 2102.709
core size 469 of 549 vol = 2061.566
core size 468 of 549 vol = 2019.258
core size 467 of 549 vol = 1978.29
core size 466 of 549 vol = 1934.788
core size 465 of 549 vol = 1898.292
core size 464 of 549 vol = 1851.537
core size 463 of 549 vol = 1814.156
core size 462 of 549 vol = 1778.773
core size 461 of 549 vol = 1747.659
core size 460 of 549 vol = 1721.848
core size 459 of 549 vol = 1696.304
core size 458 of 549 vol = 1665.789
core size 457 of 549 vol = 1634.875
core size 456 of 549 vol = 1607.911
core size 455 of 549 vol = 1576.951
core size 454 of 549 vol = 1546.36
core size 453 of 549 vol = 1517.168
core size 452 of 549 vol = 1491.31
core size 451 of 549 vol = 1464.495

core size 450 of 549 vol = 1444.334
core size 449 of 549 vol = 1418.78
core size 448 of 549 vol = 1386.969
core size 447 of 549 vol = 1356.811
core size 446 of 549 vol = 1333.629
core size 445 of 549 vol = 1300.589
core size 444 of 549 vol = 1280.904
core size 443 of 549 vol = 1249.728
core size 442 of 549 vol = 1229.532
core size 441 of 549 vol = 1205.699
core size 440 of 549 vol = 1183.69
core size 439 of 549 vol = 1164.624
core size 438 of 549 vol = 1142.88
core size 437 of 549 vol = 1117.374
core size 436 of 549 vol = 1092.248
core size 435 of 549 vol = 1073.746
core size 434 of 549 vol = 1052.639
core size 433 of 549 vol = 1036.802
core size 432 of 549 vol = 1017.351
core size 431 of 549 vol = 995.533
core size 430 of 549 vol = 975.699
core size 429 of 549 vol = 961.216
core size 428 of 549 vol = 955.268
core size 427 of 549 vol = 937.178
core size 426 of 549 vol = 921.012
core size 425 of 549 vol = 908.44
core size 424 of 549 vol = 890.574
core size 423 of 549 vol = 872.222
core size 422 of 549 vol = 855.459
core size 421 of 549 vol = 839.649
core size 420 of 549 vol = 823.711
core size 419 of 549 vol = 807.838
core size 418 of 549 vol = 791.948
core size 417 of 549 vol = 776.438
core size 416 of 549 vol = 761.226
core size 415 of 549 vol = 746.069
core size 414 of 549 vol = 730.965
core size 413 of 549 vol = 718.985
core size 412 of 549 vol = 706.163
core size 411 of 549 vol = 693.076
core size 410 of 549 vol = 679.913
core size 409 of 549 vol = 670.317
core size 408 of 549 vol = 658.6
core size 407 of 549 vol = 647.147
core size 406 of 549 vol = 634.784
core size 405 of 549 vol = 624.636
core size 404 of 549 vol = 614.899
core size 403 of 549 vol = 606.764
core size 402 of 549 vol = 598.078
core size 401 of 549 vol = 588.66

```
core size 400 of 549  vol = 578.449
core size 399 of 549  vol = 571.126
core size 398 of 549  vol = 563.662
core size 397 of 549  vol = 555.82
core size 396 of 549  vol = 548.181
core size 395 of 549  vol = 539.51
core size 394 of 549  vol = 530.113
core size 393 of 549  vol = 521.448
core size 392 of 549  vol = 513.948
core size 391 of 549  vol = 507.857
core size 390 of 549  vol = 499.963
core size 389 of 549  vol = 492.738
core size 388 of 549  vol = 484.487
core size 387 of 549  vol = 477.752
core size 386 of 549  vol = 471.432
core size 385 of 549  vol = 465.526
core size 384 of 549  vol = 459.41
core size 383 of 549  vol = 453.35
core size 382 of 549  vol = 446.668
core size 381 of 549  vol = 439.578
core size 380 of 549  vol = 431.916
core size 379 of 549  vol = 425.083
core size 378 of 549  vol = 419.541
core size 377 of 549  vol = 413.995
core size 376 of 549  vol = 408.247
core size 375 of 549  vol = 404.809
core size 374 of 549  vol = 398.661
core size 373 of 549  vol = 391.417
core size 372 of 549  vol = 383.697
core size 371 of 549  vol = 378.382
core size 370 of 549  vol = 371.965
core size 369 of 549  vol = 366.865
core size 368 of 549  vol = 361.968
core size 367 of 549  vol = 356.267
core size 366 of 549  vol = 351.749
core size 365 of 549  vol = 346.745
core size 364 of 549  vol = 340.165
core size 363 of 549  vol = 335.265
core size 362 of 549  vol = 330.816
core size 361 of 549  vol = 324.933
core size 360 of 549  vol = 320.763
core size 359 of 549  vol = 315.61
core size 358 of 549  vol = 310.889
core size 357 of 549  vol = 306.127
core size 356 of 549  vol = 300.897
core size 355 of 549  vol = 295.722
core size 354 of 549  vol = 292.082
core size 353 of 549  vol = 287.978
core size 352 of 549  vol = 285.307
core size 351 of 549  vol = 281.703
```

```
core size 350 of 549  vol = 277.265
core size 349 of 549  vol = 274.277
core size 348 of 549  vol = 270.535
core size 347 of 549  vol = 267.902
core size 346 of 549  vol = 265.058
core size 345 of 549  vol = 262.59
core size 344 of 549  vol = 259.813
core size 343 of 549  vol = 255.277
core size 342 of 549  vol = 252.031
core size 341 of 549  vol = 248.641
core size 340 of 549  vol = 247.448
core size 339 of 549  vol = 243.09
core size 338 of 549  vol = 238.753
core size 337 of 549  vol = 234.927
core size 336 of 549  vol = 230.831
core size 335 of 549  vol = 227.404
core size 334 of 549  vol = 224.89
core size 333 of 549  vol = 222.864
core size 332 of 549  vol = 220.274
core size 331 of 549  vol = 217.735
core size 330 of 549  vol = 214.715
core size 329 of 549  vol = 211.673
core size 328 of 549  vol = 209.209
core size 327 of 549  vol = 206.204
core size 326 of 549  vol = 204.268
core size 325 of 549  vol = 201.424
core size 324 of 549  vol = 198.574
core size 323 of 549  vol = 196.336
core size 322 of 549  vol = 194.324
core size 321 of 549  vol = 193.181
core size 320 of 549  vol = 190.265
core size 319 of 549  vol = 187.054
core size 318 of 549  vol = 185.427
core size 317 of 549  vol = 184.379
core size 316 of 549  vol = 181.659
core size 315 of 549  vol = 180.624
core size 314 of 549  vol = 178.681
core size 313 of 549  vol = 177.15
core size 312 of 549  vol = 175.311
core size 311 of 549  vol = 173.475
core size 310 of 549  vol = 171.428
core size 309 of 549  vol = 169.038
core size 308 of 549  vol = 167.708
core size 307 of 549  vol = 165.818
core size 306 of 549  vol = 164.518
core size 305 of 549  vol = 162.959
core size 304 of 549  vol = 161.277
core size 303 of 549  vol = 157.993
core size 302 of 549  vol = 155.784
core size 301 of 549  vol = 153.725
```

```
core size 300 of 549  vol = 152.063
core size 299 of 549  vol = 149.735
core size 298 of 549  vol = 147.607
core size 297 of 549  vol = 146.179
core size 296 of 549  vol = 144.607
core size 295 of 549  vol = 143.141
core size 294 of 549  vol = 141.474
core size 293 of 549  vol = 139.106
core size 292 of 549  vol = 137.709
core size 291 of 549  vol = 136.601
core size 290 of 549  vol = 135.889
core size 289 of 549  vol = 134.72
core size 288 of 549  vol = 132.843
core size 287 of 549  vol = 131.177
core size 286 of 549  vol = 129.994
core size 285 of 549  vol = 128.801
core size 284 of 549  vol = 127.093
core size 283 of 549  vol = 126.473
core size 282 of 549  vol = 124.646
core size 281 of 549  vol = 123.925
core size 280 of 549  vol = 122.32
core size 279 of 549  vol = 120.874
core size 278 of 549  vol = 119.497
core size 277 of 549  vol = 118.035
core size 276 of 549  vol = 116.785
core size 275 of 549  vol = 115.138
core size 274 of 549  vol = 114.049
core size 273 of 549  vol = 113.065
core size 272 of 549  vol = 111.709
core size 271 of 549  vol = 110.838
core size 270 of 549  vol = 108.771
core size 269 of 549  vol = 107.875
core size 268 of 549  vol = 106.355
core size 267 of 549  vol = 105.005
core size 266 of 549  vol = 104.069
core size 265 of 549  vol = 102.707
core size 264 of 549  vol = 101.469
core size 263 of 549  vol = 99.506
core size 262 of 549  vol = 98.427
core size 261 of 549  vol = 97.61
core size 260 of 549  vol = 96.401
core size 259 of 549  vol = 95.583
core size 258 of 549  vol = 94.177
core size 257 of 549  vol = 92.641
core size 256 of 549  vol = 91.211
core size 255 of 549  vol = 90.316
core size 254 of 549  vol = 88.78
core size 253 of 549  vol = 87.868
core size 252 of 549  vol = 86.809
core size 251 of 549  vol = 85.902
```

```
core size 250 of 549  vol = 84.546
core size 249 of 549  vol = 83.199
core size 248 of 549  vol = 82.056
core size 247 of 549  vol = 80.994
core size 246 of 549  vol = 79.986
core size 245 of 549  vol = 79.145
core size 244 of 549  vol = 78.26
core size 243 of 549  vol = 76.586
core size 242 of 549  vol = 75.487
core size 241 of 549  vol = 74.202
core size 240 of 549  vol = 73.02
core size 239 of 549  vol = 72.49
core size 238 of 549  vol = 72.292
core size 237 of 549  vol = 71.842
core size 236 of 549  vol = 71.008
core size 235 of 549  vol = 69.933
core size 234 of 549  vol = 68.574
core size 233 of 549  vol = 67.622
core size 232 of 549  vol = 66.915
core size 231 of 549  vol = 65.867
core size 230 of 549  vol = 65.298
core size 229 of 549  vol = 64.708
core size 228 of 549  vol = 63.607
core size 227 of 549  vol = 63.044
core size 226 of 549  vol = 61.98
core size 225 of 549  vol = 60.822
core size 224 of 549  vol = 60.109
core size 223 of 549  vol = 58.526
core size 222 of 549  vol = 57.26
core size 221 of 549  vol = 56.388
core size 220 of 549  vol = 55.271
core size 219 of 549  vol = 54.173
core size 218 of 549  vol = 53.351
core size 217 of 549  vol = 52.633
core size 216 of 549  vol = 52.21
core size 215 of 549  vol = 51.613
core size 214 of 549  vol = 50.764
core size 213 of 549  vol = 50.185
core size 212 of 549  vol = 49.238
core size 211 of 549  vol = 48.58
core size 210 of 549  vol = 47.092
core size 209 of 549  vol = 46.706
core size 208 of 549  vol = 45.742
core size 207 of 549  vol = 45.082
core size 206 of 549  vol = 44.814
core size 205 of 549  vol = 44.124
core size 204 of 549  vol = 43.345
core size 203 of 549  vol = 42.048
core size 202 of 549  vol = 41.578
core size 201 of 549  vol = 41.428
```

```
core size 200 of 549  vol = 41.964
core size 199 of 549  vol = 41.41
core size 198 of 549  vol = 40.66
core size 197 of 549  vol = 41.153
core size 196 of 549  vol = 40.496
core size 195 of 549  vol = 39.967
core size 194 of 549  vol = 39.37
core size 193 of 549  vol = 38.901
core size 192 of 549  vol = 38.503
core size 191 of 549  vol = 37.662
core size 190 of 549  vol = 37.104
core size 189 of 549  vol = 36.594
core size 188 of 549  vol = 36.591
core size 187 of 549  vol = 36.099
core size 186 of 549  vol = 35.525
core size 185 of 549  vol = 34.311
core size 184 of 549  vol = 33.483
core size 183 of 549  vol = 33.136
core size 182 of 549  vol = 32.626
core size 181 of 549  vol = 32.199
core size 180 of 549  vol = 31.588
core size 179 of 549  vol = 31.087
core size 178 of 549  vol = 30.764
core size 177 of 549  vol = 30.409
core size 176 of 549  vol = 29.194
core size 175 of 549  vol = 29.066
core size 174 of 549  vol = 28.53
core size 173 of 549  vol = 28.095
core size 172 of 549  vol = 27.375
core size 171 of 549  vol = 27.001
core size 170 of 549  vol = 26.6
core size 169 of 549  vol = 25.991
core size 168 of 549  vol = 25.446
core size 167 of 549  vol = 25.044
core size 166 of 549  vol = 24.724
core size 165 of 549  vol = 24.363
core size 164 of 549  vol = 23.965
core size 163 of 549  vol = 23.401
core size 162 of 549  vol = 22.823
core size 161 of 549  vol = 22.327
core size 160 of 549  vol = 21.936
core size 159 of 549  vol = 21.467
core size 158 of 549  vol = 21.067
core size 157 of 549  vol = 20.563
core size 156 of 549  vol = 20.146
core size 155 of 549  vol = 19.773
core size 154 of 549  vol = 19.375
core size 153 of 549  vol = 19.205
core size 152 of 549  vol = 18.967
core size 151 of 549  vol = 18.713
```

```
core size 150 of 549  vol = 18.222
core size 149 of 549  vol = 18.032
core size 148 of 549  vol = 17.801
core size 147 of 549  vol = 17.519
core size 146 of 549  vol = 17.15
core size 145 of 549  vol = 17.107
core size 144 of 549  vol = 16.686
core size 143 of 549  vol = 16.193
core size 142 of 549  vol = 16.157
core size 141 of 549  vol = 15.628
core size 140 of 549  vol = 15.416
core size 139 of 549  vol = 15.372
core size 138 of 549  vol = 14.733
core size 137 of 549  vol = 14.466
core size 136 of 549  vol = 14.145
core size 135 of 549  vol = 14.342
core size 134 of 549  vol = 14.227
core size 133 of 549  vol = 14.466
core size 132 of 549  vol = 14.198
core size 131 of 549  vol = 14.105
core size 130 of 549  vol = 13.922
core size 129 of 549  vol = 13.542
core size 128 of 549  vol = 13.13
core size 127 of 549  vol = 13.168
core size 126 of 549  vol = 12.959
core size 125 of 549  vol = 12.738
core size 124 of 549  vol = 12.356
core size 123 of 549  vol = 12.232
core size 122 of 549  vol = 12.264
core size 121 of 549  vol = 12.185
core size 120 of 549  vol = 11.799
core size 119 of 549  vol = 11.55
core size 118 of 549  vol = 11.006
core size 117 of 549  vol = 10.796
core size 116 of 549  vol = 10.928
core size 115 of 549  vol = 10.829
core size 114 of 549  vol = 10.843
core size 113 of 549  vol = 10.5
core size 112 of 549  vol = 10.536
core size 111 of 549  vol = 9.938
core size 110 of 549  vol = 9.878
core size 109 of 549  vol = 9.812
core size 108 of 549  vol = 9.559
core size 107 of 549  vol = 9.323
core size 106 of 549  vol = 9.168
core size 105 of 549  vol = 8.736
core size 104 of 549  vol = 8.51
core size 103 of 549  vol = 8.382
core size 102 of 549  vol = 8.161
core size 101 of 549  vol = 8.427
```

```
core size 100 of 549  vol = 8.308
core size 99 of 549  vol = 8.227
core size 98 of 549  vol = 8.264
core size 97 of 549  vol = 8.221
core size 96 of 549  vol = 7.952
core size 95 of 549  vol = 7.695
core size 94 of 549  vol = 7.59
core size 93 of 549  vol = 7.382
core size 92 of 549  vol = 7.428
core size 91 of 549  vol = 7.191
core size 90 of 549  vol = 6.825
core size 89 of 549  vol = 6.952
core size 88 of 549  vol = 6.886
core size 87 of 549  vol = 6.78
core size 86 of 549  vol = 6.536
core size 85 of 549  vol = 6.426
core size 84 of 549  vol = 6.236
core size 83 of 549  vol = 6.15
core size 82 of 549  vol = 5.88
core size 81 of 549  vol = 5.632
core size 80 of 549  vol = 5.396
core size 79 of 549  vol = 5.226
core size 78 of 549  vol = 5.203
core size 77 of 549  vol = 5.017
core size 76 of 549  vol = 4.827
core size 75 of 549  vol = 4.87
core size 74 of 549  vol = 4.673
core size 73 of 549  vol = 4.542
core size 72 of 549  vol = 4.464
core size 71 of 549  vol = 4.306
core size 70 of 549  vol = 4.279
core size 69 of 549  vol = 4.203
core size 68 of 549  vol = 4.078
core size 67 of 549  vol = 4.112
core size 66 of 549  vol = 3.966
core size 65 of 549  vol = 3.796
core size 64 of 549  vol = 3.676
core size 63 of 549  vol = 3.443
core size 62 of 549  vol = 3.315
core size 61 of 549  vol = 3.274
core size 60 of 549  vol = 3.189
core size 59 of 549  vol = 3.097
core size 58 of 549  vol = 2.825
core size 57 of 549  vol = 2.675
core size 56 of 549  vol = 2.62
core size 55 of 549  vol = 2.593
core size 54 of 549  vol = 2.479
core size 53 of 549  vol = 2.359
core size 52 of 549  vol = 2.291
core size 51 of 549  vol = 2.263
```

```
 core size 50 of 549  vol = 2.181
 core size 49 of 549  vol = 2.097
 core size 48 of 549  vol = 1.965
 core size 47 of 549  vol = 1.87
 core size 46 of 549  vol = 1.799
 core size 45 of 549  vol = 1.649
 core size 44 of 549  vol = 1.605
 core size 43 of 549  vol = 1.445
 core size 42 of 549  vol = 1.309
 core size 41 of 549  vol = 1.298
 core size 40 of 549  vol = 1.221
 core size 39 of 549  vol = 1.144
 core size 38 of 549  vol = 0.956
 core size 37 of 549  vol = 0.88
 core size 36 of 549  vol = 0.866
 core size 35 of 549  vol = 0.773
 core size 34 of 549  vol = 0.758
 core size 33 of 549  vol = 0.848
 core size 32 of 549  vol = 0.783
 core size 31 of 549  vol = 0.72
 core size 30 of 549  vol = 0.704
 core size 29 of 549  vol = 0.592
 core size 28 of 549  vol = 0.493
 FINISHED: Min vol ( 0.5 ) reached


# 39 positions (cumulative volume <= 1 Angstrom^3)
   start end length
1     19  19      1
2     60  61      2
3     65  65      1
4     95  95      1
5    152 165     14
6    180 180      1
7    216 220      5
8    234 234      1
9    260 260      1
10   262 262      1
11   320 321      2
12   323 323      1
13   386 386      1
14   418 418      1
15   439 440      2
16   443 446      4
# 49 positions (cumulative volume <= 2 Angstrom^3)
   start end length
1     19  19      1
2     55  55      1
3     60  61      2
4     63  65      3
```

```
5      92  92       1
6      95  97       3
7     100 100       1
8     151 165      15
9     180 180       1
10    216 220       5
11    234 234       1
12    260 262       3
13    320 323       4
14    386 386       1
15    418 418       1
16    439 440       2
17    443 446       4
# 59 positions (cumulative volume <= 3 Angstrom^3)
   start end length
1      13  13       1
2      18  19       2
3      55  58       4
4      60  65       6
5      92  92       1
6      95  97       3
7     100 100       1
8     115 115       1
9     122 122       1
10    151 165      15
11    180 180       1
12    216 220       5
13    234 234       1
14    260 262       3
15    320 323       4
16    386 386       1
17    418 418       1
18    439 440       2
19    443 446       4
20    448 449       2
# 67 positions (cumulative volume <= 4 Angstrom^3)
   start end length
1       9   9       1
2      13  13       1
3      18  19       2
4      55  66      12
5      71  71       1
6      92  92       1
7      95  97       3
8     100 100       1
9     115 115       1
10    122 122       1
11    151 165      15
12    180 180       1
13    216 220       5
```

	start	end	length
14	234	234	1
15	260	262	3
16	320	324	5
17	368	368	1
18	386	386	1
19	418	418	1
20	420	420	1
21	439	440	2
22	443	449	7

77 positions (cumulative volume <= 5 Angstrom^3)

	start	end	length
1	8	9	2
2	13	13	1
3	15	15	1
4	17	20	4
5	55	66	12
6	69	69	1
7	71	71	1
8	89	89	1
9	92	93	2
10	95	97	3
11	100	100	1
12	115	115	1
13	122	122	1
14	151	165	15
15	180	180	1
16	216	220	5
17	234	234	1
18	260	262	3
19	318	318	1
20	320	324	5
21	358	358	1
22	368	368	1
23	386	386	1
24	418	418	1
25	420	420	1
26	439	440	2
27	442	449	8

83 positions (cumulative volume <= 6 Angstrom^3)

	start	end	length
1	8	9	2
2	13	13	1
3	15	15	1
4	17	20	4
5	55	69	15
6	71	71	1
7	89	89	1
8	92	97	6
9	100	100	1
10	115	115	1

```
11   122 122      1
12   151 165     15
13   180 180      1
14   214 214      1
15   216 220      5
16   234 234      1
17   260 263      4
18   318 318      1
19   320 324      5
20   330 330      1
21   358 358      1
22   368 368      1
23   386 386      1
24   418 418      1
25   420 420      1
26   439 440      2
27   442 449      8
# 91 positions (cumulative volume <= 7 Angstrom^3)
   start end length
1      8   9      2
2     13  13      1
3     15  15      1
4     17  20      4
5     55  71     17
6     88  89      2
7     92  97      6
8    100 100      1
9    115 115      1
10   122 122      1
11   135 135      1
12   151 165     15
13   180 180      1
14   214 214      1
15   216 220      5
16   234 234      1
17   260 263      4
18   318 318      1
19   320 324      5
20   327 327      1
21   330 330      1
22   358 358      1
23   368 368      1
24   385 386      2
25   414 414      1
26   417 418      2
27   420 420      1
28   438 440      3
29   442 449      8
# 97 positions (cumulative volume <= 8 Angstrom^3)
   start end length
```

```
1      8   9      2
2     13  13      1
3     15  15      1
4     17  20      4
5     36  36      1
6     47  47      1
7     55  71     17
8     88  89      2
9     92  97      6
10   100 100      1
11   105 105      1
12   115 115      1
13   122 122      1
14   135 135      1
15   151 165     15
16   180 180      1
17   214 214      1
18   216 220      5
19   234 234      1
20   260 263      4
21   318 318      1
22   320 324      5
23   327 327      1
24   330 330      1
25   358 358      1
26   360 360      1
27   362 362      1
28   368 368      1
29   385 386      2
30   414 414      1
31   417 418      2
32   420 420      1
33   438 440      3
34   442 449      8
35   504 504      1
# 106 positions (cumulative volume <= 9 Angstrom^3)
   start end length
1      8   9      2
2     12  13      2
3     15  20      6
4     28  28      1
5     36  36      1
6     47  47      1
7     55  71     17
8     88  90      3
9     92  97      6
10   100 100      1
11   103 103      1
12   105 105      1
13   109 109      1
```

14	115	115	1
15	122	122	1
16	135	135	1
17	151	165	15
18	180	180	1
19	214	220	7
20	234	234	1
21	260	263	4
22	318	318	1
23	320	324	5
24	327	327	1
25	330	330	1
26	358	360	3
27	362	362	1
28	368	368	1
29	385	386	2
30	414	414	1
31	416	418	3
32	420	420	1
33	438	440	3
34	442	449	8
35	504	504	1

112 positions (cumulative volume <= 10 Angstrom^3)

	start	end	length
1	8	9	2
2	12	13	2
3	15	20	6
4	28	28	1
5	36	36	1
6	47	47	1
7	55	71	17
8	88	90	3
9	92	98	7
10	100	101	2
11	103	103	1
12	105	105	1
13	109	109	1
14	113	113	1
15	115	115	1
16	122	122	1
17	135	135	1
18	140	140	1
19	148	148	1
20	151	165	15
21	180	180	1
22	214	220	7
23	234	234	1
24	260	263	4
25	318	318	1
26	320	324	5

```
27   327 327      1
28   330 330      1
29   358 360      3
30   362 362      1
31   368 368      1
32   385 386      2
33   414 414      1
34   416 418      3
35   420 420      1
36   428 428      1
37   438 440      3
38   442 449      8
39   504 504      1
# 118 positions (cumulative volume <= 11 Angstrom^3)
   start end length
1      8   9      2
2     12  13      2
3     15  20      6
4     28  28      1
5     36  36      1
6     42  42      1
7     46  47      2
8     49  49      1
9     55  71     17
10    88  90      3
11    92  98      7
12   100 101      2
13   103 103      1
14   105 105      1
15   109 109      1
16   113 113      1
17   115 115      1
18   122 122      1
19   126 126      1
20   135 135      1
21   140 140      1
22   148 148      1
23   151 165     15
24   175 176      2
25   180 180      1
26   214 220      7
27   234 234      1
28   260 263      4
29   318 318      1
30   320 324      5
31   327 327      1
32   330 330      1
33   358 360      3
34   362 362      1
35   368 368      1
```

```
36   385 386      2
37   414 414      1
38   416 418      3
39   420 420      1
40   428 428      1
41   438 440      3
42   442 449      8
43   504 504      1
# 121 positions (cumulative volume <= 12 Angstrom^3)
   start end length
1      8   9      2
2     12  20      9
3     28  28      1
4     36  36      1
5     42  42      1
6     46  47      2
7     49  49      1
8     55  72     18
9     88  90      3
10    92  98      7
11   100 101      2
12   103 103      1
13   105 105      1
14   109 109      1
15   113 113      1
16   115 115      1
17   122 122      1
18   126 126      1
19   135 135      1
20   140 140      1
21   148 148      1
22   151 165     15
23   175 176      2
24   180 180      1
25   214 220      7
26   234 234      1
27   251 251      1
28   260 263      4
29   318 318      1
30   320 324      5
31   327 327      1
32   330 330      1
33   358 360      3
34   362 362      1
35   368 368      1
36   385 386      2
37   414 414      1
38   416 418      3
39   420 420      1
40   428 428      1
```

	start	end	length
41	438	440	3
42	442	449	8
43	504	504	1

127 positions (cumulative volume <= 13 Angstrom^3)

	start	end	length
1	8	9	2
2	12	20	9
3	28	28	1
4	36	36	1
5	42	42	1
6	45	47	3
7	49	49	1
8	55	72	18
9	88	98	11
10	100	101	2
11	103	103	1
12	105	106	2
13	109	109	1
14	113	113	1
15	115	115	1
16	122	122	1
17	126	126	1
18	135	135	1
19	140	140	1
20	148	148	1
21	151	165	15
22	175	176	2
23	180	180	1
24	214	220	7
25	234	234	1
26	251	251	1
27	260	263	4
28	318	324	7
29	327	327	1
30	330	331	2
31	356	356	1
32	358	360	3
33	362	362	1
34	368	368	1
35	385	386	2
36	414	414	1
37	416	418	3
38	420	420	1
39	428	428	1
40	438	440	3
41	442	449	8
42	504	504	1

131 positions (cumulative volume <= 14 Angstrom^3)

	start	end	length
1	8	9	2

```
2     12  20      9
3     28  28      1
4     36  36      1
5     42  42      1
6     45  47      3
7     49  49      1
8     55  72     18
9     88  98     11
10   100 101      2
11   103 103      1
12   105 106      2
13   109 109      1
14   113 113      1
15   115 115      1
16   122 122      1
17   126 126      1
18   135 135      1
19   140 140      1
20   148 148      1
21   150 165     16
22   175 176      2
23   180 180      1
24   189 189      1
25   214 221      8
26   234 234      1
27   251 251      1
28   260 263      4
29   313 313      1
30   318 324      7
31   327 327      1
32   330 331      2
33   356 356      1
34   358 360      3
35   362 362      1
36   368 368      1
37   385 386      2
38   414 414      1
39   416 418      3
40   420 420      1
41   428 428      1
42   438 440      3
43   442 449      8
44   504 504      1
# 139 positions (cumulative volume <= 15 Angstrom^3)
   start end length
1      8   9      2
2     12  20      9
3     28  28      1
4     33  33      1
5     36  36      1
```

6	42	42	1
7	45	49	5
8	55	72	18
9	88	98	11
10	100	101	2
11	103	103	1
12	105	106	2
13	109	109	1
14	113	113	1
15	115	115	1
16	122	122	1
17	126	126	1
18	135	135	1
19	140	140	1
20	148	148	1
21	150	165	16
22	175	176	2
23	180	180	1
24	189	189	1
25	214	221	8
26	234	234	1
27	251	251	1
28	260	263	4
29	313	314	2
30	318	324	7
31	327	327	1
32	330	331	2
33	356	356	1
34	358	360	3
35	362	362	1
36	364	364	1
37	366	368	3
38	381	381	1
39	385	386	2
40	388	388	1
41	414	414	1
42	416	418	3
43	420	420	1
44	428	428	1
45	438	440	3
46	442	449	8
47	504	504	1

142 positions (cumulative volume <= 16 Angstrom^3)

	start	end	length
1	8	9	2
2	12	20	9
3	28	28	1
4	33	33	1
5	36	36	1
6	42	42	1

7	45	49	5
8	55	72	18
9	88	98	11
10	100	101	2
11	103	103	1
12	105	106	2
13	109	109	1
14	113	113	1
15	115	115	1
16	122	122	1
17	126	126	1
18	135	135	1
19	140	140	1
20	148	148	1
21	150	165	16
22	175	176	2
23	180	180	1
24	189	189	1
25	214	221	8
26	234	234	1
27	251	251	1
28	260	263	4
29	313	314	2
30	318	324	7
31	327	327	1
32	329	331	3
33	333	333	1
34	356	356	1
35	358	360	3
36	362	362	1
37	364	364	1
38	366	368	3
39	381	381	1
40	383	383	1
41	385	386	2
42	388	388	1
43	414	414	1
44	416	418	3
45	420	420	1
46	428	428	1
47	438	440	3
48	442	449	8
49	504	504	1

145 positions (cumulative volume <= 17 Angstrom^3)

	start	end	length
1	8	9	2
2	12	20	9
3	28	28	1
4	33	33	1
5	36	36	1

6	42	42	1
7	45	49	5
8	55	72	18
9	88	98	11
10	100	101	2
11	103	103	1
12	105	106	2
13	109	109	1
14	113	113	1
15	115	115	1
16	122	122	1
17	126	126	1
18	135	135	1
19	140	140	1
20	148	148	1
21	150	165	16
22	175	176	2
23	180	180	1
24	189	189	1
25	214	221	8
26	234	234	1
27	251	251	1
28	260	263	4
29	311	311	1
30	313	314	2
31	318	324	7
32	327	327	1
33	329	331	3
34	333	333	1
35	352	352	1
36	356	356	1
37	358	360	3
38	362	362	1
39	364	364	1
40	366	369	4
41	381	381	1
42	383	383	1
43	385	386	2
44	388	388	1
45	414	414	1
46	416	418	3
47	420	420	1
48	428	428	1
49	438	440	3
50	442	449	8
51	504	504	1

149 positions (cumulative volume <= 18 Angstrom^3)

	start	end	length
1	8	10	3
2	12	20	9

3	28	28	1
4	33	33	1
5	36	36	1
6	42	42	1
7	45	49	5
8	55	72	18
9	88	98	11
10	100	101	2
11	103	103	1
12	105	106	2
13	109	109	1
14	113	113	1
15	115	115	1
16	117	117	1
17	122	122	1
18	126	126	1
19	135	135	1
20	140	140	1
21	148	148	1
22	150	165	16
23	175	176	2
24	180	180	1
25	182	182	1
26	189	189	1
27	214	221	8
28	234	234	1
29	251	251	1
30	260	263	4
31	311	311	1
32	313	314	2
33	318	324	7
34	327	327	1
35	329	331	3
36	333	333	1
37	352	352	1
38	356	356	1
39	358	360	3
40	362	362	1
41	364	364	1
42	366	369	4
43	381	381	1
44	383	383	1
45	385	386	2
46	388	388	1
47	412	412	1
48	414	414	1
49	416	418	3
50	420	420	1
51	428	428	1
52	438	440	3

```
53   442 449      8
54   504 504      1
# 153 positions (cumulative volume <= 19 Angstrom^3)
   start end length
1      8  10      3
2     12  20      9
3     28  28      1
4     33  33      1
5     36  36      1
6     42  42      1
7     45  49      5
8     55  72     18
9     88  98     11
10   100 101      2
11   103 103      1
12   105 106      2
13   109 109      1
14   113 113      1
15   115 115      1
16   117 117      1
17   122 122      1
18   126 126      1
19   135 135      1
20   140 140      1
21   148 148      1
22   150 165     16
23   175 176      2
24   180 180      1
25   182 182      1
26   189 189      1
27   214 221      8
28   234 234      1
29   251 252      2
30   256 256      1
31   259 263      5
32   311 311      1
33   313 314      2
34   318 324      7
35   327 327      1
36   329 331      3
37   333 333      1
38   352 352      1
39   356 356      1
40   358 360      3
41   362 362      1
42   364 364      1
43   366 369      4
44   381 381      1
45   383 383      1
46   385 386      2
```

```
47   388 388      1
48   412 412      1
49   414 418      5
50   420 420      1
51   428 428      1
52   438 440      3
53   442 449      8
54   504 504      1
# 156 positions (cumulative volume <= 20 Angstrom^3)
   start end length
1      8  10      3
2     12  20      9
3     28  28      1
4     33  33      1
5     36  36      1
6     42  42      1
7     45  49      5
8     55  73     19
9     88  98     11
10   100 101      2
11   103 103      1
12   105 106      2
13   109 109      1
14   113 113      1
15   115 115      1
16   117 117      1
17   122 123      2
18   126 126      1
19   135 135      1
20   140 140      1
21   148 148      1
22   150 165     16
23   175 176      2
24   180 180      1
25   182 182      1
26   189 189      1
27   214 221      8
28   234 234      1
29   251 252      2
30   256 256      1
31   259 263      5
32   311 311      1
33   313 314      2
34   318 324      7
35   327 327      1
36   329 331      3
37   333 333      1
38   352 352      1
39   356 356      1
40   358 360      3
```

41	362	362	1
42	364	364	1
43	366	369	4
44	381	381	1
45	383	383	1
46	385	386	2
47	388	388	1
48	412	412	1
49	414	418	5
50	420	421	2
51	428	428	1
52	438	440	3
53	442	449	8
54	504	504	1

158 positions (cumulative volume <= 21 Angstrom^3)

	start	end	length
1	8	10	3
2	12	20	9
3	28	28	1
4	33	33	1
5	36	36	1
6	42	42	1
7	45	49	5
8	55	73	19
9	88	98	11
10	100	101	2
11	103	103	1
12	105	106	2
13	109	109	1
14	113	113	1
15	115	115	1
16	117	117	1
17	122	123	2
18	126	126	1
19	135	135	1
20	140	140	1
21	148	148	1
22	150	165	16
23	175	176	2
24	178	178	1
25	180	180	1
26	182	182	1
27	189	189	1
28	214	221	8
29	234	234	1
30	251	252	2
31	256	256	1
32	259	263	5
33	311	311	1
34	313	314	2

```
35   318 324      7
36   327 327      1
37   329 331      3
38   333 333      1
39   352 352      1
40   356 356      1
41   358 360      3
42   362 364      3
43   366 369      4
44   381 381      1
45   383 383      1
46   385 386      2
47   388 388      1
48   412 412      1
49   414 418      5
50   420 421      2
51   428 428      1
52   438 440      3
53   442 449      8
54   504 504      1
# 161 positions (cumulative volume <= 22 Angstrom^3)
   start end length
1      1   1      1
2      8  10      3
3     12  20      9
4     28  28      1
5     33  33      1
6     36  36      1
7     38  38      1
8     42  42      1
9     45  49      5
10    55  73     19
11    88  98     11
12   100 101      2
13   103 103      1
14   105 106      2
15   109 109      1
16   113 113      1
17   115 115      1
18   117 117      1
19   122 123      2
20   126 126      1
21   135 135      1
22   140 140      1
23   148 148      1
24   150 165     16
25   175 176      2
26   178 178      1
27   180 180      1
28   182 182      1
```

29	189	189	1
30	214	221	8
31	234	234	1
32	251	252	2
33	256	256	1
34	259	263	5
35	311	311	1
36	313	314	2
37	318	324	7
38	327	327	1
39	329	331	3
40	333	333	1
41	352	352	1
42	356	356	1
43	358	360	3
44	362	364	3
45	366	369	4
46	378	378	1
47	381	381	1
48	383	383	1
49	385	386	2
50	388	388	1
51	412	412	1
52	414	418	5
53	420	421	2
54	428	428	1
55	438	440	3
56	442	449	8
57	504	504	1

163 positions (cumulative volume <= 23 Angstrom^3)

	start	end	length
1	1	1	1
2	8	10	3
3	12	20	9
4	28	28	1
5	33	33	1
6	36	36	1
7	38	38	1
8	42	42	1
9	45	49	5
10	55	74	20
11	88	98	11
12	100	101	2
13	103	103	1
14	105	106	2
15	109	109	1
16	113	113	1
17	115	115	1
18	117	117	1
19	122	123	2

20	126	126	1
21	135	135	1
22	140	140	1
23	148	148	1
24	150	165	16
25	175	176	2
26	178	178	1
27	180	180	1
28	182	182	1
29	189	189	1
30	214	221	8
31	234	234	1
32	251	252	2
33	256	256	1
34	259	263	5
35	289	289	1
36	311	311	1
37	313	314	2
38	318	324	7
39	327	327	1
40	329	331	3
41	333	333	1
42	352	352	1
43	356	356	1
44	358	360	3
45	362	364	3
46	366	369	4
47	378	378	1
48	381	381	1
49	383	383	1
50	385	386	2
51	388	388	1
52	412	412	1
53	414	418	5
54	420	421	2
55	428	428	1
56	438	440	3
57	442	449	8
58	504	504	1

165 positions (cumulative volume <= 24 Angstrom^3)

	start	end	length
1	1	1	1
2	8	10	3
3	12	20	9
4	28	28	1
5	33	33	1
6	36	36	1
7	38	38	1
8	42	42	1
9	45	49	5

10	55	74	20
11	78	78	1
12	88	98	11
13	100	101	2
14	103	103	1
15	105	106	2
16	109	109	1
17	113	113	1
18	115	115	1
19	117	117	1
20	122	123	2
21	126	126	1
22	135	135	1
23	140	140	1
24	148	148	1
25	150	165	16
26	175	176	2
27	178	178	1
28	180	180	1
29	182	182	1
30	189	189	1
31	214	221	8
32	234	234	1
33	251	253	3
34	256	256	1
35	259	263	5
36	289	289	1
37	311	311	1
38	313	314	2
39	318	324	7
40	327	327	1
41	329	331	3
42	333	333	1
43	352	352	1
44	356	356	1
45	358	360	3
46	362	364	3
47	366	369	4
48	378	378	1
49	381	381	1
50	383	383	1
51	385	386	2
52	388	388	1
53	412	412	1
54	414	418	5
55	420	421	2
56	428	428	1
57	438	440	3
58	442	449	8
59	504	504	1

167 positions (cumulative volume <= 25 Angstrom^3)

	start	end	length
1	1	1	1
2	8	10	3
3	12	20	9
4	28	28	1
5	33	33	1
6	36	36	1
7	38	38	1
8	42	42	1
9	45	49	5
10	55	74	20
11	78	78	1
12	86	86	1
13	88	98	11
14	100	101	2
15	103	103	1
16	105	106	2
17	109	109	1
18	112	113	2
19	115	115	1
20	117	117	1
21	122	123	2
22	126	126	1
23	135	135	1
24	140	140	1
25	148	148	1
26	150	165	16
27	175	176	2
28	178	178	1
29	180	180	1
30	182	182	1
31	189	189	1
32	214	221	8
33	234	234	1
34	251	253	3
35	256	256	1
36	259	263	5
37	289	289	1
38	311	311	1
39	313	314	2
40	318	324	7
41	327	327	1
42	329	331	3
43	333	333	1
44	352	352	1
45	356	356	1
46	358	360	3
47	362	364	3
48	366	369	4

49	378	378	1
50	381	381	1
51	383	383	1
52	385	386	2
53	388	388	1
54	412	412	1
55	414	418	5
56	420	421	2
57	428	428	1
58	438	440	3
59	442	449	8
60	504	504	1

170 positions (cumulative volume <= 26 Angstrom^3)

	start	end	length
1	1	1	1
2	8	10	3
3	12	20	9
4	28	28	1
5	33	33	1
6	36	36	1
7	38	38	1
8	42	42	1
9	45	49	5
10	55	74	20
11	78	78	1
12	86	86	1
13	88	98	11
14	100	101	2
15	103	106	4
16	109	109	1
17	112	113	2
18	115	115	1
19	117	117	1
20	122	123	2
21	126	126	1
22	135	135	1
23	140	140	1
24	148	148	1
25	150	165	16
26	175	176	2
27	178	178	1
28	180	180	1
29	182	182	1
30	189	189	1
31	214	221	8
32	234	234	1
33	251	253	3
34	256	256	1
35	259	263	5
36	289	289	1

37	311	311	1
38	313	314	2
39	318	324	7
40	326	327	2
41	329	331	3
42	333	333	1
43	352	352	1
44	356	356	1
45	358	360	3
46	362	364	3
47	366	369	4
48	378	378	1
49	381	381	1
50	383	383	1
51	385	386	2
52	388	388	1
53	412	412	1
54	414	418	5
55	420	421	2
56	428	428	1
57	438	449	12
58	504	504	1

171 positions (cumulative volume <= 27 Angstrom^3)

	start	end	length
1	1	1	1
2	8	10	3
3	12	20	9
4	28	28	1
5	33	33	1
6	36	36	1
7	38	38	1
8	42	42	1
9	45	49	5
10	55	74	20
11	78	78	1
12	86	86	1
13	88	98	11
14	100	101	2
15	103	106	4
16	109	109	1
17	112	113	2
18	115	115	1
19	117	117	1
20	122	123	2
21	126	126	1
22	135	135	1
23	140	140	1
24	148	148	1
25	150	165	16
26	175	176	2

27	178	178	1
28	180	180	1
29	182	182	1
30	189	189	1
31	214	221	8
32	234	234	1
33	251	253	3
34	256	256	1
35	259	263	5
36	289	289	1
37	311	311	1
38	313	314	2
39	318	324	7
40	326	327	2
41	329	331	3
42	333	333	1
43	352	352	1
44	356	356	1
45	358	360	3
46	362	364	3
47	366	369	4
48	378	379	2
49	381	381	1
50	383	383	1
51	385	386	2
52	388	388	1
53	412	412	1
54	414	418	5
55	420	421	2
56	428	428	1
57	438	449	12
58	504	504	1

173 positions (cumulative volume <= 28 Angstrom^3)

	start	end	length
1	1	1	1
2	8	10	3
3	12	20	9
4	28	28	1
5	33	33	1
6	36	36	1
7	38	38	1
8	42	42	1
9	45	50	6
10	55	74	20
11	78	78	1
12	86	86	1
13	88	98	11
14	100	101	2
15	103	106	4
16	109	109	1

```
17   112 113      2
18   115 115      1
19   117 117      1
20   122 123      2
21   126 126      1
22   135 135      1
23   140 140      1
24   148 148      1
25   150 165     16
26   175 176      2
27   178 178      1
28   180 180      1
29   182 182      1
30   189 189      1
31   214 221      8
32   234 234      1
33   251 253      3
34   256 257      2
35   259 263      5
36   289 289      1
37   311 311      1
38   313 314      2
39   318 324      7
40   326 327      2
41   329 331      3
42   333 333      1
43   352 352      1
44   356 356      1
45   358 360      3
46   362 364      3
47   366 369      4
48   378 379      2
49   381 381      1
50   383 383      1
51   385 386      2
52   388 388      1
53   412 412      1
54   414 418      5
55   420 421      2
56   428 428      1
57   438 449     12
58   504 504      1
# 175 positions (cumulative volume <= 29 Angstrom^3)
   start end length
1      1   1      1
2      8  10      3
3     12  20      9
4     28  28      1
5     33  33      1
6     36  36      1
```

7	38	38	1
8	42	42	1
9	45	50	6
10	55	74	20
11	78	78	1
12	86	86	1
13	88	98	11
14	100	101	2
15	103	106	4
16	109	109	1
17	112	113	2
18	115	115	1
19	117	117	1
20	121	123	3
21	126	126	1
22	135	135	1
23	140	140	1
24	148	148	1
25	150	165	16
26	175	176	2
27	178	178	1
28	180	180	1
29	182	182	1
30	189	189	1
31	214	221	8
32	234	234	1
33	251	253	3
34	256	257	2
35	259	263	5
36	289	289	1
37	311	311	1
38	313	314	2
39	318	324	7
40	326	327	2
41	329	331	3
42	333	333	1
43	352	352	1
44	356	356	1
45	358	360	3
46	362	364	3
47	366	369	4
48	377	379	3
49	381	381	1
50	383	383	1
51	385	386	2
52	388	388	1
53	412	412	1
54	414	418	5
55	420	421	2
56	428	428	1

```
57   438 449      12
58   504 504       1
# 177 positions (cumulative volume <= 30 Angstrom^3)
   start end length
1      1   1       1
2      8  10       3
3     12  20       9
4     28  28       1
5     33  33       1
6     36  36       1
7     38  38       1
8     42  42       1
9     45  50       6
10    55  74      20
11    78  78       1
12    86  86       1
13    88  98      11
14   100 101       2
15   103 106       4
16   109 109       1
17   112 113       2
18   115 115       1
19   117 117       1
20   121 123       3
21   126 126       1
22   135 135       1
23   140 140       1
24   148 148       1
25   150 165      16
26   175 176       2
27   178 178       1
28   180 180       1
29   182 182       1
30   189 189       1
31   214 221       8
32   234 234       1
33   251 253       3
34   256 257       2
35   259 263       5
36   289 289       1
37   311 311       1
38   313 314       2
39   318 327      10
40   329 331       3
41   333 333       1
42   352 352       1
43   356 356       1
44   358 360       3
45   362 364       3
46   366 369       4
```

47	377	379	3
48	381	381	1
49	383	383	1
50	385	386	2
51	388	388	1
52	412	412	1
53	414	421	8
54	428	428	1
55	438	449	12
56	504	504	1

179 positions (cumulative volume <= 31 Angstrom^3)

	start	end	length
1	1	1	1
2	8	10	3
3	12	20	9
4	28	28	1
5	33	33	1
6	36	36	1
7	38	38	1
8	42	42	1
9	45	50	6
10	55	74	20
11	78	78	1
12	84	84	1
13	86	86	1
14	88	98	11
15	100	101	2
16	103	106	4
17	109	109	1
18	112	113	2
19	115	115	1
20	117	117	1
21	121	123	3
22	126	126	1
23	135	135	1
24	140	140	1
25	148	148	1
26	150	165	16
27	175	176	2
28	178	178	1
29	180	180	1
30	182	182	1
31	189	189	1
32	214	221	8
33	234	234	1
34	251	253	3
35	256	257	2
36	259	264	6
37	289	289	1
38	311	311	1

39	313	314	2
40	318	327	10
41	329	331	3
42	333	333	1
43	352	352	1
44	356	356	1
45	358	360	3
46	362	364	3
47	366	369	4
48	377	379	3
49	381	381	1
50	383	383	1
51	385	386	2
52	388	388	1
53	412	412	1
54	414	421	8
55	428	428	1
56	438	449	12
57	504	504	1

181 positions (cumulative volume <= 32 Angstrom^3)

	start	end	length
1	1	1	1
2	8	20	13
3	28	28	1
4	33	33	1
5	36	36	1
6	38	38	1
7	42	42	1
8	45	50	6
9	55	74	20
10	78	78	1
11	84	84	1
12	86	86	1
13	88	98	11
14	100	101	2
15	103	106	4
16	109	109	1
17	112	113	2
18	115	115	1
19	117	117	1
20	121	123	3
21	126	126	1
22	135	135	1
23	140	140	1
24	148	148	1
25	150	165	16
26	175	176	2
27	178	178	1
28	180	180	1
29	182	182	1

```
30   189 189      1
31   214 221      8
32   234 234      1
33   251 253      3
34   256 257      2
35   259 264      6
36   289 289      1
37   311 311      1
38   313 314      2
39   318 327     10
40   329 331      3
41   333 333      1
42   352 352      1
43   356 356      1
44   358 360      3
45   362 364      3
46   366 369      4
47   377 379      3
48   381 381      1
49   383 383      1
50   385 386      2
51   388 388      1
52   412 412      1
53   414 421      8
54   428 428      1
55   437 449     13
56   504 504      1
# 183 positions (cumulative volume <= 33 Angstrom^3)
   start end length
1      1   1      1
2      3   3      1
3      8  20     13
4     28  28      1
5     33  33      1
6     36  36      1
7     38  38      1
8     42  42      1
9     45  50      6
10    55  74     20
11    78  78      1
12    84  84      1
13    86  86      1
14    88  98     11
15   100 101      2
16   103 106      4
17   109 109      1
18   112 113      2
19   115 115      1
20   117 117      1
21   121 123      3
```

22	126	126	1
23	135	135	1
24	140	140	1
25	148	148	1
26	150	165	16
27	167	167	1
28	175	176	2
29	178	178	1
30	180	180	1
31	182	182	1
32	189	189	1
33	214	221	8
34	234	234	1
35	251	253	3
36	256	257	2
37	259	264	6
38	289	289	1
39	311	311	1
40	313	314	2
41	318	327	10
42	329	331	3
43	333	333	1
44	352	352	1
45	356	356	1
46	358	360	3
47	362	364	3
48	366	369	4
49	377	379	3
50	381	381	1
51	383	383	1
52	385	386	2
53	388	388	1
54	412	412	1
55	414	421	8
56	428	428	1
57	437	449	13
58	504	504	1

185 positions (cumulative volume <= 34 Angstrom^3)

	start	end	length
1	1	1	1
2	3	3	1
3	8	20	13
4	28	28	1
5	33	33	1
6	36	36	1
7	38	38	1
8	42	42	1
9	44	50	7
10	55	74	20
11	78	78	1

12	84	84	1
13	86	86	1
14	88	98	11
15	100	101	2
16	103	106	4
17	109	109	1
18	112	113	2
19	115	115	1
20	117	117	1
21	121	123	3
22	126	126	1
23	135	135	1
24	139	140	2
25	148	148	1
26	150	165	16
27	167	167	1
28	175	176	2
29	178	178	1
30	180	180	1
31	182	182	1
32	189	189	1
33	214	221	8
34	234	234	1
35	251	253	3
36	256	257	2
37	259	264	6
38	289	289	1
39	311	311	1
40	313	314	2
41	318	327	10
42	329	331	3
43	333	333	1
44	352	352	1
45	356	356	1
46	358	360	3
47	362	364	3
48	366	369	4
49	377	379	3
50	381	381	1
51	383	383	1
52	385	386	2
53	388	388	1
54	412	412	1
55	414	421	8
56	428	428	1
57	437	449	13
58	504	504	1

186 positions (cumulative volume <= 35 Angstrom^3)

	start	end	length
1	1	1	1

2	3	3	1
3	8	20	13
4	28	28	1
5	33	33	1
6	36	36	1
7	38	38	1
8	42	42	1
9	44	50	7
10	55	74	20
11	78	78	1
12	84	84	1
13	86	86	1
14	88	98	11
15	100	101	2
16	103	106	4
17	109	109	1
18	112	113	2
19	115	115	1
20	117	117	1
21	121	123	3
22	126	126	1
23	135	135	1
24	139	140	2
25	148	148	1
26	150	165	16
27	167	167	1
28	175	176	2
29	178	178	1
30	180	180	1
31	182	182	1
32	189	189	1
33	214	221	8
34	234	234	1
35	240	240	1
36	251	253	3
37	256	257	2
38	259	264	6
39	289	289	1
40	311	311	1
41	313	314	2
42	318	327	10
43	329	331	3
44	333	333	1
45	352	352	1
46	356	356	1
47	358	360	3
48	362	364	3
49	366	369	4
50	377	379	3
51	381	381	1

52	383	383	1
53	385	386	2
54	388	388	1
55	412	412	1
56	414	421	8
57	428	428	1
58	437	449	13
59	504	504	1

187 positions (cumulative volume <= 36 Angstrom^3)

	start	end	length
1	1	1	1
2	3	3	1
3	8	20	13
4	28	28	1
5	33	33	1
6	36	36	1
7	38	38	1
8	42	42	1
9	44	50	7
10	55	74	20
11	78	78	1
12	84	84	1
13	86	86	1
14	88	98	11
15	100	101	2
16	103	106	4
17	109	109	1
18	112	113	2
19	115	115	1
20	117	117	1
21	121	123	3
22	126	126	1
23	135	135	1
24	139	140	2
25	148	148	1
26	150	165	16
27	167	167	1
28	175	178	4
29	180	180	1
30	182	182	1
31	189	189	1
32	214	221	8
33	234	234	1
34	240	240	1
35	251	253	3
36	256	257	2
37	259	264	6
38	289	289	1
39	311	311	1
40	313	314	2

41	318	327	10
42	329	331	3
43	333	333	1
44	352	352	1
45	356	356	1
46	358	360	3
47	362	364	3
48	366	369	4
49	377	379	3
50	381	381	1
51	383	383	1
52	385	386	2
53	388	388	1
54	412	412	1
55	414	421	8
56	428	428	1
57	437	449	13
58	504	504	1

190 positions (cumulative volume <= 37 Angstrom^3)

	start	end	length
1	1	1	1
2	3	3	1
3	8	21	14
4	28	28	1
5	33	34	2
6	36	36	1
7	38	38	1
8	42	42	1
9	44	50	7
10	55	74	20
11	78	78	1
12	84	84	1
13	86	86	1
14	88	98	11
15	100	106	7
16	109	109	1
17	112	113	2
18	115	115	1
19	117	117	1
20	121	123	3
21	126	126	1
22	135	135	1
23	139	140	2
24	148	148	1
25	150	165	16
26	167	167	1
27	175	178	4
28	180	180	1
29	182	182	1
30	189	189	1

31	214	221	8
32	234	234	1
33	240	240	1
34	251	253	3
35	256	257	2
36	259	264	6
37	289	289	1
38	311	311	1
39	313	314	2
40	318	327	10
41	329	331	3
42	333	333	1
43	352	352	1
44	356	356	1
45	358	360	3
46	362	364	3
47	366	369	4
48	377	379	3
49	381	381	1
50	383	383	1
51	385	386	2
52	388	388	1
53	412	412	1
54	414	421	8
55	428	428	1
56	437	449	13
57	504	504	1

192 positions (cumulative volume <= 38 Angstrom^3)

	start	end	length
1	1	1	1
2	3	3	1
3	8	21	14
4	28	28	1
5	33	34	2
6	36	36	1
7	38	38	1
8	42	42	1
9	44	50	7
10	55	74	20
11	78	78	1
12	84	84	1
13	86	86	1
14	88	98	11
15	100	106	7
16	109	109	1
17	112	113	2
18	115	115	1
19	117	117	1
20	121	123	3
21	126	126	1

22	135	135	1
23	139	140	2
24	148	148	1
25	150	165	16
26	167	167	1
27	175	178	4
28	180	180	1
29	182	182	1
30	189	189	1
31	214	221	8
32	234	234	1
33	240	240	1
34	251	253	3
35	255	257	3
36	259	264	6
37	289	289	1
38	311	311	1
39	313	314	2
40	318	327	10
41	329	331	3
42	333	333	1
43	352	352	1
44	356	356	1
45	358	360	3
46	362	364	3
47	366	369	4
48	377	379	3
49	381	381	1
50	383	383	1
51	385	388	4
52	412	412	1
53	414	421	8
54	428	428	1
55	437	449	13
56	504	504	1

194 positions (cumulative volume <= 39 Angstrom^3)

	start	end	length
1	1	1	1
2	3	3	1
3	8	21	14
4	28	28	1
5	33	34	2
6	36	36	1
7	38	38	1
8	42	42	1
9	44	50	7
10	55	74	20
11	78	78	1
12	84	84	1
13	86	86	1

```
14    88  98     11
15   100 106      7
16   109 109      1
17   112 113      2
18   115 115      1
19   117 117      1
20   121 123      3
21   126 126      1
22   135 135      1
23   139 140      2
24   148 148      1
25   150 165     16
26   167 167      1
27   175 178      4
28   180 180      1
29   182 182      1
30   189 189      1
31   214 221      8
32   234 234      1
33   240 240      1
34   251 253      3
35   255 257      3
36   259 264      6
37   289 289      1
38   306 306      1
39   311 311      1
40   313 314      2
41   318 327     10
42   329 331      3
43   333 333      1
44   352 352      1
45   356 356      1
46   358 360      3
47   362 364      3
48   366 369      4
49   377 379      3
50   381 381      1
51   383 383      1
52   385 388      4
53   412 412      1
54   414 421      8
55   428 428      1
56   437 449     13
57   501 501      1
58   504 504      1
# 196 positions (cumulative volume <= 40 Angstrom^3)
   start end length
1      1   1      1
2      3   3      1
3      8  21     14
```

4	28	28	1
5	31	31	1
6	33	34	2
7	36	38	3
8	42	42	1
9	44	50	7
10	55	74	20
11	78	78	1
12	84	84	1
13	86	86	1
14	88	98	11
15	100	106	7
16	109	109	1
17	112	113	2
18	115	115	1
19	117	117	1
20	121	123	3
21	126	126	1
22	135	135	1
23	139	140	2
24	148	148	1
25	150	165	16
26	167	167	1
27	175	178	4
28	180	180	1
29	182	182	1
30	189	189	1
31	214	221	8
32	234	234	1
33	240	240	1
34	251	253	3
35	255	257	3
36	259	264	6
37	289	289	1
38	306	306	1
39	311	311	1
40	313	314	2
41	318	327	10
42	329	331	3
43	333	333	1
44	352	352	1
45	356	356	1
46	358	360	3
47	362	364	3
48	366	369	4
49	377	379	3
50	381	381	1
51	383	383	1
52	385	388	4
53	412	412	1

```
54   414 421      8
55   428 428      1
56   437 449     13
57   501 501      1
58   504 504      1
# 198 positions (cumulative volume <= 41 Angstrom^3)
   start end length
1      1   1      1
2      3   3      1
3      8  21     14
4     28  28      1
5     31  31      1
6     33  34      2
7     36  38      3
8     42  42      1
9     44  50      7
10    55  74     20
11    78  78      1
12    84  84      1
13    86  86      1
14    88  98     11
15   100 106      7
16   109 109      1
17   112 113      2
18   115 115      1
19   117 117      1
20   121 123      3
21   126 126      1
22   135 135      1
23   139 140      2
24   148 148      1
25   150 165     16
26   167 167      1
27   175 178      4
28   180 180      1
29   182 182      1
30   189 189      1
31   214 221      8
32   234 234      1
33   240 240      1
34   251 253      3
35   255 257      3
36   259 264      6
37   289 289      1
38   306 306      1
39   311 311      1
40   313 314      2
41   318 327     10
42   329 331      3
43   333 333      1
```

```
44   352 352      1
45   356 356      1
46   358 360      3
47   362 364      3
48   366 369      4
49   377 379      3
50   381 381      1
51   383 383      1
52   385 388      4
53   408 408      1
54   412 412      1
55   414 421      8
56   426 426      1
57   428 428      1
58   437 449     13
59   501 501      1
60   504 504      1
# 203 positions (cumulative volume <= 42 Angstrom^3)
   start end length
1      1   1      1
2      3   3      1
3      8  21     14
4     28  28      1
5     31  31      1
6     33  34      2
7     36  38      3
8     42  50      9
9     55  74     20
10    78  78      1
11    84  84      1
12    86  86      1
13    88  98     11
14   100 109     10
15   112 113      2
16   115 115      1
17   117 117      1
18   121 123      3
19   126 126      1
20   135 136      2
21   139 140      2
22   148 148      1
23   150 165     16
24   167 167      1
25   175 178      4
26   180 180      1
27   182 182      1
28   189 189      1
29   214 221      8
30   234 234      1
31   240 240      1
```

32	251	253	3
33	255	257	3
34	259	264	6
35	289	289	1
36	306	306	1
37	311	311	1
38	313	314	2
39	318	327	10
40	329	331	3
41	333	333	1
42	352	352	1
43	356	356	1
44	358	360	3
45	362	364	3
46	366	369	4
47	377	379	3
48	381	381	1
49	383	383	1
50	385	388	4
51	408	408	1
52	412	412	1
53	414	421	8
54	426	426	1
55	428	428	1
56	437	449	13
57	457	457	1
58	501	501	1
59	504	504	1

204 positions (cumulative volume <= 43 Angstrom^3)

	start	end	length
1	1	1	1
2	3	3	1
3	8	21	14
4	28	28	1
5	31	31	1
6	33	34	2
7	36	38	3
8	42	50	9
9	55	74	20
10	78	78	1
11	84	84	1
12	86	86	1
13	88	98	11
14	100	109	10
15	112	113	2
16	115	115	1
17	117	117	1
18	121	123	3
19	126	126	1
20	135	136	2

21	139	140	2
22	148	148	1
23	150	165	16
24	167	167	1
25	175	178	4
26	180	180	1
27	182	182	1
28	189	189	1
29	214	221	8
30	234	234	1
31	240	240	1
32	251	253	3
33	255	257	3
34	259	264	6
35	289	289	1
36	306	306	1
37	310	311	2
38	313	314	2
39	318	327	10
40	329	331	3
41	333	333	1
42	352	352	1
43	356	356	1
44	358	360	3
45	362	364	3
46	366	369	4
47	377	379	3
48	381	381	1
49	383	383	1
50	385	388	4
51	408	408	1
52	412	412	1
53	414	421	8
54	426	426	1
55	428	428	1
56	437	449	13
57	457	457	1
58	501	501	1
59	504	504	1

205 positions (cumulative volume <= 44 Angstrom^3)

	start	end	length
1	1	1	1
2	3	3	1
3	8	21	14
4	28	28	1
5	31	31	1
6	33	34	2
7	36	38	3
8	42	50	9
9	55	74	20

10	78	78	1
11	84	84	1
12	86	86	1
13	88	98	11
14	100	109	10
15	112	113	2
16	115	115	1
17	117	117	1
18	121	123	3
19	126	126	1
20	135	136	2
21	139	140	2
22	148	148	1
23	150	165	16
24	167	167	1
25	175	178	4
26	180	182	3
27	189	189	1
28	214	221	8
29	234	234	1
30	240	240	1
31	251	253	3
32	255	257	3
33	259	264	6
34	289	289	1
35	306	306	1
36	310	311	2
37	313	314	2
38	318	327	10
39	329	331	3
40	333	333	1
41	352	352	1
42	356	356	1
43	358	360	3
44	362	364	3
45	366	369	4
46	377	379	3
47	381	381	1
48	383	383	1
49	385	388	4
50	408	408	1
51	412	412	1
52	414	421	8
53	426	426	1
54	428	428	1
55	437	449	13
56	457	457	1
57	501	501	1
58	504	504	1

207 positions (cumulative volume <= 45 Angstrom^3)

	start	end	length
1	1	1	1
2	3	3	1
3	8	21	14
4	28	28	1
5	31	31	1
6	33	34	2
7	36	38	3
8	42	50	9
9	55	74	20
10	78	78	1
11	84	84	1
12	86	86	1
13	88	98	11
14	100	110	11
15	112	113	2
16	115	115	1
17	117	117	1
18	121	123	3
19	126	126	1
20	135	136	2
21	139	140	2
22	148	148	1
23	150	165	16
24	167	167	1
25	175	178	4
26	180	182	3
27	189	189	1
28	214	221	8
29	234	234	1
30	240	240	1
31	251	253	3
32	255	264	10
33	289	289	1
34	306	306	1
35	310	311	2
36	313	314	2
37	318	327	10
38	329	331	3
39	333	333	1
40	352	352	1
41	356	356	1
42	358	360	3
43	362	364	3
44	366	369	4
45	377	379	3
46	381	381	1
47	383	383	1
48	385	388	4
49	408	408	1

```
50   412 412      1
51   414 421      8
52   426 426      1
53   428 428      1
54   437 449     13
55   457 457      1
56   501 501      1
57   504 504      1
# 209 positions (cumulative volume <= 46 Angstrom^3)
   start end length
1      1   1      1
2      3   3      1
3      8  21     14
4     28  28      1
5     31  31      1
6     33  38      6
7     42  50      9
8     55  74     20
9     78  78      1
10    84  84      1
11    86  86      1
12    88  98     11
13   100 110     11
14   112 113      2
15   115 115      1
16   117 117      1
17   121 123      3
18   126 126      1
19   135 136      2
20   139 140      2
21   148 148      1
22   150 165     16
23   167 167      1
24   175 178      4
25   180 182      3
26   189 189      1
27   214 221      8
28   234 234      1
29   240 240      1
30   251 253      3
31   255 264     10
32   289 289      1
33   306 306      1
34   310 311      2
35   313 314      2
36   318 331     14
37   333 333      1
38   352 352      1
39   356 356      1
40   358 360      3
```

```
41   362 364      3
42   366 369      4
43   377 379      3
44   381 381      1
45   383 383      1
46   385 388      4
47   408 408      1
48   412 412      1
49   414 421      8
50   426 426      1
51   428 428      1
52   437 449     13
53   457 457      1
54   501 501      1
55   504 504      1
# 210 positions (cumulative volume <= 47 Angstrom^3)
   start end length
1      1   1      1
2      3   3      1
3      8  21     14
4     28  28      1
5     31  31      1
6     33  38      6
7     42  50      9
8     55  74     20
9     78  78      1
10    84  84      1
11    86  86      1
12    88  98     11
13   100 110     11
14   112 113      2
15   115 115      1
16   117 117      1
17   121 123      3
18   126 126      1
19   135 136      2
20   139 140      2
21   148 148      1
22   150 165     16
23   167 167      1
24   175 178      4
25   180 182      3
26   189 189      1
27   214 221      8
28   234 234      1
29   240 240      1
30   251 253      3
31   255 264     10
32   289 289      1
33   306 306      1
```

```
34   310 311       2
35   313 314       2
36   318 331      14
37   333 333       1
38   352 352       1
39   356 356       1
40   358 360       3
41   362 364       3
42   366 369       4
43   377 379       3
44   381 381       1
45   383 383       1
46   385 388       4
47   408 408       1
48   412 412       1
49   414 421       8
50   426 426       1
51   428 428       1
52   437 450      14
53   457 457       1
54   501 501       1
55   504 504       1
# 211 positions (cumulative volume <= 48 Angstrom^3)
   start end length
1      1   1       1
2      3   3       1
3      8  22      15
4     28  28       1
5     31  31       1
6     33  38       6
7     42  50       9
8     55  74      20
9     78  78       1
10    84  84       1
11    86  86       1
12    88  98      11
13   100 110      11
14   112 113       2
15   115 115       1
16   117 117       1
17   121 123       3
18   126 126       1
19   135 136       2
20   139 140       2
21   148 148       1
22   150 165      16
23   167 167       1
24   175 178       4
25   180 182       3
26   189 189       1
```

27	214	221	8
28	234	234	1
29	240	240	1
30	251	253	3
31	255	264	10
32	289	289	1
33	306	306	1
34	310	311	2
35	313	314	2
36	318	331	14
37	333	333	1
38	352	352	1
39	356	356	1
40	358	360	3
41	362	364	3
42	366	369	4
43	377	379	3
44	381	381	1
45	383	383	1
46	385	388	4
47	408	408	1
48	412	412	1
49	414	421	8
50	426	426	1
51	428	428	1
52	437	450	14
53	457	457	1
54	501	501	1
55	504	504	1

212 positions (cumulative volume <= 49 Angstrom^3)

	start	end	length
1	1	1	1
2	3	3	1
3	8	22	15
4	28	28	1
5	31	31	1
6	33	38	6
7	42	50	9
8	55	74	20
9	78	78	1
10	84	84	1
11	86	86	1
12	88	98	11
13	100	110	11
14	112	113	2
15	115	115	1
16	117	117	1
17	121	123	3
18	126	126	1
19	130	130	1

```
20   135 136      2
21   139 140      2
22   148 148      1
23   150 165     16
24   167 167      1
25   175 178      4
26   180 182      3
27   189 189      1
28   214 221      8
29   234 234      1
30   240 240      1
31   251 253      3
32   255 264     10
33   289 289      1
34   306 306      1
35   310 311      2
36   313 314      2
37   318 331     14
38   333 333      1
39   352 352      1
40   356 356      1
41   358 360      3
42   362 364      3
43   366 369      4
44   377 379      3
45   381 381      1
46   383 383      1
47   385 388      4
48   408 408      1
49   412 412      1
50   414 421      8
51   426 426      1
52   428 428      1
53   437 450     14
54   457 457      1
55   501 501      1
56   504 504      1
# 213 positions (cumulative volume <= 50 Angstrom^3)
   start end length
1      1   1      1
2      3   3      1
3      8  22     15
4     28  28      1
5     31  31      1
6     33  38      6
7     42  50      9
8     55  74     20
9     78  78      1
10    84  84      1
11    86  86      1
```

```
12    88  98     11
13   100 110     11
14   112 113      2
15   115 115      1
16   117 117      1
17   121 123      3
18   126 126      1
19   130 130      1
20   135 136      2
21   139 140      2
22   148 148      1
23   150 165     16
24   167 167      1
25   175 178      4
26   180 182      3
27   189 189      1
28   214 221      8
29   234 234      1
30   240 240      1
31   251 253      3
32   255 264     10
33   289 289      1
34   306 306      1
35   310 311      2
36   313 314      2
37   318 331     14
38   333 333      1
39   352 352      1
40   356 356      1
41   358 360      3
42   362 364      3
43   366 369      4
44   377 379      3
45   381 381      1
46   383 383      1
47   385 388      4
48   394 394      1
49   408 408      1
50   412 412      1
51   414 421      8
52   426 426      1
53   428 428      1
54   437 450     14
55   457 457      1
56   501 501      1
57   504 504      1
# 215 positions (cumulative volume <= 51 Angstrom^3)
   start end length
1      1   1      1
2      3   3      1
```

3	8	22	15
4	28	28	1
5	31	31	1
6	33	38	6
7	42	50	9
8	55	74	20
9	78	78	1
10	84	84	1
11	86	86	1
12	88	98	11
13	100	110	11
14	112	113	2
15	115	115	1
16	117	117	1
17	121	124	4
18	126	126	1
19	130	130	1
20	135	136	2
21	139	140	2
22	148	148	1
23	150	165	16
24	167	167	1
25	175	178	4
26	180	182	3
27	189	189	1
28	214	221	8
29	234	234	1
30	240	240	1
31	251	253	3
32	255	264	10
33	289	289	1
34	306	306	1
35	310	311	2
36	313	314	2
37	318	331	14
38	333	333	1
39	352	352	1
40	356	356	1
41	358	360	3
42	362	364	3
43	366	369	4
44	377	379	3
45	381	381	1
46	383	383	1
47	385	388	4
48	394	394	1
49	408	409	2
50	412	412	1
51	414	421	8
52	426	426	1

53	428	428	1
54	437	450	14
55	457	457	1
56	501	501	1
57	504	504	1

216 positions (cumulative volume <= 52 Angstrom^3)

	start	end	length
1	1	1	1
2	3	3	1
3	8	22	15
4	28	28	1
5	31	31	1
6	33	38	6
7	42	51	10
8	55	74	20
9	78	78	1
10	84	84	1
11	86	86	1
12	88	98	11
13	100	110	11
14	112	113	2
15	115	115	1
16	117	117	1
17	121	124	4
18	126	126	1
19	130	130	1
20	135	136	2
21	139	140	2
22	148	148	1
23	150	165	16
24	167	167	1
25	175	178	4
26	180	182	3
27	189	189	1
28	214	221	8
29	234	234	1
30	240	240	1
31	251	253	3
32	255	264	10
33	289	289	1
34	306	306	1
35	310	311	2
36	313	314	2
37	318	331	14
38	333	333	1
39	352	352	1
40	356	356	1
41	358	360	3
42	362	364	3
43	366	369	4

44	377	379	3
45	381	381	1
46	383	383	1
47	385	388	4
48	394	394	1
49	408	409	2
50	412	412	1
51	414	421	8
52	426	426	1
53	428	428	1
54	437	450	14
55	457	457	1
56	501	501	1
57	504	504	1

218 positions (cumulative volume <= 53 Angstrom^3)

	start	end	length
1	1	1	1
2	3	3	1
3	8	22	15
4	28	28	1
5	31	31	1
6	33	38	6
7	42	51	10
8	55	74	20
9	78	78	1
10	84	84	1
11	86	86	1
12	88	110	23
13	112	113	2
14	115	115	1
15	117	117	1
16	121	124	4
17	126	126	1
18	130	130	1
19	135	136	2
20	139	140	2
21	148	148	1
22	150	165	16
23	167	167	1
24	175	178	4
25	180	182	3
26	189	189	1
27	214	221	8
28	234	234	1
29	240	240	1
30	251	253	3
31	255	264	10
32	289	289	1
33	306	306	1
34	310	311	2

35	313	314	2
36	318	331	14
37	333	333	1
38	352	352	1
39	356	356	1
40	358	360	3
41	362	364	3
42	366	369	4
43	377	381	5
44	383	383	1
45	385	388	4
46	394	394	1
47	408	409	2
48	412	412	1
49	414	421	8
50	426	426	1
51	428	428	1
52	437	450	14
53	457	457	1
54	501	501	1
55	504	504	1

219 positions (cumulative volume <= 54 Angstrom^3)

	start	end	length
1	1	1	1
2	3	3	1
3	8	22	15
4	28	28	1
5	31	31	1
6	33	38	6
7	42	51	10
8	55	74	20
9	78	78	1
10	84	84	1
11	86	86	1
12	88	110	23
13	112	113	2
14	115	115	1
15	117	117	1
16	121	124	4
17	126	126	1
18	130	130	1
19	135	136	2
20	139	140	2
21	148	148	1
22	150	165	16
23	167	167	1
24	175	178	4
25	180	182	3
26	189	189	1
27	214	221	8

```
28   234 234      1
29   240 240      1
30   251 253      3
31   255 264     10
32   289 289      1
33   306 306      1
34   310 311      2
35   313 314      2
36   318 331     14
37   333 333      1
38   352 352      1
39   356 356      1
40   358 364      7
41   366 369      4
42   377 381      5
43   383 383      1
44   385 388      4
45   394 394      1
46   408 409      2
47   412 412      1
48   414 421      8
49   426 426      1
50   428 428      1
51   437 450     14
52   457 457      1
53   501 501      1
54   504 504      1
# 220 positions (cumulative volume <= 55 Angstrom^3)
   start end length
1      1   1      1
2      3   3      1
3      8  22     15
4     28  28      1
5     31  31      1
6     33  38      6
7     42  51     10
8     55  74     20
9     78  78      1
10    84  84      1
11    86  86      1
12    88 110     23
13   112 113      2
14   115 115      1
15   117 117      1
16   121 124      4
17   126 126      1
18   130 130      1
19   135 136      2
20   139 140      2
21   148 148      1
```

22	150	165	16
23	167	167	1
24	175	178	4
25	180	182	3
26	189	189	1
27	214	221	8
28	234	234	1
29	240	240	1
30	251	253	3
31	255	265	11
32	289	289	1
33	306	306	1
34	310	311	2
35	313	314	2
36	318	331	14
37	333	333	1
38	352	352	1
39	356	356	1
40	358	364	7
41	366	369	4
42	377	381	5
43	383	383	1
44	385	388	4
45	394	394	1
46	408	409	2
47	412	412	1
48	414	421	8
49	426	426	1
50	428	428	1
51	437	450	14
52	457	457	1
53	501	501	1
54	504	504	1

221 positions (cumulative volume <= 56 Angstrom^3)

	start	end	length
1	1	1	1
2	3	3	1
3	8	22	15
4	28	28	1
5	31	31	1
6	33	38	6
7	42	51	10
8	54	74	21
9	78	78	1
10	84	84	1
11	86	86	1
12	88	110	23
13	112	113	2
14	115	115	1
15	117	117	1

16	121	124	4
17	126	126	1
18	130	130	1
19	135	136	2
20	139	140	2
21	148	148	1
22	150	165	16
23	167	167	1
24	175	178	4
25	180	182	3
26	189	189	1
27	214	221	8
28	234	234	1
29	240	240	1
30	251	253	3
31	255	265	11
32	289	289	1
33	306	306	1
34	310	311	2
35	313	314	2
36	318	331	14
37	333	333	1
38	352	352	1
39	356	356	1
40	358	364	7
41	366	369	4
42	377	381	5
43	383	383	1
44	385	388	4
45	394	394	1
46	408	409	2
47	412	412	1
48	414	421	8
49	426	426	1
50	428	428	1
51	437	450	14
52	457	457	1
53	501	501	1
54	504	504	1

222 positions (cumulative volume <= 57 Angstrom^3)

	start	end	length
1	1	1	1
2	3	3	1
3	8	22	15
4	28	28	1
5	31	31	1
6	33	38	6
7	42	51	10
8	54	74	21
9	78	78	1

10	84	84	1
11	86	86	1
12	88	110	23
13	112	113	2
14	115	115	1
15	117	117	1
16	121	124	4
17	126	126	1
18	130	130	1
19	135	136	2
20	138	140	3
21	148	148	1
22	150	165	16
23	167	167	1
24	175	178	4
25	180	182	3
26	189	189	1
27	214	221	8
28	234	234	1
29	240	240	1
30	251	253	3
31	255	265	11
32	289	289	1
33	306	306	1
34	310	311	2
35	313	314	2
36	318	331	14
37	333	333	1
38	352	352	1
39	356	356	1
40	358	364	7
41	366	369	4
42	377	381	5
43	383	383	1
44	385	388	4
45	394	394	1
46	408	409	2
47	412	412	1
48	414	421	8
49	426	426	1
50	428	428	1
51	437	450	14
52	457	457	1
53	501	501	1
54	504	504	1

223 positions (cumulative volume <= 58 Angstrom^3)

	start	end	length
1	1	3	3
2	8	22	15
3	28	28	1

4	31	31	1
5	33	38	6
6	42	51	10
7	54	74	21
8	78	78	1
9	84	84	1
10	86	86	1
11	88	110	23
12	112	113	2
13	115	115	1
14	117	117	1
15	121	124	4
16	126	126	1
17	130	130	1
18	135	136	2
19	138	140	3
20	148	148	1
21	150	165	16
22	167	167	1
23	175	178	4
24	180	182	3
25	189	189	1
26	214	221	8
27	234	234	1
28	240	240	1
29	251	253	3
30	255	265	11
31	289	289	1
32	306	306	1
33	310	311	2
34	313	314	2
35	318	331	14
36	333	333	1
37	352	352	1
38	356	356	1
39	358	364	7
40	366	369	4
41	377	381	5
42	383	383	1
43	385	388	4
44	394	394	1
45	408	409	2
46	412	412	1
47	414	421	8
48	426	426	1
49	428	428	1
50	437	450	14
51	457	457	1
52	501	501	1
53	504	504	1

224 positions (cumulative volume <= 59 Angstrom^3)

	start	end	length
1	1	3	3
2	8	22	15
3	28	28	1
4	31	31	1
5	33	38	6
6	42	51	10
7	54	74	21
8	78	78	1
9	81	81	1
10	84	84	1
11	86	86	1
12	88	110	23
13	112	113	2
14	115	115	1
15	117	117	1
16	121	124	4
17	126	126	1
18	130	130	1
19	135	136	2
20	138	140	3
21	148	148	1
22	150	165	16
23	167	167	1
24	175	178	4
25	180	182	3
26	189	189	1
27	214	221	8
28	234	234	1
29	240	240	1
30	251	253	3
31	255	265	11
32	289	289	1
33	306	306	1
34	310	311	2
35	313	314	2
36	318	331	14
37	333	333	1
38	352	352	1
39	356	356	1
40	358	364	7
41	366	369	4
42	377	381	5
43	383	383	1
44	385	388	4
45	394	394	1
46	408	409	2
47	412	412	1
48	414	421	8

49	426	426	1
50	428	428	1
51	437	450	14
52	457	457	1
53	501	501	1
54	504	504	1

224 positions (cumulative volume <= 60 Angstrom^3)

	start	end	length
1	1	3	3
2	8	22	15
3	28	28	1
4	31	31	1
5	33	38	6
6	42	51	10
7	54	74	21
8	78	78	1
9	81	81	1
10	84	84	1
11	86	86	1
12	88	110	23
13	112	113	2
14	115	115	1
15	117	117	1
16	121	124	4
17	126	126	1
18	130	130	1
19	135	136	2
20	138	140	3
21	148	148	1
22	150	165	16
23	167	167	1
24	175	178	4
25	180	182	3
26	189	189	1
27	214	221	8
28	234	234	1
29	240	240	1
30	251	253	3
31	255	265	11
32	289	289	1
33	306	306	1
34	310	311	2
35	313	314	2
36	318	331	14
37	333	333	1
38	352	352	1
39	356	356	1
40	358	364	7
41	366	369	4
42	377	381	5

43	383	383	1
44	385	388	4
45	394	394	1
46	408	409	2
47	412	412	1
48	414	421	8
49	426	426	1
50	428	428	1
51	437	450	14
52	457	457	1
53	501	501	1
54	504	504	1

226 positions (cumulative volume <= 61 Angstrom^3)

	start	end	length
1	1	3	3
2	8	22	15
3	28	28	1
4	31	31	1
5	33	38	6
6	42	51	10
7	54	74	21
8	77	78	2
9	81	81	1
10	84	84	1
11	86	86	1
12	88	110	23
13	112	113	2
14	115	115	1
15	117	117	1
16	121	124	4
17	126	126	1
18	130	130	1
19	135	136	2
20	138	140	3
21	148	148	1
22	150	165	16
23	167	167	1
24	175	178	4
25	180	182	3
26	189	189	1
27	214	221	8
28	234	234	1
29	240	240	1
30	251	253	3
31	255	265	11
32	289	289	1
33	306	306	1
34	310	311	2
35	313	314	2
36	318	331	14

```
37   333 333      1
38   352 352      1
39   356 356      1
40   358 364      7
41   366 370      5
42   377 381      5
43   383 383      1
44   385 388      4
45   394 394      1
46   408 409      2
47   412 412      1
48   414 421      8
49   426 426      1
50   428 428      1
51   437 450     14
52   457 457      1
53   501 501      1
54   504 504      1
# 227 positions (cumulative volume <= 62 Angstrom^3)
   start end length
1      1   3      3
2      5   5      1
3      8  22     15
4     28  28      1
5     31  31      1
6     33  38      6
7     42  51     10
8     54  74     21
9     77  78      2
10    81  81      1
11    84  84      1
12    86  86      1
13    88 110     23
14   112 113      2
15   115 115      1
16   117 117      1
17   121 124      4
18   126 126      1
19   130 130      1
20   135 136      2
21   138 140      3
22   148 148      1
23   150 165     16
24   167 167      1
25   175 178      4
26   180 182      3
27   189 189      1
28   214 221      8
29   234 234      1
30   240 240      1
```

31	251	253	3
32	255	265	11
33	289	289	1
34	306	306	1
35	310	311	2
36	313	314	2
37	318	331	14
38	333	333	1
39	352	352	1
40	356	356	1
41	358	364	7
42	366	370	5
43	377	381	5
44	383	383	1
45	385	388	4
46	394	394	1
47	408	409	2
48	412	412	1
49	414	421	8
50	426	426	1
51	428	428	1
52	437	450	14
53	457	457	1
54	501	501	1
55	504	504	1

227 positions (cumulative volume <= 63 Angstrom^3)

	start	end	length
1	1	3	3
2	5	5	1
3	8	22	15
4	28	28	1
5	31	31	1
6	33	38	6
7	42	51	10
8	54	74	21
9	77	78	2
10	81	81	1
11	84	84	1
12	86	86	1
13	88	110	23
14	112	113	2
15	115	115	1
16	117	117	1
17	121	124	4
18	126	126	1
19	130	130	1
20	135	136	2
21	138	140	3
22	148	148	1
23	150	165	16

```
24   167 167      1
25   175 178      4
26   180 182      3
27   189 189      1
28   214 221      8
29   234 234      1
30   240 240      1
31   251 253      3
32   255 265     11
33   289 289      1
34   306 306      1
35   310 311      2
36   313 314      2
37   318 331     14
38   333 333      1
39   352 352      1
40   356 356      1
41   358 364      7
42   366 370      5
43   377 381      5
44   383 383      1
45   385 388      4
46   394 394      1
47   408 409      2
48   412 412      1
49   414 421      8
50   426 426      1
51   428 428      1
52   437 450     14
53   457 457      1
54   501 501      1
55   504 504      1
# 229 positions (cumulative volume <= 64 Angstrom^3)
   start end length
1      1   3      3
2      5   5      1
3      8  22     15
4     28  28      1
5     31  31      1
6     33  38      6
7     42  51     10
8     54  74     21
9     77  78      2
10    81  81      1
11    84  84      1
12    86  86      1
13    88 110     23
14   112 113      2
15   115 115      1
16   117 117      1
```

17	121	124	4
18	126	126	1
19	130	130	1
20	135	136	2
21	138	140	3
22	148	148	1
23	150	165	16
24	167	167	1
25	175	178	4
26	180	182	3
27	189	189	1
28	214	221	8
29	223	223	1
30	234	234	1
31	240	240	1
32	251	253	3
33	255	265	11
34	288	289	2
35	306	306	1
36	310	311	2
37	313	314	2
38	318	331	14
39	333	333	1
40	352	352	1
41	356	356	1
42	358	364	7
43	366	370	5
44	377	381	5
45	383	383	1
46	385	388	4
47	394	394	1
48	408	409	2
49	412	412	1
50	414	421	8
51	426	426	1
52	428	428	1
53	437	450	14
54	457	457	1
55	501	501	1
56	504	504	1

230 positions (cumulative volume <= 65 Angstrom^3)

	start	end	length
1	1	3	3
2	5	5	1
3	8	22	15
4	28	28	1
5	31	31	1
6	33	38	6
7	42	51	10
8	54	74	21

```
9     77  78      2
10    81  81      1
11    84  84      1
12    86  86      1
13    88 110     23
14   112 113      2
15   115 115      1
16   117 117      1
17   121 124      4
18   126 126      1
19   130 130      1
20   135 136      2
21   138 140      3
22   148 148      1
23   150 165     16
24   167 167      1
25   175 178      4
26   180 182      3
27   189 189      1
28   214 223     10
29   234 234      1
30   240 240      1
31   251 253      3
32   255 265     11
33   288 289      2
34   306 306      1
35   310 311      2
36   313 314      2
37   318 331     14
38   333 333      1
39   352 352      1
40   356 356      1
41   358 364      7
42   366 370      5
43   377 381      5
44   383 383      1
45   385 388      4
46   394 394      1
47   408 409      2
48   412 412      1
49   414 421      8
50   426 426      1
51   428 428      1
52   437 450     14
53   457 457      1
54   501 501      1
55   504 504      1
# 232 positions (cumulative volume <= 66 Angstrom^3)
   start end length
1      1   3      3
```

2	5	5	1
3	8	22	15
4	28	28	1
5	31	31	1
6	33	38	6
7	42	51	10
8	54	74	21
9	77	78	2
10	81	81	1
11	84	84	1
12	86	86	1
13	88	110	23
14	112	113	2
15	115	115	1
16	117	117	1
17	121	124	4
18	126	126	1
19	130	130	1
20	135	136	2
21	138	140	3
22	148	148	1
23	150	165	16
24	167	167	1
25	175	182	8
26	189	189	1
27	212	212	1
28	214	223	10
29	234	234	1
30	240	240	1
31	251	253	3
32	255	265	11
33	288	289	2
34	306	306	1
35	310	311	2
36	313	314	2
37	318	331	14
38	333	333	1
39	352	352	1
40	356	356	1
41	358	364	7
42	366	370	5
43	377	381	5
44	383	383	1
45	385	388	4
46	394	394	1
47	408	409	2
48	412	412	1
49	414	421	8
50	426	426	1
51	428	428	1

52	437	450	14
53	457	457	1
54	501	501	1
55	504	504	1

233 positions (cumulative volume <= 67 Angstrom^3)

	start	end	length
1	1	3	3
2	5	5	1
3	8	22	15
4	28	28	1
5	31	31	1
6	33	38	6
7	42	51	10
8	54	74	21
9	77	78	2
10	81	81	1
11	84	84	1
12	86	86	1
13	88	110	23
14	112	113	2
15	115	115	1
16	117	117	1
17	121	124	4
18	126	126	1
19	130	130	1
20	135	136	2
21	138	140	3
22	148	148	1
23	150	165	16
24	167	167	1
25	175	182	8
26	189	189	1
27	212	212	1
28	214	224	11
29	234	234	1
30	240	240	1
31	251	253	3
32	255	265	11
33	288	289	2
34	306	306	1
35	310	311	2
36	313	314	2
37	318	331	14
38	333	333	1
39	352	352	1
40	356	356	1
41	358	364	7
42	366	370	5
43	377	381	5
44	383	383	1

45	385	388	4
46	394	394	1
47	408	409	2
48	412	412	1
49	414	421	8
50	426	426	1
51	428	428	1
52	437	450	14
53	457	457	1
54	501	501	1
55	504	504	1

234 positions (cumulative volume <= 68 Angstrom^3)

	start	end	length
1	1	3	3
2	5	5	1
3	8	22	15
4	28	28	1
5	31	31	1
6	33	38	6
7	42	51	10
8	54	74	21
9	77	78	2
10	81	81	1
11	84	84	1
12	86	86	1
13	88	110	23
14	112	113	2
15	115	115	1
16	117	117	1
17	121	124	4
18	126	126	1
19	130	130	1
20	135	136	2
21	138	140	3
22	148	148	1
23	150	165	16
24	167	167	1
25	175	182	8
26	184	184	1
27	189	189	1
28	212	212	1
29	214	224	11
30	234	234	1
31	240	240	1
32	251	253	3
33	255	265	11
34	288	289	2
35	306	306	1
36	310	311	2
37	313	314	2

38	318	331	14
39	333	333	1
40	352	352	1
41	356	356	1
42	358	364	7
43	366	370	5
44	377	381	5
45	383	383	1
46	385	388	4
47	394	394	1
48	408	409	2
49	412	412	1
50	414	421	8
51	426	426	1
52	428	428	1
53	437	450	14
54	457	457	1
55	501	501	1
56	504	504	1

235 positions (cumulative volume <= 69 Angstrom^3)

	start	end	length
1	1	3	3
2	5	6	2
3	8	22	15
4	28	28	1
5	31	31	1
6	33	38	6
7	42	51	10
8	54	74	21
9	77	78	2
10	81	81	1
11	84	84	1
12	86	86	1
13	88	110	23
14	112	113	2
15	115	115	1
16	117	117	1
17	121	124	4
18	126	126	1
19	130	130	1
20	135	136	2
21	138	140	3
22	148	148	1
23	150	165	16
24	167	167	1
25	175	182	8
26	184	184	1
27	189	189	1
28	212	212	1
29	214	224	11

30	234	234	1
31	240	240	1
32	251	253	3
33	255	265	11
34	288	289	2
35	306	306	1
36	310	311	2
37	313	314	2
38	318	331	14
39	333	333	1
40	352	352	1
41	356	356	1
42	358	364	7
43	366	370	5
44	377	381	5
45	383	383	1
46	385	388	4
47	394	394	1
48	408	409	2
49	412	412	1
50	414	421	8
51	426	426	1
52	428	428	1
53	437	450	14
54	457	457	1
55	501	501	1
56	504	504	1

236 positions (cumulative volume <= 70 Angstrom^3)

	start	end	length
1	1	6	6
2	8	22	15
3	28	28	1
4	31	31	1
5	33	38	6
6	42	51	10
7	54	74	21
8	77	78	2
9	81	81	1
10	84	84	1
11	86	86	1
12	88	110	23
13	112	113	2
14	115	115	1
15	117	117	1
16	121	124	4
17	126	126	1
18	130	130	1
19	135	136	2
20	138	140	3
21	148	148	1

22	150	165	16
23	167	167	1
24	175	182	8
25	184	184	1
26	189	189	1
27	212	212	1
28	214	224	11
29	234	234	1
30	240	240	1
31	251	253	3
32	255	265	11
33	288	289	2
34	306	306	1
35	310	311	2
36	313	314	2
37	318	331	14
38	333	333	1
39	352	352	1
40	356	356	1
41	358	364	7
42	366	370	5
43	377	381	5
44	383	383	1
45	385	388	4
46	394	394	1
47	408	409	2
48	412	412	1
49	414	421	8
50	426	426	1
51	428	428	1
52	437	450	14
53	457	457	1
54	501	501	1
55	504	504	1

236 positions (cumulative volume <= 71 Angstrom^3)

	start	end	length
1	1	6	6
2	8	22	15
3	28	28	1
4	31	31	1
5	33	38	6
6	42	51	10
7	54	74	21
8	77	78	2
9	81	81	1
10	84	84	1
11	86	86	1
12	88	110	23
13	112	113	2
14	115	115	1

15	117	117	1
16	121	124	4
17	126	126	1
18	130	130	1
19	135	136	2
20	138	140	3
21	148	148	1
22	150	165	16
23	167	167	1
24	175	182	8
25	184	184	1
26	189	189	1
27	212	212	1
28	214	224	11
29	234	234	1
30	240	240	1
31	251	253	3
32	255	265	11
33	288	289	2
34	306	306	1
35	310	311	2
36	313	314	2
37	318	331	14
38	333	333	1
39	352	352	1
40	356	356	1
41	358	364	7
42	366	370	5
43	377	381	5
44	383	383	1
45	385	388	4
46	394	394	1
47	408	409	2
48	412	412	1
49	414	421	8
50	426	426	1
51	428	428	1
52	437	450	14
53	457	457	1
54	501	501	1
55	504	504	1

238 positions (cumulative volume <= 72 Angstrom^3)

	start	end	length
1	1	22	22
2	28	28	1
3	31	31	1
4	33	38	6
5	42	51	10
6	54	74	21
7	77	78	2

```
8      81  81       1
9      84  84       1
10     86  86       1
11     88 110      23
12    112 113       2
13    115 115       1
14    117 117       1
15    121 124       4
16    126 127       2
17    130 130       1
18    135 136       2
19    138 140       3
20    148 148       1
21    150 165      16
22    167 167       1
23    175 182       8
24    184 184       1
25    189 189       1
26    212 212       1
27    214 224      11
28    234 234       1
29    240 240       1
30    251 253       3
31    255 265      11
32    288 289       2
33    306 306       1
34    310 311       2
35    313 314       2
36    318 331      14
37    333 333       1
38    352 352       1
39    356 356       1
40    358 364       7
41    366 370       5
42    377 381       5
43    383 383       1
44    385 388       4
45    394 394       1
46    408 409       2
47    412 412       1
48    414 421       8
49    426 426       1
50    428 428       1
51    437 450      14
52    457 457       1
53    501 501       1
54    504 504       1
# 240 positions (cumulative volume <= 73 Angstrom^3)
   start end length
1      1  22     22
```

2	28	28	1
3	31	31	1
4	33	38	6
5	42	51	10
6	54	74	21
7	77	78	2
8	81	81	1
9	84	84	1
10	86	86	1
11	88	110	23
12	112	113	2
13	115	115	1
14	117	117	1
15	121	124	4
16	126	127	2
17	130	130	1
18	135	136	2
19	138	140	3
20	148	148	1
21	150	165	16
22	167	167	1
23	175	182	8
24	184	184	1
25	189	189	1
26	212	212	1
27	214	224	11
28	234	234	1
29	240	240	1
30	251	253	3
31	255	265	11
32	288	289	2
33	306	306	1
34	310	311	2
35	313	314	2
36	318	331	14
37	333	333	1
38	352	352	1
39	356	356	1
40	358	364	7
41	366	370	5
42	377	381	5
43	383	383	1
44	385	388	4
45	392	392	1
46	394	394	1
47	408	409	2
48	412	412	1
49	414	421	8
50	426	426	1
51	428	428	1

```
52   437 450      14
53   457 457       1
54   501 502       2
55   504 504       1
# 241 positions (cumulative volume <= 74 Angstrom^3)
   start end length
1      1  22      22
2     28  28       1
3     31  31       1
4     33  38       6
5     42  51      10
6     54  74      21
7     77  78       2
8     81  81       1
9     84  84       1
10    86  86       1
11    88 110      23
12   112 113       2
13   115 115       1
14   117 117       1
15   121 124       4
16   126 127       2
17   130 130       1
18   135 136       2
19   138 140       3
20   148 148       1
21   150 165      16
22   167 167       1
23   175 182       8
24   184 184       1
25   189 189       1
26   212 212       1
27   214 224      11
28   234 234       1
29   240 240       1
30   251 253       3
31   255 265      11
32   288 289       2
33   306 306       1
34   310 311       2
35   313 314       2
36   318 331      14
37   333 333       1
38   352 352       1
39   356 356       1
40   358 364       7
41   366 370       5
42   377 381       5
43   383 383       1
44   385 388       4
```

45	392	392	1
46	394	394	1
47	408	409	2
48	412	412	1
49	414	421	8
50	426	426	1
51	428	428	1
52	437	450	14
53	457	457	1
54	501	504	4

242 positions (cumulative volume <= 75 Angstrom^3)

	start	end	length
1	1	22	22
2	28	28	1
3	31	31	1
4	33	38	6
5	42	51	10
6	54	74	21
7	77	78	2
8	81	81	1
9	84	84	1
10	86	86	1
11	88	110	23
12	112	113	2
13	115	115	1
14	117	117	1
15	121	124	4
16	126	127	2
17	130	130	1
18	135	136	2
19	138	140	3
20	148	148	1
21	150	165	16
22	167	167	1
23	175	182	8
24	184	184	1
25	189	189	1
26	212	212	1
27	214	224	11
28	234	234	1
29	240	240	1
30	251	253	3
31	255	265	11
32	288	289	2
33	306	306	1
34	310	311	2
35	313	314	2
36	318	331	14
37	333	333	1
38	352	352	1

```
39   356 356      1
40   358 370     13
41   377 381      5
42   383 383      1
43   385 388      4
44   392 392      1
45   394 394      1
46   408 409      2
47   412 412      1
48   414 421      8
49   426 426      1
50   428 428      1
51   437 450     14
52   457 457      1
53   501 504      4
# 243 positions (cumulative volume <= 76 Angstrom^3)
   start end length
1      1  22     22
2     28  28      1
3     31  31      1
4     33  38      6
5     42  51     10
6     54  74     21
7     77  78      2
8     81  81      1
9     84  84      1
10    86  86      1
11    88 110     23
12   112 113      2
13   115 115      1
14   117 117      1
15   121 124      4
16   126 127      2
17   130 130      1
18   135 136      2
19   138 140      3
20   148 148      1
21   150 165     16
22   167 167      1
23   175 182      8
24   184 184      1
25   189 189      1
26   212 224     13
27   234 234      1
28   240 240      1
29   251 253      3
30   255 265     11
31   288 289      2
32   306 306      1
33   310 311      2
```

```
34   313 314      2
35   318 331     14
36   333 333      1
37   352 352      1
38   356 356      1
39   358 370     13
40   377 381      5
41   383 383      1
42   385 388      4
43   392 392      1
44   394 394      1
45   408 409      2
46   412 412      1
47   414 421      8
48   426 426      1
49   428 428      1
50   437 450     14
51   457 457      1
52   501 504      4
# 244 positions (cumulative volume <= 77 Angstrom^3)
   start end length
1      1  22     22
2     28  28      1
3     31  31      1
4     33  38      6
5     42  51     10
6     54  74     21
7     77  78      2
8     81  81      1
9     84  84      1
10    86  86      1
11    88 110     23
12   112 113      2
13   115 115      1
14   117 117      1
15   121 124      4
16   126 127      2
17   130 130      1
18   135 136      2
19   138 140      3
20   148 148      1
21   150 165     16
22   167 167      1
23   175 184     10
24   189 189      1
25   212 224     13
26   234 234      1
27   240 240      1
28   251 253      3
29   255 265     11
```

30	288	289	2
31	306	306	1
32	310	311	2
33	313	314	2
34	318	331	14
35	333	333	1
36	352	352	1
37	356	356	1
38	358	370	13
39	377	381	5
40	383	383	1
41	385	388	4
42	392	392	1
43	394	394	1
44	408	409	2
45	412	412	1
46	414	421	8
47	426	426	1
48	428	428	1
49	437	450	14
50	457	457	1
51	501	504	4

244 positions (cumulative volume <= 78 Angstrom^3)

	start	end	length
1	1	22	22
2	28	28	1
3	31	31	1
4	33	38	6
5	42	51	10
6	54	74	21
7	77	78	2
8	81	81	1
9	84	84	1
10	86	86	1
11	88	110	23
12	112	113	2
13	115	115	1
14	117	117	1
15	121	124	4
16	126	127	2
17	130	130	1
18	135	136	2
19	138	140	3
20	148	148	1
21	150	165	16
22	167	167	1
23	175	184	10
24	189	189	1
25	212	224	13
26	234	234	1

27	240	240	1
28	251	253	3
29	255	265	11
30	288	289	2
31	306	306	1
32	310	311	2
33	313	314	2
34	318	331	14
35	333	333	1
36	352	352	1
37	356	356	1
38	358	370	13
39	377	381	5
40	383	383	1
41	385	388	4
42	392	392	1
43	394	394	1
44	408	409	2
45	412	412	1
46	414	421	8
47	426	426	1
48	428	428	1
49	437	450	14
50	457	457	1
51	501	504	4

245 positions (cumulative volume <= 79 Angstrom^3)

	start	end	length
1	1	22	22
2	28	28	1
3	31	31	1
4	33	38	6
5	42	51	10
6	54	74	21
7	77	78	2
8	81	81	1
9	84	84	1
10	86	110	25
11	112	113	2
12	115	115	1
13	117	117	1
14	121	124	4
15	126	127	2
16	130	130	1
17	135	136	2
18	138	140	3
19	148	148	1
20	150	165	16
21	167	167	1
22	175	184	10
23	189	189	1

```
24   212 224     13
25   234 234      1
26   240 240      1
27   251 253      3
28   255 265     11
29   288 289      2
30   306 306      1
31   310 311      2
32   313 314      2
33   318 331     14
34   333 333      1
35   352 352      1
36   356 356      1
37   358 370     13
38   377 381      5
39   383 383      1
40   385 388      4
41   392 392      1
42   394 394      1
43   408 409      2
44   412 412      1
45   414 421      8
46   426 426      1
47   428 428      1
48   437 450     14
49   457 457      1
50   501 504      4
# 247 positions (cumulative volume <= 80 Angstrom^3)
   start end length
1      1  22     22
2     28  28      1
3     31  31      1
4     33  38      6
5     42  51     10
6     54  74     21
7     77  78      2
8     81  81      1
9     84  84      1
10    86 110     25
11   112 113      2
12   115 117      3
13   121 124      4
14   126 127      2
15   130 130      1
16   135 136      2
17   138 140      3
18   148 148      1
19   150 165     16
20   167 167      1
21   175 184     10
```

```
22   189 189      1
23   212 224     13
24   234 234      1
25   240 240      1
26   251 253      3
27   255 265     11
28   288 289      2
29   306 306      1
30   310 311      2
31   313 314      2
32   318 331     14
33   333 333      1
34   352 352      1
35   356 356      1
36   358 370     13
37   377 381      5
38   383 383      1
39   385 388      4
40   391 392      2
41   394 394      1
42   408 409      2
43   412 412      1
44   414 421      8
45   426 426      1
46   428 428      1
47   437 450     14
48   457 457      1
49   501 504      4
# 248 positions (cumulative volume <= 81 Angstrom^3)
   start end length
1      1  22     22
2     28  28      1
3     31  31      1
4     33  38      6
5     42  51     10
6     54  74     21
7     77  78      2
8     81  81      1
9     84  84      1
10    86 110     25
11   112 113      2
12   115 117      3
13   121 124      4
14   126 127      2
15   130 130      1
16   135 136      2
17   138 140      3
18   148 148      1
19   150 165     16
20   167 167      1
```

21	175	184	10
22	189	189	1
23	212	224	13
24	234	234	1
25	240	240	1
26	251	253	3
27	255	265	11
28	288	289	2
29	306	306	1
30	310	311	2
31	313	314	2
32	318	331	14
33	333	333	1
34	352	352	1
35	356	356	1
36	358	370	13
37	377	381	5
38	383	383	1
39	385	388	4
40	391	392	2
41	394	394	1
42	408	409	2
43	412	412	1
44	414	421	8
45	426	426	1
46	428	428	1
47	437	450	14
48	457	457	1
49	500	504	5

248 positions (cumulative volume <= 82 Angstrom^3)

	start	end	length
1	1	22	22
2	28	28	1
3	31	31	1
4	33	38	6
5	42	51	10
6	54	74	21
7	77	78	2
8	81	81	1
9	84	84	1
10	86	110	25
11	112	113	2
12	115	117	3
13	121	124	4
14	126	127	2
15	130	130	1
16	135	136	2
17	138	140	3
18	148	148	1
19	150	165	16

```
20   167 167      1
21   175 184     10
22   189 189      1
23   212 224     13
24   234 234      1
25   240 240      1
26   251 253      3
27   255 265     11
28   288 289      2
29   306 306      1
30   310 311      2
31   313 314      2
32   318 331     14
33   333 333      1
34   352 352      1
35   356 356      1
36   358 370     13
37   377 381      5
38   383 383      1
39   385 388      4
40   391 392      2
41   394 394      1
42   408 409      2
43   412 412      1
44   414 421      8
45   426 426      1
46   428 428      1
47   437 450     14
48   457 457      1
49   500 504      5
# 249 positions (cumulative volume <= 83 Angstrom^3)
   start end length
1      1  22     22
2     28  28      1
3     31  31      1
4     33  38      6
5     42  51     10
6     54  74     21
7     77  78      2
8     81  81      1
9     84  84      1
10    86 110     25
11   112 113      2
12   115 117      3
13   121 124      4
14   126 127      2
15   130 130      1
16   135 136      2
17   138 140      3
18   148 148      1
```

19	150	165	16
20	167	167	1
21	175	184	10
22	189	189	1
23	212	224	13
24	234	234	1
25	240	240	1
26	251	253	3
27	255	265	11
28	288	289	2
29	306	306	1
30	310	314	5
31	318	331	14
32	333	333	1
33	352	352	1
34	356	356	1
35	358	370	13
36	377	381	5
37	383	383	1
38	385	388	4
39	391	392	2
40	394	394	1
41	408	409	2
42	412	412	1
43	414	421	8
44	426	426	1
45	428	428	1
46	437	450	14
47	457	457	1
48	500	504	5

250 positions (cumulative volume <= 84 Angstrom^3)

	start	end	length
1	1	22	22
2	28	28	1
3	31	31	1
4	33	38	6
5	42	51	10
6	54	74	21
7	77	78	2
8	81	81	1
9	84	84	1
10	86	110	25
11	112	113	2
12	115	117	3
13	121	124	4
14	126	127	2
15	130	130	1
16	135	136	2
17	138	140	3
18	148	148	1

19	150	165	16
20	167	167	1
21	175	184	10
22	189	189	1
23	212	224	13
24	234	234	1
25	240	240	1
26	251	253	3
27	255	265	11
28	288	289	2
29	306	306	1
30	310	314	5
31	318	331	14
32	333	333	1
33	352	352	1
34	356	356	1
35	358	370	13
36	377	381	5
37	383	383	1
38	385	388	4
39	391	392	2
40	394	394	1
41	408	409	2
42	412	412	1
43	414	421	8
44	426	426	1
45	428	428	1
46	431	431	1
47	437	450	14
48	457	457	1
49	500	504	5

251 positions (cumulative volume <= 85 Angstrom^3)

	start	end	length
1	1	22	22
2	28	28	1
3	31	31	1
4	33	38	6
5	42	51	10
6	54	74	21
7	77	78	2
8	81	81	1
9	84	84	1
10	86	110	25
11	112	113	2
12	115	117	3
13	121	124	4
14	126	127	2
15	130	130	1
16	135	136	2
17	138	140	3

18	148	148	1
19	150	165	16
20	167	167	1
21	175	184	10
22	189	189	1
23	212	224	13
24	234	234	1
25	240	240	1
26	251	253	3
27	255	265	11
28	288	289	2
29	306	306	1
30	310	314	5
31	316	316	1
32	318	331	14
33	333	333	1
34	352	352	1
35	356	356	1
36	358	370	13
37	377	381	5
38	383	383	1
39	385	388	4
40	391	392	2
41	394	394	1
42	408	409	2
43	412	412	1
44	414	421	8
45	426	426	1
46	428	428	1
47	431	431	1
48	437	450	14
49	457	457	1
50	500	504	5

252 positions (cumulative volume <= 86 Angstrom^3)

	start	end	length
1	1	22	22
2	28	28	1
3	31	31	1
4	33	38	6
5	42	51	10
6	54	74	21
7	77	78	2
8	81	81	1
9	84	84	1
10	86	110	25
11	112	113	2
12	115	117	3
13	121	124	4
14	126	127	2
15	130	130	1

16	135	136	2
17	138	140	3
18	148	148	1
19	150	165	16
20	167	167	1
21	175	184	10
22	189	189	1
23	212	224	13
24	234	234	1
25	240	240	1
26	251	253	3
27	255	265	11
28	288	289	2
29	306	306	1
30	310	314	5
31	316	331	16
32	333	333	1
33	352	352	1
34	356	356	1
35	358	370	13
36	377	381	5
37	383	383	1
38	385	388	4
39	391	392	2
40	394	394	1
41	408	409	2
42	412	412	1
43	414	421	8
44	426	426	1
45	428	428	1
46	431	431	1
47	437	450	14
48	457	457	1
49	500	504	5

253 positions (cumulative volume <= 87 Angstrom^3)

	start	end	length
1	1	22	22
2	28	28	1
3	31	31	1
4	33	38	6
5	42	51	10
6	54	74	21
7	77	78	2
8	81	81	1
9	84	84	1
10	86	110	25
11	112	113	2
12	115	117	3
13	121	124	4
14	126	127	2

```
15   130 130      1
16   135 136      2
17   138 140      3
18   148 148      1
19   150 165     16
20   167 167      1
21   175 184     10
22   189 189      1
23   212 224     13
24   234 234      1
25   240 240      1
26   251 253      3
27   255 265     11
28   288 289      2
29   306 306      1
30   310 314      5
31   316 331     16
32   333 333      1
33   352 352      1
34   356 356      1
35   358 370     13
36   377 381      5
37   383 383      1
38   385 388      4
39   391 392      2
40   394 394      1
41   408 409      2
42   412 412      1
43   414 421      8
44   426 426      1
45   428 428      1
46   431 431      1
47   437 450     14
48   453 453      1
49   457 457      1
50   500 504      5
# 254 positions (cumulative volume <= 88 Angstrom^3)
   start end length
1      1  22     22
2     28  28      1
3     31  31      1
4     33  38      6
5     42  51     10
6     54  74     21
7     77  78      2
8     81  81      1
9     84  84      1
10    86 110     25
11   112 113      2
12   115 117      3
```

13	121	124	4
14	126	127	2
15	130	130	1
16	135	136	2
17	138	140	3
18	148	148	1
19	150	165	16
20	167	167	1
21	175	184	10
22	189	189	1
23	212	224	13
24	234	234	1
25	240	240	1
26	251	253	3
27	255	265	11
28	288	289	2
29	306	306	1
30	310	314	5
31	316	331	16
32	333	333	1
33	352	352	1
34	356	356	1
35	358	370	13
36	377	381	5
37	383	383	1
38	385	389	5
39	391	392	2
40	394	394	1
41	408	409	2
42	412	412	1
43	414	421	8
44	426	426	1
45	428	428	1
46	431	431	1
47	437	450	14
48	453	453	1
49	457	457	1
50	500	504	5

255 positions (cumulative volume <= 89 Angstrom^3)

	start	end	length
1	1	22	22
2	28	28	1
3	31	31	1
4	33	38	6
5	42	51	10
6	54	74	21
7	77	78	2
8	81	81	1
9	84	84	1
10	86	110	25

11	112	113	2
12	115	117	3
13	121	124	4
14	126	127	2
15	130	130	1
16	135	136	2
17	138	140	3
18	148	148	1
19	150	165	16
20	167	167	1
21	175	184	10
22	189	189	1
23	212	224	13
24	234	234	1
25	240	240	1
26	251	253	3
27	255	265	11
28	288	289	2
29	306	306	1
30	310	314	5
31	316	331	16
32	333	333	1
33	352	352	1
34	356	356	1
35	358	370	13
36	377	381	5
37	383	383	1
38	385	389	5
39	391	392	2
40	394	394	1
41	408	409	2
42	412	421	10
43	426	426	1
44	428	428	1
45	431	431	1
46	437	450	14
47	453	453	1
48	457	457	1
49	500	504	5

255 positions (cumulative volume <= 90 Angstrom^3)

	start	end	length
1	1	22	22
2	28	28	1
3	31	31	1
4	33	38	6
5	42	51	10
6	54	74	21
7	77	78	2
8	81	81	1
9	84	84	1

10	86	110	25
11	112	113	2
12	115	117	3
13	121	124	4
14	126	127	2
15	130	130	1
16	135	136	2
17	138	140	3
18	148	148	1
19	150	165	16
20	167	167	1
21	175	184	10
22	189	189	1
23	212	224	13
24	234	234	1
25	240	240	1
26	251	253	3
27	255	265	11
28	288	289	2
29	306	306	1
30	310	314	5
31	316	331	16
32	333	333	1
33	352	352	1
34	356	356	1
35	358	370	13
36	377	381	5
37	383	383	1
38	385	389	5
39	391	392	2
40	394	394	1
41	408	409	2
42	412	421	10
43	426	426	1
44	428	428	1
45	431	431	1
46	437	450	14
47	453	453	1
48	457	457	1
49	500	504	5

256 positions (cumulative volume <= 91 Angstrom^3)

	start	end	length
1	1	22	22
2	28	28	1
3	31	31	1
4	33	38	6
5	42	51	10
6	54	74	21
7	77	78	2
8	81	81	1

9	84	84	1
10	86	110	25
11	112	113	2
12	115	117	3
13	121	124	4
14	126	127	2
15	130	130	1
16	135	136	2
17	138	140	3
18	148	148	1
19	150	165	16
20	167	167	1
21	175	184	10
22	189	189	1
23	212	224	13
24	234	234	1
25	240	240	1
26	251	253	3
27	255	265	11
28	288	289	2
29	306	306	1
30	310	314	5
31	316	331	16
32	333	333	1
33	352	352	1
34	356	356	1
35	358	370	13
36	377	381	5
37	383	383	1
38	385	389	5
39	391	392	2
40	394	394	1
41	408	409	2
42	412	421	10
43	426	426	1
44	428	428	1
45	431	431	1
46	437	451	15
47	453	453	1
48	457	457	1
49	500	504	5

257 positions (cumulative volume <= 92 Angstrom^3)

	start	end	length
1	1	22	22
2	28	28	1
3	31	31	1
4	33	38	6
5	42	51	10
6	54	74	21
7	77	78	2

8	81	81	1
9	84	84	1
10	86	113	28
11	115	117	3
12	121	124	4
13	126	127	2
14	130	130	1
15	135	136	2
16	138	140	3
17	148	148	1
18	150	165	16
19	167	167	1
20	175	184	10
21	189	189	1
22	212	224	13
23	234	234	1
24	240	240	1
25	251	253	3
26	255	265	11
27	288	289	2
28	306	306	1
29	310	314	5
30	316	331	16
31	333	333	1
32	352	352	1
33	356	356	1
34	358	370	13
35	377	381	5
36	383	383	1
37	385	389	5
38	391	392	2
39	394	394	1
40	408	409	2
41	412	421	10
42	426	426	1
43	428	428	1
44	431	431	1
45	437	451	15
46	453	453	1
47	457	457	1
48	500	504	5

258 positions (cumulative volume <= 93 Angstrom^3)

	start	end	length
1	1	22	22
2	28	28	1
3	31	31	1
4	33	38	6
5	42	51	10
6	54	74	21
7	77	78	2

```
8      81  81       1
9      84  84       1
10     86 113      28
11    115 117       3
12    121 124       4
13    126 127       2
14    130 130       1
15    133 133       1
16    135 136       2
17    138 140       3
18    148 148       1
19    150 165      16
20    167 167       1
21    175 184      10
22    189 189       1
23    212 224      13
24    234 234       1
25    240 240       1
26    251 253       3
27    255 265      11
28    288 289       2
29    306 306       1
30    310 314       5
31    316 331      16
32    333 333       1
33    352 352       1
34    356 356       1
35    358 370      13
36    377 381       5
37    383 383       1
38    385 389       5
39    391 392       2
40    394 394       1
41    408 409       2
42    412 421      10
43    426 426       1
44    428 428       1
45    431 431       1
46    437 451      15
47    453 453       1
48    457 457       1
49    500 504       5
# 258 positions (cumulative volume <= 94 Angstrom^3)
   start end length
1      1  22      22
2     28  28       1
3     31  31       1
4     33  38       6
5     42  51      10
6     54  74      21
```

7	77	78	2
8	81	81	1
9	84	84	1
10	86	113	28
11	115	117	3
12	121	124	4
13	126	127	2
14	130	130	1
15	133	133	1
16	135	136	2
17	138	140	3
18	148	148	1
19	150	165	16
20	167	167	1
21	175	184	10
22	189	189	1
23	212	224	13
24	234	234	1
25	240	240	1
26	251	253	3
27	255	265	11
28	288	289	2
29	306	306	1
30	310	314	5
31	316	331	16
32	333	333	1
33	352	352	1
34	356	356	1
35	358	370	13
36	377	381	5
37	383	383	1
38	385	389	5
39	391	392	2
40	394	394	1
41	408	409	2
42	412	421	10
43	426	426	1
44	428	428	1
45	431	431	1
46	437	451	15
47	453	453	1
48	457	457	1
49	500	504	5

259 positions (cumulative volume <= 95 Angstrom^3)

	start	end	length
1	1	22	22
2	28	28	1
3	31	31	1
4	33	38	6
5	42	51	10

6	54	74	21
7	77	78	2
8	81	81	1
9	84	84	1
10	86	113	28
11	115	117	3
12	121	124	4
13	126	127	2
14	130	130	1
15	133	136	4
16	138	140	3
17	148	148	1
18	150	165	16
19	167	167	1
20	175	184	10
21	189	189	1
22	212	224	13
23	234	234	1
24	240	240	1
25	251	253	3
26	255	265	11
27	288	289	2
28	306	306	1
29	310	314	5
30	316	331	16
31	333	333	1
32	352	352	1
33	356	356	1
34	358	370	13
35	377	381	5
36	383	383	1
37	385	389	5
38	391	392	2
39	394	394	1
40	408	409	2
41	412	421	10
42	426	426	1
43	428	428	1
44	431	431	1
45	437	451	15
46	453	453	1
47	457	457	1
48	500	504	5

260 positions (cumulative volume <= 96 Angstrom^3)

	start	end	length
1	1	22	22
2	28	28	1
3	31	31	1
4	33	38	6
5	42	51	10

6	54	74	21
7	77	78	2
8	81	81	1
9	84	84	1
10	86	113	28
11	115	117	3
12	121	124	4
13	126	127	2
14	130	130	1
15	133	136	4
16	138	140	3
17	148	148	1
18	150	165	16
19	167	167	1
20	175	184	10
21	189	189	1
22	212	224	13
23	234	234	1
24	240	240	1
25	251	253	3
26	255	265	11
27	288	289	2
28	306	306	1
29	310	314	5
30	316	331	16
31	333	333	1
32	352	352	1
33	356	356	1
34	358	370	13
35	377	381	5
36	383	383	1
37	385	389	5
38	391	392	2
39	394	394	1
40	408	409	2
41	412	421	10
42	426	426	1
43	428	429	2
44	431	431	1
45	437	451	15
46	453	453	1
47	457	457	1
48	500	504	5

261 positions (cumulative volume <= 97 Angstrom^3)

	start	end	length
1	1	22	22
2	28	28	1
3	31	31	1
4	33	38	6
5	42	51	10

```
6      54  74      21
7      77  78       2
8      81  81       1
9      84  84       1
10     86 113      28
11    115 117       3
12    121 127       7
13    130 130       1
14    133 136       4
15    138 140       3
16    148 148       1
17    150 165      16
18    167 167       1
19    175 184      10
20    189 189       1
21    212 224      13
22    234 234       1
23    240 240       1
24    251 253       3
25    255 265      11
26    288 289       2
27    306 306       1
28    310 314       5
29    316 331      16
30    333 333       1
31    352 352       1
32    356 356       1
33    358 370      13
34    377 381       5
35    383 383       1
36    385 389       5
37    391 392       2
38    394 394       1
39    408 409       2
40    412 421      10
41    426 426       1
42    428 429       2
43    431 431       1
44    437 451      15
45    453 453       1
46    457 457       1
47    500 504       5
# 262 positions (cumulative volume <= 98 Angstrom^3)
   start end length
1       1  22      22
2      28  28       1
3      31  31       1
4      33  38       6
5      42  51      10
6      54  74      21
```

7	77	78	2
8	81	81	1
9	84	84	1
10	86	113	28
11	115	117	3
12	121	127	7
13	130	130	1
14	133	136	4
15	138	140	3
16	148	148	1
17	150	165	16
18	167	167	1
19	175	184	10
20	189	189	1
21	212	224	13
22	234	234	1
23	240	240	1
24	251	253	3
25	255	265	11
26	288	289	2
27	306	306	1
28	310	314	5
29	316	333	18
30	352	352	1
31	356	356	1
32	358	370	13
33	377	381	5
34	383	383	1
35	385	389	5
36	391	392	2
37	394	394	1
38	408	409	2
39	412	421	10
40	426	426	1
41	428	429	2
42	431	431	1
43	437	451	15
44	453	453	1
45	457	457	1
46	500	504	5

263 positions (cumulative volume <= 99 Angstrom^3)

	start	end	length
1	1	22	22
2	28	28	1
3	31	31	1
4	33	38	6
5	42	51	10
6	54	74	21
7	77	78	2
8	81	81	1

```
9     84  84       1
10    86 113      28
11   115 117       3
12   121 127       7
13   130 130       1
14   133 136       4
15   138 140       3
16   148 148       1
17   150 165      16
18   167 167       1
19   175 184      10
20   189 189       1
21   212 224      13
22   234 234       1
23   240 240       1
24   251 253       3
25   255 265      11
26   288 289       2
27   306 306       1
28   310 314       5
29   316 333      18
30   352 352       1
31   356 356       1
32   358 370      13
33   377 381       5
34   383 383       1
35   385 389       5
36   391 392       2
37   394 394       1
38   408 410       3
39   412 421      10
40   426 426       1
41   428 429       2
42   431 431       1
43   437 451      15
44   453 453       1
45   457 457       1
46   500 504       5
# 264 positions (cumulative volume <= 100 Angstrom^3)
   start end length
1      1  22      22
2     28  28       1
3     31  31       1
4     33  38       6
5     42  51      10
6     54  74      21
7     77  78       2
8     81  81       1
9     84  84       1
10    86 113      28
```

11	115	117	3
12	121	127	7
13	130	130	1
14	133	136	4
15	138	140	3
16	148	148	1
17	150	165	16
18	167	167	1
19	175	184	10
20	189	189	1
21	212	224	13
22	234	234	1
23	240	240	1
24	251	253	3
25	255	265	11
26	283	283	1
27	288	289	2
28	306	306	1
29	310	314	5
30	316	333	18
31	352	352	1
32	356	356	1
33	358	370	13
34	377	381	5
35	383	383	1
36	385	389	5
37	391	392	2
38	394	394	1
39	408	410	3
40	412	421	10
41	426	426	1
42	428	429	2
43	431	431	1
44	437	451	15
45	453	453	1
46	457	457	1
47	500	504	5

CORE DATA FOR CHARACTERIZED AND PUTATIVE CLASS C ENZYMES (n=96)

core size 362 of 363 vol = 3050.361
core size 361 of 363 vol = 2087.572
core size 360 of 363 vol = 1064.898
core size 359 of 363 vol = 139.944
core size 358 of 363 vol = 131.587
core size 357 of 363 vol = 124.582
core size 356 of 363 vol = 118.752
core size 355 of 363 vol = 113.162
core size 354 of 363 vol = 107.56

```
core size 353 of 363  vol = 102.196
core size 352 of 363  vol = 97.655
core size 351 of 363  vol = 92.825
core size 350 of 363  vol = 89.158
core size 349 of 363  vol = 85.604
core size 348 of 363  vol = 82.166
core size 347 of 363  vol = 78.74
core size 346 of 363  vol = 74.967
core size 345 of 363  vol = 71.143
core size 344 of 363  vol = 67.594
core size 343 of 363  vol = 64.59
core size 342 of 363  vol = 61.823
core size 341 of 363  vol = 59.387
core size 340 of 363  vol = 56.728
core size 339 of 363  vol = 54.176
core size 338 of 363  vol = 51.649
core size 337 of 363  vol = 49.366
core size 336 of 363  vol = 46.966
core size 335 of 363  vol = 44.816
core size 334 of 363  vol = 42.777
core size 333 of 363  vol = 41.035
core size 332 of 363  vol = 39.574
core size 331 of 363  vol = 38.153
core size 330 of 363  vol = 36.722
core size 329 of 363  vol = 35.414
core size 328 of 363  vol = 34.218
core size 327 of 363  vol = 33.367
core size 326 of 363  vol = 32.483
core size 325 of 363  vol = 31.622
core size 324 of 363  vol = 30.833
core size 323 of 363  vol = 30.042
core size 322 of 363  vol = 29.314
core size 321 of 363  vol = 28.688
core size 320 of 363  vol = 28.096
core size 319 of 363  vol = 27.596
core size 318 of 363  vol = 27.075
core size 317 of 363  vol = 26.622
core size 316 of 363  vol = 26.111
core size 315 of 363  vol = 25.565
core size 314 of 363  vol = 25.056
core size 313 of 363  vol = 24.594
core size 312 of 363  vol = 24.163
core size 311 of 363  vol = 23.716
core size 310 of 363  vol = 23.344
core size 309 of 363  vol = 22.984
core size 308 of 363  vol = 22.675
core size 307 of 363  vol = 22.356
core size 306 of 363  vol = 22.003
core size 305 of 363  vol = 21.666
core size 304 of 363  vol = 21.296
```

```
core size 303 of 363  vol = 20.932
core size 302 of 363  vol = 20.596
core size 301 of 363  vol = 20.27
core size 300 of 363  vol = 19.925
core size 299 of 363  vol = 19.629
core size 298 of 363  vol = 19.326
core size 297 of 363  vol = 19.007
core size 296 of 363  vol = 18.689
core size 295 of 363  vol = 18.368
core size 294 of 363  vol = 18.05
core size 293 of 363  vol = 17.733
core size 292 of 363  vol = 17.442
core size 291 of 363  vol = 17.158
core size 290 of 363  vol = 16.905
core size 289 of 363  vol = 16.618
core size 288 of 363  vol = 16.365
core size 287 of 363  vol = 16.127
core size 286 of 363  vol = 15.877
core size 285 of 363  vol = 15.638
core size 284 of 363  vol = 15.384
core size 283 of 363  vol = 15.188
core size 282 of 363  vol = 14.974
core size 281 of 363  vol = 14.754
core size 280 of 363  vol = 14.523
core size 279 of 363  vol = 14.307
core size 278 of 363  vol = 14.065
core size 277 of 363  vol = 13.832
core size 276 of 363  vol = 13.588
core size 275 of 363  vol = 13.329
core size 274 of 363  vol = 13.098
core size 273 of 363  vol = 12.878
core size 272 of 363  vol = 12.666
core size 271 of 363  vol = 12.447
core size 270 of 363  vol = 12.274
core size 269 of 363  vol = 12.091
core size 268 of 363  vol = 11.872
core size 267 of 363  vol = 11.694
core size 266 of 363  vol = 11.506
core size 265 of 363  vol = 11.309
core size 264 of 363  vol = 11.095
core size 263 of 363  vol = 10.916
core size 262 of 363  vol = 10.754
core size 261 of 363  vol = 10.591
core size 260 of 363  vol = 10.44
core size 259 of 363  vol = 10.273
core size 258 of 363  vol = 10.123
core size 257 of 363  vol = 9.969
core size 256 of 363  vol = 9.836
core size 255 of 363  vol = 9.691
core size 254 of 363  vol = 9.56
```

```
core size 253 of 363  vol = 9.428
core size 252 of 363  vol = 9.32
core size 251 of 363  vol = 9.192
core size 250 of 363  vol = 9.064
core size 249 of 363  vol = 8.918
core size 248 of 363  vol = 8.797
core size 247 of 363  vol = 8.661
core size 246 of 363  vol = 8.521
core size 245 of 363  vol = 8.402
core size 244 of 363  vol = 8.289
core size 243 of 363  vol = 8.16
core size 242 of 363  vol = 8.03
core size 241 of 363  vol = 7.919
core size 240 of 363  vol = 7.82
core size 239 of 363  vol = 7.693
core size 238 of 363  vol = 7.592
core size 237 of 363  vol = 7.478
core size 236 of 363  vol = 7.391
core size 235 of 363  vol = 7.254
core size 234 of 363  vol = 7.15
core size 233 of 363  vol = 7.052
core size 232 of 363  vol = 6.935
core size 231 of 363  vol = 6.845
core size 230 of 363  vol = 6.751
core size 229 of 363  vol = 6.648
core size 228 of 363  vol = 6.554
core size 227 of 363  vol = 6.494
core size 226 of 363  vol = 6.413
core size 225 of 363  vol = 6.329
core size 224 of 363  vol = 6.255
core size 223 of 363  vol = 6.167
core size 222 of 363  vol = 6.096
core size 221 of 363  vol = 6.03
core size 220 of 363  vol = 5.954
core size 219 of 363  vol = 5.868
core size 218 of 363  vol = 5.789
core size 217 of 363  vol = 5.726
core size 216 of 363  vol = 5.65
core size 215 of 363  vol = 5.583
core size 214 of 363  vol = 5.522
core size 213 of 363  vol = 5.48
core size 212 of 363  vol = 5.404
core size 211 of 363  vol = 5.356
core size 210 of 363  vol = 5.28
core size 209 of 363  vol = 5.197
core size 208 of 363  vol = 5.131
core size 207 of 363  vol = 5.057
core size 206 of 363  vol = 4.97
core size 205 of 363  vol = 4.908
core size 204 of 363  vol = 4.837
```

core size 203 of 363 vol = 4.773
core size 202 of 363 vol = 4.709
core size 201 of 363 vol = 4.64
core size 200 of 363 vol = 4.579
core size 199 of 363 vol = 4.53
core size 198 of 363 vol = 4.483
core size 197 of 363 vol = 4.454
core size 196 of 363 vol = 4.398
core size 195 of 363 vol = 4.353
core size 194 of 363 vol = 4.307
core size 193 of 363 vol = 4.261
core size 192 of 363 vol = 4.215
core size 191 of 363 vol = 4.156
core size 190 of 363 vol = 4.099
core size 189 of 363 vol = 4.05
core size 188 of 363 vol = 3.993
core size 187 of 363 vol = 3.937
core size 186 of 363 vol = 3.896
core size 185 of 363 vol = 3.852
core size 184 of 363 vol = 3.793
core size 183 of 363 vol = 3.734
core size 182 of 363 vol = 3.684
core size 181 of 363 vol = 3.653
core size 180 of 363 vol = 3.603
core size 179 of 363 vol = 3.559
core size 178 of 363 vol = 3.525
core size 177 of 363 vol = 3.494
core size 176 of 363 vol = 3.461
core size 175 of 363 vol = 3.427
core size 174 of 363 vol = 3.394
core size 173 of 363 vol = 3.351
core size 172 of 363 vol = 3.319
core size 171 of 363 vol = 3.296
core size 170 of 363 vol = 3.268
core size 169 of 363 vol = 3.233
core size 168 of 363 vol = 3.2
core size 167 of 363 vol = 3.164
core size 166 of 363 vol = 3.125
core size 165 of 363 vol = 3.092
core size 164 of 363 vol = 3.058
core size 163 of 363 vol = 3.031
core size 162 of 363 vol = 2.979
core size 161 of 363 vol = 2.928
core size 160 of 363 vol = 2.894
core size 159 of 363 vol = 2.86
core size 158 of 363 vol = 2.817
core size 157 of 363 vol = 2.783
core size 156 of 363 vol = 2.745
core size 155 of 363 vol = 2.704
core size 154 of 363 vol = 2.665

core size 153 of 363 vol = 2.639
core size 152 of 363 vol = 2.602
core size 151 of 363 vol = 2.565
core size 150 of 363 vol = 2.524
core size 149 of 363 vol = 2.482
core size 148 of 363 vol = 2.442
core size 147 of 363 vol = 2.399
core size 146 of 363 vol = 2.365
core size 145 of 363 vol = 2.346
core size 144 of 363 vol = 2.315
core size 143 of 363 vol = 2.274
core size 142 of 363 vol = 2.246
core size 141 of 363 vol = 2.214
core size 140 of 363 vol = 2.181
core size 139 of 363 vol = 2.145
core size 138 of 363 vol = 2.112
core size 137 of 363 vol = 2.079
core size 136 of 363 vol = 2.063
core size 135 of 363 vol = 2.043
core size 134 of 363 vol = 2.006
core size 133 of 363 vol = 1.96
core size 132 of 363 vol = 1.936
core size 131 of 363 vol = 1.909
core size 130 of 363 vol = 1.882
core size 129 of 363 vol = 1.855
core size 128 of 363 vol = 1.827
core size 127 of 363 vol = 1.797
core size 126 of 363 vol = 1.766
core size 125 of 363 vol = 1.74
core size 124 of 363 vol = 1.717
core size 123 of 363 vol = 1.694
core size 122 of 363 vol = 1.665
core size 121 of 363 vol = 1.642
core size 120 of 363 vol = 1.612
core size 119 of 363 vol = 1.594
core size 118 of 363 vol = 1.575
core size 117 of 363 vol = 1.547
core size 116 of 363 vol = 1.515
core size 115 of 363 vol = 1.483
core size 114 of 363 vol = 1.46
core size 113 of 363 vol = 1.445
core size 112 of 363 vol = 1.427
core size 111 of 363 vol = 1.411
core size 110 of 363 vol = 1.387
core size 109 of 363 vol = 1.363
core size 108 of 363 vol = 1.342
core size 107 of 363 vol = 1.319
core size 106 of 363 vol = 1.296
core size 105 of 363 vol = 1.272
core size 104 of 363 vol = 1.255

```
 core size 103 of 363  vol = 1.232
 core size 102 of 363  vol = 1.208
 core size 101 of 363  vol = 1.18
 core size 100 of 363  vol = 1.162
 core size 99 of 363  vol = 1.136
 core size 98 of 363  vol = 1.102
 core size 97 of 363  vol = 1.08
 core size 96 of 363  vol = 1.059
 core size 95 of 363  vol = 1.038
 core size 94 of 363  vol = 1.019
 core size 93 of 363  vol = 0.999
 core size 92 of 363  vol = 0.976
 core size 91 of 363  vol = 0.957
 core size 90 of 363  vol = 0.938
 core size 89 of 363  vol = 0.92
 core size 88 of 363  vol = 0.9
 core size 87 of 363  vol = 0.873
 core size 86 of 363  vol = 0.853
 core size 85 of 363  vol = 0.836
 core size 84 of 363  vol = 0.822
 core size 83 of 363  vol = 0.808
 core size 82 of 363  vol = 0.802
 core size 81 of 363  vol = 0.788
 core size 80 of 363  vol = 0.772
 core size 79 of 363  vol = 0.753
 core size 78 of 363  vol = 0.732
 core size 77 of 363  vol = 0.72
 core size 76 of 363  vol = 0.698
 core size 75 of 363  vol = 0.683
 core size 74 of 363  vol = 0.674
 core size 73 of 363  vol = 0.653
 core size 72 of 363  vol = 0.634
 core size 71 of 363  vol = 0.615
 core size 70 of 363  vol = 0.599
 core size 69 of 363  vol = 0.587
 core size 68 of 363  vol = 0.575
 core size 67 of 363  vol = 0.558
 core size 66 of 363  vol = 0.542
 core size 65 of 363  vol = 0.522
 core size 64 of 363  vol = 0.501
 core size 63 of 363  vol = 0.481
 FINISHED: Min vol ( 0.5 ) reached

# 94 positions (cumulative volume <= 1 Angstrom^3)
   start end length
1      3   3      1
2      9   9      1
3     13  14      2
4     17  19      3
5     46  51      6
```

6	54	72	19
7	78	78	1
8	89	89	1
9	91	91	1
10	93	93	1
11	95	96	2
12	98	98	1
13	100	104	5
14	112	113	2
15	115	115	1
16	134	136	3
17	140	140	1
18	152	162	11
19	165	165	1
20	216	216	1
21	252	252	1
22	255	256	2
23	259	259	1
24	263	263	1
25	322	323	2
26	363	368	6
27	377	378	2
28	386	388	3
29	414	416	3
30	439	439	1
31	441	447	7
32	449	449	1

134 positions (cumulative volume <= 2 Angstrom^3)

	start	end	length
1	3	6	4
2	8	10	3
3	13	19	7
4	31	31	1
5	38	38	1
6	46	52	7
7	54	74	21
8	78	78	1
9	89	89	1
10	91	98	8
11	100	104	5
12	110	113	4
13	115	117	3
14	121	121	1
15	123	124	2
16	134	137	4
17	140	140	1
18	150	150	1
19	152	162	11
20	164	165	2
21	216	216	1

22	252	252	1
23	255	256	2
24	259	259	1
25	263	264	2
26	320	323	4
27	363	369	7
28	375	381	7
29	386	388	3
30	390	391	2
31	414	416	3
32	437	449	13

163 positions (cumulative volume <= 3 Angstrom^3)

	start	end	length
1	3	6	4
2	8	20	13
3	31	31	1
4	33	33	1
5	36	36	1
6	38	38	1
7	46	74	29
8	78	78	1
9	87	87	1
10	89	98	10
11	100	105	6
12	110	113	4
13	115	117	3
14	120	124	5
15	126	126	1
16	130	130	1
17	133	141	9
18	150	150	1
19	152	162	11
20	164	165	2
21	215	216	2
22	252	253	2
23	255	256	2
24	259	260	2
25	262	265	4
26	320	323	4
27	325	325	1
28	361	361	1
29	363	369	7
30	375	381	7
31	383	383	1
32	385	391	7
33	414	416	3
34	428	428	1
35	436	449	14

189 positions (cumulative volume <= 4 Angstrom^3)

start end length

```
1      3   6      4
2      8  20     13
3     30  31      2
4     33  34      2
5     36  38      3
6     40  40      1
7     46  74     29
8     78  78      1
9     87  87      1
10    89 105     17
11   110 113      4
12   115 117      3
13   120 130     11
14   133 141      9
15   150 165     16
16   176 176      1
17   180 180      1
18   214 216      3
19   225 225      1
20   251 253      3
21   255 256      2
22   259 260      2
23   262 265      4
24   317 325      9
25   361 369      9
26   375 383      9
27   385 392      8
28   410 410      1
29   413 416      4
30   428 429      2
31   436 449     14
# 207 positions (cumulative volume <= 5 Angstrom^3)
   start end length
1      3  20     18
2     30  31      2
3     33  34      2
4     36  41      6
5     45  74     30
6     77  78      2
7     86  87      2
8     89 106     18
9    109 117      9
10   120 130     11
11   133 141      9
12   148 165     18
13   173 173      1
14   175 176      2
15   179 180      2
16   213 216      4
17   225 225      1
```

18	251	253	3
19	255	256	2
20	259	260	2
21	262	265	4
22	316	325	10
23	361	369	9
24	375	392	18
25	409	410	2
26	413	416	4
27	428	429	2
28	436	449	14

221 positions (cumulative volume <= 6 Angstrom^3)

	start	end	length
1	2	21	20
2	30	34	5
3	36	42	7
4	45	74	30
5	76	79	4
6	86	87	2
7	89	107	19
8	109	117	9
9	119	130	12
10	133	141	9
11	148	166	19
12	173	173	1
13	175	176	2
14	179	180	2
15	213	216	4
16	225	225	1
17	251	253	3
18	255	260	6
19	262	265	4
20	316	325	10
21	361	369	9
22	375	392	18
23	408	410	3
24	413	416	4
25	428	429	2
26	433	433	1
27	436	450	15

233 positions (cumulative volume <= 7 Angstrom^3)

	start	end	length
1	2	22	21
2	30	42	13
3	44	74	31
4	76	79	4
5	86	87	2
6	89	107	19
7	109	117	9
8	119	130	12

9	133	142	10
10	148	166	19
11	173	173	1
12	175	176	2
13	179	180	2
14	212	216	5
15	225	225	1
16	227	227	1
17	234	234	1
18	251	253	3
19	255	260	6
20	262	265	4
21	316	325	10
22	358	358	1
23	361	369	9
24	375	392	18
25	407	410	4
26	412	416	5
27	426	426	1
28	428	429	2
29	433	433	1
30	435	450	16

242 positions (cumulative volume <= 8 Angstrom^3)

	start	end	length
1	2	22	21
2	30	42	13
3	44	79	36
4	86	87	2
5	89	107	19
6	109	117	9
7	119	130	12
8	133	142	10
9	148	166	19
10	173	177	5
11	179	180	2
12	212	216	5
13	225	225	1
14	227	227	1
15	234	234	1
16	251	253	3
17	255	265	11
18	316	325	10
19	358	358	1
20	361	370	10
21	375	392	18
22	407	416	10
23	426	429	4
24	432	450	19

250 positions (cumulative volume <= 9 Angstrom^3)

	start	end	length

1	2	22	21
2	29	42	14
3	44	79	36
4	86	87	2
5	89	107	19
6	109	117	9
7	119	130	12
8	133	142	10
9	144	145	2
10	148	166	19
11	173	180	8
12	212	216	5
13	225	225	1
14	227	227	1
15	231	231	1
16	233	234	2
17	251	265	15
18	316	325	10
19	358	358	1
20	360	370	11
21	375	392	18
22	407	416	10
23	426	429	4
24	432	450	19

258 positions (cumulative volume <= 10 Angstrom^3)

	start	end	length
1	1	22	22
2	29	42	14
3	44	80	37
4	86	87	2
5	89	107	19
6	109	130	22
7	133	142	10
8	144	145	2
9	147	166	20
10	173	181	9
11	211	216	6
12	225	225	1
13	227	227	1
14	231	231	1
15	233	234	2
16	251	265	15
17	316	325	10
18	358	358	1
19	360	370	11
20	375	392	18
21	407	416	10
22	426	429	4
23	431	450	20
24	452	452	1

```
# 264 positions (cumulative volume <= 11 Angstrom^3)
   start end length
1      1  22     22
2     27  27      1
3     29  42     14
4     44  80     37
5     86  87      2
6     89 107     19
7    109 130     22
8    132 142     11
9    144 145      2
10   147 167     21
11   173 181      9
12   211 216      6
13   224 227      4
14   231 231      1
15   233 234      2
16   251 265     15
17   316 325     10
18   358 370     13
19   375 392     18
20   407 416     10
21   426 429      4
22   431 450     20
23   452 452      1
# 269 positions (cumulative volume <= 12 Angstrom^3)
   start end length
1      1  22     22
2     27  27      1
3     29  42     14
4     44  80     37
5     86  87      2
6     89 107     19
7    109 142     34
8    144 145      2
9    147 167     21
10   173 181      9
11   211 216      6
12   224 227      4
13   231 231      1
14   233 234      2
15   251 266     16
16   303 303      1
17   316 325     10
18   356 356      1
19   358 370     13
20   375 392     18
21   407 416     10
22   426 429      4
23   431 450     20
```

	start	end	length
24	452	453	2

274 positions (cumulative volume <= 13 Angstrom^3)

	start	end	length
1	1	22	22
2	27	27	1
3	29	80	52
4	84	84	1
5	86	87	2
6	89	107	19
7	109	142	34
8	144	145	2
9	147	167	21
10	173	181	9
11	211	216	6
12	224	227	4
13	230	231	2
14	233	234	2
15	251	266	16
16	303	303	1
17	305	305	1
18	316	325	10
19	355	356	2
20	358	370	13
21	375	392	18
22	407	416	10
23	426	429	4
24	431	450	20
25	452	453	2

278 positions (cumulative volume <= 14 Angstrom^3)

	start	end	length
1	1	22	22
2	27	27	1
3	29	80	52
4	84	84	1
5	86	87	2
6	89	142	54
7	144	145	2
8	147	167	21
9	172	181	10
10	211	216	6
11	224	227	4
12	230	231	2
13	233	234	2
14	251	266	16
15	268	268	1
16	303	303	1
17	305	305	1
18	316	325	10
19	355	356	2
20	358	370	13

21	375	392	18
22	407	416	10
23	424	424	1
24	426	429	4
25	431	450	20
26	452	453	2

283 positions (cumulative volume <= 15 Angstrom^3)

	start	end	length
1	1	23	23
2	27	27	1
3	29	80	52
4	84	84	1
5	86	87	2
6	89	142	54
7	144	167	24
8	172	181	10
9	211	216	6
10	224	227	4
11	230	234	5
12	251	266	16
13	268	268	1
14	303	303	1
15	305	305	1
16	316	325	10
17	355	356	2
18	358	370	13
19	375	392	18
20	407	416	10
21	424	424	1
22	426	453	28

287 positions (cumulative volume <= 16 Angstrom^3)

	start	end	length
1	1	23	23
2	27	80	54
3	84	84	1
4	86	87	2
5	89	142	54
6	144	167	24
7	172	181	10
8	210	216	7
9	224	227	4
10	230	234	5
11	251	266	16
12	268	268	1
13	303	303	1
14	305	305	1
15	316	325	10
16	354	356	3
17	358	370	13
18	375	392	18

```
19   406 416     11
20   424 424      1
21   426 453     28
# 291 positions (cumulative volume <= 17 Angstrom^3)
   start end length
1      1  23     23
2     27  81     55
3     84  84      1
4     86  87      2
5     89 142     54
6    144 168     25
7    172 181     10
8    183 183      1
9    210 216      7
10   224 227      4
11   230 234      5
12   251 266     16
13   268 268      1
14   303 303      1
15   305 305      1
16   316 325     10
17   354 370     17
18   375 392     18
19   406 416     11
20   424 424      1
21   426 453     28
# 294 positions (cumulative volume <= 18 Angstrom^3)
   start end length
1      1  23     23
2     27  81     55
3     84  87      4
4     89 142     54
5    144 168     25
6    172 181     10
7    183 183      1
8    210 216      7
9    224 227      4
10   230 234      5
11   251 266     16
12   268 268      1
13   302 303      2
14   305 305      1
15   316 325     10
16   353 370     18
17   375 392     18
18   406 416     11
19   424 424      1
20   426 453     28
# 297 positions (cumulative volume <= 19 Angstrom^3)
   start end length
```

```
1       1  23      23
2      27  82      56
3      84  87       4
4      89 142      54
5     144 168      25
6     172 181      10
7     183 183       1
8     210 216       7
9     224 227       4
10    230 234       5
11    251 266      16
12    268 268       1
13    302 303       2
14    305 306       2
15    316 325      10
16    353 371      19
17    375 392      18
18    406 416      11
19    424 424       1
20    426 453      28
# 301 positions (cumulative volume <= 20 Angstrom^3)
   start end length
1       1  23      23
2      27  87      61
3      89 142      54
4     144 168      25
5     171 181      11
6     183 183       1
7     210 216       7
8     224 228       5
9     230 234       5
10    251 266      16
11    268 268       1
12    302 303       2
13    305 306       2
14    316 325      10
15    353 371      19
16    375 392      18
17    406 416      11
18    424 424       1
19    426 454      29
# 304 positions (cumulative volume <= 21 Angstrom^3)
   start end length
1       1  23      23
2      27  87      61
3      89 142      54
4     144 168      25
5     171 181      11
6     183 183       1
7     210 216       7
```

```
8    224 234     11
9    251 266     16
10   268 268      1
11   302 306      5
12   316 325     10
13   353 371     19
14   375 392     18
15   406 416     11
16   424 454     31
# 306 positions (cumulative volume <= 22 Angstrom^3)
   start end length
1      1  23     23
2     27  87     61
3     89 142     54
4    144 168     25
5    171 181     11
6    183 183      1
7    210 216      7
8    224 234     11
9    251 269     19
10   302 306      5
11   316 325     10
12   353 371     19
13   375 392     18
14   406 416     11
15   424 454     31
# 310 positions (cumulative volume <= 23 Angstrom^3)
   start end length
1      1  23     23
2     27  87     61
3     89 142     54
4    144 168     25
5    171 181     11
6    183 185      3
7    210 216      7
8    224 234     11
9    236 237      2
10   251 269     19
11   302 306      5
12   316 325     10
13   353 371     19
14   375 392     18
15   406 416     11
16   424 454     31
# 312 positions (cumulative volume <= 24 Angstrom^3)
   start end length
1      1  23     23
2     27  87     61
3     89 168     80
4    171 181     11
```

```
5    183 185      3
6    210 216      7
7    224 234     11
8    236 237      2
9    239 239      1
10   251 269     19
11   302 306      5
12   316 325     10
13   353 371     19
14   375 392     18
15   406 416     11
16   424 454     31
# 314 positions (cumulative volume <= 25 Angstrom^3)
   start end length
1      1  23     23
2     27  87     61
3     89 169     81
4    171 181     11
5    183 185      3
6    210 216      7
7    224 237     14
8    239 239      1
9    251 269     19
10   302 306      5
11   316 325     10
12   353 371     19
13   375 392     18
14   406 416     11
15   424 454     31
# 316 positions (cumulative volume <= 26 Angstrom^3)
   start end length
1      1  23     23
2     26  87     62
3     89 169     81
4    171 185     15
5    210 216      7
6    224 237     14
7    239 239      1
8    251 269     19
9    302 306      5
10   316 325     10
11   353 371     19
12   375 392     18
13   406 416     11
14   424 454     31
# 318 positions (cumulative volume <= 27 Angstrom^3)
   start end length
1      1  87     87
2     89 169     81
3    171 185     15
```

```
4    210 216      7
5    224 237     14
6    239 239      1
7    251 269     19
8    302 306      5
9    316 325     10
10   353 371     19
11   375 392     18
12   406 416     11
13   424 454     31
# 320 positions (cumulative volume <= 28 Angstrom^3)
   start end length
1      1  87     87
2     89 169     81
3    171 185     15
4    210 216      7
5    224 237     14
6    239 239      1
7    251 269     19
8    302 307      6
9    316 325     10
10   353 372     20
11   375 392     18
12   406 416     11
13   424 454     31
# 322 positions (cumulative volume <= 29 Angstrom^3)
   start end length
1      1  87     87
2     89 185     97
3    209 216      8
4    224 237     14
5    239 239      1
6    251 269     19
7    302 307      6
8    316 325     10
9    353 372     20
10   375 392     18
11   406 416     11
12   424 454     31
# 323 positions (cumulative volume <= 30 Angstrom^3)
   start end length
1      1  87     87
2     89 185     97
3    209 216      8
4    224 237     14
5    239 239      1
6    251 269     19
7    302 307      6
8    314 314      1
9    316 325     10
```

```
10   353 372     20
11   375 392     18
12   406 416     11
13   424 454     31
# 325 positions (cumulative volume <= 31 Angstrom^3)
   start end length
1      1  87     87
2     89 185     97
3    209 216      8
4    224 239     16
5    243 243      1
6    251 269     19
7    302 307      6
8    314 314      1
9    316 325     10
10   353 372     20
11   375 392     18
12   406 416     11
13   424 454     31
# 326 positions (cumulative volume <= 32 Angstrom^3)
   start end length
1      1  87     87
2     89 185     97
3    208 216      9
4    224 239     16
5    243 243      1
6    251 269     19
7    302 307      6
8    314 314      1
9    316 325     10
10   353 372     20
11   375 392     18
12   406 416     11
13   424 454     31
# 327 positions (cumulative volume <= 33 Angstrom^3)
   start end length
1      1  87     87
2     89 185     97
3    208 216      9
4    224 239     16
5    243 243      1
6    250 269     20
7    302 307      6
8    314 314      1
9    316 325     10
10   353 372     20
11   375 392     18
12   406 416     11
13   424 454     31
# 328 positions (cumulative volume <= 34 Angstrom^3)
```

	start	end	length
1	1	87	87
2	89	185	97
3	208	216	9
4	224	239	16
5	243	243	1
6	250	269	20
7	302	307	6
8	314	325	12
9	353	372	20
10	375	392	18
11	406	416	11
12	424	454	31

329 positions (cumulative volume <= 35 Angstrom^3)

	start	end	length
1	1	87	87
2	89	185	97
3	208	216	9
4	224	239	16
5	243	244	2
6	250	269	20
7	302	307	6
8	314	325	12
9	353	372	20
10	375	392	18
11	406	416	11
12	424	454	31

330 positions (cumulative volume <= 36 Angstrom^3)

	start	end	length
1	1	87	87
2	89	185	97
3	208	216	9
4	224	239	16
5	243	244	2
6	250	269	20
7	301	307	7
8	314	325	12
9	353	372	20
10	375	392	18
11	406	416	11
12	424	454	31

331 positions (cumulative volume <= 37 Angstrom^3)

	start	end	length
1	1	87	87
2	89	185	97
3	208	216	9
4	224	240	17
5	243	244	2
6	250	269	20
7	301	307	7

```
8    314 325     12
9    353 372     20
10   375 392     18
11   406 416     11
12   424 454     31
# 331 positions (cumulative volume <= 38 Angstrom^3)
   start end length
1      1  87     87
2     89 185     97
3    208 216      9
4    224 240     17
5    243 244      2
6    250 269     20
7    301 307      7
8    314 325     12
9    353 372     20
10   375 392     18
11   406 416     11
12   424 454     31
# 332 positions (cumulative volume <= 39 Angstrom^3)
   start end length
1      1  87     87
2     89 185     97
3    208 216      9
4    224 241     18
5    243 244      2
6    250 269     20
7    301 307      7
8    314 325     12
9    353 372     20
10   375 392     18
11   406 416     11
12   424 454     31
# 333 positions (cumulative volume <= 40 Angstrom^3)
   start end length
1      1  87     87
2     89 185     97
3    208 216      9
4    224 244     21
5    250 269     20
6    301 307      7
7    314 325     12
8    353 372     20
9    375 392     18
10   406 416     11
11   424 454     31
# 333 positions (cumulative volume <= 41 Angstrom^3)
   start end length
1      1  87     87
2     89 185     97
```

3	208	216	9
4	224	244	21
5	250	269	20
6	301	307	7
7	314	325	12
8	353	372	20
9	375	392	18
10	406	416	11
11	424	454	31

334 positions (cumulative volume <= 42 Angstrom^3)

	start	end	length
1	1	87	87
2	89	185	97
3	203	203	1
4	208	216	9
5	224	244	21
6	250	269	20
7	301	307	7
8	314	325	12
9	353	372	20
10	375	392	18
11	406	416	11
12	424	454	31

335 positions (cumulative volume <= 43 Angstrom^3)

	start	end	length
1	1	87	87
2	89	185	97
3	203	203	1
4	208	216	9
5	224	244	21
6	250	270	21
7	301	307	7
8	314	325	12
9	353	372	20
10	375	392	18
11	406	416	11
12	424	454	31

335 positions (cumulative volume <= 44 Angstrom^3)

	start	end	length
1	1	87	87
2	89	185	97
3	203	203	1
4	208	216	9
5	224	244	21
6	250	270	21
7	301	307	7
8	314	325	12
9	353	372	20
10	375	392	18
11	406	416	11

```
12   424 454     31
# 336 positions (cumulative volume <= 45 Angstrom^3)
   start end length
1      1  87     87
2     89 185     97
3    203 203      1
4    208 216      9
5    224 244     21
6    250 270     21
7    301 307      7
8    314 325     12
9    353 372     20
10   375 392     18
11   406 416     11
12   423 454     32
# 336 positions (cumulative volume <= 46 Angstrom^3)
   start end length
1      1  87     87
2     89 185     97
3    203 203      1
4    208 216      9
5    224 244     21
6    250 270     21
7    301 307      7
8    314 325     12
9    353 372     20
10   375 392     18
11   406 416     11
12   423 454     32
# 337 positions (cumulative volume <= 47 Angstrom^3)
   start end length
1      1  87     87
2     89 185     97
3    203 203      1
4    208 216      9
5    224 244     21
6    250 270     21
7    283 283      1
8    301 307      7
9    314 325     12
10   353 372     20
11   375 392     18
12   406 416     11
13   423 454     32
# 337 positions (cumulative volume <= 48 Angstrom^3)
   start end length
1      1  87     87
2     89 185     97
3    203 203      1
4    208 216      9
```

```
5    224 244     21
6    250 270     21
7    283 283      1
8    301 307      7
9    314 325     12
10   353 372     20
11   375 392     18
12   406 416     11
13   423 454     32
# 337 positions (cumulative volume <= 49 Angstrom^3)
   start end length
1      1  87     87
2     89 185     97
3    203 203      1
4    208 216      9
5    224 244     21
6    250 270     21
7    283 283      1
8    301 307      7
9    314 325     12
10   353 372     20
11   375 392     18
12   406 416     11
13   423 454     32
# 338 positions (cumulative volume <= 50 Angstrom^3)
   start end length
1      1  87     87
2     89 185     97
3    202 203      2
4    208 216      9
5    224 244     21
6    250 270     21
7    283 283      1
8    301 307      7
9    314 325     12
10   353 372     20
11   375 392     18
12   406 416     11
13   423 454     32
# 338 positions (cumulative volume <= 51 Angstrom^3)
   start end length
1      1  87     87
2     89 185     97
3    202 203      2
4    208 216      9
5    224 244     21
6    250 270     21
7    283 283      1
8    301 307      7
9    314 325     12
```

```
10   353 372     20
11   375 392     18
12   406 416     11
13   423 454     32
# 339 positions (cumulative volume <= 52 Angstrom^3)
   start end length
1      1  87     87
2     89 185     97
3    202 203      2
4    208 216      9
5    224 245     22
6    250 270     21
7    283 283      1
8    301 307      7
9    314 325     12
10   353 372     20
11   375 392     18
12   406 416     11
13   423 454     32
# 339 positions (cumulative volume <= 53 Angstrom^3)
   start end length
1      1  87     87
2     89 185     97
3    202 203      2
4    208 216      9
5    224 245     22
6    250 270     21
7    283 283      1
8    301 307      7
9    314 325     12
10   353 372     20
11   375 392     18
12   406 416     11
13   423 454     32
# 339 positions (cumulative volume <= 54 Angstrom^3)
   start end length
1      1  87     87
2     89 185     97
3    202 203      2
4    208 216      9
5    224 245     22
6    250 270     21
7    283 283      1
8    301 307      7
9    314 325     12
10   353 372     20
11   375 392     18
12   406 416     11
13   423 454     32
# 340 positions (cumulative volume <= 55 Angstrom^3)
```

```
   start end length
1      1  87     87
2     89 185     97
3    202 203      2
4    208 216      9
5    224 245     22
6    250 270     21
7    283 284      2
8    301 307      7
9    314 325     12
10   353 372     20
11   375 392     18
12   406 416     11
13   423 454     32
# 340 positions (cumulative volume <= 56 Angstrom^3)
   start end length
1      1  87     87
2     89 185     97
3    202 203      2
4    208 216      9
5    224 245     22
6    250 270     21
7    283 284      2
8    301 307      7
9    314 325     12
10   353 372     20
11   375 392     18
12   406 416     11
13   423 454     32
# 341 positions (cumulative volume <= 57 Angstrom^3)
   start end length
1      1  87     87
2     89 185     97
3    202 203      2
4    208 216      9
5    224 245     22
6    249 270     22
7    283 284      2
8    301 307      7
9    314 325     12
10   353 372     20
11   375 392     18
12   406 416     11
13   423 454     32
# 341 positions (cumulative volume <= 58 Angstrom^3)
   start end length
1      1  87     87
2     89 185     97
3    202 203      2
4    208 216      9
```

```
5    224 245     22
6    249 270     22
7    283 284      2
8    301 307      7
9    314 325     12
10   353 372     20
11   375 392     18
12   406 416     11
13   423 454     32
# 341 positions (cumulative volume <= 59 Angstrom^3)
   start end length
1      1  87     87
2     89 185     97
3    202 203      2
4    208 216      9
5    224 245     22
6    249 270     22
7    283 284      2
8    301 307      7
9    314 325     12
10   353 372     20
11   375 392     18
12   406 416     11
13   423 454     32
# 342 positions (cumulative volume <= 60 Angstrom^3)
   start end length
1      1  87     87
2     89 185     97
3    202 203      2
4    208 216      9
5    224 245     22
6    249 270     22
7    282 284      3
8    301 307      7
9    314 325     12
10   353 372     20
11   375 392     18
12   406 416     11
13   423 454     32
# 342 positions (cumulative volume <= 61 Angstrom^3)
   start end length
1      1  87     87
2     89 185     97
3    202 203      2
4    208 216      9
5    224 245     22
6    249 270     22
7    282 284      3
8    301 307      7
9    314 325     12
```

```
10   353 372     20
11   375 392     18
12   406 416     11
13   423 454     32
# 343 positions (cumulative volume <= 62 Angstrom^3)
   start end length
1      1  87     87
2     89 185     97
3    202 203      2
4    208 216      9
5    224 246     23
6    249 270     22
7    282 284      3
8    301 307      7
9    314 325     12
10   353 372     20
11   375 392     18
12   406 416     11
13   423 454     32
# 343 positions (cumulative volume <= 63 Angstrom^3)
   start end length
1      1  87     87
2     89 185     97
3    202 203      2
4    208 216      9
5    224 246     23
6    249 270     22
7    282 284      3
8    301 307      7
9    314 325     12
10   353 372     20
11   375 392     18
12   406 416     11
13   423 454     32
# 343 positions (cumulative volume <= 64 Angstrom^3)
   start end length
1      1  87     87
2     89 185     97
3    202 203      2
4    208 216      9
5    224 246     23
6    249 270     22
7    282 284      3
8    301 307      7
9    314 325     12
10   353 372     20
11   375 392     18
12   406 416     11
13   423 454     32
# 344 positions (cumulative volume <= 65 Angstrom^3)
```

```
   start end length
1      1  87     87
2     89 185     97
3    202 203      2
4    206 206      1
5    208 216      9
6    224 246     23
7    249 270     22
8    282 284      3
9    301 307      7
10   314 325     12
11   353 372     20
12   375 392     18
13   406 416     11
14   423 454     32
# 344 positions (cumulative volume <= 66 Angstrom^3)
   start end length
1      1  87     87
2     89 185     97
3    202 203      2
4    206 206      1
5    208 216      9
6    224 246     23
7    249 270     22
8    282 284      3
9    301 307      7
10   314 325     12
11   353 372     20
12   375 392     18
13   406 416     11
14   423 454     32
# 344 positions (cumulative volume <= 67 Angstrom^3)
   start end length
1      1  87     87
2     89 185     97
3    202 203      2
4    206 206      1
5    208 216      9
6    224 246     23
7    249 270     22
8    282 284      3
9    301 307      7
10   314 325     12
11   353 372     20
12   375 392     18
13   406 416     11
14   423 454     32
# 345 positions (cumulative volume <= 68 Angstrom^3)
   start end length
1      1  87     87
```

```
2      89 185     97
3     197 197      1
4     202 203      2
5     206 206      1
6     208 216      9
7     224 246     23
8     249 270     22
9     282 284      3
10    301 307      7
11    314 325     12
12    353 372     20
13    375 392     18
14    406 416     11
15    423 454     32
# 345 positions (cumulative volume <= 69 Angstrom^3)
   start end length
1       1  87     87
2      89 185     97
3     197 197      1
4     202 203      2
5     206 206      1
6     208 216      9
7     224 246     23
8     249 270     22
9     282 284      3
10    301 307      7
11    314 325     12
12    353 372     20
13    375 392     18
14    406 416     11
15    423 454     32
# 345 positions (cumulative volume <= 70 Angstrom^3)
   start end length
1       1  87     87
2      89 185     97
3     197 197      1
4     202 203      2
5     206 206      1
6     208 216      9
7     224 246     23
8     249 270     22
9     282 284      3
10    301 307      7
11    314 325     12
12    353 372     20
13    375 392     18
14    406 416     11
15    423 454     32
# 345 positions (cumulative volume <= 71 Angstrom^3)
   start end length
```

```
1      1  87     87
2     89 185     97
3    197 197      1
4    202 203      2
5    206 206      1
6    208 216      9
7    224 246     23
8    249 270     22
9    282 284      3
10   301 307      7
11   314 325     12
12   353 372     20
13   375 392     18
14   406 416     11
15   423 454     32
# 346 positions (cumulative volume <= 72 Angstrom^3)
   start end length
1      1  87     87
2     89 185     97
3    197 197      1
4    202 203      2
5    206 206      1
6    208 216      9
7    224 246     23
8    249 270     22
9    282 284      3
10   287 287      1
11   301 307      7
12   314 325     12
13   353 372     20
14   375 392     18
15   406 416     11
16   423 454     32
# 346 positions (cumulative volume <= 73 Angstrom^3)
   start end length
1      1  87     87
2     89 185     97
3    197 197      1
4    202 203      2
5    206 206      1
6    208 216      9
7    224 246     23
8    249 270     22
9    282 284      3
10   287 287      1
11   301 307      7
12   314 325     12
13   353 372     20
14   375 392     18
15   406 416     11
```

```
16   423 454     32
# 346 positions (cumulative volume <= 74 Angstrom^3)
   start end length
1      1  87     87
2     89 185     97
3    197 197      1
4    202 203      2
5    206 206      1
6    208 216      9
7    224 246     23
8    249 270     22
9    282 284      3
10   287 287      1
11   301 307      7
12   314 325     12
13   353 372     20
14   375 392     18
15   406 416     11
16   423 454     32
# 347 positions (cumulative volume <= 75 Angstrom^3)
   start end length
1      1  87     87
2     89 185     97
3    197 197      1
4    202 203      2
5    206 206      1
6    208 216      9
7    224 246     23
8    249 270     22
9    282 284      3
10   286 287      2
11   301 307      7
12   314 325     12
13   353 372     20
14   375 392     18
15   406 416     11
16   423 454     32
# 347 positions (cumulative volume <= 76 Angstrom^3)
   start end length
1      1  87     87
2     89 185     97
3    197 197      1
4    202 203      2
5    206 206      1
6    208 216      9
7    224 246     23
8    249 270     22
9    282 284      3
10   286 287      2
11   301 307      7
```

```
12   314 325     12
13   353 372     20
14   375 392     18
15   406 416     11
16   423 454     32
# 347 positions (cumulative volume <= 77 Angstrom^3)
   start end length
1      1  87     87
2     89 185     97
3    197 197      1
4    202 203      2
5    206 206      1
6    208 216      9
7    224 246     23
8    249 270     22
9    282 284      3
10   286 287      2
11   301 307      7
12   314 325     12
13   353 372     20
14   375 392     18
15   406 416     11
16   423 454     32
# 347 positions (cumulative volume <= 78 Angstrom^3)
   start end length
1      1  87     87
2     89 185     97
3    197 197      1
4    202 203      2
5    206 206      1
6    208 216      9
7    224 246     23
8    249 270     22
9    282 284      3
10   286 287      2
11   301 307      7
12   314 325     12
13   353 372     20
14   375 392     18
15   406 416     11
16   423 454     32
# 348 positions (cumulative volume <= 79 Angstrom^3)
   start end length
1      1  87     87
2     89 185     97
3    197 197      1
4    202 203      2
5    206 206      1
6    208 216      9
7    224 246     23
```

```
8    249 270     22
9    282 287      6
10   301 307      7
11   314 325     12
12   353 372     20
13   375 392     18
14   406 416     11
15   423 454     32
# 348 positions (cumulative volume <= 80 Angstrom^3)
   start end length
1      1  87     87
2     89 185     97
3    197 197      1
4    202 203      2
5    206 206      1
6    208 216      9
7    224 246     23
8    249 270     22
9    282 287      6
10   301 307      7
11   314 325     12
12   353 372     20
13   375 392     18
14   406 416     11
15   423 454     32
# 348 positions (cumulative volume <= 81 Angstrom^3)
   start end length
1      1  87     87
2     89 185     97
3    197 197      1
4    202 203      2
5    206 206      1
6    208 216      9
7    224 246     23
8    249 270     22
9    282 287      6
10   301 307      7
11   314 325     12
12   353 372     20
13   375 392     18
14   406 416     11
15   423 454     32
# 348 positions (cumulative volume <= 82 Angstrom^3)
   start end length
1      1  87     87
2     89 185     97
3    197 197      1
4    202 203      2
5    206 206      1
6    208 216      9
```

```
7    224 246     23
8    249 270     22
9    282 287      6
10   301 307      7
11   314 325     12
12   353 372     20
13   375 392     18
14   406 416     11
15   423 454     32
# 349 positions (cumulative volume <= 83 Angstrom^3)
   start end length
1      1  87     87
2     89 185     97
3    197 197      1
4    202 203      2
5    205 206      2
6    208 216      9
7    224 246     23
8    249 270     22
9    282 287      6
10   301 307      7
11   314 325     12
12   353 372     20
13   375 392     18
14   406 416     11
15   423 454     32
# 349 positions (cumulative volume <= 84 Angstrom^3)
   start end length
1      1  87     87
2     89 185     97
3    197 197      1
4    202 203      2
5    205 206      2
6    208 216      9
7    224 246     23
8    249 270     22
9    282 287      6
10   301 307      7
11   314 325     12
12   353 372     20
13   375 392     18
14   406 416     11
15   423 454     32
# 349 positions (cumulative volume <= 85 Angstrom^3)
   start end length
1      1  87     87
2     89 185     97
3    197 197      1
4    202 203      2
5    205 206      2
```

```
6    208 216      9
7    224 246     23
8    249 270     22
9    282 287      6
10   301 307      7
11   314 325     12
12   353 372     20
13   375 392     18
14   406 416     11
15   423 454     32
# 350 positions (cumulative volume <= 86 Angstrom^3)
   start end length
1      1  87     87
2     89 185     97
3    197 197      1
4    202 206      5
5    208 216      9
6    224 246     23
7    249 270     22
8    282 287      6
9    301 307      7
10   314 325     12
11   353 372     20
12   375 392     18
13   406 416     11
14   423 454     32
# 350 positions (cumulative volume <= 87 Angstrom^3)
   start end length
1      1  87     87
2     89 185     97
3    197 197      1
4    202 206      5
5    208 216      9
6    224 246     23
7    249 270     22
8    282 287      6
9    301 307      7
10   314 325     12
11   353 372     20
12   375 392     18
13   406 416     11
14   423 454     32
# 350 positions (cumulative volume <= 88 Angstrom^3)
   start end length
1      1  87     87
2     89 185     97
3    197 197      1
4    202 206      5
5    208 216      9
6    224 246     23
```

```
7    249 270     22
8    282 287      6
9    301 307      7
10   314 325     12
11   353 372     20
12   375 392     18
13   406 416     11
14   423 454     32
# 350 positions (cumulative volume <= 89 Angstrom^3)
   start end length
1      1  87     87
2     89 185     97
3    197 197      1
4    202 206      5
5    208 216      9
6    224 246     23
7    249 270     22
8    282 287      6
9    301 307      7
10   314 325     12
11   353 372     20
12   375 392     18
13   406 416     11
14   423 454     32
# 351 positions (cumulative volume <= 90 Angstrom^3)
   start end length
1      1  87     87
2     89 185     97
3    197 197      1
4    202 206      5
5    208 216      9
6    224 246     23
7    249 270     22
8    282 287      6
9    300 307      8
10   314 325     12
11   353 372     20
12   375 392     18
13   406 416     11
14   423 454     32
# 351 positions (cumulative volume <= 91 Angstrom^3)
   start end length
1      1  87     87
2     89 185     97
3    197 197      1
4    202 206      5
5    208 216      9
6    224 246     23
7    249 270     22
8    282 287      6
```

```
9    300 307      8
10   314 325     12
11   353 372     20
12   375 392     18
13   406 416     11
14   423 454     32
# 351 positions (cumulative volume <= 92 Angstrom^3)
   start end length
1      1  87     87
2     89 185     97
3    197 197      1
4    202 206      5
5    208 216      9
6    224 246     23
7    249 270     22
8    282 287      6
9    300 307      8
10   314 325     12
11   353 372     20
12   375 392     18
13   406 416     11
14   423 454     32
# 352 positions (cumulative volume <= 93 Angstrom^3)
   start end length
1      1  87     87
2     89 185     97
3    197 198      2
4    202 206      5
5    208 216      9
6    224 246     23
7    249 270     22
8    282 287      6
9    300 307      8
10   314 325     12
11   353 372     20
12   375 392     18
13   406 416     11
14   423 454     32
# 352 positions (cumulative volume <= 94 Angstrom^3)
   start end length
1      1  87     87
2     89 185     97
3    197 198      2
4    202 206      5
5    208 216      9
6    224 246     23
7    249 270     22
8    282 287      6
9    300 307      8
10   314 325     12
```

```
11   353 372     20
12   375 392     18
13   406 416     11
14   423 454     32
# 352 positions (cumulative volume <= 95 Angstrom^3)
   start end length
1      1  87     87
2     89 185     97
3    197 198      2
4    202 206      5
5    208 216      9
6    224 246     23
7    249 270     22
8    282 287      6
9    300 307      8
10   314 325     12
11   353 372     20
12   375 392     18
13   406 416     11
14   423 454     32
# 352 positions (cumulative volume <= 96 Angstrom^3)
   start end length
1      1  87     87
2     89 185     97
3    197 198      2
4    202 206      5
5    208 216      9
6    224 246     23
7    249 270     22
8    282 287      6
9    300 307      8
10   314 325     12
11   353 372     20
12   375 392     18
13   406 416     11
14   423 454     32
# 352 positions (cumulative volume <= 97 Angstrom^3)
   start end length
1      1  87     87
2     89 185     97
3    197 198      2
4    202 206      5
5    208 216      9
6    224 246     23
7    249 270     22
8    282 287      6
9    300 307      8
10   314 325     12
11   353 372     20
12   375 392     18
```

```
13   406 416     11
14   423 454     32
# 353 positions (cumulative volume <= 98 Angstrom^3)
   start end length
1      1  87     87
2     89 185     97
3    197 198      2
4    202 206      5
5    208 216      9
6    224 246     23
7    249 270     22
8    281 287      7
9    300 307      8
10   314 325     12
11   353 372     20
12   375 392     18
13   406 416     11
14   423 454     32
# 353 positions (cumulative volume <= 99 Angstrom^3)
   start end length
1      1  87     87
2     89 185     97
3    197 198      2
4    202 206      5
5    208 216      9
6    224 246     23
7    249 270     22
8    281 287      7
9    300 307      8
10   314 325     12
11   353 372     20
12   375 392     18
13   406 416     11
14   423 454     32
# 353 positions (cumulative volume <= 100 Angstrom^3)
   start end length
1      1  87     87
2     89 185     97
3    197 198      2
4    202 206      5
5    208 216      9
6    224 246     23
7    249 270     22
8    281 287      7
9    300 307      8
10   314 325     12
11   353 372     20
12   375 392     18
13   406 416     11
14   423 454     32
```

CORE DATA FOR CHARACTERIZED AND PUTATIVE CLASS C ENZYMES (n=42)

```
core size 446 of 447  vol = 14432.33
core size 445 of 447  vol = 13781.53
core size 444 of 447  vol = 13158.23
core size 443 of 447  vol = 12531.02
core size 442 of 447  vol = 11917.36
core size 441 of 447  vol = 11312.84
core size 440 of 447  vol = 10709.75
core size 439 of 447  vol = 9952.836
core size 438 of 447  vol = 9213.775
core size 437 of 447  vol = 8643.713
core size 436 of 447  vol = 8206.064
core size 435 of 447  vol = 7687
core size 434 of 447  vol = 7293.285
core size 433 of 447  vol = 6870.334
core size 432 of 447  vol = 6416.77
core size 431 of 447  vol = 5987.272
core size 430 of 447  vol = 5505.611
core size 429 of 447  vol = 5093.883
core size 428 of 447  vol = 4631.803
core size 427 of 447  vol = 4288.073
core size 426 of 447  vol = 3939.761
core size 425 of 447  vol = 3589.911
core size 424 of 447  vol = 3188.872
core size 423 of 447  vol = 2776.266
core size 422 of 447  vol = 2426.599
core size 421 of 447  vol = 2057.42
core size 420 of 447  vol = 1761.787
core size 419 of 447  vol = 1448.129
core size 418 of 447  vol = 1135.486
core size 417 of 447  vol = 850.328
core size 416 of 447  vol = 593.62
core size 415 of 447  vol = 334.367
core size 414 of 447  vol = 136.174
core size 413 of 447  vol = 117.403
core size 412 of 447  vol = 107.879
core size 411 of 447  vol = 99.885
core size 410 of 447  vol = 93.696
core size 409 of 447  vol = 87.854
core size 408 of 447  vol = 82.253
core size 407 of 447  vol = 76.873
core size 406 of 447  vol = 72.026
core size 405 of 447  vol = 68.566
core size 404 of 447  vol = 64.275
core size 403 of 447  vol = 60.985
core size 402 of 447  vol = 56.944
```

core size 401 of 447 vol = 53.455
core size 400 of 447 vol = 50.434
core size 399 of 447 vol = 48.158
core size 398 of 447 vol = 45.909
core size 397 of 447 vol = 43.588
core size 396 of 447 vol = 40.916
core size 395 of 447 vol = 38.876
core size 394 of 447 vol = 37.046
core size 393 of 447 vol = 35.378
core size 392 of 447 vol = 33.917
core size 391 of 447 vol = 32.592
core size 390 of 447 vol = 31.478
core size 389 of 447 vol = 30.342
core size 388 of 447 vol = 29.241
core size 387 of 447 vol = 28.364
core size 386 of 447 vol = 27.505
core size 385 of 447 vol = 26.764
core size 384 of 447 vol = 26.08
core size 383 of 447 vol = 25.397
core size 382 of 447 vol = 24.82
core size 381 of 447 vol = 24.261
core size 380 of 447 vol = 23.714
core size 379 of 447 vol = 23.174
core size 378 of 447 vol = 22.631
core size 377 of 447 vol = 22.089
core size 376 of 447 vol = 21.671
core size 375 of 447 vol = 21.201
core size 374 of 447 vol = 20.745
core size 373 of 447 vol = 20.295
core size 372 of 447 vol = 19.893
core size 371 of 447 vol = 19.459
core size 370 of 447 vol = 19.014
core size 369 of 447 vol = 18.601
core size 368 of 447 vol = 18.218
core size 367 of 447 vol = 17.86
core size 366 of 447 vol = 17.474
core size 365 of 447 vol = 17.137
core size 364 of 447 vol = 16.78
core size 363 of 447 vol = 16.416
core size 362 of 447 vol = 16.097
core size 361 of 447 vol = 15.789
core size 360 of 447 vol = 15.515
core size 359 of 447 vol = 15.224
core size 358 of 447 vol = 14.915
core size 357 of 447 vol = 14.61
core size 356 of 447 vol = 14.328
core size 355 of 447 vol = 14.026
core size 354 of 447 vol = 13.769
core size 353 of 447 vol = 13.534
core size 352 of 447 vol = 13.287

core size 351 of 447 vol = 13.061
core size 350 of 447 vol = 12.869
core size 349 of 447 vol = 12.671
core size 348 of 447 vol = 12.472
core size 347 of 447 vol = 12.264
core size 346 of 447 vol = 12.044
core size 345 of 447 vol = 11.861
core size 344 of 447 vol = 11.685
core size 343 of 447 vol = 11.384
core size 342 of 447 vol = 11.159
core size 341 of 447 vol = 10.987
core size 340 of 447 vol = 10.824
core size 339 of 447 vol = 10.654
core size 338 of 447 vol = 10.511
core size 337 of 447 vol = 10.358
core size 336 of 447 vol = 10.042
core size 335 of 447 vol = 9.865
core size 334 of 447 vol = 9.728
core size 333 of 447 vol = 9.572
core size 332 of 447 vol = 9.438
core size 331 of 447 vol = 9.335
core size 330 of 447 vol = 9.22
core size 329 of 447 vol = 9.085
core size 328 of 447 vol = 8.949
core size 327 of 447 vol = 8.832
core size 326 of 447 vol = 8.714
core size 325 of 447 vol = 8.604
core size 324 of 447 vol = 8.488
core size 323 of 447 vol = 8.328
core size 322 of 447 vol = 8.222
core size 321 of 447 vol = 8.101
core size 320 of 447 vol = 8.014
core size 319 of 447 vol = 7.85
core size 318 of 447 vol = 7.722
core size 317 of 447 vol = 7.637
core size 316 of 447 vol = 7.561
core size 315 of 447 vol = 7.465
core size 314 of 447 vol = 7.269
core size 313 of 447 vol = 7.193
core size 312 of 447 vol = 7.084
core size 311 of 447 vol = 6.999
core size 310 of 447 vol = 6.893
core size 309 of 447 vol = 6.798
core size 308 of 447 vol = 6.703
core size 307 of 447 vol = 6.59
core size 306 of 447 vol = 6.479
core size 305 of 447 vol = 6.402
core size 304 of 447 vol = 6.325
core size 303 of 447 vol = 6.238
core size 302 of 447 vol = 6.128

```
core size 301 of 447  vol = 6.081
core size 300 of 447  vol = 5.994
core size 299 of 447  vol = 5.921
core size 298 of 447  vol = 5.839
core size 297 of 447  vol = 5.761
core size 296 of 447  vol = 5.657
core size 295 of 447  vol = 5.603
core size 294 of 447  vol = 5.54
core size 293 of 447  vol = 5.461
core size 292 of 447  vol = 5.372
core size 291 of 447  vol = 5.285
core size 290 of 447  vol = 5.202
core size 289 of 447  vol = 5.131
core size 288 of 447  vol = 5.056
core size 287 of 447  vol = 4.998
core size 286 of 447  vol = 4.923
core size 285 of 447  vol = 4.868
core size 284 of 447  vol = 4.798
core size 283 of 447  vol = 4.729
core size 282 of 447  vol = 4.673
core size 281 of 447  vol = 4.623
core size 280 of 447  vol = 4.548
core size 279 of 447  vol = 4.491
core size 278 of 447  vol = 4.424
core size 277 of 447  vol = 4.351
core size 276 of 447  vol = 4.282
core size 275 of 447  vol = 4.216
core size 274 of 447  vol = 4.14
core size 273 of 447  vol = 4.072
core size 272 of 447  vol = 4.013
core size 271 of 447  vol = 3.972
core size 270 of 447  vol = 3.934
core size 269 of 447  vol = 3.894
core size 268 of 447  vol = 3.832
core size 267 of 447  vol = 3.792
core size 266 of 447  vol = 3.746
core size 265 of 447  vol = 3.711
core size 264 of 447  vol = 3.653
core size 263 of 447  vol = 3.613
core size 262 of 447  vol = 3.578
core size 261 of 447  vol = 3.52
core size 260 of 447  vol = 3.461
core size 259 of 447  vol = 3.367
core size 258 of 447  vol = 3.264
core size 257 of 447  vol = 3.212
core size 256 of 447  vol = 3.156
core size 255 of 447  vol = 3.109
core size 254 of 447  vol = 3.082
core size 253 of 447  vol = 3.02
core size 252 of 447  vol = 2.972
```

```
core size 251 of 447  vol = 2.928
core size 250 of 447  vol = 2.892
core size 249 of 447  vol = 2.853
core size 248 of 447  vol = 2.797
core size 247 of 447  vol = 2.717
core size 246 of 447  vol = 2.68
core size 245 of 447  vol = 2.641
core size 244 of 447  vol = 2.588
core size 243 of 447  vol = 2.546
core size 242 of 447  vol = 2.512
core size 241 of 447  vol = 2.461
core size 240 of 447  vol = 2.431
core size 239 of 447  vol = 2.39
core size 238 of 447  vol = 2.349
core size 237 of 447  vol = 2.322
core size 236 of 447  vol = 2.296
core size 235 of 447  vol = 2.27
core size 234 of 447  vol = 2.238
core size 233 of 447  vol = 2.186
core size 232 of 447  vol = 2.131
core size 231 of 447  vol = 2.094
core size 230 of 447  vol = 2.065
core size 229 of 447  vol = 2.034
core size 228 of 447  vol = 2.016
core size 227 of 447  vol = 1.994
core size 226 of 447  vol = 1.948
core size 225 of 447  vol = 1.926
core size 224 of 447  vol = 1.891
core size 223 of 447  vol = 1.857
core size 222 of 447  vol = 1.842
core size 221 of 447  vol = 1.821
core size 220 of 447  vol = 1.794
core size 219 of 447  vol = 1.772
core size 218 of 447  vol = 1.739
core size 217 of 447  vol = 1.694
core size 216 of 447  vol = 1.664
core size 215 of 447  vol = 1.65
core size 214 of 447  vol = 1.621
core size 213 of 447  vol = 1.598
core size 212 of 447  vol = 1.583
core size 211 of 447  vol = 1.569
core size 210 of 447  vol = 1.538
core size 209 of 447  vol = 1.513
core size 208 of 447  vol = 1.489
core size 207 of 447  vol = 1.468
core size 206 of 447  vol = 1.465
core size 205 of 447  vol = 1.447
core size 204 of 447  vol = 1.429
core size 203 of 447  vol = 1.413
core size 202 of 447  vol = 1.401
```

core size 201 of 447 vol = 1.386
core size 200 of 447 vol = 1.359
core size 199 of 447 vol = 1.338
core size 198 of 447 vol = 1.31
core size 197 of 447 vol = 1.306
core size 196 of 447 vol = 1.285
core size 195 of 447 vol = 1.25
core size 194 of 447 vol = 1.227
core size 193 of 447 vol = 1.208
core size 192 of 447 vol = 1.193
core size 191 of 447 vol = 1.177
core size 190 of 447 vol = 1.15
core size 189 of 447 vol = 1.132
core size 188 of 447 vol = 1.115
core size 187 of 447 vol = 1.11
core size 186 of 447 vol = 1.099
core size 185 of 447 vol = 1.081
core size 184 of 447 vol = 1.072
core size 183 of 447 vol = 1.05
core size 182 of 447 vol = 1.036
core size 181 of 447 vol = 1.023
core size 180 of 447 vol = 1.003
core size 179 of 447 vol = 0.991
core size 178 of 447 vol = 0.995
core size 177 of 447 vol = 0.986
core size 176 of 447 vol = 0.983
core size 175 of 447 vol = 0.965
core size 174 of 447 vol = 0.955
core size 173 of 447 vol = 0.938
core size 172 of 447 vol = 0.915
core size 171 of 447 vol = 0.899
core size 170 of 447 vol = 0.886
core size 169 of 447 vol = 0.862
core size 168 of 447 vol = 0.843
core size 167 of 447 vol = 0.827
core size 166 of 447 vol = 0.816
core size 165 of 447 vol = 0.795
core size 164 of 447 vol = 0.78
core size 163 of 447 vol = 0.765
core size 162 of 447 vol = 0.758
core size 161 of 447 vol = 0.751
core size 160 of 447 vol = 0.738
core size 159 of 447 vol = 0.731
core size 158 of 447 vol = 0.722
core size 157 of 447 vol = 0.707
core size 156 of 447 vol = 0.696
core size 155 of 447 vol = 0.68
core size 154 of 447 vol = 0.671
core size 153 of 447 vol = 0.664
core size 152 of 447 vol = 0.65

```
 core size 151 of 447  vol = 0.627
 core size 150 of 447  vol = 0.618
 core size 149 of 447  vol = 0.608
 core size 148 of 447  vol = 0.595
 core size 147 of 447  vol = 0.583
 core size 146 of 447  vol = 0.578
 core size 145 of 447  vol = 0.57
 core size 144 of 447  vol = 0.561
 core size 143 of 447  vol = 0.558
 core size 142 of 447  vol = 0.554
 core size 141 of 447  vol = 0.537
 core size 140 of 447  vol = 0.525
 core size 139 of 447  vol = 0.517
 core size 138 of 447  vol = 0.506
 core size 137 of 447  vol = 0.499
 FINISHED: Min vol ( 0.5 ) reached

# 180 positions (cumulative volume <= 1 Angstrom^3)
   start end length
1      1  13     13
2     17  17      1
3     19  20      2
4     22  22      1
5     29  29      1
6     31  31      1
7     38  38      1
8     47  47      1
9     49  50      2
10    54  66     13
11    68  69      2
12    72  72      1
13    75  78      4
14    86  87      2
15    95  98      4
16   100 105      6
17   109 113      5
18   115 117      3
19   121 127      7
20   129 130      2
21   133 140      8
22   148 166     19
23   214 225     12
24   232 232      1
25   239 239      1
26   251 252      2
27   259 265      7
28   267 267      1
29   311 311      1
30   318 333     16
31   363 370      8
```

```
32   375 375      1
33   377 379      3
34   382 386      5
35   388 388      1
36   390 391      2
37   393 393      1
38   414 419      6
39   437 437      1
40   439 439      1
41   441 441      1
42   443 451      9
43   453 453      1
# 228 positions (cumulative volume <= 2 Angstrom^3)
   start end length
1      1  20     20
2     22  22      1
3     29  29      1
4     31  32      2
5     38  38      1
6     46  50      5
7     54  73     20
8     75  78      4
9     84  84      1
10    86  87      2
11    89  89      1
12    92  98      7
13   100 105      6
14   107 107      1
15   109 117      9
16   121 127      7
17   129 130      2
18   133 140      8
19   146 146      1
20   148 166     19
21   173 174      2
22   176 176      1
23   180 180      1
24   186 186      1
25   189 189      1
26   191 191      1
27   212 212      1
28   214 225     12
29   232 232      1
30   234 234      1
31   237 237      1
32   239 239      1
33   251 252      2
34   256 256      1
35   259 267      9
36   311 311      1
```

```
37   318 334      17
38   357 357       1
39   363 370       8
40   375 379       5
41   382 386       5
42   388 393       6
43   411 411       1
44   414 419       6
45   428 429       2
46   431 432       2
47   434 453      20
# 253 positions (cumulative volume <= 3 Angstrom^3)
   start end length
1      1  22      22
2     29  29       1
3     31  33       3
4     38  38       1
5     46  51       6
6     53  78      26
7     84  84       1
8     86  87       2
9     89  89       1
10    91 105      15
11   107 107       1
12   109 117       9
13   120 130      11
14   133 141       9
15   146 166      21
16   172 174       3
17   176 176       1
18   180 180       1
19   184 184       1
20   186 186       1
21   189 189       1
22   191 191       1
23   211 225      15
24   232 232       1
25   234 234       1
26   237 237       1
27   239 239       1
28   251 252       2
29   256 267      12
30   310 312       3
31   317 334      18
32   356 357       2
33   363 370       8
34   375 379       5
35   382 394      13
36   411 411       1
37   414 420       7
```

```
38   424 424      1
39   428 429      2
40   431 432      2
41   434 453     20
# 272 positions (cumulative volume <= 4 Angstrom^3)
   start end length
1      1  22     22
2     29  29      1
3     31  33      3
4     36  36      1
5     38  39      2
6     46  78     33
7     84  87      4
8     89 107     19
9    109 117      9
10   120 130     11
11   133 141      9
12   145 166     22
13   172 176      5
14   180 180      1
15   183 184      2
16   186 186      1
17   189 191      3
18   211 225     15
19   227 227      1
20   232 232      1
21   234 234      1
22   237 237      1
23   239 239      1
24   250 253      4
25   256 267     12
26   310 313      4
27   315 315      1
28   317 334     18
29   356 358      3
30   361 361      1
31   363 370      8
32   375 379      5
33   382 394     13
34   408 408      1
35   410 411      2
36   414 420      7
37   424 424      1
38   428 429      2
39   431 432      2
40   434 453     20
# 288 positions (cumulative volume <= 5 Angstrom^3)
   start end length
1      1  22     22
2     27  27      1
```

3	29	29	1
4	31	33	3
5	36	39	4
6	42	42	1
7	46	78	33
8	83	87	5
9	89	107	19
10	109	117	9
11	120	130	11
12	132	142	11
13	145	166	22
14	172	176	5
15	180	180	1
16	183	184	2
17	186	186	1
18	189	191	3
19	211	225	15
20	227	227	1
21	232	232	1
22	234	234	1
23	237	237	1
24	239	239	1
25	250	253	4
26	255	267	13
27	310	313	4
28	315	315	1
29	317	334	18
30	356	358	3
31	361	361	1
32	363	370	8
33	375	395	21
34	402	402	1
35	405	405	1
36	408	408	1
37	410	412	3
38	414	421	8
39	424	424	1
40	426	429	4
41	431	432	2
42	434	453	20

301 positions (cumulative volume <= 6 Angstrom^3)

	start	end	length
1	1	22	22
2	27	27	1
3	29	34	6
4	36	40	5
5	42	42	1
6	45	78	34
7	83	87	5
8	89	107	19

9	109	117	9
10	120	130	11
11	132	142	11
12	144	166	23
13	172	177	6
14	179	180	2
15	183	186	4
16	189	191	3
17	211	225	15
18	227	227	1
19	232	234	3
20	237	237	1
21	239	239	1
22	250	253	4
23	255	267	13
24	310	313	4
25	315	315	1
26	317	335	19
27	356	358	3
28	361	361	1
29	363	371	9
30	375	395	21
31	402	402	1
32	405	405	1
33	407	408	2
34	410	412	3
35	414	422	9
36	424	424	1
37	426	429	4
38	431	432	2
39	434	453	20

312 positions (cumulative volume <= 7 Angstrom^3)

	start	end	length
1	1	22	22
2	27	34	8
3	36	42	7
4	45	79	35
5	83	87	5
6	89	107	19
7	109	118	10
8	120	130	11
9	132	142	11
10	144	166	23
11	172	177	6
12	179	180	2
13	182	186	5
14	189	191	3
15	211	225	15
16	227	227	1
17	232	234	3

18	237	237	1
19	239	239	1
20	250	253	4
21	255	267	13
22	302	303	2
23	310	313	4
24	315	335	21
25	356	359	4
26	361	361	1
27	363	371	9
28	375	395	21
29	402	402	1
30	405	405	1
31	407	408	2
32	410	424	15
33	426	429	4
34	431	432	2
35	434	453	20

320 positions (cumulative volume <= 8 Angstrom^3)

	start	end	length
1	1	23	23
2	27	34	8
3	36	42	7
4	45	79	35
5	83	87	5
6	89	107	19
7	109	118	10
8	120	130	11
9	132	142	11
10	144	167	24
11	172	177	6
12	179	186	8
13	189	191	3
14	211	225	15
15	227	227	1
16	231	234	4
17	237	237	1
18	239	239	1
19	250	253	4
20	255	268	14
21	302	303	2
22	310	313	4
23	315	335	21
24	338	338	1
25	355	359	5
26	361	361	1
27	363	371	9
28	375	395	21
29	402	402	1
30	405	405	1

```
31   407 408      2
32   410 424     15
33   426 429      4
34   431 453     23
# 329 positions (cumulative volume <= 9 Angstrom^3)
   start end length
1      1  23     23
2     27  42     16
3     44  79     36
4     83  87      5
5     89 107     19
6    109 130     22
7    132 142     11
8    144 167     24
9    172 191     20
10   211 225     15
11   227 227      1
12   231 234      4
13   237 237      1
14   239 239      1
15   250 253      4
16   255 268     14
17   302 303      2
18   305 305      1
19   310 313      4
20   315 335     21
21   338 338      1
22   355 359      5
23   361 371     11
24   375 395     21
25   402 402      1
26   405 405      1
27   407 424     18
28   426 429      4
29   431 453     23
# 336 positions (cumulative volume <= 10 Angstrom^3)
   start end length
1      1  23     23
2     27  42     16
3     44  80     37
4     83  87      5
5     89 107     19
6    109 167     59
7    172 191     20
8    211 225     15
9    227 227      1
10   231 234      4
11   237 237      1
12   239 240      2
13   250 268     19
```

14	302	303	2
15	305	305	1
16	310	313	4
17	315	335	21
18	338	338	1
19	355	371	17
20	375	395	21
21	402	402	1
22	405	424	20
23	426	429	4
24	431	453	23

342 positions (cumulative volume <= 11 Angstrom^3)

	start	end	length
1	1	23	23
2	26	42	17
3	44	80	37
4	83	87	5
5	89	167	79
6	172	191	20
7	211	225	15
8	227	227	1
9	231	234	4
10	237	237	1
11	239	240	2
12	250	268	19
13	302	303	2
14	305	305	1
15	309	313	5
16	315	336	22
17	338	338	1
18	354	371	18
19	375	395	21
20	402	402	1
21	405	424	20
22	426	453	28

346 positions (cumulative volume <= 12 Angstrom^3)

	start	end	length
1	1	23	23
2	26	42	17
3	44	80	37
4	83	87	5
5	89	167	79
6	172	191	20
7	211	225	15
8	227	227	1
9	229	229	1
10	231	234	4
11	237	237	1
12	239	240	2
13	250	268	19

```
14   302 303      2
15   305 306      2
16   309 336     28
17   338 338      1
18   353 371     19
19   375 395     21
20   402 402      1
21   405 424     20
22   426 453     28
# 351 positions (cumulative volume <= 13 Angstrom^3)
   start end length
1      1  23     23
2     26  42     17
3     44  80     37
4     83  87      5
5     89 167     79
6    171 191     21
7    211 225     15
8    227 227      1
9    229 229      1
10   231 234      4
11   237 237      1
12   239 240      2
13   250 268     19
14   270 270      1
15   302 303      2
16   305 306      2
17   309 336     28
18   338 338      1
19   353 371     19
20   375 397     23
21   402 402      1
22   405 424     20
23   426 454     29
# 355 positions (cumulative volume <= 14 Angstrom^3)
   start end length
1      1  23     23
2     26  42     17
3     44  80     37
4     83  87      5
5     89 167     79
6    170 191     22
7    211 225     15
8    227 227      1
9    229 234      6
10   237 237      1
11   239 241      3
12   250 268     19
13   270 270      1
14   302 303      2
```

```
15   305 306      2
16   309 336     28
17   338 338      1
18   353 371     19
19   375 397     23
20   402 402      1
21   405 454     50
# 359 positions (cumulative volume <= 15 Angstrom^3)
   start end length
1      1  23     23
2     26  42     17
3     44  80     37
4     82  87      6
5     89 167     79
6    170 191     22
7    211 225     15
8    227 234      8
9    237 237      1
10   239 241      3
11   250 270     21
12   302 303      2
13   305 306      2
14   309 336     28
15   338 338      1
16   353 371     19
17   375 397     23
18   402 403      2
19   405 454     50
# 362 positions (cumulative volume <= 16 Angstrom^3)
   start end length
1      1  24     24
2     26  42     17
3     44  87     44
4     89 167     79
5    170 191     22
6    211 225     15
7    227 234      8
8    237 241      5
9    250 270     21
10   302 303      2
11   305 306      2
12   309 336     28
13   338 338      1
14   353 371     19
15   375 397     23
16   402 403      2
17   405 454     50
# 365 positions (cumulative volume <= 17 Angstrom^3)
   start end length
1      1  24     24
```

```
2      26  87     62
3      89 167     79
4     170 191     22
5     202 202      1
6     211 225     15
7     227 234      8
8     237 241      5
9     250 270     21
10    302 306      5
11    309 336     28
12    338 338      1
13    353 371     19
14    375 397     23
15    402 403      2
16    405 454     50
# 368 positions (cumulative volume <= 18 Angstrom^3)
   start end length
1      1  87     87
2     89 167     79
3    170 191     22
4    202 202      1
5    211 225     15
6    227 234      8
7    237 243      7
8    250 270     21
9    302 306      5
10   309 336     28
11   338 338      1
12   353 371     19
13   375 397     23
14   402 403      2
15   405 454     50
# 370 positions (cumulative volume <= 19 Angstrom^3)
   start end length
1      1  87     87
2     89 167     79
3    170 191     22
4    202 202      1
5    211 234     24
6    237 243      7
7    250 270     21
8    302 306      5
9    309 338     30
10   353 371     19
11   375 397     23
12   402 403      2
13   405 454     50
# 373 positions (cumulative volume <= 20 Angstrom^3)
   start end length
1      1  87     87
```

```
2      89 167     79
3     170 191     22
4     197 197      1
5     202 202      1
6     211 234     24
7     237 243      7
8     250 270     21
9     302 306      5
10    308 338     31
11    353 372     20
12    375 397     23
13    402 403      2
14    405 454     50
# 375 positions (cumulative volume <= 21 Angstrom^3)
   start end length
1      1  87     87
2     89 167     79
3    170 191     22
4    197 197      1
5    202 202      1
6    210 234     25
7    237 243      7
8    250 270     21
9    301 306      6
10   308 338     31
11   353 372     20
12   375 397     23
13   402 403      2
14   405 454     50
# 377 positions (cumulative volume <= 22 Angstrom^3)
   start end length
1      1  87     87
2     89 168     80
3    170 191     22
4    197 197      1
5    202 202      1
6    210 234     25
7    237 243      7
8    250 270     21
9    301 338     38
10   353 372     20
11   375 397     23
12   402 403      2
13   405 454     50
# 379 positions (cumulative volume <= 23 Angstrom^3)
   start end length
1      1  87     87
2     89 168     80
3    170 191     22
4    197 197      1
```

```
5    202 202      1
6    210 234     25
7    236 243      8
8    250 270     21
9    301 338     38
10   353 372     20
11   375 397     23
12   401 403      3
13   405 454     50
# 381 positions (cumulative volume <= 24 Angstrom^3)
   start end length
1      1  87     87
2     89 168     80
3    170 191     22
4    197 197      1
5    202 202      1
6    209 234     26
7    236 243      8
8    250 270     21
9    301 338     38
10   353 372     20
11   375 397     23
12   401 454     54
# 383 positions (cumulative volume <= 25 Angstrom^3)
   start end length
1      1  87     87
2     89 168     80
3    170 191     22
4    197 197      1
5    202 202      1
6    209 234     26
7    236 243      8
8    250 270     21
9    301 339     39
10   353 372     20
11   375 397     23
12   400 454     55
# 384 positions (cumulative volume <= 26 Angstrom^3)
   start end length
1      1  87     87
2     89 191    103
3    197 197      1
4    202 202      1
5    209 234     26
6    236 243      8
7    250 270     21
8    301 339     39
9    353 372     20
10   375 397     23
11   400 454     55
```

386 positions (cumulative volume <= 27 Angstrom^3)

	start	end	length
1	1	87	87
2	89	191	103
3	197	197	1
4	202	202	1
5	209	243	35
6	250	270	21
7	301	339	39
8	353	372	20
9	375	398	24
10	400	454	55

387 positions (cumulative volume <= 28 Angstrom^3)

	start	end	length
1	1	87	87
2	89	191	103
3	197	197	1
4	202	202	1
5	208	243	36
6	250	270	21
7	301	339	39
8	353	372	20
9	375	398	24
10	400	454	55

388 positions (cumulative volume <= 29 Angstrom^3)

	start	end	length
1	1	87	87
2	89	191	103
3	196	197	2
4	202	202	1
5	208	243	36
6	250	270	21
7	301	339	39
8	353	372	20
9	375	398	24
10	400	454	55

389 positions (cumulative volume <= 30 Angstrom^3)

	start	end	length
1	1	87	87
2	89	191	103
3	196	197	2
4	202	202	1
5	208	243	36
6	250	270	21
7	301	339	39
8	353	372	20
9	375	454	80

390 positions (cumulative volume <= 31 Angstrom^3)

	start	end	length
1	1	87	87

```
2     89 191    103
3    196 197      2
4    202 202      1
5    208 243     36
6    250 270     21
7    284 284      1
8    301 339     39
9    353 372     20
10   375 454     80
# 391 positions (cumulative volume <= 32 Angstrom^3)
   start end length
1      1  87     87
2     89 191    103
3    196 197      2
4    202 203      2
5    208 243     36
6    250 270     21
7    284 284      1
8    301 339     39
9    353 372     20
10   375 454     80
# 392 positions (cumulative volume <= 33 Angstrom^3)
   start end length
1      1  87     87
2     89 191    103
3    196 197      2
4    202 203      2
5    208 244     37
6    250 270     21
7    284 284      1
8    301 339     39
9    353 372     20
10   375 454     80
# 393 positions (cumulative volume <= 34 Angstrom^3)
   start end length
1      1  87     87
2     89 191    103
3    196 197      2
4    202 204      3
5    208 244     37
6    250 270     21
7    284 284      1
8    301 339     39
9    353 372     20
10   375 454     80
# 393 positions (cumulative volume <= 35 Angstrom^3)
   start end length
1      1  87     87
2     89 191    103
3    196 197      2
```

```
4    202 204      3
5    208 244     37
6    250 270     21
7    284 284      1
8    301 339     39
9    353 372     20
10   375 454     80
# 394 positions (cumulative volume <= 36 Angstrom^3)
   start end length
1      1  87     87
2     89 191    103
3    196 197      2
4    202 205      4
5    208 244     37
6    250 270     21
7    284 284      1
8    301 339     39
9    353 372     20
10   375 454     80
# 394 positions (cumulative volume <= 37 Angstrom^3)
   start end length
1      1  87     87
2     89 191    103
3    196 197      2
4    202 205      4
5    208 244     37
6    250 270     21
7    284 284      1
8    301 339     39
9    353 372     20
10   375 454     80
# 395 positions (cumulative volume <= 38 Angstrom^3)
   start end length
1      1  87     87
2     89 191    103
3    196 197      2
4    202 205      4
5    208 244     37
6    249 270     22
7    284 284      1
8    301 339     39
9    353 372     20
10   375 454     80
# 396 positions (cumulative volume <= 39 Angstrom^3)
   start end length
1      1  87     87
2     89 191    103
3    196 197      2
4    202 205      4
5    208 244     37
```

```
6    246 246      1
7    249 270     22
8    284 284      1
9    301 339     39
10   353 372     20
11   375 454     80
# 396 positions (cumulative volume <= 40 Angstrom^3)
   start end length
1      1  87     87
2     89 191    103
3    196 197      2
4    202 205      4
5    208 244     37
6    246 246      1
7    249 270     22
8    284 284      1
9    301 339     39
10   353 372     20
11   375 454     80
# 397 positions (cumulative volume <= 41 Angstrom^3)
   start end length
1      1  87     87
2     89 191    103
3    196 198      3
4    202 205      4
5    208 244     37
6    246 246      1
7    249 270     22
8    284 284      1
9    301 339     39
10   353 372     20
11   375 454     80
# 397 positions (cumulative volume <= 42 Angstrom^3)
   start end length
1      1  87     87
2     89 191    103
3    196 198      3
4    202 205      4
5    208 244     37
6    246 246      1
7    249 270     22
8    284 284      1
9    301 339     39
10   353 372     20
11   375 454     80
# 397 positions (cumulative volume <= 43 Angstrom^3)
   start end length
1      1  87     87
2     89 191    103
3    196 198      3
```

```
4    202 205      4
5    208 244     37
6    246 246      1
7    249 270     22
8    284 284      1
9    301 339     39
10   353 372     20
11   375 454     80
# 398 positions (cumulative volume <= 44 Angstrom^3)
   start end length
1      1  87     87
2     89 191    103
3    196 198      3
4    202 205      4
5    208 244     37
6    246 246      1
7    249 270     22
8    282 282      1
9    284 284      1
10   301 339     39
11   353 372     20
12   375 454     80
# 398 positions (cumulative volume <= 45 Angstrom^3)
   start end length
1      1  87     87
2     89 191    103
3    196 198      3
4    202 205      4
5    208 244     37
6    246 246      1
7    249 270     22
8    282 282      1
9    284 284      1
10   301 339     39
11   353 372     20
12   375 454     80
# 399 positions (cumulative volume <= 46 Angstrom^3)
   start end length
1      1  87     87
2     89 191    103
3    196 198      3
4    202 205      4
5    208 244     37
6    246 247      2
7    249 270     22
8    282 282      1
9    284 284      1
10   301 339     39
11   353 372     20
12   375 454     80
```

399 positions (cumulative volume <= 47 Angstrom^3)

	start	end	length
1	1	87	87
2	89	191	103
3	196	198	3
4	202	205	4
5	208	244	37
6	246	247	2
7	249	270	22
8	282	282	1
9	284	284	1
10	301	339	39
11	353	372	20
12	375	454	80

399 positions (cumulative volume <= 48 Angstrom^3)

	start	end	length
1	1	87	87
2	89	191	103
3	196	198	3
4	202	205	4
5	208	244	37
6	246	247	2
7	249	270	22
8	282	282	1
9	284	284	1
10	301	339	39
11	353	372	20
12	375	454	80

400 positions (cumulative volume <= 49 Angstrom^3)

	start	end	length
1	1	87	87
2	89	191	103
3	195	198	4
4	202	205	4
5	208	244	37
6	246	247	2
7	249	270	22
8	282	282	1
9	284	284	1
10	301	339	39
11	353	372	20
12	375	454	80

400 positions (cumulative volume <= 50 Angstrom^3)

	start	end	length
1	1	87	87
2	89	191	103
3	195	198	4
4	202	205	4
5	208	244	37
6	246	247	2

```
7    249 270     22
8    282 282      1
9    284 284      1
10   301 339     39
11   353 372     20
12   375 454     80
# 401 positions (cumulative volume <= 51 Angstrom^3)
   start end length
1      1  87     87
2     89 191    103
3    195 198      4
4    202 206      5
5    208 244     37
6    246 247      2
7    249 270     22
8    282 282      1
9    284 284      1
10   301 339     39
11   353 372     20
12   375 454     80
# 401 positions (cumulative volume <= 52 Angstrom^3)
   start end length
1      1  87     87
2     89 191    103
3    195 198      4
4    202 206      5
5    208 244     37
6    246 247      2
7    249 270     22
8    282 282      1
9    284 284      1
10   301 339     39
11   353 372     20
12   375 454     80
# 401 positions (cumulative volume <= 53 Angstrom^3)
   start end length
1      1  87     87
2     89 191    103
3    195 198      4
4    202 206      5
5    208 244     37
6    246 247      2
7    249 270     22
8    282 282      1
9    284 284      1
10   301 339     39
11   353 372     20
12   375 454     80
# 402 positions (cumulative volume <= 54 Angstrom^3)
   start end length
```

```
1      1  87     87
2     89 191    103
3    195 198      4
4    202 206      5
5    208 244     37
6    246 247      2
7    249 270     22
8    282 282      1
9    284 284      1
10   300 339     40
11   353 372     20
12   375 454     80
# 402 positions (cumulative volume <= 55 Angstrom^3)
   start end length
1      1  87     87
2     89 191    103
3    195 198      4
4    202 206      5
5    208 244     37
6    246 247      2
7    249 270     22
8    282 282      1
9    284 284      1
10   300 339     40
11   353 372     20
12   375 454     80
# 402 positions (cumulative volume <= 56 Angstrom^3)
   start end length
1      1  87     87
2     89 191    103
3    195 198      4
4    202 206      5
5    208 244     37
6    246 247      2
7    249 270     22
8    282 282      1
9    284 284      1
10   300 339     40
11   353 372     20
12   375 454     80
# 403 positions (cumulative volume <= 57 Angstrom^3)
   start end length
1      1  87     87
2     89 191    103
3    195 198      4
4    202 206      5
5    208 244     37
6    246 270     25
7    282 282      1
8    284 284      1
```

```
9    300 339     40
10   353 372     20
11   375 454     80
# 403 positions (cumulative volume <= 58 Angstrom^3)
   start end length
1      1  87     87
2     89 191    103
3    195 198      4
4    202 206      5
5    208 244     37
6    246 270     25
7    282 282      1
8    284 284      1
9    300 339     40
10   353 372     20
11   375 454     80
# 403 positions (cumulative volume <= 59 Angstrom^3)
   start end length
1      1  87     87
2     89 191    103
3    195 198      4
4    202 206      5
5    208 244     37
6    246 270     25
7    282 282      1
8    284 284      1
9    300 339     40
10   353 372     20
11   375 454     80
# 403 positions (cumulative volume <= 60 Angstrom^3)
   start end length
1      1  87     87
2     89 191    103
3    195 198      4
4    202 206      5
5    208 244     37
6    246 270     25
7    282 282      1
8    284 284      1
9    300 339     40
10   353 372     20
11   375 454     80
# 404 positions (cumulative volume <= 61 Angstrom^3)
   start end length
1      1  87     87
2     89 191    103
3    195 198      4
4    202 206      5
5    208 244     37
6    246 270     25
```

```
7    282 282      1
8    284 284      1
9    286 286      1
10   300 339     40
11   353 372     20
12   375 454     80
# 404 positions (cumulative volume <= 62 Angstrom^3)
   start end length
1      1  87     87
2     89 191    103
3    195 198      4
4    202 206      5
5    208 244     37
6    246 270     25
7    282 282      1
8    284 284      1
9    286 286      1
10   300 339     40
11   353 372     20
12   375 454     80
# 404 positions (cumulative volume <= 63 Angstrom^3)
   start end length
1      1  87     87
2     89 191    103
3    195 198      4
4    202 206      5
5    208 244     37
6    246 270     25
7    282 282      1
8    284 284      1
9    286 286      1
10   300 339     40
11   353 372     20
12   375 454     80
# 404 positions (cumulative volume <= 64 Angstrom^3)
   start end length
1      1  87     87
2     89 191    103
3    195 198      4
4    202 206      5
5    208 244     37
6    246 270     25
7    282 282      1
8    284 284      1
9    286 286      1
10   300 339     40
11   353 372     20
12   375 454     80
# 405 positions (cumulative volume <= 65 Angstrom^3)
   start end length
```

```
1       1  87     87
2      89 191    103
3     194 198      5
4     202 206      5
5     208 244     37
6     246 270     25
7     282 282      1
8     284 284      1
9     286 286      1
10    300 339     40
11    353 372     20
12    375 454     80
# 405 positions (cumulative volume <= 66 Angstrom^3)
   start end length
1       1  87     87
2      89 191    103
3     194 198      5
4     202 206      5
5     208 244     37
6     246 270     25
7     282 282      1
8     284 284      1
9     286 286      1
10    300 339     40
11    353 372     20
12    375 454     80
# 405 positions (cumulative volume <= 67 Angstrom^3)
   start end length
1       1  87     87
2      89 191    103
3     194 198      5
4     202 206      5
5     208 244     37
6     246 270     25
7     282 282      1
8     284 284      1
9     286 286      1
10    300 339     40
11    353 372     20
12    375 454     80
# 405 positions (cumulative volume <= 68 Angstrom^3)
   start end length
1       1  87     87
2      89 191    103
3     194 198      5
4     202 206      5
5     208 244     37
6     246 270     25
7     282 282      1
8     284 284      1
```

```
9    286 286      1
10   300 339     40
11   353 372     20
12   375 454     80
# 406 positions (cumulative volume <= 69 Angstrom^3)
   start end length
1      1  87     87
2     89 191    103
3    194 198      5
4    202 206      5
5    208 244     37
6    246 270     25
7    282 282      1
8    284 286      3
9    300 339     40
10   353 372     20
11   375 454     80
# 406 positions (cumulative volume <= 70 Angstrom^3)
   start end length
1      1  87     87
2     89 191    103
3    194 198      5
4    202 206      5
5    208 244     37
6    246 270     25
7    282 282      1
8    284 286      3
9    300 339     40
10   353 372     20
11   375 454     80
# 406 positions (cumulative volume <= 71 Angstrom^3)
   start end length
1      1  87     87
2     89 191    103
3    194 198      5
4    202 206      5
5    208 244     37
6    246 270     25
7    282 282      1
8    284 286      3
9    300 339     40
10   353 372     20
11   375 454     80
# 406 positions (cumulative volume <= 72 Angstrom^3)
   start end length
1      1  87     87
2     89 191    103
3    194 198      5
4    202 206      5
5    208 244     37
```

```
6    246 270     25
7    282 282      1
8    284 286      3
9    300 339     40
10   353 372     20
11   375 454     80
# 407 positions (cumulative volume <= 73 Angstrom^3)
   start end length
1      1  87     87
2     89 191    103
3    194 198      5
4    202 206      5
5    208 270     63
6    282 282      1
7    284 286      3
8    300 339     40
9    353 372     20
10   375 454     80
# 407 positions (cumulative volume <= 74 Angstrom^3)
   start end length
1      1  87     87
2     89 191    103
3    194 198      5
4    202 206      5
5    208 270     63
6    282 282      1
7    284 286      3
8    300 339     40
9    353 372     20
10   375 454     80
# 407 positions (cumulative volume <= 75 Angstrom^3)
   start end length
1      1  87     87
2     89 191    103
3    194 198      5
4    202 206      5
5    208 270     63
6    282 282      1
7    284 286      3
8    300 339     40
9    353 372     20
10   375 454     80
# 407 positions (cumulative volume <= 76 Angstrom^3)
   start end length
1      1  87     87
2     89 191    103
3    194 198      5
4    202 206      5
5    208 270     63
6    282 282      1
```

```
7    284 286      3
8    300 339     40
9    353 372     20
10   375 454     80
# 408 positions (cumulative volume <= 77 Angstrom^3)
   start end length
1      1  87     87
2     89 191    103
3    194 198      5
4    202 206      5
5    208 270     63
6    282 282      1
7    284 286      3
8    289 289      1
9    300 339     40
10   353 372     20
11   375 454     80
# 408 positions (cumulative volume <= 78 Angstrom^3)
   start end length
1      1  87     87
2     89 191    103
3    194 198      5
4    202 206      5
5    208 270     63
6    282 282      1
7    284 286      3
8    289 289      1
9    300 339     40
10   353 372     20
11   375 454     80
# 408 positions (cumulative volume <= 79 Angstrom^3)
   start end length
1      1  87     87
2     89 191    103
3    194 198      5
4    202 206      5
5    208 270     63
6    282 282      1
7    284 286      3
8    289 289      1
9    300 339     40
10   353 372     20
11   375 454     80
# 408 positions (cumulative volume <= 80 Angstrom^3)
   start end length
1      1  87     87
2     89 191    103
3    194 198      5
4    202 206      5
5    208 270     63
```

```
6    282 282      1
7    284 286      3
8    289 289      1
9    300 339     40
10   353 372     20
11   375 454     80
# 408 positions (cumulative volume <= 81 Angstrom^3)
   start end length
1      1  87     87
2     89 191    103
3    194 198      5
4    202 206      5
5    208 270     63
6    282 282      1
7    284 286      3
8    289 289      1
9    300 339     40
10   353 372     20
11   375 454     80
# 408 positions (cumulative volume <= 82 Angstrom^3)
   start end length
1      1  87     87
2     89 191    103
3    194 198      5
4    202 206      5
5    208 270     63
6    282 282      1
7    284 286      3
8    289 289      1
9    300 339     40
10   353 372     20
11   375 454     80
# 409 positions (cumulative volume <= 83 Angstrom^3)
   start end length
1      1  87     87
2     89 191    103
3    194 198      5
4    202 206      5
5    208 270     63
6    282 286      5
7    289 289      1
8    300 339     40
9    353 372     20
10   375 454     80
# 409 positions (cumulative volume <= 84 Angstrom^3)
   start end length
1      1  87     87
2     89 191    103
3    194 198      5
4    202 206      5
```

```
5    208 270     63
6    282 286      5
7    289 289      1
8    300 339     40
9    353 372     20
10   375 454     80
# 409 positions (cumulative volume <= 85 Angstrom^3)
   start end length
1      1  87     87
2     89 191    103
3    194 198      5
4    202 206      5
5    208 270     63
6    282 286      5
7    289 289      1
8    300 339     40
9    353 372     20
10   375 454     80
# 409 positions (cumulative volume <= 86 Angstrom^3)
   start end length
1      1  87     87
2     89 191    103
3    194 198      5
4    202 206      5
5    208 270     63
6    282 286      5
7    289 289      1
8    300 339     40
9    353 372     20
10   375 454     80
# 409 positions (cumulative volume <= 87 Angstrom^3)
   start end length
1      1  87     87
2     89 191    103
3    194 198      5
4    202 206      5
5    208 270     63
6    282 286      5
7    289 289      1
8    300 339     40
9    353 372     20
10   375 454     80
# 410 positions (cumulative volume <= 88 Angstrom^3)
   start end length
1      1  87     87
2     89 191    103
3    194 198      5
4    202 206      5
5    208 270     63
6    282 286      5
```

```
7    288 289      2
8    300 339     40
9    353 372     20
10   375 454     80
# 410 positions (cumulative volume <= 89 Angstrom^3)
   start end length
1      1  87     87
2     89 191    103
3    194 198      5
4    202 206      5
5    208 270     63
6    282 286      5
7    288 289      2
8    300 339     40
9    353 372     20
10   375 454     80
# 410 positions (cumulative volume <= 90 Angstrom^3)
   start end length
1      1  87     87
2     89 191    103
3    194 198      5
4    202 206      5
5    208 270     63
6    282 286      5
7    288 289      2
8    300 339     40
9    353 372     20
10   375 454     80
# 410 positions (cumulative volume <= 91 Angstrom^3)
   start end length
1      1  87     87
2     89 191    103
3    194 198      5
4    202 206      5
5    208 270     63
6    282 286      5
7    288 289      2
8    300 339     40
9    353 372     20
10   375 454     80
# 410 positions (cumulative volume <= 92 Angstrom^3)
   start end length
1      1  87     87
2     89 191    103
3    194 198      5
4    202 206      5
5    208 270     63
6    282 286      5
7    288 289      2
8    300 339     40
```

```
9    353 372     20
10   375 454     80
# 410 positions (cumulative volume <= 93 Angstrom^3)
   start end length
1      1  87     87
2     89 191    103
3    194 198      5
4    202 206      5
5    208 270     63
6    282 286      5
7    288 289      2
8    300 339     40
9    353 372     20
10   375 454     80
# 411 positions (cumulative volume <= 94 Angstrom^3)
  start end length
1     1  87     87
2    89 191    103
3   194 198      5
4   202 206      5
5   208 270     63
6   282 289      8
7   300 339     40
8   353 372     20
9   375 454     80
# 411 positions (cumulative volume <= 95 Angstrom^3)
  start end length
1     1  87     87
2    89 191    103
3   194 198      5
4   202 206      5
5   208 270     63
6   282 289      8
7   300 339     40
8   353 372     20
9   375 454     80
# 411 positions (cumulative volume <= 96 Angstrom^3)
  start end length
1     1  87     87
2    89 191    103
3   194 198      5
4   202 206      5
5   208 270     63
6   282 289      8
7   300 339     40
8   353 372     20
9   375 454     80
# 411 positions (cumulative volume <= 97 Angstrom^3)
  start end length
1     1  87     87
```

```
2    89 191    103
3   194 198      5
4   202 206      5
5   208 270     63
6   282 289      8
7   300 339     40
8   353 372     20
9   375 454     80
# 411 positions (cumulative volume <= 98 Angstrom^3)
  start end length
1     1  87     87
2    89 191    103
3   194 198      5
4   202 206      5
5   208 270     63
6   282 289      8
7   300 339     40
8   353 372     20
9   375 454     80
# 411 positions (cumulative volume <= 99 Angstrom^3)
  start end length
1     1  87     87
2    89 191    103
3   194 198      5
4   202 206      5
5   208 270     63
6   282 289      8
7   300 339     40
8   353 372     20
9   375 454     80
# 412 positions (cumulative volume <= 100 Angstrom^3)
  start end length
1     1  87     87
2    89 191    103
3   194 198      5
4   202 206      5
5   208 270     63
6   281 289      9
7   300 339     40
8   353 372     20
9   375 454     80
```
