# Supplementary material for: Insights into the mechanism(s) of digestion of crystalline cellulose by plant class C GH9 endoglucanases
Source: J Mol Model. 2019 Jul 23;25(8):240. doi: 10.1007/s00894-019-4133-1 (PMC7385011; doi:10.1007/s00894-019-4133-1)
Supplement: Supplementary file 5 — (PDF 208 kb) [file 894_2019_4133_MOESM5_ESM.pdf]

Supplementary Text 3

```
          -------------------------------------------------------
          Amber 16 SANDER                              2016
          -------------------------------------------------------

| Run on 08/13/2017 at 00:51:42

|   Executable path: /usr/local/amber16/bin/sander
| Working directory: /Users/Swati
|          Hostname: Unknown
  [-O]verwriting output

File Assignments:
|  MDIN: minimization_file_2.in
| MDOUT: 3WY9_min3_2.out
|INPCRD: 3WY9_min3_1.rst
|  PARM: /Users/Swati/3WY9_files/3WY9.prmtop
|RESTRT: 3WY9_min3_2.rst
|  REFC: refc
| MDVEL: mdvel
| MDFRC: mdfrc
|  MDEN: mden
| MDCRD: mdcrd
|MDINFO: mdinfo
|  MTMD: mtmd
|INPDIP: inpdip
|RSTDIP: rstdip
|INPTRA: inptraj


 Here is the input file:

PROT: minimization_2
&cntrl
imin = 1,
maxcyc = 5000,
ncyc = 10000,
igb = 0,
ntpr =  100,
ntp = 0
cut = 12
&end


--------------------------------------------------------------------------------
   1.  RESOURCE   USE:
--------------------------------------------------------------------------------

| Flags:
 getting box info from netcdf restart file
| NetCDF restart box info found
|Largest sphere to fit in unit cell has radius =    43.021
| New format PARM file being parsed.
| Version =    1.000 Date = 08/08/17 Time = 03:26:06
```

```
 NATOM  =  108958 NTYPES =      17 NBONH =  104467 MBONA  =    4615
 NTHETH =    9760 MTHETA =    6270 NPHIH =   20223 MPHIA  =   19473
 NHPARM =       0 NPARM  =       0 NNB   =  182012 NRES   =   33963
 NBONA  =    4615 NTHETA =    6270 NPHIA =   19473 NUMBND =      67
 NUMANG =     152 NPTRA  =     191 NATYP =      36 NPHB   =       1
 IFBOX  =       1 NMXRS  =      24 IFCAP =       0 NEXTRA =       0
 NCOPY  =       0


|     Memory Use     Allocated
|     Real             8586309
|     Hollerith         360839
|     Integer          4397980
|     Max Pairs       99660250
|     nblistReal       1307496
|     nblist Int       3585143
|       Total           499186 kbytes

| Note: 1-4 EEL scale factors are being read from the topology file.

| Note: 1-4 VDW scale factors are being read from the topology file.
| Duplicated    0 dihedrals
| Duplicated    0 dihedrals

     BOX TYPE: RECTILINEAR

--------------------------------------------------------------------------------
   2.  CONTROL  DATA  FOR  THE  RUN
--------------------------------------------------------------------------------

default_name

General flags:
     imin    =       1, nmropt  =       0

Nature and format of input:
     ntx     =       1, irest   =       0, ntrx    =       1

Nature and format of output:
     ntxo    =       2, ntpr    =     100, ntrx    =       1, ntwr    =       1
     iwrap   =       0, ntwx    =       0, ntwv    =       0, ntwe    =       0
     ioutfm  =       1, ntwprt  =       0, idecomp =       0, rbornstat=      0

Potential function:
     ntf     =       1, ntb     =       1, igb     =       0, nsnb    =      25
     ipol    =       0, gbsa    =       0, iesp    =       0
     dielc   =   1.00000, cut     =  12.00000, intdiel =   1.00000

Frozen or restrained atoms:
     ibelly  =       0, ntr     =       0

Energy minimization:
     maxcyc  =    5000, ncyc    =   10000, ntmin   =       1
     dx0     =   0.01000, drms    =   0.00010
```

```
Ewald parameters:
     verbose =       0, ew_type =       0, nbflag  =       1, use_pme =       1
     vdwmeth =       1, eedmeth =       1, netfrc  =       0
     Box X =  123.894   Box Y =   86.041   Box Z =  120.804
     Alpha =   90.000   Beta  =   90.000   Gamma =   90.000
     NFFT1 =  128       NFFT2 =   90       NFFT3 =  120
     Cutoff=   12.000   Tol   =0.100E-04
     Ewald Coefficient =  0.22664
     Interpolation order =    4
|  INFO: Old style inpcrd file read


--------------------------------------------------------------------------------
   3.  ATOMIC COORDINATES AND VELOCITIES
--------------------------------------------------------------------------------

default_name
 begin time read from input coords =     0.000 ps

 Number of triangulated 3-point waters found:    33386

     Sum of charges from parm topology file =  -0.00000039
     Forcing neutrality...

--------------------------------------------------------------------------------
   4.  RESULTS
--------------------------------------------------------------------------------

 ---------------------------------------------------
 APPROXIMATING switch and d/dx switch using CUBIC SPLINE INTERPOLATION
 using   5000.0 points per unit in tabled values
 TESTING RELATIVE ERROR over r ranging from 0.0 to cutoff
| CHECK switch(x): max rel err =   0.2738E-14   at   2.422500
| CHECK d/dx switch(x): max rel err =   0.7967E-11   at   2.716640
 ---------------------------------------------------
| Local SIZE OF NONBOND LIST =   54068725
| TOTAL SIZE OF NONBOND LIST =   54068725


   NSTEP       ENERGY          RMS            GMAX         NAME    NUMBER
      1      -4.0743E+05     3.9773E+00     2.5249E+02     OE2      2287

 BOND    =    32679.6549  ANGLE   =     3821.6131  DIHED      =     6769.6726
 VDWAALS =    79544.4577  EEL     =  -560075.0788  HBOND      =        0.0000
 1-4 VDW =     3206.3674  1-4 EEL =    26623.1514  RESTRAINT  =        0.0000


   NSTEP       ENERGY          RMS            GMAX         NAME    NUMBER
    100      -4.1967E+05     4.3273E-01     3.5870E+01     C        7578

 BOND    =    31212.6883  ANGLE   =     1635.0353  DIHED      =     6674.1253
 VDWAALS =    73765.4432  EEL     =  -561298.5479  HBOND      =        0.0000
 1-4 VDW =     2242.9987  1-4 EEL =    26093.3367  RESTRAINT  =        0.0000
```

```
  NSTEP       ENERGY          RMS            GMAX         NAME    NUMBER
    200      -4.2130E+05     5.9209E-01     7.9308E+01     C        7276

 BOND    =     31291.4606  ANGLE   =      1387.4006  DIHED      =      6635.4671
 VDWAALS =     73779.5780  EEL     =   -562451.5931  HBOND      =         0.0000
 1-4 VDW =      2070.3073  1-4 EEL =     25983.7068  RESTRAINT  =         0.0000


  NSTEP       ENERGY          RMS            GMAX         NAME    NUMBER
    300      -4.2227E+05     4.9068E-01     6.7758E+01     CE1      7690

 BOND    =     31366.6586  ANGLE   =      1304.5645  DIHED      =      6615.0445
 VDWAALS =     74004.2024  EEL     =   -563497.4347  HBOND      =         0.0000
 1-4 VDW =      2001.7591  1-4 EEL =     25938.0316  RESTRAINT  =         0.0000


  NSTEP       ENERGY          RMS            GMAX         NAME    NUMBER
    400      -4.2300E+05     4.5303E-01     6.4055E+01     CE1      7690

 BOND    =     31450.7686  ANGLE   =      1261.3643  DIHED      =      6594.4938
 VDWAALS =     74289.8862  EEL     =   -564472.8335  HBOND      =         0.0000
 1-4 VDW =      1964.4071  1-4 EEL =     25910.4399  RESTRAINT  =         0.0000


  NSTEP       ENERGY          RMS            GMAX         NAME    NUMBER
    500      -4.2363E+05     3.7313E-01     5.3893E+01     CE1      7690

 BOND    =     31533.0108  ANGLE   =      1235.5423  DIHED      =      6575.3517
 VDWAALS =     74610.7520  EEL     =   -565413.6388  HBOND      =         0.0000
 1-4 VDW =      1940.6597  1-4 EEL =     25892.6224  RESTRAINT  =         0.0000


  NSTEP       ENERGY          RMS            GMAX         NAME    NUMBER
    600      -4.2416E+05     1.5208E-01     9.0968E+00     CE1      7690

 BOND    =     31608.1921  ANGLE   =      1217.9322  DIHED      =      6559.3107
 VDWAALS =     74935.1691  EEL     =   -566282.2412  HBOND      =         0.0000
 1-4 VDW =      1923.8000  1-4 EEL =     25878.0264  RESTRAINT  =         0.0000


  NSTEP       ENERGY          RMS            GMAX         NAME    NUMBER
    700      -4.2465E+05     1.4002E-01     6.2203E+00     CE1      7690

 BOND    =     31687.5293  ANGLE   =      1207.4636  DIHED      =      6543.6779
 VDWAALS =     75268.1089  EEL     =   -567133.1244  HBOND      =         0.0000
 1-4 VDW =      1910.5107  1-4 EEL =     25864.7429  RESTRAINT  =         0.0000


  NSTEP       ENERGY          RMS            GMAX         NAME    NUMBER
    800      -4.2510E+05     1.3456E-01     6.8662E+00     CE1      7690

 BOND    =     31764.3137  ANGLE   =      1201.0680  DIHED      =      6532.2066
```

```
VDWAALS =     75600.9159  EEL      =  -567949.6462  HBOND      =         0.0000
1-4 VDW =      1900.5196  1-4 EEL =    25852.4024  RESTRAINT  =         0.0000


  NSTEP       ENERGY          RMS            GMAX         NAME    NUMBER
    900      -4.2550E+05     5.1118E-01     8.7368E+01     CG       3213

BOND    =     31848.9242  ANGLE    =     1197.3435  DIHED      =      6523.4301
VDWAALS =     75925.7816  EEL      =  -568730.5844  HBOND      =         0.0000
1-4 VDW =      1892.5964  1-4 EEL =    25840.2627  RESTRAINT  =         0.0000


  NSTEP       ENERGY          RMS            GMAX         NAME    NUMBER
   1000      -4.2589E+05     4.1303E-01     6.6538E+01     CE1      7690

BOND    =     31914.9715  ANGLE    =     1193.4261  DIHED      =      6517.4167
VDWAALS =     76232.8797  EEL      =  -569465.1475  HBOND      =         0.0000
1-4 VDW =      1886.1382  1-4 EEL =    25830.2818  RESTRAINT  =         0.0000


  NSTEP       ENERGY          RMS            GMAX         NAME    NUMBER
   1100      -4.2625E+05     3.5107E-01     5.5756E+01     CE1      7690

BOND    =     31978.8488  ANGLE    =     1190.8181  DIHED      =      6514.2283
VDWAALS =     76531.7913  EEL      =  -570170.3587  HBOND      =         0.0000
1-4 VDW =      1880.4536  1-4 EEL =    25821.5084  RESTRAINT  =         0.0000


  NSTEP       ENERGY          RMS            GMAX         NAME    NUMBER
   1200      -4.2660E+05     3.2079E-01     5.0003E+01     CE1      7690

BOND    =     32042.4468  ANGLE    =     1188.3056  DIHED      =      6512.3061
VDWAALS =     76824.6566  EEL      =  -570852.3589  HBOND      =         0.0000
1-4 VDW =      1875.6007  1-4 EEL =    25813.8819  RESTRAINT  =         0.0000


  NSTEP       ENERGY          RMS            GMAX         NAME    NUMBER
   1300      -4.2691E+05     1.7631E-01     2.3461E+01     CE1      7690

BOND    =     32099.0501  ANGLE    =     1186.1113  DIHED      =      6510.8869
VDWAALS =     77101.2724  EEL      =  -571489.0280  HBOND      =         0.0000
1-4 VDW =      1871.2346  1-4 EEL =    25806.8266  RESTRAINT  =         0.0000


  NSTEP       ENERGY          RMS            GMAX         NAME    NUMBER
   1400      -4.2721E+05     1.1860E-01     8.1646E+00     CE1      7690

BOND    =     32154.5483  ANGLE    =     1184.6806  DIHED      =      6508.9184
VDWAALS =     77362.1524  EEL      =  -572087.1550  HBOND      =         0.0000
1-4 VDW =      1867.1360  1-4 EEL =    25800.4805  RESTRAINT  =         0.0000


  NSTEP       ENERGY          RMS            GMAX         NAME    NUMBER
   1500      -4.2749E+05     1.1479E-01     8.5025E+00     CE1      7690
```

```
BOND    =     32209.0346  ANGLE   =      1183.4895  DIHED      =      6506.9186
VDWAALS =     77614.4593  EEL     =   -572666.9289  HBOND      =         0.0000
1-4 VDW =      1863.1560  1-4 EEL =     25795.0427  RESTRAINT  =         0.0000


   NSTEP       ENERGY          RMS            GMAX         NAME    NUMBER
   1600      -4.2776E+05     4.0384E-01     6.2315E+01     CE1      7690

BOND    =     32269.0950  ANGLE   =      1182.8509  DIHED      =      6505.4034
VDWAALS =     77855.7131  EEL     =   -573226.1031  HBOND      =         0.0000
1-4 VDW =      1859.4422  1-4 EEL =     25790.2108  RESTRAINT  =         0.0000


   NSTEP       ENERGY          RMS            GMAX         NAME    NUMBER
   1700      -4.2803E+05     1.0762E-01     6.9342E+00     CE1      7690

BOND    =     32311.4581  ANGLE   =      1181.0826  DIHED      =      6503.8729
VDWAALS =     78083.4515  EEL     =   -573755.1554  HBOND      =         0.0000
1-4 VDW =      1856.2124  1-4 EEL =     25785.6835  RESTRAINT  =         0.0000


   NSTEP       ENERGY          RMS            GMAX         NAME    NUMBER
   1800      -4.2829E+05     2.8655E-01     4.5459E+01     CE1      7690

BOND    =     32364.4811  ANGLE   =      1180.3382  DIHED      =      6502.2780
VDWAALS =     78307.2847  EEL     =   -574275.4507  HBOND      =         0.0000
1-4 VDW =      1853.1415  1-4 EEL =     25781.3818  RESTRAINT  =         0.0000


   NSTEP       ENERGY          RMS            GMAX         NAME    NUMBER
   1900      -4.2854E+05     1.4462E-01     1.7709E+01     CE1      7690

BOND    =     32408.6965  ANGLE   =      1178.8896  DIHED      =      6500.5513
VDWAALS =     78517.8045  EEL     =   -574771.0444  HBOND      =         0.0000
1-4 VDW =      1850.5270  1-4 EEL =     25777.5257  RESTRAINT  =         0.0000


   NSTEP       ENERGY          RMS            GMAX         NAME    NUMBER
   2000      -4.2878E+05     1.0300E-01     6.1988E+00     CE1      7690

BOND    =     32453.6414  ANGLE   =      1177.4678  DIHED      =      6499.1494
VDWAALS =     78720.5513  EEL     =   -575252.4661  HBOND      =         0.0000
1-4 VDW =      1848.1772  1-4 EEL =     25773.9781  RESTRAINT  =         0.0000


   NSTEP       ENERGY          RMS            GMAX         NAME    NUMBER
   2100      -4.2902E+05     1.0659E-01     7.8688E+00     CE1      7690

BOND    =     32498.4418  ANGLE   =      1176.0866  DIHED      =      6498.1178
VDWAALS =     78920.8095  EEL     =   -575729.2338  HBOND      =         0.0000
1-4 VDW =      1846.1438  1-4 EEL =     25770.5828  RESTRAINT  =         0.0000
```

```
  NSTEP       ENERGY          RMS            GMAX         NAME    NUMBER
   2200      -4.2925E+05     1.1979E-01     1.2721E+01     CE1      7690

 BOND    =    32541.5566  ANGLE   =     1175.0323  DIHED      =     6497.0650
 VDWAALS =    79111.7229  EEL     =  -576186.0845  HBOND      =        0.0000
 1-4 VDW =     1844.2957  1-4 EEL =    25767.2633  RESTRAINT  =        0.0000


  NSTEP       ENERGY          RMS            GMAX         NAME    NUMBER
   2300      -4.2947E+05     3.3339E-01     5.1576E+01     CE1      7690

 BOND    =    32588.0814  ANGLE   =     1174.5852  DIHED      =     6496.1196
 VDWAALS =    79296.2536  EEL     =  -576628.6575  HBOND      =        0.0000
 1-4 VDW =     1842.5833  1-4 EEL =    25764.1456  RESTRAINT  =        0.0000


  NSTEP       ENERGY          RMS            GMAX         NAME    NUMBER
   2400      -4.2969E+05     2.7617E-01     4.3559E+01     CE1      7690

 BOND    =    32627.8686  ANGLE   =     1173.7630  DIHED      =     6495.2764
 VDWAALS =    79479.0684  EEL     =  -577067.7093  HBOND      =        0.0000
 1-4 VDW =     1841.1437  1-4 EEL =    25760.9855  RESTRAINT  =        0.0000


  NSTEP       ENERGY          RMS            GMAX         NAME    NUMBER
   2500      -4.2991E+05     2.4414E-01     3.3957E+01     CE1      7690

 BOND    =    32668.1947  ANGLE   =     1172.9840  DIHED      =     6494.3507
 VDWAALS =    79656.8698  EEL     =  -577499.4681  HBOND      =        0.0000
 1-4 VDW =     1839.6328  1-4 EEL =    25757.8188  RESTRAINT  =        0.0000


  NSTEP       ENERGY          RMS            GMAX         NAME    NUMBER
   2600      -4.3012E+05     1.3717E-01     1.6617E+01     CE1      7690

 BOND    =    32705.3280  ANGLE   =     1172.2101  DIHED      =     6493.0949
 VDWAALS =    79822.3487  EEL     =  -577907.6119  HBOND      =        0.0000
 1-4 VDW =     1838.3512  1-4 EEL =    25754.8170  RESTRAINT  =        0.0000


  NSTEP       ENERGY          RMS            GMAX         NAME    NUMBER
   2700      -4.3033E+05     1.0044E-01     7.6430E+00     CE1      7690

 BOND    =    32743.0278  ANGLE   =     1171.6378  DIHED      =     6491.7219
 VDWAALS =    79980.1719  EEL     =  -578303.3298  HBOND      =        0.0000
 1-4 VDW =     1837.0237  1-4 EEL =    25752.0809  RESTRAINT  =        0.0000


  NSTEP       ENERGY          RMS            GMAX         NAME    NUMBER
   2800      -4.3053E+05     3.8553E-01     7.7209E+01     CG       3213

 BOND    =    32788.1055  ANGLE   =     1171.6400  DIHED      =     6490.2029
 VDWAALS =    80136.0177  EEL     =  -578699.0928  HBOND      =        0.0000
 1-4 VDW =     1835.5900  1-4 EEL =    25749.6499  RESTRAINT  =        0.0000
```

```
  NSTEP       ENERGY          RMS            GMAX         NAME    NUMBER
   2900      -4.3073E+05     3.2051E-01     4.9022E+01     CE1      7690

 BOND    =     32823.3959  ANGLE   =      1171.0376  DIHED      =      6488.4719
 VDWAALS =     80284.2560  EEL     =   -579079.1115  HBOND      =         0.0000
 1-4 VDW =      1834.3608  1-4 EEL =     25747.2164  RESTRAINT  =         0.0000


  NSTEP       ENERGY          RMS            GMAX         NAME    NUMBER
   3000      -4.3093E+05     2.6854E-01     4.2445E+01     CE1      7690

 BOND    =     32857.7391  ANGLE   =      1170.5752  DIHED      =      6486.7037
 VDWAALS =     80424.2435  EEL     =   -579442.7223  HBOND      =         0.0000
 1-4 VDW =      1833.0532  1-4 EEL =     25744.9564  RESTRAINT  =         0.0000


  NSTEP       ENERGY          RMS            GMAX         NAME    NUMBER
   3100      -4.3112E+05     2.4035E-01     3.7521E+01     CE1      7690

 BOND    =     32893.1967  ANGLE   =      1169.6927  DIHED      =      6485.2343
 VDWAALS =     80561.7306  EEL     =   -579806.3702  HBOND      =         0.0000
 1-4 VDW =      1831.8022  1-4 EEL =     25743.5336  RESTRAINT  =         0.0000


  NSTEP       ENERGY          RMS            GMAX         NAME    NUMBER
   3200      -4.3132E+05     1.4333E-01     1.8055E+01     CE1      7690

 BOND    =     32927.3349  ANGLE   =      1168.2806  DIHED      =      6484.6351
 VDWAALS =     80697.6263  EEL     =   -580167.9301  HBOND      =         0.0000
 1-4 VDW =      1830.6338  1-4 EEL =     25742.4137  RESTRAINT  =         0.0000


  NSTEP       ENERGY          RMS            GMAX         NAME    NUMBER
   3300      -4.3151E+05     9.7663E-02     7.8731E+00     CE1      7690

 BOND    =     32962.0136  ANGLE   =      1167.3962  DIHED      =      6483.6509
 VDWAALS =     80830.0793  EEL     =   -580519.2542  HBOND      =         0.0000
 1-4 VDW =      1829.7813  1-4 EEL =     25741.3101  RESTRAINT  =         0.0000


  NSTEP       ENERGY          RMS            GMAX         NAME    NUMBER
   3400      -4.3169E+05     1.0844E-01     1.1271E+01     CE1      7690

 BOND    =     32998.2332  ANGLE   =      1166.8887  DIHED      =      6481.9417
 VDWAALS =     80966.9609  EEL     =   -580876.7537  HBOND      =         0.0000
 1-4 VDW =      1829.0472  1-4 EEL =     25740.2841  RESTRAINT  =         0.0000


  NSTEP       ENERGY          RMS            GMAX         NAME    NUMBER
   3500      -4.3188E+05     9.6518E-02     7.8744E+00     CE1      7690

 BOND    =     33034.5426  ANGLE   =      1166.4964  DIHED      =      6480.1533
```

```
VDWAALS =     81107.6123  EEL      =  -581234.8831  HBOND      =         0.0000
1-4 VDW =      1828.4030  1-4 EEL =    25739.4789  RESTRAINT  =         0.0000


  NSTEP       ENERGY          RMS            GMAX         NAME    NUMBER
  3600      -4.3205E+05     2.5875E-01     3.8888E+01     CE1      7690

BOND    =     33073.7323  ANGLE    =      1166.4610  DIHED      =      6478.7013
VDWAALS =     81249.5737  EEL      =  -581587.8405  HBOND      =         0.0000
1-4 VDW =      1827.7786  1-4 EEL =    25738.8230  RESTRAINT  =         0.0000


  NSTEP       ENERGY          RMS            GMAX         NAME    NUMBER
  3700      -4.3223E+05     2.3593E-01     3.5061E+01     CE1      7690

BOND    =     33109.2388  ANGLE    =      1166.3445  DIHED      =      6477.7481
VDWAALS =     81391.6173  EEL      =  -581936.8626  HBOND      =         0.0000
1-4 VDW =      1827.1398  1-4 EEL =    25737.9213  RESTRAINT  =         0.0000


  NSTEP       ENERGY          RMS            GMAX         NAME    NUMBER
  3800      -4.3240E+05     1.4182E-01     1.7990E+01     CE1      7690

BOND    =     33142.9129  ANGLE    =      1166.2205  DIHED      =      6477.1349
VDWAALS =     81532.0803  EEL      =  -582277.2856  HBOND      =         0.0000
1-4 VDW =      1826.5301  1-4 EEL =    25737.2001  RESTRAINT  =         0.0000


  NSTEP       ENERGY          RMS            GMAX         NAME    NUMBER
  3900      -4.3256E+05     1.5320E-01     2.0595E+01     CE1      7690

BOND    =     33177.9969  ANGLE    =      1166.5085  DIHED      =      6476.7605
VDWAALS =     81672.2008  EEL      =  -582612.6009  HBOND      =         0.0000
1-4 VDW =      1825.8607  1-4 EEL =    25736.2611  RESTRAINT  =         0.0000


  NSTEP       ENERGY          RMS            GMAX         NAME    NUMBER
  4000      -4.3271E+05     7.9563E-02     3.3188E+00     CE1      7690

BOND    =     33211.0011  ANGLE    =      1166.6319  DIHED      =      6476.3850
VDWAALS =     81808.8036  EEL      =  -582936.2866  HBOND      =         0.0000
1-4 VDW =      1825.2025  1-4 EEL =    25735.3734  RESTRAINT  =         0.0000


  NSTEP       ENERGY          RMS            GMAX         NAME    NUMBER
  4100      -4.3286E+05     3.3584E-01     1.0048E+02     CG       3213

BOND    =     33249.1173  ANGLE    =      1167.2749  DIHED      =      6476.0080
VDWAALS =     81944.1824  EEL      =  -583255.5236  HBOND      =         0.0000
1-4 VDW =      1824.4520  1-4 EEL =    25734.6292  RESTRAINT  =         0.0000


  NSTEP       ENERGY          RMS            GMAX         NAME    NUMBER
  4200      -4.3301E+05     2.3594E-01     3.2671E+01     CE1      7690
```

```
BOND    =     33278.9146  ANGLE   =      1167.5729  DIHED      =      6475.7748
VDWAALS =     82075.6547  EEL     =   -583562.5531  HBOND      =         0.0000
1-4 VDW =      1823.8053  1-4 EEL =     25733.3468  RESTRAINT  =         0.0000


  NSTEP       ENERGY          RMS            GMAX         NAME    NUMBER
   4300      -4.3315E+05     2.3575E-01     3.3055E+01     CG       3213

BOND    =     33310.3633  ANGLE   =      1167.7409  DIHED      =      6475.6843
VDWAALS =     82205.2476  EEL     =   -583864.5727  HBOND      =         0.0000
1-4 VDW =      1823.2308  1-4 EEL =     25732.3246  RESTRAINT  =         0.0000


  NSTEP       ENERGY          RMS            GMAX         NAME    NUMBER
   4400      -4.3329E+05     1.8235E-01     2.2265E+01     CE1      7690

BOND    =     33340.1505  ANGLE   =      1168.1683  DIHED      =      6475.7611
VDWAALS =     82332.4773  EEL     =   -584160.2654  HBOND      =         0.0000
1-4 VDW =      1822.5187  1-4 EEL =     25731.0794  RESTRAINT  =         0.0000


  NSTEP       ENERGY          RMS            GMAX         NAME    NUMBER
   4500      -4.3342E+05     1.5117E-01     1.7610E+01     CE1      7690

BOND    =     33369.3467  ANGLE   =      1168.1790  DIHED      =      6475.8601
VDWAALS =     82455.7767  EEL     =   -584445.3600  HBOND      =         0.0000
1-4 VDW =      1821.9624  1-4 EEL =     25729.7881  RESTRAINT  =         0.0000


  NSTEP       ENERGY          RMS            GMAX         NAME    NUMBER
   4600      -4.3356E+05     7.6819E-02     3.8025E+00     CG       3213

BOND    =     33397.2927  ANGLE   =      1168.3381  DIHED      =      6475.7621
VDWAALS =     82575.6560  EEL     =   -584723.2739  HBOND      =         0.0000
1-4 VDW =      1821.3715  1-4 EEL =     25728.1632  RESTRAINT  =         0.0000


  NSTEP       ENERGY          RMS            GMAX         NAME    NUMBER
   4700      -4.3369E+05     8.8181E-02     7.0531E+00     NE2      8655

BOND    =     33425.2658  ANGLE   =      1168.4641  DIHED      =      6474.2225
VDWAALS =     82691.2285  EEL     =   -584992.9022  HBOND      =         0.0000
1-4 VDW =      1820.8578  1-4 EEL =     25726.4283  RESTRAINT  =         0.0000


  NSTEP       ENERGY          RMS            GMAX         NAME    NUMBER
   4800      -4.3381E+05     7.5012E-02     3.6977E+00     NE2      8655

BOND    =     33452.1764  ANGLE   =      1168.6809  DIHED      =      6472.8166
VDWAALS =     82802.4675  EEL     =   -585254.9780  HBOND      =         0.0000
1-4 VDW =      1820.3536  1-4 EEL =     25724.6591  RESTRAINT  =         0.0000
```

```
  NSTEP       ENERGY          RMS            GMAX         NAME    NUMBER
   4900      -4.3394E+05     2.0032E-01     2.6049E+01     NE2      8655

 BOND    =     33480.3896  ANGLE   =      1169.2780  DIHED      =      6472.1110
 VDWAALS =     82910.9901  EEL     =   -585514.6411  HBOND      =         0.0000
 1-4 VDW =      1819.8707  1-4 EEL =     25722.8852  RESTRAINT  =         0.0000


  NSTEP       ENERGY          RMS            GMAX         NAME    NUMBER
   5000      -4.3407E+05     1.7436E-01     2.3542E+01     NE2      8655

 BOND    =     33506.0853  ANGLE   =      1169.2249  DIHED      =      6471.6103
 VDWAALS =     83017.0209  EEL     =   -585770.2257  HBOND      =         0.0000
 1-4 VDW =      1819.4910  1-4 EEL =     25721.3021  RESTRAINT  =         0.0000


  Maximum number of minimization cycles reached.


                    FINAL RESULTS



  NSTEP       ENERGY          RMS            GMAX         NAME    NUMBER
   5000      -4.3407E+05     1.7436E-01     2.3542E+01     NE2      8655

 BOND    =     33506.0853  ANGLE   =      1169.2249  DIHED      =      6471.6103
 VDWAALS =     83017.0209  EEL     =   -585770.2257  HBOND      =         0.0000
 1-4 VDW =      1819.4910  1-4 EEL =     25721.3021  RESTRAINT  =         0.0000

--------------------------------------------------------------------------------
   5.  TIMINGS
--------------------------------------------------------------------------------

|                Build the list            22.81 (88.24% of List )
|                Other                      3.04 (11.76% of List )
|             List time                 25.85 ( 0.25% of Nonbo)
|                   Short_ene time          9466.54 (99.66% of Direc)
|                   Other                     31.99 ( 0.34% of Direc)
|                Direct Ewald time       9498.53 (91.70% of Ewald)
|                Adjust Ewald time         35.33 ( 0.34% of Ewald)
|                   Fill Bspline coeffs       27.74 ( 3.39% of Recip)
|                   Fill charge grid         120.27 (14.71% of Recip)
|                   Scalar sum               155.67 (19.04% of Recip)
|                   Grad sum                 205.51 (25.14% of Recip)
|                   FFT time                 308.22 (37.71% of Recip)
|                Recip Ewald time         817.42 ( 7.89% of Ewald)
|                Virial junk                2.34 ( 0.02% of Ewald)
|                Other                      5.12 ( 0.05% of Ewald)
|             Ewald time             10358.76 (99.75% of Nonbo)
|          Nonbond force          10384.62 (99.51% of Force)
|          Bond/Angle/Dihedral       50.22 ( 0.48% of Force)
|          Other                      1.38 ( 0.01% of Force)
|       Force time             10436.22 (100.0% of Runmd)
```

```
|    Runmd Time            10436.22 (99.71% of Total)
|    Other                    30.36 ( 0.29% of Total)
| Total time             10466.62 (100.0% of ALL  )

| Number of list builds   :         14

| Highest rstack allocated:    5837340
| Highest istack allocated:     108958
|           Job began  at 00:51:42.048  on 08/13/2017
|           Setup done at 00:51:43.000  on 08/13/2017
|           Run   done at 03:46:08.665  on 08/13/2017
|     wallclock() was called  190076 times

          -------------------------------------------------------
          Amber 16 SANDER                              2016
          -------------------------------------------------------

| Run on 08/14/2017 at 01:34:10

|   Executable path: /usr/local/amber16/bin/sander
| Working directory: /Users/Swati
|          Hostname: Unknown
  [-O]verwriting output

File Assignments:
|  MDIN: minimization_file_2.in
| MDOUT: LJP6_min3_2.out
|INPCRD: LJP6_min3_1.rst
|  PARM: /Users/Swati/LJP6_files/LJP6.prmtop
|RESTRT: LJP6_min3_2.rst
|  REFC: refc
| MDVEL: mdvel
| MDFRC: mdfrc
|  MDEN: mden
| MDCRD: mdcrd
|MDINFO: mdinfo
|  MTMD: mtmd
|INPDIP: inpdip
|RSTDIP: rstdip
|INPTRA: inptraj


 Here is the input file:

PROT: minimization_2
&cntrl
imin = 1,
maxcyc = 5000,
ncyc = 10000,
igb = 0,
ntpr =  100,
ntp = 0
cut = 12
&end
```

```
--------------------------------------------------------------------------------
   1.  RESOURCE   USE: 
--------------------------------------------------------------------------------

| Flags:                                                                        
 getting box info from netcdf restart file
| NetCDF restart box info found
|Largest sphere to fit in unit cell has radius =    44.819
| New format PARM file being parsed.
| Version =    1.000 Date = 08/06/17 Time = 07:17:45
 NATOM  =  107805 NTYPES =      17 NBONH =  103425 MBONA  =    4491
 NTHETH =    9523 MTHETA =    6101 NPHIH =   19785 MPHIA  =   18920
 NHPARM =       0 NPARM  =       0 NNB   =  179511 NRES   =   33643
 NBONA  =    4491 NTHETA =    6101 NPHIA =   18920 NUMBND =      67
 NUMANG =     152 NPTRA  =     191 NATYP =      36 NPHB   =       1
 IFBOX  =       1 NMXRS  =      24 IFCAP =       0 NEXTRA =       0
 NCOPY  =       0


|     Memory Use     Allocated
|     Real             8496271
|     Hollerith         357060
|     Integer          4323872
|     Max Pairs       98605640
|     nblistReal       1293660
|     nblist Int       3573335
|       Total           493905 kbytes

| Note: 1-4 EEL scale factors are being read from the topology file.

| Note: 1-4 VDW scale factors are being read from the topology file.
| Duplicated    0 dihedrals
| Duplicated    0 dihedrals

     BOX TYPE: RECTILINEAR

--------------------------------------------------------------------------------
   2.  CONTROL  DATA  FOR  THE  RUN
--------------------------------------------------------------------------------

default_name                                                                    

General flags:
     imin    =       1, nmropt  =       0

Nature and format of input:
     ntx     =       1, irest   =       0, ntrx    =       1

Nature and format of output:
     ntxo    =       2, ntpr    =     100, ntrx    =       1, ntwr    =       1
     iwrap   =       0, ntwx    =       0, ntwv    =       0, ntwe    =       0
     ioutfm  =       1, ntwprt  =       0, idecomp =       0, rbornstat=      0
```

```
Potential function:
     ntf     =       1, ntb     =       1, igb     =       0, nsnb    =      25
     ipol    =       0, gbsa    =       0, iesp    =       0
     dielc   =   1.00000, cut     =  12.00000, intdiel =   1.00000

Frozen or restrained atoms:
     ibelly  =       0, ntr     =       0

Energy minimization:
     maxcyc  =    5000, ncyc    =   10000, ntmin   =       1
     dx0     =   0.01000, drms    =   0.00010

Ewald parameters:
     verbose =       0, ew_type =       0, nbflag  =       1, use_pme =       1
     vdwmeth =       1, eedmeth =       1, netfrc  =       0
     Box X =  117.910   Box Y =   89.637   Box Z =  121.218
     Alpha =   90.000   Beta  =   90.000   Gamma =   90.000
     NFFT1 =  120       NFFT2 =   90       NFFT3 =  125
     Cutoff=   12.000   Tol   =0.100E-04
     Ewald Coefficient =  0.22664
     Interpolation order =    4
|  INFO: Old style inpcrd file read


--------------------------------------------------------------------------------
   3.  ATOMIC COORDINATES AND VELOCITIES
--------------------------------------------------------------------------------

default_name
 begin time read from input coords =     0.000 ps

 Number of triangulated 3-point waters found:    33071

     Sum of charges from parm topology file =  -0.00000035
     Forcing neutrality...

--------------------------------------------------------------------------------
   4.  RESULTS
--------------------------------------------------------------------------------

 ---------------------------------------------------
 APPROXIMATING switch and d/dx switch using CUBIC SPLINE INTERPOLATION
 using   5000.0 points per unit in tabled values
 TESTING RELATIVE ERROR over r ranging from 0.0 to cutoff
| CHECK switch(x): max rel err =   0.2738E-14   at   2.422500
| CHECK d/dx switch(x): max rel err =   0.7967E-11   at   2.716640
 ---------------------------------------------------
| Local SIZE OF NONBOND LIST =   53314213
| TOTAL SIZE OF NONBOND LIST =   53314213


   NSTEP       ENERGY          RMS            GMAX         NAME    NUMBER
      1      -4.0036E+05     4.7605E+00     7.0995E+02     C        3792
```

```
BOND    =    40159.1664  ANGLE   =     4173.9815  DIHED      =     6614.0321
VDWAALS =    78057.3200  EEL     =  -556195.6061  HBOND      =        0.0000
1-4 VDW =     2790.3026  1-4 EEL =    24042.2383  RESTRAINT  =        0.0000


   NSTEP       ENERGY          RMS            GMAX         NAME    NUMBER
    100      -4.1752E+05     4.2203E-01     7.5970E+01     N        3794

BOND    =    32073.8265  ANGLE   =     1993.4333  DIHED      =     6519.7751
VDWAALS =    73667.5498  EEL     =  -557327.3895  HBOND      =        0.0000
1-4 VDW =     2081.6286  1-4 EEL =    23468.1826  RESTRAINT  =        0.0000


   NSTEP       ENERGY          RMS            GMAX         NAME    NUMBER
    200      -4.1948E+05     5.9705E-01     1.3145E+02     C        3812

BOND    =    31640.3039  ANGLE   =     1666.7976  DIHED      =     6482.3217
VDWAALS =    73787.2375  EEL     =  -558374.2134  HBOND      =        0.0000
1-4 VDW =     1947.5960  1-4 EEL =    23368.2252  RESTRAINT  =        0.0000


   NSTEP       ENERGY          RMS            GMAX         NAME    NUMBER
    300      -4.2058E+05     5.2360E-01     9.5490E+01     C        3812

BOND    =    31530.7177  ANGLE   =     1521.5410  DIHED      =     6459.1489
VDWAALS =    74055.1402  EEL     =  -559374.4469  HBOND      =        0.0000
1-4 VDW =     1890.2184  1-4 EEL =    23335.6818  RESTRAINT  =        0.0000


   NSTEP       ENERGY          RMS            GMAX         NAME    NUMBER
    400      -4.2135E+05     4.2680E-01     9.1233E+01     C        3812

BOND    =    31501.2383  ANGLE   =     1447.0847  DIHED      =     6442.6834
VDWAALS =    74359.0674  EEL     =  -560290.1587  HBOND      =        0.0000
1-4 VDW =     1860.7054  1-4 EEL =    23326.2782  RESTRAINT  =        0.0000


   NSTEP       ENERGY          RMS            GMAX         NAME    NUMBER
    500      -4.2196E+05     2.8243E-01     4.8215E+01     CD       3230

BOND    =    31526.1727  ANGLE   =     1388.3608  DIHED      =     6431.1022
VDWAALS =    74667.2365  EEL     =  -561135.3787  HBOND      =        0.0000
1-4 VDW =     1842.8537  1-4 EEL =    23320.2571  RESTRAINT  =        0.0000


   NSTEP       ENERGY          RMS            GMAX         NAME    NUMBER
    600      -4.2247E+05     2.2774E-01     4.4242E+01     C        3812

BOND    =    31561.8449  ANGLE   =     1356.3709  DIHED      =     6423.0452
VDWAALS =    74973.5376  EEL     =  -561927.1480  HBOND      =        0.0000
1-4 VDW =     1830.9076  1-4 EEL =    23316.3241  RESTRAINT  =        0.0000
```

```
   NSTEP       ENERGY          RMS            GMAX         NAME    NUMBER
    700      -4.2290E+05     1.4834E-01     1.5758E+01     C        3812

 BOND    =    31611.3304  ANGLE   =     1331.0660  DIHED      =     6415.5626
 VDWAALS =    75269.2559  EEL     =  -562665.9996  HBOND      =        0.0000
 1-4 VDW =     1821.9016  1-4 EEL =    23313.2512  RESTRAINT  =        0.0000


   NSTEP       ENERGY          RMS            GMAX         NAME    NUMBER
    800      -4.2330E+05     1.3579E-01     1.2911E+01     C        3812

 BOND    =    31663.3559  ANGLE   =     1315.7513  DIHED      =     6409.5776
 VDWAALS =    75566.3926  EEL     =  -563384.5002  HBOND      =        0.0000
 1-4 VDW =     1814.9841  1-4 EEL =    23309.8510  RESTRAINT  =        0.0000


   NSTEP       ENERGY          RMS            GMAX         NAME    NUMBER
    900      -4.2367E+05     4.8981E-01     8.8835E+01     C        3812

 BOND    =    31726.4121  ANGLE   =     1307.2666  DIHED      =     6403.8369
 VDWAALS =    75869.7140  EEL     =  -564093.4667  HBOND      =        0.0000
 1-4 VDW =     1809.5157  1-4 EEL =    23307.7011  RESTRAINT  =        0.0000


   NSTEP       ENERGY          RMS            GMAX         NAME    NUMBER
   1000      -4.2401E+05     4.4153E-01     9.6354E+01     CD       3230

 BOND    =    31785.5620  ANGLE   =     1292.2703  DIHED      =     6398.9747
 VDWAALS =    76158.5055  EEL     =  -564755.3911  HBOND      =        0.0000
 1-4 VDW =     1805.6116  1-4 EEL =    23307.8694  RESTRAINT  =        0.0000


   NSTEP       ENERGY          RMS            GMAX         NAME    NUMBER
   1100      -4.2431E+05     3.4125E-01     6.4933E+01     C        3812

 BOND    =    31832.1085  ANGLE   =     1283.9429  DIHED      =     6397.6308
 VDWAALS =    76428.8077  EEL     =  -565368.5764  HBOND      =        0.0000
 1-4 VDW =     1802.1439  1-4 EEL =    23309.2388  RESTRAINT  =        0.0000


   NSTEP       ENERGY          RMS            GMAX         NAME    NUMBER
   1200      -4.2462E+05     3.0420E-01     4.9586E+01     CZ       3806

 BOND    =    31889.3631  ANGLE   =     1225.7687  DIHED      =     6411.5608
 VDWAALS =    76685.4536  EEL     =  -565954.2588  HBOND      =        0.0000
 1-4 VDW =     1799.1220  1-4 EEL =    23319.3490  RESTRAINT  =        0.0000


   NSTEP       ENERGY          RMS            GMAX         NAME    NUMBER
   1300      -4.2491E+05     1.9717E-01     3.1292E+01     CE2      3808

 BOND    =    31937.1838  ANGLE   =     1204.2795  DIHED      =     6405.0534
 VDWAALS =    76933.9375  EEL     =  -566512.3338  HBOND      =        0.0000
 1-4 VDW =     1796.2227  1-4 EEL =    23321.8231  RESTRAINT  =        0.0000
```

```
  NSTEP       ENERGY          RMS            GMAX         NAME    NUMBER
   1400      -4.2518E+05     1.5912E-01     2.3584E+01     CZ       3806

 BOND    =     31984.2500  ANGLE   =      1198.7269  DIHED      =      6391.9452
 VDWAALS =     77164.6413  EEL     =   -567033.0362  HBOND      =         0.0000
 1-4 VDW =      1793.8397  1-4 EEL =     23322.0726  RESTRAINT  =         0.0000


  NSTEP       ENERGY          RMS            GMAX         NAME    NUMBER
   1500      -4.2542E+05     1.0919E-01     1.1430E+01     CE2      3808

 BOND    =     32029.0480  ANGLE   =      1195.3299  DIHED      =      6386.8404
 VDWAALS =     77380.7945  EEL     =   -567529.3288  HBOND      =         0.0000
 1-4 VDW =      1791.7565  1-4 EEL =     23323.5169  RESTRAINT  =         0.0000


  NSTEP       ENERGY          RMS            GMAX         NAME    NUMBER
   1600      -4.2565E+05     4.2111E-01     7.8000E+01     CD       3230

 BOND    =     32081.0993  ANGLE   =      1193.8988  DIHED      =      6381.9940
 VDWAALS =     77586.3012  EEL     =   -568011.7539  HBOND      =         0.0000
 1-4 VDW =      1789.5505  1-4 EEL =     23326.0576  RESTRAINT  =         0.0000


  NSTEP       ENERGY          RMS            GMAX         NAME    NUMBER
   1700      -4.2588E+05     3.4467E-01     6.7153E+01     CZ       3806

 BOND    =     32121.3542  ANGLE   =      1191.5326  DIHED      =      6377.1254
 VDWAALS =     77779.5714  EEL     =   -568467.5694  HBOND      =         0.0000
 1-4 VDW =      1787.7827  1-4 EEL =     23328.0820  RESTRAINT  =         0.0000


  NSTEP       ENERGY          RMS            GMAX         NAME    NUMBER
   1800      -4.2610E+05     3.1399E-01     7.2533E+01     CD       3230

 BOND    =     32160.9932  ANGLE   =      1189.7351  DIHED      =      6372.6317
 VDWAALS =     77964.8542  EEL     =   -568905.3069  HBOND      =         0.0000
 1-4 VDW =      1786.1920  1-4 EEL =     23329.3929  RESTRAINT  =         0.0000


  NSTEP       ENERGY          RMS            GMAX         NAME    NUMBER
   1900      -4.2631E+05     2.7061E-01     4.9665E+01     CE2      3808

 BOND    =     32200.7163  ANGLE   =      1188.0689  DIHED      =      6368.8913
 VDWAALS =     78140.7665  EEL     =   -569325.1557  HBOND      =         0.0000
 1-4 VDW =      1784.5532  1-4 EEL =     23330.1925  RESTRAINT  =         0.0000


  NSTEP       ENERGY          RMS            GMAX         NAME    NUMBER
   2000      -4.2652E+05     1.5137E-01     2.5248E+01     CE2      3808

 BOND    =     32237.8330  ANGLE   =      1186.8015  DIHED      =      6366.0304
```

```
VDWAALS =     78312.2094  EEL      =  -569734.2486  HBOND      =         0.0000
1-4 VDW =      1783.1387  1-4 EEL =    23330.6318  RESTRAINT  =         0.0000


  NSTEP       ENERGY          RMS            GMAX         NAME    NUMBER
   2100      -4.2672E+05     9.5443E-02     7.1025E+00     CZ       3806

BOND    =     32277.0022  ANGLE    =      1185.7779  DIHED      =      6363.7445
VDWAALS =     78483.7133  EEL      =  -570139.4495  HBOND      =         0.0000
1-4 VDW =      1781.8925  1-4 EEL =    23330.9989  RESTRAINT  =         0.0000


  NSTEP       ENERGY          RMS            GMAX         NAME    NUMBER
   2200      -4.2691E+05     9.6173E-02     9.0962E+00     CE2      3808

BOND    =     32317.5350  ANGLE    =      1184.9615  DIHED      =      6361.7807
VDWAALS =     78658.5543  EEL      =  -570548.0313  HBOND      =         0.0000
1-4 VDW =      1780.7519  1-4 EEL =    23331.4051  RESTRAINT  =         0.0000


  NSTEP       ENERGY          RMS            GMAX         NAME    NUMBER
   2300      -4.2710E+05     3.5344E-01     7.2085E+01     CD       3230

BOND    =     32363.4110  ANGLE    =      1184.7582  DIHED      =      6360.2636
VDWAALS =     78833.4293  EEL      =  -570955.2340  HBOND      =         0.0000
1-4 VDW =      1779.7350  1-4 EEL =    23331.8598  RESTRAINT  =         0.0000


  NSTEP       ENERGY          RMS            GMAX         NAME    NUMBER
   2400      -4.2729E+05     2.8641E-01     5.3787E+01     CD       3230

BOND    =     32400.7073  ANGLE    =      1183.6718  DIHED      =      6359.3655
VDWAALS =     79001.4905  EEL      =  -571348.3138  HBOND      =         0.0000
1-4 VDW =      1778.6566  1-4 EEL =    23332.4375  RESTRAINT  =         0.0000


  NSTEP       ENERGY          RMS            GMAX         NAME    NUMBER
   2500      -4.2748E+05     2.5626E-01     5.1406E+01     CE2      3808

BOND    =     32439.3989  ANGLE    =      1183.2020  DIHED      =      6355.1172
VDWAALS =     79169.7727  EEL      =  -571742.3009  HBOND      =         0.0000
1-4 VDW =      1777.4784  1-4 EEL =    23332.9304  RESTRAINT  =         0.0000


  NSTEP       ENERGY          RMS            GMAX         NAME    NUMBER
   2600      -4.2767E+05     1.5318E-01     2.3446E+01     CE2      3808

BOND    =     32476.5295  ANGLE    =      1182.5608  DIHED      =      6352.6728
VDWAALS =     79338.0036  EEL      =  -572127.9059  HBOND      =         0.0000
1-4 VDW =      1776.5026  1-4 EEL =    23333.2685  RESTRAINT  =         0.0000


  NSTEP       ENERGY          RMS            GMAX         NAME    NUMBER
   2700      -4.2784E+05     1.1493E-01     1.5842E+01     CE2      3808
```

```
BOND    =     32512.6890  ANGLE   =      1181.8367  DIHED      =      6350.0138
VDWAALS =     79495.5559  EEL     =   -572488.5002  HBOND      =         0.0000
1-4 VDW =      1775.7392  1-4 EEL =     23333.5700  RESTRAINT  =         0.0000


  NSTEP       ENERGY          RMS            GMAX         NAME    NUMBER
   2800      -4.2800E+05     8.7812E-02     7.1043E+00     CD1      3802

BOND    =     32548.0015  ANGLE   =      1180.9310  DIHED      =      6347.5716
VDWAALS =     79647.3427  EEL     =   -572835.4372  HBOND      =         0.0000
1-4 VDW =      1775.2324  1-4 EEL =     23333.6533  RESTRAINT  =         0.0000


  NSTEP       ENERGY          RMS            GMAX         NAME    NUMBER
   2900      -4.2816E+05     1.0100E-01     1.3537E+01     CE2      3808

BOND    =     32583.2789  ANGLE   =      1180.1377  DIHED      =      6345.4171
VDWAALS =     79796.9423  EEL     =   -573176.8380  HBOND      =         0.0000
1-4 VDW =      1774.7179  1-4 EEL =     23333.2584  RESTRAINT  =         0.0000


  NSTEP       ENERGY          RMS            GMAX         NAME    NUMBER
   3000      -4.2832E+05     2.8262E-01     5.0628E+01     CE2      3808

BOND    =     32621.9654  ANGLE   =      1179.5955  DIHED      =      6343.3918
VDWAALS =     79948.1785  EEL     =   -573520.3245  HBOND      =         0.0000
1-4 VDW =      1774.0978  1-4 EEL =     23332.5471  RESTRAINT  =         0.0000


  NSTEP       ENERGY          RMS            GMAX         NAME    NUMBER
   3100      -4.2848E+05     2.3012E-01     4.4178E+01     CE2      3808

BOND    =     32655.6422  ANGLE   =      1178.6979  DIHED      =      6341.3892
VDWAALS =     80097.8365  EEL     =   -573857.3012  HBOND      =         0.0000
1-4 VDW =      1773.4839  1-4 EEL =     23331.6245  RESTRAINT  =         0.0000


  NSTEP       ENERGY          RMS            GMAX         NAME    NUMBER
   3200      -4.2863E+05     2.1479E-01     3.6635E+01     CD       3230

BOND    =     32689.5754  ANGLE   =      1178.1486  DIHED      =      6338.9210
VDWAALS =     80246.8669  EEL     =   -574190.0170  HBOND      =         0.0000
1-4 VDW =      1772.8807  1-4 EEL =     23330.6770  RESTRAINT  =         0.0000


  NSTEP       ENERGY          RMS            GMAX         NAME    NUMBER
   3300      -4.2878E+05     1.7219E-01     3.1147E+01     CE2      3808

BOND    =     32721.9198  ANGLE   =      1177.3450  DIHED      =      6336.8914
VDWAALS =     80391.8714  EEL     =   -574510.0997  HBOND      =         0.0000
1-4 VDW =      1772.3983  1-4 EEL =     23329.9326  RESTRAINT  =         0.0000
```

```
  NSTEP       ENERGY          RMS            GMAX         NAME    NUMBER
   3400      -4.2893E+05     8.1284E-02     6.7763E+00     CE2      3808

 BOND    =     32753.9897  ANGLE   =      1176.7510  DIHED      =      6335.5354
 VDWAALS =     80538.5270  EEL     =   -574831.7005  HBOND      =         0.0000
 1-4 VDW =      1771.9497  1-4 EEL =     23329.1356  RESTRAINT  =         0.0000


  NSTEP       ENERGY          RMS            GMAX         NAME    NUMBER
   3500      -4.2907E+05     9.4228E-02     1.2073E+01     CE2      3808

 BOND    =     32787.0335  ANGLE   =      1176.3653  DIHED      =      6334.4890
 VDWAALS =     80683.7861  EEL     =   -575150.4336  HBOND      =         0.0000
 1-4 VDW =      1771.5115  1-4 EEL =     23328.3161  RESTRAINT  =         0.0000


  NSTEP       ENERGY          RMS            GMAX         NAME    NUMBER
   3600      -4.2921E+05     2.7081E-01     4.8061E+01     CE2      3808

 BOND    =     32822.8357  ANGLE   =      1176.3322  DIHED      =      6333.6054
 VDWAALS =     80827.5624  EEL     =   -575466.9056  HBOND      =         0.0000
 1-4 VDW =      1771.0916  1-4 EEL =     23327.4922  RESTRAINT  =         0.0000


  NSTEP       ENERGY          RMS            GMAX         NAME    NUMBER
   3700      -4.2935E+05     2.2450E-01     4.2361E+01     CE2      3808

 BOND    =     32853.8954  ANGLE   =      1176.0403  DIHED      =      6332.8344
 VDWAALS =     80967.9834  EEL     =   -575777.9413  HBOND      =         0.0000
 1-4 VDW =      1770.6226  1-4 EEL =     23326.6463  RESTRAINT  =         0.0000


  NSTEP       ENERGY          RMS            GMAX         NAME    NUMBER
   3800      -4.2949E+05     1.9314E-01     3.4823E+01     CE2      3808

 BOND    =     32884.3866  ANGLE   =      1175.5451  DIHED      =      6332.2109
 VDWAALS =     81103.3377  EEL     =   -576078.2902  HBOND      =         0.0000
 1-4 VDW =      1770.1893  1-4 EEL =     23325.7696  RESTRAINT  =         0.0000


  NSTEP       ENERGY          RMS            GMAX         NAME    NUMBER
   3900      -4.2963E+05     1.2553E-01     2.1301E+01     CE2      3808

 BOND    =     32915.3777  ANGLE   =      1175.1394  DIHED      =      6331.6896
 VDWAALS =     81242.9836  EEL     =   -576386.0594  HBOND      =         0.0000
 1-4 VDW =      1769.7879  1-4 EEL =     23324.7174  RESTRAINT  =         0.0000


  NSTEP       ENERGY          RMS            GMAX         NAME    NUMBER
   4000      -4.2976E+05     7.7552E-02     6.0963E+00     CE2      3808

 BOND    =     32945.4667  ANGLE   =      1174.7274  DIHED      =      6331.2640
 VDWAALS =     81376.7889  EEL     =   -576679.1745  HBOND      =         0.0000
 1-4 VDW =      1769.3567  1-4 EEL =     23323.6069  RESTRAINT  =         0.0000
```

```
   NSTEP       ENERGY          RMS            GMAX         NAME    NUMBER
   4100      -4.2989E+05     3.0480E-01     5.8444E+01     CE2      3808

 BOND    =     32980.3989  ANGLE   =      1174.7729  DIHED      =      6330.2750
 VDWAALS =     81511.4227  EEL     =   -576974.6184  HBOND      =         0.0000
 1-4 VDW =      1768.8466  1-4 EEL =     23322.2750  RESTRAINT  =         0.0000


   NSTEP       ENERGY          RMS            GMAX         NAME    NUMBER
   4200      -4.3002E+05     7.6980E-02     6.2645E+00     CE2      3808

 BOND    =     33005.3701  ANGLE   =      1174.3094  DIHED      =      6327.9855
 VDWAALS =     81638.7793  EEL     =   -577254.6966  HBOND      =         0.0000
 1-4 VDW =      1768.2723  1-4 EEL =     23320.6105  RESTRAINT  =         0.0000


   NSTEP       ENERGY          RMS            GMAX         NAME    NUMBER
   4300      -4.3014E+05     2.1207E-01     4.1527E+01     CE2      3808

 BOND    =     33036.4328  ANGLE   =      1174.3391  DIHED      =      6326.5923
 VDWAALS =     81766.4419  EEL     =   -577534.9707  HBOND      =         0.0000
 1-4 VDW =      1767.6381  1-4 EEL =     23318.8112  RESTRAINT  =         0.0000


   NSTEP       ENERGY          RMS            GMAX         NAME    NUMBER
   4400      -4.3027E+05     1.9147E-01     3.6979E+01     CE2      3808

 BOND    =     33064.7771  ANGLE   =      1174.3908  DIHED      =      6325.7401
 VDWAALS =     81892.4315  EEL     =   -577811.7660  HBOND      =         0.0000
 1-4 VDW =      1766.9091  1-4 EEL =     23317.1267  RESTRAINT  =         0.0000


   NSTEP       ENERGY          RMS            GMAX         NAME    NUMBER
   4500      -4.3040E+05     1.1142E-01     1.6974E+01     CE2      3808

 BOND    =     33091.7530  ANGLE   =      1174.4080  DIHED      =      6324.6254
 VDWAALS =     82016.1806  EEL     =   -578084.0010  HBOND      =         0.0000
 1-4 VDW =      1766.1100  1-4 EEL =     23315.3999  RESTRAINT  =         0.0000


   NSTEP       ENERGY          RMS            GMAX         NAME    NUMBER
   4600      -4.3052E+05     7.8601E-02     8.2571E+00     CE2      3808

 BOND    =     33119.5318  ANGLE   =      1174.4184  DIHED      =      6323.6888
 VDWAALS =     82138.9335  EEL     =   -578354.1368  HBOND      =         0.0000
 1-4 VDW =      1765.4470  1-4 EEL =     23313.3861  RESTRAINT  =         0.0000


   NSTEP       ENERGY          RMS            GMAX         NAME    NUMBER
   4700      -4.3064E+05     8.7948E-02     1.1624E+01     CE2      3808

 BOND    =     33147.5502  ANGLE   =      1174.3965  DIHED      =      6323.4818
```

```
 VDWAALS =     82261.6476  EEL     =  -578623.5613  HBOND      =        0.0000
 1-4 VDW =      1764.9577  1-4 EEL =    23311.5059  RESTRAINT  =        0.0000


   NSTEP       ENERGY          RMS            GMAX         NAME    NUMBER
   4800      -4.3075E+05     7.6503E-02     7.9890E+00     CE2      3808

 BOND    =     33173.5461  ANGLE   =     1174.4503  DIHED      =     6323.5292
 VDWAALS =     82376.7281  EEL     =  -578874.9382  HBOND      =        0.0000
 1-4 VDW =      1764.4728  1-4 EEL =    23309.8243  RESTRAINT  =        0.0000


   NSTEP       ENERGY          RMS            GMAX         NAME    NUMBER
   4900      -4.3086E+05     2.0427E-01     4.0076E+01     CE2      3808

 BOND    =     33200.9619  ANGLE   =     1174.7099  DIHED      =     6323.6537
 VDWAALS =     82490.0201  EEL     =  -579123.0490  HBOND      =        0.0000
 1-4 VDW =      1763.9750  1-4 EEL =    23308.1793  RESTRAINT  =        0.0000


   NSTEP       ENERGY          RMS            GMAX         NAME    NUMBER
   5000      -4.3097E+05     1.9520E-01     3.5763E+01     CD       3230

 BOND    =     33225.7442  ANGLE   =     1174.7834  DIHED      =     6323.8304
 VDWAALS =     82601.6250  EEL     =  -579367.2882  HBOND      =        0.0000
 1-4 VDW =      1763.4604  1-4 EEL =    23306.5890  RESTRAINT  =        0.0000

  Maximum number of minimization cycles reached.


                    FINAL RESULTS



   NSTEP       ENERGY          RMS            GMAX         NAME    NUMBER
   5000      -4.3097E+05     1.9520E-01     3.5763E+01     CD       3230

 BOND    =     33225.7442  ANGLE   =     1174.7834  DIHED      =     6323.8304
 VDWAALS =     82601.6250  EEL     =  -579367.2882  HBOND      =        0.0000
 1-4 VDW =      1763.4604  1-4 EEL =    23306.5890  RESTRAINT  =        0.0000

--------------------------------------------------------------------------------
   5.  TIMINGS
--------------------------------------------------------------------------------

|                Build the list            28.42 (90.70% of List )
|                Other                      2.91 ( 9.30% of List )
|             List time                 31.34 ( 0.31% of Nonbo)
|                   Short_ene time          9311.51 (99.67% of Direc)
|                   Other                     30.61 ( 0.33% of Direc)
|                Direct Ewald time       9342.11 (91.54% of Ewald)
|                Adjust Ewald time         34.72 ( 0.34% of Ewald)
|                   Fill Bspline coeffs       27.11 ( 3.30% of Recip)
```

```
|                    Fill charge grid         118.67 (14.44% of Recip)
|                    Scalar sum               155.71 (18.95% of Recip)
|                    Grad sum                 206.43 (25.13% of Recip)
|                    FFT time                 313.68 (38.18% of Recip)
|                 Recip Ewald time         821.60 ( 8.05% of Ewald)
|                 Virial junk                2.36 ( 0.02% of Ewald)
|                 Other                      4.97 ( 0.05% of Ewald)
|              Ewald time            10205.78 (99.69% of Nonbo)
|           Nonbond force          10237.13 (99.51% of Force)
|           Bond/Angle/Dihedral       48.98 ( 0.48% of Force)
|           Other                      1.34 ( 0.01% of Force)
|        Force time            10287.45 (100.0% of Runmd)
|     Runmd Time            10287.45 (99.71% of Total)
|     Other                    29.56 ( 0.29% of Total)
|  Total time            10317.04 (100.0% of ALL  )

|  Number of list builds   :         18

|  Highest rstack allocated:    5732293
|  Highest istack allocated:     107805
|           Job began  at 01:34:10.635  on 08/14/2017
|           Setup done at 01:34:11.587  on 08/14/2017
|           Run   done at 04:26:07.673  on 08/14/2017
|     wallclock() was called  190084 times

          -------------------------------------------------------
          Amber 16 SANDER                              2016
          -------------------------------------------------------

| Run on 08/13/2017 at 14:53:00

|   Executable path: /usr/local/amber16/bin/sander
| Working directory: /Users/Swati
|          Hostname: Unknown
  [-O]verwriting output

File Assignments:
|  MDIN: minimization_file_2.in
| MDOUT: NAT0_min3_2.out
|INPCRD: NAT0_min3_1.rst
|  PARM: /Users/Swati/NAT0_files/NAT0.prmtop
|RESTRT: NAT0_min3_2.rst
|  REFC: refc
| MDVEL: mdvel
| MDFRC: mdfrc
|  MDEN: mden
| MDCRD: mdcrd
|MDINFO: mdinfo
|  MTMD: mtmd
|INPDIP: inpdip
|RSTDIP: rstdip
|INPTRA: inptraj
```

Supplementary Text 3

Here is the input file:

```
PROT: minimization_2
&cntrl
imin = 1,
maxcyc = 5000,
ncyc = 10000,
igb = 0,
ntpr =  100,
ntp = 0
cut = 12
&end


--------------------------------------------------------------------------------
   1.  RESOURCE   USE:
--------------------------------------------------------------------------------

| Flags:
 getting box info from netcdf restart file
| NetCDF restart box info found
|Largest sphere to fit in unit cell has radius =    43.290
| New format PARM file being parsed.
| Version =    1.000 Date = 08/03/17 Time = 07:15:59
 NATOM  =  108587 NTYPES =      17 NBONH =  104265 MBONA  =    4427
 NTHETH =    9210 MTHETA =    6011 NPHIH =   19189 MPHIA  =   18495
 NHPARM =       0 NPARM  =       0 NNB   =  179552 NRES   =   33974
 NBONA  =    4427 NTHETA =    6011 NPHIA =   18495 NUMBND =      67
 NUMANG =     152 NPTRA  =     191 NATYP =      36 NPHB   =       1
 IFBOX  =       1 NMXRS  =      24 IFCAP =       0 NEXTRA =       0
 NCOPY  =       0


|     Memory Use     Allocated
|     Real             8557276
|     Hollerith         359737
|     Integer          4292225
|     Max Pairs       99320909
|     nblistReal       1303044
|     nblist Int       3569367
|       Total           497120 kbytes

| Note: 1-4 EEL scale factors are being read from the topology file.

| Note: 1-4 VDW scale factors are being read from the topology file.
| Duplicated    0 dihedrals
| Duplicated    0 dihedrals

     BOX TYPE: RECTILINEAR

--------------------------------------------------------------------------------
   2.  CONTROL  DATA  FOR  THE  RUN
--------------------------------------------------------------------------------
```

```
default_name

General flags:
     imin    =       1, nmropt  =       0

Nature and format of input:
     ntx     =       1, irest   =       0, ntrx    =       1

Nature and format of output:
     ntxo    =       2, ntpr    =     100, ntrx    =       1, ntwr    =       1
     iwrap   =       0, ntwx    =       0, ntwv    =       0, ntwe    =       0
     ioutfm  =       1, ntwprt  =       0, idecomp =       0, rbornstat=      0

Potential function:
     ntf     =       1, ntb     =       1, igb     =       0, nsnb    =      25
     ipol    =       0, gbsa    =       0, iesp    =       0
     dielc   =   1.00000, cut     =  12.00000, intdiel =   1.00000

Frozen or restrained atoms:
     ibelly  =       0, ntr     =       0

Energy minimization:
     maxcyc  =    5000, ncyc    =   10000, ntmin   =       1
     dx0     =   0.01000, drms    =   0.00010

Ewald parameters:
     verbose =       0, ew_type =       0, nbflag  =       1, use_pme =       1
     vdwmeth =       1, eedmeth =       1, netfrc  =       0
     Box X =  122.784   Box Y =   86.581   Box Z =  120.454
     Alpha =   90.000   Beta  =   90.000   Gamma =   90.000
     NFFT1 =  128       NFFT2 =   90       NFFT3 =  120
     Cutoff=   12.000   Tol   =0.100E-04
     Ewald Coefficient =  0.22664
     Interpolation order =    4
|  INFO: Old style inpcrd file read


--------------------------------------------------------------------------------
   3.  ATOMIC COORDINATES AND VELOCITIES
--------------------------------------------------------------------------------

default_name
 begin time read from input coords =     0.000 ps

 Number of triangulated 3-point waters found:    33397

     Sum of charges from parm topology file =  -0.00000039
     Forcing neutrality...

--------------------------------------------------------------------------------
   4.  RESULTS
--------------------------------------------------------------------------------

 ---------------------------------------------------
```

```
APPROXIMATING switch and d/dx switch using CUBIC SPLINE INTERPOLATION
using   5000.0 points per unit in tabled values
TESTING RELATIVE ERROR over r ranging from 0.0 to cutoff
| CHECK switch(x): max rel err =   0.2738E-14   at   2.422500
| CHECK d/dx switch(x): max rel err =   0.7967E-11   at   2.716640
---------------------------------------------------
| Local SIZE OF NONBOND LIST =   53975629
| TOTAL SIZE OF NONBOND LIST =   53975629


   NSTEP       ENERGY          RMS            GMAX         NAME    NUMBER
      1      -4.1424E+05     3.4604E+00     3.0891E+02     NE2      3896

 BOND    =     32215.3288  ANGLE   =      2894.2659  DIHED      =      6372.8222
 VDWAALS =     79840.8552  EEL     =   -562877.3862  HBOND      =         0.0000
 1-4 VDW =      2848.5805  1-4 EEL =     24464.4772  RESTRAINT  =         0.0000


   NSTEP       ENERGY          RMS            GMAX         NAME    NUMBER
    100      -4.2360E+05     4.6601E-01     3.8919E+01     C        3051

 BOND    =     31462.1947  ANGLE   =      1483.8942  DIHED      =      6296.7741
 VDWAALS =     75217.6031  EEL     =   -563905.1139  HBOND      =         0.0000
 1-4 VDW =      2065.4770  1-4 EEL =     23777.4973  RESTRAINT  =         0.0000


   NSTEP       ENERGY          RMS            GMAX         NAME    NUMBER
    200      -4.2494E+05     6.4777E-01     1.0087E+02     CG       3137

 BOND    =     31532.3712  ANGLE   =      1356.7681  DIHED      =      6270.7246
 VDWAALS =     75233.6698  EEL     =   -564884.3727  HBOND      =         0.0000
 1-4 VDW =      1909.7719  1-4 EEL =     23638.7817  RESTRAINT  =         0.0000


   NSTEP       ENERGY          RMS            GMAX         NAME    NUMBER
    300      -4.2571E+05     5.0321E-01     5.7089E+01     CZ       3037

 BOND    =     31595.4569  ANGLE   =      1317.2490  DIHED      =      6254.6531
 VDWAALS =     75441.2149  EEL     =   -565764.5486  HBOND      =         0.0000
 1-4 VDW =      1853.2864  1-4 EEL =     23590.3327  RESTRAINT  =         0.0000


   NSTEP       ENERGY          RMS            GMAX         NAME    NUMBER
    400      -4.2629E+05     4.2191E-01     4.9299E+01     CG       3032

 BOND    =     31665.5164  ANGLE   =      1299.8061  DIHED      =      6239.8487
 VDWAALS =     75698.1963  EEL     =   -566585.4903  HBOND      =         0.0000
 1-4 VDW =      1823.0413  1-4 EEL =     23564.8713  RESTRAINT  =         0.0000


   NSTEP       ENERGY          RMS            GMAX         NAME    NUMBER
    500      -4.2680E+05     3.9923E-01     5.4231E+01     CG       3137

 BOND    =     31737.6234  ANGLE   =      1290.4650  DIHED      =      6228.5845
```

```
VDWAALS =     75978.8880  EEL      =  -567391.7847  HBOND       =         0.0000
1-4 VDW =      1803.2996  1-4 EEL =    23550.0282  RESTRAINT  =         0.0000


  NSTEP       ENERGY          RMS            GMAX         NAME    NUMBER
    600      -4.2726E+05     3.1711E-01     3.2933E+01     CZ       3037

BOND    =     31807.2875  ANGLE    =      1285.3807  DIHED       =      6217.9236
VDWAALS =     76266.5985  EEL      =  -568165.5869  HBOND       =         0.0000
1-4 VDW =      1789.5714  1-4 EEL =    23540.4694  RESTRAINT  =         0.0000


  NSTEP       ENERGY          RMS            GMAX         NAME    NUMBER
    700      -4.2766E+05     1.9674E-01     1.9753E+01     NE2      7140

BOND    =     31872.9707  ANGLE    =      1282.6293  DIHED       =      6211.5126
VDWAALS =     76557.4354  EEL      =  -568897.6467  HBOND       =         0.0000
1-4 VDW =      1779.8237  1-4 EEL =    23533.4694  RESTRAINT  =         0.0000


  NSTEP       ENERGY          RMS            GMAX         NAME    NUMBER
    800      -4.2802E+05     2.0922E-01     2.3429E+01     NE2      7140

BOND    =     31938.5833  ANGLE    =      1280.8466  DIHED       =      6206.8355
VDWAALS =     76842.1489  EEL      =  -569589.7889  HBOND       =         0.0000
1-4 VDW =      1772.4628  1-4 EEL =    23526.6868  RESTRAINT  =         0.0000


  NSTEP       ENERGY          RMS            GMAX         NAME    NUMBER
    900      -4.2835E+05     1.6197E-01     1.6809E+01     NE2      7140

BOND    =     31998.7934  ANGLE    =      1279.8446  DIHED       =      6202.1679
VDWAALS =     77113.6431  EEL      =  -570233.6566  HBOND       =         0.0000
1-4 VDW =      1766.4317  1-4 EEL =    23520.2712  RESTRAINT  =         0.0000


  NSTEP       ENERGY          RMS            GMAX         NAME    NUMBER
   1000      -4.2865E+05     4.1924E-01     5.6945E+01     NE2      7140

BOND    =     32063.6454  ANGLE    =      1280.3819  DIHED       =      6197.2454
VDWAALS =     77375.5533  EEL      =  -570844.5022  HBOND       =         0.0000
1-4 VDW =      1761.6476  1-4 EEL =    23514.9436  RESTRAINT  =         0.0000


  NSTEP       ENERGY          RMS            GMAX         NAME    NUMBER
   1100      -4.2895E+05     3.4879E-01     5.0483E+01     NE2      7140

BOND    =     32119.2376  ANGLE    =      1278.7164  DIHED       =      6192.7311
VDWAALS =     77636.1601  EEL      =  -571448.4473  HBOND       =         0.0000
1-4 VDW =      1757.6165  1-4 EEL =    23510.3279  RESTRAINT  =         0.0000


  NSTEP       ENERGY          RMS            GMAX         NAME    NUMBER
   1200      -4.2923E+05     2.9712E-01     4.7271E+01     NE2      7140
```

```
BOND    =     32171.5264  ANGLE   =      1276.8961  DIHED      =      6189.0290
VDWAALS =     77877.6734  EEL     =  -572008.9744  HBOND      =         0.0000
1-4 VDW =      1754.3479  1-4 EEL =    23506.7514  RESTRAINT  =         0.0000


  NSTEP       ENERGY          RMS            GMAX         NAME    NUMBER
   1300      -4.2951E+05     2.7565E-01     4.7598E+01     CG       3137

BOND    =     32223.2166  ANGLE   =      1274.9125  DIHED      =      6185.9165
VDWAALS =     78113.3365  EEL     =  -572557.1914  HBOND      =         0.0000
1-4 VDW =      1751.4393  1-4 EEL =    23502.3356  RESTRAINT  =         0.0000


  NSTEP       ENERGY          RMS            GMAX         NAME    NUMBER
   1400      -4.2977E+05     1.5633E-01     1.8948E+01     CG       3137

BOND    =     32272.6721  ANGLE   =      1271.8728  DIHED      =      6182.8827
VDWAALS =     78344.4628  EEL     =  -573090.5664  HBOND      =         0.0000
1-4 VDW =      1748.4986  1-4 EEL =    23497.5877  RESTRAINT  =         0.0000


  NSTEP       ENERGY          RMS            GMAX         NAME    NUMBER
   1500      -4.3003E+05     1.0600E-01     7.1559E+00     NE2      7140

BOND    =     32321.8997  ANGLE   =      1269.4524  DIHED      =      6179.5991
VDWAALS =     78570.4784  EEL     =  -573607.4335  HBOND      =         0.0000
1-4 VDW =      1745.7704  1-4 EEL =    23491.7154  RESTRAINT  =         0.0000


  NSTEP       ENERGY          RMS            GMAX         NAME    NUMBER
   1600      -4.3026E+05     4.0342E-01     6.6590E+01     NE2      7140

BOND    =     32377.0753  ANGLE   =      1269.1114  DIHED      =      6176.1376
VDWAALS =     78786.7166  EEL     =  -574102.9114  HBOND      =         0.0000
1-4 VDW =      1743.2674  1-4 EEL =    23485.6133  RESTRAINT  =         0.0000


  NSTEP       ENERGY          RMS            GMAX         NAME    NUMBER
   1700      -4.3051E+05     1.0492E-01     8.0790E+00     NE2      7140

BOND    =     32416.4014  ANGLE   =      1266.5719  DIHED      =      6173.3124
VDWAALS =     78991.4158  EEL     =  -574576.7080  HBOND      =         0.0000
1-4 VDW =      1741.2259  1-4 EEL =    23480.1534  RESTRAINT  =         0.0000


  NSTEP       ENERGY          RMS            GMAX         NAME    NUMBER
   1800      -4.3074E+05     2.9284E-01     4.6794E+01     NE2      7140

BOND    =     32465.9944  ANGLE   =      1266.7569  DIHED      =      6171.2672
VDWAALS =     79194.9067  EEL     =  -575050.0222  HBOND      =         0.0000
1-4 VDW =      1739.5925  1-4 EEL =    23475.5912  RESTRAINT  =         0.0000
```

```
  NSTEP       ENERGY          RMS            GMAX         NAME    NUMBER
   1900      -4.3096E+05     2.6122E-01     4.4354E+01     NE2      7140

 BOND    =     32510.8017  ANGLE   =      1266.0380  DIHED      =      6169.5879
 VDWAALS =     79388.1708  EEL     =   -575504.3753  HBOND      =         0.0000
 1-4 VDW =      1738.0779  1-4 EEL =     23472.3048  RESTRAINT  =         0.0000


  NSTEP       ENERGY          RMS            GMAX         NAME    NUMBER
   2000      -4.3118E+05     1.7050E-01     2.4660E+01     NE2      7140

 BOND    =     32552.8917  ANGLE   =      1265.9018  DIHED      =      6168.1082
 VDWAALS =     79574.3759  EEL     =   -575945.0717  HBOND      =         0.0000
 1-4 VDW =      1736.6586  1-4 EEL =     23468.9426  RESTRAINT  =         0.0000


  NSTEP       ENERGY          RMS            GMAX         NAME    NUMBER
   2100      -4.3139E+05     9.5601E-02     5.4156E+00     NE2      7140

 BOND    =     32595.7410  ANGLE   =      1265.7782  DIHED      =      6166.6003
 VDWAALS =     79759.5186  EEL     =   -576381.2745  HBOND      =         0.0000
 1-4 VDW =      1735.3018  1-4 EEL =     23466.3394  RESTRAINT  =         0.0000


  NSTEP       ENERGY          RMS            GMAX         NAME    NUMBER
   2200      -4.3160E+05     9.4262E-02     5.8576E+00     NE2      7140

 BOND    =     32638.4719  ANGLE   =      1266.0138  DIHED      =      6165.1645
 VDWAALS =     79940.1316  EEL     =   -576805.2965  HBOND      =         0.0000
 1-4 VDW =      1734.0954  1-4 EEL =     23463.7908  RESTRAINT  =         0.0000


  NSTEP       ENERGY          RMS            GMAX         NAME    NUMBER
   2300      -4.3180E+05     1.0345E-01     1.0220E+01     NE2      7140

 BOND    =     32681.1538  ANGLE   =      1266.1527  DIHED      =      6164.2512
 VDWAALS =     80118.0348  EEL     =   -577224.8403  HBOND      =         0.0000
 1-4 VDW =      1733.0109  1-4 EEL =     23461.3600  RESTRAINT  =         0.0000


  NSTEP       ENERGY          RMS            GMAX         NAME    NUMBER
   2400      -4.3199E+05     2.8504E-01     4.4932E+01     NE2      7140

 BOND    =     32725.1000  ANGLE   =      1266.9005  DIHED      =      6163.8345
 VDWAALS =     80288.1953  EEL     =   -577628.4506  HBOND      =         0.0000
 1-4 VDW =      1732.0082  1-4 EEL =     23459.2293  RESTRAINT  =         0.0000


  NSTEP       ENERGY          RMS            GMAX         NAME    NUMBER
   2500      -4.3218E+05     2.6260E-01     4.3027E+01     NE2      7140

 BOND    =     32764.2839  ANGLE   =      1267.7179  DIHED      =      6163.5901
 VDWAALS =     80452.8748  EEL     =   -578020.8732  HBOND      =         0.0000
 1-4 VDW =      1731.0144  1-4 EEL =     23457.0857  RESTRAINT  =         0.0000
```

```
   NSTEP       ENERGY          RMS            GMAX         NAME    NUMBER
   2600      -4.3237E+05     2.0783E-01     3.5330E+01     NE2      7140

 BOND    =     32803.2099  ANGLE   =      1267.5864  DIHED      =      6163.3478
 VDWAALS =     80615.7341  EEL     =   -578406.8008  HBOND      =         0.0000
 1-4 VDW =      1730.1443  1-4 EEL =     23455.5345  RESTRAINT  =         0.0000


   NSTEP       ENERGY          RMS            GMAX         NAME    NUMBER
   2700      -4.3256E+05     1.6313E-01     2.6744E+01     NE2      7140

 BOND    =     32841.9413  ANGLE   =      1267.8450  DIHED      =      6163.1898
 VDWAALS =     80779.3081  EEL     =   -578790.3037  HBOND      =         0.0000
 1-4 VDW =      1729.4671  1-4 EEL =     23453.5036  RESTRAINT  =         0.0000


   NSTEP       ENERGY          RMS            GMAX         NAME    NUMBER
   2800      -4.3273E+05     9.0926E-02     7.5705E+00     NE2      7140

 BOND    =     32879.3537  ANGLE   =      1267.7315  DIHED      =      6163.0599
 VDWAALS =     80936.9516  EEL     =   -579159.0330  HBOND      =         0.0000
 1-4 VDW =      1728.8327  1-4 EEL =     23451.7186  RESTRAINT  =         0.0000


   NSTEP       ENERGY          RMS            GMAX         NAME    NUMBER
   2900      -4.3291E+05     9.8809E-02     1.0693E+01     NE2      7140

 BOND    =     32917.0133  ANGLE   =      1268.0488  DIHED      =      6162.9778
 VDWAALS =     81090.4213  EEL     =   -579521.7713  HBOND      =         0.0000
 1-4 VDW =      1728.2001  1-4 EEL =     23449.8094  RESTRAINT  =         0.0000


   NSTEP       ENERGY          RMS            GMAX         NAME    NUMBER
   3000      -4.3307E+05     2.6055E-01     4.5377E+01     NE2      7140

 BOND    =     32956.3690  ANGLE   =      1269.0371  DIHED      =      6162.9778
 VDWAALS =     81238.8175  EEL     =   -579875.2491  HBOND      =         0.0000
 1-4 VDW =      1727.6017  1-4 EEL =     23447.9570  RESTRAINT  =         0.0000


   NSTEP       ENERGY          RMS            GMAX         NAME    NUMBER
   3100      -4.3324E+05     2.5457E-01     4.1911E+01     NE2      7140

 BOND    =     32992.5337  ANGLE   =      1268.9003  DIHED      =      6163.0790
 VDWAALS =     81383.2390  EEL     =   -580220.6466  HBOND      =         0.0000
 1-4 VDW =      1727.0014  1-4 EEL =     23446.5979  RESTRAINT  =         0.0000


   NSTEP       ENERGY          RMS            GMAX         NAME    NUMBER
   3200      -4.3341E+05     1.9830E-01     3.3901E+01     NE2      7140

 BOND    =     33027.4064  ANGLE   =      1269.0671  DIHED      =      6163.3758
```

```
VDWAALS =    81528.3702  EEL     =  -580565.6261  HBOND      =         0.0000
1-4 VDW =     1726.4232  1-4 EEL =    23444.9010  RESTRAINT  =         0.0000


  NSTEP       ENERGY          RMS            GMAX         NAME    NUMBER
   3300      -4.3357E+05     1.1338E-01     1.3958E+01     NE2      7140

BOND    =    33061.6049  ANGLE   =     1268.6299  DIHED      =      6163.7865
VDWAALS =    81670.5939  EEL     =  -580902.2260  HBOND      =         0.0000
1-4 VDW =     1725.9052  1-4 EEL =    23443.7261  RESTRAINT  =         0.0000


  NSTEP       ENERGY          RMS            GMAX         NAME    NUMBER
   3400      -4.3373E+05     8.5630E-02     5.5514E+00     NE2      7140

BOND    =    33096.7097  ANGLE   =     1268.5578  DIHED      =      6164.3304
VDWAALS =    81814.6162  EEL     =  -581243.3917  HBOND      =         0.0000
1-4 VDW =     1725.4052  1-4 EEL =    23442.4903  RESTRAINT  =         0.0000


  NSTEP       ENERGY          RMS            GMAX         NAME    NUMBER
   3500      -4.3390E+05     8.8931E-02     8.6417E+00     NE2      7140

BOND    =    33132.1194  ANGLE   =     1268.5123  DIHED      =      6164.7963
VDWAALS =    81958.6953  EEL     =  -581586.6995  HBOND      =         0.0000
1-4 VDW =     1724.8761  1-4 EEL =    23441.4169  RESTRAINT  =         0.0000


  NSTEP       ENERGY          RMS            GMAX         NAME    NUMBER
   3600      -4.3405E+05     2.4537E-01     4.8160E+01     NE2      7140

BOND    =    33169.3626  ANGLE   =     1268.9965  DIHED      =      6165.0873
VDWAALS =    82099.5677  EEL     =  -581921.0069  HBOND      =         0.0000
1-4 VDW =     1724.2484  1-4 EEL =    23440.4662  RESTRAINT  =         0.0000


  NSTEP       ENERGY          RMS            GMAX         NAME    NUMBER
   3700      -4.3421E+05     2.4109E-01     4.3220E+01     NE2      7140

BOND    =    33203.3045  ANGLE   =     1269.2048  DIHED      =      6165.2235
VDWAALS =    82237.9050  EEL     =  -582247.7279  HBOND      =         0.0000
1-4 VDW =     1723.6580  1-4 EEL =    23439.2129  RESTRAINT  =         0.0000


  NSTEP       ENERGY          RMS            GMAX         NAME    NUMBER
   3800      -4.3436E+05     1.4181E-01     2.0819E+01     NE2      7140

BOND    =    33234.9833  ANGLE   =     1268.4792  DIHED      =      6165.2444
VDWAALS =    82372.7976  EEL     =  -582562.1995  HBOND      =         0.0000
1-4 VDW =     1723.0838  1-4 EEL =    23438.6211  RESTRAINT  =         0.0000


  NSTEP       ENERGY          RMS            GMAX         NAME    NUMBER
   3900      -4.3450E+05     1.6010E-01     2.6267E+01     NE2      7140
```

```
BOND    =     33267.3689  ANGLE   =      1268.4968  DIHED      =      6165.1575
VDWAALS =     82506.2003  EEL     =   -582868.6389  HBOND      =         0.0000
1-4 VDW =      1722.5826  1-4 EEL =     23437.8981  RESTRAINT  =         0.0000


  NSTEP       ENERGY          RMS            GMAX         NAME    NUMBER
   4000      -4.3464E+05     8.1295E-02     6.7586E+00     NE2      7140

BOND    =     33298.8828  ANGLE   =      1267.8028  DIHED      =      6164.9211
VDWAALS =     82638.9229  EEL     =   -583173.5440  HBOND      =         0.0000
1-4 VDW =      1722.1221  1-4 EEL =     23437.7997  RESTRAINT  =         0.0000


  NSTEP       ENERGY          RMS            GMAX         NAME    NUMBER
   4100      -4.3478E+05     3.0435E-01     5.2191E+01     NE2      7140

BOND    =     33334.8794  ANGLE   =      1268.1942  DIHED      =      6164.5638
VDWAALS =     82771.1504  EEL     =   -583479.4189  HBOND      =         0.0000
1-4 VDW =      1721.7529  1-4 EEL =     23438.2165  RESTRAINT  =         0.0000


  NSTEP       ENERGY          RMS            GMAX         NAME    NUMBER
   4200      -4.3492E+05     7.9363E-02     6.4143E+00     NE2      7140

BOND    =     33361.6533  ANGLE   =      1267.6805  DIHED      =      6164.1038
VDWAALS =     82896.8825  EEL     =   -583771.0315  HBOND      =         0.0000
1-4 VDW =      1721.4096  1-4 EEL =     23438.2654  RESTRAINT  =         0.0000


  NSTEP       ENERGY          RMS            GMAX         NAME    NUMBER
   4300      -4.3506E+05     2.0912E-01     3.8895E+01     NE2      7140

BOND    =     33394.6399  ANGLE   =      1268.2725  DIHED      =      6163.6830
VDWAALS =     83025.1295  EEL     =   -584068.4569  HBOND      =         0.0000
1-4 VDW =      1720.9317  1-4 EEL =     23437.9518  RESTRAINT  =         0.0000


  NSTEP       ENERGY          RMS            GMAX         NAME    NUMBER
   4400      -4.3519E+05     1.3127E-01     2.0152E+01     CG       6196

BOND    =     33422.7927  ANGLE   =      1267.6905  DIHED      =      6163.6447
VDWAALS =     83145.8726  EEL     =   -584346.1183  HBOND      =         0.0000
1-4 VDW =      1720.4557  1-4 EEL =     23437.0078  RESTRAINT  =         0.0000


  NSTEP       ENERGY          RMS            GMAX         NAME    NUMBER
   4500      -4.3532E+05     1.0463E-01     1.4210E+01     NE2      7140

BOND    =     33451.5082  ANGLE   =      1266.9235  DIHED      =      6163.7132
VDWAALS =     83264.6678  EEL     =   -584619.4069  HBOND      =         0.0000
1-4 VDW =      1720.0763  1-4 EEL =     23436.5790  RESTRAINT  =         0.0000
```

```
  NSTEP       ENERGY          RMS            GMAX         NAME    NUMBER
   4600      -4.3544E+05     7.6402E-02     6.2995E+00     NE2      7140

 BOND    =     33478.6505  ANGLE   =      1266.5204  DIHED      =      6163.8230
 VDWAALS =     83378.4579  EEL     =   -584882.5755  HBOND      =         0.0000
 1-4 VDW =      1719.7523  1-4 EEL =     23436.2583  RESTRAINT  =         0.0000


  NSTEP       ENERGY          RMS            GMAX         NAME    NUMBER
   4700      -4.3556E+05     2.8284E-01     5.4720E+01     NE2      7140

 BOND    =     33508.5333  ANGLE   =      1267.1370  DIHED      =      6163.9533
 VDWAALS =     83490.5650  EEL     =   -585142.9004  HBOND      =         0.0000
 1-4 VDW =      1719.4453  1-4 EEL =     23436.0074  RESTRAINT  =         0.0000


  NSTEP       ENERGY          RMS            GMAX         NAME    NUMBER
   4800      -4.3569E+05     7.6255E-02     6.8137E+00     NE2      7140

 BOND    =     33533.0487  ANGLE   =      1265.7543  DIHED      =      6164.0501
 VDWAALS =     83606.4810  EEL     =   -585411.3571  HBOND      =         0.0000
 1-4 VDW =      1719.1226  1-4 EEL =     23436.1273  RESTRAINT  =         0.0000


  NSTEP       ENERGY          RMS            GMAX         NAME    NUMBER
   4900      -4.3581E+05     2.4034E-01     5.2165E+01     CG       6196

 BOND    =     33562.4024  ANGLE   =      1266.0615  DIHED      =      6164.1697
 VDWAALS =     83723.1315  EEL     =   -585676.5399  HBOND      =         0.0000
 1-4 VDW =      1718.8605  1-4 EEL =     23436.1895  RESTRAINT  =         0.0000


  NSTEP       ENERGY          RMS            GMAX         NAME    NUMBER
   5000      -4.3592E+05     1.6471E-01     3.0625E+01     NE2      7140

 BOND    =     33588.0177  ANGLE   =      1265.1992  DIHED      =      6164.2564
 VDWAALS =     83838.5396  EEL     =   -585934.3952  HBOND      =         0.0000
 1-4 VDW =      1718.5470  1-4 EEL =     23436.4684  RESTRAINT  =         0.0000


  Maximum number of minimization cycles reached.


                    FINAL RESULTS



  NSTEP       ENERGY          RMS            GMAX         NAME    NUMBER
   5000      -4.3592E+05     1.6471E-01     3.0625E+01     NE2      7140

 BOND    =     33588.0177  ANGLE   =      1265.1992  DIHED      =      6164.2564
 VDWAALS =     83838.5396  EEL     =   -585934.3952  HBOND      =         0.0000
 1-4 VDW =      1718.5470  1-4 EEL =     23436.4684  RESTRAINT  =         0.0000
```

```
--------------------------------------------------------------------------------
   5.  TIMINGS
--------------------------------------------------------------------------------

|                Build the list            22.48 (88.60% of List )
|                Other                      2.89 (11.40% of List )
|             List time                 25.37 ( 0.25% of Nonbo)
|                   Short_ene time         9366.05 (99.67% of Direc)
|                   Other                    30.80 ( 0.33% of Direc)
|                Direct Ewald time       9396.85 (91.67% of Ewald)
|                Adjust Ewald time         34.49 ( 0.34% of Ewald)
|                   Fill Bspline coeffs       27.03 ( 3.33% of Recip)
|                   Fill charge grid         118.11 (14.54% of Recip)
|                   Scalar sum               155.25 (19.11% of Recip)
|                   Grad sum                 208.52 (25.67% of Recip)
|                   FFT time                 303.53 (37.36% of Recip)
|                Recip Ewald time         812.46 ( 7.93% of Ewald)
|                Virial junk                2.26 ( 0.02% of Ewald)
|                Other                      4.82 ( 0.05% of Ewald)
|             Ewald time             10250.89 (99.75% of Nonbo)
|          Nonbond force          10276.27 (99.52% of Force)
|          Bond/Angle/Dihedral       47.80 ( 0.46% of Force)
|          Other                      1.32 ( 0.01% of Force)
|       Force time             10325.39 (100.0% of Runmd)
|    Runmd Time             10325.39 (99.72% of Total)
|    Other                     29.19 ( 0.28% of Total)
| Total time             10354.60 (100.0% of ALL  )

| Number of list builds   :         14

| Highest rstack allocated:    5827323
| Highest istack allocated:     108587
|           Job began  at 14:53:00.793  on 08/13/2017
|           Setup done at 14:53:01.768  on 08/13/2017
|           Run   done at 17:45:35.434  on 08/13/2017
|     wallclock() was called  190076 times

          -------------------------------------------------------
          Amber 16 SANDER                              2016
          -------------------------------------------------------

| Run on 08/13/2017 at 07:02:43

|   Executable path: /usr/local/amber16/bin/sander
| Working directory: /Users/Swati
|          Hostname: Unknown
  [-O]verwriting output

File Assignments:
|  MDIN: minimization_file_2.in
| MDOUT: ZSP9_min3_2.out
|INPCRD: ZSP9_min3_1.rst
|  PARM: /Users/Swati/ZSP9_files/ZSP9.prmtop
|RESTRT: ZSP9_min3_2.rst
```

```
|  REFC: refc
| MDVEL: mdvel
| MDFRC: mdfrc
|  MDEN: mden
| MDCRD: mdcrd
|MDINFO: mdinfo
|  MTMD: mtmd
|INPDIP: inpdip
|RSTDIP: rstdip
|INPTRA: inptraj


 Here is the input file:

PROT: minimization_2
&cntrl
imin = 1,
maxcyc = 5000,
ncyc = 10000,
igb = 0,
ntpr =  100,
ntp = 0
cut = 12
&end


--------------------------------------------------------------------------------
   1.  RESOURCE   USE:
--------------------------------------------------------------------------------

| Flags:
 getting box info from netcdf restart file
| NetCDF restart box info found
|Largest sphere to fit in unit cell has radius =    42.894
| New format PARM file being parsed.
| Version =    1.000 Date = 08/07/17 Time = 06:43:25
 NATOM  =  107718 NTYPES =      17 NBONH =  103122 MBONA  =    4716
 NTHETH =    9972 MTHETA =    6416 NPHIH =   20783 MPHIA  =   19788
 NHPARM =       0 NPARM  =       0 NNB   =  181165 NRES   =   33503
 NBONA  =    4716 NTHETA =    6416 NPHIA =   19788 NUMBND =      67
 NUMANG =     152 NPTRA  =     191 NATYP =      36 NPHB   =       1
 IFBOX  =       1 NMXRS  =      24 IFCAP =       0 NEXTRA =       0
 NCOPY  =       0


|     Memory Use     Allocated
|     Real             8489530
|     Hollerith         356659
|     Integer          4411905
|     Max Pairs       98526064
|     nblistReal       1292616
|     nblist Int       3561915
|       Total           493831 kbytes
```

```
| Note: 1-4 EEL scale factors are being read from the topology file.

| Note: 1-4 VDW scale factors are being read from the topology file.
| Duplicated    0 dihedrals
| Duplicated    0 dihedrals

     BOX TYPE: RECTILINEAR

--------------------------------------------------------------------------------
   2.  CONTROL  DATA  FOR  THE  RUN
--------------------------------------------------------------------------------

default_name

General flags:
     imin    =       1, nmropt  =       0

Nature and format of input:
     ntx     =       1, irest   =       0, ntrx    =       1

Nature and format of output:
     ntxo    =       2, ntpr    =     100, ntrx    =       1, ntwr    =       1
     iwrap   =       0, ntwx    =       0, ntwv    =       0, ntwe    =       0
     ioutfm  =       1, ntwprt  =       0, idecomp =       0, rbornstat=      0

Potential function:
     ntf     =       1, ntb     =       1, igb     =       0, nsnb    =      25
     ipol    =       0, gbsa    =       0, iesp    =       0
     dielc   =   1.00000, cut     =  12.00000, intdiel =   1.00000

Frozen or restrained atoms:
     ibelly  =       0, ntr     =       0

Energy minimization:
     maxcyc  =    5000, ncyc    =   10000, ntmin   =       1
     dx0     =   0.01000, drms    =   0.00010

Ewald parameters:
     verbose =       0, ew_type =       0, nbflag  =       1, use_pme =       1
     vdwmeth =       1, eedmeth =       1, netfrc  =       0
     Box X =  119.635   Box Y =   85.788   Box Z =  124.094
     Alpha =   90.000   Beta  =   90.000   Gamma =   90.000
     NFFT1 =  120       NFFT2 =   90       NFFT3 =  125
     Cutoff=   12.000   Tol   =0.100E-04
     Ewald Coefficient =  0.22664
     Interpolation order =    4
|  INFO: Old style inpcrd file read


--------------------------------------------------------------------------------
   3.  ATOMIC COORDINATES AND VELOCITIES
--------------------------------------------------------------------------------

default_name
```

```
 begin time read from input coords =     0.000 ps

 Number of triangulated 3-point waters found:    32903

     Sum of charges from parm topology file =  -0.00000034
     Forcing neutrality...

--------------------------------------------------------------------------------
   4.  RESULTS
--------------------------------------------------------------------------------

 ---------------------------------------------------
 APPROXIMATING switch and d/dx switch using CUBIC SPLINE INTERPOLATION
 using   5000.0 points per unit in tabled values
 TESTING RELATIVE ERROR over r ranging from 0.0 to cutoff
| CHECK switch(x): max rel err =   0.2738E-14   at   2.422500
| CHECK d/dx switch(x): max rel err =   0.7967E-11   at   2.716640
 ---------------------------------------------------
| Local SIZE OF NONBOND LIST =   53421490
| TOTAL SIZE OF NONBOND LIST =   53421490


   NSTEP       ENERGY          RMS            GMAX         NAME    NUMBER
      1      -3.9968E+05     4.4082E+00     3.2481E+02     CE1      5221

 BOND    =    32433.8734  ANGLE   =     4402.9534  DIHED      =     7075.4076
 VDWAALS =    78355.6999  EEL     =  -549978.1784  HBOND      =        0.0000
 1-4 VDW =     3444.5249  1-4 EEL =    24584.7867  RESTRAINT  =        0.0000


   NSTEP       ENERGY          RMS            GMAX         NAME    NUMBER
    100      -4.1405E+05     7.2624E-01     5.7842E+01     CZ       8770

 BOND    =    30580.8131  ANGLE   =     1750.2106  DIHED      =     6867.4645
 VDWAALS =    71793.1882  EEL     =  -551249.0705  HBOND      =        0.0000
 1-4 VDW =     2303.2207  1-4 EEL =    23904.3005  RESTRAINT  =        0.0000


   NSTEP       ENERGY          RMS            GMAX         NAME    NUMBER
    200      -4.1581E+05     5.7657E-01     9.8645E+01     CZ       8770

 BOND    =    30636.4204  ANGLE   =     1493.6331  DIHED      =     6803.0545
 VDWAALS =    71887.9490  EEL     =  -552522.0640  HBOND      =        0.0000
 1-4 VDW =     2118.9609  1-4 EEL =    23776.7070  RESTRAINT  =        0.0000


   NSTEP       ENERGY          RMS            GMAX         NAME    NUMBER
    300      -4.1684E+05     5.5339E-01     9.8778E+01     CG       3232

 BOND    =    30734.2105  ANGLE   =     1392.4934  DIHED      =     6771.3743
 VDWAALS =    72213.9485  EEL     =  -553720.4885  HBOND      =        0.0000
 1-4 VDW =     2044.2571  1-4 EEL =    23719.8506  RESTRAINT  =        0.0000
```

```
  NSTEP       ENERGY          RMS            GMAX         NAME    NUMBER
    400      -4.1761E+05     4.6208E-01     9.9316E+01     CG       3232

 BOND    =    30827.3013  ANGLE   =     1343.4935  DIHED      =     6749.2181
 VDWAALS =    72582.4226  EEL     =  -554807.1539  HBOND      =        0.0000
 1-4 VDW =     2004.4568  1-4 EEL =    23685.4693  RESTRAINT  =        0.0000


  NSTEP       ENERGY          RMS            GMAX         NAME    NUMBER
    500      -4.1827E+05     3.7544E-01     7.0650E+01     CZ       8770

 BOND    =    30917.9788  ANGLE   =     1315.6533  DIHED      =     6730.8288
 VDWAALS =    72964.3157  EEL     =  -555835.1679  HBOND      =        0.0000
 1-4 VDW =     1979.1137  1-4 EEL =    23660.1041  RESTRAINT  =        0.0000


  NSTEP       ENERGY          RMS            GMAX         NAME    NUMBER
    600      -4.1884E+05     3.1620E-01     5.6210E+01     CZ       8770

 BOND    =    31006.3388  ANGLE   =     1297.8864  DIHED      =     6712.6602
 VDWAALS =    73336.7723  EEL     =  -556793.5465  HBOND      =        0.0000
 1-4 VDW =     1960.7565  1-4 EEL =    23641.8617  RESTRAINT  =        0.0000


  NSTEP       ENERGY          RMS            GMAX         NAME    NUMBER
    700      -4.1934E+05     1.5491E-01     1.5797E+01     CZ       8770

 BOND    =    31086.9947  ANGLE   =     1285.6745  DIHED      =     6698.6341
 VDWAALS =    73701.3199  EEL     =  -557688.8535  HBOND      =        0.0000
 1-4 VDW =     1946.6607  1-4 EEL =    23627.1187  RESTRAINT  =        0.0000


  NSTEP       ENERGY          RMS            GMAX         NAME    NUMBER
    800      -4.1979E+05     5.7790E-01     1.0820E+02     CZ       8770

 BOND    =    31181.7987  ANGLE   =     1279.0035  DIHED      =     6684.3977
 VDWAALS =    74054.8172  EEL     =  -558537.0950  HBOND      =        0.0000
 1-4 VDW =     1935.4130  1-4 EEL =    23613.7034  RESTRAINT  =        0.0000


  NSTEP       ENERGY          RMS            GMAX         NAME    NUMBER
    900      -4.2021E+05     5.6034E-01     1.5607E+02     CG       3232

 BOND    =    31255.1347  ANGLE   =     1273.8222  DIHED      =     6672.4587
 VDWAALS =    74387.0763  EEL     =  -559325.1971  HBOND      =        0.0000
 1-4 VDW =     1926.4883  1-4 EEL =    23600.8920  RESTRAINT  =        0.0000


  NSTEP       ENERGY          RMS            GMAX         NAME    NUMBER
   1000      -4.2060E+05     1.3018E-01     1.0063E+01     CZ       8770

 BOND    =    31315.0988  ANGLE   =     1269.3347  DIHED      =     6663.9999
 VDWAALS =    74702.1636  EEL     =  -560063.8571  HBOND      =        0.0000
 1-4 VDW =     1919.1231  1-4 EEL =    23589.5280  RESTRAINT  =        0.0000
```

```
  NSTEP       ENERGY          RMS            GMAX         NAME    NUMBER
   1100      -4.2096E+05     3.4872E-01     6.9535E+01     CZ       8770

 BOND    =     31389.1657  ANGLE   =      1267.8813  DIHED      =      6657.4656
 VDWAALS =     75005.5362  EEL     =   -560769.4472  HBOND      =         0.0000
 1-4 VDW =      1912.8305  1-4 EEL =     23579.5959  RESTRAINT  =         0.0000


  NSTEP       ENERGY          RMS            GMAX         NAME    NUMBER
   1200      -4.2130E+05     3.2014E-01     6.3435E+01     CZ       8770

 BOND    =     31454.8264  ANGLE   =      1266.5995  DIHED      =      6651.7771
 VDWAALS =     75297.8929  EEL     =   -561452.5029  HBOND      =         0.0000
 1-4 VDW =      1907.1498  1-4 EEL =     23571.2336  RESTRAINT  =         0.0000


  NSTEP       ENERGY          RMS            GMAX         NAME    NUMBER
   1300      -4.2163E+05     2.0882E-01     3.5305E+01     CZ       8770

 BOND    =     31513.6706  ANGLE   =      1265.7256  DIHED      =      6646.9245
 VDWAALS =     75565.8721  EEL     =   -562084.2102  HBOND      =         0.0000
 1-4 VDW =      1902.3406  1-4 EEL =     23564.0265  RESTRAINT  =         0.0000


  NSTEP       ENERGY          RMS            GMAX         NAME    NUMBER
   1400      -4.2194E+05     1.9980E-01     3.5723E+01     CZ       8770

 BOND    =     31573.0022  ANGLE   =      1265.5867  DIHED      =      6642.4846
 VDWAALS =     75825.9113  EEL     =   -562699.3791  HBOND      =         0.0000
 1-4 VDW =      1898.1290  1-4 EEL =     23557.2946  RESTRAINT  =         0.0000


  NSTEP       ENERGY          RMS            GMAX         NAME    NUMBER
   1500      -4.2224E+05     1.1496E-01     7.9267E+00     CZ       8770

 BOND    =     31629.6175  ANGLE   =      1265.4450  DIHED      =      6638.3776
 VDWAALS =     76076.3709  EEL     =   -563294.9112  HBOND      =         0.0000
 1-4 VDW =      1894.3553  1-4 EEL =     23551.3741  RESTRAINT  =         0.0000


  NSTEP       ENERGY          RMS            GMAX         NAME    NUMBER
   1600      -4.2252E+05     4.1203E-01     7.5173E+01     CZ       8770

 BOND    =     31693.5323  ANGLE   =      1266.3129  DIHED      =      6635.1195
 VDWAALS =     76317.9240  EEL     =   -563870.8327  HBOND      =         0.0000
 1-4 VDW =      1891.0067  1-4 EEL =     23546.8349  RESTRAINT  =         0.0000


  NSTEP       ENERGY          RMS            GMAX         NAME    NUMBER
   1700      -4.2280E+05     3.4146E-01     6.2231E+01     CZ       8770

 BOND    =     31745.8074  ANGLE   =      1266.4467  DIHED      =      6632.3513
```

```
VDWAALS =     76559.1520  EEL      =  -564437.7905  HBOND      =         0.0000
1-4 VDW =      1888.1556  1-4 EEL =    23542.9638  RESTRAINT  =         0.0000


  NSTEP       ENERGY          RMS            GMAX         NAME    NUMBER
   1800      -4.2307E+05     2.9358E-01     5.4976E+01     CZ       8770

BOND    =     31797.0727  ANGLE    =      1266.3760  DIHED      =      6629.6371
VDWAALS =     76793.5209  EEL      =  -564983.5788  HBOND      =         0.0000
1-4 VDW =      1885.7719  1-4 EEL =    23539.6328  RESTRAINT  =         0.0000


  NSTEP       ENERGY          RMS            GMAX         NAME    NUMBER
   1900      -4.2333E+05     2.6644E-01     4.4735E+01     CZ       8770

BOND    =     31847.8514  ANGLE    =      1265.9773  DIHED      =      6627.1711
VDWAALS =     77017.5452  EEL      =  -565508.9095  HBOND      =         0.0000
1-4 VDW =      1883.5268  1-4 EEL =    23537.2525  RESTRAINT  =         0.0000


  NSTEP       ENERGY          RMS            GMAX         NAME    NUMBER
   2000      -4.2358E+05     1.5134E-01     2.2192E+01     CZ       8770

BOND    =     31894.1486  ANGLE    =      1265.6912  DIHED      =      6625.4577
VDWAALS =     77230.4850  EEL      =  -566013.2263  HBOND      =         0.0000
1-4 VDW =      1881.6298  1-4 EEL =    23534.8619  RESTRAINT  =         0.0000


  NSTEP       ENERGY          RMS            GMAX         NAME    NUMBER
   2100      -4.2382E+05     1.0003E-01     6.0654E+00     CZ       8770

BOND    =     31941.1326  ANGLE    =      1265.1742  DIHED      =      6624.2051
VDWAALS =     77435.9029  EEL      =  -566502.6719  HBOND      =         0.0000
1-4 VDW =      1879.9426  1-4 EEL =    23532.8216  RESTRAINT  =         0.0000


  NSTEP       ENERGY          RMS            GMAX         NAME    NUMBER
   2200      -4.2406E+05     1.2015E-01     1.6229E+01     CZ       8770

BOND    =     31987.6150  ANGLE    =      1264.6596  DIHED      =      6622.8719
VDWAALS =     77632.6149  EEL      =  -566977.3769  HBOND      =         0.0000
1-4 VDW =      1878.4129  1-4 EEL =    23530.3655  RESTRAINT  =         0.0000


  NSTEP       ENERGY          RMS            GMAX         NAME    NUMBER
   2300      -4.2428E+05     1.0258E-01     9.5560E+00     CZ       8770

BOND    =     32030.2945  ANGLE    =      1264.2582  DIHED      =      6621.4300
VDWAALS =     77811.5446  EEL      =  -567416.0252  HBOND      =         0.0000
1-4 VDW =      1876.9808  1-4 EEL =    23527.8248  RESTRAINT  =         0.0000


  NSTEP       ENERGY          RMS            GMAX         NAME    NUMBER
   2400      -4.2450E+05     3.0176E-01     6.5946E+01     CG       3232
```

```
BOND    =     32075.4729  ANGLE   =      1264.2987  DIHED      =      6620.0146
VDWAALS =     77983.1149  EEL     =   -567843.1782  HBOND      =         0.0000
1-4 VDW =      1875.6714  1-4 EEL =     23525.0918  RESTRAINT  =         0.0000


  NSTEP       ENERGY          RMS            GMAX         NAME    NUMBER
   2500      -4.2472E+05     2.4011E-01     4.7700E+01     CZ       8770

BOND    =     32115.6095  ANGLE   =      1263.7981  DIHED      =      6618.7517
VDWAALS =     78146.8588  EEL     =   -568258.1858  HBOND      =         0.0000
1-4 VDW =      1874.2964  1-4 EEL =     23522.6782  RESTRAINT  =         0.0000


  NSTEP       ENERGY          RMS            GMAX         NAME    NUMBER
   2600      -4.2493E+05     1.3877E-01     2.1257E+01     CZ       8770

BOND    =     32154.0198  ANGLE   =      1263.3594  DIHED      =      6617.6831
VDWAALS =     78307.0140  EEL     =   -568666.1240  HBOND      =         0.0000
1-4 VDW =      1873.0558  1-4 EEL =     23520.0227  RESTRAINT  =         0.0000


  NSTEP       ENERGY          RMS            GMAX         NAME    NUMBER
   2700      -4.2513E+05     1.6024E-01     2.9621E+01     CZ       8770

BOND    =     32193.0622  ANGLE   =      1263.1761  DIHED      =      6616.9484
VDWAALS =     78459.9217  EEL     =   -569054.4248  HBOND      =         0.0000
1-4 VDW =      1871.9028  1-4 EEL =     23517.3045  RESTRAINT  =         0.0000


  NSTEP       ENERGY          RMS            GMAX         NAME    NUMBER
   2800      -4.2533E+05     1.1155E-01     1.5362E+01     CZ       8770

BOND    =     32230.1978  ANGLE   =      1262.9511  DIHED      =      6616.4419
VDWAALS =     78606.8973  EEL     =   -569428.7949  HBOND      =         0.0000
1-4 VDW =      1870.7755  1-4 EEL =     23514.5940  RESTRAINT  =         0.0000


  NSTEP       ENERGY          RMS            GMAX         NAME    NUMBER
   2900      -4.2551E+05     3.1444E-01     6.7749E+01     CZ       8770

BOND    =     32271.4215  ANGLE   =      1263.2161  DIHED      =      6616.1796
VDWAALS =     78747.6580  EEL     =   -569791.9180  HBOND      =         0.0000
1-4 VDW =      1869.6984  1-4 EEL =     23511.7658  RESTRAINT  =         0.0000


  NSTEP       ENERGY          RMS            GMAX         NAME    NUMBER
   3000      -4.2570E+05     2.8699E-01     5.7515E+01     CG       3232

BOND    =     32307.0646  ANGLE   =      1263.2544  DIHED      =      6616.1606
VDWAALS =     78885.0426  EEL     =   -570153.6433  HBOND      =         0.0000
1-4 VDW =      1868.6803  1-4 EEL =     23508.7907  RESTRAINT  =         0.0000
```

```
  NSTEP       ENERGY          RMS            GMAX         NAME    NUMBER
   3100      -4.2589E+05     2.4908E-01     4.9826E+01     CZ       8770

 BOND    =     32341.2695  ANGLE   =      1263.2754  DIHED      =      6616.2173
 VDWAALS =     79011.7911  EEL     =   -570494.0503  HBOND      =         0.0000
 1-4 VDW =      1867.6754  1-4 EEL =     23506.1864  RESTRAINT  =         0.0000


  NSTEP       ENERGY          RMS            GMAX         NAME    NUMBER
   3200      -4.2607E+05     1.9836E-01     4.0496E+01     CZ       8770

 BOND    =     32375.4351  ANGLE   =      1263.4432  DIHED      =      6616.4433
 VDWAALS =     79137.6124  EEL     =   -570837.3433  HBOND      =         0.0000
 1-4 VDW =      1866.7113  1-4 EEL =     23503.5005  RESTRAINT  =         0.0000


  NSTEP       ENERGY          RMS            GMAX         NAME    NUMBER
   3300      -4.2625E+05     1.5616E-01     2.9536E+01     CZ       8770

 BOND    =     32408.5616  ANGLE   =      1263.6488  DIHED      =      6616.5889
 VDWAALS =     79258.6925  EEL     =   -571169.0227  HBOND      =         0.0000
 1-4 VDW =      1865.8489  1-4 EEL =     23500.8772  RESTRAINT  =         0.0000


  NSTEP       ENERGY          RMS            GMAX         NAME    NUMBER
   3400      -4.2643E+05     3.6594E-01     6.9534E+01     CZ       8770

 BOND    =     32447.5500  ANGLE   =      1264.2796  DIHED      =      6616.5170
 VDWAALS =     79378.7844  EEL     =   -571499.0024  HBOND      =         0.0000
 1-4 VDW =      1865.0241  1-4 EEL =     23498.4710  RESTRAINT  =         0.0000


  NSTEP       ENERGY          RMS            GMAX         NAME    NUMBER
   3500      -4.2661E+05     3.0422E-01     6.4807E+01     CZ       8770

 BOND    =     32478.4387  ANGLE   =      1264.4189  DIHED      =      6616.1881
 VDWAALS =     79495.7339  EEL     =   -571820.0529  HBOND      =         0.0000
 1-4 VDW =      1864.3610  1-4 EEL =     23495.8511  RESTRAINT  =         0.0000


  NSTEP       ENERGY          RMS            GMAX         NAME    NUMBER
   3600      -4.2678E+05     2.7596E-01     6.3445E+01     CG       3232

 BOND    =     32510.2506  ANGLE   =      1264.5700  DIHED      =      6615.4842
 VDWAALS =     79612.4280  EEL     =   -572140.8481  HBOND      =         0.0000
 1-4 VDW =      1863.6485  1-4 EEL =     23493.4272  RESTRAINT  =         0.0000


  NSTEP       ENERGY          RMS            GMAX         NAME    NUMBER
   3700      -4.2695E+05     2.4080E-01     4.7797E+01     CZ       8770

 BOND    =     32541.6134  ANGLE   =      1264.8384  DIHED      =      6614.6970
 VDWAALS =     79724.4035  EEL     =   -572450.0645  HBOND      =         0.0000
 1-4 VDW =      1862.8613  1-4 EEL =     23491.2777  RESTRAINT  =         0.0000
```

```
  NSTEP       ENERGY          RMS            GMAX         NAME    NUMBER
   3800      -4.2712E+05     1.5149E-01     2.9079E+01     CZ       8770

 BOND    =     32571.7818  ANGLE   =      1265.0748  DIHED      =      6613.7944
 VDWAALS =     79836.4213  EEL     =   -572757.0903  HBOND      =         0.0000
 1-4 VDW =      1862.0373  1-4 EEL =     23489.1915  RESTRAINT  =         0.0000


  NSTEP       ENERGY          RMS            GMAX         NAME    NUMBER
   3900      -4.2729E+05     1.5089E-01     2.8782E+01     CZ       8770

 BOND    =     32603.9725  ANGLE   =      1265.5565  DIHED      =      6612.8637
 VDWAALS =     79951.8510  EEL     =   -573069.3684  HBOND      =         0.0000
 1-4 VDW =      1861.2330  1-4 EEL =     23487.0234  RESTRAINT  =         0.0000


  NSTEP       ENERGY          RMS            GMAX         NAME    NUMBER
   4000      -4.2745E+05     8.3240E-02     5.8736E+00     CZ       8770

 BOND    =     32633.3962  ANGLE   =      1265.8517  DIHED      =      6611.8438
 VDWAALS =     80062.0119  EEL     =   -573364.3710  HBOND      =         0.0000
 1-4 VDW =      1860.5512  1-4 EEL =     23485.0477  RESTRAINT  =         0.0000


  NSTEP       ENERGY          RMS            GMAX         NAME    NUMBER
   4100      -4.2760E+05     3.3192E-01     9.2775E+01     CG       3232

 BOND    =     32668.8279  ANGLE   =      1266.6559  DIHED      =      6610.5334
 VDWAALS =     80176.2413  EEL     =   -573664.9225  HBOND      =         0.0000
 1-4 VDW =      1859.8365  1-4 EEL =     23483.3216  RESTRAINT  =         0.0000


  NSTEP       ENERGY          RMS            GMAX         NAME    NUMBER
   4200      -4.2775E+05     2.4405E-01     4.8546E+01     CZ       8770

 BOND    =     32696.0130  ANGLE   =      1267.0561  DIHED      =      6608.8537
 VDWAALS =     80287.1886  EEL     =   -573953.4938  HBOND      =         0.0000
 1-4 VDW =      1859.3439  1-4 EEL =     23481.2563  RESTRAINT  =         0.0000


  NSTEP       ENERGY          RMS            GMAX         NAME    NUMBER
   4300      -4.2790E+05     2.3221E-01     4.5121E+01     CZ       8770

 BOND    =     32724.7778  ANGLE   =      1267.3376  DIHED      =      6606.5795
 VDWAALS =     80395.4680  EEL     =   -574235.9642  HBOND      =         0.0000
 1-4 VDW =      1858.6716  1-4 EEL =     23479.9656  RESTRAINT  =         0.0000


  NSTEP       ENERGY          RMS            GMAX         NAME    NUMBER
   4400      -4.2806E+05     1.4525E-01     2.7806E+01     CZ       8770

 BOND    =     32751.9228  ANGLE   =      1266.7909  DIHED      =      6604.9175
```

```
VDWAALS =    80503.7824  EEL     =  -574521.7083  HBOND      =        0.0000
1-4 VDW =     1858.0279  1-4 EEL =    23479.0393  RESTRAINT  =        0.0000


  NSTEP       ENERGY          RMS            GMAX         NAME    NUMBER
  4500      -4.2821E+05     1.4245E-01     2.6434E+01     CZ       8770

BOND    =    32780.8036  ANGLE   =     1265.9738  DIHED      =     6604.2400
VDWAALS =    80611.6085  EEL     =  -574809.5537  HBOND      =        0.0000
1-4 VDW =     1857.4086  1-4 EEL =    23478.3815  RESTRAINT  =        0.0000


  NSTEP       ENERGY          RMS            GMAX         NAME    NUMBER
  4600      -4.2836E+05     1.0197E-01     1.5087E+01     CZ       8770

BOND    =    32808.6095  ANGLE   =     1265.5495  DIHED      =     6603.8858
VDWAALS =    80717.7924  EEL     =  -575092.5015  HBOND      =        0.0000
1-4 VDW =     1856.8678  1-4 EEL =    23477.6491  RESTRAINT  =        0.0000


  NSTEP       ENERGY          RMS            GMAX         NAME    NUMBER
  4700      -4.2851E+05     8.7249E-02     9.6396E+00     CZ       8770

BOND    =    32836.9438  ANGLE   =     1265.3513  DIHED      =     6603.4892
VDWAALS =    80827.2761  EEL     =  -575377.9045  HBOND      =        0.0000
1-4 VDW =     1856.3565  1-4 EEL =    23476.8394  RESTRAINT  =        0.0000


  NSTEP       ENERGY          RMS            GMAX         NAME    NUMBER
  4800      -4.2865E+05     2.3059E-01     4.8289E+01     CZ       8770

BOND    =    32867.2165  ANGLE   =     1265.4592  DIHED      =     6602.9873
VDWAALS =    80938.2322  EEL     =  -575659.6539  HBOND      =        0.0000
1-4 VDW =     1855.9070  1-4 EEL =    23475.9226  RESTRAINT  =        0.0000


  NSTEP       ENERGY          RMS            GMAX         NAME    NUMBER
  4900      -4.2880E+05     2.2264E-01     4.2970E+01     CZ       8770

BOND    =    32894.8902  ANGLE   =     1265.5494  DIHED      =     6602.4111
VDWAALS =    81051.7878  EEL     =  -575943.0636  HBOND      =        0.0000
1-4 VDW =     1855.4865  1-4 EEL =    23474.8693  RESTRAINT  =        0.0000


  NSTEP       ENERGY          RMS            GMAX         NAME    NUMBER
  5000      -4.2894E+05     1.7728E-01     3.5294E+01     CZ       8770

BOND    =    32921.1156  ANGLE   =     1265.3979  DIHED      =     6601.7029
VDWAALS =    81161.7586  EEL     =  -576215.3173  HBOND      =        0.0000
1-4 VDW =     1855.0540  1-4 EEL =    23473.9436  RESTRAINT  =        0.0000


 Maximum number of minimization cycles reached.
```

```
                    FINAL RESULTS



   NSTEP       ENERGY          RMS            GMAX         NAME    NUMBER
   5000      -4.2894E+05     1.7728E-01     3.5294E+01     CZ       8770

 BOND    =    32921.1156  ANGLE   =     1265.3979  DIHED      =     6601.7029
 VDWAALS =    81161.7586  EEL     =  -576215.3173  HBOND      =        0.0000
 1-4 VDW =     1855.0540  1-4 EEL =    23473.9436  RESTRAINT  =        0.0000

--------------------------------------------------------------------------------
   5.  TIMINGS
--------------------------------------------------------------------------------

|                Build the list            21.09 (87.47% of List )
|                Other                      3.02 (12.53% of List )
|             List time                 24.11 ( 0.23% of Nonbo)
|                   Short_ene time          9420.14 (99.67% of Direc)
|                   Other                     30.85 ( 0.33% of Direc)
|                Direct Ewald time       9450.99 (91.62% of Ewald)
|                Adjust Ewald time         35.53 ( 0.34% of Ewald)
|                   Fill Bspline coeffs       27.13 ( 3.30% of Recip)
|                   Fill charge grid         119.99 (14.61% of Recip)
|                   Scalar sum               156.59 (19.06% of Recip)
|                   Grad sum                 202.58 (24.66% of Recip)
|                   FFT time                 315.14 (38.36% of Recip)
|                Recip Ewald time         821.46 ( 7.96% of Ewald)
|                Virial junk                2.33 ( 0.02% of Ewald)
|                Other                      5.27 ( 0.05% of Ewald)
|             Ewald time             10315.60 (99.77% of Nonbo)
|          Nonbond force          10339.72 (99.49% of Force)
|          Bond/Angle/Dihedral       51.30 ( 0.49% of Force)
|          Other                      1.36 ( 0.01% of Force)
|       Force time             10392.37 (100.0% of Runmd)
|    Runmd Time             10392.37 (99.69% of Total)
|    Other                     32.01 ( 0.31% of Total)
| Total time             10424.41 (100.0% of ALL  )

| Number of list builds   :         13

| Highest rstack allocated:    5729944
| Highest istack allocated:     107718
|           Job began  at 07:02:43.142  on 08/13/2017
|           Setup done at 07:02:44.005  on 08/13/2017
|           Run   done at 09:56:27.585  on 08/13/2017
|     wallclock() was called  190074 times

          -------------------------------------------------------
          Amber 16 SANDER                              2016
          -------------------------------------------------------

| Run on 02/21/2019 at 04:29:15
```

```
|   Executable path: /usr/local/amber16/bin/sander
| Working directory: /Users/Swati
|          Hostname: Unknown
  [-O]verwriting output

File Assignments:
|  MDIN: /Users/Swati/minimization_file_2.in
| MDOUT: /Users/Swati/3WY9_files_2/truncated_3WY9_min3_2.out
|INPCRD: /Users/Swati/3WY9_files_2/truncated_3WY9_min3_1.rst
|  PARM: /Users/Swati/3WY9_files_2/3WY9.prmtop
|RESTRT: /Users/Swati/3WY9_files_2/truncated_3WY9_min3_2.rst
|  REFC: refc
| MDVEL: mdvel
| MDFRC: mdfrc
|  MDEN: mden
| MDCRD: mdcrd
|MDINFO: mdinfo
|  MTMD: mtmd
|INPDIP: inpdip
|RSTDIP: rstdip
|INPTRA: inptraj


 Here is the input file:

PROT: minimization_2
&cntrl
imin = 1,
maxcyc = 5000,
ncyc = 10000,
igb = 0,
ntpr =  100,
ntp = 0
cut = 12
&end


--------------------------------------------------------------------------------
   1.  RESOURCE   USE: 
--------------------------------------------------------------------------------

| Flags:                                                                        
 getting box info from netcdf restart file
| NetCDF restart box info found
|Largest sphere to fit in unit cell has radius =    43.417
| New format PARM file being parsed.
| Version =    1.000 Date = 08/09/17 Time = 18:38:06
 NATOM  =   89944 NTYPES =      17 NBONH =   86305 MBONA  =    3731
 NTHETH =    7791 MTHETA =    5069 NPHIH =   16227 MPHIA  =   15704
 NHPARM =       0 NPARM  =       0 NNB   =  149382 NRES   =   28096
 NBONA  =    3731 NTHETA =    5069 NPHIA =   15704 NUMBND =      67
 NUMANG =     152 NPTRA  =     191 NATYP =      36 NPHB   =       1
 IFBOX  =       1 NMXRS  =      24 IFCAP =       0 NEXTRA =       0
```

```
 NCOPY  =       0


|     Memory Use     Allocated
|     Real             7102941
|     Hollerith         297930
|     Integer          3593065
|     Max Pairs       82268778
|     nblistReal       1079328
|     nblist Int       2979057
|       Total           412122 kbytes

| Note: 1-4 EEL scale factors are being read from the topology file.

| Note: 1-4 VDW scale factors are being read from the topology file.
| Duplicated    0 dihedrals
| Duplicated    0 dihedrals

     BOX TYPE: RECTILINEAR

--------------------------------------------------------------------------------
   2.  CONTROL  DATA  FOR  THE  RUN
--------------------------------------------------------------------------------

default_name

General flags:
     imin    =       1, nmropt  =       0

Nature and format of input:
     ntx     =       1, irest   =       0, ntrx    =       1

Nature and format of output:
     ntxo    =       2, ntpr    =     100, ntrx    =       1, ntwr    =       1
     iwrap   =       0, ntwx    =       0, ntwv    =       0, ntwe    =       0
     ioutfm  =       1, ntwprt  =       0, idecomp =       0, rbornstat=      0

Potential function:
     ntf     =       1, ntb     =       1, igb     =       0, nsnb    =      25
     ipol    =       0, gbsa    =       0, iesp    =       0
     dielc   =   1.00000, cut     =  12.00000, intdiel =   1.00000

Frozen or restrained atoms:
     ibelly  =       0, ntr     =       0

Energy minimization:
     maxcyc  =    5000, ncyc    =   10000, ntmin   =       1
     dx0     =   0.01000, drms    =   0.00010

Ewald parameters:
     verbose =       0, ew_type =       0, nbflag  =       1, use_pme =       1
     vdwmeth =       1, eedmeth =       1, netfrc  =       0
     Box X =  117.210   Box Y =   86.834   Box Z =  105.320
     Alpha =   90.000   Beta  =   90.000   Gamma =   90.000
```

```
     NFFT1 =  120       NFFT2 =   90       NFFT3 =  108
     Cutoff=   12.000   Tol   =0.100E-04
     Ewald Coefficient =  0.22664
     Interpolation order =    4
|  INFO: Old style inpcrd file read


--------------------------------------------------------------------------------
   3.  ATOMIC COORDINATES AND VELOCITIES
--------------------------------------------------------------------------------

default_name
 begin time read from input coords =     0.000 ps

 Number of triangulated 3-point waters found:    27622

     Sum of charges from parm topology file =  -0.00000030
     Forcing neutrality...

--------------------------------------------------------------------------------
   4.  RESULTS
--------------------------------------------------------------------------------

 ---------------------------------------------------
 APPROXIMATING switch and d/dx switch using CUBIC SPLINE INTERPOLATION
 using   5000.0 points per unit in tabled values
 TESTING RELATIVE ERROR over r ranging from 0.0 to cutoff
| CHECK switch(x): max rel err =   0.2738E-14   at   2.422500
| CHECK d/dx switch(x): max rel err =   0.7967E-11   at   2.716640
 ---------------------------------------------------
| Local SIZE OF NONBOND LIST =   44435104
| TOTAL SIZE OF NONBOND LIST =   44435104


   NSTEP       ENERGY          RMS            GMAX         NAME    NUMBER
      1      -3.3570E+05     4.4705E+00     7.3544E+02     C        3773

 BOND    =    36377.0014  ANGLE   =     2650.3300  DIHED      =     5381.3421
 VDWAALS =    65807.3845  EEL     =  -469077.7931  HBOND      =        0.0000
 1-4 VDW =     2197.8628  1-4 EEL =    20962.0641  RESTRAINT  =        0.0000


   NSTEP       ENERGY          RMS            GMAX         NAME    NUMBER
    100      -3.5025E+05     6.9449E-01     1.2712E+02     N        3789

 BOND    =    27031.5879  ANGLE   =     1516.8236  DIHED      =     5402.5154
 VDWAALS =    63530.8221  EEL     =  -469847.9188  HBOND      =        0.0000
 1-4 VDW =     1690.4601  1-4 EEL =    20426.3791  RESTRAINT  =        0.0000


   NSTEP       ENERGY          RMS            GMAX         NAME    NUMBER
    200      -3.5175E+05     6.1494E-01     9.4068E+01     C        3787

 BOND    =    26640.0580  ANGLE   =     1283.0204  DIHED      =     5371.9422
```

```
VDWAALS =     63592.7272  EEL      =  -470584.0847  HBOND      =         0.0000
1-4 VDW =      1589.9694  1-4 EEL =    20353.6026  RESTRAINT  =         0.0000


   NSTEP       ENERGY          RMS            GMAX         NAME    NUMBER
    300      -3.5250E+05     5.8636E-01     1.2914E+02     CD       3234

BOND    =     26552.7552  ANGLE    =     1204.3470  DIHED      =      5347.5305
VDWAALS =     63746.9670  EEL      =  -471238.9004  HBOND      =         0.0000
1-4 VDW =      1555.3613  1-4 EEL =    20335.4100  RESTRAINT  =         0.0000


   NSTEP       ENERGY          RMS            GMAX         NAME    NUMBER
    400      -3.5302E+05     4.7726E-01     7.9582E+01     CD       3234

BOND    =     26545.3261  ANGLE    =     1145.6396  DIHED      =      5333.5248
VDWAALS =     63922.7691  EEL      =  -471837.0130  HBOND      =         0.0000
1-4 VDW =      1534.5648  1-4 EEL =    20333.0602  RESTRAINT  =         0.0000


   NSTEP       ENERGY          RMS            GMAX         NAME    NUMBER
    500      -3.5346E+05     4.2275E-01     6.4251E+01     C        3787

BOND    =     26566.4500  ANGLE    =     1082.2853  DIHED      =      5324.1443
VDWAALS =     64111.0034  EEL      =  -472400.4148  HBOND      =         0.0000
1-4 VDW =      1521.8673  1-4 EEL =    20338.5228  RESTRAINT  =         0.0000


   NSTEP       ENERGY          RMS            GMAX         NAME    NUMBER
    600      -3.5384E+05     3.5467E-01     6.1094E+01     N        3789

BOND    =     26593.6258  ANGLE    =     1019.4151  DIHED      =      5320.7823
VDWAALS =     64310.9792  EEL      =  -472942.4822  HBOND      =         0.0000
1-4 VDW =      1511.0032  1-4 EEL =    20342.3286  RESTRAINT  =         0.0000


   NSTEP       ENERGY          RMS            GMAX         NAME    NUMBER
    700      -3.5418E+05     2.8741E-01     4.7548E+01     C        3787

BOND    =     26626.0208  ANGLE    =      990.4963  DIHED      =      5315.1192
VDWAALS =     64503.3558  EEL      =  -473465.7901  HBOND      =         0.0000
1-4 VDW =      1502.9418  1-4 EEL =    20345.6433  RESTRAINT  =         0.0000


   NSTEP       ENERGY          RMS            GMAX         NAME    NUMBER
    800      -3.5447E+05     1.2379E-01     8.5597E+00     C        3787

BOND    =     26657.1895  ANGLE    =      977.7999  DIHED      =      5310.7030
VDWAALS =     64694.8466  EEL      =  -473961.6221  HBOND      =         0.0000
1-4 VDW =      1498.4252  1-4 EEL =    20348.4099  RESTRAINT  =         0.0000


   NSTEP       ENERGY          RMS            GMAX         NAME    NUMBER
    900      -3.5474E+05     1.2717E-01     1.1239E+01     N        3789
```

```
BOND    =     26696.4335  ANGLE   =       968.5064  DIHED      =      5308.0440
VDWAALS =     64889.9751  EEL     =   -474452.5659  HBOND      =         0.0000
1-4 VDW =      1494.9843  1-4 EEL =     20350.5116  RESTRAINT  =         0.0000


  NSTEP       ENERGY          RMS            GMAX         NAME    NUMBER
   1000      -3.5498E+05     4.5368E-01     6.7079E+01     N        3789

BOND    =     26745.0513  ANGLE   =       963.1718  DIHED      =      5306.3550
VDWAALS =     65082.6645  EEL     =   -474925.2180  HBOND      =         0.0000
1-4 VDW =      1491.8941  1-4 EEL =     20351.5484  RESTRAINT  =         0.0000


  NSTEP       ENERGY          RMS            GMAX         NAME    NUMBER
   1100      -3.5522E+05     3.8712E-01     5.2983E+01     C        3787

BOND    =     26780.8717  ANGLE   =       961.2304  DIHED      =      5305.6539
VDWAALS =     65273.0402  EEL     =   -475381.6141  HBOND      =         0.0000
1-4 VDW =      1489.3339  1-4 EEL =     20353.0157  RESTRAINT  =         0.0000


  NSTEP       ENERGY          RMS            GMAX         NAME    NUMBER
   1200      -3.5544E+05     4.0197E-01     6.6361E+01     CD       3234

BOND    =     26824.4265  ANGLE   =       955.4600  DIHED      =      5306.7788
VDWAALS =     65459.9214  EEL     =   -475824.7086  HBOND      =         0.0000
1-4 VDW =      1486.8931  1-4 EEL =     20353.5044  RESTRAINT  =         0.0000


  NSTEP       ENERGY          RMS            GMAX         NAME    NUMBER
   1300      -3.5565E+05     3.0666E-01     3.8369E+01     C        3787

BOND    =     26858.8046  ANGLE   =       954.2594  DIHED      =      5303.4012
VDWAALS =     65636.7127  EEL     =   -476245.2930  HBOND      =         0.0000
1-4 VDW =      1484.6364  1-4 EEL =     20355.1162  RESTRAINT  =         0.0000


  NSTEP       ENERGY          RMS            GMAX         NAME    NUMBER
   1400      -3.5585E+05     2.4090E-01     3.1480E+01     C        3787

BOND    =     26898.5396  ANGLE   =       950.7193  DIHED      =      5301.7393
VDWAALS =     65810.8529  EEL     =   -476650.4426  HBOND      =         0.0000
1-4 VDW =      1482.4716  1-4 EEL =     20356.0021  RESTRAINT  =         0.0000


  NSTEP       ENERGY          RMS            GMAX         NAME    NUMBER
   1500      -3.5604E+05     1.2237E-01     1.1506E+01     C        3802

BOND    =     26934.9643  ANGLE   =       949.7724  DIHED      =      5300.1586
VDWAALS =     65979.5224  EEL     =   -477046.6295  HBOND      =         0.0000
1-4 VDW =      1480.7263  1-4 EEL =     20357.7625  RESTRAINT  =         0.0000
```

```
   NSTEP       ENERGY          RMS            GMAX         NAME    NUMBER
   1600      -3.5623E+05     1.0447E-01     6.5359E+00     C        3787

 BOND    =     26973.5337  ANGLE   =       948.3894  DIHED      =     5298.8699
 VDWAALS =     66143.2977  EEL     =   -477433.7001  HBOND      =        0.0000
 1-4 VDW =      1479.1077  1-4 EEL =     20359.4025  RESTRAINT  =        0.0000


   NSTEP       ENERGY          RMS            GMAX         NAME    NUMBER
   1700      -3.5641E+05     3.5904E-01     4.9246E+01     C        3787

 BOND    =     27016.3501  ANGLE   =       947.6783  DIHED      =     5297.6234
 VDWAALS =     66304.1595  EEL     =   -477813.7997  HBOND      =        0.0000
 1-4 VDW =      1477.7307  1-4 EEL =     20360.9338  RESTRAINT  =        0.0000


   NSTEP       ENERGY          RMS            GMAX         NAME    NUMBER
   1800      -3.5659E+05     3.0895E-01     4.3408E+01     CG       3219

 BOND    =     27051.3977  ANGLE   =       946.7535  DIHED      =     5295.9649
 VDWAALS =     66459.2936  EEL     =   -478186.0448  HBOND      =        0.0000
 1-4 VDW =      1476.5156  1-4 EEL =     20362.9189  RESTRAINT  =        0.0000


   NSTEP       ENERGY          RMS            GMAX         NAME    NUMBER
   1900      -3.5677E+05     2.6565E-01     3.0004E+01     C        3787

 BOND    =     27088.3226  ANGLE   =       943.6513  DIHED      =     5295.6954
 VDWAALS =     66604.8802  EEL     =   -478540.7310  HBOND      =        0.0000
 1-4 VDW =      1475.4424  1-4 EEL =     20363.6225  RESTRAINT  =        0.0000


   NSTEP       ENERGY          RMS            GMAX         NAME    NUMBER
   2000      -3.5695E+05     2.3324E-01     2.8154E+01     CG       3219

 BOND    =     27123.8935  ANGLE   =       942.8663  DIHED      =     5294.8963
 VDWAALS =     66750.9726  EEL     =   -478897.0472  HBOND      =        0.0000
 1-4 VDW =      1474.5281  1-4 EEL =     20364.7091  RESTRAINT  =        0.0000


   NSTEP       ENERGY          RMS            GMAX         NAME    NUMBER
   2100      -3.5711E+05     1.2024E-01     1.0887E+01     C        3756

 BOND    =     27159.1501  ANGLE   =       940.8324  DIHED      =     5294.8052
 VDWAALS =     66892.3006  EEL     =   -479240.0677  HBOND      =        0.0000
 1-4 VDW =      1473.5660  1-4 EEL =     20365.2272  RESTRAINT  =        0.0000


   NSTEP       ENERGY          RMS            GMAX         NAME    NUMBER
   2200      -3.5728E+05     1.0093E-01     6.8957E+00     C        3756

 BOND    =     27194.4651  ANGLE   =       940.0978  DIHED      =     5294.2600
 VDWAALS =     67031.2324  EEL     =   -479575.1310  HBOND      =        0.0000
 1-4 VDW =      1472.7296  1-4 EEL =     20365.8945  RESTRAINT  =        0.0000
```

```
   NSTEP       ENERGY          RMS            GMAX         NAME    NUMBER
   2300      -3.5743E+05     3.5229E-01     4.5395E+01     C         3756

 BOND    =     27233.9260  ANGLE   =       940.7672  DIHED      =      5293.4005
 VDWAALS =     67170.3450  EEL     =  -479910.3779  HBOND      =         0.0000
 1-4 VDW =      1472.1051  1-4 EEL =     20366.5537  RESTRAINT  =         0.0000


   NSTEP       ENERGY          RMS            GMAX         NAME    NUMBER
   2400      -3.5759E+05     3.4363E-01     7.7904E+01     CG        3219

 BOND    =     27269.6344  ANGLE   =       938.4750  DIHED      =      5293.1087
 VDWAALS =     67304.5438  EEL     =  -480235.5482  HBOND      =         0.0000
 1-4 VDW =      1471.5003  1-4 EEL =     20366.9713  RESTRAINT  =         0.0000


   NSTEP       ENERGY          RMS            GMAX         NAME    NUMBER
   2500      -3.5774E+05     2.4859E-01     3.2101E+01     C         3756

 BOND    =     27300.1420  ANGLE   =       938.0541  DIHED      =      5292.2229
 VDWAALS =     67434.7792  EEL     =  -480548.9865  HBOND      =         0.0000
 1-4 VDW =      1470.9216  1-4 EEL =     20368.1478  RESTRAINT  =         0.0000


   NSTEP       ENERGY          RMS            GMAX         NAME    NUMBER
   2600      -3.5789E+05     2.4858E-01     4.3231E+01     CG        3219

 BOND    =     27333.6367  ANGLE   =       936.5515  DIHED      =      5292.0147
 VDWAALS =     67561.9958  EEL     =  -480854.3612  HBOND      =         0.0000
 1-4 VDW =      1470.3106  1-4 EEL =     20368.6210  RESTRAINT  =         0.0000


   NSTEP       ENERGY          RMS            GMAX         NAME    NUMBER
   2700      -3.5804E+05     1.8594E-01     2.2011E+01     C         3756

 BOND    =     27363.4995  ANGLE   =       936.2022  DIHED      =      5291.3768
 VDWAALS =     67685.7290  EEL     =  -481151.1435  HBOND      =         0.0000
 1-4 VDW =      1469.6473  1-4 EEL =     20369.5776  RESTRAINT  =         0.0000


   NSTEP       ENERGY          RMS            GMAX         NAME    NUMBER
   2800      -3.5817E+05     1.2001E-01     1.1859E+01     C         3756

 BOND    =     27393.5415  ANGLE   =       935.1194  DIHED      =      5291.2036
 VDWAALS =     67803.2085  EEL     =  -481436.7262  HBOND      =         0.0000
 1-4 VDW =      1468.9505  1-4 EEL =     20370.0416  RESTRAINT  =         0.0000


   NSTEP       ENERGY          RMS            GMAX         NAME    NUMBER
   2900      -3.5831E+05     1.0956E-01     8.9988E+00     C         3745

 BOND    =     27422.4813  ANGLE   =       934.8567  DIHED      =      5290.8932
```

```
VDWAALS =     67915.6454  EEL      =  -481712.6968  HBOND      =         0.0000
1-4 VDW =      1468.3352  1-4 EEL =    20370.5190  RESTRAINT  =         0.0000


  NSTEP       ENERGY          RMS            GMAX         NAME    NUMBER
   3000      -3.5844E+05     3.5022E-01     9.0234E+01     CG       3219

BOND    =     27453.8586  ANGLE    =       934.9934  DIHED      =      5290.8182
VDWAALS =     68023.0113  EEL      =  -481979.6135  HBOND      =         0.0000
1-4 VDW =      1467.7146  1-4 EEL =    20371.2099  RESTRAINT  =         0.0000


  NSTEP       ENERGY          RMS            GMAX         NAME    NUMBER
   3100      -3.5857E+05     2.4742E-01     3.2119E+01     CG       3219

BOND    =     27480.4746  ANGLE    =       934.2605  DIHED      =      5290.9548
VDWAALS =     68128.4966  EEL      =  -482245.9187  HBOND      =         0.0000
1-4 VDW =      1467.1954  1-4 EEL =    20371.4153  RESTRAINT  =         0.0000


  NSTEP       ENERGY          RMS            GMAX         NAME    NUMBER
   3200      -3.5871E+05     2.2581E-01     1.9924E+01     NE2      2687

BOND    =     27506.5788  ANGLE    =       934.3734  DIHED      =      5290.8974
VDWAALS =     68230.6592  EEL      =  -482506.2406  HBOND      =         0.0000
1-4 VDW =      1466.8148  1-4 EEL =    20371.8743  RESTRAINT  =         0.0000


  NSTEP       ENERGY          RMS            GMAX         NAME    NUMBER
   3300      -3.5884E+05     1.8625E-01     1.8575E+01     NE2      2687

BOND    =     27533.0392  ANGLE    =       933.4891  DIHED      =      5291.1381
VDWAALS =     68331.2722  EEL      =  -482763.5724  HBOND      =         0.0000
1-4 VDW =      1466.3332  1-4 EEL =    20372.2477  RESTRAINT  =         0.0000


  NSTEP       ENERGY          RMS            GMAX         NAME    NUMBER
   3400      -3.5897E+05     8.5762E-02     4.4971E+00     NE2      2687

BOND    =     27557.7019  ANGLE    =       933.1223  DIHED      =      5291.3123
VDWAALS =     68430.8792  EEL      =  -483016.7188  HBOND      =         0.0000
1-4 VDW =      1465.9792  1-4 EEL =    20372.6957  RESTRAINT  =         0.0000


  NSTEP       ENERGY          RMS            GMAX         NAME    NUMBER
   3500      -3.5909E+05     8.1721E-02     3.5374E+00     NE2      2687

BOND    =     27583.3052  ANGLE    =       932.6942  DIHED      =      5291.6138
VDWAALS =     68530.8927  EEL      =  -483270.1362  HBOND      =         0.0000
1-4 VDW =      1465.6215  1-4 EEL =    20373.1603  RESTRAINT  =         0.0000


  NSTEP       ENERGY          RMS            GMAX         NAME    NUMBER
   3600      -3.5921E+05     2.9391E-01     4.1567E+01     CG       3219
```

```
BOND    =    27610.4751  ANGLE   =      932.7623  DIHED      =     5292.0204
VDWAALS =    68626.6159  EEL     =  -483512.8218  HBOND      =        0.0000
1-4 VDW =     1465.2609  1-4 EEL =    20373.7782  RESTRAINT  =        0.0000


  NSTEP       ENERGY          RMS            GMAX         NAME    NUMBER
   3700      -3.5933E+05     2.5350E-01     4.9543E+01     CG       3219

BOND    =    27633.1467  ANGLE   =      932.4384  DIHED      =     5292.4257
VDWAALS =    68720.6220  EEL     =  -483750.9871  HBOND      =        0.0000
1-4 VDW =     1464.9873  1-4 EEL =    20374.3681  RESTRAINT  =        0.0000


  NSTEP       ENERGY          RMS            GMAX         NAME    NUMBER
   3800      -3.5945E+05     2.2008E-01     2.6801E+01     NE2      2687

BOND    =    27656.5848  ANGLE   =      931.8160  DIHED      =     5292.8884
VDWAALS =    68815.3417  EEL     =  -483986.0734  HBOND      =        0.0000
1-4 VDW =     1464.6792  1-4 EEL =    20374.8191  RESTRAINT  =        0.0000


  NSTEP       ENERGY          RMS            GMAX         NAME    NUMBER
   3900      -3.5957E+05     1.0382E-01     9.9506E+00     NE2      2687

BOND    =    27678.8134  ANGLE   =      931.2730  DIHED      =     5293.2151
VDWAALS =    68912.7542  EEL     =  -484221.9820  HBOND      =        0.0000
1-4 VDW =     1464.4012  1-4 EEL =    20375.4670  RESTRAINT  =        0.0000


  NSTEP       ENERGY          RMS            GMAX         NAME    NUMBER
   4000      -3.5967E+05     1.5135E-01     1.7563E+01     NE2      2687

BOND    =    27701.9718  ANGLE   =      930.8378  DIHED      =     5293.1598
VDWAALS =    69006.7114  EEL     =  -484445.1331  HBOND      =        0.0000
1-4 VDW =     1464.0853  1-4 EEL =    20376.1478  RESTRAINT  =        0.0000


  NSTEP       ENERGY          RMS            GMAX         NAME    NUMBER
   4100      -3.5977E+05     7.4426E-02     3.5786E+00     NE2      2687

BOND    =    27723.3847  ANGLE   =      930.4472  DIHED      =     5292.9322
VDWAALS =    69098.4270  EEL     =  -484660.4658  HBOND      =        0.0000
1-4 VDW =     1463.8385  1-4 EEL =    20376.8030  RESTRAINT  =        0.0000


  NSTEP       ENERGY          RMS            GMAX         NAME    NUMBER
   4200      -3.5987E+05     2.8711E-01     3.3497E+01     NE2      2687

BOND    =    27748.2469  ANGLE   =      930.9931  DIHED      =     5292.9934
VDWAALS =    69191.8753  EEL     =  -484877.0549  HBOND      =        0.0000
1-4 VDW =     1463.6234  1-4 EEL =    20377.5125  RESTRAINT  =        0.0000
```

```
  NSTEP       ENERGY          RMS            GMAX         NAME    NUMBER
   4300      -3.5997E+05     7.3571E-02     4.2971E+00     NE2      2687

 BOND    =     27766.8385  ANGLE   =       929.8444  DIHED      =      5293.2808
 VDWAALS =     69283.0656  EEL     =   -485084.9309  HBOND      =         0.0000
 1-4 VDW =      1463.3472  1-4 EEL =     20378.3734  RESTRAINT  =         0.0000


  NSTEP       ENERGY          RMS            GMAX         NAME    NUMBER
   4400      -3.6006E+05     2.1425E-01     3.4098E+01     CD       3234

 BOND    =     27790.2077  ANGLE   =       930.0020  DIHED      =      5293.4777
 VDWAALS =     69377.4692  EEL     =   -485298.0072  HBOND      =         0.0000
 1-4 VDW =      1463.1633  1-4 EEL =     20379.1465  RESTRAINT  =         0.0000


  NSTEP       ENERGY          RMS            GMAX         NAME    NUMBER
   4500      -3.6016E+05     1.7655E-01     2.2437E+01     NE2      2687

 BOND    =     27810.8422  ANGLE   =       929.4957  DIHED      =      5293.5194
 VDWAALS =     69468.2680  EEL     =   -485502.9157  HBOND      =         0.0000
 1-4 VDW =      1463.0261  1-4 EEL =     20379.6328  RESTRAINT  =         0.0000


  NSTEP       ENERGY          RMS            GMAX         NAME    NUMBER
   4600      -3.6025E+05     9.2804E-02     8.5069E+00     NE2      2687

 BOND    =     27830.5447  ANGLE   =       929.0364  DIHED      =      5293.5466
 VDWAALS =     69556.9879  EEL     =   -485703.9807  HBOND      =         0.0000
 1-4 VDW =      1462.9877  1-4 EEL =     20380.0500  RESTRAINT  =         0.0000


  NSTEP       ENERGY          RMS            GMAX         NAME    NUMBER
   4700      -3.6034E+05     7.3819E-02     5.0223E+00     NE2      2687

 BOND    =     27850.0203  ANGLE   =       928.7309  DIHED      =      5293.7118
 VDWAALS =     69642.4630  EEL     =   -485897.1561  HBOND      =         0.0000
 1-4 VDW =      1462.8310  1-4 EEL =     20380.3379  RESTRAINT  =         0.0000


  NSTEP       ENERGY          RMS            GMAX         NAME    NUMBER
   4800      -3.6043E+05     7.9689E-02     6.7168E+00     NE2      2687

 BOND    =     27869.5112  ANGLE   =       928.5959  DIHED      =      5293.9324
 VDWAALS =     69728.2261  EEL     =   -486090.7836  HBOND      =         0.0000
 1-4 VDW =      1462.6960  1-4 EEL =     20380.6114  RESTRAINT  =         0.0000


  NSTEP       ENERGY          RMS            GMAX         NAME    NUMBER
   4900      -3.6051E+05     2.2971E-01     2.8571E+01     NE2      2687

 BOND    =     27890.1278  ANGLE   =       928.8873  DIHED      =      5294.1834
 VDWAALS =     69812.6451  EEL     =   -486279.9148  HBOND      =         0.0000
 1-4 VDW =      1462.6257  1-4 EEL =     20380.8848  RESTRAINT  =         0.0000
```

```
  NSTEP       ENERGY          RMS            GMAX         NAME    NUMBER
   5000      -3.6059E+05     1.9451E-01     2.4554E+01     NE2      2687

 BOND    =    27908.1261  ANGLE   =      928.1343  DIHED      =     5294.5330
 VDWAALS =    69895.8320  EEL     =  -486464.6504  HBOND      =        0.0000
 1-4 VDW =     1462.5575  1-4 EEL =    20381.2479  RESTRAINT  =        0.0000


  Maximum number of minimization cycles reached.


                    FINAL RESULTS



  NSTEP       ENERGY          RMS            GMAX         NAME    NUMBER
   5000      -3.6059E+05     1.9451E-01     2.4554E+01     NE2      2687

 BOND    =    27908.1261  ANGLE   =      928.1343  DIHED      =     5294.5330
 VDWAALS =    69895.8320  EEL     =  -486464.6504  HBOND      =        0.0000
 1-4 VDW =     1462.5575  1-4 EEL =    20381.2479  RESTRAINT  =        0.0000

--------------------------------------------------------------------------------
   5.  TIMINGS
--------------------------------------------------------------------------------

|                Build the list            26.24 (91.22% of List )
|                Other                      2.53 ( 8.78% of List )
|             List time                 28.77 ( 0.17% of Nonbo)
|                   Short_ene time         8205.70 (99.68% of Direc)
|                   Other                    26.35 ( 0.32% of Direc)
|                Direct Ewald time       8232.05 (47.70% of Ewald)
|                Adjust Ewald time         30.72 ( 0.18% of Ewald)
|                   Fill Bspline coeffs      24.31 ( 0.27% of Recip)
|                   Fill charge grid        102.29 ( 1.14% of Recip)
|                   Scalar sum              140.77 ( 1.57% of Recip)
|                   Grad sum               8437.62 (93.87% of Recip)
|                   FFT time                283.53 ( 3.15% of Recip)
|                Recip Ewald time        8988.53 (52.08% of Ewald)
|                Virial junk                1.87 ( 0.01% of Ewald)
|                Other                      4.33 ( 0.03% of Ewald)
|             Ewald time             17257.52 (99.83% of Nonbo)
|          Nonbond force          17286.30 (99.75% of Force)
|          Bond/Angle/Dihedral       42.12 ( 0.24% of Force)
|          Other                      1.04 ( 0.01% of Force)
|       Force time             17329.46 (100.0% of Runmd)
|    Runmd Time             17329.46 (99.85% of Total)
|    Other                     26.33 ( 0.15% of Total)
| Total time             17355.86 (100.0% of ALL  )

| Number of list builds   :         19
```

```
| Highest rstack allocated:    4877674
| Highest istack allocated:      89944
|           Job began  at 04:29:15.661  on 02/21/2019
|           Setup done at 04:29:16.533  on 02/21/2019
|           Run   done at 09:18:31.519  on 02/21/2019
|     wallclock() was called  190086 times

          -------------------------------------------------------
          Amber 16 SANDER                              2016
          -------------------------------------------------------

| Run on 02/21/2019 at 12:02:52

|   Executable path: /usr/local/amber16/bin/sander
| Working directory: /Users/Swati
|          Hostname: Unknown
  [-O]verwriting output

File Assignments:
|  MDIN: /Users/Swati/minimization_file_2.in
| MDOUT: /Users/Swati/LJP6_files_2/truncated_LJP6_min3_2.out
|INPCRD: /Users/Swati/LJP6_files_2/truncated_LJP6_min3_1.rst
|  PARM: /Users/Swati/LJP6_files_2/LJP6.prmtop
|RESTRT: /Users/Swati/LJP6_files_2/truncated_LJP6_min3_2.rst
|  REFC: refc
| MDVEL: mdvel
| MDFRC: mdfrc
|  MDEN: mden
| MDCRD: mdcrd
|MDINFO: mdinfo
|  MTMD: mtmd
|INPDIP: inpdip
|RSTDIP: rstdip
|INPTRA: inptraj


 Here is the input file:

PROT: minimization_2
&cntrl
imin = 1,
maxcyc = 5000,
ncyc = 10000,
igb = 0,
ntpr =  100,
ntp = 0
cut = 12
&end


--------------------------------------------------------------------------------
   1.  RESOURCE   USE:
--------------------------------------------------------------------------------
```

```
| Flags:
 getting box info from netcdf restart file
| NetCDF restart box info found
|Largest sphere to fit in unit cell has radius =    42.185
| New format PARM file being parsed.
| Version =    1.000 Date = 08/09/17 Time = 04:08:03
 NATOM  =   87372 NTYPES =      17 NBONH =   83756 MBONA  =    3718
 NTHETH =    7789 MTHETA =    5057 NPHIH =   16221 MPHIA  =   15711
 NHPARM =       0 NPARM  =       0 NNB   =  145906 NRES   =   27236
 NBONA  =    3718 NTHETA =    5057 NPHIA =   15711 NUMBND =      67
 NUMANG =     152 NPTRA  =     191 NATYP =      36 NPHB   =       1
 IFBOX  =       1 NMXRS  =      24 IFCAP =       0 NEXTRA =       0
 NCOPY  =       0


|     Memory Use     Allocated
|     Real             6902375
|     Hollerith         289354
|     Integer          3534516
|     Max Pairs       79916256
|     nblistReal       1048464
|     nblist Int       2924001
|       Total           400647 kbytes

| Note: 1-4 EEL scale factors are being read from the topology file.

| Note: 1-4 VDW scale factors are being read from the topology file.
| Duplicated    0 dihedrals
| Duplicated    0 dihedrals

     BOX TYPE: RECTILINEAR

--------------------------------------------------------------------------------
   2.  CONTROL  DATA  FOR  THE  RUN
--------------------------------------------------------------------------------

default_name

General flags:
     imin    =       1, nmropt  =       0

Nature and format of input:
     ntx     =       1, irest   =       0, ntrx    =       1

Nature and format of output:
     ntxo    =       2, ntpr    =     100, ntrx    =       1, ntwr    =       1
     iwrap   =       0, ntwx    =       0, ntwv    =       0, ntwe    =       0
     ioutfm  =       1, ntwprt  =       0, idecomp =       0, rbornstat=      0

Potential function:
     ntf     =       1, ntb     =       1, igb     =       0, nsnb    =      25
     ipol    =       0, gbsa    =       0, iesp    =       0
     dielc   =   1.00000, cut     =  12.00000, intdiel =   1.00000
```

```
Frozen or restrained atoms:
     ibelly  =       0, ntr     =       0

Energy minimization:
     maxcyc  =    5000, ncyc    =   10000, ntmin   =       1
     dx0     =   0.01000, drms    =   0.00010

Ewald parameters:
     verbose =       0, ew_type =       0, nbflag  =       1, use_pme =       1
     vdwmeth =       1, eedmeth =       1, netfrc  =       0
     Box X =  117.910   Box Y =   84.370   Box Z =  105.320
     Alpha =   90.000   Beta  =   90.000   Gamma =   90.000
     NFFT1 =  120       NFFT2 =   90       NFFT3 =  108
     Cutoff=   12.000   Tol   =0.100E-04
     Ewald Coefficient =  0.22664
     Interpolation order =    4
|  INFO: Old style inpcrd file read


--------------------------------------------------------------------------------
   3.  ATOMIC COORDINATES AND VELOCITIES
--------------------------------------------------------------------------------

default_name
 begin time read from input coords =     0.000 ps

 Number of triangulated 3-point waters found:    26771

     Sum of charges from parm topology file =  -0.00000030
     Forcing neutrality...

--------------------------------------------------------------------------------
   4.  RESULTS
--------------------------------------------------------------------------------

 ---------------------------------------------------
 APPROXIMATING switch and d/dx switch using CUBIC SPLINE INTERPOLATION
 using   5000.0 points per unit in tabled values
 TESTING RELATIVE ERROR over r ranging from 0.0 to cutoff
| CHECK switch(x): max rel err =   0.2738E-14   at   2.422500
| CHECK d/dx switch(x): max rel err =   0.7967E-11   at   2.716640
 ---------------------------------------------------
| Local SIZE OF NONBOND LIST =   42990561
| TOTAL SIZE OF NONBOND LIST =   42990561


   NSTEP       ENERGY          RMS            GMAX         NAME    NUMBER
      1      -3.2648E+05     4.4620E+00     7.3662E+02     C        3764

 BOND    =    36149.0277  ANGLE   =     2659.3115  DIHED      =     5394.6458
 VDWAALS =    66891.9642  EEL     =  -459857.9824  HBOND      =        0.0000
 1-4 VDW =     2117.0330  1-4 EEL =    20167.4574  RESTRAINT  =        0.0000
```

```
  NSTEP       ENERGY          RMS            GMAX         NAME    NUMBER
    100      -3.4060E+05     6.2106E-01     1.3272E+02     CD       3231

 BOND    =     26923.6423  ANGLE   =      1568.8696  DIHED      =      5408.8239
 VDWAALS =     64557.1993  EEL     =   -460414.3375  HBOND      =         0.0000
 1-4 VDW =      1698.2596  1-4 EEL =     19660.0467  RESTRAINT  =         0.0000


  NSTEP       ENERGY          RMS            GMAX         NAME    NUMBER
    200      -3.4200E+05     7.7236E-01     2.1460E+02     CD       3231

 BOND    =     26497.6987  ANGLE   =      1352.0073  DIHED      =      5349.4163
 VDWAALS =     64510.0292  EEL     =   -460926.7872  HBOND      =         0.0000
 1-4 VDW =      1605.2364  1-4 EEL =     19614.0185  RESTRAINT  =         0.0000


  NSTEP       ENERGY          RMS            GMAX         NAME    NUMBER
    300      -3.4271E+05     6.8305E-01     1.6928E+02     CD       3231

 BOND    =     26395.6662  ANGLE   =      1232.6552  DIHED      =      5329.3898
 VDWAALS =     64569.7896  EEL     =   -461403.9473  HBOND      =         0.0000
 1-4 VDW =      1564.1965  1-4 EEL =     19604.0078  RESTRAINT  =         0.0000


  NSTEP       ENERGY          RMS            GMAX         NAME    NUMBER
    400      -3.4320E+05     5.8303E-01     1.0341E+02     CD       2309

 BOND    =     26363.0651  ANGLE   =      1147.6336  DIHED      =      5319.7943
 VDWAALS =     64671.5429  EEL     =   -461845.3620  HBOND      =         0.0000
 1-4 VDW =      1539.1826  1-4 EEL =     19605.4762  RESTRAINT  =         0.0000


  NSTEP       ENERGY          RMS            GMAX         NAME    NUMBER
    500      -3.4359E+05     1.4666E-01     1.2756E+01     CD       2309

 BOND    =     26352.1294  ANGLE   =      1087.3992  DIHED      =      5311.7066
 VDWAALS =     64788.8793  EEL     =   -462265.5709  HBOND      =         0.0000
 1-4 VDW =      1521.9374  1-4 EEL =     19611.5782  RESTRAINT  =         0.0000


  NSTEP       ENERGY          RMS            GMAX         NAME    NUMBER
    600      -3.4392E+05     3.7980E-01     3.8033E+01     C        3778

 BOND    =     26367.8209  ANGLE   =      1052.6143  DIHED      =      5304.2604
 VDWAALS =     64918.2934  EEL     =   -462689.8453  HBOND      =         0.0000
 1-4 VDW =      1510.4032  1-4 EEL =     19618.0880  RESTRAINT  =         0.0000


  NSTEP       ENERGY          RMS            GMAX         NAME    NUMBER
    700      -3.4422E+05     3.2621E-01     5.7659E+01     C        3778

 BOND    =     26382.9183  ANGLE   =      1033.6388  DIHED      =      5297.1536
 VDWAALS =     65062.0022  EEL     =   -463116.9104  HBOND      =         0.0000
 1-4 VDW =      1502.4715  1-4 EEL =     19622.0635  RESTRAINT  =         0.0000
```

```
   NSTEP       ENERGY          RMS            GMAX         NAME    NUMBER
    800      -3.4448E+05     2.8480E-01     4.7931E+01     C        3778

 BOND    =     26409.3702  ANGLE   =      1017.7793  DIHED      =      5290.2470
 VDWAALS =     65204.5772  EEL     =   -463519.3186  HBOND      =         0.0000
 1-4 VDW =      1496.7465  1-4 EEL =     19625.1903  RESTRAINT  =         0.0000


   NSTEP       ENERGY          RMS            GMAX         NAME    NUMBER
    900      -3.4471E+05     1.0826E-01     6.4968E+00     C        3764

 BOND    =     26431.1516  ANGLE   =      1009.4331  DIHED      =      5284.9138
 VDWAALS =     65344.0906  EEL     =   -463895.7200  HBOND      =         0.0000
 1-4 VDW =      1493.1978  1-4 EEL =     19626.4434  RESTRAINT  =         0.0000


   NSTEP       ENERGY          RMS            GMAX         NAME    NUMBER
   1000      -3.4492E+05     1.6565E-01     2.3569E+01     CE3      3293

 BOND    =     26460.0928  ANGLE   =      1002.5586  DIHED      =      5281.1643
 VDWAALS =     65482.6495  EEL     =   -464261.7446  HBOND      =         0.0000
 1-4 VDW =      1490.4363  1-4 EEL =     19627.4617  RESTRAINT  =         0.0000


   NSTEP       ENERGY          RMS            GMAX         NAME    NUMBER
   1100      -3.4511E+05     1.2459E-01     1.3834E+01     CE3      3293

 BOND    =     26486.7742  ANGLE   =       998.1471  DIHED      =      5278.3816
 VDWAALS =     65611.5460  EEL     =   -464597.9856  HBOND      =         0.0000
 1-4 VDW =      1488.1282  1-4 EEL =     19627.7368  RESTRAINT  =         0.0000


   NSTEP       ENERGY          RMS            GMAX         NAME    NUMBER
   1200      -3.4528E+05     3.4377E-01     4.2185E+01     CE3      3293

 BOND    =     26518.0861  ANGLE   =       995.0684  DIHED      =      5276.5006
 VDWAALS =     65736.9188  EEL     =   -464923.8084  HBOND      =         0.0000
 1-4 VDW =      1486.1351  1-4 EEL =     19627.4402  RESTRAINT  =         0.0000


   NSTEP       ENERGY          RMS            GMAX         NAME    NUMBER
   1300      -3.4545E+05     3.0200E-01     5.4765E+01     CD       3231

 BOND    =     26544.7795  ANGLE   =       991.0255  DIHED      =      5275.3691
 VDWAALS =     65854.4218  EEL     =   -465231.0297  HBOND      =         0.0000
 1-4 VDW =      1484.6102  1-4 EEL =     19626.8976  RESTRAINT  =         0.0000


   NSTEP       ENERGY          RMS            GMAX         NAME    NUMBER
   1400      -3.4562E+05     2.8895E-01     5.1999E+01     CD       3231

 BOND    =     26571.3771  ANGLE   =       988.7225  DIHED      =      5274.1737
```

```
VDWAALS =     65972.2163  EEL     =  -465537.4058  HBOND      =         0.0000
1-4 VDW =      1483.2770  1-4 EEL =    19625.7474  RESTRAINT  =         0.0000


  NSTEP       ENERGY          RMS            GMAX         NAME    NUMBER
  1500      -3.4579E+05     1.6689E-01     1.6758E+01     CG       1665

BOND    =     26596.9559  ANGLE   =       985.7913  DIHED      =      5273.0063
VDWAALS =     66085.0737  EEL     =  -465831.9439  HBOND      =         0.0000
1-4 VDW =      1482.3057  1-4 EEL =    19623.7843  RESTRAINT  =         0.0000


  NSTEP       ENERGY          RMS            GMAX         NAME    NUMBER
  1600      -3.4594E+05     1.2292E-01     1.1621E+01     CD       3231

BOND    =     26622.8836  ANGLE   =       983.6852  DIHED      =      5271.8127
VDWAALS =     66196.0323  EEL     =  -466116.7374  HBOND      =         0.0000
1-4 VDW =      1481.4584  1-4 EEL =    19621.2646  RESTRAINT  =         0.0000


  NSTEP       ENERGY          RMS            GMAX         NAME    NUMBER
  1700      -3.4609E+05     9.3302E-02     4.7774E+00     CZ       1670

BOND    =     26648.7818  ANGLE   =       981.7191  DIHED      =      5270.7631
VDWAALS =     66303.5196  EEL     =  -466392.7296  HBOND      =         0.0000
1-4 VDW =      1480.6247  1-4 EEL =    19618.8036  RESTRAINT  =         0.0000


  NSTEP       ENERGY          RMS            GMAX         NAME    NUMBER
  1800      -3.4623E+05     3.6171E-01     7.6343E+01     CD       3231

BOND    =     26679.2044  ANGLE   =       980.2070  DIHED      =      5270.1468
VDWAALS =     66408.6158  EEL     =  -466664.8559  HBOND      =         0.0000
1-4 VDW =      1479.8266  1-4 EEL =    19616.3067  RESTRAINT  =         0.0000


  NSTEP       ENERGY          RMS            GMAX         NAME    NUMBER
  1900      -3.4638E+05     9.1529E-02     4.2000E+00     CZ       1670

BOND    =     26699.7213  ANGLE   =       978.4234  DIHED      =      5269.5707
VDWAALS =     66510.7294  EEL     =  -466929.3312  HBOND      =         0.0000
1-4 VDW =      1479.0883  1-4 EEL =    19613.8837  RESTRAINT  =         0.0000


  NSTEP       ENERGY          RMS            GMAX         NAME    NUMBER
  2000      -3.4651E+05     2.3658E-01     2.2007E+01     CG       1665

BOND    =     26726.2894  ANGLE   =       977.0274  DIHED      =      5268.5579
VDWAALS =     66611.0936  EEL     =  -467187.6254  HBOND      =         0.0000
1-4 VDW =      1478.3391  1-4 EEL =    19611.9851  RESTRAINT  =         0.0000


  NSTEP       ENERGY          RMS            GMAX         NAME    NUMBER
  2100      -3.4665E+05     2.0572E-01     1.7530E+01     NE2      4277
```

```
BOND    =     26749.4764  ANGLE   =       975.7397  DIHED      =      5267.5818
VDWAALS =     66711.0546  EEL     =   -467442.3161  HBOND      =         0.0000
1-4 VDW =      1477.5832  1-4 EEL =     19610.6054  RESTRAINT  =         0.0000


  NSTEP       ENERGY          RMS            GMAX         NAME    NUMBER
   2200      -3.4678E+05     1.2300E-01     1.3916E+01     CD       3231

BOND    =     26771.4785  ANGLE   =       974.3260  DIHED      =      5266.9597
VDWAALS =     66806.6277  EEL     =   -467683.7994  HBOND      =         0.0000
1-4 VDW =      1476.8559  1-4 EEL =     19609.7682  RESTRAINT  =         0.0000


  NSTEP       ENERGY          RMS            GMAX         NAME    NUMBER
   2300      -3.4690E+05     8.8033E-02     4.0194E+00     CE1      3805

BOND    =     26793.8936  ANGLE   =       973.2777  DIHED      =      5266.7557
VDWAALS =     66903.1107  EEL     =   -467925.5079  HBOND      =         0.0000
1-4 VDW =      1476.0618  1-4 EEL =     19609.0067  RESTRAINT  =         0.0000


  NSTEP       ENERGY          RMS            GMAX         NAME    NUMBER
   2400      -3.4703E+05     9.9168E-02     6.3925E+00     NE2      4277

BOND    =     26816.7042  ANGLE   =       972.3388  DIHED      =      5267.1167
VDWAALS =     66999.3025  EEL     =   -468166.7445  HBOND      =         0.0000
1-4 VDW =      1475.2995  1-4 EEL =     19608.4316  RESTRAINT  =         0.0000


  NSTEP       ENERGY          RMS            GMAX         NAME    NUMBER
   2500      -3.4715E+05     2.7355E-01     2.4149E+01     CE1      3805

BOND    =     26841.0923  ANGLE   =       971.8678  DIHED      =      5268.2662
VDWAALS =     67091.7688  EEL     =   -468400.7551  HBOND      =         0.0000
1-4 VDW =      1474.5809  1-4 EEL =     19608.0848  RESTRAINT  =         0.0000


  NSTEP       ENERGY          RMS            GMAX         NAME    NUMBER
   2600      -3.4726E+05     2.4630E-01     4.3352E+01     CD       3231

BOND    =     26861.4176  ANGLE   =       971.4684  DIHED      =      5269.5810
VDWAALS =     67183.9304  EEL     =   -468630.2856  HBOND      =         0.0000
1-4 VDW =      1473.9361  1-4 EEL =     19606.9981  RESTRAINT  =         0.0000


  NSTEP       ENERGY          RMS            GMAX         NAME    NUMBER
   2700      -3.4738E+05     2.0281E-01     1.8575E+01     CE1      3805

BOND    =     26882.3431  ANGLE   =       970.7817  DIHED      =      5270.3541
VDWAALS =     67274.2643  EEL     =   -468857.4501  HBOND      =         0.0000
1-4 VDW =      1473.3011  1-4 EEL =     19606.4151  RESTRAINT  =         0.0000
```

```
  NSTEP       ENERGY          RMS            GMAX         NAME    NUMBER
   2800      -3.4750E+05     1.2340E-01     1.0326E+01     CG       3216

 BOND    =     26902.3913  ANGLE   =       970.4593  DIHED      =      5268.0970
 VDWAALS =     67365.1851  EEL     =   -469084.1496  HBOND      =         0.0000
 1-4 VDW =      1472.4758  1-4 EEL =     19605.8274  RESTRAINT  =         0.0000


  NSTEP       ENERGY          RMS            GMAX         NAME    NUMBER
   2900      -3.4761E+05     1.3480E-01     1.2941E+01     NE2      4277

 BOND    =     26923.4534  ANGLE   =       969.9944  DIHED      =      5267.7358
 VDWAALS =     67454.3995  EEL     =   -469300.8215  HBOND      =         0.0000
 1-4 VDW =      1471.8941  1-4 EEL =     19605.5292  RESTRAINT  =         0.0000


  NSTEP       ENERGY          RMS            GMAX         NAME    NUMBER
   3000      -3.4771E+05     7.4287E-02     3.1739E+00     NE2      4277

 BOND    =     26942.9708  ANGLE   =       969.7152  DIHED      =      5267.4567
 VDWAALS =     67541.6150  EEL     =   -469510.7864  HBOND      =         0.0000
 1-4 VDW =      1471.4196  1-4 EEL =     19605.1413  RESTRAINT  =         0.0000


  NSTEP       ENERGY          RMS            GMAX         NAME    NUMBER
   3100      -3.4781E+05     2.5958E-01     3.0057E+01     NE2      4277

 BOND    =     26964.7716  ANGLE   =       969.8831  DIHED      =      5267.0999
 VDWAALS =     67626.5072  EEL     =   -469713.9406  HBOND      =         0.0000
 1-4 VDW =      1471.0129  1-4 EEL =     19604.7974  RESTRAINT  =         0.0000


  NSTEP       ENERGY          RMS            GMAX         NAME    NUMBER
   3200      -3.4791E+05     2.2658E-01     3.6526E+01     CD       3231

 BOND    =     26984.1144  ANGLE   =       968.9749  DIHED      =      5266.6164
 VDWAALS =     67709.5848  EEL     =   -469913.4681  HBOND      =         0.0000
 1-4 VDW =      1470.6812  1-4 EEL =     19604.7350  RESTRAINT  =         0.0000


  NSTEP       ENERGY          RMS            GMAX         NAME    NUMBER
   3300      -3.4800E+05     2.0546E-01     2.2739E+01     NE2      4277

 BOND    =     27002.1510  ANGLE   =       968.9363  DIHED      =      5266.1040
 VDWAALS =     67788.4473  EEL     =   -470104.9372  HBOND      =         0.0000
 1-4 VDW =      1470.3544  1-4 EEL =     19604.6139  RESTRAINT  =         0.0000


  NSTEP       ENERGY          RMS            GMAX         NAME    NUMBER
   3400      -3.4810E+05     1.3043E-01     1.3502E+01     NE2      4277

 BOND    =     27020.4102  ANGLE   =       968.2175  DIHED      =      5265.5456
 VDWAALS =     67866.0296  EEL     =   -470295.0620  HBOND      =         0.0000
 1-4 VDW =      1470.0864  1-4 EEL =     19604.8411  RESTRAINT  =         0.0000
```

```
  NSTEP       ENERGY          RMS            GMAX         NAME    NUMBER
   3500      -3.4819E+05     6.9864E-02     2.7520E+00     NE2      4277

 BOND    =     27038.5407  ANGLE   =       967.9475  DIHED      =      5264.8434
 VDWAALS =     67941.9742  EEL     =   -470481.1142  HBOND      =         0.0000
 1-4 VDW =      1469.8078  1-4 EEL =     19604.7990  RESTRAINT  =         0.0000


  NSTEP       ENERGY          RMS            GMAX         NAME    NUMBER
   3600      -3.4829E+05     7.0653E-02     2.8802E+00     NE2      4277

 BOND    =     27057.2044  ANGLE   =       967.6987  DIHED      =      5264.1806
 VDWAALS =     68016.8331  EEL     =   -470665.9241  HBOND      =         0.0000
 1-4 VDW =      1469.4970  1-4 EEL =     19604.6754  RESTRAINT  =         0.0000


  NSTEP       ENERGY          RMS            GMAX         NAME    NUMBER
   3700      -3.4837E+05     2.5765E-01     3.8031E+01     CD       3231

 BOND    =     27077.9999  ANGLE   =       967.7479  DIHED      =      5263.6106
 VDWAALS =     68090.4415  EEL     =   -470848.0506  HBOND      =         0.0000
 1-4 VDW =      1469.1901  1-4 EEL =     19604.5269  RESTRAINT  =         0.0000


  NSTEP       ENERGY          RMS            GMAX         NAME    NUMBER
   3800      -3.4847E+05     2.2048E-01     3.1478E+01     CD       3231

 BOND    =     27094.9451  ANGLE   =       967.8412  DIHED      =      5263.1438
 VDWAALS =     68162.2888  EEL     =   -471026.4877  HBOND      =         0.0000
 1-4 VDW =      1468.8254  1-4 EEL =     19604.1827  RESTRAINT  =         0.0000


  NSTEP       ENERGY          RMS            GMAX         NAME    NUMBER
   3900      -3.4855E+05     1.8172E-01     1.7269E+01     CD       3231

 BOND    =     27112.9626  ANGLE   =       967.5811  DIHED      =      5262.7221
 VDWAALS =     68235.8586  EEL     =   -471206.2842  HBOND      =         0.0000
 1-4 VDW =      1468.5240  1-4 EEL =     19604.0975  RESTRAINT  =         0.0000


  NSTEP       ENERGY          RMS            GMAX         NAME    NUMBER
   4000      -3.4864E+05     1.0695E-01     9.6503E+00     NE2      4277

 BOND    =     27130.2050  ANGLE   =       967.7138  DIHED      =      5262.3512
 VDWAALS =     68310.2895  EEL     =   -471384.8043  HBOND      =         0.0000
 1-4 VDW =      1468.1709  1-4 EEL =     19603.9577  RESTRAINT  =         0.0000


  NSTEP       ENERGY          RMS            GMAX         NAME    NUMBER
   4100      -3.4873E+05     1.2404E-01     1.2289E+01     NE2      4277

 BOND    =     27148.4205  ANGLE   =       967.5636  DIHED      =      5262.0253
```

```
VDWAALS =    68383.0841  EEL      =  -471559.1192  HBOND      =        0.0000
1-4 VDW =     1467.9159  1-4 EEL =    19604.0203  RESTRAINT  =        0.0000


  NSTEP       ENERGY          RMS            GMAX         NAME    NUMBER
  4200      -3.4881E+05     9.6300E-02     9.0546E+00     NE2      4277

BOND    =    27165.1744  ANGLE    =      967.6734  DIHED      =     5261.8014
VDWAALS =    68452.7510  EEL      =  -471726.1793  HBOND      =        0.0000
1-4 VDW =     1467.7097  1-4 EEL =    19603.9879  RESTRAINT  =        0.0000


  NSTEP       ENERGY          RMS            GMAX         NAME    NUMBER
  4300      -3.4888E+05     2.5289E-01     3.7627E+01     CD       3231

BOND    =    27183.7208  ANGLE    =      967.9507  DIHED      =     5261.6963
VDWAALS =    68520.3363  EEL      =  -471889.0819  HBOND      =        0.0000
1-4 VDW =     1467.5138  1-4 EEL =    19603.9510  RESTRAINT  =        0.0000


  NSTEP       ENERGY          RMS            GMAX         NAME    NUMBER
  4400      -3.4896E+05     6.5908E-02     3.0517E+00     NE2      4277

BOND    =    27197.8904  ANGLE    =      967.5960  DIHED      =     5261.7182
VDWAALS =    68587.2483  EEL      =  -472048.7466  HBOND      =        0.0000
1-4 VDW =     1467.3635  1-4 EEL =    19603.9363  RESTRAINT  =        0.0000


  NSTEP       ENERGY          RMS            GMAX         NAME    NUMBER
  4500      -3.4904E+05     1.8788E-01     1.9282E+01     CE1      3805

BOND    =    27214.7405  ANGLE    =      967.9593  DIHED      =     5261.9303
VDWAALS =    68652.7409  EEL      =  -472204.3973  HBOND      =        0.0000
1-4 VDW =     1467.1902  1-4 EEL =    19603.7123  RESTRAINT  =        0.0000


  NSTEP       ENERGY          RMS            GMAX         NAME    NUMBER
  4600      -3.4911E+05     1.4908E-01     1.5097E+01     NE2      4277

BOND    =    27229.8672  ANGLE    =      967.7202  DIHED      =     5262.3635
VDWAALS =    68715.6209  EEL      =  -472355.8674  HBOND      =        0.0000
1-4 VDW =     1467.0266  1-4 EEL =    19603.4986  RESTRAINT  =        0.0000


  NSTEP       ENERGY          RMS            GMAX         NAME    NUMBER
  4700      -3.4918E+05     6.2037E-02     2.5142E+00     H2      50427

BOND    =    27243.9090  ANGLE    =      967.9383  DIHED      =     5262.9719
VDWAALS =    68777.0871  EEL      =  -472505.3085  HBOND      =        0.0000
1-4 VDW =     1466.8564  1-4 EEL =    19603.0514  RESTRAINT  =        0.0000


  NSTEP       ENERGY          RMS            GMAX         NAME    NUMBER
  4800      -3.4926E+05     6.3348E-02     2.9948E+00     NE2      4277
```

```
 BOND    =    27258.7432  ANGLE   =      967.9374  DIHED      =     5263.4251
 VDWAALS =    68838.9422  EEL     =  -472654.0098  HBOND      =        0.0000
 1-4 VDW =     1466.7231  1-4 EEL =    19602.7008  RESTRAINT  =        0.0000


   NSTEP       ENERGY          RMS            GMAX         NAME    NUMBER
   4900      -3.4932E+05     2.2864E-01     2.7341E+01     NE2      4277

 BOND    =    27275.5046  ANGLE   =      967.8371  DIHED      =     5263.5070
 VDWAALS =    68900.5214  EEL     =  -472800.6770  HBOND      =        0.0000
 1-4 VDW =     1466.6135  1-4 EEL =    19602.5027  RESTRAINT  =        0.0000


   NSTEP       ENERGY          RMS            GMAX         NAME    NUMBER
   5000      -3.4939E+05     1.8539E-01     2.3386E+01     NE2      4277

 BOND    =    27288.8469  ANGLE   =      968.1360  DIHED      =     5263.3696
 VDWAALS =    68961.7778  EEL     =  -472945.1878  HBOND      =        0.0000
 1-4 VDW =     1466.5092  1-4 EEL =    19602.1465  RESTRAINT  =        0.0000


  Maximum number of minimization cycles reached.


                    FINAL RESULTS



   NSTEP       ENERGY          RMS            GMAX         NAME    NUMBER
   5000      -3.4939E+05     1.8539E-01     2.3386E+01     NE2      4277

 BOND    =    27288.8469  ANGLE   =      968.1360  DIHED      =     5263.3696
 VDWAALS =    68961.7778  EEL     =  -472945.1878  HBOND      =        0.0000
 1-4 VDW =     1466.5092  1-4 EEL =    19602.1465  RESTRAINT  =        0.0000

--------------------------------------------------------------------------------
   5.  TIMINGS
--------------------------------------------------------------------------------

|                Build the list            27.75 (92.22% of List )
|                Other                      2.34 ( 7.78% of List )
|             List time                 30.09 ( 0.32% of Nonbo)
|                   Short_ene time         8417.60 (99.68% of Direc)
|                   Other                    26.69 ( 0.32% of Direc)
|                Direct Ewald time      8444.29 (91.34% of Ewald)
|                Adjust Ewald time        32.43 ( 0.35% of Ewald)
|                   Fill Bspline coeffs      26.41 ( 3.47% of Recip)
|                   Fill charge grid        104.54 (13.72% of Recip)
|                   Scalar sum              148.86 (19.54% of Recip)
|                   Grad sum                184.30 (24.19% of Recip)
|                   FFT time                297.80 (39.08% of Recip)
|                Recip Ewald time        761.93 ( 8.24% of Ewald)
|                Virial junk               1.92 ( 0.02% of Ewald)
```

```
|                Other                      4.58 ( 0.05% of Ewald)
|             Ewald time             9245.17 (99.68% of Nonbo)
|          Nonbond force          9275.27 (99.52% of Force)
|          Bond/Angle/Dihedral       43.86 ( 0.47% of Force)
|          Other                      1.00 ( 0.01% of Force)
|       Force time             9320.14 (100.0% of Runmd)
|    Runmd Time             9320.14 (99.72% of Total)
|    Other                    26.07 ( 0.28% of Total)
| Total time             9346.23 (100.0% of ALL  )

| Number of list builds   :         20

| Highest rstack allocated:    4808230
| Highest istack allocated:      87372
|           Job began  at 12:02:52.833  on 02/21/2019
|           Setup done at 12:02:53.500  on 02/21/2019
|           Run   done at 14:38:39.064  on 02/21/2019
|     wallclock() was called  190088 times

          -------------------------------------------------------
          Amber 16 SANDER                              2016
          -------------------------------------------------------

| Run on 02/21/2019 at 17:36:44

|   Executable path: /usr/local/amber16/bin/sander
| Working directory: /Users/Swati
|          Hostname: Unknown
  [-O]verwriting output

File Assignments:
|  MDIN: /Users/Swati/minimization_file_2.in
| MDOUT: /Users/Swati/NAT0_files_2/truncated_NAT0_min3_2.out
|INPCRD: /Users/Swati/NAT0_files_2/truncated_NAT0_min3_1.rst
|  PARM: /Users/Swati/NAT0_files_2/NAT0.prmtop
|RESTRT: /Users/Swati/NAT0_files_2/truncated_NAT0_min3_2.rst
|  REFC: refc
| MDVEL: mdvel
| MDFRC: mdfrc
|  MDEN: mden
| MDCRD: mdcrd
|MDINFO: mdinfo
|  MTMD: mtmd
|INPDIP: inpdip
|RSTDIP: rstdip
|INPTRA: inptraj


 Here is the input file:

PROT: minimization_2
&cntrl
imin = 1,
maxcyc = 5000,
```

```
ncyc = 10000,
igb = 0,
ntpr =  100,
ntp = 0
cut = 12
&end


--------------------------------------------------------------------------------
   1.  RESOURCE   USE:
--------------------------------------------------------------------------------

| Flags:
 getting box info from netcdf restart file
| NetCDF restart box info found
|Largest sphere to fit in unit cell has radius =    44.861
| New format PARM file being parsed.
| Version =    1.000 Date = 08/09/17 Time = 07:09:32
 NATOM  =   65080 NTYPES =      17 NBONH =   61577 MBONA  =    3583
 NTHETH =    7353 MTHETA =    4865 NPHIH =   15405 MPHIA  =   14966
 NHPARM =       0 NPARM  =       0 NNB   =  114677 NRES   =   19909
 NBONA  =    3583 NTHETA =    4865 NPHIA =   14966 NUMBND =      67
 NUMANG =     151 NPTRA  =     191 NATYP =      36 NPHB   =       1
 IFBOX  =       1 NMXRS  =      24 IFCAP =       0 NEXTRA =       0
 NCOPY  =       0


|     Memory Use     Allocated
|     Real             5163211
|     Hollerith         215151
|     Integer          2953777
|     Max Pairs       59526506
|     nblistReal        780960
|     nblist Int       2187281
|       Total           299886 kbytes

| Note: 1-4 EEL scale factors are being read from the topology file.

| Note: 1-4 VDW scale factors are being read from the topology file.
| Duplicated    0 dihedrals
| Duplicated    0 dihedrals

     BOX TYPE: RECTILINEAR

--------------------------------------------------------------------------------
   2.  CONTROL  DATA  FOR  THE  RUN
--------------------------------------------------------------------------------

default_name

General flags:
     imin    =       1, nmropt  =       0

Nature and format of input:
```

```
     ntx     =       1, irest   =       0, ntrx    =       1

Nature and format of output:
     ntxo    =       2, ntpr    =     100, ntrx    =       1, ntwr    =       1
     iwrap   =       0, ntwx    =       0, ntwv    =       0, ntwe    =       0
     ioutfm  =       1, ntwprt  =       0, idecomp =       0, rbornstat=      0

Potential function:
     ntf     =       1, ntb     =       1, igb     =       0, nsnb    =      25
     ipol    =       0, gbsa    =       0, iesp    =       0
     dielc   =   1.00000, cut     =  12.00000, intdiel =   1.00000

Frozen or restrained atoms:
     ibelly  =       0, ntr     =       0

Energy minimization:
     maxcyc  =    5000, ncyc    =   10000, ntmin   =       1
     dx0     =   0.01000, drms    =   0.00010

Ewald parameters:
     verbose =       0, ew_type =       0, nbflag  =       1, use_pme =       1
     vdwmeth =       1, eedmeth =       1, netfrc  =       0
     Box X =   97.446   Box Y =   89.722   Box Z =   90.199
     Alpha =   90.000   Beta  =   90.000   Gamma =   90.000
     NFFT1 =  100       NFFT2 =   90       NFFT3 =   90
     Cutoff=   12.000   Tol   =0.100E-04
     Ewald Coefficient =  0.22664
     Interpolation order =    4
|  INFO: Old style inpcrd file read


--------------------------------------------------------------------------------
   3.  ATOMIC COORDINATES AND VELOCITIES
--------------------------------------------------------------------------------

default_name
 begin time read from input coords =     0.000 ps

 Number of triangulated 3-point waters found:    19440

     Sum of charges from parm topology file =  -0.00000033
     Forcing neutrality...

--------------------------------------------------------------------------------
   4.  RESULTS
--------------------------------------------------------------------------------

 ---------------------------------------------------
 APPROXIMATING switch and d/dx switch using CUBIC SPLINE INTERPOLATION
 using   5000.0 points per unit in tabled values
 TESTING RELATIVE ERROR over r ranging from 0.0 to cutoff
| CHECK switch(x): max rel err =   0.2738E-14   at   2.422500
| CHECK d/dx switch(x): max rel err =   0.7967E-11   at   2.716640
 ---------------------------------------------------
```

```
| Local SIZE OF NONBOND LIST =   31615604
| TOTAL SIZE OF NONBOND LIST =   31615604


   NSTEP       ENERGY          RMS            GMAX         NAME    NUMBER
      1      -2.3729E+05     5.0938E+00     7.4715E+02     C        3693

 BOND    =     28585.6938  ANGLE   =      2442.4317  DIHED      =      4978.7907
 VDWAALS =     47953.8265  EEL     =   -342825.6076  HBOND      =         0.0000
 1-4 VDW =      2043.3985  1-4 EEL =     19532.0386  RESTRAINT  =         0.0000


   NSTEP       ENERGY          RMS            GMAX         NAME    NUMBER
    100      -2.5048E+05     4.5926E-01     3.5916E+01     C        3693

 BOND    =     19947.3321  ANGLE   =      1548.1980  DIHED      =      5039.9904
 VDWAALS =     45641.8821  EEL     =   -343221.3344  HBOND      =         0.0000
 1-4 VDW =      1622.3101  1-4 EEL =     18938.1554  RESTRAINT  =         0.0000


   NSTEP       ENERGY          RMS            GMAX         NAME    NUMBER
    200      -2.5210E+05     7.5401E-01     1.1553E+02     C        3693

 BOND    =     19341.2335  ANGLE   =      1278.7278  DIHED      =      5025.6976
 VDWAALS =     45582.9834  EEL     =   -343690.6421  HBOND      =         0.0000
 1-4 VDW =      1516.2520  1-4 EEL =     18848.3444  RESTRAINT  =         0.0000


   NSTEP       ENERGY          RMS            GMAX         NAME    NUMBER
    300      -2.5281E+05     2.0389E-01     1.5748E+01     C        3693

 BOND    =     19180.7572  ANGLE   =      1176.6028  DIHED      =      5009.2266
 VDWAALS =     45630.1284  EEL     =   -344104.9118  HBOND      =         0.0000
 1-4 VDW =      1478.9464  1-4 EEL =     18820.9000  RESTRAINT  =         0.0000


   NSTEP       ENERGY          RMS            GMAX         NAME    NUMBER
    400      -2.5324E+05     1.7517E-01     1.4872E+01     C        3693

 BOND    =     19133.7180  ANGLE   =      1121.6991  DIHED      =      4997.7892
 VDWAALS =     45701.8446  EEL     =   -344461.4214  HBOND      =         0.0000
 1-4 VDW =      1459.8386  1-4 EEL =     18808.5995  RESTRAINT  =         0.0000


   NSTEP       ENERGY          RMS            GMAX         NAME    NUMBER
    500      -2.5355E+05     4.7329E-01     7.2942E+01     C        3693

 BOND    =     19128.0608  ANGLE   =      1088.6742  DIHED      =      4993.6844
 VDWAALS =     45786.1896  EEL     =   -344799.4563  HBOND      =         0.0000
 1-4 VDW =      1447.9195  1-4 EEL =     18801.6533  RESTRAINT  =         0.0000


   NSTEP       ENERGY          RMS            GMAX         NAME    NUMBER
    600      -2.5382E+05     3.9844E-01     5.4750E+01     C        3693
```

```
BOND    =    19126.5551  ANGLE   =     1070.1137  DIHED      =     4990.8832
VDWAALS =    45875.1357  EEL     =  -345117.7008  HBOND      =        0.0000
1-4 VDW =     1439.4902  1-4 EEL =    18795.7758  RESTRAINT  =        0.0000


  NSTEP       ENERGY          RMS            GMAX         NAME    NUMBER
    700      -2.5405E+05     3.7197E-01     5.1310E+01     C        3693

BOND    =    19139.6031  ANGLE   =     1054.0001  DIHED      =     4987.3849
VDWAALS =    45965.5959  EEL     =  -345425.9048  HBOND      =        0.0000
1-4 VDW =     1432.7050  1-4 EEL =    18792.7068  RESTRAINT  =        0.0000


  NSTEP       ENERGY          RMS            GMAX         NAME    NUMBER
    800      -2.5426E+05     3.0274E-01     3.8816E+01     C        3693

BOND    =    19151.1553  ANGLE   =     1044.8119  DIHED      =     4988.4247
VDWAALS =    46057.9873  EEL     =  -345722.8119  HBOND      =        0.0000
1-4 VDW =     1427.2656  1-4 EEL =    18790.0595  RESTRAINT  =        0.0000


  NSTEP       ENERGY          RMS            GMAX         NAME    NUMBER
    900      -2.5446E+05     2.0526E-01     2.2304E+01     CA       3697

BOND    =    19168.1819  ANGLE   =     1036.7133  DIHED      =     4987.6238
VDWAALS =    46148.3345  EEL     =  -346010.0011  HBOND      =        0.0000
1-4 VDW =     1422.9260  1-4 EEL =    18788.2307  RESTRAINT  =        0.0000


  NSTEP       ENERGY          RMS            GMAX         NAME    NUMBER
   1000      -2.5464E+05     1.1422E-01     5.8816E+00     C        3707

BOND    =    19185.3412  ANGLE   =     1031.1800  DIHED      =     4985.2251
VDWAALS =    46237.2713  EEL     =  -346284.0687  HBOND      =        0.0000
1-4 VDW =     1419.5525  1-4 EEL =    18785.8113  RESTRAINT  =        0.0000


  NSTEP       ENERGY          RMS            GMAX         NAME    NUMBER
   1100      -2.5481E+05     1.0674E-01     4.6692E+00     N        3695

BOND    =    19204.4187  ANGLE   =     1026.4402  DIHED      =     4983.5029
VDWAALS =    46323.8608  EEL     =  -346544.6668  HBOND      =        0.0000
1-4 VDW =     1417.1359  1-4 EEL =    18783.6136  RESTRAINT  =        0.0000


  NSTEP       ENERGY          RMS            GMAX         NAME    NUMBER
   1200      -2.5496E+05     4.3881E-01     8.3362E+01     CD       3153

BOND    =    19229.2372  ANGLE   =     1023.2889  DIHED      =     4983.4150
VDWAALS =    46411.2339  EEL     =  -346802.2012  HBOND      =        0.0000
1-4 VDW =     1415.4418  1-4 EEL =    18781.7179  RESTRAINT  =        0.0000
```

```
  NSTEP       ENERGY          RMS            GMAX         NAME    NUMBER
   1300      -2.5511E+05     3.2445E-01     3.9888E+01     CA       3697

 BOND    =     19246.4908  ANGLE   =      1020.4848  DIHED      =      4982.4567
 VDWAALS =     46498.8281  EEL     =   -347054.0654  HBOND      =         0.0000
 1-4 VDW =      1413.9343  1-4 EEL =     18779.6000  RESTRAINT  =         0.0000


  NSTEP       ENERGY          RMS            GMAX         NAME    NUMBER
   1400      -2.5526E+05     2.8238E-01     3.7812E+01     CA       3697

 BOND    =     19267.7220  ANGLE   =      1017.7156  DIHED      =      4979.3180
 VDWAALS =     46586.3043  EEL     =   -347302.0486  HBOND      =         0.0000
 1-4 VDW =      1412.3957  1-4 EEL =     18778.7959  RESTRAINT  =         0.0000


  NSTEP       ENERGY          RMS            GMAX         NAME    NUMBER
   1500      -2.5540E+05     2.7796E-01     3.6376E+01     CD       3153

 BOND    =     19286.8217  ANGLE   =      1016.5958  DIHED      =      4976.5127
 VDWAALS =     46671.3788  EEL     =   -347535.2549  HBOND      =         0.0000
 1-4 VDW =      1410.8885  1-4 EEL =     18776.9996  RESTRAINT  =         0.0000


  NSTEP       ENERGY          RMS            GMAX         NAME    NUMBER
   1600      -2.5553E+05     1.5224E-01     1.7244E+01     CA       3697

 BOND    =     19306.3614  ANGLE   =      1014.7036  DIHED      =      4973.6169
 VDWAALS =     46752.1717  EEL     =   -347757.3156  HBOND      =         0.0000
 1-4 VDW =      1409.4291  1-4 EEL =     18775.7831  RESTRAINT  =         0.0000


  NSTEP       ENERGY          RMS            GMAX         NAME    NUMBER
   1700      -2.5565E+05     9.9662E-02     6.1878E+00     C        3707

 BOND    =     19324.9297  ANGLE   =      1013.5467  DIHED      =      4972.4166
 VDWAALS =     46827.9712  EEL     =   -347968.0408  HBOND      =         0.0000
 1-4 VDW =      1408.3049  1-4 EEL =     18774.2395  RESTRAINT  =         0.0000


  NSTEP       ENERGY          RMS            GMAX         NAME    NUMBER
   1800      -2.5577E+05     1.1762E-01     1.1245E+01     C        3707

 BOND    =     19344.7276  ANGLE   =      1012.5963  DIHED      =      4971.7632
 VDWAALS =     46903.5360  EEL     =   -348179.3329  HBOND      =         0.0000
 1-4 VDW =      1407.3290  1-4 EEL =     18773.0393  RESTRAINT  =         0.0000


  NSTEP       ENERGY          RMS            GMAX         NAME    NUMBER
   1900      -2.5587E+05     3.9047E-01     9.1599E+01     CD       3153

 BOND    =     19367.4021  ANGLE   =      1011.8896  DIHED      =      4970.7499
 VDWAALS =     46976.9559  EEL     =   -348379.6915  HBOND      =         0.0000
 1-4 VDW =      1406.5087  1-4 EEL =     18771.9684  RESTRAINT  =         0.0000
```

```
   NSTEP       ENERGY          RMS            GMAX         NAME    NUMBER
   2000      -2.5598E+05     2.8806E-01     4.1529E+01     CD       3153

 BOND    =     19383.8279  ANGLE   =      1011.3198  DIHED      =      4969.4916
 VDWAALS =     47050.7344  EEL     =  -348574.9661  HBOND      =         0.0000
 1-4 VDW =      1405.7827  1-4 EEL =    18770.7187  RESTRAINT  =         0.0000


   NSTEP       ENERGY          RMS            GMAX         NAME    NUMBER
   2100      -2.5609E+05     2.5927E-01     3.8279E+01     CD       3153

 BOND    =     19402.7588  ANGLE   =      1010.5311  DIHED      =      4967.2599
 VDWAALS =     47123.3933  EEL     =  -348768.7741  HBOND      =         0.0000
 1-4 VDW =      1405.0345  1-4 EEL =    18770.3972  RESTRAINT  =         0.0000


   NSTEP       ENERGY          RMS            GMAX         NAME    NUMBER
   2200      -2.5619E+05     2.0463E-01     1.9622E+01     CE1      3736

 BOND    =     19419.6568  ANGLE   =      1009.5154  DIHED      =      4964.6032
 VDWAALS =     47194.1160  EEL     =  -348954.8121  HBOND      =         0.0000
 1-4 VDW =      1404.4610  1-4 EEL =    18769.9712  RESTRAINT  =         0.0000


   NSTEP       ENERGY          RMS            GMAX         NAME    NUMBER
   2300      -2.5629E+05     1.2962E-01     1.3693E+01     CE1      3736

 BOND    =     19437.0025  ANGLE   =      1008.2553  DIHED      =      4963.0635
 VDWAALS =     47263.4346  EEL     =  -349137.2948  HBOND      =         0.0000
 1-4 VDW =      1403.9515  1-4 EEL =    18770.3257  RESTRAINT  =         0.0000


   NSTEP       ENERGY          RMS            GMAX         NAME    NUMBER
   2400      -2.5639E+05     8.7337E-02     5.1149E+00     CE1      3736

 BOND    =     19453.9932  ANGLE   =      1007.3400  DIHED      =      4961.9996
 VDWAALS =     47332.4261  EEL     =  -349316.3724  HBOND      =         0.0000
 1-4 VDW =      1403.4338  1-4 EEL =    18770.3249  RESTRAINT  =         0.0000


   NSTEP       ENERGY          RMS            GMAX         NAME    NUMBER
   2500      -2.5648E+05     9.8944E-02     8.6679E+00     CE1      3736

 BOND    =     19470.8559  ANGLE   =      1006.4447  DIHED      =      4959.9416
 VDWAALS =     47398.2040  EEL     =  -349489.6870  HBOND      =         0.0000
 1-4 VDW =      1403.0594  1-4 EEL =    18770.0532  RESTRAINT  =         0.0000


   NSTEP       ENERGY          RMS            GMAX         NAME    NUMBER
   2600      -2.5657E+05     8.3405E-02     4.7471E+00     CE1      3736

 BOND    =     19486.9804  ANGLE   =      1005.7509  DIHED      =      4958.1882
```

```
VDWAALS =     47462.1862  EEL      =  -349658.1287  HBOND      =         0.0000
1-4 VDW =      1402.5573  1-4 EEL =    18770.7885  RESTRAINT  =         0.0000


  NSTEP       ENERGY          RMS            GMAX         NAME    NUMBER
  2700      -2.5666E+05     2.4412E-01     3.9325E+01     CD       3153

BOND    =     19504.8897  ANGLE    =     1005.3800  DIHED      =      4957.1746
VDWAALS =     47527.9992  EEL      =  -349828.5323  HBOND      =         0.0000
1-4 VDW =      1402.1534  1-4 EEL =    18771.5747  RESTRAINT  =         0.0000


  NSTEP       ENERGY          RMS            GMAX         NAME    NUMBER
  2800      -2.5675E+05     1.4581E-01     1.6354E+01     CD       1360

BOND    =     19520.6758  ANGLE    =     1004.6567  DIHED      =      4956.2857
VDWAALS =     47593.9899  EEL      =  -349999.0338  HBOND      =         0.0000
1-4 VDW =      1401.7628  1-4 EEL =    18772.4518  RESTRAINT  =         0.0000


  NSTEP       ENERGY          RMS            GMAX         NAME    NUMBER
  2900      -2.5683E+05     1.2988E-01     1.4694E+01     CE1      3736

BOND    =     19536.4943  ANGLE    =     1004.2739  DIHED      =      4955.2191
VDWAALS =     47655.9546  EEL      =  -350160.1285  HBOND      =         0.0000
1-4 VDW =      1401.4328  1-4 EEL =    18773.3257  RESTRAINT  =         0.0000


  NSTEP       ENERGY          RMS            GMAX         NAME    NUMBER
  3000      -2.5691E+05     1.3161E-01     1.5703E+01     CE1      3736

BOND    =     19552.5116  ANGLE    =     1003.8994  DIHED      =      4954.4691
VDWAALS =     47719.7273  EEL      =  -350318.5405  HBOND      =         0.0000
1-4 VDW =      1401.1979  1-4 EEL =    18773.7737  RESTRAINT  =         0.0000


  NSTEP       ENERGY          RMS            GMAX         NAME    NUMBER
  3100      -2.5699E+05     7.3400E-02     3.1449E+00     CE1      3736

BOND    =     19567.5627  ANGLE    =     1003.5576  DIHED      =      4953.9054
VDWAALS =     47783.6941  EEL      =  -350472.4012  HBOND      =         0.0000
1-4 VDW =      1400.9552  1-4 EEL =    18773.5300  RESTRAINT  =         0.0000


  NSTEP       ENERGY          RMS            GMAX         NAME    NUMBER
  3200      -2.5706E+05     2.7807E-01     4.7568E+01     CD       1360

BOND    =     19584.9666  ANGLE    =     1003.5340  DIHED      =      4953.4875
VDWAALS =     47847.5830  EEL      =  -350625.8682  HBOND      =         0.0000
1-4 VDW =      1400.7372  1-4 EEL =    18773.0712  RESTRAINT  =         0.0000


  NSTEP       ENERGY          RMS            GMAX         NAME    NUMBER
  3300      -2.5714E+05     2.3348E-01     4.5663E+01     CD       1360
```

```
BOND    =     19598.8921  ANGLE   =      1003.2048  DIHED      =      4953.2211
VDWAALS =     47908.1524  EEL     =  -350771.9947  HBOND      =         0.0000
1-4 VDW =      1400.5471  1-4 EEL =     18772.6409  RESTRAINT  =         0.0000


  NSTEP       ENERGY          RMS            GMAX         NAME    NUMBER
   3400      -2.5721E+05     2.0717E-01     3.0334E+01     CD       1360

BOND    =     19612.3822  ANGLE   =      1002.7484  DIHED      =      4953.0651
VDWAALS =     47967.9718  EEL     =  -350915.6156  HBOND      =         0.0000
1-4 VDW =      1400.3012  1-4 EEL =     18772.1066  RESTRAINT  =         0.0000


  NSTEP       ENERGY          RMS            GMAX         NAME    NUMBER
   3500      -2.5728E+05     1.6313E-01     1.9731E+01     CE1      3736

BOND    =     19626.5341  ANGLE   =      1002.4136  DIHED      =      4952.9153
VDWAALS =     48028.2555  EEL     =  -351058.2297  HBOND      =         0.0000
1-4 VDW =      1400.0877  1-4 EEL =     18771.6304  RESTRAINT  =         0.0000


  NSTEP       ENERGY          RMS            GMAX         NAME    NUMBER
   3600      -2.5734E+05     9.9984E-02     1.0084E+01     CE1      3736

BOND    =     19639.7743  ANGLE   =      1001.9611  DIHED      =      4952.6233
VDWAALS =     48088.0464  EEL     =  -351195.8917  HBOND      =         0.0000
1-4 VDW =      1399.8750  1-4 EEL =     18771.3380  RESTRAINT  =         0.0000


  NSTEP       ENERGY          RMS            GMAX         NAME    NUMBER
   3700      -2.5741E+05     6.7959E-02     2.9889E+00     CE1      3736

BOND    =     19652.9327  ANGLE   =      1001.6696  DIHED      =      4952.2857
VDWAALS =     48145.3015  EEL     =  -351329.5753  HBOND      =         0.0000
1-4 VDW =      1399.6939  1-4 EEL =     18771.3666  RESTRAINT  =         0.0000


  NSTEP       ENERGY          RMS            GMAX         NAME    NUMBER
   3800      -2.5747E+05     2.8797E-01     6.5180E+01     CD       1360

BOND    =     19668.0652  ANGLE   =      1001.7359  DIHED      =      4952.0943
VDWAALS =     48199.8708  EEL     =  -351460.1990  HBOND      =         0.0000
1-4 VDW =      1399.5403  1-4 EEL =     18771.6102  RESTRAINT  =         0.0000


  NSTEP       ENERGY          RMS            GMAX         NAME    NUMBER
   3900      -2.5753E+05     6.9890E-02     3.7067E+00     CE1      3736

BOND    =     19678.3132  ANGLE   =      1001.2382  DIHED      =      4952.1442
VDWAALS =     48254.4272  EEL     =  -351590.8492  HBOND      =         0.0000
1-4 VDW =      1399.3669  1-4 EEL =     18772.1790  RESTRAINT  =         0.0000
```

```
  NSTEP       ENERGY          RMS            GMAX         NAME    NUMBER
   4000      -2.5760E+05     1.7862E-01     2.0855E+01     CE1      3736

 BOND    =     19691.7732  ANGLE   =      1001.1391  DIHED      =      4952.1422
 VDWAALS =     48310.0288  EEL     =   -351722.8566  HBOND      =         0.0000
 1-4 VDW =      1399.1573  1-4 EEL =     18772.9077  RESTRAINT  =         0.0000


  NSTEP       ENERGY          RMS            GMAX         NAME    NUMBER
   4100      -2.5766E+05     1.6401E-01     2.4464E+01     CD       1360

 BOND    =     19703.8431  ANGLE   =      1000.8625  DIHED      =      4951.9531
 VDWAALS =     48363.6775  EEL     =   -351848.6962  HBOND      =         0.0000
 1-4 VDW =      1398.9136  1-4 EEL =     18773.3828  RESTRAINT  =         0.0000


  NSTEP       ENERGY          RMS            GMAX         NAME    NUMBER
   4200      -2.5772E+05     1.3145E-01     1.4750E+01     CE1      3736

 BOND    =     19715.5384  ANGLE   =      1000.6629  DIHED      =      4951.7359
 VDWAALS =     48416.7486  EEL     =   -351972.3506  HBOND      =         0.0000
 1-4 VDW =      1398.6927  1-4 EEL =     18773.8971  RESTRAINT  =         0.0000


  NSTEP       ENERGY          RMS            GMAX         NAME    NUMBER
   4300      -2.5777E+05     6.7613E-02     3.9060E+00     CE1      3736

 BOND    =     19726.7826  ANGLE   =      1000.4054  DIHED      =      4951.5450
 VDWAALS =     48467.8076  EEL     =   -352090.8392  HBOND      =         0.0000
 1-4 VDW =      1398.4779  1-4 EEL =     18774.2564  RESTRAINT  =         0.0000


  NSTEP       ENERGY          RMS            GMAX         NAME    NUMBER
   4400      -2.5783E+05     2.3647E-01     2.9786E+01     CE1      3736

 BOND    =     19739.9542  ANGLE   =      1000.3457  DIHED      =      4951.3716
 VDWAALS =     48519.7107  EEL     =   -352211.9185  HBOND      =         0.0000
 1-4 VDW =      1398.2677  1-4 EEL =     18774.5800  RESTRAINT  =         0.0000


  NSTEP       ENERGY          RMS            GMAX         NAME    NUMBER
   4500      -2.5789E+05     6.5272E-02     3.1731E+00     CE1      3736

 BOND    =     19749.9589  ANGLE   =       999.9141  DIHED      =      4951.1892
 VDWAALS =     48569.2097  EEL     =   -352328.6791  HBOND      =         0.0000
 1-4 VDW =      1398.1150  1-4 EEL =     18774.9336  RESTRAINT  =         0.0000


  NSTEP       ENERGY          RMS            GMAX         NAME    NUMBER
   4600      -2.5794E+05     1.8523E-01     2.2717E+01     CE1      3736

 BOND    =     19761.9385  ANGLE   =       999.6354  DIHED      =      4950.9861
 VDWAALS =     48617.9748  EEL     =   -352443.8519  HBOND      =         0.0000
 1-4 VDW =      1397.9960  1-4 EEL =     18775.2182  RESTRAINT  =         0.0000
```

```
   NSTEP       ENERGY          RMS            GMAX         NAME    NUMBER
   4700      -2.5800E+05     8.4924E-02     7.7125E+00     CE1      3736

 BOND    =    19772.3444  ANGLE   =      999.1556  DIHED      =     4950.7728
 VDWAALS =    48666.2314  EEL     =  -352558.9612  HBOND      =        0.0000
 1-4 VDW =     1397.9158  1-4 EEL =    18775.5280  RESTRAINT  =        0.0000


   NSTEP       ENERGY          RMS            GMAX         NAME    NUMBER
   4800      -2.5805E+05     1.2121E-01     1.2972E+01     CE1      3736

 BOND    =    19783.5534  ANGLE   =      998.6850  DIHED      =     4950.5749
 VDWAALS =    48714.4218  EEL     =  -352674.0511  HBOND      =        0.0000
 1-4 VDW =     1397.8214  1-4 EEL =    18775.6940  RESTRAINT  =        0.0000


   NSTEP       ENERGY          RMS            GMAX         NAME    NUMBER
   4900      -2.5811E+05     2.6278E-01     3.0804E+01     CE1      3736

 BOND    =    19795.9177  ANGLE   =      998.4538  DIHED      =     4950.3923
 VDWAALS =    48761.7961  EEL     =  -352787.2207  HBOND      =        0.0000
 1-4 VDW =     1397.7154  1-4 EEL =    18775.7614  RESTRAINT  =        0.0000


   NSTEP       ENERGY          RMS            GMAX         NAME    NUMBER
   5000      -2.5816E+05     7.1486E-02     4.6857E+00     CE1      3736

 BOND    =    19804.7813  ANGLE   =      997.8405  DIHED      =     4950.2105
 VDWAALS =    48807.9069  EEL     =  -352897.2595  HBOND      =        0.0000
 1-4 VDW =     1397.5974  1-4 EEL =    18775.8161  RESTRAINT  =        0.0000

  Maximum number of minimization cycles reached.


                    FINAL RESULTS



   NSTEP       ENERGY          RMS            GMAX         NAME    NUMBER
   5000      -2.5816E+05     7.1486E-02     4.6857E+00     CE1      3736

 BOND    =    19804.7813  ANGLE   =      997.8405  DIHED      =     4950.2105
 VDWAALS =    48807.9069  EEL     =  -352897.2595  HBOND      =        0.0000
 1-4 VDW =     1397.5974  1-4 EEL =    18775.8161  RESTRAINT  =        0.0000

--------------------------------------------------------------------------------
   5.  TIMINGS
--------------------------------------------------------------------------------

|                Build the list            18.37 (91.58% of List )
|                Other                      1.69 ( 8.42% of List )
```

```
|              List time                 20.05 ( 0.29% of Nonbo)
|                    Short_ene time          6236.74 (99.68% of Direc)
|                    Other                     19.96 ( 0.32% of Direc)
|                 Direct Ewald time       6256.70 (91.62% of Ewald)
|                 Adjust Ewald time         25.77 ( 0.38% of Ewald)
|                    Fill Bspline coeffs       18.76 ( 3.47% of Recip)
|                    Fill charge grid          75.46 (13.95% of Recip)
|                    Scalar sum               103.82 (19.19% of Recip)
|                    Grad sum                 129.38 (23.91% of Recip)
|                    FFT time                 213.62 (39.48% of Recip)
|                 Recip Ewald time         541.06 ( 7.92% of Ewald)
|                 Virial junk                1.42 ( 0.02% of Ewald)
|                 Other                      4.37 ( 0.06% of Ewald)
|              Ewald time              6829.33 (99.71% of Nonbo)
|           Nonbond force           6849.39 (99.41% of Force)
|           Bond/Angle/Dihedral       40.05 ( 0.58% of Force)
|           Other                      0.66 ( 0.01% of Force)
|        Force time              6890.10 (100.0% of Runmd)
|     Runmd Time              6890.10 (99.72% of Total)
|     Other                     19.65 ( 0.28% of Total)
| Total time              6909.77 (100.0% of ALL  )

| Number of list builds   :         18

| Highest rstack allocated:    3470642
| Highest istack allocated:      65080
|           Job began  at 17:36:44.410  on 02/21/2019
|           Setup done at 17:36:44.947  on 02/21/2019
|           Run   done at 19:31:54.181  on 02/21/2019
|     wallclock() was called  190084 times

          -------------------------------------------------------
          Amber 16 SANDER                              2016
          -------------------------------------------------------

| Run on 02/20/2019 at 17:17:31

|   Executable path: /usr/local/amber16/bin/sander
| Working directory: /Users/Swati
|          Hostname: Unknown
  [-O]verwriting output

File Assignments:
|  MDIN: /Users/Swati/minimization_file_2.in
| MDOUT: /Users/Swati/ZSP9_files_2/truncated_ZSP9_min3_2.out
|INPCRD: /Users/Swati/ZSP9_files_2/truncated_ZSP9_min3_1.rst
|  PARM: /Users/Swati/ZSP9_files_2/ZSP9.prmtop
|RESTRT: /Users/Swati/ZSP9_files_2/truncated_ZSP9_min3_2.rst
|  REFC: refc
| MDVEL: mdvel
| MDFRC: mdfrc
|  MDEN: mden
| MDCRD: mdcrd
|MDINFO: mdinfo
```

```
|  MTMD: mtmd
|INPDIP: inpdip
|RSTDIP: rstdip
|INPTRA: inptraj


 Here is the input file:

PROT: minimization_2
&cntrl
imin = 1,
maxcyc = 5000,
ncyc = 10000,
igb = 0,
ntpr =  100,
ntp = 0
cut = 12
&end


--------------------------------------------------------------------------------
   1.  RESOURCE   USE:
--------------------------------------------------------------------------------

| Flags:
 getting box info from netcdf restart file
| NetCDF restart box info found
|Largest sphere to fit in unit cell has radius =    43.290
| New format PARM file being parsed.
| Version =    1.000 Date = 08/10/17 Time = 02:13:56
 NATOM  =   92322 NTYPES =      17 NBONH =   88587 MBONA  =    3843
 NTHETH =    8001 MTHETA =    5230 NPHIH =   16775 MPHIA  =   16079
 NHPARM =       0 NPARM  =       0 NNB   =  153390 NRES   =   28827
 NBONA  =    3843 NTHETA =    5230 NPHIA =   16079 NUMBND =      67
 NUMANG =     152 NPTRA  =     191 NATYP =      36 NPHB   =       1
 IFBOX  =       1 NMXRS  =      24 IFCAP =       0 NEXTRA =       0
 NCOPY  =       0


|     Memory Use     Allocated
|     Real             7288505
|     Hollerith         305795
|     Integer          3691274
|     Max Pairs       84443856
|     nblistReal       1107864
|     nblist Int       3033137
|       Total           422917 kbytes

| Note: 1-4 EEL scale factors are being read from the topology file.

| Note: 1-4 VDW scale factors are being read from the topology file.
| Duplicated    0 dihedrals
| Duplicated    0 dihedrals
```

```
     BOX TYPE: RECTILINEAR

--------------------------------------------------------------------------------
   2.  CONTROL  DATA  FOR  THE  RUN
--------------------------------------------------------------------------------

default_name

General flags:
     imin    =       1, nmropt  =       0

Nature and format of input:
     ntx     =       1, irest   =       0, ntrx    =       1

Nature and format of output:
     ntxo    =       2, ntpr    =     100, ntrx    =       1, ntwr    =       1
     iwrap   =       0, ntwx    =       0, ntwv    =       0, ntwe    =       0
     ioutfm  =       1, ntwprt  =       0, idecomp =       0, rbornstat=      0

Potential function:
     ntf     =       1, ntb     =       1, igb     =       0, nsnb    =      25
     ipol    =       0, gbsa    =       0, iesp    =       0
     dielc   =   1.00000, cut     =  12.00000, intdiel =   1.00000

Frozen or restrained atoms:
     ibelly  =       0, ntr     =       0

Energy minimization:
     maxcyc  =    5000, ncyc    =   10000, ntmin   =       1
     dx0     =   0.01000, drms    =   0.00010

Ewald parameters:
     verbose =       0, ew_type =       0, nbflag  =       1, use_pme =       1
     vdwmeth =       1, eedmeth =       1, netfrc  =       0
     Box X =  118.606   Box Y =   86.581   Box Z =  106.952
     Alpha =   90.000   Beta  =   90.000   Gamma =   90.000
     NFFT1 =  120       NFFT2 =   90       NFFT3 =  108
     Cutoff=   12.000   Tol   =0.100E-04
     Ewald Coefficient =  0.22664
     Interpolation order =    4
|  INFO: Old style inpcrd file read


--------------------------------------------------------------------------------
   3.  ATOMIC COORDINATES AND VELOCITIES
--------------------------------------------------------------------------------

default_name
 begin time read from input coords =     0.000 ps

 Number of triangulated 3-point waters found:    28349

     Sum of charges from parm topology file =  -0.00000026
     Forcing neutrality...
```

```
--------------------------------------------------------------------------------
   4.  RESULTS
--------------------------------------------------------------------------------

 ---------------------------------------------------
 APPROXIMATING switch and d/dx switch using CUBIC SPLINE INTERPOLATION
 using   5000.0 points per unit in tabled values
 TESTING RELATIVE ERROR over r ranging from 0.0 to cutoff
| CHECK switch(x): max rel err =   0.2738E-14   at   2.422500
| CHECK d/dx switch(x): max rel err =   0.7967E-11   at   2.716640
 ---------------------------------------------------
| Local SIZE OF NONBOND LIST =   45680654
| TOTAL SIZE OF NONBOND LIST =   45680654


   NSTEP       ENERGY          RMS            GMAX         NAME    NUMBER
      1      -3.4663E+05     4.4204E+00     7.3532E+02     C        3786

 BOND    =    37478.5623  ANGLE   =     2727.8166  DIHED      =     5547.3663
 VDWAALS =    69428.2816  EEL     =  -483213.4724  HBOND      =        0.0000
 1-4 VDW =     2150.0887  1-4 EEL =    19251.9883  RESTRAINT  =        0.0000


   NSTEP       ENERGY          RMS            GMAX         NAME    NUMBER
    100      -3.6114E+05     7.0015E-01     1.3230E+02     C        3800

 BOND    =    28167.2649  ANGLE   =     1601.1037  DIHED      =     5565.4396
 VDWAALS =    67040.0381  EEL     =  -483880.1840  HBOND      =        0.0000
 1-4 VDW =     1705.8561  1-4 EEL =    18658.3330  RESTRAINT  =        0.0000


   NSTEP       ENERGY          RMS            GMAX         NAME    NUMBER
    200      -3.6261E+05     7.4260E-01     9.7903E+01     C        3800

 BOND    =    27790.1455  ANGLE   =     1359.9399  DIHED      =     5530.1852
 VDWAALS =    67051.7976  EEL     =  -484551.8178  HBOND      =        0.0000
 1-4 VDW =     1613.4361  1-4 EEL =    18600.9306  RESTRAINT  =        0.0000


   NSTEP       ENERGY          RMS            GMAX         NAME    NUMBER
    300      -3.6334E+05     1.8066E-01     1.4320E+01     C        3800

 BOND    =    27685.3367  ANGLE   =     1275.8406  DIHED      =     5507.8262
 VDWAALS =    67168.2024  EEL     =  -485147.3485  HBOND      =        0.0000
 1-4 VDW =     1582.0614  1-4 EEL =    18592.2689  RESTRAINT  =        0.0000


   NSTEP       ENERGY          RMS            GMAX         NAME    NUMBER
    400      -3.6383E+05     1.5222E-01     1.1507E+01     N        3802

 BOND    =    27674.7094  ANGLE   =     1227.5164  DIHED      =     5492.1901
 VDWAALS =    67309.2572  EEL     =  -485690.7491  HBOND      =        0.0000
 1-4 VDW =     1565.2225  1-4 EEL =    18595.3366  RESTRAINT  =        0.0000
```

```
  NSTEP       ENERGY          RMS            GMAX         NAME    NUMBER
    500      -3.6421E+05     3.9962E-01     6.3379E+01     CD       3248

 BOND    =     27696.1168  ANGLE   =      1182.9635  DIHED      =      5486.9177
 VDWAALS =     67459.1429  EEL     =   -486192.2990  HBOND      =         0.0000
 1-4 VDW =      1554.0706  1-4 EEL =     18599.8108  RESTRAINT  =         0.0000


  NSTEP       ENERGY          RMS            GMAX         NAME    NUMBER
    600      -3.6458E+05     4.0079E-01     8.8124E+01     CD       3248

 BOND    =     27722.9446  ANGLE   =      1124.0107  DIHED      =      5482.9957
 VDWAALS =     67615.6574  EEL     =   -486669.9080  HBOND      =         0.0000
 1-4 VDW =      1541.0633  1-4 EEL =     18606.7278  RESTRAINT  =         0.0000


  NSTEP       ENERGY          RMS            GMAX         NAME    NUMBER
    700      -3.6488E+05     2.9902E-01     4.8884E+01     C        3800

 BOND    =     27747.6299  ANGLE   =      1104.1064  DIHED      =      5477.3560
 VDWAALS =     67769.6794  EEL     =   -487120.3763  HBOND      =         0.0000
 1-4 VDW =      1535.2137  1-4 EEL =     18611.2458  RESTRAINT  =         0.0000


  NSTEP       ENERGY          RMS            GMAX         NAME    NUMBER
    800      -3.6514E+05     1.9460E-01     3.2227E+01     C        3800

 BOND    =     27773.3351  ANGLE   =      1094.2684  DIHED      =      5471.4371
 VDWAALS =     67921.0933  EEL     =   -487545.6466  HBOND      =         0.0000
 1-4 VDW =      1531.3028  1-4 EEL =     18613.1607  RESTRAINT  =         0.0000


  NSTEP       ENERGY          RMS            GMAX         NAME    NUMBER
    900      -3.6539E+05     1.1548E-01     8.6733E+00     C        3800

 BOND    =     27805.0273  ANGLE   =      1084.4201  DIHED      =      5466.8761
 VDWAALS =     68073.5225  EEL     =   -487959.4723  HBOND      =         0.0000
 1-4 VDW =      1527.8796  1-4 EEL =     18614.1880  RESTRAINT  =         0.0000


  NSTEP       ENERGY          RMS            GMAX         NAME    NUMBER
   1000      -3.6561E+05     1.1159E-01     9.6165E+00     C        3800

 BOND    =     27835.8712  ANGLE   =      1078.2597  DIHED      =      5464.7244
 VDWAALS =     68221.5710  EEL     =   -488351.6049  HBOND      =         0.0000
 1-4 VDW =      1525.0294  1-4 EEL =     18615.0592  RESTRAINT  =         0.0000


  NSTEP       ENERGY          RMS            GMAX         NAME    NUMBER
   1100      -3.6582E+05     1.2129E-01     1.1991E+01     C        3800

 BOND    =     27867.1875  ANGLE   =      1072.0871  DIHED      =      5462.3794
```

```
VDWAALS =     68362.3188  EEL     =  -488720.9874  HBOND      =         0.0000
1-4 VDW =      1522.4596  1-4 EEL =    18615.8866  RESTRAINT  =         0.0000


  NSTEP       ENERGY          RMS            GMAX         NAME    NUMBER
   1200      -3.6601E+05     1.0463E-01     8.0162E+00     C        3800

BOND    =     27895.9711  ANGLE   =     1067.5870  DIHED      =      5459.0860
VDWAALS =     68494.3449  EEL     =  -489067.2985  HBOND      =         0.0000
1-4 VDW =      1520.2909  1-4 EEL =    18617.1296  RESTRAINT  =         0.0000


  NSTEP       ENERGY          RMS            GMAX         NAME    NUMBER
   1300      -3.6620E+05     2.7121E-01     4.1542E+01     C        3786

BOND    =     27926.4790  ANGLE   =     1064.5321  DIHED      =      5456.1348
VDWAALS =     68620.4396  EEL     =  -489400.6047  HBOND      =         0.0000
1-4 VDW =      1518.5528  1-4 EEL =    18618.4072  RESTRAINT  =         0.0000


  NSTEP       ENERGY          RMS            GMAX         NAME    NUMBER
   1400      -3.6638E+05     2.7349E-01     5.5638E+01     CD       3248

BOND    =     27957.4091  ANGLE   =     1058.2656  DIHED      =      5454.1160
VDWAALS =     68741.3593  EEL     =  -489721.9453  HBOND      =         0.0000
1-4 VDW =      1516.9460  1-4 EEL =    18618.8287  RESTRAINT  =         0.0000


  NSTEP       ENERGY          RMS            GMAX         NAME    NUMBER
   1500      -3.6655E+05     2.0681E-01     2.8565E+01     C        3786

BOND    =     27983.4766  ANGLE   =     1055.7493  DIHED      =      5450.7975
VDWAALS =     68859.0686  EEL     =  -490033.8112  HBOND      =         0.0000
1-4 VDW =      1515.5989  1-4 EEL =    18618.8380  RESTRAINT  =         0.0000


  NSTEP       ENERGY          RMS            GMAX         NAME    NUMBER
   1600      -3.6672E+05     1.5971E-01     2.0657E+01     C        3786

BOND    =     28012.8247  ANGLE   =     1050.9059  DIHED      =      5448.9518
VDWAALS =     68974.4986  EEL     =  -490337.3630  HBOND      =         0.0000
1-4 VDW =      1514.3189  1-4 EEL =    18618.2802  RESTRAINT  =         0.0000


  NSTEP       ENERGY          RMS            GMAX         NAME    NUMBER
   1700      -3.6688E+05     1.2484E-01     1.2174E+01     C        3786

BOND    =     28039.7998  ANGLE   =     1048.4214  DIHED      =      5447.3351
VDWAALS =     69089.1193  EEL     =  -490633.8849  HBOND      =         0.0000
1-4 VDW =      1513.1617  1-4 EEL =    18618.0611  RESTRAINT  =         0.0000


  NSTEP       ENERGY          RMS            GMAX         NAME    NUMBER
   1800      -3.6703E+05     3.6876E-01     9.4931E+01     CD       3248
```

```
BOND    =     28072.2979  ANGLE   =      1045.9289  DIHED      =      5446.5784
VDWAALS =     69204.5488  EEL     =  -490927.5841  HBOND      =         0.0000
1-4 VDW =      1512.0119  1-4 EEL =     18618.0587  RESTRAINT  =         0.0000


  NSTEP       ENERGY          RMS            GMAX         NAME    NUMBER
   1900      -3.6718E+05     2.7025E-01     4.1302E+01     CD       3248

BOND    =     28098.7350  ANGLE   =      1043.0701  DIHED      =      5446.5324
VDWAALS =     69321.2035  EEL     =  -491218.0143  HBOND      =         0.0000
1-4 VDW =      1510.9964  1-4 EEL =     18618.3152  RESTRAINT  =         0.0000


  NSTEP       ENERGY          RMS            GMAX         NAME    NUMBER
   2000      -3.6733E+05     2.3184E-01     3.6436E+01     CE1      4295

BOND    =     28125.6654  ANGLE   =      1041.0862  DIHED      =      5445.9318
VDWAALS =     69437.8749  EEL     =  -491506.1993  HBOND      =         0.0000
1-4 VDW =      1510.0575  1-4 EEL =     18618.2840  RESTRAINT  =         0.0000


  NSTEP       ENERGY          RMS            GMAX         NAME    NUMBER
   2100      -3.6747E+05     1.9943E-01     3.1100E+01     CE1      4295

BOND    =     28152.6367  ANGLE   =      1039.0362  DIHED      =      5444.8202
VDWAALS =     69551.4856  EEL     =  -491784.9647  HBOND      =         0.0000
1-4 VDW =      1509.2391  1-4 EEL =     18618.6103  RESTRAINT  =         0.0000


  NSTEP       ENERGY          RMS            GMAX         NAME    NUMBER
   2200      -3.6761E+05     8.1550E-02     4.3438E+00     CE1      4295

BOND    =     28178.0119  ANGLE   =      1037.6783  DIHED      =      5443.7066
VDWAALS =     69663.8008  EEL     =  -492058.0224  HBOND      =         0.0000
1-4 VDW =      1508.5560  1-4 EEL =     18619.3298  RESTRAINT  =         0.0000


  NSTEP       ENERGY          RMS            GMAX         NAME    NUMBER
   2300      -3.6774E+05     8.6683E-02     6.9881E+00     CE1      4295

BOND    =     28203.9160  ANGLE   =      1036.5201  DIHED      =      5442.5775
VDWAALS =     69772.4943  EEL     =  -492319.6287  HBOND      =         0.0000
1-4 VDW =      1508.0854  1-4 EEL =     18620.1604  RESTRAINT  =         0.0000


  NSTEP       ENERGY          RMS            GMAX         NAME    NUMBER
   2400      -3.6786E+05     9.6730E-02     1.0718E+01     CE1      4295

BOND    =     28229.0495  ANGLE   =      1035.3760  DIHED      =      5441.6269
VDWAALS =     69879.1572  EEL     =  -492572.5971  HBOND      =         0.0000
1-4 VDW =      1507.7375  1-4 EEL =     18621.1848  RESTRAINT  =         0.0000
```

```
   NSTEP       ENERGY          RMS            GMAX         NAME    NUMBER
   2500      -3.6797E+05     2.6478E-01     4.2194E+01     CE1      4295

 BOND    =     28255.9595  ANGLE   =      1034.3797  DIHED      =      5440.7486
 VDWAALS =     69983.7137  EEL     =   -492818.0215  HBOND      =         0.0000
 1-4 VDW =      1507.4789  1-4 EEL =     18622.2806  RESTRAINT  =         0.0000


   NSTEP       ENERGY          RMS            GMAX         NAME    NUMBER
   2600      -3.6809E+05     2.6669E-01     4.7175E+01     CD       3248

 BOND    =     28279.2279  ANGLE   =      1033.4382  DIHED      =      5439.8894
 VDWAALS =     70085.3300  EEL     =   -493056.5576  HBOND      =         0.0000
 1-4 VDW =      1507.2935  1-4 EEL =     18623.3776  RESTRAINT  =         0.0000


   NSTEP       ENERGY          RMS            GMAX         NAME    NUMBER
   2700      -3.6820E+05     1.9430E-01     2.8482E+01     CE1      4295

 BOND    =     28300.3820  ANGLE   =      1032.0697  DIHED      =      5439.0808
 VDWAALS =     70180.1453  EEL     =   -493281.5462  HBOND      =         0.0000
 1-4 VDW =      1507.0786  1-4 EEL =     18624.3268  RESTRAINT  =         0.0000


   NSTEP       ENERGY          RMS            GMAX         NAME    NUMBER
   2800      -3.6831E+05     1.2155E-01     1.5814E+01     CE1      4295

 BOND    =     28321.5353  ANGLE   =      1031.1002  DIHED      =      5438.3562
 VDWAALS =     70273.5559  EEL     =   -493504.6618  HBOND      =         0.0000
 1-4 VDW =      1506.9063  1-4 EEL =     18625.2379  RESTRAINT  =         0.0000


   NSTEP       ENERGY          RMS            GMAX         NAME    NUMBER
   2900      -3.6841E+05     1.2971E-01     1.8693E+01     CE1      4295

 BOND    =     28343.5533  ANGLE   =      1030.1451  DIHED      =      5437.7478
 VDWAALS =     70364.3602  EEL     =   -493722.7234  HBOND      =         0.0000
 1-4 VDW =      1506.7125  1-4 EEL =     18626.0308  RESTRAINT  =         0.0000


   NSTEP       ENERGY          RMS            GMAX         NAME    NUMBER
   3000      -3.6852E+05     7.2695E-02     4.1749E+00     CE1      4295

 BOND    =     28363.9884  ANGLE   =      1029.3344  DIHED      =      5437.2615
 VDWAALS =     70450.3790  EEL     =   -493930.3167  HBOND      =         0.0000
 1-4 VDW =      1506.5222  1-4 EEL =     18626.6680  RESTRAINT  =         0.0000


   NSTEP       ENERGY          RMS            GMAX         NAME    NUMBER
   3100      -3.6861E+05     2.5753E-01     3.8613E+01     CE1      4295

 BOND    =     28386.7906  ANGLE   =      1028.9284  DIHED      =      5436.8264
 VDWAALS =     70533.5734  EEL     =   -494130.9366  HBOND      =         0.0000
 1-4 VDW =      1506.3472  1-4 EEL =     18627.1935  RESTRAINT  =         0.0000
```

```
  NSTEP       ENERGY          RMS            GMAX         NAME    NUMBER
   3200      -3.6871E+05     2.2447E-01     3.2015E+01     CD       3248

 BOND    =     28406.3276  ANGLE   =      1028.1899  DIHED      =      5436.4316
 VDWAALS =     70615.5617  EEL     =   -494329.6855  HBOND      =         0.0000
 1-4 VDW =      1506.1184  1-4 EEL =     18627.6294  RESTRAINT  =         0.0000


  NSTEP       ENERGY          RMS            GMAX         NAME    NUMBER
   3300      -3.6881E+05     1.9700E-01     2.9960E+01     CE1      4295

 BOND    =     28425.4591  ANGLE   =      1027.7356  DIHED      =      5436.1076
 VDWAALS =     70694.5066  EEL     =   -494523.2472  HBOND      =         0.0000
 1-4 VDW =      1505.8997  1-4 EEL =     18628.0232  RESTRAINT  =         0.0000


  NSTEP       ENERGY          RMS            GMAX         NAME    NUMBER
   3400      -3.6890E+05     1.1264E-01     1.5029E+01     CE1      4295

 BOND    =     28443.6111  ANGLE   =      1027.0876  DIHED      =      5435.8257
 VDWAALS =     70771.1237  EEL     =   -494713.3948  HBOND      =         0.0000
 1-4 VDW =      1505.6911  1-4 EEL =     18628.3762  RESTRAINT  =         0.0000


  NSTEP       ENERGY          RMS            GMAX         NAME    NUMBER
   3500      -3.6900E+05     6.8921E-02     3.2235E+00     CE1      4295

 BOND    =     28462.2049  ANGLE   =      1026.5364  DIHED      =      5435.4973
 VDWAALS =     70845.4748  EEL     =   -494901.3085  HBOND      =         0.0000
 1-4 VDW =      1505.5177  1-4 EEL =     18628.7444  RESTRAINT  =         0.0000


  NSTEP       ENERGY          RMS            GMAX         NAME    NUMBER
   3600      -3.6909E+05     6.9897E-02     3.7045E+00     CE1      4295

 BOND    =     28481.0985  ANGLE   =      1026.0004  DIHED      =      5435.1262
 VDWAALS =     70918.3720  EEL     =   -495088.2440  HBOND      =         0.0000
 1-4 VDW =      1505.3579  1-4 EEL =     18629.1116  RESTRAINT  =         0.0000


  NSTEP       ENERGY          RMS            GMAX         NAME    NUMBER
   3700      -3.6919E+05     2.4359E-01     3.8109E+01     CE1      4295

 BOND    =     28502.6700  ANGLE   =      1025.6823  DIHED      =      5434.7467
 VDWAALS =     70992.5797  EEL     =   -495278.0821  HBOND      =         0.0000
 1-4 VDW =      1505.2094  1-4 EEL =     18629.3990  RESTRAINT  =         0.0000


  NSTEP       ENERGY          RMS            GMAX         NAME    NUMBER
   3800      -3.6928E+05     1.9925E-01     3.1286E+01     CE1      4295

 BOND    =     28520.6148  ANGLE   =      1025.2592  DIHED      =      5434.3808
```

```
VDWAALS =     71065.1410  EEL     =  -495460.7795  HBOND      =        0.0000
1-4 VDW =      1505.0690  1-4 EEL =    18629.5592  RESTRAINT  =        0.0000


  NSTEP       ENERGY          RMS            GMAX         NAME    NUMBER
   3900      -3.6937E+05     1.9398E-01     2.8176E+01     CE1      4295

BOND    =     28539.1807  ANGLE   =     1024.8723  DIHED      =     5434.0431
VDWAALS =     71137.9021  EEL     =  -495641.1939  HBOND      =        0.0000
1-4 VDW =      1504.9204  1-4 EEL =    18629.6226  RESTRAINT  =        0.0000


  NSTEP       ENERGY          RMS            GMAX         NAME    NUMBER
   4000      -3.6946E+05     1.1106E-01     1.4786E+01     CE1      4295

BOND    =     28556.2450  ANGLE   =     1024.4932  DIHED      =     5433.7193
VDWAALS =     71209.0657  EEL     =  -495816.1178  HBOND      =        0.0000
1-4 VDW =      1504.7553  1-4 EEL =    18629.6141  RESTRAINT  =        0.0000


  NSTEP       ENERGY          RMS            GMAX         NAME    NUMBER
   4100      -3.6954E+05     8.9660E-02     9.7689E+00     CE1      4295

BOND    =     28574.1840  ANGLE   =     1024.1973  DIHED      =     5433.4208
VDWAALS =     71281.4450  EEL     =  -495991.7591  HBOND      =        0.0000
1-4 VDW =      1504.5755  1-4 EEL =    18629.5400  RESTRAINT  =        0.0000


  NSTEP       ENERGY          RMS            GMAX         NAME    NUMBER
   4200      -3.6963E+05     9.1967E-02     1.1361E+01     CE1      4295

BOND    =     28592.2865  ANGLE   =     1024.0040  DIHED      =     5433.1436
VDWAALS =     71354.8166  EEL     =  -496166.2822  HBOND      =        0.0000
1-4 VDW =      1504.4007  1-4 EEL =    18629.4276  RESTRAINT  =        0.0000


  NSTEP       ENERGY          RMS            GMAX         NAME    NUMBER
   4300      -3.6971E+05     2.3073E-01     3.7107E+01     CE1      4295

BOND    =     28613.0665  ANGLE   =     1023.9930  DIHED      =     5432.8856
VDWAALS =     71431.9416  EEL     =  -496347.3792  HBOND      =        0.0000
1-4 VDW =      1504.2191  1-4 EEL =    18629.2536  RESTRAINT  =        0.0000


  NSTEP       ENERGY          RMS            GMAX         NAME    NUMBER
   4400      -3.6980E+05     6.5940E-02     4.1776E+00     CE1      4295

BOND    =     28629.5197  ANGLE   =     1023.6695  DIHED      =     5432.6823
VDWAALS =     71508.2030  EEL     =  -496523.9302  HBOND      =        0.0000
1-4 VDW =      1504.0178  1-4 EEL =    18629.0171  RESTRAINT  =        0.0000


  NSTEP       ENERGY          RMS            GMAX         NAME    NUMBER
   4500      -3.6988E+05     1.8210E-01     2.8073E+01     CE1      4295
```

```
BOND    =     28649.3638  ANGLE   =      1023.6940  DIHED      =      5432.5193
VDWAALS =     71586.1405  EEL     =  -496700.2771  HBOND      =         0.0000
1-4 VDW =      1503.8322  1-4 EEL =     18628.7284  RESTRAINT  =         0.0000


  NSTEP       ENERGY          RMS            GMAX         NAME    NUMBER
  4600      -3.6995E+05     1.4625E-01     2.2198E+01     CE1      4295

BOND    =     28667.1274  ANGLE   =      1023.5582  DIHED      =      5432.4164
VDWAALS =     71663.9946  EEL     =  -496872.8122  HBOND      =         0.0000
1-4 VDW =      1503.6264  1-4 EEL =     18628.3877  RESTRAINT  =         0.0000


  NSTEP       ENERGY          RMS            GMAX         NAME    NUMBER
  4700      -3.7003E+05     1.1834E-01     1.6957E+01     CE1      4295

BOND    =     28684.6762  ANGLE   =      1023.5645  DIHED      =      5432.3493
VDWAALS =     71742.1966  EEL     =  -497043.9552  HBOND      =         0.0000
1-4 VDW =      1503.4356  1-4 EEL =     18628.0332  RESTRAINT  =         0.0000


  NSTEP       ENERGY          RMS            GMAX         NAME    NUMBER
  4800      -3.7010E+05     8.9065E-02     1.1228E+01     CE1      4295

BOND    =     28701.7054  ANGLE   =      1023.5616  DIHED      =      5432.3279
VDWAALS =     71818.2087  EEL     =  -497209.9717  HBOND      =         0.0000
1-4 VDW =      1503.2390  1-4 EEL =     18627.6473  RESTRAINT  =         0.0000


  NSTEP       ENERGY          RMS            GMAX         NAME    NUMBER
  4900      -3.7018E+05     2.2767E-01     3.5888E+01     CE1      4295

BOND    =     28721.0842  ANGLE   =      1023.7886  DIHED      =      5432.3323
VDWAALS =     71895.7751  EEL     =  -497379.6785  HBOND      =         0.0000
1-4 VDW =      1503.0453  1-4 EEL =     18627.2209  RESTRAINT  =         0.0000


  NSTEP       ENERGY          RMS            GMAX         NAME    NUMBER
  5000      -3.7025E+05     6.2254E-02     4.2246E+00     CE1      4295

BOND    =     28735.8677  ANGLE   =      1023.7488  DIHED      =      5432.3510
VDWAALS =     71970.5825  EEL     =  -497542.7263  HBOND      =         0.0000
1-4 VDW =      1502.8557  1-4 EEL =     18626.7793  RESTRAINT  =         0.0000

 Maximum number of minimization cycles reached.


                  FINAL RESULTS



  NSTEP       ENERGY          RMS            GMAX         NAME    NUMBER
```

```
  5000      -3.7025E+05     6.2254E-02     4.2246E+00     CE1      4295

 BOND    =    28735.8677  ANGLE   =     1023.7488  DIHED      =     5432.3510
 VDWAALS =    71970.5825  EEL     =  -497542.7263  HBOND      =        0.0000
 1-4 VDW =     1502.8557  1-4 EEL =    18626.7793  RESTRAINT  =        0.0000

--------------------------------------------------------------------------------
   5.  TIMINGS
--------------------------------------------------------------------------------

|                Build the list            26.60 (91.36% of List )
|                Other                      2.52 ( 8.64% of List )
|             List time                 29.12 ( 0.30% of Nonbo)
|                   Short_ene time         8705.69 (99.69% of Direc)
|                   Other                    27.42 ( 0.31% of Direc)
|                Direct Ewald time       8733.11 (91.72% of Ewald)
|                Adjust Ewald time         32.32 ( 0.34% of Ewald)
|                   Fill Bspline coeffs       24.45 ( 3.26% of Recip)
|                   Fill charge grid         106.50 (14.21% of Recip)
|                   Scalar sum               143.78 (19.19% of Recip)
|                   Grad sum                 186.72 (24.92% of Recip)
|                   FFT time                 287.87 (38.42% of Recip)
|                Recip Ewald time         749.33 ( 7.87% of Ewald)
|                Virial junk                2.02 ( 0.02% of Ewald)
|                Other                      4.56 ( 0.05% of Ewald)
|             Ewald time               9521.36 (99.70% of Nonbo)
|          Nonbond force          9550.48 (99.53% of Force)
|          Bond/Angle/Dihedral       44.35 ( 0.46% of Force)
|          Other                      1.10 ( 0.01% of Force)
|       Force time             9595.94 (100.0% of Runmd)
|    Runmd Time             9595.94 (99.72% of Total)
|    Other                    26.75 ( 0.28% of Total)
| Total time             9622.71 (100.0% of ALL  )

| Number of list builds   :         18

| Highest rstack allocated:    4941880
| Highest istack allocated:      92322
|           Job began  at 17:17:31.941  on 02/20/2019
|           Setup done at 17:17:32.739  on 02/20/2019
|           Run   done at 19:57:54.656  on 02/20/2019
|     wallclock() was called  190084 times
```
