# Supplementary material for: Insights into the mechanism(s) of digestion of crystalline cellulose by plant class C GH9 endoglucanases
Source: J Mol Model. 2019 Jul 23;25(8):240. doi: 10.1007/s00894-019-4133-1 (PMC7385011; doi:10.1007/s00894-019-4133-1)
Supplement: Supplementary file 6 — (PDF 215 kb) [file 894_2019_4133_MOESM6_ESM.pdf]

Supplementary Text 4

ENSEMBLE FREQUENCY DATA (FULL LENGTH MINIMIZED CHARACTERIZED CLASS C)

```
  [,1]     [,2]     [,3]     [,4]     [,5]     [,6]     [,7]     [,8]     [,9]
 [,10]    [,11]    [,12]    [,13]    [,14]    [,15]    [,16]
[1,] 0.000364 0.000466 0.001258 0.003716 0.005726 0.006855 0.007277 0.010009
0.010339 0.012131 0.014230 0.015547 0.016808 0.019403 0.020951 0.021778
[2,] 0.000420 0.000575 0.001249 0.003826 0.004993 0.006780 0.007087 0.008667
0.008990 0.011130 0.011834 0.012549 0.013127 0.015028 0.015487 0.016363
[3,] 0.000502 0.000648 0.001497 0.003840 0.005918 0.006475 0.007749 0.009656
0.010144 0.011277 0.013026 0.015548 0.015808 0.016205 0.017694 0.021884
[4,] 0.000455 0.000712 0.002008 0.003958 0.006390 0.007638 0.008366 0.009941
0.010514 0.011037 0.013560 0.015833 0.016429 0.019737 0.020690 0.021781
        [,17]    [,18]    [,19]    [,20]    [,21]    [,22]    [,23]    [,24]
[,25]    [,26]    [,27]    [,28]    [,29]    [,30]    [,31]    [,32]
[1,] 0.024115 0.024604 0.026986 0.027399 0.028319 0.029132 0.029657 0.030548
0.031502 0.032794 0.033736 0.035257 0.037578 0.037974 0.038572 0.039389
[2,] 0.017275 0.018238 0.019608 0.020642 0.021266 0.021898 0.022356 0.025342
0.026227 0.026886 0.028069 0.028415 0.029753 0.030345 0.031908 0.032882
[3,] 0.023065 0.023982 0.026379 0.027171 0.029478 0.030001 0.030400 0.031363
0.032585 0.033486 0.035555 0.036828 0.037360 0.038059 0.039898 0.040264
[4,] 0.022571 0.024101 0.025549 0.027434 0.027836 0.029221 0.029813 0.031510
0.034214 0.034562 0.036801 0.037973 0.038998 0.040907 0.041307 0.042598
        [,33]    [,34]    [,35]    [,36]    [,37]    [,38]    [,39]    [,40]
[,41]    [,42]    [,43]    [,44]    [,45]    [,46]    [,47]    [,48]
[1,] 0.040604 0.041550 0.041706 0.042929 0.043690 0.045365 0.045543 0.046779
0.047974 0.048662 0.050003 0.050473 0.051240 0.052290 0.052749 0.053767
[2,] 0.033398 0.033989 0.035627 0.036844 0.037134 0.038145 0.038533 0.039393
0.040940 0.041136 0.042093 0.042474 0.043677 0.044598 0.045840 0.046485
[3,] 0.041127 0.042026 0.043289 0.043634 0.044849 0.045545 0.046586 0.047360
0.047726 0.049646 0.051021 0.051260 0.052379 0.054800 0.055239 0.056510
[4,] 0.043067 0.043276 0.045381 0.046319 0.047752 0.048544 0.049907 0.050440
0.050724 0.052383 0.053244 0.054146 0.054875 0.055644 0.056308 0.058106
        [,49]    [,50]    [,51]    [,52]    [,53]    [,54]    [,55]    [,56]
[,57]    [,58]    [,59]    [,60]    [,61]    [,62]    [,63]    [,64]
[1,] 0.055135 0.056275 0.056824 0.056899 0.058225 0.058723 0.059938 0.061429
0.062209 0.063963 0.065380 0.066278 0.066757 0.068433 0.069360 0.069671
[2,] 0.047048 0.047485 0.047685 0.048278 0.049323 0.049752 0.051113 0.051530
0.052028 0.053272 0.053824 0.054186 0.055209 0.055607 0.056100 0.057020
[3,] 0.058070 0.058735 0.059205 0.059977 0.060491 0.061682 0.062651 0.063994
0.064556 0.065502 0.066072 0.066740 0.068699 0.069781 0.070468 0.071300
[4,] 0.058475 0.060611 0.060888 0.061030 0.061907 0.064439 0.064680 0.066550
0.068129 0.068516 0.068944 0.069418 0.070717 0.071488 0.071838 0.074079
        [,65]    [,66]    [,67]    [,68]    [,69]    [,70]    [,71]    [,72]
[,73]    [,74]    [,75]    [,76]    [,77]    [,78]    [,79]    [,80]
[1,] 0.071881 0.072867 0.074042 0.074847 0.076202 0.076743 0.077079 0.078404
0.078870 0.080178 0.080719 0.081063 0.082557 0.084564 0.084771 0.085552
[2,] 0.057277 0.058663 0.059338 0.059811 0.061067 0.061597 0.062262 0.063578
0.064842 0.065668 0.067151 0.068483 0.069400 0.070362 0.070750 0.071578
[3,] 0.071512 0.072363 0.073429 0.074202 0.075028 0.075560 0.076005 0.077229
0.077949 0.078765 0.079959 0.081182 0.081738 0.082650 0.083822 0.084338
[4,] 0.074648 0.075138 0.076245 0.076666 0.078181 0.078993 0.079756 0.081644
0.082212 0.083162 0.083402 0.084681 0.084766 0.085573 0.086923 0.088463
        [,81]    [,82]    [,83]    [,84]    [,85]    [,86]    [,87]    [,88]
[,89]    [,90]    [,91]    [,92]    [,93]    [,94]    [,95]    [,96]
```

```
[1,] 0.086915 0.087542 0.089154 0.090141 0.091899 0.092360 0.093943 0.094842
0.096887 0.098032 0.098986 0.099790 0.100897 0.101229 0.101669 0.102867
[2,] 0.071741 0.072880 0.073742 0.074672 0.076097 0.077754 0.078260 0.078832
0.080755 0.082595 0.083019 0.084174 0.084348 0.084939 0.087766 0.089204
[3,] 0.085092 0.085853 0.086721 0.088363 0.088793 0.089707 0.089991 0.091004
0.092893 0.093778 0.094072 0.095311 0.096172 0.097141 0.098289 0.098830
[4,] 0.089370 0.090253 0.092658 0.093838 0.094280 0.094697 0.095610 0.096896
0.097330 0.099012 0.099340 0.100309 0.102243 0.102501 0.103225 0.104354
        [,97]    [,98]    [,99]   [,100]   [,101]   [,102]   [,103]   [,104]
[,105]   [,106]   [,107]   [,108]   [,109]   [,110]   [,111]   [,112]
[1,] 0.103626 0.105057 0.105495 0.106588 0.107689 0.108684 0.108981 0.111051
0.112122 0.112419 0.114070 0.114501 0.114693 0.115820 0.116348 0.116551
[2,] 0.089977 0.090506 0.091156 0.092566 0.093478 0.094614 0.095001 0.095477
0.096092 0.097753 0.099249 0.099526 0.100061 0.101121 0.102168 0.103434
[3,] 0.099790 0.100908 0.101201 0.103032 0.103269 0.103888 0.104491 0.105535
0.106517 0.108151 0.108290 0.108828 0.110845 0.111811 0.111938 0.112431
[4,] 0.104711 0.105828 0.106441 0.107990 0.108356 0.108887 0.109271 0.110787
0.111558 0.112395 0.113081 0.114002 0.115212 0.115734 0.116621 0.117877
       [,113]   [,114]   [,115]   [,116]   [,117]   [,118]   [,119]   [,120]
[,121]   [,122]   [,123]   [,124]   [,125]   [,126]   [,127]   [,128]
[1,] 0.117695 0.119453 0.120309 0.122132 0.122244 0.123148 0.123748 0.126100
0.126465 0.126704 0.128634 0.129771 0.130496 0.131449 0.132449 0.133293
[2,] 0.104040 0.104827 0.105309 0.106200 0.106716 0.108852 0.109020 0.109744
0.110681 0.111876 0.112232 0.112938 0.113559 0.114602 0.114825 0.115612
[3,] 0.113449 0.115172 0.115746 0.116824 0.117322 0.118603 0.119855 0.120739
0.121357 0.121713 0.123651 0.124141 0.124976 0.125883 0.126478 0.126612
[4,] 0.118775 0.119553 0.120326 0.121251 0.122031 0.123041 0.123336 0.124710
0.125646 0.126643 0.127195 0.128253 0.129528 0.130531 0.131031 0.131646
       [,129]   [,130]   [,131]   [,132]   [,133]   [,134]   [,135]   [,136]
[,137]   [,138]   [,139]   [,140]   [,141]   [,142]   [,143]   [,144]
[1,] 0.133818 0.134961 0.135992 0.136717 0.137021 0.138151 0.138745 0.140438
0.140812 0.142111 0.142296 0.143181 0.144012 0.146069 0.146727 0.147876
[2,] 0.116233 0.117520 0.118904 0.119945 0.120816 0.121841 0.122079 0.123020
0.124666 0.125329 0.126680 0.127696 0.128222 0.128596 0.129419 0.131241
[3,] 0.127823 0.128397 0.129129 0.131468 0.131980 0.133338 0.135451 0.135856
0.136788 0.137538 0.138465 0.138802 0.140213 0.140726 0.141150 0.142446
[4,] 0.132455 0.133316 0.134736 0.135394 0.136124 0.136454 0.137353 0.137528
0.138564 0.140386 0.141568 0.142494 0.143110 0.145262 0.145544 0.146925
       [,145]   [,146]   [,147]   [,148]   [,149]   [,150]   [,151]   [,152]
[,153]   [,154]   [,155]   [,156]   [,157]   [,158]   [,159]   [,160]
[1,] 0.149614 0.149828 0.150380 0.151346 0.152851 0.154207 0.154607 0.155658
0.155955 0.156617 0.157496 0.158157 0.158805 0.159448 0.160828 0.161885
[2,] 0.132389 0.133113 0.133745 0.135150 0.136246 0.136883 0.137459 0.138867
0.139464 0.140191 0.142399 0.144110 0.144867 0.145961 0.146645 0.147551
[3,] 0.142659 0.143551 0.145217 0.145403 0.146530 0.147604 0.148004 0.149109
0.151656 0.152333 0.152890 0.154049 0.156325 0.156806 0.157449 0.158163
[4,] 0.147916 0.148507 0.149227 0.150983 0.151439 0.153674 0.154208 0.155505
0.155728 0.156870 0.158246 0.159036 0.159618 0.160521 0.161763 0.163500
       [,161]   [,162]   [,163]   [,164]   [,165]   [,166]   [,167]   [,168]
[,169]   [,170]   [,171]   [,172]   [,173]   [,174]   [,175]   [,176]
[1,] 0.163293 0.165406 0.166122 0.167141 0.168897 0.169981 0.170891 0.171781
0.173026 0.173930 0.174691 0.174924 0.176058 0.176544 0.177580 0.180098
[2,] 0.148467 0.148495 0.149902 0.150638 0.151408 0.151930 0.153132 0.153564
0.154571 0.155563 0.157251 0.157652 0.158336 0.159917 0.160803 0.162046
```

[3,] 0.158284 0.160099 0.161388 0.162722 0.163947 0.165044 0.165216 0.165674
0.167151 0.167390 0.167998 0.168889 0.170326 0.172252 0.174044 0.176061
[4,] 0.163890 0.164622 0.165226 0.166085 0.166928 0.167523 0.169110 0.169854
0.169947 0.170887 0.171872 0.172760 0.173959 0.175587 0.176109 0.177505
[,177] [,178] [,179] [,180] [,181] [,182] [,183] [,184]
[,185] [,186] [,187] [,188] [,189] [,190] [,191] [,192]
[1,] 0.182374 0.182768 0.183164 0.184774 0.185873 0.187133 0.187350 0.188912
0.189489 0.191881 0.192658 0.193755 0.195186 0.196719 0.198817 0.199526
[2,] 0.163003 0.163320 0.164182 0.165789 0.166491 0.168052 0.168244 0.168442
0.168925 0.169488 0.170932 0.173370 0.173794 0.174932 0.176171 0.177016
[3,] 0.176325 0.176730 0.177435 0.178101 0.178995 0.179194 0.179531 0.180416
0.181428 0.182865 0.183257 0.184287 0.185616 0.185977 0.187631 0.188745
[4,] 0.178803 0.179840 0.180535 0.182094 0.183034 0.183393 0.184644 0.186105
0.186991 0.187808 0.188845 0.189902 0.190299 0.191806 0.192047 0.192981
[,193] [,194] [,195] [,196] [,197] [,198] [,199] [,200]
[,201] [,202] [,203] [,204] [,205] [,206] [,207] [,208]
[1,] 0.200011 0.201818 0.202734 0.204074 0.204680 0.205282 0.207197 0.208629
0.208910 0.209466 0.211315 0.213024 0.213156 0.214947 0.215871 0.216236
[2,] 0.178142 0.179060 0.180055 0.181325 0.182320 0.183212 0.185298 0.186345
0.187892 0.188874 0.190400 0.190835 0.191020 0.192484 0.192834 0.193296
[3,] 0.189414 0.190273 0.191595 0.192551 0.193408 0.194086 0.194490 0.195112
0.196197 0.197342 0.199387 0.200124 0.200859 0.202120 0.202861 0.204370
[4,] 0.194132 0.194720 0.195993 0.197010 0.197662 0.198661 0.198820 0.200297
0.201533 0.202741 0.203560 0.205088 0.205573 0.206781 0.207568 0.208026
[,209] [,210] [,211] [,212] [,213] [,214] [,215] [,216]
[,217] [,218] [,219] [,220] [,221] [,222] [,223] [,224]
[1,] 0.218746 0.219551 0.220627 0.221423 0.223487 0.225119 0.225309 0.226064
0.226559 0.228012 0.228237 0.229960 0.230912 0.231317 0.232061 0.232885
[2,] 0.195011 0.196608 0.197676 0.198038 0.200219 0.200877 0.201382 0.201661
0.202169 0.202878 0.203313 0.204011 0.205365 0.206604 0.206880 0.207400
[3,] 0.205635 0.205909 0.207198 0.207541 0.209506 0.210556 0.211161 0.211344
0.212745 0.214152 0.214992 0.216751 0.216914 0.217963 0.218326 0.219031
[4,] 0.208705 0.209979 0.211445 0.212482 0.213862 0.214714 0.215267 0.216952
0.218930 0.219701 0.220724 0.220922 0.221564 0.222430 0.223575 0.224039
[,225] [,226] [,227] [,228] [,229] [,230] [,231] [,232]
[,233] [,234] [,235] [,236] [,237] [,238] [,239] [,240]
[1,] 0.233029 0.234423 0.236139 0.237529 0.238037 0.239359 0.239845 0.241391
0.242192 0.242825 0.243771 0.246664 0.248684 0.249617 0.249774 0.251765
[2,] 0.209353 0.210503 0.212331 0.213776 0.214452 0.215150 0.215921 0.217267
0.218265 0.219212 0.220181 0.221595 0.222745 0.223258 0.224019 0.224724
[3,] 0.220356 0.221338 0.222570 0.223530 0.224332 0.224423 0.225062 0.225440
0.226581 0.228143 0.228974 0.229702 0.230155 0.232147 0.233511 0.233605
[4,] 0.225407 0.225564 0.226611 0.228011 0.228606 0.229217 0.232403 0.233592
0.234014 0.235352 0.235540 0.236380 0.237072 0.238324 0.240990 0.241722
[,241] [,242] [,243] [,244] [,245] [,246] [,247] [,248]
[,249] [,250] [,251] [,252] [,253] [,254] [,255] [,256]
[1,] 0.252200 0.252861 0.254592 0.254954 0.256968 0.259177 0.259255 0.260739
0.262048 0.262927 0.263780 0.264959 0.266457 0.267477 0.268444 0.269410
[2,] 0.225533 0.226011 0.226306 0.227878 0.229104 0.229999 0.230588 0.232549
0.233507 0.235353 0.236082 0.237937 0.238744 0.239763 0.240221 0.240649
[3,] 0.234321 0.236753 0.237060 0.237763 0.238577 0.239671 0.241657 0.242927
0.243928 0.244536 0.244671 0.246194 0.246943 0.248327 0.248934 0.250097
[4,] 0.241852 0.242819 0.243937 0.244529 0.245282 0.245705 0.247054 0.247987
0.249030 0.250119 0.251322 0.252486 0.253768 0.254198 0.255168 0.256318

```
       [,257]   [,258]   [,259]   [,260]   [,261]   [,262]   [,263]   [,264]
[,265]   [,266]   [,267]   [,268]   [,269]   [,270]   [,271]   [,272]
[1,] 0.272170 0.272930 0.274881 0.275476 0.276344 0.277829 0.278445 0.280150
0.280782 0.282487 0.284493 0.285878 0.286077 0.287016 0.287748 0.289400
[2,] 0.241777 0.243825 0.245208 0.245931 0.247462 0.249345 0.250062 0.250869
0.252014 0.252771 0.253434 0.254361 0.255775 0.255925 0.256586 0.258698
[3,] 0.251905 0.253181 0.254687 0.255050 0.255572 0.256509 0.257199 0.258424
0.259400 0.260776 0.261287 0.262009 0.263775 0.265376 0.267419 0.268547
[4,] 0.257358 0.257818 0.259341 0.260046 0.262188 0.263306 0.263811 0.264870
0.265500 0.267974 0.269490 0.270466 0.270781 0.271807 0.272561 0.273395
       [,273]   [,274]   [,275]   [,276]   [,277]   [,278]   [,279]   [,280]
[,281]   [,282]   [,283]   [,284]   [,285]   [,286]   [,287]   [,288]
[1,] 0.289716 0.291963 0.293113 0.295233 0.298141 0.299191 0.299687 0.300885
0.301246 0.303193 0.304353 0.305042 0.305409 0.305801 0.308980 0.309961
[2,] 0.259717 0.260881 0.261724 0.262035 0.263175 0.264512 0.266893 0.267292
0.268583 0.270338 0.271536 0.273046 0.273644 0.274135 0.275511 0.276612
[3,] 0.269858 0.271652 0.272690 0.274299 0.274595 0.275169 0.275451 0.276787
0.277447 0.278154 0.281541 0.282199 0.282684 0.283517 0.284955 0.286193
[4,] 0.274260 0.274961 0.276625 0.277110 0.278781 0.279658 0.280273 0.282324
0.283276 0.284608 0.285171 0.286645 0.287047 0.287529 0.290077 0.291475
       [,289]   [,290]   [,291]   [,292]   [,293]   [,294]   [,295]   [,296]
[,297]   [,298]   [,299]   [,300]   [,301]   [,302]   [,303]   [,304]
[1,] 0.311671 0.312742 0.313015 0.314124 0.315583 0.315751 0.316395 0.318233
0.319687 0.321007 0.321380 0.323054 0.323992 0.324270 0.327457 0.328126
[2,] 0.277105 0.278602 0.280449 0.281064 0.281479 0.283564 0.285095 0.285860
0.286792 0.287439 0.288819 0.290352 0.292245 0.292926 0.293613 0.295613
[3,] 0.287571 0.288386 0.289005 0.290054 0.291310 0.292936 0.293413 0.294360
0.295433 0.296265 0.298959 0.299616 0.300599 0.301895 0.302422 0.303735
[4,] 0.292537 0.293951 0.295169 0.296415 0.297054 0.298349 0.300367 0.300881
0.300956 0.302291 0.303742 0.304067 0.305903 0.307174 0.308096 0.309071
       [,305]   [,306]   [,307]   [,308]   [,309]   [,310]   [,311]   [,312]
[,313]   [,314]   [,315]   [,316]   [,317]   [,318]   [,319]   [,320]
[1,] 0.329666 0.331391 0.332524 0.333429 0.334833 0.335573 0.336401 0.336579
0.337674 0.337966 0.339946 0.341285 0.341737 0.342921 0.344417 0.345404
[2,] 0.296444 0.297331 0.298627 0.298948 0.301417 0.302533 0.303488 0.303941
0.305152 0.306250 0.307613 0.309520 0.309885 0.311751 0.311985 0.313456
[3,] 0.304770 0.305358 0.305889 0.307399 0.308032 0.309379 0.310484 0.310519
0.311867 0.313322 0.314586 0.315349 0.316363 0.317677 0.318367 0.319218
[4,] 0.309691 0.311636 0.312687 0.313388 0.314565 0.315152 0.316309 0.318254
0.319225 0.320246 0.320558 0.321668 0.323674 0.325549 0.325603 0.327106
       [,321]   [,322]   [,323]   [,324]   [,325]   [,326]   [,327]   [,328]
[,329]   [,330]   [,331]   [,332]   [,333]   [,334]   [,335]   [,336]
[1,] 0.346566 0.348248 0.349494 0.350702 0.351207 0.352084 0.353613 0.356698
0.357276 0.357328 0.359614 0.359822 0.361243 0.362682 0.363861 0.365752
[2,] 0.314717 0.316432 0.317464 0.317503 0.319572 0.320973 0.321717 0.322456
0.326450 0.326961 0.327502 0.328824 0.330107 0.330673 0.332934 0.333790
[3,] 0.321701 0.322665 0.323666 0.325395 0.326852 0.328627 0.329703 0.330336
0.330922 0.334421 0.335256 0.338268 0.339246 0.339934 0.340512 0.341524
[4,] 0.328118 0.330163 0.331181 0.332074 0.332769 0.333881 0.334101 0.336414
0.337674 0.338080 0.339637 0.341395 0.343590 0.344054 0.345192 0.346785
       [,337]   [,338]   [,339]   [,340]   [,341]   [,342]   [,343]   [,344]
[,345]   [,346]   [,347]   [,348]   [,349]   [,350]   [,351]   [,352]
[1,] 0.366438 0.367667 0.369275 0.370390 0.371983 0.373546 0.374693 0.375637
0.377908 0.378138 0.380156 0.380327 0.381455 0.383457 0.384989 0.385615
```

```
[2,] 0.336810 0.337218 0.339869 0.340721 0.341785 0.342863 0.343445 0.343933
0.344826 0.347813 0.348575 0.349867 0.351582 0.352262 0.353013 0.355196
[3,] 0.343769 0.343861 0.345208 0.347103 0.348796 0.349696 0.349714 0.350598
0.351235 0.353953 0.354828 0.355988 0.356908 0.357698 0.359087 0.360524
[4,] 0.347807 0.349029 0.349715 0.350458 0.351533 0.351752 0.353460 0.355040
0.355353 0.356650 0.358272 0.359526 0.360853 0.361864 0.362409 0.364672
       [,353]   [,354]   [,355]   [,356]   [,357]   [,358]   [,359]   [,360]
[,361]   [,362]   [,363]   [,364]   [,365]   [,366]   [,367]   [,368]
[1,] 0.386563 0.388784 0.389059 0.391329 0.393402 0.394528 0.395408 0.397508
0.398813 0.399387 0.400482 0.401343 0.402796 0.403335 0.404365 0.406596
[2,] 0.355431 0.356483 0.357960 0.359385 0.360421 0.361916 0.363487 0.364840
0.365154 0.365455 0.368695 0.369987 0.370460 0.371386 0.372834 0.374979
[3,] 0.362343 0.363003 0.364102 0.365172 0.365680 0.367767 0.368883 0.370301
0.370762 0.372489 0.373227 0.374267 0.375591 0.376523 0.377926 0.379368
[4,] 0.365276 0.366231 0.367063 0.368638 0.369326 0.370649 0.373206 0.375707
0.375957 0.377486 0.379872 0.380986 0.382728 0.384517 0.385181 0.385643
       [,369]   [,370]   [,371]   [,372]   [,373]   [,374]   [,375]   [,376]
[,377]   [,378]   [,379]   [,380]   [,381]   [,382]   [,383]   [,384]
[1,] 0.407310 0.408189 0.410886 0.412104 0.412662 0.413512 0.414384 0.416018
0.416672 0.417891 0.418962 0.419782 0.422185 0.422805 0.424020 0.424852
[2,] 0.375781 0.376242 0.377933 0.379271 0.380314 0.382344 0.383615 0.385506
0.386756 0.388098 0.388476 0.389590 0.390927 0.391557 0.392634 0.394283
[3,] 0.381464 0.382385 0.383408 0.384924 0.386119 0.387169 0.388801 0.389635
0.390293 0.391985 0.392920 0.393620 0.393942 0.395344 0.396338 0.398053
[4,] 0.387071 0.387606 0.389824 0.393132 0.393708 0.394845 0.395192 0.396663
0.397025 0.397875 0.400399 0.400884 0.402396 0.403369 0.403786 0.405111
       [,385]   [,386]   [,387]   [,388]   [,389]   [,390]   [,391]   [,392]
[,393]   [,394]   [,395]   [,396]   [,397]   [,398]   [,399]   [,400]
[1,] 0.429270 0.431746 0.432323 0.433708 0.435194 0.436169 0.436691 0.438029
0.439637 0.440931 0.442243 0.443968 0.446960 0.447082 0.448132 0.449128
[2,] 0.395351 0.396019 0.396977 0.397433 0.400371 0.400565 0.402415 0.404723
0.405705 0.408004 0.409506 0.410356 0.410558 0.412150 0.412897 0.415171
[3,] 0.399007 0.399589 0.400553 0.400815 0.402146 0.403734 0.407463 0.407620
0.409152 0.410954 0.411275 0.412694 0.414376 0.415388 0.416315 0.416423
[4,] 0.406122 0.407799 0.410000 0.411175 0.411749 0.412343 0.414080 0.416021
0.416844 0.417782 0.420228 0.420875 0.422638 0.423654 0.425030 0.426737
       [,401]   [,402]   [,403]   [,404]   [,405]   [,406]   [,407]   [,408]
[,409]   [,410]   [,411]   [,412]   [,413]   [,414]   [,415]   [,416]
[1,] 0.452721 0.453442 0.455121 0.455768 0.456668 0.458259 0.459793 0.459899
0.460455 0.461942 0.463005 0.464727 0.466279 0.467068 0.469640 0.469990
[2,] 0.416750 0.417995 0.419362 0.420482 0.421890 0.424763 0.426713 0.427313
0.428651 0.429123 0.429871 0.431703 0.432084 0.433183 0.435202 0.435861
[3,] 0.418147 0.418996 0.420174 0.422416 0.423802 0.425685 0.428950 0.430524
0.431612 0.432455 0.433739 0.434467 0.435061 0.435924 0.437868 0.438234
[4,] 0.427286 0.428166 0.428593 0.431198 0.432969 0.433302 0.434435 0.434630
0.435692 0.437434 0.439502 0.440598 0.441594 0.441993 0.443106 0.446309
       [,417]   [,418]   [,419]   [,420]   [,421]   [,422]   [,423]   [,424]
[,425]   [,426]   [,427]   [,428]   [,429]   [,430]   [,431]   [,432]
[1,] 0.474501 0.474997 0.478533 0.479417 0.480432 0.482256 0.484549 0.485809
0.486797 0.487450 0.490462 0.491203 0.492162 0.493257 0.494597 0.496670
[2,] 0.437536 0.438849 0.440731 0.441096 0.444221 0.445026 0.445834 0.446669
0.447350 0.449002 0.449565 0.451739 0.452641 0.454460 0.455193 0.455939
[3,] 0.440801 0.441799 0.442845 0.444689 0.445602 0.447265 0.449335 0.450683
0.451186 0.452111 0.454737 0.456491 0.458556 0.459626 0.460865 0.462335
```

[4,] 0.448219 0.449512 0.449872 0.450450 0.451682 0.454523 0.454892 0.457298
0.458064 0.458436 0.459726 0.460894 0.462804 0.463774 0.465600 0.466777
 [,433] [,434] [,435] [,436] [,437] [,438] [,439] [,440]
[,441] [,442] [,443] [,444] [,445] [,446] [,447] [,448]
[1,] 0.498854 0.500995 0.502317 0.503233 0.505296 0.506429 0.508661 0.509935
0.510848 0.512730 0.513369 0.515640 0.517200 0.518285 0.519873 0.520511
[2,] 0.458215 0.459781 0.461888 0.464418 0.465987 0.468636 0.469634 0.471110
0.473473 0.475339 0.476171 0.478638 0.479445 0.481638 0.481884 0.483421
[3,] 0.463980 0.465012 0.466619 0.466710 0.468828 0.471619 0.472512 0.472942
0.475143 0.476807 0.477495 0.478989 0.480168 0.480888 0.481803 0.482610
[4,] 0.468960 0.469758 0.471121 0.472303 0.473246 0.474660 0.475256 0.476554
0.478589 0.481178 0.483333 0.485208 0.487263 0.488422 0.491582 0.491744
 [,449] [,450] [,451] [,452] [,453] [,454] [,455] [,456]
[,457] [,458] [,459] [,460] [,461] [,462] [,463] [,464]
[1,] 0.521380 0.523386 0.524426 0.525502 0.527923 0.530212 0.530925 0.531996
0.533320 0.535839 0.537392 0.539768 0.542079 0.543280 0.543929 0.546667
[2,] 0.484585 0.486255 0.487355 0.487947 0.488820 0.490259 0.491824 0.493651
0.494209 0.495634 0.496426 0.498139 0.499439 0.500694 0.502710 0.505335
[3,] 0.485400 0.485895 0.487167 0.488517 0.488967 0.489746 0.491568 0.494587
0.495397 0.496839 0.497436 0.498388 0.501524 0.502349 0.504780 0.505511
[4,] 0.492228 0.493759 0.494307 0.495546 0.497230 0.501629 0.502379 0.503663
0.504541 0.505977 0.506734 0.509422 0.510825 0.510938 0.514555 0.515064
 [,465] [,466] [,467] [,468] [,469] [,470] [,471] [,472]
[,473] [,474] [,475] [,476] [,477] [,478] [,479] [,480]
[1,] 0.547218 0.548869 0.551996 0.553321 0.555042 0.555832 0.557305 0.558450
0.560670 0.563294 0.564757 0.566377 0.567528 0.569510 0.571344 0.574585
[2,] 0.505686 0.506972 0.509900 0.511818 0.513014 0.513799 0.515344 0.516876
0.517642 0.518352 0.520778 0.522311 0.523933 0.524709 0.525759 0.527158
[3,] 0.511372 0.512683 0.515708 0.516307 0.517050 0.518546 0.519420 0.520452
0.521417 0.521631 0.524745 0.525998 0.526647 0.529501 0.531341 0.533112
[4,] 0.516584 0.517922 0.518490 0.519850 0.520899 0.526034 0.528726 0.530430
0.531113 0.532471 0.533899 0.534678 0.538583 0.540378 0.543302 0.544032
 [,481] [,482] [,483] [,484] [,485] [,486] [,487] [,488]
[,489] [,490] [,491] [,492] [,493] [,494] [,495] [,496]
[1,] 0.575076 0.577113 0.578482 0.580355 0.582507 0.584815 0.585398 0.587748
0.587909 0.590478 0.592518 0.592661 0.595354 0.596360 0.598536 0.600580
[2,] 0.527910 0.530553 0.531752 0.533528 0.534141 0.537263 0.539833 0.540225
0.541756 0.545440 0.547855 0.549963 0.551611 0.552356 0.554031 0.555890
[3,] 0.534582 0.537039 0.539332 0.542860 0.543120 0.545822 0.547732 0.549686
0.551273 0.551729 0.553794 0.554142 0.555779 0.557604 0.559051 0.562330
[4,] 0.546182 0.546351 0.548783 0.551301 0.552333 0.554072 0.554974 0.557075
0.559065 0.559990 0.562318 0.567140 0.569902 0.570720 0.572723 0.573869
 [,497] [,498] [,499] [,500] [,501] [,502] [,503] [,504]
[,505] [,506] [,507] [,508] [,509] [,510] [,511] [,512]
[1,] 0.601185 0.602735 0.604810 0.607240 0.608325 0.609013 0.612813 0.614310
0.615357 0.618541 0.619343 0.620918 0.622423 0.623709 0.624589 0.626076
[2,] 0.556116 0.558146 0.559739 0.561629 0.562770 0.565060 0.565507 0.570247
0.571432 0.572630 0.574813 0.575342 0.578702 0.579455 0.580651 0.583454
[3,] 0.563071 0.564107 0.564736 0.566084 0.566696 0.568163 0.569176 0.570617
0.571914 0.575859 0.577625 0.577784 0.580365 0.581927 0.583576 0.584529
[4,] 0.574886 0.576027 0.577378 0.581173 0.583134 0.584679 0.586163 0.586878
0.587750 0.588870 0.589738 0.591676 0.593293 0.594230 0.595332 0.596780
 [,513] [,514] [,515] [,516] [,517] [,518] [,519] [,520]
[,521] [,522] [,523] [,524] [,525] [,526] [,527] [,528]

[1,] 0.627334 0.628328 0.629796 0.631220 0.634488 0.635631 0.636798 0.638162
0.639627 0.640766 0.642927 0.643833 0.645501 0.648013 0.648715 0.649481
[2,] 0.584648 0.585815 0.587798 0.592355 0.592653 0.596617 0.596744 0.598804
0.603507 0.605427 0.606898 0.609523 0.610057 0.612193 0.614296 0.617887
[3,] 0.585017 0.587776 0.589154 0.590746 0.592389 0.595548 0.596338 0.599821
0.600980 0.601096 0.602625 0.603375 0.606990 0.607475 0.609621 0.611442
[4,] 0.598219 0.598557 0.599956 0.601326 0.601776 0.603700 0.604904 0.605966
0.607741 0.609236 0.609548 0.611926 0.615933 0.617648 0.618830 0.621058
[,529] [,530] [,531] [,532] [,533] [,534] [,535] [,536]
[,537] [,538] [,539] [,540] [,541] [,542] [,543] [,544]
[1,] 0.651786 0.652946 0.654339 0.654668 0.657458 0.657882 0.659404 0.660617
0.664358 0.667034 0.668107 0.671355 0.674493 0.675333 0.676224 0.678571
[2,] 0.621520 0.623917 0.625137 0.626205 0.628332 0.628400 0.630308 0.632491
0.633025 0.633921 0.635735 0.639446 0.640043 0.642980 0.644966 0.647956
[3,] 0.612890 0.617731 0.618305 0.618553 0.618943 0.620397 0.620720 0.623315
0.623798 0.624035 0.626785 0.627627 0.628911 0.632285 0.635071 0.636082
[4,] 0.623583 0.624336 0.625490 0.626809 0.629047 0.632968 0.633912 0.634343
0.638656 0.640860 0.641573 0.643113 0.646856 0.648697 0.650903 0.652333
[,545] [,546] [,547] [,548] [,549] [,550] [,551] [,552]
[,553] [,554] [,555] [,556] [,557] [,558] [,559] [,560]
[1,] 0.681792 0.684435 0.685430 0.687259 0.690255 0.691159 0.693109 0.694854
0.696944 0.698373 0.700206 0.702660 0.705096 0.705291 0.708021 0.710781
[2,] 0.648577 0.649477 0.651515 0.653111 0.656071 0.656894 0.659365 0.663189
0.665217 0.666623 0.669289 0.669762 0.672479 0.674880 0.676944 0.679414
[3,] 0.636224 0.637459 0.638828 0.639805 0.641650 0.644306 0.646722 0.649702
0.652713 0.655230 0.657596 0.661034 0.662318 0.662715 0.664086 0.664897
[4,] 0.655756 0.656281 0.659317 0.663076 0.664208 0.666256 0.668256 0.671047
0.672474 0.674029 0.676503 0.677891 0.679791 0.681540 0.684816 0.685321
[,561] [,562] [,563] [,564] [,565] [,566] [,567] [,568]
[,569] [,570] [,571] [,572] [,573] [,574] [,575] [,576]
[1,] 0.712855 0.717434 0.718966 0.719750 0.723165 0.727352 0.729003 0.732058
0.737449 0.741185 0.744359 0.745745 0.747595 0.749144 0.750966 0.752525
[2,] 0.681282 0.681904 0.684798 0.685980 0.689591 0.691037 0.693182 0.697373
0.698071 0.699494 0.702862 0.703923 0.704856 0.706655 0.706697 0.707954
[3,] 0.668315 0.671278 0.674607 0.678342 0.679803 0.680805 0.683244 0.684874
0.685899 0.689136 0.691763 0.694144 0.696391 0.698258 0.702324 0.703717
[4,] 0.687154 0.689835 0.690435 0.694315 0.695395 0.696880 0.699850 0.702236
0.704654 0.705302 0.705436 0.707017 0.707346 0.709402 0.711567 0.715658
[,577] [,578] [,579] [,580] [,581] [,582] [,583] [,584]
[,585] [,586] [,587] [,588] [,589] [,590] [,591] [,592]
[1,] 0.757198 0.758665 0.759529 0.760869 0.763598 0.768215 0.770165 0.770617
0.773309 0.774600 0.778186 0.779099 0.781835 0.786531 0.787700 0.790667
[2,] 0.711924 0.714761 0.715587 0.717435 0.719878 0.720878 0.724751 0.725830
0.727677 0.729245 0.729344 0.731218 0.734908 0.736969 0.737756 0.738094
[3,] 0.704626 0.707377 0.710227 0.710893 0.713219 0.714680 0.717966 0.719948
0.723443 0.723852 0.725039 0.726845 0.732265 0.732573 0.734177 0.735824
[4,] 0.717722 0.719427 0.721789 0.723008 0.724539 0.726315 0.728606 0.730614
0.731681 0.733761 0.735280 0.738260 0.739713 0.741616 0.744167 0.744528
[,593] [,594] [,595] [,596] [,597] [,598] [,599] [,600]
[,601] [,602] [,603] [,604] [,605] [,606] [,607] [,608]
[1,] 0.792368 0.792861 0.797933 0.798528 0.801538 0.804851 0.807286 0.809118
0.810230 0.812724 0.813543 0.816719 0.819035 0.820596 0.821643 0.823855
[2,] 0.743453 0.743973 0.744901 0.748392 0.750067 0.751847 0.756006 0.760325
0.761082 0.761923 0.764722 0.768709 0.771545 0.773013 0.774768 0.775700

[3,] 0.738428 0.740455 0.742039 0.743508 0.746256 0.747817 0.748434 0.752744
0.754940 0.756071 0.761093 0.764714 0.765773 0.766880 0.767781 0.771750
[4,] 0.747584 0.749259 0.750636 0.755716 0.759895 0.760815 0.763816 0.765821
0.767818 0.768666 0.769108 0.773778 0.777861 0.783514 0.784686 0.784798
[,609] [,610] [,611] [,612] [,613] [,614] [,615] [,616]
[,617] [,618] [,619] [,620] [,621] [,622] [,623] [,624]
[1,] 0.829610 0.830580 0.831485 0.832468 0.835832 0.839833 0.840452 0.842180
0.846366 0.855127 0.856327 0.857866 0.858499 0.863435 0.868642 0.870927
[2,] 0.777808 0.778261 0.783424 0.785937 0.786582 0.788288 0.790181 0.793600
0.795279 0.798507 0.799866 0.801599 0.804741 0.807831 0.810871 0.811100
[3,] 0.772675 0.777523 0.778624 0.781282 0.782126 0.783528 0.785949 0.790642
0.792980 0.793986 0.795354 0.796308 0.798013 0.800752 0.801362 0.805404
[4,] 0.785991 0.790130 0.791407 0.793911 0.797723 0.801444 0.805451 0.807698
0.810700 0.812705 0.813352 0.815151 0.819557 0.822214 0.822727 0.825523
[,625] [,626] [,627] [,628] [,629] [,630] [,631] [,632]
[,633] [,634] [,635] [,636] [,637] [,638] [,639] [,640]
[1,] 0.871964 0.872560 0.874650 0.878776 0.882641 0.884008 0.887426 0.890982
0.892251 0.894825 0.897463 0.898371 0.901144 0.902442 0.903576 0.906129
[2,] 0.813121 0.815353 0.818715 0.819303 0.823183 0.825066 0.828419 0.829893
0.833358 0.836108 0.838034 0.840504 0.843801 0.845703 0.848710 0.850535
[3,] 0.809645 0.811475 0.813455 0.814374 0.815885 0.817793 0.818860 0.824604
0.825375 0.826009 0.830799 0.833235 0.834287 0.835739 0.838450 0.840991
[4,] 0.829694 0.830933 0.834906 0.835250 0.837050 0.840428 0.842802 0.844851
0.849038 0.850226 0.855433 0.857791 0.859320 0.860534 0.862261 0.866567
[,641] [,642] [,643] [,644] [,645] [,646] [,647] [,648]
[,649] [,650] [,651] [,652] [,653] [,654] [,655] [,656]
[1,] 0.907877 0.910938 0.913361 0.918944 0.921058 0.922688 0.929453 0.933952
0.937323 0.938175 0.940362 0.941186 0.943467 0.946780 0.948517 0.950696
[2,] 0.852089 0.854317 0.855011 0.858231 0.859831 0.864225 0.866790 0.868945
0.871366 0.876563 0.879342 0.882323 0.882747 0.885236 0.888154 0.889795
[3,] 0.842522 0.843863 0.844182 0.848870 0.850178 0.854300 0.855411 0.859090
0.860660 0.864511 0.868355 0.871209 0.874447 0.878127 0.878432 0.880739
[4,] 0.868425 0.869500 0.872551 0.876490 0.877908 0.882260 0.884500 0.885619
0.887420 0.887852 0.890606 0.892584 0.894718 0.897167 0.897774 0.899467
[,657] [,658] [,659] [,660] [,661] [,662] [,663] [,664]
[,665] [,666] [,667] [,668] [,669] [,670] [,671] [,672]
[1,] 0.952651 0.959918 0.962365 0.966206 0.969673 0.971317 0.973645 0.979114
0.981198 0.983909 0.986958 0.988383 0.989988 0.994526 0.995748 0.997639
[2,] 0.891505 0.891715 0.894500 0.896226 0.899838 0.900693 0.902739 0.904099
0.904432 0.909101 0.911725 0.913955 0.919700 0.923744 0.926053 0.933199
[3,] 0.881939 0.884772 0.889544 0.890364 0.891751 0.897888 0.899451 0.900443
0.901349 0.901604 0.908332 0.914748 0.917303 0.917899 0.921344 0.924604
[4,] 0.902067 0.904863 0.909900 0.913475 0.916439 0.919402 0.926998 0.927095
0.930435 0.933114 0.934875 0.939588 0.939836 0.950313 0.950812 0.951332
[,673] [,674] [,675] [,676] [,677] [,678] [,679] [,680]
[,681] [,682] [,683] [,684] [,685] [,686] [,687] [,688]
[1,] 1.000747 1.004707 1.005088 1.006570 1.015114 1.016663 1.020368 1.022493
1.025949 1.027277 1.031857 1.032792 1.038213 1.040251 1.041358 1.042887
[2,] 0.934069 0.935303 0.937480 0.943699 0.945018 0.947084 0.949565 0.952368
0.953518 0.954513 0.955858 0.959773 0.964745 0.966107 0.969660 0.972509
[3,] 0.927781 0.929468 0.930877 0.932453 0.934075 0.936267 0.938734 0.941626
0.944110 0.949027 0.949925 0.954194 0.957127 0.957776 0.964962 0.966462
[4,] 0.954812 0.957316 0.961137 0.964233 0.964406 0.966324 0.968862 0.973649
0.977685 0.979524 0.982418 0.985023 0.987110 0.990095 0.991424 0.993288

```
       [,689]   [,690]   [,691]   [,692]   [,693]   [,694]   [,695]   [,696]
[,697]   [,698]   [,699]   [,700]   [,701]   [,702]   [,703]   [,704]
[1,] 1.044585 1.045682 1.051047 1.052323 1.056106 1.060172 1.060941 1.063407
1.066786 1.070975 1.071921 1.074517 1.079782 1.081124 1.084019 1.086935
[2,] 0.979379 0.985563 0.986763 0.992192 0.994922 0.997143 0.998667 1.002073
1.002923 1.004152 1.007208 1.009456 1.017077 1.019467 1.024518 1.029816
[3,] 0.970695 0.974004 0.979617 0.984141 0.987569 0.988841 0.990056 0.992799
0.996241 0.997414 1.002193 1.003644 1.004718 1.012856 1.016545 1.019056
[4,] 0.995128 0.998436 1.002617 1.004384 1.006530 1.006835 1.012786 1.019692
1.020638 1.023573 1.025152 1.027677 1.029622 1.033505 1.036364 1.038214
       [,705]   [,706]   [,707]   [,708]   [,709]   [,710]   [,711]   [,712]
[,713]   [,714]   [,715]   [,716]   [,717]   [,718]   [,719]   [,720]
[1,] 1.088116 1.090803 1.094719 1.096478 1.098042 1.102812 1.106033 1.108217
1.109308 1.112901 1.114504 1.119037 1.123390 1.128199 1.130106 1.133276
[2,] 1.035519 1.040451 1.046491 1.050393 1.052859 1.058157 1.060794 1.063378
1.067961 1.068763 1.070156 1.071336 1.072550 1.078014 1.080154 1.083811
[3,] 1.020978 1.022569 1.024477 1.027155 1.030253 1.030347 1.034637 1.037285
1.038414 1.043203 1.043978 1.049068 1.050351 1.051214 1.053689 1.056857
[4,] 1.039384 1.040946 1.043539 1.045202 1.047000 1.050787 1.053225 1.060266
1.064712 1.066255 1.072373 1.074472 1.078080 1.083202 1.089731 1.091485
       [,721]   [,722]   [,723]   [,724]   [,725]   [,726]   [,727]   [,728]
[,729]   [,730]   [,731]   [,732]   [,733]   [,734]   [,735]   [,736]
[1,] 1.133979 1.138382 1.144529 1.149840 1.150991 1.151937 1.154686 1.156207
1.159132 1.166390 1.169756 1.172577 1.173356 1.175487 1.178307 1.181122
[2,] 1.086527 1.089109 1.091302 1.100459 1.104214 1.105201 1.111789 1.114597
1.116104 1.119447 1.121032 1.123005 1.130778 1.133643 1.135231 1.140037
[3,] 1.058379 1.059994 1.060500 1.063750 1.066207 1.073103 1.078200 1.083375
1.092390 1.094403 1.095260 1.100474 1.105533 1.107446 1.111361 1.116255
[4,] 1.092522 1.093358 1.101017 1.101395 1.109162 1.110677 1.110952 1.113954
1.115621 1.121695 1.124862 1.125317 1.129993 1.134030 1.135589 1.144277
       [,737]   [,738]   [,739]   [,740]   [,741]   [,742]   [,743]   [,744]
[,745]   [,746]   [,747]   [,748]   [,749]   [,750]   [,751]   [,752]
[1,] 1.184490 1.188447 1.192882 1.193785 1.204896 1.207446 1.210360 1.220810
1.222988 1.225753 1.229155 1.233283 1.234847 1.243251 1.243545 1.247506
[2,] 1.152558 1.153993 1.156003 1.159968 1.166207 1.167453 1.175909 1.178081
1.179552 1.181093 1.184995 1.190362 1.194086 1.196316 1.198444 1.203854
[3,] 1.120988 1.125176 1.128336 1.134669 1.136615 1.140524 1.141995 1.146327
1.146885 1.150027 1.152826 1.156295 1.159959 1.163051 1.164504 1.166195
[4,] 1.147602 1.149333 1.151259 1.157202 1.161522 1.163834 1.165029 1.168434
1.169435 1.170082 1.171224 1.176192 1.176407 1.180510 1.181252 1.190243
       [,753]   [,754]   [,755]   [,756]   [,757]   [,758]   [,759]   [,760]
[,761]   [,762]   [,763]   [,764]   [,765]   [,766]   [,767]   [,768]
[1,] 1.249296 1.254698 1.256663 1.261170 1.264382 1.268072 1.269358 1.272286
1.280727 1.289523 1.293834 1.294604 1.300468 1.305049 1.307861 1.310964
[2,] 1.208339 1.213271 1.215774 1.222399 1.226300 1.229454 1.231287 1.236066
1.238559 1.241223 1.249423 1.252622 1.253940 1.256423 1.258089 1.258929
[3,] 1.167516 1.170275 1.172607 1.175727 1.181375 1.184548 1.186533 1.187318
1.189206 1.197287 1.199613 1.201619 1.204449 1.208907 1.216196 1.227821
[4,] 1.195975 1.199336 1.202062 1.203640 1.206467 1.207701 1.210848 1.213001
1.216026 1.219518 1.221059 1.224072 1.224939 1.231292 1.235143 1.238497
       [,769]   [,770]   [,771]   [,772]   [,773]   [,774]   [,775]   [,776]
[,777]   [,778]   [,779]   [,780]   [,781]   [,782]   [,783]   [,784]
[1,] 1.312957 1.322660 1.324618 1.325104 1.327871 1.329686 1.335078 1.337073
1.339982 1.342033 1.348601 1.351119 1.354870 1.359072 1.361521 1.372982
```

[2,] 1.261237 1.261950 1.265769 1.267024 1.277416 1.279580 1.279605 1.281348
1.286303 1.289431 1.296013 1.297212 1.298469 1.301085 1.304512 1.305568
[3,] 1.229394 1.231581 1.232912 1.234543 1.237219 1.240444 1.240694 1.245404
1.247647 1.251608 1.257639 1.262763 1.265913 1.273259 1.277931 1.280824
[4,] 1.240644 1.242016 1.245130 1.249477 1.254994 1.260913 1.262523 1.264722
1.273374 1.278384 1.279027 1.284415 1.285867 1.287511 1.290281 1.291910
[,785] [,786] [,787] [,788] [,789] [,790] [,791] [,792]
[,793] [,794] [,795] [,796] [,797] [,798] [,799] [,800]
[1,] 1.380551 1.381523 1.384197 1.384542 1.385417 1.386719 1.393377 1.397790
1.400819 1.405863 1.409685 1.413762 1.418898 1.419846 1.424084 1.431620
[2,] 1.308108 1.312614 1.313931 1.314006 1.318215 1.319460 1.321755 1.324552
1.326776 1.330513 1.335138 1.336878 1.341968 1.345963 1.353590 1.359363
[3,] 1.282078 1.284268 1.287599 1.290222 1.292685 1.293046 1.294739 1.296805
1.297626 1.305127 1.310177 1.310640 1.314151 1.315090 1.315857 1.328201
[4,] 1.294499 1.299674 1.301998 1.303964 1.310255 1.311198 1.313833 1.318247
1.320486 1.327785 1.330997 1.337634 1.342029 1.347780 1.353705 1.356622
[,801] [,802] [,803] [,804] [,805] [,806] [,807] [,808]
[,809] [,810] [,811] [,812] [,813] [,814] [,815] [,816]
[1,] 1.437848 1.440078 1.443609 1.451068 1.458897 1.464120 1.465552 1.469817
1.472193 1.478885 1.483214 1.486822 1.489719 1.496146 1.500875 1.502495
[2,] 1.363843 1.370284 1.372514 1.374298 1.379482 1.384165 1.390686 1.395048
1.396470 1.401785 1.402884 1.408060 1.413058 1.416649 1.418368 1.425317
[3,] 1.332710 1.337727 1.338381 1.340918 1.343838 1.351021 1.357711 1.360547
1.363508 1.363865 1.365037 1.371552 1.375048 1.375512 1.376603 1.378989
[4,] 1.360058 1.366558 1.371285 1.376566 1.380043 1.385747 1.387039 1.387939
1.388814 1.389600 1.395367 1.401610 1.409816 1.414610 1.416361 1.417593
[,817] [,818] [,819] [,820] [,821] [,822] [,823] [,824]
[,825] [,826] [,827] [,828] [,829] [,830] [,831] [,832]
[1,] 1.504770 1.509501 1.511766 1.515004 1.516395 1.520821 1.523214 1.524719
1.527812 1.531067 1.534590 1.538252 1.544043 1.551124 1.551847 1.554811
[2,] 1.427699 1.429999 1.430692 1.442157 1.443992 1.444622 1.446815 1.448538
1.449988 1.458007 1.467992 1.471120 1.472321 1.476487 1.479776 1.485969
[3,] 1.379661 1.383106 1.387421 1.390698 1.391521 1.398748 1.403758 1.407401
1.409110 1.413280 1.414831 1.426242 1.428227 1.430833 1.436633 1.442826
[4,] 1.421465 1.425898 1.428827 1.435492 1.437035 1.438308 1.439013 1.444145
1.451421 1.454095 1.461396 1.464886 1.468559 1.476189 1.478156 1.478941
[,833] [,834] [,835] [,836] [,837] [,838] [,839] [,840]
[,841] [,842] [,843] [,844] [,845] [,846] [,847] [,848]
[1,] 1.559397 1.562380 1.566122 1.568937 1.572086 1.576723 1.584791 1.589197
1.595493 1.598685 1.603472 1.606258 1.617748 1.621850 1.634248 1.634679
[2,] 1.488539 1.494027 1.505006 1.513510 1.514068 1.515854 1.517938 1.521198
1.523820 1.532062 1.532518 1.533474 1.547662 1.549989 1.552919 1.555937
[3,] 1.444459 1.446971 1.451516 1.454664 1.457834 1.464794 1.474872 1.475999
1.479677 1.481361 1.488514 1.491583 1.498629 1.502685 1.510866 1.521570
[4,] 1.485913 1.492035 1.506507 1.510415 1.512644 1.515788 1.518257 1.524225
1.526816 1.531872 1.542777 1.549838 1.551506 1.555134 1.566291 1.572928
[,849] [,850] [,851] [,852] [,853] [,854] [,855] [,856]
[,857] [,858] [,859] [,860] [,861] [,862] [,863] [,864]
[1,] 1.639045 1.643158 1.647622 1.656985 1.667770 1.672104 1.676625 1.680964
1.690803 1.693841 1.698934 1.703692 1.705468 1.712768 1.716169 1.722486
[2,] 1.557347 1.565907 1.574306 1.578884 1.582085 1.587608 1.590300 1.595878
1.600449 1.603007 1.606141 1.608883 1.611824 1.616064 1.618584 1.623911
[3,] 1.525676 1.526455 1.530854 1.543870 1.544994 1.553474 1.561156 1.564059
1.565560 1.573271 1.574962 1.576666 1.579678 1.594898 1.597724 1.601496

[4,] 1.577547 1.581281 1.582528 1.584268 1.586711 1.592614 1.596295 1.607301
1.618666 1.621985 1.625306 1.625525 1.628862 1.634386 1.637922 1.644299
[,865] [,866] [,867] [,868] [,869] [,870] [,871] [,872]
[,873] [,874] [,875] [,876] [,877] [,878] [,879] [,880]
[1,] 1.723594 1.732489 1.735841 1.736059 1.741213 1.752872 1.756048 1.759661
1.766353 1.772263 1.774465 1.776832 1.789583 1.794928 1.796082 1.800445
[2,] 1.632968 1.638414 1.644249 1.649046 1.653734 1.663943 1.666526 1.676980
1.678955 1.681575 1.697461 1.698375 1.705018 1.705745 1.714736 1.718383
[3,] 1.606556 1.611891 1.617431 1.620772 1.629469 1.639163 1.641189 1.645172
1.646868 1.653842 1.655365 1.657095 1.657485 1.662076 1.664791 1.665584
[4,] 1.645239 1.647871 1.654491 1.657377 1.666123 1.670445 1.682374 1.693635
1.696896 1.701735 1.703309 1.706994 1.722416 1.731847 1.737588 1.738761
[,881] [,882] [,883] [,884] [,885] [,886] [,887] [,888]
[,889] [,890] [,891] [,892] [,893] [,894] [,895] [,896]
[1,] 1.803395 1.805223 1.811399 1.826817 1.827510 1.834334 1.840409 1.842667
1.849879 1.860624 1.862584 1.862619 1.866430 1.879655 1.880523 1.887549
[2,] 1.723277 1.735377 1.745033 1.757343 1.760176 1.772932 1.778187 1.781144
1.800789 1.818266 1.822096 1.825694 1.830515 1.833496 1.834630 1.838422
[3,] 1.676553 1.679606 1.693100 1.701828 1.721931 1.733560 1.736286 1.740508
1.745947 1.748515 1.754967 1.759765 1.761245 1.767269 1.768977 1.772530
[4,] 1.742373 1.747286 1.753525 1.756497 1.761958 1.763425 1.771655 1.778843
1.783486 1.791287 1.795538 1.802563 1.803624 1.813621 1.814468 1.818102
[,897] [,898] [,899] [,900] [,901] [,902] [,903] [,904]
[,905] [,906] [,907] [,908] [,909] [,910] [,911] [,912]
[1,] 1.898568 1.901192 1.904929 1.909751 1.925343 1.946135 1.956420 1.961453
1.964707 1.967194 1.973154 1.973789 1.983671 1.993272 1.996596 2.008133
[2,] 1.842073 1.843049 1.843337 1.853364 1.856145 1.875730 1.881263 1.888370
1.893289 1.895924 1.900012 1.909081 1.911853 1.914122 1.919065 1.921997
[3,] 1.776990 1.783126 1.790402 1.794830 1.796402 1.803799 1.811244 1.819265
1.828443 1.832250 1.836098 1.844833 1.860582 1.871217 1.875750 1.886842
[4,] 1.823312 1.834335 1.840474 1.842468 1.849558 1.858493 1.861191 1.863227
1.868697 1.881756 1.884432 1.890040 1.903943 1.911497 1.921420 1.925274
[,913] [,914] [,915] [,916] [,917] [,918] [,919] [,920]
[,921] [,922] [,923] [,924] [,925] [,926] [,927] [,928]
[1,] 2.014712 2.016969 2.017118 2.031504 2.040268 2.042872 2.043928 2.054603
2.068381 2.070640 2.073802 2.093376 2.097840 2.099249 2.105737 2.108155
[2,] 1.931093 1.942602 1.950968 1.956349 1.959174 1.968488 1.969773 1.978433
1.982857 1.993869 2.004507 2.025924 2.037339 2.047151 2.051243 2.068510
[3,] 1.898426 1.903270 1.918011 1.921399 1.927378 1.937983 1.942929 1.948152
1.955822 1.969473 1.983694 1.986176 1.989917 1.997863 2.013017 2.015204
[4,] 1.928010 1.930718 1.934108 1.936940 1.941827 1.945241 1.955870 1.961915
1.975029 1.982250 2.009142 2.010701 2.020022 2.026077 2.033083 2.036352
[,929] [,930] [,931] [,932] [,933] [,934] [,935] [,936]
[,937] [,938] [,939] [,940] [,941] [,942] [,943] [,944]
[1,] 2.119826 2.134125 2.145073 2.155668 2.160051 2.162974 2.165540 2.169438
2.174715 2.194218 2.207526 2.209707 2.217385 2.228625 2.237691 2.242940
[2,] 2.070928 2.075509 2.077946 2.081393 2.087901 2.093884 2.097144 2.106769
2.122587 2.133437 2.140956 2.157558 2.158234 2.164675 2.170645 2.178028
[3,] 2.028000 2.034654 2.039112 2.042577 2.049795 2.059206 2.071785 2.073466
2.083821 2.092033 2.097844 2.110562 2.111243 2.122717 2.127095 2.132096
[4,] 2.039321 2.047036 2.048909 2.067702 2.069836 2.075684 2.079747 2.105971
2.109912 2.110945 2.122262 2.130672 2.141293 2.146873 2.157151 2.160730
[,945] [,946] [,947] [,948] [,949] [,950] [,951] [,952]
[,953] [,954] [,955] [,956] [,957] [,958] [,959] [,960]

[1,] 2.247288 2.257394 2.261812 2.281048 2.281356 2.282695 2.311298 2.319645
2.323806 2.327779 2.336695 2.337913 2.344776 2.347147 2.364811 2.372198
[2,] 2.185179 2.194715 2.203571 2.205021 2.221056 2.231302 2.244407 2.247612
2.258771 2.262377 2.274008 2.278507 2.280395 2.285364 2.300184 2.307668
[3,] 2.142689 2.153507 2.165806 2.169435 2.174973 2.183834 2.196705 2.200100
2.207244 2.207943 2.220419 2.232962 2.258351 2.275065 2.286762 2.293667
[4,] 2.163933 2.168098 2.176267 2.182958 2.195092 2.195414 2.198358 2.200521
2.224267 2.231854 2.239745 2.255130 2.262959 2.265679 2.284240 2.292416
[,961] [,962] [,963] [,964] [,965] [,966] [,967] [,968]
[,969] [,970] [,971] [,972] [,973] [,974] [,975] [,976]
[1,] 2.380991 2.401949 2.418106 2.423760 2.438781 2.439643 2.462238 2.466208
2.484114 2.484237 2.510393 2.542248 2.551474 2.557444 2.558219 2.559320
[2,] 2.313469 2.345025 2.363835 2.367010 2.369306 2.371103 2.400380 2.401986
2.407909 2.409123 2.420633 2.442099 2.443604 2.470187 2.473979 2.480599
[3,] 2.299407 2.304417 2.304810 2.327074 2.330157 2.345656 2.349769 2.358120
2.369211 2.385533 2.390036 2.407047 2.410248 2.410930 2.414291 2.445532
[4,] 2.305321 2.337373 2.345770 2.349887 2.356226 2.385689 2.397963 2.407593
2.410020 2.421133 2.425473 2.440062 2.444083 2.449169 2.455258 2.462065
[,977] [,978] [,979] [,980] [,981] [,982] [,983] [,984]
[,985] [,986] [,987] [,988] [,989] [,990] [,991] [,992]
[1,] 2.563184 2.570447 2.577142 2.578918 2.581359 2.597751 2.614705 2.622403
2.644534 2.651434 2.667918 2.671503 2.675091 2.687730 2.705681 2.720167
[2,] 2.501685 2.503184 2.525576 2.531927 2.541989 2.552170 2.572481 2.582387
2.593413 2.606452 2.606863 2.614904 2.619064 2.654228 2.657368 2.665325
[3,] 2.452160 2.466091 2.469643 2.474333 2.485490 2.498583 2.513561 2.524448
2.541259 2.556127 2.578691 2.584752 2.590280 2.610238 2.625323 2.634743
[4,] 2.481738 2.490541 2.503118 2.510397 2.514260 2.517954 2.531489 2.541997
2.548748 2.560295 2.575660 2.589190 2.595146 2.626798 2.648466 2.652256
[,993] [,994] [,995] [,996] [,997] [,998] [,999] [,1000]
[,1001] [,1002] [,1003] [,1004] [,1005] [,1006] [,1007] [,1008]
[1,] 2.767769 2.775074 2.790925 2.792246 2.804803 2.813039 2.821365 2.862497
2.869724 2.894895 2.925984 2.950159 2.961255 2.975964 2.977000 3.005047
[2,] 2.666256 2.673112 2.709639 2.748281 2.751245 2.759403 2.759533 2.766801
2.780298 2.807681 2.816625 2.820950 2.834740 2.844265 2.861570 2.891365
[3,] 2.641790 2.671612 2.679597 2.683354 2.697585 2.700754 2.702755 2.713944
2.734555 2.748824 2.752965 2.759207 2.783404 2.794324 2.805092 2.812228
[4,] 2.670076 2.674664 2.680029 2.703468 2.715577 2.717309 2.728909 2.730455
2.764052 2.778954 2.797164 2.828091 2.856692 2.865340 2.870428 2.883999
[,1009] [,1010] [,1011] [,1012] [,1013] [,1014] [,1015] [,1016]
[,1017] [,1018] [,1019] [,1020] [,1021] [,1022] [,1023] [,1024]
[1,] 3.028786 3.033409 3.073375 3.077466 3.083454 3.093600 3.102071 3.120562
3.152774 3.160497 3.164728 3.171571 3.195509 3.206460 3.212671 3.263559
[2,] 2.906799 2.915033 2.927615 2.958873 2.963133 2.972805 2.985110 2.996023
3.013745 3.031885 3.051946 3.055460 3.099080 3.104380 3.117796 3.186126
[3,] 2.842487 2.879367 2.879906 2.947417 2.959126 2.961164 2.963785 3.007626
3.031336 3.039370 3.045898 3.050543 3.065628 3.071741 3.091835 3.131945
[4,] 2.886209 2.912073 2.916844 2.937331 2.942614 2.950601 2.957313 2.969350
2.971194 3.015785 3.037761 3.038953 3.063564 3.076983 3.088632 3.091533
[,1025] [,1026] [,1027] [,1028] [,1029] [,1030] [,1031] [,1032]
[,1033] [,1034] [,1035] [,1036] [,1037] [,1038] [,1039] [,1040]
[1,] 3.273322 3.274731 3.283270 3.293941 3.297246 3.337590 3.369691 3.396619
3.424684 3.443157 3.501806 3.506114 3.508895 3.513207 3.534158 3.561838
[2,] 3.193406 3.201367 3.227073 3.227621 3.256712 3.259953 3.282770 3.297415
3.301332 3.307605 3.335214 3.419102 3.451524 3.469805 3.480589 3.493880

[3,] 3.167362 3.174748 3.231361 3.243103 3.244429 3.282863 3.313487 3.316642
3.360416 3.377370 3.388711 3.405721 3.418254 3.461053 3.464307 3.473196
[4,] 3.133887 3.147061 3.169845 3.195781 3.245752 3.248168 3.250604 3.251704
3.278165 3.297107 3.310076 3.374653 3.407917 3.414766 3.451105 3.472221
[,1041] [,1042] [,1043] [,1044] [,1045] [,1046] [,1047] [,1048]
[,1049] [,1050] [,1051] [,1052] [,1053] [,1054] [,1055] [,1056]
[1,] 3.593167 3.598027 3.609450 3.635484 3.660475 3.665893 3.675018 3.691656
3.757020 3.778879 3.798320 3.830172 3.876176 3.879068 3.882926 3.920390
[2,] 3.527823 3.528247 3.536504 3.578760 3.608149 3.626008 3.666790 3.669008
3.674419 3.756062 3.831243 3.835704 3.838567 3.878219 3.935562 3.941718
[3,] 3.528225 3.561635 3.577696 3.627206 3.632635 3.707521 3.723808 3.729416
3.766647 3.796199 3.811724 3.817811 3.831004 3.893798 3.903368 3.910177
[4,] 3.578368 3.580116 3.584163 3.585664 3.589120 3.651065 3.671919 3.683896
3.712805 3.729374 3.732756 3.741928 3.814315 3.821526 3.823315 3.831350
[,1057] [,1058] [,1059] [,1060] [,1061] [,1062] [,1063] [,1064]
[,1065] [,1066] [,1067] [,1068] [,1069] [,1070] [,1071] [,1072]
[1,] 3.955644 3.962149 3.977090 4.107287 4.121704 4.134202 4.156603 4.200823
4.208807 4.293391 4.343838 4.418727 4.453515 4.469351 4.557397 4.587394
[2,] 4.031295 4.048014 4.173562 4.208804 4.373547 4.398648 4.475588 4.503483
4.503939 4.509127 4.515605 4.519945 4.596510 4.694299 4.694717 4.701719
[3,] 3.955408 4.002831 4.024336 4.090199 4.174100 4.188110 4.229968 4.301416
4.370263 4.448249 4.448888 4.489380 4.509117 4.573074 4.631831 4.667253
[4,] 3.837941 3.913371 3.915736 3.996332 4.060422 4.063897 4.072509 4.125158
4.127581 4.130775 4.141523 4.190454 4.235083 4.286511 4.309863 4.465758
[,1073] [,1074] [,1075] [,1076] [,1077] [,1078] [,1079] [,1080]
[,1081] [,1082] [,1083] [,1084] [,1085] [,1086] [,1087] [,1088]
[1,] 4.655840 4.715070 4.964339 5.091839 5.110773 5.374099 5.390804 5.424436
5.476810 5.497804 5.532953 5.672594 5.681136 5.854882 6.088399 6.246119
[2,] 4.861429 4.911563 5.082563 5.213718 5.371194 5.377822 5.510802 5.547538
5.565095 5.614945 5.669307 5.823483 5.828548 5.893095 6.001749 6.016262
[3,] 4.709250 4.712235 4.778412 4.801292 4.913808 4.939086 5.092572 5.179479
5.193685 5.292683 5.315480 5.326660 5.603091 5.629499 5.646551 5.926286
[4,] 4.553858 4.630532 4.761352 4.826019 4.827847 4.832207 4.907645 5.063358
5.092675 5.129558 5.135286 5.231234 5.329446 5.408253 5.714595 6.098093
[,1089] [,1090] [,1091] [,1092] [,1093] [,1094] [,1095] [,1096]
[,1097] [,1098] [,1099] [,1100] [,1101] [,1102] [,1103] [,1104]
[1,] 6.419527 6.477648 6.645738 6.688029 6.691488 6.940196 6.991746 7.137576
7.510130 7.526881 7.573702 7.735502 7.742791 7.820110 8.427038 8.685282
[2,] 6.097126 6.626564 6.679343 6.906376 7.132438 7.209681 7.427801 7.549435
7.586627 7.758160 7.910948 7.988159 8.007590 8.052098 8.108070 8.408891
[3,] 6.024090 6.357933 6.489985 6.493645 6.588968 6.614640 6.738340 6.786860
6.795009 7.228057 7.464797 7.535624 7.723636 7.749610 7.840289 7.844412
[4,] 6.198020 6.251382 6.273764 6.524179 6.524279 6.598573 7.262207 7.337279
7.355242 7.460747 7.626085 7.675600 7.858999 7.868136 8.034713 8.088677
[,1105] [,1106] [,1107] [,1108] [,1109] [,1110] [,1111] [,1112]
[,1113] [,1114] [,1115] [,1116] [,1117] [,1118] [,1119] [,1120]
[1,] 8.719657 9.045413 9.209628 9.227136 9.238416 9.614638 9.681154 9.734347
9.744338 9.837638 9.912884 9.933479 10.028429 10.080323 10.128555 10.170994
[2,] 8.527113 8.575351 8.606436 8.649892 8.799283 8.823829 8.850515 8.978387
8.983746 9.175449 9.258692 9.442143 9.581283 9.646919 9.671434 9.724040
[3,] 7.971710 8.235056 8.501842 8.704423 8.748301 8.871735 8.934066 9.048965
9.061045 9.176801 9.179204 9.247578 9.264420 9.435368 9.474415 9.523799
[4,] 8.130655 8.646838 8.704771 8.735817 8.867078 8.897237 8.951884 9.079582
9.133587 9.270208 9.533316 9.549717 9.591173 9.671700 9.698678 9.757981

```
      [,1121]   [,1122]   [,1123]   [,1124]   [,1125]   [,1126]   [,1127]
[,1128]  [,1129]  [,1130]  [,1131]  [,1132]  [,1133]  [,1134]  [,1135]  [,1136]
[1,] 10.229270 10.426601 10.490232 10.521509 10.616884 10.739450 11.028005
11.07866 11.16846 11.24377 11.33041 11.36616 11.39400 11.40020 11.46070 11.56217
[2,]  9.975688 10.266675 10.298938 10.357734 10.405234 10.491577 10.539829
10.62932 10.74291 10.74780 10.83018 10.95283 11.02940 11.04978 11.12726 11.18047
[3,]  9.539504  9.577675  9.588479  9.631621  9.780816  9.831636  9.892903
10.16048 10.32995 10.36571 10.40685 10.45200 10.57916 10.57991 10.81786 10.86153
[4,]  9.782712  9.791288  9.966414  9.975157 10.002245 10.090044 10.218412
10.35121 10.35254 10.43037 10.53841 10.60268 10.61028 10.67433 10.71625 10.79376
      [,1137]  [,1138]  [,1139]  [,1140]  [,1141]  [,1142]  [,1143]  [,1144]
[,1145]  [,1146]  [,1147]  [,1148]  [,1149]  [,1150]  [,1151]  [,1152]
[1,] 11.56603 11.65767 11.69085 11.87388 11.90982 11.99358 12.03341 12.08632
12.09549 12.20776 12.26660 12.29586 12.33682 12.35006 12.46022 12.56340
[2,] 11.19684 11.40591 11.48819 11.52758 11.61292 11.65494 11.68698 11.72955
11.74559 11.76741 11.77942 11.84391 11.84519 11.85834 12.07331 12.08987
[3,] 10.88424 10.90742 11.03810 11.05603 11.10373 11.12434 11.22695 11.27150
11.33232 11.34621 11.40315 11.50441 11.56387 11.56715 11.59878 11.75174
[4,] 10.85204 10.97788 11.00331 11.13230 11.14693 11.20610 11.27960 11.32351
11.33102 11.35592 11.41357 11.44631 11.48837 11.58264 11.71907 11.73653
      [,1153]  [,1154]  [,1155]  [,1156]  [,1157]  [,1158]  [,1159]  [,1160]
[,1161]  [,1162]  [,1163]  [,1164]  [,1165]  [,1166]  [,1167]  [,1168]
[1,] 12.57996 12.61861 12.64425 12.66382 12.70716 12.73807 12.73852 12.74896
12.78745 12.83810 12.89820 12.90399 12.91324 12.94556 13.03910 13.08632
[2,] 12.09542 12.17763 12.19618 12.24985 12.26083 12.27004 12.39893 12.55597
12.60239 12.60530 12.63975 12.69117 12.74394 12.88564 12.90887 12.92817
[3,] 11.75756 11.78890 11.85517 11.91344 11.94830 11.95677 11.97090 11.97224
12.04718 12.08251 12.08722 12.11953 12.13979 12.15839 12.23877 12.37080
[4,] 11.74483 11.81495 11.95596 12.01583 12.05138 12.18748 12.20181 12.36564
12.40470 12.46864 12.64995 12.66856 12.75033 12.75397 12.78948 12.79580
      [,1169]  [,1170]  [,1171]  [,1172]  [,1173]  [,1174]  [,1175]  [,1176]
[,1177]  [,1178]  [,1179]  [,1180]  [,1181]  [,1182]  [,1183]  [,1184]
[1,] 13.12091 13.16249 13.19440 13.29037 13.39774 13.45613 13.54362 13.58119
13.62662 13.70515 13.75128 13.75779 13.82930 13.85975 13.89736 13.99894
[2,] 12.96745 12.98204 13.03600 13.04959 13.09630 13.19741 13.22025 13.22784
13.22935 13.25075 13.26737 13.26832 13.27035 13.28318 13.35155 13.52200
[3,] 12.41971 12.46545 12.52934 12.59066 12.68044 12.72383 12.73905 12.79503
12.81927 12.82170 12.87269 12.94600 12.95110 13.04970 13.05557 13.16659
[4,] 12.80966 12.82008 12.84280 12.89032 12.94779 12.96521 12.98650 12.99729
13.04006 13.10252 13.11679 13.16032 13.21815 13.28305 13.29102 13.33466
      [,1185]  [,1186]  [,1187]  [,1188]  [,1189]  [,1190]  [,1191]  [,1192]
[,1193]  [,1194]  [,1195]  [,1196]  [,1197]  [,1198]  [,1199]  [,1200]
[1,] 14.00230 14.02634 14.03524 14.06762 14.09726 14.12521 14.17182 14.20961
14.23363 14.24326 14.26844 14.28348 14.29595 14.30234 14.31701 14.35536
[2,] 13.53506 13.56114 13.57852 13.61653 13.62333 13.63857 13.72771 13.75312
13.75842 13.78783 13.79374 13.87262 13.93013 14.03108 14.05879 14.05945
[3,] 13.32925 13.35711 13.36559 13.40359 13.40906 13.50348 13.52253 13.62465
13.66237 13.68466 13.73706 13.75749 13.81310 13.81650 13.83094 13.83886
[4,] 13.51098 13.54076 13.56411 13.57781 13.63489 13.68446 13.69008 13.71470
13.73255 13.80790 13.85943 13.96627 13.97015 14.01387 14.01403 14.01688
      [,1201]  [,1202]  [,1203]  [,1204]  [,1205]  [,1206]  [,1207]  [,1208]
[,1209]  [,1210]  [,1211]  [,1212]  [,1213]  [,1214]  [,1215]  [,1216]
[1,] 14.35860 14.47838 14.48509 14.50875 14.52185 14.56372 14.59545 14.69383
14.74154 14.82006 14.84058 14.90985 14.95507 14.99857 14.99930 15.00525
```

[2,] 14.07165 14.13323 14.14597 14.19075 14.20732 14.22117 14.22480 14.22601
14.23976 14.32471 14.34149 14.36149 14.38350 14.39847 14.40431 14.40666
[3,] 13.87357 13.90623 13.92404 14.09348 14.13606 14.14533 14.22976 14.24203
14.26809 14.27359 14.27874 14.31456 14.31627 14.33445 14.42360 14.43022
[4,] 14.04115 14.05121 14.05197 14.05407 14.07490 14.11507 14.13126 14.14633
14.17620 14.19421 14.23411 14.23411 14.24927 14.27161 14.28483 14.36233
[,1217] [,1218] [,1219] [,1220] [,1221] [,1222] [,1223] [,1224]
[,1225] [,1226] [,1227] [,1228] [,1229] [,1230] [,1231] [,1232]
[1,] 15.07353 15.08579 15.11301 15.11957 15.14833 15.16416 15.16469 15.18323
15.19940 15.29915 15.31829 15.34802 15.35603 15.41288 15.44330 15.44698
[2,] 14.42271 14.46474 14.47260 14.47285 14.56390 14.62038 14.64369 14.67164
14.68222 14.68613 14.69295 14.75526 14.76212 14.78762 14.79883 14.87782
[3,] 14.47108 14.48358 14.48954 14.50267 14.50673 14.52746 14.53542 14.54663
14.57099 14.60227 14.60782 14.61330 14.62670 14.64385 14.65168 14.67387
[4,] 14.40573 14.46483 14.49492 14.51636 14.54542 14.55518 14.62313 14.63487
14.65658 14.69474 14.70777 14.72780 14.76317 14.78381 14.80142 14.80559
[,1233] [,1234] [,1235] [,1236] [,1237] [,1238] [,1239] [,1240]
[,1241] [,1242] [,1243] [,1244] [,1245] [,1246] [,1247] [,1248]
[1,] 15.45945 15.46389 15.51009 15.52160 15.52761 15.61920 15.65377 15.66331
15.66434 15.67035 15.68630 15.70321 15.70928 15.73855 15.78133 15.78907
[2,] 14.88474 14.90766 14.91442 14.94313 14.98838 14.99504 15.00271 15.01687
15.01947 15.07401 15.10853 15.12009 15.21354 15.22051 15.26517 15.26870
[3,] 14.71353 14.79198 14.79490 14.80446 14.82164 14.85389 14.85523 14.86140
14.86269 14.88327 14.96678 14.99225 14.99634 15.01248 15.02072 15.05404
[4,] 14.82217 14.84949 14.85402 14.91080 14.92677 14.94429 14.96097 15.01366
15.03130 15.04997 15.10010 15.12027 15.15962 15.16812 15.17452 15.17617
[,1249] [,1250] [,1251] [,1252] [,1253] [,1254] [,1255] [,1256]
[,1257] [,1258] [,1259] [,1260] [,1261] [,1262] [,1263] [,1264]
[1,] 15.83359 15.94067 15.97780 15.97898 15.99677 16.03738 16.09674 16.09918
16.10713 16.14897 16.14966 16.15107 16.15356 16.22511 16.24532 16.24935
[2,] 15.27098 15.27983 15.30156 15.34300 15.34308 15.35530 15.40109 15.40776
15.46022 15.47333 15.47530 15.50652 15.57324 15.61032 15.62534 15.65593
[3,] 15.07913 15.14674 15.19601 15.19761 15.27594 15.36703 15.38316 15.42901
15.45315 15.45780 15.46875 15.47756 15.52782 15.58230 15.62719 15.64179
[4,] 15.19996 15.21478 15.22235 15.23763 15.24220 15.25446 15.29399 15.37670
15.39150 15.40179 15.42214 15.49194 15.49272 15.50810 15.51771 15.52912
[,1265] [,1266] [,1267] [,1268] [,1269] [,1270] [,1271] [,1272]
[,1273] [,1274] [,1275] [,1276] [,1277] [,1278] [,1279] [,1280]
[1,] 16.25256 16.33766 16.36753 16.38956 16.39230 16.40250 16.42721 16.46805
16.49738 16.50864 16.53786 16.55398 16.58386 16.59875 16.62396 16.70825
[2,] 15.70492 15.71762 15.73113 15.79744 15.83307 15.83675 15.85250 15.85755
15.87983 15.92375 15.93612 15.95536 16.01437 16.06330 16.08275 16.10478
[3,] 15.65525 15.72652 15.74041 15.74594 15.82856 15.90370 15.93318 15.95411
15.95808 15.97472 15.98071 15.99681 16.00262 16.00818 16.02983 16.07456
[4,] 15.53452 15.55950 15.56089 15.59365 15.60639 15.61082 15.63102 15.64460
15.66825 15.71228 15.72324 15.73419 15.76352 15.80664 15.84185 15.88597
[,1281] [,1282] [,1283] [,1284] [,1285] [,1286] [,1287] [,1288]
[,1289] [,1290] [,1291] [,1292] [,1293] [,1294] [,1295] [,1296]
[1,] 16.71784 16.73814 16.78511 16.82035 16.84481 16.84839 16.89113 16.91265
16.95222 16.95614 16.96402 16.99361 16.99792 17.09957 17.10151 17.12994
[2,] 16.11016 16.13165 16.25421 16.25552 16.26347 16.26477 16.28805 16.30361
16.35100 16.35377 16.36659 16.43248 16.44616 16.45399 16.49073 16.50740
[3,] 16.11855 16.14241 16.21514 16.23884 16.25114 16.28294 16.28678 16.29617
16.29971 16.32614 16.32845 16.36188 16.38224 16.40938 16.46511 16.50275

[4,] 15.95543 15.98430 16.04533 16.06006 16.15522 16.16308 16.19433 16.20063
16.20338 16.24071 16.26839 16.29418 16.31954 16.32974 16.34435 16.37116
[,1297] [,1298] [,1299] [,1300] [,1301] [,1302] [,1303] [,1304]
[,1305] [,1306] [,1307] [,1308] [,1309] [,1310] [,1311] [,1312]
[1,] 17.15226 17.16249 17.16666 17.21084 17.21418 17.21653 17.23053 17.32881
17.34991 17.39977 17.45108 17.45834 17.47190 17.50706 17.50944 17.56891
[2,] 16.52475 16.55308 16.59292 16.59956 16.61214 16.61557 16.67763 16.68381
16.69946 16.73240 16.74435 16.74772 16.75455 16.76235 16.76795 16.78863
[3,] 16.51752 16.53454 16.57028 16.57111 16.57904 16.58847 16.60029 16.61298
16.73607 16.78052 16.78598 16.83863 16.87551 16.88544 16.91433 16.95422
[4,] 16.40883 16.45203 16.45295 16.46291 16.49420 16.51852 16.51952 16.53332
16.60580 16.66170 16.67718 16.69302 16.70479 16.73686 16.74218 16.74740
[,1313] [,1314] [,1315] [,1316] [,1317] [,1318] [,1319] [,1320]
[,1321] [,1322] [,1323] [,1324] [,1325] [,1326] [,1327] [,1328]
[1,] 17.58085 17.66776 17.71161 17.80106 17.80594 17.84589 17.85678 17.86755
17.87180 17.87611 17.91814 18.00376 18.03683 18.04771 18.05135 18.05864
[2,] 16.82450 16.90365 16.96358 16.99427 17.01462 17.02606 17.03211 17.05201
17.05796 17.07623 17.08439 17.10532 17.12225 17.13535 17.14227 17.19694
[3,] 16.97214 16.98066 17.01683 17.05839 17.09222 17.09567 17.10091 17.10362
17.14687 17.15027 17.15693 17.16552 17.17635 17.21915 17.22645 17.26051
[4,] 16.74833 16.81154 16.84917 16.88098 16.89255 16.92350 16.92417 16.94930
16.96332 16.99698 17.00510 17.01319 17.02755 17.06118 17.07955 17.10061
[,1329] [,1330] [,1331] [,1332] [,1333] [,1334] [,1335] [,1336]
[,1337] [,1338] [,1339] [,1340] [,1341] [,1342] [,1343] [,1344]
[1,] 18.09176 18.09789 18.10323 18.10941 18.11904 18.13762 18.19264 18.20416
18.26704 18.32309 18.33934 18.38514 18.42110 18.45273 18.46198 18.48241
[2,] 17.24851 17.26788 17.29315 17.33953 17.36774 17.39241 17.43389 17.44664
17.44846 17.45010 17.49252 17.52201 17.56225 17.57883 17.62734 17.64068
[3,] 17.27137 17.30806 17.31053 17.31224 17.37681 17.39534 17.46798 17.48345
17.50414 17.53713 17.63550 17.65681 17.66450 17.67622 17.68574 17.70746
[4,] 17.12536 17.14985 17.16204 17.19373 17.21449 17.35114 17.36944 17.37515
17.37657 17.43480 17.50040 17.51890 17.52632 17.52742 17.59522 17.65154
[,1345] [,1346] [,1347] [,1348] [,1349] [,1350] [,1351] [,1352]
[,1353] [,1354] [,1355] [,1356] [,1357] [,1358] [,1359] [,1360]
[1,] 18.50592 18.52830 18.57138 18.58926 18.66846 18.68413 18.69764 18.71492
18.73189 18.78279 18.81943 18.83076 18.93942 18.95794 18.98218 19.03910
[2,] 17.67623 17.68704 17.70580 17.73003 17.77321 17.77578 17.84721 17.87046
17.89462 17.95209 17.97609 18.02637 18.03134 18.08987 18.10931 18.19631
[3,] 17.71398 17.71514 17.75339 17.77789 17.78808 17.80324 17.86049 17.87207
17.90900 17.93437 18.00906 18.01815 18.03101 18.08387 18.11427 18.14989
[4,] 17.68346 17.70536 17.75873 17.79588 17.80365 17.83535 17.84612 17.90755
17.92830 17.93295 17.95389 17.97956 18.02905 18.04023 18.05980 18.10172
[,1361] [,1362] [,1363] [,1364] [,1365] [,1366] [,1367] [,1368]
[,1369] [,1370] [,1371] [,1372] [,1373] [,1374] [,1375] [,1376]
[1,] 19.06621 19.10164 19.11208 19.11437 19.16412 19.19567 19.20767 19.24083
19.31872 19.32263 19.34453 19.37773 19.38419 19.49700 19.50515 19.55867
[2,] 18.24299 18.32540 18.37717 18.38660 18.44628 18.51021 18.51291 18.52617
18.53154 18.53378 18.56094 18.60996 18.63866 18.64574 18.64823 18.65277
[3,] 18.18649 18.18674 18.20776 18.23606 18.34214 18.36873 18.40031 18.45087
18.48212 18.48283 18.52052 18.55360 18.56322 18.57632 18.58325 18.58426
[4,] 18.12633 18.19800 18.21773 18.22491 18.23290 18.33704 18.39407 18.41977
18.43184 18.46280 18.48026 18.50672 18.61375 18.62144 18.62876 18.65636
[,1377] [,1378] [,1379] [,1380] [,1381] [,1382] [,1383] [,1384]
[,1385] [,1386] [,1387] [,1388] [,1389] [,1390] [,1391] [,1392]

[1,] 19.57645 19.58363 19.67610 19.82323 19.85847 19.87972 19.88581 19.88917
19.91500 19.92593 19.93407 19.94728 19.95670 20.07830 20.08354 20.09709
[2,] 18.67800 18.68047 18.68174 18.74706 18.75266 18.82446 18.82816 18.85825
18.91806 18.97346 19.00098 19.03844 19.04973 19.06213 19.08477 19.17623
[3,] 18.63057 18.64562 18.67110 18.67866 18.70499 18.74498 18.87315 18.89908
18.91081 18.91812 18.92479 18.94310 18.98756 19.07098 19.08657 19.11645
[4,] 18.67729 18.68336 18.70763 18.72661 18.76366 18.82003 18.85796 18.91017
18.92411 18.93289 18.97685 19.00787 19.01777 19.09880 19.11496 19.15148
[,1393] [,1394] [,1395] [,1396] [,1397] [,1398] [,1399] [,1400]
[,1401] [,1402] [,1403] [,1404] [,1405] [,1406] [,1407] [,1408]
[1,] 20.16849 20.18600 20.19887 20.24855 20.26383 20.33176 20.45294 20.45679
20.47543 20.49817 20.51821 20.53749 20.56407 20.57659 20.62135 20.63052
[2,] 19.20834 19.20843 19.21349 19.22694 19.26317 19.33721 19.34258 19.34272
19.39128 19.44663 19.46078 19.49526 19.49816 19.49899 19.52109 19.60590
[3,] 19.11746 19.12721 19.19312 19.20155 19.21352 19.31380 19.34111 19.35532
19.37017 19.42052 19.43634 19.44778 19.47169 19.48284 19.49090 19.51569
[4,] 19.18415 19.21379 19.30356 19.31149 19.31873 19.31927 19.33194 19.38301
19.41584 19.41939 19.41975 19.43138 19.43412 19.48145 19.48358 19.50968
[,1409] [,1410] [,1411] [,1412] [,1413] [,1414] [,1415] [,1416]
[,1417] [,1418] [,1419] [,1420] [,1421] [,1422] [,1423] [,1424]
[1,] 20.64808 20.68020 20.69602 20.69629 20.71162 20.72328 20.75483 20.82856
20.83581 20.84267 20.88444 20.90086 21.00557 21.01861 21.09750 21.10397
[2,] 19.61796 19.67406 19.70777 19.80069 19.80997 19.83050 19.83293 19.94384
19.97724 20.01973 20.16963 20.19575 20.24767 20.25726 20.29567 20.31084
[3,] 19.54505 19.56732 19.60959 19.65777 19.74832 19.78751 19.81041 19.82070
19.94692 19.94999 19.97098 19.97249 20.04618 20.07792 20.09961 20.10953
[4,] 19.53354 19.58993 19.61037 19.75224 19.78833 19.81874 19.86179 19.92129
19.93009 19.94492 19.97064 20.02836 20.05465 20.11716 20.12344 20.21459
[,1425] [,1426] [,1427] [,1428] [,1429] [,1430] [,1431] [,1432]
[,1433] [,1434] [,1435] [,1436] [,1437] [,1438] [,1439] [,1440]
[1,] 21.13192 21.19252 21.19819 21.24753 21.28599 21.29802 21.31269 21.37518
21.40323 21.42489 21.55471 21.60395 21.66191 21.71825 21.73497 21.74348
[2,] 20.32951 20.34042 20.34117 20.37814 20.42674 20.44256 20.44799 20.55857
20.59933 20.61857 20.62080 20.62263 20.65312 20.67569 20.75083 20.77142
[3,] 20.12453 20.14097 20.15211 20.20485 20.20611 20.25491 20.25826 20.26183
20.31073 20.34454 20.38259 20.41095 20.41205 20.45603 20.48407 20.57797
[4,] 20.22367 20.24385 20.25710 20.26731 20.27963 20.30702 20.35942 20.36758
20.37030 20.47952 20.54473 20.57768 20.65656 20.77014 20.77608 20.79134
[,1441] [,1442] [,1443] [,1444] [,1445] [,1446] [,1447] [,1448]
[,1449] [,1450] [,1451] [,1452] [,1453] [,1454] [,1455] [,1456]
[1,] 21.75307 21.77190 21.81408 21.83406 21.84570 21.85035 21.86058 21.86926
21.87820 21.91444 21.92679 21.97342 22.02309 22.09909 22.11620 22.17701
[2,] 20.79426 20.88845 20.93485 21.03497 21.06848 21.10330 21.17957 21.24912
21.25416 21.28890 21.32155 21.36732 21.37572 21.38698 21.39362 21.43101
[3,] 20.67294 20.67711 20.69954 20.76241 20.88133 20.95417 21.00317 21.06861
21.07402 21.14949 21.16883 21.17032 21.18480 21.24147 21.40057 21.42203
[4,] 20.86807 20.91512 20.92908 20.93644 20.98192 21.00166 21.00334 21.02056
21.02099 21.09270 21.10595 21.11488 21.12868 21.21884 21.25223 21.29791
[,1457] [,1458] [,1459] [,1460] [,1461] [,1462] [,1463] [,1464]
[,1465] [,1466] [,1467] [,1468] [,1469] [,1470] [,1471] [,1472]
[1,] 22.19338 22.20278 22.21203 22.23575 22.30847 22.37021 22.41800 22.44842
22.45117 22.48040 22.54724 22.59132 22.60882 22.62165 22.62313 22.66497
[2,] 21.45720 21.60759 21.64018 21.66231 21.69068 21.69791 21.74023 21.74537
21.76259 21.82485 21.92151 21.98452 21.98765 22.01089 22.08365 22.09840

[3,] 21.50128 21.55852 21.60430 21.68856 21.76089 21.78305 21.81172 21.85430
21.88254 21.88274 21.94586 21.94721 21.96949 22.06760 22.17818 22.20780
[4,] 21.30146 21.35772 21.36110 21.38506 21.43393 21.47169 21.48705 21.52578
21.67889 21.68605 21.79367 21.79909 21.88184 21.89684 21.90489 21.93362
[,1473] [,1474] [,1475] [,1476] [,1477] [,1478] [,1479] [,1480]
[,1481] [,1482] [,1483] [,1484] [,1485] [,1486] [,1487] [,1488]
[1,] 22.67834 22.71465 22.71759 22.74921 22.75391 22.76665 22.82238 22.86242
22.88293 22.90801 22.97208 22.99101 22.99468 23.04881 23.07374 23.08612
[2,] 22.13770 22.15229 22.15804 22.19161 22.20104 22.20847 22.24958 22.28054
22.33794 22.37236 22.37776 22.40645 22.51126 22.52818 22.57642 22.64655
[3,] 22.24669 22.25575 22.28333 22.29255 22.30900 22.31240 22.38984 22.39820
22.40997 22.41399 22.41977 22.42296 22.58740 22.60272 22.68329 22.68747
[4,] 21.94375 21.98330 22.01336 22.33432 22.35278 22.35893 22.40640 22.44767
22.51988 22.59633 22.61639 22.62794 22.63720 22.66430 22.68431 22.69765
[,1489] [,1490] [,1491] [,1492] [,1493] [,1494] [,1495] [,1496]
[,1497] [,1498] [,1499] [,1500] [,1501] [,1502] [,1503] [,1504]
[1,] 23.26281 23.34261 23.44208 23.53917 23.55356 23.58355 23.60690 23.62770
23.71804 23.72828 23.77899 23.79209 23.85511 23.87751 23.94901 23.95562
[2,] 22.65932 22.68642 22.82840 22.91936 22.95693 22.96904 22.98536 23.02514
23.17341 23.18507 23.24675 23.28241 23.34670 23.40240 23.47745 23.48217
[3,] 22.69013 22.76654 22.76841 22.78177 22.80267 22.80441 22.80723 22.82513
22.89288 22.92660 22.93728 22.99153 23.06099 23.12780 23.14321 23.24048
[4,] 22.73253 22.75270 22.75888 22.78851 22.79763 22.80638 22.82432 22.84561
22.95529 22.96752 22.98658 23.04418 23.05117 23.09481 23.10195 23.17273
[,1505] [,1506] [,1507] [,1508] [,1509] [,1510] [,1511] [,1512]
[,1513] [,1514] [,1515] [,1516] [,1517] [,1518] [,1519] [,1520]
[1,] 24.00322 24.05374 24.17120 24.17736 24.28567 24.30473 24.31956 24.33650
24.33970 24.37684 24.45450 24.47675 24.57294 24.69191 24.69201 24.69409
[2,] 23.48411 23.63624 23.72252 23.73241 23.73438 23.76456 23.76586 23.83224
23.84463 23.87999 23.92555 23.97124 24.03226 24.04060 24.04109 24.14943
[3,] 23.30843 23.30983 23.36633 23.65766 23.68042 23.69634 23.77357 23.82395
23.86189 23.87221 23.90529 23.92197 23.93788 24.02477 24.12064 24.15742
[4,] 23.26133 23.59169 23.68210 23.86950 23.88999 23.90627 23.94384 23.94931
23.97892 23.98233 23.99964 24.11287 24.21999 24.27332 24.30718 24.34404
[,1521] [,1522] [,1523] [,1524] [,1525] [,1526] [,1527] [,1528]
[,1529] [,1530] [,1531] [,1532] [,1533] [,1534] [,1535] [,1536]
[1,] 24.73552 24.86397 24.86940 24.87881 24.88118 24.89597 24.96040 24.99182
25.04582 25.07664 25.09376 25.21683 25.24008 25.25923 25.27432 25.27508
[2,] 24.15252 24.17156 24.18326 24.26019 24.34492 24.38640 24.40474 24.45931
24.52481 24.56426 24.58013 24.66101 24.76999 24.80192 24.89199 24.90856
[3,] 24.16025 24.28209 24.28538 24.33709 24.34421 24.35120 24.37381 24.38044
24.47943 24.71982 24.72621 24.80860 24.85348 24.87393 24.89599 25.00948
[4,] 24.44157 24.56336 24.62206 24.62811 24.63828 24.67102 24.70154 24.70956
24.72709 24.77071 24.78081 24.80510 24.83276 24.85143 24.85188 24.92001
[,1537] [,1538] [,1539] [,1540] [,1541] [,1542] [,1543] [,1544]
[,1545] [,1546] [,1547] [,1548] [,1549] [,1550] [,1551] [,1552]
[1,] 25.49628 25.53093 25.58527 25.62871 25.64919 25.69075 25.72214 25.75426
25.92546 25.98142 26.03294 26.34659 26.47021 26.56914 26.57435 26.66211
[2,] 25.09065 25.09692 25.13163 25.16747 25.34288 25.37565 25.45890 25.54042
25.56023 25.58573 25.59131 25.61761 25.83818 25.95376 25.96155 25.99006
[3,] 25.09276 25.11584 25.25557 25.34139 25.39588 25.40705 25.44425 25.60650
25.66472 25.67790 25.72039 25.72444 25.72848 25.74002 25.80557 25.82898
[4,] 24.92493 24.95487 25.03929 25.14702 25.20735 25.26881 25.32848 25.38751
25.49331 25.50392 25.55614 25.60247 25.66527 25.70039 25.70366 25.96289

```
      [,1553]  [,1554]  [,1555]  [,1556]  [,1557]  [,1558]  [,1559]  [,1560]
[,1561]  [,1562]  [,1563]  [,1564]  [,1565]  [,1566]  [,1567]  [,1568]
[1,] 26.68356 26.68485 26.74354 26.79499 26.83248 26.86168 26.98455 27.04257
27.06920 27.12606 27.15289 27.19193 27.19907 27.27507 27.34580 27.39940
[2,] 26.02346 26.22026 26.28420 26.37118 26.39536 26.42431 26.51993 26.54984
26.58260 26.59017 26.61651 26.62905 26.80816 26.89710 27.00788 27.08214
[3,] 25.84190 25.88406 26.19176 26.20246 26.21074 26.30562 26.42685 26.43852
26.52671 26.64472 26.77935 26.78015 27.12516 27.13920 27.29649 27.32519
[4,] 26.03846 26.17917 26.21735 26.24490 26.25741 26.26536 26.52885 26.53167
26.56078 26.58316 26.64921 26.70519 26.81021 26.83916 26.86437 26.98988
      [,1569]  [,1570]  [,1571]  [,1572]  [,1573]  [,1574]  [,1575]  [,1576]
[,1577]  [,1578]  [,1579]  [,1580]  [,1581]  [,1582]  [,1583]  [,1584]
[1,] 27.43377 27.44376 27.48220 27.68661 27.71551 27.72115 27.83480 27.96259
28.00279 28.18516 28.42692 28.64542 28.70143 28.90218 28.92814 28.96130
[2,] 27.15542 27.18795 27.32235 27.46487 27.47408 27.52739 27.59578 27.61820
27.67569 27.70538 28.14675 28.26192 28.29282 28.31247 28.33207 28.35015
[3,] 27.33944 27.42680 27.46524 27.50553 27.56359 27.59342 27.62832 27.80263
27.93502 27.94148 27.96363 27.97509 27.97578 28.17137 28.30903 28.41988
[4,] 27.01878 27.09339 27.11554 27.11721 27.20644 27.31091 27.39522 27.40188
27.40762 27.40929 27.59477 27.62260 27.67191 27.72318 27.80194 28.04332
      [,1585]  [,1586]  [,1587]  [,1588]  [,1589]  [,1590]  [,1591]  [,1592]
[,1593]  [,1594]  [,1595]  [,1596]  [,1597]  [,1598]  [,1599]  [,1600]
[1,] 29.16721 29.22465 29.24927 29.36262 29.47686 29.57946 29.65740 29.71683
29.74143 29.82356 29.92253 29.98661 30.29306 30.40950 30.52306 30.57802
[2,] 28.35892 28.40392 28.69665 28.73672 28.96883 29.19057 29.86495 30.05196
30.06200 30.09626 30.17406 30.20071 30.50903 30.79154 30.91911 31.00718
[3,] 28.43819 28.56268 28.61807 28.67075 28.72134 28.77278 28.82863 29.07500
29.22031 29.25006 29.53394 29.64333 29.69591 29.85152 30.03333 30.13361
[4,] 28.20963 28.21731 28.25699 28.31384 28.36732 28.42970 28.73057 28.94496
29.14491 29.61058 29.63751 29.74845 29.77567 29.87230 29.88117 30.44677
      [,1601]  [,1602]  [,1603]  [,1604]  [,1605]  [,1606]  [,1607]  [,1608]
[,1609]  [,1610]  [,1611]  [,1612]  [,1613]  [,1614]  [,1615]  [,1616]
[1,] 30.64258 31.33167 31.42411 31.42540 31.44618 31.52432 31.62029 31.81152
32.36092 32.61528 32.68368 33.28449 33.49680 33.65115 33.83872 34.75047
[2,] 31.18554 31.33011 31.38138 31.42144 31.44826 31.52339 31.53392 31.90210
31.96240 32.14362 32.88480 33.00581 33.02078 33.16324 33.18044 33.57804
[3,] 30.20241 30.34424 30.93285 31.08813 31.09439 31.56253 31.79440 31.83950
32.31723 32.43049 32.52697 32.58913 32.61509 32.74731 32.93778 33.03155
[4,] 30.69919 30.71828 30.80065 31.10567 31.71450 31.73416 31.74090 32.38628
32.57337 32.62469 32.84147 33.44829 33.49442 33.49458 33.79547 34.17114
      [,1617]  [,1618]  [,1619]  [,1620]  [,1621]  [,1622]  [,1623]  [,1624]
[,1625]  [,1626]  [,1627]  [,1628]  [,1629]  [,1630]  [,1631]  [,1632]
[1,] 34.78396 35.27187 35.82951 35.84268 36.18872 36.69250 36.86700 37.06049
37.08756 37.16773 37.32320 37.36670 37.65296 37.72624 37.99955 38.11475
[2,] 34.09323 34.33632 34.52777 34.81119 34.84858 34.90965 35.40747 35.80302
35.89897 35.95408 36.32079 36.85616 37.64426 37.94378 38.46757 38.66559
[3,] 33.71985 33.93314 33.94114 34.14912 34.67247 35.06287 35.62537 35.72747
36.27121 36.58984 36.68500 36.93084 37.23443 38.28827 38.78507 39.15980
[4,] 34.18019 34.48680 34.94913 35.02950 35.10225 35.21012 35.39019 35.40161
35.63056 35.84048 35.93979 36.09992 36.31262 36.83247 37.30965 38.24818
      [,1633]  [,1634]  [,1635]  [,1636]  [,1637]  [,1638]  [,1639]  [,1640]
[,1641]
[1,] 39.95018 40.17559 40.53910 42.62173 42.83818 43.17693 44.69009 46.56155
46.68403
```

```
[2,] 39.11469 39.40311 40.50702 40.97838 41.64801 44.67057 44.95737 45.30978
45.52053
[3,] 39.32811 41.43319 41.48425 44.03696 44.67561 44.84295 46.62989 47.45336
52.26427
[4,] 38.57350 39.62850 42.31144 42.44367 42.83738 45.86228 46.68057 47.83299
53.95470
```

ENSEMBLE FLUCTUATION DATA (FULL LENGTH MINIMIZED CHARACTERIZED CLASS C)

```
                                [,1]      [,2]      [,3]      [,4]      [,5]
  [,6]      [,7]      [,8]      [,9]     [,10]     [,11]     [,12]
full_length_NAT0_min3_2.pdb 0.3196883 0.2232239 0.1545854 0.1561112 0.1787880
0.1479947 0.1307400 0.1539423 0.1415609 0.1128160 0.1420726 0.1656465
full_length_LJP6_min3_2.pdb 0.3571856 0.2375279 0.1583190 0.1545050 0.1818254
0.1520698 0.1344166 0.1570454 0.1463246 0.1139684 0.1503097 0.1645586
full_length_3WY9_min3_2.pdb 0.3481567 0.2559911 0.1815551 0.1497023 0.1690659
0.1471666 0.1340629 0.1514703 0.1311907 0.1065059 0.1378035 0.1508002
full_length_ZSP9_min3_2.pdb 0.3173784 0.2308521 0.1519285 0.1465663 0.1643808
0.1358226 0.1209944 0.1373037 0.1248568 0.1012153 0.1308126 0.1492657
                               [,13]     [,14]     [,15]     [,16]     [,17]
 [,18]     [,19]     [,20]     [,21]     [,22]     [,23]     [,24]
full_length_NAT0_min3_2.pdb 0.1468837 0.1441886 0.1853621 0.2001398 0.1754705
0.1934311 0.2005835 0.2236689 0.3260988 0.3840125 0.4992762 0.8606003
full_length_LJP6_min3_2.pdb 0.1466754 0.1420203 0.1682390 0.1808545 0.1671676
0.1871562 0.1845880 0.2056680 0.2952795 0.3517449 0.6223474 0.8378657
full_length_3WY9_min3_2.pdb 0.1363147 0.1333722 0.1634204 0.1762523 0.1573457
0.1725799 0.1691923 0.1893345 0.2732467 0.3311142 0.4297773 0.7259664
full_length_ZSP9_min3_2.pdb 0.1351241 0.1354901 0.1738703 0.1810634 0.1584898
0.1729080 0.1693952 0.1912764 0.2745783 0.3255493 0.4252642 0.7583563
                               [,25]     [,26]     [,27]     [,28]     [,29]
 [,30]     [,31]     [,32]     [,33]     [,34]     [,35]     [,36]
full_length_NAT0_min3_2.pdb 0.6993454 0.4713915 0.4338513 0.3395530 0.3956608
0.3505418 0.3492005 0.3905368 0.3290432 0.2794946 0.2656402 0.3193043
full_length_LJP6_min3_2.pdb 0.7120482 0.4092856 0.3469848 0.2858257 0.3544627
0.3048290 0.2970382 0.3305780 0.2885586 0.2461263 0.2513429 0.3042105
full_length_3WY9_min3_2.pdb 0.6669924 0.4196245 0.3455474 0.2733379 0.3165930
0.3013039 0.2986600 0.3310508 0.2842775 0.2331438 0.2352446 0.2808779
full_length_ZSP9_min3_2.pdb 0.6621184 0.4236385 0.3693460 0.3037867 0.3195784
0.2793174 0.3012740 0.3327706 0.2799590 0.2353484 0.2376726 0.2892603
                               [,37]     [,38]     [,39]     [,40]     [,41]
 [,42]     [,43]     [,44]     [,45]     [,46]     [,47]     [,48]
full_length_NAT0_min3_2.pdb 0.3556406 0.2481690 0.2477753 0.3655055 0.4366645
0.4359033 0.4515755 0.3655805 0.3303875 0.2226349 0.2459569 0.2025737
full_length_LJP6_min3_2.pdb 0.3526383 0.2405269 0.2476133 0.3641436 0.4083933
0.4130006 0.4692369 0.3977740 0.3326215 0.2251198 0.2448243 0.1922890
full_length_3WY9_min3_2.pdb 0.3375881 0.2252787 0.2210604 0.3211285 0.3555462
0.3596943 0.3919072 0.3291919 0.2992048 0.2020295 0.2230669 0.1822818
full_length_ZSP9_min3_2.pdb 0.3364455 0.2290780 0.2287595 0.3356412 0.3974273
0.3915119 0.4014096 0.3356316 0.3100502 0.2074522 0.2266238 0.1830055
                               [,49]     [,50]     [,51]     [,52]     [,53]
 [,54]     [,55]     [,56]     [,57]     [,58]     [,59]     [,60]
full_length_NAT0_min3_2.pdb 0.1616169 0.1465335 0.1508275 0.1389680 0.1786573
0.2065200 0.2180453 0.2032642 0.1887584 0.1502672 0.1405319 0.1226331
```

full_length_LJP6_min3_2.pdb 0.1600677 0.1434811 0.1394187 0.1367476 0.1750194
0.2015058 0.2111719 0.1891475 0.1846376 0.1479320 0.1391913 0.1283807
full_length_3WY9_min3_2.pdb 0.1485762 0.1314739 0.1367631 0.1287425 0.1823465
0.1985815 0.1985027 0.1822218 0.1653304 0.1353288 0.1245898 0.1143613
full_length_ZSP9_min3_2.pdb 0.1469989 0.1316041 0.1350062 0.1258677 0.1703367
0.1996043 0.1956918 0.1781692 0.1666738 0.1369491 0.1257081 0.1162998
[,61] [,62] [,63] [,64]
[,65] [,66] [,67] [,68] [,69] [,70] [,71]
full_length_NAT0_min3_2.pdb 0.10008939 0.08762135 0.08840737 0.07828815
0.07668575 0.07762335 0.07890102 0.07166728 0.07218994 0.08890492 0.09253132
full_length_LJP6_min3_2.pdb 0.10206264 0.09937556 0.09497032 0.08286848
0.07871699 0.07893929 0.08090042 0.06975773 0.07592788 0.08700427 0.09116539
full_length_3WY9_min3_2.pdb 0.09476385 0.08617302 0.08584639 0.07673886
0.07657040 0.07598235 0.07877535 0.06940872 0.07150234 0.08508582 0.08721158
full_length_ZSP9_min3_2.pdb 0.09399595 0.08499766 0.08502086 0.07713667
0.07618207 0.07569626 0.07672307 0.06834513 0.07052531 0.08417388 0.08531236
[,72] [,73] [,74] [,75] [,76]
[,77] [,78] [,79] [,80] [,81] [,82] [,83]
full_length_NAT0_min3_2.pdb 0.10003419 0.1090555 0.1284989 0.1537887 0.1745473
0.1646314 0.1816855 0.2270194 0.1834902 0.1706271 0.2286842 0.2665217
full_length_LJP6_min3_2.pdb 0.09910786 0.1063162 0.1218680 0.1496173 0.1721420
0.1532628 0.1659223 0.2104270 0.1638032 0.1628122 0.2190658 0.2373030
full_length_3WY9_min3_2.pdb 0.09665789 0.1027889 0.1185584 0.1444783 0.1658230
0.1601808 0.1648085 0.2029895 0.1850676 0.1603727 0.2111310 0.2639838
full_length_ZSP9_min3_2.pdb 0.09565777 0.1018089 0.1132475 0.1369165 0.1743836
0.1536273 0.1539782 0.1960381 0.1652570 0.1477665 0.1932746 0.2339648
[,84] [,85] [,86] [,87] [,88]
[,89] [,90] [,91] [,92] [,93] [,94] [,95]
full_length_NAT0_min3_2.pdb 0.2378051 0.2604722 0.2335851 0.2043446 0.2350950
0.2028793 0.1542886 0.1663535 0.1893671 0.1524530 0.1284861 0.1479045
full_length_LJP6_min3_2.pdb 0.2120601 0.2259742 0.2072419 0.1898935 0.2489077
0.2071545 0.1601107 0.1740243 0.1895226 0.1614289 0.1410228 0.1569521
full_length_3WY9_min3_2.pdb 0.2330191 0.2390187 0.2037850 0.1816519 0.2197927
0.1882351 0.1481129 0.1578840 0.1771465 0.1472548 0.1241084 0.1384713
full_length_ZSP9_min3_2.pdb 0.2217295 0.2274805 0.1928023 0.1664972 0.2074993
0.1804025 0.1388652 0.1469815 0.1692455 0.1417007 0.1206408 0.1361090
[,96] [,97] [,98] [,99] [,100]
[,101] [,102] [,103] [,104] [,105] [,106] [,107]
full_length_NAT0_min3_2.pdb 0.1613675 0.1246133 0.1276144 0.1679361 0.1550204
0.1398281 0.1757814 0.1877944 0.1763517 0.1961819 0.3863130 0.3994325
full_length_LJP6_min3_2.pdb 0.1698018 0.1337036 0.1389379 0.1835908 0.1594692
0.1505690 0.1832040 0.1912371 0.1783362 0.1891400 0.3536686 0.3056758
full_length_3WY9_min3_2.pdb 0.1514048 0.1175416 0.1217324 0.1594293 0.1434923
0.1324246 0.1631771 0.1703447 0.1647122 0.1852500 0.3661232 0.3940274
full_length_ZSP9_min3_2.pdb 0.1488365 0.1167993 0.1227629 0.1586679 0.1445989
0.1344562 0.1670381 0.1735122 0.1681582 0.1888724 0.3645007 0.4699058
[,108] [,109] [,110] [,111] [,112]
[,113] [,114] [,115] [,116] [,117] [,118] [,119]
full_length_NAT0_min3_2.pdb 0.3454319 0.2609574 0.2323626 0.1736243 0.1752331
0.1647375 0.1767861 0.1873204 0.2268780 0.2761920 0.2519213 0.3475499
full_length_LJP6_min3_2.pdb 0.2601560 0.2224007 0.2113690 0.1654635 0.1625904
0.1572997 0.1626359 0.1785612 0.2130848 0.2517954 0.2568468 0.3346860
full_length_3WY9_min3_2.pdb 0.3179675 0.2534538 0.2232601 0.1595074 0.1561986
0.1506377 0.1550040 0.1685244 0.2014984 0.2382031 0.2396591 0.3062926

full_length_ZSP9_min3_2.pdb 0.3406083 0.2550940 0.2257503 0.1649842 0.1602997
0.1539685 0.1567050 0.1723242 0.2039922 0.2439571 0.2444846 0.3001813
[,120] [,121] [,122] [,123] [,124]
[,125] [,126] [,127] [,128] [,129] [,130] [,131]
full_length_NAT0_min3_2.pdb 0.3296586 0.2688816 0.2943371 0.3835587 0.3605248
0.2858334 0.2876476 0.2848095 0.2779504 0.3192896 0.3386772 0.3693850
full_length_LJP6_min3_2.pdb 0.3316055 0.2585317 0.2719698 0.3692452 0.3314841
0.2738849 0.2717322 0.2512421 0.2509220 0.2712574 0.2930777 0.3312446
full_length_3WY9_min3_2.pdb 0.2984437 0.2411717 0.2676186 0.3595661 0.3480983
0.2708830 0.2598456 0.2449289 0.2468975 0.2732237 0.3189743 0.3646283
full_length_ZSP9_min3_2.pdb 0.3006035 0.2369477 0.2540461 0.3418798 0.3137615
0.2513104 0.2500119 0.2350519 0.2364522 0.2762180 0.2873823 0.3305879
[,132] [,133] [,134] [,135] [,136]
[,137] [,138] [,139] [,140] [,141] [,142] [,143]
full_length_NAT0_min3_2.pdb 0.5365120 0.4342103 0.4417758 0.3005118 0.2968430
0.2318007 0.2253858 0.2130404 0.2074657 0.2565186 0.3194285 0.4596938
full_length_LJP6_min3_2.pdb 0.4998995 0.4202946 0.5006126 0.2973789 0.2767567
0.2197573 0.2003761 0.1870158 0.1868150 0.2180212 0.2767060 0.4569681
full_length_3WY9_min3_2.pdb 0.5650614 0.4068225 0.4306841 0.2754609 0.2622780
0.2043561 0.1948757 0.1871036 0.1841208 0.2410577 0.3054881 0.4471092
full_length_ZSP9_min3_2.pdb 0.5360737 0.4022353 0.4481232 0.2763177 0.2698092
0.2114109 0.2051898 0.2002165 0.1909468 0.2485114 0.3166520 0.4421914
[,144] [,145] [,146] [,147] [,148]
[,149] [,150] [,151] [,152] [,153] [,154] [,155]
full_length_NAT0_min3_2.pdb 0.2434871 0.1952443 0.1418791 0.1121879 0.09977407
0.09637321 0.09294732 0.07728541 0.07197192 0.08280979 0.08341047 0.07067412
full_length_LJP6_min3_2.pdb 0.2688042 0.1772320 0.1299703 0.1096802 0.09480796
0.09398721 0.09008488 0.07574676 0.07305077 0.08407578 0.08381655 0.07156825
full_length_3WY9_min3_2.pdb 0.2224096 0.1774204 0.1365966 0.1098087 0.09349746
0.09262989 0.08936759 0.07383564 0.07122935 0.08335552 0.08135068 0.06882460
full_length_ZSP9_min3_2.pdb 0.2340860 0.1834637 0.1374051 0.1161181 0.09723754
0.09563267 0.09329681 0.07678869 0.07208001 0.08403600 0.08253856 0.06872620
[,156] [,157] [,158] [,159]
[,160] [,161] [,162] [,163] [,164] [,165] [,166] [,167]
full_length_NAT0_min3_2.pdb 0.06861091 0.08757696 0.08597975 0.09019373
0.1064090 0.1230109 0.1489518 0.1766416 0.1949334 0.2337121 0.4179950 0.4652846
full_length_LJP6_min3_2.pdb 0.06881692 0.08981210 0.08915911 0.08819829
0.1055561 0.1262479 0.1525866 0.1646772 0.1942502 0.2387112 0.4544579 0.4541354
full_length_3WY9_min3_2.pdb 0.06717630 0.08524517 0.08404811 0.08599619
0.1024609 0.1173650 0.1428997 0.1643837 0.1776778 0.2274040 0.4010092 0.4138021
full_length_ZSP9_min3_2.pdb 0.06738536 0.08656654 0.08457569 0.08374694
0.1003444 0.1174854 0.1406880 0.1566831 0.1747754 0.2167255 0.4103178 0.4211202
[,168] [,169] [,170] [,171] [,172]
[,173] [,174] [,175] [,176] [,177] [,178] [,179]
full_length_NAT0_min3_2.pdb 0.3823836 0.3098074 0.3650247 0.2768742 0.1873769
0.2262145 0.2235673 0.1463380 0.1482533 0.2021974 0.1668029 0.1214262
full_length_LJP6_min3_2.pdb 0.3894454 0.3310796 0.3782941 0.2880926 0.1920419
0.2443459 0.2323802 0.1496085 0.1501455 0.1972697 0.1655308 0.1203039
full_length_3WY9_min3_2.pdb 0.3515268 0.2885648 0.3483479 0.2647482 0.1809137
0.2192568 0.2212300 0.1451896 0.1446563 0.2051002 0.1683046 0.1226216
full_length_ZSP9_min3_2.pdb 0.3388700 0.2948298 0.3490219 0.2615722 0.1799238
0.2083950 0.2277627 0.1487052 0.1449087 0.2025665 0.1702642 0.1241679
[,180] [,181] [,182] [,183] [,184]
[,185] [,186] [,187] [,188] [,189] [,190] [,191]

full_length_NAT0_min3_2.pdb 0.1613385 0.1686201 0.1241703 0.1278884 0.1855128
0.1692012 0.1310254 0.1919351 0.2535562 0.2495806 0.2183766 0.2194902
full_length_LJP6_min3_2.pdb 0.1549673 0.1625702 0.1209949 0.1232709 0.1723888
0.1546452 0.1232095 0.1876444 0.2321808 0.2185696 0.2175892 0.1931683
full_length_3WY9_min3_2.pdb 0.1554896 0.1648571 0.1219626 0.1227076 0.1746150
0.1623066 0.1286877 0.1803350 0.2578450 0.2505629 0.1991026 0.2197051
full_length_ZSP9_min3_2.pdb 0.1609376 0.1730492 0.1273400 0.1279283 0.1798727
0.1679570 0.1319670 0.1915247 0.2568105 0.2544830 0.2047017 0.2194046
[,192] [,193] [,194] [,195] [,196]
[,197] [,198] [,199] [,200] [,201] [,202] [,203]
full_length_NAT0_min3_2.pdb 0.1866673 0.2898891 0.2449882 0.2280059 0.3140019
0.2985051 0.2692667 0.2981483 0.3748672 0.3671069 0.5177073 0.4062048
full_length_LJP6_min3_2.pdb 0.1584260 0.1539385 0.1692688 0.2555603 0.2338326
0.2379171 0.2847308 0.2346199 0.1600115 0.1982907 0.2595587 0.2218552
full_length_3WY9_min3_2.pdb 0.1890023 0.3004550 0.2282653 0.1953788 0.2654711
0.2468130 0.2010208 0.2189422 0.3741722 0.7372896 0.3784034 0.2449740
full_length_ZSP9_min3_2.pdb 0.1823256 0.2742170 0.2288988 0.1976182 0.2489923
0.2488763 0.1980770 0.2150917 0.3595893 0.5482589 0.3415142 0.2387206
[,204] [,205] [,206] [,207] [,208]
[,209] [,210] [,211] [,212] [,213] [,214] [,215]
full_length_NAT0_min3_2.pdb 0.5324502 1.2196275 0.6557838 0.2731366 0.1765072
0.1217177 0.1439180 0.1137319 0.08755393 0.1042233 0.1117117 0.09003226
full_length_LJP6_min3_2.pdb 0.1971927 0.1879839 0.2771696 0.1890907 0.1679328
0.1419249 0.1486731 0.1099980 0.09124985 0.1181058 0.1147320 0.09587453
full_length_3WY9_min3_2.pdb 0.2168315 0.2418716 0.2453572 0.2588817 0.1552630
0.1219351 0.1361838 0.1022757 0.08490684 0.1037010 0.1029952 0.08532272
full_length_ZSP9_min3_2.pdb 0.2111103 0.2317161 0.2103421 0.2301465 0.1501846
0.1255726 0.1355395 0.1008240 0.08602133 0.1151304 0.1041380 0.08427442
[,216] [,217] [,218] [,219] [,220]
[,221] [,222] [,223] [,224] [,225] [,226] [,227]
full_length_NAT0_min3_2.pdb 0.08221563 0.1135033 0.1200940 0.10637324 0.1245592
0.1605191 0.1827010 0.1747440 0.2619650 0.3294792 0.3605301 0.3542147
full_length_LJP6_min3_2.pdb 0.08496510 0.1144763 0.1171977 0.10398461 0.1210511
0.1496623 0.1732651 0.1773387 0.2472985 0.2968717 0.3350471 0.3369360
full_length_3WY9_min3_2.pdb 0.07844964 0.1058930 0.1133051 0.10255013 0.1176050
0.1530351 0.1775933 0.1680014 0.2486031 0.3016814 0.3630223 0.3523868
full_length_ZSP9_min3_2.pdb 0.07824628 0.1053350 0.1077170 0.09674758 0.1167827
0.1424803 0.1705193 0.1684431 0.2510264 0.2856304 0.3315243 0.3342762
[,228] [,229] [,230] [,231] [,232]
[,233] [,234] [,235] [,236] [,237] [,238] [,239]
full_length_NAT0_min3_2.pdb 0.3524559 0.1970606 0.2232085 0.2821584 0.2162002
0.1765977 0.2563741 0.2993337 0.2563249 0.3002670 0.4593023 0.4723820
full_length_LJP6_min3_2.pdb 0.3207277 0.1952504 0.2093143 0.2704191 0.2403830
0.1839404 0.2551470 0.3048373 0.2232343 0.2493905 0.3675359 0.3153598
full_length_3WY9_min3_2.pdb 0.3219024 0.1818950 0.2083776 0.2612864 0.2054655
0.1659958 0.2390230 0.2724812 0.2229895 0.2471556 0.3686815 0.3139440
full_length_ZSP9_min3_2.pdb 0.3140090 0.1862862 0.2016315 0.2587164 0.2170695
0.1659492 0.2420414 0.2725689 0.2210173 0.2431803 0.3676126 0.3100403
[,240] [,241] [,242] [,243] [,244]
[,245] [,246] [,247] [,248] [,249] [,250] [,251]
full_length_NAT0_min3_2.pdb 0.5612077 1.2258366 1.3773290 0.6650069 0.3468417
0.3188941 0.2268168 0.2367599 0.2357999 0.1517210 0.10369672 0.1345207
full_length_LJP6_min3_2.pdb 0.2956225 0.3187921 0.3689689 0.5045111 1.5161053
1.8127747 0.9012147 0.4273299 0.3148820 0.2307318 0.14824945 0.1731237

full_length_3WY9_min3_2.pdb 0.2869361 0.3970212 0.4532171 0.5698855 0.5743212
0.2890708 0.3153485 0.2578360 0.2180856 0.1287890 0.10683224 0.1487535
full_length_ZSP9_min3_2.pdb 0.2825840 0.3548302 0.4567147 0.5667292 0.5615838
0.2780376 0.3118348 0.2503711 0.2202812 0.1427123 0.09956139 0.1326110
[,252] [,253] [,254] [,255] [,256]
[,257] [,258] [,259] [,260] [,261] [,262] [,263]
full_length_NAT0_min3_2.pdb 0.1476506 0.1640734 0.1343415 0.1029649 0.1235516
0.1195623 0.1122042 0.1182411 0.1342413 0.1364007 0.1508958 0.1728415
full_length_LJP6_min3_2.pdb 0.1829138 0.1772459 0.1185531 0.1163591 0.1225878
0.1270772 0.1260202 0.1236475 0.1342695 0.1368968 0.1478027 0.1770050
full_length_3WY9_min3_2.pdb 0.1538580 0.1656143 0.1270510 0.1128041 0.1195777
0.1175910 0.1096504 0.1097301 0.1201842 0.1263109 0.1474149 0.1628836
full_length_ZSP9_min3_2.pdb 0.1470890 0.1487556 0.1263044 0.1077575 0.1180836
0.1139918 0.1112065 0.1107185 0.1227339 0.1264356 0.1471203 0.1676359
[,264] [,265] [,266] [,267] [,268]
[,269] [,270] [,271] [,272] [,273] [,274] [,275]
full_length_NAT0_min3_2.pdb 0.1885753 0.2157460 0.2491608 0.2896825 0.3443700
0.4330131 0.5437863 0.9017508 0.9826421 0.7547387 0.6438823 0.5493778
full_length_LJP6_min3_2.pdb 0.1996284 0.2113933 0.2351902 0.2843113 0.4239725
0.5226386 0.4485517 0.4439024 0.7824616 1.0940147 0.6878617 0.7150412
full_length_3WY9_min3_2.pdb 0.1730064 0.2420182 0.3064377 0.3783683 0.3880348
0.6845393 0.7858304 0.9994164 1.8247172 2.6247137 1.4339856 0.7621819
full_length_ZSP9_min3_2.pdb 0.1729088 0.2152514 0.2640781 0.3193233 0.3615502
0.5954095 0.6750753 1.0340120 1.5037288 2.0943029 1.5963914 1.2874334
[,276] [,277] [,278] [,279] [,280]
[,281] [,282] [,283] [,284] [,285] [,286] [,287]
full_length_NAT0_min3_2.pdb 0.5210536 0.5663250 0.3887627 0.3105386 0.3630185
0.3182907 0.2123821 0.2285531 0.2690830 0.2181671 0.1741657 0.2161404
full_length_LJP6_min3_2.pdb 0.6522853 0.5534438 0.4028002 0.3276883 0.3527281
0.3191552 0.2108196 0.2236189 0.2693408 0.2136496 0.1702703 0.2144649
full_length_3WY9_min3_2.pdb 0.4425266 0.3655690 0.2542011 0.2824706 0.3699680
0.2942341 0.1908303 0.1975243 0.2604497 0.2167220 0.1633129 0.1966876
full_length_ZSP9_min3_2.pdb 0.8314671 0.4512584 0.2949528 0.2417765 0.3327327
0.2856033 0.1783226 0.1807767 0.2351668 0.2010423 0.1568704 0.1820802
[,288] [,289] [,290] [,291] [,292]
[,293] [,294] [,295] [,296] [,297] [,298] [,299]
full_length_NAT0_min3_2.pdb 0.2209540 0.1658051 0.1736202 0.2114580 0.1906856
0.1923915 0.2584151 0.3253847 0.3235853 0.8821547 0.8145363 0.5714279
full_length_LJP6_min3_2.pdb 0.2362051 0.1918143 0.1853592 0.2188139 0.2230889
0.2129519 0.2632392 0.3750563 0.5768349 0.9550438 0.9510411 0.7796372
full_length_3WY9_min3_2.pdb 0.2157132 0.1783419 0.1684294 0.2027534 0.1973190
0.1794221 0.2443584 0.3384765 0.6058613 0.9515670 0.7485705 0.5972338
full_length_ZSP9_min3_2.pdb 0.2165520 0.1759960 0.1627489 0.1995395 0.1906125
0.1788183 0.2471901 0.3395544 0.5993972 0.9361321 0.7341188 0.5489407
[,300] [,301] [,302] [,303] [,304]
[,305] [,306] [,307] [,308] [,309] [,310] [,311]
full_length_NAT0_min3_2.pdb 0.3138861 0.2664286 0.2465426 0.2183586 0.2327058
0.3493401 0.3410967 0.2595173 0.2040906 0.1754986 0.1818308 0.2125164
full_length_LJP6_min3_2.pdb 0.3291583 0.2844972 0.2464757 0.2493883 0.2607761
0.3846175 0.3070715 0.2522175 0.2112331 0.1847412 0.2139460 0.2703803
full_length_3WY9_min3_2.pdb 0.2504978 0.2241606 0.2311749 0.2265967 0.2204957
0.3367734 0.3181753 0.2566895 0.1999186 0.1718267 0.1752378 0.1878163
full_length_ZSP9_min3_2.pdb 0.2386916 0.2123109 0.2172055 0.2063991 0.2166531
0.3373596 0.3217039 0.2515069 0.1933735 0.1640083 0.1711386 0.2152072

```
                              [,312]    [,313]    [,314]    [,315]    [,316]
 [,317]     [,318]     [,319]     [,320]     [,321]     [,322]     [,323]
full_length_NAT0_min3_2.pdb 0.2414486 0.2283307 0.1682893 0.1173220 0.1174488
0.10175734 0.09268723 0.09401411 0.09792324 0.07618005 0.07542024 0.09429517
full_length_LJP6_min3_2.pdb 0.2787958 0.2234321 0.1759360 0.1225008 0.1272809
0.10838595 0.10294133 0.09989265 0.09979649 0.08078950 0.08226748 0.09996246
full_length_3WY9_min3_2.pdb 0.2364674 0.2208098 0.1761308 0.1166271 0.1175748
0.09948961 0.09191895 0.08571864 0.09084718 0.07600825 0.06973039 0.08720780
full_length_ZSP9_min3_2.pdb 0.2286588 0.2265034 0.1595759 0.1100743 0.1127273
0.09994155 0.09149876 0.09455725 0.09078184 0.07442813 0.07095279 0.08518972
                               [,324]     [,325]     [,326]    [,327]    [,328]
   [,329]    [,330]    [,331]    [,332]    [,333]    [,334]    [,335]
full_length_NAT0_min3_2.pdb 0.09358183 0.08562380 0.10264704 0.1169391 0.1195472
0.1469750 0.1619687 0.1864932 0.2766296 0.2720494 0.4233353 0.4044409
full_length_LJP6_min3_2.pdb 0.09518286 0.08838814 0.09803826 0.1104623 0.1178582
0.1421808 0.1454830 0.1652985 0.2436093 0.2634219 0.3001857 0.6647977
full_length_3WY9_min3_2.pdb 0.08640634 0.07796134 0.09320773 0.1018613 0.1050095
0.1320294 0.1452098 0.1773607 0.2716352 0.2934010 0.3967891 0.3326480
full_length_ZSP9_min3_2.pdb 0.08409375 0.07554672 0.09198760 0.1005182 0.1010903
0.1292192 0.1448106 0.1650155 0.2406462 0.2629745 0.3310616 0.3221582
                              [,336]    [,337]    [,338]    [,339]    [,340]
[,341]    [,342]    [,343]    [,344]    [,345]    [,346]    [,347]
full_length_NAT0_min3_2.pdb 0.2919457 0.3143556 0.3958034 0.6754753 0.7617202
1.1718183 1.6571198 2.5306997 2.1758173 0.4637586 0.4770311 0.2849911
full_length_LJP6_min3_2.pdb 0.6894427 0.5050267 0.6517782 0.9979131 1.1899075
1.0769221 0.9674503 0.6744161 0.7151862 0.5326054 0.3617873 0.2698526
full_length_3WY9_min3_2.pdb 0.3774864 0.6995759 0.8593886 0.7744411 0.7706800
1.6192820 2.1619894 3.2887422 1.5189812 1.0930510 0.4163075 0.2806865
full_length_ZSP9_min3_2.pdb 0.3218516 0.5855571 0.5550949 0.8467519 0.9141060
0.9667708 1.4685788 1.2583708 0.8552622 0.7171351 0.3465868 0.2478656
                              [,348]    [,349]    [,350]    [,351]    [,352]
[,353]    [,354]    [,355]    [,356]    [,357]    [,358]    [,359]
full_length_NAT0_min3_2.pdb 0.2334017 0.2731438 0.2423602 0.1668180 0.1729708
0.2020863 0.1607497 0.1276503 0.1480245 0.1615102 0.1372141 0.1369468
full_length_LJP6_min3_2.pdb 0.2208872 0.2359922 0.2137513 0.1506865 0.1589591
0.1986176 0.1638017 0.1258277 0.1457753 0.1562051 0.1351065 0.1330977
full_length_3WY9_min3_2.pdb 0.2183242 0.2649382 0.2194584 0.1505455 0.1614980
0.1885072 0.1479185 0.1194406 0.1419927 0.1581543 0.1409274 0.1374248
full_length_ZSP9_min3_2.pdb 0.2023418 0.2434137 0.2113994 0.1427161 0.1502656
0.1834325 0.1464615 0.1135791 0.1351346 0.1538369 0.1330103 0.1284159
                              [,360]    [,361]    [,362]    [,363]    [,364]
[,365]    [,366]    [,367]    [,368]    [,369]    [,370]    [,371]
full_length_NAT0_min3_2.pdb 0.1567080 0.1533256 0.1525568 0.1607723 0.2003611
0.2407954 0.2889836 0.3974003 0.4916526 0.3409232 0.2280434 0.1698976
full_length_LJP6_min3_2.pdb 0.1496280 0.1469089 0.1475769 0.1470772 0.1729550
0.2048724 0.2674560 0.3517166 0.3707064 0.2680658 0.2294958 0.1560522
full_length_3WY9_min3_2.pdb 0.1569837 0.1517114 0.1508115 0.1516627 0.1787329
0.2241454 0.2714470 0.4280722 0.5247509 0.3394221 0.2237061 0.1589920
full_length_ZSP9_min3_2.pdb 0.1436861 0.1475704 0.1435521 0.1463127 0.1826573
0.2251255 0.2712883 0.4258260 0.5057400 0.3294837 0.2159857 0.1566501
                              [,372]    [,373]    [,374]    [,375]    [,376]
[,377]    [,378]    [,379]    [,380]    [,381]    [,382]    [,383]
full_length_NAT0_min3_2.pdb 0.1450885 0.1416329 0.1722536 0.2259433 0.2190511
0.2842111 0.3586944 0.2910577 0.2070482 0.2262944 0.3051769 0.2348114
```

full_length_LJP6_min3_2.pdb 0.1484424 0.1381706 0.1607516 0.2063200 0.2044452
0.2360227 0.2892570 0.3032601 0.2115752 0.2366411 0.2856864 0.2360280
full_length_3WY9_min3_2.pdb 0.1429081 0.1371921 0.1611263 0.2077111 0.2097937
0.2425633 0.3034839 0.2989956 0.1963395 0.2275755 0.3054255 0.2306504
full_length_ZSP9_min3_2.pdb 0.1384039 0.1332355 0.1541072 0.1976879 0.2004013
0.2476645 0.3002164 0.2900759 0.1853764 0.2128848 0.2964219 0.2206670
[,384] [,385] [,386] [,387] [,388]
[,389] [,390] [,391] [,392] [,393] [,394] [,395]
full_length_NAT0_min3_2.pdb 0.1921964 0.1864954 0.2000791 0.2296918 0.2472939
0.2458650 0.2525678 0.2923279 0.3118989 0.3416197 0.3724065 0.3403229
full_length_LJP6_min3_2.pdb 0.2020802 0.1859861 0.1996147 0.2225206 0.2322167
0.2361137 0.2460875 0.2743932 0.3252386 0.3271875 0.3486249 0.3334171
full_length_3WY9_min3_2.pdb 0.1903859 0.1810137 0.1982929 0.2184759 0.2211964
0.2340732 0.2393422 0.2552440 0.2546778 0.3287345 0.3663275 0.3227375
full_length_ZSP9_min3_2.pdb 0.1818779 0.1705150 0.1898191 0.2085239 0.2145682
0.2257551 0.2328509 0.2495401 0.2475161 0.3218007 0.3501096 0.2965943
[,396] [,397] [,398] [,399] [,400]
[,401] [,402] [,403] [,404] [,405] [,406] [,407]
full_length_NAT0_min3_2.pdb 0.3995665 0.3723693 0.4437251 0.4292490 0.4313815
0.4784891 0.3906918 0.2922486 0.3612939 0.3098175 0.3087551 0.2371757
full_length_LJP6_min3_2.pdb 0.3790464 0.3932192 0.4478589 0.4201777 0.4235698
0.4671620 0.3745649 0.2763548 0.3466248 0.2975788 0.2740057 0.2151758
full_length_3WY9_min3_2.pdb 0.3771951 0.3770130 0.4415107 0.4266757 0.4202790
0.5480952 0.4341126 0.2964440 0.3248459 0.2846703 0.2812827 0.2095489
full_length_ZSP9_min3_2.pdb 0.3565085 0.3630832 0.4257734 0.4086308 0.4260231
0.4941382 0.4283097 0.2905594 0.3365285 0.2743274 0.2746827 0.2064335
[,408] [,409] [,410] [,411] [,412]
[,413] [,414] [,415] [,416] [,417] [,418] [,419]
full_length_NAT0_min3_2.pdb 0.1800746 0.1285022 0.1361129 0.1130092 0.1183540
0.1066585 0.1386243 0.2144600 0.2519164 0.2598152 0.2934099 0.2634932
full_length_LJP6_min3_2.pdb 0.1729230 0.1262948 0.1312156 0.1102780 0.1196256
0.1109008 0.1491226 0.2254392 0.2611799 0.3503969 0.3330826 0.3134083
full_length_3WY9_min3_2.pdb 0.1622399 0.1184042 0.1294863 0.1121629 0.1179604
0.1078086 0.1371503 0.2060968 0.2357654 0.2460774 0.2836182 0.2542697
full_length_ZSP9_min3_2.pdb 0.1560842 0.1139346 0.1246454 0.1059647 0.1104620
0.1009663 0.1295965 0.1987463 0.2330761 0.2417042 0.2749481 0.2600123
[,420] [,421] [,422] [,423] [,424]
[,425] [,426] [,427] [,428] [,429] [,430] [,431]
full_length_NAT0_min3_2.pdb 0.3263829 0.3584908 0.3338612 0.2886836 0.2763231
0.2391208 0.2722287 0.2424929 0.1844475 0.2051575 0.1469907 0.1470828
full_length_LJP6_min3_2.pdb 0.3855441 0.4019431 0.4018356 0.3249097 0.3021208
0.2446238 0.3092643 0.2605094 0.2009640 0.2316358 0.1518640 0.1497900
full_length_3WY9_min3_2.pdb 0.2999651 0.2787382 0.3210419 0.2825439 0.2657330
0.2316843 0.2953518 0.2366540 0.1803755 0.2163546 0.1491281 0.1425270
full_length_ZSP9_min3_2.pdb 0.3039039 0.2799376 0.2915350 0.2625598 0.2480036
0.2209710 0.2756160 0.2240070 0.1685077 0.1952122 0.1346201 0.1329828
[,432] [,433] [,434] [,435] [,436]
[,437] [,438] [,439] [,440] [,441] [,442] [,443]
full_length_NAT0_min3_2.pdb 0.1409748 0.1280213 0.1448553 0.1122225 0.08764260
0.08450463 0.09404560 0.07841642 0.07600624 0.08330723 0.09185806 0.08863836
full_length_LJP6_min3_2.pdb 0.1431114 0.1259721 0.1354400 0.1054965 0.08858623
0.08480836 0.09465783 0.07870600 0.07584912 0.08516850 0.09099756 0.07784313
full_length_3WY9_min3_2.pdb 0.1389205 0.1258926 0.1336207 0.1097072 0.08523299
0.08390862 0.09628782 0.07872967 0.07292499 0.08374233 0.09336213 0.08060178

full_length_ZSP9_min3_2.pdb 0.1322896 0.1208733 0.1379329 0.1073343 0.08644267
0.08188767 0.09191787 0.07652105 0.07388065 0.07864567 0.08586370 0.07891208
[,444] [,445] [,446] [,447] [,448]
[,449] [,450] [,451] [,452] [,453] [,454] [,455]
full_length_NAT0_min3_2.pdb 0.08946350 0.10356883 0.1216836 0.1390408 0.1499716
0.1580496 0.2052600 0.2738827 0.3237569 0.2498270 0.2566564 0.3311427
full_length_LJP6_min3_2.pdb 0.08644216 0.09776621 0.1123607 0.1325951 0.1436794
0.1506023 0.2010362 0.2735335 0.2755034 0.2547911 0.2302414 0.6110722
full_length_3WY9_min3_2.pdb 0.09118232 0.13017198 0.1402256 0.1685965 0.2617823
0.6099037 0.3308077 0.2312693 0.2843327 0.2142809 0.3626122 0.5678479
full_length_ZSP9_min3_2.pdb 0.07940523 0.09498944 0.1081161 0.1229663 0.1371127
0.1442871 0.1682919 0.2242683 0.2757583 0.3124984 0.6337256 0.3294781
[,456] [,457] [,458] [,459] [,460]
[,461] [,462] [,463] [,464] [,465] [,466] [,467]
full_length_NAT0_min3_2.pdb 0.2349760 0.3797328 0.5589767 0.7382005 0.5278705
0.4346329 0.3810145 0.4680260 0.5022146 0.7539091 0.6546761 0.7362656
full_length_LJP6_min3_2.pdb 0.6748617 0.7103659 0.6518287 1.1931463 0.8920020
0.4293344 0.3936280 0.5131101 0.4308950 0.6996821 0.8634043 1.3667374
full_length_3WY9_min3_2.pdb 0.5894828 0.6102353 0.5560284 0.3673902 0.3355826
0.4963009 0.4568555 0.6368495 0.6955609 0.9736330 0.9711785 1.0153500
full_length_ZSP9_min3_2.pdb 0.3720462 0.4530249 0.6362316 0.7735260 0.8338651
0.7775086 0.5297464 0.4829244 0.5437954 0.4447606 0.3380750 0.3179922
[,468] [,469] [,470] [,471] [,472]
[,473] [,474] [,475] [,476] [,477] [,478] [,479] [,480]
full_length_NAT0_min3_2.pdb 0.6973481 0.7075207 1.638630 1.667005 1.761859
2.317088 2.644650 1.6328615 1.2956617 0.9239979 1.0337038 0.7258282 0.7311667
full_length_LJP6_min3_2.pdb 1.3980201 1.6275076 2.825181 3.037508 4.143364
3.451972 2.048884 1.4196337 1.1110480 0.8217429 0.8085582 0.5278862 0.5607573
full_length_3WY9_min3_2.pdb 1.3867636 2.3163870 2.914814 2.262081 2.527977
1.757412 1.227107 0.9310849 0.6802129 0.5083104 0.4890419 0.4079737 0.3664078
full_length_ZSP9_min3_2.pdb 0.4598244 0.4467830 1.585611 1.921554 2.271355
1.738254 1.294807 0.9607062 0.7273669 0.5604316 0.4842068 0.3503581 0.3583083
[,481] [,482] [,483] [,484] [,485]
[,486] [,487] [,488] [,489] [,490] [,491] [,492]
full_length_NAT0_min3_2.pdb 0.5065194 0.5100970 0.4278061 0.3925724 0.4061619
0.4062478 0.4034743 0.5068475 0.5937166 0.7487160 0.6454965 0.6982453
full_length_LJP6_min3_2.pdb 0.3940171 0.4254505 0.3523713 0.3557924 0.3596012
0.3204467 0.3631707 0.3288250 0.3222679 0.3772179 0.3949513 0.4900359
full_length_3WY9_min3_2.pdb 0.2845021 0.3337182 0.3308372 0.3136693 0.3368761
0.3264328 0.3401496 0.4227551 0.4607701 0.6973104 0.5913345 0.6206864
full_length_ZSP9_min3_2.pdb 0.2853848 0.3106522 0.3064096 0.3120056 0.3244627
0.3398146 0.3229285 0.4144052 0.5045538 0.8181930 0.7278643 0.6677258
[,493] [,494] [,495] [,496] [,497]
[,498] [,499] [,500] [,501] [,502] [,503] [,504] [,505]
full_length_NAT0_min3_2.pdb 0.6180556 0.5518858 0.5618348 0.6019247 0.6529105
1.0594308 1.446952 2.256145 2.508393 3.742370 3.893818 3.853175 5.415547
full_length_LJP6_min3_2.pdb 0.5965823 0.5951662 0.6319787 0.6950454 0.7490130
1.2121591 1.583770 2.266967 2.612376 3.553327 5.098370 5.215503 3.387958
full_length_3WY9_min3_2.pdb 0.4955497 0.4695758 0.4939271 0.5949857 0.6526753
0.9804259 1.276697 1.815664 1.747756 2.545709 2.650453 2.323065 2.741738
full_length_ZSP9_min3_2.pdb 0.5406598 0.5297424 0.5575984 0.6269512 0.6610797
1.0209210 1.361748 1.877835 1.940264 2.828043 3.070138 3.597140 3.786238
[,506] [,507] [,508] [,509] [,510]
[,511] [,512] [,513] [,514] [,515] [,516] [,517] [,518]

```
full_length_NAT0_min3_2.pdb 5.180925 4.173286 3.577222 2.467427 2.389042
2.763174 2.039813 2.132556 1.5076036 1.6621768 1.2325474 1.330614 1.1372702
full_length_LJP6_min3_2.pdb 3.554711 2.691283 3.028247 2.778708 1.903099
1.977261 1.300398 1.361317 1.3002192 2.1934332 1.7369556 1.197032 1.1916655
full_length_3WY9_min3_2.pdb 3.240164 2.542770 2.429789 1.808200 1.614639
1.704477 1.310441 1.145277 0.7878547 0.8459413 0.7556141 1.028763 0.7424966
full_length_ZSP9_min3_2.pdb 2.804801 2.671869 2.496157 1.813372 1.675560
1.803831 1.403816 1.154433 0.7906925 0.8344382 0.7959697 1.008235 0.7841107
                              [,519]    [,520]    [,521]    [,522]   [,523]
[,524]    [,525]    [,526]   [,527]   [,528]   [,529]   [,530]   [,531]
full_length_NAT0_min3_2.pdb 0.7355202 0.9041625 1.1749435 1.2645546 1.444030
2.1312157 2.2355549 3.3364955 3.799554 5.416810 4.458846 3.317396 2.277998
full_length_LJP6_min3_2.pdb 0.7917551 0.7690283 0.7802671 0.6256356 0.968987
1.1579626 1.7273077 2.3227743 2.771366 3.991400 5.016451 3.373177 2.498458
full_length_3WY9_min3_2.pdb 0.6110084 0.7547093 0.6079178 0.6381749 0.627818
0.8281570 0.9731539 0.9684481 1.262278 1.865969 2.327039 2.266245 1.968945
full_length_ZSP9_min3_2.pdb 0.5959874 0.7271007 0.7943244 0.7965218 0.604015
0.8142092 1.0049623 1.0170329 1.297532 1.899267 2.390044 2.261404 1.913872
                             [,532]    [,533]    [,534]    [,535]    [,536]
[,537]    [,538]    [,539]    [,540]    [,541]    [,542]    [,543]
full_length_NAT0_min3_2.pdb 0.924822 0.6107717 0.5337644 0.4480679 0.5739998
0.5096938 0.6839057 1.0016973 0.7373792 0.7603165 0.5065300 0.4278187
full_length_LJP6_min3_2.pdb 1.675837 1.0728670 0.6883206 0.5970188 0.4853311
0.5884350 0.6303360 1.0013463 0.8746468 0.6292079 0.3711316 0.3561321
full_length_3WY9_min3_2.pdb 1.347930 0.8945131 0.5805275 0.4874732 0.3452246
0.4195395 0.4019494 0.5915172 0.8225195 0.5793124 0.6468452 0.4659401
full_length_ZSP9_min3_2.pdb 1.355475 0.8896444 0.5940054 0.5373143 0.4034580
0.4934888 0.4594652 0.6571901 0.8436131 0.7194017 0.7447892 0.5393900
                              [,544]    [,545]    [,546]    [,547]    [,548]
[,549]
full_length_NAT0_min3_2.pdb 0.3614892 0.3427763 0.4135060 0.5842817 0.6635542
0.4804471
full_length_LJP6_min3_2.pdb 0.3160837 0.3155660 0.3721032 0.5825975 0.7259901
0.5160531
full_length_3WY9_min3_2.pdb 0.5159347 0.5862409 0.4390097 0.3911238 0.4079572
0.4253828
full_length_ZSP9_min3_2.pdb 0.6932788 0.5245696 0.3856878 0.3671026 0.3894689
0.4347121
```

ENSEMBLE FREQUENCY DATA (TRUNCATED MINIMIZED CHARACTERIZED CLASS C)

```
  [,1]     [,2]     [,3]     [,4]     [,5]     [,6]     [,7]     [,8]     [,9]
 [,10]    [,11]    [,12]    [,13]    [,14]    [,15]    [,16]
[1,] 0.005423 0.008604 0.010389 0.011791 0.013958 0.018328 0.020979 0.021412
0.022090 0.022919 0.024907 0.025605 0.026657 0.028015 0.030685 0.031446
[2,] 0.009348 0.009980 0.013403 0.019114 0.020712 0.021412 0.022050 0.023406
0.025812 0.026792 0.029493 0.030238 0.033364 0.034080 0.037005 0.037924
[3,] 0.008415 0.009812 0.011954 0.018095 0.020286 0.020757 0.021807 0.022971
0.024126 0.025357 0.026323 0.028216 0.030358 0.030935 0.032848 0.034850
[4,] 0.008369 0.009800 0.010786 0.012080 0.017443 0.019344 0.019683 0.020775
0.021679 0.023307 0.024899 0.025318 0.028185 0.029876 0.031931 0.032413
        [,17]    [,18]    [,19]    [,20]    [,21]    [,22]    [,23]    [,24]
```

```
[,25]    [,26]    [,27]    [,28]    [,29]    [,30]    [,31]    [,32]
[1,] 0.035726 0.036823 0.038051 0.039966 0.040708 0.041405 0.044170 0.044856
0.046800 0.047160 0.049151 0.050794 0.052429 0.053715 0.055091 0.055915
[2,] 0.038308 0.039498 0.040099 0.042202 0.044554 0.045566 0.047891 0.048787
0.050356 0.052032 0.053051 0.055004 0.055783 0.058270 0.059578 0.060190
[3,] 0.036224 0.037532 0.038441 0.041247 0.042014 0.044095 0.045129 0.046864
0.048731 0.050284 0.052035 0.053818 0.055376 0.056289 0.056961 0.058524
[4,] 0.034466 0.035137 0.036071 0.036805 0.038515 0.040586 0.040950 0.044933
0.045784 0.046586 0.048124 0.048617 0.048982 0.052121 0.054450 0.055647
        [,33]    [,34]    [,35]    [,36]    [,37]    [,38]    [,39]    [,40]
[,41]    [,42]    [,43]    [,44]    [,45]    [,46]    [,47]    [,48]
[1,] 0.057857 0.058170 0.061621 0.063324 0.064320 0.065118 0.066062 0.067206
0.070175 0.070665 0.072068 0.072927 0.074724 0.076109 0.076686 0.077866
[2,] 0.060715 0.062927 0.064641 0.065783 0.067889 0.068635 0.070250 0.071235
0.071838 0.073788 0.075268 0.075824 0.076983 0.080532 0.080853 0.082151
[3,] 0.058851 0.060005 0.062461 0.063041 0.064535 0.064733 0.066664 0.067336
0.069102 0.069919 0.070352 0.072375 0.074936 0.075858 0.077358 0.077585
[4,] 0.057056 0.058228 0.058448 0.060260 0.061107 0.062109 0.062773 0.066241
0.066640 0.068246 0.068934 0.070097 0.070605 0.073455 0.074633 0.075736
        [,49]    [,50]    [,51]    [,52]    [,53]    [,54]    [,55]    [,56]
[,57]    [,58]    [,59]    [,60]    [,61]    [,62]    [,63]    [,64]
[1,] 0.079576 0.080916 0.083861 0.084825 0.085828 0.086504 0.087460 0.089724
0.091665 0.092770 0.093751 0.095550 0.096394 0.098627 0.100021 0.100479
[2,] 0.084473 0.084973 0.087507 0.089553 0.090425 0.090781 0.094235 0.094925
0.095783 0.097352 0.098387 0.100937 0.102457 0.103623 0.104360 0.107017
[3,] 0.079341 0.080923 0.081688 0.084389 0.086243 0.087995 0.089380 0.090242
0.091001 0.092287 0.094585 0.095939 0.096252 0.098261 0.100503 0.100840
[4,] 0.076742 0.079080 0.080138 0.081370 0.082354 0.083716 0.086147 0.087439
0.088326 0.088930 0.089868 0.090672 0.094033 0.095228 0.096177 0.097599
        [,65]    [,66]    [,67]    [,68]    [,69]    [,70]    [,71]    [,72]
[,73]    [,74]    [,75]    [,76]    [,77]    [,78]    [,79]    [,80]
[1,] 0.102842 0.104221 0.104776 0.106037 0.106837 0.108481 0.109650 0.110896
0.111578 0.112359 0.113209 0.116272 0.117816 0.119598 0.121400 0.122492
[2,] 0.108149 0.109024 0.110376 0.111541 0.112442 0.113043 0.114035 0.116947
0.118559 0.119209 0.119898 0.120774 0.122187 0.124256 0.125844 0.127420
[3,] 0.102644 0.103584 0.105112 0.106445 0.107421 0.109038 0.109582 0.111994
0.113513 0.114722 0.115015 0.117220 0.119817 0.121508 0.122300 0.123408
[4,] 0.098496 0.099472 0.101772 0.103479 0.103787 0.105140 0.106487 0.107416
0.110096 0.110575 0.111885 0.113064 0.114754 0.115310 0.118133 0.118485
        [,81]    [,82]    [,83]    [,84]    [,85]    [,86]    [,87]    [,88]
[,89]    [,90]    [,91]    [,92]    [,93]    [,94]    [,95]    [,96]
[1,] 0.123960 0.126078 0.127575 0.128180 0.129360 0.132039 0.132719 0.133604
0.134944 0.135563 0.137855 0.139582 0.139913 0.141117 0.142387 0.144142
[2,] 0.127521 0.130173 0.131149 0.132154 0.134487 0.135060 0.136337 0.137769
0.138105 0.140951 0.141272 0.143253 0.144377 0.145664 0.148036 0.149615
[3,] 0.124200 0.126426 0.127566 0.129363 0.129609 0.132409 0.133029 0.134498
0.136277 0.137459 0.138696 0.140125 0.140203 0.141264 0.141745 0.145873
[4,] 0.119601 0.121068 0.123497 0.124631 0.126375 0.127457 0.128114 0.129050
0.129850 0.131585 0.133058 0.135701 0.137155 0.137383 0.139302 0.140317
        [,97]    [,98]    [,99]   [,100]   [,101]   [,102]   [,103]   [,104]
[,105]   [,106]   [,107]   [,108]   [,109]   [,110]   [,111]   [,112]
[1,] 0.145148 0.146484 0.148400 0.150831 0.151634 0.152616 0.153731 0.155481
0.156470 0.156863 0.157873 0.158815 0.161287 0.161575 0.163234 0.164927
[2,] 0.150090 0.150833 0.152228 0.155049 0.155544 0.156683 0.157953 0.159642
```

0.160511 0.160793 0.161677 0.164224 0.166664 0.167799 0.168606 0.169228
[3,] 0.146331 0.147181 0.149570 0.151899 0.153432 0.154533 0.155262 0.157653
0.158586 0.159435 0.160343 0.162130 0.164318 0.164800 0.167220 0.168372
[4,] 0.140465 0.143322 0.144501 0.145501 0.146704 0.147668 0.147971 0.149512
0.151664 0.153788 0.155033 0.156410 0.157624 0.158455 0.160122 0.162493
[,113] [,114] [,115] [,116] [,117] [,118] [,119] [,120]
[,121] [,122] [,123] [,124] [,125] [,126] [,127] [,128]
[1,] 0.166473 0.166930 0.168290 0.169487 0.170552 0.171554 0.172806 0.173777
0.174465 0.177944 0.179613 0.179817 0.183047 0.184011 0.185461 0.189191
[2,] 0.171093 0.173240 0.173338 0.175557 0.176319 0.177481 0.178084 0.180209
0.181081 0.182116 0.183961 0.184753 0.187420 0.188242 0.188427 0.190056
[3,] 0.169293 0.169665 0.170749 0.171817 0.173915 0.175067 0.176548 0.177068
0.179592 0.180901 0.182222 0.182930 0.184334 0.185874 0.186876 0.188294
[4,] 0.163084 0.165163 0.166466 0.166826 0.167456 0.169419 0.170334 0.171362
0.172692 0.173050 0.174048 0.177336 0.177926 0.178362 0.179018 0.180499
[,129] [,130] [,131] [,132] [,133] [,134] [,135] [,136]
[,137] [,138] [,139] [,140] [,141] [,142] [,143] [,144]
[1,] 0.189681 0.190820 0.191834 0.193736 0.194637 0.196388 0.197863 0.198395
0.198560 0.201742 0.203198 0.203785 0.204991 0.206541 0.208413 0.209157
[2,] 0.192126 0.194120 0.195626 0.196784 0.197551 0.199587 0.200215 0.202050
0.203829 0.204089 0.205476 0.207144 0.209770 0.210583 0.210866 0.212986
[3,] 0.189018 0.190153 0.190739 0.192432 0.194508 0.195039 0.195295 0.197487
0.198934 0.200642 0.202854 0.203718 0.205473 0.205999 0.207408 0.208684
[4,] 0.183567 0.187053 0.187479 0.188627 0.190441 0.191993 0.192584 0.193178
0.194772 0.195494 0.197449 0.198634 0.199939 0.200685 0.203067 0.203902
[,145] [,146] [,147] [,148] [,149] [,150] [,151] [,152]
[,153] [,154] [,155] [,156] [,157] [,158] [,159] [,160]
[1,] 0.212145 0.213393 0.213803 0.214497 0.215836 0.218098 0.219718 0.220618
0.221945 0.223408 0.224703 0.226028 0.226900 0.228390 0.231408 0.233172
[2,] 0.213205 0.214481 0.215674 0.219033 0.220161 0.220760 0.222327 0.223712
0.227143 0.227738 0.228559 0.229734 0.231061 0.231637 0.232625 0.234926
[3,] 0.209839 0.210071 0.212138 0.212941 0.214788 0.215330 0.215837 0.217559
0.219144 0.220648 0.221642 0.223527 0.224676 0.226657 0.228000 0.228327
[4,] 0.205285 0.207732 0.208951 0.210169 0.210639 0.214038 0.215186 0.215850
0.217752 0.219934 0.220879 0.221867 0.222166 0.223523 0.224438 0.226729
[,161] [,162] [,163] [,164] [,165] [,166] [,167] [,168]
[,169] [,170] [,171] [,172] [,173] [,174] [,175] [,176]
[1,] 0.234385 0.235236 0.235653 0.238773 0.239211 0.240664 0.241640 0.242664
0.243156 0.243593 0.245300 0.247521 0.248619 0.249568 0.250677 0.252436
[2,] 0.235803 0.237813 0.237967 0.238789 0.240307 0.241808 0.243086 0.244068
0.245928 0.248019 0.248537 0.251200 0.252299 0.252858 0.255222 0.257290
[3,] 0.230218 0.232217 0.233429 0.233504 0.235766 0.236501 0.237331 0.238336
0.241069 0.241713 0.244124 0.245425 0.246565 0.247428 0.249080 0.250060
[4,] 0.227432 0.229080 0.229961 0.231542 0.232428 0.233451 0.235693 0.236755
0.237864 0.238331 0.239689 0.240738 0.241021 0.243094 0.244166 0.244892
[,177] [,178] [,179] [,180] [,181] [,182] [,183] [,184]
[,185] [,186] [,187] [,188] [,189] [,190] [,191] [,192]
[1,] 0.253666 0.254974 0.255585 0.257020 0.258185 0.259542 0.260143 0.261031
0.261694 0.261984 0.263648 0.264276 0.265415 0.269626 0.270735 0.271676
[2,] 0.257992 0.258407 0.259254 0.262872 0.263579 0.265905 0.267534 0.268725
0.270166 0.270956 0.271699 0.274155 0.274478 0.275919 0.277251 0.279094
[3,] 0.250491 0.253833 0.254562 0.255269 0.256769 0.258178 0.259302 0.260373
0.261201 0.264218 0.264788 0.265817 0.267295 0.267496 0.270755 0.271373
[4,] 0.246526 0.247930 0.248212 0.249690 0.250210 0.250611 0.251708 0.252723

```
0.252840 0.255877 0.256796 0.257609 0.259842 0.260925 0.262625 0.262856
       [,193]   [,194]   [,195]   [,196]   [,197]   [,198]   [,199]   [,200]
[,201]   [,202]   [,203]   [,204]   [,205]   [,206]   [,207]   [,208]
[1,] 0.272697 0.274614 0.278217 0.281987 0.283176 0.283698 0.285874 0.287217
0.288917 0.289761 0.291364 0.291691 0.293119 0.294216 0.296761 0.297185
[2,] 0.280051 0.281332 0.282071 0.283230 0.284463 0.286169 0.288057 0.290686
0.291316 0.292428 0.293212 0.294618 0.295023 0.295695 0.297289 0.300017
[3,] 0.272539 0.273714 0.275079 0.276530 0.277923 0.278399 0.281290 0.281562
0.283515 0.284543 0.285283 0.285490 0.288380 0.290190 0.292135 0.293181
[4,] 0.264783 0.266275 0.267634 0.270188 0.271249 0.272679 0.275470 0.276705
0.279934 0.282044 0.282404 0.283837 0.285261 0.286711 0.287503 0.290522
       [,209]   [,210]   [,211]   [,212]   [,213]   [,214]   [,215]   [,216]
[,217]   [,218]   [,219]   [,220]   [,221]   [,222]   [,223]   [,224]
[1,] 0.300384 0.301621 0.303745 0.305277 0.306754 0.309178 0.309777 0.310756
0.313052 0.314868 0.315567 0.317071 0.318441 0.319811 0.320604 0.321755
[2,] 0.301786 0.302811 0.306825 0.307520 0.307848 0.308731 0.313171 0.314647
0.316175 0.317251 0.318868 0.319379 0.320750 0.321365 0.323239 0.326694
[3,] 0.295699 0.296848 0.297079 0.299239 0.301753 0.302901 0.304250 0.305937
0.307260 0.309990 0.310637 0.311889 0.312425 0.313847 0.317291 0.317850
[4,] 0.292254 0.293621 0.294671 0.295655 0.296711 0.297435 0.300381 0.302338
0.303472 0.304205 0.306975 0.307855 0.308846 0.310845 0.313115 0.313987
       [,225]   [,226]   [,227]   [,228]   [,229]   [,230]   [,231]   [,232]
[,233]   [,234]   [,235]   [,236]   [,237]   [,238]   [,239]   [,240]
[1,] 0.323420 0.325086 0.325850 0.326683 0.328846 0.330317 0.330875 0.332759
0.33300 0.334707 0.336883 0.338309 0.339120 0.341186 0.342291 0.345190
[2,] 0.327732 0.329367 0.331780 0.332939 0.334906 0.335341 0.337581 0.339704
0.34202 0.342645 0.343986 0.344856 0.345439 0.348726 0.349507 0.351725
[3,] 0.318630 0.319613 0.320085 0.322710 0.323308 0.324389 0.325854 0.326762
0.32891 0.329299 0.331643 0.332035 0.335116 0.337176 0.339547 0.340329
[4,] 0.317108 0.318617 0.319966 0.320858 0.321542 0.321712 0.324071 0.326304
0.32863 0.330322 0.330616 0.332677 0.332827 0.336131 0.337207 0.337747
       [,241]   [,242]   [,243]   [,244]   [,245]   [,246]   [,247]   [,248]
[,249]   [,250]   [,251]   [,252]   [,253]   [,254]   [,255]   [,256]
[1,] 0.347479 0.348182 0.350217 0.350911 0.353362 0.355706 0.357996 0.358778
0.361431 0.362812 0.364219 0.366220 0.366537 0.369480 0.370596 0.371719
[2,] 0.353305 0.354759 0.357224 0.358453 0.359371 0.360976 0.361613 0.363815
0.365117 0.367556 0.368713 0.370321 0.370701 0.372556 0.374487 0.375475
[3,] 0.340992 0.341911 0.343513 0.345215 0.346165 0.346800 0.350804 0.351151
0.352484 0.353876 0.355893 0.358170 0.359269 0.359783 0.362193 0.364526
[4,] 0.340556 0.343631 0.344650 0.346691 0.347025 0.348536 0.349591 0.352181
0.352278 0.353291 0.355886 0.357927 0.360175 0.362058 0.363842 0.364560
       [,257]   [,258]   [,259]   [,260]   [,261]   [,262]   [,263]   [,264]
[,265]   [,266]   [,267]   [,268]   [,269]   [,270]   [,271]   [,272]
[1,] 0.373684 0.375288 0.376890 0.378762 0.380312 0.382018 0.382670 0.384265
0.385037 0.386942 0.387936 0.389713 0.390944 0.392657 0.393508 0.395307
[2,] 0.375711 0.378478 0.380730 0.382285 0.383589 0.386273 0.387566 0.387973
0.390074 0.391472 0.391758 0.394552 0.395314 0.396377 0.397332 0.398536
[3,] 0.365846 0.366945 0.368184 0.368932 0.369438 0.370246 0.371169 0.372469
0.373090 0.376347 0.378055 0.379443 0.382940 0.384675 0.385461 0.386777
[4,] 0.364959 0.367541 0.368169 0.369203 0.370818 0.371443 0.374044 0.375073
0.375807 0.378689 0.379382 0.381650 0.383006 0.385001 0.387793 0.388768
       [,273]   [,274]   [,275]   [,276]   [,277]   [,278]   [,279]   [,280]
[,281]   [,282]   [,283]   [,284]   [,285]   [,286]   [,287]   [,288]
[1,] 0.397353 0.400075 0.401069 0.402609 0.403643 0.403815 0.405649 0.405991
```

0.408826 0.410594 0.412257 0.413271 0.415216 0.416370 0.418245 0.419458
[2,] 0.401500 0.403717 0.404409 0.406939 0.408216 0.409047 0.411305 0.412066
0.414418 0.415634 0.416611 0.418133 0.419750 0.421919 0.423983 0.424201
[3,] 0.387526 0.388769 0.390411 0.391860 0.393668 0.394830 0.396426 0.397047
0.397707 0.398558 0.400909 0.403307 0.404288 0.405581 0.406807 0.407926
[4,] 0.390553 0.391660 0.393389 0.394174 0.395712 0.397478 0.399466 0.400252
0.401648 0.403508 0.404577 0.405603 0.407856 0.410371 0.412073 0.413378
[,289] [,290] [,291] [,292] [,293] [,294] [,295] [,296]
[,297] [,298] [,299] [,300] [,301] [,302] [,303] [,304]
[1,] 0.420799 0.422728 0.423208 0.423997 0.425708 0.426409 0.429087 0.432339
0.433095 0.433709 0.434516 0.437065 0.438735 0.439814 0.440373 0.443363
[2,] 0.425506 0.427157 0.427713 0.431510 0.432197 0.433953 0.434668 0.435692
0.436907 0.439771 0.440762 0.442614 0.445404 0.447893 0.449771 0.453794
[3,] 0.409489 0.411831 0.414712 0.415024 0.416026 0.418994 0.419446 0.421293
0.421424 0.422735 0.426453 0.426787 0.427552 0.431679 0.432421 0.432956
[4,] 0.414721 0.415082 0.416436 0.418128 0.419721 0.422160 0.422397 0.423914
0.425648 0.426006 0.429124 0.431799 0.432727 0.433914 0.436388 0.438365
[,305] [,306] [,307] [,308] [,309] [,310] [,311] [,312]
[,313] [,314] [,315] [,316] [,317] [,318] [,319] [,320]
[1,] 0.444084 0.445815 0.451743 0.453452 0.455909 0.458576 0.460005 0.461501
0.462455 0.464395 0.465632 0.467215 0.469890 0.471837 0.473434 0.474558
[2,] 0.454288 0.456555 0.458227 0.458869 0.460331 0.460797 0.461805 0.464003
0.465572 0.466931 0.467075 0.469841 0.470683 0.472265 0.473655 0.474559
[3,] 0.434909 0.436328 0.438683 0.439557 0.442218 0.445265 0.446686 0.449948
0.450232 0.451678 0.452197 0.455673 0.458211 0.458653 0.459595 0.459949
[4,] 0.440498 0.443396 0.443968 0.445560 0.446653 0.446971 0.449780 0.451904
0.453327 0.455097 0.456806 0.459588 0.460712 0.463181 0.463846 0.466888
[,321] [,322] [,323] [,324] [,325] [,326] [,327] [,328]
[,329] [,330] [,331] [,332] [,333] [,334] [,335] [,336]
[1,] 0.475148 0.476375 0.478172 0.480177 0.482574 0.483544 0.485408 0.487953
0.490031 0.494681 0.494941 0.496358 0.500123 0.502286 0.503626 0.504907
[2,] 0.476175 0.477712 0.477880 0.481244 0.483186 0.484316 0.488069 0.488806
0.491285 0.493772 0.494505 0.497194 0.500146 0.501804 0.502602 0.504738
[3,] 0.462416 0.465314 0.466659 0.469052 0.469856 0.471178 0.473270 0.473959
0.476159 0.480150 0.481408 0.482929 0.484409 0.485127 0.488346 0.489931
[4,] 0.469074 0.471173 0.472415 0.475523 0.477969 0.478399 0.480723 0.482170
0.483677 0.487099 0.488842 0.489402 0.493104 0.493424 0.494629 0.495870
[,337] [,338] [,339] [,340] [,341] [,342] [,343] [,344]
[,345] [,346] [,347] [,348] [,349] [,350] [,351] [,352]
[1,] 0.506157 0.507772 0.508715 0.512315 0.514746 0.515840 0.518105 0.518702
0.521136 0.522162 0.526069 0.526726 0.528168 0.529941 0.531789 0.535460
[2,] 0.505471 0.508768 0.509428 0.512123 0.513282 0.513901 0.517639 0.519078
0.520801 0.523307 0.524722 0.525208 0.527913 0.530426 0.533710 0.534130
[3,] 0.490466 0.491843 0.492692 0.495606 0.498405 0.499048 0.501289 0.503020
0.505162 0.507617 0.509327 0.509889 0.515035 0.517780 0.519769 0.522601
[4,] 0.496689 0.499698 0.503298 0.504407 0.506337 0.507996 0.511751 0.513516
0.514970 0.516856 0.517448 0.518431 0.519671 0.521722 0.522557 0.526263
[,353] [,354] [,355] [,356] [,357] [,358] [,359] [,360]
[,361] [,362] [,363] [,364] [,365] [,366] [,367] [,368]
[1,] 0.535493 0.540462 0.541159 0.542560 0.544275 0.546186 0.547580 0.548724
0.549341 0.550610 0.553319 0.553836 0.558597 0.559867 0.561790 0.562545
[2,] 0.535614 0.537417 0.538461 0.541716 0.542501 0.546476 0.547105 0.548692
0.549942 0.552683 0.554395 0.556651 0.556886 0.558228 0.560207 0.565359
[3,] 0.525527 0.526321 0.527609 0.528270 0.529722 0.531728 0.533563 0.535165

```
0.539658 0.541442 0.542326 0.543188 0.545305 0.547746 0.549446 0.549997
[4,] 0.527430 0.530203 0.532217 0.534009 0.536141 0.536639 0.538244 0.540820
0.542133 0.544492 0.546017 0.548969 0.550331 0.552230 0.554632 0.557305
       [,369]   [,370]   [,371]   [,372]   [,373]   [,374]   [,375]   [,376]
[,377]   [,378]   [,379]   [,380]   [,381]   [,382]   [,383]   [,384]
[1,] 0.563517 0.566688 0.568951 0.570555 0.570713 0.572528 0.573983 0.576165
0.579717 0.580359 0.581473 0.584362 0.585309 0.587377 0.590313 0.590530
[2,] 0.566003 0.566970 0.569577 0.573118 0.574267 0.575340 0.577231 0.579662
0.580586 0.584012 0.586141 0.587624 0.588403 0.588712 0.594647 0.599062
[3,] 0.553396 0.554378 0.558588 0.559443 0.561082 0.562267 0.562670 0.566689
0.568223 0.569956 0.571375 0.573405 0.575350 0.577049 0.579048 0.579959
[4,] 0.558437 0.561587 0.563203 0.565558 0.566536 0.567978 0.570568 0.573332
0.573759 0.576585 0.577582 0.580505 0.581806 0.584002 0.584685 0.586993
       [,385]   [,386]   [,387]   [,388]   [,389]   [,390]   [,391]   [,392]
[,393]   [,394]   [,395]   [,396]   [,397]   [,398]   [,399]   [,400]
[1,] 0.593095 0.594302 0.596678 0.600800 0.601109 0.605613 0.605711 0.608156
0.609414 0.611612 0.615279 0.616788 0.617440 0.619462 0.622338 0.625249
[2,] 0.599929 0.602694 0.606346 0.607614 0.611773 0.614289 0.616111 0.618139
0.618991 0.622082 0.622894 0.625513 0.631471 0.634407 0.637456 0.638544
[3,] 0.581822 0.585085 0.585763 0.589949 0.592115 0.598104 0.599060 0.600522
0.602584 0.603517 0.606613 0.610662 0.612590 0.617161 0.618627 0.620290
[4,] 0.589954 0.591636 0.592848 0.595928 0.597631 0.601601 0.603128 0.603428
0.609028 0.609131 0.613059 0.616391 0.617881 0.620406 0.621186 0.624530
       [,401]   [,402]   [,403]   [,404]   [,405]   [,406]   [,407]   [,408]
[,409]   [,410]   [,411]   [,412]   [,413]   [,414]   [,415]   [,416]
[1,] 0.625848 0.627536 0.628924 0.630758 0.635251 0.638299 0.639940 0.642866
0.650399 0.651435 0.653092 0.654890 0.657852 0.660882 0.662479 0.666978
[2,] 0.641895 0.642985 0.643958 0.648450 0.651931 0.655376 0.658041 0.662055
0.664453 0.664930 0.668562 0.669235 0.671465 0.674807 0.676522 0.679694
[3,] 0.623937 0.626874 0.630153 0.634546 0.635574 0.639197 0.641166 0.642118
0.643476 0.647249 0.650189 0.653008 0.655483 0.656436 0.658698 0.660852
[4,] 0.626193 0.630714 0.632971 0.637217 0.639796 0.642205 0.644314 0.647886
0.650966 0.651620 0.654260 0.655409 0.658846 0.662925 0.664271 0.665851
       [,417]   [,418]   [,419]   [,420]   [,421]   [,422]   [,423]   [,424]
[,425]   [,426]   [,427]   [,428]   [,429]   [,430]   [,431]   [,432]
[1,] 0.669131 0.671271 0.673591 0.676799 0.680038 0.681982 0.685645 0.688213
0.695824 0.698156 0.701997 0.706079 0.710067 0.711479 0.712659 0.716776
[2,] 0.681330 0.683006 0.684547 0.687335 0.689789 0.690868 0.692779 0.693691
0.696185 0.700873 0.704588 0.705942 0.710091 0.715793 0.718890 0.721905
[3,] 0.663405 0.666343 0.667430 0.669015 0.671853 0.674807 0.676140 0.677282
0.679849 0.680728 0.681829 0.685669 0.687706 0.688187 0.691361 0.692868
[4,] 0.667695 0.671036 0.673608 0.673686 0.675792 0.676091 0.680338 0.683416
0.684042 0.686609 0.686894 0.692123 0.695780 0.696060 0.697449 0.699894
       [,433]   [,434]   [,435]   [,436]   [,437]   [,438]   [,439]   [,440]
[,441]   [,442]   [,443]   [,444]   [,445]   [,446]   [,447]   [,448]
[1,] 0.722859 0.723808 0.726700 0.728204 0.730435 0.732585 0.734293 0.734791
0.739263 0.742039 0.744312 0.744859 0.746911 0.748761 0.753021 0.754111
[2,] 0.724036 0.726855 0.728037 0.731981 0.733597 0.735916 0.737809 0.744132
0.744865 0.745467 0.747909 0.748531 0.752164 0.753340 0.756240 0.757617
[3,] 0.695728 0.699587 0.702282 0.704819 0.707651 0.709311 0.715757 0.719613
0.719993 0.722033 0.724581 0.729663 0.730317 0.733499 0.737439 0.739391
[4,] 0.705165 0.707101 0.710356 0.713598 0.714698 0.717911 0.718472 0.721633
0.724709 0.728055 0.729896 0.731871 0.734518 0.734787 0.739639 0.743287
       [,449]   [,450]   [,451]   [,452]   [,453]   [,454]   [,455]   [,456]
```

[,457] [,458] [,459] [,460] [,461] [,462] [,463] [,464]
[1,] 0.756809 0.759420 0.761709 0.763800 0.765496 0.773232 0.774019 0.775489
0.777976 0.784027 0.787676 0.789238 0.789720 0.793885 0.794847 0.796540
[2,] 0.759681 0.759943 0.764848 0.766125 0.766561 0.768290 0.770856 0.773523
0.776471 0.778213 0.782497 0.785472 0.787044 0.787820 0.792005 0.796540
[3,] 0.743532 0.747001 0.749820 0.752310 0.753178 0.754587 0.757930 0.760868
0.764548 0.766730 0.770734 0.773819 0.775111 0.777475 0.781763 0.782723
[4,] 0.745429 0.748785 0.749865 0.751681 0.754983 0.759079 0.761713 0.764294
0.765681 0.771404 0.774004 0.776027 0.776791 0.778018 0.779998 0.782438
[,465] [,466] [,467] [,468] [,469] [,470] [,471] [,472]
[,473] [,474] [,475] [,476] [,477] [,478] [,479] [,480]
[1,] 0.798755 0.799448 0.801813 0.806019 0.809372 0.812523 0.814193 0.818894
0.821206 0.823694 0.826758 0.830657 0.831889 0.838008 0.841772 0.843505
[2,] 0.798477 0.801732 0.804353 0.805553 0.809516 0.810513 0.814268 0.817762
0.818685 0.822287 0.823635 0.828299 0.831817 0.835599 0.839482 0.842593
[3,] 0.788303 0.789742 0.791997 0.797252 0.798931 0.800142 0.802986 0.806411
0.809535 0.810914 0.816192 0.817762 0.821437 0.824200 0.824907 0.826943
[4,] 0.783507 0.785465 0.786229 0.789015 0.791667 0.795620 0.797278 0.801016
0.806575 0.811815 0.812640 0.814046 0.818595 0.823774 0.824242 0.827910
[,481] [,482] [,483] [,484] [,485] [,486] [,487] [,488]
[,489] [,490] [,491] [,492] [,493] [,494] [,495] [,496]
[1,] 0.844704 0.846718 0.848157 0.851703 0.853289 0.858557 0.863559 0.867838
0.869613 0.873955 0.874677 0.878121 0.879761 0.884534 0.891605 0.895416
[2,] 0.845768 0.848603 0.851956 0.856182 0.856867 0.857840 0.859674 0.865346
0.867389 0.869137 0.874067 0.876705 0.878461 0.880449 0.886909 0.887887
[3,] 0.831704 0.833051 0.837471 0.839308 0.841165 0.842897 0.845809 0.850987
0.852485 0.856877 0.858630 0.861837 0.864994 0.873019 0.879507 0.882160
[4,] 0.832051 0.834560 0.835118 0.837635 0.840406 0.840577 0.846802 0.849257
0.849676 0.852072 0.854359 0.856781 0.862643 0.867971 0.871848 0.875874
[,497] [,498] [,499] [,500] [,501] [,502] [,503] [,504]
[,505] [,506] [,507] [,508] [,509] [,510] [,511] [,512]
[1,] 0.899240 0.900661 0.908144 0.913072 0.916505 0.917924 0.921977 0.922385
0.924124 0.931626 0.932996 0.938726 0.943366 0.947544 0.951560 0.953124
[2,] 0.892500 0.895082 0.895850 0.900305 0.904188 0.906711 0.910016 0.910624
0.915279 0.917575 0.918446 0.919986 0.921443 0.924287 0.929783 0.931564
[3,] 0.886686 0.887671 0.890507 0.894013 0.896756 0.898316 0.902267 0.903097
0.907689 0.918242 0.919920 0.921639 0.922392 0.924730 0.926684 0.929596
[4,] 0.879491 0.884221 0.890932 0.893261 0.896209 0.900281 0.902669 0.905186
0.907752 0.911965 0.915000 0.917172 0.919209 0.922092 0.924754 0.930538
[,513] [,514] [,515] [,516] [,517] [,518] [,519] [,520]
[,521] [,522] [,523] [,524] [,525] [,526] [,527] [,528]
[1,] 0.957516 0.960114 0.961621 0.963643 0.964108 0.968369 0.973095 0.974184
0.977838 0.980411 0.982995 0.985614 0.986236 0.991038 0.993520 0.998376
[2,] 0.933755 0.934152 0.936837 0.938647 0.945690 0.947366 0.950208 0.955244
0.958215 0.960166 0.964824 0.967393 0.968761 0.970750 0.971772 0.975197
[3,] 0.932114 0.936866 0.937664 0.939686 0.944529 0.947593 0.952655 0.955847
0.961072 0.963254 0.967175 0.969837 0.970560 0.974841 0.978525 0.982141
[4,] 0.931464 0.934398 0.936490 0.941352 0.945186 0.946603 0.951577 0.957783
0.958673 0.961069 0.964440 0.967120 0.971161 0.972510 0.972750 0.975361
[,529] [,530] [,531] [,532] [,533] [,534] [,535] [,536]
[,537] [,538] [,539] [,540] [,541] [,542] [,543] [,544]
[1,] 0.999216 1.001144 1.002939 1.006360 1.008213 1.011249 1.016676 1.020242
1.027271 1.031729 1.033613 1.036415 1.039661 1.046494 1.048786 1.053710
[2,] 0.978757 0.981616 0.985372 0.988158 0.992232 0.994224 1.000162 1.005316

```
1.008092 1.014653 1.016752 1.020494 1.025449 1.031573 1.035389 1.042124
[3,] 0.983684 0.996006 0.998117 1.001659 1.006156 1.007099 1.009389 1.012359
1.014277 1.016544 1.017411 1.027270 1.029488 1.033166 1.035100 1.038635
[4,] 0.977801 0.981465 0.984763 0.985475 0.988271 0.993295 1.001361 1.003900
1.007749 1.010509 1.014088 1.016294 1.022360 1.027231 1.029264 1.034140
        [,545]   [,546]   [,547]   [,548]   [,549]   [,550]   [,551]   [,552]
[,553]   [,554]   [,555]   [,556]   [,557]   [,558]   [,559]   [,560]
[1,] 1.055721 1.057311 1.063941 1.066303 1.067388 1.073128 1.074716 1.078618
1.079610 1.081514 1.087760 1.088250 1.095413 1.097580 1.109424 1.113196
[2,] 1.045539 1.051296 1.053222 1.055089 1.060106 1.063775 1.066537 1.072329
1.076897 1.082437 1.083625 1.088098 1.090360 1.093969 1.102674 1.104991
[3,] 1.039509 1.043490 1.044193 1.051852 1.053700 1.056719 1.058908 1.061156
1.064749 1.073817 1.076479 1.082338 1.086228 1.091463 1.093286 1.097527
[4,] 1.038607 1.046069 1.050777 1.053866 1.058722 1.064652 1.069094 1.072498
1.077245 1.080326 1.081912 1.083963 1.088709 1.092527 1.095331 1.097104
        [,561]   [,562]   [,563]   [,564]   [,565]   [,566]   [,567]   [,568]
[,569]   [,570]   [,571]   [,572]   [,573]   [,574]   [,575]   [,576]
[1,] 1.121967 1.122677 1.125737 1.129394 1.133221 1.138761 1.148395 1.150548
1.161843 1.169845 1.175635 1.177022 1.178063 1.184725 1.190374 1.197511
[2,] 1.106549 1.108926 1.113522 1.117601 1.124207 1.127949 1.129726 1.138198
1.143842 1.146917 1.152279 1.156039 1.159807 1.165087 1.168281 1.172637
[3,] 1.100320 1.101769 1.102839 1.107213 1.118178 1.120058 1.122097 1.125941
1.126966 1.132163 1.136355 1.136956 1.139887 1.147006 1.150964 1.153562
[4,] 1.101551 1.105312 1.110177 1.111427 1.118302 1.118879 1.121759 1.126137
1.129091 1.134789 1.142616 1.146922 1.148864 1.152128 1.155987 1.159404
        [,577]   [,578]   [,579]   [,580]   [,581]   [,582]   [,583]   [,584]
[,585]   [,586]   [,587]   [,588]   [,589]   [,590]   [,591]   [,592]
[1,] 1.199761 1.202034 1.203972 1.205763 1.208995 1.213636 1.218746 1.228083
1.232275 1.235426 1.238621 1.240087 1.242516 1.246209 1.248313 1.250027
[2,] 1.178848 1.182460 1.184586 1.192235 1.196556 1.200093 1.210728 1.214043
1.214914 1.218057 1.220093 1.223271 1.228641 1.234187 1.237690 1.238910
[3,] 1.156162 1.159998 1.162772 1.172195 1.174611 1.177506 1.185971 1.191564
1.196029 1.197863 1.201055 1.203684 1.206027 1.208254 1.212131 1.213017
[4,] 1.161222 1.164158 1.165563 1.170391 1.173993 1.176802 1.178056 1.185490
1.192382 1.197371 1.199894 1.208374 1.209083 1.210421 1.219334 1.222922
        [,593]   [,594]   [,595]   [,596]   [,597]   [,598]   [,599]   [,600]
[,601]   [,602]   [,603]   [,604]   [,605]   [,606]   [,607]   [,608]
[1,] 1.254635 1.262425 1.265388 1.270992 1.272598 1.275690 1.287012 1.288819
1.294010 1.298019 1.300523 1.302316 1.305151 1.307980 1.309819 1.320131
[2,] 1.241485 1.246257 1.252183 1.254770 1.261197 1.264475 1.267658 1.270716
1.273242 1.283207 1.285674 1.291322 1.295365 1.301181 1.305693 1.307583
[3,] 1.215259 1.224100 1.227285 1.231153 1.234613 1.236491 1.251414 1.254973
1.258234 1.264376 1.265426 1.269275 1.273738 1.276435 1.279255 1.282475
[4,] 1.227998 1.230135 1.233779 1.239599 1.241692 1.245463 1.248694 1.251787
1.259799 1.264707 1.265946 1.273097 1.275054 1.281767 1.284545 1.287485
        [,609]   [,610]   [,611]   [,612]   [,613]   [,614]   [,615]   [,616]
[,617]   [,618]   [,619]   [,620]   [,621]   [,622]   [,623]   [,624]
[1,] 1.321739 1.326719 1.331035 1.338044 1.340590 1.343509 1.350336 1.352917
1.357901 1.359262 1.362721 1.365237 1.377254 1.380523 1.384994 1.388572
[2,] 1.313820 1.319135 1.322622 1.324869 1.328766 1.335203 1.337746 1.342651
1.346767 1.349036 1.350084 1.358627 1.370623 1.372953 1.374110 1.378752
[3,] 1.285286 1.288341 1.291974 1.300525 1.304262 1.306357 1.315665 1.324315
1.325945 1.332109 1.332598 1.339965 1.342198 1.343323 1.349015 1.357193
[4,] 1.293712 1.297148 1.299292 1.303761 1.306998 1.311867 1.317615 1.319719
```

1.324376 1.325123 1.336079 1.342530 1.345212 1.349650 1.350389 1.353524
       [,625]   [,626]   [,627]   [,628]   [,629]   [,630]   [,631]   [,632]
[,633]   [,634]   [,635]   [,636]   [,637]   [,638]   [,639]   [,640]
[1,] 1.390741 1.395198 1.398035 1.402065 1.405687 1.407930 1.412191 1.414237
1.420847 1.425463 1.431604 1.435506 1.436639 1.440279 1.445484 1.449216
[2,] 1.384211 1.388061 1.394856 1.399959 1.402482 1.405492 1.412340 1.415264
1.419693 1.422041 1.423115 1.426085 1.430587 1.434340 1.436672 1.438835
[3,] 1.361940 1.364703 1.367858 1.370340 1.374123 1.378911 1.382200 1.384936
1.390924 1.393971 1.398990 1.409880 1.412304 1.417517 1.420161 1.420712
[4,] 1.356204 1.358818 1.364592 1.375111 1.380146 1.382489 1.384518 1.387001
1.400255 1.404193 1.407592 1.409568 1.420165 1.426297 1.427336 1.439387
       [,641]   [,642]   [,643]   [,644]   [,645]   [,646]   [,647]   [,648]
[,649]   [,650]   [,651]   [,652]   [,653]   [,654]   [,655]   [,656]
[1,] 1.452946 1.456656 1.461239 1.462600 1.468247 1.471447 1.477417 1.480034
1.482194 1.496083 1.497883 1.502050 1.510010 1.512820 1.516490 1.520029
[2,] 1.449076 1.456017 1.458741 1.460149 1.468772 1.469451 1.473517 1.484638
1.490949 1.495786 1.507922 1.515181 1.516099 1.523436 1.525556 1.528494
[3,] 1.428816 1.430663 1.436468 1.443047 1.449271 1.451408 1.461020 1.465105
1.468682 1.471381 1.477072 1.479747 1.481221 1.485147 1.488061 1.492355
[4,] 1.443916 1.449528 1.457084 1.461228 1.464200 1.466795 1.475902 1.480335
1.486389 1.488290 1.494330 1.500588 1.503108 1.506374 1.513989 1.518301
       [,657]   [,658]   [,659]   [,660]   [,661]   [,662]   [,663]   [,664]
[,665]   [,666]   [,667]   [,668]   [,669]   [,670]   [,671]   [,672]
[1,] 1.522665 1.530334 1.530881 1.538339 1.540156 1.543001 1.545620 1.550733
1.556520 1.559317 1.566893 1.574098 1.578542 1.586260 1.594634 1.602141
[2,] 1.532943 1.534219 1.536871 1.541084 1.542919 1.549104 1.556761 1.560978
1.568821 1.574131 1.577084 1.582411 1.590471 1.595630 1.596908 1.601915
[3,] 1.498744 1.501625 1.504024 1.506587 1.508685 1.513004 1.515968 1.517640
1.536875 1.537554 1.546190 1.556466 1.557469 1.564771 1.572057 1.576524
[4,] 1.523844 1.530429 1.538048 1.538572 1.543756 1.552813 1.553815 1.554642
1.558569 1.561108 1.566098 1.581165 1.585100 1.595116 1.596549 1.599550
       [,673]   [,674]   [,675]   [,676]   [,677]   [,678]   [,679]   [,680]
[,681]   [,682]   [,683]   [,684]   [,685]   [,686]   [,687]   [,688]
[1,] 1.604861 1.611255 1.619856 1.627223 1.637529 1.639470 1.646680 1.648013
1.650207 1.653893 1.662659 1.673283 1.677876 1.681157 1.688115 1.698221
[2,] 1.603151 1.607064 1.610694 1.618977 1.623519 1.629732 1.629988 1.636122
1.637788 1.641338 1.642965 1.649418 1.661185 1.671364 1.679267 1.686746
[3,] 1.577534 1.580200 1.583816 1.587487 1.591005 1.599200 1.599872 1.602190
1.607619 1.612911 1.629120 1.636677 1.638507 1.646341 1.648513 1.652784
[4,] 1.607971 1.609289 1.613385 1.615244 1.621425 1.627050 1.633311 1.636863
1.641568 1.647193 1.650423 1.653423 1.663399 1.668675 1.680226 1.693774
       [,689]   [,690]   [,691]   [,692]   [,693]   [,694]   [,695]   [,696]
[,697]   [,698]   [,699]   [,700]   [,701]   [,702]   [,703]   [,704]
[1,] 1.699115 1.701634 1.707410 1.712182 1.725596 1.728265 1.738209 1.743328
1.748716 1.753174 1.761317 1.765874 1.772071 1.776679 1.785745 1.790781
[2,] 1.695393 1.707521 1.709430 1.712749 1.715443 1.722918 1.735233 1.738860
1.741904 1.748118 1.755132 1.770196 1.778718 1.787785 1.792837 1.804859
[3,] 1.655145 1.661388 1.662438 1.670609 1.677500 1.684538 1.688827 1.689963
1.698373 1.701365 1.708805 1.710079 1.711988 1.726104 1.735658 1.736987
[4,] 1.694810 1.697646 1.700662 1.707457 1.719931 1.721921 1.726554 1.733501
1.735454 1.738197 1.744614 1.751968 1.758356 1.767181 1.770560 1.779708
       [,705]   [,706]   [,707]   [,708]   [,709]   [,710]   [,711]   [,712]
[,713]   [,714]   [,715]   [,716]   [,717]   [,718]   [,719]   [,720]
[1,] 1.791911 1.795566 1.799432 1.805202 1.816910 1.820681 1.824373 1.844166

1.850639 1.857505 1.861575 1.870597 1.891172 1.895890 1.898320 1.912238
[2,] 1.806960 1.814633 1.816305 1.835283 1.840855 1.849722 1.854603 1.861661
1.864432 1.865034 1.871044 1.873253 1.886197 1.888479 1.896918 1.901939
[3,] 1.747190 1.752419 1.759649 1.766193 1.767596 1.780736 1.787689 1.800966
1.806544 1.813409 1.819099 1.824197 1.828083 1.841871 1.847084 1.853469
[4,] 1.783777 1.788644 1.791232 1.796295 1.801345 1.808116 1.817312 1.831114
1.847447 1.850354 1.852677 1.860634 1.881166 1.886830 1.887532 1.891293
[,721] [,722] [,723] [,724] [,725] [,726] [,727] [,728]
[,729] [,730] [,731] [,732] [,733] [,734] [,735] [,736]
[1,] 1.913053 1.934794 1.942051 1.943853 1.953148 1.968591 1.971221 1.989110
1.993930 2.000380 2.004985 2.008902 2.015918 2.026217 2.035339 2.050088
[2,] 1.917268 1.923008 1.926209 1.937117 1.951233 1.960141 1.964878 1.974599
1.977736 1.988874 1.989521 2.020333 2.027054 2.030340 2.032980 2.036252
[3,] 1.858046 1.860818 1.865613 1.873495 1.879452 1.882072 1.886113 1.905757
1.920320 1.923450 1.928868 1.940369 1.965361 1.973675 1.981638 1.985458
[4,] 1.895318 1.899033 1.910726 1.914112 1.923141 1.928516 1.934132 1.937011
1.947318 1.961638 1.962657 1.966147 1.981398 1.989845 1.997525 2.004348
[,737] [,738] [,739] [,740] [,741] [,742] [,743] [,744]
[,745] [,746] [,747] [,748] [,749] [,750] [,751] [,752]
[1,] 2.058933 2.073504 2.078091 2.078789 2.084243 2.096313 2.112098 2.113077
2.121520 2.125568 2.131530 2.141500 2.162964 2.164478 2.174627 2.180746
[2,] 2.045197 2.053290 2.061443 2.071354 2.078371 2.081828 2.095402 2.105633
2.116909 2.120622 2.122237 2.133719 2.140480 2.153200 2.155563 2.167306
[3,] 1.988525 1.994024 2.000281 2.001093 2.012279 2.022483 2.027561 2.038847
2.040817 2.054394 2.082833 2.089384 2.094898 2.101942 2.107438 2.108512
[4,] 2.009825 2.016010 2.019091 2.021671 2.026493 2.050506 2.064546 2.067890
2.076862 2.086126 2.090823 2.118569 2.133342 2.144512 2.158551 2.160870
[,753] [,754] [,755] [,756] [,757] [,758] [,759] [,760]
[,761] [,762] [,763] [,764] [,765] [,766] [,767] [,768]
[1,] 2.192047 2.199752 2.220254 2.239070 2.245503 2.251921 2.254253 2.258909
2.269859 2.280834 2.285085 2.312635 2.313772 2.317262 2.326496 2.349242
[2,] 2.182892 2.192640 2.203213 2.215203 2.235020 2.251725 2.253844 2.259369
2.271482 2.274828 2.280751 2.300017 2.302698 2.310542 2.329977 2.349124
[3,] 2.114135 2.138991 2.140297 2.147050 2.153444 2.161122 2.162002 2.176078
2.180646 2.211784 2.220485 2.234607 2.260289 2.260908 2.263676 2.273664
[4,] 2.164322 2.166562 2.168179 2.171986 2.175374 2.181923 2.189406 2.193156
2.195195 2.216780 2.222313 2.228395 2.235362 2.257537 2.261286 2.266235
[,769] [,770] [,771] [,772] [,773] [,774] [,775] [,776]
[,777] [,778] [,779] [,780] [,781] [,782] [,783] [,784]
[1,] 2.351569 2.352179 2.370561 2.382367 2.383576 2.418484 2.446178 2.461629
2.463861 2.466682 2.503927 2.513590 2.522172 2.536073 2.544268 2.549413
[2,] 2.356586 2.381520 2.387887 2.394229 2.403912 2.407692 2.415409 2.433590
2.450103 2.452532 2.458998 2.462712 2.472994 2.475806 2.502642 2.503929
[3,] 2.287911 2.293818 2.294099 2.302565 2.314405 2.327928 2.350405 2.356096
2.387330 2.391910 2.426217 2.449249 2.456641 2.464218 2.493157 2.511893
[4,] 2.320358 2.345447 2.347085 2.351584 2.358109 2.369517 2.385081 2.409306
2.416535 2.455299 2.470115 2.473671 2.482141 2.498250 2.511621 2.523348
[,785] [,786] [,787] [,788] [,789] [,790] [,791] [,792]
[,793] [,794] [,795] [,796] [,797] [,798] [,799] [,800]
[1,] 2.565546 2.581001 2.595372 2.616227 2.622834 2.632609 2.643429 2.646625
2.650232 2.651649 2.677842 2.680860 2.692784 2.698827 2.700604 2.731753
[2,] 2.513360 2.529579 2.530977 2.542490 2.572413 2.581405 2.583237 2.600025
2.606430 2.613287 2.646332 2.650483 2.688909 2.705336 2.719155 2.727279
[3,] 2.531990 2.543597 2.551891 2.570803 2.587764 2.588952 2.595654 2.606087

2.616855 2.622392 2.641187 2.648498 2.664951 2.666225 2.674108 2.695332
[4,] 2.530539 2.541327 2.548087 2.552716 2.571532 2.581287 2.618859 2.631314
2.639419 2.641355 2.680722 2.693449 2.702432 2.709301 2.716565 2.720313
[,801] [,802] [,803] [,804] [,805] [,806] [,807] [,808]
[,809] [,810] [,811] [,812] [,813] [,814] [,815] [,816]
[1,] 2.741753 2.754149 2.759355 2.769574 2.784809 2.790119 2.801361 2.832042
2.849558 2.885531 2.893832 2.934880 2.966301 3.010912 3.021245 3.031425
[2,] 2.734988 2.756744 2.766159 2.779991 2.780121 2.813495 2.822959 2.867989
2.882304 2.887503 2.894335 2.920280 2.940907 2.956715 2.964729 2.973736
[3,] 2.701642 2.707439 2.723852 2.742138 2.742729 2.774952 2.779902 2.801677
2.806683 2.820326 2.843726 2.847104 2.851746 2.865024 2.882608 2.908628
[4,] 2.730719 2.733279 2.749215 2.762618 2.769149 2.782022 2.803679 2.805785
2.822152 2.833445 2.852273 2.885912 2.894362 2.900261 2.921669 2.942124
[,817] [,818] [,819] [,820] [,821] [,822] [,823] [,824]
[,825] [,826] [,827] [,828] [,829] [,830] [,831] [,832]
[1,] 3.054602 3.054869 3.079265 3.114339 3.153893 3.167795 3.174072 3.177557
3.203014 3.206411 3.245336 3.274625 3.323890 3.345744 3.371664 3.396530
[2,] 2.988853 3.012732 3.045322 3.073378 3.074163 3.079022 3.090546 3.098351
3.102070 3.109175 3.169496 3.184413 3.205548 3.251195 3.258535 3.284859
[3,] 2.911488 2.913659 2.928427 2.965412 3.012030 3.042397 3.106252 3.118736
3.135065 3.141951 3.165991 3.199676 3.206025 3.235600 3.258913 3.285534
[4,] 2.958839 2.997404 3.005170 3.018082 3.023906 3.042158 3.046941 3.049873
3.057862 3.067520 3.127657 3.132616 3.149232 3.171248 3.178047 3.200972
[,833] [,834] [,835] [,836] [,837] [,838] [,839] [,840]
[,841] [,842] [,843] [,844] [,845] [,846] [,847] [,848]
[1,] 3.412609 3.429601 3.476508 3.477903 3.545081 3.549204 3.572869 3.589627
3.621346 3.648136 3.660278 3.683358 3.698891 3.752654 3.756804 3.790939
[2,] 3.292078 3.342205 3.355977 3.382534 3.400217 3.426052 3.431870 3.489103
3.504311 3.578625 3.598946 3.606084 3.617591 3.680087 3.711262 3.717395
[3,] 3.319774 3.328406 3.345326 3.382032 3.409052 3.458576 3.490318 3.509012
3.517539 3.684009 3.711835 3.722628 3.737309 3.770400 3.781948 3.792414
[4,] 3.224060 3.341232 3.372289 3.409618 3.411644 3.430213 3.462381 3.493031
3.519544 3.526574 3.532857 3.571527 3.588349 3.597647 3.632208 3.697935
[,849] [,850] [,851] [,852] [,853] [,854] [,855] [,856]
[,857] [,858] [,859] [,860] [,861] [,862] [,863] [,864]
[1,] 3.881167 3.897306 3.977362 4.004025 4.019477 4.091555 4.113996 4.128879
4.196370 4.196465 4.221719 4.258379 4.384851 4.464526 4.494107 4.522305
[2,] 3.721566 3.740585 3.790704 3.823181 3.893548 3.968604 4.008927 4.021672
4.087275 4.096729 4.312190 4.430271 4.460093 4.485457 4.523152 4.643320
[3,] 3.818739 3.865704 3.904699 3.936251 3.950596 3.957096 4.003031 4.050247
4.244088 4.299258 4.314510 4.363980 4.398707 4.447465 4.498088 4.587395
[4,] 3.700154 3.739151 3.740955 3.750794 3.764782 3.848747 3.849042 3.860421
3.876801 3.944919 3.971295 4.068017 4.167264 4.290909 4.302451 4.387756
[,865] [,866] [,867] [,868] [,869] [,870] [,871] [,872]
[,873] [,874] [,875] [,876] [,877] [,878] [,879] [,880]
[1,] 4.550875 4.599177 4.653197 4.690138 4.751529 4.807293 4.852652 4.905280
5.077230 5.155261 5.180423 5.215384 5.237488 5.392901 5.430719 5.534095
[2,] 4.687608 4.694299 4.700495 4.759318 4.792534 4.995668 5.059772 5.142835
5.296802 5.330309 5.448843 5.486993 5.766315 5.911060 6.004699 6.038925
[3,] 4.602075 4.628278 4.638248 4.763053 4.766086 4.780138 4.823873 5.119692
5.160538 5.173476 5.315390 5.497087 5.604695 5.648878 5.673928 5.781183
[4,] 4.426936 4.432215 4.463469 4.568987 4.605133 4.703517 4.946794 4.986292
4.988660 5.058539 5.104155 5.455213 5.485000 5.504880 5.676558 5.881281
[,881] [,882] [,883] [,884] [,885] [,886] [,887] [,888]

```
[,889]   [,890]   [,891]   [,892]   [,893]   [,894]   [,895]   [,896]
[1,] 5.589965 5.767867 6.052346 6.078269 6.085933 6.393687 6.487933 6.641742
6.738408 6.969921 7.036731 7.140404 7.610609 7.899896 8.344070 8.435703
[2,] 6.262782 6.395895 6.487005 6.785208 7.098957 7.293727 7.310551 7.365915
7.404848 7.449041 7.723867 8.207970 8.420599 8.491463 8.537327 8.907593
[3,] 5.966968 6.106890 6.424964 6.598189 6.681010 7.118384 7.190721 7.219088
7.895013 8.072613 8.085137 8.411818 8.428674 8.650435 8.779201 8.901919
[4,] 5.972497 6.265774 6.350889 6.458562 6.512274 6.517672 6.663337 6.818611
7.474427 7.762308 7.952604 7.975595 8.133127 8.145657 8.169175 8.222715
       [,897]   [,898]   [,899]   [,900]   [,901]   [,902]   [,903]    [,904]
[,905]    [,906]    [,907]   [,908]   [,909]   [,910]   [,911]   [,912]
[1,] 8.671456 9.036462 9.148956 9.153180 9.211363 9.262603 9.441913  9.672174
9.846933 10.221835 10.234272 10.28064 10.50622 10.58955 10.79782 10.98169
[2,] 8.936631 9.148497 9.529522 9.556449 9.628405 9.672999 9.677895  9.915883
10.271418 10.435723 10.569179 10.60457 10.63879 10.76614 10.77615 10.96440
[3,] 8.989596 8.991283 9.240579 9.633358 9.648136 9.654826 9.839453 10.003221
10.350040 10.537079 10.559595 10.56356 10.59809 10.65525 10.88509 10.91682
[4,] 8.416475 8.692710 8.892249 9.064702 9.204264 9.205689 9.305720  9.315372
9.503633  9.677054  9.833476 10.03766 10.23263 10.35013 10.49814 10.58428
       [,913]   [,914]   [,915]   [,916]   [,917]   [,918]   [,919]   [,920]
[,921]   [,922]   [,923]   [,924]   [,925]   [,926]   [,927]   [,928]
[1,] 11.09261 11.20552 11.31983 11.56842 11.57700 11.61765 11.64611 12.00462
12.04777 12.12590 12.12691 12.21266 12.22715 12.24914 12.34720 12.35527
[2,] 10.99462 11.04093 11.05092 11.12447 11.15378 11.29200 11.45986 11.48429
11.59333 11.73500 11.85036 11.90537 11.94516 11.96451 11.98884 12.01442
[3,] 11.09900 11.20834 11.41233 11.42383 11.45045 11.48889 11.59081 11.60456
11.61407 11.63018 11.70087 11.74779 11.88908 11.91667 11.97351 11.97814
[4,] 10.71805 10.73333 10.74124 11.02386 11.04173 11.12858 11.19039 11.32171
11.32598 11.38251 11.39596 11.61712 11.61919 11.66518 11.78379 11.93064
       [,929]   [,930]   [,931]   [,932]   [,933]   [,934]   [,935]   [,936]
[,937]   [,938]   [,939]   [,940]   [,941]   [,942]   [,943]   [,944]
[1,] 12.48413 12.49688 12.55555 12.56860 12.59643 12.60368 12.60831 12.62613
12.71297 12.73688 12.75301 12.77492 12.80505 12.82755 12.83605 12.83913
[2,] 12.06075 12.11583 12.24406 12.32634 12.35389 12.42489 12.43949 12.53486
12.56525 12.65438 12.74999 12.83416 12.90602 12.91124 13.04141 13.04472
[3,] 12.00921 12.02564 12.11332 12.29557 12.35068 12.40504 12.40718 12.44653
12.59372 12.62151 12.65018 12.80158 12.88349 12.89790 12.94506 13.00767
[4,] 11.97425 12.14235 12.15490 12.26029 12.28392 12.41116 12.51240 12.52374
12.63341 12.63857 12.65264 12.71027 12.74375 12.76485 12.77019 12.85741
       [,945]   [,946]   [,947]   [,948]   [,949]   [,950]   [,951]   [,952]
[,953]   [,954]   [,955]   [,956]   [,957]   [,958]   [,959]   [,960]
[1,] 12.92103 12.93476 13.06629 13.08822 13.13631 13.15088 13.27448 13.40996
13.41702 13.42911 13.45082 13.54154 13.62512 13.63403 13.63999 13.65272
[2,] 13.17161 13.17279 13.20559 13.22319 13.28236 13.41760 13.43084 13.44709
13.47292 13.56733 13.61585 13.66905 13.79268 13.84866 13.87428 13.89574
[3,] 13.01989 13.02487 13.04520 13.25126 13.33253 13.34027 13.48227 13.48972
13.50415 13.62654 13.64102 13.64795 13.65144 13.72236 13.84273 13.85074
[4,] 12.88621 12.92462 12.94309 13.02454 13.05455 13.08398 13.11650 13.13083
13.24874 13.29300 13.32253 13.37042 13.41956 13.43457 13.43856 13.46879
       [,961]   [,962]   [,963]   [,964]   [,965]   [,966]   [,967]   [,968]
[,969]   [,970]   [,971]   [,972]   [,973]   [,974]   [,975]   [,976]
[1,] 13.69797 13.88546 13.95388 13.95719 14.04772 14.17342 14.20013 14.23458
14.24269 14.32651 14.38298 14.42990 14.49937 14.51021 14.59647 14.61468
[2,] 13.93708 13.94279 13.94329 13.98435 13.98860 14.01638 14.08783 14.16470
```

```
14.19331 14.22055 14.25471 14.26264 14.34145 14.37537 14.39640 14.40097
[3,] 13.89803 13.95768 13.98368 14.02089 14.02905 14.04802 14.11136 14.15529
14.22867 14.24591 14.35336 14.46305 14.49214 14.49291 14.49885 14.51138
[4,] 13.48405 13.60718 13.65361 13.70448 13.75671 13.83797 13.90875 13.95571
14.00944 14.03985 14.09277 14.18415 14.21219 14.27157 14.27164 14.30939
      [,977]   [,978]   [,979]   [,980]   [,981]   [,982]   [,983]   [,984]
[,985]   [,986]   [,987]   [,988]   [,989]   [,990]   [,991]   [,992]
[1,] 14.64057 14.67435 14.74154 14.75928 14.78451 14.83727 14.88106 14.90054
14.90375 14.91929 14.95609 14.97610 15.07633 15.10980 15.14880 15.16770
[2,] 14.46156 14.46877 14.49981 14.51824 14.52195 14.55349 14.58299 14.61268
14.63270 14.64845 14.68349 14.72086 14.74324 14.78390 14.80019 14.83034
[3,] 14.59724 14.61709 14.64085 14.65460 14.65572 14.68254 14.70203 14.71902
14.73117 14.73495 14.77238 14.79218 14.79487 14.83034 14.90712 14.91572
[4,] 14.31421 14.33844 14.34718 14.46254 14.48511 14.49536 14.56432 14.61919
14.63090 14.63863 14.64031 14.67155 14.70549 14.73435 14.74425 14.74767
      [,993]   [,994]   [,995]   [,996]   [,997]   [,998]   [,999]  [,1000]
[,1001]  [,1002]  [,1003]  [,1004]  [,1005]  [,1006]  [,1007]  [,1008]
[1,] 15.19425 15.29375 15.32667 15.35650 15.36709 15.39286 15.45447 15.47003
15.52079 15.56443 15.56821 15.57483 15.58522 15.74338 15.78243 15.78769
[2,] 14.85679 14.85806 14.88485 14.91822 14.91895 14.97528 14.98974 15.08514
15.08675 15.14178 15.19240 15.20150 15.24360 15.26146 15.26710 15.30508
[3,] 14.99606 15.02640 15.04552 15.05039 15.08875 15.09162 15.13195 15.15842
15.17640 15.18154 15.25747 15.26562 15.26794 15.30839 15.36740 15.36814
[4,] 14.76392 14.78203 14.79273 14.82620 14.84925 14.85514 14.86104 14.87003
14.93848 14.96987 15.00607 15.05431 15.06625 15.11095 15.12651 15.15327
     [,1009]  [,1010]  [,1011]  [,1012]  [,1013]  [,1014]  [,1015]  [,1016]
[,1017]  [,1018]  [,1019]  [,1020]  [,1021]  [,1022]  [,1023]  [,1024]
[1,] 15.82237 15.89714 15.89798 15.96816 15.97822 16.06245 16.06656 16.09959
16.11921 16.12347 16.27705 16.29889 16.31007 16.32090 16.33124 16.33191
[2,] 15.31226 15.34416 15.36973 15.44689 15.49578 15.49877 15.50841 15.51185
15.51374 15.52587 15.59695 15.65121 15.66355 15.73579 15.74138 15.76995
[3,] 15.39669 15.39765 15.41995 15.42499 15.44754 15.45985 15.57723 15.58131
15.58647 15.61881 15.71517 15.76088 15.84233 15.86651 15.91053 15.96317
[4,] 15.17619 15.17824 15.18562 15.23114 15.25548 15.30410 15.31934 15.35541
15.38532 15.38932 15.45318 15.46471 15.49234 15.50509 15.64051 15.64058
     [,1025]  [,1026]  [,1027]  [,1028]  [,1029]  [,1030]  [,1031]  [,1032]
[,1033]  [,1034]  [,1035]  [,1036]  [,1037]  [,1038]  [,1039]  [,1040]
[1,] 16.43137 16.49535 16.52238 16.52888 16.54409 16.55951 16.56098 16.58085
16.59993 16.68939 16.72146 16.73146 16.73414 16.73807 16.81258 16.82540
[2,] 15.77077 15.81739 15.83903 15.83999 15.88324 15.99802 16.02069 16.05553
16.08665 16.10367 16.11664 16.12921 16.19559 16.19593 16.24008 16.28542
[3,] 15.96889 15.98710 15.99005 16.02350 16.10886 16.14574 16.16386 16.17620
16.19611 16.22952 16.24944 16.26679 16.29887 16.32180 16.32241 16.33935
[4,] 15.67272 15.68244 15.70550 15.75940 15.76359 15.76359 15.86759 15.87938
15.88242 15.98967 16.02928 16.11043 16.13720 16.16831 16.22136 16.22667
     [,1041]  [,1042]  [,1043]  [,1044]  [,1045]  [,1046]  [,1047]  [,1048]
[,1049]  [,1050]  [,1051]  [,1052]  [,1053]  [,1054]  [,1055]  [,1056]
[1,] 16.85136 16.86689 16.92447 16.96128 17.00057 17.00381 17.03936 17.04328
17.05715 17.07464 17.11042 17.13052 17.27314 17.27947 17.28378 17.30415
[2,] 16.30012 16.34444 16.45740 16.48843 16.50135 16.55264 16.55816 16.61414
16.62159 16.66758 16.67404 16.67713 16.68441 16.73145 16.75879 16.78104
[3,] 16.44418 16.48953 16.55824 16.57939 16.61197 16.61257 16.62062 16.64036
16.66071 16.68256 16.69529 16.82611 16.83803 16.84266 16.88196 16.92272
[4,] 16.24476 16.25885 16.29448 16.34352 16.37150 16.43724 16.44381 16.46574
```

```
16.55021 16.55643 16.57039 16.59620 16.61581 16.64295 16.66316 16.74204
      [,1057]  [,1058]  [,1059]  [,1060]  [,1061]  [,1062]  [,1063]  [,1064]
[,1065]  [,1066]  [,1067]  [,1068]  [,1069]  [,1070]  [,1071]  [,1072]
[1,] 17.31853 17.37357 17.39953 17.40069 17.41059 17.44583 17.52636 17.52693
17.54520 17.60595 17.64235 17.69224 17.70162 17.74875 17.89002 17.92569
[2,] 16.90247 16.94892 16.98845 17.03018 17.03431 17.05083 17.07505 17.10245
17.14052 17.14857 17.20848 17.25467 17.29052 17.29813 17.30882 17.35552
[3,] 16.96295 16.96669 17.00851 17.02654 17.03770 17.04840 17.06026 17.10161
17.10977 17.11605 17.12293 17.14191 17.29952 17.32579 17.33788 17.39357
[4,] 16.75951 16.83032 16.88722 16.88774 16.93298 17.01346 17.02392 17.03281
17.04160 17.04826 17.05845 17.06266 17.08851 17.10013 17.13781 17.21620
      [,1073]  [,1074]  [,1075]  [,1076]  [,1077]  [,1078]  [,1079]  [,1080]
[,1081]  [,1082]  [,1083]  [,1084]  [,1085]  [,1086]  [,1087]  [,1088]
[1,] 17.93594 17.95472 17.97215 18.14944 18.15115 18.19016 18.23161 18.25798
18.26486 18.26980 18.32735 18.35535 18.36423 18.38663 18.39742 18.41076
[2,] 17.37998 17.38903 17.44109 17.44177 17.48362 17.53598 17.62078 17.62833
17.63950 17.67321 17.71108 17.71590 17.73041 17.81708 17.82621 17.84366
[3,] 17.39865 17.43330 17.48975 17.52160 17.52227 17.53601 17.54092 17.54916
17.54956 17.67423 17.69080 17.76238 17.78578 17.84401 17.87543 17.91772
[4,] 17.32493 17.32962 17.35577 17.36073 17.36933 17.40116 17.43105 17.43588
17.46699 17.56162 17.59656 17.61130 17.62939 17.64906 17.70730 17.73110
      [,1089]  [,1090]  [,1091]  [,1092]  [,1093]  [,1094]  [,1095]  [,1096]
[,1097]  [,1098]  [,1099]  [,1100]  [,1101]  [,1102]  [,1103]  [,1104]
[1,] 18.42459 18.45403 18.47186 18.48767 18.49714 18.76341 18.89249 18.91580
18.94226 18.94266 18.99208 18.99662 19.01463 19.07854 19.10598 19.16793
[2,] 17.90163 17.92549 17.94990 17.98924 18.05811 18.07618 18.17801 18.17921
18.18770 18.19898 18.27024 18.31932 18.32397 18.33649 18.37824 18.44858
[3,] 17.93132 17.98275 18.07669 18.10026 18.21889 18.22241 18.23652 18.32929
18.37277 18.38741 18.40747 18.43957 18.47042 18.50888 18.52501 18.53229
[4,] 17.73265 17.77931 17.79086 17.85023 17.89223 17.94552 17.96523 17.97678
17.99455 18.07663 18.10633 18.12880 18.14014 18.18942 18.33675 18.46265
      [,1105]  [,1106]  [,1107]  [,1108]  [,1109]  [,1110]  [,1111]  [,1112]
[,1113]  [,1114]  [,1115]  [,1116]  [,1117]  [,1118]  [,1119]  [,1120]
[1,] 19.21328 19.24741 19.33097 19.34475 19.38136 19.50686 19.50772 19.56468
19.60928 19.71679 19.80448 19.80906 19.82768 19.83402 19.86354 19.87216
[2,] 18.49350 18.53666 18.58941 18.77342 18.77637 18.78904 18.79157 18.79904
18.85010 18.89364 18.90471 18.96003 18.97136 19.02830 19.06750 19.08036
[3,] 18.56099 18.62210 18.62373 18.69081 18.77624 18.88292 18.94767 18.94843
18.98870 18.99264 19.00071 19.02908 19.03220 19.03474 19.06313 19.08451
[4,] 18.49296 18.51989 18.55585 18.59317 18.63149 18.63885 18.72608 18.72799
18.73619 18.73735 18.78870 18.80315 18.81885 18.87946 18.88153 18.89540
      [,1121]  [,1122]  [,1123]  [,1124]  [,1125]  [,1126]  [,1127]  [,1128]
[,1129]  [,1130]  [,1131]  [,1132]  [,1133]  [,1134]  [,1135]  [,1136]
[1,] 19.98609 19.99356 19.99624 20.01716 20.06793 20.10213 20.13527 20.15148
20.19935 20.20004 20.21369 20.22140 20.25545 20.31400 20.31768 20.43232
[2,] 19.10169 19.11542 19.13360 19.14717 19.19498 19.29929 19.31720 19.32353
19.36376 19.39325 19.40765 19.47438 19.54895 19.63783 19.67904 19.70168
[3,] 19.09592 19.10230 19.11163 19.17155 19.18040 19.20846 19.21419 19.34253
19.37244 19.40307 19.43772 19.48491 19.49306 19.51033 19.51779 19.56651
[4,] 18.90549 18.94938 18.98353 19.02786 19.07276 19.17192 19.24424 19.26978
19.27083 19.38698 19.40905 19.43666 19.46967 19.58035 19.58108 19.59127
      [,1137]  [,1138]  [,1139]  [,1140]  [,1141]  [,1142]  [,1143]  [,1144]
[,1145]  [,1146]  [,1147]  [,1148]  [,1149]  [,1150]  [,1151]  [,1152]
[1,] 20.44607 20.45320 20.47429 20.51600 20.56787 20.65773 20.71728 20.73187
```

20.76194 20.78163 20.80336 20.83890 20.87583 20.92016 20.93424 21.20723
[2,] 19.71414 19.76824 19.78659 19.78857 19.83121 19.84440 19.85711 19.88549
19.94278 20.09304 20.12324 20.14381 20.20381 20.21333 20.22207 20.27511
[3,] 19.59166 19.62852 19.63034 19.64955 19.68941 19.73640 19.84115 19.84157
19.88632 19.89307 19.89394 19.95673 19.99076 19.99334 20.03728 20.03807
[4,] 19.61425 19.67615 19.70905 19.74883 19.75904 19.79568 19.83086 19.83320
19.85012 19.91466 19.91497 19.94137 19.94699 19.96155 20.00437 20.03646
[,1153] [,1154] [,1155] [,1156] [,1157] [,1158] [,1159] [,1160]
[,1161] [,1162] [,1163] [,1164] [,1165] [,1166] [,1167] [,1168]
[1,] 21.30693 21.31571 21.33150 21.36622 21.37844 21.38252 21.38383 21.40973
21.43750 21.48988 21.63335 21.65155 21.65268 21.70072 21.74012 21.81613
[2,] 20.29375 20.29446 20.31034 20.32851 20.43936 20.49612 20.53684 20.54769
20.55757 20.60778 20.66938 20.67843 20.69922 20.70453 20.70915 20.81942
[3,] 20.19071 20.24564 20.26044 20.31025 20.36815 20.36850 20.44043 20.49811
20.52296 20.58573 20.60455 20.66177 20.77362 20.86326 20.90139 20.90611
[4,] 20.06080 20.07888 20.14247 20.17235 20.20483 20.35776 20.37987 20.40094
20.40803 20.58619 20.58631 20.58859 20.63789 20.66652 20.76945 20.78092
[,1169] [,1170] [,1171] [,1172] [,1173] [,1174] [,1175] [,1176]
[,1177] [,1178] [,1179] [,1180] [,1181] [,1182] [,1183] [,1184]
[1,] 21.84152 21.84858 21.91531 21.93276 21.99412 22.01532 22.03031 22.07078
22.09175 22.15175 22.16977 22.19857 22.26365 22.26773 22.33195 22.34479
[2,] 20.83977 20.85167 20.85961 20.94263 21.09347 21.14562 21.17137 21.28727
21.30563 21.34593 21.35491 21.36663 21.45093 21.53245 21.57544 21.64414
[3,] 20.94472 20.96581 21.00816 21.14989 21.16461 21.17414 21.21183 21.23940
21.30377 21.31783 21.43116 21.46342 21.48376 21.54125 21.55060 21.55355
[4,] 20.79096 20.80152 20.81111 20.81495 20.85091 20.85954 20.86152 20.92479
20.93158 20.94387 20.97299 21.00479 21.03383 21.06908 21.08925 21.13119
[,1185] [,1186] [,1187] [,1188] [,1189] [,1190] [,1191] [,1192]
[,1193] [,1194] [,1195] [,1196] [,1197] [,1198] [,1199] [,1200]
[1,] 22.37850 22.38867 22.42698 22.45451 22.52982 22.56849 22.64683 22.68914
22.72371 22.84066 22.86660 22.87466 22.91611 22.93220 22.93255 22.95030
[2,] 21.65405 21.67163 21.82832 21.84815 21.87070 21.87404 21.88070 21.94212
21.97397 22.04990 22.08583 22.08982 22.14180 22.19114 22.19882 22.22000
[3,] 21.60031 21.87143 21.87900 21.95200 22.00311 22.01356 22.05491 22.06604
22.07281 22.14207 22.17145 22.19325 22.20488 22.22930 22.25001 22.30717
[4,] 21.15475 21.31326 21.35898 21.39136 21.53241 21.55102 21.63825 21.65608
21.69164 21.81121 21.83981 21.93582 21.95880 21.99668 22.00446 22.04416
[,1201] [,1202] [,1203] [,1204] [,1205] [,1206] [,1207] [,1208]
[,1209] [,1210] [,1211] [,1212] [,1213] [,1214] [,1215] [,1216]
[1,] 23.03183 23.09207 23.13396 23.18950 23.26190 23.27609 23.38117 23.43722
23.53108 23.63409 23.68771 23.80646 23.88863 23.92739 24.02533 24.03384
[2,] 22.23572 22.31033 22.39209 22.40536 22.45257 22.48074 22.56252 22.59390
22.60340 22.66768 22.69384 22.74183 22.78173 22.83126 22.91988 22.96789
[3,] 22.37287 22.37974 22.41554 22.44024 22.45397 22.46086 22.53988 22.54933
22.60740 22.61927 22.73669 22.79476 22.79820 22.90192 22.91013 22.95434
[4,] 22.06558 22.07460 22.10312 22.15498 22.20814 22.21254 22.21441 22.24458
22.26664 22.44722 22.46397 22.48223 22.51283 22.52959 22.67741 22.83762
[,1217] [,1218] [,1219] [,1220] [,1221] [,1222] [,1223] [,1224]
[,1225] [,1226] [,1227] [,1228] [,1229] [,1230] [,1231] [,1232]
[1,] 24.04593 24.06350 24.07236 24.13259 24.22000 24.25792 24.26888 24.32791
24.36761 24.53476 24.60171 24.64199 24.67568 24.67596 24.71579 24.83091
[2,] 23.02845 23.11578 23.13693 23.17625 23.23437 23.34343 23.37655 23.42497
23.43901 23.46861 23.50366 23.64385 23.70322 23.77736 23.78900 23.78920
[3,] 22.97957 23.00269 23.00975 23.13727 23.14107 23.15826 23.40353 23.50868

23.53778 23.57635 23.60678 23.62069 23.66697 23.70275 23.70289 23.82514
[4,] 22.92557 22.97720 22.98761 23.07104 23.08199 23.11147 23.18214 23.28465
23.33314 23.35336 23.51148 23.54719 23.64354 23.70447 23.73052 23.78412
[,1233] [,1234] [,1235] [,1236] [,1237] [,1238] [,1239] [,1240]
[,1241] [,1242] [,1243] [,1244] [,1245] [,1246] [,1247] [,1248]
[1,] 24.86157 24.89096 25.08994 25.27593 25.29511 25.35608 25.36170 25.37438
25.39999 25.40946 25.42027 25.50850 25.51393 25.59596 25.66631 25.68776
[2,] 23.81124 23.83184 23.93844 24.19433 24.20287 24.22964 24.26061 24.27234
24.27420 24.39402 24.46781 24.48492 24.49204 24.51186 24.51391 24.51530
[3,] 24.03728 24.06739 24.12132 24.12269 24.19734 24.39236 24.45276 24.45353
24.45573 24.47510 24.59575 24.61262 24.73606 24.75487 24.87106 24.93693
[4,] 23.78629 23.86517 23.91828 24.09571 24.14369 24.26700 24.35246 24.41091
24.45667 24.50297 24.50802 24.57492 24.72922 24.73251 24.74274 24.76630
[,1249] [,1250] [,1251] [,1252] [,1253] [,1254] [,1255] [,1256]
[,1257] [,1258] [,1259] [,1260] [,1261] [,1262] [,1263] [,1264]
[1,] 25.69221 25.79264 25.80847 25.91344 26.11383 26.21487 26.28646 26.42778
26.43275 26.49739 26.52834 26.62948 26.75866 26.88212 26.97150 27.05719
[2,] 24.57649 24.61844 24.64808 24.74291 24.90736 25.04962 25.13119 25.14292
25.15613 25.36663 25.37854 25.52804 25.61229 25.66106 25.71104 25.81538
[3,] 24.96679 25.12653 25.35558 25.51054 25.52634 25.57259 25.62197 25.69612
25.69629 25.73719 25.85034 25.85491 25.87627 25.93342 25.93601 25.96420
[4,] 24.98291 25.01641 25.12385 25.13038 25.43316 25.43715 25.58807 25.62378
25.63565 25.75897 25.80929 25.93013 25.94316 26.02170 26.05495 26.13637
[,1265] [,1266] [,1267] [,1268] [,1269] [,1270] [,1271] [,1272]
[,1273] [,1274] [,1275] [,1276] [,1277] [,1278] [,1279] [,1280]
[1,] 27.11931 27.13566 27.22264 27.35724 27.39861 27.47500 27.50120 27.50284
27.50710 27.51871 27.54799 27.59214 27.61977 27.69356 27.70851 27.73962
[2,] 25.85998 25.98149 26.11186 26.15969 26.17190 26.17388 26.44443 26.52674
26.67028 26.89152 26.91444 27.04995 27.06668 27.12057 27.14842 27.31934
[3,] 26.01817 26.03570 26.04881 26.17560 26.30549 26.33587 26.36163 26.43190
26.59857 26.62761 26.67763 26.80922 26.98219 27.07932 27.43297 27.50376
[4,] 26.15726 26.20727 26.21319 26.22993 26.34092 26.53407 26.55201 26.58919
26.65687 26.75665 26.94895 27.00333 27.10182 27.11959 27.42839 27.45849
[,1281] [,1282] [,1283] [,1284] [,1285] [,1286] [,1287] [,1288]
[,1289] [,1290] [,1291] [,1292] [,1293] [,1294] [,1295] [,1296]
[1,] 27.83053 28.01498 28.18163 28.24330 28.30799 28.47991 28.52155 28.63152
28.69547 28.73567 29.03603 29.23597 29.63332 29.64861 29.90026 29.95644
[2,] 27.35749 27.52101 27.59582 27.66073 27.83848 27.86359 27.92655 28.16689
28.24195 28.38819 28.42765 28.46177 28.64378 28.65190 28.71982 29.91302
[3,] 27.56756 27.85110 28.00928 28.09212 28.11704 28.18516 28.23516 28.24017
28.30369 28.36964 29.07755 29.16273 29.25933 29.53739 29.61597 29.71644
[4,] 27.53714 27.63108 27.83617 27.84164 28.01573 28.04499 28.35661 28.56958
28.76441 28.97388 29.05692 29.06780 29.09409 29.22740 29.44556 29.64467
[,1297] [,1298] [,1299] [,1300] [,1301] [,1302] [,1303] [,1304]
[,1305] [,1306] [,1307] [,1308] [,1309] [,1310] [,1311] [,1312]
[1,] 30.00884 30.02520 30.12150 30.73423 30.97565 31.11815 31.15495 31.36157
31.72649 31.77824 31.98878 32.42618 33.50836 34.19352 34.21581 34.50928
[2,] 29.94928 29.99261 30.23352 30.26367 30.28538 30.48946 30.53183 30.71551
30.98991 31.32473 31.42473 31.57738 32.00851 32.46519 32.93594 33.39811
[3,] 29.81019 30.01787 30.26872 30.30014 30.59119 30.62253 30.85811 30.91643
32.43476 32.87499 33.52264 33.59476 33.87131 34.03332 34.60232 34.93696
[4,] 29.85226 29.93761 29.97282 30.55356 30.64969 30.90626 30.96085 31.57545
31.69969 32.28988 32.29380 33.05169 33.17590 33.41001 34.04613 34.09562
[,1313] [,1314] [,1315] [,1316] [,1317] [,1318] [,1319] [,1320]

```
[,1321]  [,1322]  [,1323]  [,1324]  [,1325]  [,1326]  [,1327]  [,1328]
[1,] 35.29025 35.74843 36.05069 36.17639 36.94872 37.00226 37.73972 38.23532
38.25010 38.66615 39.69011 40.37664 42.62678 44.70101 46.83309 46.86925
[2,] 34.08801 34.59588 34.82207 35.67207 35.69463 36.98905 37.05487 38.82427
38.94277 39.09743 41.70490 42.24012 43.51904 43.56965 45.20974 46.22865
[3,] 35.15453 35.39830 35.57540 35.89390 35.89760 36.40016 36.97205 37.00448
37.43931 37.70909 37.93995 38.42348 45.21771 45.67352 45.89973 46.05780
[4,] 34.13663 34.79815 35.11297 35.38020 35.48676 36.24775 36.33705 36.64639
36.94341 37.56133 37.93534 38.11885 39.33115 45.59601 46.13697 46.27463
      [,1329]
[1,] 48.56459
[2,] 46.57173
[3,] 47.03335
[4,] 48.92600
```

ENSEMBLE FLUCTUATION DATA (TRUNCATED MINIMIZED CHARACTERIZED CLASS C)

```
                               [,1]      [,2]      [,3]      [,4]       [,5]
  [,6]       [,7]       [,8]       [,9]      [,10]      [,11]      [,12]
truncated_NAT0_min3_2.pdb 0.2686120 0.3159591 0.2031525 0.1301167 0.08449354
0.07728656 0.09122293 0.08435095 0.07492049 0.07532367 0.06180074 0.06255590
truncated_LJP6_min3_2.pdb 0.2877811 0.3271260 0.2037915 0.1287195 0.09208894
0.08388374 0.09726197 0.08971073 0.08277357 0.08445301 0.06709366 0.06190006
truncated_3WY9_min3_2.pdb 0.2905862 0.2714322 0.1982417 0.1306096 0.08977540
0.08474345 0.09949776 0.08731032 0.08137949 0.08057904 0.06255494 0.06004946
truncated_ZSP9_min3_2.pdb 0.2695810 0.4161586 0.2133221 0.1304265 0.09091895
0.08323717 0.09342747 0.08909486 0.08212028 0.08016562 0.06595004 0.06311620
                               [,13]      [,14]      [,15]      [,16]      [,17]
     [,18]      [,19]      [,20]      [,21]      [,22]     [,23]     [,24]
truncated_NAT0_min3_2.pdb 0.09019069 0.08609215 0.07945822 0.08696955 0.09313863
0.09146197 0.09022025 0.09717740 0.08972347 0.08830529 0.1623856 0.2304689
truncated_LJP6_min3_2.pdb 0.09234245 0.08379574 0.07783368 0.08277631 0.08629546
0.08481543 0.08640986 0.09689111 0.08552417 0.08583342 0.1539300 0.2290774
truncated_3WY9_min3_2.pdb 0.08691951 0.08465812 0.07662810 0.08379442 0.08925975
0.08740776 0.08778938 0.09719148 0.08852958 0.08643517 0.1594846 0.2429677
truncated_ZSP9_min3_2.pdb 0.08982278 0.08523186 0.07670428 0.08327990 0.09071973
0.08885493 0.08865433 0.09654066 0.08754311 0.08759966 0.1534706 0.2262190
                              [,25]     [,26]     [,27]     [,28]     [,29]
[,30]     [,31]     [,32]     [,33]     [,34]     [,35]     [,36]     [,37]
truncated_NAT0_min3_2.pdb 0.3573638 0.7423731 0.5845159 0.3055955 0.2444315
0.1876411 0.2365323 0.2452859 0.1936218 0.2198958 0.1495749 0.1136738 0.1107844
truncated_LJP6_min3_2.pdb 0.3608252 0.6916603 0.5580086 0.2908151 0.2379749
0.1845353 0.2239360 0.2412568 0.1851295 0.2130263 0.1457016 0.1120302 0.1193249
truncated_3WY9_min3_2.pdb 0.3469702 0.7231355 0.5288983 0.2883561 0.2379384
0.1787985 0.2161762 0.2178282 0.1823451 0.2141649 0.1515373 0.1141058 0.1201814
truncated_ZSP9_min3_2.pdb 0.3627680 0.8010653 0.6190711 0.2946309 0.2411108
0.1810355 0.2166985 0.2247755 0.1813853 0.2088168 0.1436791 0.1123590 0.1079254
                              [,38]     [,39]     [,40]     [,41]     [,42]
[,43]     [,44]     [,45]     [,46]     [,47]     [,48]     [,49]
truncated_NAT0_min3_2.pdb 0.1577473 0.1819203 0.1106717 0.1140148 0.1909778
0.3005952 0.3142297 0.3073622 0.2493300 0.1935721 0.1155648 0.1292841
truncated_LJP6_min3_2.pdb 0.1460414 0.2001416 0.1142078 0.1160066 0.1927309
0.2801997 0.3314022 0.3283650 0.2627233 0.1951923 0.1165111 0.1297846
```

truncated_3WY9_min3_2.pdb 0.1407166 0.1964862 0.1193980 0.1217353 0.1959144
0.2772054 0.3146073 0.3124329 0.2706675 0.2082149 0.1208917 0.1320033
truncated_ZSP9_min3_2.pdb 0.1406807 0.1858418 0.1135551 0.1188536 0.1934450
0.2943423 0.3267280 0.3207666 0.2677378 0.2089317 0.1193021 0.1274233
[,50] [,51] [,52] [,53] [,54]
[,55] [,56] [,57] [,58] [,59] [,60] [,61]
truncated_NAT0_min3_2.pdb 0.09669973 0.09187585 0.08139909 0.07546209 0.08450696
0.1263103 0.1231539 0.1182606 0.09812859 0.09894122 0.07958106 0.08863907
truncated_LJP6_min3_2.pdb 0.09757863 0.09005185 0.07837148 0.07014041 0.08594555
0.1355788 0.1233058 0.1201541 0.09454183 0.09552642 0.07903082 0.08927727
truncated_3WY9_min3_2.pdb 0.09982974 0.09062791 0.07907229 0.07130768 0.08612635
0.1145617 0.1252348 0.1154853 0.09233353 0.09894265 0.08156423 0.08963279
truncated_ZSP9_min3_2.pdb 0.09993375 0.09045105 0.07985490 0.07150883 0.08608821
0.1146564 0.1281114 0.1168072 0.09402061 0.10057920 0.08149543 0.08921958
[,62] [,63] [,64] [,65] [,66]
[,67] [,68] [,69] [,70] [,71] [,72]
truncated_NAT0_min3_2.pdb 0.09223453 0.08131647 0.07903866 0.06866571 0.06316109
0.06844582 0.06670948 0.06261164 0.05890787 0.07011668 0.07167501
truncated_LJP6_min3_2.pdb 0.08848963 0.07887071 0.07352542 0.06449634 0.06190336
0.06621199 0.06258515 0.06021919 0.05684112 0.06608144 0.07042316
truncated_3WY9_min3_2.pdb 0.08891605 0.08141111 0.07510585 0.06598543 0.06226474
0.06742098 0.06433456 0.06078458 0.05762695 0.06761884 0.07104137
truncated_ZSP9_min3_2.pdb 0.08937591 0.08036136 0.07595258 0.06638456 0.06235131
0.06749913 0.06477864 0.06156537 0.05757076 0.06776937 0.07116326
[,73] [,74] [,75] [,76] [,77]
[,78] [,79] [,80] [,81] [,82] [,83] [,84]
truncated_NAT0_min3_2.pdb 0.07065972 0.08317331 0.08645824 0.09617869 0.1215854
0.1325457 0.1187546 0.1320532 0.1397920 0.09778438 0.09967885 0.1233547
truncated_LJP6_min3_2.pdb 0.06983286 0.08282271 0.08811889 0.09181172 0.1158127
0.1415655 0.1198444 0.1198733 0.1511791 0.10915286 0.10967507 0.1401048
truncated_3WY9_min3_2.pdb 0.07078445 0.08511801 0.08869073 0.09078412 0.1176756
0.1388234 0.1189539 0.1194904 0.1450797 0.10794226 0.10576085 0.1373660
truncated_ZSP9_min3_2.pdb 0.07097880 0.08556787 0.08684984 0.09173655 0.1161791
0.1434993 0.1179013 0.1174801 0.1391977 0.10415190 0.10252929 0.1326600
[,85] [,86] [,87] [,88] [,89]
[,90] [,91] [,92] [,93] [,94] [,95] [,96]
truncated_NAT0_min3_2.pdb 0.1400251 0.1265017 0.1337110 0.1350727 0.1224247
0.1462513 0.1221606 0.1029208 0.1109718 0.1120920 0.10208866 0.09751979
truncated_LJP6_min3_2.pdb 0.1507534 0.1379030 0.1385344 0.1373559 0.1234002
0.1448548 0.1228466 0.1034885 0.1092446 0.1098082 0.09959289 0.09411577
truncated_3WY9_min3_2.pdb 0.1540137 0.1494673 0.1435327 0.1356146 0.1206612
0.1473509 0.1185593 0.1055449 0.1123770 0.1090746 0.09947309 0.09498133
truncated_ZSP9_min3_2.pdb 0.1508308 0.1386006 0.1432179 0.1291762 0.1176711
0.1412757 0.1198311 0.1033036 0.1104050 0.1097659 0.10012960 0.09585234
[,97] [,98] [,99] [,100] [,101]
[,102] [,103] [,104] [,105] [,106] [,107] [,108]
truncated_NAT0_min3_2.pdb 0.09169371 0.09457920 0.08155261 0.08640012 0.09776672
0.08761522 0.09383325 0.1064549 0.09733085 0.09723079 0.1047954 0.2377562
truncated_LJP6_min3_2.pdb 0.08994306 0.09428143 0.08128741 0.08567113 0.09981591
0.08604247 0.09418057 0.1061091 0.09734703 0.10166140 0.1068019 0.2355929
truncated_3WY9_min3_2.pdb 0.08943838 0.09293232 0.08217921 0.08562024 0.09896487
0.08574296 0.09248656 0.1074127 0.09643489 0.09944387 0.1044185 0.2319530
truncated_ZSP9_min3_2.pdb 0.08886799 0.09299248 0.08160787 0.08611546 0.09948208
0.08548383 0.09356743 0.1082378 0.09656457 0.10051113 0.1057813 0.2365320

```
                              [,109]    [,110]    [,111]    [,112]     [,113]
[,114]    [,115]     [,116]     [,117]    [,118]    [,119]    [,120]
truncated_NAT0_min3_2.pdb 0.2075683 0.1695089 0.1484952 0.1351191 0.09898613
0.09304846 0.1048134 0.08877026 0.08933654 0.1094723 0.1564367 0.1715134
truncated_LJP6_min3_2.pdb 0.1980074 0.1681288 0.1441961 0.1343825 0.10532055
0.09383380 0.1049872 0.09118188 0.09076263 0.1073815 0.1512228 0.1661146
truncated_3WY9_min3_2.pdb 0.1964417 0.1605090 0.1446940 0.1325748 0.10163987
0.09275269 0.1021945 0.09129559 0.09219573 0.1122067 0.1502926 0.1706771
truncated_ZSP9_min3_2.pdb 0.1960550 0.1623590 0.1423840 0.1345042 0.10177799
0.09197260 0.1010503 0.09096486 0.09120976 0.1117049 0.1600674 0.1712602
                              [,121]    [,122]    [,123]    [,124]    [,125]
[,126]    [,127]    [,128]    [,129]    [,130]    [,131]    [,132]    [,133]
truncated_NAT0_min3_2.pdb 0.2049532 0.1842956 0.1300009 0.1769774 0.2496943
0.2087167 0.1387728 0.1326096 0.1257390 0.1236555 0.1336157 0.1875095 0.2235900
truncated_LJP6_min3_2.pdb 0.2198363 0.1853696 0.1305393 0.1810777 0.2461163
0.2064646 0.1392420 0.1308304 0.1114540 0.1188816 0.1368059 0.1649414 0.2148153
truncated_3WY9_min3_2.pdb 0.2165792 0.1851279 0.1366457 0.1754764 0.2506203
0.2108910 0.1490996 0.1289300 0.1084153 0.1166767 0.1293753 0.1684905 0.2104650
truncated_ZSP9_min3_2.pdb 0.2470375 0.1937220 0.1357804 0.1774165 0.2476356
0.2005185 0.1473174 0.1338052 0.1128679 0.1201034 0.1364304 0.1736737 0.2283677
                              [,134]    [,135]    [,136]    [,137]    [,138]
[,139]    [,140]    [,141]    [,142]    [,143]    [,144]    [,145]    [,146]
truncated_NAT0_min3_2.pdb 0.3785678 0.2678273 0.3194278 0.1575077 0.1570737
0.1114231 0.1064895 0.1051874 0.1066000 0.1372778 0.1810673 0.3448535 0.1809126
truncated_LJP6_min3_2.pdb 0.3813042 0.2647363 0.3176591 0.1537291 0.1548016
0.1112834 0.1078941 0.1109689 0.1208648 0.1344524 0.1880027 0.3555445 0.1824759
truncated_3WY9_min3_2.pdb 0.4091268 0.2759712 0.3407028 0.1653689 0.1634416
0.1151135 0.1101454 0.1035366 0.1040964 0.1303553 0.1775122 0.3250583 0.1547690
truncated_ZSP9_min3_2.pdb 0.4561016 0.3132840 0.3762857 0.1766561 0.1630401
0.1141811 0.1081787 0.1014643 0.1060459 0.1327825 0.1741540 0.3402254 0.1562512
                              [,147]     [,148]     [,149]     [,150]     [,151]
   [,152]     [,153]     [,154]     [,155]     [,156]     [,157]
truncated_NAT0_min3_2.pdb 0.1035554 0.07495852 0.06592299 0.06548330 0.06583898
0.05634442 0.04929218 0.05658499 0.05726726 0.05393186 0.05197507
truncated_LJP6_min3_2.pdb 0.1089330 0.08317113 0.06951303 0.06729218 0.06788102
0.05651978 0.04963625 0.05590482 0.05689959 0.05311364 0.05115351
truncated_3WY9_min3_2.pdb 0.1008385 0.07366669 0.06633904 0.06408836 0.06630597
0.05648636 0.04944718 0.05683383 0.05761642 0.05410006 0.05187278
truncated_ZSP9_min3_2.pdb 0.0992648 0.06987115 0.06283868 0.06243684 0.06450304
0.05482577 0.04875888 0.05585654 0.05643596 0.05276643 0.05099871
                               [,158]     [,159]     [,160]     [,161]     [,162]
    [,163]    [,164]    [,165]    [,166]    [,167]    [,168]    [,169]
truncated_NAT0_min3_2.pdb 0.04917741 0.05563583 0.05466691 0.06389513 0.07038098
0.08019165 0.1101439 0.1235640 0.1304695 0.1593705 0.3825810 0.3700569
truncated_LJP6_min3_2.pdb 0.04770298 0.05456993 0.05381173 0.06115614 0.06886587
0.07480319 0.1004569 0.1132255 0.1234980 0.1520186 0.3331405 0.3419008
truncated_3WY9_min3_2.pdb 0.04854104 0.05503977 0.05397201 0.06188264 0.06894978
0.07642362 0.1026399 0.1126064 0.1232753 0.1652356 0.3827028 0.3317116
truncated_ZSP9_min3_2.pdb 0.04807003 0.05496386 0.05334952 0.06124102 0.06927373
0.07700672 0.1041344 0.1185819 0.1268862 0.1615717 0.3618603 0.3303862
                              [,170]    [,171]    [,172]    [,173]    [,174]
[,175]    [,176]     [,177]     [,178]    [,179]     [,180]     [,181]
truncated_NAT0_min3_2.pdb 0.2692032 0.2140664 0.2866265 0.2030527 0.1232500
0.1476909 0.1396321 0.08615813 0.08996765 0.1189478 0.09887427 0.07308994
```

truncated_LJP6_min3_2.pdb 0.2602052 0.2153610 0.2540220 0.1901206 0.1150139
0.1460660 0.1462434 0.08926661 0.08784924 0.1210903 0.10030537 0.07335904
truncated_3WY9_min3_2.pdb 0.2662243 0.2108887 0.2651450 0.1970194 0.1189560
0.1526129 0.1401629 0.08739189 0.08444130 0.1126247 0.10003755 0.07364542
truncated_ZSP9_min3_2.pdb 0.2786818 0.2204453 0.2637366 0.1941355 0.1163066
0.1457277 0.1440033 0.08877227 0.08505891 0.1140736 0.09855782 0.07249773
[,182] [,183] [,184] [,185] [,186]
[,187] [,188] [,189] [,190] [,191] [,192] [,193]
truncated_NAT0_min3_2.pdb 0.09370317 0.09375575 0.07278638 0.07731852 0.1137970
0.09846006 0.07808354 0.1265892 0.1563974 0.1539979 0.1513621 0.1237684
truncated_LJP6_min3_2.pdb 0.09340736 0.09358982 0.07096841 0.07697556 0.1075429
0.09290971 0.07578568 0.1259829 0.1571645 0.1431649 0.1487868 0.1331858
truncated_3WY9_min3_2.pdb 0.09256376 0.09447793 0.07238922 0.07865014 0.1122288
0.09611248 0.07960990 0.1277737 0.1630149 0.1532812 0.1604894 0.1333406
truncated_ZSP9_min3_2.pdb 0.09038212 0.09314385 0.07105094 0.07579439 0.1076439
0.09267643 0.07600387 0.1217419 0.1537718 0.1490310 0.1517857 0.1274171
[,194] [,195] [,196] [,197] [,198]
[,199] [,200] [,201] [,202] [,203] [,204] [,205]
truncated_NAT0_min3_2.pdb 0.1055586 0.10467432 0.1259738 0.1858444 0.1509615
0.1652424 0.1797826 0.2748360 0.2535398 0.1996948 0.1660286 0.1505169
truncated_LJP6_min3_2.pdb 0.1146724 0.11038050 0.1296779 0.1746169 0.1530403
0.1585270 0.1810169 0.1530080 0.1479112 0.2231777 0.2067350 0.1790877
truncated_3WY9_min3_2.pdb 0.1087016 0.09846074 0.1247405 0.1790189 0.1389387
0.1593762 0.1768232 0.2587471 0.2435577 0.2074096 0.1747719 0.1722757
truncated_ZSP9_min3_2.pdb 0.1010600 0.09094903 0.1133609 0.1658053 0.1363865
0.1494683 0.1733832 0.1355245 0.2717435 0.2331981 0.1854367 0.1585277
[,206] [,207] [,208] [,209] [,210]
[,211] [,212] [,213] [,214] [,215] [,216] [,217]
truncated_NAT0_min3_2.pdb 0.2112859 0.1529761 0.1333042 0.10680988 0.09770305
0.08002660 0.06589856 0.07435992 0.07594338 0.07225690 0.05745492 0.07054864
truncated_LJP6_min3_2.pdb 0.1618585 0.1345677 0.1220709 0.10077066 0.09399663
0.07495743 0.06528689 0.08055210 0.07714287 0.07202517 0.05851141 0.07196709
truncated_3WY9_min3_2.pdb 0.2371197 0.1446082 0.1421224 0.09491192 0.09423700
0.07824549 0.06491554 0.07565813 0.08062812 0.07431689 0.05942846 0.07367989
truncated_ZSP9_min3_2.pdb 0.1524771 0.1502174 0.1338281 0.09333595 0.09473330
0.07939390 0.06416400 0.08045568 0.07867164 0.07199483 0.05785174 0.07140452
[,218] [,219] [,220] [,221] [,222]
[,223] [,224] [,225] [,226] [,227] [,228] [,229]
truncated_NAT0_min3_2.pdb 0.08023804 0.07430628 0.07800825 0.09682973 0.1300839
0.1248754 0.1802624 0.2501032 0.2705166 0.2692136 0.2333643 0.1183848
truncated_LJP6_min3_2.pdb 0.08011971 0.07405462 0.07921654 0.09508556 0.1305693
0.1287230 0.1873729 0.2477171 0.2517355 0.2632502 0.2361189 0.1223993
truncated_3WY9_min3_2.pdb 0.08246693 0.07533507 0.07837444 0.09551227 0.1294812
0.1214830 0.1830149 0.2318309 0.2395322 0.2502319 0.2429651 0.1231941
truncated_ZSP9_min3_2.pdb 0.08005219 0.07259702 0.07667162 0.09544065 0.1237608
0.1183256 0.1753485 0.2406825 0.2757574 0.2897007 0.2455634 0.1226132
[,230] [,231] [,232] [,233] [,234]
[,235] [,236] [,237] [,238] [,239] [,240] [,241] [,242]
truncated_NAT0_min3_2.pdb 0.1355800 0.1798984 0.1500934 0.1117636 0.1690822
0.2059635 0.1647737 0.1866075 0.2991791 0.2789752 0.2311345 0.2827907 0.3445316
truncated_LJP6_min3_2.pdb 0.1361938 0.1837851 0.1515688 0.1109346 0.1630966
0.1979012 0.1529472 0.1725395 0.2719433 0.2296197 0.2193846 0.2604253 0.3307600
truncated_3WY9_min3_2.pdb 0.1397834 0.1831330 0.1506077 0.1171783 0.1762143
0.1992468 0.1639358 0.1853518 0.2959250 0.2508466 0.2102340 0.2996974 0.4353912

truncated_ZSP9_min3_2.pdb 0.1395332 0.1866225 0.1504927 0.1127834 0.1715680
0.1935324 0.1565712 0.1914143 0.3118947 0.2898417 0.2216639 0.2715223 0.3219575
[,243] [,244] [,245] [,246] [,247]
[,248] [,249] [,250] [,251] [,252] [,253] [,254]
truncated_NAT0_min3_2.pdb 0.4466746 0.9579352 1.529860 0.6946875 0.3090072
0.3264733 0.1872513 0.10825874 0.1487796 0.1613702 0.1561135 0.09822503
truncated_LJP6_min3_2.pdb 0.4551334 0.8991528 1.155731 0.5803382 0.2750675
0.2684778 0.1382449 0.08879658 0.1441864 0.1411581 0.1616687 0.09924718
truncated_3WY9_min3_2.pdb 0.4999261 1.1686636 1.561481 0.7143802 0.3189190
0.3120323 0.1554913 0.09395286 0.1552655 0.1540940 0.1659230 0.09973019
truncated_ZSP9_min3_2.pdb 0.4289078 0.8919035 1.576790 0.6870902 0.3120258
0.3105781 0.1581365 0.10055808 0.1567830 0.1545586 0.1602395 0.09819157
[,255] [,256] [,257] [,258] [,259]
[,260] [,261] [,262] [,263] [,264] [,265] [,266]
truncated_NAT0_min3_2.pdb 0.09123125 0.09536569 0.09514062 0.1012024 0.09370671
0.08603974 0.09331216 0.1143687 0.1177321 0.1163896 0.1576626 0.2227396
truncated_LJP6_min3_2.pdb 0.08538959 0.09050392 0.08313150 0.0983110 0.09024187
0.08329448 0.09443741 0.1113269 0.1177004 0.1150925 0.1568294 0.2079227
truncated_3WY9_min3_2.pdb 0.08709242 0.10051705 0.09369617 0.1030085 0.10120047
0.08838528 0.10248759 0.1191052 0.1193593 0.1208224 0.1694336 0.2228789
truncated_ZSP9_min3_2.pdb 0.08768583 0.09833615 0.09669146 0.1055762 0.10183075
0.09225169 0.10513013 0.1232047 0.1273480 0.1237405 0.1739321 0.2252497
[,267] [,268] [,269] [,270] [,271]
[,272] [,273] [,274] [,275] [,276] [,277] [,278] [,279]
truncated_NAT0_min3_2.pdb 0.2498731 0.2337461 0.2951083 0.2875824 0.2435987
0.1294873 0.1413648 0.1769187 0.1391202 0.1111261 0.1297526 0.1473590 0.1326868
truncated_LJP6_min3_2.pdb 0.2549661 0.2339436 0.3229288 0.3005296 0.2616171
0.1301531 0.1358644 0.1628516 0.1307994 0.1100103 0.1249776 0.1356218 0.1247331
truncated_3WY9_min3_2.pdb 0.2559213 0.2495269 0.2927968 0.2802331 0.2677748
0.1344142 0.1476145 0.1902895 0.1424392 0.1149758 0.1391062 0.1500845 0.1356685
truncated_ZSP9_min3_2.pdb 0.2787467 0.2595661 0.3186547 0.2906324 0.2805213
0.1420767 0.1430415 0.1929851 0.1458979 0.1176451 0.1401155 0.1471050 0.1334500
[,280] [,281] [,282] [,283] [,284]
[,285] [,286] [,287] [,288] [,289] [,290] [,291] [,292]
truncated_NAT0_min3_2.pdb 0.1253945 0.1307211 0.1328853 0.1613217 0.2073194
0.2345360 0.2164879 0.7341382 0.5631539 0.5212324 0.2835056 0.2196368 0.1622782
truncated_LJP6_min3_2.pdb 0.1214724 0.1194566 0.1195014 0.1479773 0.1919774
0.2379293 0.1909369 0.2344625 0.5132295 0.4393620 0.2632329 0.2152143 0.1707330
truncated_3WY9_min3_2.pdb 0.1354998 0.1337620 0.1317998 0.1617585 0.2038743
0.2622867 0.2872526 0.6950085 0.6737631 0.5115841 0.2210423 0.1868661 0.1708879
truncated_ZSP9_min3_2.pdb 0.1296416 0.1274653 0.1282647 0.1478228 0.1893452
0.2408116 0.2602491 0.3692869 0.5556511 0.5568699 0.2550246 0.1812830 0.1708013
[,293] [,294] [,295] [,296] [,297]
[,298] [,299] [,300] [,301] [,302] [,303] [,304]
truncated_NAT0_min3_2.pdb 0.1695658 0.1763288 0.2850631 0.2174601 0.1801303
0.1600808 0.1358143 0.1451290 0.2040465 0.2567553 0.1750246 0.1288274
truncated_LJP6_min3_2.pdb 0.1686409 0.1709885 0.2882097 0.2183656 0.1839729
0.1518506 0.1376948 0.1454614 0.1901152 0.2194542 0.1571258 0.1349341
truncated_3WY9_min3_2.pdb 0.1721047 0.1629489 0.2637678 0.2024554 0.1773857
0.1548993 0.1328071 0.1419194 0.1629136 0.2056444 0.1669929 0.1345944
truncated_ZSP9_min3_2.pdb 0.1755144 0.1747357 0.2794815 0.2019589 0.1734649
0.1470261 0.1303962 0.1435435 0.1690728 0.2112372 0.1589564 0.1263350
[,305] [,306] [,307] [,308] [,309]
[,310] [,311] [,312] [,313] [,314] [,315]

truncated_NAT0_min3_2.pdb 0.08430270 0.09093378 0.08194044 0.08184055 0.08394334
0.07891083 0.06655747 0.07364052 0.08231937 0.07747646 0.07847997
truncated_LJP6_min3_2.pdb 0.08601849 0.09153386 0.07910193 0.08277414 0.08432588
0.07772506 0.06338902 0.07099074 0.08035726 0.07257090 0.07536938
truncated_3WY9_min3_2.pdb 0.08667092 0.08950323 0.07968298 0.08260946 0.08107955
0.07590606 0.06285680 0.06899052 0.07877175 0.07304703 0.07309591
truncated_ZSP9_min3_2.pdb 0.08421055 0.08917806 0.07824884 0.08097869 0.08146894
0.07356602 0.06225621 0.06784960 0.07740923 0.07079503 0.06933691
[,316] [,317] [,318] [,319] [,320]
[,321] [,322] [,323] [,324] [,325] [,326] [,327]
truncated_NAT0_min3_2.pdb 0.08578950 0.08455040 0.08458299 0.1152886 0.1066052
0.1115104 0.2072835 0.2232380 0.2173207 0.4487902 0.3577847 0.3462450
truncated_LJP6_min3_2.pdb 0.08026033 0.08041124 0.08824773 0.1150786 0.1071293
0.1145122 0.2125355 0.2295213 0.2375932 0.4703038 0.4274002 0.3517427
truncated_3WY9_min3_2.pdb 0.08087155 0.08274250 0.08739705 0.1170382 0.1049628
0.1131282 0.2100311 0.2361167 0.2328490 0.4647255 0.3910277 0.3661965
truncated_ZSP9_min3_2.pdb 0.07833306 0.08062553 0.08437707 0.1112385 0.1106659
0.1186126 0.2327214 0.2429011 0.2476097 0.5084777 0.4524319 0.3958618
[,328] [,329] [,330] [,331] [,332]
[,333] [,334] [,335] [,336] [,337] [,338] [,339]
truncated_NAT0_min3_2.pdb 0.3342685 0.2185366 0.1551371 0.1754874 0.1657845
0.1066376 0.1089936 0.1423811 0.1218894 0.09902331 0.10643530 0.1101946
truncated_LJP6_min3_2.pdb 0.3185622 0.1996421 0.1475681 0.1678918 0.1474245
0.1000140 0.1120474 0.1367355 0.1164514 0.09306377 0.10314474 0.1056552
truncated_3WY9_min3_2.pdb 0.3316253 0.2156880 0.1553474 0.1818864 0.1601237
0.1081230 0.1148696 0.1369275 0.1193861 0.09450382 0.10350103 0.1086202
truncated_ZSP9_min3_2.pdb 0.3501325 0.2161778 0.1560414 0.1802631 0.1543712
0.1029432 0.1069922 0.1288058 0.1147717 0.09062271 0.09957853 0.1027472
[,340] [,341] [,342] [,343] [,344]
[,345] [,346] [,347] [,348] [,349] [,350] [,351]
truncated_NAT0_min3_2.pdb 0.09865677 0.09856429 0.09717386 0.08909442 0.10114689
0.08887613 0.09767312 0.1167209 0.1795055 0.2314703 0.2401132 0.1510997
truncated_LJP6_min3_2.pdb 0.09524585 0.09683682 0.09490613 0.08842072 0.10067864
0.08662756 0.09649470 0.1171867 0.1790856 0.2515452 0.2551110 0.1535789
truncated_3WY9_min3_2.pdb 0.09415773 0.09597737 0.09237079 0.08651620 0.09926233
0.08706567 0.09380023 0.1120812 0.1730279 0.2208767 0.2337945 0.1427053
truncated_ZSP9_min3_2.pdb 0.08917869 0.09382731 0.09488622 0.08654185 0.09939120
0.08792384 0.09682289 0.1178619 0.1734937 0.2267066 0.2404539 0.1423779
[,352] [,353] [,354] [,355] [,356]
[,357] [,358] [,359] [,360] [,361] [,362] [,363]
truncated_NAT0_min3_2.pdb 0.1358815 0.08047874 0.08721186 0.07847692 0.07660652
0.1013752 0.1201194 0.1419599 0.1698599 0.1759596 0.1023067 0.1217433
truncated_LJP6_min3_2.pdb 0.1330952 0.08040197 0.08617851 0.07925054 0.07729908
0.1059897 0.1207157 0.1352291 0.1651174 0.1812863 0.1064020 0.1250016
truncated_3WY9_min3_2.pdb 0.1324762 0.07963540 0.08463149 0.07866285 0.07853664
0.1042134 0.1220509 0.1396184 0.1689053 0.1821543 0.1068461 0.1251260
truncated_ZSP9_min3_2.pdb 0.1331959 0.08131250 0.08721222 0.07921158 0.07915247
0.1077150 0.1237266 0.1338895 0.1636577 0.1809382 0.1062020 0.1254703
[,364] [,365] [,366] [,367] [,368]
[,369] [,370] [,371] [,372] [,373] [,374] [,375]
truncated_NAT0_min3_2.pdb 0.1545213 0.10474884 0.09774898 0.08907046 0.08848289
0.09388740 0.10689111 0.10167423 0.09434537 0.1195958 0.1406192 0.2602449
truncated_LJP6_min3_2.pdb 0.1461072 0.09983707 0.09239387 0.08643679 0.09054367
0.09152181 0.09847275 0.10524988 0.10013888 0.1190409 0.1498176 0.2587476

truncated_3WY9_min3_2.pdb 0.1591630 0.10574688 0.10134151 0.08801450 0.09016784
0.09462252 0.09331187 0.09922522 0.09929248 0.1114284 0.1818024 0.2625397
truncated_ZSP9_min3_2.pdb 0.1589871 0.10754072 0.10091840 0.08821149 0.09229383
0.09730622 0.09482504 0.10352320 0.10063604 0.1118145 0.1802659 0.2359741
[,376] [,377] [,378] [,379] [,380]
[,381] [,382] [,383] [,384] [,385] [,386] [,387] [,388]
truncated_NAT0_min3_2.pdb 0.3041623 0.1903516 0.2283643 0.2323065 0.2883386
0.2635030 0.2704568 0.3283489 0.1892110 0.1190602 0.1645823 0.1457424 0.1574584
truncated_LJP6_min3_2.pdb 0.3017553 0.2044738 0.2508279 0.2321403 0.2735181
0.2544773 0.2566171 0.2930540 0.1842632 0.1316078 0.1974299 0.1565060 0.1510294
truncated_3WY9_min3_2.pdb 0.3043532 0.2318418 0.2639452 0.2579396 0.2834316
0.2426860 0.2414657 0.2852043 0.1886712 0.1276748 0.1816045 0.1290399 0.1565823
truncated_ZSP9_min3_2.pdb 0.3079053 0.2279315 0.2678646 0.2581335 0.2995013
0.2554481 0.2621251 0.3081226 0.1968686 0.1271891 0.1879907 0.1426875 0.1589490
[,389] [,390] [,391] [,392] [,393]
[,394] [,395] [,396] [,397] [,398] [,399] [,400]
truncated_NAT0_min3_2.pdb 0.1204348 0.07970519 0.06194994 0.07449881 0.06744763
0.06804804 0.07059852 0.09931365 0.1702947 0.1911623 0.2010709 0.2186556
truncated_LJP6_min3_2.pdb 0.1130255 0.07946660 0.06096401 0.07434723 0.06785891
0.06753713 0.06966779 0.09485279 0.1574549 0.1787791 0.1988054 0.2143831
truncated_3WY9_min3_2.pdb 0.1153675 0.07868771 0.06082064 0.07630492 0.06921490
0.06992444 0.07102154 0.10076583 0.1657148 0.1797512 0.1904166 0.2076219
truncated_ZSP9_min3_2.pdb 0.1172273 0.08175175 0.06172095 0.07689610 0.06856122
0.06967360 0.06819198 0.09484648 0.1565601 0.1823735 0.2069538 0.2145658
[,401] [,402] [,403] [,404] [,405]
[,406] [,407] [,408] [,409] [,410] [,411] [,412]
truncated_NAT0_min3_2.pdb 0.1993619 0.2382938 0.2959271 0.2707251 0.1917515
0.1729180 0.1176149 0.1788888 0.1531242 0.1118420 0.1616245 0.09431833
truncated_LJP6_min3_2.pdb 0.1994390 0.2423577 0.2865553 0.2437267 0.1842832
0.1519158 0.1069372 0.1839982 0.1476651 0.1261584 0.1620593 0.09264110
truncated_3WY9_min3_2.pdb 0.1898464 0.2419930 0.2895201 0.2689853 0.2022348
0.1692077 0.1151240 0.1857671 0.1549611 0.1110075 0.1656376 0.09452924
truncated_ZSP9_min3_2.pdb 0.1956604 0.2314823 0.2748979 0.2534959 0.1872510
0.1611223 0.1184013 0.1798964 0.1524993 0.1093363 0.1566663 0.08883917
[,413] [,414] [,415] [,416] [,417]
[,418] [,419] [,420] [,421] [,422] [,423]
truncated_NAT0_min3_2.pdb 0.07493119 0.08480518 0.07727075 0.09818767 0.08322049
0.07169741 0.06471988 0.07144674 0.06754021 0.07171830 0.06945023
truncated_LJP6_min3_2.pdb 0.07518844 0.08076936 0.07628957 0.10044287 0.07433371
0.07032007 0.06340437 0.07212193 0.06347758 0.06820727 0.06840530
truncated_3WY9_min3_2.pdb 0.07381656 0.08632515 0.07855149 0.10324034 0.07861203
0.06997920 0.06554094 0.07467691 0.06668955 0.06628565 0.06620596
truncated_ZSP9_min3_2.pdb 0.07342577 0.08599892 0.07867268 0.10169462 0.07842412
0.07135751 0.06462859 0.07228392 0.06567992 0.06405025 0.06506868
[,424] [,425] [,426] [,427] [,428]
[,429] [,430] [,431] [,432] [,433] [,434] [,435]
truncated_NAT0_min3_2.pdb 0.07053294 0.07263665 0.06934686 0.06724454 0.07661396
0.09450711 0.09821288 0.08898385 0.1409955 0.1749973 0.1767889 0.1496157
truncated_LJP6_min3_2.pdb 0.06876470 0.06392101 0.06710804 0.06975575 0.08320444
0.09882768 0.10143471 0.09864541 0.1361861 0.1804721 0.2002087 0.1522454
truncated_3WY9_min3_2.pdb 0.06931196 0.06410859 0.06600633 0.06736726 0.08269815
0.09632355 0.09468069 0.09216112 0.1216185 0.1531309 0.1790573 0.1673531
truncated_ZSP9_min3_2.pdb 0.06727587 0.06177655 0.06540717 0.06680464 0.08225203
0.09725864 0.09746161 0.09137853 0.1200910 0.1755757 0.1938585 0.1517736

```
                              [,436]    [,437]    [,438]    [,439]    [,440]
[,441]    [,442]    [,443]    [,444]    [,445]
truncated_NAT0_min3_2.pdb 0.1345061 0.8410219 0.4643539 0.3595612 0.4796191
0.8092008 0.9812930 1.7597183 2.1595574 2.8240312
truncated_LJP6_min3_2.pdb 0.1336010 0.1976106 0.1580230 0.3165870 0.6935557
0.8154912 0.4906173 0.3865080 0.5162733 0.4260776
truncated_3WY9_min3_2.pdb 0.1457332 0.3707547 0.7142533 0.7295207 0.4677534
0.3498115 0.5011035 0.4419466 0.4099050 0.9035446
truncated_ZSP9_min3_2.pdb 0.1414303 0.8183968 0.4225198 0.3250661 0.4741645
0.7056796 0.7895093 1.2710228 1.3196872 1.2900109
```
