# Supplementary material for: Insights into the mechanism(s) of digestion of crystalline cellulose by plant class C GH9 endoglucanases
Source: J Mol Model. 2019 Jul 23;25(8):240. doi: 10.1007/s00894-019-4133-1 (PMC7385011; doi:10.1007/s00894-019-4133-1)
Supplement: Supplementary file 7 — (PDF 282 kb) [file 894_2019_4133_MOESM7_ESM.pdf]

Supplementary Text 5

```
Call:
  nma.pdb(pdb = pdbFL4_NAT0, mass = TRUE)

Class:
  VibrationalModes (nma)

Number of modes:
  1704 (6 trivial)

Frequencies:
  Mode 7:       0.003
  Mode 8:       0.003
  Mode 9:       0.005
  Mode 10:      0.009
  Mode 11:      0.011
  Mode 12:      0.012

+ attr: modes, frequencies, force.constants, fluctuations,
        U, L, xyz, mass, temp, triv.modes, natoms, call

   NORMAL MODES for FULL LENGTH MINIMIZED CHARACTERIZED Q5NAT0

   [1] 0.000000000 0.000000000 0.000000000 0.000000000 0.000000000 0.000000000
0.002824720 0.003202931 0.004874404 0.009218630 0.010806140 0.012158438
  [13] 0.012574248 0.012958184 0.014676813 0.015545943 0.016296876 0.017709161
0.018905303 0.019111851 0.019711454 0.021021109 0.022410915 0.022970199
  [25] 0.023486007 0.024069785 0.024506761 0.025032396 0.025280098 0.025513982
0.025906103 0.026265870 0.026575063 0.026913190 0.027804328 0.027903906
  [37] 0.028474950 0.029031711 0.029841870 0.030498423 0.030799669 0.030881391
0.031460790 0.032154813 0.032236243 0.032485547 0.032953145 0.033189816
  [49] 0.033938462 0.034213871 0.034388150 0.034649753 0.034787645 0.035280383
0.035476561 0.035691538 0.036156907 0.036185969 0.036388055 0.036953434
  [61] 0.037108708 0.037506577 0.037596966 0.037975717 0.038729302 0.038881067
0.039004977 0.039110040 0.039404270 0.040027389 0.040364530 0.040488905
  [73] 0.040640021 0.041111912 0.041466812 0.041797191 0.042216880 0.042485129
0.042565542 0.042866191 0.043162985 0.043440262 0.043576496 0.043922728
  [85] 0.044102579 0.044652252 0.044708944 0.044953031 0.045123163 0.045725966
0.045977877 0.046166731 0.046320104 0.046403970 0.046614470 0.046825105
  [97] 0.047593715 0.047709332 0.048005205 0.048323907 0.048538344 0.048942151
0.049325966 0.049459559 0.049567503 0.049901610 0.050252149 0.050345818
 [109] 0.050519098 0.050884053 0.051078076 0.051306676 0.051479182 0.051713357
0.051936967 0.052061185 0.052272405 0.052475294 0.052682450 0.052892865
 [121] 0.052972781 0.053333057 0.053435783 0.053637813 0.053985441 0.054173967
0.054212528 0.054509813 0.054666190 0.054798552 0.055408097 0.055446256
 [133] 0.055593166 0.055664882 0.055723325 0.056220005 0.056428676 0.056710549
0.056980137 0.057137511 0.057446778 0.057762282 0.057936334 0.058071275
 [145] 0.058157141 0.058397503 0.058742837 0.058834612 0.058895501 0.059261018
0.059386977 0.059790172 0.059912905 0.060209390 0.060478669 0.060676452
 [157] 0.060842793 0.061046451 0.061069475 0.061347779 0.061450505 0.061605094
0.061824270 0.061847210 0.062077365 0.062319272 0.062473333 0.062747833
 [169] 0.062837790 0.062964037 0.063225791 0.063373252 0.063538712 0.063783416
0.063994142 0.064100925 0.064399762 0.064706027 0.064824142 0.065337127
 [181] 0.065435912 0.065550007 0.065749101 0.065831110 0.066078825 0.066314729
```

0.066411488 0.066696176 0.066875384 0.067225209 0.067501212 0.067655267
[193] 0.067751607 0.067887559 0.067950401 0.068121848 0.068428112 0.068665352
0.069158271 0.069357964 0.069614780 0.069834026 0.070059818 0.070254424
[205] 0.070596117 0.070641670 0.070792469 0.070858812 0.071059608 0.071106289
0.071537624 0.071563291 0.071770056 0.071871807 0.072326773 0.072510580
[217] 0.072767930 0.072862899 0.072981870 0.073240331 0.073516320 0.073703690
0.073768273 0.073951066 0.074192326 0.074334219 0.074565237 0.074651812
[229] 0.074954206 0.075126359 0.075432224 0.075615349 0.075663572 0.075749059
0.075922913 0.076102695 0.076377958 0.076450388 0.076571889 0.076874803
[241] 0.076927176 0.077065348 0.077180466 0.077360272 0.077440452 0.077487212
0.077736397 0.077989982 0.078069515 0.078346274 0.078551631 0.078588384
[253] 0.078646218 0.079161467 0.079287600 0.079385299 0.079579381 0.079698655
0.079945066 0.080167658 0.080320755 0.080583340 0.080769213 0.080982815
[265] 0.081125007 0.081428248 0.081628800 0.081794957 0.081851764 0.081931567
0.082081684 0.082289108 0.082515967 0.082644490 0.082898492 0.083064854
[277] 0.083126126 0.083358304 0.083476578 0.083639822 0.083897755 0.084043606
0.084353321 0.084649486 0.084693163 0.084810173 0.085042370 0.085163214
[289] 0.085241254 0.085336589 0.085671496 0.085711106 0.085948385 0.086157967
0.086424505 0.086769518 0.086933280 0.087000853 0.087237672 0.087572395
[301] 0.087579482 0.087834497 0.087995702 0.088099270 0.088254537 0.088369842
0.088555532 0.088887573 0.088958075 0.089162287 0.089253292 0.089451591
[313] 0.089636595 0.089753370 0.089873797 0.089948032 0.090290382 0.090487808
0.090576783 0.090706590 0.091053460 0.091070567 0.091311534 0.091393054
[325] 0.091602346 0.091861498 0.091976962 0.092098058 0.092234375 0.092266226
0.092383652 0.092530499 0.092732854 0.092772453 0.092796886 0.092988582
[337] 0.093078296 0.093303491 0.093536946 0.093618017 0.093869580 0.094068785
0.094170112 0.094319549 0.094527050 0.094777938 0.094899196 0.094988438
[349] 0.095196477 0.095243563 0.095421585 0.095706658 0.095765395 0.096072652
0.096334240 0.096453805 0.096709516 0.096791986 0.096986233 0.097157673
[361] 0.097332584 0.097521729 0.097721394 0.097865020 0.098043450 0.098059596
0.098352089 0.098447979 0.098554699 0.098663614 0.098825737 0.098950866
[373] 0.099124147 0.099250048 0.099557873 0.099775299 0.099979205 0.100149568
0.100284034 0.100430426 0.100539451 0.100637913 0.100790452 0.100828268
[385] 0.101086445 0.101115633 0.101377576 0.101492697 0.101641336 0.101682448
0.101969393 0.102259126 0.102391934 0.102460808 0.102648893 0.102949631
[397] 0.103068156 0.103097521 0.103189737 0.103319633 0.103418510 0.103653865
0.103671214 0.104073582 0.104333319 0.104673993 0.104716938 0.104999933
[409] 0.105154575 0.105208761 0.105406361 0.105516607 0.105785371 0.105943769
0.106192610 0.106312166 0.106437181 0.106492855 0.106928791 0.106965502
[421] 0.107171209 0.107280467 0.107357766 0.107412491 0.107683816 0.107762824
0.107862206 0.107924421 0.108059526 0.108182404 0.108275554 0.108468268
[433] 0.108531769 0.108741618 0.108956061 0.109245581 0.109295536 0.109407998
0.109602881 0.109757154 0.110161207 0.110295294 0.110386087 0.110671752
[445] 0.110774470 0.110839621 0.111215033 0.111375374 0.111440400 0.111716681
0.111863510 0.111955746 0.112210330 0.112344001 0.112457694 0.112519843
[457] 0.112742603 0.113067798 0.113116962 0.113355867 0.113598395 0.113758828
0.113898243 0.114058254 0.114349365 0.114427534 0.114551542 0.114802135
[469] 0.114886390 0.115156493 0.115271696 0.115313440 0.115376906 0.115670173
0.115810678 0.115900428 0.116012928 0.116100122 0.116232589 0.116451400
[481] 0.116510115 0.116706704 0.116944557 0.117037225 0.117265336 0.117339296
0.117489555 0.117538916 0.117583086 0.118049739 0.118127067 0.118380790
[493] 0.118503547 0.118629273 0.118721694 0.118867435 0.119013423 0.119392836
0.119478200 0.119619102 0.119719010 0.119865123 0.120056957 0.120370284
[505] 0.120386802 0.120626218 0.120799021 0.120952207 0.121203255 0.121364176

0.121567188 0.121874866 0.122055447 0.122293979 0.122395222 0.122501447
[517] 0.122689055 0.122883077 0.123040874 0.123120931 0.123204328 0.123393742
0.123440742 0.123757066 0.123799530 0.123950131 0.124288904 0.124656020
[529] 0.124752300 0.124886948 0.124998858 0.125143361 0.125224197 0.125495262
0.125526544 0.125688781 0.125739456 0.125902425 0.125994234 0.126105563
[541] 0.126367421 0.126579719 0.126742607 0.126853878 0.126916363 0.127043633
0.127197065 0.127579738 0.127592147 0.127881665 0.128093234 0.128257161
[553] 0.128403127 0.128808570 0.128899096 0.128951456 0.129151074 0.129294365
0.129566592 0.129736077 0.129760090 0.130062409 0.130253615 0.130504918
[565] 0.130689566 0.130760388 0.130879469 0.130987516 0.131170808 0.131282088
0.131327808 0.131463813 0.131820748 0.131849184 0.131994536 0.132337981
[577] 0.132515678 0.132793128 0.133055436 0.133167995 0.133263448 0.133308583
0.133550155 0.133909757 0.134114555 0.134252360 0.134701790 0.134760072
[589] 0.134931388 0.135046322 0.135097143 0.135307070 0.135716059 0.135821098
0.136243975 0.136597135 0.136926539 0.137097088 0.137151028 0.137448977
[601] 0.137503608 0.137681353 0.137941986 0.137951443 0.138477331 0.138631904
0.138836213 0.138969062 0.139081115 0.139549664 0.139620981 0.139893574
[613] 0.140069823 0.140335136 0.140483157 0.140744542 0.140989722 0.141237257
0.141371881 0.141586996 0.141636454 0.141768109 0.142145680 0.142555839
[625] 0.142760121 0.142991926 0.143166750 0.143269420 0.143381645 0.143433221
0.143889360 0.144070655 0.144163633 0.144378449 0.144724885 0.145082978
[637] 0.145193017 0.145247264 0.145460653 0.145779151 0.145885366 0.146059414
0.146496138 0.146697092 0.147133731 0.147290312 0.147548564 0.147679065
[649] 0.148161883 0.148478341 0.148511007 0.148678660 0.148733594 0.149189232
0.149296414 0.149683934 0.149711346 0.149759135 0.149966780 0.150254826
[661] 0.150387526 0.150713933 0.150758885 0.150966413 0.151040137 0.151097901
0.151669734 0.151722083 0.151746456 0.151875601 0.152406710 0.152486051
[673] 0.152542769 0.152804826 0.153388957 0.153782964 0.153929984 0.154139323
0.154286578 0.154547973 0.154809335 0.154985293 0.155132316 0.155291924
[685] 0.155379084 0.155935137 0.156085160 0.156498440 0.156676139 0.156779495
0.156985195 0.157209076 0.157648816 0.157970642 0.158238036 0.158345252
[697] 0.158399152 0.158787667 0.158915394 0.159140221 0.159287305 0.159423132
0.159589241 0.160325599 0.160410577 0.160590177 0.160792972 0.161150019
[709] 0.161385467 0.161775343 0.161817066 0.162023172 0.162398416 0.162439199
0.162606433 0.162704154 0.163063230 0.163370126 0.163627150 0.163892812
[721] 0.163955549 0.164151254 0.164315204 0.164333702 0.164715986 0.164927070
0.165321388 0.165444070 0.165683277 0.165824787 0.165883510 0.166154026
[733] 0.166360544 0.166586347 0.166743422 0.166750258 0.167075398 0.167367144
0.167480162 0.167521673 0.167594992 0.167611767 0.167822907 0.167901978
[745] 0.168100096 0.168355841 0.168480373 0.168887919 0.169284981 0.169482903
0.169837416 0.170304862 0.170568960 0.170811963 0.171073205 0.171186216
[757] 0.171320076 0.171831404 0.171932648 0.172024260 0.172068503 0.172413072
0.172528510 0.172848643 0.173022359 0.173231730 0.173410613 0.173842300
[769] 0.174044281 0.174323347 0.174936696 0.175052929 0.175663299 0.175840573
0.175957648 0.176231673 0.176640406 0.176850005 0.177132373 0.177232661
[781] 0.177454122 0.177669390 0.177847798 0.178250978 0.178464008 0.178612481
0.179021439 0.179127174 0.179450074 0.179689173 0.179914791 0.179946536
[793] 0.180517491 0.181042368 0.181072307 0.181144896 0.181480956 0.181823430
0.181900732 0.182411076 0.182637284 0.183036557 0.183409137 0.183562856
[805] 0.183826715 0.184034598 0.184121497 0.184446091 0.184622135 0.185228253
0.185473834 0.185518487 0.185677076 0.186223254 0.186480971 0.186706318
[817] 0.186749524 0.186988500 0.187165942 0.187261465 0.187400874 0.187447973
0.187484050 0.187662238 0.187780981 0.188237443 0.188669442 0.188785136
[829] 0.189005793 0.189247943 0.189634367 0.189925603 0.190458600 0.190733106

0.190847284 0.191028700 0.191293119 0.191916577 0.192129683 0.192332806
 [841] 0.192596881 0.192725662 0.193182241 0.193785310 0.193837065 0.194047343
0.194475618 0.194750771 0.195092607 0.195251398 0.195452768 0.195742787
 [853] 0.195911977 0.196050273 0.196520010 0.196779753 0.196982068 0.197224700
0.197422325 0.197681780 0.197962520 0.198229957 0.198322323 0.198546350
 [865] 0.198601520 0.198794208 0.198906751 0.199142078 0.199314991 0.199742434
0.200016989 0.200889540 0.201040414 0.201321126 0.201492924 0.202174769
 [877] 0.202228824 0.202667370 0.203064868 0.203157653 0.203463395 0.203814108
0.203968159 0.204426145 0.205115159 0.205599359 0.206026366 0.206329514
 [889] 0.206465862 0.206949840 0.207190335 0.207456377 0.207844778 0.208298740
0.208615161 0.208842338 0.208964440 0.209194991 0.209508486 0.209704319
 [901] 0.209860624 0.210256511 0.210336971 0.210762853 0.210907504 0.211145411
0.211687143 0.212034229 0.212164941 0.212231669 0.212920407 0.213254261
 [913] 0.213580537 0.213781586 0.213792308 0.213862023 0.214211143 0.214764720
0.215115201 0.215151407 0.215407146 0.216042327 0.216450606 0.217049009
 [925] 0.217105427 0.217390389 0.217431808 0.217498900 0.218123461 0.218250178
0.218663891 0.219296858 0.219457527 0.219674644 0.219942515 0.220183199
 [937] 0.220845707 0.221839954 0.222590749 0.222902279 0.223089307 0.223580012
0.223921446 0.224042171 0.224665623 0.224888863 0.225481723 0.225534067
 [949] 0.226016270 0.226059302 0.226855874 0.227179953 0.227529735 0.227617945
0.228131554 0.228900942 0.229191960 0.229214394 0.230297533 0.230517571
 [961] 0.230543613 0.230673445 0.231015524 0.231733095 0.233252795 0.233671871
0.233915700 0.234073856 0.234194971 0.234419510 0.234705466 0.235762895
 [973] 0.236484270 0.236843732 0.237910024 0.238051374 0.238236450 0.238590194
0.238972830 0.239122821 0.239352050 0.240371969 0.240452571 0.241167481
 [985] 0.242098577 0.242400398 0.242669957 0.242824862 0.243321572 0.243464618
0.243753368 0.244010689 0.244645584 0.244755103 0.245207000 0.245638524
 [997] 0.246664028 0.247494227 0.247779406 0.248545197 0.249738804 0.249943705
0.250850962 0.250851568 0.251688233 0.252188578 0.253201959 0.253774740
[1009] 0.254342447 0.254595581 0.255106707 0.255507338 0.255587874 0.255744562
0.256561866 0.257370570 0.257737954 0.258817453 0.259496119 0.259960393
[1021] 0.260139180 0.260311861 0.260922819 0.261793143 0.262492005 0.264779397
0.265158106 0.265257151 0.265885231 0.265948053 0.266543336 0.266934061
[1033] 0.267327200 0.269272903 0.269578501 0.270792396 0.271034840 0.273357731
0.274361750 0.274604871 0.275891364 0.276400592 0.276986397 0.277195144
[1045] 0.279031706 0.279320417 0.279470551 0.279931648 0.280121607 0.280264850
0.281148908 0.282601506 0.283148855 0.284504497 0.284876278 0.284984336
[1057] 0.285267644 0.285699614 0.287947537 0.288010384 0.288384629 0.288853419
0.288998470 0.290730213 0.291760400 0.292156243 0.293321265 0.295323651
[1069] 0.297225297 0.297826446 0.298011586 0.298139182 0.298311561 0.298336779
0.299201176 0.300370329 0.301338104 0.301682214 0.301688931 0.302371984
[1081] 0.302623112 0.304500967 0.304534157 0.304726360 0.305795298 0.308380024
0.309386607 0.309612738 0.310181592 0.313011124 0.313461024 0.313617067
[1093] 0.315126385 0.316800263 0.317399172 0.322550454 0.323114757 0.323809366
0.324481100 0.326203422 0.326512407 0.329776992 0.331708761 0.331826263
[1105] 0.334555956 0.336466913 0.339764940 0.340881281 0.343414953 0.345592416
0.354609893 0.359134778 0.359801879 0.361370823 0.368936538 0.369527618
[1117] 0.370678559 0.371614932 0.373176939 0.374367916 0.379062172 0.379403353
0.385105042 0.392747256 0.397763802 0.403042728 0.405069060 0.410290763
[1129] 0.411594161 0.411700584 0.419280550 0.420836368 0.425182810 0.436157987
0.436644161 0.437994548 0.440611226 0.442862492 0.445067458 0.448835394
[1141] 0.451375961 0.462016602 0.468989003 0.469969671 0.478667904 0.482993377
0.483452179 0.484752933 0.493498674 0.495115015 0.496562071 0.496821217
[1153] 0.499189549 0.501146063 0.501615101 0.501886451 0.504007018 0.505309375

0.505407492 0.506516801 0.507576879 0.512863809 0.513915279 0.518494807
[1165] 0.519369207 0.520350971 0.521561967 0.528528597 0.528635533 0.529740719
0.530287958 0.532254107 0.533674101 0.536570704 0.537227457 0.537370114
[1177] 0.538797711 0.541177663 0.541243375 0.542315535 0.544180751 0.548388121
0.549253301 0.551181395 0.552095835 0.553308380 0.553518172 0.556080994
[1189] 0.557419689 0.557685915 0.559012860 0.559312612 0.561801687 0.562455258
0.564122827 0.564600876 0.565363006 0.565935172 0.567341432 0.568030926
[1201] 0.568040915 0.568273706 0.569133982 0.569699671 0.570256781 0.571590016
0.571718317 0.571923327 0.572638512 0.574722581 0.575743265 0.576503759
[1213] 0.577416459 0.578115976 0.580218723 0.582553554 0.583821659 0.585716485
0.586765985 0.587508524 0.589199123 0.590189986 0.590329499 0.591864494
[1225] 0.592513013 0.593316265 0.595480809 0.595503907 0.596063267 0.596252260
0.596912264 0.597571518 0.598160219 0.599146325 0.599944749 0.600451525
[1237] 0.600654656 0.600786658 0.601185420 0.601642420 0.601764693 0.601898997
0.602207836 0.603013863 0.603081720 0.605591899 0.606226819 0.606500460
[1249] 0.606804661 0.607374049 0.607860485 0.608035437 0.610081276 0.611070818
0.612696075 0.613120080 0.614549378 0.615480666 0.615737298 0.616375020
[1261] 0.616390164 0.616512264 0.617908711 0.618653382 0.618735940 0.618787849
0.618856452 0.619433778 0.619773953 0.619888113 0.620157748 0.620487932
[1273] 0.622910225 0.623416413 0.623697093 0.624131989 0.624830125 0.625156322
0.625446487 0.625525983 0.625773476 0.625936461 0.627051167 0.627152108
[1285] 0.628385095 0.629559160 0.629694174 0.629886004 0.629906574 0.630347935
0.630689673 0.630777383 0.631397026 0.631463398 0.631649237 0.632254324
[1297] 0.632370758 0.633300537 0.635438303 0.636177924 0.636201515 0.636555849
0.637488683 0.638549863 0.638589947 0.638747599 0.639577956 0.639590293
[1309] 0.639618371 0.639667357 0.641083598 0.641481713 0.641623456 0.642206229
0.643302193 0.643889977 0.644323254 0.644377150 0.645023706 0.645062917
[1321] 0.645867741 0.647114291 0.647231789 0.647547190 0.647910703 0.648071952
0.648131887 0.648422123 0.648914305 0.650557311 0.650744101 0.651139032
[1333] 0.652051897 0.653210491 0.653281006 0.654107977 0.654524524 0.655289801
0.655365600 0.655518889 0.656089358 0.656172515 0.658168829 0.658674726
[1345] 0.659341204 0.659421415 0.660269334 0.660333494 0.660363932 0.660646935
0.662014508 0.662528633 0.662931713 0.663883554 0.664861674 0.664999939
[1357] 0.665258124 0.665927828 0.665972444 0.667044227 0.667103046 0.667329009
0.668976561 0.669806183 0.670067714 0.671495490 0.671618962 0.672340389
[1369] 0.672545723 0.672748359 0.672927897 0.673700017 0.675307927 0.675973950
0.676131466 0.676278026 0.676336304 0.676956453 0.677116826 0.677177052
[1381] 0.677286287 0.677466324 0.677813660 0.678840890 0.679055823 0.680227650
0.681270277 0.681572306 0.682422861 0.683091940 0.683690587 0.683857437
[1393] 0.684339124 0.684660758 0.685074609 0.685870744 0.686200667 0.687660919
0.688104606 0.688198194 0.688516204 0.688929467 0.689763550 0.690435862
[1405] 0.690643684 0.692633535 0.692972027 0.693414982 0.694127021 0.694947922
0.695593557 0.695678654 0.695825175 0.697303469 0.697521282 0.697787310
[1417] 0.698123085 0.699534806 0.699605539 0.700719108 0.700794240 0.701390905
0.702755081 0.702902000 0.704185466 0.704278157 0.704353592 0.705975467
[1429] 0.708610016 0.708689423 0.709239750 0.709618957 0.709727731 0.709787670
0.710249579 0.710462389 0.710590016 0.710823893 0.710991732 0.713154493
[1441] 0.713247528 0.713488006 0.715292407 0.716172981 0.716441788 0.716584611
0.717641657 0.718718824 0.719777117 0.719844841 0.720734157 0.720924618
[1453] 0.720945840 0.721273363 0.721730799 0.721949751 0.722681466 0.723202671
0.723764921 0.724041685 0.724046478 0.724318762 0.724573990 0.725069572
[1465] 0.726103033 0.726463588 0.726482835 0.726602297 0.727621031 0.729436309
0.729852536 0.731168063 0.731626843 0.732514390 0.732675100 0.732821517
[1477] 0.733625604 0.734289285 0.734496527 0.734540997 0.734749374 0.735839691

0.736308495 0.736683413 0.739017482 0.740744636 0.741707218 0.741992805
[1489] 0.742137981 0.742301675 0.742623667 0.743682293 0.743880516 0.743959461
0.744133766 0.744288359 0.744433530 0.745049813 0.745259739 0.746031458
[1501] 0.746052067 0.746576109 0.746894478 0.747961181 0.748169004 0.748471703
0.749499916 0.749776555 0.749935390 0.750091489 0.750471132 0.751543696
[1513] 0.751718130 0.752757690 0.754000738 0.754118511 0.754609276 0.755730484
0.756607116 0.756767797 0.756976213 0.757000975 0.757376787 0.757700781
[1525] 0.757924097 0.758530689 0.758579877 0.759131841 0.759185961 0.759398384
0.760327340 0.760994014 0.761335816 0.761752386 0.762816941 0.763131173
[1537] 0.763584897 0.764091279 0.764708001 0.767147463 0.768027652 0.768055042
0.768944157 0.770580112 0.771080684 0.772174975 0.772528946 0.773285112
[1549] 0.773625641 0.774140163 0.775270422 0.776092558 0.776883339 0.777339072
0.777482127 0.777704600 0.778878031 0.779749169 0.780569314 0.781371659
[1561] 0.782472827 0.782576783 0.784324279 0.784631174 0.784870452 0.785146872
0.785195440 0.785794294 0.787044946 0.787403925 0.788948631 0.790856126
[1573] 0.790857696 0.790891085 0.791554145 0.793606826 0.793693398 0.793881352
0.794117189 0.794166389 0.795144260 0.795374356 0.795644564 0.796503582
[1585] 0.796993593 0.797265540 0.798433969 0.799218190 0.799889849 0.800128679
0.800158136 0.802848951 0.803634427 0.804180383 0.805035767 0.806040630
[1597] 0.806693467 0.807186080 0.810370007 0.811247638 0.812048136 0.816925300
0.818839530 0.820368350 0.820448718 0.821802340 0.821859838 0.822132864
[1609] 0.822152782 0.823056505 0.823847757 0.824423773 0.824872218 0.826757111
0.827672577 0.828052610 0.828921711 0.829331486 0.829927456 0.830036425
[1621] 0.831195265 0.832272345 0.833087668 0.833609943 0.833762970 0.837442535
0.837879478 0.838929906 0.838954754 0.839680710 0.841605967 0.842238876
[1633] 0.844978325 0.845452255 0.848529635 0.851819737 0.852652225 0.853051016
0.855628905 0.856013082 0.856503472 0.859542952 0.860388943 0.860751206
[1645] 0.862417389 0.863777557 0.864093520 0.865595979 0.867962758 0.870601324
0.870639555 0.873255095 0.874681685 0.875974985 0.877667896 0.879294361
[1657] 0.880085434 0.881014030 0.890864971 0.892178279 0.892197457 0.893599675
0.894961219 0.897661773 0.899380598 0.905379301 0.908930550 0.909883131
[1669] 0.918208082 0.920386734 0.921131862 0.925821212 0.938210951 0.938416064
0.938662825 0.943512170 0.945223230 0.952665755 0.952844442 0.957429364
[1681] 0.958247237 0.966360178 0.968892759 0.969246482 0.970294138 0.971390262
0.972320728 0.972887246 0.976606828 0.977556570 0.977574100 0.981091099
[1693] 0.982723986 1.005957184 1.008791187 1.013344709 1.022655917 1.039048101
1.041683851 1.045793713 1.063961068 1.086010090 1.087437513 1.107100042

FLUCTUATION DATA for FULL LENGTH MINIMIZED CHARACTERIZED Q5NAT0

[1] 0.33763368 0.24146425 0.16758703 0.17059228 0.19602990 0.16087099
0.14082389 0.16782632 0.15615656 0.12212089 0.15166817 0.18032675 0.15961736
[14] 0.15418767 0.20008316 0.21812907 0.18929028 0.20845080 0.21782902
0.24494704 0.35255651 0.40945460 0.52652965 0.89613919 0.73075673 0.49920553
[27] 0.46191422 0.36889118 0.43326002 0.38654210 0.37963153 0.42421857
0.36088256 0.30750439 0.29028075 0.34777886 0.38428068 0.26886506 0.26724064
[40] 0.39216989 0.46357243 0.45883309 0.47802276 0.38601428 0.35069521
0.23800048 0.26421171 0.21702935 0.17151375 0.15621972 0.16230857 0.14708004
[53] 0.18917208 0.22278688 0.23571920 0.22110767 0.20251928 0.16016502
0.14721645 0.12704097 0.10275581 0.09003299 0.09143940 0.08001517 0.07750836
[66] 0.07923716 0.08114797 0.07326601 0.07354916 0.09182074 0.09631079
0.10365121 0.11322341 0.13509757 0.16133921 0.18250263 0.17346563 0.19266675
[79] 0.24170691 0.19601896 0.18214821 0.24490663 0.28503131 0.25441087
0.27958265 0.24875076 0.21707987 0.25056563 0.21661201 0.16317092 0.17611126

[92] 0.20155491 0.16123290 0.13452092 0.15689942 0.17134772 0.13086037
0.13391033 0.17803318 0.16429093 0.14633863 0.18511587 0.19974044 0.18589826
[105] 0.20647431 0.40093278 0.41517227 0.36008787 0.27401314 0.24411281
0.18228433 0.18463423 0.17196521 0.18628983 0.20130436 0.24491579 0.29593696
[118] 0.27025769 0.37252499 0.35884806 0.29393831 0.31839945 0.41577895
0.39471885 0.31958470 0.32411887 0.31935115 0.30662132 0.35492408 0.37854324
[131] 0.40369444 0.57752144 0.47024440 0.47358577 0.32521327 0.31859915
0.24823338 0.23885200 0.22464316 0.21790182 0.26942963 0.33502031 0.47765582
[144] 0.25788764 0.20671000 0.15001989 0.11996392 0.10492029 0.10033006
0.09823649 0.08220401 0.07452737 0.08607158 0.08826605 0.07433587 0.07124536
[157] 0.09247248 0.09227033 0.09513915 0.11225988 0.13196413 0.15893752
0.18650840 0.20734832 0.24933054 0.43651080 0.48472894 0.40125197 0.32890189
[170] 0.38482600 0.29169455 0.20033430 0.24179766 0.23719001 0.15573819
0.15918912 0.21532134 0.17647968 0.12923074 0.17283366 0.18023979 0.13232583
[183] 0.13738347 0.19911861 0.18091628 0.14067532 0.20556903 0.27043752
0.26499778 0.23251658 0.23190018 0.19663157 0.30326663 0.25737627 0.23689656
[196] 0.32572957 0.30977600 0.28064841 0.31305206 0.39229563 0.38373213
0.44903689 0.53215562 0.41937967 0.54276205 1.23320968 0.66638524 0.28051881
[209] 0.18329684 0.12915341 0.15449926 0.12160804 0.09263859 0.11248484
0.12126768 0.09556638 0.08837638 0.12405755 0.12980446 0.11386822 0.13488950
[222] 0.17528790 0.19601143 0.18691340 0.27903601 0.35123832 0.38457862
0.38096843 0.37808038 0.21536814 0.24425397 0.30797966 0.23573820 0.19320140
[235] 0.27967955 0.32447258 0.27514076 0.32020844 0.48483797 0.49258287
0.58457454 1.25795176 1.40896353 0.69075384 0.36536403 0.33270109 0.23878311
[248] 0.24818520 0.24331412 0.15621936 0.10623261 0.13645955 0.14949581
0.16787537 0.13845573 0.10627429 0.13027755 0.12609592 0.11687579 0.12567523
[261] 0.14528776 0.14582787 0.16057985 0.18762023 0.20597861 0.23220983
0.26834365 0.31524272 0.37311325 0.46109362 0.57499200 0.94256222 1.02366015
[274] 0.79464578 0.68639235 0.59022020 0.55920207 0.60439470 0.42013866
0.33891074 0.39447842 0.34609818 0.23227001 0.24952013 0.29256319 0.23546991
[287] 0.18738494 0.23300685 0.23802187 0.17708546 0.18463750 0.22613462
0.20374414 0.20204458 0.27020560 0.34127539 0.34123359 0.50918210 0.90792395
[300] 0.84043064 0.59172230 0.32972572 0.27898613 0.26087356 0.23278673
0.24939605 0.37058880 0.35955490 0.27426496 0.21532351 0.18495256 0.19239492
[313] 0.22214561 0.25142252 0.24121868 0.17842959 0.12505255 0.12475988
0.10793957 0.09624939 0.09795128 0.10276023 0.07950411 0.07754413 0.09825230
[326] 0.09804817 0.08906489 0.10709535 0.12376170 0.12639786 0.15421403
0.17239295 0.19856900 0.29056623 0.28862420 0.44676138 0.42980362 0.31188004
[339] 0.33542380 0.41873636 0.70474872 0.78810441 1.20121773 1.68329328
2.56019534 2.19339287 1.86265556 1.65368212 0.88391573 0.48318748 0.49820882
[352] 0.30232341 0.24855842 0.28784616 0.25564970 0.17712747 0.18314204
0.21348489 0.16991701 0.13473121 0.15746984 0.17220332 0.14541318 0.14516154
[365] 0.16872912 0.16582262 0.16345693 0.17473404 0.21871354 0.25917899
0.30972706 0.42501757 0.52116084 0.36584698 0.25028920 0.18834445 0.15947258
[378] 0.15555629 0.19124648 0.24941985 0.23908329 0.30767170 0.38738713
0.32044487 0.23101664 0.24993201 0.33589077 0.26429486 0.21446278 0.20875329
[391] 0.22175225 0.25660607 0.27371098 0.27423474 0.28609620 0.33115383
0.35485237 0.39018984 0.42396035 0.38424980 0.44625740 0.41143711 0.48875773
[404] 0.47955024 0.48138386 0.52741021 0.43299780 0.32803414 0.40230207
0.34456522 0.33792132 0.25907802 0.19940854 0.14180145 0.14831121 0.12282830
[417] 0.13029029 0.11667368 0.15092584 0.23029201 0.26903663 0.27746037
0.31563300 0.28356818 0.35062007 0.38200887 0.36179642 0.31653691 0.30745743
[430] 0.26931997 0.30070432 0.26682522 0.20284294 0.21999444 0.16238044
0.16474132 0.15533852 0.13940081 0.15267106 0.11756031 0.09025302 0.08840334

[443] 0.09845355 0.08064056 0.07772344 0.08688080 0.09623028 0.09244214
0.09435987 0.11062969 0.12942983 0.14714306 0.15978748 0.17031380 0.21846872
[456] 0.28847377 0.34021406 0.26695255 0.27690948 0.35369568 0.25568825
0.40626520 0.58858689 0.76889646 0.55528253 0.46348611 0.41306976 0.50934224
[469] 0.54452608 0.80427789 0.70872377 0.79130806 0.75453639 0.76615172
0.51378698 0.51988768 0.69691936 0.61384895 0.92678413 0.96955407 1.57334214
[482] 1.54435387 1.64713948 2.13295759 2.38339744 3.76341912 5.28949181
3.69177598 2.11825842 1.42326329 1.14940462 0.83069455 0.98448733 0.72346863
[495] 0.75425241 0.55059118 0.56210583 0.48377905 0.44553105 0.45814064
0.45193411 0.44809489 0.55534216 0.64661806 0.80302903 0.70201454 0.75469457
[508] 0.66952897 0.58960915 0.58214029 0.58435635 0.59573802 0.95937195
1.28139730 2.02397532 2.23340342 3.39831352 3.49364622 3.46610699 4.94342330
[521] 5.63668918 4.79951731 5.69465699 4.73966784 3.79750167 3.28412838
2.23147556 2.20093154 2.53449617 1.82382533 1.94521009 1.37762932 1.55684355
[534] 1.16889572 1.30708142 1.12365781 0.70709219 0.86223364 1.08527006
1.14185301 1.35317259 2.00920085 2.12713913 3.17422055 3.57717743 5.15983858
[547] 4.20348234 3.06195208 2.09461071 1.38152087 0.82960892 0.54901793
0.51765010 0.45876058 0.60668570 0.55483604 0.73690426 1.06103202 0.79455368
[560] 0.82228551 0.56446908 0.48003397 0.41131545 0.38999050 0.46549411
0.64183545 0.72393321 0.53704263

MASSES for FULL LENGTH MINIMIZED CHARACTERIZED Q5NAT0

[1] 138.14694 114.07900 163.17300 57.05100 131.19600 71.07800 113.15800
87.07700 129.18000 87.07700 113.15800 113.15800 163.17300 147.17400 128.10600
[16] 71.07800 117.12600 157.19400 87.07700 57.05100 99.13100 113.15800
97.11500 57.05100 87.07700 117.12600 157.19400 113.15800 71.07800 186.21000
[31] 157.19400 71.07800 114.10300 87.07700 57.05100 113.15800 71.07800
114.07900 57.05100 129.18000 71.07800 114.10300 57.05100 99.13100 114.07900
[46] 113.15800 99.13100 57.05100 57.05100 163.17300 163.17300 114.07900
71.07800 57.05100 114.07900 114.10300 99.13100 129.18000 147.17400 57.05100
[61] 113.15800 97.11500 131.19600 71.07800 147.17400 101.10400 99.13100
101.10400 131.19600 131.19600 71.07800 186.21000 87.07700 99.13100 113.15800
[76] 128.10600 163.17300 57.05100 128.10600 128.10600 131.19600 71.07800
71.07800 71.07800 57.05100 128.10600 113.15800 57.05100 137.13900 71.07800
[91] 99.13100 128.10600 71.07800 113.15800 129.18000 186.21000 57.05100
101.10400 114.07900 163.17300 147.17400 71.07800 129.18000 71.07800 137.13900
[106] 97.11500 128.10600 97.11500 114.10300 99.13100 113.15800 163.17300
71.07800 128.10600 99.13100 57.05100 114.07900 57.05100 114.07900 87.07700
[121] 114.07900 137.13900 114.10300 103.14300 186.21000 117.12600 157.19400
97.11500 128.10600 114.07900 131.19600 101.10400 101.10400 87.07700 157.19400
[136] 117.12600 71.07800 163.17300 157.19400 113.15800 114.07900 97.11500
117.12600 114.10300 97.11500 57.05100 87.07700 114.07900 113.15800 71.07800
[151] 57.05100 128.10600 101.10400 71.07800 71.07800 71.07800 131.19600
71.07800 71.07800 71.07800 87.07700 113.15800 99.13100 147.17400 157.19400
[166] 87.07700 87.07700 114.10300 97.11500 57.05100 163.17300 71.07800
114.07900 117.12600 113.15800 113.15800 117.12600 137.13900 87.07700 129.18000
[181] 117.12600 113.15800 147.17400 114.07900 147.17400 71.07800 114.07900
129.18000 163.17300 157.19400 57.05100 157.19400 163.17300 114.07900 114.10300
[196] 87.07700 113.15800 101.10400 99.13100 71.07800 157.19400 114.10300
163.17300 163.17300 57.05100 87.07700 147.17400 87.07700 57.05100 163.17300
[211] 57.05100 114.07900 128.10600 113.15800 113.15800 186.21000 71.07800
87.07700 71.07800 186.21000 113.15800 163.17300 117.12600 71.07800 87.07700
[226] 114.07900 114.07900 157.19400 157.19400 163.17300 113.15800 114.07900

```
163.17300 113.15800  71.07800 114.10300 114.10300  71.07800 114.07900  71.07800
[241] 113.15800  57.05100  57.05100 101.10400  57.05100 186.21000  87.07700
113.15800 114.10300 117.12600 147.17400  57.05100 186.21000 114.07900  99.13100
[256] 129.18000 163.17300  97.11500  57.05100  99.13100 117.12600 113.15800
113.15800  71.07800  71.07800 129.18000 147.17400 113.15800 113.15800 117.12600
[271]  57.05100 129.18000  71.07800  57.05100 128.10600 137.13900  71.07800
57.05100  99.13100 113.15800 117.12600  57.05100 163.17300 157.19400 157.19400
[286] 129.18000  71.07800 114.07900 147.17400 147.17400  71.07800 103.14300
87.07700 103.14300 113.15800  57.05100 129.18000 114.07900  71.07800  71.07800
[301] 114.07900 114.10300  99.13100  57.05100 157.19400 101.10400  97.11500
57.05100  57.05100 131.19600 113.15800 163.17300 137.13900 117.12600 157.19400
[316] 186.21000 114.10300 114.10300 113.15800 117.12600 147.17400  99.13100
101.10400  87.07700  71.07800  87.07700 147.17400 113.15800 113.15800  71.07800
[331]  99.13100 163.17300  87.07700 114.07900 137.13900 113.15800  71.07800
57.05100  57.05100  71.07800  99.13100 157.19400 103.14300  87.07700  57.05100
[346]  57.05100  57.05100  57.05100  71.07800  99.13100  71.07800  57.05100
71.07800  71.07800 128.10600 113.15800 113.15800  71.07800 147.17400  71.07800
[361] 129.18000  87.07700 117.12600  99.13100 114.07900 163.17300 113.15800
113.15800  57.05100  87.07700 114.10300  97.11500 157.19400  57.05100 101.10400
[376]  87.07700 163.17300 131.19600  99.13100  57.05100 163.17300  57.05100
71.07800  99.13100 163.17300  97.11500 157.19400 117.12600  71.07800 137.13900
[391] 137.13900 157.19400  57.05100  87.07700  87.07700 113.15800  71.07800
87.07700 113.15800 157.19400  71.07800  87.07700  97.11500  87.07700 147.17400
[406]  57.05100  57.05100 114.10300  97.11500 114.10300 113.15800 113.15800
114.07900  57.05100  71.07800  99.13100  99.13100  57.05100  57.05100  97.11500
[421] 114.07900 128.10600 137.13900 114.07900 114.07900 147.17400  71.07800
114.07900 128.10600 157.19400 114.10300 114.10300 163.17300 128.10600 117.12600
[436] 101.10400 128.10600  71.07800  71.07800 101.10400 163.17300 114.10300
114.10300  71.07800  97.11500 113.15800 131.19600  57.05100 113.15800 113.15800
[451]  71.07800 157.19400 113.15800  71.07800  71.07800  57.05100 137.13900
57.05100  71.07800 157.19400  71.07800 157.19400  57.05100 157.19400 113.15800
[466]  57.05100 117.12600  87.07700 113.15800 117.12600 137.13900  57.05100
113.15800  71.07800  71.07800 114.10300 137.13900 101.10400  87.07700 113.15800
[481]  97.11500 137.13900  57.05100  71.07800 114.10300 137.13900 117.12600
137.13900  71.07800  87.07700  97.11500  99.13100 128.10600 113.15800 128.10600
[496] 117.12600 129.18000  71.07800 101.10400  71.07800  87.07700 186.21000
128.10600 129.18000 114.07900  57.05100 157.19400 101.10400 163.17300 137.13900
[511] 157.19400 163.17300  71.07800  99.13100 101.10400  99.13100  87.07700
114.10300 157.19400  87.07700  97.11500  71.07800  57.05100  57.05100 129.18000
[526] 101.10400  99.13100 128.10600 128.10600 113.15800 137.13900 113.15800
57.05100 113.15800  57.05100 129.18000 113.15800 163.17300  57.05100  97.11500
[541]  99.13100 186.21000  57.05100 113.15800 128.10600 129.18000  71.07800
71.07800 157.19400 163.17300  57.05100 163.17300  99.13100 113.15800  97.11500
[556]  87.07700 186.21000 101.10400  97.11500  87.07700 113.15800  97.11500
71.07800  57.05100 128.10600  87.07700  71.07800  88.08534

Call:
  nma.pdb(pdb = pdbFL4_LJP6, mass = TRUE)

Class:
  VibrationalModes (nma)

Number of modes:
  1680 (6 trivial)
```

```
Frequencies:
  Mode 7:       0.003
  Mode 8:       0.004
  Mode 9:       0.006
  Mode 10:      0.01
  Mode 11:      0.011
  Mode 12:      0.013

+ attr: modes, frequencies, force.constants, fluctuations,
        U, L, xyz, mass, temp, triv.modes, natoms, call
```

NORMAL MODES for FULL LENGTH MINIMIZED CHARACTERIZED Q8LJP6

```
  [1] 0.000000000 0.000000000 0.000000000 0.000000000 0.000000000 0.000000000
0.003230493 0.003786416 0.005510991 0.009773461 0.011185094 0.012877789
  [13] 0.013293964 0.014675950 0.014895236 0.016610165 0.017228477 0.017674800
0.018029523 0.019404506 0.019681232 0.020287105 0.020734169 0.021285746
  [25] 0.022022122 0.022389995 0.022724629 0.023381697 0.023565683 0.023979110
0.025248014 0.025575957 0.025975432 0.026487227 0.026761704 0.027308874
  [37] 0.027519542 0.028081265 0.028392546 0.028547801 0.028756000 0.029527424
0.029793873 0.030158917 0.030495100 0.030744929 0.031253184 0.031869163
  [49] 0.032090152 0.032263341 0.032551758 0.032829927 0.032992708 0.033266810
0.033825200 0.034005568 0.034241623 0.034437835 0.034604399 0.034866922
  [61] 0.035035422 0.035378608 0.035816228 0.036014059 0.036118706 0.036195067
0.036630540 0.036931835 0.037211637 0.037339391 0.037397348 0.037650826
  [73] 0.037856468 0.038133471 0.038441108 0.038787794 0.038893443 0.038937380
0.039217083 0.039865375 0.039988451 0.040248583 0.040291356 0.041016301
  [85] 0.041496428 0.041874086 0.041960500 0.042277437 0.042446059 0.042479167
0.042712870 0.043096913 0.043312081 0.043452506 0.044142191 0.044321723
  [97] 0.044395957 0.044613376 0.045244534 0.045409112 0.045602266 0.045779392
0.046323385 0.046572337 0.047093999 0.047361099 0.047549520 0.047819373
 [109] 0.048189794 0.048460785 0.048500755 0.048659005 0.048868343 0.048941892
0.049098720 0.049468521 0.049644098 0.049902372 0.050300768 0.050388566
 [121] 0.050490510 0.050887538 0.051183101 0.051311613 0.051491236 0.051717031
0.051875967 0.052160104 0.052388576 0.052494116 0.052916087 0.052963456
 [133] 0.053075489 0.053263907 0.053469903 0.053608762 0.053889638 0.054066787
0.054235652 0.054463324 0.054561137 0.054744674 0.054815190 0.054970237
 [145] 0.055493748 0.055668522 0.055918450 0.056204459 0.056225637 0.056410718
0.056662960 0.056878467 0.056917199 0.057028128 0.057427814 0.057578246
 [157] 0.057785300 0.057852548 0.058144727 0.058357584 0.058709409 0.058921301
0.058966638 0.059173328 0.059412777 0.059825960 0.060000147 0.060144356
 [169] 0.060501491 0.060632185 0.060857154 0.060902922 0.061023002 0.061306889
0.061388024 0.061613933 0.061709033 0.061995907 0.062204952 0.062297319
 [181] 0.062486104 0.062564697 0.062729665 0.062756310 0.063154238 0.063216576
0.063364058 0.063788578 0.063857239 0.064061792 0.064267468 0.064418442
 [193] 0.064482701 0.064865940 0.065014161 0.065043960 0.065284768 0.065394866
0.065584390 0.065865730 0.066116189 0.066285311 0.066374481 0.066793710
 [205] 0.066826505 0.067099052 0.067215223 0.067507404 0.067802995 0.067925793
0.068034410 0.068249084 0.068550715 0.068652071 0.068877692 0.069124200
 [217] 0.069212640 0.069299505 0.069409257 0.069488222 0.069553988 0.069631879
0.070077893 0.070262536 0.070430153 0.070700631 0.070840042 0.070915985
 [229] 0.071120359 0.071297684 0.071398334 0.071545591 0.071636699 0.071691485
0.071816629 0.072233028 0.072291742 0.072354610 0.072494509 0.072721967
```

[241] 0.072988811 0.073007896 0.073253991 0.073536129 0.073626494 0.073750415
0.073959629 0.074129309 0.074278654 0.074488934 0.074603784 0.074867981
[253] 0.075017881 0.075079310 0.075164787 0.075262621 0.075560559 0.075874855
0.075910234 0.075990444 0.076165909 0.076292180 0.076394373 0.076680977
[265] 0.076688905 0.076921413 0.077089667 0.077173081 0.077358144 0.077629771
0.077847432 0.077915407 0.078216842 0.078358720 0.078484045 0.078611587
[277] 0.078847582 0.079229115 0.079297663 0.079549137 0.079659076 0.079829175
0.080137477 0.080144589 0.080352443 0.080412159 0.080684650 0.080742551
[289] 0.080809345 0.081353556 0.081375192 0.081488885 0.081526799 0.081730828
0.081951970 0.082002181 0.082299574 0.082532235 0.082630389 0.082905061
[301] 0.082959123 0.083033744 0.083347667 0.083521020 0.083620740 0.083887187
0.084164077 0.084237029 0.084348666 0.084453108 0.084651281 0.084902562
[313] 0.085177341 0.085263687 0.085326051 0.085525015 0.085804296 0.085840599
0.086095028 0.086244506 0.086326410 0.086453223 0.086706731 0.086932551
[325] 0.087157205 0.087308346 0.087495855 0.087650891 0.087763525 0.087945888
0.088010813 0.088080866 0.088238750 0.088429729 0.088578555 0.088950102
[337] 0.088974731 0.089205032 0.089343637 0.089437148 0.089624443 0.089781587
0.090071856 0.090087181 0.090382633 0.090807345 0.090954091 0.090998779
[349] 0.091117143 0.091380997 0.091493746 0.091608015 0.091813920 0.091911394
0.092314532 0.092447927 0.092505037 0.092786922 0.092874785 0.093152016
[361] 0.093191709 0.093216304 0.093415139 0.093466645 0.093863104 0.093967887
0.094038217 0.094245935 0.094360629 0.094499981 0.094592278 0.094799316
[373] 0.095036559 0.095125406 0.095177983 0.095489915 0.095711686 0.095766850
0.096010409 0.096185168 0.096298605 0.096418345 0.096675067 0.096863665
[385] 0.096981532 0.097226868 0.097565225 0.097618953 0.097729818 0.097903449
0.098063212 0.098131897 0.098295541 0.098607887 0.098818560 0.099026354
[397] 0.099130663 0.099216099 0.099279139 0.099449937 0.099548204 0.099622477
0.099766032 0.099923704 0.100142486 0.100173087 0.100343878 0.100636529
[409] 0.100826635 0.101029672 0.101144187 0.101236932 0.101679583 0.101854439
0.101961444 0.102046123 0.102185530 0.102327098 0.102402447 0.102673320
[421] 0.102916409 0.103113489 0.103253663 0.103405773 0.103614635 0.103749249
0.103934880 0.104133439 0.104231543 0.104280864 0.104363905 0.104561648
[433] 0.104631878 0.104793832 0.104844096 0.105135664 0.105195277 0.105294437
0.105591719 0.105709678 0.105970902 0.106256517 0.106296082 0.106414451
[445] 0.106675374 0.106854619 0.106994507 0.107088217 0.107375696 0.107396925
0.107605456 0.107825800 0.107885336 0.108162852 0.108283975 0.108647119
[457] 0.108974774 0.109012888 0.109205924 0.109341299 0.109437166 0.109691245
0.109917432 0.110152354 0.110217987 0.110386546 0.110487466 0.110658590
[469] 0.110776185 0.110978709 0.111288916 0.111430853 0.111468923 0.111742979
0.111804054 0.111846412 0.112121466 0.112220375 0.112407002 0.112821211
[481] 0.112832885 0.112922759 0.113166327 0.113168790 0.113183225 0.113234911
0.113466313 0.113679308 0.113901245 0.114019050 0.114219372 0.114341944
[493] 0.114485849 0.114718812 0.114988428 0.115037321 0.115196519 0.115307839
0.115346934 0.115562600 0.115648163 0.115969142 0.115988034 0.116248169
[505] 0.116361095 0.116534136 0.116779065 0.117086885 0.117321053 0.117424642
0.117688274 0.117827339 0.118187629 0.118404646 0.118455337 0.118529729
[517] 0.118636319 0.118930388 0.119038748 0.119194940 0.119408005 0.119477034
0.119644404 0.119960815 0.120237636 0.120438656 0.120559108 0.120663906
[529] 0.120899106 0.121109696 0.121130087 0.121261341 0.121377011 0.121416343
0.121734494 0.122182494 0.122342644 0.122559020 0.122830399 0.122969932
[541] 0.123127411 0.123393639 0.123829399 0.123875007 0.124021228 0.124255884
0.124294712 0.124728643 0.124997439 0.125084952 0.125695431 0.125816185
[553] 0.125902224 0.126089392 0.126175144 0.126330633 0.126370027 0.126608132
0.126762191 0.126858970 0.127150556 0.127305949 0.127390285 0.127789527

[565] 0.128091058 0.128113600 0.128258544 0.128440406 0.128503302 0.128621912 0.128877969 0.129095459 0.129316501 0.129556816 0.129748670 0.129887986
[577] 0.130222399 0.130267713 0.130456191 0.130688984 0.130917397 0.131072865 0.131206432 0.131379489 0.131658371 0.132163399 0.132278823 0.132382954
[589] 0.132525426 0.132928110 0.133111965 0.133241112 0.133443424 0.133554043 0.133642492 0.133769895 0.133797065 0.133921106 0.134093683 0.134552680
[601] 0.134751989 0.134804894 0.134896560 0.135150194 0.135317554 0.135458248 0.135481901 0.135708313 0.135915712 0.136160100 0.136437008 0.136589810
[613] 0.136712799 0.136717709 0.136893792 0.137181498 0.137192853 0.137356709 0.137719339 0.137855745 0.138011106 0.138232917 0.138602209 0.138838493
[625] 0.138923577 0.139176334 0.139646650 0.139720728 0.139862698 0.140088177 0.140148015 0.140330804 0.140357967 0.140883503 0.141082935 0.141406099
[637] 0.141487402 0.141752653 0.141960410 0.142107451 0.142289504 0.142397521 0.142555927 0.142596434 0.142877710 0.143316795 0.143328725 0.143480807
[649] 0.143680778 0.144041730 0.144352305 0.144480871 0.144798027 0.144974080 0.145263917 0.145346460 0.145649296 0.145772809 0.145983348 0.146149221
[661] 0.146467000 0.146737837 0.146913720 0.146926478 0.147146212 0.147239226 0.147614301 0.147686097 0.147999978 0.148267160 0.148543837 0.148683260
[673] 0.148947600 0.149070589 0.149350103 0.149470558 0.149674457 0.149686557 0.149852303 0.150141159 0.150239822 0.150397632 0.150431818 0.150554015
[685] 0.150894582 0.151035274 0.151209006 0.151312916 0.151365388 0.151618871 0.151905952 0.152157120 0.152629424 0.152970836 0.153164205 0.153526372
[697] 0.153624262 0.153780494 0.153945616 0.154050722 0.154563133 0.154841647 0.154888181 0.154956852 0.155098186 0.155338730 0.155448190 0.155617403
[709] 0.155680627 0.156225636 0.156404211 0.156643477 0.156905385 0.157310552 0.157637006 0.158101592 0.158139638 0.158634691 0.158813747 0.158943445
[721] 0.159152954 0.159350345 0.159485007 0.159799647 0.159903519 0.160123873 0.160583788 0.160729709 0.161117872 0.161586950 0.161962032 0.162319941
[733] 0.162807728 0.163189007 0.163335702 0.163847521 0.164120775 0.164458200 0.164539658 0.164671844 0.164825826 0.165047359 0.165242615 0.165374470
[745] 0.165540881 0.165895420 0.166268781 0.166420905 0.166948604 0.167016639 0.167289032 0.167371231 0.167836264 0.167965027 0.168030012 0.168265317
[757] 0.168512845 0.168657162 0.168983281 0.169352377 0.169570536 0.169871716 0.170859040 0.171059434 0.171444598 0.171902369 0.171967708 0.172100077
[769] 0.172587007 0.172815374 0.173193781 0.173356778 0.173855924 0.174016917 0.174089101 0.174565260 0.174729224 0.174950307 0.175195763 0.175604456
[781] 0.175722842 0.176046016 0.176407729 0.176516322 0.176592361 0.176975860 0.177037180 0.177224014 0.177984475 0.178236909 0.178251902 0.178516233
[793] 0.178581917 0.178750912 0.178929657 0.179258495 0.179363031 0.179929995 0.179952167 0.180034774 0.180129157 0.180405340 0.180555864 0.180862560
[805] 0.181025298 0.181275594 0.181464346 0.181716058 0.181844604 0.181886806 0.182010013 0.182335588 0.182480564 0.182806687 0.182910441 0.182965619
[817] 0.183170123 0.183340830 0.183780824 0.183919427 0.184254826 0.184389708 0.184468955 0.185130108 0.185552277 0.185968039 0.186289212 0.186484367
[829] 0.186673483 0.186949889 0.187364105 0.187815713 0.188122691 0.188371356 0.188439520 0.188724211 0.189178799 0.189392042 0.189525138 0.189721504
[841] 0.190286358 0.190308854 0.190351574 0.190744328 0.190971408 0.191415499 0.191486944 0.191933339 0.192410428 0.192466633 0.192660986 0.192806935
[853] 0.193133655 0.193167686 0.193317249 0.193526914 0.193992184 0.194122061 0.194420320 0.194610315 0.195045535 0.195755728 0.195832769 0.196049111
[865] 0.196304703 0.196574202 0.196911459 0.197052460 0.197267849 0.198006276 0.198125851 0.198236794 0.198576073 0.198819308 0.199200516 0.199392626
[877] 0.199731781 0.200178644 0.200575771 0.200694636 0.200757038 0.201016032 0.201345157 0.201361762 0.201751978 0.201853523 0.202227008 0.202415369

[889] 0.202535968 0.202646059 0.203378845 0.203651048 0.204150323 0.204364553
0.204902022 0.205300191 0.205478770 0.205810296 0.206162116 0.206430465
[901] 0.206811974 0.207396357 0.207454790 0.207822962 0.208435137 0.208633798
0.208989167 0.209664515 0.210269943 0.210981294 0.211153449 0.211913656
[913] 0.212302374 0.212457185 0.213578818 0.214612754 0.214835475 0.215070804
0.215426548 0.215493853 0.215550797 0.215798668 0.216034941 0.216093031
[925] 0.216668807 0.216978042 0.217488767 0.218106912 0.218644313 0.219144914
0.219385088 0.219871099 0.220028011 0.220070429 0.220193553 0.220528399
[937] 0.221087298 0.221150704 0.221816774 0.222302369 0.222595357 0.222784632
0.223166787 0.223298582 0.223300624 0.223851074 0.224255810 0.224748589
[949] 0.225332544 0.226533064 0.226737262 0.227171089 0.227723195 0.228803927
0.228987628 0.229134537 0.229417862 0.229613207 0.229972064 0.230162701
[961] 0.230472404 0.231055487 0.231928892 0.232538662 0.233710133 0.233780897
0.234132122 0.234199135 0.234493678 0.234930271 0.235545283 0.236332497
[973] 0.237033011 0.237265433 0.238358160 0.238460901 0.239013847 0.239172391
0.239364590 0.239385912 0.240247958 0.240334135 0.240598955 0.240910540
[985] 0.241379972 0.242076186 0.243193753 0.243436161 0.243727388 0.244768816
0.244871970 0.245068278 0.245879759 0.246592082 0.246682923 0.247009138
[997] 0.247619110 0.248789773 0.248983958 0.250141096 0.250445310 0.250670551
0.251730851 0.251806308 0.253134874 0.253291479 0.253571188 0.253750884
[1009] 0.254287666 0.255277087 0.256112647 0.256374161 0.256949482 0.257103764
0.257369930 0.257660105 0.259302529 0.259542726 0.259867029 0.259911668
[1021] 0.260213270 0.261984990 0.263857153 0.264084287 0.264386973 0.265132407
0.265395580 0.266683567 0.267120225 0.267326679 0.268054489 0.269148044
[1033] 0.269783450 0.271349050 0.271741814 0.272318945 0.273768339 0.273961369
0.274406154 0.274979489 0.275457668 0.276295227 0.277120338 0.278044278
[1045] 0.278200883 0.280179654 0.280406530 0.281031804 0.284088542 0.284411531
0.284765911 0.285906828 0.285931102 0.287216824 0.287361731 0.288356520
[1057] 0.288879156 0.289014641 0.289452264 0.291509864 0.294288593 0.295685642
0.296464308 0.297015704 0.297490987 0.298945225 0.298959417 0.299300170
[1069] 0.301004792 0.301084221 0.302544044 0.304763639 0.304855758 0.305080515
0.308451190 0.311523513 0.311704215 0.311820563 0.313426962 0.315484321
[1081] 0.315982410 0.319552649 0.320214604 0.325142408 0.326489094 0.326587999
0.332841163 0.334296538 0.336701639 0.337436893 0.337764551 0.337960828
[1093] 0.338203543 0.338365992 0.341220478 0.344830368 0.344845720 0.345102787
0.350915176 0.358807504 0.360716454 0.363407512 0.368854929 0.369082406
[1105] 0.373617780 0.374861013 0.375453730 0.377131566 0.379096050 0.384237973
0.384696000 0.386359729 0.389905217 0.390376028 0.392991101 0.409698459
[1117] 0.411326798 0.418258978 0.425049133 0.427344566 0.433760760 0.437297865
0.438373707 0.443301803 0.447645679 0.449824890 0.450372045 0.451621551
[1129] 0.453188495 0.461518902 0.464751892 0.466064565 0.466908525 0.468085808
0.472105850 0.472768653 0.473483013 0.476891182 0.477037148 0.481661262
[1141] 0.482096296 0.484278234 0.489052427 0.492642602 0.494327311 0.494954831
0.496299293 0.502679947 0.507311692 0.509958739 0.510758118 0.512213870
[1153] 0.513375282 0.515514064 0.516698183 0.521652366 0.521771100 0.523766900
0.526724288 0.528565235 0.529050183 0.530901935 0.532169699 0.532559290
[1165] 0.537508280 0.539443723 0.540367573 0.541033014 0.542363945 0.543345372
0.543753277 0.545080772 0.545251038 0.545453340 0.545959870 0.546238383
[1177] 0.547731500 0.547761142 0.548064969 0.553010434 0.553389519 0.553516593
0.555394406 0.555817713 0.557038799 0.557288573 0.557497707 0.560815996
[1189] 0.563955991 0.564997515 0.565062787 0.565834368 0.566984140 0.568161837
0.569070178 0.571311647 0.571825505 0.572253809 0.573121856 0.573432411
[1201] 0.574633699 0.574934736 0.575962916 0.578181936 0.578682317 0.578848125
0.578881032 0.579347253 0.579712437 0.579766856 0.580057893 0.581546587

[1213] 0.585248975 0.585531490 0.586095290 0.586470741 0.587353571 0.587766143
0.589776253 0.590192540 0.590343036 0.590971408 0.591100366 0.592788028
[1225] 0.593594471 0.594015480 0.596163868 0.596752218 0.596766289 0.597025174
0.598330197 0.598599810 0.599535868 0.599896320 0.600188674 0.600265396
[1237] 0.600290799 0.600613770 0.602369628 0.602722291 0.603142493 0.603604349
0.603918397 0.604041173 0.604090277 0.604426493 0.605306632 0.605471069
[1249] 0.605476549 0.607412938 0.608554477 0.609039450 0.609620359 0.609840083
0.609921239 0.610483737 0.611355115 0.611497317 0.612025072 0.612257068
[1261] 0.613889561 0.614031651 0.614504222 0.614643594 0.615231646 0.616168254
0.616460062 0.616750992 0.616804320 0.617685050 0.617929166 0.618630289
[1273] 0.618866972 0.620776547 0.620946065 0.621750004 0.621900847 0.621947238
0.622127491 0.622569546 0.623412228 0.623415113 0.623661941 0.624591322
[1285] 0.624726416 0.625788999 0.626054373 0.626094286 0.626680304 0.626725491
0.628079308 0.628819529 0.629121936 0.629737456 0.630724293 0.630977033
[1297] 0.632577254 0.632759303 0.633290198 0.633363770 0.633678739 0.633779404
0.634224600 0.635101235 0.635347609 0.635731107 0.636905630 0.637984535
[1309] 0.638241386 0.638263968 0.638448066 0.638703302 0.639233580 0.641186557
0.641657269 0.641683105 0.641839920 0.642324881 0.642630767 0.643619245
[1321] 0.643871526 0.645008783 0.645166399 0.645434814 0.645588422 0.646308863
0.646635487 0.646975062 0.647537528 0.647823202 0.648308240 0.648438119
[1333] 0.648683548 0.648750533 0.650126358 0.650386242 0.651027434 0.651263097
0.651325383 0.651459988 0.651609862 0.651722546 0.652125333 0.652816677
[1345] 0.654350273 0.655509557 0.656101983 0.656494694 0.656707818 0.656832103
0.657181846 0.657324539 0.657687039 0.657839325 0.658242218 0.658568154
[1357] 0.658820229 0.658952823 0.660540874 0.660991690 0.661362540 0.661846438
0.662733299 0.663271843 0.663743893 0.664534174 0.664777090 0.664811725
[1369] 0.664843044 0.665650571 0.666211509 0.667290822 0.668210925 0.668465293
0.668714622 0.669136821 0.669341492 0.669696371 0.670154333 0.670975571
[1381] 0.671018889 0.672365084 0.672803121 0.673257446 0.674337973 0.674788656
0.675731667 0.675824682 0.676920868 0.677284361 0.678909340 0.679779678
[1393] 0.681310041 0.682135542 0.682451051 0.683556566 0.684740112 0.684792195
0.685035230 0.685134561 0.685194932 0.685677823 0.687111981 0.687288375
[1405] 0.687295967 0.687371885 0.687836676 0.687882154 0.687905518 0.689107179
0.689210020 0.690528052 0.690595984 0.691147598 0.691305668 0.691648106
[1417] 0.692242902 0.693255603 0.693758241 0.694441805 0.694752326 0.694873671
0.695286198 0.696950181 0.697533557 0.697535136 0.697626968 0.697871160
[1429] 0.698528362 0.699869399 0.699967927 0.700006882 0.700847299 0.702102142
0.702103819 0.702790151 0.702791016 0.703020262 0.704715102 0.704911707
[1441] 0.705938976 0.706545726 0.707954534 0.708207111 0.708372942 0.708740104
0.708783473 0.710762491 0.711423699 0.712113516 0.714823569 0.715237197
[1453] 0.716156092 0.716325812 0.717004341 0.717272324 0.717601877 0.717795128
0.717807691 0.718459754 0.719315798 0.719424919 0.719594378 0.719689959
[1465] 0.721633347 0.722368380 0.722685549 0.722724647 0.722756680 0.723290859
0.723685997 0.724999821 0.725359457 0.725758006 0.727366403 0.728207385
[1477] 0.729946566 0.730527812 0.731131271 0.732451176 0.733652828 0.733739864
0.734339390 0.734631875 0.735690570 0.735835147 0.736028911 0.736143090
[1489] 0.736786146 0.737236276 0.739815229 0.740372938 0.740751526 0.741241675
0.741359920 0.742082515 0.742170217 0.742463899 0.743525342 0.743797937
[1501] 0.745170072 0.746258368 0.746342083 0.746687505 0.747486961 0.747920626
0.748170426 0.748835474 0.749082196 0.749289387 0.749465899 0.749746943
[1513] 0.750031461 0.750725195 0.751014621 0.751247966 0.752214400 0.752794115
0.752884675 0.753367181 0.755410726 0.755451684 0.756219114 0.757295395
[1525] 0.757606283 0.758059137 0.758557671 0.760427695 0.761942368 0.762565262
0.762766350 0.763350147 0.763697329 0.766152340 0.766345000 0.767640176

[1537] 0.767951957 0.769011619 0.769928779 0.771162034 0.771239483 0.771271258
0.773703449 0.775176449 0.775340257 0.775370198 0.775862897 0.775884167
[1549] 0.776967015 0.777168971 0.777745068 0.778487382 0.779190289 0.779606075
0.780228341 0.780363904 0.782120328 0.782174979 0.782478670 0.782667909
[1561] 0.783927426 0.785279618 0.785948605 0.786746302 0.787122296 0.788175500
0.788233073 0.788809261 0.789063917 0.790361749 0.792105502 0.792615937
[1573] 0.794053774 0.794318117 0.797216150 0.797315690 0.797867017 0.801213189
0.801731063 0.803045171 0.804442752 0.804641621 0.805043004 0.805130738
[1585] 0.805544345 0.809004756 0.809180435 0.810812167 0.810933793 0.811379018
0.811900330 0.814973598 0.815957499 0.817306425 0.817681038 0.818129263
[1597] 0.819608229 0.820070304 0.820586820 0.820693000 0.821099262 0.821292726
0.823819870 0.825415880 0.827470365 0.828250352 0.829397960 0.829866702
[1609] 0.831915417 0.834195053 0.834222108 0.835031113 0.836067790 0.836407304
0.837277323 0.837729788 0.844372893 0.846098559 0.846560911 0.846854958
[1621] 0.847148035 0.847418189 0.847549356 0.848221491 0.852581087 0.853176255
0.856614826 0.859887013 0.869763227 0.872482114 0.872627888 0.873124917
[1633] 0.874252749 0.874638810 0.875222973 0.879106544 0.883152715 0.884980299
0.886239835 0.888785101 0.890842878 0.891683718 0.892141128 0.892520984
[1645] 0.893586508 0.893735699 0.898938073 0.899787197 0.902334477 0.912678264
0.914356049 0.914646828 0.916534101 0.922248226 0.929296268 0.931606985
[1657] 0.932603641 0.935200337 0.939074331 0.939534362 0.940357213 0.947038406
0.952313533 0.953588792 0.954320365 0.959174832 0.966218111 0.976493866
[1669] 0.980370925 0.987114460 0.989651812 0.995382613 0.999045712 1.012944120
1.027110676 1.059333245 1.063728704 1.067138012 1.071312378 1.073800980

FLUCTUATION DATA for FULL LENGTH MINIMIZED CHARACTERIZED Q8LJP6

[1] 0.35180956 0.23100155 0.15332905 0.14828642 0.17474003 0.14737318
0.13054205 0.15164589 0.14157909 0.11081393 0.14695989 0.16061506 0.14393678
[14] 0.13980138 0.16506679 0.17790087 0.16525440 0.18494798 0.18271844
0.20336635 0.29172115 0.34759078 0.61642130 0.83055721 0.70450626 0.40283048
[27] 0.34004924 0.28025753 0.34811529 0.30016714 0.29316297 0.32672212
0.28533129 0.24392441 0.24984549 0.30214875 0.35128777 0.23970518 0.24708826
[40] 0.36348792 0.40838141 0.41297482 0.46873740 0.39702193 0.33139487
0.22416374 0.24322742 0.19058486 0.15880642 0.14232662 0.13865487 0.13638520
[53] 0.17473499 0.20107234 0.21085918 0.18866319 0.18465715 0.14779024
0.13948280 0.12860126 0.10244579 0.09958434 0.09479292 0.08282449 0.07878801
[66] 0.07861765 0.08037385 0.06954334 0.07560865 0.08613080 0.09035833
0.09852618 0.10525316 0.12026989 0.14848423 0.17105303 0.15153620 0.16324508
[79] 0.20707317 0.16003104 0.15837372 0.21331632 0.23080909 0.20558680
0.21905989 0.20208714 0.18538019 0.24402177 0.20291366 0.15703737 0.17103472
[92] 0.18635719 0.15902952 0.13927813 0.15488886 0.16774932 0.13244254
0.13778420 0.18209599 0.15835803 0.14995295 0.18242166 0.19042201 0.17800716
[105] 0.18900499 0.35345506 0.30576910 0.26041130 0.22275867 0.21170839
0.16572431 0.16280002 0.15767166 0.16303018 0.17865910 0.21339778 0.25264173
[118] 0.25785847 0.33608082 0.33272538 0.25901645 0.27262667 0.37016888
0.33163275 0.27343148 0.27048958 0.24942440 0.24927465 0.26893816 0.29119484
[131] 0.33019869 0.49926913 0.42049444 0.50092377 0.29750519 0.27722849
0.22000573 0.20098955 0.18752144 0.18740593 0.21855133 0.27715825 0.45754616
[144] 0.26948833 0.17779613 0.13052043 0.11009680 0.09531515 0.09444964
0.09046351 0.07609856 0.07335730 0.08421395 0.08399051 0.07172557 0.06869679
[157] 0.08957116 0.08908238 0.08796074 0.10490110 0.12571624 0.15205531
0.16361210 0.19295305 0.23777257 0.45305319 0.45207725 0.38768000 0.32995614
[170] 0.37723696 0.28698079 0.19132954 0.24392009 0.23191054 0.14923086

0.15011952 0.19729709 0.16551376 0.12043441 0.15526001 0.16284542 0.12127270
[183] 0.12360573 0.17269857 0.15497958 0.12354852 0.18783667 0.23229958
0.21880084 0.21775862 0.19355543 0.15905884 0.15475642 0.17031598 0.25660272
[196] 0.23482924 0.23896683 0.28602401 0.23574646 0.16103265 0.19961167
0.26080347 0.22280102 0.19823862 0.18879179 0.27796158 0.18960256 0.16836668
[209] 0.14230585 0.14882906 0.11030359 0.09163899 0.11837662 0.11500196
0.09622390 0.08528033 0.11480615 0.11756052 0.10423324 0.12134860 0.15012076
[222] 0.17364188 0.17749567 0.24770296 0.29758676 0.33578391 0.33767432
0.32122257 0.19566826 0.20976500 0.27063686 0.24048390 0.18411714 0.25508220
[235] 0.30446231 0.22305623 0.24920246 0.36689728 0.31484915 0.29541289
0.31865206 0.36867909 0.50444226 1.51600856 1.81227351 0.90051766 0.42684341
[248] 0.31480672 0.23091588 0.14867583 0.17368132 0.18345639 0.17787974
0.11906321 0.11676684 0.12285361 0.12732106 0.12626300 0.12386352 0.13436587
[261] 0.13700148 0.14796137 0.17716601 0.19970562 0.21150925 0.23547423
0.28465842 0.42443487 0.52329740 0.44924458 0.44460311 0.78345213 1.09486313
[274] 0.68842207 0.71515010 0.65215090 0.55289040 0.40254137 0.32758711
0.35229719 0.31865307 0.21064266 0.22344402 0.26891890 0.21343953 0.17025356
[287] 0.21426772 0.23596568 0.19188388 0.18541504 0.21870681 0.22317149
0.21318076 0.26329952 0.37506665 0.57711164 0.95505420 0.95105543 0.77984530
[300] 0.32950659 0.28496742 0.24698741 0.24969541 0.26090753 0.38435592
0.30647273 0.25188099 0.21111399 0.18500461 0.21452963 0.27111428 0.27974138
[313] 0.22443371 0.17680295 0.12293354 0.12772214 0.10833646 0.10299500
0.10019073 0.09982442 0.08046590 0.08223126 0.10001420 0.09487347 0.08790104
[326] 0.09784816 0.11022001 0.11727574 0.14178222 0.14509194 0.16449968
0.24264419 0.26250591 0.29839079 0.54423575 0.42378652 0.66066990 0.68545920
[339] 0.50233727 0.64916381 0.99427658 1.18623890 1.07437965 0.96556812
0.67303875 0.71389834 0.53149318 0.35986412 0.26875600 0.21974676 0.23499895
[352] 0.21315506 0.15018420 0.15814036 0.19777319 0.16330803 0.12516880
0.14449300 0.15505080 0.13429181 0.13166772 0.14756765 0.14534844 0.14599355
[365] 0.14448088 0.17021933 0.20243552 0.26575226 0.35030681 0.36889369
0.26533053 0.22684830 0.15334787 0.14659666 0.13573637 0.15778970 0.20198054
[378] 0.20012138 0.23157977 0.28483469 0.29945397 0.20834743 0.23435028
0.28294536 0.23430675 0.20077685 0.18543187 0.19848981 0.22132966 0.23021252
[391] 0.23364239 0.24397614 0.27206796 0.32313094 0.32507733 0.34719973
0.33257135 0.37861652 0.39256100 0.44703340 0.41865148 0.42130609 0.46503264
[404] 0.37168988 0.27355207 0.34268888 0.29358739 0.26978890 0.21192753
0.16898119 0.12316421 0.12913783 0.10901424 0.11887620 0.11086379 0.14902316
[417] 0.22573645 0.26198313 0.35120628 0.33365148 0.31348897 0.38572430
0.40172090 0.40113941 0.32368209 0.30161829 0.24416481 0.30960803 0.26102047
[430] 0.20149254 0.23247296 0.15223120 0.14959254 0.14299664 0.12531956
0.13498803 0.10482615 0.08841782 0.08442770 0.09363453 0.07790329 0.07531748
[443] 0.08410965 0.08940636 0.07675346 0.08524572 0.09561025 0.11024841
0.13082058 0.14128601 0.14690639 0.19762678 0.27013492 0.27109099 0.24907641
[456] 0.22232313 0.30606870 0.25237965 0.43592539 0.91992496 1.02906149
0.55932606 0.45800428 0.63566467 0.60071291 0.66670492 0.70323199 0.64627148
[469] 1.18680245 0.88727500 0.42578650 0.39113022 0.51130402 0.42910607
0.69592957 0.85927646 1.35864494 1.38826686 1.61254992 2.33483100 2.81042578
[482] 3.03084991 4.14574319 3.45930845 2.05288603 1.42046651 1.10571080
0.81758079 0.80038295 0.52367818 0.55535255 0.39091872 0.42198028 0.34936016
[495] 0.35250809 0.35605834 0.31695842 0.35839566 0.32443499 0.31906712
0.37430282 0.39269760 0.48934468 0.59733249 0.59855164 0.63676059 0.70377214
[508] 0.75784556 1.22731192 1.60209393 2.29367203 2.64211487 3.58421521
5.13617957 5.25107821 3.41711152 3.58678920 2.72043598 3.05567432 2.79981323
[521] 1.91934365 1.98701890 1.30678231 1.36234143 1.29594588 2.18450730

1.72838456 1.19223999 1.18887433 0.79092007 0.77129086 0.78345481 0.63228351
[534] 0.98140653 1.17497270 1.75125760 2.35378275 2.80468538 4.03374560
5.06402907 3.41204479 2.53057486 1.70111598 1.09069834 0.70012806 0.60660931
[547] 0.48977004 0.59222011 0.63144560 1.00261158 0.87484363 0.62799831
0.36925145 0.35400271 0.31373741 0.31401030 0.37079546 0.58135217 0.72431960
[560] 0.51375996

MASSES for FULL LENGTH MINIMIZED CHARACTERIZED Q8LJP6

[1] 138.1469 114.0790 163.1730 114.1030 117.1260 71.0780 113.1580 87.0770
129.1800 87.0770 113.1580 113.1580 147.1740 147.1740 128.1060 71.0780
[17] 117.1260 157.1940 87.0770 57.0510 163.1730 113.1580 97.1150 137.1390
114.1030 117.1260 157.1940 99.1310 101.1040 186.2100 157.1940 71.0780
[33] 114.1030 87.0770 57.0510 113.1580 114.1030 114.0790 57.0510 129.1800
71.0780 87.0770 57.0510 99.1310 114.0790 113.1580 99.1310 57.0510
[49] 57.0510 163.1730 163.1730 114.0790 71.0780 57.0510 114.0790 114.1030
99.1310 129.1800 147.1740 57.0510 113.1580 97.1150 131.1960 71.0780
[65] 147.1740 101.1040 113.1580 101.1040 131.1960 131.1960 87.0770 186.2100
87.0770 113.1580 113.1580 128.1060 163.1730 57.0510 129.1800 117.1260
[81] 131.1960 57.0510 71.0780 101.1040 57.0510 128.1060 113.1580 57.0510
137.1390 71.0780 131.1960 114.0790 71.0780 99.1310 129.1800 186.2100
[97] 57.0510 101.1040 114.0790 163.1730 113.1580 113.1580 129.1800 71.0780
137.1390 97.1150 128.1060 97.1150 163.1730 99.1310 113.1580 163.1730
[113] 57.0510 128.1060 99.1310 57.0510 114.0790 57.0510 114.1030 87.0770
114.0790 137.1390 163.1730 103.1430 186.2100 117.1260 157.1940 97.1150
[129] 128.1060 114.0790 113.1580 101.1040 101.1040 114.1030 157.1940 137.1390
71.0780 163.1730 129.1800 129.1800 114.0790 97.1150 87.0770 114.1030
[145] 97.1150 57.0510 87.0770 114.0790 113.1580 71.0780 57.0510 128.1060
101.1040 71.0780 71.0780 71.0780 131.1960 71.0780 71.0780 71.0780
[161] 87.0770 113.1580 99.1310 147.1740 157.1940 157.1940 87.0770 114.1030
97.1150 71.0780 163.1730 87.0770 101.1040 128.1060 113.1580 113.1580
[177] 157.1940 137.1390 71.0780 163.1730 117.1260 113.1580 147.1740 128.1060
147.1740 71.0780 114.0790 129.1800 163.1730 157.1940 57.0510 129.1800
[193] 163.1730 114.0790 87.0770 87.0770 113.1580 101.1040 99.1310 71.0780
117.1260 163.1730 163.1730 157.1940 87.0770 99.1310 87.0770 57.0510
[209] 163.1730 114.1030 114.0790 128.1060 113.1580 113.1580 186.2100 71.0780
71.0780 71.0780 186.2100 113.1580 163.1730 117.1260 71.0780 87.0770
[225] 114.1030 114.1030 117.1260 163.1730 163.1730 113.1580 114.1030 163.1730
113.1580 57.0510 129.1800 114.1030 57.0510 114.0790 87.0770 131.1960
[241] 57.0510 57.0510 101.1040 57.0510 186.2100 71.0780 131.1960 101.1040
128.1060 147.1740 57.0510 186.2100 114.0790 99.1310 129.1800 163.1730
[257] 101.1040 57.0510 99.1310 117.1260 101.1040 113.1580 99.1310 71.0780
129.1800 147.1740 113.1580 131.1960 117.1260 57.0510 129.1800 71.0780
[273] 57.0510 131.1960 137.1390 71.0780 97.1150 99.1310 147.1740 128.1060
157.1940 163.1730 137.1390 117.1260 129.1800 71.0780 128.1060 137.1390
[289] 147.1740 131.1960 103.1430 87.0770 113.1580 113.1580 57.0510 129.1800
57.0510 113.1580 157.1940 114.1030 99.1310 117.1260 129.1800 101.1040
[305] 97.1150 57.0510 57.0510 113.1580 131.1960 147.1740 157.1940 117.1260
129.1800 186.2100 114.1030 114.1030 131.1960 117.1260 147.1740 99.1310
[321] 101.1040 87.0770 71.0780 87.0770 147.1740 113.1580 71.0780 101.1040
99.1310 163.1730 87.0770 114.0790 163.1730 113.1580 99.1310 87.0770
[337] 87.0770 157.1940 57.0510 101.1040 113.1580 157.1940 103.1430 71.0780
71.0780 57.0510 114.1030 99.1310 71.0780 97.1150 101.1040 117.1260
[353] 113.1580 113.1580 87.0770 147.1740 71.0780 129.1800 87.0770 117.1260

```
99.1310 114.0790 163.1730 113.1580 113.1580  57.0510 114.0790 114.1030
[369]  97.1150 157.1940  57.0510 101.1040  87.0770 163.1730 131.1960  99.1310
57.0510 163.1730  57.0510 114.1030 114.1030 147.1740  97.1150 157.1940
[385] 117.1260  99.1310 137.1390 137.1390 157.1940  57.0510  87.0770 157.1940
157.1940  57.0510 163.1730  71.0780  71.0780 186.2100 163.1730 101.1040
[401] 157.1940 129.1800  71.0780  87.0770 114.0790  97.1150 114.1030  99.1310
113.1580 101.1040  57.0510  71.0780  99.1310  99.1310  57.0510  57.0510
[417]  97.1150 114.0790  71.0780 163.1730 114.0790 114.1030 147.1740  71.0780
114.0790 128.1060 157.1940 114.0790 114.1030 163.1730 128.1060 117.1260
[433] 101.1040 114.0790  97.1150  57.0510 101.1040 163.1730 114.1030 114.1030
71.0780  97.1150 113.1580 113.1580  57.0510 113.1580 113.1580  71.0780
[449] 157.1940 113.1580 114.1030  71.0780  57.0510 137.1390  57.0510  57.0510
163.1730 114.1030 117.1260 113.1580 113.1580  97.1150  99.1310  99.1310
[465] 101.1040 101.1040 117.1260  97.1150 129.1800  99.1310  71.0780  97.1150
113.1580  97.1150 129.1800  99.1310  71.0780  97.1150  71.0780  87.0770
[481]  97.1150  71.0780  97.1150  87.0770  87.0770  87.0770  97.1150 113.1580
71.0780 113.1580  87.0770 117.1260 157.1940 129.1800 101.1040  87.0770
[497]  87.0770 186.2100 113.1580  87.0770 129.1800  57.0510  99.1310 101.1040
163.1730 163.1730 157.1940 163.1730  87.0770  71.0780 113.1580  99.1310
[513] 101.1040 114.1030 129.1800  87.0770  71.0780 129.1800 128.1060 113.1580
101.1040 114.1030 113.1580 129.1800 113.1580  87.0770 113.1580  87.0770
[529] 129.1800 113.1580 163.1730  57.0510  97.1150 113.1580 186.2100  57.0510
113.1580 101.1040 129.1800  71.0780  57.0510 114.1030  87.0770 163.1730
[545]  57.0510 147.1740  97.1150  87.0770 186.2100 113.1580 114.1030  87.0770
113.1580  97.1150  99.1310  57.0510 129.1800  87.0770 131.1960 145.1133

Call:
  nma.pdb(pdb = pdbFL4_3WY9, mass = TRUE)

Class:
  VibrationalModes (nma)

Number of modes:
  1728 (6 trivial)

Frequencies:
  Mode 7:       0.003
  Mode 8:       0.004
  Mode 9:       0.006
  Mode 10:      0.009
  Mode 11:      0.011
  Mode 12:      0.012

+ attr: modes, frequencies, force.constants, fluctuations,
        U, L, xyz, mass, temp, triv.modes, natoms, call

 NORMAL MODES for FULL LENGTH MINIMIZED CHARACTERIZED Q93WY9

  [1] 0.000000000 0.000000000 0.000000000 0.000000000 0.000000000 0.000000000
0.003323253 0.003752818 0.005579510 0.009460018 0.011499977 0.012460927
  [13] 0.013505683 0.014941929 0.015864483 0.016578109 0.017472285 0.019057434
0.019738421 0.019966811 0.020482196 0.023096119 0.023238802 0.024298118
  [25] 0.025255538 0.025664937 0.026034363 0.026751764 0.027038547 0.027659096
0.028380499 0.028631970 0.028965325 0.029278454 0.029913933 0.030087862
```

 [37] 0.030671928 0.030798846 0.031071513 0.031646631 0.031876713 0.031994102
0.032165840 0.032674087 0.033103846 0.033258814 0.033690511 0.033822204
 [49] 0.033916811 0.034343188 0.034966671 0.035218224 0.035773415 0.035956339
0.036354975 0.036441269 0.037016785 0.037364822 0.038017049 0.038036367
 [61] 0.038240929 0.038420016 0.038609428 0.038771137 0.039262270 0.039473955
0.039696319 0.039746378 0.040063444 0.040530174 0.040662453 0.040953570
 [73] 0.041067527 0.041417609 0.041680975 0.041980718 0.042314268 0.042532800
0.042659463 0.042885096 0.043068104 0.043187919 0.043532587 0.043655188
 [85] 0.043867907 0.044004255 0.044185781 0.044353716 0.044726220 0.044833979
0.044992176 0.045137195 0.045479622 0.045725412 0.045801242 0.045971265
 [97] 0.046476786 0.046821318 0.047119541 0.047307318 0.047456738 0.047527674
0.047721011 0.047957956 0.048493704 0.048637136 0.049102331 0.049458023
[109] 0.049560604 0.049666033 0.049734583 0.050118900 0.050178250 0.050311090
0.050565707 0.050760198 0.050930079 0.051131606 0.051290875 0.051540652
[121] 0.051987177 0.052073104 0.052450187 0.052815467 0.053124862 0.053217282
0.053380293 0.053606163 0.053847788 0.054039607 0.054245926 0.054546047
[133] 0.054627717 0.054834826 0.055008010 0.055142996 0.055417696 0.055686493
0.055967125 0.056001059 0.056096643 0.056302172 0.056463903 0.056563631
[145] 0.056870673 0.057095824 0.057117115 0.057528733 0.057647493 0.057935678
0.057988120 0.058376896 0.058432844 0.058533977 0.058839348 0.058942792
[157] 0.059157060 0.059438991 0.059599436 0.059750123 0.059904661 0.059948409
0.060086208 0.060266999 0.060407007 0.060556103 0.060715890 0.060962368
[169] 0.061192333 0.061311227 0.061569928 0.061881808 0.061979561 0.062347921
0.062399090 0.062683412 0.062967859 0.063026362 0.063134180 0.063264640
[181] 0.063643274 0.063891542 0.064063967 0.064534925 0.064596519 0.064650807
0.064903417 0.065001108 0.065139690 0.065341972 0.065530876 0.065611806
[193] 0.065815909 0.066012858 0.066370474 0.066508678 0.066687820 0.066838065
0.066977007 0.067003664 0.067160746 0.067185445 0.067378391 0.067498960
[205] 0.067849303 0.068081678 0.068198404 0.068445508 0.068519484 0.068712923
0.069069761 0.069200562 0.069231668 0.069482025 0.069494054 0.069759992
[217] 0.069849621 0.069959596 0.070203027 0.070211506 0.070397957 0.070472040
0.070641312 0.070830387 0.070960798 0.071082418 0.071116441 0.071468544
[229] 0.071640058 0.071842019 0.072045173 0.072196549 0.072298049 0.072368787
0.072551964 0.072747216 0.072963646 0.072987423 0.073134597 0.073264710
[241] 0.073437374 0.073581239 0.073700941 0.073812212 0.074098549 0.074383613
0.074478562 0.074752181 0.074913473 0.075130574 0.075244444 0.075353269
[253] 0.075471670 0.075490798 0.075688342 0.075837122 0.076014274 0.076070902
0.076161585 0.076548564 0.076625461 0.076699639 0.076890453 0.077073729
[265] 0.077232468 0.077496855 0.077945473 0.077971629 0.078058807 0.078147994
0.078299703 0.078417855 0.078556629 0.078668922 0.078706585 0.078806450
[277] 0.079033371 0.079383065 0.079598318 0.079636655 0.079720900 0.079761719
0.079898001 0.080070123 0.080151384 0.080375295 0.080577053 0.080719490
[289] 0.080912564 0.081145300 0.081463392 0.081512195 0.081586121 0.081691459
0.082209036 0.082337576 0.082392625 0.082481272 0.082704847 0.083025964
[301] 0.083137247 0.083243208 0.083465502 0.083550888 0.083737585 0.083818917
0.083901378 0.084064400 0.084317429 0.084521765 0.084662950 0.084803752
[313] 0.084967427 0.085014963 0.085150721 0.085415212 0.085525459 0.085716573
0.085803853 0.086003037 0.086059862 0.086313645 0.086443700 0.086624894
[325] 0.086817672 0.086950032 0.087106621 0.087211971 0.087388673 0.087529576
0.087634706 0.087730039 0.087789786 0.087841274 0.088095244 0.088338592
[337] 0.088343897 0.088516909 0.088762525 0.088991668 0.089093794 0.089252724
0.089351008 0.089472119 0.089698602 0.089746596 0.089985338 0.090078464
[349] 0.090151547 0.090376187 0.090463031 0.090707288 0.090993907 0.091109498
0.091268942 0.091394025 0.091472287 0.091550345 0.091613268 0.091695073

[361] 0.091897062 0.092363086 0.092536111 0.092588790 0.092802618 0.092925909
0.093090949 0.093207337 0.093539247 0.093611929 0.093756447 0.093911801
[373] 0.093993088 0.094134061 0.094358750 0.094571389 0.094674853 0.094906669
0.094983105 0.095019633 0.095318266 0.095570257 0.095682702 0.095864929
[385] 0.095961588 0.096050766 0.096141179 0.096190435 0.096360005 0.096475730
0.096713183 0.096966512 0.097026054 0.097218661 0.097386050 0.097566653
[397] 0.097689247 0.097919101 0.098074706 0.098283300 0.098620216 0.098687744
0.098935250 0.098979277 0.099169878 0.099262681 0.099292405 0.099467510
[409] 0.099634935 0.099713207 0.099895562 0.100026191 0.100062651 0.100147544
0.100373914 0.100476698 0.100544994 0.100664212 0.100732758 0.100857908
[421] 0.101028043 0.101218790 0.101366082 0.101641461 0.101731884 0.101886142
0.102089926 0.102195569 0.102460808 0.102483673 0.102616685 0.102628162
[433] 0.102933021 0.102964638 0.103107716 0.103207164 0.103335935 0.103650321
0.103754376 0.103791722 0.104006142 0.104328706 0.104454033 0.104496947
[445] 0.104784646 0.104931881 0.105165174 0.105274348 0.105427266 0.105647718
0.105701291 0.105889002 0.106008782 0.106050350 0.106399454 0.106542080
[457] 0.106569061 0.106854382 0.106967160 0.107202403 0.107295813 0.107509370
0.107626640 0.107848937 0.107984488 0.108172453 0.108349221 0.108391995
[469] 0.108472004 0.108713778 0.108868961 0.109127265 0.109156624 0.109379285
0.109526588 0.109632806 0.109761654 0.109794765 0.110206955 0.110330886
[481] 0.110573520 0.110616464 0.110810365 0.110909072 0.111033816 0.111157167
0.111237123 0.111350240 0.111504253 0.111608929 0.111919766 0.112064973
[493] 0.112131180 0.112256033 0.112468617 0.112548992 0.112705975 0.112801452
0.113212875 0.113387928 0.113483724 0.113676745 0.113864211 0.114058809
[505] 0.114190760 0.114388235 0.114477884 0.114685908 0.114822764 0.115086303
0.115148904 0.115270708 0.115387553 0.115462167 0.115773270 0.116161304
[517] 0.116259064 0.116350101 0.116613881 0.116845962 0.117078448 0.117221802
0.117287907 0.117383433 0.117673207 0.117905458 0.118169732 0.118233592
[529] 0.118328355 0.118533896 0.118628739 0.118678267 0.118860402 0.118879261
0.119224475 0.119330021 0.119429322 0.119556511 0.119944027 0.120012114
[541] 0.120332294 0.120526957 0.120639867 0.120701161 0.120971682 0.121053631
0.121207331 0.121365115 0.121411231 0.121479539 0.121587294 0.121878815
[553] 0.122041023 0.122102237 0.122239285 0.122382700 0.122397912 0.122840709
0.122908326 0.122979201 0.123169475 0.123374547 0.123632457 0.123654071
[565] 0.123860283 0.124032358 0.124185227 0.124320693 0.124501186 0.124808227
0.125107023 0.125251907 0.125289517 0.125360864 0.125394906 0.125640001
[577] 0.125828466 0.125962264 0.126239570 0.126346773 0.126459294 0.126557605
0.126965650 0.126992481 0.127073238 0.127298388 0.127380442 0.127538831
[589] 0.127624998 0.127862153 0.128045864 0.128222792 0.128371757 0.128687967
0.128719457 0.128807980 0.129202940 0.129624544 0.129645745 0.129764872
[601] 0.129857463 0.130318843 0.130508606 0.130769008 0.130970497 0.131049672
0.131237414 0.131498395 0.131590246 0.131864361 0.132233624 0.132634233
[613] 0.132725299 0.132809054 0.132933255 0.133096741 0.133430515 0.133560112
0.133852997 0.134150247 0.134377488 0.134525004 0.134739300 0.134868297
[625] 0.135157691 0.135276740 0.135426081 0.135682366 0.136079524 0.136301133
0.136429303 0.136524145 0.136788003 0.136941523 0.137033238 0.137209285
[637] 0.137302574 0.137469339 0.137588781 0.138075421 0.138254446 0.138328995
0.138420614 0.138900966 0.139215004 0.139356217 0.139435263 0.139585237
[649] 0.139834442 0.140013570 0.140196535 0.140428333 0.140595625 0.140695940
0.140772885 0.140842053 0.141096580 0.141615618 0.141753993 0.141760425
[661] 0.141839292 0.141964871 0.141999660 0.142262709 0.142338362 0.142753911
0.143031158 0.143363006 0.143449821 0.143596748 0.143646131 0.143737886
[673] 0.143955536 0.144158450 0.144603893 0.144785518 0.145065169 0.145323541
0.145369985 0.145729187 0.145766380 0.145805213 0.146119407 0.146141161

 [685] 0.146292485 0.146673089 0.146738872 0.146889752 0.147171084 0.147515084
0.147652734 0.147999122 0.148280997 0.148547588 0.148636402 0.148944369
 [697] 0.149226835 0.149297517 0.149483352 0.149734693 0.149989834 0.150089862
0.150151787 0.150228778 0.150351898 0.150700319 0.150752332 0.151031920
 [709] 0.151314841 0.151800446 0.152200813 0.152439532 0.152755004 0.152997245
0.153200419 0.153327678 0.153455905 0.153526042 0.153730082 0.153799600
 [721] 0.153983703 0.154088865 0.154142774 0.154421145 0.154762204 0.155099820
0.155404921 0.155544301 0.155730652 0.155753666 0.155857064 0.156265274
 [733] 0.156322241 0.156662881 0.157082381 0.157487093 0.157741338 0.158082205
0.158147727 0.158308135 0.158435608 0.158787906 0.159048512 0.159159001
 [745] 0.159371326 0.159469123 0.159937573 0.160138347 0.160387441 0.160502532
0.160695192 0.160860224 0.161208560 0.161405242 0.161496866 0.161604428
 [757] 0.161776987 0.162105462 0.162557044 0.162643036 0.162988727 0.163295221
0.163421749 0.163483660 0.163660120 0.163861743 0.163942261 0.164078172
 [769] 0.164322373 0.164862321 0.165277332 0.165563679 0.165657055 0.166233738
0.166515322 0.166568784 0.166836973 0.166961804 0.167673188 0.167777318
 [781] 0.168087061 0.168502999 0.168747776 0.168778396 0.169186720 0.169522354
0.169590253 0.169866496 0.169996106 0.170092783 0.170420019 0.170710055
 [793] 0.171124354 0.171260185 0.171436693 0.171567109 0.171665041 0.171743081
0.171903769 0.171946717 0.172196014 0.172341436 0.172591630 0.172988977
 [805] 0.173216595 0.173400753 0.173575887 0.174174563 0.174379572 0.174531810
0.174664991 0.175091199 0.175587073 0.176338003 0.176531245 0.176751507
 [817] 0.176891322 0.177077953 0.177257599 0.177271317 0.177612282 0.177785119
0.178055477 0.178484233 0.178846893 0.179116356 0.179593501 0.179924223
 [829] 0.180125993 0.180291433 0.180447387 0.180473074 0.180660421 0.180843512
0.180967639 0.181140840 0.181295924 0.181379316 0.181494634 0.182190010
 [841] 0.182454049 0.182516513 0.182550234 0.182991368 0.183170469 0.183524835
0.183861505 0.183876314 0.184018563 0.184292971 0.184503899 0.184681259
 [853] 0.185004187 0.185504286 0.185652519 0.185840232 0.186049542 0.186247124
0.186381651 0.186733382 0.186850004 0.186959102 0.187032453 0.187281010
 [865] 0.187559289 0.187734640 0.188238385 0.188636076 0.188830013 0.188979255
0.189252762 0.189651263 0.189989809 0.190161454 0.190419561 0.190782171
 [877] 0.191166487 0.191336414 0.191494616 0.191755355 0.191966528 0.192295794
0.192710547 0.193258866 0.193454454 0.193628848 0.194026065 0.194210120
 [889] 0.194823723 0.195033457 0.195319885 0.195651535 0.195728099 0.195751910
0.196321219 0.196633018 0.196813090 0.197430921 0.197736166 0.197982544
 [901] 0.198553239 0.198948517 0.199075225 0.199232685 0.199663857 0.199778953
0.199809698 0.200072577 0.201056856 0.201259402 0.201570160 0.201812108
 [913] 0.202080780 0.202436579 0.202980277 0.203035427 0.203164075 0.203770916
0.203942823 0.204153735 0.204677960 0.204746819 0.204773972 0.205006024
 [925] 0.205177390 0.205353794 0.205406519 0.206077505 0.206273095 0.207085046
0.207307424 0.208832149 0.209535869 0.209605429 0.209959997 0.210254101
 [937] 0.210437925 0.210742300 0.210990778 0.211124716 0.211314436 0.211698510
0.211881739 0.212167150 0.212563388 0.212586267 0.212934445 0.213031076
 [949] 0.213557587 0.213788458 0.214209369 0.214347263 0.214670344 0.215234216
0.215461526 0.215696113 0.216171378 0.216885563 0.217099185 0.217795093
 [961] 0.218059697 0.218635276 0.219293624 0.219569458 0.220420575 0.220617629
0.220966392 0.221571120 0.221844693 0.222163484 0.223350927 0.224153564
 [973] 0.224162265 0.224207969 0.224468399 0.224960375 0.225818880 0.225932705
0.227021514 0.227272311 0.227462260 0.227863528 0.228393665 0.229092139
 [985] 0.229176155 0.229751172 0.230098806 0.230201932 0.230521417 0.231218502
0.231254378 0.231918462 0.232091731 0.232371994 0.232969677 0.233528195
 [997] 0.233804841 0.234231147 0.234424210 0.234828413 0.235239351 0.236070187
0.236204167 0.236485287 0.236542639 0.237160572 0.237814022 0.239192195

[1009] 0.240068020 0.240666114 0.241325135 0.241602918 0.241622418 0.242758456
0.242946463 0.243165733 0.243759240 0.243968020 0.244406137 0.244975288
[1021] 0.245818352 0.246051174 0.246499513 0.246923188 0.247092957 0.247123197
0.247373837 0.248896604 0.249228917 0.249935648 0.250114107 0.250351181
[1033] 0.250915074 0.251575036 0.252339748 0.252874367 0.253714246 0.254457399
0.255585842 0.255885712 0.256156457 0.257135929 0.257885184 0.258358087
[1045] 0.258681134 0.259853919 0.260140641 0.260536496 0.260711585 0.261430973
0.261563971 0.261803351 0.262197468 0.263186460 0.263896222 0.264082321
[1057] 0.264370925 0.265529263 0.266048184 0.266586715 0.266901084 0.268327588
0.270069854 0.270095223 0.273240579 0.273780274 0.273874444 0.274003435
[1069] 0.276014824 0.277101782 0.277468009 0.277577308 0.277977082 0.278658490
0.278951582 0.279852234 0.281668864 0.283249434 0.283965514 0.286096715
[1081] 0.286616092 0.286674680 0.288368554 0.289709695 0.289847676 0.291754583
0.292488510 0.292998761 0.293714098 0.294254463 0.296090395 0.296229551
[1093] 0.296609352 0.298838910 0.300363287 0.301038577 0.303114548 0.303340971
0.306451717 0.307124548 0.307355558 0.308885632 0.310096120 0.310728333
[1105] 0.310976378 0.311513186 0.313932107 0.314458754 0.314715690 0.316530774
0.318422508 0.319100819 0.319278581 0.321880121 0.325163871 0.325708870
[1117] 0.327332196 0.330085050 0.331402977 0.332717472 0.335569092 0.335672000
0.335713916 0.337248536 0.337961015 0.340349041 0.342527063 0.343835571
[1129] 0.344929007 0.345379061 0.347906044 0.352800525 0.353717555 0.357530944
0.359176847 0.362212315 0.362708668 0.366149152 0.366936890 0.367346779
[1141] 0.371902263 0.376733265 0.377006651 0.378199701 0.387446226 0.390630237
0.401309875 0.405454332 0.405568674 0.408534588 0.410775732 0.413234186
[1153] 0.413481969 0.414624408 0.415063215 0.427888797 0.434853625 0.436897683
0.443070325 0.443352883 0.443631921 0.445644385 0.445759271 0.448688835
[1165] 0.452506361 0.459701607 0.464072890 0.469559034 0.470741150 0.474050312
0.478765502 0.482131813 0.482201552 0.483431771 0.483987487 0.484428013
[1177] 0.489134074 0.491153366 0.491567350 0.492549837 0.492859227 0.493935462
0.497746442 0.499048311 0.500586372 0.507314913 0.510938408 0.513428346
[1189] 0.514002932 0.514540596 0.515579312 0.515835755 0.517661208 0.517679729
0.522373436 0.523468665 0.524524278 0.525631048 0.527313593 0.528553734
[1201] 0.529893112 0.530302909 0.530340117 0.530832104 0.531851196 0.533274068
0.533615148 0.534339345 0.535771448 0.536099718 0.537443314 0.539824053
[1213] 0.541218640 0.541294900 0.542034282 0.545731160 0.547135275 0.547992033
0.549373355 0.550139936 0.550364467 0.550660164 0.552303037 0.553220957
[1225] 0.554067939 0.554530904 0.554956056 0.556787640 0.559781036 0.560887265
0.561412672 0.561427719 0.562069286 0.563357827 0.564765638 0.565558921
[1237] 0.566744495 0.567713289 0.568052776 0.569775541 0.569860980 0.569901895
0.571041437 0.572704904 0.572762952 0.574937886 0.575066388 0.577506579
[1249] 0.581062840 0.581669927 0.581858675 0.582681353 0.582895870 0.584848270
0.585260466 0.587466055 0.588279816 0.588758450 0.589884625 0.591515335
[1261] 0.591586738 0.592066314 0.592809948 0.593508437 0.593885535 0.594547548
0.596129196 0.596864628 0.597488027 0.598586311 0.598962749 0.600371684
[1273] 0.601178025 0.601293905 0.601402623 0.602182850 0.602192083 0.602574235
0.604445372 0.604583523 0.605439273 0.606099700 0.606196860 0.606315561
[1285] 0.606617724 0.606783642 0.607017579 0.607785618 0.608177541 0.608453053
0.608538577 0.608685994 0.609049120 0.609205665 0.609666729 0.610489982
[1297] 0.612164036 0.612194862 0.612604267 0.612728796 0.613394672 0.613422587
0.613550129 0.613576592 0.614001433 0.615721459 0.616126486 0.616255236
[1309] 0.616558115 0.616663547 0.616830007 0.617513570 0.618029814 0.618924537
0.619412616 0.620418835 0.620451558 0.622051430 0.623903698 0.624270571
[1321] 0.625122974 0.625212964 0.625645936 0.625740161 0.626024169 0.626139819
0.627155925 0.628254860 0.628820737 0.629159278 0.629631197 0.629723820

[1333] 0.631155691 0.631434194 0.631545264 0.633199916 0.634701073 0.635304111
0.635785732 0.636117002 0.636196976 0.636235894 0.636556406 0.636672570
[1345] 0.637213025 0.638101293 0.638973857 0.639446673 0.640888578 0.641390550
0.641596571 0.642227508 0.642332315 0.642490685 0.642554965 0.643083248
[1357] 0.643120844 0.643412317 0.643778991 0.644712630 0.644882003 0.645803085
0.646833474 0.646951297 0.647166663 0.647870492 0.647882007 0.648043927
[1369] 0.648221280 0.648452299 0.648700046 0.651098923 0.651962737 0.652068892
0.653096027 0.653839170 0.653997969 0.654557051 0.655328475 0.655678440
[1381] 0.655839324 0.656537348 0.657342208 0.657990595 0.658056613 0.658157245
0.658209584 0.659045899 0.659106546 0.659234702 0.659399520 0.659540043
[1393] 0.659613452 0.660428793 0.660568675 0.661428030 0.661923839 0.662131677
0.662178806 0.662225875 0.662268408 0.662784723 0.663441747 0.663798903
[1405] 0.665482905 0.665900459 0.666511126 0.668365494 0.668769355 0.668915275
0.669136613 0.669317385 0.669727669 0.669872870 0.670337514 0.670595741
[1417] 0.671058846 0.671276175 0.671536700 0.671951736 0.672216766 0.672833541
0.673528469 0.674066843 0.675408107 0.675577808 0.675818647 0.676826133
[1429] 0.677393110 0.678050249 0.678152583 0.678730880 0.679122553 0.679650402
0.679921671 0.681412795 0.681624538 0.682704362 0.683553379 0.683641754
[1441] 0.684220687 0.684233626 0.684926233 0.685542307 0.685719862 0.685720250
0.685961996 0.686108671 0.686151237 0.686962846 0.687243336 0.687709651
[1453] 0.688333390 0.688452003 0.689127065 0.691420529 0.691895432 0.692113849
0.692244054 0.692365857 0.692714608 0.693513204 0.695035049 0.695319131
[1465] 0.695863033 0.696058790 0.697257043 0.697420847 0.697627531 0.699445687
0.699940749 0.700197085 0.700465857 0.701375754 0.701722717 0.701867471
[1477] 0.702298846 0.702500544 0.702647877 0.703091870 0.703703828 0.704072022
0.704781308 0.705753784 0.707289438 0.707506808 0.707971583 0.708381005
[1489] 0.708565564 0.709395520 0.710817389 0.710872497 0.711246311 0.711277988
0.712583871 0.713157850 0.713709061 0.713768311 0.713986814 0.714266816
[1501] 0.714464426 0.715398442 0.715420642 0.716284155 0.716343846 0.716406803
0.717867114 0.718549105 0.718808038 0.719037955 0.719831434 0.720042995
[1513] 0.720427644 0.721975363 0.723637798 0.723710725 0.724103203 0.725378139
0.727276619 0.728544212 0.729394706 0.730571309 0.731450562 0.731660824
[1525] 0.731930950 0.732265546 0.732291333 0.732541622 0.733520909 0.736262671
0.736631818 0.738020829 0.739081541 0.739759195 0.740018151 0.741200188
[1537] 0.742435019 0.742812966 0.743282347 0.743301670 0.744026839 0.744507465
0.745492830 0.745583869 0.745645308 0.745959082 0.747340570 0.747659088
[1549] 0.749519822 0.750020097 0.750841036 0.751294411 0.751449891 0.751562773
0.751727009 0.751784441 0.752316679 0.753087977 0.753426305 0.753493777
[1561] 0.753591025 0.753644602 0.753991248 0.756662873 0.758007109 0.758115573
0.758121253 0.759407390 0.759429354 0.759989101 0.760027929 0.760074971
[1573] 0.760373147 0.760620272 0.761500938 0.762070281 0.762238867 0.762416717
0.763139820 0.764291600 0.765405625 0.765652863 0.767260230 0.768386830
[1585] 0.768404829 0.769336047 0.774115966 0.774492565 0.776010044 0.776938113
0.777450263 0.777619048 0.778156857 0.778428405 0.778441795 0.778736712
[1597] 0.780099070 0.781111074 0.781654015 0.782250221 0.782296881 0.784318789
0.784955180 0.785153356 0.785276457 0.785380832 0.785745343 0.785852186
[1609] 0.785895135 0.787446034 0.791318617 0.791405264 0.792556413 0.792725784
0.793439398 0.793773515 0.794118529 0.795925548 0.797249638 0.797616172
[1621] 0.799831896 0.801314081 0.802050641 0.802537518 0.802968361 0.805369650
0.806284597 0.806491665 0.807158731 0.807222277 0.807285661 0.807468757
[1633] 0.808494297 0.808860777 0.809065762 0.809722667 0.814521351 0.814687757
0.814816981 0.816289815 0.817030639 0.818168613 0.819716762 0.819791725
[1645] 0.821535124 0.823607301 0.823619542 0.828907853 0.829122346 0.831521660
0.831958576 0.832649442 0.833453743 0.833503995 0.834088541 0.834699445

[1657] 0.835580003 0.836032039 0.836560485 0.839194933 0.841191797 0.841288423
0.841622340 0.841805069 0.841886670 0.844742069 0.846803449 0.848459838
[1669] 0.848732992 0.850588655 0.851413020 0.852196298 0.852947869 0.854538283
0.856712299 0.858183333 0.860380700 0.860762918 0.865088066 0.866635684
[1681] 0.867304929 0.869567687 0.872211664 0.873666575 0.874663397 0.876487311
0.876714593 0.885176971 0.887395970 0.887485210 0.888650173 0.894140983
[1693] 0.897421139 0.898056739 0.904903852 0.906352244 0.907700533 0.908566030
0.908927819 0.910768567 0.913413236 0.914712516 0.919104528 0.926224190
[1705] 0.927111888 0.927221305 0.930057694 0.937157378 0.942419207 0.949947913
0.951308490 0.958521569 0.962720805 0.963972652 0.967196558 0.971163984
[1717] 0.984811217 0.991181374 0.995956448 0.998094580 1.024458314 1.025089400
1.056157779 1.063790532 1.065779505 1.086806773 1.096361153 1.150595204

FLUCTUATION DATA for FULL LENGTH MINIMIZED CHARACTERIZED Q93WY9

[1] 0.36042698 0.26762505 0.18938083 0.15673412 0.17795884 0.15406573
0.13889426 0.15864196 0.13971139 0.11173749 0.14284848 0.15979184 0.14514198
[14] 0.14034858 0.17352699 0.18999248 0.16867203 0.18457432 0.18367869
0.20707444 0.29488192 0.35020897 0.44795675 0.75037755 0.68717819 0.43591248
[27] 0.36071025 0.29059322 0.34090614 0.32810906 0.32228445 0.35861364
0.31021758 0.25717000 0.25776035 0.30694312 0.36513720 0.24589749 0.24100211
[40] 0.34818860 0.38255263 0.38304540 0.41821527 0.34982933 0.31878152
0.21716479 0.24009690 0.19509637 0.15722647 0.13975448 0.14725081 0.13640346
[53] 0.19232261 0.21385927 0.21534728 0.19938344 0.17896882 0.14515243
0.13168328 0.11921544 0.09779635 0.08871080 0.08872939 0.07848832 0.07730096
[66] 0.07687775 0.08017344 0.07038361 0.07195478 0.08619529 0.08929325
0.09887552 0.10500428 0.12224414 0.14973100 0.17173858 0.16595428 0.17057491
[79] 0.21148406 0.19172413 0.16610444 0.21977088 0.27396783 0.24133301
0.24813860 0.21078796 0.18784227 0.22813705 0.19567162 0.15246972 0.16329134
[92] 0.18474105 0.15282780 0.12778372 0.14479627 0.15893291 0.12245640
0.12694192 0.16812107 0.15205052 0.13862049 0.17192602 0.18197431 0.17463517
[105] 0.19633834 0.38166580 0.41078221 0.33350051 0.26749560 0.23603062
0.16903998 0.16669715 0.15863940 0.16521060 0.18281353 0.21970837 0.25790335
[118] 0.25712785 0.33024116 0.32719614 0.26584808 0.29139175 0.39103684
0.38172531 0.30326522 0.29333992 0.27543712 0.27246601 0.30398375 0.35514840
[131] 0.39803405 0.60499585 0.44143521 0.46224612 0.30020292 0.28411140
0.22149544 0.20938419 0.19970892 0.19535167 0.25490763 0.32161568 0.46538718
[144] 0.23722892 0.18906660 0.14495906 0.11746729 0.09859560 0.09708465
0.09504662 0.07874275 0.07393065 0.08695843 0.08630702 0.07226954 0.06952329
[157] 0.08973568 0.08980306 0.09001957 0.10698139 0.12497977 0.15117161
0.17170892 0.18714442 0.24034161 0.41551509 0.42791560 0.36608545 0.30438990
[170] 0.36550376 0.27737829 0.19206732 0.23350688 0.23404537 0.15395822
0.15497912 0.21824134 0.17856846 0.13047671 0.16670958 0.17689119 0.13062324
[183] 0.13208103 0.18802101 0.17417561 0.13827326 0.19331274 0.27414815
0.26538245 0.21162650 0.23137196 0.19915608 0.31379734 0.24172502 0.20538525
[196] 0.27808269 0.26031619 0.21103944 0.23141742 0.39085573 0.75409123
0.49644166 0.39113168 0.25553353 0.22373857 0.24964788 0.25175642 0.26669361
[209] 0.16150710 0.12845722 0.14535623 0.10922429 0.08976751 0.11158419
0.11167881 0.09044516 0.08427854 0.11565460 0.12231145 0.10940268 0.12726187
[222] 0.16681451 0.18993590 0.17934534 0.26457029 0.32171149 0.38578829
0.37770817 0.34555149 0.19864354 0.22763694 0.28474741 0.22341001 0.18117515
[235] 0.25991835 0.29416431 0.23921514 0.26386323 0.38945962 0.33175641
0.30069388 0.41131794 0.47118179 0.58969342 0.59055840 0.30095637 0.32899359
[248] 0.26931877 0.22538811 0.13358253 0.10957826 0.15084798 0.15598028

0.16944502 0.13115344 0.11645423 0.12641688 0.12418824 0.11402013 0.11624458
[261] 0.13046815 0.13495591 0.15614919 0.17629639 0.18947797 0.25967471
0.32656290 0.40568034 0.41877258 0.72531486 0.83523309 1.05194354 1.89100430
[274] 2.69723304 1.49330548 0.80975549 0.48084245 0.39814582 0.27916056
0.31097665 0.40313768 0.32043750 0.20975180 0.21756785 0.28409494 0.23429709
[287] 0.17666901 0.21336397 0.23367896 0.19072466 0.17998790 0.21928870
0.21273058 0.19088857 0.25827143 0.35782978 0.62910787 0.97822378 0.77451257
[300] 0.61936265 0.26814679 0.23764707 0.24640615 0.24188453 0.23728021
0.35780626 0.33614075 0.27146538 0.21124502 0.18174183 0.18580129 0.19706628
[313] 0.24660821 0.23347742 0.18619419 0.12386885 0.12464706 0.10497337
0.09509447 0.08937351 0.09541523 0.07878891 0.07168316 0.09127009 0.09057554
[326] 0.08071065 0.09733892 0.10861552 0.11103544 0.13822147 0.15482535
0.18975438 0.28570308 0.30971313 0.42013856 0.38311475 0.30630871 0.35865553
[339] 0.40603920 0.73680501 0.89852288 0.81448350 0.81278163 1.67309188
2.22031260 3.34535148 1.56492803 1.12826202 0.44415054 0.30097590 0.23518013
[352] 0.28346265 0.23549743 0.16208474 0.17252295 0.20096645 0.15756008
0.12625969 0.15046809 0.16811294 0.14843361 0.14390973 0.16666794 0.16225519
[365] 0.15925945 0.16126279 0.19245477 0.23943786 0.28928377 0.45197250
0.55075745 0.36052725 0.24159584 0.17322272 0.15435264 0.14731530 0.17521142
[378] 0.22443813 0.22364665 0.25833707 0.32415326 0.32228269 0.21491577
0.24711968 0.33127218 0.25683043 0.21037074 0.20152987 0.21848888 0.24345307
[391] 0.24572081 0.25841072 0.26960064 0.28931146 0.29130740 0.37175777
0.41316175 0.36485651 0.42098390 0.41337680 0.48271124 0.47105178 0.46468081
[404] 0.59259379 0.47215871 0.32852896 0.35912431 0.31199557 0.30257397
0.22561212 0.17557940 0.12703707 0.13823528 0.11956024 0.12795775 0.11674095
[417] 0.14798278 0.22071253 0.25217223 0.26288809 0.30501353 0.27310845
0.32231875 0.29910134 0.34602739 0.30748977 0.29403667 0.25950273 0.32394911
[430] 0.26005127 0.19830591 0.23169355 0.16373929 0.15858960 0.15208773
0.13560137 0.14022963 0.11404580 0.08737027 0.08701847 0.09899467 0.07966492
[443] 0.07372784 0.08567012 0.09502386 0.08218850 0.09414842 0.13399664
0.14529631 0.17528271 0.26951800 0.62099933 0.34092196 0.24151778 0.29561893
[456] 0.22398739 0.37267861 0.58127686 0.79279353 0.50493191 0.38939060
0.33983455 0.41646825 0.46766416 0.73234215 0.60704771 0.63042169 0.65641102
[469] 0.60320802 0.41348943 0.38126785 0.53201355 0.47566559 0.63854171
0.66832135 0.91095171 0.86726917 0.90771194 1.22812132 2.13546100 2.67947947
[482] 2.00986114 2.20713026 1.47106028 1.00492067 0.75884212 0.55790480
0.42861719 0.44206405 0.39589072 0.37949738 0.31507589 0.37335421 0.37794951
[495] 0.35846380 0.38174269 0.36379396 0.37636520 0.46183673 0.50656640
0.74751034 0.64288231 0.66814774 0.53576145 0.49578488 0.50573496 0.57336440
[508] 0.59879761 0.88904710 1.13596350 1.62799781 1.53925877 2.29194181
2.35143851 2.04490132 2.40841592 2.89949875 2.25878320 2.19142274 1.61031089
[521] 1.45627538 1.51708650 1.12560913 0.97956250 0.66970483 0.74598081
0.68244266 0.95999866 0.70894212 0.60449065 0.76670876 0.62732966 0.64338210
[534] 0.60867848 0.78532768 0.89187469 0.86792434 1.17895427 1.76278677
2.17701546 2.09897111 1.82989363 1.23069761 0.81476982 0.52556903 0.46885053
[547] 0.34590949 0.43926749 0.43772112 0.63499521 0.87452015 0.62930217
0.70137218 0.51660667 0.56297379 0.63554052 0.48621607 0.43053113 0.44219216
[560] 0.43347477 0.36540820 0.40568222 0.40686782 0.51230579 0.58444715
0.88323064 1.25032426 1.84032963 1.91281230 2.22953823 1.95650967 1.56994314
[573] 1.46163865 1.82034304 1.70077763 3.02443641

MASSES for FULL LENGTH MINIMIZED CHARACTERIZED Q93WY9

[1] 138.1469 114.1030 163.1730 57.0510 128.1060 71.0780 113.1580 87.0770

```
129.1800  87.0770 147.1740 113.1580 147.1740 163.1730 128.1060  71.0780
 [17] 117.1260 157.1940  87.0770  57.0510 163.1730 113.1580  97.1150 137.1390
114.0790 117.1260 157.1940  99.1310 117.1260 186.2100 157.1940  57.0510
 [33] 114.1030  87.0770  57.0510 113.1580 113.1580 114.0790  57.0510 129.1800
71.0780  87.0770  57.0510  99.1310 114.0790 113.1580  99.1310  57.0510
 [49]  57.0510 163.1730 163.1730 114.0790  71.0780  57.0510 114.0790 114.1030
99.1310 129.1800 147.1740  57.0510 113.1580  97.1150 131.1960  71.0780
 [65] 147.1740 101.1040  99.1310 101.1040 131.1960 131.1960  87.0770 186.2100
87.0770 113.1580 113.1580 128.1060 163.1730  57.0510 129.1800 117.1260
 [81] 131.1960  57.0510 128.1060  87.0770  57.0510 128.1060 113.1580  87.0770
114.1030  71.0780 113.1580 114.0790  71.0780  99.1310 129.1800 186.2100
 [97]  57.0510 101.1040 114.0790 163.1730 113.1580 113.1580 129.1800  71.0780
137.1390  97.1150 128.1060  97.1150 114.1030  99.1310 113.1580 163.1730
[113]  57.0510 128.1060  99.1310  57.0510 114.0790  57.0510 101.1040 101.1040
114.0790 137.1390 163.1730 103.1430 186.2100 117.1260 157.1940  97.1150
[129] 128.1060 114.0790 131.1960 101.1040 101.1040  87.0770 157.1940  71.0780
71.0780 163.1730 157.1940 113.1580 114.0790  97.1150  87.0770 157.1940
[145]  97.1150  57.0510  87.0770 114.0790 113.1580  71.0780  57.0510 128.1060
101.1040  71.0780  71.0780  71.0780 131.1960  71.0780  71.0780  71.0780
[161]  87.0770 113.1580  99.1310 147.1740 157.1940 114.1030  87.0770 114.1030
97.1150  71.0780 163.1730  71.0780 129.1800 128.1060 113.1580 113.1580
[177] 101.1040 137.1390  71.0780 163.1730 117.1260 113.1580 147.1740 128.1060
147.1740  71.0780 114.0790 129.1800 163.1730 157.1940  57.0510 129.1800
[193] 163.1730 114.0790  87.0770  87.0770 113.1580 101.1040  99.1310  71.0780
117.1260 129.1800 163.1730 163.1730 157.1940  87.0770  99.1310  87.0770
[209]  57.0510 163.1730  71.0780 114.0790 128.1060 113.1580 113.1580 186.2100
71.0780  71.0780  71.0780 186.2100 113.1580 163.1730 129.1800  71.0780
[225]  87.0770 114.1030 129.1800 128.1060 163.1730 163.1730 113.1580 114.1030
163.1730 113.1580  57.0510 128.1060 114.1030  57.0510 114.0790  71.0780
[241] 113.1580  57.0510  57.0510 101.1040  57.0510 186.2100  87.0770 131.1960
101.1040 128.1060 147.1740  57.0510 186.2100 114.0790  99.1310 129.1800
[257] 163.1730  71.0780  57.0510  99.1310 117.1260 101.1040 113.1580  71.0780
71.0780 129.1800 147.1740 113.1580 131.1960 117.1260  57.0510 114.1030
[273]  71.0780  57.0510 114.1030 137.1390  71.0780  97.1150  99.1310 147.1740
128.1060 129.1800 163.1730 117.1260 128.1060 129.1800  71.0780 128.1060
[289] 114.1030 147.1740 131.1960 103.1430  71.0780 103.1430 113.1580  57.0510
129.1800  57.0510 114.1030 117.1260 114.1030 113.1580 137.1390 129.1800
[305]  87.0770  97.1150  57.0510  57.0510 113.1580 113.1580 147.1740 157.1940
117.1260 157.1940 186.2100 114.1030 114.1030 131.1960 117.1260 147.1740
[321]  99.1310 101.1040  87.0770  71.0780  87.0770 147.1740 113.1580  71.0780
101.1040  99.1310 163.1730  87.0770 114.0790 163.1730 113.1580  71.0780
[337]  87.0770  71.0780 157.1940 129.1800  87.0770 113.1580 129.1800 103.1430
87.0770  87.0770  57.0510 101.1040  99.1310 113.1580  97.1150  87.0770
[353] 128.1060 113.1580 113.1580  87.0770 147.1740  71.0780 129.1800  87.0770
117.1260  99.1310 114.0790 163.1730 113.1580 113.1580  57.0510 114.0790
[369] 114.1030  97.1150 157.1940  71.0780 101.1040  87.0770 163.1730 131.1960
99.1310  57.0510 163.1730  57.0510 114.1030 114.1030 163.1730  97.1150
[385] 157.1940 117.1260  99.1310 137.1390 137.1390 157.1940  57.0510  87.0770
157.1940  57.0510  57.0510 163.1730  71.0780 101.1040 186.2100 147.1740
[401]  87.0770 157.1940 129.1800  71.0780  87.0770 114.0790  97.1150 114.1030
113.1580 113.1580  71.0780  57.0510  71.0780 113.1580  99.1310  57.0510
[417]  57.0510  97.1150 114.0790  71.0780 163.1730 114.0790 114.1030 147.1740
71.0780 114.0790 117.1260 157.1940 114.0790 114.1030 163.1730 128.1060
[433] 117.1260 101.1040 128.1060  97.1150  71.0780 101.1040 163.1730 114.1030
```

```
114.1030  71.0780  97.1150 113.1580 113.1580  57.0510  99.1310 113.1580
[449]  71.0780 157.1940 113.1580 137.1390  57.0510  57.0510 117.1260  87.0770
129.1800 163.1730  87.0770 117.1260 113.1580 113.1580  97.1150  99.1310
[465]  71.0780 113.1580  97.1150 117.1260  97.1150 129.1800  97.1150 114.0790
97.1150 128.1060 117.1260 129.1800  99.1310 101.1040  97.1150  71.0780
[481]  97.1150  71.0780  87.0770  87.0770 101.1040  71.0780 114.0790 113.1580
101.1040 113.1580 128.1060 117.1260 129.1800 128.1060 101.1040  71.0780
[497]  87.0770 186.2100  99.1310  97.1150 129.1800  57.0510 129.1800 101.1040
163.1730 163.1730 157.1940 163.1730  87.0770  99.1310 113.1580  99.1310
[513] 101.1040 114.1030 129.1800  87.0770  71.0780 131.1960 101.1040 131.1960
129.1800 114.1030 113.1580 129.1800 113.1580  87.0770 113.1580 163.1730
[529] 117.1260 113.1580 163.1730  57.0510  87.0770 113.1580 186.2100  57.0510
113.1580  87.0770 129.1800 163.1730  57.0510 114.0790  87.0770 163.1730
[545]  99.1310 147.1740  97.1150  71.0780 186.2100 113.1580 114.1030  87.0770
113.1580  97.1150  71.0780  57.0510 129.1800 101.1040 113.1580 128.1060
[561] 147.1740  99.1310 163.1730  99.1310 137.1390  87.0770  71.0780  87.0770
97.1150  71.0780 101.1040  99.1310  87.0770 113.1580  87.0770 104.0843

Call:
  nma.pdb(pdb = pdbFL4_ZSP9, mass = TRUE)

Class:
  VibrationalModes (nma)

Number of modes:
  1755 (6 trivial)

Frequencies:
  Mode 7:       0.003
  Mode 8:       0.004
  Mode 9:       0.006
  Mode 10:      0.009
  Mode 11:      0.012
  Mode 12:      0.013

+ attr: modes, frequencies, force.constants, fluctuations,
        U, L, xyz, mass, temp, triv.modes, natoms, call
```

NORMAL MODES for FULL LENGTH MINIMIZED CHARACTERIZED Q9ZSP9

```
  [1] 0.000000000 0.000000000 0.000000000 0.000000000 0.000000000 0.000000000
0.003122845 0.003865860 0.006010075 0.009255650 0.011538459 0.013348157
  [13] 0.013975762 0.014973258 0.015913903 0.016204131 0.018309045 0.019183288
0.019817826 0.019919181 0.022224208 0.022445926 0.022788632 0.023375197
  [25] 0.024440520 0.024916264 0.025305135 0.026183770 0.026261048 0.026873631
0.027174512 0.027484543 0.028439790 0.029169241 0.029411816 0.029572427
  [37] 0.029738560 0.030062174 0.030284221 0.031167561 0.031403573 0.031846900
0.032202831 0.032240172 0.033026856 0.033223380 0.033649887 0.033901124
  [49] 0.034200912 0.034766158 0.035071553 0.035322359 0.035447633 0.036022499
0.036356368 0.036599063 0.036939379 0.036967140 0.037267074 0.037665624
  [61] 0.038106559 0.038231654 0.038514839 0.038666791 0.039027700 0.039154380
0.039384337 0.039796693 0.040177404 0.040628177 0.041086643 0.041315655
  [73] 0.041385488 0.041580276 0.041789918 0.042287322 0.042592016 0.042668666
0.042896908 0.043148898 0.043171200 0.043506394 0.043668532 0.043899365
```

[85] 0.044030151 0.044289421 0.044423050 0.044676071 0.045061652 0.045166367 0.045599488 0.045715162 0.045913650 0.046019178 0.046048616 0.046857821
[97] 0.047149098 0.047322843 0.047549520 0.047927576 0.048176125 0.048319713 0.048427844 0.048483517 0.048883373 0.049221867 0.049474409 0.049549103
[109] 0.049986055 0.050212817 0.050537396 0.050675294 0.051061708 0.051181122 0.051372790 0.051428969 0.051598612 0.051784088 0.051809029 0.052190689
[121] 0.052531982 0.052797239 0.052937623 0.053207761 0.053392629 0.053437916 0.053678175 0.053915249 0.053965731 0.054297733 0.054385831 0.054538848
[133] 0.054648116 0.054841292 0.055002254 0.055125308 0.055399182 0.055516110 0.055708322 0.055965767 0.056341299 0.056602354 0.056696477 0.056819206
[145] 0.057001693 0.057227435 0.057423183 0.057583525 0.057756362 0.057997075 0.058102236 0.058365614 0.058555394 0.058727527 0.058772367 0.058994553
[157] 0.059122580 0.059422156 0.059472222 0.059595185 0.059741220 0.059930026 0.060314474 0.060530163 0.060584331 0.060897515 0.060970886 0.061510451
[169] 0.061656058 0.061808289 0.062004486 0.062251763 0.062458937 0.062510016 0.062754090 0.062905475 0.063178098 0.063240613 0.063317469 0.063479084
[181] 0.063523362 0.063694397 0.063742101 0.064029756 0.064296627 0.064379306 0.064518045 0.064623767 0.064785251 0.064952768 0.065094372 0.065231396
[193] 0.065378983 0.065508066 0.065661204 0.065709215 0.066134959 0.066366275 0.066441423 0.066673385 0.067042402 0.067123208 0.067222948 0.067400944
[205] 0.067531976 0.067679037 0.067917589 0.067958602 0.068051348 0.068493047 0.068579161 0.068684532 0.069048488 0.069113389 0.069329837 0.069428961
[217] 0.069648247 0.069770521 0.069891856 0.070091085 0.070166936 0.070481026 0.070548739 0.070602396 0.070695436 0.070910270 0.071101302 0.071146888
[229] 0.071385915 0.071523636 0.071576563 0.071758762 0.072014578 0.072153381 0.072223735 0.072377887 0.072610420 0.072632569 0.072769671 0.072852295
[241] 0.073236181 0.073335206 0.073551456 0.073608946 0.073732210 0.073951238 0.074061107 0.074199666 0.074365222 0.074569484 0.074693366 0.074811119
[253] 0.075077623 0.075308547 0.075334442 0.075583183 0.075637455 0.076044758 0.076227575 0.076561468 0.076639509 0.076743220 0.076886499 0.076984120
[265] 0.077218364 0.077300165 0.077379752 0.077504209 0.077691417 0.077706902 0.077760017 0.078117034 0.078142484 0.078341909 0.078476299 0.078587578
[277] 0.078812236 0.078882109 0.079060769 0.079341094 0.079397263 0.079706601 0.079767435 0.079806960 0.080012051 0.080056993 0.080205723 0.080341882
[289] 0.080596698 0.080713527 0.080916946 0.081037221 0.081164184 0.081227513 0.081441157 0.081469144 0.081701070 0.081769250 0.081937132 0.082336345
[301] 0.082513204 0.082702703 0.082982936 0.083024134 0.083207446 0.083287623 0.083343716 0.083481888 0.083633007 0.083846111 0.083987075 0.084095279
[313] 0.084420109 0.084517719 0.084706471 0.085029562 0.085150423 0.085298290 0.085487837 0.085751437 0.085800310 0.085958110 0.086063247 0.086302052
[325] 0.086311591 0.086422160 0.086492622 0.086562733 0.086649891 0.086965325 0.087098479 0.087130318 0.087191493 0.087501645 0.087668662 0.087822961
[337] 0.087841995 0.087978140 0.088255972 0.088425003 0.088622869 0.088737124 0.088805606 0.088833697 0.089080573 0.089286065 0.089370567 0.089696060
[349] 0.089721896 0.089864919 0.089996175 0.090212358 0.090292907 0.090313664 0.090643735 0.090880538 0.090937240 0.091141743 0.091154109 0.091309731
[361] 0.091461071 0.091586306 0.091734568 0.091895822 0.092090219 0.092147551 0.092294499 0.092495042 0.092652649 0.093007649 0.093075846 0.093263710
[373] 0.093352888 0.093451739 0.093564022 0.093859865 0.094012489 0.094042931 0.094166211 0.094201309 0.094392568 0.094496497 0.094719390 0.095014168
[385] 0.095033761 0.095222151 0.095385210 0.095490446 0.095577943 0.095642322 0.095936509 0.096029667 0.096191489 0.096309126 0.096342917 0.096643620
[397] 0.096936597 0.096993415 0.097236637 0.097304474 0.097396193 0.097612985 0.097946388 0.098052104 0.098127509 0.098227228 0.098396635 0.098690567

[409] 0.098753302 0.098798692 0.098887232 0.099121847 0.099152763 0.099425610 0.099650569 0.099803348 0.099972364 0.100147039 0.100347160 0.100424373
[421] 0.100593101 0.100897707 0.101044715 0.101136423 0.101161341 0.101208654 0.101362209 0.101423415 0.101765742 0.101923427 0.102259745 0.102279559
[433] 0.102353458 0.102689354 0.102838481 0.102896225 0.103036448 0.103221153 0.103478623 0.103586273 0.103650810 0.103757794 0.103882712 0.104000662
[445] 0.104089158 0.104162746 0.104467370 0.104551231 0.104655358 0.104757447 0.105017301 0.105150480 0.105300091 0.105436997 0.105668215 0.105704886
[457] 0.105829062 0.106030882 0.106073158 0.106223853 0.106254253 0.106654951 0.106754535 0.106946793 0.106984327 0.107012498 0.107128066 0.107221895
[469] 0.107423810 0.107722386 0.107851168 0.108114598 0.108186970 0.108222553 0.108509828 0.108853604 0.109046342 0.109118444 0.109359367 0.109492239
[481] 0.109589707 0.109669651 0.109848045 0.109886778 0.110158562 0.110232465 0.110525517 0.110736963 0.110803735 0.110928255 0.111162066 0.111186217
[493] 0.111341481 0.111727450 0.111878341 0.112157946 0.112181545 0.112283671 0.112449472 0.112583084 0.112832324 0.112963465 0.113283555 0.113494103
[505] 0.113528468 0.113708939 0.113794560 0.113927262 0.114022160 0.114305054 0.114316133 0.114487951 0.114699159 0.114776096 0.114878452 0.115005168
[517] 0.115270048 0.115433425 0.115867094 0.115935500 0.116200440 0.116331486 0.116359353 0.116506310 0.116938492 0.117207324 0.117336273 0.117604626
[529] 0.117842494 0.118063686 0.118156012 0.118253836 0.118496280 0.118690326 0.118898117 0.119053217 0.119166460 0.119473112 0.119841981 0.119920900
[541] 0.120003460 0.120118338 0.120276182 0.120823238 0.120864007 0.120955244 0.121074867 0.121287762 0.121521235 0.121760709 0.121927853 0.122001166
[553] 0.122265496 0.122375456 0.122497001 0.122570904 0.122707223 0.122848545 0.122950567 0.122995986 0.123055696 0.123315608 0.123410882 0.123480647
[565] 0.123639218 0.123797893 0.123841569 0.123964844 0.124133204 0.124374573 0.124555598 0.124815736 0.125024388 0.125066522 0.125340757 0.125402077
[577] 0.125652903 0.125946679 0.126208164 0.126301957 0.126462098 0.126615035 0.126720021 0.126796257 0.126971037 0.127258087 0.127377757 0.127710314
[589] 0.128006293 0.128128230 0.128252619 0.128498571 0.128655780 0.128726639 0.129116845 0.129211173 0.129533352 0.129598454 0.129977176 0.130150408
[601] 0.130398025 0.130559642 0.130921944 0.130963244 0.131158931 0.131231334 0.131289999 0.131363871 0.131545484 0.131921208 0.132107423 0.132140878
[613] 0.132284855 0.132437188 0.132709076 0.132779775 0.132921822 0.133099881 0.133293951 0.133444658 0.133623347 0.133720085 0.133853565 0.134027457
[625] 0.134359297 0.134511352 0.134935893 0.134943871 0.135115329 0.135150663 0.135325696 0.135588243 0.135699914 0.136021993 0.136106605 0.136203625
[637] 0.136407764 0.136500580 0.136710854 0.136991271 0.137129233 0.137323327 0.137584086 0.137617314 0.137797578 0.138118892 0.138231909 0.138481355
[649] 0.138802000 0.138865675 0.139188801 0.139390476 0.139518350 0.139713023 0.139830638 0.140109874 0.140391711 0.140539132 0.141010381 0.141157605
[661] 0.141375285 0.141533405 0.141937391 0.142203316 0.142352420 0.142830544 0.143030804 0.143292225 0.143355762 0.143463770 0.143671698 0.143726695
[673] 0.143967325 0.144276919 0.144526360 0.144831435 0.144909855 0.145178623 0.145324325 0.145348290 0.145584151 0.145957145 0.146069819 0.146138735
[685] 0.146330226 0.146463109 0.146730155 0.147042889 0.147270533 0.147415628 0.147749629 0.148035316 0.148134014 0.148378848 0.148605042 0.148832424
[697] 0.149227599 0.149422506 0.149718282 0.149749663 0.149968638 0.150158197 0.150345917 0.150422556 0.150513293 0.150632061 0.150732839 0.150964399
[709] 0.151365220 0.151424784 0.151543172 0.151745037 0.151984221 0.152295730 0.152499838 0.152588178 0.152708567 0.152907651 0.153475547 0.153547407
[721] 0.153667951 0.153775387 0.154264331 0.154463214 0.155085774 0.155273328 0.155429612 0.155497230 0.155634737 0.155899314 0.156032246 0.156148277

[733] 0.156390931 0.156602884 0.156893357 0.156985034 0.157346215 0.157687855 0.157959016 0.157971443 0.158156216 0.158188645 0.158422498 0.158543729
[745] 0.158946951 0.159283568 0.159369816 0.159734564 0.159842044 0.160015475 0.160191249 0.160417051 0.160821640 0.160963333 0.161258047 0.161333663
[757] 0.161371026 0.161484083 0.161640239 0.162035209 0.162197316 0.162263440 0.162341007 0.162414559 0.162622945 0.162776452 0.162967745 0.163007375
[769] 0.163144685 0.163469017 0.164187359 0.164392188 0.164743510 0.164904107 0.164941967 0.165268366 0.165481807 0.166079155 0.166341967 0.166424253
[781] 0.166988807 0.167084874 0.167651357 0.167730056 0.168027299 0.168210286 0.168279918 0.168450753 0.168629826 0.168917538 0.169075593 0.169291715
[793] 0.169584726 0.169610042 0.170133359 0.170480948 0.170616994 0.170640598 0.170797355 0.171078831 0.171260111 0.171583718 0.171749423 0.171929922
[805] 0.172126936 0.172386331 0.172581356 0.172616725 0.172669691 0.172919850 0.173257390 0.173592887 0.174074987 0.174283747 0.174449790 0.174546540
[817] 0.174653244 0.174959500 0.175021599 0.175167712 0.175231688 0.175553818 0.175860667 0.175897752 0.176126573 0.176155190 0.176542939 0.176697757
[829] 0.176988168 0.177400514 0.177536750 0.178037765 0.178756084 0.178817998 0.178877837 0.179050938 0.179955756 0.180151093 0.180400215 0.180573821
[841] 0.180655654 0.180926832 0.181205015 0.181337695 0.181597882 0.181725118 0.182173534 0.182179513 0.182406771 0.182534068 0.182544892 0.182728520
[853] 0.182934051 0.183214370 0.183470378 0.183660943 0.184372398 0.184539384 0.185056208 0.185164379 0.185316232 0.185631165 0.186304916 0.186446127
[865] 0.187204577 0.187292642 0.187531872 0.187585015 0.187608779 0.188029088 0.188591288 0.188820153 0.189047669 0.189321478 0.189440451 0.189470868
[877] 0.189769363 0.190062657 0.190218190 0.190724341 0.190871040 0.191052301 0.191377847 0.191446660 0.191743598 0.191944622 0.192430305 0.192598131
[889] 0.193246021 0.193494189 0.193643042 0.193816156 0.194398691 0.194571523 0.195392560 0.195627129 0.195841693 0.195950051 0.196162196 0.196624064
[901] 0.196679065 0.197106056 0.197291153 0.197790538 0.198511071 0.198658205 0.198907707 0.198933493 0.199407235 0.199759553 0.199969557 0.200232606
[913] 0.200339473 0.200454245 0.200660745 0.200897043 0.201783551 0.202178966 0.202547724 0.202693177 0.202914251 0.202923676 0.203151357 0.203203719
[925] 0.203801618 0.204126189 0.204247142 0.204423109 0.205106576 0.205286064 0.205588209 0.205703562 0.205886835 0.206646989 0.207358247 0.207609007
[937] 0.207692322 0.207830153 0.208190301 0.208726656 0.209084351 0.209515317 0.209821815 0.210244583 0.210771446 0.210932604 0.211295256 0.211612762
[949] 0.212027718 0.212283044 0.212987553 0.213362383 0.213447310 0.213745326 0.214329241 0.214384367 0.214503846 0.214657777 0.214988698 0.215590689
[961] 0.215608195 0.215800018 0.216365277 0.216554792 0.216990007 0.217220086 0.217460988 0.217782707 0.218454002 0.218752376 0.218832722 0.219606025
[973] 0.219883484 0.220323218 0.220610281 0.221015221 0.221063122 0.221383382 0.221394995 0.221647644 0.221817745 0.222018978 0.222610435 0.222952899
[985] 0.223666722 0.224188932 0.225477791 0.225675761 0.226107031 0.226507680 0.226948308 0.227109531 0.227744551 0.228217089 0.228794904 0.229351164
[997] 0.229541489 0.229573930 0.230548062 0.230981586 0.231172486 0.231470552 0.232432431 0.232938579 0.233143740 0.233838478 0.233970488 0.234359693
[1009] 0.234803063 0.234823181 0.235164072 0.235801839 0.235842226 0.236325637 0.236839133 0.237205053 0.237694964 0.237808536 0.238073931 0.238192853
[1021] 0.239013476 0.239443627 0.239682377 0.240875683 0.241161074 0.241654757 0.243323602 0.243759707 0.243969629 0.244301282 0.245835817 0.246464521
[1033] 0.246959192 0.247075837 0.247777515 0.248172472 0.248464876 0.248745378 0.248997031 0.249385791 0.249462872 0.249799298 0.250956814 0.251266342
[1045] 0.251806459 0.252166982 0.252551515 0.253093193 0.253486363 0.253841956 0.254184396 0.255437932 0.256034501 0.256376680 0.257989279 0.259019279

[1057] 0.259184399 0.260072034 0.260288506 0.260380454 0.261621004 0.262263250
0.262271604 0.262902240 0.263071663 0.264345772 0.264968891 0.265409181
[1069] 0.266270881 0.267386131 0.268863060 0.269426944 0.269635483 0.269725278
0.270285910 0.270382327 0.271611655 0.272830012 0.272977082 0.273379135
[1081] 0.273545079 0.273690931 0.274095310 0.274267563 0.276389273 0.277397021
0.277449340 0.278658126 0.279182495 0.279711813 0.279838294 0.282196301
[1093] 0.282403797 0.283362135 0.284517540 0.284933224 0.286732107 0.286824542
0.286947050 0.288164430 0.288980370 0.289528914 0.290670133 0.291165432
[1105] 0.292371356 0.294171425 0.295487558 0.295664696 0.296574464 0.301066680
0.301140205 0.301310825 0.301449756 0.301530496 0.303803900 0.304109865
[1117] 0.306670472 0.307352756 0.307492828 0.307871241 0.308940325 0.310833312
0.311128660 0.311201721 0.311524976 0.311943364 0.314843839 0.314939282
[1129] 0.317147804 0.318297432 0.320743887 0.320931237 0.321182010 0.323251408
0.323307392 0.323472271 0.325799615 0.327212306 0.327530007 0.328735296
[1141] 0.329608043 0.330409418 0.336335706 0.339645300 0.340317111 0.342480285
0.347284437 0.349634794 0.349709951 0.349858912 0.352896434 0.355934721
[1153] 0.357012138 0.358129143 0.359164259 0.360462511 0.364017521 0.365784425
0.367418795 0.370125180 0.371931549 0.380465842 0.393022264 0.396230735
[1165] 0.397931346 0.398643072 0.406521075 0.408162525 0.408832248 0.419599383
0.428898417 0.431109555 0.431636950 0.434733788 0.439512074 0.440936752
[1177] 0.446173532 0.451143770 0.451940688 0.451980900 0.452646311 0.454180191
0.467276907 0.468004548 0.469571927 0.470355259 0.473980095 0.474731130
[1189] 0.476186802 0.477383839 0.479571161 0.480995282 0.484579314 0.490912616
0.491830415 0.492898182 0.494672638 0.494961638 0.495153870 0.495744809
[1201] 0.497180759 0.497794149 0.498012322 0.502120074 0.502666366 0.503253467
0.505552990 0.508758685 0.512058046 0.512086934 0.514001922 0.516665043
[1213] 0.518236502 0.518422230 0.519984604 0.521004518 0.522154746 0.523887911
0.524295252 0.527326250 0.531022084 0.531379792 0.532560217 0.532779320
[1225] 0.535720409 0.536329087 0.536862203 0.537689070 0.538077183 0.538473390
0.539549670 0.541662425 0.543974925 0.544837351 0.545247716 0.545435879
[1237] 0.547061381 0.548673896 0.550316875 0.551695660 0.552508465 0.554502286
0.554913811 0.555647243 0.555945872 0.559665432 0.560548613 0.561991474
[1249] 0.566070841 0.566478971 0.568304395 0.568385376 0.569184784 0.569417553
0.569873070 0.570361112 0.571548757 0.572688473 0.573543230 0.573783722
[1261] 0.574724807 0.576099477 0.576412822 0.577380858 0.578636289 0.580229070
0.580417370 0.580821263 0.581180660 0.582908515 0.585010403 0.585654639
[1273] 0.586083297 0.587686798 0.588754535 0.588859223 0.589404326 0.589578655
0.589790276 0.591403557 0.592504655 0.594785425 0.594878726 0.595798203
[1285] 0.595801647 0.595842035 0.596371793 0.596591428 0.596670205 0.597094603
0.597950438 0.598290930 0.598607808 0.599238905 0.599619453 0.600461544
[1297] 0.600781472 0.600892581 0.601262625 0.601574379 0.602520593 0.604070736
0.605308306 0.606385743 0.606798148 0.606992353 0.607196070 0.608611665
[1309] 0.608856008 0.608948901 0.609329870 0.610100001 0.610199969 0.610370495
0.610786037 0.611735994 0.611946224 0.612306878 0.612397386 0.612739813
[1321] 0.613304229 0.613397625 0.613840066 0.614639988 0.614898413 0.615258654
0.615601918 0.616688438 0.617048025 0.617430233 0.617639078 0.618457760
[1333] 0.619675529 0.619979864 0.620013672 0.620182214 0.620727886 0.620801824
0.620956161 0.621267839 0.621360955 0.621433758 0.621611064 0.622598860
[1345] 0.624096517 0.624396769 0.624617175 0.625018139 0.626430662 0.626446431
0.626757359 0.626951664 0.627223068 0.627271003 0.627795363 0.627825785
[1357] 0.628483847 0.628740449 0.628829740 0.629236272 0.629510776 0.630165290
0.630601690 0.630876704 0.631089829 0.631309503 0.631898641 0.632829955
[1369] 0.633465745 0.634347241 0.635734215 0.636307334 0.637520966 0.637813549
0.639700244 0.639987140 0.640474394 0.640596115 0.640653092 0.641412686

[1381] 0.641937174 0.642445621 0.642946357 0.643146878 0.643436725 0.643961472
0.644701963 0.645549989 0.645568019 0.645768862 0.646376839 0.646853171
[1393] 0.646876255 0.647143394 0.648561177 0.649650339 0.649952310 0.650277085
0.651114174 0.651279646 0.651319257 0.651339091 0.651814226 0.652565253
[1405] 0.653297911 0.653911515 0.654135587 0.654734498 0.654747671 0.655233362
0.655510620 0.656157575 0.656467704 0.656744344 0.657392281 0.657746926
[1417] 0.658151491 0.658627749 0.659098245 0.659682783 0.659941052 0.660339421
0.662957236 0.663276502 0.663413684 0.663440774 0.664549307 0.665476244
[1429] 0.665800427 0.666152307 0.666314425 0.667589785 0.667716540 0.668669469
0.669273635 0.669482010 0.669688050 0.670696492 0.671397745 0.671544414
[1441] 0.672130055 0.672471635 0.672851875 0.673501033 0.673933652 0.673979092
0.674372304 0.674856371 0.675392861 0.675781971 0.675991581 0.676357914
[1453] 0.676639992 0.677142451 0.677447105 0.679591663 0.680097751 0.681529584
0.682317381 0.682588573 0.683065964 0.683357098 0.683711853 0.683862789
[1465] 0.684196383 0.684675668 0.686652676 0.686861051 0.686929328 0.687438066
0.687823547 0.687938492 0.687944494 0.688382000 0.688731243 0.689412094
[1477] 0.690447034 0.691142375 0.692229418 0.692357022 0.692519717 0.693325972
0.693895566 0.694064616 0.695150459 0.695599838 0.695836587 0.696511456
[1489] 0.697094090 0.697632397 0.699260424 0.699425551 0.699535041 0.699545053
0.700560523 0.701198623 0.701291005 0.701355042 0.701371168 0.701571562
[1501] 0.701680266 0.702474889 0.702513308 0.702983618 0.703414238 0.704795882
0.705013392 0.707007872 0.707343621 0.707986091 0.708529815 0.709298962
[1513] 0.710360556 0.710517471 0.710788096 0.711240114 0.712580725 0.712734343
0.713844216 0.713959602 0.715570748 0.715732449 0.715986722 0.716104238
[1525] 0.716189746 0.716324839 0.716503304 0.717204888 0.717670194 0.718129625
0.718273664 0.718321482 0.720244788 0.721390334 0.721980135 0.723351040
[1537] 0.725337037 0.725707170 0.727044918 0.727864156 0.727947360 0.728106903
0.729026846 0.729369094 0.729397572 0.729660170 0.729711217 0.730947593
[1549] 0.731332965 0.731570978 0.731858548 0.733706549 0.734105190 0.734494665
0.734785296 0.735525164 0.735639318 0.735995803 0.736836373 0.737485058
[1561] 0.737755387 0.738038934 0.738413522 0.741034969 0.741157212 0.742994069
0.743095571 0.744303674 0.744495438 0.744750553 0.744892590 0.745375852
[1573] 0.745547873 0.746457366 0.746729416 0.746814419 0.752397499 0.752464289
0.752568015 0.752600646 0.753366391 0.754062902 0.755271690 0.756552527
[1585] 0.756888302 0.757081562 0.757236389 0.757676610 0.757953272 0.758023884
0.758246704 0.759037172 0.759164741 0.759268786 0.759780044 0.759918439
[1597] 0.760060690 0.760359622 0.760515763 0.760714229 0.762538539 0.762741177
0.763057565 0.763995019 0.764128838 0.764851862 0.764970034 0.766141050
[1609] 0.767604307 0.769284699 0.773035907 0.773571320 0.777471881 0.777573769
0.777911591 0.778179822 0.778680698 0.778801732 0.779170751 0.779356373
[1621] 0.779409739 0.779495484 0.779694998 0.781528027 0.783262065 0.784123924
0.784672576 0.785667599 0.786836816 0.788796111 0.789393099 0.789736787
[1633] 0.789834207 0.790080405 0.790521513 0.791011756 0.791139484 0.791772376
0.792120963 0.792675185 0.793110746 0.793406611 0.793486598 0.794500647
[1645] 0.794579089 0.795056157 0.796339181 0.796500052 0.799072131 0.800128885
0.800986098 0.803021166 0.803587603 0.803754776 0.805306272 0.806293315
[1657] 0.806844755 0.806896114 0.807292925 0.810954815 0.812134162 0.814325594
0.814919142 0.815347263 0.815541532 0.815664984 0.819746133 0.819789654
[1669] 0.819928794 0.820239197 0.820585955 0.821603493 0.822466997 0.824972213
0.825625897 0.826298285 0.826838390 0.827280872 0.828422286 0.828786404
[1681] 0.829089580 0.830148934 0.831741174 0.833023981 0.833212502 0.833313609
0.836052520 0.836474518 0.837220370 0.837995422 0.839184912 0.842378743
[1693] 0.842820025 0.845432436 0.846019759 0.846875386 0.847674796 0.848606291
0.853084837 0.856262646 0.856783788 0.859554120 0.866051227 0.866445021

[1705] 0.868065114 0.868462121 0.869950455 0.869999355 0.875229962 0.878194608
0.879425295 0.881617701 0.881875558 0.882101537 0.883289358 0.887646242
[1717] 0.888733059 0.896291120 0.896664020 0.905735595 0.908346397 0.909061577
0.913938679 0.917998924 0.920464602 0.921101282 0.921376351 0.922428014
[1729] 0.925230038 0.930357451 0.930480738 0.934644704 0.940889538 0.941970077
0.942947694 0.944395406 0.946807794 0.947054227 0.950017226 0.952811636
[1741] 0.954130738 0.956254129 0.959066895 0.965907547 0.972144553 0.984295544
0.988472658 1.001899114 1.035259048 1.036875668 1.041673382 1.077824301
[1753] 1.087397168 1.100737851 1.169054518

FLUCTUATION DATA for FULL LENGTH MINIMIZED CHARACTERIZED Q9ZSP9

[1] 0.33032251 0.24101651 0.15794716 0.15201492 0.17307511 0.14331973
0.12560418 0.14448045 0.13492783 0.10750468 0.13654149 0.16006733 0.14609871
[14] 0.14472677 0.18768454 0.19912942 0.17328814 0.18898301 0.18871902
0.21513774 0.30381013 0.35117253 0.44963353 0.79013059 0.68845796 0.44585546
[27] 0.39031917 0.32987270 0.35514766 0.31509616 0.33295651 0.36838031
0.31443256 0.26684714 0.26730540 0.32413680 0.37311995 0.25655415 0.25570537
[40] 0.37185175 0.43386287 0.42325621 0.43788480 0.36476663 0.33790283
0.22866742 0.25008323 0.20060821 0.15887759 0.14279699 0.14877366 0.13587480
[53] 0.18303487 0.21886257 0.21682426 0.19980688 0.18407004 0.14988405
0.13497740 0.12298356 0.09809517 0.08848591 0.08908452 0.07981704 0.07730613
[66] 0.07699322 0.07868717 0.06957748 0.07085601 0.08517595 0.08744580
0.09765874 0.10327698 0.11604961 0.14171406 0.17962188 0.15757932 0.15831775
[79] 0.20207599 0.16919695 0.15114170 0.19857243 0.23927639 0.22659840
0.23410297 0.19892329 0.17153003 0.21596010 0.18866737 0.14341289 0.15292070
[92] 0.17845186 0.14852881 0.12538869 0.14459122 0.15901019 0.12370516
0.13031042 0.17103277 0.15674526 0.14363004 0.18007531 0.19042459 0.18251772
[105] 0.20515591 0.38701039 0.49496869 0.36373063 0.27486708 0.24394336
0.17852688 0.17475362 0.16464992 0.17008001 0.19084965 0.22711557 0.26861477
[118] 0.26607000 0.32937205 0.33571295 0.26720529 0.28237916 0.37917917
0.35360904 0.29040056 0.29101593 0.27264375 0.26889797 0.31645024 0.33285678
[131] 0.37116081 0.58507306 0.44554763 0.48804100 0.30773117 0.29785070
0.23378455 0.22427750 0.21733098 0.20595231 0.26746522 0.33839401 0.46573348
[144] 0.25302786 0.19823439 0.14798053 0.12597011 0.10375245 0.10162060
0.10129278 0.08362013 0.07596550 0.08936891 0.08975599 0.07352266 0.07075147
[157] 0.09298974 0.09250199 0.08886731 0.10624984 0.12760705 0.15083121
0.16483850 0.18630131 0.23271378 0.42795730 0.43872427 0.35768149 0.31604207
[170] 0.37230197 0.27882920 0.19493028 0.22760503 0.24615866 0.16135328
0.15951672 0.22156128 0.18533892 0.13570786 0.17743121 0.19073330 0.13994421
[183] 0.14104150 0.19839877 0.18415505 0.14472536 0.20880158 0.27814781
0.27319689 0.22013129 0.23342852 0.19386090 0.28911937 0.24464219 0.20945894
[196] 0.26321965 0.26505184 0.21028230 0.22976698 0.37973725 0.56767557
0.40800841 0.35708396 0.25172449 0.21953644 0.24083007 0.21732107 0.23880558
[209] 0.15739652 0.13341158 0.14655145 0.10903810 0.09236801 0.12575245
0.11483391 0.09063707 0.08579802 0.11767372 0.11857323 0.10504779 0.12937032
[222] 0.15928930 0.18457158 0.18278681 0.27188373 0.31080267 0.36031878
0.36672955 0.34464632 0.20792167 0.22569384 0.28863588 0.24051326 0.18468986
[235] 0.26808498 0.29934516 0.24084916 0.26247604 0.39227497 0.33093921
0.29832814 0.37068698 0.47737869 0.59004099 0.58037410 0.29201436 0.32802383
[248] 0.26453595 0.22946030 0.14832693 0.10260228 0.13512538 0.14982696
0.15344845 0.13104329 0.11184847 0.12580374 0.12151319 0.11615780 0.11852087
[261] 0.13497924 0.13687050 0.15801389 0.18469478 0.19327894 0.23501761
0.28729471 0.35044621 0.39781196 0.64325324 0.73124265 1.09536583 1.58090589

[274] 2.16847269 1.66071205 1.35839408 0.88796327 0.49698165 0.32879950
0.27292650 0.37073108 0.31647229 0.20032374 0.20338000 0.26269805 0.22194843
[287] 0.17223519 0.20181577 0.23852821 0.19122037 0.17674603 0.21990323
0.21021033 0.19386567 0.26547234 0.36503009 0.63045437 0.97174618 0.76849088
[300] 0.57737237 0.26172044 0.23035034 0.23832014 0.22756846 0.24053163
0.36752932 0.34713230 0.27214439 0.20911947 0.17785900 0.18632961 0.22912860
[313] 0.24289613 0.24473717 0.17353857 0.12001335 0.12288897 0.10813984
0.09624520 0.09990995 0.09710938 0.07810697 0.07322604 0.08997396 0.08887749
[326] 0.07810536 0.09638574 0.10798062 0.10702785 0.13550532 0.15561338
0.17801839 0.25476978 0.28016563 0.35667448 0.32423702 0.28037800 0.35318172
[339] 0.35158691 0.62652463 0.60019225 0.90161588 0.96595763 1.01656603
1.52876541 1.31113060 0.90673228 0.75659499 0.37727507 0.27039785 0.22057248
[352] 0.26372287 0.23042168 0.15562297 0.16167510 0.19769483 0.15823410
0.12101452 0.14437528 0.16627968 0.14287006 0.13620357 0.15562039 0.16170026
[365] 0.15478949 0.15899675 0.20100495 0.24528547 0.29610127 0.45875941
0.54186413 0.35862546 0.24035675 0.17578919 0.15448092 0.14670082 0.17247617
[378] 0.21893620 0.21750512 0.26758316 0.32703095 0.32093422 0.20987056
0.23873337 0.33026884 0.25472142 0.20785399 0.19689618 0.21558596 0.23991632
[391] 0.24568479 0.25692157 0.27121703 0.29242252 0.29271192 0.37441361
0.40573829 0.34601943 0.40898808 0.40744774 0.47685052 0.46525842 0.48249524
[404] 0.54933781 0.47766203 0.33171210 0.38019946 0.30978703 0.30252682
0.22714439 0.17285069 0.12483447 0.13587952 0.11564134 0.12384376 0.11317923
[417] 0.14504798 0.21941079 0.25636170 0.26607939 0.30566902 0.28768923
0.33605316 0.30848393 0.32528134 0.29528286 0.28505963 0.25687287 0.31181898
[430] 0.25461107 0.19224783 0.21470359 0.15416918 0.15416915 0.14948129
0.13352545 0.14662933 0.11302956 0.08937929 0.08610229 0.09552408 0.07768148
[443] 0.07458743 0.08082538 0.08741582 0.07989509 0.08185600 0.09793334
0.11044186 0.12661764 0.14154442 0.14812462 0.17250669 0.22998222 0.27904079
[456] 0.31409043 0.63473007 0.72206957 0.60949031 0.35254249 0.30325847
0.26837017 0.25125625 0.34693811 0.54037330 0.59579699 0.44284145 0.38002794
[469] 0.35458929 0.40609910 0.49501349 0.68464189 0.82584817 0.89549172
0.83776438 0.58181403 0.53770415 0.60340335 0.49941602 0.39245363 0.36733002
[482] 0.49589366 0.46000364 0.67954450 0.58663865 0.88490478 0.91939513
0.90233539 1.08072188 1.29657487 1.57034445 1.86293126 1.37615340 1.01123639
[495] 0.73287957 0.55227492 0.43423319 0.40661345 0.31773087 0.36208237
0.31794489 0.35373603 0.36065262 0.36519615 0.37916679 0.38806969 0.37115689
[508] 0.46481428 0.56003831 0.87676531 0.78610809 0.72432130 0.59027538
0.55908848 0.56490196 0.59192738 0.58562335 0.90541945 1.18516379 1.64200858
[521] 1.67648295 2.50361555 2.69977079 3.19120844 3.32768758 2.40816526
2.32339905 2.20584361 1.57260583 1.48245377 1.57409241 1.17261340 0.95412096
[534] 0.64400696 0.70403730 0.69588906 0.90743668 0.72650966 0.56886323
0.73100181 0.78876781 0.76619282 0.55597715 0.75683288 0.90099133 0.88962053
[547] 1.19017374 1.77001404 2.20198562 2.04680973 1.72618662 1.20854340
0.78939428 0.51590819 0.50462984 0.39516520 0.51345915 0.49833586 0.70750392
[560] 0.89343206 0.77309936 0.80577849 0.59494427 0.75388129 0.58182658
0.44258228 0.41164067 0.42651984 0.43821739 0.36116177 0.38714879 0.38622618
[573] 0.50428976 0.57833180 0.89808445 1.22881451 1.76329688 2.22251414
2.00705666 2.54497933 2.12667711 1.62157236 1.88708668 1.69405660 3.22317179

MASSES for FULL LENGTH MINIMIZED CHARACTERIZED Q9ZSP9

[1] 138.1469 114.1030 163.1730 57.0510 128.1060 71.0780 113.1580 87.0770
129.1800 87.0770 147.1740 113.1580 147.1740 163.1730 128.1060 71.0780
[17] 117.1260 157.1940 87.0770 57.0510 163.1730 113.1580 97.1150 157.1940

```
114.1030 117.1260 157.1940  99.1310 117.1260 186.2100 157.1940  57.0510
 [33] 114.1030  87.0770  57.0510 113.1580 114.1030 114.0790  57.0510 129.1800
71.0780  87.0770  57.0510 113.1580 114.0790 113.1580  99.1310  57.0510
 [49]  57.0510 163.1730 163.1730 114.0790  71.0780  57.0510 114.0790 114.1030
99.1310 129.1800 147.1740  57.0510 113.1580  97.1150 131.1960  71.0780
 [65] 147.1740 101.1040  99.1310 101.1040 131.1960 113.1580  87.0770 186.2100
87.0770 113.1580 113.1580 128.1060 163.1730  57.0510 157.1940 117.1260
 [81] 131.1960  71.0780  71.0780  87.0770  57.0510 128.1060 113.1580  87.0770
137.1390  71.0780 131.1960 114.0790  71.0780  99.1310 129.1800 186.2100
 [97]  57.0510 101.1040 114.0790 163.1730 113.1580 113.1580 129.1800  71.0780
137.1390  97.1150 128.1060  97.1150 163.1730  99.1310 113.1580 163.1730
[113]  57.0510 128.1060  99.1310  57.0510 114.0790  57.0510 114.1030 101.1040
114.0790 137.1390 163.1730 103.1430 186.2100 117.1260 157.1940  97.1150
[129] 128.1060 114.0790 131.1960 101.1040 101.1040  87.0770 157.1940  71.0780
71.0780 163.1730 157.1940 113.1580 114.0790  97.1150 114.1030 137.1390
[145]  97.1150  57.0510  87.0770 114.0790 113.1580  71.0780  57.0510 128.1060
101.1040  71.0780  71.0780  71.0780 131.1960  71.0780  71.0780  71.0780
[161]  87.0770 113.1580  99.1310 147.1740 157.1940 157.1940 163.1730 114.1030
97.1150  57.0510 163.1730  87.0770 114.1030 128.1060 113.1580 113.1580
[177] 114.1030 137.1390  71.0780 137.1390 117.1260 113.1580 147.1740 128.1060
147.1740  71.0780 114.0790 129.1800 163.1730 157.1940  57.0510 129.1800
[193] 163.1730 114.0790  87.0770  87.0770 113.1580 101.1040  99.1310  71.0780
117.1260 129.1800 163.1730 163.1730 157.1940  87.0770  99.1310  87.0770
[209]  57.0510 163.1730  71.0780 114.0790 128.1060 113.1580 113.1580 186.2100
57.0510  71.0780  71.0780 186.2100 113.1580 163.1730 129.1800  71.0780
[225]  87.0770 114.1030 114.1030 117.1260 147.1740 163.1730 113.1580 114.1030
163.1730 113.1580  57.0510 157.1940 114.1030  57.0510 114.0790  71.0780
[241] 113.1580  57.0510  57.0510 101.1040  57.0510 186.2100  87.0770 131.1960
101.1040 128.1060 147.1740  57.0510 186.2100 114.0790  99.1310 129.1800
[257] 163.1730  71.0780  57.0510  99.1310 117.1260 101.1040 113.1580  99.1310
71.0780 117.1260 147.1740 113.1580 131.1960  87.0770  57.0510 129.1800
[273]  71.0780  57.0510 137.1390 114.1030  71.0780  97.1150  99.1310 147.1740
128.1060 129.1800 163.1730 117.1260 117.1260 129.1800  71.0780 128.1060
[289] 114.1030 147.1740 131.1960 103.1430  87.0770 131.1960 113.1580  57.0510
129.1800  57.0510 114.1030 157.1940 114.1030 101.1040 117.1260 129.1800
[305] 101.1040  97.1150  57.0510  57.0510 113.1580 113.1580 163.1730 157.1940
117.1260 157.1940 186.2100 114.1030 114.1030 131.1960 117.1260 147.1740
[321]  99.1310 101.1040  87.0770  71.0780  71.0780 147.1740 113.1580  71.0780
101.1040 101.1040 163.1730  87.0770 114.0790 163.1730 113.1580  71.0780
[337]  87.0770  71.0780  57.0510 129.1800 163.1730 113.1580 129.1800 103.1430
87.0770  87.0770  57.0510 147.1740  99.1310  87.0770  97.1150 114.1030
[353] 128.1060 113.1580 113.1580  87.0770 147.1740  71.0780 129.1800  87.0770
117.1260  99.1310 114.0790 163.1730 113.1580 113.1580  57.0510 114.0790
[369] 114.1030  97.1150 157.1940  71.0780 101.1040  87.0770 163.1730 131.1960
99.1310  57.0510 163.1730  57.0510 114.1030 114.1030 163.1730  97.1150
[385] 157.1940 117.1260  99.1310 137.1390 137.1390 157.1940  71.0780  87.0770
157.1940  57.0510  57.0510 163.1730  71.0780 101.1040 186.2100 163.1730
[401]  87.0770 157.1940 129.1800  71.0780  87.0770 114.0790  97.1150 114.1030
113.1580 113.1580 101.1040  57.0510  71.0780 113.1580  99.1310  57.0510
[417]  57.0510  97.1150 114.0790  71.0780 163.1730 114.0790 114.1030 147.1740
71.0780 114.0790 117.1260 157.1940 114.0790 114.1030 163.1730 128.1060
[433] 117.1260 101.1040 128.1060  97.1150  71.0780 101.1040 163.1730 114.1030
114.1030  71.0780  97.1150 113.1580 113.1580  57.0510  99.1310 113.1580
[449]  71.0780 157.1940 113.1580 137.1390  71.0780  57.0510 137.1390  87.0770
```

```
57.0510 163.1730 114.1030 117.1260 113.1580 113.1580  97.1150  99.1310
[465]  99.1310  97.1150 114.0790  97.1150 129.1800  97.1150 101.1040  97.1150
129.1800  97.1150  71.0780  97.1150 157.1940 101.1040 129.1800  99.1310
[481] 101.1040  97.1150  71.0780  97.1150 157.1940  97.1150 157.1940  99.1310
113.1580  97.1150  99.1310  97.1150  71.0780 114.1030  71.0780 137.1390
[497]  99.1310 101.1040 113.1580 117.1260 117.1260 157.1940  71.0780 101.1040
87.0770  87.0770 186.2100  71.0780 113.1580 114.1030  57.0510 129.1800
[513] 101.1040 163.1730 163.1730 157.1940 163.1730  87.0770  71.0780  99.1310
99.1310 101.1040 114.1030 129.1800  87.0770  57.0510 129.1800 101.1040
[529]  99.1310 129.1800 114.1030 113.1580 129.1800 113.1580  87.0770 113.1580
99.1310 129.1800 113.1580 163.1730  57.0510  97.1150 113.1580 186.2100
[545]  57.0510 113.1580 101.1040 129.1800 163.1730  57.0510 114.1030  87.0770
147.1740 113.1580 147.1740  97.1150  71.0780 186.2100 113.1580 114.1030
[561]  87.0770 113.1580  97.1150  71.0780  57.0510 129.1800  87.0770 113.1580
128.1060 147.1740  99.1310 163.1730 113.1580 137.1390 101.1040  71.0780
[577]  87.0770  97.1150  71.0780 113.1580  99.1310  87.0770  99.1310  87.0770
104.0843

Call:
  nma.pdb(pdb = pdbT4_NAT0, mass = TRUE)

Class:
  VibrationalModes (nma)

Number of modes:
  1368 (6 trivial)

Frequencies:
  Mode 7:       0.012
  Mode 8:       0.015
  Mode 9:       0.016
  Mode 10:      0.017
  Mode 11:      0.018
  Mode 12:      0.021

+ attr: modes, frequencies, force.constants, fluctuations,
        U, L, xyz, mass, temp, triv.modes, natoms, call

 NORMAL MODES for TRUNCATED MINIMIZED CHARACTERIZED Q5NAT0

  [1] 0.00000000 0.00000000 0.00000000 0.00000000 0.00000000 0.00000000
0.01170952 0.01463620 0.01588841 0.01680498 0.01800984 0.02097587 0.02219113
  [14] 0.02242504 0.02318642 0.02333778 0.02385520 0.02486233 0.02489134
0.02611014 0.02640869 0.02786394 0.02918357 0.02978197 0.03045105 0.03086170
  [27] 0.03143340 0.03163342 0.03196400 0.03270353 0.03309390 0.03405953
0.03432696 0.03491665 0.03549690 0.03584557 0.03655751 0.03687830 0.03738414
  [40] 0.03762290 0.03804569 0.03861664 0.03900920 0.03964779 0.03976103
0.04006755 0.04068892 0.04101383 0.04127456 0.04191762 0.04205005 0.04275881
  [53] 0.04302132 0.04321342 0.04353870 0.04383209 0.04424164 0.04450110
0.04520813 0.04558865 0.04594371 0.04615658 0.04636929 0.04691779 0.04744393
  [66] 0.04811115 0.04847829 0.04883568 0.04932134 0.04949949 0.04990085
0.05039334 0.05044760 0.05063929 0.05114127 0.05153672 0.05175571 0.05191770
  [79] 0.05224866 0.05242603 0.05269495 0.05293092 0.05313106 0.05356764
0.05371827 0.05387601 0.05437419 0.05482374 0.05502136 0.05539095 0.05564486
```

[92] 0.05626752 0.05643676 0.05667145 0.05700791 0.05736625 0.05751156
0.05797829 0.05807149 0.05835672 0.05854544 0.05903061 0.05922917 0.05952629
[105] 0.05970029 0.06033631 0.06049186 0.06069127 0.06112420 0.06174555
0.06186400 0.06232923 0.06243703 0.06266603 0.06283557 0.06301370 0.06328205
[118] 0.06344176 0.06381002 0.06388619 0.06405151 0.06416886 0.06461181
0.06482004 0.06494594 0.06502117 0.06547732 0.06572772 0.06595662 0.06622012
[131] 0.06649249 0.06669504 0.06708640 0.06723990 0.06734605 0.06756366
0.06781345 0.06823015 0.06845735 0.06889277 0.06893688 0.06917347 0.06954233
[144] 0.06971840 0.06993696 0.07001895 0.07046863 0.07062553 0.07081376
0.07130248 0.07139035 0.07166339 0.07195687 0.07200297 0.07239171 0.07279438
[157] 0.07287107 0.07324292 0.07349495 0.07353561 0.07383640 0.07403083
0.07423499 0.07433269 0.07481467 0.07486832 0.07536066 0.07572314 0.07584681
[170] 0.07591073 0.07608738 0.07653748 0.07664926 0.07668395 0.07743015
0.07752186 0.07773884 0.07782107 0.07789118 0.07812530 0.07838215 0.07841656
[183] 0.07864445 0.07894294 0.07915571 0.07926076 0.07941656 0.07968197
0.07981632 0.07985360 0.07996803 0.08022325 0.08062686 0.08090395 0.08096561
[196] 0.08115420 0.08130528 0.08137893 0.08143587 0.08150489 0.08182282
0.08199477 0.08222552 0.08257703 0.08272690 0.08289681 0.08308589 0.08341693
[209] 0.08367964 0.08425026 0.08429429 0.08455982 0.08463826 0.08491330
0.08508272 0.08538496 0.08558615 0.08562506 0.08588308 0.08616296 0.08636968
[222] 0.08659243 0.08661203 0.08701468 0.08727033 0.08733054 0.08786823
0.08814914 0.08833845 0.08842500 0.08866602 0.08878293 0.08903549 0.08933116
[235] 0.08937680 0.08956889 0.08968279 0.08990973 0.09001348 0.09009379
0.09041093 0.09076298 0.09099419 0.09118898 0.09142381 0.09162875 0.09178936
[248] 0.09196732 0.09234416 0.09237611 0.09265757 0.09302181 0.09310360
0.09314658 0.09346055 0.09350431 0.09387012 0.09411684 0.09420588 0.09462186
[261] 0.09492829 0.09501057 0.09543592 0.09552081 0.09561716 0.09564020
0.09592383 0.09615053 0.09621282 0.09656614 0.09693202 0.09719430 0.09722335
[274] 0.09727271 0.09766461 0.09772749 0.09799086 0.09818751 0.09830907
0.09851061 0.09876138 0.09889748 0.09892526 0.09902137 0.09916081 0.09940612
[287] 0.09971118 0.09996350 0.10015842 0.10053479 0.10064685 0.10086155
0.10095280 0.10108294 0.10116322 0.10131984 0.10145688 0.10181986 0.10191150
[300] 0.10211759 0.10226160 0.10246686 0.10280584 0.10291026 0.10295996
0.10337086 0.10342525 0.10358383 0.10366730 0.10380649 0.10416250 0.10440079
[313] 0.10450482 0.10481148 0.10492416 0.10504106 0.10530683 0.10546738
0.10556353 0.10576585 0.10580943 0.10603936 0.10659734 0.10680103 0.10708372
[326] 0.10748274 0.10785763 0.10805238 0.10816976 0.10836348 0.10855814
0.10877632 0.10883615 0.10904053 0.10934130 0.10944770 0.10963315 0.10970221
[339] 0.10986096 0.11001637 0.11029438 0.11062013 0.11082762 0.11086213
0.11123109 0.11169695 0.11184030 0.11213163 0.11217195 0.11248078 0.11280527
[352] 0.11285702 0.11313532 0.11332055 0.11343528 0.11367151 0.11405259
0.11428067 0.11431403 0.11452887 0.11463090 0.11484195 0.11497664 0.11515902
[365] 0.11557509 0.11562210 0.11580488 0.11592731 0.11598541 0.11625590
0.11648739 0.11660345 0.11694575 0.11719068 0.11725594 0.11745311 0.11776928
[378] 0.11795346 0.11811088 0.11819738 0.11839331 0.11875263 0.11897894
0.11932960 0.11943314 0.11952833 0.11965658 0.11971510 0.12001644 0.12011771
[391] 0.12028471 0.12061887 0.12083844 0.12109610 0.12118549 0.12130395
0.12146974 0.12151383 0.12174979 0.12223421 0.12229367 0.12241323 0.12248149
[404] 0.12271847 0.12278832 0.12288658 0.12327627 0.12364352 0.12377395
0.12394819 0.12406401 0.12422591 0.12447433 0.12483339 0.12503128 0.12504627
[417] 0.12553502 0.12572958 0.12576806 0.12591661 0.12627398 0.12649484
0.12673301 0.12707932 0.12739188 0.12770972 0.12821459 0.12841733 0.12864485
[430] 0.12870981 0.12881673 0.12909624 0.12920196 0.12941440 0.12968433
0.13014097 0.13029134 0.13065661 0.13082885 0.13122863 0.13150957 0.13216723

[443] 0.13245258 0.13263471 0.13294402 0.13310026 0.13340754 0.13377387
0.13387457 0.13434553 0.13460604 0.13527786 0.13537492 0.13544039 0.13564260
[456] 0.13586371 0.13588626 0.13611358 0.13629296 0.13686798 0.13698443
0.13707981 0.13722396 0.13744032 0.13756678 0.13778324 0.13795640 0.13811027
[469] 0.13825051 0.13855450 0.13861793 0.13898483 0.13920536 0.13979667
0.14003356 0.14015235 0.14048757 0.14095450 0.14132260 0.14136471 0.14164459
[482] 0.14176722 0.14210683 0.14220839 0.14233089 0.14273004 0.14283941
0.14320585 0.14347657 0.14358361 0.14395483 0.14440266 0.14456158 0.14480424
[495] 0.14500579 0.14508979 0.14539908 0.14576577 0.14602611 0.14613596
0.14631136 0.14644996 0.14671686 0.14696466 0.14704952 0.14719569 0.14730656
[508] 0.14799536 0.14835307 0.14850043 0.14874219 0.14887378 0.14906592
0.14960116 0.15007383 0.15044647 0.15060944 0.15075351 0.15100366 0.15115196
[521] 0.15178201 0.15200380 0.15227735 0.15246753 0.15278087 0.15315800
0.15348561 0.15372703 0.15417654 0.15446649 0.15486258 0.15519974 0.15545194
[534] 0.15573634 0.15582122 0.15591191 0.15613084 0.15655112 0.15670952
0.15693912 0.15706021 0.15720811 0.15750993 0.15780315 0.15788002 0.15821963
[547] 0.15837900 0.15840283 0.15870908 0.15910838 0.15921462 0.15936139
0.15949779 0.15982120 0.16016879 0.16025662 0.16049859 0.16076162 0.16129613
[560] 0.16161360 0.16184681 0.16197806 0.16204545 0.16233016 0.16254427
0.16298950 0.16327994 0.16344825 0.16393175 0.16430380 0.16482075 0.16495487
[573] 0.16525787 0.16550905 0.16577009 0.16597747 0.16618215 0.16626063
0.16655677 0.16732181 0.16748160 0.16788704 0.16855884 0.16873434 0.16891964
[586] 0.16907454 0.16937548 0.16993053 0.17024290 0.17089425 0.17155655
0.17194009 0.17245934 0.17256117 0.17266852 0.17307659 0.17336518 0.17392927
[599] 0.17423825 0.17441683 0.17462148 0.17472930 0.17488898 0.17501559
0.17538976 0.17565472 0.17602091 0.17666937 0.17675925 0.17705943 0.17724252
[612] 0.17732868 0.17761920 0.17783904 0.17788198 0.17820365 0.17885100
0.17900651 0.17921066 0.17954342 0.17972025 0.18052984 0.18069722 0.18105692
[625] 0.18124143 0.18149470 0.18154550 0.18179076 0.18196756 0.18218181
0.18245252 0.18308326 0.18334463 0.18363743 0.18391302 0.18433970 0.18451481
[638] 0.18489832 0.18524562 0.18540273 0.18548223 0.18591580 0.18593937
0.18676817 0.18701072 0.18728270 0.18750999 0.18783082 0.18796576 0.18830680
[651] 0.18851740 0.18855851 0.18872488 0.18913588 0.18935359 0.18973379
0.18999508 0.19049909 0.19065673 0.19078064 0.19134767 0.19149594 0.19155400
[664] 0.19185321 0.19261141 0.19284148 0.19322682 0.19335865 0.19373354
0.19448773 0.19472983 0.19506547 0.19520073 0.19552429 0.19558822 0.19571011
[677] 0.19598805 0.19601965 0.19619745 0.19635231 0.19690290 0.19709166
0.19732614 0.19760072 0.19809037 0.19867357 0.19900268 0.19931588 0.19961596
[690] 0.19991527 0.20006118 0.20014422 0.20094646 0.20096663 0.20139723
0.20190528 0.20244678 0.20290926 0.20360141 0.20377800 0.20387481 0.20423753
[703] 0.20443296 0.20448047 0.20469850 0.20523787 0.20584328 0.20610749
0.20623981 0.20713128 0.20754470 0.20782705 0.20818063 0.20882038 0.20912402
[716] 0.20921739 0.21004528 0.21039043 0.21048047 0.21080137 0.21114655
0.21142804 0.21205848 0.21225715 0.21285312 0.21287930 0.21319902 0.21341966
[729] 0.21361190 0.21424573 0.21433332 0.21452032 0.21473854 0.21612233
0.21650402 0.21693163 0.21708005 0.21767533 0.21885917 0.21920518 0.21937441
[742] 0.22008390 0.22026872 0.22110397 0.22138069 0.22163496 0.22242705
0.22300135 0.22324997 0.22447635 0.22473213 0.22508583 0.22533912 0.22548762
[755] 0.22587350 0.22656247 0.22689065 0.22783090 0.22834298 0.22915770
0.22933658 0.22946119 0.22978099 0.23039117 0.23111397 0.23131379 0.23170762
[768] 0.23193266 0.23236235 0.23274162 0.23401909 0.23412779 0.23466337
0.23478844 0.23550893 0.23562426 0.23714872 0.23763176 0.23832399 0.23850174
[781] 0.23883160 0.23899419 0.23920400 0.23964486 0.24034915 0.24055384
0.24209026 0.24216584 0.24230670 0.24275908 0.24334041 0.24403851 0.24506053

[794] 0.24520442 0.24538420 0.24571621 0.24640311 0.24872547 0.24896646
0.24978865 0.24995060 0.25001813 0.25185454 0.25234266 0.25280744 0.25337057
[807] 0.25412794 0.25453682 0.25492404 0.25629881 0.25643837 0.25780654
0.25811443 0.25885376 0.25909745 0.25914257 0.25920859 0.26048127 0.26060279
[820] 0.26140016 0.26147084 0.26216380 0.26284071 0.26307186 0.26412831
0.26435507 0.26452811 0.26497319 0.26560242 0.26639005 0.26773627 0.26792604
[833] 0.26817839 0.26867066 0.27047173 0.27074824 0.27290284 0.27414396
0.27620596 0.27694103 0.27755609 0.27816346 0.27818345 0.27929462 0.28086925
[846] 0.28264807 0.28351111 0.28466698 0.28482110 0.28499634 0.28673511
0.28796781 0.29016439 0.29111632 0.29226334 0.29331297 0.29401092 0.29474120
[859] 0.29674972 0.29681019 0.29966970 0.30041295 0.30083547 0.30154205
0.30286905 0.30398752 0.30449302 0.30545138 0.30609534 0.30831200 0.30860521
[872] 0.30987815 0.31061843 0.31354715 0.31741262 0.31845453 0.31908391
0.32191813 0.32232562 0.32321912 0.32340997 0.32603274 0.32701353 0.32842998
[885] 0.33628532 0.33651110 0.33739748 0.33845480 0.33952189 0.34131882
0.34339319 0.34467762 0.34692598 0.34900269 0.35059829 0.35249550 0.35862020
[898] 0.36136458 0.36224787 0.36346585 0.36423498 0.36959948 0.37090147
0.37629173 0.37827621 0.38223266 0.39154523 0.39238298 0.39499827 0.40243513
[911] 0.40539032 0.41016739 0.41317521 0.42017873 0.42218869 0.42528705
0.43906603 0.44733797 0.45973662 0.46237009 0.46866891 0.47843151 0.48139980
[924] 0.48150770 0.48294253 0.48303892 0.48433768 0.48904647 0.49497379
0.49942542 0.50880220 0.50892931 0.51082256 0.51587359 0.51791547 0.52298387
[937] 0.52744018 0.53007461 0.53276565 0.53547604 0.54132382 0.54152455
0.54247682 0.54314324 0.54403755 0.55143522 0.55242529 0.55421346 0.55423652
[950] 0.55621064 0.55653163 0.55702268 0.55924930 0.55932935 0.55993247
0.56234027 0.56262749 0.56394654 0.56424003 0.56486406 0.56502614 0.56512913
[963] 0.56553026 0.56747089 0.56800428 0.56836394 0.56885196 0.56952253
0.57002251 0.57021120 0.57027963 0.57209222 0.57240132 0.57530294 0.57578515
[976] 0.57684185 0.57716227 0.57848086 0.58134057 0.58281914 0.58297256
0.58323549 0.58370654 0.58567151 0.58747632 0.58766156 0.58779681 0.58807142
[989] 0.58904461 0.59306219 0.59452164 0.59459220 0.59651729 0.59918015
0.59974455 0.60047167 0.60064257 0.60240821 0.60359371 0.60457720 0.60603084
[1002] 0.60625724 0.60805675 0.60843588 0.60897486 0.60967822 0.61107080
0.61143880 0.61206551 0.61305174 0.61396500 0.61435919 0.61442749 0.61474385
[1015] 0.61544552 0.61591320 0.61639649 0.61797081 0.61865866 0.61937422
0.61984059 0.62038280 0.62241254 0.62308148 0.62368631 0.62390187 0.62442419
[1028] 0.62567290 0.62598793 0.62701370 0.62789467 0.62797095 0.62831378
0.63149380 0.63227774 0.63238219 0.63362079 0.63457018 0.63458694 0.63598602
[1041] 0.63618641 0.63786106 0.63794251 0.63859790 0.63898700 0.63907137
0.64210783 0.64253853 0.64275894 0.64297208 0.64317588 0.64318910 0.64514461
[1054] 0.64521202 0.64640958 0.64692876 0.64705604 0.64735422 0.64765522
0.64768409 0.64807242 0.64844554 0.65019027 0.65081451 0.65100917 0.65106119
[1067] 0.65113767 0.65258528 0.65284997 0.65335335 0.65364685 0.65475336
0.65489300 0.65546630 0.65628608 0.65697185 0.65704733 0.65731477 0.65765162
[1080] 0.65834044 0.65872697 0.65913035 0.66146343 0.66158455 0.66166703
0.66205686 0.66233180 0.66315360 0.66338356 0.66359027 0.66388041 0.66408037
[1093] 0.66476177 0.66629414 0.66630492 0.66665188 0.66780496 0.66781152
0.66943984 0.66961733 0.67051324 0.67317123 0.67384192 0.67403445 0.67414615
[1106] 0.67471470 0.67803450 0.67806637 0.67879453 0.67956755 0.68005980
0.68018784 0.68027894 0.68134958 0.68187173 0.68203652 0.68245053 0.68265088
[1119] 0.68287876 0.68315461 0.68369520 0.68403054 0.68432326 0.68449695
0.68940752 0.69177474 0.69220139 0.69269278 0.69275933 0.69359823 0.69367897
[1132] 0.69400730 0.69517321 0.69567241 0.69679944 0.69769045 0.69824259
0.69975654 0.70000598 0.70064849 0.70293283 0.70298342 0.70397392 0.70477584

[1145] 0.70670499 0.70827487 0.70835680 0.70869771 0.70880297 0.70933016
0.70948412 0.71151495 0.71162790 0.71164935 0.71174836 0.71206807 0.71357764
[1158] 0.71416540 0.71468996 0.71531420 0.71555480 0.71569138 0.71586149
0.71629377 0.71732966 0.71739389 0.71941414 0.71965626 0.72015262 0.72088579
[1171] 0.72172708 0.72180095 0.72337151 0.72441341 0.72466666 0.72541924
0.72554029 0.72591772 0.72653663 0.72717559 0.72795270 0.72799124 0.73292953
[1184] 0.73433544 0.73480148 0.73494213 0.73536587 0.73567038 0.73588203
0.73595214 0.73599446 0.73642067 0.73689765 0.73779750 0.74025840 0.74058675
[1197] 0.74140796 0.74208062 0.74209330 0.74215924 0.74337868 0.74380916
0.74392941 0.74506768 0.74536122 0.74542846 0.74640319 0.74676273 0.74701690
[1210] 0.74770294 0.74805790 0.74907307 0.74937765 0.74986415 0.75096261
0.75103154 0.75211367 0.75232991 0.75289702 0.75307360 0.75371222 0.75417464
[1223] 0.75543829 0.75608641 0.75739756 0.75810490 0.75862791 0.75993365
0.76095221 0.76106354 0.76120006 0.76215442 0.76216032 0.76225836 0.76254259
[1236] 0.76380833 0.76480682 0.76641828 0.76701391 0.76761439 0.76788521
0.76957903 0.77050102 0.77204388 0.77373026 0.77511725 0.77550375 0.77654681
[1249] 0.77788608 0.77851693 0.78024634 0.78044320 0.78091450 0.78291380
0.78326975 0.78387514 0.78405225 0.78500516 0.78564529 0.78774322 0.78833502
[1262] 0.78941030 0.79005633 0.79060059 0.79070687 0.79123851 0.79307900
0.79356389 0.79356923 0.79403747 0.79720485 0.80015418 0.80096969 0.80120758
[1275] 0.80142444 0.80170783 0.80211912 0.80226499 0.80243588 0.80282904
0.80391282 0.80520415 0.80636692 0.80664662 0.80671642 0.80829163 0.80853957
[1288] 0.80867588 0.81018213 0.81333408 0.81488068 0.81622609 0.81825988
0.81862800 0.81925981 0.81973825 0.82129934 0.82328895 0.82518613 0.82708301
[1301] 0.82787252 0.82881857 0.82906835 0.83043728 0.83244637 0.83307564
0.83463380 0.83465870 0.83472369 0.83489950 0.83534341 0.83601265 0.83616175
[1314] 0.83643109 0.83776243 0.83824388 0.83961638 0.84205626 0.84239420
0.84489591 0.84581984 0.84686064 0.84935533 0.84997607 0.85161307 0.85316089
[1327] 0.85457296 0.85760793 0.86055565 0.86638367 0.86660723 0.86669697
0.87027727 0.87109444 0.87185617 0.87209360 0.87349126 0.88233055 0.88578920
[1340] 0.88782433 0.88834906 0.88980436 0.89169204 0.89667303 0.89719125
0.90015873 0.90629176 0.92129078 0.93066249 0.93494934 0.94546961 0.95158727
[1353] 0.95560188 0.95726628 0.96743065 0.96813122 0.97773121 0.98413006
0.98432030 0.98965920 1.00267762 1.01131215 1.02132159 1.06409111 1.08917220
[1366] 1.08959257 1.10671565 1.10912367

FLUCTUATION DATA for TRUNCATED MINIMIZED CHARACTERIZED Q5NAT0

[1] 0.51997966 0.26663928 0.31464325 0.20187998 0.12860179 0.08346960
0.07576905 0.08934334 0.08354220 0.07449898 0.07453790 0.06133781 0.06237938
[14] 0.08997126 0.08582535 0.07941264 0.08704734 0.09312616 0.09154202
0.09037601 0.09747112 0.09008117 0.08879760 0.16266032 0.23063794 0.35707352
[27] 0.74210603 0.58375856 0.30470129 0.24315798 0.18626020 0.23373002
0.24345555 0.19273832 0.21981659 0.14967302 0.11422376 0.11129366 0.15844546
[40] 0.18260642 0.11117093 0.11449729 0.19170806 0.30132424 0.31485878
0.30812669 0.24996779 0.19421593 0.11595715 0.12976365 0.09712296 0.09217372
[53] 0.08163066 0.07575464 0.08475435 0.12655837 0.12333451 0.11860965
0.09855519 0.09925317 0.07979125 0.08869799 0.09230108 0.08134885 0.07912675
[66] 0.06881699 0.06328879 0.06854631 0.06688949 0.06285423 0.05908912
0.07029353 0.07192618 0.07095056 0.08334523 0.08663065 0.09629778 0.12166172
[79] 0.13247767 0.11864202 0.13161835 0.13920276 0.09717115 0.09889682
0.12212161 0.13841921 0.12486993 0.13217325 0.13442577 0.12183425 0.14591982
[92] 0.12211834 0.10295895 0.11107180 0.11239014 0.10237312 0.09778594
0.09210077 0.09501880 0.08184181 0.08671035 0.09825466 0.08797529 0.09406944

[105] 0.10687649 0.09781794 0.09754155 0.10516093 0.23831406 0.20815122
0.17005303 0.14895482 0.13550033 0.09924419 0.09331360 0.10494197 0.08886902
[118] 0.08965470 0.10989908 0.15682069 0.17179627 0.20545778 0.18497901
0.13056155 0.17747246 0.25034458 0.20944342 0.13942610 0.13322758 0.12623126
[131] 0.12417923 0.13421948 0.18839358 0.22442939 0.37966420 0.26874222
0.32035648 0.15819286 0.15764679 0.11184370 0.10669419 0.10543434 0.10679767
[144] 0.13762654 0.18149762 0.34536537 0.18120738 0.10377021 0.07506042
0.06611103 0.06558762 0.06591619 0.05650682 0.04946252 0.05670570 0.05744076
[157] 0.05418441 0.05220439 0.04941999 0.05601586 0.05506951 0.06428027
0.07087582 0.08086522 0.11081169 0.12429638 0.13145121 0.16047295 0.38401052
[170] 0.37166374 0.27076756 0.21549918 0.28819350 0.20431813 0.12423270
0.14873738 0.14059269 0.08683547 0.09064563 0.11970952 0.09944962 0.07352204
[183] 0.09425327 0.09430115 0.07315197 0.07770700 0.11433310 0.09890597
0.07840559 0.12705671 0.15699974 0.15451663 0.15180210 0.12404907 0.10565766
[196] 0.10462301 0.12573964 0.18584141 0.15097619 0.16504912 0.17925247
0.15114710 0.13778469 0.27427214 0.25311006 0.19930276 0.16568131 0.15041303
[209] 0.21133813 0.15310753 0.13350269 0.10702716 0.09801864 0.08028291
0.06608852 0.07467441 0.07632399 0.07252836 0.05773122 0.07101084 0.08071882
[222] 0.07472776 0.07852120 0.09759109 0.13088250 0.12559308 0.18113936
0.25135930 0.27177222 0.27061535 0.23462614 0.11924957 0.13658303 0.18112515
[235] 0.15105309 0.11251965 0.17013379 0.20713892 0.16562693 0.18739370
0.30022957 0.27988190 0.23172181 0.28318576 0.34498747 0.44735321 0.95911150
[248] 1.53141173 0.69541001 0.30939819 0.32663874 0.18735594 0.10837995
0.14887501 0.16145143 0.15617511 0.09832618 0.09136889 0.09555003 0.09531429
[261] 0.10138815 0.09396441 0.08634522 0.09368494 0.11481676 0.11830112
0.11708199 0.15847024 0.22366167 0.25091993 0.23489160 0.29624181 0.28867703
[274] 0.24441456 0.13006843 0.14199673 0.17742479 0.13937396 0.11133403
0.12980783 0.14712401 0.13260792 0.12532682 0.13000641 0.13225781 0.16147290
[287] 0.20713213 0.23322597 0.21436051 0.37078027 0.72913831 0.55985820
0.51930965 0.28305908 0.21971320 0.16249630 0.17001815 0.17692523 0.28583374
[300] 0.21822024 0.18073196 0.16055477 0.13616318 0.14545019 0.20427223
0.25696547 0.17533113 0.12905435 0.08453214 0.09121784 0.08226232 0.08207552
[313] 0.08413639 0.07915938 0.06679546 0.07378860 0.08245048 0.07769275
0.07865532 0.08590424 0.08473424 0.08490817 0.11567412 0.10700380 0.11201630
[326] 0.20804708 0.22405844 0.21808118 0.44989350 0.35857442 0.34703751
0.33470779 0.21882966 0.15562042 0.17592746 0.16596284 0.10684376 0.10936581
[339] 0.14271799 0.12209175 0.09924842 0.10673645 0.11053533 0.09896945
0.09884283 0.09748052 0.08948170 0.10144221 0.08913045 0.09809574 0.11729752
[352] 0.18016946 0.23237672 0.24095020 0.15189815 0.13638613 0.08078145
0.08745849 0.07855956 0.07659978 0.10118319 0.11996108 0.14200671 0.17016848
[365] 0.17628432 0.10250825 0.12190219 0.15453945 0.10482280 0.09794932
0.08927706 0.08859278 0.09413157 0.10702245 0.10146327 0.09429603 0.11971822
[378] 0.14098781 0.26082933 0.30489366 0.19112009 0.22903307 0.23274103
0.28860593 0.26370575 0.27046001 0.32817487 0.18877548 0.11853888 0.16370263
[391] 0.14451620 0.15659064 0.12024132 0.07950353 0.06181041 0.07449489
0.06762435 0.06831407 0.07087665 0.09969888 0.17083727 0.19165661 0.20164121
[404] 0.21942797 0.20015827 0.23912825 0.29663618 0.27103972 0.19200586
0.17320120 0.11787894 0.17916369 0.15342779 0.11209307 0.16188252 0.09463164
[417] 0.07521749 0.08503482 0.07746891 0.09840398 0.08342307 0.07187625
0.06490586 0.07163242 0.06770885 0.07187150 0.06963464 0.07063510 0.07272841
[430] 0.06942962 0.06711618 0.07639721 0.09433852 0.09771468 0.08800750
0.14000791 0.17395356 0.17495442 0.14730460 0.13196394 0.18640732 0.13760548
[443] 0.29724494 0.69215360 0.82989887 0.45908550 0.35553335 0.47299089
0.37385141 0.35909441 0.85611534 0.79626452 0.97280959 1.75539450 2.15749803

[456] 2.82380533

MASSES for TRUNCATED MINIMIZED CHARACTERIZED Q5NAT0

```
 [1]  58.05894  57.05100  57.05100 137.13900 114.07900 163.17300  57.05100
131.19600  71.07800 113.15800  87.07700 129.18000  87.07700 113.15800 113.15800
 [16] 163.17300 147.17400 128.10600  71.07800 117.12600 157.19400  87.07700
57.05100  99.13100 113.15800  97.11500  57.05100  87.07700 117.12600 157.19400
 [31] 113.15800  71.07800 186.21000 157.19400  71.07800 114.10300  87.07700
57.05100 113.15800  71.07800 114.07900  57.05100 129.18000  71.07800 114.10300
 [46]  57.05100  99.13100 114.07900 113.15800  99.13100  57.05100  57.05100
163.17300 163.17300 114.07900  71.07800  57.05100 114.07900 114.10300  99.13100
 [61] 129.18000 147.17400  57.05100 113.15800  97.11500 131.19600  71.07800
147.17400 101.10400  99.13100 101.10400 131.19600 131.19600  71.07800 186.21000
 [76]  87.07700  99.13100 113.15800 128.10600 163.17300  57.05100 128.10600
128.10600 131.19600  71.07800  71.07800  71.07800  57.05100 128.10600 113.15800
 [91]  57.05100 137.13900  71.07800  99.13100 128.10600  71.07800 113.15800
129.18000 186.21000  57.05100 101.10400 114.07900 163.17300 147.17400  71.07800
[106] 129.18000  71.07800 137.13900  97.11500 128.10600  97.11500 114.10300
99.13100 113.15800 163.17300  71.07800 128.10600  99.13100  57.05100 114.07900
[121]  57.05100 114.07900  87.07700 114.07900 137.13900 114.10300 103.14300
186.21000 117.12600 157.19400  97.11500 128.10600 114.07900 131.19600 101.10400
[136] 101.10400  87.07700 157.19400 117.12600  71.07800 163.17300 157.19400
113.15800 114.07900  97.11500 117.12600 114.10300  97.11500  57.05100  87.07700
[151] 114.07900 113.15800  71.07800  57.05100 128.10600 101.10400  71.07800
71.07800  71.07800 131.19600  71.07800  71.07800  71.07800  87.07700 113.15800
[166]  99.13100 147.17400 157.19400  87.07700  87.07700 114.10300  97.11500
57.05100 163.17300  71.07800 114.07900 117.12600 113.15800 113.15800 117.12600
[181] 137.13900  87.07700 129.18000 117.12600 113.15800 147.17400 114.07900
147.17400  71.07800 114.07900 129.18000 163.17300 157.19400  57.05100 157.19400
[196] 163.17300 114.07900 114.10300  87.07700 113.15800 101.10400  99.13100
71.07800 157.19400 114.10300 163.17300 163.17300  57.05100  87.07700  87.07700
[211]  57.05100 163.17300  57.05100 114.07900 128.10600 113.15800 113.15800
186.21000  71.07800  87.07700  71.07800 186.21000 113.15800 163.17300 117.12600
[226]  71.07800  87.07700 114.07900 114.07900 157.19400 157.19400 163.17300
113.15800 114.07900 163.17300 113.15800  71.07800 114.10300 114.10300  71.07800
[241] 114.07900  71.07800 113.15800  57.05100  57.05100 101.10400  57.05100
186.21000  87.07700 113.15800 114.10300 117.12600 147.17400  57.05100 186.21000
[256] 114.07900  99.13100 129.18000 163.17300  97.11500  57.05100  99.13100
117.12600 113.15800 113.15800  71.07800  71.07800 129.18000 147.17400 113.15800
[271] 113.15800 117.12600 117.12600  57.05100 163.17300 157.19400 157.19400
129.18000  71.07800 114.07900 147.17400 147.17400  71.07800 103.14300  87.07700
[286] 103.14300 113.15800  57.05100 129.18000 114.07900  71.07800  71.07800
114.07900 114.10300  99.13100  57.05100 157.19400 101.10400  97.11500  57.05100
[301]  57.05100 131.19600 113.15800 163.17300 137.13900 117.12600 157.19400
186.21000 114.10300 114.10300 113.15800 117.12600 147.17400  99.13100 101.10400
[316]  87.07700  71.07800  87.07700 147.17400 113.15800 113.15800  71.07800
99.13100 163.17300  87.07700 114.07900 137.13900 113.15800  71.07800  57.05100
[331]  57.05100  71.07800  57.05100  71.07800  71.07800 128.10600 113.15800
113.15800  71.07800 147.17400  71.07800 129.18000  87.07700 117.12600  99.13100
[346] 114.07900 163.17300 113.15800 113.15800  57.05100  87.07700 114.10300
97.11500 157.19400  57.05100 101.10400  87.07700 163.17300 131.19600  99.13100
[361]  57.05100 163.17300  57.05100  71.07800  99.13100 163.17300  97.11500
157.19400 117.12600  71.07800 137.13900 137.13900 157.19400  57.05100  87.07700
```

```
[376]  87.07700 113.15800  71.07800 163.17300  71.07800  87.07700 186.21000
163.17300  57.05100 157.19400 157.19400  57.05100  57.05100 114.10300  97.11500
[391] 114.10300 113.15800 113.15800 114.07900  57.05100  71.07800  99.13100
99.13100  57.05100  57.05100  97.11500 114.07900 128.10600 137.13900 114.07900
[406] 114.07900 147.17400  71.07800 114.07900 128.10600 157.19400 114.10300
114.10300 163.17300 128.10600 117.12600 101.10400 128.10600  71.07800  71.07800
[421] 101.10400 163.17300 114.10300 114.10300  71.07800  97.11500 113.15800
131.19600  57.05100 113.15800 113.15800  71.07800 157.19400 113.15800  71.07800
[436]  71.07800  57.05100 137.13900  57.05100  57.05100 117.12600  87.07700
113.15800 117.12600 137.13900  57.05100 113.15800  71.07800  71.07800 114.10300
[451] 137.13900 101.10400  87.07700 113.15800  97.11500 154.14634

Call:
  nma.pdb(pdb = pdbT4_LJP6, mass = TRUE)

Class:
  VibrationalModes (nma)

Number of modes:
  1392 (6 trivial)

Frequencies:
  Mode 7:       0.001
  Mode 8:       0.001
  Mode 9:       0.001
  Mode 10:      0.004
  Mode 11:      0.004
  Mode 12:      0.006

+ attr: modes, frequencies, force.constants, fluctuations,
        U, L, xyz, mass, temp, triv.modes, natoms, call

 NORMAL MODES for TRUNCATED MINIMIZED CHARACTERIZED Q8LJP6

  [1] 0.0000000000 0.0000000000 0.0000000000 0.0000000000 0.0000000000
0.0000000000 0.0007632803 0.0013124253 0.0013965790 0.0037359059 0.0044163704
  [12] 0.0064668631 0.0072673012 0.0087027343 0.0093698882 0.0109689975
0.0137813845 0.0149062858 0.0157120675 0.0158708684 0.0176029972 0.0180737239
  [23] 0.0192551173 0.0207494347 0.0217722566 0.0228041877 0.0229795709
0.0234190428 0.0239743560 0.0243106245 0.0244922859 0.0255462276 0.0260635354
  [34] 0.0266478801 0.0268500568 0.0276426067 0.0283282386 0.0293937249
0.0301274044 0.0306756445 0.0310702898 0.0312535897 0.0318174576 0.0323104134
  [45] 0.0331676763 0.0335971524 0.0339141968 0.0345604510 0.0350415671
0.0355332792 0.0356464439 0.0360073770 0.0366554255 0.0368487518 0.0373275178
  [56] 0.0375676469 0.0380782983 0.0386464777 0.0388745519 0.0390053017
0.0393881957 0.0398930048 0.0403475830 0.0405017279 0.0407694578 0.0415132109
  [67] 0.0416481453 0.0421144550 0.0424746944 0.0427407333 0.0433570900
0.0436119394 0.0439359897 0.0440905155 0.0451335470 0.0451958006 0.0455750399
  [78] 0.0462234839 0.0463643782 0.0468367336 0.0474166890 0.0477050847
0.0479183263 0.0487196132 0.0488660106 0.0491108418 0.0493939616 0.0495212338
  [89] 0.0498856184 0.0501709295 0.0506957837 0.0509295818 0.0511380460
0.0513831432 0.0514213341 0.0521202672 0.0522762818 0.0524105710 0.0528061135
 [100] 0.0528660401 0.0531899058 0.0534184783 0.0535964757 0.0540152275
0.0546448711 0.0548726915 0.0550043260 0.0550641603 0.0554759434 0.0555181628
```

[111] 0.0557674015 0.0561178614 0.0565038154 0.0568100666 0.0569761359
0.0574073005 0.0575386392 0.0579070339 0.0583649626 0.0584404295 0.0586662478
[122] 0.0590537763 0.0591521352 0.0597556342 0.0599950808 0.0603096442
0.0604617040 0.0608906512 0.0612335070 0.0615240395 0.0616765962 0.0618588809
[133] 0.0623527957 0.0626623956 0.0627179532 0.0631048848 0.0633574619
0.0636100317 0.0637035429 0.0640267894 0.0642349429 0.0646167109 0.0649270245
[144] 0.0652319790 0.0654395891 0.0657070946 0.0657699016 0.0662071173
0.0664174001 0.0666815528 0.0669488252 0.0670939555 0.0671833717 0.0676095743
[155] 0.0677044829 0.0679533832 0.0681010221 0.0685944880 0.0686884045
0.0689859116 0.0691427031 0.0693680065 0.0695711023 0.0698387414 0.0701192681
[166] 0.0705316818 0.0707375241 0.0709491958 0.0711732293 0.0713101178
0.0714997271 0.0718309123 0.0720029698 0.0722097044 0.0727150005 0.0727555720
[177] 0.0728604655 0.0729737129 0.0731387532 0.0733878612 0.0735528332
0.0736969881 0.0738837291 0.0742378910 0.0744615548 0.0747264237 0.0747952037
[188] 0.0750845388 0.0755505011 0.0758491450 0.0759916105 0.0761165067
0.0763318456 0.0765018924 0.0765576636 0.0768573376 0.0771633982 0.0772477170
[199] 0.0775231622 0.0775935438 0.0777842817 0.0779012642 0.0781731105
0.0783676095 0.0785754901 0.0787805715 0.0789241644 0.0790932816 0.0793088421
[210] 0.0796016594 0.0798194965 0.0801079178 0.0802759610 0.0804595533
0.0806804114 0.0808655913 0.0811000245 0.0813667875 0.0819366683 0.0819655683
[221] 0.0823038824 0.0824454866 0.0827106664 0.0827129633 0.0829264456
0.0833380936 0.0834113125 0.0834852256 0.0837976092 0.0839160191 0.0840096921
[232] 0.0843261406 0.0844196586 0.0845461857 0.0847557978 0.0849221009
0.0850654511 0.0854549413 0.0858017864 0.0859561944 0.0860969403 0.0862073441
[243] 0.0862715221 0.0865336125 0.0865859939 0.0868707569 0.0871628723
0.0874922363 0.0875842536 0.0880834546 0.0882591294 0.0883123517 0.0887628107
[254] 0.0889735920 0.0892532918 0.0893758103 0.0896148332 0.0897211906
0.0898995822 0.0901013791 0.0901720559 0.0903449311 0.0905841932 0.0909185759
[265] 0.0910816916 0.0913473120 0.0914931922 0.0918002621 0.0920847175
0.0921189581 0.0923730957 0.0926566126 0.0927978415 0.0929955284 0.0933469181
[276] 0.0935430386 0.0936412829 0.0939259608 0.0940551853 0.0944016916
0.0945317395 0.0947971786 0.0949909715 0.0952959410 0.0954337949 0.0955775458
[287] 0.0958119367 0.0959204013 0.0961793745 0.0964791437 0.0965783354
0.0967821719 0.0969133380 0.0971079943 0.0973053851 0.0975741821 0.0976355583
[298] 0.0979132800 0.0979652651 0.0984544107 0.0984742192 0.0988716059
0.0990635649 0.0991319404 0.0993738791 0.0994445884 0.0997233682 0.0998469923
[309] 0.0999301684 0.1000806222 0.1001744779 0.1002660984 0.1005027866
0.1006375354 0.1008880410 0.1011687271 0.1012444378 0.1015822555 0.1017213018
[320] 0.1018162573 0.1020503431 0.1021289966 0.1023233853 0.1026368004
0.1027517428 0.1029154245 0.1031118927 0.1033921764 0.1036625395 0.1037082235
[331] 0.1038603991 0.1040850209 0.1042653173 0.1045635864 0.1046553580
0.1047463238 0.1050351485 0.1051219303 0.1053110351 0.1056762456 0.1060038831
[342] 0.1061582559 0.1063931451 0.1065393460 0.1067717361 0.1072810572
0.1075172629 0.1076880498 0.1078363710 0.1078578618 0.1080487424 0.1083015182
[353] 0.1084988559 0.1086841827 0.1087869150 0.1090421610 0.1091764628
0.1092789642 0.1094156377 0.1096242565 0.1098352466 0.1100017440 0.1100147536
[364] 0.1102437237 0.1106272260 0.1107695537 0.1107911614 0.1111583061
0.1114615371 0.1115092506 0.1118886417 0.1120699451 0.1121998326 0.1125743089
[375] 0.1126502240 0.1128480377 0.1129364418 0.1131265902 0.1135343809
0.1136084282 0.1137307682 0.1139911657 0.1140661379 0.1145098525 0.1147013675
[386] 0.1148930043 0.1151975088 0.1152986119 0.1153871142 0.1156214382
0.1159666305 0.1162444650 0.1163049180 0.1164267090 0.1165292454 0.1167942476
[397] 0.1169884104 0.1172323902 0.1174525739 0.1177071052 0.1178530264
0.1179254360 0.1181969518 0.1184662418 0.1184721217 0.1187414276 0.1188121232

[408] 0.1189058925 0.1191631651 0.1197216547 0.1198437778 0.1200473569
0.1202037134 0.1204683074 0.1205885191 0.1207102899 0.1209125148 0.1211585232
[419] 0.1212238392 0.1216161438 0.1218056358 0.1219902653 0.1220825273
0.1220976728 0.1226965910 0.1230812175 0.1232052533 0.1235790734 0.1236116597
[430] 0.1239358253 0.1242249956 0.1245443107 0.1247756483 0.1248755891
0.1251876807 0.1253390396 0.1255880754 0.1258791851 0.1261904005 0.1262748799
[441] 0.1265029527 0.1268108395 0.1271225637 0.1271975625 0.1275418104
0.1276655798 0.1280835435 0.1283130406 0.1287837899 0.1290296127 0.1295191740
[452] 0.1296042198 0.1297933688 0.1301717178 0.1301962340 0.1302126729
0.1305086061 0.1308777267 0.1312225508 0.1313657997 0.1314929047 0.1316570247
[463] 0.1319491427 0.1321775814 0.1322185856 0.1323768309 0.1324754351
0.1326317506 0.1332023238 0.1334281424 0.1336025883 0.1339400193 0.1341252257
[474] 0.1346058518 0.1349495025 0.1352538935 0.1354354329 0.1356684570
0.1357887366 0.1361763771 0.1363285420 0.1365334220 0.1367955021 0.1371954382
[485] 0.1373170550 0.1374526626 0.1376057172 0.1377388339 0.1379098475
0.1381397979 0.1384080785 0.1385325620 0.1387224102 0.1388115801 0.1391954429
[496] 0.1392702151 0.1393847516 0.1396283287 0.1397809944 0.1399794642
0.1402716766 0.1404144429 0.1405690479 0.1409389587 0.1412082893 0.1412716868
[507] 0.1417639098 0.1420470127 0.1422449029 0.1424831461 0.1428194598
0.1428823197 0.1432331709 0.1434936060 0.1438751883 0.1439438343 0.1442820100
[518] 0.1444479952 0.1448206790 0.1450271856 0.1452868456 0.1454726677
0.1460667847 0.1461133395 0.1464258345 0.1467924616 0.1469056164 0.1472661471
[529] 0.1473455913 0.1474149410 0.1476608822 0.1480601251 0.1483353954
0.1483967722 0.1488121697 0.1490332020 0.1491759884 0.1493804590 0.1498916826
[540] 0.1499840921 0.1503477705 0.1505906881 0.1506718258 0.1510500316
0.1513230435 0.1518248067 0.1518565028 0.1519663036 0.1523092017 0.1524702693
[551] 0.1525271592 0.1527155331 0.1528827174 0.1531354259 0.1534819010
0.1536278074 0.1538152451 0.1539157495 0.1541177941 0.1546846862 0.1548262689
[562] 0.1549508038 0.1551957382 0.1557411425 0.1559409846 0.1562560342
0.1565301609 0.1566247992 0.1566874555 0.1568537167 0.1571564604 0.1572389623
[573] 0.1575189365 0.1577086561 0.1581880043 0.1583633271 0.1585633789
0.1590418233 0.1593849945 0.1595980502 0.1602340161 0.1604199721 0.1607806836
[584] 0.1610969604 0.1615438353 0.1616716562 0.1624504257 0.1626725468
0.1631421236 0.1633051482 0.1634316691 0.1638683903 0.1640947666 0.1644769124
[595] 0.1647916289 0.1651596633 0.1655224416 0.1656935199 0.1659378623
0.1660584878 0.1662126325 0.1668853988 0.1670965466 0.1673573067 0.1674279743
[606] 0.1675916665 0.1679470047 0.1682261726 0.1687519044 0.1689852295
0.1690433793 0.1697942330 0.1700298522 0.1704457304 0.1709366322 0.1711160645
[617] 0.1714008591 0.1718073741 0.1720244077 0.1723069659 0.1728732611
0.1730691276 0.1733247751 0.1738832397 0.1740984130 0.1744892081 0.1751245417
[628] 0.1753633269 0.1754261490 0.1756615681 0.1758084459 0.1760545047
0.1764148370 0.1768250812 0.1770645772 0.1771492466 0.1773376053 0.1776805100
[639] 0.1780945947 0.1782815283 0.1787278832 0.1789705650 0.1791981474
0.1794133703 0.1795903278 0.1802903795 0.1804607924 0.1808619295 0.1811615353
[650] 0.1815456375 0.1818629905 0.1820221206 0.1824433592 0.1828002435
0.1830355886 0.1831930778 0.1834600920 0.1838994562 0.1840813207 0.1844177986
[661] 0.1846992257 0.1848553662 0.1849240728 0.1855286586 0.1862793539
0.1864686102 0.1865258588 0.1868811818 0.1872607889 0.1875106642 0.1879752615
[672] 0.1882973156 0.1884815218 0.1886849478 0.1891450537 0.1893523152
0.1896392424 0.1898464980 0.1900364671 0.1901937529 0.1904497553 0.1906344063
[683] 0.1907941864 0.1909223918 0.1918664823 0.1921422069 0.1922404617
0.1923367568 0.1929020941 0.1929259257 0.1932141009 0.1939574479 0.1943379614
[694] 0.1946563203 0.1954645609 0.1959122360 0.1959684063 0.1964425937
0.1965836089 0.1967686829 0.1970756608 0.1971363822 0.1973734987 0.1976555740

[705] 0.1980520045 0.1985538767 0.1988174608 0.1990358401 0.1993487297
0.1998572316 0.2001629528 0.2006188935 0.2008925664 0.2010623363 0.2013841525
[716] 0.2014973864 0.2017360946 0.2019843026 0.2024950051 0.2026965514
0.2031807185 0.2031954913 0.2034962596 0.2036190798 0.2038541853 0.2040429684
[727] 0.2043732911 0.2051270755 0.2057544739 0.2062105808 0.2066394503
0.2072356875 0.2079547379 0.2080853955 0.2081459476 0.2084488691 0.2088800551
[738] 0.2093881530 0.2099228360 0.2100505811 0.2103836318 0.2107586461
0.2115534416 0.2120522671 0.2124377502 0.2130953342 0.2133738392 0.2138910986
[749] 0.2143165950 0.2144845968 0.2155365769 0.2159019371 0.2164016248
0.2165830967 0.2170966768 0.2173057788 0.2174507958 0.2176427407 0.2177119787
[760] 0.2185632596 0.2187108016 0.2192050686 0.2194731084 0.2202546862
0.2206838102 0.2208482874 0.2212459799 0.2223047623 0.2228709696 0.2231035559
[771] 0.2236260048 0.2237258307 0.2244493835 0.2244936184 0.2262206549
0.2265939962 0.2267811065 0.2269308958 0.2271241971 0.2276030876 0.2280249923
[782] 0.2284981704 0.2290589657 0.2295486067 0.2297163300 0.2304115085
0.2309502748 0.2315586283 0.2317669234 0.2318084507 0.2325071244 0.2328454767
[793] 0.2335402346 0.2336596752 0.2342912128 0.2349564164 0.2356282886
0.2358945258 0.2363427862 0.2377485605 0.2388128274 0.2389353040 0.2392276686
[804] 0.2397463596 0.2400373670 0.2403617999 0.2413565690 0.2415120019
0.2418843611 0.2429377566 0.2439329241 0.2443148647 0.2456170229 0.2456763666
[815] 0.2462645429 0.2467624897 0.2469520124 0.2473519235 0.2482736003
0.2491220758 0.2492371999 0.2494380947 0.2497572635 0.2498908989 0.2504253341
[826] 0.2517776370 0.2518436761 0.2523177135 0.2530890893 0.2531389264
0.2535896673 0.2552681563 0.2557110331 0.2558011108 0.2566316087 0.2569471654
[837] 0.2572845371 0.2589055697 0.2591054205 0.2608391231 0.2617975454
0.2624481419 0.2628357030 0.2632074404 0.2642522329 0.2646933797 0.2653568750
[848] 0.2653647978 0.2669574041 0.2674045555 0.2695308110 0.2702028643
0.2704465864 0.2707664369 0.2719767991 0.2728440774 0.2736649693 0.2740366673
[859] 0.2744551199 0.2751505897 0.2762122460 0.2777388739 0.2781678752
0.2790155925 0.2792699455 0.2797934394 0.2801467443 0.2803146905 0.2806354340
[870] 0.2833448374 0.2840107808 0.2849517141 0.2869733984 0.2872988311
0.2884551516 0.2887720726 0.2909614414 0.2915591717 0.2927119890 0.2934630716
[881] 0.2945894368 0.2948394613 0.2972878013 0.2979361831 0.3010774488
0.3019310636 0.3022305854 0.3027121579 0.3053160996 0.3066062458 0.3068594804
[892] 0.3070315424 0.3078151235 0.3098740202 0.3111949647 0.3140452500
0.3170582181 0.3186642861 0.3191711022 0.3217691814 0.3221356203 0.3304981826
[903] 0.3349926498 0.3361182463 0.3370726229 0.3384860095 0.3429528679
0.3445803024 0.3448303681 0.3450579004 0.3472102148 0.3483998039 0.3557270705
[914] 0.3580021257 0.3609287432 0.3662916352 0.3674483695 0.3715116852
0.3728098126 0.3821811943 0.3869481864 0.3900007366 0.3911108623 0.3982825342
[925] 0.4025045502 0.4053612663 0.4145736683 0.4240503289 0.4298282082
0.4303238285 0.4319500341 0.4330900495 0.4343804931 0.4423209943 0.4560611207
[936] 0.4564235513 0.4618400853 0.4637793338 0.4650301272 0.4750100794
0.4757809451 0.4813861669 0.4840161924 0.4913100977 0.4920038438 0.4938525567
[947] 0.4949948757 0.4951201310 0.4988854558 0.5011708796 0.5100981723
0.5141400363 0.5146913375 0.5174170770 0.5191182667 0.5222159873 0.5224587004
[958] 0.5270022724 0.5277281529 0.5288529331 0.5290774969 0.5308353727
0.5315345745 0.5348174231 0.5387779650 0.5393521744 0.5419064318 0.5452072742
[969] 0.5480030803 0.5491508873 0.5500677123 0.5505129911 0.5510723989
0.5515749629 0.5527227536 0.5539834021 0.5569071846 0.5587753034 0.5594438876
[980] 0.5610044911 0.5610457582 0.5613340242 0.5634836425 0.5641644719
0.5661617831 0.5682965724 0.5701694238 0.5717632858 0.5718788582 0.5747545781
[991] 0.5748275338 0.5770350730 0.5776165512 0.5776423582 0.5783611142
0.5787462434 0.5800398353 0.5829852952 0.5832699054 0.5838627016 0.5841857362

[1002] 0.5862290152 0.5872763917 0.5884224288 0.5910775892 0.5922758702
0.5928240485 0.5932190672 0.5941656909 0.5942959378 0.5943826467 0.5951704380
[1013] 0.5952607216 0.5958515573 0.5973682292 0.5989959041 0.5996005489
0.6001755906 0.6010632188 0.6027218708 0.6034825749 0.6038749209 0.6039617644
[1024] 0.6048961410 0.6052354463 0.6053909692 0.6060399343 0.6064249451
0.6065024232 0.6071600253 0.6077757196 0.6083941215 0.6088108461 0.6091383651
[1035] 0.6098664783 0.6106421548 0.6111059896 0.6119479001 0.6122853040
0.6129085168 0.6134548984 0.6134812003 0.6140426449 0.6147217466 0.6147649703
[1046] 0.6158964202 0.6161936182 0.6181511197 0.6181660148 0.6182232564
0.6193106792 0.6203450144 0.6205308157 0.6213880229 0.6217531819 0.6218683232
[1057] 0.6226412932 0.6227872838 0.6234357737 0.6240382108 0.6255191998
0.6265082937 0.6265687754 0.6267677458 0.6268332537 0.6268712581 0.6271164432
[1068] 0.6285524808 0.6296425213 0.6298906893 0.6313416009 0.6314537704
0.6320265026 0.6320461004 0.6329765166 0.6334093207 0.6334291956 0.6335388763
[1079] 0.6342927126 0.6365862296 0.6370313135 0.6377235299 0.6383418473
0.6386844240 0.6389361170 0.6391878099 0.6404991318 0.6405057560 0.6413783075
[1090] 0.6422721737 0.6425626716 0.6434357603 0.6456553156 0.6462638298
0.6465167838 0.6475209422 0.6476287840 0.6487226936 0.6488680081 0.6497652152
[1101] 0.6498926406 0.6498928745 0.6499511606 0.6500930834 0.6510090687
0.6515405665 0.6519729551 0.6543275877 0.6552260559 0.6559876568 0.6567948489
[1112] 0.6568747924 0.6571930047 0.6576595775 0.6581870904 0.6589191673
0.6590739750 0.6602245627 0.6611096910 0.6617960895 0.6619420162 0.6621462329
[1123] 0.6630323871 0.6635058867 0.6636785554 0.6640349540 0.6646713253
0.6646842443 0.6654812876 0.6664771492 0.6680864622 0.6682296885 0.6684413253
[1134] 0.6690797334 0.6697960665 0.6698873716 0.6701616093 0.6717975206
0.6719696796 0.6722985874 0.6733896424 0.6738381779 0.6742969523 0.6750353860
[1145] 0.6763263230 0.6766645868 0.6785827029 0.6785903551 0.6787486812
0.6789591097 0.6802871160 0.6812002431 0.6812878262 0.6815240829 0.6822948279
[1156] 0.6835993465 0.6844309876 0.6852292932 0.6862035831 0.6895914074
0.6896457691 0.6898787046 0.6899453978 0.6901053766 0.6909983080 0.6917958639
[1167] 0.6920440710 0.6930102970 0.6932172185 0.6942518860 0.6949715591
0.6952057707 0.6955943761 0.6958443588 0.6961752312 0.6964188039 0.6972908638
[1178] 0.6976947182 0.6991830622 0.6995071771 0.7003496765 0.7008828622
0.7011432788 0.7023473377 0.7036907078 0.7052886612 0.7060283156 0.7064341850
[1189] 0.7066575539 0.7076265386 0.7079549814 0.7079903665 0.7087527554
0.7089876842 0.7092228030 0.7097220916 0.7107032575 0.7134162176 0.7137072335
[1200] 0.7143169241 0.7153799591 0.7155483759 0.7157031980 0.7166411310
0.7169705317 0.7169830206 0.7172634251 0.7175841919 0.7181817385 0.7195381424
[1211] 0.7205364210 0.7212519224 0.7214424227 0.7216157957 0.7224964287
0.7235798986 0.7237338251 0.7240975881 0.7241905290 0.7242713048 0.7261978627
[1222] 0.7265517808 0.7266344379 0.7267591903 0.7268975292 0.7283456578
0.7309608477 0.7318639125 0.7323095449 0.7343111386 0.7346270822 0.7354768622
[1233] 0.7356786394 0.7368855990 0.7371284514 0.7385279499 0.7392646199
0.7404407275 0.7406630404 0.7409108256 0.7435844813 0.7439234882 0.7443059031
[1244] 0.7443627346 0.7444759591 0.7455202330 0.7460610304 0.7473491623
0.7479576254 0.7480251002 0.7489047136 0.7497386995 0.7498684245 0.7502232634
[1255] 0.7504914341 0.7517495004 0.7531256304 0.7533490418 0.7541420905
0.7546150660 0.7559863147 0.7565112765 0.7566709068 0.7571368828 0.7577467795
[1266] 0.7581831959 0.7589843596 0.7591043962 0.7596499445 0.7604752276
0.7619496982 0.7627474869 0.7637522859 0.7651989917 0.7655491236 0.7661991708
[1277] 0.7671593494 0.7686368822 0.7689990528 0.7695031225 0.7702995992
0.7705304081 0.7710167053 0.7715923562 0.7738925105 0.7748609393 0.7760720281
[1288] 0.7762621602 0.7762653417 0.7766245909 0.7769605108 0.7786962143
0.7828470883 0.7829853036 0.7834181040 0.7839187022 0.7841081269 0.7841381373

[1299] 0.7860723543 0.7872590211 0.7875342327 0.7876487766 0.7879674116
0.7880005217 0.7880232318 0.7890055700 0.7896787140 0.7901539479 0.7916723632
[1310] 0.7942989675 0.7965640508 0.7978600006 0.7980461480 0.7982556705
0.8015885438 0.8017767952 0.8041347718 0.8054606818 0.8062272131 0.8070119904
[1321] 0.8086477221 0.8093460300 0.8112452810 0.8132780684 0.8140225824
0.8142176187 0.8142433618 0.8184197977 0.8184406888 0.8197135478 0.8219283491
[1332] 0.8253303793 0.8256819784 0.8277579585 0.8280139735 0.8288377176
0.8292632956 0.8318695152 0.8324501585 0.8349342795 0.8360683655 0.8370510594
[1343] 0.8397362753 0.8401148159 0.8410635089 0.8446748684 0.8457995925
0.8479865878 0.8485757251 0.8490874001 0.8505003240 0.8517953671 0.8519161524
[1354] 0.8529252240 0.8704641443 0.8709902399 0.8716201067 0.8751137404
0.8755499219 0.8758639665 0.8788100158 0.8794203557 0.8820618225 0.8859929254
[1365] 0.8893195277 0.8921870659 0.8932764540 0.8943513311 0.9004360081
0.9010664173 0.9068367241 0.9133877086 0.9197740692 0.9244624299 0.9292251369
[1376] 0.9361216810 0.9391769198 0.9505704285 0.9508709111 0.9534530710
0.9679585001 0.9688193058 0.9916804914 0.9931929532 0.9951631012 1.0278120129
[1387] 1.0343861775 1.0499286577 1.0505389337 1.0701290428 1.0821207339
1.0861287919

FLUCTUATION DATA for TRUNCATED MINIMIZED CHARACTERIZED Q8LJP6

[1] 0.53407862 0.57730432 0.42927461 0.34865747 0.24215254
0.24287086 0.29705526 0.24125685 0.19176073 0.23950782 0.24266843
[12] 0.17447595 0.19966504 0.25619341 0.23634347 0.20565492
0.26424010 0.32202486 0.28393973 0.31257906 0.34876664 0.40859471
[23] 0.53597582 0.57092710 0.69233970 1.10922165 0.90462839
0.60301599 0.55400089 0.52006921 0.67850395 0.72528391 0.60043835
[34] 0.68086483 0.60086149 0.53879778 0.52816478 0.61265917
0.69479778 0.49627380 0.49561974 0.69461297 0.80296620 0.80577603
[45] 0.83862779 0.66201160 0.55318504 0.40045296 0.42997395
0.32108934 0.24548795 0.22894743 0.25853688 0.22198316 0.30769095
[56] 0.38423430 0.40907906 0.39807608 0.34134214 0.26348190
0.22814117 0.18980194 0.14257808 0.12267193 0.12208281 0.10100559
[67] 0.08214158 0.08039605 0.08180126 0.06808110 0.06878207
0.08260111 0.08857656 0.10358397 0.11309117 0.13261176 0.17290277
[78] 0.21369208 0.19831545 0.19948528 0.27422204 0.21726430
0.20324191 0.27776140 0.31521906 0.28801017 0.29894933 0.26130428
[89] 0.22063383 0.27112250 0.24910116 0.17803047 0.18837569
0.22566819 0.18964285 0.15081829 0.18218994 0.22004569 0.17202436
[100] 0.17052545 0.23847827 0.23849072 0.21134639 0.25973758
0.30605266 0.28748277 0.29571642 0.49771934 0.47457564 0.41673875
[111] 0.39873781 0.37910933 0.29396508 0.29825508 0.26657825
0.29399204 0.35530128 0.44325600 0.51455278 0.48444400 0.63311224
[122] 0.68155407 0.55033818 0.58022513 0.77213681 0.77216974
0.69503857 0.71661272 0.63084096 0.56130264 0.67879925 0.80980374
[133] 0.79668769 1.08693293 0.89103947 0.90051174 0.60687106
0.56762556 0.44031048 0.39567396 0.36436947 0.35476668 0.41512884
[144] 0.51749224 0.73674011 0.49261890 0.34609962 0.25726246
0.21318951 0.16316665 0.15344467 0.16507644 0.13527595 0.10362827
[155] 0.12010030 0.13065439 0.09887251 0.08018076 0.11341652
0.12250660 0.10216998 0.11345026 0.15220854 0.18035425 0.17687854
[166] 0.21302532 0.27412495 0.48202621 0.48614279 0.41004623
0.38111565 0.44249055 0.33583211 0.23170119 0.30784890 0.30792492
[177] 0.20121398 0.21805702 0.29710417 0.24529131 0.18760457

0.26212049   0.28779816   0.21756497   0.23091925   0.33094378   0.30436959
[188]   0.24412969   0.34347069   0.43949254   0.40771170   0.38407940
0.34361629   0.28355962   0.27876439   0.32621130   0.42721079   0.41536985
[199]   0.40060856   0.44808627   0.34137035   0.34431664   0.40028742
0.31982001   0.30690546   0.26947011   0.31640469   0.22911668   0.24641599
[210]   0.21610148   0.23250865   0.16780683   0.14290508   0.20914455
0.20333267   0.14513057   0.14108726   0.20762310   0.19490439   0.15385096
[221]   0.20154721   0.26501938   0.26614373   0.25347899   0.37629959
0.49018825   0.53485487   0.59836884   0.57260495   0.35918251   0.39496359
[232]   0.52377046   0.42996492   0.32374385   0.45562363   0.52817033
0.39774604   0.39995892   0.55772334   0.47639209   0.40217608   0.42570137
[243]   0.53513968   0.65008208   1.16179504   1.46335101   0.82977000
0.45479223   0.38269898   0.20788492   0.12352774   0.16314640   0.16367525
[254]   0.20579075   0.14784427   0.13018486   0.17609436   0.16129220
0.14836211   0.18108604   0.21792279   0.21324145   0.22694162   0.30312028
[265]   0.33465513   0.35238088   0.43783999   0.56905125   0.57399646
0.71644697   0.71326651   0.61677169   0.38337302   0.40300748   0.45803232
[276]   0.33829020   0.27238129   0.34849501   0.35624207   0.26850051
0.26207352   0.32291075   0.29476660   0.28154839   0.36642488   0.48287625
[287]   0.43199461   0.49168171   0.81967780   0.67903557   0.45701993
0.35703615   0.33144216   0.34478800   0.39018253   0.56651434   0.46782783
[298]   0.38262268   0.30096474   0.25583728   0.26120914   0.28606514
0.32796009   0.30082509   0.26082123   0.18222750   0.17860335   0.15667685
[309]   0.12611584   0.12312528   0.13198721   0.10028791   0.08989589
0.12214304   0.12673278   0.11208339   0.12783954   0.16777035   0.18389534
[320]   0.20345843   0.24342660   0.28900265   0.41133110   0.46358936
0.51710704   0.85706594   0.83919003   0.74005989   0.68266688   0.47735442
[331]   0.38710381   0.43272138   0.37390017   0.25829223   0.27233249
0.32472160   0.25936756   0.19271282   0.23620201   0.26211889   0.20709238
[342]   0.20478815   0.26055858   0.26265018   0.24439740   0.26748040
0.34140443   0.37072869   0.44253697   0.58069286   0.61244285   0.50768740
[353]   0.44796928   0.32555407   0.28264976   0.26065655   0.32402228
0.40648183   0.37902472   0.42434947   0.53081084   0.58953929   0.43650997
[364]   0.46392676   0.57584706   0.52302929   0.41351564   0.40056833
0.42170244   0.51023353   0.50755369   0.52160869   0.60997967   0.70855648
[375]   0.82578938   1.05901564   1.16857378   0.94136481   1.00328323
0.84368586   0.96364115   1.03496311   1.02308170   1.00708630   0.79251733
[386]   0.67845365   0.82127148   0.65586070   0.53855717   0.40057983
0.33178377   0.23051457   0.23102004   0.19096402   0.22720532   0.20084364
[397]   0.25830978   0.35455807   0.37904944   0.39492443   0.47572240
0.44751853   0.54805327   0.58844480   0.62235230   0.58489627   0.58034404
[408]   0.52167409   0.62485529   0.49304476   0.40298497   0.37931753
0.30488234   0.32010729   0.27584433   0.22941027   0.20434579   0.14248028
[419]   0.10589933   0.11675124   0.12438810   0.08345680   0.08119352
0.10573081   0.10416554   0.08849283   0.11541981   0.13881432   0.14233921
[430]   0.18092477   0.21281728   0.23342574   0.28263752   0.36218004
0.40513004   0.35334137   0.35500662   0.44322378   0.39245203   0.62313607
[441]   1.04069306   1.22836930   0.82112465   0.72838021   0.96205809
0.94159616   1.06057842   1.95477032   1.93140190   2.28115066   2.90051746
[452]   2.75955102   4.05328640   5.01705987 10.18511913 19.77254427
32.38665122 60.96758937 52.25396596 185.43053200 168.58300356 167.64836271
[463] 343.30386323 757.94613231

MASSES for TRUNCATED MINIMIZED CHARACTERIZED Q8LJP6

```
 [1]  72.08594  57.05100 137.13900 114.07900 163.17300 114.10300 117.12600
71.07800 113.15800  87.07700 129.18000  87.07700 113.15800 113.15800 147.17400
 [16] 147.17400 128.10600  71.07800 117.12600 157.19400  87.07700  57.05100
163.17300 113.15800  97.11500 137.13900 114.10300 117.12600 157.19400  99.13100
 [31] 101.10400 186.21000 157.19400  71.07800 114.10300  87.07700  57.05100
113.15800 114.10300 114.07900  57.05100 129.18000  71.07800  87.07700  57.05100
 [46]  99.13100 114.07900 113.15800  99.13100  57.05100  57.05100 163.17300
163.17300 114.07900  71.07800  57.05100 114.07900 114.10300  99.13100 129.18000
 [61] 147.17400  57.05100 113.15800  97.11500 131.19600  71.07800 147.17400
101.10400 113.15800 101.10400 131.19600 131.19600  87.07700 186.21000  87.07700
 [76] 113.15800 113.15800 128.10600 163.17300  57.05100 129.18000 117.12600
131.19600  57.05100  71.07800 101.10400  57.05100 128.10600 113.15800  57.05100
 [91] 137.13900  71.07800 131.19600 114.07900  71.07800  99.13100 129.18000
186.21000  57.05100 101.10400 114.07900 163.17300 113.15800 113.15800 129.18000
[106]  71.07800 137.13900  97.11500 128.10600  97.11500 163.17300  99.13100
113.15800 163.17300  57.05100 128.10600  99.13100  57.05100 114.07900  57.05100
[121] 114.10300  87.07700 114.07900 137.13900 163.17300 103.14300 186.21000
117.12600 157.19400  97.11500 128.10600 114.07900 113.15800 101.10400 101.10400
[136] 114.10300 157.19400 137.13900  71.07800 163.17300 129.18000 129.18000
114.07900  97.11500  87.07700 114.10300  97.11500  57.05100  87.07700 114.07900
[151] 113.15800  71.07800  57.05100 128.10600 101.10400  71.07800  71.07800
71.07800 131.19600  71.07800  71.07800  71.07800  87.07700 113.15800  99.13100
[166] 147.17400 157.19400 157.19400  87.07700 114.10300  97.11500  71.07800
163.17300  87.07700 101.10400 128.10600 113.15800 113.15800 157.19400 137.13900
[181]  71.07800 163.17300 117.12600 113.15800 147.17400 128.10600 147.17400
71.07800 114.07900 129.18000 163.17300 157.19400  57.05100 129.18000 163.17300
[196] 114.07900  87.07700  87.07700 113.15800 101.10400  71.07800 117.12600
163.17300 163.17300 157.19400  87.07700  99.13100  87.07700  57.05100 163.17300
[211] 114.10300 114.07900 128.10600 113.15800 113.15800 186.21000  71.07800
71.07800  71.07800 186.21000 113.15800 163.17300 117.12600  71.07800  87.07700
[226] 114.10300 114.10300 117.12600 163.17300 163.17300 113.15800 114.10300
163.17300 113.15800  57.05100 129.18000 114.10300  57.05100 114.07900  87.07700
[241] 131.19600  57.05100  57.05100 101.10400  57.05100 186.21000  71.07800
131.19600 101.10400 128.10600 147.17400  57.05100 186.21000 114.07900  99.13100
[256] 129.18000 163.17300 101.10400  57.05100  99.13100 117.12600 101.10400
113.15800  99.13100  71.07800 129.18000 147.17400 113.15800 131.19600 117.12600
[271] 128.10600 157.19400 163.17300 137.13900 117.12600 129.18000  71.07800
128.10600 137.13900 147.17400 131.19600 103.14300  87.07700 113.15800 113.15800
[286]  57.05100 129.18000  57.05100 113.15800 157.19400 114.10300  99.13100
117.12600 129.18000 101.10400  97.11500  57.05100  57.05100 113.15800 131.19600
[301] 147.17400 157.19400 117.12600 129.18000 186.21000 114.10300 114.10300
131.19600 117.12600 147.17400  99.13100 101.10400  87.07700  71.07800  87.07700
[316] 147.17400 113.15800  71.07800 101.10400  99.13100 163.17300  87.07700
114.07900 163.17300 113.15800  99.13100  87.07700  87.07700  99.13100  71.07800
[331]  97.11500 101.10400 117.12600 113.15800 113.15800  87.07700 147.17400
71.07800 129.18000  87.07700 117.12600  99.13100 114.07900 163.17300 113.15800
[346] 113.15800  57.05100 114.07900 114.10300  97.11500 157.19400  57.05100
101.10400  87.07700 163.17300 131.19600  99.13100  57.05100 163.17300  57.05100
[361] 114.10300 114.10300 147.17400  97.11500 157.19400 117.12600  99.13100
137.13900 137.13900 157.19400  57.05100  87.07700  87.07700 113.15800  99.13100
[376]  87.07700 113.15800  71.07800 186.21000 163.17300 101.10400 157.19400
129.18000  71.07800  87.07700 114.07900  97.11500 114.10300  99.13100 113.15800
[391] 101.10400  57.05100  71.07800  99.13100  99.13100  57.05100  57.05100
```

```
97.11500 114.07900  71.07800 163.17300 114.07900 114.10300 147.17400  71.07800
[406] 114.07900 128.10600 157.19400 114.07900 114.10300 163.17300 128.10600
117.12600 101.10400 114.07900  97.11500  57.05100 101.10400 163.17300 114.10300
[421] 114.10300  71.07800  97.11500 113.15800 113.15800  57.05100 113.15800
113.15800  71.07800 157.19400 113.15800 114.10300  71.07800  57.05100 137.13900
[436]  57.05100  57.05100 113.15800  97.11500  99.13100  99.13100 101.10400
101.10400 117.12600  97.11500 129.18000  99.13100  71.07800  97.11500 113.15800
[451]  97.11500 129.18000  99.13100  71.07800  97.11500  71.07800  87.07700
97.11500  71.07800  97.11500  87.07700  87.07700  87.07700 114.12234

Call:
  nma.pdb(pdb = pdbT4_3WY9, mass = TRUE)

Class:
  VibrationalModes (nma)

Number of modes:
  1398 (6 trivial)

Frequencies:
  Mode 7:       0.001
  Mode 8:       0.001
  Mode 9:       0.001
  Mode 10:      0.004
  Mode 11:      0.005
  Mode 12:      0.006

+ attr: modes, frequencies, force.constants, fluctuations,
        U, L, xyz, mass, temp, triv.modes, natoms, call

 NORMAL MODES for TRUNCATED MINIMIZED CHARACTERIZED Q93WY9

  [1] 0.0000000000 0.0000000000 0.0000000000 0.0000000000 0.0000000000
0.0000000000 0.0007957747 0.0012328089 0.0014146001 0.0037291195 0.0051227128
  [12] 0.0064649043 0.0071176254 0.0086076196 0.0095386804 0.0110975627
0.0136289053 0.0143539760 0.0146655904 0.0157249594 0.0169251386 0.0189514713
  [23] 0.0197480438 0.0200067323 0.0215993840 0.0225691586 0.0231120159
0.0234876247 0.0237012652 0.0242072523 0.0246227666 0.0249523280 0.0255422611
  [34] 0.0264810104 0.0267380304 0.0271889565 0.0286169262 0.0288426347
0.0294862184 0.0300764946 0.0306487960 0.0308033692 0.0313442316 0.0321965375
  [45] 0.0322586301 0.0328422694 0.0337017873 0.0341416103 0.0349648595
0.0353331143 0.0359957678 0.0363835301 0.0366568075 0.0371346376 0.0375582061
  [56] 0.0376461161 0.0378414097 0.0382024911 0.0384032006 0.0388236945
0.0392822644 0.0396036857 0.0397333113 0.0402690313 0.0404547948 0.0409826296
  [67] 0.0411248489 0.0414726145 0.0417865842 0.0420024339 0.0427712439
0.0429122576 0.0433871671 0.0434976603 0.0441562475 0.0442665377 0.0447321666
  [78] 0.0448608071 0.0454079959 0.0460832581 0.0462974042 0.0466910261
0.0471874948 0.0476474389 0.0478606727 0.0480368535 0.0482441662 0.0485323421
  [89] 0.0489087568 0.0492650752 0.0496298093 0.0498873955 0.0504282632
0.0505549357 0.0508417221 0.0511620640 0.0513853615 0.0517216836 0.0519137955
 [100] 0.0524098460 0.0525286061 0.0529184801 0.0533693775 0.0536781752
0.0538696577 0.0540152275 0.0540576506 0.0549301331 0.0551657298 0.0555573866
 [111] 0.0557151425 0.0558010032 0.0560112352 0.0564618844 0.0566309877
0.0569730238 0.0571089098 0.0574465573 0.0578304329 0.0580586238 0.0583601884
```

[122] 0.0586351521 0.0591962257 0.0592729847 0.0594937271 0.0596015606
0.0597577537 0.0604285980 0.0606146360 0.0610188512 0.0612200614 0.0613993698
[133] 0.0620398135 0.0622786125 0.0624905625 0.0627169435 0.0630020426
0.0633110682 0.0634890593 0.0638032694 0.0640661415 0.0641789225 0.0644215881
[144] 0.0648872185 0.0651560199 0.0653299539 0.0655525188 0.0656133505
0.0660297397 0.0662134297 0.0663007860 0.0666954166 0.0668623157 0.0669730355
[155] 0.0673913600 0.0674807575 0.0677372113 0.0680068532 0.0681439690
0.0687495932 0.0688407224 0.0689271376 0.0691159546 0.0693241736 0.0694196571
[166] 0.0697714287 0.0698597746 0.0700191315 0.0702210655 0.0704236794
0.0706206907 0.0708055282 0.0708647102 0.0711262354 0.0715112400 0.0716103513
[177] 0.0721275733 0.0723202933 0.0724978281 0.0725219323 0.0728754131
0.0729271846 0.0732225176 0.0734746168 0.0737332405 0.0738767005 0.0739568892
[188] 0.0742588721 0.0745995398 0.0748449705 0.0750971886 0.0753130877
0.0756490081 0.0759140717 0.0760460905 0.0762308982 0.0764237112 0.0766819681
[199] 0.0767346375 0.0768840285 0.0773026225 0.0774031539 0.0775303503
0.0779597708 0.0780576712 0.0782166802 0.0786768098 0.0787953605 0.0789421353
[210] 0.0790240755 0.0791873816 0.0792887181 0.0795478632 0.0796824446
0.0797455209 0.0800758168 0.0805113246 0.0808124798 0.0808499278 0.0809667045
[221] 0.0810494106 0.0812501183 0.0815462156 0.0818237527 0.0819315673
0.0820271978 0.0822924943 0.0825253295 0.0828590654 0.0828923804 0.0829024639
[232] 0.0832876232 0.0835003948 0.0837516501 0.0838666516 0.0840354678
0.0843208837 0.0844877430 0.0846511315 0.0848106213 0.0849957433 0.0850721507
[243] 0.0855574401 0.0856154486 0.0860557415 0.0862625664 0.0863975361
0.0866219694 0.0867595915 0.0870501886 0.0872160375 0.0874502466 0.0876211202
[254] 0.0878628986 0.0881846217 0.0883804466 0.0886323004 0.0886971512
0.0888458147 0.0890152900 0.0891753540 0.0894421044 0.0897853960 0.0899012727
[265] 0.0900216432 0.0901484563 0.0902492732 0.0903816526 0.0905806977
0.0907056122 0.0908660438 0.0910912858 0.0914251991 0.0915946031 0.0918535009
[276] 0.0919126342 0.0921905609 0.0923980461 0.0927365412 0.0928892388
0.0931033292 0.0931938833 0.0933025408 0.0935429032 0.0936038106 0.0937650916
[287] 0.0943106857 0.0944712962 0.0945356247 0.0948482011 0.0950274969
0.0951606823 0.0952172297 0.0953621039 0.0956318602 0.0957189637 0.0958926693
[298] 0.0962903190 0.0963999533 0.0967046704 0.0967360974 0.0968627496
0.0970273592 0.0971046033 0.0974238875 0.0976306289 0.0978514308 0.0981006591
[309] 0.0985187096 0.0985960698 0.0987431693 0.0988602046 0.0990676560
0.0991795836 0.0994363097 0.0996580674 0.0998313891 0.1001299643 0.1002065862
[320] 0.1003118141 0.1005307587 0.1005415926 0.1007869333 0.1010244072
0.1012435621 0.1013288415 0.1014425192 0.1015817568 0.1018558063 0.1020550591
[331] 0.1024638980 0.1025595244 0.1029432331 0.1030699994 0.1032698528
0.1033403475 0.1034134885 0.1036553308 0.1038291768 0.1041311277 0.1043347753
[342] 0.1045490505 0.1046404718 0.1048945786 0.1049613275 0.1051935918
0.1053283517 0.1058870886 0.1060074674 0.1061268742 0.1066683690 0.1067803950
[353] 0.1069168271 0.1071441432 0.1073470301 0.1076003950 0.1077182712
0.1078862748 0.1079389716 0.1085060929 0.1086030463 0.1087427823 0.1088786166
[364] 0.1090673625 0.1091976899 0.1095367636 0.1095571117 0.1100620586
0.1101569528 0.1105345689 0.1106232189 0.1106949803 0.1107961912 0.1111654839
[375] 0.1113684369 0.1114735810 0.1116672411 0.1118533195 0.1119546145
0.1122510686 0.1123344181 0.1126821493 0.1129534864 0.1131422629 0.1134252292
[386] 0.1136191299 0.1141898730 0.1143789341 0.1145465668 0.1148099679
0.1150566958 0.1151727690 0.1152842211 0.1154490035 0.1155373906 0.1157446049
[397] 0.1158194262 0.1160530955 0.1162036006 0.1165400049 0.1169434740
0.1171092742 0.1171907896 0.1172612319 0.1173137120 0.1174725212 0.1176656724
[408] 0.1180103585 0.1182564068 0.1183300680 0.1186736781 0.1189769161
0.1190587484 0.1192301053 0.1194205198 0.1195103147 0.1197960007 0.1198452573

[419] 0.1199661990 0.1202550148 0.1203960596 0.1206440660 0.1209668659
0.1210418074 0.1211935370 0.1214608756 0.1217110827 0.1218537682 0.1222149347
[430] 0.1225432085 0.1230694863 0.1232303333 0.1233750600 0.1235816355
0.1238043377 0.1239677049 0.1242793248 0.1245771529 0.1250530532 0.1251240290
[441] 0.1254665972 0.1256281052 0.1259965464 0.1262694636 0.1264843295
0.1268734453 0.1270039493 0.1273801437 0.1274226917 0.1274513142 0.1276726232
[452] 0.1278168780 0.1282942853 0.1284332071 0.1286459358 0.1289170758
0.1290305943 0.1292435156 0.1294578477 0.1296284525 0.1297549168 0.1300222831
[463] 0.1302161744 0.1304323067 0.1307412086 0.1309029815 0.1310029850
0.1311353674 0.1312911562 0.1315243970 0.1318655132 0.1320168907 0.1323226678
[474] 0.1324144258 0.1324922603 0.1328381372 0.1330970264 0.1333880797
0.1336419233 0.1337650667 0.1341777172 0.1346125321 0.1347275500 0.1351227341
[485] 0.1352026628 0.1354400150 0.1357410667 0.1359472979 0.1361978596
0.1363846431 0.1366949184 0.1371786359 0.1373388202 0.1376558696 0.1378773336
[496] 0.1379770554 0.1380813831 0.1384948903 0.1388103940 0.1390974140
0.1391648675 0.1393244954 0.1396153571 0.1401016475 0.1401276802 0.1403454242
[507] 0.1407267231 0.1407854796 0.1413148023 0.1414435329 0.1416854478
0.1421098578 0.1422687630 0.1424306921 0.1426093123 0.1428854220 0.1429992771
[518] 0.1433076923 0.1437392077 0.1439078432 0.1441556385 0.1443784485
0.1444978763 0.1445748120 0.1447914664 0.1451695501 0.1453385303 0.1456697297
[529] 0.1458236267 0.1461209671 0.1461936700 0.1466678214 0.1468929426
0.1470534832 0.1473984444 0.1475297646 0.1478145054 0.1481656445 0.1488826227
[540] 0.1492474577 0.1495358730 0.1498458792 0.1500472423 0.1500686804
0.1504487392 0.1506446726 0.1506656053 0.1512328759 0.1513022854 0.1516640556
[551] 0.1523933305 0.1525796285 0.1527279726 0.1527761451 0.1530550977
0.1532240606 0.1533994425 0.1536062890 0.1539953819 0.1541071928 0.1544146652
[562] 0.1546833761 0.1549820240 0.1553845449 0.1557877330 0.1560804540
0.1561765001 0.1564902663 0.1567549345 0.1567683460 0.1571950580 0.1574159858
[573] 0.1575536672 0.1578526615 0.1586036304 0.1589572294 0.1592201832
0.1594200336 0.1596366922 0.1598757153 0.1600017818 0.1603024515 0.1603947061
[584] 0.1605123958 0.1613986503 0.1616460374 0.1617000123 0.1618098650
0.1619522570 0.1621721708 0.1623026183 0.1626024607 0.1628809136 0.1633750104
[595] 0.1637313004 0.1639384760 0.1641592780 0.1649120175 0.1651269159
0.1655561053 0.1658734318 0.1662810447 0.1663812504 0.1665857387 0.1668802381
[606] 0.1669692377 0.1670110275 0.1673616959 0.1682971527 0.1683500484
0.1684406774 0.1688556692 0.1688904683 0.1693378678 0.1695378188 0.1696648427
[617] 0.1697900559 0.1703773551 0.1705015993 0.1708717888 0.1709861930
0.1713651656 0.1714584113 0.1718452605 0.1723864782 0.1726812797 0.1732271237
[628] 0.1735211538 0.1737683377 0.1740222302 0.1741899775 0.1743741243
0.1746061745 0.1749115721 0.1751280131 0.1752637040 0.1753300292 0.1759027923
[639] 0.1761656150 0.1764433361 0.1767214090 0.1768535858 0.1779775013
0.1782256109 0.1784952311 0.1788915012 0.1789566234 0.1790446424 0.1794174646
[650] 0.1796046433 0.1797685905 0.1799680014 0.1802352962 0.1804023211
0.1806319559 0.1808568875 0.1814769778 0.1816584090 0.1819635942 0.1822709088
[661] 0.1830766166 0.1836216316 0.1836651491 0.1840227610 0.1840500820
0.1843092580 0.1845200981 0.1848291920 0.1851620537 0.1853882523 0.1857743871
[672] 0.1859487648 0.1860008624 0.1864006088 0.1867892615 0.1869265152
0.1872593010 0.1873081942 0.1878222541 0.1882405384 0.1885796020 0.1891371523
[683] 0.1894915890 0.1896763047 0.1898670444 0.1902709825 0.1903710681
0.1904852637 0.1909657047 0.1912066317 0.1924436656 0.1925617623 0.1926398177
[694] 0.1928907353 0.1929268448 0.1932622085 0.1936318571 0.1939325023
0.1940808883 0.1942022285 0.1945709374 0.1947690439 0.1950100780 0.1952194169
[705] 0.1952890818 0.1953362893 0.1957511340 0.1959586471 0.1961430839
0.1972731137 0.1973376253 0.1977736965 0.1982813193 0.1984549823 0.1991604571

[716] 0.1997911881 0.1998377123 0.1999595495 0.2003543922 0.2006265953
0.2007834700 0.2011326851 0.2012389489 0.2013663537 0.2016241882 0.2017688007
[727] 0.2023176735 0.2031633888 0.2037269686 0.2038152270 0.2041719111
0.2044371728 0.2046725761 0.2047577054 0.2051953518 0.2055485320 0.2057143363
[738] 0.2064545751 0.2067636499 0.2068536128 0.2071744415 0.2074752409
0.2076732346 0.2080806480 0.2082450444 0.2087153700 0.2093118657 0.2096627025
[749] 0.2101381123 0.2103753842 0.2109848355 0.2114959012 0.2115540403
0.2122721260 0.2126123001 0.2134878921 0.2138726826 0.2143125765 0.2145717357
[760] 0.2149281293 0.2151099611 0.2153213454 0.2161014116 0.2166627863
0.2167499845 0.2169494382 0.2173257688 0.2177499047 0.2178926499 0.2182584181
[771] 0.2185113327 0.2188544246 0.2198170039 0.2205478677 0.2206772102
0.2208919247 0.2215077765 0.2231518603 0.2236249853 0.2241410204 0.2243027366
[782] 0.2244453207 0.2249122899 0.2251087313 0.2251377045 0.2257847212
0.2263383065 0.2266208235 0.2272555370 0.2273810640 0.2281146207 0.2296078561
[793] 0.2300467301 0.2303711588 0.2307683561 0.2310394263 0.2314122177
0.2327342166 0.2327822091 0.2328396566 0.2331757340 0.2335551477 0.2339717867
[804] 0.2340170359 0.2347753362 0.2350334330 0.2366968452 0.2371554451
0.2379155070 0.2392863209 0.2393094497 0.2394712358 0.2399885033 0.2407628205
[815] 0.2410444042 0.2410607444 0.2415042405 0.2421324742 0.2428311727
0.2440012940 0.2443088512 0.2459587617 0.2463096934 0.2479101737 0.2491884121
[826] 0.2494542914 0.2498722469 0.2513078222 0.2527504269 0.2532665769
0.2538516353 0.2542662977 0.2552309919 0.2560472141 0.2561238225 0.2564151112
[837] 0.2570073425 0.2574619857 0.2577312711 0.2586658582 0.2590121401
0.2598164352 0.2598775074 0.2602613538 0.2615231003 0.2617445665 0.2621252439
[848] 0.2626711426 0.2635592402 0.2638288790 0.2651244775 0.2653595955
0.2663968033 0.2666335544 0.2672823778 0.2683891299 0.2685553742 0.2687672289
[859] 0.2693917328 0.2702143010 0.2714319781 0.2715843751 0.2716716605
0.2724742387 0.2740708657 0.2762165561 0.2776054592 0.2805036227 0.2810686659
[870] 0.2818357687 0.2821132593 0.2831881251 0.2846909152 0.2849722033
0.2863005001 0.2873138191 0.2884848750 0.2899841934 0.2903609975 0.2910982632
[881] 0.2926727420 0.2938579996 0.2959845089 0.2979127593 0.2981404142
0.2985369606 0.3054783758 0.3068379349 0.3070754708 0.3076811252 0.3090393951
[892] 0.3095122151 0.3110141297 0.3110688961 0.3129209605 0.3144950807
0.3157632065 0.3163380560 0.3165981880 0.3184304630 0.3200093815 0.3203029904
[903] 0.3278783426 0.3300022768 0.3305870763 0.3324875515 0.3338036190
0.3356421534 0.3375472985 0.3408814292 0.3414263047 0.3423969207 0.3427655093
[914] 0.3473464657 0.3474570391 0.3479690896 0.3593113099 0.3601156944
0.3615517977 0.3620047667 0.3669345775 0.3731527735 0.3767943447 0.3782695502
[925] 0.3791073083 0.3826735895 0.3887737969 0.3933103469 0.4034181941
0.4088203825 0.4113795087 0.4246303990 0.4267822521 0.4276232398 0.4348887488
[936] 0.4471946059 0.4521998345 0.4525471386 0.4540671002 0.4615992191
0.4620614749 0.4675491434 0.4681065067 0.4715715844 0.4755906027 0.4771888544
[947] 0.4772338130 0.4838108443 0.4939795631 0.4943583368 0.4945296751
0.4992356719 0.5019945214 0.5033731697 0.5088063304 0.5120250006 0.5166309435
[958] 0.5171824301 0.5172794944 0.5181243156 0.5195196931 0.5250929455
0.5258577586 0.5302271950 0.5328327563 0.5376596965 0.5379302170 0.5385581045
[969] 0.5395429331 0.5418517632 0.5421688532 0.5423958897 0.5444137883
0.5461586174 0.5487754985 0.5494141592 0.5507236635 0.5509146918 0.5515405192
[980] 0.5519175842 0.5539259012 0.5543817886 0.5570919777 0.5580775285
0.5593269685 0.5605628693 0.5606046434 0.5614928649 0.5648032668 0.5654259296
[991] 0.5660679100 0.5694453326 0.5712639828 0.5715495991 0.5726246002
0.5740116916 0.5742800186 0.5743838393 0.5748388587 0.5793599109 0.5811341103
[1002] 0.5813029009 0.5843884524 0.5845499334 0.5848624974 0.5875068429
0.5878184809 0.5880433137 0.5880558486 0.5889395716 0.5895686226 0.5921497561

[1013] 0.5923203684 0.5933305244 0.5946025905 0.5959473058 0.5961213353
0.5965237460 0.5978668942 0.5987968429 0.6007105513 0.6025601311 0.6026433602
[1024] 0.6034593211 0.6052713752 0.6058796866 0.6058957195 0.6060198926
0.6062879244 0.6080735125 0.6084859191 0.6089803469 0.6092655775 0.6092678433
[1035] 0.6098468946 0.6102513371 0.6106051315 0.6108560784 0.6109342177
0.6111226315 0.6117098239 0.6121194910 0.6121752290 0.6129085582 0.6144931338
[1046] 0.6146703810 0.6163235459 0.6169466210 0.6173832363 0.6174389915
0.6182253665 0.6182840774 0.6191096396 0.6196509621 0.6200184515 0.6201233968
[1057] 0.6216720427 0.6218381803 0.6218853084 0.6227088217 0.6239076770
0.6239225971 0.6245020131 0.6245215023 0.6249735976 0.6250757051 0.6255324009
[1068] 0.6257833728 0.6281535311 0.6282349823 0.6283401073 0.6309278541
0.6318398723 0.6334753614 0.6339584309 0.6348373080 0.6358866008 0.6360005375
[1079] 0.6363618493 0.6364217727 0.6370877149 0.6387815244 0.6395125851
0.6398713794 0.6401155422 0.6405094537 0.6410787778 0.6415629724 0.6419055270
[1090] 0.6425380726 0.6429899096 0.6430019248 0.6433355826 0.6453953322
0.6455678421 0.6462853278 0.6476316588 0.6480437897 0.6486802683 0.6486918267
[1101] 0.6488490745 0.6492343513 0.6496312726 0.6497875135 0.6500570406
0.6503050136 0.6528468254 0.6530790976 0.6531689391 0.6539305343 0.6547194869
[1112] 0.6554971917 0.6555693340 0.6563775808 0.6568595410 0.6569399201
0.6572429740 0.6573764445 0.6581708110 0.6583278340 0.6584486783 0.6585810199
[1123] 0.6589459417 0.6619708113 0.6624708222 0.6627017853 0.6637910416
0.6638620727 0.6641549315 0.6645228150 0.6655997295 0.6662036952 0.6662164514
[1134] 0.6665707901 0.6667274494 0.6667376882 0.6678429865 0.6690990598
0.6694126532 0.6707654703 0.6712071928 0.6723049736 0.6728966345 0.6736921772
[1145] 0.6739476530 0.6750291007 0.6766740762 0.6771151240 0.6793304234
0.6793959338 0.6796589929 0.6813856398 0.6821932645 0.6824650997 0.6828372891
[1156] 0.6834324150 0.6840038413 0.6847156593 0.6851484435 0.6851728251
0.6856788206 0.6868065061 0.6868366928 0.6878913049 0.6880726164 0.6915995416
[1167] 0.6927941183 0.6928011748 0.6935339862 0.6936079058 0.6937532204
0.6942710590 0.6943332809 0.6943743031 0.6948919157 0.6952814799 0.6954892735
[1178] 0.6956060107 0.6957753732 0.6968650935 0.6970259371 0.6975356085
0.6976453407 0.6999655929 0.7005066826 0.7010602718 0.7016858609 0.7017492122
[1189] 0.7025371592 0.7027888533 0.7028902697 0.7031296790 0.7040067568
0.7040762850 0.7044591702 0.7051214760 0.7051541834 0.7054990366 0.7062156951
[1200] 0.7070563987 0.7089303572 0.7089377891 0.7097369209 0.7098572920
0.7098729034 0.7110339485 0.7115981251 0.7116444522 0.7124256150 0.7124396236
[1211] 0.7128397816 0.7151480156 0.7161201557 0.7163817515 0.7172618359
0.7182835206 0.7182897096 0.7205760042 0.7210080261 0.7221099005 0.7224398406
[1222] 0.7234424134 0.7253977468 0.7269612618 0.7276252087 0.7277072740
0.7283789220 0.7294812952 0.7307844752 0.7319378545 0.7321928649 0.7323572597
[1233] 0.7330853765 0.7335766246 0.7345957215 0.7348382965 0.7367887417
0.7373438617 0.7376923637 0.7378952172 0.7387751826 0.7388390734 0.7388896404
[1244] 0.7396907264 0.7443182907 0.7444909467 0.7456881268 0.7465556156
0.7467329613 0.7474327899 0.7476224975 0.7477370747 0.7489092797 0.7494060412
[1255] 0.7497777545 0.7499809361 0.7503822060 0.7503831174 0.7510863743
0.7516974228 0.7528022920 0.7529182004 0.7535199311 0.7539349739 0.7541707743
[1266] 0.7542812337 0.7556068781 0.7557650403 0.7566968670 0.7567378391
0.7569446740 0.7588986672 0.7598678709 0.7599244552 0.7616520560 0.7617876495
[1277] 0.7625223612 0.7626502771 0.7627088137 0.7629411388 0.7633248774
0.7644883565 0.7655550628 0.7656177445 0.7659022373 0.7699469066 0.7716830563
[1288] 0.7721521269 0.7727846069 0.7732831462 0.7735112638 0.7742444363
0.7748533715 0.7748556435 0.7768512871 0.7803021632 0.7808311010 0.7816650331
[1299] 0.7816872306 0.7843958596 0.7860442868 0.7870180073 0.7870298996
0.7870647226 0.7873763878 0.7893145754 0.7895852851 0.7915627696 0.7918636758

[1310] 0.7937199985 0.7952459703 0.7955781507 0.7977859931 0.8014170316
0.8038591305 0.8041080436 0.8048361456 0.8056177503 0.8067804882 0.8067812417
[1321] 0.8074222026 0.8091950537 0.8092666407 0.8096008902 0.8104944967
0.8105349370 0.8109752892 0.8118212992 0.8120911032 0.8122955374 0.8134631009
[1332] 0.8157261597 0.8162878604 0.8167590400 0.8171716127 0.8182472009
0.8208131473 0.8208225132 0.8212705502 0.8220414747 0.8243404889 0.8267205894
[1343] 0.8282537619 0.8335977734 0.8346726333 0.8356401149 0.8399266074
0.8417348948 0.8423084668 0.8435532089 0.8439271560 0.8449487813 0.8456979267
[1354] 0.8457729380 0.8467236080 0.8477094581 0.8534407913 0.8594819770
0.8608992758 0.8649802185 0.8661301202 0.8677047657 0.8689654534 0.8719870720
[1365] 0.8756230268 0.8760773438 0.8802748398 0.8807258369 0.8841139030
0.8849420443 0.9064116721 0.9096623956 0.9125422602 0.9204475125 0.9214871228
[1376] 0.9224790199 0.9262669197 0.9288856521 0.9362088874 0.9366078279
0.9407249781 0.9436495887 0.9469157106 0.9492815626 0.9535221823 0.9535711005
[1387] 0.9602222520 0.9677360399 0.9681603058 0.9738329163 0.9773343898
0.9803226592 0.9865486274 1.0702233057 1.0756038773 1.0782642035 1.0801193238
[1398] 1.0914983383

FLUCTUATION DATA for TRUNCATED MINIMIZED CHARACTERIZED Q93WY9

[1] 0.52856563 0.51225619 0.42253854 0.35274440 0.24116484
0.25106423 0.30569137 0.24110193 0.19246019 0.23754284 0.24282649
[12] 0.17361563 0.19893636 0.26733980 0.24254815 0.21333621
0.27837848 0.33597208 0.29257638 0.32120748 0.36377135 0.42156850
[23] 0.55018202 0.59315736 0.68498498 1.15161166 0.89081643
0.61905006 0.58150836 0.53822180 0.70476353 0.72280676 0.61856885
[34] 0.70922590 0.62331405 0.56185338 0.54434999 0.61680531
0.69797773 0.50360448 0.50346908 0.70138048 0.80618294 0.78167800
[45] 0.81631273 0.66654193 0.56181234 0.40516695 0.43740158
0.32780555 0.25006623 0.23517690 0.26540163 0.22435634 0.28859096
[56] 0.38659344 0.41124789 0.40549772 0.35563393 0.27166005
0.23314787 0.19223925 0.14833048 0.12635839 0.12444116 0.10062534
[67] 0.08370640 0.08173858 0.08191278 0.06934085 0.07119211
0.08520555 0.09349957 0.11137019 0.11979448 0.14155058 0.18668849
[78] 0.22403942 0.21096952 0.21462815 0.29280457 0.23945916
0.22054501 0.30396064 0.35054335 0.33099327 0.33248977 0.28200098
[89] 0.23564666 0.29695555 0.25557692 0.18720040 0.20108685
0.23266842 0.19432693 0.15479766 0.18633681 0.22037520 0.17183304
[100] 0.17032565 0.23806836 0.23667701 0.20689455 0.25363608
0.29864152 0.28222979 0.28433018 0.48040085 0.45723280 0.39454322
[111] 0.39324215 0.37007494 0.28606892 0.29792352 0.26780334
0.30208648 0.36842582 0.46080685 0.54235326 0.49774189 0.64703674
[122] 0.70869453 0.58686282 0.58607873 0.79129568 0.79383733
0.70852685 0.72607111 0.64680150 0.57474217 0.68377845 0.84300499
[133] 0.81602996 1.14261323 0.92549043 0.95563623 0.64060051
0.59760658 0.45641511 0.40729000 0.35851893 0.33727938 0.41139713
[144] 0.50831957 0.71269694 0.47288661 0.34469833 0.24945213
0.22203266 0.17019171 0.15983564 0.16671615 0.13818989 0.10582827
[155] 0.11958499 0.12997731 0.10059690 0.08041155 0.11203486
0.12390238 0.10741517 0.11627517 0.15793502 0.19181906 0.18673547
[166] 0.22276545 0.30220834 0.55587728 0.49296570 0.42660147
0.38293937 0.45589833 0.34298927 0.24003160 0.31532496 0.29335778
[177] 0.19311398 0.20804738 0.27719010 0.23850770 0.18275352
0.25406912 0.28058739 0.21650269 0.23182444 0.33507302 0.31218834

[188]   0.25529742   0.35671234   0.45757961   0.43409583   0.41975789
0.36926320   0.29737989   0.27738058   0.33287403   0.45805966   0.41737288
[199]   0.41930124   0.46767345   0.35554618   0.36598607   0.51671167
0.43152426   0.33520139   0.32601011   0.30031173   0.37004682   0.26045281
[210]   0.28764871   0.23143317   0.25503212   0.18233469   0.14801844
0.20861285   0.21798301   0.15360746   0.14610464   0.21651309   0.20595152
[221]   0.16052344   0.20405609   0.27489989   0.27768645   0.25258991
0.37910532   0.48726780   0.53103995   0.59329792   0.59353807   0.37209908
[232]   0.41506626   0.54313380   0.44358170   0.35133728   0.50304382
0.55782882   0.43557747   0.44014596   0.63365653   0.54531874   0.42640835
[243]   0.51241410   0.66691176   0.72602317   1.47752020   1.91345466
0.99408222   0.51898148   0.44061425   0.23419269   0.13475547   0.17831153
[254]   0.18037991   0.21864655   0.15556972   0.13992278   0.19817662
0.18322798   0.16054865   0.20201829   0.23689865   0.23432615   0.24921487
[265]   0.32166477   0.36687666   0.38870037   0.47950916   0.59898923
0.63352453   0.71729489   0.72802376   0.65638342   0.41639415   0.45405109
[276]   0.52719509   0.38286361   0.30239096   0.37886042   0.38821020
0.29177042   0.27187184   0.32772067   0.29783137   0.28430122   0.37854550
[287]   0.49760807   0.55204839   1.02948221   0.99236083   0.75687383
0.40274311   0.31818665   0.32274599   0.33592945   0.35702757   0.51293454
[298]   0.42324704   0.35520356   0.28468024   0.23790124   0.25021404
0.25178006   0.31348171   0.31287145   0.26267961   0.18051029   0.17012871
[309]   0.14875294   0.12079543   0.11614648   0.12333212   0.09508851
0.08604094   0.11932786   0.12381738   0.10934257   0.13100484   0.17275529
[320]   0.18670467   0.21383374   0.25163080   0.29810534   0.41636608
0.48763341   0.52862753   0.87436522   0.80710615   0.77120752   0.70249609
[331]   0.50085341   0.40690405   0.45029182   0.38562725   0.26734010
0.27625442   0.31858891   0.25335239   0.18907978   0.23415659   0.25425709
[342]   0.19518745   0.19302446   0.24388295   0.24251494   0.23005004
0.25616587   0.32016321   0.34126483   0.40659291   0.51651651   0.56259071
[353]   0.46719253   0.42973290   0.31341332   0.26993474   0.25601717
0.32542063   0.40955864   0.38430379   0.42829166   0.52724781   0.58156434
[364]   0.43034870   0.45933066   0.59497764   0.54202938   0.43508852
0.41973171   0.42988727   0.52555361   0.52616309   0.52183513   0.61825197
[375]   0.70443553   0.90620435   1.09482912   1.20814739   1.00132624
1.04504905   0.89596685   1.00511961   1.06030389   1.03180360   1.02259970
[386]   0.81642982   0.67747908   0.80018383   0.62844236   0.55856512
0.41039768   0.33622695   0.23068017   0.23289861   0.19175906   0.22912386
[397]   0.20053328   0.25571310   0.35283458   0.36987668   0.37408668
0.45075870   0.41660118   0.53229470   0.58122797   0.64281969   0.59820234
[408]   0.60389264   0.54066703   0.63313707   0.50731298   0.39490666
0.38639883   0.30015975   0.31304196   0.29312549   0.23626801   0.21165932
[419]   0.15079504   0.10585826   0.11779768   0.12450908   0.08533204
0.07773657   0.10154621   0.10664104   0.09042621   0.11400235   0.14050740
[430]   0.15359958   0.18806150   0.21197363   0.23812195   0.28530060
0.34048800   0.39799150   0.40267179   0.37447919   0.46076790   0.45359045
[441]   0.71320978   1.10054807   1.15736482   0.82515450   0.71541749
0.97295646   1.00378535   1.00676285   1.66370424   1.55962786   1.81391576
[452]   2.48794621   2.33914334   2.42327754   3.78451108   4.65931303
9.50337172 15.60807998 23.78112353 55.41148960 52.40315731 159.86502217
[463] 173.67516011 173.00040601 342.13753507 673.68123621

MASSES for TRUNCATED MINIMIZED CHARACTERIZED Q93WY9

```
 [1]  88.08494  87.07700 137.13900 114.10300 163.17300  57.05100 128.10600
71.07800 113.15800  87.07700 129.18000  87.07700 147.17400 113.15800 147.17400
 [16] 163.17300 128.10600  71.07800 117.12600 157.19400  87.07700  57.05100
163.17300 113.15800  97.11500 137.13900 114.07900 117.12600 157.19400  99.13100
 [31] 117.12600 186.21000 157.19400  57.05100 114.10300  87.07700  57.05100
113.15800 113.15800 114.07900  57.05100 129.18000  71.07800  87.07700  57.05100
 [46]  99.13100 114.07900 113.15800  99.13100  57.05100  57.05100 163.17300
163.17300 114.07900  71.07800  57.05100 114.07900 114.10300  99.13100 129.18000
 [61] 147.17400  57.05100 113.15800  97.11500 131.19600  71.07800 147.17400
101.10400  99.13100 101.10400 131.19600 131.19600  87.07700 186.21000  87.07700
 [76] 113.15800 113.15800 128.10600 163.17300  57.05100 129.18000 117.12600
131.19600  57.05100 128.10600  87.07700  57.05100 128.10600 113.15800  87.07700
 [91] 114.10300  71.07800 113.15800 114.07900  71.07800  99.13100 129.18000
186.21000  57.05100 101.10400 114.07900 163.17300 113.15800 113.15800 129.18000
[106]  71.07800 137.13900  97.11500 128.10600  97.11500 114.10300  99.13100
113.15800 163.17300  57.05100 128.10600  99.13100  57.05100 114.07900  57.05100
[121] 101.10400 101.10400 114.07900 137.13900 163.17300 103.14300 186.21000
117.12600 157.19400  97.11500 128.10600 114.07900 131.19600 101.10400 101.10400
[136]  87.07700 157.19400  71.07800  71.07800 163.17300 157.19400 113.15800
114.07900  97.11500  87.07700 157.19400  97.11500  57.05100  87.07700 114.07900
[151] 113.15800  71.07800  57.05100 128.10600 101.10400  71.07800  71.07800
71.07800 131.19600  71.07800  71.07800  71.07800  87.07700 113.15800  99.13100
[166] 147.17400 157.19400 114.10300  87.07700 114.10300  97.11500  71.07800
163.17300  71.07800 129.18000 128.10600 113.15800 113.15800 101.10400 137.13900
[181]  71.07800 163.17300 117.12600 113.15800 147.17400 128.10600 147.17400
71.07800 114.07900 129.18000 163.17300 157.19400  57.05100 129.18000 163.17300
[196] 114.07900  87.07700  87.07700 113.15800 101.10400  99.13100  71.07800
117.12600 129.18000 163.17300 163.17300 157.19400  87.07700  87.07700  57.05100
[211] 163.17300  71.07800 114.07900 128.10600 113.15800 113.15800 186.21000
71.07800  71.07800  71.07800 186.21000 113.15800 163.17300 129.18000  71.07800
[226]  87.07700 114.10300 129.18000 128.10600 163.17300 163.17300 113.15800
114.10300 163.17300 113.15800  57.05100 128.10600 114.10300  57.05100 114.07900
[241]  71.07800 113.15800  57.05100  57.05100 101.10400  57.05100 186.21000
87.07700 131.19600 101.10400 128.10600 147.17400  57.05100 186.21000 114.07900
[256]  99.13100 129.18000 163.17300  71.07800  57.05100  99.13100 117.12600
101.10400 113.15800  71.07800  71.07800 129.18000 147.17400 113.15800 131.19600
[271] 117.12600 128.10600 129.18000 163.17300 117.12600 128.10600 129.18000
71.07800 128.10600 114.10300 147.17400 131.19600 103.14300  71.07800 103.14300
[286] 113.15800  57.05100 129.18000  57.05100 114.10300 117.12600 114.10300
113.15800 137.13900 129.18000  87.07700  97.11500  57.05100  57.05100 113.15800
[301] 113.15800 147.17400 157.19400 117.12600 157.19400 186.21000 114.10300
114.10300 131.19600 117.12600 147.17400  99.13100 101.10400  87.07700  71.07800
[316]  87.07700 147.17400 113.15800  71.07800 101.10400  99.13100 163.17300
87.07700 114.07900 163.17300 113.15800  71.07800  87.07700 101.10400  99.13100
[331] 113.15800  97.11500  87.07700 128.10600 113.15800 113.15800  87.07700
147.17400  71.07800 129.18000  87.07700 117.12600  99.13100 114.07900 163.17300
[346] 113.15800 113.15800  57.05100 114.07900 114.10300  97.11500 157.19400
71.07800 101.10400  87.07700 163.17300 131.19600  99.13100  57.05100 163.17300
[361]  57.05100 114.10300 114.10300 163.17300  97.11500 157.19400 117.12600
99.13100 137.13900 137.13900 157.19400  57.05100  87.07700 157.19400  57.05100
[376]  57.05100 163.17300  71.07800 101.10400 186.21000 147.17400  87.07700
157.19400 129.18000  71.07800  87.07700 114.07900  97.11500 114.10300 113.15800
[391] 113.15800  71.07800  57.05100  71.07800 113.15800  99.13100  57.05100
57.05100  97.11500 114.07900  71.07800 163.17300 114.07900 114.10300 147.17400
```

```
[406]  71.07800 114.07900 117.12600 157.19400 114.07900 114.10300 163.17300
128.10600 117.12600 101.10400 128.10600  97.11500  71.07800 101.10400 163.17300
[421] 114.10300 114.10300  71.07800  97.11500 113.15800 113.15800  57.05100
99.13100 113.15800  71.07800 157.19400 113.15800 137.13900  57.05100  57.05100
[436] 117.12600  87.07700  87.07700 117.12600 113.15800 113.15800  97.11500
99.13100  71.07800 113.15800  97.11500 117.12600  97.11500 129.18000  97.11500
[451] 114.07900  97.11500 128.10600 117.12600 129.18000  99.13100 101.10400
97.11500  71.07800  97.11500  71.07800  87.07700  87.07700 101.10400  71.07800
[466] 131.08634

Call:
  nma.pdb(pdb = pdbT4_ZSP9, mass = TRUE)

Class:
  VibrationalModes (nma)

Number of modes:
  1428 (6 trivial)

Frequencies:
  Mode 7:       0.002
  Mode 8:       0.002
  Mode 9:       0.004
  Mode 10:      0.007
  Mode 11:      0.009
  Mode 12:      0.011

+ attr: modes, frequencies, force.constants, fluctuations,
        U, L, xyz, mass, temp, triv.modes, natoms, call

 NORMAL MODES for TRUNCATED MINIMIZED CHARACTERIZED Q9ZSP9

  [1] 0.000000000 0.000000000 0.000000000 0.000000000 0.000000000 0.000000000
0.001583572 0.002434602 0.003839561 0.006951993 0.009438572 0.011348093
  [13] 0.014597207 0.015132279 0.015921859 0.017307688 0.017961959 0.020092011
0.021217210 0.022170575 0.022515784 0.022932123 0.023272567 0.023944225
  [25] 0.024069258 0.024412521 0.025007086 0.025290116 0.026317414 0.027638483
0.028277225 0.028894403 0.029010764 0.029678023 0.029923669 0.030303034
  [37] 0.030396928 0.031569297 0.031756895 0.032583647 0.032932000 0.033224524
0.033767488 0.033902992 0.034505438 0.034882174 0.035055660 0.036060450
  [49] 0.036348703 0.036780635 0.036893407 0.037813285 0.037881886 0.038262119
0.038344452 0.038676943 0.039268076 0.039430296 0.039910462 0.040149338
  [61] 0.040680825 0.040875563 0.041109140 0.041191009 0.041748074 0.041929097
0.042317860 0.042609556 0.043241552 0.043355922 0.044183201 0.044289421
  [73] 0.044548034 0.044844147 0.045317257 0.045855685 0.046174686 0.046546223
0.046778018 0.046862686 0.047069519 0.047542595 0.047798180 0.048411365
  [85] 0.048981986 0.049248362 0.049340856 0.049629554 0.050042526 0.050303286
0.050520101 0.050900727 0.050993204 0.051335796 0.051669500 0.051892322
  [97] 0.052296144 0.052486878 0.052570542 0.052836565 0.053257962 0.053388122
0.053985910 0.054353452 0.054611254 0.054755777 0.055110832 0.055655325
 [109] 0.055833224 0.056112670 0.056197248 0.056660277 0.056710996 0.056794905
0.057086728 0.057212826 0.057397372 0.057664628 0.057897410 0.058305909
 [121] 0.058878940 0.058917002 0.059190877 0.059640010 0.059958126 0.060119081
0.060482648 0.060653905 0.060986671 0.061283540 0.061331055 0.061459366
```

[133] 0.061651539 0.062046754 0.062505153 0.062636115 0.063004254 0.063112310
0.063432979 0.063899273 0.064090057 0.064290521 0.064573968 0.064812810
[145] 0.065014161 0.065094955 0.065244404 0.065456038 0.065643649 0.065910710
0.066262379 0.066501251 0.066579860 0.066913061 0.066964903 0.067089236
[157] 0.067491830 0.067840716 0.068201561 0.068576022 0.068723797 0.068909312
0.069187932 0.069262578 0.069585664 0.069674246 0.069826046 0.069963760
[169] 0.070008097 0.070541557 0.070645794 0.070788175 0.070891871 0.071242773
0.071595493 0.071662330 0.071916200 0.072221630 0.072259149 0.072526473
[181] 0.072755050 0.072962257 0.073248112 0.073488405 0.073584682 0.073789388
0.073866928 0.074254949 0.074607179 0.074778777 0.074872210 0.074981912
[193] 0.075385869 0.075694867 0.075899222 0.075957103 0.076163248 0.076197829
0.076401667 0.076654215 0.077070442 0.077148296 0.077415425 0.077499796
[205] 0.077633034 0.077909068 0.078006057 0.078196275 0.078442400 0.078603370
0.078784269 0.079079509 0.079213607 0.079300857 0.079467898 0.079622659
[217] 0.079875488 0.079927004 0.080158494 0.080422081 0.080637859 0.080742551
0.080996420 0.081013462 0.081293597 0.081420937 0.081801151 0.081953360
[229] 0.082209036 0.082403077 0.082618280 0.082848518 0.083185525 0.083419056
0.083617408 0.083964000 0.084266643 0.084536898 0.084871826 0.084908827
[241] 0.085097307 0.085280470 0.085502207 0.085738439 0.085869955 0.086179426
0.086219243 0.086395044 0.086507556 0.086694606 0.087063718 0.087140783
[253] 0.087384326 0.087558655 0.087758619 0.087986490 0.088239468 0.088302025
0.088546808 0.088955370 0.089040612 0.089186005 0.089337683 0.089664850
[265] 0.089848428 0.090007854 0.090037962 0.090260359 0.090387818 0.090662457
0.090916626 0.091125205 0.091335110 0.091429909 0.091555463 0.091712751
[277] 0.091767421 0.092223664 0.092438062 0.092568269 0.092723293 0.092959567
0.093312721 0.093433847 0.093592309 0.093819645 0.093894268 0.094014106
[289] 0.094264075 0.094537366 0.094648897 0.094781947 0.094890654 0.095152164
0.095479039 0.095838238 0.095946937 0.096230849 0.096321355 0.096466803
[301] 0.096666289 0.096851635 0.096993807 0.097227519 0.097283516 0.097504584
0.097715951 0.098140931 0.098151126 0.098403456 0.098621115 0.098697112
[313] 0.098936402 0.099309751 0.099398219 0.099564870 0.099652475 0.099841284
0.100145395 0.100303860 0.100481235 0.100671761 0.100812942 0.100868204
[325] 0.101202647 0.101295339 0.101616910 0.101761510 0.102028746 0.102144745
0.102320043 0.102460808 0.102592244 0.102657406 0.102742005 0.103202501
[337] 0.103440551 0.103574046 0.103667793 0.104021606 0.104094877 0.104277707
0.104461672 0.104668427 0.104959035 0.105180106 0.105327029 0.105502922
[349] 0.105724773 0.105834327 0.106015472 0.106276659 0.106338134 0.106534115
0.106621459 0.106975211 0.107173927 0.107397869 0.107628994 0.107763411
[361] 0.107964431 0.108201485 0.108299062 0.108439424 0.108837663 0.109191543
0.109383222 0.109575029 0.109856231 0.110125215 0.110184658 0.110413964
[373] 0.110504659 0.110690861 0.111192482 0.111241905 0.111511976 0.111678129
0.111825122 0.112072318 0.112129034 0.112265848 0.112627286 0.112762260
[385] 0.113002363 0.113111699 0.113341676 0.113541855 0.113831728 0.113986499
0.113991388 0.114202295 0.114332972 0.114393660 0.114555854 0.114735151
[397] 0.114852431 0.115088614 0.115177278 0.115459864 0.115684406 0.115707833
0.116056151 0.116127391 0.116333990 0.116598784 0.116698239 0.116975310
[409] 0.117102569 0.117368974 0.117490309 0.117856465 0.118000162 0.118201881
0.118388279 0.118866689 0.118953388 0.119299450 0.119443850 0.119697744
[421] 0.119808053 0.119881500 0.120150177 0.120565306 0.120635247 0.120856777
0.121023809 0.121088779 0.121359584 0.121645300 0.121887439 0.121921101
[433] 0.122199286 0.122437330 0.122533080 0.122800699 0.122995471 0.123385838
0.123505877 0.123674042 0.124181250 0.124215514 0.124357362 0.124619133
[445] 0.124847695 0.124935110 0.125393593 0.125444085 0.125804709 0.125835612
0.125936723 0.126349881 0.126351384 0.126544194 0.127054698 0.127260774

[457] 0.127434122 0.127570406 0.127823319 0.128139400 0.128384976 0.128695053
0.128854185 0.129001831 0.129172254 0.129185196 0.129475750 0.129698878
[469] 0.129788490 0.129921135 0.130359849 0.130607651 0.130820813 0.131014489
0.131049286 0.131432018 0.131529886 0.131669049 0.131863976 0.132260535
[481] 0.132487289 0.132694378 0.132880272 0.133036873 0.133104544 0.133177695
0.133354273 0.133820539 0.133995514 0.134067423 0.134571504 0.134727456
[493] 0.135227484 0.135389229 0.135572362 0.135647171 0.135781834 0.136025345
0.136163914 0.136475805 0.136717987 0.136818091 0.137299714 0.137658446
[505] 0.137805849 0.138159508 0.138186732 0.138278903 0.138912272 0.139001321
0.139263031 0.139738765 0.139764050 0.140032294 0.140222099 0.140311940
[517] 0.140486673 0.140554361 0.140828025 0.140939138 0.141303420 0.141372150
0.141460634 0.141543427 0.141924809 0.141953897 0.142289237 0.142465990
[529] 0.142864502 0.143115431 0.143444170 0.143616062 0.144075051 0.144227225
0.144482888 0.144678847 0.145128627 0.145392897 0.145523155 0.145756474
[541] 0.145897346 0.145911408 0.146481008 0.146724199 0.146935788 0.147055636
0.147422329 0.147857512 0.148003144 0.148760841 0.148862886 0.149097435
[553] 0.149336280 0.149772835 0.149959264 0.150408831 0.150454127 0.150911956
0.151265366 0.151397264 0.151666060 0.151864426 0.152368812 0.152395242
[565] 0.152591830 0.152869793 0.153124178 0.153257120 0.153591529 0.153843897
0.154033785 0.154206850 0.154375783 0.154556659 0.154580176 0.154916716
[577] 0.155274877 0.155655324 0.155777652 0.155923034 0.156024048 0.156077046
0.156450281 0.156778525 0.156939848 0.157081494 0.157192157 0.157292839
[589] 0.157573361 0.157693879 0.158036531 0.158130027 0.158214823 0.158669017
0.159310441 0.159465152 0.159671755 0.159811218 0.160300713 0.160434182
[601] 0.160881245 0.161291894 0.161337667 0.161800629 0.161856039 0.162355595
0.162775285 0.163314455 0.163413379 0.163724725 0.164297938 0.164636923
[613] 0.164788324 0.165135889 0.165710641 0.165786365 0.166049717 0.166181160
0.166540572 0.166579884 0.166952625 0.167107158 0.167204217 0.167695167
[625] 0.168242133 0.168335077 0.168425263 0.168768490 0.168953298 0.169199670
0.170270278 0.170464009 0.170650914 0.170678521 0.171031889 0.171332865
[637] 0.171398938 0.171742859 0.171792114 0.171852409 0.171912168 0.172339525
0.172581796 0.173016943 0.173465089 0.173857089 0.174168745 0.174340783
[649] 0.175008718 0.175029342 0.175116659 0.175773431 0.176144980 0.176437019
0.176680554 0.177115069 0.177265887 0.177355388 0.177560362 0.177868520
[661] 0.178771600 0.179110982 0.179308016 0.179670776 0.179933937 0.180410535
0.180648362 0.180923892 0.181292291 0.181396772 0.181505729 0.181652064
[673] 0.181903796 0.182248533 0.182310858 0.182779596 0.183145783 0.183204208
0.184006656 0.184094186 0.184679339 0.184845568 0.184936743 0.184948658
[685] 0.185204320 0.185509816 0.185916137 0.186542969 0.186968044 0.187101308
0.187253349 0.187405334 0.188261462 0.188611702 0.188778629 0.188961896
[697] 0.189610923 0.190080715 0.190142071 0.190823326 0.191303050 0.191586262
0.192145569 0.192399895 0.192587675 0.192756940 0.193555249 0.193687185
[709] 0.194035660 0.194212076 0.194694509 0.194885991 0.195020274 0.195410709
0.195845962 0.196396684 0.196701474 0.197080802 0.197300141 0.197472678
[721] 0.197546742 0.197842334 0.198311274 0.198388025 0.198492696 0.198562615
0.198860710 0.199426732 0.200278774 0.200456520 0.200687757 0.201272931
[733] 0.201614577 0.201830244 0.201989758 0.202367998 0.202659058 0.203268716
0.203407614 0.203667155 0.203808702 0.204262892 0.204742241 0.205305003
[745] 0.205465949 0.206094098 0.207032687 0.207140510 0.207330760 0.207725066
0.208166452 0.208602290 0.208774708 0.208885209 0.209147884 0.209465320
[757] 0.209631168 0.209813063 0.210223920 0.210638546 0.211142232 0.211589419
0.211922860 0.212042054 0.212522808 0.212856334 0.213016986 0.213306103
[769] 0.213561976 0.213892224 0.214323568 0.214557982 0.216262288 0.216376925
0.216820033 0.216854669 0.217412235 0.218421359 0.218548541 0.218768934

[781] 0.219032014 0.219276643 0.219982129 0.220178655 0.220634564 0.221000665
0.221228176 0.221441098 0.222252855 0.222384053 0.223027928 0.223236013
[793] 0.223563980 0.224081286 0.224642734 0.225238772 0.225644387 0.225902880
0.226136549 0.226379319 0.226563309 0.228044765 0.228682338 0.228868627
[805] 0.229412949 0.229654241 0.230163361 0.231851882 0.232048071 0.233096963
0.233844165 0.233992843 0.234157761 0.234330293 0.234488871 0.234743344
[817] 0.235089361 0.235233213 0.235674026 0.235800550 0.236673301 0.237032743
0.237307866 0.237790694 0.237957132 0.239132090 0.239347763 0.239645438
[829] 0.242484869 0.243753576 0.243824905 0.244060356 0.244396239 0.245000154
0.245715749 0.247026571 0.247411005 0.248224827 0.249504347 0.250137045
[841] 0.250317688 0.250737993 0.250981593 0.252162362 0.252234175 0.253149683
0.253679450 0.254281290 0.254350862 0.255310921 0.255709002 0.256510718
[853] 0.257556516 0.258213237 0.258707327 0.258943038 0.260584034 0.261276871
0.261645257 0.261966281 0.262365946 0.262575270 0.263001942 0.263129092
[865] 0.263905148 0.264534523 0.264945755 0.265467583 0.266493962 0.267004653
0.267566205 0.268779716 0.270368274 0.270774997 0.270970767 0.272078157
[877] 0.272190458 0.273470155 0.274534665 0.275605173 0.276126717 0.276498358
0.276766328 0.277635021 0.277875005 0.277987926 0.278311442 0.278748967
[889] 0.281499296 0.281692380 0.282443799 0.283414067 0.283735771 0.283791785
0.284757772 0.285773636 0.291086429 0.292270319 0.293881876 0.293898209
[901] 0.294774376 0.296148605 0.297481962 0.298570983 0.298850437 0.299145931
0.300778716 0.301488742 0.301877157 0.303323184 0.306055866 0.306147968
[913] 0.307811873 0.308234849 0.308809317 0.309317785 0.310722586 0.312233281
0.312245652 0.312707104 0.313372284 0.316110766 0.317165534 0.321004789
[925] 0.325014736 0.328717958 0.330124798 0.333381400 0.334867598 0.336254058
0.340196697 0.341541687 0.345168693 0.353982706 0.355393095 0.355475122
[937] 0.355737111 0.357959494 0.359568848 0.371728814 0.372745090 0.373416978
0.379197499 0.385972493 0.388953939 0.396254995 0.398389175 0.401085930
[949] 0.404471614 0.406150037 0.406316460 0.410833970 0.415592871 0.435128014
0.443420324 0.448830428 0.449471114 0.453888301 0.454240058 0.454892977
[961] 0.456381205 0.461527190 0.461727033 0.469314465 0.471694521 0.474598039
0.476322960 0.479178030 0.482852727 0.482862642 0.485311390 0.485758668
[973] 0.490642269 0.495098618 0.495671302 0.499084170 0.504239382 0.509112384
0.512027326 0.515675377 0.517786704 0.521048370 0.521419625 0.521611745
[985] 0.528429477 0.528903342 0.530244823 0.532405686 0.535525923 0.535603135
0.536958328 0.537273757 0.542462924 0.542510271 0.542696069 0.543637106
[997] 0.546339581 0.549733200 0.550737071 0.552653022 0.554589279 0.554875968
0.556034530 0.557276209 0.557815189 0.560694417 0.563011033 0.563231759
[1009] 0.565693605 0.565808022 0.566122768 0.567410743 0.568157579 0.568627760
0.568747087 0.570685664 0.571324327 0.572175213 0.572583946 0.574382582
[1021] 0.575043989 0.575691854 0.576406780 0.576721539 0.579292358 0.580271982
0.580916218 0.581958575 0.583061109 0.583245281 0.583258419 0.583621133
[1033] 0.584096234 0.584427158 0.587102264 0.588090157 0.589194329 0.590306885
0.592047254 0.593558390 0.594560628 0.595703941 0.596955674 0.597472913
[1045] 0.599406886 0.600000141 0.601251018 0.601252978 0.602096949 0.602148735
0.602658218 0.602841761 0.605260578 0.605732778 0.605946261 0.606780303
[1057] 0.607386790 0.608529669 0.608773379 0.608934196 0.608969075 0.609618448
0.610323266 0.610349433 0.610854565 0.611127357 0.611197960 0.611484455
[1069] 0.611909486 0.612130809 0.612822900 0.612920419 0.613299128 0.613420853
0.613542615 0.613727958 0.615139146 0.615785140 0.616529212 0.617519333
[1081] 0.617764232 0.617768804 0.618679913 0.618998466 0.619545671 0.620058426
0.620206719 0.621135866 0.621631724 0.622621359 0.622931329 0.623740101
[1093] 0.624355430 0.625449909 0.625642717 0.625879864 0.626455893 0.626696471
0.629427397 0.629429590 0.630075123 0.630270495 0.630674029 0.631815297

[1105] 0.631899102 0.631899443 0.633980067 0.634216393 0.634276259 0.636310519
0.636414151 0.637201457 0.638814694 0.638883229 0.639305655 0.639959513
[1117] 0.641006743 0.641065324 0.641470578 0.641748670 0.642451496 0.643419462
0.643968120 0.645259953 0.645388739 0.645818892 0.647473391 0.647603928
[1129] 0.647867912 0.648372294 0.648755316 0.648879426 0.649678938 0.651214925
0.651554990 0.652931947 0.654032497 0.654042780 0.654918027 0.656472334
[1141] 0.656674182 0.656845639 0.657015008 0.657142993 0.657339857 0.657420890
0.657918603 0.658142504 0.658866999 0.660372179 0.662454610 0.662543868
[1153] 0.663043504 0.663138337 0.663231379 0.663302413 0.663910071 0.664479969
0.665164622 0.665669692 0.666551922 0.666963604 0.667627176 0.667928756
[1165] 0.668249627 0.669724681 0.670174536 0.670203884 0.671085022 0.671303023
0.672422176 0.673160296 0.674214623 0.674584774 0.674801569 0.675135175
[1177] 0.676672935 0.677228670 0.677648910 0.677860727 0.678185134 0.678780831
0.681524269 0.682682805 0.683859955 0.684428304 0.684919188 0.685583818
[1189] 0.686242304 0.686289679 0.686979697 0.687115336 0.688744391 0.688764195
0.688768204 0.688907204 0.688928511 0.689920909 0.690137272 0.690425333
[1201] 0.691574105 0.691839856 0.691873575 0.693439585 0.694248712 0.695067429
0.696871600 0.698185181 0.698640277 0.698667342 0.700769643 0.701168387
[1213] 0.701672342 0.702262633 0.704258537 0.704268878 0.704452123 0.704885043
0.705278749 0.705998932 0.706566447 0.707279052 0.707461911 0.707854488
[1225] 0.708117491 0.708774199 0.709090643 0.710069649 0.710254697 0.710718476
0.710818583 0.711078050 0.711840655 0.712374041 0.712843566 0.713384209
[1237] 0.714293431 0.714822931 0.715398088 0.718100295 0.718490109 0.718861476
0.718986397 0.722117881 0.722120354 0.722160376 0.722972641 0.723024143
[1249] 0.723525846 0.725325024 0.725700032 0.725884985 0.726060071 0.726118993
0.726746015 0.726896292 0.726930912 0.728034069 0.728150458 0.728364246
[1261] 0.728844080 0.729422766 0.729641684 0.729931904 0.730538196 0.730888522
0.731625995 0.732021910 0.734759337 0.735546911 0.735563338 0.736104224
[1273] 0.738527161 0.738846102 0.740339939 0.740466641 0.740655619 0.741252781
0.743231466 0.743780143 0.745413080 0.745803494 0.746117185 0.746446456
[1285] 0.746579264 0.747614620 0.747767579 0.748250339 0.749127642 0.750025771
0.750100185 0.750131775 0.750640922 0.750990708 0.751013036 0.751405597
[1297] 0.752242417 0.754052892 0.754640006 0.755153426 0.755171807 0.755434399
0.757912015 0.760244648 0.760581441 0.762044687 0.762901828 0.763209736
[1309] 0.764458155 0.764639564 0.765128347 0.766296773 0.767990498 0.769148764
0.770575773 0.771721838 0.772306425 0.773884933 0.774881468 0.775307047
[1321] 0.776182308 0.776217536 0.776419752 0.777503564 0.778368252 0.781250002
0.782027422 0.784022531 0.785443221 0.786342972 0.787079865 0.787824460
[1333] 0.787984674 0.788559181 0.788980320 0.791453433 0.791505999 0.791669708
0.792046675 0.794201378 0.795502832 0.795920536 0.796046585 0.797743494
[1345] 0.798362505 0.802639144 0.802701959 0.805079738 0.805641332 0.805827866
0.807763769 0.808552318 0.810442928 0.810646590 0.811875230 0.812391234
[1357] 0.812758014 0.813984400 0.814762514 0.814854518 0.815379152 0.817503436
0.817690842 0.819826701 0.820103879 0.820677769 0.821721672 0.822533518
[1369] 0.823258031 0.826211112 0.827044263 0.828551202 0.828822727 0.831886156
0.833528231 0.833985448 0.835186427 0.836602360 0.839701404 0.839784356
[1381] 0.842405295 0.842845165 0.847514837 0.850691478 0.853587221 0.856869994
0.857916553 0.858076891 0.858464915 0.860429423 0.863640085 0.866549587
[1393] 0.868615277 0.869578435 0.870820543 0.871333080 0.879733263 0.881116148
0.884796366 0.885580766 0.894324014 0.902873059 0.904439929 0.907032873
[1405] 0.914991706 0.916709082 0.919937792 0.928654337 0.929328867 0.932219032
0.932376943 0.938854335 0.943091709 0.946673589 0.948098219 0.958209977
[1417] 0.959389773 0.963464636 0.967361053 0.975417955 0.980262001 0.982630159
0.989174335 0.998133142 1.074690804 1.081047260 1.082658762 1.113243002

Supplementary Text 5

FLUCTUATION DATA for TRUNCATED MINIMIZED CHARACTERIZED Q9ZSP9

[1] 0.38132767 0.54733740 0.32694749 0.23151536 0.15763097 0.15053162
0.18726737 0.16616411 0.13471286 0.16191801 0.16912021 0.13093495
[13] 0.15958088 0.20298766 0.18847680 0.17505992 0.22534062 0.26229347
0.23390004 0.26044221 0.28863280 0.34140185 0.46271489 0.50149396
[25] 0.63659784 1.14191012 0.89980534 0.53592948 0.47106186 0.42483582
0.54717405 0.57269969 0.48489202 0.56261332 0.49516127 0.43112292
[37] 0.39838580 0.48702530 0.53906483 0.37838270 0.38137736 0.54480285
0.64825545 0.63982888 0.68381298 0.56280127 0.48559811 0.33212667
[49] 0.35999425 0.27834607 0.21261013 0.19345570 0.20551814 0.17959508
0.23474253 0.30940567 0.31615032 0.30551900 0.26741688 0.20651027
[61] 0.17648399 0.15003555 0.11402800 0.10601490 0.10576310 0.08798591
0.07631474 0.07791631 0.08117956 0.06836283 0.07066271 0.08140382
[73] 0.09098907 0.10357418 0.10194492 0.12100092 0.16105084 0.19192203
0.16136440 0.16627350 0.20664968 0.15439888 0.15176936 0.20736286
[85] 0.22924527 0.21138161 0.23413273 0.20461073 0.18454971 0.24089273
0.21297686 0.15918787 0.18246862 0.21210022 0.17582998 0.15018258
[97] 0.18056744 0.20405797 0.15879386 0.16699528 0.22913004 0.21067370
0.18769675 0.24045044 0.26419046 0.23552662 0.24225331 0.43054088
[109] 0.39098429 0.32824963 0.29207381 0.27866358 0.21383079 0.21780468
0.19474490 0.21433729 0.26638752 0.33385653 0.38541144 0.37321514
[121] 0.51473270 0.51733651 0.42076255 0.44559888 0.60050580 0.58393930
0.52288625 0.54202126 0.48059905 0.43960417 0.52982524 0.63458297
[133] 0.64506701 0.95585030 0.74668101 0.77641883 0.48435173 0.42707187
0.32349393 0.27447067 0.23919552 0.22082215 0.27486616 0.32976827
[145] 0.51165101 0.29097025 0.20232608 0.14074671 0.12621717 0.10253741
0.10554785 0.11034768 0.09360318 0.08042259 0.09658645 0.10682195
[157] 0.08576355 0.07624093 0.11085953 0.11788005 0.10368474 0.11957471
0.16202388 0.18742485 0.18847511 0.23034519 0.29666170 0.51315693
[169] 0.48650593 0.45490261 0.40918557 0.47812358 0.35828975 0.24817170
0.31691623 0.31015346 0.20162475 0.20702233 0.27001818 0.22133705
[181] 0.16276093 0.21701884 0.23060086 0.16620378 0.16950981 0.24248071
0.21074105 0.16171262 0.23537900 0.29901299 0.27336504 0.25663043
[193] 0.21512986 0.16967921 0.16157135 0.19514178 0.27209202 0.25296524
0.26904676 0.31693191 0.24676059 0.24151705 0.40774432 0.34209673
[205] 0.25189283 0.22582309 0.20332511 0.28434644 0.20444374 0.19069058
0.14622810 0.16776581 0.13038026 0.10338347 0.15074715 0.15556637
[217] 0.11763461 0.11094672 0.16321747 0.16258515 0.13526254 0.17308029
0.22768586 0.23433969 0.23030356 0.33826600 0.43767965 0.50382388
[229] 0.54695231 0.48739256 0.29120660 0.32644516 0.42270196 0.33204958
0.25601055 0.37404612 0.40549079 0.30682272 0.34328338 0.51843509
[241] 0.45661755 0.34220647 0.38497459 0.45467110 0.58262358 1.09483907
1.82988777 0.89175760 0.45800839 0.41070996 0.22653481 0.13570669
[253] 0.17680826 0.16860345 0.18582991 0.12748195 0.11658300 0.15851457
0.16120987 0.14454015 0.16634969 0.19336766 0.19533071 0.20964397
[265] 0.26363894 0.28855607 0.32463521 0.40114027 0.51700139 0.51872275
0.60957679 0.59775962 0.55011038 0.33357267 0.35036522 0.43673892
[277] 0.32145573 0.25607461 0.32603119 0.33828610 0.26278070 0.24876211
0.30272305 0.29205531 0.27304544 0.34670315 0.45976452 0.50519127
[289] 0.63544664 0.86643558 0.80918538 0.45228150 0.33016524 0.34904036
0.36324810 0.39405780 0.54120221 0.41433056 0.34978729 0.28383480
[301] 0.25035262 0.27035207 0.27200301 0.32598955 0.29969506 0.23775244

0.16803851 0.17289802 0.14760622 0.12029840 0.12242275 0.12763431
[313] 0.09457142 0.08748278 0.11867916 0.11473056 0.09444987 0.11544647
0.14869228 0.14901887 0.16859434 0.20636706 0.23818153 0.36486515
[325] 0.40623795 0.44833385 0.78386009 0.75568320 0.67562149 0.62286269
0.42902434 0.32602929 0.36485984 0.32959009 0.22472302 0.21353790
[337] 0.25943560 0.22870936 0.16351816 0.18417002 0.21550582 0.17532399
0.16369688 0.19869037 0.21205141 0.20166723 0.20120316 0.25820472
[349] 0.28735883 0.37102205 0.48863298 0.51235698 0.38723117 0.35227684
0.24485975 0.22543462 0.19979633 0.24593198 0.30008343 0.27952295
[361] 0.30476829 0.39276733 0.45425066 0.33015037 0.36539645 0.46088419
0.42081643 0.33906715 0.32709660 0.33084702 0.39247109 0.39418547
[373] 0.39644047 0.45743140 0.51268289 0.66383831 0.77520523 0.90519928
0.73495671 0.78328073 0.69593289 0.80064285 0.81098319 0.80679644
[385] 0.82058964 0.63534788 0.50656866 0.60726817 0.48224434 0.42615951
0.31846380 0.24814090 0.17154148 0.18489930 0.15771839 0.19272974
[397] 0.17840736 0.23457018 0.33678762 0.38250735 0.41451749 0.47342617
0.42127641 0.50489802 0.52768907 0.55357923 0.48341247 0.48788929
[409] 0.43483696 0.49968262 0.42547745 0.32304120 0.32838494 0.26003509
0.26007915 0.23938694 0.19380636 0.17934694 0.13024355 0.09846229
[421] 0.10589765 0.10799598 0.07886344 0.07293740 0.08813391 0.08541255
0.07238070 0.08923658 0.09743992 0.10841394 0.13564272 0.14743905
[433] 0.14557719 0.18170319 0.25725062 0.28236973 0.23566062 0.23233915
0.26594601 0.25478846 0.47799471 0.93310176 1.02622289 0.60219518
[445] 0.53293517 0.73576809 0.68601272 0.73101076 1.17894611 1.16731778
1.29185624 1.76420313 1.77412814 1.76790402 1.99726037 1.85491142
[457] 1.82873376 3.05694144 5.26789954 10.99064358 13.65164393 17.54534655
26.03843444 29.13527259 33.64072126 43.63177576 46.35561621 32.64996876
[469] 20.34887804 14.11727858 7.91804241 7.13552136 3.81023812 3.70758125
2.63756164 2.79993840

MASSES for TRUNCATED MINIMIZED CHARACTERIZED Q9ZSP9

[1] 72.08594 57.05100 137.13900 114.10300 163.17300 57.05100 128.10600
71.07800 113.15800 87.07700 129.18000 87.07700 147.17400 113.15800 147.17400
[16] 163.17300 128.10600 71.07800 117.12600 157.19400 87.07700 57.05100
163.17300 113.15800 97.11500 157.19400 114.10300 117.12600 157.19400 99.13100
[31] 117.12600 186.21000 157.19400 57.05100 114.10300 87.07700 57.05100
113.15800 114.10300 114.07900 57.05100 129.18000 71.07800 87.07700 57.05100
[46] 113.15800 114.07900 113.15800 99.13100 57.05100 57.05100 163.17300
163.17300 114.07900 71.07800 57.05100 114.07900 114.10300 99.13100 129.18000
[61] 147.17400 57.05100 113.15800 97.11500 131.19600 71.07800 147.17400
101.10400 99.13100 101.10400 131.19600 113.15800 87.07700 186.21000 87.07700
[76] 113.15800 113.15800 128.10600 163.17300 57.05100 157.19400 117.12600
131.19600 71.07800 71.07800 87.07700 57.05100 128.10600 113.15800 87.07700
[91] 137.13900 71.07800 131.19600 114.07900 71.07800 99.13100 129.18000
186.21000 57.05100 101.10400 114.07900 163.17300 113.15800 113.15800 129.18000
[106] 71.07800 137.13900 97.11500 128.10600 97.11500 163.17300 99.13100
113.15800 163.17300 57.05100 128.10600 99.13100 57.05100 114.07900 57.05100
[121] 114.10300 101.10400 114.07900 137.13900 163.17300 103.14300 186.21000
117.12600 157.19400 97.11500 128.10600 114.07900 131.19600 101.10400 101.10400
[136] 87.07700 157.19400 71.07800 71.07800 163.17300 157.19400 113.15800
114.07900 97.11500 114.10300 137.13900 97.11500 57.05100 87.07700 114.07900
[151] 113.15800 71.07800 57.05100 128.10600 101.10400 71.07800 71.07800
71.07800 131.19600 71.07800 71.07800 71.07800 87.07700 113.15800 99.13100

```
[166] 147.17400 157.19400 157.19400 163.17300 114.10300  97.11500  57.05100
163.17300  87.07700 114.10300 128.10600 113.15800 113.15800 114.10300 137.13900
[181]  71.07800 137.13900 117.12600 113.15800 147.17400 128.10600 147.17400
71.07800 114.07900 129.18000 163.17300 157.19400  57.05100 129.18000 163.17300
[196] 114.07900  87.07700  87.07700 113.15800 101.10400  71.07800 117.12600
129.18000 163.17300 163.17300 157.19400  87.07700  99.13100  87.07700  57.05100
[211] 163.17300  71.07800 114.07900 128.10600 113.15800 113.15800 186.21000
57.05100  71.07800  71.07800 186.21000 113.15800 163.17300 129.18000  71.07800
[226]  87.07700 114.10300 114.10300 117.12600 147.17400 163.17300 113.15800
114.10300 163.17300 113.15800  57.05100 157.19400 114.10300  57.05100 114.07900
[241]  71.07800 113.15800  57.05100  57.05100 101.10400  57.05100 186.21000
87.07700 131.19600 101.10400 128.10600 147.17400  57.05100 186.21000 114.07900
[256]  99.13100 129.18000 163.17300  71.07800  57.05100  99.13100 117.12600
101.10400 113.15800  99.13100  71.07800 117.12600 147.17400 113.15800 131.19600
[271]  87.07700 128.10600 129.18000 163.17300 117.12600 117.12600 129.18000
71.07800 128.10600 114.10300 147.17400 131.19600 103.14300  87.07700 131.19600
[286] 113.15800  57.05100 129.18000  57.05100 114.10300 157.19400 114.10300
101.10400 117.12600 129.18000 101.10400  97.11500  57.05100  57.05100 113.15800
[301] 113.15800 163.17300 157.19400 117.12600 157.19400 186.21000 114.10300
114.10300 131.19600 117.12600 147.17400  99.13100 101.10400  87.07700  71.07800
[316]  71.07800 147.17400 113.15800  71.07800 101.10400 101.10400 163.17300
87.07700 114.07900 163.17300 113.15800  71.07800  87.07700  71.07800  99.13100
[331]  87.07700  97.11500 114.10300 128.10600 113.15800 113.15800  87.07700
147.17400  71.07800 129.18000  87.07700 117.12600  99.13100 114.07900 163.17300
[346] 113.15800 113.15800  57.05100 114.07900 114.10300  97.11500 157.19400
71.07800 101.10400  87.07700 163.17300 131.19600  99.13100  57.05100 163.17300
[361]  57.05100 114.10300 114.10300 163.17300  97.11500 157.19400 117.12600
99.13100 137.13900 137.13900 157.19400  71.07800  87.07700 157.19400  57.05100
[376]  57.05100 163.17300  71.07800 101.10400 186.21000 163.17300  87.07700
157.19400 129.18000  71.07800  87.07700 114.07900  97.11500 114.10300 113.15800
[391] 113.15800 101.10400  57.05100  71.07800 113.15800  99.13100  57.05100
57.05100  97.11500 114.07900  71.07800 163.17300 114.07900 114.10300 147.17400
[406]  71.07800 114.07900 117.12600 157.19400 114.07900 114.10300 163.17300
128.10600 117.12600 101.10400 128.10600  97.11500  71.07800 101.10400 163.17300
[421] 114.10300 114.10300  71.07800  97.11500 113.15800 113.15800  57.05100
99.13100 113.15800  71.07800 157.19400 113.15800 137.13900  71.07800  57.05100
[436] 137.13900  87.07700  57.05100 163.17300 114.10300 117.12600 113.15800
113.15800  97.11500  99.13100  99.13100  97.11500 114.07900  97.11500 129.18000
[451]  97.11500 101.10400  97.11500 129.18000  97.11500  71.07800  97.11500
157.19400 101.10400 129.18000  99.13100 101.10400  97.11500  71.07800  97.11500
[466]  97.11500 157.19400  99.13100 113.15800  97.11500  99.13100  97.11500
71.07800 114.10300  71.07800 154.14634
```
