# Supplementary material for: Insights into the mechanism(s) of digestion of crystalline cellulose by plant class C GH9 endoglucanases
Source: J Mol Model. 2019 Jul 23;25(8):240. doi: 10.1007/s00894-019-4133-1 (PMC7385011; doi:10.1007/s00894-019-4133-1)
Supplement: Supplementary file 8 — (PDF 2217 kb) [file 894_2019_4133_MOESM8_ESM.pdf]

Supplementary Text 6

40.1ns Q93WY9 SIMULATION DATA

	TS	BOND	ANGLE	DIHED	IMPRP
	ELECT	VDW	BOUNDARY	MISC	KINETIC
	TOTAL	TEMP	POTENTIAL	TOTAL3	TEMPAVG
	PRESSURE	GPRESSURE	VOLUME	PRESSAVG	GPRESSAVG
[191]ENERGY:	0	4999.8078	5466.3194	5772.4511	305.5429
	-8134.0996	-1843.7802	0.0000	0.0000	7775.6378
	14341.8792	296.4639	6566.2414	14454.0148	296.4639
	2906.9481	466.8796	728456.8384	2906.9481	466.8796
[279]ENERGY:	10000	3189.6290	5372.8645	5772.7262	352.9152
	-13312.4858	-1563.0828	0.0000	0.0000	7870.9213
	7683.4877	300.0968	-187.4337	7747.4660	321.8834
	144.3399	115.6437	735475.2369	-0.1284	-0.0147
[318]ENERGY:	20000	3310.7142	5348.7964	5717.9680	311.8514
	-13460.3565	-1639.6275	0.0000	0.0000	7741.3509
	7330.6969	295.1566	-410.6541	7392.8458	298.4916
	-3.6076	1.8687	733208.9482	0.4936	0.5063
[370]ENERGY:	30000	3302.4411	5332.2753	5771.1947	343.8806
	-13640.7224	-1565.0050	0.0000	0.0000	7816.0831
	7360.1475	298.0060	-455.9356	7423.0826	300.4791
	6.5075	30.1525	728537.4003	0.3582	0.3695
[408]ENERGY:	40000	3328.1596	5339.2590	5771.9647	355.1878
	-13780.5478	-1570.1671	0.0000	0.0000	7826.2029
	7270.0592	298.3918	-556.1438	7336.5472	297.1183
	-8.6407	-20.8424	711892.0438	3.9213	3.9026
[460]ENERGY:	50000	3307.5300	5307.8902	5759.1695	331.4808
	-13748.2327	-1568.5930	0.0000	0.0000	7821.5762
	7210.8211	298.2154	-610.7551	7276.9016	300.3920
	-19.3502	1.6552	706270.9176	-0.2569	-0.2358
[498]ENERGY:	60000	3149.7592	5420.0209	5734.5158	329.9383
	-13670.4110	-1623.8743	0.0000	0.0000	7902.4475
	7242.3965	301.2988	-660.0510	7308.5010	296.6690
	124.6850	97.9537	715705.2994	0.6945	0.6735
[549]ENERGY:	70000	3287.2150	5366.8327	5734.7017	335.7602
	-13647.8582	-1724.9439	0.0000	0.0000	7834.8640
	7186.5715	298.7220	-648.2926	7248.7653	299.3904
	-164.3345	-89.9533	712000.6865	-0.8037	-0.7991
[586]ENERGY:	80000	3241.9688	5273.0821	5778.4420	351.7036
	-13716.4847	-1674.4155	0.0000	0.0000	7964.2153
	7218.5116	303.6539	-745.7037	7283.3567	298.5708
	-74.3919	-92.3141	734696.9518	-0.7541	-0.7668
[637]ENERGY:	90000	3238.6281	5310.1939	5824.2163	337.9477
	-13765.8102	-1631.1465	0.0000	0.0000	7746.5284
	7060.5577	295.3540	-685.9707	7129.2999	298.5867
	-2.7227	-80.7479	736230.1953	0.8356	0.8478
[677]ENERGY:	100000	3236.3599	5307.7785	5747.8780	332.6728
	-13771.0064	-1711.6380	0.0000	0.0000	7850.5704
	6992.6153	299.3209	-857.9551	7057.1860	296.9325
	89.1388	-14.9513	705965.2972	-1.7908	-1.7687
[728]ENERGY:	110000	3222.0286	5327.8896	5788.5150	313.7196
	-13879.8678	-1597.8366	0.0000	0.0000	7863.3914
	7037.8400	299.8097	-825.5515	7103.8962	297.0375
	-18.0528	-89.7528	680241.2341	3.4264	3.4213

```
[764]ENERGY:  120000      3245.8473      5352.3219      5717.1681       347.0089
        -13854.5421     -1645.5611         0.0000         0.0000      7695.9106
         6858.1536       293.4241      -837.7571      6920.7460       297.1428
        -188.2022      -151.4988    666180.6792        -2.1118        -2.1215
[814]ENERGY:  130000      3271.2315      5411.9784      5720.3573       326.8963
        -13866.7089     -1679.5346         0.0000         0.0000      7765.0022
         6949.2222       296.0584      -815.7800      7011.3369       296.7823
          -4.8745       -58.4113    675134.1117         4.3739         4.3867
[850]ENERGY:  140000      3299.8419      5403.6674      5734.4972       344.6452
        -13848.4665     -1689.7605         0.0000         0.0000      7824.5560
         7068.9806       298.3290      -755.5753      7133.7471       297.7924
          19.1598        -6.9990    673243.8452         0.4809         0.4586
[900]ENERGY:  150000      3241.4089      5433.0005      5742.7048       321.9516
        -13872.8761     -1653.3276         0.0000         0.0000      7866.2599
         7079.1220       299.9191      -787.1380      7145.8130       299.7096
          98.5067       133.0162    691319.4090         3.7687         3.7796
[936]ENERGY:  160000      3220.4833      5350.9896      5765.7775       320.9108
        -13780.5275     -1602.3484         0.0000         0.0000      7806.5467
         7081.8319       297.6424      -724.7148      7142.7774       298.4003
          49.6024        81.1187    688945.9496         1.7483         1.7652
[986]ENERGY:  170000      3322.8100      5345.3153      5776.6333       325.2419
        -13905.9005     -1645.0953         0.0000         0.0000      7851.8029
         7070.8075       299.3679      -780.9954      7132.7989       298.9419
          44.6247        39.1822    707705.8740        -1.8875        -1.8784
[1022]ENERGY:  180000      3304.3984      5383.4209      5663.6439
339.4317         -13886.9689     -1594.7025         0.0000         0.0000
7975.4606           7184.6841       304.0826      -790.7764      7254.6895
300.0251              60.1850        22.4443    712211.8218         0.7402
 0.7168
[1072]ENERGY:  190000      3298.2445      5425.2535      5766.6753
324.3027         -13928.3065     -1644.1822         0.0000         0.0000
7846.2674           7088.2547       299.1568      -758.0127      7150.6231
299.9245             -72.3984       -84.1196    692626.9100         3.6727
 3.6821
[1111]ENERGY:  200000      3303.6705      5329.4833      5763.5689
334.6463         -13898.3785     -1577.8792         0.0000         0.0000
7669.0404           6924.1518       292.3996      -744.8886      6988.4459
298.8470             -20.5065        43.0158    672354.0153         4.4785
 4.4616
[1161]ENERGY:  210000      3270.7582      5466.8753      5659.8662
339.9722         -13958.2659     -1587.0602         0.0000         0.0000
7750.6770           6942.8227       295.5122      -807.8543      7006.6020
299.1548              -5.3515         6.3538    690244.7411         2.1653
 2.1694
[1197]ENERGY:  220000      3234.2794      5437.9984      5678.3187
336.3131         -13876.8442     -1623.9096         0.0000         0.0000
7766.9305           6953.0864       296.1319      -813.8441      7014.7317
299.6224             133.2029         1.3342    663258.2835         0.5533
 0.5398
[1247]ENERGY:  230000      3260.9418      5487.7609      5679.7587
336.5154         -13924.7825     -1660.1468         0.0000         0.0000
7877.2753           7057.3228       300.3391      -819.9525      7122.5849
297.1957              58.3036        43.6604    644431.2248        -3.0436
-3.0354
```

```
[1283]ENERGY:  240000      3282.0198      5392.5443      5684.4089
357.4245        -13955.4977     -1597.2995         0.0000         0.0000
7769.8185          6933.4189       296.2420      -836.3996      6991.7494
299.2619           114.1427        23.3173    667654.0578         1.5756
 1.5592
[1333]ENERGY:  250000      3235.9803      5410.8250      5689.0952
341.0679        -13956.6865     -1613.8646         0.0000         0.0000
7813.1439          6919.5611       297.8939      -893.5828      6984.4207
296.8334           135.1558         1.1311    652624.1953         2.7407
 2.7459
[1369]ENERGY:  260000      3259.2161      5383.3947      5719.5083
339.6894        -13926.3447     -1633.9289         0.0000         0.0000
7896.0904          7037.6254       301.0564      -858.4650      7101.9220
298.5331           -64.7558       -82.8163    672280.3830         0.1235
 0.1345
[1419]ENERGY:  270000      3236.5942      5466.9394      5722.2395
338.4589        -13957.4198     -1655.6909         0.0000         0.0000
7851.1847          7002.3059       299.3443      -848.8788      7070.0726
299.5463            65.2941       -14.7453    665605.2257         2.0154
 1.9898
[1455]ENERGY:  280000      3315.8239      5445.7966      5726.1096
303.3124        -14018.3714     -1673.8364         0.0000         0.0000
7847.8863          6946.7209       299.2186      -901.1654      7010.1424
299.7178          -193.3945      -105.9713    670041.0728        -3.9643
-3.9421
[1505]ENERGY:  290000      3214.1677      5421.9023      5715.5375
335.8627        -13908.8344     -1604.9225         0.0000         0.0000
7777.8188          6951.5321       296.5471      -826.2867      7014.7788
298.3414           120.9856        93.7366    654907.7751         1.4212
 1.4079
[1544]ENERGY:  300000      3301.5510      5437.6849      5688.7394
336.5595        -13909.9324     -1682.1126         0.0000         0.0000
7781.8448          6954.3346       296.7006      -827.5101      7017.5132
297.7865           -12.6179       -89.2738    670824.8626         4.4300
 4.4153
[1594]ENERGY:  310000      3291.7837      5367.5769      5749.7393
326.9678        -13914.1023     -1612.3211         0.0000         0.0000
7773.0371          6982.6815       296.3648      -790.3556      7042.4926
298.9349            99.5621        12.7929    642216.2702        -0.3007
-0.2513
[1630]ENERGY:  320000      3361.9638      5360.7632      5737.8385
336.6761        -13969.6801     -1692.3265         0.0000         0.0000
7788.5288          6923.7638       296.9554      -864.7650      6989.3741
298.0580           -74.4160      -128.1646    648221.4198         1.6609
 1.6289
[1680]ENERGY:  330000      3304.0422      5388.9137      5704.1670
325.7263        -13977.2603     -1631.9901         0.0000         0.0000
7877.2909          6990.8898       300.3397      -886.4012      7057.1005
297.3733            74.0362        27.4887    642829.7681         1.9855
 2.0001
[1716]ENERGY:  340000      3250.8072      5324.0818      5710.1313
345.1865        -14055.7796     -1629.8709         0.0000         0.0000
7828.8611          6773.4175       298.4932     -1055.4437      6836.7832
299.3579            83.6228        47.8290    642015.6588         2.3969
```

```
 2.3803
[1766]ENERGY:  350000      3217.6963      5404.0071      5678.2123
335.1649        -13984.1365     -1657.2724         0.0000         0.0000
7769.8528          6763.5245       296.2433     -1006.3283      6827.2906
297.4133            -68.9166       -21.1554    648346.6755        -2.3845
-2.3718
[1802]ENERGY:  360000      3306.1552      5408.5819      5706.5150
321.9875        -13946.9753     -1708.7793         0.0000         0.0000
7775.6491          6863.1340       296.4643      -912.5151      6922.3080
299.5995             44.2840        -1.1775    642139.2257         3.2545
 3.2627
[1852]ENERGY:  370000      3181.2185      5420.2693      5718.6141
347.1248        -13932.6976     -1690.7947         0.0000         0.0000
7875.7680          6919.5024       300.2816      -956.2656      6985.3413
297.4473             47.0914       -53.3378    674614.8953        -1.2324
-1.2489
[1888]ENERGY:  380000      3287.5787      5470.4857      5703.5595
342.3122        -13934.9289     -1643.2190         0.0000         0.0000
7781.6225          7007.4107       296.6921      -774.2118      7068.8710
298.2815             52.1228        10.8856    703524.3334         0.6326
 0.6454
[1938]ENERGY:  390000      3280.5097      5327.8737      5686.4201
321.3249        -13937.7121     -1655.9780         0.0000         0.0000
7813.2555          6835.6938       297.8982      -977.5617      6903.0777
299.2872             -8.0749       -56.6040    722412.6307        -1.0304
-1.0323
[1977]ENERGY:  400000      3182.4184      5436.7659      5729.3467
332.7414        -13983.9860     -1627.8365         0.0000         0.0000
7793.6403          6863.0902       297.1503      -930.5501      6925.0803
298.9829            -51.7531       -62.6404    726932.9185         2.5828
 2.5814
[2027]ENERGY:  410000      3235.9592      5359.1469      5727.0011
323.2636        -13942.7613     -1611.6800         0.0000         0.0000
7926.4547          7017.3842       302.2141      -909.0705      7084.9219
298.1497            119.4820        90.7809    715121.3958         2.4100
 2.4218
[2063]ENERGY:  420000      3312.3825      5417.5419      5675.9026
335.8374        -13996.0908     -1604.6852         0.0000         0.0000
7868.3569          7009.2452       299.9990      -859.1117      7076.3410
298.4195            -69.9731       -26.4544    751271.7299         2.1821
 2.1589
[2113]ENERGY:  430000      3228.8452      5422.3213      5692.0887
331.0327        -13977.2814     -1607.4655         0.0000         0.0000
7939.4322          7028.9732       302.7089      -910.4589      7094.1267
300.8373            130.1503        99.6312    748868.5549         4.6291
 4.6387
[2149]ENERGY:  440000      3317.0937      5459.0389      5684.4332
331.1039        -14079.7984     -1556.6470         0.0000         0.0000
7955.5099          7110.7342       303.3219      -844.7757      7172.5279
301.0266             56.8317        77.3616    775569.8950         1.4706
 1.4809
[2199]ENERGY:  450000      3199.9456      5415.2450      5694.0876
329.8241        -13922.2607     -1655.0573         0.0000         0.0000
7869.1907          6930.9749       300.0308      -938.2158      6998.6283
```

298.8155 -109.5392 -117.9133 763203.5692 -0.1502
-0.1648
[2235]ENERGY: 460000 3284.8034 5345.5300 5650.8951
340.3515 -13867.5213 -1704.4346 0.0000 0.0000
7747.5544 6797.1786 295.3932 -950.3759 6860.8472
298.3193 -110.9667 -107.0490 771921.1152 0.9352
0.9228
[2285]ENERGY: 470000 3208.1614 5369.0488 5707.6233
332.5892 -14026.5240 -1575.8828 0.0000 0.0000
7887.5670 6902.5829 300.7315 -984.9841 6963.2897
297.3935 138.2403 115.1405 750029.1887 2.5443
2.5425
[2321]ENERGY: 480000 3317.7552 5385.2364 5709.5449
319.6456 -13957.4189 -1610.7283 0.0000 0.0000
7816.1561 6980.1910 298.0088 -835.9651 7043.4918
298.7276 -4.8237 24.2269 740907.4338 0.7009
0.7000
[2371]ENERGY: 490000 3222.0445 5361.4632 5640.6155
305.5886 -13956.6233 -1597.9446 0.0000 0.0000
7765.4219 6740.5659 296.0744 -1024.8560 6804.6173
297.9561 93.0425 79.8546 732734.2057 0.1310
0.1207
[2410]ENERGY: 500000 3249.7353 5376.5026 5718.9523
342.3969 -13976.0588 -1621.7013 0.0000 0.0000
7766.6257 6856.4527 296.1203 -910.1730 6920.0987
296.6705 14.0501 -25.5571 737264.6681 -0.1526
-0.1563
[2460]ENERGY: 510000 3230.6175 5442.8395 5653.7768
351.3087 -13996.1414 -1642.5414 0.0000 0.0000
7842.2638 6882.1234 299.0042 -960.1403 6945.0475
298.4663 -39.0496 -48.3517 715952.9136 0.6846
0.7016
[2496]ENERGY: 520000 3231.5730 5387.1996 5722.8011
330.0320 -14040.4860 -1641.2392 0.0000 0.0000
7792.3520 6782.2324 297.1012 -1010.1196 6846.3878
297.0418 73.4665 0.2830 727123.7940 4.6682
4.6607
[2546]ENERGY: 530000 3259.5345 5550.0297 5719.5473
303.8027 -14066.7780 -1659.3348 0.0000 0.0000
7906.2764 7013.0777 301.4448 -893.1988 7079.2904
300.2209 -19.4291 11.8341 780115.6322 -3.1171
-3.1137
[2582]ENERGY: 540000 3235.5371 5402.8813 5692.9369
314.8675 -14003.4274 -1646.9159 0.0000 0.0000
7899.3017 6895.1814 301.1789 -1004.1203 6961.0198
299.2561 122.1470 126.4585 755425.4658 2.3829
2.3739
[2632]ENERGY: 550000 3215.8780 5348.6171 5700.1722
346.0287 -13903.2201 -1694.5235 0.0000 0.0000
7780.2355 6793.1879 296.6392 -987.0477 6856.3338
299.0800 -2.6654 12.6989 758446.2796 2.7832
2.7679
[2668]ENERGY: 560000 3264.7875 5332.1288 5721.2256
329.8433 -14025.6564 -1671.8250 0.0000 0.0000

```
7796.2336           6746.7373      297.2492     -1049.4963      6811.9738
297.5905           -36.4256       -48.3576    759825.3431        -0.6703
-0.6691
[2718]ENERGY:  570000      3223.7730      5340.8040      5730.4644
311.9277        -14022.3019     -1601.0836         0.0000          0.0000
7928.8285           6912.4121      302.3047     -1016.4163      6977.6452
298.3046             1.0875        57.7949    746617.1774        -2.4779
-2.4760
[2754]ENERGY:  580000      3215.1177      5338.0845      5737.4726
330.7121        -14044.4279     -1613.5983         0.0000          0.0000
7882.1702           6845.5309      300.5257     -1036.6393      6914.2155
299.6784             5.3637        87.8232    747721.2050        -2.7072
-2.7013
[2804]ENERGY:  590000      3226.5214      5342.2645      5738.8081
312.6248        -13980.0761     -1570.7331         0.0000          0.0000
7629.4797           6698.8893      290.8913      -930.5904      6760.9874
298.0199           205.3331       186.5452    704012.3720        -0.3842
-0.3969
[2843]ENERGY:  600000      3266.7187      5475.4902      5732.6939
348.6558        -14092.7820     -1698.4657         0.0000          0.0000
7857.2037           6889.5146      299.5738      -967.6891      6953.4634
297.6639           -15.7694       -24.5487    725539.2592         3.5216
 3.5033
[2893]ENERGY:  610000      3302.6022      5298.5061      5681.2670
313.8317        -14045.4501     -1645.5659         0.0000          0.0000
7810.2571           6715.4481      297.7839     -1094.8090      6780.2727
297.3322            55.7743        21.4891    756778.9675         0.1689
 0.1706
[2929]ENERGY:  620000      3199.3516      5390.8425      5676.3784
340.2000        -14005.7476     -1703.7780         0.0000          0.0000
7703.5396           6600.7864      293.7150     -1102.7532      6664.4538
297.8343           -54.6534       -60.6829    780190.4471         3.1727
 3.1957
[2979]ENERGY:  630000      3227.9160      5350.5511      5733.6949
342.4140        -14021.3379     -1650.9258         0.0000          0.0000
7926.1866           6908.4991      302.2039     -1017.6876      6976.8855
298.3260            54.4327        20.5465    779945.2728        -0.7909
-0.7867
[3015]ENERGY:  640000      3224.3778      5393.5915      5644.7400
337.2225        -14081.7057     -1649.1929         0.0000          0.0000
7853.0404           6722.0735      299.4151     -1130.9668      6784.7613
297.6203            93.7497        64.3105    794131.4452         0.4322
 0.4319
[3065]ENERGY:  650000      3211.2961      5384.2846      5649.7980
323.2831        -14087.9994     -1653.6166         0.0000          0.0000
7825.8856           6652.9313      298.3797     -1172.9543      6712.1038
299.3709            38.0915         8.7455    807527.2540         1.7360
 1.7491
[3101]ENERGY:  660000      3304.8021      5351.7865      5642.5121
345.9410        -14066.3151     -1624.0891         0.0000          0.0000
7840.2974           6794.9350      298.9292     -1045.3624      6856.3939
298.7503            52.5558         5.7695    786396.2731        -0.5361
-0.5521
[3151]ENERGY:  670000      3270.7216      5406.3859      5605.0217
```

```
343.5747          -13969.6026     -1678.3608         0.0000         0.0000
7868.5888           6846.3293      300.0079     -1022.2595      6908.6877
299.2083            -120.2489      -135.5122    778878.3072        -0.7877
-0.7615
[3187]ENERGY:  680000      3268.5043      5441.2502      5687.9078
319.8395          -14018.0024     -1662.9773         0.0000         0.0000
7857.7386           6894.2608      299.5942      -963.4779      6959.2690
299.6854             -99.7076      -108.3319    740106.6255         2.2166
 2.2037
[3237]ENERGY:  690000      3264.2183      5378.9984      5718.5831
348.2574          -14125.0227     -1630.1571         0.0000         0.0000
7840.2173           6795.0946      298.9262     -1045.1227      6858.3988
298.1286             -25.0743        20.6720    724938.4926        -0.5169
-0.5313
[3276]ENERGY:  700000      3273.7671      5301.5310      5718.7966
338.8485          -14031.0057     -1686.5259         0.0000         0.0000
7847.1948           6762.6065      299.1922     -1084.5883      6824.7635
298.7304              50.6020       -10.7668    724194.0809         3.6772
 3.6852
[3326]ENERGY:  710000      3219.6100      5436.3716      5704.1009
338.9782          -14055.7003     -1665.6235         0.0000         0.0000
7859.3799           6837.1167      299.6568     -1022.2632      6901.8822
298.3270             -24.9984       -20.7040    710720.2061        -0.0236
-0.0081
[3362]ENERGY:  720000      3282.1918      5347.9030      5712.0321
358.7092          -13977.0497     -1725.2190         0.0000         0.0000
7848.1292           6846.6964      299.2278     -1001.4327      6909.5410
298.8771              -7.3693         9.1479    693383.8657        -1.3991
-1.4213
[3412]ENERGY:  730000      3205.5226      5402.4972      5657.6895
333.1609          -14094.0439     -1602.9395         0.0000         0.0000
7904.8648           6806.7516      301.3910     -1098.1132      6874.3970
299.9713              79.8184        88.7274    690716.4583         1.7037
 1.7168
[3448]ENERGY:  740000      3281.3515      5422.5087      5632.3714
347.6282          -14066.2596     -1711.5042         0.0000         0.0000
7922.0177           6828.1138      302.0450     -1093.9040      6893.0338
299.4270              48.6284       -17.4648    707241.4812         3.5943
 3.5861
[3498]ENERGY:  750000      3224.2971      5354.2414      5670.9839
331.5271          -14110.3028     -1649.7442         0.0000         0.0000
7867.0429           6688.0452      299.9489     -1178.9976      6751.5102
298.8985             -31.4413       -21.5830    679316.7253         4.5655
 4.5655
[3534]ENERGY:  760000      3242.3149      5523.6384      5651.6948
343.3278          -14080.5905     -1676.2750         0.0000         0.0000
7776.9372           6781.0476      296.5135      -995.8895      6844.7851
300.0697             179.7338       109.6929    672587.6303        -0.2739
-0.2620
[3584]ENERGY:  770000      3221.0673      5406.3014      5647.4158
319.6749          -13975.3458     -1676.0278         0.0000         0.0000
7927.5026           6870.5884      302.2541     -1056.9142      6932.2409
299.5592              87.5886        19.7775    676157.6194         0.7502
 0.7323
```

[3620]ENERGY: 780000   3278.3228   5432.2522   5674.0608
356.2722   -14060.3074   -1695.7914   0.0000   0.0000
7947.7278   6932.5369   303.0252   -1015.1909   7003.2488
299.7970   89.4942   21.9896   666165.4678   3.2304
 3.2285
[3670]ENERGY: 790000   3256.7714   5452.4393   5681.6510
343.8038   -14147.2693   -1666.0795   0.0000   0.0000
7739.6199   6660.9365   295.0906   -1078.6834   6723.6447
299.9063   77.8376   62.5178   636173.1521   -1.0018
-0.9833
[3709]ENERGY: 800000   3221.0457   5343.7904   5663.2167
318.5267   -14104.1157   -1623.3563   0.0000   0.0000
7819.5126   6638.6203   298.1367   -1180.8924   6704.2450
296.8575   50.4465   22.8922   621753.5797   -0.2462
-0.2353
[3759]ENERGY: 810000   3293.9612   5364.3481   5648.4852
321.5073   -14089.7297   -1676.2118   0.0000   0.0000
7797.3674   6659.7277   297.2924   -1137.6397   6722.4222
297.8053   82.4238   52.3176   632578.3560   3.2397
 3.2321
[3795]ENERGY: 820000   3189.2135   5349.0251   5649.6275
328.1309   -14125.1694   -1682.5152   0.0000   0.0000
7826.7339   6535.0463   298.4121   -1291.6876   6598.4541
297.4417   34.1952   -20.1875   613491.0019   -3.4198
-3.4026
[3845]ENERGY: 830000   3262.8894   5348.1664   5663.7297
350.3915   -14008.3684   -1681.2495   0.0000   0.0000
7800.9829   6736.5420   297.4302   -1064.4408   6799.2468
298.5060   -12.6289   -86.4981   606184.7576   -4.0246
-4.0265
[3881]ENERGY: 840000   3226.7039   5402.7689   5728.4437
319.9525   -14006.9618   -1652.0100   0.0000   0.0000
7824.3048   6843.2021   298.3195   -981.1028   6907.4383
299.0259   97.4687   -8.4565   597684.2275   -0.5867
-0.5841
[3931]ENERGY: 850000   3279.1309   5406.3615   5701.5200
336.0104   -13999.5694   -1762.6223   0.0000   0.0000
7905.6138   6866.4449   301.4195   -1039.1689   6932.2042
299.9421   -252.2892   -176.3382   584424.3981   -1.4104
-1.3483
[3967]ENERGY: 860000   3212.1434   5454.0055   5693.1302
311.3594   -14034.4729   -1695.2572   0.0000   0.0000
7910.5776   6851.4860   301.6088   -1059.0916   6916.6576
300.8483   107.1295   69.6068   580673.4491   -2.1506
-2.1768
[4017]ENERGY: 870000   3189.6716   5389.1667   5640.2651
331.7346   -14057.0513   -1714.7529   0.0000   0.0000
7786.5456   6565.5794   296.8798   -1220.9662   6632.5225
299.2433   -133.9249   -161.6964   559853.5206   1.2363
 1.2608
[4053]ENERGY: 880000   3260.9378   5417.3891   5642.7908
318.7754   -13984.8006   -1705.7354   0.0000   0.0000
7837.9497   6787.3068   298.8397   -1050.6429   6852.3929
297.3539   -120.8846   -60.5289   567294.4042   -0.0543

```
-0.0592
[4103]ENERGY:  890000      3275.4669      5362.1317      5642.0377
349.1689        -13979.3511     -1687.3731         0.0000         0.0000
7710.8590          6672.9400      293.9941     -1037.9190      6732.8473
298.1196           -88.0577       -98.6335    593166.0846         1.5359
 1.5132
[4142]ENERGY:  900000      3340.1615      5290.1428      5649.2525
338.3317        -13980.4817     -1720.5052         0.0000         0.0000
7863.3335          6780.2351      299.8075     -1083.0984      6844.5363
298.2437            -6.2099         7.1920    621066.2771         0.5049
 0.5225
[4192]ENERGY:  910000      3203.5313      5383.3922      5650.5137
308.8976        -14055.0337     -1720.0620         0.0000         0.0000
7759.1776          6530.4167      295.8363     -1228.7609      6596.1768
298.4935           227.4135        74.9976    626611.0917         8.8564
 8.8623
[4228]ENERGY:  920000      3176.9391      5333.3923      5679.5058
329.1833        -13999.8382     -1697.3261         0.0000         0.0000
7724.0272          6545.8836      294.4961     -1178.1437      6608.2253
297.0285           209.6455        67.2341    648813.0554         4.5145
 4.5288
[4278]ENERGY:  930000      3267.6177      5341.8617      5729.4090
312.4129        -14047.2215     -1738.9713         0.0000         0.0000
7803.3099          6668.4183      297.5190     -1134.8916      6731.4620
296.4609            25.7245       165.6629    649171.0619        -3.3962
-3.3851
[4314]ENERGY:  940000      3237.6544      5388.2327      5702.7501
335.2091        -14044.4042     -1697.8784         0.0000         0.0000
7767.1117          6688.6755      296.1388     -1078.4362      6747.5136
297.4293            12.4632       -10.2105    679527.1381         1.7844
 1.7969
[4364]ENERGY:  950000      3238.1719      5412.7025      5716.2576
336.5048        -14084.7956     -1674.5447         0.0000         0.0000
7867.7278          6812.0244      299.9751     -1055.7034      6876.2287
297.6650            79.4139        93.8653    699268.9735         0.2295
 0.2477
[4400]ENERGY:  960000      3189.2775      5424.4183      5669.0132
334.9287        -14065.6018     -1725.4981         0.0000         0.0000
7820.0239          6646.5617      298.1562     -1173.4622      6709.9534
298.7645            -3.3488       -82.8217    699368.1179         0.4565
 0.4540
[4450]ENERGY:  970000      3244.5325      5369.0285      5729.5760
337.1085        -14200.5800     -1646.8741         0.0000         0.0000
7838.8022          6671.5937      298.8722     -1167.2085      6733.9812
298.0225           136.0881        93.0758    678882.1484         3.4071
 3.3918
[4486]ENERGY:  980000      3182.2751      5350.1287      5705.9557
318.2984        -14082.5612     -1754.8582         0.0000         0.0000
7940.6109          6659.8494      302.7539     -1280.7615      6728.0708
296.9105            51.2341         0.7363    663954.5152        -3.6318
-3.6273
[4536]ENERGY:  990000      3304.8242      5336.1974      5706.8806
309.3838        -13991.1156     -1712.9476         0.0000         0.0000
7834.5683          6787.7911      298.7108     -1046.7772      6850.0224
```

```
297.1302            -58.7813       -14.2871    678044.6499         0.1673
 0.1879
[4575]ENERGY: 1000000      3312.1413      5316.0854      5680.2132
346.7949         -14044.9965     -1740.1730         0.0000         0.0000
7781.5025           6651.5677       296.6875     -1129.9348      6718.3571
299.5392            -63.5255       -44.1165    680285.8938        -0.3585
-0.3588
[268]ENERGY: 1010000      3272.9194      5382.7451      5684.1767       343.9548
        -14046.7669     -1766.1922         0.0000         0.0000      7778.7189
         6649.5557      296.6151     -1129.1632      6715.6748      297.5243
         129.2834       19.8355    680285.8938        1.1530        1.1379
[307]ENERGY: 1020000      3225.6458      5337.2926      5702.9259       319.2071
        -13981.5448     -1697.2839         0.0000         0.0000      7748.5471
         6654.7898      295.4646     -1093.7573      6715.8053      297.6042
         108.5357       53.6129    680285.8938       -2.3898       -2.3891
[359]ENERGY: 1030000      3227.7852      5279.6463      5730.1135       343.0516
        -14042.5697     -1696.8883         0.0000         0.0000      7808.3973
         6649.5358      297.7468     -1158.8615      6715.1163      297.5940
         -71.3604      -33.7209    680285.8938        1.2778        1.2860
[397]ENERGY: 1040000      3264.3479      5358.0692      5713.7550       356.0034
        -14042.4322     -1705.8477         0.0000         0.0000      7712.0329
         6655.9284      294.0722     -1056.1045      6716.3438      297.4642
        -154.4198     -135.8219    680285.8938        0.1889        0.1875
[449]ENERGY: 1050000      3198.4383      5430.8157      5652.4053       330.0917
        -13988.2129     -1755.0251         0.0000         0.0000      7785.2945
         6653.8074      296.8658     -1131.4870      6716.2917      297.4187
          48.8103      -12.4780    680285.8938       -1.7985       -1.8090
[487]ENERGY: 1060000      3265.9425      5347.3148      5687.5245       330.3672
        -14001.6926     -1700.0647         0.0000         0.0000      7723.4438
         6652.8355      294.5074     -1070.6082      6717.5677      297.4894
          99.2200      125.0836    680285.8938        0.6468        0.6603
[538]ENERGY: 1070000      3270.3471      5422.0737      5654.1689       310.7094
        -14119.8430     -1633.9929         0.0000         0.0000      7750.2552
         6653.7185      295.5297     -1096.5368      6717.5067      297.5562
          87.8024      167.9633    680285.8938        0.6554        0.6678
[575]ENERGY: 1080000      3139.5932      5364.2400      5639.2814       322.5109
        -14093.8709     -1672.9380         0.0000         0.0000      7952.4779
         6651.2944      303.2408     -1301.1835      6716.5509      297.6607
         178.0171      146.3261    680285.8938       -0.9001       -0.9209
[626]ENERGY: 1090000      3272.2008      5344.9419      5660.1477       337.6950
        -13975.8920     -1752.7699         0.0000         0.0000      7770.3281
         6656.6517      296.2951     -1113.6764      6717.3922      297.7352
         -75.5723      -20.3781    680285.8938       -0.4792       -0.4781
[666]ENERGY: 1100000      3157.2030      5368.1728      5692.8733       315.1590
        -14037.1312     -1649.6861         0.0000         0.0000      7805.2263
         6651.8171      297.6259     -1153.4092      6716.5854      297.7141
         119.1789       77.4351    680285.8938        1.5141        1.5057
[717]ENERGY: 1110000      3249.4776      5306.7149      5749.3735       350.4405
        -14052.2199     -1722.5757         0.0000         0.0000      7772.7098
         6653.9207      296.3860     -1118.7892      6717.5524      297.9462
         -31.3451       -9.6658    680285.8938        0.4761        0.4755
[753]ENERGY: 1120000      3200.7451      5404.2183      5653.2680       332.9931
        -14096.6629     -1703.0657         0.0000         0.0000      7861.7895
         6653.2854      299.7827     -1208.5041      6717.0625      298.0004
```

91.2148 95.8344 680285.8938 0.5893 0.6025
[803]ENERGY: 1130000 3173.0303 5393.2928 5655.8264 316.1367
-14123.6718 -1679.4207 0.0000 0.0000 7917.6024
6652.7961 301.9109 -1264.8063 6717.2028 297.9790
125.6450 127.0497 680285.8938 -0.9608 -0.9555
[839]ENERGY: 1140000 3185.5060 5265.3488 5719.5400 333.5665
-13952.5284 -1751.2525 0.0000 0.0000 7853.4098
6653.5902 299.4632 -1199.8196 6716.8233 297.9872
34.4170 -41.1491 680285.8938 0.7487 0.7441
[889]ENERGY: 1150000 3250.1318 5349.8187 5728.7503 319.3475
-14104.2608 -1676.8769 0.0000 0.0000 7787.3584
6654.2690 296.9445 -1133.0894 6715.9176 297.9400
0.7192 -4.4171 680285.8938 0.3747 0.3782
[925]ENERGY: 1160000 3233.2499 5385.9394 5726.5583 332.4843
-14146.8756 -1684.0720 0.0000 0.0000 7805.6790
6652.9632 297.6431 -1152.7158 6718.0922 297.8930
75.3928 13.8103 680285.8938 -1.6426 -1.6436
[975]ENERGY: 1170000 3178.8957 5444.4943 5721.6181 351.0226
-14123.3254 -1723.2718 0.0000 0.0000 7801.7888
6651.2223 297.4948 -1150.5665 6717.5592 297.8618
20.2473 -13.1399 680285.8938 0.5170 0.5259
[1011]ENERGY: 1180000 3155.4534 5430.0743 5696.9581
327.8736 -14025.7471 -1725.7938 0.0000 0.0000
7795.6722 6654.4907 297.2616 -1141.1815 6718.5976
297.7763 90.6659 82.0859 680285.8938 -0.8084
-0.8085
[1061]ENERGY: 1190000 3264.4363 5330.7345 5700.3991
335.4259 -14129.2425 -1713.7638 0.0000 0.0000
7865.2361 6653.2256 299.9141 -1212.0105 6717.7648
297.8318 110.7022 -12.7245 680285.8938 0.7213
0.7032
[1100]ENERGY: 1200000 3262.6017 5377.0471 5686.3092
328.4500 -14006.2403 -1784.9086 0.0000 0.0000
7788.1997 6651.4588 296.9766 -1136.7409 6718.6561
298.0358 112.4585 11.6492 680285.8938 0.3544
0.3732
[1150]ENERGY: 1210000 3229.6409 5349.4887 5706.7094
334.4625 -14084.2662 -1742.0762 0.0000 0.0000
7858.6627 6652.6220 299.6635 -1206.0408 6717.8006
298.0213 -109.3712 3.5023 680285.8938 -0.8718
-0.8799
[1186]ENERGY: 1220000 3171.7044 5457.2234 5680.3139
338.5091 -14097.1335 -1742.0490 0.0000 0.0000
7846.5842 6655.1525 299.2029 -1191.4317 6718.6998
298.1310 52.5628 -29.5889 680285.8938 2.1164
2.1092
[1236]ENERGY: 1230000 3111.1770 5478.2201 5724.8969
314.8995 -14113.0225 -1707.0100 0.0000 0.0000
7847.5708 6656.7317 299.2405 -1190.8391 6719.0038
298.0902 172.0253 94.1982 680285.8938 -1.2035
-1.2026
[1272]ENERGY: 1240000 3181.9996 5453.8594 5708.7953
347.2111 -14153.4735 -1755.2922 0.0000 0.0000
7869.8871 6652.9867 300.0915 -1216.9003 6719.2916

297.9452 57.1392 -27.9507 680285.8938 0.1265
0.1208
[1322]ENERGY: 1250000 3232.7986 5415.8146 5698.3256
307.9360 -14030.6693 -1700.6700 0.0000 0.0000
7733.5610 6657.0965 294.8932 -1076.4645 6718.5760
297.9616 119.3616 176.8703 680285.8938 -0.5198
-0.5168
[1358]ENERGY: 1260000 3243.4574 5495.5760 5718.7860
331.0663 -14120.2862 -1781.0650 0.0000 0.0000
7769.7663 6657.3008 296.2737 -1112.4655 6719.8572
298.0534 37.2449 27.9544 680285.8938 -0.8107
-0.8095
[1408]ENERGY: 1270000 3126.9500 5391.7811 5707.6534
331.1525 -14025.6078 -1720.4985 0.0000 0.0000
7842.4336 6653.8643 299.0446 -1188.5694 6718.8750
298.2713 60.9287 131.5401 680285.8938 1.9194
1.9278
[1444]ENERGY: 1280000 3263.7516 5326.2988 5690.3423
304.2728 -14026.6150 -1683.2816 0.0000 0.0000
7781.7092 6656.4781 296.7291 -1125.2310 6718.4387
298.0905 30.7290 2.0084 680285.8938 -1.2752
-1.2737
[1494]ENERGY: 1290000 3159.4293 5457.3482 5625.9533
322.2337 -14087.9028 -1725.0028 0.0000 0.0000
7901.8306 6653.8895 301.3095 -1247.9412 6719.2240
298.4214 5.0555 16.8925 680285.8938 1.2377
1.2482
[1533]ENERGY: 1300000 3184.7104 5336.4634 5729.1061
333.9880 -14128.3741 -1705.4085 0.0000 0.0000
7899.7715 6650.2567 301.2310 -1249.5148 6718.5802
298.3255 93.0867 95.0853 680285.8938 0.0058
-0.0019
[1583]ENERGY: 1310000 3151.4056 5314.3040 5726.2109
340.0689 -14000.2724 -1726.2687 0.0000 0.0000
7850.6782 6656.1265 299.3590 -1194.5517 6717.9500
298.5389 -34.6349 -28.3500 680285.8938 3.5540
3.5482
[1619]ENERGY: 1320000 3184.0201 5461.4396 5732.4319
329.4166 -14024.8567 -1810.3901 0.0000 0.0000
7782.6233 6654.6847 296.7640 -1127.9386 6720.0067
298.2954 -17.6440 -16.0420 680285.8938 -4.0587
-4.0504
[1669]ENERGY: 1330000 3228.6925 5435.7260 5699.3010
331.0508 -14055.6402 -1769.3954 0.0000 0.0000
7789.2205 6658.9552 297.0155 -1130.2653 6719.3163
298.5037 -87.6665 -129.5686 680285.8938 -1.8661
-1.8813
[1705]ENERGY: 1340000 3204.9174 5359.8980 5668.7455
355.1809 -14055.8973 -1741.9910 0.0000 0.0000
7860.4833 6651.3367 299.7329 -1209.1467 6718.8197
298.3613 51.5841 3.3051 680285.8938 -2.7649
-2.7671
[1755]ENERGY: 1350000 3235.1891 5355.9872 5696.2845
339.2340 -14077.4351 -1699.5771 0.0000 0.0000

```
7807.5463            6657.2287       297.7143     -1150.3175      6720.2006
298.4347            -102.4258        -31.2616    680285.8938        -1.9035
-1.8987
[1791]ENERGY: 1360000      3278.0907      5336.7861      5719.0171
347.2500          -14060.1488     -1733.9438         0.0000         0.0000
7771.3945            6658.4458       296.3358     -1112.9487      6720.7628
298.4039              67.8265         38.5037    680285.8938        -4.2439
-4.2412
[1841]ENERGY: 1370000      3181.0407      5419.0970      5678.8982
331.5864          -14007.0528     -1787.4074         0.0000         0.0000
7841.1232            6657.2853       298.9947     -1183.8379      6721.0731
298.3674              76.9093        -17.1033    680285.8938         0.2562
 0.2495
[1877]ENERGY: 1380000      3237.2396      5389.0003      5678.6927
338.3095          -14158.5928     -1665.1685         0.0000         0.0000
7837.4980            6656.9788       298.8564     -1180.5193      6720.2955
298.5453             129.7983        156.0896    680285.8938        -2.8244
-2.8139
[1927]ENERGY: 1390000      3219.6624      5390.7906      5683.1418
321.3720          -14126.9929     -1678.2466         0.0000         0.0000
7844.7316            6654.4589       299.1323     -1190.2728      6721.2555
298.7720            -100.3024        -56.5766    680285.8938         2.4049
 2.4158
[1966]ENERGY: 1400000      3242.0740      5365.6570      5695.2196
350.1132          -13973.9807     -1824.2084         0.0000         0.0000
7806.0778            6660.9525       297.6583     -1145.1253      6722.2285
298.8128              34.5710        -66.8621    680285.8938        -2.3998
-2.4173
[2016]ENERGY: 1410000      3219.9297      5318.4600      5694.8403
337.1267          -14050.6603     -1712.4609         0.0000         0.0000
7853.1470            6660.3824       299.4532     -1192.7646      6722.0194
298.4774             -21.5795        -37.9365    680285.8938        -5.1040
-5.0976
[2052]ENERGY: 1420000      3247.8390      5365.6272      5700.9636
336.5113          -14021.7761     -1815.1006         0.0000         0.0000
7844.5019            6658.5664       299.1235     -1185.9355      6721.2470
298.3864            -117.2650       -115.9506    680285.8938        -1.2236
-1.2250
[2102]ENERGY: 1430000      3209.4104      5353.1185      5738.5737
325.8432          -14056.8428     -1735.2571         0.0000         0.0000
7822.3332            6657.1791       298.2782     -1165.1541      6721.6857
298.4965             195.6906        121.3226    680285.8938         0.9124
 0.9076
[2138]ENERGY: 1440000      3274.7188      5411.9285      5636.5723
315.7109          -14125.4494     -1712.0451         0.0000         0.0000
7856.4913            6657.9273       299.5807     -1198.5640      6720.5967
298.5528            -151.3197       -102.7445    680285.8938         0.1387
 0.1341
[2188]ENERGY: 1450000      3235.9586      5455.7832      5630.6644
349.6392          -14090.1043     -1702.5277         0.0000         0.0000
7777.1188            6656.5323       296.5541     -1120.5865      6720.5580
298.5913             -96.8896        -92.8696    680285.8938        -3.9159
-3.9162
[2224]ENERGY: 1460000      3307.5657      5350.4768      5705.7654
```

316.7408 -14074.2044 -1761.9439 0.0000 0.0000
7815.5396 6659.9401 298.0191 -1155.5995 6720.6927
298.5602 -194.6656 -137.2739 680285.8938 -6.2237
-6.2252
[2274]ENERGY: 1470000 3149.7976 5440.9526 5683.3048
349.7944 -14079.0967 -1689.2982 0.0000 0.0000
7800.4869 6655.9414 297.4451 -1144.5455 6719.8002
298.3144 163.0257 153.3641 680285.8938 -10.4562
-10.4536
[2310]ENERGY: 1480000 3307.7754 5377.8788 5667.9661
346.2541 -14056.7768 -1755.1598 0.0000 0.0000
7771.2244 6659.1622 296.3293 -1112.0622 6720.3756
298.3622 -68.9212 -99.0793 680285.8938 -7.7330
-7.7225
[2360]ENERGY: 1490000 3143.9966 5405.6099 5695.6730
307.2975 -14030.5143 -1699.9191 0.0000 0.0000
7834.3902 6656.5338 298.7379 -1177.8564 6719.5454
298.6241 44.1513 65.2632 680285.8938 -11.3061
-11.3014
[2399]ENERGY: 1500000 3203.5238 5381.3578 5723.3110
317.3186 -14031.1832 -1775.3581 0.0000 0.0000
7837.7496 6656.7195 298.8660 -1181.0301 6720.1326
298.5472 21.6190 -32.6739 680285.8938 -7.1648
-7.1794
[2449]ENERGY: 1510000 3227.2815 5364.1836 5666.6062
337.7421 -14039.1096 -1762.3200 0.0000 0.0000
7860.7217 6655.1055 299.7420 -1205.6162 6720.4682
298.5137 -140.8812 -118.6625 680285.8938 -5.7473
-5.7380
[2485]ENERGY: 1520000 3229.0267 5404.0746 5668.5595
367.4359 -14040.9428 -1752.1171 0.0000 0.0000
7780.2431 6656.2797 296.6732 -1123.9633 6720.0121
298.5356 75.2863 30.9851 680285.8938 -4.7564
-4.7830
[2535]ENERGY: 1530000 3305.4621 5316.5243 5636.5819
345.2985 -14096.6948 -1667.6648 0.0000 0.0000
7816.4050 6655.9120 298.0521 -1160.4930 6719.5029
298.7543 -2.9982 54.4168 680285.8938 0.0552
0.0913
[2571]ENERGY: 1540000 3240.0718 5397.5934 5657.1666
316.5625 -13989.5624 -1785.8307 0.0000 0.0000
7821.8226 6657.8239 298.2587 -1163.9987 6720.5811
298.7506 -41.6706 -106.3842 680285.8938 -3.7482
-3.7604
[2621]ENERGY: 1550000 3242.1534 5314.5032 5648.4633
350.6408 -14108.9993 -1652.3752 0.0000 0.0000
7861.4925 6655.8788 299.7714 -1205.6137 6719.7712
298.5133 -42.3115 -88.9053 680285.8938 -4.1505
-4.1526
[2657]ENERGY: 1560000 3204.2024 5318.7822 5682.2310
309.6335 -14047.9863 -1614.4591 0.0000 0.0000
7806.3477 6658.7514 297.6686 -1147.5962 6721.0773
298.7114 49.9454 64.9969 680285.8938 -4.4147
-4.4073

[2707]ENERGY: 1570000 3204.4597 5334.3692 5677.2698
342.6491 -14005.9196 -1708.4749 0.0000 0.0000
7811.1304 6655.4838 297.8510 -1155.6466 6721.3814
298.6115 80.0651 37.7484 680285.8938 -4.8934
-4.9152
[2743]ENERGY: 1580000 3266.9969 5382.2789 5655.0797
313.6797 -13993.7172 -1771.0690 0.0000 0.0000
7807.6801 6660.9292 297.7194 -1146.7509 6723.6721
298.7706 -14.7879 -66.4696 680285.8938 -4.2297
-4.2220
[2793]ENERGY: 1590000 3245.5075 5441.4392 5642.2008
292.9615 -14107.4808 -1738.1365 0.0000 0.0000
7883.3143 6659.8060 300.6035 -1223.5083 6722.6895
298.8344 67.7438 -45.4253 680285.8938 -4.1969
-4.2073
[2832]ENERGY: 1600000 3286.8643 5438.6772 5644.6476
319.8318 -14059.7982 -1729.5878 0.0000 0.0000
7760.3129 6660.9478 295.9132 -1099.3651 6722.2379
298.7349 -123.8304 -87.8160 680285.8938 -3.1877
-3.1768
[2882]ENERGY: 1610000 3230.0556 5362.3512 5654.0116
329.9583 -14023.6671 -1720.8318 0.0000 0.0000
7828.7092 6660.5872 298.5213 -1168.1220 6723.2775
298.6633 33.6578 -37.9116 680285.8938 -5.7508
-5.7472
[2918]ENERGY: 1620000 3204.2328 5319.5739 5706.7613
341.4377 -14015.6732 -1750.5198 0.0000 0.0000
7854.3837 6660.1963 299.5003 -1194.1874 6721.9594
298.6029 66.7141 63.7924 680285.8938 -5.0492
-5.0407
[2968]ENERGY: 1630000 3270.1090 5386.7555 5685.3559
332.6955 -14061.9630 -1772.1086 0.0000 0.0000
7816.7750 6657.6194 298.0662 -1159.1557 6722.0156
298.7846 -70.6949 -81.5343 680285.8938 -9.0341
-9.0463
[3004]ENERGY: 1640000 3288.1767 5342.1590 5662.8377
316.9436 -13995.0616 -1747.5182 0.0000 0.0000
7793.9080 6661.4452 297.1943 -1132.4628 6723.3359
298.7063 36.7019 -39.7161 680285.8938 -2.5277
-2.5176
[3054]ENERGY: 1650000 3204.2607 5403.6352 5672.2728
341.7004 -14014.9860 -1785.6422 0.0000 0.0000
7837.6548 6658.8957 298.8624 -1178.7590 6722.9919
298.6206 11.6396 -13.7768 680285.8938 -3.4714
-3.4742
[3090]ENERGY: 1660000 3238.5695 5445.7180 5697.4600
312.4727 -14060.9496 -1711.8981 0.0000 0.0000
7739.6292 6661.0017 295.1245 -1078.6275 6723.6606
298.7760 20.9102 -33.0301 680285.8938 -6.6590
-6.6590
[3140]ENERGY: 1670000 3282.4234 5432.5784 5663.0546
345.0452 -14093.6496 -1756.1975 0.0000 0.0000
7789.6513 6662.9058 297.0320 -1126.7455 6724.8315
298.7487 5.0900 -19.0726 680285.8938 -4.8301

-4.8255
[3176]ENERGY: 1680000 3152.5679 5349.0778 5692.1905
321.7117 -14031.8564 -1712.1564 0.0000 0.0000
7885.9564 6657.4913 300.7042 -1228.4650 6723.1305
298.7993 128.4767 88.3500 680285.8938 -7.6567
-7.6702
[3226]ENERGY: 1690000 3219.7758 5394.1565 5674.3842
319.9615 -14123.0928 -1677.3530 0.0000 0.0000
7854.1436 6661.9756 299.4912 -1192.1679 6723.4050
298.9097 -35.6913 -1.6491 680285.8938 -3.9864
-3.9758
[3265]ENERGY: 1700000 3234.6306 5449.1678 5674.6235
313.4904 -14137.1672 -1705.9699 0.0000 0.0000
7830.5597 6659.3348 298.5919 -1171.2249 6723.4310
298.8473 110.8040 59.2748 680285.8938 -8.0678
-8.0773
[3315]ENERGY: 1710000 3254.8682 5303.7486 5654.1728
343.3185 -14025.2718 -1690.2664 0.0000 0.0000
7820.7506 6661.3206 298.2178 -1159.4300 6723.6282
298.7147 127.7638 74.0278 680285.8938 -5.3616
-5.3484
[3351]ENERGY: 1720000 3260.6702 5464.2595 5688.8971
356.8274 -14144.8853 -1675.3905 0.0000 0.0000
7711.1880 6661.5664 294.0400 -1049.6215 6723.3811
298.7409 -16.7144 -26.1026 680285.8938 -5.6650
-5.6955
[3401]ENERGY: 1730000 3223.3770 5371.1218 5657.7241
314.9728 -14065.8517 -1720.8632 0.0000 0.0000
7877.7396 6658.2204 300.3909 -1219.5192 6723.0973
298.7913 -16.8871 11.6973 680285.8938 -4.2750
-4.2586
[3437]ENERGY: 1740000 3202.3057 5444.4932 5686.2345
342.1213 -14104.5701 -1789.4851 0.0000 0.0000
7880.3729 6661.4723 300.4913 -1218.9006 6723.0504
298.9949 133.5127 83.6205 680285.8938 -5.9379
-5.9311
[3487]ENERGY: 1750000 3242.7503 5337.0821 5694.9427
310.2285 -14054.7132 -1760.3434 0.0000 0.0000
7888.6883 6658.6353 300.8084 -1230.0530 6722.6835
299.0250 29.7505 -63.0324 680285.8938 -2.9965
-2.9914
[3523]ENERGY: 1760000 3175.7301 5324.8581 5695.9168
343.7622 -14025.6514 -1746.7224 0.0000 0.0000
7892.1734 6660.0669 300.9413 -1232.1065 6723.8080
299.1259 -12.2112 -29.2171 680285.8938 -4.9016
-4.9187
[3573]ENERGY: 1770000 3244.1179 5342.2677 5684.7257
337.9630 -14036.7877 -1726.1755 0.0000 0.0000
7817.6451 6663.7562 298.0994 -1153.8889 6723.6765
299.1876 70.5337 55.3621 680285.8938 -3.5597
-3.5630
[3609]ENERGY: 1780000 3191.0110 5323.0023 5704.1804
330.0572 -13965.5588 -1820.0013 0.0000 0.0000
7898.1144 6660.8052 301.1678 -1237.3092 6724.1736

299.0766 -81.0057 -125.0687 680285.8938 -10.3940
-10.3971
[3659]ENERGY: 1790000 3274.2704 5465.5437 5678.7780
335.3993 -14148.0184 -1796.3832 0.0000 0.0000
7847.9869 6657.5766 299.2564 -1190.4103 6723.1944
299.5904 -63.0025 -80.3472 680285.8938 -6.6740
-6.6604
[3698]ENERGY: 1800000 3279.4237 5373.8551 5720.1128
323.4563 -14134.8300 -1830.6924 0.0000 0.0000
7924.1845 6655.5100 302.1619 -1268.6745 6723.1299
299.7654 -169.9109 -89.3253 680285.8938 -15.8563
-15.8448
[3748]ENERGY: 1810000 3211.9109 5350.8998 5714.3072
337.2091 -13996.7371 -1896.1282 0.0000 0.0000
7939.6320 6661.0937 302.7510 -1278.5383 6724.1192
299.8223 79.5972 36.1001 680285.8938 -14.9961
-15.0103
[3784]ENERGY: 1820000 3236.8417 5365.5471 5710.1173
322.3250 -14135.2086 -1749.2013 0.0000 0.0000
7907.4788 6657.9001 301.5249 -1249.5787 6722.3556
299.8948 139.4691 136.7510 680285.8938 -23.4279
-23.4177
[3834]ENERGY: 1830000 3119.7272 5489.6424 5680.0391
338.1086 -14106.5077 -1762.7579 0.0000 0.0000
7900.2963 6658.5479 301.2510 -1241.7484 6722.1946
299.9441 4.3040 8.9168 680285.8938 -21.2221
-21.2246
[3870]ENERGY: 1840000 3194.4287 5349.2745 5710.3282
324.8113 -14150.5626 -1776.8380 0.0000 0.0000
8004.0395 6655.4816 305.2069 -1348.5579 6722.7541
300.1176 67.8791 23.2774 680285.8938 -19.0422
-19.0604
[3920]ENERGY: 1850000 3297.4440 5334.6040 5724.7694
341.0169 -14147.5087 -1764.2158 0.0000 0.0000
7872.3057 6658.4156 300.1837 -1213.8901 6724.5912
300.1716 41.2460 -7.7438 680285.8938 -18.6442
-18.6123
[3956]ENERGY: 1860000 3279.0016 5314.9713 5715.9988
325.9160 -14067.2940 -1822.8014 0.0000 0.0000
7908.1646 6653.9569 301.5511 -1254.2078 6722.1647
300.1101 -3.7518 -3.6357 680285.8938 -18.7316
-18.7499
[4006]ENERGY: 1870000 3243.2908 5377.9316 5678.7447
348.7317 -14137.1613 -1765.3402 0.0000 0.0000
7912.9803 6659.1776 301.7347 -1253.8027 6720.7203
299.9289 -50.4015 6.0141 680285.8938 -19.5620
-19.5495
[4042]ENERGY: 1880000 3223.9123 5401.0666 5720.2742
331.5384 -14107.6517 -1799.0564 0.0000 0.0000
7886.9988 6657.0823 300.7440 -1229.9166 6722.7633
300.1205 107.5546 66.8510 680285.8938 -20.5668
-20.5826
[4092]ENERGY: 1890000 3286.0278 5348.4262 5644.4122
321.1753 -14107.0190 -1729.5724 0.0000 0.0000

7893.4957 6656.9459 300.9917 -1236.5498 6721.3232
300.0504 112.2919 -3.6792 680285.8938 -18.0090
-18.0177
[4131]ENERGY: 1900000 3223.2864 5407.5134 5660.5267
342.7023 -14062.2548 -1780.1749 0.0000 0.0000
7868.6011 6660.2003 300.0425 -1208.4008 6722.5289
300.1416 -32.3041 42.8455 680285.8938 -18.4202
-18.4002
[4181]ENERGY: 1910000 3214.4906 5359.2186 5710.0440
340.0774 -14109.6010 -1734.4180 0.0000 0.0000
7879.6890 6659.5004 300.4652 -1220.1886 6723.7451
300.3128 140.6522 55.7300 680285.8938 -18.7200
-18.7259
[4217]ENERGY: 1920000 3224.5769 5379.3887 5713.3663
337.5569 -14082.4941 -1748.7789 0.0000 0.0000
7836.9614 6660.5773 298.8360 -1176.3841 6723.8913
300.0920 157.2679 19.9261 680285.8938 -16.5315
-16.5519
[4267]ENERGY: 1930000 3136.6912 5301.3810 5667.2122
365.6627 -13986.7293 -1767.1186 0.0000 0.0000
7943.0212 6660.1203 302.8802 -1282.9009 6724.1069
300.0710 77.1344 44.7145 680285.8938 -18.0556
-18.0392
[4303]ENERGY: 1940000 3198.8290 5345.6425 5662.0181
333.0184 -14023.0798 -1785.0433 0.0000 0.0000
7928.6643 6660.0491 302.3328 -1268.6152 6723.9118
300.4163 -40.7045 -53.8174 680285.8938 -19.2161
-19.2186
[4353]ENERGY: 1950000 3274.0497 5366.4059 5746.0938
328.7355 -14209.6005 -1695.3797 0.0000 0.0000
7847.9435 6658.2483 299.2547 -1189.6952 6723.3653
300.4319 -49.3843 72.4690 680285.8938 -20.6453
-20.6190
[4389]ENERGY: 1960000 3207.3606 5369.7335 5676.7163
317.9893 -14128.2320 -1725.2846 0.0000 0.0000
7941.1081 6659.3911 302.8073 -1281.7170 6723.6533
300.5053 102.8896 77.6187 680285.8938 -20.4273
-20.4458
[4439]ENERGY: 1970000 3230.2311 5377.8639 5714.8963
333.3134 -14232.0062 -1675.8991 0.0000 0.0000
7910.7758 6659.1751 301.6506 -1251.6007 6724.0092
300.4345 291.6387 254.6083 680285.8938 -21.8200
-21.8214
[4475]ENERGY: 1980000 3270.7988 5420.8616 5695.8159
360.9722 -14114.7858 -1739.7167 0.0000 0.0000
7768.1852 6662.1312 296.2134 -1106.0540 6724.0593
300.2664 139.8252 23.0120 680285.8938 -22.2707
-22.2597
[4525]ENERGY: 1990000 3271.2176 5350.5374 5680.6534
337.1454 -14092.7087 -1748.1453 0.0000 0.0000
7862.0067 6660.7064 299.7910 -1201.3003 6725.6752
300.3802 107.2367 55.7048 680285.8938 -16.9065
-16.9158
[4564]ENERGY: 2000000 3247.4889 5381.3306 5685.0846

349.2871 -14041.5618 -1825.6361 0.0000 0.0000
7865.9741 6661.9674 299.9423 -1204.0067 6725.0738
300.3038 218.0084 99.7029 680285.8938 -20.6777
-20.6804
[4614]ENERGY: 2010000 3211.5785 5380.5482 5595.4507
330.1805 -13960.3042 -1770.0753 0.0000 0.0000
7873.4962 6660.8746 300.2291 -1212.6215 6725.4298
300.4311 -33.2692 -44.2933 680285.8938 -16.9240
-16.9135
[4650]ENERGY: 2020000 3251.9348 5499.4491 5716.3003
323.5107 -14215.3670 -1741.7796 0.0000 0.0000
7829.6839 6663.7321 298.5585 -1165.9518 6725.8350
300.1613 -15.1371 -10.3656 680285.8938 -20.5973
-20.5905
[4700]ENERGY: 2030000 3312.8740 5359.0987 5653.3533
339.7776 -14080.9756 -1780.1399 0.0000 0.0000
7857.1086 6661.0968 299.6042 -1196.0118 6724.6137
300.2000 -71.6316 -12.1017 680285.8938 -19.7669
-19.7871
[4736]ENERGY: 2040000 3333.9502 5340.5488 5675.0027
337.6362 -14082.9386 -1793.7566 0.0000 0.0000
7856.3019 6666.7446 299.5735 -1189.5573 6726.9604
300.5452 -24.0334 -22.2532 680285.8938 -18.8646
-18.8430
[4786]ENERGY: 2050000 3199.9159 5407.9587 5651.8796
352.4624 -14148.2631 -1768.8544 0.0000 0.0000
7962.6520 6657.7511 303.6288 -1304.9009 6726.6854
300.3719 -96.6571 -22.6618 680285.8938 -18.1959
-18.2048
[4822]ENERGY: 2060000 3266.9979 5314.5992 5670.3217
341.1655 -14066.9197 -1736.4271 0.0000 0.0000
7871.3070 6661.0446 300.1456 -1210.2624 6726.8195
300.4344 148.4265 79.6070 680285.8938 -19.7112
-19.7291
[4872]ENERGY: 2070000 3231.0787 5435.9935 5735.4354
349.3257 -14212.6144 -1709.6021 0.0000 0.0000
7834.8573 6664.4741 298.7557 -1170.3832 6726.7117
300.4412 -7.8106 -39.0289 680285.8938 -22.4109
-22.4137
[4908]ENERGY: 2080000 3287.3535 5369.2006 5744.8161
340.3847 -14164.3624 -1807.5632 0.0000 0.0000
7891.4045 6661.2337 300.9120 -1230.1708 6727.1052
300.3559 -155.7066 -158.0319 680285.8938 -20.1161
-20.1095
[4958]ENERGY: 2090000 3286.8665 5407.5234 5712.1693
321.6727 -14134.3038 -1778.4286 0.0000 0.0000
7843.7186 6659.2182 299.0936 -1184.5005 6726.3349
300.3637 99.1845 36.5597 680285.8938 -16.4251
-16.4297
[4997]ENERGY: 2100000 3295.2573 5434.7082 5622.4432
325.8135 -14130.0484 -1835.3320 0.0000 0.0000
7946.9769 6659.8187 303.0310 -1287.1582 6726.2906
300.4349 -180.2000 -202.4652 680285.8938 -15.9110
-15.9215

[5047]ENERGY: 2110000 3256.2977 5392.3566 5665.7388
336.6709 -14115.3663 -1796.9079 0.0000 0.0000
7921.6550 6660.4447 302.0655 -1261.2103 6724.1804
300.2692 -91.0068 -49.3878 680285.8938 -16.2240
-16.1992
[5083]ENERGY: 2120000 3245.9678 5346.1293 5685.9485
345.3887 -14099.7786 -1769.6544 0.0000 0.0000
7908.6898 6662.6910 301.5711 -1245.9987 6725.5013
300.3188 67.7701 -74.5061 680285.8938 -16.2074
-16.2062
[5133]ENERGY: 2130000 3237.2745 5421.3547 5726.4476
307.4984 -14146.2491 -1787.3731 0.0000 0.0000
7898.8595 6657.8127 301.1963 -1241.0469 6726.2143
300.2167 -34.6944 -49.0825 680285.8938 -20.4352
-20.4622
[5169]ENERGY: 2140000 3312.4072 5333.8108 5700.3755
354.8024 -14159.1382 -1751.5095 0.0000 0.0000
7870.9131 6661.6612 300.1306 -1209.2518 6725.7240
300.2215 79.5936 76.0507 680285.8938 -19.9603
-19.9383
[5219]ENERGY: 2150000 3244.8752 5437.2214 5723.8178
326.4441 -14202.2139 -1755.2083 0.0000 0.0000
7883.8023 6658.7385 300.6221 -1225.0638 6725.5318
300.2641 87.8807 37.4327 680285.8938 -21.1918
-21.2116
[5255]ENERGY: 2160000 3280.5560 5393.9261 5779.6627
330.2170 -14247.6001 -1746.5490 0.0000 0.0000
7872.3654 6662.5782 300.1860 -1209.7873 6725.5086
300.1909 87.0566 24.0104 680285.8938 -20.3519
-20.3410
[5305]ENERGY: 2170000 3213.2529 5380.9848 5735.7577
351.1335 -14172.9287 -1720.6711 0.0000 0.0000
7875.8588 6663.3879 300.3192 -1212.4709 6725.6787
300.1236 74.5558 22.8191 680285.8938 -19.7950
-19.7845
[5341]ENERGY: 2180000 3290.0552 5339.3048 5722.7020
316.4418 -14113.4551 -1800.6128 0.0000 0.0000
7907.0171 6661.4530 301.5073 -1245.5641 6726.7994
300.2492 -201.9225 -141.1467 680285.8938 -16.3116
-16.2960
[5391]ENERGY: 2190000 3254.4652 5415.3816 5674.9982
334.5786 -14141.7790 -1752.6506 0.0000 0.0000
7877.7673 6662.7612 300.3920 -1215.0061 6725.5542
300.2024 -82.2924 -82.3068 680285.8938 -17.6033
-17.6011
[5430]ENERGY: 2200000 3309.1996 5353.5560 5732.3161
314.4571 -14058.4391 -1771.7018 0.0000 0.0000
7787.3104 6666.6983 296.9427 -1120.6121 6727.0939
300.1949 44.7297 -41.7566 680285.8938 -21.6440
-21.6646
[5480]ENERGY: 2210000 3184.9733 5420.1440 5734.9468
338.2878 -14106.2295 -1788.1757 0.0000 0.0000
7878.4199 6662.3666 300.4169 -1216.0532 6725.9803
300.0975 60.3271 -1.1196 680285.8938 -24.5363

-24.5311
[5516]ENERGY: 2220000 3285.7263 5419.4494 5721.1701
355.5297 -14195.8571 -1717.8428 0.0000 0.0000
7794.6335 6662.8091 297.2219 -1131.8244 6726.0017
300.3139 6.8139 -68.3018 680285.8938 -26.7335
-26.7434
[5566]ENERGY: 2230000 3313.1119 5340.8504 5706.6694
333.4633 -14146.1084 -1751.7239 0.0000 0.0000
7865.4642 6661.7268 299.9228 -1203.7374 6726.1344
300.3739 -58.6294 -75.9070 680285.8938 -26.6698
-26.6676
[5602]ENERGY: 2240000 3346.3410 5332.6458 5683.9383
325.5625 -14100.0164 -1757.8012 0.0000 0.0000
7834.3846 6665.0545 298.7377 -1169.3301 6726.2266
300.2523 -78.5861 -80.5079 680285.8938 -25.3058
-25.3109
[5652]ENERGY: 2250000 3220.6067 5392.3310 5726.8194
321.3538 -14146.4340 -1752.0401 0.0000 0.0000
7900.3856 6663.0224 301.2544 -1237.3632 6727.3294
300.5993 -33.9564 -55.2354 680285.8938 -27.8941
-27.8902
[5688]ENERGY: 2260000 3311.5370 5406.8249 5693.0157
328.4548 -14160.2155 -1770.6550 0.0000 0.0000
7851.1896 6660.1515 299.3785 -1191.0380 6725.5411
300.5394 54.7898 21.3295 680285.8938 -23.1143
-23.1046
[5738]ENERGY: 2270000 3248.5546 5370.4252 5650.9116
335.4384 -14122.2295 -1760.5656 0.0000 0.0000
7939.2920 6661.8267 302.7380 -1277.4654 6726.5783
300.4178 -33.2783 -56.6541 680285.8938 -25.9346
-25.9388
[5774]ENERGY: 2280000 3213.1469 5369.3251 5665.5143
330.9136 -14157.9360 -1729.7822 0.0000 0.0000
7971.6727 6662.8543 303.9727 -1308.8183 6726.8897
300.4930 111.1400 8.3489 680285.8938 -24.9106
-24.9139
[5824]ENERGY: 2290000 3280.1869 5419.4505 5678.4074
317.1124 -14138.3522 -1803.9078 0.0000 0.0000
7910.5329 6663.4302 301.6414 -1247.1027 6728.1933
300.5835 111.3678 99.5131 680285.8938 -29.3395
-29.3335
[5863]ENERGY: 2300000 3163.7890 5454.9649 5692.2311
324.1418 -14065.3707 -1772.9635 0.0000 0.0000
7866.6302 6663.4228 299.9673 -1203.2073 6727.4431
300.6081 100.4771 45.5867 680285.8938 -27.5126
-27.5190
[5913]ENERGY: 2310000 3321.8926 5360.4496 5678.3858
328.2197 -14060.0655 -1735.8031 0.0000 0.0000
7773.8157 6666.8950 296.4281 -1106.9207 6727.3234
300.5085 -107.8365 -38.4268 680285.8938 -24.7102
-24.6921
[5949]ENERGY: 2320000 3291.5437 5383.9553 5636.9210
325.6881 -14149.7988 -1756.0291 0.0000 0.0000
7930.9435 6663.2237 302.4197 -1267.7199 6726.6558

```
300.4573           -136.2576      -159.1405    680285.8938       -23.8850
-23.8847
[5999]ENERGY: 2330000      3157.4016      5429.0671      5674.9908
333.0222         -14091.5200     -1847.8456         0.0000          0.0000
8004.6340           6659.7500       305.2296     -1344.8839      6726.6789
300.5291            14.9713        70.1100    680285.8938       -27.0161
-27.0241
[6035]ENERGY: 2340000      3229.3055      5445.6528      5720.1769
335.1174         -14157.2923     -1787.8321         0.0000          0.0000
7877.0411           6662.1694       300.3643     -1214.8717      6726.2975
300.6368            15.3042        -1.3168    680285.8938       -23.5962
-23.6044
[6085]ENERGY: 2350000      3233.9432      5486.9297      5688.2738
334.7811         -14162.6944     -1839.8046         0.0000          0.0000
7919.8751           6661.3040       301.9976     -1258.5712      6727.6945
300.7292           -23.9389       -66.5680    680285.8938       -29.2515
-29.2530
[6121]ENERGY: 2360000      3231.9233      5434.0179      5695.3544
329.0870         -14197.0090     -1708.9888         0.0000          0.0000
7881.4779           6665.8628       300.5335     -1215.6150      6728.7376
300.6887            84.2930        31.1557    680285.8938       -20.2826
-20.2773
[6171]ENERGY: 2370000      3258.7103      5328.3649      5710.4721
333.3461         -14115.4754     -1758.5032         0.0000          0.0000
7905.3858           6662.3007       301.4451     -1243.0851      6727.8094
300.7559           -89.2130       -29.3044    680285.8938       -30.6053
-30.6097
[6207]ENERGY: 2380000      3233.7200      5428.4465      5662.5125
321.8327         -14092.4532     -1753.4218         0.0000          0.0000
7864.2624           6664.8991       299.8770     -1199.3633      6727.7042
300.7681            91.6291        24.5316    680285.8938       -24.1549
-24.1712
[6257]ENERGY: 2390000      3242.9300      5334.1238      5678.9466
335.3573         -14090.6419     -1761.4253         0.0000          0.0000
7923.2697           6662.5602       302.1271     -1260.7096      6728.1838
300.7379            52.5610       -27.1962    680285.8938       -23.3884
-23.3762
[6296]ENERGY: 2400000      3221.3991      5477.5546      5667.5504
327.0907         -14104.4791     -1829.8880         0.0000          0.0000
7907.0527           6666.2803       301.5087     -1240.7724      6728.5927
300.6180            11.2951        36.8624    680285.8938       -23.1770
-23.1696
[6346]ENERGY: 2410000      3301.4173      5399.6543      5685.2995
336.0158         -14111.1449     -1731.1129         0.0000          0.0000
7787.5759           6667.7050       296.9528     -1119.8710      6728.8834
300.4726            65.0396        26.7128    680285.8938       -26.4799
-26.4864
[6382]ENERGY: 2420000      3188.4490      5475.4085      5718.0266
337.5680         -14180.9188     -1725.6077         0.0000          0.0000
7854.9958           6667.9215       299.5237     -1187.0743      6730.0271
300.7202           177.2840        54.1968    680285.8938       -23.1808
-23.1808
[6432]ENERGY: 2430000      3236.9445      5366.1959      5744.8600
322.0956         -14143.7501     -1754.6075         0.0000          0.0000
```

7895.6621 6667.4006 301.0743 -1228.2616 6730.0034
300.8953 279.5538 206.7533 680285.8938 -24.7229
-24.7258
[6468]ENERGY: 2440000 3305.5937 5479.4365 5655.7209
327.0423 -14131.4654 -1798.1745 0.0000 0.0000
7828.6603 6666.8140 298.5194 -1161.8463 6729.8461
300.9059 -166.6567 -144.8705 680285.8938 -24.1808
-24.1586
[6518]ENERGY: 2450000 3279.3936 5299.0034 5669.5526
329.0265 -14101.8725 -1691.3831 0.0000 0.0000
7882.6025 6666.3228 300.5763 -1216.2797 6728.9423
300.9094 -7.5830 32.0958 680285.8938 -19.1104
-19.1215
[6554]ENERGY: 2460000 3209.5741 5290.8751 5681.8238
313.5956 -14044.4580 -1755.3734 0.0000 0.0000
7967.0501 6663.0872 303.7965 -1303.9629 6729.6623
301.1327 75.9021 42.0056 680285.8938 -21.3471
-21.3593
[6604]ENERGY: 2470000 3292.0190 5497.6076 5704.0393
340.0591 -14205.1535 -1753.1257 0.0000 0.0000
7791.4109 6666.8566 297.0991 -1124.5543 6730.6265
300.8863 -24.0856 -41.6303 680285.8938 -18.2816
-18.2699
[6640]ENERGY: 2480000 3223.3250 5341.0459 5672.9680
332.5084 -14143.1131 -1743.3598 0.0000 0.0000
7977.1738 6660.5482 304.1825 -1316.6256 6729.9036
300.7860 10.6651 -23.9131 680285.8938 -22.4628
-22.4667
[6690]ENERGY: 2490000 3230.7824 5331.7656 5725.8539
334.5818 -14121.1784 -1731.0535 0.0000 0.0000
7893.6086 6664.3604 300.9960 -1229.2482 6730.4183
300.9503 31.2781 -21.0698 680285.8938 -17.6746
-17.6626
[6729]ENERGY: 2500000 3289.1030 5368.9118 5722.5283
312.9915 -14132.4633 -1820.9980 0.0000 0.0000
7923.9914 6664.0646 302.1546 -1259.9268 6730.1022
300.8109 -91.8866 -139.4196 680285.8938 -27.2209
-27.2401
[6779]ENERGY: 2510000 3262.1350 5239.6396 5719.4457
330.3285 -14095.5048 -1673.2707 0.0000 0.0000
7881.4194 6664.1926 300.5312 -1217.2268 6729.6491
300.9045 80.9287 -6.4844 680285.8938 -25.0545
-25.0537
[6815]ENERGY: 2520000 3235.6704 5347.1669 5688.0420
309.7628 -14179.6861 -1679.9921 0.0000 0.0000
7942.3084 6663.2722 302.8530 -1279.0362 6728.4640
300.7041 82.2655 39.3475 680285.8938 -26.7042
-26.7022
[6865]ENERGY: 2530000 3189.2209 5450.9110 5706.7413
334.5539 -14047.6051 -1833.6665 0.0000 0.0000
7865.1827 6665.3383 299.9121 -1199.8444 6729.9083
300.8647 90.4197 43.6594 680285.8938 -21.7706
-21.7615
[6901]ENERGY: 2540000 3284.4725 5434.0251 5728.9827

327.7802 -14150.6241 -1811.0708 0.0000 0.0000
7851.9604 6665.5260 299.4079 -1186.4344 6730.4827
300.7681 -47.1641 -9.9730 680285.8938 -20.9248
-20.9378
[6951]ENERGY: 2550000 3337.1994 5423.3751 5687.6710
330.1772 -14205.1289 -1747.7211 0.0000 0.0000
7842.4972 6668.0701 299.0471 -1174.4272 6730.6522
300.8917 20.4258 -29.6973 680285.8938 -21.3728
-21.3752
[6987]ENERGY: 2560000 3337.7466 5335.4695 5695.4346
336.1057 -14101.4832 -1833.7214 0.0000 0.0000
7898.9947 6668.5463 301.2014 -1230.4484 6732.4305
301.1080 21.4518 -0.2053 680285.8938 -20.9789
-20.9710
[7037]ENERGY: 2570000 3310.0369 5423.0725 5742.2164
358.5029 -14268.3782 -1733.2102 0.0000 0.0000
7835.6634 6667.9038 298.7865 -1167.7596 6731.8250
300.9498 93.8808 51.2251 680285.8938 -24.2891
-24.2841
[7073]ENERGY: 2580000 3296.5399 5436.6335 5680.3145
331.6235 -14215.8857 -1752.3036 0.0000 0.0000
7891.5205 6668.4426 300.9164 -1223.0779 6731.1491
300.9708 55.7669 -7.0252 680285.8938 -24.2911
-24.2850
[7123]ENERGY: 2590000 3232.4632 5389.3311 5712.0527
330.1178 -14079.6781 -1805.4003 0.0000 0.0000
7887.7324 6666.6188 300.7720 -1221.1135 6730.7549
301.1220 70.7867 32.7811 680285.8938 -28.6690
-28.6663
[7162]ENERGY: 2600000 3310.8690 5305.5399 5624.7046
320.6358 -14116.8892 -1772.6898 0.0000 0.0000
7993.4008 6665.5709 304.8013 -1327.8299 6731.2384
301.0249 -120.2083 -32.0830 680285.8938 -26.2457
-26.2290
[7212]ENERGY: 2610000 3185.8188 5399.8234 5683.5137
348.8793 -14101.6556 -1770.1356 0.0000 0.0000
7919.2436 6665.4876 301.9735 -1253.7560 6731.1458
301.1575 17.4256 -17.4240 680285.8938 -27.5003
-27.5139
[7248]ENERGY: 2620000 3201.0941 5421.1726 5705.1325
342.5566 -14179.7350 -1732.3045 0.0000 0.0000
7907.9025 6665.8188 301.5411 -1242.0838 6731.9552
301.0651 92.9768 38.0084 680285.8938 -30.2454
-30.2624
[7298]ENERGY: 2630000 3300.6887 5344.4810 5714.2949
337.8298 -14126.8433 -1785.1165 0.0000 0.0000
7884.3980 6669.7326 300.6448 -1214.6654 6733.0765
301.0902 -15.7029 0.0310 680285.8938 -25.1026
-25.0830
[7334]ENERGY: 2640000 3218.6528 5401.2489 5740.7151
326.2883 -14106.6091 -1780.3426 0.0000 0.0000
7870.9300 6670.8834 300.1313 -1200.0466 6732.8757
301.0739 201.6096 19.0467 680285.8938 -29.2165
-29.2308

[7384]ENERGY: 2650000 3245.8963 5333.2795 5806.4586
354.8067 -14137.0548 -1760.5876 0.0000 0.0000
7828.0949 6670.8936 298.4979 -1157.2012 6733.9244
301.3070 119.8824 70.3745 680285.8938 -25.5884
-25.5866
[7420]ENERGY: 2660000 3276.8789 5430.5140 5663.4525
326.5028 -14162.8926 -1780.1998 0.0000 0.0000
7916.8174 6671.0733 301.8810 -1245.7442 6731.7297
301.2872 -54.6725 -35.2426 680285.8938 -28.2387
-28.2395
[7470]ENERGY: 2670000 3245.1100 5467.4628 5672.0450
318.1876 -14133.3981 -1764.1473 0.0000 0.0000
7862.8797 6668.1397 299.8243 -1194.7400 6732.7953
301.2050 -67.2673 12.0859 680285.8938 -27.7463
-27.7334
[7506]ENERGY: 2680000 3271.0955 5378.3366 5730.8701
320.4947 -14204.4077 -1726.9236 0.0000 0.0000
7897.2752 6666.7409 301.1358 -1230.5343 6732.1642
301.1082 -25.0735 -4.2123 680285.8938 -27.4074
-27.4264
[7556]ENERGY: 2690000 3198.5745 5447.8261 5729.9479
351.4550 -14128.2967 -1820.2684 0.0000 0.0000
7890.5922 6669.8305 300.8810 -1220.7616 6733.6486
301.1946 21.4727 -65.4332 680285.8938 -21.9532
-21.9500
[7595]ENERGY: 2700000 3272.5790 5344.8215 5728.8829
336.0232 -14127.5432 -1771.3603 0.0000 0.0000
7888.9715 6672.3746 300.8192 -1216.5969 6733.8832
301.3314 -35.2323 -47.3642 680285.8938 -26.3319
-26.3269
[7645]ENERGY: 2710000 3191.6071 5504.1674 5674.6198
336.2629 -14257.6089 -1705.7873 0.0000 0.0000
7927.9701 6671.2311 302.3063 -1256.7390 6733.4781
301.2163 11.9012 -6.0561 680285.8938 -27.0721
-27.0964
[7681]ENERGY: 2720000 3180.5971 5418.3519 5724.9547
346.2597 -14117.0774 -1772.9598 0.0000 0.0000
7888.6970 6668.8232 300.8087 -1219.8738 6733.7886
301.1809 -3.0108 14.6180 680285.8938 -24.6603
-24.6315
[7731]ENERGY: 2730000 3228.8625 5474.0272 5659.5869
324.7858 -14157.4995 -1766.3800 0.0000 0.0000
7904.2633 6667.6462 301.4023 -1236.6171 6734.4313
301.1359 -48.0441 -18.7086 680285.8938 -25.0919
-25.1009
[7767]ENERGY: 2740000 3195.8635 5415.7300 5769.6703
317.5661 -14222.4476 -1725.5995 0.0000 0.0000
7919.0634 6669.8463 301.9667 -1249.2171 6734.2358
301.2049 110.1035 17.8545 680285.8938 -26.1425
-26.1478
[7817]ENERGY: 2750000 3294.2653 5335.2124 5703.7557
320.9882 -14168.9822 -1740.9146 0.0000 0.0000
7926.6390 6670.9637 302.2555 -1255.6753 6734.4324
301.2071 -17.1964 -47.4678 680285.8938 -23.0095

-23.0107
[7853]ENERGY: 2760000 3204.7539 5480.9314 5706.5756
342.3942 -14217.0706 -1774.7816 0.0000 0.0000
7926.3051 6669.1080 302.2428 -1257.1970 6734.1367
301.4018 -107.3096 -125.1888 680285.8938 -23.4526
-23.4373
[7903]ENERGY: 2770000 3298.8727 5459.9358 5697.9185
348.6350 -14193.6971 -1842.2346 0.0000 0.0000
7902.7829 6672.2132 301.3459 -1230.5697 6734.3799
301.4578 -63.6515 -63.2451 680285.8938 -26.3589
-26.3536
[7939]ENERGY: 2780000 3258.0979 5453.7362 5723.4462
326.4421 -14222.3690 -1813.9371 0.0000 0.0000
7943.6346 6669.0508 302.9036 -1274.5838 6734.1614
301.6843 -53.1076 -23.4529 680285.8938 -26.4703
-26.4683
[7989]ENERGY: 2790000 3310.1923 5433.0459 5708.2650
324.7771 -14267.9760 -1777.2846 0.0000 0.0000
7944.1662 6675.1859 302.9239 -1268.9803 6737.3119
301.6949 -44.1979 -54.1722 680285.8938 -23.9951
-24.0001
[8028]ENERGY: 2800000 3224.3337 5393.5792 5735.5956
334.2089 -14124.2967 -1727.4954 0.0000 0.0000
7834.9136 6670.8389 298.7579 -1164.0747 6735.9548
301.6274 19.0941 -56.7308 680285.8938 -22.8954
-22.9184
[8078]ENERGY: 2810000 3267.7431 5405.0309 5698.3491
333.2643 -14180.2967 -1720.4536 0.0000 0.0000
7869.1618 6672.7990 300.0638 -1196.3628 6736.0817
301.7073 -68.9734 11.1380 680285.8938 -22.4772
-22.4867
[8114]ENERGY: 2820000 3255.9070 5370.4328 5727.5928
337.6408 -14113.8997 -1787.3165 0.0000 0.0000
7883.2617 6673.6189 300.6015 -1209.6428 6735.4227
301.6353 -210.1092 -179.4922 680285.8938 -25.6567
-25.6244
[8164]ENERGY: 2830000 3337.9455 5348.0926 5717.5736
342.6440 -14096.5591 -1852.3539 0.0000 0.0000
7878.0864 6675.4292 300.4041 -1202.6572 6737.1586
301.4789 -34.0511 -78.0152 680285.8938 -24.5920
-24.6065
[8200]ENERGY: 2840000 3314.9025 5423.5688 5708.7336
347.6051 -14168.4220 -1854.7382 0.0000 0.0000
7903.7062 6675.3560 301.3811 -1228.3502 6735.6855
301.7338 -98.9867 -122.9422 680285.8938 -29.0627
-29.0624
[8250]ENERGY: 2850000 3257.9040 5483.1555 5670.7629
332.4951 -14182.3559 -1761.0913 0.0000 0.0000
7869.1677 6670.0381 300.0641 -1199.1296 6736.2759
301.7380 71.8643 -54.7967 680285.8938 -28.7427
-28.7584
[8286]ENERGY: 2860000 3296.4450 5422.1096 5707.0214
335.0418 -14187.0184 -1768.5199 0.0000 0.0000
7866.3650 6671.4446 299.9572 -1194.9204 6735.8030

301.7411 -154.9791 -170.4817 680285.8938 -27.4831
-27.4656
[8336]ENERGY: 2870000 3277.8137 5430.1408 5719.7337
331.0683 -14221.0936 -1839.1769 0.0000 0.0000
7969.6226 6668.1086 303.8946 -1301.5140 6736.6436
301.6597 -74.5522 -82.2984 680285.8938 -25.1974
-25.1996
[8372]ENERGY: 2880000 3259.1511 5412.9318 5732.8171
321.2703 -14122.9774 -1767.1295 0.0000 0.0000
7838.2386 6674.3019 298.8847 -1163.9367 6736.5679
301.5855 -55.0279 -64.8628 680285.8938 -24.0206
-23.9999
[8422]ENERGY: 2890000 3217.3264 5433.6663 5714.3607
338.2184 -14205.0002 -1792.1712 0.0000 0.0000
7965.5230 6671.9234 303.7382 -1293.5996 6735.9152
301.7194 -21.3897 -73.1689 680285.8938 -26.3611
-26.3824
[8461]ENERGY: 2900000 3301.9642 5405.2204 5701.2232
338.0469 -14155.2124 -1815.0679 0.0000 0.0000
7897.1159 6673.2903 301.1298 -1223.8256 6737.0493
301.7216 -2.8262 -128.5458 680285.8938 -29.2380
-29.2452
[8511]ENERGY: 2910000 3230.3995 5394.8502 5689.1937
332.5508 -14071.5043 -1777.8132 0.0000 0.0000
7875.2031 6672.8797 300.2942 -1202.3233 6736.6056
301.6748 21.9701 30.8085 680285.8938 -24.6926
-24.6915
[8547]ENERGY: 2920000 3228.3190 5353.3124 5720.0646
326.0864 -14206.3802 -1758.9026 0.0000 0.0000
8005.6817 6668.1814 305.2696 -1337.5003 6735.4182
301.6508 241.7229 96.9366 680285.8938 -27.4983
-27.4915
[8597]ENERGY: 2930000 3305.2974 5409.2372 5660.2363
328.4527 -14159.5405 -1776.4787 0.0000 0.0000
7905.3164 6672.5208 301.4425 -1232.7956 6736.9039
301.6194 -134.3592 -167.0333 680285.8938 -29.4981
-29.4943
[8633]ENERGY: 2940000 3260.1046 5413.6360 5655.4530
328.8157 -14180.4358 -1756.7310 0.0000 0.0000
7951.6942 6672.5366 303.2109 -1279.1576 6738.4193
301.7785 -99.1172 -74.5560 680285.8938 -27.7598
-27.7663
[8683]ENERGY: 2950000 3226.9365 5418.9797 5756.0322
330.6125 -14270.3013 -1716.4566 0.0000 0.0000
7928.0123 6673.8153 302.3079 -1254.1970 6738.1253
301.8628 105.7885 26.9203 680285.8938 -23.6979
-23.6991
[8719]ENERGY: 2960000 3221.3784 5403.9357 5696.5599
352.2534 -14172.2126 -1754.8478 0.0000 0.0000
7925.1761 6672.2432 302.1997 -1252.9329 6737.6352
301.8646 -127.8129 -82.2292 680285.8938 -22.4327
-22.4108
[8769]ENERGY: 2970000 3251.1455 5529.5155 5681.7406
347.7049 -14215.4466 -1819.8111 0.0000 0.0000

7895.4062 6670.2551 301.0646 -1225.1510 6739.3686
301.7235 -56.7343 -80.9032 680285.8938 -25.1183
-25.0991
[8805]ENERGY: 2980000 3326.7671 5456.1337 5731.2514
342.7100 -14226.3377 -1816.3599 0.0000 0.0000
7861.9346 6676.0992 299.7882 -1185.8354 6739.2606
301.6731 -68.1578 -112.1838 680285.8938 -23.0779
-23.1062
[8855]ENERGY: 2990000 3322.9228 5449.3895 5689.4182
331.3690 -14169.5721 -1818.4304 0.0000 0.0000
7868.6735 6673.7705 300.0452 -1194.9030 6738.3913
301.5412 -78.9801 -57.0779 680285.8938 -24.5535
-24.5553
[8894]ENERGY: 3000000 3319.9294 5405.7358 5698.6499
360.9079 -14205.7395 -1763.2641 0.0000 0.0000
7863.6530 6679.8723 299.8538 -1183.7807 6739.1453
301.6292 53.2016 -27.1104 680285.8938 -26.4583
-26.4663
[8944]ENERGY: 3010000 3327.4501 5428.5227 5681.2987
340.4762 -14139.6396 -1846.9303 0.0000 0.0000
7882.6669 6673.8446 300.5788 -1208.8223 6738.0561
301.7163 -44.2173 -58.8911 680285.8938 -22.2223
-22.2085
[8980]ENERGY: 3020000 3246.7131 5410.6340 5698.1097
343.2709 -14175.4108 -1744.9524 0.0000 0.0000
7897.3085 6675.6730 301.1371 -1221.6355 6739.0134
301.6999 -87.2076 -134.7390 680285.8938 -26.7558
-26.7603
[9030]ENERGY: 3030000 3303.9420 5391.6052 5704.9029
328.6121 -14195.7849 -1877.1144 0.0000 0.0000
8017.6422 6673.8051 305.7256 -1343.8371 6739.2842
301.7816 -130.6435 -127.1231 680285.8938 -24.2433
-24.2415
[9066]ENERGY: 3040000 3240.0259 5370.5253 5752.7562
351.5461 -14116.8920 -1789.5166 0.0000 0.0000
7867.9945 6676.4394 300.0193 -1191.5551 6739.6138
301.8567 16.3732 -17.5192 680285.8938 -27.2252
-27.2289
[9116]ENERGY: 3050000 3208.7029 5453.7632 5676.4159
337.1009 -14205.6795 -1721.6764 0.0000 0.0000
7924.8041 6673.4313 302.1856 -1251.3729 6737.3967
301.8078 167.6894 136.6962 680285.8938 -23.0366
-23.0351
[9152]ENERGY: 3060000 3259.6914 5410.5961 5685.2934
330.5101 -14253.1288 -1726.2349 0.0000 0.0000
7963.8889 6670.6164 303.6759 -1293.2726 6737.6229
301.7195 -79.4085 -44.6596 680285.8938 -26.8726
-26.8689
[9202]ENERGY: 3070000 3261.6322 5436.2987 5682.0263
340.7706 -14171.3715 -1841.1290 0.0000 0.0000
7966.8490 6675.0762 303.7888 -1291.7728 6738.7775
301.8934 -4.3322 27.0755 680285.8938 -26.4574
-26.4553
[9238]ENERGY: 3080000 3312.7321 5313.3554 5676.9663

309.0691 -14161.6382 -1752.6217 0.0000 0.0000
7976.5944 6674.4575 304.1604 -1302.1369 6738.7042
301.7991 -213.7417 -199.8228 680285.8938 -25.9270
-25.9275
[9288]ENERGY: 3090000 3279.8707 5388.2776 5763.1192
356.4637 -14218.6353 -1742.8844 0.0000 0.0000
7848.8841 6675.0956 299.2906 -1173.7885 6738.7368
301.6937 -22.2980 -24.6872 680285.8938 -27.7624
-27.7589
[9327]ENERGY: 3100000 3288.0398 5334.2582 5726.2732
313.8130 -14123.9685 -1775.0518 0.0000 0.0000
7911.0373 6674.4011 301.6606 -1236.6361 6738.7897
301.6424 -39.4955 -52.5863 680285.8938 -23.9817
-23.9867
[9377]ENERGY: 3110000 3298.7411 5415.1879 5730.4697
334.7914 -14269.3488 -1720.5678 0.0000 0.0000
7883.6250 6672.8986 300.6153 -1210.7265 6739.0467
301.7338 82.1870 -75.8756 680285.8938 -25.4325
-25.4510
[9413]ENERGY: 3120000 3268.2737 5411.8898 5683.4499
348.7264 -14163.3769 -1733.2450 0.0000 0.0000
7861.5249 6677.2429 299.7726 -1184.2821 6739.1058
301.7554 65.5229 35.6464 680285.8938 -24.3614
-24.3569
[9463]ENERGY: 3130000 3251.1926 5388.0198 5769.1256
333.6533 -14207.3627 -1733.7046 0.0000 0.0000
7874.3793 6675.3032 300.2628 -1199.0761 6740.1870
301.7097 230.4668 164.9622 680285.8938 -25.6176
-25.6382
[9499]ENERGY: 3140000 3243.1635 5366.5486 5728.1038
331.3780 -14329.2160 -1640.4580 0.0000 0.0000
7974.1599 6673.6798 304.0676 -1300.4801 6738.3645
301.6596 91.2830 85.0058 680285.8938 -24.6480
-24.6193
[9549]ENERGY: 3150000 3242.2839 5553.7923 5634.9906
349.2301 -14250.3541 -1715.0959 0.0000 0.0000
7861.1079 6675.9548 299.7567 -1185.1531 6739.3902
301.8552 92.4151 79.6594 680285.8938 -30.7585
-30.7406
[9585]ENERGY: 3160000 3313.3628 5356.2012 5677.0595
332.4302 -14122.8665 -1792.8813 0.0000 0.0000
7918.4528 6681.7587 301.9434 -1236.6942 6740.5781
301.9253 -204.1384 -185.5670 680285.8938 -30.9354
-30.9363
[9635]ENERGY: 3170000 3269.3778 5489.2843 5664.4154
334.2763 -14248.2380 -1765.7433 0.0000 0.0000
7932.1362 6675.5086 302.4651 -1256.6276 6741.2877
301.8204 -161.3725 -86.5056 680285.8938 -28.0017
-28.0188
[9671]ENERGY: 3180000 3218.1353 5450.9364 5710.2930
354.4454 -14235.7940 -1776.3624 0.0000 0.0000
7954.7131 6676.3667 303.3260 -1278.3464 6741.1724
301.9534 140.6957 77.8856 680285.8938 -21.8691
-21.8699

[9721]ENERGY: 3190000 3309.3262 5343.4027 5707.1874
327.2889 -14154.1050 -1783.3871 0.0000 0.0000
7927.5588 6677.2720 302.2906 -1250.2868 6742.0705
301.9707 -195.5996 -167.2166 680285.8938 -25.7099
-25.7191
[9760]ENERGY: 3200000 3280.6665 5546.7241 5732.2960
326.9288 -14335.9351 -1729.8736 0.0000 0.0000
7857.9839 6678.7905 299.6376 -1179.1934 6741.4768
301.8108 -42.9931 -39.1059 680285.8938 -27.7203
-27.7117
[9810]ENERGY: 3210000 3293.2167 5405.6561 5741.9863
331.8821 -14116.2781 -1835.0565 0.0000 0.0000
7859.0083 6680.4150 299.6767 -1178.5933 6743.0107
301.9853 -110.5749 -106.5363 680285.8938 -24.9241
-24.9298
[9846]ENERGY: 3220000 3251.8381 5416.5740 5732.1399
333.0843 -14196.9087 -1758.9306 0.0000 0.0000
7900.6748 6678.4718 301.2655 -1222.2030 6742.3568
302.1722 86.8143 44.3524 680285.8938 -23.6853
-23.6840
[9896]ENERGY: 3230000 3213.3323 5460.5571 5692.0000
351.8060 -14163.3471 -1746.6423 0.0000 0.0000
7873.7801 6681.4861 300.2399 -1192.2940 6744.0186
302.1231 37.8656 -29.8680 680285.8938 -26.0393
-26.0222
[9932]ENERGY: 3240000 3321.7131 5367.0175 5745.0806
325.4846 -14129.5431 -1800.0647 0.0000 0.0000
7854.1008 6683.7888 299.4895 -1170.3121 6743.8891
302.1750 115.2479 7.4445 680285.8938 -20.9897
-21.0086
[9982]ENERGY: 3250000 3219.4671 5451.4864 5666.2208
335.7023 -14249.2113 -1765.7807 0.0000 0.0000
8018.1795 6676.0640 305.7461 -1342.1155 6744.8809
302.0226 77.1222 76.7893 680285.8938 -21.5264
-21.5306
[10018]ENERGY: 3260000 3250.3576 5420.3229 5708.2888
347.5391 -14192.0110 -1779.1571 0.0000 0.0000
7924.7086 6680.0489 302.1819 -1244.6597 6744.8372
302.0402 33.5993 -80.7991 680285.8938 -21.2086
-21.1932
[10068]ENERGY: 3270000 3224.2719 5564.6840 5713.4867
325.4809 -14264.3730 -1748.3314 0.0000 0.0000
7868.2846 6683.5037 300.0304 -1184.7809 6744.9943
302.1065 -34.7933 -5.9795 680285.8938 -23.4181
-23.4220
[10104]ENERGY: 3280000 3320.8176 5500.5592 5694.6615
338.8941 -14212.2765 -1783.1792 0.0000 0.0000
7823.0622 6682.5390 298.3060 -1140.5232 6744.8883
302.1954 -67.9656 -29.0278 680285.8938 -20.5330
-20.5389
[10154]ENERGY: 3290000 3224.2026 5466.8164 5670.0709
311.6760 -14172.0057 -1772.8092 0.0000 0.0000
7950.5612 6678.5121 303.1677 -1272.0491 6743.7361
302.1250 -197.0100 -73.9148 680285.8938 -23.9015

-23.8832
[10193]ENERGY: 3300000 3292.3345 5433.3165 5756.9671
339.0678 -14091.6565 -1857.9457 0.0000 0.0000
7811.2106 6683.2944 297.8541 -1127.9161 6745.6426
302.2540 135.9484 120.8069 680285.8938 -21.4015
-21.4182
[10243]ENERGY: 3310000 3269.6049 5424.1305 5731.8333
337.2537 -14192.6980 -1829.1750 0.0000 0.0000
7937.0277 6677.9770 302.6517 -1259.0507 6744.3753
302.3059 64.9911 70.4039 680285.8938 -21.6131
-21.6048
[10279]ENERGY: 3320000 3308.3521 5365.9902 5726.0419
333.6123 -14226.2802 -1749.7641 0.0000 0.0000
7922.3164 6680.2686 302.0907 -1242.0478 6743.7180
302.1810 164.2150 86.2970 680285.8938 -22.2582
-22.2589
[10329]ENERGY: 3330000 3263.5066 5434.1091 5690.2402
344.6756 -14150.5982 -1801.3516 0.0000 0.0000
7897.1686 6677.7504 301.1318 -1219.4182 6741.7831
302.2432 -3.8106 -131.4538 680285.8938 -26.5430
-26.5464
[10365]ENERGY: 3340000 3239.1703 5462.1521 5706.8485
334.2053 -14207.9984 -1763.7651 0.0000 0.0000
7910.7522 6681.3648 301.6497 -1229.3873 6743.6043
302.2787 112.1202 15.1558 680285.8938 -24.7455
-24.7449
[10415]ENERGY: 3350000 3241.5615 5388.1814 5736.7048
341.4730 -14194.0085 -1709.9185 0.0000 0.0000
7877.9056 6681.8993 300.3972 -1196.0063 6742.9433
302.0649 94.3721 94.4984 680285.8938 -26.7948
-26.7990
[10451]ENERGY: 3360000 3309.1738 5468.9842 5708.7581
315.8059 -14206.3064 -1788.7462 0.0000 0.0000
7873.5212 6681.1906 300.2301 -1192.3306 6742.6442
302.1672 52.6841 41.2132 680285.8938 -22.2882
-22.2728
[10501]ENERGY: 3370000 3286.9129 5353.2536 5723.1626
318.6123 -14231.4226 -1726.8600 0.0000 0.0000
7956.2732 6679.9319 303.3855 -1276.3413 6743.7931
301.9507 -52.5944 -45.2260 680285.8938 -28.0496
-28.0723
[10537]ENERGY: 3380000 3299.5167 5390.1481 5696.3444
322.9504 -14179.6930 -1789.1275 0.0000 0.0000
7937.9648 6678.1039 302.6874 -1259.8610 6743.8553
302.1179 -65.1825 -56.9491 680285.8938 -22.6723
-22.6780
[10587]ENERGY: 3390000 3248.4128 5455.1508 5739.6545
333.7826 -14283.5826 -1728.4980 0.0000 0.0000
7918.5778 6683.4978 301.9481 -1235.0800 6745.0131
302.3335 169.7329 149.1519 680285.8938 -27.9074
-27.9012
[10626]ENERGY: 3400000 3311.5052 5500.0759 5707.5799
322.8925 -14228.2707 -1804.2314 0.0000 0.0000
7871.3265 6680.8780 300.1464 -1190.4485 6743.8297

302.2451 -100.9465 -111.6964 680285.8938 -22.4106
-22.4007
[10676]ENERGY: 3410000 3295.8087 5445.6436 5669.1595
332.2822 -14160.1531 -1772.1475 0.0000 0.0000
7870.3693 6680.9626 300.1099 -1189.4067 6745.1694
302.3945 72.0061 82.4492 680285.8938 -23.8216
-23.8231
[10712]ENERGY: 3420000 3217.9798 5487.6014 5687.4495
335.8153 -14233.9586 -1719.8958 0.0000 0.0000
7904.7926 6679.7841 301.4225 -1225.0085 6745.3723
302.3798 178.4769 53.5115 680285.8938 -24.7390
-24.7280
[10762]ENERGY: 3430000 3218.5668 5507.1722 5737.8021
341.9551 -14296.0204 -1768.3675 0.0000 0.0000
7937.6245 6678.7328 302.6744 -1258.8917 6745.9374
302.3667 34.0241 -31.3433 680285.8938 -25.2389
-25.2616
[10798]ENERGY: 3440000 3319.9545 5409.1037 5762.5783
328.9498 -14253.6418 -1765.1361 0.0000 0.0000
7882.7763 6684.5847 300.5830 -1198.1917 6745.9113
302.3174 -80.2011 -100.4280 680285.8938 -28.3058
-28.2889
[10848]ENERGY: 3450000 3264.2394 5456.1788 5722.0629
335.3453 -14250.7217 -1742.2772 0.0000 0.0000
7897.9162 6682.7438 301.1603 -1215.1724 6746.3054
302.4229 54.3388 4.0207 680285.8938 -30.3329
-30.3529
[10884]ENERGY: 3460000 3275.9313 5374.9215 5712.1521
335.5534 -14221.7403 -1731.5776 0.0000 0.0000
7937.5578 6682.7982 302.6719 -1254.7596 6746.1555
302.4824 -42.0895 19.7717 680285.8938 -28.3305
-28.3052
[10934]ENERGY: 3470000 3321.6221 5400.7044 5703.1826
342.8402 -14260.3987 -1785.3037 0.0000 0.0000
7960.1226 6682.7695 303.5323 -1277.3531 6745.9772
302.3453 -75.8349 26.3402 680285.8938 -28.1956
-28.1935
[10970]ENERGY: 3480000 3290.7224 5369.4732 5692.0968
329.5222 -14200.2194 -1779.8833 0.0000 0.0000
7984.4322 6686.1440 304.4593 -1298.2882 6746.1293
302.3808 9.7013 -100.2234 680285.8938 -28.8158
-28.8298
[11020]ENERGY: 3490000 3273.3974 5361.7520 5755.4493
333.7271 -14119.5965 -1917.0399 0.0000 0.0000
7997.0711 6684.7605 304.9412 -1312.3106 6746.6955
302.4374 -229.1939 -224.5202 680285.8938 -26.2187
-26.2140
[11059]ENERGY: 3500000 3247.0707 5464.8978 5708.9915
323.7214 -14227.2812 -1783.9865 0.0000 0.0000
7949.6451 6683.0589 303.1328 -1266.5863 6746.4702
302.5261 14.5575 -11.4591 680285.8938 -27.5925
-27.5855
[11109]ENERGY: 3510000 3264.8078 5357.4408 5663.3572
333.5268 -14148.4780 -1753.5793 0.0000 0.0000

7963.4539 6680.5290 303.6593 -1282.9249 6744.4056
302.5561 -0.5591 30.0823 680285.8938 -28.4549
-28.4670
[11145]ENERGY: 3520000 3284.6306 5485.4782 5692.5257
324.5246 -14172.6257 -1829.8688 0.0000 0.0000
7898.4978 6683.1625 301.1825 -1215.3354 6747.5295
302.5483 56.5859 -15.8056 680285.8938 -25.5099
-25.5188
[11195]ENERGY: 3530000 3284.2921 5418.1749 5699.1592
355.8590 -14268.8745 -1701.1720 0.0000 0.0000
7894.3456 6681.7843 301.0241 -1212.5613 6746.7404
302.4340 -15.1176 11.8754 680285.8938 -28.7443
-28.7236
[11231]ENERGY: 3540000 3332.4372 5377.5241 5676.0621
324.2793 -14180.0169 -1783.3599 0.0000 0.0000
7934.7723 6681.6981 302.5657 -1253.0742 6747.4077
302.5343 93.5141 1.8228 680285.8938 -25.4831
-25.4920
[11281]ENERGY: 3550000 3263.7121 5491.8945 5715.4752
314.1793 -14215.5414 -1837.0431 0.0000 0.0000
7946.7782 6679.4548 303.0235 -1267.3234 6746.4755
302.6414 -60.1321 -5.4666 680285.8938 -29.2796
-29.2681
[11317]ENERGY: 3560000 3310.8507 5429.6536 5698.7461
345.5782 -14155.1291 -1805.4049 0.0000 0.0000
7860.9768 6685.2714 299.7517 -1175.7054 6747.5081
302.5402 -4.7370 -82.2061 680285.8938 -31.1694
-31.1939
[11367]ENERGY: 3570000 3243.3431 5464.8228 5712.6261
336.0068 -14218.4636 -1733.5994 0.0000 0.0000
7880.5149 6685.2505 300.4967 -1195.2644 6748.7214
302.5816 151.1748 215.7027 680285.8938 -28.9827
-28.9654
[11403]ENERGY: 3580000 3271.6021 5552.6273 5636.4273
338.1990 -14193.6927 -1791.0170 0.0000 0.0000
7868.9133 6683.0594 300.0544 -1185.8539 6748.2207
302.4374 -1.1700 -20.5874 680285.8938 -28.2828
-28.2872
[11453]ENERGY: 3590000 3293.1988 5428.2587 5697.0567
306.9785 -14214.1514 -1777.1580 0.0000 0.0000
7946.5189 6680.7023 303.0136 -1265.8167 6749.5012
302.4342 -86.9447 -17.4646 680285.8938 -29.0578
-29.0370
[11492]ENERGY: 3600000 3353.0443 5447.5646 5720.4466
327.9389 -14210.9604 -1790.0076 0.0000 0.0000
7839.6308 6687.6573 298.9378 -1151.9736 6750.5488
302.3984 -216.5047 -167.8892 680285.8938 -23.3293
-23.3503
[11542]ENERGY: 3610000 3252.2038 5487.4154 5704.2695
314.6701 -14222.9814 -1823.8836 0.0000 0.0000
7972.9398 6684.6337 304.0211 -1288.3061 6750.8743
302.4319 176.8144 123.0355 680285.8938 -22.2776
-22.2954
[11578]ENERGY: 3620000 3306.5497 5403.2369 5723.9347

355.6674 -14141.1569 -1839.2466 0.0000 0.0000
7877.8374 6686.8227 300.3946 -1191.0147 6749.8522
302.4659 13.9510 51.1920 680285.8938 -21.0835
-21.0746
[11628]ENERGY: 3630000 3329.0778 5416.0023 5719.8462
327.2065 -14212.8784 -1818.7082 0.0000 0.0000
7926.8703 6687.4164 302.2643 -1239.4539 6751.5950
302.6964 -36.2551 -79.4413 680285.8938 -25.1092
-25.1003
[11664]ENERGY: 3640000 3188.7545 5499.1751 5715.9492
312.0201 -14267.2794 -1726.3187 0.0000 0.0000
7961.4435 6683.7443 303.5827 -1277.6992 6750.5745
302.7046 -38.5421 -4.6287 680285.8938 -27.0325
-27.0212
[11714]ENERGY: 3650000 3232.0061 5348.2160 5722.5781
340.8420 -14161.2728 -1789.6692 0.0000 0.0000
7991.8252 6684.5256 304.7412 -1307.2996 6749.5932
302.4769 63.9986 35.1999 680285.8938 -19.6337
-19.6533
[11750]ENERGY: 3660000 3324.4752 5370.1089 5734.3706
322.4461 -14214.3146 -1774.4012 0.0000 0.0000
7920.8463 6683.5313 302.0346 -1237.3150 6749.3464
302.3799 -156.6960 -186.6303 680285.8938 -24.0157
-24.0029
[11800]ENERGY: 3670000 3218.6973 5568.6514 5671.0026
325.8932 -14117.7023 -1833.7114 0.0000 0.0000
7853.4299 6686.2606 299.4639 -1167.1693 6750.2511
302.3800 -43.0001 -73.8874 680285.8938 -21.1111
-21.1305
[11836]ENERGY: 3680000 3343.5976 5353.6123 5689.7238
353.4566 -14148.2561 -1792.5384 0.0000 0.0000
7888.0055 6687.6013 300.7824 -1200.4043 6749.0846
302.4100 3.4625 -53.3387 680285.8938 -21.8009
-21.7924
[11886]ENERGY: 3690000 3310.2416 5439.4170 5680.9887
346.6430 -14099.5970 -1859.9371 0.0000 0.0000
7869.1785 6686.9347 300.0645 -1182.2438 6750.4350
302.3744 -90.7010 -114.9231 680285.8938 -24.3138
-24.3276
[11925]ENERGY: 3700000 3236.8271 5448.9633 5711.9562
358.8602 -14192.8071 -1745.2870 0.0000 0.0000
7870.8176 6689.3303 300.1270 -1181.4874 6748.6544
302.7278 -68.7459 -30.6802 680285.8938 -26.0595
-26.0363
[11975]ENERGY: 3710000 3261.8653 5385.8457 5753.6372
344.3457 -14136.3822 -1840.8619 0.0000 0.0000
7919.7665 6688.2162 301.9935 -1231.5504 6748.9374
302.9231 -49.2960 -145.3022 680285.8938 -22.3410
-22.3511
[12011]ENERGY: 3720000 3314.3307 5360.9778 5721.9427
325.7947 -14178.7855 -1763.6170 0.0000 0.0000
7907.6863 6688.3297 301.5328 -1219.3566 6748.6331
302.9081 54.1556 -11.1513 680285.8938 -26.0355
-26.0239

[12061]ENERGY: 3730000 3295.5083 5426.8155 5748.9104
353.1154 -14233.7515 -1797.6798 0.0000 0.0000
7893.3750 6686.2933 300.9871 -1207.0817 6748.9200
302.9538 -85.7233 -90.2166 680285.8938 -26.6638
-26.6761
[12097]ENERGY: 3740000 3284.0804 5492.5118 5700.6876
343.6084 -14253.6292 -1817.2614 0.0000 0.0000
7937.4223 6687.4200 302.6667 -1250.0023 6748.6039
303.0156 -97.3558 -127.6005 680285.8938 -23.8553
-23.8599
[12147]ENERGY: 3750000 3277.5089 5388.2800 5686.1356
330.3857 -14144.4194 -1824.4913 0.0000 0.0000
7971.0061 6684.4055 303.9473 -1286.6006 6749.2510
302.9128 -56.0345 -89.5740 680285.8938 -24.7068
-24.7015
[12183]ENERGY: 3760000 3347.4435 5437.0694 5749.8511
324.1451 -14235.8876 -1789.1194 0.0000 0.0000
7855.4254 6688.9274 299.5400 -1166.4980 6749.0303
303.0860 46.7603 -49.2540 680285.8938 -21.6775
-21.6872
[12233]ENERGY: 3770000 3259.9381 5416.9699 5696.4955
327.2657 -14144.2748 -1812.6534 0.0000 0.0000
7943.6624 6687.4034 302.9047 -1256.2590 6749.3649
302.9362 11.1935 -15.3537 680285.8938 -20.6133
-20.5839
[12269]ENERGY: 3780000 3297.3302 5289.4419 5696.9643
342.0499 -14160.4112 -1752.7210 0.0000 0.0000
7973.2982 6685.9524 304.0347 -1287.3459 6749.7304
303.0373 3.2844 47.7777 680285.8938 -22.7547
-22.7443
[12319]ENERGY: 3790000 3296.3927 5418.9175 5711.1885
334.8887 -14186.4643 -1812.0967 0.0000 0.0000
7924.5690 6687.3954 302.1766 -1237.1736 6750.0101
303.0413 -10.6766 -66.9819 680285.8938 -24.6023
-24.6245
[12358]ENERGY: 3800000 3255.7275 5474.9968 5660.7641
313.2304 -14207.7044 -1779.6507 0.0000 0.0000
7965.7856 6683.1493 303.7482 -1282.6363 6750.1942
303.0639 96.1550 0.1058 680285.8938 -22.9030
-22.9090
[12408]ENERGY: 3810000 3250.5265 5466.3689 5737.5051
329.0945 -14238.3954 -1818.7134 0.0000 0.0000
7956.9909 6683.3771 303.4129 -1273.6138 6750.3336
303.1970 -46.8471 -58.1241 680285.8938 -22.3522
-22.3385
[12444]ENERGY: 3820000 3298.9050 5512.1949 5689.4991
332.0813 -14227.7915 -1814.4984 0.0000 0.0000
7894.0220 6684.4124 301.0118 -1209.6096 6750.4298
303.0532 -34.6924 -54.8925 680285.8938 -22.5947
-22.5994
[12494]ENERGY: 3830000 3264.4315 5485.0386 5752.1592
336.9480 -14281.2515 -1828.2297 0.0000 0.0000
7956.6716 6685.7676 303.4007 -1270.9040 6749.7975
303.2143 55.2248 11.3241 680285.8938 -20.2914

-20.2960
[12530]ENERGY: 3840000 3246.2915 5470.4378 5720.4630
342.3300 -14188.7804 -1783.9635 0.0000 0.0000
7880.7030 6687.4815 300.5039 -1193.2215 6750.9766
303.1938 -71.6865 -112.8447 680285.8938 -19.7463
-19.7328
[12580]ENERGY: 3850000 3230.9796 5490.2878 5707.1771
317.7844 -14184.4495 -1812.1109 0.0000 0.0000
7935.4538 6685.1224 302.5917 -1250.3314 6749.4275
303.1415 -52.0967 -72.7979 680285.8938 -18.2027
-18.2200
[12616]ENERGY: 3860000 3285.3790 5475.3624 5686.1338
350.2642 -14143.8277 -1841.5571 0.0000 0.0000
7879.8925 6691.6470 300.4730 -1188.2455 6751.4708
303.2353 -125.7819 -119.0317 680285.8938 -19.2320
-19.2265
[12666]ENERGY: 3870000 3371.3791 5399.6528 5695.6996
327.5356 -14234.2583 -1783.3621 0.0000 0.0000
7911.4615 6688.1082 301.6768 -1223.3533 6751.7829
303.2069 -70.0349 -120.4284 680285.8938 -24.3974
-24.4102
[12702]ENERGY: 3880000 3254.3442 5408.9583 5738.2594
330.7799 -14182.2125 -1739.2748 0.0000 0.0000
7879.6594 6690.5140 300.4641 -1189.1455 6751.2319
303.1272 -9.2836 29.5230 680285.8938 -16.4641
-16.4399
[12752]ENERGY: 3890000 3312.7632 5418.8696 5670.4474
334.9984 -14195.7358 -1820.2870 0.0000 0.0000
7961.8358 6682.8916 303.5976 -1278.9441 6751.1349
302.9519 -279.7253 -236.1476 680285.8938 -22.8667
-22.8790
[12791]ENERGY: 3900000 3358.4686 5347.6859 5714.6621
328.7202 -14244.7231 -1824.1794 0.0000 0.0000
8004.6793 6685.3136 305.2313 -1319.3657 6751.0054
303.2683 -76.1916 -99.1502 680285.8938 -18.4895
-18.4955
[12841]ENERGY: 3910000 3307.3217 5495.4770 5733.8788
316.1752 -14195.3270 -1848.1143 0.0000 0.0000
7877.7111 6687.1224 300.3898 -1190.5887 6750.7150
303.2350 -96.7064 -138.9317 680285.8938 -18.7733
-18.7549
[12877]ENERGY: 3920000 3313.7516 5469.2425 5688.5152
327.2183 -14228.6165 -1786.5846 0.0000 0.0000
7903.1593 6686.6858 301.3602 -1216.4735 6750.1252
303.3465 -81.7757 -52.1890 680285.8938 -24.0111
-24.0102
[12927]ENERGY: 3930000 3242.2278 5371.4015 5702.7719
346.4733 -14096.5446 -1830.0312 0.0000 0.0000
7947.5801 6683.8787 303.0540 -1263.7013 6751.2133
303.0216 3.9838 -64.9509 680285.8938 -24.4021
-24.4240
[12963]ENERGY: 3940000 3342.1670 5381.8098 5721.0317
326.0268 -14170.2136 -1810.3434 0.0000 0.0000
7894.6243 6685.1026 301.0348 -1209.5218 6749.2906

303.3296 94.6629 -48.3097 680285.8938 -22.7121
-22.7194
[13013]ENERGY: 3950000 3294.5829 5473.4561 5748.6175
329.6600 -14182.3920 -1843.3708 0.0000 0.0000
7870.6131 6691.1668 300.1192 -1179.4463 6751.9071
303.2336 -18.8090 -61.5819 680285.8938 -23.8686
-23.8528
[13049]ENERGY: 3960000 3264.3999 5457.6423 5717.3882
328.0451 -14198.1678 -1851.7215 0.0000 0.0000
7970.3473 6687.9334 303.9222 -1282.4139 6750.1390
303.4048 -114.8850 -70.1073 680285.8938 -21.4050
-21.4048
[13099]ENERGY: 3970000 3230.3879 5373.5211 5711.0397
340.8793 -14160.3405 -1779.5117 0.0000 0.0000
7973.5681 6689.5440 304.0450 -1284.0241 6750.9522
303.2991 -67.1824 -104.7018 680285.8938 -21.0921
-21.0974
[13135]ENERGY: 3980000 3286.6826 5484.9406 5720.2372
337.8305 -14318.8374 -1769.6861 0.0000 0.0000
7944.8491 6686.0166 302.9499 -1258.8325 6750.1750
303.1224 -100.5497 -84.5324 680285.8938 -25.3819
-25.3676
[13185]ENERGY: 3990000 3251.1489 5415.6968 5673.1103
345.4362 -14199.5021 -1781.8298 0.0000 0.0000
7984.7376 6688.7979 304.4709 -1295.9397 6751.2257
303.3324 87.3734 -1.7445 680285.8938 -21.3239
-21.3328
[13224]ENERGY: 4000000 3185.3275 5445.0355 5764.3337
334.7140 -14228.8909 -1779.3612 0.0000 0.0000
7964.5985 6685.7570 303.7030 -1278.8415 6750.6612
303.4057 133.5599 85.9653 680285.8938 -19.7591
-19.7632
[13274]ENERGY: 4010000 3294.1061 5460.7904 5670.9410
344.9614 -14268.9003 -1785.1245 0.0000 0.0000
7969.7845 6686.5587 303.9007 -1283.2258 6749.5304
303.4921 -109.7532 -72.9814 680285.8938 -20.7610
-20.7424
[13310]ENERGY: 4020000 3257.2884 5397.6653 5741.4043
328.3356 -14262.3221 -1796.1023 0.0000 0.0000
8018.7453 6685.0145 305.7677 -1333.7307 6750.4372
303.6254 -103.5004 -66.5156 680285.8938 -26.2146
-26.2269
[13360]ENERGY: 4030000 3340.2773 5483.1062 5702.0617
331.4548 -14283.7784 -1767.1491 0.0000 0.0000
7882.5458 6688.5184 300.5742 -1194.0275 6751.6479
303.5481 -88.8200 -54.7263 680285.8938 -20.9586
-20.9596
[13396]ENERGY: 4040000 3240.2182 5440.5336 5762.0039
351.3555 -14203.1577 -1730.2846 0.0000 0.0000
7828.4786 6689.1474 298.5125 -1139.3311 6752.0770
303.4807 34.5016 36.2926 680285.8938 -20.0668
-20.0674
[13446]ENERGY: 4050000 3216.8837 5393.4695 5754.5620
317.9460 -14144.0201 -1865.9124 0.0000 0.0000

8013.0387 6685.9673 305.5501 -1327.0713 6753.3772
303.3128 -44.5747 -55.8984 680285.8938 -20.7190
-20.6988
[13482]ENERGY: 4060000 3309.2427 5532.2967 5703.1546
319.5612 -14208.5745 -1841.4246 0.0000 0.0000
7876.1638 6690.4199 300.3308 -1185.7439 6754.0918
303.4532 3.3095 -20.1159 680285.8938 -23.9125
-23.9246
[13532]ENERGY: 4070000 3339.2706 5314.8065 5705.0950
352.6604 -14198.2329 -1768.9696 0.0000 0.0000
7945.4347 6690.0648 302.9722 -1255.3699 6753.9616
303.5295 59.0112 -21.1026 680285.8938 -17.4037
-17.4187
[13568]ENERGY: 4080000 3314.9513 5349.3006 5722.7036
330.2578 -14235.0397 -1747.9291 0.0000 0.0000
7954.8892 6689.1336 303.3328 -1265.7556 6754.4600
303.5500 89.8698 85.7959 680285.8938 -19.1752
-19.1607
[13618]ENERGY: 4090000 3274.7937 5363.9604 5740.8067
326.0477 -14204.5228 -1749.6568 0.0000 0.0000
7939.3914 6690.8203 302.7418 -1248.5711 6754.2207
303.3709 19.9041 -0.5482 680285.8938 -21.8047
-21.8157
[13657]ENERGY: 4100000 3312.0560 5387.8442 5753.2533
343.8958 -14276.8368 -1641.0825 0.0000 0.0000
7812.4062 6691.5363 297.8996 -1120.8699 6755.0804
303.4894 74.8129 132.3706 680285.8938 -20.1912
-20.1800
[13707]ENERGY: 4110000 3297.6383 5467.2407 5680.1244
322.1334 -14225.2975 -1731.3150 0.0000 0.0000
7879.7990 6690.3233 300.4694 -1189.4757 6754.8401
303.5050 15.4288 19.8875 680285.8938 -21.9118
-21.9256
[13743]ENERGY: 4120000 3287.8036 5432.2974 5715.1316
335.7687 -14190.1968 -1807.6870 0.0000 0.0000
7914.2179 6687.3354 301.7819 -1226.8825 6753.3005
303.4476 -69.1083 21.1161 680285.8938 -20.2835
-20.2794
[13793]ENERGY: 4130000 3315.8964 5354.2431 5745.2237
325.7543 -14183.4634 -1806.4391 0.0000 0.0000
7941.3153 6692.5303 302.8152 -1248.7850 6757.0772
303.4819 -13.4627 -54.4622 680285.8938 -21.1770
-21.1606
[13829]ENERGY: 4140000 3290.4491 5471.2298 5701.5169
335.4178 -14243.4675 -1810.6282 0.0000 0.0000
7947.8329 6692.3508 303.0637 -1255.4821 6755.6212
303.5045 3.8848 -13.2149 680285.8938 -20.1835
-20.1907
[13879]ENERGY: 4150000 3332.9911 5398.3011 5748.7834
332.0508 -14252.9657 -1858.2465 0.0000 0.0000
7992.4087 6693.3229 304.7634 -1299.0858 6756.2654
303.3926 -125.7769 -166.1817 680285.8938 -22.3084
-22.3152
[13915]ENERGY: 4160000 3277.0179 5367.0200 5702.2583

345.0322 -14102.8235 -1804.5459 0.0000 0.0000
7908.4872 6692.4463 301.5634 -1216.0409 6756.5904
303.5255 -174.1861 -83.1445 680285.8938 -21.5030
-21.4952
[13965]ENERGY: 4170000 3273.3455 5422.0422 5735.4587
337.9049 -14289.4730 -1785.6500 0.0000 0.0000
7996.8248 6690.4531 304.9318 -1306.3716 6756.2211
303.4521 39.2413 -18.8039 680285.8938 -17.8057
-17.8093
[14001]ENERGY: 4180000 3250.4766 5335.1654 5772.9067
326.6050 -14177.2689 -1746.8011 0.0000 0.0000
7928.5346 6689.6182 302.3278 -1238.9163 6755.7118
303.5497 -32.0121 37.1413 680285.8938 -21.3379
-21.3331
[14051]ENERGY: 4190000 3291.7452 5380.5041 5754.8233
338.4014 -14227.1744 -1756.0026 0.0000 0.0000
7911.1855 6693.4824 301.6663 -1217.7031 6755.9782
303.5249 -135.3590 -100.4948 680285.8938 -19.9202
-19.9289
[14090]ENERGY: 4200000 3268.9151 5369.3235 5696.9764
333.9563 -14133.3109 -1818.4029 0.0000 0.0000
7975.4883 6692.9457 304.1182 -1282.5426 6757.9361
303.5865 55.3133 -1.4044 680285.8938 -24.7278
-24.7322
[14140]ENERGY: 4210000 3320.7659 5396.1591 5749.6872
334.0161 -14235.0089 -1800.1467 0.0000 0.0000
7926.9592 6692.4319 302.2677 -1234.5273 6757.7320
303.7158 17.3011 -17.7294 680285.8938 -20.8050
-20.8103
[14176]ENERGY: 4220000 3322.1417 5372.4611 5723.6151
325.4702 -14198.9035 -1748.2580 0.0000 0.0000
7903.1647 6699.6914 301.3604 -1203.4733 6758.8817
303.7953 -10.5430 -49.1505 680285.8938 -22.8011
-22.7992
[14226]ENERGY: 4230000 3261.3201 5342.2094 5779.2991
323.6247 -14248.1568 -1750.7885 0.0000 0.0000
7987.7195 6695.2275 304.5846 -1292.4921 6758.7147
303.8065 1.6672 18.8671 680285.8938 -22.8504
-22.8579
[14262]ENERGY: 4240000 3310.7237 5450.6174 5688.7577
349.6355 -14216.2770 -1784.7238 0.0000 0.0000
7897.3027 6696.0360 301.1369 -1201.2667 6759.5081
303.7869 37.0457 9.6248 680285.8938 -21.8455
-21.8221
[14312]ENERGY: 4250000 3253.2191 5452.4176 5749.0748
327.9697 -14267.1175 -1781.1252 0.0000 0.0000
7961.1569 6695.5955 303.5718 -1265.5614 6759.6730
303.6232 41.2977 -38.7817 680285.8938 -23.2973
-23.3049
[14348]ENERGY: 4260000 3286.8735 5292.5637 5743.2052
344.2831 -14198.0327 -1782.3746 0.0000 0.0000
8007.9096 6694.4278 305.3545 -1313.4817 6759.9020
303.6534 2.2562 32.7343 680285.8938 -20.3945
-20.3889

[14398]ENERGY: 4270000 3257.3045 5361.6168 5722.5725
322.4632 -14195.1231 -1752.1784 0.0000 0.0000
7981.2326 6697.8881 304.3373 -1283.3445 6759.7006
303.6326 -167.8303 -84.2461 680285.8938 -19.3595
-19.3610
[14434]ENERGY: 4280000 3210.5656 5483.1877 5688.7060
371.0432 -14151.2262 -1821.9314 0.0000 0.0000
7914.6849 6695.0297 301.7997 -1219.6552 6759.8746
303.5446 39.1165 -25.0831 680285.8938 -20.5042
-20.4984
[14484]ENERGY: 4290000 3287.1732 5337.0663 5710.5485
334.8818 -14241.7137 -1775.9069 0.0000 0.0000
8041.1654 6693.2146 306.6226 -1347.9507 6760.5554
303.4525 10.4291 34.7674 680285.8938 -19.6231
-19.6309
[14523]ENERGY: 4300000 3296.2149 5353.8555 5686.6268
357.2700 -14199.0380 -1736.9075 0.0000 0.0000
7939.0028 6697.0244 302.7270 -1241.9783 6759.9307
303.6106 83.8483 23.7623 680285.8938 -17.4463
-17.4459
[14573]ENERGY: 4310000 3372.9032 5352.2815 5706.0290
361.1821 -14140.4453 -1832.9541 0.0000 0.0000
7879.7336 6698.7300 300.4669 -1181.0036 6760.8239
303.5020 39.0027 26.0019 680285.8938 -19.3751
-19.3880
[14609]ENERGY: 4320000 3317.0914 5444.5341 5722.1009
344.5645 -14244.2816 -1826.0840 0.0000 0.0000
7938.1188 6696.0442 302.6933 -1242.0746 6761.8371
303.6993 -100.9924 -85.6038 680285.8938 -21.1885
-21.1621
[14659]ENERGY: 4330000 3317.2033 5406.6644 5728.8804
344.2515 -14096.2694 -1905.4423 0.0000 0.0000
7903.3742 6698.6622 301.3684 -1204.7121 6762.6144
303.6147 -143.6947 -219.6924 680285.8938 -20.4682
-20.4948
[14695]ENERGY: 4340000 3244.3248 5475.1626 5649.8770
342.1960 -14226.7417 -1799.1192 0.0000 0.0000
8010.0673 6695.7669 305.4368 -1314.3004 6761.1841
303.3511 5.5377 28.9034 680285.8938 -24.7059
-24.6803
[14745]ENERGY: 4350000 3278.0676 5463.1066 5713.7222
325.8957 -14247.8701 -1850.7999 0.0000 0.0000
8013.8210 6695.9432 305.5799 -1317.8779 6760.8645
303.5521 14.8290 -5.9449 680285.8938 -23.0302
-23.0597
[14781]ENERGY: 4360000 3291.4679 5406.5440 5772.6170
310.2809 -14202.1525 -1858.2086 0.0000 0.0000
7978.8357 6699.3844 304.2459 -1279.4513 6759.9758
303.7242 130.2715 15.5788 680285.8938 -20.8550
-20.8556
[14831]ENERGY: 4370000 3225.4647 5349.3998 5720.0618
326.2463 -14134.1252 -1757.1067 0.0000 0.0000
7969.4210 6699.3618 303.8869 -1270.0592 6760.7154
303.4103 111.3694 64.6787 680285.8938 -22.9462

-22.9266
[14867]ENERGY: 4380000 3348.2654 5440.4416 5715.9758
330.5905 -14268.5303 -1771.7661 0.0000 0.0000
7904.7115 6699.6884 301.4194 -1205.0230 6760.2468
303.7118 -15.6711 -63.6853 680285.8938 -20.6653
-20.6759
[14917]ENERGY: 4390000 3246.9568 5446.3869 5747.2398
326.7060 -14237.6108 -1820.0658 0.0000 0.0000
7987.4686 6697.0815 304.5751 -1290.3871 6762.3767
303.6867 -109.5599 -69.5778 680285.8938 -17.1787
-17.1659
[14956]ENERGY: 4400000 3366.1354 5428.1969 5699.9493
330.2275 -14248.6580 -1814.1159 0.0000 0.0000
7936.6261 6698.3613 302.6364 -1238.2648 6764.0146
303.8219 -84.0253 -78.6791 680285.8938 -19.5285
-19.5234
[15006]ENERGY: 4410000 3274.1463 5400.9083 5642.7868
329.9945 -14212.3489 -1802.2429 0.0000 0.0000
8065.4127 6698.6568 307.5472 -1366.7558 6761.9516
303.8717 -86.1128 -103.5693 680285.8938 -19.2829
-19.3074
[15042]ENERGY: 4420000 3314.7254 5369.5064 5676.2863
350.4887 -14198.8908 -1768.3618 0.0000 0.0000
7955.4854 6699.2396 303.3555 -1256.2458 6763.1383
303.7987 -30.9380 -81.6786 680285.8938 -16.6466
-16.6572
[15092]ENERGY: 4430000 3309.7639 5379.1837 5749.7420
338.7237 -14307.0645 -1766.2155 0.0000 0.0000
7994.4293 6698.5624 304.8405 -1295.8668 6761.6440
303.9031 3.9661 120.9926 680285.8938 -16.6320
-16.5815
[15128]ENERGY: 4440000 3268.5244 5488.4096 5708.7980
335.8702 -14198.7453 -1762.5664 0.0000 0.0000
7863.1105 6703.4011 299.8331 -1159.7094 6763.1782
303.7572 91.2287 16.7919 680285.8938 -15.9393
-15.9715
[15178]ENERGY: 4450000 3339.0900 5399.5595 5745.6879
347.0902 -14323.0201 -1757.8558 0.0000 0.0000
7948.0769 6698.6286 303.0730 -1249.4483 6763.2420
303.8039 -71.2934 -86.4815 680285.8938 -11.7860
-11.7799
[15214]ENERGY: 4460000 3317.5963 5390.5148 5739.4409
318.5450 -14234.6839 -1740.2128 0.0000 0.0000
7910.9737 6702.1740 301.6582 -1208.7997 6763.1679
303.8339 56.3009 0.0625 680285.8938 -17.8452
-17.8528
[15264]ENERGY: 4470000 3326.2055 5481.6748 5721.4665
343.0302 -14190.3463 -1846.3438 0.0000 0.0000
7863.5257 6699.2126 299.8489 -1164.3131 6764.2186
303.7874 -171.8865 -108.2690 680285.8938 -17.9844
-17.9783
[15300]ENERGY: 4480000 3397.0561 5435.3151 5673.0692
320.9448 -14326.0336 -1767.8920 0.0000 0.0000
7967.7955 6700.2551 303.8249 -1267.5405 6763.1761

303.9290 -23.6692 -88.2827 680285.8938 -13.9118
-13.9096
[15350]ENERGY: 4490000 3312.1987 5507.2494 5727.6902
327.7311 -14277.1001 -1761.7475 0.0000 0.0000
7861.9249 6697.9465 299.7879 -1163.9783 6763.9494
303.9288 8.2300 23.2107 680285.8938 -15.5273
-15.5258
[15389]ENERGY: 4500000 3248.6011 5463.4068 5686.7784
330.4621 -14200.2101 -1795.7054 0.0000 0.0000
7968.0971 6701.4300 303.8364 -1266.6671 6764.6997
303.8677 108.7837 99.4364 680285.8938 -17.6019
-17.6109
[15439]ENERGY: 4510000 3215.6645 5437.8695 5695.8705
334.1280 -14232.4476 -1801.5518 0.0000 0.0000
8050.0726 6699.6057 306.9623 -1350.4670 6764.7855
303.7089 -41.0379 -108.4530 680285.8938 -16.4991
-16.5177
[15475]ENERGY: 4520000 3277.0249 5449.3908 5718.6918
341.4159 -14172.9215 -1798.4625 0.0000 0.0000
7883.1823 6698.3217 300.5985 -1184.8606 6764.3259
303.7630 83.5946 50.8428 680285.8938 -16.8958
-16.8669
[15525]ENERGY: 4530000 3290.4806 5479.6068 5655.7765
324.5484 -14229.2716 -1817.9644 0.0000 0.0000
7997.8621 6701.0383 304.9714 -1296.8237 6764.2813
303.8084 -55.5863 -177.7452 680285.8938 -17.2535
-17.2556
[15561]ENERGY: 4540000 3290.0319 5379.8764 5706.9605
319.7570 -14262.5230 -1730.5661 0.0000 0.0000
7996.5867 6700.1234 304.9227 -1296.4633 6764.0796
304.0009 -65.7342 -48.5312 680285.8938 -15.4516
-15.4556
[15611]ENERGY: 4550000 3343.2317 5425.3508 5672.1525
336.9252 -14283.8840 -1768.0743 0.0000 0.0000
7974.4475 6700.1496 304.0785 -1274.2980 6765.8089
303.8216 13.0044 -7.3608 680285.8938 -12.9181
-12.9115
[15647]ENERGY: 4560000 3253.3278 5398.7778 5754.5627
355.0460 -14232.6759 -1803.4799 0.0000 0.0000
7974.7716 6700.3301 304.0909 -1274.4415 6766.0818
303.7231 26.5971 -3.2168 680285.8938 -13.7754
-13.7784
[15697]ENERGY: 4570000 3340.1890 5400.3463 5745.0037
356.6984 -14225.2623 -1820.1818 0.0000 0.0000
7907.5703 6704.3636 301.5284 -1203.2067 6765.9117
303.8407 23.5453 -15.7850 680285.8938 -15.6229
-15.6281
[15733]ENERGY: 4580000 3244.3971 5493.3764 5711.2323
325.1914 -14173.2513 -1863.0863 0.0000 0.0000
7963.5356 6701.3953 303.6625 -1262.1404 6765.4864
303.9509 84.6100 18.7553 680285.8938 -13.5526
-13.5487
[15783]ENERGY: 4590000 3299.2213 5393.8451 5706.1283
318.9668 -14227.9142 -1761.5765 0.0000 0.0000

7973.5478 6702.2186 304.0442 -1271.3292 6764.6207
303.9333 -84.2990 -50.7883 680285.8938 -11.7201
-11.7165
[15822]ENERGY: 4600000 3277.7834 5452.6270 5689.5270
345.9983 -14189.5019 -1820.4097 0.0000 0.0000
7944.8734 6700.8975 302.9508 -1243.9759 6765.4580
303.8994 -43.8113 -69.0379 680285.8938 -17.7434
-17.7630
[15872]ENERGY: 4610000 3286.8169 5330.8959 5762.0548
343.9552 -14196.5202 -1819.8801 0.0000 0.0000
7992.8734 6700.1959 304.7812 -1292.6775 6765.8547
303.8428 161.5504 85.9033 680285.8938 -17.4464
-17.4395
[15908]ENERGY: 4620000 3260.6685 5425.5742 5699.8264
348.7563 -14216.4116 -1835.7329 0.0000 0.0000
8014.3652 6697.0461 305.6007 -1317.3191 6765.9045
303.8659 -124.1855 -93.0397 680285.8938 -17.5779
-17.5749
[15958]ENERGY: 4630000 3210.2302 5417.0166 5768.9892
327.8703 -14234.7332 -1821.2101 0.0000 0.0000
8030.9994 6699.1625 306.2350 -1331.8369 6765.8575
303.8941 -60.3370 46.2671 680285.8938 -12.9735
-12.9652
[15994]ENERGY: 4640000 3208.4287 5429.5389 5732.1022
329.4041 -14190.6506 -1850.0629 0.0000 0.0000
8039.8178 6698.5782 306.5712 -1341.2396 6765.6005
303.7553 155.8783 56.9839 680285.8938 -13.8307
-13.8348
[16044]ENERGY: 4650000 3253.1108 5378.2851 5730.0721
304.5874 -14226.0543 -1735.0791 0.0000 0.0000
7997.1711 6702.0931 304.9450 -1295.0780 6766.4607
303.8463 137.1269 41.1825 680285.8938 -12.0916
-12.1051
[16080]ENERGY: 4660000 3313.1857 5454.5832 5676.3740
326.3596 -14234.1510 -1821.6042 0.0000 0.0000
7986.8686 6701.6158 304.5522 -1285.2528 6766.2307
303.8900 31.7130 -20.9532 680285.8938 -9.0114
-8.9789
[16130]ENERGY: 4670000 3223.2270 5428.2611 5722.8859
345.9115 -14208.8731 -1800.6570 0.0000 0.0000
7991.8087 6702.5641 304.7406 -1289.2446 6765.8078
303.8979 45.8960 37.4275 680285.8938 -15.4108
-15.4363
[16166]ENERGY: 4680000 3290.1572 5461.5098 5742.5450
327.3746 -14203.1172 -1795.9152 0.0000 0.0000
7882.9716 6705.5258 300.5904 -1177.4459 6766.7908
304.1098 -88.0702 -61.8995 680285.8938 -14.1200
-14.1056
[16216]ENERGY: 4690000 3300.2084 5444.2507 5732.3734
337.8976 -14207.8607 -1827.9563 0.0000 0.0000
7922.3836 6701.2967 302.0933 -1221.0869 6767.0324
303.9065 -3.5580 -64.0760 680285.8938 -9.6810
-9.6892
[16255]ENERGY: 4700000 3279.7070 5418.7106 5684.4154

```
337.0858          -14250.2014     -1816.4985         0.0000         0.0000
8042.3015            6695.5204      306.6659     -1346.7811      6765.8131
303.9101             -73.1553       -94.0238    680285.8938       -11.7731
-11.7626
[16305]ENERGY: 4710000      3240.8614      5432.2314      5682.5636
342.2015          -14102.1974     -1815.3606         0.0000         0.0000
7925.0128            6705.3127      302.1935     -1219.7002      6767.6907
303.8742             -69.4258       -59.9508    680285.8938       -14.5796
-14.6015
[16341]ENERGY: 4720000      3360.1679      5480.6205      5725.5666
360.1734          -14301.4162     -1861.4564         0.0000         0.0000
7942.7346            6706.3903      302.8693     -1236.3443      6768.3106
303.9881            -197.0799      -106.7953    680285.8938        -7.7800
-7.7643
[16391]ENERGY: 4730000      3232.0433      5472.9807      5763.4306
328.8589          -14228.4500     -1835.2030         0.0000         0.0000
7969.3303            6702.9909      303.8834     -1266.3394      6768.0704
304.0286             125.2571        30.4761    680285.8938       -10.2619
-10.2676
[16427]ENERGY: 4740000      3296.6594      5476.7065      5702.4951
315.9747          -14261.0610     -1886.3334         0.0000         0.0000
8055.6646            6700.1060      307.1755     -1355.5586      6768.3295
303.8866            -113.7066      -200.0972    680285.8938       -12.5068
-12.5115
[16477]ENERGY: 4750000      3285.6822      5373.5596      5710.7790
334.1271          -14197.5010     -1747.1523         0.0000         0.0000
7942.4889            6701.9835      302.8599     -1240.5054      6767.1897
303.8224              -9.3469        19.3509    680285.8938       -11.3423
-11.3353
[16513]ENERGY: 4760000      3269.3958      5374.9987      5777.5239
344.8446          -14271.5771     -1806.7756         0.0000         0.0000
8013.2049            6701.6151      305.5564     -1311.5898      6767.2205
303.8211             202.1595        96.9262    680285.8938        -7.5967
-7.6015
[16563]ENERGY: 4770000      3373.3522      5458.6386      5773.3219
339.4801          -14248.2774     -1895.7678         0.0000         0.0000
7903.2415            6703.9891      301.3633     -1199.2523      6768.2653
303.8735             -14.8668        28.2176    680285.8938       -12.5036
-12.4860
[16599]ENERGY: 4780000      3208.2657      5439.6528      5669.9834
345.8501          -14189.0311     -1771.0736         0.0000         0.0000
8000.5861            6704.2334      305.0752     -1296.3527      6768.0263
303.8350             -22.1254       -50.6351    680285.8938        -9.8359
-9.8624
[16649]ENERGY: 4790000      3227.3008      5361.6920      5780.2216
345.4564          -14156.9945     -1834.2981         0.0000         0.0000
7978.0858            6701.4641      304.2173     -1276.6218      6767.2742
304.0265             124.0566        77.4988    680285.8938       -10.0008
-9.9990
[16688]ENERGY: 4800000      3304.5676      5452.0318      5747.3646
334.2560          -14303.4671     -1753.6296         0.0000         0.0000
7919.2958            6700.4190      301.9755     -1218.8768      6766.6899
303.9588            -132.0984      -112.6174    680285.8938       -10.4645
-10.4473
```

[16738]ENERGY: 4810000 3180.5607 5454.0862 5729.4084
342.1319 -14267.9871 -1724.0743 0.0000 0.0000
7988.2608 6702.3866 304.6053 -1285.8742 6767.3011
304.0234 -75.6058 -57.1107 680285.8938 -12.8947
-12.9047
[16774]ENERGY: 4820000 3248.3232 5382.2425 5750.1638
349.3663 -14204.4160 -1799.4001 0.0000 0.0000
7980.2424 6706.5222 304.2995 -1273.7202 6767.7745
303.9611 43.2705 63.1585 680285.8938 -17.5736
-17.5618
[16824]ENERGY: 4830000 3293.0445 5463.5854 5687.6986
351.1928 -14220.6463 -1892.0204 0.0000 0.0000
8018.4525 6701.3072 305.7565 -1317.1453 6768.5003
304.0101 -207.3410 -170.2634 680285.8938 -18.0873
-18.0973
[16860]ENERGY: 4840000 3287.1233 5408.2015 5755.7917
355.7623 -14238.1910 -1799.5403 0.0000 0.0000
7933.6016 6702.7491 302.5210 -1230.8525 6768.2276
303.9776 106.1513 156.6727 680285.8938 -14.1015
-14.0996
[16910]ENERGY: 4850000 3272.6819 5440.9350 5742.5597
337.4877 -14292.4712 -1825.4522 0.0000 0.0000
8026.0844 6701.8254 306.0475 -1324.2591 6767.8291
304.1011 -47.7488 -63.1906 680285.8938 -15.5002
-15.4964
[16946]ENERGY: 4860000 3229.1532 5398.2288 5755.1844
334.5909 -14230.9694 -1815.8141 0.0000 0.0000
8030.8995 6701.2733 306.2311 -1329.6263 6767.8745
304.3139 119.7522 71.1038 680285.8938 -13.1916
-13.1837
[16996]ENERGY: 4870000 3300.2865 5330.8144 5690.1919
330.4548 -14106.7051 -1851.1545 0.0000 0.0000
8006.6024 6700.4904 305.3047 -1306.1120 6767.0672
304.3694 -93.3327 -89.2700 680285.8938 -13.3723
-13.3831
[17032]ENERGY: 4880000 3160.0121 5448.2817 5703.4762
360.4703 -14226.2436 -1754.4576 0.0000 0.0000
8011.0264 6702.5655 305.4734 -1308.4609 6766.9129
304.1216 108.9469 51.9312 680285.8938 -14.2995
-14.3026
[17082]ENERGY: 4890000 3191.1605 5291.6702 5764.6225
342.7787 -14189.5979 -1762.3907 0.0000 0.0000
8065.4221 6703.6652 307.5476 -1361.7569 6768.6786
304.2114 200.4672 77.5935 680285.8938 -12.9775
-12.9863
[17121]ENERGY: 4900000 3268.9763 5320.0724 5734.8812
317.7386 -14109.5571 -1829.6025 0.0000 0.0000
8002.8053 6705.3142 305.1599 -1297.4911 6768.6873
304.1832 130.0225 14.1797 680285.8938 -14.6667
-14.6657
[17171]ENERGY: 4910000 3295.3800 5456.4326 5708.9999
369.6413 -14281.8388 -1779.3882 0.0000 0.0000
7929.7241 6698.9509 302.3732 -1230.7732 6767.2117
304.1307 46.8093 -27.5963 680285.8938 -14.0150

-14.0048
[17207]ENERGY: 4920000 3229.9223 5426.8905 5685.3260
357.4397 -14182.0315 -1782.5239 0.0000 0.0000
7965.8490 6700.8722 303.7507 -1264.9769 6766.8442
304.0992 41.4004 -23.8775 680285.8938 -10.7716
-10.7698
[17257]ENERGY: 4930000 3311.3156 5462.6367 5694.8376
347.6633 -14252.9274 -1790.5872 0.0000 0.0000
7932.5375 6705.4761 302.4804 -1227.0613 6768.8605
304.2659 -58.0986 -30.5537 680285.8938 -16.3168
-16.3057
[17293]ENERGY: 4940000 3266.8427 5470.9072 5712.2222
324.5167 -14188.6359 -1808.4729 0.0000 0.0000
7929.8790 6707.2589 302.3791 -1222.6201 6770.9284
304.2608 62.2779 -34.1934 680285.8938 -18.1826
-18.2138
[17343]ENERGY: 4950000 3329.1499 5453.8622 5706.0433
331.3030 -14187.6533 -1846.3034 0.0000 0.0000
7918.4103 6704.8120 301.9418 -1213.5982 6770.2364
304.2680 -128.4598 -74.1066 680285.8938 -15.6161
-15.6007
[17379]ENERGY: 4960000 3239.7354 5487.5979 5722.6316
342.3752 -14221.1234 -1822.7432 0.0000 0.0000
7954.3826 6702.8560 303.3134 -1251.5266 6769.3243
303.9430 -62.2934 -80.8827 680285.8938 -12.8178
-12.8163
[17429]ENERGY: 4970000 3212.5472 5420.5039 5741.9705
327.8161 -14153.2382 -1845.5122 0.0000 0.0000
8003.3881 6707.4755 305.1821 -1295.9126 6769.5410
303.8933 -69.4660 -114.8278 680285.8938 -14.1818
-14.1865
[17465]ENERGY: 4980000 3268.6963 5416.1749 5698.5471
343.9133 -14205.9843 -1844.8938 0.0000 0.0000
8027.4910 6703.9444 306.1012 -1323.5466 6769.8121
304.1324 48.5273 8.0129 680285.8938 -12.6882
-12.6861
[17515]ENERGY: 4990000 3370.8057 5419.6198 5721.9552
351.6465 -14315.2898 -1766.1244 0.0000 0.0000
7921.7910 6704.4039 302.0707 -1217.3871 6768.2879
304.0891 -82.8188 -69.0566 680285.8938 -13.4485
-13.4343
[17554]ENERGY: 5000000 3316.7967 5453.9257 5783.7490
334.9787 -14355.7714 -1807.9806 0.0000 0.0000
7973.9744 6699.6725 304.0605 -1274.3019 6769.0730
304.2121 -83.4338 -64.6575 680285.8938 -12.8661
-12.8864
[17604]ENERGY: 5010000 3289.0208 5405.6976 5720.7844
327.5081 -14186.7065 -1778.4740 0.0000 0.0000
7927.1905 6705.0209 302.2766 -1222.1696 6769.9634
304.0905 -29.2042 -34.7870 680285.8938 -15.1342
-15.1243
[17640]ENERGY: 5020000 3317.5380 5493.2195 5682.6076
317.9586 -14252.4379 -1800.9925 0.0000 0.0000
7948.9353 6706.8285 303.1057 -1242.1068 6770.3944

303.9661 -39.3797 -51.8309 680285.8938 -12.0393
-12.0479
[17690]ENERGY: 5030000 3235.5844 5461.6463 5735.6715
326.1001 -14220.1467 -1822.2656 0.0000 0.0000
7988.7469 6705.3369 304.6238 -1283.4100 6770.2772
304.1290 41.7103 -26.4263 680285.8938 -8.3874
-8.3808
[17726]ENERGY: 5040000 3296.5775 5538.4184 5679.3080
325.0053 -14269.5669 -1782.3864 0.0000 0.0000
7919.5435 6706.8995 301.9850 -1212.6440 6771.6788
304.1066 157.2876 143.4673 680285.8938 -13.2959
-13.2995
[17776]ENERGY: 5050000 3263.5418 5336.2704 5769.6937
318.9173 -14209.0600 -1726.5575 0.0000 0.0000
7954.3530 6707.1586 303.3123 -1247.1943 6771.7837
304.2208 -4.0252 78.4931 680285.8938 -8.6842
-8.6769
[17812]ENERGY: 5060000 3239.4523 5444.9134 5738.3220
353.5938 -14204.3351 -1843.0769 0.0000 0.0000
7977.9065 6706.7760 304.2104 -1271.1305 6772.9325
304.1469 145.4297 24.7561 680285.8938 -13.7126
-13.7221
[17862]ENERGY: 5070000 3254.9183 5451.5966 5712.5762
353.9569 -14129.4947 -1885.0290 0.0000 0.0000
7947.4227 6705.9469 303.0480 -1241.4758 6772.6581
304.1984 40.1682 -4.8916 680285.8938 -11.0702
-11.0591
[17898]ENERGY: 5080000 3301.8809 5362.3173 5759.1187
337.8153 -14195.8779 -1848.2583 0.0000 0.0000
7989.1449 6706.1409 304.6390 -1283.0041 6771.3198
304.1223 -90.2265 -77.6318 680285.8938 -16.3464
-16.3613
[17948]ENERGY: 5090000 3312.7234 5321.5228 5677.5391
345.6278 -14146.1750 -1789.2150 0.0000 0.0000
7988.1206 6710.1438 304.5999 -1277.9769 6772.4951
304.2036 124.9699 100.0942 680285.8938 -11.3405
-11.3409
[17987]ENERGY: 5100000 3349.1198 5386.5116 5722.5732
338.7536 -14173.0470 -1857.8789 0.0000 0.0000
7942.3377 6708.3699 302.8541 -1233.9678 6772.9577
304.1702 -116.9010 -131.0356 680285.8938 -14.3722
-14.3651
[18037]ENERGY: 5110000 3281.0597 5457.6817 5678.1419
350.1578 -14191.4078 -1832.9357 0.0000 0.0000
7968.0663 6710.7639 303.8352 -1257.3024 6773.9404
304.4373 106.3408 -1.8102 680285.8938 -11.1096
-11.0926
[18073]ENERGY: 5120000 3243.2792 5389.2803 5776.8861
355.1225 -14203.8649 -1814.3828 0.0000 0.0000
7960.6456 6706.9661 303.5523 -1253.6795 6771.9178
304.2048 -107.7914 -71.3285 680285.8938 -15.0968
-15.1116
[18123]ENERGY: 5130000 3285.1919 5431.7293 5771.0665
324.1178 -14221.8836 -1855.7545 0.0000 0.0000

7974.8028 6709.2702 304.0921 -1265.5326 6774.6000
304.2152 73.1001 -15.6233 680285.8938 -15.9198
-15.9193
[18159]ENERGY: 5140000 3261.6572 5528.6460 5714.1187
335.5962 -14294.7059 -1830.3172 0.0000 0.0000
7990.9673 6705.9623 304.7085 -1285.0049 6774.1925
304.4181 -25.9916 0.5487 680285.8938 -14.4622
-14.4527
[18209]ENERGY: 5150000 3300.6489 5424.0425 5716.2836
322.6629 -14200.5456 -1823.4686 0.0000 0.0000
7967.3795 6707.0033 303.8090 -1260.3763 6773.2118
304.2747 51.6693 8.9056 680285.8938 -15.1274
-15.1245
[18245]ENERGY: 5160000 3263.3844 5396.8983 5698.8393
327.4421 -14255.6999 -1757.0210 0.0000 0.0000
8033.5103 6707.3535 306.3307 -1326.1568 6774.5717
304.3716 80.0536 16.0549 680285.8938 -16.1056
-16.1130
[18295]ENERGY: 5170000 3279.8766 5492.2126 5688.8618
327.5259 -14200.5868 -1815.8973 0.0000 0.0000
7940.0477 6712.0406 302.7668 -1228.0072 6774.5132
304.3869 27.1714 -23.2047 680285.8938 -16.8819
-16.8777
[18331]ENERGY: 5180000 3296.1748 5434.4469 5716.0798
327.5600 -14240.5515 -1767.1491 0.0000 0.0000
7944.8507 6711.4115 302.9500 -1233.4392 6773.7929
304.1412 121.1180 81.1874 680285.8938 -14.6414
-14.6449
[18381]ENERGY: 5190000 3258.8909 5483.9712 5766.1675
369.4049 -14216.6377 -1848.4073 0.0000 0.0000
7898.7536 6712.1432 301.1922 -1186.6104 6774.9604
304.4182 2.2160 -36.5464 680285.8938 -15.0915
-15.0955
[18420]ENERGY: 5200000 3264.0963 5410.8766 5712.7209
360.2979 -14152.9247 -1892.3356 0.0000 0.0000
8008.5672 6711.2987 305.3796 -1297.2685 6775.0061
304.4361 -132.0025 -167.6371 680285.8938 -15.1637
-15.1816
[18470]ENERGY: 5210000 3371.5415 5403.7006 5692.6184
334.1310 -14171.8264 -1839.6744 0.0000 0.0000
7922.4830 6712.9735 302.0971 -1209.5095 6775.3043
304.3184 -189.7687 -157.8501 680285.8938 -14.2645
-14.2464
[18506]ENERGY: 5220000 3285.2493 5381.2270 5735.8567
325.3352 -14101.6200 -1884.0845 0.0000 0.0000
7970.5022 6712.4658 303.9281 -1258.0364 6775.3555
304.3791 -90.7124 -29.0722 680285.8938 -14.5140
-14.5161
[18556]ENERGY: 5230000 3273.7027 5487.4577 5713.0429
340.1038 -14328.8548 -1716.4202 0.0000 0.0000
7943.1777 6712.2098 302.8862 -1230.9679 6774.3527
304.4189 35.3880 87.6950 680285.8938 -17.5613
-17.5635
[18592]ENERGY: 5240000 3319.2232 5458.6098 5751.0176

```
326.6163          -14206.4040     -1883.7766         0.0000         0.0000
7949.5693            6714.8557      303.1299     -1234.7137      6776.8429
304.5317              23.7974       -30.4296    680285.8938       -15.3276
-15.3256
[18642]ENERGY: 5250000      3314.8777      5401.9716      5747.0367
333.0428          -14271.7319     -1781.3237         0.0000         0.0000
7966.2399            6710.1131      303.7656     -1256.1269      6775.7353
304.4288             -47.2795       -30.5255    680285.8938       -20.9217
-20.9187
[18678]ENERGY: 5260000      3200.6349      5467.3474      5744.9881
317.7197          -14242.3246     -1810.5107         0.0000         0.0000
8028.1420            6705.9967      306.1260     -1322.1453      6775.3816
304.5078             -31.6122       -42.5002    680285.8938       -20.5924
-20.6004
[18728]ENERGY: 5270000      3237.9677      5437.2927      5767.5503
342.6503          -14266.1117     -1794.5125         0.0000         0.0000
7985.6547            6710.4916      304.5059     -1275.1631      6776.5562
304.5564             128.4559        72.7079    680285.8938       -16.9833
-16.9851
[18764]ENERGY: 5280000      3291.0420      5411.9370      5679.2639
320.1729          -14143.4466     -1822.7909         0.0000         0.0000
7978.1334            6714.3117      304.2191     -1263.8217      6775.9363
304.5913              11.4539       -13.7056    680285.8938       -16.2381
-16.2280
[18814]ENERGY: 5290000      3279.9311      5508.4498      5732.9044
334.3252          -14197.1976     -1834.4440         0.0000         0.0000
7891.5847            6715.5536      300.9188     -1176.0311      6776.9619
304.4986               2.6817        -9.7268    680285.8938       -16.5588
-16.5495
[18853]ENERGY: 5300000      3251.6544      5442.5711      5704.1070
339.5949          -14269.4008     -1754.7059         0.0000         0.0000
7995.3958            6709.2166      304.8773     -1286.1792      6776.4954
304.5634              18.2213        -4.1991    680285.8938       -15.8492
-15.8611
[18903]ENERGY: 5310000      3251.9385      5421.7772      5756.2110
311.5193          -14218.2385     -1801.3422         0.0000         0.0000
7991.0876            6712.9529      304.7131     -1278.1347      6777.0043
304.4837              87.0808        55.0746    680285.8938       -14.5644
-14.5636
[18939]ENERGY: 5320000      3332.7415      5363.9903      5741.3897
337.7688          -14257.9429     -1791.9940         0.0000         0.0000
7986.7392            6712.6927      304.5472     -1274.0465      6776.3264
304.4119             -99.8989         9.0502    680285.8938       -15.0865
-15.0728
[18989]ENERGY: 5330000      3281.3387      5463.3251      5726.7456
351.2263          -14280.7937     -1856.3901         0.0000         0.0000
8028.7060            6714.1578      306.1475     -1314.5481      6776.7777
304.4618             -28.1458       -46.0384    680285.8938       -16.1112
-16.1370
[19025]ENERGY: 5340000      3310.7968      5386.8651      5725.2162
324.4885          -14128.5590     -1908.4513         0.0000         0.0000
7999.2428            6709.5991      305.0240     -1289.6437      6777.1648
304.6241            -245.0045      -249.9489    680285.8938       -15.4832
-15.4801
```

[19075]ENERGY: 5350000 3223.6116 5457.0463 5690.1504
333.8830 -14228.6032 -1699.4958 0.0000 0.0000
7940.6860 6717.2783 302.7912 -1223.4077 6777.5180
304.3122 117.5763 35.2505 680285.8938 -16.7347
-16.7361
[19111]ENERGY: 5360000 3265.2646 5493.4264 5735.7948
351.7074 -14260.2044 -1841.7502 0.0000 0.0000
7969.3270 6713.5657 303.8833 -1255.7613 6780.0723
304.2828 11.3976 -41.8205 680285.8938 -15.7677
-15.7551
[19161]ENERGY: 5370000 3376.0802 5376.3403 5687.7548
321.5037 -14228.8779 -1850.4967 0.0000 0.0000
8031.0748 6713.3791 306.2378 -1317.6956 6780.1202
304.3408 -90.1237 -103.8343 680285.8938 -8.9793
-8.9629
[19197]ENERGY: 5380000 3266.8138 5442.7340 5722.3481
327.2356 -14243.5237 -1802.4163 0.0000 0.0000
8000.9013 6714.0928 305.0873 -1286.8085 6779.2231
304.3572 69.5289 -6.1089 680285.8938 -18.7337
-18.7574
[19247]ENERGY: 5390000 3238.1981 5434.4047 5751.1104
340.7741 -14143.1023 -1920.7862 0.0000 0.0000
8014.4424 6715.0412 305.6036 -1299.4012 6780.3280
304.5662 -19.2294 -47.6470 680285.8938 -11.7864
-11.7830
[19286]ENERGY: 5400000 3281.0201 5521.0522 5720.7201
335.6585 -14308.1732 -1781.8251 0.0000 0.0000
7948.8195 6717.2721 303.1013 -1231.5474 6780.2339
304.3995 173.0326 66.8971 680285.8938 -13.5391
-13.5304
[19336]ENERGY: 5410000 3321.2001 5550.8413 5744.3258
351.3104 -14265.0497 -1831.0958 0.0000 0.0000
7846.5335 6718.0657 299.2010 -1128.4678 6780.0666
304.4656 -84.1798 -81.1386 680285.8938 -12.8032
-12.7945
[19372]ENERGY: 5420000 3269.6395 5386.3796 5702.8303
328.2839 -14164.7900 -1785.1305 0.0000 0.0000
7980.7037 6717.9165 304.3171 -1262.7871 6780.0159
304.4666 152.0175 124.4078 680285.8938 -11.7606
-11.7787
[19422]ENERGY: 5430000 3297.1791 5472.3600 5743.8917
339.0650 -14197.8864 -1828.4723 0.0000 0.0000
7891.9240 6718.0611 300.9318 -1173.8629 6781.5144
304.3705 -43.2117 -68.6920 680285.8938 -12.1746
-12.1814
[19458]ENERGY: 5440000 3310.2972 5401.9696 5677.8548
333.2882 -14217.4607 -1821.8140 0.0000 0.0000
8033.4201 6717.5553 306.3273 -1315.8648 6781.4148
304.4397 -19.7089 -48.5912 680285.8938 -11.0213
-11.0053
[19508]ENERGY: 5450000 3237.6458 5437.8967 5728.2002
362.3297 -14201.9823 -1838.8947 0.0000 0.0000
7993.1844 6718.3796 304.7930 -1274.8047 6781.3793
304.5842 48.0475 -30.3364 680285.8938 -8.4363

-8.4388
[19544]ENERGY: 5460000 3267.2645 5494.8988 5681.7376
319.0099 -14253.7350 -1796.6321 0.0000 0.0000
8005.4828 6718.0265 305.2620 -1287.4563 6780.8311
304.6238 96.0920 83.9173 680285.8938 -12.7154
-12.7295
[19594]ENERGY: 5470000 3343.8732 5439.9929 5733.1219
328.2310 -14284.0349 -1807.3863 0.0000 0.0000
7962.5545 6716.3523 303.6250 -1246.2023 6781.9849
304.6267 127.0316 137.4205 680285.8938 -9.8801
-9.8622
[19630]ENERGY: 5480000 3255.8179 5390.3981 5765.0210
333.4783 -14228.0794 -1775.4180 0.0000 0.0000
7975.8857 6717.1035 304.1334 -1258.7821 6782.1232
304.6394 4.5175 -19.6215 680285.8938 -13.5003
-13.5128
[19680]ENERGY: 5490000 3248.3751 5522.4787 5689.5534
336.8648 -14318.6477 -1718.0849 0.0000 0.0000
7957.4663 6718.0057 303.4310 -1239.4607 6782.3891
304.7719 41.1621 81.3977 680285.8938 -13.2612
-13.2637
[19719]ENERGY: 5500000 3330.0661 5430.3889 5752.3560
340.0750 -14315.5060 -1774.7292 0.0000 0.0000
7955.2094 6717.8603 303.3450 -1237.3491 6782.9859
304.7755 39.8499 -5.2739 680285.8938 -16.2160
-16.2075
[19769]ENERGY: 5510000 3267.4575 5472.7607 5741.5410
339.9000 -14260.9887 -1824.0221 0.0000 0.0000
7984.4723 6721.1207 304.4608 -1263.3516 6783.5168
304.8050 -26.2845 -91.3864 680285.8938 -14.2076
-14.2129
[19805]ENERGY: 5520000 3315.5470 5479.6087 5667.3796
319.0729 -14177.5200 -1826.4915 0.0000 0.0000
7944.3944 6721.9911 302.9326 -1222.4032 6783.5286
304.7714 -51.6259 -49.6027 680285.8938 -15.6531
-15.6475
[19855]ENERGY: 5530000 3375.3325 5351.7888 5696.1014
335.3263 -14217.7962 -1787.8454 0.0000 0.0000
7968.0079 6720.9152 303.8330 -1247.0927 6783.3428
304.6536 130.2882 76.1452 680285.8938 -11.7832
-11.7845
[19891]ENERGY: 5540000 3359.3778 5498.4709 5739.5610
340.7697 -14304.9073 -1813.1609 0.0000 0.0000
7900.0010 6720.1122 301.2398 -1179.8888 6783.1656
304.5465 -130.7609 -10.5448 680285.8938 -15.2193
-15.2233
[19941]ENERGY: 5550000 3245.5954 5360.4950 5768.7382
349.0015 -14259.9639 -1801.1519 0.0000 0.0000
8056.5305 6719.2447 307.2085 -1337.2858 6783.6971
304.7283 70.4914 19.1828 680285.8938 -17.9640
-17.9550
[19977]ENERGY: 5560000 3278.5224 5536.8987 5780.6100
352.0793 -14327.6997 -1859.1146 0.0000 0.0000
7955.0155 6716.3117 303.3376 -1238.7039 6784.0083

304.7498 -134.6850 -143.1351 680285.8938 -18.3087
-18.3086
[20027]ENERGY: 5570000 3238.8496 5471.2325 5757.8711
326.8149 -14318.2985 -1769.4314 0.0000 0.0000
8012.0675 6719.1057 305.5131 -1292.9618 6784.5727
304.8776 -50.7983 -20.2228 680285.8938 -12.3167
-12.3185
[20063]ENERGY: 5580000 3313.8542 5373.0942 5700.3450
342.8407 -14300.2526 -1782.6506 0.0000 0.0000
8069.1897 6716.4206 307.6912 -1352.7691 6783.7114
304.7615 55.1760 -4.2022 680285.8938 -15.6490
-15.6596
[20113]ENERGY: 5590000 3329.8235 5409.3746 5745.2797
350.6149 -14266.4394 -1826.5658 0.0000 0.0000
7979.5193 6721.6067 304.2719 -1257.9126 6784.7437
304.8210 104.8022 21.9776 680285.8938 -14.4223
-14.4322
[20152]ENERGY: 5600000 3318.6237 5400.3120 5736.2695
330.2245 -14287.8115 -1790.9146 0.0000 0.0000
8012.7764 6719.4800 305.5401 -1293.2964 6784.2650
304.9373 -31.1006 -82.5984 680285.8938 -10.1901
-10.1766
[20202]ENERGY: 5610000 3314.0792 5487.7659 5738.4651
340.5804 -14294.8454 -1832.5140 0.0000 0.0000
7966.5383 6720.0696 303.7770 -1246.4687 6784.5910
305.0154 -138.2785 -160.2527 680285.8938 -9.9579
-9.9456
[20238]ENERGY: 5620000 3288.4560 5548.6419 5681.6601
332.7200 -14309.2199 -1812.8660 0.0000 0.0000
7991.3049 6720.6969 304.7213 -1270.6080 6785.6737
305.0411 116.9259 -7.1634 680285.8938 -9.5609
-9.5807
[20288]ENERGY: 5630000 3341.1260 5386.6445 5751.5944
308.1212 -14166.2037 -1827.4962 0.0000 0.0000
7929.8736 6723.6597 302.3789 -1206.2139 6785.5705
304.9770 -194.5882 -65.2418 680285.8938 -16.2554
-16.2235
[20324]ENERGY: 5640000 3310.3651 5525.1083 5703.1527
352.9092 -14300.7413 -1845.9351 0.0000 0.0000
7971.3506 6716.2095 303.9605 -1255.1411 6785.9513
304.9510 -36.6371 9.7996 680285.8938 -12.6001
-12.5978
[20374]ENERGY: 5650000 3326.7397 5476.9120 5734.3887
329.1487 -14291.2485 -1816.2824 0.0000 0.0000
7963.3840 6723.0421 303.6567 -1240.3419 6785.8825
305.1004 -53.8632 -59.7785 680285.8938 -12.4074
-12.4410
[20410]ENERGY: 5660000 3293.2850 5455.7541 5761.0394
336.5422 -14209.9992 -1820.0235 0.0000 0.0000
7905.2386 6721.8367 301.4395 -1183.4020 6786.1306
304.8845 17.5743 17.0684 680285.8938 -13.4492
-13.4383
[20460]ENERGY: 5670000 3332.7008 5489.6672 5737.4765
329.4794 -14211.8929 -1838.3248 0.0000 0.0000

7885.2035 6724.3097 300.6755 -1160.8937 6786.7568
304.6552 -28.7440 -34.2149 680285.8938 -18.0412
-18.0265
[20496]ENERGY: 5680000 3262.7427 5464.7662 5702.5645
331.6652 -14250.8126 -1849.6672 0.0000 0.0000
8055.5443 6716.8030 307.1709 -1338.7412 6786.3435
304.6683 -130.3824 -108.7906 680285.8938 -15.5254
-15.5416
[20546]ENERGY: 5690000 3360.6565 5410.1167 5733.5659
361.7513 -14352.8155 -1775.0710 0.0000 0.0000
7986.9801 6725.1839 304.5564 -1261.7962 6785.8148
304.5840 31.4936 -70.5995 680285.8938 -14.5874
-14.5973
[20585]ENERGY: 5700000 3309.4968 5390.2868 5711.8071
353.5286 -14201.2571 -1786.7958 0.0000 0.0000
7945.5306 6722.5970 302.9759 -1222.9336 6786.6111
304.6722 -128.2405 -32.2491 680285.8938 -11.3100
-11.2843
[20635]ENERGY: 5710000 3300.4284 5476.1944 5715.8791
337.8863 -14294.3210 -1829.0887 0.0000 0.0000
8015.1504 6722.1288 305.6306 -1293.0216 6785.7927
304.7748 -145.0238 -122.0475 680285.8938 -16.9940
-17.0059
[20671]ENERGY: 5720000 3194.5169 5485.5864 5707.3926
349.5839 -14191.9209 -1832.1778 0.0000 0.0000
8005.7025 6718.6836 305.2703 -1287.0189 6786.5592
304.7890 19.1695 27.0950 680285.8938 -9.4588
-9.4598
[20721]ENERGY: 5730000 3370.0458 5432.8331 5701.1777
321.6506 -14271.6353 -1849.9853 0.0000 0.0000
8020.9926 6725.0792 305.8534 -1295.9134 6788.9564
304.7647 -126.0716 -122.8959 680285.8938 -18.8396
-18.8439
[20757]ENERGY: 5740000 3318.0447 5482.0563 5738.0427
352.4317 -14289.4285 -1895.5062 0.0000 0.0000
8019.6432 6725.2837 305.8019 -1294.3594 6790.4330
304.9668 103.2145 57.3633 680285.8938 -15.2454
-15.2572
[20807]ENERGY: 5750000 3299.4279 5470.1448 5770.3128
348.2713 -14361.6438 -1852.1332 0.0000 0.0000
8049.6117 6723.9915 306.9447 -1325.6202 6789.8022
305.0304 75.2631 1.1555 680285.8938 -15.6727
-15.6614
[20843]ENERGY: 5760000 3271.4054 5440.5236 5768.2474
332.8815 -14258.7579 -1860.6906 0.0000 0.0000
8031.0960 6724.7054 306.2386 -1306.3906 6788.4885
305.1797 -57.4675 -63.9296 680285.8938 -12.2081
-12.1937
[20893]ENERGY: 5770000 3377.9488 5343.1084 5716.3720
348.0742 -14238.5616 -1802.5519 0.0000 0.0000
7984.2224 6728.6122 304.4513 -1255.6102 6789.7093
305.1021 187.9335 84.1672 680285.8938 -12.3500
-12.3552
[20929]ENERGY: 5780000 3280.2794 5552.5463 5771.8375

345.9695 -14338.6912 -1855.4876 0.0000 0.0000
7970.9210 6727.3748 303.9441 -1243.5461 6790.9546
305.0884 -8.0503 -17.2053 680285.8938 -9.1780
-9.1759
[20979]ENERGY: 5790000 3195.2873 5387.8397 5757.0364
339.1610 -14157.0599 -1886.9488 0.0000 0.0000
8090.3816 6725.6972 308.4993 -1364.6844 6790.3920
305.0767 11.3388 35.9125 680285.8938 -13.5622
-13.5692
[21018]ENERGY: 5800000 3231.5178 5442.5278 5715.0636
346.7546 -14184.1190 -1863.0676 0.0000 0.0000
8038.0970 6726.7741 306.5056 -1311.3228 6790.1589
305.3668 109.5214 27.9411 680285.8938 -12.1999
-12.1971
[21068]ENERGY: 5810000 3344.4412 5535.6836 5688.2574
328.5797 -14267.7238 -1855.9988 0.0000 0.0000
7952.4443 6725.6836 303.2395 -1226.7608 6791.3837
305.4216 45.3258 55.9209 680285.8938 -13.9554
-13.9531
[21104]ENERGY: 5820000 3292.5422 5492.2206 5764.8051
322.0928 -14276.1716 -1772.6423 0.0000 0.0000
7904.7032 6727.5500 301.4191 -1177.1532 6791.2242
305.4058 142.3498 57.4153 680285.8938 -13.3667
-13.3684
[21154]ENERGY: 5830000 3295.6955 5454.1865 5721.2916
342.8522 -14244.9710 -1831.9045 0.0000 0.0000
7989.6403 6726.7905 304.6579 -1262.8497 6791.1294
305.3847 -3.3615 -65.8529 680285.8938 -9.5915
-9.6020
[21190]ENERGY: 5840000 3243.6922 5464.0108 5742.9567
323.6036 -14304.6693 -1759.3104 0.0000 0.0000
8015.5019 6725.7856 305.6440 -1289.7164 6792.0768
305.2200 0.7037 1.3814 680285.8938 -15.0506
-15.0477
[21240]ENERGY: 5850000 3259.8947 5463.3772 5657.7929
346.9533 -14248.9138 -1792.1099 0.0000 0.0000
8037.4345 6724.4291 306.4803 -1313.0055 6792.1570
305.3070 139.6408 148.0179 680285.8938 -19.5623
-19.5413
[21276]ENERGY: 5860000 3236.7613 5374.0153 5731.2700
340.5493 -14265.5468 -1765.5166 0.0000 0.0000
8077.9699 6729.5024 308.0260 -1348.4675 6792.5925
305.4706 87.8365 101.4354 680285.8938 -9.7778
-9.7960
[21326]ENERGY: 5870000 3205.3574 5465.8297 5742.8309
356.3285 -14267.3958 -1813.1983 0.0000 0.0000
8040.1568 6729.9093 306.5841 -1310.2475 6793.3641
305.3611 239.6148 188.1138 680285.8938 -15.8357
-15.8165
[21362]ENERGY: 5880000 3244.5668 5355.1510 5760.2113
337.7494 -14271.4774 -1733.5560 0.0000 0.0000
8035.6442 6728.2894 306.4121 -1307.3548 6793.2792
305.4137 24.8337 6.8882 680285.8938 -11.6360
-11.6409

[21412]ENERGY: 5890000 3303.0628 5400.9516 5720.8993
332.8722 -14263.2159 -1821.5919 0.0000 0.0000
8059.0951 6732.0732 307.3063 -1327.0219 6794.3043
305.3100 -46.5663 -32.3131 680285.8938 -10.1531
-10.1710
[21451]ENERGY: 5900000 3331.2498 5434.9181 5707.9898
333.5626 -14265.7421 -1834.2252 0.0000 0.0000
8018.3901 6726.1432 305.7541 -1292.2469 6793.2468
305.2495 140.3180 139.2217 680285.8938 -8.4226
-8.4109
[21501]ENERGY: 5910000 3293.6472 5565.5312 5703.5412
343.8890 -14335.3656 -1813.0071 0.0000 0.0000
7971.6674 6729.9031 303.9725 -1241.7643 6793.8405
305.4148 43.8719 13.1113 680285.8938 -13.7780
-13.7908
[21537]ENERGY: 5920000 3331.6434 5412.3602 5711.1307
349.9557 -14195.4317 -1805.6251 0.0000 0.0000
7929.6242 6733.6572 302.3694 -1195.9670 6794.6045
305.3884 107.7796 67.2222 680285.8938 -11.6772
-11.6782
[21587]ENERGY: 5930000 3214.3642 5573.5439 5726.2887
317.0203 -14336.4369 -1836.8969 0.0000 0.0000
8070.3267 6728.2099 307.7346 -1342.1168 6793.8830
305.6053 -16.7055 3.2389 680285.8938 -13.1654
-13.1402
[21623]ENERGY: 5940000 3328.9818 5412.3554 5674.5758
328.2862 -14261.8738 -1806.7825 0.0000 0.0000
8053.2267 6728.7696 307.0825 -1324.4571 6794.1769
305.6794 133.3877 76.5724 680285.8938 -13.1651
-13.1806
[21673]ENERGY: 5950000 3293.3894 5442.6882 5699.1072
333.6907 -14200.6701 -1814.3729 0.0000 0.0000
7977.4175 6731.2501 304.1918 -1246.1674 6795.1522
305.6685 -22.8228 0.3717 680285.8938 -14.0652
-14.0700
[21709]ENERGY: 5960000 3304.6716 5437.7626 5728.3469
329.9355 -14320.7857 -1818.1424 0.0000 0.0000
8068.6129 6730.4014 307.6692 -1338.2115 6794.7341
305.6656 -94.8825 -89.5917 680285.8938 -10.6767
-10.6849
[21759]ENERGY: 5970000 3378.6103 5436.1085 5715.6870
349.6439 -14319.6368 -1796.4243 0.0000 0.0000
7971.0247 6735.0133 303.9480 -1236.0114 6795.8887
305.7599 -23.6546 12.0248 680285.8938 -10.4118
-10.4131
[21795]ENERGY: 5980000 3283.9000 5341.4026 5747.0893
317.4734 -14247.7022 -1781.7251 0.0000 0.0000
8070.1944 6730.6324 307.7295 -1339.5620 6795.4747
305.7023 128.2101 31.5155 680285.8938 -11.1018
-11.1012
[21845]ENERGY: 5990000 3328.3782 5368.7224 5723.5009
314.4497 -14215.9161 -1817.5503 0.0000 0.0000
8029.8711 6731.4559 306.1919 -1298.4152 6796.2975
305.5705 -12.3951 -38.0204 680285.8938 -15.6143

-15.6132
[21884]ENERGY: 6000000 3267.0205 5407.6350 5741.2131
328.4195 -14161.2594 -1802.7680 0.0000 0.0000
7950.7907 6731.0515 303.1765 -1219.7393 6796.1214
305.3300 -86.1474 -67.1391 680285.8938 -10.8120
-10.8191
[21934]ENERGY: 6010000 3268.0784 5513.2212 5662.1939
330.4218 -14226.6816 -1756.9922 0.0000 0.0000
7945.6949 6735.9364 302.9822 -1209.7585 6797.5456
305.6182 135.7156 21.2826 680285.8938 -11.6571
-11.6555
[21970]ENERGY: 6020000 3294.1237 5397.4853 5727.6972
345.4872 -14255.6470 -1807.8873 0.0000 0.0000
8030.9400 6732.1991 306.2327 -1298.7409 6798.3995
305.5072 210.0864 136.8275 680285.8938 -15.1787
-15.1574
[22020]ENERGY: 6030000 3181.0972 5404.2132 5714.3535
328.9826 -14120.3116 -1827.7174 0.0000 0.0000
8050.4206 6731.0381 306.9755 -1319.3825 6796.3191
305.4880 236.2480 200.3982 680285.8938 -10.1349
-10.1571
[22056]ENERGY: 6040000 3303.5236 5396.6894 5716.1377
334.6958 -14278.7236 -1833.2255 0.0000 0.0000
8090.4119 6729.5093 308.5005 -1360.9026 6796.5942
305.3694 112.0975 -79.5643 680285.8938 -11.9824
-11.9536
[22106]ENERGY: 6050000 3311.3555 5445.2400 5704.2605
334.5922 -14252.5213 -1783.1555 0.0000 0.0000
7973.5894 6733.3608 304.0458 -1240.2286 6796.9688
305.2583 126.3987 40.8558 680285.8938 -17.8592
-17.8667
[22142]ENERGY: 6060000 3316.8957 5413.8758 5688.9058
318.6937 -14200.9596 -1816.5671 0.0000 0.0000
8013.1293 6733.9735 305.5535 -1279.1558 6796.0069
305.3717 21.4461 9.4296 680285.8938 -13.2931
-13.3042
[22192]ENERGY: 6070000 3251.6985 5560.1480 5693.7836
349.6780 -14344.0376 -1747.2392 0.0000 0.0000
7966.5344 6730.5658 303.7768 -1235.9687 6797.5911
305.3972 61.7455 31.3395 680285.8938 -8.1504
-8.1551
[22228]ENERGY: 6080000 3293.4552 5399.1374 5758.5885
345.1242 -14299.6847 -1740.0734 0.0000 0.0000
7974.7785 6731.3257 304.0912 -1243.4528 6796.9030
305.4966 -38.6561 -28.6369 680285.8938 -11.4652
-11.4641
[22278]ENERGY: 6090000 3210.0476 5511.3545 5735.2519
336.4810 -14117.4272 -1861.3548 0.0000 0.0000
7919.7998 6734.1528 301.9947 -1185.6470 6796.7614
305.4394 -100.0734 -114.7663 680285.8938 -9.3161
-9.3061
[22317]ENERGY: 6100000 3344.4095 5382.5286 5716.8726
323.4208 -14155.6593 -1803.2466 0.0000 0.0000
7926.1907 6734.5162 302.2384 -1191.6745 6797.5183

305.4761 26.2546 -31.2021 680285.8938 -13.5453
-13.5368
[22367]ENERGY: 6110000 3373.6560 5426.8456 5688.2387
353.7767 -14302.5429 -1817.4170 0.0000 0.0000
8010.3567 6732.9138 305.4478 -1277.4429 6798.6285
305.3043 -37.4957 -53.5473 680285.8938 -16.3540
-16.3647
[22403]ENERGY: 6120000 3246.2206 5499.6069 5690.2607
321.5646 -14189.4378 -1833.5078 0.0000 0.0000
7997.0692 6731.7765 304.9411 -1265.2927 6797.6175
305.5269 98.3143 66.6666 680285.8938 -14.7665
-14.7648
[22453]ENERGY: 6130000 3301.4417 5452.6983 5690.9037
322.4369 -14261.8626 -1807.3469 0.0000 0.0000
8035.4189 6733.6900 306.4035 -1301.7290 6798.3516
305.6443 -53.9521 -64.7205 680285.8938 -14.2306
-14.2187
[22489]ENERGY: 6140000 3258.1225 5589.7403 5677.0973
341.4940 -14308.6112 -1774.5782 0.0000 0.0000
7951.3689 6734.6334 303.1985 -1216.7355 6798.6660
305.5830 65.4322 33.8223 680285.8938 -11.2485
-11.2597
[22539]ENERGY: 6150000 3318.2655 5476.4229 5722.2947
324.7706 -14264.7498 -1814.6762 0.0000 0.0000
7971.2732 6733.6008 303.9575 -1237.6724 6798.5999
305.6889 78.2501 16.9881 680285.8938 -14.5208
-14.5271
[22575]ENERGY: 6160000 3306.8825 5446.8338 5715.3970
333.0688 -14320.9119 -1772.3130 0.0000 0.0000
8023.4143 6732.3714 305.9457 -1291.0429 6798.2272
305.6373 71.2603 17.8556 680285.8938 -11.9459
-11.9427
[22625]ENERGY: 6170000 3400.6373 5440.4140 5725.9343
335.0374 -14290.9608 -1831.9648 0.0000 0.0000
7951.0670 6730.1644 303.1870 -1220.9026 6798.1091
305.8422 -131.0103 -106.5070 680285.8938 -7.7728
-7.7750
[22661]ENERGY: 6180000 3300.5682 5418.0999 5672.5323
334.7945 -14224.2264 -1838.1532 0.0000 0.0000
8070.5432 6734.1584 307.7428 -1336.3848 6798.4291
305.8233 28.5034 -18.0146 680285.8938 -10.6546
-10.6448
[22711]ENERGY: 6190000 3294.4757 5488.9293 5733.9874
315.2489 -14272.9341 -1710.5938 0.0000 0.0000
7888.8033 6737.9168 300.8128 -1150.8866 6799.2133
305.7474 175.6833 78.2681 680285.8938 -8.2384
-8.2474
[22750]ENERGY: 6200000 3288.0020 5424.0128 5793.4439
350.8587 -14293.8458 -1808.8963 0.0000 0.0000
7979.6155 6733.1910 304.2756 -1246.4246 6797.8768
306.0403 -17.6665 -28.8381 680285.8938 -8.6593
-8.6372
[22800]ENERGY: 6210000 3278.1212 5504.0910 5703.1823
359.4933 -14343.7550 -1714.5678 0.0000 0.0000

7949.1126 6735.6776 303.1125 -1213.4350 6799.1332
306.1454 86.2596 16.7690 680285.8938 -7.4301
-7.4533
[22836]ENERGY: 6220000 3252.8211 5471.7577 5678.6413
322.3966 -14252.7423 -1841.9024 0.0000 0.0000
8105.4825 6736.4545 309.0751 -1369.0280 6800.1388
306.0592 30.8245 -32.3672 680285.8938 -11.0055
-10.9972
[22886]ENERGY: 6230000 3254.5624 5471.2881 5684.8904
337.2828 -14234.5989 -1842.0540 0.0000 0.0000
8063.3313 6734.7022 307.4678 -1328.6291 6799.6244
306.0420 -69.1189 -8.1232 680285.8938 -8.8311
-8.8265
[22922]ENERGY: 6240000 3361.8474 5438.2862 5696.9714
362.9552 -14226.9091 -1844.8998 0.0000 0.0000
7949.3691 6737.6204 303.1223 -1211.7487 6801.0683
306.0652 79.2793 -5.2868 680285.8938 -6.8610
-6.8635
[22972]ENERGY: 6250000 3418.3298 5463.0685 5606.5780
327.1896 -14317.1801 -1747.4021 0.0000 0.0000
7985.6756 6736.2593 304.5067 -1249.4163 6801.2049
306.0240 74.6329 19.7340 680285.8938 -3.8673
-3.8845
[23008]ENERGY: 6260000 3255.9135 5300.3522 5701.2892
315.7577 -14204.4126 -1730.4713 0.0000 0.0000
8097.4286 6735.8573 308.7680 -1361.5713 6801.3260
305.7640 1.7369 -24.8197 680285.8938 -8.8356
-8.8205
[23058]ENERGY: 6270000 3248.4480 5460.0355 5754.8398
349.4679 -14223.7880 -1861.8936 0.0000 0.0000
8011.9207 6739.0303 305.5075 -1272.8904 6803.4936
305.8212 88.2113 26.2014 680285.8938 -10.0424
-10.0563
[23094]ENERGY: 6280000 3299.4899 5396.6287 5681.2976
341.9095 -14185.5507 -1785.3601 0.0000 0.0000
7987.1080 6735.5227 304.5613 -1251.5853 6802.4325
306.0066 -38.6016 -77.5081 680285.8938 -11.7207
-11.7241
[23144]ENERGY: 6290000 3397.8661 5454.6495 5720.1700
329.1192 -14359.0097 -1794.9291 0.0000 0.0000
7989.8222 6737.6883 304.6648 -1252.1340 6800.4580
305.8011 -56.2427 -64.2043 680285.8938 -12.8644
-12.8602
[23183]ENERGY: 6300000 3319.0241 5428.4513 5732.7461
364.3074 -14328.3428 -1801.7859 0.0000 0.0000
8020.9239 6735.3240 305.8508 -1285.5999 6802.0362
306.2714 108.0819 81.9932 680285.8938 -11.6608
-11.6423
[23233]ENERGY: 6310000 3284.3192 5429.8110 5718.2993
374.9141 -14228.6614 -1868.4248 0.0000 0.0000
8026.0601 6736.3175 306.0466 -1289.7426 6801.8265
306.1120 136.3633 120.7742 680285.8938 -16.9361
-16.9444
[23269]ENERGY: 6320000 3287.5657 5441.0218 5728.2679

340.2755 -14267.6430 -1874.6986 0.0000 0.0000
8082.8284 6737.6178 308.2113 -1345.2106 6802.8212
305.9943 -94.2000 -110.9926 680285.8938 -15.5438
-15.5340
[23319]ENERGY: 6330000 3300.3247 5381.2160 5701.7777
331.9516 -14214.0798 -1869.1948 0.0000 0.0000
8102.7747 6734.7700 308.9719 -1368.0047 6801.7638
306.1651 -59.7789 -13.6902 680285.8938 -13.1591
-13.1543
[23355]ENERGY: 6340000 3291.2112 5446.0468 5707.9580
336.3269 -14344.1897 -1744.1450 0.0000 0.0000
8040.5756 6733.7839 306.6001 -1306.7918 6802.7292
306.0524 -28.2767 -76.8579 680285.8938 -12.3758
-12.3849
[23405]ENERGY: 6350000 3300.8702 5455.9495 5671.0783
362.4655 -14232.3283 -1813.5399 0.0000 0.0000
7993.0193 6737.5146 304.7867 -1255.5047 6803.0642
306.1383 -21.8315 -57.0726 680285.8938 -12.7521
-12.7634
[23441]ENERGY: 6360000 3343.6187 5368.0914 5649.7969
329.2220 -14198.6457 -1835.6702 0.0000 0.0000
8079.6530 6736.0661 308.0902 -1343.5869 6802.3641
306.1477 -100.6380 -78.2544 680285.8938 -11.7405
-11.7384
[23491]ENERGY: 6370000 3367.1301 5494.1833 5712.2544
329.3328 -14284.1886 -1862.1568 0.0000 0.0000
7983.2133 6739.7685 304.4128 -1243.4448 6803.5910
306.0633 -3.4296 3.7292 680285.8938 -15.2753
-15.2783
[23527]ENERGY: 6380000 3315.8071 5500.1989 5726.2072
352.8041 -14345.4505 -1825.3167 0.0000 0.0000
8016.0552 6740.3053 305.6651 -1275.7499 6803.0299
306.1221 -180.8532 -130.2917 680285.8938 -10.6315
-10.6120
[23577]ENERGY: 6390000 3336.6794 5423.7974 5760.2179
357.9815 -14315.2081 -1861.4342 0.0000 0.0000
8036.0478 6738.0817 306.4275 -1297.9661 6802.3778
305.9920 -11.3891 -83.0677 680285.8938 -10.3675
-10.3976
[23616]ENERGY: 6400000 3350.5985 5550.2066 5731.6912
323.7695 -14316.6089 -1911.3661 0.0000 0.0000
8007.5354 6735.8262 305.3402 -1271.7092 6802.8259
305.8504 -160.4361 -68.4899 680285.8938 -16.2913
-16.2575
[23666]ENERGY: 6410000 3247.1689 5521.2654 5754.5417
333.3902 -14303.3135 -1852.2638 0.0000 0.0000
8035.6529 6736.4419 306.4124 -1299.2111 6803.6723
305.7013 99.9359 92.1609 680285.8938 -19.1341
-19.1692
[23702]ENERGY: 6420000 3314.6121 5499.5009 5721.9393
343.9166 -14328.8889 -1840.3556 0.0000 0.0000
8028.8788 6739.6032 306.1541 -1289.2756 6803.1174
305.9125 75.3126 -1.5429 680285.8938 -12.8803
-12.8635

[23752]ENERGY: 6430000 3327.3799 5447.7885 5748.7734
357.7833 -14287.5615 -1814.7972 0.0000 0.0000
7959.6611 6739.0274 303.5147 -1220.6337 6802.2896
305.9931 69.5237 5.3498 680285.8938 -13.4468
-13.4338
[23788]ENERGY: 6440000 3294.0591 5327.1250 5648.5334
298.9905 -14192.0235 -1804.7728 0.0000 0.0000
8160.9415 6732.8531 311.1899 -1428.0884 6801.9129
305.8395 -9.2388 6.9947 680285.8938 -19.7114
-19.7265
[23838]ENERGY: 6450000 3333.1671 5485.1984 5728.3827
318.6264 -14280.7438 -1820.2707 0.0000 0.0000
7974.6626 6739.0227 304.0867 -1235.6399 6802.8452
305.9028 87.4057 35.7639 680285.8938 -17.2018
-17.1937
[23874]ENERGY: 6460000 3255.3849 5418.8501 5701.6404
352.4700 -14341.4193 -1751.7117 0.0000 0.0000
8099.9050 6735.1193 308.8624 -1364.7857 6803.1928
306.0357 -28.8634 -36.2831 680285.8938 -18.6351
-18.6528
[23924]ENERGY: 6470000 3334.6693 5430.9363 5750.7978
336.6538 -14184.1217 -1904.1386 0.0000 0.0000
7975.8476 6740.6446 304.1319 -1235.2030 6804.1551
305.9404 -115.4886 -135.9166 680285.8938 -14.9875
-14.9927
[23960]ENERGY: 6480000 3267.8380 5469.2586 5668.5848
342.6496 -14248.0351 -1775.3917 0.0000 0.0000
8015.6228 6740.5269 305.6486 -1275.0959 6803.7965
305.8200 -56.1353 32.0512 680285.8938 -19.5838
-19.5566
[24010]ENERGY: 6490000 3318.3032 5473.1759 5747.0562
345.0284 -14337.7826 -1770.2764 0.0000 0.0000
7961.6010 6737.1057 303.5887 -1224.4953 6803.3282
305.9405 37.3100 26.6228 680285.8938 -16.4258
-16.4389
[24049]ENERGY: 6500000 3320.4939 5426.5214 5732.2709
345.6163 -14264.8066 -1779.4291 0.0000 0.0000
7958.6141 6739.2808 303.4748 -1219.3333 6803.7619
305.8200 90.6657 58.0120 680285.8938 -15.3941
-15.3909
[24099]ENERGY: 6510000 3254.0034 5423.8705 5721.0153
347.6913 -14314.7371 -1798.8376 0.0000 0.0000
8103.7933 6736.7992 309.0107 -1366.9941 6802.8224
305.8377 -9.6766 -73.0892 680285.8938 -15.9593
-15.9806
[24135]ENERGY: 6520000 3292.6340 5366.0397 5729.4145
343.7440 -14225.2553 -1808.8441 0.0000 0.0000
8038.6588 6736.3914 306.5270 -1302.2673 6803.5479
305.8841 -40.1980 -18.9093 680285.8938 -11.9457
-11.9214
[24185]ENERGY: 6530000 3297.6419 5520.9814 5705.8752
334.3667 -14258.9935 -1851.4046 0.0000 0.0000
7993.6455 6742.1126 304.8106 -1251.5330 6802.7283
305.9808 24.0714 -8.8310 680285.8938 -13.5638

-13.5626
[24221]ENERGY: 6540000 3258.7190 5426.3960 5686.0751
360.7291 -14249.9926 -1773.9154 0.0000 0.0000
8033.9442 6741.9553 306.3472 -1291.9889 6803.4517
306.2508 11.8485 -0.9607 680285.8938 -16.6461
-16.6550
[24271]ENERGY: 6550000 3207.4406 5497.1143 5687.2969
361.5028 -14274.6913 -1825.5396 0.0000 0.0000
8084.1611 6737.2849 308.2621 -1346.8763 6803.5786
306.1654 88.8609 63.0708 680285.8938 -14.1446
-14.1291
[24307]ENERGY: 6560000 3283.8094 5491.5706 5708.5513
353.2482 -14306.1023 -1865.8492 0.0000 0.0000
8068.4433 6733.6713 307.6628 -1334.7720 6803.2635
306.0838 -141.6667 -42.6650 680285.8938 -18.8226
-18.8121
[24357]ENERGY: 6570000 3350.7631 5490.3510 5672.6806
332.8220 -14254.1495 -1815.1016 0.0000 0.0000
7964.7198 6742.0853 303.7076 -1222.6344 6804.8406
306.2494 -73.8615 -73.6156 680285.8938 -13.6115
-13.6290
[24393]ENERGY: 6580000 3309.3311 5496.6080 5740.3468
352.4177 -14246.9996 -1866.3293 0.0000 0.0000
7953.9918 6739.3665 303.2985 -1214.6253 6803.7210
306.3427 -18.8048 -27.1537 680285.8938 -13.5031
-13.4962
[24443]ENERGY: 6590000 3357.6212 5409.6616 5698.1406
324.2975 -14201.3150 -1817.6345 0.0000 0.0000
7970.0012 6740.7727 303.9090 -1229.2285 6802.8620
306.3348 -61.3174 -49.7040 680285.8938 -15.0585
-15.0657
[24482]ENERGY: 6600000 3377.0044 5485.7011 5714.3109
341.3308 -14418.5714 -1834.8463 0.0000 0.0000
8072.7250 6737.6547 307.8260 -1335.0704 6804.0190
306.2615 133.2667 56.9373 680285.8938 -12.6071
-12.5971
[24532]ENERGY: 6610000 3347.0414 5469.9929 5674.4331
323.5585 -14256.3535 -1849.7622 0.0000 0.0000
8031.2827 6740.1929 306.2458 -1291.0898 6804.4381
306.4305 -8.0459 -21.5720 680285.8938 -12.3580
-12.3596
[24568]ENERGY: 6620000 3283.9428 5561.3121 5710.8499
300.8329 -14234.9747 -1913.1826 0.0000 0.0000
8031.6235 6740.4040 306.2588 -1291.2195 6805.6575
306.2161 -19.1993 -21.6948 680285.8938 -13.3558
-13.3514
[24618]ENERGY: 6630000 3254.0589 5355.7009 5697.3628
334.4602 -14201.6804 -1894.3003 0.0000 0.0000
8191.7552 6737.3573 312.3648 -1454.3980 6804.8109
306.3799 -125.0716 -192.2820 680285.8938 -12.7962
-12.8184
[24654]ENERGY: 6640000 3333.6452 5396.2756 5697.3292
352.7124 -14279.0395 -1827.0036 0.0000 0.0000
8062.5997 6736.5190 307.4399 -1326.0808 6805.0321

306.2522 -93.6396 -105.9790 680285.8938 -13.9507
-13.9528
[24704]ENERGY: 6650000 3259.1650 5465.2266 5686.7066
331.0538 -14257.8151 -1726.8311 0.0000 0.0000
7981.8746 6739.3805 304.3618 -1242.4942 6805.1260
306.0941 116.7811 86.5845 680285.8938 -14.4009
-14.4043
[24740]ENERGY: 6660000 3284.4356 5557.7893 5704.5213
343.0901 -14305.1668 -1818.4957 0.0000 0.0000
7972.7417 6738.9156 304.0135 -1233.8261 6804.9287
305.9569 88.3392 -10.4453 680285.8938 -14.8539
-14.8598
[24790]ENERGY: 6670000 3336.8716 5527.1828 5665.7548
324.6971 -14284.7499 -1860.6848 0.0000 0.0000
8029.0485 6738.1203 306.1606 -1290.9283 6805.5820
306.0550 26.8017 54.5761 680285.8938 -14.6603
-14.6302
[24826]ENERGY: 6680000 3254.0850 5385.1087 5692.2426
333.0035 -14200.7988 -1831.2784 0.0000 0.0000
8108.2269 6740.5894 309.1798 -1367.6375 6805.8634
305.9097 0.9370 -1.7318 680285.8938 -15.5999
-15.6126
[24876]ENERGY: 6690000 3396.3383 5449.9281 5689.6000
333.1055 -14280.7164 -1763.0162 0.0000 0.0000
7920.9810 6746.2203 302.0398 -1174.7607 6806.5042
306.1996 -54.8423 -63.3925 680285.8938 -9.4706
-9.4576
[24915]ENERGY: 6700000 3305.6212 5319.5673 5733.9805
334.2469 -14269.3332 -1730.6425 0.0000 0.0000
8050.7564 6744.1967 306.9883 -1306.5598 6807.8711
306.3169 258.2193 89.4731 680285.8938 -14.4147
-14.4224
[24965]ENERGY: 6710000 3294.9155 5438.7434 5713.6072
327.7746 -14299.2571 -1779.1426 0.0000 0.0000
8045.1311 6741.7720 306.7738 -1303.3591 6807.7004
306.3019 -73.5925 -89.8108 680285.8938 -14.0751
-14.0843
[25001]ENERGY: 6720000 3303.1824 5426.5033 5726.1171
342.4455 -14210.5744 -1877.5558 0.0000 0.0000
8034.7748 6744.8928 306.3789 -1289.8820 6808.0908
306.0404 67.4529 37.6265 680285.8938 -9.3267
-9.3110
[25051]ENERGY: 6730000 3390.1249 5459.0785 5644.1274
311.4298 -14250.7579 -1794.4475 0.0000 0.0000
7983.7595 6743.3147 304.4336 -1240.4449 6807.6349
306.3010 -44.0651 -26.1170 680285.8938 -12.0298
-12.0448
[25087]ENERGY: 6740000 3338.7814 5423.8440 5754.1329
374.0536 -14229.8036 -1883.0538 0.0000 0.0000
7968.2412 6746.1957 303.8419 -1222.0455 6808.3880
306.4127 7.2081 -10.7928 680285.8938 -14.6615
-14.6490
[25137]ENERGY: 6750000 3319.8042 5407.2374 5719.8063
329.6018 -14270.0479 -1799.0371 0.0000 0.0000

8036.2208 6743.5857 306.4341 -1292.6352 6809.4137
306.4110 -3.7403 15.0635 680285.8938 -9.5917
-9.6025
[25173]ENERGY: 6760000 3399.5605 5410.1159 5745.9106
342.3587 -14360.2341 -1737.6073 0.0000 0.0000
7946.8424 6746.9468 303.0259 -1199.8957 6810.4961
306.3791 95.6410 39.1435 680285.8938 -13.6008
-13.5887
[25223]ENERGY: 6770000 3344.3436 5486.0136 5729.1001
339.3046 -14328.6602 -1846.9414 0.0000 0.0000
8022.0437 6745.2040 305.8935 -1276.8397 6809.8541
306.1998 -10.0290 -40.0352 680285.8938 -12.9656
-12.9834
[25259]ENERGY: 6780000 3244.1211 5584.5906 5721.7904
321.0310 -14252.8344 -1822.8110 0.0000 0.0000
7950.5331 6746.4209 303.1666 -1204.1122 6811.1019
306.3933 229.4470 151.6046 680285.8938 -15.1333
-15.1184
[25309]ENERGY: 6790000 3306.9545 5534.9060 5711.6147
351.5630 -14242.3343 -1920.2792 0.0000 0.0000
8003.7436 6746.1683 305.1957 -1257.5753 6812.2923
306.2551 -100.0125 -138.9291 680285.8938 -13.0364
-13.0447
[25348]ENERGY: 6800000 3343.0096 5402.0851 5677.3459
312.9293 -14287.2627 -1766.5080 0.0000 0.0000
8063.4562 6745.0554 307.4726 -1318.4008 6810.1309
306.3201 127.2151 113.6911 680285.8938 -13.4540
-13.4406
[25398]ENERGY: 6810000 3304.0254 5512.0311 5715.0712
319.5011 -14270.3927 -1805.2722 0.0000 0.0000
7973.9325 6748.8963 304.0589 -1225.0361 6811.6478
306.4332 17.2773 26.9924 680285.8938 -15.7584
-15.7754
[25434]ENERGY: 6820000 3261.3227 5379.0804 5749.0523
356.8055 -14273.7110 -1733.3437 0.0000 0.0000
8007.4606 6746.6668 305.3374 -1260.7939 6811.1941
306.2560 89.4367 51.3952 680285.8938 -11.6190
-11.6196
[25484]ENERGY: 6830000 3275.5877 5436.8285 5742.7590
350.0688 -14342.3379 -1734.4586 0.0000 0.0000
8018.4850 6746.9326 305.7578 -1271.5524 6811.4851
306.1873 70.4174 13.0879 680285.8938 -13.8630
-13.8660
[25520]ENERGY: 6840000 3271.3252 5463.7297 5702.7016
328.1100 -14338.2740 -1720.2501 0.0000 0.0000
8036.6768 6744.0190 306.4514 -1292.6578 6811.9672
306.1017 17.8139 28.2708 680285.8938 -13.5622
-13.5608
[25570]ENERGY: 6850000 3263.2589 5488.8400 5717.9585
346.3614 -14321.6022 -1781.9930 0.0000 0.0000
8033.7794 6746.6031 306.3410 -1287.1764 6812.9065
306.3194 89.4141 63.7877 680285.8938 -13.8183
-13.8164
[25606]ENERGY: 6860000 3342.4764 5532.4302 5774.2828

352.9581 -14282.3341 -1895.9822 0.0000 0.0000
7927.5617 6751.3929 302.2907 -1176.1687 6815.3486
306.1937 -58.4090 -97.9747 680285.8938 -12.8544
-12.8516
[25656]ENERGY: 6870000 3240.7498 5462.5767 5724.0935
331.7667 -14176.1894 -1883.8548 0.0000 0.0000
8051.2422 6750.3846 307.0068 -1300.8576 6813.3194
306.4074 139.5975 114.5771 680285.8938 -10.9234
-10.9202
[25692]ENERGY: 6880000 3322.1694 5486.8445 5751.1783
326.8131 -14281.0696 -1846.1009 0.0000 0.0000
7989.1133 6748.9481 304.6378 -1240.1652 6812.0290
306.5048 -14.5917 13.2412 680285.8938 -17.0566
-17.0634
[25742]ENERGY: 6890000 3282.1058 5467.3238 5741.8682
350.8496 -14230.7327 -1879.8375 0.0000 0.0000
8016.0405 6747.6177 305.6646 -1268.4228 6812.7623
306.5713 -23.2400 -17.1755 680285.8938 -9.9526
-9.9543
[25781]ENERGY: 6900000 3348.4098 5366.7030 5741.5834
336.6788 -14300.3251 -1814.1549 0.0000 0.0000
8068.4783 6747.3732 307.6641 -1321.1051 6812.6139
306.2888 -27.1378 -43.6274 680285.8938 -10.5785
-10.5710
[25831]ENERGY: 6910000 3341.0218 5472.7318 5721.3090
345.3482 -14244.6192 -1856.5250 0.0000 0.0000
7971.0646 6750.3312 303.9495 -1220.7334 6812.6615
306.3995 79.3714 34.5939 680285.8938 -14.7204
-14.7112
[25867]ENERGY: 6920000 3291.0712 5505.1331 5715.3013
318.2482 -14238.3044 -1847.8594 0.0000 0.0000
8004.4185 6748.0085 305.2214 -1256.4100 6811.6438
306.3874 -42.7036 -31.0238 680285.8938 -14.2414
-14.2476
[25917]ENERGY: 6930000 3318.8507 5440.2292 5674.9986
349.2429 -14215.6143 -1859.0739 0.0000 0.0000
8038.4886 6747.1217 306.5205 -1291.3669 6811.9537
306.5138 201.0567 125.3710 680285.8938 -13.0540
-13.0478
[25953]ENERGY: 6940000 3345.8780 5355.2801 5731.2175
338.5049 -14267.6371 -1785.6162 0.0000 0.0000
8030.8930 6748.5202 306.2309 -1282.3727 6811.7478
306.6000 -4.7731 -22.7308 680285.8938 -16.8462
-16.8303
[26003]ENERGY: 6950000 3340.8839 5365.9746 5766.3256
341.7559 -14182.3257 -1848.9814 0.0000 0.0000
7967.3228 6750.9558 303.8069 -1216.3671 6812.0662
306.6488 -37.6263 -49.6674 680285.8938 -12.2298
-12.2580
[26039]ENERGY: 6960000 3307.0234 5486.4186 5737.5132
341.4928 -14275.4667 -1820.7397 0.0000 0.0000
7973.0513 6749.2929 304.0253 -1223.7584 6811.8401
306.7167 -123.1602 -7.7181 680285.8938 -13.3857
-13.3533

[26089]ENERGY: 6970000 3308.2002 5392.9331 5700.0292
349.1254 -14271.6222 -1788.6629 0.0000 0.0000
8056.0469 6746.0498 307.1901 -1309.9971 6812.0304
306.5744 35.0742 54.6281 680285.8938 -14.9075
-14.9426
[26125]ENERGY: 6980000 3327.2541 5515.7402 5704.0772
338.8360 -14353.9197 -1787.8554 0.0000 0.0000
8002.1394 6746.2719 305.1345 -1255.8676 6811.9599
306.6353 -22.9209 -46.4509 680285.8938 -12.1484
-12.1442
[26175]ENERGY: 6990000 3288.1156 5596.2423 5726.3902
329.8060 -14291.3159 -1865.9643 0.0000 0.0000
7962.7520 6746.0260 303.6326 -1216.7260 6812.9673
306.6640 -170.4113 -164.2055 680285.8938 -15.1287
-15.1164
[26214]ENERGY: 7000000 3357.6873 5443.4384 5721.5112
350.9739 -14329.7573 -1807.0847 0.0000 0.0000
8008.9455 6745.7143 305.3940 -1263.2312 6812.8722
306.7096 98.0848 -48.5774 680285.8938 -13.8369
-13.8409
[26264]ENERGY: 7010000 3349.2429 5500.5967 5746.7685
340.2720 -14355.0459 -1733.5341 0.0000 0.0000
7898.4007 6746.7007 301.1788 -1151.7000 6811.5475
306.6292 154.3809 149.5480 680285.8938 -17.6784
-17.6767
[26300]ENERGY: 7020000 3268.1673 5486.3247 5713.1298
326.7386 -14319.3159 -1834.3988 0.0000 0.0000
8105.2930 6745.9387 309.0679 -1359.3543 6813.4771
306.6709 13.8482 0.2253 680285.8938 -14.1126
-14.1252
[26350]ENERGY: 7030000 3296.8657 5403.5853 5674.6102
333.3028 -14205.0507 -1869.8626 0.0000 0.0000
8112.5130 6745.9638 309.3432 -1366.5492 6812.8531
306.6819 -140.8825 -119.6170 680285.8938 -13.8931
-13.8760
[26386]ENERGY: 7040000 3324.4579 5383.5820 5681.5359
337.0992 -14287.2506 -1815.4275 0.0000 0.0000
8121.1882 6745.1850 309.6740 -1376.0032 6811.9222
306.5739 -93.6689 -145.7001 680285.8938 -13.8813
-13.8957
[26436]ENERGY: 7050000 3257.8411 5474.5555 5753.1953
336.3622 -14277.0015 -1842.1652 0.0000 0.0000
8040.4439 6743.2313 306.5951 -1297.2126 6811.6659
306.7140 5.3521 -81.3183 680285.8938 -13.0880
-13.0908
[26472]ENERGY: 7060000 3282.6583 5514.1285 5752.5581
346.8123 -14302.2627 -1821.2534 0.0000 0.0000
7975.5030 6748.1442 304.1188 -1227.3589 6813.0906
306.6226 93.7327 -6.5858 680285.8938 -12.2203
-12.2167
[26522]ENERGY: 7070000 3293.4573 5446.3705 5751.0029
348.5950 -14343.6988 -1786.4949 0.0000 0.0000
8038.1046 6747.3365 306.5059 -1290.7681 6812.3891
306.5138 38.2680 46.8580 680285.8938 -15.2381

-15.2427
[26558]ENERGY: 7080000 3282.3771 5417.2965 5750.0302
345.1909 -14231.5290 -1830.9136 0.0000 0.0000
8013.7402 6746.1924 305.5768 -1267.5479 6811.6879
306.5297 -39.2584 -60.2619 680285.8938 -15.5702
-15.5765
[26608]ENERGY: 7090000 3398.6681 5456.6941 5739.5402
322.8778 -14306.8294 -1842.5004 0.0000 0.0000
7982.1956 6750.6461 304.3740 -1231.5495 6811.1840
306.7265 -28.9133 -4.6749 680285.8938 -17.6913
-17.6838
[26647]ENERGY: 7100000 3279.2405 5384.1446 5704.5848
338.4436 -14243.8148 -1883.6596 0.0000 0.0000
8162.7902 6741.7293 311.2604 -1421.0609 6810.3073
306.6349 -101.3910 -58.7475 680285.8938 -15.2208
-15.2139
[26697]ENERGY: 7110000 3290.3139 5485.8600 5657.4926
325.3859 -14344.3913 -1765.6035 0.0000 0.0000
8095.5402 6744.5979 308.6960 -1350.9424 6811.8456
306.6546 62.7979 6.0527 680285.8938 -11.8766
-11.8664
[26733]ENERGY: 7120000 3266.0312 5451.7966 5795.2241
349.6020 -14258.4204 -1877.7294 0.0000 0.0000
8020.9214 6747.4255 305.8507 -1273.4959 6811.2104
306.5634 -90.8409 -51.8953 680285.8938 -11.9305
-11.9332
[26783]ENERGY: 7130000 3193.1702 5486.4578 5770.9850
330.2058 -14266.2295 -1810.2618 0.0000 0.0000
8038.6080 6742.9355 306.5251 -1295.6725 6811.8469
306.5399 134.8973 72.0253 680285.8938 -12.0225
-12.0373
[26819]ENERGY: 7140000 3347.2852 5475.7913 5681.3154
324.5665 -14317.2425 -1797.3762 0.0000 0.0000
8034.2387 6748.5785 306.3585 -1285.6602 6810.7169
306.6365 -171.5579 -90.6858 680285.8938 -12.6924
-12.6698
[26869]ENERGY: 7150000 3299.7958 5512.9206 5747.2094
325.4218 -14275.9322 -1810.7782 0.0000 0.0000
7949.0644 6747.7015 303.1106 -1201.3629 6810.6748
306.4542 -39.5729 -6.4512 680285.8938 -14.7469
-14.7629
[26905]ENERGY: 7160000 3341.6634 5398.2694 5765.0304
319.3695 -14214.5464 -1867.6995 0.0000 0.0000
8004.5210 6746.6079 305.2253 -1257.9131 6810.2565
306.6359 -89.2134 -107.3654 680285.8938 -14.1917
-14.2057
[26955]ENERGY: 7170000 3271.2134 5424.4314 5706.1192
333.4151 -14272.1652 -1813.6347 0.0000 0.0000
8099.7069 6749.0861 308.8549 -1350.6208 6812.5565
306.7599 133.9277 145.0264 680285.8938 -15.2764
-15.2519
[26991]ENERGY: 7180000 3297.5463 5364.1800 5741.8957
329.0102 -14226.2609 -1840.6692 0.0000 0.0000
8079.7288 6745.4311 308.0931 -1334.2978 6813.2767

306.7683 -127.6270 -159.8208 680285.8938 -11.5803
-11.5993
[27041]ENERGY: 7190000 3344.3022 5485.7679 5739.5118
344.2024 -14347.1921 -1780.7759 0.0000 0.0000
7964.9635 6750.7798 303.7169 -1214.1837 6814.2299
306.8897 0.1487 4.6604 680285.8938 -10.3843
-10.3668
[27080]ENERGY: 7200000 3325.7978 5452.7228 5727.5864
313.3046 -14282.5194 -1883.5305 0.0000 0.0000
8097.7753 6751.1371 308.7812 -1346.6382 6814.9626
306.7406 -168.3558 -145.1243 680285.8938 -13.8582
-13.8493
[27130]ENERGY: 7210000 3326.5780 5407.8941 5682.3320
346.2214 -14326.6409 -1719.1714 0.0000 0.0000
8035.0326 6752.2458 306.3887 -1282.7868 6814.3794
306.4468 -52.3633 -14.0067 680285.8938 -14.0234
-14.0457
[27166]ENERGY: 7220000 3180.6002 5420.4452 5781.1377
319.5536 -14378.9868 -1742.0152 0.0000 0.0000
8164.2857 6745.0204 311.3174 -1419.2653 6814.5316
306.4666 160.3046 123.1425 680285.8938 -11.4451
-11.4497
[27216]ENERGY: 7230000 3317.2990 5351.1097 5751.9326
319.3777 -14242.9597 -1855.5472 0.0000 0.0000
8104.5962 6745.8084 309.0413 -1358.7878 6814.8525
306.3528 -95.6815 -57.8383 680285.8938 -14.0574
-14.0380
[27252]ENERGY: 7240000 3367.6036 5470.8256 5692.5472
314.1425 -14286.3548 -1791.4752 0.0000 0.0000
7985.3309 6752.6197 304.4935 -1232.7112 6815.4041
306.2682 -18.8449 -90.5928 680285.8938 -16.8886
-16.8833
[27302]ENERGY: 7250000 3300.0781 5433.7772 5759.6268
362.9617 -14330.9082 -1851.8698 0.0000 0.0000
8076.6350 6750.3008 307.9751 -1326.3343 6816.6670
306.5031 49.4119 -41.4672 680285.8938 -12.6560
-12.6644
[27338]ENERGY: 7260000 3343.2740 5329.8666 5738.4759
325.2491 -14253.7386 -1804.9709 0.0000 0.0000
8072.7839 6750.9400 307.8283 -1321.8439 6815.1644
306.5621 45.6226 -0.6019 680285.8938 -8.5305
-8.5464
[27388]ENERGY: 7270000 3333.5059 5451.7395 5755.6063
339.5042 -14295.6425 -1791.8701 0.0000 0.0000
7961.1834 6754.0268 303.5728 -1207.1566 6815.1173
306.4128 -45.0403 -88.9045 680285.8938 -14.4413
-14.4357
[27424]ENERGY: 7280000 3195.1032 5467.1117 5723.3713
312.9817 -14298.9837 -1788.8559 0.0000 0.0000
8138.6905 6749.4187 310.3414 -1389.2718 6814.9508
306.3217 97.0800 61.5473 680285.8938 -10.8831
-10.8779
[27474]ENERGY: 7290000 3293.5719 5399.4663 5716.0144
328.7180 -14182.8390 -1800.6190 0.0000 0.0000

7999.3792 6753.6918 305.0292 -1245.6874 6815.6382
306.3433 155.5452 59.8870 680285.8938 -15.3492
-15.3548
[27513]ENERGY: 7300000 3329.5052 5382.3360 5740.1213
329.7795 -14269.0855 -1821.6617 0.0000 0.0000
8058.8171 6749.8119 307.2957 -1309.0052 6814.7631
306.3675 -45.2909 -123.8975 680285.8938 -11.8919
-11.9013
[27563]ENERGY: 7310000 3312.6026 5435.9912 5719.2882
349.8391 -14233.5148 -1784.2520 0.0000 0.0000
7953.7096 6753.6639 303.2878 -1200.0457 6816.0040
306.2945 102.2908 1.9683 680285.8938 -13.4001
-13.3940
[27599]ENERGY: 7320000 3370.9375 5417.3136 5710.4399
331.6773 -14337.8319 -1767.3134 0.0000 0.0000
8025.0369 6750.2600 306.0076 -1274.7770 6816.3827
306.4553 -115.6774 -128.6107 680285.8938 -14.3026
-14.2852
[27649]ENERGY: 7330000 3362.0836 5426.6839 5734.4636
355.2635 -14279.9493 -1814.3782 0.0000 0.0000
7967.7132 6751.8804 303.8218 -1215.8328 6815.8567
306.3644 39.2197 80.1188 680285.8938 -14.0267
-14.0452
[27685]ENERGY: 7340000 3309.8367 5478.6352 5698.3649
334.9942 -14251.8828 -1803.5731 0.0000 0.0000
7983.4377 6749.8127 304.4214 -1233.6250 6816.1442
306.3527 7.7862 -6.9533 680285.8938 -17.4311
-17.4124
[27735]ENERGY: 7350000 3218.6185 5441.5595 5780.0480
319.0755 -14171.8000 -1912.4970 0.0000 0.0000
8077.1840 6752.1884 307.9960 -1324.9955 6819.1365
306.3775 107.7183 -29.6918 680285.8938 -16.1390
-16.1305
[27771]ENERGY: 7360000 3293.9479 5511.6871 5722.8282
362.0144 -14230.5496 -1869.5511 0.0000 0.0000
7965.9110 6756.2879 303.7530 -1209.6232 6819.0365
306.6070 -61.7826 -31.6652 680285.8938 -11.6158
-11.6325
[27821]ENERGY: 7370000 3366.1538 5491.6430 5729.8516
317.7452 -14274.9461 -1861.3777 0.0000 0.0000
7993.0922 6762.1621 304.7895 -1230.9301 6820.9172
306.6569 144.7160 -36.5798 680285.8938 -15.6002
-15.6006
[27857]ENERGY: 7380000 3211.3872 5411.8671 5699.0078
326.0087 -14203.0232 -1840.0011 0.0000 0.0000
8145.4147 6750.6611 310.5978 -1394.7535 6819.4648
306.7225 -69.1011 -99.0307 680285.8938 -15.7779
-15.7870
[27907]ENERGY: 7390000 3255.3517 5494.7492 5716.0270
336.5479 -14279.6567 -1828.3979 0.0000 0.0000
8060.0810 6754.7021 307.3439 -1305.3788 6821.1406
306.6476 134.0252 36.2204 680285.8938 -17.0712
-17.0537
[27946]ENERGY: 7400000 3308.1911 5407.6777 5692.5489

321.7093 -14257.7509 -1846.6640 0.0000 0.0000
8123.6041 6749.3162 309.7661 -1374.2879 6819.3138
306.7426 -59.1927 -77.2474 680285.8938 -16.1414
-16.1629
[27996]ENERGY: 7410000 3268.9313 5452.7624 5784.5185
369.5692 -14263.7018 -1817.4169 0.0000 0.0000
7958.3243 6752.9871 303.4637 -1205.3372 6821.1345
306.5861 34.1079 -50.0893 680285.8938 -18.7536
-18.7541
[28032]ENERGY: 7420000 3339.6108 5468.8527 5750.9368
350.8949 -14284.2890 -1833.0597 0.0000 0.0000
7967.0172 6759.9637 303.7952 -1207.0535 6820.2952
306.5503 -36.4561 -23.6300 680285.8938 -15.0673
-15.0556
[28082]ENERGY: 7430000 3312.8281 5404.8931 5745.7762
343.3649 -14331.6613 -1802.1294 0.0000 0.0000
8080.5961 6753.6676 308.1262 -1326.9285 6821.3335
306.7231 -63.8035 -69.7142 680285.8938 -17.5508
-17.5646
[28118]ENERGY: 7440000 3349.1445 5440.1830 5707.2766
337.6131 -14319.4500 -1852.7826 0.0000 0.0000
8091.0603 6753.0450 308.5252 -1338.0153 6821.3642
306.7719 -130.7410 -161.4301 680285.8938 -17.1938
-17.1855
[28168]ENERGY: 7450000 3304.5967 5466.2811 5748.7484
332.1467 -14281.4243 -1825.1011 0.0000 0.0000
8012.9081 6758.1556 305.5451 -1254.7525 6821.5152
306.5943 63.7865 63.8824 680285.8938 -17.1140
-17.1023
[28204]ENERGY: 7460000 3268.5148 5456.3442 5755.7869
311.4704 -14367.6718 -1790.4625 0.0000 0.0000
8121.9822 6755.9643 309.7043 -1366.0179 6822.2874
306.5102 126.8599 52.1499 680285.8938 -18.6541
-18.6816
[28254]ENERGY: 7470000 3337.9994 5407.0950 5652.2877
336.7042 -14280.9827 -1798.1585 0.0000 0.0000
8098.3070 6753.2521 308.8015 -1345.0549 6820.1575
306.6317 96.2668 31.9037 680285.8938 -17.8453
-17.8225
[28290]ENERGY: 7480000 3322.5581 5379.2193 5748.0950
337.6223 -14307.7810 -1805.9635 0.0000 0.0000
8081.5927 6755.3428 308.1642 -1326.2499 6820.3092
306.6135 -109.9654 -114.0403 680285.8938 -16.7431
-16.7431
[28340]ENERGY: 7490000 3337.5393 5418.0061 5700.2389
333.5297 -14210.1902 -1804.2182 0.0000 0.0000
7983.0286 6757.9342 304.4058 -1225.0944 6821.9069
306.6359 48.7413 -33.7440 680285.8938 -14.0382
-14.0426
[28379]ENERGY: 7500000 3329.4951 5547.6197 5689.3792
364.1041 -14329.1801 -1867.5283 0.0000 0.0000
8026.2382 6760.1277 306.0534 -1266.1105 6823.0433
306.8490 -65.7460 -68.9375 680285.8938 -15.0805
-15.0906

[28429]ENERGY: 7510000   3342.9437   5478.0261   5711.4165
323.1322   -14345.5915   -1824.0998   0.0000   0.0000
8070.4129   6756.2401   307.7379   -1314.1728   6823.8582
306.7911   -13.4925   -7.0831   680285.8938   -13.4575
-13.4391
[28465]ENERGY: 7520000   3332.2289   5466.6409   5704.8895
343.8265   -14385.3889   -1779.5500   0.0000   0.0000
8076.9588   6759.6057   307.9875   -1317.3531   6823.9834
306.8430   21.2909   -56.3517   680285.8938   -13.1083
-13.1109
[28515]ENERGY: 7530000   3231.2550   5527.1407   5737.1673
352.1619   -14332.9568   -1829.5013   0.0000   0.0000
8074.7951   6760.0620   307.9050   -1314.7331   6824.3761
306.9349   157.6341   20.3887   680285.8938   -14.2152
-14.2364
[28551]ENERGY: 7540000   3272.4551   5499.8004   5731.4233
318.4598   -14404.9570   -1787.2173   0.0000   0.0000
8129.0082   6758.9724   309.9722   -1370.0357   6823.2504
306.7224   -21.2257   10.2770   680285.8938   -16.6464
-16.6172
[28601]ENERGY: 7550000   3306.5495   5493.8431   5744.5553
348.6764   -14359.9091   -1786.4127   0.0000   0.0000
8011.8300   6759.1325   305.5040   -1252.6975   6824.5133
306.9544   54.7753   -3.8521   680285.8938   -15.0865
-15.0888
[28637]ENERGY: 7560000   3336.9826   5439.3866   5750.8936
348.2870   -14338.6518   -1834.3505   0.0000   0.0000
8052.8081   6755.3556   307.0666   -1297.4526   6825.0798
306.9432   53.8801   -1.9892   680285.8938   -17.2046
-17.2171
[28687]ENERGY: 7570000   3304.2141   5525.1076   5754.6660
335.8867   -14302.8354   -1918.3881   0.0000   0.0000
8060.6531   6759.3041   307.3657   -1301.3491   6825.2027
306.8679   59.6576   -94.1058   680285.8938   -13.4230
-13.4199
[28723]ENERGY: 7580000   3278.4521   5478.0176   5785.5393
349.9280   -14273.0873   -1868.1743   0.0000   0.0000
8007.0321   6757.7075   305.3210   -1249.3246   6824.9338
306.9041   -22.9687   -85.3720   680285.8938   -11.1347
-11.1353
[28773]ENERGY: 7590000   3284.1868   5486.4598   5728.3649
341.8076   -14312.1726   -1871.2110   0.0000   0.0000
8105.9320   6763.3674   309.0923   -1342.5646   6824.9054
306.9407   104.9732   42.6784   680285.8938   -10.5138
-10.5112
[28812]ENERGY: 7600000   3317.6977   5412.4458   5795.8198
333.1492   -14289.2815   -1811.5213   0.0000   0.0000
8001.2570   6759.5667   305.1008   -1241.6903   6824.8436
307.0101   163.7439   87.4985   680285.8938   -12.1522
-12.1595
[28862]ENERGY: 7610000   3363.4786   5441.7513   5692.4578
344.8383   -14283.4139   -1893.9016   0.0000   0.0000
8093.8390   6759.0496   308.6311   -1334.7894   6824.4594
306.9562   -127.9908   -106.2521   680285.8938   -11.3917

-11.3883
[28898]ENERGY: 7620000 3340.7818 5401.2444 5713.5451
356.3794 -14388.9222 -1809.1846 0.0000 0.0000
8142.9431 6756.7870 310.5036 -1386.1561 6825.1428
307.0100 148.0706 81.0676 680285.8938 -12.5973
-12.5941
[28948]ENERGY: 7630000 3283.0193 5450.9710 5779.1266
350.2322 -14263.1628 -1814.0077 0.0000 0.0000
7975.5062 6761.6850 304.1189 -1213.8212 6823.3958
306.9665 23.1534 10.5073 680285.8938 -13.2970
-13.3003
[28984]ENERGY: 7640000 3296.2974 5522.6829 5721.0717
345.6755 -14322.6560 -1808.5477 0.0000 0.0000
8006.3759 6760.8996 305.2960 -1245.4762 6824.2698
306.9014 -85.7009 -30.2911 680285.8938 -10.2016
-10.1894
[29034]ENERGY: 7650000 3304.5394 5506.9507 5784.6675
320.7240 -14357.7486 -1802.5532 0.0000 0.0000
8003.5519 6760.1317 305.1883 -1243.4201 6823.9926
306.9834 230.5362 141.7772 680285.8938 -10.8513
-10.8845
[29070]ENERGY: 7660000 3296.0779 5457.9158 5727.5956
338.4888 -14322.2590 -1846.4471 0.0000 0.0000
8105.8043 6757.1762 309.0874 -1348.6281 6824.3197
306.9191 134.6523 70.8572 680285.8938 -9.7159
-9.7098
[29120]ENERGY: 7670000 3330.0906 5477.2644 5709.2718
335.3841 -14307.9893 -1849.7157 0.0000 0.0000
8066.0538 6760.3596 307.5716 -1305.6941 6824.5318
306.7470 -194.6418 -183.7791 680285.8938 -8.6281
-8.6061
[29156]ENERGY: 7680000 3382.2051 5468.9815 5684.0186
315.6617 -14277.7323 -1917.1120 0.0000 0.0000
8101.8395 6757.8623 308.9362 -1343.9772 6825.3584
306.9713 -105.1473 -83.9386 680285.8938 -6.5882
-6.6145
[29206]ENERGY: 7690000 3268.7604 5387.3703 5729.9753
335.4403 -14306.0312 -1772.1513 0.0000 0.0000
8115.8970 6759.2608 309.4722 -1356.6362 6824.5679
307.0246 -0.4882 -66.2259 680285.8938 -6.5655
-6.5560
[29245]ENERGY: 7700000 3264.9013 5382.5389 5740.3934
326.0152 -14210.1432 -1891.3008 0.0000 0.0000
8149.4878 6761.8927 310.7531 -1387.5952 6826.6030
306.9784 118.2441 -9.0096 680285.8938 -8.9076
-8.9066
[29295]ENERGY: 7710000 3381.5858 5409.4469 5719.8699
343.3480 -14374.2109 -1782.5843 0.0000 0.0000
8064.7339 6762.1893 307.5213 -1302.5445 6826.0524
306.9026 -163.6924 -75.9908 680285.8938 -9.0423
-9.0265
[29331]ENERGY: 7720000 3342.2407 5465.9102 5650.5194
350.0503 -14353.9728 -1787.5238 0.0000 0.0000
8096.8265 6764.0505 308.7451 -1332.7760 6826.7846

306.9486 100.5202 30.1739 680285.8938 -7.0489
-7.0652
[29381]ENERGY: 7730000 3397.3277 5431.4008 5705.7898
345.7569 -14334.4724 -1777.6113 0.0000 0.0000
7996.4636 6764.6552 304.9181 -1231.8085 6826.4542
306.9485 -27.6982 1.9364 680285.8938 -9.2745
-9.2598
[29417]ENERGY: 7740000 3284.4266 5472.2431 5814.0362
333.9631 -14409.1550 -1766.1396 0.0000 0.0000
8035.0100 6764.3844 306.3879 -1270.6256 6827.2632
306.9425 -8.9506 -20.6663 680285.8938 -9.1382
-9.1355
[29467]ENERGY: 7750000 3370.0687 5493.7100 5723.7258
335.6031 -14330.7422 -1807.3559 0.0000 0.0000
7978.1834 6763.1929 304.2210 -1214.9905 6828.5049
306.8958 -106.9312 -132.0125 680285.8938 -7.8373
-7.8499
[29503]ENERGY: 7760000 3338.1870 5408.1328 5678.5360
317.8816 -14214.6430 -1918.3631 0.0000 0.0000
8149.4785 6759.2098 310.7528 -1390.2687 6826.6529
306.8297 91.8379 130.1248 680285.8938 -7.4170
-7.4090
[29553]ENERGY: 7770000 3309.8641 5505.9143 5712.7152
330.6789 -14299.3717 -1826.0666 0.0000 0.0000
8032.0894 6765.8235 306.2765 -1266.2659 6828.4090
306.8404 -24.1969 -84.3996 680285.8938 -10.6631
-10.6525
[29589]ENERGY: 7780000 3318.3897 5520.9092 5725.2033
323.7118 -14249.6308 -1891.5230 0.0000 0.0000
8016.9088 6763.9688 305.6977 -1252.9400 6827.6493
306.8208 -40.4427 -6.8020 680285.8938 -6.4852
-6.4832
[29639]ENERGY: 7790000 3275.9851 5533.7584 5688.6220
324.2691 -14356.5892 -1804.8640 0.0000 0.0000
8098.8666 6760.0480 308.8228 -1338.8186 6826.8292
307.1627 4.9618 -37.6831 680285.8938 -7.0560
-7.0777
[29678]ENERGY: 7800000 3249.0059 5420.7227 5716.9366
340.2778 -14277.9351 -1784.0365 0.0000 0.0000
8096.5515 6761.5229 308.7346 -1335.0286 6826.9463
307.1216 -25.4266 -20.5253 680285.8938 -9.3314
-9.3321
[29728]ENERGY: 7810000 3280.3336 5494.2886 5699.5232
330.8385 -14278.4750 -1861.3775 0.0000 0.0000
8094.6671 6759.7986 308.6627 -1334.8685 6827.1658
307.1151 164.5258 80.2986 680285.8938 -7.7200
-7.7316
[29764]ENERGY: 7820000 3395.3918 5509.7196 5734.1928
315.2529 -14371.7272 -1829.1261 0.0000 0.0000
8010.9007 6764.6046 305.4686 -1246.2962 6828.1134
307.2333 -30.6858 11.8099 680285.8938 -12.7033
-12.6759
[29814]ENERGY: 7830000 3228.6912 5481.6964 5679.5011
344.3013 -14209.9941 -1772.6044 0.0000 0.0000

8015.9441 6767.5355 305.6609 -1248.4086 6829.0285
307.1872 87.0664 109.7657 680285.8938 -10.0118
-10.0107
[29850]ENERGY: 7840000 3282.2503 5471.6422 5680.3968
317.6238 -14361.5881 -1776.7786 0.0000 0.0000
8149.8413 6763.3878 310.7666 -1386.4535 6829.1661
307.1910 0.9342 -1.7573 680285.8938 -6.2171
-6.2111
[29900]ENERGY: 7850000 3277.7706 5540.6562 5720.0526
344.5236 -14360.2509 -1853.8055 0.0000 0.0000
8095.2647 6764.2113 308.6855 -1331.0534 6829.5078
307.2500 -16.3658 -64.6482 680285.8938 -7.9477
-7.9646
[29936]ENERGY: 7860000 3295.9558 5436.4394 5743.1468
340.2479 -14318.2074 -1814.0265 0.0000 0.0000
8078.7218 6762.2778 308.0547 -1316.4440 6828.9477
307.1336 -73.6972 -75.0002 680285.8938 -6.1155
-6.1153
[29986]ENERGY: 7870000 3232.9638 5466.5025 5741.8438
318.3471 -14265.7458 -1841.7316 0.0000 0.0000
8110.9628 6763.1427 309.2841 -1347.8201 6828.5373
306.9322 25.3410 10.2685 680285.8938 -10.3997
-10.3916
[30022]ENERGY: 7880000 3282.3380 5481.7410 5679.5464
339.9078 -14349.1935 -1786.4874 0.0000 0.0000
8114.4349 6762.2872 309.4165 -1352.1477 6828.4103
306.9176 162.9172 162.9826 680285.8938 -5.6709
-5.7019
[30072]ENERGY: 7890000 3267.7756 5489.0701 5739.5300
353.6272 -14251.0927 -1830.3693 0.0000 0.0000
7996.1098 6764.6509 304.9046 -1231.4589 6828.6956
306.8574 -32.3209 -86.5848 680285.8938 -8.8398
-8.8246
[30111]ENERGY: 7900000 3360.1869 5546.1227 5770.8334
337.4287 -14418.8694 -1854.2285 0.0000 0.0000
8021.5839 6763.0576 305.8759 -1258.5263 6828.4410
307.1254 61.9838 23.2253 680285.8938 -9.8624
-9.8493
[30161]ENERGY: 7910000 3188.2460 5592.3470 5693.6789
349.8106 -14354.2451 -1794.3574 0.0000 0.0000
8086.6236 6762.1037 308.3560 -1324.5199 6829.3116
307.0902 48.6822 -39.4074 680285.8938 -4.9844
-4.9953
[30197]ENERGY: 7920000 3324.5127 5421.0769 5753.1488
344.6811 -14316.0859 -1810.9694 0.0000 0.0000
8048.6607 6765.0249 306.9084 -1283.6358 6829.3736
307.1318 116.7663 14.5901 680285.8938 -10.4001
-10.3922
[30247]ENERGY: 7930000 3290.3328 5429.0858 5744.6888
354.4470 -14238.1532 -1876.4468 0.0000 0.0000
8058.1902 6762.1445 307.2718 -1296.0456 6828.6600
307.1921 165.7077 125.5704 680285.8938 -7.4810
-7.4987
[30283]ENERGY: 7940000 3288.7784 5548.0506 5753.1218

330.3445 -14420.8611 -1807.4010 0.0000 0.0000
8070.4728 6762.5061 307.7401 -1307.9667 6829.8857
307.0922 61.1721 -9.9176 680285.8938 -8.0195
-8.0034
[30333]ENERGY: 7950000 3276.8371 5467.0068 5756.7923
373.3206 -14335.7145 -1801.6759 0.0000 0.0000
8026.4913 6763.0576 306.0631 -1263.4337 6829.1906
307.1021 64.2060 68.5898 680285.8938 -9.1470
-9.1448
[30369]ENERGY: 7960000 3327.4571 5506.6209 5711.8277
327.7819 -14382.9749 -1779.7759 0.0000 0.0000
8053.6591 6764.5959 307.0990 -1289.0632 6828.9358
306.9466 73.7431 -19.5578 680285.8938 -11.0493
-11.0512
[30419]ENERGY: 7970000 3272.3082 5451.0257 5701.7237
344.8977 -14206.1728 -1830.0034 0.0000 0.0000
8035.9667 6769.7460 306.4244 -1266.2208 6830.6381
307.0046 2.6011 0.0950 680285.8938 -9.2800
-9.2702
[30455]ENERGY: 7980000 3314.5809 5549.3142 5733.4628
342.5898 -14424.6083 -1802.1081 0.0000 0.0000
8050.0147 6763.2460 306.9600 -1286.7686 6828.4670
307.0618 -153.0862 -31.2780 680285.8938 -8.1167
-8.1236
[30505]ENERGY: 7990000 3326.7745 5482.0726 5699.9983
347.7946 -14349.3375 -1786.8459 0.0000 0.0000
8046.4575 6766.9141 306.8244 -1279.5434 6829.6458
306.9480 134.7446 68.3197 680285.8938 -8.5374
-8.5298
[30544]ENERGY: 8000000 3309.7223 5500.2943 5741.9187
351.1498 -14288.7508 -1826.3900 0.0000 0.0000
7982.9561 6770.9002 304.4030 -1212.0558 6830.7017
307.1641 -93.9191 -87.5102 680285.8938 -11.2149
-11.2318
[30594]ENERGY: 8010000 3326.6048 5583.1567 5713.2929
347.5697 -14345.2571 -1869.7449 0.0000 0.0000
8013.8801 6769.5022 305.5822 -1244.3779 6831.7987
307.0814 -47.1916 -56.0510 680285.8938 -6.8114
-6.8015
[30630]ENERGY: 8020000 3338.1087 5444.1586 5700.7144
352.5358 -14251.2424 -1828.3735 0.0000 0.0000
8010.8125 6766.7141 305.4652 -1244.0984 6831.1945
307.0402 -14.5293 -38.4035 680285.8938 -7.3499
-7.3617
[30680]ENERGY: 8030000 3289.5852 5416.1248 5776.1436
349.7058 -14318.1104 -1816.1588 0.0000 0.0000
8066.3637 6763.6539 307.5835 -1302.7099 6831.1691
306.8423 -30.9132 -40.0318 680285.8938 -8.9596
-8.9563
[30716]ENERGY: 8040000 3252.6704 5474.4981 5672.1838
315.7470 -14179.2503 -1850.5296 0.0000 0.0000
8081.7427 6767.0622 308.1699 -1314.6805 6831.9582
306.9028 111.8597 86.4382 680285.8938 -4.1431
-4.1438

[30766]ENERGY: 8050000 3318.9774 5542.5147 5749.8390
342.2524 -14330.9234 -1808.5260 0.0000 0.0000
7956.1447 6770.2787 303.3806 -1185.8659 6832.3646
307.0627 74.7381 15.5756 680285.8938 -3.9091
-3.9082
[30802]ENERGY: 8060000 3288.0852 5462.4755 5723.1050
338.0175 -14292.9430 -1795.6852 0.0000 0.0000
8046.1960 6769.2509 306.8144 -1276.9450 6832.0608
307.1840 95.2864 6.8883 680285.8938 -9.2159
-9.2269
[30852]ENERGY: 8070000 3311.0739 5588.7922 5705.4997
319.6226 -14324.0445 -1780.9006 0.0000 0.0000
7953.3780 6773.4214 303.2751 -1179.9566 6834.0378
307.0038 108.5060 26.9614 680285.8938 -5.9910
-5.9792
[30888]ENERGY: 8080000 3349.5348 5442.1083 5696.6077
326.5928 -14335.6329 -1752.6717 0.0000 0.0000
8042.1046 6768.6435 306.6584 -1273.4610 6834.3658
307.0794 -131.1316 -74.4564 680285.8938 -7.6422
-7.6287
[30938]ENERGY: 8090000 3356.3246 5526.8156 5751.1739
341.3276 -14310.5272 -1820.4624 0.0000 0.0000
7928.5859 6773.2379 302.3298 -1155.3479 6833.3097
307.0473 70.7755 46.7511 680285.8938 -4.0297
-4.0452
[30977]ENERGY: 8100000 3283.2026 5480.7282 5717.1544
331.6234 -14273.7958 -1838.4572 0.0000 0.0000
8071.4909 6771.9465 307.7790 -1299.5445 6833.9017
307.1637 45.4158 -11.3434 680285.8938 -8.0339
-8.0208
[31027]ENERGY: 8110000 3350.7437 5559.1434 5723.6966
355.6527 -14324.0348 -1852.8169 0.0000 0.0000
7956.4581 6768.8429 303.3926 -1187.6152 6833.1223
307.1593 -48.4605 -98.1595 680285.8938 -10.7592
-10.7760
[31063]ENERGY: 8120000 3282.5484 5455.8298 5703.2814
344.3934 -14303.9250 -1801.8569 0.0000 0.0000
8088.2028 6768.4740 308.4162 -1319.7288 6833.3619
307.1716 96.2225 94.7634 680285.8938 -9.4780
-9.4737
[31113]ENERGY: 8130000 3361.8152 5412.8773 5728.8422
332.7716 -14319.2766 -1831.5770 0.0000 0.0000
8082.1103 6767.5630 308.1839 -1314.5473 6834.0172
307.2662 52.7015 -17.4447 680285.8938 -5.3236
-5.3317
[31149]ENERGY: 8140000 3305.7711 5490.8508 5719.2753
331.0258 -14234.6028 -1901.5055 0.0000 0.0000
8055.9700 6766.7847 307.1871 -1289.1852 6833.4387
307.1463 -22.3558 8.7783 680285.8938 -15.2464
-15.2265
[31199]ENERGY: 8150000 3236.4968 5479.6093 5754.6799
356.9262 -14315.2859 -1839.9214 0.0000 0.0000
8095.7695 6768.2744 308.7047 -1327.4950 6833.5156
307.4613 174.6824 96.5661 680285.8938 -4.2344

-4.2465
[31235]ENERGY: 8160000 3336.5582 5502.5507 5703.0151
336.9666 -14324.8708 -1856.3801 0.0000 0.0000
8072.6985 6770.5383 307.8250 -1302.1602 6834.7954
307.3512 92.2552 8.4917 680285.8938 -11.5997
-11.6056
[31285]ENERGY: 8170000 3259.4188 5508.3603 5686.0672
325.7576 -14245.8212 -1832.4071 0.0000 0.0000
8068.5845 6769.9600 307.6681 -1298.6245 6834.2951
307.1818 114.4949 -11.8562 680285.8938 -8.3441
-8.3522
[31321]ENERGY: 8180000 3313.8906 5446.7866 5698.7256
320.2638 -14322.8846 -1783.6299 0.0000 0.0000
8094.4493 6767.6015 308.6544 -1326.8478 6833.4746
307.0191 -67.8386 16.8556 680285.8938 -6.3513
-6.3272
[31371]ENERGY: 8190000 3346.1176 5442.8038 5726.7776
332.3534 -14353.0187 -1803.2098 0.0000 0.0000
8074.8574 6766.6813 307.9073 -1308.1761 6833.4700
307.1029 9.9052 53.6806 680285.8938 -1.9564
-1.9500
[31410]ENERGY: 8200000 3273.7862 5506.1250 5746.9361
336.6045 -14368.6800 -1765.3549 0.0000 0.0000
8035.5702 6764.9870 306.4092 -1270.5831 6833.7495
307.1125 104.5588 65.9484 680285.8938 -4.2068
-4.2070
[31460]ENERGY: 8210000 3296.9704 5466.8293 5719.7888
296.7944 -14243.7730 -1812.2990 0.0000 0.0000
8044.9364 6769.2474 306.7664 -1275.6890 6833.7634
307.2474 25.0896 22.5689 680285.8938 -6.6634
-6.6824
[31496]ENERGY: 8220000 3334.6435 5534.1044 5665.4838
323.0738 -14277.9147 -1898.4730 0.0000 0.0000
8088.5579 6769.4757 308.4298 -1319.0822 6834.9173
307.2878 -58.8320 -103.0578 680285.8938 -1.0789
-1.0679
[31546]ENERGY: 8230000 3311.7839 5498.9143 5708.7567
331.0996 -14279.8355 -1835.1786 0.0000 0.0000
8035.8927 6771.4330 306.4215 -1264.4596 6834.1281
307.2200 53.2430 50.6183 680285.8938 -9.1911
-9.2008
[31582]ENERGY: 8240000 3293.5323 5486.7402 5688.9947
346.7526 -14210.3630 -1882.8191 0.0000 0.0000
8043.5683 6766.4059 306.7142 -1277.1624 6834.0717
307.2540 36.1013 -0.0780 680285.8938 -3.3353
-3.3309
[31632]ENERGY: 8250000 3358.4498 5439.3833 5742.8251
343.4353 -14350.2193 -1853.6564 0.0000 0.0000
8086.9649 6767.1828 308.3690 -1319.7822 6835.1497
307.2756 44.5826 -15.6991 680285.8938 -3.6517
-3.6397
[31668]ENERGY: 8260000 3218.6829 5512.2320 5708.2240
318.7156 -14267.8751 -1789.0680 0.0000 0.0000
8067.2814 6768.1927 307.6184 -1299.0887 6835.2886

307.3988 201.0909 95.0420 680285.8938 -1.5382
-1.5619
[31718]ENERGY: 8270000 3294.4777 5500.8126 5694.8654
349.1719 -14273.9284 -1824.2254 0.0000 0.0000
8031.6058 6772.7797 306.2581 -1258.8262 6836.8919
307.4477 60.0113 -30.2153 680285.8938 -4.6216
-4.6112
[31754]ENERGY: 8280000 3263.6582 5393.3773 5765.4929
345.6829 -14331.1360 -1784.5121 0.0000 0.0000
8116.7533 6769.3164 309.5049 -1347.4368 6836.3064
307.4737 -139.2169 -105.8512 680285.8938 -2.4668
-2.4631
[31804]ENERGY: 8290000 3286.9336 5485.0673 5729.8918
336.6414 -14299.9935 -1864.6333 0.0000 0.0000
8097.6247 6771.5321 308.7755 -1326.0926 6835.5656
307.5969 26.7085 18.5244 680285.8938 -6.8582
-6.8684
[31843]ENERGY: 8300000 3330.1248 5559.5365 5695.3434
324.1182 -14340.1197 -1830.4104 0.0000 0.0000
8037.1191 6775.7119 306.4683 -1261.4072 6836.4166
307.6715 87.1843 95.1737 680285.8938 -4.4406
-4.4335
[31893]ENERGY: 8310000 3327.4123 5340.7374 5744.4766
347.6810 -14346.3244 -1763.8261 0.0000 0.0000
8118.8630 6769.0198 309.5853 -1349.8432 6835.2655
307.5547 -189.8755 -64.2445 680285.8938 -8.2953
-8.2687
[31929]ENERGY: 8320000 3288.3203 5451.6755 5684.2094
302.1985 -14240.5748 -1805.1926 0.0000 0.0000
8089.1516 6769.7879 308.4524 -1319.3637 6835.8653
307.2985 -2.8502 -22.7005 680285.8938 -6.7173
-6.7320
[31979]ENERGY: 8330000 3284.5680 5503.7798 5739.7941
347.9811 -14235.2879 -1861.1112 0.0000 0.0000
7992.4351 6772.1592 304.7644 -1220.2759 6837.6033
307.5223 -12.1727 -53.5466 680285.8938 -2.7853
-2.7951
[32015]ENERGY: 8340000 3318.0216 5411.7422 5771.7311
334.6831 -14303.5117 -1837.3978 0.0000 0.0000
8074.1210 6769.3895 307.8793 -1304.7315 6835.8866
307.5782 -107.2918 -86.6953 680285.8938 -2.5452
-2.5427
[32065]ENERGY: 8350000 3363.5729 5379.6408 5748.7521
335.4642 -14275.8307 -1832.5332 0.0000 0.0000
8047.5113 6766.5775 306.8646 -1280.9339 6834.7070
307.5647 92.8605 41.3744 680285.8938 -6.1953
-6.1912
[32101]ENERGY: 8360000 3369.4048 5331.4269 5754.8154
365.7389 -14266.8496 -1781.0599 0.0000 0.0000
7997.0608 6770.5373 304.9408 -1226.5235 6836.2512
307.4059 41.9411 18.9493 680285.8938 -3.8522
-3.8492
[32151]ENERGY: 8370000 3299.9520 5446.9302 5736.4914
346.3750 -14355.6231 -1790.6735 0.0000 0.0000

8089.9103 6773.3624 308.4813 -1316.5479 6836.8625
307.5200 75.8847 14.6398 680285.8938 -1.1319
-1.1469
[32187]ENERGY: 8380000 3277.3903 5485.6115 5725.0485
320.4559 -14290.2298 -1788.2868 0.0000 0.0000
8043.5126 6773.5022 306.7121 -1270.0105 6836.7986
307.5938 189.8673 107.4879 680285.8938 -6.0964
-6.0951
[32237]ENERGY: 8390000 3325.2268 5589.6035 5737.8195
330.7746 -14397.8059 -1895.1734 0.0000 0.0000
8080.2835 6770.7285 308.1142 -1309.5550 6836.5419
307.4627 -154.0945 -160.1804 680285.8938 -3.3810
-3.3763
[32276]ENERGY: 8400000 3286.8358 5398.6850 5759.5177
323.0456 -14311.4856 -1828.9629 0.0000 0.0000
8145.3648 6773.0005 310.5959 -1372.3643 6837.2347
307.5159 -51.5254 -121.7341 680285.8938 -8.6031
-8.5903
[32326]ENERGY: 8410000 3351.3540 5519.6044 5741.2544
348.7909 -14277.3631 -1882.5216 0.0000 0.0000
7968.5220 6769.6410 303.8526 -1198.8810 6837.5205
307.7216 -44.0656 -40.0214 680285.8938 -2.1770
-2.1767
[32362]ENERGY: 8420000 3356.5299 5438.8530 5702.6648
347.8829 -14311.4739 -1829.4983 0.0000 0.0000
8065.5175 6770.4760 307.5512 -1295.0415 6837.0631
307.6027 19.4727 -20.7590 680285.8938 -8.0152
-8.0345
[32412]ENERGY: 8430000 3367.0725 5478.1957 5687.5357
333.0979 -14305.4159 -1800.2568 0.0000 0.0000
8017.6451 6777.8741 305.7257 -1239.7710 6837.7112
307.8096 -51.7018 -8.6326 680285.8938 -3.4882
-3.4919
[32448]ENERGY: 8440000 3324.7940 5461.1332 5750.1988
345.3414 -14286.3569 -1772.9354 0.0000 0.0000
7951.9022 6774.0775 303.2189 -1177.8247 6838.1800
307.7697 -71.4729 -58.6820 680285.8938 -7.6810
-7.6771
[32498]ENERGY: 8450000 3293.0235 5450.1513 5748.9789
356.1730 -14298.7906 -1800.1705 0.0000 0.0000
8021.1194 6770.4849 305.8582 -1250.6345 6836.8917
307.6113 50.2248 60.1118 680285.8938 -2.8652
-2.8545
[32534]ENERGY: 8460000 3257.6491 5433.9583 5719.8562
313.6525 -14232.2535 -1836.8326 0.0000 0.0000
8118.4429 6774.4729 309.5693 -1343.9700 6836.8046
307.7240 87.1883 16.1994 680285.8938 -6.1921
-6.2027
[32584]ENERGY: 8470000 3322.1865 5559.2431 5690.5310
342.7262 -14362.4995 -1852.1361 0.0000 0.0000
8073.7247 6773.7758 307.8641 -1299.9489 6836.4150
307.6320 61.0348 97.6989 680285.8938 -7.5621
-7.5570
[32620]ENERGY: 8480000 3301.9719 5393.0197 5740.3284

```
339.0631          -14229.7494     -1865.7417         0.0000         0.0000
8093.6100           6772.5020      308.6224     -1321.1080      6838.2843
307.6432             -34.2775      -20.9824    680285.8938        -8.2308
-8.2199
[32670]ENERGY: 8490000      3313.7382      5480.8662      5746.0501
327.6867          -14383.5084     -1785.4619         0.0000         0.0000
8072.2450           6771.6159      307.8077     -1300.6291      6837.8993
307.6146              99.7205       37.6332    680285.8938        -6.8680
-6.8702
[32709]ENERGY: 8500000      3309.6744      5508.6268      5741.6524
350.7616          -14376.0014     -1769.5996         0.0000         0.0000
8011.5589           6776.6732      305.4937     -1234.8857      6839.1727
307.6941             133.3582       67.1730    680285.8938        -6.3579
-6.3726
[32759]ENERGY: 8510000      3277.9956      5523.7645      5734.5947
318.5989          -14296.6804     -1844.0053         0.0000         0.0000
8061.6870           6775.9550      307.4051     -1285.7320      6839.2767
307.8459              28.0436       51.6543    680285.8938        -9.6455
-9.6377
[32795]ENERGY: 8520000      3266.8926      5430.5641      5749.4546
352.0250          -14260.8979     -1839.4738         0.0000         0.0000
8074.9128           6773.4773      307.9094     -1301.4355      6839.6868
307.9285              42.2992      -14.0623    680285.8938        -6.6450
-6.6530
[32845]ENERGY: 8530000      3343.9990      5536.3351      5721.2584
342.4928          -14320.0078     -1869.6781         0.0000         0.0000
8018.9194           6773.3189      305.7743     -1245.6005      6839.9382
307.8499              69.1205       40.0665    680285.8938        -6.9152
-6.9141
[32881]ENERGY: 8540000      3295.4433      5535.2985      5717.7837
337.3369          -14277.3311     -1888.0475         0.0000         0.0000
8052.4886           6772.9725      307.0544     -1279.5162      6839.6536
307.7699              81.1643       17.3851    680285.8938        -7.5582
-7.5632
[32931]ENERGY: 8550000      3360.1915      5438.0540      5666.5608
331.6747          -14270.3350     -1844.4678         0.0000         0.0000
8094.9960           6776.6741      308.6753     -1318.3219      6839.7074
307.7676              35.9211       24.2182    680285.8938        -6.9631
-6.9640
[32967]ENERGY: 8560000      3294.3923      5469.7538      5712.7469
354.2695          -14277.5963     -1885.0393         0.0000         0.0000
8104.7439           6773.2708      309.0470     -1331.4731      6839.7971
307.8769             -86.5170      -78.0294    680285.8938        -9.0776
-9.0548
[33017]ENERGY: 8570000      3289.8320      5485.7367      5718.7402
360.3640          -14280.2709     -1859.2718         0.0000         0.0000
8060.5658           6775.6961      307.3624     -1284.8698      6840.2965
307.8156              75.3665       14.5746    680285.8938        -9.6277
-9.6452
[33053]ENERGY: 8580000      3379.8472      5476.9148      5716.1409
327.3033          -14404.4088     -1820.3136         0.0000         0.0000
8097.0494           6772.5332      308.7535     -1324.5162      6840.8679
307.7940             -42.2331      -53.6471    680285.8938        -6.4406
-6.4266
```

[33103]ENERGY: 8590000 3372.2209 5420.2764 5726.3714
341.4297 -14240.0846 -1885.9377 0.0000 0.0000
8043.3256 6777.6018 306.7050 -1265.7238 6840.7348
307.8416 -37.7752 -56.7267 680285.8938 -7.0020
-7.0237
[33142]ENERGY: 8600000 3280.8919 5483.1084 5734.1192
336.3029 -14242.7389 -1844.5935 0.0000 0.0000
8030.3381 6777.4281 306.2097 -1252.9100 6841.3198
307.7486 -160.7502 -91.1631 680285.8938 -3.8564
-3.8383
[33192]ENERGY: 8610000 3274.8147 5521.3735 5719.1690
351.2894 -14226.3089 -1889.1415 0.0000 0.0000
8022.1679 6773.3642 305.8982 -1248.8038 6841.4936
307.6662 -34.3739 -38.1281 680285.8938 -6.7821
-6.7738
[33228]ENERGY: 8620000 3365.8034 5425.1840 5715.7058
318.9042 -14260.1580 -1863.4734 0.0000 0.0000
8073.1796 6775.1456 307.8434 -1298.0340 6841.6150
307.8005 55.0094 9.6091 680285.8938 -6.1705
-6.1785
[33278]ENERGY: 8630000 3336.2314 5473.6213 5688.7357
332.5310 -14386.6496 -1740.2810 0.0000 0.0000
8069.8946 6774.0834 307.7181 -1295.8112 6839.6201
307.8032 165.8162 130.2257 680285.8938 -6.7911
-6.7903
[33314]ENERGY: 8640000 3345.8959 5493.9130 5691.1503
321.5629 -14297.0015 -1919.5011 0.0000 0.0000
8138.4820 6774.5014 310.3334 -1363.9805 6841.6745
307.7413 -49.9216 -10.1689 680285.8938 -6.1733
-6.1812
[33364]ENERGY: 8650000 3293.0170 5410.6351 5734.2144
331.4552 -14344.4565 -1828.1538 0.0000 0.0000
8176.6268 6773.3382 311.7880 -1403.2887 6841.3474
307.6857 162.1697 72.4745 680285.8938 -2.1617
-2.1599
[33400]ENERGY: 8660000 3320.7787 5395.2395 5737.1800
340.1815 -14314.0513 -1844.6901 0.0000 0.0000
8140.6602 6775.2986 310.4165 -1365.3616 6841.3815
307.7360 165.1877 76.2172 680285.8938 -12.5811
-12.5767
[33450]ENERGY: 8670000 3319.7024 5405.3458 5764.0383
349.3334 -14264.0966 -1816.9032 0.0000 0.0000
8020.4319 6777.8519 305.8320 -1242.5799 6841.5590
307.8106 56.5666 30.6923 680285.8938 -6.2523
-6.2427
[33486]ENERGY: 8680000 3401.4327 5441.1331 5760.8887
330.6721 -14378.3112 -1821.2832 0.0000 0.0000
8039.5325 6774.0646 306.5603 -1265.4678 6841.3726
307.6923 -75.7233 -17.8771 680285.8938 -8.8884
-8.9104
[33536]ENERGY: 8690000 3369.5909 5416.4442 5728.5711
349.6961 -14253.4271 -1789.5678 0.0000 0.0000
7954.3072 6775.6145 303.3106 -1178.6926 6840.5388
307.7038 22.7069 13.2839 680285.8938 -7.6956

-7.6912
[33575]ENERGY: 8700000 3348.4265 5576.1572 5707.2224
327.6072 -14341.6413 -1852.4149 0.0000 0.0000
8012.4220 6777.7789 305.5266 -1234.6431 6839.8173
307.5876 -198.1858 -33.1887 680285.8938 -6.3915
-6.3658
[33625]ENERGY: 8710000 3281.9894 5456.4925 5706.1502
337.8530 -14283.9233 -1854.1562 0.0000 0.0000
8129.0265 6773.4321 309.9729 -1355.5944 6841.3942
307.7673 -117.2230 -55.7004 680285.8938 -9.3822
-9.3874
[33661]ENERGY: 8720000 3404.7229 5436.8461 5706.7291
340.5255 -14303.8191 -1838.5961 0.0000 0.0000
8031.6418 6778.0503 306.2595 -1253.5915 6843.8707
307.6885 -8.0067 67.6011 680285.8938 -7.2786
-7.2951
[33711]ENERGY: 8730000 3352.5197 5462.7619 5701.2790
353.8158 -14284.8155 -1867.8891 0.0000 0.0000
8060.7950 6778.4670 307.3711 -1282.3281 6844.2759
307.7672 -66.5566 -91.8772 680285.8938 -10.6384
-10.6345
[33747]ENERGY: 8740000 3299.3303 5477.5948 5727.7987
334.4068 -14267.8274 -1853.9535 0.0000 0.0000
8062.4978 6779.8476 307.4360 -1282.6502 6844.3632
307.9049 -37.5398 -52.2757 680285.8938 -5.7974
-5.7918
[33797]ENERGY: 8750000 3307.3209 5469.9834 5646.8945
339.1350 -14222.4039 -1825.0624 0.0000 0.0000
8068.2822 6784.1497 307.6566 -1284.1325 6845.6402
307.8338 -11.7721 -108.0643 680285.8938 -9.1074
-9.1210
[33833]ENERGY: 8760000 3354.5437 5481.9830 5706.8626
325.1228 -14257.5659 -1860.2179 0.0000 0.0000
8028.2105 6778.9389 306.1286 -1249.2716 6844.6517
307.8094 48.9241 32.4214 680285.8938 -9.3775
-9.3719
[33883]ENERGY: 8770000 3334.9485 5466.2682 5778.6867
331.8179 -14327.8753 -1847.3627 0.0000 0.0000
8046.5697 6783.0530 306.8287 -1263.5167 6845.0042
308.0239 -101.3797 -63.9746 680285.8938 -6.3919
-6.3882
[33919]ENERGY: 8780000 3327.8934 5439.0465 5694.0760
332.8390 -14235.5535 -1857.9265 0.0000 0.0000
8080.1836 6780.5586 308.1104 -1299.6251 6845.5184
307.8084 18.5777 32.1390 680285.8938 -7.7270
-7.7369
[33969]ENERGY: 8790000 3362.6186 5413.8485 5716.0043
346.5518 -14255.2289 -1885.5072 0.0000 0.0000
8081.9805 6780.2676 308.1789 -1301.7129 6846.2623
307.8691 72.7017 11.0796 680285.8938 -6.1066
-6.1089
[34008]ENERGY: 8800000 3306.6660 5458.4889 5686.9460
337.9371 -14271.8305 -1842.1764 0.0000 0.0000
8104.2415 6780.2724 309.0278 -1323.9691 6845.7990

307.8888 99.6998 31.7372 680285.8938 -7.9190
-7.9004
[34058]ENERGY: 8810000 3357.2751 5470.5324 5750.7344
320.0553 -14320.8370 -1818.3022 0.0000 0.0000
8023.3487 6782.8067 305.9432 -1240.5420 6846.0317
307.9983 -49.3247 -59.7520 680285.8938 -8.8267
-8.8446
[34094]ENERGY: 8820000 3330.2523 5516.9765 5762.3043
344.9354 -14339.6047 -1835.5472 0.0000 0.0000
8003.7240 6783.0406 305.1949 -1220.6834 6846.6607
307.9453 -135.6354 -54.1733 680285.8938 -7.4705
-7.4600
[34144]ENERGY: 8830000 3331.7417 5410.7942 5711.3824
349.9557 -14277.3472 -1838.6336 0.0000 0.0000
8095.9349 6783.8281 308.7111 -1312.1068 6848.8672
307.7385 167.1247 108.5133 680285.8938 -8.3548
-8.3578
[34180]ENERGY: 8840000 3394.8299 5434.1545 5687.6720
317.8312 -14231.4519 -1866.4303 0.0000 0.0000
8045.3547 6781.9603 306.7823 -1263.3945 6848.6972
307.8376 -10.0418 -70.1993 680285.8938 -9.1393
-9.1362
[34230]ENERGY: 8850000 3366.1766 5485.1388 5673.8079
335.2938 -14253.3294 -1883.0076 0.0000 0.0000
8056.3488 6780.4288 307.2016 -1275.9199 6847.5184
307.9302 -181.3306 -82.9441 680285.8938 -6.9603
-6.9660
[34266]ENERGY: 8860000 3389.3836 5482.1308 5722.3538
325.5253 -14374.1671 -1804.7099 0.0000 0.0000
8041.3039 6781.8204 306.6279 -1259.4835 6847.6653
308.0511 -68.2871 -32.5162 680285.8938 -5.4148
-5.4141
[34316]ENERGY: 8870000 3356.3402 5438.0338 5729.8189
342.9763 -14332.4956 -1787.1958 0.0000 0.0000
8036.9094 6784.3872 306.4603 -1252.5222 6847.3625
307.9639 -104.5334 -158.4269 680285.8938 -7.7848
-7.8018
[34352]ENERGY: 8880000 3345.0235 5454.8933 5691.2017
332.1017 -14207.2015 -1802.1044 0.0000 0.0000
7971.8185 6785.7328 303.9783 -1186.0857 6848.5895
307.9104 94.8144 41.8549 680285.8938 -4.6939
-4.6762
[34402]ENERGY: 8890000 3302.5427 5520.5942 5691.1081
350.8377 -14275.6644 -1842.1094 0.0000 0.0000
8038.1645 6785.4733 306.5082 -1252.6911 6849.5471
308.0345 -8.5459 -40.9391 680285.8938 -6.1016
-6.1156
[34441]ENERGY: 8900000 3367.6457 5432.2863 5681.5870
326.7770 -14295.1553 -1811.2596 0.0000 0.0000
8082.4568 6784.3379 308.1971 -1298.1189 6850.5417
307.8066 -70.7446 -107.0244 680285.8938 -9.8227
-9.8065
[34491]ENERGY: 8910000 3251.9710 5451.1941 5713.8866
339.4117 -14346.2692 -1834.9410 0.0000 0.0000

8206.8120 6782.0652 312.9390 -1424.7468 6850.4681
307.7450 61.0898 25.8372 680285.8938 -12.5008
-12.4970
[34527]ENERGY: 8920000 3304.7656 5558.7729 5663.2641
337.6843 -14401.2910 -1732.4288 0.0000 0.0000
8055.3433 6786.1103 307.1632 -1269.2329 6849.9301
307.8740 92.9785 80.9558 680285.8938 -9.9922
-9.9938
[34577]ENERGY: 8930000 3350.5731 5469.3965 5707.4996
309.5467 -14272.3569 -1846.2442 0.0000 0.0000
8067.3223 6785.7372 307.6200 -1281.5852 6850.2884
307.9834 -65.7548 -33.1091 680285.8938 -10.9510
-10.9529
[34613]ENERGY: 8940000 3241.5553 5543.1839 5703.0323
347.6691 -14306.8426 -1763.4907 0.0000 0.0000
8021.2918 6786.3992 305.8648 -1234.8926 6850.7739
308.0537 82.5683 125.8667 680285.8938 -10.2979
-10.3135
[34663]ENERGY: 8950000 3332.4937 5458.9200 5676.0075
347.4773 -14314.2146 -1816.4827 0.0000 0.0000
8097.7359 6781.9372 308.7797 -1315.7987 6849.5843
307.9440 40.8311 52.6018 680285.8938 -9.1039
-9.0777
[34699]ENERGY: 8960000 3371.9228 5443.2325 5701.0885
335.2097 -14355.9258 -1787.9307 0.0000 0.0000
8079.0032 6786.6002 308.0654 -1292.4031 6848.9186
307.9530 -49.4100 -33.1497 680285.8938 -11.8591
-11.8696
[34749]ENERGY: 8970000 3297.1894 5476.7268 5693.4089
350.1268 -14228.7286 -1934.5619 0.0000 0.0000
8131.1433 6785.3048 310.0536 -1345.8385 6851.0317
307.9894 89.8605 48.6949 680285.8938 -12.4096
-12.4104
[34785]ENERGY: 8980000 3353.6349 5490.2446 5744.7957
330.2773 -14311.2630 -1801.0807 0.0000 0.0000
7979.7674 6786.3763 304.2814 -1193.3912 6850.6858
307.8716 -163.6494 -96.8211 680285.8938 -12.0305
-12.0173
[34835]ENERGY: 8990000 3279.7027 5402.4080 5795.7413
344.7934 -14281.4237 -1771.4487 0.0000 0.0000
8013.9259 6783.6989 305.5839 -1230.2270 6849.8737
307.7120 -28.9712 -38.4141 680285.8938 -10.3350
-10.3534
[34874]ENERGY: 9000000 3372.5597 5438.4235 5787.5858
329.9529 -14328.3413 -1879.5728 0.0000 0.0000
8066.7754 6787.3831 307.5992 -1279.3923 6851.3584
307.9794 81.5908 45.2720 680285.8938 -14.5684
-14.5746
[34924]ENERGY: 9010000 3284.9649 5594.8038 5684.4551
354.6334 -14468.2313 -1713.2467 0.0000 0.0000
8045.6995 6783.0787 306.7955 -1262.6208 6849.9341
307.9844 155.7862 27.1648 680285.8938 -9.2602
-9.2633
[34960]ENERGY: 9020000 3294.9608 5572.7717 5704.8763

341.4219 -14347.8206 -1808.3663 0.0000 0.0000
8029.1162 6786.9600 306.1631 -1242.1562 6850.8048
307.9569 23.7316 56.0065 680285.8938 -10.6731
-10.6719
[35010]ENERGY: 9030000 3377.5831 5399.4306 5767.4109
373.7037 -14343.8250 -1818.1685 0.0000 0.0000
8029.8093 6785.9441 306.1896 -1243.8652 6850.1102
307.9048 -74.2013 -149.3545 680285.8938 -9.7635
-9.7674
[35046]ENERGY: 9040000 3373.8383 5414.8822 5694.0908
353.9161 -14305.2977 -1784.9776 0.0000 0.0000
8039.5572 6786.0092 306.5613 -1253.5479 6848.8218
307.8533 -44.5750 -63.6302 680285.8938 -8.1117
-8.0989
[35096]ENERGY: 9050000 3322.5969 5458.8757 5722.6624
344.5893 -14315.5063 -1839.2919 0.0000 0.0000
8089.4701 6783.3961 308.4645 -1306.0739 6850.9744
307.7499 36.4771 -3.6826 680285.8938 -12.2873
-12.2640
[35132]ENERGY: 9060000 3286.8750 5437.9527 5781.6597
358.9987 -14269.7340 -1833.3805 0.0000 0.0000
8018.2037 6780.5754 305.7470 -1237.6284 6850.9910
307.6902 -16.2488 -76.8120 680285.8938 -11.1213
-11.1344
[35182]ENERGY: 9070000 3273.2750 5455.2053 5734.0069
320.3666 -14309.6206 -1774.3998 0.0000 0.0000
8086.9775 6785.8107 308.3695 -1301.1667 6850.3384
308.0439 122.3519 15.0679 680285.8938 -13.1481
-13.1436
[35218]ENERGY: 9080000 3358.7851 5528.8056 5677.5549
333.2576 -14377.3161 -1817.4063 0.0000 0.0000
8080.4723 6784.1531 308.1214 -1296.3192 6850.5057
308.0771 -19.6879 29.8310 680285.8938 -9.3046
-9.3209
[35268]ENERGY: 9090000 3328.5740 5514.0036 5722.3552
347.3293 -14300.7202 -1869.2289 0.0000 0.0000
8041.1703 6783.4833 306.6228 -1257.6870 6850.1159
308.0111 -250.4952 -211.8951 680285.8938 -9.9532
-9.9595
[35307]ENERGY: 9100000 3275.7127 5374.8684 5745.0949
361.4087 -14284.9069 -1782.3879 0.0000 0.0000
8092.5893 6782.3792 308.5835 -1310.2101 6850.1430
308.1078 135.9988 114.7621 680285.8938 -10.6902
-10.6870
[35357]ENERGY: 9110000 3310.7326 5540.7443 5744.9113
339.8838 -14412.8606 -1820.7401 0.0000 0.0000
8081.5516 6784.2229 308.1626 -1297.3287 6851.2648
308.1468 12.2298 9.2322 680285.8938 -12.8639
-12.8667
[35393]ENERGY: 9120000 3322.8528 5355.6353 5717.1663
326.1751 -14276.6783 -1787.5847 0.0000 0.0000
8125.9694 6783.5360 309.8563 -1342.4335 6850.8472
308.0917 125.5499 108.5509 680285.8938 -12.4990
-12.4812

[35443]ENERGY: 9130000 3306.0317 5597.5490 5710.0461
338.1879 -14356.2400 -1829.9602 0.0000 0.0000
8016.6549 6782.2693 305.6880 -1234.3856 6851.0739
308.2409 61.5526 33.5216 680285.8938 -11.9488
-11.9455
[35479]ENERGY: 9140000 3318.1100 5516.0125 5755.4409
339.6506 -14359.8294 -1816.3966 0.0000 0.0000
8035.6144 6788.6024 306.4109 -1247.0120 6850.7099
308.2814 53.1380 -49.5167 680285.8938 -11.9449
-11.9545
[35529]ENERGY: 9150000 3302.4025 5448.6952 5781.8661
328.3865 -14353.3853 -1817.3159 0.0000 0.0000
8094.8373 6785.4863 308.6692 -1309.3510 6850.3264
308.3415 26.3110 -60.5046 680285.8938 -12.3844
-12.3742
[35565]ENERGY: 9160000 3324.1178 5521.0018 5692.5314
341.5990 -14323.5550 -1836.2299 0.0000 0.0000
8065.2896 6784.7548 307.5425 -1280.5348 6850.8801
308.4380 -24.8730 -4.8809 680285.8938 -16.1642
-16.1845
[35615]ENERGY: 9170000 3285.1701 5473.8518 5762.6878
330.2733 -14378.7369 -1769.3733 0.0000 0.0000
8080.0179 6783.8908 308.1041 -1296.1271 6850.8991
308.2077 105.1129 42.6585 680285.8938 -16.6986
-16.6882
[35651]ENERGY: 9180000 3322.9884 5479.1649 5702.2676
335.6748 -14356.2715 -1843.6659 0.0000 0.0000
8145.4234 6785.5816 310.5981 -1359.8417 6850.5883
308.2060 55.5572 91.6599 680285.8938 -17.8390
-17.8409
[35701]ENERGY: 9190000 3334.5677 5446.6598 5688.5268
331.7799 -14269.2188 -1859.7096 0.0000 0.0000
8112.8352 6785.4410 309.3555 -1327.3941 6850.9862
308.3437 60.7423 107.6596 680285.8938 -13.2596
-13.2579
[35740]ENERGY: 9200000 3356.5970 5565.7523 5681.8191
338.7915 -14347.0676 -1844.4452 0.0000 0.0000
8037.9076 6789.3546 306.4984 -1248.5529 6852.4586
308.3065 17.3756 -60.9653 680285.8938 -13.8293
-13.8361
[35790]ENERGY: 9210000 3344.4748 5462.0930 5729.7987
368.1211 -14418.4778 -1800.4862 0.0000 0.0000
8099.7899 6785.3137 308.8581 -1314.4762 6850.5947
308.5417 -30.6495 1.4785 680285.8938 -12.0616
-12.0524
[35826]ENERGY: 9220000 3357.2340 5528.1178 5725.9653
348.3547 -14349.2502 -1857.2305 0.0000 0.0000
8035.4038 6788.5949 306.4029 -1246.8089 6853.0603
308.3798 -77.4910 -145.2502 680285.8938 -14.1815
-14.1926
[35876]ENERGY: 9230000 3369.6288 5517.0715 5696.9092
347.5937 -14387.3923 -1762.0089 0.0000 0.0000
8009.0751 6790.8771 305.3989 -1218.1980 6854.1373
308.4838 144.0914 78.1043 680285.8938 -17.5726

-17.5661
[35912]ENERGY: 9240000 3299.4350 5437.9513 5753.8109
312.7214 -14309.8684 -1829.4877 0.0000 0.0000
8124.4818 6789.0442 309.7996 -1335.4376 6854.0507
308.3938 -100.8505 -122.0954 680285.8938 -10.1439
-10.1593
[35962]ENERGY: 9250000 3362.7376 5410.1772 5710.4435
343.7786 -14300.6279 -1830.9833 0.0000 0.0000
8091.2844 6786.8101 308.5337 -1304.4743 6852.7104
308.3718 -74.1168 -97.5587 680285.8938 -14.0552
-14.0398
[35998]ENERGY: 9260000 3316.1925 5418.1915 5760.8055
352.7598 -14362.2002 -1830.9849 0.0000 0.0000
8129.4315 6784.1956 309.9883 -1345.2360 6854.3719
308.3665 88.8646 -4.6356 680285.8938 -15.1326
-15.1208
[36048]ENERGY: 9270000 3269.0369 5524.9169 5745.5957
338.8275 -14278.3488 -1828.8484 0.0000 0.0000
8016.9905 6788.1703 305.7008 -1228.8203 6852.4329
308.5525 35.4274 46.1929 680285.8938 -10.9027
-10.9071
[36084]ENERGY: 9280000 3386.9377 5508.0050 5718.7591
309.6522 -14407.3401 -1828.4543 0.0000 0.0000
8095.6961 6783.2557 308.7019 -1312.4404 6852.0713
308.3329 -108.5975 -79.7890 680285.8938 -11.2446
-11.2508
[36134]ENERGY: 9290000 3361.1452 5461.9327 5785.7330
333.4891 -14424.4888 -1832.8624 0.0000 0.0000
8105.7606 6790.7093 309.0857 -1315.0513 6852.9003
308.5168 -17.2085 -21.2863 680285.8938 -11.9468
-11.9540
[36173]ENERGY: 9300000 3347.4443 5461.4751 5712.7198
321.3632 -14350.3391 -1806.0214 0.0000 0.0000
8102.1714 6788.8133 308.9489 -1313.3581 6853.3765
308.4458 -13.3741 -4.7799 680285.8938 -14.1494
-14.1475
[36223]ENERGY: 9310000 3381.4467 5451.2980 5758.3317
372.8398 -14311.9357 -1853.5584 0.0000 0.0000
7992.8144 6791.2364 304.7789 -1201.5780 6854.1847
308.5400 69.9942 -12.7506 680285.8938 -10.7348
-10.7139
[36259]ENERGY: 9320000 3290.1529 5470.6771 5747.1172
310.0610 -14349.9445 -1837.2174 0.0000 0.0000
8154.0177 6784.8640 310.9258 -1369.1537 6853.4396
308.5357 150.2102 129.4957 680285.8938 -13.4464
-13.4769
[36309]ENERGY: 9330000 3319.9142 5565.7001 5735.3589
339.3507 -14427.7884 -1794.4347 0.0000 0.0000
8049.4560 6787.5567 306.9387 -1261.8993 6853.3734
308.6216 -57.7348 -87.6053 680285.8938 -15.6930
-15.6936
[36345]ENERGY: 9340000 3338.7574 5484.6149 5697.6197
339.3610 -14341.1502 -1789.7516 0.0000 0.0000
8060.6364 6790.0876 307.3651 -1270.5488 6853.8067

308.4882 -31.9707 -11.5335 680285.8938 -13.9876
-13.9727
[36395]ENERGY: 9350000 3362.8567 5426.7904 5753.9086
355.7012 -14310.1336 -1867.5978 0.0000 0.0000
8069.2544 6790.7798 307.6937 -1278.4746 6854.3827
308.4738 -79.5634 -67.1527 680285.8938 -17.5000
-17.4900
[36431]ENERGY: 9360000 3295.2569 5448.9878 5738.4335
329.3368 -14355.8083 -1764.4682 0.0000 0.0000
8097.3669 6789.1053 308.7657 -1308.2616 6853.6564
308.4332 26.5592 22.7638 680285.8938 -18.2546
-18.2659
[36481]ENERGY: 9370000 3323.4108 5345.2653 5681.7963
348.6283 -14291.8492 -1758.4695 0.0000 0.0000
8141.6088 6790.3909 310.4527 -1351.2179 6852.6400
308.4721 125.1614 94.7765 680285.8938 -17.5597
-17.5618
[36517]ENERGY: 9380000 3257.1025 5551.7922 5740.0870
339.6651 -14421.4842 -1807.8050 0.0000 0.0000
8124.2620 6783.6197 309.7912 -1340.6423 6852.3580
308.4653 83.0569 95.8601 680285.8938 -11.3403
-11.3296
[36567]ENERGY: 9390000 3297.9992 5461.0322 5719.2157
363.7541 -14255.9036 -1876.3646 0.0000 0.0000
8081.0458 6790.7788 308.1433 -1290.2670 6854.3021
308.2568 -150.5969 -73.0836 680285.8938 -19.3477
-19.3364
[36606]ENERGY: 9400000 3362.9607 5435.1422 5729.8416
347.4834 -14311.0890 -1853.4629 0.0000 0.0000
8077.4928 6788.3687 308.0078 -1289.1241 6853.8858
308.3363 -37.9699 -101.0738 680285.8938 -16.3267
-16.3454
[36656]ENERGY: 9410000 3237.2656 5470.2910 5752.0196
331.7002 -14311.4104 -1808.7877 0.0000 0.0000
8113.9090 6784.9873 309.3964 -1328.9217 6853.1955
308.4925 91.9036 84.9852 680285.8938 -17.7187
-17.7004
[36692]ENERGY: 9420000 3420.3992 5388.6517 5740.2687
353.0107 -14347.4982 -1774.8197 0.0000 0.0000
8009.4224 6789.4348 305.4122 -1219.9876 6853.2795
308.3784 72.3663 -20.3369 680285.8938 -18.0548
-18.0617
[36742]ENERGY: 9430000 3232.4958 5482.5994 5761.6723
374.9713 -14334.6745 -1802.6225 0.0000 0.0000
8073.9019 6788.3438 307.8709 -1285.5581 6855.1296
308.4145 197.4069 147.6214 680285.8938 -14.2303
-14.2399
[36778]ENERGY: 9440000 3286.6605 5497.1970 5775.1016
331.3879 -14333.5392 -1801.1853 0.0000 0.0000
8039.3465 6794.9690 306.5532 -1244.3775 6854.9632
308.3952 129.5823 129.9832 680285.8938 -18.3150
-18.3297
[36828]ENERGY: 9450000 3343.0981 5379.6164 5754.4010
356.9473 -14288.3514 -1810.4146 0.0000 0.0000

8054.1544 6789.4511 307.1179 -1264.7033 6855.3989
308.3625 -81.1717 -118.1860 680285.8938 -17.3298
-17.3129
[36864]ENERGY: 9460000 3273.5679 5444.9122 5724.4876
325.3728 -14321.6243 -1827.4133 0.0000 0.0000
8169.0514 6788.3542 311.4991 -1380.6972 6855.8254
308.4994 152.2092 78.6101 680285.8938 -16.5170
-16.5363
[36914]ENERGY: 9470000 3295.1011 5437.1537 5679.1498
356.0173 -14336.4723 -1767.2285 0.0000 0.0000
8127.2402 6790.9612 309.9048 -1336.2789 6856.4448
308.5182 -26.1263 -22.8335 680285.8938 -15.7879
-15.7576
[36950]ENERGY: 9480000 3340.9777 5494.5879 5681.5081
341.3276 -14322.0705 -1836.8382 0.0000 0.0000
8095.4243 6794.9169 308.6916 -1300.5074 6856.4016
308.5174 109.8081 -67.9174 680285.8938 -19.3899
-19.4059
[37000]ENERGY: 9490000 3333.2454 5415.2431 5818.0299
336.5662 -14226.5512 -1879.9684 0.0000 0.0000
7997.9525 6794.5175 304.9748 -1203.4350 6858.2403
308.4052 -116.1246 -91.6073 680285.8938 -15.5859
-15.5744
[37039]ENERGY: 9500000 3301.8234 5433.6776 5751.6182
321.9571 -14255.1556 -1845.6472 0.0000 0.0000
8088.1862 6796.4595 308.4156 -1291.7266 6859.5654
308.4166 59.1551 -22.1143 680285.8938 -13.8951
-13.9133
[37089]ENERGY: 9510000 3297.9387 5403.8502 5720.3746
345.3547 -14231.7205 -1899.6717 0.0000 0.0000
8158.2971 6794.4231 311.0890 -1363.8740 6858.0057
308.4058 -8.1800 -1.1771 680285.8938 -15.8380
-15.8331
[37125]ENERGY: 9520000 3343.7514 5501.3971 5701.8093
327.0387 -14384.1913 -1777.5298 0.0000 0.0000
8083.1056 6795.3809 308.2219 -1287.7247 6859.5211
308.5660 43.5518 15.2489 680285.8938 -15.1723
-15.1860
[37175]ENERGY: 9530000 3450.5599 5473.5763 5717.0070
341.7327 -14277.6465 -1922.2512 0.0000 0.0000
8014.5369 6797.5151 305.6072 -1217.0219 6861.2238
308.4852 -139.9662 -140.6414 680285.8938 -12.3921
-12.3797
[37211]ENERGY: 9540000 3400.0112 5473.7381 5730.5901
332.0254 -14261.2068 -1930.1157 0.0000 0.0000
8053.4344 6798.4767 307.0904 -1254.9577 6862.4081
308.4138 -147.1516 -234.3431 680285.8938 -11.5807
-11.5722
[37261]ENERGY: 9550000 3288.5726 5456.8928 5765.9873
363.6179 -14360.5832 -1778.4686 0.0000 0.0000
8060.6859 6796.7048 307.3670 -1263.9812 6862.0371
308.1667 53.7410 41.5922 680285.8938 -15.9412
-15.9497
[37297]ENERGY: 9560000 3318.4926 5439.9278 5737.5611

342.2583 -14331.0039 -1765.0001 0.0000 0.0000
8053.8020 6796.0377 307.1045 -1257.7642 6862.1803
308.2727 86.4378 66.0284 680285.8938 -11.6913
-11.6849
[37347]ENERGY: 9570000 3338.0504 5426.8108 5685.6261
304.3908 -14272.7480 -1808.6731 0.0000 0.0000
8123.6528 6797.1098 309.7680 -1326.5430 6862.7018
308.2810 66.5400 -2.4217 680285.8938 -13.3215
-13.3041
[37383]ENERGY: 9580000 3320.5711 5502.6269 5699.4257
325.1760 -14376.5573 -1776.9654 0.0000 0.0000
8103.6884 6797.9654 309.0067 -1305.7230 6861.9666
308.1709 -10.3290 -51.4570 680285.8938 -13.8902
-13.9068
[37433]ENERGY: 9590000 3385.5873 5510.2259 5778.3632
366.6873 -14368.7281 -1847.7663 0.0000 0.0000
7972.6408 6797.0100 304.0096 -1175.6308 6861.9955
308.3220 -83.5518 -114.9420 680285.8938 -12.8629
-12.8591
[37472]ENERGY: 9600000 3353.2396 5565.3359 5807.6837
357.1848 -14416.4627 -1866.9542 0.0000 0.0000
7997.4119 6797.4389 304.9542 -1199.9730 6861.9471
308.3538 -52.3734 -83.1600 680285.8938 -14.0261
-14.0240
[37522]ENERGY: 9610000 3297.4204 5527.3775 5757.8450
320.1178 -14342.9634 -1810.5881 0.0000 0.0000
8051.6067 6800.8158 307.0207 -1250.7908 6862.6160
308.2063 6.4408 -105.6927 680285.8938 -11.8476
-11.8657
[37558]ENERGY: 9620000 3306.3199 5433.7075 5676.4131
345.0708 -14259.6560 -1778.7917 0.0000 0.0000
8075.9000 6798.9636 307.9471 -1276.9364 6862.3403
308.3163 60.2153 18.7910 680285.8938 -15.2484
-15.2432
[37608]ENERGY: 9630000 3318.5313 5441.1120 5703.5031
343.0404 -14283.4217 -1821.7467 0.0000 0.0000
8095.3829 6796.4013 308.6900 -1298.9816 6862.1996
308.4753 90.1660 51.0424 680285.8938 -12.9012
-12.8918
[37644]ENERGY: 9640000 3302.1592 5428.8516 5730.5233
345.1130 -14243.1708 -1821.0352 0.0000 0.0000
8052.8019 6795.2430 307.0663 -1257.5588 6861.2881
308.5976 -94.8983 -83.7014 680285.8938 -14.7827
-14.7602
[37694]ENERGY: 9650000 3279.0192 5540.6662 5707.6218
329.0336 -14281.8585 -1801.0562 0.0000 0.0000
8024.5382 6797.9643 305.9886 -1226.5739 6862.0810
308.4405 94.8673 89.2308 680285.8938 -16.8563
-16.8954
[37730]ENERGY: 9660000 3278.9233 5459.2646 5693.0619
335.1074 -14414.3485 -1762.3645 0.0000 0.0000
8204.3271 6793.9714 312.8442 -1410.3557 6861.8235
308.5870 11.5602 -14.4099 680285.8938 -11.4503
-11.4298

[37780]ENERGY: 9670000 3328.2707 5436.6794 5736.6798
343.7993 -14293.1101 -1852.8119 0.0000 0.0000
8098.1816 6797.6889 308.7967 -1300.4928 6863.1469
308.4162 28.4794 6.6389 680285.8938 -12.6228
-12.6350
[37816]ENERGY: 9680000 3326.9904 5408.5906 5713.5176
339.6730 -14345.0909 -1785.9715 0.0000 0.0000
8142.3757 6800.0850 310.4819 -1342.2907 6863.8148
308.5827 -144.6142 -168.8772 680285.8938 -15.1926
-15.1762
[37866]ENERGY: 9690000 3311.2599 5449.5328 5709.8525
326.7525 -14384.9767 -1767.7079 0.0000 0.0000
8149.7338 6794.4469 310.7625 -1355.2869 6862.6245
308.4825 27.8076 30.2978 680285.8938 -15.8926
-15.9000
[37905]ENERGY: 9700000 3276.8324 5527.8256 5742.6615
351.0411 -14266.0531 -1897.5113 0.0000 0.0000
8065.5058 6800.3021 307.5507 -1265.2037 6864.4503
308.4561 -39.4979 -77.4749 680285.8938 -13.3094
-13.3098
[37955]ENERGY: 9710000 3322.9988 5522.0052 5692.1146
356.5762 -14303.7501 -1870.4395 0.0000 0.0000
8078.0730 6797.5781 308.0299 -1280.4949 6864.0328
308.3022 -33.6506 -117.3635 680285.8938 -12.3660
-12.3637
[37991]ENERGY: 9720000 3384.9030 5457.5163 5729.4892
362.6840 -14297.1470 -1843.8952 0.0000 0.0000
8008.3851 6801.9355 305.3726 -1206.4497 6863.8421
308.5553 17.4012 -3.6270 680285.8938 -12.1768
-12.1894
[38041]ENERGY: 9730000 3289.7036 5450.8256 5685.2614
349.1122 -14302.9805 -1779.2234 0.0000 0.0000
8105.4123 6798.1111 309.0724 -1307.3012 6863.1206
308.5421 -32.6301 -33.9135 680285.8938 -13.9874
-13.9855
[38077]ENERGY: 9740000 3315.7820 5539.5482 5690.0995
357.5171 -14400.7918 -1781.0646 0.0000 0.0000
8078.4743 6799.5647 308.0453 -1278.9096 6865.7871
308.4905 2.7561 -57.4236 680285.8938 -19.3549
-19.3518
[38127]ENERGY: 9750000 3300.1555 5440.3330 5756.9217
348.7431 -14364.2003 -1755.4337 0.0000 0.0000
8074.0603 6800.5795 307.8769 -1273.4807 6865.5755
308.6114 80.3770 48.2783 680285.8938 -10.4256
-10.4346
[38163]ENERGY: 9760000 3311.0114 5566.9600 5746.7835
325.9262 -14409.7836 -1722.0160 0.0000 0.0000
7984.1073 6802.9888 304.4469 -1181.1185 6865.0551
308.6553 28.9490 67.5209 680285.8938 -13.3293
-13.3044
[38213]ENERGY: 9770000 3325.5009 5467.4081 5749.4053
354.8283 -14369.1941 -1779.5095 0.0000 0.0000
8050.8936 6799.3326 306.9936 -1251.5610 6864.3256
308.5264 -7.2504 -19.2027 680285.8938 -16.9662

-16.9988
[38249]ENERGY: 9780000 3300.2168 5473.9785 5750.9505
341.6332 -14319.1485 -1790.1437 0.0000 0.0000
8041.7250 6799.2118 306.6439 -1242.5132 6864.7634
308.3866 65.3055 -37.6118 680285.8938 -16.3863
-16.3864
[38299]ENERGY: 9790000 3341.9058 5467.3108 5717.4442
315.0340 -14293.2194 -1749.3521 0.0000 0.0000
8002.3779 6801.5013 305.1436 -1200.8766 6864.2458
308.3494 -4.8869 -7.3263 680285.8938 -15.7517
-15.7425
[38338]ENERGY: 9800000 3223.1031 5504.0057 5757.4958
340.8532 -14362.9914 -1795.7812 0.0000 0.0000
8131.7160 6798.4012 310.0754 -1333.3148 6865.3087
308.2843 24.1502 21.9741 680285.8938 -17.2993
-17.2884
[38388]ENERGY: 9810000 3340.2879 5484.5948 5718.6066
378.3383 -14376.8663 -1766.0191 0.0000 0.0000
8022.4751 6801.4174 305.9099 -1221.0577 6864.2395
308.3789 -62.9728 17.3093 680285.8938 -14.0343
-14.0303
[38424]ENERGY: 9820000 3293.6865 5474.5176 5754.8956
360.9223 -14359.7271 -1861.4019 0.0000 0.0000
8134.7619 6797.6549 310.1916 -1337.1071 6866.4156
308.4837 20.0057 -48.0335 680285.8938 -12.7413
-12.7517
[38474]ENERGY: 9830000 3365.2819 5401.1495 5731.2454
322.4388 -14306.6490 -1830.1728 0.0000 0.0000
8117.8523 6801.1462 309.5468 -1316.7062 6864.5418
308.6337 -21.9394 -46.6519 680285.8938 -11.9993
-11.9779
[38510]ENERGY: 9840000 3318.2868 5393.6281 5796.4934
345.9298 -14280.8399 -1805.2787 0.0000 0.0000
8031.5481 6799.7676 306.2559 -1231.7806 6865.2784
308.3495 58.9826 32.4561 680285.8938 -9.4740
-9.4765
[38560]ENERGY: 9850000 3351.2193 5406.3351 5747.2068
361.2644 -14314.0037 -1748.9400 0.0000 0.0000
7997.3949 6800.4769 304.9536 -1196.9180 6865.7155
308.4521 -111.1149 -51.2141 680285.8938 -16.2520
-16.2528
[38596]ENERGY: 9860000 3303.4348 5466.9668 5737.4138
328.7727 -14396.9006 -1728.5385 0.0000 0.0000
8089.9415 6801.0905 308.4825 -1288.8510 6865.6174
308.5317 160.8240 162.0398 680285.8938 -11.8595
-11.8684
[38646]ENERGY: 9870000 3323.3675 5413.1981 5731.7033
343.8337 -14236.0457 -1857.5628 0.0000 0.0000
8081.1318 6799.6259 308.1466 -1281.5060 6866.1278
308.5891 -101.7726 -101.8635 680285.8938 -13.5349
-13.5429
[38682]ENERGY: 9880000 3429.9239 5432.0785 5739.6868
344.1802 -14401.7027 -1780.7522 0.0000 0.0000
8037.3458 6800.7603 306.4770 -1236.5855 6866.3022

308.5551 -105.4965 -135.4083 680285.8938 -13.6322
-13.6227
[38732]ENERGY: 9890000 3402.3077 5446.4024 5727.0035
347.4098 -14413.2160 -1757.4744 0.0000 0.0000
8048.6973 6801.1303 306.9098 -1247.5670 6864.9162
308.7688 -105.4983 -32.1514 680285.8938 -15.6251
-15.6247
[38771]ENERGY: 9900000 3150.1823 5502.9721 5766.0834
328.8663 -14231.7846 -1841.3220 0.0000 0.0000
8123.3816 6798.3792 309.7576 -1325.0024 6866.1794
308.6942 52.5420 13.1666 680285.8938 -9.8900
-9.8929
[38821]ENERGY: 9910000 3310.2704 5467.3263 5707.7195
348.0657 -14368.9521 -1780.7348 0.0000 0.0000
8118.1713 6801.8663 309.5590 -1316.3050 6867.9559
308.7196 -89.6619 -111.1533 680285.8938 -12.2593
-12.2646
[38857]ENERGY: 9920000 3340.6351 5471.6848 5712.5705
329.0140 -14314.1819 -1863.9118 0.0000 0.0000
8124.8381 6800.6489 309.8132 -1324.1892 6868.6224
308.4945 -146.5383 -97.3907 680285.8938 -14.8699
-14.8654
[38907]ENERGY: 9930000 3340.1544 5507.7426 5720.1466
342.3173 -14257.3180 -1884.0680 0.0000 0.0000
8037.3559 6806.3307 306.4773 -1231.0252 6869.1258
308.7849 -74.7021 -32.9900 680285.8938 -11.4410
-11.4466
[38943]ENERGY: 9940000 3376.6802 5461.8969 5689.5827
342.5006 -14342.6086 -1794.2201 0.0000 0.0000
8070.7699 6804.6017 307.7515 -1266.1683 6869.9038
308.6141 -5.6179 33.0316 680285.8938 -14.1894
-14.2031
[38993]ENERGY: 9950000 3298.4076 5465.3237 5697.5949
378.0301 -14382.7863 -1689.3181 0.0000 0.0000
8034.4888 6801.7406 306.3680 -1232.7482 6867.4955
308.8025 122.8048 158.8356 680285.8938 -12.1151
-12.1083
[39029]ENERGY: 9960000 3368.3519 5420.5969 5697.5340
328.8480 -14259.7543 -1789.9124 0.0000 0.0000
8037.5959 6803.2600 306.4865 -1234.3358 6868.1536
308.7114 82.6521 7.8605 680285.8938 -15.0437
-15.0300
[39079]ENERGY: 9970000 3270.4374 5425.6341 5713.9680
337.6001 -14312.3302 -1714.1792 0.0000 0.0000
8084.6599 6805.7901 308.2811 -1278.8698 6868.0747
308.4952 201.2725 170.1501 680285.8938 -11.3694
-11.3798
[39115]ENERGY: 9980000 3368.0916 5419.3865 5713.1120
323.3945 -14333.7016 -1770.7190 0.0000 0.0000
8084.8265 6804.3906 308.2875 -1280.4360 6868.3259
308.3718 104.1722 -3.6155 680285.8938 -11.6848
-11.6986
[39165]ENERGY: 9990000 3306.2693 5495.0828 5693.2613
316.5293 -14264.4038 -1849.3964 0.0000 0.0000

8105.6350 6802.9774 309.0809 -1302.6576 6868.5061
308.3943 113.4287 46.0478 680285.8938 -14.6481
-14.6404
[39204]ENERGY: 10000000 3371.6592 5439.8667 5672.2979
359.8772 -14296.1487 -1794.6216 0.0000 0.0000
8049.0025 6801.9331 306.9214 -1247.0694 6868.5464
308.5440 18.7403 13.0819 680285.8938 -13.5504
-13.5435
[39254]ENERGY: 10010000 3316.4079 5337.4063 5665.4768
317.3690 -14187.7469 -1780.0205 0.0000 0.0000
8133.3103 6802.2030 310.1362 -1331.1073 6868.8953
308.5224 105.3684 33.7249 680285.8938 -8.9128
-8.9028
[39290]ENERGY: 10020000 3281.0820 5407.4619 5699.7502
318.3877 -14277.0929 -1749.0829 0.0000 0.0000
8122.0711 6802.5770 309.7077 -1319.4941 6868.5926
308.2977 1.6533 2.6745 680285.8938 -11.6898
-11.6894
[39340]ENERGY: 10030000 3431.5729 5497.0834 5680.7241
337.7855 -14312.8946 -1818.2349 0.0000 0.0000
7993.3965 6809.4330 304.8011 -1183.9635 6869.2510
308.3598 -52.0155 -57.5377 680285.8938 -13.2754
-13.2769
[39376]ENERGY: 10040000 3419.8946 5520.7793 5717.8260
334.8019 -14389.4276 -1762.2878 0.0000 0.0000
7964.6749 6806.2613 303.7059 -1158.4136 6869.3294
308.4269 105.8191 10.3358 680285.8938 -10.3329
-10.3341
[39426]ENERGY: 10050000 3289.5607 5415.3125 5695.9802
346.1690 -14286.5479 -1768.1368 0.0000 0.0000
8111.3103 6803.6479 309.2973 -1307.6623 6869.5650
308.4798 118.5437 71.5189 680285.8938 -14.5864
-14.5786
[39462]ENERGY: 10060000 3391.9800 5527.3491 5644.7148
330.6721 -14263.8656 -1906.3369 0.0000 0.0000
8078.8086 6803.3221 308.0580 -1275.4865 6870.1604
308.3676 -205.2341 -108.2323 680285.8938 -12.7220
-12.7301
[39512]ENERGY: 10070000 3375.6019 5441.5543 5691.1110
342.1090 -14298.6693 -1815.6436 0.0000 0.0000
8070.9751 6807.0384 307.7593 -1263.9366 6871.0633
308.3079 -116.9029 -105.3794 680285.8938 -14.6373
-14.6535
[39548]ENERGY: 10080000 3254.4615 5437.7498 5688.1474
359.5229 -14227.8885 -1798.0902 0.0000 0.0000
8088.4935 6802.3964 308.4273 -1286.0970 6869.9438
308.2506 93.6827 0.8915 680285.8938 -13.2197
-13.2028
[39598]ENERGY: 10090000 3302.2963 5576.6996 5727.5192
332.5799 -14292.9225 -1840.2251 0.0000 0.0000
8002.5421 6808.4895 305.1498 -1194.0526 6871.4070
308.5097 -7.7705 37.6720 680285.8938 -15.1495
-15.1600
[39637]ENERGY: 10100000 3345.9153 5457.1267 5715.0023

```
350.8648          -14298.0947     -1826.3998         0.0000         0.0000
8060.8797           6805.2944       307.3743     -1255.5853      6872.9731
308.4805           -116.4856      -138.0096    680285.8938       -13.8198
-13.8140
[39687]ENERGY: 10110000      3369.0874      5417.7830      5649.1022
344.0671          -14273.1462     -1744.5309         0.0000         0.0000
8044.2306           6806.5933       306.7395     -1237.6373      6872.3277
308.3742            -13.5531        -4.7700    680285.8938       -14.8683
-14.8611
[39723]ENERGY: 10120000      3303.1965      5493.2888      5752.7922
368.2926          -14249.0825     -1878.4359         0.0000         0.0000
8020.3864           6810.4382       305.8303     -1209.9482      6871.8381
308.4978            -90.9411       -29.8954    680285.8938       -11.6999
-11.7153
[39773]ENERGY: 10130000      3355.1873      5411.4311      5677.6940
354.1883          -14196.2603     -1830.5108         0.0000         0.0000
8035.6526           6807.3821       306.4124     -1228.2705      6872.6268
308.4214             -9.5030       -43.4800    680285.8938       -15.9378
-15.9260
[39809]ENERGY: 10140000      3314.6930      5385.9309      5704.1193
352.6287          -14307.4836     -1786.1552         0.0000         0.0000
8143.1594           6806.8924       310.5118     -1336.2671      6872.5281
308.5046            217.0865       103.0741    680285.8938       -12.9847
-12.9944
[39859]ENERGY: 10150000      3384.1129      5441.1482      5735.1342
331.1544          -14291.7192     -1836.5173         0.0000         0.0000
8046.5912           6809.9044       306.8295     -1236.6868      6872.7040
308.6245            -79.9213       -40.3348    680285.8938       -12.7253
-12.7172
[39895]ENERGY: 10160000      3389.2402      5372.5445      5714.8279
342.5183          -14309.9551     -1772.5745         0.0000         0.0000
8074.3121           6810.9136       307.8865     -1263.3986      6872.6537
308.6852            -91.6693      -113.1437    680285.8938       -16.0542
-16.0613
[39945]ENERGY: 10170000      3288.8448      5417.9910      5745.2072
344.7782          -14301.4385     -1768.8894         0.0000         0.0000
8079.6719           6806.1653       308.0909     -1273.5067      6872.2238
308.5950            -79.6739       -63.2322    680285.8938       -14.2421
-14.2243
[39981]ENERGY: 10180000      3368.1951      5525.4784      5750.9278
330.8862          -14356.6125     -1812.8100         0.0000         0.0000
8003.8112           6809.8763       305.1982     -1193.9349      6874.0992
308.3782            -25.9413        -0.3151    680285.8938       -15.3845
-15.3850
[40031]ENERGY: 10190000      3314.0664      5443.8782      5783.1792
336.7961          -14348.3302     -1795.5071         0.0000         0.0000
8075.6431           6809.7256       307.9373     -1265.9175      6872.6618
308.3669             24.7265        13.4740    680285.8938       -15.5928
-15.5979
[40070]ENERGY: 10200000      3375.5860      5374.2239      5753.5527
361.2829          -14363.8629     -1806.9185         0.0000         0.0000
8115.2815           6809.1457       309.4488     -1306.1358      6873.3470
308.5909            -49.5955       -77.0149    680285.8938       -10.6795
-10.6816
```

[40120]ENERGY: 10210000 3327.4167 5494.6947 5731.4989
359.2151 -14395.5474 -1794.8375 0.0000 0.0000
8084.1773 6806.6179 308.2627 -1277.5594 6873.0276
308.6779 123.4524 1.6099 680285.8938 -9.4965
-9.5166
[40156]ENERGY: 10220000 3244.1555 5445.0141 5744.1295
351.3253 -14352.0245 -1791.7201 0.0000 0.0000
8164.8314 6805.7112 311.3382 -1359.1202 6873.1276
308.6385 1.1329 22.0338 680285.8938 -13.5179
-13.5099
[40206]ENERGY: 10230000 3294.3243 5538.6452 5690.9198
344.6708 -14321.2863 -1852.6481 0.0000 0.0000
8112.8583 6807.4840 309.3564 -1305.3743 6873.0846
308.7086 120.3737 110.4737 680285.8938 -11.6687
-11.6629
[40242]ENERGY: 10240000 3352.9090 5405.5127 5734.1934
342.9218 -14283.8152 -1846.8590 0.0000 0.0000
8104.4283 6809.2909 309.0349 -1295.1374 6872.1944
308.7735 -73.9996 -68.0256 680285.8938 -9.2784
-9.2796
[40292]ENERGY: 10250000 3221.0226 5450.3803 5773.9835
317.4961 -14299.2129 -1821.3512 0.0000 0.0000
8164.8331 6807.1516 311.3383 -1357.6815 6872.6603
308.6972 28.7081 -5.6059 680285.8938 -11.0912
-11.0839
[40328]ENERGY: 10260000 3321.6017 5402.8704 5770.5773
328.9400 -14291.0220 -1843.6995 0.0000 0.0000
8117.9473 6807.2154 309.5504 -1310.7319 6872.0875
308.5199 -95.1878 -78.3910 680285.8938 -5.2565
-5.2628
[40378]ENERGY: 10270000 3402.3293 5395.8277 5736.6333
333.2192 -14360.3009 -1860.0124 0.0000 0.0000
8159.4119 6807.1081 311.1315 -1352.3038 6872.3009
308.6509 14.1548 -13.7978 680285.8938 -9.3565
-9.3461
[40414]ENERGY: 10280000 3337.4869 5515.3834 5705.4838
319.3194 -14328.4631 -1805.4705 0.0000 0.0000
8064.5528 6808.2926 307.5144 -1256.2602 6873.2839
308.6980 -101.6666 -80.1110 680285.8938 -13.6255
-13.6454
[40464]ENERGY: 10290000 3331.2596 5495.8729 5747.1376
346.3026 -14407.5216 -1744.3620 0.0000 0.0000
8041.3748 6810.0638 306.6306 -1231.3110 6873.8784
308.9381 102.4224 88.4447 680285.8938 -10.6882
-10.6793
[40503]ENERGY: 10300000 3348.5971 5470.3950 5757.4330
331.3743 -14275.6768 -1851.5182 0.0000 0.0000
8029.1668 6809.7713 306.1651 -1219.3955 6873.6282
308.8292 -10.7183 -85.4411 680285.8938 -12.7632
-12.7624
[40553]ENERGY: 10310000 3365.7650 5386.5130 5695.6276
368.2860 -14301.4468 -1769.4366 0.0000 0.0000
8065.7616 6811.0698 307.5605 -1254.6918 6872.9377
308.6835 127.2941 41.5101 680285.8938 -15.9324

-15.9257
[40589]ENERGY: 10320000 3304.8832 5426.0972 5756.3580
335.6095 -14312.9565 -1804.6954 0.0000 0.0000
8103.8162 6809.1122 309.0116 -1294.7040 6873.5195
308.5575 171.7943 113.2790 680285.8938 -11.2813
-11.2832
[40639]ENERGY: 10330000 3356.6484 5380.7453 5755.4249
323.5051 -14315.7010 -1774.1002 0.0000 0.0000
8082.1777 6808.7002 308.1865 -1273.4775 6874.6636
308.6066 148.3407 34.6537 680285.8938 -10.8582
-10.8766
[40675]ENERGY: 10340000 3258.8216 5410.9567 5799.2692
325.3137 -14262.3707 -1860.8286 0.0000 0.0000
8138.4279 6809.5899 310.3314 -1328.8380 6875.2259
308.7050 10.7415 -92.3650 680285.8938 -12.4707
-12.4598
[40725]ENERGY: 10350000 3325.2269 5464.5527 5775.9325
345.3314 -14406.5847 -1769.4139 0.0000 0.0000
8070.4390 6805.4840 307.7389 -1264.9550 6873.7767
308.7517 -39.9760 -58.2610 680285.8938 -12.8168
-12.8166
[40761]ENERGY: 10360000 3311.5115 5509.5703 5757.7137
342.5771 -14353.6682 -1837.8005 0.0000 0.0000
8080.8088 6810.7127 308.1343 -1270.0962 6874.3265
308.7605 58.8710 13.7527 680285.8938 -11.6713
-11.6732
[40811]ENERGY: 10370000 3308.0772 5440.7478 5778.0765
337.5001 -14293.4177 -1876.7303 0.0000 0.0000
8117.6278 6811.8814 309.5382 -1305.7464 6874.3711
308.5879 -74.8114 -44.3108 680285.8938 -15.2553
-15.2537
[40847]ENERGY: 10380000 3293.9297 5423.3512 5765.5949
354.5017 -14308.7740 -1802.5801 0.0000 0.0000
8083.0183 6809.0417 308.2185 -1273.9766 6873.3071
308.6845 -8.6104 -17.7419 680285.8938 -12.8772
-12.8655
[40897]ENERGY: 10390000 3312.1094 5389.0128 5713.7393
322.6062 -14319.2401 -1794.2250 0.0000 0.0000
8181.5676 6805.5703 311.9764 -1375.9973 6874.5462
308.5888 -64.0304 -26.0714 680285.8938 -16.9566
-16.9511
[40936]ENERGY: 10400000 3311.5161 5443.5601 5724.2081
314.9542 -14321.1627 -1748.6248 0.0000 0.0000
8087.3227 6811.7737 308.3827 -1275.5489 6873.4624
308.8115 17.2645 50.7718 680285.8938 -11.4488
-11.4776
[40986]ENERGY: 10410000 3267.7313 5558.9364 5710.0571
328.6154 -14340.7954 -1804.8812 0.0000 0.0000
8085.7819 6805.4455 308.3239 -1280.3363 6874.4323
308.7053 -84.0171 -32.8472 680285.8938 -17.0901
-17.0762
[41022]ENERGY: 10420000 3264.9287 5461.5286 5678.0086
359.9040 -14255.0682 -1828.9800 0.0000 0.0000
8129.6125 6809.9343 309.9952 -1319.6782 6876.1609

308.6816 -41.4377 -41.5723 680285.8938 -13.4345
-13.4404
[41072]ENERGY: 10430000 3337.1171 5408.7819 5723.5844
334.8586 -14293.7827 -1835.2922 0.0000 0.0000
8133.3984 6808.6654 310.1396 -1324.7330 6876.2473
308.7441 -18.1774 -72.3847 680285.8938 -13.1112
-13.1105
[41108]ENERGY: 10440000 3293.8327 5504.6311 5730.8418
330.7826 -14305.9866 -1798.4551 0.0000 0.0000
8056.6155 6812.2620 307.2117 -1244.3535 6876.8969
308.8691 -1.4136 -43.8053 680285.8938 -11.4957
-11.5016
[41158]ENERGY: 10450000 3317.0918 5512.7915 5706.5891
332.4532 -14380.6820 -1830.2250 0.0000 0.0000
8151.0892 6809.1079 310.8142 -1341.9813 6876.1972
308.7256 18.3053 -8.3193 680285.8938 -14.3430
-14.3282
[41194]ENERGY: 10460000 3343.2479 5464.8790 5695.1076
325.5884 -14312.9583 -1761.8239 0.0000 0.0000
8057.1914 6811.2322 307.2337 -1245.9592 6876.2843
308.7297 154.8103 17.4032 680285.8938 -11.6891
-11.6979
[41244]ENERGY: 10470000 3396.9201 5404.4493 5724.8389
338.8982 -14372.2659 -1769.0879 0.0000 0.0000
8089.3204 6813.0730 308.4588 -1276.2473 6877.1424
308.7192 -40.0118 -52.9259 680285.8938 -12.6149
-12.6124
[41280]ENERGY: 10480000 3288.9967 5472.5812 5733.3599
361.8425 -14334.9027 -1783.2392 0.0000 0.0000
8074.2984 6812.9368 307.8860 -1261.3616 6877.5887
308.8339 58.3381 106.5279 680285.8938 -13.0169
-12.9972
[41330]ENERGY: 10490000 3293.9321 5419.3336 5722.4320
343.2685 -14286.4525 -1815.4254 0.0000 0.0000
8134.6213 6811.7097 310.1862 -1322.9116 6878.4099
308.8320 48.6472 -18.4329 680285.8938 -11.7805
-11.7923
[41369]ENERGY: 10500000 3372.4426 5471.7899 5688.8358
341.3369 -14337.7395 -1794.5584 0.0000 0.0000
8071.9737 6814.0810 307.7974 -1257.8928 6878.2406
308.6515 33.1584 -43.2977 680285.8938 -16.3030
-16.3040
[41419]ENERGY: 10510000 3314.7141 5530.5216 5776.7798
343.2896 -14370.5534 -1852.4283 0.0000 0.0000
8069.3961 6811.7193 307.6991 -1257.6768 6878.9469
308.8351 -140.1342 -194.0855 680285.8938 -16.1774
-16.1748
[41455]ENERGY: 10520000 3378.6492 5380.8891 5704.7430
343.0373 -14323.3403 -1814.3614 0.0000 0.0000
8140.1934 6809.8103 310.3987 -1330.3831 6876.5215
308.8385 -89.1465 -73.0222 680285.8938 -16.2564
-16.2590
[41505]ENERGY: 10530000 3320.8733 5391.1260 5697.0540
347.9723 -14339.8480 -1776.4080 0.0000 0.0000

8167.4892 6808.2589 311.4395 -1359.2304 6878.0749
308.8208 -57.8266 -125.7428 680285.8938 -12.3868
-12.3901
[41541]ENERGY: 10540000 3374.7484 5465.6631 5701.0005
349.7573 -14335.0493 -1814.6037 0.0000 0.0000
8069.8738 6811.3900 307.7173 -1258.4837 6878.6885
308.7137 90.5227 40.4200 680285.8938 -12.1258
-12.1236
[41591]ENERGY: 10550000 3244.7889 5530.7712 5694.4203
310.8649 -14315.3730 -1782.4844 0.0000 0.0000
8128.7346 6811.7226 309.9618 -1317.0120 6879.3519
308.8160 132.6875 103.1718 680285.8938 -12.9539
-12.9538
[41627]ENERGY: 10560000 3316.8537 5458.1779 5761.3826
356.5622 -14334.4245 -1824.4152 0.0000 0.0000
8078.7152 6812.8519 308.0544 -1265.8633 6878.4151
308.8318 -87.9289 -14.8680 680285.8938 -13.0526
-13.0613
[41677]ENERGY: 10570000 3361.3141 5424.1160 5715.9126
376.3811 -14297.8263 -1820.2103 0.0000 0.0000
8056.1097 6815.7969 307.1925 -1240.3128 6879.8177
308.6694 34.0468 13.1009 680285.8938 -14.1632
-14.1492
[41713]ENERGY: 10580000 3299.7174 5430.1437 5726.4653
312.1373 -14278.4256 -1769.4088 0.0000 0.0000
8094.0225 6814.6520 308.6381 -1279.3706 6880.4206
308.6784 -32.4319 -21.7650 680285.8938 -14.5655
-14.5614
[41763]ENERGY: 10590000 3332.7449 5522.3971 5735.3398
340.9414 -14298.1060 -1854.7318 0.0000 0.0000
8038.6556 6817.2412 306.5269 -1221.4144 6881.4802
308.7535 20.5130 45.1508 680285.8938 -14.0117
-14.0040
[41802]ENERGY: 10600000 3308.1271 5342.3886 5724.4618
341.3873 -14293.9765 -1685.6099 0.0000 0.0000
8084.0864 6820.8648 308.2593 -1263.2216 6880.8600
308.7292 73.7746 84.9664 680285.8938 -15.7041
-15.7315
[41852]ENERGY: 10610000 3377.4872 5412.2274 5722.1604
341.4816 -14366.0203 -1744.9511 0.0000 0.0000
8072.5982 6814.9834 307.8212 -1257.6148 6880.6343
308.5193 -7.6933 99.2621 680285.8938 -12.6975
-12.6818
[41888]ENERGY: 10620000 3348.4207 5391.6635 5751.2940
344.6399 -14367.0182 -1724.8598 0.0000 0.0000
8068.0631 6812.2032 307.6483 -1255.8599 6880.4574
308.6282 -55.1104 -63.2305 680285.8938 -9.5805
-9.5794
[41938]ENERGY: 10630000 3271.9498 5346.0474 5694.8576
344.6064 -14244.9198 -1742.7272 0.0000 0.0000
8145.3192 6815.1334 310.5942 -1330.1858 6880.7793
308.6220 51.9611 9.1311 680285.8938 -9.8934
-9.9053
[41974]ENERGY: 10640000 3251.7926 5406.5608 5711.7739

332.3586 -14179.8642 -1795.8591 0.0000 0.0000
8088.6992 6815.4618 308.4351 -1273.2374 6881.4963
308.4804 -42.7926 -21.8835 680285.8938 -11.3897
-11.3916
[42024]ENERGY: 10650000 3187.1467 5360.8218 5756.2110
350.3083 -14238.2380 -1795.4150 0.0000 0.0000
8196.1407 6816.9756 312.5321 -1379.1651 6881.0155
308.5252 53.1048 43.5228 680285.8938 -9.6827
-9.6753
[42060]ENERGY: 10660000 3376.9204 5458.4465 5724.4563
328.9953 -14342.5615 -1791.5859 0.0000 0.0000
8063.1449 6817.8160 307.4607 -1245.3289 6881.7377
308.7314 13.6694 36.2576 680285.8938 -9.3829
-9.3790
[42110]ENERGY: 10670000 3346.9934 5389.9527 5671.0807
332.6317 -14199.1520 -1825.1486 0.0000 0.0000
8101.1663 6817.5242 308.9105 -1283.6421 6881.7194
308.7021 52.5950 -37.8609 680285.8938 -10.2179
-10.2138
[42146]ENERGY: 10680000 3380.9795 5330.8423 5758.1322
311.6288 -14170.6114 -1877.6774 0.0000 0.0000
8083.2762 6816.5701 308.2284 -1266.7061 6883.2039
308.7954 -81.2759 -128.6364 680285.8938 -11.5868
-11.5859
[42196]ENERGY: 10690000 3301.1788 5314.7782 5744.3664
350.5147 -14257.8463 -1828.8652 0.0000 0.0000
8192.6056 6816.7321 312.3973 -1375.8735 6884.0394
308.8714 9.8196 -0.1095 680285.8938 -10.1057
-10.1042
[42235]ENERGY: 10700000 3325.6894 5380.5795 5760.6722
334.7319 -14294.7304 -1790.3254 0.0000 0.0000
8102.5528 6819.1700 308.9634 -1283.3827 6882.5738
308.9795 45.6137 45.5750 680285.8938 -11.6596
-11.6661
[42285]ENERGY: 10710000 3330.0936 5447.6233 5787.5619
367.8801 -14354.6915 -1857.3466 0.0000 0.0000
8094.8865 6816.0073 308.6711 -1278.8791 6883.7824
308.9232 -149.2367 -131.1838 680285.8938 -12.2893
-12.3002
[42321]ENERGY: 10720000 3228.0926 5451.0008 5736.9245
345.7889 -14263.3559 -1857.1046 0.0000 0.0000
8172.8450 6814.1912 311.6438 -1358.6538 6883.6228
308.9142 -18.8187 11.2268 680285.8938 -8.5889
-8.5751
[42371]ENERGY: 10730000 3416.0325 5367.6904 5775.7612
339.3018 -14327.5339 -1779.4617 0.0000 0.0000
8032.6515 6824.4419 306.2980 -1208.2096 6884.2664
308.8555 11.6773 -53.1491 680285.8938 -10.9070
-10.9041
[42407]ENERGY: 10740000 3483.6586 5452.2467 5710.4849
359.6505 -14441.9186 -1755.7373 0.0000 0.0000
8011.1341 6819.5189 305.4775 -1191.6152 6883.7146
308.8741 -91.7014 0.1446 680285.8938 -13.1386
-13.1506

[42457]ENERGY: 10750000 3315.6845 5479.6398 5736.6528
333.9087 -14298.7652 -1795.3756 0.0000 0.0000
8047.8938 6819.6388 306.8792 -1228.2550 6882.9121
308.6531 5.4050 -27.8181 680285.8938 -13.2178
-13.2162
[42493]ENERGY: 10760000 3311.8514 5481.2081 5709.4698
346.6325 -14347.3270 -1759.7597 0.0000 0.0000
8078.3242 6820.3993 308.0395 -1257.9249 6882.7870
309.0125 16.5591 85.9098 680285.8938 -10.3232
-10.3214
[42543]ENERGY: 10770000 3247.9927 5567.8444 5736.2362
340.1693 -14323.3706 -1843.1621 0.0000 0.0000
8093.7550 6819.4649 308.6279 -1274.2901 6882.8615
308.9895 -39.2145 -37.2054 680285.8938 -11.8868
-11.8727
[42579]ENERGY: 10780000 3355.7496 5362.4983 5714.9011
336.0381 -14276.5400 -1768.3471 0.0000 0.0000
8094.0624 6818.3624 308.6396 -1275.6999 6882.8389
308.6666 -166.9246 -183.5063 680285.8938 -15.8446
-15.8633
[42629]ENERGY: 10790000 3373.4578 5423.8512 5729.5247
360.6471 -14325.4223 -1806.1224 0.0000 0.0000
8060.0911 6816.0273 307.3443 -1244.0639 6882.7127
308.6887 -239.3582 -223.4032 680285.8938 -14.6982
-14.6949
[42668]ENERGY: 10800000 3327.3985 5448.3803 5710.0061
335.6572 -14335.4930 -1800.0554 0.0000 0.0000
8131.0359 6816.9296 310.0495 -1314.1063 6884.2245
308.7971 44.9054 -28.7769 680285.8938 -16.9532
-16.9500
[42718]ENERGY: 10810000 3320.0289 5486.1470 5705.2587
346.4643 -14329.6984 -1772.9834 0.0000 0.0000
8060.0224 6815.2395 307.3417 -1244.7829 6882.7621
308.7461 141.7130 110.9269 680285.8938 -17.7717
-17.7699
[42754]ENERGY: 10820000 3330.5209 5457.1561 5731.6452
325.5769 -14331.4998 -1803.5432 0.0000 0.0000
8106.7973 6816.6534 309.1253 -1290.1440 6882.8682
308.8906 17.3547 -59.7580 680285.8938 -19.4193
-19.4228
[42804]ENERGY: 10830000 3326.0893 5478.1145 5707.2873
330.0903 -14288.2253 -1762.2468 0.0000 0.0000
8029.2494 6820.3587 306.1682 -1208.8907 6882.9142
308.6612 -19.7002 0.7949 680285.8938 -15.2384
-15.2404
[42840]ENERGY: 10840000 3376.5694 5462.5563 5736.4280
347.4620 -14297.5858 -1870.0570 0.0000 0.0000
8064.5967 6819.9696 307.5161 -1244.6271 6884.0255
308.8356 -48.8493 -90.1180 680285.8938 -13.7297
-13.7284
[42890]ENERGY: 10850000 3329.7885 5455.5602 5737.8602
347.1715 -14336.5675 -1748.6968 0.0000 0.0000
8036.0665 6821.1825 306.4282 -1214.8840 6882.6084
308.9357 75.5230 87.3285 680285.8938 -17.0095

-16.9992
[42926]ENERGY: 10860000 3262.2420 5450.2477 5648.0730
350.4858 -14194.7974 -1804.2559 0.0000 0.0000
8105.0343 6817.0295 309.0580 -1288.0048 6883.2521
308.6971 105.4039 81.9242 680285.8938 -19.7987
-19.8218
[42976]ENERGY: 10870000 3367.0507 5474.2677 5754.8818
335.6898 -14360.4620 -1796.9903 0.0000 0.0000
8044.4529 6818.8906 306.7480 -1225.5622 6884.4972
308.6762 -127.6109 -138.9860 680285.8938 -17.1858
-17.1518
[43012]ENERGY: 10880000 3340.0673 5478.3042 5716.5458
338.4465 -14333.4048 -1788.4191 0.0000 0.0000
8067.8998 6819.4397 307.6420 -1248.4601 6883.1965
308.7172 117.5682 41.2024 680285.8938 -13.9447
-13.9690
[43062]ENERGY: 10890000 3298.0321 5449.7920 5726.9949
322.8690 -14362.9095 -1703.5197 0.0000 0.0000
8089.3120 6820.5709 308.4585 -1268.7410 6882.8568
308.6372 100.2382 102.6090 680285.8938 -16.1194
-16.1171
[43101]ENERGY: 10900000 3317.5512 5436.2969 5682.0487
369.4056 -14317.3261 -1856.4859 0.0000 0.0000
8183.3135 6814.8039 312.0429 -1368.5096 6884.6232
308.7906 -23.0740 -28.2252 680285.8938 -10.0898
-10.0935
[43151]ENERGY: 10910000 3375.6814 5419.6241 5707.9331
332.3632 -14298.5659 -1782.2634 0.0000 0.0000
8064.9612 6819.7337 307.5300 -1245.2275 6884.3191
308.8820 18.6947 -30.9554 680285.8938 -13.8078
-13.8070
[43187]ENERGY: 10920000 3277.1204 5384.6789 5723.5237
315.1305 -14258.9039 -1753.3288 0.0000 0.0000
8126.8297 6815.0505 309.8891 -1311.7792 6882.9883
308.8314 64.2098 71.0942 680285.8938 -14.8597
-14.8422
[43237]ENERGY: 10930000 3321.6192 5494.2187 5721.5200
344.4184 -14332.5148 -1844.3271 0.0000 0.0000
8111.2579 6816.1922 309.2953 -1295.0657 6882.6056
308.6069 -176.4113 -75.0655 680285.8938 -20.8190
-20.8443
[43273]ENERGY: 10940000 3333.0772 5494.3331 5770.6004
354.9219 -14379.3479 -1791.4446 0.0000 0.0000
8037.4696 6819.6098 306.4817 -1217.8598 6883.6104
308.8553 10.0517 -73.3409 680285.8938 -15.7141
-15.7141
[43323]ENERGY: 10950000 3243.8957 5465.8466 5762.0209
369.5008 -14341.2295 -1777.9283 0.0000 0.0000
8095.7124 6817.8185 308.7026 -1277.8939 6882.7826
308.7622 -56.4048 -115.9830 680285.8938 -16.5140
-16.4986
[43359]ENERGY: 10960000 3357.0811 5452.9489 5715.0933
347.3939 -14429.6685 -1731.1720 0.0000 0.0000
8106.1974 6817.8741 309.1024 -1288.3233 6883.6366

308.9043 200.1176 45.5874 680285.8938 -15.4225
-15.4265
[43409]ENERGY: 10970000 3370.6640 5489.1944 5720.1392
327.6487 -14425.5467 -1777.8762 0.0000 0.0000
8113.2947 6817.5181 309.3730 -1295.7766 6885.1100
308.9692 -100.0975 -50.7046 680285.8938 -11.2091
-11.2008
[43445]ENERGY: 10980000 3351.5819 5416.2243 5723.1060
360.2832 -14303.8492 -1799.5788 0.0000 0.0000
8070.9066 6818.6741 307.7567 -1252.2325 6883.8049
308.7577 -47.5294 -95.3763 680285.8938 -9.9867
-10.0086
[43495]ENERGY: 10990000 3381.1509 5454.8131 5786.6725
324.0349 -14388.0644 -1756.0958 0.0000 0.0000
8017.3756 6819.8867 305.7155 -1197.4889 6885.6524
308.9278 -19.9059 -55.1728 680285.8938 -13.3563
-13.3456
[43534]ENERGY: 11000000 3294.6098 5452.3133 5711.3377
365.6265 -14311.0760 -1799.6283 0.0000 0.0000
8105.4169 6818.5999 309.0726 -1286.8170 6885.1089
308.6374 86.0328 65.6667 680285.8938 -18.9896
-18.9948
[268]ENERGY: 11010000 3320.5106 5448.1170 5734.4918
318.0675 -14357.9019 -1751.0344 0.0000 0.0000
8106.2321 6818.4827 309.1037 -1287.7494 6884.8622
308.8820 39.5606 3.4171 680285.8938 -15.6539
-15.6620
[307]ENERGY: 11020000 3376.4314 5374.6354 5725.5252
315.3077 -14249.1423 -1838.1849 0.0000 0.0000
8114.5583 6819.1309 309.4212 -1295.4274 6885.5044
308.7539 -14.4047 -44.0385 680285.8938 -13.5385
-13.5099
[359]ENERGY: 11030000 3337.3965 5418.2168 5706.7587
334.7096 -14329.4808 -1727.1089 0.0000 0.0000
8080.7815 6821.2735 308.1332 -1259.5079 6884.8608
308.8359 83.9647 16.6910 680285.8938 -15.4840
-15.5043
[397]ENERGY: 11040000 3323.3114 5455.7089 5760.1204
326.5673 -14325.4030 -1834.7451 0.0000 0.0000
8116.4789 6822.0388 309.4944 -1294.4401 6886.2816
308.7298 21.6582 17.9814 680285.8938 -17.2724
-17.2578
[449]ENERGY: 11050000 3277.3529 5548.7865 5695.6170
344.7476 -14382.3101 -1726.0660 0.0000 0.0000
8062.7498 6820.8779 307.4457 -1241.8720 6885.4460
308.8321 91.0200 85.5271 680285.8938 -14.0331
-14.0240
[487]ENERGY: 11060000 3334.3216 5436.9391 5685.3184
323.4914 -14357.3725 -1820.2321 0.0000 0.0000
8215.3174 6817.7832 313.2633 -1397.5342 6886.8068
308.9768 100.2241 0.6784 680285.8938 -13.7824
-13.7989
[538]ENERGY: 11070000 3274.8645 5409.0722 5723.9850
351.2697 -14337.2258 -1732.3077 0.0000 0.0000

8130.6112 6820.2690 310.0333 -1310.3422 6886.3964
308.9379 147.0256 105.1415 680285.8938 -13.6866
-13.6857
[575]ENERGY: 11080000 3358.0929 5493.8629 5736.4514
333.3438 -14369.3537 -1762.8152 0.0000 0.0000
8031.8025 6821.3846 306.2656 -1210.4179 6886.2620
308.9402 28.6117 13.9070 680285.8938 -17.0268
-17.0429
[626]ENERGY: 11090000 3379.7842 5434.8489 5699.4440
357.4433 -14404.3072 -1772.5292 0.0000 0.0000
8123.3315 6818.0155 309.7557 -1305.3160 6886.0463
308.9764 -86.2667 -60.1166 680285.8938 -13.0833
-13.0618
[666]ENERGY: 11100000 3360.4135 5476.1985 5703.4595
339.8463 -14354.4994 -1764.2910 0.0000 0.0000
8060.0269 6821.1543 307.3418 -1238.8726 6886.7739
308.9744 66.3717 40.2309 680285.8938 -16.6000
-16.5936
[717]ENERGY: 11110000 3355.9813 5487.2725 5647.6169
307.1635 -14264.1226 -1741.6846 0.0000 0.0000
8032.1315 6824.3585 306.2781 -1207.7730 6887.4236
308.9927 55.4396 142.8623 680285.8938 -19.4265
-19.4257
[753]ENERGY: 11120000 3335.7624 5390.6248 5742.1022
336.3099 -14323.7308 -1785.6438 0.0000 0.0000
8125.3549 6820.7796 309.8329 -1304.5753 6887.4811
309.0184 -57.6947 -8.4970 680285.8938 -13.0353
-13.0442
[803]ENERGY: 11130000 3260.9402 5458.7310 5701.9091
358.0477 -14243.6010 -1783.5362 0.0000 0.0000
8072.1147 6824.6054 307.8027 -1247.5093 6889.4148
308.9963 255.2630 124.9644 680285.8938 -11.9903
-12.0131
[839]ENERGY: 11140000 3293.8430 5376.7203 5704.8375
342.4486 -14261.8720 -1805.0907 0.0000 0.0000
8169.8449 6820.7316 311.5294 -1349.1133 6888.4634
308.9231 -46.8553 -82.1899 680285.8938 -14.2410
-14.2249
[889]ENERGY: 11150000 3371.0907 5411.3810 5677.6493
382.8085 -14309.6243 -1793.0323 0.0000 0.0000
8084.1997 6824.4726 308.2636 -1259.7271 6887.5590
309.1649 -24.5869 -20.8170 680285.8938 -13.6282
-13.6191
[925]ENERGY: 11160000 3318.4602 5543.2539 5664.1906
345.3077 -14399.4558 -1713.2451 0.0000 0.0000
8064.9002 6823.4118 307.5277 -1241.4885 6888.1995
309.3124 174.5437 27.7365 680285.8938 -12.4526
-12.4749
[975]ENERGY: 11170000 3363.5110 5449.7006 5698.5956
342.6959 -14348.8200 -1784.1662 0.0000 0.0000
8098.8117 6820.3286 308.8207 -1278.4830 6888.2686
309.2294 -98.4571 -52.5776 680285.8938 -14.5666
-14.5527
[1011]ENERGY: 11180000 3356.8657 5382.1545 5658.1571

336.2644 -14272.5680 -1770.0032 0.0000 0.0000
8133.8779 6824.7484 310.1579 -1309.1295 6888.8764
309.2166 -42.0239 -126.0820 680285.8938 -14.3005
-14.3002
[1061]ENERGY: 11190000 3372.3172 5412.3043 5752.9723
339.6431 -14271.8614 -1840.1794 0.0000 0.0000
8059.9496 6825.1458 307.3389 -1234.8039 6888.9834
309.1519 -71.3452 -38.4655 680285.8938 -16.2491
-16.2508
[1100]ENERGY: 11200000 3367.2437 5501.9656 5716.2143
350.0024 -14400.9916 -1787.5515 0.0000 0.0000
8076.0869 6822.9698 307.9542 -1253.1171 6889.2812
309.1204 -50.3838 -81.4713 680285.8938 -10.8427
-10.8419
[1150]ENERGY: 11210000 3371.8629 5464.7823 5698.6384
355.7156 -14313.5425 -1804.8353 0.0000 0.0000
8052.2958 6824.9172 307.0470 -1227.3786 6889.5222
309.0959 -30.9775 32.1563 680285.8938 -14.7836
-14.7888
[1186]ENERGY: 11220000 3307.1087 5445.7753 5702.6225
354.8591 -14386.4044 -1749.1074 0.0000 0.0000
8147.6980 6822.5519 310.6849 -1325.1461 6889.2029
309.1424 209.8605 133.2372 680285.8938 -15.5606
-15.5501
[1236]ENERGY: 11230000 3348.2180 5428.1347 5769.9362
337.8298 -14299.0493 -1862.7991 0.0000 0.0000
8100.3501 6822.6203 308.8794 -1277.7298 6888.6466
309.1161 77.9794 -3.0004 680285.8938 -14.1424
-14.1569
[1272]ENERGY: 11240000 3299.3712 5397.8126 5764.8693
341.1973 -14217.1111 -1834.9876 0.0000 0.0000
8074.3988 6825.5506 307.8898 -1248.8483 6889.5071
308.9335 -58.5082 -108.3714 680285.8938 -12.5328
-12.5089
[1322]ENERGY: 11250000 3362.2721 5429.6244 5685.0068
347.3377 -14359.4407 -1765.5744 0.0000 0.0000
8124.0415 6823.2674 309.7828 -1300.7741 6888.5573
308.9721 -58.6499 -2.7699 680285.8938 -15.9495
-15.9827
[1358]ENERGY: 11260000 3263.5615 5459.0587 5779.1286
345.7573 -14317.4858 -1802.3494 0.0000 0.0000
8095.7812 6823.4520 308.7052 -1272.3291 6888.5923
309.2891 -53.6925 19.9559 680285.8938 -14.5430
-14.5345
[1408]ENERGY: 11270000 3346.8551 5496.4809 5717.6892
328.6975 -14333.4734 -1799.9529 0.0000 0.0000
8070.7780 6827.0743 307.7518 -1243.7036 6889.4259
309.2913 -38.2132 -32.5251 680285.8938 -15.3473
-15.3349
[1444]ENERGY: 11280000 3332.6452 5439.2362 5748.0883
333.5117 -14395.5930 -1731.9701 0.0000 0.0000
8097.3611 6823.2793 308.7654 -1274.0818 6890.5527
308.9542 5.1083 49.8542 680285.8938 -15.1198
-15.1303

[1494]ENERGY: 11290000 3403.5252 5513.4426 5725.9765
344.1493 -14268.9026 -1836.9910 0.0000 0.0000
7954.0182 6835.2183 303.2995 -1118.8000 6891.2018
309.0524 32.2326 12.6999 680285.8938 -18.2816
-18.2703
[1533]ENERGY: 11300000 3350.0079 5400.4149 5692.3261
324.0131 -14339.7698 -1724.7572 0.0000 0.0000
8123.9521 6826.1871 309.7794 -1297.7650 6890.4550
309.0843 51.3861 -28.9720 680285.8938 -15.3425
-15.3443
[1583]ENERGY: 11310000 3331.6617 5436.1057 5740.6444
350.6043 -14220.7621 -1887.4963 0.0000 0.0000
8073.2439 6824.0015 307.8458 -1249.2424 6890.5480
309.1214 -161.2366 -231.6127 680285.8938 -16.0427
-16.0331
[1619]ENERGY: 11320000 3239.0338 5480.1042 5762.9946
346.6042 -14349.9933 -1784.7414 0.0000 0.0000
8131.5550 6825.5573 310.0693 -1305.9978 6891.3957
309.1896 66.9972 -13.6478 680285.8938 -15.4583
-15.4704
[1669]ENERGY: 11330000 3256.0731 5513.6156 5744.4285
339.8170 -14321.1622 -1760.3802 0.0000 0.0000
8058.4348 6830.8266 307.2811 -1227.6082 6892.4694
309.0845 91.9885 37.5111 680285.8938 -18.4857
-18.4732
[1705]ENERGY: 11340000 3292.3750 5502.7186 5774.3874
341.3405 -14338.8887 -1770.9899 0.0000 0.0000
8028.2042 6829.1471 306.1284 -1199.0571 6892.1045
309.0666 27.6404 -38.2034 680285.8938 -16.2819
-16.2898
[1755]ENERGY: 11350000 3335.3195 5510.3221 5699.3296
347.1702 -14327.2677 -1855.0815 0.0000 0.0000
8118.8062 6828.5985 309.5832 -1290.2077 6892.7528
309.1197 8.5354 36.4117 680285.8938 -18.2743
-18.2604
[1791]ENERGY: 11360000 3389.2153 5496.9926 5734.0224
340.5613 -14409.2546 -1790.6077 0.0000 0.0000
8068.8661 6829.7955 307.6789 -1239.0707 6892.1181
308.9758 145.7549 68.4302 680285.8938 -15.0480
-15.0727
[1841]ENERGY: 11370000 3359.4653 5467.4122 5729.5101
352.8801 -14317.2102 -1807.5754 0.0000 0.0000
8045.0264 6829.5084 306.7698 -1215.5180 6893.2422
308.9870 121.8283 83.9783 680285.8938 -15.7399
-15.7446
[1877]ENERGY: 11380000 3302.8918 5477.6200 5723.1230
335.8811 -14369.9971 -1789.1615 0.0000 0.0000
8146.5219 6826.8792 310.6400 -1319.6428 6893.9381
308.9460 -105.1648 -47.3744 680285.8938 -16.0505
-16.0349
[1927]ENERGY: 11390000 3316.0643 5513.5584 5740.5491
387.7312 -14407.8127 -1776.6642 0.0000 0.0000
8056.5446 6829.9707 307.2090 -1226.5739 6894.4164
309.0770 58.1178 75.0092 680285.8938 -14.5471

-14.5482
[1966]ENERGY: 11400000 3284.6674 5545.2573 5747.0819
334.4272 -14353.9003 -1801.8449 0.0000 0.0000
8072.9263 6828.6150 307.8337 -1244.3113 6893.6091
309.0683 -7.3972 -32.6845 680285.8938 -16.3164
-16.3235
[2016]ENERGY: 11410000 3286.8431 5425.2238 5736.4709
358.6401 -14340.5282 -1765.0344 0.0000 0.0000
8123.2651 6824.8804 309.7532 -1298.3847 6893.4588
309.1424 114.0045 106.4729 680285.8938 -13.9474
-13.9340
[2052]ENERGY: 11420000 3336.3782 5501.1617 5744.8401
345.5804 -14308.8510 -1814.4829 0.0000 0.0000
8027.1347 6831.7612 306.0876 -1195.3735 6894.8009
308.8179 111.9488 27.6507 680285.8938 -21.3645
-21.3932
[2102]ENERGY: 11430000 3385.3710 5524.4898 5711.3220
338.4479 -14430.0229 -1769.1636 0.0000 0.0000
8067.9042 6828.3485 307.6422 -1239.5558 6893.0792
308.9476 -55.3445 -13.2561 680285.8938 -19.6273
-19.5957
[2138]ENERGY: 11440000 3263.0888 5373.4922 5760.7308
354.7981 -14307.7850 -1840.4404 0.0000 0.0000
8220.1585 6824.0430 313.4479 -1396.1156 6894.1859
309.1762 -43.7150 -138.4314 680285.8938 -19.6277
-19.6448
[2188]ENERGY: 11450000 3365.8251 5408.8587 5681.9161
359.6182 -14357.8211 -1702.1864 0.0000 0.0000
8076.0821 6832.2927 307.9540 -1243.7894 6894.7104
309.1266 29.9713 8.6379 680285.8938 -17.8480
-17.8425
[2224]ENERGY: 11460000 3317.8626 5383.0856 5708.8931
342.2130 -14238.7805 -1782.1213 0.0000 0.0000
8100.3552 6831.5077 308.8796 -1268.8475 6895.1054
308.9543 95.4078 48.0380 680285.8938 -18.1246
-18.1232
[2274]ENERGY: 11470000 3302.3602 5478.7386 5722.7073
325.8737 -14251.9342 -1814.7945 0.0000 0.0000
8066.8834 6829.8345 307.6033 -1237.0490 6896.2526
309.0343 118.9317 14.8704 680285.8938 -14.3935
-14.3864
[2310]ENERGY: 11480000 3338.1582 5519.1908 5696.7992
340.8877 -14283.4440 -1808.7018 0.0000 0.0000
8026.0356 6828.9258 306.0457 -1197.1098 6895.7066
308.9784 -78.2287 -42.6187 680285.8938 -13.9948
-13.9951
[2360]ENERGY: 11490000 3373.4736 5480.2643 5707.2402
355.1781 -14402.9889 -1759.3402 0.0000 0.0000
8074.8311 6828.6582 307.9063 -1246.1729 6895.2823
309.0925 25.8183 -74.2250 680285.8938 -15.0605
-15.0669
[2399]ENERGY: 11500000 3319.9022 5354.1049 5754.9140
322.9389 -14376.5047 -1752.9402 0.0000 0.0000
8204.2101 6826.6251 312.8398 -1377.5850 6896.2467

309.1473 -16.2674 -37.1287 680285.8938 -15.2631
-15.2599
[2449]ENERGY: 11510000 3279.0079 5465.5801 5658.4287
327.7480 -14302.7129 -1754.9948 0.0000 0.0000
8155.0580 6828.1150 310.9655 -1326.9430 6894.9303
309.0994 -19.5643 -2.0163 680285.8938 -12.1624
-12.1811
[2485]ENERGY: 11520000 3368.5586 5486.0568 5701.7340
347.2333 -14341.9921 -1785.8508 0.0000 0.0000
8056.1861 6831.9259 307.1954 -1224.2602 6894.3791
308.9859 99.1359 58.1199 680285.8938 -10.4047
-10.3901
[2535]ENERGY: 11530000 3330.3253 5461.0295 5709.6775
334.6625 -14399.4786 -1703.7183 0.0000 0.0000
8098.7316 6831.2295 308.8177 -1267.5021 6896.1771
309.2160 97.6587 76.0194 680285.8938 -13.1817
-13.1815
[2571]ENERGY: 11540000 3330.6799 5489.2498 5683.0810
345.0574 -14341.7968 -1760.4607 0.0000 0.0000
8089.0316 6834.8422 308.4478 -1254.1894 6896.5789
309.0585 -25.2383 -21.2003 680285.8938 -13.7180
-13.7146
[2621]ENERGY: 11550000 3300.3915 5559.5190 5676.3493
344.4370 -14386.8837 -1699.0707 0.0000 0.0000
8037.8607 6832.6030 306.4966 -1205.2577 6897.3047
309.2766 -21.5454 -21.5853 680285.8938 -13.8918
-13.8943
[2657]ENERGY: 11560000 3285.9307 5490.4423 5700.9185
338.8975 -14329.3796 -1730.8425 0.0000 0.0000
8080.2685 6836.2353 308.1137 -1244.0332 6899.4595
309.1347 38.3783 -45.0559 680285.8938 -12.0632
-12.0608
[2707]ENERGY: 11570000 3361.7349 5418.2195 5745.3799
340.6612 -14347.7088 -1802.8442 0.0000 0.0000
8118.2241 6833.6666 309.5610 -1284.5575 6898.6769
309.2522 100.4932 13.6196 680285.8938 -16.3766
-16.3863
[2743]ENERGY: 11580000 3382.8065 5411.1318 5665.5537
355.9263 -14340.8682 -1789.8569 0.0000 0.0000
8149.3442 6834.0374 310.7476 -1315.3068 6899.0588
309.0773 -1.7555 -119.0495 680285.8938 -17.5806
-17.5655
[2793]ENERGY: 11590000 3377.7194 5394.4692 5667.6796
338.5903 -14300.1530 -1767.5288 0.0000 0.0000
8122.6897 6833.4665 309.7313 -1289.2233 6897.5696
309.1820 -53.2608 6.0698 680285.8938 -14.4338
-14.4117
[2832]ENERGY: 11600000 3352.6613 5423.0101 5702.1745
344.1536 -14350.0337 -1767.9989 0.0000 0.0000
8131.7867 6835.7537 310.0781 -1296.0330 6900.4854
309.2217 23.7982 42.5102 680285.8938 -15.6191
-15.6508
[2882]ENERGY: 11610000 3343.8143 5496.1095 5703.3196
329.8928 -14386.2708 -1779.2085 0.0000 0.0000

8125.7574 6833.4143 309.8482 -1292.3431 6899.4088
309.0361 97.7388 22.8067 680285.8938 -12.3009
-12.3127
[2918]ENERGY: 11620000 3335.2895 5505.4075 5711.2639
326.2694 -14418.5005 -1735.0549 0.0000 0.0000
8105.6376 6830.3125 309.0810 -1275.3251 6899.3744
309.1023 22.0032 47.4431 680285.8938 -10.2725
-10.2616
[2968]ENERGY: 11630000 3351.7471 5481.1961 5693.4362
314.4345 -14324.6849 -1883.1800 0.0000 0.0000
8198.4790 6831.4279 312.6212 -1367.0511 6899.2613
309.2059 -173.2269 -101.3799 680285.8938 -14.7864
-14.7840
[3004]ENERGY: 11640000 3268.2466 5478.2340 5693.0658
358.2170 -14250.1073 -1825.8725 0.0000 0.0000
8112.2260 6834.0095 309.3323 -1278.2164 6901.2100
308.9999 5.0991 -30.9036 680285.8938 -15.1875
-15.1826
[3054]ENERGY: 11650000 3414.5246 5528.6062 5731.5903
341.5309 -14424.0453 -1761.0081 0.0000 0.0000
8008.2100 6839.4087 305.3660 -1168.8014 6901.8127
308.8775 17.4594 29.1422 680285.8938 -15.9255
-15.9277
[3090]ENERGY: 11660000 3263.7084 5500.7642 5734.3973
345.6518 -14328.3274 -1785.7131 0.0000 0.0000
8104.5120 6834.9931 309.0381 -1269.5189 6902.1945
309.0215 85.8495 30.4674 680285.8938 -13.8731
-13.8904
[3140]ENERGY: 11670000 3309.8221 5340.3018 5701.8995
341.6917 -14290.5406 -1751.9103 0.0000 0.0000
8182.4301 6833.6944 312.0093 -1348.7357 6901.4639
309.1137 117.0014 64.0455 680285.8938 -14.1165
-14.1000
[3176]ENERGY: 11680000 3363.2946 5486.9095 5753.7808
366.5492 -14413.7745 -1807.0289 0.0000 0.0000
8089.6350 6839.3657 308.4708 -1250.2693 6903.1343
309.0113 -37.1612 -77.8819 680285.8938 -15.6836
-15.6733
[3226]ENERGY: 11690000 3280.0806 5446.4941 5760.9446
334.1188 -14226.8447 -1845.6609 0.0000 0.0000
8090.6797 6839.8122 308.5107 -1250.8675 6903.8033
309.1020 153.0728 56.4418 680285.8938 -14.1957
-14.2253
[3265]ENERGY: 11700000 3316.8645 5522.3058 5680.7473
332.6634 -14159.8887 -1858.1517 0.0000 0.0000
8008.3238 6842.8642 305.3703 -1165.4595 6903.0145
309.1789 -86.4641 -86.7969 680285.8938 -13.4361
-13.4168
[3315]ENERGY: 11710000 3322.9568 5348.7327 5775.1612
325.5304 -14283.6567 -1842.3799 0.0000 0.0000
8189.5700 6835.9145 312.2815 -1353.6555 6902.3035
308.9140 -4.6967 -47.1018 680285.8938 -18.0681
-18.0672
[3351]ENERGY: 11720000 3318.4892 5451.1597 5673.4163

321.9012 -14315.7748 -1777.4360 0.0000 0.0000
8166.5932 6838.3489 311.4054 -1328.2444 6902.8745
309.0104 -98.1401 -46.6930 680285.8938 -14.1375
-14.1415
[3401]ENERGY: 11730000 3358.8546 5422.1488 5694.9437
331.1809 -14388.4851 -1719.8203 0.0000 0.0000
8139.0752 6837.8978 310.3561 -1301.1774 6903.6734
309.1415 -87.8382 -19.2566 680285.8938 -14.3349
-14.3129
[3437]ENERGY: 11740000 3297.6257 5542.1901 5684.7962
334.3075 -14355.0725 -1735.2885 0.0000 0.0000
8071.3368 6839.8954 307.7731 -1231.4415 6902.8920
309.2917 15.6979 -40.7188 680285.8938 -14.6001
-14.6367
[3487]ENERGY: 11750000 3366.7587 5391.8059 5750.2958
317.4947 -14266.8581 -1805.4817 0.0000 0.0000
8083.1196 6837.1349 308.2224 -1245.9847 6902.8400
309.0994 -26.2906 -85.6148 680285.8938 -17.1737
-17.1615
[3523]ENERGY: 11760000 3349.6478 5422.1140 5797.2616
350.5034 -14375.1600 -1799.3130 0.0000 0.0000
8092.6799 6837.7338 308.5869 -1254.9461 6904.1727
309.1303 -7.0967 -65.2261 680285.8938 -13.9022
-13.9036
[3573]ENERGY: 11770000 3353.5356 5405.8226 5717.8957
335.7050 -14362.2689 -1723.3960 0.0000 0.0000
8111.6389 6838.9329 309.3099 -1272.7060 6903.4708
309.0199 -2.9744 -70.0344 680285.8938 -14.8461
-14.8312
[3609]ENERGY: 11780000 3379.2284 5493.5316 5735.6027
337.9179 -14383.4266 -1739.8439 0.0000 0.0000
8017.3775 6840.3875 305.7155 -1176.9900 6903.8314
309.0300 -42.5205 13.9992 680285.8938 -18.4936
-18.4875
[3659]ENERGY: 11790000 3351.9302 5340.0380 5701.1695
337.0987 -14258.9268 -1775.8693 0.0000 0.0000
8141.5735 6837.0139 310.4513 -1304.5596 6904.1114
309.0250 -33.7155 -20.4908 680285.8938 -17.5466
-17.5528
[3698]ENERGY: 11800000 3411.7070 5473.0879 5714.1749
323.5368 -14332.0283 -1783.5368 0.0000 0.0000
8034.3730 6841.3145 306.3636 -1193.0585 6905.0550
309.2111 -92.1991 -41.1388 680285.8938 -19.3576
-19.3588
[3748]ENERGY: 11810000 3374.5113 5419.2321 5676.6063
350.2531 -14339.2024 -1824.0293 0.0000 0.0000
8178.7819 6836.1530 311.8701 -1342.6289 6905.1241
309.3706 25.9860 -24.5022 680285.8938 -19.0853
-19.0895
[3784]ENERGY: 11820000 3354.0367 5453.7420 5755.6581
339.6244 -14292.6734 -1777.5686 0.0000 0.0000
8008.6615 6841.4807 305.3832 -1167.1808 6904.3698
309.3354 -24.3355 -4.0933 680285.8938 -16.4086
-16.4099

[3834]ENERGY: 11830000 3303.1011 5593.5444 5793.5313
354.4581 -14448.3996 -1757.6221 0.0000 0.0000
8001.8865 6840.4997 305.1248 -1161.3868 6904.6185
309.3110 -98.2787 -86.5455 680285.8938 -21.2466
-21.2436
[3870]ENERGY: 11840000 3362.6322 5354.5350 5716.4616
319.5507 -14305.6826 -1793.8629 0.0000 0.0000
8181.9856 6835.6195 311.9923 -1346.3660 6904.7475
309.3243 144.2748 111.1070 680285.8938 -16.0850
-16.0934
[3920]ENERGY: 11850000 3334.7700 5435.3310 5703.5035
339.6956 -14370.4416 -1655.4046 0.0000 0.0000
8054.4868 6841.9407 307.1306 -1212.5461 6904.9732
309.3527 88.3176 70.5728 680285.8938 -13.9453
-13.9487
[3956]ENERGY: 11860000 3405.7675 5525.8396 5675.3368
353.8199 -14408.7728 -1748.9679 0.0000 0.0000
8041.5182 6844.5413 306.6361 -1196.9769 6907.5108
309.3976 -28.5128 1.6186 680285.8938 -20.4408
-20.4478
[4006]ENERGY: 11870000 3370.9550 5500.3108 5729.7741
337.2974 -14448.2199 -1805.8793 0.0000 0.0000
8157.7536 6841.9916 311.0683 -1315.7620 6907.7445
309.4043 77.7777 62.9413 680285.8938 -14.4257
-14.4038
[4042]ENERGY: 11880000 3386.8073 5434.6625 5746.5453
365.0217 -14259.1925 -1847.3952 0.0000 0.0000
8020.3769 6846.8260 305.8299 -1173.5509 6908.7722
309.3508 5.9343 -55.7346 680285.8938 -15.7053
-15.7039
[4092]ENERGY: 11890000 3297.0491 5436.5617 5744.5426
344.9426 -14291.4710 -1804.1979 0.0000 0.0000
8118.0105 6845.4376 309.5528 -1272.5729 6908.4712
309.4428 29.8691 95.3117 680285.8938 -15.3112
-15.3088
[4131]ENERGY: 11900000 3361.2123 5417.8572 5675.4880
334.4200 -14322.5939 -1750.2600 0.0000 0.0000
8129.1751 6845.2986 309.9786 -1283.8765 6908.7754
309.7161 120.2599 144.5519 680285.8938 -14.7821
-14.8031
[4181]ENERGY: 11910000 3355.6982 5446.8918 5738.5125
334.4240 -14262.8649 -1819.5463 0.0000 0.0000
8050.0088 6843.1242 306.9598 -1206.8847 6908.8497
309.4343 -28.8875 -53.0924 680285.8938 -17.6114
-17.6042
[4217]ENERGY: 11920000 3301.4237 5477.3840 5772.5980
372.5179 -14368.4877 -1804.5220 0.0000 0.0000
8095.5441 6846.4579 308.6961 -1249.0862 6910.2036
309.5388 177.0990 70.5829 680285.8938 -14.0219
-14.0194
[4267]ENERGY: 11930000 3372.0528 5517.0385 5707.2740
328.1649 -14371.7418 -1827.9579 0.0000 0.0000
8116.5628 6841.3933 309.4976 -1275.1695 6910.3282
309.6226 -16.8542 2.8786 680285.8938 -15.9396

-15.9373
[4303]ENERGY: 11940000 3400.5542 5473.6944 5695.1403
345.4467 -14347.9981 -1748.5758 0.0000 0.0000
8025.8395 6844.1011 306.0382 -1181.7384 6908.7135
309.4819 -85.3696 -105.3194 680285.8938 -16.3208
-16.3229
[4353]ENERGY: 11950000 3297.1498 5419.7762 5744.7912
323.5402 -14239.4150 -1820.7773 0.0000 0.0000
8120.7350 6845.8001 309.6567 -1274.9349 6909.9061
309.6078 -23.2271 -75.6071 680285.8938 -18.5601
-18.5659
[4389]ENERGY: 11960000 3349.9474 5373.1703 5737.8897
336.9590 -14409.0686 -1672.6157 0.0000 0.0000
8126.2386 6842.5207 309.8666 -1283.7180 6908.1404
309.3885 28.8626 51.8206 680285.8938 -17.4524
-17.4373
[4439]ENERGY: 11970000 3304.5959 5489.6815 5724.7753
354.2372 -14293.3307 -1786.4196 0.0000 0.0000
8051.2445 6844.7842 307.0069 -1206.4603 6909.4245
309.3876 73.1521 63.0781 680285.8938 -14.4326
-14.4630
[4475]ENERGY: 11980000 3334.7327 5571.6895 5712.1921
334.9189 -14413.1839 -1792.3640 0.0000 0.0000
8094.5426 6842.5278 308.6580 -1252.0147 6909.9594
309.5215 53.8105 42.9784 680285.8938 -18.9611
-18.9404
[4525]ENERGY: 11990000 3341.8564 5486.5930 5685.4665
332.1888 -14339.8614 -1797.6855 0.0000 0.0000
8135.4568 6844.0146 310.2181 -1291.4422 6909.4457
309.5158 -163.6281 -99.5159 680285.8938 -19.3488
-19.3500
[4564]ENERGY: 12000000 3318.0706 5514.4437 5708.1136
359.9336 -14393.2433 -1831.3339 0.0000 0.0000
8167.2869 6843.2713 311.4318 -1324.0156 6910.5170
309.4817 123.6842 69.7441 680285.8938 -16.8423
-16.8381
[4614]ENERGY: 12010000 3326.8808 5503.3667 5718.8047
330.3234 -14328.9048 -1773.6969 0.0000 0.0000
8068.1959 6844.9698 307.6533 -1223.2260 6910.2338
309.4493 -86.1640 -114.3784 680285.8938 -20.2613
-20.2586
[4650]ENERGY: 12020000 3305.2793 5378.9032 5735.4983
329.0357 -14322.4212 -1763.6818 0.0000 0.0000
8180.5137 6843.1273 311.9362 -1337.3864 6909.2318
309.3564 202.1434 216.6141 680285.8938 -15.0011
-15.0165
[4700]ENERGY: 12030000 3381.6890 5525.0166 5672.9865
334.7290 -14364.3543 -1832.5287 0.0000 0.0000
8127.6437 6845.1819 309.9202 -1282.4618 6909.3656
309.3475 -58.2674 -52.3449 680285.8938 -18.9090
-18.8908
[4736]ENERGY: 12040000 3309.9076 5457.7060 5733.0324
327.5636 -14390.5827 -1748.1073 0.0000 0.0000
8151.2327 6840.7524 310.8196 -1310.4804 6909.3542

309.4675 158.8914 96.6384 680285.8938 -17.2092
-17.2160
[4786]ENERGY: 12050000 3221.1939 5431.1906 5753.0276
351.6786 -14288.0266 -1746.2805 0.0000 0.0000
8120.4780 6843.2617 309.6469 -1277.2163 6908.5237
309.5003 90.4242 5.2994 680285.8938 -15.4619
-15.4720
[4822]ENERGY: 12060000 3262.5165 5454.8677 5708.5025
337.9167 -14253.9116 -1775.7187 0.0000 0.0000
8110.0542 6844.2273 309.2494 -1265.8269 6909.6465
309.4860 -24.0462 -22.2526 680285.8938 -17.7365
-17.7174
[4872]ENERGY: 12070000 3342.0318 5422.8498 5702.7797
347.6042 -14343.0436 -1773.3794 0.0000 0.0000
8143.4428 6842.2853 310.5226 -1301.1575 6908.0334
309.4276 119.9590 44.9735 680285.8938 -20.6249
-20.6311
[4908]ENERGY: 12080000 3219.7874 5403.4827 5752.3472
348.9074 -14253.9958 -1824.6121 0.0000 0.0000
8199.4198 6845.3367 312.6571 -1354.0831 6910.5604
309.6186 53.5005 -14.2510 680285.8938 -20.2604
-20.2831
[4958]ENERGY: 12090000 3248.1054 5374.5677 5738.8187
360.0069 -14329.4221 -1723.3960 0.0000 0.0000
8177.6626 6846.3432 311.8275 -1331.3194 6910.8869
309.5391 129.9173 26.8464 680285.8938 -21.6461
-21.6401
[4997]ENERGY: 12100000 3294.7826 5432.1879 5726.6140
347.1189 -14316.4178 -1771.7762 0.0000 0.0000
8129.1379 6841.6473 309.9771 -1287.4906 6909.5567
309.5839 82.6477 75.9789 680285.8938 -13.6471
-13.6519
[5047]ENERGY: 12110000 3300.2389 5496.2324 5688.2529
342.6830 -14360.7318 -1766.0333 0.0000 0.0000
8145.0642 6845.7063 310.5844 -1299.3579 6909.3375
309.4537 32.1457 -43.9921 680285.8938 -16.6268
-16.6172
[5083]ENERGY: 12120000 3335.6681 5393.2014 5735.2074
334.0708 -14294.4836 -1841.0808 0.0000 0.0000
8181.3443 6843.9276 311.9678 -1337.4166 6909.6479
309.5130 -52.8807 -44.1807 680285.8938 -16.4415
-16.4454
[5133]ENERGY: 12130000 3309.8809 5392.6134 5734.8250
333.7259 -14253.7857 -1823.8941 0.0000 0.0000
8149.7902 6843.1557 310.7646 -1306.6345 6908.4285
309.4526 -43.0068 -54.5777 680285.8938 -18.8315
-18.8150
[5169]ENERGY: 12140000 3237.7724 5406.7948 5696.7940
329.4611 -14229.2013 -1775.0029 0.0000 0.0000
8171.2330 6837.8510 311.5823 -1333.3820 6907.5029
309.3073 53.4209 42.6270 680285.8938 -20.0897
-20.1118
[5219]ENERGY: 12150000 3382.4268 5401.6015 5720.0051
354.4503 -14349.5025 -1806.1779 0.0000 0.0000

8141.0016 6843.8049 310.4295 -1297.1968 6907.4268
309.2508 -211.7923 -183.8701 680285.8938 -16.0612
-16.0244
[5255]ENERGY: 12160000 3317.8082 5479.2670 5806.5812
334.7270 -14330.6817 -1881.6948 0.0000 0.0000
8115.6882 6841.6951 309.4643 -1273.9931 6908.1771
309.2739 40.8301 -27.1826 680285.8938 -16.2503
-16.2847
[5305]ENERGY: 12170000 3323.5728 5491.2014 5660.3002
332.1144 -14292.7825 -1718.5357 0.0000 0.0000
8049.3390 6845.2097 306.9343 -1204.1293 6908.0199
309.2971 20.6419 28.4115 680285.8938 -15.7292
-15.7050
[5341]ENERGY: 12180000 3299.1266 5537.1396 5684.2399
326.5353 -14230.6025 -1853.3712 0.0000 0.0000
8081.0485 6844.1163 308.1434 -1236.9322 6910.0923
309.2923 108.8915 -22.6281 680285.8938 -13.6336
-13.6523
[5391]ENERGY: 12190000 3324.1521 5440.3501 5741.7621
331.7611 -14330.3999 -1841.7638 0.0000 0.0000
8176.9257 6842.7874 311.7994 -1334.1383 6908.5165
309.4928 -51.4788 -38.7426 680285.8938 -17.7166
-17.6993
[5430]ENERGY: 12200000 3347.5275 5452.3682 5716.6100
329.1491 -14392.4058 -1753.0878 0.0000 0.0000
8142.5371 6842.6981 310.4881 -1299.8389 6908.8673
309.4048 -46.6682 -28.2485 680285.8938 -22.2188
-22.2119
[5480]ENERGY: 12210000 3343.1742 5465.5616 5740.3636
332.4863 -14305.3378 -1787.8709 0.0000 0.0000
8058.7248 6847.1019 307.2922 -1211.6229 6909.6431
309.3748 54.0580 7.1912 680285.8938 -16.9600
-16.9684
[5516]ENERGY: 12220000 3300.0764 5513.1925 5698.1600
339.2294 -14295.9496 -1830.9759 0.0000 0.0000
8118.1141 6841.8469 309.5568 -1276.2673 6907.8423
309.4596 -158.6481 -69.7525 680285.8938 -14.4860
-14.4984
[5566]ENERGY: 12230000 3309.7631 5398.2353 5713.2130
347.1923 -14272.7632 -1840.1231 0.0000 0.0000
8188.1653 6843.6828 312.2279 -1344.4825 6909.1530
309.4132 -19.3962 21.0394 680285.8938 -20.2021
-20.2016
[5602]ENERGY: 12240000 3383.8213 5505.7614 5681.9785
344.7137 -14272.6906 -1840.1894 0.0000 0.0000
8043.8002 6847.1951 306.7231 -1196.6051 6910.6845
309.5914 -108.7211 -14.5259 680285.8938 -17.1084
-17.1108
[5652]ENERGY: 12250000 3390.6182 5356.8570 5692.2061
368.1559 -14244.9147 -1836.7617 0.0000 0.0000
8116.7457 6842.9065 309.5046 -1273.8392 6908.9135
309.7540 -143.8090 -42.4444 680285.8938 -16.4879
-16.4682
[5688]ENERGY: 12260000 3342.2794 5478.7411 5702.7369

369.0840 -14310.8524 -1847.8090 0.0000 0.0000
8108.0660 6842.2461 309.1736 -1265.8200 6910.6933
309.6129 -3.7048 -63.5042 680285.8938 -15.2817
-15.3090
[5738]ENERGY: 12270000 3347.9813 5385.3136 5694.0980
365.1945 -14282.4539 -1812.3544 0.0000 0.0000
8143.9739 6841.7529 310.5429 -1302.2209 6910.1061
309.6163 30.2488 68.9242 680285.8938 -19.7128
-19.6962
[5774]ENERGY: 12280000 3274.0083 5468.2323 5758.6916
365.1315 -14389.8587 -1755.9286 0.0000 0.0000
8125.5637 6845.8401 309.8408 -1279.7236 6910.6846
309.7444 183.7725 177.7394 680285.8938 -11.1672
-11.1674
[5824]ENERGY: 12290000 3272.7075 5480.3145 5709.5642
328.1855 -14417.4818 -1695.4570 0.0000 0.0000
8164.3001 6842.1329 311.3179 -1322.1672 6909.4777
309.5005 106.9175 36.5098 680285.8938 -15.4596
-15.4495
[5863]ENERGY: 12300000 3337.3220 5497.1610 5694.5628
335.6946 -14339.0059 -1800.0297 0.0000 0.0000
8117.1013 6842.8060 309.5182 -1274.2953 6910.8498
309.4721 -91.6045 -167.7141 680285.8938 -16.9343
-16.9407
[5913]ENERGY: 12310000 3353.9344 5429.8048 5712.9498
347.3253 -14326.4653 -1759.0397 0.0000 0.0000
8085.1215 6843.6307 308.2987 -1241.4908 6909.6337
309.4507 -45.9523 6.4571 680285.8938 -15.8529
-15.8507
[5949]ENERGY: 12320000 3291.2245 5597.7152 5727.2830
362.5486 -14336.2349 -1841.0841 0.0000 0.0000
8045.4227 6846.8750 306.7849 -1198.5477 6911.1128
309.5591 88.5593 45.1876 680285.8938 -12.7549
-12.7646
[5999]ENERGY: 12330000 3349.9009 5443.0560 5655.6720
344.1989 -14341.0497 -1769.2432 0.0000 0.0000
8159.4751 6842.0100 311.1339 -1317.4652 6910.2345
309.5589 23.5861 -12.9257 680285.8938 -21.7384
-21.7514
[6035]ENERGY: 12340000 3253.8620 5392.3367 5723.1086
322.1370 -14320.7592 -1719.8000 0.0000 0.0000
8194.2851 6845.1701 312.4613 -1349.1150 6911.9930
309.6057 89.7908 94.9753 680285.8938 -14.4626
-14.4499
[6085]ENERGY: 12350000 3423.6437 5480.8751 5715.1713
330.8764 -14268.1907 -1857.2678 0.0000 0.0000
8023.2305 6848.3384 305.9387 -1174.8921 6910.8205
309.5910 -94.6875 -136.2224 680285.8938 -20.0178
-20.0243
[6121]ENERGY: 12360000 3357.2329 5495.1117 5719.2316
335.7279 -14342.3834 -1738.5825 0.0000 0.0000
8021.0818 6847.4200 305.8568 -1173.6618 6910.9039
309.6486 -98.7172 -43.0170 680285.8938 -17.5229
-17.5134

[6171]ENERGY: 12370000 3261.9700 5545.7114 5717.7494
314.1256 -14255.5171 -1798.9640 0.0000 0.0000
8061.1358 6846.2111 307.3841 -1214.9247 6910.0710
309.5629 -52.9900 2.5240 680285.8938 -19.2198
-19.2160
[6207]ENERGY: 12380000 3418.4596 5485.3822 5762.5513
334.5191 -14406.7982 -1786.2188 0.0000 0.0000
8039.8585 6847.7536 306.5728 -1192.1049 6911.2446
309.6604 143.2966 52.5452 680285.8938 -17.5265
-17.5337
[6257]ENERGY: 12390000 3376.5454 5566.4032 5714.1743
317.1384 -14324.9017 -1873.2445 0.0000 0.0000
8069.0213 6845.1364 307.6848 -1223.8849 6910.8437
309.4713 -116.5938 -111.8520 680285.8938 -20.1780
-20.1659
[6296]ENERGY: 12400000 3431.5477 5420.7429 5694.8199
349.0892 -14391.2467 -1733.4933 0.0000 0.0000
8078.5776 6850.0374 308.0492 -1228.5403 6910.9678
309.6661 -66.7798 -87.9362 680285.8938 -14.7619
-14.7870
[6346]ENERGY: 12410000 3398.6076 5418.2508 5711.8239
351.4480 -14352.7497 -1800.6706 0.0000 0.0000
8118.8384 6845.5484 309.5844 -1273.2900 6912.0718
309.6211 -54.6007 -35.1520 680285.8938 -14.7760
-14.7525
[6382]ENERGY: 12420000 3327.8817 5428.9785 5746.8382
350.8030 -14238.1150 -1846.7734 0.0000 0.0000
8079.4201 6849.0331 308.0813 -1230.3870 6911.4322
309.7005 58.0485 -9.8094 680285.8938 -20.8034
-20.8027
[6432]ENERGY: 12430000 3311.6184 5434.7617 5706.3025
331.6743 -14317.0554 -1759.8212 0.0000 0.0000
8141.3460 6848.8262 310.4427 -1292.5198 6911.2209
309.6818 61.2421 89.8780 680285.8938 -16.6037
-16.5906
[6468]ENERGY: 12440000 3354.4174 5419.1709 5687.2252
334.3080 -14239.3227 -1838.0175 0.0000 0.0000
8129.1186 6846.8999 309.9764 -1282.2187 6911.9390
309.5137 60.2495 -25.5423 680285.8938 -18.2095
-18.2378
[6518]ENERGY: 12450000 3348.5629 5384.5383 5676.6055
339.2421 -14278.5926 -1836.6679 0.0000 0.0000
8208.1604 6841.8487 312.9904 -1366.3117 6911.0109
309.4181 -106.1513 -18.7005 680285.8938 -19.2875
-19.2844
[6554]ENERGY: 12460000 3353.3217 5539.7289 5642.6667
339.8894 -14263.3678 -1813.3259 0.0000 0.0000
8049.6505 6848.5634 306.9462 -1201.0870 6911.2008
309.4532 -220.4924 -189.5793 680285.8938 -18.9749
-18.9803
[6604]ENERGY: 12470000 3292.4204 5568.7590 5721.3690
338.0451 -14259.9085 -1883.6193 0.0000 0.0000
8068.1546 6845.2203 307.6517 -1222.9343 6910.8073
309.3949 -150.9841 -48.7131 680285.8938 -17.5793

-17.5516
[6640]ENERGY: 12480000 3343.1934 5482.4005 5715.9175
340.0963 -14320.1317 -1798.9484 0.0000 0.0000
8081.6806 6844.2083 308.1675 -1237.4724 6911.8479
309.5562 -36.6729 -92.8908 680285.8938 -18.5335
-18.5516
[6690]ENERGY: 12490000 3307.2788 5467.7445 5733.7247
356.2859 -14292.3705 -1854.6699 0.0000 0.0000
8126.3563 6844.3498 309.8711 -1282.0065 6911.1645
309.4795 -2.3085 9.4116 680285.8938 -18.4321
-18.4363
[6729]ENERGY: 12500000 3244.6607 5518.6724 5661.5073
333.0359 -14281.8036 -1767.6509 0.0000 0.0000
8136.2387 6844.6605 310.2479 -1291.5783 6911.2034
309.4909 101.3767 69.0288 680285.8938 -19.7847
-19.7789
[6779]ENERGY: 12510000 3359.2817 5433.6191 5714.2362
332.1984 -14336.8680 -1804.1898 0.0000 0.0000
8149.2860 6847.5637 310.7454 -1301.7223 6911.5839
309.5034 8.1635 -42.1050 680285.8938 -17.1606
-17.1680
[6815]ENERGY: 12520000 3380.9030 5368.8811 5741.0866
340.4657 -14211.8948 -1844.4217 0.0000 0.0000
8075.4292 6850.4491 307.9291 -1224.9801 6912.9793
309.4338 -4.7460 -1.6593 680285.8938 -21.0627
-21.0431
[6865]ENERGY: 12530000 3286.6096 5483.4178 5710.0819
343.3968 -14360.2706 -1766.0638 0.0000 0.0000
8148.2441 6845.4157 310.7057 -1302.8283 6912.3412
309.4927 -60.3203 -121.8089 680285.8938 -15.4919
-15.5003
[6901]ENERGY: 12540000 3433.5425 5515.2927 5710.0850
353.2384 -14393.7490 -1818.7265 0.0000 0.0000
8048.0345 6847.7178 306.8845 -1200.3167 6914.7486
309.5080 -78.6810 -174.1033 680285.8938 -21.0458
-21.0486
[6951]ENERGY: 12550000 3332.2650 5562.5976 5684.8358
321.1572 -14215.5615 -1820.5537 0.0000 0.0000
7986.1171 6850.8574 304.5235 -1135.2597 6914.5723
309.3329 -3.5124 -25.3954 680285.8938 -17.3205
-17.3178
[6987]ENERGY: 12560000 3312.7648 5426.5686 5708.0018
309.5769 -14326.1182 -1804.6071 0.0000 0.0000
8220.5763 6846.7632 313.4638 -1373.8131 6913.3653
309.5758 26.3406 -0.6180 680285.8938 -21.0449
-21.0349
[7037]ENERGY: 12570000 3396.7192 5418.5318 5737.1389
368.4480 -14335.7642 -1829.0341 0.0000 0.0000
8092.3030 6848.3426 308.5726 -1243.9604 6914.7419
309.3770 -46.2182 13.2556 680285.8938 -14.8676
-14.8806
[7073]ENERGY: 12580000 3289.1544 5586.8885 5674.7583
358.0998 -14333.1868 -1858.8712 0.0000 0.0000
8132.6938 6849.5367 310.1127 -1283.1571 6914.8245

309.4278 -12.2351 -53.7428 680285.8938 -13.5991
-13.6073
[7123]ENERGY: 12590000 3285.0015 5595.0899 5701.9522
340.7627 -14379.6716 -1764.3447 0.0000 0.0000
8069.7932 6848.5831 307.7142 -1221.2101 6915.3037
309.4220 -4.1409 -50.6076 680285.8938 -17.6189
-17.6228
[7162]ENERGY: 12600000 3273.8934 5506.9534 5677.4099
335.4559 -14256.4037 -1811.9509 0.0000 0.0000
8121.2227 6846.5806 309.6753 -1274.6421 6913.5212
309.4665 -15.4725 3.7335 680285.8938 -16.0465
-16.0312
[7212]ENERGY: 12610000 3330.7851 5347.7671 5757.4588
332.1967 -14255.8527 -1808.5058 0.0000 0.0000
8143.5824 6847.4316 310.5279 -1296.1508 6913.0963
309.3027 -100.2354 -70.5307 680285.8938 -13.9561
-13.9682
[7248]ENERGY: 12620000 3346.3110 5499.5174 5694.9090
327.5015 -14268.2300 -1857.1698 0.0000 0.0000
8106.4757 6849.3148 309.1130 -1257.1609 6914.6809
309.6658 -19.0844 -47.1029 680285.8938 -16.6949
-16.6833
[7298]ENERGY: 12630000 3416.5534 5409.2576 5710.9031
346.6051 -14377.4379 -1757.0877 0.0000 0.0000
8099.8197 6848.6133 308.8592 -1251.2064 6915.1556
309.7308 111.5001 125.4453 680285.8938 -18.2098
-18.2082
[7334]ENERGY: 12640000 3276.3454 5548.2570 5687.0917
369.2717 -14299.3746 -1697.6214 0.0000 0.0000
7967.7520 6851.7217 303.8232 -1116.0303 6916.1372
309.5322 75.3710 102.9224 680285.8938 -19.1219
-19.1244
[7384]ENERGY: 12650000 3373.2905 5493.6495 5696.8048
323.1169 -14340.0175 -1798.9124 0.0000 0.0000
8101.9657 6849.8975 308.9410 -1252.0682 6916.3231
309.4630 -44.4082 -4.4544 680285.8938 -17.0595
-17.0743
[7420]ENERGY: 12660000 3315.4048 5515.2407 5648.6379
351.8519 -14259.1999 -1825.2563 0.0000 0.0000
8105.2400 6851.9189 309.0659 -1253.3211 6916.1650
309.4623 118.6814 52.5944 680285.8938 -21.3098
-21.3060
[7470]ENERGY: 12670000 3316.6775 5500.5762 5701.8545
316.4298 -14320.8228 -1795.9483 0.0000 0.0000
8132.8607 6851.6275 310.1191 -1281.2332 6916.4082
309.4779 102.2178 91.2459 680285.8938 -14.4583
-14.4593
[7506]ENERGY: 12680000 3320.3651 5496.5589 5626.0986
353.8598 -14380.9562 -1788.0715 0.0000 0.0000
8219.9963 6847.8508 313.4417 -1372.1454 6916.2195
309.5326 27.9310 -11.3251 680285.8938 -16.9412
-16.9394
[7556]ENERGY: 12690000 3356.6031 5460.5744 5725.0631
341.6059 -14281.4142 -1849.3219 0.0000 0.0000

8099.7733 6852.8838 308.8574 -1246.8895 6916.4950
309.6696 62.7093 7.5136 680285.8938 -19.1690
-19.1687
[7595]ENERGY: 12700000 3274.2006 5463.9939 5713.6970
362.5384 -14339.2171 -1798.2981 0.0000 0.0000
8172.2599 6849.1746 311.6214 -1323.0853 6916.6696
309.6274 -6.0130 -127.5006 680285.8938 -20.0951
-20.0952
[7645]ENERGY: 12710000 3276.2429 5589.6078 5684.5423
364.0823 -14311.7289 -1798.6280 0.0000 0.0000
8048.2414 6852.3597 306.8924 -1195.8817 6918.1210
309.7687 50.2548 81.4010 680285.8938 -15.3092
-15.3101
[7681]ENERGY: 12720000 3353.5888 5384.4876 5722.1976
353.7120 -14336.6618 -1778.8723 0.0000 0.0000
8153.9830 6852.4350 310.9245 -1301.5480 6917.6965
309.8909 62.9203 -3.4601 680285.8938 -19.3873
-19.3856
[7731]ENERGY: 12730000 3381.6110 5391.6761 5685.9178
329.4871 -14285.8594 -1825.1070 0.0000 0.0000
8174.6987 6852.4242 311.7144 -1322.2745 6916.9757
309.8706 -90.3556 -70.5621 680285.8938 -15.4455
-15.4367
[7767]ENERGY: 12740000 3287.8157 5515.1602 5698.9728
336.7719 -14299.4257 -1837.4815 0.0000 0.0000
8150.3351 6852.1486 310.7854 -1298.1865 6919.4419
309.8589 -21.2470 -39.2699 680285.8938 -16.4738
-16.4661
[7817]ENERGY: 12750000 3347.2107 5530.5421 5681.5235
336.7100 -14377.3063 -1816.4127 0.0000 0.0000
8148.6239 6850.8913 310.7202 -1297.7327 6919.3028
309.8068 204.6912 34.3314 680285.8938 -14.5352
-14.5681
[7853]ENERGY: 12760000 3347.7621 5416.8437 5721.8488
333.8972 -14319.4394 -1774.7237 0.0000 0.0000
8128.4112 6854.5999 309.9494 -1273.8113 6919.3208
309.8634 90.4874 -1.8183 680285.8938 -17.3562
-17.3588
[7903]ENERGY: 12770000 3293.5277 5465.0064 5711.0655
353.5193 -14361.0225 -1769.3061 0.0000 0.0000
8159.4096 6852.1998 311.1314 -1307.2098 6918.4829
309.9727 123.3817 75.4089 680285.8938 -20.6748
-20.6508
[7939]ENERGY: 12780000 3434.5977 5497.2519 5718.5663
325.8435 -14335.4238 -1865.2192 0.0000 0.0000
8079.4633 6855.0796 308.0830 -1224.3837 6919.1139
310.1007 -53.9964 -82.1328 680285.8938 -16.1368
-16.1359
[7989]ENERGY: 12790000 3416.3817 5467.6380 5683.6319
352.1500 -14355.0809 -1831.4517 0.0000 0.0000
8116.9797 6850.2487 309.5135 -1266.7309 6918.7453
309.9689 -169.5930 -188.0869 680285.8938 -19.0247
-19.0140
[8028]ENERGY: 12800000 3294.0776 5560.7422 5703.6214

354.9795 -14300.4533 -1864.2596 0.0000 0.0000
8108.4450 6857.1528 309.1881 -1251.2923 6919.7544
310.1844 60.8601 10.4339 680285.8938 -20.0510
-20.0655
[8078]ENERGY: 12810000 3276.9422 5501.7922 5717.2807
335.1017 -14353.6938 -1749.5161 0.0000 0.0000
8125.4494 6853.3562 309.8365 -1272.0932 6919.4909
309.9096 84.5927 60.1220 680285.8938 -15.5915
-15.5863
[8114]ENERGY: 12820000 3370.3200 5533.8519 5691.8114
348.6530 -14372.2042 -1774.4866 0.0000 0.0000
8058.5056 6856.4510 307.2838 -1202.0546 6920.3674
309.8332 -60.6265 -69.1000 680285.8938 -20.3181
-20.3316
[8164]ENERGY: 12830000 3430.5312 5443.9464 5665.6160
331.2254 -14310.7442 -1855.1034 0.0000 0.0000
8150.1401 6855.6114 310.7780 -1294.5286 6920.3169
310.1081 -146.2613 -113.7324 680285.8938 -18.9587
-18.9454
[8200]ENERGY: 12840000 3361.4448 5546.1247 5721.4025
360.5314 -14329.0686 -1812.5663 0.0000 0.0000
8012.4795 6860.3480 305.5288 -1152.1316 6921.7897
310.1002 -133.6447 -78.0358 680285.8938 -15.2218
-15.2364
[8250]ENERGY: 12850000 3334.4465 5430.2541 5723.3927
337.9390 -14320.4176 -1738.8687 0.0000 0.0000
8087.8812 6854.6273 308.4040 -1233.2539 6920.6878
310.0314 131.9889 117.3725 680285.8938 -22.3862
-22.3676
[8286]ENERGY: 12860000 3306.2620 5472.9597 5726.5089
367.7048 -14285.1696 -1829.9490 0.0000 0.0000
8098.0345 6856.3512 308.7911 -1241.6833 6921.9931
310.0687 1.8318 -127.6849 680285.8938 -19.0991
-19.1268
[8336]ENERGY: 12870000 3266.1100 5636.8378 5704.2279
365.7937 -14323.6057 -1789.9060 0.0000 0.0000
8000.1979 6859.6555 305.0604 -1140.5424 6921.8411
310.0536 70.9512 38.6460 680285.8938 -21.3306
-21.3019
[8372]ENERGY: 12880000 3202.5256 5558.0782 5758.3482
344.1636 -14283.1338 -1851.8825 0.0000 0.0000
8130.6857 6858.7850 310.0362 -1271.9007 6923.6803
309.9635 144.4548 48.0625 680285.8938 -20.7731
-20.7838
[8422]ENERGY: 12890000 3361.7304 5495.6794 5733.2882
323.8947 -14302.0202 -1845.2822 0.0000 0.0000
8089.9682 6857.2586 308.4835 -1232.7096 6923.4911
310.1339 22.9055 -44.3185 680285.8938 -19.2504
-19.2494
[8461]ENERGY: 12900000 3370.7625 5454.2445 5694.2938
350.6976 -14348.6393 -1787.1400 0.0000 0.0000
8123.4847 6857.7037 309.7616 -1265.7810 6923.2685
310.1414 -77.1004 -83.5619 680285.8938 -19.2203
-19.2260

[8511]ENERGY: 12910000 3376.6538 5491.2006 5729.9953
324.1446 -14290.7816 -1798.6819 0.0000 0.0000
8030.1750 6862.7057 306.2035 -1167.4693 6924.0563
310.0169 47.1510 2.5273 680285.8938 -17.5132
-17.5223
[8547]ENERGY: 12920000 3385.8416 5405.6462 5666.0007
313.8804 -14317.4373 -1811.5124 0.0000 0.0000
8213.8467 6856.2661 313.2072 -1357.5806 6923.2254
310.0173 49.6904 13.5407 680285.8938 -15.4432
-15.4525
[8597]ENERGY: 12930000 3366.9783 5416.3754 5679.0619
346.1893 -14282.0407 -1815.7606 0.0000 0.0000
8149.4717 6860.2753 310.7525 -1289.1964 6925.0774
310.0458 101.6246 103.6158 680285.8938 -17.0555
-17.0379
[8633]ENERGY: 12940000 3315.7702 5567.1243 5647.4502
333.3178 -14345.4712 -1813.8342 0.0000 0.0000
8153.3071 6857.6640 310.8987 -1295.6431 6924.0214
310.0910 28.2444 91.6070 680285.8938 -14.2713
-14.2627
[8683]ENERGY: 12950000 3328.6752 5507.6181 5676.4970
339.8804 -14286.0806 -1764.3376 0.0000 0.0000
8056.5147 6858.7673 307.2079 -1197.7474 6924.2565
310.0820 -188.5147 -180.5039 680285.8938 -11.7127
-11.7024
[8719]ENERGY: 12960000 3311.6000 5541.2028 5698.1247
319.5797 -14414.8606 -1756.8389 0.0000 0.0000
8154.1846 6852.9923 310.9322 -1301.1923 6922.9702
310.1734 -179.8656 -123.9211 680285.8938 -10.2980
-10.2994
[8769]ENERGY: 12970000 3389.6574 5487.8077 5667.9549
338.6162 -14338.4548 -1766.9042 0.0000 0.0000
8077.2434 6855.9206 307.9983 -1221.3228 6924.2502
310.1206 28.2391 -12.4039 680285.8938 -16.7453
-16.7626
[8805]ENERGY: 12980000 3387.4862 5434.0367 5709.0546
343.2907 -14344.7849 -1766.4372 0.0000 0.0000
8096.4650 6859.1112 308.7313 -1237.3538 6923.4245
309.8541 -21.6668 25.1830 680285.8938 -14.0955
-14.0954
[8855]ENERGY: 12990000 3365.1708 5430.7153 5671.0324
337.3033 -14332.8648 -1774.1168 0.0000 0.0000
8162.4033 6859.6434 311.2456 -1302.7598 6924.5751
310.0155 45.1158 -27.8502 680285.8938 -10.3557
-10.3556
[8894]ENERGY: 13000000 3339.0907 5458.6074 5748.2811
329.7228 -14379.2663 -1782.5141 0.0000 0.0000
8148.0993 6862.0208 310.7002 -1286.0785 6925.3117
310.0139 12.4891 -15.8235 680285.8938 -20.3678
-20.3561
[8944]ENERGY: 13010000 3425.6382 5518.7782 5683.3503
324.7700 -14227.6313 -1835.4109 0.0000 0.0000
7976.8278 6866.3223 304.1693 -1110.5055 6925.9010
309.9978 -143.8715 -114.1106 680285.8938 -16.7221

-16.7335
[8980]ENERGY: 13020000 3326.4904 5467.5205 5765.2286
345.1743 -14365.3475 -1814.0067 0.0000 0.0000
8133.7145 6858.7742 310.1516 -1274.9403 6925.1186
310.0883 -8.8293 -73.6782 680285.8938 -15.0511
-15.0535
[9030]ENERGY: 13030000 3361.8465 5408.5216 5666.4728
333.1853 -14300.5786 -1796.6023 0.0000 0.0000
8184.5476 6857.3929 312.0900 -1327.1546 6924.9649
310.1456 104.5796 38.8327 680285.8938 -16.0880
-16.0722
[9066]ENERGY: 13040000 3404.8015 5417.7977 5701.6049
334.0657 -14388.1514 -1771.5899 0.0000 0.0000
8159.9652 6858.4937 311.1526 -1301.4715 6924.6846
310.1698 83.4985 75.3328 680285.8938 -16.6963
-16.7044
[9116]ENERGY: 13050000 3375.0261 5482.6088 5739.0739
315.4423 -14325.1898 -1813.0310 0.0000 0.0000
8084.4529 6858.3832 308.2732 -1226.0696 6925.8516
310.0248 35.7812 -38.2384 680285.8938 -16.5050
-16.5192
[9152]ENERGY: 13060000 3365.2642 5423.6736 5725.3935
339.6509 -14348.6338 -1737.0521 0.0000 0.0000
8094.9024 6863.1987 308.6717 -1231.7037 6924.9571
310.0981 -74.4357 -134.2371 680285.8938 -15.1701
-15.1432
[9202]ENERGY: 13070000 3340.0012 5449.5627 5713.8732
376.4525 -14337.2259 -1834.6003 0.0000 0.0000
8152.7396 6860.8029 310.8771 -1291.9367 6926.4677
310.2514 -91.7617 -107.1898 680285.8938 -11.9569
-11.9493
[9238]ENERGY: 13080000 3355.1488 5495.2343 5742.2304
361.2098 -14313.7531 -1855.2840 0.0000 0.0000
8079.0561 6863.8423 308.0674 -1215.2138 6927.9095
310.1402 4.8998 22.0093 680285.8938 -12.5525
-12.5714
[9288]ENERGY: 13090000 3353.0949 5414.7554 5709.6357
333.8366 -14303.9403 -1791.4739 0.0000 0.0000
8144.2358 6860.1442 310.5528 -1284.0916 6927.8730
310.2079 -86.5841 -139.2750 680285.8938 -14.2283
-14.2388
[9327]ENERGY: 13100000 3339.9648 5508.7223 5753.5180
354.6171 -14351.0381 -1864.7556 0.0000 0.0000
8121.6190 6862.6476 309.6904 -1258.9715 6928.1422
310.1140 66.7293 39.2625 680285.8938 -16.9904
-16.9750
[9377]ENERGY: 13110000 3335.3834 5434.4087 5721.0681
344.4220 -14375.3194 -1735.5237 0.0000 0.0000
8139.4662 6863.9053 310.3710 -1275.5608 6926.9973
310.1274 48.8334 43.2633 680285.8938 -16.1628
-16.1602
[9413]ENERGY: 13120000 3378.2809 5480.4271 5668.6333
341.8595 -14306.5330 -1769.1042 0.0000 0.0000
8073.5973 6867.1609 307.8593 -1206.4364 6929.2080

310.0953 -198.2479 -135.8236 680285.8938 -15.5451
-15.5549
[9463]ENERGY: 13130000 3269.4741 5480.7427 5708.3487
337.1175 -14295.5637 -1812.9299 0.0000 0.0000
8174.2159 6861.4054 311.6960 -1312.8105 6929.6937
310.1502 40.6577 27.3023 680285.8938 -14.0331
-14.0418
[9499]ENERGY: 13140000 3260.5085 5444.5636 5705.6418
344.4345 -14266.4649 -1794.8040 0.0000 0.0000
8168.3721 6862.2515 311.4732 -1306.1206 6929.3879
310.0571 26.4647 -17.4677 680285.8938 -12.8945
-12.8948
[9549]ENERGY: 13150000 3365.6965 5466.1452 5665.2129
331.3587 -14335.3932 -1747.6656 0.0000 0.0000
8114.8699 6860.2244 309.4331 -1254.6455 6929.3239
310.1648 -125.4065 -77.1345 680285.8938 -14.4299
-14.4023
[9585]ENERGY: 13160000 3353.2225 5395.3269 5679.3905
334.9875 -14211.7577 -1831.3023 0.0000 0.0000
8146.6048 6866.4722 310.6432 -1280.1326 6930.6543
310.0785 7.0974 -60.3329 680285.8938 -14.2847
-14.2980
[9635]ENERGY: 13170000 3353.1018 5498.0952 5714.4984
317.9706 -14264.8227 -1794.6812 0.0000 0.0000
8040.6772 6864.8394 306.6040 -1175.8378 6930.8491
309.9509 47.9373 22.6641 680285.8938 -10.2066
-10.2099
[9671]ENERGY: 13180000 3430.8298 5419.0925 5648.7721
316.7849 -14218.0612 -1832.8363 0.0000 0.0000
8104.3341 6868.9160 309.0313 -1235.4181 6932.1079
310.1555 -12.4284 -21.1363 680285.8938 -18.9645
-18.9611
[9721]ENERGY: 13190000 3369.0851 5404.9566 5663.9242
355.9788 -14259.2863 -1857.7035 0.0000 0.0000
8188.0490 6865.0039 312.2235 -1323.0451 6931.3528
310.1910 18.5339 4.5723 680285.8938 -22.7265
-22.7298
[9760]ENERGY: 13200000 3292.9907 5516.0534 5744.0011
334.0066 -14264.7319 -1893.4706 0.0000 0.0000
8136.6364 6865.4857 310.2631 -1271.1507 6932.4374
310.3379 -60.1231 -36.6985 680285.8938 -21.4712
-21.4677
[9810]ENERGY: 13210000 3286.7999 5440.6824 5721.1795
340.8584 -14321.2044 -1815.6126 0.0000 0.0000
8214.9440 6867.6472 313.2491 -1347.2968 6931.5122
310.3427 -7.9332 28.0995 680285.8938 -14.1462
-14.1459
[9846]ENERGY: 13220000 3337.1047 5474.0665 5719.0180
330.9063 -14330.0923 -1834.0423 0.0000 0.0000
8166.3162 6863.2771 311.3948 -1303.0391 6931.6901
310.0584 114.2646 45.6484 680285.8938 -18.6235
-18.6455
[9896]ENERGY: 13230000 3380.1939 5558.9167 5664.7958
322.7035 -14419.9555 -1753.9557 0.0000 0.0000

8113.1101 6865.8088 309.3660 -1247.3013 6932.0429
309.9249 -83.6131 -47.3570 680285.8938 -12.8624
-12.8559
[9932]ENERGY: 13240000 3357.9161 5498.1426 5719.9155
349.8257 -14383.8080 -1810.4413 0.0000 0.0000
8135.6337 6867.1842 310.2248 -1268.4494 6933.1869
310.3007 -98.2934 -52.0825 680285.8938 -12.0865
-12.0683
[9982]ENERGY: 13250000 3369.4975 5442.9739 5676.4610
309.0515 -14282.1273 -1779.5166 0.0000 0.0000
8132.0798 6868.4197 310.0893 -1263.6601 6933.2628
310.4036 58.0018 -77.0101 680285.8938 -16.6181
-16.6265
[10018]ENERGY: 13260000 3260.9361 5483.2933 5753.2725
337.9843 -14284.2623 -1846.9192 0.0000 0.0000
8161.6524 6865.9570 311.2170 -1295.6954 6933.5054
310.2227 31.4777 1.7251 680285.8938 -14.3411
-14.3285
[10068]ENERGY: 13270000 3304.6758 5518.8559 5704.6784
339.3982 -14295.0657 -1815.0403 0.0000 0.0000
8111.4631 6868.9655 309.3032 -1242.4976 6933.4620
310.2816 -2.6076 -22.7471 680285.8938 -17.0903
-17.1137
[10104]ENERGY: 13280000 3347.3447 5404.6562 5712.7274
336.6229 -14297.7742 -1808.2348 0.0000 0.0000
8173.6613 6869.0034 311.6749 -1304.6579 6933.4476
310.3401 78.5821 34.5716 680285.8938 -14.3687
-14.3501
[10154]ENERGY: 13290000 3316.3053 5481.7806 5638.9757
323.7936 -14292.5916 -1860.7523 0.0000 0.0000
8259.0443 6866.5557 314.9307 -1392.4886 6934.5019
310.3691 177.0614 18.1161 680285.8938 -16.5992
-16.6037
[10193]ENERGY: 13300000 3309.9587 5443.8077 5710.7184
343.2333 -14239.6246 -1888.2772 0.0000 0.0000
8189.8445 6869.6608 312.2920 -1320.1837 6934.5561
310.3722 -116.1375 -94.7813 680285.8938 -18.1486
-18.1568
[10243]ENERGY: 13310000 3303.0000 5496.1874 5717.9472
355.7194 -14346.2351 -1743.4342 0.0000 0.0000
8086.5218 6869.7065 308.3521 -1216.8154 6933.2543
310.3326 31.5492 63.7482 680285.8938 -17.9262
-17.9169
[10279]ENERGY: 13320000 3290.7523 5368.4571 5745.3556
352.1304 -14251.9869 -1821.6996 0.0000 0.0000
8186.3918 6869.4008 312.1603 -1316.9910 6933.8178
310.4070 61.6384 18.0158 680285.8938 -16.8361
-16.8322
[10329]ENERGY: 13330000 3274.2678 5530.2161 5685.3007
315.8192 -14297.7063 -1821.9887 0.0000 0.0000
8181.9567 6867.8655 311.9912 -1314.0913 6934.5674
310.3821 20.4677 -48.0283 680285.8938 -16.6352
-16.6599
[10365]ENERGY: 13340000 3385.3022 5542.3481 5695.9549

327.4107 -14362.3919 -1827.2629 0.0000 0.0000
8108.3994 6869.7606 309.1863 -1238.6388 6934.1814
310.3855 17.2278 -90.2816 680285.8938 -16.3400
-16.3238
[10415]ENERGY: 13350000 3332.3766 5453.3174 5709.0677
347.5588 -14203.3307 -1823.3868 0.0000 0.0000
8054.9684 6870.5714 307.1489 -1184.3970 6934.8236
310.4330 -51.2567 -71.6479 680285.8938 -20.4296
-20.4345
[10451]ENERGY: 13360000 3403.9711 5511.4957 5710.5678
332.7319 -14397.0147 -1771.5083 0.0000 0.0000
8082.3843 6872.6279 308.1943 -1209.7564 6934.6901
310.2563 -22.2185 20.8266 680285.8938 -15.9046
-15.9054
[10501]ENERGY: 13370000 3329.0040 5492.7730 5736.4415
333.4378 -14297.3781 -1797.9959 0.0000 0.0000
8072.5328 6868.8150 307.8187 -1203.7178 6935.4259
310.2035 32.7475 -123.3595 680285.8938 -17.5043
-17.5244
[10537]ENERGY: 13380000 3305.4130 5528.3091 5606.5673
350.1741 -14282.7846 -1718.8984 0.0000 0.0000
8082.9686 6871.7492 308.2166 -1211.2194 6934.4625
310.2775 -2.1152 -61.6797 680285.8938 -21.8228
-21.8040
[10587]ENERGY: 13390000 3333.6088 5489.0666 5680.2913
326.0432 -14245.2077 -1806.5618 0.0000 0.0000
8094.6857 6871.9260 308.6634 -1222.7597 6933.6294
310.1808 -31.9258 -48.9203 680285.8938 -19.7795
-19.7546
[10626]ENERGY: 13400000 3358.5465 5489.4732 5707.2245
354.3665 -14323.3400 -1851.6764 0.0000 0.0000
8132.4056 6867.0000 310.1017 -1265.4056 6935.4537
310.2097 25.3662 -109.7359 680285.8938 -22.8844
-22.9003
[10676]ENERGY: 13410000 3303.2949 5503.6625 5679.6332
325.4512 -14243.1808 -1809.8518 0.0000 0.0000
8109.0382 6868.0474 309.2107 -1240.9907 6934.1892
310.2313 106.0500 94.2436 680285.8938 -19.6549
-19.6525
[10712]ENERGY: 13420000 3375.2068 5469.0094 5713.9580
340.3722 -14398.2965 -1797.4272 0.0000 0.0000
8165.5145 6868.3371 311.3642 -1297.1773 6935.0495
310.2201 -88.1364 -30.0543 680285.8938 -19.0820
-19.0648
[10762]ENERGY: 13430000 3342.5543 5525.6038 5678.0544
340.6803 -14362.5645 -1782.9364 0.0000 0.0000
8129.2808 6870.6729 309.9826 -1258.6079 6934.9984
310.0118 -8.1295 -15.4193 680285.8938 -23.0050
-23.0380
[10798]ENERGY: 13440000 3354.8463 5406.1780 5704.1608
329.0265 -14356.9455 -1752.3212 0.0000 0.0000
8183.5421 6868.4872 312.0517 -1315.0549 6935.2602
310.0150 -18.5718 -132.4182 680285.8938 -21.0353
-21.0347

[10848]ENERGY: 13450000 3422.4454 5477.8110 5681.1978
330.0824 -14331.1511 -1787.8578 0.0000 0.0000
8078.2850 6870.8127 308.0380 -1207.4723 6934.4595
310.1066 -68.9384 -90.0620 680285.8938 -20.6511
-20.6325
[10884]ENERGY: 13460000 3295.0825 5522.0052 5715.5158
345.5758 -14319.3976 -1773.9451 0.0000 0.0000
8085.5936 6870.4302 308.3167 -1215.1634 6934.0197
310.0603 51.7354 4.5104 680285.8938 -19.4800
-19.4832
[10934]ENERGY: 13470000 3403.7335 5541.7604 5661.7658
328.0484 -14305.4045 -1801.7788 0.0000 0.0000
8044.2540 6872.3789 306.7404 -1171.8751 6935.3753
310.1830 -58.6570 -78.3026 680285.8938 -10.2540
-10.2563
[10970]ENERGY: 13480000 3267.3390 5552.0164 5675.5937
352.2906 -14219.4356 -1812.3118 0.0000 0.0000
8055.5787 6871.0710 307.1722 -1184.5077 6936.1856
310.0060 137.6075 124.0081 680285.8938 -17.7364
-17.7428
[11020]ENERGY: 13490000 3275.0641 5508.1501 5714.7817
367.1097 -14272.4440 -1843.1474 0.0000 0.0000
8123.4351 6872.9494 309.7597 -1250.4857 6935.2406
310.2440 76.4517 74.8613 680285.8938 -18.5705
-18.5524
[11059]ENERGY: 13500000 3346.8297 5413.9414 5752.9571
351.8320 -14288.8931 -1815.1492 0.0000 0.0000
8110.6722 6872.1901 309.2730 -1238.4821 6937.4716
310.3768 27.0908 1.1430 680285.8938 -16.0293
-16.0446
[11109]ENERGY: 13510000 3306.7178 5440.1924 5705.7285
323.5755 -14213.5658 -1827.6557 0.0000 0.0000
8136.4183 6871.4111 310.2548 -1265.0073 6936.0315
310.1892 -9.7069 23.4920 680285.8938 -20.5136
-20.5114
[11145]ENERGY: 13520000 3390.6384 5479.4693 5699.3709
324.5278 -14318.6754 -1792.2175 0.0000 0.0000
8087.0547 6870.1682 308.3724 -1216.8864 6934.5205
310.2086 6.0267 15.2675 680285.8938 -15.6551
-15.6505
[11195]ENERGY: 13530000 3326.5853 5486.2152 5711.6272
351.6923 -14241.0515 -1805.7465 0.0000 0.0000
8040.9710 6870.2930 306.6152 -1170.6780 6934.8933
310.2082 -76.0505 -75.8263 680285.8938 -19.2964
-19.2991
[11231]ENERGY: 13540000 3293.7386 5535.8579 5731.2449
324.6612 -14291.7422 -1810.7136 0.0000 0.0000
8083.4975 6866.5444 308.2368 -1216.9531 6935.7937
310.1881 119.8424 27.5431 680285.8938 -18.8112
-18.8033
[11281]ENERGY: 13550000 3346.2480 5403.3562 5685.3951
348.1182 -14313.7564 -1756.8228 0.0000 0.0000
8156.6532 6869.1915 311.0263 -1287.4617 6934.0974
310.1919 39.2875 -57.9504 680285.8938 -23.1991

-23.2305
[11317]ENERGY: 13560000   3280.6641   5389.8622   5758.6915
341.3200   -14309.9788   -1800.2636   0.0000   0.0000
8207.1299   6867.4252   312.9511   -1339.7047   6935.0601
310.1767   -134.6117   -117.7806   680285.8938   -18.1629
-18.1327
[11367]ENERGY: 13570000   3347.6515   5545.7831   5742.6941
335.0949   -14298.4326   -1837.0218   0.0000   0.0000
8033.3754   6869.1446   306.3256   -1164.2308   6936.6601
310.2659   -15.7765   -113.9256   680285.8938   -18.8875
-18.9038
[11403]ENERGY: 13580000   3307.9971   5332.9445   5757.1354
341.8663   -14253.5390   -1826.1453   0.0000   0.0000
8211.5402   6871.7993   313.1193   -1339.7409   6936.7133
310.2829   46.2363   -91.0735   680285.8938   -20.4020
-20.3967
[11453]ENERGY: 13590000   3318.8391   5517.3055   5665.9931
334.5989   -14322.0482   -1815.9324   0.0000   0.0000
8169.7196   6868.4756   311.5246   -1301.2440   6934.6420
310.3534   112.0615   81.8421   680285.8938   -20.1499
-20.1362
[11492]ENERGY: 13600000   3368.2864   5463.9185   5673.2291
354.6319   -14291.4150   -1854.3658   0.0000   0.0000
8153.8903   6868.1754   310.9210   -1285.7149   6934.8793
310.3509   -58.8134   -32.6259   680285.8938   -18.5732
-18.5828
[11542]ENERGY: 13610000   3291.4319   5532.6451   5710.1926
353.8989   -14263.7950   -1816.4448   0.0000   0.0000
8065.0271   6872.9559   307.5325   -1192.0712   6935.7696
310.2546   59.2154   34.5622   680285.8938   -16.7620
-16.7690
[11578]ENERGY: 13620000   3380.0213   5470.2604   5712.1157
358.2146   -14304.7934   -1794.9168   0.0000   0.0000
8052.5804   6873.4821   307.0579   -1179.0983   6937.0291
310.1371   -122.7368   -137.3571   680285.8938   -20.8863
-20.8811
[11628]ENERGY: 13630000   3295.3516   5380.4733   5682.8511
331.2955   -14245.9419   -1771.9793   0.0000   0.0000
8199.9497   6872.0001   312.6773   -1327.9496   6937.6593
310.2037   43.4892   23.3827   680285.8938   -19.1783
-19.1942
[11664]ENERGY: 13640000   3376.1605   5472.4734   5698.6731
315.7026   -14283.2435   -1809.7961   0.0000   0.0000
8106.9410   6876.9111   309.1307   -1230.0299   6938.4503
310.1904   -128.5571   -132.5238   680285.8938   -23.5874
-23.5649
[11714]ENERGY: 13650000   3338.5629   5452.9112   5718.3178
344.8124   -14327.1169   -1804.9373   0.0000   0.0000
8148.4821   6871.0322   310.7148   -1277.4499   6937.4700
310.2096   51.8864   18.6618   680285.8938   -26.4115
-26.4203
[11750]ENERGY: 13660000   3365.2716   5449.7872   5685.6859
356.3992   -14422.9292   -1745.1781   0.0000   0.0000
8184.1908   6873.2274   312.0764   -1310.9634   6938.2161

310.1216 51.5060 15.7508 680285.8938 -23.2542
-23.2530
[11800]ENERGY: 13670000 3284.8025 5476.7710 5714.0351
361.8732 -14365.9714 -1757.5388 0.0000 0.0000
8155.2309 6869.2025 310.9721 -1286.0284 6938.2777
310.3687 128.4004 75.6210 680285.8938 -15.3240
-15.3240
[11836]ENERGY: 13680000 3325.8835 5505.2567 5692.5306
343.8897 -14261.8930 -1890.9391 0.0000 0.0000
8159.6135 6874.3418 311.1392 -1285.2717 6938.3670
309.9703 -110.6732 -53.5251 680285.8938 -17.9634
-17.9658
[11886]ENERGY: 13690000 3330.1127 5533.1079 5721.2240
319.2616 -14333.4002 -1848.4184 0.0000 0.0000
8151.7519 6873.6395 310.8394 -1278.1124 6939.3419
310.0486 2.8652 -6.0639 680285.8938 -19.4273
-19.4217
[11925]ENERGY: 13700000 3256.1626 5561.7183 5735.9503
355.7004 -14335.3786 -1745.0407 0.0000 0.0000
8044.9251 6874.0374 306.7660 -1170.8877 6939.8675
310.0473 214.0255 139.4862 680285.8938 -18.5016
-18.4972
[11975]ENERGY: 13710000 3376.4038 5475.7598 5726.8562
344.3578 -14407.0913 -1736.0604 0.0000 0.0000
8092.8821 6873.1080 308.5946 -1219.7741 6937.7237
310.1805 -80.2165 5.2970 680285.8938 -21.7599
-21.7570
[12011]ENERGY: 13720000 3284.0017 5478.4425 5739.2698
346.3672 -14324.8983 -1708.8333 0.0000 0.0000
8060.5044 6874.8540 307.3600 -1185.6504 6939.8450
310.0404 147.6465 113.9127 680285.8938 -20.9118
-20.9245
[12061]ENERGY: 13730000 3305.2963 5490.5853 5672.9582
344.9515 -14204.1767 -1922.6213 0.0000 0.0000
8188.5245 6875.5177 312.2416 -1313.0068 6939.5789
310.2439 -81.9037 -165.8801 680285.8938 -18.9429
-18.9263
[12097]ENERGY: 13740000 3400.8534 5559.4036 5722.3726
303.3186 -14415.0749 -1795.8306 0.0000 0.0000
8103.5531 6878.5960 309.0015 -1224.9572 6940.8013
310.2906 -35.1552 -85.5532 680285.8938 -17.5307
-17.5456
[12147]ENERGY: 13750000 3318.0440 5440.4926 5742.6575
325.9140 -14291.9121 -1788.4857 0.0000 0.0000
8124.0595 6870.7698 309.7835 -1253.2896 6940.3799
310.0687 110.5453 29.3427 680285.8938 -15.0261
-15.0064
[12183]ENERGY: 13760000 3418.4794 5504.6315 5736.5046
317.0979 -14341.4211 -1865.4343 0.0000 0.0000
8105.2247 6875.0827 309.0653 -1230.1420 6938.9116
310.1905 -82.2675 -109.5037 680285.8938 -17.0768
-17.0934
[12233]ENERGY: 13770000 3423.5006 5487.5985 5703.5625
336.9154 -14302.6434 -1826.9030 0.0000 0.0000

8054.9309 6876.9616 307.1475 -1177.9693 6938.7722
310.3938 -150.8548 -86.0037 680285.8938 -16.8264
-16.8156
[12269]ENERGY: 13780000 3348.9925 5446.9251 5653.2714
329.7089 -14285.2287 -1815.7240 0.0000 0.0000
8195.4074 6873.3526 312.5041 -1322.0548 6940.2926
310.3260 14.8810 -13.4085 680285.8938 -14.2079
-14.2376
[12319]ENERGY: 13790000 3338.4300 5490.7076 5711.3835
342.4720 -14335.6640 -1833.1252 0.0000 0.0000
8158.0050 6872.2088 311.0779 -1285.7961 6942.0082
310.3181 23.9263 73.1920 680285.8938 -14.5563
-14.5399
[12358]ENERGY: 13800000 3350.4743 5487.1824 5720.0231
348.7932 -14328.9638 -1826.8504 0.0000 0.0000
8125.9168 6876.5756 309.8543 -1249.3412 6940.7641
310.3713 -70.9010 -132.5315 680285.8938 -18.3156
-18.3231
[12408]ENERGY: 13810000 3399.3485 5562.9295 5646.5841
358.8868 -14417.9010 -1845.8728 0.0000 0.0000
8169.2430 6873.2182 311.5064 -1296.0248 6941.4702
310.3864 -40.7696 -76.3614 680285.8938 -18.4762
-18.4735
[12444]ENERGY: 13820000 3335.6391 5358.0488 5720.7801
357.4185 -14277.6701 -1880.1053 0.0000 0.0000
8257.4931 6871.6043 314.8715 -1385.8889 6941.8931
310.5351 49.9168 18.5971 680285.8938 -15.9676
-15.9629
[12494]ENERGY: 13830000 3406.5812 5541.7939 5711.3675
346.2285 -14352.2272 -1804.6497 0.0000 0.0000
8031.8049 6880.8991 306.2657 -1150.9058 6944.0200
310.3671 70.8983 24.5963 680285.8938 -20.0321
-20.0366
[12530]ENERGY: 13840000 3369.9602 5485.3039 5722.2629
367.1169 -14416.9490 -1833.2321 0.0000 0.0000
8179.8337 6874.2964 311.9102 -1305.5372 6942.9712
310.5000 -136.2542 -125.1199 680285.8938 -16.9265
-16.9211
[12580]ENERGY: 13850000 3323.8831 5518.0176 5684.5076
341.5266 -14332.9896 -1861.7076 0.0000 0.0000
8205.9865 6879.2242 312.9075 -1326.7623 6944.8104
310.5856 -59.6812 -134.1345 680285.8938 -17.5174
-17.5151
[12616]ENERGY: 13860000 3374.9806 5591.2939 5666.9142
354.1237 -14430.7091 -1817.2801 0.0000 0.0000
8138.1024 6877.4256 310.3190 -1260.6768 6944.0591
310.6432 -41.7216 -23.9388 680285.8938 -19.1780
-19.1784
[12666]ENERGY: 13870000 3389.0640 5416.1553 5685.2472
339.5240 -14273.5090 -1862.8050 0.0000 0.0000
8185.4799 6879.1564 312.1255 -1306.3236 6944.0218
310.6120 -210.2818 -196.5565 680285.8938 -18.7328
-18.7468
[12702]ENERGY: 13880000 3279.9304 5613.8975 5718.0654

334.0522 -14342.6350 -1858.1593 0.0000 0.0000
8133.9472 6879.0984 310.1605 -1254.8488 6944.8817
310.5864 20.1758 14.1938 680285.8938 -20.8196
-20.8138
[12752]ENERGY: 13890000 3416.4841 5485.9108 5683.5854
353.6215 -14305.7250 -1819.8879 0.0000 0.0000
8068.5661 6882.5550 307.6674 -1186.0111 6944.8970
310.4368 -110.4598 -103.9595 680285.8938 -19.4778
-19.4660
[12791]ENERGY: 13900000 3321.6736 5492.7372 5701.1855
340.9759 -14303.8480 -1795.5493 0.0000 0.0000
8118.5349 6875.7098 309.5728 -1242.8251 6943.4238
310.6697 5.8468 10.6488 680285.8938 -19.1027
-19.0990
[12841]ENERGY: 13910000 3341.9092 5604.8973 5728.1279
340.5566 -14370.5135 -1837.7136 0.0000 0.0000
8072.3756 6879.6394 307.8127 -1192.7362 6943.3432
310.6626 4.7313 26.8309 680285.8938 -17.3187
-17.3274
[12877]ENERGY: 13920000 3428.8786 5433.0282 5760.4312
337.2390 -14342.6057 -1874.9177 0.0000 0.0000
8137.9279 6879.9815 310.3123 -1257.9463 6944.3372
310.7372 -96.4835 -122.5466 680285.8938 -19.4124
-19.4188
[12927]ENERGY: 13930000 3290.5919 5531.6901 5680.1031
329.6292 -14353.2758 -1813.1701 0.0000 0.0000
8217.3459 6882.9142 313.3406 -1334.4317 6945.4589
310.5391 98.5469 75.8521 680285.8938 -16.8111
-16.7851
[12963]ENERGY: 13940000 3294.4793 5520.9109 5732.3923
359.4655 -14351.8249 -1829.0468 0.0000 0.0000
8155.9977 6882.3740 311.0013 -1273.6237 6945.7956
310.8094 -57.7422 -11.1813 680285.8938 -12.4309
-12.4476
[13013]ENERGY: 13950000 3303.6410 5514.0970 5725.6002
330.8228 -14317.7619 -1829.9897 0.0000 0.0000
8148.8727 6875.2822 310.7297 -1273.5905 6944.2334
310.7270 -73.9809 28.9250 680285.8938 -17.4085
-17.4047
[13049]ENERGY: 13960000 3410.6594 5504.4009 5717.5475
356.2201 -14316.2172 -1943.0758 0.0000 0.0000
8150.6697 6880.2046 310.7982 -1270.4651 6945.0022
310.6538 -126.7791 -116.4931 680285.8938 -16.0529
-16.0553
[13099]ENERGY: 13970000 3312.7874 5450.6958 5730.4179
346.0341 -14367.5737 -1795.4618 0.0000 0.0000
8199.6964 6876.5961 312.6676 -1323.1003 6945.7080
310.7783 -45.1930 -104.1934 680285.8938 -13.5213
-13.5137
[13135]ENERGY: 13980000 3326.7885 5534.6813 5777.1046
351.4124 -14361.5732 -1835.9681 0.0000 0.0000
8090.3773 6882.8229 308.4991 -1207.5544 6946.6570
310.7896 104.0715 -63.5203 680285.8938 -16.5975
-16.6176

[13185]ENERGY: 13990000 3331.2903 5531.8269 5737.5932
350.8388 -14360.7813 -1823.2210 0.0000 0.0000
8114.8457 6882.3926 309.4322 -1232.4531 6947.0963
310.9796 -16.3508 -22.5678 680285.8938 -16.2540
-16.2446
[13224]ENERGY: 14000000 3360.3950 5536.6556 5694.7868
354.8604 -14436.2257 -1762.3072 0.0000 0.0000
8133.9984 6882.1634 310.1625 -1251.8351 6946.3995
310.8752 100.1507 96.4822 680285.8938 -15.3363
-15.3405
[13274]ENERGY: 14010000 3387.2979 5536.2982 5694.8809
325.8803 -14348.4972 -1820.5710 0.0000 0.0000
8107.5129 6882.8020 309.1525 -1224.7109 6946.4183
310.6566 -182.6748 -135.9689 680285.8938 -15.7155
-15.7011
[13310]ENERGY: 14020000 3324.3859 5547.0213 5694.2929
330.6372 -14321.8734 -1778.4769 0.0000 0.0000
8086.3597 6882.3468 308.3459 -1204.0129 6948.4957
310.7966 93.2278 36.3513 680285.8938 -15.8140
-15.8345
[13360]ENERGY: 14030000 3333.6258 5439.2047 5773.1419
360.5313 -14330.4749 -1794.5967 0.0000 0.0000
8104.7304 6886.1625 309.0464 -1218.5679 6947.9892
310.8802 46.4755 4.8915 680285.8938 -15.0847
-15.0825
[13396]ENERGY: 14040000 3345.4860 5488.1419 5701.2215
339.8240 -14312.3863 -1796.1742 0.0000 0.0000
8113.4435 6879.5564 309.3787 -1233.8871 6947.5190
310.7962 -59.6869 -121.7208 680285.8938 -14.4702
-14.4643
[13446]ENERGY: 14050000 3319.9129 5445.8327 5731.3436
337.9693 -14319.6331 -1862.7608 0.0000 0.0000
8228.7644 6881.4290 313.7761 -1347.3354 6948.9133
310.8717 -71.2617 -41.4461 680285.8938 -11.2547
-11.2630
[13482]ENERGY: 14060000 3412.0009 5472.4597 5704.1591
330.7106 -14366.2723 -1837.4303 0.0000 0.0000
8168.2494 6883.8772 311.4685 -1284.3722 6949.4734
310.7761 -153.6822 -140.3089 680285.8938 -15.9820
-15.9728
[13532]ENERGY: 14070000 3265.7476 5578.5190 5783.5774
334.8591 -14386.6941 -1820.5746 0.0000 0.0000
8129.4136 6884.8480 309.9876 -1244.5656 6950.5293
310.7811 129.4260 64.7491 680285.8938 -15.3702
-15.3575
[13568]ENERGY: 14080000 3257.7878 5542.8453 5771.0019
342.2113 -14341.2451 -1804.8035 0.0000 0.0000
8118.0392 6885.8370 309.5539 -1232.2022 6951.5011
310.9061 160.2832 121.8781 680285.8938 -11.0363
-11.0380
[13618]ENERGY: 14090000 3374.4789 5432.2678 5667.0621
330.6753 -14345.8180 -1777.4002 0.0000 0.0000
8204.3907 6885.6567 312.8466 -1318.7341 6951.6159
310.8884 112.7868 124.4092 680285.8938 -16.1685

-16.1687
[13657]ENERGY: 14100000 3371.7705 5472.8681 5723.5739
346.9867 -14340.9566 -1799.4236 0.0000 0.0000
8109.9054 6884.7242 309.2438 -1225.1812 6950.3577
310.9780 10.5093 -91.9389 680285.8938 -7.4474
-7.4464
[13707]ENERGY: 14110000 3385.7947 5479.8580 5654.0736
343.4475 -14304.7327 -1828.8370 0.0000 0.0000
8153.2223 6882.8264 310.8955 -1270.3959 6951.9990
311.0253 105.2039 62.6255 680285.8938 -13.9792
-13.9861
[13743]ENERGY: 14120000 3335.4170 5441.6103 5738.5377
339.7538 -14369.2557 -1752.1307 0.0000 0.0000
8152.3412 6886.2736 310.8619 -1266.0677 6950.0712
310.9156 -17.3769 23.4408 680285.8938 -8.6252
-8.6336
[13793]ENERGY: 14130000 3355.9954 5573.6317 5656.2891
334.1977 -14389.2052 -1768.5998 0.0000 0.0000
8125.3828 6887.6918 309.8340 -1237.6911 6950.6709
310.8864 -71.6453 -125.7896 680285.8938 -8.9261
-8.9364
[13829]ENERGY: 14140000 3340.5050 5512.3998 5734.6511
342.4271 -14365.3528 -1804.4093 0.0000 0.0000
8125.5817 6885.8026 309.8415 -1239.7791 6951.6565
311.0765 71.5406 5.0013 680285.8938 -11.0128
-11.0051
[13879]ENERGY: 14150000 3378.0225 5497.3060 5709.3187
363.8316 -14344.3195 -1811.3893 0.0000 0.0000
8093.6539 6886.4238 308.6241 -1207.2300 6952.0240
311.0091 -76.8016 -92.1680 680285.8938 -10.7872
-10.7739
[13915]ENERGY: 14160000 3329.6744 5516.0135 5713.4980
353.2201 -14305.4962 -1836.5626 0.0000 0.0000
8115.6157 6885.9629 309.4615 -1229.6528 6951.3087
310.9073 94.7888 80.9745 680285.8938 -15.4806
-15.4851
[13965]ENERGY: 14170000 3391.8026 5459.5563 5671.3204
331.1970 -14328.8686 -1796.3479 0.0000 0.0000
8158.5142 6887.1740 311.0973 -1271.3402 6951.2670
311.1042 -70.5608 -158.7315 680285.8938 -10.1227
-10.1363
[14001]ENERGY: 14180000 3292.9254 5516.8514 5712.7324
345.2413 -14341.0597 -1804.1772 0.0000 0.0000
8164.0719 6886.5855 311.3092 -1277.4864 6952.9962
310.9573 -66.4119 -65.0197 680285.8938 -11.0379
-11.0272
[14051]ENERGY: 14190000 3307.2406 5515.2570 5734.6780
339.0172 -14353.1827 -1829.0475 0.0000 0.0000
8171.3051 6885.2677 311.5850 -1286.0374 6952.1153
310.8316 86.0442 93.3167 680285.8938 -10.6201
-10.6250
[14090]ENERGY: 14200000 3334.8624 5509.3195 5715.8013
333.5881 -14333.5441 -1826.2022 0.0000 0.0000
8151.7525 6885.5776 310.8395 -1266.1749 6953.9753

310.7801 -77.1673 -48.4250 680285.8938 -14.2539
-14.2424
[14140]ENERGY: 14210000 3426.7779 5486.8924 5744.8063
350.7964 -14331.1308 -1868.8530 0.0000 0.0000
8077.4733 6886.7625 308.0071 -1190.7108 6953.1197
310.7078 -164.2207 -204.1407 680285.8938 -10.9970
-11.0087
[14176]ENERGY: 14220000 3305.0816 5505.5962 5724.7939
341.4535 -14299.4161 -1820.9025 0.0000 0.0000
8129.6165 6886.2231 309.9954 -1243.3934 6953.3293
310.7017 -3.1773 -8.8393 680285.8938 -12.8485
-12.8519
[14226]ENERGY: 14230000 3325.7966 5430.1242 5725.8830
319.1834 -14362.0356 -1699.0548 0.0000 0.0000
8148.2208 6888.1175 310.7048 -1260.1032 6952.3990
310.7272 152.1004 129.0210 680285.8938 -13.2927
-13.2741
[14262]ENERGY: 14240000 3399.7337 5487.5814 5711.9350
348.0737 -14353.7870 -1773.9361 0.0000 0.0000
8071.6836 6891.2843 307.7863 -1180.3993 6953.2442
310.6170 -30.8397 -52.0856 680285.8938 -14.0336
-14.0433
[14312]ENERGY: 14250000 3352.4798 5522.0497 5722.0780
343.0601 -14381.8655 -1822.4303 0.0000 0.0000
8151.4666 6886.8384 310.8286 -1264.6282 6953.6384
310.7956 92.5913 65.3486 680285.8938 -14.5174
-14.5153
[14348]ENERGY: 14260000 3417.4895 5552.2334 5679.5263
328.8826 -14324.8496 -1860.3225 0.0000 0.0000
8097.0131 6889.9729 308.7522 -1207.0402 6953.9997
310.7915 -126.4832 -130.5449 680285.8938 -9.8203
-9.8188
[14398]ENERGY: 14270000 3385.3732 5527.3028 5708.5044
340.2496 -14379.6517 -1769.9269 0.0000 0.0000
8077.1983 6889.0496 307.9966 -1188.1487 6953.4916
311.0093 -32.7084 -45.7006 680285.8938 -14.3204
-14.3202
[14434]ENERGY: 14280000 3435.3558 5450.7303 5679.5541
362.2358 -14342.0793 -1779.6914 0.0000 0.0000
8084.7107 6890.8160 308.2831 -1193.8946 6953.2430
311.0501 -11.4686 -76.1496 680285.8938 -13.5556
-13.5666
[14484]ENERGY: 14290000 3356.8652 5481.6435 5667.0344
347.8158 -14372.6098 -1769.1217 0.0000 0.0000
8175.8999 6887.5273 311.7602 -1288.3726 6952.6834
311.0583 57.1816 8.9773 680285.8938 -13.1128
-13.1061
[14523]ENERGY: 14300000 3348.1118 5518.3845 5713.8047
343.3506 -14417.6316 -1787.2014 0.0000 0.0000
8169.6869 6888.5055 311.5233 -1281.1815 6955.4544
310.7097 69.5811 44.0205 680285.8938 -16.7868
-16.7855
[14573]ENERGY: 14310000 3301.6841 5504.3654 5784.8622
368.6437 -14353.5455 -1843.0360 0.0000 0.0000

8125.1029 6888.0768 309.8233 -1237.0261 6954.6337
310.9676 -233.3687 -123.8697 680285.8938 -15.1378
-15.1484
[14609]ENERGY: 14320000 3309.4976 5449.8030 5726.3746
334.0192 -14333.7309 -1750.3567 0.0000 0.0000
8150.7537 6886.3605 310.8014 -1264.3932 6954.4050
311.0682 -41.1065 -45.4595 680285.8938 -11.2199
-11.2129
[14659]ENERGY: 14330000 3394.8852 5427.4328 5766.2920
340.4628 -14399.3731 -1802.0508 0.0000 0.0000
8160.9348 6888.5835 311.1896 -1272.3512 6953.5068
311.0537 -43.7370 15.0812 680285.8938 -17.6857
-17.6919
[14695]ENERGY: 14340000 3300.9487 5429.0450 5767.1970
363.6241 -14448.2169 -1703.6174 0.0000 0.0000
8174.8249 6883.8053 311.7193 -1291.0196 6953.3895
310.6635 44.6092 -2.4155 680285.8938 -19.2386
-19.2259
[14745]ENERGY: 14350000 3334.5047 5537.9788 5715.5434
355.6196 -14358.3880 -1751.0939 0.0000 0.0000
8056.5586 6890.7231 307.2096 -1165.8355 6954.8307
310.6830 67.8178 22.6108 680285.8938 -16.3429
-16.3444
[14781]ENERGY: 14360000 3366.1976 5519.0643 5696.2228
336.1533 -14363.0670 -1771.1593 0.0000 0.0000
8106.2207 6889.6325 309.1033 -1216.5883 6955.2702
310.8969 21.5964 11.1919 680285.8938 -16.7535
-16.7386
[14831]ENERGY: 14370000 3410.6224 5432.8303 5689.5103
357.1183 -14280.4660 -1860.9559 0.0000 0.0000
8139.0020 6887.6614 310.3533 -1251.3405 6956.4378
310.9621 34.2715 -89.0266 680285.8938 -14.7632
-14.7931
[14867]ENERGY: 14380000 3337.3204 5470.1410 5704.4409
340.8676 -14329.5910 -1812.5051 0.0000 0.0000
8177.9021 6888.5758 311.8366 -1289.3263 6955.3202
310.9301 -76.7763 -92.6348 680285.8938 -17.4277
-17.4114
[14917]ENERGY: 14390000 3425.8159 5577.8789 5711.3855
341.0304 -14424.3142 -1826.9020 0.0000 0.0000
8084.1211 6889.0156 308.2606 -1195.1055 6954.7049
310.9980 -26.7685 -140.0087 680285.8938 -15.0164
-15.0212
[14956]ENERGY: 14400000 3363.1284 5503.1525 5676.3457
343.9111 -14406.8825 -1782.8179 0.0000 0.0000
8193.2172 6890.0544 312.4206 -1303.1627 6953.6864
311.0704 72.6926 63.5053 680285.8938 -12.9863
-12.9899
[15006]ENERGY: 14410000 3272.5042 5603.1048 5705.5888
335.6835 -14355.7055 -1810.5880 0.0000 0.0000
8142.2460 6892.8338 310.4770 -1249.4122 6955.5164
310.8170 55.5170 -11.6734 680285.8938 -10.9228
-10.9008
[15042]ENERGY: 14420000 3434.9040 5524.7520 5705.9540

362.2257 -14344.7203 -1857.4918 0.0000 0.0000
8063.8799 6889.5036 307.4887 -1174.3763 6955.2718
310.9076 -191.3986 -187.8507 680285.8938 -15.4508
-15.4668
[15092]ENERGY: 14430000 3394.0941 5385.0773 5701.4763
369.2895 -14350.6231 -1757.6677 0.0000 0.0000
8148.8193 6890.4657 310.7276 -1258.3536 6955.2292
310.8748 -80.4737 -82.8158 680285.8938 -14.5045
-14.5076
[15128]ENERGY: 14440000 3402.5214 5467.8287 5705.3968
354.1320 -14252.2871 -1901.1628 0.0000 0.0000
8112.3365 6888.7654 309.3365 -1223.5710 6955.6096
310.9200 -136.6062 -180.7085 680285.8938 -17.9365
-17.9404
[15178]ENERGY: 14450000 3278.0507 5419.1365 5742.4224
334.0442 -14298.8388 -1816.1085 0.0000 0.0000
8227.4266 6886.1332 313.7250 -1341.2935 6954.4487
310.7809 88.9553 90.8459 680285.8938 -14.1592
-14.1615
[15214]ENERGY: 14460000 3354.5802 5534.9312 5735.3482
342.5345 -14387.3644 -1811.1969 0.0000 0.0000
8120.2026 6889.0354 309.6364 -1231.1672 6954.8951
310.6752 80.7756 21.6557 680285.8938 -21.7312
-21.7213
[15264]ENERGY: 14470000 3351.5758 5528.5521 5630.3002
353.9597 -14340.9985 -1749.5811 0.0000 0.0000
8118.7816 6892.5897 309.5822 -1226.1919 6954.8191
310.8233 -104.5056 2.5600 680285.8938 -16.3602
-16.3496
[15300]ENERGY: 14480000 3261.9500 5523.2356 5681.5312
366.6831 -14338.8094 -1796.3245 0.0000 0.0000
8191.9413 6890.2072 312.3719 -1301.7341 6956.4138
311.0377 93.5891 37.9875 680285.8938 -13.7001
-13.7022
[15350]ENERGY: 14490000 3339.1252 5432.0158 5697.8421
367.4725 -14259.6440 -1837.8623 0.0000 0.0000
8153.2312 6892.1805 310.8959 -1261.0508 6956.0669
310.8999 -142.2408 -158.9261 680285.8938 -16.7653
-16.7760
[15389]ENERGY: 14500000 3314.2398 5539.4211 5736.1516
342.8189 -14329.9357 -1739.0359 0.0000 0.0000
8027.3340 6890.9939 306.0952 -1136.3401 6955.7722
310.8467 -107.1240 -57.1215 680285.8938 -14.8196
-14.8064
[15439]ENERGY: 14510000 3390.9741 5378.5105 5680.7955
349.5375 -14225.7091 -1793.1785 0.0000 0.0000
8113.8290 6894.7591 309.3934 -1219.0699 6956.0656
310.7689 34.7783 -44.6858 680285.8938 -21.2725
-21.2838
[15475]ENERGY: 14520000 3388.4254 5543.8296 5716.3636
343.6428 -14368.1056 -1815.3824 0.0000 0.0000
8084.0335 6892.8068 308.2572 -1191.2267 6955.2902
310.8355 -137.4724 -20.9902 680285.8938 -18.3949
-18.3996

[15525]ENERGY: 14530000 3279.5994 5558.8249 5711.6579
327.2094 -14314.8947 -1809.7240 0.0000 0.0000
8140.2592 6892.9322 310.4012 -1247.3271 6956.6043
310.9935 39.3175 -6.5170 680285.8938 -16.6325
-16.6424
[15561]ENERGY: 14540000 3306.5668 5599.6807 5643.2427
363.3235 -14409.8327 -1823.9618 0.0000 0.0000
8209.9094 6888.9287 313.0571 -1320.9808 6956.9866
311.0292 -103.7366 -129.3607 680285.8938 -18.2938
-18.2760
[15611]ENERGY: 14550000 3307.7088 5521.3120 5755.9492
340.0942 -14401.0248 -1810.8608 0.0000 0.0000
8176.9031 6890.0817 311.7985 -1286.8214 6955.4801
311.0977 -96.2460 -0.3622 680285.8938 -15.9968
-15.9872
[15647]ENERGY: 14560000 3250.2927 5536.3376 5678.8962
330.3540 -14256.8866 -1748.4125 0.0000 0.0000
8103.3716 6893.9530 308.9946 -1209.4186 6956.3951
311.1265 187.4563 131.0429 680285.8938 -19.0789
-19.1084
[15697]ENERGY: 14570000 3307.9104 5522.8237 5670.0840
365.7589 -14289.1530 -1812.9335 0.0000 0.0000
8128.3238 6892.8143 309.9461 -1235.5096 6956.6327
311.1926 40.5167 -53.4148 680285.8938 -17.9331
-17.9236
[15733]ENERGY: 14580000 3360.9055 5391.9902 5728.3895
340.3394 -14295.7429 -1790.1238 0.0000 0.0000
8154.6453 6890.4032 310.9498 -1264.2421 6955.9656
311.0298 42.6098 -34.1086 680285.8938 -21.2270
-21.2377
[15783]ENERGY: 14590000 3327.2824 5504.9232 5681.3922
373.9671 -14242.1144 -1882.1941 0.0000 0.0000
8129.6427 6892.8990 309.9964 -1236.7437 6958.4276
310.8555 52.9678 8.1116 680285.8938 -14.5729
-14.5550
[15822]ENERGY: 14600000 3273.9260 5528.5154 5690.0573
339.8233 -14337.9207 -1774.4438 0.0000 0.0000
8173.7441 6893.7016 311.6780 -1280.0425 6958.0513
311.0009 131.3740 104.5935 680285.8938 -15.8714
-15.8711
[15872]ENERGY: 14610000 3345.0610 5494.9093 5707.0415
345.0634 -14335.6882 -1833.6944 0.0000 0.0000
8166.2141 6888.9068 311.3909 -1277.3073 6958.0703
310.9500 67.9059 50.4016 680285.8938 -16.4051
-16.4126
[15908]ENERGY: 14620000 3383.3359 5493.1276 5615.4451
342.9862 -14378.5653 -1780.1564 0.0000 0.0000
8216.5023 6892.6754 313.3085 -1323.8269 6958.0873
310.9411 66.3380 -63.8170 680285.8938 -17.8749
-17.8718
[15958]ENERGY: 14630000 3292.5808 5490.8112 5724.8184
355.8048 -14289.6615 -1765.7081 0.0000 0.0000
8087.2306 6895.8762 308.3791 -1191.3544 6958.8665
311.0609 80.2274 3.3334 680285.8938 -12.9302

-12.9405
[15994]ENERGY: 14640000 3308.6342 5573.9774 5682.3817
356.6540 -14329.0285 -1811.7361 0.0000 0.0000
8117.0056 6897.8884 309.5145 -1219.1172 6958.6790
311.0005 66.2482 66.8309 680285.8938 -16.1420
-16.1109
[16044]ENERGY: 14650000 3322.8512 5458.3707 5735.1478
361.9018 -14335.0051 -1838.4664 0.0000 0.0000
8186.7465 6891.5466 312.1738 -1295.1999 6958.3400
311.1531 -51.2439 -84.5976 680285.8938 -18.1553
-18.1931
[16080]ENERGY: 14660000 3300.0017 5392.0296 5743.3999
341.5813 -14301.8645 -1774.7471 0.0000 0.0000
8192.3798 6892.7807 312.3887 -1299.5991 6957.4802
310.9613 106.7037 88.0140 680285.8938 -18.4537
-18.4300
[16130]ENERGY: 14670000 3367.7252 5396.5477 5770.6866
335.6524 -14433.2405 -1737.1676 0.0000 0.0000
8195.2035 6895.4073 312.4963 -1299.7962 6957.2737
310.9536 186.5532 93.0792 680285.8938 -16.9878
-17.0058
[16166]ENERGY: 14680000 3399.8440 5458.6024 5683.6476
334.3034 -14318.1866 -1792.3294 0.0000 0.0000
8128.0401 6893.9214 309.9353 -1234.1186 6958.7763
311.0217 -58.8131 -62.7282 680285.8938 -16.7005
-16.6764
[16216]ENERGY: 14690000 3294.0271 5545.0576 5692.2604
329.4483 -14416.7861 -1772.8482 0.0000 0.0000
8217.6831 6888.8422 313.3535 -1328.8410 6958.4049
311.0655 61.4396 -19.3123 680285.8938 -12.9560
-12.9582
[16255]ENERGY: 14700000 3274.1979 5533.9195 5733.0738
345.9001 -14382.7605 -1726.1960 0.0000 0.0000
8116.2194 6894.3543 309.4845 -1221.8651 6959.2636
311.0096 164.5037 87.4829 680285.8938 -16.4328
-16.4414
[16305]ENERGY: 14710000 3347.1205 5448.7469 5731.4862
359.7345 -14287.5869 -1859.2746 0.0000 0.0000
8155.9387 6896.1654 310.9991 -1259.7734 6959.6124
311.0596 -128.4416 -193.3489 680285.8938 -16.5331
-16.5426
[16341]ENERGY: 14720000 3302.6438 5474.2428 5741.4604
332.5659 -14304.6609 -1795.9160 0.0000 0.0000
8146.0682 6896.4042 310.6227 -1249.6641 6959.2549
311.1625 -138.9990 -44.8907 680285.8938 -18.9638
-18.9460
[16391]ENERGY: 14730000 3333.0701 5537.4907 5774.3918
343.5856 -14427.1510 -1815.3719 0.0000 0.0000
8145.6014 6891.6167 310.6049 -1253.9847 6960.0009
311.2559 -106.2842 -98.5848 680285.8938 -9.2537
-9.2786
[16427]ENERGY: 14740000 3317.7436 5444.2319 5719.6950
346.5942 -14373.7742 -1721.5355 0.0000 0.0000
8161.1171 6894.0722 311.1966 -1267.0449 6959.5513

310.9875 78.2470 32.5448 680285.8938 -13.0137
-13.0063
[16477]ENERGY: 14750000 3339.3501 5505.2533 5704.6221
354.4233 -14344.5222 -1875.8123 0.0000 0.0000
8212.7360 6896.0504 313.1649 -1316.6856 6960.7169
310.9103 37.5258 16.5088 680285.8938 -14.0284
-14.0395
[16513]ENERGY: 14760000 3294.5018 5530.5921 5681.1211
349.9426 -14276.6587 -1888.2231 0.0000 0.0000
8202.4787 6893.7546 312.7737 -1308.7241 6960.8796
310.8811 63.9521 35.9142 680285.8938 -13.6209
-13.6017
[16563]ENERGY: 14770000 3430.5310 5519.1837 5721.5038
314.8469 -14364.5909 -1794.0923 0.0000 0.0000
8070.6203 6898.0025 307.7458 -1172.6178 6960.4986
310.9883 -12.3079 -46.7572 680285.8938 -15.1636
-15.1732
[16599]ENERGY: 14780000 3327.9438 5535.4689 5794.6668
329.2756 -14368.7641 -1777.2482 0.0000 0.0000
8057.1875 6898.5304 307.2336 -1158.6572 6962.1732
310.8062 -4.8269 -61.8536 680285.8938 -16.0590
-16.0527
[16649]ENERGY: 14790000 3350.1036 5556.9578 5701.5144
341.7119 -14277.8595 -1830.0835 0.0000 0.0000
8057.3504 6899.6951 307.2398 -1157.6553 6962.0863
310.6741 -4.3496 -64.9356 680285.8938 -11.7638
-11.7610
[16688]ENERGY: 14800000 3351.3642 5433.3961 5675.7979
352.0926 -14276.1251 -1835.2112 0.0000 0.0000
8195.2511 6896.5657 312.4981 -1298.6855 6961.7521
310.8067 61.2250 25.8846 680285.8938 -13.6707
-13.6574
[16738]ENERGY: 14810000 3328.1055 5532.8547 5678.3667
339.0474 -14348.4889 -1751.9011 0.0000 0.0000
8120.8650 6898.8493 309.6617 -1222.0157 6962.5406
310.7180 43.6502 36.2376 680285.8938 -15.9540
-15.9473
[16774]ENERGY: 14820000 3274.3949 5548.1372 5749.9422
342.1550 -14372.1754 -1769.6611 0.0000 0.0000
8124.3338 6897.1266 309.7939 -1227.2072 6962.8915
310.8287 18.4366 0.3734 680285.8938 -13.7527
-13.7806
[16824]ENERGY: 14830000 3323.0137 5519.8400 5727.2249
328.7709 -14354.6846 -1809.7290 0.0000 0.0000
8162.1155 6896.5515 311.2346 -1265.5641 6962.2690
310.8290 97.3886 27.3664 680285.8938 -10.0330
-10.0478
[16860]ENERGY: 14840000 3265.0636 5485.7973 5716.2594
373.3371 -14280.8745 -1840.6392 0.0000 0.0000
8179.0548 6897.9985 311.8805 -1281.0563 6963.9486
311.1134 99.3952 -52.1266 680285.8938 -10.1597
-10.1272
[16910]ENERGY: 14850000 3367.9824 5494.0749 5701.3340
339.1194 -14433.1219 -1830.1013 0.0000 0.0000

8252.1275 6891.4149 314.6669 -1360.7126 6963.8710
311.0692 -16.7271 -89.8419 680285.8938 -15.5123
-15.5504
[16946]ENERGY: 14860000 3332.3434 5507.7333 5707.9989
359.8569 -14421.9958 -1784.1183 0.0000 0.0000
8198.3622 6900.1807 312.6168 -1298.1815 6964.5125
311.0809 -67.0825 -95.8110 680285.8938 -7.1460
-7.1048
[16996]ENERGY: 14870000 3296.5501 5466.1617 5718.1845
329.0585 -14297.5819 -1775.3841 0.0000 0.0000
8163.3231 6900.3121 311.2807 -1263.0111 6964.7833
310.9299 67.9354 101.6742 680285.8938 -12.4457
-12.4407
[17032]ENERGY: 14880000 3330.0383 5489.4256 5729.1112
345.6180 -14375.8567 -1791.3105 0.0000 0.0000
8173.8201 6900.8461 311.6809 -1272.9740 6965.7825
311.1807 150.1112 4.6083 680285.8938 -14.3917
-14.4177
[17082]ENERGY: 14890000 3314.8595 5506.8896 5701.6345
339.2367 -14338.1932 -1773.7259 0.0000 0.0000
8148.1412 6898.8422 310.7018 -1249.2989 6966.1490
311.0621 20.5063 -33.6073 680285.8938 -11.1593
-11.1380
[17121]ENERGY: 14900000 3386.2903 5536.0710 5709.0624
346.6833 -14420.0415 -1792.2785 0.0000 0.0000
8132.3518 6898.1388 310.0997 -1234.2130 6965.8531
311.2030 -176.1787 -122.6371 680285.8938 -13.7226
-13.7325
[17171]ENERGY: 14910000 3353.5160 5536.2354 5724.6530
329.4025 -14306.9858 -1794.1238 0.0000 0.0000
8060.1894 6902.8867 307.3480 -1157.3028 6966.4304
311.0699 91.1127 22.2376 680285.8938 -9.5739
-9.5773
[17207]ENERGY: 14920000 3361.6311 5529.7444 5724.4373
357.1146 -14375.2163 -1801.2443 0.0000 0.0000
8105.3922 6901.8590 309.0717 -1203.5332 6966.5911
311.0053 -68.7329 -97.3382 680285.8938 -11.7127
-11.7164
[17257]ENERGY: 14930000 3364.5912 5481.1095 5702.5225
338.4264 -14367.8281 -1784.9920 0.0000 0.0000
8166.6657 6900.4953 311.4081 -1266.1704 6967.3688
311.0887 29.8009 32.8535 680285.8938 -12.3413
-12.3288
[17293]ENERGY: 14940000 3313.4069 5420.8875 5669.9693
366.3772 -14336.2790 -1728.9923 0.0000 0.0000
8197.2631 6902.6327 312.5749 -1294.6304 6967.7818
311.1220 34.5840 49.6492 680285.8938 -10.1420
-10.1601
[17343]ENERGY: 14950000 3323.4848 5549.4134 5668.5171
354.0375 -14285.6838 -1867.1656 0.0000 0.0000
8160.1193 6902.7227 311.1585 -1257.3966 6969.0789
311.1269 -16.5909 -57.9641 680285.8938 -11.3479
-11.3462
[17379]ENERGY: 14960000 3388.0347 5453.0262 5666.8216

347.0746 -14297.3353 -1816.8211 0.0000 0.0000
8162.2763 6903.0770 311.2408 -1259.1993 6969.1598
311.1725 106.2659 87.5587 680285.8938 -11.6984
-11.7074
[17429]ENERGY: 14970000 3321.3679 5491.9509 5666.7264
343.1393 -14262.7040 -1873.1254 0.0000 0.0000
8220.4161 6907.7713 313.4577 -1312.6448 6969.7168
311.2892 -76.0329 -97.9157 680285.8938 -11.1190
-11.0958
[17465]ENERGY: 14980000 3321.2384 5584.0299 5714.4511
356.7376 -14369.1157 -1841.1240 0.0000 0.0000
8136.3681 6902.5854 310.2528 -1233.7826 6969.2231
311.4300 -76.9367 -62.6620 680285.8938 -10.2715
-10.2844
[17515]ENERGY: 14990000 3374.3861 5484.0830 5678.9407
352.1496 -14349.3259 -1842.4330 0.0000 0.0000
8204.1398 6901.9403 312.8371 -1302.1995 6969.7176
311.2207 -103.2006 -80.8255 680285.8938 -13.7244
-13.7167
[17554]ENERGY: 15000000 3360.9263 5506.2267 5670.4904
361.7056 -14362.3494 -1816.1096 0.0000 0.0000
8183.5401 6904.4302 312.0516 -1279.1099 6970.2793
311.2416 -112.6731 -125.2698 680285.8938 -11.7115
-11.7102
[17604]ENERGY: 15010000 3332.5659 5422.5633 5735.7666
334.9071 -14301.7081 -1824.4681 0.0000 0.0000
8203.9027 6903.5294 312.8280 -1300.3733 6970.9410
311.2514 51.1747 4.9681 680285.8938 -10.6155
-10.6133
[17640]ENERGY: 15020000 3321.4393 5410.6881 5693.2325
360.7518 -14238.2462 -1792.6380 0.0000 0.0000
8148.7277 6903.9551 310.7241 -1244.7726 6971.4638
311.3114 27.1645 64.8671 680285.8938 -14.3919
-14.3995
[17690]ENERGY: 15030000 3367.2743 5539.2714 5690.0858
324.5299 -14362.0824 -1796.2337 0.0000 0.0000
8140.9572 6903.8025 310.4278 -1237.1547 6969.6416
311.2347 -11.3313 74.0844 680285.8938 -16.7654
-16.7599
[17726]ENERGY: 15040000 3282.6273 5532.9628 5695.7759
350.9917 -14304.4963 -1814.0464 0.0000 0.0000
8160.3210 6904.1360 311.1662 -1256.1849 6969.8110
311.1001 105.8152 69.0633 680285.8938 -17.1520
-17.1684
[17776]ENERGY: 15050000 3298.0400 5450.4485 5686.0101
339.4018 -14244.0846 -1814.6461 0.0000 0.0000
8189.3473 6904.5170 312.2730 -1284.8303 6969.9351
311.1245 127.6985 120.9014 680285.8938 -13.0401
-13.0182
[17812]ENERGY: 15060000 3371.6095 5576.8196 5690.2322
345.6912 -14387.2325 -1794.3439 0.0000 0.0000
8102.5112 6905.2874 308.9618 -1197.2239 6970.1548
311.0832 -4.8221 38.7750 680285.8938 -18.7777
-18.7779

[17862]ENERGY: 15070000 3268.2581 5572.2287 5667.5083
344.4573 -14316.1076 -1821.3430 0.0000 0.0000
8187.2305 6902.2323 312.1923 -1284.9982 6969.5102
311.0452 18.7048 -19.5758 680285.8938 -14.6686
-14.6839
[17898]ENERGY: 15080000 3348.0980 5504.6814 5666.3309
341.2105 -14306.5932 -1793.1758 0.0000 0.0000
8143.2074 6903.7591 310.5136 -1239.4483 6971.1537
311.3302 145.2675 84.9785 680285.8938 -15.3561
-15.3663
[17948]ENERGY: 15090000 3308.4912 5504.2974 5698.5164
343.4973 -14261.8407 -1850.1423 0.0000 0.0000
8162.2838 6905.1030 311.2410 -1257.1808 6971.5208
311.1763 -81.7453 -118.3345 680285.8938 -13.3028
-13.2882
[17987]ENERGY: 15100000 3327.9420 5465.2894 5670.4944
343.4007 -14299.5117 -1819.2221 0.0000 0.0000
8217.6917 6906.0843 313.3538 -1311.6074 6970.2947
311.2410 155.3247 30.2293 680285.8938 -9.8755
-9.8753
[18037]ENERGY: 15110000 3242.7533 5465.1888 5673.1212
335.1441 -14293.4723 -1783.9102 0.0000 0.0000
8262.4791 6901.3039 315.0616 -1361.1751 6970.9651
311.2572 -55.4436 -34.4006 680285.8938 -14.9044
-14.8987
[18073]ENERGY: 15120000 3403.0030 5532.7095 5712.8912
352.6075 -14368.0937 -1837.8211 0.0000 0.0000
8113.5410 6908.8372 309.3824 -1204.7037 6970.2662
311.1491 -82.3137 -82.8138 680285.8938 -11.0868
-11.0904
[18123]ENERGY: 15130000 3356.0054 5519.0547 5741.0368
352.3159 -14428.4073 -1783.7582 0.0000 0.0000
8148.1807 6904.4280 310.7033 -1243.7527 6970.6598
311.3064 -18.8149 28.6187 680285.8938 -12.5801
-12.5616
[18159]ENERGY: 15140000 3313.5548 5548.1030 5708.0158
327.4465 -14308.8437 -1802.9989 0.0000 0.0000
8117.8882 6903.1657 309.5482 -1214.7225 6970.7026
311.3429 112.4427 158.1238 680285.8938 -8.7525
-8.7591
[18209]ENERGY: 15150000 3275.3545 5400.7788 5745.6343
326.5157 -14263.9059 -1814.4779 0.0000 0.0000
8235.2267 6905.1261 314.0225 -1330.1006 6972.2952
311.2934 81.9636 57.4514 680285.8938 -10.9520
-10.9666
[18245]ENERGY: 15160000 3328.1870 5482.9453 5709.6467
350.7966 -14384.5949 -1768.9061 0.0000 0.0000
8187.6893 6905.7639 312.2098 -1281.9254 6973.0704
311.3757 -64.7669 -23.8697 680285.8938 -13.1890
-13.1750
[18295]ENERGY: 15170000 3354.2156 5479.1975 5677.8062
328.7103 -14464.5083 -1704.6104 0.0000 0.0000
8236.5549 6907.3658 314.0731 -1329.1891 6974.0828
311.5049 -27.6299 -12.9902 680285.8938 -8.8954

-8.8825
[18331]ENERGY: 15180000 3416.4547 5537.7346 5695.4336
320.0222 -14339.3542 -1829.1396 0.0000 0.0000
8111.4230 6912.5742 309.3016 -1198.8488 6975.0371
311.3311 -146.6626 -92.1103 680285.8938 -8.5988
-8.6079
[18381]ENERGY: 15190000 3387.6812 5481.9116 5719.5709
334.9938 -14404.3227 -1764.8414 0.0000 0.0000
8154.4162 6909.4095 310.9410 -1245.0066 6974.5188
311.4581 133.3221 55.8531 680285.8938 -13.8583
-13.8778
[18420]ENERGY: 15200000 3402.3593 5474.2453 5730.7469
331.8260 -14311.4593 -1833.0494 0.0000 0.0000
8112.4565 6907.1253 309.3410 -1205.3312 6973.7861
311.4480 -19.4056 -11.5905 680285.8938 -11.6039
-11.5919
[18470]ENERGY: 15210000 3350.5800 5411.5306 5699.2260
344.5725 -14358.8254 -1740.1126 0.0000 0.0000
8203.3020 6910.2731 312.8051 -1293.0289 6974.7167
311.3575 213.5304 181.0617 680285.8938 -11.3476
-11.3457
[18506]ENERGY: 15220000 3255.5978 5613.0743 5700.0610
339.7116 -14364.1853 -1802.8227 0.0000 0.0000
8168.5568 6909.9935 311.4802 -1258.5633 6975.9651
311.6951 145.8573 82.3529 680285.8938 -13.0336
-13.0288
[18556]ENERGY: 15230000 3349.5672 5473.8197 5701.4752
364.3887 -14313.0569 -1786.1954 0.0000 0.0000
8120.0336 6910.0322 309.6300 -1210.0015 6974.1372
311.3979 -22.9650 22.5380 680285.8938 -14.1499
-14.1553
[18592]ENERGY: 15240000 3458.0136 5399.6633 5694.6239
336.5888 -14303.9593 -1807.1160 0.0000 0.0000
8130.0820 6907.8962 310.0131 -1222.1857 6975.9214
311.3502 -162.8796 -168.6449 680285.8938 -13.6570
-13.6684
[18642]ENERGY: 15250000 3277.5845 5515.5869 5711.5958
333.6069 -14263.6973 -1819.6250 0.0000 0.0000
8154.1953 6909.2470 310.9326 -1244.9483 6976.6155
311.2186 -69.2591 -58.9255 680285.8938 -16.3623
-16.3671
[18678]ENERGY: 15260000 3305.9244 5443.2822 5717.3165
345.3966 -14267.0889 -1803.4670 0.0000 0.0000
8169.1740 6910.5378 311.5038 -1258.6362 6975.8346
311.4612 88.9323 -9.0043 680285.8938 -14.8233
-14.8128
[18728]ENERGY: 15270000 3354.3174 5480.1311 5666.0756
356.5074 -14289.0661 -1854.2344 0.0000 0.0000
8193.2538 6906.9847 312.4220 -1286.2690 6974.5360
311.3142 -127.8218 -81.8906 680285.8938 -17.9231
-17.9320
[18764]ENERGY: 15280000 3312.9967 5543.5274 5686.7491
339.3314 -14313.4916 -1846.7907 0.0000 0.0000
8186.4313 6908.7535 312.1618 -1277.6778 6975.1511

311.3960 -106.8707 -127.4218 680285.8938 -14.3672
-14.3499
[18814]ENERGY: 15290000 3352.5711 5436.7598 5731.2731
318.5696 -14337.1643 -1766.3058 0.0000 0.0000
8173.2191 6908.9226 311.6580 -1264.2965 6975.5218
311.4536 47.2034 -49.1901 680285.8938 -14.3965
-14.4079
[18853]ENERGY: 15300000 3451.1898 5509.7730 5694.2616
325.4560 -14409.4850 -1804.4310 0.0000 0.0000
8146.7377 6913.5021 310.6482 -1233.2355 6975.2218
311.2546 12.7314 52.3719 680285.8938 -14.0150
-14.0005
[18903]ENERGY: 15310000 3373.9509 5533.7244 5690.2094
349.5886 -14330.8140 -1817.3381 0.0000 0.0000
8110.5887 6909.9100 309.2698 -1200.6787 6975.4903
311.1849 5.4129 -26.4324 680285.8938 -11.2420
-11.2612
[18939]ENERGY: 15320000 3337.5106 5551.2425 5661.1322
313.1386 -14298.6890 -1797.7506 0.0000 0.0000
8143.0417 6909.6261 310.5073 -1233.4156 6976.5558
311.0742 69.0267 47.2327 680285.8938 -16.1453
-16.1414
[18989]ENERGY: 15330000 3377.7927 5469.7834 5714.7847
326.5815 -14392.5905 -1776.0129 0.0000 0.0000
8188.7048 6909.0437 312.2485 -1279.6611 6975.6695
311.0461 -4.1181 -65.3720 680285.8938 -18.6445
-18.6484
[19025]ENERGY: 15340000 3421.1024 5491.6801 5664.1984
334.9431 -14341.4866 -1873.2066 0.0000 0.0000
8209.8527 6907.0835 313.0549 -1302.7692 6975.4221
311.2091 -21.5340 -37.5203 680285.8938 -12.7206
-12.7267
[19075]ENERGY: 15350000 3281.9073 5514.3946 5658.2882
342.8395 -14339.1620 -1769.4308 0.0000 0.0000
8217.7908 6906.6275 313.3576 -1311.1633 6974.3812
311.1805 49.0376 -66.8214 680285.8938 -13.9597
-13.9528
[19111]ENERGY: 15360000 3376.5323 5549.9547 5652.2959
342.9732 -14394.0968 -1783.5487 0.0000 0.0000
8166.2100 6910.3205 311.3908 -1255.8894 6974.9459
311.3612 -134.6458 -83.8625 680285.8938 -11.8192
-11.7919
[19161]ENERGY: 15370000 3373.5381 5477.7435 5687.5076
339.7296 -14295.7395 -1792.3973 0.0000 0.0000
8124.0653 6914.4472 309.7837 -1209.6180 6974.9772
311.3539 183.4511 68.4806 680285.8938 -12.7084
-12.7307
[19197]ENERGY: 15380000 3351.7296 5532.7107 5680.5688
348.1871 -14383.4333 -1790.6699 0.0000 0.0000
8168.4363 6907.5293 311.4757 -1260.9070 6974.8522
311.4930 -16.4382 -20.8180 680285.8938 -11.0284
-11.0085
[19247]ENERGY: 15390000 3343.8281 5476.6517 5683.9160
346.5171 -14268.7185 -1815.9839 0.0000 0.0000

8144.8941 6911.1047 310.5779 -1233.7894 6974.5830
311.3059 -93.6940 -121.3920 680285.8938 -9.3692
-9.3721
[19286]ENERGY: 15400000 3325.4173 5403.8081 5727.5418
357.1958 -14374.0208 -1696.9245 0.0000 0.0000
8165.0681 6908.0858 311.3472 -1256.9823 6974.7452
311.2431 163.3052 112.0061 680285.8938 -10.8255
-10.8382
[19336]ENERGY: 15410000 3287.5282 5434.0781 5752.0532
321.4223 -14297.7580 -1817.6596 0.0000 0.0000
8225.5188 6905.1830 313.6523 -1320.3358 6974.6546
311.2727 2.9766 74.4863 680285.8938 -10.0699
-10.0670
[19372]ENERGY: 15420000 3319.8873 5639.7768 5642.1188
340.4358 -14394.8488 -1740.7202 0.0000 0.0000
8104.0536 6910.7033 309.0206 -1193.3503 6974.3319
311.0677 -12.5185 -59.8355 680285.8938 -14.0355
-14.0448
[19422]ENERGY: 15430000 3335.2058 5536.4353 5698.7274
340.7646 -14405.8855 -1713.9488 0.0000 0.0000
8118.6389 6909.9377 309.5768 -1208.7012 6974.4495
311.1645 73.5205 110.9538 680285.8938 -8.5119
-8.5053
[19458]ENERGY: 15440000 3326.9084 5544.8850 5709.0704
346.3987 -14342.7746 -1818.6429 0.0000 0.0000
8144.3956 6910.2406 310.5589 -1234.1550 6974.9838
311.1919 4.0974 -11.5387 680285.8938 -15.8883
-15.8865
[19508]ENERGY: 15450000 3242.8472 5490.1145 5675.8284
367.9480 -14341.9389 -1723.8541 0.0000 0.0000
8198.1067 6909.0520 312.6070 -1289.0548 6974.9543
311.0971 118.9397 102.4855 680285.8938 -8.1730
-8.1731
[19544]ENERGY: 15460000 3303.7206 5499.9618 5695.6437
327.0175 -14235.5356 -1753.2503 0.0000 0.0000
8072.1686 6909.7262 307.8048 -1162.4423 6976.2800
311.0197 -76.9779 -65.0171 680285.8938 -8.4429
-8.4400
[19594]ENERGY: 15470000 3384.4839 5436.7534 5674.6163
347.7226 -14274.0415 -1743.2079 0.0000 0.0000
8088.5191 6914.8460 308.4283 -1173.6731 6978.2028
310.9213 88.9934 10.7637 680285.8938 -13.7788
-13.7820
[19630]ENERGY: 15480000 3348.7992 5528.1359 5672.3223
347.4103 -14285.3899 -1815.4804 0.0000 0.0000
8118.2417 6914.0391 309.5616 -1204.2026 6979.5103
311.3281 -219.3867 -181.4077 680285.8938 -12.8819
-12.8749
[19680]ENERGY: 15490000 3327.5732 5455.2179 5715.8539
341.5005 -14310.7623 -1725.3112 0.0000 0.0000
8108.8346 6912.9065 309.2029 -1195.9281 6978.5726
311.2413 67.4606 122.8105 680285.8938 -12.7197
-12.7344
[19719]ENERGY: 15500000 3378.0784 5445.7363 5676.9294

355.9122 -14222.4633 -1835.7520 0.0000 0.0000
8117.2980 6915.7390 309.5257 -1201.5590 6979.3159
311.2698 91.3953 20.4220 680285.8938 -9.6933
-9.7017
[19769]ENERGY: 15510000 3421.3441 5503.2891 5737.1328
366.1034 -14302.8699 -1848.8195 0.0000 0.0000
8041.7421 6917.9221 306.6446 -1123.8200 6979.4657
311.4077 -20.2592 -7.6121 680285.8938 -11.2855
-11.2731
[19805]ENERGY: 15520000 3388.3616 5481.1679 5698.2334
352.4140 -14326.7148 -1853.1497 0.0000 0.0000
8175.8009 6916.1133 311.7565 -1259.6875 6978.3850
311.4368 -140.0083 -120.8838 680285.8938 -12.7563
-12.7463
[19855]ENERGY: 15530000 3343.5647 5602.9261 5623.5970
356.1934 -14340.1204 -1812.9271 0.0000 0.0000
8135.1223 6908.3560 310.2053 -1226.7663 6979.4615
311.4040 -109.2101 -22.1351 680285.8938 -10.4150
-10.4042
[19891]ENERGY: 15540000 3353.8404 5494.6471 5682.3327
353.8076 -14377.8703 -1721.3918 0.0000 0.0000
8128.2569 6913.6226 309.9435 -1214.6343 6981.1870
311.2770 39.6560 72.0112 680285.8938 -14.8843
-14.8895
[19941]ENERGY: 15550000 3335.9178 5592.5989 5625.6539
325.8706 -14337.5655 -1839.7822 0.0000 0.0000
8213.8485 6916.5420 313.2073 -1297.3065 6982.9913
311.4280 -89.3286 -10.1714 680285.8938 -12.0094
-12.0028
[19977]ENERGY: 15560000 3358.9665 5498.5206 5670.2395
358.1809 -14329.2001 -1816.2556 0.0000 0.0000
8173.9468 6914.3987 311.6858 -1259.5481 6980.9847
311.4056 39.0160 34.1374 680285.8938 -8.3423
-8.3676
[20027]ENERGY: 15570000 3360.8035 5466.3126 5704.6278
334.0942 -14317.2939 -1836.3780 0.0000 0.0000
8205.8649 6918.0312 312.9029 -1287.8337 6982.6883
311.3306 -8.9111 -90.8635 680285.8938 -11.0387
-11.0285
[20063]ENERGY: 15580000 3358.4051 5454.6120 5680.9321
338.2076 -14387.3852 -1683.3980 0.0000 0.0000
8154.3883 6915.7618 310.9400 -1238.6265 6982.2770
311.3445 -47.7784 -15.3966 680285.8938 -10.0146
-10.0207
[20113]ENERGY: 15590000 3344.9312 5532.5984 5688.4628
349.5951 -14295.1565 -1830.2945 0.0000 0.0000
8126.3395 6916.4761 309.8704 -1209.8634 6983.1818
311.2439 -72.2747 -36.8345 680285.8938 -9.9378
-9.9205
[20152]ENERGY: 15600000 3342.3074 5531.1997 5713.5487
364.4609 -14255.0273 -1814.6851 0.0000 0.0000
8036.7200 6918.5243 306.4531 -1118.1956 6984.3418
311.1349 0.9132 -20.2597 680285.8938 -13.2845
-13.3172

[20202]ENERGY: 15610000 3361.7941 5430.2493 5678.4750
358.4241 -14325.1000 -1691.9293 0.0000 0.0000
8108.8884 6920.8016 309.2050 -1188.0868 6983.7964
311.3647 153.1503 167.5438 680285.8938 -9.6265
-9.6053
[20238]ENERGY: 15620000 3402.6768 5500.0283 5677.3413
337.0508 -14401.8512 -1768.3842 0.0000 0.0000
8170.4808 6917.3426 311.5536 -1253.1382 6984.1783
311.5007 94.0720 123.0975 680285.8938 -7.8660
-7.8612
[20288]ENERGY: 15630000 3389.1391 5530.0569 5709.4050
351.5560 -14356.9163 -1785.1410 0.0000 0.0000
8083.5841 6921.6837 308.2401 -1161.9004 6985.2979
311.5236 -120.8941 -85.4602 680285.8938 -7.7922
-7.7895
[20324]ENERGY: 15640000 3368.0975 5570.4122 5683.5495
345.6847 -14275.1411 -1895.3958 0.0000 0.0000
8120.8400 6918.0469 309.6607 -1202.7931 6985.3753
311.4081 -136.2081 -170.6429 680285.8938 -7.6120
-7.6070
[20374]ENERGY: 15650000 3340.5561 5423.0986 5729.2667
328.3346 -14308.5002 -1726.4140 0.0000 0.0000
8136.5940 6922.9359 310.2614 -1213.6581 6987.0358
311.5223 5.4885 -37.7782 680285.8938 -9.4494
-9.4714
[20410]ENERGY: 15660000 3356.2909 5528.9244 5710.0454
356.0462 -14285.3189 -1831.9084 0.0000 0.0000
8088.9711 6923.0506 308.4455 -1165.9205 6988.0914
311.4332 -34.6365 -114.3043 680285.8938 -9.3062
-9.3093
[20460]ENERGY: 15670000 3334.4066 5533.8336 5645.0475
316.9664 -14371.3194 -1758.7825 0.0000 0.0000
8218.1874 6918.3397 313.3727 -1299.8477 6988.1641
311.4575 107.5975 3.9056 680285.8938 -14.7813
-14.7886
[20496]ENERGY: 15680000 3349.8229 5526.7627 5722.3341
344.7451 -14333.3403 -1828.2567 0.0000 0.0000
8142.2907 6924.3585 310.4787 -1217.9322 6988.4487
311.5551 90.7880 28.0120 680285.8938 -11.0426
-11.0289
[20546]ENERGY: 15690000 3312.9465 5480.7617 5708.1671
357.9761 -14283.3510 -1826.1884 0.0000 0.0000
8170.2277 6920.5397 311.5440 -1249.6880 6988.3225
311.5022 8.6889 35.7649 680285.8938 -11.4540
-11.4412
[20585]ENERGY: 15700000 3361.3009 5532.1576 5741.7046
339.5848 -14359.5735 -1784.4109 0.0000 0.0000
8095.2925 6926.0560 308.6866 -1169.2365 6988.6802
311.3748 91.2920 58.1859 680285.8938 -11.5165
-11.5318
[20635]ENERGY: 15710000 3374.4529 5550.0123 5678.5607
332.7396 -14291.7544 -1815.9378 0.0000 0.0000
8096.4105 6924.4838 308.7292 -1171.9267 6990.1914
311.2862 -11.1308 -104.8578 680285.8938 -13.3266

-13.3229
[20671]ENERGY: 15720000 3375.0373 5408.7658 5724.0501
346.0801 -14353.4033 -1790.0277 0.0000 0.0000
8214.2440 6924.7462 313.2224 -1289.4978 6989.4807
311.4185 -48.1174 -138.7322 680285.8938 -13.2423
-13.2543
[20721]ENERGY: 15730000 3283.7426 5472.0163 5725.9195
354.4681 -14384.0052 -1722.4646 0.0000 0.0000
8194.2645 6923.9412 312.4605 -1270.3233 6989.4075
311.4835 205.9927 140.5775 680285.8938 -4.6177
-4.6066
[20757]ENERGY: 15740000 3409.6632 5587.6361 5685.1477
344.3977 -14380.6289 -1790.4646 0.0000 0.0000
8068.6895 6924.4406 307.6721 -1144.2489 6989.2134
311.4048 32.4097 -5.1434 680285.8938 -7.4698
-7.4667
[20807]ENERGY: 15750000 3378.3782 5552.6275 5666.2893
362.0312 -14409.8756 -1758.3409 0.0000 0.0000
8131.9360 6923.0458 310.0838 -1208.8902 6988.1966
311.4378 -121.6540 -50.1984 680285.8938 -10.1203
-10.1128
[20843]ENERGY: 15760000 3337.9560 5522.7534 5666.1811
336.6883 -14293.0318 -1792.6024 0.0000 0.0000
8146.7979 6924.7425 310.6505 -1222.0554 6989.9642
311.4180 57.9066 3.2694 680285.8938 -11.3058
-11.3192
[20893]ENERGY: 15770000 3278.4933 5512.9277 5723.0741
338.7932 -14328.5421 -1747.7233 0.0000 0.0000
8146.5634 6923.5863 310.6416 -1222.9770 6989.8104
311.3178 -76.5802 -17.2239 680285.8938 -10.2847
-10.2768
[20929]ENERGY: 15780000 3321.6596 5522.1747 5732.0187
334.6275 -14349.2190 -1742.4225 0.0000 0.0000
8104.9086 6923.7477 309.0532 -1181.1609 6989.5037
311.2029 78.0974 56.6489 680285.8938 -11.5574
-11.5580
[20979]ENERGY: 15790000 3269.6423 5510.3912 5662.8338
336.4179 -14309.6058 -1735.9025 0.0000 0.0000
8188.7130 6922.4899 312.2488 -1266.2232 6991.3913
311.3280 28.1961 46.4431 680285.8938 -10.1439
-10.1439
[21018]ENERGY: 15800000 3357.9730 5517.2035 5683.4030
346.1028 -14385.1887 -1754.0933 0.0000 0.0000
8159.1454 6924.5458 311.1214 -1234.5997 6991.3745
311.4465 43.2054 -25.0145 680285.8938 -13.1727
-13.1758
[21068]ENERGY: 15810000 3297.0727 5651.3235 5647.2792
344.1717 -14395.8513 -1781.4748 0.0000 0.0000
8162.7835 6925.3045 311.2601 -1237.4789 6991.3275
311.5281 36.5370 -10.4395 680285.8938 -8.7605
-8.7665
[21104]ENERGY: 15820000 3295.9468 5533.5622 5697.5584
334.4361 -14269.5515 -1807.2885 0.0000 0.0000
8139.8376 6924.5011 310.3851 -1215.3365 6991.2536

311.6339 26.4252 19.9264 680285.8938 -9.8406
-9.8415
[21154]ENERGY: 15830000 3433.3016 5456.7779 5694.8575
328.8123 -14353.9601 -1760.5608 0.0000 0.0000
8127.9163 6927.1447 309.9306 -1200.7715 6991.8150
311.7216 -153.8275 -126.7827 680285.8938 -8.5258
-8.5217
[21190]ENERGY: 15840000 3352.0546 5534.4762 5713.9946
343.9610 -14357.6220 -1781.1724 0.0000 0.0000
8119.6608 6925.3528 309.6158 -1194.3080 6990.8878
311.4886 2.6301 -71.4832 680285.8938 -12.4003
-12.4025
[21240]ENERGY: 15850000 3452.2575 5362.3010 5659.8206
357.5136 -14277.9728 -1821.6794 0.0000 0.0000
8191.4786 6923.7191 312.3543 -1267.7595 6990.7783
311.4918 -190.6165 -108.4733 680285.8938 -9.3816
-9.3677
[21276]ENERGY: 15860000 3389.3861 5507.4958 5634.5032
314.2432 -14297.1685 -1766.5333 0.0000 0.0000
8140.6363 6922.5628 310.4156 -1218.0735 6991.2224
311.3882 -97.4388 -64.4289 680285.8938 -8.0696
-8.0892
[21326]ENERGY: 15870000 3268.4622 5511.4276 5685.2826
332.3735 -14252.3067 -1779.1249 0.0000 0.0000
8156.3908 6922.5052 311.0163 -1233.8856 6990.9550
311.3726 157.4527 127.4855 680285.8938 -4.9618
-4.9550
[21362]ENERGY: 15880000 3327.9320 5443.2637 5681.8751
319.3602 -14294.6574 -1723.8215 0.0000 0.0000
8173.8485 6927.8006 311.6820 -1246.0480 6991.8256
311.2475 41.8422 39.4426 680285.8938 -5.7389
-5.7469
[21412]ENERGY: 15890000 3223.2559 5515.6068 5671.0463
353.5909 -14179.7832 -1890.2686 0.0000 0.0000
8234.5017 6927.9497 313.9948 -1306.5520 6994.5221
311.4614 103.9008 -53.9543 680285.8938 -9.8983
-9.9082
[21451]ENERGY: 15900000 3366.4287 5545.6252 5710.0529
351.8188 -14292.7667 -1909.7953 0.0000 0.0000
8155.5664 6926.9300 310.9849 -1228.6364 6993.7912
311.5994 73.1956 0.4582 680285.8938 -7.9003
-7.8878
[21501]ENERGY: 15910000 3248.0186 5507.7417 5720.4459
319.9046 -14328.3416 -1731.4457 0.0000 0.0000
8188.1721 6924.4957 312.2282 -1263.6764 6993.3041
311.6276 34.1916 13.9231 680285.8938 -6.7657
-6.7719
[21537]ENERGY: 15920000 3389.1929 5512.2239 5697.4728
351.7135 -14327.9020 -1844.6185 0.0000 0.0000
8150.0260 6928.1085 310.7736 -1221.9175 6992.9948
311.7541 39.6211 -34.7199 680285.8938 -1.7378
-1.7209
[21587]ENERGY: 15930000 3298.0514 5550.3237 5687.6006
362.1421 -14343.8408 -1838.9620 0.0000 0.0000

8214.5104 6929.8255 313.2325 -1284.6849 6993.9402
311.8141 203.7475 156.6470 680285.8938 -5.8813
-5.8788
[21623]ENERGY: 15940000 3308.6043 5487.5600 5672.1198
323.5121 -14302.6986 -1753.2485 0.0000 0.0000
8190.4782 6926.3274 312.3161 -1264.1509 6991.7080
311.8836 46.4079 18.4201 680285.8938 -6.2457
-6.2373
[21673]ENERGY: 15950000 3383.3312 5459.4095 5695.9889
326.8975 -14353.2750 -1717.4049 0.0000 0.0000
8132.2829 6927.2300 310.0971 -1205.0529 6992.1468
311.8556 -67.4482 -83.5872 680285.8938 -8.6116
-8.6279
[21709]ENERGY: 15960000 3260.0617 5454.3080 5676.4754
337.6895 -14293.9032 -1777.8067 0.0000 0.0000
8267.4126 6924.2373 315.2498 -1343.1753 6994.0152
311.9946 217.5326 157.2120 680285.8938 -5.6574
-5.6530
[21759]ENERGY: 15970000 3323.7293 5612.9794 5645.0700
335.8152 -14380.1451 -1765.2641 0.0000 0.0000
8152.6953 6924.8800 310.8754 -1227.8153 6994.3427
311.8582 15.1274 -14.9940 680285.8938 -11.5791
-11.5597
[21795]ENERGY: 15980000 3336.8907 5514.9922 5648.3719
338.7399 -14286.6718 -1859.0339 0.0000 0.0000
8235.7528 6929.0418 314.0425 -1306.7110 6995.6844
312.1395 -42.9554 -98.2115 680285.8938 -12.5785
-12.6062
[21845]ENERGY: 15990000 3366.8436 5579.6538 5680.1181
332.6189 -14425.1564 -1805.1991 0.0000 0.0000
8199.5673 6928.4462 312.6627 -1271.1211 6994.8919
312.1872 -163.8637 -186.3405 680285.8938 -7.3375
-7.3366
[21884]ENERGY: 16000000 3334.1694 5479.9518 5650.3983
337.1444 -14309.8983 -1805.6385 0.0000 0.0000
8241.2784 6927.4054 314.2532 -1313.8730 6995.4544
312.0210 114.4102 19.9861 680285.8938 -9.7881
-9.7790
[21934]ENERGY: 16010000 3339.3603 5444.5581 5658.7195
332.0276 -14315.2869 -1760.4941 0.0000 0.0000
8231.5069 6930.3914 313.8806 -1301.1155 6994.8490
311.8613 85.8370 -9.5280 680285.8938 -9.8682
-9.8703
[21970]ENERGY: 16020000 3404.9863 5448.5798 5664.2456
337.1879 -14321.6987 -1835.5422 0.0000 0.0000
8228.6428 6926.4016 313.7714 -1302.2413 6994.4379
311.8954 -149.4712 -154.4478 680285.8938 -6.1099
-6.1136
[22020]ENERGY: 16030000 3364.6118 5505.0937 5630.4122
347.9450 -14340.1410 -1814.9049 0.0000 0.0000
8234.8118 6927.8287 314.0067 -1306.9831 6993.7442
312.0551 28.2252 -7.8623 680285.8938 -9.3372
-9.3354
[22056]ENERGY: 16040000 3398.8318 5475.2363 5647.5905

342.8599 -14364.0464 -1776.6842 0.0000 0.0000
8205.9654 6929.7532 312.9067 -1276.2122 6995.9150
312.0595 -21.6009 -69.3740 680285.8938 -7.0826
-7.0903
[22106]ENERGY: 16050000 3323.0413 5578.7355 5691.0282
334.8055 -14392.3919 -1751.3536 0.0000 0.0000
8145.7993 6929.6642 310.6125 -1216.1350 6995.2570
312.0236 -39.1536 -64.1335 680285.8938 -10.4662
-10.4738
[22142]ENERGY: 16060000 3361.8560 5606.1422 5656.1530
354.5203 -14325.1444 -1833.7810 0.0000 0.0000
8109.1245 6928.8707 309.2140 -1180.2538 6995.0179
311.9873 -14.2367 -58.0313 680285.8938 -8.2040
-8.1865
[22192]ENERGY: 16070000 3320.0419 5546.9328 5652.3438
331.3035 -14347.0035 -1810.9417 0.0000 0.0000
8233.7693 6926.4460 313.9669 -1307.3232 6994.5400
312.1015 13.5857 -57.6251 680285.8938 -10.2013
-10.2020
[22228]ENERGY: 16080000 3413.7064 5417.2998 5706.5061
354.8738 -14365.8306 -1740.8482 0.0000 0.0000
8147.2783 6932.9856 310.6689 -1214.2927 6995.1482
312.0194 63.2531 11.3082 680285.8938 -8.0127
-8.0252
[22278]ENERGY: 16090000 3348.8112 5601.4395 5644.6397
360.9609 -14343.0340 -1829.5497 0.0000 0.0000
8146.6340 6929.9015 310.6443 -1216.7325 6996.0241
311.8255 24.0626 -54.3384 680285.8938 -10.5473
-10.5406
[22317]ENERGY: 16100000 3340.3324 5541.8787 5654.4309
351.5418 -14388.9972 -1830.2975 0.0000 0.0000
8257.0894 6925.9783 314.8561 -1331.1111 6995.1762
312.0966 -68.0559 -100.5689 680285.8938 -8.7066
-8.6995
[22367]ENERGY: 16110000 3351.0302 5562.8414 5656.1013
341.8585 -14375.5338 -1760.5836 0.0000 0.0000
8152.3363 6928.0502 310.8617 -1224.2861 6995.5936
312.2017 12.0317 -13.3267 680285.8938 -4.9937
-4.9919
[22403]ENERGY: 16120000 3328.3612 5462.0414 5646.1720
347.5596 -14305.7444 -1755.1619 0.0000 0.0000
8203.5985 6926.8265 312.8164 -1276.7720 6995.4025
312.1473 20.3536 -27.7150 680285.8938 -10.0350
-10.0417
[22453]ENERGY: 16130000 3343.5450 5485.7929 5666.1192
351.3782 -14340.9999 -1748.4887 0.0000 0.0000
8173.4695 6930.8161 311.6676 -1242.6533 6995.9878
312.0232 8.6036 -54.7105 680285.8938 -5.9415
-5.9479
[22489]ENERGY: 16140000 3408.4447 5491.8209 5671.6840
328.0388 -14370.8796 -1770.2456 0.0000 0.0000
8168.9725 6927.8356 311.4961 -1241.1368 6995.9879
312.0222 -129.0202 -133.1536 680285.8938 -5.9210
-5.8999

[22539]ENERGY: 16150000 3379.8964 5525.0065 5656.6542
353.5150 -14379.4847 -1735.8153 0.0000 0.0000
8134.9981 6934.7703 310.2006 -1200.2278 6996.4576
312.0960 22.4209 -8.9632 680285.8938 -7.1525
-7.1501
[22575]ENERGY: 16160000 3299.6918 5554.1324 5668.1948
323.0058 -14356.2650 -1806.7730 0.0000 0.0000
8248.9408 6930.9276 314.5454 -1318.0132 6996.3875
312.3987 -38.3185 13.7617 680285.8938 -7.5672
-7.5887
[22625]ENERGY: 16170000 3388.4948 5417.3975 5680.3527
337.2902 -14320.1139 -1807.0896 0.0000 0.0000
8235.6513 6931.9830 314.0387 -1303.6683 6997.2961
312.2575 -35.9397 -40.9670 680285.8938 -13.5728
-13.5611
[22661]ENERGY: 16180000 3325.9464 5456.9158 5719.7980
318.2513 -14255.9393 -1836.4855 0.0000 0.0000
8202.6726 6931.1593 312.7811 -1271.5134 6996.9550
312.1887 -174.8653 -142.4946 680285.8938 -9.9199
-9.9261
[22711]ENERGY: 16190000 3326.4241 5440.9686 5672.2329
343.9261 -14248.6029 -1834.8998 0.0000 0.0000
8230.4451 6930.4939 313.8401 -1299.9511 6997.8103
312.0787 -54.6213 -97.6786 680285.8938 -7.9425
-7.9358
[22750]ENERGY: 16200000 3292.3483 5588.0682 5757.2670
338.7219 -14418.3550 -1838.5709 0.0000 0.0000
8210.2104 6929.6900 313.0686 -1280.5204 6998.2789
312.1346 154.3034 92.4838 680285.8938 -9.5606
-9.5775
[22800]ENERGY: 16210000 3331.4777 5462.8452 5718.3458
368.6281 -14329.5977 -1760.0748 0.0000 0.0000
8138.7913 6930.4155 310.3452 -1208.3758 6997.9218
312.1246 -113.5729 -138.1928 680285.8938 -11.3271
-11.3166
[22836]ENERGY: 16220000 3435.9567 5540.4333 5684.7527
338.0830 -14371.8059 -1829.7798 0.0000 0.0000
8134.1742 6931.8142 310.1692 -1202.3600 6997.4932
312.0884 -67.3588 -72.0062 680285.8938 -12.1167
-12.1204
[22886]ENERGY: 16230000 3294.3460 5584.7020 5704.2439
344.1301 -14348.6124 -1841.7014 0.0000 0.0000
8194.1073 6931.2155 312.4545 -1262.8918 6997.5050
312.0063 -54.5276 -156.8842 680285.8938 -7.5962
-7.5923
[22922]ENERGY: 16240000 3305.0911 5514.9801 5667.6927
346.5320 -14279.5046 -1791.9921 0.0000 0.0000
8168.9695 6931.7688 311.4960 -1237.2008 6997.3840
312.1503 262.3729 237.9441 680285.8938 -16.0365
-16.0345
[22972]ENERGY: 16250000 3290.6657 5488.3001 5674.9482
345.9761 -14294.1706 -1724.2753 0.0000 0.0000
8152.3342 6933.7784 310.8616 -1218.5558 6997.2968
312.1052 160.9462 61.3245 680285.8938 -10.7481

-10.7640
[23008]ENERGY: 16260000 3311.7188 5518.2071 5660.4087
340.2454 -14275.0864 -1789.2509 0.0000 0.0000
8166.4695 6932.7123 311.4007 -1233.7572 6997.5308
311.8800 -62.6896 -61.9924 680285.8938 -9.7778
-9.7533
[23058]ENERGY: 16270000 3371.9020 5518.2337 5664.2551
339.5556 -14368.2045 -1774.7317 0.0000 0.0000
8179.9475 6930.9577 311.9146 -1248.9898 6997.4031
311.9604 -43.5732 -12.3612 680285.8938 -11.2103
-11.2151
[23094]ENERGY: 16280000 3420.0061 5533.0686 5681.9515
363.5023 -14374.7743 -1835.5262 0.0000 0.0000
8145.6276 6933.8554 310.6059 -1211.7722 6998.1315
312.0060 -137.7653 -80.4252 680285.8938 -13.7371
-13.7397
[23144]ENERGY: 16290000 3372.0012 5441.7491 5681.0173
313.1562 -14260.3399 -1775.1938 0.0000 0.0000
8163.1923 6935.5823 311.2757 -1227.6100 6997.3708
312.0570 -55.8836 -19.8882 680285.8938 -9.8477
-9.8687
[23183]ENERGY: 16300000 3359.8680 5534.8219 5696.3676
361.6300 -14428.6017 -1785.2097 0.0000 0.0000
8190.7271 6929.6032 312.3256 -1261.1239 6997.2856
312.0677 63.9870 51.7017 680285.8938 -8.3426
-8.3300
[23233]ENERGY: 16310000 3287.7217 5503.1175 5666.9605
345.6493 -14337.2878 -1777.9726 0.0000 0.0000
8239.2856 6927.4742 314.1772 -1311.8114 6996.7899
312.0487 157.8818 56.8461 680285.8938 -9.9924
-9.9894
[23269]ENERGY: 16320000 3325.4589 5418.1104 5687.2039
337.2401 -14252.0593 -1804.8055 0.0000 0.0000
8220.3239 6931.4725 313.4542 -1288.8515 6997.7258
312.1277 1.8021 -26.6170 680285.8938 -7.8982
-7.8949
[23319]ENERGY: 16330000 3278.8813 5472.6328 5691.4243
332.0711 -14329.4530 -1729.2832 0.0000 0.0000
8215.3676 6931.6409 313.2652 -1283.7267 6996.7948
311.8641 212.7492 77.7634 680285.8938 -8.7821
-8.7770
[23355]ENERGY: 16340000 3396.0130 5467.7220 5693.7574
318.9162 -14351.6708 -1743.0817 0.0000 0.0000
8147.6938 6929.3500 310.6847 -1218.3438 6995.6849
311.9778 117.4266 59.6177 680285.8938 -8.9803
-8.9759
[23405]ENERGY: 16350000 3369.3707 5486.2297 5645.3149
364.9352 -14272.1104 -1834.2634 0.0000 0.0000
8172.6106 6932.0873 311.6348 -1240.5233 6996.7185
311.9403 -38.5486 -9.5391 680285.8938 -13.3235
-13.3274
[23441]ENERGY: 16360000 3370.5513 5553.0362 5631.7340
339.4428 -14336.3102 -1783.2680 0.0000 0.0000
8159.4297 6934.6158 311.1322 -1224.8139 6998.4595

311.9614 22.9070 54.6595 680285.8938 -9.6243
-9.6278
[23491]ENERGY: 16370000 3295.3188 5581.6383 5678.4977
343.0455 -14333.3317 -1798.4249 0.0000 0.0000
8168.4165 6935.1602 311.4749 -1233.2563 6999.7901
312.0371 0.0033 33.8856 680285.8938 -14.5560
-14.5620
[23527]ENERGY: 16380000 3392.4607 5538.8204 5663.0619
331.3541 -14308.8848 -1782.4161 0.0000 0.0000
8100.0458 6934.4419 308.8678 -1165.6039 6999.6010
312.1229 41.3538 18.3137 680285.8938 -13.0709
-13.0834
[23577]ENERGY: 16390000 3288.8217 5464.4070 5722.4134
355.7083 -14319.5827 -1776.5179 0.0000 0.0000
8198.9702 6934.2201 312.6400 -1264.7502 7000.3781
312.0370 -29.0282 -26.1154 680285.8938 -11.3034
-11.2904
[23616]ENERGY: 16400000 3361.2873 5499.1408 5702.5433
335.1598 -14344.1629 -1778.3694 0.0000 0.0000
8160.7806 6936.3795 311.1837 -1224.4011 7001.7498
312.1689 40.9530 36.0733 680285.8938 -10.1751
-10.1624
[23666]ENERGY: 16410000 3358.7737 5578.0061 5664.5146
338.8166 -14290.5076 -1818.1370 0.0000 0.0000
8105.4804 6936.9467 309.0750 -1168.5337 7002.1075
312.1712 -152.8146 -143.5001 680285.8938 -10.4991
-10.4945
[23702]ENERGY: 16420000 3352.5185 5505.7555 5665.9713
350.1535 -14297.2071 -1860.0955 0.0000 0.0000
8219.3544 6936.4507 313.4172 -1282.9037 7002.6669
312.1467 20.3348 -18.3478 680285.8938 -8.1886
-8.2140
[23752]ENERGY: 16430000 3372.2001 5460.5586 5689.2843
335.9372 -14342.6778 -1821.9896 0.0000 0.0000
8240.0130 6933.3259 314.2050 -1306.6871 7002.6712
312.3336 -27.8713 -13.7602 680285.8938 -8.9666
-8.9586
[23788]ENERGY: 16440000 3312.0213 5543.3032 5702.1860
322.0305 -14324.9440 -1761.0492 0.0000 0.0000
8146.6598 6940.2076 310.6453 -1206.4522 7003.7098
312.1394 -35.3644 -54.7365 680285.8938 -7.5330
-7.5274
[23838]ENERGY: 16450000 3364.5511 5450.9935 5650.4658
336.0796 -14259.1597 -1761.8784 0.0000 0.0000
8157.5347 6938.5865 311.0600 -1218.9482 7002.9684
312.0357 37.1673 36.6856 680285.8938 -7.6938
-7.7030
[23874]ENERGY: 16460000 3340.6657 5420.2942 5732.7233
323.9456 -14267.3333 -1784.9372 0.0000 0.0000
8170.9065 6936.2647 311.5698 -1234.6417 7004.7627
312.0932 -166.8093 -126.3386 680285.8938 -4.7987
-4.7709
[23924]ENERGY: 16470000 3334.0089 5537.4508 5720.1073
312.1087 -14341.1774 -1764.6186 0.0000 0.0000

8144.7793 6942.6591 310.5736 -1202.1202 7004.7838
312.0806 84.2090 64.6006 680285.8938 -9.0529
-9.0788
[23960]ENERGY: 16480000 3417.3339 5520.9063 5670.8274
337.1460 -14299.0613 -1793.9225 0.0000 0.0000
8089.3380 6942.5678 308.4595 -1146.7702 7004.4306
312.0980 -29.5001 -17.1320 680285.8938 -11.3828
-11.3681
[24010]ENERGY: 16490000 3353.3795 5526.0044 5693.3226
338.4989 -14324.5837 -1775.5996 0.0000 0.0000
8131.3277 6942.3497 310.0606 -1188.9780 7004.7246
312.1347 42.1490 28.4389 680285.8938 -10.7051
-10.7143
[24049]ENERGY: 16500000 3408.6479 5428.2081 5718.0196
353.7742 -14263.2778 -1844.3478 0.0000 0.0000
8141.5871 6942.6113 310.4518 -1198.9758 7003.8456
312.2339 -6.8341 -22.1450 680285.8938 -6.3654
-6.3693
[24099]ENERGY: 16510000 3295.7060 5477.0123 5638.7853
333.0726 -14294.8124 -1772.1691 0.0000 0.0000
8258.0939 6935.6886 314.8944 -1322.4053 7002.3976
312.4249 -36.9152 -81.0904 680285.8938 -5.4252
-5.4301
[24135]ENERGY: 16520000 3361.7141 5498.2688 5687.5988
332.3169 -14337.5508 -1783.6445 0.0000 0.0000
8178.9808 6937.6840 311.8777 -1241.2968 7004.1830
312.3899 -6.1115 -67.8577 680285.8938 -5.1560
-5.1446
[24185]ENERGY: 16530000 3344.9710 5534.3404 5612.1358
350.3114 -14393.7199 -1778.3309 0.0000 0.0000
8263.9152 6933.6231 315.1164 -1330.2922 7003.0150
312.3170 -53.7139 -54.4499 680285.8938 -4.9431
-4.9428
[24221]ENERGY: 16540000 3290.2422 5492.9169 5705.3616
341.3869 -14259.1171 -1833.7747 0.0000 0.0000
8203.8838 6940.8996 312.8273 -1262.9842 7003.9030
312.2545 -34.3677 -83.6205 680285.8938 -7.1147
-7.1114
[24271]ENERGY: 16550000 3287.5635 5593.7198 5668.9423
327.9792 -14410.9458 -1831.5747 0.0000 0.0000
8297.3852 6933.0695 316.3927 -1364.3158 7003.9443
312.2415 16.3369 -60.0077 680285.8938 -8.0099
-8.0274
[24307]ENERGY: 16560000 3288.9887 5427.3762 5662.8692
354.1331 -14301.7780 -1775.1549 0.0000 0.0000
8280.2513 6936.6858 315.7393 -1343.5655 7004.6923
312.2865 73.9173 -1.6921 680285.8938 -7.2077
-7.2051
[24357]ENERGY: 16570000 3343.0395 5425.1182 5618.0163
325.9965 -14303.2386 -1684.2023 0.0000 0.0000
8217.9955 6942.7251 313.3654 -1275.2704 7004.7083
312.3509 153.8813 40.7340 680285.8938 -5.4308
-5.4107
[24393]ENERGY: 16580000 3289.5479 5416.0364 5655.7625

355.4477 -14265.9961 -1774.4750 0.0000 0.0000
8262.9853 6939.3088 315.0810 -1323.6765 7005.9485
312.2380 68.9262 17.1962 680285.8938 -9.9244
-9.9450
[24443]ENERGY: 16590000 3367.3390 5453.6757 5668.9029
337.4416 -14298.4913 -1749.2506 0.0000 0.0000
8159.8137 6939.4309 311.1469 -1220.3828 7004.2595
312.2514 -14.4338 -46.8310 680285.8938 -10.0630
-10.0573
[24482]ENERGY: 16600000 3351.2954 5537.2480 5656.0696
329.2135 -14393.9640 -1763.7976 0.0000 0.0000
8221.0140 6937.0789 313.4805 -1283.9351 7005.4778
312.3276 -48.3448 -42.0844 680285.8938 -10.9248
-10.9216
[24532]ENERGY: 16610000 3330.4822 5524.6261 5670.4360
375.8251 -14363.9830 -1751.9370 0.0000 0.0000
8157.3293 6942.7786 311.0521 -1214.5507 7005.8897
312.4175 156.5346 116.7938 680285.8938 -12.3537
-12.3517
[24568]ENERGY: 16620000 3334.8944 5452.3366 5663.2952
319.5583 -14282.0238 -1726.0220 0.0000 0.0000
8177.6431 6939.6818 311.8267 -1237.9613 7006.5026
312.2917 -9.4338 -10.7181 680285.8938 -9.3484
-9.3432
[24618]ENERGY: 16630000 3347.9745 5535.4615 5651.0715
349.8590 -14306.7296 -1862.8335 0.0000 0.0000
8227.3841 6942.1876 313.7234 -1285.1965 7006.3599
312.2868 -2.3562 25.4473 680285.8938 -6.6506
-6.6770
[24654]ENERGY: 16640000 3375.5947 5542.9951 5670.3515
342.3700 -14436.4565 -1816.8016 0.0000 0.0000
8262.9655 6941.0187 315.0802 -1321.9468 7007.0517
312.4182 23.4497 34.8435 680285.8938 -12.0445
-12.0140
[24704]ENERGY: 16650000 3293.3052 5508.5807 5672.3446
346.1545 -14295.8836 -1785.7649 0.0000 0.0000
8205.7977 6944.5341 312.9003 -1261.2636 7009.7894
312.2934 -30.8645 -33.1342 680285.8938 -14.2667
-14.2775
[24740]ENERGY: 16660000 3414.2319 5552.0341 5665.2303
331.8134 -14418.8636 -1728.5577 0.0000 0.0000
8130.3975 6946.2859 310.0252 -1184.1116 7009.8033
312.2800 -73.2102 -118.2213 680285.8938 -5.8345
-5.8296
[24790]ENERGY: 16670000 3347.7050 5453.5603 5672.4672
364.7421 -14320.8841 -1873.7967 0.0000 0.0000
8299.7646 6943.5583 316.4834 -1356.2063 7009.9752
312.1374 -16.8135 -28.9314 680285.8938 -7.9962
-7.9951
[24826]ENERGY: 16680000 3392.0985 5483.2754 5644.4211
339.4148 -14380.0160 -1741.6355 0.0000 0.0000
8206.3341 6943.8924 312.9208 -1262.4417 7009.3884
312.4123 6.4988 -34.1828 680285.8938 -6.0688
-6.0616

[24876]ENERGY: 16690000 3292.8504 5511.4064 5657.6388
362.3785 -14310.9604 -1840.0970 0.0000 0.0000
8267.3216 6940.5383 315.2463 -1326.7833 7010.6100
312.2762 60.0079 -37.0622 680285.8938 -8.8805
-8.8970
[24915]ENERGY: 16700000 3408.0426 5412.0515 5657.2300
337.2521 -14322.3283 -1785.5931 0.0000 0.0000
8233.9235 6940.5783 313.9728 -1293.3453 7009.8248
312.2291 86.8973 91.1623 680285.8938 -12.5834
-12.5858
[24965]ENERGY: 16710000 3402.8607 5499.1999 5661.5955
326.5880 -14304.3384 -1804.0507 0.0000 0.0000
8163.5447 6945.3996 311.2891 -1218.1451 7009.1917
312.1579 -53.4688 1.8594 680285.8938 -7.4787
-7.4750
[25001]ENERGY: 16720000 3439.4767 5465.7694 5697.3130
315.8417 -14390.5873 -1788.3460 0.0000 0.0000
8203.0450 6942.5125 312.7953 -1260.5325 7009.2000
312.3145 -101.4224 -161.5988 680285.8938 -9.4201
-9.4255
[25051]ENERGY: 16730000 3334.1809 5468.3655 5625.5221
338.3609 -14259.4932 -1801.1160 0.0000 0.0000
8234.1453 6939.9655 313.9812 -1294.1797 7009.7496
312.4251 -115.5673 -126.1898 680285.8938 -11.5415
-11.5487
[25087]ENERGY: 16740000 3372.9803 5504.6044 5678.6038
366.1360 -14337.9397 -1771.7077 0.0000 0.0000
8132.1547 6944.8317 310.0922 -1187.3229 7011.1053
312.2895 141.1280 56.6962 680285.8938 -8.3839
-8.3672
[25137]ENERGY: 16750000 3401.7513 5450.2116 5661.0821
346.5861 -14353.9303 -1780.9424 0.0000 0.0000
8225.0554 6949.8137 313.6346 -1275.2417 7012.0414
312.2886 -54.9283 -40.8870 680285.8938 -12.5083
-12.5151
[25173]ENERGY: 16760000 3347.4040 5632.5033 5698.7118
328.3394 -14368.3147 -1794.1691 0.0000 0.0000
8099.5024 6943.9772 308.8471 -1155.5252 7011.4090
312.4195 -95.1731 -57.4218 680285.8938 -7.6058
-7.5873
[25223]ENERGY: 16770000 3437.2085 5530.7958 5644.2409
349.7293 -14428.0457 -1772.8573 0.0000 0.0000
8186.0270 6947.0987 312.1464 -1238.9284 7013.7293
312.5266 -92.3545 -57.9335 680285.8938 -6.7357
-6.7474
[25259]ENERGY: 16780000 3310.5648 5511.4231 5623.4150
335.9878 -14331.6430 -1725.5113 0.0000 0.0000
8223.8618 6948.0983 313.5891 -1275.7635 7014.7417
312.4933 4.1427 65.4715 680285.8938 -5.3711
-5.3635
[25309]ENERGY: 16790000 3377.5173 5558.1037 5670.0351
333.2209 -14294.6589 -1834.8787 0.0000 0.0000
8143.3761 6952.7155 310.5201 -1190.6607 7016.5986
312.4522 -121.8710 -134.3218 680285.8938 -6.7222

-6.7348
[25348]ENERGY: 16800000 3368.3414 5510.8165 5692.6029
368.0549 -14340.6370 -1833.6342 0.0000 0.0000
8179.8942 6945.4388 311.9126 -1234.4554 7015.0794
312.2722 -218.9205 -139.7488 680285.8938 -9.3322
-9.3247
[25398]ENERGY: 16810000 3321.5179 5498.5450 5653.7877
338.1832 -14236.1260 -1850.1786 0.0000 0.0000
8223.1585 6948.8877 313.5623 -1274.2708 7015.3826
312.5441 -34.3782 -76.6081 680285.8938 -8.2787
-8.2876
[25434]ENERGY: 16820000 3309.7991 5498.6499 5677.0560
315.0381 -14395.2074 -1676.1924 0.0000 0.0000
8220.5055 6949.6488 313.4611 -1270.8567 7014.8888
312.2535 71.3025 42.2328 680285.8938 -7.7043
-7.7053
[25484]ENERGY: 16830000 3350.1966 5549.4294 5699.5827
332.1413 -14381.4184 -1783.8080 0.0000 0.0000
8185.5495 6951.6730 312.1282 -1233.8764 7015.7609
312.4408 66.8195 -100.9064 680285.8938 -7.3568
-7.3387
[25520]ENERGY: 16840000 3292.5654 5556.3835 5674.0541
370.1906 -14377.2259 -1800.6719 0.0000 0.0000
8232.5762 6947.8720 313.9214 -1284.7043 7016.5889
312.4876 -97.9912 -111.3748 680285.8938 -9.4212
-9.4271
[25570]ENERGY: 16850000 3387.3839 5574.4960 5638.5426
354.6282 -14421.6522 -1759.8280 0.0000 0.0000
8179.9254 6953.4959 311.9137 -1226.4295 7016.2225
312.4770 -38.1560 -26.1700 680285.8938 -8.1170
-8.1216
[25606]ENERGY: 16860000 3387.7155 5455.0955 5685.2821
346.6617 -14274.1523 -1837.1019 0.0000 0.0000
8188.0982 6951.5987 312.2254 -1236.4995 7016.7081
312.3814 -80.9617 -118.6686 680285.8938 -5.4097
-5.4090
[25656]ENERGY: 16870000 3378.0794 5500.6007 5641.4717
325.2318 -14367.1639 -1767.0364 0.0000 0.0000
8235.7208 6946.9039 314.0413 -1288.8169 7016.2829
312.4452 -183.8789 -176.1389 680285.8938 -5.5440
-5.5434
[25692]ENERGY: 16880000 3352.6306 5476.6466 5645.8364
339.1235 -14315.1660 -1813.3151 0.0000 0.0000
8265.9746 6951.7305 315.1949 -1314.2440 7017.3334
312.2966 84.2243 -6.1868 680285.8938 -7.0215
-7.0327
[25742]ENERGY: 16890000 3407.4464 5577.6294 5692.8028
324.2102 -14429.0049 -1767.3388 0.0000 0.0000
8144.4759 6950.2209 310.5620 -1194.2549 7017.2847
312.3662 39.8819 23.3172 680285.8938 -9.3569
-9.3399
[25781]ENERGY: 16900000 3400.0886 5462.7056 5707.1759
366.4354 -14396.3707 -1735.5046 0.0000 0.0000
8149.3584 6953.8885 310.7482 -1195.4699 7018.2999

312.3753 55.5450 26.0756 680285.8938 -15.3079
-15.3171
[25831]ENERGY: 16910000 3416.1314 5490.9339 5670.0211
335.9702 -14282.2301 -1832.5930 0.0000 0.0000
8153.9495 6952.1830 310.9232 -1201.7665 7017.5173
312.7235 -105.9812 -28.1539 680285.8938 -8.4692
-8.4757
[25867]ENERGY: 16920000 3403.8094 5517.9642 5672.9375
347.2678 -14400.1099 -1775.7240 0.0000 0.0000
8189.1825 6955.3274 312.2667 -1233.8551 7017.5621
312.6874 16.3501 21.4976 680285.8938 -4.9743
-4.9538
[25917]ENERGY: 16930000 3333.1536 5426.3913 5635.5172
354.1217 -14330.8114 -1746.7295 0.0000 0.0000
8276.5122 6948.1550 315.5968 -1328.3572 7017.7136
312.3150 37.7349 41.8814 680285.8938 -12.3161
-12.3205
[25953]ENERGY: 16940000 3352.6988 5537.4506 5698.9784
318.8099 -14387.5791 -1782.3781 0.0000 0.0000
8214.2961 6952.2765 313.2244 -1262.0196 7018.7663
312.2796 -25.2697 -70.8524 680285.8938 -10.7715
-10.7844
[26003]ENERGY: 16950000 3394.8017 5551.9986 5642.0371
344.5001 -14363.0309 -1814.7171 0.0000 0.0000
8198.7076 6954.2971 312.6299 -1244.4105 7018.2601
312.3668 -275.4549 -164.2505 680285.8938 -14.5325
-14.5324
[26039]ENERGY: 16960000 3338.8110 5481.3644 5678.2879
371.5520 -14276.3186 -1802.2211 0.0000 0.0000
8159.6012 6951.0768 311.1388 -1208.5244 7017.1120
312.3492 123.6005 63.5016 680285.8938 -6.7362
-6.7278
[26089]ENERGY: 16970000 3370.3974 5437.8353 5697.8312
355.6977 -14330.7230 -1786.9870 0.0000 0.0000
8206.5111 6950.5627 312.9275 -1255.9484 7016.4293
312.3347 -71.6482 -99.8153 680285.8938 -9.3265
-9.3320
[26125]ENERGY: 16980000 3324.6998 5502.7471 5698.6704
314.5826 -14319.0609 -1840.9780 0.0000 0.0000
8268.3386 6948.9998 315.2851 -1319.3388 7016.7111
312.3571 -124.6408 -73.7771 680285.8938 -6.1389
-6.1304
[26175]ENERGY: 16990000 3352.3382 5447.7328 5700.0613
340.0853 -14302.3326 -1769.4621 0.0000 0.0000
8182.0460 6950.4688 311.9946 -1231.5771 7016.7199
312.4840 67.9020 -26.8014 680285.8938 -10.1556
-10.1787
[26214]ENERGY: 17000000 3406.1237 5431.9606 5739.5197
342.5558 -14331.2160 -1830.0153 0.0000 0.0000
8196.5398 6955.4683 312.5473 -1241.0715 7017.8471
312.6926 -101.6091 -107.4267 680285.8938 -6.6173
-6.5826
[26264]ENERGY: 17010000 3392.6639 5592.0215 5651.8115
330.6426 -14377.9067 -1778.5657 0.0000 0.0000

8139.8285 6950.4954 310.3848 -1189.3331 7018.0282
312.7373 86.0068 -39.7496 680285.8938 -5.6266
-5.6570
[26300]ENERGY: 17020000 3330.4131 5399.2061 5671.3144
345.3979 -14260.4867 -1800.1286 0.0000 0.0000
8268.2123 6953.9285 315.2803 -1314.2839 7017.5066
312.6034 34.3385 -28.2382 680285.8938 -5.5223
-5.5229
[26350]ENERGY: 17030000 3387.2958 5530.2329 5618.6742
334.2037 -14245.0899 -1799.9621 0.0000 0.0000
8131.1640 6956.5185 310.0544 -1174.6455 7017.7888
312.5632 -232.6638 -205.2246 680285.8938 -2.3758
-2.3663
[26386]ENERGY: 17040000 3342.6399 5423.3177 5704.0293
346.5971 -14244.6254 -1790.0616 0.0000 0.0000
8172.0144 6953.9114 311.6121 -1218.1030 7017.5606
312.3985 -124.6244 -49.5216 680285.8938 -10.8046
-10.7940
[26436]ENERGY: 17050000 3326.0557 5465.3015 5704.0208
341.1144 -14333.7195 -1729.2829 0.0000 0.0000
8180.3105 6953.8006 311.9284 -1226.5099 7018.6566
312.2710 68.1825 57.2758 680285.8938 -8.6504
-8.6703
[26472]ENERGY: 17060000 3368.8060 5577.4547 5633.8699
324.8392 -14225.4842 -1894.8599 0.0000 0.0000
8165.3877 6950.0133 311.3594 -1215.3743 7017.4301
312.3942 -226.0240 -145.9153 680285.8938 -17.9942
-17.9789
[26522]ENERGY: 17070000 3351.5283 5452.0101 5677.0983
340.1631 -14306.3379 -1806.8418 0.0000 0.0000
8243.8521 6951.4723 314.3514 -1292.3798 7018.8335
312.3809 84.6820 -13.7453 680285.8938 -8.4305
-8.4289
[26558]ENERGY: 17080000 3313.1797 5496.0180 5688.5524
339.7092 -14398.2389 -1719.1971 0.0000 0.0000
8232.0298 6952.0532 313.9006 -1279.9766 7017.2098
312.4071 83.7825 27.0859 680285.8938 -14.7228
-14.7220
[26608]ENERGY: 17090000 3345.2541 5580.0165 5661.0923
343.7247 -14391.9559 -1811.7556 0.0000 0.0000
8221.6621 6948.0381 313.5052 -1273.6240 7016.5489
312.4203 -91.8685 -94.1168 680285.8938 -5.0778
-5.0785
[26647]ENERGY: 17100000 3418.5068 5410.2464 5689.8190
331.7945 -14369.8676 -1766.2487 0.0000 0.0000
8235.8083 6950.0586 314.0446 -1285.7496 7018.0130
312.3211 169.0796 68.6673 680285.8938 -7.3985
-7.4015
[26697]ENERGY: 17110000 3322.6101 5505.3732 5693.4438
329.9131 -14358.8572 -1741.3719 0.0000 0.0000
8196.7675 6947.8787 312.5560 -1248.8887 7017.8255
312.2904 128.4257 145.5607 680285.8938 -6.3349
-6.3330
[26733]ENERGY: 17120000 3326.2705 5542.3734 5676.8119

326.2984 -14372.5631 -1699.7811 0.0000 0.0000
8154.7058 6954.1158 310.9521 -1200.5899 7018.9757
312.3136 83.9461 -2.4986 680285.8938 -11.3628
-11.3842
[26783]ENERGY: 17130000 3330.6020 5434.8329 5664.3642
341.8717 -14313.0329 -1745.2037 0.0000 0.0000
8239.3155 6952.7496 314.1784 -1286.5659 7018.0253
312.1359 233.2850 183.0083 680285.8938 -6.8487
-6.8337
[26819]ENERGY: 17140000 3365.3191 5505.0312 5734.4296
366.0007 -14379.0411 -1877.8477 0.0000 0.0000
8238.2795 6952.1712 314.1389 -1286.1083 7019.0559
312.3057 -57.0843 -123.9842 680285.8938 -9.5494
-9.5469
[26869]ENERGY: 17150000 3393.3217 5408.3434 5677.1655
345.2855 -14303.4474 -1794.2113 0.0000 0.0000
8225.6428 6952.1003 313.6570 -1273.5426 7016.5334
312.2990 79.8122 -18.5319 680285.8938 -13.3116
-13.3262
[26905]ENERGY: 17160000 3376.6024 5467.5351 5688.1390
325.3920 -14382.7313 -1689.8245 0.0000 0.0000
8170.9596 6956.0723 311.5719 -1214.8873 7017.5299
312.2681 140.0219 99.0871 680285.8938 -10.3446
-10.3412
[26955]ENERGY: 17170000 3279.6168 5483.5672 5718.0106
340.7616 -14232.7600 -1841.5592 0.0000 0.0000
8204.5241 6952.1612 312.8517 -1252.3629 7018.5015
312.3734 34.9803 25.9902 680285.8938 -15.4915
-15.4788
[26991]ENERGY: 17180000 3261.2465 5545.7662 5747.4313
332.9303 -14317.5827 -1806.4028 0.0000 0.0000
8188.4196 6951.8084 312.2376 -1236.6112 7019.0589
312.4111 127.8021 74.7734 680285.8938 -10.1328
-10.1440
[27041]ENERGY: 17190000 3370.5185 5511.7332 5726.0256
329.6917 -14477.9499 -1741.2709 0.0000 0.0000
8234.5520 6953.3003 313.9967 -1281.2517 7018.5214
312.2571 80.2519 71.8140 680285.8938 -10.6770
-10.6625
[27080]ENERGY: 17200000 3342.2313 5533.9722 5651.9430
316.6421 -14335.5294 -1781.5433 0.0000 0.0000
8221.3577 6949.0734 313.4936 -1272.2843 7018.1857
312.3561 -70.1049 -104.7169 680285.8938 -10.2004
-10.2100
[27130]ENERGY: 17210000 3396.5780 5462.2211 5667.3340
354.4782 -14334.4954 -1770.0138 0.0000 0.0000
8173.7852 6949.8873 311.6796 -1223.8979 7017.9345
312.4528 -88.9543 -26.9888 680285.8938 -10.2538
-10.2518
[27166]ENERGY: 17220000 3361.8505 5470.2055 5749.4524
334.9956 -14377.8901 -1772.9941 0.0000 0.0000
8188.2610 6953.8808 312.2316 -1234.3802 7017.8188
312.4825 129.1795 114.7239 680285.8938 -9.4528
-9.4405

[27216]ENERGY: 17230000 3382.2590 5545.0360 5674.4721
332.0091 -14278.8700 -1827.7196 0.0000 0.0000
8126.1183 6953.3049 309.8620 -1172.8134 7019.9619
312.4310 -41.6023 -52.2449 680285.8938 -10.2553
-10.2786
[27252]ENERGY: 17240000 3390.3467 5460.4458 5700.7155
336.8129 -14341.0803 -1746.1175 0.0000 0.0000
8153.2724 6954.3955 310.8974 -1198.8769 7019.2337
312.4434 -1.6301 25.3266 680285.8938 -8.4653
-8.4512
[27302]ENERGY: 17250000 3339.6169 5444.3395 5731.7094
352.4625 -14358.9890 -1731.4648 0.0000 0.0000
8176.2325 6953.9070 311.7729 -1222.3255 7017.7872
312.2773 -36.9656 0.4234 680285.8938 -10.9085
-10.9185
[27338]ENERGY: 17260000 3286.1619 5509.4271 5671.1630
337.2336 -14276.8925 -1762.8096 0.0000 0.0000
8192.6499 6956.9334 312.3990 -1235.7165 7019.5400
312.2374 51.4681 91.1084 680285.8938 -9.5028
-9.4817
[27388]ENERGY: 17270000 3412.3437 5437.7026 5706.0285
333.6637 -14273.6832 -1843.6568 0.0000 0.0000
8182.9913 6955.3897 312.0307 -1227.6016 7020.2032
312.3857 -70.9876 -113.4254 680285.8938 -7.3442
-7.3736
[27424]ENERGY: 17280000 3415.6889 5539.5702 5708.9328
325.8069 -14334.0720 -1824.5050 0.0000 0.0000
8122.2380 6953.6598 309.7140 -1168.5782 7021.3422
312.4292 -94.1375 -153.8705 680285.8938 -12.2338
-12.2273
[27474]ENERGY: 17290000 3472.0760 5565.1988 5609.9341
353.1383 -14353.1859 -1842.0245 0.0000 0.0000
8154.5849 6959.7217 310.9475 -1194.8632 7023.3070
312.4135 -117.5354 -110.8863 680285.8938 -7.7438
-7.7431
[27513]ENERGY: 17300000 3334.1882 5624.7624 5673.3635
330.1610 -14301.4686 -1778.7989 0.0000 0.0000
8079.7202 6961.9277 308.0928 -1117.7925 7024.2383
312.4670 46.5753 6.4918 680285.8938 -9.8546
-9.8241
[27563]ENERGY: 17310000 3357.0253 5522.7478 5685.0666
354.1694 -14406.6131 -1740.4160 0.0000 0.0000
8185.7952 6957.7753 312.1376 -1228.0200 7022.8873
312.5601 121.9006 52.5330 680285.8938 -11.7208
-11.7581
[27599]ENERGY: 17320000 3354.5453 5497.9092 5684.0123
346.2852 -14346.2407 -1765.2032 0.0000 0.0000
8184.8590 6956.1671 312.1019 -1228.6919 7022.7729
312.5202 24.2677 27.2377 680285.8938 -12.4513
-12.4423
[27649]ENERGY: 17330000 3343.0470 5446.0564 5647.5033
332.1540 -14288.4297 -1697.4795 0.0000 0.0000
8175.2011 6958.0527 311.7336 -1217.1484 7022.0828
312.5027 122.4760 51.1058 680285.8938 -10.5353

-10.5302
[27685]ENERGY: 17340000 3324.9021 5632.0736 5703.1224
336.7755 -14381.6451 -1831.5348 0.0000 0.0000
8171.2039 6954.8974 311.5812 -1216.3064 7022.9003
312.4532 -91.3563 -111.9309 680285.8938 -8.8991
-8.9157
[27735]ENERGY: 17350000 3410.1372 5440.8488 5658.1226
327.0895 -14281.9621 -1794.6472 0.0000 0.0000
8197.2408 6956.8296 312.5740 -1240.4111 7021.5687
312.5691 -52.2441 2.7446 680285.8938 -10.6169
-10.5799
[27771]ENERGY: 17360000 3407.4511 5461.9342 5682.8514
349.6029 -14408.9952 -1784.2822 0.0000 0.0000
8248.3210 6956.8831 314.5218 -1291.4378 7022.3056
312.6093 -65.1854 -65.2962 680285.8938 -10.1397
-10.1650
[27821]ENERGY: 17370000 3228.8339 5592.5503 5684.1934
341.9543 -14329.4022 -1766.2099 0.0000 0.0000
8205.2134 6957.1332 312.8780 -1248.0802 7023.1312
312.5385 201.4204 70.9288 680285.8938 -16.8522
-16.8454
[27857]ENERGY: 17380000 3300.6962 5566.8731 5713.5702
316.9928 -14367.1688 -1757.0682 0.0000 0.0000
8187.2639 6961.1593 312.1936 -1226.1047 7022.4752
312.4444 273.1275 221.1023 680285.8938 -10.1537
-10.1600
[27907]ENERGY: 17390000 3350.5886 5492.4834 5671.9547
331.6603 -14397.9507 -1656.7489 0.0000 0.0000
8163.6122 6955.5996 311.2917 -1208.0126 7022.1162
312.3916 36.2427 106.4968 680285.8938 -10.2696
-10.2627
[27946]ENERGY: 17400000 3338.8067 5570.4420 5650.8290
357.4241 -14287.0161 -1835.9666 0.0000 0.0000
8162.7012 6957.2203 311.2570 -1205.4809 7023.3833
312.3359 94.1614 61.3874 680285.8938 -8.9690
-8.9715
[27996]ENERGY: 17410000 3361.0543 5486.6510 5631.4286
367.0751 -14336.5680 -1773.6255 0.0000 0.0000
8220.0254 6956.0407 313.4428 -1263.9846 7023.2751
312.4965 -84.5689 -80.6552 680285.8938 -11.6604
-11.6680
[28032]ENERGY: 17420000 3375.7672 5589.9430 5676.8074
337.8740 -14327.7450 -1822.2632 0.0000 0.0000
8127.9725 6958.3559 309.9327 -1169.6166 7023.8944
312.7703 -52.4687 3.0055 680285.8938 -14.3003
-14.2829
[28082]ENERGY: 17430000 3333.4089 5459.9378 5679.9207
330.5402 -14283.1680 -1732.7320 0.0000 0.0000
8174.1559 6962.0635 311.6937 -1212.0924 7025.4813
312.8967 -1.2004 -8.9951 680285.8938 -8.1642
-8.1759
[28118]ENERGY: 17440000 3358.7664 5431.6444 5743.2062
349.3381 -14336.0340 -1716.7598 0.0000 0.0000
8128.6072 6958.7685 309.9569 -1169.8387 7024.5402

312.6341 22.3798 23.5474 680285.8938 -9.8779
-9.8968
[28168]ENERGY: 17450000 3411.9085 5453.2060 5640.4272
343.9618 -14393.4743 -1764.3103 0.0000 0.0000
8263.0975 6954.8165 315.0852 -1308.2810 7023.1522
312.6864 -108.5331 -85.9157 680285.8938 -12.7205
-12.7081
[28204]ENERGY: 17460000 3443.4274 5424.6731 5708.4256
350.7230 -14369.1341 -1722.7973 0.0000 0.0000
8123.6167 6958.9343 309.7666 -1164.6824 7022.2523
312.6824 -58.7295 -46.0576 680285.8938 -9.7822
-9.7829
[28254]ENERGY: 17470000 3400.9855 5476.4466 5652.1940
360.3409 -14325.2934 -1806.2740 0.0000 0.0000
8200.3609 6958.7606 312.6930 -1241.6003 7024.2570
312.7585 -97.8526 -181.7233 680285.8938 -10.1613
-10.1610
[28290]ENERGY: 17480000 3374.6408 5509.6797 5658.5058
329.2108 -14258.8480 -1851.5795 0.0000 0.0000
8196.5273 6958.1368 312.5468 -1238.3904 7023.4614
312.6501 -40.4637 -114.7787 680285.8938 -9.1167
-9.0956
[28340]ENERGY: 17490000 3361.9007 5525.7131 5648.4197
324.6808 -14337.4962 -1733.5111 0.0000 0.0000
8164.7645 6954.4716 311.3356 -1210.2929 7022.9946
312.4231 -66.8502 -31.1554 680285.8938 -13.8515
-13.8644
[28379]ENERGY: 17500000 3422.5993 5446.5994 5656.7648
332.0466 -14359.9213 -1760.7198 0.0000 0.0000
8220.5402 6957.9092 313.4625 -1262.6310 7022.6181
312.4257 -26.6139 -32.4156 680285.8938 -8.7827
-8.7907
[28429]ENERGY: 17510000 3379.8991 5492.9974 5698.8058
345.9753 -14289.8336 -1759.6524 0.0000 0.0000
8090.1670 6958.3587 308.4911 -1131.8083 7021.5040
312.4653 -84.7252 -65.1570 680285.8938 -16.6822
-16.6723
[28465]ENERGY: 17520000 3375.3606 5511.5832 5641.5374
358.5986 -14286.2774 -1756.0524 0.0000 0.0000
8114.0056 6958.7556 309.4001 -1155.2499 7022.3795
312.3876 -30.0777 2.2965 680285.8938 -15.0560
-15.0428
[28515]ENERGY: 17530000 3325.8903 5543.6799 5685.2309
337.8817 -14289.8059 -1767.2594 0.0000 0.0000
8124.3492 6959.9668 309.7945 -1164.3824 7023.5143
312.5277 203.7764 170.2652 680285.8938 -12.8190
-12.8399
[28551]ENERGY: 17540000 3384.0187 5568.5895 5707.2148
335.1701 -14391.3314 -1766.7432 0.0000 0.0000
8117.0869 6954.0054 309.5176 -1163.0816 7024.3921
312.5144 -20.1445 -22.4389 680285.8938 -16.3664
-16.3578
[28601]ENERGY: 17550000 3289.9636 5514.7025 5686.0986
325.3878 -14275.2914 -1762.5921 0.0000 0.0000

8177.4311 6955.7001 311.8186 -1221.7309 7023.6127
312.4884 33.4393 101.9611 680285.8938 -11.6919
-11.6949
[28637]ENERGY: 17560000 3327.9189 5437.3276 5687.2905
344.0868 -14301.3612 -1740.7982 0.0000 0.0000
8203.0655 6957.5298 312.7961 -1245.5357 7026.0060
312.4889 -7.9681 -19.0705 680285.8938 -11.4587
-11.4536
[28687]ENERGY: 17570000 3379.3912 5544.0837 5727.5854
331.8041 -14415.5164 -1802.3254 0.0000 0.0000
8193.3369 6958.3594 312.4251 -1234.9774 7024.1247
312.7160 -58.5721 -83.0742 680285.8938 -13.5340
-13.5567
[28723]ENERGY: 17580000 3400.7954 5468.4598 5676.8527
343.1121 -14296.3631 -1840.3816 0.0000 0.0000
8205.0902 6957.5654 312.8733 -1247.5248 7024.6719
312.4617 -201.7043 -178.1318 680285.8938 -12.9300
-12.9039
[28773]ENERGY: 17590000 3395.4164 5518.5019 5739.5223
353.4821 -14375.0067 -1771.3514 0.0000 0.0000
8101.2795 6961.8442 308.9149 -1139.4354 7025.5034
312.4408 -33.8467 -29.6290 680285.8938 -15.9813
-15.9878
[28812]ENERGY: 17600000 3379.2566 5501.8858 5702.1704
367.4375 -14338.1987 -1804.9125 0.0000 0.0000
8155.4298 6963.0689 310.9797 -1192.3609 7026.9255
312.6979 25.4704 3.1830 680285.8938 -14.0896
-14.0810
[28862]ENERGY: 17610000 3365.9024 5484.6399 5700.0883
331.7607 -14318.0500 -1794.2294 0.0000 0.0000
8187.0840 6957.1961 312.1867 -1229.8880 7025.7472
312.5060 -182.0122 -124.1604 680285.8938 -8.9720
-8.9787
[28898]ENERGY: 17620000 3374.3526 5488.8812 5696.6869
342.2460 -14319.9822 -1841.8738 0.0000 0.0000
8221.4103 6961.7211 313.4956 -1259.6892 7028.2074
312.7165 15.0781 -65.6804 680285.8938 -15.8397
-15.8346
[28948]ENERGY: 17630000 3343.0792 5516.6543 5711.2282
339.8495 -14383.2534 -1759.9311 0.0000 0.0000
8194.6154 6962.2420 312.4739 -1232.3734 7027.6901
312.7114 -67.4250 -97.1439 680285.8938 -14.7522
-14.7627
[28984]ENERGY: 17640000 3357.5184 5469.8034 5672.3597
332.0145 -14306.3748 -1795.3243 0.0000 0.0000
8229.3091 6959.3059 313.7968 -1270.0032 7027.5532
312.9167 45.5332 37.2332 680285.8938 -9.4459
-9.4487
[29034]ENERGY: 17650000 3320.2331 5564.1428 5645.5301
345.3225 -14351.1116 -1843.4017 0.0000 0.0000
8278.0378 6958.7530 315.6549 -1319.2848 7027.2611
312.9820 0.5450 30.6107 680285.8938 -10.9061
-10.8958
[29070]ENERGY: 17660000 3325.4392 5536.1974 5685.1686

336.7346 -14363.3683 -1796.2673 0.0000 0.0000
8236.8790 6960.7832 314.0855 -1276.0957 7027.7272
312.9433 -125.2628 -103.3101 680285.8938 -12.5555
-12.5550
[29120]ENERGY: 17670000 3334.4009 5546.0683 5663.1378
323.7771 -14376.4416 -1812.1430 0.0000 0.0000
8279.4071 6958.2065 315.7071 -1321.2005 7026.5158
312.7204 -45.5177 18.9805 680285.8938 -11.5963
-11.5915
[29156]ENERGY: 17680000 3363.8828 5455.3714 5647.5894
330.7982 -14297.0910 -1796.1229 0.0000 0.0000
8256.3746 6960.8024 314.8289 -1295.5722 7027.4204
312.7149 -19.9897 9.6707 680285.8938 -12.4061
-12.4028
[29206]ENERGY: 17690000 3354.5485 5502.1444 5668.9922
357.5258 -14351.4164 -1769.7738 0.0000 0.0000
8197.6420 6959.6628 312.5893 -1237.9792 7027.5081
312.4346 -87.9761 -73.7179 680285.8938 -14.1917
-14.2075
[29245]ENERGY: 17700000 3362.0817 5537.7960 5648.7646
334.7243 -14369.2694 -1739.6556 0.0000 0.0000
8187.3925 6961.8342 312.1985 -1225.5583 7026.0760
312.5690 -65.1931 -76.0969 680285.8938 -14.2258
-14.2177
[29295]ENERGY: 17710000 3336.2250 5478.6624 5700.1539
338.9818 -14385.0559 -1798.3626 0.0000 0.0000
8290.6781 6961.2828 316.1369 -1329.3953 7027.1320
312.5469 -42.6604 -20.9373 680285.8938 -13.3244
-13.3356
[29331]ENERGY: 17720000 3377.8646 5454.1288 5712.8355
316.7839 -14269.4948 -1816.3405 0.0000 0.0000
8181.9642 6957.7417 311.9915 -1224.2225 7026.2707
312.7245 -11.8902 70.6233 680285.8938 -10.1688
-10.1729
[29381]ENERGY: 17730000 3337.1931 5484.0511 5650.6133
354.7481 -14294.7334 -1747.9126 0.0000 0.0000
8180.1368 6964.0965 311.9218 -1216.0404 7026.7153
312.6873 -116.8112 -71.6000 680285.8938 -12.7790
-12.7658
[29417]ENERGY: 17740000 3373.1934 5430.0552 5657.5519
354.6874 -14303.7087 -1720.2450 0.0000 0.0000
8169.9486 6961.4828 311.5333 -1208.4658 7026.6838
312.7879 -71.7167 -59.6220 680285.8938 -12.3614
-12.3721
[29467]ENERGY: 17750000 3421.9588 5499.8631 5665.3249
333.9399 -14363.6790 -1791.8354 0.0000 0.0000
8196.3728 6961.9450 312.5409 -1234.4278 7028.2167
312.7731 52.2874 24.3202 680285.8938 -10.3334
-10.3215
[29503]ENERGY: 17760000 3370.7982 5463.2461 5692.0733
348.4911 -14337.9355 -1732.3545 0.0000 0.0000
8157.9240 6962.2426 311.0748 -1195.6814 7028.0105
312.8007 -75.0521 -65.4842 680285.8938 -12.8486
-12.8427

[29553]ENERGY: 17770000 3316.4871 5598.6197 5672.0808
347.1143 -14443.2302 -1729.2509 0.0000 0.0000
8203.3910 6965.2118 312.8085 -1238.1792 7028.4763
312.8721 85.6823 80.9410 680285.8938 -14.2364
-14.2486
[29589]ENERGY: 17780000 3362.0902 5466.2973 5708.0374
339.9385 -14313.7588 -1776.9063 0.0000 0.0000
8177.0580 6962.7564 311.8044 -1214.3017 7028.3897
313.0383 17.6781 -47.4311 680285.8938 -11.2848
-11.2931
[29639]ENERGY: 17790000 3387.4204 5522.4336 5673.5389
346.0880 -14322.3397 -1834.2894 0.0000 0.0000
8190.6883 6963.5401 312.3242 -1227.1482 7029.3571
313.1195 -90.5202 -115.5634 680285.8938 -12.8142
-12.8030
[29678]ENERGY: 17800000 3337.0990 5499.9196 5691.4824
324.6972 -14337.9244 -1748.3495 0.0000 0.0000
8193.3848 6960.3091 312.4270 -1233.0757 7028.9482
313.1247 30.8078 -4.4766 680285.8938 -11.6006
-11.6248
[29728]ENERGY: 17810000 3392.7899 5472.1048 5662.8819
361.3869 -14381.1423 -1779.8406 0.0000 0.0000
8234.6199 6962.8006 313.9993 -1271.8193 7030.2787
313.1965 24.1700 -33.0706 680285.8938 -6.8204
-6.7954
[29764]ENERGY: 17820000 3315.8747 5505.5149 5675.9408
347.5576 -14418.4267 -1719.0582 0.0000 0.0000
8257.0952 6964.4982 314.8564 -1292.5970 7031.0038
313.0892 273.5173 100.2741 680285.8938 -10.0289
-10.0256
[29814]ENERGY: 17830000 3364.0339 5412.6317 5679.7783
325.6652 -14352.9111 -1761.7412 0.0000 0.0000
8294.7833 6962.2402 316.2935 -1332.5431 7030.7991
313.0998 50.3222 27.6872 680285.8938 -10.3636
-10.3678
[29850]ENERGY: 17840000 3342.8707 5505.0823 5647.1720
326.7627 -14374.6001 -1719.5156 0.0000 0.0000
8239.0110 6966.7830 314.1668 -1272.2280 7031.9627
312.9553 135.5109 23.9259 680285.8938 -12.9836
-12.9978
[29900]ENERGY: 17850000 3402.6350 5488.0475 5690.9461
356.1780 -14399.3745 -1782.7212 0.0000 0.0000
8215.1211 6970.8321 313.2558 -1244.2890 7031.6492
313.0417 130.0146 39.4693 680285.8938 -11.6595
-11.6464
[29936]ENERGY: 17860000 3323.2855 5485.0441 5677.8631
363.8373 -14326.6824 -1771.9110 0.0000 0.0000
8213.4983 6964.9349 313.1939 -1248.5634 7030.8938
313.1817 108.8319 104.4510 680285.8938 -10.6053
-10.6140
[29986]ENERGY: 17870000 3412.1538 5519.9839 5678.9758
337.5410 -14387.0465 -1754.5331 0.0000 0.0000
8159.7476 6966.8224 311.1443 -1192.9252 7031.5070
313.0057 56.5645 85.1057 680285.8938 -5.8493

-5.8256
[30022]ENERGY: 17880000 3424.5097 5439.8043 5676.6838
350.5812 -14315.1104 -1755.9044 0.0000 0.0000
8145.9860 6966.5502 310.6196 -1179.4358 7030.5257
312.9841 -21.9729 -2.6138 680285.8938 -10.0782
-10.0920
[30072]ENERGY: 17890000 3341.1605 5525.1608 5654.2229
329.7551 -14338.8636 -1693.1173 0.0000 0.0000
8152.1325 6970.4510 310.8540 -1181.6815 7031.0924
312.9034 57.0861 11.9113 680285.8938 -7.9650
-7.9659
[30111]ENERGY: 17900000 3352.1935 5534.0915 5637.6740
333.1349 -14330.7172 -1751.4902 0.0000 0.0000
8190.8565 6965.7431 312.3306 -1225.1134 7031.0167
312.9302 10.6404 -17.7020 680285.8938 -13.3312
-13.3355
[30161]ENERGY: 17910000 3470.4181 5559.7427 5700.0818
360.0501 -14410.3853 -1767.2059 0.0000 0.0000
8056.5992 6969.3008 307.2111 -1087.2984 7032.4722
313.0849 35.9565 21.3588 680285.8938 -11.7788
-11.7717
[30197]ENERGY: 17920000 3416.0397 5454.9601 5634.0108
316.9686 -14291.6406 -1789.0906 0.0000 0.0000
8225.5566 6966.8046 313.6537 -1258.7520 7033.1546
312.9684 -12.0563 -53.4054 680285.8938 -11.9518
-11.9625
[30247]ENERGY: 17930000 3352.5042 5453.5629 5638.0062
358.5880 -14292.1764 -1759.4689 0.0000 0.0000
8214.4126 6965.4287 313.2288 -1248.9839 7032.9093
312.9870 25.1673 39.7122 680285.8938 -13.5368
-13.5287
[30283]ENERGY: 17940000 3336.6582 5380.3161 5701.7953
354.1266 -14335.0687 -1766.8073 0.0000 0.0000
8296.7120 6967.7323 316.3670 -1328.9797 7033.9948
312.9011 159.0405 80.4038 680285.8938 -14.6371
-14.6453
[30333]ENERGY: 17950000 3448.2907 5548.7601 5701.3529
358.0281 -14377.1611 -1809.3676 0.0000 0.0000
8101.8196 6971.7226 308.9354 -1130.0970 7033.7826
312.9904 88.8342 -10.8283 680285.8938 -10.7852
-10.7767
[30369]ENERGY: 17960000 3396.1414 5562.9154 5704.7134
324.4090 -14426.2681 -1831.7287 0.0000 0.0000
8231.3684 6961.5508 313.8754 -1269.8176 7032.8259
313.0323 -251.5640 -184.4323 680285.8938 -13.0956
-13.0990
[30419]ENERGY: 17970000 3374.6011 5430.0972 5690.6094
338.0130 -14349.7907 -1771.0381 0.0000 0.0000
8254.7262 6967.2180 314.7660 -1287.5082 7032.2433
313.0485 -89.5415 -100.8899 680285.8938 -10.1898
-10.1751
[30455]ENERGY: 17980000 3302.7917 5531.6148 5710.3072
329.8023 -14296.8093 -1809.0067 0.0000 0.0000
8194.3797 6963.0795 312.4649 -1231.3002 7032.1494

312.9702 57.9351 42.1531 680285.8938 -13.6772
-13.6954
[30505]ENERGY: 17990000 3344.3010 5412.5093 5690.0188
356.9889 -14293.3044 -1816.9555 0.0000 0.0000
8270.5340 6964.0922 315.3688 -1306.4418 7032.0073
312.9214 9.2389 -20.4913 680285.8938 -16.0718
-16.0700
[30544]ENERGY: 18000000 3357.5670 5512.7625 5667.7366
338.6491 -14331.7950 -1801.9036 0.0000 0.0000
8221.3226 6964.3392 313.4923 -1256.9834 7031.6349
312.8020 -16.9692 -40.9806 680285.8938 -8.3696
-8.3795
[30594]ENERGY: 18010000 3359.6674 5433.2911 5661.3646
351.1745 -14370.4917 -1759.1860 0.0000 0.0000
8287.8709 6963.6909 316.0299 -1324.1801 7032.4807
312.7902 18.0528 -116.0171 680285.8938 -10.4623
-10.4480
[30630]ENERGY: 18020000 3359.0286 5469.7633 5624.3350
328.4172 -14304.7538 -1780.2690 0.0000 0.0000
8269.5283 6966.0496 315.3305 -1303.4787 7032.0212
312.8511 19.1161 11.8734 680285.8938 -10.0472
-10.0415
[30680]ENERGY: 18030000 3298.5261 5487.7070 5645.2097
327.0928 -14287.7747 -1779.8584 0.0000 0.0000
8275.8868 6966.7894 315.5729 -1309.0974 7033.3711
312.9277 73.8177 41.5167 680285.8938 -17.0584
-17.0699
[30716]ENERGY: 18040000 3438.0181 5537.9531 5682.9904
352.5684 -14409.6903 -1841.3699 0.0000 0.0000
8205.6136 6966.0834 312.8933 -1239.5302 7032.2657
313.0828 -23.5412 -69.1437 680285.8938 -10.6044
-10.6080
[30766]ENERGY: 18050000 3428.9482 5615.4318 5669.5508
322.2216 -14419.5434 -1770.0292 0.0000 0.0000
8122.3480 6968.9278 309.7182 -1153.4202 7032.9490
313.2277 -57.6412 -56.5710 680285.8938 -12.4243
-12.4261
[30802]ENERGY: 18060000 3309.4106 5526.6678 5673.4278
339.1779 -14346.3926 -1756.6269 0.0000 0.0000
8219.1995 6964.8642 313.4113 -1254.3354 7032.1062
313.0034 229.7018 118.9308 680285.8938 -9.3515
-9.3422
[30852]ENERGY: 18070000 3266.3078 5474.1694 5711.0917
315.2362 -14282.7696 -1790.4538 0.0000 0.0000
8273.5868 6967.1685 315.4852 -1306.4183 7033.5802
312.8978 110.2935 110.5656 680285.8938 -14.0363
-14.0532
[30888]ENERGY: 18080000 3368.1667 5542.4306 5688.3790
361.9315 -14299.9612 -1837.1467 0.0000 0.0000
8143.1892 6966.9892 310.5129 -1176.2000 7033.3709
312.9382 -90.1857 -38.6684 680285.8938 -13.9364
-13.9170
[30938]ENERGY: 18090000 3477.6077 5484.2097 5646.6847
336.2028 -14278.7729 -1827.8306 0.0000 0.0000

8129.8425 6967.9439 310.0040 -1161.8986 7034.2246
312.9658 -71.4651 -103.0802 680285.8938 -8.2146
-8.2234
[30977]ENERGY: 18100000 3382.2025 5542.2991 5622.5314
337.3588 -14243.1340 -1773.1841 0.0000 0.0000
8100.4824 6968.5562 308.8845 -1131.9262 7033.9432
312.9421 12.3331 -0.2208 680285.8938 -13.7118
-13.7000
[31027]ENERGY: 18110000 3344.8057 5550.8685 5619.1415
338.0701 -14489.7104 -1701.6958 0.0000 0.0000
8302.3223 6963.8021 316.5809 -1338.5203 7033.9205
312.9928 149.1417 123.3248 680285.8938 -12.4158
-12.4306
[31063]ENERGY: 18120000 3340.4135 5496.2085 5674.4489
352.4922 -14325.5083 -1849.6168 0.0000 0.0000
8279.9351 6968.3731 315.7273 -1311.5620 7034.1590
313.0583 77.3355 24.0422 680285.8938 -15.6628
-15.6523
[31113]ENERGY: 18130000 3278.6926 5498.7609 5700.0273
336.3434 -14327.3472 -1825.3257 0.0000 0.0000
8301.5081 6962.6594 316.5499 -1338.8487 7034.5168
313.0587 26.4487 -51.5605 680285.8938 -10.4893
-10.4796
[31149]ENERGY: 18140000 3235.1729 5527.4670 5667.9245
351.8660 -14270.5907 -1832.3300 0.0000 0.0000
8289.3723 6968.8820 316.0871 -1320.4904 7035.5418
313.1021 176.5619 51.2880 680285.8938 -13.0482
-13.0620
[31199]ENERGY: 18150000 3318.4193 5467.6347 5651.4146
366.6660 -14332.6639 -1731.7678 0.0000 0.0000
8226.5730 6966.2758 313.6925 -1260.2972 7034.2360
313.1120 28.6504 11.9799 680285.8938 -18.0474
-18.0374
[31235]ENERGY: 18160000 3357.8334 5593.8807 5648.8286
348.8636 -14351.9563 -1783.9850 0.0000 0.0000
8155.8975 6969.3626 310.9975 -1186.5349 7034.8884
313.1940 11.8960 56.0966 680285.8938 -14.0594
-14.0602
[31285]ENERGY: 18170000 3330.0827 5563.2400 5695.7080
337.6582 -14386.0240 -1771.0725 0.0000 0.0000
8202.6068 6972.1990 312.7786 -1230.4078 7036.3060
313.2219 117.8029 172.5479 680285.8938 -15.4950
-15.4936
[31321]ENERGY: 18180000 3284.4194 5477.0750 5684.2375
348.2345 -14263.5179 -1786.3860 0.0000 0.0000
8227.7540 6971.8166 313.7375 -1255.9375 7036.8848
313.0822 -71.7821 -33.0461 680285.8938 -15.0096
-15.0064
[31371]ENERGY: 18190000 3395.7346 5572.2613 5688.2821
332.1771 -14277.8159 -1881.5229 0.0000 0.0000
8143.1812 6972.2976 310.5126 -1170.8836 7035.9244
312.9615 -221.2317 -174.2072 680285.8938 -16.4219
-16.4312
[31410]ENERGY: 18200000 3268.0192 5479.7340 5621.8666

345.0737 -14199.8082 -1806.5245 0.0000 0.0000
8265.7523 6974.1130 315.1865 -1291.6393 7037.8142
313.1146 156.0449 40.5862 680285.8938 -16.0898
-16.0925
[31460]ENERGY: 18210000 3342.3840 5599.3878 5666.6824
330.2101 -14316.2286 -1810.2583 0.0000 0.0000
8158.3626 6970.5400 311.0915 -1187.8227 7038.2362
312.8633 -90.9150 -196.1605 680285.8938 -14.7233
-14.7241
[31496]ENERGY: 18220000 3319.7985 5492.9696 5686.9943
342.5678 -14399.0599 -1768.7780 0.0000 0.0000
8298.1315 6972.6238 316.4211 -1325.5077 7038.3300
313.1256 38.6165 23.6803 680285.8938 -14.8110
-14.8102
[31546]ENERGY: 18230000 3388.0382 5513.5160 5721.3857
361.7292 -14398.3459 -1839.9627 0.0000 0.0000
8224.0192 6970.3796 313.5951 -1253.6395 7038.3659
313.1592 -31.4053 -81.5625 680285.8938 -14.6504
-14.6519
[31582]ENERGY: 18240000 3356.5507 5630.1234 5683.8847
353.2084 -14299.4552 -1862.5737 0.0000 0.0000
8112.6457 6974.3840 309.3483 -1138.2617 7039.4347
313.1475 -8.5930 -39.5004 680285.8938 -14.2049
-14.1910
[31632]ENERGY: 18250000 3306.7684 5549.1394 5691.2115
339.3534 -14249.8118 -1812.2287 0.0000 0.0000
8150.0551 6974.4873 310.7747 -1175.5677 7038.4481
313.0530 -29.4627 23.4718 680285.8938 -14.8907
-14.8999
[31668]ENERGY: 18260000 3369.8948 5560.5967 5681.9096
332.4504 -14309.9221 -1787.3891 0.0000 0.0000
8126.0159 6973.5563 309.8581 -1152.4596 7037.5235
313.0266 -70.0308 -23.4130 680285.8938 -15.6169
-15.6175
[31718]ENERGY: 18270000 3423.0441 5466.6650 5667.2490
342.2743 -14345.0449 -1810.3271 0.0000 0.0000
8226.9046 6970.7649 313.7051 -1256.1397 7039.2456
312.8867 -77.9966 -115.7201 680285.8938 -16.1440
-16.1499
[31754]ENERGY: 18280000 3404.4714 5456.9461 5639.6889
337.1005 -14437.4431 -1661.1347 0.0000 0.0000
8232.2872 6971.9165 313.9104 -1260.3707 7039.0750
312.9626 93.3778 28.9111 680285.8938 -12.2407
-12.2279
[31804]ENERGY: 18290000 3298.2921 5582.9068 5654.3265
354.7421 -14370.4709 -1766.1617 0.0000 0.0000
8217.8599 6971.4949 313.3602 -1246.3650 7039.5691
313.0580 -113.1872 -124.7288 680285.8938 -12.6352
-12.6464
[31843]ENERGY: 18300000 3362.4384 5573.7036 5654.6973
339.5948 -14407.4884 -1772.1018 0.0000 0.0000
8222.6803 6973.5242 313.5441 -1249.1561 7039.1234
313.0691 133.7689 63.8925 680285.8938 -13.0739
-13.0661

[31893]ENERGY: 18310000 3297.5255 5571.8048 5658.6294
359.1872 -14360.2859 -1758.1253 0.0000 0.0000
8203.4499 6972.1856 312.8108 -1231.2642 7039.0829
313.1044 98.7343 49.8441 680285.8938 -15.0010
-15.0010
[31929]ENERGY: 18320000 3360.1755 5507.3306 5657.9467
357.5839 -14393.0765 -1813.1662 0.0000 0.0000
8294.5940 6971.3881 316.2862 -1323.2059 7037.5571
313.2215 14.3446 -28.0483 680285.8938 -11.4982
-11.5072
[31979]ENERGY: 18330000 3324.0640 5499.3209 5688.6169
352.6020 -14261.8899 -1822.0711 0.0000 0.0000
8195.4811 6976.1240 312.5069 -1219.3571 7037.5534
313.0659 -52.4428 -49.4548 680285.8938 -9.5341
-9.5193
[32015]ENERGY: 18340000 3331.4506 5454.2500 5653.2184
322.6650 -14281.8167 -1771.6474 0.0000 0.0000
8262.6469 6970.7669 315.0681 -1291.8800 7038.8099
313.0145 129.4113 24.8200 680285.8938 -10.6719
-10.6761
[32065]ENERGY: 18350000 3420.4993 5510.6547 5681.6166
365.9105 -14339.4318 -1783.0418 0.0000 0.0000
8119.2986 6975.5060 309.6019 -1143.7925 7038.3376
312.9371 73.9190 -27.2573 680285.8938 -14.1858
-14.1871
[32101]ENERGY: 18360000 3346.1649 5562.1691 5697.6787
347.3973 -14360.2664 -1765.0420 0.0000 0.0000
8147.5611 6975.6627 310.6796 -1171.8984 7037.3439
313.1398 -153.5359 -99.6763 680285.8938 -12.5382
-12.5371
[32151]ENERGY: 18370000 3392.8409 5494.1331 5671.5104
334.3497 -14277.1664 -1862.6057 0.0000 0.0000
8219.2489 6972.3110 313.4132 -1246.9379 7038.3325
313.2114 -119.6308 -119.6264 680285.8938 -13.6328
-13.6369
[32187]ENERGY: 18380000 3377.1640 5537.8183 5639.1261
341.7657 -14283.1760 -1826.4224 0.0000 0.0000
8188.9945 6975.2702 312.2596 -1213.7243 7039.2252
313.0599 -127.9779 -102.2330 680285.8938 -14.2022
-14.2031
[32237]ENERGY: 18390000 3363.6511 5556.1861 5691.6065
341.2036 -14325.4849 -1846.7177 0.0000 0.0000
8194.1055 6974.5503 312.4545 -1219.5552 7040.1185
313.0616 145.4768 75.4949 680285.8938 -13.1855
-13.1766
[32276]ENERGY: 18400000 3396.6330 5494.5124 5704.1774
328.0670 -14256.3267 -1876.2740 0.0000 0.0000
8183.6500 6974.4391 312.0558 -1209.2109 7040.9426
312.9519 75.8979 -1.4516 680285.8938 -12.5706
-12.5699
[32326]ENERGY: 18410000 3387.4103 5497.5303 5676.0852
347.6902 -14272.6365 -1860.9997 0.0000 0.0000
8198.4432 6973.5231 312.6199 -1224.9201 7041.4566
312.9624 -26.8748 -39.9170 680285.8938 -15.6586

-15.6453
[32362]ENERGY: 18420000 3256.1138 5448.0641 5703.9854
354.8964 -14261.1780 -1781.8945 0.0000 0.0000
8252.7201 6972.7073 314.6895 -1280.0128 7042.1434
313.0904 123.8688 62.0632 680285.8938 -10.1134
-10.1255
[32412]ENERGY: 18430000 3470.1150 5529.0378 5655.4150
326.8636 -14320.0124 -1792.6118 0.0000 0.0000
8109.4047 6978.2120 309.2247 -1131.1927 7041.8089
312.9224 -140.3678 -85.5951 680285.8938 -17.8983
-17.9074
[32448]ENERGY: 18440000 3373.0793 5444.7328 5631.7583
334.6077 -14314.0062 -1729.6778 0.0000 0.0000
8233.0206 6973.5145 313.9384 -1259.5061 7040.6165
313.0946 24.5708 30.5658 680285.8938 -13.2065
-13.2087
[32498]ENERGY: 18450000 3441.0880 5568.0553 5677.2728
353.1617 -14352.4834 -1902.4230 0.0000 0.0000
8191.3918 6976.0632 312.3510 -1215.3286 7042.3803
313.0687 -142.5355 -174.6777 680285.8938 -12.7631
-12.7647
[32534]ENERGY: 18460000 3320.5819 5532.5214 5638.2786
356.9474 -14296.8112 -1782.7603 0.0000 0.0000
8208.9691 6977.7270 313.0212 -1231.2421 7041.8579
313.1268 87.4723 9.0274 680285.8938 -13.6246
-13.6483
[32584]ENERGY: 18470000 3291.9436 5652.7218 5664.7978
320.6841 -14396.9600 -1833.6965 0.0000 0.0000
8272.5233 6972.0140 315.4447 -1300.5092 7041.6070
313.1496 104.0776 -6.3497 680285.8938 -12.8375
-12.8169
[32620]ENERGY: 18480000 3349.5831 5452.2906 5676.7941
358.6345 -14365.1103 -1781.6511 0.0000 0.0000
8281.1827 6971.7236 315.7748 -1309.4591 7042.0370
312.9816 -145.7589 -121.5133 680285.8938 -17.0168
-17.0168
[32670]ENERGY: 18490000 3384.6142 5501.4084 5693.2333
346.8417 -14339.0812 -1819.0841 0.0000 0.0000
8208.1215 6976.0539 312.9889 -1232.0676 7041.0267
312.8683 -124.6335 -190.4461 680285.8938 -14.4500
-14.4485
[32709]ENERGY: 18500000 3346.9998 5489.9720 5648.9152
336.8998 -14292.9321 -1778.1747 0.0000 0.0000
8223.3806 6975.0605 313.5708 -1248.3201 7042.3121
312.9632 -151.1339 -116.5352 680285.8938 -12.3793
-12.3683
[32759]ENERGY: 18510000 3379.2603 5544.2302 5694.4711
322.9303 -14372.8887 -1785.5296 0.0000 0.0000
8193.7310 6976.2047 312.4402 -1217.5263 7042.9206
312.9601 146.4267 53.0338 680285.8938 -11.6456
-11.6696
[32795]ENERGY: 18520000 3351.9102 5571.1094 5682.4568
340.0032 -14339.9388 -1789.8288 0.0000 0.0000
8162.7770 6978.4890 311.2599 -1184.2880 7043.2460

312.9947 -15.8136 41.8662 680285.8938 -14.0054
-13.9860
[32845]ENERGY: 18530000 3263.8523 5546.2773 5687.1519
344.6200 -14351.4126 -1805.6434 0.0000 0.0000
8288.2572 6973.1026 316.0446 -1315.1546 7042.4996
313.1103 92.5967 46.9917 680285.8938 -12.0804
-12.0872
[32881]ENERGY: 18540000 3479.0791 5480.4788 5663.5614
339.2775 -14364.7841 -1786.4041 0.0000 0.0000
8167.3961 6978.6047 311.4360 -1188.7914 7043.8695
313.1439 -211.5185 -211.0159 680285.8938 -12.1157
-12.1020
[32931]ENERGY: 18550000 3338.9754 5558.9238 5649.6239
331.7121 -14309.1479 -1829.0311 0.0000 0.0000
8236.3645 6977.4206 314.0659 -1258.9439 7043.2659
313.0829 -126.5601 -98.9443 680285.8938 -11.9740
-11.9869
[32967]ENERGY: 18560000 3341.2290 5454.6652 5700.3480
322.8933 -14322.9273 -1790.2773 0.0000 0.0000
8271.4479 6977.3787 315.4036 -1294.0692 7043.4645
313.0861 57.1289 78.3522 680285.8938 -10.1958
-10.1925
[33017]ENERGY: 18570000 3347.9583 5603.9095 5627.8145
348.6426 -14302.2819 -1791.1097 0.0000 0.0000
8142.2978 6977.2311 310.4789 -1165.0667 7042.6281
313.0307 89.9022 110.0664 680285.8938 -13.4105
-13.4124
[33053]ENERGY: 18580000 3336.9285 5612.2695 5681.9756
373.6323 -14290.4156 -1849.0720 0.0000 0.0000
8112.3473 6977.6655 309.3369 -1134.6818 7042.4514
313.0920 45.7861 22.6960 680285.8938 -14.4728
-14.4732
[33103]ENERGY: 18590000 3405.8813 5447.9177 5662.2785
352.1250 -14244.0625 -1882.2571 0.0000 0.0000
8235.1820 6977.0648 314.0208 -1258.1172 7043.5987
313.0718 -93.1960 -75.0535 680285.8938 -15.9836
-15.9702
[33142]ENERGY: 18600000 3374.7252 5522.3296 5673.1560
361.1261 -14307.4486 -1815.0526 0.0000 0.0000
8173.1747 6982.0104 311.6563 -1191.1643 7044.6469
313.0808 -94.4392 -59.6947 680285.8938 -12.5645
-12.5750
[33192]ENERGY: 18610000 3316.8880 5614.9473 5675.8160
338.1807 -14334.0680 -1845.3360 0.0000 0.0000
8210.0216 6976.4495 313.0614 -1233.5721 7044.2314
312.9525 21.2711 -39.1372 680285.8938 -16.6967
-16.7065
[33228]ENERGY: 18620000 3400.5807 5487.2617 5655.2691
325.2619 -14221.9971 -1856.8999 0.0000 0.0000
8187.2241 6976.7006 312.1921 -1210.5235 7043.5238
313.0623 -272.8356 -175.9326 680285.8938 -13.0855
-13.0792
[33278]ENERGY: 18630000 3319.9382 5507.2780 5645.7286
322.5770 -14228.6989 -1831.9173 0.0000 0.0000

8244.1964 6979.1020 314.3645 -1265.0944 7044.6961
312.9675 -36.7339 36.6922 680285.8938 -14.1726
-14.1681
[33314]ENERGY: 18640000 3413.9335 5409.5088 5694.7666
344.4862 -14343.8526 -1760.4275 0.0000 0.0000
8218.1536 6976.5686 313.3714 -1241.5850 7044.4774
313.1002 81.2899 18.1928 680285.8938 -14.6408
-14.6521
[33364]ENERGY: 18650000 3412.1774 5421.6457 5678.4072
335.7471 -14305.8574 -1820.5948 0.0000 0.0000
8257.1457 6978.6709 314.8583 -1278.4748 7043.1794
313.0186 -29.7739 -85.1333 680285.8938 -11.0395
-11.0350
[33400]ENERGY: 18660000 3433.5617 5491.6400 5667.6428
360.2466 -14361.1326 -1762.9769 0.0000 0.0000
8152.7485 6981.7299 310.8774 -1171.0186 7044.7600
312.9894 -60.0791 -84.3370 680285.8938 -9.6766
-9.6641
[33450]ENERGY: 18670000 3386.5832 5399.6034 5702.9782
355.1941 -14416.8860 -1730.5772 0.0000 0.0000
8277.9516 6974.8473 315.6516 -1303.1043 7043.8881
312.9775 -52.9773 15.1734 680285.8938 -15.0122
-15.0120
[33486]ENERGY: 18680000 3335.0023 5568.2486 5670.7919
332.6464 -14330.2903 -1831.3449 0.0000 0.0000
8231.8336 6976.8875 313.8931 -1254.9461 7044.3371
313.0523 38.8145 12.8674 680285.8938 -15.0723
-15.0912
[33536]ENERGY: 18690000 3390.9855 5440.6022 5662.3070
336.3624 -14303.9869 -1814.8588 0.0000 0.0000
8266.4883 6977.8998 315.2145 -1288.5885 7045.2934
312.9894 -49.4864 -47.8383 680285.8938 -19.5701
-19.5607
[33575]ENERGY: 18700000 3358.5455 5472.4175 5706.5402
342.3654 -14230.7049 -1905.5485 0.0000 0.0000
8236.6911 6980.3062 314.0783 -1256.3849 7047.0066
313.2419 -143.1470 -171.3252 680285.8938 -15.5176
-15.5214
[33625]ENERGY: 18710000 3392.0471 5582.6458 5662.8102
319.1530 -14326.1854 -1794.7676 0.0000 0.0000
8147.4893 6983.1924 310.6769 -1164.2969 7046.1966
313.2977 77.1823 88.0463 680285.8938 -16.1390
-16.1311
[33661]ENERGY: 18720000 3382.6874 5516.7867 5719.2651
328.9737 -14310.8986 -1834.7965 0.0000 0.0000
8180.6307 6982.6485 311.9406 -1197.9821 7046.6973
313.2683 -166.3333 -73.1157 680285.8938 -17.5883
-17.5843
[33711]ENERGY: 18730000 3370.2798 5529.7240 5640.8891
351.5811 -14302.8854 -1806.3387 0.0000 0.0000
8198.9579 6982.2078 312.6395 -1216.7501 7047.1877
313.1915 67.2465 62.8006 680285.8938 -18.8209
-18.8305
[33747]ENERGY: 18740000 3334.8241 5508.0401 5718.4566

347.4016 -14448.1328 -1701.1203 0.0000 0.0000
8216.0814 6975.5507 313.2924 -1240.5307 7045.6756
313.1294 161.8702 120.2224 680285.8938 -19.2039
-19.1952
[33797]ENERGY: 18750000 3381.0956 5427.5215 5647.5840
340.4026 -14259.5411 -1876.7987 0.0000 0.0000
8317.8636 6978.1274 317.1736 -1339.7362 7047.2993
313.0510 -110.8900 -82.7546 680285.8938 -21.2041
-21.2193
[33833]ENERGY: 18760000 3408.8576 5479.7139 5662.6222
331.0209 -14266.2533 -1817.9162 0.0000 0.0000
8184.3249 6982.3698 312.0815 -1201.9551 7047.3043
313.0743 98.1271 25.3569 680285.8938 -19.7215
-19.7091
[33883]ENERGY: 18770000 3375.6963 5526.1947 5672.8799
335.8859 -14293.6876 -1833.9568 0.0000 0.0000
8199.7136 6982.7259 312.6683 -1216.9877 7048.2003
313.1070 -77.1710 -105.4652 680285.8938 -14.6624
-14.6594
[33919]ENERGY: 18780000 3285.6767 5579.7010 5654.0920
331.8519 -14335.7123 -1762.9046 0.0000 0.0000
8225.5595 6978.2641 313.6538 -1247.2954 7045.4110
312.8986 -134.1231 -146.5489 680285.8938 -15.8189
-15.8137
[33969]ENERGY: 18790000 3377.9651 5470.6665 5664.9556
354.7088 -14370.1193 -1839.3268 0.0000 0.0000
8320.1334 6978.9834 317.2601 -1341.1500 7046.0084
313.0266 -153.1042 -143.1099 680285.8938 -18.0822
-18.0973
[34008]ENERGY: 18800000 3321.3997 5576.0784 5716.8577
316.6795 -14353.7413 -1777.1589 0.0000 0.0000
8183.3779 6983.4930 312.0454 -1199.8849 7046.7196
313.1935 151.5689 96.7748 680285.8938 -14.2432
-14.2619
[34058]ENERGY: 18810000 3348.4249 5566.6126 5687.7010
362.0103 -14334.7753 -1816.6826 0.0000 0.0000
8166.7008 6979.9917 311.4095 -1186.7090 7046.9343
313.2029 -104.5251 -131.1879 680285.8938 -13.9476
-13.9198
[34094]ENERGY: 18820000 3437.3100 5522.7560 5686.4789
345.1560 -14342.2248 -1869.0411 0.0000 0.0000
8199.7780 6980.2131 312.6708 -1219.5649 7045.4726
313.3082 -87.6761 -82.1891 680285.8938 -15.8231
-15.8177
[34144]ENERGY: 18830000 3359.4744 5570.1413 5665.9418
344.2813 -14391.0597 -1765.3006 0.0000 0.0000
8197.9537 6981.4323 312.6012 -1216.5214 7046.3736
313.3132 50.5974 111.0559 680285.8938 -12.7586
-12.7698
[34180]ENERGY: 18840000 3377.4556 5460.7385 5677.5252
334.4179 -14277.3568 -1801.1264 0.0000 0.0000
8210.1944 6981.8484 313.0680 -1228.3460 7046.3477
313.1932 -21.6145 -76.1100 680285.8938 -16.6438
-16.6526

[34230]ENERGY: 18850000 3329.1429 5472.0195 5611.8214
334.3685 -14283.4090 -1697.8750 0.0000 0.0000
8219.8474 6985.9157 313.4360 -1233.9317 7047.5263
313.0692 108.9208 86.4478 680285.8938 -16.7834
-16.7572
[34266]ENERGY: 18860000 3352.4239 5519.8524 5672.9476
348.3126 -14248.9532 -1829.8987 0.0000 0.0000
8168.4110 6983.0957 311.4747 -1185.3153 7048.2438
312.9176 52.1401 59.2215 680285.8938 -16.0550
-16.0670
[34316]ENERGY: 18870000 3373.4362 5503.9757 5724.9882
347.7483 -14334.3217 -1830.0436 0.0000 0.0000
8195.8604 6981.6435 312.5214 -1214.2169 7047.0635
313.0803 -36.9207 -66.2848 680285.8938 -15.6904
-15.6928
[34352]ENERGY: 18880000 3337.0880 5555.5894 5680.2287
355.5227 -14319.3082 -1796.7535 0.0000 0.0000
8168.9438 6981.3108 311.4950 -1187.6330 7048.2932
313.0565 -107.8912 -100.9658 680285.8938 -17.7371
-17.7374
[34402]ENERGY: 18890000 3316.0138 5592.6662 5715.9451
329.3763 -14299.7608 -1798.9185 0.0000 0.0000
8123.8888 6979.2109 309.7770 -1144.6780 7048.2899
312.9446 -28.5393 -42.3677 680285.8938 -13.1237
-13.1493
[34441]ENERGY: 18900000 3318.9100 5511.6440 5647.0372
347.9028 -14315.0147 -1782.9685 0.0000 0.0000
8254.6890 6982.1997 314.7646 -1272.4893 7048.9778
313.0084 35.5637 -29.5108 680285.8938 -12.5291
-12.4994
[34491]ENERGY: 18910000 3403.2065 5585.7002 5630.3914
363.2241 -14272.6034 -1844.2091 0.0000 0.0000
8116.5411 6982.2508 309.4968 -1134.2903 7047.7495
313.2278 -106.7882 -125.2810 680285.8938 -11.0093
-11.0062
[34527]ENERGY: 18920000 3398.9073 5468.7219 5648.2550
341.0386 -14409.5282 -1685.3630 0.0000 0.0000
8219.9204 6981.9520 313.4388 -1237.9684 7048.0831
313.3387 135.2686 118.2797 680285.8938 -12.1766
-12.1927
[34577]ENERGY: 18930000 3413.0916 5487.3815 5673.6944
362.6304 -14377.1305 -1782.5624 0.0000 0.0000
8202.5369 6979.6419 312.7760 -1222.8950 7048.4184
313.5046 -33.3656 -32.1704 680285.8938 -12.8515
-12.8504
[34613]ENERGY: 18940000 3335.7246 5610.0592 5710.7463
360.2210 -14355.7203 -1846.3025 0.0000 0.0000
8167.9524 6982.6806 311.4572 -1185.2718 7049.5406
313.4479 -98.9071 -108.1198 680285.8938 -17.1340
-17.1212
[34663]ENERGY: 18950000 3364.0079 5558.2964 5655.0611
351.3000 -14350.0967 -1725.4313 0.0000 0.0000
8131.8078 6984.9452 310.0789 -1146.8626 7048.9636
313.2734 16.5944 62.0927 680285.8938 -14.4827

-14.4842
[34699]ENERGY: 18960000 3440.3079 5560.7847 5645.1234
363.1237 -14348.2263 -1799.8001 0.0000 0.0000
8122.9140 6984.2274 309.7398 -1138.6866 7049.4812
313.2543 -87.3779 -103.2090 680285.8938 -17.4661
-17.4743
[34749]ENERGY: 18970000 3337.4149 5359.2909 5736.6273
342.1657 -14312.9715 -1781.9127 0.0000 0.0000
8300.1788 6980.7934 316.4992 -1319.3854 7049.0406
313.3128 7.3115 43.7221 680285.8938 -13.2140
-13.2046
[34785]ENERGY: 18980000 3346.7184 5450.2444 5705.4025
335.0486 -14323.3546 -1804.6778 0.0000 0.0000
8274.9432 6984.3248 315.5369 -1290.6185 7049.7427
313.2662 64.0060 1.0011 680285.8938 -18.0724
-18.0690
[34835]ENERGY: 18990000 3341.1965 5440.1958 5662.5401
332.2269 -14181.9482 -1803.0292 0.0000 0.0000
8193.0856 6984.2674 312.4156 -1208.8182 7048.3296
313.2530 -33.2841 -60.4437 680285.8938 -14.5229
-14.5402
[34874]ENERGY: 19000000 3337.1342 5424.3487 5594.8231
321.9932 -14293.3808 -1703.1792 0.0000 0.0000
8300.0840 6981.8232 316.4956 -1318.2608 7048.9561
313.0611 108.4683 68.0295 680285.8938 -17.6325
-17.6153
[34924]ENERGY: 19010000 3380.1483 5550.5499 5664.6570
343.1577 -14337.2946 -1765.5327 0.0000 0.0000
8152.1456 6987.8311 310.8545 -1164.3145 7049.2645
313.1361 -164.3354 -114.7087 680285.8938 -17.1882
-17.2135
[34960]ENERGY: 19020000 3365.5275 5517.2767 5678.5209
334.4841 -14358.2809 -1778.2082 0.0000 0.0000
8222.5853 6981.9054 313.5404 -1240.6799 7050.6774
313.3325 -74.8295 -45.4972 680285.8938 -12.8606
-12.8443
[35010]ENERGY: 19030000 3371.1145 5471.6406 5646.3054
330.1763 -14296.4495 -1772.5043 0.0000 0.0000
8234.9249 6985.2080 314.0110 -1249.7169 7049.6850
313.2345 -38.5220 32.7266 680285.8938 -15.6172
-15.6029
[35046]ENERGY: 19040000 3406.7861 5513.1150 5689.1710
361.3058 -14299.4507 -1854.3885 0.0000 0.0000
8166.5369 6983.0756 311.4032 -1183.4613 7050.8958
313.2038 -112.5662 -190.7330 680285.8938 -13.6759
-13.6865
[35096]ENERGY: 19050000 3293.5155 5372.3681 5677.8963
341.1703 -14329.1024 -1703.6329 0.0000 0.0000
8328.0269 6980.2419 317.5611 -1347.7850 7049.1144
313.0876 111.5629 108.7694 680285.8938 -14.9898
-14.9987
[35132]ENERGY: 19060000 3355.2558 5644.3616 5634.7479
336.5683 -14345.8951 -1857.6435 0.0000 0.0000
8215.4280 6982.8230 313.2675 -1232.6050 7049.8853

313.3443 20.7989 -51.2202 680285.8938 -21.7794
-21.7777
[35182]ENERGY: 19070000 3436.8255 5494.9236 5608.8307
345.2848 -14385.7314 -1750.8593 0.0000 0.0000
8235.7097 6984.9836 314.0409 -1250.7260 7050.0109
313.3093 28.9361 20.3715 680285.8938 -17.0880
-17.0833
[35218]ENERGY: 19080000 3425.9314 5526.6182 5686.8511
341.3281 -14327.6538 -1812.4414 0.0000 0.0000
8141.6397 6982.2733 310.4539 -1159.3664 7050.7950
313.1888 -75.1722 -84.6499 680285.8938 -14.1728
-14.1801
[35268]ENERGY: 19090000 3340.7116 5576.6723 5690.6703
338.9929 -14325.4684 -1797.7853 0.0000 0.0000
8161.4998 6985.2932 311.2111 -1176.2066 7050.9238
313.2723 175.0680 151.4152 680285.8938 -12.8605
-12.8687
[35307]ENERGY: 19100000 3366.0600 5452.2085 5661.9392
350.7199 -14243.2663 -1773.2625 0.0000 0.0000
8166.7990 6981.1977 311.4132 -1185.6013 7049.4325
313.1818 46.8232 26.3618 680285.8938 -14.9623
-14.9503
[35357]ENERGY: 19110000 3321.8308 5517.3568 5655.2352
356.8684 -14292.0691 -1850.6736 0.0000 0.0000
8277.1387 6985.6871 315.6206 -1291.4516 7050.9655
313.3101 43.1789 31.7649 680285.8938 -16.0834
-16.0874
[35393]ENERGY: 19120000 3404.9355 5463.1430 5664.0978
326.3249 -14271.4184 -1800.0581 0.0000 0.0000
8197.4366 6984.4613 312.5815 -1212.9753 7051.4390
313.2102 -33.2948 -38.1238 680285.8938 -16.5390
-16.5252
[35443]ENERGY: 19130000 3269.2910 5604.4438 5687.3275
332.8585 -14342.4916 -1793.2925 0.0000 0.0000
8226.5856 6984.7223 313.6930 -1241.8633 7051.2176
313.4286 79.6008 132.6126 680285.8938 -18.9443
-18.9460
[35479]ENERGY: 19140000 3360.5967 5520.6132 5655.7468
332.3655 -14300.3866 -1778.3872 0.0000 0.0000
8192.9672 6983.5156 312.4111 -1209.4516 7051.9336
313.2655 -23.6217 -26.9372 680285.8938 -16.7869
-16.7949
[35529]ENERGY: 19150000 3369.9656 5505.1021 5663.7416
356.6557 -14239.9812 -1880.0602 0.0000 0.0000
8213.6047 6989.0283 313.1980 -1224.5764 7052.5541
313.3026 -18.3550 -42.6639 680285.8938 -18.7246
-18.7179
[35565]ENERGY: 19160000 3304.7673 5544.6530 5713.2453
336.9164 -14354.4762 -1744.1424 0.0000 0.0000
8186.5662 6987.5296 312.1670 -1199.0366 7050.8827
313.2462 -12.1824 43.4011 680285.8938 -20.8657
-20.8621
[35615]ENERGY: 19170000 3404.4062 5327.5737 5693.8443
347.2035 -14280.7067 -1806.1724 0.0000 0.0000

8298.5886 6984.7373 316.4386 -1313.8513 7051.7722
313.2513 -11.6605 -50.8062 680285.8938 -18.9781
-18.9751
[35651]ENERGY: 19180000 3368.8493 5513.7758 5723.8813
334.0283 -14350.8029 -1748.5384 0.0000 0.0000
8146.8432 6988.0366 310.6523 -1158.8066 7051.2981
313.1806 -53.2533 -68.0318 680285.8938 -20.1662
-20.1711
[35701]ENERGY: 19190000 3287.6158 5490.7663 5702.4047
327.7866 -14272.4562 -1759.9678 0.0000 0.0000
8210.4776 6986.6272 313.0788 -1223.8504 7052.9177
313.0120 110.2744 63.4999 680285.8938 -16.3426
-16.3459
[35740]ENERGY: 19200000 3344.4245 5585.6534 5683.6525
350.6910 -14263.3182 -1850.9005 0.0000 0.0000
8138.1838 6988.3865 310.3221 -1149.7973 7053.1592
313.1673 -26.2699 -61.0111 680285.8938 -18.3431
-18.3449
[35790]ENERGY: 19210000 3426.6957 5462.1079 5718.5596
347.2871 -14272.3680 -1804.4435 0.0000 0.0000
8111.4808 6989.3196 309.3038 -1122.1611 7053.0556
313.0401 -250.4888 -223.0975 680285.8938 -15.0720
-15.0861
[35826]ENERGY: 19220000 3235.5916 5617.1188 5705.4105
332.7471 -14329.7948 -1780.3707 0.0000 0.0000
8205.3945 6986.0970 312.8849 -1219.2975 7054.0005
313.1354 141.3559 90.6769 680285.8938 -21.4996
-21.4813
[35876]ENERGY: 19230000 3332.3820 5622.2810 5650.6577
349.5402 -14415.5164 -1776.7848 0.0000 0.0000
8224.3488 6986.9086 313.6077 -1237.4403 7053.1965
313.1025 -134.4259 -91.4059 680285.8938 -16.9911
-16.9914
[35912]ENERGY: 19240000 3428.3701 5466.3683 5707.5863
351.4680 -14266.9881 -1814.0849 0.0000 0.0000
8115.1693 6987.8890 309.4445 -1127.2803 7052.4633
313.1825 7.6549 4.4195 680285.8938 -18.2396
-18.2627
[35962]ENERGY: 19250000 3337.6721 5509.2921 5694.8481
339.5997 -14262.3965 -1859.9679 0.0000 0.0000
8226.6402 6985.6876 313.6951 -1240.9526 7052.3714
313.0964 -38.3386 -87.4489 680285.8938 -21.1497
-21.1275
[35998]ENERGY: 19260000 3351.9196 5441.0232 5611.1975
342.1257 -14203.1191 -1784.7445 0.0000 0.0000
8228.1912 6986.5935 313.7542 -1241.5977 7050.9087
313.1909 43.4195 29.2978 680285.8938 -15.2691
-15.2651
[36048]ENERGY: 19270000 3319.2888 5403.0649 5675.9984
370.3041 -14300.9762 -1812.5839 0.0000 0.0000
8326.1849 6981.2809 317.4909 -1344.9039 7052.9592
313.2113 -38.6063 -59.9998 680285.8938 -16.0862
-16.0933
[36084]ENERGY: 19280000 3322.3151 5607.0103 5714.8339

323.6026 -14323.5542 -1817.6729 0.0000 0.0000
8160.1122 6986.6469 311.1582 -1173.4652 7053.7691
313.3425 -85.0155 -130.3698 680285.8938 -13.3316
-13.3264
[36134]ENERGY: 19290000 3366.1207 5523.5370 5683.8786
341.3080 -14348.1690 -1789.9760 0.0000 0.0000
8211.9900 6988.6893 313.1364 -1223.3006 7053.7278
313.3908 -93.0626 -81.7853 680285.8938 -16.3394
-16.3404
[36173]ENERGY: 19300000 3316.9399 5457.8449 5681.6864
345.5313 -14197.5788 -1836.8988 0.0000 0.0000
8218.4580 6985.9828 313.3831 -1232.4752 7054.3425
313.4807 74.9108 90.4562 680285.8938 -17.9390
-17.9457
[36223]ENERGY: 19310000 3329.3617 5519.6325 5685.8895
344.2417 -14238.9694 -1830.0530 0.0000 0.0000
8180.2514 6990.3544 311.9262 -1189.8970 7055.8596
313.2706 -36.8770 -30.1523 680285.8938 -13.5821
-13.5657
[36259]ENERGY: 19320000 3266.7886 5482.2580 5632.7073
353.6361 -14226.8315 -1807.5540 0.0000 0.0000
8286.4879 6987.4924 315.9771 -1298.9955 7056.3434
313.2916 68.5011 -10.5043 680285.8938 -14.8729
-14.8943
[36309]ENERGY: 19330000 3384.2684 5521.7122 5670.8176
360.7440 -14337.9443 -1749.2263 0.0000 0.0000
8139.9821 6990.3536 310.3906 -1149.6284 7054.5335
313.1738 0.9775 -28.9925 680285.8938 -13.5950
-13.5979
[36345]ENERGY: 19340000 3306.8714 5519.0059 5696.3659
367.8896 -14262.3155 -1828.8444 0.0000 0.0000
8195.2194 6994.1923 312.4969 -1201.0271 7055.6342
313.1932 26.1061 8.3372 680285.8938 -17.5116
-17.4920
[36395]ENERGY: 19350000 3316.4395 5560.8830 5714.9783
348.6931 -14373.1310 -1770.3644 0.0000 0.0000
8190.8880 6988.3864 312.3318 -1202.5016 7055.3601
313.4281 16.2069 12.4519 680285.8938 -16.0225
-16.0223
[36431]ENERGY: 19360000 3336.6031 5625.0083 5656.7806
359.5427 -14331.5463 -1805.5176 0.0000 0.0000
8149.8508 6990.7215 310.7670 -1159.1293 7055.0024
313.4250 142.1371 91.6799 680285.8938 -17.4634
-17.4784
[36481]ENERGY: 19370000 3276.5248 5556.1393 5671.2041
343.5603 -14219.1103 -1830.3709 0.0000 0.0000
8191.1898 6989.1371 312.3433 -1202.0527 7056.3390
313.3376 246.2151 112.6473 680285.8938 -14.5620
-14.5750
[36517]ENERGY: 19380000 3320.5284 5454.1035 5741.3427
347.1710 -14333.3102 -1774.5074 0.0000 0.0000
8233.5306 6988.8586 313.9578 -1244.6721 7055.7575
313.3569 97.0945 29.9634 680285.8938 -14.3396
-14.3327

[36567]ENERGY: 19390000 3310.6889 5541.4412 5660.6147
331.6831 -14297.4474 -1781.5442 0.0000 0.0000
8224.7138 6990.1501 313.6216 -1234.5637 7056.2796
313.3383 26.5277 -13.4743 680285.8938 -19.3265
-19.3103
[36606]ENERGY: 19400000 3360.3290 5524.7194 5675.4916
333.3911 -14315.0645 -1832.2226 0.0000 0.0000
8243.0657 6989.7097 314.3214 -1253.3560 7057.0005
313.3625 -142.3229 -130.0213 680285.8938 -19.5229
-19.5277
[36656]ENERGY: 19410000 3357.0412 5494.4931 5707.5736
335.3995 -14313.1916 -1779.5044 0.0000 0.0000
8190.0130 6991.8244 312.2984 -1198.1886 7055.7993
313.2746 55.7250 89.3917 680285.8938 -18.8117
-18.8261
[36692]ENERGY: 19420000 3374.3372 5590.0049 5714.2294
339.8788 -14370.2111 -1749.7496 0.0000 0.0000
8094.5996 6993.0892 308.6601 -1101.5103 7056.6591
313.2289 45.4132 75.6594 680285.8938 -14.6953
-14.6765
[36742]ENERGY: 19430000 3291.7122 5571.3971 5654.8942
342.9991 -14288.4639 -1772.4908 0.0000 0.0000
8190.1895 6990.2375 312.3051 -1199.9521 7056.9143
313.2117 -25.4809 -18.0574 680285.8938 -13.9621
-13.9831
[36778]ENERGY: 19440000 3358.8691 5488.1833 5655.0742
332.8606 -14299.1830 -1750.9991 0.0000 0.0000
8210.3407 6995.1458 313.0735 -1215.1949 7057.1727
313.3722 151.2346 91.2570 680285.8938 -15.6624
-15.6571
[36828]ENERGY: 19450000 3372.1229 5552.2536 5679.3727
330.9096 -14312.8592 -1842.0031 0.0000 0.0000
8212.2583 6992.0548 313.1467 -1220.2034 7057.3078
313.4529 -125.5085 -131.0329 680285.8938 -13.6192
-13.6039
[36864]ENERGY: 19460000 3470.1207 5433.2463 5658.8725
351.1935 -14308.8073 -1844.7806 0.0000 0.0000
8231.0355 6990.8806 313.8627 -1240.1549 7057.6443
313.3556 -74.2023 -144.3905 680285.8938 -17.7320
-17.7354
[36914]ENERGY: 19470000 3441.6090 5561.1928 5705.7249
339.6676 -14316.1438 -1820.7347 0.0000 0.0000
8084.0832 6995.3989 308.2591 -1088.6843 7057.3442
313.3184 -152.3734 -169.4063 680285.8938 -19.0972
-19.0807
[36950]ENERGY: 19480000 3418.0936 5542.7115 5618.4306
343.6094 -14324.4860 -1804.6942 0.0000 0.0000
8199.2190 6992.8839 312.6494 -1206.3351 7057.4723
313.3673 -104.5801 -56.4526 680285.8938 -19.1558
-19.1705
[37000]ENERGY: 19490000 3430.2189 5559.7377 5707.2104
326.3061 -14361.5013 -1864.7742 0.0000 0.0000
8195.7225 6992.9200 312.5161 -1202.8025 7057.1169
313.4361 -124.9972 -58.3213 680285.8938 -18.7755

-18.7773
[37039]ENERGY: 19500000 3384.6350 5436.3999 5661.5360
324.6276 -14298.6403 -1768.9990 0.0000 0.0000
8248.3360 6987.8952 314.5224 -1260.4408 7056.9317
313.3526 43.0789 2.9913 680285.8938 -18.5461
-18.5380
[37089]ENERGY: 19510000 3286.8302 5534.2502 5676.6070
345.7393 -14324.9978 -1759.6021 0.0000 0.0000
8237.1069 6995.9337 314.0942 -1241.1732 7057.4853
313.3258 77.7585 83.2157 680285.8938 -16.3273
-16.3387
[37125]ENERGY: 19520000 3379.1540 5424.0182 5656.3658
314.4864 -14161.0254 -1815.4662 0.0000 0.0000
8197.3942 6994.9269 312.5799 -1202.4672 7058.2989
313.1728 -59.1967 -47.5822 680285.8938 -18.0301
-18.0206
[37175]ENERGY: 19530000 3372.7544 5524.8789 5658.4861
334.7710 -14277.9446 -1847.0110 0.0000 0.0000
8221.7292 6987.6640 313.5078 -1234.0653 7057.7241
313.2150 -168.4851 -91.9347 680285.8938 -12.3668
-12.3725
[37211]ENERGY: 19540000 3383.2991 5547.3224 5651.6671
356.0891 -14328.8045 -1814.3634 0.0000 0.0000
8197.7303 6992.9401 312.5927 -1204.7902 7057.2979
313.3845 65.6135 50.6423 680285.8938 -14.3273
-14.3496
[37261]ENERGY: 19550000 3392.9954 5498.2037 5660.7637
350.6150 -14269.9026 -1842.0151 0.0000 0.0000
8201.3247 6991.9848 312.7297 -1209.3400 7058.2248
313.1945 -143.2055 -137.2137 680285.8938 -19.7062
-19.6818
[37297]ENERGY: 19560000 3291.4511 5527.7571 5648.9518
345.9729 -14270.3670 -1806.8872 0.0000 0.0000
8253.9528 6990.8315 314.7365 -1263.1213 7056.5645
313.3259 66.3640 72.9516 680285.8938 -17.4857
-17.4856
[37347]ENERGY: 19570000 3395.7345 5512.9882 5647.6576
325.8123 -14227.1291 -1859.9177 0.0000 0.0000
8193.7467 6988.8923 312.4408 -1204.8543 7058.1645
313.2935 7.4302 -77.0002 680285.8938 -16.5700
-16.5678
[37383]ENERGY: 19580000 3283.7156 5457.6407 5636.1704
334.3046 -14283.0646 -1767.0412 0.0000 0.0000
8329.3088 6991.0344 317.6100 -1338.2745 7056.6996
313.2673 95.3754 86.8004 680285.8938 -19.7729
-19.7783
[37433]ENERGY: 19590000 3318.7992 5462.8816 5678.2063
328.7992 -14302.5628 -1781.8918 0.0000 0.0000
8280.5627 6984.7945 315.7512 -1295.7683 7057.4865
313.2691 63.0098 -16.1387 680285.8938 -17.0861
-17.0867
[37472]ENERGY: 19600000 3357.6064 5499.3641 5686.6537
342.7775 -14267.2190 -1841.0632 0.0000 0.0000
8211.5896 6989.7091 313.1212 -1221.8805 7058.0908

313.2719 153.2302 -3.2299 680285.8938 -17.7738
-17.7667
[37522]ENERGY: 19610000 3368.9546 5477.1713 5639.3048
339.5538 -14292.5241 -1769.1611 0.0000 0.0000
8231.7768 6995.0761 313.8909 -1236.7008 7059.0195
313.3496 196.6178 116.6656 680285.8938 -17.5411
-17.5384
[37558]ENERGY: 19620000 3392.6479 5436.8796 5725.7035
360.6531 -14223.5103 -1879.5991 0.0000 0.0000
8181.8140 6994.5888 311.9858 -1187.2252 7059.5814
313.3174 -6.9962 -75.5339 680285.8938 -16.8989
-16.9240
[37608]ENERGY: 19630000 3410.9026 5529.9132 5677.6125
349.6377 -14331.3521 -1815.3218 0.0000 0.0000
8172.1047 6993.4968 311.6155 -1178.6078 7057.7238
313.2992 -65.6241 -31.5385 680285.8938 -13.3547
-13.3289
[37644]ENERGY: 19640000 3295.5689 5520.1084 5702.3820
352.5303 -14327.2821 -1686.6956 0.0000 0.0000
8136.6742 6993.2862 310.2645 -1143.3880 7057.5075
313.3422 12.7438 8.1269 680285.8938 -17.7051
-17.7140
[37694]ENERGY: 19650000 3382.4120 5545.2278 5644.2687
333.5055 -14335.1333 -1711.4087 0.0000 0.0000
8133.8218 6992.6937 310.1557 -1141.1280 7058.3077
313.2100 20.9487 3.3770 680285.8938 -17.9056
-17.8937
[37730]ENERGY: 19660000 3421.6836 5471.3333 5665.9713
327.7661 -14251.3388 -1839.5413 0.0000 0.0000
8200.2029 6996.0773 312.6870 -1204.1256 7059.8108
313.1206 -1.2987 -24.9277 680285.8938 -13.8323
-13.8658
[37780]ENERGY: 19670000 3413.0101 5546.5655 5682.7763
357.6862 -14318.5162 -1808.7813 0.0000 0.0000
8122.8627 6995.6034 309.7379 -1127.2593 7060.2932
312.9862 -38.6494 -61.9485 680285.8938 -19.8394
-19.8174
[37816]ENERGY: 19680000 3443.9368 5466.2548 5654.3548
358.8450 -14314.1055 -1828.8793 0.0000 0.0000
8213.5682 6993.9748 313.1966 -1219.5934 7060.7514
313.0994 0.8920 -63.3587 680285.8938 -17.3113
-17.3080
[37866]ENERGY: 19690000 3322.3748 5484.4782 5674.2419
332.1046 -14228.3593 -1787.1033 0.0000 0.0000
8197.5523 6995.2890 312.5859 -1202.2632 7060.9820
313.0263 195.6917 79.3284 680285.8938 -15.5095
-15.5072
[37905]ENERGY: 19700000 3469.4415 5493.3832 5753.6408
339.6896 -14286.4714 -1916.4690 0.0000 0.0000
8140.3800 6993.5948 310.4058 -1146.7852 7061.3219
312.9436 -87.9910 -149.2145 680285.8938 -16.9506
-16.9536
[37955]ENERGY: 19710000 3325.9743 5382.8423 5749.1415
351.5888 -14243.3511 -1813.2862 0.0000 0.0000

8239.1741 6992.0837 314.1730 -1247.0903 7061.6654
313.0326 -8.2895 -74.3060 680285.8938 -17.3029
-17.2898
[37991]ENERGY: 19720000 3353.3067 5468.3458 5741.0927
344.3985 -14399.0841 -1741.6484 0.0000 0.0000
8228.9680 6995.3792 313.7838 -1233.5888 7061.4617
313.1582 145.9020 112.8103 680285.8938 -21.2564
-21.2706
[38041]ENERGY: 19730000 3385.2454 5533.4137 5654.4005
327.2091 -14315.3165 -1848.8129 0.0000 0.0000
8256.7976 6992.9370 314.8450 -1263.8606 7062.4355
313.2289 51.3464 -68.8570 680285.8938 -19.0144
-19.0150
[38077]ENERGY: 19740000 3324.0089 5486.8253 5686.7272
323.0299 -14321.4604 -1747.1126 0.0000 0.0000
8241.6396 6993.6578 314.2670 -1247.9818 7061.3134
313.3415 14.7822 60.2203 680285.8938 -17.0445
-17.0381
[38127]ENERGY: 19750000 3342.2880 5526.9359 5697.7041
327.1404 -14204.1800 -1814.7925 0.0000 0.0000
8124.7517 6999.8475 309.8099 -1124.9042 7061.8180
313.2754 89.8063 -30.7627 680285.8938 -19.4155
-19.4270
[38163]ENERGY: 19760000 3376.9625 5496.9438 5662.2407
322.2409 -14273.4410 -1764.5326 0.0000 0.0000
8176.1597 6996.5741 311.7702 -1179.5857 7061.8766
313.3599 128.0667 81.1759 680285.8938 -18.5226
-18.5203
[38213]ENERGY: 19770000 3315.7277 5553.3374 5710.5029
330.2731 -14298.0967 -1811.1945 0.0000 0.0000
8193.9353 6994.4852 312.4480 -1199.4501 7062.9187
313.0989 67.2520 32.1992 680285.8938 -18.7648
-18.7624
[38249]ENERGY: 19780000 3274.1819 5534.7281 5645.6660
339.6741 -14324.7872 -1728.2475 0.0000 0.0000
8252.9892 6994.2046 314.6998 -1258.7846 7061.5880
313.3024 44.7223 25.3944 680285.8938 -18.8772
-18.8706
[38299]ENERGY: 19790000 3389.3720 5596.9768 5692.6779
347.5111 -14380.7553 -1787.7196 0.0000 0.0000
8137.5978 6995.6607 310.2997 -1141.9371 7061.6224
313.3801 -238.5599 -189.1849 680285.8938 -16.3807
-16.3807
[38338]ENERGY: 19800000 3311.5775 5508.2105 5698.2225
324.9951 -14227.9236 -1857.3103 0.0000 0.0000
8240.9693 6998.7410 314.2414 -1242.2283 7061.4162
313.2139 45.9257 -0.8681 680285.8938 -18.2184
-18.2229
[38388]ENERGY: 19810000 3414.5016 5575.7419 5656.4780
338.9184 -14370.3204 -1758.7376 0.0000 0.0000
8143.3403 6999.9221 310.5187 -1143.4182 7062.0307
313.3273 7.2464 -68.2285 680285.8938 -16.1681
-16.1510
[38424]ENERGY: 19820000 3347.2760 5486.1810 5711.6310

```
355.5508          -14310.4443     -1839.3966         0.0000         0.0000
8245.8560           6996.6539      314.4278     -1249.2021      7062.8441
313.5598           -185.9496      -134.3820    680285.8938       -18.1113
-18.1276
[38474]ENERGY: 19830000      3402.7661      5523.9346      5646.1498
332.4598          -14299.1263     -1805.1326         0.0000         0.0000
8200.9667           7002.0181      312.7161     -1198.9486      7063.2618
313.5726             -2.2735       -73.2597    680285.8938       -18.0483
-18.0460
[38510]ENERGY: 19840000      3308.9397      5486.2508      5723.0285
345.0765          -14317.4500     -1825.0446         0.0000         0.0000
8273.5306           6994.3316      315.4831     -1279.1990      7063.0493
313.3781            -21.9804       -52.6573    680285.8938       -20.1607
-20.1647
[38560]ENERGY: 19850000      3314.1453      5598.7790      5677.3295
326.6335          -14326.3782     -1765.1924         0.0000         0.0000
8174.1194           6999.4362      311.6924     -1174.6832      7061.9721
313.3446            151.9090       121.9282    680285.8938       -18.4744
-18.4843
[38596]ENERGY: 19860000      3372.3103      5520.0311      5683.0315
322.1633          -14302.4618     -1781.7683         0.0000         0.0000
8185.7468           6999.0529      312.1357     -1186.6939      7063.9208
313.5157             21.6816       -79.2960    680285.8938       -15.9649
-15.9569
[38646]ENERGY: 19870000      3314.9510      5485.1135      5704.9538
339.7699          -14258.7675     -1860.5070         0.0000         0.0000
8268.6954           6994.2091      315.2987     -1274.4864      7063.0205
313.6017             -4.6975       -75.2222    680285.8938       -16.9992
-17.0019
[38682]ENERGY: 19880000      3332.9874      5491.4798      5684.9150
368.9790          -14321.0109     -1833.1402         0.0000         0.0000
8267.8039           6992.0140      315.2647     -1275.7899      7062.7832
313.5227             93.4416        11.6285    680285.8938       -19.8042
-19.8012
[38732]ENERGY: 19890000      3358.7054      5456.9419      5697.0633
345.1498          -14342.9098     -1781.2721         0.0000         0.0000
8265.9289           6999.6074      315.1932     -1266.3215      7063.2059
313.4430           -106.9774       -66.1412    680285.8938       -17.2625
-17.2574
[38771]ENERGY: 19900000      3382.8797      5557.8890      5619.6076
317.6656          -14309.9305     -1796.1827         0.0000         0.0000
8223.3041           6995.2327      313.5678     -1228.0713      7065.1231
313.3410            -79.3824       -70.9985    680285.8938       -19.6839
-19.6873
[38821]ENERGY: 19910000      3320.2087      5469.8253      5648.0320
357.1371          -14338.0306     -1802.1869         0.0000         0.0000
8340.7241           6995.7097      318.0453     -1345.0144      7065.0114
313.3389             -7.8685       -17.7984    680285.8938       -18.6273
-18.6366
[38857]ENERGY: 19920000      3367.6584      5555.9640      5711.9199
350.1238          -14419.2223     -1745.4392         0.0000         0.0000
8181.2992           7002.3038      311.9661     -1178.9954      7065.3979
313.5938             74.8261        75.4230    680285.8938       -14.9264
-14.9252
```

```
[38907]ENERGY: 19930000      3375.5420      5499.2803      5686.8278
354.8375         -14316.4676     -1832.3583         0.0000         0.0000
8228.8625           6996.5242       313.7798     -1232.3383      7065.3988
313.3361            -79.8627       -39.7559    680285.8938       -15.8546
-15.8351
[38943]ENERGY: 19940000      3426.6488      5483.3888      5673.9594
338.7669         -14378.5350     -1764.2862         0.0000         0.0000
8218.5766           6998.5193       313.3876     -1220.0574      7064.2667
313.4854            -96.6654      -128.9283    680285.8938       -13.9583
-13.9957
[38993]ENERGY: 19950000      3342.6746      5610.3571      5675.7942
328.0389         -14373.3914     -1802.9296         0.0000         0.0000
8219.9172           7000.4610       313.4387     -1219.4562      7064.8902
313.5459             -5.2780       -14.0102    680285.8938       -15.9811
-15.9603
[39029]ENERGY: 19960000      3378.6296      5511.0533      5643.9327
351.5816         -14401.5421     -1707.0146         0.0000         0.0000
8224.8407           7001.4811       313.6264     -1223.3596      7065.5264
313.4776             43.4573       -14.5538    680285.8938       -11.4871
-11.4874
[39079]ENERGY: 19970000      3335.5675      5532.1497      5627.0179
365.5352         -14297.2205     -1738.8833         0.0000         0.0000
8177.5803           7001.7467       311.8243     -1175.8336      7066.0082
313.4959             93.8188        74.1461    680285.8938       -11.7768
-11.7689
[39115]ENERGY: 19980000      3298.8616      5496.7805      5674.2880
338.1849         -14227.4544     -1785.9114         0.0000         0.0000
8205.2920           7000.0411       312.8810     -1205.2508      7065.2333
313.5759             30.0263        69.8218    680285.8938       -17.3011
-17.2999
[39165]ENERGY: 19990000      3334.5185      5540.8533      5701.0846
350.7088         -14310.1089     -1818.3988         0.0000         0.0000
8196.3170           6994.9745       312.5388     -1201.3425      7064.2955
313.4313             25.9149       -37.2285    680285.8938       -15.6209
-15.6200
[39204]ENERGY: 20000000      3315.8443      5414.0699      5699.4810
353.2916         -14209.1049     -1817.4286         0.0000         0.0000
8242.8199           6998.9732       314.3120     -1243.8467      7065.5496
313.5060            115.1911        31.4431    680285.8938       -17.6691
-17.6759
```

40.1ns Q8LJP6 SIMULATION DATA

```
                  TS            BOND           ANGLE           DIHED           IMPRP
              ELECT             VDW        BOUNDARY            MISC         KINETIC
              TOTAL            TEMP       POTENTIAL          TOTAL3         TEMPAVG
          PRESSURE       GPRESSURE          VOLUME        PRESSAVG       GPRESSAVG
[191]ENERGY:       0       4825.7375       5324.7236       5641.4695        295.6590
         -9244.6331      -1762.6473          0.0000          0.0000       7582.2299
         12662.5391        296.4687       5080.3092      12770.8391        296.4687
          2869.8628        504.4981     718822.6829       2869.8628        504.4981
[279]ENERGY:   10000       3207.6936       5275.1286       5637.9631        340.6081
        -13675.3958      -1595.4022          0.0000          0.0000       7715.6098
          6906.2051        301.6839       -809.4047       6966.9136        321.4630
```

```
        -19.1770        26.0408    671341.7472        -5.2476        -5.1047
[318]ENERGY:   20000     3149.8881      5202.4290      5596.9617       331.1071
       -13824.9602     -1580.2266         0.0000         0.0000      7591.4949
        6466.6941       296.8309     -1124.8008      6527.5675       300.1132
        183.2514        78.5816    671614.5889         0.3868         0.3722
[370]ENERGY:   30000     3181.1358      5135.8029      5566.2936       330.6999
       -13948.4391     -1570.7178         0.0000         0.0000      7573.9534
        6268.7287       296.1450     -1305.2247      6328.9724       298.2765
        -54.6593       -45.8716    674320.6968         0.0007         0.0294
[408]ENERGY:   40000     3190.0070      5237.7604      5577.0545       331.3075
       -13949.8435     -1639.1522         0.0000         0.0000      7663.2848
        6410.4185       299.6379     -1252.8663      6474.2867       298.5818
         78.2373        55.4913    630912.4250         1.3771         1.3418
[460]ENERGY:   50000     3156.4891      5190.3730      5563.2384       339.9715
       -13931.2816     -1672.2100         0.0000         0.0000      7600.6186
        6247.1991       297.1877     -1353.4196      6309.6681       298.6943
        -19.1232       -55.3864    628905.1138         1.5852         1.6226
[498]ENERGY:   60000     3217.1413      5254.1283      5473.0069       308.1163
       -13901.9547     -1637.8486         0.0000         0.0000      7676.1919
        6388.7814       300.1426     -1287.4105      6449.6946       298.5016
         52.3279        38.3390    661707.3109         3.2082         3.1954
[549]ENERGY:   70000     3238.3290      5159.3004      5556.0584       302.1582
       -13921.0565     -1599.7070         0.0000         0.0000      7629.2459
        6364.3283       298.3070     -1264.9176      6426.3273       300.3755
         34.2303         6.6902    655110.8926        -1.9675        -1.9664
[586]ENERGY:   80000     3133.3989      5266.8915      5537.7790       326.8946
       -13945.0575     -1642.0673         0.0000         0.0000      7650.8094
        6328.6486       299.1501     -1322.1608      6390.4326       299.8738
        323.2783       237.2277    665564.1026        -2.8420        -2.8502
[637]ENERGY:   90000     3148.0606      5283.1731      5508.2306       324.1648
       -14006.7121     -1640.1755         0.0000         0.0000      7768.3559
        6385.0974       303.7463     -1383.2585      6448.0192       299.6132
         56.8380        59.4680    683543.2700         3.2391         3.2776
[677]ENERGY:  100000     3134.2633      5310.4758      5509.6777       316.9759
       -13993.6709     -1679.1044         0.0000         0.0000      7639.5057
        6238.1232       298.7082     -1401.3825      6302.6979       301.5901
          3.6846        13.4831    704957.5497         4.7689         4.7837
[728]ENERGY:  110000     3272.8075      5285.3915      5501.4445       329.5293
       -14020.7761     -1650.6279         0.0000         0.0000      7693.3443
        6411.1131       300.8133     -1282.2312      6470.9468       300.7788
         41.3354        86.9024    693546.0255         2.6557         2.6355
[764]ENERGY:  120000     3173.4677      5233.4588      5484.4159       318.6371
       -13934.6420     -1679.6000         0.0000         0.0000      7787.2173
        6382.9547       304.4838     -1404.2626      6443.7592       300.6300
        134.0875        -8.1535    698191.8440         0.2774         0.2643
[814]ENERGY:  130000     3159.5513      5308.2470      5524.5601       300.4078
       -13951.6726     -1728.5142         0.0000         0.0000      7615.6493
        6228.2287       297.7754     -1387.4206      6290.5889       299.1196
         83.2206       -27.4734    690565.0632         1.3824         1.3677
[850]ENERGY:  140000     3220.7511      5187.5435      5476.4798       317.8856
       -13940.3662     -1708.5407         0.0000         0.0000      7703.1248
        6256.8779       301.1957     -1446.2469      6319.2648       299.8096
         29.1188        20.8814    692871.8460         2.2105         2.2157
[900]ENERGY:  150000     3172.2852      5161.2838      5555.2567       303.9391
```

-14046.9971 -1634.7064 0.0000 0.0000 7727.7767
6238.8380 302.1596 -1488.9387 6302.6868 301.4051
40.6683 60.6253 692819.0048 2.0534 2.0474
[936]ENERGY: 160000 3197.7610 5312.4626 5501.2550 318.5999
-13950.3584 -1751.7483 0.0000 0.0000 7548.8722
6176.8441 295.1644 -1372.0281 6235.9428 300.3564
1.9669 19.4204 709437.4397 3.0320 3.0190
[986]ENERGY: 170000 3216.0505 5237.6703 5472.0475 323.9349
-13981.7234 -1712.0234 0.0000 0.0000 7696.7279
6252.6843 300.9456 -1444.0436 6316.4098 300.3456
101.8253 -27.5106 730521.5215 -1.7520 -1.7511
[1022]ENERGY: 180000 3203.4625 5183.6633 5503.4093
305.6517 -13994.2809 -1776.9255 0.0000 0.0000
7627.4884 6052.4688 298.2383 -1575.0196 6113.0599
299.2014 -11.0809 50.2572 716815.8351 -0.0458
-0.0727
[1072]ENERGY: 190000 3183.1978 5195.2756 5541.3142
319.5725 -14125.3550 -1590.1547 0.0000 0.0000
7673.8879 6197.7383 300.0525 -1476.1495 6262.0125
298.0152 28.7415 47.8967 699454.4790 4.0254
4.0155
[1111]ENERGY: 200000 3154.8594 5230.3512 5529.4449
316.7139 -14069.2097 -1667.0550 0.0000 0.0000
7627.3103 6122.4150 298.2313 -1504.8953 6182.3669
298.9073 11.5123 91.2973 676773.0766 5.1370
5.1453
[1161]ENERGY: 210000 3167.0488 5209.1065 5501.7613
337.7381 -14099.8470 -1670.7570 0.0000 0.0000
7699.1177 6144.1684 301.0390 -1554.9492 6210.7260
298.2521 93.6728 45.6057 681432.2371 -0.7892
-0.7954
[1197]ENERGY: 220000 3230.1740 5264.5169 5503.4116
343.2371 -14125.1510 -1659.9842 0.0000 0.0000
7632.9412 6189.1456 298.4515 -1443.7955 6250.5148
298.9734 147.7541 116.6700 656028.4447 2.9393
2.9439
[1247]ENERGY: 230000 3247.9918 5163.9306 5480.2357
313.2978 -14082.9978 -1681.6638 0.0000 0.0000
7629.5805 6070.3747 298.3201 -1559.2058 6130.7319
300.2094 1.7180 15.1082 670832.8216 -0.6506
-0.6455
[1283]ENERGY: 240000 3171.2147 5284.1186 5482.6760
314.2209 -14088.8646 -1703.9184 0.0000 0.0000
7642.8284 6102.2756 298.8381 -1540.5528 6167.0340
298.1940 -34.8040 -33.3967 673632.0742 -1.0367
-1.0342
[1333]ENERGY: 250000 3149.3999 5294.5800 5436.4876
346.2894 -14161.2286 -1610.4402 0.0000 0.0000
7608.2592 6063.3473 297.4864 -1544.9120 6127.1941
298.5357 83.7497 68.3522 685066.8939 5.8604
5.8278
[1369]ENERGY: 260000 3225.5925 5118.3850 5419.8981
311.5559 -14000.5955 -1697.8359 0.0000 0.0000
7662.8957 6039.8957 299.6227 -1623.0000 6101.9254

299.1289 -212.8030 -135.6024 698988.1237 -1.7838
-1.7814
[1419]ENERGY: 270000 3262.2448 5211.7726 5439.9850
312.9182 -14109.2568 -1668.5242 0.0000 0.0000
7627.8134 6076.9530 298.2510 -1550.8605 6136.7628
297.8302 32.6460 -60.1177 691315.3461 2.2743
2.2921
[1455]ENERGY: 280000 3179.5923 5205.0978 5457.7145
310.5933 -14129.6538 -1663.8158 0.0000 0.0000
7570.3249 5929.8532 296.0032 -1640.4717 5997.3552
298.0936 7.9115 3.6230 716556.8775 -0.5323
-0.5316
[1505]ENERGY: 290000 3208.9937 5289.9287 5511.5684
317.7886 -14124.0019 -1735.2212 0.0000 0.0000
7691.7707 6160.8269 300.7518 -1530.9438 6222.5134
298.2721 64.3110 8.4665 719242.1461 2.2057
2.2027
[1544]ENERGY: 300000 3127.8994 5159.0971 5512.5592
315.8513 -14080.5476 -1672.0867 0.0000 0.0000
7750.3024 6113.0751 303.0404 -1637.2274 6180.3636
300.3918 67.8732 57.6109 663216.7458 -4.3288
-4.3437
[1594]ENERGY: 310000 3166.6262 5159.0435 5540.7864
326.1186 -14004.9791 -1705.4744 0.0000 0.0000
7709.3739 6191.4950 301.4400 -1517.8788 6255.4469
298.7531 57.0864 -48.6293 652600.6578 4.4120
4.4101
[1630]ENERGY: 320000 3194.6519 5137.7509 5463.7066
320.6484 -14069.0620 -1691.0534 0.0000 0.0000
7529.9565 5886.5990 294.4247 -1643.3575 5947.1280
297.0757 31.6550 -61.2296 657862.8409 -0.2126
-0.2063
[1680]ENERGY: 330000 3151.7368 5158.0687 5473.0229
321.0034 -14084.2482 -1641.5155 0.0000 0.0000
7568.9167 5946.9847 295.9481 -1621.9320 6012.2274
297.0223 80.9591 -7.4396 662343.6741 3.8245
3.8056
[1716]ENERGY: 340000 3220.1281 5317.5182 5496.4321
304.5560 -14133.4557 -1715.8649 0.0000 0.0000
7526.3629 6015.6767 294.2842 -1510.6862 6075.3067
298.0336 73.1866 -24.8080 642678.2909 0.4703
0.4821
[1766]ENERGY: 350000 3266.3500 5296.4363 5468.4957
330.9260 -14161.7808 -1772.9119 0.0000 0.0000
7673.4188 6100.9341 300.0342 -1572.4847 6164.6111
298.4271 -83.1604 -143.5563 666252.9139 0.7581
0.7676
[1802]ENERGY: 360000 3146.7320 5170.9279 5409.7478
324.0193 -14099.7295 -1601.3462 0.0000 0.0000
7569.4728 5919.8241 295.9699 -1649.6487 5978.3047
298.2576 115.7125 90.9189 657272.3717 1.4032
1.3960
[1852]ENERGY: 370000 3165.3502 5271.5517 5479.7902
301.1891 -14112.9218 -1649.2654 0.0000 0.0000

7643.8164 6099.5105 298.8767 -1544.3059 6162.6860
298.4695 55.7563 27.5310 682108.5685 -1.1780
-1.1651
[1888]ENERGY: 380000 3100.8534 5216.8210 5494.3445
342.3288 -14112.6829 -1718.7477 0.0000 0.0000
7672.6555 5995.5726 300.0043 -1677.0829 6059.3475
298.9415 208.2194 121.3972 678227.1882 -3.8919
-3.9079
[1938]ENERGY: 390000 3132.0725 5122.0896 5507.6832
306.3184 -14093.7759 -1670.0501 0.0000 0.0000
7501.9028 5806.2405 293.3278 -1695.6623 5862.7003
295.0688 81.9529 -0.4604 682021.0348 -1.8560
-1.8823
[1977]ENERGY: 400000 3188.2410 5179.2022 5482.7744
310.0823 -14045.9924 -1770.0990 0.0000 0.0000
7755.2696 6099.4781 303.2346 -1655.7915 6165.0416
297.1081 -138.5724 -29.8745 667169.3578 1.8998
1.9149
[2027]ENERGY: 410000 3227.9930 5212.5200 5533.9786
335.9363 -14138.9613 -1726.0647 0.0000 0.0000
7504.5778 5949.9797 293.4324 -1554.5981 6011.4630
297.1169 41.7281 1.3475 672218.6170 2.8310
2.8404
[2063]ENERGY: 420000 3165.4822 5192.0029 5535.6506
306.7445 -14130.9667 -1645.2822 0.0000 0.0000
7567.8759 5991.5072 295.9074 -1576.3687 6056.5162
299.0656 -99.8361 -96.0929 683823.1495 0.3041
0.3017
[2113]ENERGY: 430000 3215.9323 5196.7794 5482.6209
322.7668 -14259.1607 -1572.0358 0.0000 0.0000
7564.6061 5951.5090 295.7796 -1613.0971 6015.1601
296.9798 91.9531 115.3779 649135.0679 0.4941
0.4542
[2149]ENERGY: 440000 3176.6525 5313.8393 5514.0539
326.1144 -14214.8401 -1654.8835 0.0000 0.0000
7642.7309 6103.6674 298.8343 -1539.0636 6168.4071
298.7760 -95.5689 -48.2877 641225.6748 -1.1752
-1.1321
[2199]ENERGY: 450000 3202.7286 5122.8624 5478.0199
335.8013 -14136.0024 -1691.9034 0.0000 0.0000
7586.3812 5897.8876 296.6310 -1688.4936 5962.8081
298.4343 24.6386 -41.5415 629154.2351 0.4058
0.3928
[2235]ENERGY: 460000 3194.1707 5247.7174 5476.8390
315.3845 -14034.1001 -1748.4748 0.0000 0.0000
7734.9805 6186.5172 302.4413 -1548.4632 6250.7580
298.1630 1.1290 -134.3915 649005.4468 0.6558
0.6136
[2285]ENERGY: 470000 3253.5079 5246.2612 5483.5793
328.7802 -14204.5052 -1661.4596 0.0000 0.0000
7661.1827 6107.3465 299.5558 -1553.8363 6170.6054
301.3081 6.0064 8.4037 632383.9201 3.1167
3.1301
[2321]ENERGY: 480000 3170.9905 5251.9664 5494.4038

310.8398 -14128.5598 -1639.3572 0.0000 0.0000
7623.7021 6083.9856 298.0902 -1539.7166 6141.9058
300.0841 110.6643 35.1574 618088.9235 1.0781
1.0696
[2371]ENERGY: 490000 3201.3230 5191.3811 5536.2684
322.2892 -14212.2729 -1685.0878 0.0000 0.0000
7566.6715 5920.5724 295.8603 -1646.0991 5981.9096
298.4466 -76.5195 -61.1883 603134.7622 -2.0537
-2.0402
[2410]ENERGY: 500000 3230.7809 5147.2431 5528.2240
296.9720 -14128.7693 -1651.6630 0.0000 0.0000
7579.4397 6002.2274 296.3596 -1577.2123 6061.2263
296.8549 152.1187 -15.2777 603948.5095 0.0796
0.0365
[2460]ENERGY: 510000 3159.3759 5275.8924 5521.0978
323.2543 -14160.0011 -1668.7438 0.0000 0.0000
7600.0692 6050.9448 297.1662 -1549.1244 6116.5791
297.7435 207.1361 171.6650 591018.1983 1.5024
1.5328
[2496]ENERGY: 520000 3150.3025 5238.4652 5545.4200
312.8538 -14146.0530 -1717.7446 0.0000 0.0000
7619.0204 6002.2643 297.9072 -1616.7561 6066.6875
297.7474 -21.0754 -117.5450 595239.4883 4.1867
4.1825
[2546]ENERGY: 530000 3110.0141 5225.7586 5478.9499
281.8321 -14190.8002 -1636.1457 0.0000 0.0000
7659.5464 5929.1553 299.4918 -1730.3912 5994.4300
297.6570 221.6747 125.7922 611440.6140 -7.4699
-7.4803
[2582]ENERGY: 540000 3246.5003 5254.4550 5523.0099
316.1619 -14175.3017 -1711.9310 0.0000 0.0000
7678.5853 6131.4797 300.2362 -1547.1056 6195.7335
298.5582 -37.8575 -83.5111 592836.3056 2.4713
2.4741
[2632]ENERGY: 550000 3196.7366 5241.7333 5504.6677
323.7017 -14083.2223 -1675.8282 0.0000 0.0000
7543.7395 6051.5282 294.9637 -1492.2113 6112.3310
299.7362 139.8902 56.7691 597548.4805 1.2361
1.2492
[2668]ENERGY: 560000 3160.9721 5269.1701 5558.4655
327.2357 -14175.3073 -1712.2117 0.0000 0.0000
7545.7628 5974.0872 295.0428 -1571.6757 6034.8187
297.8485 25.2916 -14.5210 612584.9942 3.3438
3.3818
[2718]ENERGY: 570000 3178.4423 5200.5543 5548.2792
307.0462 -14143.1066 -1671.5394 0.0000 0.0000
7607.6741 6027.3500 297.4635 -1580.3241 6091.5436
298.1492 21.2374 -17.6019 607933.7578 -1.8291
-1.8532
[2754]ENERGY: 580000 3211.3373 5257.4790 5507.6691
298.8225 -14090.8230 -1685.8960 0.0000 0.0000
7601.3608 6099.9497 297.2167 -1501.4111 6160.9906
299.2976 139.2734 116.8220 646665.9374 -0.6408
-0.6449

[2804]ENERGY: 590000   3180.2494   5246.4579   5493.1731
312.8397   -14101.7262   -1742.9409   0.0000   0.0000
7605.0991   5993.1522   297.3629   -1611.9470   6052.6279
299.9419   -53.3330   -140.1889   625943.8043   2.5141
 2.4936
[2843]ENERGY: 600000   3139.6611   5250.0079   5458.2493
310.4929   -14143.3432   -1703.8135   0.0000   0.0000
7557.0342   5868.2888   295.4835   -1688.7454   5930.9823
299.1656   -26.0180   -8.1851   644026.0798   3.7310
 3.7591
[2893]ENERGY: 610000   3217.2688   5114.3993   5507.2074
332.1408   -14096.7760   -1801.5053   0.0000   0.0000
7713.4490   5986.1841   301.5994   -1727.2649   6048.4138
297.5501   53.0706   -79.4223   647182.2944   -1.8298
-1.8423
[2929]ENERGY: 620000   3210.6548   5294.9019   5513.3782
341.4890   -14086.1075   -1776.4620   0.0000   0.0000
7588.5840   6086.4383   296.7171   -1502.1457   6149.8715
298.1988   -143.1521   -2.4047   656516.4358   -0.3907
-0.3793
[2979]ENERGY: 630000   3254.9755   5226.3642   5523.9240
326.5812   -14155.1582   -1663.3466   0.0000   0.0000
7643.3796   6156.7196   298.8596   -1486.6600   6218.6955
298.7348   30.2154   -3.5412   663513.7250   1.0703
 1.0922
[3015]ENERGY: 640000   3162.6554   5177.5220   5517.3976
322.9595   -14141.7063   -1664.9902   0.0000   0.0000
7647.7969   6021.6350   299.0324   -1626.1620   6083.5519
298.8410   32.2018   92.7894   652712.0362   -1.3109
-1.2985
[3065]ENERGY: 650000   3141.5643   5152.4374   5525.7948
319.9497   -14097.4902   -1729.8477   0.0000   0.0000
7644.8776   5957.2858   298.9182   -1687.5918   6018.1044
298.9213   119.1363   -15.5433   657779.4320   0.6602
 0.6312
[3101]ENERGY: 660000   3178.5661   5213.6609   5431.1324
311.7142   -14183.1129   -1612.7737   0.0000   0.0000
7606.9900   5946.1769   297.4368   -1660.8131   6007.1291
297.2168   7.2777   30.1774   639252.1170   -2.0630
-2.0638
[3151]ENERGY: 670000   3237.1552   5247.5467   5482.6286
311.6235   -14162.4850   -1684.0767   0.0000   0.0000
7511.5750   5943.9674   293.7060   -1567.6076   6004.2360
298.0805   -38.4109   -16.8664   617790.6021   -2.9196
-2.9114
[3187]ENERGY: 680000   3150.5209   5225.0376   5473.8021
320.0579   -14092.9812   -1759.0590   0.0000   0.0000
7538.5617   5855.9401   294.7612   -1682.6216   5918.5995
297.4763   -36.3602   -68.2010   583842.9402   5.2118
 5.2143
[3237]ENERGY: 690000   3159.4901   5272.9820   5513.8699
313.0658   -14213.5114   -1670.3568   0.0000   0.0000
7669.3224   6044.8620   299.8740   -1624.4604   6107.5991
298.5108   159.5797   104.8781   589601.5533   0.6852

0.6544
[3276]ENERGY: 700000   3179.6756   5241.2386   5518.7736
322.3114   -14204.2760   -1684.6371   0.0000   0.0000
7580.8332   5953.9193   296.4140   -1626.9139   6017.0115
297.8344   -54.5929   -66.0844   572892.7831   0.3040
0.3476
[3326]ENERGY: 710000   3187.4944   5181.7025   5507.4651
336.3667   -14156.8944   -1700.0440   0.0000   0.0000
7669.0675   6025.1578   299.8640   -1643.9097   6088.5649
298.1604   -98.6580   -130.1614   565673.4489   1.1296
1.1028
[3362]ENERGY: 720000   3175.7428   5222.1522   5421.5118
309.1209   -14063.6557   -1749.1710   0.0000   0.0000
7732.4581   6048.1590   302.3426   -1684.2990   6111.8369
299.6518   32.4442   -67.6525   568525.6612   0.0355
0.0444
[3412]ENERGY: 730000   3178.0021   5150.5679   5462.3951
324.8491   -14243.3236   -1691.1954   0.0000   0.0000
7625.9542   5807.2494   298.1783   -1818.7048   5872.4242
298.2450   -12.4343   -14.8937   556473.5261   0.4922
0.4808
[3448]ENERGY: 740000   3211.6268   5154.3371   5447.9762
313.5540   -14149.2952   -1661.2088   0.0000   0.0000
7475.0406   5792.0306   292.2775   -1683.0100   5851.7211
298.7068   71.4434   71.1251   538945.2063   0.7503
0.7495
[3498]ENERGY: 750000   3157.5150   5293.6925   5521.2970
312.2221   -14157.7117   -1740.4561   0.0000   0.0000
7607.3507   5993.9095   297.4509   -1613.4412   6053.8539
296.4732   -153.3649   -110.4382   511540.4688   3.8760
3.8782
[266]ENERGY: 760000   3197.8384   5216.7852   5542.7027   334.9515
-14173.9059   -1667.6730   0.0000   0.0000   7542.9030
5993.6020   294.9653   -1549.3010   6054.9761   298.7688
-113.6951   -227.3699   511540.4688   -2.2748   -2.2676
[305]ENERGY: 770000   3225.5945   5195.6890   5478.5354   330.7931
-14174.7753   -1661.4917   0.0000   0.0000   7601.0488
5995.3937   297.2391   -1605.6550   6056.6821   298.6155
-130.7157   -119.3894   511540.4688   0.1474   0.1563
[357]ENERGY: 780000   3230.5901   5114.4164   5496.4802   335.4343
-14169.1010   -1686.3511   0.0000   0.0000   7671.4698
5992.9387   299.9929   -1678.5311   6055.1181   298.7200
-99.3304   -53.5613   511540.4688   -1.3898   -1.3946
[395]ENERGY: 790000   3158.4628   5299.4381   5430.7599   316.8697
-14236.3479   -1688.3892   0.0000   0.0000   7712.5594
5993.3528   301.5998   -1719.2067   6056.0208   298.8345
214.0871   73.7267   511540.4688   0.9678   0.9461
[449]ENERGY: 800000   3048.0276   5135.7370   5525.5240   338.8113
-14148.7100   -1621.5955   0.0000   0.0000   7713.1703
5990.9647   301.6236   -1722.2056   6056.4928   298.8690
361.2124   235.6887   511540.4688   1.3379   1.3543
[487]ENERGY: 810000   3148.3092   5218.0582   5505.9913   324.8584
-14153.0022   -1708.5775   0.0000   0.0000   7665.8278
6001.4652   299.7723   -1664.3626   6057.4837   298.7552

```
         24.8755        -84.1389    511540.4688         1.3439         1.3359
[538]ENERGY:  820000       3283.3956       5059.2874       5520.7991       331.9711
       -14060.5760     -1767.5295         0.0000         0.0000      7625.8451
        5993.1928       298.2088     -1632.6523      6056.4956       298.8599
        -57.0940        -26.9767    511540.4688        -1.7212        -1.7222
[575]ENERGY:  830000       3194.1267       5178.0068       5446.9890       325.9645
       -14077.2006     -1710.8084         0.0000         0.0000      7638.4752
        5995.5533       298.7027     -1642.9219      6055.5498       298.7464
       -250.4178       -172.6658    511540.4688         0.1642         0.2005
[626]ENERGY:  840000       3119.3366       5326.9482       5490.4198       309.4861
       -14160.7073     -1712.8549         0.0000         0.0000      7620.6463
        5993.2749       298.0055     -1627.3715      6055.8796       298.9456
       -144.0461       -148.0715    511540.4688        -1.7187        -1.7574
[663]ENERGY:  850000       3175.4798       5178.7180       5423.4485       322.0752
       -14038.8326     -1718.7560         0.0000         0.0000      7651.7926
        5993.9256       299.2235     -1657.8670      6055.8081       298.9841
       -129.1660        -11.0812    511540.4688         2.7433         2.7842
[714]ENERGY:  860000       3217.1063       5176.7442       5492.1934       303.7678
       -14175.5415     -1663.6608         0.0000         0.0000      7643.2842
        5993.8936       298.8907     -1649.3906      6056.0356       299.2231
        -26.8345         10.6633    511540.4688        -0.7814        -0.7886
[750]ENERGY:  870000       3235.3609       5253.6397       5428.2635       325.6029
       -14149.5964     -1698.9618         0.0000         0.0000      7600.6878
        5994.9966       297.2250     -1605.6912      6056.1866       299.1644
       -241.3918       -251.2092    511540.4688        -1.1243        -1.1316
[800]ENERGY:  880000       3256.0524       5189.9114       5475.0814       330.4329
       -14120.7140     -1724.3149         0.0000         0.0000      7589.6983
        5996.1474       296.7953     -1593.5509      6056.9617       299.3418
        -17.1987        -89.1222    511540.4688         1.5015         1.4838
[836]ENERGY:  890000       3189.3874       5249.9633       5477.2788       301.3267
       -14100.5516     -1699.0427         0.0000         0.0000      7580.2697
        5998.6316       296.4266     -1581.6380      6055.9997       299.2596
        138.5789        145.4097    511540.4688        -2.7253        -2.7090
[889]ENERGY:  900000       3199.2526       5200.7205       5527.2691       316.1408
       -14226.5085     -1681.8363         0.0000         0.0000      7658.9712
        5994.0093       299.5042     -1664.9619      6056.3762       299.3049
       -116.0650        -85.0733    511540.4688         3.7667         3.7854
[925]ENERGY:  910000       3170.3267       5178.9258       5493.9404       327.8482
       -14125.0815     -1646.6921         0.0000         0.0000      7597.1735
        5996.4410       297.0876     -1600.7326      6056.8510       299.1060
        190.3628         21.9411    511540.4688         0.5845         0.5422
[975]ENERGY:  920000       3190.5878       5199.5378       5491.9828       295.5269
       -14179.2840     -1660.5753         0.0000         0.0000      7658.1762
        5995.9523       299.4731     -1662.2239      6056.8455       299.1058
         -8.4160        -13.1341    511540.4688        -3.5077        -3.4875
[1011]ENERGY:  930000       3142.2966       5221.5418       5501.0759
291.4699          -14138.3749     -1702.1865         0.0000         0.0000
7678.9775            5994.8004       300.2865      -1684.1771      6057.9988
299.2440             -98.6732      -117.8735    511540.4688         2.8386
 2.8230
[1061]ENERGY:  940000       3206.5629       5176.7234       5491.8602
314.1993          -14230.1194     -1590.3773         0.0000         0.0000
7628.3430            5997.1922       298.3065      -1631.1508      6056.8235
299.0567             294.0727       192.5879    511540.4688         0.0522
```

```
 0.0443
[1097]ENERGY:  950000      3173.5836      5178.2456      5490.1169
335.9457         -14156.5473     -1683.3870         0.0000         0.0000
7654.5370           5992.4945       299.3308     -1662.0425      6055.5883
299.1291            119.0863       133.5968    511540.4688         1.1990
 1.2292
[1147]ENERGY:  960000      3114.6129      5120.6527      5513.5546
301.5498         -14033.2200     -1689.7148         0.0000         0.0000
7665.9756           5993.4109       299.7781     -1672.5647      6055.5359
299.0726             38.4540        66.4380    511540.4688         2.3172
 2.3209
[1183]ENERGY:  970000      3179.4548      5242.4013      5487.4061
319.2903         -14174.6919     -1741.7611         0.0000         0.0000
7683.0393           5995.1389       300.4454     -1687.9005      6056.0704
299.0150           -126.5395      -184.9954    511540.4688         1.0868
 1.0711
[1233]ENERGY:  980000      3199.8026      5136.1107      5461.2766
312.9185         -14141.0034     -1625.4811         0.0000         0.0000
7650.4234           5994.0474       299.1699     -1656.3760      6055.8676
299.0049            164.2714        85.6511    511540.4688        -0.5558
-0.5612
[1269]ENERGY:  990000      3220.6008      5239.7783      5446.7169
330.1018         -14233.7689     -1678.2108         0.0000         0.0000
7667.6532           5992.8713       299.8437     -1674.7819      6056.0383
299.4782             60.4684        80.3062    511540.4688        -1.8808
-1.8813
[1322]ENERGY: 1000000      3126.4032      5213.9271      5456.4955
317.0790         -14122.4047     -1669.0260         0.0000         0.0000
7671.4461           5993.9202       299.9920     -1677.5260      6056.8849
299.5114            -45.0164       -67.4676    511540.4688        -1.6660
-1.6738
[1358]ENERGY: 1010000      3189.9665      5186.6858      5487.8501
324.7463         -14185.3643     -1603.7844         0.0000         0.0000
7594.6316           5994.7315       296.9882     -1599.9001      6055.7495
299.6354             70.3534       -41.4593    511540.4688       -13.9550
-13.9482
[1408]ENERGY: 1020000      3177.0235      5153.4447      5481.6129
316.5996         -14102.6109     -1710.6425         0.0000         0.0000
7678.3184           5993.7456       300.2608     -1684.5727      6056.3583
300.0143           -190.7220      -226.2630    511540.4688       -22.1472
-22.1376
[1444]ENERGY: 1030000      3239.0269      5088.4119      5473.6677
331.9881         -14184.2589     -1691.6564         0.0000         0.0000
7735.5869           5992.7662       302.5002     -1742.8207      6056.3992
300.2631             66.0232       -52.1891    511540.4688       -24.3887
-24.3996
[1494]ENERGY: 1040000      3094.0823      5250.6395      5450.0159
318.1249         -14220.9764     -1612.6208         0.0000         0.0000
7712.1203           5991.3857       301.5826     -1720.7346      6056.7263
300.4083            169.5541       154.2758    511540.4688       -21.9800
-21.9517
[1530]ENERGY: 1050000      3234.7339      5283.3431      5388.1613
320.3056         -14242.0927     -1639.3300         0.0000         0.0000
7647.6195           5992.7408       299.0603     -1654.8787      6055.6975
```

300.3406 124.1266 90.2139 511540.4688 -24.5584
-24.5662
[1580]ENERGY: 1060000 3234.9128 5101.3625 5472.9714
311.7782 -14165.2726 -1709.7531 0.0000 0.0000
7744.4349 5990.4341 302.8462 -1754.0008 6056.4676
300.5224 90.1953 80.3949 511540.4688 -17.1418
-17.1318
[1616]ENERGY: 1070000 3242.9074 5250.1353 5472.8057
331.8446 -14192.4092 -1709.6361 0.0000 0.0000
7599.4782 5995.1260 297.1777 -1604.3522 6057.1955
300.6387 -84.9079 15.8433 511540.4688 -21.3446
-21.3614
[1666]ENERGY: 1080000 3208.8623 5281.2133 5429.7572
319.3899 -14237.0567 -1734.5979 0.0000 0.0000
7726.3465 5993.9147 302.1389 -1732.4319 6057.5518
300.8772 14.9907 43.3483 511540.4688 -16.1087
-16.1099
[1702]ENERGY: 1090000 3167.9812 5266.6048 5472.9827
307.6174 -14231.5105 -1677.9617 0.0000 0.0000
7690.8671 5996.5809 300.7515 -1694.2862 6057.7454
300.8915 -8.0459 -42.1898 511540.4688 -17.8384
-17.8369
[1755]ENERGY: 1100000 3155.9341 5245.0937 5414.0877
325.2440 -14166.3543 -1698.1197 0.0000 0.0000
7718.6967 5994.5822 301.8398 -1724.1145 6056.7682
300.9507 91.3333 133.0140 511540.4688 -17.1765
-17.1745
[1791]ENERGY: 1110000 3203.3522 5187.3741 5445.7953
330.5336 -14195.3518 -1678.5657 0.0000 0.0000
7702.6800 5995.8177 301.2134 -1706.8624 6057.1549
300.9881 -21.3634 -67.6729 511540.4688 -19.5560
-19.5506
[1841]ENERGY: 1120000 3146.6842 5301.3076 5467.1988
327.8612 -14246.5520 -1693.6184 0.0000 0.0000
7691.3611 5994.2424 300.7708 -1697.1186 6057.2959
300.9209 184.5880 43.4034 511540.4688 -23.3504
-23.3784
[1877]ENERGY: 1130000 3178.4929 5235.2227 5478.4612
325.0295 -14347.6254 -1668.2820 0.0000 0.0000
7790.4033 5991.7022 304.6438 -1798.7010 6056.4861
300.8712 23.4275 65.0725 511540.4688 -17.9434
-17.9215
[1927]ENERGY: 1140000 3193.2684 5253.8529 5492.7235
322.7969 -14245.7753 -1689.6436 0.0000 0.0000
7667.7636 5994.9864 299.8480 -1672.7771 6056.9106
300.9092 219.0596 108.2001 511540.4688 -21.7608
-21.7703
[1963]ENERGY: 1150000 3227.4783 5214.1432 5482.1403
302.8554 -14086.5917 -1798.6533 0.0000 0.0000
7653.1306 5994.5028 299.2758 -1658.6278 6056.7648
300.9477 -168.0160 -227.4965 511540.4688 -17.8468
-17.8499
[2013]ENERGY: 1160000 3228.3403 5256.7215 5424.6529
317.2166 -14130.8753 -1735.6225 0.0000 0.0000

7633.8634 5994.2969 298.5223 -1639.5665 6056.4079
301.0767 32.7468 -45.3241 511540.4688 -20.6259
-20.6119
[2049]ENERGY: 1170000 3212.1243 5215.3513 5410.3641
313.0006 -14158.8215 -1720.7485 0.0000 0.0000
7724.0332 5995.3036 302.0484 -1728.7297 6058.5658
301.0302 -117.2274 -169.2773 511540.4688 -22.1560
-22.1482
[2099]ENERGY: 1180000 3180.0582 5237.4276 5476.7069
336.9315 -14225.2846 -1727.8025 0.0000 0.0000
7718.7155 5996.7526 301.8405 -1721.9629 6059.0256
301.0011 40.3021 32.9924 511540.4688 -15.4808
-15.4912
[2135]ENERGY: 1190000 3161.6422 5207.8344 5470.0237
339.9636 -14167.6821 -1752.8935 0.0000 0.0000
7733.8552 5992.7434 302.4325 -1741.1118 6057.3041
301.1656 126.0927 116.9674 511540.4688 -18.6136
-18.6061
[2188]ENERGY: 1200000 3171.1238 5226.0128 5425.1215
316.7777 -14175.0702 -1734.7085 0.0000 0.0000
7765.5842 5994.8414 303.6733 -1770.7428 6058.0598
301.2003 -58.2011 2.9238 511540.4688 -13.3629
-13.3549
[2224]ENERGY: 1210000 3229.9569 5208.0383 5466.4541
304.8867 -14204.9286 -1704.3929 0.0000 0.0000
7697.9092 5997.9236 301.0269 -1699.9856 6057.7422
301.0936 -48.4060 -112.6910 511540.4688 -20.6860
-20.6978
[2274]ENERGY: 1220000 3210.5375 5327.0689 5451.3401
313.2199 -14221.8024 -1754.2257 0.0000 0.0000
7672.2636 5998.4019 300.0240 -1673.8617 6059.2551
301.4352 169.5717 63.6241 511540.4688 -21.0970
-21.0868
[2310]ENERGY: 1230000 3123.1477 5283.7729 5442.6387
316.6112 -14260.7071 -1644.9938 0.0000 0.0000
7735.6113 5996.0810 302.5012 -1739.5304 6059.2486
301.0796 60.2581 93.3988 511540.4688 -20.7919
-20.7990
[2360]ENERGY: 1240000 3182.4578 5181.5907 5441.6622
334.0246 -14153.8347 -1768.6170 0.0000 0.0000
7776.2424 5993.5260 304.0901 -1782.7165 6058.8163
301.0385 -139.8188 -156.4869 511540.4688 -15.1005
-15.1338
[2396]ENERGY: 1250000 3241.3007 5285.5492 5401.4571
334.9859 -14183.9989 -1733.5134 0.0000 0.0000
7650.7086 5996.4892 299.1811 -1654.2195 6059.1540
300.8533 -191.9290 -61.7403 511540.4688 -22.3564
-22.3346
[2446]ENERGY: 1260000 3230.0261 5308.2249 5406.5372
323.5975 -14119.5805 -1761.5400 0.0000 0.0000
7611.4068 5998.6720 297.6442 -1612.7348 6059.5390
300.9707 -88.6444 -193.3539 511540.4688 -20.5162
-20.5072
[2482]ENERGY: 1270000 3222.8359 5188.5512 5429.6254

342.0445 -14145.9046 -1725.7677 0.0000 0.0000
7685.8618 5997.2466 300.5557 -1688.6152 6059.1807
301.1162 -118.7129 -119.5480 511540.4688 -18.6080
-18.6182
[2532]ENERGY: 1280000 3165.2620 5291.8903 5419.6654
311.6633 -14160.6473 -1696.9966 0.0000 0.0000
7666.5204 5997.3574 299.7994 -1669.1630 6059.1447
301.2947 49.1566 -60.9685 511540.4688 -19.3938
-19.3966
[2568]ENERGY: 1290000 3168.0594 5268.8406 5475.4270
305.7762 -14252.4435 -1708.2124 0.0000 0.0000
7739.0740 5996.5213 302.6366 -1742.5527 6059.7027
301.5177 179.5985 122.9271 511540.4688 -19.8975
-19.8937
[2621]ENERGY: 1300000 3145.8645 5290.1714 5450.8904
340.5416 -14207.8486 -1759.4638 0.0000 0.0000
7738.3247 5998.4802 302.6073 -1739.8445 6059.7489
301.6030 -11.7838 39.7472 511540.4688 -18.6090
-18.5872
[2657]ENERGY: 1310000 3199.4147 5296.1935 5392.8237
285.6905 -14196.2015 -1727.8250 0.0000 0.0000
7746.5441 5996.6399 302.9287 -1749.9042 6059.7154
301.7849 33.7667 56.4955 511540.4688 -22.5925
-22.6044
[2707]ENERGY: 1320000 3187.0527 5188.7000 5463.5639
317.1958 -14148.1673 -1766.8796 0.0000 0.0000
7756.9030 5998.3684 303.3338 -1758.5346 6060.9116
301.6389 25.9170 -118.8963 511540.4688 -18.3987
-18.4216
[2743]ENERGY: 1330000 3145.8990 5198.9092 5454.9976
326.3551 -14122.1382 -1691.9829 0.0000 0.0000
7690.3969 6002.4366 300.7331 -1687.9602 6060.9627
301.6087 55.1839 -41.2265 511540.4688 -19.6823
-19.6601
[2793]ENERGY: 1340000 3248.7882 5172.7614 5467.4396
313.3783 -14238.6302 -1692.4065 0.0000 0.0000
7729.1993 6000.5302 302.2505 -1728.6691 6060.9231
301.5517 44.6521 -57.5257 511540.4688 -19.6325
-19.6601
[2829]ENERGY: 1350000 3133.0838 5160.4954 5488.0559
345.3815 -14160.2191 -1750.8875 0.0000 0.0000
7781.4673 5997.3774 304.2944 -1784.0899 6060.8158
301.7496 -38.0125 -142.9660 511540.4688 -18.0794
-18.0788
[2879]ENERGY: 1360000 3176.6978 5268.1444 5504.3910
333.5971 -14294.9076 -1743.0218 0.0000 0.0000
7754.2261 5999.1270 303.2291 -1755.0991 6061.3779
301.5195 -227.7315 -186.2794 511540.4688 -19.1566
-19.1457
[2915]ENERGY: 1370000 3214.2892 5259.6977 5533.4330
327.1848 -14230.8175 -1749.5258 0.0000 0.0000
7646.5401 6000.8014 299.0181 -1645.7387 6061.6933
301.8465 -82.6403 -22.8277 511540.4688 -21.7612
-21.7616

[2965]ENERGY: 1380000 3169.1999 5179.6933 5453.4137
328.9201 -14161.0144 -1689.8629 0.0000 0.0000
7720.0287 6000.3784 301.8918 -1719.6503 6062.0924
301.9045 342.0122 140.8653 511540.4688 -17.9161
-17.8906
[3001]ENERGY: 1390000 3186.9244 5258.4443 5489.1194
328.3660 -14193.7729 -1765.7960 0.0000 0.0000
7698.2615 6001.5467 301.0406 -1696.7147 6062.3614
302.0048 -49.3211 -87.6556 511540.4688 -15.7055
-15.7141
[3054]ENERGY: 1400000 3178.0767 5172.9347 5478.1562
312.3265 -14186.7216 -1739.9153 0.0000 0.0000
7782.7652 5997.6222 304.3452 -1785.1429 6061.6637
301.8370 -48.7386 -71.8452 511540.4688 -18.6184
-18.6141
[3090]ENERGY: 1410000 3218.8771 5271.5913 5475.4721
331.5664 -14256.6581 -1731.8973 0.0000 0.0000
7689.5221 5998.4735 300.6989 -1691.0485 6062.8265
301.7862 184.9249 186.2453 511540.4688 -19.4179
-19.4232
[3140]ENERGY: 1420000 3169.7066 5245.7656 5492.5428
317.9903 -14235.1984 -1745.7611 0.0000 0.0000
7753.4692 5998.5150 303.1995 -1754.9542 6062.2923
302.0134 114.3326 94.3088 511540.4688 -19.0676
-19.0625
[3176]ENERGY: 1430000 3286.1234 5217.5812 5447.2554
298.6632 -14187.6499 -1716.9327 0.0000 0.0000
7658.8544 6003.8951 299.4996 -1654.9594 6063.4962
302.3807 -144.2542 -103.9839 511540.4688 -15.2819
-15.2752
[3226]ENERGY: 1440000 3204.9738 5283.4691 5418.8970
311.4633 -14282.0582 -1723.7535 0.0000 0.0000
7783.5419 5996.5336 304.3755 -1787.0084 6062.0497
302.2054 -111.2351 -216.3763 511540.4688 -15.2343
-15.2687
[3262]ENERGY: 1450000 3192.9843 5235.5162 5479.0917
339.3556 -14309.9481 -1709.6388 0.0000 0.0000
7769.4190 5996.7800 303.8233 -1772.6391 6062.8577
302.2408 73.9539 38.1596 511540.4688 -17.8097
-17.7797
[3312]ENERGY: 1460000 3184.9824 5278.9695 5462.0073
298.0215 -14259.3336 -1713.6453 0.0000 0.0000
7748.2319 5999.2338 302.9947 -1748.9982 6062.8704
302.3137 74.2403 -0.3250 511540.4688 -15.4270
-15.4389
[3348]ENERGY: 1470000 3158.7964 5179.3714 5394.5606
341.2657 -14175.0564 -1652.0236 0.0000 0.0000
7753.6582 6000.5723 303.2069 -1753.0859 6063.7231
302.3583 110.0120 102.8663 511540.4688 -13.4737
-13.4723
[3398]ENERGY: 1480000 3158.9216 5256.6541 5460.9298
335.4608 -14237.7075 -1791.3425 0.0000 0.0000
7816.8606 5999.7770 305.6785 -1817.0836 6064.5013
302.3581 230.6349 189.9663 511540.4688 -16.0026

-16.0083
[3434]ENERGY: 1490000 3192.6154 5189.6918 5504.1858
343.9495 -14182.7512 -1757.9196 0.0000 0.0000
7713.5083 6003.2800 301.6369 -1710.2283 6064.7937
302.3879 175.4558 103.1537 511540.4688 -11.5513
-11.5460
[3487]ENERGY: 1500000 3170.9388 5283.9234 5535.1435
328.0882 -14364.5466 -1649.3549 0.0000 0.0000
7697.4746 6001.6671 301.0099 -1695.8075 6063.8382
302.3630 186.1410 129.5556 511540.4688 -19.1438
-19.1531
[3523]ENERGY: 1510000 3181.2024 5234.1482 5441.6958
327.7818 -14125.8972 -1748.6341 0.0000 0.0000
7695.0992 6005.3962 300.9170 -1689.7030 6064.4884
302.4755 -190.0188 -238.5069 511540.4688 -14.9895
-14.9875
[3573]ENERGY: 1520000 3167.8562 5234.9748 5455.5481
316.4882 -14153.9242 -1778.4284 0.0000 0.0000
7759.5409 6002.0556 303.4370 -1757.4853 6065.3530
302.3808 55.4619 -10.8067 511540.4688 -20.0963
-20.0904
[3609]ENERGY: 1530000 3157.1267 5264.3942 5498.9472
329.6770 -14224.7162 -1730.6641 0.0000 0.0000
7707.4202 6002.1851 301.3988 -1705.2351 6064.5204
302.4179 -89.8582 -72.6176 511540.4688 -15.0560
-15.0456
[3659]ENERGY: 1540000 3264.9597 5220.5762 5386.4567
289.2069 -14281.7904 -1690.3124 0.0000 0.0000
7810.9426 6000.0393 305.4470 -1810.9033 6064.4385
302.6226 -100.4378 -131.3864 511540.4688 -18.0321
-18.0399
[3695]ENERGY: 1550000 3176.6767 5408.7912 5399.8044
350.3595 -14310.1622 -1751.0437 0.0000 0.0000
7727.1085 6001.5345 302.1687 -1725.5740 6065.1296
302.6854 85.4154 77.7487 511540.4688 -15.3991
-15.3991
[3745]ENERGY: 1560000 3171.2097 5187.9371 5442.7199
317.6722 -14212.2879 -1675.2519 0.0000 0.0000
7767.4573 5999.4564 303.7465 -1768.0009 6064.0976
302.6852 -103.3125 -89.4814 511540.4688 -15.2626
-15.2574
[3781]ENERGY: 1570000 3229.4720 5179.9322 5448.4045
329.0770 -14284.7687 -1660.1519 0.0000 0.0000
7761.3224 6003.2876 303.5066 -1758.0348 6064.5451
302.7120 -52.3412 -89.3881 511540.4688 -10.8233
-10.8527
[3831]ENERGY: 1580000 3249.3363 5304.5571 5409.6323
315.7612 -14281.8996 -1714.0834 0.0000 0.0000
7717.2052 6000.5090 301.7814 -1716.6962 6065.2285
302.6564 11.4409 -22.3457 511540.4688 -16.7998
-16.7868
[3867]ENERGY: 1590000 3198.6193 5232.8819 5448.7304
308.6357 -14183.5149 -1733.8942 0.0000 0.0000
7734.5479 6006.0061 302.4596 -1728.5418 6065.9237

302.7550 -37.8617 -80.5851 511540.4688 -15.4411
-15.4264
[3920]ENERGY: 1600000 3232.6908 5306.1044 5440.7020
329.6147 -14328.7870 -1715.4706 0.0000 0.0000
7734.4612 5999.3155 302.4562 -1735.1457 6065.7971
302.8107 -65.8157 -169.0732 511540.4688 -17.7822
-17.8172
[3956]ENERGY: 1610000 3211.5615 5262.4320 5437.2413
312.9369 -14290.0950 -1688.5646 0.0000 0.0000
7755.8547 6001.3667 303.2928 -1754.4880 6065.1433
302.7055 16.5010 47.6742 511540.4688 -18.1920
-18.1659
[4006]ENERGY: 1620000 3216.9845 5224.9938 5446.4733
329.9935 -14266.8412 -1714.2032 0.0000 0.0000
7763.7074 6001.1080 303.5999 -1762.5994 6066.6108
302.8131 -86.6507 -122.3680 511540.4688 -17.4766
-17.4690
[4042]ENERGY: 1630000 3198.6050 5319.1739 5470.8484
347.3414 -14341.2930 -1744.8587 0.0000 0.0000
7751.9733 6001.7903 303.1410 -1750.1830 6065.1224
302.7580 -349.4644 -242.6611 511540.4688 -19.9121
-19.9037
[4092]ENERGY: 1640000 3183.8503 5243.1686 5450.8566
317.2717 -14265.3570 -1686.5914 0.0000 0.0000
7761.1019 6004.3006 303.4980 -1756.8012 6065.3983
302.6182 -32.6688 -26.8587 511540.4688 -19.9750
-19.9730
[4128]ENERGY: 1650000 3143.1103 5313.0303 5491.8471
329.9997 -14353.8000 -1668.0802 0.0000 0.0000
7746.4422 6002.5494 302.9247 -1743.8928 6066.1737
302.5834 40.1110 180.3064 511540.4688 -20.0224
-20.0364
[4178]ENERGY: 1660000 3210.1352 5202.8231 5436.3366
318.9019 -14195.4342 -1741.9884 0.0000 0.0000
7772.8611 6003.6352 303.9579 -1769.2259 6066.2759
302.7262 111.0839 106.1174 511540.4688 -20.3044
-20.3093
[4214]ENERGY: 1670000 3154.1608 5149.1437 5498.4309
306.6975 -14183.2374 -1712.2768 0.0000 0.0000
7789.2790 6002.1978 304.5999 -1787.0813 6066.3733
302.6555 9.6220 -20.2765 511540.4688 -16.1217
-16.1076
[4264]ENERGY: 1680000 3222.8452 5330.7091 5425.5102
323.9160 -14304.9328 -1713.4221 0.0000 0.0000
7719.2990 6003.9247 301.8633 -1715.3744 6066.3079
302.5937 -192.0688 -119.1749 511540.4688 -20.6474
-20.6632
[4300]ENERGY: 1690000 3169.1607 5255.7458 5437.2820
314.7984 -14289.7066 -1711.1329 0.0000 0.0000
7828.0474 6004.1948 306.1159 -1823.8526 6066.5311
303.1410 90.6348 33.3767 511540.4688 -17.3828
-17.3748
[4353]ENERGY: 1700000 3221.8950 5305.0468 5494.9442
301.1932 -14298.6706 -1687.4857 0.0000 0.0000

7669.1792 6006.1020 299.9034 -1663.0772 6067.7341
303.0179 172.3648 27.7006 511540.4688 -16.7322
-16.7279
[4389]ENERGY: 1710000 3206.3515 5237.7934 5458.8998
317.8020 -14291.5748 -1678.7239 0.0000 0.0000
7752.7349 6003.2829 303.1708 -1749.4520 6068.0578
302.9540 124.7153 69.4049 511540.4688 -23.1473
-23.1624
[4439]ENERGY: 1720000 3249.9979 5246.8673 5477.2256
350.0167 -14279.8293 -1737.0487 0.0000 0.0000
7697.7136 6004.9430 301.0192 -1692.7706 6067.8314
302.8739 33.2499 -32.9574 511540.4688 -19.5329
-19.5257
[4475]ENERGY: 1730000 3241.9915 5267.8291 5435.4856
310.7640 -14310.1653 -1752.7853 0.0000 0.0000
7810.4858 6003.6055 305.4292 -1806.8804 6068.3456
302.7757 127.4045 125.3885 511540.4688 -19.2169
-19.2261
[4525]ENERGY: 1740000 3259.5556 5257.0136 5452.2198
322.3784 -14211.7206 -1784.1592 0.0000 0.0000
7714.3878 6009.6754 301.6713 -1704.7125 6068.6105
302.9594 -65.4003 -179.6029 511540.4688 -10.5387
-10.5387
[4561]ENERGY: 1750000 3228.5764 5314.5844 5467.2120
299.1813 -14290.8242 -1759.5856 0.0000 0.0000
7745.9063 6005.0507 302.9038 -1740.8556 6068.6449
303.0832 -200.0629 -83.6747 511540.4688 -17.8792
-17.8500
[4611]ENERGY: 1760000 3176.1560 5259.3865 5483.6223
310.2715 -14291.9612 -1695.6476 0.0000 0.0000
7760.8177 6002.6452 303.4869 -1758.1725 6068.8223
303.0379 -107.5377 42.5433 511540.4688 -17.2647
-17.2802
[4647]ENERGY: 1770000 3254.0398 5252.5002 5453.5280
309.9540 -14256.7058 -1773.2674 0.0000 0.0000
7766.4507 6006.4995 303.7072 -1759.9512 6068.3805
303.0574 -291.4431 -198.9290 511540.4688 -16.8898
-16.8904
[4697]ENERGY: 1780000 3245.0748 5359.4270 5450.2823
289.9055 -14286.6263 -1758.9003 0.0000 0.0000
7707.0619 6006.2248 301.3848 -1700.8371 6068.6963
303.0241 -113.3431 -200.9240 511540.4688 -12.7551
-12.7624
[4733]ENERGY: 1790000 3218.3055 5338.1001 5514.2522
334.3759 -14319.1316 -1744.5209 0.0000 0.0000
7665.2969 6006.6781 299.7516 -1658.6188 6068.9276
302.8060 -53.1075 -68.0302 511540.4688 -20.5275
-20.5152
[4786]ENERGY: 1800000 3213.6494 5338.6080 5461.1651
327.0389 -14234.9557 -1759.7581 0.0000 0.0000
7661.6002 6007.3476 299.6070 -1654.2526 6068.8383
303.0744 1.6174 -3.0749 511540.4688 -17.0409
-17.0496
[4822]ENERGY: 1810000 3249.6578 5339.5479 5387.0194

337.1313 -14250.5694 -1744.5808 0.0000 0.0000
7686.1040 6004.3102 300.5652 -1681.7938 6068.3094
303.1607 -21.5374 57.4155 511540.4688 -17.5042
-17.5013
[4872]ENERGY: 1820000 3168.6557 5301.8832 5481.9554
331.9036 -14345.7550 -1679.8112 0.0000 0.0000
7745.4572 6004.2889 302.8862 -1741.1683 6068.8596
303.1053 56.4113 89.6534 511540.4688 -14.6055
-14.5917
[4908]ENERGY: 1830000 3211.3651 5164.5291 5484.0301
341.3767 -14260.9911 -1650.3304 0.0000 0.0000
7714.4578 6004.4373 301.6740 -1710.0205 6068.2020
303.1735 219.6908 85.7238 511540.4688 -20.6779
-20.7165
[4958]ENERGY: 1840000 3245.8585 5292.3643 5445.0350
344.9019 -14378.2250 -1661.3551 0.0000 0.0000
7719.6908 6008.2705 301.8786 -1711.4204 6069.9861
303.2434 82.2201 -31.8623 511540.4688 -17.3619
-17.3495
[4994]ENERGY: 1850000 3285.2591 5288.4037 5463.9608
322.5195 -14275.1474 -1742.5777 0.0000 0.0000
7666.4176 6008.8355 299.7954 -1657.5821 6070.0415
303.0090 -69.8544 -77.2973 511540.4688 -17.9635
-17.9543
[5044]ENERGY: 1860000 3224.6215 5255.8176 5499.1442
337.4200 -14333.8757 -1726.3752 0.0000 0.0000
7749.6206 6006.3731 303.0490 -1743.2475 6070.1218
302.9108 -14.6097 58.6739 511540.4688 -14.2258
-14.2224
[5080]ENERGY: 1870000 3193.7511 5294.6475 5463.1155
311.3713 -14319.9662 -1688.3368 0.0000 0.0000
7751.9134 6006.4957 303.1387 -1745.4177 6070.0160
302.8664 132.9527 83.4919 511540.4688 -18.9712
-18.9791
[5130]ENERGY: 1880000 3240.9375 5288.4977 5430.1209
324.5127 -14227.4083 -1731.8563 0.0000 0.0000
7684.1088 6008.9131 300.4872 -1675.1957 6070.8576
302.9921 77.7810 13.0729 511540.4688 -10.0694
-10.0713
[5166]ENERGY: 1890000 3210.3341 5293.7150 5417.2865
321.8225 -14281.0672 -1689.3398 0.0000 0.0000
7731.5640 6004.3151 302.3429 -1727.2489 6069.4826
302.8921 105.6918 73.3600 511540.4688 -16.0135
-16.0176
[5219]ENERGY: 1900000 3284.7795 5208.7672 5484.4524
308.5822 -14338.7170 -1705.9745 0.0000 0.0000
7766.3247 6008.2144 303.7022 -1758.1102 6068.9927
303.1102 -104.9822 -68.6137 511540.4688 -13.0619
-13.0573
[5255]ENERGY: 1910000 3202.4366 5279.3209 5490.6256
321.1363 -14410.2659 -1683.6074 0.0000 0.0000
7805.4125 6005.0587 305.2308 -1800.3538 6069.2534
303.3282 -147.4000 -146.7370 511540.4688 -15.5006
-15.4807

[5305]ENERGY: 1920000 3315.8790 5230.2382 5492.2573
336.2112 -14369.7110 -1740.0687 0.0000 0.0000
7747.6863 6012.4923 302.9734 -1735.1940 6071.7482
303.3681 -185.4130 -251.3752 511540.4688 -12.4575
-12.4816
[5341]ENERGY: 1930000 3260.7336 5222.2660 5420.5983
335.1129 -14232.0195 -1713.4010 0.0000 0.0000
7716.2836 6009.5739 301.7454 -1706.7097 6072.0118
303.1554 -146.6659 -144.1662 511540.4688 -16.0743
-16.0740
[5391]ENERGY: 1940000 3247.3270 5221.3246 5478.7589
329.0850 -14306.1715 -1738.2482 0.0000 0.0000
7777.8402 6009.9160 304.1526 -1767.9242 6072.2768
303.1563 133.7355 -37.6060 511540.4688 -15.5798
-15.5994
[5427]ENERGY: 1950000 3106.4312 5215.2113 5464.7207
331.6043 -14183.3573 -1666.3357 0.0000 0.0000
7738.7718 6007.0463 302.6248 -1731.7255 6071.7343
302.8863 209.9866 198.0644 511540.4688 -13.9548
-13.9209
[5477]ENERGY: 1960000 3215.5226 5229.6851 5455.6235
326.1056 -14245.7423 -1753.3538 0.0000 0.0000
7780.2426 6008.0834 304.2465 -1772.1592 6071.1713
302.9259 11.7728 -58.1578 511540.4688 -16.6810
-16.6864
[5513]ENERGY: 1970000 3232.0589 5293.9322 5460.6402
324.0081 -14319.0699 -1669.2941 0.0000 0.0000
7684.7644 6007.0397 300.5128 -1677.7246 6071.6129
303.2760 39.0516 54.1757 511540.4688 -17.4318
-17.4145
[5563]ENERGY: 1980000 3264.6707 5266.4248 5449.2767
319.7704 -14365.8994 -1676.7145 0.0000 0.0000
7750.1085 6007.6372 303.0681 -1742.4713 6070.8655
303.4641 -98.2339 -97.0357 511540.4688 -16.0606
-16.0927
[5599]ENERGY: 1990000 3218.4221 5278.6344 5476.7859
330.0772 -14363.0008 -1685.9327 0.0000 0.0000
7757.9286 6012.9146 303.3739 -1745.0140 6071.7906
303.5268 233.3385 190.6009 511540.4688 -18.8924
-18.8688
[5652]ENERGY: 2000000 3241.1755 5329.9372 5472.7034
328.4418 -14373.4077 -1664.5417 0.0000 0.0000
7678.3258 6012.6343 300.2610 -1665.6915 6072.0233
303.4764 262.9744 101.2648 511540.4688 -12.3134
-12.3153
[5688]ENERGY: 2010000 3210.2510 5259.1467 5491.0470
316.6426 -14265.7670 -1742.8827 0.0000 0.0000
7740.8216 6009.2592 302.7049 -1731.5625 6071.3049
303.4567 -3.6657 22.8940 511540.4688 -17.5756
-17.5828
[5738]ENERGY: 2020000 3165.0202 5282.9834 5447.6381
314.8446 -14279.8026 -1719.6856 0.0000 0.0000
7795.2740 6006.2721 304.8343 -1789.0019 6070.7668
303.3204 60.9846 50.0399 511540.4688 -16.4765

-16.4666
[5774]ENERGY: 2030000 3173.0745 5336.1473 5473.3794
324.6064 -14379.5639 -1649.3247 0.0000 0.0000
7729.0133 6007.3323 302.2432 -1721.6810 6072.0840
303.2465 156.7363 193.8214 511540.4688 -12.6849
-12.7025
[5824]ENERGY: 2040000 3184.1455 5324.1452 5480.7748
324.2663 -14293.1772 -1719.0896 0.0000 0.0000
7707.8672 6008.9323 301.4163 -1698.9349 6070.7582
303.1090 140.3170 105.7038 511540.4688 -16.7494
-16.7309
[5860]ENERGY: 2050000 3222.9019 5384.6426 5417.0345
325.6396 -14402.0677 -1722.3654 0.0000 0.0000
7779.5117 6005.2972 304.2179 -1774.2144 6072.2644
303.2421 -47.0158 1.6245 511540.4688 -14.2274
-14.2253
[5910]ENERGY: 2060000 3218.5270 5277.6365 5459.7397
322.2139 -14291.6980 -1781.9559 0.0000 0.0000
7806.8225 6011.2857 305.2859 -1795.5368 6072.5639
303.2558 96.4734 -88.6367 511540.4688 -18.0303
-18.0840
[5946]ENERGY: 2070000 3225.7495 5304.4825 5462.7248
324.9278 -14332.6600 -1680.9264 0.0000 0.0000
7709.7459 6014.0441 301.4897 -1695.7017 6073.1697
303.3329 -121.3599 -69.2135 511540.4688 -13.4710
-13.4204
[5996]ENERGY: 2080000 3234.9944 5311.0449 5445.3878
326.7102 -14344.6485 -1642.2031 0.0000 0.0000
7678.3116 6009.5973 300.2605 -1668.7144 6072.7885
303.2851 -5.1187 17.6599 511540.4688 -17.3294
-17.3312
[6032]ENERGY: 2090000 3168.3621 5349.8776 5500.9203
328.3365 -14366.5810 -1662.2147 0.0000 0.0000
7690.2222 6008.9229 300.7263 -1681.2993 6072.8008
303.4116 60.7070 85.6050 511540.4688 -15.7231
-15.7147
[6085]ENERGY: 2100000 3175.6009 5275.6530 5443.1627
334.2701 -14226.1907 -1756.0156 0.0000 0.0000
7763.4560 6009.9364 303.5901 -1753.5196 6074.5862
303.4343 53.6834 -35.9852 511540.4688 -16.0709
-16.0928
[6121]ENERGY: 2110000 3264.1027 5370.7720 5452.2478
340.7548 -14468.3657 -1652.9113 0.0000 0.0000
7705.6345 6012.2348 301.3290 -1693.3997 6074.0261
303.5106 -122.4595 -89.4370 511540.4688 -17.3073
-17.2686
[6171]ENERGY: 2120000 3162.1433 5247.5971 5468.5557
338.8517 -14270.3214 -1695.8370 0.0000 0.0000
7759.4161 6010.4054 303.4321 -1749.0107 6073.9241
303.6189 110.5848 32.2261 511540.4688 -14.2102
-14.2393
[6207]ENERGY: 2130000 3192.2841 5300.4340 5418.2415
318.1054 -14343.8901 -1670.9689 0.0000 0.0000
7795.8392 6010.0452 304.8564 -1785.7940 6074.8685

303.5621 7.8815 5.9085 511540.4688 -14.7926
-14.7892
[6257]ENERGY: 2140000 3216.8486 5271.6217 5420.2168
314.2899 -14332.4831 -1634.0982 0.0000 0.0000
7755.8173 6012.2131 303.2914 -1743.6042 6074.5451
303.4103 33.8372 -6.6437 511540.4688 -21.9240
-21.9447
[6293]ENERGY: 2150000 3239.7992 5252.3824 5431.8692
339.0028 -14236.8226 -1749.4273 0.0000 0.0000
7736.6647 6013.4684 302.5424 -1723.1964 6076.3376
303.4296 -165.7940 -232.0076 511540.4688 -17.6122
-17.5954
[6343]ENERGY: 2160000 3173.1109 5296.8705 5482.5564
289.6801 -14264.8476 -1695.4333 0.0000 0.0000
7730.9179 6012.8549 302.3177 -1718.0630 6075.8791
303.4976 297.4487 101.8584 511540.4688 -16.8535
-16.8718
[6379]ENERGY: 2170000 3194.8262 5302.9368 5467.5451
318.0816 -14301.9836 -1741.3400 0.0000 0.0000
7772.0858 6012.1518 303.9275 -1759.9339 6076.2112
303.5429 -67.4222 -6.4634 511540.4688 -14.2794
-14.2524
[6429]ENERGY: 2180000 3174.2753 5252.5125 5426.0265
331.1167 -14380.7973 -1636.6096 0.0000 0.0000
7843.8038 6010.3279 306.7321 -1833.4759 6073.9507
303.5016 215.3228 88.5924 511540.4688 -18.0933
-18.1248
[6465]ENERGY: 2190000 3242.8592 5302.0252 5477.7289
346.6983 -14353.0845 -1726.2863 0.0000 0.0000
7724.0492 6013.9900 302.0491 -1710.0592 6075.2978
303.3872 -193.0257 -268.7829 511540.4688 -14.5810
-14.5735
[6518]ENERGY: 2200000 3289.7890 5346.1660 5472.3830
346.8262 -14368.8705 -1739.7077 0.0000 0.0000
7668.3487 6014.9346 299.8709 -1653.4141 6075.6330
303.3373 -180.9904 -127.9047 511540.4688 -17.4776
-17.4491
[6554]ENERGY: 2210000 3275.9198 5284.7428 5479.8596
330.4375 -14334.0932 -1807.6396 0.0000 0.0000
7782.0770 6011.3038 304.3182 -1770.7732 6075.9365
303.3043 -203.3319 -272.7280 511540.4688 -14.9278
-14.9319
[6604]ENERGY: 2220000 3223.3749 5286.4768 5428.8880
309.8924 -14319.9786 -1705.4955 0.0000 0.0000
7790.9867 6014.1446 304.6667 -1776.8420 6076.7813
303.6664 5.9672 -70.7838 511540.4688 -19.9740
-20.0001
[6640]ENERGY: 2230000 3228.4261 5209.0998 5428.1390
326.3050 -14323.7186 -1637.5767 0.0000 0.0000
7781.5928 6012.2674 304.2993 -1769.3254 6076.0601
303.6505 -43.1171 -17.2531 511540.4688 -13.8154
-13.7958
[6690]ENERGY: 2240000 3162.1898 5266.8452 5499.4822
348.3659 -14266.0376 -1712.0739 0.0000 0.0000

7717.8534 6016.6249 301.8068 -1701.2284 6077.4206
303.7559 210.0327 76.0211 511540.4688 -12.9611
-12.9612
[6726]ENERGY: 2250000 3220.3937 5194.5040 5467.5288
334.6198 -14293.3941 -1652.3793 0.0000 0.0000
7744.9173 6016.1903 302.8651 -1728.7270 6075.2390
303.6102 -163.5937 -114.7512 511540.4688 -15.8788
-15.8526
[6776]ENERGY: 2260000 3180.5106 5310.0637 5424.9992
332.6449 -14356.4215 -1700.6534 0.0000 0.0000
7821.7006 6012.8440 305.8677 -1808.8565 6075.6492
303.6272 9.5219 -111.8324 511540.4688 -14.1379
-14.1552
[6812]ENERGY: 2270000 3160.7628 5320.5222 5486.0352
330.0354 -14370.5967 -1716.2326 0.0000 0.0000
7798.5820 6009.1083 304.9637 -1789.4737 6074.8536
303.7803 -69.2448 -112.8528 511540.4688 -14.2659
-14.2671
[6862]ENERGY: 2280000 3240.2407 5266.2532 5423.6536
321.3810 -14294.7314 -1733.7693 0.0000 0.0000
7788.6666 6011.6944 304.5759 -1776.9722 6075.3633
303.5915 -6.2131 -23.9474 511540.4688 -12.6344
-12.6418
[6898]ENERGY: 2290000 3225.2097 5302.4201 5449.3099
336.3389 -14269.3875 -1762.8677 0.0000 0.0000
7734.2731 6015.2965 302.4489 -1718.9766 6076.6168
303.4936 -83.4444 -76.6833 511540.4688 -14.3350
-14.3225
[6951]ENERGY: 2300000 3211.6977 5297.4193 5463.3117
312.2385 -14322.6039 -1687.3615 0.0000 0.0000
7735.0238 6009.7257 302.4782 -1725.2981 6074.7086
303.4137 -21.9157 -62.8860 511540.4688 -11.7353
-11.7403
[6987]ENERGY: 2310000 3169.2523 5345.4424 5435.6009
310.4689 -14313.9664 -1736.3476 0.0000 0.0000
7800.4504 6010.9008 305.0367 -1789.5496 6075.0676
303.5924 -155.2978 -129.7903 511540.4688 -18.3820
-18.3827
[7037]ENERGY: 2320000 3201.6619 5276.4906 5495.8144
331.0900 -14314.4641 -1685.1578 0.0000 0.0000
7712.3834 6017.8186 301.5929 -1694.5648 6076.5464
303.6184 192.5353 115.6993 511540.4688 -14.5262
-14.5587
[7073]ENERGY: 2330000 3240.0201 5248.6843 5427.1119
323.4702 -14295.1003 -1696.0898 0.0000 0.0000
7766.1858 6014.2823 303.6968 -1751.9035 6076.6727
303.3292 -76.2987 -121.2942 511540.4688 -10.8157
-10.7889
[7123]ENERGY: 2340000 3184.8438 5261.0456 5469.0646
321.3272 -14233.0555 -1784.6730 0.0000 0.0000
7794.2764 6012.8291 304.7953 -1781.4472 6077.5954
303.3920 18.7121 39.2958 511540.4688 -17.5437
-17.5559
[7159]ENERGY: 2350000 3216.8360 5228.6066 5479.0094

349.2089 -14251.0969 -1779.5817 0.0000 0.0000
7771.9154 6014.8978 303.9209 -1757.0176 6078.9826
303.4191 -220.2319 -238.9752 511540.4688 -7.9645
-7.9622
[7209]ENERGY: 2360000 3214.7936 5275.9617 5481.0638
322.0010 -14281.2181 -1707.9969 0.0000 0.0000
7711.2888 6015.8939 301.5501 -1695.3949 6077.4456
303.6073 -127.3145 -38.3457 511540.4688 -16.3547
-16.3427
[7245]ENERGY: 2370000 3271.0725 5324.2738 5465.2883
319.3740 -14351.7061 -1737.3332 0.0000 0.0000
7723.8761 6014.8455 302.0423 -1709.0305 6078.9348
303.6592 18.8008 -135.0775 511540.4688 -13.4900
-13.4914
[7295]ENERGY: 2380000 3219.4014 5212.5050 5457.6196
308.7447 -14358.9005 -1645.6516 0.0000 0.0000
7820.6327 6014.3513 305.8260 -1806.2814 6077.5681
303.5157 195.4152 51.3700 511540.4688 -12.6459
-12.6523
[7331]ENERGY: 2390000 3152.2360 5252.2344 5446.3683
331.3123 -14291.7943 -1686.8309 0.0000 0.0000
7810.1952 6013.7210 305.4178 -1796.4742 6078.4099
303.6340 171.2457 106.9920 511540.4688 -8.4575
-8.4523
[7384]ENERGY: 2400000 3207.0303 5331.7515 5454.3643
328.6462 -14409.8175 -1693.4603 0.0000 0.0000
7793.9947 6012.5091 304.7843 -1781.4855 6078.2415
303.6393 57.0739 -15.6318 511540.4688 -14.8425
-14.8403
[7420]ENERGY: 2410000 3137.6518 5215.0625 5493.6075
345.0791 -14337.2451 -1676.1948 0.0000 0.0000
7836.0656 6014.0265 306.4295 -1822.0391 6078.4263
303.4949 177.0763 121.3207 511540.4688 -10.2809
-10.2810
[7470]ENERGY: 2420000 3172.1507 5350.5314 5501.5782
341.2738 -14348.5912 -1738.8817 0.0000 0.0000
7738.6507 6016.7119 302.6201 -1721.9388 6079.8922
303.5045 -91.0541 -61.4430 511540.4688 -14.1699
-14.1645
[7506]ENERGY: 2430000 3230.9379 5305.7947 5499.6219
331.8175 -14345.7493 -1666.6688 0.0000 0.0000
7664.9539 6020.7078 299.7381 -1644.2460 6080.9744
303.3108 107.5851 -8.1536 511540.4688 -7.7171
-7.7477
[7556]ENERGY: 2440000 3180.0516 5327.4666 5377.3454
313.1599 -14384.4244 -1600.5693 0.0000 0.0000
7800.8082 6013.8380 305.0507 -1786.9701 6078.7348
303.2425 59.6086 157.8165 511540.4688 -10.5334
-10.5059
[7592]ENERGY: 2450000 3162.9530 5352.8757 5549.8096
302.7813 -14341.5235 -1727.8740 0.0000 0.0000
7717.7969 6016.8191 301.8046 -1700.9778 6080.3197
303.2677 121.8975 92.1044 511540.4688 -10.4094
-10.4294

[7642]ENERGY: 2460000 3260.9250 5294.0361 5527.9795
319.9392 -14360.1695 -1689.1808 0.0000 0.0000
7666.9653 6020.4949 299.8168 -1646.4704 6080.4149
303.4061 97.4391 75.5212 511540.4688 -6.7200
-6.7357
[7678]ENERGY: 2470000 3155.4198 5298.6540 5490.1811
310.3403 -14291.2281 -1702.7966 0.0000 0.0000
7754.9543 6015.5247 303.2576 -1739.4295 6080.7195
303.4770 59.8311 -132.9274 511540.4688 -6.5777
-6.5398
[7728]ENERGY: 2480000 3268.2178 5257.8652 5457.9794
320.4187 -14328.2917 -1680.8138 0.0000 0.0000
7725.5293 6020.9050 302.1069 -1704.6243 6080.6981
303.5198 -7.8650 -68.1073 511540.4688 -11.6084
-11.6059
[7764]ENERGY: 2490000 3195.4820 5333.4957 5474.5453
330.1754 -14373.5899 -1652.0266 0.0000 0.0000
7708.1746 6016.2566 301.4283 -1691.9180 6080.4047
303.6730 177.0181 157.1366 511540.4688 -10.7142
-10.7160
[7817]ENERGY: 2500000 3186.3890 5255.2256 5448.3468
320.8935 -14253.1310 -1698.0901 0.0000 0.0000
7760.4325 6020.0661 303.4718 -1740.3663 6081.3859
303.6188 -82.6019 -86.2815 511540.4688 -9.2493
-9.2859
[7853]ENERGY: 2510000 3251.7912 5264.6403 5455.3136
326.2291 -14317.9912 -1678.0158 0.0000 0.0000
7719.3514 6021.3187 301.8654 -1698.0327 6082.1644
303.7109 -36.0977 -100.7278 511540.4688 -9.6234
-9.5835
[7903]ENERGY: 2520000 3158.8234 5328.2193 5469.7691
360.5800 -14327.5101 -1787.2918 0.0000 0.0000
7816.7200 6019.3099 305.6730 -1797.4101 6081.7290
303.6296 -83.9441 -143.7639 511540.4688 -13.1388
-13.1535
[7939]ENERGY: 2530000 3276.7468 5256.5050 5438.1188
303.0808 -14305.2620 -1761.8752 0.0000 0.0000
7811.4485 6018.7626 305.4668 -1792.6859 6082.7856
303.5327 -138.2893 -168.2877 511540.4688 -12.3635
-12.3519
[7989]ENERGY: 2540000 3181.2661 5254.9721 5460.0617
304.9659 -14352.7956 -1656.4982 0.0000 0.0000
7826.5746 6018.5465 306.0583 -1808.0280 6082.6047
303.7825 68.3765 -9.8606 511540.4688 -13.4467
-13.4650
[8025]ENERGY: 2550000 3240.7419 5292.6451 5508.6557
326.2985 -14345.0068 -1742.2134 0.0000 0.0000
7741.7515 6022.8725 302.7413 -1718.8790 6082.9408
303.6502 94.1117 40.7739 511540.4688 -12.5858
-12.5915
[8075]ENERGY: 2560000 3253.1115 5248.4076 5500.6796
339.6395 -14427.0132 -1680.1674 0.0000 0.0000
7784.4534 6019.1111 304.4112 -1765.3423 6082.8345
303.7458 -79.7699 -109.9590 511540.4688 -11.1998

-11.1860
[8111]ENERGY: 2570000 3189.3523 5193.1357 5435.9978
352.4779 -14322.0199 -1718.8553 0.0000 0.0000
7887.0051 6017.0935 308.4215 -1869.9116 6083.2405
303.8989 52.7228 -103.5781 511540.4688 -10.3834
-10.3985
[8161]ENERGY: 2580000 3222.8961 5306.1972 5454.7697
322.6157 -14346.3424 -1728.4938 0.0000 0.0000
7786.7999 6018.4424 304.5029 -1768.3576 6083.6975
303.8200 -69.4408 -136.7138 511540.4688 -11.4389
-11.4286
[8197]ENERGY: 2590000 3231.0758 5321.4973 5547.2260
329.3196 -14351.3110 -1752.9361 0.0000 0.0000
7696.7686 6021.6403 300.9823 -1675.1283 6084.1832
303.7252 -41.0177 -80.7122 511540.4688 -11.7791
-11.7855
[8250]ENERGY: 2600000 3156.3410 5338.0459 5481.1262
330.1378 -14370.3496 -1678.3183 0.0000 0.0000
7765.6304 6022.6135 303.6751 -1743.0170 6084.1577
303.6845 37.2259 80.8785 511540.4688 -13.5462
-13.5435
[8286]ENERGY: 2610000 3211.6818 5314.4114 5535.9775
318.8734 -14348.7747 -1738.4223 0.0000 0.0000
7728.0433 6021.7904 302.2053 -1706.2529 6084.6729
303.9685 -28.9322 -132.9045 511540.4688 -10.3607
-10.3684
[8336]ENERGY: 2620000 3190.3375 5272.0988 5490.9880
349.7242 -14356.3549 -1657.8199 0.0000 0.0000
7734.6958 6023.6696 302.4654 -1711.0262 6084.5384
303.8687 131.0901 79.4483 511540.4688 -10.0030
-9.9910
[8372]ENERGY: 2630000 3282.8771 5241.3389 5468.2297
340.0911 -14356.5953 -1736.4871 0.0000 0.0000
7781.0854 6020.5398 304.2795 -1760.5456 6083.6068
303.7907 -180.5729 -142.5433 511540.4688 -12.8694
-12.8669
[8422]ENERGY: 2640000 3250.3331 5289.5807 5469.1046
328.2435 -14394.7539 -1663.6414 0.0000 0.0000
7741.8860 6020.7526 302.7466 -1721.1334 6085.1236
303.8060 56.4724 -1.1028 511540.4688 -16.5043
-16.5096
[8458]ENERGY: 2650000 3252.5940 5335.6571 5473.5496
350.9712 -14362.3357 -1696.3388 0.0000 0.0000
7672.0939 6026.1914 300.0173 -1645.9024 6084.4916
303.7070 112.7483 32.7645 511540.4688 -12.2793
-12.2490
[8508]ENERGY: 2660000 3230.4584 5233.5115 5427.6502
335.1166 -14335.0479 -1649.7140 0.0000 0.0000
7779.4436 6021.4186 304.2153 -1758.0250 6083.0000
303.7550 -133.2665 -81.7725 511540.4688 -13.6193
-13.6351
[8544]ENERGY: 2670000 3225.1451 5263.5169 5446.3059
314.3221 -14219.8906 -1773.9673 0.0000 0.0000
7766.8657 6022.2978 303.7234 -1744.5680 6086.3050

303.6892 -7.2188 -45.7979 511540.4688 -10.0569
-10.0860
[8594]ENERGY: 2680000 3248.4758 5295.5137 5458.9593
308.8287 -14351.5736 -1740.4464 0.0000 0.0000
7798.4678 6018.2253 304.9592 -1780.2425 6084.5033
303.7419 -12.4515 -94.3170 511540.4688 -14.5561
-14.5296
[8630]ENERGY: 2690000 3108.7655 5352.0305 5488.6404
342.4620 -14409.6804 -1631.5393 0.0000 0.0000
7768.2160 6018.8946 303.7762 -1749.3213 6083.8785
303.8723 142.4661 113.5475 511540.4688 -11.1121
-11.1167
[8683]ENERGY: 2700000 3199.0138 5291.3418 5403.0656
328.7691 -14334.9612 -1645.6908 0.0000 0.0000
7778.7144 6020.2526 304.1867 -1758.4617 6085.4919
303.7600 327.6358 280.2836 511540.4688 -11.5869
-11.5758
[8719]ENERGY: 2710000 3244.2458 5245.6432 5465.1597
334.3459 -14263.7900 -1664.7881 0.0000 0.0000
7665.7408 6026.5572 299.7689 -1639.1835 6085.7094
303.7784 77.1355 49.9472 511540.4688 -12.0844
-12.0986
[8769]ENERGY: 2720000 3185.6171 5345.1684 5523.7797
306.9310 -14480.1563 -1646.0221 0.0000 0.0000
7786.2278 6021.5458 304.4806 -1764.6821 6084.8239
303.8627 67.2214 132.9108 511540.4688 -13.6685
-13.6534
[8805]ENERGY: 2730000 3207.5171 5280.2063 5451.6225
345.2477 -14316.2953 -1643.1066 0.0000 0.0000
7697.1893 6022.3811 300.9987 -1674.8083 6083.8896
303.8024 -132.7569 -13.5124 511540.4688 -11.2682
-11.2753
[8855]ENERGY: 2740000 3167.8107 5212.7559 5486.6807
309.2004 -14227.5447 -1688.4836 0.0000 0.0000
7762.3052 6022.7247 303.5451 -1739.5806 6084.9432
303.4499 58.0758 -22.9287 511540.4688 -16.4475
-16.4626
[8891]ENERGY: 2750000 3193.6186 5301.2365 5501.3444
336.5523 -14248.5145 -1742.6510 0.0000 0.0000
7681.7013 6023.2875 300.3930 -1658.4138 6084.9568
303.7689 31.4458 -23.0214 511540.4688 -10.1262
-10.1158
[8941]ENERGY: 2760000 3207.6455 5345.8360 5436.6479
333.3237 -14316.9523 -1703.7209 0.0000 0.0000
7723.5595 6026.3394 302.0299 -1697.2201 6086.0646
303.8454 -17.3590 -119.7568 511540.4688 -13.8866
-13.9053
[8977]ENERGY: 2770000 3227.3128 5166.7952 5445.4651
294.5721 -14317.7176 -1649.7287 0.0000 0.0000
7853.4814 6020.1802 307.1105 -1833.3012 6085.7284
303.8530 68.6934 -121.9300 511540.4688 -12.9946
-13.0082
[9027]ENERGY: 2780000 3170.6302 5280.5261 5450.5674
311.2896 -14273.6980 -1698.0877 0.0000 0.0000

7782.5160 6023.7436 304.3354 -1758.7725 6085.7433
303.8114 -90.2818 13.5148 511540.4688 -13.1617
-13.1411
[9063]ENERGY: 2790000 3242.6642 5314.4986 5448.1959
326.7704 -14303.2216 -1743.4772 0.0000 0.0000
7738.5676 6023.9981 302.6168 -1714.5696 6086.6940
303.9065 -46.4421 22.7739 511540.4688 -10.8304
-10.8186
[9116]ENERGY: 2800000 3201.3701 5258.4788 5431.9845
314.6184 -14259.7717 -1692.5290 0.0000 0.0000
7769.6145 6023.7655 303.8309 -1745.8490 6086.8257
303.7917 -169.1800 -142.6301 511540.4688 -14.9029
-14.9155
[9152]ENERGY: 2810000 3202.8190 5313.3404 5473.6358
325.3949 -14335.3982 -1699.1622 0.0000 0.0000
7744.6404 6025.2701 302.8543 -1719.3703 6086.7703
303.7798 -199.5593 -144.9483 511540.4688 -12.3561
-12.3472
[9202]ENERGY: 2820000 3230.6854 5355.4937 5433.9902
336.3496 -14356.3970 -1693.6816 0.0000 0.0000
7718.8276 6025.2679 301.8449 -1693.5597 6086.9485
303.8743 201.7065 226.5665 511540.4688 -15.7702
-15.7817
[9238]ENERGY: 2830000 3260.7464 5284.4100 5447.8069
327.4082 -14332.4032 -1742.6051 0.0000 0.0000
7776.7577 6022.1210 304.1102 -1754.6367 6087.1562
304.0031 -135.1222 -261.3490 511540.4688 -13.9607
-13.9543
[9288]ENERGY: 2840000 3165.6750 5290.7422 5473.1779
334.6217 -14245.9403 -1723.8731 0.0000 0.0000
7729.7935 6024.1969 302.2737 -1705.5965 6088.2192
303.9354 126.1879 57.2654 511540.4688 -16.6393
-16.6291
[9324]ENERGY: 2850000 3216.9521 5331.2605 5445.7271
310.2402 -14332.8525 -1680.1010 0.0000 0.0000
7734.4007 6025.6271 302.4539 -1708.7736 6087.0474
303.9084 -25.9508 -33.7329 511540.4688 -16.0588
-16.0532
[9374]ENERGY: 2860000 3215.3320 5385.2235 5496.3382
315.5419 -14387.2609 -1724.8977 0.0000 0.0000
7727.6272 6027.9042 302.1890 -1699.7230 6088.8978
303.9027 -119.1498 -172.6268 511540.4688 -18.0516
-18.0459
[9410]ENERGY: 2870000 3196.2284 5325.9850 5441.8678
314.7981 -14307.8651 -1705.6098 0.0000 0.0000
7761.0394 6026.4438 303.4956 -1734.5956 6088.4225
303.8897 302.4218 158.4159 511540.4688 -14.9812
-15.0064
[9460]ENERGY: 2880000 3124.7157 5393.2066 5481.7224
323.9992 -14327.9229 -1712.2368 0.0000 0.0000
7743.2404 6026.7246 302.7995 -1716.5158 6088.8511
304.1203 35.3561 -7.5498 511540.4688 -16.6671
-16.6409
[9496]ENERGY: 2890000 3187.1621 5316.4026 5462.0114

303.7098 -14237.3310 -1753.6721 0.0000 0.0000
7748.6374 6026.9203 303.0106 -1721.7171 6089.6378
303.9577 26.0660 -23.0024 511540.4688 -17.5460
-17.5860
[9549]ENERGY: 2900000 3302.7013 5283.1137 5466.9419
339.3333 -14380.9563 -1717.0532 0.0000 0.0000
7732.3376 6026.4183 302.3732 -1705.9194 6087.9192
304.1241 -187.3446 -157.8434 511540.4688 -14.7760
-14.7624
[9585]ENERGY: 2910000 3202.7463 5344.0151 5472.5187
326.4546 -14215.3425 -1751.9674 0.0000 0.0000
7650.1026 6028.5274 299.1574 -1621.5752 6088.8551
304.0520 -248.0963 -153.5176 511540.4688 -14.8377
-14.8279
[9635]ENERGY: 2920000 3183.6429 5362.6388 5497.9098
336.4744 -14394.2037 -1627.5744 0.0000 0.0000
7669.2092 6028.0968 299.9045 -1641.1123 6088.9570
304.0085 183.4123 164.9136 511540.4688 -13.0089
-13.0294
[9671]ENERGY: 2930000 3254.2806 5284.1783 5453.9049
310.0865 -14376.2322 -1635.8843 0.0000 0.0000
7737.1372 6027.4709 302.5609 -1709.6663 6088.6385
304.0881 55.8359 -29.4771 511540.4688 -14.3732
-14.3699
[9721]ENERGY: 2940000 3232.5935 5391.0959 5466.8784
326.1793 -14345.6759 -1712.3545 0.0000 0.0000
7667.6814 6026.3981 299.8448 -1641.2833 6087.7316
304.1130 -27.9684 -40.8522 511540.4688 -20.1210
-20.0958
[9757]ENERGY: 2950000 3141.0484 5293.6839 5472.6036
325.2434 -14368.6873 -1630.8540 0.0000 0.0000
7792.4325 6025.4706 304.7232 -1766.9620 6088.8776
304.1202 241.8516 120.6474 511540.4688 -11.4910
-11.5058
[9807]ENERGY: 2960000 3194.3313 5335.4676 5444.2155
311.5744 -14337.9152 -1659.1259 0.0000 0.0000
7733.1792 6021.7269 302.4061 -1711.4524 6089.2706
303.9660 8.2422 69.8511 511540.4688 -18.0178
-18.0137
[9843]ENERGY: 2970000 3163.9029 5328.7483 5510.3662
319.8766 -14300.4187 -1756.0646 0.0000 0.0000
7760.0940 6026.5047 303.4586 -1733.5893 6089.3191
304.2341 100.8354 37.3410 511540.4688 -12.8068
-12.8072
[9893]ENERGY: 2980000 3218.0155 5343.1922 5455.3329
328.6098 -14374.3585 -1698.4450 0.0000 0.0000
7754.5612 6026.9081 303.2422 -1727.6531 6089.4453
304.2972 -46.7967 -36.2940 511540.4688 -16.5021
-16.5008
[9929]ENERGY: 2990000 3250.3200 5190.0167 5473.0386
318.1071 -14374.3099 -1596.8237 0.0000 0.0000
7768.3012 6028.6501 303.7795 -1739.6511 6088.5405
304.2039 138.1943 103.3154 511540.4688 -15.6601
-15.6716

[9982]ENERGY: 3000000 3242.8510 5316.3742 5497.0429
323.5909 -14302.8507 -1772.2835 0.0000 0.0000
7721.2037 6025.9283 301.9378 -1695.2754 6090.1700
304.2994 -9.2326 -43.3238 511540.4688 -14.8950
-14.8688
[10018]ENERGY: 3010000 3197.1511 5304.7326 5483.4850
318.4660 -14433.7605 -1663.0179 0.0000 0.0000
7817.4038 6024.4602 305.6997 -1792.9437 6090.0877
304.3097 178.4358 93.9845 511540.4688 -15.4331
-15.4525
[10068]ENERGY: 3020000 3215.8622 5201.8047 5475.4278
319.0872 -14324.3823 -1715.0138 0.0000 0.0000
7850.6867 6023.4725 307.0012 -1827.2142 6089.2813
304.2682 -237.4033 -124.9491 511540.4688 -17.8574
-17.8644
[10104]ENERGY: 3030000 3205.7529 5205.8967 5431.6522
317.9969 -14290.3562 -1690.0138 0.0000 0.0000
7845.4035 6026.3321 306.7946 -1819.0713 6090.4647
304.2712 -116.8664 -34.9078 511540.4688 -18.3228
-18.2862
[10154]ENERGY: 3040000 3264.8675 5225.8665 5436.0636
322.2852 -14345.2747 -1684.6252 0.0000 0.0000
7806.5868 6025.7698 305.2767 -1780.8170 6089.3181
304.2402 24.7553 -37.5126 511540.4688 -11.0340
-11.0685
[10190]ENERGY: 3050000 3219.9965 5226.6653 5439.3250
324.3761 -14328.9578 -1691.4440 0.0000 0.0000
7838.2478 6028.2088 306.5148 -1810.0390 6091.1592
304.3847 -173.9300 -183.8452 511540.4688 -19.7942
-19.7800
[10240]ENERGY: 3060000 3281.8461 5271.2994 5436.6489
339.5545 -14413.6080 -1592.1075 0.0000 0.0000
7710.3395 6033.9728 301.5129 -1676.3667 6091.0904
304.3235 146.7140 41.1207 511540.4688 -13.2629
-13.2797
[10276]ENERGY: 3070000 3190.9383 5316.0563 5458.8827
296.8558 -14317.3049 -1704.9962 0.0000 0.0000
7785.8374 6026.2695 304.4653 -1759.5680 6090.4788
304.2770 54.2585 46.0997 511540.4688 -18.9628
-18.9440
[10326]ENERGY: 3080000 3217.9807 5261.5426 5489.5802
300.0744 -14387.6293 -1618.6251 0.0000 0.0000
7767.1957 6030.1192 303.7363 -1737.0765 6090.3946
303.9749 -105.8194 -68.5529 511540.4688 -11.9892
-11.9998
[10362]ENERGY: 3090000 3201.2752 5248.3660 5442.7881
320.8708 -14303.1041 -1698.2726 0.0000 0.0000
7815.7748 6027.6982 305.6360 -1788.0766 6092.0704
304.2269 91.4609 26.5899 511540.4688 -17.1237
-17.1112
[10415]ENERGY: 3100000 3254.8958 5321.1368 5488.9303
343.8860 -14349.1633 -1736.5071 0.0000 0.0000
7708.9998 6032.1783 301.4606 -1676.8215 6091.8423
304.2308 -91.1016 -141.3350 511540.4688 -16.3104

-16.3283
[10451]ENERGY: 3110000 3129.7797 5253.5828 5497.9610
343.9797 -14309.6946 -1710.1881 0.0000 0.0000
7821.0952 6026.5157 305.8440 -1794.5795 6092.9884
304.1338 40.9320 -55.7367 511540.4688 -19.1758
-19.1649
[10501]ENERGY: 3120000 3221.8274 5263.7956 5460.4010
318.9547 -14381.5686 -1706.4920 0.0000 0.0000
7853.6538 6030.5718 307.1173 -1823.0820 6092.9606
304.3404 113.0046 33.4418 511540.4688 -12.7014
-12.6977
[10537]ENERGY: 3130000 3248.8805 5308.0287 5432.5505
320.9485 -14319.0496 -1667.7432 0.0000 0.0000
7709.6052 6033.2206 301.4842 -1676.3846 6093.4166
304.3737 81.8511 35.0038 511540.4688 -22.9182
-22.9272
[10587]ENERGY: 3140000 3224.2972 5301.0311 5444.5442
327.4201 -14368.7716 -1669.3275 0.0000 0.0000
7774.8737 6034.0673 304.0366 -1740.8064 6092.7585
304.3850 13.3825 12.1583 511540.4688 -15.0633
-15.0399
[10623]ENERGY: 3150000 3229.6600 5232.3377 5474.9425
320.5880 -14283.2945 -1735.8751 0.0000 0.0000
7789.5378 6027.8964 304.6100 -1761.6414 6093.3066
304.4820 29.9378 -174.8534 511540.4688 -14.7951
-14.8227
[10673]ENERGY: 3160000 3219.3002 5276.5495 5472.2690
317.7082 -14308.1126 -1696.8929 0.0000 0.0000
7751.4593 6032.2807 303.1209 -1719.1786 6092.0347
304.3709 107.4485 133.0961 511540.4688 -22.6049
-22.5606
[10709]ENERGY: 3170000 3250.3437 5259.8255 5446.4083
328.6113 -14279.2037 -1740.4601 0.0000 0.0000
7762.6148 6028.1397 303.5572 -1734.4751 6091.3645
304.3430 -30.4373 -96.6939 511540.4688 -13.5252
-13.5641
[10759]ENERGY: 3180000 3207.2783 5299.7987 5494.2931
298.5236 -14305.6043 -1732.2379 0.0000 0.0000
7766.6547 6028.7063 303.7152 -1737.9484 6092.2562
304.3901 -147.8836 -90.9070 511540.4688 -17.1993
-17.1948
[10795]ENERGY: 3190000 3277.6054 5246.5950 5426.0340
338.1956 -14335.3878 -1743.6157 0.0000 0.0000
7821.4053 6030.8318 305.8562 -1790.5735 6092.7860
304.3755 299.8345 118.5132 511540.4688 -16.8122
-16.8374
[10848]ENERGY: 3200000 3279.1294 5326.1784 5487.0103
332.8223 -14415.1415 -1688.2115 0.0000 0.0000
7710.3720 6032.1594 301.5142 -1678.2126 6093.0225
304.4321 -73.5138 -64.9521 511540.4688 -15.7193
-15.6798
[10884]ENERGY: 3210000 3210.3487 5293.7571 5384.9135
337.7881 -14297.2223 -1773.5766 0.0000 0.0000
7870.9235 6026.9320 307.7926 -1843.9915 6092.8083

304.4736 -0.8374 -15.0306 511540.4688 -12.1509
-12.1542
[10934]ENERGY: 3220000 3235.5363 5326.9831 5427.9622
330.0521 -14346.1220 -1694.7198 0.0000 0.0000
7751.0251 6030.7171 303.1040 -1720.3080 6092.4986
304.1915 -82.1257 -127.3083 511540.4688 -17.6650
-17.6658
[10970]ENERGY: 3230000 3210.0663 5192.3704 5487.7121
311.3187 -14333.2797 -1656.4480 0.0000 0.0000
7814.1038 6025.8435 305.5707 -1788.2603 6091.0932
304.0730 0.2185 0.7277 511540.4688 -14.7666
-14.7524
[11020]ENERGY: 3240000 3223.7609 5238.6293 5473.9084
327.2641 -14288.5024 -1754.2471 0.0000 0.0000
7807.6561 6028.4693 305.3185 -1779.1868 6091.8855
304.3204 86.4801 11.2080 511540.4688 -12.1964
-12.2249
[11056]ENERGY: 3250000 3265.6240 5213.7463 5437.9818
290.1433 -14278.2388 -1730.3559 0.0000 0.0000
7831.8942 6030.7949 306.2663 -1801.0993 6092.9430
304.4615 1.0755 -23.8369 511540.4688 -11.8539
-11.8461
[11106]ENERGY: 3260000 3194.8290 5250.9335 5516.0803
323.0122 -14372.6037 -1699.0710 0.0000 0.0000
7815.5257 6028.7059 305.6263 -1786.8197 6093.1886
304.3565 18.9016 -27.5359 511540.4688 -13.7954
-13.7817
[11142]ENERGY: 3270000 3247.3492 5308.5148 5449.1883
318.1203 -14336.3041 -1771.2357 0.0000 0.0000
7814.8466 6030.4794 305.5997 -1784.3671 6093.5193
304.4718 57.7898 -41.9856 511540.4688 -12.5401
-12.5556
[11192]ENERGY: 3280000 3204.9057 5286.9226 5489.8953
315.4691 -14282.5979 -1749.8827 0.0000 0.0000
7768.2992 6033.0112 303.7795 -1735.2879 6094.1413
304.3668 -92.1536 -57.2301 511540.4688 -17.1923
-17.1944
[11228]ENERGY: 3290000 3267.6116 5299.3968 5496.2464
320.0017 -14373.5656 -1686.2456 0.0000 0.0000
7709.5372 6032.9825 301.4816 -1676.5546 6093.3178
304.4496 54.0625 14.8633 511540.4688 -9.5803
-9.6030
[11281]ENERGY: 3300000 3214.5836 5358.2403 5459.3933
289.6485 -14404.2082 -1682.9725 0.0000 0.0000
7793.2236 6027.9087 304.7541 -1765.3149 6092.6606
304.4562 -95.5678 -12.3580 511540.4688 -16.5215
-16.4914
[11317]ENERGY: 3310000 3238.0105 5259.7684 5412.2430
311.3945 -14267.8128 -1752.2840 0.0000 0.0000
7830.4002 6031.7198 306.2079 -1798.6804 6094.1822
304.4078 -59.1684 -115.9796 511540.4688 -11.1408
-11.1540
[11367]ENERGY: 3320000 3230.9807 5275.1429 5443.2523
310.6264 -14357.9701 -1624.0779 0.0000 0.0000

7755.2683 6033.2226 303.2699 -1722.0456 6094.2231
304.3604 42.5149 -21.5984 511540.4688 -14.1087
-14.0962
[11403]ENERGY: 3330000 3204.2507 5242.5566 5453.4539
316.6578 -14301.9100 -1740.3543 0.0000 0.0000
7856.3118 6030.9664 307.2212 -1825.3454 6093.8684
304.7100 -30.8507 46.3760 511540.4688 -15.4727
-15.4795
[11453]ENERGY: 3340000 3193.4086 5382.1266 5405.5412
331.9816 -14346.2289 -1694.2299 0.0000 0.0000
7758.9427 6031.5420 303.4136 -1727.4007 6096.2458
304.7125 144.9808 61.4096 511540.4688 -16.9083
-16.8983
[11489]ENERGY: 3350000 3321.8699 5191.7201 5465.0927
317.2155 -14285.1079 -1695.1756 0.0000 0.0000
7716.6856 6032.3003 301.7611 -1684.3853 6094.1864
304.5689 -63.6194 -59.8375 511540.4688 -18.7806
-18.7883
[11539]ENERGY: 3360000 3275.9947 5274.3659 5425.6651
346.0888 -14336.8456 -1763.4927 0.0000 0.0000
7810.4307 6032.2069 305.4270 -1778.2238 6095.7896
304.5435 -74.2614 -39.4713 511540.4688 -16.5631
-16.5669
[11575]ENERGY: 3370000 3201.3068 5308.1272 5482.5602
321.4455 -14291.2762 -1706.4781 0.0000 0.0000
7720.7267 6036.4120 301.9191 -1684.3148 6096.1052
304.7711 220.3999 241.9594 511540.4688 -18.1653
-18.1471
[11625]ENERGY: 3380000 3258.5812 5257.2328 5471.3856
341.7156 -14390.3899 -1666.9690 0.0000 0.0000
7762.3275 6033.8838 303.5459 -1728.4437 6095.1044
304.6567 193.1679 168.8347 511540.4688 -15.7719
-15.7810
[11661]ENERGY: 3390000 3231.7765 5193.1135 5486.2674
325.1083 -14346.0007 -1748.2396 0.0000 0.0000
7892.2669 6034.2924 308.6272 -1857.9745 6094.2974
304.7243 -81.4061 -114.2481 511540.4688 -16.2279
-16.2460
[11714]ENERGY: 3400000 3106.9165 5261.7553 5431.1290
319.2043 -14390.8532 -1620.9860 0.0000 0.0000
7921.7638 6028.9297 309.7807 -1892.8341 6094.5689
304.5812 52.7829 142.6376 511540.4688 -14.3286
-14.3309
[11750]ENERGY: 3410000 3240.7891 5253.4184 5470.9270
346.2109 -14352.1184 -1718.6006 0.0000 0.0000
7791.8419 6032.4684 304.7001 -1759.3735 6095.5718
304.6892 11.0507 -78.5356 511540.4688 -15.3865
-15.3736
[11800]ENERGY: 3420000 3237.1358 5335.2722 5461.2842
323.3059 -14388.9119 -1726.2760 0.0000 0.0000
7792.3621 6034.1723 304.7204 -1758.1898 6096.0785
304.8200 124.9858 24.5569 511540.4688 -16.1089
-16.1157
[11836]ENERGY: 3430000 3291.2681 5295.6316 5416.1522

322.3107 -14254.8155 -1767.9474 0.0000 0.0000
7731.9722 6034.5720 302.3589 -1697.4002 6095.5793
304.6739 -208.2880 -193.7004 511540.4688 -17.4405
-17.4291
[11886]ENERGY: 3440000 3212.8582 5289.0632 5497.0746
310.1054 -14286.5291 -1743.5210 0.0000 0.0000
7755.2226 6034.2739 303.2681 -1720.9487 6095.8127
304.7151 -138.8297 -50.3834 511540.4688 -18.1388
-18.1350
[11922]ENERGY: 3450000 3226.6828 5349.2532 5433.6332
314.9677 -14397.5104 -1692.3302 0.0000 0.0000
7797.1441 6031.8405 304.9074 -1765.3036 6096.5127
304.6180 91.6110 53.4345 511540.4688 -13.9752
-13.9670
[11972]ENERGY: 3460000 3290.8118 5321.4492 5430.0776
326.8920 -14423.7102 -1696.2120 0.0000 0.0000
7784.1824 6033.4909 304.4006 -1750.6916 6095.8805
304.9763 -213.8140 -232.7198 511540.4688 -13.7851
-13.7944
[12008]ENERGY: 3470000 3272.4144 5293.5317 5482.5069
319.2867 -14400.9948 -1759.5524 0.0000 0.0000
7824.6579 6031.8504 305.9834 -1792.8075 6096.9193
304.7772 -13.5061 -118.0295 511540.4688 -16.4811
-16.5026
[12058]ENERGY: 3480000 3197.3915 5400.8371 5454.6820
326.4001 -14424.3167 -1667.6455 0.0000 0.0000
7747.2600 6034.6086 302.9567 -1712.6514 6095.6189
304.7063 -59.4071 -134.9503 511540.4688 -17.7212
-17.7045
[12094]ENERGY: 3490000 3187.1464 5312.6866 5519.0332
343.8364 -14356.5506 -1711.2605 0.0000 0.0000
7738.5954 6033.4870 302.6179 -1705.1085 6094.7945
304.6267 160.7776 34.9624 511540.4688 -12.6834
-12.6721
[12147]ENERGY: 3500000 3218.9075 5254.4858 5438.9128
335.1779 -14322.3218 -1683.3475 0.0000 0.0000
7788.3165 6030.1312 304.5622 -1758.1853 6095.4045
304.6128 195.9961 80.1266 511540.4688 -18.7031
-18.7326
[12183]ENERGY: 3510000 3199.0516 5253.8646 5488.4695
341.2870 -14331.0679 -1684.5747 0.0000 0.0000
7762.2521 6029.2821 303.5430 -1732.9700 6096.3771
304.5960 -120.2091 -48.1988 511540.4688 -14.7766
-14.7873
[12233]ENERGY: 3520000 3209.6106 5250.2998 5465.9300
317.3421 -14315.1582 -1715.1003 0.0000 0.0000
7820.3647 6033.2888 305.8155 -1787.0760 6095.4742
304.6768 -34.4189 -3.4535 511540.4688 -18.8993
-18.8748
[12269]ENERGY: 3530000 3180.9898 5282.3017 5450.0020
332.5277 -14296.3686 -1743.6929 0.0000 0.0000
7826.8260 6032.5857 306.0682 -1794.2403 6095.5298
304.4699 -200.9936 -147.0321 511540.4688 -12.7182
-12.7161

[12319]ENERGY: 3540000 3149.2113 5398.3156 5425.5003
318.7681 -14335.3839 -1687.0958 0.0000 0.0000
7763.3084 6032.6239 303.5843 -1730.6845 6096.8620
304.5818 180.5669 104.3605 511540.4688 -14.4855
-14.4816
[12355]ENERGY: 3550000 3183.4685 5336.4174 5467.3430
321.7703 -14395.5411 -1703.1854 0.0000 0.0000
7823.3541 6033.6268 305.9324 -1789.7273 6097.5489
304.6958 -15.6407 34.8964 511540.4688 -14.3595
-14.3722
[12405]ENERGY: 3560000 3186.4544 5354.3281 5433.5993
301.3120 -14269.9422 -1797.3129 0.0000 0.0000
7824.1269 6032.5656 305.9626 -1791.5613 6097.8364
304.5717 1.5419 -3.5466 511540.4688 -14.8297
-14.8230
[12441]ENERGY: 3570000 3303.3480 5242.8155 5505.2848
333.8748 -14280.6601 -1784.5816 0.0000 0.0000
7715.4570 6035.5384 301.7131 -1679.9186 6097.3367
304.5569 123.8353 68.2819 511540.4688 -15.5728
-15.5543
[12491]ENERGY: 3580000 3190.2452 5339.1967 5496.5174
323.5644 -14367.6538 -1742.1442 0.0000 0.0000
7794.5205 6034.2463 304.8048 -1760.2742 6097.7782
304.5695 346.2959 168.5330 511540.4688 -19.8922
-19.9172
[12527]ENERGY: 3590000 3255.1868 5291.2403 5445.8923
312.0435 -14367.7685 -1708.5129 0.0000 0.0000
7805.6950 6033.7765 305.2418 -1771.9184 6098.0880
304.6855 -82.1159 -105.0066 511540.4688 -15.9595
-15.9476
[12580]ENERGY: 3600000 3238.7271 5363.1906 5485.2268
319.5040 -14393.2930 -1654.7048 0.0000 0.0000
7677.8229 6036.4737 300.2414 -1641.3492 6098.9730
304.6158 -83.9381 -154.4156 511540.4688 -18.0178
-18.0124
[12616]ENERGY: 3610000 3229.5388 5333.0067 5460.3679
316.8697 -14245.0826 -1764.4724 0.0000 0.0000
7709.9555 6040.1838 301.4979 -1669.7717 6099.1380
304.6213 -206.0896 -228.1481 511540.4688 -14.9032
-14.9102
[12666]ENERGY: 3620000 3271.5631 5274.6525 5426.1108
304.4020 -14268.9042 -1774.0549 0.0000 0.0000
7801.4368 6035.2061 305.0753 -1766.2307 6100.4386
304.7444 93.8593 -52.2405 511540.4688 -15.5539
-15.5691
[12702]ENERGY: 3630000 3193.4499 5323.1039 5475.0071
327.0974 -14287.9961 -1729.0807 0.0000 0.0000
7739.6365 6041.2180 302.6586 -1698.4184 6101.3326
304.5999 -78.7283 -100.8746 511540.4688 -18.0263
-17.9919
[12752]ENERGY: 3640000 3175.1443 5307.0746 5500.6124
336.6324 -14299.6989 -1736.5362 0.0000 0.0000
7756.2367 6039.4653 303.3078 -1716.7714 6101.4641
304.5509 89.3030 -32.4179 511540.4688 -19.0406

-19.0872
[12788]ENERGY: 3650000 3316.3269 5280.9054 5431.1416
314.5743 -14375.0332 -1763.1693 0.0000 0.0000
7834.7342 6039.4799 306.3774 -1795.2543 6100.7994
304.8173 -234.6231 -237.8482 511540.4688 -16.4977
-16.4729
[12838]ENERGY: 3660000 3152.6570 5271.0096 5433.7948
323.5701 -14297.3590 -1706.2528 0.0000 0.0000
7859.1025 6036.5221 307.3303 -1822.5804 6101.6202
304.7591 290.3523 49.4643 511540.4688 -16.4360
-16.4393
[12874]ENERGY: 3670000 3215.6840 5338.3353 5442.3469
300.1567 -14273.7624 -1748.3711 0.0000 0.0000
7765.3485 6039.7380 303.6641 -1725.6106 6102.2531
304.8076 -84.3547 -143.6728 511540.4688 -19.8074
-19.8068
[12924]ENERGY: 3680000 3266.3541 5280.0064 5424.0625
304.2363 -14373.7516 -1717.6048 0.0000 0.0000
7855.7885 6039.0913 307.2007 -1816.6972 6102.5507
304.6515 146.6178 -22.6755 511540.4688 -13.7312
-13.7264
[12960]ENERGY: 3690000 3347.5329 5373.0612 5452.4814
313.8357 -14420.1793 -1724.3595 0.0000 0.0000
7696.2351 6038.6075 300.9614 -1657.6276 6102.1548
304.9514 -229.2076 -78.3263 511540.4688 -13.8499
-13.8245
[13013]ENERGY: 3700000 3164.0331 5329.9410 5416.9367
335.1205 -14303.7391 -1729.0253 0.0000 0.0000
7826.3516 6039.6184 306.0496 -1786.7332 6103.1672
304.8494 -225.1320 -263.9273 511540.4688 -17.7098
-17.7508
[13049]ENERGY: 3710000 3241.1207 5269.0823 5455.4464
308.7878 -14355.3660 -1713.5573 0.0000 0.0000
7837.7323 6043.2463 306.4946 -1794.4860 6103.2036
304.7969 -11.5795 -86.2053 511540.4688 -16.7656
-16.7492
[13099]ENERGY: 3720000 3245.1146 5263.1865 5495.3218
314.4798 -14256.2063 -1746.4748 0.0000 0.0000
7727.0569 6042.4785 302.1667 -1684.5784 6102.3572
305.0085 -57.8134 -129.3978 511540.4688 -18.5353
-18.5346
[13135]ENERGY: 3730000 3173.5248 5238.9057 5431.4826
329.4970 -14356.1239 -1649.1469 0.0000 0.0000
7870.6982 6038.8375 307.7838 -1831.8607 6102.7786
304.8622 146.7864 105.5262 511540.4688 -17.6419
-17.6354
[13185]ENERGY: 3740000 3234.3303 5344.7636 5460.5033
302.1348 -14333.1605 -1709.4016 0.0000 0.0000
7743.1951 6042.3651 302.7978 -1700.8301 6104.4860
304.8832 -73.1324 -29.9243 511540.4688 -17.3481
-17.3618
[13221]ENERGY: 3750000 3244.1081 5375.8949 5437.4895
331.3818 -14347.6416 -1734.9486 0.0000 0.0000
7733.7946 6040.0787 302.4302 -1693.7159 6103.8965

304.9139 120.8916 69.1477 511540.4688 -14.7048
-14.7107
[13271]ENERGY: 3760000 3272.8144 5330.8358 5460.5300
314.1860 -14379.6884 -1726.4385 0.0000 0.0000
7770.1153 6042.3547 303.8505 -1727.7607 6103.9158
304.8883 132.5079 75.2770 511540.4688 -19.4888
-19.4683
[13307]ENERGY: 3770000 3221.0599 5297.4273 5472.2724
301.3866 -14403.9579 -1659.8687 0.0000 0.0000
7812.8260 6041.1456 305.5207 -1771.6804 6104.6177
304.8222 89.7574 2.8651 511540.4688 -16.6052
-16.6105
[13357]ENERGY: 3780000 3211.6839 5282.3852 5465.9953
308.4880 -14366.7091 -1698.0698 0.0000 0.0000
7839.1672 6042.9405 306.5508 -1796.2266 6105.3022
304.6270 -87.9183 -133.5604 511540.4688 -15.6588
-15.6381
[13393]ENERGY: 3790000 3200.1729 5322.5139 5436.6080
322.3042 -14343.2883 -1668.7442 0.0000 0.0000
7771.9385 6041.5049 303.9218 -1730.4335 6104.2827
304.6214 -4.0044 -99.3550 511540.4688 -12.9045
-12.9292
[13446]ENERGY: 3800000 3234.8785 5269.3944 5437.1092
343.3883 -14323.6537 -1714.2851 0.0000 0.0000
7792.5095 6039.3412 304.7262 -1753.1684 6105.5557
304.6369 1.1811 -140.5981 511540.4688 -16.0771
-16.0934
[13482]ENERGY: 3810000 3204.0501 5265.6355 5463.8312
317.5053 -14309.6710 -1695.4639 0.0000 0.0000
7798.8850 6044.7723 304.9755 -1754.1127 6105.0251
304.5877 97.3170 0.0416 511540.4688 -16.7469
-16.7520
[13532]ENERGY: 3820000 3163.7659 5280.7795 5447.8729
321.4180 -14341.9129 -1727.4627 0.0000 0.0000
7895.9092 6040.3699 308.7696 -1855.5393 6105.8524
304.7612 253.0449 174.9963 511540.4688 -15.0944
-15.0825
[13568]ENERGY: 3830000 3222.1465 5365.5070 5488.7118
304.5399 -14399.2754 -1680.3957 0.0000 0.0000
7741.9809 6043.2150 302.7503 -1698.7659 6106.2826
304.9238 159.3837 27.6903 511540.4688 -20.9061
-20.8987
[13618]ENERGY: 3840000 3181.7001 5267.1485 5435.7172
327.5227 -14245.1504 -1702.6049 0.0000 0.0000
7779.0827 6043.4158 304.2011 -1735.6669 6106.8852
304.9208 35.1557 40.1344 511540.4688 -15.1769
-15.1659
[13654]ENERGY: 3850000 3265.7811 5344.1621 5470.9232
317.3425 -14389.9470 -1730.7068 0.0000 0.0000
7765.3782 6042.9333 303.6652 -1722.4449 6106.9205
304.9505 66.3141 -73.7999 511540.4688 -13.0837
-13.1064
[13704]ENERGY: 3860000 3188.5959 5398.3075 5535.3896
324.4968 -14412.6669 -1748.6388 0.0000 0.0000

7756.2093 6041.6933 303.3067 -1714.5160 6105.4933
304.8843 -77.7237 -50.6021 511540.4688 -17.1117
-17.0931
[13740]ENERGY: 3870000 3244.0213 5289.4085 5474.7649
305.8276 -14343.8683 -1716.6746 0.0000 0.0000
7788.6516 6042.1311 304.5753 -1746.5205 6106.2744
305.0747 -149.1716 -168.8591 511540.4688 -16.3273
-16.3164
[13790]ENERGY: 3880000 3112.1414 5281.3937 5431.5599
317.5189 -14281.8948 -1701.6852 0.0000 0.0000
7881.0366 6040.0705 308.1881 -1840.9661 6105.3154
305.0581 52.5539 112.5526 511540.4688 -16.0734
-16.0734
[13826]ENERGY: 3890000 3240.7024 5313.7190 5412.5119
332.4084 -14298.7852 -1801.2719 0.0000 0.0000
7842.5170 6041.8016 306.6817 -1800.7154 6104.3498
304.8789 -47.7958 -122.6504 511540.4688 -18.1857
-18.2006
[13879]ENERGY: 3900000 3205.9211 5335.4002 5437.7825
327.4068 -14312.3380 -1779.5979 0.0000 0.0000
7826.8077 6041.3824 306.0674 -1785.4252 6105.2561
305.1838 -43.7734 -54.9622 511540.4688 -15.4968
-15.4865
[13915]ENERGY: 3910000 3221.5922 5281.5235 5474.8497
315.4105 -14380.6032 -1756.8333 0.0000 0.0000
7881.2937 6037.2332 308.1981 -1844.0605 6104.8590
305.0496 93.0616 -9.1839 511540.4688 -18.7700
-18.7889
[13965]ENERGY: 3920000 3212.2015 5272.8724 5453.9419
312.7952 -14306.0650 -1706.9192 0.0000 0.0000
7802.2066 6041.0334 305.1054 -1761.1733 6104.3188
304.9030 43.3344 -10.3164 511540.4688 -19.3050
-19.2964
[14001]ENERGY: 3930000 3258.1127 5325.3350 5517.4148
327.5451 -14392.1867 -1682.7003 0.0000 0.0000
7687.4675 6040.9882 300.6185 -1646.4794 6103.8777
304.7935 -191.6788 -35.5749 511540.4688 -14.5842
-14.5868
[14051]ENERGY: 3940000 3224.4893 5323.5030 5463.3168
315.2616 -14343.7456 -1706.9612 0.0000 0.0000
7765.0711 6040.9351 303.6532 -1724.1360 6104.5119
304.8596 -355.7286 -256.2187 511540.4688 -18.0095
-17.9904
[14087]ENERGY: 3950000 3174.7925 5322.1698 5441.4253
331.3758 -14431.2449 -1635.1522 0.0000 0.0000
7835.3157 6038.6820 306.4001 -1796.6337 6104.4742
304.9031 60.9676 115.2386 511540.4688 -13.9612
-13.9787
[14137]ENERGY: 3960000 3250.8142 5313.0723 5483.9305
305.8924 -14326.9758 -1732.8370 0.0000 0.0000
7745.9695 6039.8661 302.9063 -1706.1034 6105.3416
305.0132 -5.8594 53.4876 511540.4688 -15.5005
-15.4762
[14173]ENERGY: 3970000 3181.4626 5276.2588 5460.5575

315.9443 -14324.5218 -1741.2843 0.0000 0.0000
7873.3418 6041.7588 307.8872 -1831.5830 6105.6194
304.9395 -16.1964 -135.1479 511540.4688 -15.2547
-15.2942
[14223]ENERGY: 3980000 3207.9900 5423.9294 5464.0428
311.9534 -14301.0898 -1743.7075 0.0000 0.0000
7682.8599 6045.9782 300.4384 -1636.8817 6106.7216
304.9447 89.6688 11.3344 511540.4688 -11.9672
-11.9538
[14259]ENERGY: 3990000 3249.4694 5360.2744 5426.0072
303.3590 -14347.7135 -1738.3571 0.0000 0.0000
7792.4188 6045.4581 304.7227 -1746.9607 6106.4783
304.9144 -13.3583 -103.5156 511540.4688 -11.6405
-11.6516
[14312]ENERGY: 4000000 3221.3080 5257.6159 5488.0306
338.1267 -14314.7887 -1712.0553 0.0000 0.0000
7768.8082 6047.0454 303.7994 -1721.7628 6107.9048
304.8248 192.8945 69.6412 511540.4688 -13.9610
-13.9725
[14348]ENERGY: 4010000 3253.3165 5355.8803 5401.2360
331.4850 -14328.8508 -1762.1601 0.0000 0.0000
7793.5111 6044.4179 304.7654 -1749.0932 6107.5523
304.8692 -109.1446 -166.2865 511540.4688 -19.0620
-19.0091
[14398]ENERGY: 4020000 3221.9139 5357.3869 5505.8188
317.1618 -14336.3177 -1788.3619 0.0000 0.0000
7764.4576 6042.0594 303.6292 -1722.3983 6106.2110
305.1061 -88.4895 -70.1379 511540.4688 -9.7386
-9.7683
[14434]ENERGY: 4030000 3211.6380 5332.7649 5490.6250
331.1660 -14369.1378 -1740.7644 0.0000 0.0000
7784.6011 6040.8928 304.4169 -1743.7083 6106.8672
305.1361 55.1513 43.1252 511540.4688 -12.4017
-12.3947
[14484]ENERGY: 4040000 3105.7959 5270.7942 5509.7589
310.4960 -14331.8377 -1651.0775 0.0000 0.0000
7830.4347 6044.3646 306.2093 -1786.0702 6106.2995
305.1181 146.3992 127.4942 511540.4688 -11.6705
-11.6638
[14520]ENERGY: 4050000 3230.9994 5373.0334 5504.9328
308.4876 -14466.0705 -1698.2602 0.0000 0.0000
7785.7806 6038.9031 304.4631 -1746.8775 6107.0072
304.9504 44.0769 60.4280 511540.4688 -13.8025
-13.8184
[14570]ENERGY: 4060000 3195.3970 5305.7890 5479.8091
338.3509 -14283.5785 -1728.7342 0.0000 0.0000
7736.2545 6043.2877 302.5264 -1692.9668 6106.7191
304.8002 7.8107 -118.7212 511540.4688 -11.7356
-11.7378
[14606]ENERGY: 4070000 3205.0210 5310.4382 5491.7955
291.2390 -14228.9133 -1769.8582 0.0000 0.0000
7746.4896 6046.2118 302.9266 -1700.2778 6107.3226
304.7227 36.7560 -3.0717 511540.4688 -13.3803
-13.3718

[14656]ENERGY: 4080000 3190.2017 5298.0378 5487.2337
320.8098 -14376.6216 -1701.6344 0.0000 0.0000
7822.9165 6040.9435 305.9153 -1781.9730 6107.6976
304.6738 197.8097 78.3379 511540.4688 -14.9803
-14.9759
[14692]ENERGY: 4090000 3235.4107 5317.8195 5490.9406
332.7188 -14384.7916 -1694.8545 0.0000 0.0000
7749.0775 6046.3209 303.0278 -1702.7566 6107.9149
304.8615 39.0645 33.6472 511540.4688 -14.1765
-14.1761
[14745]ENERGY: 4100000 3266.4135 5373.8107 5483.4338
313.7750 -14288.0607 -1796.0966 0.0000 0.0000
7694.3402 6047.6159 300.8873 -1646.7243 6108.4911
304.8309 -214.8144 -140.0010 511540.4688 -12.4019
-12.4104
[14781]ENERGY: 4110000 3224.0547 5366.0078 5455.1705
339.9823 -14438.6654 -1703.8915 0.0000 0.0000
7802.1386 6044.7970 305.1028 -1757.3416 6107.9466
304.9208 291.1732 224.0529 511540.4688 -14.6935
-14.6930
[14831]ENERGY: 4120000 3206.3283 5214.5938 5491.2014
310.7916 -14253.7622 -1689.1693 0.0000 0.0000
7762.8937 6042.8773 303.5681 -1720.0164 6106.9932
304.7254 35.7586 2.9024 511540.4688 -17.2325
-17.2373
[14867]ENERGY: 4130000 3167.2036 5275.9937 5437.3315
326.6114 -14258.8524 -1702.0314 0.0000 0.0000
7799.2524 6045.5089 304.9899 -1753.7435 6107.3508
304.8255 -44.6756 -122.9925 511540.4688 -13.7748
-13.7835
[14917]ENERGY: 4140000 3232.8894 5353.5918 5392.4107
322.1997 -14334.2537 -1687.5283 0.0000 0.0000
7761.6093 6040.9190 303.5179 -1720.6903 6108.1705
304.7523 -47.8917 24.0598 511540.4688 -17.6431
-17.6043
[14953]ENERGY: 4150000 3257.4980 5235.5452 5528.5946
351.1694 -14279.9401 -1804.4494 0.0000 0.0000
7757.4451 6045.8627 303.3550 -1711.5824 6107.9944
304.9262 -396.8850 -255.3032 511540.4688 -17.4271
-17.4478
[15003]ENERGY: 4160000 3187.0449 5215.9923 5441.2436
322.0258 -14336.7916 -1680.0432 0.0000 0.0000
7893.2689 6042.7407 308.6664 -1850.5283 6109.1161
304.7131 90.5120 -2.2436 511540.4688 -18.8066
-18.8219
[15039]ENERGY: 4170000 3245.9220 5240.5398 5522.1001
304.4061 -14355.0204 -1684.9406 0.0000 0.0000
7772.0086 6045.0157 303.9245 -1726.9929 6108.7056
304.7303 48.3822 8.6909 511540.4688 -18.0779
-18.0675
[15089]ENERGY: 4180000 3207.6910 5357.8875 5425.0980
310.9402 -14245.0859 -1791.8747 0.0000 0.0000
7781.1154 6045.7715 304.2806 -1735.3439 6109.0956
305.0744 -59.5847 -46.2728 511540.4688 -17.1455

-17.1453
[15125]ENERGY: 4190000 3252.3789 5336.9573 5453.2567
293.9923 -14347.1297 -1761.2554 0.0000 0.0000
7814.7364 6042.9365 305.5954 -1771.7998 6110.6296
304.9925 -62.9059 -82.4478 511540.4688 -17.8260
-17.8292
[15178]ENERGY: 4200000 3276.5543 5231.9229 5426.0808
316.5269 -14291.6067 -1779.2480 0.0000 0.0000
7864.7512 6044.9815 307.5512 -1819.7697 6109.3312
305.0352 -115.3457 -168.7040 511540.4688 -17.5207
-17.5117
[15214]ENERGY: 4210000 3217.7588 5292.9010 5464.0416
329.2153 -14377.2936 -1651.1967 0.0000 0.0000
7770.8251 6046.2516 303.8782 -1724.5736 6109.2023
304.8666 123.9648 88.7612 511540.4688 -18.4293
-18.4099
[15264]ENERGY: 4220000 3170.9750 5202.1557 5504.7149
335.4599 -14314.5732 -1699.7720 0.0000 0.0000
7848.5246 6047.4848 306.9167 -1801.0398 6109.0546
304.8412 205.6385 8.9286 511540.4688 -11.8030
-11.8239
[15300]ENERGY: 4230000 3226.3041 5329.4777 5445.1563
325.9914 -14371.4903 -1727.6265 0.0000 0.0000
7815.1514 6042.9640 305.6116 -1772.1873 6109.6265
304.7938 162.5627 68.1878 511540.4688 -22.8964
-22.9015
[15350]ENERGY: 4240000 3118.4838 5279.3645 5508.4310
310.6429 -14306.9028 -1663.3680 0.0000 0.0000
7799.7313 6046.3827 305.0086 -1753.3486 6110.2954
304.8211 67.0823 -18.3613 511540.4688 -18.5287
-18.5294
[15386]ENERGY: 4250000 3155.5841 5255.1729 5473.4878
332.0224 -14343.3267 -1704.3345 0.0000 0.0000
7874.1990 6042.8050 307.9207 -1831.3940 6109.2178
304.8462 14.4453 29.3422 511540.4688 -15.2624
-15.2648
[15436]ENERGY: 4260000 3240.2302 5308.2759 5445.3540
317.2974 -14277.7751 -1775.0374 0.0000 0.0000
7789.7480 6048.0931 304.6182 -1741.6549 6110.8832
304.7821 -196.7982 -221.2930 511540.4688 -15.9699
-15.9708
[15472]ENERGY: 4270000 3210.5135 5340.8061 5478.8756
324.7293 -14372.9301 -1717.8491 0.0000 0.0000
7783.5842 6047.7294 304.3772 -1735.8548 6110.5572
304.8626 -236.1865 -170.8471 511540.4688 -12.6144
-12.6028
[15522]ENERGY: 4280000 3256.6675 5250.4328 5473.5519
326.8754 -14266.6871 -1749.3668 0.0000 0.0000
7757.5827 6049.0563 303.3604 -1708.5264 6110.8329
304.9798 -37.3740 11.0951 511540.4688 -19.3815
-19.3796
[15558]ENERGY: 4290000 3204.1496 5248.2137 5458.0568
321.2884 -14199.9284 -1777.7390 0.0000 0.0000
7794.3373 6048.3783 304.7977 -1745.9590 6110.8248

305.1394 -261.7339 -190.9515 511540.4688 -15.6733
-15.6856
[15611]ENERGY: 4300000 3228.5093 5348.3264 5446.3816
320.6529 -14387.0008 -1720.7417 0.0000 0.0000
7810.7638 6046.8915 305.4400 -1763.8723 6110.9748
304.9877 -122.1982 -69.5901 511540.4688 -20.1380
-20.1137
[15647]ENERGY: 4310000 3286.4770 5295.1806 5502.0276
328.1017 -14339.8611 -1740.9291 0.0000 0.0000
7718.9074 6049.9041 301.8480 -1669.0033 6111.5184
305.1233 -102.6735 -93.4922 511540.4688 -15.5484
-15.5620
[15697]ENERGY: 4320000 3229.8581 5344.9957 5448.0841
311.0696 -14399.3230 -1722.6315 0.0000 0.0000
7836.6012 6048.6542 306.4504 -1787.9470 6111.5824
305.2727 -75.5253 -62.2509 511540.4688 -14.5869
-14.6035
[15733]ENERGY: 4330000 3280.9280 5263.6269 5454.6852
322.9161 -14391.1962 -1683.9384 0.0000 0.0000
7803.9443 6050.9659 305.1734 -1752.9784 6111.0765
305.0121 44.9705 0.1446 511540.4688 -16.7444
-16.7642
[15783]ENERGY: 4340000 3261.2792 5311.5768 5465.8961
306.3797 -14361.8059 -1679.1497 0.0000 0.0000
7744.5129 6048.6891 302.8493 -1695.8237 6112.3388
304.8076 -18.9134 16.9048 511540.4688 -15.5086
-15.4616
[15819]ENERGY: 4350000 3208.5356 5410.9791 5443.1569
336.8104 -14380.8583 -1722.6914 0.0000 0.0000
7755.7701 6051.7024 303.2895 -1704.0677 6111.9309
305.0093 -77.0575 -83.1712 511540.4688 -15.9258
-15.9425
[15869]ENERGY: 4360000 3293.1155 5372.7433 5477.6466
331.6495 -14364.3308 -1730.4295 0.0000 0.0000
7672.5284 6052.9229 300.0343 -1619.6055 6113.0658
305.0876 -67.4447 -152.8898 511540.4688 -17.3480
-17.3462
[15905]ENERGY: 4370000 3277.3704 5291.1950 5479.9263
321.8807 -14367.5384 -1737.2675 0.0000 0.0000
7787.3265 6052.8930 304.5235 -1734.4335 6113.4355
305.0404 -24.9622 -49.5116 511540.4688 -15.5782
-15.5703
[15955]ENERGY: 4380000 3182.9191 5410.9194 5375.6577
340.3779 -14364.2119 -1708.8569 0.0000 0.0000
7809.7741 6046.5794 305.4013 -1763.1947 6112.8037
304.9971 -117.4058 -57.6681 511540.4688 -17.3778
-17.3907
[15991]ENERGY: 4390000 3331.3627 5251.6732 5466.7838
344.1015 -14437.5070 -1659.4373 0.0000 0.0000
7751.8965 6048.8734 303.1380 -1703.0231 6112.4462
305.1115 -7.2832 26.2438 511540.4688 -16.5232
-16.4857
[16044]ENERGY: 4400000 3262.4413 5301.9537 5468.5678
325.1835 -14394.3204 -1695.7439 0.0000 0.0000

7782.6034 6050.6855 304.3388 -1731.9179 6112.9478
305.0767 -172.7036 -153.9021 511540.4688 -18.1718
-18.2000
[16080]ENERGY: 4410000 3322.4492 5305.8563 5509.8008
319.9046 -14467.8443 -1723.1299 0.0000 0.0000
7780.4211 6047.4578 304.2535 -1732.9633 6112.9274
304.9860 -180.5874 -153.3815 511540.4688 -15.7949
-15.8028
[16130]ENERGY: 4420000 3262.6929 5364.5464 5443.7195
319.7871 -14295.5684 -1803.9686 0.0000 0.0000
7759.6671 6050.8759 303.4419 -1708.7911 6112.2531
304.8818 -281.2951 -202.0532 511540.4688 -15.1634
-15.1455
[16166]ENERGY: 4430000 3217.2363 5270.9077 5397.0106
330.1983 -14383.5727 -1693.3322 0.0000 0.0000
7908.0274 6046.4755 309.2435 -1861.5519 6112.7661
305.0188 190.7688 59.8016 511540.4688 -12.0850
-12.1066
[16216]ENERGY: 4440000 3258.2154 5309.6992 5444.3012
337.5371 -14346.7560 -1689.2375 0.0000 0.0000
7735.0798 6048.8393 302.4804 -1686.2406 6113.0441
304.9570 96.4212 9.0901 511540.4688 -12.6337
-12.6284
[16252]ENERGY: 4450000 3289.6310 5196.9181 5434.4819
338.9314 -14321.1221 -1715.8746 0.0000 0.0000
7828.0347 6051.0005 306.1154 -1777.0343 6113.3526
305.0953 -141.9796 -214.9004 511540.4688 -13.2179
-13.2061
[16302]ENERGY: 4460000 3171.5415 5246.4549 5474.6183
343.9074 -14299.2684 -1710.2831 0.0000 0.0000
7823.4543 6050.4249 305.9363 -1773.0294 6113.6672
305.0015 -34.1161 2.2830 511540.4688 -11.6412
-11.6557
[16338]ENERGY: 4470000 3231.4817 5397.4529 5442.8342
347.0312 -14482.8842 -1645.5549 0.0000 0.0000
7760.3296 6050.6905 303.4678 -1709.6391 6113.0131
304.9673 75.7188 83.8012 511540.4688 -13.2550
-13.2387
[16388]ENERGY: 4480000 3284.4539 5322.3553 5459.6738
323.9502 -14402.5406 -1719.0695 0.0000 0.0000
7782.1442 6050.9674 304.3209 -1731.1768 6114.5475
304.9946 103.4072 16.1197 511540.4688 -12.2681
-12.2934
[16424]ENERGY: 4490000 3218.0272 5281.2354 5482.8363
314.4219 -14319.2447 -1709.0550 0.0000 0.0000
7783.1109 6051.3319 304.3587 -1731.7790 6113.9163
305.1078 -91.7432 8.2668 511540.4688 -18.5195
-18.4995
[16477]ENERGY: 4500000 3202.9722 5186.8873 5456.8396
333.2284 -14299.4874 -1683.7168 0.0000 0.0000
7855.0370 6051.7602 307.1713 -1803.2768 6114.0166
305.0046 109.7143 82.8695 511540.4688 -13.4526
-13.4717
[16513]ENERGY: 4510000 3260.4663 5324.4830 5481.3362

315.4182 -14357.3861 -1705.4070 0.0000 0.0000
7734.0130 6052.9237 302.4387 -1681.0894 6113.7718
304.9343 -156.4552 -62.5472 511540.4688 -15.7777
-15.7597
[16563]ENERGY: 4520000 3264.9659 5350.6132 5437.6592
318.3735 -14365.8480 -1707.7417 0.0000 0.0000
7753.1181 6051.1401 303.1858 -1701.9780 6114.2550
304.9952 75.0022 -14.9491 511540.4688 -13.1636
-13.1960
[16599]ENERGY: 4530000 3177.2010 5294.0371 5434.4621
325.7982 -14308.4958 -1684.0160 0.0000 0.0000
7810.9529 6049.9395 305.4474 -1761.0134 6113.8213
304.8392 257.7271 140.6676 511540.4688 -15.2643
-15.2457
[16649]ENERGY: 4540000 3208.9360 5363.0702 5483.7458
306.7412 -14370.2924 -1672.0298 0.0000 0.0000
7734.0149 6054.1858 302.4388 -1679.8290 6114.8008
305.0369 45.3056 34.9928 511540.4688 -10.8769
-10.8717
[16685]ENERGY: 4550000 3256.9026 5356.6349 5445.0376
325.9797 -14255.1327 -1802.1405 0.0000 0.0000
7722.1581 6049.4396 301.9751 -1672.7185 6113.6770
305.2341 48.6596 73.9961 511540.4688 -9.4881
-9.4885
[16735]ENERGY: 4560000 3225.8854 5268.3374 5485.0433
322.0760 -14399.5383 -1666.0573 0.0000 0.0000
7814.0425 6049.7890 305.5683 -1764.2534 6114.8172
304.9291 94.8874 6.8444 511540.4688 -15.2880
-15.2968
[16771]ENERGY: 4570000 3243.6440 5260.7491 5480.2840
311.6046 -14298.8950 -1748.3248 0.0000 0.0000
7801.6440 6050.7059 305.0834 -1750.9381 6115.0218
305.1379 82.8396 9.7093 511540.4688 -9.8372
-9.8577
[16821]ENERGY: 4580000 3187.2082 5316.2309 5467.6871
317.1800 -14416.3071 -1636.3527 0.0000 0.0000
7814.6486 6050.2951 305.5920 -1764.3535 6114.9611
305.1575 8.5657 -22.3297 511540.4688 -11.6139
-11.5905
[16857]ENERGY: 4590000 3239.8028 5259.9653 5437.6220
315.5869 -14399.9410 -1637.9081 0.0000 0.0000
7837.6784 6052.8062 306.4925 -1784.8721 6114.0527
304.9192 96.4527 66.4390 511540.4688 -13.9401
-13.9476
[16910]ENERGY: 4600000 3209.3362 5277.3560 5413.9113
333.1263 -14343.0786 -1656.3181 0.0000 0.0000
7820.3599 6054.6930 305.8153 -1765.6669 6116.1981
304.7930 324.9190 112.9312 511540.4688 -15.1239
-15.1255
[16946]ENERGY: 4610000 3306.4003 5255.7689 5465.6972
323.9590 -14357.4607 -1701.6685 0.0000 0.0000
7762.3462 6055.0423 303.5467 -1707.3039 6114.8942
304.8848 37.1503 -21.1593 511540.4688 -9.9518
-9.9454

[16996]ENERGY: 4620000 3211.8108 5313.0377 5483.6892
320.3410 -14408.2208 -1641.1981 0.0000 0.0000
7776.2877 6055.7475 304.0919 -1720.5402 6115.8600
304.9264 46.2773 -18.4749 511540.4688 -20.5484
-20.5515
[17032]ENERGY: 4630000 3195.4630 5366.1769 5437.4115
314.3812 -14379.3398 -1711.5283 0.0000 0.0000
7829.0460 6051.6106 306.1550 -1777.4354 6116.5442
304.9115 17.2656 -84.9602 511540.4688 -14.2485
-14.2397
[17082]ENERGY: 4640000 3250.7431 5264.8834 5440.6344
309.8929 -14283.7064 -1700.4872 0.0000 0.0000
7773.9154 6055.8755 303.9991 -1718.0399 6117.9031
305.0667 47.5560 12.2894 511540.4688 -13.7427
-13.7344
[17118]ENERGY: 4650000 3294.7448 5306.6951 5485.0361
341.5561 -14402.5135 -1764.7365 0.0000 0.0000
7793.6014 6054.3834 304.7689 -1739.2180 6116.0494
305.0187 -28.4076 -42.2621 511540.4688 -19.8440
-19.8422
[17168]ENERGY: 4660000 3233.3653 5230.7697 5437.8505
319.8298 -14345.6940 -1662.5644 0.0000 0.0000
7838.8421 6052.3990 306.5380 -1786.4431 6117.3707
305.1638 -120.2821 -43.7496 511540.4688 -13.9251
-13.9249
[17204]ENERGY: 4670000 3249.7569 5324.5835 5425.9617
307.7353 -14382.1318 -1721.3374 0.0000 0.0000
7847.1828 6051.7510 306.8642 -1795.4317 6116.6732
304.9804 61.2087 -27.4070 511540.4688 -12.2498
-12.2487
[17254]ENERGY: 4680000 3214.1104 5358.7463 5453.0104
338.0968 -14431.3015 -1706.3858 0.0000 0.0000
7828.7133 6054.9898 306.1420 -1773.7235 6117.8405
305.0077 301.3649 250.4912 511540.4688 -11.7072
-11.7211
[17290]ENERGY: 4690000 3176.8080 5279.1527 5458.8194
345.6315 -14369.5908 -1684.1228 0.0000 0.0000
7842.2079 6048.9060 306.6697 -1793.3019 6117.6751
305.1737 215.7876 129.8599 511540.4688 -14.4587
-14.4340
[17343]ENERGY: 4700000 3259.1555 5309.3110 5450.5277
331.2898 -14377.3305 -1720.1398 0.0000 0.0000
7802.0835 6054.8974 305.1006 -1747.1862 6117.4950
304.9064 151.4847 147.3636 511540.4688 -11.4604
-11.4735
[17379]ENERGY: 4710000 3231.5370 5311.0254 5453.9584
339.4182 -14459.0106 -1661.3968 0.0000 0.0000
7837.8500 6053.3816 306.4992 -1784.4684 6118.0175
305.1961 219.5321 89.1468 511540.4688 -18.3409
-18.3256
[17429]ENERGY: 4720000 3233.7984 5251.5454 5412.0956
318.4195 -14308.2861 -1691.0624 0.0000 0.0000
7838.9229 6055.4334 306.5412 -1783.4895 6119.5911
305.2144 21.1548 -123.2261 511540.4688 -13.0647

-13.1135
[17465]ENERGY: 4730000 3274.8103 5274.1018 5487.4375
316.1339 -14415.7241 -1627.8894 0.0000 0.0000
7748.6580 6057.5279 303.0114 -1691.1301 6118.9374
305.1590 101.9294 92.6436 511540.4688 -19.6503
-19.6170
[17515]ENERGY: 4740000 3194.5617 5372.3616 5434.9187
324.4534 -14307.1232 -1788.4616 0.0000 0.0000
7825.1202 6055.8308 306.0014 -1769.2893 6119.6523
305.1909 -29.9342 -122.3200 511540.4688 -13.9169
-13.9453
[17551]ENERGY: 4750000 3265.1743 5351.3377 5423.5854
323.2555 -14505.0310 -1653.7545 0.0000 0.0000
7848.2291 6052.7964 306.9051 -1795.4327 6119.6106
305.1499 77.3879 103.8359 511540.4688 -14.6343
-14.6359
[17601]ENERGY: 4760000 3245.6432 5254.3062 5450.3734
319.1806 -14237.3076 -1747.1173 0.0000 0.0000
7773.9347 6059.0132 303.9998 -1714.9215 6120.3573
305.1677 27.6524 9.4995 511540.4688 -17.4068
-17.3837
[17637]ENERGY: 4770000 3215.3535 5311.6180 5460.2899
296.2393 -14387.5808 -1693.2389 0.0000 0.0000
7855.7988 6058.4797 307.2011 -1797.3191 6120.0962
305.3680 -75.0094 -121.7184 511540.4688 -11.6941
-11.7029
[17687]ENERGY: 4780000 3232.5837 5393.4536 5433.5326
317.5433 -14307.8415 -1742.5425 0.0000 0.0000
7730.5445 6057.2737 302.3031 -1673.2708 6119.6786
305.1111 -193.0061 -247.5128 511540.4688 -14.2679
-14.2566
[17723]ENERGY: 4790000 3197.8267 5302.1515 5425.3428
309.6973 -14376.1388 -1625.7428 0.0000 0.0000
7824.5293 6057.6659 305.9783 -1766.8635 6120.1097
305.0260 201.1109 197.8657 511540.4688 -17.6629
-17.6851
[17776]ENERGY: 4800000 3292.1448 5200.9071 5414.3944
329.0243 -14362.0218 -1665.1214 0.0000 0.0000
7847.6490 6056.9764 306.8824 -1790.6725 6119.5847
305.3636 -209.2071 -133.3673 511540.4688 -13.1746
-13.1430
[17812]ENERGY: 4810000 3186.1630 5319.6246 5466.3407
315.6234 -14310.5834 -1725.3264 0.0000 0.0000
7806.1155 6057.9573 305.2583 -1748.1582 6120.6624
305.1293 97.7736 25.8032 511540.4688 -11.2271
-11.2460
[17862]ENERGY: 4820000 3244.8934 5346.8769 5430.7645
317.8717 -14410.3813 -1682.0566 0.0000 0.0000
7803.9239 6051.8926 305.1726 -1752.0313 6120.0097
305.1889 -163.7078 -221.3222 511540.4688 -12.7266
-12.7365
[17898]ENERGY: 4830000 3158.6892 5358.7706 5421.6876
326.8140 -14427.8940 -1605.4506 0.0000 0.0000
7821.1731 6053.7899 305.8471 -1767.3832 6119.3470

305.0263 89.7077 77.3132 511540.4688 -13.9304
-13.9095
[17948]ENERGY: 4840000 3242.0396 5330.5702 5424.2494
306.4317 -14332.5656 -1757.4030 0.0000 0.0000
7840.1526 6053.4749 306.5893 -1786.6777 6120.4941
305.3268 -82.0132 -99.1638 511540.4688 -14.4903
-14.5007
[17984]ENERGY: 4850000 3270.6392 5375.1878 5494.8551
334.7796 -14398.8573 -1764.3099 0.0000 0.0000
7748.1810 6060.4755 302.9927 -1687.7055 6121.3355
305.2803 -305.8113 -255.6119 511540.4688 -13.8439
-13.8532
[18034]ENERGY: 4860000 3140.0860 5290.4379 5455.0652
351.4921 -14347.2737 -1648.9058 0.0000 0.0000
7813.3609 6054.2626 305.5416 -1759.0983 6118.8631
305.3487 -55.1209 11.4331 511540.4688 -19.3732
-19.3480
[18070]ENERGY: 4870000 3231.3220 5367.6409 5411.7222
329.4703 -14385.2548 -1652.7672 0.0000 0.0000
7758.2052 6060.3387 303.3847 -1697.8665 6121.5090
305.1744 31.8841 11.6333 511540.4688 -16.4150
-16.4183
[18120]ENERGY: 4880000 3211.8841 5265.1538 5500.3056
342.8139 -14437.5832 -1665.9615 0.0000 0.0000
7840.0074 6056.6201 306.5836 -1783.3872 6121.3826
305.1037 32.6843 83.5598 511540.4688 -15.9656
-15.9696
[18156]ENERGY: 4890000 3271.8221 5358.4218 5428.5960
327.0989 -14427.8858 -1655.4686 0.0000 0.0000
7754.1770 6056.7615 303.2272 -1697.4156 6122.0068
305.3371 -145.9581 -224.6557 511540.4688 -18.8528
-18.8518
[18209]ENERGY: 4900000 3312.9802 5361.6606 5439.1724
299.6200 -14378.0349 -1725.6915 0.0000 0.0000
7751.7135 6061.4203 303.1309 -1690.2931 6122.5224
305.6073 -76.9843 -40.3172 511540.4688 -14.7585
-14.7536
[18245]ENERGY: 4910000 3172.4320 5321.9920 5423.6385
328.4243 -14316.8759 -1722.5584 0.0000 0.0000
7848.6599 6055.7123 306.9220 -1792.9476 6123.0938
305.4949 -242.5023 -109.6179 511540.4688 -16.7533
-16.7643
[18295]ENERGY: 4920000 3157.6457 5310.1732 5485.1888
325.9998 -14338.1670 -1718.0401 0.0000 0.0000
7833.4813 6056.2816 306.3284 -1777.1997 6122.0379
305.3412 -14.5169 23.0037 511540.4688 -20.5074
-20.5015
[18331]ENERGY: 4930000 3209.9605 5277.7553 5412.3968
332.4125 -14365.4664 -1639.8210 0.0000 0.0000
7832.6529 6059.8907 306.2960 -1772.7622 6122.7190
305.1148 -114.0048 -1.7368 511540.4688 -15.8756
-15.8805
[18381]ENERGY: 4940000 3325.6723 5355.7680 5473.2580
326.5339 -14335.3201 -1723.0003 0.0000 0.0000

7641.8063 6064.7181 298.8330 -1577.0882 6123.1604
305.0459 -126.9651 -136.9235 511540.4688 -14.7597
-14.7779
[18417]ENERGY: 4950000 3183.8445 5279.4271 5453.8137
328.0054 -14342.0854 -1713.6445 0.0000 0.0000
7869.5585 6058.9193 307.7392 -1810.6393 6122.5599
305.2453 60.2641 18.4825 511540.4688 -16.0604
-16.0413
[18467]ENERGY: 4960000 3206.9350 5303.9439 5482.9551
327.7859 -14373.5645 -1708.5528 0.0000 0.0000
7818.1612 6057.6638 305.7293 -1760.4975 6123.2060
305.2681 1.4737 -154.0735 511540.4688 -16.4263
-16.4116
[18503]ENERGY: 4970000 3258.0308 5369.0681 5471.7609
321.4491 -14316.4492 -1804.3497 0.0000 0.0000
7756.7569 6056.2668 303.3281 -1700.4901 6123.4624
305.3923 -43.7887 -41.4122 511540.4688 -14.9019
-14.9258
[18553]ENERGY: 4980000 3298.9846 5312.5967 5428.4765
314.2697 -14354.4718 -1790.2220 0.0000 0.0000
7849.5966 6059.2303 306.9586 -1790.3664 6125.3364
305.3589 -25.2350 -63.3564 511540.4688 -15.3446
-15.3176
[18589]ENERGY: 4990000 3264.0781 5240.8666 5477.1805
322.6365 -14316.1442 -1764.1441 0.0000 0.0000
7837.6010 6062.0745 306.4895 -1775.5265 6124.6074
305.4109 11.6820 -65.1939 511540.4688 -20.0246
-20.0365
[18642]ENERGY: 5000000 3258.3273 5316.0132 5419.2336
332.4553 -14339.2402 -1702.8894 0.0000 0.0000
7780.7089 6064.6086 304.2647 -1716.1003 6124.8147
305.4859 -253.4686 -310.6234 511540.4688 -13.5910
-13.5768
[18678]ENERGY: 5010000 3218.3423 5313.7143 5465.5936
340.0447 -14387.1050 -1703.4942 0.0000 0.0000
7817.3093 6064.4050 305.6960 -1752.9042 6124.8281
305.5067 61.7549 -82.6236 511540.4688 -16.8989
-16.9187
[18728]ENERGY: 5020000 3212.6387 5267.0160 5462.8008
303.0286 -14361.6821 -1717.1984 0.0000 0.0000
7893.2220 6059.8256 308.6646 -1833.3964 6124.0631
305.5809 -35.9106 72.8698 511540.4688 -17.4680
-17.4558
[18764]ENERGY: 5030000 3213.0174 5304.7074 5433.3831
298.5506 -14368.4315 -1672.2331 0.0000 0.0000
7849.8548 6058.8487 306.9687 -1791.0061 6125.5774
305.5513 203.7562 109.7288 511540.4688 -14.0462
-14.0700
[18814]ENERGY: 5040000 3235.8604 5308.1024 5443.8824
322.4471 -14320.2315 -1668.2428 0.0000 0.0000
7742.8587 6064.6767 302.7846 -1678.1820 6124.4578
305.4307 88.2817 105.7722 511540.4688 -14.2935
-14.2872
[18850]ENERGY: 5050000 3244.4750 5298.9968 5461.0320

318.2751 -14421.9916 -1704.0519 0.0000 0.0000
7865.3486 6062.0840 307.5746 -1803.2646 6125.0833
305.3617 32.4088 -37.2307 511540.4688 -13.7041
-13.6872
[18900]ENERGY: 5060000 3284.0767 5250.1425 5447.9101
328.8781 -14348.0332 -1746.8575 0.0000 0.0000
7842.3552 6058.4720 306.6754 -1783.8832 6125.3178
305.3343 -69.8242 -126.2697 511540.4688 -8.4282
-8.4353
[18936]ENERGY: 5070000 3174.6149 5247.4821 5486.9983
336.8113 -14286.8210 -1677.1737 0.0000 0.0000
7781.8746 6063.7865 304.3103 -1718.0881 6125.8872
305.2140 7.0278 -69.7083 511540.4688 -12.9430
-12.9477
[18986]ENERGY: 5080000 3212.2938 5332.7162 5470.9675
328.4341 -14380.4916 -1670.8906 0.0000 0.0000
7768.9632 6061.9926 303.8054 -1706.9706 6125.1763
305.2623 30.3569 35.9473 511540.4688 -5.6048
-5.6084
[19022]ENERGY: 5090000 3340.4261 5276.7052 5470.5382
311.9986 -14347.7066 -1705.6660 0.0000 0.0000
7719.7040 6065.9995 301.8791 -1653.7045 6125.7313
305.4528 -269.1892 -160.3176 511540.4688 -11.4899
-11.4815
[19075]ENERGY: 5100000 3268.8094 5291.6216 5461.5641
338.9646 -14317.9240 -1786.2415 0.0000 0.0000
7806.6058 6063.4000 305.2774 -1743.2058 6125.7274
305.5384 -219.6605 -260.0782 511540.4688 -11.5704
-11.5674
[19111]ENERGY: 5110000 3226.4546 5427.1182 5475.4184
320.3266 -14473.0833 -1721.8560 0.0000 0.0000
7805.2582 6059.6367 305.2247 -1745.6215 6124.9387
305.6882 304.1299 181.8593 511540.4688 -10.0450
-10.0597
[19161]ENERGY: 5120000 3168.8916 5261.5313 5500.8140
320.4526 -14201.1375 -1718.1910 0.0000 0.0000
7733.6098 6065.9707 302.4229 -1667.6391 6125.7083
305.6659 117.2595 36.6995 511540.4688 -10.0474
-10.0403
[19197]ENERGY: 5130000 3207.6140 5285.7918 5405.9276
329.6917 -14292.9608 -1693.5154 0.0000 0.0000
7816.4549 6059.0039 305.6626 -1757.4510 6125.1556
305.6828 -16.4183 -74.7028 511540.4688 -11.4992
-11.5069
[19247]ENERGY: 5140000 3208.0370 5327.3220 5441.9048
309.1443 -14388.9519 -1661.4008 0.0000 0.0000
7825.2300 6061.2854 306.0057 -1763.9447 6125.9832
305.7778 82.0589 -14.1630 511540.4688 -12.6222
-12.6099
[19283]ENERGY: 5150000 3302.8545 5244.1555 5479.1644
312.9031 -14400.5537 -1661.4196 0.0000 0.0000
7787.3871 6064.4913 304.5259 -1722.8958 6125.1602
305.4931 -112.7276 -170.1470 511540.4688 -13.0079
-13.0247

[19333]ENERGY: 5160000 3194.6894 5253.9272 5455.5326
311.3414 -14319.9280 -1704.8305 0.0000 0.0000
7872.1703 6062.9024 307.8413 -1809.2679 6127.0852
305.6922 281.2005 196.9872 511540.4688 -11.6651
-11.6644
[19369]ENERGY: 5170000 3232.4337 5337.7193 5452.1423
322.6244 -14303.9715 -1740.9424 0.0000 0.0000
7760.5451 6060.5509 303.4762 -1699.9942 6126.5579
305.5693 -153.6694 1.6175 511540.4688 -10.4642
-10.4553
[19419]ENERGY: 5180000 3261.0518 5294.2179 5446.5449
336.8131 -14403.1661 -1675.9482 0.0000 0.0000
7803.2483 6062.7617 305.1461 -1740.4866 6127.7428
305.4207 140.7455 86.8765 511540.4688 -12.1675
-12.1682
[19455]ENERGY: 5190000 3271.0354 5205.5580 5492.2681
349.5435 -14355.6802 -1734.2982 0.0000 0.0000
7833.8733 6062.2999 306.3437 -1771.5735 6128.0356
305.5496 129.0366 87.4642 511540.4688 -6.8390
-6.8344
[19508]ENERGY: 5200000 3244.2009 5288.7880 5494.9673
314.6558 -14431.7624 -1683.6155 0.0000 0.0000
7835.1302 6062.3642 306.3929 -1772.7660 6127.6323
305.5626 109.8966 40.9076 511540.4688 -13.4242
-13.4339
[19544]ENERGY: 5210000 3149.2739 5342.2788 5473.7165
311.0853 -14344.2701 -1699.2007 0.0000 0.0000
7829.7296 6062.6133 306.1817 -1767.1163 6128.9824
305.5411 203.0288 132.5681 511540.4688 -9.3336
-9.3184
[19594]ENERGY: 5220000 3228.0068 5301.5165 5468.9584
308.1674 -14376.3629 -1675.1505 0.0000 0.0000
7809.2585 6064.3941 305.3812 -1744.8643 6127.3861
305.3131 138.6008 98.0094 511540.4688 -10.9970
-11.0073
[19630]ENERGY: 5230000 3265.7744 5388.7310 5490.9367
323.6558 -14426.2506 -1779.6436 0.0000 0.0000
7802.4664 6065.6701 305.1156 -1736.7963 6127.8199
305.1926 -28.8585 22.2093 511540.4688 -9.3695
-9.3700
[19680]ENERGY: 5240000 3201.9646 5313.2022 5513.2558
324.2632 -14336.4606 -1766.0372 0.0000 0.0000
7817.9067 6068.0947 305.7194 -1749.8120 6129.5305
305.5812 -5.6355 -16.1373 511540.4688 -13.7540
-13.7628
[19716]ENERGY: 5250000 3238.9742 5315.1950 5443.5000
335.1148 -14263.2207 -1788.2565 0.0000 0.0000
7783.4063 6064.7131 304.3702 -1718.6932 6129.5626
305.4669 50.7136 -35.1851 511540.4688 -10.7862
-10.7770
[19766]ENERGY: 5260000 3231.7240 5319.4321 5500.2459
313.0842 -14339.3785 -1761.3815 0.0000 0.0000
7805.2295 6068.9556 305.2236 -1736.2739 6129.9708
305.5861 180.5899 48.6724 511540.4688 -13.7102

-13.7174
[19802]ENERGY: 5270000 3275.7962 5309.8921 5437.7028
313.3998 -14456.1687 -1603.8917 0.0000 0.0000
7792.2810 6069.0116 304.7173 -1723.2694 6128.2041
305.4710 48.6791 87.1075 511540.4688 -10.2766
-10.2587
[19852]ENERGY: 5280000 3211.6494 5387.6577 5458.8068
322.6747 -14358.5434 -1724.3224 0.0000 0.0000
7769.1136 6067.0364 303.8113 -1702.0772 6130.5251
305.6420 3.4088 62.6216 511540.4688 -10.7958
-10.7906
[19888]ENERGY: 5290000 3185.4734 5283.5518 5473.1274
326.2680 -14250.5382 -1768.0840 0.0000 0.0000
7817.0988 6066.8972 305.6878 -1750.2016 6129.6568
305.6359 11.9190 34.7758 511540.4688 -16.7922
-16.8087
[19941]ENERGY: 5300000 3285.9576 5276.0824 5456.2157
312.4493 -14336.1676 -1715.3930 0.0000 0.0000
7791.7439 6070.8882 304.6963 -1720.8556 6131.3945
305.4786 3.3478 -118.9806 511540.4688 -6.9022
-6.9036
[19977]ENERGY: 5310000 3216.2921 5312.2191 5437.2274
335.1663 -14398.1809 -1643.3663 0.0000 0.0000
7805.8985 6065.2564 305.2498 -1740.6421 6130.1504
305.3877 35.1192 22.0049 511540.4688 -11.0176
-11.0106
[20027]ENERGY: 5320000 3280.4868 5262.9857 5475.9046
331.1579 -14387.1402 -1628.2843 0.0000 0.0000
7731.7903 6066.9007 302.3518 -1664.8896 6128.9182
305.3104 50.6076 85.0593 511540.4688 -9.9394
-9.9313
[20063]ENERGY: 5330000 3186.6294 5402.4208 5420.6089
316.7358 -14384.0245 -1681.2975 0.0000 0.0000
7804.8397 6065.9126 305.2084 -1738.9271 6130.1507
305.1433 152.4599 50.6784 511540.4688 -14.2485
-14.2481
[20113]ENERGY: 5340000 3246.8739 5311.9496 5459.6516
313.6604 -14317.0755 -1767.1491 0.0000 0.0000
7819.8624 6067.7732 305.7958 -1752.0891 6130.1444
305.4029 -56.6069 -18.9118 511540.4688 -10.8844
-10.8926
[20149]ENERGY: 5350000 3235.1602 5332.9206 5452.7204
312.0022 -14251.6091 -1769.4110 0.0000 0.0000
7757.3436 6069.1269 303.3510 -1688.2167 6129.5801
305.4546 29.2269 59.5448 511540.4688 -13.0139
-13.0126
[20199]ENERGY: 5360000 3222.8249 5244.2161 5436.6928
307.2597 -14277.0435 -1753.3758 0.0000 0.0000
7885.7238 6066.2980 308.3714 -1819.4258 6131.3715
305.4980 47.1685 74.4116 511540.4688 -14.8428
-14.8281
[20235]ENERGY: 5370000 3163.3840 5307.7840 5500.9384
315.4780 -14301.9418 -1761.9869 0.0000 0.0000
7841.7181 6065.3737 306.6505 -1776.3443 6131.5921

305.4632 183.2084 129.9032 511540.4688 -10.7714
-10.8154
[20285]ENERGY: 5380000 3305.4610 5286.4545 5529.3998
316.6651 -14298.1912 -1839.2396 0.0000 0.0000
7771.5626 6072.1123 303.9071 -1699.4504 6130.6999
305.4510 22.5344 -67.5217 511540.4688 -15.3129
-15.2719
[20321]ENERGY: 5390000 3262.6177 5481.4868 5430.8307
323.2904 -14451.0775 -1707.2802 0.0000 0.0000
7729.1856 6069.0535 302.2499 -1660.1321 6130.8838
305.4325 45.8044 9.3724 511540.4688 -16.1582
-16.1794
[20374]ENERGY: 5400000 3290.5259 5292.1397 5414.1909
327.2415 -14363.1317 -1771.9760 0.0000 0.0000
7878.2540 6067.2444 308.0792 -1811.0097 6132.8537
305.6155 -192.5861 -172.6668 511540.4688 -10.5230
-10.5160
[20410]ENERGY: 5410000 3229.2591 5242.6065 5398.3187
304.0927 -14314.1201 -1651.8259 0.0000 0.0000
7859.8113 6068.1423 307.3580 -1791.6690 6132.2975
305.5013 -169.3908 2.9017 511540.4688 -14.5842
-14.5724
[20460]ENERGY: 5420000 3161.3378 5261.6653 5472.7669
330.0865 -14282.3289 -1703.8048 0.0000 0.0000
7827.9991 6067.7219 306.1140 -1760.2772 6132.7842
305.3134 32.1383 23.6043 511540.4688 -8.9240
-8.9354
[20496]ENERGY: 5430000 3168.2940 5385.3634 5416.2834
313.1619 -14308.8578 -1678.0312 0.0000 0.0000
7776.1786 6072.3922 304.0876 -1703.7864 6133.6325
305.5494 173.5936 145.4711 511540.4688 -10.7696
-10.7680
[20546]ENERGY: 5440000 3253.7349 5246.3136 5459.8332
346.0803 -14313.2581 -1709.5319 0.0000 0.0000
7784.5247 6067.6966 304.4140 -1716.8281 6133.4268
305.6046 -23.8532 -80.2318 511540.4688 -13.5468
-13.5338
[20582]ENERGY: 5450000 3172.9307 5423.8139 5430.1796
314.7710 -14353.0253 -1688.5656 0.0000 0.0000
7771.9954 6072.0999 303.9240 -1699.8955 6134.6286
305.4665 -9.3619 -83.0459 511540.4688 -14.0327
-14.0329
[20632]ENERGY: 5460000 3236.0168 5362.2162 5451.1853
322.8263 -14444.9065 -1619.8850 0.0000 0.0000
7766.3718 6073.8248 303.7041 -1692.5470 6135.1846
305.5307 66.4704 139.7900 511540.4688 -12.0957
-12.1111
[20668]ENERGY: 5470000 3192.5149 5266.7895 5499.8637
323.3185 -14305.8211 -1738.8237 0.0000 0.0000
7836.0294 6073.8712 306.4281 -1762.1582 6136.0441
305.4396 106.1637 22.3836 511540.4688 -10.5251
-10.5355
[20718]ENERGY: 5480000 3279.0525 5320.6884 5542.3276
339.7029 -14348.4181 -1754.4383 0.0000 0.0000

7698.8065 6077.7216 301.0619 -1621.0849 6136.9415
305.5704 -49.9462 47.4135 511540.4688 -13.4954
-13.4942
[20754]ENERGY: 5490000 3267.0571 5228.7799 5474.4111
330.5581 -14339.8403 -1635.4693 0.0000 0.0000
7750.3721 6075.8688 303.0784 -1674.5034 6136.9522
305.5111 -166.0070 -55.2117 511540.4688 -11.8690
-11.8609
[20807]ENERGY: 5500000 3250.1757 5352.7444 5459.6240
332.9130 -14333.4971 -1759.9750 0.0000 0.0000
7773.0726 6075.0576 303.9661 -1698.0150 6137.4240
305.4505 115.7214 60.1418 511540.4688 -15.7424
-15.7289
[20843]ENERGY: 5510000 3251.6729 5295.3655 5457.1236
308.3905 -14245.5185 -1722.8327 0.0000 0.0000
7736.6757 6080.8771 302.5428 -1655.7987 6138.5892
305.5913 63.3769 23.8876 511540.4688 -11.1644
-11.1687
[20893]ENERGY: 5520000 3196.7865 5304.2461 5479.8585
323.0525 -14367.1146 -1757.3146 0.0000 0.0000
7896.1628 6075.6772 308.7796 -1820.4856 6139.1163
305.5853 -107.5310 -35.9144 511540.4688 -16.2295
-16.2294
[20929]ENERGY: 5530000 3242.6640 5325.8551 5456.3002
307.1654 -14246.8888 -1774.0787 0.0000 0.0000
7767.8087 6078.8259 303.7603 -1688.9828 6138.8580
305.7861 -207.6447 -202.1277 511540.4688 -13.8377
-13.8391
[20979]ENERGY: 5540000 3215.1096 5268.4505 5450.7778
330.8219 -14266.0458 -1730.6126 0.0000 0.0000
7805.8607 6074.3622 305.2483 -1731.4985 6138.6756
305.5708 20.4595 33.6822 511540.4688 -15.3670
-15.3778
[21015]ENERGY: 5550000 3306.9232 5285.8700 5445.2514
327.1373 -14382.4268 -1737.2324 0.0000 0.0000
7830.8966 6076.4192 306.2273 -1754.4773 6137.3529
305.7937 -92.4692 -60.6097 511540.4688 -18.4764
-18.4584
[21065]ENERGY: 5560000 3233.9607 5323.9445 5481.3931
333.4352 -14397.8985 -1713.9798 0.0000 0.0000
7814.1827 6075.0378 305.5737 -1739.1449 6138.7671
305.5645 -100.8624 -132.5725 511540.4688 -17.6733
-17.6920
[21101]ENERGY: 5570000 3254.1787 5307.8881 5441.7976
323.2190 -14234.1623 -1808.4563 0.0000 0.0000
7789.5665 6074.0312 304.6111 -1715.5352 6137.8575
305.7003 -350.6904 -263.9094 511540.4688 -11.8607
-11.8625
[21151]ENERGY: 5580000 3276.4865 5332.8977 5450.4691
328.3772 -14314.3536 -1735.3812 0.0000 0.0000
7739.8662 6078.3620 302.6676 -1661.5042 6137.8390
305.8220 -56.9912 70.6801 511540.4688 -15.5328
-15.5000
[21187]ENERGY: 5590000 3263.7755 5289.8214 5418.3435

317.7703 -14330.3883 -1659.5299 0.0000 0.0000
7776.1841 6075.9767 304.0878 -1700.2074 6136.7033
305.7183 72.5889 -58.3400 511540.4688 -18.3880
-18.4157
[21240]ENERGY: 5600000 3212.4685 5271.1762 5473.4019
324.6429 -14312.4408 -1706.1873 0.0000 0.0000
7811.5028 6074.5640 305.4689 -1736.9387 6137.3178
305.8395 3.2711 45.3950 511540.4688 -12.4027
-12.4054
[21276]ENERGY: 5610000 3145.6704 5273.0023 5417.3523
321.9601 -14341.9657 -1662.9864 0.0000 0.0000
7920.0497 6073.0826 309.7137 -1846.9670 6137.5414
305.8648 247.9873 142.2431 511540.4688 -20.1871
-20.2023
[21326]ENERGY: 5620000 3175.6365 5392.6993 5418.9267
323.9232 -14345.1918 -1732.9226 0.0000 0.0000
7843.4978 6076.5691 306.7201 -1766.9288 6137.6930
306.0079 -20.6740 -6.0454 511540.4688 -21.8980
-21.8813
[21362]ENERGY: 5630000 3334.9276 5371.1461 5387.1415
309.4439 -14337.1584 -1757.2718 0.0000 0.0000
7766.4972 6074.7261 303.7090 -1691.7711 6138.2284
305.9234 -18.4753 17.7167 511540.4688 -16.2349
-16.2245
[21412]ENERGY: 5640000 3196.9199 5350.3162 5472.3158
331.3809 -14297.0044 -1704.4980 0.0000 0.0000
7729.5181 6078.9484 302.2629 -1650.5696 6138.0865
305.8410 -62.8038 -115.3282 511540.4688 -19.7191
-19.7352
[21448]ENERGY: 5650000 3291.9711 5276.5781 5454.6483
340.8497 -14387.3817 -1710.1980 0.0000 0.0000
7808.8708 6075.3384 305.3660 -1733.5324 6137.5791
305.9467 39.2573 49.4714 511540.4688 -17.1953
-17.1847
[21498]ENERGY: 5660000 3193.3887 5288.8795 5458.1015
314.6177 -14299.7881 -1706.5657 0.0000 0.0000
7825.4153 6074.0488 306.0130 -1751.3665 6138.4024
305.9214 58.8726 -63.1076 511540.4688 -19.1344
-19.1374
[21534]ENERGY: 5670000 3189.4420 5357.6649 5443.1465
305.3575 -14371.1218 -1672.9811 0.0000 0.0000
7824.5062 6076.0142 305.9774 -1748.4919 6138.3452
305.9506 12.5862 24.5755 511540.4688 -21.9142
-21.9051
[21584]ENERGY: 5680000 3228.6047 5317.1613 5398.9084
304.8397 -14346.7157 -1699.0051 0.0000 0.0000
7870.3721 6074.1655 307.7710 -1796.2066 6137.4650
305.8051 -3.3329 -43.8875 511540.4688 -18.3247
-18.3360
[21620]ENERGY: 5690000 3261.6132 5348.4770 5443.8442
318.6105 -14399.3099 -1667.3694 0.0000 0.0000
7769.4844 6075.3500 303.8258 -1694.1344 6137.6214
305.7855 75.4757 86.9759 511540.4688 -16.1433
-16.1544

[21673]ENERGY: 5700000 3209.0705 5368.0474 5500.6827
340.6747 -14361.5648 -1707.2350 0.0000 0.0000
7729.0053 6078.6808 302.2429 -1650.3245 6138.3003
305.9151 -223.2544 -196.1957 511540.4688 -20.5380
-20.5447
[21709]ENERGY: 5710000 3224.8248 5390.9038 5418.2583
325.8495 -14393.2707 -1682.0978 0.0000 0.0000
7791.3486 6075.8164 304.6808 -1715.5322 6137.8710
306.0657 102.0123 189.3761 511540.4688 -20.1179
-20.0977
[21759]ENERGY: 5720000 3314.7229 5322.2621 5427.8610
338.1449 -14408.9574 -1706.9718 0.0000 0.0000
7789.6584 6076.7202 304.6147 -1712.9382 6138.4351
306.0146 -124.9006 -201.0617 511540.4688 -19.0262
-19.0481
[21795]ENERGY: 5730000 3178.9767 5412.2648 5432.0326
333.5366 -14383.6705 -1739.6629 0.0000 0.0000
7839.2556 6072.7329 306.5542 -1766.5227 6138.9628
305.9486 69.0276 -23.3315 511540.4688 -19.8185
-19.7876
[21845]ENERGY: 5740000 3236.0902 5393.7451 5436.9976
323.1388 -14340.3520 -1746.4536 0.0000 0.0000
7769.1635 6072.3295 303.8133 -1696.8339 6139.0352
305.9171 -63.2335 -52.7116 511540.4688 -21.2086
-21.2144
[21881]ENERGY: 5750000 3260.3788 5239.2579 5471.4670
322.0083 -14332.4180 -1757.2650 0.0000 0.0000
7869.5582 6072.9873 307.7392 -1796.5710 6139.3296
306.0477 -2.7287 -71.2966 511540.4688 -17.4235
-17.4140
[21931]ENERGY: 5760000 3310.0212 5212.1397 5427.0587
338.2617 -14356.8226 -1763.8228 0.0000 0.0000
7908.2454 6075.0811 309.2521 -1833.1642 6139.4818
306.0118 -113.7108 -154.9840 511540.4688 -20.1946
-20.1928
[21967]ENERGY: 5770000 3273.0162 5282.6316 5482.4783
315.6057 -14321.9699 -1747.5947 0.0000 0.0000
7792.8365 6077.0037 304.7390 -1715.8328 6139.5570
305.8530 -152.3458 -203.9113 511540.4688 -17.7895
-17.8187
[22017]ENERGY: 5780000 3268.0019 5283.9677 5433.3536
310.9953 -14386.7279 -1681.8068 0.0000 0.0000
7848.9618 6076.7456 306.9338 -1772.2162 6139.9500
305.8230 141.5892 98.1929 511540.4688 -18.4279
-18.4256
[22053]ENERGY: 5790000 3221.4976 5364.5840 5460.2386
345.2952 -14339.6395 -1736.2601 0.0000 0.0000
7763.9310 6079.6467 303.6086 -1684.2843 6140.5777
305.8988 144.1647 44.8379 511540.4688 -24.2157
-24.2073
[22106]ENERGY: 5800000 3221.9814 5304.3154 5460.6553
313.6470 -14356.2136 -1719.0254 0.0000 0.0000
7846.2513 6071.6113 306.8278 -1774.6400 6139.8918
305.9916 -96.3778 -110.1803 511540.4688 -15.4694

-15.4700
[22142]ENERGY: 5810000 3094.7839 5313.4473 5402.8883
316.6431 -14291.5864 -1690.8011 0.0000 0.0000
7930.3981 6075.7733 310.1183 -1854.6249 6139.3563
306.1722 197.7352 93.4484 511540.4688 -22.4130
-22.4116
[22192]ENERGY: 5820000 3242.6374 5321.8297 5402.7920
312.0373 -14358.7790 -1712.6820 0.0000 0.0000
7867.1062 6074.9416 307.6433 -1792.1646 6139.7473
306.0043 -118.6134 -200.1717 511540.4688 -16.6136
-16.6180
[22228]ENERGY: 5830000 3267.4399 5244.7813 5450.5277
328.5304 -14447.6697 -1591.2204 0.0000 0.0000
7823.1539 6075.5433 305.9246 -1747.6107 6139.4754
305.8029 71.3667 91.7056 511540.4688 -18.1062
-18.0930
[22278]ENERGY: 5840000 3242.5001 5262.4542 5428.8378
325.1658 -14288.6785 -1749.0055 0.0000 0.0000
7852.9872 6074.2610 307.0912 -1778.7261 6139.6317
306.0405 -76.9841 -121.1426 511540.4688 -15.9701
-15.9865
[22314]ENERGY: 5850000 3318.1838 5295.5151 5458.4772
312.5885 -14348.4418 -1713.7723 0.0000 0.0000
7753.2675 6075.8179 303.1916 -1677.4496 6140.5177
306.0967 -11.7218 46.1064 511540.4688 -20.5492
-20.5383
[22364]ENERGY: 5860000 3264.1636 5380.7605 5432.1479
326.6687 -14416.8284 -1728.1453 0.0000 0.0000
7814.6994 6073.4665 305.5939 -1741.2330 6140.2651
305.8716 -37.2646 -63.2184 511540.4688 -18.1627
-18.1697
[22400]ENERGY: 5870000 3219.1092 5292.2235 5431.5647
328.0301 -14312.1256 -1730.3554 0.0000 0.0000
7847.7645 6076.2109 306.8870 -1771.5535 6141.1827
305.7637 118.7068 28.5096 511540.4688 -18.4799
-18.4626
[22450]ENERGY: 5880000 3244.7396 5305.7390 5433.7573
311.5968 -14253.3361 -1754.6010 0.0000 0.0000
7789.0074 6076.9030 304.5893 -1712.1044 6140.4999
305.9275 17.4757 -34.0399 511540.4688 -19.5602
-19.5664
[22486]ENERGY: 5890000 3227.9960 5224.7763 5470.1385
330.3117 -14369.2890 -1638.1554 0.0000 0.0000
7831.6800 6077.4582 306.2580 -1754.2219 6140.0122
305.9698 101.6859 91.9292 511540.4688 -19.4881
-19.5022
[22539]ENERGY: 5900000 3185.9137 5278.7238 5481.1747
328.5615 -14393.8274 -1684.6197 0.0000 0.0000
7875.5958 6071.5224 307.9753 -1804.0734 6139.9513
305.9826 75.6019 59.4099 511540.4688 -17.0557
-17.0437
[22575]ENERGY: 5910000 3253.7407 5417.3473 5438.5917
305.2183 -14408.2818 -1717.9429 0.0000 0.0000
7791.5855 6080.2588 304.6901 -1711.3267 6141.1844

306.1350 95.7679 93.7054 511540.4688 -19.6069
-19.6173
[22625]ENERGY: 5920000 3266.8405 5376.7767 5470.8025
321.3305 -14474.4384 -1685.9563 0.0000 0.0000
7800.0929 6075.4484 305.0228 -1724.6445 6140.0926
305.9332 124.8883 110.4317 511540.4688 -12.1243
-12.1196
[22661]ENERGY: 5930000 3235.8161 5222.0834 5457.5133
328.8527 -14295.9439 -1689.1771 0.0000 0.0000
7819.3391 6078.4836 305.7754 -1740.8556 6139.7861
305.9910 16.8691 -58.9219 511540.4688 -13.8576
-13.8632
[22711]ENERGY: 5940000 3300.3473 5336.7459 5442.5135
323.2713 -14448.6981 -1673.0325 0.0000 0.0000
7800.1250 6081.2724 305.0240 -1718.8526 6142.7486
305.7126 86.2143 -32.7618 511540.4688 -13.5398
-13.5288
[22747]ENERGY: 5950000 3269.4866 5300.2497 5384.3246
320.7602 -14314.5823 -1725.1817 0.0000 0.0000
7844.5841 6079.6412 306.7626 -1764.9429 6141.3066
305.8488 62.8231 88.3195 511540.4688 -16.4474
-16.4320
[22797]ENERGY: 5960000 3182.1555 5336.7457 5455.0566
322.7054 -14303.5727 -1718.7478 0.0000 0.0000
7802.9556 6077.2983 305.1347 -1725.6573 6142.4230
306.0384 71.9954 98.8997 511540.4688 -19.2483
-19.2683
[22833]ENERGY: 5970000 3309.3206 5268.4826 5458.5137
314.7808 -14320.7128 -1779.4579 0.0000 0.0000
7829.2773 6080.2043 306.1640 -1749.0730 6143.4645
306.0759 -119.0428 -163.0637 511540.4688 -16.7498
-16.7356
[22883]ENERGY: 5980000 3262.5430 5324.7607 5459.0421
318.8990 -14356.6399 -1717.6328 0.0000 0.0000
7789.8986 6080.8706 304.6241 -1709.0280 6142.6062
306.0360 -106.8974 -151.7336 511540.4688 -11.6411
-11.6473
[22919]ENERGY: 5990000 3257.9631 5352.1103 5453.8125
303.8047 -14355.8541 -1695.6871 0.0000 0.0000
7761.0178 6077.1673 303.4947 -1683.8505 6142.1186
306.0027 47.0358 30.8798 511540.4688 -18.4540
-18.4767
[22972]ENERGY: 6000000 3198.9940 5374.3055 5445.4211
337.5293 -14405.7022 -1733.3383 0.0000 0.0000
7862.7991 6080.0085 307.4749 -1782.7906 6143.1472
305.8932 -34.4620 -35.7652 511540.4688 -14.9833
-14.9642
[23008]ENERGY: 6010000 3195.0452 5400.4568 5417.9359
339.1554 -14327.6409 -1729.4637 0.0000 0.0000
7787.1380 6082.6266 304.5162 -1704.5114 6144.0978
306.0077 326.1235 267.6465 511540.4688 -17.6912
-17.6978
[23058]ENERGY: 6020000 3267.0685 5359.0828 5452.4283
336.5812 -14373.3691 -1689.4399 0.0000 0.0000

7732.7562 6085.1079 302.3896 -1647.6483 6144.6281
306.0681 -32.3738 -136.9579 511540.4688 -14.7680
-14.7688
[23094]ENERGY: 6030000 3279.8612 5342.6920 5435.3280
324.6134 -14522.6639 -1647.0256 0.0000 0.0000
7863.5969 6076.4021 307.5061 -1787.1948 6142.3122
306.0240 33.0453 20.9662 511540.4688 -16.7152
-16.7015
[23144]ENERGY: 6040000 3272.7253 5390.8133 5434.3619
322.9888 -14508.0217 -1661.0054 0.0000 0.0000
7829.9718 6081.8340 306.1912 -1748.1378 6143.6669
305.7743 -9.8756 30.7211 511540.4688 -13.5212
-13.5160
[23180]ENERGY: 6050000 3322.1513 5363.3042 5470.7153
320.5980 -14481.1420 -1704.0126 0.0000 0.0000
7786.8563 6078.4706 304.5051 -1708.3857 6142.4835
305.9279 -122.1205 -107.3840 511540.4688 -13.9334
-13.9386
[23230]ENERGY: 6060000 3229.6433 5417.4426 5438.2139
339.4939 -14404.8326 -1644.1936 0.0000 0.0000
7707.2696 6083.0370 301.3929 -1624.2326 6142.3399
305.7559 34.2469 60.7940 511540.4688 -6.7958
-6.8045
[23266]ENERGY: 6070000 3244.8313 5273.7031 5485.4890
323.1085 -14424.0394 -1651.7592 0.0000 0.0000
7827.2702 6078.6035 306.0855 -1748.6667 6142.6077
305.8349 41.5124 52.5274 511540.4688 -10.8679
-10.8616
[23316]ENERGY: 6080000 3230.4615 5269.5461 5471.3831
335.3063 -14371.4431 -1694.6723 0.0000 0.0000
7839.5928 6080.1742 306.5674 -1759.4185 6142.5577
306.0503 -34.7135 -36.3292 511540.4688 -12.9803
-12.9838
[23352]ENERGY: 6090000 3206.2533 5287.0212 5411.9276
331.1739 -14293.2764 -1677.9321 0.0000 0.0000
7814.4420 6079.6095 305.5839 -1734.8325 6142.2265
305.7558 158.4129 41.1429 511540.4688 -14.0349
-14.0473
[23405]ENERGY: 6100000 3179.0361 5352.2316 5507.3488
335.1653 -14386.4277 -1708.1866 0.0000 0.0000
7803.8780 6083.0455 305.1708 -1720.8325 6143.5123
305.7208 201.3890 174.1269 511540.4688 -13.4275
-13.4109
[23441]ENERGY: 6110000 3280.3728 5366.8070 5447.4115
306.1684 -14458.0986 -1723.6007 0.0000 0.0000
7863.4132 6082.4736 307.4989 -1780.9396 6143.4470
305.8967 -237.3075 -187.9404 511540.4688 -9.1972
-9.2044
[23491]ENERGY: 6120000 3247.2509 5365.1592 5481.7497
309.9575 -14427.4918 -1684.6350 0.0000 0.0000
7789.1604 6081.1508 304.5952 -1708.0096 6144.2656
306.0141 -28.7848 30.4321 511540.4688 -11.1810
-11.1713
[23527]ENERGY: 6130000 3296.3398 5395.8157 5500.9439

320.1513 -14479.5009 -1716.5416 0.0000 0.0000
7763.3302 6080.5384 303.5851 -1682.7918 6143.8744
305.8559 26.1906 -7.1998 511540.4688 -15.2782
-15.3050
[23577]ENERGY: 6140000 3148.1364 5359.3440 5439.9312
304.9025 -14399.0673 -1650.6771 0.0000 0.0000
7873.2374 6075.8072 307.8831 -1797.4302 6141.8494
305.9614 48.0091 33.1303 511540.4688 -10.9387
-10.9343
[23613]ENERGY: 6150000 3280.9465 5258.3510 5432.6801
330.6351 -14335.0354 -1727.0880 0.0000 0.0000
7834.8276 6075.3169 306.3811 -1759.5107 6142.1224
305.9352 91.6738 54.9023 511540.4688 -12.7530
-12.7424
[23663]ENERGY: 6160000 3263.6103 5339.5150 5433.9685
309.5162 -14361.6378 -1722.9725 0.0000 0.0000
7819.3852 6081.3850 305.7772 -1738.0001 6143.2994
305.9275 -125.3985 15.9425 511540.4688 -11.2416
-11.2416
[23699]ENERGY: 6170000 3198.8692 5307.7895 5503.3799
311.9050 -14384.3064 -1772.6144 0.0000 0.0000
7914.1698 6079.1926 309.4837 -1834.9772 6143.7476
305.8270 -2.9692 -108.4340 511540.4688 -15.3784
-15.3662
[23749]ENERGY: 6180000 3251.5938 5347.5773 5418.6490
313.7008 -14392.8234 -1642.2137 0.0000 0.0000
7785.6448 6082.1286 304.4578 -1703.5162 6143.6959
305.9238 222.0606 197.8575 511540.4688 -9.9302
-9.9422
[23785]ENERGY: 6190000 3201.2865 5357.0021 5467.9117
335.3740 -14416.3851 -1692.5906 0.0000 0.0000
7825.5295 6078.1281 306.0175 -1747.4014 6142.8939
305.8075 149.9290 205.3480 511540.4688 -14.2613
-14.2459
[23838]ENERGY: 6200000 3187.1925 5369.4084 5437.8738
326.2745 -14351.0293 -1691.5462 0.0000 0.0000
7802.1832 6080.3570 305.1045 -1721.8262 6144.0485
305.8522 226.2322 55.0109 511540.4688 -12.7232
-12.7302
[23874]ENERGY: 6210000 3292.4643 5293.4525 5447.6662
324.8400 -14434.6780 -1655.0685 0.0000 0.0000
7810.2443 6078.9208 305.4197 -1731.3235 6142.7786
305.8490 112.5949 33.6080 511540.4688 -13.1053
-13.1314
[23924]ENERGY: 6220000 3278.4261 5355.7051 5469.7715
313.1033 -14417.9453 -1659.1901 0.0000 0.0000
7742.3867 6082.2573 302.7662 -1660.1295 6143.3729
305.8666 153.4818 81.8438 511540.4688 -10.2149
-10.2140
[23960]ENERGY: 6230000 3212.1218 5286.2647 5494.3748
314.5935 -14377.6008 -1725.6662 0.0000 0.0000
7873.8298 6077.9176 307.9062 -1795.9121 6145.0301
305.9115 -111.7480 -148.8359 511540.4688 -9.6294
-9.6178

[24010]ENERGY: 6240000 3217.6050 5349.8733 5476.5773
333.5469 -14362.1281 -1691.0547 0.0000 0.0000
7757.7527 6082.1724 303.3670 -1675.5803 6144.6518
305.7484 205.2354 36.8210 511540.4688 -7.6254
-7.6273
[24046]ENERGY: 6250000 3157.2450 5338.1716 5493.0980
333.2677 -14409.5828 -1706.2340 0.0000 0.0000
7874.0591 6080.0247 307.9152 -1794.0344 6144.9656
305.9234 35.7278 47.2138 511540.4688 -10.8828
-10.8539
[24096]ENERGY: 6260000 3288.6200 5355.6577 5447.8892
313.0771 -14446.8043 -1704.3137 0.0000 0.0000
7825.7431 6079.8691 306.0258 -1745.8740 6144.2723
305.7970 -81.4007 -2.9841 511540.4688 -8.8402
-8.8763
[24132]ENERGY: 6270000 3284.6118 5294.4823 5466.3576
312.6449 -14385.8417 -1744.7808 0.0000 0.0000
7853.7113 6081.1855 307.1195 -1772.5259 6145.2343
305.8411 -25.3290 -5.9760 511540.4688 -14.6825
-14.6504
[24182]ENERGY: 6280000 3288.3270 5289.6623 5439.6559
317.2276 -14330.8369 -1731.6829 0.0000 0.0000
7813.0359 6085.3890 305.5289 -1727.6470 6145.6487
305.8185 64.6097 54.0550 511540.4688 -14.3286
-14.3543
[24218]ENERGY: 6290000 3252.9860 5260.1523 5481.9500
330.3256 -14473.7136 -1590.5458 0.0000 0.0000
7820.3739 6081.5283 305.8158 -1738.8456 6144.3984
306.0008 169.1121 168.3428 511540.4688 -10.9148
-10.8868
[24271]ENERGY: 6300000 3201.2998 5278.0611 5403.3736
327.7921 -14387.2889 -1756.1658 0.0000 0.0000
8008.5592 6075.6311 313.1748 -1932.9281 6144.3479
306.0609 -81.5320 -57.8172 511540.4688 -15.9668
-15.9862
[24307]ENERGY: 6310000 3204.4037 5402.4414 5464.0112
329.1212 -14410.7788 -1704.4555 0.0000 0.0000
7795.5838 6080.3270 304.8464 -1715.2568 6143.3960
306.0187 177.4414 150.1460 511540.4688 -15.2026
-15.1901
[24357]ENERGY: 6320000 3204.3928 5310.0844 5455.9320
324.4027 -14349.4848 -1650.8830 0.0000 0.0000
7788.9963 6083.4404 304.5888 -1705.5559 6145.8208
306.1217 296.4325 130.9457 511540.4688 -14.8726
-14.8743
[24393]ENERGY: 6330000 3204.3971 5409.3029 5414.2266
326.6411 -14396.2524 -1655.0420 0.0000 0.0000
7783.3967 6086.6701 304.3698 -1696.7266 6144.6747
305.9731 161.1914 198.3007 511540.4688 -16.1328
-16.1425
[24443]ENERGY: 6340000 3147.9541 5369.9915 5513.5757
323.9395 -14399.0680 -1714.1531 0.0000 0.0000
7840.2679 6082.5077 306.5938 -1757.7602 6144.3299
306.1513 175.3000 87.1860 511540.4688 -13.7836

-13.7784
[24479]ENERGY: 6350000 3215.7392 5469.4825 5421.6256
310.8972 -14340.7441 -1770.4535 0.0000 0.0000
7774.4122 6080.9591 304.0185 -1693.4531 6145.1329
305.9545 72.6757 51.0247 511540.4688 -16.9190
-16.8955
[24529]ENERGY: 6360000 3213.2956 5360.9966 5478.1308
303.5904 -14356.5484 -1703.3147 0.0000 0.0000
7788.4635 6084.6138 304.5680 -1703.8496 6145.1075
305.9405 -16.5815 -59.1256 511540.4688 -16.3523
-16.3706
[24565]ENERGY: 6370000 3150.9978 5385.5701 5473.9436
337.0385 -14312.4811 -1777.6583 0.0000 0.0000
7824.1520 6081.5626 305.9636 -1742.5893 6145.3743
306.1142 78.9562 130.0795 511540.4688 -12.6934
-12.6815
[24615]ENERGY: 6380000 3283.3755 5343.5382 5435.0293
312.5907 -14421.4419 -1726.0489 0.0000 0.0000
7854.3271 6081.3701 307.1436 -1772.9570 6145.8784
306.0951 -41.2119 7.4257 511540.4688 -17.8023
-17.8188
[24651]ENERGY: 6390000 3209.3659 5336.0392 5451.8103
320.6560 -14382.8602 -1665.6771 0.0000 0.0000
7811.4548 6080.7890 305.4671 -1730.6658 6146.1118
306.0795 76.9243 -5.7757 511540.4688 -13.6897
-13.6962
[24704]ENERGY: 6400000 3219.9907 5309.5646 5412.2735
314.4138 -14349.1067 -1684.0217 0.0000 0.0000
7860.0786 6083.1928 307.3685 -1776.8858 6145.9006
305.9273 140.8679 45.2755 511540.4688 -13.5550
-13.5457
[24740]ENERGY: 6410000 3275.3025 5324.5076 5472.4714
318.2972 -14437.4048 -1680.8215 0.0000 0.0000
7808.8888 6081.2412 305.3667 -1727.6476 6145.3161
305.9371 -53.1097 71.0122 511540.4688 -13.5423
-13.5488
[24790]ENERGY: 6420000 3261.8904 5368.2797 5462.7290
320.0252 -14403.5863 -1755.1245 0.0000 0.0000
7831.4985 6085.7119 306.2509 -1745.7866 6147.0059
306.0601 -173.2998 -124.1240 511540.4688 -14.6851
-14.6850
[24826]ENERGY: 6430000 3213.2903 5325.9839 5500.4164
343.1222 -14384.5718 -1725.3691 0.0000 0.0000
7812.1816 6085.0535 305.4955 -1727.1281 6145.9372
305.9162 86.2878 123.0533 511540.4688 -10.7660
-10.7632
[24876]ENERGY: 6440000 3283.8853 5305.9343 5440.1595
333.3148 -14245.4507 -1826.5807 0.0000 0.0000
7792.1413 6083.4037 304.7118 -1708.7376 6147.1398
305.8278 -38.6522 -165.9357 511540.4688 -13.3327
-13.3443
[24912]ENERGY: 6450000 3171.4763 5417.6415 5470.5238
316.6261 -14360.6962 -1740.0255 0.0000 0.0000
7804.6312 6080.1771 305.2002 -1724.4541 6146.3057

305.9644 125.6599 151.2508 511540.4688 -15.6773
-15.6550
[24962]ENERGY: 6460000 3187.3348 5344.7423 5485.9371
317.7658 -14359.9439 -1685.0223 0.0000 0.0000
7789.4470 6080.2607 304.6064 -1709.1863 6146.4197
305.9965 -27.2237 15.3884 511540.4688 -16.9640
-16.9946
[24998]ENERGY: 6470000 3268.8478 5270.4625 5462.7350
314.3916 -14354.6581 -1692.0317 0.0000 0.0000
7814.8823 6084.6293 305.6011 -1730.2529 6146.0402
306.1048 -37.4787 42.3219 511540.4688 -13.3519
-13.3304
[25048]ENERGY: 6480000 3250.9857 5308.2643 5492.7601
318.4270 -14365.3772 -1716.0683 0.0000 0.0000
7795.9725 6084.9642 304.8616 -1711.0083 6146.3994
306.1735 54.0648 -74.7477 511540.4688 -16.9822
-16.9836
[25084]ENERGY: 6490000 3235.3213 5330.9798 5436.3032
330.0160 -14372.8484 -1787.6738 0.0000 0.0000
7914.9974 6087.0955 309.5161 -1827.9019 6147.5760
306.0231 -41.9185 -67.2130 511540.4688 -15.6093
-15.6299
[25137]ENERGY: 6500000 3221.4647 5254.1576 5458.6861
318.1858 -14363.7822 -1704.5507 0.0000 0.0000
7899.0356 6083.1969 308.8919 -1815.8387 6147.2264
305.8789 -141.4775 -185.0690 511540.4688 -15.9298
-15.9067
[25173]ENERGY: 6510000 3175.1472 5413.5955 5443.3393
314.6368 -14317.6360 -1752.2817 0.0000 0.0000
7809.5080 6086.3091 305.3909 -1723.1989 6147.3138
306.0368 29.8455 9.9727 511540.4688 -14.2809
-14.2745
[25223]ENERGY: 6520000 3263.3363 5231.1993 5458.4243
337.1941 -14392.4483 -1673.6786 0.0000 0.0000
7861.2549 6085.2821 307.4145 -1775.9728 6147.6838
305.9481 -42.5742 -69.4588 511540.4688 -18.9530
-18.9664
[25259]ENERGY: 6530000 3145.5700 5263.2848 5487.8949
326.0217 -14276.3615 -1726.5218 0.0000 0.0000
7864.2068 6084.0949 307.5299 -1780.1119 6147.6818
306.0626 78.2070 29.2055 511540.4688 -15.0862
-15.1131
[25309]ENERGY: 6540000 3239.0704 5308.6000 5423.7135
312.7489 -14293.5436 -1711.9024 0.0000 0.0000
7805.8968 6084.5835 305.2497 -1721.3133 6147.2291
306.2303 97.0529 26.0039 511540.4688 -17.7787
-17.7429
[25345]ENERGY: 6550000 3237.0503 5312.3573 5423.2041
322.1839 -14318.9916 -1726.9326 0.0000 0.0000
7834.8919 6083.7632 306.3836 -1751.1287 6147.7104
306.1897 -115.4693 -117.9688 511540.4688 -16.8791
-16.8848
[25395]ENERGY: 6560000 3168.8267 5226.2841 5412.8233
308.5569 -14318.0353 -1614.3252 0.0000 0.0000

7899.5516 6083.6821 308.9121 -1815.8696 6147.1756
305.9666 137.3472 68.6372 511540.4688 -19.8038
-19.8149
[25431]ENERGY: 6570000 3198.7174 5427.0443 5404.8331
314.1750 -14402.6880 -1699.4299 0.0000 0.0000
7838.9458 6081.5978 306.5421 -1757.3480 6148.7816
306.1727 103.8361 -98.7391 511540.4688 -13.5496
-13.5408
[25481]ENERGY: 6580000 3189.4129 5437.6707 5438.7363
322.2350 -14412.3552 -1716.7833 0.0000 0.0000
7821.0111 6079.9274 305.8408 -1741.0837 6146.6100
306.3731 31.0228 25.5119 511540.4688 -19.3399
-19.3427
[25517]ENERGY: 6590000 3225.5713 5293.7218 5486.2325
334.7555 -14353.9031 -1676.9715 0.0000 0.0000
7773.7778 6083.1842 303.9937 -1690.5936 6147.2492
306.3321 -80.6633 -81.2601 511540.4688 -14.9524
-14.9428
[25570]ENERGY: 6600000 3258.4078 5363.0220 5457.5656
316.4437 -14373.6654 -1732.8770 0.0000 0.0000
7797.1642 6086.0610 304.9082 -1711.1032 6147.2091
306.6248 -151.7428 -136.2446 511540.4688 -15.4240
-15.4369
[25606]ENERGY: 6610000 3194.5240 5328.1987 5452.5958
308.8168 -14366.8508 -1712.2477 0.0000 0.0000
7877.0883 6082.1250 308.0337 -1794.9632 6147.1542
306.4218 6.2351 25.9422 511540.4688 -17.4402
-17.4369
[25656]ENERGY: 6620000 3298.1754 5288.2788 5392.3028
297.8196 -14388.4145 -1686.0136 0.0000 0.0000
7881.4865 6083.6348 308.2056 -1797.8516 6147.8036
306.6155 109.2119 9.7522 511540.4688 -16.7697
-16.7765
[25692]ENERGY: 6630000 3120.1569 5347.0033 5439.0930
320.8685 -14356.7634 -1648.0824 0.0000 0.0000
7862.1401 6084.4161 307.4491 -1777.7240 6145.4790
306.7187 84.5065 102.7001 511540.4688 -16.4045
-16.3861
[25742]ENERGY: 6640000 3213.8567 5334.8930 5401.3297
318.9058 -14392.3099 -1672.5270 0.0000 0.0000
7876.7266 6080.8750 308.0195 -1795.8515 6147.2586
306.5050 87.6666 126.6022 511540.4688 -18.7955
-18.8002
[25778]ENERGY: 6650000 3171.9570 5330.9581 5523.8168
311.5459 -14447.8513 -1671.0155 0.0000 0.0000
7862.8913 6082.3023 307.4785 -1780.5890 6147.2492
306.5015 93.8596 74.3950 511540.4688 -17.7778
-17.7833
[25828]ENERGY: 6660000 3199.6408 5321.9849 5497.0026
312.6678 -14332.4785 -1744.8962 0.0000 0.0000
7831.9295 6085.8509 306.2677 -1746.0787 6149.1900
306.5501 93.1387 4.3020 511540.4688 -15.9437
-15.9119
[25864]ENERGY: 6670000 3292.4524 5282.4493 5415.9804

325.1179 -14265.9017 -1779.7052 0.0000 0.0000
7813.4232 6083.8164 305.5440 -1729.6068 6148.4772
306.5030 27.3945 8.1588 511540.4688 -18.2610
-18.3062
[25914]ENERGY: 6680000 3265.7185 5304.5672 5422.1480
319.7081 -14353.6226 -1724.3039 0.0000 0.0000
7851.8202 6086.0356 307.0455 -1765.7846 6149.4329
306.4704 14.6155 45.4312 511540.4688 -19.4272
-19.3982
[25950]ENERGY: 6690000 3269.0251 5321.6499 5449.5553
319.1113 -14391.8902 -1675.1929 0.0000 0.0000
7794.4315 6086.6899 304.8014 -1707.7416 6149.6760
306.4402 48.5587 2.8289 511540.4688 -17.1610
-17.1576
[26003]ENERGY: 6700000 3137.8756 5376.2341 5427.4168
336.5635 -14368.8756 -1652.5375 0.0000 0.0000
7829.0427 6085.7195 306.1548 -1743.3231 6149.3723
306.3603 -69.6390 -86.6436 511540.4688 -21.1134
-21.1265
[26039]ENERGY: 6710000 3237.0006 5260.4408 5457.0096
304.3650 -14342.4310 -1676.8367 0.0000 0.0000
7847.7100 6087.2583 306.8848 -1760.4518 6149.4095
306.2507 231.8626 142.2769 511540.4688 -16.7055
-16.7165
[26089]ENERGY: 6720000 3253.7156 5195.9679 5453.7604
333.1174 -14285.7170 -1697.8390 0.0000 0.0000
7834.6337 6087.6390 306.3735 -1746.9946 6148.6553
306.4709 28.9817 -8.8595 511540.4688 -16.0804
-16.0723
[26125]ENERGY: 6730000 3208.0435 5275.5862 5473.6420
321.5364 -14482.1572 -1659.3709 0.0000 0.0000
7941.9155 6079.1954 310.5687 -1862.7201 6147.9344
306.4188 -44.0667 35.5987 511540.4688 -14.8923
-14.8808
[26175]ENERGY: 6740000 3113.5242 5410.6816 5501.6713
337.0721 -14398.9866 -1763.5009 0.0000 0.0000
7880.9495 6081.4111 308.1847 -1799.5384 6148.6694
306.1027 78.0403 100.9165 511540.4688 -21.6445
-21.6545
[26211]ENERGY: 6750000 3186.9459 5420.7540 5473.5594
329.3697 -14443.5731 -1712.6377 0.0000 0.0000
7829.0734 6083.4917 306.1560 -1745.5817 6147.8108
306.2326 -260.0481 -157.1129 511540.4688 -16.3440
-16.3467
[26261]ENERGY: 6760000 3276.6116 5335.7381 5449.7021
320.9385 -14442.9731 -1685.2466 0.0000 0.0000
7831.6822 6086.4527 306.2581 -1745.2295 6148.8931
306.2352 27.7014 31.3688 511540.4688 -16.9684
-16.9615
[26297]ENERGY: 6770000 3215.2018 5352.3058 5440.0854
338.2663 -14291.1679 -1758.9735 0.0000 0.0000
7791.2405 6086.9584 304.6766 -1704.2821 6149.6402
306.4465 -125.0296 -102.6798 511540.4688 -19.1255
-19.1402

[26347]ENERGY: 6780000 3213.9260 5386.7165 5384.3676
310.8723 -14355.5066 -1683.6528 0.0000 0.0000
7830.3848 6087.1077 306.2073 -1743.2770 6148.7703
306.2986 54.4413 60.7166 511540.4688 -15.1371
-15.1157
[26383]ENERGY: 6790000 3269.1049 5389.0753 5418.9411
305.3343 -14405.0164 -1701.4059 0.0000 0.0000
7808.8628 6084.8961 305.3657 -1723.9667 6149.6539
306.3907 148.7613 23.5456 511540.4688 -18.9722
-18.9917
[26436]ENERGY: 6800000 3221.2204 5277.4790 5455.3248
331.8158 -14312.7732 -1718.4663 0.0000 0.0000
7829.3100 6083.9104 306.1653 -1745.3996 6149.3324
306.3534 -3.3613 -20.0220 511540.4688 -14.7484
-14.7233
[26472]ENERGY: 6810000 3274.3508 5382.7679 5463.3176
312.7939 -14349.7060 -1787.7906 0.0000 0.0000
7790.0142 6085.7478 304.6286 -1704.2663 6150.2132
306.3424 -162.9591 -172.0065 511540.4688 -15.8384
-15.8488
[26522]ENERGY: 6820000 3263.4363 5356.7024 5502.6594
319.8826 -14457.4708 -1743.8806 0.0000 0.0000
7845.5778 6086.9072 306.8014 -1758.6706 6151.1132
306.4570 -72.4874 -210.6989 511540.4688 -15.1094
-15.1113
[26558]ENERGY: 6830000 3278.5003 5316.0861 5435.8379
307.3181 -14381.8490 -1672.6175 0.0000 0.0000
7805.7771 6089.0529 305.2450 -1716.7241 6150.5207
306.2093 39.2182 93.0794 511540.4688 -17.2305
-17.2308
[26608]ENERGY: 6840000 3257.4507 5352.4357 5466.7206
326.3046 -14392.2648 -1682.7511 0.0000 0.0000
7758.6876 6086.5834 303.4036 -1672.1042 6149.7707
306.1980 200.4990 173.3432 511540.4688 -16.2167
-16.2328
[26644]ENERGY: 6850000 3294.3781 5285.3176 5450.0501
342.1162 -14387.4818 -1716.8079 0.0000 0.0000
7820.5632 6088.1355 305.8232 -1732.4277 6149.7868
306.2041 -40.4351 -59.1640 511540.4688 -15.4089
-15.3877
[26694]ENERGY: 6860000 3166.0398 5260.2037 5497.6498
328.6364 -14364.7400 -1671.8287 0.0000 0.0000
7864.7331 6080.6941 307.5505 -1784.0390 6150.0237
306.3151 148.9361 180.1020 511540.4688 -12.0702
-12.0855
[26730]ENERGY: 6870000 3181.7588 5464.9425 5396.7718
317.8393 -14344.3798 -1793.0224 0.0000 0.0000
7861.9474 6085.8576 307.4416 -1776.0898 6151.2195
306.2810 -39.7909 -8.7497 511540.4688 -15.9650
-15.9721
[26780]ENERGY: 6880000 3246.9345 5321.9958 5471.4253
327.5666 -14372.2621 -1792.7924 0.0000 0.0000
7884.1672 6087.0349 308.3105 -1797.1322 6150.6919
306.3700 -37.9048 -94.7167 511540.4688 -15.4929

-15.4705
[26816]ENERGY: 6890000 3240.6420 5397.0562 5523.6380
304.5575 -14328.5856 -1790.0800 0.0000 0.0000
7742.8878 6090.1159 302.7857 -1652.7719 6151.5217
306.3747 -66.3918 -77.0498 511540.4688 -20.0864
-20.1098
[26869]ENERGY: 6900000 3204.3245 5384.9346 5399.5161
297.9733 -14380.0798 -1645.3142 0.0000 0.0000
7825.8142 6087.1686 306.0286 -1738.6456 6151.4369
306.3585 -195.6751 -129.6414 511540.4688 -10.8191
-10.7944
[26905]ENERGY: 6910000 3211.5839 5371.3514 5436.1667
301.4826 -14413.8157 -1681.0558 0.0000 0.0000
7861.8144 6087.5277 307.4364 -1774.2867 6151.0756
306.1431 290.9978 210.0517 511540.4688 -19.3294
-19.3394
[26955]ENERGY: 6920000 3221.3519 5407.3333 5444.9118
345.0287 -14367.7351 -1764.1640 0.0000 0.0000
7801.8170 6088.5436 305.0902 -1713.2733 6150.9852
306.0998 -212.1392 -127.3165 511540.4688 -14.7318
-14.7212
[26991]ENERGY: 6930000 3230.8034 5234.1991 5473.9583
330.5549 -14378.6792 -1669.7718 0.0000 0.0000
7866.6844 6087.7491 307.6268 -1778.9353 6150.1568
306.0561 -71.7254 -2.8663 511540.4688 -12.1956
-12.1931
[27041]ENERGY: 6940000 3314.8763 5229.5976 5465.9361
365.5196 -14356.3436 -1761.0066 0.0000 0.0000
7832.3974 6090.9770 306.2860 -1741.4204 6151.8920
306.2806 -150.0245 -207.5461 511540.4688 -22.4194
-22.4710
[27077]ENERGY: 6950000 3230.1648 5324.2754 5445.7508
311.1356 -14349.4859 -1696.4805 0.0000 0.0000
7821.2505 6086.6107 305.8501 -1734.6398 6150.8634
306.4167 -47.3606 -56.7155 511540.4688 -19.2217
-19.1748
[27127]ENERGY: 6960000 3308.1256 5389.3456 5424.4552
328.4280 -14414.3619 -1706.9812 0.0000 0.0000
7760.6570 6089.6683 303.4806 -1670.9887 6150.7724
306.2512 -37.0032 -33.9232 511540.4688 -20.7332
-20.7194
[27163]ENERGY: 6970000 3245.8233 5298.3804 5453.6236
333.9064 -14450.5726 -1662.2282 0.0000 0.0000
7869.6993 6088.6323 307.7447 -1781.0671 6149.9613
306.1799 34.2649 2.0833 511540.4688 -17.5545
-17.5830
[27213]ENERGY: 6980000 3350.5372 5247.0076 5534.1932
297.4254 -14334.8103 -1791.9945 0.0000 0.0000
7787.7014 6090.0601 304.5382 -1697.6413 6150.6006
306.1220 3.0210 -83.8064 511540.4688 -18.5499
-18.5535
[27249]ENERGY: 6990000 3213.4969 5331.0162 5443.7889
326.1832 -14312.8378 -1737.3561 0.0000 0.0000
7822.3427 6086.6340 305.8928 -1735.7087 6150.5847

306.2566 76.4616 36.7742 511540.4688 -20.5780
-20.5636
[27302]ENERGY: 7000000 3256.2809 5286.2893 5438.3612
312.4868 -14317.4315 -1684.8335 0.0000 0.0000
7796.7205 6087.8737 304.8909 -1708.8468 6150.4420
306.4362 94.9356 -1.3193 511540.4688 -17.0125
-17.0062
[27338]ENERGY: 7010000 3199.4871 5357.3018 5504.9534
334.1861 -14420.5090 -1737.0613 0.0000 0.0000
7847.2776 6085.6357 306.8679 -1761.6419 6151.4968
306.3588 -32.5253 -57.9462 511540.4688 -24.6823
-24.7088
[27388]ENERGY: 7020000 3231.0464 5199.6552 5479.2214
323.3589 -14371.4741 -1685.7544 0.0000 0.0000
7907.2913 6083.3448 309.2147 -1823.9465 6150.7603
306.1532 -39.4959 -6.7880 511540.4688 -17.7376
-17.7263
[27424]ENERGY: 7030000 3222.3627 5337.3083 5429.9881
331.7842 -14407.6616 -1683.3543 0.0000 0.0000
7854.7987 6085.2261 307.1620 -1769.5726 6149.8368
306.1437 -17.7104 90.2988 511540.4688 -20.2801
-20.2832
[27474]ENERGY: 7040000 3232.0908 5299.2601 5494.3946
309.4213 -14303.1653 -1799.6080 0.0000 0.0000
7856.9845 6089.3779 307.2475 -1767.6066 6150.6835
306.1404 -48.1418 -195.6481 511540.4688 -20.1577
-20.1622
[27510]ENERGY: 7050000 3227.4942 5350.4269 5462.7089
317.6665 -14335.7889 -1751.6526 0.0000 0.0000
7818.3026 6089.1575 305.7348 -1729.1450 6151.0775
306.2781 -47.4590 -32.6737 511540.4688 -19.2213
-19.1973
[27560]ENERGY: 7060000 3234.8520 5239.8390 5465.1240
312.9149 -14255.8532 -1703.6891 0.0000 0.0000
7795.8283 6089.0159 304.8560 -1706.8123 6149.6305
306.2365 -178.6012 -176.9011 511540.4688 -20.4860
-20.4920
[27596]ENERGY: 7070000 3284.2586 5383.8215 5405.9966
325.1619 -14407.1577 -1700.1552 0.0000 0.0000
7795.5264 6087.4520 304.8442 -1708.0744 6151.4565
306.4311 112.4937 132.8794 511540.4688 -19.3170
-19.3392
[27646]ENERGY: 7080000 3191.2127 5267.2511 5455.5648
299.4646 -14285.3780 -1708.5767 0.0000 0.0000
7866.2477 6085.7863 307.6097 -1780.4614 6150.7913
306.2552 93.8975 73.0149 511540.4688 -22.3968
-22.3756
[27682]ENERGY: 7090000 3209.0917 5356.9742 5444.1479
328.8648 -14421.7935 -1697.7519 0.0000 0.0000
7865.8390 6085.3721 307.5938 -1780.4669 6151.3185
306.2228 -14.8243 6.2993 511540.4688 -18.6012
-18.6060
[27735]ENERGY: 7100000 3257.3423 5346.2652 5452.9015
338.4029 -14449.1991 -1710.9056 0.0000 0.0000

7857.3942 6092.2013 307.2635 -1765.1929 6153.1146
306.3131 -186.5349 -109.8056 511540.4688 -22.0531
-22.0418
[27771]ENERGY: 7110000 3235.6585 5274.3178 5415.9288
317.9050 -14261.2188 -1753.0565 0.0000 0.0000
7863.4832 6093.0179 307.5016 -1770.4653 6153.7457
306.3984 18.1318 -65.9688 511540.4688 -22.8009
-22.8429
[27821]ENERGY: 7120000 3297.9311 5330.8633 5521.6547
331.5674 -14344.4732 -1767.5908 0.0000 0.0000
7726.6288 6096.5813 302.1499 -1630.0476 6154.0839
306.3730 -202.8476 -221.5940 511540.4688 -23.6352
-23.5908
[27857]ENERGY: 7130000 3170.4429 5287.6689 5447.5449
345.6160 -14292.6742 -1678.6412 0.0000 0.0000
7808.9173 6088.8746 305.3678 -1720.0426 6154.9551
306.4033 324.1351 99.4815 511540.4688 -21.6416
-21.6733
[27907]ENERGY: 7140000 3253.8624 5372.0842 5480.4714
326.4974 -14412.3638 -1668.8759 0.0000 0.0000
7739.1530 6090.8286 302.6397 -1648.3244 6154.7240
306.3626 113.9092 46.8052 511540.4688 -19.6499
-19.6490
[27943]ENERGY: 7150000 3250.4071 5319.4510 5461.6738
301.3908 -14392.7098 -1683.2564 0.0000 0.0000
7835.3866 6092.3430 306.4029 -1743.0435 6154.4690
306.4440 76.7437 27.2450 511540.4688 -21.4124
-21.3807
[27993]ENERGY: 7160000 3295.0356 5236.8477 5439.3507
316.3701 -14351.9089 -1699.1624 0.0000 0.0000
7858.5006 6095.0333 307.3068 -1763.4673 6154.9136
306.5021 135.4935 83.5128 511540.4688 -18.1475
-18.1824
[28029]ENERGY: 7170000 3305.1199 5360.4498 5473.3292
336.8142 -14425.8867 -1707.2714 0.0000 0.0000
7751.7683 6094.3232 303.1330 -1657.4451 6156.8696
306.4768 -107.1695 -66.0348 511540.4688 -22.8312
-22.8116
[28079]ENERGY: 7180000 3278.9416 5384.6881 5465.7663
328.0260 -14429.0084 -1672.8621 0.0000 0.0000
7740.0295 6095.5809 302.6740 -1644.4486 6156.8028
306.5866 193.4463 65.2327 511540.4688 -21.6436
-21.6525
[28115]ENERGY: 7190000 3229.7626 5428.1669 5450.6004
307.9016 -14372.4973 -1722.2445 0.0000 0.0000
7772.1465 6093.8362 303.9299 -1678.3103 6157.1409
306.4552 -105.0755 -22.2759 511540.4688 -20.5570
-20.5582
[28168]ENERGY: 7200000 3248.7124 5404.0530 5447.8351
329.7101 -14359.9628 -1740.4165 0.0000 0.0000
7763.9775 6093.9087 303.6105 -1670.0688 6156.8556
306.5228 -32.5343 -109.8388 511540.4688 -15.8606
-15.8592
[28204]ENERGY: 7210000 3243.9024 5362.8753 5464.9692

329.0378 -14510.3109 -1681.2700 0.0000 0.0000
7883.7115 6092.9153 308.2927 -1790.7963 6157.5655
306.6363 19.1295 -25.2705 511540.4688 -20.2234
-20.2235
[28254]ENERGY: 7220000 3298.2408 5319.6608 5424.2974
298.6691 -14404.0544 -1690.4798 0.0000 0.0000
7849.3858 6095.7198 306.9504 -1753.6660 6158.1583
306.6351 -110.1912 -153.7661 511540.4688 -18.6325
-18.6233
[28290]ENERGY: 7230000 3275.5703 5396.5799 5452.8988
330.6359 -14433.4325 -1727.0655 0.0000 0.0000
7800.8921 6096.0790 305.0540 -1704.8131 6157.8955
306.6491 146.9209 86.4946 511540.4688 -18.3569
-18.3544
[28340]ENERGY: 7240000 3257.6153 5279.6086 5462.8639
352.9048 -14319.3597 -1706.2193 0.0000 0.0000
7767.0945 6094.5081 303.7324 -1672.5864 6157.8989
306.6988 -192.6056 -97.2239 511540.4688 -19.3579
-19.3821
[28376]ENERGY: 7250000 3259.6141 5287.6982 5499.8616
316.0296 -14356.0666 -1807.4084 0.0000 0.0000
7897.2760 6097.0045 308.8231 -1800.2715 6158.3864
306.5680 -164.7917 -234.2221 511540.4688 -18.3590
-18.3238
[28426]ENERGY: 7260000 3254.1415 5335.1427 5439.3168
302.9298 -14270.1685 -1717.0939 0.0000 0.0000
7751.5308 6095.7992 303.1237 -1655.7317 6158.2571
306.5391 4.1358 -64.4070 511540.4688 -15.1369
-15.1600
[28462]ENERGY: 7270000 3273.4661 5389.1862 5468.6974
329.8231 -14382.3116 -1743.1148 0.0000 0.0000
7761.8268 6097.5732 303.5264 -1664.2536 6158.9960
306.5551 -166.2368 -94.0494 511540.4688 -21.3364
-21.3141
[28512]ENERGY: 7280000 3234.9030 5283.6864 5397.4628
350.8723 -14337.5915 -1752.8995 0.0000 0.0000
7919.3201 6095.7536 309.6851 -1823.5665 6158.9193
306.6253 9.5755 -5.5537 511540.4688 -14.0419
-14.0397
[28548]ENERGY: 7290000 3213.0341 5288.2521 5484.4842
331.6264 -14349.8127 -1691.7174 0.0000 0.0000
7818.9978 6094.8645 305.7620 -1724.1332 6158.3037
306.6073 -1.9895 84.5727 511540.4688 -22.4728
-22.4792
[28601]ENERGY: 7300000 3278.7919 5348.9561 5479.7831
321.4257 -14385.8602 -1749.0727 0.0000 0.0000
7801.6621 6095.6859 305.0841 -1705.9762 6160.2845
306.4910 -18.5748 -86.0167 511540.4688 -17.1326
-17.1435
[28637]ENERGY: 7310000 3266.1469 5329.3026 5450.4714
304.6068 -14385.7164 -1752.9855 0.0000 0.0000
7882.1719 6093.9977 308.2325 -1788.1742 6158.5982
306.4713 -124.5207 -252.9975 511540.4688 -15.9254
-15.9342

[28687]ENERGY: 7320000 3200.6986 5296.8389 5436.7396
330.5696 -14265.5200 -1753.6747 0.0000 0.0000
7850.5827 6096.2347 306.9972 -1754.3480 6159.2711
306.4794 41.5092 -40.7390 511540.4688 -18.3787
-18.3746
[28723]ENERGY: 7330000 3249.9244 5279.8691 5461.8071
333.4784 -14305.8846 -1792.6933 0.0000 0.0000
7866.8235 6093.3247 307.6323 -1773.4988 6159.0177
306.5674 -50.4239 -139.8364 511540.4688 -20.1892
-20.1720
[28773]ENERGY: 7340000 3249.0592 5313.0177 5461.7350
316.1551 -14321.2761 -1770.1146 0.0000 0.0000
7846.7363 6095.3126 306.8467 -1751.4238 6160.2507
306.6903 -217.7438 -235.8946 511540.4688 -19.7361
-19.7382
[28809]ENERGY: 7350000 3301.5557 5360.8053 5441.6003
316.0969 -14445.2327 -1685.1813 0.0000 0.0000
7808.9073 6098.5515 305.3674 -1710.3557 6160.0798
306.7816 -198.7150 -179.0313 511540.4688 -21.0576
-21.0655
[28859]ENERGY: 7360000 3272.8512 5291.2044 5461.2741
323.7865 -14330.2725 -1745.8838 0.0000 0.0000
7823.8786 6096.8385 305.9529 -1727.0402 6161.6725
306.7390 3.4688 -89.1507 511540.4688 -16.2569
-16.2690
[28895]ENERGY: 7370000 3199.1896 5441.0213 5441.9777
329.0768 -14334.7934 -1742.5627 0.0000 0.0000
7764.6947 6098.6040 303.6385 -1666.0906 6160.9143
306.5972 36.6040 -50.4432 511540.4688 -18.5832
-18.5698
[28945]ENERGY: 7380000 3226.9293 5270.2982 5448.7503
336.8499 -14368.7713 -1628.1254 0.0000 0.0000
7814.1110 6100.0420 305.5709 -1714.0690 6160.6048
306.6424 66.6982 -1.1140 511540.4688 -21.2126
-21.2246
[28981]ENERGY: 7390000 3204.4925 5340.5662 5403.0075
336.0991 -14258.7034 -1719.0118 0.0000 0.0000
7793.5574 6100.0075 304.7672 -1693.5499 6161.7458
306.7768 47.4441 78.0344 511540.4688 -20.9491
-20.9324
[29034]ENERGY: 7400000 3267.4488 5313.8920 5458.4424
312.4856 -14337.5715 -1737.0706 0.0000 0.0000
7821.1240 6098.7507 305.8452 -1722.3733 6162.3868
306.7104 34.5648 74.9293 511540.4688 -18.4142
-18.4180
[29070]ENERGY: 7410000 3210.5478 5309.8929 5458.1203
354.9973 -14315.3836 -1709.3331 0.0000 0.0000
7790.7152 6099.5567 304.6560 -1691.1585 6162.8779
306.6123 107.5938 -0.4676 511540.4688 -20.2705
-20.2669
[29120]ENERGY: 7420000 3168.3317 5277.7291 5440.2692
338.5658 -14293.7071 -1690.8201 0.0000 0.0000
7861.5191 6101.8876 307.4248 -1759.6315 6163.7922
306.7388 70.9619 89.3761 511540.4688 -20.7701

-20.7592
[29156]ENERGY: 7430000 3255.0119 5440.1449 5421.6076
334.2936 -14418.6774 -1691.6303 0.0000 0.0000
7760.0184 6100.7687 303.4556 -1659.2497 6163.7905
306.8748 -6.5854 100.5288 511540.4688 -14.3543
-14.3725
[29206]ENERGY: 7440000 3214.5516 5374.0916 5425.6067
312.8692 -14309.4114 -1652.5868 0.0000 0.0000
7739.0948 6104.2158 302.6374 -1634.8791 6164.1014
306.8772 191.1753 229.1303 511540.4688 -15.5086
-15.5130
[29242]ENERGY: 7450000 3221.7235 5289.8830 5431.9518
301.6566 -14360.6016 -1724.3739 0.0000 0.0000
7940.9273 6101.1667 310.5301 -1839.7606 6163.1550
306.8406 353.7863 241.1786 511540.4688 -17.0888
-17.0813
[29292]ENERGY: 7460000 3292.1802 5242.2741 5428.4342
338.6754 -14326.0099 -1666.4260 0.0000 0.0000
7795.1579 6104.2861 304.8298 -1690.8719 6164.2675
306.9965 286.4037 262.2341 511540.4688 -15.7745
-15.7865
[29328]ENERGY: 7470000 3251.9678 5349.3702 5440.4626
313.7095 -14307.3010 -1737.8043 0.0000 0.0000
7792.5099 6102.9147 304.7262 -1689.5953 6165.1387
307.0611 -132.5294 -125.8306 511540.4688 -14.9410
-14.9381
[29378]ENERGY: 7480000 3300.3949 5203.9052 5450.7284
334.3998 -14368.6248 -1679.2558 0.0000 0.0000
7858.3070 6099.8546 307.2992 -1758.4524 6164.0058
306.8670 97.8919 3.1668 511540.4688 -12.3844
-12.3729
[29414]ENERGY: 7490000 3255.7537 5333.3545 5389.1166
311.2124 -14364.3702 -1749.9174 0.0000 0.0000
7925.9451 6101.0947 309.9442 -1824.8504 6163.6924
307.0848 -110.6514 -55.0326 511540.4688 -16.6437
-16.6373
[29467]ENERGY: 7500000 3207.6547 5280.9190 5447.1469
326.1978 -14407.0166 -1678.6225 0.0000 0.0000
7922.0283 6098.3075 309.7910 -1823.7208 6164.3058
307.0633 -132.2045 -186.2191 511540.4688 -15.0091
-15.0156
[29503]ENERGY: 7510000 3267.6517 5290.4799 5478.7629
318.3524 -14339.3821 -1711.2735 0.0000 0.0000
7797.1566 6101.7479 304.9079 -1695.4086 6165.0943
306.8245 37.2398 -31.5491 511540.4688 -18.5426
-18.5516
[29553]ENERGY: 7520000 3261.9358 5381.3923 5429.9387
311.5462 -14331.1463 -1762.5703 0.0000 0.0000
7812.0580 6103.1544 305.4906 -1708.9036 6165.2801
307.0627 -73.2819 -134.7175 511540.4688 -13.5445
-13.5475
[29589]ENERGY: 7530000 3277.4069 5297.1683 5476.1611
327.0263 -14354.9821 -1782.0133 0.0000 0.0000
7857.3417 6098.1090 307.2615 -1759.2327 6164.9782

307.0049 65.0481 97.2657 511540.4688 -16.1341
-16.0973
[29639]ENERGY: 7540000 3192.2711 5365.1352 5467.4888
310.7872 -14274.1027 -1736.8897 0.0000 0.0000
7777.2854 6101.9753 304.1309 -1675.3101 6165.6558
307.0008 58.4241 13.8908 511540.4688 -17.4737
-17.4911
[29675]ENERGY: 7550000 3253.5460 5300.1382 5479.8710
335.0897 -14387.1667 -1717.2267 0.0000 0.0000
7840.5365 6104.7881 306.6043 -1735.7484 6165.9360
307.0183 12.4386 -58.3736 511540.4688 -13.0107
-13.0166
[29725]ENERGY: 7560000 3204.8897 5337.5590 5445.0688
329.6273 -14392.1471 -1744.3446 0.0000 0.0000
7921.4478 6102.1009 309.7683 -1819.3469 6167.0255
307.1335 175.0602 92.6203 511540.4688 -18.5565
-18.5647
[29761]ENERGY: 7570000 3302.3987 5430.4342 5408.9956
308.6708 -14470.2150 -1733.2668 0.0000 0.0000
7854.5141 6101.5315 307.1509 -1752.9826 6165.9849
307.0205 -42.8941 -57.9161 511540.4688 -15.1071
-15.1050
[29811]ENERGY: 7580000 3201.9505 5309.1622 5406.9819
341.3315 -14411.7022 -1648.1248 0.0000 0.0000
7901.1671 6100.7663 308.9753 -1800.4008 6166.0134
307.0412 72.6109 -20.4907 511540.4688 -12.4112
-12.4016
[29847]ENERGY: 7590000 3178.0004 5437.4993 5394.7967
312.4578 -14381.3686 -1671.0508 0.0000 0.0000
7835.2532 6105.5881 306.3977 -1729.6651 6165.6733
306.9017 210.2417 68.6203 511540.4688 -17.2038
-17.2334
[29900]ENERGY: 7600000 3249.7943 5384.5876 5442.7601
306.4769 -14399.4079 -1739.9284 0.0000 0.0000
7858.0031 6102.2857 307.2873 -1755.7174 6166.4778
307.0200 -70.3985 -85.0361 511540.4688 -17.7235
-17.7206
[29936]ENERGY: 7610000 3232.0893 5355.2083 5450.1310
314.2383 -14381.5359 -1752.6379 0.0000 0.0000
7884.2594 6101.7524 308.3141 -1782.5070 6165.8233
307.0270 -89.6037 -92.0690 511540.4688 -16.3269
-16.3092
[29986]ENERGY: 7620000 3231.0980 5348.5660 5409.4645
296.7373 -14409.9411 -1695.7971 0.0000 0.0000
7920.5265 6100.6541 309.7323 -1819.8724 6165.2118
306.8823 150.0779 92.6990 511540.4688 -17.3586
-17.3582
[30022]ENERGY: 7630000 3221.7403 5401.6490 5425.4114
305.3163 -14288.5420 -1786.1564 0.0000 0.0000
7823.0000 6102.4186 305.9185 -1720.5814 6166.5004
306.7499 -178.9871 -109.1174 511540.4688 -19.1983
-19.1852
[30072]ENERGY: 7640000 3220.4128 5357.2443 5413.8299
330.8306 -14364.5198 -1688.0192 0.0000 0.0000

7830.3874 6100.1661 306.2074 -1730.2213 6165.3679
306.7333 161.6860 113.1110 511540.4688 -14.6415
-14.6450
[30108]ENERGY: 7650000 3304.4694 5293.7204 5429.7557
338.2588 -14460.2020 -1696.7785 0.0000 0.0000
7889.9171 6099.1410 308.5353 -1790.7761 6165.2821
306.6504 5.3855 28.6553 511540.4688 -13.2958
-13.3187
[30158]ENERGY: 7660000 3268.6364 5397.1000 5454.8206
329.7659 -14341.0842 -1778.6924 0.0000 0.0000
7771.1806 6101.7269 303.8921 -1669.4537 6166.2247
306.8485 48.0172 -16.1288 511540.4688 -15.4038
-15.4128
[30194]ENERGY: 7670000 3244.8619 5358.0440 5437.1364
302.3586 -14319.4087 -1722.6721 0.0000 0.0000
7800.6489 6100.9689 305.0445 -1699.6800 6164.0204
306.8951 155.3558 94.7264 511540.4688 -8.9061
-8.8927
[30244]ENERGY: 7680000 3240.4654 5317.1769 5419.2741
313.6858 -14339.9840 -1710.5699 0.0000 0.0000
7858.1591 6098.2073 307.2934 -1759.9518 6164.9587
306.8244 6.9956 -63.9370 511540.4688 -12.9932
-12.9804
[30280]ENERGY: 7690000 3281.2222 5437.5781 5410.5797
320.2317 -14427.8478 -1702.7207 0.0000 0.0000
7781.8230 6100.8662 304.3083 -1680.9568 6165.5548
307.0490 33.5079 -32.2963 511540.4688 -11.5669
-11.5613
[30333]ENERGY: 7700000 3207.8662 5456.2281 5444.6801
325.8907 -14425.7475 -1681.7055 0.0000 0.0000
7775.8298 6103.0420 304.0739 -1672.7879 6165.4290
307.0849 -40.0044 7.6049 511540.4688 -17.4892
-17.5077
[30369]ENERGY: 7710000 3218.1895 5331.3920 5394.9851
313.8630 -14314.0886 -1649.6747 0.0000 0.0000
7810.1960 6104.8622 305.4178 -1705.3338 6166.4001
306.9735 -65.2075 -111.5094 511540.4688 -12.8413
-12.8483
[30419]ENERGY: 7720000 3199.3223 5373.6891 5425.2685
327.2047 -14397.4146 -1706.5945 0.0000 0.0000
7878.7449 6100.2205 308.0984 -1778.5245 6166.3346
306.8946 -24.6482 -53.9764 511540.4688 -16.3195
-16.2826
[30455]ENERGY: 7730000 3332.8525 5270.5381 5471.8526
329.7152 -14429.3934 -1668.9985 0.0000 0.0000
7799.2240 6105.7907 304.9888 -1693.4334 6166.9172
306.9319 -85.8915 -53.2764 511540.4688 -16.2472
-16.2577
[30505]ENERGY: 7740000 3339.7393 5229.3583 5453.3546
312.0808 -14344.6901 -1716.6832 0.0000 0.0000
7827.6135 6100.7732 306.0989 -1726.8403 6166.1095
306.8130 -75.7901 -6.4683 511540.4688 -17.0674
-17.0676
[30541]ENERGY: 7750000 3337.8445 5208.3972 5494.7987

334.3446 -14326.3387 -1696.3876 0.0000 0.0000
7752.2493 6104.9080 303.1518 -1647.3413 6167.2029
306.6161 -20.6313 26.7978 511540.4688 -10.8731
-10.8845
[30591]ENERGY: 7760000 3218.6154 5271.6017 5479.2510
329.2026 -14270.9661 -1756.4571 0.0000 0.0000
7831.3862 6102.6336 306.2465 -1728.7525 6167.7266
306.8102 36.5773 -5.3250 511540.4688 -14.5772
-14.5906
[30627]ENERGY: 7770000 3238.8235 5260.6392 5382.7106
317.8355 -14238.7595 -1733.2688 0.0000 0.0000
7877.9056 6105.8862 308.0656 -1772.0194 6168.9127
306.9565 5.5515 -74.3828 511540.4688 -16.8268
-16.8385
[30677]ENERGY: 7780000 3219.3545 5298.1123 5417.6124
329.6708 -14313.2846 -1760.5588 0.0000 0.0000
7913.1955 6104.1022 309.4456 -1809.0933 6168.4006
306.9996 -94.8862 -60.2658 511540.4688 -16.9787
-16.9446
[30713]ENERGY: 7790000 3255.1009 5267.6383 5426.4549
317.7065 -14376.3060 -1655.9420 0.0000 0.0000
7873.6366 6108.2892 307.8987 -1765.3474 6168.3728
306.9809 75.1334 58.1997 511540.4688 -15.0381
-15.0434
[30766]ENERGY: 7800000 3216.4661 5378.3079 5390.7466
336.4480 -14348.8213 -1703.7560 0.0000 0.0000
7835.8427 6105.2340 306.4207 -1730.6087 6168.2058
306.9414 33.6613 17.2859 511540.4688 -13.0423
-13.0630
[30802]ENERGY: 7810000 3277.1531 5397.4367 5448.2399
349.8272 -14412.7592 -1684.0470 0.0000 0.0000
7733.4069 6109.2577 302.4150 -1624.1492 6168.7212
306.9475 23.0356 -90.2639 511540.4688 -13.1103
-13.0933
[30852]ENERGY: 7820000 3244.6622 5247.2285 5445.8341
339.8074 -14386.2203 -1685.6423 0.0000 0.0000
7899.4561 6105.1256 308.9084 -1794.3305 6167.5178
306.7607 105.7680 -30.5278 511540.4688 -16.6978
-16.7068
[30888]ENERGY: 7830000 3257.9289 5218.0359 5492.1478
327.7436 -14429.3679 -1645.6897 0.0000 0.0000
7881.3487 6102.1473 308.2003 -1779.2014 6167.7046
306.9035 -74.1170 -122.3279 511540.4688 -14.6275
-14.6189
[30938]ENERGY: 7840000 3220.1574 5292.3996 5449.3258
322.7898 -14310.1192 -1749.7109 0.0000 0.0000
7880.6579 6105.5004 308.1732 -1775.1575 6167.6488
306.8900 -60.6239 -76.9193 511540.4688 -13.1341
-13.1590
[30974]ENERGY: 7850000 3245.2241 5352.1407 5425.0445
319.2587 -14251.7039 -1755.7248 0.0000 0.0000
7770.9012 6105.1407 303.8812 -1665.7606 6167.5902
306.8323 146.6337 34.6281 511540.4688 -13.5845
-13.5456

[31024]ENERGY: 7860000 3286.5774 5331.2347 5427.4485
335.5441 -14350.5143 -1721.4795 0.0000 0.0000
7798.9501 6107.7610 304.9781 -1691.1891 6167.7514
306.7728 121.5101 118.0907 511540.4688 -16.8753
-16.8891
[31060]ENERGY: 7870000 3249.4026 5329.1113 5473.9101
314.7848 -14339.7363 -1702.5986 0.0000 0.0000
7779.4555 6104.3294 304.2157 -1675.1261 6167.6285
306.7944 -34.2333 -96.8533 511540.4688 -10.6055
-10.6122
[31110]ENERGY: 7880000 3343.3546 5287.7234 5499.6782
312.4816 -14395.2628 -1735.5355 0.0000 0.0000
7788.4993 6100.9388 304.5694 -1687.5605 6167.2172
306.9194 -153.4439 -138.0646 511540.4688 -13.7782
-13.7576
[31146]ENERGY: 7890000 3251.0109 5423.1752 5465.3023
332.5665 -14469.3553 -1689.5657 0.0000 0.0000
7790.6446 6103.7784 304.6533 -1686.8661 6166.8008
306.7410 54.7748 -25.5137 511540.4688 -15.7550
-15.7811
[31199]ENERGY: 7900000 3306.0797 5273.8251 5460.3661
330.4258 -14354.3898 -1766.1121 0.0000 0.0000
7854.0942 6104.2889 307.1345 -1749.8053 6167.9787
306.6951 -318.2013 -207.0396 511540.4688 -12.9885
-12.9978
[31235]ENERGY: 7910000 3244.0574 5392.3938 5491.6447
336.1444 -14471.2837 -1658.4505 0.0000 0.0000
7767.3572 6101.8632 303.7426 -1665.4940 6167.2420
306.8525 10.5025 24.6958 511540.4688 -13.1464
-13.1136
[31285]ENERGY: 7920000 3225.5977 5277.8364 5473.3136
317.9680 -14428.3270 -1664.3134 0.0000 0.0000
7898.0170 6100.0922 308.8521 -1797.9247 6167.5301
306.9576 112.9254 110.3757 511540.4688 -14.0759
-14.0971
[31321]ENERGY: 7930000 3210.9440 5352.4117 5465.2586
295.5002 -14371.4447 -1705.4234 0.0000 0.0000
7858.4135 6105.6601 307.3034 -1752.7534 6168.7399
306.9699 -21.5759 -78.4458 511540.4688 -13.0729
-13.0561
[31371]ENERGY: 7940000 3182.5320 5314.2541 5461.2859
335.6358 -14359.0219 -1660.0829 0.0000 0.0000
7829.6455 6104.2485 306.1784 -1725.3970 6167.3171
306.8690 211.0090 207.3353 511540.4688 -13.8796
-13.8772
[31407]ENERGY: 7950000 3253.3786 5412.8567 5402.7716
334.9956 -14338.4455 -1722.4722 0.0000 0.0000
7767.3984 6110.4832 303.7442 -1656.9152 6169.2629
306.9589 24.4007 16.2872 511540.4688 -11.9863
-12.0036
[31457]ENERGY: 7960000 3299.4157 5363.6295 5441.3299
309.7089 -14385.3576 -1744.4506 0.0000 0.0000
7825.7497 6110.0255 306.0261 -1715.7242 6168.9891
307.0112 -114.4973 -82.5815 511540.4688 -17.9951

-17.9979
[31493]ENERGY: 7970000 3244.7437 5311.3694 5449.7340
330.4696 -14318.4071 -1710.9309 0.0000 0.0000
7802.7409 6109.7195 305.1263 -1693.0214 6169.2585
307.0298 43.9073 27.7999 511540.4688 -13.6946
-13.7076
[31543]ENERGY: 7980000 3245.5800 5328.3228 5400.3292
339.9793 -14356.0004 -1706.2053 0.0000 0.0000
7851.0401 6103.0458 307.0150 -1747.9942 6168.2356
306.9918 -111.6696 -143.5210 511540.4688 -15.8450
-15.8041
[31579]ENERGY: 7990000 3322.9504 5303.8027 5441.5405
346.7198 -14456.1517 -1653.5715 0.0000 0.0000
7803.4831 6108.7732 305.1553 -1694.7099 6170.1421
307.0294 -99.4718 -115.4169 511540.4688 -14.0599
-14.0563
[31632]ENERGY: 8000000 3259.8241 5298.8411 5435.8181
321.7700 -14366.6083 -1780.3331 0.0000 0.0000
7934.1199 6103.4318 310.2639 -1830.6881 6169.1284
307.1207 70.7388 55.3239 511540.4688 -17.1764
-17.1828
[31668]ENERGY: 8010000 3268.1198 5367.9477 5389.4475
312.7775 -14363.0983 -1746.4721 0.0000 0.0000
7871.5453 6100.2673 307.8169 -1771.2780 6169.4436
307.1454 -33.9384 -73.8806 511540.4688 -11.6549
-11.6573
[31718]ENERGY: 8020000 3290.9700 5355.3560 5388.6261
319.5903 -14438.3311 -1713.4822 0.0000 0.0000
7903.2626 6105.9916 309.0572 -1797.2710 6169.2936
307.1968 -91.7445 -25.6332 511540.4688 -18.6076
-18.6184
[31754]ENERGY: 8030000 3199.7715 5327.2132 5412.6745
335.1964 -14344.6097 -1707.0308 0.0000 0.0000
7879.5701 6102.7853 308.1307 -1776.7848 6167.3555
307.0006 -18.6401 -49.2611 511540.4688 -14.3987
-14.4222
[31804]ENERGY: 8040000 3299.8193 5322.9711 5400.2274
322.9247 -14359.7126 -1720.3237 0.0000 0.0000
7838.8316 6104.7378 306.5376 -1734.0938 6168.1368
306.9477 -159.3570 -144.4578 511540.4688 -19.8573
-19.8383
[31840]ENERGY: 8050000 3271.8350 5305.4493 5490.5751
329.8808 -14381.0596 -1696.8144 0.0000 0.0000
7783.0032 6102.8695 304.3545 -1680.1337 6167.5794
307.2380 -181.5235 -154.2335 511540.4688 -16.7063
-16.7049
[31890]ENERGY: 8060000 3227.0658 5231.6404 5406.3661
337.5229 -14356.3785 -1704.6755 0.0000 0.0000
7962.4389 6103.9802 311.3713 -1858.4588 6168.6362
307.2611 -140.9575 -14.8776 511540.4688 -17.4048
-17.3968
[31926]ENERGY: 8070000 3195.5763 5431.9310 5433.1524
341.7171 -14446.5450 -1646.8940 0.0000 0.0000
7796.5085 6105.4463 304.8826 -1691.0622 6169.1959

307.0161 93.5935 79.7572 511540.4688 -19.6576
-19.6736
[31976]ENERGY: 8080000 3239.6842 5301.2646 5382.0454
341.5282 -14408.2122 -1715.9092 0.0000 0.0000
7963.3629 6103.7638 311.4074 -1859.5991 6169.2736
306.8857 164.3412 70.7961 511540.4688 -18.8621
-18.8589
[32012]ENERGY: 8090000 3286.9073 5309.1102 5505.2825
317.6905 -14398.9078 -1723.5996 0.0000 0.0000
7813.1896 6109.6728 305.5349 -1703.5168 6171.1965
307.0535 -176.4549 -178.9390 511540.4688 -20.6512
-20.6588
[32065]ENERGY: 8100000 3261.5522 5239.5599 5490.5570
330.1668 -14375.3672 -1718.4135 0.0000 0.0000
7880.9674 6109.0225 308.1854 -1771.9449 6171.2728
307.0930 34.0568 -59.5018 511540.4688 -18.0449
-18.0306
[32101]ENERGY: 8110000 3229.3117 5363.4197 5442.9250
320.6974 -14377.9888 -1727.8868 0.0000 0.0000
7857.5544 6108.0326 307.2698 -1749.5218 6171.0943
307.2652 -53.7402 -38.4688 511540.4688 -19.4228
-19.4245
[32151]ENERGY: 8120000 3236.6121 5414.9291 5413.9491
308.8315 -14376.8926 -1690.3886 0.0000 0.0000
7806.6979 6113.7384 305.2810 -1692.9595 6171.7233
307.0639 -47.6429 -104.5991 511540.4688 -20.8657
-20.8716
[32187]ENERGY: 8130000 3239.6295 5352.5688 5409.4723
341.5078 -14379.0541 -1672.0881 0.0000 0.0000
7815.6943 6107.7304 305.6328 -1707.9638 6172.6824
307.2773 87.5250 -27.1414 511540.4688 -18.2900
-18.2967
[32237]ENERGY: 8140000 3261.6413 5347.3980 5445.2695
344.3051 -14352.1305 -1734.5981 0.0000 0.0000
7800.2409 6112.1262 305.0285 -1688.1147 6170.6196
307.2407 -46.8219 4.4511 511540.4688 -20.2757
-20.2474
[32273]ENERGY: 8150000 3287.4485 5301.2614 5415.0738
317.5399 -14369.5803 -1723.4359 0.0000 0.0000
7875.5093 6103.8167 307.9719 -1771.6926 6170.9196
307.1778 -85.9128 -44.8518 511540.4688 -20.3451
-20.3488
[32323]ENERGY: 8160000 3321.8763 5338.4437 5432.4839
316.5609 -14378.6230 -1731.1852 0.0000 0.0000
7808.4008 6107.9574 305.3476 -1700.4434 6171.3081
307.1335 25.0555 -3.3436 511540.4688 -20.5278
-20.5433
[32359]ENERGY: 8170000 3315.5993 5247.6854 5462.4494
323.2064 -14359.6398 -1726.5941 0.0000 0.0000
7842.1510 6104.8576 306.6674 -1737.2934 6172.1314
307.2614 -42.5860 -97.1648 511540.4688 -19.0471
-19.0382
[32409]ENERGY: 8180000 3281.7677 5344.3389 5391.2935
325.7480 -14277.7029 -1754.6061 0.0000 0.0000

7795.4100 6106.2492 304.8396 -1689.1608 6171.4829
307.2297 -115.7306 -111.4319 511540.4688 -17.0513
-17.0675
[32445]ENERGY: 8190000 3260.4618 5327.9362 5402.0445
344.0407 -14253.8199 -1763.2975 0.0000 0.0000
7795.3920 6112.7578 304.8389 -1682.6342 6171.9245
307.0722 -122.9975 -196.3733 511540.4688 -16.5224
-16.5277
[32498]ENERGY: 8200000 3215.4144 5325.5433 5469.1297
340.1800 -14372.7581 -1693.2181 0.0000 0.0000
7824.1392 6108.4305 305.9631 -1715.7088 6171.7719
306.8998 113.7755 32.7350 511540.4688 -19.4689
-19.4736
[32534]ENERGY: 8210000 3148.1194 5324.6709 5462.5520
349.1540 -14344.2347 -1722.3729 0.0000 0.0000
7890.5452 6108.4340 308.5599 -1782.1112 6171.9057
306.8306 108.3200 94.1076 511540.4688 -13.8215
-13.7873
[32584]ENERGY: 8220000 3164.2710 5349.5005 5486.9450
315.8897 -14289.3921 -1739.8796 0.0000 0.0000
7819.8052 6107.1397 305.7936 -1712.6655 6172.5506
306.8285 -13.0139 -96.2363 511540.4688 -18.7420
-18.7619
[32620]ENERGY: 8230000 3278.8185 5173.3359 5410.5860
327.5183 -14346.1146 -1615.1786 0.0000 0.0000
7880.7837 6109.7491 308.1782 -1771.0345 6171.6846
306.8829 -46.5069 -94.0386 511540.4688 -17.7950
-17.8006
[32670]ENERGY: 8240000 3209.1101 5385.6041 5412.5506
304.2737 -14357.0087 -1752.1251 0.0000 0.0000
7906.8031 6109.2078 309.1957 -1797.5953 6172.3557
306.8803 -72.8884 -68.0773 511540.4688 -19.2953
-19.2931
[32706]ENERGY: 8250000 3249.7630 5406.2176 5434.3665
309.9508 -14471.2301 -1685.9309 0.0000 0.0000
7864.3200 6107.4569 307.5344 -1756.8631 6171.4937
306.9197 89.2458 48.0964 511540.4688 -13.3375
-13.3184
[32756]ENERGY: 8260000 3233.4997 5301.8107 5481.8452
335.7784 -14441.1635 -1694.0969 0.0000 0.0000
7890.1727 6107.8464 308.5453 -1782.3263 6170.7807
306.7583 254.1355 195.4122 511540.4688 -18.8592
-18.8695
[32792]ENERGY: 8270000 3195.3482 5316.8638 5476.0884
326.5723 -14328.0314 -1739.5644 0.0000 0.0000
7859.8001 6107.0769 307.3576 -1752.7231 6171.2720
306.7176 -144.0614 -283.2084 511540.4688 -19.2087
-19.2208
[32842]ENERGY: 8280000 3180.1290 5401.2649 5427.3114
339.9413 -14390.1449 -1695.3100 0.0000 0.0000
7846.6526 6109.8443 306.8435 -1736.8083 6170.6873
307.0354 -61.1551 -37.6821 511540.4688 -15.1858
-15.1804
[32878]ENERGY: 8290000 3348.0012 5300.1455 5456.1053

347.6134 -14431.6049 -1700.7359 0.0000 0.0000
7789.7146 6109.2391 304.6169 -1680.4755 6170.7864
306.9941 126.3406 102.9081 511540.4688 -17.8123
-17.7782
[32931]ENERGY: 8300000 3213.2697 5289.7798 5439.3571
331.8822 -14348.6963 -1710.5499 0.0000 0.0000
7894.3638 6109.4064 308.7092 -1784.9573 6172.3076
306.9618 233.4330 192.5554 511540.4688 -15.4586
-15.4897
[32967]ENERGY: 8310000 3188.0256 5261.0706 5454.3961
314.0407 -14333.0215 -1699.4154 0.0000 0.0000
7921.8770 6106.9731 309.7851 -1814.9039 6171.6074
306.8226 -14.2153 -9.7064 511540.4688 -16.1235
-16.1328
[33017]ENERGY: 8320000 3252.2597 5352.9357 5440.1200
326.5716 -14350.2201 -1751.4822 0.0000 0.0000
7839.1987 6109.3834 306.5520 -1729.8153 6172.9018
306.9541 14.0703 69.3584 511540.4688 -14.1140
-14.1009
[33053]ENERGY: 8330000 3215.1091 5386.3687 5454.0387
315.9651 -14397.7928 -1691.3494 0.0000 0.0000
7826.3011 6108.6405 306.0476 -1717.6606 6171.8388
306.7427 14.9372 -29.1829 511540.4688 -20.5484
-20.5577
[33103]ENERGY: 8340000 3248.5904 5353.5936 5439.3177
302.8625 -14323.6104 -1732.2854 0.0000 0.0000
7818.3515 6106.8197 305.7368 -1711.5317 6170.9699
307.0334 -199.2479 -138.1263 511540.4688 -17.7003
-17.6795
[33139]ENERGY: 8350000 3257.7746 5338.2130 5508.4249
326.1629 -14417.2403 -1745.4489 0.0000 0.0000
7842.4636 6110.3498 306.6797 -1732.1138 6171.6436
307.0320 -38.2402 -67.1137 511540.4688 -17.1906
-17.1997
[33189]ENERGY: 8360000 3202.1244 5309.0063 5501.4476
328.5892 -14346.3684 -1681.1701 0.0000 0.0000
7795.1921 6108.8211 304.8311 -1686.3710 6172.6555
307.0084 -3.8426 -50.9474 511540.4688 -16.3153
-16.3334
[33225]ENERGY: 8370000 3219.1560 5237.9485 5477.1345
325.2433 -14375.3549 -1677.5927 0.0000 0.0000
7902.5360 6109.0707 309.0288 -1793.4653 6173.3271
306.9591 106.1476 2.8947 511540.4688 -15.6465
-15.6258
[33275]ENERGY: 8380000 3218.9825 5287.1874 5438.3454
311.8320 -14364.0602 -1678.2646 0.0000 0.0000
7894.7963 6108.8188 308.7261 -1785.9774 6173.9848
307.0293 219.9762 89.9355 511540.4688 -12.8974
-12.9042
[33311]ENERGY: 8390000 3255.0637 5292.3416 5462.3343
319.7129 -14293.6098 -1721.5980 0.0000 0.0000
7796.0036 6110.2483 304.8628 -1685.7553 6172.1490
307.0411 -37.8034 -73.5957 511540.4688 -16.3600
-16.3456

[33364]ENERGY: 8400000 3288.5456 5295.6428 5444.4858
326.9811 -14361.8580 -1747.7154 0.0000 0.0000
7862.3997 6108.4817 307.4593 -1753.9180 6173.1249
307.1608 -72.1970 -46.8412 511540.4688 -16.1067
-16.1184
[33400]ENERGY: 8410000 3176.0447 5329.1422 5451.7326
333.0757 -14393.3150 -1619.2164 0.0000 0.0000
7834.1040 6111.5678 306.3528 -1722.5362 6171.9985
307.1226 249.2581 161.5223 511540.4688 -17.3321
-17.3560
[33450]ENERGY: 8420000 3190.9800 5328.6380 5465.5411
324.0647 -14358.2479 -1720.5333 0.0000 0.0000
7879.5341 6109.9768 308.1293 -1769.5574 6173.3847
307.1583 107.7463 -67.7999 511540.4688 -16.7831
-16.7619
[33486]ENERGY: 8430000 3239.3247 5408.2330 5409.7059
331.2513 -14402.7624 -1755.3305 0.0000 0.0000
7881.1509 6111.5730 308.1925 -1769.5779 6173.3936
307.0985 172.9968 158.0643 511540.4688 -17.1786
-17.1635
[33536]ENERGY: 8440000 3228.9081 5274.4972 5507.9321
324.3735 -14323.1089 -1737.5700 0.0000 0.0000
7834.5591 6109.5911 306.3706 -1724.9680 6173.9460
307.2178 -42.8044 -95.5585 511540.4688 -17.0479
-17.0594
[33572]ENERGY: 8450000 3205.1765 5443.6760 5475.1211
316.4068 -14387.0953 -1715.4429 0.0000 0.0000
7770.9970 6108.8393 303.8850 -1662.1577 6173.4991
307.0936 -110.6239 -145.9108 511540.4688 -17.1052
-17.1203
[33622]ENERGY: 8460000 3217.7224 5299.1490 5456.5758
334.0359 -14436.2798 -1641.4448 0.0000 0.0000
7883.1214 6112.8798 308.2696 -1770.2415 6175.1317
307.1977 82.5567 25.4958 511540.4688 -19.6119
-19.6152
[33658]ENERGY: 8470000 3236.7490 5365.4992 5417.6707
322.1673 -14343.1500 -1654.5538 0.0000 0.0000
7773.9057 6118.2880 303.9987 -1655.6177 6175.9956
307.1970 174.7128 102.7642 511540.4688 -19.5872
-19.5733
[33708]ENERGY: 8480000 3231.2283 5389.6104 5451.3816
329.7039 -14408.2559 -1703.7594 0.0000 0.0000
7824.4229 6114.3317 305.9742 -1710.0912 6175.4862
307.1245 -53.3775 -91.5024 511540.4688 -14.4701
-14.4601
[33744]ENERGY: 8490000 3296.4365 5299.8596 5457.1639
357.2479 -14355.3187 -1761.8310 0.0000 0.0000
7820.3165 6113.8746 305.8136 -1706.4419 6175.6874
307.2101 91.7447 -71.6078 511540.4688 -16.4814
-16.4870
[33797]ENERGY: 8500000 3201.0230 5368.7760 5442.8064
311.8142 -14347.2942 -1690.0735 0.0000 0.0000
7826.2105 6113.2623 306.0441 -1712.9482 6175.7830
307.3481 -7.9968 -2.2970 511540.4688 -19.8108

-19.8049
[33833]ENERGY: 8510000 3307.9811 5326.1080 5500.6321
327.2204 -14475.6424 -1677.7687 0.0000 0.0000
7801.0590 6109.5895 305.0605 -1691.4695 6175.7801
307.1765 55.6802 58.3749 511540.4688 -18.8862
-18.8944
[33883]ENERGY: 8520000 3308.6517 5293.3002 5434.3011
337.5427 -14348.7458 -1720.4622 0.0000 0.0000
7811.2005 6115.7882 305.4571 -1695.4123 6176.6416
307.1581 -136.2316 -207.5679 511540.4688 -16.8924
-16.8888
[33919]ENERGY: 8530000 3227.9883 5411.8881 5427.4841
311.6123 -14346.8870 -1765.7719 0.0000 0.0000
7847.0798 6113.3937 306.8602 -1733.6861 6177.5872
307.0744 -33.0266 -6.5387 511540.4688 -20.7841
-20.7916
[33969]ENERGY: 8540000 3265.8184 5344.6870 5457.0913
337.4718 -14449.7476 -1649.7439 0.0000 0.0000
7807.9258 6113.5027 305.3291 -1694.4231 6176.2642
306.9883 189.4583 91.5777 511540.4688 -18.9271
-18.9279
[34005]ENERGY: 8550000 3173.7267 5191.2097 5431.0621
325.1104 -14300.7015 -1681.0765 0.0000 0.0000
7974.3184 6113.6492 311.8358 -1860.6691 6176.9580
307.2141 237.1659 159.6134 511540.4688 -20.1464
-20.1433
[34055]ENERGY: 8560000 3196.7671 5263.3650 5471.8911
320.5656 -14389.5340 -1661.8803 0.0000 0.0000
7910.4365 6111.6110 309.3377 -1798.8255 6177.5893
306.9287 107.8199 33.2053 511540.4688 -19.3898
-19.3871
[34091]ENERGY: 8570000 3186.5265 5412.4139 5459.1597
340.7433 -14396.1668 -1706.0829 0.0000 0.0000
7818.6377 6115.2313 305.7479 -1703.4064 6179.0187
307.1001 -31.2186 -54.8709 511540.4688 -15.3877
-15.3914
[34141]ENERGY: 8580000 3270.6116 5361.2566 5480.8529
324.3291 -14451.8998 -1669.7084 0.0000 0.0000
7799.7360 6115.1780 305.0088 -1684.5580 6178.7915
307.2069 125.2885 54.2702 511540.4688 -14.7724
-14.7579
[34177]ENERGY: 8590000 3289.5897 5274.9640 5476.2606
330.8676 -14475.1117 -1681.5091 0.0000 0.0000
7901.7145 6116.7756 308.9967 -1784.9388 6180.5114
307.1272 9.6150 -31.4824 511540.4688 -19.7930
-19.7973
[34230]ENERGY: 8600000 3237.5240 5350.4715 5430.5926
318.4201 -14412.4223 -1671.5917 0.0000 0.0000
7866.5661 6119.5604 307.6222 -1747.0057 6178.6482
307.1556 -104.2852 -83.1202 511540.4688 -13.5029
-13.5110
[34266]ENERGY: 8610000 3233.9798 5323.4775 5488.4549
320.5059 -14302.7586 -1763.2920 0.0000 0.0000
7818.5702 6118.9377 305.7453 -1699.6325 6179.2560

307.0198 -106.8690 -105.6665 511540.4688 -20.3027
-20.2907
[34316]ENERGY: 8620000 3247.9236 5348.7757 5442.9030
318.0064 -14353.3950 -1742.5636 0.0000 0.0000
7851.9509 6113.6008 307.0507 -1738.3500 6179.2316
307.0924 15.7915 -27.8459 511540.4688 -18.7917
-18.8051
[34352]ENERGY: 8630000 3217.4555 5360.0205 5454.6471
327.6544 -14348.5106 -1742.3192 0.0000 0.0000
7843.8173 6112.7650 306.7326 -1731.0523 6178.5762
307.2742 -56.6191 -75.9894 511540.4688 -18.6179
-18.6096
[34402]ENERGY: 8640000 3260.2485 5294.0991 5448.6494
315.0857 -14341.3542 -1750.0885 0.0000 0.0000
7889.9440 6116.5840 308.5364 -1773.3600 6179.4942
307.3228 -61.5461 -85.4128 511540.4688 -18.3111
-18.3198
[34438]ENERGY: 8650000 3283.5709 5351.0125 5426.7456
320.6630 -14482.9656 -1658.3636 0.0000 0.0000
7876.5433 6117.2060 308.0123 -1759.3372 6180.5919
307.4475 69.9294 19.6605 511540.4688 -18.8877
-18.9021
[34488]ENERGY: 8660000 3162.3288 5385.2078 5460.9067
317.9292 -14283.9199 -1770.4884 0.0000 0.0000
7846.2242 6118.1884 306.8267 -1728.0358 6180.5373
307.3554 -100.4878 -62.2105 511540.4688 -19.6607
-19.6209
[34524]ENERGY: 8670000 3259.2607 5316.3530 5481.1270
306.6368 -14322.1879 -1697.3342 0.0000 0.0000
7776.7331 6120.5885 304.1093 -1656.1446 6181.0817
307.2141 2.3318 118.2189 511540.4688 -17.4104
-17.4291
[34574]ENERGY: 8680000 3246.8158 5271.3836 5415.2048
329.8248 -14318.8038 -1672.7144 0.0000 0.0000
7849.2844 6120.9952 306.9464 -1728.2892 6182.6805
307.1952 161.0435 -11.9331 511540.4688 -17.0481
-17.0556
[34610]ENERGY: 8690000 3247.9538 5355.6880 5483.4969
318.8076 -14422.4009 -1694.2002 0.0000 0.0000
7832.9911 6122.3363 306.3092 -1710.6547 6183.4895
307.3494 -110.6242 -68.0974 511540.4688 -18.8949
-18.8804
[34663]ENERGY: 8700000 3271.3929 5476.6068 5452.4041
333.0614 -14463.1715 -1709.4832 0.0000 0.0000
7762.7102 6123.5208 303.5609 -1639.1894 6184.7217
307.4055 172.5574 97.1740 511540.4688 -15.7984
-15.8268
[34699]ENERGY: 8710000 3215.2304 5308.5698 5498.7386
315.1636 -14382.6233 -1696.7032 0.0000 0.0000
7861.5077 6119.8835 307.4244 -1741.6241 6184.4615
307.4427 -56.0633 -116.3326 511540.4688 -16.3174
-16.3086
[34749]ENERGY: 8720000 3242.1656 5365.0391 5424.2486
321.9226 -14406.4062 -1652.0806 0.0000 0.0000

7826.7954 6121.6846 306.0670 -1705.1109 6184.0600
307.3513 -33.0404 -32.8990 511540.4688 -16.8680
-16.8528
[34785]ENERGY: 8730000 3179.7657 5287.8193 5530.0548
327.5279 -14360.0218 -1711.0640 0.0000 0.0000
7866.0665 6120.1483 307.6027 -1745.9182 6183.9888
307.2982 144.1894 64.8370 511540.4688 -14.9706
-14.9707
[34835]ENERGY: 8740000 3236.9299 5327.2826 5467.1737
329.7050 -14387.3073 -1642.1193 0.0000 0.0000
7788.6692 6120.3338 304.5760 -1668.3354 6182.2699
307.0003 -60.7642 28.2904 511540.4688 -19.7457
-19.7454
[34871]ENERGY: 8750000 3282.3276 5305.7449 5416.8472
309.0458 -14370.2713 -1671.5952 0.0000 0.0000
7846.8099 6118.9089 306.8496 -1727.9010 6183.7036
307.2191 230.5784 87.7743 511540.4688 -16.4209
-16.4327
[34921]ENERGY: 8760000 3362.4940 5382.6234 5419.1216
325.5453 -14424.4574 -1735.5008 0.0000 0.0000
7797.0016 6126.8277 304.9019 -1670.1739 6184.4625
307.2273 -89.5089 -181.3633 511540.4688 -17.5991
-17.6083
[34957]ENERGY: 8770000 3301.9194 5355.2086 5457.7520
323.6757 -14463.2510 -1696.8950 0.0000 0.0000
7845.1343 6123.5441 306.7841 -1721.5902 6185.1023
307.2675 118.4907 87.6472 511540.4688 -21.0036
-20.9888
[35007]ENERGY: 8780000 3348.5513 5301.1647 5440.9876
320.6135 -14356.3060 -1742.3649 0.0000 0.0000
7810.2711 6122.9172 305.4208 -1687.3539 6184.7103
307.0769 -121.5651 -112.2150 511540.4688 -18.8904
-18.8965
[35043]ENERGY: 8790000 3218.4282 5390.7365 5454.8984
330.9368 -14448.4763 -1669.2884 0.0000 0.0000
7844.0318 6121.2669 306.7410 -1722.7649 6186.6780
307.3013 136.7298 42.6589 511540.4688 -18.8319
-18.8179
[35096]ENERGY: 8800000 3274.3959 5326.3987 5491.3187
324.3949 -14427.8246 -1711.3724 0.0000 0.0000
7847.0716 6124.3828 306.8599 -1722.6889 6187.4027
307.4624 109.3562 35.3853 511540.4688 -19.8305
-19.8574
[35132]ENERGY: 8810000 3279.4933 5262.3587 5438.1033
312.7730 -14313.6900 -1724.1656 0.0000 0.0000
7869.1964 6124.0691 307.7250 -1745.1272 6186.4890
307.4566 240.1065 220.7002 511540.4688 -15.9984
-15.9827
[35182]ENERGY: 8820000 3293.6628 5372.0606 5418.7176
305.2522 -14325.2660 -1769.9459 0.0000 0.0000
7832.9856 6127.4669 306.3090 -1705.5187 6187.1631
307.2860 -141.4316 -102.5031 511540.4688 -20.9769
-20.9934
[35218]ENERGY: 8830000 3189.8084 5265.0804 5517.7613

298.0804 -14297.1035 -1717.5904 0.0000 0.0000
7868.1323 6124.1689 307.6834 -1743.9634 6187.1253
307.2025 39.6868 101.7684 511540.4688 -16.3869
-16.3681
[35268]ENERGY: 8840000 3235.6683 5386.4858 5431.8672
312.5202 -14426.4547 -1719.1817 0.0000 0.0000
7902.1666 6123.0718 309.0143 -1779.0948 6187.1218
307.3035 -77.6224 41.4956 511540.4688 -12.3856
-12.3564
[35304]ENERGY: 8850000 3283.8880 5369.4481 5448.1020
327.8135 -14360.5913 -1753.0349 0.0000 0.0000
7812.6315 6128.2571 305.5131 -1684.3745 6188.7953
307.3191 -126.2699 -127.5710 511540.4688 -16.0618
-16.0957
[35354]ENERGY: 8860000 3284.7224 5340.2780 5414.9563
322.5553 -14419.2034 -1652.2662 0.0000 0.0000
7834.7714 6125.8139 306.3789 -1708.9575 6188.2669
307.1817 82.8892 75.0836 511540.4688 -15.2445
-15.2508
[35390]ENERGY: 8870000 3267.8588 5349.5797 5428.2842
321.7304 -14352.0592 -1757.2544 0.0000 0.0000
7866.9805 6125.1201 307.6384 -1741.8604 6188.7894
307.2602 -23.2924 14.3402 511540.4688 -14.4011
-14.3810
[35440]ENERGY: 8880000 3246.8707 5439.9977 5465.3522
332.3863 -14359.7468 -1761.4809 0.0000 0.0000
7763.1882 6126.5672 303.5796 -1636.6209 6188.1884
307.1529 19.8203 -35.9273 511540.4688 -19.2885
-19.2915
[35476]ENERGY: 8890000 3196.2272 5279.1755 5416.8341
352.4179 -14310.2621 -1693.4545 0.0000 0.0000
7882.2254 6123.1635 308.2345 -1759.0619 6186.8382
307.2867 -91.9229 -44.9649 511540.4688 -18.8110
-18.8044
[35529]ENERGY: 8900000 3257.5678 5293.8489 5488.6445
312.8130 -14351.9139 -1707.7327 0.0000 0.0000
7834.4193 6127.6468 306.3651 -1706.7725 6188.7662
307.4440 -118.2116 -11.1059 511540.4688 -16.9707
-16.9668
[35565]ENERGY: 8910000 3333.8335 5287.9346 5443.5087
314.2074 -14387.5349 -1678.6149 0.0000 0.0000
7812.0131 6125.3475 305.4889 -1686.6656 6189.2039
307.3415 -185.9688 -89.0698 511540.4688 -18.7404
-18.7410
[35615]ENERGY: 8920000 3246.6962 5387.8491 5469.9718
312.8869 -14408.9546 -1685.0863 0.0000 0.0000
7801.0646 6124.4277 305.0608 -1676.6369 6186.9648
307.3200 -32.6736 57.7351 511540.4688 -16.6307
-16.6415
[35651]ENERGY: 8930000 3187.5854 5374.4641 5452.5257
317.3893 -14372.1130 -1624.2675 0.0000 0.0000
7794.0529 6129.6369 304.7866 -1664.4160 6189.4295
307.4145 342.9132 244.6935 511540.4688 -17.2679
-17.2732

[35701]ENERGY: 8940000 3296.1820 5312.5366 5487.0948
315.5233 -14359.9922 -1732.6869 0.0000 0.0000
7809.8301 6128.4877 305.4035 -1681.3424 6189.3881
307.5664 -131.8163 -93.1451 511540.4688 -15.3122
-15.3069
[35737]ENERGY: 8950000 3273.5988 5338.8236 5417.6302
330.9404 -14324.2212 -1770.8922 0.0000 0.0000
7861.1388 6127.0183 307.4100 -1734.1205 6189.4608
307.3982 174.0525 42.1110 511540.4688 -17.5485
-17.5664
[35787]ENERGY: 8960000 3263.5571 5355.8850 5509.5654
307.5702 -14329.1071 -1756.8606 0.0000 0.0000
7777.7082 6128.3182 304.1474 -1649.3900 6190.1507
307.4080 -100.1783 -85.4387 511540.4688 -13.4934
-13.4935
[35823]ENERGY: 8970000 3203.4965 5346.1560 5473.9214
318.3779 -14370.6086 -1708.2195 0.0000 0.0000
7861.7982 6124.9221 307.4357 -1736.8762 6189.9424
307.4559 247.2667 201.3922 511540.4688 -12.4602
-12.4474
[35873]ENERGY: 8980000 3212.8514 5360.6685 5475.7214
332.8086 -14318.6990 -1765.6973 0.0000 0.0000
7828.2581 6125.9117 306.1242 -1702.3464 6190.3586
307.4778 190.4382 -9.7323 511540.4688 -19.8597
-19.8605
[35909]ENERGY: 8990000 3258.3781 5318.3172 5431.9548
322.4064 -14357.8065 -1744.4445 0.0000 0.0000
7892.3651 6121.1705 308.6311 -1771.1946 6189.3288
307.3676 -47.8260 -28.9016 511540.4688 -17.7665
-17.7531
[35962]ENERGY: 9000000 3298.9106 5367.8559 5427.7364
322.2336 -14426.3195 -1724.6382 0.0000 0.0000
7858.4776 6124.2564 307.3059 -1734.2212 6189.5860
307.4779 -156.2830 -133.5547 511540.4688 -13.4414
-13.4550
[35998]ENERGY: 9010000 3212.1604 5295.5450 5495.1111
338.4132 -14387.4550 -1688.4025 0.0000 0.0000
7860.8306 6126.2027 307.3979 -1734.6279 6190.9194
307.3544 231.9439 137.5037 511540.4688 -19.2507
-19.2439
[36048]ENERGY: 9020000 3229.1443 5339.9680 5413.1994
308.7609 -14385.3163 -1658.5415 0.0000 0.0000
7880.5140 6127.7287 308.1676 -1752.7853 6191.5116
307.2332 142.3386 109.2290 511540.4688 -14.6548
-14.6594
[36084]ENERGY: 9030000 3253.3914 5379.5880 5499.8563
323.5192 -14321.3414 -1762.0578 0.0000 0.0000
7754.9323 6127.8881 303.2567 -1627.0442 6190.3100
307.2635 -19.5437 -33.6545 511540.4688 -18.6727
-18.6863
[36134]ENERGY: 9040000 3197.2150 5322.6061 5532.1944
323.5563 -14323.9200 -1748.3167 0.0000 0.0000
7824.8283 6128.1633 305.9900 -1696.6649 6191.0457
307.2671 -69.9168 14.5744 511540.4688 -17.3320

-17.3068
[36170]ENERGY: 9050000 3279.9509 5363.9874 5483.3298
304.3232 -14330.1155 -1773.6490 0.0000 0.0000
7801.1890 6129.0158 305.0656 -1672.1732 6191.2268
307.1636 -159.5533 -153.0240 511540.4688 -15.0783
-15.0899
[36220]ENERGY: 9060000 3243.7986 5304.5280 5438.3899
338.5577 -14385.3027 -1685.8570 0.0000 0.0000
7873.0399 6127.1545 307.8753 -1745.8855 6190.6301
307.2960 79.7510 50.6427 511540.4688 -19.5868
-19.5812
[36256]ENERGY: 9070000 3199.8412 5313.5653 5488.5695
308.8809 -14397.9400 -1708.6437 0.0000 0.0000
7920.3464 6124.6195 309.7253 -1795.7269 6189.5330
307.4809 101.8561 87.4673 511540.4688 -15.9371
-15.9604
[36306]ENERGY: 9080000 3304.6782 5380.6146 5500.4515
326.6170 -14407.6712 -1767.0058 0.0000 0.0000
7788.6052 6126.2894 304.5735 -1662.3157 6189.6368
307.3787 -207.7668 -150.5759 511540.4688 -15.7769
-15.7517
[36342]ENERGY: 9090000 3248.3810 5426.4277 5474.8278
331.9171 -14380.1964 -1680.7687 0.0000 0.0000
7707.8786 6128.4671 301.4167 -1579.4114 6190.6597
307.3572 -3.5672 -45.6558 511540.4688 -13.7106
-13.7280
[36395]ENERGY: 9100000 3176.6734 5307.5471 5500.7148
335.4186 -14341.5939 -1714.0239 0.0000 0.0000
7862.6848 6127.4209 307.4704 -1735.2639 6190.3741
307.4349 149.2849 118.6831 511540.4688 -16.1496
-16.1308
[36431]ENERGY: 9110000 3192.0990 5271.3765 5449.3778
314.1639 -14327.5683 -1731.9028 0.0000 0.0000
7957.4396 6124.9856 311.1758 -1832.4540 6189.2706
307.3613 -44.3283 -67.8627 511540.4688 -14.5822
-14.5923
[36481]ENERGY: 9120000 3217.3257 5302.1441 5452.1014
332.0076 -14456.8228 -1713.7370 0.0000 0.0000
7990.4868 6123.5059 312.4681 -1866.9809 6189.8087
307.3827 68.3645 28.9882 511540.4688 -14.8578
-14.8508
[36517]ENERGY: 9130000 3231.6078 5250.3857 5463.0736
320.1696 -14367.2282 -1751.1656 0.0000 0.0000
7976.1451 6122.9881 311.9073 -1853.1570 6189.5897
307.4902 8.4311 -93.7919 511540.4688 -14.4375
-14.4406
[36567]ENERGY: 9140000 3267.3720 5360.6375 5456.7924
355.5231 -14350.1756 -1730.3981 0.0000 0.0000
7766.4783 6126.2294 303.7083 -1640.2488 6189.9419
307.4621 -131.9947 -70.6834 511540.4688 -14.0892
-14.0860
[36603]ENERGY: 9150000 3206.7802 5313.3851 5417.4964
324.4760 -14312.2454 -1731.9525 0.0000 0.0000
7907.6313 6125.5712 309.2280 -1782.0601 6190.9986

307.4829 -78.1706 4.9036 511540.4688 -11.8709
-11.8593
[36653]ENERGY: 9160000 3381.8190 5295.0616 5473.8762
311.3767 -14410.1008 -1752.1082 0.0000 0.0000
7829.3059 6129.2304 306.1651 -1700.0755 6191.9242
307.3905 -18.9195 -95.4437 511540.4688 -21.9352
-21.9530
[36689]ENERGY: 9170000 3263.9351 5257.4528 5504.2708
323.9109 -14399.2648 -1676.0352 0.0000 0.0000
7855.9334 6130.2030 307.2064 -1725.7304 6191.3782
307.4710 -40.2844 -63.9432 511540.4688 -18.0007
-17.9974
[36739]ENERGY: 9180000 3159.0858 5383.0905 5415.9698
319.3973 -14404.5265 -1684.6439 0.0000 0.0000
7937.7796 6126.1526 310.4070 -1811.6270 6191.8275
307.2553 106.9331 127.0940 511540.4688 -14.5766
-14.5825
[36775]ENERGY: 9190000 3287.0567 5378.5211 5450.7280
315.4911 -14387.3557 -1737.3297 0.0000 0.0000
7821.5707 6128.6821 305.8626 -1692.8886 6192.4042
307.2415 -116.2223 -93.0533 511540.4688 -18.7471
-18.7447
[36828]ENERGY: 9200000 3236.2923 5289.5237 5481.4114
312.7053 -14284.0972 -1745.4739 0.0000 0.0000
7839.0545 6129.4160 306.5463 -1709.6385 6192.1391
307.2611 -131.5576 5.6112 511540.4688 -16.1958
-16.1675
[36864]ENERGY: 9210000 3248.5038 5307.2613 5427.2244
330.7679 -14448.2931 -1670.0526 0.0000 0.0000
7929.5903 6125.0020 310.0868 -1804.5883 6190.8480
307.2982 -37.3865 -56.5530 511540.4688 -14.6050
-14.6403
[36914]ENERGY: 9220000 3234.4025 5343.7780 5401.7573
315.2723 -14326.3579 -1693.9757 0.0000 0.0000
7853.5931 6128.4696 307.1149 -1725.1235 6191.3061
307.2256 30.1242 41.8217 511540.4688 -16.0528
-16.0322
[36950]ENERGY: 9230000 3306.0297 5349.1343 5413.4867
311.3397 -14342.6979 -1729.6834 0.0000 0.0000
7819.1171 6126.7262 305.7667 -1692.3909 6191.8774
307.3091 -106.3041 -131.1083 511540.4688 -13.8040
-13.8097
[37000]ENERGY: 9240000 3296.5541 5337.2453 5444.3617
342.4295 -14384.0343 -1659.7471 0.0000 0.0000
7753.4447 6130.2538 303.1986 -1623.1908 6190.2450
307.0958 47.8197 111.4716 511540.4688 -25.4982
-25.5081
[37036]ENERGY: 9250000 3232.1194 5298.4306 5461.1809
306.4489 -14388.8275 -1673.5920 0.0000 0.0000
7892.8385 6128.5987 308.6496 -1764.2397 6191.5973
307.3921 50.3357 16.3080 511540.4688 -16.1087
-16.1023
[37086]ENERGY: 9260000 3220.1485 5217.3767 5497.0255
321.3274 -14364.4727 -1668.9570 0.0000 0.0000

7906.1052 6128.5536 309.1684 -1777.5516 6191.8723
307.4809 72.1517 40.6796 511540.4688 -19.7225
-19.7257
[37122]ENERGY: 9270000 3290.5167 5344.0145 5495.4677
304.5606 -14401.0374 -1698.1484 0.0000 0.0000
7791.6650 6127.0386 304.6932 -1664.6263 6191.5049
307.4422 -103.4770 106.2646 511540.4688 -17.8110
-17.8159
[37172]ENERGY: 9280000 3247.1930 5228.7398 5519.4219
304.4632 -14355.7309 -1692.3871 0.0000 0.0000
7875.3619 6127.0617 307.9661 -1748.3003 6192.2461
307.2292 -86.0449 -43.1589 511540.4688 -17.4954
-17.5067
[37208]ENERGY: 9290000 3218.4109 5327.9864 5478.7848
317.7772 -14384.2225 -1719.9658 0.0000 0.0000
7889.0116 6127.7827 308.4999 -1761.2289 6191.7331
307.1855 128.2884 14.6550 511540.4688 -18.3779
-18.3803
[37261]ENERGY: 9300000 3238.4053 5338.2102 5450.1171
309.9450 -14350.0242 -1652.6417 0.0000 0.0000
7797.8163 6131.8281 304.9337 -1665.9882 6194.7311
307.3351 5.6840 -18.8630 511540.4688 -18.0906
-18.0755
[37297]ENERGY: 9310000 3211.7166 5389.3459 5456.1847
312.7876 -14379.2405 -1685.7686 0.0000 0.0000
7825.0106 6130.0364 305.9972 -1694.9742 6191.7860
307.2368 152.4499 90.3834 511540.4688 -18.4528
-18.4418
[37347]ENERGY: 9320000 3226.8303 5306.2683 5446.5025
316.5407 -14337.5462 -1731.9276 0.0000 0.0000
7903.7284 6130.3964 309.0754 -1773.3320 6194.4149
307.5686 155.7637 21.4981 511540.4688 -16.5231
-16.5438
[37383]ENERGY: 9330000 3292.8547 5270.6192 5465.5454
321.2403 -14396.3695 -1672.9831 0.0000 0.0000
7845.8392 6126.7463 306.8117 -1719.0929 6192.8756
307.5272 -185.5091 -187.3246 511540.4688 -14.8729
-14.8496
[37433]ENERGY: 9340000 3262.1787 5350.5367 5442.7746
324.8857 -14353.8905 -1664.9209 0.0000 0.0000
7771.2621 6132.8264 303.8953 -1638.4357 6194.3038
307.3116 214.6410 172.4144 511540.4688 -16.7908
-16.8065
[37469]ENERGY: 9350000 3282.8456 5230.4928 5444.0726
331.3393 -14412.9561 -1674.8665 0.0000 0.0000
7929.8896 6130.8173 310.0985 -1799.0723 6193.4770
307.3096 51.8685 7.9829 511540.4688 -18.9545
-18.9556
[37519]ENERGY: 9360000 3316.1278 5261.2555 5453.1615
330.9423 -14265.5250 -1792.8346 0.0000 0.0000
7831.7731 6134.9007 306.2616 -1696.8724 6193.3718
307.3545 -144.8454 -152.5929 511540.4688 -20.9106
-20.8982
[37555]ENERGY: 9370000 3232.6091 5300.0009 5475.7049

334.4799 -14461.9672 -1657.5291 0.0000 0.0000
7905.4833 6128.7817 309.1440 -1776.7015 6194.5398
307.4654 41.6823 -19.1117 511540.4688 -18.5993
-18.6129
[37605]ENERGY: 9380000 3279.0120 5335.0418 5469.8938
329.6757 -14391.3508 -1729.4267 0.0000 0.0000
7837.4216 6130.2674 306.4825 -1707.1542 6194.6388
307.3669 93.1633 41.3494 511540.4688 -19.8164
-19.8178
[37641]ENERGY: 9390000 3228.6071 5238.1962 5421.9957
329.1353 -14345.0772 -1693.7654 0.0000 0.0000
7952.1314 6131.2231 310.9682 -1820.9083 6194.7110
307.2966 -0.5879 -45.6729 511540.4688 -18.5703
-18.5387
[37694]ENERGY: 9400000 3243.0164 5342.8943 5472.1055
340.5108 -14333.2794 -1761.5359 0.0000 0.0000
7826.5487 6130.2605 306.0573 -1696.2882 6195.8106
307.2315 214.0785 -0.0822 511540.4688 -18.8414
-18.8552
[37730]ENERGY: 9410000 3239.7158 5266.9838 5460.1205
337.0278 -14341.3917 -1666.2034 0.0000 0.0000
7836.6655 6132.9185 306.4529 -1703.7471 6195.9599
307.4183 55.2961 59.7126 511540.4688 -20.1416
-20.1403
[37780]ENERGY: 9420000 3350.5205 5315.7298 5420.6054
310.5204 -14331.9219 -1777.9600 0.0000 0.0000
7847.3628 6134.8570 306.8712 -1712.5059 6195.4203
307.2819 -167.8281 -51.8011 511540.4688 -22.7414
-22.7578
[37816]ENERGY: 9430000 3310.9400 5325.2559 5440.7784
307.9094 -14342.5582 -1744.7671 0.0000 0.0000
7835.3437 6132.9021 306.4012 -1702.4417 6195.9382
307.2908 -258.3162 -201.6313 511540.4688 -14.7640
-14.7374
[37866]ENERGY: 9440000 3290.0275 5372.2106 5464.8053
343.0500 -14348.9431 -1740.9670 0.0000 0.0000
7753.3734 6133.5567 303.1958 -1619.8167 6195.1226
307.3430 -251.3546 -250.4667 511540.4688 -20.8677
-20.8815
[37902]ENERGY: 9450000 3249.4730 5366.2552 5477.5662
334.9666 -14442.5831 -1668.2033 0.0000 0.0000
7815.5171 6132.9917 305.6259 -1682.5254 6193.9834
307.2850 53.2298 19.7802 511540.4688 -17.8030
-17.7982
[37952]ENERGY: 9460000 3266.7774 5309.6167 5452.9204
308.0589 -14400.7517 -1671.2890 0.0000 0.0000
7863.1708 6128.5035 307.4894 -1734.6673 6194.2696
307.2606 5.7411 -5.5310 511540.4688 -22.4523
-22.4545
[37988]ENERGY: 9470000 3282.2421 5283.8485 5462.9818
335.2113 -14352.1910 -1705.9853 0.0000 0.0000
7827.3770 6133.4844 306.0897 -1693.8926 6194.2049
307.2892 -76.3996 -61.5878 511540.4688 -22.7120
-22.7112

[38038]ENERGY: 9480000 3294.7698 5319.5384 5405.9344
306.1155 -14331.6705 -1733.2149 0.0000 0.0000
7867.0757 6128.5485 307.6421 -1738.5272 6193.6452
307.2279 -79.4615 -122.3456 511540.4688 -22.7727
-22.7845
[38074]ENERGY: 9490000 3331.5682 5360.3365 5409.8816
331.7118 -14376.7126 -1750.6962 0.0000 0.0000
7828.5560 6134.6453 306.1358 -1693.9106 6195.0732
307.4084 -257.2582 -248.3808 511540.4688 -22.8486
-22.8550
[38127]ENERGY: 9500000 3292.2138 5307.8598 5456.2959
319.0874 -14450.2012 -1665.9779 0.0000 0.0000
7869.5914 6128.8692 307.7405 -1740.7222 6194.2286
307.3052 111.5739 103.6881 511540.4688 -19.6872
-19.6806
[38163]ENERGY: 9510000 3222.5367 5351.2759 5466.3568
335.9071 -14326.9563 -1702.7118 0.0000 0.0000
7784.8447 6131.2532 304.4265 -1653.5916 6195.2394
307.5000 113.0156 153.5277 511540.4688 -24.0584
-24.0318
[38213]ENERGY: 9520000 3256.4728 5324.8255 5474.2150
324.1225 -14441.4252 -1669.8103 0.0000 0.0000
7863.1714 6131.5717 307.4894 -1731.5997 6193.8542
307.6155 -202.7616 -134.6931 511540.4688 -22.1733
-22.1879
[38249]ENERGY: 9530000 3300.0622 5372.9810 5421.9765
310.3737 -14299.9191 -1778.6296 0.0000 0.0000
7802.8738 6129.7185 305.1315 -1673.1552 6194.7597
307.5539 81.8995 54.5597 511540.4688 -18.1762
-18.2035
[38299]ENERGY: 9540000 3249.4096 5287.1362 5471.1742
325.9597 -14379.3019 -1753.6330 0.0000 0.0000
7931.2548 6131.9996 310.1518 -1799.2553 6196.1468
307.3247 -260.1040 -248.3984 511540.4688 -16.7174
-16.7070
[38335]ENERGY: 9550000 3132.0273 5183.5798 5483.1353
327.8993 -14248.2413 -1718.8120 0.0000 0.0000
7969.3744 6128.9629 311.6425 -1840.4115 6195.9485
307.4584 131.9589 90.2854 511540.4688 -17.9924
-17.9828
[38385]ENERGY: 9560000 3243.6430 5353.1718 5495.7864
309.2896 -14366.2028 -1705.9661 0.0000 0.0000
7804.7284 6134.4503 305.2040 -1670.2781 6196.7785
307.4916 23.6201 -4.8094 511540.4688 -17.4169
-17.4220
[38421]ENERGY: 9570000 3232.8774 5332.8011 5461.9198
299.3216 -14377.2241 -1696.8789 0.0000 0.0000
7879.6516 6132.4685 308.1339 -1747.1831 6195.5900
307.7215 0.0595 76.5258 511540.4688 -18.9709
-18.9404
[38471]ENERGY: 9580000 3295.6455 5261.6694 5439.5384
320.4710 -14337.8857 -1702.6480 0.0000 0.0000
7856.5383 6133.3288 307.2301 -1723.2095 6195.3964
307.7810 -17.9715 -95.4535 511540.4688 -21.2216

-21.2417
[38507]ENERGY: 9590000 3285.0236 5322.0912 5479.6497
297.5682 -14437.4116 -1696.5652 0.0000 0.0000
7879.4926 6129.8486 308.1277 -1749.6440 6196.6767
307.4428 101.8894 102.8899 511540.4688 -17.7557
-17.7982
[38560]ENERGY: 9600000 3196.8417 5292.5515 5441.3758
329.8793 -14382.9658 -1610.0258 0.0000 0.0000
7865.6223 6133.2791 307.5853 -1732.3433 6195.4419
307.5196 145.2261 168.1447 511540.4688 -19.2597
-19.2175
[38596]ENERGY: 9610000 3289.3911 5295.3227 5481.5977
314.7608 -14412.4454 -1686.5622 0.0000 0.0000
7850.9317 6132.9963 307.0108 -1717.9353 6196.9142
307.5762 155.1345 35.8130 511540.4688 -16.5958
-16.5916
[38646]ENERGY: 9620000 3194.0118 5278.5741 5398.9610
325.1981 -14248.6633 -1776.7957 0.0000 0.0000
7962.8163 6134.1023 311.3861 -1828.7140 6196.8578
307.6230 48.3444 -104.0927 511540.4688 -17.5080
-17.5342
[38682]ENERGY: 9630000 3353.2844 5335.1069 5441.5790
299.3631 -14307.8240 -1773.4887 0.0000 0.0000
7787.0660 6135.0867 304.5133 -1651.9793 6196.1300
307.7543 -209.9053 -229.5033 511540.4688 -21.6547
-21.6334
[38732]ENERGY: 9640000 3380.2584 5294.4443 5494.5797
345.6032 -14451.4989 -1767.1122 0.0000 0.0000
7834.7551 6131.0296 306.3782 -1703.7255 6196.0367
307.6344 -283.3722 -297.4211 511540.4688 -24.6857
-24.6792
[38768]ENERGY: 9650000 3303.7072 5337.3097 5461.6735
311.6011 -14329.9584 -1753.7909 0.0000 0.0000
7804.8173 6135.3596 305.2075 -1669.4577 6196.5546
307.3814 35.6319 19.8987 511540.4688 -19.1532
-19.1711
[38818]ENERGY: 9660000 3308.6497 5317.0268 5456.2473
332.3205 -14374.5442 -1720.6594 0.0000 0.0000
7817.0280 6136.0688 305.6850 -1680.9592 6197.0571
307.5154 98.6506 -5.8678 511540.4688 -21.9426
-21.9265
[38854]ENERGY: 9670000 3199.6888 5346.5554 5493.4092
314.5197 -14373.5294 -1735.1015 0.0000 0.0000
7885.5982 6131.1404 308.3664 -1754.4578 6196.3851
307.5073 -38.6982 -36.4215 511540.4688 -20.1386
-20.1459
[38904]ENERGY: 9680000 3299.2214 5319.8721 5454.0792
336.4419 -14423.3326 -1708.6419 0.0000 0.0000
7854.5904 6132.2304 307.1539 -1722.3600 6196.7879
307.5818 73.6829 114.1449 511540.4688 -19.4805
-19.4604
[38940]ENERGY: 9690000 3335.5617 5329.2300 5436.5720
333.5858 -14342.4872 -1808.8167 0.0000 0.0000
7850.7479 6134.3935 307.0036 -1716.3544 6197.4505

307.8010 -372.0382 -304.3765 511540.4688 -18.8203
-18.8232
[38993]ENERGY: 9700000 3276.9924 5304.4034 5427.5284
330.8324 -14304.1769 -1669.4182 0.0000 0.0000
7768.3857 6134.5473 303.7828 -1633.8384 6197.2601
307.7437 -35.3937 -0.8679 511540.4688 -23.7785
-23.7899
[39029]ENERGY: 9710000 3261.5540 5281.9446 5477.3621
336.5118 -14296.1830 -1799.5524 0.0000 0.0000
7873.6468 6135.2840 307.8991 -1738.3629 6197.7441
307.3863 -62.6939 -88.5736 511540.4688 -18.3419
-18.3317
[39079]ENERGY: 9720000 3252.7837 5356.6398 5440.5940
330.4467 -14307.5966 -1756.4257 0.0000 0.0000
7817.2977 6133.7397 305.6955 -1683.5580 6197.2364
307.3823 -2.6516 54.8870 511540.4688 -20.1945
-20.2073
[39115]ENERGY: 9730000 3274.3248 5319.2522 5448.0805
327.8218 -14266.7703 -1795.9076 0.0000 0.0000
7827.2402 6134.0417 306.0844 -1693.1986 6197.0754
307.3324 -176.2592 -110.9795 511540.4688 -20.5148
-20.5291
[39165]ENERGY: 9740000 3333.1088 5287.7989 5436.6237
323.1925 -14386.6706 -1646.9812 0.0000 0.0000
7789.8751 6136.9472 304.6232 -1652.9279 6196.5227
307.6510 -130.5890 -146.6053 511540.4688 -16.5142
-16.5084
[39201]ENERGY: 9750000 3285.3069 5239.3301 5481.9213
342.9980 -14389.8075 -1731.2697 0.0000 0.0000
7902.8566 6131.3356 309.0413 -1771.5210 6197.4535
307.6759 -30.8471 -44.3701 511540.4688 -14.5707
-14.5569
[39251]ENERGY: 9760000 3163.0285 5304.0698 5495.4973
302.6502 -14418.9978 -1689.3072 0.0000 0.0000
7979.1161 6136.0569 312.0235 -1843.0591 6197.7930
307.6393 68.9372 17.8115 511540.4688 -12.2234
-12.2541
[39287]ENERGY: 9770000 3305.2330 5316.4715 5440.4601
316.7301 -14423.6077 -1703.4811 0.0000 0.0000
7882.2850 6134.0910 308.2369 -1748.1941 6198.3839
307.8242 -101.7939 -55.3556 511540.4688 -13.5543
-13.5289
[39337]ENERGY: 9780000 3286.2112 5284.1032 5452.7756
312.8710 -14336.7353 -1728.2265 0.0000 0.0000
7867.1657 6138.1649 307.6456 -1729.0007 6198.6850
307.7634 -115.4131 -142.0771 511540.4688 -12.4617
-12.4437
[39373]ENERGY: 9790000 3314.1938 5313.1813 5442.3793
318.9636 -14379.9588 -1691.9368 0.0000 0.0000
7819.2280 6136.0505 305.7710 -1683.1775 6198.7508
307.6809 217.8606 83.4797 511540.4688 -15.9247
-15.9528
[39426]ENERGY: 9800000 3294.8122 5368.7854 5394.8191
325.2471 -14344.9786 -1728.5794 0.0000 0.0000

7829.0750 6139.1808 306.1561 -1689.8942 6199.5352
307.7340 5.3279 12.7924 511540.4688 -17.6241
-17.6226
[39462]ENERGY: 9810000 3286.2460 5294.5511 5393.4009
309.0122 -14352.2274 -1699.5052 0.0000 0.0000
7900.9366 6132.4141 308.9662 -1768.5225 6198.8115
307.7481 21.9049 -135.3792 511540.4688 -14.5194
-14.5081
[39512]ENERGY: 9820000 3118.8066 5423.6963 5436.6662
318.7173 -14407.6903 -1729.3005 0.0000 0.0000
7970.7736 6131.6692 311.6972 -1839.1044 6198.7584
307.6920 91.8928 83.3867 511540.4688 -9.0537
-9.0718
[39548]ENERGY: 9830000 3267.7113 5339.5029 5455.4300
317.8703 -14327.6512 -1769.5558 0.0000 0.0000
7853.3593 6136.6668 307.1057 -1716.6925 6198.2640
307.4608 -123.4138 -147.7022 511540.4688 -18.4717
-18.4707
[39598]ENERGY: 9840000 3226.7116 5343.3586 5474.1866
341.9377 -14471.2793 -1661.3034 0.0000 0.0000
7879.8421 6133.4539 308.1413 -1746.3882 6199.2027
307.4517 -48.8542 -64.0722 511540.4688 -12.4734
-12.4731
[39634]ENERGY: 9850000 3254.4559 5338.1556 5430.8708
307.8221 -14293.6540 -1674.5582 0.0000 0.0000
7777.6555 6140.7477 304.1453 -1636.9078 6201.9811
307.5065 128.2004 13.1589 511540.4688 -13.6048
-13.5975
[39684]ENERGY: 9860000 3313.9813 5297.2381 5430.6254
299.4085 -14379.6328 -1674.9512 0.0000 0.0000
7853.4467 6140.1159 307.1092 -1713.3308 6202.3252
307.4829 -38.9455 -36.5737 511540.4688 -12.6450
-12.6338
[39720]ENERGY: 9870000 3180.1284 5280.8707 5494.3149
334.6166 -14300.8216 -1710.8807 0.0000 0.0000
7859.2022 6137.4304 307.3342 -1721.7717 6202.5528
307.5007 257.0650 107.6377 511540.4688 -13.2992
-13.3192
[39770]ENERGY: 9880000 3284.5320 5242.6180 5450.6813
309.8499 -14318.3392 -1742.6944 0.0000 0.0000
7912.1801 6138.8278 309.4059 -1773.3523 6202.8335
307.3204 19.3309 4.2389 511540.4688 -12.1161
-12.1113
[39806]ENERGY: 9890000 3294.6827 5275.6645 5473.7062
320.9579 -14445.0970 -1689.0460 0.0000 0.0000
7910.2408 6141.1090 309.3301 -1769.1318 6202.8201
307.5656 76.6588 79.8610 511540.4688 -12.5260
-12.5107
[39859]ENERGY: 9900000 3254.1648 5297.4182 5501.8762
332.8024 -14341.6454 -1739.2191 0.0000 0.0000
7834.7939 6140.1911 306.3797 -1694.6028 6203.6822
307.4405 -17.0522 -48.1992 511540.4688 -8.9403
-8.9520
[39895]ENERGY: 9910000 3155.5488 5361.8460 5364.4159

316.4572 -14270.3711 -1689.7919 0.0000 0.0000
7900.2969 6138.4019 308.9412 -1761.8950 6203.7312
307.5026 27.0999 25.8103 511540.4688 -15.4289
-15.4232
[39945]ENERGY: 9920000 3269.3981 5172.8039 5434.4729
314.4013 -14339.8798 -1719.3981 0.0000 0.0000
8006.8575 6138.6558 313.1083 -1868.2017 6203.4555
307.3898 -7.1545 -29.3806 511540.4688 -13.9719
-13.9760
[39981]ENERGY: 9930000 3237.7846 5297.6955 5465.2039
345.0559 -14381.7247 -1681.2297 0.0000 0.0000
7855.8417 6138.6272 307.2028 -1717.2145 6202.5956
307.5113 219.5050 167.1770 511540.4688 -15.3471
-15.3389
[40031]ENERGY: 9940000 3193.1349 5379.2533 5526.0669
322.0517 -14366.8133 -1767.8389 0.0000 0.0000
7854.3655 6140.2199 307.1451 -1714.1455 6204.0161
307.6087 -44.1337 -8.4313 511540.4688 -14.9536
-14.9513
[40067]ENERGY: 9950000 3160.3474 5293.1349 5507.9227
325.7819 -14344.9964 -1654.3187 0.0000 0.0000
7852.3121 6140.1839 307.0648 -1712.1282 6204.0457
307.4243 95.1921 74.5281 511540.4688 -13.4878
-13.4802
[40117]ENERGY: 9960000 3300.5388 5331.6889 5503.2155
306.9971 -14406.4474 -1714.7636 0.0000 0.0000
7819.9102 6141.1395 305.7977 -1678.7707 6203.2378
307.1898 69.1421 -62.9680 511540.4688 -16.3069
-16.3256
[40153]ENERGY: 9970000 3249.9267 5340.4633 5474.2514
342.6233 -14447.9400 -1646.7497 0.0000 0.0000
7828.3273 6140.9024 306.1269 -1687.4249 6204.0301
307.0164 184.3305 85.3423 511540.4688 -18.1400
-18.1288
[40203]ENERGY: 9980000 3256.2339 5352.1589 5467.4315
304.7646 -14375.0382 -1772.4827 0.0000 0.0000
7905.3722 6138.4402 309.1397 -1766.9320 6203.1766
306.9952 -309.5442 -190.1213 511540.4688 -12.8357
-12.8414
[40239]ENERGY: 9990000 3257.9410 5310.6951 5461.7967
318.2240 -14417.0681 -1706.3648 0.0000 0.0000
7913.4669 6138.6908 309.4562 -1774.7761 6203.4784
307.2411 -71.8259 -205.4141 511540.4688 -12.5390
-12.5418
[40292]ENERGY: 10000000 3291.0531 5334.3926 5401.7287
320.4149 -14481.0015 -1684.4437 0.0000 0.0000
7955.0259 6137.1699 311.0814 -1817.8559 6203.5289
307.4629 -175.2098 -73.2942 511540.4688 -13.3965
-13.3746
[40328]ENERGY: 10010000 3242.7823 5386.0860 5453.8510
303.9347 -14422.1585 -1717.4411 0.0000 0.0000
7893.6864 6140.7407 308.6827 -1752.9457 6203.0552
307.7465 -23.7022 89.4588 511540.4688 -13.2943
-13.3147

[40378]ENERGY: 10020000 3292.3219 5332.8653 5501.3294
317.2695 -14390.4632 -1738.9724 0.0000 0.0000
7824.6020 6138.9524 305.9812 -1685.6496 6202.7879
307.5971 -419.8545 -373.3456 511540.4688 -11.6946
-11.7139
[40414]ENERGY: 10030000 3231.0893 5324.5896 5450.1573
342.5051 -14361.3594 -1706.2147 0.0000 0.0000
7859.9090 6140.6761 307.3619 -1719.2329 6204.5986
307.3423 32.3841 65.3371 511540.4688 -13.4248
-13.4127
[40464]ENERGY: 10040000 3223.9443 5259.1573 5436.8012
315.4442 -14391.3442 -1580.8124 0.0000 0.0000
7882.5192 6145.7096 308.2460 -1736.8096 6204.4999
307.4683 177.0693 147.9133 511540.4688 -15.0240
-15.0267
[40500]ENERGY: 10050000 3180.8846 5291.7553 5433.4507
310.4978 -14408.9639 -1588.1591 0.0000 0.0000
7920.1742 6139.6396 309.7185 -1780.5346 6205.0537
307.4785 18.5702 -55.1722 511540.4688 -18.2297
-18.2317
[40550]ENERGY: 10060000 3240.5322 5260.0606 5461.2739
330.0755 -14425.7329 -1691.2335 0.0000 0.0000
7967.9236 6142.8993 311.5858 -1825.0243 6205.3872
307.5222 30.5594 -61.0951 511540.4688 -9.8819
-9.8585
[40586]ENERGY: 10070000 3283.6482 5403.6878 5475.6743
337.3823 -14334.7483 -1780.5354 0.0000 0.0000
7758.2696 6143.3785 303.3873 -1614.8911 6206.7972
307.4413 88.4471 101.8524 511540.4688 -14.2527
-14.2713
[40636]ENERGY: 10080000 3287.6139 5273.9192 5486.3939
323.6977 -14333.0587 -1727.0696 0.0000 0.0000
7830.8473 6142.3437 306.2254 -1688.5036 6206.5686
307.6611 -162.6022 -202.3219 511540.4688 -15.4696
-15.4537
[40672]ENERGY: 10090000 3256.6958 5449.4829 5443.9560
322.3827 -14418.8455 -1706.1917 0.0000 0.0000
7791.3759 6138.8561 304.6819 -1652.5198 6206.0329
307.6478 44.9464 -11.6100 511540.4688 -15.2175
-15.2423
[40725]ENERGY: 10100000 3321.6468 5355.9258 5472.1718
338.8660 -14388.6325 -1687.3307 0.0000 0.0000
7732.5141 6145.1613 302.3801 -1587.3528 6207.0291
307.5793 152.8001 78.2007 511540.4688 -14.7076
-14.7025
[40761]ENERGY: 10110000 3262.8147 5329.4539 5523.3869
327.7983 -14353.0163 -1785.2968 0.0000 0.0000
7840.5831 6145.7238 306.6061 -1694.8593 6208.2348
307.7130 -190.0576 -91.8330 511540.4688 -17.2999
-17.3130
[40811]ENERGY: 10120000 3283.2472 5415.2550 5466.9046
326.4939 -14406.5092 -1762.7077 0.0000 0.0000
7818.2514 6140.9352 305.7328 -1677.3161 6207.6794
307.6202 -139.4893 -175.0269 511540.4688 -10.8157

```
-10.7796
[40847]ENERGY: 10130000      3262.2382      5307.6840      5443.8494
320.1649         -14317.7080     -1742.9957         0.0000         0.0000
7870.5445           6143.7771       307.7778     -1726.7674      6207.8484
307.7490            -39.1511       -50.8969    511540.4688       -20.5322
-20.5480
[40897]ENERGY: 10140000      3301.9026      5282.3910      5440.8668
328.0620         -14375.1055     -1713.9029         0.0000         0.0000
7881.6586           6145.8726       308.2124     -1735.7860      6208.1100
307.8782             74.0146        93.4552    511540.4688       -13.2344
-13.2376
[40933]ENERGY: 10150000      3213.1586      5309.6420      5456.3555
318.5697         -14323.4739     -1692.1131         0.0000         0.0000
7862.5164           6144.6552       307.4638     -1717.8612      6208.4522
307.7878             54.8310        26.2821    511540.4688       -13.4339
-13.4285
[40983]ENERGY: 10160000      3178.3824      5428.3340      5513.5307
323.8414         -14345.0156     -1756.5484         0.0000         0.0000
7801.8329           6144.3575       305.0908     -1657.4754      6208.3161
307.8127            191.1526        37.0635    511540.4688       -12.0465
-12.0564
[41019]ENERGY: 10170000      3221.3911      5295.3367      5442.2924
307.6778         -14230.4915     -1799.3267         0.0000         0.0000
7904.1619           6141.0418       309.0924     -1763.1201      6208.1037
307.7868           -204.0310      -163.1666    511540.4688       -13.1362
-13.1117
[41069]ENERGY: 10180000      3244.4659      5399.4951      5472.1574
307.9513         -14333.4715     -1789.3857         0.0000         0.0000
7840.8712           6142.0836       306.6174     -1698.7875      6209.3525
307.9138           -155.6890      -103.3250    511540.4688       -13.7554
-13.7657
[41105]ENERGY: 10190000      3307.4611      5403.3715      5470.7549
312.5260         -14463.5012     -1702.4888         0.0000         0.0000
7818.0745           6146.1980       305.7259     -1671.8765      6209.7662
308.0376            -17.3599      -127.1290    511540.4688        -9.4475
-9.4662
[41158]ENERGY: 10200000      3214.9914      5504.1755      5457.5642
307.1487         -14433.8145     -1695.2347         0.0000         0.0000
7792.6129           6147.4434       304.7302     -1645.1695      6209.3530
308.0868           -185.5439      -178.4854    511540.4688       -13.3081
-13.2855
[41194]ENERGY: 10210000      3274.5773      5294.6521      5476.8559
320.1240         -14408.2682     -1683.8174         0.0000         0.0000
7871.7230           6145.8467       307.8238     -1725.8763      6209.8913
307.9969            -86.5074       -94.4228    511540.4688       -13.6509
-13.6562
[41244]ENERGY: 10220000      3233.7341      5342.2952      5447.0725
311.5418         -14406.4590     -1712.3924         0.0000         0.0000
7928.2510           6144.0432       310.0344     -1784.2078      6209.8754
307.9278            -50.8308       -26.0746    511540.4688       -12.7158
-12.7027
[41280]ENERGY: 10230000      3249.5072      5444.6874      5428.5798
331.6559         -14339.1619     -1742.4094         0.0000         0.0000
7773.4947           6146.3536       303.9826     -1627.1411      6211.2889
```

308.0146 83.3172 31.2034 511540.4688 -10.9212
-10.9390
[41330]ENERGY: 10240000 3291.3308 5269.7053 5463.4749
351.3068 -14365.1176 -1747.1466 0.0000 0.0000
7884.8556 6148.4094 308.3374 -1736.4463 6211.9258
307.9289 -59.6943 -33.8577 511540.4688 -15.3952
-15.3933
[41366]ENERGY: 10250000 3251.4374 5401.6357 5432.1483
332.2351 -14434.7449 -1687.0673 0.0000 0.0000
7851.6428 6147.2871 307.0386 -1704.3557 6211.6131
307.9700 -18.1435 -80.5485 511540.4688 -14.4369
-14.4352
[41416]ENERGY: 10260000 3279.7006 5354.6135 5456.4811
319.5933 -14425.7744 -1725.6545 0.0000 0.0000
7886.0107 6144.9703 308.3826 -1741.0404 6210.6745
308.0067 -91.0827 -127.7002 511540.4688 -11.7116
-11.7224
[41452]ENERGY: 10270000 3211.2536 5337.1543 5465.0458
329.3348 -14470.2067 -1677.0362 0.0000 0.0000
7948.3346 6143.8802 310.8197 -1804.4544 6212.2364
308.0416 2.5523 -20.9409 511540.4688 -12.5149
-12.5124
[41502]ENERGY: 10280000 3275.8962 5383.9943 5448.7860
324.2394 -14432.9702 -1701.9786 0.0000 0.0000
7855.7472 6153.7144 307.1991 -1702.0329 6214.2862
308.0790 -44.5453 -117.2532 511540.4688 -11.7931
-11.7821
[41538]ENERGY: 10290000 3298.4013 5358.7304 5448.0606
306.6417 -14328.1972 -1775.7766 0.0000 0.0000
7842.9824 6150.8426 306.6999 -1692.1398 6214.3576
307.8574 -158.1134 -84.8001 511540.4688 -20.5669
-20.5764
[41591]ENERGY: 10300000 3278.9758 5323.3787 5491.8766
340.9707 -14386.2357 -1690.9705 0.0000 0.0000
7794.1531 6152.1487 304.7905 -1642.0044 6215.0686
307.9557 -24.7982 -45.3930 511540.4688 -14.0998
-14.0809
[41627]ENERGY: 10310000 3199.0439 5336.2345 5453.9920
331.0578 -14320.6782 -1739.0384 0.0000 0.0000
7888.5688 6149.1803 308.4826 -1739.3885 6215.3738
308.0222 80.1015 25.2876 511540.4688 -15.1392
-15.1568
[41677]ENERGY: 10320000 3284.9116 5240.6701 5473.2795
296.0001 -14304.6735 -1751.3471 0.0000 0.0000
7910.6116 6149.4522 309.3446 -1761.1594 6215.1103
308.0880 -255.1813 -217.4171 511540.4688 -15.4575
-15.4800
[41713]ENERGY: 10330000 3344.5258 5326.8653 5445.4453
325.4048 -14352.6063 -1770.7158 0.0000 0.0000
7834.9486 6153.8676 306.3858 -1681.0810 6216.2725
308.3119 -251.2653 -243.3860 511540.4688 -14.1666
-14.1509
[41763]ENERGY: 10340000 3262.0846 5332.2817 5425.9842
330.5417 -14443.8160 -1713.5812 0.0000 0.0000

7957.0717 6150.5667 311.1614 -1806.5050 6214.9775
308.2367 -22.1245 29.9514 511540.4688 -13.0568
-13.0671
[41799]ENERGY: 10350000 3246.6623 5383.5710 5462.6279
324.4964 -14326.3220 -1759.8434 0.0000 0.0000
7824.4451 6155.6372 305.9750 -1668.8079 6218.3152
307.9794 31.9266 -91.3946 511540.4688 -14.5930
-14.6016
[41849]ENERGY: 10360000 3290.6525 5353.2084 5461.7976
316.6568 -14354.0319 -1712.7795 0.0000 0.0000
7799.6732 6155.1771 305.0063 -1644.4961 6217.3815
308.0055 33.9437 79.3182 511540.4688 -15.2491
-15.2382
[41885]ENERGY: 10370000 3292.8285 5313.7497 5479.0234
343.2606 -14409.2075 -1723.9695 0.0000 0.0000
7857.2279 6152.9131 307.2570 -1704.3148 6215.0850
308.0975 -73.0143 -56.8639 511540.4688 -13.9880
-13.9538
[41935]ENERGY: 10380000 3297.1099 5304.5988 5437.7629
339.0388 -14397.3912 -1734.3901 0.0000 0.0000
7905.4170 6152.1462 309.1415 -1753.2708 6216.1304
308.1973 46.5758 -37.8568 511540.4688 -15.5831
-15.6085
[41971]ENERGY: 10390000 3281.9616 5298.9226 5500.1234
322.1436 -14405.8031 -1723.4377 0.0000 0.0000
7875.3273 6149.2377 307.9648 -1726.0896 6215.8147
308.1298 3.5124 -51.6278 511540.4688 -13.0282
-13.0115
[42024]ENERGY: 10400000 3320.9553 5282.7986 5439.2451
315.9814 -14410.9116 -1666.7843 0.0000 0.0000
7868.7249 6150.0094 307.7066 -1718.7155 6215.2788
307.8801 30.5049 -27.4410 511540.4688 -15.5841
-15.6017
[42060]ENERGY: 10410000 3239.5013 5454.4112 5448.3394
310.5255 -14402.1323 -1681.7671 0.0000 0.0000
7786.2612 6155.1392 304.4819 -1631.1220 6217.5181
308.0529 25.0484 80.0426 511540.4688 -14.5043
-14.5059
[42110]ENERGY: 10420000 3270.9212 5403.6476 5438.5861
294.9044 -14419.8818 -1706.9866 0.0000 0.0000
7873.1000 6154.2908 307.8777 -1718.8091 6217.2558
308.1642 -94.6387 -164.1744 511540.4688 -16.0153
-16.0122
[42146]ENERGY: 10430000 3222.5291 5285.7385 5460.6087
335.1573 -14358.8684 -1720.2899 0.0000 0.0000
7927.5025 6152.3779 310.0051 -1775.1246 6218.2565
308.0100 155.2186 114.4652 511540.4688 -13.9624
-13.9397
[42196]ENERGY: 10440000 3323.9923 5353.5799 5459.8918
326.1325 -14457.2690 -1710.9019 0.0000 0.0000
7860.0342 6155.4598 307.3668 -1704.5743 6217.7259
307.9596 -107.2666 -56.8857 511540.4688 -15.4031
-15.4062
[42232]ENERGY: 10450000 3304.8418 5261.3579 5455.1059

331.6628 -14352.8317 -1706.6557 0.0000 0.0000
7861.1961 6154.6771 307.4122 -1706.5190 6217.3879
307.8440 -255.2227 -198.8429 511540.4688 -11.9010
-11.9087
[42282]ENERGY: 10460000 3235.5502 5383.3565 5532.7798
337.9860 -14427.6429 -1752.5077 0.0000 0.0000
7847.0627 6156.5846 306.8595 -1690.4781 6218.6028
307.9322 310.0440 150.6363 511540.4688 -15.7847
-15.7988
[42318]ENERGY: 10470000 3223.6398 5366.2349 5451.7380
310.2803 -14369.2219 -1717.6623 0.0000 0.0000
7888.2832 6153.2919 308.4714 -1734.9913 6217.9010
308.0314 114.1981 78.6926 511540.4688 -12.2202
-12.2198
[42368]ENERGY: 10480000 3331.5420 5342.3308 5382.2502
317.0873 -14393.2329 -1716.5595 0.0000 0.0000
7889.2646 6152.6826 308.5098 -1736.5820 6217.5216
307.8775 -288.3625 -213.9520 511540.4688 -14.1276
-14.1046
[42404]ENERGY: 10490000 3268.5857 5337.2390 5489.7950
307.2707 -14415.6320 -1708.8497 0.0000 0.0000
7875.2852 6153.6939 307.9631 -1721.5913 6216.8116
307.9822 58.2037 -21.8217 511540.4688 -15.2827
-15.2836
[42457]ENERGY: 10500000 3173.6428 5352.6436 5470.7327
324.3003 -14366.8685 -1651.0481 0.0000 0.0000
7849.1111 6152.5139 306.9396 -1696.5972 6216.2185
308.0354 305.8322 215.2341 511540.4688 -16.5634
-16.5626
[42493]ENERGY: 10510000 3220.5353 5389.6778 5480.4798
335.0806 -14409.0794 -1695.0215 0.0000 0.0000
7831.1431 6152.8157 306.2370 -1678.3275 6216.2117
307.9319 66.0146 14.9274 511540.4688 -12.7675
-12.7877
[42543]ENERGY: 10520000 3224.8439 5302.6955 5461.9057
294.8941 -14327.7262 -1674.6048 0.0000 0.0000
7868.3592 6150.3674 307.6923 -1717.9918 6214.9832
307.8353 -28.8261 -87.1134 511540.4688 -17.3039
-17.3061
[42579]ENERGY: 10530000 3296.9075 5339.5879 5478.9524
339.4420 -14354.9594 -1742.9752 0.0000 0.0000
7797.7032 6154.6584 304.9293 -1643.0449 6214.7990
307.8556 -84.5518 -233.0852 511540.4688 -16.0135
-16.0180
[42629]ENERGY: 10540000 3261.7898 5313.3200 5503.3762
342.7664 -14350.7872 -1735.1423 0.0000 0.0000
7819.7064 6155.0293 305.7897 -1664.6771 6215.9073
307.7178 13.8262 105.4283 511540.4688 -18.8687
-18.8641
[42665]ENERGY: 10550000 3247.6409 5348.1472 5460.8734
309.6413 -14369.0209 -1673.3061 0.0000 0.0000
7827.1079 6151.0838 306.0792 -1676.0241 6214.6654
308.0736 -31.5759 -32.1713 511540.4688 -11.7900
-11.7858

[42715]ENERGY: 10560000 3267.9561 5269.7104 5417.8392
332.7591 -14345.1107 -1752.4442 0.0000 0.0000
7963.8003 6154.5102 311.4245 -1809.2900 6216.4493
307.9891 99.6892 3.8447 511540.4688 -16.8857
-16.8672
[42751]ENERGY: 10570000 3258.8474 5330.9776 5452.5198
299.1411 -14394.9953 -1720.6735 0.0000 0.0000
7924.7615 6150.5787 309.8979 -1774.1828 6215.9879
308.0659 -62.9977 -13.2875 511540.4688 -16.3943
-16.3972
[42801]ENERGY: 10580000 3280.7965 5295.5214 5419.3932
333.3827 -14448.3163 -1669.4472 0.0000 0.0000
7940.3453 6151.6755 310.5073 -1788.6697 6217.0918
308.0193 258.0559 258.4885 511540.4688 -16.1714
-16.1905
[42837]ENERGY: 10590000 3298.9195 5344.9418 5394.7165
342.9838 -14277.1599 -1761.7360 0.0000 0.0000
7812.4545 6155.1202 305.5062 -1657.3343 6216.5238
307.9726 -183.3165 -213.0445 511540.4688 -16.4571
-16.4560
[42890]ENERGY: 10600000 3268.8826 5349.0437 5426.5781
333.5354 -14398.3777 -1675.9823 0.0000 0.0000
7848.9047 6152.5846 306.9315 -1696.3201 6216.1247
307.9292 113.3081 147.4974 511540.4688 -16.0203
-16.0094
[42926]ENERGY: 10610000 3289.0189 5220.4833 5482.5115
322.7130 -14319.5271 -1712.6880 0.0000 0.0000
7873.1709 6155.6825 307.8805 -1717.4884 6217.4879
307.9343 28.8508 -6.7775 511540.4688 -15.2189
-15.2366
[42976]ENERGY: 10620000 3153.6309 5276.7019 5437.7841
334.2061 -14309.2117 -1659.5424 0.0000 0.0000
7919.5596 6153.1284 309.6945 -1766.4312 6217.2898
308.0892 265.7694 134.5569 511540.4688 -14.9553
-14.9364
[43012]ENERGY: 10630000 3204.2394 5288.9925 5448.2357
328.7116 -14370.4779 -1688.2353 0.0000 0.0000
7943.3004 6154.7664 310.6229 -1788.5340 6218.0460
307.9566 172.4365 104.6356 511540.4688 -13.7563
-13.7729
[43062]ENERGY: 10640000 3202.4354 5409.1379 5404.2583
315.7186 -14347.1942 -1676.6001 0.0000 0.0000
7847.1359 6154.8918 306.8624 -1692.2441 6218.4603
308.0852 292.4253 220.5339 511540.4688 -15.9020
-15.9015
[43098]ENERGY: 10650000 3243.1666 5343.6514 5426.5745
329.7465 -14395.1069 -1673.8051 0.0000 0.0000
7881.7385 6155.9654 308.2155 -1725.7730 6219.2792
308.0445 198.0429 153.6244 511540.4688 -16.1229
-16.1089
[43148]ENERGY: 10660000 3252.9459 5412.9843 5474.3859
322.5881 -14405.6810 -1717.5071 0.0000 0.0000
7815.0570 6154.7730 305.6079 -1660.2840 6219.5099
308.1379 -104.9451 -182.5242 511540.4688 -14.9139

-14.9217
[43184]ENERGY: 10670000 3353.8959 5247.2749 5412.2339
299.7346 -14251.0875 -1786.6514 0.0000 0.0000
7880.6902 6156.0906 308.1745 -1724.5996 6220.1775
308.1293 -91.8326 -42.7378 511540.4688 -14.5735
-14.5744
[43234]ENERGY: 10680000 3252.8588 5289.4044 5402.3051
315.7891 -14275.3284 -1730.0313 0.0000 0.0000
7900.5612 6155.5589 308.9516 -1745.0023 6218.8763
308.1419 134.3302 7.7408 511540.4688 -17.5567
-17.5456
[43270]ENERGY: 10690000 3236.3298 5349.2816 5434.6031
313.5202 -14356.1520 -1749.3504 0.0000 0.0000
7927.7688 6156.0010 310.0155 -1771.7677 6219.8617
308.0812 146.1047 36.9159 511540.4688 -10.1255
-10.1474
[43323]ENERGY: 10700000 3242.2289 5366.3343 5449.7759
322.2697 -14441.9054 -1710.3720 0.0000 0.0000
7925.3341 6153.6656 309.9203 -1771.6685 6220.0175
308.2065 174.8943 13.6156 511540.4688 -15.5065
-15.4773
[43359]ENERGY: 10710000 3305.5845 5278.8415 5514.6484
309.7817 -14341.6836 -1774.8079 0.0000 0.0000
7863.5461 6155.9108 307.5041 -1707.6353 6219.2788
308.1982 -228.7269 -284.3541 511540.4688 -22.3267
-22.3556
[43409]ENERGY: 10720000 3271.7766 5379.4429 5452.0897
319.2707 -14327.8173 -1763.3477 0.0000 0.0000
7825.1927 6156.6077 306.0043 -1668.5850 6220.5147
308.2562 -60.4807 -41.2294 511540.4688 -11.9714
-11.9711
[43445]ENERGY: 10730000 3325.1268 5331.0385 5412.1498
331.4267 -14391.7598 -1705.2658 0.0000 0.0000
7856.9742 6159.6905 307.2471 -1697.2837 6220.2039
308.2841 -118.3314 -116.8968 511540.4688 -15.5046
-15.4938
[43495]ENERGY: 10740000 3319.1789 5262.4296 5512.7453
332.6225 -14467.5779 -1694.8747 0.0000 0.0000
7892.1585 6156.6821 308.6230 -1735.4764 6220.2198
308.2571 211.8481 229.1036 511540.4688 -15.4114
-15.3944
[43531]ENERGY: 10750000 3282.9241 5381.0768 5435.7420
323.4271 -14392.0053 -1699.4824 0.0000 0.0000
7826.7161 6158.3985 306.0639 -1668.3176 6220.3045
308.3169 58.3549 -8.6986 511540.4688 -16.2473
-16.2598
[43581]ENERGY: 10760000 3157.1088 5353.4055 5469.9386
305.0474 -14368.0336 -1727.5235 0.0000 0.0000
7964.4397 6154.3829 311.4495 -1810.0569 6219.9245
308.4097 47.8965 18.8745 511540.4688 -16.5172
-16.5123
[43617]ENERGY: 10770000 3250.1902 5361.3378 5474.7736
331.3980 -14434.0334 -1689.2661 0.0000 0.0000
7863.0309 6157.4310 307.4839 -1705.5999 6220.6856

308.4174 -177.7605 -163.5046 511540.4688 -14.8304
-14.8320
[43667]ENERGY: 10780000 3300.2447 5346.6233 5495.3760
330.0337 -14489.9730 -1698.8093 0.0000 0.0000
7873.1625 6156.6577 307.8801 -1716.5047 6220.3170
308.3448 -94.0523 -105.4148 511540.4688 -13.9035
-13.9030
[43703]ENERGY: 10790000 3264.6523 5344.7771 5441.9540
325.3112 -14391.1862 -1692.3174 0.0000 0.0000
7864.2352 6157.4262 307.5310 -1706.8090 6220.3514
308.4118 99.9210 51.4105 511540.4688 -17.0230
-17.0129
[43756]ENERGY: 10800000 3260.6842 5191.8201 5462.6117
303.0404 -14306.3855 -1672.1584 0.0000 0.0000
7919.4670 6159.0795 309.6909 -1760.3875 6220.4836
308.2212 -34.3096 -138.8264 511540.4688 -17.0411
-17.0573
[43792]ENERGY: 10810000 3230.6930 5351.7201 5454.3551
319.5896 -14388.0969 -1632.3203 0.0000 0.0000
7824.3319 6160.2725 305.9706 -1664.0595 6220.9964
308.2421 -27.6697 -57.7780 511540.4688 -15.2553
-15.2574
[43842]ENERGY: 10820000 3289.2866 5260.8809 5461.3921
334.1423 -14348.5746 -1669.4308 0.0000 0.0000
7831.4815 6159.1780 306.2502 -1672.3036 6221.6414
308.2698 -95.9529 -86.5034 511540.4688 -15.4912
-15.5040
[43878]ENERGY: 10830000 3299.3419 5334.5365 5473.1754
335.9656 -14401.9408 -1715.5708 0.0000 0.0000
7833.8918 6159.3996 306.3445 -1674.4923 6221.0987
308.3614 18.8702 -74.0954 511540.4688 -18.8827
-18.8862
[43928]ENERGY: 10840000 3352.6117 5333.0409 5399.5133
312.0108 -14408.9712 -1751.3719 0.0000 0.0000
7922.0836 6158.9173 309.7932 -1763.1663 6222.0132
308.3728 123.8822 58.9494 511540.4688 -11.9272
-11.9290
[43964]ENERGY: 10850000 3208.2984 5366.7956 5491.4014
313.5538 -14397.6919 -1705.7724 0.0000 0.0000
7881.5532 6158.1382 308.2083 -1723.4150 6221.9271
308.1665 52.4350 25.5482 511540.4688 -14.3302
-14.3229
[44014]ENERGY: 10860000 3308.8027 5309.3886 5466.1023
299.3083 -14461.1757 -1710.0154 0.0000 0.0000
7946.4154 6158.8261 310.7447 -1787.5893 6221.9834
308.1502 -178.6270 -134.2023 511540.4688 -14.6152
-14.5937
[44050]ENERGY: 10870000 3310.5192 5279.1007 5448.4326
318.6282 -14393.7689 -1686.8024 0.0000 0.0000
7885.9536 6162.0631 308.3803 -1723.8905 6223.0885
308.2007 -10.6967 -24.1551 511540.4688 -13.8051
-13.8133
[44100]ENERGY: 10880000 3302.4946 5384.2245 5456.8714
343.9262 -14451.4287 -1705.8312 0.0000 0.0000

7829.5060 6159.7628 306.1730 -1669.7432 6222.0708
308.1007 54.6893 42.3457 511540.4688 -16.7323
-16.7256
[44136]ENERGY: 10890000 3248.6259 5352.7297 5431.5660
305.3852 -14416.7183 -1721.9977 0.0000 0.0000
7958.7281 6158.3189 311.2262 -1800.4092 6222.9957
308.2183 -127.6622 -71.6280 511540.4688 -15.2320
-15.2402
[44189]ENERGY: 10900000 3283.2408 5309.2677 5424.8886
319.1603 -14362.8708 -1727.7888 0.0000 0.0000
7913.7675 6159.6651 309.4680 -1754.1023 6223.4390
308.4378 -118.5118 -188.5428 511540.4688 -14.4828
-14.4706
[44225]ENERGY: 10910000 3201.7715 5412.8143 5445.3949
312.6818 -14516.2371 -1646.1362 0.0000 0.0000
7947.3475 6157.6367 310.7811 -1789.7108 6223.6994
308.2633 66.8570 31.6442 511540.4688 -14.4592
-14.4746
[44275]ENERGY: 10920000 3291.6612 5275.5629 5477.9305
331.6051 -14412.2619 -1743.5571 0.0000 0.0000
7937.6755 6158.6163 310.4029 -1779.0592 6224.1279
308.2291 116.7002 10.2402 511540.4688 -16.7277
-16.7070
[44311]ENERGY: 10930000 3188.7472 5334.0705 5485.0975
327.1586 -14311.0831 -1731.9167 0.0000 0.0000
7869.7974 6161.8714 307.7485 -1707.9260 6224.3064
308.2600 190.8601 145.8439 511540.4688 -13.4871
-13.4934
[44361]ENERGY: 10940000 3289.2718 5331.0957 5461.2427
311.6920 -14385.8762 -1725.7651 0.0000 0.0000
7880.4601 6162.1209 308.1655 -1718.3392 6225.8892
308.5124 -211.5557 -66.0049 511540.4688 -13.5984
-13.5971
[44397]ENERGY: 10950000 3263.0739 5386.3029 5432.9191
317.2701 -14344.4113 -1753.7397 0.0000 0.0000
7860.3956 6161.8106 307.3809 -1698.5850 6225.6969
308.5162 57.8104 -21.2568 511540.4688 -16.7434
-16.7442
[44447]ENERGY: 10960000 3204.3548 5378.7732 5479.9874
304.7901 -14262.9401 -1769.4022 0.0000 0.0000
7829.8762 6165.4394 306.1874 -1664.4368 6226.6773
308.3942 185.5733 5.2523 511540.4688 -15.8728
-15.8932
[44483]ENERGY: 10970000 3215.2930 5309.0644 5486.9073
328.7931 -14389.0928 -1765.7410 0.0000 0.0000
7970.9889 6156.2129 311.7056 -1814.7760 6225.8882
308.4103 219.0224 127.0493 511540.4688 -15.7289
-15.7180
[44533]ENERGY: 10980000 3306.5929 5412.2298 5425.8823
318.6167 -14409.1077 -1769.0758 0.0000 0.0000
7875.1993 6160.3373 307.9598 -1714.8620 6226.0703
308.4412 -57.9536 -136.3472 511540.4688 -12.9599
-12.9725
[44569]ENERGY: 10990000 3198.0773 5398.2162 5489.1174

325.9869 -14422.0445 -1723.3449 0.0000 0.0000
7897.0992 6163.1077 308.8162 -1733.9915 6226.5902
308.5305 89.1492 41.2023 511540.4688 -16.5385
-16.5292
[44622]ENERGY: 11000000 3277.3743 5343.4959 5416.0465
326.7226 -14327.9902 -1795.1666 0.0000 0.0000
7924.7994 6165.2818 309.8994 -1759.5176 6226.4324
308.4407 66.6178 19.2754 511540.4688 -20.2341
-20.2372
[268]ENERGY: 11010000 3249.1400 5331.7286 5448.6366
332.8110 -14358.8280 -1716.7647 0.0000 0.0000
7876.1513 6162.8749 307.9970 -1713.2764 6226.5272
308.4357 -24.0556 -22.5812 511540.4688 -13.3994
-13.3997
[307]ENERGY: 11020000 3258.6197 5342.5401 5456.3713
334.2784 -14396.0908 -1705.1842 0.0000 0.0000
7873.7815 6164.3159 307.9043 -1709.4656 6225.5669
308.4174 88.9598 108.5173 511540.4688 -15.9513
-15.9421
[359]ENERGY: 11030000 3251.7775 5359.5856 5460.2914
300.6926 -14437.3193 -1697.2369 0.0000 0.0000
7926.2126 6164.0035 309.9547 -1762.2091 6227.1145
308.2601 -11.5122 -34.1313 511540.4688 -20.5427
-20.5588
[397]ENERGY: 11040000 3291.7795 5353.4501 5455.0129
329.0559 -14473.7127 -1660.6686 0.0000 0.0000
7865.9131 6160.8303 307.5967 -1705.0829 6225.5678
308.2739 143.7704 182.7055 511540.4688 -18.0119
-18.0129
[449]ENERGY: 11050000 3243.2609 5360.1763 5435.1948
315.8221 -14342.7871 -1759.6009 0.0000 0.0000
7909.3841 6161.4502 309.2966 -1747.9339 6226.1999
308.4004 54.9039 84.4520 511540.4688 -18.4357
-18.4045
[487]ENERGY: 11060000 3340.6738 5290.9771 5381.2740
311.5701 -14316.9374 -1800.5117 0.0000 0.0000
7954.9063 6161.9522 311.0767 -1792.9541 6225.5743
308.5833 -397.3912 -252.2644 511540.4688 -10.9434
-10.9320
[538]ENERGY: 11070000 3237.8276 5373.5960 5443.0012
331.2168 -14358.9634 -1743.5798 0.0000 0.0000
7882.5698 6165.6682 308.2480 -1716.9016 6226.9988
308.3582 197.5438 116.4608 511540.4688 -17.2271
-17.2700
[575]ENERGY: 11080000 3306.9764 5312.4558 5419.6285
339.9862 -14363.0770 -1680.5626 0.0000 0.0000
7833.8182 6169.2255 306.3416 -1664.5928 6227.4137
308.4137 101.5456 148.6654 511540.4688 -14.0087
-13.9775
[626]ENERGY: 11090000 3254.9603 5392.0187 5418.8855
357.0776 -14404.7019 -1749.3609 0.0000 0.0000
7894.2634 6163.1428 308.7053 -1731.1206 6227.1860
308.4192 234.4900 180.0654 511540.4688 -16.7801
-16.8049

[666]ENERGY: 11100000 3282.7263 5308.7767 5471.5876
354.3714 -14313.1980 -1785.5748 0.0000 0.0000
7845.8965 6164.5858 306.8139 -1681.3107 6226.7603
308.3743 -143.1947 -265.2030 511540.4688 -15.5219
-15.5032
[717]ENERGY: 11110000 3236.1701 5300.0284 5455.9927
316.7941 -14405.7430 -1729.7427 0.0000 0.0000
7985.3935 6158.8932 312.2689 -1826.5003 6225.4336
308.3709 0.1709 -102.1721 511540.4688 -17.7989
-17.8129
[753]ENERGY: 11120000 3211.1288 5293.1053 5457.4905
346.5104 -14223.2619 -1718.2877 0.0000 0.0000
7798.0754 6164.7607 304.9439 -1633.3147 6225.3908
308.3951 170.8905 154.5131 511540.4688 -13.4747
-13.4530
[803]ENERGY: 11130000 3333.5539 5356.2936 5478.1910
315.1471 -14413.0347 -1735.0279 0.0000 0.0000
7828.9816 6164.1047 306.1524 -1664.8769 6226.9765
308.5886 -56.8652 -100.0888 511540.4688 -16.1244
-16.1616
[839]ENERGY: 11140000 3232.5619 5281.4038 5453.9236
327.8073 -14330.2307 -1750.4332 0.0000 0.0000
7947.3406 6162.3735 310.7809 -1784.9672 6226.2356
308.6025 76.7919 58.8559 511540.4688 -14.6452
-14.6320
[889]ENERGY: 11150000 3245.0386 5242.3351 5438.1613
340.6538 -14231.2398 -1754.6023 0.0000 0.0000
7881.3510 6161.6978 308.2004 -1719.6532 6225.5489
308.3432 -116.7788 -174.5995 511540.4688 -12.9172
-12.9103
[925]ENERGY: 11160000 3282.2801 5362.8756 5460.6627
327.8390 -14464.5003 -1702.7218 0.0000 0.0000
7897.2596 6163.6949 308.8225 -1733.5647 6226.3429
308.5492 -93.9929 -15.8546 511540.4688 -20.0483
-20.0372
[975]ENERGY: 11170000 3248.9927 5266.2809 5438.2874
341.0713 -14290.9510 -1725.2980 0.0000 0.0000
7886.8970 6165.2803 308.4172 -1721.6167 6226.2798
308.6812 152.0896 -12.6789 511540.4688 -10.5082
-10.4943
[1011]ENERGY: 11180000 3257.7062 5328.6904 5505.9111
328.3507 -14362.4882 -1737.9260 0.0000 0.0000
7845.8761 6166.1203 306.8131 -1679.7558 6226.8384
308.7039 -36.2437 -171.7911 511540.4688 -18.6122
-18.6467
[1061]ENERGY: 11190000 3280.6911 5442.8487 5468.7745
312.0999 -14485.9792 -1740.6650 0.0000 0.0000
7885.0370 6162.8071 308.3445 -1722.2299 6226.7000
308.6219 176.9733 49.0152 511540.4688 -14.0933
-14.1075
[1100]ENERGY: 11200000 3253.3252 5397.6845 5431.2068
306.5942 -14375.2735 -1769.1719 0.0000 0.0000
7921.0273 6165.3925 309.7519 -1755.6347 6227.0699
308.6381 -156.8775 -31.1324 511540.4688 -14.4980

-14.4627
[1150]ENERGY: 11210000 3283.8421 5323.1849 5450.5674
314.0403 -14363.4291 -1652.5110 0.0000 0.0000
7809.1440 6164.8387 305.3767 -1644.3053 6225.7417
308.5662 216.8679 153.5203 511540.4688 -16.3380
-16.3587
[1186]ENERGY: 11220000 3289.4411 5336.0203 5421.3013
300.7201 -14228.9211 -1767.1205 0.0000 0.0000
7814.9885 6166.4297 305.6052 -1648.5587 6226.9813
308.5869 -17.1564 -44.4025 511540.4688 -15.6077
-15.5907
[1236]ENERGY: 11230000 3307.5731 5490.9508 5410.5652
326.6778 -14385.5384 -1813.4528 0.0000 0.0000
7826.5207 6163.2965 306.0562 -1663.2242 6226.3792
308.5334 -98.6058 -92.0933 511540.4688 -18.5682
-18.5709
[1272]ENERGY: 11240000 3242.0212 5298.3999 5437.0329
328.6584 -14405.9022 -1727.1276 0.0000 0.0000
7987.3809 6160.4636 312.3467 -1826.9173 6227.1161
308.5825 36.9001 -1.5926 511540.4688 -15.8891
-15.9133
[1322]ENERGY: 11250000 3288.3917 5305.1640 5473.0028
326.9545 -14360.4322 -1729.2971 0.0000 0.0000
7858.9190 6162.7027 307.3231 -1696.2163 6226.0055
308.4455 35.4619 -17.3640 511540.4688 -14.9706
-14.9373
[1358]ENERGY: 11260000 3236.4472 5257.6358 5472.1709
333.0197 -14376.2868 -1683.0567 0.0000 0.0000
7925.1047 6165.0348 309.9113 -1760.0699 6226.7844
308.5372 89.1295 61.6720 511540.4688 -15.6664
-15.6740
[1408]ENERGY: 11270000 3187.0212 5352.3944 5476.7221
339.8023 -14335.2312 -1717.4114 0.0000 0.0000
7857.2266 6160.5239 307.2570 -1696.7027 6226.1630
308.3853 97.4032 97.3096 511540.4688 -15.2012
-15.2152
[1444]ENERGY: 11280000 3246.6923 5387.1652 5476.1759
339.5339 -14524.5933 -1620.5350 0.0000 0.0000
7861.5398 6165.9789 307.4256 -1695.5609 6227.9163
308.3750 198.6856 224.6670 511540.4688 -18.7447
-18.7388
[1494]ENERGY: 11290000 3322.9945 5389.2728 5443.5785
311.6873 -14369.0911 -1755.4594 0.0000 0.0000
7820.1900 6163.1726 305.8087 -1657.0174 6228.0440
308.5670 44.8421 -60.7007 511540.4688 -15.8113
-15.8156
[1533]ENERGY: 11300000 3200.3031 5366.8530 5412.0989
312.4286 -14366.2410 -1703.2540 0.0000 0.0000
7943.4339 6165.6225 310.6281 -1777.8114 6228.6388
308.5360 134.9444 50.2074 511540.4688 -17.8339
-17.8296
[1583]ENERGY: 11310000 3287.3590 5395.5061 5467.5767
311.0096 -14336.5337 -1797.6589 0.0000 0.0000
7840.1252 6167.3840 306.5882 -1672.7412 6230.3676

308.5525 62.4783 -79.3698 511540.4688 -13.6759
-13.6561
[1619]ENERGY: 11320000 3248.3635 5343.1878 5475.6371
318.7826 -14446.5601 -1670.3862 0.0000 0.0000
7897.6285 6166.6532 308.8369 -1730.9754 6229.7157
308.6008 83.1055 35.0537 511540.4688 -17.9101
-17.9293
[1669]ENERGY: 11330000 3305.6419 5327.5190 5432.9279
326.9725 -14511.6808 -1680.8907 0.0000 0.0000
7964.5556 6165.0454 311.4541 -1799.5102 6229.2563
308.5308 270.2835 152.4854 511540.4688 -15.8350
-15.8168
[1705]ENERGY: 11340000 3307.3902 5331.0373 5481.2455
330.0167 -14381.7089 -1691.7671 0.0000 0.0000
7792.9142 6169.1279 304.7420 -1623.7863 6230.5033
308.4826 -30.6068 -119.3097 511540.4688 -12.4994
-12.5242
[1755]ENERGY: 11350000 3318.5609 5209.0284 5437.1471
326.9806 -14308.4168 -1725.3327 0.0000 0.0000
7906.0693 6164.0369 309.1670 -1742.0324 6229.6453
308.4339 -40.7946 -87.2003 511540.4688 -12.7922
-12.7911
[1791]ENERGY: 11360000 3149.2661 5364.8786 5446.5448
298.2892 -14288.1137 -1774.0771 0.0000 0.0000
7969.3087 6166.0966 311.6399 -1803.2121 6230.6513
308.3274 94.1670 135.3351 511540.4688 -13.8965
-13.9072
[1841]ENERGY: 11370000 3265.8759 5348.3952 5468.7124
329.0919 -14337.7067 -1716.4442 0.0000 0.0000
7809.9898 6167.9142 305.4098 -1642.0756 6231.1338
308.5673 112.0854 45.9530 511540.4688 -15.2750
-15.2489
[1877]ENERGY: 11380000 3202.1731 5378.6403 5449.7695
338.6100 -14435.8400 -1684.2362 0.0000 0.0000
7918.4150 6167.5317 309.6497 -1750.8833 6229.8000
308.5943 191.7274 35.2723 511540.4688 -18.0674
-18.0924
[1927]ENERGY: 11390000 3264.5513 5328.8170 5449.5655
325.6635 -14350.8619 -1745.3279 0.0000 0.0000
7895.8906 6168.2980 308.7689 -1727.5926 6231.1115
308.7836 37.4524 85.7410 511540.4688 -15.6766
-15.6491
[1966]ENERGY: 11400000 3236.5200 5301.8713 5409.0942
333.5648 -14385.7592 -1729.3136 0.0000 0.0000
8000.8084 6166.7860 312.8717 -1834.0225 6230.1929
308.9120 104.6339 79.6440 511540.4688 -13.0468
-13.0716
[2016]ENERGY: 11410000 3281.4645 5276.8146 5449.7524
304.4299 -14340.0458 -1723.2090 0.0000 0.0000
7918.6178 6167.8245 309.6577 -1750.7934 6230.3480
308.6078 1.4010 20.2397 511540.4688 -17.0244
-17.0100
[2052]ENERGY: 11420000 3247.3684 5422.7340 5409.6712
324.5387 -14357.3686 -1714.3325 0.0000 0.0000

7834.5968 6167.2080 306.3720 -1667.3887 6230.0068
308.6700 -36.7808 -16.0089 511540.4688 -12.1982
-12.2019
[2102]ENERGY: 11430000 3181.1933 5327.8992 5455.6804
325.4426 -14374.9462 -1713.6076 0.0000 0.0000
7963.4695 6165.1312 311.4116 -1798.3383 6230.7734
308.6668 169.8191 132.4729 511540.4688 -16.6060
-16.6233
[2138]ENERGY: 11440000 3287.3752 5395.0947 5456.3232
325.2377 -14324.7500 -1800.3886 0.0000 0.0000
7827.4885 6166.3808 306.0941 -1661.1077 6231.0513
308.7394 -184.4532 -318.1371 511540.4688 -16.5174
-16.4743
[2188]ENERGY: 11450000 3277.4371 5338.6399 5405.6423
322.3503 -14408.1344 -1678.4810 0.0000 0.0000
7909.3555 6166.8096 309.2955 -1742.5458 6230.7599
308.4968 36.5214 -3.9625 511540.4688 -19.5667
-19.5920
[2224]ENERGY: 11460000 3298.8264 5313.4530 5449.8031
317.4833 -14379.3989 -1685.0001 0.0000 0.0000
7853.8029 6168.9698 307.1231 -1684.8331 6231.0371
308.3600 -4.5133 1.5833 511540.4688 -15.9758
-15.9715
[2274]ENERGY: 11470000 3209.3921 5338.6262 5425.3437
319.7942 -14375.8962 -1680.9238 0.0000 0.0000
7930.5704 6166.9065 310.1251 -1763.6639 6230.1176
308.5929 90.0605 36.1853 511540.4688 -21.6960
-21.7041
[2310]ENERGY: 11480000 3287.0431 5370.0845 5452.9397
317.1461 -14349.1874 -1766.2803 0.0000 0.0000
7855.5869 6167.3325 307.1928 -1688.2544 6231.6630
308.6497 -157.2666 -228.2025 511540.4688 -18.0997
-18.0859
[2360]ENERGY: 11490000 3234.1762 5338.6611 5391.9811
316.2976 -14358.5457 -1708.8839 0.0000 0.0000
7955.4061 6169.0925 311.0963 -1786.3135 6231.3604
308.2739 -143.1092 -117.1587 511540.4688 -15.4765
-15.4766
[2399]ENERGY: 11500000 3275.8856 5282.7933 5502.3880
327.3258 -14364.1361 -1730.2023 0.0000 0.0000
7874.1371 6168.1914 307.9183 -1705.9457 6232.5455
308.5184 150.3462 1.9399 511540.4688 -19.4550
-19.4685
[2449]ENERGY: 11510000 3224.9887 5387.5042 5436.0598
331.7085 -14351.5204 -1724.4359 0.0000 0.0000
7867.6660 6171.9709 307.6652 -1695.6951 6232.1943
308.5956 -9.1781 3.8951 511540.4688 -17.6164
-17.6249
[2485]ENERGY: 11520000 3232.0964 5371.0615 5449.4856
295.0178 -14366.9025 -1708.0406 0.0000 0.0000
7895.1722 6167.8906 308.7408 -1727.2817 6231.4376
308.5712 128.3588 53.9812 511540.4688 -19.7113
-19.7111
[2535]ENERGY: 11530000 3258.2467 5387.4283 5440.6005

333.6179 -14402.0943 -1726.5963 0.0000 0.0000
7874.8813 6166.0841 307.9474 -1708.7972 6231.2311
308.4944 -109.2344 -114.9267 511540.4688 -15.8511
-15.8204
[2571]ENERGY: 11540000 3265.0473 5408.5943 5425.4330
312.5120 -14408.4198 -1740.5219 0.0000 0.0000
7905.3983 6168.0433 309.1407 -1737.3550 6231.3454
308.5561 8.5722 -38.6257 511540.4688 -16.8069
-16.8294
[2621]ENERGY: 11550000 3237.2854 5314.1362 5482.4305
316.3550 -14360.8651 -1716.7858 0.0000 0.0000
7895.2481 6167.8044 308.7438 -1727.4437 6229.7425
308.7137 -162.4433 -82.9273 511540.4688 -16.6392
-16.6419
[2657]ENERGY: 11560000 3249.2356 5325.4356 5442.0056
344.1628 -14292.4363 -1725.5135 0.0000 0.0000
7823.8111 6166.7009 305.9503 -1657.1102 6231.2977
308.7812 -31.7805 31.3969 511540.4688 -17.7718
-17.7594
[2707]ENERGY: 11570000 3343.6167 5330.0742 5443.4110
313.4765 -14416.1852 -1758.6545 0.0000 0.0000
7911.2260 6166.9645 309.3686 -1744.2615 6231.1568
308.7023 -24.2685 -43.2746 511540.4688 -18.1069
-18.1102
[2743]ENERGY: 11580000 3306.2344 5289.5484 5447.8844
315.4341 -14367.9996 -1710.8227 0.0000 0.0000
7890.1284 6170.4074 308.5436 -1719.7210 6230.5057
308.7026 -25.9566 -58.1202 511540.4688 -19.2388
-19.2596
[2793]ENERGY: 11590000 3281.3752 5297.2538 5404.9086
337.4062 -14357.4614 -1744.5748 0.0000 0.0000
7950.5069 6169.4145 310.9047 -1781.0925 6230.9513
308.7004 -80.9535 -12.3299 511540.4688 -17.2690
-17.2594
[2832]ENERGY: 11600000 3253.2543 5344.8525 5455.7554
320.0763 -14395.0136 -1741.7055 0.0000 0.0000
7929.2798 6166.4993 310.0746 -1762.7806 6231.2033
308.7035 79.8668 -21.0588 511540.4688 -18.9616
-18.9623
[2882]ENERGY: 11610000 3244.6750 5379.4173 5423.0963
325.5939 -14336.9517 -1782.4664 0.0000 0.0000
7914.8875 6168.2519 309.5118 -1746.6356 6232.4300
308.7631 -40.8219 -120.8374 511540.4688 -15.8980
-15.8689
[2918]ENERGY: 11620000 3310.3642 5307.6959 5452.9397
325.5188 -14352.8425 -1683.5626 0.0000 0.0000
7808.9058 6169.0193 305.3674 -1639.8865 6231.9393
308.8895 -239.6746 -241.7359 511540.4688 -15.5376
-15.5684
[2968]ENERGY: 11630000 3301.0556 5257.5103 5444.3195
343.5067 -14296.1501 -1794.6458 0.0000 0.0000
7910.5079 6166.1039 309.3405 -1744.4040 6232.3646
308.6108 -98.0521 -95.7482 511540.4688 -20.0299
-20.0219

[3004]ENERGY: 11640000 3271.3541 5384.3397 5463.1125
314.7923 -14367.7749 -1723.9053 0.0000 0.0000
7828.0962 6170.0146 306.1178 -1658.0816 6231.3205
308.5211 -21.1536 -95.7281 511540.4688 -17.8262
-17.8092
[3054]ENERGY: 11650000 3204.1001 5436.6995 5478.2572
321.1437 -14395.5079 -1775.0830 0.0000 0.0000
7898.5081 6168.1176 308.8713 -1730.3905 6231.1328
308.5903 -39.7341 -35.2737 511540.4688 -17.5682
-17.5782
[3090]ENERGY: 11660000 3290.4094 5293.4925 5460.3058
341.9887 -14406.3782 -1655.2864 0.0000 0.0000
7844.1968 6168.7286 306.7474 -1675.4683 6231.6429
308.8054 -12.4960 5.4320 511540.4688 -17.6061
-17.6115
[3140]ENERGY: 11670000 3261.7874 5256.2921 5423.6163
315.5060 -14319.0702 -1712.0336 0.0000 0.0000
7944.2068 6170.3047 310.6583 -1773.9020 6233.3037
308.7714 91.9007 18.3824 511540.4688 -16.9167
-16.9480
[3176]ENERGY: 11680000 3204.7350 5324.4362 5454.7684
323.6391 -14371.6530 -1687.4417 0.0000 0.0000
7915.3978 6163.8819 309.5318 -1751.5159 6231.8294
308.6399 -22.7523 28.5613 511540.4688 -15.6218
-15.5995
[3226]ENERGY: 11690000 3362.3970 5313.7589 5491.8308
326.1635 -14367.9600 -1762.1077 0.0000 0.0000
7810.1119 6174.1944 305.4145 -1635.9175 6233.7886
308.6876 -88.7420 -189.8748 511540.4688 -18.3244
-18.3116
[3265]ENERGY: 11700000 3324.6219 5298.5927 5461.0898
320.0381 -14351.2594 -1767.2545 0.0000 0.0000
7886.2580 6172.0866 308.3922 -1714.1714 6233.1906
308.7816 104.6667 132.8069 511540.4688 -16.1348
-16.1367
[3315]ENERGY: 11710000 3288.7683 5315.1666 5456.0039
338.1116 -14379.3886 -1717.5179 0.0000 0.0000
7868.5942 6169.7381 307.7015 -1698.8561 6232.3836
308.6818 -24.1921 -75.5374 511540.4688 -21.1705
-21.1686
[3351]ENERGY: 11720000 3274.3529 5366.0917 5478.8129
328.7389 -14471.2236 -1656.2205 0.0000 0.0000
7846.6982 6167.2506 306.8453 -1679.4477 6231.6834
308.7426 127.2803 144.9498 511540.4688 -15.9728
-15.9941
[3401]ENERGY: 11730000 3311.3494 5364.0273 5456.0009
313.5512 -14331.8345 -1763.1208 0.0000 0.0000
7819.9737 6169.9472 305.8002 -1650.0265 6232.6200
308.6731 -71.7496 -91.2850 511540.4688 -16.9476
-16.9339
[3437]ENERGY: 11740000 3324.3823 5284.6830 5433.0459
302.5221 -14376.7173 -1710.9692 0.0000 0.0000
7914.1735 6171.1204 309.4839 -1743.0532 6232.7842
308.5401 -50.9750 -106.8280 511540.4688 -17.9431

-17.9295
[3487]ENERGY: 11750000 3279.3559 5358.3768 5512.1819
308.6586 -14311.0376 -1787.5208 0.0000 0.0000
7811.0587 6171.0735 305.4516 -1639.9852 6233.0482
308.7188 -124.0537 -100.2774 511540.4688 -17.7330
-17.7519
[3523]ENERGY: 11760000 3250.0465 5352.5281 5469.0915
342.1592 -14352.1836 -1741.9145 0.0000 0.0000
7850.6916 6170.4188 307.0014 -1680.2728 6232.5514
308.6739 69.0759 -52.4980 511540.4688 -21.1019
-21.0863
[3573]ENERGY: 11770000 3232.6696 5271.7299 5430.2394
322.0222 -14281.9194 -1726.7327 0.0000 0.0000
7922.7660 6170.7750 309.8199 -1751.9909 6232.8702
308.4990 -1.0776 -44.5095 511540.4688 -17.2805
-17.2723
[3609]ENERGY: 11780000 3306.3474 5332.5201 5490.1832
324.3004 -14360.2284 -1782.2765 0.0000 0.0000
7861.0060 6171.8522 307.4048 -1689.1539 6233.0321
308.5152 -269.0003 -185.1403 511540.4688 -17.2827
-17.2929
[3659]ENERGY: 11790000 3156.1997 5409.0842 5461.6818
329.0797 -14324.0049 -1713.0241 0.0000 0.0000
7845.7751 6164.7915 306.8092 -1680.9836 6234.4934
308.4657 260.7497 199.1925 511540.4688 -15.2282
-15.2200
[3698]ENERGY: 11800000 3316.4172 5368.2067 5452.6426
340.6054 -14418.7854 -1753.0525 0.0000 0.0000
7866.5886 6172.6226 307.6231 -1693.9659 6233.9118
308.3541 -78.6658 -87.7452 511540.4688 -15.7937
-15.7995
[3748]ENERGY: 11810000 3240.1419 5356.1367 5433.9625
313.2437 -14336.3396 -1712.8632 0.0000 0.0000
7876.3970 6170.6791 308.0066 -1705.7179 6232.6290
308.3992 -59.2724 -37.6672 511540.4688 -19.5642
-19.5682
[3784]ENERGY: 11820000 3223.2268 5363.2434 5412.3179
331.5787 -14262.7057 -1777.4051 0.0000 0.0000
7880.7764 6171.0323 308.1779 -1709.7440 6234.3491
308.4859 43.0712 37.2871 511540.4688 -14.6037
-14.6072
[3834]ENERGY: 11830000 3253.8428 5341.8365 5494.4804
330.7029 -14458.0267 -1690.8004 0.0000 0.0000
7899.1879 6171.2235 308.8979 -1727.9644 6234.9577
308.3907 -94.1610 -92.5118 511540.4688 -15.7360
-15.7352
[3870]ENERGY: 11840000 3226.1870 5386.5667 5468.6134
311.2222 -14329.4438 -1708.2996 0.0000 0.0000
7818.3043 6173.1501 305.7349 -1645.1541 6235.0155
308.4423 -101.4649 -103.3511 511540.4688 -14.2701
-14.2677
[3920]ENERGY: 11850000 3200.8837 5181.8712 5471.9168
309.8000 -14280.4284 -1659.1945 0.0000 0.0000
7941.7328 6166.5816 310.5616 -1775.1512 6232.8561

308.4237 173.0606 119.2381 511540.4688 -15.5080
-15.5084
[3956]ENERGY: 11860000 3230.8313 5422.9363 5432.3258
320.4548 -14415.6450 -1678.7129 0.0000 0.0000
7858.3842 6170.5745 307.3022 -1687.8097 6233.1806
308.2500 100.9210 85.1819 511540.4688 -16.8754
-16.9069
[4006]ENERGY: 11870000 3284.9022 5288.7906 5479.0467
313.2516 -14447.7617 -1651.8652 0.0000 0.0000
7900.2619 6166.6261 308.9399 -1733.6357 6234.4538
308.5075 110.9484 40.0031 511540.4688 -16.1512
-16.1107
[4042]ENERGY: 11880000 3258.9961 5366.7352 5431.5381
339.6655 -14388.6337 -1699.7450 0.0000 0.0000
7863.3605 6171.9167 307.4968 -1691.4438 6234.9615
308.8543 48.9686 49.9251 511540.4688 -17.2987
-17.3007
[4092]ENERGY: 11890000 3276.9864 5347.7690 5434.2062
322.0408 -14365.8119 -1720.0116 0.0000 0.0000
7874.1294 6169.3083 307.9180 -1704.8211 6234.7072
308.5706 -15.3847 -31.8458 511540.4688 -18.9076
-18.9019
[4131]ENERGY: 11900000 3262.0405 5320.7913 5494.4684
339.3284 -14383.2525 -1701.7140 0.0000 0.0000
7840.0077 6171.6697 306.5836 -1668.3380 6234.6695
308.9116 32.1007 109.9277 511540.4688 -11.2704
-11.2849
[4181]ENERGY: 11910000 3254.3719 5299.5657 5476.1136
330.4087 -14333.6680 -1700.4878 0.0000 0.0000
7848.4514 6174.7555 306.9138 -1673.6959 6236.1272
308.5814 -73.6539 -92.7964 511540.4688 -15.7584
-15.7419
[4217]ENERGY: 11920000 3293.5786 5374.5893 5443.1487
304.5312 -14365.6163 -1731.7267 0.0000 0.0000
7856.4890 6174.9939 307.2281 -1681.4952 6235.7111
308.7402 -106.4826 -153.0310 511540.4688 -17.7768
-17.7798
[4267]ENERGY: 11930000 3314.2674 5308.5642 5446.4377
290.5970 -14304.8749 -1806.3695 0.0000 0.0000
7923.8704 6172.4921 309.8631 -1751.3782 6235.8720
308.7166 -75.8751 -124.0108 511540.4688 -14.4060
-14.4138
[4303]ENERGY: 11940000 3273.8470 5317.6959 5465.3050
329.5442 -14411.1866 -1706.6212 0.0000 0.0000
7902.9109 6171.4952 309.0435 -1731.4157 6235.3243
308.6995 114.1227 -28.9692 511540.4688 -14.6926
-14.7023
[4353]ENERGY: 11950000 3378.4175 5261.4660 5476.3553
296.0504 -14394.4470 -1694.3286 0.0000 0.0000
7851.8564 6175.3699 307.0470 -1676.4865 6234.5129
308.6242 75.5151 22.0832 511540.4688 -16.1250
-16.1253
[4389]ENERGY: 11960000 3320.0454 5270.6751 5468.3064
315.8915 -14424.3141 -1710.8360 0.0000 0.0000

7929.3015 6169.0699 310.0755 -1760.2316 6236.1519
308.5927 -17.6044 -126.1369 511540.4688 -13.8006
-13.8034
[4439]ENERGY: 11970000 3249.4702 5384.6747 5417.4446
325.9220 -14288.6066 -1753.8684 0.0000 0.0000
7838.5460 6173.5826 306.5265 -1664.9634 6235.1479
308.6229 -111.1313 -0.0980 511540.4688 -15.4655
-15.4306
[4475]ENERGY: 11980000 3209.7306 5326.7782 5452.4733
319.7185 -14307.4928 -1741.6053 0.0000 0.0000
7911.0258 6170.6282 309.3608 -1740.3976 6236.1016
308.7377 -12.9870 17.0887 511540.4688 -16.4499
-16.4819
[4525]ENERGY: 11990000 3304.7763 5350.2340 5478.0442
317.7195 -14404.9203 -1729.8984 0.0000 0.0000
7850.6294 6166.5847 306.9990 -1684.0447 6235.1959
308.6110 -7.4606 -75.2663 511540.4688 -18.2370
-18.2231
[4564]ENERGY: 12000000 3271.6873 5295.6104 5452.0315
313.1627 -14344.6415 -1753.0522 0.0000 0.0000
7938.8719 6173.6702 310.4497 -1765.2017 6235.8370
308.6664 42.5231 -4.9783 511540.4688 -16.3224
-16.3226
[4614]ENERGY: 12010000 3290.6318 5339.6379 5465.1453
315.0076 -14409.8057 -1708.0461 0.0000 0.0000
7876.6109 6169.1817 308.0150 -1707.4292 6235.5252
308.8811 13.3296 -61.4501 511540.4688 -17.6559
-17.6679
[4650]ENERGY: 12020000 3313.8284 5426.0869 5424.4142
329.9317 -14489.4743 -1648.5716 0.0000 0.0000
7818.8356 6175.0508 305.7557 -1643.7847 6236.5801
308.7610 157.2436 90.0753 511540.4688 -16.1796
-16.1756
[4700]ENERGY: 12030000 3290.7285 5341.0180 5489.0582
344.9661 -14404.5350 -1719.2885 0.0000 0.0000
7831.9266 6173.8740 306.2676 -1658.0526 6236.6105
308.7816 -73.8369 -56.7305 511540.4688 -11.7445
-11.7465
[4736]ENERGY: 12040000 3238.3265 5357.9282 5464.3161
315.8663 -14333.5623 -1742.9095 0.0000 0.0000
7870.2558 6170.2210 307.7665 -1700.0348 6236.9803
308.7338 89.4117 -31.1899 511540.4688 -16.4077
-16.4015
[4786]ENERGY: 12050000 3253.1212 5423.4383 5487.7372
327.6158 -14395.8751 -1696.1582 0.0000 0.0000
7780.6192 6180.4984 304.2612 -1600.1208 6237.9835
308.6988 275.0514 116.6630 511540.4688 -18.5590
-18.5681
[4822]ENERGY: 12060000 3193.9563 5337.2049 5474.2631
341.6835 -14401.5868 -1671.3476 0.0000 0.0000
7896.1237 6170.2971 308.7780 -1725.8266 6236.9711
308.6996 -39.4127 -11.3214 511540.4688 -19.1725
-19.1684
[4872]ENERGY: 12070000 3250.2829 5292.8981 5454.1332

349.2148 -14338.5955 -1741.0011 0.0000 0.0000
7905.2834 6172.2157 309.1362 -1733.0677 6237.0082
308.6415 133.3163 28.6960 511540.4688 -20.8400
-20.7937
[4908]ENERGY: 12080000 3249.7996 5336.0831 5376.8834
337.9972 -14288.1810 -1754.3579 0.0000 0.0000
7914.0556 6172.2800 309.4793 -1741.7757 6237.7495
308.6769 -27.2543 -79.4303 511540.4688 -20.3261
-20.3688
[4958]ENERGY: 12090000 3213.7962 5275.8853 5444.4984
323.8718 -14356.7518 -1720.5019 0.0000 0.0000
7989.7137 6170.5116 312.4379 -1819.2021 6237.5444
308.6078 112.3765 -29.8480 511540.4688 -20.5468
-20.5623
[4997]ENERGY: 12100000 3198.7187 5370.9456 5429.9745
310.5105 -14330.8382 -1712.0369 0.0000 0.0000
7909.7976 6177.0718 309.3128 -1732.7258 6237.9664
308.8539 -74.6866 -72.9286 511540.4688 -16.5984
-16.5726
[5047]ENERGY: 12110000 3224.9816 5331.6383 5515.2810
329.3289 -14370.7131 -1765.1142 0.0000 0.0000
7910.6215 6176.0240 309.3450 -1734.5975 6238.3734
308.6865 -62.2267 -33.2240 511540.4688 -18.9654
-18.9556
[5083]ENERGY: 12120000 3311.5878 5364.4539 5435.5258
301.0123 -14368.6188 -1737.4981 0.0000 0.0000
7868.8673 6175.3302 307.7122 -1693.5371 6237.8897
308.6983 -15.2336 -125.2395 511540.4688 -19.4444
-19.4656
[5133]ENERGY: 12130000 3353.3969 5411.8338 5389.1642
302.3936 -14411.5193 -1734.6270 0.0000 0.0000
7864.2228 6174.8649 307.5306 -1689.3579 6238.9511
308.5938 0.3592 -49.4062 511540.4688 -15.0213
-15.0294
[5169]ENERGY: 12140000 3325.4275 5303.6189 5474.6425
328.9304 -14398.2826 -1782.5213 0.0000 0.0000
7924.4033 6176.2187 309.8839 -1748.1846 6237.3588
308.5697 -196.1735 -139.3871 511540.4688 -23.7452
-23.7269
[5219]ENERGY: 12150000 3322.2788 5327.6230 5476.6618
318.7749 -14358.8188 -1757.6587 0.0000 0.0000
7849.3666 6178.2276 306.9496 -1671.1391 6237.5319
308.5412 -145.4873 -69.3319 511540.4688 -16.2716
-16.2721
[5255]ENERGY: 12160000 3202.7264 5386.1325 5456.5461
323.2339 -14426.6350 -1733.3993 0.0000 0.0000
7960.7101 6169.3147 311.3037 -1791.3954 6237.4369
308.6756 70.7833 -3.8766 511540.4688 -19.0377
-19.0746
[5305]ENERGY: 12170000 3281.4428 5322.9419 5406.3835
301.8382 -14279.4481 -1713.3498 0.0000 0.0000
7858.9221 6178.7305 307.3233 -1680.1915 6237.3797
308.5837 52.6089 103.8918 511540.4688 -20.5634
-20.5270

[5341]ENERGY: 12180000 3241.0651 5373.2862 5524.0632
319.3029 -14393.1452 -1766.4109 0.0000 0.0000
7878.2668 6176.4280 308.0797 -1701.8388 6238.1112
308.7519 36.0774 3.2058 511540.4688 -13.4949
-13.5174
[5391]ENERGY: 12190000 3307.5766 5347.9018 5452.3257
336.9674 -14398.0107 -1717.7425 0.0000 0.0000
7848.5844 6177.6027 306.9190 -1670.9817 6238.1666
308.8502 14.9135 77.3482 511540.4688 -20.9992
-20.9987
[5430]ENERGY: 12200000 3240.1615 5294.7877 5442.6211
321.0414 -14260.2106 -1698.2423 0.0000 0.0000
7836.8009 6176.9597 306.4582 -1659.8412 6237.5157
308.7678 37.3769 47.5452 511540.4688 -19.8831
-19.8690
[5480]ENERGY: 12210000 3295.6224 5423.2444 5453.4961
323.2139 -14382.1323 -1737.6570 0.0000 0.0000
7800.8836 6176.6711 305.0537 -1624.2125 6237.8214
308.8973 118.6801 58.7854 511540.4688 -16.9679
-16.9699
[5516]ENERGY: 12220000 3290.7937 5360.9002 5489.3535
305.0143 -14446.3354 -1691.6950 0.0000 0.0000
7867.0899 6175.1212 307.6427 -1691.9687 6238.3927
308.7195 -95.4265 -2.5130 511540.4688 -19.7960
-19.8058
[5566]ENERGY: 12230000 3181.1944 5374.7817 5437.7439
329.8109 -14382.8847 -1688.9765 0.0000 0.0000
7921.6751 6173.3449 309.7772 -1748.3302 6238.6401
308.8443 196.1046 138.5509 511540.4688 -19.4602
-19.4425
[5602]ENERGY: 12240000 3231.1769 5337.0472 5415.2310
318.6147 -14338.1593 -1720.1030 0.0000 0.0000
7930.3387 6174.1462 310.1160 -1756.1925 6238.0458
308.8855 -163.3919 -85.0076 511540.4688 -12.9846
-12.9975
[5652]ENERGY: 12250000 3256.0000 5391.3571 5521.1362
320.8856 -14439.6750 -1704.2224 0.0000 0.0000
7827.1686 6172.6501 306.0815 -1654.5185 6237.6274
308.7260 -61.7002 -106.0764 511540.4688 -19.8271
-19.8342
[5688]ENERGY: 12260000 3277.0819 5308.7277 5457.6459
339.0541 -14346.2268 -1766.0666 0.0000 0.0000
7904.6230 6174.8393 309.1104 -1729.7837 6238.0264
308.6800 -227.5907 -154.3129 511540.4688 -15.6856
-15.6571
[5738]ENERGY: 12270000 3292.1298 5348.5501 5412.1808
341.6855 -14298.4172 -1739.3484 0.0000 0.0000
7818.8527 6175.6333 305.7564 -1643.2195 6237.5035
308.7696 33.5310 -30.5965 511540.4688 -15.7787
-15.7940
[5774]ENERGY: 12280000 3262.9648 5376.1676 5428.6547
311.7520 -14375.1284 -1702.8088 0.0000 0.0000
7872.2989 6173.9008 307.8464 -1698.3981 6238.0134
308.5548 -206.8291 -121.1057 511540.4688 -18.0234

-18.0041
[5824]ENERGY: 12290000 3227.4434 5345.3039 5445.3510
325.4155 -14425.9091 -1714.1327 0.0000 0.0000
7967.7926 6171.2645 311.5807 -1796.5281 6237.3548
308.6548 28.9084 -18.6553 511540.4688 -20.4554
-20.4923
[5863]ENERGY: 12300000 3335.6413 5445.6677 5419.3782
338.2025 -14500.1792 -1668.8402 0.0000 0.0000
7807.9340 6177.8043 305.3294 -1630.1297 6237.5017
308.4750 -26.0297 -98.5851 511540.4688 -14.7718
-14.7620
[5913]ENERGY: 12310000 3243.8798 5234.0732 5482.3185
330.6990 -14285.0638 -1756.6941 0.0000 0.0000
7925.4683 6174.6807 309.9256 -1750.7875 6238.0616
308.4982 -255.7085 -195.0960 511540.4688 -11.4642
-11.4369
[5949]ENERGY: 12320000 3314.9937 5327.3766 5401.2426
335.9826 -14337.0820 -1733.3657 0.0000 0.0000
7869.1639 6178.3116 307.7238 -1690.8523 6238.5242
308.5837 62.5562 78.4094 511540.4688 -20.9754
-20.9858
[5999]ENERGY: 12330000 3319.0338 5320.0711 5396.6177
300.0379 -14390.1012 -1674.8633 0.0000 0.0000
7905.8633 6176.6592 309.1589 -1729.2041 6238.5900
308.8326 -133.7382 -168.5937 511540.4688 -14.6655
-14.6758
[6035]ENERGY: 12340000 3293.9266 5277.5188 5421.2602
318.3401 -14331.3089 -1749.3242 0.0000 0.0000
7942.6436 6173.0561 310.5972 -1769.5875 6238.8557
308.7284 -58.3613 -74.7851 511540.4688 -15.6851
-15.6797
[6085]ENERGY: 12350000 3299.8475 5218.6206 5424.8713
314.7597 -14199.8264 -1806.2635 0.0000 0.0000
7921.8427 6173.8519 309.7838 -1747.9908 6238.7706
308.9698 -104.5076 -235.6943 511540.4688 -14.4180
-14.4349
[6121]ENERGY: 12360000 3245.6774 5289.1589 5458.9134
341.4063 -14349.6560 -1737.1334 0.0000 0.0000
7926.1469 6174.5134 309.9521 -1751.6335 6238.3273
308.5603 26.5127 -40.9436 511540.4688 -20.3296
-20.3109
[6171]ENERGY: 12370000 3176.8836 5319.8336 5421.7745
327.0963 -14331.7957 -1723.1856 0.0000 0.0000
7978.8405 6169.4472 312.0127 -1809.3933 6238.3300
308.6440 158.4403 87.3371 511540.4688 -19.4697
-19.4676
[6207]ENERGY: 12380000 3193.5772 5416.6921 5459.1340
297.8183 -14366.0837 -1741.7890 0.0000 0.0000
7915.2617 6174.6106 309.5264 -1740.6510 6238.9555
308.9324 11.7108 3.1501 511540.4688 -17.9320
-17.9291
[6257]ENERGY: 12390000 3304.7706 5343.5183 5445.1505
329.2144 -14347.6319 -1722.9600 0.0000 0.0000
7825.2030 6177.2650 306.0047 -1647.9380 6239.1347

308.8268 36.4272 -35.3788 511540.4688 -12.7726
-12.7917
[6296]ENERGY: 12400000 3275.1953 5392.2704 5453.8312
308.9483 -14347.7526 -1774.5754 0.0000 0.0000
7867.7273 6175.6444 307.6676 -1692.0828 6239.7785
308.7968 -152.7960 -266.3310 511540.4688 -17.0122
-17.0055
[6346]ENERGY: 12410000 3185.2599 5396.1309 5459.4274
337.2548 -14411.2033 -1679.7036 0.0000 0.0000
7886.8478 6174.0139 308.4153 -1712.8339 6239.0997
308.6042 200.1393 155.9477 511540.4688 -13.9082
-13.9235
[6382]ENERGY: 12420000 3316.7608 5239.1823 5434.1635
317.1342 -14339.8044 -1734.9875 0.0000 0.0000
7942.5054 6174.9543 310.5918 -1767.5511 6239.6601
308.5911 112.5779 44.6716 511540.4688 -19.3817
-19.3485
[6432]ENERGY: 12430000 3229.7406 5349.0779 5478.4593
321.9167 -14355.9530 -1697.8481 0.0000 0.0000
7850.2106 6175.6040 306.9826 -1674.6066 6240.6640
308.7117 150.5393 27.3729 511540.4688 -17.0624
-17.0687
[6468]ENERGY: 12440000 3351.8464 5276.2239 5481.2677
323.9099 -14405.0728 -1699.1631 0.0000 0.0000
7850.9597 6179.9716 307.0119 -1670.9880 6240.3042
308.6318 31.3886 -71.6896 511540.4688 -19.4223
-19.4581
[6518]ENERGY: 12450000 3340.9125 5265.6184 5421.0360
312.3685 -14355.2050 -1752.7440 0.0000 0.0000
7943.9320 6175.9184 310.6476 -1768.0136 6240.9865
308.7173 -98.9260 -77.9948 511540.4688 -13.5888
-13.5466
[6554]ENERGY: 12460000 3229.3198 5377.6852 5456.1297
323.3910 -14446.9784 -1763.9075 0.0000 0.0000
7999.0996 6174.7394 312.8049 -1824.3601 6239.9200
308.6631 -105.3653 -82.9705 511540.4688 -21.3178
-21.3114
[6604]ENERGY: 12470000 3257.6060 5313.8196 5470.3196
308.4480 -14391.5140 -1661.0931 0.0000 0.0000
7880.4184 6178.0045 308.1639 -1702.4139 6239.7039
308.8386 180.1279 70.1184 511540.4688 -17.0008
-17.0393
[6640]ENERGY: 12480000 3212.0776 5351.9852 5425.7924
320.1856 -14374.5709 -1698.6927 0.0000 0.0000
7939.2574 6176.0346 310.4648 -1763.2228 6240.5546
308.7711 145.6174 74.4160 511540.4688 -16.3296
-16.3263
[6690]ENERGY: 12490000 3262.1761 5274.1550 5482.4446
332.5754 -14363.6834 -1748.8752 0.0000 0.0000
7934.6030 6173.3956 310.2828 -1761.2074 6241.2177
308.7476 116.5102 74.9269 511540.4688 -12.6877
-12.6839
[6729]ENERGY: 12500000 3246.0994 5291.6578 5478.8175
315.5910 -14420.6229 -1700.4312 0.0000 0.0000

7964.0411 6175.1527 311.4339 -1788.8884 6240.6951
308.6174 -21.1200 26.1928 511540.4688 -18.4679
-18.4602
[6779]ENERGY: 12510000 3319.2733 5328.2229 5491.0526
320.7774 -14385.6947 -1752.9820 0.0000 0.0000
7858.5562 6179.2057 307.3090 -1679.3505 6241.8177
308.6845 -51.3230 -132.2556 511540.4688 -17.3295
-17.3053
[6815]ENERGY: 12520000 3264.7316 5313.2846 5392.4707
332.0378 -14331.4671 -1690.5347 0.0000 0.0000
7894.9285 6175.4513 308.7313 -1719.4772 6239.9764
308.6338 -104.0779 -37.2950 511540.4688 -20.6522
-20.6919
[6865]ENERGY: 12530000 3286.5372 5258.3455 5427.7880
314.7008 -14323.6339 -1735.6924 0.0000 0.0000
7946.1979 6174.2432 310.7362 -1771.9547 6240.0198
308.7626 125.4475 -15.0795 511540.4688 -19.5067
-19.4801
[6901]ENERGY: 12540000 3295.3561 5337.5638 5458.7325
325.2630 -14322.0489 -1778.6397 0.0000 0.0000
7863.3042 6179.5310 307.4946 -1683.7732 6240.9120
308.7681 -82.8542 -47.7465 511540.4688 -15.3887
-15.3897
[6951]ENERGY: 12550000 3230.7470 5327.1191 5499.2808
313.1159 -14414.8142 -1707.7333 0.0000 0.0000
7929.7697 6177.4851 310.0938 -1752.2846 6241.0352
308.6860 -77.6768 -55.3388 511540.4688 -18.5116
-18.5316
[6987]ENERGY: 12560000 3350.4434 5277.8092 5469.1760
329.7558 -14300.2365 -1776.8859 0.0000 0.0000
7830.3672 6180.4292 306.2066 -1649.9380 6240.8377
308.7781 -254.4588 -237.5636 511540.4688 -13.2578
-13.2476
[7037]ENERGY: 12570000 3187.6970 5252.6257 5482.9078
320.7589 -14283.9996 -1682.7536 0.0000 0.0000
7901.2372 6178.4734 308.9780 -1722.7638 6239.3025
308.8733 -46.7474 113.6923 511540.4688 -16.2524
-16.2463
[7073]ENERGY: 12580000 3297.0453 5340.9994 5436.2570
317.5502 -14431.7190 -1642.3326 0.0000 0.0000
7860.2290 6178.0293 307.3744 -1682.1997 6241.7580
308.7899 73.5653 12.0134 511540.4688 -12.4593
-12.4634
[7123]ENERGY: 12590000 3287.0233 5283.1590 5485.3031
335.4099 -14344.3710 -1717.9060 0.0000 0.0000
7849.8556 6178.4739 306.9687 -1671.3817 6239.9639
308.8514 37.9883 51.6727 511540.4688 -15.8500
-15.8401
[7162]ENERGY: 12600000 3344.5061 5366.6670 5452.3839
343.3640 -14477.1775 -1676.8416 0.0000 0.0000
7826.6184 6179.5203 306.0600 -1647.0980 6240.6059
308.8359 -129.1756 7.2629 511540.4688 -15.8749
-15.8827
[7212]ENERGY: 12610000 3203.9573 5391.6573 5479.7753

329.6340 -14410.2029 -1749.6528 0.0000 0.0000
7928.5710 6173.7391 310.0469 -1754.8319 6240.6219
308.8289 -23.1633 -83.0419 511540.4688 -12.7816
-12.7891
[7248]ENERGY: 12620000 3236.9346 5245.2203 5418.3413
327.5861 -14310.7865 -1667.9503 0.0000 0.0000
7930.2184 6179.5639 310.1113 -1750.6545 6242.4915
308.6943 169.4711 16.9316 511540.4688 -14.9818
-14.9787
[7298]ENERGY: 12630000 3249.1089 5361.9369 5450.3685
319.9925 -14311.2206 -1720.3435 0.0000 0.0000
7833.2067 6183.0493 306.3177 -1650.1574 6241.4220
308.5744 198.1484 31.0866 511540.4688 -17.0223
-17.0375
[7334]ENERGY: 12640000 3324.7624 5333.9786 5424.9891
329.2035 -14371.2046 -1756.4614 0.0000 0.0000
7892.8023 6178.0698 308.6482 -1714.7324 6241.5163
308.6298 -253.5543 -206.7366 511540.4688 -18.5688
-18.5669
[7384]ENERGY: 12650000 3254.5087 5451.0248 5429.4713
303.4300 -14335.0068 -1754.7687 0.0000 0.0000
7832.3831 6181.0423 306.2855 -1651.3408 6242.7869
308.7859 -87.2744 -80.0061 511540.4688 -16.1846
-16.1512
[7420]ENERGY: 12660000 3275.9291 5281.0527 5450.4468
328.6016 -14408.8014 -1682.9842 0.0000 0.0000
7933.5672 6177.8118 310.2423 -1755.7554 6241.5476
308.8719 100.1543 83.3565 511540.4688 -12.3350
-12.3478
[7470]ENERGY: 12670000 3295.2988 5396.7917 5451.4991
336.7188 -14411.7227 -1748.1423 0.0000 0.0000
7856.3413 6176.7846 307.2223 -1679.5566 6241.5394
308.7377 -188.2662 -234.8477 511540.4688 -17.1195
-17.1351
[7506]ENERGY: 12680000 3305.1633 5364.0328 5457.5429
328.1869 -14432.4598 -1659.2178 0.0000 0.0000
7817.6488 6180.8972 305.7093 -1636.7516 6241.5937
308.8441 -11.2558 -8.4797 511540.4688 -15.1727
-15.1676
[7556]ENERGY: 12690000 3231.6274 5343.0706 5460.2403
353.1679 -14387.0838 -1687.7613 0.0000 0.0000
7865.2449 6178.5061 307.5705 -1686.7388 6241.8750
308.9389 264.3761 129.4509 511540.4688 -19.8379
-19.8471
[7595]ENERGY: 12700000 3201.8186 5324.4590 5466.3549
337.0855 -14380.8318 -1686.6051 0.0000 0.0000
7913.2824 6175.5635 309.4490 -1737.7189 6241.6730
309.0123 14.4705 45.6179 511540.4688 -17.8525
-17.8767
[7645]ENERGY: 12710000 3318.0654 5397.7641 5490.9228
346.5978 -14534.4069 -1724.6378 0.0000 0.0000
7884.9298 6179.2353 308.3403 -1705.6945 6242.0581
308.9737 -199.3610 -89.6346 511540.4688 -17.8538
-17.7970

[7681]ENERGY: 12720000 3283.8166 5424.1596 5411.8031
308.3580 -14374.5927 -1735.9772 0.0000 0.0000
7861.4960 6179.0633 307.4239 -1682.4326 6244.1829
308.8188 118.9720 53.6109 511540.4688 -18.3958
-18.4257
[7731]ENERGY: 12730000 3251.8434 5296.6409 5437.4304
303.9817 -14413.3136 -1608.9960 0.0000 0.0000
7914.0563 6181.6430 309.4793 -1732.4133 6244.2770
309.0167 238.7029 279.8512 511540.4688 -19.3809
-19.3700
[7767]ENERGY: 12740000 3302.0351 5327.5803 5433.1552
338.3922 -14429.3047 -1677.6132 0.0000 0.0000
7885.6845 6179.9295 308.3698 -1705.7550 6243.3983
308.8082 110.6558 48.8019 511540.4688 -21.1381
-21.1422
[7817]ENERGY: 12750000 3355.8983 5296.5061 5399.0761
328.3848 -14427.1000 -1677.5492 0.0000 0.0000
7905.1676 6180.3836 309.1317 -1724.7840 6243.5492
308.8946 48.9943 -15.6326 511540.4688 -22.1204
-22.1314
[7853]ENERGY: 12760000 3235.2848 5323.4235 5479.6881
345.8794 -14345.6668 -1718.8214 0.0000 0.0000
7862.9954 6182.7830 307.4826 -1680.2124 6244.5357
309.1499 -66.1155 -62.4341 511540.4688 -14.5742
-14.5592
[7903]ENERGY: 12770000 3306.4368 5415.1160 5479.7730
327.6854 -14405.8844 -1756.1615 0.0000 0.0000
7817.6865 6184.6518 305.7108 -1633.0347 6244.8882
309.0859 -8.0103 -53.4055 511540.4688 -23.0497
-23.0476
[7939]ENERGY: 12780000 3286.7529 5313.8808 5435.9938
320.7603 -14348.7254 -1752.3088 0.0000 0.0000
7923.6228 6179.9762 309.8534 -1743.6466 6245.0388
308.8517 -145.8158 -103.4721 511540.4688 -15.5473
-15.5404
[7989]ENERGY: 12790000 3266.5397 5320.4000 5464.7243
307.5662 -14345.7143 -1725.7482 0.0000 0.0000
7892.0171 6179.7849 308.6175 -1712.2322 6245.5901
308.7762 27.9265 -1.2484 511540.4688 -16.4856
-16.4933
[8028]ENERGY: 12800000 3277.9731 5398.6267 5404.4703
334.9941 -14411.4215 -1710.9957 0.0000 0.0000
7887.1843 6180.8313 308.4285 -1706.3529 6245.1739
308.6668 -103.1412 -111.1501 511540.4688 -16.1524
-16.1580
[8078]ENERGY: 12810000 3208.7553 5389.1977 5444.5754
302.0284 -14350.5115 -1682.4620 0.0000 0.0000
7871.4641 6183.0475 307.8137 -1688.4166 6245.1801
308.8722 179.4655 129.1969 511540.4688 -17.5045
-17.5010
[8114]ENERGY: 12820000 3206.5577 5302.8125 5436.4313
322.2481 -14309.9944 -1710.4009 0.0000 0.0000
7934.1437 6181.7981 310.2648 -1752.3456 6245.2102
308.8847 192.6534 189.5969 511540.4688 -16.7391

```
-16.7460
[8164]ENERGY: 12830000      3259.4655      5440.2787      5449.1036
299.4907         -14366.4066     -1736.0244         0.0000         0.0000
7835.8879           6181.7955       306.4225     -1654.0924      6245.9981
308.9091             16.9313        52.8944    511540.4688       -17.8135
-17.7893
[8200]ENERGY: 12840000      3233.5614      5440.2762      5368.2403
357.8915         -14353.8075     -1732.5000         0.0000         0.0000
7869.5129           6183.1747       307.7374     -1686.3382      6245.8750
308.7147            146.5044        -6.4306    511540.4688       -18.7059
-18.7190
[8250]ENERGY: 12850000      3255.2771      5383.1063      5447.3580
315.8758         -14307.3434     -1830.3943         0.0000         0.0000
7918.3742           6182.2538       309.6481     -1736.1203      6246.1088
308.5945            -59.6883      -151.5708    511540.4688       -12.4890
-12.5001
[8286]ENERGY: 12860000      3294.1057      5389.6953      5407.6363
297.3801         -14351.9756     -1733.8944         0.0000         0.0000
7880.9106           6183.8579       308.1831     -1697.0526      6245.3584
308.6914            -75.6666       -89.5073    511540.4688       -17.0803
-17.0586
[8336]ENERGY: 12870000      3265.2774      5290.8317      5454.4955
330.7366         -14366.9835     -1694.9167         0.0000         0.0000
7900.9981           6180.4391       308.9687     -1720.5590      6246.3444
308.8210             -7.4336      -100.1991    511540.4688       -13.5970
-13.6280
[8372]ENERGY: 12880000      3302.9289      5363.4584      5417.1221
316.5754         -14498.4558     -1663.6426         0.0000         0.0000
7945.1299           6183.1163       310.6944     -1762.0136      6247.4243
308.8720            107.7431        44.4459    511540.4688       -13.7334
-13.7329
[8422]ENERGY: 12890000      3244.2521      5380.3226      5474.2586
306.5553         -14442.9147     -1718.3157         0.0000         0.0000
7937.7636           6181.9217       310.4064     -1755.8418      6246.6727
308.7071             41.5874       -16.3328    511540.4688       -19.9797
-19.9389
[8461]ENERGY: 12900000      3261.4352      5309.5705      5427.0128
347.5859         -14352.5498     -1674.2891         0.0000         0.0000
7866.0615           6184.8271       307.6025     -1681.2344      6245.8086
308.6881             18.5636       -73.7127    511540.4688       -16.3386
-16.3669
[8511]ENERGY: 12910000      3246.9712      5278.0980      5479.6139
348.3387         -14316.0214     -1726.7036         0.0000         0.0000
7871.3632           6181.6602       307.8098     -1689.7031      6246.7260
308.8765             -5.8141       -16.8227    511540.4688       -18.0126
-17.9967
[8547]ENERGY: 12920000      3320.0581      5358.0392      5479.7740
323.7298         -14543.0357     -1642.3598         0.0000         0.0000
7886.6710           6182.8765       308.4084     -1703.7945      6245.5740
308.7791            -17.3272       146.5948    511540.4688       -15.4171
-15.4140
[8597]ENERGY: 12930000      3236.2573      5406.7765      5485.5199
329.5422         -14441.2731     -1631.3094         0.0000         0.0000
7799.3423           6184.8557       304.9934     -1614.4866      6246.4112
```

308.7428 345.8971 293.3449 511540.4688 -16.0348
-16.0634
[8633]ENERGY: 12940000 3256.9482 5331.4726 5427.2667
327.9983 -14354.6896 -1669.5923 0.0000 0.0000
7863.9015 6183.3055 307.5180 -1680.5960 6246.8469
308.9329 -2.3639 -94.1983 511540.4688 -14.8318
-14.8298
[8683]ENERGY: 12950000 3253.3240 5443.3159 5450.0883
337.0845 -14301.5668 -1822.0149 0.0000 0.0000
7823.8055 6184.0365 305.9500 -1639.7690 6247.5617
308.9733 -334.4582 -250.6407 511540.4688 -14.2020
-14.1834
[8719]ENERGY: 12960000 3247.3785 5394.8673 5461.3277
338.0594 -14357.8140 -1753.0654 0.0000 0.0000
7856.7125 6187.4660 307.2369 -1669.2465 6247.9987
309.0565 24.9882 -24.9809 511540.4688 -11.9150
-11.9286
[8769]ENERGY: 12970000 3260.4950 5263.2880 5464.3162
295.8803 -14333.5453 -1684.1979 0.0000 0.0000
7917.7212 6183.9576 309.6226 -1733.7637 6246.8957
308.9239 -93.0672 -49.2096 511540.4688 -15.3700
-15.3554
[8805]ENERGY: 12980000 3283.4075 5296.0654 5447.1647
327.2815 -14339.5573 -1728.9367 0.0000 0.0000
7898.0882 6183.5133 308.8549 -1714.5749 6246.7870
308.9985 -60.7418 -2.2558 511540.4688 -11.8794
-11.8778
[8855]ENERGY: 12990000 3289.9186 5457.3884 5395.7942
337.8182 -14452.5313 -1720.3594 0.0000 0.0000
7874.2793 6182.3081 307.9238 -1691.9712 6248.3154
309.1249 -68.6930 -78.1943 511540.4688 -12.0007
-12.0144
[8894]ENERGY: 13000000 3273.1683 5463.8807 5434.5188
307.3344 -14459.3818 -1748.4318 0.0000 0.0000
7914.0893 6185.1779 309.4806 -1728.9114 6249.1934
308.9285 40.0229 -79.4341 511540.4688 -15.4609
-15.4753
[8944]ENERGY: 13010000 3291.3904 5333.9309 5416.5591
349.0685 -14362.1254 -1725.3570 0.0000 0.0000
7880.1320 6183.5984 308.1527 -1696.5336 6248.2643
308.9763 -26.6411 56.3684 511540.4688 -15.1441
-15.1249
[8980]ENERGY: 13020000 3236.1205 5425.4485 5470.6160
328.8491 -14388.2757 -1737.1401 0.0000 0.0000
7848.5926 6184.2110 306.9193 -1664.3816 6249.4813
309.1742 12.2174 74.3711 511540.4688 -11.1025
-11.1205
[9030]ENERGY: 13030000 3269.4958 5300.7914 5434.7422
317.7295 -14445.1921 -1644.5257 0.0000 0.0000
7952.1378 6185.1789 310.9685 -1766.9589 6248.9590
308.8928 -71.3157 -69.9795 511540.4688 -16.2253
-16.2184
[9066]ENERGY: 13040000 3219.0841 5354.3105 5529.9156
314.2086 -14371.6652 -1673.6174 0.0000 0.0000

7812.9210 6185.1571 305.5244 -1627.7639 6249.5294
308.9165 294.1176 211.4465 511540.4688 -16.9303
-16.9292
[9116]ENERGY: 13050000 3331.1612 5303.9491 5436.2843
327.8328 -14386.4365 -1762.1810 0.0000 0.0000
7937.8104 6188.4202 310.4082 -1749.3902 6250.0324
308.8980 -123.7033 -152.0937 511540.4688 -22.0011
-21.9949
[9152]ENERGY: 13060000 3279.5214 5300.4580 5453.8228
312.6367 -14314.4059 -1697.2511 0.0000 0.0000
7853.4600 6188.2419 307.1097 -1665.2181 6249.3963
308.8751 -244.9099 -176.8813 511540.4688 -21.1768
-21.1841
[9202]ENERGY: 13070000 3244.2150 5376.1633 5385.6666
327.0836 -14436.5873 -1691.9074 0.0000 0.0000
7982.6098 6187.2436 312.1601 -1795.3662 6249.7917
308.8158 -59.0330 -89.6742 511540.4688 -23.6302
-23.6231
[9238]ENERGY: 13080000 3214.8338 5260.1709 5434.6317
297.9058 -14285.5704 -1657.8135 0.0000 0.0000
7920.2995 6184.4577 309.7234 -1735.8418 6249.1616
308.9997 217.0000 151.4179 511540.4688 -20.2971
-20.2897
[9288]ENERGY: 13090000 3250.3341 5352.1184 5462.3806
308.3723 -14415.7634 -1717.6770 0.0000 0.0000
7943.9668 6183.7317 310.6489 -1760.2351 6249.9194
308.8521 92.3560 52.5118 511540.4688 -27.0559
-27.0356
[9327]ENERGY: 13100000 3356.7322 5406.6068 5401.3641
327.8843 -14414.5179 -1717.8590 0.0000 0.0000
7832.0650 6192.2757 306.2730 -1639.7894 6249.8313
309.1033 -226.4904 -108.0722 511540.4688 -20.1581
-20.1550
[9377]ENERGY: 13110000 3368.8320 5433.7411 5436.1465
311.6000 -14374.8902 -1759.5098 0.0000 0.0000
7773.9101 6189.8297 303.9989 -1584.0805 6251.0966
309.1903 -246.0613 -187.4225 511540.4688 -21.6242
-21.6565
[9413]ENERGY: 13120000 3333.5901 5332.7315 5408.7439
306.2115 -14401.7558 -1627.6099 0.0000 0.0000
7837.2500 6189.1611 306.4758 -1648.0889 6251.2722
308.9474 133.1781 114.6108 511540.4688 -22.7132
-22.7218
[9463]ENERGY: 13130000 3242.9638 5332.5073 5396.3957
309.3326 -14328.9811 -1736.9208 0.0000 0.0000
7971.2297 6186.5273 311.7151 -1784.7024 6252.0682
309.0180 -96.7273 -109.7469 511540.4688 -20.9136
-20.8985
[9499]ENERGY: 13140000 3269.8584 5389.2697 5359.7047
328.6398 -14383.9124 -1636.3950 0.0000 0.0000
7860.8917 6188.0567 307.4003 -1672.8350 6250.8568
308.9816 -41.0850 -38.6685 511540.4688 -18.2934
-18.2784
[9549]ENERGY: 13150000 3311.0463 5440.3557 5418.9043

321.9441 -14348.1413 -1764.2514 0.0000 0.0000
7810.4546 6190.3124 305.4279 -1620.1423 6252.2295
308.9214 47.7806 -135.4874 511540.4688 -23.7704
-23.7908
[9585]ENERGY: 13160000 3276.1191 5349.3639 5369.8298
324.9492 -14388.1484 -1718.4683 0.0000 0.0000
7970.5386 6184.1840 311.6880 -1786.3546 6250.8824
308.7233 -138.7833 -198.4270 511540.4688 -23.3840
-23.3683
[9635]ENERGY: 13170000 3303.1147 5468.9051 5419.2940
322.2400 -14453.3520 -1721.4459 0.0000 0.0000
7845.9810 6184.7368 306.8172 -1661.2441 6250.3186
308.7680 -114.4544 51.8267 511540.4688 -22.5634
-22.5776
[9671]ENERGY: 13180000 3310.6398 5401.1862 5431.6634
310.5295 -14409.4509 -1744.6720 0.0000 0.0000
7887.1393 6187.0354 308.4267 -1700.1039 6249.7326
309.0786 -68.2658 -74.2775 511540.4688 -13.7833
-13.7845
[9721]ENERGY: 13190000 3246.7208 5378.7640 5417.3576
308.9421 -14312.1176 -1684.6287 0.0000 0.0000
7835.6618 6190.7000 306.4137 -1644.9617 6253.0078
308.9732 220.1561 22.9269 511540.4688 -16.4754
-16.4880
[9760]ENERGY: 13200000 3241.2844 5392.2088 5421.5166
323.8713 -14352.5405 -1771.0781 0.0000 0.0000
7931.5682 6186.8308 310.1641 -1744.7375 6252.9508
309.0511 74.4030 91.8930 511540.4688 -16.3799
-16.3503
[9810]ENERGY: 13210000 3277.7741 5391.9683 5401.5535
322.0722 -14384.3623 -1767.7281 0.0000 0.0000
7949.9956 6191.2733 310.8847 -1758.7223 6253.2077
309.1266 56.7412 34.7705 511540.4688 -17.9717
-17.9904
[9846]ENERGY: 13220000 3255.7326 5383.0657 5443.6819
324.8404 -14263.9454 -1806.6732 0.0000 0.0000
7859.4945 6196.1966 307.3457 -1663.2979 6253.5184
309.0510 -76.5465 -53.2187 511540.4688 -19.0387
-19.0380
[9896]ENERGY: 13230000 3236.7281 5411.4998 5417.3348
342.1143 -14368.5717 -1733.6357 0.0000 0.0000
7885.6002 6191.0697 308.3665 -1694.5305 6253.5567
309.0417 -65.5963 -20.2189 511540.4688 -18.2903
-18.2576
[9932]ENERGY: 13240000 3199.2901 5416.9159 5410.3331
328.0464 -14376.3261 -1715.7241 0.0000 0.0000
7925.3158 6187.8511 309.9196 -1737.4647 6253.0764
309.1219 110.5102 154.4966 511540.4688 -18.5887
-18.6133
[9982]ENERGY: 13250000 3203.8646 5403.8685 5473.2206
327.9582 -14348.5502 -1758.5504 0.0000 0.0000
7887.6749 6189.4863 308.4476 -1698.1887 6254.5060
309.0335 -37.0815 10.4397 511540.4688 -13.3125
-13.3392

[10018]ENERGY: 13260000 3283.9713 5376.6961 5382.5780
300.2396 -14338.0473 -1730.0177 0.0000 0.0000
7916.7250 6192.1449 309.5837 -1724.5801 6253.7191
308.8686 5.9622 -177.6180 511540.4688 -15.9962
-15.9892
[10068]ENERGY: 13270000 3277.1536 5302.1226 5448.8288
353.9570 -14347.4134 -1764.4541 0.0000 0.0000
7923.7867 6193.9811 309.8598 -1729.8056 6255.0800
309.0679 -5.8108 -96.1088 511540.4688 -16.1098
-16.0955
[10104]ENERGY: 13280000 3285.3981 5291.4762 5439.1718
288.2942 -14306.4959 -1761.5791 0.0000 0.0000
7953.0688 6189.3341 311.0049 -1763.7347 6255.1145
309.0505 -48.1988 -82.7824 511540.4688 -19.9328
-19.9491
[10154]ENERGY: 13290000 3268.1520 5353.4264 5443.0366
325.2513 -14362.6090 -1723.8072 0.0000 0.0000
7889.1638 6192.6140 308.5059 -1696.5498 6256.2341
308.9772 -22.0658 56.9667 511540.4688 -17.8987
-17.8722
[10193]ENERGY: 13300000 3204.8401 5309.2186 5502.0105
319.5650 -14319.2213 -1805.0227 0.0000 0.0000
7977.9077 6189.2979 311.9762 -1788.6098 6256.1779
309.0125 121.7015 13.2647 511540.4688 -17.0544
-17.0741
[10243]ENERGY: 13310000 3338.5042 5312.9203 5444.0782
321.9289 -14340.7175 -1723.8485 0.0000 0.0000
7839.6392 6192.5047 306.5692 -1647.1345 6255.7364
308.9661 -239.0425 -129.1730 511540.4688 -18.8090
-18.8062
[10279]ENERGY: 13320000 3272.5413 5276.3863 5443.9359
316.1451 -14311.9414 -1793.6598 0.0000 0.0000
7988.2036 6191.6108 312.3788 -1796.5927 6257.4302
309.0156 126.5214 -91.6789 511540.4688 -20.3507
-20.3645
[10329]ENERGY: 13330000 3301.7960 5464.1129 5432.4850
305.4801 -14352.8749 -1690.6704 0.0000 0.0000
7734.2671 6194.5958 302.4486 -1539.6713 6256.2847
309.1916 -1.7217 4.3833 511540.4688 -19.7456
-19.7077
[10365]ENERGY: 13340000 3217.4453 5355.5961 5445.6957
340.9011 -14377.6644 -1703.7948 0.0000 0.0000
7915.5030 6193.6820 309.5359 -1721.8211 6256.3250
309.0385 59.4149 19.0131 511540.4688 -15.1654
-15.1778
[10415]ENERGY: 13350000 3334.8352 5324.1627 5446.5890
313.7564 -14328.5030 -1719.4666 0.0000 0.0000
7825.9898 6197.3633 306.0355 -1628.6265 6256.5415
309.0420 142.7148 109.2486 511540.4688 -17.0579
-17.0578
[10451]ENERGY: 13360000 3260.4485 5339.7440 5431.5257
349.3103 -14287.0676 -1758.7367 0.0000 0.0000
7863.8269 6199.0511 307.5151 -1664.7758 6257.7267
308.9415 -61.1013 -134.3592 511540.4688 -18.0629

-18.0616
[10501]ENERGY: 13370000 3320.2269 5290.0687 5423.3679
325.4871 -14328.1398 -1772.3410 0.0000 0.0000
7934.8819 6193.5516 310.2937 -1741.3302 6257.0364
308.9535 209.6189 107.8601 511540.4688 -15.6961
-15.7113
[10537]ENERGY: 13380000 3195.1126 5234.9564 5431.6143
338.5291 -14347.9175 -1691.5745 0.0000 0.0000
8028.5325 6189.2530 313.9559 -1839.2795 6257.6324
309.1847 40.7910 -26.5209 511540.4688 -17.7242
-17.7151
[10587]ENERGY: 13390000 3189.7103 5327.9133 5431.6293
341.2333 -14295.4902 -1725.3546 0.0000 0.0000
7921.6346 6191.2759 309.7756 -1730.3587 6257.9909
309.0978 -15.5045 16.3797 511540.4688 -12.9711
-12.9451
[10626]ENERGY: 13400000 3302.7814 5366.3378 5408.5587
322.5893 -14328.9519 -1718.2594 0.0000 0.0000
7841.5455 6194.6014 306.6438 -1646.9441 6257.4267
308.8482 5.0913 -16.5383 511540.4688 -17.7239
-17.7432
[10676]ENERGY: 13410000 3263.7071 5367.6324 5430.7155
325.0176 -14349.2403 -1759.6403 0.0000 0.0000
7914.1173 6192.3092 309.4817 -1721.8081 6257.6592
309.0911 -108.0995 -125.0204 511540.4688 -13.4351
-13.4377
[10712]ENERGY: 13420000 3245.9088 5378.5125 5433.5091
332.3069 -14403.4992 -1687.7986 0.0000 0.0000
7895.9680 6194.9075 308.7720 -1701.0605 6258.1218
308.9728 -38.9463 -51.5904 511540.4688 -16.2554
-16.2656
[10762]ENERGY: 13430000 3258.0481 5394.4792 5452.7125
325.4278 -14434.9442 -1714.1968 0.0000 0.0000
7913.5805 6195.1071 309.4607 -1718.4734 6257.9411
308.9609 52.4486 30.9044 511540.4688 -14.2456
-14.2311
[10798]ENERGY: 13440000 3258.5216 5471.6477 5438.2186
342.8946 -14398.3866 -1731.9522 0.0000 0.0000
7812.2527 6193.1963 305.4983 -1619.0563 6258.0627
309.0808 16.4059 -36.6122 511540.4688 -14.0532
-14.0480
[10848]ENERGY: 13450000 3302.5142 5313.2156 5478.0931
299.0285 -14356.9049 -1701.5096 0.0000 0.0000
7860.6470 6195.0838 307.3907 -1665.5631 6258.0289
308.8436 210.0724 78.6020 511540.4688 -19.3062
-19.3374
[10884]ENERGY: 13460000 3273.0504 5246.7739 5447.8670
333.7425 -14333.7935 -1705.2209 0.0000 0.0000
7933.5632 6195.9825 310.2421 -1737.5807 6258.6248
308.8616 25.6209 80.6450 511540.4688 -12.4899
-12.4698
[10934]ENERGY: 13470000 3255.2642 5373.6764 5421.4437
325.9197 -14387.6992 -1668.3562 0.0000 0.0000
7876.4253 6196.6739 308.0077 -1679.7514 6258.1262

308.9384 67.1736 -94.0797 511540.4688 -12.6847
-12.7123
[10970]ENERGY: 13480000 3249.7252 5421.0800 5450.2337
316.7013 -14429.6429 -1720.5529 0.0000 0.0000
7907.1205 6194.6648 309.2081 -1712.4557 6258.0805
308.8820 34.8574 -82.7609 511540.4688 -18.1891
-18.1682
[11020]ENERGY: 13490000 3271.2422 5443.1528 5491.6125
313.5288 -14432.0386 -1721.6671 0.0000 0.0000
7830.1233 6195.9538 306.1971 -1634.1695 6257.6136
309.0162 -60.0533 -79.6609 511540.4688 -15.4009
-15.3799
[11059]ENERGY: 13500000 3226.3166 5396.7629 5458.7289
314.5537 -14377.6625 -1720.8902 0.0000 0.0000
7892.4123 6190.2218 308.6329 -1702.1905 6258.3669
309.0202 201.1535 75.5953 511540.4688 -17.6241
-17.6537
[11109]ENERGY: 13510000 3218.7827 5344.9719 5462.3356
323.0132 -14360.8367 -1679.3089 0.0000 0.0000
7884.1000 6193.0578 308.3079 -1691.0422 6256.3137
309.0669 -78.6560 -84.2032 511540.4688 -15.2165
-15.2056
[11145]ENERGY: 13520000 3238.0640 5370.1322 5481.3602
339.2075 -14417.2973 -1684.3770 0.0000 0.0000
7866.8499 6193.9395 307.6333 -1672.9104 6257.4093
309.0746 114.8044 109.7969 511540.4688 -18.3811
-18.3612
[11195]ENERGY: 13530000 3303.0050 5376.3135 5424.4234
304.9683 -14387.4233 -1795.0690 0.0000 0.0000
7968.6192 6194.8371 311.6130 -1773.7821 6257.3785
309.0612 -12.3053 -109.3357 511540.4688 -15.7903
-15.8113
[11231]ENERGY: 13540000 3272.4423 5443.1060 5433.6543
334.3152 -14378.7433 -1785.5041 0.0000 0.0000
7872.8181 6192.0886 307.8667 -1680.7295 6258.1008
309.0647 9.1115 -62.7001 511540.4688 -18.5950
-18.6022
[11281]ENERGY: 13550000 3271.2650 5310.5562 5441.0124
317.1483 -14423.7276 -1653.0199 0.0000 0.0000
7929.8799 6193.1144 310.0981 -1736.7655 6256.6645
309.1093 -36.9428 13.0521 511540.4688 -15.4779
-15.4582
[11317]ENERGY: 13560000 3207.7417 5334.5352 5474.7571
321.5041 -14326.2957 -1786.6092 0.0000 0.0000
7966.0557 6191.6889 311.5127 -1774.3668 6257.3356
309.0215 67.5621 35.5883 511540.4688 -16.3168
-16.3331
[11367]ENERGY: 13570000 3256.1708 5385.3130 5450.6548
313.3571 -14320.5299 -1756.5674 0.0000 0.0000
7864.9202 6193.3186 307.5578 -1671.6016 6257.3346
309.1405 238.1745 158.3454 511540.4688 -13.8682
-13.8681
[11403]ENERGY: 13580000 3247.0710 5258.2465 5438.1288
319.4367 -14370.5475 -1668.9333 0.0000 0.0000

7972.8364 6196.2385 311.7779 -1776.5979 6256.9010
308.7959 147.3219 87.8730 511540.4688 -14.2545
-14.2428
[11453]ENERGY: 13590000 3234.2756 5452.7354 5455.8857
313.9573 -14433.6565 -1762.9837 0.0000 0.0000
7927.4179 6187.6318 310.0018 -1739.7861 6256.6176
309.0941 -61.0409 -136.6125 511540.4688 -18.9226
-18.9262
[11492]ENERGY: 13600000 3306.6098 5393.4500 5458.2049
328.7318 -14425.5364 -1713.4700 0.0000 0.0000
7847.4596 6195.4498 306.8750 -1652.0098 6257.4902
309.2872 -62.8375 -39.6787 511540.4688 -16.9599
-16.9510
[11542]ENERGY: 13610000 3304.9838 5322.6480 5431.8575
345.6933 -14364.1610 -1747.9640 0.0000 0.0000
7900.1090 6193.1666 308.9339 -1706.9424 6258.4142
309.1604 75.4568 -42.0086 511540.4688 -20.6891
-20.6767
[11578]ENERGY: 13620000 3304.5605 5383.9440 5422.8593
310.1798 -14308.3697 -1697.7866 0.0000 0.0000
7783.9398 6199.3271 304.3911 -1584.6128 6258.3582
308.9458 121.1097 38.4691 511540.4688 -17.6942
-17.7267
[11628]ENERGY: 13630000 3345.1345 5320.2035 5409.8853
325.6741 -14317.6900 -1750.9150 0.0000 0.0000
7865.0420 6197.3345 307.5626 -1667.7075 6258.6766
309.1927 -328.2054 -262.1751 511540.4688 -13.1744
-13.1604
[11664]ENERGY: 13640000 3250.3331 5296.4245 5434.5208
334.9905 -14325.7995 -1728.0607 0.0000 0.0000
7933.8497 6196.2585 310.2533 -1737.5912 6260.4372
309.2038 103.0357 32.6224 511540.4688 -18.0993
-18.0834
[11714]ENERGY: 13650000 3282.1323 5340.5350 5422.4098
331.1050 -14436.7963 -1666.6771 0.0000 0.0000
7920.9726 6193.6813 309.7498 -1727.2913 6260.0623
308.9420 -69.7013 -48.5746 511540.4688 -22.5646
-22.5983
[11750]ENERGY: 13660000 3321.4807 5349.0405 5467.0476
333.9259 -14415.6525 -1675.8790 0.0000 0.0000
7818.3685 6198.3317 305.7374 -1620.0368 6261.3507
309.1295 130.7114 66.4683 511540.4688 -19.6628
-19.6573
[11800]ENERGY: 13670000 3229.1330 5376.5301 5448.5015
317.1708 -14393.8792 -1715.1605 0.0000 0.0000
7932.8956 6195.1914 310.2160 -1737.7043 6260.9459
309.0670 46.2950 13.6643 511540.4688 -14.8398
-14.8293
[11836]ENERGY: 13680000 3293.1217 5373.9866 5409.8561
355.7572 -14424.1623 -1778.0040 0.0000 0.0000
7968.7002 6199.2553 311.6161 -1769.4449 6262.7330
308.9317 -2.8690 -145.7440 511540.4688 -20.4023
-20.4365
[11886]ENERGY: 13690000 3223.8259 5292.2233 5469.4703

302.8026 -14345.0649 -1630.9385 0.0000 0.0000
7886.2745 6198.5931 308.3929 -1687.6814 6261.7411
308.9547 155.9735 164.7895 511540.4688 -18.4418
-18.4000
[11925]ENERGY: 13700000 3341.3964 5400.5512 5455.6971
326.5281 -14414.0251 -1738.3017 0.0000 0.0000
7821.6391 6193.4852 305.8653 -1628.1539 6261.6336
308.9595 -159.7281 -162.5605 511540.4688 -14.3063
-14.2935
[11975]ENERGY: 13710000 3304.4796 5291.0605 5471.0618
310.7340 -14370.8437 -1696.5797 0.0000 0.0000
7890.3677 6200.2802 308.5530 -1690.0876 6263.2008
308.9317 126.8212 55.8279 511540.4688 -19.3991
-19.4272
[12011]ENERGY: 13720000 3334.5238 5233.0479 5470.7026
300.8606 -14233.1536 -1787.4006 0.0000 0.0000
7882.6364 6201.2170 308.2506 -1681.4194 6262.9593
309.0890 96.0054 -2.8759 511540.4688 -14.4847
-14.4723
[12061]ENERGY: 13730000 3207.3468 5383.2232 5438.3035
321.6546 -14325.3936 -1748.8808 0.0000 0.0000
7920.2808 6196.5344 309.7227 -1723.7464 6263.0845
308.9989 212.0326 87.9208 511540.4688 -15.9377
-15.9475
[12097]ENERGY: 13740000 3280.1236 5481.5952 5424.7434
316.4594 -14358.8477 -1812.0213 0.0000 0.0000
7865.3252 6197.3778 307.5737 -1667.9475 6264.0261
309.0953 -16.4954 -33.8664 511540.4688 -12.6683
-12.6512
[12147]ENERGY: 13750000 3256.0639 5350.6329 5464.7062
312.1126 -14334.4593 -1722.1088 0.0000 0.0000
7873.9043 6200.8517 307.9092 -1673.0526 6263.7629
309.2530 60.7051 -71.4846 511540.4688 -12.3276
-12.3266
[12183]ENERGY: 13760000 3274.8676 5325.7593 5465.0865
326.0807 -14452.8658 -1652.5659 0.0000 0.0000
7913.9155 6200.2779 309.4738 -1713.6376 6263.4306
309.1591 170.1654 108.1952 511540.4688 -17.9124
-17.9376
[12233]ENERGY: 13770000 3423.4457 5379.3759 5399.0278
325.4402 -14449.4764 -1762.2863 0.0000 0.0000
7888.1032 6203.6300 308.4644 -1684.4732 6264.2956
309.2336 99.5744 -64.7452 511540.4688 -14.5003
-14.5084
[12269]ENERGY: 13780000 3230.4619 5424.9932 5451.9446
329.0621 -14417.3608 -1745.7056 0.0000 0.0000
7926.4022 6199.7976 309.9621 -1726.6046 6263.9633
309.2079 28.2100 -85.5037 511540.4688 -16.8757
-16.8646
[12319]ENERGY: 13790000 3305.9333 5341.9188 5507.5365
355.2592 -14468.3446 -1743.1561 0.0000 0.0000
7904.7923 6203.9394 309.1170 -1700.8529 6264.7498
309.3583 239.4449 167.3659 511540.4688 -11.5158
-11.5081

[12358]ENERGY: 13800000 3274.8971 5321.6895 5424.7260
342.3285 -14339.7519 -1740.6686 0.0000 0.0000
7914.8683 6198.0890 309.5110 -1716.7793 6264.4468
309.3672 -125.2163 -110.0706 511540.4688 -15.8662
-15.8611
[12408]ENERGY: 13810000 3292.8216 5298.1104 5487.9997
321.0214 -14385.8498 -1696.5243 0.0000 0.0000
7885.2976 6202.8766 308.3547 -1682.4211 6265.0019
309.3172 -97.0767 -72.4952 511540.4688 -15.2998
-15.2937
[12444]ENERGY: 13820000 3266.3270 5289.2437 5441.6593
336.1584 -14279.9239 -1759.2814 0.0000 0.0000
7907.1577 6201.3409 309.2095 -1705.8168 6264.8691
309.3121 -92.9132 -131.4232 511540.4688 -14.0066
-14.0379
[12494]ENERGY: 13830000 3237.0361 5267.4139 5422.1254
315.2389 -14301.0476 -1717.6323 0.0000 0.0000
7978.4584 6201.5929 311.9977 -1776.8656 6264.9021
309.3681 -25.0851 -108.4558 511540.4688 -15.2519
-15.2230
[12530]ENERGY: 13840000 3268.2712 5377.0373 5410.7622
302.4205 -14373.5992 -1777.0012 0.0000 0.0000
7990.6335 6198.5244 312.4738 -1792.1091 6264.9868
309.4436 -105.5795 -122.7738 511540.4688 -11.6930
-11.6975
[12580]ENERGY: 13850000 3292.5357 5287.9199 5438.5116
310.4432 -14333.4532 -1756.4149 0.0000 0.0000
7963.7236 6203.2660 311.4215 -1760.4576 6264.8897
309.3096 241.1342 49.8853 511540.4688 -17.5808
-17.5948
[12616]ENERGY: 13860000 3296.3599 5283.6616 5457.6466
326.1150 -14464.0221 -1637.1650 0.0000 0.0000
7940.3415 6202.9375 310.5072 -1737.4040 6264.4284
309.2506 261.8477 254.9817 511540.4688 -13.5945
-13.5831
[12666]ENERGY: 13870000 3237.8178 5438.9955 5406.0673
318.4064 -14384.2820 -1674.2877 0.0000 0.0000
7858.4631 6201.1806 307.3053 -1657.2825 6264.2889
309.2284 110.1420 110.4227 511540.4688 -18.2778
-18.2861
[12702]ENERGY: 13880000 3266.4547 5362.1867 5465.8485
314.3743 -14386.0917 -1745.3730 0.0000 0.0000
7926.6082 6204.0078 309.9701 -1722.6005 6264.6194
309.3821 113.3975 -1.5779 511540.4688 -13.0405
-13.0251
[12752]ENERGY: 13890000 3306.2696 5376.7196 5430.9315
331.0341 -14421.9596 -1714.9542 0.0000 0.0000
7892.0878 6200.1287 308.6202 -1691.9592 6264.9964
309.5117 -211.3120 -150.4667 511540.4688 -18.6093
-18.5992
[12791]ENERGY: 13900000 3336.1509 5393.7699 5392.7006
331.3130 -14391.9481 -1744.6032 0.0000 0.0000
7883.6937 6201.0768 308.2920 -1682.6169 6265.4962
309.5324 -104.0964 -52.0141 511540.4688 -13.2996

-13.3345
[12841]ENERGY: 13910000 3229.8258 5437.4647 5458.8040
319.5662 -14336.6992 -1787.1401 0.0000 0.0000
7881.6996 6203.5211 308.2140 -1678.1785 6265.6195
309.3023 123.9742 16.3841 511540.4688 -15.2126
-15.1927
[12877]ENERGY: 13920000 3298.5747 5354.3419 5453.9053
325.7933 -14367.0903 -1738.6244 0.0000 0.0000
7877.4741 6204.3746 308.0487 -1673.0995 6265.9692
309.3892 -130.1745 -160.5236 511540.4688 -11.2939
-11.3138
[12927]ENERGY: 13930000 3307.7108 5278.4255 5485.4543
316.2034 -14332.5404 -1718.5701 0.0000 0.0000
7865.7944 6202.4780 307.5920 -1663.3164 6265.9319
309.5892 -58.4978 -165.2744 511540.4688 -16.2288
-16.1949
[12963]ENERGY: 13940000 3291.7010 5382.4713 5448.6336
302.4091 -14340.4017 -1768.5824 0.0000 0.0000
7884.6311 6200.8620 308.3286 -1683.7691 6265.9166
309.4954 -14.0560 -63.8452 511540.4688 -17.4473
-17.4672
[13013]ENERGY: 13950000 3386.8119 5440.8611 5428.0049
326.3620 -14355.6159 -1809.0793 0.0000 0.0000
7785.0460 6202.3908 304.4343 -1582.6553 6265.1159
309.5314 -143.5326 -129.3783 511540.4688 -13.7530
-13.7416
[13049]ENERGY: 13960000 3220.0242 5449.6733 5416.3927
312.9828 -14440.2125 -1688.5271 0.0000 0.0000
7931.9379 6202.2713 310.1786 -1729.6666 6266.8015
309.6556 134.6705 119.8043 511540.4688 -14.2640
-14.2729
[13099]ENERGY: 13970000 3206.6225 5323.9679 5460.7184
312.7014 -14356.1282 -1704.0520 0.0000 0.0000
7956.2276 6200.0576 311.1284 -1756.1700 6266.0310
309.5997 -22.5701 18.3117 511540.4688 -18.6242
-18.6357
[13135]ENERGY: 13980000 3278.1441 5422.4855 5447.7133
323.1044 -14396.5858 -1725.1407 0.0000 0.0000
7850.9855 6200.7063 307.0129 -1650.2791 6266.1987
309.5089 -24.1168 -21.3334 511540.4688 -11.2980
-11.2955
[13185]ENERGY: 13990000 3280.7723 5283.8779 5485.0012
346.7864 -14415.8469 -1676.8903 0.0000 0.0000
7900.0019 6203.7024 308.9297 -1696.2995 6266.4149
309.5729 40.8151 -40.5277 511540.4688 -19.2414
-19.2305
[13224]ENERGY: 14000000 3305.4618 5375.9509 5398.1831
297.4546 -14293.7916 -1760.8284 0.0000 0.0000
7883.6297 6206.0602 308.2895 -1677.5695 6266.8490
309.3863 23.2841 -17.2702 511540.4688 -17.9866
-17.9876
[13274]ENERGY: 14010000 3239.4520 5347.7773 5452.5839
320.7563 -14373.0298 -1735.8944 0.0000 0.0000
7950.1673 6201.8126 310.8914 -1748.3547 6267.0001

309.4061 185.8528 112.9694 511540.4688 -12.9143
-12.9244
[13310]ENERGY: 14020000 3256.1527 5354.1845 5403.0317
309.5921 -14348.4820 -1746.7306 0.0000 0.0000
7974.3483 6202.0967 311.8370 -1772.2516 6266.7042
309.5861 -83.7582 -95.9907 511540.4688 -13.9276
-13.9103
[13360]ENERGY: 14030000 3299.2581 5222.2201 5427.4935
336.7159 -14379.7576 -1690.0974 0.0000 0.0000
7986.9354 6202.7680 312.3292 -1784.1674 6266.8798
309.6435 26.6622 -82.2924 511540.4688 -14.5844
-14.5963
[13396]ENERGY: 14040000 3260.6337 5218.7557 5429.0363
322.2543 -14313.6996 -1716.2535 0.0000 0.0000
8001.0358 6201.7628 312.8806 -1799.2731 6266.7206
309.4237 16.9437 -32.4719 511540.4688 -16.1787
-16.1743
[13446]ENERGY: 14050000 3238.2299 5273.0190 5436.0171
309.4364 -14322.0490 -1708.2716 0.0000 0.0000
7980.0583 6206.4402 312.0603 -1773.6181 6266.4814
309.5134 182.6383 121.5574 511540.4688 -10.8650
-10.8569
[13482]ENERGY: 14060000 3247.0713 5295.4200 5488.0517
336.0239 -14404.8401 -1633.3149 0.0000 0.0000
7873.6173 6202.0293 307.8979 -1671.5880 6266.1754
309.3537 11.6605 67.0346 511540.4688 -14.0738
-14.0735
[13532]ENERGY: 14070000 3225.7497 5397.3266 5390.6355
308.9924 -14396.7478 -1682.3273 0.0000 0.0000
7958.7476 6202.3768 311.2269 -1756.3708 6266.4225
309.3199 -91.0880 -93.4415 511540.4688 -18.3355
-18.3444
[13568]ENERGY: 14080000 3237.4451 5403.2409 5443.8579
309.8937 -14352.1060 -1702.7759 0.0000 0.0000
7864.0018 6203.5576 307.5219 -1660.4443 6267.2352
309.4507 -83.3626 -78.7385 511540.4688 -11.0419
-11.0131
[13618]ENERGY: 14090000 3249.1656 5303.5546 5470.0549
336.4251 -14348.3116 -1750.0325 0.0000 0.0000
7943.7165 6204.5726 310.6392 -1739.1439 6268.5493
309.4084 -50.2538 -39.1789 511540.4688 -16.2027
-16.2284
[13657]ENERGY: 14100000 3281.3006 5330.7401 5435.2693
325.2906 -14390.8423 -1699.7264 0.0000 0.0000
7924.6972 6206.7290 309.8954 -1717.9682 6267.7875
309.6086 289.4324 142.4397 511540.4688 -12.6172
-12.6284
[13707]ENERGY: 14110000 3304.5145 5312.6353 5488.4309
327.7427 -14472.1104 -1723.5457 0.0000 0.0000
7970.8360 6208.5032 311.6997 -1762.3328 6269.0276
309.5944 115.9217 93.2756 511540.4688 -16.2103
-16.1857
[13743]ENERGY: 14120000 3375.2894 5336.9300 5409.0378
334.8900 -14398.5575 -1713.4085 0.0000 0.0000

7866.3669 6210.5481 307.6144 -1655.8188 6268.0791
309.6192 -87.8783 -40.8662 511540.4688 -17.4501
-17.4482
[13793]ENERGY: 14130000 3277.0255 5344.0755 5455.0595
327.3300 -14384.4604 -1782.5747 0.0000 0.0000
7969.4408 6205.8962 311.6451 -1763.5446 6270.2257
309.5242 -80.3530 -95.9472 511540.4688 -16.1776
-16.2014
[13829]ENERGY: 14140000 3258.3546 5450.8372 5447.1705
327.3167 -14395.8706 -1723.3642 0.0000 0.0000
7844.9108 6209.3551 306.7754 -1635.5557 6271.4878
309.5902 -20.7691 36.7954 511540.4688 -18.1449
-18.1364
[13879]ENERGY: 14150000 3340.1305 5349.2851 5436.4091
311.3519 -14317.9847 -1793.4806 0.0000 0.0000
7884.3976 6210.1087 308.3195 -1674.2888 6271.2227
309.5069 -43.4036 -125.2853 511540.4688 -16.5282
-16.5285
[13915]ENERGY: 14160000 3192.6687 5381.0072 5409.5452
319.5603 -14406.6421 -1694.9811 0.0000 0.0000
8000.3655 6201.5237 312.8544 -1798.8418 6270.7529
309.6772 184.1516 70.6965 511540.4688 -18.5703
-18.5581
[13965]ENERGY: 14170000 3281.0459 5329.6975 5453.6785
313.1596 -14393.9818 -1701.3129 0.0000 0.0000
7924.0833 6206.3702 309.8714 -1717.7131 6270.2586
309.7499 142.4763 106.5021 511540.4688 -15.0011
-15.0126
[14001]ENERGY: 14180000 3332.4171 5364.9186 5469.2386
314.1956 -14384.9202 -1731.2096 0.0000 0.0000
7846.9627 6211.6027 306.8556 -1635.3600 6271.5166
309.6329 13.2414 -26.9085 511540.4688 -14.6682
-14.6783
[14051]ENERGY: 14190000 3266.8753 5446.6316 5452.0867
324.8742 -14343.6879 -1828.1316 0.0000 0.0000
7890.5581 6209.2065 308.5604 -1681.3516 6271.2647
309.5838 -35.3043 -105.9246 511540.4688 -13.4202
-13.4363
[14090]ENERGY: 14200000 3231.4162 5327.8369 5397.7535
327.8152 -14344.7978 -1702.7745 0.0000 0.0000
7970.2549 6207.5044 311.6769 -1762.7505 6270.4890
309.5709 -167.4715 -94.8971 511540.4688 -14.5860
-14.5633
[14140]ENERGY: 14210000 3280.3384 5367.2556 5406.4667
318.2164 -14358.1326 -1713.7131 0.0000 0.0000
7906.8917 6207.3231 309.1991 -1699.5686 6270.8080
309.6543 -176.8656 -50.5780 511540.4688 -13.0180
-13.0010
[14176]ENERGY: 14220000 3339.6216 5414.7241 5410.0109
322.8518 -14467.0028 -1669.6756 0.0000 0.0000
7859.7151 6210.2451 307.3543 -1649.4700 6270.3429
309.6082 1.4621 114.7680 511540.4688 -19.0820
-19.1016
[14226]ENERGY: 14230000 3217.2656 5372.2062 5438.8897

319.4323 -14373.6267 -1691.3949 0.0000 0.0000
7924.9811 6207.7534 309.9065 -1717.2277 6271.1232
309.7448 262.8980 208.7453 511540.4688 -16.4287
-16.4432
[14262]ENERGY: 14240000 3245.6874 5504.0994 5420.4625
321.7935 -14482.2829 -1745.1188 0.0000 0.0000
7941.4404 6206.0815 310.5502 -1735.3589 6271.4364
309.5223 76.5379 -90.4138 511540.4688 -13.6278
-13.6071
[14312]ENERGY: 14250000 3252.1022 5439.7421 5445.1456
315.9775 -14442.8955 -1757.3381 0.0000 0.0000
7955.9063 6208.6402 311.1158 -1747.2662 6272.2011
309.7707 127.9012 42.9822 511540.4688 -17.1917
-17.1902
[14348]ENERGY: 14260000 3278.6368 5405.7354 5476.0131
322.2794 -14426.8995 -1731.6615 0.0000 0.0000
7883.4783 6207.5820 308.2835 -1675.8963 6271.3645
309.8410 38.7616 62.2136 511540.4688 -15.9331
-15.9312
[14398]ENERGY: 14270000 3251.2225 5402.7114 5424.0700
329.5703 -14325.7462 -1780.7137 0.0000 0.0000
7907.9046 6209.0189 309.2387 -1698.8857 6273.2928
309.7189 -96.8969 -148.8104 511540.4688 -13.5310
-13.5485
[14434]ENERGY: 14280000 3271.9498 5356.6032 5454.5560
314.6200 -14359.2857 -1738.3260 0.0000 0.0000
7909.4418 6209.5591 309.2988 -1699.8827 6272.5006
309.5631 39.2914 -50.1239 511540.4688 -19.5203
-19.5258
[14484]ENERGY: 14290000 3265.9341 5360.7542 5456.6220
305.9415 -14360.7674 -1687.1653 0.0000 0.0000
7867.7963 6209.1153 307.6703 -1658.6810 6272.6396
309.6999 -4.3754 -85.2649 511540.4688 -13.4769
-13.4689
[14523]ENERGY: 14300000 3283.1667 5409.6275 5461.8445
322.1014 -14482.7136 -1733.9428 0.0000 0.0000
7947.9363 6208.0201 310.8042 -1739.9162 6272.8383
309.5101 167.5815 16.7835 511540.4688 -14.2571
-14.2434
[14573]ENERGY: 14310000 3288.1116 5305.4925 5432.2624
321.2550 -14398.7392 -1740.0733 0.0000 0.0000
8000.0415 6208.3505 312.8417 -1791.6910 6272.3209
309.4866 -111.8219 -100.6126 511540.4688 -17.3921
-17.4029
[14609]ENERGY: 14320000 3151.7122 5362.1222 5419.5420
325.1019 -14389.9068 -1628.1304 0.0000 0.0000
7967.5853 6208.0264 311.5725 -1759.5588 6271.9190
309.6630 195.4718 158.9229 511540.4688 -12.5272
-12.5215
[14659]ENERGY: 14330000 3216.2780 5270.6042 5451.0494
331.4196 -14305.2896 -1768.9886 0.0000 0.0000
8011.1588 6206.2318 313.2765 -1804.9270 6273.5850
309.7670 14.8849 -44.5487 511540.4688 -16.0080
-16.0228

[14695]ENERGY: 14340000 3205.2466 5254.6619 5494.6304
326.5062 -14277.0511 -1717.7460 0.0000 0.0000
7922.6603 6208.9083 309.8158 -1713.7521 6272.2157
309.6237 226.0356 150.5896 511540.4688 -14.2828
-14.2796
[14745]ENERGY: 14350000 3286.9795 5390.3568 5448.1074
337.6648 -14387.5258 -1816.7097 0.0000 0.0000
7948.5667 6207.4396 310.8288 -1741.1271 6274.3080
309.6369 129.0383 -45.1919 511540.4688 -17.1851
-17.1909
[14781]ENERGY: 14360000 3335.2444 5360.7181 5445.9914
324.7698 -14369.8304 -1761.1802 0.0000 0.0000
7878.8441 6214.5572 308.1023 -1664.2869 6273.1001
309.6660 26.1931 20.7455 511540.4688 -19.6681
-19.6573
[14831]ENERGY: 14370000 3329.7887 5390.4389 5448.6811
320.6316 -14363.7726 -1799.9084 0.0000 0.0000
7883.7258 6209.5850 308.2932 -1674.1407 6273.0016
309.5555 -150.9843 -214.2177 511540.4688 -13.4160
-13.3922
[14867]ENERGY: 14380000 3220.3199 5390.4813 5489.1668
317.7435 -14375.2149 -1749.0889 0.0000 0.0000
7915.9996 6209.4074 309.5553 -1706.5922 6272.8455
309.4373 41.8644 -46.0099 511540.4688 -17.2956
-17.3114
[14917]ENERGY: 14390000 3330.2532 5397.0881 5433.2763
341.8349 -14372.3633 -1765.7156 0.0000 0.0000
7845.2172 6209.5908 306.7873 -1635.6264 6272.9495
309.5731 -205.7485 -144.1444 511540.4688 -11.7927
-11.7890
[14956]ENERGY: 14400000 3314.4650 5438.5796 5357.8222
342.2805 -14359.4935 -1760.6378 0.0000 0.0000
7875.5776 6208.5936 307.9746 -1666.9840 6273.3433
309.6436 -114.5475 -70.7390 511540.4688 -18.8841
-18.8843
[15006]ENERGY: 14410000 3205.4190 5360.3008 5482.4433
326.4488 -14398.2818 -1685.1014 0.0000 0.0000
7915.5147 6206.7435 309.5363 -1708.7712 6272.1697
309.7670 194.3994 142.5159 511540.4688 -13.8469
-13.8529
[15042]ENERGY: 14420000 3336.9739 5380.0626 5406.1787
333.1275 -14371.7046 -1739.3340 0.0000 0.0000
7867.8229 6213.1269 307.6713 -1654.6960 6273.7576
309.7677 -62.3187 -82.4316 511540.4688 -11.1611
-11.1524
[15092]ENERGY: 14430000 3341.5724 5368.0801 5430.9877
318.4484 -14432.3792 -1766.9706 0.0000 0.0000
7947.4024 6207.1412 310.7833 -1740.2612 6272.9834
309.7677 -132.4250 -114.1407 511540.4688 -17.6835
-17.6822
[15128]ENERGY: 14440000 3275.7686 5337.9119 5492.3753
311.3306 -14434.2334 -1655.9973 0.0000 0.0000
7883.6047 6210.7605 308.2885 -1672.8442 6272.6312
309.8724 -6.9881 87.3518 511540.4688 -8.7614

-8.7570
[15178]ENERGY: 14450000 3326.8566 5405.7597 5500.5255
307.8100 -14432.7075 -1709.1468 0.0000 0.0000
7810.5739 6209.6713 305.4326 -1600.9026 6272.5479
309.7253 -238.6573 -249.1926 511540.4688 -12.3356
-12.3509
[15214]ENERGY: 14460000 3231.4433 5426.6638 5419.7108
303.5326 -14296.9379 -1739.1546 0.0000 0.0000
7867.3457 6212.6038 307.6527 -1654.7419 6272.4433
309.8211 137.2981 -40.9370 511540.4688 -12.5045
-12.5142
[15264]ENERGY: 14470000 3272.5948 5305.6995 5419.3976
330.8369 -14400.0848 -1731.9057 0.0000 0.0000
8008.8090 6205.3473 313.1846 -1803.4617 6271.4883
309.7967 37.5496 6.0797 511540.4688 -9.9258
-9.9160
[15300]ENERGY: 14480000 3282.5105 5281.9351 5444.4634
341.0085 -14320.1968 -1691.5079 0.0000 0.0000
7868.5218 6206.7345 307.6987 -1661.7872 6273.7763
309.7788 43.7952 -147.0392 511540.4688 -13.5464
-13.5488
[15350]ENERGY: 14490000 3283.9532 5341.4846 5468.1237
338.1057 -14416.1404 -1784.2176 0.0000 0.0000
7980.1491 6211.4583 312.0639 -1768.6908 6275.3014
309.6356 -33.7645 -76.9007 511540.4688 -11.1116
-11.1014
[15389]ENERGY: 14500000 3342.6898 5405.1618 5500.4090
313.5198 -14498.6404 -1753.1679 0.0000 0.0000
7899.3280 6209.3000 308.9033 -1690.0281 6274.0232
309.6053 -46.4492 -13.5797 511540.4688 -14.5609
-14.5601
[15439]ENERGY: 14510000 3311.7495 5376.3025 5482.7594
313.6440 -14366.0407 -1743.2284 0.0000 0.0000
7836.1529 6211.3394 306.4329 -1624.8136 6274.3146
309.8029 -167.3734 -173.5721 511540.4688 -14.1860
-14.2002
[15475]ENERGY: 14520000 3346.4491 5266.5110 5462.9505
313.6191 -14384.2323 -1728.7885 0.0000 0.0000
7932.3772 6208.8862 310.1957 -1723.4910 6273.0437
309.8727 -116.9579 -150.4552 511540.4688 -12.3576
-12.3535
[15525]ENERGY: 14530000 3342.7428 5358.3258 5411.3321
313.8278 -14451.4271 -1722.4960 0.0000 0.0000
7960.8835 6213.1890 311.3105 -1747.6945 6274.7374
309.8986 77.1252 -95.4956 511540.4688 -14.4539
-14.4377
[15561]ENERGY: 14540000 3177.7327 5435.5409 5432.5924
301.4717 -14393.8473 -1740.2669 0.0000 0.0000
7992.0801 6205.3037 312.5304 -1786.7764 6273.9685
309.6070 60.1196 8.3255 511540.4688 -10.2214
-10.2365
[15611]ENERGY: 14550000 3234.0094 5350.8504 5446.7266
320.3275 -14372.7608 -1748.3834 0.0000 0.0000
7978.2987 6209.0685 311.9915 -1769.2302 6274.4195

309.8398 -18.4739 -24.9277 511540.4688 -13.5502
-13.5604
[15647]ENERGY: 14560000 3293.7127 5403.1355 5465.7016
331.6725 -14325.8332 -1801.2188 0.0000 0.0000
7844.0253 6211.1956 306.7407 -1632.8297 6276.0022
309.9456 33.4692 -29.4089 511540.4688 -11.8773
-11.8742
[15697]ENERGY: 14570000 3277.1620 5389.5590 5417.9003
322.3342 -14363.5766 -1770.2318 0.0000 0.0000
7937.4038 6210.5509 310.3923 -1726.8529 6275.1724
309.7156 42.6887 -96.4976 511540.4688 -16.9072
-16.9332
[15733]ENERGY: 14580000 3251.2160 5368.0074 5435.9942
321.3476 -14369.5436 -1721.7492 0.0000 0.0000
7924.5761 6209.8484 309.8907 -1714.7276 6274.5202
309.7482 178.9285 -38.8502 511540.4688 -10.7684
-10.7570
[15783]ENERGY: 14590000 3336.9176 5249.9477 5468.0213
330.2883 -14373.5659 -1716.4460 0.0000 0.0000
7911.4685 6206.6315 309.3781 -1704.8370 6273.6818
309.7861 -228.7839 -117.8317 511540.4688 -15.2212
-15.1801
[15822]ENERGY: 14600000 3258.4861 5363.6468 5373.9275
319.0636 -14376.9094 -1644.8692 0.0000 0.0000
7919.6969 6213.0424 309.6999 -1706.6546 6274.2523
309.8977 249.1465 288.2131 511540.4688 -16.0318
-16.0366
[15872]ENERGY: 14610000 3193.7539 5442.1730 5393.1287
341.0840 -14350.7615 -1722.5851 0.0000 0.0000
7912.2695 6209.0626 309.4094 -1703.2070 6275.2468
309.9145 -84.1155 -11.9754 511540.4688 -12.0745
-12.0747
[15908]ENERGY: 14620000 3298.7024 5373.4638 5470.7334
314.5157 -14460.5579 -1720.9191 0.0000 0.0000
7934.9577 6210.8959 310.2966 -1724.0618 6275.1429
309.7947 97.2120 -16.9992 511540.4688 -15.0715
-15.0899
[15958]ENERGY: 14630000 3207.0342 5381.2040 5435.0143
308.6070 -14426.5086 -1700.9365 0.0000 0.0000
8004.1021 6208.5165 313.0005 -1795.5856 6275.5343
309.8904 175.8199 58.0391 511540.4688 -12.8304
-12.8096
[15994]ENERGY: 14640000 3294.4717 5326.7746 5466.9154
324.4778 -14365.7975 -1706.7233 0.0000 0.0000
7877.0897 6217.2083 308.0337 -1659.8814 6275.9229
309.8961 101.0176 56.4550 511540.4688 -8.3966
-8.4062
[16044]ENERGY: 14650000 3287.9193 5423.4738 5430.8963
331.9391 -14447.3321 -1777.0687 0.0000 0.0000
7961.5719 6211.3996 311.3374 -1750.1723 6276.9442
309.9607 -166.7165 -158.7077 511540.4688 -13.1697
-13.1833
[16080]ENERGY: 14660000 3243.0934 5407.0362 5428.1813
324.2536 -14381.4022 -1753.0459 0.0000 0.0000

7945.7703 6213.8867 310.7195 -1731.8837 6277.2744
309.9151 24.0135 56.5836 511540.4688 -15.1302
-15.1053
[16130]ENERGY: 14670000 3238.5303 5388.2761 5392.1761
321.1484 -14386.5635 -1710.5670 0.0000 0.0000
7969.6257 6212.6261 311.6523 -1756.9997 6277.4973
309.8382 17.6182 30.8780 511540.4688 -14.1693
-14.1962
[16166]ENERGY: 14680000 3243.4217 5196.1260 5489.6561
305.5880 -14320.4426 -1631.9045 0.0000 0.0000
7930.2230 6212.6677 310.1115 -1717.5553 6277.7806
309.8983 93.5183 71.3620 511540.4688 -14.1776
-14.1663
[16216]ENERGY: 14690000 3236.3675 5352.2492 5475.2868
321.5728 -14376.2225 -1737.0955 0.0000 0.0000
7944.1702 6216.3286 310.6569 -1727.8416 6278.6652
309.5994 231.7676 92.7593 511540.4688 -12.9887
-12.9827
[16255]ENERGY: 14700000 3290.5706 5516.8945 5415.3263
307.4847 -14468.8981 -1743.8075 0.0000 0.0000
7897.0806 6214.6511 308.8155 -1682.4295 6277.7095
309.5644 -166.7192 -190.2810 511540.4688 -14.3325
-14.3349
[16305]ENERGY: 14710000 3304.4244 5246.8359 5489.3362
331.1249 -14377.9332 -1745.6011 0.0000 0.0000
7964.0146 6212.2017 311.4329 -1751.8129 6278.7918
309.6671 93.8059 -47.7912 511540.4688 -7.7486
-7.7771
[16341]ENERGY: 14720000 3241.5208 5345.4468 5475.4243
306.3421 -14416.3580 -1671.0364 0.0000 0.0000
7935.1800 6216.5195 310.3053 -1718.6605 6278.3969
309.4740 170.5524 43.1774 511540.4688 -13.5087
-13.4983
[16391]ENERGY: 14730000 3310.3123 5327.3165 5437.2794
305.0714 -14381.1244 -1739.2920 0.0000 0.0000
7955.4540 6215.0172 311.0982 -1740.4368 6278.6059
309.6032 8.9516 17.8746 511540.4688 -14.8262
-14.8207
[16427]ENERGY: 14740000 3221.1695 5274.6680 5452.2705
327.7432 -14320.9505 -1693.1929 0.0000 0.0000
7948.5365 6210.2444 310.8276 -1738.2921 6276.6687
309.7392 126.7985 54.2946 511540.4688 -13.8122
-13.8205
[16477]ENERGY: 14750000 3307.5291 5432.6263 5443.0381
323.6123 -14426.4166 -1698.7144 0.0000 0.0000
7833.9491 6215.6239 306.3467 -1618.3252 6278.2034
309.8293 -87.6254 28.8231 511540.4688 -13.1715
-13.1605
[16513]ENERGY: 14760000 3226.9124 5452.6220 5442.1529
312.6742 -14405.7344 -1712.1803 0.0000 0.0000
7901.9133 6218.3601 309.0044 -1683.5531 6278.3509
309.9048 114.0134 14.4030 511540.4688 -15.5554
-15.5538
[16563]ENERGY: 14770000 3236.2633 5459.1210 5465.4446

327.8973 -14441.2049 -1726.0580 0.0000 0.0000
7894.7374 6216.2007 308.7238 -1678.5367 6279.3347
309.6651 -142.0577 -114.7015 511540.4688 -13.7751
-13.7898
[16599]ENERGY: 14780000 3300.9373 5314.8658 5405.4717
308.7611 -14303.8857 -1761.8501 0.0000 0.0000
7953.2191 6217.5192 311.0108 -1735.6999 6279.3930
309.6153 2.5556 36.6366 511540.4688 -16.9231
-16.9258
[16649]ENERGY: 14790000 3221.8287 5429.5905 5422.7423
309.1211 -14329.0624 -1751.8371 0.0000 0.0000
7910.1622 6212.5454 309.3270 -1697.6168 6279.4551
309.7149 -69.2111 -108.6791 511540.4688 -14.0623
-14.0277
[16688]ENERGY: 14800000 3287.6503 5373.7246 5422.4591
310.6404 -14345.0114 -1703.0161 0.0000 0.0000
7869.7112 6216.1581 307.7452 -1653.5531 6279.3039
309.7747 148.1713 44.0630 511540.4688 -12.6765
-12.6974
[16738]ENERGY: 14810000 3266.5073 5385.5691 5452.8160
324.6718 -14334.4005 -1682.4877 0.0000 0.0000
7809.8788 6222.5547 305.4054 -1587.3241 6280.0579
309.6804 68.9507 78.3655 511540.4688 -17.2614
-17.2671
[16774]ENERGY: 14820000 3215.9174 5242.6106 5439.3544
328.9807 -14326.3304 -1729.1016 0.0000 0.0000
8042.2401 6213.6712 314.4919 -1828.5688 6280.0686
309.7988 -6.2949 -81.4126 511540.4688 -15.1031
-15.1034
[16824]ENERGY: 14830000 3249.2662 5436.9073 5436.4071
333.6078 -14457.7043 -1717.1904 0.0000 0.0000
7938.9354 6220.2291 310.4522 -1718.7064 6280.5185
310.0190 -109.6975 -86.5153 511540.4688 -15.3429
-15.3501
[16860]ENERGY: 14840000 3223.7544 5382.4983 5493.6093
328.1283 -14448.4096 -1693.6379 0.0000 0.0000
7931.0858 6217.0286 310.1452 -1714.0572 6280.6599
310.0537 201.3127 140.7162 511540.4688 -15.2788
-15.2628
[16910]ENERGY: 14850000 3270.8715 5355.3156 5501.4783
314.6053 -14392.9180 -1692.4779 0.0000 0.0000
7859.1595 6216.0343 307.3326 -1643.1252 6281.6244
309.9185 60.8525 32.4681 511540.4688 -22.5780
-22.5679
[16946]ENERGY: 14860000 3340.8037 5328.4898 5398.0272
319.9717 -14368.3697 -1745.2679 0.0000 0.0000
7946.5522 6220.2070 310.7500 -1726.3452 6281.4261
309.7941 -140.6023 -95.8752 511540.4688 -14.9295
-14.9513
[16996]ENERGY: 14870000 3290.9928 5308.7710 5457.2798
329.3516 -14383.2026 -1733.2001 0.0000 0.0000
7946.4915 6216.4840 310.7477 -1730.0074 6281.6037
309.7794 106.7071 33.1161 511540.4688 -16.6610
-16.6526

[17032]ENERGY: 14880000 3218.6738 5458.5544 5474.5407
352.9934 -14481.7350 -1734.9168 0.0000 0.0000
7930.8857 6218.9961 310.1374 -1711.8896 6281.2764
309.7293 8.6879 25.0925 511540.4688 -12.5306
-12.5261
[17082]ENERGY: 14890000 3316.2834 5283.8432 5471.9621
324.4624 -14380.1607 -1707.3998 0.0000 0.0000
7910.8490 6219.8396 309.3539 -1691.0095 6280.9469
309.5539 -47.5403 8.7613 511540.4688 -14.7117
-14.7077
[17121]ENERGY: 14900000 3303.4026 5302.4579 5457.0676
331.5832 -14325.4149 -1766.1329 0.0000 0.0000
7912.1918 6215.1552 309.4064 -1697.0365 6281.6020
309.6901 -92.2315 -43.6706 511540.4688 -15.0586
-15.0655
[17171]ENERGY: 14910000 3256.7457 5375.0015 5483.2407
313.2001 -14455.1066 -1720.8321 0.0000 0.0000
7964.8076 6217.0568 311.4639 -1747.7508 6281.7233
309.6921 91.3608 70.0321 511540.4688 -18.7369
-18.7414
[17207]ENERGY: 14920000 3358.8835 5364.6710 5424.8476
323.5538 -14407.3173 -1696.3178 0.0000 0.0000
7847.9058 6216.2266 306.8925 -1631.6792 6279.5136
309.6821 10.7730 -116.7635 511540.4688 -16.0271
-16.0287
[17257]ENERGY: 14930000 3283.8768 5416.5509 5406.7167
344.7388 -14480.1889 -1766.3379 0.0000 0.0000
8006.0189 6211.3753 313.0755 -1794.6436 6279.9808
309.5856 7.1728 -7.7918 511540.4688 -13.6855
-13.6859
[17293]ENERGY: 14940000 3211.5254 5428.9048 5492.4119
348.0288 -14402.2965 -1730.6169 0.0000 0.0000
7868.4199 6216.3774 307.6947 -1652.0425 6280.7569
309.5594 179.6704 84.2584 511540.4688 -11.6035
-11.5976
[17343]ENERGY: 14950000 3274.1224 5388.0364 5462.1229
342.0135 -14287.6556 -1751.5920 0.0000 0.0000
7791.1273 6218.1748 304.6722 -1572.9524 6280.6765
309.5106 78.5696 -49.6578 511540.4688 -12.6223
-12.6221
[17379]ENERGY: 14960000 3247.1806 5390.3578 5462.6836
315.5619 -14405.5404 -1787.6604 0.0000 0.0000
7993.3965 6215.9796 312.5819 -1777.4170 6281.3723
309.6704 100.0889 -35.3191 511540.4688 -14.9559
-14.9725
[17429]ENERGY: 14970000 3366.4340 5359.1712 5493.6550
323.0526 -14482.2774 -1711.1552 0.0000 0.0000
7869.8384 6218.7187 307.7502 -1651.1197 6282.2982
309.7099 -311.3127 -287.8406 511540.4688 -11.2837
-11.2579
[17465]ENERGY: 14980000 3339.5121 5283.5727 5444.3767
318.4445 -14346.3254 -1770.9465 0.0000 0.0000
7950.7019 6219.3359 310.9123 -1731.3660 6281.4520
309.7370 -64.0712 35.6213 511540.4688 -11.9351

-11.9340
[17515]ENERGY: 14990000 3305.1300 5343.1257 5438.8922
342.3233 -14381.4151 -1710.2533 0.0000 0.0000
7883.0197 6220.8225 308.2656 -1662.1972 6283.1820
309.7782 -120.3534 -214.3940 511540.4688 -15.0296
-15.0190
[17554]ENERGY: 15000000 3258.7505 5390.5012 5471.7117
324.3753 -14373.1542 -1745.2216 0.0000 0.0000
7888.0812 6215.0440 308.4635 -1673.0372 6282.2943
309.8488 -149.0256 -118.9459 511540.4688 -17.1462
-17.1623
[17604]ENERGY: 15010000 3277.9179 5375.8589 5473.2270
314.5050 -14366.8624 -1765.0700 0.0000 0.0000
7908.6844 6218.2609 309.2692 -1690.4235 6284.0867
309.9432 -242.3821 -290.0283 511540.4688 -10.6653
-10.6740
[17640]ENERGY: 15020000 3256.0785 5455.7218 5498.6458
310.3563 -14419.1401 -1744.4450 0.0000 0.0000
7861.7423 6218.9597 307.4336 -1642.7826 6283.1644
309.7602 -101.1150 40.2863 511540.4688 -11.5737
-11.5582
[17690]ENERGY: 15030000 3286.7896 5293.3970 5436.3802
309.3074 -14357.7313 -1697.7259 0.0000 0.0000
7947.2072 6217.6242 310.7757 -1729.5830 6283.1639
309.7279 253.7352 193.3756 511540.4688 -17.3947
-17.4132
[17726]ENERGY: 15040000 3308.9782 5275.7568 5441.1360
327.9207 -14369.2244 -1718.9771 0.0000 0.0000
7951.7096 6217.2998 310.9517 -1734.4098 6284.1117
309.7101 -141.4062 -138.2751 511540.4688 -12.4937
-12.4964
[17776]ENERGY: 15050000 3265.1203 5278.0083 5467.5630
326.9181 -14358.7686 -1685.5009 0.0000 0.0000
7924.3166 6217.6568 309.8805 -1706.6597 6284.0323
309.8234 -159.4077 -79.1647 511540.4688 -14.3246
-14.3035
[17812]ENERGY: 15060000 3155.0549 5395.0477 5455.0696
335.0463 -14370.0788 -1750.6383 0.0000 0.0000
7996.5203 6216.0217 312.7040 -1780.4986 6283.7151
309.6028 57.7464 53.9272 511540.4688 -15.7704
-15.7713
[17862]ENERGY: 15070000 3228.2643 5483.1965 5423.8443
315.6092 -14338.4934 -1796.3814 0.0000 0.0000
7906.7809 6222.8204 309.1948 -1683.9605 6285.6421
309.5685 154.2176 -30.6424 511540.4688 -10.8508
-10.8756
[17898]ENERGY: 15080000 3269.3059 5360.2112 5458.2455
333.2862 -14476.0186 -1641.9847 0.0000 0.0000
7914.8634 6217.9091 309.5109 -1696.9544 6283.4367
309.5358 -15.5441 -12.8138 511540.4688 -11.2416
-11.2249
[17948]ENERGY: 15090000 3143.0728 5353.0048 5463.7407
297.1495 -14299.1447 -1676.3361 0.0000 0.0000
7936.7679 6218.2549 310.3674 -1718.5130 6284.9034

309.6251 44.1288 -33.4584 511540.4688 -12.4548
-12.4615
[17987]ENERGY: 15100000 3245.3584 5370.9870 5461.2991
331.1613 -14437.2928 -1693.2365 0.0000 0.0000
7939.7582 6218.0347 310.4844 -1721.7235 6283.4472
309.9256 75.6538 33.8641 511540.4688 -10.3408
-10.3439
[18037]ENERGY: 15110000 3304.8049 5384.0728 5408.9789
336.8062 -14442.2856 -1664.2375 0.0000 0.0000
7895.5540 6223.6937 308.7558 -1671.8603 6284.2494
309.9028 79.1670 -10.4846 511540.4688 -12.6364
-12.5972
[18073]ENERGY: 15120000 3302.8817 5267.5870 5451.1075
313.2182 -14406.5662 -1709.5150 0.0000 0.0000
8000.1277 6218.8410 312.8451 -1781.2867 6284.7037
309.8575 -77.2491 -33.2209 511540.4688 -13.0920
-13.1211
[18123]ENERGY: 15130000 3262.3130 5387.2583 5430.7836
339.7181 -14532.2781 -1666.3405 0.0000 0.0000
7996.8858 6218.3401 312.7183 -1778.5457 6284.3763
309.8621 91.6183 108.2024 511540.4688 -12.2523
-12.2677
[18159]ENERGY: 15140000 3333.3740 5341.7343 5427.5869
343.7699 -14389.0487 -1691.3831 0.0000 0.0000
7853.6341 6219.6674 307.1165 -1633.9667 6283.8309
309.8132 65.7855 106.6011 511540.4688 -10.2520
-10.2407
[18209]ENERGY: 15150000 3344.7757 5296.0133 5441.6516
318.6892 -14415.8076 -1712.4347 0.0000 0.0000
7948.0791 6220.9665 310.8098 -1727.1126 6284.8971
309.9611 29.2557 -49.2826 511540.4688 -7.2587
-7.2599
[18245]ENERGY: 15160000 3218.3063 5398.6640 5459.5121
332.3290 -14278.3553 -1739.6864 0.0000 0.0000
7831.0225 6221.7922 306.2323 -1609.2303 6284.5514
309.9757 226.7464 135.0201 511540.4688 -9.8983
-9.9061
[18295]ENERGY: 15170000 3315.9090 5393.0532 5436.7939
354.0519 -14381.5213 -1717.8401 0.0000 0.0000
7825.5773 6226.0239 306.0193 -1599.5534 6285.4504
309.9217 7.1364 -134.1672 511540.4688 -12.4984
-12.4960
[18331]ENERGY: 15180000 3276.8266 5354.6164 5449.6035
330.8426 -14404.7775 -1738.5052 0.0000 0.0000
7951.9027 6220.5091 310.9593 -1731.3936 6285.1465
310.0341 -46.2327 -93.7348 511540.4688 -9.1367
-9.1353
[18381]ENERGY: 15190000 3313.0279 5351.9496 5416.1104
339.5553 -14468.2976 -1696.5902 0.0000 0.0000
7963.7064 6219.4617 311.4209 -1744.2447 6285.5322
309.7603 208.2555 209.2078 511540.4688 -17.6393
-17.6137
[18420]ENERGY: 15200000 3325.7851 5434.2535 5477.1587
309.1787 -14372.9408 -1736.0189 0.0000 0.0000

7786.6806 6224.0969 304.4983 -1562.5838 6285.8007
309.7965 -160.8632 -119.8170 511540.4688 -10.8262
-10.8210
[18470]ENERGY: 15210000 3286.9071 5352.3255 5417.0202
321.2542 -14276.5929 -1755.8332 0.0000 0.0000
7878.8343 6223.9153 308.1019 -1654.9190 6285.6117
309.6865 -183.8179 -88.7255 511540.4688 -10.0411
-10.0446
[18506]ENERGY: 15220000 3281.7571 5366.4887 5499.2860
311.2140 -14411.0512 -1696.7104 0.0000 0.0000
7871.2830 6222.2672 307.8066 -1649.0157 6285.5104
309.7032 52.0124 37.2041 511540.4688 -11.3950
-11.4323
[18556]ENERGY: 15230000 3288.8555 5450.1563 5467.6909
317.0693 -14370.5123 -1737.1715 0.0000 0.0000
7808.0194 6224.1077 305.3327 -1583.9117 6285.3542
309.7614 -54.8798 -46.6735 511540.4688 -14.8914
-14.8749
[18592]ENERGY: 15240000 3245.4064 5326.1688 5510.3025
349.0302 -14500.1082 -1656.5871 0.0000 0.0000
7945.8279 6220.0405 310.7217 -1725.7874 6286.2091
309.7616 -134.0934 -151.6466 511540.4688 -12.4873
-12.4918
[18642]ENERGY: 15250000 3197.4543 5366.3793 5450.8201
329.9478 -14415.5772 -1666.3381 0.0000 0.0000
7957.4711 6220.1572 311.1770 -1737.3139 6286.0416
309.5482 125.8222 153.4340 511540.4688 -8.4200
-8.4211
[18678]ENERGY: 15260000 3358.0318 5392.7877 5375.7866
327.6598 -14340.7898 -1777.5035 0.0000 0.0000
7887.5844 6223.5571 308.4441 -1664.0273 6286.8273
309.6724 -260.8594 -185.6907 511540.4688 -13.4193
-13.4048
[18728]ENERGY: 15270000 3237.2234 5264.8781 5479.0233
324.4265 -14437.2532 -1635.0350 0.0000 0.0000
7986.4411 6219.7041 312.3099 -1766.7370 6286.6027
310.0978 146.3284 68.3499 511540.4688 -9.6139
-9.6277
[18764]ENERGY: 15280000 3350.5202 5427.0941 5442.9593
317.3117 -14371.2621 -1791.2788 0.0000 0.0000
7849.1674 6224.5117 306.9418 -1624.6556 6288.0826
309.9809 -233.4919 -169.5770 511540.4688 -15.8493
-15.8588
[18814]ENERGY: 15290000 3296.7409 5344.0448 5459.3977
323.8304 -14350.4773 -1747.5210 0.0000 0.0000
7898.7774 6224.7928 308.8818 -1673.9846 6287.5024
310.0067 16.1992 -101.0066 511540.4688 -11.7966
-11.7713
[18853]ENERGY: 15300000 3296.0313 5431.9800 5465.1039
335.8448 -14437.6665 -1755.6944 0.0000 0.0000
7887.4145 6223.0136 308.4375 -1664.4009 6286.8795
309.9963 7.5723 -27.1299 511540.4688 -16.5547
-16.5531
[18903]ENERGY: 15310000 3333.7231 5354.5605 5488.9840

329.3113 -14498.0901 -1652.6888 0.0000 0.0000
7869.2976 6225.0975 307.7290 -1644.2001 6285.7708
309.9042 -168.6256 -91.2510 511540.4688 -10.0115
-10.0177
[18939]ENERGY: 15320000 3283.8347 5217.1664 5449.7708
310.0478 -14329.7141 -1703.5575 0.0000 0.0000
7992.3390 6219.8871 312.5405 -1772.4519 6287.7719
309.9338 341.8599 260.6695 511540.4688 -15.6803
-15.6946
[18989]ENERGY: 15330000 3288.8381 5328.3297 5436.6595
309.7596 -14361.6193 -1676.8480 0.0000 0.0000
7896.2865 6221.4062 308.7844 -1674.8803 6286.3522
309.8560 124.1530 85.5227 511540.4688 -8.5752
-8.5552
[19025]ENERGY: 15340000 3302.7698 5445.2413 5497.1236
343.5862 -14541.5880 -1721.8239 0.0000 0.0000
7894.9321 6220.2412 308.7314 -1674.6909 6286.3657
309.8497 -44.0680 -93.7323 511540.4688 -12.1175
-12.1296
[19075]ENERGY: 15350000 3348.4972 5391.6160 5448.0264
347.5175 -14475.0650 -1646.6044 0.0000 0.0000
7810.9003 6224.8881 305.4454 -1586.0123 6285.5352
309.8213 55.7870 12.9249 511540.4688 -12.4316
-12.4319
[19111]ENERGY: 15360000 3307.4155 5340.7917 5416.9281
348.7945 -14326.5434 -1739.6421 0.0000 0.0000
7878.6316 6226.3759 308.0940 -1652.2557 6287.0521
309.8388 -21.5854 -145.0229 511540.4688 -9.7283
-9.7222
[19161]ENERGY: 15370000 3244.9244 5371.1192 5476.6287
308.0158 -14389.0475 -1706.5612 0.0000 0.0000
7919.7254 6224.8047 309.7010 -1694.9207 6285.7286
309.9051 145.8759 116.4662 511540.4688 -9.7531
-9.7457
[19197]ENERGY: 15380000 3204.4228 5318.2479 5445.9366
323.1728 -14357.3704 -1744.3680 0.0000 0.0000
8029.6053 6219.6470 313.9978 -1809.9582 6286.3306
309.9369 108.8196 89.3418 511540.4688 -12.5912
-12.6088
[19247]ENERGY: 15390000 3241.6767 5374.2815 5475.3488
305.5380 -14471.9928 -1694.3223 0.0000 0.0000
7989.2389 6219.7688 312.4193 -1769.4701 6285.7765
309.7478 -134.8656 -132.8696 511540.4688 -15.9813
-15.9746
[19286]ENERGY: 15400000 3293.4414 5303.2761 5449.4944
322.3468 -14342.4310 -1702.0539 0.0000 0.0000
7898.2408 6222.3145 308.8608 -1675.9263 6285.9792
309.9184 -101.8035 -166.9447 511540.4688 -16.2455
-16.2501
[19336]ENERGY: 15410000 3240.5982 5408.6527 5458.0427
338.2554 -14408.3488 -1708.8744 0.0000 0.0000
7893.8634 6222.1893 308.6897 -1671.6741 6286.5554
309.9821 -8.1456 -7.8253 511540.4688 -14.0863
-14.0782

[19372]ENERGY: 15420000 3217.1272 5531.4196 5426.8034
326.6336 -14376.6049 -1758.6362 0.0000 0.0000
7855.8611 6222.6040 307.2036 -1633.2572 6286.4976
310.1932 -104.8699 -93.5892 511540.4688 -13.1455
-13.1347
[19422]ENERGY: 15430000 3204.8260 5425.9326 5493.9626
302.6877 -14374.8424 -1812.5798 0.0000 0.0000
7977.1921 6217.1787 311.9482 -1760.0134 6286.9555
309.7528 -58.6672 -123.9199 511540.4688 -12.7033
-12.7104
[19458]ENERGY: 15440000 3271.3587 5357.4188 5385.3756
326.6320 -14352.4872 -1684.9875 0.0000 0.0000
7920.5504 6223.8609 309.7332 -1696.6895 6286.2529
309.9031 -84.2007 -53.4581 511540.4688 -14.1793
-14.1782
[19508]ENERGY: 15450000 3299.5297 5401.5200 5416.9578
325.3038 -14407.2430 -1730.2944 0.0000 0.0000
7911.4480 6217.2219 309.3773 -1694.2261 6286.3920
309.8850 113.2734 69.5077 511540.4688 -10.2588
-10.2696
[19544]ENERGY: 15460000 3318.8298 5342.3089 5373.9838
322.6764 -14387.3135 -1708.0189 0.0000 0.0000
7958.1358 6220.6023 311.2030 -1737.5335 6286.7831
309.8537 53.4333 -85.8714 511540.4688 -16.8927
-16.9038
[19594]ENERGY: 15470000 3287.2046 5266.3421 5476.2596
327.9473 -14374.9430 -1728.0416 0.0000 0.0000
7965.2188 6219.9877 311.4800 -1745.2311 6285.1003
310.1803 -109.3135 -124.0460 511540.4688 -14.3939
-14.3681
[19630]ENERGY: 15480000 3136.6595 5394.9134 5440.7804
324.5796 -14367.2380 -1767.1709 0.0000 0.0000
8054.8252 6217.3493 314.9841 -1837.4759 6284.9519
310.0816 58.9165 -15.1466 511540.4688 -15.4137
-15.4231
[19680]ENERGY: 15490000 3270.8930 5471.5597 5480.6648
344.7065 -14378.5657 -1754.4708 0.0000 0.0000
7791.6988 6226.4862 304.6945 -1565.2126 6285.3854
309.9175 -118.9882 -69.6472 511540.4688 -12.0927
-12.0750
[19719]ENERGY: 15500000 3273.8748 5471.5874 5448.8247
328.1305 -14480.6362 -1697.0660 0.0000 0.0000
7877.2596 6221.9747 308.0404 -1655.2849 6285.9054
309.8777 192.2229 50.7730 511540.4688 -16.8269
-16.8138
[19769]ENERGY: 15510000 3292.7503 5292.6491 5464.7124
346.9173 -14304.8083 -1758.5808 0.0000 0.0000
7886.8197 6220.4597 308.4142 -1666.3599 6285.4697
310.0476 -85.1608 -70.8444 511540.4688 -14.3440
-14.3663
[19805]ENERGY: 15520000 3277.6994 5350.9335 5455.2709
313.5917 -14380.8229 -1730.5994 0.0000 0.0000
7935.7019 6221.7750 310.3257 -1713.9269 6285.2854
309.9656 89.9790 68.0657 511540.4688 -16.5495

-16.5631
[19855]ENERGY: 15530000 3279.3581 5384.1827 5478.2286
309.7731 -14370.8105 -1750.6895 0.0000 0.0000
7894.4718 6224.5143 308.7134 -1669.9575 6285.3426
309.7923 309.1316 274.4000 511540.4688 -11.8909
-11.8877
[19891]ENERGY: 15540000 3274.5905 5396.7215 5453.3728
353.8761 -14316.4289 -1767.4623 0.0000 0.0000
7827.8195 6222.4892 306.1070 -1605.3303 6285.2121
309.7937 49.5278 -58.2825 511540.4688 -14.6287
-14.6527
[19941]ENERGY: 15550000 3213.2640 5379.7821 5424.1097
341.0864 -14379.2513 -1723.9066 0.0000 0.0000
7962.8475 6217.9319 311.3873 -1744.9156 6284.3112
309.8715 156.1322 43.9754 511540.4688 -13.6207
-13.5890
[19977]ENERGY: 15560000 3275.7181 5298.7459 5479.8188
363.2500 -14368.4854 -1719.0261 0.0000 0.0000
7892.0865 6222.1077 308.6202 -1669.9788 6285.6107
310.1170 -106.2680 -141.5904 511540.4688 -9.9818
-10.0006
[20027]ENERGY: 15570000 3214.2276 5345.1602 5471.8841
284.5479 -14321.8853 -1688.8358 0.0000 0.0000
7915.8930 6220.9917 309.5511 -1694.9014 6282.9563
309.9693 93.0820 2.7416 511540.4688 -13.5210
-13.4917
[20063]ENERGY: 15580000 3328.9589 5335.2814 5452.7987
323.8268 -14289.1063 -1825.5863 0.0000 0.0000
7896.2906 6222.4639 308.7846 -1673.8267 6284.4473
309.7986 -239.2682 -123.7123 511540.4688 -17.5177
-17.5273
[20113]ENERGY: 15590000 3267.8612 5542.8073 5416.5807
320.6411 -14465.9707 -1767.9935 0.0000 0.0000
7907.4479 6221.3740 309.2209 -1686.0739 6284.0503
309.7764 156.6147 79.0894 511540.4688 -13.7845
-13.8134
[20152]ENERGY: 15600000 3243.9770 5421.0320 5435.6526
328.9720 -14435.1231 -1695.1994 0.0000 0.0000
7921.5966 6220.9077 309.7742 -1700.6889 6284.4529
309.9997 260.2392 187.1508 511540.4688 -14.9517
-14.9506
[20202]ENERGY: 15610000 3221.6362 5397.8212 5496.9466
305.8662 -14412.3699 -1643.8486 0.0000 0.0000
7855.3142 6221.3657 307.1822 -1633.9485 6285.4982
309.8900 116.9347 74.1954 511540.4688 -12.7780
-12.7534
[20238]ENERGY: 15620000 3276.7828 5361.3421 5466.8699
342.8578 -14438.1026 -1686.1974 0.0000 0.0000
7896.6561 6220.2087 308.7989 -1676.4474 6285.7723
309.6898 249.6057 210.9944 511540.4688 -15.0635
-15.0628
[20288]ENERGY: 15630000 3288.8018 5456.6095 5434.6171
310.0899 -14373.2600 -1725.0485 0.0000 0.0000
7830.1153 6221.9251 306.1968 -1608.1902 6285.8748

309.8665 -183.1219 -175.1682 511540.4688 -15.4098
-15.4232
[20324]ENERGY: 15640000 3253.1814 5333.6582 5484.5098
334.6150 -14416.2837 -1730.4846 0.0000 0.0000
7965.9300 6225.1261 311.5078 -1740.8039 6287.8366
309.8074 -59.8849 20.8978 511540.4688 -10.8349
-10.8340
[20374]ENERGY: 15650000 3254.4649 5294.2853 5447.0937
340.5118 -14370.5624 -1724.2550 0.0000 0.0000
7982.1028 6223.6411 312.1403 -1758.4617 6287.5322
309.8860 -5.4118 17.7653 511540.4688 -16.3767
-16.3658
[20410]ENERGY: 15660000 3173.7566 5419.8932 5482.8594
313.7068 -14376.6570 -1701.9498 0.0000 0.0000
7910.1030 6221.7123 309.3247 -1688.3907 6287.4995
310.1512 32.8698 18.4167 511540.4688 -9.2508
-9.2620
[20460]ENERGY: 15670000 3331.5170 5312.5161 5471.9554
322.2738 -14412.9690 -1726.5254 0.0000 0.0000
7923.5473 6222.3153 309.8504 -1701.2320 6286.7166
310.1299 65.6053 33.6075 511540.4688 -12.4189
-12.4206
[20496]ENERGY: 15680000 3254.4787 5384.6345 5413.8790
324.7114 -14409.8449 -1728.2516 0.0000 0.0000
7981.2852 6220.8923 312.1083 -1760.3929 6287.9140
310.0346 -13.6789 -38.1177 511540.4688 -14.9909
-14.9612
[20546]ENERGY: 15690000 3271.6374 5384.1378 5426.8052
329.3687 -14414.9945 -1749.9552 0.0000 0.0000
7976.3611 6223.3605 311.9157 -1753.0006 6286.9232
309.9308 7.9361 40.0754 511540.4688 -10.7798
-10.7950
[20585]ENERGY: 15700000 3333.4308 5329.0985 5443.5167
312.4971 -14418.0126 -1739.7681 0.0000 0.0000
7961.5843 6222.3466 311.3379 -1739.2377 6288.3304
310.0935 82.2963 -105.8313 511540.4688 -15.9352
-15.9569
[20635]ENERGY: 15710000 3244.3242 5403.6743 5469.2056
335.6424 -14504.0590 -1626.9825 0.0000 0.0000
7901.1097 6222.9147 308.9730 -1678.1950 6287.2410
309.9948 113.3659 30.4126 511540.4688 -10.0384
-10.0224
[20671]ENERGY: 15720000 3295.3272 5390.0154 5445.0795
331.2902 -14423.5106 -1724.2369 0.0000 0.0000
7908.5483 6222.5132 309.2639 -1686.0351 6287.9301
309.9425 -42.9401 -94.3797 511540.4688 -11.8713
-11.8776
[20721]ENERGY: 15730000 3286.6049 5356.4226 5462.6398
311.9611 -14311.4219 -1771.2762 0.0000 0.0000
7892.0279 6226.9583 308.6179 -1665.0696 6288.4940
309.9313 -113.1330 -69.3803 511540.4688 -20.4680
-20.4589
[20757]ENERGY: 15740000 3265.0266 5347.2652 5472.6808
325.1370 -14373.0152 -1683.7788 0.0000 0.0000

7874.9111 6228.2268 307.9485 -1646.6843 6289.1125
309.9674 -212.8902 -159.9462 511540.4688 -13.5592
-13.5499
[20807]ENERGY: 15750000 3282.5765 5342.9517 5406.6706
316.3270 -14355.3335 -1714.0861 0.0000 0.0000
7943.8220 6222.9282 310.6433 -1720.8938 6289.2920
309.9358 263.0330 176.8333 511540.4688 -19.1398
-19.1274
[20843]ENERGY: 15760000 3361.1970 5342.9601 5457.7116
314.5305 -14440.0608 -1717.1287 0.0000 0.0000
7904.9036 6224.1132 309.1214 -1680.7904 6287.8693
309.9204 -142.6267 -120.3305 511540.4688 -11.6410
-11.6582
[20893]ENERGY: 15770000 3295.7794 5398.7406 5464.9603
326.0002 -14375.6787 -1759.9059 0.0000 0.0000
7875.4871 6225.3830 307.9710 -1650.1040 6288.5849
310.0516 -149.5169 -83.8490 511540.4688 -15.9107
-15.9106
[20929]ENERGY: 15780000 3277.4384 5353.3234 5495.5964
303.3137 -14447.4210 -1679.3192 0.0000 0.0000
7922.5684 6225.5002 309.8122 -1697.0682 6288.1566
309.8605 -179.6648 -114.3089 511540.4688 -15.3443
-15.3325
[20979]ENERGY: 15790000 3252.5901 5334.7918 5440.4170
339.6176 -14392.9335 -1751.1282 0.0000 0.0000
7997.7716 6221.1264 312.7530 -1776.6451 6288.5283
309.9679 149.7189 183.5352 511540.4688 -14.3307
-14.3596
[21018]ENERGY: 15800000 3273.2724 5245.6633 5467.0985
332.4811 -14380.8657 -1754.0102 0.0000 0.0000
8037.5265 6221.1659 314.3076 -1816.3606 6288.1552
310.1332 43.5517 -100.9067 511540.4688 -16.2060
-16.1926
[21068]ENERGY: 15810000 3365.9760 5457.5470 5427.5689
303.8845 -14407.7244 -1744.8569 0.0000 0.0000
7823.1013 6225.4964 305.9225 -1597.6050 6288.2721
309.9293 107.9791 29.8495 511540.4688 -11.2729
-11.2945
[21104]ENERGY: 15820000 3246.7562 5383.6805 5474.3493
321.1267 -14415.1657 -1738.9459 0.0000 0.0000
7952.3231 6224.1242 310.9757 -1728.1989 6288.0417
310.0195 -132.3357 -120.8904 511540.4688 -16.1151
-16.0840
[21154]ENERGY: 15830000 3288.6187 5298.4165 5463.5876
317.9051 -14417.4878 -1668.4146 0.0000 0.0000
7942.3288 6224.9542 310.5849 -1717.3745 6288.3441
310.0460 18.9850 -27.2324 511540.4688 -12.5358
-12.5715
[21190]ENERGY: 15840000 3271.4241 5383.4918 5447.3416
339.6693 -14409.5202 -1704.1790 0.0000 0.0000
7897.0257 6225.2533 308.8133 -1671.7724 6288.7533
310.0190 28.7108 -97.2538 511540.4688 -11.2551
-11.2350
[21240]ENERGY: 15850000 3230.7579 5382.4439 5485.4210

325.6390 -14363.1982 -1776.1708 0.0000 0.0000
7939.1335 6224.0263 310.4599 -1715.1073 6289.2679
309.9719 -0.0811 -67.1757 511540.4688 -16.4293
-16.4449
[21276]ENERGY: 15860000 3307.8627 5334.8381 5403.7273
305.9566 -14396.4871 -1670.8889 0.0000 0.0000
7940.0887 6225.0973 310.4973 -1714.9914 6288.8603
310.0758 204.0769 192.5066 511540.4688 -11.0437
-11.0355
[21326]ENERGY: 15870000 3251.9544 5390.3736 5480.5257
328.8498 -14406.6540 -1678.9351 0.0000 0.0000
7857.2329 6223.3474 307.2572 -1633.8856 6288.1319
310.1575 147.1498 70.5288 511540.4688 -12.4439
-12.4306
[21362]ENERGY: 15880000 3256.4133 5454.0712 5444.2938
358.5456 -14465.1723 -1711.7190 0.0000 0.0000
7891.0461 6227.4787 308.5795 -1663.5674 6288.7869
310.2540 -71.7367 -111.8265 511540.4688 -14.6472
-14.6405
[21412]ENERGY: 15890000 3206.8890 5383.1151 5413.8871
327.3458 -14395.1699 -1682.4875 0.0000 0.0000
7972.8147 6226.3944 311.7770 -1746.4203 6289.0357
310.1233 229.4026 85.4802 511540.4688 -12.0891
-12.0950
[21451]ENERGY: 15900000 3210.3945 5304.7290 5449.6441
336.1397 -14327.7887 -1771.1734 0.0000 0.0000
8019.6743 6221.6194 313.6095 -1798.0549 6288.4271
310.2352 135.8377 148.4652 511540.4688 -9.4412
-9.4544
[21501]ENERGY: 15910000 3348.2216 5393.0860 5463.2054
327.5010 -14408.1363 -1720.5711 0.0000 0.0000
7826.2869 6229.5934 306.0471 -1596.6935 6290.4082
310.0246 -171.2745 -154.8294 511540.4688 -15.6957
-15.6813
[21537]ENERGY: 15920000 3294.2820 5407.1714 5459.2669
339.5923 -14432.0346 -1753.8095 0.0000 0.0000
7909.7580 6224.2264 309.3112 -1685.5315 6289.9686
310.1094 24.8684 -20.5332 511540.4688 -13.2991
-13.3285
[21587]ENERGY: 15930000 3368.5826 5327.2841 5396.7028
322.1627 -14380.7496 -1724.4899 0.0000 0.0000
7916.4324 6225.9251 309.5722 -1690.5073 6289.9362
309.9897 -165.7181 -103.8572 511540.4688 -15.8165
-15.7806
[21623]ENERGY: 15940000 3257.9056 5379.9778 5417.4736
314.9355 -14255.3879 -1800.6188 0.0000 0.0000
7915.8330 6230.1188 309.5488 -1685.7142 6290.2821
309.9499 3.4391 -158.3102 511540.4688 -15.6294
-15.6284
[21673]ENERGY: 15950000 3307.0488 5334.1854 5468.4847
315.7108 -14364.6133 -1722.9470 0.0000 0.0000
7891.6889 6229.5583 308.6046 -1662.1307 6289.7851
310.0307 8.7634 -49.8228 511540.4688 -13.6187
-13.6279

[21709]ENERGY: 15960000 3285.2657 5362.7383 5446.4456
355.9589 -14497.8981 -1685.7603 0.0000 0.0000
7955.7624 6222.5126 311.1102 -1733.2498 6289.3632
310.0761 41.5940 -10.9101 511540.4688 -12.9185
-12.9114
[21759]ENERGY: 15970000 3274.0384 5400.9112 5464.7464
323.8062 -14390.7891 -1765.6782 0.0000 0.0000
7921.1293 6228.1643 309.7559 -1692.9650 6289.3896
310.2219 -78.7921 -99.9262 511540.4688 -9.8561
-9.8678
[21795]ENERGY: 15980000 3257.7154 5371.6567 5434.7907
331.0665 -14486.5704 -1635.2465 0.0000 0.0000
7952.1381 6225.5506 310.9685 -1726.5875 6289.7319
310.1427 -30.3108 112.8377 511540.4688 -14.4466
-14.4316
[21845]ENERGY: 15990000 3217.5559 5342.8970 5427.8068
313.0780 -14376.9931 -1670.2230 0.0000 0.0000
7970.3387 6224.4602 311.6802 -1745.8785 6290.5211
310.0160 33.1794 131.6130 511540.4688 -9.2614
-9.2573
[21884]ENERGY: 16000000 3207.6387 5380.6752 5470.7101
321.7231 -14457.8130 -1715.3678 0.0000 0.0000
8013.5006 6221.0669 313.3681 -1792.4337 6290.9433
310.0659 148.0621 78.9647 511540.4688 -13.2537
-13.2589
[21934]ENERGY: 16010000 3340.5920 5378.0842 5485.7306
345.8847 -14439.1670 -1679.5039 0.0000 0.0000
7801.1473 6232.7681 305.0640 -1568.3792 6290.1559
309.9323 -5.8998 -14.0630 511540.4688 -17.6930
-17.6854
[21970]ENERGY: 16020000 3282.4687 5347.8273 5412.6220
333.6556 -14331.2741 -1782.8676 0.0000 0.0000
7965.1511 6227.5831 311.4774 -1737.5680 6290.8335
309.8192 -11.8985 -73.9524 511540.4688 -10.7629
-10.7812
[22020]ENERGY: 16030000 3260.6163 5356.9550 5491.1894
353.1484 -14383.3289 -1752.8589 0.0000 0.0000
7902.6591 6228.3804 309.0336 -1674.2787 6290.7325
309.9341 17.6756 -67.0749 511540.4688 -13.4899
-13.4906
[22056]ENERGY: 16040000 3256.9889 5318.7627 5428.1432
320.0914 -14300.9944 -1765.8430 0.0000 0.0000
7968.5580 6225.7069 311.6106 -1742.8511 6292.1727
309.9197 -44.3920 -185.5568 511540.4688 -10.8505
-10.8434
[22106]ENERGY: 16050000 3297.7544 5359.9191 5429.6676
327.5381 -14382.8894 -1758.6794 0.0000 0.0000
7954.5960 6227.9063 311.0646 -1726.6897 6292.0389
309.9004 -28.2896 -114.5660 511540.4688 -12.0634
-12.0718
[22142]ENERGY: 16060000 3209.4308 5433.1115 5430.9059
336.4324 -14448.0751 -1662.8273 0.0000 0.0000
7930.1040 6229.0822 310.1068 -1701.0219 6292.0870
309.8727 16.4862 13.2297 511540.4688 -8.2953

-8.3122
[22192]ENERGY: 16070000 3285.5180 5471.9001 5458.1500
325.3213 -14493.5248 -1707.9695 0.0000 0.0000
7888.3949 6227.7899 308.4758 -1660.6050 6292.5474
310.1005 -87.4982 -22.6680 511540.4688 -10.2804
-10.2528
[22228]ENERGY: 16080000 3251.2018 5354.7492 5440.2701
341.1743 -14388.1902 -1726.5398 0.0000 0.0000
7957.1024 6229.7678 311.1626 -1727.3346 6293.2912
310.0727 -111.9997 -79.2591 511540.4688 -12.0283
-12.0395
[22278]ENERGY: 16090000 3207.3385 5408.7423 5478.0730
324.6985 -14310.9925 -1768.0156 0.0000 0.0000
7892.1423 6231.9864 308.6223 -1660.1559 6293.7740
310.1255 158.6458 53.4356 511540.4688 -12.7129
-12.7190
[22317]ENERGY: 16100000 3270.8794 5440.8691 5455.2112
336.8962 -14432.4323 -1788.8453 0.0000 0.0000
7948.4093 6230.9877 310.8227 -1717.4216 6294.9858
310.0688 6.7564 -163.3296 511540.4688 -11.6204
-11.6254
[22367]ENERGY: 16110000 3309.9815 5432.8744 5410.9721
324.3986 -14324.8846 -1736.4543 0.0000 0.0000
7822.0698 6238.9577 305.8822 -1583.1122 6294.8352
310.2961 -19.4250 -79.3191 511540.4688 -13.4220
-13.4079
[22403]ENERGY: 16120000 3355.6251 5399.9797 5411.2083
347.4172 -14431.8848 -1785.5277 0.0000 0.0000
7933.7206 6230.5384 310.2483 -1703.1822 6294.1719
310.1346 -55.3076 -110.2667 511540.4688 -7.2171
-7.2047
[22453]ENERGY: 16130000 3377.6291 5448.5039 5431.6594
315.1218 -14411.7481 -1788.2788 0.0000 0.0000
7856.4768 6229.3640 307.2276 -1627.1128 6295.3923
310.1601 -254.1149 -337.7079 511540.4688 -15.6059
-15.6247
[22489]ENERGY: 16140000 3275.7031 5427.7051 5395.7943
330.5952 -14363.7939 -1760.7675 0.0000 0.0000
7924.0043 6229.2407 309.8683 -1694.7636 6295.4678
310.1988 -73.7138 -10.3311 511540.4688 -12.4180
-12.4267
[22539]ENERGY: 16150000 3306.3012 5331.6897 5426.6541
329.8068 -14355.8270 -1738.7490 0.0000 0.0000
7934.3067 6234.1825 310.2712 -1700.1242 6296.8342
310.0454 -115.1973 -152.6738 511540.4688 -12.0525
-12.0447
[22575]ENERGY: 16160000 3298.0804 5450.1095 5400.3729
344.0576 -14398.9511 -1796.6099 0.0000 0.0000
7935.7295 6232.7889 310.3268 -1702.9406 6296.7232
310.1399 -139.9725 -87.0474 511540.4688 -13.0553
-13.0274
[22625]ENERGY: 16170000 3286.5678 5394.4277 5443.5180
345.9933 -14447.6107 -1716.1423 0.0000 0.0000
7924.9341 6231.6880 309.9047 -1693.2462 6297.5077

310.2219 136.8021 142.1915 511540.4688 -9.7542
-9.7664
[22661]ENERGY: 16180000 3318.8147 5510.4032 5424.2867
318.6574 -14478.1206 -1675.3841 0.0000 0.0000
7812.9430 6231.6004 305.5253 -1581.3427 6297.4283
310.1199 66.1033 101.0597 511540.4688 -14.5942
-14.5885
[22711]ENERGY: 16190000 3310.7221 5388.3889 5394.1518
325.4624 -14375.8847 -1781.9392 0.0000 0.0000
7971.3495 6232.2510 311.7197 -1739.0986 6296.1868
310.0558 -150.9141 -204.3823 511540.4688 -14.4066
-14.4404
[22750]ENERGY: 16200000 3200.6325 5414.9605 5425.6677
303.6684 -14381.9638 -1665.9759 0.0000 0.0000
7934.1370 6231.1263 310.2646 -1703.0107 6295.0799
310.2736 121.4981 -15.8022 511540.4688 -13.8591
-13.8615
[22800]ENERGY: 16210000 3246.6969 5381.9811 5481.9056
306.1597 -14383.2741 -1754.9910 0.0000 0.0000
7952.7169 6231.1950 310.9911 -1721.5219 6297.3358
310.1427 5.2344 -117.5853 511540.4688 -13.2848
-13.2745
[22836]ENERGY: 16220000 3261.9435 5390.7726 5443.7171
333.9207 -14375.5574 -1704.7903 0.0000 0.0000
7884.6751 6234.6813 308.3303 -1649.9938 6296.9583
310.2494 159.9688 82.7407 511540.4688 -14.9998
-15.0001
[22886]ENERGY: 16230000 3325.3706 5362.7381 5462.0273
332.0266 -14441.4111 -1716.2953 0.0000 0.0000
7906.0853 6230.5414 309.1676 -1675.5439 6296.3064
310.0096 6.7122 19.9012 511540.4688 -13.3770
-13.3625
[22922]ENERGY: 16240000 3353.8988 5301.7313 5423.2591
328.1192 -14383.1785 -1708.9785 0.0000 0.0000
7919.7749 6234.6263 309.7029 -1685.1486 6296.6290
310.0929 172.4155 156.7677 511540.4688 -12.0810
-12.0734
[22972]ENERGY: 16250000 3364.8937 5376.8916 5470.3402
323.0742 -14443.6156 -1815.1019 0.0000 0.0000
7953.7398 6230.2221 311.0311 -1723.5178 6297.6169
310.0754 -69.7337 -79.4773 511540.4688 -16.1900
-16.2023
[23008]ENERGY: 16260000 3327.7359 5402.1270 5457.6497
316.7598 -14399.8493 -1748.1797 0.0000 0.0000
7879.0990 6235.3424 308.1123 -1643.7566 6298.8316
310.2327 -16.6852 -78.4476 511540.4688 -13.1214
-13.1347
[23058]ENERGY: 16270000 3328.0492 5380.5718 5410.2881
300.4225 -14356.8232 -1786.1474 0.0000 0.0000
7953.4758 6229.8368 311.0208 -1723.6390 6297.1554
310.1708 -164.9351 -291.4803 511540.4688 -18.9363
-18.9545
[23094]ENERGY: 16280000 3319.2677 5429.8719 5444.3935
324.4683 -14344.9695 -1770.0196 0.0000 0.0000

7834.0966 6237.1088 306.3525 -1596.9878 6297.1865
310.2245 -145.4665 -180.8863 511540.4688 -14.8266
-14.8220
[23144]ENERGY: 16290000 3319.8975 5470.3100 5438.5368
323.7693 -14406.7810 -1792.4685 0.0000 0.0000
7878.1149 6231.3790 308.0738 -1646.7359 6297.3062
310.2953 -2.9202 -57.3953 511540.4688 -11.9528
-11.9306
[23183]ENERGY: 16300000 3246.3131 5379.3647 5489.1937
314.0062 -14404.8764 -1727.7388 0.0000 0.0000
7937.7679 6234.0304 310.4065 -1703.7375 6296.3682
310.3045 -89.3568 -195.6157 511540.4688 -14.5033
-14.4994
[23233]ENERGY: 16310000 3338.9824 5494.4639 5438.5329
321.4916 -14457.8347 -1728.3598 0.0000 0.0000
7828.6219 6235.8982 306.1384 -1592.7237 6297.2730
310.2504 75.0909 -34.1130 511540.4688 -10.3981
-10.3952
[23269]ENERGY: 16320000 3242.5756 5447.7328 5439.4475
334.8038 -14448.4018 -1707.0608 0.0000 0.0000
7921.2876 6230.3845 309.7621 -1690.9030 6295.9596
310.2617 -75.9624 42.3141 511540.4688 -12.6955
-12.6696
[23319]ENERGY: 16330000 3287.5210 5436.1430 5425.1224
310.5839 -14395.6744 -1721.2219 0.0000 0.0000
7889.8710 6232.3451 308.5335 -1657.5260 6296.8863
310.1168 -70.4473 -52.9124 511540.4688 -12.2837
-12.2922
[23355]ENERGY: 16340000 3365.2790 5333.0018 5447.1819
288.7601 -14398.0622 -1750.4819 0.0000 0.0000
7948.0888 6233.7674 310.8101 -1714.3214 6296.8181
310.0554 -295.3764 -246.4255 511540.4688 -12.6205
-12.6206
[23405]ENERGY: 16350000 3200.2951 5406.1956 5472.5964
323.8428 -14422.7970 -1708.5219 0.0000 0.0000
7962.4012 6234.0122 311.3698 -1728.3890 6296.1089
310.1489 96.8154 -14.6745 511540.4688 -11.0835
-11.1089
[23441]ENERGY: 16360000 3306.6448 5391.5103 5479.0158
310.4136 -14446.7866 -1737.1428 0.0000 0.0000
7931.0240 6234.6792 310.1428 -1696.3448 6296.1567
310.1982 -91.7914 -75.6544 511540.4688 -10.0208
-9.9956
[23491]ENERGY: 16370000 3263.5194 5366.6832 5430.8374
301.4444 -14415.2662 -1648.8060 0.0000 0.0000
7933.5010 6231.9132 310.2397 -1701.5878 6296.2462
310.2435 117.3891 87.0628 511540.4688 -14.7643
-14.7788
[23527]ENERGY: 16380000 3347.6598 5325.3879 5489.8535
338.1017 -14459.2611 -1730.9576 0.0000 0.0000
7922.2519 6233.0361 309.7998 -1689.2158 6295.8614
310.4173 -113.1824 -74.9915 511540.4688 -12.1706
-12.1758
[23577]ENERGY: 16390000 3280.7887 5363.9913 5414.5231

304.7390 -14466.5885 -1653.5707 0.0000 0.0000
7990.0287 6233.9117 312.4502 -1756.1171 6296.2131
310.4826 -115.4394 -36.9903 511540.4688 -12.3166
-12.3093
[23616]ENERGY: 16400000 3377.7087 5356.1227 5417.7505
316.4663 -14356.1900 -1737.6640 0.0000 0.0000
7859.7591 6233.9534 307.3560 -1625.8058 6297.2991
310.5399 -70.0886 -14.7916 511540.4688 -11.7621
-11.7483
[23666]ENERGY: 16410000 3277.3121 5406.8885 5515.7795
312.0256 -14486.8765 -1678.0105 0.0000 0.0000
7889.2319 6236.3505 308.5085 -1652.8813 6296.7734
310.6289 30.2264 1.4162 511540.4688 -9.1295
-9.1535
[23702]ENERGY: 16420000 3250.6301 5459.2125 5457.3594
312.7813 -14355.0596 -1785.2015 0.0000 0.0000
7894.9732 6234.6954 308.7330 -1660.2778 6297.8808
310.6069 -180.0249 -180.7482 511540.4688 -18.3841
-18.3803
[23752]ENERGY: 16430000 3254.9102 5377.1893 5474.5356
331.4398 -14455.8209 -1676.3260 0.0000 0.0000
7928.0103 6233.9383 310.0250 -1694.0720 6296.7863
310.5263 244.4820 118.2622 511540.4688 -11.7614
-11.7756
[23788]ENERGY: 16440000 3347.7380 5438.2783 5440.1745
309.9368 -14296.8552 -1796.0386 0.0000 0.0000
7794.0102 6237.2441 304.7849 -1556.7661 6297.0998
310.3852 0.7961 -84.9913 511540.4688 -9.2370
-9.2073
[23838]ENERGY: 16450000 3243.3870 5445.2368 5476.7865
311.6249 -14445.4294 -1683.1854 0.0000 0.0000
7884.5654 6232.9858 308.3261 -1651.5796 6297.0723
310.3923 31.4151 37.8789 511540.4688 -12.0675
-12.0904
[23874]ENERGY: 16460000 3295.5702 5361.9076 5457.4616
305.7920 -14360.2156 -1716.7855 0.0000 0.0000
7891.3938 6235.1241 308.5931 -1656.2697 6297.7732
310.4269 41.8043 44.9907 511540.4688 -14.5176
-14.4807
[23924]ENERGY: 16470000 3307.5187 5393.9044 5473.1932
350.1706 -14401.8867 -1726.8928 0.0000 0.0000
7840.1302 6236.1377 306.5884 -1603.9925 6297.3232
310.6820 -79.8760 39.5551 511540.4688 -10.0845
-10.1231
[23960]ENERGY: 16480000 3294.8555 5427.6446 5426.0415
327.3677 -14372.2414 -1773.0620 0.0000 0.0000
7906.8061 6237.4120 309.1958 -1669.3941 6299.3461
310.5869 188.1132 74.1875 511540.4688 -14.5947
-14.5844
[24010]ENERGY: 16490000 3322.3263 5373.6626 5506.8185
331.1341 -14413.6333 -1686.0843 0.0000 0.0000
7800.0639 6234.2878 305.0216 -1565.7761 6298.5431
310.6021 236.9522 116.0523 511540.4688 -9.2963
-9.2901

[24049]ENERGY: 16500000 3357.6404 5427.4424 5469.1162
342.3489 -14440.6353 -1765.5530 0.0000 0.0000
7847.6355 6237.9951 306.8819 -1609.6404 6299.3875
310.7559 -155.1723 -199.6807 511540.4688 -13.3822
-13.3841
[24099]ENERGY: 16510000 3222.4675 5371.3379 5459.5162
344.5958 -14420.1982 -1698.0815 0.0000 0.0000
7951.9903 6231.6281 310.9627 -1720.3623 6298.9276
310.6702 92.0029 88.5218 511540.4688 -16.2370
-16.2544
[24135]ENERGY: 16520000 3252.6901 5464.6500 5465.9591
335.4417 -14445.7734 -1756.4896 0.0000 0.0000
7919.8528 6236.3307 309.7060 -1683.5221 6299.6808
310.7380 42.9660 -60.3580 511540.4688 -8.0901
-8.0819
[24185]ENERGY: 16530000 3287.8674 5450.0372 5471.3690
317.2229 -14436.0865 -1685.3917 0.0000 0.0000
7831.0468 6236.0651 306.2332 -1594.9817 6299.2099
310.6531 -44.4772 -93.8199 511540.4688 -15.9100
-15.9135
[24221]ENERGY: 16540000 3366.5635 5297.9350 5507.6204
348.3329 -14399.3408 -1773.5438 0.0000 0.0000
7887.9230 6235.4902 308.4574 -1652.4328 6299.3405
310.5338 -133.7822 -120.8906 511540.4688 -15.5287
-15.5198
[24271]ENERGY: 16550000 3309.9894 5366.5139 5432.7986
328.7547 -14399.0249 -1754.4839 0.0000 0.0000
7949.5228 6234.0706 310.8662 -1715.4522 6298.8710
310.4578 -30.2052 -121.6182 511540.4688 -21.2390
-21.2418
[24307]ENERGY: 16560000 3342.0007 5380.1848 5498.9722
344.7877 -14402.6245 -1802.9979 0.0000 0.0000
7876.1287 6236.4517 307.9961 -1639.6771 6299.7971
310.5418 -57.6097 8.2490 511540.4688 -13.9891
-13.9727
[24357]ENERGY: 16570000 3262.7905 5317.6760 5488.7916
324.9481 -14382.9087 -1756.3270 0.0000 0.0000
7980.6772 6235.6477 312.0845 -1745.0295 6299.9495
310.5275 94.2564 40.6828 511540.4688 -18.5443
-18.5475
[24393]ENERGY: 16580000 3325.4338 5443.4679 5442.4598
306.5522 -14353.2908 -1818.9053 0.0000 0.0000
7893.7318 6239.4493 308.6845 -1654.2825 6301.5342
310.3240 -222.9135 -231.3171 511540.4688 -13.1050
-13.0960
[24443]ENERGY: 16590000 3222.3604 5436.7149 5437.7932
332.0543 -14440.4108 -1710.3193 0.0000 0.0000
7962.2715 6240.4642 311.3648 -1721.8074 6301.2060
310.3531 73.3750 154.0892 511540.4688 -16.0992
-16.1177
[24482]ENERGY: 16600000 3252.8778 5326.3832 5491.6223
318.8291 -14327.3807 -1766.6386 0.0000 0.0000
7943.4941 6239.1872 310.6305 -1704.3070 6301.5732
310.5767 -44.2649 -129.3380 511540.4688 -12.5271

-12.5363
[24532]ENERGY: 16610000 3348.9234 5338.7293 5445.1898
347.7237 -14443.1378 -1734.8815 0.0000 0.0000
7934.4337 6236.9805 310.2762 -1697.4531 6300.9780
310.5851 1.0601 44.9168 511540.4688 -14.9588
-14.9599
[24568]ENERGY: 16620000 3270.3227 5328.0850 5474.7376
289.8796 -14451.1646 -1699.6875 0.0000 0.0000
8020.3677 6232.5405 313.6366 -1787.8271 6299.4242
310.7339 155.6899 196.3861 511540.4688 -15.1722
-15.1608
[24618]ENERGY: 16630000 3335.1433 5381.9102 5461.1966
325.5266 -14414.5656 -1734.9059 0.0000 0.0000
7883.1057 6237.4110 308.2690 -1645.6947 6300.1232
310.6465 -242.7964 -241.6925 511540.4688 -10.4642
-10.4396
[24654]ENERGY: 16640000 3293.5794 5368.1464 5483.5025
345.5880 -14399.3351 -1738.8174 0.0000 0.0000
7888.2371 6240.9008 308.4696 -1647.3363 6300.4181
310.5647 97.0724 -22.7292 511540.4688 -14.2871
-14.3051
[24704]ENERGY: 16650000 3285.1149 5401.9825 5491.9192
340.0045 -14437.3669 -1790.1820 0.0000 0.0000
7946.0433 6237.5155 310.7301 -1708.5278 6301.3275
310.5959 22.7144 -83.6049 511540.4688 -13.8284
-13.8431
[24740]ENERGY: 16660000 3326.4459 5307.7213 5485.9539
342.6864 -14414.3961 -1713.7705 0.0000 0.0000
7904.4887 6239.1295 309.1052 -1665.3591 6301.1522
310.4192 -41.0522 -46.5447 511540.4688 -13.7193
-13.7145
[24790]ENERGY: 16670000 3292.5392 5372.8020 5429.9781
333.5543 -14374.4150 -1731.0027 0.0000 0.0000
7913.2937 6236.7496 309.4495 -1676.5442 6301.9559
310.5264 165.8006 67.7995 511540.4688 -13.5239
-13.5273
[24826]ENERGY: 16680000 3285.9578 5428.6411 5421.7431
313.6121 -14412.0110 -1745.9187 0.0000 0.0000
7948.2611 6240.2855 310.8169 -1707.9756 6301.5344
310.5155 73.5600 182.8991 511540.4688 -18.8417
-18.8565
[24876]ENERGY: 16690000 3288.9636 5377.1743 5449.1079
324.1565 -14347.5167 -1772.6950 0.0000 0.0000
7918.0887 6237.2794 309.6370 -1680.8093 6301.9890
310.7335 107.1698 -10.7855 511540.4688 -14.8908
-14.8886
[24915]ENERGY: 16700000 3211.4811 5491.5913 5479.4290
347.4069 -14513.4466 -1717.0257 0.0000 0.0000
7937.8400 6237.2761 310.4094 -1700.5639 6300.5672
310.5574 127.6341 81.2575 511540.4688 -11.8699
-11.8491
[24965]ENERGY: 16710000 3288.4694 5426.4127 5471.3576
314.7035 -14343.5936 -1829.0244 0.0000 0.0000
7910.2976 6238.6228 309.3323 -1671.6748 6302.8469

310.4803 -321.4938 -327.0906 511540.4688 -16.0251
-16.0281
[25001]ENERGY: 16720000 3265.1773 5463.2705 5451.3516
336.9787 -14415.7731 -1754.5607 0.0000 0.0000
7889.1776 6235.6218 308.5064 -1653.5558 6301.0438
310.4596 17.6248 40.6143 511540.4688 -14.5476
-14.5605
[25051]ENERGY: 16730000 3326.3403 5309.2836 5457.6565
337.6708 -14417.1097 -1695.1623 0.0000 0.0000
7918.4134 6237.0926 309.6497 -1681.3208 6300.5422
310.3649 -132.6205 -133.4430 511540.4688 -9.7197
-9.7061
[25087]ENERGY: 16740000 3241.3326 5445.3063 5439.9072
330.0337 -14363.4921 -1765.8591 0.0000 0.0000
7909.7941 6237.0227 309.3126 -1672.7714 6301.3943
310.5873 -83.5864 -78.2371 511540.4688 -11.3958
-11.3997
[25137]ENERGY: 16750000 3283.7289 5333.0202 5511.9352
339.6526 -14445.6038 -1742.2287 0.0000 0.0000
7955.7865 6236.2910 311.1112 -1719.4955 6302.0416
310.5935 0.3674 -23.6799 511540.4688 -9.2968
-9.2661
[25173]ENERGY: 16760000 3270.6945 5410.5085 5430.8984
322.7949 -14396.6711 -1724.5163 0.0000 0.0000
7921.6038 6235.3128 309.7744 -1686.2910 6300.8300
310.4910 305.4789 253.0389 511540.4688 -9.4506
-9.4875
[25223]ENERGY: 16770000 3285.8295 5336.5359 5483.0802
325.4249 -14370.7009 -1802.8884 0.0000 0.0000
7980.6301 6237.9113 312.0827 -1742.7188 6301.5984
310.5033 -140.4264 -162.1209 511540.4688 -11.6664
-11.6662
[25259]ENERGY: 16780000 3319.2357 5322.4293 5436.1417
334.8410 -14383.2855 -1754.5160 0.0000 0.0000
7962.0051 6236.8514 311.3543 -1725.1537 6301.5277
310.6061 -73.8817 -52.1142 511540.4688 -13.4224
-13.4021
[25309]ENERGY: 16790000 3290.7167 5431.5335 5484.5465
313.8586 -14401.3078 -1807.9567 0.0000 0.0000
7926.6254 6238.0162 309.9708 -1688.6092 6301.7209
310.6225 -130.6160 -217.4617 511540.4688 -10.2692
-10.3019
[25348]ENERGY: 16800000 3274.7643 5393.2409 5463.3339
347.9933 -14435.2490 -1732.7679 0.0000 0.0000
7927.7891 6239.1045 310.0163 -1688.6846 6302.3125
310.8188 -162.0399 -149.9100 511540.4688 -10.8573
-10.8352
[25398]ENERGY: 16810000 3296.3128 5477.2269 5440.3963
338.2099 -14455.8375 -1717.5131 0.0000 0.0000
7859.8686 6238.6639 307.3603 -1621.2047 6302.8707
310.7377 -59.7339 -58.1445 511540.4688 -6.9802
-7.0007
[25434]ENERGY: 16820000 3252.5587 5433.3109 5418.4979
333.2685 -14413.5931 -1696.0556 0.0000 0.0000

7910.6308 6238.6180 309.3453 -1672.0128 6303.1041
310.8861 32.6248 18.3863 511540.4688 -13.7118
-13.7004
[25484]ENERGY: 16830000 3309.3877 5364.7338 5436.2232
320.1558 -14341.4681 -1713.6338 0.0000 0.0000
7865.6371 6241.0357 307.5859 -1624.6014 6302.9987
310.8057 -55.7434 63.7184 511540.4688 -14.0217
-14.0007
[25520]ENERGY: 16840000 3362.2473 5360.7293 5415.6757
333.6943 -14438.4184 -1844.8495 0.0000 0.0000
8045.1251 6234.2038 314.6047 -1810.9213 6302.5650
310.4177 -269.3591 -270.0285 511540.4688 -14.3264
-14.3508
[25570]ENERGY: 16850000 3266.9312 5409.2892 5435.9628
301.4239 -14424.1397 -1720.0201 0.0000 0.0000
7965.7853 6235.2326 311.5022 -1730.5527 6301.1365
310.4449 44.8978 146.9230 511540.4688 -12.5580
-12.5790
[25606]ENERGY: 16860000 3314.8170 5377.5457 5411.1224
333.9641 -14298.2656 -1746.8177 0.0000 0.0000
7846.5317 6238.8977 306.8387 -1607.6340 6302.6172
310.4055 50.1998 -85.3615 511540.4688 -15.4295
-15.4255
[25656]ENERGY: 16870000 3287.1302 5472.3252 5435.0935
343.3988 -14465.4106 -1680.2732 0.0000 0.0000
7850.2849 6242.5489 306.9855 -1607.7360 6302.8642
310.3805 72.7795 -4.3712 511540.4688 -12.5527
-12.5283
[25692]ENERGY: 16880000 3257.6533 5444.9384 5438.5846
286.3882 -14386.6302 -1736.2522 0.0000 0.0000
7935.1253 6239.8072 310.3032 -1695.3181 6302.9904
310.5008 -38.1189 -61.5735 511540.4688 -13.1676
-13.1658
[25742]ENERGY: 16890000 3201.0435 5389.5701 5455.3394
333.7233 -14411.3053 -1721.2048 0.0000 0.0000
7990.3754 6237.5415 312.4638 -1752.8339 6301.9213
310.6743 263.5110 238.8863 511540.4688 -10.1571
-10.1518
[25781]ENERGY: 16900000 3198.7583 5392.6601 5468.9952
322.2762 -14442.3877 -1686.5732 0.0000 0.0000
7984.0292 6237.7581 312.2156 -1746.2711 6301.5769
310.4511 288.5207 244.6447 511540.4688 -12.6809
-12.6952
[25831]ENERGY: 16910000 3316.0478 5377.4588 5464.9697
318.4632 -14448.4640 -1699.1522 0.0000 0.0000
7910.8771 6240.2002 309.3550 -1670.6768 6302.7644
310.4792 45.1725 20.7627 511540.4688 -16.3989
-16.3929
[25867]ENERGY: 16920000 3218.6893 5459.3292 5485.6357
332.0533 -14452.0891 -1688.8539 0.0000 0.0000
7885.8164 6240.5808 308.3750 -1645.2356 6303.2046
310.3892 67.7934 19.1160 511540.4688 -16.7969
-16.8030
[25917]ENERGY: 16930000 3244.4536 5343.8626 5466.0735

308.9661 -14327.8917 -1734.0521 0.0000 0.0000
7937.7571 6239.1691 310.4061 -1698.5880 6302.4192
310.4735 -64.8343 -114.4169 511540.4688 -12.2937
-12.2887
[25953]ENERGY: 16940000 3288.8545 5261.5608 5465.4735
326.3983 -14343.2448 -1735.9612 0.0000 0.0000
7974.2512 6237.3324 311.8332 -1736.9188 6301.5953
310.3853 -40.0585 -39.8210 511540.4688 -9.7326
-9.7179
[26003]ENERGY: 16950000 3239.6647 5337.1342 5506.2089
314.6105 -14424.7523 -1721.2798 0.0000 0.0000
7986.5194 6238.1056 312.3130 -1748.4138 6301.7171
310.4456 19.2320 15.2980 511540.4688 -5.5879
-5.5860
[26039]ENERGY: 16960000 3220.5307 5357.7755 5477.1943
339.1587 -14375.6728 -1742.5566 0.0000 0.0000
7962.7240 6239.1539 311.3824 -1723.5701 6303.5001
310.3827 105.7533 36.9561 511540.4688 -10.8467
-10.8559
[26089]ENERGY: 16970000 3238.8686 5402.6357 5445.3481
333.4719 -14482.2988 -1682.9256 0.0000 0.0000
7979.5201 6234.6199 312.0393 -1744.9001 6301.6264
310.4387 89.2308 66.3133 511540.4688 -10.0396
-10.0413
[26125]ENERGY: 16980000 3301.1518 5332.2090 5437.5378
323.9899 -14404.6967 -1698.5538 0.0000 0.0000
7948.6112 6240.2492 310.8306 -1708.3621 6302.0107
310.4445 121.4248 51.3894 511540.4688 -7.4428
-7.4539
[26175]ENERGY: 16990000 3271.1209 5407.6064 5481.7220
322.5949 -14438.4221 -1698.6954 0.0000 0.0000
7893.2726 6239.1992 308.6665 -1654.0734 6303.2190
310.3957 185.4130 87.5445 511540.4688 -11.5080
-11.5036
[26214]ENERGY: 17000000 3288.7340 5373.4413 5427.1143
317.2906 -14437.9819 -1677.8347 0.0000 0.0000
7947.0013 6237.7648 310.7676 -1709.2365 6302.0276
310.4427 -94.4568 -83.0060 511540.4688 -8.5626
-8.5442
[26264]ENERGY: 17010000 3204.2656 5281.2234 5427.6022
310.1218 -14319.8394 -1736.5081 0.0000 0.0000
8069.8585 6236.7241 315.5719 -1833.1344 6301.4926
310.5140 175.7288 164.0338 511540.4688 -6.5222
-6.5262
[26300]ENERGY: 17020000 3257.9892 5376.8637 5461.7094
319.3894 -14422.2801 -1712.1906 0.0000 0.0000
7955.8076 6237.2886 311.1120 -1718.5190 6302.6714
310.5107 74.4817 36.3756 511540.4688 -10.6234
-10.6263
[26350]ENERGY: 17030000 3352.4341 5422.3101 5446.3455
329.3428 -14465.2538 -1808.3767 0.0000 0.0000
7958.5355 6235.3375 311.2187 -1723.1979 6301.8059
310.5792 -217.8080 -229.2440 511540.4688 -10.0537
-10.0964

[26386]ENERGY: 17040000 3298.4325 5266.8903 5490.2580
329.1856 -14322.5308 -1737.6231 0.0000 0.0000
7913.3098 6237.9223 309.4501 -1675.3875 6301.1615
310.4297 -23.0561 -80.0694 511540.4688 -11.8877
-11.8447
[26436]ENERGY: 17050000 3180.2836 5418.9243 5483.1127
306.8388 -14436.8395 -1686.9481 0.0000 0.0000
7973.5497 6238.9216 311.8058 -1734.6281 6301.3329
310.3892 41.5147 56.4952 511540.4688 -10.8974
-10.8913
[26472]ENERGY: 17060000 3295.0908 5304.9753 5476.4705
319.8623 -14354.8423 -1712.4895 0.0000 0.0000
7908.3788 6237.4458 309.2573 -1670.9329 6301.1896
310.4801 100.6945 6.1871 511540.4688 -9.9069
-9.9197
[26522]ENERGY: 17070000 3288.8626 5431.9730 5438.1130
317.4790 -14450.5339 -1758.4587 0.0000 0.0000
7972.1038 6239.5387 311.7492 -1732.5651 6301.6440
310.4366 -13.4736 -141.4579 511540.4688 -11.2686
-11.2553
[26558]ENERGY: 17080000 3285.9983 5337.0206 5473.7347
300.6975 -14398.6896 -1709.1006 0.0000 0.0000
7948.9860 6238.6470 310.8452 -1710.3390 6300.6186
310.7236 159.7408 201.9556 511540.4688 -11.7050
-11.7109
[26608]ENERGY: 17090000 3244.6234 5479.8611 5460.6762
333.6443 -14461.0237 -1701.3637 0.0000 0.0000
7882.3741 6238.7918 308.2404 -1643.5823 6301.3829
310.4539 103.7106 108.5297 511540.4688 -10.8289
-10.8413
[26647]ENERGY: 17100000 3264.0039 5379.2392 5491.1860
332.7930 -14389.3960 -1775.6104 0.0000 0.0000
7931.8018 6234.0174 310.1732 -1697.7844 6301.2257
310.4471 8.3252 -35.1015 511540.4688 -12.8358
-12.8188
[26697]ENERGY: 17110000 3224.0836 5343.1867 5469.0866
340.7729 -14363.7686 -1721.8452 0.0000 0.0000
7945.9617 6237.4778 310.7270 -1708.4839 6301.1936
310.5480 119.0481 130.1151 511540.4688 -9.4795
-9.5139
[26733]ENERGY: 17120000 3185.0652 5369.6853 5467.9915
316.3293 -14390.5185 -1714.1708 0.0000 0.0000
8001.6458 6236.0278 312.9045 -1765.6180 6300.8656
310.4257 -103.2194 -117.6752 511540.4688 -12.9198
-12.9108
[26783]ENERGY: 17130000 3230.5805 5389.9253 5434.4145
349.0170 -14349.2834 -1697.2736 0.0000 0.0000
7879.3939 6236.7742 308.1238 -1642.6197 6300.4765
310.3672 33.3362 -72.9660 511540.4688 -7.6543
-7.6317
[26819]ENERGY: 17140000 3237.5331 5326.0988 5463.8429
342.1573 -14394.9695 -1733.6846 0.0000 0.0000
7994.5424 6235.5204 312.6267 -1759.0220 6300.8495
310.3230 220.3730 48.5208 511540.4688 -9.3406

-9.3580
[26869]ENERGY: 17150000 3338.2043 5342.2230 5386.3179
323.4700 -14372.5149 -1779.6457 0.0000 0.0000
7994.0950 6232.1495 312.6092 -1761.9455 6299.2231
310.4263 75.8779 -12.1682 511540.4688 -10.4718
-10.4608
[26905]ENERGY: 17160000 3296.5412 5293.7840 5486.0083
319.8065 -14337.6249 -1693.9295 0.0000 0.0000
7875.1063 6239.6920 307.9562 -1635.4143 6299.4257
310.5625 134.6670 24.9002 511540.4688 -10.6543
-10.6713
[26955]ENERGY: 17170000 3282.6519 5303.7775 5454.7648
329.6333 -14367.3288 -1712.9781 0.0000 0.0000
7945.2163 6235.7368 310.6978 -1709.4795 6301.0256
310.3185 -96.2281 -39.7950 511540.4688 -11.4402
-11.4297
[26991]ENERGY: 17180000 3317.5729 5342.8548 5446.0039
342.2818 -14349.6056 -1766.2065 0.0000 0.0000
7904.3115 6237.2128 309.0982 -1667.0987 6300.2359
310.4691 -300.8976 -267.5973 511540.4688 -10.1025
-10.1072
[27041]ENERGY: 17190000 3274.2504 5410.5301 5450.2761
315.5901 -14348.8304 -1783.1248 0.0000 0.0000
7919.8275 6238.5190 309.7050 -1681.3085 6301.8610
310.3777 -69.3431 -159.2384 511540.4688 -7.4884
-7.4611
[27080]ENERGY: 17200000 3278.1590 5435.6373 5488.7804
328.9866 -14421.9652 -1766.5324 0.0000 0.0000
7892.4789 6235.5447 308.6355 -1656.9343 6300.7127
310.4006 -5.7985 85.3973 511540.4688 -10.4828
-10.5025
[27130]ENERGY: 17210000 3215.8583 5425.2528 5431.4778
319.9564 -14354.6508 -1745.5523 0.0000 0.0000
7944.1671 6236.5094 310.6568 -1707.6578 6301.7186
310.5815 121.8166 26.2590 511540.4688 -10.4453
-10.4592
[27166]ENERGY: 17220000 3339.0482 5415.9890 5415.6636
321.1687 -14432.5040 -1702.8415 0.0000 0.0000
7882.5483 6239.0722 308.2472 -1643.4761 6301.1760
310.4199 43.0967 -3.0240 511540.4688 -9.7946
-9.7847
[27216]ENERGY: 17230000 3300.1288 5350.1663 5500.7845
348.8034 -14461.6099 -1716.7673 0.0000 0.0000
7918.9542 6240.4599 309.6708 -1678.4943 6300.0233
310.4978 58.9951 -10.7863 511540.4688 -11.5033
-11.5176
[27252]ENERGY: 17240000 3273.5723 5293.2883 5512.1139
331.9499 -14393.8871 -1718.6839 0.0000 0.0000
7936.8846 6235.2379 310.3720 -1701.6466 6301.0529
310.4276 -41.8764 -127.2451 511540.4688 -6.1363
-6.1275
[27302]ENERGY: 17250000 3243.2172 5325.0274 5423.7172
318.8255 -14370.0099 -1691.3168 0.0000 0.0000
7988.0634 6237.5239 312.3733 -1750.5395 6301.4711

310.4677 167.6669 101.4408 511540.4688 -8.4963
-8.4859
[27338]ENERGY: 17260000 3258.1703 5284.2126 5457.0858
320.5339 -14362.8926 -1729.6271 0.0000 0.0000
8007.1546 6234.6374 313.1199 -1772.5172 6300.7347
310.1503 -12.5408 -57.8735 511540.4688 -2.5930
-2.5964
[27388]ENERGY: 17270000 3311.3444 5230.4137 5492.8185
360.3649 -14376.8442 -1705.7529 0.0000 0.0000
7920.6775 6233.0218 309.7382 -1687.6556 6301.6054
310.2620 -5.7570 -33.2007 511540.4688 -7.0990
-7.0793
[27424]ENERGY: 17280000 3247.4702 5408.9621 5444.1369
325.5614 -14383.0347 -1778.5309 0.0000 0.0000
7971.5709 6236.1360 311.7284 -1735.4349 6300.8527
310.2090 -234.2527 -92.5168 511540.4688 -8.7295
-8.7408
[27474]ENERGY: 17290000 3176.7823 5384.5801 5486.2808
335.5242 -14379.1158 -1712.6654 0.0000 0.0000
7946.2431 6237.6293 310.7380 -1708.6138 6300.0203
310.2505 14.0184 -122.5981 511540.4688 -3.8075
-3.8046
[27513]ENERGY: 17300000 3235.0367 5435.5696 5472.6277
335.5056 -14356.8005 -1743.0861 0.0000 0.0000
7857.8148 6236.6677 307.2800 -1621.1471 6300.5743
310.2150 85.3652 0.3397 511540.4688 -9.3354
-9.3662
[27563]ENERGY: 17310000 3240.3135 5361.2660 5478.7765
315.2919 -14359.6838 -1797.6026 0.0000 0.0000
7996.8795 6235.2409 312.7181 -1761.6386 6300.7950
310.2899 -40.3556 33.6345 511540.4688 -8.8316
-8.8103
[27599]ENERGY: 17320000 3265.2978 5383.2949 5445.4770
335.5600 -14422.7732 -1685.9049 0.0000 0.0000
7918.8743 6239.8258 309.6677 -1679.0485 6302.1440
310.5551 246.9575 194.6944 511540.4688 -1.9411
-1.9503
[27649]ENERGY: 17330000 3181.1966 5423.3237 5430.6734
334.3497 -14363.5036 -1735.4675 0.0000 0.0000
7966.6122 6237.1846 311.5345 -1729.4276 6302.6555
310.4790 -2.7493 82.1238 511540.4688 -4.6363
-4.5950
[27685]ENERGY: 17340000 3262.4012 5463.6322 5451.4216
326.8239 -14384.6313 -1770.7381 0.0000 0.0000
7893.7868 6242.6963 308.6867 -1651.0905 6304.0421
310.4590 105.5493 -92.9463 511540.4688 -6.7963
-6.8624
[27735]ENERGY: 17350000 3259.8421 5259.5005 5499.2836
322.0326 -14374.0667 -1740.4112 0.0000 0.0000
8012.6023 6238.7833 313.3329 -1773.8191 6301.9201
310.5138 272.6125 175.1538 511540.4688 -2.7710
-2.7393
[27771]ENERGY: 17360000 3281.3616 5345.4594 5499.7755
336.5330 -14493.1767 -1713.1124 0.0000 0.0000

7977.8244 6234.6648 311.9729 -1743.1596 6302.9811
310.5285 137.9663 66.2612 511540.4688 -7.0741
-7.0743
[27821]ENERGY: 17370000 3297.3049 5266.1979 5501.8483
314.8481 -14429.2581 -1733.1708 0.0000 0.0000
8021.1941 6238.9645 313.6689 -1782.2296 6303.9877
310.4579 -53.0293 -32.2177 511540.4688 -10.7626
-10.7628
[27857]ENERGY: 17380000 3277.3756 5341.4577 5473.6379
326.5594 -14348.9416 -1784.4768 0.0000 0.0000
7953.4064 6239.0186 311.0181 -1714.3878 6303.7591
310.4077 -209.3542 -137.8337 511540.4688 -6.5160
-6.4897
[27907]ENERGY: 17390000 3266.3653 5371.9430 5445.3714
336.5151 -14421.2496 -1705.6020 0.0000 0.0000
7946.0498 6239.3929 310.7304 -1706.6569 6302.8721
310.4761 79.7812 51.8523 511540.4688 -6.2625
-6.2607
[27946]ENERGY: 17400000 3384.2412 5351.6510 5446.8178
330.7234 -14410.0075 -1740.3118 0.0000 0.0000
7873.1011 6236.2152 307.8777 -1636.8859 6302.4540
310.4254 -3.3063 -45.5278 511540.4688 -3.6603
-3.6993
[27996]ENERGY: 17410000 3259.0484 5374.8613 5463.7863
313.4040 -14436.1771 -1725.9835 0.0000 0.0000
7988.6434 6237.5828 312.3960 -1751.0606 6303.6738
310.4365 -50.4188 -31.5645 511540.4688 -6.1274
-6.1192
[28032]ENERGY: 17420000 3355.9935 5321.1221 5489.9738
325.1295 -14431.6998 -1769.1304 0.0000 0.0000
7952.0488 6243.4376 310.9650 -1708.6112 6304.2934
310.4073 -120.7079 -21.4461 511540.4688 -6.4156
-6.3923
[28082]ENERGY: 17430000 3251.7065 5265.3001 5468.7694
359.0941 -14380.7477 -1696.0602 0.0000 0.0000
7971.3192 6239.3814 311.7186 -1731.9378 6303.1248
310.4618 202.4102 180.9605 511540.4688 -5.1472
-5.1467
[28118]ENERGY: 17440000 3305.4707 5351.2128 5510.5200
304.7434 -14395.3728 -1708.7372 0.0000 0.0000
7878.1268 6245.9636 308.0743 -1632.1631 6305.3943
310.4649 171.9607 82.8846 511540.4688 -6.0469
-6.0774
[28168]ENERGY: 17450000 3282.3391 5500.2756 5426.3871
330.3893 -14479.2864 -1728.3117 0.0000 0.0000
7908.5948 6240.3879 309.2657 -1668.2069 6305.4497
310.4647 19.5064 46.9214 511540.4688 -6.6166
-6.6122
[28204]ENERGY: 17460000 3220.5441 5376.2809 5453.0147
323.6677 -14366.6248 -1662.1263 0.0000 0.0000
7894.8972 6239.6535 308.7301 -1655.2437 6304.5217
310.3706 34.9072 129.8070 511540.4688 -3.9776
-3.9591
[28254]ENERGY: 17470000 3195.4576 5343.5118 5476.3486

335.5189 -14399.4051 -1636.0592 0.0000 0.0000
7927.4835 6242.8561 310.0044 -1684.6274 6306.2962
310.6554 170.1925 117.2427 511540.4688 -6.4111
-6.4260
[28290]ENERGY: 17480000 3313.2618 5384.1215 5460.5354
328.6562 -14437.1822 -1735.1938 0.0000 0.0000
7929.3202 6243.5190 310.0762 -1685.8012 6306.6528
310.5374 91.4980 8.0601 511540.4688 -9.7490
-9.7326
[28340]ENERGY: 17490000 3217.7447 5324.4729 5499.4093
330.7929 -14485.3753 -1657.4295 0.0000 0.0000
8010.0914 6239.7063 313.2347 -1770.3850 6307.1349
310.6802 150.4069 88.1488 511540.4688 -2.8038
-2.8152
[28379]ENERGY: 17500000 3343.2164 5389.8903 5477.9356
324.6240 -14350.3639 -1837.2185 0.0000 0.0000
7897.2719 6245.3558 308.8229 -1651.9160 6308.3849
310.6132 -70.0394 -66.1144 511540.4688 -7.7557
-7.7544
[28429]ENERGY: 17510000 3286.5170 5438.8946 5378.7870
309.4462 -14324.1490 -1776.1306 0.0000 0.0000
7929.9240 6243.2892 310.0998 -1686.6348 6308.4071
310.6392 -66.1399 -122.3541 511540.4688 -2.1194
-2.1151
[28465]ENERGY: 17520000 3263.3188 5437.5144 5458.1033
325.3581 -14432.7483 -1659.7599 0.0000 0.0000
7852.2476 6244.0340 307.0623 -1608.2136 6305.3159
310.7495 -19.8581 22.5873 511540.4688 -4.3510
-4.3470
[28515]ENERGY: 17530000 3247.5647 5368.0216 5458.8363
318.5455 -14381.5207 -1739.6721 0.0000 0.0000
7970.3933 6242.1686 311.6824 -1728.2247 6306.1673
310.8424 -92.0118 -50.2210 511540.4688 -6.4308
-6.4378
[28551]ENERGY: 17540000 3325.3270 5332.3904 5472.2772
331.4233 -14373.7262 -1782.8059 0.0000 0.0000
7936.3688 6241.2547 310.3518 -1695.1142 6307.0757
310.5992 -197.2693 -188.9700 511540.4688 -4.1936
-4.2154
[28601]ENERGY: 17550000 3338.3490 5374.5998 5507.8610
335.4658 -14380.8105 -1809.5143 0.0000 0.0000
7881.7073 6247.6582 308.2143 -1634.0491 6307.9271
310.8120 -166.6271 -39.6030 511540.4688 -8.0229
-7.9989
[28637]ENERGY: 17560000 3206.0436 5379.0302 5480.7016
328.2833 -14423.2298 -1706.3188 0.0000 0.0000
7977.1503 6241.6605 311.9466 -1735.4898 6307.3599
310.7228 161.8871 -28.2571 511540.4688 -0.9962
-1.0288
[28687]ENERGY: 17570000 3259.8015 5430.4677 5413.7190
294.6411 -14379.3682 -1756.8438 0.0000 0.0000
7983.1900 6245.6074 312.1828 -1737.5826 6309.3081
310.7338 4.6263 -90.1318 511540.4688 -3.6208
-3.6107

[28723]ENERGY: 17580000 3239.0492 5403.5173 5434.4889
341.8754 -14388.4603 -1771.4954 0.0000 0.0000
7982.9372 6241.9122 312.1729 -1741.0250 6307.7662
310.6428 -112.8563 -139.3795 511540.4688 -5.4050
-5.3905
[28773]ENERGY: 17590000 3238.8232 5414.0373 5472.7469
328.5773 -14369.4833 -1724.9399 0.0000 0.0000
7882.5876 6242.3491 308.2487 -1640.2385 6307.7050
310.4778 69.7871 90.7356 511540.4688 -4.5056
-4.5070
[28812]ENERGY: 17600000 3300.5926 5345.4588 5435.9102
336.2407 -14415.9057 -1754.0948 0.0000 0.0000
7995.6962 6243.8981 312.6718 -1751.7981 6307.1369
310.6592 45.2167 -43.5464 511540.4688 2.8025
2.7949
[28862]ENERGY: 17610000 3237.8805 5346.0676 5493.0947
337.5098 -14376.9294 -1715.8212 0.0000 0.0000
7921.0765 6242.8785 309.7538 -1678.1980 6307.1783
310.8716 122.5489 41.7077 511540.4688 -10.1802
-10.1833
[28898]ENERGY: 17620000 3254.8818 5378.8748 5478.7379
302.9955 -14372.8579 -1680.8844 0.0000 0.0000
7881.3681 6243.1159 308.2010 -1638.2522 6307.5633
310.6261 60.8097 -66.2180 511540.4688 -6.0108
-6.0113
[28948]ENERGY: 17630000 3299.7135 5443.8042 5492.7856
338.6658 -14478.7350 -1725.0870 0.0000 0.0000
7877.4178 6248.5650 308.0465 -1628.8528 6307.2534
310.6390 -63.1710 4.2946 511540.4688 -6.7946
-6.7806
[28984]ENERGY: 17640000 3246.5164 5357.2464 5424.6146
326.0940 -14281.1826 -1747.3368 0.0000 0.0000
7916.7710 6242.7230 309.5855 -1674.0480 6306.9269
310.6767 -62.1785 -14.0874 511540.4688 -7.1128
-7.0970
[29034]ENERGY: 17650000 3247.7216 5331.0500 5441.0620
337.0254 -14302.2526 -1735.0340 0.0000 0.0000
7925.9413 6245.5137 309.9441 -1680.4276 6308.2369
310.8865 72.0538 18.1306 511540.4688 -5.2045
-5.1986
[29070]ENERGY: 17660000 3246.1951 5348.6110 5453.1366
323.1318 -14249.0768 -1768.4897 0.0000 0.0000
7891.9594 6245.4674 308.6152 -1646.4920 6308.7442
310.5493 90.8258 86.1621 511540.4688 -9.7079
-9.7309
[29120]ENERGY: 17670000 3234.1894 5254.6080 5493.7758
319.6994 -14341.2578 -1726.7916 0.0000 0.0000
8008.4089 6242.6321 313.1690 -1765.7768 6307.5862
310.6243 188.3238 198.4238 511540.4688 -10.3354
-10.3395
[29156]ENERGY: 17680000 3246.1073 5338.5362 5457.3934
311.7495 -14340.9846 -1709.1269 0.0000 0.0000
7939.4540 6243.1290 310.4725 -1696.3250 6308.2725
310.6761 52.5248 45.4195 511540.4688 -11.3076

-11.2942
[29206]ENERGY: 17690000 3279.7342 5377.6428 5425.2527
329.0051 -14443.7692 -1719.8376 0.0000 0.0000
7996.3266 6244.3546 312.6965 -1751.9720 6307.6695
310.8384 201.5016 90.2702 511540.4688 -8.7105
-8.7287
[29245]ENERGY: 17700000 3296.3942 5420.8969 5437.1647
324.2139 -14368.5947 -1843.8196 0.0000 0.0000
7976.7828 6243.0382 311.9322 -1733.7446 6307.2394
310.8335 -185.4794 -152.9722 511540.4688 -9.7775
-9.7699
[29295]ENERGY: 17710000 3276.3384 5414.2527 5420.0701
336.2511 -14422.2680 -1726.3129 0.0000 0.0000
7945.9416 6244.2730 310.7262 -1701.6686 6306.5006
310.7202 61.9923 54.2417 511540.4688 -14.1802
-14.1593
[29331]ENERGY: 17720000 3288.6761 5343.6005 5451.1234
343.7254 -14465.2537 -1671.7498 0.0000 0.0000
7954.5655 6244.6874 311.0634 -1709.8780 6308.0216
310.6193 162.6125 98.1463 511540.4688 -6.2902
-6.3182
[29381]ENERGY: 17730000 3274.8244 5328.7396 5476.1336
327.2065 -14383.6009 -1818.8688 0.0000 0.0000
8040.4561 6244.8905 314.4222 -1795.5656 6308.2294
310.6178 -123.1154 -46.3730 511540.4688 -13.2418
-13.2235
[29417]ENERGY: 17740000 3266.1789 5435.5650 5416.2199
318.8717 -14396.6748 -1746.3433 0.0000 0.0000
7951.8443 6245.6617 310.9570 -1706.1826 6308.9915
310.5510 -35.3545 5.0337 511540.4688 -10.9065
-10.9279
[29467]ENERGY: 17750000 3264.7231 5502.1730 5459.9355
322.7505 -14440.6458 -1780.2546 0.0000 0.0000
7911.9823 6240.6639 309.3982 -1671.3184 6307.9011
310.8657 204.2209 218.0472 511540.4688 -8.1707
-8.1534
[29503]ENERGY: 17760000 3350.8143 5311.0858 5429.0432
343.2368 -14358.9498 -1817.7951 0.0000 0.0000
7983.8965 6241.3317 312.2104 -1742.5648 6308.2000
310.6061 -34.0510 -186.0687 511540.4688 -11.5052
-11.5272
[29553]ENERGY: 17770000 3254.4491 5342.3802 5506.4652
324.0801 -14443.5162 -1713.2973 0.0000 0.0000
7973.7846 6244.3457 311.8150 -1729.4389 6307.3380
310.7968 81.1793 47.5368 511540.4688 -9.4631
-9.4358
[29589]ENERGY: 17780000 3285.2199 5519.2304 5465.8086
323.5792 -14519.5465 -1730.6451 0.0000 0.0000
7899.6876 6243.3341 308.9174 -1656.3535 6307.1828
310.8703 83.2179 58.0990 511540.4688 -10.0836
-10.1016
[29639]ENERGY: 17790000 3295.1468 5387.3043 5456.8564
321.3651 -14422.4290 -1700.8233 0.0000 0.0000
7905.8547 6243.2749 309.1586 -1662.5797 6307.3711

310.9056 36.9252 3.4193 511540.4688 -9.5500
-9.5390
[29678]ENERGY: 17800000 3253.0290 5322.7453 5479.3316
327.9417 -14369.4273 -1756.1474 0.0000 0.0000
7990.1179 6247.5908 312.4537 -1742.5271 6309.9108
310.9757 69.2065 32.2010 511540.4688 -5.5292
-5.5514
[29728]ENERGY: 17810000 3316.1874 5309.3515 5477.6935
322.5083 -14409.3734 -1655.6009 0.0000 0.0000
7887.4631 6248.2294 308.4394 -1639.2337 6309.2176
311.0317 47.0421 9.8780 511540.4688 -9.2963
-9.2786
[29764]ENERGY: 17820000 3303.1653 5414.1805 5464.7007
334.6155 -14417.0672 -1770.9411 0.0000 0.0000
7919.0145 6247.6682 309.6732 -1671.3462 6308.2146
310.9933 -169.1235 -117.5812 511540.4688 -5.8870
-5.8651
[29814]ENERGY: 17830000 3270.4303 5444.6427 5480.9814
328.1703 -14474.2285 -1827.7248 0.0000 0.0000
8018.6598 6240.9312 313.5698 -1777.7287 6307.5952
310.8975 -114.3095 -17.8803 511540.4688 -8.0403
-8.0304
[29850]ENERGY: 17840000 3301.7494 5483.3176 5431.3358
337.1259 -14324.7136 -1849.6843 0.0000 0.0000
7866.9860 6246.1168 307.6386 -1620.8693 6309.1792
310.8121 -177.7034 -196.1756 511540.4688 -5.6346
-5.6671
[29900]ENERGY: 17850000 3247.8985 5297.0849 5477.5944
351.9873 -14354.5409 -1769.0912 0.0000 0.0000
7991.2335 6242.1664 312.4973 -1749.0671 6309.5349
310.8239 41.5854 30.5501 511540.4688 -3.9084
-3.9223
[29936]ENERGY: 17860000 3331.3977 5344.3489 5515.0242
356.2766 -14432.5579 -1736.2573 0.0000 0.0000
7870.0731 6248.3052 307.7593 -1621.7679 6309.6050
310.6891 -165.2362 -67.6178 511540.4688 -8.5002
-8.4905
[29986]ENERGY: 17870000 3193.7196 5384.4276 5507.4024
340.0659 -14442.3419 -1747.7696 0.0000 0.0000
8008.5028 6244.0069 313.1726 -1764.4959 6309.7180
310.8339 167.4750 83.6670 511540.4688 -3.6930
-3.6718
[30022]ENERGY: 17880000 3218.4927 5320.2251 5516.4058
333.2996 -14418.1734 -1751.4680 0.0000 0.0000
8022.9124 6241.6942 313.7361 -1781.2182 6309.4148
310.7998 341.8186 205.8206 511540.4688 -10.2475
-10.2544
[30072]ENERGY: 17890000 3309.8527 5457.7443 5489.3097
348.0294 -14421.6448 -1832.2145 0.0000 0.0000
7897.7345 6248.8112 308.8410 -1648.9233 6310.5438
310.9627 -132.0716 -82.7171 511540.4688 -4.4126
-4.4224
[30111]ENERGY: 17900000 3229.4883 5306.3200 5502.1805
340.3892 -14379.6507 -1752.2494 0.0000 0.0000

7998.1796 6244.6575 312.7689 -1753.5221 6310.4635
310.9340 2.2786 -0.5472 511540.4688 -0.1646
-0.1540
[30161]ENERGY: 17910000 3244.6478 5451.3705 5494.8838
333.1268 -14439.9431 -1778.3189 0.0000 0.0000
7942.2275 6247.9944 310.5809 -1694.2331 6310.6447
311.0296 15.6088 -164.6960 511540.4688 -5.3901
-5.4035
[30197]ENERGY: 17920000 3274.5485 5453.3966 5460.4287
324.5337 -14393.7393 -1780.2976 0.0000 0.0000
7909.7566 6248.6272 309.3112 -1661.1294 6310.1570
311.0070 -2.9185 32.8287 511540.4688 -6.5598
-6.5476
[30247]ENERGY: 17930000 3296.3141 5414.1273 5463.1745
327.7741 -14390.8411 -1750.1653 0.0000 0.0000
7883.6403 6244.0239 308.2899 -1639.6163 6310.6764
310.9918 -11.2510 -123.1319 511540.4688 -8.3415
-8.3559
[30283]ENERGY: 17940000 3255.7543 5452.8727 5411.0942
328.9706 -14407.8733 -1781.8802 0.0000 0.0000
7988.0359 6246.9742 312.3723 -1741.0617 6311.0604
311.0828 68.7596 134.5346 511540.4688 -9.6653
-9.6486
[30333]ENERGY: 17950000 3317.5292 5357.4251 5454.6047
321.9476 -14426.3965 -1749.5783 0.0000 0.0000
7974.3792 6249.9110 311.8382 -1724.4682 6311.1347
311.1049 -224.9336 -141.1068 511540.4688 -3.6870
-3.7088
[30369]ENERGY: 17960000 3263.0188 5404.9037 5463.0511
315.0044 -14377.6602 -1743.2086 0.0000 0.0000
7923.9839 6249.0930 309.8675 -1674.8909 6311.7576
311.1321 228.6018 198.2453 511540.4688 -8.2294
-8.2295
[30419]ENERGY: 17970000 3341.7076 5352.9680 5511.1899
328.5897 -14435.5387 -1764.7788 0.0000 0.0000
7914.6999 6248.8376 309.5045 -1665.8622 6310.5257
311.0582 -47.8761 -110.2972 511540.4688 -7.6195
-7.6009
[30455]ENERGY: 17980000 3234.2928 5430.6268 5492.9601
322.3908 -14406.8480 -1762.4030 0.0000 0.0000
7936.9443 6247.9638 310.3743 -1688.9805 6311.9918
310.8426 -78.6418 -10.3184 511540.4688 -5.4412
-5.4386
[30505]ENERGY: 17990000 3277.0281 5380.7720 5505.4475
356.7793 -14495.0020 -1735.5776 0.0000 0.0000
7958.6139 6248.0612 311.2217 -1710.5527 6312.3252
311.0388 251.9196 130.4654 511540.4688 -6.3643
-6.3642
[30544]ENERGY: 18000000 3190.7451 5506.6251 5501.2052
330.6656 -14464.0164 -1706.7502 0.0000 0.0000
7892.4363 6250.9108 308.6338 -1641.5255 6312.0162
311.0533 272.2339 78.6677 511540.4688 -3.6207
-3.6314
[30594]ENERGY: 18010000 3282.7770 5386.7230 5461.9413

328.9208 -14365.5870 -1847.4222 0.0000 0.0000
8002.1176 6249.4705 312.9229 -1752.6472 6312.9993
311.0016 38.7110 -66.6810 511540.4688 -10.0638
-10.0558
[30630]ENERGY: 18020000 3286.4957 5478.1144 5456.9744
318.1460 -14539.3073 -1660.0980 0.0000 0.0000
7907.1398 6247.4651 309.2088 -1659.6748 6311.8551
310.8990 -22.8525 -39.6833 511540.4688 -7.7369
-7.7305
[30680]ENERGY: 18030000 3225.5459 5355.2747 5422.5141
338.3119 -14411.2440 -1633.2923 0.0000 0.0000
7950.6582 6247.7684 310.9106 -1702.8898 6312.9067
310.7406 257.9958 157.0609 511540.4688 -3.4515
-3.4519
[30716]ENERGY: 18040000 3270.6241 5337.6878 5478.2236
331.2534 -14426.5804 -1718.0980 0.0000 0.0000
7976.8285 6249.9389 311.9340 -1726.8896 6312.9903
310.7528 31.7160 75.8992 511540.4688 -3.3924
-3.4043
[30766]ENERGY: 18050000 3289.9209 5361.4647 5452.6223
308.4136 -14383.8297 -1767.9214 0.0000 0.0000
7989.0917 6249.7621 312.4136 -1739.3296 6312.7039
310.8221 -25.3770 -2.8757 511540.4688 -4.4780
-4.4870
[30802]ENERGY: 18060000 3192.7051 5507.3543 5526.1963
322.7807 -14415.8060 -1750.7909 0.0000 0.0000
7867.2658 6249.7053 307.6495 -1617.5605 6313.2053
310.9158 -72.5384 -130.7554 511540.4688 -3.4077
-3.4053
[30852]ENERGY: 18070000 3312.6213 5383.9141 5475.9932
303.6089 -14484.9309 -1669.8316 0.0000 0.0000
7928.1013 6249.4764 310.0285 -1678.6249 6312.7423
310.8999 -102.7433 -19.6329 511540.4688 -9.7604
-9.7191
[30888]ENERGY: 18080000 3274.5990 5416.9607 5480.6097
340.1343 -14408.8088 -1755.8600 0.0000 0.0000
7903.8454 6251.4803 309.0800 -1652.3651 6312.9705
310.8110 146.1825 53.5122 511540.4688 -5.5916
-5.6237
[30938]ENERGY: 18090000 3318.0000 5378.2167 5444.5137
336.4354 -14337.3014 -1770.2244 0.0000 0.0000
7879.6019 6249.2420 308.1320 -1630.3600 6312.7686
310.7751 93.2455 -87.7250 511540.4688 -6.7535
-6.7661
[30977]ENERGY: 18100000 3290.2640 5396.9985 5514.4391
307.3428 -14409.5526 -1763.2978 0.0000 0.0000
7914.4040 6250.5979 309.4929 -1663.8061 6312.2005
310.9835 59.9343 -2.9883 511540.4688 -7.2128
-7.2126
[31027]ENERGY: 18110000 3250.6788 5491.1682 5502.2086
328.1868 -14447.0151 -1730.3379 0.0000 0.0000
7855.6270 6250.5165 307.1944 -1605.1105 6312.9602
310.6164 79.2551 -12.6557 511540.4688 -7.8168
-7.8095

[31063]ENERGY: 18120000 3295.9586 5363.9107 5463.1851
342.7515 -14441.5648 -1739.0149 0.0000 0.0000
7962.9673 6248.1936 311.3920 -1714.7737 6311.8327
310.9088 70.4056 50.4533 511540.4688 -3.3497
-3.3413
[31113]ENERGY: 18130000 3223.9507 5435.4380 5487.2109
346.1296 -14537.1565 -1708.7375 0.0000 0.0000
8000.3368 6247.1720 312.8533 -1753.1649 6313.5934
310.9866 40.7330 31.8014 511540.4688 -7.6315
-7.6205
[31149]ENERGY: 18140000 3370.9701 5374.3057 5489.1175
334.7818 -14505.4834 -1701.9377 0.0000 0.0000
7885.4687 6247.2227 308.3614 -1638.2460 6313.9689
310.9140 4.9286 -37.2497 511540.4688 -9.9564
-9.9742
[31199]ENERGY: 18150000 3258.9331 5392.7507 5442.2412
323.0660 -14452.4722 -1720.0703 0.0000 0.0000
8003.2007 6247.6492 312.9653 -1755.5515 6313.3899
311.0335 127.6009 47.1193 511540.4688 -5.7512
-5.7551
[31235]ENERGY: 18160000 3411.3613 5306.1946 5481.6747
322.9800 -14447.0962 -1719.2731 0.0000 0.0000
7892.5625 6248.4038 308.6388 -1644.1587 6314.1501
310.9457 -47.2564 -91.6777 511540.4688 -10.4702
-10.4570
[31285]ENERGY: 18170000 3335.8405 5435.6508 5488.8011
317.6794 -14482.3109 -1735.6939 0.0000 0.0000
7889.9053 6249.8723 308.5349 -1640.0330 6314.1482
310.9892 45.6532 18.2497 511540.4688 -10.4104
-10.4091
[31321]ENERGY: 18180000 3216.2471 5468.8030 5525.7047
328.1832 -14540.7653 -1710.5580 0.0000 0.0000
7962.3537 6249.9685 311.3680 -1712.3852 6314.5025
310.9613 230.7678 62.3606 511540.4688 -6.9826
-7.0154
[31371]ENERGY: 18190000 3246.4608 5438.6017 5495.2380
341.4413 -14457.3819 -1741.4566 0.0000 0.0000
7927.5469 6250.4502 310.0068 -1677.0967 6315.2012
311.3717 103.7090 42.0840 511540.4688 -7.1909
-7.1531
[31410]ENERGY: 18200000 3397.2785 5332.1975 5478.5097
333.3534 -14466.5636 -1823.0607 0.0000 0.0000
7998.1230 6249.8377 312.7667 -1748.2852 6316.1886
311.5435 -130.8255 -138.8109 511540.4688 -6.4398
-6.4377
[31460]ENERGY: 18210000 3290.1056 5360.4219 5491.2116
327.6880 -14519.2938 -1709.0481 0.0000 0.0000
8006.4309 6247.5161 313.0916 -1758.9147 6314.9019
311.8660 328.3736 252.0112 511540.4688 -7.6452
-7.6722
[31496]ENERGY: 18220000 3306.0052 5434.0541 5487.2809
325.9785 -14444.8551 -1786.5282 0.0000 0.0000
7932.0273 6253.9626 310.1820 -1678.0646 6316.1818
311.9803 -152.5711 -113.3504 511540.4688 -7.3182

-7.3018
[31546]ENERGY: 18230000 3297.0557 5427.3677 5480.9010
331.1300 -14506.7856 -1761.8231 0.0000 0.0000
7987.1770 6255.0227 312.3387 -1732.1543 6315.9094
311.7926 144.9439 85.2507 511540.4688 -11.3638
-11.3595
[31582]ENERGY: 18240000 3304.1506 5465.2779 5465.5248
326.4395 -14571.3190 -1722.4277 0.0000 0.0000
7984.8472 6252.4934 312.2476 -1732.3539 6316.5745
311.9419 29.8304 21.2290 511540.4688 -4.6056
-4.6190
[31632]ENERGY: 18250000 3303.1447 5383.9702 5440.2456
314.5769 -14518.0021 -1731.6695 0.0000 0.0000
8056.9484 6249.2143 315.0671 -1807.7341 6316.3176
311.9156 134.2600 34.9411 511540.4688 -8.7157
-8.7040
[31668]ENERGY: 18260000 3292.8995 5408.7900 5483.1694
332.5478 -14575.0446 -1736.2016 0.0000 0.0000
8045.2587 6251.4192 314.6100 -1793.8396 6316.5835
311.7456 19.5420 106.0206 511540.4688 -6.8948
-6.8896
[31718]ENERGY: 18270000 3249.4755 5417.2229 5541.9042
307.3579 -14532.6666 -1748.0339 0.0000 0.0000
8017.5417 6252.8018 313.5261 -1764.7399 6317.6685
311.9646 57.4561 15.8252 511540.4688 -12.9402
-12.9310
[31754]ENERGY: 18280000 3312.4752 5391.1449 5476.7020
322.0442 -14428.7799 -1749.3498 0.0000 0.0000
7931.5944 6255.8310 310.1651 -1675.7634 6317.7708
311.7855 100.0614 17.0083 511540.4688 -2.3764
-2.4015
[31804]ENERGY: 18290000 3240.3370 5433.4425 5427.8606
309.7260 -14371.4586 -1797.5800 0.0000 0.0000
8013.0606 6255.3882 313.3509 -1757.6724 6318.2913
311.8648 28.6435 -24.8926 511540.4688 -8.7788
-8.7732
[31843]ENERGY: 18300000 3243.2078 5450.5653 5494.5317
304.6646 -14530.4505 -1729.6654 0.0000 0.0000
8021.8530 6254.7063 313.6947 -1767.1466 6318.3886
311.7970 222.9468 149.1392 511540.4688 -0.3546
-0.3509
[31893]ENERGY: 18310000 3334.9864 5406.8867 5451.3952
331.1387 -14447.8299 -1744.1848 0.0000 0.0000
7923.0214 6255.4137 309.8299 -1667.6076 6319.9555
311.8477 150.8570 63.1619 511540.4688 -7.0339
-7.0433
[31929]ENERGY: 18320000 3278.0740 5382.7417 5495.3124
345.7475 -14534.3972 -1701.2169 0.0000 0.0000
7990.4649 6256.7265 312.4673 -1733.7384 6317.4099
311.5853 -16.7281 -30.3410 511540.4688 -14.2536
-14.2471
[31979]ENERGY: 18330000 3280.5399 5394.4075 5536.6141
305.1122 -14496.6674 -1755.9877 0.0000 0.0000
7989.7939 6253.8126 312.4410 -1735.9813 6319.3633

311.8180 10.3902 -25.5231 511540.4688 -11.0529
-11.0788
[32015]ENERGY: 18340000 3230.5906 5439.6883 5462.0899
319.4904 -14486.0425 -1725.9762 0.0000 0.0000
8014.7551 6254.5956 313.4171 -1760.1594 6318.9818
312.1370 -9.4191 -19.7000 511540.4688 -15.1700
-15.1200
[32065]ENERGY: 18350000 3323.6301 5471.3785 5500.0191
324.9891 -14479.0500 -1740.2518 0.0000 0.0000
7855.6472 6256.3622 307.1952 -1599.2850 6320.3756
311.9892 -24.0456 -45.0583 511540.4688 -9.2356
-9.2753
[32101]ENERGY: 18360000 3303.2258 5416.4922 5516.4708
318.6180 -14560.4830 -1689.8714 0.0000 0.0000
7951.4885 6255.9409 310.9431 -1695.5476 6320.2607
311.9673 121.6292 33.9773 511540.4688 -16.0984
-16.0921
[32151]ENERGY: 18370000 3241.0494 5322.1254 5484.5598
326.2260 -14452.5759 -1740.8090 0.0000 0.0000
8072.9222 6253.4979 315.6917 -1819.4243 6319.0840
311.7833 248.4168 223.3756 511540.4688 -15.2955
-15.3096
[32187]ENERGY: 18380000 3265.1963 5417.0332 5430.3453
333.8546 -14401.4465 -1758.9445 0.0000 0.0000
7971.3536 6257.3919 311.7199 -1713.9617 6319.9429
311.8562 -12.0967 1.6883 511540.4688 -14.8738
-14.8492
[32237]ENERGY: 18390000 3354.7696 5425.0758 5523.5073
326.8490 -14563.6089 -1766.3774 0.0000 0.0000
7958.0782 6258.2935 311.2008 -1699.7846 6319.5058
311.8685 78.4428 41.2471 511540.4688 -17.0954
-17.1154
[32276]ENERGY: 18400000 3246.1676 5387.6965 5513.4931
347.6298 -14431.5703 -1795.1057 0.0000 0.0000
7988.4566 6256.7675 312.3887 -1731.6891 6320.1286
311.8973 -113.6601 -105.3620 511540.4688 -10.0643
-10.0526
[32326]ENERGY: 18410000 3240.8539 5418.4171 5520.6607
351.5913 -14437.2740 -1773.0603 0.0000 0.0000
7936.4363 6257.6250 310.3545 -1678.8113 6320.2302
312.0244 53.6274 -86.3821 511540.4688 -12.5405
-12.5608
[32362]ENERGY: 18420000 3254.2403 5392.8911 5459.6322
324.4460 -14532.8761 -1683.2196 0.0000 0.0000
8038.6666 6253.7805 314.3522 -1784.8861 6319.3076
312.0238 11.6919 -12.2564 511540.4688 -19.8663
-19.8445
[32412]ENERGY: 18430000 3271.5316 5386.1205 5528.6033
355.2885 -14449.8330 -1722.6147 0.0000 0.0000
7891.6632 6260.7594 308.6036 -1630.9038 6322.2155
312.1095 188.7172 126.9199 511540.4688 -17.4588
-17.4653
[32448]ENERGY: 18440000 3284.6558 5450.8084 5450.4128
315.1292 -14545.6301 -1723.9275 0.0000 0.0000

8023.5883 6255.0370 313.7625 -1768.5514 6320.9094
312.0462 86.0726 38.4626 511540.4688 -17.4394
-17.4216
[32498]ENERGY: 18450000 3325.6433 5462.9680 5476.9399
349.8683 -14577.0309 -1690.0774 0.0000 0.0000
7911.2322 6259.5435 309.3689 -1651.6888 6320.3672
312.1071 82.6869 94.1077 511540.4688 -18.8958
-18.9156
[32534]ENERGY: 18460000 3267.1374 5540.6109 5459.9851
311.8238 -14484.7226 -1738.1045 0.0000 0.0000
7898.2157 6254.9457 308.8598 -1643.2700 6319.5559
312.1041 -94.7104 25.5683 511540.4688 -18.0376
-18.0035
[32584]ENERGY: 18470000 3296.0525 5464.1304 5458.2702
329.9413 -14580.6850 -1724.5755 0.0000 0.0000
8013.8361 6256.9701 313.3812 -1756.8660 6321.3925
312.1842 64.4808 -56.7914 511540.4688 -16.3587
-16.3766
[32620]ENERGY: 18480000 3286.6908 5519.2391 5510.7265
352.6487 -14537.1787 -1730.2299 0.0000 0.0000
7859.5571 6261.4536 307.3481 -1598.1034 6322.7499
312.0921 252.8033 207.7496 511540.4688 -11.0551
-11.0615
[32670]ENERGY: 18490000 3314.0699 5359.6022 5502.3279
356.8719 -14532.5086 -1736.5169 0.0000 0.0000
7994.7043 6258.5508 312.6330 -1736.1534 6323.0210
311.9090 -24.4775 -112.1540 511540.4688 -7.1808
-7.1807
[32709]ENERGY: 18500000 3285.8031 5503.6242 5455.6287
320.2102 -14524.2291 -1706.1422 0.0000 0.0000
7925.6380 6260.5328 309.9322 -1665.1053 6322.9395
311.9685 227.2218 80.2517 511540.4688 -13.8046
-13.7921
[32759]ENERGY: 18510000 3349.1232 5386.3967 5509.1094
323.0495 -14470.3727 -1738.8090 0.0000 0.0000
7904.1284 6262.6256 309.0911 -1641.5028 6323.2271
311.9922 -52.1116 -72.4442 511540.4688 -18.6780
-18.6952
[32795]ENERGY: 18520000 3301.5931 5434.7619 5483.9777
331.3684 -14557.8848 -1702.7746 0.0000 0.0000
7969.2247 6260.2662 311.6367 -1708.9584 6322.2542
311.9415 58.5993 -37.9011 511540.4688 -9.5039
-9.4942
[32845]ENERGY: 18530000 3229.8815 5443.4161 5504.0022
322.1601 -14484.5010 -1654.4934 0.0000 0.0000
7902.1915 6262.6570 309.0153 -1639.5345 6322.4857
312.0298 55.2784 30.2285 511540.4688 -12.2221
-12.2275
[32881]ENERGY: 18540000 3348.1335 5431.7248 5503.6273
349.2924 -14526.4202 -1746.9508 0.0000 0.0000
7903.9179 6263.3248 309.0828 -1640.5930 6321.9134
311.8701 -27.3196 -139.5731 511540.4688 -11.6250
-11.6182
[32931]ENERGY: 18550000 3321.9662 5305.9279 5548.5364

334.9355 -14474.2378 -1785.8140 0.0000 0.0000
8002.4805 6253.7946 312.9371 -1748.6859 6322.8781
311.7140 -57.3410 -21.8566 511540.4688 -11.7341
-11.7171
[32967]ENERGY: 18560000 3328.1979 5440.4572 5542.7823
326.1248 -14501.3934 -1752.3621 0.0000 0.0000
7876.8012 6260.6079 308.0224 -1616.1933 6323.3688
311.7454 53.6021 20.7177 511540.4688 -13.4301
-13.4473
[33017]ENERGY: 18570000 3309.3930 5435.3979 5483.1643
331.3634 -14477.6898 -1785.0656 0.0000 0.0000
7964.7741 6261.3374 311.4626 -1703.4367 6324.7429
311.8679 -79.1768 -204.8586 511540.4688 -13.1247
-13.1369
[33053]ENERGY: 18580000 3275.4819 5493.3126 5529.0520
344.2885 -14532.8090 -1769.0282 0.0000 0.0000
7921.5366 6261.8345 309.7718 -1659.7021 6324.5917
311.9364 30.8134 121.6444 511540.4688 -10.0472
-10.0186
[33103]ENERGY: 18590000 3264.2713 5442.3716 5476.0951
313.9900 -14447.5690 -1680.5395 0.0000 0.0000
7898.0564 6266.6758 308.8536 -1631.3806 6326.2968
311.8736 330.1584 176.9410 511540.4688 -9.3140
-9.3536
[33142]ENERGY: 18600000 3314.9490 5421.0806 5482.6750
351.6953 -14485.0075 -1756.1024 0.0000 0.0000
7933.5100 6262.8000 310.2400 -1670.7100 6326.2293
311.9713 26.1118 -16.7763 511540.4688 -13.7760
-13.7554
[33192]ENERGY: 18610000 3356.4244 5370.5876 5499.7653
321.8150 -14596.1771 -1694.8050 0.0000 0.0000
8004.4377 6262.0479 313.0137 -1742.3897 6327.0318
312.1300 165.6787 105.1525 511540.4688 -10.1663
-10.1506
[33228]ENERGY: 18620000 3275.1276 5435.5014 5517.2182
328.9026 -14466.4170 -1764.9608 0.0000 0.0000
7939.4134 6264.7853 310.4709 -1674.6280 6326.8269
312.0269 -149.4775 -189.1304 511540.4688 -16.9283
-16.9361
[33278]ENERGY: 18630000 3222.4654 5468.1431 5490.2342
356.7478 -14475.8372 -1734.3821 0.0000 0.0000
7932.6068 6259.9780 310.2047 -1672.6289 6326.9727
312.1501 -197.9371 -144.6106 511540.4688 -7.2567
-7.2726
[33314]ENERGY: 18640000 3255.1627 5415.2510 5507.6781
315.0119 -14496.4542 -1748.5468 0.0000 0.0000
8014.8635 6262.9662 313.4214 -1751.8973 6326.8415
312.0168 132.8885 210.7379 511540.4688 -8.1646
-8.1381
[33364]ENERGY: 18650000 3267.7107 5492.3699 5512.5530
294.7125 -14485.3818 -1715.0312 0.0000 0.0000
7897.7132 6264.6462 308.8402 -1633.0670 6327.3859
312.0498 -176.6486 -111.5959 511540.4688 -7.8238
-7.8395

[33400]ENERGY: 18660000 3289.6014 5364.2160 5493.7293
323.6721 -14458.1323 -1693.1278 0.0000 0.0000
7945.3211 6265.2798 310.7019 -1680.0413 6329.1416
312.1255 222.6781 78.7672 511540.4688 -13.1080
-13.1205
[33450]ENERGY: 18670000 3337.5417 5474.6365 5448.6568
325.4520 -14464.3104 -1753.6810 0.0000 0.0000
7895.6013 6263.8969 308.7576 -1631.7045 6329.7151
312.2643 -1.0859 -42.3998 511540.4688 -9.5510
-9.5494
[33486]ENERGY: 18680000 3307.4175 5440.8675 5555.3208
348.8607 -14555.3172 -1756.8638 0.0000 0.0000
7925.1477 6265.4331 309.9130 -1659.7146 6330.1332
312.2139 -93.4994 -52.0744 511540.4688 -12.7346
-12.7157
[33536]ENERGY: 18690000 3383.6633 5466.0000 5461.2365
335.1010 -14495.3374 -1867.0358 0.0000 0.0000
7983.4455 6267.0731 312.1928 -1716.3724 6330.6310
312.2070 -248.6163 -141.3744 511540.4688 -16.8272
-16.8510
[33575]ENERGY: 18700000 3236.3238 5452.0824 5509.5267
307.7933 -14464.4287 -1740.6714 0.0000 0.0000
7963.6134 6264.2394 311.4172 -1699.3740 6330.2745
311.9917 -91.5087 -6.6490 511540.4688 -15.7680
-15.7389
[33625]ENERGY: 18710000 3276.3447 5403.8274 5493.7062
317.7361 -14448.8949 -1707.8005 0.0000 0.0000
7931.3286 6266.2476 310.1547 -1665.0809 6329.8619
311.9907 -39.9556 85.4800 511540.4688 -20.2215
-20.2464
[33661]ENERGY: 18720000 3379.8916 5365.2583 5535.2437
314.7474 -14470.0027 -1725.9782 0.0000 0.0000
7871.2116 6270.3717 307.8039 -1600.8400 6331.9270
312.3124 7.2275 -49.4284 511540.4688 -13.4567
-13.4603
[33711]ENERGY: 18730000 3327.4042 5440.2042 5477.6198
338.6304 -14525.2509 -1739.3608 0.0000 0.0000
7947.7970 6267.0439 310.7987 -1680.7531 6332.9161
312.0917 -143.9895 -111.9702 511540.4688 -12.9066
-12.8939
[33747]ENERGY: 18740000 3392.7898 5377.1261 5486.0327
337.9579 -14488.6710 -1723.7781 0.0000 0.0000
7884.2371 6265.6945 308.3132 -1618.5426 6331.8226
312.3346 -130.5871 -135.1653 511540.4688 -14.3325
-14.3308
[33797]ENERGY: 18750000 3229.7147 5411.4327 5484.0848
322.6467 -14467.5642 -1689.2672 0.0000 0.0000
7974.7223 6265.7698 311.8516 -1708.9525 6331.1896
312.3674 190.1629 223.1561 511540.4688 -18.4058
-18.4095
[33833]ENERGY: 18760000 3311.2294 5429.1393 5498.5577
350.8217 -14490.4674 -1711.8427 0.0000 0.0000
7880.1762 6267.6142 308.1544 -1612.5619 6332.3634
312.2676 21.8402 16.1952 511540.4688 -18.0856

-18.0785
[33883]ENERGY: 18770000 3272.9222 5473.4882 5500.7209
320.1075 -14519.9949 -1697.0905 0.0000 0.0000
7917.9765 6268.1298 309.6326 -1649.8466 6332.7725
312.2704 121.4018 112.5330 511540.4688 -17.1218
-17.1322
[33919]ENERGY: 18780000 3336.9937 5398.6715 5507.7274
334.6612 -14522.5467 -1761.2580 0.0000 0.0000
7976.0172 6270.2664 311.9023 -1705.7508 6333.1824
312.2771 -130.1232 -85.3133 511540.4688 -19.6694
-19.6642
[33969]ENERGY: 18790000 3387.8205 5323.1921 5549.3542
317.8420 -14434.5030 -1789.0760 0.0000 0.0000
7916.2794 6270.9093 309.5662 -1645.3702 6332.8715
312.2650 -66.3661 -3.4344 511540.4688 -22.1600
-22.1753
[34008]ENERGY: 18800000 3300.6542 5410.6652 5520.0817
334.0939 -14522.2243 -1756.0511 0.0000 0.0000
7982.5196 6269.7393 312.1566 -1712.7803 6334.2656
312.1162 249.2711 85.5895 511540.4688 -20.0442
-20.0503
[34058]ENERGY: 18810000 3322.8879 5402.6057 5528.0371
349.1598 -14549.3843 -1739.8565 0.0000 0.0000
7955.5983 6269.0480 311.1038 -1686.5503 6334.1506
312.4232 -70.3095 -31.4614 511540.4688 -12.7266
-12.6986
[34094]ENERGY: 18820000 3252.7004 5387.9346 5439.6396
339.0220 -14466.3865 -1715.1158 0.0000 0.0000
8026.6470 6264.4413 313.8822 -1762.2058 6334.2089
312.3595 59.4637 30.2790 511540.4688 -14.1813
-14.2092
[34144]ENERGY: 18830000 3168.7049 5436.0552 5540.2695
321.7423 -14516.5917 -1765.5532 0.0000 0.0000
8081.1403 6265.7673 316.0131 -1815.3731 6333.1580
312.2746 90.5604 -10.2896 511540.4688 -17.0542
-17.0304
[34180]ENERGY: 18840000 3385.7213 5363.5231 5472.1361
323.3332 -14444.9507 -1763.4313 0.0000 0.0000
7934.8591 6271.1908 310.2928 -1663.6683 6334.0306
312.1446 -216.2525 -218.5611 511540.4688 -18.7896
-18.8140
[34230]ENERGY: 18850000 3383.1852 5396.1860 5540.2586
313.3268 -14488.2836 -1796.5351 0.0000 0.0000
7920.2586 6268.3966 309.7218 -1651.8620 6332.9382
312.2004 135.3300 14.2495 511540.4688 -20.2072
-20.1959
[34266]ENERGY: 18860000 3276.0861 5344.0877 5556.0710
335.8007 -14482.1994 -1769.6518 0.0000 0.0000
8010.4636 6270.6578 313.2493 -1739.8058 6333.5835
312.2981 184.3535 84.2982 511540.4688 -19.5533
-19.5478
[34316]ENERGY: 18870000 3247.0719 5336.9670 5572.0648
338.0869 -14493.5466 -1726.9514 0.0000 0.0000
7994.3126 6268.0053 312.6177 -1726.3073 6332.4542

312.1806 63.4643 -27.7155 511540.4688 -20.5050
-20.4967
[34352]ENERGY: 18880000 3349.4856 5389.7401 5531.8747
336.9949 -14467.8272 -1769.0192 0.0000 0.0000
7898.4840 6269.7330 308.8703 -1628.7510 6334.9028
312.0694 -177.8308 -217.4994 511540.4688 -17.7860
-17.7868
[34402]ENERGY: 18890000 3314.8275 5398.0998 5507.4354
330.8006 -14517.7155 -1706.6314 0.0000 0.0000
7944.8271 6271.6434 310.6826 -1673.1837 6334.3093
312.1490 -24.4877 -47.9010 511540.4688 -21.1121
-21.1218
[34441]ENERGY: 18900000 3260.1243 5396.0528 5489.4613
325.3588 -14447.4814 -1689.2987 0.0000 0.0000
7937.9600 6272.1770 310.4140 -1665.7829 6334.7909
312.3181 256.5011 163.0970 511540.4688 -17.4115
-17.4181
[34491]ENERGY: 18910000 3344.5252 5416.7596 5502.0865
329.7433 -14610.7322 -1668.2433 0.0000 0.0000
7959.7057 6273.8448 311.2644 -1685.8609 6334.9986
312.3533 -117.5563 -31.4535 511540.4688 -21.7656
-21.7351
[34527]ENERGY: 18920000 3344.7187 5443.3365 5494.1947
334.0727 -14456.0703 -1838.9931 0.0000 0.0000
7949.8174 6271.0766 310.8777 -1678.7408 6335.8594
312.2391 -148.0041 -191.5330 511540.4688 -22.0837
-22.1112
[34577]ENERGY: 18930000 3231.4800 5477.5196 5487.7107
328.4751 -14471.7076 -1704.7187 0.0000 0.0000
7922.0975 6270.8566 309.7937 -1651.2409 6335.2631
312.3879 -22.0956 -6.4288 511540.4688 -20.9113
-20.9172
[34613]ENERGY: 18940000 3247.1533 5437.4851 5545.3065
309.8454 -14430.0396 -1845.2133 0.0000 0.0000
8009.8512 6274.3888 313.2254 -1735.4625 6336.4537
312.2421 7.9768 -50.5525 511540.4688 -19.9235
-19.8925
[34663]ENERGY: 18950000 3234.9575 5477.8173 5510.3801
322.3012 -14515.2345 -1750.9915 0.0000 0.0000
7993.2562 6272.4864 312.5764 -1720.7699 6335.5767
312.1272 -27.1162 55.8385 511540.4688 -23.6063
-23.6131
[34699]ENERGY: 18960000 3242.7329 5463.2380 5547.9801
345.6511 -14471.4478 -1803.2353 0.0000 0.0000
7945.7880 6270.7068 310.7202 -1675.0812 6335.3250
312.2218 -115.6354 -34.0118 511540.4688 -21.4912
-21.5065
[34749]ENERGY: 18970000 3251.9201 5284.6807 5496.6905
316.7344 -14389.8253 -1739.9554 0.0000 0.0000
8051.7656 6272.0105 314.8644 -1779.7551 6335.0989
312.3708 52.0235 0.0231 511540.4688 -18.8790
-18.8928
[34785]ENERGY: 18980000 3176.6010 5433.9569 5491.6161
331.6690 -14464.0532 -1713.8148 0.0000 0.0000

8012.0649 6268.0397 313.3119 -1744.0252 6336.2619
312.5193 179.6567 86.9394 511540.4688 -22.2691
-22.2510
[34835]ENERGY: 18990000 3331.9179 5348.3810 5512.8696
298.6427 -14520.6415 -1766.2938 0.0000 0.0000
8067.3147 6272.1906 315.4725 -1795.1241 6336.0425
312.5596 -93.2068 -148.3865 511540.4688 -16.4056
-16.4242
[34874]ENERGY: 19000000 3250.4510 5405.9051 5484.2569
336.2402 -14475.4244 -1776.7465 0.0000 0.0000
8043.7611 6268.4434 314.5514 -1775.3178 6335.1531
312.5550 -254.7963 -291.0894 511540.4688 -20.7468
-20.7303
[34924]ENERGY: 19010000 3260.0126 5461.7772 5517.5418
324.4290 -14426.2545 -1825.0488 0.0000 0.0000
7957.9998 6270.4571 311.1977 -1687.5428 6337.0646
312.6140 -410.2070 -384.6288 511540.4688 -20.6324
-20.6136
[34960]ENERGY: 19020000 3278.9052 5399.7841 5508.9083
328.7105 -14501.9152 -1698.5989 0.0000 0.0000
7958.7504 6274.5443 311.2271 -1684.2061 6336.9167
312.4681 170.6390 123.3560 511540.4688 -18.7951
-18.8087
[35010]ENERGY: 19030000 3190.2842 5407.2336 5486.0245
337.8895 -14479.3795 -1692.0580 0.0000 0.0000
8022.8839 6272.8781 313.7350 -1750.0058 6337.4062
312.4488 3.9521 -99.6136 511540.4688 -18.2055
-18.2266
[35046]ENERGY: 19040000 3310.5741 5393.1002 5493.2010
320.0869 -14408.9866 -1801.9503 0.0000 0.0000
7970.0939 6276.1194 311.6706 -1693.9746 6337.3985
312.5428 39.7018 -29.8419 511540.4688 -22.2858
-22.2589
[35096]ENERGY: 19050000 3327.8088 5363.5714 5465.1123
333.0725 -14494.8916 -1762.8174 0.0000 0.0000
8037.2052 6269.0612 314.2950 -1768.1440 6337.0850
312.2224 -53.4578 -131.2234 511540.4688 -20.6470
-20.6517
[35132]ENERGY: 19060000 3383.8658 5465.8617 5544.8223
338.2031 -14550.2857 -1741.6023 0.0000 0.0000
7834.9047 6275.7696 306.3841 -1559.1351 6338.0512
312.3099 -0.9688 -29.5567 511540.4688 -18.3999
-18.3883
[35182]ENERGY: 19070000 3300.2157 5478.6759 5452.1207
343.6547 -14640.2079 -1675.9864 0.0000 0.0000
8014.2453 6272.7179 313.3972 -1741.5274 6338.3764
312.3352 -20.3547 -42.2013 511540.4688 -21.3707
-21.3939
[35218]ENERGY: 19080000 3224.6383 5446.8103 5511.6612
310.6846 -14536.2201 -1747.1220 0.0000 0.0000
8057.6635 6268.1158 315.0951 -1789.5476 6338.1065
312.5427 -71.1166 -96.6608 511540.4688 -21.6930
-21.6866
[35268]ENERGY: 19090000 3258.5656 5356.8183 5505.1667

344.2663 -14470.8303 -1830.8428 0.0000 0.0000
8107.8514 6270.9951 317.0577 -1836.8563 6339.0937
312.4627 30.7370 -103.9081 511540.4688 -18.8676
-18.8711
[35307]ENERGY: 19100000 3285.2216 5406.6664 5495.9281
332.2512 -14532.1444 -1699.8313 0.0000 0.0000
7987.1356 6275.2271 312.3371 -1711.9085 6337.5467
312.6424 -23.4119 -104.1394 511540.4688 -19.1777
-19.1782
[35357]ENERGY: 19110000 3310.1551 5393.2824 5453.9517
324.2313 -14475.8343 -1752.6666 0.0000 0.0000
8020.2679 6273.3875 313.6327 -1746.8804 6337.5729
312.8486 115.2574 106.7532 511540.4688 -14.6338
-14.6206
[35393]ENERGY: 19120000 3269.9669 5509.1494 5508.4959
315.3390 -14569.8164 -1761.8654 0.0000 0.0000
7999.4551 6270.7245 312.8188 -1728.7305 6337.8377
312.8248 -1.0040 -42.0199 511540.4688 -16.8913
-16.9005
[35443]ENERGY: 19130000 3319.2888 5414.3417 5513.4509
327.1060 -14536.3483 -1720.1248 0.0000 0.0000
7955.6355 6273.3498 311.1052 -1682.2857 6337.7849
312.6041 57.7096 -0.0736 511540.4688 -14.2012
-14.2309
[35479]ENERGY: 19140000 3320.9864 5414.8829 5463.9028
340.7973 -14536.8718 -1723.1770 0.0000 0.0000
7989.6236 6270.1443 312.4344 -1719.4793 6336.6359
312.8575 -46.8984 -92.9387 511540.4688 -18.4385
-18.4098
[35529]ENERGY: 19150000 3317.5662 5415.9019 5470.9024
326.8647 -14412.3710 -1831.7030 0.0000 0.0000
7986.6973 6273.8585 312.3199 -1712.8388 6338.1298
312.9933 -46.7131 -46.2283 511540.4688 -14.8481
-14.8340
[35565]ENERGY: 19160000 3295.4401 5485.4140 5504.1747
326.3021 -14543.8931 -1784.5077 0.0000 0.0000
7987.6554 6270.5854 312.3574 -1717.0700 6338.3036
313.0096 -13.6331 -42.8763 511540.4688 -17.3365
-17.3482
[35615]ENERGY: 19170000 3286.1840 5469.2213 5453.3297
300.4907 -14546.3872 -1735.8293 0.0000 0.0000
8047.3191 6274.3283 314.6905 -1772.9909 6338.7535
312.8406 -75.3601 -23.9111 511540.4688 -11.7398
-11.7430
[35651]ENERGY: 19180000 3280.9257 5385.3026 5514.2518
348.9234 -14496.6187 -1811.3083 0.0000 0.0000
8049.3713 6270.8478 314.7708 -1778.5236 6339.0625
312.8447 -123.5408 -68.2527 511540.4688 -13.7958
-13.7870
[35701]ENERGY: 19190000 3345.3521 5473.0541 5465.1502
309.8840 -14495.6702 -1769.0646 0.0000 0.0000
7946.7115 6275.4171 310.7563 -1671.2943 6339.0165
312.9412 -1.7196 -34.5368 511540.4688 -14.5623
-14.5782

[35740]ENERGY: 19200000 3341.5141 5402.9324 5492.6058
320.4917 -14479.6236 -1741.5915 0.0000 0.0000
7937.7475 6274.0763 310.4057 -1663.6711 6337.4883
312.8803 -162.7575 -182.7522 511540.4688 -18.9457
-18.9215
[35790]ENERGY: 19210000 3282.6366 5360.8115 5492.5449
299.2371 -14567.9441 -1707.5146 0.0000 0.0000
8109.3374 6269.1088 317.1158 -1840.2286 6338.0381
312.7799 -51.3278 -48.1277 511540.4688 -17.4216
-17.4366
[35826]ENERGY: 19220000 3326.0238 5370.7298 5508.5460
332.5398 -14458.6253 -1743.6311 0.0000 0.0000
7940.1728 6275.7558 310.5006 -1664.4170 6338.6540
312.6775 26.4004 -94.4379 511540.4688 -16.7134
-16.7372
[35876]ENERGY: 19230000 3339.3736 5453.5273 5508.4472
333.9255 -14574.1933 -1802.8322 0.0000 0.0000
8013.1774 6271.4254 313.3554 -1741.7520 6338.8165
312.7808 -200.1752 -167.1522 511540.4688 -18.5824
-18.5478
[35912]ENERGY: 19240000 3269.0404 5410.3016 5506.1748
323.8922 -14552.3058 -1679.4255 0.0000 0.0000
7997.0369 6274.7145 312.7243 -1722.3224 6338.4729
312.7226 -68.2972 37.5808 511540.4688 -9.7050
-9.7173
[35962]ENERGY: 19250000 3333.0101 5345.6749 5484.7862
323.8367 -14506.0168 -1750.8338 0.0000 0.0000
8039.9667 6270.4240 314.4030 -1769.5427 6338.8086
312.8209 21.8593 30.1964 511540.4688 -14.1613
-14.1558
[35998]ENERGY: 19260000 3375.8352 5411.9104 5501.9801
335.0428 -14596.0127 -1734.7252 0.0000 0.0000
7980.1609 6274.1915 312.0643 -1705.9695 6339.8169
313.0045 -34.3261 -13.1655 511540.4688 -13.9898
-13.9847
[36048]ENERGY: 19270000 3343.4393 5395.0218 5482.5191
325.3715 -14470.8491 -1725.3778 0.0000 0.0000
7927.9510 6278.0757 310.0226 -1649.8753 6339.1959
312.8578 130.4134 142.5603 511540.4688 -15.5969
-15.6006
[36084]ENERGY: 19280000 3296.6757 5446.7291 5500.0555
299.6604 -14545.7611 -1748.5692 0.0000 0.0000
8026.2323 6275.0226 313.8659 -1751.2097 6339.8640
312.7840 226.4179 121.9703 511540.4688 -9.7207
-9.7125
[36134]ENERGY: 19290000 3342.6976 5372.4630 5524.1263
332.5330 -14517.9308 -1731.7985 0.0000 0.0000
7951.1946 6273.2854 310.9316 -1677.9093 6339.5232
312.6577 24.6212 -64.5170 511540.4688 -12.6222
-12.6245
[36173]ENERGY: 19300000 3333.7441 5423.4904 5461.5852
330.7218 -14439.0358 -1834.3634 0.0000 0.0000
7999.3359 6275.4781 312.8142 -1723.8578 6339.5051
312.7751 -10.0955 -46.9603 511540.4688 -12.1207

-12.1309
[36223]ENERGY: 19310000 3264.9610 5421.5025 5518.4652
348.5467 -14503.9808 -1766.3870 0.0000 0.0000
7991.3151 6274.4227 312.5005 -1716.8925 6338.9466
312.9235 147.4654 9.3542 511540.4688 -11.6335
-11.6239
[36259]ENERGY: 19320000 3287.8844 5453.0378 5478.4270
347.8641 -14442.9818 -1789.1449 0.0000 0.0000
7940.2044 6275.2912 310.5018 -1664.9132 6339.8177
312.9197 -86.7478 -115.2956 511540.4688 -11.8022
-11.8066
[36309]ENERGY: 19330000 3293.7475 5446.8718 5508.2985
317.0487 -14442.0883 -1832.2266 0.0000 0.0000
7984.5541 6276.2057 312.2361 -1708.3483 6340.0932
312.8600 8.9809 37.5778 511540.4688 -11.2054
-11.1874
[36345]ENERGY: 19340000 3287.4152 5345.7780 5466.0066
343.1691 -14404.8606 -1804.9094 0.0000 0.0000
8045.0251 6277.6240 314.6008 -1767.4012 6340.8964
312.7051 -84.4511 -137.2181 511540.4688 -11.6904
-11.7277
[36395]ENERGY: 19350000 3264.2644 5468.5521 5542.1054
333.5265 -14445.1103 -1836.6800 0.0000 0.0000
7948.2340 6274.8921 310.8158 -1673.3419 6340.8616
312.8275 -105.8341 -185.9291 511540.4688 -7.1298
-7.1232
[36431]ENERGY: 19360000 3311.2950 5495.1603 5516.5584
345.2803 -14549.7583 -1792.0271 0.0000 0.0000
7952.3552 6278.8638 310.9770 -1673.4914 6340.0898
312.7551 3.8139 19.6182 511540.4688 -12.3899
-12.3712
[36481]ENERGY: 19370000 3291.2395 5481.7436 5503.0360
337.5004 -14506.2885 -1750.4408 0.0000 0.0000
7920.0557 6276.8458 309.7139 -1643.2099 6338.9824
312.8272 94.2892 -25.7765 511540.4688 -9.4712
-9.4870
[36517]ENERGY: 19380000 3254.4463 5425.4166 5527.7950
315.0160 -14482.8394 -1760.5890 0.0000 0.0000
7992.7540 6271.9996 312.5568 -1720.7544 6338.7023
312.8753 -9.6635 12.8805 511540.4688 -9.4028
-9.3957
[36567]ENERGY: 19390000 3308.3246 5458.2366 5538.1910
329.4144 -14474.1177 -1812.1399 0.0000 0.0000
7927.9288 6275.8378 310.0218 -1652.0910 6339.6038
312.9941 58.7034 -10.4027 511540.4688 -11.4577
-11.4619
[36606]ENERGY: 19400000 3265.0125 5384.1508 5463.9895
328.9893 -14519.9494 -1671.4903 0.0000 0.0000
8024.1485 6274.8509 313.7844 -1749.2976 6338.3818
312.8980 87.6009 104.9093 511540.4688 -12.8287
-12.8205
[36656]ENERGY: 19410000 3269.9944 5355.3531 5504.5322
315.8511 -14428.8816 -1772.2602 0.0000 0.0000
8031.0048 6275.5938 314.0526 -1755.4110 6339.1324

312.9531 -52.8102 -81.9747 511540.4688 -8.9147
-8.9215
[36692]ENERGY: 19420000 3292.4797 5422.5678 5533.6384
336.9204 -14577.5244 -1734.4771 0.0000 0.0000
7999.5951 6273.2000 312.8243 -1726.3951 6338.3677
313.0721 178.1092 91.4844 511540.4688 -12.3061
-12.3109
[36742]ENERGY: 19430000 3257.6723 5408.6551 5553.3443
328.7334 -14464.2475 -1794.3038 0.0000 0.0000
7984.3721 6274.2258 312.2290 -1710.1462 6339.4018
312.9542 16.6739 -37.9264 511540.4688 -15.5364
-15.5334
[36778]ENERGY: 19440000 3278.3168 5445.2260 5444.6232
330.4331 -14482.9052 -1761.5596 0.0000 0.0000
8019.0117 6273.1461 313.5836 -1745.8656 6338.5735
312.9320 235.2552 88.4667 511540.4688 -3.9250
-3.9424
[36828]ENERGY: 19450000 3305.5838 5434.9055 5469.4610
323.3534 -14459.7540 -1884.5335 0.0000 0.0000
8080.2421 6269.2582 315.9780 -1810.9838 6340.6740
312.8231 -49.0039 55.6601 511540.4688 -15.4818
-15.4400
[36864]ENERGY: 19460000 3299.0995 5362.0352 5515.6678
325.9355 -14510.1254 -1757.9370 0.0000 0.0000
8033.6617 6268.3375 314.1565 -1765.3242 6339.6900
312.8640 135.1028 49.4972 511540.4688 -10.3258
-10.3545
[36914]ENERGY: 19470000 3294.7329 5388.4475 5470.4718
315.0857 -14446.2864 -1816.0245 0.0000 0.0000
8067.4503 6273.8773 315.4778 -1793.5729 6340.3094
312.8173 -159.1642 -100.3429 511540.4688 -10.0495
-10.0519
[36950]ENERGY: 19480000 3259.7491 5366.9096 5479.9594
348.4955 -14485.4961 -1708.2623 0.0000 0.0000
8017.5071 6278.8623 313.5247 -1738.6448 6340.6586
312.9205 207.9216 98.2389 511540.4688 -7.6425
-7.6440
[37000]ENERGY: 19490000 3185.6887 5350.8683 5543.1174
313.1165 -14463.1966 -1683.8201 0.0000 0.0000
8029.8663 6275.6404 314.0080 -1754.2259 6339.7554
313.0316 142.6191 56.8887 511540.4688 -7.8236
-7.8223
[37039]ENERGY: 19500000 3321.2303 5338.9123 5502.1106
332.9028 -14513.9372 -1765.2776 0.0000 0.0000
8061.1092 6277.0505 315.2298 -1784.0587 6340.1752
312.9971 15.3384 16.3890 511540.4688 -9.1771
-9.1627
[37089]ENERGY: 19510000 3295.0835 5403.1678 5484.9515
305.5352 -14560.8932 -1735.3245 0.0000 0.0000
8083.3646 6275.8849 316.1001 -1807.4798 6340.6190
313.1131 75.4226 54.2235 511540.4688 -8.9108
-8.9035
[37125]ENERGY: 19520000 3320.1213 5397.8866 5471.2717
323.5609 -14468.1176 -1690.8394 0.0000 0.0000

7926.8907 6280.7741 309.9812 -1646.1166 6341.3411
313.2459 65.2881 103.8221 511540.4688 -7.1934
-7.1861
[37175]ENERGY: 19530000 3252.2219 5458.9779 5474.0781
341.9569 -14500.3528 -1732.6398 0.0000 0.0000
7983.2681 6277.5103 312.1858 -1705.7578 6341.4114
313.0831 -80.0249 6.1189 511540.4688 -9.1222
-9.1232
[37211]ENERGY: 19540000 3228.3590 5368.9013 5462.2040
329.3424 -14504.1121 -1680.9370 0.0000 0.0000
8072.9480 6276.7054 315.6928 -1796.2425 6342.1887
313.3083 183.5685 97.3307 511540.4688 -7.0938
-7.1073
[37261]ENERGY: 19550000 3265.3876 5469.6448 5507.9257
354.9694 -14542.0434 -1721.1135 0.0000 0.0000
7940.1205 6274.8911 310.4985 -1665.2294 6340.8932
313.2251 14.4080 -42.3275 511540.4688 -12.2419
-12.2440
[37297]ENERGY: 19560000 3253.0213 5369.2434 5486.6397
322.2446 -14539.6352 -1705.3244 0.0000 0.0000
8087.2557 6273.4451 316.2523 -1813.8106 6341.1394
313.3217 146.0244 76.6321 511540.4688 -4.8415
-4.8442
[37347]ENERGY: 19570000 3282.2877 5425.8070 5447.8982
308.1144 -14484.5238 -1776.0490 0.0000 0.0000
8069.2759 6272.8104 315.5492 -1796.4654 6341.0537
313.2716 2.8819 -13.8681 511540.4688 -12.0853
-12.0709
[37383]ENERGY: 19580000 3307.7981 5397.8395 5424.0188
312.6772 -14508.1914 -1712.7565 0.0000 0.0000
8054.0620 6275.4476 314.9542 -1778.6144 6341.3242
313.4193 -43.3858 112.2257 511540.4688 -5.5303
-5.5406
[37433]ENERGY: 19590000 3340.9030 5409.3134 5490.8849
338.1472 -14534.2995 -1785.3002 0.0000 0.0000
8018.8342 6278.4830 313.5766 -1740.3511 6341.6916
313.2856 -71.5379 -63.0084 511540.4688 -9.6151
-9.6090
[37472]ENERGY: 19600000 3208.5520 5418.6733 5452.4167
319.0451 -14481.2244 -1703.6432 0.0000 0.0000
8061.7131 6275.5326 315.2534 -1786.1806 6342.3217
313.2211 176.7461 37.4657 511540.4688 -6.7871
-6.8152
[37522]ENERGY: 19610000 3273.8003 5396.6360 5503.4644
331.6778 -14457.5284 -1770.0999 0.0000 0.0000
8000.1768 6278.1270 312.8470 -1722.0498 6341.9634
313.3381 -142.5698 -250.0814 511540.4688 -9.1928
-9.1817
[37558]ENERGY: 19620000 3265.5740 5394.6030 5463.4709
300.0607 -14438.6004 -1816.6591 0.0000 0.0000
8105.2823 6273.7314 316.9572 -1831.5509 6341.3679
313.3908 -203.9543 -109.4073 511540.4688 -11.6472
-11.6379
[37608]ENERGY: 19630000 3309.0410 5415.3600 5488.3405

343.9572 -14508.1345 -1772.1928 0.0000 0.0000
8002.8416 6279.2129 312.9512 -1723.6286 6342.1939
313.2801 61.9989 -46.1482 511540.4688 -6.7869
-6.7884
[37644]ENERGY: 19640000 3328.3277 5454.4764 5452.1852
316.4285 -14539.4635 -1750.7490 0.0000 0.0000
8012.4200 6273.6253 313.3258 -1738.7946 6341.6209
313.3107 -112.8846 -63.6989 511540.4688 -6.2525
-6.2573
[37694]ENERGY: 19650000 3220.1929 5413.9695 5494.0053
325.2934 -14452.1462 -1714.3431 0.0000 0.0000
7991.8874 6278.8591 312.5229 -1713.0283 6341.9837
313.2836 -128.4037 -83.3408 511540.4688 -6.4967
-6.4715
[37730]ENERGY: 19660000 3299.7982 5430.6115 5501.7664
353.4649 -14546.4381 -1786.2099 0.0000 0.0000
8022.7062 6275.6993 313.7280 -1747.0069 6342.6070
313.2248 -93.6826 -144.3482 511540.4688 -8.9828
-8.9743
[37780]ENERGY: 19670000 3316.6900 5408.8159 5439.7758
327.3549 -14436.0237 -1800.8149 0.0000 0.0000
8019.8083 6275.6062 313.6147 -1744.2021 6341.1835
313.1927 -31.9553 -18.5514 511540.4688 -6.9663
-6.9872
[37816]ENERGY: 19680000 3397.1901 5393.4828 5507.6585
310.4354 -14468.1638 -1732.9303 0.0000 0.0000
7872.5536 6280.2262 307.8563 -1592.3273 6341.8812
313.0741 33.5031 111.0854 511540.4688 -9.3689
-9.3771
[37866]ENERGY: 19690000 3314.2520 5449.7816 5434.2014
326.6309 -14583.7120 -1692.5991 0.0000 0.0000
8028.7474 6277.3024 313.9643 -1751.4451 6342.7849
313.2677 186.9702 111.5709 511540.4688 -6.7132
-6.7177
[37905]ENERGY: 19700000 3267.5534 5430.0676 5439.7517
334.8203 -14436.9195 -1729.9296 0.0000 0.0000
7970.8348 6276.1786 311.6996 -1694.6562 6342.2699
313.1153 -168.7134 -109.2283 511540.4688 -10.1901
-10.1764
[37955]ENERGY: 19710000 3344.4597 5411.5363 5508.1089
348.4748 -14495.1944 -1734.3484 0.0000 0.0000
7898.0193 6281.0561 308.8522 -1616.9632 6342.6847
313.1594 74.1125 46.0778 511540.4688 -10.6648
-10.6740
[37991]ENERGY: 19720000 3359.8772 5409.0731 5494.3497
341.3469 -14443.7701 -1736.2423 0.0000 0.0000
7856.4216 6281.0562 307.2255 -1575.3655 6344.3555
313.1275 11.3069 45.5886 511540.4688 -7.5492
-7.5590
[38041]ENERGY: 19730000 3251.6429 5448.4910 5481.9240
327.5517 -14468.5051 -1760.1379 0.0000 0.0000
7998.9021 6279.8687 312.7972 -1719.0335 6343.6197
313.2718 -55.2248 -13.6354 511540.4688 -7.5870
-7.5771

[38077]ENERGY: 19740000 3290.6718 5428.6461 5479.2757
327.5151 -14539.6491 -1685.9273 0.0000 0.0000
7978.5512 6279.0837 312.0014 -1699.4675 6344.5301
313.0439 322.0595 197.4161 511540.4688 -10.8676
-10.8841
[38127]ENERGY: 19750000 3421.4653 5386.1328 5495.1431
320.6825 -14598.5357 -1755.6100 0.0000 0.0000
8010.7060 6279.9839 313.2588 -1730.7220 6344.3236
313.3769 -7.1669 -43.8233 511540.4688 -11.2035
-11.1927
[38163]ENERGY: 19760000 3348.0350 5433.9049 5495.9519
313.0079 -14541.5506 -1732.3790 0.0000 0.0000
7964.6465 6281.6167 311.4576 -1683.0298 6343.6020
313.3155 134.1117 1.6592 511540.4688 -7.7567
-7.7706
[38213]ENERGY: 19770000 3339.4812 5434.1579 5432.6414
348.4336 -14448.5044 -1700.8032 0.0000 0.0000
7878.4242 6283.8306 308.0859 -1594.5935 6343.4468
313.2486 53.0417 -33.0350 511540.4688 -9.8980
-9.9047
[38249]ENERGY: 19780000 3298.3313 5361.3762 5430.9579
319.5976 -14527.8544 -1699.3204 0.0000 0.0000
8094.9284 6278.0166 316.5523 -1816.9118 6343.4458
313.1707 116.4069 66.0934 511540.4688 -9.7597
-9.7344
[38299]ENERGY: 19790000 3321.9172 5392.7372 5478.8182
330.8436 -14523.3532 -1727.4559 0.0000 0.0000
8006.5084 6280.0155 313.0946 -1726.4929 6345.7256
313.3934 -14.4298 -3.1936 511540.4688 -9.3508
-9.3486
[38338]ENERGY: 19800000 3240.0579 5425.0041 5471.6620
325.7426 -14492.9452 -1734.8176 0.0000 0.0000
8048.0622 6282.7659 314.7196 -1765.2962 6346.4086
313.1742 -31.9915 -57.2793 511540.4688 -11.5986
-11.6003
[38388]ENERGY: 19810000 3278.8474 5475.9397 5480.1033
333.7345 -14476.7739 -1744.6109 0.0000 0.0000
7940.8516 6288.0918 310.5271 -1652.7598 6347.3323
313.1133 132.5600 50.7229 511540.4688 -8.1969
-8.1925
[38424]ENERGY: 19820000 3266.5447 5362.0415 5437.5639
322.1917 -14399.4708 -1725.6651 0.0000 0.0000
8020.3856 6283.5914 313.6373 -1736.7941 6346.7359
313.1311 290.2345 177.7850 511540.4688 -12.2070
-12.2241
[38474]ENERGY: 19830000 3262.3069 5434.6990 5452.1268
331.7101 -14540.0720 -1667.7448 0.0000 0.0000
8009.0751 6282.1011 313.1950 -1726.9739 6347.1150
313.0322 105.3354 144.7432 511540.4688 -8.7169
-8.6908
[38510]ENERGY: 19840000 3234.0132 5431.6690 5392.1474
315.6961 -14477.4749 -1698.6708 0.0000 0.0000
8085.6295 6283.0095 316.1887 -1802.6200 6346.8627
313.1057 33.1267 1.0263 511540.4688 -9.6398

```
-9.6730
[38560]ENERGY: 19850000      3266.3101      5422.9977      5457.6613
313.4761         -14522.4486     -1760.6811         0.0000         0.0000
8099.3563          6276.6719       316.7255     -1822.6845      6347.3462
312.8957           -192.3175      -202.4579    511540.4688        -9.3761
-9.3535
[38596]ENERGY: 19860000      3363.4815      5385.3501      5467.4367
330.6980         -14501.1324     -1757.1668         0.0000         0.0000
7995.9053          6284.5724       312.6800     -1711.3329      6349.1180
313.2722            -20.3090       -74.9329    511540.4688        -5.9894
-5.9785
[38646]ENERGY: 19870000      3291.2985      5360.9779      5512.6636
357.4143         -14480.7152     -1741.0189         0.0000         0.0000
7984.6796          6285.3000       312.2410     -1699.3796      6347.8297
313.1938            -76.5125       -48.4054    511540.4688        -7.4797
-7.4769
[38682]ENERGY: 19880000      3338.1678      5426.2753      5515.4684
306.3684         -14551.4526     -1749.6305         0.0000         0.0000
7997.4645          6282.6612       312.7410     -1714.8033      6347.6565
313.0909             54.4506        10.7777    511540.4688        -7.8549
-7.8884
[38732]ENERGY: 19890000      3334.0258      5354.1621      5531.8007
321.5773         -14594.4372     -1691.7416         0.0000         0.0000
8024.8074          6280.1944       313.8102     -1744.6130      6347.4223
312.9846           -203.5945      -149.5055    511540.4688        -7.3394
-7.3254
[38771]ENERGY: 19900000      3327.1174      5472.4450      5439.9215
337.6913         -14486.5507     -1773.1535         0.0000         0.0000
7969.9983          6287.4692       311.6669     -1682.5291      6348.0169
313.2179             -6.7699       -13.3770    511540.4688        -7.3749
-7.3853
[38821]ENERGY: 19910000      3366.8001      5366.1406      5542.8974
322.1939         -14536.3866     -1761.4172         0.0000         0.0000
7984.2135          6284.4418       312.2228     -1699.7717      6347.0100
313.3263           -173.8696        -5.0099    511540.4688        -4.3410
-4.3268
[38857]ENERGY: 19920000      3264.1718      5366.2550      5482.6732
325.2803         -14501.1949     -1675.7892         0.0000         0.0000
8021.4319          6282.8282       313.6782     -1738.6037      6346.5448
313.2303            162.1848       212.0061    511540.4688        -5.6638
-5.6780
[38907]ENERGY: 19930000      3239.5623      5365.8819      5534.5929
346.0860         -14529.9213     -1743.3731         0.0000         0.0000
8067.2587          6280.0874       315.4703     -1787.1713      6346.4171
313.2006            -15.1980       -82.3019    511540.4688        -9.9478
-9.9501
[38943]ENERGY: 19940000      3295.9541      5373.9427      5477.1966
333.0617         -14529.5821     -1751.9804         0.0000         0.0000
8084.6088          6283.2014       316.1488     -1801.4074      6348.7690
313.3016            -10.8421        25.1344    511540.4688       -12.0348
-12.0201
[38993]ENERGY: 19950000      3283.1376      5460.4273      5489.0294
323.6608         -14554.4299     -1733.0587         0.0000         0.0000
8011.0544          6279.8209       313.2724     -1731.2335      6347.9608
```

```
313.3701              9.9561       -11.1576    511540.4688        -8.0893
-8.0950
[39029]ENERGY: 19960000      3298.1547      5414.1389      5492.9488
319.0960          -14514.6728     -1790.0019         0.0000         0.0000
8062.8552            6282.5189       315.2981     -1780.3363      6348.2417
313.3849             25.3539       -54.7394    511540.4688       -10.4924
-10.4827
[39079]ENERGY: 19970000      3273.7679      5409.6799      5475.8623
334.9780          -14423.9228     -1842.3267         0.0000         0.0000
8053.2784            6281.3171       314.9236     -1771.9613      6346.9244
313.3732           -209.4287      -141.8078    511540.4688        -8.3008
-8.3110
[39115]ENERGY: 19980000      3335.5807      5437.4711      5454.4191
330.7873          -14425.2756     -1846.5869         0.0000         0.0000
8000.3306            6286.7264       312.8531     -1713.6042      6347.3191
313.3555            -43.5071       -84.1041    511540.4688        -9.2114
-9.1993
[39165]ENERGY: 19990000      3350.1422      5392.3097      5491.6825
325.0803          -14479.3541     -1753.8957         0.0000         0.0000
7954.5387            6280.5036       311.0624     -1674.0351      6346.8094
313.2877              2.1243       -31.2519    511540.4688       -11.0988
-11.1120
[39204]ENERGY: 20000000      3352.8457      5374.7234      5461.8607
331.4245          -14437.3686     -1778.3423         0.0000         0.0000
7981.8801            6287.0235       312.1315     -1694.8566      6347.0740
313.0697              1.4789      -128.9643    511540.4688        -9.9421
-9.9520
```

40.1ns Q5NAT0 SIMULATION DATA

```
                  TS           BOND          ANGLE          DIHED          IMPRP
               ELECT            VDW       BOUNDARY           MISC        KINETIC
               TOTAL           TEMP      POTENTIAL         TOTAL3        TEMPAVG
            PRESSURE      GPRESSURE         VOLUME       PRESSAVG      GPRESSAVG
[192]ENERGY:       0      4683.0396      5161.8033      5250.3882       306.8334
         -10081.5245     -1805.4586         0.0000         0.0000      7393.9912
          10909.0727       295.7613      3515.0815     11012.7278       295.7613
           2697.6206       398.5367    686386.8863      2697.6206       398.5367
[280]ENERGY:   10000      3179.2743      5058.8267      5311.6943       355.7430
         -15021.8723     -1674.5847         0.0000         0.0000      7587.1364
           4796.2178       303.4872     -2790.9186      4860.7764       322.3989
            -82.5392       -18.9135    672387.9418        -1.2609        -1.1551
[319]ENERGY:   20000      3141.5211      4981.9891      5279.4834       369.4766
         -15202.3680     -1655.7454         0.0000         0.0000      7500.6846
           4415.0413       300.0291     -3085.6433      4472.6539       300.4789
             37.9836        53.0530    698024.9507         1.8096         1.8279
[371]ENERGY:   30000      3128.2969      5047.9974      5351.8320       339.2491
         -15294.7699     -1723.0906         0.0000         0.0000      7479.8344
           4329.3493       299.1951     -3150.4850      4392.0901       298.2180
             45.0358        55.3733    692726.1275         1.0559         1.0596
[409]ENERGY:   40000      3062.2959      4915.5486      5367.8251       318.7022
         -15242.8011     -1637.7519         0.0000         0.0000      7480.6200
           4264.4389       299.2265     -3216.1811      4322.8023       297.5825
            248.5802       223.1176    701542.2779         6.2008         6.2109
```

```
[461]ENERGY:   50000      3114.1617      5034.4429      5274.3527       332.5935
        -15208.4339     -1743.8920         0.0000         0.0000      7411.4709
         4214.6959       296.4605     -3196.7750      4272.8341       298.4510
         -103.0185      -100.7097    704384.8430        -0.3675        -0.3619
[499]ENERGY:   60000      3097.8642      5036.8064      5240.2388       343.9810
        -15265.4221     -1744.6155         0.0000         0.0000      7442.5076
         4151.3604       297.7020     -3291.1472      4212.9153       298.0233
          -34.7921         3.0120    699868.2769         0.6415         0.6211
[550]ENERGY:   70000      3074.5542      5053.4172      5273.9797       317.6805
        -15226.1987     -1767.4160         0.0000         0.0000      7367.8019
         4093.8189       294.7138     -3273.9831      4153.0377       298.1102
           40.0759        34.8112    698526.4767        -1.2876        -1.2894
[587]ENERGY:   80000      3070.6400      5057.7975      5284.0248       321.8735
        -15316.1261     -1737.8328         0.0000         0.0000      7377.5988
         4057.9757       295.1056     -3319.6230      4114.9641       297.7017
          -22.7557        72.0622    720049.7578        -1.6671        -1.6594
[638]ENERGY:   90000      3188.7484      5057.0386      5268.2942       339.2538
        -15338.4012     -1686.8652         0.0000         0.0000      7427.4300
         4255.4986       297.0989     -3171.9314      4315.6071       297.4097
          -52.0524        -1.5799    714893.7784         0.4332         0.4270
[678]ENERGY:  100000      3122.6054      5003.7403      5249.5625       336.5185
        -15303.4820     -1710.5339         0.0000         0.0000      7404.7576
         4103.1684       296.1920     -3301.5892      4163.1204       299.3055
          -80.0537       -24.8176    696932.0826         1.7566         1.7731
[729]ENERGY:  110000      3160.8026      4973.3056      5281.4846       316.5356
        -15270.1713     -1847.1071         0.0000         0.0000      7406.1703
         4021.0204       296.2485     -3385.1499      4082.4161       296.8630
          -77.0907       -27.8147    686244.0339         2.4133         2.4224
[765]ENERGY:  120000      3138.1472      5022.4709      5280.5085       327.5227
        -15341.0587     -1783.2466         0.0000         0.0000      7410.1731
         4054.5172       296.4086     -3355.6559      4118.8734       297.6333
         -197.2839      -156.7220    665377.0122        -1.6327        -1.6432
[815]ENERGY:  130000      3202.6548      5138.8816      5252.4392       333.9543
        -15459.1582     -1698.9895         0.0000         0.0000      7407.6073
         4177.3895       296.3060     -3230.2178      4236.0669       299.6500
           56.8709       -14.8516    671776.5077         2.9286         2.9160
[851]ENERGY:  140000      3085.8142      5076.2766      5272.1700       335.3563
        -15394.3227     -1796.4673         0.0000         0.0000      7419.4002
         3998.2273       296.7777     -3421.1729      4060.8329       298.0591
          159.9578       101.7084    684619.4366         1.9825         1.9630
[901]ENERGY:  150000      3111.8361      5083.1119      5194.7035       333.0186
        -15333.2478     -1807.0807         0.0000         0.0000      7503.4813
         4085.8229       300.1410     -3417.6584      4147.4784       297.8895
          -52.3318       -29.5616    700702.1112         4.4062         4.4006
[937]ENERGY:  160000      3130.7430      5115.6523      5278.8343       321.1254
        -15377.8524     -1812.1028         0.0000         0.0000      7395.2483
         4051.6481       295.8116     -3343.6002      4107.9063       296.5258
           31.8291       -22.9406    717273.6389         2.4444         2.4440
[987]ENERGY:  170000      3058.7526      5124.1746      5265.0265       336.1663
        -15400.3537     -1725.4467         0.0000         0.0000      7484.3084
         4142.6279       299.3741     -3341.6805      4203.1832       297.4357
          -13.0242       -23.6936    733087.6888        -0.4243        -0.4048
[1023]ENERGY:  180000      3125.6147      5082.3773      5267.5084
314.0244         -15325.5238     -1823.3447         0.0000         0.0000
```

7431.5927 4072.2489 297.2654 -3359.3438 4132.3864
297.9681 83.7870 3.7261 740883.1004 0.6615
0.6711
[1073]ENERGY: 190000 3118.9140 5050.7932 5263.2028
348.3429 -15375.2489 -1761.8486 0.0000 0.0000
7512.2367 4156.3922 300.4912 -3355.8446 4216.1917
297.0861 56.8162 67.3044 722809.3811 3.2182
3.2120
[1112]ENERGY: 200000 3080.3335 5033.9149 5233.2114
336.0872 -15348.5494 -1798.2025 0.0000 0.0000
7492.5896 4029.3847 299.7053 -3463.2050 4090.8770
298.6231 112.9594 84.1613 702906.6414 4.3992
4.3810
[1162]ENERGY: 210000 3089.8266 5110.4777 5239.0001
311.0779 -15420.6888 -1768.1098 0.0000 0.0000
7431.4155 3992.9992 297.2583 -3438.4163 4054.4464
298.5238 46.5186 60.7693 689125.7304 -0.9311
-0.9430
[1198]ENERGY: 220000 3074.2429 5041.4207 5255.1024
316.9341 -15347.5610 -1718.6998 0.0000 0.0000
7529.2702 4150.7095 301.1725 -3378.5607 4209.1234
299.4646 224.1866 194.7159 685239.4691 5.3086
5.3269
[1248]ENERGY: 230000 3137.3814 4989.3466 5235.1423
326.2866 -15306.5878 -1862.3171 0.0000 0.0000
7382.5812 3901.8331 295.3049 -3480.7481 3961.5159
298.2712 131.0423 38.5305 709309.1164 1.0928
1.0767
[1284]ENERGY: 240000 3140.2591 5170.8556 5235.1706
333.4220 -15492.2768 -1731.9710 0.0000 0.0000
7425.2765 4080.7360 297.0128 -3344.5405 4139.1248
298.8658 71.1658 49.1631 732596.3059 -0.3714
-0.3433
[1334]ENERGY: 250000 3125.4599 5118.9266 5226.9230
328.7232 -15407.3719 -1774.1848 0.0000 0.0000
7545.7005 4164.1764 301.8298 -3381.5240 4225.3742
298.0997 -32.8910 -8.0237 695916.6936 -0.5836
-0.5843
[1370]ENERGY: 260000 3133.2764 5045.5796 5239.1577
326.3608 -15324.1771 -1869.6407 0.0000 0.0000
7408.5020 3959.0588 296.3418 -3449.4433 4021.7467
299.4157 -61.9046 -101.5359 702353.2128 -0.0716
-0.0793
[1420]ENERGY: 270000 3029.6619 5008.3538 5245.3589
369.8971 -15451.0902 -1777.6385 0.0000 0.0000
7374.6956 3799.2386 294.9895 -3575.4571 3860.5254
296.7146 32.1197 28.0131 697952.2055 2.3330
2.3323
[1456]ENERGY: 280000 3093.3491 5071.8494 5274.8198
327.0725 -15405.6705 -1828.8256 0.0000 0.0000
7516.9480 4049.5428 300.6796 -3467.4052 4110.4772
298.2562 51.1884 -24.5381 701994.0217 -3.7611
-3.7694
[1506]ENERGY: 290000 3180.1234 5189.8434 5221.1853

352.0418 -15401.5479 -1825.6905 0.0000 0.0000
7508.2830 4224.2385 300.3330 -3284.0445 4286.2596
300.9571 91.2755 33.3868 698064.6628 2.6793
2.6574
[1545]ENERGY: 300000 3153.6723 5052.9039 5224.9574
338.5462 -15450.4421 -1755.0664 0.0000 0.0000
7521.6768 4086.2482 300.8688 -3435.4286 4151.1479
300.0045 -1.3354 3.2928 687814.6668 -0.2884
-0.2634
[1595]ENERGY: 310000 3128.9202 5194.0378 5247.8622
329.1210 -15454.7060 -1808.6787 0.0000 0.0000
7515.2216 4151.7783 300.6106 -3363.4434 4212.5856
299.3496 58.0041 44.3335 675003.2078 3.9818
3.9646
[1631]ENERGY: 320000 3191.8297 5072.2861 5309.0604
322.9730 -15474.3037 -1724.8493 0.0000 0.0000
7396.0688 4093.0649 295.8445 -3303.0039 4152.2915
301.6515 36.2039 -10.0398 661515.3969 -0.9377
-0.9499
[1681]ENERGY: 330000 3188.1577 5142.1591 5220.0213
330.6006 -15448.3503 -1746.4110 0.0000 0.0000
7405.6963 4091.8737 296.2296 -3313.8226 4151.8937
298.0389 5.3475 -100.1743 671585.1337 4.1113
4.1347
[1717]ENERGY: 340000 3107.7792 5034.0935 5263.9061
339.0191 -15382.0802 -1771.9517 0.0000 0.0000
7481.1202 4071.8863 299.2465 -3409.2339 4131.3843
298.5296 90.7662 51.4247 671626.4256 1.0178
1.0321
[1767]ENERGY: 350000 3196.6530 5083.8554 5203.3836
345.5323 -15417.8760 -1788.1398 0.0000 0.0000
7394.0026 4017.4111 295.7618 -3376.5915 4073.0743
298.9525 -64.2600 -84.0474 673934.2969 -1.2356
-1.2541
[1803]ENERGY: 360000 3135.6357 5064.8411 5258.4824
331.4628 -15384.1400 -1756.6055 0.0000 0.0000
7437.0930 4086.7695 297.4854 -3350.3235 4145.6040
300.0044 -52.7826 -66.1945 645732.6009 -0.3870
-0.3815
[1853]ENERGY: 370000 3091.8282 5096.0485 5253.2554
348.4179 -15444.4315 -1768.3599 0.0000 0.0000
7468.8392 4045.5978 298.7553 -3423.2415 4108.6778
297.7511 98.3014 -17.5770 638783.4087 -5.1491
-5.1625
[1889]ENERGY: 380000 3198.6707 5006.3495 5239.2580
313.1935 -15388.0393 -1831.6789 0.0000 0.0000
7591.7990 4129.5524 303.6737 -3462.2465 4191.7022
298.0483 -48.0668 -57.5298 652389.9513 -0.6791
-0.6615
[1939]ENERGY: 390000 3128.8689 5147.8211 5259.6669
318.3621 -15581.7435 -1787.0239 0.0000 0.0000
7395.8570 3881.8085 295.8360 -3514.0485 3942.0817
299.1600 -142.3356 -94.9207 686284.3115 1.5374
1.5360

[1978]ENERGY: 400000   3121.1737   5141.9525   5237.8007
341.7890   -15493.6921   -1747.4277   0.0000   0.0000
7368.9459   3970.5422   294.7595   -3398.4037   4029.6973
298.9845   25.4521   28.8310   705941.6149   3.5616
 3.5573
[2028]ENERGY: 410000   3142.2449   5044.7495   5244.1139
321.6315   -15396.8693   -1736.1777   0.0000   0.0000
7612.2967   4231.9896   304.4936   -3380.3071   4296.8475
299.1721   -18.1874   -108.3123   679499.8009   -0.5217
-0.5209
[2064]ENERGY: 420000   3216.8908   5130.8109   5290.4419
321.6035   -15498.0018   -1733.2487   0.0000   0.0000
7488.2068   4216.7034   299.5300   -3271.5034   4278.5936
300.7439   -139.4307   -177.5957   680666.3954   -1.0010
-1.0069
[2114]ENERGY: 430000   3121.0415   5164.8624   5265.1284
332.6614   -15579.2907   -1724.8834   0.0000   0.0000
7515.0701   4094.5898   300.6045   -3420.4803   4155.0956
300.3176   27.2165   64.1419   667615.0500   5.4422
 5.4634
[2150]ENERGY: 440000   3092.0472   5183.9058   5198.9928
330.7777   -15535.6322   -1732.6821   0.0000   0.0000
7426.2896   3963.6987   297.0533   -3462.5909   4024.8097
299.5429   47.6377   -8.5732   674988.9697   -1.6516
-1.6698
[2200]ENERGY: 450000   3077.3846   5092.2759   5267.3630
321.1853   -15492.0090   -1804.4830   0.0000   0.0000
7453.5859   3915.3028   298.1451   -3538.2831   3977.8881
297.9475   -5.2406   -53.3322   658647.5438   0.2443
 0.2519
[2236]ENERGY: 460000   3133.7603   5018.5541   5289.9176
316.7362   -15472.1638   -1740.7415   0.0000   0.0000
7427.2223   3973.2852   297.0906   -3453.9371   4030.1727
299.0902   13.3217   -62.7911   674876.2122   1.3911
 1.4016
[2286]ENERGY: 470000   3127.4913   4992.2781   5201.0203
309.6305   -15466.0376   -1726.5359   0.0000   0.0000
7374.8192   3812.6660   294.9945   -3562.1532   3873.8736
298.9193   105.6638   96.6128   673444.7289   5.0763
 5.0788
[2322]ENERGY: 480000   3036.5908   5115.9841   5217.8342
313.4169   -15567.1400   -1665.2517   0.0000   0.0000
7488.7517   3940.1861   299.5518   -3548.5657   4001.5793
297.5428   199.3978   96.8170   673403.7469   2.0426
 2.0107
[2372]ENERGY: 490000   3074.1220   5148.1432   5240.8117
333.3690   -15618.8598   -1711.9101   0.0000   0.0000
7516.0583   3981.7343   300.6441   -3534.3240   4044.2000
298.1546   -40.3821   -32.6725   681281.5782   1.2225
 1.2551
[2411]ENERGY: 500000   3097.6311   4922.4368   5213.7887
329.8594   -15453.6425   -1798.3853   0.0000   0.0000
7469.8322   3781.5204   298.7950   -3688.3119   3839.3250
298.4082   -28.0602   -16.0500   688492.8974   0.1499

0.1439
[2461]ENERGY: 510000   3083.0804   5012.2147   5229.6451
307.1805   -15452.2272   -1735.2215   0.0000   0.0000
7376.4206   3821.0925   295.0585   -3555.3281   3878.9534
297.1383   101.6399   94.9606   688405.5946   2.9463
2.9756
[2497]ENERGY: 520000   3068.9549   5124.1922   5224.8445
335.1759   -15577.0118   -1785.5000   0.0000   0.0000
7415.5809   3806.2366   296.6249   -3609.3443   3866.3422
297.4389   100.6106   44.0728   694027.3584   3.6587
3.6335
[2547]ENERGY: 530000   3061.9368   5019.8254   5254.1827
326.8375   -15434.7492   -1807.2652   0.0000   0.0000
7441.6178   3862.3858   297.6664   -3579.2319   3920.7241
297.1332   56.6951   54.0381   727824.8496   -5.1175
-5.1007
[2583]ENERGY: 540000   3194.2206   5090.9538   5272.8668
333.1087   -15515.4852   -1782.5055   0.0000   0.0000
7632.1911   4225.3503   305.2894   -3406.8408   4290.6255
301.0082   -38.4270   -0.6722   709960.0054   2.2609
2.2506
[2633]ENERGY: 550000   3132.9713   4994.0708   5317.8419
335.4273   -15495.3285   -1805.4549   0.0000   0.0000
7424.1332   3903.6611   296.9670   -3520.4721   3969.6982
300.0941   -127.8158   -117.9040   698332.9392   0.2879
0.2845
[2669]ENERGY: 560000   3221.2659   5099.6914   5243.8712
326.3126   -15433.6642   -1877.9119   0.0000   0.0000
7461.3530   4040.9180   298.4558   -3420.4350   4098.7261
298.8873   -35.9170   -81.4857   695842.3292   0.1598
0.1422
[2719]ENERGY: 570000   3060.1780   5159.7786   5248.4677
346.1091   -15516.2960   -1805.7228   0.0000   0.0000
7346.1233   3838.6380   293.8466   -3507.4853   3898.8865
300.3268   72.3303   72.5222   686379.1208   -0.4547
-0.4636
[2755]ENERGY: 580000   3140.3712   5090.5282   5285.9250
320.6720   -15555.7305   -1807.5421   0.0000   0.0000
7457.2172   3931.4411   298.2904   -3525.7761   3990.7002
297.4480   -80.0786   -48.5354   702888.1075   -4.1345
-4.1205
[2805]ENERGY: 590000   3072.7276   5144.5042   5269.4252
338.4690   -15552.6688   -1757.2563   0.0000   0.0000
7485.6410   4000.8419   299.4274   -3484.7991   4061.9260
297.3207   41.2457   -38.4816   694238.8761   4.7380
4.7319
[2844]ENERGY: 600000   3134.1521   5099.2271   5260.4997
354.6530   -15569.6431   -1785.8289   0.0000   0.0000
7444.7570   3937.8169   297.7920   -3506.9401   4000.7552
299.5258   -62.8926   -72.2141   682707.5143   -0.1467
-0.1464
[2894]ENERGY: 610000   3145.1552   5080.5676   5310.3160
326.3685   -15596.2202   -1801.5025   0.0000   0.0000
7361.9771   3826.6616   294.4808   -3535.3155   3887.0152

299.3837 -65.7452 -31.4421 686235.4425 -1.4283
-1.4276
[2930]ENERGY: 620000 3164.7786 5143.6284 5291.1578
316.4663 -15638.2498 -1724.2813 0.0000 0.0000
7444.6669 3998.1669 297.7884 -3446.5000 4054.7716
297.9952 -1.9774 79.8357 693794.7112 1.7361
1.7609
[2980]ENERGY: 630000 3119.3063 5214.7017 5266.5190
311.5646 -15569.0838 -1820.3797 0.0000 0.0000
7394.5355 3917.1635 295.7831 -3477.3720 3980.4682
299.6749 46.0007 -8.5482 710997.0600 -0.6284
-0.6517
[3016]ENERGY: 640000 3174.7675 5031.1634 5271.0624
318.9766 -15585.7465 -1737.1738 0.0000 0.0000
7409.8712 3882.9207 296.3965 -3526.9504 3942.5428
298.7167 116.9771 61.2754 698601.6393 -1.3210
-1.3145
[3066]ENERGY: 650000 3177.0533 5132.7307 5286.5408
340.8901 -15503.2876 -1789.6163 0.0000 0.0000
7430.2905 4074.6015 297.2133 -3355.6890 4134.1177
298.6829 -6.0652 -51.3619 684283.4684 -1.4557
-1.4616
[3102]ENERGY: 660000 3157.3254 5104.2407 5296.4471
340.7365 -15562.8822 -1724.5104 0.0000 0.0000
7467.1329 4078.4900 298.6870 -3388.6430 4135.9954
300.3581 92.1610 30.8259 683870.1097 1.9933
2.0015
[3152]ENERGY: 670000 3089.3575 5114.8798 5281.5975
331.4598 -15559.5396 -1728.7316 0.0000 0.0000
7445.9934 3975.0167 297.8414 -3470.9767 4037.3958
299.3014 87.5926 -22.6748 687287.8922 -0.0462
-0.0598
[3188]ENERGY: 680000 3085.1129 4969.3597 5249.2296
317.5620 -15570.1568 -1759.7263 0.0000 0.0000
7377.4651 3668.8463 295.1003 -3708.6189 3731.8391
297.6035 -75.7937 -43.8626 666151.5232 4.1600
4.1813
[3238]ENERGY: 690000 3102.9781 5040.9401 5256.1336
344.0963 -15601.4491 -1781.1087 0.0000 0.0000
7420.0614 3781.6516 296.8042 -3638.4097 3844.6507
296.9483 -170.7987 -55.8693 663287.8371 0.2688
0.2735
[3277]ENERGY: 700000 3118.9327 5052.0453 5258.5682
325.2747 -15600.4720 -1800.9939 0.0000 0.0000
7469.3541 3822.7092 298.7759 -3646.6449 3881.4868
298.6444 -29.7427 -90.9349 653356.5542 1.5502
1.5562
[3327]ENERGY: 710000 3179.5555 5070.6584 5272.9611
359.1280 -15511.5614 -1754.4067 0.0000 0.0000
7393.8983 4010.2332 295.7576 -3383.6651 4067.2059
296.1479 -71.1332 -86.9386 626891.9138 -0.3440
-0.3508
[3363]ENERGY: 720000 3103.1979 5079.5094 5250.9275
343.0918 -15637.0600 -1737.8415 0.0000 0.0000

7506.3051 3908.1301 300.2539 -3598.1750 3972.1141
298.9742 109.9135 78.9654 618407.9429 -2.6763
-2.6934
[3413]ENERGY: 730000 3132.0804 5136.5958 5322.1565
313.3612 -15625.6487 -1745.6477 0.0000 0.0000
7298.1017 3830.9992 291.9257 -3467.1025 3885.7272
298.4104 203.9190 157.9265 641397.5686 7.1437
7.1609
[3449]ENERGY: 740000 3103.4202 5059.2642 5267.9758
322.4721 -15678.5302 -1805.5239 0.0000 0.0000
7448.7751 3717.8533 297.9527 -3730.9218 3779.3021
297.9642 28.8116 53.9967 621221.5827 -0.9396
-0.9131
[3499]ENERGY: 750000 3184.4887 5157.9876 5250.2888
354.1707 -15667.1871 -1738.8487 0.0000 0.0000
7462.1009 4003.0008 298.4858 -3459.1001 4062.8674
298.8223 -23.9161 -149.1651 598798.9646 5.1931
5.2049
[3535]ENERGY: 760000 3074.9927 5143.0871 5199.2593
335.9872 -15548.8272 -1796.6240 0.0000 0.0000
7466.5369 3874.4120 298.6632 -3592.1249 3939.5927
299.4873 -73.5628 -20.6852 587003.6180 -1.4957
-1.5196
[3585]ENERGY: 770000 3021.5819 5088.0804 5238.2836
347.2035 -15562.3278 -1778.8406 0.0000 0.0000
7437.2795 3791.2605 297.4929 -3646.0191 3850.0204
298.2558 56.3357 32.1641 588565.3355 0.2428
0.2504
[3621]ENERGY: 780000 3128.7049 5044.5780 5221.2300
335.0084 -15462.5257 -1787.9726 0.0000 0.0000
7366.8157 3845.8388 294.6743 -3520.9769 3902.0810
297.4929 -34.0066 -69.9849 572471.9140 3.6503
3.6510
[3671]ENERGY: 790000 3158.9555 5138.7105 5231.9086
342.1291 -15689.9207 -1718.4646 0.0000 0.0000
7585.0219 4048.3401 303.4026 -3536.6817 4115.8385
301.1857 -118.1325 -107.7669 581207.2866 2.0623
2.0862
[3710]ENERGY: 800000 3109.3253 5117.0824 5263.7548
320.5087 -15700.9071 -1714.9292 0.0000 0.0000
7541.5991 3936.4339 301.6657 -3605.1652 4006.9187
301.4937 -105.0339 -76.2315 576041.9977 2.6303
2.6237
[3760]ENERGY: 810000 3145.3001 5037.6537 5278.2738
325.0220 -15582.5449 -1805.6051 0.0000 0.0000
7507.0316 3905.1313 300.2830 -3601.9004 3966.4459
298.1440 -129.7316 -166.0159 572015.2389 2.1397
2.1465
[3796]ENERGY: 820000 3118.0676 5152.6129 5287.9136
334.9788 -15675.6172 -1774.5878 0.0000 0.0000
7440.2699 3883.6377 297.6125 -3556.6322 3944.1103
298.2845 -188.0620 -176.5159 579804.4380 -1.7902
-1.8076
[3846]ENERGY: 830000 3119.2520 5153.5045 5235.6748

333.9058 -15612.9916 -1806.6763 0.0000 0.0000
7440.1258 3862.7950 297.6067 -3577.3308 3925.6701
299.1036 -6.6282 12.1214 590742.1042 -1.6203
-1.6360
[3882]ENERGY: 840000 3111.9001 5053.6886 5213.5331
331.3233 -15673.4079 -1725.3709 0.0000 0.0000
7395.3912 3707.0575 295.8173 -3688.3337 3766.2147
299.1782 -123.6952 -144.5526 599607.0270 1.9048
1.9083
[3932]ENERGY: 850000 3099.8319 5159.0361 5207.0059
351.5159 -15628.1456 -1697.1629 0.0000 0.0000
7562.6466 4054.7279 302.5076 -3507.9187 4112.5872
299.9441 -36.7523 -53.3497 608231.2720 1.1001
1.0939
[3968]ENERGY: 860000 3165.5765 5130.1721 5253.6396
318.5936 -15637.4389 -1780.3599 0.0000 0.0000
7587.1571 4037.3401 303.4880 -3549.8170 4103.3866
300.7874 -126.3864 -112.2356 635059.3687 1.6425
1.6777
[4018]ENERGY: 870000 3085.6251 5046.5693 5282.4229
349.3944 -15734.1253 -1710.6595 0.0000 0.0000
7503.9812 3823.2080 300.1610 -3680.7732 3884.5757
299.5773 -59.0729 -59.4872 638560.8523 4.6718
4.6641
[4054]ENERGY: 880000 3204.2029 5106.6024 5276.2232
362.3494 -15672.3741 -1785.8709 0.0000 0.0000
7412.8624 3903.9953 296.5162 -3508.8671 3965.1868
298.3971 20.1930 6.2761 648394.6854 -1.0150
-0.9885
[4104]ENERGY: 890000 3129.0806 5123.6061 5287.3198
337.2269 -15675.5439 -1759.9174 0.0000 0.0000
7459.5184 3901.2904 298.3824 -3558.2280 3964.2316
300.2189 56.6201 80.3820 657940.7285 0.7625
0.7611
[4143]ENERGY: 900000 3157.3628 5104.1606 5278.8508
315.6052 -15668.6179 -1702.2780 0.0000 0.0000
7492.6129 3977.6964 299.7062 -3514.9165 4040.7213
298.7781 131.0939 95.4240 668517.9627 -0.2763
-0.2766
[4193]ENERGY: 910000 3052.3556 5048.3192 5210.3962
338.0524 -15609.0228 -1699.7287 0.0000 0.0000
7435.1625 3775.5345 297.4082 -3659.6280 3839.5701
299.3335 57.2086 141.0280 649747.5354 4.0159
4.0167
[4229]ENERGY: 920000 3139.6230 5147.5743 5196.5671
312.7321 -15648.5065 -1748.2678 0.0000 0.0000
7433.0229 3832.7451 297.3226 -3600.2779 3890.6953
298.0999 -53.8105 -25.9730 657553.5451 1.6576
1.6467
[4279]ENERGY: 930000 3105.2252 5180.2862 5231.3569
316.9823 -15718.0245 -1737.9304 0.0000 0.0000
7387.6688 3765.5644 295.5084 -3622.1044 3823.9493
297.2416 -106.1315 -60.2365 679305.0425 -0.8180
-0.8152

```
[4315]ENERGY:  940000      3231.9699      5064.2342      5258.6796
317.6068         -15714.4221     -1779.4844         0.0000         0.0000
7435.3571           3813.9411       297.4160     -3621.4160      3870.8461
298.1927             -82.0115      -110.4575    694607.3580         0.7375
 0.7214
[4365]ENERGY:  950000      3058.7383      5072.9182      5269.7776
338.2606         -15512.1562     -1787.9044         0.0000         0.0000
7492.3472           3931.9813       299.6956     -3560.3659      3991.6569
299.2385              98.9738        89.9541    701291.3609         0.7675
 0.7420
[4401]ENERGY:  960000      3106.5409      4963.9316      5244.8190
327.5458         -15610.0498     -1729.4746         0.0000         0.0000
7408.9043           3712.2173       296.3579     -3696.6870      3773.4159
298.1141              38.7373        89.3275    680065.9889        -2.7848
-2.7673
[4451]ENERGY:  970000      3163.8488      4989.5316      5221.6318
300.8973         -15611.3858     -1789.9242         0.0000         0.0000
7566.1797           3840.7791       302.6489     -3725.4006      3902.5568
297.0335             131.2596        83.1796    652764.0069         1.5058
 1.4911
[4487]ENERGY:  980000      3134.8613      5148.7357      5249.8069
329.9036         -15690.6120     -1759.7066         0.0000         0.0000
7411.2375           3824.2263       296.4512     -3587.0111      3883.6367
297.8708             -24.9599       -20.3240    665697.6024        -0.2226
-0.2105
[4537]ENERGY:  990000      3138.3679      5141.0050      5228.5475
312.6811         -15659.4855     -1749.3609         0.0000         0.0000
7435.1838           3846.9388       297.4091     -3588.2450      3907.4947
298.2929             -21.6855       -50.1959    663143.5011        -0.5846
-0.5865
[4576]ENERGY: 1000000      3141.4388      5130.6049      5277.5644
330.6152         -15760.7694     -1711.3107         0.0000         0.0000
7516.9058           3925.0491       300.6780     -3591.8567      3985.9368
299.2967              29.1699        54.3248    656151.2420        -1.2910
-1.2907
[269]ENERGY: 1010000      3118.5446      5075.5672      5274.7883        331.3344
        -15628.8916     -1715.9387         0.0000         0.0000       7474.5614
         3929.9656       299.0198     -3544.5958      3988.2025       300.1142
         -10.1897        -5.1528    656151.2420         0.1777         0.1687
[308]ENERGY: 1020000      3193.0267      5076.9738      5268.0610        348.6188
        -15710.1920     -1727.6348         0.0000         0.0000       7481.2238
         3930.0772       299.2864     -3551.1466      3988.2718       300.1884
         118.9233        80.6427    656151.2420        -0.1369        -0.1333
[360]ENERGY: 1030000      3092.3392      5039.1446      5261.4103        309.6746
        -15639.6431     -1660.4838         0.0000         0.0000       7523.7115
         3926.1534       300.9861     -3597.5581      3987.3758       300.1528
         -50.7301       -68.5227    656151.2420        -1.4696        -1.4777
[398]ENERGY: 1040000      3171.7749      5107.0066      5315.3158        338.0733
        -15753.6268     -1767.8602         0.0000         0.0000       7516.2281
         3926.9116       300.6867     -3589.3164      3989.0822       300.2051
          87.9361        29.3912    656151.2420         1.8201         1.8264
[450]ENERGY: 1050000      3132.5272      5105.9343      5239.9259        323.8519
        -15669.6168     -1764.5640         0.0000         0.0000       7561.5842
         3929.6428       302.5012     -3631.9414      3989.5633       300.3358
```

```
        48.2273          3.8100    656151.2420        -1.3954        -1.4103
[488]ENERGY: 1060000       3185.0776       5113.0898       5236.5396        328.9191
       -15630.3543     -1745.4366         0.0000         0.0000       7444.0451
         3931.8803      297.7990    -3512.1648      3989.3764       300.4848
           2.0938      -44.0894    656151.2420         2.5044         2.5229
[539]ENERGY: 1070000       3045.6216       5033.6439       5238.9268        331.4349
       -15573.8816     -1742.9768         0.0000         0.0000       7594.0880
         3926.8567      303.8015    -3667.2313      3989.0907       300.4605
         -31.6628       42.5827    656151.2420        -2.3652        -2.3540
[576]ENERGY: 1080000       3164.3185       5047.0736       5228.5049        311.9635
       -15627.3653     -1712.0613         0.0000         0.0000       7519.7121
         3932.1459      300.8261    -3587.5662      3991.2400       300.4826
         106.0794        2.4271    656151.2420         0.8860         0.8805
[627]ENERGY: 1090000       3104.9405       5167.5023       5250.0484        334.7215
       -15629.5037     -1842.9717         0.0000         0.0000       7541.8912
         3926.6285      301.7134    -3615.2627      3990.6706       300.4734
         101.3561       43.0360    656151.2420         1.1652         1.1421
[667]ENERGY: 1100000       3124.2040       5181.2254       5223.8442        356.3144
       -15661.1873     -1784.1089         0.0000         0.0000       7490.8892
         3931.1810      299.6730    -3559.7082      3991.3821       300.3982
           8.3877       21.8072    656151.2420        -0.3465        -0.3270
[718]ENERGY: 1110000       3157.6805       5145.9613       5265.9942        312.5239
       -15587.1714     -1813.6826         0.0000         0.0000       7448.6271
         3929.9330      297.9823    -3518.6941      3992.5651       300.4745
         -92.8301     -129.0754    656151.2420         0.8505         0.8431
[754]ENERGY: 1120000       3160.4423       5069.0065       5285.0351        354.2103
       -15682.9936     -1713.4202         0.0000         0.0000       7462.1708
         3934.4513      298.5241    -3527.7195      3991.4907       300.4934
          19.9002       27.9187    656151.2420        -0.7996        -0.7943
[804]ENERGY: 1130000       3209.3782       5130.7589       5280.7692        330.8008
       -15768.0905     -1759.7389         0.0000         0.0000       7502.5226
         3926.4003      300.1384    -3576.1223      3992.9218       300.6672
           0.1463      -16.2160    656151.2420         1.4995         1.5130
[840]ENERGY: 1140000       3187.2797       5207.6356       5245.4209        328.0342
       -15756.5004     -1764.0744         0.0000         0.0000       7482.4012
         3930.1967      299.3335    -3552.2045      3992.2482       300.7003
        -185.8115     -163.1702    656151.2420        -1.0098        -1.0242
[890]ENERGY: 1150000       3125.9820       5123.8735       5247.6602        344.8588
       -15707.2332     -1792.0783         0.0000         0.0000       7588.1585
         3931.2214      303.5643    -3656.9370      3992.8840       300.5328
          -4.5459      -60.0908    656151.2420        -1.5687        -1.5701
[926]ENERGY: 1160000       3108.8290       5240.8077       5303.3476        306.9555
       -15732.9059     -1743.1260         0.0000         0.0000       7449.5409
         3933.4487      298.0189    -3516.0922      3992.8152       300.8292
          15.7935       14.0724    656151.2420         1.4967         1.4997
[976]ENERGY: 1170000       3203.4710       5195.1639       5293.9336        327.0240
       -15705.3408     -1806.6793         0.0000         0.0000       7423.7038
         3931.2762      296.9853    -3492.4276      3991.6024       300.8024
          -9.6948        9.2784    656151.2420        -2.6899        -2.6825
[1012]ENERGY: 1180000      3166.6177       5126.8889       5251.6964
330.0288         -15663.0329     -1736.3078         0.0000         0.0000
7458.3151           3934.2063       298.3699     -3524.1088      3991.6520
300.7396            -152.3701      -104.6227    656151.2420         2.6984
 2.6953
```

[1062]ENERGY: 1190000 3187.7277 5087.8116 5274.2794
325.7112 -15700.5926 -1768.8652 0.0000 0.0000
7527.4005 3933.4725 301.1337 -3593.9280 3991.6251
300.6332 -53.2105 -60.0923 656151.2420 -0.6512
-0.6597
[1101]ENERGY: 1200000 3124.8917 5054.6546 5303.5005
335.5070 -15633.0663 -1781.3133 0.0000 0.0000
7527.0394 3931.2136 301.1192 -3595.8259 3991.2501
300.8150 43.8428 -14.1783 656151.2420 -0.7250
-0.7247
[1151]ENERGY: 1210000 3069.2737 5137.9784 5250.0484
344.6018 -15685.6980 -1725.5376 0.0000 0.0000
7539.2963 3929.9630 301.6095 -3609.3333 3992.1413
300.8082 -82.5831 -114.4971 656151.2420 -1.2651
-1.2486
[1187]ENERGY: 1220000 3101.7655 5022.4202 5333.4346
332.7915 -15639.9201 -1734.0355 0.0000 0.0000
7512.9955 3929.4517 300.5574 -3583.5438 3991.0248
300.9533 36.0516 21.5662 656151.2420 1.4734
1.4650
[1237]ENERGY: 1230000 3103.7455 5055.8053 5202.3414
336.8952 -15677.3514 -1690.6787 0.0000 0.0000
7600.2963 3931.0535 304.0499 -3669.2427 3992.2642
300.7596 88.6856 -6.0119 656151.2420 -0.2738
-0.2615
[1273]ENERGY: 1240000 3079.5259 5126.8010 5205.7971
350.5200 -15648.9079 -1738.9795 0.0000 0.0000
7557.7723 3932.5288 302.3487 -3625.2435 3991.9950
300.6867 -34.9153 -50.8640 656151.2420 1.7827
1.7733
[1323]ENERGY: 1250000 3166.1027 5069.5940 5240.5757
314.7782 -15708.1715 -1697.7984 0.0000 0.0000
7548.1229 3933.2037 301.9627 -3614.9192 3992.9418
300.9567 -38.0565 -67.4142 656151.2420 -1.2282
-1.2424
[1359]ENERGY: 1260000 3126.3571 5052.1778 5277.9735
326.9479 -15739.4444 -1725.2962 0.0000 0.0000
7613.4785 3932.1943 304.5772 -3681.2842 3993.2271
301.2066 30.6448 15.2658 656151.2420 -1.0025
-0.9879
[1409]ENERGY: 1270000 3186.7795 5117.7206 5250.9870
347.1520 -15727.0976 -1769.5436 0.0000 0.0000
7525.4932 3931.4911 301.0574 -3594.0021 3993.6041
301.1243 -35.0756 -65.7970 656151.2420 1.5760
1.5761
[1445]ENERGY: 1280000 3184.6205 5070.6611 5222.7999
330.8682 -15620.3638 -1713.1598 0.0000 0.0000
7462.1614 3937.5875 298.5238 -3524.5738 3993.6673
300.8761 23.3350 21.8410 656151.2420 -1.2571
-1.2670
[1495]ENERGY: 1290000 3165.7844 5096.2674 5227.3057
359.8039 -15766.9611 -1702.2568 0.0000 0.0000
7553.4498 3933.3932 302.1758 -3620.0567 3993.7284
301.1089 31.7780 16.6942 656151.2420 2.4470

```
 2.4341
[1534]ENERGY: 1300000      3092.2028      5120.0240      5307.3247
329.4836         -15721.2808     -1766.6691         0.0000         0.0000
7570.1987           3931.2839       302.8458     -3638.9148      3994.2972
301.2549             60.2977        32.7408    656151.2420        -1.3890
-1.3848
[1584]ENERGY: 1310000      3119.2535      5085.3368      5288.9054
326.4813         -15720.2603     -1707.6237         0.0000         0.0000
7541.9736           3934.0667       301.7167     -3607.9069      3994.0717
301.1964            127.3238        99.7741    656151.2420        -1.7378
-1.7286
[1620]ENERGY: 1320000      3093.7106      5145.5057      5249.6797
339.5724         -15747.2907     -1695.9058         0.0000         0.0000
7548.9434           3934.2152       301.9955     -3614.7282      3995.8637
301.1275            122.8876        -4.0803    656151.2420         1.3400
 1.3388
[1670]ENERGY: 1330000      3129.5508      5073.7137      5321.0238
334.2743         -15718.5594     -1788.5604         0.0000         0.0000
7582.3951           3933.8379       303.3337     -3648.5571      3995.8339
300.9519            -80.0610       -77.0076    656151.2420        -0.0003
 0.0031
[1706]ENERGY: 1340000      3042.1725      5099.2504      5287.4190
337.1003         -15642.8362     -1697.9865         0.0000         0.0000
7507.6434           3932.7629       300.3433     -3574.8804      3994.5900
301.1841            -30.6884        37.0408    656151.2420        -0.6162
-0.6156
[1756]ENERGY: 1350000      3178.3367      5064.3092      5270.8475
337.6608         -15732.3425     -1721.7198         0.0000         0.0000
7540.2236           3937.3155       301.6466     -3602.9081      3995.9921
301.4323            124.1931        54.5352    656151.2420        -0.4457
-0.4608
[1792]ENERGY: 1360000      3167.5103      5111.2411      5255.3469
328.8455         -15727.9508     -1754.7989         0.0000         0.0000
7554.8324           3935.0265       302.2311     -3619.8060      3995.6638
301.2498           -112.7605       -59.9524    656151.2420         1.5894
 1.6102
[1842]ENERGY: 1370000      3120.6887      5045.3195      5256.2097
332.3217         -15682.4049     -1738.3741         0.0000         0.0000
7603.0301           3936.7907       304.1592     -3666.2394      3995.6852
301.5272            144.8537        50.3519    656151.2420         0.0234
 0.0069
[1878]ENERGY: 1380000      3184.2826      5097.7385      5286.9223
344.1554         -15744.6954     -1714.7214         0.0000         0.0000
7482.7615           3936.4435       299.3479     -3546.3180      3995.4789
301.2462            -59.9761       -66.3143    656151.2420        -0.4990
-0.4744
[1928]ENERGY: 1390000      3198.5600      5157.4490      5280.4024
311.5238         -15755.4931     -1720.3160         0.0000         0.0000
7467.0631           3939.1892       298.7199     -3527.8740      3995.3739
301.4271            -33.9930       -86.4707    656151.2420         0.9403
 0.9469
[1967]ENERGY: 1400000      3144.9789      5072.5644      5257.7541
331.3486         -15712.1559     -1710.2985         0.0000         0.0000
7550.7170           3934.9088       302.0664     -3615.8083      3996.3414
```

301.5900 -15.0458 64.4352 656151.2420 -0.2359
-0.2534
[2017]ENERGY: 1410000 3160.5877 5123.2947 5290.5244
339.2333 -15621.6876 -1812.1626 0.0000 0.0000
7458.9854 3938.7752 298.3967 -3520.2102 3996.5596
301.3866 -100.2792 -180.7632 656151.2420 -0.4419
-0.4496
[2053]ENERGY: 1420000 3029.9497 5174.7437 5307.6297
312.4446 -15701.4260 -1719.7560 0.0000 0.0000
7529.0080 3932.5937 301.1980 -3596.4143 3996.3828
301.5138 149.2735 96.8417 656151.2420 -0.1406
-0.1281
[2103]ENERGY: 1430000 3131.3548 5096.2903 5221.1383
352.7365 -15719.2081 -1711.8529 0.0000 0.0000
7562.6766 3933.1354 302.5449 -3629.5412 3996.5634
301.4552 69.5466 -26.4306 656151.2420 0.7142
0.6830
[2139]ENERGY: 1440000 3116.1853 5009.9323 5310.9272
354.0495 -15631.9521 -1768.7974 0.0000 0.0000
7543.8666 3934.2114 301.7924 -3609.6552 3996.6916
301.3315 -7.0683 -21.5582 656151.2420 0.0862
0.1109
[2189]ENERGY: 1450000 3073.2160 5077.9175 5235.1414
332.6808 -15702.2017 -1684.6644 0.0000 0.0000
7602.0801 3934.1697 304.1212 -3667.9103 3997.2514
301.5020 31.7654 87.7517 656151.2420 0.4486
0.4308
[2225]ENERGY: 1460000 3122.4731 5094.5564 5272.8319
352.6459 -15753.6037 -1707.2994 0.0000 0.0000
7554.8302 3936.4345 302.2310 -3618.3957 3997.3223
301.3957 -172.4570 -90.2803 656151.2420 -1.2930
-1.2684
[2275]ENERGY: 1470000 3133.1869 5159.6372 5232.3436
339.1830 -15691.4828 -1757.9858 0.0000 0.0000
7521.7477 3936.6298 300.9075 -3585.1179 3998.1988
301.6756 18.0719 -3.9715 656151.2420 1.0807
1.0657
[2311]ENERGY: 1480000 3134.7065 5083.7288 5319.1768
314.2807 -15724.1334 -1734.0298 0.0000 0.0000
7543.0028 3936.7324 301.7578 -3606.2704 3998.7304
301.6028 72.9195 -11.1271 656151.2420 -0.6209
-0.6092
[2361]ENERGY: 1490000 3110.3615 5071.7306 5277.9648
343.7396 -15679.4592 -1748.4654 0.0000 0.0000
7560.3568 3936.2288 302.4521 -3624.1280 3999.0294
301.5579 34.6465 -21.5684 656151.2420 -0.4641
-0.4659
[2400]ENERGY: 1500000 3172.6596 5094.8279 5290.6828
339.7068 -15712.1362 -1648.3216 0.0000 0.0000
7404.3266 3941.7459 296.2101 -3462.5807 3999.6528
301.3344 93.9347 201.1722 656151.2420 1.0311
1.0238
[2450]ENERGY: 1510000 3195.3170 5070.0486 5251.4353
347.5939 -15706.7699 -1679.3776 0.0000 0.0000

7466.3955 3944.6428 298.6931 -3521.7527 4002.0325
301.4199 4.2737 -7.2083 656151.2420 0.1116
0.1142
[2486]ENERGY: 1520000 3044.2511 5122.7220 5295.3318
337.2040 -15707.9288 -1707.1123 0.0000 0.0000
7553.8537 3938.3215 302.1919 -3615.5322 4000.9525
301.4743 -1.7613 -23.2669 656151.2420 -1.4422
-1.4506
[2536]ENERGY: 1530000 3136.1963 5063.0961 5278.6558
326.1116 -15654.4085 -1763.6457 0.0000 0.0000
7552.7255 3938.7312 302.1468 -3613.9943 4001.3416
301.4430 29.6048 53.2090 656151.2420 0.9144
0.9168
[2572]ENERGY: 1540000 3108.8359 5049.2745 5328.3901
336.9242 -15695.9062 -1712.8469 0.0000 0.0000
7526.1778 3940.8495 301.0847 -3585.3284 4002.5363
301.3137 -17.7958 -20.9396 656151.2420 -3.0590
-3.0345
[2622]ENERGY: 1550000 3106.3656 5176.4882 5197.6299
339.4438 -15701.7910 -1758.3667 0.0000 0.0000
7578.1993 3937.9690 303.1659 -3640.2303 4001.8375
301.3978 -36.7296 -28.8458 656151.2420 2.1731
2.1657
[2658]ENERGY: 1560000 3127.0973 5086.0708 5291.0576
338.3608 -15689.3842 -1713.6026 0.0000 0.0000
7502.7799 3942.3796 300.1487 -3560.4003 4001.2573
301.5652 85.0345 -14.0353 656151.2420 0.5906
0.5874
[2708]ENERGY: 1570000 3058.6967 5178.5954 5234.7069
335.2297 -15781.1694 -1721.6455 0.0000 0.0000
7633.1466 3937.5604 305.3640 -3695.5862 4001.2126
301.6031 137.9398 31.0791 656151.2420 -0.5187
-0.5393
[2744]ENERGY: 1580000 3217.5721 5107.6365 5279.0218
343.4268 -15724.9703 -1770.3668 0.0000 0.0000
7491.8638 3944.1840 299.7120 -3547.6798 4001.9414
301.7767 -150.9771 -134.2512 656151.2420 1.9681
1.9955
[2794]ENERGY: 1590000 3155.6460 5061.3783 5290.4332
352.3602 -15620.3994 -1780.0153 0.0000 0.0000
7483.8506 3943.2535 299.3914 -3540.5971 4002.6238
301.5885 32.1802 15.8458 656151.2420 -1.9370
-1.9529
[2833]ENERGY: 1600000 3101.7312 5124.0448 5313.9448
326.4631 -15685.5813 -1742.7406 0.0000 0.0000
7502.3303 3940.1923 300.1307 -3562.1380 4001.7317
301.5778 -56.5336 -29.1028 656151.2420 -0.5509
-0.5614
[2883]ENERGY: 1610000 3121.8907 5149.5729 5278.4289
313.7179 -15742.6769 -1733.6251 0.0000 0.0000
7552.7067 3940.0150 302.1460 -3612.6917 4001.5518
301.5519 -45.1462 -49.7382 656151.2420 0.7974
0.8125
[2919]ENERGY: 1620000 3152.3830 5045.0918 5302.9083

344.1710 -15712.4361 -1703.1138 0.0000 0.0000
7514.0165 3943.0207 300.5982 -3570.9958 4003.5360
301.8126 63.9146 41.4730 656151.2420 -1.1653
-1.1668
[2969]ENERGY: 1630000 3050.8998 5124.2475 5292.7266
341.4259 -15836.0834 -1672.1088 0.0000 0.0000
7634.4632 3935.5708 305.4167 -3698.8924 4002.6403
301.9037 51.2771 56.5106 656151.2420 0.2039
0.2101
[3005]ENERGY: 1640000 3032.8364 5071.1137 5336.7453
330.1876 -15614.9688 -1761.8118 0.0000 0.0000
7547.9169 3942.0194 301.9544 -3605.8975 4002.6160
301.8122 9.9398 -21.7186 656151.2420 1.5884
1.5898
[3055]ENERGY: 1650000 3215.1148 5170.0860 5259.2074
354.1419 -15774.7367 -1739.7728 0.0000 0.0000
7458.3656 3942.4061 298.3719 -3515.9595 4003.1848
301.8832 62.9361 -48.5371 656151.2420 -0.2910
-0.3119
[3091]ENERGY: 1660000 3102.4322 5040.6194 5322.6325
327.2535 -15627.0581 -1814.1462 0.0000 0.0000
7590.4778 3942.2111 303.6571 -3648.2667 4002.7298
301.7912 31.4788 -26.4984 656151.2420 0.1260
0.1289
[3141]ENERGY: 1670000 3138.0338 5056.0277 5289.7561
351.4223 -15644.0974 -1770.5998 0.0000 0.0000
7518.3395 3938.8823 300.7712 -3579.4572 4000.9578
301.8049 65.5763 10.3378 656151.2420 -2.2907
-2.2823
[3177]ENERGY: 1680000 3112.6163 5121.4851 5278.2181
335.9558 -15696.0446 -1762.8265 0.0000 0.0000
7549.6979 3939.1021 302.0257 -3610.5958 4001.5477
302.0756 -17.2812 -37.0689 656151.2420 3.0822
3.0746
[3227]ENERGY: 1690000 3163.7459 5052.4784 5292.6217
323.5012 -15709.4928 -1735.0511 0.0000 0.0000
7552.3241 3940.1276 302.1307 -3612.1965 4000.8985
301.8542 59.4426 60.1142 656151.2420 -1.7278
-1.7065
[3266]ENERGY: 1700000 3191.1916 5124.6331 5273.2064
353.8559 -15853.7181 -1697.3141 0.0000 0.0000
7544.5120 3936.3668 301.8182 -3608.1452 4000.1043
301.7626 -10.1117 85.8286 656151.2420 0.4128
0.3948
[3316]ENERGY: 1710000 3127.1249 5068.2920 5288.0342
347.4041 -15750.8373 -1744.3936 0.0000 0.0000
7603.7161 3939.3405 304.1867 -3664.3757 4001.0396
302.0366 -49.8446 -42.9743 656151.2420 -0.2132
-0.2184
[3352]ENERGY: 1720000 3126.6602 5163.1473 5292.1021
319.3422 -15678.9190 -1803.5738 0.0000 0.0000
7523.7987 3942.5578 300.9896 -3581.2409 4000.9318
301.8903 10.8589 -59.5494 656151.2420 -0.6551
-0.6590

[3402]ENERGY: 1730000 3108.2360 5138.9104 5231.6838
330.0156 -15690.6904 -1749.7300 0.0000 0.0000
7571.6452 3940.0706 302.9037 -3631.5746 4000.6040
301.8414 63.0104 -23.3931 656151.2420 0.8656
0.8995
[3438]ENERGY: 1740000 3213.9203 5146.3495 5278.9667
326.1769 -15860.7757 -1691.4216 0.0000 0.0000
7525.3912 3938.6072 301.0533 -3586.7840 3998.6094
301.8913 31.1415 119.4196 656151.2420 0.7338
0.7125
[3488]ENERGY: 1750000 3132.3752 5145.3628 5306.1793
356.3386 -15762.7211 -1752.2833 0.0000 0.0000
7511.7498 3937.0012 300.5075 -3574.7485 3999.9086
302.2275 -131.2147 -59.4601 656151.2420 -1.5193
-1.5143
[3524]ENERGY: 1760000 3145.9480 5155.2912 5295.5878
326.4194 -15734.9129 -1744.7835 0.0000 0.0000
7496.9322 3940.4823 299.9148 -3556.4499 3999.8810
302.4024 197.7511 106.1376 656151.2420 -0.2241
-0.2166
[3574]ENERGY: 1770000 3197.3057 5135.9879 5281.3644
308.6581 -15773.5485 -1762.7478 0.0000 0.0000
7552.9175 3939.9373 302.1545 -3612.9802 4000.4577
302.2942 184.9469 113.7402 656151.2420 -1.4378
-1.4553
[3610]ENERGY: 1780000 3132.7855 4997.0156 5292.5314
343.2720 -15701.5724 -1758.2292 0.0000 0.0000
7629.9262 3935.7291 305.2352 -3694.1972 4000.4693
302.2393 -11.9419 -54.6613 656151.2420 3.1372
3.1467
[3660]ENERGY: 1790000 3183.6371 5101.3534 5269.2453
345.5131 -15778.1712 -1729.7976 0.0000 0.0000
7547.7068 3939.4868 301.9460 -3608.2199 4001.5487
302.2044 -15.0291 -1.9234 656151.2420 -0.1076
-0.1086
[3699]ENERGY: 1800000 3143.6471 5126.4161 5238.7519
302.2961 -15785.7426 -1726.1733 0.0000 0.0000
7641.2813 3940.4765 305.6895 -3700.8047 4000.4445
302.2978 -46.7762 -40.9150 656151.2420 0.7462
0.7357
[3749]ENERGY: 1810000 3132.6437 5154.3488 5315.1322
337.1721 -15737.3121 -1720.3776 0.0000 0.0000
7460.5795 3942.1866 298.4605 -3518.3930 4001.2432
302.1240 31.9670 67.9515 656151.2420 -0.9728
-0.9631
[3785]ENERGY: 1820000 3085.6018 5181.4329 5282.8352
346.0784 -15770.8122 -1707.4715 0.0000 0.0000
7523.2711 3940.9358 300.9685 -3582.3353 4001.5826
301.9943 42.9772 16.5497 656151.2420 -1.0739
-1.0824
[3835]ENERGY: 1830000 3101.8102 5187.0989 5284.4175
336.6790 -15767.2198 -1738.4440 0.0000 0.0000
7532.6399 3936.9816 301.3433 -3595.6583 4001.8322
302.0236 19.7647 28.5755 656151.2420 1.6970

 1.6971
[3871]ENERGY: 1840000   3146.5383   5141.3499   5296.0111
334.9195   -15782.7768   -1745.8729   0.0000   0.0000
7547.4468   3937.6159   301.9356   -3609.8310   4000.5194
301.9513   -72.0605   -122.0535   656151.2420   -2.5316
-2.5177
[3921]ENERGY: 1850000   3206.9136   5079.0581   5284.7830
336.5049   -15798.1807   -1748.0134   0.0000   0.0000
7576.7771   3937.8425   303.1090   -3638.9347   4000.9887
301.9462   12.9131   1.8567   656151.2420   1.8899
 1.8934
[3957]ENERGY: 1860000   3123.2590   5142.7743   5264.8005
349.7420   -15727.0407   -1717.3945   0.0000   0.0000
7507.8548   3943.9953   300.3517   -3563.8595   4001.7217
301.9549   65.2586   -1.1913   656151.2420   1.9537
 1.9553
[4007]ENERGY: 1870000   3115.3578   5114.6111   5287.5377
336.8478   -15793.5390   -1663.2392   0.0000   0.0000
7540.9963   3938.5726   301.6776   -3602.4237   4000.5055
301.8869   104.3655   51.3044   656151.2420   -2.5125
-2.5263
[4043]ENERGY: 1880000   3186.9140   5095.8429   5267.0836
353.1228   -15732.9651   -1747.6919   0.0000   0.0000
7515.4462   3937.7525   300.6554   -3577.6937   4000.7413
302.1046   86.1644   84.8527   656151.2420   0.0208
 0.0303
[4093]ENERGY: 1890000   3161.6146   5182.8796   5256.8276
338.0380   -15800.5753   -1678.5428   0.0000   0.0000
7478.8446   3939.0862   299.1912   -3539.7584   4001.5415
302.1529   -48.7557   14.5006   656151.2420   0.1560
 0.1603
[4132]ENERGY: 1900000   3174.8723   5121.8920   5234.3643
308.8725   -15682.8963   -1773.7065   0.0000   0.0000
7556.0986   3939.4969   302.2817   -3616.6016   4002.0940
302.1280   -195.7344   -135.7212   656151.2420   0.5689
 0.5675
[4182]ENERGY: 1910000   3168.1697   5000.4033   5269.3314
334.4816   -15613.5654   -1770.4835   0.0000   0.0000
7557.4083   3945.7455   302.3341   -3611.6628   4001.9160
301.9811   -95.3639   -108.6229   656151.2420   -0.2399
-0.2396
[4218]ENERGY: 1920000   3166.7697   5245.3643   5277.5496
335.7481   -15764.5760   -1798.9838   0.0000   0.0000
7481.5904   3943.4623   299.3010   -3538.1281   4002.9538
302.0675   -91.3368   -57.9696   656151.2420   1.0369
 1.0219
[4268]ENERGY: 1930000   3136.1754   5103.6423   5327.0137
358.9530   -15720.5233   -1792.6431   0.0000   0.0000
7527.3869   3940.0050   301.1331   -3587.3819   4003.3573
302.1214   -14.4507   39.7001   656151.2420   -0.3912
-0.3857
[4304]ENERGY: 1940000   3142.9184   5148.1692   5282.7945
349.2916   -15691.8603   -1806.1295   0.0000   0.0000
7515.7389   3940.9229   300.6671   -3574.8160   4002.9513

302.0691 -76.7266 -32.2486 656151.2420 0.6723
0.6759
[4354]ENERGY: 1950000 3180.7617 5114.9695 5304.4261
338.9161 -15774.5928 -1738.4698 0.0000 0.0000
7515.9315 3941.9425 300.6748 -3573.9890 4004.0787
302.0653 -48.4588 -113.1975 656151.2420 -2.1968
-2.2072
[4390]ENERGY: 1960000 3104.7124 5105.4622 5272.6698
334.8431 -15769.1157 -1674.9850 0.0000 0.0000
7570.8988 3944.4857 302.8738 -3626.4131 4001.9747
301.9607 96.9628 151.0964 656151.2420 -0.3070
-0.2738
[4440]ENERGY: 1970000 3184.5432 5054.4733 5237.5538
348.3751 -15762.4625 -1714.4691 0.0000 0.0000
7593.3474 3941.3613 303.7719 -3651.9861 4001.7694
301.8936 -116.6191 -67.9287 656151.2420 0.0781
0.0657
[4476]ENERGY: 1980000 3098.5690 5218.3547 5271.0912
341.9321 -15768.6169 -1751.0248 0.0000 0.0000
7530.4679 3940.7733 301.2564 -3589.6946 4001.1852
302.1833 -24.4965 -42.8394 656151.2420 -0.0532
-0.0653
[4526]ENERGY: 1990000 3129.6986 5151.7789 5244.8073
326.4080 -15742.9989 -1749.6387 0.0000 0.0000
7582.1132 3942.1682 303.3224 -3639.9450 4001.1861
302.0410 -63.6983 11.3554 656151.2420 0.6181
0.6311
[4565]ENERGY: 2000000 3210.1056 5070.2962 5324.5510
339.6444 -15818.2580 -1752.7236 0.0000 0.0000
7566.9096 3940.5252 302.7142 -3626.3844 3999.8689
302.1343 -61.5178 -72.9907 656151.2420 2.1028
2.0890
[4615]ENERGY: 2010000 3131.2707 5026.3245 5258.6605
329.3715 -15750.9094 -1699.0321 0.0000 0.0000
7642.6725 3938.3582 305.7451 -3704.3143 4001.0238
302.2061 27.9115 13.3034 656151.2420 -2.4936
-2.5037
[4651]ENERGY: 2020000 3090.1899 5112.8239 5264.0030
338.6124 -15700.5807 -1738.5783 0.0000 0.0000
7573.5934 3940.0635 302.9816 -3633.5299 4001.9368
302.2342 67.8924 24.8182 656151.2420 1.1845
1.1967
[4701]ENERGY: 2030000 3123.6371 5106.6571 5220.8829
340.1108 -15768.6231 -1629.8818 0.0000 0.0000
7550.1303 3942.9135 302.0430 -3607.2168 4001.2705
302.1750 116.9326 85.8052 656151.2420 -1.7287
-1.7383
[4737]ENERGY: 2040000 3091.8154 5077.0325 5232.8556
303.7594 -15717.8531 -1729.6347 0.0000 0.0000
7678.6244 3936.5994 307.1834 -3742.0250 4002.1427
302.2457 -8.7319 -49.7137 656151.2420 1.6215
1.6259
[4787]ENERGY: 2050000 3087.1063 5048.9859 5269.3534
322.1354 -15692.1594 -1765.9525 0.0000 0.0000

7668.4550 3937.9240 306.7765 -3730.5311 4002.2427
302.1353 11.8708 9.4098 656151.2420 -1.3037
-1.2935
[4823]ENERGY: 2060000 3160.6188 5128.1176 5244.3893
302.0829 -15768.2646 -1751.5010 0.0000 0.0000
7626.9027 3942.3456 305.1142 -3684.5571 4003.4570
302.1508 -19.5915 -43.2914 656151.2420 0.3603
0.3524
[4873]ENERGY: 2070000 3066.4038 5207.4369 5204.1679
335.3907 -15657.8547 -1757.4362 0.0000 0.0000
7541.4162 3939.5246 301.6944 -3601.8916 4002.5081
302.0451 92.7479 36.8650 656151.2420 0.7586
0.7569
[4909]ENERGY: 2080000 3168.8348 5118.1974 5228.4351
327.9501 -15717.2629 -1742.3616 0.0000 0.0000
7556.3307 3940.1236 302.2910 -3616.2071 4001.9765
302.4393 -99.4729 -96.0981 656151.2420 0.7462
0.7498
[4959]ENERGY: 2090000 3118.8735 5158.9874 5172.7596
329.6900 -15799.9147 -1736.3970 0.0000 0.0000
7693.2342 3937.2330 307.7678 -3756.0013 4002.4175
302.4963 148.8438 117.7937 656151.2420 -1.3021
-1.3147
[4998]ENERGY: 2100000 3195.5487 5196.4897 5260.8943
357.1556 -15812.8926 -1718.1172 0.0000 0.0000
7463.7137 3942.7921 298.5859 -3520.9215 4001.2607
302.4630 -170.5126 -89.2159 656151.2420 1.9025
1.8977
[5048]ENERGY: 2110000 3175.0792 5198.0966 5245.5446
336.1280 -15771.6011 -1687.8208 0.0000 0.0000
7449.8417 3945.2682 298.0309 -3504.5735 4002.1054
302.4458 200.4947 196.4669 656151.2420 -2.2332
-2.1989
[5084]ENERGY: 2120000 3176.2410 5102.3133 5254.2555
333.5848 -15704.8117 -1770.1943 0.0000 0.0000
7548.4873 3939.8758 301.9772 -3608.6115 4002.5655
302.6341 -75.3028 -74.6976 656151.2420 0.5149
0.4943
[5134]ENERGY: 2130000 3203.2909 5087.0059 5270.4374
314.1177 -15705.7795 -1833.4998 0.0000 0.0000
7604.9765 3940.5491 304.2371 -3664.4274 4003.4630
302.6250 -154.8311 -108.2528 656151.2420 -0.2428
-0.2263
[5170]ENERGY: 2140000 3210.5659 5162.2971 5241.4636
319.6246 -15748.5103 -1796.1818 0.0000 0.0000
7553.1185 3942.3776 302.1625 -3610.7409 4002.2086
302.6207 -88.0895 -98.3808 656151.2420 0.5678
0.5489
[5220]ENERGY: 2150000 3099.4997 5110.3384 5234.8120
342.0024 -15718.6486 -1722.6660 0.0000 0.0000
7595.2076 3940.5456 303.8463 -3654.6620 4002.5330
302.6421 148.5102 11.9985 656151.2420 -0.9109
-0.9114
[5256]ENERGY: 2160000 3246.2360 5137.1389 5242.2220

323.4080   -15734.2783   -1773.5761   0.0000   0.0000
7498.5196   3939.6700   299.9783   -3558.8496   4004.3197
302.6532   -52.2991   -69.8987   656151.2420   0.3117
 0.3270
[5306]ENERGY: 2170000   3226.2762   5202.4087   5190.6034
318.2896   -15765.7604   -1760.3025   0.0000   0.0000
7530.4237   3941.9388   301.2546   -3588.4849   4002.5784
302.6050   -15.3734   -108.0749   656151.2420   0.6718
 0.6418
[5342]ENERGY: 2180000   3089.0484   5189.6619   5256.2904
326.3850   -15802.9253   -1750.9074   0.0000   0.0000
7629.2714   3936.8245   305.2090   -3692.4469   4003.2569
302.7941   66.7716   16.3292   656151.2420   -1.6804
-1.6754
[5392]ENERGY: 2190000   3195.5020   5042.5912   5216.9565
325.1379   -15757.6534   -1736.8358   0.0000   0.0000
7654.3468   3940.0454   306.2121   -3714.3015   4003.3395
302.6053   -102.3698   -106.6809   656151.2420   1.7577
 1.7756
[5431]ENERGY: 2200000   3205.4792   5202.5705   5230.0297
337.7623   -15852.4070   -1680.1632   0.0000   0.0000
7500.7259   3943.9972   300.0665   -3556.7287   4002.8657
302.7850   69.1633   75.9002   656151.2420   0.6337
 0.6290
[5481]ENERGY: 2210000   3195.1925   5177.3236   5201.3621
346.6085   -15737.5368   -1728.7476   0.0000   0.0000
7490.5180   3944.7202   299.6582   -3545.7977   4002.8968
302.7125   -21.9702   -33.7878   656151.2420   -0.7592
-0.7667
[5517]ENERGY: 2220000   3141.1278   5156.9512   5210.4246
343.4263   -15752.3167   -1712.9392   0.0000   0.0000
7555.0902   3941.7642   302.2414   -3613.3260   4002.9805
302.5024   136.2451   54.9678   656151.2420   -1.5597
-1.5559
[5567]ENERGY: 2230000   3257.6442   5172.1769   5237.0479
350.6302   -15811.7204   -1801.1296   0.0000   0.0000
7534.7153   3939.3645   301.4263   -3595.3508   4003.2787
302.6077   -89.5324   -157.9186   656151.2420   0.3523
 0.3361
[5603]ENERGY: 2240000   3177.1449   5115.4621   5224.9256
341.2695   -15817.9337   -1657.3671   0.0000   0.0000
7557.1383   3940.6395   302.3233   -3616.4988   4002.7007
302.6586   102.6752   29.3581   656151.2420   -3.7356
-3.7255
[5653]ENERGY: 2250000   3132.8800   5165.2777   5295.0537
331.3875   -15802.8227   -1716.5587   0.0000   0.0000
7538.6374   3943.8549   301.5832   -3594.7825   4004.0015
303.4477   28.2203   -8.3677   656151.2420   -2.0371
-2.0295
[5689]ENERGY: 2260000   3108.4819   5086.1812   5259.3758
350.4885   -15760.2797   -1762.7710   0.0000   0.0000
7657.1185   3938.5952   306.3230   -3718.5233   4004.4651
303.3032   24.8646   48.2427   656151.2420   -12.1146
-12.1047

[5739]ENERGY: 2270000 3134.7698 5097.7581 5227.3242
322.5484 -15804.2229 -1702.7994 0.0000 0.0000
7663.6234 3939.0016 306.5833 -3724.6219 4003.7310
303.3342 101.0289 101.6134 656151.2420 -20.1282
-20.1401
[5775]ENERGY: 2280000 3207.3796 5117.4534 5228.3865
338.4008 -15780.1046 -1748.0418 0.0000 0.0000
7582.6121 3946.0859 303.3424 -3636.5261 4004.5158
303.5390 10.9608 -63.0946 656151.2420 -8.1724
-8.1694
[5825]ENERGY: 2290000 3166.9422 5222.8530 5230.2281
323.4173 -15735.8819 -1802.1102 0.0000 0.0000
7539.9213 3945.3698 301.6345 -3594.5515 4003.5398
303.5616 -91.3545 -27.4800 656151.2420 -11.6930
-11.6978
[5864]ENERGY: 2300000 3254.1805 5191.4367 5228.5185
353.8740 -15921.8516 -1750.5822 0.0000 0.0000
7585.7341 3941.3101 303.4673 -3644.4240 4003.8028
303.4788 -50.7503 -30.3028 656151.2420 -14.9534
-14.9444
[5914]ENERGY: 2310000 3062.2500 5209.6856 5218.4696
337.7124 -15781.2385 -1765.6821 0.0000 0.0000
7656.8660 3938.0630 306.3129 -3718.8029 4002.4446
303.5187 114.1866 135.6266 656151.2420 -10.9785
-10.9736
[5950]ENERGY: 2320000 3278.6750 5198.1663 5239.0918
332.6940 -15935.5081 -1732.3540 0.0000 0.0000
7561.1984 3941.9635 302.4857 -3619.2349 4003.5019
303.3314 -134.3948 -99.7047 656151.2420 -16.1806
-16.1953
[6000]ENERGY: 2330000 3150.5684 5114.7155 5243.3964
345.2353 -15766.9428 -1742.8458 0.0000 0.0000
7596.7456 3940.8725 303.9078 -3655.8730 4002.8330
303.5670 -29.9106 -29.0853 656151.2420 -6.1073
-6.0953
[6036]ENERGY: 2340000 3085.6990 5187.8735 5311.5634
334.4501 -15705.8098 -1785.8649 0.0000 0.0000
7513.0395 3940.9508 300.5591 -3572.0887 4003.6437
303.7054 -13.3238 -55.7282 656151.2420 -15.4372
-15.4374
[6086]ENERGY: 2350000 3108.0777 5214.8109 5215.5118
315.3994 -15793.2455 -1730.4779 0.0000 0.0000
7608.4945 3938.5709 304.3778 -3669.9236 4002.7987
303.4817 -145.4004 -153.7805 656151.2420 -11.3612
-11.3585
[6122]ENERGY: 2360000 3139.3696 5234.5458 5238.3699
349.1377 -15724.2268 -1781.4766 0.0000 0.0000
7488.4101 3944.1298 299.5738 -3544.2803 4002.8815
303.4674 19.3285 -1.0628 656151.2420 -9.8396
-9.8481
[6172]ENERGY: 2370000 3140.1914 5132.9616 5242.9826
321.3774 -15735.5593 -1732.4172 0.0000 0.0000
7574.4716 3944.0082 303.0167 -3630.4634 4001.6703
303.6291 -35.3803 56.1541 656151.2420 -10.1125

-10.1148
[6208]ENERGY: 2380000 3149.2437 5222.6201 5259.3151
330.2510 -15867.3046 -1702.4582 0.0000 0.0000
7548.7959 3940.4629 301.9896 -3608.3330 4001.1655
303.6890 100.4501 33.9899 656151.2420 -11.0325
-11.0225
[6258]ENERGY: 2390000 3158.7909 5213.5435 5279.9211
300.7634 -15799.6795 -1759.5115 0.0000 0.0000
7550.7325 3944.5604 302.0671 -3606.1721 4002.7922
303.7253 146.5380 43.1227 656151.2420 -13.5642
-13.5850
[6297]ENERGY: 2400000 3249.7838 5208.1729 5278.4430
315.6764 -15882.7624 -1743.6652 0.0000 0.0000
7516.2446 3941.8930 300.6874 -3574.3516 4002.0714
303.7689 102.7185 64.0213 656151.2420 -19.9847
-19.9776
[6347]ENERGY: 2410000 3203.1306 5111.4271 5253.8155
348.9482 -15687.8630 -1906.9561 0.0000 0.0000
7619.3232 3941.8254 304.8110 -3677.4978 4002.8606
303.8017 -49.4767 -138.3456 656151.2420 -19.5819
-19.5801
[6383]ENERGY: 2420000 3132.3158 5058.5118 5224.1339
350.7016 -15743.4411 -1713.9505 0.0000 0.0000
7631.8728 3940.1442 305.3131 -3691.7286 4000.8728
303.7666 182.0528 128.6366 656151.2420 -16.8757
-16.8642
[6433]ENERGY: 2430000 3184.9268 5197.4857 5292.5949
352.7490 -15889.4619 -1770.8176 0.0000 0.0000
7575.5199 3942.9969 303.0587 -3632.5230 4003.0005
303.7374 -9.7919 -63.2916 656151.2420 -20.6712
-20.6784
[6469]ENERGY: 2440000 3107.2641 5173.1274 5308.1244
344.3945 -15916.7077 -1652.5700 0.0000 0.0000
7579.6692 3943.3019 303.2247 -3636.3674 4003.0043
303.7920 167.6702 82.7078 656151.2420 -17.9965
-18.0117
[6519]ENERGY: 2450000 3065.6582 5116.8449 5321.4641
331.4253 -15803.7558 -1705.2531 0.0000 0.0000
7615.9895 3942.3732 304.6777 -3673.6163 4003.2039
303.7460 40.6605 -26.4443 656151.2420 -14.4294
-14.4196
[6555]ENERGY: 2460000 3135.9717 5217.5639 5279.2644
334.0823 -15796.8327 -1826.6376 0.0000 0.0000
7598.9765 3942.3885 303.9971 -3656.5880 4003.5086
303.7907 70.9342 -16.8022 656151.2420 -19.8021
-19.8043
[6605]ENERGY: 2470000 3167.0493 5217.6431 5269.6632
321.7337 -15902.7376 -1743.1261 0.0000 0.0000
7611.4780 3941.7036 304.4972 -3669.7744 4002.0636
303.9245 7.2189 41.6153 656151.2420 -18.0422
-18.0274
[6641]ENERGY: 2480000 3189.0556 5114.5316 5221.1582
357.2565 -15769.9154 -1757.1686 0.0000 0.0000
7585.9946 3940.9125 303.4777 -3645.0821 4003.0902

303.8463 -42.6548 -82.5761 656151.2420 -20.6323
-20.6421
[6691]ENERGY: 2490000 3121.8998 5279.8720 5274.7923
312.2904 -15757.3986 -1787.9173 0.0000 0.0000
7501.5423 3945.0809 300.0992 -3556.4614 4002.5714
303.7346 41.5317 8.5729 656151.2420 -16.7883
-16.7808
[6730]ENERGY: 2500000 3155.3811 5103.7586 5240.2491
309.6914 -15683.6101 -1766.7566 0.0000 0.0000
7584.6672 3943.3807 303.4246 -3641.2864 4003.4966
303.6752 -9.4746 4.5628 656151.2420 -14.3410
-14.3428
[6780]ENERGY: 2510000 3100.2506 5239.6014 5238.5357
333.6457 -15801.8293 -1779.8353 0.0000 0.0000
7612.4602 3942.8290 304.5365 -3669.6312 4002.8669
303.8739 60.8943 101.4629 656151.2420 -16.1407
-16.1483
[6816]ENERGY: 2520000 3282.4376 5214.9810 5211.5000
323.2964 -15799.7360 -1866.2596 0.0000 0.0000
7576.9389 3943.1584 303.1154 -3633.7805 4001.8454
303.9822 -253.3885 -219.3876 656151.2420 -15.9081
-15.8857
[6866]ENERGY: 2530000 3190.2924 5109.3753 5249.8790
340.3672 -15756.9765 -1833.5259 0.0000 0.0000
7641.0894 3940.5009 305.6818 -3700.5885 4003.3073
303.9390 11.3449 -23.2533 656151.2420 -15.9548
-15.9712
[6902]ENERGY: 2540000 3160.7817 5152.4038 5266.7623
319.5078 -15720.7839 -1818.7770 0.0000 0.0000
7579.5306 3939.4252 303.2191 -3640.1054 4003.6855
304.0137 89.6818 30.3302 656151.2420 -19.9455
-19.9406
[6952]ENERGY: 2550000 3103.2927 5254.2276 5250.1213
343.0612 -15851.4015 -1774.5763 0.0000 0.0000
7617.9324 3942.6574 304.7554 -3675.2750 4004.2081
304.0696 -76.3393 -61.2962 656151.2420 -14.2384
-14.2349
[6988]ENERGY: 2560000 3098.8388 5158.4779 5256.9361
335.8229 -15763.2944 -1821.8213 0.0000 0.0000
7674.5151 3939.4752 307.0190 -3735.0399 4003.1940
303.9632 -31.7193 -0.8599 656151.2420 -20.5145
-20.5235
[7038]ENERGY: 2570000 3128.5483 5176.2264 5236.3031
348.0838 -15768.0370 -1777.9152 0.0000 0.0000
7595.6255 3938.8349 303.8630 -3656.7906 4003.6738
303.7942 148.7436 86.7086 656151.2420 -15.5015
-15.5091
[7074]ENERGY: 2580000 3175.3071 5187.0035 5282.3779
351.9794 -15821.8028 -1783.9781 0.0000 0.0000
7552.8407 3943.7278 302.1514 -3609.1129 4004.0228
303.7480 90.0577 18.8843 656151.2420 -15.9034
-15.8987
[7124]ENERGY: 2590000 3128.5811 5229.1024 5247.5452
357.0105 -15824.0957 -1830.1000 0.0000 0.0000

7632.1215 3940.1650 305.3230 -3691.9565 4002.7828
303.7871 2.6263 -44.7818 656151.2420 -15.4872
-15.4700
[7163]ENERGY: 2600000 3161.7352 5188.8594 5237.1707
333.2387 -15855.0819 -1701.0485 0.0000 0.0000
7576.9092 3941.7828 303.1143 -3635.1264 4002.8916
303.6752 127.3027 111.6301 656151.2420 -17.4887
-17.5051
[7213]ENERGY: 2610000 3161.2003 5162.1648 5270.5169
322.1879 -15766.4269 -1748.7574 0.0000 0.0000
7539.8110 3940.6966 301.6301 -3599.1144 4002.2854
303.7699 -17.5688 4.1711 656151.2420 -18.2021
-18.2096
[7249]ENERGY: 2620000 3152.7857 5190.0596 5284.1066
339.4255 -15778.1075 -1777.7486 0.0000 0.0000
7536.8742 3947.3956 301.5127 -3589.4787 4003.3716
303.7440 84.2294 9.4900 656151.2420 -20.3405
-20.3279
[7299]ENERGY: 2630000 3210.0399 5128.4339 5248.2915
339.4770 -15751.7212 -1790.3507 0.0000 0.0000
7556.5632 3940.7337 302.3003 -3615.8295 4001.3485
303.8169 -77.1483 -94.3878 656151.2420 -19.4171
-19.4068
[7335]ENERGY: 2640000 3112.1294 5165.3137 5286.5112
348.4694 -15742.5554 -1806.9531 0.0000 0.0000
7577.1731 3940.0883 303.1248 -3637.0848 4002.2308
303.7017 -137.2808 -105.6673 656151.2420 -18.2698
-18.2709
[7385]ENERGY: 2650000 3031.7110 5226.9504 5249.9020
338.3445 -15688.9801 -1786.5798 0.0000 0.0000
7571.6118 3942.9598 302.9023 -3628.6520 4001.3067
303.8751 -66.1988 -43.3419 656151.2420 -18.1531
-18.1553
[7421]ENERGY: 2660000 3090.9894 5236.3353 5216.1025
341.4232 -15866.9141 -1746.6802 0.0000 0.0000
7667.8310 3939.0872 306.7516 -3728.7438 4001.4058
303.7877 -15.9562 -9.8661 656151.2420 -13.7924
-13.7870
[7471]ENERGY: 2670000 3152.7300 5177.9650 5229.5321
344.3152 -15785.9299 -1798.8445 0.0000 0.0000
7618.4911 3938.2590 304.7777 -3680.2321 4001.6827
303.7519 -46.6472 -75.0376 656151.2420 -21.7012
-21.7127
[7507]ENERGY: 2680000 3111.8205 5188.2269 5268.0587
329.5929 -15737.9934 -1784.7718 0.0000 0.0000
7565.4690 3940.4029 302.6566 -3625.0661 4002.6846
303.8955 -50.6914 -21.6951 656151.2420 -13.3678
-13.3748
[7557]ENERGY: 2690000 3173.6798 5170.6171 5218.0916
347.7057 -15765.0042 -1764.8302 0.0000 0.0000
7562.4441 3942.7039 302.5356 -3619.7402 4004.2125
303.6710 210.4740 33.8848 656151.2420 -21.1352
-21.1194
[7596]ENERGY: 2700000 3109.0230 5176.0979 5237.5329

336.3589 -15813.1026 -1798.7137 0.0000 0.0000
7692.2478 3939.4443 307.7284 -3752.8035 4002.3716
303.5183 5.2784 -57.2415 656151.2420 -13.8440
-13.8619
[7646]ENERGY: 2710000 3228.3691 5217.8505 5238.0560
337.9008 -15840.6239 -1768.5375 0.0000 0.0000
7527.9274 3940.9423 301.1547 -3586.9851 4001.2441
303.6083 0.6940 17.6192 656151.2420 -21.9489
-21.9448
[7682]ENERGY: 2720000 3121.9174 5230.9762 5303.7744
333.8166 -15853.8276 -1744.2880 0.0000 0.0000
7547.6899 3940.0590 301.9453 -3607.6310 4001.7809
303.9750 -103.9768 -29.8602 656151.2420 -17.4003
-17.3935
[7732]ENERGY: 2730000 3120.6795 5184.4515 5267.9106
341.4438 -15765.6391 -1815.4895 0.0000 0.0000
7608.6936 3942.0503 304.3858 -3666.6433 4002.5566
303.9160 -43.7655 -52.7125 656151.2420 -15.2256
-15.2365
[7768]ENERGY: 2740000 3199.8684 5283.8089 5261.5657
344.8194 -15946.7476 -1797.5971 0.0000 0.0000
7594.5873 3940.3051 303.8215 -3654.2822 4002.1771
303.9965 -15.6775 -143.1812 656151.2420 -20.6918
-20.6952
[7818]ENERGY: 2750000 3104.5966 5250.6541 5261.0432
340.9119 -15791.2532 -1775.9701 0.0000 0.0000
7547.1391 3937.1215 301.9233 -3610.0176 4001.6878
303.9062 -37.1344 -72.4468 656151.2420 -17.7211
-17.7136
[7854]ENERGY: 2760000 3098.6399 5136.0499 5278.3749
337.2294 -15794.1598 -1744.5766 0.0000 0.0000
7628.0985 3939.6563 305.1621 -3688.4422 4002.1685
303.9198 -18.4339 -15.8414 656151.2420 -19.3517
-19.3569
[7904]ENERGY: 2770000 3149.2592 5112.6775 5305.7957
328.2712 -15822.2763 -1782.7605 0.0000 0.0000
7649.0326 3939.9993 305.9995 -3709.0333 4003.2665
303.9358 -74.5953 -17.1102 656151.2420 -17.7554
-17.7448
[7940]ENERGY: 2780000 3234.8720 5162.7446 5306.3520
328.9194 -15896.8258 -1794.0529 0.0000 0.0000
7597.6254 3939.6347 303.9430 -3657.9907 4004.0251
303.9446 -34.7951 23.2730 656151.2420 -20.3506
-20.3532
[7990]ENERGY: 2790000 3128.8725 5236.1486 5252.3445
346.1051 -15834.0108 -1750.9226 0.0000 0.0000
7563.1002 3941.6374 302.5618 -3621.4628 4005.3672
304.0093 -112.8641 -106.5476 656151.2420 -17.5085
-17.5166
[8029]ENERGY: 2800000 3163.7388 5178.5958 5263.8565
338.1367 -15789.1605 -1792.2651 0.0000 0.0000
7580.7415 3943.6437 303.2676 -3637.0978 4005.4075
304.1312 -77.5625 -79.4347 656151.2420 -25.3907
-25.3666

[8079]ENERGY: 2810000 3183.2179 5209.9740 5229.6786
342.5530 -15853.9634 -1751.5280 0.0000 0.0000
7582.8752 3942.8073 303.3529 -3640.0679 4003.1202
304.1292 -20.9462 -34.7495 656151.2420 -15.8699
-15.8984
[8115]ENERGY: 2820000 3125.2497 5165.8880 5305.4875
339.5760 -15878.6984 -1723.8466 0.0000 0.0000
7609.4976 3943.1538 304.4179 -3666.3438 4004.2314
304.0037 104.6584 67.6635 656151.2420 -19.6327
-19.6090
[8165]ENERGY: 2830000 3119.1781 5218.9876 5281.0918
316.9236 -15766.2603 -1836.7912 0.0000 0.0000
7609.4737 3942.6032 304.4170 -3666.8705 4005.0417
304.1939 50.0102 14.3450 656151.2420 -17.5944
-17.6130
[8201]ENERGY: 2840000 3183.6770 5189.1199 5252.5498
345.7999 -15832.5839 -1781.6495 0.0000 0.0000
7585.3786 3942.2917 303.4531 -3643.0868 4003.9008
304.1160 63.7558 87.9742 656151.2420 -21.2763
-21.2772
[8251]ENERGY: 2850000 3151.8200 5220.3432 5228.9708
328.9897 -15694.4697 -1820.9086 0.0000 0.0000
7528.1828 3942.9283 301.1650 -3585.2545 4003.6926
304.2162 -85.1667 -108.8215 656151.2420 -19.8479
-19.8391
[8287]ENERGY: 2860000 3075.5606 5287.2606 5260.3527
348.4361 -15806.3242 -1805.9678 0.0000 0.0000
7582.4722 3941.7902 303.3368 -3640.6820 4004.5437
304.1993 -179.0917 -185.0232 656151.2420 -18.2880
-18.2935
[8337]ENERGY: 2870000 3133.1266 5228.3307 5313.4445
345.7975 -15857.4392 -1832.0681 0.0000 0.0000
7609.1598 3940.3518 304.4044 -3668.8080 4003.5120
304.1905 -5.2162 -14.4162 656151.2420 -17.3997
-17.3949
[8373]ENERGY: 2880000 3138.5263 5216.0475 5264.4696
359.4606 -15849.0313 -1858.3752 0.0000 0.0000
7673.4707 3944.5681 306.9772 -3728.9025 4004.7777
304.3642 39.5829 -47.3414 656151.2420 -23.2737
-23.2705
[8423]ENERGY: 2890000 3142.7367 5162.2737 5276.2075
354.8350 -15811.0865 -1754.3176 0.0000 0.0000
7575.3925 3946.0414 303.0536 -3629.3511 4004.4364
304.2929 95.7400 -7.4502 656151.2420 -23.1872
-23.1874
[8462]ENERGY: 2900000 3219.3661 5109.6922 5229.6222
325.1670 -15789.8065 -1740.0045 0.0000 0.0000
7592.5650 3946.6014 303.7406 -3645.9636 4004.7395
304.2761 -97.2347 -121.7193 656151.2420 -25.3448
-25.3579
[8512]ENERGY: 2910000 3157.5266 5162.6146 5295.8457
332.4922 -15753.0171 -1771.1948 0.0000 0.0000
7520.3560 3944.6232 300.8518 -3575.7328 4005.0591
304.4839 13.1720 18.1564 656151.2420 -22.2292

-22.2188
[8548]ENERGY: 2920000 3171.3089 5224.2457 5243.0699
358.7529 -15840.2378 -1799.7551 0.0000 0.0000
7587.0830 3944.4674 303.5213 -3642.6156 4005.0611
304.6044 151.7954 38.0286 656151.2420 -25.9874
-25.9876
[8598]ENERGY: 2930000 3128.0475 5121.6787 5265.7409
338.0178 -15793.9484 -1769.9579 0.0000 0.0000
7654.4885 3944.0671 306.2178 -3710.4215 4004.6565
304.7949 87.3450 5.4850 656151.2420 -21.5313
-21.5464
[8634]ENERGY: 2940000 3239.5991 5229.1966 5243.1191
335.0239 -15799.2715 -1901.6627 0.0000 0.0000
7602.6674 3948.6719 304.1447 -3653.9955 4005.1815
304.6016 -36.0834 -129.0893 656151.2420 -25.9267
-25.9192
[8684]ENERGY: 2950000 3235.4086 5137.0305 5301.9087
345.4041 -15829.9429 -1811.3068 0.0000 0.0000
7566.2456 3944.7477 302.6877 -3621.4979 4006.0155
304.6801 -44.6056 -110.0614 656151.2420 -22.5881
-22.5892
[8720]ENERGY: 2960000 3083.0649 5148.0275 5254.8404
348.3278 -15814.2348 -1747.1175 0.0000 0.0000
7668.6440 3941.5523 306.7841 -3727.0917 4004.5556
304.6340 -73.8493 7.9033 656151.2420 -18.1994
-18.1838
[8770]ENERGY: 2970000 3135.8677 5224.4857 5296.6328
345.9954 -15905.3113 -1750.5517 0.0000 0.0000
7594.1109 3941.2294 303.8024 -3652.8815 4003.8693
304.5384 3.5529 21.7688 656151.2420 -20.9048
-20.9167
[8806]ENERGY: 2980000 3157.2791 5128.6791 5259.0466
342.7465 -15802.1401 -1795.2120 0.0000 0.0000
7652.9000 3943.2991 306.1543 -3709.6009 4005.6849
304.5442 -10.4127 -115.1732 656151.2420 -21.8267
-21.8194
[8856]ENERGY: 2990000 3164.3028 5180.4661 5236.6191
321.6603 -15754.2819 -1853.6283 0.0000 0.0000
7645.2817 3940.4199 305.8495 -3704.8618 4004.4382
304.5397 -64.3728 -23.8583 656151.2420 -25.1738
-25.1848
[8895]ENERGY: 3000000 3134.8832 5218.4065 5259.6314
336.8943 -15933.9696 -1678.6961 0.0000 0.0000
7607.8932 3945.0430 304.3538 -3662.8502 4005.8767
304.7237 28.3086 -4.0991 656151.2420 -21.8323
-21.8199
[8945]ENERGY: 3010000 3160.6014 5219.2792 5242.7626
352.0568 -15750.5580 -1872.8142 0.0000 0.0000
7593.6617 3944.9896 303.7844 -3648.6721 4006.2033
304.7521 41.9357 -23.1044 656151.2420 -24.4170
-24.4231
[8981]ENERGY: 3020000 3188.4412 5165.6502 5238.9643
343.7717 -15812.6145 -1763.8886 0.0000 0.0000
7584.5025 3944.8268 303.4180 -3639.6756 4004.6680

304.6855 -61.1477 -29.5183 656151.2420 -23.6071
-23.6029
[9031]ENERGY: 3030000 3118.1301 5233.5350 5296.0174
319.8208 -15831.6642 -1790.7044 0.0000 0.0000
7599.7872 3944.9219 304.0295 -3654.8654 4005.5882
304.8308 41.1903 -17.0207 656151.2420 -24.6970
-24.7030
[9067]ENERGY: 3040000 3096.9864 5229.3026 5246.4710
342.0724 -15854.5073 -1703.9873 0.0000 0.0000
7584.8251 3941.1629 303.4309 -3643.6622 4004.1902
304.5336 99.1074 97.0211 656151.2420 -26.9289
-26.9247
[9117]ENERGY: 3050000 3246.4243 5111.6412 5225.2778
339.5582 -15803.5045 -1766.0431 0.0000 0.0000
7590.7182 3944.0721 303.6667 -3646.6460 4005.2672
304.7237 23.2243 -43.0094 656151.2420 -23.8052
-23.8167
[9153]ENERGY: 3060000 3174.7673 5149.3063 5302.3156
353.1017 -15947.7908 -1732.4740 0.0000 0.0000
7645.0015 3944.2276 305.8383 -3700.7739 4005.3926
304.7286 89.8061 67.8715 656151.2420 -23.6110
-23.6002
[9203]ENERGY: 3070000 3154.3175 5137.5228 5286.0394
335.8543 -15825.1108 -1758.2165 0.0000 0.0000
7615.1390 3945.5457 304.6436 -3669.5933 4006.7130
304.7561 75.6916 76.4573 656151.2420 -22.5864
-22.5824
[9239]ENERGY: 3080000 3177.2631 5185.7226 5302.8066
329.3244 -15770.8535 -1797.7688 0.0000 0.0000
7521.4593 3947.9536 300.8960 -3573.5057 4007.2111
304.9450 194.7479 86.1940 656151.2420 -26.6097
-26.6051
[9289]ENERGY: 3090000 3139.8538 5100.8445 5249.4423
344.8013 -15740.1809 -1832.4800 0.0000 0.0000
7679.1888 3941.4698 307.2059 -3737.7190 4005.6519
304.7766 -49.7189 -1.9702 656151.2420 -24.4381
-24.4573
[9328]ENERGY: 3100000 3178.8443 5189.6625 5303.9836
317.0634 -15826.5679 -1805.6753 0.0000 0.0000
7590.8948 3948.2053 303.6737 -3642.6894 4006.4280
304.7536 -128.3396 -111.8829 656151.2420 -29.2489
-29.2248
[9378]ENERGY: 3110000 3165.7764 5136.2601 5298.4335
358.2446 -15797.5965 -1814.5771 0.0000 0.0000
7600.3644 3946.9053 304.0526 -3653.4590 4008.1170
304.9153 51.0842 -52.4816 656151.2420 -26.7779
-26.7966
[9414]ENERGY: 3120000 3185.7758 5194.0272 5252.5087
328.9333 -15845.8815 -1799.9562 0.0000 0.0000
7634.9323 3950.3396 305.4355 -3684.5927 4008.4956
304.8288 -79.8182 -183.9699 656151.2420 -21.9334
-21.9440
[9464]ENERGY: 3130000 3183.3332 5284.1825 5237.3966
327.5761 -15852.2931 -1857.3033 0.0000 0.0000

7622.0028 3944.8948 304.9182 -3677.1080 4008.3524
304.9616 -38.7109 -103.5909 656151.2420 -24.2491
-24.2359
[9500]ENERGY: 3140000 3165.1707 5217.2269 5250.7474
346.2330 -15899.5589 -1753.8983 0.0000 0.0000
7619.5770 3945.4978 304.8212 -3674.0792 4007.9060
304.9293 -29.2321 -154.3200 656151.2420 -26.3119
-26.3182
[9550]ENERGY: 3150000 3144.7309 5203.9844 5260.1187
344.0903 -15854.0794 -1796.5988 0.0000 0.0000
7639.5243 3941.7704 305.6192 -3697.7540 4007.0499
304.7647 -164.4352 -153.1108 656151.2420 -27.0261
-26.9929
[9586]ENERGY: 3160000 3235.6689 5247.0547 5185.6083
322.8535 -15860.1503 -1777.1647 0.0000 0.0000
7594.0690 3947.9395 303.8007 -3646.1295 4006.2314
304.6037 -142.1366 -112.7918 656151.2420 -29.2882
-29.3105
[9636]ENERGY: 3170000 3198.0441 5243.6507 5284.4802
334.0814 -15836.9503 -1810.3701 0.0000 0.0000
7531.4647 3944.4008 301.2962 -3587.0640 4006.6831
304.5232 90.7558 37.1497 656151.2420 -24.1293
-24.1179
[9672]ENERGY: 3180000 3180.7526 5119.8725 5262.4255
332.8077 -15883.0777 -1765.5676 0.0000 0.0000
7693.9847 3941.1976 307.7979 -3752.7871 4007.0935
304.6370 38.4466 -107.1928 656151.2420 -25.5664
-25.5786
[9722]ENERGY: 3190000 3238.4894 5061.5873 5311.0150
343.6035 -15871.9998 -1793.2911 0.0000 0.0000
7655.8868 3945.2909 306.2737 -3710.5958 4005.6137
304.6532 -178.9264 -103.4353 656151.2420 -27.0981
-27.1043
[9761]ENERGY: 3200000 3199.3614 5103.6825 5298.1455
353.1506 -15841.3806 -1774.1008 0.0000 0.0000
7606.8032 3945.6617 304.3102 -3661.1414 4005.8987
304.7095 -139.4935 -183.5431 656151.2420 -23.1241
-23.1233
[9811]ENERGY: 3210000 3186.8782 5229.1041 5274.2941
333.8472 -15907.8704 -1839.4734 0.0000 0.0000
7666.1732 3942.9529 306.6853 -3723.2202 4006.7686
304.8118 74.3467 17.3690 656151.2420 -27.2732
-27.2651
[9847]ENERGY: 3220000 3221.2561 5260.7358 5285.2020
326.1895 -15860.9331 -1821.1089 0.0000 0.0000
7530.8842 3942.2255 301.2730 -3588.6587 4007.2295
304.6320 -2.4615 -73.6180 656151.2420 -24.0299
-24.0471
[9897]ENERGY: 3230000 3200.8185 5241.0485 5246.1358
333.0345 -15896.0442 -1712.7870 0.0000 0.0000
7536.0702 3948.2763 301.4805 -3587.7939 4006.6502
304.6340 152.1579 137.9109 656151.2420 -23.4307
-23.4204
[9933]ENERGY: 3240000 3253.6599 5113.3045 5293.4881

334.6740 -15865.8396 -1787.6909 0.0000 0.0000
7605.8246 3947.4206 304.2710 -3658.4040 4006.3715
304.5405 -113.4530 -110.8394 656151.2420 -21.3567
-21.3543
[9983]ENERGY: 3250000 3168.1838 5227.8306 5233.4695
332.5047 -15907.9518 -1706.2517 0.0000 0.0000
7597.7752 3945.5604 303.9490 -3652.2148 4006.7231
304.7118 96.1063 115.0356 656151.2420 -21.5522
-21.5554
[10019]ENERGY: 3260000 3112.0844 5159.7506 5243.3749
322.0833 -15757.6102 -1783.3530 0.0000 0.0000
7646.9366 3943.2666 305.9157 -3703.6699 4005.2764
304.8051 -124.4262 -136.9393 656151.2420 -22.9045
-22.9186
[10069]ENERGY: 3270000 3164.5144 5178.0076 5228.2935
327.6079 -15890.2177 -1752.4348 0.0000 0.0000
7687.2109 3942.9817 307.5269 -3744.2292 4007.0572
304.6227 -94.3300 -156.9853 656151.2420 -20.2903
-20.2787
[10105]ENERGY: 3280000 3175.4142 5221.1777 5268.2460
339.8588 -15885.5397 -1761.1433 0.0000 0.0000
7587.9397 3945.9534 303.5555 -3641.9863 4006.2696
304.6353 -23.8081 28.1038 656151.2420 -21.0002
-20.9967
[10155]ENERGY: 3290000 3246.3213 5219.4865 5269.5629
316.9740 -15924.1886 -1761.7533 0.0000 0.0000
7581.9564 3948.3593 303.3162 -3633.5970 4007.3462
304.6175 -57.9541 -92.9896 656151.2420 -23.7082
-23.7112
[10194]ENERGY: 3300000 3133.1179 5051.5968 5315.9260
342.0013 -15834.4440 -1726.7471 0.0000 0.0000
7663.4259 3944.8769 306.5754 -3718.5490 4007.2865
304.7741 213.5900 92.4480 656151.2420 -25.6824
-25.6751
[10244]ENERGY: 3310000 3199.5269 5108.7088 5250.1398
330.2078 -15775.6288 -1804.7937 0.0000 0.0000
7638.3377 3946.4985 305.5717 -3691.8392 4008.6767
304.7940 -134.6758 -151.7494 656151.2420 -25.4215
-25.4190
[10280]ENERGY: 3320000 3143.7249 5228.3548 5306.5680
331.2686 -15841.0423 -1803.7352 0.0000 0.0000
7583.3640 3948.5028 303.3725 -3634.8612 4007.5476
304.6377 -129.9734 -48.9135 656151.2420 -26.1415
-26.1380
[10330]ENERGY: 3330000 3201.4000 5267.4281 5247.3408
337.1759 -15887.1021 -1752.6214 0.0000 0.0000
7538.3283 3951.9496 301.5708 -3586.3788 4008.6210
304.6275 123.4768 58.2434 656151.2420 -25.6319
-25.6329
[10366]ENERGY: 3340000 3211.2329 5153.4024 5267.5300
356.9288 -15868.1571 -1783.6919 0.0000 0.0000
7611.7107 3948.9558 304.5065 -3662.7549 4010.6310
304.8447 142.7883 59.0070 656151.2420 -24.4572
-24.4626

[10416]ENERGY: 3350000 3191.7153 5172.2980 5307.5036
348.1545 -15846.9908 -1759.4996 0.0000 0.0000
7535.5981 3948.7791 301.4616 -3586.8190 4009.8554
304.7870 28.3539 7.6518 656151.2420 -23.2244
-23.2256
[10452]ENERGY: 3360000 3100.4563 5211.6233 5362.3867
328.8132 -15978.6014 -1734.2919 0.0000 0.0000
7656.3789 3946.7652 306.2934 -3709.6137 4009.6310
304.8954 93.1828 91.7249 656151.2420 -23.2821
-23.2719
[10502]ENERGY: 3370000 3102.4889 5176.3252 5299.2758
340.3344 -15854.7815 -1823.6136 0.0000 0.0000
7704.6580 3944.6872 308.2248 -3759.9708 4009.8871
304.7876 -58.5374 -70.5165 656151.2420 -23.4122
-23.4222
[10538]ENERGY: 3380000 3154.8644 5205.3172 5269.4461
339.8188 -15899.9482 -1765.1369 0.0000 0.0000
7640.8505 3945.2120 305.6722 -3695.6385 4009.6204
304.7874 -65.4875 -145.4016 656151.2420 -24.6604
-24.6530
[10588]ENERGY: 3390000 3222.9221 5205.2905 5310.3416
353.2767 -15953.9286 -1778.2093 0.0000 0.0000
7592.6424 3952.3353 303.7437 -3640.3071 4010.6761
304.8748 -159.2000 -162.4692 656151.2420 -25.0944
-25.1047
[10627]ENERGY: 3400000 3148.6597 5071.3391 5315.3722
328.5976 -15864.9027 -1773.1182 0.0000 0.0000
7718.4775 3944.4253 308.7777 -3774.0522 4008.7858
305.0049 -142.9050 -89.9778 656151.2420 -22.7292
-22.7133
[10677]ENERGY: 3410000 3229.2770 5279.9478 5314.6594
329.7950 -15963.3842 -1848.9180 0.0000 0.0000
7609.0119 3950.3890 304.3985 -3658.6230 4009.5910
304.8351 -27.4081 -114.6887 656151.2420 -25.6456
-25.6638
[10713]ENERGY: 3420000 3245.8238 5153.2176 5290.4678
334.5944 -15864.4863 -1847.0275 0.0000 0.0000
7637.8078 3950.3976 305.5505 -3687.4102 4009.9920
304.9236 -91.3464 -76.9961 656151.2420 -23.1424
-23.1523
[10763]ENERGY: 3430000 3151.6478 5201.7317 5330.0656
335.1852 -15877.7817 -1791.4409 0.0000 0.0000
7600.1343 3949.5420 304.0434 -3650.5922 4011.3478
304.8726 3.5570 -62.3860 656151.2420 -24.6222
-24.6039
[10799]ENERGY: 3440000 3206.0308 5173.6959 5285.2724
338.5517 -15804.6550 -1777.0207 0.0000 0.0000
7531.2701 3953.1452 301.2885 -3578.1250 4010.5073
304.9236 -41.6953 19.2912 656151.2420 -25.7161
-25.7199
[10849]ENERGY: 3450000 3118.9282 5212.6045 5271.2457
329.0351 -15798.4518 -1780.5629 0.0000 0.0000
7596.5526 3949.3515 303.9001 -3647.2012 4010.2879
304.8226 -110.8613 -102.6489 656151.2420 -26.2817

-26.2849
[10885]ENERGY: 3460000 3196.0325 5182.5546 5280.4784
313.0168 -15874.5547 -1791.4095 0.0000 0.0000
7642.6902 3948.8082 305.7458 -3693.8820 4011.6488
304.8839 -57.2467 -90.5788 656151.2420 -20.2933
-20.2926
[10935]ENERGY: 3470000 3070.8192 5190.0266 5262.1773
352.5509 -15868.5947 -1704.7449 0.0000 0.0000
7646.9679 3949.2022 305.9169 -3697.7657 4010.7904
304.9135 36.8738 24.5097 656151.2420 -26.1565
-26.1554
[10971]ENERGY: 3480000 3193.5871 5237.7159 5294.6754
341.0375 -15798.5923 -1850.2861 0.0000 0.0000
7532.8151 3950.9526 301.3503 -3581.8625 4012.0080
304.8387 21.4843 -32.1455 656151.2420 -24.4770
-24.4759
[11021]ENERGY: 3490000 3140.0847 5213.8273 5280.7368
348.5295 -15872.2044 -1764.0952 0.0000 0.0000
7603.1253 3950.0039 304.1630 -3653.1214 4011.9374
304.8088 118.0830 67.8290 656151.2420 -21.0388
-21.0589
[11060]ENERGY: 3500000 3215.4277 5262.6977 5258.2094
327.7490 -15925.2034 -1799.9111 0.0000 0.0000
7610.4094 3949.3787 304.4544 -3661.0307 4010.4846
304.9300 -110.4228 -117.1267 656151.2420 -22.6301
-22.6164
[11110]ENERGY: 3510000 3186.3934 5261.8392 5258.0736
364.2002 -15782.5515 -1830.9260 0.0000 0.0000
7492.1325 3949.1615 299.7228 -3542.9710 4011.7868
305.0948 119.6226 19.8642 656151.2420 -21.9382
-21.9316
[11146]ENERGY: 3520000 3089.4350 5151.5335 5267.0387
339.3332 -15815.9194 -1735.7255 0.0000 0.0000
7651.8294 3947.5249 306.1114 -3704.3045 4010.6450
304.9075 -1.1636 5.7136 656151.2420 -22.7296
-22.7380
[11196]ENERGY: 3530000 3169.5367 5211.1948 5274.4145
333.3687 -15880.7190 -1695.5761 0.0000 0.0000
7538.6551 3950.8747 301.5839 -3587.7805 4011.4396
304.8658 -76.9209 -64.9111 656151.2420 -21.9151
-21.8928
[11232]ENERGY: 3540000 3129.7012 5244.6959 5256.7530
348.6401 -15867.9690 -1773.2188 0.0000 0.0000
7611.4175 3950.0200 304.4948 -3661.3975 4011.8839
305.0847 102.2571 93.0183 656151.2420 -22.4005
-22.4174
[11282]ENERGY: 3550000 3254.1771 5141.8475 5252.5814
325.1177 -15950.1410 -1697.1408 0.0000 0.0000
7622.9220 3949.3639 304.9550 -3673.5581 4012.1770
305.1836 0.3768 26.8386 656151.2420 -20.9134
-20.9193
[11318]ENERGY: 3560000 3204.7030 5142.0468 5286.0630
351.5160 -15858.4695 -1784.5527 0.0000 0.0000
7608.9562 3950.2627 304.3963 -3658.6935 4012.5115

304.9536 73.9599 -65.4294 656151.2420 -28.1095
-28.1054
[11368]ENERGY: 3570000 3198.1810 5142.0409 5262.9387
335.7735 -15850.9649 -1766.5980 0.0000 0.0000
7627.6569 3949.0280 305.1444 -3678.6289 4012.3408
304.8543 -15.4237 -41.7553 656151.2420 -23.4985
-23.4935
[11404]ENERGY: 3580000 3229.7754 5191.2833 5271.6390
327.5126 -15933.3991 -1755.0347 0.0000 0.0000
7624.4720 3956.2484 305.0170 -3668.2235 4013.3764
305.0052 97.6417 18.1163 656151.2420 -19.5242
-19.5351
[11454]ENERGY: 3590000 3162.9093 5126.5403 5233.5486
331.7409 -15862.8184 -1750.6898 0.0000 0.0000
7705.8133 3947.0442 308.2711 -3758.7691 4013.3132
304.9589 118.2450 116.3172 656151.2420 -25.1307
-25.1218
[11493]ENERGY: 3600000 3188.1887 5170.0250 5322.7564
351.4749 -15838.9882 -1821.6936 0.0000 0.0000
7581.5576 3953.3208 303.3002 -3628.2368 4014.6553
304.9934 -24.9083 -72.1294 656151.2420 -21.5128
-21.5208
[11543]ENERGY: 3610000 3136.4806 5194.9279 5280.5227
311.0070 -15953.8992 -1703.3130 0.0000 0.0000
7685.4273 3951.1533 307.4555 -3734.2740 4012.4664
305.0963 45.6127 60.9749 656151.2420 -23.5312
-23.5143
[11579]ENERGY: 3620000 3243.1799 5119.8998 5284.2723
329.5615 -15896.9838 -1782.0181 0.0000 0.0000
7652.7264 3950.6379 306.1473 -3702.0886 4013.2925
305.1877 -67.2957 -65.9414 656151.2420 -25.2929
-25.2951
[11629]ENERGY: 3630000 3243.9541 5098.5588 5313.9313
342.2223 -15939.2026 -1792.2885 0.0000 0.0000
7685.4989 3952.6743 307.4584 -3732.8246 4014.0823
305.1825 87.8293 83.8989 656151.2420 -22.5587
-22.5486
[11665]ENERGY: 3640000 3168.1499 5286.6139 5224.6736
357.9339 -15939.8739 -1734.9915 0.0000 0.0000
7591.4967 3954.0026 303.6978 -3637.4941 4013.5048
305.3055 -65.8853 -31.7532 656151.2420 -22.0365
-22.0486
[11715]ENERGY: 3650000 3177.4733 5164.5245 5315.5585
316.9503 -15905.0433 -1732.9695 0.0000 0.0000
7614.8293 3951.3230 304.6312 -3663.5063 4013.8864
305.2291 122.3795 43.0883 656151.2420 -28.5372
-28.5325
[11751]ENERGY: 3660000 3201.4129 5222.9660 5225.3574
351.7784 -15906.2126 -1803.3285 0.0000 0.0000
7660.5255 3952.4991 306.4593 -3708.0264 4013.8526
305.2887 12.5616 15.9974 656151.2420 -23.7531
-23.7595
[11801]ENERGY: 3670000 3116.1193 5298.6224 5289.8523
346.9931 -15948.7819 -1779.5987 0.0000 0.0000

7627.0406 3950.2471 305.1198 -3676.7935 4013.6733
305.1452 69.6024 19.4553 656151.2420 -26.4411
-26.4228
[11837]ENERGY: 3680000 3133.2257 5205.3648 5279.0913
346.5340 -15874.0178 -1779.3833 0.0000 0.0000
7642.5369 3953.3516 305.7397 -3689.1853 4014.1330
305.0425 135.0214 43.0358 656151.2420 -20.9252
-20.9639
[11887]ENERGY: 3690000 3172.7073 5138.5710 5292.9352
341.0185 -15908.8021 -1761.8097 0.0000 0.0000
7675.8809 3950.5011 307.0736 -3725.3798 4012.6064
305.0869 33.5700 -6.4664 656151.2420 -24.8773
-24.8535
[11926]ENERGY: 3700000 3140.5403 5267.5273 5257.7518
337.5003 -15856.9215 -1790.7619 0.0000 0.0000
7596.3033 3951.9395 303.8901 -3644.3638 4013.3863
305.0870 -37.4603 -45.7591 656151.2420 -19.4805
-19.4888
[11976]ENERGY: 3710000 3171.4960 5232.7336 5284.8061
337.3919 -15885.3162 -1772.3860 0.0000 0.0000
7585.6759 3954.4012 303.4650 -3631.2746 4013.9343
305.3078 104.8961 34.0208 656151.2420 -20.4593
-20.4563
[12012]ENERGY: 3720000 3138.4457 5136.5224 5238.8080
315.1905 -15808.2692 -1731.9834 0.0000 0.0000
7665.0673 3953.7814 306.6410 -3711.2859 4014.0625
305.2493 71.6070 -43.3902 656151.2420 -25.6126
-25.6156
[12062]ENERGY: 3730000 3045.0822 5158.5687 5251.5121
348.8245 -15768.9604 -1681.6255 0.0000 0.0000
7596.7049 3950.1064 303.9062 -3646.5985 4013.3388
305.1047 210.8950 197.0839 656151.2420 -21.9930
-22.0027
[12098]ENERGY: 3740000 3173.5014 5179.4875 5306.9346
347.7274 -15955.0589 -1748.3292 0.0000 0.0000
7649.1006 3953.3634 306.0023 -3695.7372 4013.0807
305.0920 -152.7809 -109.9089 656151.2420 -22.1802
-22.1543
[12148]ENERGY: 3750000 3172.4988 5092.8606 5334.7514
335.0576 -15849.8080 -1696.0877 0.0000 0.0000
7565.8312 3955.1039 302.6711 -3610.7273 4012.9059
304.9482 22.2979 -42.3246 656151.2420 -21.1047
-21.1159
[12184]ENERGY: 3760000 3161.6001 5172.2245 5309.0368
335.8349 -15870.9809 -1741.0532 0.0000 0.0000
7590.1051 3956.7673 303.6422 -3633.3377 4013.7555
305.0553 -48.5573 29.7745 656151.2420 -23.1078
-23.0939
[12234]ENERGY: 3770000 3099.3442 5150.8515 5256.4378
340.1999 -15781.2300 -1756.8454 0.0000 0.0000
7644.4510 3953.2089 305.8163 -3691.2420 4014.8675
305.1522 -29.6025 -61.7773 656151.2420 -18.1679
-18.1889
[12270]ENERGY: 3780000 3091.6106 5243.0499 5247.6790

321.3351 -15837.1982 -1714.3735 0.0000 0.0000
7601.7945 3953.8974 304.1098 -3647.8971 4015.4721
305.0889 132.6997 96.5182 656151.2420 -23.5301
-23.5326
[12320]ENERGY: 3790000 3118.3790 5197.7707 5270.0540
351.0138 -15870.9816 -1783.1182 0.0000 0.0000
7666.2111 3949.3288 306.6868 -3716.8823 4016.0379
305.2733 -7.6614 4.0590 656151.2420 -17.8985
-17.8848
[12359]ENERGY: 3800000 3104.6295 5182.7037 5271.7213
334.4531 -15893.8774 -1694.9587 0.0000 0.0000
7647.8172 3952.4889 305.9509 -3695.3283 4016.1351
305.2654 154.3647 106.4511 656151.2420 -21.3178
-21.3168
[12409]ENERGY: 3810000 3133.2983 5183.9170 5250.9269
346.3604 -15799.8546 -1846.4964 0.0000 0.0000
7684.1381 3952.2897 307.4039 -3731.8484 4016.6390
305.1151 -17.3534 -93.7801 656151.2420 -19.3496
-19.3518
[12445]ENERGY: 3820000 3205.4945 5245.2367 5245.5330
317.7390 -15850.4387 -1778.7551 0.0000 0.0000
7573.1768 3957.9863 302.9649 -3615.1905 4016.2682
305.1487 -83.9633 -126.1854 656151.2420 -29.1750
-29.1741
[12495]ENERGY: 3830000 3157.8717 5189.5507 5280.9725
338.4176 -15928.7130 -1773.2969 0.0000 0.0000
7687.1086 3951.9112 307.5228 -3735.1974 4015.8416
305.3001 103.7699 -4.2192 656151.2420 -20.4267
-20.4399
[12531]ENERGY: 3840000 3275.4753 5193.9284 5267.3615
351.3041 -15911.8849 -1802.9655 0.0000 0.0000
7583.1339 3956.3529 303.3633 -3626.7811 4015.2350
305.3112 -123.2069 -102.1322 656151.2420 -24.3390
-24.3303
[12581]ENERGY: 3850000 3174.2384 5117.3768 5308.5153
340.3030 -15846.5553 -1796.2366 0.0000 0.0000
7655.6372 3953.2787 306.2638 -3702.3584 4015.8606
305.1292 43.0354 -50.1769 656151.2420 -25.1349
-25.1292
[12617]ENERGY: 3860000 3160.9164 5142.6924 5284.2284
343.1094 -15798.7975 -1745.9964 0.0000 0.0000
7567.7711 3953.9238 302.7487 -3613.8473 4015.8795
305.1558 5.3493 -42.2557 656151.2420 -22.7198
-22.7230
[12667]ENERGY: 3870000 3219.6701 5181.3342 5269.0398
341.1165 -15817.0889 -1783.1441 0.0000 0.0000
7546.7102 3957.6378 301.9061 -3589.0724 4015.9500
305.1167 -98.8794 -71.5335 656151.2420 -23.5808
-23.5766
[12703]ENERGY: 3880000 3266.4580 5040.3031 5232.2524
335.6071 -15793.4490 -1740.3653 0.0000 0.0000
7615.3555 3956.1620 304.6523 -3659.1935 4017.2077
305.0718 24.6515 -103.7677 656151.2420 -24.3472
-24.3540

[12753]ENERGY: 3890000 3221.5593 5175.3946 5248.2804
331.1931 -15914.5392 -1674.2342 0.0000 0.0000
7569.4233 3957.0773 302.8148 -3612.3460 4016.4471
305.0170 -26.0319 36.8312 656151.2420 -27.5453
-27.5721
[12792]ENERGY: 3900000 3161.6369 5119.3812 5263.8906
337.2594 -15833.5521 -1751.7730 0.0000 0.0000
7657.9686 3954.8116 306.3570 -3703.1570 4016.8931
305.0447 52.1731 62.1634 656151.2420 -22.3505
-22.3229
[12842]ENERGY: 3910000 3127.3490 5176.5518 5283.7259
336.2066 -15848.5881 -1787.0592 0.0000 0.0000
7665.9031 3954.0891 306.6745 -3711.8140 4018.0666
305.0159 85.5833 18.7972 656151.2420 -22.0133
-22.0187
[12878]ENERGY: 3920000 3197.3787 5097.3175 5275.6028
351.6359 -15853.9675 -1747.5616 0.0000 0.0000
7634.9603 3955.3662 305.4366 -3679.5942 4016.8436
305.0314 -44.1471 -7.5048 656151.2420 -25.6810
-25.6775
[12928]ENERGY: 3930000 3190.1671 5170.3612 5320.4352
337.5447 -15861.5129 -1826.2522 0.0000 0.0000
7624.2309 3954.9738 305.0074 -3669.2570 4017.1180
305.1570 -70.0463 -102.4436 656151.2420 -20.5601
-20.5548
[12964]ENERGY: 3940000 3145.2685 5216.2490 5291.1659
323.9578 -15880.9529 -1789.5789 0.0000 0.0000
7648.5654 3954.6748 305.9809 -3693.8906 4017.9061
305.0372 -186.7507 -126.3660 656151.2420 -24.9940
-24.9974
[13014]ENERGY: 3950000 3208.4917 5179.6520 5264.8735
320.8165 -15841.9556 -1755.8209 0.0000 0.0000
7579.2847 3955.3419 303.2093 -3623.9428 4017.1069
305.1341 80.7037 -2.7502 656151.2420 -22.1382
-22.1363
[13050]ENERGY: 3960000 3205.8050 5184.5238 5278.3246
360.7207 -15996.4069 -1614.0156 0.0000 0.0000
7538.9482 3957.8996 301.5956 -3581.0486 4016.0559
304.9834 157.4051 101.8814 656151.2420 -23.3545
-23.3557
[13100]ENERGY: 3970000 3192.0590 5179.7271 5262.3803
350.9218 -15855.8974 -1791.9588 0.0000 0.0000
7620.1486 3957.3806 304.8440 -3662.7680 4018.4927
305.0927 -86.8390 -147.9947 656151.2420 -22.1089
-22.1215
[13136]ENERGY: 3980000 3120.9497 5135.0969 5309.2973
350.6304 -15824.6217 -1786.7658 0.0000 0.0000
7652.5856 3957.1724 306.1417 -3695.4132 4018.3093
305.2343 61.6223 77.7593 656151.2420 -22.5401
-22.5195
[13186]ENERGY: 3990000 3179.9200 5245.2081 5254.4577
340.1201 -15865.3150 -1838.9403 0.0000 0.0000
7640.2858 3955.7364 305.6496 -3684.5494 4018.0248
305.2818 -72.0715 -41.7631 656151.2420 -25.1200

```
-25.1376
[13225]ENERGY: 4000000      3167.8008      5222.7417      5274.7997
346.2924         -15898.0437     -1726.0043         0.0000         0.0000
7568.6775           3956.2642        302.7849     -3612.4133      4017.6939
305.4209            -14.3113         23.2625    656151.2420       -19.4503
-19.4473
[13275]ENERGY: 4010000      3190.6770      5198.6286      5269.4093
335.6620         -15854.2769     -1766.4765         0.0000         0.0000
7583.5386           3957.1621        303.3795     -3626.3765      4017.2719
305.3147            -31.4998        -96.9843    656151.2420       -25.0943
-25.1070
[13311]ENERGY: 4020000      3161.0259      5105.5176      5254.0731
316.6770         -15800.2637     -1789.7266         0.0000         0.0000
7707.6667           3954.9700        308.3452     -3752.6967      4018.2383
305.3866            102.1304         29.2000    656151.2420       -25.5666
-25.5380
[13361]ENERGY: 4030000      3183.5406      5199.4676      5298.6119
344.1985         -15889.9646     -1793.4245         0.0000         0.0000
7613.1771           3955.6065        304.5651     -3657.5705      4018.4687
305.3911             26.2078          0.8916    656151.2420       -26.0630
-26.0784
[13397]ENERGY: 4040000      3206.7567      5042.4134      5294.9369
312.9182         -15851.5118     -1755.7421         0.0000         0.0000
7705.3349           3955.1062        308.2519     -3750.2287      4017.1808
305.4258           -123.1013       -114.5448    656151.2420       -22.5797
-22.5862
[13447]ENERGY: 4050000      3153.2606      5211.9604      5292.4447
321.8313         -15895.1310     -1753.8860         0.0000         0.0000
7626.4110           3956.8910        305.0946     -3669.5200      4019.2951
305.3041             50.1520        -18.4557    656151.2420       -24.7638
-24.7536
[13483]ENERGY: 4060000      3146.5599      5122.5572      5249.8801
336.6324         -15740.7419     -1747.1331         0.0000         0.0000
7588.2087           3955.9631        303.5663     -3632.2455      4017.7876
305.4803            -18.9219        -89.3951    656151.2420       -25.5771
-25.5864
[13533]ENERGY: 4070000      3201.4888      5165.3202      5268.2097
315.6432         -15781.8056     -1781.1014         0.0000         0.0000
7570.6856           3958.4405        302.8653     -3612.2451      4019.7717
305.5119              6.9574         -7.5424    656151.2420       -20.9055
-20.8810
[13569]ENERGY: 4080000      3266.4747      5171.3457      5225.5270
336.5059         -15848.6387     -1799.8207         0.0000         0.0000
7608.0770           3959.4710        304.3611     -3648.6060      4019.3276
305.5403            -69.0993        -84.4711    656151.2420       -27.2206
-27.2363
[13619]ENERGY: 4090000      3178.7956      5107.3239      5245.4400
366.5555         -15793.1599     -1752.3005         0.0000         0.0000
7610.2286           3962.8832        304.4472     -3647.3454      4022.5742
305.5274             85.4737         39.0394    656151.2420       -21.1271
-21.1146
[13658]ENERGY: 4100000      3112.2143      5181.0111      5261.4755
322.1193         -15811.0756     -1762.2844         0.0000         0.0000
7655.0868           3958.5470        306.2417     -3696.5398      4022.1269
```

305.4684 92.7951 61.1640 656151.2420 -24.4748
-24.4823
[13708]ENERGY: 4110000 3175.7578 5152.4186 5301.9946
351.4052 -15849.6436 -1793.8435 0.0000 0.0000
7620.0278 3958.1169 304.8392 -3661.9109 4022.6237
305.4236 -8.0261 -71.3964 656151.2420 -20.2678
-20.2737
[13744]ENERGY: 4120000 3153.1076 5176.8270 5234.8508
340.1033 -15782.9480 -1792.0288 0.0000 0.0000
7629.7249 3959.6367 305.2271 -3670.0882 4021.6190
305.5466 -66.3156 -45.8755 656151.2420 -19.9474
-19.9386
[13794]ENERGY: 4130000 3168.8081 5116.4948 5271.1898
329.7410 -15896.4781 -1732.2002 0.0000 0.0000
7701.3130 3958.8683 308.0910 -3742.4448 4022.5444
305.2788 93.7234 0.6597 656151.2420 -20.6411
-20.6536
[13830]ENERGY: 4140000 3178.8165 5212.0801 5264.7010
341.3266 -15799.5069 -1808.6227 0.0000 0.0000
7573.8297 3962.6242 302.9911 -3611.2055 4022.7004
305.4165 97.4526 61.3084 656151.2420 -21.0871
-21.0737
[13880]ENERGY: 4150000 3197.8541 5057.2448 5333.4350
332.6281 -15857.7093 -1764.7369 0.0000 0.0000
7660.7664 3959.4822 306.4690 -3701.2842 4022.6382
305.3368 -0.6508 -66.1567 656151.2420 -22.8729
-22.8944
[13916]ENERGY: 4160000 3203.5889 5058.6881 5254.4779
352.5764 -15796.9602 -1758.0499 0.0000 0.0000
7646.7916 3961.1127 305.9099 -3685.6789 4023.0873
305.3210 39.0282 -42.9404 656151.2420 -20.4509
-20.4432
[13966]ENERGY: 4170000 3183.3704 5161.5890 5287.9006
328.8400 -15883.2791 -1760.3873 0.0000 0.0000
7645.9315 3963.9650 305.8755 -3681.9664 4023.7131
305.4568 -93.1558 -139.3840 656151.2420 -22.8723
-22.8778
[14002]ENERGY: 4180000 3230.5152 5104.3965 5333.7138
343.8279 -15875.9785 -1687.7619 0.0000 0.0000
7516.3464 3965.0594 300.6914 -3551.2870 4023.5868
305.5710 158.0916 43.5115 656151.2420 -23.0952
-23.0891
[14052]ENERGY: 4190000 3150.9969 5182.1565 5250.1773
319.8310 -15858.2669 -1747.8666 0.0000 0.0000
7663.7043 3960.7326 306.5865 -3702.9717 4023.9260
305.6349 37.3930 30.4347 656151.2420 -26.4069
-26.3966
[14091]ENERGY: 4200000 3164.4961 5152.2430 5227.7247
326.3376 -15854.8346 -1740.5257 0.0000 0.0000
7686.2149 3961.6560 307.4870 -3724.5590 4023.9942
305.6041 -126.0859 -37.2232 656151.2420 -21.3288
-21.3144
[14141]ENERGY: 4210000 3206.1642 5152.3275 5293.4378
337.5295 -15853.7869 -1814.3042 0.0000 0.0000

7641.2822 3962.6502 305.6895 -3678.6320 4025.1298
305.6818 -33.7598 -71.5753 656151.2420 -24.8440
-24.8715
[14177]ENERGY: 4220000 3218.2921 5211.1074 5306.3205
340.5254 -15842.8368 -1847.5440 0.0000 0.0000
7580.4481 3966.3127 303.2558 -3614.1355 4024.7197
305.5671 -49.9823 -83.2487 656151.2420 -17.3083
-17.2998
[14227]ENERGY: 4230000 3177.1353 5132.2737 5258.5462
334.5894 -15918.3061 -1748.0188 0.0000 0.0000
7723.4768 3959.6966 308.9777 -3763.7802 4025.0102
305.5207 31.4766 -54.4505 656151.2420 -23.9815
-23.9876
[14263]ENERGY: 4240000 3206.6835 5191.8181 5272.1236
354.6527 -15894.0159 -1710.8695 0.0000 0.0000
7541.3916 3961.7840 301.6934 -3579.6076 4023.7778
305.5298 -8.5123 41.2530 656151.2420 -21.2532
-21.2342
[14313]ENERGY: 4250000 3252.8305 5200.6855 5292.4093
336.0136 -15926.2905 -1701.0655 0.0000 0.0000
7510.5268 3965.1096 300.4586 -3545.4172 4023.9202
305.4212 13.9901 -20.8176 656151.2420 -21.8165
-21.8307
[14349]ENERGY: 4260000 3153.0086 5153.3277 5291.3788
361.1064 -15778.3312 -1808.6112 0.0000 0.0000
7590.8607 3962.7397 303.6724 -3628.1209 4023.3351
305.4191 -255.8809 -207.5528 656151.2420 -23.7448
-23.7483
[14399]ENERGY: 4270000 3163.8278 5175.2881 5294.7273
361.7614 -15822.8050 -1776.1561 0.0000 0.0000
7566.3032 3962.9467 302.6900 -3603.3565 4023.7897
305.5150 22.2487 13.4696 656151.2420 -24.1605
-24.1526
[14435]ENERGY: 4280000 3182.4528 5200.2033 5274.4954
338.9830 -15842.8999 -1775.5822 0.0000 0.0000
7586.2092 3963.8616 303.4863 -3622.3476 4024.2545
305.5519 -162.6040 -100.7818 656151.2420 -23.7334
-23.7300
[14485]ENERGY: 4290000 3184.7010 5105.9233 5283.0472
331.6899 -15863.3243 -1657.1371 0.0000 0.0000
7578.8680 3963.7680 303.1926 -3615.1001 4024.2148
305.5382 -1.5134 -48.1129 656151.2420 -24.7366
-24.7368
[14524]ENERGY: 4300000 3220.8268 5154.7291 5291.3045
330.1618 -15873.4363 -1767.0647 0.0000 0.0000
7606.7773 3963.2986 304.3091 -3643.4787 4024.5255
305.4316 22.5290 -56.2778 656151.2420 -23.8058
-23.7983
[14574]ENERGY: 4310000 3163.7419 5163.6994 5255.2443
364.9235 -15873.4065 -1761.7994 0.0000 0.0000
7651.9578 3964.3609 306.1166 -3687.5969 4023.8181
305.4395 -37.1236 -44.6673 656151.2420 -26.7647
-26.7830
[14610]ENERGY: 4320000 3180.0833 5136.8712 5282.6262

344.5198 -15943.7880 -1718.3543 0.0000 0.0000
7681.8314 3963.7896 307.3117 -3718.0417 4023.2738
305.4153 -87.7220 -116.0743 656151.2420 -22.4472
-22.4438
[14660]ENERGY: 4330000 3166.4568 5218.7311 5319.1652
332.1702 -15859.1910 -1785.3348 0.0000 0.0000
7570.5393 3962.5367 302.8594 -3608.0026 4024.1194
305.3252 101.6762 50.0145 656151.2420 -25.4512
-25.4556
[14696]ENERGY: 4340000 3214.5285 5093.8987 5287.3130
335.7881 -15873.7453 -1731.4555 0.0000 0.0000
7638.6549 3964.9824 305.5844 -3673.6724 4024.0973
305.2530 41.9529 -46.8583 656151.2420 -21.3985
-21.4020
[14746]ENERGY: 4350000 3158.6244 5207.5492 5232.9174
327.3988 -15791.5810 -1846.6810 0.0000 0.0000
7671.7644 3959.9922 306.9089 -3711.7722 4023.9032
305.3355 -58.1167 -73.3965 656151.2420 -20.8132
-20.8159
[14782]ENERGY: 4360000 3162.2064 5174.0587 5265.4664
343.8812 -15898.6627 -1799.5232 0.0000 0.0000
7712.2211 3959.6479 308.5274 -3752.5732 4024.4015
305.2311 -4.6941 7.1977 656151.2420 -25.5028
-25.4844
[14832]ENERGY: 4370000 3184.3469 5025.3786 5300.1853
352.5448 -15788.3655 -1746.7375 0.0000 0.0000
7635.8553 3963.2080 305.4724 -3672.6473 4024.7177
305.1565 -47.7593 -41.3782 656151.2420 -17.7446
-17.7556
[14868]ENERGY: 4380000 3133.9576 5168.2817 5237.1874
330.4794 -15866.6884 -1731.3540 0.0000 0.0000
7692.4955 3964.3592 307.7383 -3728.1363 4023.8763
305.3190 47.3573 -58.1095 656151.2420 -25.9415
-25.9389
[14918]ENERGY: 4390000 3187.0195 5108.3687 5266.4552
330.9916 -15845.9917 -1680.1170 0.0000 0.0000
7597.9220 3964.6484 303.9549 -3633.2736 4024.6975
305.4074 -20.6420 -1.2470 656151.2420 -20.6718
-20.6497
[14957]ENERGY: 4400000 3154.2814 5195.3200 5298.3988
339.5754 -15898.7388 -1786.1885 0.0000 0.0000
7659.5180 3962.1663 306.4190 -3697.3517 4025.4519
305.4510 -58.7675 -128.2341 656151.2420 -25.5938
-25.6073
[15007]ENERGY: 4410000 3185.3391 5155.8850 5295.8056
325.0465 -15865.6082 -1765.1209 0.0000 0.0000
7628.0698 3959.4171 305.1609 -3668.6527 4025.1962
305.3810 21.1602 27.6167 656151.2420 -28.6668
-28.6704
[15043]ENERGY: 4420000 3219.9327 5193.3404 5273.1772
328.2757 -15932.7247 -1714.4815 0.0000 0.0000
7595.3671 3962.8869 303.8527 -3632.4801 4025.0851
305.5635 -24.4920 1.3261 656151.2420 -18.6245
-18.6198

[15093]ENERGY: 4430000 3165.0126 5258.9338 5254.2250
329.7845 -15761.7502 -1786.8385 0.0000 0.0000
7504.4813 3963.8486 300.2168 -3540.6327 4025.6293
305.7266 -19.7424 -93.4868 656151.2420 -27.7298
-27.7311
[15129]ENERGY: 4440000 3108.3144 5249.4888 5282.0183
334.4292 -15877.0621 -1779.3603 0.0000 0.0000
7644.2998 3962.1281 305.8102 -3682.1716 4023.9689
305.6428 121.6863 17.3029 656151.2420 -25.0895
-25.0878
[15179]ENERGY: 4450000 3176.1101 5204.4157 5293.0004
327.0472 -15878.0306 -1754.2544 0.0000 0.0000
7591.4075 3959.6959 303.6943 -3631.7116 4024.3637
305.5974 -112.7537 -150.4223 656151.2420 -24.1377
-24.1368
[15215]ENERGY: 4460000 3217.8032 5193.4023 5200.4961
326.6551 -15832.5462 -1770.2489 0.0000 0.0000
7631.3376 3966.8991 305.2917 -3664.4385 4025.2778
305.2316 -11.1259 -51.3998 656151.2420 -26.9354
-26.9519
[15265]ENERGY: 4470000 3202.6631 5211.8635 5231.7417
334.8865 -15885.2107 -1775.4526 0.0000 0.0000
7642.4344 3962.9260 305.7356 -3679.5084 4023.7610
305.2906 -120.3279 -143.5269 656151.2420 -26.9304
-26.9226
[15301]ENERGY: 4480000 3106.6853 5123.5603 5277.2129
347.0737 -15802.2582 -1790.3156 0.0000 0.0000
7700.0104 3961.9688 308.0389 -3738.0416 4024.6798
305.6168 -67.5477 -140.4192 656151.2420 -24.6399
-24.6427
[15351]ENERGY: 4490000 3179.5515 5136.7449 5236.7074
316.7117 -15726.5901 -1826.7760 0.0000 0.0000
7644.0435 3960.3929 305.8000 -3683.6506 4024.6505
305.5719 53.4015 1.9547 656151.2420 -26.5285
-26.5401
[15390]ENERGY: 4500000 3147.5141 5110.6896 5278.0579
338.0806 -15913.1324 -1675.9077 0.0000 0.0000
7675.9234 3961.2256 307.0753 -3714.6978 4022.8896
305.5802 -39.0011 -41.2648 656151.2420 -26.2339
-26.2278
[15440]ENERGY: 4510000 3234.7402 5243.3508 5275.2717
327.2656 -15970.5127 -1721.2558 0.0000 0.0000
7578.5824 3967.4423 303.1812 -3611.1401 4025.0162
305.4044 142.9808 75.3702 656151.2420 -20.7234
-20.7245
[15476]ENERGY: 4520000 3157.2451 5144.0332 5277.2566
311.6162 -15865.8236 -1697.6191 0.0000 0.0000
7638.1885 3964.8969 305.5657 -3673.2916 4023.2164
305.4536 176.8157 59.1345 656151.2420 -21.5666
-21.5544
[15526]ENERGY: 4530000 3150.1217 5226.5405 5244.7966
319.5322 -15764.7289 -1837.9583 0.0000 0.0000
7624.3982 3962.7021 305.0140 -3661.6961 4024.3793
305.3617 -6.7118 -87.1715 656151.2420 -18.5382

-18.5315
[15562]ENERGY: 4540000 3165.8403 5181.9894 5308.6380
338.7414 -15933.9318 -1728.2574 0.0000 0.0000
7629.2331 3962.2530 305.2075 -3666.9801 4023.1690
305.3796 -130.3592 -36.3302 656151.2420 -24.0557
-24.0677
[15612]ENERGY: 4550000 3131.2568 5156.6823 5217.7579
339.7719 -15854.3426 -1734.2242 0.0000 0.0000
7701.5004 3958.4026 308.0985 -3743.0979 4024.1615
305.4793 35.5277 -19.1140 656151.2420 -16.4591
-16.4581
[15648]ENERGY: 4560000 3189.1372 5173.6164 5264.4075
353.7692 -15860.0928 -1761.8170 0.0000 0.0000
7600.1360 3959.1566 304.0434 -3640.9794 4022.9759
305.3887 -70.4123 -98.6936 656151.2420 -26.6425
-26.6367
[15698]ENERGY: 4570000 3180.0307 5153.8387 5246.7854
350.8853 -15914.1271 -1747.3161 0.0000 0.0000
7690.6375 3960.7344 307.6640 -3729.9031 4023.9137
305.4848 77.6368 22.0234 656151.2420 -22.6289
-22.6366
[15734]ENERGY: 4580000 3209.4649 5158.6431 5220.4432
327.9255 -15701.9382 -1832.7298 0.0000 0.0000
7583.3170 3965.1258 303.3706 -3618.1912 4025.3666
305.4062 -50.6899 -72.3578 656151.2420 -23.2132
-23.2075
[15784]ENERGY: 4590000 3235.2315 5229.3320 5253.8478
327.9622 -15835.9258 -1810.9393 0.0000 0.0000
7564.4600 3963.9683 302.6162 -3600.4917 4024.1895
305.0926 -250.3856 -191.6809 656151.2420 -25.0935
-25.0961
[15823]ENERGY: 4600000 3185.9118 5159.2111 5213.4235
360.1054 -15908.8686 -1687.6649 0.0000 0.0000
7638.8160 3960.9344 305.5908 -3677.8816 4024.4362
305.5199 167.6388 101.5190 656151.2420 -22.1811
-22.1854
[15873]ENERGY: 4610000 3171.5423 5218.4684 5260.8757
327.1829 -15780.7065 -1858.9612 0.0000 0.0000
7622.6737 3961.0753 304.9451 -3661.5984 4025.1853
305.3979 -59.4682 -63.4748 656151.2420 -22.3400
-22.3417
[15909]ENERGY: 4620000 3170.0476 5107.8878 5229.5646
340.1682 -15894.0374 -1677.5682 0.0000 0.0000
7686.9316 3962.9941 307.5157 -3723.9375 4024.0157
305.3374 91.6364 111.1114 656151.2420 -17.7192
-17.7075
[15959]ENERGY: 4630000 3195.1724 5215.9398 5300.9414
333.5308 -15853.7519 -1794.1353 0.0000 0.0000
7567.9601 3965.6572 302.7562 -3602.3030 4025.2677
305.4455 -64.1378 -0.5155 656151.2420 -20.1372
-20.1494
[15995]ENERGY: 4640000 3173.7769 5179.7249 5246.8000
331.4142 -15837.2733 -1812.4131 0.0000 0.0000
7682.8218 3964.8514 307.3513 -3717.9703 4024.6312

305.4202 -93.6113 -102.2083 656151.2420 -19.8814
-19.8609
[16045]ENERGY: 4650000 3106.6881 5118.4542 5339.4649
355.4669 -15973.0173 -1720.5982 0.0000 0.0000
7733.1643 3959.6230 309.3652 -3773.5413 4024.4518
305.1879 125.6606 93.9729 656151.2420 -21.7602
-21.7684
[16081]ENERGY: 4660000 3171.0956 5236.9185 5256.4394
314.5978 -15899.9067 -1792.6661 0.0000 0.0000
7679.2976 3965.7761 307.2103 -3713.5215 4025.1637
305.3289 95.7070 -3.9867 656151.2420 -24.8862
-24.8947
[16131]ENERGY: 4670000 3174.9169 5176.3687 5275.1201
310.5952 -15857.7985 -1737.2978 0.0000 0.0000
7623.1506 3965.0551 304.9641 -3658.0955 4026.3264
305.4738 9.4092 -24.4478 656151.2420 -20.2053
-20.2073
[16167]ENERGY: 4680000 3186.4141 5131.9449 5311.2169
327.3892 -15804.1703 -1813.2684 0.0000 0.0000
7623.0817 3962.6080 304.9614 -3660.4737 4026.2348
305.4514 -41.4921 -60.6807 656151.2420 -21.8334
-21.8477
[16217]ENERGY: 4690000 3143.1719 5146.2169 5248.8491
338.0038 -15811.3429 -1708.1896 0.0000 0.0000
7609.7693 3966.4785 304.4288 -3643.2908 4025.8708
305.5285 -40.2300 -41.2808 656151.2420 -24.6780
-24.6505
[16256]ENERGY: 4700000 3138.0439 5263.0960 5258.1091
321.4026 -15844.3120 -1787.1633 0.0000 0.0000
7617.4075 3966.5840 304.7344 -3650.8235 4026.6547
305.7617 -18.6962 12.6517 656151.2420 -17.9372
-17.9447
[16306]ENERGY: 4710000 3147.5715 5149.4583 5224.0177
344.2000 -15871.9134 -1668.9223 0.0000 0.0000
7638.0183 3962.4300 305.5589 -3675.5882 4026.1490
305.5339 91.7929 25.0602 656151.2420 -19.6943
-19.7094
[16342]ENERGY: 4720000 3157.8974 5233.6459 5259.1162
332.6119 -15838.4212 -1767.8582 0.0000 0.0000
7588.4580 3965.4501 303.5763 -3623.0080 4026.4269
305.4574 38.0507 -13.3533 656151.2420 -24.3838
-24.3628
[16392]ENERGY: 4730000 3214.5798 5095.6680 5232.8453
348.8837 -15837.9500 -1666.4967 0.0000 0.0000
7577.0151 3964.5452 303.1185 -3612.4698 4026.2699
305.5844 199.3476 102.2080 656151.2420 -21.7577
-21.7663
[16428]ENERGY: 4740000 3122.5285 5220.8721 5262.8029
362.9159 -15833.8057 -1814.1788 0.0000 0.0000
7641.5802 3962.7151 305.7014 -3678.8651 4027.2494
305.6645 -127.5315 -154.2207 656151.2420 -24.3728
-24.3664
[16478]ENERGY: 4750000 3202.3316 5166.8740 5231.2939
338.1729 -15851.2146 -1742.5885 0.0000 0.0000

7618.5912 3963.4605 304.7817 -3655.1307 4026.6763
305.4483 -59.1335 45.0304 656151.2420 -23.1457
-23.1529
[16514]ENERGY: 4760000 3164.2350 5126.7071 5277.3073
343.4120 -15813.2511 -1749.6672 0.0000 0.0000
7617.9304 3966.6735 304.7553 -3651.2569 4027.3413
305.5295 -35.2228 -104.3341 656151.2420 -18.8406
-18.8460
[16564]ENERGY: 4770000 3127.4558 5137.0713 5258.4083
331.5529 -15886.3958 -1715.2429 0.0000 0.0000
7710.7156 3963.5653 308.4672 -3747.1503 4028.2642
305.3957 81.8580 55.4165 656151.2420 -21.1643
-21.1500
[16600]ENERGY: 4780000 3239.3978 5183.6467 5274.2224
358.2566 -15873.4050 -1806.0422 0.0000 0.0000
7593.2574 3969.3337 303.7683 -3623.9236 4027.7854
305.5933 -244.2121 -200.8856 656151.2420 -19.3505
-19.3595
[16650]ENERGY: 4790000 3193.8106 5105.0024 5299.8048
351.0182 -15890.3027 -1726.8468 0.0000 0.0000
7638.9307 3971.4172 305.5954 -3667.5136 4027.8321
305.7206 28.6438 -18.9721 656151.2420 -22.4810
-22.4718
[16689]ENERGY: 4800000 3188.4312 5220.2399 5291.5642
316.5784 -15898.8167 -1760.1339 0.0000 0.0000
7609.6416 3967.5046 304.4237 -3642.1370 4028.2126
305.7185 -18.1303 -78.5075 656151.2420 -24.5260
-24.5224
[16739]ENERGY: 4810000 3198.3444 5184.5517 5226.7450
330.3655 -15838.5480 -1733.1872 0.0000 0.0000
7597.1392 3965.4107 303.9236 -3631.7284 4029.1751
305.5230 -24.0121 -161.6925 656151.2420 -25.6912
-25.7026
[16775]ENERGY: 4820000 3130.7848 5061.0794 5309.5204
324.3028 -15728.1714 -1747.9043 0.0000 0.0000
7617.3677 3966.9792 304.7328 -3650.3885 4028.0989
305.6995 -50.6548 -33.1529 656151.2420 -24.7073
-24.7148
[16825]ENERGY: 4830000 3160.3373 5113.9291 5255.1224
336.8313 -15753.5853 -1784.2323 0.0000 0.0000
7638.8782 3967.2807 305.5933 -3671.5975 4028.7179
305.7246 113.1613 35.8064 656151.2420 -22.4539
-22.4397
[16861]ENERGY: 4840000 3104.6795 5190.4514 5284.7853
345.7149 -15886.4321 -1698.1845 0.0000 0.0000
7622.5319 3963.5464 304.9394 -3658.9856 4027.3940
305.5054 114.3621 44.1822 656151.2420 -27.3587
-27.3730
[16911]ENERGY: 4850000 3189.8829 5148.8924 5277.1791
315.1487 -15814.9007 -1722.2584 0.0000 0.0000
7574.6089 3968.5530 303.0222 -3606.0559 4027.9290
305.6335 38.9046 18.5693 656151.2420 -22.7808
-22.7591
[16947]ENERGY: 4860000 3230.8260 5265.0991 5279.1287

324.2427 -15982.9394 -1715.4513 0.0000 0.0000
7567.0598 3967.9657 302.7202 -3599.0941 4027.0426
305.5235 -48.3836 -101.5694 656151.2420 -24.6063
-24.6289
[16997]ENERGY: 4870000 3200.3301 5115.2942 5290.4207
358.8707 -15818.9777 -1837.0090 0.0000 0.0000
7656.9994 3965.9286 306.3183 -3691.0708 4028.3865
305.5596 -56.5215 -164.0185 656151.2420 -24.3700
-24.3409
[17033]ENERGY: 4880000 3219.7567 5240.8136 5233.0195
354.0964 -15837.4596 -1804.4689 0.0000 0.0000
7562.1845 3967.9422 302.5252 -3594.2423 4029.0566
305.5700 40.5529 -26.3782 656151.2420 -26.0608
-26.0696
[17083]ENERGY: 4890000 3143.9178 5141.5428 5293.1562
354.1056 -15832.1410 -1772.2920 0.0000 0.0000
7640.7497 3969.0392 305.6682 -3671.7106 4028.3068
305.4029 -11.1197 7.8288 656151.2420 -25.1789
-25.1865
[17122]ENERGY: 4900000 3190.7470 5170.7840 5281.2269
352.7986 -15860.7651 -1763.9516 0.0000 0.0000
7597.4412 3968.2812 303.9356 -3629.1601 4028.7453
305.4849 -56.4751 -1.4649 656151.2420 -25.1199
-25.1392
[17172]ENERGY: 4910000 3131.6865 5219.7020 5282.3193
332.7260 -15826.8734 -1853.1398 0.0000 0.0000
7678.8848 3965.3055 307.1938 -3713.5793 4029.8618
305.7628 -7.2139 -42.1804 656151.2420 -25.5327
-25.5250
[17208]ENERGY: 4920000 3163.6032 5113.2931 5230.8345
354.2042 -15871.5033 -1749.0560 0.0000 0.0000
7723.3120 3964.6877 308.9711 -3758.6243 4028.0149
305.6525 187.6679 175.0924 656151.2420 -26.1514
-26.1559
[17258]ENERGY: 4930000 3222.8108 5227.7430 5277.5557
354.4223 -15941.9262 -1730.5842 0.0000 0.0000
7560.6257 3970.6470 302.4628 -3589.9786 4028.7837
305.7700 -78.3808 -45.1002 656151.2420 -22.1824
-22.1584
[17294]ENERGY: 4940000 3245.5337 5197.8429 5253.1053
304.9226 -15830.6373 -1794.9866 0.0000 0.0000
7592.5160 3968.2967 303.7386 -3624.2194 4029.0324
305.8029 -124.9848 -145.0291 656151.2420 -23.1446
-23.1500
[17344]ENERGY: 4950000 3121.7174 5176.1596 5263.4263
321.1638 -15805.1744 -1751.6038 0.0000 0.0000
7640.0396 3965.7284 305.6398 -3674.3113 4029.1021
305.6669 -30.2931 -31.9338 656151.2420 -25.2968
-25.3058
[17380]ENERGY: 4960000 3266.2654 5220.8810 5248.5508
340.8275 -15925.8211 -1791.9828 0.0000 0.0000
7610.2748 3968.9956 304.4490 -3641.2792 4029.3977
305.7168 -139.2718 -153.5968 656151.2420 -21.5547
-21.5455

[17430]ENERGY: 4970000 3147.5966 5255.0883 5258.0541
338.8421 -15870.2254 -1832.7398 0.0000 0.0000
7667.8253 3964.4412 306.7513 -3703.3841 4029.1867
305.7072 -135.5933 -149.0330 656151.2420 -23.7148
-23.7264
[17466]ENERGY: 4980000 3195.8193 5214.1316 5284.7496
319.2780 -15840.6019 -1812.8530 0.0000 0.0000
7609.8681 3970.3918 304.4328 -3639.4763 4031.6715
305.5813 -9.2769 -53.6365 656151.2420 -19.5501
-19.5478
[17516]ENERGY: 4990000 3161.6141 5136.0222 5302.3553
330.6064 -15839.4786 -1744.0138 0.0000 0.0000
7621.8681 3968.9736 304.9128 -3652.8945 4030.3717
305.7286 59.8467 18.5985 656151.2420 -25.5171
-25.5022
[17555]ENERGY: 5000000 3169.1068 5180.3586 5290.7885
335.8401 -15822.5602 -1799.9922 0.0000 0.0000
7613.9525 3967.4941 304.5962 -3646.4584 4029.5971
305.6476 -81.0819 -88.0469 656151.2420 -17.8755
-17.8846
[17605]ENERGY: 5010000 3104.3882 5331.3645 5207.5921
324.8799 -15887.7948 -1708.3240 0.0000 0.0000
7597.7855 3969.8914 303.9494 -3627.8941 4030.5646
305.5616 93.0162 107.3341 656151.2420 -21.4358
-21.4239
[17641]ENERGY: 5020000 3228.8551 5185.8807 5291.1267
310.1849 -15830.9855 -1750.5096 0.0000 0.0000
7537.7693 3972.3216 301.5485 -3565.4477 4031.0247
305.5123 73.7675 3.3241 656151.2420 -21.2926
-21.2888
[17691]ENERGY: 5030000 3178.2215 5159.8910 5223.3259
326.7910 -15841.8752 -1770.1748 0.0000 0.0000
7690.1472 3966.3266 307.6443 -3723.8206 4031.0425
305.6838 -98.4789 -103.9856 656151.2420 -24.8075
-24.8326
[17727]ENERGY: 5040000 3205.8233 5191.1457 5247.7658
315.2164 -15835.9662 -1715.5081 0.0000 0.0000
7565.6416 3974.1185 302.6635 -3591.5231 4031.4986
305.5826 36.8419 -103.3596 656151.2420 -26.7031
-26.7121
[17777]ENERGY: 5050000 3129.5723 5141.4303 5297.0710
344.4270 -15825.6033 -1773.5544 0.0000 0.0000
7654.9483 3968.2913 306.2362 -3686.6570 4031.0682
305.4731 77.8552 -19.7558 656151.2420 -24.1573
-24.1446
[17813]ENERGY: 5060000 3197.3141 5192.8077 5306.0123
329.1897 -15887.6475 -1799.9445 0.0000 0.0000
7633.1327 3970.8646 305.3635 -3662.2681 4030.4220
305.3086 -196.3423 -142.7683 656151.2420 -25.7889
-25.7975
[17863]ENERGY: 5070000 3070.0522 5155.7127 5281.0415
351.2184 -15814.1575 -1782.9756 0.0000 0.0000
7705.8609 3966.7526 308.2730 -3739.1083 4031.9271
305.4814 152.0307 29.6226 656151.2420 -19.6413

-19.6391
[17899]ENERGY: 5080000 3172.2682 5229.6115 5237.1005
329.7593 -15780.2223 -1761.6021 0.0000 0.0000
7544.5998 3971.5150 301.8217 -3573.0848 4032.0481
305.6440 96.9672 76.4047 656151.2420 -26.5111
-26.5132
[17949]ENERGY: 5090000 3173.7698 5149.8489 5290.9604
328.8702 -15787.8547 -1747.5424 0.0000 0.0000
7564.8183 3972.8705 302.6306 -3591.9478 4032.1535
305.4753 -85.8768 -82.0943 656151.2420 -21.5527
-21.5367
[17988]ENERGY: 5100000 3232.9844 5150.5492 5239.9974
343.1702 -15808.0807 -1803.6044 0.0000 0.0000
7617.1555 3972.1716 304.7243 -3644.9839 4031.5900
305.4712 -48.9455 -1.4686 656151.2420 -24.8859
-24.8851
[18038]ENERGY: 5110000 3114.2603 5146.0692 5299.6639
337.0870 -15887.9994 -1745.5662 0.0000 0.0000
7704.8099 3968.3246 308.2309 -3736.4853 4031.5620
305.6630 95.7307 58.2979 656151.2420 -18.1163
-18.1227
[18074]ENERGY: 5120000 3196.8355 5192.6201 5321.2946
346.0690 -15946.4163 -1741.2297 0.0000 0.0000
7600.9732 3970.1464 304.0769 -3630.8267 4031.6789
305.7010 69.0707 -35.5824 656151.2420 -27.0226
-27.0399
[18124]ENERGY: 5130000 3262.3613 5144.7009 5310.4122
320.2873 -15870.2702 -1825.3359 0.0000 0.0000
7631.5720 3973.7277 305.3010 -3657.8443 4031.8389
305.5897 -186.4684 -115.8369 656151.2420 -19.8838
-19.8539
[18160]ENERGY: 5140000 3138.6003 5124.3073 5267.9314
343.6134 -15831.6539 -1759.4818 0.0000 0.0000
7683.0093 3966.3260 307.3588 -3716.6833 4031.2521
305.7544 -79.2398 -108.6832 656151.2420 -27.2428
-27.2652
[18210]ENERGY: 5150000 3166.4321 5253.6838 5289.0987
299.5651 -15840.3052 -1757.7672 0.0000 0.0000
7559.3820 3970.0892 302.4131 -3589.2927 4031.3889
305.6266 -32.2432 -15.1545 656151.2420 -22.4085
-22.3916
[18246]ENERGY: 5160000 3194.9623 5198.4141 5272.6565
308.8627 -15856.0834 -1832.6827 0.0000 0.0000
7683.4777 3969.6073 307.3775 -3713.8705 4032.0630
305.7352 10.1002 -15.3267 656151.2420 -23.5872
-23.5923
[18296]ENERGY: 5170000 3182.2490 5153.6953 5255.4373
359.7968 -15828.2566 -1779.0239 0.0000 0.0000
7626.0380 3969.9361 305.0796 -3656.1020 4030.4991
305.5869 -27.7353 -28.0707 656151.2420 -25.1644
-25.1708
[18332]ENERGY: 5180000 3212.7072 5207.1347 5241.6113
358.5848 -15890.4905 -1728.3651 0.0000 0.0000
7571.1515 3972.3339 302.8839 -3598.8176 4030.5324

305.6430 -121.5729 -159.8978 656151.2420 -19.6125
-19.6196
[18382]ENERGY: 5190000 3162.0418 5254.9456 5250.4374
329.1076 -15864.9591 -1819.0725 0.0000 0.0000
7657.9471 3970.4478 306.3562 -3687.4992 4032.7392
305.6213 -0.7367 -23.2741 656151.2420 -23.8879
-23.8908
[18421]ENERGY: 5200000 3150.1509 5168.9711 5279.9791
342.9331 -15885.2815 -1755.0342 0.0000 0.0000
7670.3534 3972.0718 306.8525 -3698.2816 4033.5738
305.7682 50.2106 -24.2957 656151.2420 -23.1334
-23.1274
[18471]ENERGY: 5210000 3137.7628 5284.2028 5205.3750
359.9324 -15799.5821 -1775.9560 0.0000 0.0000
7559.8598 3971.5947 302.4322 -3588.2651 4032.9987
305.7672 -2.9213 8.1510 656151.2420 -22.3905
-22.3780
[18507]ENERGY: 5220000 3167.8240 5199.5066 5255.2238
323.4806 -15811.5929 -1825.6424 0.0000 0.0000
7664.3952 3973.1951 306.6141 -3691.2002 4034.8831
305.6936 -11.6572 -52.5590 656151.2420 -24.2668
-24.2538
[18557]ENERGY: 5230000 3122.1468 5246.5447 5278.3285
351.8570 -15932.1127 -1729.4549 0.0000 0.0000
7635.0797 3972.3891 305.4414 -3662.6906 4033.6567
305.7877 120.2709 30.5134 656151.2420 -22.5577
-22.5731
[18593]ENERGY: 5240000 3167.4593 5185.0687 5279.3871
329.9376 -15824.3240 -1826.1804 0.0000 0.0000
7658.9545 3970.3028 306.3965 -3688.6518 4032.6037
305.6570 -43.3676 -103.1480 656151.2420 -23.6798
-23.6844
[18643]ENERGY: 5250000 3141.5205 5155.9625 5297.0388
315.8625 -15785.9022 -1786.3716 0.0000 0.0000
7631.7558 3969.8663 305.3084 -3661.8896 4032.5869
305.6173 -54.3747 -61.6948 656151.2420 -24.1223
-24.1061
[18679]ENERGY: 5260000 3235.4508 5212.0141 5324.5568
359.8817 -15893.6635 -1825.9768 0.0000 0.0000
7562.5020 3974.7650 302.5379 -3587.7369 4033.6682
305.9384 -100.1107 -138.9998 656151.2420 -21.1918
-21.1892
[18729]ENERGY: 5270000 3179.5073 5155.3179 5262.6078
344.1925 -15747.1681 -1905.2643 0.0000 0.0000
7681.2035 3970.3965 307.2865 -3710.8069 4034.5503
305.8739 -3.2979 -155.2980 656151.2420 -22.9137
-22.9264
[18765]ENERGY: 5280000 3187.8508 5209.9260 5243.3769
331.0031 -15841.4634 -1784.0102 0.0000 0.0000
7624.6170 3971.3001 305.0228 -3653.3169 4032.9426
305.8635 20.5226 49.1042 656151.2420 -24.8370
-24.8277
[18815]ENERGY: 5290000 3201.6838 5224.9434 5285.6565
340.6808 -15920.7522 -1718.5074 0.0000 0.0000

7561.1560 3974.8609 302.4840 -3586.2951 4034.2226
305.9247 20.8889 10.7230 656151.2420 -24.4692
-24.4848
[18854]ENERGY: 5300000 3167.2885 5149.8281 5247.4082
337.6953 -15783.8641 -1730.3146 0.0000 0.0000
7586.8491 3974.8906 303.5119 -3611.9585 4033.7841
305.7786 -80.8809 -90.5077 656151.2420 -23.5746
-23.5634
[18904]ENERGY: 5310000 3123.9620 5225.8544 5273.7932
347.7182 -15852.6327 -1764.1475 0.0000 0.0000
7619.3772 3973.9248 304.8132 -3645.4524 4034.2408
305.7866 64.1323 1.4906 656151.2420 -22.3961
-22.4003
[18940]ENERGY: 5320000 3212.7222 5226.3877 5231.0358
323.7248 -15763.4298 -1834.4573 0.0000 0.0000
7579.4491 3975.4324 303.2159 -3604.0166 4033.7732
305.7586 14.0129 -39.1610 656151.2420 -24.3697
-24.3736
[18990]ENERGY: 5330000 3270.1258 5095.9913 5300.5615
324.0938 -15804.7473 -1841.1576 0.0000 0.0000
7628.0825 3972.9500 305.1614 -3655.1325 4034.7505
305.9384 -133.7306 -143.6081 656151.2420 -21.5136
-21.5201
[19026]ENERGY: 5340000 3181.8473 5155.8343 5278.0486
344.9045 -15874.1000 -1716.2399 0.0000 0.0000
7602.2998 3972.5947 304.1300 -3629.7050 4033.2120
305.9797 31.1183 -24.5959 656151.2420 -25.2041
-25.1904
[19076]ENERGY: 5350000 3178.7779 5172.0578 5331.0283
339.9221 -15928.5696 -1711.8242 0.0000 0.0000
7592.9712 3974.3636 303.7568 -3618.6076 4035.0326
305.9378 -9.5857 -62.6514 656151.2420 -24.0294
-24.0378
[19112]ENERGY: 5360000 3181.3131 5177.4012 5191.2007
321.1465 -15771.4372 -1721.5037 0.0000 0.0000
7597.5157 3975.6362 303.9386 -3621.8794 4035.6083
305.7438 -12.5216 -50.7587 656151.2420 -24.9546
-24.9303
[19162]ENERGY: 5370000 3190.1570 5192.8220 5225.2504
336.7664 -15872.3289 -1731.8237 0.0000 0.0000
7631.8505 3972.6936 305.3122 -3659.1569 4033.9296
305.9785 -41.3686 -63.1677 656151.2420 -25.9083
-25.9217
[19198]ENERGY: 5380000 3146.5641 5238.3621 5276.1244
336.5572 -15955.3125 -1691.0714 0.0000 0.0000
7621.1258 3972.3497 304.8831 -3648.7761 4034.4009
305.8383 81.4687 25.3928 656151.2420 -26.1792
-26.1936
[19248]ENERGY: 5390000 3155.8455 5256.1507 5209.4157
364.9621 -15769.3655 -1885.2404 0.0000 0.0000
7639.7696 3971.5377 305.6290 -3668.2318 4036.2388
305.9971 -19.6644 -82.9745 656151.2420 -23.0983
-23.1058
[19287]ENERGY: 5400000 3166.3134 5136.4964 5297.9878

319.2711 -15788.4763 -1826.4105 0.0000 0.0000
7667.6820 3972.8638 306.7456 -3694.8182 4035.4676
305.9338 5.0331 6.7812 656151.2420 -25.5772
-25.5579
[19337]ENERGY: 5410000 3169.1842 5176.2620 5238.8317
335.5477 -15804.4942 -1837.2630 0.0000 0.0000
7696.8598 3974.9282 307.9129 -3721.9316 4036.9932
305.9553 -67.7119 -94.6837 656151.2420 -22.2445
-22.2640
[19373]ENERGY: 5420000 3145.2432 5136.6696 5297.1236
299.3874 -15905.9517 -1708.1172 0.0000 0.0000
7707.8310 3972.1858 308.3518 -3735.6451 4035.6256
305.7929 -77.9903 -160.5312 656151.2420 -26.4235
-26.4247
[19423]ENERGY: 5430000 3140.2903 5074.1761 5273.3291
358.4340 -15857.9505 -1760.4540 0.0000 0.0000
7742.8725 3970.6977 309.7536 -3772.1748 4036.5605
305.6028 -65.6603 -5.3075 656151.2420 -30.4428
-30.4108
[19459]ENERGY: 5440000 3265.7792 5118.7152 5261.8910
350.4736 -15860.0969 -1779.9859 0.0000 0.0000
7620.0205 3976.7967 304.8389 -3643.2238 4037.3175
305.7287 -63.6991 -70.3165 656151.2420 -27.0755
-27.0822
[19509]ENERGY: 5450000 3228.6539 5101.8554 5260.5614
333.3797 -15875.9572 -1780.3932 0.0000 0.0000
7706.5697 3974.6697 308.3013 -3731.9000 4036.5775
305.9566 -12.3256 -27.9286 656151.2420 -24.8118
-24.8303
[19545]ENERGY: 5460000 3191.4190 5214.4751 5290.8541
302.0145 -15841.3914 -1782.1074 0.0000 0.0000
7604.1669 3979.4307 304.2047 -3624.7362 4038.5457
305.7808 31.9217 -58.9675 656151.2420 -31.6386
-31.6322
[19595]ENERGY: 5470000 3160.9887 5115.4261 5252.7743
295.1969 -15849.1351 -1748.2386 0.0000 0.0000
7746.3773 3973.3896 309.8938 -3772.9877 4036.9010
305.8893 18.0002 36.3108 656151.2420 -22.6757
-22.6638
[19631]ENERGY: 5480000 3093.3623 5160.2226 5255.5871
339.7621 -15825.4396 -1747.2108 0.0000 0.0000
7695.9067 3972.1906 307.8747 -3723.7161 4037.3313
306.0344 135.1180 84.3821 656151.2420 -24.1087
-24.1068
[19681]ENERGY: 5490000 3185.4644 5256.5309 5246.3512
345.1009 -15886.9751 -1758.7991 0.0000 0.0000
7586.4750 3974.1482 303.4969 -3612.3267 4037.3573
306.0428 -69.8316 -7.4686 656151.2420 -24.4207
-24.4322
[19720]ENERGY: 5500000 3149.7604 5150.3698 5242.7323
331.9755 -15798.9719 -1744.6900 0.0000 0.0000
7643.8966 3975.0726 305.7941 -3668.8240 4038.2961
306.0046 65.4669 49.8184 656151.2420 -20.9782
-20.9742

[19770]ENERGY: 5510000 3215.3265 5105.9774 5253.0037
316.1094 -15780.2642 -1746.3921 0.0000 0.0000
7614.5596 3978.3202 304.6205 -3636.2394 4038.5159
305.9300 -147.2035 -136.8776 656151.2420 -27.7647
-27.7809
[19806]ENERGY: 5520000 3154.0611 5125.8049 5308.2136
343.7785 -15878.7002 -1763.8715 0.0000 0.0000
7683.1491 3972.4354 307.3644 -3710.7137 4038.8594
305.8410 -111.4880 -123.7228 656151.2420 -26.2980
-26.2799
[19856]ENERGY: 5530000 3130.5165 5152.7117 5278.9242
331.6190 -15864.2439 -1681.7424 0.0000 0.0000
7631.1603 3978.9454 305.2846 -3652.2149 4039.1719
305.8759 -77.3963 -18.0149 656151.2420 -22.0837
-22.0742
[19892]ENERGY: 5540000 3192.3922 5248.7017 5247.1145
349.1190 -15889.6828 -1741.5368 0.0000 0.0000
7570.6723 3976.7802 302.8647 -3593.8921 4039.5802
306.0003 -28.7498 -41.0214 656151.2420 -27.3188
-27.3375
[19942]ENERGY: 5550000 3168.0216 5211.3282 5270.0686
317.8744 -15884.1587 -1701.8084 0.0000 0.0000
7599.5367 3980.8624 304.0195 -3618.6743 4040.8790
306.0582 74.6785 -14.0880 656151.2420 -22.0504
-22.0588
[19978]ENERGY: 5560000 3252.3826 5152.9032 5278.2935
355.8212 -15922.6419 -1752.6517 0.0000 0.0000
7618.4488 3982.5557 304.7760 -3635.8931 4041.4216
305.8619 -12.7704 -14.3815 656151.2420 -29.5597
-29.5413
[20028]ENERGY: 5570000 3213.7138 5169.5587 5244.1601
335.8094 -15876.6168 -1745.4839 0.0000 0.0000
7637.7131 3978.8544 305.5467 -3658.8587 4040.9768
306.0062 -36.4419 -116.1500 656151.2420 -21.0679
-21.0709
[20064]ENERGY: 5580000 3146.7167 5301.5689 5283.1177
332.4767 -15894.8164 -1768.9713 0.0000 0.0000
7581.5183 3981.6107 303.2986 -3599.9077 4041.2099
306.0391 27.6002 49.5842 656151.2420 -19.7210
-19.7326
[20114]ENERGY: 5590000 3171.5731 5298.4523 5281.1068
348.4924 -15900.1785 -1801.6182 0.0000 0.0000
7580.5932 3978.4212 303.2616 -3602.1720 4039.9234
306.0774 -131.1123 -100.5881 656151.2420 -24.5184
-24.5096
[20153]ENERGY: 5600000 3135.2847 5224.8862 5280.4264
334.1923 -15867.9717 -1803.8782 0.0000 0.0000
7673.1238 3976.0634 306.9633 -3697.0603 4041.6010
306.1531 5.1193 14.7656 656151.2420 -25.1304
-25.1448
[20203]ENERGY: 5610000 3148.6022 5156.7258 5301.4104
314.2288 -15802.3441 -1760.9053 0.0000 0.0000
7620.4274 3978.1451 304.8552 -3642.2823 4040.7574
306.0918 -5.8461 -49.4410 656151.2420 -23.4273

-23.4179
[20239]ENERGY: 5620000 3179.9507 5270.1194 5263.5230
349.9271 -15868.7335 -1812.7158 0.0000 0.0000
7596.6304 3978.7011 303.9032 -3617.9293 4040.7007
306.2324 -74.7203 -67.9415 656151.2420 -23.7587
-23.7484
[20289]ENERGY: 5630000 3179.9887 5162.4548 5266.4553
343.6334 -15843.6608 -1759.2683 0.0000 0.0000
7630.9399 3980.5430 305.2757 -3650.3969 4040.6032
306.0883 -55.6313 -153.6231 656151.2420 -20.8602
-20.8789
[20325]ENERGY: 5640000 3241.4897 5192.9664 5298.9738
347.7105 -15852.2278 -1796.8459 0.0000 0.0000
7552.6315 3984.6982 302.1430 -3567.9333 4042.1056
305.8833 49.6864 72.9737 656151.2420 -25.5953
-25.5686
[20375]ENERGY: 5650000 3134.4371 5155.3528 5260.5916
365.4742 -15840.7666 -1767.6368 0.0000 0.0000
7669.3817 3976.8339 306.8136 -3692.5478 4042.2149
305.9730 78.0471 -2.7049 656151.2420 -23.3546
-23.3873
[20411]ENERGY: 5660000 3173.2616 5202.8410 5283.6991
330.3215 -15821.1086 -1797.4124 0.0000 0.0000
7610.4192 3982.0215 304.4548 -3628.3978 4043.1579
306.1895 124.4804 29.7265 656151.2420 -23.6600
-23.6406
[20461]ENERGY: 5670000 3227.5374 5206.2502 5263.2681
366.2294 -15950.5504 -1795.9266 0.0000 0.0000
7663.7924 3980.6007 306.5900 -3683.1917 4041.4150
306.1137 22.8823 -123.6637 656151.2420 -23.1201
-23.1265
[20497]ENERGY: 5680000 3152.4441 5134.3293 5269.0103
344.3719 -15764.3185 -1744.6785 0.0000 0.0000
7590.2820 3981.4407 303.6492 -3608.8413 4042.5127
306.0870 101.9973 -7.9829 656151.2420 -23.7786
-23.7732
[20547]ENERGY: 5690000 3239.2483 5099.3349 5250.0110
317.4759 -15837.1245 -1782.2785 0.0000 0.0000
7690.8427 3977.5098 307.6722 -3713.3330 4041.3516
306.0412 -67.9032 -103.6703 656151.2420 -22.5705
-22.5715
[20586]ENERGY: 5700000 3158.2963 5241.6821 5253.9275
332.5444 -15837.3701 -1802.0147 0.0000 0.0000
7636.2856 3983.3510 305.4896 -3652.9345 4043.1541
306.1375 54.8913 4.5473 656151.2420 -26.8068
-26.8059
[20636]ENERGY: 5710000 3169.8321 5187.8248 5233.2563
358.4793 -15843.9715 -1782.2379 0.0000 0.0000
7658.5697 3981.7529 306.3811 -3676.8169 4043.7311
306.1768 30.7208 58.0330 656151.2420 -25.5856
-25.5745
[20672]ENERGY: 5720000 3222.0498 5174.3553 5269.0354
346.3431 -15857.7525 -1816.1742 0.0000 0.0000
7646.9191 3984.7760 305.9150 -3662.1431 4044.9151

306.0550 18.4719 -38.1766 656151.2420 -28.4631
-28.4772
[20722]ENERGY: 5730000 3092.5506 5097.1239 5221.7163
339.1938 -15754.9977 -1729.9463 0.0000 0.0000
7714.2352 3979.8759 308.6080 -3734.3593 4043.5975
305.9729 96.2272 -38.2259 656151.2420 -24.4466
-24.4395
[20758]ENERGY: 5740000 3198.6029 5168.4589 5277.7484
326.1789 -15852.6332 -1781.7885 0.0000 0.0000
7647.2908 3983.8581 305.9299 -3663.4327 4044.2270
305.8980 32.2083 26.2651 656151.2420 -26.3674
-26.3773
[20808]ENERGY: 5750000 3198.3646 5257.3160 5233.8480
343.4723 -15935.2057 -1766.8724 0.0000 0.0000
7651.7825 3982.7053 306.1096 -3669.0772 4045.5052
305.9020 -62.1947 -82.3958 656151.2420 -20.8511
-20.8397
[20844]ENERGY: 5760000 3196.1811 5189.3396 5218.2864
330.1970 -15797.7549 -1737.4885 0.0000 0.0000
7588.8986 3987.6592 303.5939 -3601.2393 4045.6289
306.0830 98.8994 -58.5432 656151.2420 -22.5629
-22.5765
[20894]ENERGY: 5770000 3099.5191 5253.5714 5238.7187
299.8851 -15809.9661 -1775.4653 0.0000 0.0000
7674.5670 3980.8299 307.0211 -3693.7371 4045.5821
305.9901 7.9276 -91.6755 656151.2420 -25.4880
-25.4774
[20930]ENERGY: 5780000 3206.2841 5177.8624 5293.2648
338.0012 -15819.7322 -1801.8514 0.0000 0.0000
7589.8696 3983.6986 303.6327 -3606.1710 4045.3876
305.9922 51.2266 11.3004 656151.2420 -19.3276
-19.3291
[20980]ENERGY: 5790000 3093.8700 5192.4966 5338.7204
359.1280 -15886.8889 -1712.0151 0.0000 0.0000
7599.8541 3985.1651 304.0322 -3614.6890 4045.2421
305.9311 84.0400 19.5446 656151.2420 -19.9863
-19.9886
[21019]ENERGY: 5800000 3219.2335 5063.7620 5284.0235
348.2246 -15837.5313 -1774.3114 0.0000 0.0000
7678.9620 3982.3630 307.1969 -3696.5990 4044.1996
305.8864 95.6644 -30.3541 656151.2420 -27.6575
-27.6601
[21069]ENERGY: 5810000 3097.2558 5214.0416 5253.9911
354.8247 -15839.0107 -1696.2885 0.0000 0.0000
7598.5074 3983.3215 303.9783 -3615.1859 4046.0763
305.9791 146.8631 53.8540 656151.2420 -22.5450
-22.5367
[21105]ENERGY: 5820000 3215.7642 5134.6891 5290.2222
344.0955 -15913.3608 -1758.2517 0.0000 0.0000
7669.5530 3982.7115 306.8205 -3686.8415 4045.2727
305.9188 -141.1296 -124.9248 656151.2420 -27.2014
-27.2065
[21155]ENERGY: 5830000 3116.6700 5098.9824 5303.0023
353.1536 -15888.6682 -1739.7809 0.0000 0.0000

7738.7684 3982.1274 309.5894 -3756.6410 4044.9616
305.9576 -57.3532 -141.1580 656151.2420 -27.1789
-27.1717
[21191]ENERGY: 5840000 3207.2782 5163.7264 5268.1580
337.4436 -15836.0569 -1771.5575 0.0000 0.0000
7615.7560 3984.7479 304.6683 -3631.0081 4045.8524
306.0429 28.5814 29.9045 656151.2420 -23.2215
-23.2305
[21241]ENERGY: 5850000 3281.6380 5134.2892 5274.4082
345.3073 -15825.5516 -1829.0780 0.0000 0.0000
7606.2810 3987.2941 304.2893 -3618.9869 4047.0929
305.9660 -153.9742 -142.1350 656151.2420 -28.3344
-28.3432
[21277]ENERGY: 5860000 3202.9386 5156.6572 5264.0379
337.8957 -15878.3157 -1777.1865 0.0000 0.0000
7677.3597 3983.3870 307.1328 -3693.9727 4045.9898
305.9581 42.3763 17.4283 656151.2420 -24.2852
-24.2761
[21327]ENERGY: 5870000 3180.4919 5187.4406 5267.3858
321.9872 -15845.0975 -1768.0884 0.0000 0.0000
7641.0412 3985.1607 305.6798 -3655.8804 4045.5380
305.8141 -94.8630 -144.4066 656151.2420 -20.6279
-20.6262
[21363]ENERGY: 5880000 3081.4789 5195.9813 5255.2805
325.5165 -15843.0894 -1736.8585 0.0000 0.0000
7702.6321 3980.9414 308.1438 -3721.6907 4045.1255
305.7541 93.3082 83.0611 656151.2420 -27.8005
-27.8150
[21413]ENERGY: 5890000 3180.3516 5178.9930 5253.6605
331.8655 -15828.0557 -1797.2288 0.0000 0.0000
7662.5241 3982.1103 306.5393 -3680.4138 4044.4146
305.9903 -112.0761 -119.4224 656151.2420 -23.9206
-23.8851
[21452]ENERGY: 5900000 3114.4136 5195.0819 5255.0235
335.1840 -15850.9358 -1736.8622 0.0000 0.0000
7670.3638 3982.2688 306.8529 -3688.0950 4045.1137
305.9371 40.8257 43.2863 656151.2420 -26.9955
-27.0249
[21502]ENERGY: 5910000 3194.0473 5181.2221 5296.8055
330.6105 -15913.8349 -1709.9812 0.0000 0.0000
7604.5895 3983.4587 304.2216 -3621.1308 4046.0290
305.9569 53.3629 33.5722 656151.2420 -22.5040
-22.4883
[21538]ENERGY: 5920000 3174.0491 5178.4932 5281.5619
360.6166 -15866.6275 -1756.1651 0.0000 0.0000
7609.6732 3981.6015 304.4250 -3628.0717 4046.0181
305.9287 71.4280 76.1633 656151.2420 -22.9191
-22.9173
[21588]ENERGY: 5930000 3146.5462 5113.8473 5261.9836
329.4647 -15798.8045 -1757.6360 0.0000 0.0000
7689.9575 3985.3588 307.6367 -3704.5987 4045.9109
306.0376 -36.2126 1.6630 656151.2420 -21.5961
-21.5973
[21624]ENERGY: 5940000 3222.9384 5120.6813 5307.6288

323.1400 -15816.3957 -1809.3500 0.0000 0.0000
7633.5339 3982.1767 305.3795 -3651.3572 4046.7728
306.1075 -73.8836 -106.3432 656151.2420 -19.8085
-19.8117
[21674]ENERGY: 5950000 3235.2986 5018.2439 5247.9051
310.1902 -15667.2055 -1806.5743 0.0000 0.0000
7642.6049 3980.4629 305.7424 -3662.1419 4046.6329
305.9254 -147.2697 -163.2953 656151.2420 -27.6309
-27.6369
[21710]ENERGY: 5960000 3226.5964 5087.5350 5248.6399
347.5611 -15840.0608 -1774.5176 0.0000 0.0000
7690.7268 3986.4809 307.6675 -3704.2459 4045.9943
305.9764 -56.8180 -78.4330 656151.2420 -19.4644
-19.4562
[21760]ENERGY: 5970000 3179.0032 5183.6683 5268.0628
327.4675 -15896.9318 -1781.0174 0.0000 0.0000
7702.1145 3982.3672 308.1231 -3719.7473 4046.4508
305.9259 -79.5512 -79.5261 656151.2420 -26.5800
-26.5947
[21796]ENERGY: 5980000 3164.1631 5208.4186 5253.1509
334.1454 -15859.9356 -1789.3062 0.0000 0.0000
7673.4282 3984.0643 306.9755 -3689.3639 4046.5335
306.0189 119.7971 29.6729 656151.2420 -25.0255
-25.0273
[21846]ENERGY: 5990000 3166.7773 5236.5826 5207.5239
350.5085 -15822.8934 -1811.9827 0.0000 0.0000
7659.5746 3986.0908 306.4213 -3673.4838 4047.0916
305.8896 -63.2836 -114.6673 656151.2420 -20.5205
-20.5059
[21885]ENERGY: 6000000 3214.8587 5203.1279 5251.4972
337.0106 -15864.9482 -1821.5074 0.0000 0.0000
7664.1357 3984.1745 306.6037 -3679.9611 4048.6080
305.9857 40.1487 -14.9110 656151.2420 -26.1752
-26.1745
[269]ENERGY: 6010000 3205.4871 5226.0774 5246.3142 330.7224
-15859.9301 -1811.7618 0.0000 0.0000 7649.5096
3986.4188 306.0186 -3663.0909 4047.0334 305.9160
-154.6696 -210.7610 656151.2420 -20.4726 -20.4877
[308]ENERGY: 6020000 3100.2048 5221.3589 5293.6319 325.2728
-15830.3539 -1750.4830 0.0000 0.0000 7626.5651
3986.1966 305.1007 -3640.3684 4047.8077 305.9052
154.9497 77.4490 656151.2420 -22.9825 -22.9629
[360]ENERGY: 6030000 3075.9574 5168.5804 5245.3407 330.8950
-15780.9250 -1786.1180 0.0000 0.0000 7732.8945
3986.6249 309.3544 -3746.2696 4048.0536 305.8227
23.6603 -25.1819 656151.2420 -23.5038 -23.5146
[398]ENERGY: 6040000 3158.7682 5107.6497 5329.6173 368.0921
-15780.3176 -1756.5007 0.0000 0.0000 7562.5661
3989.8751 302.5405 -3572.6910 4047.4741 305.9919
76.2838 -13.7040 656151.2420 -19.4065 -19.4066
[450]ENERGY: 6050000 3186.5968 5229.6870 5275.8576 344.0884
-15850.4331 -1779.5574 0.0000 0.0000 7584.2238
3990.4630 303.4069 -3593.7608 4048.1370 305.8632
-174.2035 -159.2081 656151.2420 -28.2877 -28.3044

[488]ENERGY: 6060000 3196.9129 5145.3340 5248.2817 319.6035
-15837.3809 -1755.2690 0.0000 0.0000 7668.0465
3985.5286 306.7602 -3682.5179 4047.8607 305.9026
-14.0183 -71.7402 656151.2420 -19.2153 -19.1976
[539]ENERGY: 6070000 3145.9140 5222.7323 5250.4812 347.8123
-15836.1139 -1809.8600 0.0000 0.0000 7661.5246
3982.4906 306.4993 -3679.0341 4047.7544 305.9101
-127.3552 -132.5014 656151.2420 -25.8553 -25.8700
[576]ENERGY: 6080000 3178.2014 5066.3197 5252.6870 338.3305
-15834.5970 -1671.6615 0.0000 0.0000 7655.8569
3985.1370 306.2726 -3670.7199 4047.2334 306.0852
-29.8889 3.5183 656151.2420 -19.0088 -18.9927
[627]ENERGY: 6090000 3217.7221 5221.9934 5290.2059 342.6304
-15923.4223 -1750.2821 0.0000 0.0000 7591.3212
3990.1687 303.6908 -3601.1525 4049.4452 306.1867
57.7866 1.7184 656151.2420 -24.9620 -24.9585
[667]ENERGY: 6100000 3177.8788 5084.2730 5275.5293 349.4836
-15775.1835 -1756.5042 0.0000 0.0000 7632.0710
3987.5480 305.3210 -3644.5230 4048.3862 306.0928
-92.3749 -123.9169 656151.2420 -22.5185 -22.5375
[718]ENERGY: 6110000 3263.3047 5157.3931 5288.8378 322.3194
-15846.4404 -1864.4497 0.0000 0.0000 7667.6077
3988.5725 306.7426 -3679.0352 4050.0585 306.0323
-91.4519 -96.0166 656151.2420 -24.9551 -24.9465
[754]ENERGY: 6120000 3180.6249 5271.6129 5289.7562 320.5576
-16026.0604 -1713.4342 0.0000 0.0000 7666.2540
3989.3109 306.6885 -3676.9431 4051.3767 306.0983
68.5503 42.0304 656151.2420 -24.1294 -24.1261
[804]ENERGY: 6130000 3254.5858 5238.5614 5283.9341 316.3139
-15870.1861 -1812.9174 0.0000 0.0000 7581.6681
3991.9599 303.3046 -3589.7083 4051.4547 306.2822
-147.1473 -114.8592 656151.2420 -22.9335 -22.9393
[840]ENERGY: 6140000 3127.0019 5262.2151 5207.0929 330.6533
-15827.2077 -1735.6521 0.0000 0.0000 7625.6789
3989.7825 305.0653 -3635.8964 4050.9045 306.1223
247.4967 142.1579 656151.2420 -22.6448 -22.6480
[890]ENERGY: 6150000 3195.3848 5280.3409 5260.8503 333.0434
-15853.8689 -1831.7035 0.0000 0.0000 7606.0022
3990.0492 304.2781 -3615.9529 4050.4765 306.3193
-100.8156 -59.1583 656151.2420 -23.4604 -23.4515
[926]ENERGY: 6160000 3230.8459 5194.7799 5227.9463 329.8402
-15917.8349 -1696.1387 0.0000 0.0000 7620.2021
3989.6408 304.8462 -3630.5613 4050.0903 306.1764
-18.1231 -18.7695 656151.2420 -26.5837 -26.5811
[976]ENERGY: 6170000 3128.8899 5210.2277 5284.9226 347.2839
-15852.8129 -1781.5800 0.0000 0.0000 7656.0570
3992.9884 306.2806 -3663.0687 4050.7207 306.0535
19.5887 45.9608 656151.2420 -21.7902 -21.7869
[1012]ENERGY: 6180000 3141.0684 5143.2280 5266.9248
339.7176 -15814.9342 -1768.7999 0.0000 0.0000
7681.7070 3988.9118 307.3067 -3692.7952 4050.9918
306.2077 12.0865 -60.3353 656151.2420 -27.2328
-27.2579
[1062]ENERGY: 6190000 3215.2091 5201.8003 5316.0368

348.4701 -15947.8174 -1741.7950 0.0000 0.0000
7600.8610 3992.7649 304.0724 -3608.0961 4050.2642
306.2663 -118.5517 -90.3740 656151.2420 -22.4682
-22.4357
[1101]ENERGY: 6200000 3138.0362 5122.3801 5256.0582
347.3249 -15771.0068 -1764.5859 0.0000 0.0000
7659.9645 3988.1713 306.4369 -3671.7932 4050.3403
306.1985 46.8985 -16.8594 656151.2420 -27.8560
-27.8582
[1151]ENERGY: 6210000 3189.0442 5261.6703 5266.8238
330.7783 -15821.8868 -1800.5727 0.0000 0.0000
7566.0757 3991.9328 302.6809 -3574.1429 4051.3098
306.1706 14.1242 -30.5095 656151.2420 -21.8605
-21.8581
[1187]ENERGY: 6220000 3210.2803 5155.1573 5262.7923
338.0054 -15853.6979 -1757.2513 0.0000 0.0000
7636.0071 3991.2932 305.4785 -3644.7139 4050.4795
305.9743 -62.6706 -18.0572 656151.2420 -26.5937
-26.5889
[1237]ENERGY: 6230000 3194.8777 5160.5176 5290.9710
337.0747 -15938.7186 -1727.7803 0.0000 0.0000
7670.5790 3987.5212 306.8615 -3683.0579 4051.5654
306.1617 40.4001 58.2858 656151.2420 -23.9352
-23.9361
[1273]ENERGY: 6240000 3080.3985 5183.6537 5253.6994
332.3696 -15824.3676 -1771.7452 0.0000 0.0000
7729.1848 3983.1932 309.2060 -3745.9916 4052.2647
306.1055 -40.2493 -50.7206 656151.2420 -22.7607
-22.7721
[1323]ENERGY: 6250000 3221.7154 5218.1775 5247.3835
337.1129 -15859.2357 -1831.0598 0.0000 0.0000
7655.1351 3989.2290 306.2437 -3665.9062 4052.0702
306.0580 -129.2490 -106.2284 656151.2420 -18.8525
-18.8569
[1359]ENERGY: 6260000 3268.9974 5139.6641 5278.9812
316.7260 -15870.2968 -1781.3740 0.0000 0.0000
7638.4195 3991.1174 305.5750 -3647.3021 4051.7616
306.2032 41.3063 2.4560 656151.2420 -25.4234
-25.4218
[1409]ENERGY: 6270000 3172.7575 5197.0240 5241.8909
300.3220 -15803.1115 -1780.2928 0.0000 0.0000
7659.0547 3987.6448 306.4005 -3671.4099 4051.2166
305.9632 -52.9999 -92.9382 656151.2420 -23.8583
-23.8590
[1445]ENERGY: 6280000 3211.7592 5126.6963 5252.2680
337.2441 -15831.3652 -1835.9622 0.0000 0.0000
7726.2059 3986.8461 309.0869 -3739.3599 4051.3269
306.1369 -67.5976 -164.6507 656151.2420 -21.4717
-21.4812
[1495]ENERGY: 6290000 3108.0051 5169.5197 5234.0504
339.0978 -15877.8882 -1745.5244 0.0000 0.0000
7756.2898 3983.5503 310.2904 -3772.7395 4049.7457
306.1161 96.4496 125.9365 656151.2420 -27.3303
-27.3253

[1534]ENERGY: 6300000 3149.1164 5067.6607 5312.6936
336.2317 -15855.0936 -1737.0371 0.0000 0.0000
7714.5807 3988.1524 308.6218 -3726.4283 4050.0951
306.1535 69.5163 5.3507 656151.2420 -22.3819
-22.3806
[1584]ENERGY: 6310000 3208.3813 5144.4004 5226.5633
340.3632 -15927.2209 -1707.7919 0.0000 0.0000
7703.5669 3988.2622 308.1812 -3715.3047 4050.7977
305.9698 105.1072 38.7032 656151.2420 -22.0743
-22.0575
[1620]ENERGY: 6320000 3154.9165 5241.5656 5252.1772
361.8700 -15877.3196 -1734.1010 0.0000 0.0000
7588.7746 3987.8833 303.5889 -3600.8913 4050.2800
305.9403 -137.2198 -99.6924 656151.2420 -20.0056
-20.0182
[1670]ENERGY: 6330000 3187.0859 5225.0263 5239.6284
336.6146 -15888.0631 -1805.0533 0.0000 0.0000
7690.1684 3985.4072 307.6452 -3704.7612 4050.5545
305.9782 -81.9929 -131.9671 656151.2420 -25.9978
-26.0130
[1706]ENERGY: 6340000 3137.9674 5194.1154 5291.9670
329.3081 -15813.5390 -1807.2375 0.0000 0.0000
7656.1404 3988.7218 306.2839 -3667.4186 4051.4278
306.1570 -99.1609 -80.2165 656151.2420 -23.6629
-23.6232
[1756]ENERGY: 6350000 3192.0841 5228.3626 5266.3715
314.9046 -15832.7964 -1776.2826 0.0000 0.0000
7600.5264 3993.1702 304.0591 -3607.3562 4052.8685
306.1125 -145.8763 -177.1465 656151.2420 -25.9427
-25.9633
[1792]ENERGY: 6360000 3141.9885 5158.5645 5247.5046
316.7376 -15783.4020 -1810.2742 0.0000 0.0000
7718.1352 3989.2544 308.7640 -3728.8809 4051.7160
306.0270 -217.6379 -177.8882 656151.2420 -24.2997
-24.3190
[1842]ENERGY: 6370000 3084.3331 5240.5743 5278.9172
358.5080 -15817.3453 -1749.9658 0.0000 0.0000
7593.5160 3988.5375 303.7786 -3604.9785 4051.9862
305.9048 95.7380 43.5331 656151.2420 -24.0684
-24.0571
[1878]ENERGY: 6380000 3188.1541 5238.6517 5279.3352
337.8428 -15836.9005 -1786.2966 0.0000 0.0000
7570.5892 3991.3760 302.8614 -3579.2132 4051.7946
305.9003 -137.5141 -68.9543 656151.2420 -22.1344
-22.1151
[1928]ENERGY: 6390000 3200.6628 5199.9854 5214.6360
349.5172 -15860.8173 -1648.9978 0.0000 0.0000
7539.4508 3994.4372 301.6157 -3545.0137 4052.4066
305.7370 144.6198 125.7761 656151.2420 -21.7194
-21.7423
[1967]ENERGY: 6400000 3109.2512 5250.9647 5275.0742
338.9263 -15875.0004 -1753.5846 0.0000 0.0000
7646.1430 3991.7744 305.8839 -3654.3686 4051.7434
305.7341 134.9246 79.9816 656151.2420 -23.5161

-23.5044
[2017]ENERGY: 6410000 3146.7005 5132.6071 5278.0682
354.1562 -15814.1141 -1805.2744 0.0000 0.0000
7696.2372 3988.3806 307.8880 -3707.8566 4052.2163
305.9919 -153.4249 -155.1005 656151.2420 -17.2893
-17.2775
[2053]ENERGY: 6420000 3112.1208 5158.1043 5273.3350
335.3431 -15818.1087 -1761.9264 0.0000 0.0000
7690.5716 3989.4395 307.6613 -3701.1321 4053.6755
305.9414 217.2800 83.5660 656151.2420 -25.7665
-25.7938
[2103]ENERGY: 6430000 3129.9538 5140.4111 5286.3278
328.3085 -15816.3023 -1780.8644 0.0000 0.0000
7704.4154 3992.2500 308.2151 -3712.1655 4054.8107
305.9114 27.0870 -67.0794 656151.2420 -24.5557
-24.5501
[2139]ENERGY: 6440000 3206.2912 5168.8827 5282.8639
337.7410 -15844.2615 -1744.4731 0.0000 0.0000
7584.9052 3991.9494 303.4341 -3592.9558 4053.5969
306.1815 -118.6934 -50.9474 656151.2420 -20.9905
-20.9852
[2189]ENERGY: 6450000 3188.6296 5190.4901 5285.5613
337.4941 -15900.8922 -1758.6036 0.0000 0.0000
7648.2876 3990.9669 305.9697 -3657.3208 4054.2496
306.0746 -142.3077 -149.8469 656151.2420 -24.3247
-24.3203
[2225]ENERGY: 6460000 3198.1604 5198.1455 5303.3697
344.6309 -15926.3705 -1799.7067 0.0000 0.0000
7674.8615 3993.0908 307.0328 -3681.7706 4055.3471
306.2497 -158.1024 -138.9556 656151.2420 -25.0348
-25.0375
[2275]ENERGY: 6470000 3174.3166 5210.7977 5274.5026
351.6620 -15927.2845 -1806.0486 0.0000 0.0000
7713.7937 3991.7393 308.5903 -3722.0543 4055.2433
306.1798 23.4966 -53.2913 656151.2420 -28.5335
-28.5593
[2311]ENERGY: 6480000 3201.1573 5227.8901 5306.8628
330.1585 -15950.2114 -1675.6308 0.0000 0.0000
7553.7385 3993.9651 302.1873 -3559.7734 4054.7847
306.3504 107.3496 94.8225 656151.2420 -28.1451
-28.1161
[2361]ENERGY: 6490000 3271.0366 5136.9307 5260.0245
348.8577 -15869.3474 -1722.1853 0.0000 0.0000
7569.5737 3994.8905 302.8208 -3574.6832 4054.2349
306.1318 -163.2615 -145.2064 656151.2420 -22.9319
-22.9428
[2400]ENERGY: 6500000 3108.9276 5265.8485 5320.7853
351.6399 -15973.3608 -1726.0192 0.0000 0.0000
7646.4644 3994.2856 305.8968 -3652.1788 4055.0598
306.1177 -5.5560 -0.2178 656151.2420 -26.5299
-26.5394
[2450]ENERGY: 6510000 3260.3494 5267.4628 5234.4729
345.5304 -15928.1144 -1804.4219 0.0000 0.0000
7615.4671 3990.7463 304.6568 -3624.7208 4055.2600

306.3399 -207.2333 -176.1836 656151.2420 -21.6661
-21.6446
[2486]ENERGY: 6520000 3102.0756 5165.7285 5255.0902
325.4714 -15813.7954 -1794.7241 0.0000 0.0000
7751.7039 3991.5502 310.1069 -3760.1537 4056.4429
306.1861 154.8730 56.5279 656151.2420 -25.1750
-25.1929
[2536]ENERGY: 6530000 3168.0093 5231.9995 5283.3377
329.9716 -15877.4775 -1785.5841 0.0000 0.0000
7641.3567 3991.6133 305.6925 -3649.7434 4055.6709
306.2404 -54.4073 -18.4011 656151.2420 -28.0941
-28.0823
[2572]ENERGY: 6540000 3216.1216 5064.7821 5293.6080
353.2241 -15833.5119 -1692.8438 0.0000 0.0000
7596.5841 3997.9641 303.9013 -3598.6200 4055.8241
306.1131 66.8225 33.5978 656151.2420 -23.9371
-23.9364
[2622]ENERGY: 6550000 3138.2979 5292.1470 5276.2104
349.1878 -15908.8798 -1792.8737 0.0000 0.0000
7642.9007 3996.9903 305.7542 -3645.9104 4056.9015
306.1905 23.4940 -23.9943 656151.2420 -29.1007
-29.0967
[2658]ENERGY: 6560000 3206.0452 5194.3708 5228.3586
334.5764 -15776.1017 -1774.1273 0.0000 0.0000
7582.9278 3996.0497 303.3550 -3586.8780 4056.3300
305.9799 -132.9182 -80.4854 656151.2420 -22.3589
-22.3703
[2708]ENERGY: 6570000 3133.5854 5102.5352 5331.5410
350.3501 -15852.5191 -1760.4566 0.0000 0.0000
7686.6778 3991.7138 307.5055 -3694.9640 4055.8884
306.2253 -130.3084 -112.1351 656151.2420 -26.5607
-26.5483
[2744]ENERGY: 6580000 3184.1004 5138.1036 5290.6267
359.8095 -15909.8204 -1683.5055 0.0000 0.0000
7615.1064 3994.4206 304.6423 -3620.6858 4057.3416
306.3577 -46.6984 -47.5029 656151.2420 -22.6698
-22.6750
[2794]ENERGY: 6590000 3158.9965 5195.4685 5282.2648
352.2456 -15905.2969 -1801.0956 0.0000 0.0000
7713.1207 3995.7038 308.5634 -3717.4169 4058.0152
306.2321 -42.4536 -91.6578 656151.2420 -30.5911
-30.5867
[2833]ENERGY: 6600000 3173.0310 5279.1752 5305.2101
324.5590 -15908.5419 -1828.2179 0.0000 0.0000
7647.5963 3992.8117 305.9421 -3654.7845 4057.5144
306.2616 41.8109 -21.7702 656151.2420 -23.7781
-23.7748
[2883]ENERGY: 6610000 3255.3318 5208.1674 5233.8461
350.4866 -15878.4342 -1816.7220 0.0000 0.0000
7646.0558 3998.7314 305.8805 -3647.3243 4058.1670
306.3071 62.6523 -35.1123 656151.2420 -26.4114
-26.4108
[2919]ENERGY: 6620000 3116.3536 5264.7927 5245.0343
333.9982 -15884.7749 -1762.1673 0.0000 0.0000

7683.6591 3996.8958 307.3848 -3686.7633 4057.9968
306.4739 58.7970 -35.7289 656151.2420 -25.7714
-25.7904
[2969]ENERGY: 6630000 3155.1062 5207.6544 5214.3442
331.4261 -15843.3088 -1724.9014 0.0000 0.0000
7653.5428 3993.8634 306.1800 -3659.6794 4057.5859
306.4849 34.6609 0.3403 656151.2420 -24.1627
-24.1515
[3005]ENERGY: 6640000 3140.9980 5230.5243 5239.8780
338.6586 -15864.2169 -1782.7362 0.0000 0.0000
7693.0290 3996.1348 307.7596 -3696.8942 4059.0738
306.5273 200.9693 74.2250 656151.2420 -26.8499
-26.8727
[3055]ENERGY: 6650000 3124.4097 5210.7128 5242.6966
344.0518 -15940.0244 -1726.1589 0.0000 0.0000
7739.4797 3995.1674 309.6179 -3744.3123 4059.4439
306.2782 43.6971 20.5147 656151.2420 -26.1280
-26.1056
[3091]ENERGY: 6660000 3125.8083 5357.4302 5267.2532
339.5903 -15907.7781 -1815.4976 0.0000 0.0000
7631.0424 3997.8487 305.2798 -3633.1937 4059.6481
306.4791 -39.1863 -10.3090 656151.2420 -27.1560
-27.1504
[3141]ENERGY: 6670000 3193.6261 5269.8627 5250.5148
320.0079 -15855.1316 -1741.8497 0.0000 0.0000
7563.3317 4000.3619 302.5711 -3562.9699 4059.3634
306.2819 -107.3123 -90.6075 656151.2420 -26.2838
-26.2977
[3177]ENERGY: 6680000 3234.9385 5075.8909 5288.8407
328.2939 -15851.4478 -1727.0607 0.0000 0.0000
7652.8557 4002.3112 306.1525 -3650.5446 4060.9579
306.2227 66.5913 -15.9191 656151.2420 -27.8298
-27.8213
[3227]ENERGY: 6690000 3227.3420 5162.0624 5282.3402
353.1192 -15871.4771 -1784.8078 0.0000 0.0000
7630.7111 3999.2902 305.2666 -3631.4210 4059.3389
306.2746 4.2143 -29.5216 656151.2420 -25.7837
-25.7935
[3266]ENERGY: 6700000 3178.2795 5183.5919 5271.4122
333.7118 -15964.1803 -1743.1359 0.0000 0.0000
7733.1748 3992.8540 309.3657 -3740.3207 4059.4534
306.3414 -70.8513 -64.4453 656151.2420 -25.8039
-25.8003
[3316]ENERGY: 6710000 3228.2368 5068.4529 5315.7064
324.6827 -15835.3156 -1794.4279 0.0000 0.0000
7691.2531 3998.5883 307.6886 -3692.6648 4060.6432
306.3161 -11.1250 -42.2566 656151.2420 -27.2849
-27.2664
[3352]ENERGY: 6720000 3145.4592 5167.9102 5229.2925
345.7862 -15824.0879 -1744.7087 0.0000 0.0000
7678.3064 3997.9579 307.1706 -3680.3484 4060.5079
306.3438 26.6384 8.5615 656151.2420 -27.9603
-27.9834
[3402]ENERGY: 6730000 3115.9487 5100.0754 5287.0153

348.0599 -15736.8587 -1780.5121 0.0000 0.0000
7666.4511 4000.1797 306.6964 -3666.2714 4061.0301
306.2870 84.6462 44.2096 656151.2420 -28.2225
-28.2213
[3438]ENERGY: 6740000 3180.1050 5233.6141 5258.7161
352.6306 -15899.9097 -1746.4993 0.0000 0.0000
7618.8758 3997.5326 304.7931 -3621.3433 4059.9790
306.3140 150.1047 59.7711 656151.2420 -27.8891
-27.8750
[3488]ENERGY: 6750000 3180.1940 5212.1605 5261.2311
347.7025 -15914.9070 -1742.9490 0.0000 0.0000
7656.1649 3999.5969 306.2849 -3656.5679 4060.0178
306.3170 77.4824 16.8462 656151.2420 -28.8930
-28.8820
[3524]ENERGY: 6760000 3218.6597 5218.8009 5292.7866
364.6608 -15913.0873 -1732.1387 0.0000 0.0000
7553.4623 4003.1444 302.1763 -3550.3179 4061.0940
306.3055 180.8514 159.2945 656151.2420 -21.5212
-21.5281
[3574]ENERGY: 6770000 3214.4385 5172.7207 5271.0463
349.4796 -15929.3923 -1731.1345 0.0000 0.0000
7653.5635 4000.7218 306.1808 -3652.8417 4061.4151
306.3538 -1.8292 -51.4551 656151.2420 -27.1691
-27.1962
[3610]ENERGY: 6780000 3184.8045 5173.9594 5222.8692
341.8707 -15869.3540 -1746.5766 0.0000 0.0000
7689.0694 3996.6425 307.6012 -3692.4269 4060.2067
306.2262 177.9665 154.4153 656151.2420 -26.7960
-26.7770
[3660]ENERGY: 6790000 3162.1603 5139.7499 5248.8269
352.9385 -15817.6052 -1692.7575 0.0000 0.0000
7609.4088 4002.7219 304.4144 -3606.6869 4060.9652
306.4265 208.0386 202.6216 656151.2420 -20.0736
-20.0629
[3699]ENERGY: 6800000 3171.4369 5148.5810 5295.0200
355.9102 -15787.2436 -1827.7052 0.0000 0.0000
7642.9440 3998.9432 305.7560 -3644.0007 4062.2786
306.1008 -64.7615 -93.7680 656151.2420 -28.5923
-28.6099
[3749]ENERGY: 6810000 3172.1413 5176.4282 5290.8808
340.8087 -15849.4237 -1800.5051 0.0000 0.0000
7668.7197 3999.0498 306.7871 -3669.6698 4060.9276
306.1869 -31.2777 -61.9453 656151.2420 -24.5413
-24.5408
[3785]ENERGY: 6820000 3251.3392 5153.2665 5289.9237
337.6852 -15861.5337 -1778.1854 0.0000 0.0000
7611.9083 4004.4037 304.5144 -3607.5046 4061.5012
306.1670 -156.4921 -137.7107 656151.2420 -27.3644
-27.3519
[3835]ENERGY: 6830000 3204.4079 5192.5777 5259.6202
329.2347 -15860.0328 -1779.1361 0.0000 0.0000
7654.0626 4000.7342 306.2008 -3653.3285 4060.6380
306.1092 -92.8333 -92.2594 656151.2420 -27.5821
-27.5858

[3871]ENERGY: 6840000 3230.6505 5233.1736 5274.6128
341.5954 -15970.8975 -1773.0410 0.0000 0.0000
7666.5886 4002.6823 306.7019 -3663.9063 4062.2352
306.1331 -117.8487 -63.8831 656151.2420 -32.2526
-32.2512
[3921]ENERGY: 6850000 3214.8313 5128.9613 5275.2681
335.4722 -15884.3290 -1729.7180 0.0000 0.0000
7661.3386 4001.8244 306.4918 -3659.5142 4062.1990
306.1935 187.0260 182.5270 656151.2420 -25.6734
-25.6722
[3957]ENERGY: 6860000 3143.7470 5072.7021 5285.4470
333.2454 -15839.1581 -1727.4481 0.0000 0.0000
7733.4063 4001.9416 309.3749 -3731.4646 4063.1574
306.1367 123.0777 -6.9699 656151.2420 -25.9780
-25.9924
[4007]ENERGY: 6870000 3141.7827 5253.4401 5241.9648
363.2591 -15861.3725 -1764.1412 0.0000 0.0000
7628.3874 4003.3203 305.1736 -3625.0671 4063.0124
306.1408 -136.0076 -115.4713 656151.2420 -29.0942
-29.0859
[4043]ENERGY: 6880000 3127.6633 5190.5795 5278.4786
314.8226 -15827.2883 -1777.1296 0.0000 0.0000
7690.6795 3997.8056 307.6656 -3692.8739 4061.6395
306.3228 62.5344 1.9791 656151.2420 -21.6916
-21.7042
[4093]ENERGY: 6890000 3183.7196 5217.0867 5269.4795
351.7749 -15794.4121 -1843.5542 0.0000 0.0000
7615.8764 3999.9708 304.6731 -3615.9056 4061.9708
306.4035 -61.9271 -88.5634 656151.2420 -30.5456
-30.5286
[4132]ENERGY: 6900000 3156.8519 5156.8379 5228.6180
341.0406 -15862.4674 -1728.3539 0.0000 0.0000
7705.9221 3998.4492 308.2754 -3707.4728 4061.4914
306.5208 3.3602 -64.3980 656151.2420 -22.3301
-22.3408
[4182]ENERGY: 6910000 3147.9392 5187.5569 5275.1154
331.5556 -15895.9816 -1746.0234 0.0000 0.0000
7696.1679 3996.3300 307.8852 -3699.8379 4060.4014
306.4639 36.2814 35.6980 656151.2420 -28.8263
-28.8200
[4218]ENERGY: 6920000 3115.6605 5211.4137 5259.7646
347.2841 -15871.3847 -1729.7123 0.0000 0.0000
7667.0762 4000.1021 306.7214 -3666.9740 4061.5517
306.2633 -39.5999 -53.3137 656151.2420 -25.8487
-25.8613
[4268]ENERGY: 6930000 3113.9088 5251.6103 5257.6162
314.3978 -15918.8092 -1727.8567 0.0000 0.0000
7706.8452 3997.7124 308.3123 -3709.1327 4061.6516
306.1329 232.3353 201.0583 656151.2420 -24.5769
-24.5676
[4304]ENERGY: 6940000 3146.4162 5146.6997 5239.2084
326.6937 -15841.6707 -1697.3874 0.0000 0.0000
7681.7917 4001.7515 307.3101 -3680.0402 4061.1831
306.1678 54.6326 49.2008 656151.2420 -21.1551

-21.1504
[4354]ENERGY: 6950000 3148.8865 5231.4683 5270.9496
366.9261 -15939.7518 -1742.7439 0.0000 0.0000
7661.6860 3997.4209 306.5057 -3664.2652 4061.1540
306.1438 244.1208 129.4781 656151.2420 -26.7890
-26.7997
[4390]ENERGY: 6960000 3164.6845 5161.8153 5264.3754
332.9828 -15814.2889 -1794.4583 0.0000 0.0000
7682.3690 3997.4798 307.3332 -3684.8892 4062.4607
306.0391 2.7265 -88.8408 656151.2420 -23.9948
-23.9755
[4440]ENERGY: 6970000 3186.0924 5154.2300 5278.3793
339.6159 -15859.4343 -1763.6346 0.0000 0.0000
7664.8250 4000.0737 306.6313 -3664.7513 4061.5627
306.0989 33.3511 -89.2328 656151.2420 -24.3788
-24.4033
[4476]ENERGY: 6980000 3184.4807 5143.1003 5273.5894
361.3828 -15872.8296 -1801.5178 0.0000 0.0000
7710.3186 3998.5243 308.4513 -3711.7943 4061.3419
306.0395 -23.9586 -16.5248 656151.2420 -24.9584
-24.9356
[4526]ENERGY: 6990000 3168.0620 5208.1574 5314.4379
332.8155 -15880.4690 -1761.3430 0.0000 0.0000
7620.8702 4002.5310 304.8729 -3618.3392 4062.9655
305.9841 99.0275 67.6152 656151.2420 -24.4807
-24.4706
[4565]ENERGY: 7000000 3164.3408 5253.9544 5259.6636
360.7595 -15936.2589 -1716.1438 0.0000 0.0000
7613.5583 3999.8739 304.5804 -3613.6843 4060.7814
306.2982 99.4131 135.1755 656151.2420 -22.9019
-22.9131
[4615]ENERGY: 7010000 3215.1978 5110.8371 5301.2671
323.7426 -15753.0999 -1847.3732 0.0000 0.0000
7651.5976 4002.1692 306.1022 -3649.4285 4062.6382
306.1892 -111.1487 -52.4644 656151.2420 -25.9838
-25.9867
[4651]ENERGY: 7020000 3227.1019 5177.7211 5285.5040
338.9686 -15850.6481 -1799.9630 0.0000 0.0000
7622.7231 4001.4075 304.9470 -3621.3156 4061.5764
306.3509 -54.4227 -104.8705 656151.2420 -24.6118
-24.6128
[4701]ENERGY: 7030000 3214.7515 5158.5663 5243.8280
340.9208 -15852.2528 -1761.3043 0.0000 0.0000
7656.6095 4001.1190 306.3027 -3655.4905 4063.7736
306.4072 115.5590 29.9974 656151.2420 -26.1382
-26.1427
[4737]ENERGY: 7040000 3138.0222 5139.5455 5227.9873
340.0121 -15797.1945 -1751.0572 0.0000 0.0000
7701.6516 3998.9670 308.1046 -3702.6846 4063.5508
306.3458 53.4649 91.6729 656151.2420 -25.8113
-25.8134
[4787]ENERGY: 7050000 3134.0945 5219.0461 5268.3030
323.3569 -15768.5634 -1792.5280 0.0000 0.0000
7615.7780 3999.4870 304.6692 -3616.2909 4062.9252

306.4424 22.0492 14.8604 656151.2420 -22.6249
-22.6121
[4823]ENERGY: 7060000 3147.1268 5231.8612 5254.8255
339.9611 -15868.3639 -1793.3873 0.0000 0.0000
7687.1703 3999.1938 307.5252 -3687.9765 4063.5060
306.2789 -39.8979 -125.7711 656151.2420 -29.5718
-29.5952
[4873]ENERGY: 7070000 3159.2020 5150.0298 5317.4636
345.4222 -15916.6520 -1781.2834 0.0000 0.0000
7725.1062 3999.2885 309.0429 -3725.8177 4064.0577
306.2234 -48.7725 -42.3239 656151.2420 -24.5202
-24.5060
[4909]ENERGY: 7080000 3205.9116 5173.9252 5283.6499
353.5400 -15910.1559 -1694.4649 0.0000 0.0000
7594.0460 4006.4520 303.7998 -3587.5940 4065.1054
306.1780 25.3392 41.4147 656151.2420 -28.5948
-28.5857
[4959]ENERGY: 7090000 3180.6203 5151.8119 5215.8079
346.1917 -15820.5917 -1762.1552 0.0000 0.0000
7693.1035 4004.7884 307.7626 -3688.3150 4064.9853
306.1867 76.4991 50.0723 656151.2420 -26.4497
-26.4507
[4998]ENERGY: 7100000 3157.8793 5201.3848 5273.4863
347.7276 -15844.3314 -1746.5270 0.0000 0.0000
7617.7243 4007.3439 304.7471 -3610.3804 4065.7718
306.3043 92.8829 86.9213 656151.2420 -25.5200
-25.5507
[5048]ENERGY: 7110000 3241.6314 5230.2724 5295.7408
336.5968 -15953.9694 -1762.6083 0.0000 0.0000
7615.5830 4003.2467 304.6614 -3612.3363 4065.5139
306.5811 -104.8366 -95.6177 656151.2420 -23.4684
-23.4708
[5084]ENERGY: 7120000 3201.4911 5162.3030 5263.1392
345.0711 -15913.4620 -1692.4419 0.0000 0.0000
7637.2667 4003.3672 305.5289 -3633.8994 4064.5498
306.5812 165.5060 138.6878 656151.2420 -24.0587
-24.0278
[5134]ENERGY: 7130000 3182.8076 5137.7379 5297.5037
335.3880 -15931.7979 -1751.5940 0.0000 0.0000
7729.0017 3999.0470 309.1987 -3729.9547 4064.6040
306.5659 -29.4380 -37.7728 656151.2420 -19.1831
-19.1949
[5170]ENERGY: 7140000 3144.1627 5241.2216 5249.1236
323.5467 -15855.3223 -1736.1521 0.0000 0.0000
7634.6040 4001.1843 305.4223 -3633.4197 4064.8253
306.3976 72.6503 78.0994 656151.2420 -25.0501
-25.0430
[5220]ENERGY: 7150000 3167.0342 5192.7929 5238.2779
337.4537 -15836.8050 -1794.0915 0.0000 0.0000
7697.1903 4001.8524 307.9261 -3695.3379 4063.7514
306.2958 4.8855 58.5069 656151.2420 -22.0590
-22.0560
[5256]ENERGY: 7160000 3199.1181 5114.9436 5272.2218
323.7701 -15784.8790 -1731.6672 0.0000 0.0000

7615.4227 4008.9301 304.6550 -3606.4926 4065.6828
306.2798 186.8884 48.1022 656151.2420 -24.0793
-24.1126
[5306]ENERGY: 7170000 3218.6987 5224.4660 5287.8040
343.3173 -15919.1081 -1815.0841 0.0000 0.0000
7665.3209 4005.4147 306.6512 -3659.9062 4065.2001
306.1129 -97.1262 -110.5901 656151.2420 -24.9306
-24.9027
[5342]ENERGY: 7180000 3181.9337 5139.3294 5254.0958
322.1243 -15873.7996 -1694.3040 0.0000 0.0000
7675.3192 4004.6987 307.0511 -3670.6204 4065.3129
306.3378 158.5189 131.4743 656151.2420 -25.4963
-25.4872
[5392]ENERGY: 7190000 3186.6711 5222.3538 5299.2751
363.2695 -15844.6661 -1782.6975 0.0000 0.0000
7560.7100 4004.9159 302.4662 -3555.7941 4065.3762
306.3424 -67.4726 -79.5915 656151.2420 -26.1967
-26.2040
[5431]ENERGY: 7200000 3151.9926 5275.0035 5243.8974
334.6415 -15808.5390 -1835.2424 0.0000 0.0000
7642.3666 4004.1201 305.7329 -3638.2465 4066.9906
306.5837 126.3620 71.9598 656151.2420 -22.8372
-22.8433
[5481]ENERGY: 7210000 3136.3908 5162.7863 5293.2469
325.9126 -15779.8820 -1793.1928 0.0000 0.0000
7658.8023 4004.0640 306.3904 -3654.7382 4067.0708
306.5273 -65.4004 -98.4617 656151.2420 -27.0821
-27.0749
[5517]ENERGY: 7220000 3185.4974 5188.6319 5268.9204
330.3868 -15850.3645 -1764.8547 0.0000 0.0000
7646.9292 4005.1465 305.9154 -3641.7827 4066.8521
306.5364 -48.3063 5.9447 656151.2420 -18.4215
-18.4282
[5567]ENERGY: 7230000 3115.9882 5281.0578 5298.9705
337.5740 -15877.9638 -1800.3655 0.0000 0.0000
7652.1671 4007.4283 306.1249 -3644.7388 4068.4883
306.5736 -112.3183 -65.3707 656151.2420 -23.8737
-23.8801
[5603]ENERGY: 7240000 3196.6605 5200.2852 5242.1640
336.0987 -15846.0886 -1815.8943 0.0000 0.0000
7691.5004 4004.7259 307.6985 -3686.7745 4069.6554
306.5015 -65.6130 -46.4566 656151.2420 -21.1406
-21.1233
[5653]ENERGY: 7250000 3175.4445 5157.4038 5297.1360
357.6388 -15843.9883 -1735.3687 0.0000 0.0000
7602.6034 4010.8695 304.1421 -3591.7339 4069.2773
306.4055 -22.6332 -48.6308 656151.2420 -24.0041
-24.0223
[5689]ENERGY: 7260000 3169.8318 5249.4956 5325.6774
344.3721 -15974.9308 -1747.2224 0.0000 0.0000
7639.0140 4006.2377 305.5988 -3632.7763 4068.6564
306.4457 -79.2454 -85.5006 656151.2420 -21.9661
-21.9635
[5739]ENERGY: 7270000 3136.0349 5220.9412 5275.6061

356.4420 -15750.9614 -1883.7335 0.0000 0.0000
7652.8392 4007.1687 306.1518 -3645.6706 4069.9579
306.4617 -59.8385 -63.6277 656151.2420 -17.5299
-17.5030
[5775]ENERGY: 7280000 3211.4889 5202.8562 5288.6482
338.8907 -15863.7019 -1794.8210 0.0000 0.0000
7623.4293 4006.7905 304.9753 -3616.6388 4070.3049
306.3871 -39.7643 -95.4896 656151.2420 -26.3572
-26.3768
[5825]ENERGY: 7290000 3164.2777 5119.7361 5329.0554
365.6777 -15914.1920 -1701.4506 0.0000 0.0000
7645.1969 4008.3012 305.8461 -3636.8957 4070.3840
306.5547 126.8398 97.4919 656151.2420 -22.9313
-22.9158
[5864]ENERGY: 7300000 3190.2982 5164.9755 5273.9333
320.6540 -15834.4093 -1818.1006 0.0000 0.0000
7709.8939 4007.2450 308.4343 -3702.6489 4071.5532
306.7324 -118.3534 -134.4439 656151.2420 -23.5168
-23.5445
[5914]ENERGY: 7310000 3180.5688 5185.4183 5281.8884
343.9073 -15861.1380 -1709.5095 0.0000 0.0000
7590.8500 4011.9853 303.6720 -3578.8647 4071.2551
306.7481 165.6433 99.2220 656151.2420 -25.6363
-25.6172
[5950]ENERGY: 7320000 3165.4933 5116.6743 5263.6176
338.4083 -15817.6036 -1703.4052 0.0000 0.0000
7646.7845 4009.9692 305.9096 -3636.8153 4070.4112
306.6269 39.1240 101.0316 656151.2420 -26.3618
-26.3645
[6000]ENERGY: 7330000 3075.4712 5160.8463 5273.7818
332.0089 -15814.6865 -1810.8404 0.0000 0.0000
7791.0551 4007.6363 311.6812 -3783.4187 4071.4189
306.5975 50.6126 -0.4734 656151.2420 -27.9536
-27.9504
[6036]ENERGY: 7340000 3097.8630 5197.4520 5309.8420
337.4777 -15819.3294 -1776.3560 0.0000 0.0000
7663.2381 4010.1874 306.5678 -3653.0507 4072.4672
306.4735 82.1888 -49.1743 656151.2420 -25.0418
-25.0534
[6086]ENERGY: 7350000 3229.0075 5135.0608 5309.9045
353.9833 -15820.0589 -1803.7933 0.0000 0.0000
7603.1632 4007.2671 304.1645 -3595.8961 4070.7500
306.3424 -183.7880 -119.7988 656151.2420 -25.8207
-25.8196
[6122]ENERGY: 7360000 3208.0245 5216.7210 5283.7567
335.0269 -15843.8475 -1779.5161 0.0000 0.0000
7592.2254 4012.3910 303.7270 -3579.8344 4071.9449
306.3262 -38.7065 0.3139 656151.2420 -26.2168
-26.2080
[6172]ENERGY: 7370000 3190.4824 5138.7245 5259.6540
330.0484 -15862.3306 -1746.7595 0.0000 0.0000
7699.7140 4009.5333 308.0271 -3690.1807 4070.3780
306.1980 160.1402 136.9431 656151.2420 -24.0125
-24.0161

[6208]ENERGY: 7380000 3164.9235 5190.4774 5293.7011
344.8957 -15863.9747 -1751.2269 0.0000 0.0000
7632.5441 4011.3401 305.3399 -3621.2039 4071.9667
306.2708 83.3843 -31.2844 656151.2420 -18.0917
-18.0989
[6258]ENERGY: 7390000 3255.3255 5164.5911 5283.1668
333.6812 -15859.0604 -1786.8653 0.0000 0.0000
7619.0379 4009.8769 304.7996 -3609.1610 4071.6108
306.4613 -73.0886 -4.2150 656151.2420 -24.7138
-24.7090
[6297]ENERGY: 7400000 3222.2558 5231.1983 5266.5071
326.8049 -15904.6396 -1728.8681 0.0000 0.0000
7597.5860 4010.8445 303.9414 -3586.7416 4071.4351
306.4623 -23.2941 0.2500 656151.2420 -25.0844
-25.0670
[6347]ENERGY: 7410000 3180.6469 5076.3105 5267.7734
334.0576 -15815.5614 -1733.6079 0.0000 0.0000
7697.1803 4006.7993 307.9257 -3690.3810 4071.8112
306.3442 -206.4323 -160.3455 656151.2420 -28.7795
-28.7834
[6383]ENERGY: 7420000 3167.2228 5229.2085 5235.3021
339.0513 -15788.4323 -1787.7932 0.0000 0.0000
7618.5927 4013.1519 304.7818 -3605.4407 4072.2096
306.5668 -12.9390 -0.2511 656151.2420 -25.3717
-25.3940
[6433]ENERGY: 7430000 3182.2874 5174.8951 5252.6870
328.6492 -15825.3683 -1783.9645 0.0000 0.0000
7679.5537 4008.7396 307.2205 -3670.8140 4072.2643
306.6062 -86.0164 -35.5971 656151.2420 -27.1555
-27.1373
[6469]ENERGY: 7440000 3178.2179 5180.4400 5303.6582
343.3676 -15816.9027 -1800.2151 0.0000 0.0000
7624.2268 4012.7926 305.0072 -3611.4342 4072.8476
306.4886 114.6935 70.2488 656151.2420 -23.4710
-23.4788
[6519]ENERGY: 7450000 3172.4134 5233.8088 5313.4520
368.0485 -15875.0327 -1777.1791 0.0000 0.0000
7574.1998 4009.7107 303.0059 -3564.4891 4073.1495
306.5239 77.1141 -44.6784 656151.2420 -23.1201
-23.1105
[6555]ENERGY: 7460000 3098.0510 5128.3234 5269.2016
326.9043 -15756.5438 -1755.3784 0.0000 0.0000
7698.2019 4008.7599 307.9666 -3689.4420 4072.7546
306.4794 84.8765 69.9497 656151.2420 -26.9675
-26.9727
[6605]ENERGY: 7470000 3151.2466 5211.4194 5271.3289
299.9117 -15735.5439 -1797.6144 0.0000 0.0000
7611.0755 4011.8237 304.4811 -3599.2518 4074.3100
306.5220 -63.2540 -69.7909 656151.2420 -22.1909
-22.1897
[6641]ENERGY: 7480000 3065.8306 5147.0225 5293.2894
323.2151 -15890.8719 -1666.6632 0.0000 0.0000
7739.4345 4011.2569 309.6161 -3728.1776 4073.9081
306.5726 270.9031 123.3987 656151.2420 -26.6357

-26.6531
[6691]ENERGY: 7490000 3182.7035 5205.7203 5280.0807
339.3890 -15875.0411 -1767.0078 0.0000 0.0000
7647.8063 4013.6509 305.9505 -3634.1554 4073.2189
306.4566 -49.1026 -20.3053 656151.2420 -25.4469
-25.4342
[6730]ENERGY: 7500000 3143.4112 5172.9852 5219.3481
341.3786 -15766.4739 -1788.4428 0.0000 0.0000
7688.8572 4011.0637 307.5927 -3677.7935 4075.0361
306.6097 -138.1195 -84.1889 656151.2420 -23.7577
-23.7625
[6780]ENERGY: 7510000 3105.1642 5151.5461 5280.7231
325.7240 -15875.4830 -1692.5827 0.0000 0.0000
7715.5339 4010.6256 308.6599 -3704.9082 4074.4087
306.4444 186.0521 104.8310 656151.2420 -28.2541
-28.2578
[6816]ENERGY: 7520000 3186.3116 5233.4644 5270.6607
346.0387 -15850.0015 -1784.0012 0.0000 0.0000
7613.7197 4016.1924 304.5869 -3597.5273 4075.8031
306.5969 95.0169 23.6103 656151.2420 -22.1493
-22.1326
[6866]ENERGY: 7530000 3105.8622 5185.6197 5311.5031
350.8169 -15753.1887 -1796.2830 0.0000 0.0000
7606.8803 4011.2104 304.3132 -3595.6699 4075.7934
306.5074 191.3733 126.4354 656151.2420 -29.5943
-29.5919
[6902]ENERGY: 7540000 3177.3361 5169.3869 5294.0740
339.2640 -15814.5854 -1858.3156 0.0000 0.0000
7704.4902 4011.6500 308.2181 -3692.8401 4075.6363
306.6147 -14.1050 -76.4710 656151.2420 -23.6725
-23.6792
[6952]ENERGY: 7550000 3179.9518 5293.4068 5236.6624
342.2642 -15896.5899 -1754.7066 0.0000 0.0000
7615.4631 4016.4519 304.6566 -3599.0113 4075.8480
306.5914 123.3699 88.1999 656151.2420 -28.5872
-28.5682
[6988]ENERGY: 7560000 3148.4018 5085.6486 5206.7413
326.9200 -15853.9973 -1674.0935 0.0000 0.0000
7772.9303 4012.5511 310.9561 -3760.3792 4074.1066
306.3546 135.3363 14.6945 656151.2420 -25.0826
-25.0983
[7038]ENERGY: 7570000 3145.1269 5224.2044 5274.4881
322.9386 -15842.1154 -1797.2050 0.0000 0.0000
7685.8068 4013.2444 307.4707 -3672.5624 4075.9183
306.5546 -28.6992 -85.5542 656151.2420 -23.8205
-23.8122
[7074]ENERGY: 7580000 3199.1258 5213.3869 5289.0700
346.2669 -15879.3859 -1818.5642 0.0000 0.0000
7663.6855 4013.5850 306.5857 -3650.1005 4075.9231
306.5835 -19.0100 -46.1227 656151.2420 -22.1415
-22.1600
[7124]ENERGY: 7590000 3171.0508 5204.8084 5300.4008
328.3366 -15777.4238 -1709.2996 0.0000 0.0000
7502.1606 4020.0337 300.1239 -3482.1269 4074.9178

306.4945 177.9439 186.7664 656151.2420 -26.7771
-26.7732
[7163]ENERGY: 7600000 3234.7860 5225.0700 5234.5835
365.9936 -15873.3476 -1793.8521 0.0000 0.0000
7620.2758 4013.5091 304.8491 -3606.7667 4075.4961
306.4738 -99.8114 -82.4409 656151.2420 -21.5543
-21.5515
[7213]ENERGY: 7610000 3262.1252 5255.7793 5279.0388
333.4188 -15899.0928 -1767.9846 0.0000 0.0000
7554.9337 4018.2182 302.2351 -3536.7155 4075.4798
306.5501 -66.2533 -204.5683 656151.2420 -25.7379
-25.7381
[7249]ENERGY: 7620000 3197.8442 5179.9696 5263.2959
318.9784 -15855.6152 -1742.6602 0.0000 0.0000
7650.8034 4012.6160 306.0704 -3638.1874 4076.0405
306.5169 -38.0123 61.9256 656151.2420 -25.7182
-25.7046
[7299]ENERGY: 7630000 3184.9372 5178.5699 5334.8177
341.7891 -15911.0502 -1739.3807 0.0000 0.0000
7623.9963 4013.6793 304.9980 -3610.3170 4075.3161
306.4936 -10.3918 7.9380 656151.2420 -24.1003
-24.1106
[7335]ENERGY: 7640000 3262.2384 5226.5214 5240.6092
312.0666 -15907.6076 -1770.4235 0.0000 0.0000
7653.6180 4017.0225 306.1830 -3636.5955 4075.9478
306.6698 -74.0247 -62.0028 656151.2420 -25.1791
-25.1891
[7385]ENERGY: 7650000 3187.1587 5166.2511 5307.2548
331.3656 -15888.2371 -1798.2862 0.0000 0.0000
7711.5391 4017.0461 308.5001 -3694.4930 4078.2648
306.5579 -32.3796 -45.7027 656151.2420 -26.5622
-26.5592
[7421]ENERGY: 7660000 3176.5828 5081.4436 5234.7472
365.8136 -15835.5133 -1703.8571 0.0000 0.0000
7696.2323 4015.4490 307.8878 -3680.7833 4076.4292
306.5918 -66.2160 19.6122 656151.2420 -25.0521
-25.0399
[7471]ENERGY: 7670000 3184.4447 5109.8427 5263.0388
352.8431 -15906.1781 -1708.7174 0.0000 0.0000
7719.9345 4015.2083 308.8360 -3704.7262 4078.0733
306.3750 104.7590 74.9088 656151.2420 -21.8430
-21.8403
[7507]ENERGY: 7680000 3194.3752 5230.0991 5249.1521
316.3017 -15824.6783 -1786.9794 0.0000 0.0000
7638.4604 4016.7307 305.5766 -3621.7296 4078.5579
306.6846 68.6343 7.4646 656151.2420 -22.9584
-22.9884
[7557]ENERGY: 7690000 3251.4878 5191.7763 5239.3925
351.4972 -15938.1815 -1713.8658 0.0000 0.0000
7633.8822 4015.9887 305.3935 -3617.8935 4077.9365
306.6992 114.0947 26.8238 656151.2420 -26.7286
-26.7145
[7596]ENERGY: 7700000 3163.7961 5223.0899 5248.0585
360.7699 -15874.7119 -1793.2431 0.0000 0.0000

7689.2971 4017.0565 307.6103 -3672.2407 4080.1170
306.7739 -31.3841 -33.0468 656151.2420 -24.2737
-24.2686
[7646]ENERGY: 7710000 3175.5600 5252.4094 5230.4186
329.3147 -15838.8085 -1790.5772 0.0000 0.0000
7658.6326 4016.9495 306.3836 -3641.6831 4078.6280
306.7585 -149.9776 -122.2921 656151.2420 -28.0407
-28.0238
[7682]ENERGY: 7720000 3124.1452 5222.5846 5280.3119
338.8679 -15841.8579 -1706.2478 0.0000 0.0000
7601.6372 4019.4411 304.1035 -3582.1961 4078.6735
306.7994 141.2149 126.4288 656151.2420 -23.9668
-23.9794
[7732]ENERGY: 7730000 3158.9163 5203.0182 5258.0587
326.7957 -15770.4737 -1789.8712 0.0000 0.0000
7630.6878 4017.1318 305.2657 -3613.5561 4078.3282
306.7815 -199.3611 -149.1831 656151.2420 -29.0792
-29.0785
[7768]ENERGY: 7740000 3179.2109 5135.6525 5287.2943
329.3273 -15869.9271 -1700.8815 0.0000 0.0000
7662.2389 4022.9152 306.5279 -3639.3237 4079.5485
306.8890 153.7325 133.3348 656151.2420 -21.0444
-21.0515
[7818]ENERGY: 7750000 3194.6428 5228.4700 5267.3454
356.6492 -15919.2009 -1738.9318 0.0000 0.0000
7630.1542 4019.1289 305.2443 -3611.0253 4079.9819
306.7132 6.0280 -42.4009 656151.2420 -30.1756
-30.1855
[7854]ENERGY: 7760000 3133.7403 5178.0448 5234.1745
344.1394 -15862.5347 -1754.1634 0.0000 0.0000
7740.5186 4013.9196 309.6594 -3726.5991 4079.9881
306.8460 -40.6017 -70.1329 656151.2420 -24.7050
-24.6923
[7904]ENERGY: 7770000 3167.4252 5166.0669 5257.3514
332.3282 -15877.4591 -1720.1018 0.0000 0.0000
7689.3996 4015.0105 307.6144 -3674.3891 4079.8159
306.8917 -70.0037 -73.1464 656151.2420 -28.3019
-28.3070
[7940]ENERGY: 7780000 3177.2014 5213.1954 5286.4644
332.4110 -15878.4842 -1713.3767 0.0000 0.0000
7603.1769 4020.5883 304.1651 -3582.5886 4080.9810
306.8758 -72.5676 -2.8523 656151.2420 -25.2572
-25.2467
[7990]ENERGY: 7790000 3171.6860 5216.1843 5242.5679
327.7095 -15866.3610 -1769.5919 0.0000 0.0000
7695.9700 4018.1648 307.8773 -3677.8052 4082.8600
306.8867 -14.7129 -46.5576 656151.2420 -26.4481
-26.4598
[8029]ENERGY: 7800000 3201.9120 5195.0291 5257.4217
344.5257 -15850.6772 -1760.9282 0.0000 0.0000
7637.5481 4024.8313 305.5401 -3612.7168 4081.3435
306.9749 38.5328 -31.5329 656151.2420 -19.7435
-19.7575
[8079]ENERGY: 7810000 3147.2070 5195.5270 5286.2303

337.9762 -15932.4513 -1766.6899 0.0000 0.0000
7751.6384 4019.4376 310.1043 -3732.2007 4081.3075
306.9296 35.6649 20.6403 656151.2420 -27.6017
-27.5835
[8115]ENERGY: 7820000 3136.6731 5259.2940 5250.6672
330.9258 -15868.4018 -1783.9170 0.0000 0.0000
7693.9891 4019.2303 307.7980 -3674.7588 4079.5746
306.9005 -28.3638 -6.2896 656151.2420 -25.5537
-25.5550
[8165]ENERGY: 7830000 3172.8465 5243.0366 5271.2497
334.3391 -15878.4397 -1683.4832 0.0000 0.0000
7561.2507 4020.7997 302.4878 -3540.4510 4079.1621
306.9473 -62.5604 -36.4314 656151.2420 -22.3340
-22.3580
[8201]ENERGY: 7840000 3178.9823 5138.9721 5289.2584
344.9393 -15953.0642 -1730.2538 0.0000 0.0000
7749.3329 4018.1669 310.0121 -3731.1660 4079.5625
307.0323 63.3469 43.8338 656151.2420 -27.8610
-27.8343
[8251]ENERGY: 7850000 3176.2353 5164.3433 5267.3405
326.1643 -15834.4425 -1753.3975 0.0000 0.0000
7671.4979 4017.7414 306.8983 -3653.7565 4078.9383
306.9871 -72.7291 -4.5474 656151.2420 -24.4813
-24.4699
[8287]ENERGY: 7860000 3178.5531 5214.3995 5258.8359
347.6250 -15846.4609 -1771.2162 0.0000 0.0000
7635.2190 4016.9554 305.4469 -3618.2636 4079.4705
306.9230 -163.3239 -153.0735 656151.2420 -26.7014
-26.7195
[8337]ENERGY: 7870000 3149.2098 5127.0464 5283.2048
354.0405 -15890.1883 -1764.7143 0.0000 0.0000
7756.8564 4015.4552 310.3130 -3741.4012 4079.3528
306.9410 118.0224 20.1838 656151.2420 -22.8006
-22.7911
[8373]ENERGY: 7880000 3279.2862 5257.5945 5212.2091
308.0437 -15877.1090 -1808.5947 0.0000 0.0000
7645.3206 4016.7506 305.8510 -3628.5701 4079.6372
306.9370 -23.2194 -44.2143 656151.2420 -25.0581
-25.0575
[8423]ENERGY: 7890000 3207.3512 5126.2827 5262.1780
364.0042 -15910.2131 -1779.6463 0.0000 0.0000
7748.9530 4018.9097 309.9969 -3730.0433 4079.4906
307.0469 71.8388 -44.1981 656151.2420 -24.8325
-24.8481
[8462]ENERGY: 7900000 3162.3733 5215.9002 5257.3302
325.6365 -15881.0298 -1774.7136 0.0000 0.0000
7711.7524 4017.2491 308.5087 -3694.5032 4080.5738
307.0146 98.3201 59.0830 656151.2420 -23.4147
-23.4012
[8512]ENERGY: 7910000 3161.2491 5240.0357 5225.8228
336.1152 -15832.2358 -1823.5539 0.0000 0.0000
7711.8366 4019.2698 308.5120 -3692.5669 4082.1463
307.0150 34.7966 -44.2399 656151.2420 -25.5246
-25.5266

[8548]ENERGY: 7920000 3184.9597 5104.3918 5238.4314
344.0119 -15845.2985 -1767.8882 0.0000 0.0000
7758.5193 4017.1275 310.3796 -3741.3918 4081.5074
306.8799 17.2429 -5.3030 656151.2420 -24.3577
-24.3610
[8598]ENERGY: 7930000 3216.9734 5092.8085 5226.7844
343.0855 -15830.8048 -1732.9109 0.0000 0.0000
7706.3874 4022.3235 308.2940 -3684.0639 4081.0825
306.9506 -78.8659 -110.5851 656151.2420 -26.6468
-26.6569
[8634]ENERGY: 7940000 3152.7552 5184.6821 5281.9237
345.6670 -15857.2905 -1771.6825 0.0000 0.0000
7683.9055 4019.9606 307.3946 -3663.9448 4082.1114
306.9996 -43.2302 -33.3806 656151.2420 -25.5369
-25.5083
[8684]ENERGY: 7950000 3166.2927 5220.8015 5277.5034
353.8355 -15871.9795 -1842.9772 0.0000 0.0000
7716.5164 4019.9929 308.6992 -3696.5235 4082.6842
306.9187 -184.0602 -183.3137 656151.2420 -23.8340
-23.8594
[8720]ENERGY: 7960000 3122.0300 5154.4274 5255.0615
338.0562 -15825.9247 -1719.8883 0.0000 0.0000
7693.1053 4016.8674 307.7627 -3676.2379 4081.5778
306.6972 -243.4510 -116.0378 656151.2420 -18.8959
-18.8918
[8770]ENERGY: 7970000 3150.8187 5263.3652 5230.9819
371.6066 -15931.9519 -1763.8250 0.0000 0.0000
7697.6410 4018.6364 307.9441 -3679.0046 4082.7760
306.8805 -21.7397 -16.0024 656151.2420 -25.1863
-25.1765
[8806]ENERGY: 7980000 3081.8924 5192.9016 5249.7787
338.7493 -15839.4232 -1761.9057 0.0000 0.0000
7754.0347 4016.0277 310.2002 -3738.0069 4082.3568
306.8571 -66.4135 -65.6305 656151.2420 -23.9223
-23.9335
[8856]ENERGY: 7990000 3241.1077 5184.8405 5249.8073
340.3692 -15969.4379 -1723.3316 0.0000 0.0000
7695.9589 4019.3142 307.8768 -3676.6447 4083.0597
306.7441 -23.9246 -89.1451 656151.2420 -23.3888
-23.3890
[8895]ENERGY: 8000000 3105.9169 5163.7836 5262.0419
328.9854 -15772.2743 -1725.9768 0.0000 0.0000
7660.7044 4023.1812 306.4665 -3637.5232 4082.5025
306.7796 66.0510 67.5065 656151.2420 -25.7829
-25.7707
[8945]ENERGY: 8010000 3204.9312 5173.7055 5235.4946
335.1634 -15777.2973 -1740.6999 0.0000 0.0000
7593.6329 4024.9305 303.7833 -3568.7024 4083.7785
306.8689 -37.0093 45.8441 656151.2420 -21.8339
-21.8441
[8981]ENERGY: 8020000 3168.7124 5209.9957 5234.7367
319.3823 -15829.7250 -1715.5166 0.0000 0.0000
7634.7023 4022.2878 305.4263 -3612.4145 4082.7840
306.8577 364.6388 221.0678 656151.2420 -26.6690

-26.6771
[9031]ENERGY: 8030000 3192.7870 5182.5712 5260.1584
334.1999 -15804.5310 -1784.7906 0.0000 0.0000
7642.1520 4022.5469 305.7243 -3619.6051 4082.0323
306.8626 -19.4057 -5.2354 656151.2420 -24.3250
-24.3256
[9067]ENERGY: 8040000 3162.2307 5326.1490 5201.7864
343.9367 -15769.6886 -1771.3306 0.0000 0.0000
7530.7829 4023.8665 301.2690 -3506.9164 4082.3464
306.8016 -107.8967 10.7812 656151.2420 -26.3029
-26.2759
[9117]ENERGY: 8050000 3160.8642 5086.2462 5240.1800
329.5642 -15795.7425 -1726.3811 0.0000 0.0000
7725.3557 4020.0868 309.0529 -3705.2689 4083.1706
306.8092 128.1721 69.2583 656151.2420 -25.3353
-25.3476
[9153]ENERGY: 8060000 3089.3830 5211.6800 5243.3702
325.3329 -15800.5327 -1739.0368 0.0000 0.0000
7689.7289 4019.9255 307.6276 -3669.8034 4084.2726
306.9448 122.8602 73.2868 656151.2420 -26.0697
-26.0847
[9203]ENERGY: 8070000 3163.5522 5182.5006 5229.2294
326.3509 -15799.1661 -1741.8613 0.0000 0.0000
7660.1473 4020.7529 306.4442 -3639.3944 4083.5860
306.9897 116.3871 -0.2184 656151.2420 -23.2269
-23.2190
[9239]ENERGY: 8080000 3181.0175 5116.1882 5283.1235
363.0333 -15823.0125 -1772.1490 0.0000 0.0000
7675.4427 4023.6437 307.0561 -3651.7990 4083.4439
306.8234 118.2857 -9.5220 656151.2420 -26.4332
-26.4356
[9289]ENERGY: 8090000 3164.0713 5171.8055 5219.7469
343.0016 -15803.3647 -1771.4176 0.0000 0.0000
7697.6816 4021.5246 307.9457 -3676.1569 4082.9753
306.9531 14.4521 -52.8870 656151.2420 -25.6088
-25.6036
[9328]ENERGY: 8100000 3162.8373 5292.8772 5296.7159
324.3492 -15890.4778 -1761.6538 0.0000 0.0000
7599.0370 4023.6850 303.9995 -3575.3520 4083.6939
306.8299 58.1458 -5.0202 656151.2420 -23.3207
-23.3181
[9378]ENERGY: 8110000 3206.6466 5191.4992 5297.7045
354.5753 -15770.5018 -1848.3399 0.0000 0.0000
7593.3043 4024.8881 303.7701 -3568.4161 4082.7011
306.6773 -52.0261 -161.5308 656151.2420 -22.1767
-22.2001
[9414]ENERGY: 8120000 3080.5859 5149.7370 5295.8073
340.0469 -15732.1073 -1781.5673 0.0000 0.0000
7668.2982 4020.8007 306.7703 -3647.4975 4082.9288
306.8961 124.0249 58.3546 656151.2420 -32.4415
-32.4457
[9464]ENERGY: 8130000 3213.2569 5192.1038 5266.9612
350.3854 -15780.9333 -1830.7745 0.0000 0.0000
7613.9611 4024.9606 304.5965 -3589.0005 4085.0978

306.8104 -162.3811 -115.8036 656151.2420 -23.4149
-23.3786
[9500]ENERGY: 8140000 3183.0513 5182.7600 5241.6926
328.5047 -15768.1966 -1838.2961 0.0000 0.0000
7693.8817 4023.3976 307.7937 -3670.4841 4085.3700
307.0192 -33.0088 -75.3999 656151.2420 -28.3207
-28.3209
[9550]ENERGY: 8150000 3233.5488 5102.7428 5242.9109
339.4348 -15820.9144 -1720.5161 0.0000 0.0000
7649.7584 4026.9653 306.0286 -3622.7932 4085.6860
306.9932 60.8175 42.0026 656151.2420 -20.4386
-20.4480
[9586]ENERGY: 8160000 3175.5877 5153.3989 5290.2987
343.9398 -15854.9595 -1756.6646 0.0000 0.0000
7669.8042 4021.4053 306.8305 -3648.3989 4084.2224
307.1408 -34.8057 -26.3664 656151.2420 -30.4385
-30.4335
[9636]ENERGY: 8170000 3128.0587 5287.6295 5260.2952
331.7053 -15925.6000 -1730.6372 0.0000 0.0000
7671.9253 4023.3769 306.9154 -3648.5485 4085.6682
307.1333 -19.6232 -105.1670 656151.2420 -25.3026
-25.3209
[9672]ENERGY: 8180000 3210.3434 5168.1861 5263.5893
358.3405 -15919.4957 -1786.3820 0.0000 0.0000
7727.5132 4022.0948 309.1392 -3705.4184 4086.0871
307.0070 12.1393 18.7757 656151.2420 -28.3210
-28.2989
[9722]ENERGY: 8190000 3262.6855 5122.7725 5253.9730
331.2991 -15753.3274 -1880.8785 0.0000 0.0000
7690.0226 4026.5467 307.6394 -3663.4759 4087.1340
307.0410 -106.5984 -170.7965 656151.2420 -26.0075
-26.0180
[9761]ENERGY: 8200000 3167.1962 5105.1644 5271.9470
329.4055 -15743.8849 -1815.7675 0.0000 0.0000
7712.1053 4026.1660 308.5228 -3685.9393 4086.1186
306.8274 -10.6781 -47.8142 656151.2420 -21.3480
-21.3486
[9811]ENERGY: 8210000 3200.6745 5257.3616 5283.7493
341.2528 -15839.0852 -1855.9332 0.0000 0.0000
7637.0788 4025.0987 305.5213 -3611.9801 4086.4014
306.8152 -26.7545 -110.4467 656151.2420 -25.7213
-25.7075
[9847]ENERGY: 8220000 3193.8075 5250.2635 5275.6377
353.7754 -15983.7409 -1764.9609 0.0000 0.0000
7699.3790 4024.1613 308.0137 -3675.2177 4084.3847
306.9193 -88.7549 -15.1415 656151.2420 -21.5010
-21.5052
[9897]ENERGY: 8230000 3184.6039 5236.7898 5279.5573
345.7264 -15860.2210 -1783.3802 0.0000 0.0000
7618.7047 4021.7810 304.7863 -3596.9238 4085.6582
306.9666 56.3093 -79.6124 656151.2420 -27.4625
-27.4634
[9933]ENERGY: 8240000 3194.1397 5157.6933 5230.7955
345.8986 -15859.0013 -1755.0087 0.0000 0.0000

7709.3995 4023.9166 308.4145 -3685.4829 4085.1235
306.8638 -23.3152 -81.1626 656151.2420 -19.5576
-19.5643
[9983]ENERGY: 8250000 3124.8905 5182.3997 5280.4239
334.7998 -15861.6498 -1706.7528 0.0000 0.0000
7669.4026 4023.5140 306.8144 -3645.8886 4087.2678
306.6091 82.5956 24.2084 656151.2420 -29.9903
-30.0002
[10019]ENERGY: 8260000 3249.0757 5191.7971 5215.4918
349.7418 -15782.2139 -1816.1264 0.0000 0.0000
7620.4091 4028.1751 304.8545 -3592.2340 4087.6628
307.0457 -161.9058 -249.7864 656151.2420 -19.1716
-19.1517
[10069]ENERGY: 8270000 3253.0681 5169.6720 5224.4339
333.8330 -15807.6749 -1769.4789 0.0000 0.0000
7625.4364 4029.2896 305.0556 -3596.1468 4087.2461
306.8198 -123.5261 -81.6444 656151.2420 -26.0535
-26.0711
[10105]ENERGY: 8280000 3279.6943 5180.6863 5262.4093
344.4958 -15908.5591 -1757.8031 0.0000 0.0000
7626.1335 4027.0570 305.0835 -3599.0765 4087.1634
306.7626 105.4823 18.1818 656151.2420 -23.7466
-23.7359
[10155]ENERGY: 8290000 3165.5197 5239.7441 5290.4776
345.0010 -15856.0837 -1784.3802 0.0000 0.0000
7629.3908 4029.6692 305.2138 -3599.7215 4087.6557
306.7467 -116.7335 -180.9736 656151.2420 -25.8330
-25.8428
[10194]ENERGY: 8300000 3180.8265 5231.6259 5259.2238
343.1273 -15834.6376 -1842.0208 0.0000 0.0000
7685.3048 4023.4499 307.4506 -3661.8550 4088.3992
306.7577 -100.8852 -110.6441 656151.2420 -23.6645
-23.6582
[10244]ENERGY: 8310000 3247.6707 5062.1511 5245.9413
338.1506 -15858.9761 -1775.8594 0.0000 0.0000
7763.2065 4022.2848 310.5671 -3740.9218 4086.9771
306.8634 -153.7060 -94.0096 656151.2420 -21.7483
-21.7634
[10280]ENERGY: 8320000 3226.1120 5242.0783 5226.0314
329.3263 -15847.2928 -1774.0417 0.0000 0.0000
7624.9267 4027.1401 305.0352 -3597.7866 4087.9148
306.8422 0.9739 -13.8825 656151.2420 -28.7675
-28.7434
[10330]ENERGY: 8330000 3161.1750 5267.8740 5283.6667
331.1101 -15955.8305 -1774.3394 0.0000 0.0000
7710.7665 4024.4224 308.4692 -3686.3441 4087.9163
306.8701 87.9208 -11.2787 656151.2420 -21.5654
-21.5697
[10366]ENERGY: 8340000 3182.7569 5187.7447 5294.0073
348.9464 -15899.4595 -1730.5712 0.0000 0.0000
7640.0920 4023.5167 305.6419 -3616.5754 4086.6540
306.8548 21.5126 33.7624 656151.2420 -25.1932
-25.2039
[10416]ENERGY: 8350000 3202.2357 5203.6168 5262.5231

352.5758 -15829.6380 -1855.1267 0.0000 0.0000
7689.5655 4025.7522 307.6211 -3663.8133 4088.2122
306.8166 -119.1423 -209.9213 656151.2420 -20.9837
-20.9827
[10452]ENERGY: 8360000 3199.5701 5162.5155 5257.0811
318.0891 -15750.7622 -1826.7114 0.0000 0.0000
7662.3591 4022.1413 306.5327 -3640.2178 4087.4860
306.8439 53.1615 57.2062 656151.2420 -25.8513
-25.8571
[10502]ENERGY: 8370000 3153.8684 5201.7336 5261.4215
331.5102 -15775.1564 -1775.4492 0.0000 0.0000
7630.3519 4028.2799 305.2522 -3602.0719 4088.2174
306.8419 -10.1703 -9.4263 656151.2420 -22.5849
-22.5623
[10538]ENERGY: 8380000 3210.3983 5137.5654 5252.3910
344.5986 -15834.4042 -1788.6354 0.0000 0.0000
7705.1923 4027.1059 308.2462 -3678.0864 4087.5174
306.9013 202.2251 93.9786 656151.2420 -28.4199
-28.4312
[10588]ENERGY: 8390000 3204.7718 5208.3787 5242.2240
346.6457 -15903.1807 -1775.4771 0.0000 0.0000
7699.2578 4022.6201 308.0088 -3676.6377 4088.2542
306.9213 1.3576 -20.8929 656151.2420 -23.4132
-23.4106
[10627]ENERGY: 8400000 3136.7369 5199.1126 5281.5305
325.7154 -15778.9606 -1752.5322 0.0000 0.0000
7615.6402 4027.2428 304.6637 -3588.3974 4087.9527
307.0507 126.8880 -6.3844 656151.2420 -24.8085
-24.8248
[10677]ENERGY: 8410000 3160.0076 5253.9336 5249.5044
315.4355 -15849.3080 -1833.5925 0.0000 0.0000
7727.0193 4023.0000 309.1194 -3704.0193 4088.6601
307.0153 -134.4022 -211.5011 656151.2420 -27.3403
-27.3402
[10713]ENERGY: 8420000 3120.2458 5197.0945 5268.3239
345.9969 -15821.9024 -1697.8066 0.0000 0.0000
7617.4458 4029.3978 304.7359 -3588.0479 4089.6801
306.9852 139.1532 2.9713 656151.2420 -25.3442
-25.3486
[10763]ENERGY: 8430000 3215.3215 5158.7004 5300.3977
331.0564 -15850.1991 -1829.4684 0.0000 0.0000
7698.3965 4024.2050 307.9743 -3674.1915 4089.8660
306.8117 125.3086 21.7154 656151.2420 -23.2604
-23.2382
[10799]ENERGY: 8440000 3145.9113 5173.2262 5301.3632
342.4513 -15805.5609 -1693.9056 0.0000 0.0000
7564.7470 4028.2324 302.6277 -3536.5146 4088.4575
306.9743 120.1529 64.3397 656151.2420 -23.2804
-23.2974
[10849]ENERGY: 8450000 3179.3564 5228.9465 5264.4307
337.4090 -15803.7209 -1771.5774 0.0000 0.0000
7592.3479 4027.1922 303.7319 -3565.1557 4089.9969
306.9896 187.6664 86.6196 656151.2420 -23.7817
-23.7808

[10885]ENERGY: 8460000 3192.6616 5176.3071 5244.5510
347.5381 -15769.7845 -1845.2391 0.0000 0.0000
7682.8627 4028.8969 307.3529 -3653.9658 4089.7537
306.8158 -95.5679 -123.0717 656151.2420 -23.0276
-23.0262
[10935]ENERGY: 8470000 3183.9870 5162.6798 5267.5766
350.6595 -15893.2688 -1750.1954 0.0000 0.0000
7701.6454 4023.0842 308.1043 -3678.5613 4088.4577
306.9608 -165.2695 -85.0450 656151.2420 -27.2131
-27.1960
[10971]ENERGY: 8480000 3214.5154 5171.0997 5254.3388
365.8622 -15803.7182 -1827.8599 0.0000 0.0000
7657.4479 4031.6858 306.3362 -3625.7620 4089.6300
306.9231 -18.6522 -102.4730 656151.2420 -19.8287
-19.8508
[11021]ENERGY: 8490000 3200.7237 5189.7049 5301.5993
334.9556 -15843.0681 -1824.7340 0.0000 0.0000
7668.0744 4027.2558 306.7613 -3640.8186 4089.6061
306.9968 -172.7309 -157.1070 656151.2420 -24.4397
-24.4287
[11060]ENERGY: 8500000 3251.2581 5136.8376 5288.8049
346.9766 -15799.3755 -1822.1694 0.0000 0.0000
7625.1275 4027.4598 305.0432 -3597.6676 4091.0304
306.8026 -79.7327 -90.0451 656151.2420 -25.3254
-25.3145
[11110]ENERGY: 8510000 3191.0141 5127.5431 5253.1035
320.7246 -15783.1929 -1797.2738 0.0000 0.0000
7715.3207 4027.2393 308.6514 -3688.0814 4090.4337
306.7678 46.6727 -37.7739 656151.2420 -24.0522
-24.0680
[11146]ENERGY: 8520000 3217.5020 5216.2366 5261.0994
329.1903 -15841.7427 -1802.5426 0.0000 0.0000
7647.4899 4027.2328 305.9378 -3620.2571 4091.0283
306.6726 121.3664 31.8772 656151.2420 -25.1186
-25.1100
[11196]ENERGY: 8530000 3285.8819 5057.4746 5275.9108
341.7153 -15847.2611 -1748.3279 0.0000 0.0000
7660.9612 4026.3547 306.4768 -3634.6065 4090.9347
306.7024 43.2923 30.8905 656151.2420 -24.5095
-24.5118
[11232]ENERGY: 8540000 3139.3082 5153.8267 5253.1255
337.8869 -15795.0696 -1836.4422 0.0000 0.0000
7771.1145 4023.7501 310.8834 -3747.3645 4090.3286
306.8406 4.5102 -56.3701 656151.2420 -23.8574
-23.8515
[11282]ENERGY: 8550000 3127.9085 5229.6955 5256.6624
326.6636 -15893.6184 -1723.9696 0.0000 0.0000
7702.7016 4026.0436 308.1466 -3676.6581 4089.4049
306.8762 90.0030 86.5296 656151.2420 -24.6773
-24.6690
[11318]ENERGY: 8560000 3210.6146 5183.9070 5230.6920
324.9663 -15783.6998 -1726.8905 0.0000 0.0000
7591.0120 4030.6016 303.6784 -3560.4105 4089.4681
306.8915 38.9943 -43.5184 656151.2420 -21.6886

-21.7212
[11368]ENERGY: 8570000 3242.3543 5180.7203 5279.8993
351.1904 -15858.0011 -1799.0923 0.0000 0.0000
7629.0983 4026.1691 305.2021 -3602.9292 4089.0260
306.8525 -113.9762 -72.5549 656151.2420 -25.3323
-25.3025
[11404]ENERGY: 8580000 3208.8273 5227.2206 5237.6099
343.4979 -15859.1013 -1721.1965 0.0000 0.0000
7590.4309 4027.2888 303.6552 -3563.1421 4089.3123
306.8719 -2.9098 -8.2590 656151.2420 -21.6170
-21.6156
[11454]ENERGY: 8590000 3201.2911 5238.8339 5339.1493
309.4757 -15904.2888 -1798.3321 0.0000 0.0000
7640.5500 4026.6790 305.6602 -3613.8710 4089.7560
306.8579 30.5799 -33.3118 656151.2420 -25.5384
-25.5495
[11493]ENERGY: 8600000 3146.8665 5198.3214 5281.4255
329.7315 -15844.1798 -1755.5647 0.0000 0.0000
7671.2539 4027.8542 306.8885 -3643.3996 4088.9173
306.8479 32.0606 -26.6767 656151.2420 -24.4463
-24.4474
[11543]ENERGY: 8610000 3177.2448 5217.8876 5306.6557
355.0353 -15859.4733 -1822.6832 0.0000 0.0000
7647.9263 4022.5932 305.9553 -3625.3331 4089.3163
306.7596 -9.7502 -111.2272 656151.2420 -27.2419
-27.2411
[11579]ENERGY: 8620000 3205.8899 5092.0247 5307.7995
335.5659 -15890.6551 -1757.7711 0.0000 0.0000
7732.3802 4025.2340 309.3339 -3707.1462 4088.2354
306.9189 -23.2883 -10.1805 656151.2420 -23.3468
-23.3508
[11629]ENERGY: 8630000 3202.2872 5112.3958 5273.4585
376.5007 -15866.1332 -1757.9590 0.0000 0.0000
7688.3462 4028.8961 307.5723 -3659.4501 4088.4704
306.9274 59.8177 -89.7866 656151.2420 -26.5886
-26.5855
[11665]ENERGY: 8640000 3136.8486 5193.1647 5342.8659
339.8705 -15852.1723 -1841.1586 0.0000 0.0000
7706.1078 4025.5266 308.2828 -3680.5812 4088.8283
306.7942 30.2146 43.8339 656151.2420 -23.8030
-23.7920
[11715]ENERGY: 8650000 3245.2201 5184.0805 5284.1205
341.9025 -15885.3170 -1743.1119 0.0000 0.0000
7600.9128 4027.8075 304.0745 -3573.1053 4089.4777
307.0263 -69.3119 -91.7106 656151.2420 -19.1977
-19.2036
[11751]ENERGY: 8660000 3178.5076 5185.6990 5258.5413
356.9569 -15885.7967 -1764.3423 0.0000 0.0000
7699.8029 4029.3688 308.0306 -3670.4341 4089.6783
306.7506 -40.7761 -58.9908 656151.2420 -25.1803
-25.1911
[11801]ENERGY: 8670000 3109.8489 5137.5547 5290.1618
342.3865 -15792.7473 -1784.3255 0.0000 0.0000
7725.9201 4028.7992 309.0754 -3697.1209 4090.3803

306.8667 -43.2120 -46.6311 656151.2420 -19.3137
-19.2991
[11837]ENERGY: 8680000 3185.9715 5177.7321 5269.2712
341.9391 -15779.7490 -1794.1589 0.0000 0.0000
7629.5935 4030.5994 305.2219 -3598.9941 4091.7795
306.7869 -197.4434 -121.3172 656151.2420 -23.1379
-23.1380
[11887]ENERGY: 8690000 3190.1358 5130.0044 5322.4462
337.2552 -15832.1691 -1795.5124 0.0000 0.0000
7675.6964 4027.8565 307.0662 -3647.8399 4091.7980
306.8802 81.8156 38.3048 656151.2420 -19.3560
-19.3591
[11926]ENERGY: 8700000 3127.1071 5197.4227 5261.4065
342.9456 -15898.9758 -1698.5021 0.0000 0.0000
7698.1776 4029.5816 307.9656 -3668.5961 4091.2979
306.8412 127.8327 51.1357 656151.2420 -22.1547
-22.1515
[11976]ENERGY: 8710000 3212.4472 5274.5301 5198.2616
326.0253 -15808.3040 -1798.7308 0.0000 0.0000
7625.6266 4029.8560 305.0632 -3595.7706 4091.4041
307.0217 88.7431 63.3797 656151.2420 -29.0343
-29.0277
[12012]ENERGY: 8720000 3114.1226 5219.7329 5226.4670
327.5476 -15787.9850 -1809.9681 0.0000 0.0000
7736.8639 4026.7809 309.5132 -3710.0830 4091.4617
307.0418 -21.5742 -39.7806 656151.2420 -21.8259
-21.8512
[12062]ENERGY: 8730000 3231.0185 5197.6973 5209.8843
334.6283 -15826.0743 -1774.4204 0.0000 0.0000
7658.6347 4031.3684 306.3837 -3627.2663 4092.0405
306.9972 23.2837 -1.6523 656151.2420 -26.5910
-26.5752
[12098]ENERGY: 8740000 3164.7113 5174.3436 5254.6488
310.7343 -15838.7289 -1793.3363 0.0000 0.0000
7754.9771 4027.3498 310.2379 -3727.6272 4091.2014
306.8463 -288.9287 -203.7770 656151.2420 -22.7335
-22.7317
[12148]ENERGY: 8750000 3289.4209 5087.7580 5248.5988
341.4113 -15832.3536 -1778.8347 0.0000 0.0000
7673.4787 4029.4793 306.9775 -3643.9994 4090.9000
306.9380 -139.6902 -142.0348 656151.2420 -25.1489
-25.1612
[12184]ENERGY: 8760000 3216.2774 5236.9836 5284.6617
322.5886 -15881.6105 -1784.1414 0.0000 0.0000
7633.6046 4028.3641 305.3823 -3605.2405 4092.0503
306.9883 50.5927 7.8956 656151.2420 -23.8453
-23.8293
[12234]ENERGY: 8770000 3159.7957 5125.5660 5297.0909
344.7088 -15829.4342 -1711.8879 0.0000 0.0000
7645.6938 4031.5332 305.8660 -3614.1606 4092.3519
306.8872 131.6973 69.9015 656151.2420 -23.9243
-23.9388
[12270]ENERGY: 8780000 3199.9096 5201.5115 5257.6248
345.9297 -15872.8402 -1750.5211 0.0000 0.0000

7647.2849 4028.8991 305.9296 -3618.3857 4091.4182
306.8658 -45.9826 -6.8153 656151.2420 -24.9830
-24.9805
[12320]ENERGY: 8790000 3213.1082 5092.9481 5242.0228
340.5724 -15850.3738 -1690.5780 0.0000 0.0000
7682.1505 4029.8502 307.3244 -3652.3003 4091.4205
306.9120 85.1939 86.2243 656151.2420 -23.4606
-23.4532
[12359]ENERGY: 8800000 3205.9179 5191.4396 5225.3568
321.3518 -15767.3435 -1809.3484 0.0000 0.0000
7665.3217 4032.6959 306.6512 -3632.6258 4092.8008
306.8112 -27.2524 -68.5424 656151.2420 -26.2763
-26.2834
[12409]ENERGY: 8810000 3164.1815 5229.7149 5269.6419
317.8718 -15835.3567 -1723.5360 0.0000 0.0000
7609.6713 4032.1888 304.4249 -3577.4825 4093.4206
306.8848 283.2556 153.1582 656151.2420 -21.8722
-21.8693
[12445]ENERGY: 8820000 3095.2322 5210.8425 5316.6951
333.0645 -15839.3279 -1756.1505 0.0000 0.0000
7671.5570 4031.9129 306.9006 -3639.6441 4093.8969
306.6017 -75.8693 -111.3207 656151.2420 -26.8111
-26.8265
[12495]ENERGY: 8830000 3130.3841 5259.7700 5252.0597
345.2206 -15844.8267 -1756.9578 0.0000 0.0000
7643.6797 4029.3294 305.7854 -3614.3502 4093.0548
306.8569 -6.1787 -18.2737 656151.2420 -23.5886
-23.5765
[12531]ENERGY: 8840000 3127.7560 5152.4762 5286.5888
346.4429 -15851.2026 -1700.6687 0.0000 0.0000
7666.2259 4027.6186 306.6874 -3638.6073 4092.3281
306.7412 -23.2198 -41.1553 656151.2420 -23.5997
-23.6089
[12581]ENERGY: 8850000 3267.6898 5204.0566 5281.2922
338.4805 -15851.3290 -1827.3562 0.0000 0.0000
7618.2284 4031.0623 304.7672 -3587.1661 4091.9263
306.8258 -191.3223 -125.6953 656151.2420 -28.5200
-28.5077
[12617]ENERGY: 8860000 3173.1451 5214.7958 5256.5051
338.0253 -15740.0186 -1893.3572 0.0000 0.0000
7682.2385 4031.3340 307.3279 -3650.9045 4093.2616
306.8513 -231.8937 -197.2019 656151.2420 -21.0417
-21.0395
[12667]ENERGY: 8870000 3111.4502 5182.5425 5274.1268
341.9200 -15795.7752 -1742.5706 0.0000 0.0000
7659.4609 4031.1545 306.4167 -3628.3065 4092.5081
306.6071 127.8232 80.9535 656151.2420 -26.2344
-26.2482
[12703]ENERGY: 8880000 3188.2696 5085.9122 5284.9221
325.2492 -15837.7033 -1757.3007 0.0000 0.0000
7740.9442 4030.2934 309.6765 -3710.6508 4092.6969
306.5580 108.9280 -1.2549 656151.2420 -32.6649
-32.6662
[12753]ENERGY: 8890000 3146.9955 5193.5200 5276.3990

352.3633 -15925.3273 -1696.7170 0.0000 0.0000
7683.0977 4030.3312 307.3623 -3652.7665 4092.8376
306.8716 83.9126 53.3693 656151.2420 -31.0205
-31.0195
[12792]ENERGY: 8900000 3174.6395 5215.2294 5304.6500
334.6365 -15945.2965 -1747.2640 0.0000 0.0000
7694.2862 4030.8810 307.8099 -3663.4052 4093.4343
307.0014 -3.6079 14.7862 656151.2420 -24.7734
-24.7675
[12842]ENERGY: 8910000 3116.7647 5136.0472 5263.1365
365.0332 -15797.5045 -1741.5481 0.0000 0.0000
7690.5663 4032.4952 307.6611 -3658.0711 4093.9771
306.9795 126.7971 64.9038 656151.2420 -25.6994
-25.6958
[12878]ENERGY: 8920000 3160.0789 5162.5044 5297.0080
346.5099 -15779.9764 -1764.2803 0.0000 0.0000
7611.9301 4033.7747 304.5153 -3578.1554 4093.4898
306.9695 -87.2742 -104.1213 656151.2420 -29.6784
-29.6883
[12928]ENERGY: 8930000 3205.1385 5207.0357 5293.1638
322.4827 -15888.6068 -1748.4092 0.0000 0.0000
7646.2535 4037.0582 305.8884 -3609.1953 4095.0619
306.8036 -35.2665 -31.7947 656151.2420 -30.4240
-30.3926
[12964]ENERGY: 8940000 3229.4180 5166.1376 5260.4900
357.5706 -15896.7081 -1731.7932 0.0000 0.0000
7647.8054 4032.9202 305.9505 -3614.8852 4094.2012
306.8243 -50.3836 -45.7247 656151.2420 -21.1794
-21.2065
[13014]ENERGY: 8950000 3263.0609 5146.0783 5254.7939
337.5657 -15856.2975 -1721.5452 0.0000 0.0000
7608.7902 4032.4464 304.3897 -3576.3439 4093.7725
306.7200 -1.0842 -99.6220 656151.2420 -25.4217
-25.4157
[13050]ENERGY: 8960000 3215.9374 5172.2446 5205.6666
363.5219 -15912.8414 -1685.4533 0.0000 0.0000
7675.2871 4034.3629 307.0499 -3640.9242 4094.6081
306.7478 90.4859 -13.1131 656151.2420 -21.5010
-21.5031
[13100]ENERGY: 8970000 3168.8564 5131.1603 5282.5517
322.1368 -15800.0934 -1684.9572 0.0000 0.0000
7612.2522 4031.9069 304.5281 -3580.3453 4095.2609
306.7378 146.7916 50.9329 656151.2420 -28.7475
-28.7590
[13136]ENERGY: 8980000 3238.8200 5216.3934 5258.0688
331.9300 -15903.3650 -1789.8683 0.0000 0.0000
7683.3350 4035.3138 307.3718 -3648.0212 4094.3828
306.7334 109.7141 38.4760 656151.2420 -21.7545
-21.7116
[13186]ENERGY: 8990000 3243.6313 5250.6295 5299.5216
325.3831 -15926.3115 -1813.2804 0.0000 0.0000
7654.4161 4033.9897 306.2149 -3620.4265 4096.0790
306.6005 -3.7415 -71.4044 656151.2420 -26.5632
-26.6023

[13225]ENERGY: 9000000 3138.9800 5150.3903 5262.1925
357.6284 -15805.2260 -1743.4437 0.0000 0.0000
7673.6410 4034.1623 306.9840 -3639.4786 4097.5156
306.7438 -38.0461 -26.9372 656151.2420 -28.1542
-28.1549
[13275]ENERGY: 9010000 3166.2968 5154.1364 5256.3751
343.4498 -15882.9356 -1764.0061 0.0000 0.0000
7758.1076 4031.4239 310.3631 -3726.6836 4097.7645
306.8163 -8.7613 11.4454 656151.2420 -24.7276
-24.7194
[13311]ENERGY: 9020000 3203.2899 5143.0578 5222.2482
327.4151 -15860.5475 -1669.1365 0.0000 0.0000
7670.3243 4036.6513 306.8513 -3633.6730 4098.2759
306.8114 -9.4276 -29.4920 656151.2420 -28.5348
-28.5295
[13361]ENERGY: 9030000 3260.9197 5206.2784 5235.0533
336.3430 -15885.2213 -1804.1502 0.0000 0.0000
7687.9106 4037.1334 307.5549 -3650.7773 4099.0866
306.8112 -107.4858 -92.8662 656151.2420 -24.8395
-24.8455
[13397]ENERGY: 9040000 3191.9385 5226.1732 5288.0020
346.5610 -15849.7490 -1827.6593 0.0000 0.0000
7660.7240 4035.9904 306.4673 -3624.7336 4099.8352
306.8648 -85.6970 -81.7097 656151.2420 -21.1331
-21.1223
[13447]ENERGY: 9050000 3168.0839 5214.2540 5294.2627
340.3868 -15926.1519 -1745.3750 0.0000 0.0000
7685.2757 4030.7361 307.4495 -3654.5396 4098.1922
306.8218 33.4245 7.6127 656151.2420 -25.7298
-25.7278
[13483]ENERGY: 9060000 3228.5715 5126.7803 5277.4533
351.6428 -15881.2384 -1698.9225 0.0000 0.0000
7637.4281 4041.7151 305.5353 -3595.7130 4099.0317
306.8776 165.2390 40.5708 656151.2420 -26.3170
-26.3260
[13533]ENERGY: 9070000 3230.6279 5088.8746 5299.9356
337.7736 -15804.3728 -1748.2772 0.0000 0.0000
7630.5235 4035.0852 305.2591 -3595.4383 4098.7918
307.1665 -78.8437 -35.0967 656151.2420 -23.5828
-23.5813
[13569]ENERGY: 9080000 3164.9491 5247.3616 5241.6370
330.0719 -15884.5373 -1749.6294 0.0000 0.0000
7689.4217 4039.2746 307.6153 -3650.1471 4099.9292
307.3288 61.8945 -5.9302 656151.2420 -26.3660
-26.3710
[13619]ENERGY: 9090000 3241.5527 5231.1547 5231.2142
330.5429 -15952.1847 -1736.0189 0.0000 0.0000
7688.7708 4035.0318 307.5893 -3653.7389 4098.2392
307.4171 -123.1938 -124.2731 656151.2420 -18.5701
-18.5474
[13658]ENERGY: 9100000 3109.3043 5161.9194 5322.0005
344.3760 -15856.4421 -1740.3096 0.0000 0.0000
7697.9951 4038.8437 307.9583 -3659.1514 4098.4312
307.0471 74.9042 -14.9053 656151.2420 -28.6106

-28.6170
[13708]ENERGY: 9110000 3207.7617 5152.8784 5265.7656
357.2680 -15833.5500 -1764.8386 0.0000 0.0000
7653.6864 4038.9715 306.1857 -3614.7148 4099.7826
306.7723 31.8720 -4.9621 656151.2420 -29.2271
-29.2219
[13744]ENERGY: 9120000 3181.3162 5208.3268 5273.3365
334.6453 -15790.8204 -1842.9146 0.0000 0.0000
7675.4936 4039.3834 307.0581 -3636.1102 4100.1876
306.9344 -33.5460 -59.5726 656151.2420 -24.1991
-24.2121
[13794]ENERGY: 9130000 3225.5009 5096.0127 5231.3385
347.1480 -15817.8971 -1731.4518 0.0000 0.0000
7689.0317 4039.6829 307.5997 -3649.3487 4100.0834
307.0772 -90.3632 -134.2584 656151.2420 -31.7751
-31.7693
[13830]ENERGY: 9140000 3239.6856 5149.3567 5312.3100
327.1013 -15882.1624 -1801.7713 0.0000 0.0000
7694.8685 4039.3884 307.8332 -3655.4801 4100.9572
307.0581 129.5620 -72.0871 656151.2420 -23.5272
-23.5626
[13880]ENERGY: 9150000 3145.3091 5196.0351 5248.8976
346.6536 -15781.3256 -1862.9519 0.0000 0.0000
7745.1522 4037.7701 309.8448 -3707.3821 4101.6297
307.1512 33.6977 -85.4538 656151.2420 -30.0364
-30.0191
[13916]ENERGY: 9160000 3148.2049 5147.6624 5201.6431
332.5861 -15785.1996 -1808.5316 0.0000 0.0000
7801.1872 4037.5525 312.0865 -3763.6347 4101.2422
307.0842 78.1675 -27.1632 656151.2420 -24.2402
-24.2292
[13966]ENERGY: 9170000 3296.5018 5089.4577 5283.3106
355.3031 -15866.5737 -1735.6410 0.0000 0.0000
7616.9296 4039.2883 304.7153 -3577.6413 4100.1892
307.1597 -85.6474 -66.5005 656151.2420 -28.9000
-28.8960
[14002]ENERGY: 9180000 3138.3233 5161.7229 5270.3997
347.4248 -15906.4332 -1667.8023 0.0000 0.0000
7696.6401 4040.2753 307.9041 -3656.3648 4100.7566
307.0832 -2.6071 -50.3406 656151.2420 -27.6508
-27.6528
[14052]ENERGY: 9190000 3201.0281 5174.5508 5304.3749
323.8177 -15796.7581 -1781.4335 0.0000 0.0000
7614.0789 4039.6588 304.6012 -3574.4201 4099.8055
307.0837 -110.3172 -148.7014 656151.2420 -27.1571
-27.1522
[14091]ENERGY: 9200000 3195.2672 5275.3947 5270.5465
318.8968 -15920.7504 -1664.7884 0.0000 0.0000
7565.3612 4039.9277 302.6523 -3525.4335 4099.7085
307.0763 110.9100 75.9930 656151.2420 -27.1766
-27.1795
[14141]ENERGY: 9210000 3221.8271 5210.2187 5256.0377
350.9205 -15847.0104 -1795.2260 0.0000 0.0000
7643.9309 4040.6985 305.7955 -3603.2325 4101.5739

307.1839 -199.1135 -157.0692 656151.2420 -25.4846
-25.4838
[14177]ENERGY: 9220000 3223.4735 5264.8014 5282.0599
343.2195 -15864.1406 -1800.8542 0.0000 0.0000
7595.3947 4043.9541 303.8538 -3551.4406 4102.2077
307.1014 103.4642 56.9972 656151.2420 -24.5732
-24.5895
[14227]ENERGY: 9230000 3170.6515 5178.8969 5276.0569
360.4228 -15840.4047 -1734.3882 0.0000 0.0000
7628.8307 4040.0659 305.1914 -3588.7648 4102.2834
307.1958 0.8297 -47.1989 656151.2420 -25.8412
-25.8396
[14263]ENERGY: 9240000 3221.9234 5251.6983 5223.8007
329.9882 -15761.8505 -1799.4012 0.0000 0.0000
7578.0347 4044.1936 303.1593 -3533.8411 4102.1992
307.0018 103.0194 19.1195 656151.2420 -23.2766
-23.2622
[14313]ENERGY: 9250000 3162.0794 5185.9341 5221.3507
329.5275 -15821.7727 -1788.0740 0.0000 0.0000
7752.0364 4041.0814 310.1202 -3710.9550 4102.5563
307.0187 -51.4140 -27.6842 656151.2420 -24.9259
-24.9251
[14349]ENERGY: 9260000 3197.6231 5170.0583 5302.7716
344.2582 -15792.9315 -1828.3634 0.0000 0.0000
7652.4329 4045.8492 306.1356 -3606.5837 4103.7203
307.1913 98.3226 22.3489 656151.2420 -20.5094
-20.5225
[14399]ENERGY: 9270000 3163.9266 5255.4603 5233.2686
329.5259 -15845.4514 -1755.9103 0.0000 0.0000
7659.6892 4040.5089 306.4259 -3619.1803 4102.1645
307.2631 -94.1815 -75.1593 656151.2420 -26.2298
-26.2219
[14435]ENERGY: 9280000 3186.3670 5148.0313 5280.7894
321.1542 -15834.6619 -1711.6612 0.0000 0.0000
7649.9109 4039.9295 306.0347 -3609.9813 4102.3043
307.0943 59.3516 -46.5371 656151.2420 -27.1894
-27.1829
[14485]ENERGY: 9290000 3231.2796 5222.2421 5232.0869
329.6186 -15851.7192 -1765.6242 0.0000 0.0000
7643.7344 4041.6182 305.7876 -3602.1162 4101.9118
307.2027 -140.0790 -163.2346 656151.2420 -25.0387
-25.0416
[14524]ENERGY: 9300000 3233.6983 5210.0644 5275.0365
349.1486 -15871.6359 -1771.4437 0.0000 0.0000
7615.5086 4040.3768 304.6584 -3575.1318 4102.1535
307.2985 -200.7235 -189.7437 656151.2420 -27.5892
-27.6145
[14574]ENERGY: 9310000 3133.9861 5207.0655 5275.4255
327.9560 -15892.4632 -1746.6785 0.0000 0.0000
7734.1047 4039.3961 309.4029 -3694.7086 4101.7062
307.2700 -95.8898 -19.9400 656151.2420 -20.5342
-20.4927
[14610]ENERGY: 9320000 3231.8850 5131.9580 5283.5163
340.5982 -15982.1573 -1640.9334 0.0000 0.0000

7674.9498 4039.8166 307.0364 -3635.1332 4100.9103
307.3329 -71.0274 -7.4253 656151.2420 -22.7343
-22.7419
[14660]ENERGY: 9330000 3173.7436 5156.1148 5261.0987
332.0105 -15771.8957 -1804.5979 0.0000 0.0000
7697.8261 4044.3001 307.9515 -3653.5259 4102.5583
307.1976 -61.5825 -105.7582 656151.2420 -28.8876
-28.9000
[14696]ENERGY: 9340000 3266.8364 5065.3030 5233.4933
338.7395 -15805.9487 -1656.3572 0.0000 0.0000
7604.4472 4046.5135 304.2159 -3557.9337 4104.3124
307.3202 134.2335 48.7409 656151.2420 -23.7953
-23.7995
[14746]ENERGY: 9350000 3223.4962 5363.7535 5262.3643
320.3453 -15921.4215 -1778.0222 0.0000 0.0000
7573.0578 4043.5736 302.9602 -3529.4842 4104.6488
307.2633 -16.4571 -69.8942 656151.2420 -29.8342
-29.8264
[14782]ENERGY: 9360000 3174.4684 5201.3294 5222.2537
348.7227 -15812.0501 -1783.6320 0.0000 0.0000
7692.7553 4043.8475 307.7487 -3648.9079 4104.0044
307.4042 -37.5967 -17.6076 656151.2420 -27.4589
-27.4594
[14832]ENERGY: 9370000 3284.5646 5234.4727 5246.5982
331.9261 -15889.5455 -1777.3535 0.0000 0.0000
7614.9369 4045.5995 304.6355 -3569.3374 4103.6809
307.4368 -109.1555 -174.3866 656151.2420 -24.1759
-24.1847
[14868]ENERGY: 9380000 3175.9879 5220.3198 5267.5847
341.5041 -15858.6311 -1793.8417 0.0000 0.0000
7687.9772 4040.9010 307.5575 -3647.0763 4104.2116
307.1968 31.0116 -90.0704 656151.2420 -21.2835
-21.2783
[14918]ENERGY: 9390000 3234.6530 5178.1374 5301.9250
333.2377 -15937.2957 -1718.5987 0.0000 0.0000
7649.5736 4041.6323 306.0212 -3607.9413 4104.0009
307.2478 18.8459 7.9720 656151.2420 -23.3368
-23.3483
[14957]ENERGY: 9400000 3201.7367 5149.2233 5279.2828
329.6999 -15808.0539 -1780.2221 0.0000 0.0000
7670.5322 4042.1987 306.8596 -3628.3334 4104.4244
307.1197 24.0367 -16.8199 656151.2420 -27.5011
-27.4774
[15007]ENERGY: 9410000 3152.2569 5204.2599 5307.3527
362.8059 -15861.7767 -1762.5077 0.0000 0.0000
7640.8948 4043.2859 305.6740 -3597.6089 4106.1281
307.2829 48.2216 -55.7402 656151.2420 -23.9745
-23.9917
[15043]ENERGY: 9420000 3185.7104 5207.5542 5225.7328
357.8745 -15782.7983 -1782.5610 0.0000 0.0000
7630.1849 4041.6975 305.2455 -3588.4874 4105.9231
307.4289 -183.6521 -160.2266 656151.2420 -19.9443
-19.9361
[15093]ENERGY: 9430000 3155.7166 5220.4595 5228.7960

331.5901 -15804.1806 -1747.5453 0.0000 0.0000
7657.7528 4042.5891 306.3484 -3615.1638 4104.1163
307.3907 13.5255 -37.3720 656151.2420 -22.9442
-22.9496
[15129]ENERGY: 9440000 3201.3624 5165.1063 5260.4769
325.7451 -15869.6495 -1711.7360 0.0000 0.0000
7671.3032 4042.6084 306.8905 -3628.6948 4105.8756
307.3548 61.2636 79.3421 656151.2420 -21.9848
-21.9638
[15179]ENERGY: 9450000 3184.9239 5171.5693 5287.5545
333.1209 -15747.9850 -1775.9148 0.0000 0.0000
7588.0557 4041.3245 303.5602 -3546.7312 4106.4926
307.3083 -10.3787 -14.7716 656151.2420 -23.5370
-23.5527
[15215]ENERGY: 9460000 3266.9227 5203.3251 5292.4755
344.0366 -15780.7249 -1877.2384 0.0000 0.0000
7594.3865 4043.1829 303.8134 -3551.2035 4107.1011
307.2437 23.4660 -49.1305 656151.2420 -27.6068
-27.6217
[15265]ENERGY: 9470000 3206.8709 5222.7926 5296.8044
325.4034 -15812.6833 -1761.3320 0.0000 0.0000
7572.4648 4050.3209 302.9365 -3522.1440 4108.5124
307.2303 -88.0880 -145.0252 656151.2420 -27.4965
-27.4851
[15301]ENERGY: 9480000 3180.9103 5203.3805 5247.8116
336.1562 -15775.7084 -1756.8319 0.0000 0.0000
7611.2643 4046.9826 304.4886 -3564.2817 4108.5860
307.3829 148.1129 5.8185 656151.2420 -24.1556
-24.1594
[15351]ENERGY: 9490000 3147.7805 5224.6617 5226.0808
336.4198 -15785.9869 -1750.3018 0.0000 0.0000
7646.7094 4045.3634 305.9066 -3601.3459 4108.7355
307.2639 146.5833 108.7727 656151.2420 -23.0975
-23.0896
[15390]ENERGY: 9500000 3186.3539 5159.3110 5270.9866
317.8148 -15778.1511 -1782.4535 0.0000 0.0000
7670.6189 4044.4805 306.8631 -3626.1384 4108.4690
307.1939 -41.2263 -121.4950 656151.2420 -27.9446
-27.9471
[15440]ENERGY: 9510000 3174.8025 5174.2318 5269.7414
329.8397 -15814.7245 -1817.0370 0.0000 0.0000
7726.2599 4043.1140 309.0890 -3683.1459 4109.1033
307.1879 -180.2073 -154.0042 656151.2420 -26.5908
-26.5824
[15476]ENERGY: 9520000 3128.9652 5127.8090 5210.0438
337.1095 -15835.7197 -1724.3317 0.0000 0.0000
7802.8675 4046.7436 312.1537 -3756.1239 4109.1918
307.1778 -96.8506 -92.9114 656151.2420 -22.5400
-22.5351
[15526]ENERGY: 9530000 3241.8046 5176.4110 5245.6584
340.6435 -15830.2082 -1771.4889 0.0000 0.0000
7648.1494 4050.9697 305.9642 -3597.1797 4108.9584
307.3190 93.0131 -41.8957 656151.2420 -27.9594
-27.9849

[15562]ENERGY: 9540000 3105.1730 5194.8620 5269.5539
329.9582 -15861.4939 -1750.0383 0.0000 0.0000
7755.0019 4043.0168 310.2388 -3711.9851 4110.0952
307.4395 74.1320 -10.9172 656151.2420 -22.7195
-22.7134
[15612]ENERGY: 9550000 3184.0000 5262.7688 5274.1090
341.7100 -15891.9643 -1778.1306 0.0000 0.0000
7656.1792 4048.6721 306.2854 -3607.5071 4110.3742
307.3354 165.9094 101.1308 656151.2420 -26.2157
-26.2145
[15648]ENERGY: 9560000 3226.1846 5197.7691 5247.9380
311.4181 -15781.4286 -1815.2035 0.0000 0.0000
7663.0205 4049.6982 306.5591 -3613.3223 4110.5622
307.3274 -70.4617 -61.3823 656151.2420 -27.3199
-27.3063
[15698]ENERGY: 9570000 3110.8235 5106.3849 5266.7011
362.6489 -15818.2817 -1720.4572 0.0000 0.0000
7735.6872 4043.5066 309.4662 -3692.1806 4110.3223
307.4110 -15.2103 -39.7824 656151.2420 -21.6079
-21.6249
[15734]ENERGY: 9580000 3157.2811 5128.3325 5266.9878
329.2495 -15835.9550 -1744.3568 0.0000 0.0000
7746.8708 4048.4099 309.9136 -3698.4609 4110.2910
307.3471 -65.6831 -71.2022 656151.2420 -26.0067
-26.0029
[15784]ENERGY: 9590000 3242.3127 5209.5096 5238.5392
307.7129 -15901.8723 -1734.6210 0.0000 0.0000
7687.9589 4049.5400 307.5568 -3638.4189 4111.5117
307.3913 -150.3588 -111.8379 656151.2420 -21.6643
-21.6513
[15823]ENERGY: 9600000 3209.8060 5154.5573 5241.4604
351.5507 -15881.1183 -1754.6255 0.0000 0.0000
7723.0062 4044.6369 308.9589 -3678.3693 4110.5922
307.3730 26.7398 -27.5500 656151.2420 -26.8307
-26.8418
[15873]ENERGY: 9610000 3119.9500 5235.6348 5292.3526
348.2034 -15862.1939 -1771.0312 0.0000 0.0000
7688.4796 4051.3954 307.5776 -3637.0842 4112.1202
307.2836 182.7790 81.5953 656151.2420 -24.6758
-24.6716
[15909]ENERGY: 9620000 3141.3719 5265.9751 5271.1984
342.9549 -15841.7519 -1771.1891 0.0000 0.0000
7641.1515 4049.7109 305.6843 -3591.4406 4113.6317
307.2517 53.0867 14.0913 656151.2420 -27.7805
-27.8007
[15959]ENERGY: 9630000 3221.4503 5279.9487 5305.0883
351.8351 -15837.2958 -1822.9587 0.0000 0.0000
7554.6913 4052.7593 302.2254 -3501.9320 4113.2413
307.2466 78.0547 24.8681 656151.2420 -23.3563
-23.3564
[15995]ENERGY: 9640000 3211.1316 5220.5157 5241.6026
349.8406 -15850.4044 -1787.1908 0.0000 0.0000
7663.3837 4048.8789 306.5737 -3614.5047 4112.3871
307.4053 13.4815 -108.1070 656151.2420 -22.5739

-22.5558
[16045]ENERGY: 9650000 3168.2539 5125.9042 5240.9691
333.6616 -15795.2414 -1700.3891 0.0000 0.0000
7678.7404 4051.8987 307.1880 -3626.8417 4112.7135
307.2486 -6.1081 -50.4110 656151.2420 -25.2436
-25.2459
[16081]ENERGY: 9660000 3220.3844 5099.6014 5280.1764
323.6019 -15824.8575 -1683.1689 0.0000 0.0000
7632.8536 4048.5912 305.3523 -3584.2624 4111.6213
307.3610 128.7573 119.3101 656151.2420 -23.6025
-23.6195
[16131]ENERGY: 9670000 3171.6122 5156.6406 5299.8251
374.1284 -15865.1282 -1737.7158 0.0000 0.0000
7653.2190 4052.5812 306.1670 -3600.6377 4113.6633
307.3678 213.4981 77.7212 656151.2420 -19.8624
-19.8491
[16167]ENERGY: 9680000 3148.6530 5214.6394 5271.0446
350.2150 -15859.0835 -1781.1305 0.0000 0.0000
7705.8012 4050.1391 308.2706 -3655.6620 4113.8736
307.5834 -39.8881 -53.6146 656151.2420 -21.6687
-21.6799
[16217]ENERGY: 9690000 3116.3576 5183.9882 5303.5175
316.4640 -15717.6246 -1802.2080 0.0000 0.0000
7650.0628 4050.5574 306.0408 -3599.5054 4112.4813
307.4708 51.9334 73.1126 656151.2420 -27.1877
-27.1688
[16256]ENERGY: 9700000 3160.2525 5148.4840 5255.6184
341.0392 -15857.4476 -1703.0525 0.0000 0.0000
7709.4604 4054.3545 308.4170 -3655.1059 4112.5845
307.5172 108.1881 95.7421 656151.2420 -23.2128
-23.2134
[16306]ENERGY: 9710000 3204.2086 5297.6188 5228.5547
346.6077 -15835.6750 -1813.1306 0.0000 0.0000
7625.5282 4053.7124 305.0593 -3571.8158 4112.9931
307.5759 -35.5325 11.9830 656151.2420 -21.4801
-21.4866
[16342]ENERGY: 9720000 3167.1665 5130.8754 5227.8617
346.9156 -15813.2822 -1722.1446 0.0000 0.0000
7714.6194 4052.0118 308.6233 -3662.6076 4112.9918
307.4782 33.4425 30.1130 656151.2420 -29.4901
-29.4976
[16392]ENERGY: 9730000 3141.6179 5109.7803 5257.4701
354.8548 -15702.6379 -1822.3268 0.0000 0.0000
7712.8232 4051.5815 308.5515 -3661.2416 4114.0225
307.5859 189.5789 99.6371 656151.2420 -21.7265
-21.7213
[16428]ENERGY: 9740000 3186.3353 5120.7301 5246.4273
330.6326 -15816.9442 -1735.4329 0.0000 0.0000
7722.5668 4054.3150 308.9413 -3668.2518 4114.8438
307.6060 19.2943 -41.4818 656151.2420 -26.7934
-26.8013
[16478]ENERGY: 9750000 3160.2676 5258.7842 5294.6872
346.0694 -15859.6938 -1803.8721 0.0000 0.0000
7654.7380 4050.9807 306.2278 -3603.7573 4114.6682

307.5305 -27.9932 -36.1108 656151.2420 -25.5704
-25.5457
[16514]ENERGY: 9760000 3207.0235 5141.9381 5220.4902
342.0417 -15800.9120 -1803.7885 0.0000 0.0000
7742.6247 4049.4176 309.7437 -3693.2071 4113.7581
307.4415 -28.1348 -83.0945 656151.2420 -25.8697
-25.8915
[16564]ENERGY: 9770000 3220.0890 5236.8340 5253.5917
336.0148 -15868.4132 -1787.5670 0.0000 0.0000
7663.0941 4053.6433 306.5621 -3609.4507 4115.0231
307.4073 106.9431 9.5748 656151.2420 -24.7456
-24.7348
[16600]ENERGY: 9780000 3216.5327 5237.9965 5270.7915
330.1334 -15888.4407 -1779.3515 0.0000 0.0000
7664.4112 4052.0732 306.6148 -3612.3380 4114.1973
307.4019 -172.0667 -128.0887 656151.2420 -24.7709
-24.7560
[16650]ENERGY: 9790000 3157.5652 5113.0449 5209.8112
332.2880 -15753.4994 -1706.9119 0.0000 0.0000
7704.8240 4057.1221 308.2315 -3647.7019 4116.0938
307.3706 0.2243 -16.0119 656151.2420 -22.2508
-22.2532
[16689]ENERGY: 9800000 3231.2032 5226.2357 5230.2625
333.3148 -15894.9814 -1768.7117 0.0000 0.0000
7697.9441 4055.2673 307.9563 -3642.6769 4116.3954
307.4637 -62.4210 -34.1015 656151.2420 -22.2265
-22.2190
[16739]ENERGY: 9810000 3144.6281 5189.0960 5285.5401
348.1163 -15850.0747 -1787.4767 0.0000 0.0000
7722.4457 4052.2750 308.9364 -3670.1707 4116.5884
307.6450 8.3681 -30.9875 656151.2420 -28.5102
-28.5164
[16775]ENERGY: 9820000 3204.4141 5172.8677 5196.5049
322.6151 -15893.6015 -1706.8285 0.0000 0.0000
7754.4315 4050.4034 310.2160 -3704.0281 4116.9044
307.6043 82.3770 46.8862 656151.2420 -26.7329
-26.7509
[16825]ENERGY: 9830000 3196.3925 5188.0652 5240.6001
344.4646 -15880.9717 -1741.8410 0.0000 0.0000
7706.3004 4053.0103 308.2905 -3653.2901 4115.1979
307.6999 -2.3125 -50.9540 656151.2420 -27.2506
-27.2591
[16861]ENERGY: 9840000 3195.1424 5212.7412 5207.3262
377.9181 -15925.7736 -1663.9434 0.0000 0.0000
7649.8674 4053.2784 306.0329 -3596.5891 4115.9878
307.6871 177.9308 117.8959 656151.2420 -23.7019
-23.6846
[16911]ENERGY: 9850000 3078.0942 5261.9323 5246.7659
330.9417 -15890.1630 -1734.5185 0.0000 0.0000
7761.3622 4054.4148 310.4933 -3706.9474 4117.1670
307.6308 131.4057 14.4857 656151.2420 -24.3741
-24.3846
[16947]ENERGY: 9860000 3154.3084 5271.7419 5273.2130
326.4961 -15870.1802 -1710.0822 0.0000 0.0000

7610.9212 4056.4181 304.4749 -3554.5031 4117.4616
307.6229 -47.6868 30.8313 656151.2420 -23.2459
-23.2138
[16997]ENERGY: 9870000 3100.4245 5172.1216 5224.2912
345.0746 -15772.1367 -1765.2066 0.0000 0.0000
7751.0035 4055.5719 310.0789 -3695.4315 4118.1424
307.5832 75.0779 38.7180 656151.2420 -22.2727
-22.2939
[17033]ENERGY: 9880000 3199.3332 5139.4821 5267.5845
350.0614 -15804.3155 -1797.6482 0.0000 0.0000
7701.6924 4056.1900 308.1062 -3645.5024 4118.5804
307.4578 -49.6718 -102.0145 656151.2420 -26.3612
-26.3727
[17083]ENERGY: 9890000 3188.0215 5176.6425 5258.3861
320.7156 -15760.9828 -1839.4639 0.0000 0.0000
7712.2642 4055.5831 308.5291 -3656.6810 4117.6580
307.5881 -157.4314 -182.5016 656151.2420 -21.5772
-21.5836
[17122]ENERGY: 9900000 3276.7388 5108.8964 5266.0922
326.8157 -15824.9571 -1806.3913 0.0000 0.0000
7707.8967 4055.0915 308.3544 -3652.8052 4117.8007
307.7030 -191.3165 -132.0599 656151.2420 -21.9309
-21.9090
[17172]ENERGY: 9910000 3149.8317 5145.2749 5232.3633
335.3443 -15740.8178 -1818.3120 0.0000 0.0000
7753.3084 4056.9928 310.1711 -3696.3156 4119.0953
307.7012 71.7270 6.4059 656151.2420 -25.8193
-25.8257
[17208]ENERGY: 9920000 3152.4032 5233.9747 5262.4371
328.9889 -15925.5787 -1690.1893 0.0000 0.0000
7693.7171 4055.7530 307.7871 -3637.9641 4117.8713
307.6467 13.7536 0.5949 656151.2420 -19.4040
-19.4040
[17258]ENERGY: 9930000 3140.5184 5130.3374 5223.3311
312.3341 -15793.8915 -1748.4203 0.0000 0.0000
7790.3555 4054.5647 311.6532 -3735.7908 4118.8875
307.5252 99.2271 -21.9730 656151.2420 -23.6523
-23.6548
[17294]ENERGY: 9940000 3195.4435 5143.4917 5240.9601
339.1508 -15867.9290 -1678.6737 0.0000 0.0000
7683.1064 4055.5499 307.3627 -3627.5565 4116.6638
307.5530 -62.4951 -78.5949 656151.2420 -20.3589
-20.3519
[17344]ENERGY: 9950000 3223.8142 5229.1517 5240.9718
325.9097 -15922.3096 -1761.8390 0.0000 0.0000
7716.9862 4052.6850 308.7180 -3664.3012 4118.2114
307.6622 -134.2431 -83.3709 656151.2420 -25.7360
-25.7348
[17380]ENERGY: 9960000 3113.2950 5133.5316 5240.7337
350.8057 -15835.8403 -1766.8535 0.0000 0.0000
7819.0500 4054.7223 312.8011 -3764.3277 4119.4689
307.6409 22.0774 -18.3296 656151.2420 -27.6671
-27.6699
[17430]ENERGY: 9970000 3210.3764 5175.0767 5258.2912

316.4888 -15881.0982 -1715.3994 0.0000 0.0000
7694.8233 4058.5588 307.8314 -3636.2645 4120.2254
307.6475 -45.5372 -4.3619 656151.2420 -23.0556
-23.0664
[17466]ENERGY: 9980000 3231.8586 5213.9711 5269.7176
342.1361 -15883.1362 -1770.3788 0.0000 0.0000
7655.4996 4059.6680 306.2583 -3595.8317 4119.2848
307.6670 36.8323 -15.3476 656151.2420 -26.8453
-26.8246
[17516]ENERGY: 9990000 3224.0030 5231.9255 5295.8911
336.4382 -15896.7335 -1751.5379 0.0000 0.0000
7617.5689 4057.5554 304.7408 -3560.0135 4120.0003
307.7555 112.3914 108.2396 656151.2420 -25.7405
-25.7616
[17555]ENERGY: 10000000 3191.8929 5276.4509 5254.8585
354.9996 -15860.7184 -1742.8913 0.0000 0.0000
7586.2080 4060.8003 303.4862 -3525.4077 4119.9890
307.8785 -18.6017 -66.4992 656151.2420 -22.9161
-22.9060
[17605]ENERGY: 10010000 3202.5763 5167.6654 5306.5194
340.7576 -15873.1110 -1803.4756 0.0000 0.0000
7716.0426 4056.9747 308.6803 -3659.0678 4121.0879
307.8486 -39.6789 -16.3668 656151.2420 -25.8976
-25.9039
[17641]ENERGY: 10020000 3210.2396 5307.8570 5246.3895
352.9647 -15909.0778 -1810.2334 0.0000 0.0000
7660.6105 4058.7501 306.4627 -3601.8603 4121.8488
307.7864 -52.8575 -72.3278 656151.2420 -17.0779
-17.0894
[17691]ENERGY: 10030000 3189.4475 5251.9175 5269.0292
333.8925 -15863.1572 -1770.8805 0.0000 0.0000
7645.6325 4055.8816 305.8635 -3589.7509 4121.7539
307.7613 -98.2068 -43.7708 656151.2420 -25.8537
-25.8333
[17727]ENERGY: 10040000 3130.6883 5240.3082 5272.1235
350.0308 -15801.0081 -1813.3676 0.0000 0.0000
7684.3047 4063.0798 307.4106 -3621.2249 4121.9184
307.7524 72.1766 -84.8618 656151.2420 -23.7128
-23.7295
[17777]ENERGY: 10050000 3140.1325 5122.1426 5282.3847
363.9907 -15800.0738 -1763.7226 0.0000 0.0000
7712.0760 4056.9301 308.5216 -3655.1459 4121.5316
307.7905 54.6986 20.2201 656151.2420 -24.3483
-24.3322
[17813]ENERGY: 10060000 3214.1060 5166.7295 5226.0235
329.3666 -15815.0837 -1738.5984 0.0000 0.0000
7676.4393 4058.9829 307.0960 -3617.4564 4121.5970
307.6137 -155.5575 -72.5375 656151.2420 -24.7071
-24.7124
[17863]ENERGY: 10070000 3147.2383 5175.6565 5252.3692
332.3472 -15840.8234 -1693.3124 0.0000 0.0000
7687.3470 4060.8225 307.5323 -3626.5246 4122.5028
307.5038 -10.6692 4.2499 656151.2420 -25.2911
-25.2903

[17899]ENERGY: 10080000 3158.1851 5147.7383 5256.5813
352.2093 -15769.4613 -1731.0376 0.0000 0.0000
7646.2346 4060.4496 305.8876 -3585.7850 4122.1211
307.4243 -5.2449 -27.0369 656151.2420 -24.9434
-24.9513
[17949]ENERGY: 10090000 3142.7959 5235.1005 5250.0095
341.1718 -15847.5500 -1747.6674 0.0000 0.0000
7686.6922 4060.5524 307.5061 -3626.1398 4122.3920
307.5085 -68.7637 0.7698 656151.2420 -22.3282
-22.3212
[17988]ENERGY: 10100000 3213.8523 5245.1750 5270.1351
334.7973 -15817.2297 -1830.3765 0.0000 0.0000
7644.5028 4060.8562 305.8183 -3583.6466 4122.3964
307.6630 -85.9796 -189.5952 656151.2420 -26.6311
-26.6405
[18038]ENERGY: 10110000 3153.9377 5234.8861 5207.9762
331.2776 -15788.9704 -1794.9798 0.0000 0.0000
7712.0772 4056.2047 308.5216 -3655.8726 4121.5489
307.8158 -219.0328 -206.6374 656151.2420 -21.5969
-21.5794
[18074]ENERGY: 10120000 3181.3302 5235.6674 5241.3578
331.2491 -15873.6929 -1772.9297 0.0000 0.0000
7718.5668 4061.5488 308.7813 -3657.0180 4122.7971
307.8062 9.6976 9.0641 656151.2420 -23.9014
-23.9053
[18124]ENERGY: 10130000 3261.3872 5212.1885 5263.8463
326.7351 -15852.3650 -1812.0978 0.0000 0.0000
7661.8309 4061.5252 306.5115 -3600.3057 4123.7973
307.8170 123.7105 -22.3745 656151.2420 -26.5238
-26.5330
[18160]ENERGY: 10140000 3178.5935 5258.2856 5216.3539
334.8202 -15881.9933 -1815.8050 0.0000 0.0000
7768.2049 4058.4598 310.7670 -3709.7451 4122.8373
307.9047 -39.4013 -79.1354 656151.2420 -23.4832
-23.4839
[18210]ENERGY: 10150000 3222.2922 5110.8560 5231.4071
331.0320 -15771.3716 -1771.9738 0.0000 0.0000
7706.9674 4059.2094 308.3172 -3647.7581 4122.1359
307.9660 95.4625 60.7004 656151.2420 -22.9328
-22.9425
[18246]ENERGY: 10160000 3278.8356 5085.2238 5220.6662
340.2750 -15795.0871 -1758.1920 0.0000 0.0000
7688.0551 4059.7767 307.5606 -3628.2784 4122.0751
307.7997 -172.6503 -126.2570 656151.2420 -22.3650
-22.3590
[18296]ENERGY: 10170000 3159.1903 5235.9105 5301.6549
371.1563 -15800.8665 -1823.5727 0.0000 0.0000
7616.7268 4060.1996 304.7072 -3556.5272 4122.6086
307.7577 -33.0513 -63.5705 656151.2420 -25.7879
-25.7876
[18332]ENERGY: 10180000 3252.1945 5249.4319 5247.2937
333.5080 -15870.5895 -1797.4788 0.0000 0.0000
7647.6030 4061.9627 305.9424 -3585.6403 4123.2350
307.8968 82.5591 -15.3561 656151.2420 -22.0284

-22.0315
[18382]ENERGY: 10190000 3171.3571 5138.2726 5266.8518
339.4701 -15799.2477 -1758.7718 0.0000 0.0000
7700.2316 4058.1637 308.0478 -3642.0679 4122.2526
307.8612 78.8675 43.9713 656151.2420 -24.1608
-24.1508
[18421]ENERGY: 10200000 3250.3769 5122.8222 5284.0787
318.5914 -15905.5244 -1768.7073 0.0000 0.0000
7762.4580 4064.0956 310.5371 -3698.3625 4122.0973
307.9346 -1.4191 -36.6672 656151.2420 -25.7507
-25.7443
[18471]ENERGY: 10210000 3235.0544 5195.9782 5246.5719
332.4744 -15848.6500 -1765.5911 0.0000 0.0000
7665.7681 4061.6060 306.6691 -3604.1622 4122.2035
307.8383 1.7175 37.9337 656151.2420 -24.1741
-24.1712
[18507]ENERGY: 10220000 3286.7176 5193.1824 5275.2610
361.5878 -15900.6352 -1781.6294 0.0000 0.0000
7630.0277 4064.5119 305.2393 -3565.5158 4122.5013
307.6223 -105.7679 -176.6430 656151.2420 -25.1533
-25.1825
[18557]ENERGY: 10230000 3216.6587 5184.2336 5284.8936
331.0518 -15875.2706 -1788.2218 0.0000 0.0000
7703.7128 4057.0579 308.1870 -3646.6548 4121.9563
307.6318 -83.1649 -46.3800 656151.2420 -25.6745
-25.6476
[18593]ENERGY: 10240000 3238.3715 5296.0658 5320.7734
337.1615 -15860.7323 -1794.9831 0.0000 0.0000
7527.0740 4063.7308 301.1206 -3463.3432 4122.2286
307.8796 -61.8493 -49.0135 656151.2420 -24.9511
-24.9647
[18643]ENERGY: 10250000 3156.8823 5263.1322 5250.8429
331.7856 -15860.8127 -1719.2397 0.0000 0.0000
7641.2922 4063.8828 305.6899 -3577.4093 4123.8684
307.7293 130.3588 47.8599 656151.2420 -25.7456
-25.7475
[18679]ENERGY: 10260000 3221.8811 5102.5484 5264.4243
346.2357 -15853.7861 -1699.7490 0.0000 0.0000
7679.0661 4060.6205 307.2010 -3618.4456 4123.3061
307.8067 56.5282 -17.1070 656151.2420 -23.1659
-23.1619
[18729]ENERGY: 10270000 3220.2277 5191.7253 5240.3542
356.1997 -15892.1922 -1788.7156 0.0000 0.0000
7733.4195 4061.0184 309.3754 -3672.4011 4122.9777
307.8575 69.7396 -8.3586 656151.2420 -25.5250
-25.5286
[18765]ENERGY: 10280000 3136.4877 5231.3462 5270.5623
339.1778 -15805.5384 -1770.9517 0.0000 0.0000
7660.1467 4061.2306 306.4442 -3598.9161 4125.0242
307.7011 148.3817 26.8554 656151.2420 -24.5423
-24.5322
[18815]ENERGY: 10290000 3184.4277 5179.0193 5289.5518
352.9945 -15862.0614 -1767.3794 0.0000 0.0000
7684.0810 4060.6335 307.4017 -3623.4475 4123.3933

307.5826 46.8654 -52.9126 656151.2420 -23.8567
-23.8614
[18854]ENERGY: 10300000 3291.7510 5167.5361 5305.7392
354.6112 -15931.2767 -1780.9055 0.0000 0.0000
7656.0518 4063.5072 306.2803 -3592.5446 4121.8830
307.8208 -103.9773 -39.0806 656151.2420 -25.6848
-25.6707
[18904]ENERGY: 10310000 3237.9019 5129.6497 5251.0154
358.1071 -15809.6861 -1796.1374 0.0000 0.0000
7687.2310 4058.0815 307.5277 -3629.1495 4122.7235
307.9972 -25.5361 -41.5220 656151.2420 -20.9717
-20.9704
[18940]ENERGY: 10320000 3140.5091 5275.4130 5243.7699
345.7247 -15885.6916 -1746.7999 0.0000 0.0000
7686.7277 4059.6528 307.5075 -3627.0749 4123.0035
307.8214 62.4289 25.2730 656151.2420 -20.0233
-20.0277
[18990]ENERGY: 10330000 3264.6834 5229.0354 5264.7384
333.3258 -15857.2160 -1761.1606 0.0000 0.0000
7586.9200 4060.3261 303.5147 -3526.5938 4123.8744
307.7016 107.7800 58.8910 656151.2420 -18.4133
-18.4063
[19026]ENERGY: 10340000 3171.8370 5181.0435 5264.2263
325.7107 -15815.2593 -1745.9677 0.0000 0.0000
7684.1429 4065.7335 307.4041 -3618.4095 4123.0740
307.8070 19.9456 -48.8470 656151.2420 -22.1983
-22.2076
[19076]ENERGY: 10350000 3176.2456 5226.8464 5248.4727
342.1678 -15793.1208 -1771.8617 0.0000 0.0000
7638.1339 4066.8840 305.5635 -3571.2499 4125.3551
307.8677 -24.6811 -84.1511 656151.2420 -21.8172
-21.8326
[19112]ENERGY: 10360000 3253.4191 5209.8487 5222.7410
326.2965 -15730.6003 -1844.1354 0.0000 0.0000
7627.7309 4065.3005 305.1474 -3562.4304 4124.5677
307.6969 -154.3119 -124.4305 656151.2420 -23.3344
-23.3025
[19162]ENERGY: 10370000 3171.2125 5223.2944 5262.6706
357.5336 -15858.6322 -1739.7103 0.0000 0.0000
7645.2510 4061.6195 305.8483 -3583.6314 4124.1457
307.6787 17.4179 -20.8846 656151.2420 -25.3446
-25.3531
[19198]ENERGY: 10380000 3207.4448 5173.9444 5238.5281
326.2599 -15866.8601 -1699.6557 0.0000 0.0000
7683.6619 4063.3234 307.3849 -3620.3385 4124.3072
307.7848 8.6087 11.4899 656151.2420 -23.0032
-23.0260
[19248]ENERGY: 10390000 3176.5162 5236.4382 5266.9054
334.9592 -15906.2010 -1765.0159 0.0000 0.0000
7719.1437 4062.7460 308.8043 -3656.3978 4124.5660
307.9477 -129.0155 -158.6245 656151.2420 -18.0646
-18.0618
[19287]ENERGY: 10400000 3154.6457 5213.3037 5231.7782
347.9662 -15776.0650 -1766.1476 0.0000 0.0000

7657.6177 4063.0989 306.3430 -3594.5188 4125.5993
307.9158 153.1213 76.8024 656151.2420 -26.7706
-26.7660
[19337]ENERGY: 10410000 3151.4864 5174.9438 5280.0908
345.3004 -15828.8529 -1798.2952 0.0000 0.0000
7742.8789 4067.5523 309.7539 -3675.3266 4126.8371
307.7427 -49.0101 26.4417 656151.2420 -25.6997
-25.6968
[19373]ENERGY: 10420000 3210.4361 5099.0105 5246.0235
348.7116 -15841.3195 -1728.3614 0.0000 0.0000
7729.4832 4063.9840 309.2180 -3665.4992 4125.5127
307.8371 -53.6776 30.3566 656151.2420 -21.3467
-21.3525
[19423]ENERGY: 10430000 3181.9599 5097.2280 5314.7884
315.1867 -15819.2032 -1774.9149 0.0000 0.0000
7750.9059 4065.9507 310.0750 -3684.9552 4126.4723
307.8736 -75.5173 -23.6268 656151.2420 -21.6605
-21.6562
[19459]ENERGY: 10440000 3218.2165 5107.6383 5275.0206
351.7309 -15749.7731 -1828.9323 0.0000 0.0000
7689.5968 4063.4978 307.6223 -3626.0991 4127.2864
307.8143 -98.5629 -123.0930 656151.2420 -22.7605
-22.7655
[19509]ENERGY: 10450000 3151.7720 5165.8990 5259.2843
325.2786 -15880.7317 -1689.4738 0.0000 0.0000
7734.0779 4066.1063 309.4018 -3667.9716 4127.0807
307.6128 131.8385 90.4671 656151.2420 -24.6064
-24.5833
[19545]ENERGY: 10460000 3173.9149 5167.5785 5272.3748
344.8385 -15791.2169 -1767.5050 0.0000 0.0000
7666.2383 4066.2231 306.6879 -3600.0152 4126.0533
307.6650 38.3868 42.0053 656151.2420 -24.7416
-24.7648
[19595]ENERGY: 10470000 3087.2347 5188.4171 5307.2993
354.5143 -15830.6573 -1775.5698 0.0000 0.0000
7732.6980 4063.9364 309.3466 -3668.7616 4126.5710
307.4773 -22.5584 23.2966 656151.2420 -21.4153
-21.4157
[19631]ENERGY: 10480000 3202.0616 5133.4715 5250.7710
349.3796 -15861.7306 -1724.0245 0.0000 0.0000
7714.7918 4064.7203 308.6302 -3650.0715 4126.6446
307.6249 120.2244 145.4216 656151.2420 -23.2760
-23.2665
[19681]ENERGY: 10490000 3181.2841 5176.7546 5294.6621
344.1289 -15928.9856 -1681.0916 0.0000 0.0000
7678.5680 4065.3205 307.1811 -3613.2475 4126.6742
307.7450 26.0870 12.8486 656151.2420 -26.7688
-26.7614
[19720]ENERGY: 10500000 3149.7109 5161.8840 5295.1630
331.8375 -15743.4675 -1779.6105 0.0000 0.0000
7650.7015 4066.2190 306.0663 -3584.4825 4127.4832
307.7969 -134.4189 -122.2403 656151.2420 -18.6078
-18.6112
[19770]ENERGY: 10510000 3220.6034 5161.9629 5293.8671

337.2980 -15801.9347 -1834.8717 0.0000 0.0000
7688.7619 4065.6870 307.5889 -3623.0749 4126.2590
307.6951 -144.6896 -128.3573 656151.2420 -25.0176
-25.0233
[19806]ENERGY: 10520000 3155.0105 5203.4737 5263.2513
346.1566 -15867.7301 -1732.5473 0.0000 0.0000
7696.8006 4064.4153 307.9105 -3632.3853 4125.6702
307.5602 107.4074 55.1159 656151.2420 -25.0971
-25.0984
[19856]ENERGY: 10530000 3198.4446 5136.2003 5279.1692
347.3371 -15772.0620 -1796.2562 0.0000 0.0000
7671.5792 4064.4124 306.9015 -3607.1668 4126.9878
307.5956 -170.6580 -103.6004 656151.2420 -24.5453
-24.5275
[19892]ENERGY: 10540000 3201.9478 5207.5871 5268.5761
355.5242 -15794.4861 -1772.9115 0.0000 0.0000
7603.3876 4069.6252 304.1735 -3533.7624 4127.4477
307.8679 82.8278 67.0686 656151.2420 -26.3102
-26.3188
[19942]ENERGY: 10550000 3223.2711 5145.5153 5256.4552
334.1979 -15835.5894 -1741.4858 0.0000 0.0000
7680.2319 4062.5960 307.2477 -3617.6359 4125.6045
307.5879 -79.4550 -46.3923 656151.2420 -22.7896
-22.7979
[19978]ENERGY: 10560000 3177.4554 5174.3782 5248.0097
327.0419 -15784.1261 -1745.6042 0.0000 0.0000
7668.7777 4065.9327 306.7895 -3602.8451 4126.9881
307.6170 -109.8685 -35.1442 656151.2420 -25.9895
-25.9926
[20028]ENERGY: 10570000 3108.0241 5140.0005 5283.9856
368.5603 -15760.1972 -1811.7934 0.0000 0.0000
7736.2135 4064.7934 309.4872 -3671.4201 4128.2670
307.6266 -14.5429 -90.4253 656151.2420 -21.8682
-21.8619
[20064]ENERGY: 10580000 3221.4950 5204.3757 5283.1538
344.1876 -15889.5649 -1740.3949 0.0000 0.0000
7642.5867 4065.8388 305.7417 -3576.7479 4126.1393
307.6958 -259.6714 -198.5235 656151.2420 -23.3494
-23.3596
[20114]ENERGY: 10590000 3081.1502 5254.4318 5246.6622
346.2282 -15914.2159 -1654.3202 0.0000 0.0000
7706.3296 4066.2659 308.2917 -3640.0638 4127.4523
307.4508 -0.4022 57.2332 656151.2420 -24.7756
-24.7619
[20153]ENERGY: 10600000 3214.7900 5202.9714 5257.0743
340.7408 -15881.3503 -1799.0260 0.0000 0.0000
7731.5527 4066.7529 309.3008 -3664.7998 4127.8910
307.5403 -78.3677 -98.1773 656151.2420 -20.2606
-20.2597
[20203]ENERGY: 10610000 3150.7387 5084.6961 5243.9764
331.6774 -15673.2763 -1810.9035 0.0000 0.0000
7737.7756 4064.6844 309.5497 -3673.0912 4126.9213
307.4553 16.9135 10.1697 656151.2420 -26.1162
-26.1267

[20239]ENERGY: 10620000 3219.9922 5120.6876 5276.9906
359.4474 -15859.1811 -1712.5684 0.0000 0.0000
7659.0718 4064.4401 306.4012 -3594.6317 4126.6289
307.4115 14.3995 1.1122 656151.2420 -19.3758
-19.3667
[20289]ENERGY: 10630000 3180.3500 5139.9792 5300.2175
369.9172 -15778.3391 -1788.8382 0.0000 0.0000
7640.8424 4064.1290 305.6719 -3576.7134 4127.0997
307.3602 -34.8440 -10.3262 656151.2420 -27.4257
-27.4409
[20325]ENERGY: 10640000 3102.8697 5112.7947 5328.7670
356.1193 -15837.3775 -1770.3684 0.0000 0.0000
7772.1182 4064.9231 310.9236 -3707.1951 4127.3835
307.3373 28.1735 -3.2433 656151.2420 -19.7480
-19.7442
[20375]ENERGY: 10650000 3179.2129 5198.0184 5238.0472
363.4413 -15872.1768 -1717.1274 0.0000 0.0000
7675.6448 4065.0603 307.0642 -3610.5845 4126.4225
307.2611 -24.6523 -31.1741 656151.2420 -23.4710
-23.4602
[20411]ENERGY: 10660000 3136.0595 5235.0891 5256.3467
345.2897 -15720.9044 -1811.5284 0.0000 0.0000
7626.6085 4066.9608 305.1025 -3559.6478 4128.1198
307.4815 -40.1502 -55.6263 656151.2420 -23.5679
-23.5734
[20461]ENERGY: 10670000 3158.8238 5162.5579 5244.2443
350.7784 -15793.9515 -1758.6159 0.0000 0.0000
7700.7988 4064.6359 308.0705 -3636.1630 4128.5378
307.4600 101.8444 -29.8903 656151.2420 -22.4684
-22.4557
[20497]ENERGY: 10680000 3147.2542 5265.6742 5276.7244
339.4754 -15822.2442 -1813.5739 0.0000 0.0000
7672.6857 4065.9958 306.9458 -3606.6900 4128.0919
307.5027 73.4658 9.4136 656151.2420 -25.3037
-25.3141
[20547]ENERGY: 10690000 3217.2275 5124.9021 5291.5037
362.2890 -15899.4123 -1720.7698 0.0000 0.0000
7690.5033 4066.2435 307.6586 -3624.2598 4128.6607
307.3674 86.6365 -1.0069 656151.2420 -22.9882
-22.9926
[20586]ENERGY: 10700000 3168.2666 5256.0715 5301.9430
344.6821 -15952.7978 -1743.4956 0.0000 0.0000
7690.7546 4065.4245 307.6686 -3625.3301 4128.2978
307.4623 62.9939 -12.4348 656151.2420 -24.8880
-24.9045
[20636]ENERGY: 10710000 3189.4474 5163.2212 5305.5402
320.2294 -15842.3977 -1747.7802 0.0000 0.0000
7678.5284 4066.7887 307.1795 -3611.7398 4127.5402
307.4788 -40.9699 -47.5228 656151.2420 -22.8665
-22.8377
[20672]ENERGY: 10720000 3227.5335 5200.0648 5296.2930
334.7262 -15788.3317 -1866.2154 0.0000 0.0000
7662.3428 4066.4132 306.5320 -3595.9297 4127.2127
307.4449 -24.7428 -127.5010 656151.2420 -24.2862

-24.2905
[20722]ENERGY: 10730000 3247.1988 5165.0580 5216.0796
336.2441 -15855.5207 -1715.7208 0.0000 0.0000
7675.7828 4069.1217 307.0697 -3606.6611 4128.1516
307.3918 -8.4573 17.7859 656151.2420 -22.9414
-22.9436
[20758]ENERGY: 10740000 3225.6156 5168.4074 5275.1016
359.0034 -15807.6201 -1790.6659 0.0000 0.0000
7639.3493 4069.1913 305.6122 -3570.1580 4128.8929
307.6503 -24.3355 -37.4953 656151.2420 -26.4053
-26.4170
[20808]ENERGY: 10750000 3161.9341 5147.5206 5268.1802
320.3325 -15737.9612 -1791.4142 0.0000 0.0000
7700.0385 4068.6305 308.0400 -3631.4079 4128.6585
307.7491 -42.9842 -59.2547 656151.2420 -27.4051
-27.3986
[20844]ENERGY: 10760000 3187.1459 5234.6700 5208.0701
360.8515 -15815.0196 -1785.0646 0.0000 0.0000
7673.0526 4063.7058 306.9605 -3609.3468 4127.0851
307.7003 47.4585 27.1744 656151.2420 -22.0431
-22.0497
[20894]ENERGY: 10770000 3174.4428 5144.1748 5236.1464
349.2481 -15807.5729 -1808.6357 0.0000 0.0000
7776.7856 4064.5890 311.1103 -3712.1965 4126.6559
307.5219 45.9031 -25.9220 656151.2420 -26.0370
-26.0378
[20930]ENERGY: 10780000 3164.7309 5180.0045 5294.0043
341.2955 -15822.0053 -1733.8820 0.0000 0.0000
7641.7446 4065.8925 305.7080 -3575.8521 4127.1140
307.5731 49.8343 13.6988 656151.2420 -28.4161
-28.4039
[20980]ENERGY: 10790000 3257.2449 5241.1299 5247.6931
321.5932 -15841.1205 -1794.2606 0.0000 0.0000
7635.6790 4067.9591 305.4653 -3567.7199 4129.3078
307.5968 147.0855 94.9232 656151.2420 -22.9136
-22.9204
[21019]ENERGY: 10800000 3127.3262 5180.9705 5292.2707
335.8622 -15790.7778 -1802.1235 0.0000 0.0000
7720.5885 4064.1168 308.8621 -3656.4717 4128.2459
307.7743 -98.2921 -90.3319 656151.2420 -28.4353
-28.4088
[21069]ENERGY: 10810000 3188.3260 5155.0477 5298.9941
336.8085 -15809.7215 -1707.8368 0.0000 0.0000
7606.6731 4068.2911 304.3050 -3538.3820 4129.4817
307.6954 105.1750 100.4540 656151.2420 -19.4397
-19.4634
[21105]ENERGY: 10820000 3129.3868 5230.1403 5302.8978
344.5421 -15861.1153 -1734.8222 0.0000 0.0000
7653.5939 4064.6235 306.1820 -3588.9704 4127.6716
307.7954 -54.5686 63.6642 656151.2420 -23.8001
-23.7947
[21155]ENERGY: 10830000 3232.2363 5281.9081 5258.8240
334.0851 -15896.6726 -1739.7602 0.0000 0.0000
7598.3870 4069.0077 303.9735 -3529.3793 4129.6287

307.5928 -89.2549 -67.0940 656151.2420 -22.3589
-22.3500
[21191]ENERGY: 10840000 3169.4076 5132.6030 5290.0464
321.5373 -15810.5368 -1715.0702 0.0000 0.0000
7678.8098 4066.7971 307.1908 -3612.0127 4129.5250
307.6242 30.1376 51.9993 656151.2420 -23.1759
-23.1935
[21241]ENERGY: 10850000 3150.7897 5150.9006 5268.8704
355.9325 -15767.3584 -1834.5318 0.0000 0.0000
7742.9520 4067.5550 309.7568 -3675.3970 4130.0000
307.5749 38.1511 -42.7914 656151.2420 -27.3819
-27.3930
[21277]ENERGY: 10860000 3264.4389 5172.1166 5269.2264
323.4496 -15932.4469 -1764.2123 0.0000 0.0000
7734.9778 4067.5502 309.4378 -3667.4276 4129.2278
307.6960 -31.6209 55.6852 656151.2420 -23.7750
-23.7478
[21327]ENERGY: 10870000 3308.6574 5153.2306 5303.0664
339.2895 -15932.6179 -1777.8913 0.0000 0.0000
7674.0700 4067.8047 307.0012 -3606.2652 4129.6675
307.7183 -9.4260 0.5097 656151.2420 -26.2151
-26.2123
[21363]ENERGY: 10880000 3232.0912 5201.3870 5248.6649
345.0622 -15847.0921 -1777.0778 0.0000 0.0000
7668.1320 4071.1673 306.7636 -3596.9646 4130.4416
307.8236 -36.9265 -53.7617 656151.2420 -26.9665
-26.9940
[21413]ENERGY: 10890000 3221.3445 5123.8634 5239.1554
345.0171 -15895.5045 -1728.2255 0.0000 0.0000
7760.6327 4066.2830 310.4641 -3694.3497 4129.7932
307.8677 88.2335 81.3319 656151.2420 -28.2992
-28.2888
[21452]ENERGY: 10900000 3234.6083 5238.2929 5260.8430
331.6604 -15848.0886 -1794.6075 0.0000 0.0000
7648.7530 4071.4615 305.9884 -3577.2915 4129.7968
307.8005 28.7798 -47.9031 656151.2420 -24.8106
-24.8142
[21502]ENERGY: 10910000 3265.1857 5200.2824 5286.5719
355.2315 -15800.9778 -1845.3805 0.0000 0.0000
7610.5820 4071.4952 304.4613 -3539.0868 4130.9729
307.7088 148.3516 -4.3389 656151.2420 -28.8458
-28.8576
[21538]ENERGY: 10920000 3171.7343 5175.7705 5323.9117
328.1079 -15793.8269 -1846.9024 0.0000 0.0000
7709.6815 4068.4766 308.4258 -3641.2049 4131.1363
307.6207 -9.5912 -32.4367 656151.2420 -23.4921
-23.4817
[21588]ENERGY: 10930000 3215.5749 5298.5402 5296.2238
342.5503 -15910.2218 -1813.9572 0.0000 0.0000
7642.2600 4070.9701 305.7286 -3571.2899 4130.8252
307.6733 -4.2589 -78.3474 656151.2420 -26.4524
-26.4439
[21624]ENERGY: 10940000 3209.6182 5166.4348 5316.8437
323.4727 -15866.9158 -1717.8423 0.0000 0.0000

7639.3749 4070.9862 305.6132 -3568.3887 4130.8754
307.7228 111.5525 -27.2116 656151.2420 -24.8760
-24.8812
[21674]ENERGY: 10950000 3310.8079 5242.6969 5273.9858
347.1047 -15924.0371 -1732.8290 0.0000 0.0000
7554.0101 4071.7393 302.1982 -3482.2708 4132.2692
307.7736 -10.6592 -68.6816 656151.2420 -24.1979
-24.1924
[21710]ENERGY: 10960000 3198.5226 5183.2267 5311.6383
354.0436 -15922.7607 -1718.6307 0.0000 0.0000
7664.4896 4070.5294 306.6179 -3593.9602 4131.9352
307.8202 51.6314 71.2426 656151.2420 -23.4685
-23.4755
[21760]ENERGY: 10970000 3159.3043 5211.3744 5302.5293
322.1139 -15896.0243 -1718.9761 0.0000 0.0000
7690.5329 4070.8542 307.6598 -3619.6787 4132.8678
307.6604 45.6071 0.0854 656151.2420 -24.7918
-24.7946
[21796]ENERGY: 10980000 3169.1733 5257.4095 5263.5238
354.6016 -15955.9359 -1692.4531 0.0000 0.0000
7671.5568 4067.8760 306.9006 -3603.6808 4133.2201
307.5875 46.3435 32.4858 656151.2420 -18.2445
-18.2381
[21846]ENERGY: 10990000 3238.4481 5126.7631 5226.5588
344.3792 -15886.8806 -1740.8183 0.0000 0.0000
7763.5333 4071.9837 310.5801 -3691.5496 4133.7071
307.5568 -175.2283 -214.6176 656151.2420 -28.1422
-28.1420
[21885]ENERGY: 11000000 3262.1916 5146.3962 5275.5321
359.5396 -15871.7490 -1794.9994 0.0000 0.0000
7696.8343 4073.7454 307.9119 -3623.0889 4133.5861
307.4421 -48.8558 -23.9664 656151.2420 -21.2862
-21.2842
[21935]ENERGY: 11010000 3211.0543 5204.6539 5242.3544
334.3357 -15823.4336 -1786.4451 0.0000 0.0000
7692.9415 4075.4611 307.7561 -3617.4804 4134.6457
307.8484 59.5481 3.1199 656151.2420 -23.9091
-23.9055
[21971]ENERGY: 11020000 3226.8785 5235.7671 5274.9180
345.3293 -15800.7370 -1803.7294 0.0000 0.0000
7596.7739 4075.2005 303.9089 -3521.5734 4134.1742
307.6296 -87.6964 -84.8746 656151.2420 -21.9212
-21.9197
[22021]ENERGY: 11030000 3185.7511 5139.8043 5274.9393
336.4499 -15819.5668 -1751.5175 0.0000 0.0000
7707.1746 4073.0348 308.3255 -3634.1398 4132.4367
307.6775 67.9464 118.9223 656151.2420 -25.2880
-25.2857
[22057]ENERGY: 11040000 3286.5332 5197.8957 5264.8033
339.3792 -15803.9056 -1820.5740 0.0000 0.0000
7611.0842 4075.2161 304.4814 -3535.8681 4133.9785
307.8511 -111.9577 -76.0003 656151.2420 -22.8963
-22.8972
[22107]ENERGY: 11050000 3189.9836 5221.5718 5322.2496

329.2988 -15956.3227 -1777.8366 0.0000 0.0000
7736.5500 4065.4945 309.5007 -3671.0555 4132.7285
307.8563 -80.9997 -69.9801 656151.2420 -27.0602
-27.0647
[22143]ENERGY: 11060000 3172.1672 5135.1695 5299.6027
322.4682 -15776.5012 -1777.7524 0.0000 0.0000
7702.0852 4077.2391 308.1219 -3624.8461 4133.6552
307.9836 202.3258 169.4202 656151.2420 -23.9225
-23.9314
[22193]ENERGY: 11070000 3195.9244 5135.3282 5273.2179
345.5688 -15802.3838 -1743.1867 0.0000 0.0000
7664.7251 4069.1938 306.6273 -3595.5313 4134.4014
307.8685 -0.1346 -61.1414 656151.2420 -26.1794
-26.1690
[22229]ENERGY: 11080000 3272.2662 5182.2463 5280.7458
320.2498 -15876.4197 -1779.9805 0.0000 0.0000
7673.6366 4072.7446 306.9838 -3600.8920 4134.3805
307.9434 -126.2888 -117.3282 656151.2420 -28.5627
-28.5544
[22279]ENERGY: 11090000 3284.4719 5151.2613 5308.4704
342.8655 -15857.2256 -1832.4094 0.0000 0.0000
7672.2147 4069.6489 306.9269 -3602.5658 4135.2319
307.8601 -204.0990 -189.4672 656151.2420 -26.8550
-26.8619
[22318]ENERGY: 11100000 3159.6229 5265.5498 5285.3788
352.2665 -15871.0689 -1799.5983 0.0000 0.0000
7679.2515 4071.4023 307.2085 -3607.8492 4133.4346
307.7946 -4.7650 -3.6154 656151.2420 -30.0537
-30.0702
[22368]ENERGY: 11110000 3189.3656 5171.6103 5317.2942
344.4555 -15940.4945 -1750.7911 0.0000 0.0000
7741.6390 4073.0790 309.7043 -3668.5600 4133.7374
307.7087 20.3074 20.1617 656151.2420 -20.7438
-20.7305
[22404]ENERGY: 11120000 3199.2360 5161.5326 5253.6044
323.2912 -15745.1194 -1832.0698 0.0000 0.0000
7711.8496 4072.3246 308.5125 -3639.5250 4135.4543
307.7602 -65.0175 -201.2351 656151.2420 -26.6576
-26.6804
[22454]ENERGY: 11130000 3219.6392 5236.1011 5246.3882
361.5130 -15864.6875 -1754.3231 0.0000 0.0000
7629.0633 4073.6942 305.2007 -3555.3691 4135.1649
307.6904 -76.8331 -54.7549 656151.2420 -20.4516
-20.4284
[22490]ENERGY: 11140000 3222.8714 5234.2897 5255.5267
326.3410 -15910.9296 -1727.1120 0.0000 0.0000
7672.0297 4073.0168 306.9195 -3599.0129 4135.9603
307.7957 86.9346 59.3156 656151.2420 -20.8120
-20.8124
[22540]ENERGY: 11150000 3247.6959 5147.8752 5252.2023
343.5264 -15840.6723 -1743.8127 0.0000 0.0000
7667.1940 4074.0088 306.7261 -3593.1852 4136.2512
307.8673 82.2694 -4.7194 656151.2420 -29.3168
-29.3287

[22576]ENERGY: 11160000 3254.6947 5269.0938 5258.5154
337.5229 -15861.2799 -1845.8921 0.0000 0.0000
7663.2512 4075.9059 306.5684 -3587.3452 4135.4687
307.8953 -116.1702 -109.4698 656151.2420 -22.5923
-22.5891
[22626]ENERGY: 11170000 3181.8864 5220.5653 5284.0284
342.2891 -15809.0290 -1815.5193 0.0000 0.0000
7670.1124 4074.3334 306.8428 -3595.7790 4135.3727
307.7710 -109.4383 -32.9111 656151.2420 -26.0397
-26.0296
[22662]ENERGY: 11180000 3170.5117 5224.2910 5232.2518
327.4961 -15770.2696 -1739.7845 0.0000 0.0000
7632.2971 4076.7937 305.3300 -3555.5034 4136.4198
308.0473 -36.1891 0.1391 656151.2420 -20.0995
-20.1054
[22712]ENERGY: 11190000 3180.8564 5210.1371 5285.7210
351.8138 -15833.9561 -1775.7699 0.0000 0.0000
7658.1373 4076.9395 306.3638 -3581.1978 4136.1066
308.0429 -82.2860 -31.5428 656151.2420 -27.1095
-27.1073
[22751]ENERGY: 11200000 3145.0410 5228.0376 5282.2636
335.9261 -15839.4968 -1757.9247 0.0000 0.0000
7682.6813 4076.5282 307.3457 -3606.1532 4136.9143
307.9507 30.3024 130.0741 656151.2420 -25.0723
-25.0617
[22801]ENERGY: 11210000 3118.8663 5280.2008 5238.2873
337.5139 -15822.9457 -1800.9775 0.0000 0.0000
7729.7103 4080.6554 309.2271 -3649.0548 4139.1512
307.7300 67.8916 -36.5265 656151.2420 -27.0252
-27.0327
[22837]ENERGY: 11220000 3267.5934 5193.2199 5267.2634
316.8946 -15857.6767 -1708.1453 0.0000 0.0000
7597.6777 4076.8269 303.9451 -3520.8507 4138.8687
307.5976 -29.4282 -92.5932 656151.2420 -27.0210
-27.0286
[22887]ENERGY: 11230000 3180.2557 5106.3074 5271.8785
332.4488 -15852.1348 -1765.7325 0.0000 0.0000
7803.5623 4076.5855 312.1815 -3726.9768 4138.6499
307.8076 86.8874 31.2726 656151.2420 -22.4099
-22.3996
[22923]ENERGY: 11240000 3185.6892 5161.3936 5277.1663
324.0779 -15803.1159 -1723.4299 0.0000 0.0000
7659.0729 4080.8540 306.4012 -3578.2189 4138.9191
307.7998 49.9386 -34.5068 656151.2420 -25.3063
-25.3158
[22973]ENERGY: 11250000 3217.4998 5232.1812 5273.9401
353.7075 -15844.6189 -1775.4486 0.0000 0.0000
7617.2265 4074.4875 304.7271 -3542.7389 4138.2818
307.8992 51.1256 32.4451 656151.2420 -24.0349
-24.0381
[23009]ENERGY: 11260000 3165.7830 5179.2782 5253.2866
332.6653 -15798.5965 -1758.5920 0.0000 0.0000
7700.5270 4074.3516 308.0596 -3626.1753 4138.2448
307.8442 -80.5685 -90.7908 656151.2420 -21.8555

-21.8350
[23059]ENERGY: 11270000 3243.0485 5267.1729 5270.7819
339.1510 -15844.8590 -1794.9759 0.0000 0.0000
7599.6057 4079.9252 304.0222 -3519.6806 4139.4960
307.7450 95.9843 48.6874 656151.2420 -27.4151
-27.4337
[23095]ENERGY: 11280000 3210.5982 5239.7355 5241.0688
370.8100 -15763.9715 -1805.9415 0.0000 0.0000
7584.6104 4076.9098 303.4223 -3507.7006 4138.7731
307.9650 -22.2843 -20.6881 656151.2420 -24.5566
-24.5588
[23145]ENERGY: 11290000 3184.2916 5159.3962 5289.3968
344.9374 -15897.7193 -1713.4314 0.0000 0.0000
7707.1765 4074.0479 308.3256 -3633.1286 4139.6783
308.1838 -83.7400 -57.7183 656151.2420 -29.8706
-29.8706
[23184]ENERGY: 11300000 3151.4603 5271.5158 5269.7581
327.8978 -15801.1107 -1845.7050 0.0000 0.0000
7707.2181 4081.0344 308.3273 -3626.1837 4140.5279
307.9978 -123.4670 -116.4210 656151.2420 -24.8398
-24.8292
[23234]ENERGY: 11310000 3175.8794 5279.1880 5234.4969
327.1517 -15877.6315 -1763.0960 0.0000 0.0000
7705.9278 4081.9163 308.2756 -3624.0115 4141.1684
308.2425 0.7336 -73.1447 656151.2420 -25.6633
-25.6711
[23270]ENERGY: 11320000 3146.1611 5180.1079 5267.3900
326.8541 -15789.3456 -1738.5108 0.0000 0.0000
7684.5423 4077.1989 307.4201 -3607.3434 4139.5277
308.2980 57.5930 77.6935 656151.2420 -24.9659
-24.9446
[23320]ENERGY: 11330000 3291.3135 5174.3878 5274.6092
345.2134 -15862.8305 -1800.5007 0.0000 0.0000
7654.9430 4077.1356 306.2360 -3577.8074 4138.0979
308.0319 -246.3700 -108.4303 656151.2420 -26.0068
-26.0057
[23356]ENERGY: 11340000 3235.7567 5183.4699 5252.4267
326.5862 -15841.9913 -1800.0188 0.0000 0.0000
7718.3731 4074.6024 308.7735 -3643.7706 4138.0578
308.0380 -167.3199 -169.4234 656151.2420 -27.4747
-27.5028
[23406]ENERGY: 11350000 3162.7186 5282.6974 5296.1119
344.4056 -15828.9470 -1821.3677 0.0000 0.0000
7642.0836 4077.7023 305.7216 -3564.3813 4138.5892
308.1192 -40.0848 -10.2962 656151.2420 -26.0784
-26.0728
[23442]ENERGY: 11360000 3202.3202 5258.0686 5226.7241
338.9505 -15779.2971 -1799.3268 0.0000 0.0000
7631.0718 4078.5113 305.2810 -3552.5605 4138.9777
308.1466 42.4611 46.8848 656151.2420 -27.3527
-27.3579
[23492]ENERGY: 11370000 3256.3542 5279.9381 5268.5540
343.8559 -15903.9013 -1794.0497 0.0000 0.0000
7625.9877 4076.7389 305.0776 -3549.2488 4139.1668

308.2604 -149.7369 -61.0443 656151.2420 -24.1332
-24.1237
[23528]ENERGY: 11380000 3209.2494 5225.4978 5279.8599
353.0406 -15838.0978 -1846.7890 0.0000 0.0000
7694.4824 4077.2433 307.8178 -3617.2391 4139.7667
308.2184 -44.7802 -101.5593 656151.2420 -28.5691
-28.5670
[23578]ENERGY: 11390000 3218.6942 5316.6261 5305.6361
349.9156 -15887.9670 -1850.8818 0.0000 0.0000
7625.6957 4077.7189 305.0660 -3547.9768 4139.4195
308.0667 -172.0576 -93.7685 656151.2420 -28.7281
-28.7308
[23617]ENERGY: 11400000 3165.5580 5238.6166 5303.4693
351.1527 -15880.4288 -1874.7132 0.0000 0.0000
7772.6094 4076.2639 310.9432 -3696.3455 4140.8641
307.9109 -45.9988 -69.7391 656151.2420 -22.5018
-22.5021
[23667]ENERGY: 11410000 3109.4808 5264.7515 5281.4604
332.7777 -15804.8273 -1857.2566 0.0000 0.0000
7750.2186 4076.6050 310.0475 -3673.6136 4141.2340
307.9701 62.7239 -33.9954 656151.2420 -23.1580
-23.1680
[23703]ENERGY: 11420000 3171.2298 5263.6366 5253.6171
333.5565 -15843.3542 -1786.1095 0.0000 0.0000
7684.8108 4077.3871 307.4309 -3607.4237 4139.7576
307.8202 -44.8253 -47.8078 656151.2420 -21.5272
-21.5156
[23753]ENERGY: 11430000 3212.1197 5232.3789 5285.0560
343.9109 -15827.5377 -1813.2299 0.0000 0.0000
7648.5321 4081.2300 305.9795 -3567.3021 4140.2106
307.7669 -222.1261 -237.4975 656151.2420 -24.7036
-24.6999
[23789]ENERGY: 11440000 3162.6228 5237.0857 5283.7364
349.5855 -15809.4320 -1812.4380 0.0000 0.0000
7667.7186 4078.8790 306.7471 -3588.8395 4140.8954
307.8011 60.7124 -21.9834 656151.2420 -26.1228
-26.1228
[23839]ENERGY: 11450000 3178.1919 5174.3868 5214.9488
370.1628 -15893.2824 -1671.0494 0.0000 0.0000
7706.4514 4079.8098 308.2966 -3626.6415 4141.6841
307.8519 179.0456 104.7238 656151.2420 -16.1834
-16.2020
[23875]ENERGY: 11460000 3226.6332 5242.1252 5227.7103
328.7175 -15888.1436 -1723.1450 0.0000 0.0000
7665.3062 4079.2037 306.6506 -3586.1024 4141.3383
307.8255 -110.7852 -1.5619 656151.2420 -28.3253
-28.3003
[23925]ENERGY: 11470000 3263.6364 5196.5157 5238.8751
338.5953 -15801.6533 -1749.2433 0.0000 0.0000
7597.9201 4084.6461 303.9548 -3513.2741 4141.6540
307.8256 -19.0569 -45.6568 656151.2420 -24.5768
-24.5731
[23961]ENERGY: 11480000 3242.1821 5204.9442 5316.7495
340.4319 -15858.6487 -1787.4158 0.0000 0.0000

7623.4259 4081.6691 304.9752 -3541.7569 4143.5295
307.7180 126.0914 -38.8148 656151.2420 -26.8176
-26.8421
[24011]ENERGY: 11490000 3178.5040 5200.6343 5240.4112
344.4561 -15807.6522 -1765.6499 0.0000 0.0000
7689.3922 4080.0959 307.6141 -3609.2963 4141.4463
307.8881 16.5123 24.2047 656151.2420 -24.5241
-24.5019
[24050]ENERGY: 11500000 3123.3919 5207.2772 5245.8880
346.2330 -15735.0210 -1746.2763 0.0000 0.0000
7639.8367 4081.3295 305.6317 -3558.5072 4141.5826
307.9003 33.5975 -14.9142 656151.2420 -22.1167
-22.1307
[24100]ENERGY: 11510000 3168.0506 5155.9934 5248.8549
343.6874 -15805.2676 -1763.5556 0.0000 0.0000
7731.2593 4079.0224 309.2890 -3652.2370 4141.7331
307.9171 93.1377 64.5061 656151.2420 -24.5893
-24.5712
[24136]ENERGY: 11520000 3148.1407 5228.7045 5259.0336
363.2282 -15896.8480 -1758.5436 0.0000 0.0000
7736.4667 4080.1822 309.4973 -3656.2845 4141.0122
308.0072 35.0312 58.9985 656151.2420 -25.6108
-25.6178
[24186]ENERGY: 11530000 3216.3167 5292.9932 5273.1707
344.6645 -15860.0609 -1767.4864 0.0000 0.0000
7583.0966 4082.6943 303.3618 -3500.4024 4141.6191
308.0243 51.4136 -20.0234 656151.2420 -26.0877
-26.1006
[24222]ENERGY: 11540000 3165.1311 5188.2715 5292.9524
342.9772 -15773.3049 -1850.3703 0.0000 0.0000
7713.4210 4079.0779 308.5754 -3634.3431 4140.8762
307.8487 -56.9454 -91.5009 656151.2420 -24.5382
-24.5249
[24272]ENERGY: 11550000 3188.9564 5261.6610 5272.5152
331.2818 -15812.2930 -1836.7811 0.0000 0.0000
7674.2425 4079.5829 307.0081 -3594.6596 4141.3952
307.8482 74.1018 66.2775 656151.2420 -24.6442
-24.6578
[24308]ENERGY: 11560000 3213.6836 5219.4322 5271.5866
360.7655 -15869.9291 -1744.4798 0.0000 0.0000
7628.4593 4079.5182 305.1765 -3548.9411 4141.6001
307.9611 -83.7669 -20.4132 656151.2420 -24.8697
-24.8498
[24358]ENERGY: 11570000 3157.6088 5203.5388 5221.4097
343.0329 -15845.0606 -1754.2472 0.0000 0.0000
7750.5312 4076.8135 310.0600 -3673.7177 4140.7204
307.9448 144.1046 87.1401 656151.2420 -24.9811
-24.9955
[24394]ENERGY: 11580000 3242.4453 5218.1735 5277.1974
354.3657 -15894.7024 -1776.1847 0.0000 0.0000
7659.1170 4080.4118 306.4030 -3578.7052 4141.1310
307.9605 -88.0255 -75.8471 656151.2420 -24.9783
-24.9774
[24444]ENERGY: 11590000 3111.0570 5226.4339 5290.8468

345.7483 -15744.5118 -1815.7271 0.0000 0.0000
7667.4311 4081.2782 306.7356 -3586.1529 4141.9151
307.9570 56.3844 53.4441 656151.2420 -26.8207
-26.8197
[24483]ENERGY: 11600000 3200.9979 5171.5769 5225.8123
317.3623 -15794.5908 -1718.1460 0.0000 0.0000
7675.5070 4078.5198 307.0587 -3596.9872 4141.7982
307.9676 43.8231 20.3684 656151.2420 -24.4074
-24.4132
[24533]ENERGY: 11610000 3186.2026 5177.9263 5293.2451
333.3954 -15854.5395 -1749.5931 0.0000 0.0000
7692.9478 4079.5845 307.7564 -3613.3633 4142.0449
307.8022 -40.7585 -126.5359 656151.2420 -23.8786
-23.8691
[24569]ENERGY: 11620000 3251.6024 5106.2740 5256.1840
317.7387 -15834.4756 -1799.0648 0.0000 0.0000
7780.3395 4078.5981 311.2525 -3701.7414 4142.2603
307.9244 -2.6920 -93.6222 656151.2420 -27.4554
-27.4719
[24619]ENERGY: 11630000 3199.7531 5234.2361 5229.0247
328.4016 -15850.4882 -1747.2444 0.0000 0.0000
7685.3312 4079.0142 307.4517 -3606.3170 4142.1368
307.8692 76.8598 48.6574 656151.2420 -23.7886
-23.7664
[24655]ENERGY: 11640000 3227.2825 5242.3378 5230.6034
319.3787 -15888.7026 -1826.2195 0.0000 0.0000
7771.4032 4076.0834 310.8950 -3695.3198 4142.8922
307.8684 1.8874 -29.1743 656151.2420 -23.4490
-23.4537
[24705]ENERGY: 11650000 3143.0700 5224.2324 5296.2695
354.4682 -15884.0909 -1794.0826 0.0000 0.0000
7742.6655 4082.5321 309.7453 -3660.1333 4142.8818
308.0719 89.1597 34.0005 656151.2420 -22.9447
-22.9474
[24741]ENERGY: 11660000 3224.8489 5183.0809 5307.8381
337.3809 -15852.1403 -1758.5688 0.0000 0.0000
7640.5201 4082.9597 305.6590 -3557.5604 4143.8628
307.8539 -1.3318 -67.8213 656151.2420 -24.3126
-24.3178
[24791]ENERGY: 11670000 3208.7005 5229.9563 5281.4247
349.2305 -15806.2981 -1799.6580 0.0000 0.0000
7620.3817 4083.7376 304.8534 -3536.6441 4143.9731
307.7099 19.2233 23.0684 656151.2420 -25.7256
-25.7194
[24827]ENERGY: 11680000 3175.6684 5044.4435 5238.5172
338.1867 -15729.1063 -1802.3650 0.0000 0.0000
7815.9639 4081.3082 312.6776 -3734.6556 4143.8959
307.7310 -50.4629 -39.4315 656151.2420 -23.1479
-23.1507
[24877]ENERGY: 11690000 3230.3094 5205.0666 5291.3897
343.7616 -15842.7724 -1778.5048 0.0000 0.0000
7635.8064 4085.0565 305.4704 -3550.7499 4143.9041
307.7943 -72.5914 -22.9178 656151.2420 -25.8384
-25.8390

[24916]ENERGY: 11700000 3153.1389 5175.9587 5292.9922
343.4171 -15885.4472 -1705.7297 0.0000 0.0000
7711.0866 4085.4166 308.4820 -3625.6700 4143.3701
307.8544 129.9522 138.1965 656151.2420 -20.3797
-20.3752
[24966]ENERGY: 11710000 3226.3210 5166.6762 5251.0162
343.5222 -15761.8427 -1761.0296 0.0000 0.0000
7619.7683 4084.4316 304.8288 -3535.3367 4142.8602
307.7949 189.8609 95.9499 656151.2420 -26.1296
-26.1735
[25002]ENERGY: 11720000 3201.8863 5181.7839 5284.5665
332.9894 -15901.9931 -1731.1090 0.0000 0.0000
7710.9575 4079.0815 308.4769 -3631.8760 4142.5503
307.7421 127.5856 130.7401 656151.2420 -20.2982
-20.2693
[25052]ENERGY: 11730000 3183.5838 5168.5583 5256.4485
333.8906 -15812.4695 -1776.9974 0.0000 0.0000
7730.0094 4083.0237 309.2390 -3646.9858 4144.1211
307.8704 147.1514 103.3716 656151.2420 -24.5160
-24.5295
[25088]ENERGY: 11740000 3179.7040 5278.5991 5254.4874
340.1176 -15836.9837 -1769.9111 0.0000 0.0000
7635.3582 4081.3715 305.4525 -3553.9868 4143.6698
307.9899 -91.2971 -23.6962 656151.2420 -22.1623
-22.1332
[25138]ENERGY: 11750000 3217.7422 5266.2238 5250.6183
353.2443 -15837.8185 -1862.9511 0.0000 0.0000
7695.0615 4082.1206 307.8409 -3612.9409 4144.7819
307.8407 -68.0857 -64.0503 656151.2420 -22.5355
-22.5635
[25174]ENERGY: 11760000 3145.8593 5178.0226 5248.9236
312.8174 -15790.9132 -1860.5540 0.0000 0.0000
7846.0394 4080.1952 313.8808 -3765.8442 4144.2650
307.8881 -57.6530 -24.7499 656151.2420 -25.5078
-25.4813
[25224]ENERGY: 11770000 3264.8644 5191.9439 5234.2782
353.9419 -15821.6107 -1780.6240 0.0000 0.0000
7642.6227 4085.4163 305.7431 -3557.2064 4145.6483
307.5537 -15.0427 -64.7999 656151.2420 -20.6465
-20.6622
[25260]ENERGY: 11780000 3171.8810 5311.1021 5296.3246
324.1614 -15813.2734 -1794.3313 0.0000 0.0000
7587.3322 4083.1966 303.5312 -3504.1355 4144.6304
307.7511 -80.1867 -32.6907 656151.2420 -26.2392
-26.2315
[25310]ENERGY: 11790000 3225.7684 5302.6520 5249.9515
324.0966 -15871.5312 -1784.4834 0.0000 0.0000
7641.0490 4087.5029 305.6802 -3553.5461 4144.6201
307.8459 28.1711 22.0889 656151.2420 -22.9879
-22.9885
[25349]ENERGY: 11800000 3246.7809 5230.2899 5253.4181
311.2479 -15836.8631 -1826.0408 0.0000 0.0000
7702.3333 4081.1661 308.1318 -3621.1672 4145.4546
307.9089 25.2419 -93.6489 656151.2420 -25.1523

-25.1464
[25399]ENERGY: 11810000 3261.0959 5178.5572 5263.4098
328.9226 -15903.3558 -1734.5895 0.0000 0.0000
7690.2517 4084.2919 307.6485 -3605.9598 4144.6417
308.0434 -118.7903 -59.9312 656151.2420 -21.4730
-21.4753
[25435]ENERGY: 11820000 3084.9571 5281.6679 5263.8615
333.6182 -15852.4914 -1746.9458 0.0000 0.0000
7716.1795 4080.8471 308.6858 -3635.3324 4144.8643
307.7787 2.3308 -24.5845 656151.2420 -25.2714
-25.2751
[25485]ENERGY: 11830000 3212.2688 5245.1325 5211.7496
323.8038 -15871.0606 -1781.6443 0.0000 0.0000
7741.7755 4082.0253 309.7097 -3659.7502 4144.9762
308.0037 -13.6885 -36.5979 656151.2420 -23.8105
-23.8224
[25521]ENERGY: 11840000 3183.2965 5193.5307 5289.4906
333.1435 -15842.3997 -1742.1989 0.0000 0.0000
7675.4104 4090.2731 307.0548 -3585.1373 4147.9877
308.0325 191.9918 84.9405 656151.2420 -24.3479
-24.3456
[25571]ENERGY: 11850000 3191.1144 5203.3798 5255.2888
346.5184 -15910.1000 -1709.2508 0.0000 0.0000
7705.3628 4082.3135 308.2530 -3623.0494 4146.0092
307.9729 36.6773 26.1179 656151.2420 -25.9892
-25.9782
[25607]ENERGY: 11860000 3190.4127 5184.7495 5263.4345
337.2511 -15812.3294 -1831.7252 0.0000 0.0000
7754.2080 4086.0013 310.2071 -3668.2067 4147.6913
307.9393 108.4167 -29.7275 656151.2420 -22.2835
-22.2871
[25657]ENERGY: 11870000 3210.2012 5313.8777 5265.0952
352.0486 -15941.8651 -1776.8732 0.0000 0.0000
7660.5662 4083.0505 306.4609 -3577.5157 4148.4996
308.0235 -70.0584 -131.0422 656151.2420 -26.5801
-26.5802
[25693]ENERGY: 11880000 3137.0323 5250.4894 5237.7338
348.7553 -15854.1657 -1731.9530 0.0000 0.0000
7696.6717 4084.5639 307.9053 -3612.1078 4147.7086
307.8466 -24.3930 -4.7676 656151.2420 -23.7182
-23.6956
[25743]ENERGY: 11890000 3223.9106 5185.9780 5274.4657
341.6939 -15819.0361 -1719.5995 0.0000 0.0000
7599.5082 4086.9209 304.0183 -3512.5874 4148.2432
307.8539 -130.8996 -95.5469 656151.2420 -25.1701
-25.1899
[25782]ENERGY: 11900000 3161.9212 5206.7223 5289.5775
348.5708 -15857.0087 -1754.5763 0.0000 0.0000
7695.2556 4090.4622 307.8487 -3604.7934 4149.4325
307.9661 176.7142 59.3259 656151.2420 -24.3762
-24.3842
[25832]ENERGY: 11910000 3261.7509 5210.7457 5213.5098
352.2617 -15833.8464 -1715.4608 0.0000 0.0000
7601.1545 4090.1154 304.0842 -3511.0392 4150.1510

307.9881 17.0376 -60.6362 656151.2420 -23.5095
-23.4977
[25868]ENERGY: 11920000 3232.4428 5167.4408 5293.7403
331.6660 -15783.0972 -1786.1472 0.0000 0.0000
7632.3562 4088.4018 305.3324 -3543.9545 4150.1930
308.0141 -40.2912 -82.2341 656151.2420 -23.2970
-23.2981
[25918]ENERGY: 11930000 3240.7566 5146.5903 5324.1469
329.3701 -15828.2846 -1786.0829 0.0000 0.0000
7661.1305 4087.6269 306.4835 -3573.5035 4149.8322
308.0740 -41.0827 -103.4111 656151.2420 -26.4470
-26.4399
[25954]ENERGY: 11940000 3217.4724 5189.0346 5277.5003
332.9030 -15781.4500 -1767.4400 0.0000 0.0000
7623.8429 4091.8632 304.9918 -3531.9797 4150.8365
308.0743 -53.6425 -69.4786 656151.2420 -23.1074
-23.1091
[26004]ENERGY: 11950000 3179.5079 5207.0785 5247.0913
316.4564 -15876.7177 -1723.6029 0.0000 0.0000
7736.1180 4085.9315 309.4834 -3650.1865 4149.5668
307.9502 -109.3203 -144.2388 656151.2420 -24.0813
-24.0868
[26040]ENERGY: 11960000 3205.1164 5291.0353 5279.2388
320.3471 -15838.5024 -1779.5939 0.0000 0.0000
7611.0675 4088.7089 304.4808 -3522.3586 4149.3135
307.7875 174.9705 74.5911 656151.2420 -22.6501
-22.6573
[26090]ENERGY: 11970000 3140.1300 5342.5392 5267.6490
323.5975 -15910.3961 -1766.4918 0.0000 0.0000
7688.9384 4085.9662 307.5960 -3602.9722 4150.8646
307.9618 105.0418 47.0067 656151.2420 -24.1067
-24.1019
[26126]ENERGY: 11980000 3231.7248 5149.7805 5301.2632
326.7730 -15866.5542 -1747.0671 0.0000 0.0000
7691.9172 4087.8375 307.7151 -3604.0797 4150.2290
307.8575 85.0213 -17.5822 656151.2420 -22.8300
-22.8320
[26176]ENERGY: 11990000 3202.6975 5289.3824 5233.7796
349.8921 -15937.0275 -1695.8490 0.0000 0.0000
7645.6032 4088.4784 305.8624 -3557.1248 4149.1686
307.9378 73.7329 89.6278 656151.2420 -22.8775
-22.8815
[26215]ENERGY: 12000000 3202.5399 5242.3716 5285.6956
344.7025 -15848.6670 -1800.5809 0.0000 0.0000
7662.7201 4088.7818 306.5471 -3573.9384 4149.7716
307.8289 79.4565 18.3222 656151.2420 -25.0636
-25.0603
[26265]ENERGY: 12010000 3239.0108 5290.2908 5343.8948
375.1809 -15893.4543 -1872.7587 0.0000 0.0000
7607.9389 4090.1031 304.3556 -3517.8357 4149.9713
307.8760 -24.6232 -107.9400 656151.2420 -25.2839
-25.2833
[26301]ENERGY: 12020000 3192.3812 5128.5425 5299.9884
347.3290 -15802.3027 -1739.3156 0.0000 0.0000

7662.3366 4088.9595 306.5318 -3573.3771 4149.1622
307.8510 85.0492 37.3372 656151.2420 -24.7677
-24.7566
[26351]ENERGY: 12030000 3175.6511 5281.0137 5217.8956
353.4083 -15906.1097 -1710.0078 0.0000 0.0000
7676.1015 4087.9527 307.0824 -3588.1488 4148.4789
307.6631 42.2641 19.2744 656151.2420 -24.7251
-24.7280
[26387]ENERGY: 12040000 3150.0742 5201.1208 5269.2101
342.1796 -15771.1910 -1767.5617 0.0000 0.0000
7661.4696 4085.3015 306.4971 -3576.1681 4148.6481
307.6107 80.9098 106.3820 656151.2420 -24.9266
-24.9296
[26437]ENERGY: 12050000 3204.5594 5281.1606 5259.0922
330.5424 -15899.5489 -1782.8786 0.0000 0.0000
7693.1568 4086.0839 307.7647 -3607.0729 4148.6729
307.8751 -32.4579 -58.6546 656151.2420 -24.2576
-24.2500
[26473]ENERGY: 12060000 3185.9239 5170.1663 5293.8770
335.2598 -15787.4409 -1792.4660 0.0000 0.0000
7682.3835 4087.7034 307.3337 -3594.6800 4148.7339
307.8467 3.6275 -37.2732 656151.2420 -23.2526
-23.2421
[26523]ENERGY: 12070000 3195.5292 5220.6851 5257.7115
326.9536 -15843.2019 -1814.3553 0.0000 0.0000
7745.7595 4089.0817 309.8691 -3656.6778 4150.1031
308.0842 144.6244 72.2244 656151.2420 -24.7021
-24.7085
[26559]ENERGY: 12080000 3255.0682 5223.9915 5198.5549
356.7262 -15818.7643 -1826.8375 0.0000 0.0000
7700.6773 4089.4162 308.0656 -3611.2611 4149.9119
308.2022 -154.4737 -110.7724 656151.2420 -24.0337
-24.0337
[26609]ENERGY: 12090000 3195.3194 5248.9745 5275.1357
340.5166 -15911.8294 -1719.8282 0.0000 0.0000
7663.7359 4092.0246 306.5878 -3571.7113 4150.3110
308.1032 -102.1773 -98.7215 656151.2420 -21.6993
-21.7195
[26648]ENERGY: 12100000 3191.1468 5152.3603 5287.5966
312.4272 -15810.0715 -1750.6156 0.0000 0.0000
7702.2881 4085.1320 308.1300 -3617.1561 4150.0061
307.9762 -18.6664 25.2951 656151.2420 -22.2900
-22.2820
[26698]ENERGY: 12110000 3215.5069 5219.8879 5306.2840
339.6396 -15843.3400 -1773.3578 0.0000 0.0000
7623.7130 4088.3337 304.9866 -3535.3793 4151.0997
308.0540 118.3670 47.5026 656151.2420 -28.7254
-28.7294
[26734]ENERGY: 12120000 3117.4459 5178.3700 5339.7554
354.4174 -15799.0730 -1772.4003 0.0000 0.0000
7671.1832 4089.6986 306.8857 -3581.4846 4150.0789
308.3044 69.9616 37.4305 656151.2420 -22.5760
-22.5938
[26784]ENERGY: 12130000 3177.6183 5245.0862 5242.3600

350.6072 -15929.5792 -1637.3561 0.0000 0.0000
7641.1188 4089.8552 305.6830 -3551.2636 4149.8687
308.2750 112.8753 41.4615 656151.2420 -21.9867
-21.9635
[26820]ENERGY: 12140000 3172.7550 5142.1627 5281.2847
343.6008 -15818.2802 -1769.0274 0.0000 0.0000
7737.8819 4090.3776 309.5540 -3647.5044 4151.2623
308.0713 46.9562 1.8643 656151.2420 -24.0373
-24.0534
[26870]ENERGY: 12150000 3217.9775 5173.2846 5281.2193
336.3266 -15778.8346 -1860.3188 0.0000 0.0000
7717.3904 4087.0449 308.7342 -3630.3455 4151.0394
308.2810 -26.0828 -97.6895 656151.2420 -21.2301
-21.2077
[26906]ENERGY: 12160000 3234.1042 5108.7911 5247.3073
332.8470 -15843.3599 -1731.1562 0.0000 0.0000
7741.5359 4090.0695 309.7001 -3651.4664 4151.1023
308.1056 31.0801 23.7167 656151.2420 -27.2285
-27.2302
[26956]ENERGY: 12170000 3171.2744 5178.4634 5268.6539
345.6605 -15842.0230 -1722.4103 0.0000 0.0000
7689.8734 4089.4923 307.6334 -3600.3810 4150.3751
308.1153 -20.7362 -41.0404 656151.2420 -22.1224
-22.1377
[267]ENERGY: 12180000 3179.8214 5207.1626 5229.1277
360.9288 -15789.5185 -1830.6518 0.0000 0.0000
7731.2470 4088.1171 309.2885 -3643.1298 4151.8209
308.1838 -1.2009 9.9172 656151.2420 -26.7415
-26.7456
[306]ENERGY: 12190000 3258.7886 5126.1966 5300.6821
350.6770 -15928.1623 -1744.8676 0.0000 0.0000
7723.9835 4087.2979 308.9980 -3636.6855 4149.9292
308.1419 -53.3362 -49.6766 656151.2420 -25.7225
-25.7081
[360]ENERGY: 12200000 3281.6807 5149.4118 5202.5815
312.3226 -15922.6551 -1696.6006 0.0000 0.0000
7761.0892 4087.8302 310.4824 -3673.2590 4150.0195
308.1961 19.8066 -31.6790 656151.2420 -25.1249
-25.1295
[398]ENERGY: 12210000 3171.7394 5222.2752 5269.8215
350.2902 -15881.8609 -1722.8771 0.0000 0.0000
7679.5391 4088.9274 307.2200 -3590.6117 4149.3250
308.4217 60.5550 -17.3766 656151.2420 -22.0896
-22.0956
[450]ENERGY: 12220000 3179.9563 5184.4371 5267.5048
345.5902 -15884.2541 -1736.1117 0.0000 0.0000
7730.3183 4087.4410 309.2514 -3642.8773 4150.2261
308.1803 8.1725 35.7317 656151.2420 -24.8793
-24.8738
[488]ENERGY: 12230000 3237.4080 5163.4004 5237.6636
328.5782 -15817.9057 -1746.1119 0.0000 0.0000
7685.4908 4088.5234 307.4581 -3596.9675 4151.4918
308.3105 179.9875 38.3255 656151.2420 -26.1767
-26.1827

[539]ENERGY: 12240000 3195.3812 5260.5317 5253.2640
334.5297 -15877.8020 -1754.8377 0.0000 0.0000
7676.3418 4087.4086 307.0921 -3588.9331 4150.6945
308.2185 -24.0904 -10.7509 656151.2420 -23.1382
-23.1231
[576]ENERGY: 12250000 3211.0154 5213.4291 5250.9197
356.9000 -15922.4864 -1735.5492 0.0000 0.0000
7714.7391 4088.9678 308.6281 -3625.7713 4150.4605
308.3210 41.3350 31.1024 656151.2420 -26.4577
-26.4644
[627]ENERGY: 12260000 3137.2971 5196.3075 5233.0853
348.9393 -15882.6648 -1712.2988 0.0000 0.0000
7768.9046 4089.5703 310.7950 -3679.3342 4150.6033
308.3271 118.8751 49.8046 656151.2420 -22.6499
-22.6719
[664]ENERGY: 12270000 3264.9613 5211.5167 5233.6938
349.7407 -15777.3542 -1788.4709 0.0000 0.0000
7598.3068 4092.3942 303.9703 -3505.9126 4150.5594
308.3507 -124.7351 -178.5000 656151.2420 -24.5364
-24.5023
[715]ENERGY: 12280000 3243.6960 5252.6803 5247.5622
330.4018 -15957.2672 -1739.0976 0.0000 0.0000
7706.9178 4084.8932 308.3152 -3622.0245 4149.4149
308.4162 130.3417 119.7183 656151.2420 -19.0614
-19.0797
[751]ENERGY: 12290000 3168.9264 5234.1212 5291.0454
342.1039 -15830.0062 -1816.9008 0.0000 0.0000
7699.3207 4088.6107 308.0113 -3610.7100 4151.2970
308.4277 176.2119 89.9022 656151.2420 -26.0547
-26.0467
[804]ENERGY: 12300000 3216.7546 5114.0159 5299.9481
357.1702 -15851.5468 -1735.2891 0.0000 0.0000
7687.2602 4088.3131 307.5288 -3598.9471 4150.3784
308.4146 17.6414 27.5348 656151.2420 -19.8978
-19.9025
[840]ENERGY: 12310000 3243.2986 5167.9494 5253.6894
338.8209 -15792.8410 -1812.4702 0.0000 0.0000
7692.6988 4091.1460 307.7464 -3601.5529 4150.5570
308.3394 -71.4324 -115.3212 656151.2420 -29.8484
-29.8223
[890]ENERGY: 12320000 3212.4653 5246.5741 5274.6795
332.4584 -15837.9494 -1786.5783 0.0000 0.0000
7646.7797 4088.4292 305.9094 -3558.3505 4150.8652
308.2420 -6.5140 9.4887 656151.2420 -20.1848
-20.2144
[926]ENERGY: 12330000 3150.8870 5268.0550 5262.8208
339.2413 -15866.1134 -1736.3950 0.0000 0.0000
7668.8001 4087.2959 306.7903 -3581.5043 4150.8431
308.2460 -25.7601 -22.6607 656151.2420 -25.3322
-25.3310
[976]ENERGY: 12340000 3181.3958 5176.8095 5229.0934
328.3308 -15783.6431 -1755.3702 0.0000 0.0000
7713.3378 4089.9541 308.5721 -3623.3837 4152.2287
308.2441 121.7083 43.0736 656151.2420 -22.8468

-22.8418
[1012]ENERGY: 12350000 3194.9625 5091.3218 5304.3129
333.7759 -15749.4689 -1773.4294 0.0000 0.0000
7688.3376 4089.8124 307.5719 -3598.5252 4149.7858
308.0184 55.5201 -29.0257 656151.2420 -24.6093
-24.6086
[1062]ENERGY: 12360000 3072.7959 5257.2186 5214.2224
334.4905 -15841.4178 -1736.1367 0.0000 0.0000
7785.4377 4086.6108 311.4564 -3698.8270 4149.5664
308.1830 -5.1773 -61.5099 656151.2420 -24.9850
-24.9974
[1098]ENERGY: 12370000 3253.2594 5254.1466 5235.6829
308.7887 -15841.1623 -1759.6205 0.0000 0.0000
7635.5729 4086.6677 305.4611 -3548.9052 4149.6021
308.0775 2.4199 -99.0952 656151.2420 -21.3196
-21.3002
[1148]ENERGY: 12380000 3187.4657 5228.9992 5261.4208
328.1043 -15888.3097 -1736.3263 0.0000 0.0000
7703.3621 4084.7161 308.1730 -3618.6460 4150.0001
308.0426 9.6453 -0.7306 656151.2420 -21.3254
-21.3441
[1184]ENERGY: 12390000 3162.9065 5265.5878 5309.7480
345.1681 -15945.9418 -1779.0544 0.0000 0.0000
7729.3086 4087.7228 309.2110 -3641.5858 4149.5220
308.1411 67.4076 -16.5263 656151.2420 -22.3979
-22.3773
[1237]ENERGY: 12400000 3167.0975 5155.0361 5271.4450
342.4811 -15828.5950 -1792.2156 0.0000 0.0000
7769.3390 4084.5880 310.8124 -3684.7510 4150.1166
308.0901 3.2928 -92.7446 656151.2420 -25.9276
-25.9340
[1273]ENERGY: 12410000 3172.5902 5185.1001 5219.6032
343.8834 -15923.0129 -1687.4077 0.0000 0.0000
7777.7532 4088.5095 311.1490 -3689.2437 4150.7179
308.0694 121.4538 155.4214 656151.2420 -19.6457
-19.6322
[1323]ENERGY: 12420000 3185.3303 5250.8533 5265.4072
333.3297 -15782.6489 -1794.5343 0.0000 0.0000
7633.9187 4091.6560 305.3949 -3542.2627 4151.5206
308.1943 -159.8084 -123.4740 656151.2420 -22.1467
-22.1629
[1359]ENERGY: 12430000 3151.1957 5237.7683 5304.0179
338.8716 -15930.1251 -1737.0387 0.0000 0.0000
7724.5650 4089.2546 309.0212 -3635.3104 4151.0914
308.2361 69.0015 -52.7086 656151.2420 -20.3271
-20.3433
[1409]ENERGY: 12440000 3183.3964 5272.3297 5221.8167
323.7365 -15884.5128 -1715.1116 0.0000 0.0000
7690.5042 4092.1592 307.6586 -3598.3451 4152.1730
308.2261 88.8146 51.7946 656151.2420 -21.2694
-21.2488
[1445]ENERGY: 12450000 3164.6179 5248.7808 5249.4591
335.0285 -15910.2560 -1737.3783 0.0000 0.0000
7738.1056 4088.3576 309.5629 -3649.7480 4151.0338

308.2172 19.8689 -1.8158 656151.2420 -21.6883
-21.6859
[1495]ENERGY: 12460000 3216.4719 5262.0175 5259.1168
330.0209 -15831.8753 -1771.7087 0.0000 0.0000
7626.5335 4090.5767 305.0995 -3535.9569 4151.8137
308.1422 -15.3792 -96.1544 656151.2420 -21.7033
-21.7181
[1531]ENERGY: 12470000 3251.7143 5288.6522 5249.5680
369.9854 -15880.1812 -1758.2840 0.0000 0.0000
7568.9385 4090.3930 302.7954 -3478.5454 4153.0150
308.3324 -113.6888 -83.0103 656151.2420 -24.2622
-24.2576
[1581]ENERGY: 12480000 3250.8181 5223.1459 5259.6553
348.7798 -15910.3454 -1738.8364 0.0000 0.0000
7658.5703 4091.7876 306.3811 -3566.7827 4152.9553
308.0818 27.3225 21.5083 656151.2420 -20.7426
-20.7330
[1617]ENERGY: 12490000 3195.1968 5138.8478 5246.4835
362.4907 -15846.1515 -1705.4468 0.0000 0.0000
7698.4010 4089.8215 307.9745 -3608.5795 4152.2115
308.0497 -24.3919 -80.2319 656151.2420 -24.8973
-24.9041
[1670]ENERGY: 12500000 3232.6886 5258.7747 5231.7332
340.7153 -15912.9276 -1721.0050 0.0000 0.0000
7665.1949 4095.1741 306.6461 -3570.0208 4153.8395
308.0340 12.2510 -27.5207 656151.2420 -22.1442
-22.1412
[1706]ENERGY: 12510000 3153.7429 5253.2880 5285.3857
349.2460 -15863.3735 -1749.9225 0.0000 0.0000
7662.5242 4090.8909 306.5393 -3571.6333 4153.0751
308.0266 208.5262 137.6987 656151.2420 -25.1052
-25.1025
[1756]ENERGY: 12520000 3185.2018 5172.5037 5315.2200
342.8942 -15890.2846 -1751.7332 0.0000 0.0000
7718.5603 4092.3623 308.7810 -3626.1980 4153.8775
307.8375 -24.8152 -83.1533 656151.2420 -22.5723
-22.5767
[1792]ENERGY: 12530000 3281.7867 5209.3892 5285.2661
318.5212 -15942.5357 -1705.3924 0.0000 0.0000
7646.7440 4093.7791 305.9080 -3552.9649 4154.4976
308.0263 72.4302 60.9477 656151.2420 -21.7463
-21.7501
[1842]ENERGY: 12540000 3261.2472 5234.8088 5291.7499
333.5018 -15840.5854 -1809.1885 0.0000 0.0000
7623.9701 4095.5039 304.9969 -3528.4662 4155.7691
308.0046 -43.8075 -52.9964 656151.2420 -18.8758
-18.8669
[1878]ENERGY: 12550000 3159.6676 5127.0049 5257.0574
346.6881 -15747.1974 -1739.8727 0.0000 0.0000
7692.9255 4096.2733 307.7555 -3596.6522 4155.5521
307.8706 211.5863 95.9999 656151.2420 -21.2153
-21.2170
[1928]ENERGY: 12560000 3134.1585 5151.6091 5227.7937
337.0403 -15741.2235 -1749.7068 0.0000 0.0000

7732.5310 4092.2022 309.3399 -3640.3288 4155.8168
307.9365 -12.6678 -61.3234 656151.2420 -22.9418
-22.9356
[1964]ENERGY: 12570000 3187.6293 5247.7020 5274.4417
351.4037 -15835.4231 -1823.4353 0.0000 0.0000
7688.8546 4091.1730 307.5926 -3597.6816 4156.4051
308.1175 21.1806 -57.8160 656151.2420 -23.6360
-23.6505
[2014]ENERGY: 12580000 3139.3299 5234.2528 5235.2309
328.8084 -15744.7050 -1757.1056 0.0000 0.0000
7658.2513 4094.0627 306.3683 -3564.1886 4154.8806
308.1150 -52.4929 30.9729 656151.2420 -25.0905
-25.0751
[2050]ENERGY: 12590000 3177.5748 5271.4544 5259.9560
320.9866 -15831.6251 -1833.9372 0.0000 0.0000
7726.3071 4090.7166 309.0909 -3635.5905 4156.5961
308.0138 0.8418 -22.6821 656151.2420 -22.1539
-22.1796
[2103]ENERGY: 12600000 3195.6166 5142.4990 5259.2589
322.3443 -15762.4613 -1785.3604 0.0000 0.0000
7723.9080 4095.8052 308.9949 -3628.1028 4156.1640
308.1222 -193.6594 -158.4334 656151.2420 -24.0527
-24.0232
[2139]ENERGY: 12610000 3151.9489 5197.5508 5297.1514
343.5770 -15864.5401 -1738.9509 0.0000 0.0000
7707.1166 4093.8537 308.3232 -3613.2629 4156.1290
308.1026 -35.9103 -2.5907 656151.2420 -21.5756
-21.5845
[2189]ENERGY: 12620000 3209.7956 5163.8882 5248.3804
342.2901 -15801.2052 -1714.1662 0.0000 0.0000
7649.6889 4098.6718 306.0258 -3551.0170 4157.2972
308.0482 -42.0122 -98.9517 656151.2420 -19.2465
-19.2676
[2225]ENERGY: 12630000 3151.6103 5241.9788 5243.5647
323.1694 -15845.7831 -1755.1956 0.0000 0.0000
7733.6253 4092.9698 309.3837 -3640.6555 4156.2183
308.0410 -10.8314 19.6767 656151.2420 -24.3340
-24.3305
[2275]ENERGY: 12640000 3278.0667 5206.7384 5233.1310
330.3276 -15823.7776 -1746.4684 0.0000 0.0000
7619.4010 4097.4187 304.8141 -3521.9823 4157.2661
307.9725 -170.6636 -155.1006 656151.2420 -18.6897
-18.6598
[2311]ENERGY: 12650000 3131.1142 5230.6592 5271.6528
363.6805 -15887.3652 -1707.8235 0.0000 0.0000
7693.8340 4095.7520 307.7918 -3598.0821 4156.7585
308.1777 160.7551 7.9308 656151.2420 -21.1190
-21.1416
[2361]ENERGY: 12660000 3274.4570 5131.4912 5254.8823
335.6994 -15843.8975 -1710.9888 0.0000 0.0000
7657.6986 4099.3423 306.3462 -3558.3563 4157.6066
308.1116 52.3622 -32.0240 656151.2420 -16.7986
-16.7993
[2397]ENERGY: 12670000 3234.6203 5218.8212 5260.4888

323.0527 -15886.4105 -1722.1847 0.0000 0.0000
7669.7480 4098.1357 306.8283 -3571.6122 4158.1348
308.0768 93.5736 114.8040 656151.2420 -19.2244
-19.2084
[2447]ENERGY: 12680000 3262.9019 5207.6305 5249.2257
336.1836 -15804.3825 -1815.4706 0.0000 0.0000
7660.0690 4096.1575 306.4411 -3563.9115 4158.5914
308.0887 -113.0767 -106.9038 656151.2420 -17.1653
-17.1752
[2483]ENERGY: 12690000 3203.6420 5233.8311 5232.0687
348.7268 -15927.7830 -1738.1703 0.0000 0.0000
7742.2104 4094.5257 309.7271 -3647.6847 4157.0530
308.1025 37.2534 -7.1520 656151.2420 -18.9440
-18.9520
[2536]ENERGY: 12700000 3211.6274 5203.2454 5237.2606
341.6590 -15842.1105 -1805.9773 0.0000 0.0000
7749.2854 4094.9899 310.0102 -3654.2955 4158.1344
308.2085 -49.8376 45.2481 656151.2420 -20.9892
-20.9679
[2572]ENERGY: 12710000 3226.5813 5091.2602 5281.3389
365.8946 -15770.8718 -1799.0033 0.0000 0.0000
7701.2263 4096.4262 308.0876 -3604.8000 4158.4493
308.1553 -79.5754 -128.3973 656151.2420 -17.1544
-17.1593
[2622]ENERGY: 12720000 3141.3428 5175.1437 5308.3149
355.7782 -15831.8739 -1734.5223 0.0000 0.0000
7683.3180 4097.5013 307.3711 -3585.8167 4157.9267
308.1333 41.6856 11.5913 656151.2420 -17.5695
-17.5645
[2658]ENERGY: 12730000 3173.0565 5163.1565 5304.7803
336.1187 -15809.4309 -1814.3965 0.0000 0.0000
7741.3882 4094.6728 309.6942 -3646.7154 4158.5906
308.0030 33.1031 24.7452 656151.2420 -20.7937
-20.8186
[2708]ENERGY: 12740000 3149.5212 5187.0428 5301.7860
348.6694 -15865.4044 -1700.0656 0.0000 0.0000
7674.3253 4095.8746 307.0114 -3578.4508 4157.4477
308.0949 -39.4340 -57.5107 656151.2420 -16.8138
-16.8066
[2744]ENERGY: 12750000 3203.2832 5238.1327 5242.0571
332.1393 -15846.6109 -1780.3494 0.0000 0.0000
7707.9850 4096.6371 308.3579 -3611.3479 4158.4876
308.1293 -34.1120 -82.9201 656151.2420 -16.5010
-16.5033
[2794]ENERGY: 12760000 3213.0462 5157.8241 5306.1594
351.8984 -15869.8267 -1794.7447 0.0000 0.0000
7730.3133 4094.6701 309.2512 -3635.6433 4158.7616
308.1002 -74.8991 -79.8097 656151.2420 -21.1154
-21.1123
[2830]ENERGY: 12770000 3148.3654 5235.0698 5214.1387
343.2364 -15905.6279 -1765.8954 0.0000 0.0000
7824.0522 4093.3391 313.0012 -3730.7131 4159.5509
308.3249 18.6467 -9.3640 656151.2420 -15.4703
-15.4740

[2880]ENERGY: 12780000 3243.1170 5321.2753 5278.6381
342.4133 -15885.1846 -1826.2559 0.0000 0.0000
7621.9645 4095.9675 304.9167 -3525.9970 4159.8172
308.2789 -80.2753 -170.7837 656151.2420 -19.7711
-19.7809
[2916]ENERGY: 12790000 3261.8580 5213.8464 5270.8500
336.2667 -15841.4342 -1785.0458 0.0000 0.0000
7644.0998 4100.4409 305.8022 -3543.6589 4160.2721
308.1456 74.9615 5.8008 656151.2420 -20.9863
-20.9768
[2969]ENERGY: 12800000 3133.5243 5231.4865 5254.1412
342.0888 -15779.8509 -1767.3896 0.0000 0.0000
7685.0007 4099.0011 307.4385 -3585.9996 4160.5302
308.0296 92.1681 -21.9356 656151.2420 -21.0209
-21.0185
[3005]ENERGY: 12810000 3231.8758 5179.8421 5226.2635
353.0893 -15888.3932 -1732.9959 0.0000 0.0000
7729.1683 4098.8499 309.2054 -3630.3184 4159.6477
308.0967 32.7823 5.7164 656151.2420 -20.1804
-20.1750
[3055]ENERGY: 12820000 3212.7395 5219.3994 5236.6019
348.5949 -15875.3980 -1722.2334 0.0000 0.0000
7679.8105 4099.5148 307.2308 -3580.2957 4161.5961
308.1090 23.9421 1.3238 656151.2420 -19.0932
-19.0941
[3091]ENERGY: 12830000 3204.5852 5179.1407 5262.9640
323.7722 -15763.8876 -1792.4053 0.0000 0.0000
7685.0601 4099.2291 307.4408 -3585.8310 4162.3525
307.9651 41.4978 19.7660 656151.2420 -18.7502
-18.7445
[3141]ENERGY: 12840000 3218.6277 5252.7638 5248.6404
322.0298 -15902.3996 -1735.6456 0.0000 0.0000
7696.4922 4100.5087 307.8982 -3595.9836 4163.1352
308.2178 55.7945 68.1021 656151.2420 -20.6455
-20.6437
[3177]ENERGY: 12850000 3221.8965 5204.7805 5245.3145
333.4373 -15831.5654 -1791.8745 0.0000 0.0000
7716.8861 4098.8749 308.7140 -3618.0112 4162.8761
308.0983 43.4151 33.5674 656151.2420 -22.3845
-22.3880
[3227]ENERGY: 12860000 3153.6893 5145.5100 5276.0373
350.7033 -15790.9621 -1746.9660 0.0000 0.0000
7713.8507 4101.8624 308.5926 -3611.9883 4163.4899
308.1105 37.9553 -23.9139 656151.2420 -19.1644
-19.1767
[3263]ENERGY: 12870000 3177.6783 5220.6418 5321.6575
351.4139 -15854.8081 -1801.1131 0.0000 0.0000
7687.3799 4102.8502 307.5336 -3584.5297 4164.2289
308.1009 32.2319 -56.6546 656151.2420 -17.2180
-17.2216
[3313]ENERGY: 12880000 3258.5030 5181.3833 5268.9665
339.8060 -15872.2229 -1768.7421 0.0000 0.0000
7696.4823 4104.1760 307.8978 -3592.3063 4163.3442
308.1299 18.8452 -1.5448 656151.2420 -17.4376

-17.4150
[3349]ENERGY: 12890000 3136.2261 5244.9341 5298.2150
333.5625 -15841.6011 -1730.0279 0.0000 0.0000
7662.8236 4104.1323 306.5513 -3558.6912 4165.6502
308.1808 32.4189 -72.6931 656151.2420 -24.3640
-24.3868
[3402]ENERGY: 12900000 3259.3699 5242.0258 5256.9337
321.6947 -15932.9803 -1683.9133 0.0000 0.0000
7641.2345 4104.3650 305.6876 -3536.8695 4164.9484
307.9433 205.0444 92.5762 656151.2420 -17.8185
-17.7845
[3438]ENERGY: 12910000 3275.3463 5331.5926 5256.6293
317.5158 -15914.9078 -1778.0655 0.0000 0.0000
7619.3115 4107.4224 304.8106 -3511.8892 4164.9822
308.2012 50.7292 -70.3699 656151.2420 -21.5155
-21.5683
[3488]ENERGY: 12920000 3200.8293 5155.1227 5318.1164
350.1481 -15933.0204 -1760.0646 0.0000 0.0000
7771.7297 4102.8612 310.9080 -3668.8685 4165.7862
308.3199 64.2781 36.8794 656151.2420 -21.5092
-21.4602
[3524]ENERGY: 12930000 3228.5708 5189.3593 5295.2657
338.2114 -15869.2748 -1807.6437 0.0000 0.0000
7729.6561 4104.1447 309.2249 -3625.5114 4165.4645
308.2180 -121.6736 -226.1134 656151.2420 -21.5595
-21.5681
[3574]ENERGY: 12940000 3226.7964 5221.6949 5287.6317
326.8596 -15844.1387 -1821.5756 0.0000 0.0000
7709.4347 4106.7029 308.4159 -3602.7317 4166.5031
308.1769 -148.7876 -152.0746 656151.2420 -20.0969
-20.1141
[3610]ENERGY: 12950000 3145.7918 5331.7218 5267.8932
346.0927 -15874.4491 -1805.0660 0.0000 0.0000
7694.1030 4106.0875 307.8026 -3588.0155 4167.3235
308.0665 -103.4612 -158.8276 656151.2420 -19.5820
-19.5828
[3660]ENERGY: 12960000 3212.2853 5260.4369 5273.0227
350.3307 -15944.6580 -1760.6859 0.0000 0.0000
7712.8706 4103.6023 308.5534 -3609.2683 4166.1713
308.1497 -60.7206 -72.9668 656151.2420 -23.0953
-23.0778
[3696]ENERGY: 12970000 3263.9491 5180.8155 5285.1803
343.8719 -15831.7240 -1783.4842 0.0000 0.0000
7648.7734 4107.3820 305.9892 -3541.3914 4167.3268
307.9808 -74.4859 -126.5066 656151.2420 -19.7834
-19.7920
[3746]ENERGY: 12980000 3182.2202 5205.7646 5279.4158
339.5344 -15828.6632 -1776.1028 0.0000 0.0000
7703.1696 4105.3387 308.1653 -3597.8309 4167.3969
307.8256 2.7340 -76.5173 656151.2420 -24.4341
-24.4252
[267]ENERGY: 12990000 3205.3060 5293.2516 5301.9845
373.4929 -15858.0837 -1838.4873 0.0000 0.0000
7627.5983 4105.0623 305.1421 -3522.5360 4168.3919

308.1119 -2.0812 -38.3020 656151.2420 -19.6073
-19.6224
[308]ENERGY: 13000000 3213.2335 5186.6025 5257.4542
340.6402 -15805.7317 -1794.2734 0.0000 0.0000
7707.4353 4105.3606 308.3359 -3602.0747 4166.6923
308.0377 93.6560 57.1789 656151.2420 -19.6120
-19.6156
[360]ENERGY: 13010000 3176.1395 5176.5957 5294.3253
349.0092 -15827.1321 -1768.8478 0.0000 0.0000
7706.6764 4106.7663 308.3056 -3599.9102 4168.0216
308.1970 41.5737 41.2290 656151.2420 -19.3629
-19.3455
[398]ENERGY: 13020000 3230.6311 5245.2170 5323.6056
332.0260 -15800.6470 -1822.8862 0.0000 0.0000
7602.4614 4110.4079 304.1365 -3492.0535 4168.3279
308.1861 50.4406 34.2649 656151.2420 -20.3830
-20.4047
[450]ENERGY: 13030000 3171.0632 5287.2016 5280.1857
332.5454 -15908.1761 -1814.7690 0.0000 0.0000
7757.1801 4105.2310 310.3260 -3651.9492 4166.9714
308.1640 4.5017 -13.0152 656151.2420 -18.7991
-18.7857
[488]ENERGY: 13040000 3230.7496 5243.1159 5291.4502
339.1054 -15929.6117 -1756.3942 0.0000 0.0000
7686.7841 4105.1993 307.5098 -3581.5848 4167.3539
308.1860 102.8594 36.8051 656151.2420 -18.7851
-18.7975
[539]ENERGY: 13050000 3214.4592 5229.5809 5276.2692
332.2683 -15924.1269 -1796.0394 0.0000 0.0000
7769.1549 4101.5662 310.8050 -3667.5887 4167.3978
308.2460 -203.2716 -277.1457 656151.2420 -19.4663
-19.4596
[576]ENERGY: 13060000 3232.7945 5181.4075 5263.7685
331.6954 -15815.0624 -1741.3833 0.0000 0.0000
7654.2445 4107.4647 306.2081 -3546.7798 4167.7604
308.3489 0.3300 -29.4128 656151.2420 -21.4439
-21.4406
[627]ENERGY: 13070000 3217.9369 5275.3212 5250.1998
331.7426 -15899.0505 -1745.6125 0.0000 0.0000
7676.7881 4107.3255 307.1099 -3569.4626 4166.5986
308.2567 -68.5922 -91.6771 656151.2420 -21.8791
-21.8632
[664]ENERGY: 13080000 3137.1432 5169.5805 5265.6401
347.7297 -15823.7603 -1797.0876 0.0000 0.0000
7801.9594 4101.2050 312.1174 -3700.7544 4166.4495
308.3455 -40.5590 -27.6181 656151.2420 -16.7745
-16.8104
[715]ENERGY: 13090000 3281.7589 5219.3467 5276.8114
362.7164 -15853.0148 -1795.9459 0.0000 0.0000
7617.2120 4108.8847 304.7266 -3508.3273 4166.7013
308.2025 -109.1570 -113.8468 656151.2420 -24.1412
-24.1169
[754]ENERGY: 13100000 3234.8125 5223.3062 5286.6931
334.5909 -15968.0545 -1740.3328 0.0000 0.0000

7733.9357 4104.9512 309.3961 -3628.9845 4167.3250
308.1899 -95.4077 -186.5933 656151.2420 -21.8836
-21.8926
[804]ENERGY: 13110000 3224.6079 5195.4472 5221.7695
337.5385 -15854.2572 -1686.7073 0.0000 0.0000
7664.9587 4103.3572 306.6367 -3561.6015 4166.6944
308.2822 32.5854 -0.2009 656151.2420 -23.8773
-23.8569
[840]ENERGY: 13120000 3274.7910 5285.4031 5272.9307
338.3657 -15956.0691 -1698.8511 0.0000 0.0000
7596.3285 4112.8988 303.8911 -3483.4297 4167.6307
308.4309 30.6812 -102.5592 656151.2420 -17.7170
-17.7436
[890]ENERGY: 13130000 3169.0273 5215.6275 5282.3328
356.6975 -15810.6016 -1838.7141 0.0000 0.0000
7730.6811 4105.0505 309.2659 -3625.6306 4169.2689
308.2192 -90.6966 -96.8365 656151.2420 -20.1712
-20.1552
[926]ENERGY: 13140000 3195.9109 5190.1507 5310.2061
336.4091 -15779.2059 -1823.4280 0.0000 0.0000
7677.8472 4107.8902 307.1523 -3569.9570 4169.3604
308.4095 -102.2882 -164.8239 656151.2420 -20.5798
-20.5933
[976]ENERGY: 13150000 3175.5947 5265.6010 5335.9556
327.8425 -15877.5770 -1784.9794 0.0000 0.0000
7664.8265 4107.2638 306.6314 -3557.5627 4169.5056
308.4962 -116.3431 -122.6509 656151.2420 -19.4878
-19.4774
[1012]ENERGY: 13160000 3194.1939 5252.3155 5351.9033
348.4146 -15873.4168 -1831.4167 0.0000 0.0000
7663.0566 4105.0504 306.5606 -3558.0061 4169.6211
308.5872 9.6826 -65.0318 656151.2420 -18.9675
-18.9525
[1062]ENERGY: 13170000 3074.5009 5332.0350 5234.9213
353.2200 -15867.4695 -1738.9619 0.0000 0.0000
7716.1117 4104.3575 308.6830 -3611.7542 4169.5635
308.6222 79.1424 97.2080 656151.2420 -19.5286
-19.5372
[1098]ENERGY: 13180000 3130.7192 5169.4421 5267.0540
339.3334 -15823.4306 -1707.4329 0.0000 0.0000
7733.6322 4109.3175 309.3840 -3624.3148 4169.4669
308.7194 232.8941 167.3217 656151.2420 -22.1534
-22.1698
[1148]ENERGY: 13190000 3169.5789 5222.2126 5230.0967
351.0638 -15790.8317 -1766.8497 0.0000 0.0000
7695.2645 4110.5351 307.8491 -3584.7294 4170.6198
308.4946 -94.7057 -53.7570 656151.2420 -18.7879
-18.7637
[1187]ENERGY: 13200000 3167.0161 5156.1845 5269.3947
353.3055 -15853.3639 -1682.7579 0.0000 0.0000
7701.0547 4110.8336 308.0807 -3590.2210 4170.4603
308.2942 91.2316 5.6296 656151.2420 -24.2936
-24.3129
[1237]ENERGY: 13210000 3159.3928 5181.1258 5297.4335

323.5394 -15815.0488 -1769.8481 0.0000 0.0000
7730.2503 4106.8450 309.2487 -3623.4054 4169.9410
308.4218 -36.5855 -12.6324 656151.2420 -20.2714
-20.2636
[1273]ENERGY: 13220000 3210.5647 5138.0718 5269.6540
346.4476 -15839.1975 -1759.2414 0.0000 0.0000
7741.4970 4107.7962 309.6986 -3633.7008 4170.0344
308.1146 81.9716 74.5154 656151.2420 -20.2497
-20.2350
[1323]ENERGY: 13230000 3289.1389 5194.8351 5255.7540
327.7024 -15846.4984 -1796.7703 0.0000 0.0000
7684.8378 4108.9995 307.4319 -3575.8382 4171.0714
308.2604 -149.2590 -131.8218 656151.2420 -19.3383
-19.3378
[1359]ENERGY: 13240000 3234.3894 5123.1888 5278.3335
361.5268 -15849.4453 -1745.7708 0.0000 0.0000
7703.8200 4106.0423 308.1913 -3597.7776 4170.7580
308.3327 70.4202 55.7934 656151.2420 -20.5135
-20.5368
[1409]ENERGY: 13250000 3228.4414 5197.1673 5310.2638
343.6477 -15835.4736 -1750.9256 0.0000 0.0000
7616.9211 4110.0422 304.7149 -3506.8789 4170.8838
308.3766 19.7541 -42.7591 656151.2420 -23.7315
-23.7124
[1445]ENERGY: 13260000 3181.5606 5260.7249 5229.9919
328.5916 -15853.4873 -1754.0449 0.0000 0.0000
7716.5741 4109.9111 308.7015 -3606.6630 4171.3435
308.5762 77.2690 38.5039 656151.2420 -19.5000
-19.5078
[1495]ENERGY: 13270000 3179.2824 5279.5501 5275.5982
339.3263 -15907.9827 -1739.3876 0.0000 0.0000
7681.3042 4107.6908 307.2906 -3573.6134 4171.0297
308.6091 -38.5997 -104.2186 656151.2420 -20.8769
-20.8902
[1531]ENERGY: 13280000 3176.6654 5243.1721 5244.9917
338.8113 -15832.6830 -1770.9647 0.0000 0.0000
7711.9018 4111.8946 308.5146 -3600.0071 4170.6834
308.8069 -72.1746 -58.0185 656151.2420 -17.7732
-17.7496
[1581]ENERGY: 13290000 3137.3053 5181.7158 5313.2333
351.1339 -15886.0623 -1724.0734 0.0000 0.0000
7735.7972 4109.0497 309.4706 -3626.7475 4170.9658
308.6741 34.1831 -52.8406 656151.2420 -16.5872
-16.6011
[1620]ENERGY: 13300000 3225.5984 5278.7056 5310.6260
341.3424 -15881.6047 -1756.2244 0.0000 0.0000
7595.1901 4113.6333 303.8456 -3481.5568 4171.5218
308.8214 63.2158 26.2143 656151.2420 -20.4239
-20.4351
[1670]ENERGY: 13310000 3160.2125 5195.7954 5255.9665
351.9936 -15862.4000 -1719.3640 0.0000 0.0000
7725.3229 4107.5268 309.0515 -3617.7960 4170.2535
308.7046 -48.0717 -43.9824 656151.2420 -24.5565
-24.5371

[1706]ENERGY: 13320000 3143.1474 5145.4957 5283.2196
330.5857 -15770.8044 -1789.1176 0.0000 0.0000
7768.9067 4111.4331 310.7951 -3657.4736 4172.3593
308.7237 47.3115 37.2547 656151.2420 -16.4642
-16.4796
[1756]ENERGY: 13330000 3187.7385 5267.6240 5297.8437
319.7058 -15911.0853 -1762.3056 0.0000 0.0000
7708.9592 4108.4804 308.3969 -3600.4788 4172.8740
308.8503 -42.0075 -87.9090 656151.2420 -23.2485
-23.2456
[1792]ENERGY: 13340000 3185.5181 5221.9621 5258.2633
345.7574 -15849.2971 -1821.7421 0.0000 0.0000
7767.8103 4108.2719 310.7512 -3659.5383 4172.5639
308.8420 -75.5247 -71.1211 656151.2420 -22.9978
-22.9920
[1842]ENERGY: 13350000 3232.9504 5149.5415 5218.8801
372.0723 -15833.4812 -1738.2988 0.0000 0.0000
7710.6764 4112.3407 308.4656 -3598.3357 4173.2104
308.7810 16.7253 88.3196 656151.2420 -18.4974
-18.5111
[1878]ENERGY: 13360000 3198.3295 5309.1725 5259.4214
345.2739 -15845.0962 -1781.4230 0.0000 0.0000
7627.7216 4113.3997 305.1470 -3514.3220 4173.3688
308.6697 4.4729 17.4526 656151.2420 -14.9054
-14.8971
[1928]ENERGY: 13370000 3192.2036 5266.5202 5245.7546
336.2965 -15821.1438 -1861.9119 0.0000 0.0000
7754.8703 4112.5895 310.2336 -3642.2808 4174.6400
308.7040 -189.2374 -121.2531 656151.2420 -14.4339
-14.4157
[1964]ENERGY: 13380000 3161.0041 5252.9691 5286.1280
349.5050 -15826.6365 -1765.7135 0.0000 0.0000
7657.0392 4114.2955 306.3199 -3542.7438 4174.9802
308.6470 -84.1723 -55.3601 656151.2420 -15.7793
-15.7939
[2014]ENERGY: 13390000 3230.8394 5139.6880 5241.6588
335.0422 -15770.8021 -1766.9923 0.0000 0.0000
7701.8482 4111.2822 308.1124 -3590.5660 4174.9921
308.6512 -23.6619 -47.1545 656151.2420 -13.3139
-13.3101
[2053]ENERGY: 13400000 3258.6863 5249.2735 5283.2447
350.1893 -15931.7179 -1767.6506 0.0000 0.0000
7670.6531 4112.6784 306.8645 -3557.9747 4175.2515
308.7733 -22.6826 -2.0164 656151.2420 -18.6494
-18.6487
[2103]ENERGY: 13410000 3205.9862 5294.9626 5221.5002
326.3722 -15906.2384 -1760.2249 0.0000 0.0000
7730.9570 4113.3149 309.2769 -3617.6421 4173.9392
309.0206 217.9411 85.6053 656151.2420 -18.4619
-18.4606
[2139]ENERGY: 13420000 3198.9811 5254.3173 5285.3672
348.3272 -15838.5078 -1796.0151 0.0000 0.0000
7663.4614 4115.9312 306.5768 -3547.5302 4174.7834
308.9053 -147.3566 -112.8391 656151.2420 -12.0706

-12.0943
[2189]ENERGY: 13430000 3247.9968 5205.4587 5276.9785
340.3364 -15915.7718 -1727.7900 0.0000 0.0000
7685.8413 4113.0499 307.4721 -3572.7914 4173.3427
308.9447 34.7678 3.7460 656151.2420 -19.6386
-19.6279
[2225]ENERGY: 13440000 3164.1275 5300.1939 5273.5597
334.7047 -15869.9883 -1761.2449 0.0000 0.0000
7670.5414 4111.8939 306.8600 -3558.6475 4173.8207
308.7770 78.8943 73.7256 656151.2420 -17.5334
-17.5203
[2275]ENERGY: 13450000 3201.0613 5312.8080 5239.1347
348.1793 -15914.8198 -1817.5982 0.0000 0.0000
7741.4393 4110.2046 309.6963 -3631.2346 4174.0611
308.7280 -131.3309 -110.9023 656151.2420 -11.8281
-11.8420
[2311]ENERGY: 13460000 3183.4912 5159.6317 5275.8799
296.7533 -15773.0519 -1774.7103 0.0000 0.0000
7742.7247 4110.7186 309.7477 -3632.0061 4174.5997
308.8648 209.8748 108.2280 656151.2420 -15.5518
-15.5661
[2361]ENERGY: 13470000 3264.7359 5272.8501 5211.5354
342.8631 -15886.7273 -1778.2133 0.0000 0.0000
7687.7036 4114.7475 307.5466 -3572.9561 4175.2638
308.7166 -159.1899 -114.2008 656151.2420 -18.5535
-18.5212
[2397]ENERGY: 13480000 3169.7767 5222.1456 5248.1380
344.5181 -15836.3090 -1783.3608 0.0000 0.0000
7746.3888 4111.2975 309.8943 -3635.0914 4174.8574
308.9391 99.5296 99.8787 656151.2420 -12.0987
-12.1175
[2447]ENERGY: 13490000 3262.0438 5246.5639 5231.0218
346.5043 -15857.4901 -1790.0390 0.0000 0.0000
7673.7982 4112.4029 306.9903 -3561.3953 4175.4110
308.8775 -101.3850 -146.9543 656151.2420 -15.0873
-15.0724
[2486]ENERGY: 13500000 3279.6901 5200.1724 5227.4499
323.4507 -15804.3385 -1845.4873 0.0000 0.0000
7734.2876 4115.2249 309.4102 -3619.0626 4175.4595
308.8284 -109.0421 -130.5288 656151.2420 -17.6619
-17.6909
[2536]ENERGY: 13510000 3175.0447 5242.5129 5268.9224
342.2747 -15844.5354 -1780.3468 0.0000 0.0000
7711.2020 4115.0746 308.4866 -3596.1275 4175.1424
308.6676 147.6775 89.1865 656151.2420 -10.8903
-10.8608
[2572]ENERGY: 13520000 3192.7086 5249.5045 5236.5217
315.6242 -15786.6486 -1807.9042 0.0000 0.0000
7712.8664 4112.6727 308.5532 -3600.1938 4175.5312
308.7411 53.9616 35.7773 656151.2420 -16.7587
-16.7471
[2622]ENERGY: 13530000 3202.6513 5180.4791 5281.3411
351.4857 -15859.4539 -1739.9178 0.0000 0.0000
7696.6957 4113.2811 307.9063 -3583.4146 4174.5160

308.6732 -0.7413 -31.6768 656151.2420 -14.4103
-14.4387
[2658]ENERGY: 13540000 3147.8738 5168.4968 5274.2852
345.2393 -15775.8364 -1732.8525 0.0000 0.0000
7685.1010 4112.3073 307.4425 -3572.7938 4174.3525
308.8249 70.8417 70.4866 656151.2420 -13.1408
-13.1353
[2708]ENERGY: 13550000 3155.8350 5240.8759 5219.9815
316.2312 -15794.0048 -1761.5748 0.0000 0.0000
7734.9814 4112.3255 309.4379 -3622.6560 4175.8755
308.7164 -8.9604 6.9367 656151.2420 -16.4525
-16.4335
[2744]ENERGY: 13560000 3238.0406 5216.2739 5212.5165
324.9865 -15731.0809 -1826.9314 0.0000 0.0000
7683.7528 4117.5581 307.3885 -3566.1947 4177.0497
309.2258 -63.3262 -36.0713 656151.2420 -17.2768
-17.2896
[2794]ENERGY: 13570000 3217.8488 5182.6680 5262.3820
317.1749 -15847.1364 -1746.2867 0.0000 0.0000
7729.2363 4115.8868 309.2081 -3613.3495 4176.8477
309.1115 114.4898 73.5608 656151.2420 -12.6942
-12.7000
[2830]ENERGY: 13580000 3148.8749 5271.2119 5295.9062
353.3621 -15967.4655 -1722.8202 0.0000 0.0000
7735.3295 4114.3988 309.4519 -3620.9306 4176.4244
309.0968 138.0941 83.0807 656151.2420 -15.5171
-15.5200
[2880]ENERGY: 13590000 3258.4037 5211.3384 5313.4522
348.1888 -15880.6774 -1789.4738 0.0000 0.0000
7654.4956 4115.7276 306.2181 -3538.7681 4177.1277
309.2037 -35.2996 -104.7651 656151.2420 -17.7022
-17.7145
[2919]ENERGY: 13600000 3284.5264 5230.9634 5221.1233
350.4187 -15931.3597 -1748.3172 0.0000 0.0000
7708.6491 4116.0040 308.3845 -3592.6451 4177.1627
309.1598 -61.0425 -43.8476 656151.2420 -14.9240
-14.9105
[2969]ENERGY: 13610000 3164.6681 5186.5507 5274.5148
359.8289 -15861.9022 -1725.1914 0.0000 0.0000
7718.5467 4117.0157 308.7805 -3601.5310 4176.6561
309.1156 143.1671 80.5176 656151.2420 -16.5580
-16.5530
[3005]ENERGY: 13620000 3252.5246 5265.5753 5267.1715
339.0656 -15929.0714 -1823.8349 0.0000 0.0000
7744.4175 4115.8482 309.8154 -3628.5693 4176.9029
309.1468 79.0005 -54.5251 656151.2420 -14.8506
-14.8376
[3055]ENERGY: 13630000 3190.2537 5184.4690 5248.1123
359.3307 -15856.8878 -1757.4849 0.0000 0.0000
7746.6982 4114.4912 309.9067 -3632.2070 4177.3894
309.0122 39.9587 77.3180 656151.2420 -13.8623
-13.8783
[3091]ENERGY: 13640000 3177.0564 5250.0924 5311.2248
352.7305 -15877.0426 -1761.5561 0.0000 0.0000

7664.6394 4117.1449 306.6239 -3547.4945 4177.8674
309.1114 50.4491 -18.8109 656151.2420 -14.6885
-14.6896
[3141]ENERGY: 13650000 3213.0586 5205.9698 5223.3391
329.6677 -15815.4349 -1763.5719 0.0000 0.0000
7721.9481 4114.9764 308.9165 -3606.9716 4177.3140
309.0906 -71.9631 -77.3424 656151.2420 -14.1849
-14.1757
[3177]ENERGY: 13660000 3145.7284 5284.6295 5249.0319
339.1040 -15785.8910 -1864.8779 0.0000 0.0000
7744.6868 4112.4116 309.8262 -3632.2752 4177.1719
309.0930 -150.2011 -190.8600 656151.2420 -19.0836
-19.0850
[3227]ENERGY: 13670000 3211.3181 5267.1322 5206.2698
324.9417 -15807.4916 -1770.1614 0.0000 0.0000
7685.4349 4117.4437 307.4558 -3567.9912 4176.6139
309.0341 104.8763 -2.7312 656151.2420 -14.3796
-14.3938
[3263]ENERGY: 13680000 3116.2744 5210.2295 5263.1456
362.0196 -15859.2705 -1752.1844 0.0000 0.0000
7771.6123 4111.8265 310.9033 -3659.7858 4177.7977
308.9330 67.1193 21.7565 656151.2420 -13.4431
-13.4422
[3313]ENERGY: 13690000 3124.6406 5168.3221 5244.8943
332.8220 -15813.2507 -1707.0929 0.0000 0.0000
7762.7221 4113.0576 310.5477 -3649.6645 4177.7671
308.8005 66.8800 -38.8201 656151.2420 -16.0599
-16.0567
[3352]ENERGY: 13700000 3224.7036 5225.3627 5268.2560
349.6767 -15878.5056 -1746.8877 0.0000 0.0000
7671.6907 4114.2962 306.9060 -3557.3944 4176.5429
308.6923 -80.3856 -54.9976 656151.2420 -17.2611
-17.2510
[3402]ENERGY: 13710000 3232.5854 5210.5655 5244.4635
345.3087 -15837.2425 -1769.3405 0.0000 0.0000
7686.6669 4113.0068 307.5051 -3573.6601 4177.5041
308.5846 -36.7910 -6.3826 656151.2420 -17.2512
-17.2603
[3438]ENERGY: 13720000 3114.0425 5254.7826 5259.3017
341.2838 -15859.5877 -1751.8038 0.0000 0.0000
7757.8645 4115.8837 310.3534 -3641.9808 4178.3809
308.6934 43.8764 2.1482 656151.2420 -15.6933
-15.6891
[3488]ENERGY: 13730000 3217.1627 5337.5902 5179.4114
357.1962 -15841.5373 -1816.6371 0.0000 0.0000
7685.6825 4118.8686 307.4657 -3566.8139 4178.2865
308.8213 -56.9230 -68.2727 656151.2420 -15.1686
-15.1891
[3524]ENERGY: 13740000 3117.0804 5249.1179 5268.4058
339.9655 -15722.5916 -1767.9695 0.0000 0.0000
7634.1544 4118.1630 305.4043 -3515.9914 4177.6234
308.7848 -47.8096 -23.8180 656151.2420 -16.1254
-16.0902
[3574]ENERGY: 13750000 3138.9027 5208.3960 5267.3559

336.2432 -15822.4747 -1750.7231 0.0000 0.0000
7739.1482 4116.8481 309.6046 -3622.3001 4178.8555
308.9070 177.9376 90.2034 656151.2420 -18.9845
-18.9968
[3610]ENERGY: 13760000 3181.0354 5308.2398 5228.6217
308.9971 -15765.9953 -1825.2369 0.0000 0.0000
7682.5477 4118.2095 307.3403 -3564.3382 4178.1769
308.9264 -142.3575 -52.7472 656151.2420 -13.1447
-13.1328
[3660]ENERGY: 13770000 3147.3033 5261.0209 5310.1901
308.6025 -15811.4373 -1813.2956 0.0000 0.0000
7715.9627 4118.3467 308.6771 -3597.6160 4179.1537
308.8907 130.0995 76.7140 656151.2420 -15.3503
-15.3746
[3696]ENERGY: 13780000 3271.7646 5210.8221 5290.5411
347.7449 -16021.4190 -1712.4830 0.0000 0.0000
7728.7860 4115.7567 309.1901 -3613.0293 4178.9306
308.8653 74.1552 37.7207 656151.2420 -18.3846
-18.3826
[3746]ENERGY: 13790000 3218.4934 5234.3545 5281.3641
327.0837 -15794.9927 -1864.3678 0.0000 0.0000
7714.8393 4116.7744 308.6321 -3598.0649 4179.1775
308.9491 -134.7618 -140.1016 656151.2420 -18.2789
-18.2743
[3785]ENERGY: 13800000 3158.8375 5223.7631 5249.7883
323.2918 -15796.0762 -1772.0291 0.0000 0.0000
7732.2624 4119.8378 309.3292 -3612.4246 4180.0550
308.9495 -67.0861 -15.5448 656151.2420 -17.1503
-17.1484
[3835]ENERGY: 13810000 3216.4077 5218.8370 5220.1693
339.8094 -15804.1554 -1797.5105 0.0000 0.0000
7723.6345 4117.1921 308.9840 -3606.4425 4181.5188
309.1358 34.5440 -79.3469 656151.2420 -18.2412
-18.2452
[3871]ENERGY: 13820000 3154.3025 5204.6347 5297.3292
343.8053 -15821.5677 -1776.3540 0.0000 0.0000
7717.0899 4119.2399 308.7222 -3597.8501 4180.8470
309.1641 165.0149 19.1672 656151.2420 -18.4232
-18.4229
[3921]ENERGY: 13830000 3185.0311 5217.8429 5238.1614
336.0002 -15867.6375 -1747.6285 0.0000 0.0000
7759.7328 4121.5025 310.4281 -3638.2303 4181.5337
308.8551 83.0666 103.6440 656151.2420 -15.4105
-15.4063
[3957]ENERGY: 13840000 3158.8018 5212.1917 5336.8186
349.9853 -15886.4139 -1750.6246 0.0000 0.0000
7697.9370 4118.6960 307.9560 -3579.2410 4179.9374
308.8162 21.8530 53.2508 656151.2420 -17.9306
-17.9241
[4007]ENERGY: 13850000 3158.7151 5171.9146 5311.9205
354.8339 -15856.2758 -1728.9961 0.0000 0.0000
7707.2107 4119.3228 308.3270 -3587.8879 4181.1115
308.9756 -77.3710 -79.1702 656151.2420 -21.0502
-21.0445

[4043]ENERGY: 13860000 3199.5928 5166.9288 5229.6922
316.6822 -15758.5761 -1780.0143 0.0000 0.0000
7749.9897 4124.2954 310.0383 -3625.6943 4182.2276
308.9343 -42.7593 -19.7091 656151.2420 -12.6486
-12.6621
[4093]ENERGY: 13870000 3166.1547 5285.0246 5282.5283
326.0734 -15882.8763 -1769.1450 0.0000 0.0000
7713.2580 4121.0178 308.5689 -3592.2403 4183.4229
309.0736 -58.3473 -34.1411 656151.2420 -18.3789
-18.3859
[4129]ENERGY: 13880000 3227.8106 5232.1374 5205.5626
335.6330 -15879.0376 -1778.7885 0.0000 0.0000
7774.8391 4118.1565 311.0324 -3656.6826 4183.1710
309.0526 -16.3421 -69.4060 656151.2420 -14.6413
-14.6366
[4179]ENERGY: 13890000 3180.3619 5181.2979 5281.5529
345.2285 -15800.9450 -1776.3910 0.0000 0.0000
7715.3005 4126.4057 308.6506 -3588.8948 4183.6094
309.0633 78.5443 75.5864 656151.2420 -15.8078
-15.8002
[4218]ENERGY: 13900000 3186.6877 5153.9938 5254.3806
332.2089 -15842.3462 -1746.9265 0.0000 0.0000
7783.7847 4121.7830 311.3903 -3662.0017 4184.1445
309.0264 75.3544 -28.9120 656151.2420 -15.0542
-15.0626
[4268]ENERGY: 13910000 3260.7090 5246.4834 5275.8191
336.5479 -15851.0227 -1817.3331 0.0000 0.0000
7674.4169 4125.6205 307.0150 -3548.7964 4185.4798
308.9961 55.8214 6.8357 656151.2420 -15.8128
-15.8078
[4304]ENERGY: 13920000 3174.6848 5215.2226 5255.8138
330.7970 -15873.0018 -1737.1701 0.0000 0.0000
7752.0387 4118.3850 310.1203 -3633.6537 4183.9741
309.0718 -5.8903 41.4298 656151.2420 -14.2137
-14.1985
[4354]ENERGY: 13930000 3201.7957 5372.7967 5231.4451
323.1804 -15895.1202 -1830.9647 0.0000 0.0000
7716.1463 4119.2792 308.6844 -3596.8671 4185.8699
308.9669 -140.3993 -154.5807 656151.2420 -15.6719
-15.6870
[4390]ENERGY: 13940000 3143.9047 5291.0973 5276.1107
351.3267 -15790.0082 -1856.1734 0.0000 0.0000
7707.1365 4123.3942 308.3240 -3583.7422 4185.4216
309.0587 -53.1052 -86.4574 656151.2420 -12.0373
-12.0330
[4440]ENERGY: 13950000 3237.9374 5192.6943 5242.3252
314.2025 -15882.8048 -1733.8443 0.0000 0.0000
7750.0905 4120.6008 310.0424 -3629.4897 4184.6263
308.8605 -80.6271 -140.7353 656151.2420 -15.5506
-15.5669
[4476]ENERGY: 13960000 3220.2517 5194.1246 5277.4923
342.9906 -15863.0200 -1758.0152 0.0000 0.0000
7708.9615 4122.7854 308.3970 -3586.1760 4185.7411
309.0220 -84.7172 -110.6006 656151.2420 -13.3733

-13.3591
[4526]ENERGY: 13970000 3286.6847 5210.1509 5259.8565
341.1823 -15810.2355 -1754.5992 0.0000 0.0000
7591.4815 4124.5212 303.6972 -3466.9603 4185.0105
309.2058 -63.6314 -77.6545 656151.2420 -17.7814
-17.7780
[4562]ENERGY: 13980000 3195.5175 5240.3387 5238.8091
336.5088 -15800.3463 -1824.2977 0.0000 0.0000
7737.8565 4124.3866 309.5529 -3613.4700 4185.1348
309.0858 -132.4764 -157.0889 656151.2420 -16.9664
-16.9692
[4612]ENERGY: 13990000 3182.7859 5294.0687 5278.7556
346.3580 -15797.8469 -1737.1205 0.0000 0.0000
7561.6632 4128.6639 302.5043 -3432.9993 4186.6465
309.0064 181.4858 173.2635 656151.2420 -13.4591
-13.4693
[4651]ENERGY: 14000000 3130.8038 5238.0015 5305.7121
328.7077 -15874.3894 -1799.7096 0.0000 0.0000
7795.1537 4124.2798 311.8451 -3670.8739 4187.2419
308.9899 -45.0615 -95.1919 656151.2420 -14.6462
-14.6281
[4701]ENERGY: 14010000 3168.3766 5202.6711 5258.9017
326.0845 -15799.8887 -1778.4099 0.0000 0.0000
7743.4882 4121.2236 309.7782 -3622.2647 4185.8406
308.9151 57.5294 68.3367 656151.2420 -14.0547
-14.0528
[4737]ENERGY: 14020000 3160.5539 5179.8774 5277.4060
372.5947 -15818.1133 -1753.1331 0.0000 0.0000
7704.6442 4123.8298 308.2243 -3580.8144 4185.5210
309.0998 42.6301 25.4852 656151.2420 -12.1865
-12.2096
[4787]ENERGY: 14030000 3194.7229 5221.0484 5272.8563
325.2708 -15878.6838 -1809.5571 0.0000 0.0000
7795.2067 4120.8642 311.8472 -3674.3425 4184.5324
309.1262 -185.7926 -95.3613 656151.2420 -18.1985
-18.1930
[4823]ENERGY: 14040000 3215.9267 5260.8042 5262.4723
340.0132 -15906.6766 -1766.4715 0.0000 0.0000
7716.1947 4122.2630 308.6864 -3593.9317 4185.6040
308.9487 140.3411 57.3396 656151.2420 -19.0128
-19.0095
[4873]ENERGY: 14050000 3169.8174 5216.4413 5288.0086
373.8309 -15907.6027 -1778.2444 0.0000 0.0000
7758.9562 4121.2072 310.3970 -3637.7490 4186.0427
309.1097 59.9407 -11.6213 656151.2420 -12.4704
-12.4616
[4909]ENERGY: 14060000 3160.9463 5119.7532 5246.4801
339.7796 -15759.6774 -1790.6979 0.0000 0.0000
7805.3349 4121.9188 312.2524 -3683.4161 4184.7259
309.3209 -37.0361 -50.2426 656151.2420 -18.4585
-18.4682
[4959]ENERGY: 14070000 3204.4152 5216.3817 5221.3157
335.5733 -15827.6744 -1779.3039 0.0000 0.0000
7752.1017 4122.8092 310.1228 -3629.2924 4184.9446

309.3411 48.4211 37.9292 656151.2420 -18.9322
-18.9190
[4995]ENERGY: 14080000 3188.5674 5214.0124 5246.4965
346.7356 -15814.4580 -1758.8460 0.0000 0.0000
7703.2740 4125.7819 308.1695 -3577.4922 4185.5865
309.1746 -49.0727 -108.3943 656151.2420 -16.7247
-16.7310
[5045]ENERGY: 14090000 3255.4691 5186.1184 5221.3292
344.3830 -15868.6371 -1730.3851 0.0000 0.0000
7715.5853 4123.8628 308.6620 -3591.7225 4187.3422
309.2204 203.5602 97.3086 656151.2420 -19.7478
-19.7598
[5084]ENERGY: 14100000 3231.3360 5114.2496 5257.8636
322.7053 -15824.7928 -1708.0189 0.0000 0.0000
7733.4446 4126.7874 309.3764 -3606.6571 4187.3197
309.3831 -0.4428 15.5295 656151.2420 -26.1283
-26.1083
[5134]ENERGY: 14110000 3190.1038 5222.4540 5281.0873
330.1505 -15891.7234 -1736.6782 0.0000 0.0000
7730.8773 4126.2713 309.2737 -3604.6060 4186.0899
309.1776 103.9830 97.5937 656151.2420 -16.0117
-16.0276
[5170]ENERGY: 14120000 3222.4023 5225.8028 5308.1463
339.3288 -15824.7663 -1797.3815 0.0000 0.0000
7652.5954 4126.1279 306.1421 -3526.4675 4186.7796
309.3299 -158.4094 -119.9951 656151.2420 -14.1881
-14.1763
[5220]ENERGY: 14130000 3193.6973 5201.8875 5271.7506
341.0180 -15796.7274 -1832.7912 0.0000 0.0000
7747.3796 4126.2144 309.9339 -3621.1652 4186.9700
309.3391 -46.8502 -29.5053 656151.2420 -16.6336
-16.6328
[5256]ENERGY: 14140000 3302.5540 5301.3951 5295.1880
343.9507 -15905.2492 -1880.9277 0.0000 0.0000
7670.2686 4127.1795 306.8491 -3543.0891 4186.4468
309.3751 -248.9252 -227.5581 656151.2420 -12.7297
-12.7315
[5306]ENERGY: 14150000 3218.5331 5213.5191 5268.7758
338.5247 -15828.1168 -1767.5731 0.0000 0.0000
7680.3398 4124.0026 307.2520 -3556.3372 4185.0910
309.4202 46.9496 -50.7088 656151.2420 -15.3822
-15.3903
[5342]ENERGY: 14160000 3192.9592 5217.9421 5284.1016
342.0198 -15851.0770 -1778.9940 0.0000 0.0000
7715.7688 4122.7205 308.6693 -3593.0484 4184.9430
309.2598 221.5150 178.4623 656151.2420 -17.0889
-17.0713
[5392]ENERGY: 14170000 3154.6936 5140.5336 5291.0070
346.7243 -15776.0118 -1738.4189 0.0000 0.0000
7702.8314 4121.3592 308.1518 -3581.4722 4185.1829
309.3294 120.0371 39.3197 656151.2420 -17.0514
-17.0691
[5428]ENERGY: 14180000 3246.4671 5175.7967 5249.0902
339.0905 -15864.4338 -1784.7544 0.0000 0.0000

7758.9752 4120.2315 310.3978 -3638.7437 4185.7091
309.4262 -155.7909 -122.1099 656151.2420 -17.8979
-17.8891
[5478]ENERGY: 14190000 3242.0625 5191.3756 5231.8666
343.4453 -15789.2890 -1845.7410 0.0000 0.0000
7750.9610 4124.6810 310.0772 -3626.2799 4185.3410
309.3707 -44.7900 -37.3396 656151.2420 -17.3421
-17.3404
[5517]ENERGY: 14200000 3255.8255 5232.1774 5289.2516
363.7433 -15894.6648 -1787.8588 0.0000 0.0000
7665.7315 4124.2056 306.6676 -3541.5258 4185.7211
309.3589 -45.8821 -147.6018 656151.2420 -16.8588
-16.8678
[5567]ENERGY: 14210000 3168.5566 5240.9801 5232.7209
332.1427 -15809.2367 -1795.4572 0.0000 0.0000
7754.7441 4124.4504 310.2285 -3630.2937 4185.9291
309.2302 -96.8963 -10.3620 656151.2420 -18.8026
-18.7932
[5603]ENERGY: 14220000 3274.9844 5144.0091 5256.7887
330.2416 -15702.9843 -1888.1065 0.0000 0.0000
7710.2788 4125.2119 308.4497 -3585.0670 4186.2215
309.2845 -110.6473 -153.5177 656151.2420 -16.4434
-16.4407
[5653]ENERGY: 14230000 3171.1749 5254.4411 5217.0349
349.0569 -15894.9259 -1787.5445 0.0000 0.0000
7810.8319 4120.0694 312.4723 -3690.7625 4186.6500
309.4043 -6.2445 -17.9451 656151.2420 -18.8949
-18.9061
[5689]ENERGY: 14240000 3235.0552 5264.9855 5242.3421
354.9173 -15850.1609 -1773.4320 0.0000 0.0000
7652.7520 4126.4592 306.1483 -3526.2928 4186.4192
309.2522 -144.8675 -75.3089 656151.2420 -15.8817
-15.8753
[5739]ENERGY: 14250000 3190.6058 5107.4073 5221.0543
317.9567 -15815.2763 -1744.7072 0.0000 0.0000
7844.5335 4121.5742 313.8206 -3722.9593 4185.4826
309.1843 -57.8870 -44.0145 656151.2420 -16.9347
-16.9477
[5775]ENERGY: 14260000 3224.4683 5313.8982 5234.1870
336.7483 -15903.9667 -1761.2943 0.0000 0.0000
7679.4303 4123.4712 307.2156 -3555.9590 4186.3286
309.3405 50.8391 28.1366 656151.2420 -15.6625
-15.6522
[5825]ENERGY: 14270000 3134.1672 5199.5623 5254.5540
338.3088 -15792.8722 -1758.9784 0.0000 0.0000
7751.5113 4126.2530 310.0992 -3625.2583 4187.7383
309.3593 157.3350 66.2435 656151.2420 -19.0506
-19.0570
[5861]ENERGY: 14280000 3093.7932 5131.9416 5284.1457
352.1154 -15823.0835 -1774.5881 0.0000 0.0000
7857.4548 4121.7790 314.3375 -3735.6758 4188.1708
309.1749 76.6845 7.7948 656151.2420 -19.2857
-19.2825
[5911]ENERGY: 14290000 3149.3190 5197.2651 5248.6128

314.8419 -15758.9662 -1753.3729 0.0000 0.0000
7730.3632 4128.0629 309.2532 -3602.3003 4188.0437
309.1284 64.0783 31.9496 656151.2420 -14.5186
-14.5262
[5950]ENERGY: 14300000 3164.8026 5226.8368 5240.4568
354.1976 -15803.7332 -1811.3874 0.0000 0.0000
7753.0635 4124.2366 310.1613 -3628.8269 4188.3896
309.2536 3.7678 -78.0757 656151.2420 -21.2303
-21.2286
[6000]ENERGY: 14310000 3192.8666 5175.6131 5292.0940
338.7393 -15880.6113 -1718.3560 0.0000 0.0000
7725.0521 4125.3979 309.0407 -3599.6543 4186.4800
309.1574 74.0009 45.2993 656151.2420 -19.0536
-19.0482
[6036]ENERGY: 14320000 3152.4988 5177.1166 5301.0351
331.7817 -15794.9675 -1716.6832 0.0000 0.0000
7677.8678 4128.6492 307.1531 -3549.2186 4188.7738
309.0720 178.7196 77.6907 656151.2420 -17.1074
-17.1145
[6086]ENERGY: 14330000 3206.6975 5315.2074 5268.5380
349.7838 -15908.0264 -1816.0939 0.0000 0.0000
7708.6006 4124.7071 308.3826 -3583.8935 4189.4312
309.2491 -220.3468 -142.8849 656151.2420 -18.2286
-18.2056
[6122]ENERGY: 14340000 3176.9709 5240.8580 5272.7152
344.5234 -15866.8599 -1788.4607 0.0000 0.0000
7747.4925 4127.2395 309.9384 -3620.2530 4190.3512
309.1733 0.2879 -49.6315 656151.2420 -14.7166
-14.7473
[6172]ENERGY: 14350000 3215.6114 5223.0644 5279.8187
320.7588 -15871.4074 -1752.9178 0.0000 0.0000
7714.5498 4129.4780 308.6206 -3585.0719 4189.6344
309.2869 162.7820 93.8713 656151.2420 -21.0260
-21.0063
[6208]ENERGY: 14360000 3214.4408 5210.7720 5251.1257
337.4481 -15834.3816 -1784.0815 0.0000 0.0000
7732.6349 4127.9583 309.3441 -3604.6766 4189.8776
309.2878 -68.0082 -84.4713 656151.2420 -18.7236
-18.7263
[6258]ENERGY: 14370000 3222.2988 5188.9060 5295.9016
317.0195 -15871.3075 -1729.7314 0.0000 0.0000
7706.1828 4129.2699 308.2858 -3576.9129 4189.7056
309.1411 -32.8325 -124.9147 656151.2420 -22.1013
-22.0983
[6294]ENERGY: 14380000 3175.2670 5259.7708 5237.5912
357.7122 -15800.7953 -1801.8905 0.0000 0.0000
7699.7917 4127.4470 308.0302 -3572.3447 4191.2775
309.0881 156.5464 26.8432 656151.2420 -14.3280
-14.3328
[6344]ENERGY: 14390000 3174.1183 5216.2730 5245.2248
357.8380 -15806.9348 -1829.3226 0.0000 0.0000
7766.7788 4123.9756 310.7100 -3642.8032 4190.2329
309.0824 81.0992 105.6167 656151.2420 -18.2218
-18.2186

[6383]ENERGY: 14400000 3171.0805 5335.3566 5229.2965
343.4491 -15917.0102 -1782.0509 0.0000 0.0000
7747.3889 4127.5105 309.9343 -3619.8784 4189.4250
309.2789 156.7735 134.4340 656151.2420 -14.9838
-14.9782
[6433]ENERGY: 14410000 3163.2464 5359.6987 5231.0680
340.6817 -15869.8231 -1814.1795 0.0000 0.0000
7717.7657 4128.4579 308.7492 -3589.3078 4190.3141
309.3213 51.9711 8.6760 656151.2420 -14.0671
-14.0752
[6469]ENERGY: 14420000 3208.3749 5210.2620 5240.3219
337.8659 -15797.7740 -1811.2434 0.0000 0.0000
7737.9039 4125.7113 309.5548 -3612.1926 4190.7189
309.5121 83.8972 17.6090 656151.2420 -15.7887
-15.8060
[6519]ENERGY: 14430000 3176.3008 5155.7768 5270.3115
341.1353 -15848.1098 -1732.7396 0.0000 0.0000
7761.4192 4124.0942 310.4956 -3637.3249 4188.8465
309.4058 78.8751 67.1931 656151.2420 -17.0326
-17.0346
[6555]ENERGY: 14440000 3225.5889 5151.3636 5304.9659
357.8229 -15885.1843 -1819.6836 0.0000 0.0000
7791.2029 4126.0762 311.6871 -3665.1267 4188.7262
309.3317 -73.7170 -62.4989 656151.2420 -15.3094
-15.2830
[6605]ENERGY: 14450000 3197.3405 5246.9269 5256.4723
325.7236 -15823.5026 -1802.8853 0.0000 0.0000
7726.2366 4126.3119 309.0881 -3599.9248 4188.0125
309.3156 -45.0099 -43.9513 656151.2420 -15.6974
-15.6948
[6641]ENERGY: 14460000 3205.4099 5219.1098 5271.8235
316.3965 -15837.1043 -1780.4459 0.0000 0.0000
7727.5050 4122.6944 309.1388 -3604.8105 4187.1340
309.3768 -190.9115 -143.2877 656151.2420 -16.3934
-16.3994
[6691]ENERGY: 14470000 3200.5203 5177.0278 5301.8373
363.5226 -15820.4245 -1843.5254 0.0000 0.0000
7747.0220 4125.9800 309.9196 -3621.0419 4186.7460
309.3218 38.7072 -29.6133 656151.2420 -17.5776
-17.5928
[6727]ENERGY: 14480000 3152.9381 5133.3088 5273.6755
358.3459 -15893.0414 -1657.3481 0.0000 0.0000
7757.8068 4125.6854 310.3511 -3632.1213 4187.3043
309.2694 308.0049 271.3607 656151.2420 -16.1585
-16.1602
[6777]ENERGY: 14490000 3207.8906 5243.6552 5280.7718
329.4491 -15799.8429 -1774.9563 0.0000 0.0000
7639.4240 4126.3916 305.6152 -3513.0324 4187.0368
309.2638 26.6066 -0.6625 656151.2420 -17.0655
-17.0588
[6816]ENERGY: 14500000 3158.6783 5301.9073 5260.5919
328.2754 -15917.4994 -1789.4366 0.0000 0.0000
7780.7846 4123.3016 311.2703 -3657.4831 4189.1905
309.3745 51.3453 15.8412 656151.2420 -14.4418

-14.4301
[6866]ENERGY: 14510000 3215.6101 5248.3501 5244.1663
342.0602 -15963.3045 -1698.5049 0.0000 0.0000
7739.6335 4128.0108 309.6240 -3611.6227 4188.1840
309.2255 179.6576 119.1930 656151.2420 -20.0329
-20.0306
[6902]ENERGY: 14520000 3219.3456 5238.4750 5252.4942
339.2658 -15815.2772 -1797.0265 0.0000 0.0000
7693.0434 4130.3204 307.7602 -3562.7230 4189.8833
309.5014 4.2828 -47.5297 656151.2420 -15.5209
-15.5290
[6952]ENERGY: 14530000 3129.7253 5251.2036 5309.0444
345.5056 -15840.0154 -1792.8800 0.0000 0.0000
7727.6637 4130.2471 309.1452 -3597.4166 4189.5174
309.3090 -27.9989 -55.1102 656151.2420 -19.5229
-19.5300
[6988]ENERGY: 14540000 3224.6202 5195.1665 5292.0321
344.9964 -15933.1335 -1713.2182 0.0000 0.0000
7717.9389 4128.4025 308.7561 -3589.5364 4189.4538
309.2220 64.1689 -3.0873 656151.2420 -17.7993
-17.8016
[7038]ENERGY: 14550000 3305.8621 5202.6061 5253.4909
339.4111 -15849.8800 -1833.7348 0.0000 0.0000
7707.7499 4125.5055 308.3485 -3582.2445 4188.6657
309.2076 7.6913 -38.9190 656151.2420 -17.5646
-17.5736
[7074]ENERGY: 14560000 3184.7696 5112.3323 5287.5108
352.4224 -15895.3051 -1703.5821 0.0000 0.0000
7786.4663 4124.6143 311.4976 -3661.8521 4188.2488
309.2879 104.9740 138.5435 656151.2420 -17.5298
-17.5201
[7124]ENERGY: 14570000 3158.5809 5184.8051 5304.4841
360.7349 -15803.6093 -1809.7065 0.0000 0.0000
7729.1799 4124.4690 309.2058 -3604.7109 4189.4536
309.2860 53.2150 48.4115 656151.2420 -13.9903
-13.9876
[7160]ENERGY: 14580000 3152.3736 5225.6580 5327.0103
317.1516 -15886.7781 -1803.5159 0.0000 0.0000
7794.7034 4126.6030 311.8271 -3668.1005 4189.4381
309.3258 -16.9721 -94.5012 656151.2420 -18.4095
-18.4025
[7210]ENERGY: 14590000 3138.6512 5222.3853 5269.9390
332.2547 -15836.1046 -1746.1880 0.0000 0.0000
7746.3569 4127.2944 309.8930 -3619.0625 4189.8762
309.2165 -16.1593 18.0935 656151.2420 -12.2000
-12.1944
[7249]ENERGY: 14600000 3214.5609 5180.0773 5309.7329
338.1959 -15853.0897 -1711.8118 0.0000 0.0000
7649.5963 4127.2618 306.0221 -3522.3345 4189.4391
309.2101 173.3057 107.3203 656151.2420 -15.9238
-15.9324
[7299]ENERGY: 14610000 3172.6583 5278.6044 5233.5852
335.1786 -15821.7126 -1784.6002 0.0000 0.0000
7710.1933 4123.9071 308.4463 -3586.2862 4188.6815

309.2693 129.9664 55.4121 656151.2420 -19.3242
-19.3278
[7335]ENERGY: 14620000 3195.9847 5213.4566 5303.9858
330.5860 -15812.6336 -1817.2972 0.0000 0.0000
7713.0649 4127.1472 308.5612 -3585.9177 4189.7449
309.0908 28.2940 37.9305 656151.2420 -13.8576
-13.8532
[7385]ENERGY: 14630000 3202.9654 5330.5866 5289.0391
345.9707 -15896.8894 -1782.5719 0.0000 0.0000
7640.3531 4129.4537 305.6523 -3510.8994 4189.4474
309.2580 -17.2094 -80.3083 656151.2420 -16.8389
-16.8315
[7421]ENERGY: 14640000 3258.0493 5209.0445 5277.0961
345.9915 -15832.1659 -1808.8756 0.0000 0.0000
7681.2692 4130.4092 307.2892 -3550.8600 4189.2304
309.2285 107.2372 56.9387 656151.2420 -15.3933
-15.4072
[7471]ENERGY: 14650000 3293.9827 5166.8189 5234.1137
364.8216 -15801.5773 -1810.0932 0.0000 0.0000
7681.7527 4129.8193 307.3085 -3551.9334 4189.2660
309.1374 -165.7949 -61.2906 656151.2420 -15.3758
-15.3645
[7507]ENERGY: 14660000 3233.9044 5201.2339 5303.7664
368.7789 -15853.5400 -1848.5763 0.0000 0.0000
7723.2562 4128.8234 308.9689 -3594.4328 4190.9491
309.3542 -108.1849 -164.1346 656151.2420 -12.6282
-12.6259
[7557]ENERGY: 14670000 3195.9115 5300.9267 5274.0681
343.5701 -15809.8675 -1881.4889 0.0000 0.0000
7704.0678 4127.1878 308.2012 -3576.8800 4191.5439
309.2419 -204.3511 -176.7575 656151.2420 -14.1067
-14.1017
[7593]ENERGY: 14680000 3156.0166 5279.2349 5266.8742
326.0178 -15970.4799 -1715.7012 0.0000 0.0000
7783.7397 4125.7020 311.3885 -3658.0377 4190.9791
309.2917 78.1770 71.6889 656151.2420 -14.5174
-14.5247
[7643]ENERGY: 14690000 3211.0045 5206.2466 5318.8762
347.2336 -15832.7121 -1821.3018 0.0000 0.0000
7699.2842 4128.6312 308.0099 -3570.6530 4190.4911
309.1159 7.1279 -116.9607 656151.2420 -15.7066
-15.7223
[7682]ENERGY: 14700000 3288.5682 5088.9179 5296.0161
348.1760 -15828.3815 -1794.0090 0.0000 0.0000
7730.2336 4129.5214 309.2480 -3600.7123 4189.1126
309.1450 -28.7633 41.4127 656151.2420 -15.4399
-15.4230
[7732]ENERGY: 14710000 3239.7769 5249.5610 5258.5266
344.5154 -15865.4833 -1801.2141 0.0000 0.0000
7702.8300 4128.5125 308.1517 -3574.3175 4190.7879
309.1789 25.6337 66.7161 656151.2420 -16.0514
-16.0550
[7768]ENERGY: 14720000 3118.8108 5333.7110 5248.8454
333.7441 -15882.4507 -1738.0315 0.0000 0.0000

7716.3876 4131.0167 308.6941 -3585.3709 4190.0174
309.2836 28.6332 23.7767 656151.2420 -16.9619
-16.9448
[7818]ENERGY: 14730000 3189.4050 5193.1013 5268.9562
339.8943 -15848.2496 -1751.8600 0.0000 0.0000
7735.1266 4126.3739 309.4437 -3608.7527 4189.9130
309.2476 -100.3296 -121.7460 656151.2420 -12.2777
-12.3158
[7854]ENERGY: 14740000 3188.8315 5217.6284 5274.4669
324.7402 -15838.4346 -1810.8967 0.0000 0.0000
7768.3053 4124.6411 310.7711 -3643.6642 4190.7432
309.2562 60.3123 40.2502 656151.2420 -17.3322
-17.2960
[7904]ENERGY: 14750000 3234.3931 5256.4807 5278.5582
327.5707 -15903.9992 -1803.3530 0.0000 0.0000
7740.8189 4130.4695 309.6715 -3610.3494 4190.5699
309.2767 33.6779 28.2070 656151.2420 -12.7577
-12.7692
[7940]ENERGY: 14760000 3212.8698 5211.0149 5322.7198
313.9200 -15824.6268 -1808.4349 0.0000 0.0000
7702.6228 4130.0855 308.1434 -3572.5372 4189.9730
309.2246 69.3941 29.0168 656151.2420 -15.1479
-15.1474
[7990]ENERGY: 14770000 3165.4893 5274.0143 5248.8782
319.3131 -15924.6305 -1772.6576 0.0000 0.0000
7815.9565 4126.3633 312.6773 -3689.5932 4190.4435
309.3327 -11.7172 30.7135 656151.2420 -12.8711
-12.8861
[8026]ENERGY: 14780000 3128.7465 5227.3612 5259.5949
326.3087 -15731.5587 -1835.2674 0.0000 0.0000
7751.5483 4126.7336 310.1007 -3624.8147 4191.4590
309.2896 -55.1629 -119.8620 656151.2420 -13.1626
-13.1582
[8076]ENERGY: 14790000 3121.6819 5295.5321 5280.9611
344.2775 -15867.5302 -1781.3692 0.0000 0.0000
7736.5474 4130.1007 309.5006 -3606.4467 4192.1806
309.3735 112.6936 105.1518 656151.2420 -17.2745
-17.2608
[8115]ENERGY: 14800000 3162.5856 5297.7191 5303.5631
349.5201 -15836.0344 -1804.6622 0.0000 0.0000
7659.4122 4132.1034 306.4148 -3527.3089 4190.9473
309.3449 14.0716 -65.1096 656151.2420 -22.5482
-22.5619
[8165]ENERGY: 14810000 3139.8242 5251.9052 5268.8408
349.3447 -15818.5532 -1761.7648 0.0000 0.0000
7699.4865 4129.0834 308.0180 -3570.4031 4190.8723
309.2718 12.8781 19.2111 656151.2420 -18.1018
-18.0982
[8201]ENERGY: 14820000 3251.1630 5206.8424 5261.4769
332.2980 -15790.2069 -1819.8684 0.0000 0.0000
7690.4174 4132.1224 307.6551 -3558.2950 4191.3806
309.3644 -174.2865 -160.9988 656151.2420 -16.9923
-16.9958
[8251]ENERGY: 14830000 3129.5523 5297.7934 5297.2207

347.5133 -15779.7046 -1853.3462 0.0000 0.0000
7690.2212 4129.2500 307.6473 -3560.9712 4191.5554
309.3587 77.1940 17.1290 656151.2420 -12.3615
-12.3702
[8287]ENERGY: 14840000 3181.0640 5215.1179 5268.8454
348.5848 -15865.0180 -1756.1235 0.0000 0.0000
7736.5649 4129.0356 309.5013 -3607.5294 4191.6515
309.2739 190.2864 129.0483 656151.2420 -13.9702
-13.9592
[8337]ENERGY: 14850000 3203.5646 5173.6891 5284.1871
337.3077 -15809.7539 -1850.1196 0.0000 0.0000
7791.8251 4130.7001 311.7120 -3661.1249 4192.0118
309.2363 -85.4458 -90.4445 656151.2420 -12.9872
-12.9996
[8373]ENERGY: 14860000 3189.8777 5266.5327 5270.9352
343.9612 -15834.2582 -1823.4186 0.0000 0.0000
7717.1412 4130.7712 308.7242 -3586.3700 4191.7686
309.2237 -120.4226 -70.1457 656151.2420 -16.9000
-16.8871
[8423]ENERGY: 14870000 3181.6994 5274.5137 5340.1553
334.5678 -15934.0919 -1800.0714 0.0000 0.0000
7735.3166 4132.0895 309.4513 -3603.2271 4192.2330
309.0096 32.5972 -43.7802 656151.2420 -15.4510
-15.4486
[8459]ENERGY: 14880000 3261.2360 5247.3238 5298.2824
343.3980 -15903.8082 -1790.2369 0.0000 0.0000
7674.1283 4130.3233 307.0035 -3543.8050 4192.1132
309.0553 -150.7376 -137.6514 656151.2420 -14.8382
-14.8237
[8509]ENERGY: 14890000 3168.4150 5211.8555 5267.5855
344.8516 -15849.4732 -1762.0317 0.0000 0.0000
7748.8154 4130.0180 309.9914 -3618.7974 4193.4333
309.4245 49.4308 8.1982 656151.2420 -14.4432
-14.4672
[8548]ENERGY: 14900000 3220.7224 5228.4518 5233.4747
334.3258 -15828.9549 -1763.8466 0.0000 0.0000
7705.9016 4130.0749 308.2746 -3575.8267 4192.6295
309.2337 -4.3315 -19.2442 656151.2420 -16.2970
-16.2970
[8598]ENERGY: 14910000 3097.8054 5326.2708 5227.2212
351.7281 -15852.6252 -1761.6299 0.0000 0.0000
7742.1925 4130.9629 309.7264 -3611.2296 4192.6628
309.3347 140.5589 57.8506 656151.2420 -13.0223
-13.0294
[8634]ENERGY: 14920000 3090.0389 5232.9082 5262.0101
350.4444 -15825.9974 -1688.1947 0.0000 0.0000
7708.3784 4129.5879 308.3737 -3578.7905 4191.6681
309.3361 70.8892 59.0358 656151.2420 -15.4166
-15.3908
[8684]ENERGY: 14930000 3153.9364 5245.5242 5275.6900
341.1713 -15800.4976 -1775.1825 0.0000 0.0000
7686.1205 4126.7622 307.4832 -3559.3583 4191.6288
309.1833 68.4082 69.3682 656151.2420 -12.4820
-12.4814

[8720]ENERGY: 14940000 3266.7374 5305.6127 5226.3664
322.7905 -15862.4576 -1809.1279 0.0000 0.0000
7681.3875 4131.3090 307.2939 -3550.0785 4193.0776
309.2282 -50.9851 -82.9199 656151.2420 -14.5945
-14.6287
[8770]ENERGY: 14950000 3324.8985 5268.0007 5209.8287
333.6602 -15896.6267 -1741.2620 0.0000 0.0000
7636.8536 4135.3530 305.5123 -3501.5006 4193.0445
309.2008 -189.2625 -221.1751 656151.2420 -13.9970
-13.9712
[8806]ENERGY: 14960000 3250.0062 5309.8100 5265.7094
328.5304 -15980.9798 -1738.6748 0.0000 0.0000
7696.3364 4130.7377 307.8919 -3565.5987 4191.3855
309.3972 -81.1369 -58.3620 656151.2420 -14.0426
-14.0371
[8856]ENERGY: 14970000 3145.6237 5280.6708 5256.3201
333.9962 -15856.1878 -1796.6637 0.0000 0.0000
7764.0890 4127.8483 310.6024 -3636.2407 4192.1053
309.5003 -121.5072 -6.4556 656151.2420 -12.8460
-12.8503
[8892]ENERGY: 14980000 3154.3008 5274.0539 5262.5684
354.9710 -15894.5570 -1728.7194 0.0000 0.0000
7709.9855 4132.6032 308.4380 -3577.3823 4193.1003
309.5832 61.3501 -3.6153 656151.2420 -15.2554
-15.2673
[8942]ENERGY: 14990000 3181.7784 5191.1112 5260.8207
359.7053 -15843.0694 -1779.3981 0.0000 0.0000
7760.8777 4131.8258 310.4739 -3629.0519 4192.8227
309.7935 125.4354 62.7299 656151.2420 -13.9821
-13.9615
[8981]ENERGY: 15000000 3146.3691 5200.5828 5250.9751
353.2710 -15872.7361 -1818.9619 0.0000 0.0000
7866.1594 4125.6594 314.6857 -3740.5001 4192.5623
309.5566 -103.9507 -57.8508 656151.2420 -16.8204
-16.8381
[9031]ENERGY: 15010000 3227.1187 5182.6507 5256.9055
339.9228 -15819.1810 -1749.6616 0.0000 0.0000
7698.5076 4136.2627 307.9788 -3562.2450 4193.0842
309.5164 -55.4072 -28.5564 656151.2420 -13.2433
-13.2287
[9067]ENERGY: 15020000 3229.6510 5242.6345 5235.1935
348.2886 -15839.0201 -1767.9064 0.0000 0.0000
7685.8420 4134.6832 307.4721 -3551.1588 4193.9829
309.5356 -16.7093 -3.6253 656151.2420 -16.1389
-16.1703
[9117]ENERGY: 15030000 3193.5336 5243.1534 5292.4603
354.6708 -15896.4917 -1775.1753 0.0000 0.0000
7716.9433 4129.0945 308.7163 -3587.8489 4193.7474
309.4385 -62.2666 -16.2639 656151.2420 -16.3528
-16.3322
[9153]ENERGY: 15040000 3231.7994 5288.1857 5318.0913
370.1439 -15969.1152 -1748.7046 0.0000 0.0000
7639.2890 4129.6897 305.6098 -3509.5993 4193.6527
309.5557 8.6642 44.7531 656151.2420 -12.6624

-12.6596
[9203]ENERGY: 15050000 3156.4517 5166.1453 5272.6968
315.2398 -15891.1137 -1721.0891 0.0000 0.0000
7826.3230 4124.6537 313.0920 -3701.6693 4193.2792
309.4778 -124.2967 -18.8485 656151.2420 -14.3861
-14.3835
[9239]ENERGY: 15060000 3179.8819 5229.0229 5243.4104
340.1193 -15872.3954 -1768.2861 0.0000 0.0000
7778.5240 4130.2771 311.1799 -3648.2469 4195.1355
309.5111 34.3449 -23.2281 656151.2420 -14.8300
-14.8403
[9289]ENERGY: 15070000 3164.3061 5200.0569 5262.4687
350.1162 -15848.0843 -1781.5210 0.0000 0.0000
7784.4482 4131.7908 311.4168 -3652.6573 4193.9903
309.6078 38.0953 0.8820 656151.2420 -16.2556
-16.2570
[9325]ENERGY: 15080000 3233.4290 5225.1091 5268.4032
348.7973 -15903.4469 -1760.0756 0.0000 0.0000
7719.2127 4131.4287 308.8071 -3587.7840 4195.0347
309.5559 148.2119 30.2601 656151.2420 -14.9811
-14.9926
[9375]ENERGY: 15090000 3256.3085 5283.0349 5240.5525
326.3720 -15879.5781 -1788.5464 0.0000 0.0000
7694.7012 4132.8445 307.8265 -3561.8566 4194.4150
309.4076 44.8557 -1.5648 656151.2420 -13.5686
-13.5457
[9414]ENERGY: 15100000 3151.0401 5181.1852 5303.2246
321.6367 -15822.5529 -1805.7248 0.0000 0.0000
7804.7958 4133.6048 312.2309 -3671.1910 4194.7994
309.4265 -110.8819 -102.3407 656151.2420 -14.8277
-14.8295
[9464]ENERGY: 15110000 3222.4232 5155.3792 5262.9619
340.4457 -15684.2986 -1853.0523 0.0000 0.0000
7687.4309 4131.2900 307.5357 -3556.1409 4194.8753
309.1444 -269.8509 -171.2700 656151.2420 -12.0862
-12.0759
[9500]ENERGY: 15120000 3272.2549 5265.8284 5220.6090
337.4274 -15855.3292 -1794.3524 0.0000 0.0000
7687.1592 4133.5972 307.5248 -3553.5619 4194.7559
309.2298 -28.9728 -7.4010 656151.2420 -12.9204
-12.9309
[9550]ENERGY: 15130000 3185.9746 5330.2492 5221.4115
347.3616 -15858.0243 -1827.9193 0.0000 0.0000
7735.7877 4134.8411 309.4702 -3600.9466 4196.5419
309.4616 155.8440 118.8171 656151.2420 -15.6893
-15.6938
[9586]ENERGY: 15140000 3233.0449 5249.5260 5259.8557
354.4005 -15827.4741 -1861.0063 0.0000 0.0000
7724.2299 4132.5766 309.0078 -3591.6533 4195.7945
309.4616 -39.0605 -65.5660 656151.2420 -11.9921
-11.9933
[9636]ENERGY: 15150000 3246.6632 5227.4198 5225.2459
368.9836 -15850.1894 -1793.8147 0.0000 0.0000
7712.3837 4136.6921 308.5339 -3575.6916 4195.8766

309.3888 -94.6615 -105.2746 656151.2420 -13.8272
-13.8231
[9672]ENERGY: 15160000 3253.4453 5242.3451 5270.2662
320.6381 -15824.4903 -1807.2278 0.0000 0.0000
7678.1062 4133.0829 307.1626 -3545.0234 4196.4016
309.5355 -54.3457 -34.1453 656151.2420 -15.0096
-15.0120
[9722]ENERGY: 15170000 3161.2386 5215.4498 5209.4945
360.7052 -15921.8081 -1701.0739 0.0000 0.0000
7809.1985 4133.2048 312.4070 -3675.9938 4196.4854
309.7185 104.2999 47.2779 656151.2420 -14.6036
-14.6131
[9758]ENERGY: 15180000 3223.4059 5179.5277 5276.7636
323.9523 -15836.1415 -1781.4102 0.0000 0.0000
7745.4686 4131.5666 309.8575 -3613.9021 4196.4794
309.4069 -115.1923 -65.3326 656151.2420 -13.9969
-13.9981
[9808]ENERGY: 15190000 3140.4379 5151.7156 5274.3368
318.0708 -15829.3713 -1773.9807 0.0000 0.0000
7850.0758 4131.2848 314.0423 -3718.7910 4196.2421
309.6347 -61.7695 -26.4713 656151.2420 -14.7740
-14.7566
[9847]ENERGY: 15200000 3188.7622 5206.9254 5280.6022
348.8060 -15830.6362 -1727.2539 0.0000 0.0000
7670.4525 4137.6581 306.8565 -3532.7944 4197.5407
309.6544 63.2061 64.0544 656151.2420 -16.5773
-16.5864
[9897]ENERGY: 15210000 3181.8451 5194.8227 5223.0457
341.6019 -15898.0557 -1757.2033 0.0000 0.0000
7843.1422 4129.1986 313.7649 -3713.9436 4196.6214
309.3593 12.1647 49.8012 656151.2420 -14.8621
-14.8562
[9933]ENERGY: 15220000 3236.0131 5236.9472 5309.0313
330.7793 -15812.7703 -1810.9542 0.0000 0.0000
7648.9058 4137.9522 305.9945 -3510.9535 4197.9093
309.5545 96.5533 88.2100 656151.2420 -13.0976
-13.0950
[9983]ENERGY: 15230000 3197.6751 5204.3299 5253.9122
326.9015 -15923.3614 -1733.9594 0.0000 0.0000
7808.1054 4133.6032 312.3633 -3674.5021 4197.8225
309.8327 142.1899 48.2276 656151.2420 -12.9037
-12.9215
[10019]ENERGY: 15240000 3161.0633 5193.6789 5299.5015
351.4214 -15816.8531 -1789.1715 0.0000 0.0000
7736.3954 4136.0359 309.4945 -3600.3595 4197.7452
309.8687 -94.4237 -46.4995 656151.2420 -20.6611
-20.6414
[10069]ENERGY: 15250000 3232.2071 5205.6441 5233.2831
351.5115 -15853.6720 -1845.0239 0.0000 0.0000
7815.3061 4139.2560 312.6513 -3676.0501 4199.1688
309.7956 -9.4753 -11.9381 656151.2420 -15.9663
-15.9726
[10105]ENERGY: 15260000 3302.4975 5188.5826 5301.8116
324.3568 -15937.8976 -1735.7605 0.0000 0.0000

7689.2623 4132.8528 307.6089 -3556.4096 4197.7323
309.6221 3.0749 -34.8337 656151.2420 -21.1605
-21.1592
[10155]ENERGY: 15270000 3213.2552 5148.2979 5295.0638
318.9304 -15773.7309 -1784.0682 0.0000 0.0000
7715.9835 4133.7317 308.6779 -3582.2518 4197.3608
309.4758 27.7780 -43.8973 656151.2420 -17.6371
-17.6436
[10191]ENERGY: 15280000 3303.2234 5202.9866 5275.7521
356.2383 -15771.1451 -1853.4712 0.0000 0.0000
7625.1637 4138.7478 305.0447 -3486.4159 4198.4973
309.5687 -104.4969 -198.8177 656151.2420 -18.4113
-18.4245
[10241]ENERGY: 15290000 3180.5207 5157.2940 5267.0592
372.5689 -15831.0067 -1750.0499 0.0000 0.0000
7737.6586 4134.0447 309.5450 -3603.6139 4196.6285
309.4743 -124.4484 -152.7741 656151.2420 -16.6198
-16.6035
[10280]ENERGY: 15300000 3208.0202 5221.9086 5268.3487
326.2269 -15785.8151 -1761.9902 0.0000 0.0000
7660.3555 4137.0546 306.4525 -3523.3009 4197.1261
309.4694 -17.8521 11.8552 656151.2420 -22.4719
-22.4754
[10330]ENERGY: 15310000 3241.4102 5288.5093 5297.9588
313.0597 -15985.6210 -1757.6932 0.0000 0.0000
7735.2069 4132.8308 309.4469 -3602.3761 4196.0259
309.4957 -161.4664 -112.1753 656151.2420 -14.5352
-14.5359
[10366]ENERGY: 15320000 3166.9850 5278.4891 5313.2618
329.0617 -15864.6450 -1780.6592 0.0000 0.0000
7691.5044 4133.9978 307.6986 -3557.5067 4195.8709
309.6150 -197.8583 -138.4283 656151.2420 -11.7397
-11.7349
[10416]ENERGY: 15330000 3174.2381 5141.0022 5286.9433
332.7902 -15833.5243 -1743.4174 0.0000 0.0000
7773.2082 4131.2403 310.9672 -3641.9680 4197.4780
309.5749 -28.4677 37.6592 656151.2420 -14.8862
-14.8880
[10452]ENERGY: 15340000 3270.9539 5270.0262 5220.6785
330.6336 -15866.5504 -1777.9216 0.0000 0.0000
7690.4455 4138.2656 307.6563 -3552.1799 4197.2144
309.7380 3.6407 -14.8062 656151.2420 -11.9134
-11.9075
[10502]ENERGY: 15350000 3166.7609 5087.5403 5288.6679
339.1304 -15764.8609 -1808.9103 0.0000 0.0000
7824.3368 4132.6650 313.0126 -3691.6718 4197.1485
309.7143 19.6899 16.1625 656151.2420 -15.6131
-15.6178
[10538]ENERGY: 15360000 3132.2140 5273.9342 5295.5960
343.5479 -15881.7753 -1751.0706 0.0000 0.0000
7724.0884 4136.5346 309.0022 -3587.5538 4196.9297
309.8790 108.4125 82.6596 656151.2420 -17.3295
-17.3161
[10588]ENERGY: 15370000 3122.7672 5250.3724 5249.2436

327.3262 -15815.2371 -1794.0410 0.0000 0.0000
7793.5451 4133.9764 311.7808 -3659.5687 4195.4766
309.7406 -29.8582 -29.7049 656151.2420 -10.9192
-10.9332
[10624]ENERGY: 15380000 3254.6900 5281.0606 5294.4169
355.5291 -15965.5328 -1829.4466 0.0000 0.0000
7742.2169 4132.9341 309.7274 -3609.2828 4197.3017
309.7908 -43.5907 -44.6079 656151.2420 -13.8934
-13.8859
[10674]ENERGY: 15390000 3232.2644 5265.5240 5281.5275
353.1827 -15947.0165 -1815.4883 0.0000 0.0000
7763.0840 4133.0778 310.5622 -3630.0062 4198.0881
309.7554 -49.1380 -156.3780 656151.2420 -15.4152
-15.4332
[10713]ENERGY: 15400000 3174.2930 5162.7990 5226.5333
295.6391 -15905.9591 -1697.2689 0.0000 0.0000
7875.9444 4131.9807 315.0772 -3743.9637 4197.9365
309.9062 164.2346 124.4880 656151.2420 -15.9799
-15.9728
[10763]ENERGY: 15410000 3229.7848 5312.8085 5273.5594
322.0290 -15853.8992 -1801.2083 0.0000 0.0000
7653.4830 4136.5573 306.1776 -3516.9258 4198.6663
309.8665 70.4484 63.6472 656151.2420 -19.7376
-19.7300
[10799]ENERGY: 15420000 3279.9906 5206.4901 5269.8500
336.6479 -15792.8853 -1825.8345 0.0000 0.0000
7666.7757 4141.0345 306.7094 -3525.7413 4198.6009
309.8612 -29.1883 -40.2633 656151.2420 -16.1927
-16.1688
[10849]ENERGY: 15430000 3106.1761 5203.1685 5293.3145
340.3542 -15815.3439 -1768.4186 0.0000 0.0000
7777.1599 4136.4108 311.1253 -3640.7491 4199.7291
309.9159 79.1875 -5.7981 656151.2420 -15.6428
-15.6718
[10885]ENERGY: 15440000 3141.7860 5193.9828 5265.2637
339.6187 -15830.7066 -1781.9361 0.0000 0.0000
7806.2204 4134.2289 312.2878 -3671.9915 4198.8013
310.0477 138.1709 147.0522 656151.2420 -14.8304
-14.8429
[10935]ENERGY: 15450000 3199.4287 5189.5403 5212.9679
313.4749 -15857.3451 -1751.5948 0.0000 0.0000
7829.3900 4135.8621 313.2147 -3693.5280 4197.5523
309.9666 107.6778 19.5424 656151.2420 -17.7856
-17.7720
[10971]ENERGY: 15460000 3200.6656 5248.5754 5249.7882
339.2226 -15934.0116 -1766.7152 0.0000 0.0000
7799.5213 4137.0462 312.0198 -3662.4751 4199.0208
310.0212 54.4917 7.7225 656151.2420 -17.4177
-17.4291
[11021]ENERGY: 15470000 3184.9022 5187.6246 5291.8653
355.1029 -15957.9016 -1733.9928 0.0000 0.0000
7804.8246 4132.4252 312.2320 -3672.3994 4198.2984
309.9443 76.0279 21.6653 656151.2420 -20.6060
-20.6044

[11057]ENERGY: 15480000 3201.4965 5252.6855 5287.1786
350.6876 -15867.3570 -1846.8294 0.0000 0.0000
7757.9952 4135.8569 310.3586 -3622.1383 4200.0184
310.0883 -79.3125 -119.7407 656151.2420 -12.9386
-12.9370
[11107]ENERGY: 15490000 3180.8327 5207.7369 5212.2969
336.9803 -15765.0599 -1733.7538 0.0000 0.0000
7696.0606 4135.0937 307.8809 -3560.9669 4199.4365
309.9010 -89.1218 -73.2931 656151.2420 -21.3684
-21.3545
[11146]ENERGY: 15500000 3236.2907 5204.3270 5305.1847
338.7180 -15930.3246 -1709.7037 0.0000 0.0000
7694.6613 4139.1534 307.8249 -3555.5079 4201.0255
309.7710 -76.6967 -33.2622 656151.2420 -19.0901
-19.0997
[11196]ENERGY: 15510000 3236.3674 5256.5759 5255.3410
336.4916 -15798.0851 -1836.8262 0.0000 0.0000
7687.6118 4137.4764 307.5429 -3550.1354 4201.5411
310.0279 -60.3637 -122.9863 656151.2420 -17.8424
-17.8449
[11232]ENERGY: 15520000 3198.1678 5277.2845 5296.5492
346.0378 -15916.2549 -1803.1186 0.0000 0.0000
7738.6197 4137.2856 309.5835 -3601.3341 4199.3673
309.9813 -222.6930 -151.5936 656151.2420 -19.3527
-19.3248
[11282]ENERGY: 15530000 3164.9627 5314.6950 5264.1147
351.8830 -15950.1535 -1764.3721 0.0000 0.0000
7755.6063 4136.7363 310.2630 -3618.8700 4202.0588
310.2421 68.1923 18.9181 656151.2420 -16.2367
-16.2521
[11318]ENERGY: 15540000 3211.3638 5190.6474 5273.0595
349.3031 -15848.1168 -1757.4792 0.0000 0.0000
7719.9527 4138.7304 308.8367 -3581.2223 4202.3939
310.1249 16.7450 -20.5204 656151.2420 -16.6503
-16.6731
[11368]ENERGY: 15550000 3256.7685 5165.9542 5239.8495
324.3054 -15883.6665 -1817.7831 0.0000 0.0000
7852.2840 4137.7118 314.1306 -3714.5721 4202.8719
310.3021 -0.0181 -58.9627 656151.2420 -16.6707
-16.6430
[11404]ENERGY: 15560000 3266.4233 5242.4691 5228.8872
324.1398 -15868.5003 -1813.9480 0.0000 0.0000
7762.4192 4141.8903 310.5356 -3620.5289 4203.6038
310.2476 -154.2227 -167.8101 656151.2420 -14.6181
-14.6235
[11454]ENERGY: 15570000 3142.4788 5252.9261 5344.7506
338.8671 -15822.0659 -1823.9156 0.0000 0.0000
7709.4016 4142.4428 308.4146 -3566.9589 4203.4366
310.1240 48.6890 71.3696 656151.2420 -20.4908
-20.4931
[11490]ENERGY: 15580000 3235.3707 5178.2149 5261.7162
335.9854 -15853.3600 -1746.2226 0.0000 0.0000
7730.1778 4141.8823 309.2458 -3588.2955 4203.8556
310.3515 146.9470 149.6891 656151.2420 -14.5930

-14.5966
[11540]ENERGY: 15590000 3279.3185 5292.0221 5254.8499
331.3829 -15928.4642 -1743.0724 0.0000 0.0000
7656.7573 4142.7941 306.3086 -3513.9632 4202.1869
310.1933 64.0655 37.8008 656151.2420 -22.9038
-22.8966
[11579]ENERGY: 15600000 3129.8310 5228.2219 5302.7099
318.2142 -15857.0427 -1748.7003 0.0000 0.0000
7767.7775 4141.0114 310.7499 -3626.7661 4202.1542
310.1205 148.7463 127.1929 656151.2420 -14.9942
-14.9866
[11629]ENERGY: 15610000 3272.0665 5240.3336 5253.2611
328.2097 -15874.5751 -1774.3207 0.0000 0.0000
7695.5872 4140.5623 307.8620 -3555.0249 4202.8155
310.0817 -69.9049 -69.3827 656151.2420 -11.4174
-11.4406
[11665]ENERGY: 15620000 3159.8634 5294.0675 5281.0135
344.7142 -15887.3675 -1820.9557 0.0000 0.0000
7769.2382 4140.5735 310.8084 -3628.6647 4204.1555
310.2276 -53.5223 -81.9443 656151.2420 -17.1966
-17.1732
[11715]ENERGY: 15630000 3290.5741 5200.4686 5268.3093
343.5858 -15905.4753 -1793.8928 0.0000 0.0000
7737.4508 4141.0205 309.5367 -3596.4303 4204.6220
310.1895 -207.0468 -154.3625 656151.2420 -16.9965
-17.0088
[11751]ENERGY: 15640000 3195.3788 5217.3244 5285.2506
339.3730 -15866.6809 -1774.6296 0.0000 0.0000
7747.2240 4143.2403 309.9277 -3603.9837 4204.9601
310.3007 82.3083 36.8888 656151.2420 -16.7473
-16.7611
[11801]ENERGY: 15650000 3246.8528 5255.7471 5271.0370
316.5139 -15964.5924 -1766.3623 0.0000 0.0000
7779.3026 4138.4986 311.2110 -3640.8039 4204.2338
310.3151 46.7442 16.9912 656151.2420 -17.3017
-17.3160
[11837]ENERGY: 15660000 3211.1489 5198.2603 5265.9203
340.8786 -15911.2255 -1754.2211 0.0000 0.0000
7789.6834 4140.4449 311.6263 -3649.2385 4204.9761
310.3667 88.8413 30.1210 656151.2420 -11.9406
-11.9392
[11887]ENERGY: 15670000 3214.5538 5125.2080 5274.9926
326.0042 -15844.9501 -1778.8906 0.0000 0.0000
7824.3911 4141.3089 313.0148 -3683.0822 4205.3840
310.1074 38.7070 46.1110 656151.2420 -17.2651
-17.2366
[11923]ENERGY: 15680000 3166.7413 5282.8659 5271.6444
353.7825 -15861.8950 -1777.0342 0.0000 0.0000
7709.1469 4145.2517 308.4044 -3563.8952 4205.8578
310.2439 134.1898 93.4336 656151.2420 -12.6480
-12.6481
[11973]ENERGY: 15690000 3157.5220 5286.7141 5262.9960
319.7882 -15923.4855 -1744.8788 0.0000 0.0000
7784.5598 4143.2160 311.4213 -3641.3439 4204.7405

310.2109 -114.7501 -118.7371 656151.2420 -17.6428
-17.6436
[12012]ENERGY: 15700000 3203.7807 5215.7129 5213.2650
323.3984 -15860.8420 -1735.2088 0.0000 0.0000
7781.9249 4142.0312 311.3159 -3639.8937 4205.4781
310.2428 -71.6613 3.9417 656151.2420 -12.1539
-12.1511
[12062]ENERGY: 15710000 3171.5450 5132.4786 5301.1605
341.9587 -15825.1288 -1800.9125 0.0000 0.0000
7821.3405 4142.4420 312.8927 -3678.8985 4205.9269
310.2067 -53.2847 19.8599 656151.2420 -16.1175
-16.1199
[12098]ENERGY: 15720000 3171.1419 5233.3901 5271.3914
335.6478 -15840.8218 -1807.8118 0.0000 0.0000
7777.5661 4140.5037 311.1415 -3637.0624 4206.1344
310.1113 -123.0890 -10.6508 656151.2420 -16.1349
-16.1324
[12148]ENERGY: 15730000 3224.8867 5195.7430 5241.2180
325.7980 -15841.0864 -1844.1051 0.0000 0.0000
7842.8560 4145.3103 313.7535 -3697.5458 4207.0614
310.0998 -135.4974 -127.6669 656151.2420 -18.0876
-18.0852
[12184]ENERGY: 15740000 3259.9262 5235.6330 5237.8152
328.7388 -15848.3882 -1810.0042 0.0000 0.0000
7735.8727 4139.5935 309.4736 -3596.2792 4206.1249
310.1987 -129.7306 -109.1490 656151.2420 -17.5167
-17.5243
[12234]ENERGY: 15750000 3190.4319 5236.7658 5298.0838
371.0626 -15938.9493 -1716.8012 0.0000 0.0000
7702.1705 4142.7640 308.1253 -3559.4064 4205.6983
310.4291 42.3225 24.9694 656151.2420 -16.0620
-16.0671
[12270]ENERGY: 15760000 3191.3863 5237.5991 5271.6435
349.7180 -15860.6804 -1774.1825 0.0000 0.0000
7730.2062 4145.6902 309.2469 -3584.5160 4207.0969
310.5035 67.9215 -9.9616 656151.2420 -18.3968
-18.3914
[12320]ENERGY: 15770000 3232.5135 5218.4131 5304.3078
359.3269 -15884.2032 -1769.5007 0.0000 0.0000
7689.6545 4150.5118 307.6246 -3539.1426 4207.9085
310.1756 53.1951 -7.8352 656151.2420 -13.6231
-13.6233
[12356]ENERGY: 15780000 3157.7406 5276.8231 5277.8430
349.7022 -15848.2933 -1771.9432 0.0000 0.0000
7702.5753 4144.4478 308.1415 -3558.1275 4208.6091
310.2822 100.4339 83.5128 656151.2420 -17.7171
-17.7178
[12406]ENERGY: 15790000 3179.4557 5245.1848 5284.3395
323.5706 -15792.4344 -1825.0181 0.0000 0.0000
7733.0836 4148.1817 309.3620 -3584.9018 4208.4560
310.1833 151.1118 20.3719 656151.2420 -17.4475
-17.4492
[12445]ENERGY: 15800000 3245.9259 5225.9102 5310.4113
346.9632 -16025.5509 -1652.8565 0.0000 0.0000

7693.8863 4144.6895 307.7939 -3549.1968 4207.5308
310.2981 20.3327 -7.3791 656151.2420 -13.7771
-13.7770
[12495]ENERGY: 15810000 3246.1578 5191.9488 5274.7952
344.1938 -15857.1817 -1772.9316 0.0000 0.0000
7720.7236 4147.7059 308.8675 -3573.0177 4208.4157
310.2439 -94.7861 -84.5576 656151.2420 -16.4830
-16.4898
[12531]ENERGY: 15820000 3180.9429 5154.3688 5307.7051
340.6395 -15757.6779 -1855.0534 0.0000 0.0000
7774.6641 4145.5891 311.0254 -3629.0750 4209.3397
310.2754 -82.9351 -32.1413 656151.2420 -17.8863
-17.8749
[12581]ENERGY: 15830000 3260.8482 5189.7534 5304.9902
356.0052 -15809.6504 -1778.0302 0.0000 0.0000
7626.0460 4149.9624 305.0800 -3476.0836 4208.4905
310.2466 97.9659 41.9625 656151.2420 -15.0428
-15.0345
[12617]ENERGY: 15840000 3212.2374 5232.9432 5294.3772
347.2538 -15829.9428 -1810.7337 0.0000 0.0000
7700.4463 4146.5815 308.0564 -3553.8648 4207.0230
310.3069 -32.3724 -85.8382 656151.2420 -16.0341
-16.0431
[12667]ENERGY: 15850000 3234.2870 5234.0673 5292.7782
338.8473 -15861.8296 -1773.7832 0.0000 0.0000
7682.6617 4147.0285 307.3449 -3535.6332 4208.3854
310.0284 -98.3756 -92.9850 656151.2420 -17.3777
-17.3730
[12703]ENERGY: 15860000 3187.5045 5272.0694 5242.9410
337.3831 -15850.5612 -1737.7097 0.0000 0.0000
7694.5689 4146.1961 307.8212 -3548.3729 4208.8523
310.2159 110.1428 182.4128 656151.2420 -14.9731
-14.9789
[12753]ENERGY: 15870000 3201.9036 5154.6742 5314.7510
341.4754 -15792.5307 -1800.1270 0.0000 0.0000
7730.0353 4150.1819 309.2401 -3579.8534 4210.8885
310.2448 74.7569 -14.7167 656151.2420 -19.3924
-19.3960
[12789]ENERGY: 15880000 3236.2363 5314.8028 5324.4532
357.4800 -16000.7275 -1756.1764 0.0000 0.0000
7671.6573 4147.7257 306.9046 -3523.9316 4210.6815
310.1464 -61.6729 -81.1576 656151.2420 -23.1854
-23.1837
[12839]ENERGY: 15890000 3176.0824 5152.7562 5236.2460
352.6313 -15774.6266 -1812.8448 0.0000 0.0000
7817.0852 4147.3295 312.7225 -3669.7557 4210.7865
310.4019 -67.7327 -56.3118 656151.2420 -16.5338
-16.5140
[12878]ENERGY: 15900000 3239.4665 5267.2842 5237.8708
356.2474 -15819.8613 -1763.0227 0.0000 0.0000
7636.1541 4154.1389 305.4843 -3482.0151 4211.3322
310.3082 -161.8566 -113.0841 656151.2420 -14.5448
-14.5750
[12928]ENERGY: 15910000 3190.0465 5214.3967 5247.0703

342.3653 -15885.0068 -1690.6924 0.0000 0.0000
7732.3974 4150.5772 309.3346 -3581.8203 4210.2160
310.0944 82.0270 9.9135 656151.2420 -18.2430
-18.2138
[12964]ENERGY: 15920000 3169.0607 5178.4762 5253.7526
346.5287 -15858.7694 -1751.3286 0.0000 0.0000
7807.8892 4145.6094 312.3546 -3662.2798 4210.3840
310.2036 34.8264 77.7325 656151.2420 -11.1765
-11.2041
[13014]ENERGY: 15930000 3209.0426 5234.7941 5306.4755
330.4125 -15848.7733 -1804.0129 0.0000 0.0000
7721.1199 4149.0585 308.8834 -3572.0614 4211.3255
310.1362 -73.5577 -90.7440 656151.2420 -19.4254
-19.4117
[13050]ENERGY: 15940000 3254.2007 5231.5298 5243.2142
328.4639 -15801.7814 -1781.0918 0.0000 0.0000
7674.5035 4149.0388 307.0185 -3525.4647 4211.5812
310.2787 -83.4127 -62.2990 656151.2420 -12.1705
-12.1671
[13100]ENERGY: 15950000 3195.1226 5134.1667 5240.0338
346.8476 -15817.3022 -1753.9846 0.0000 0.0000
7802.9245 4147.8083 312.1560 -3655.1162 4210.7423
310.2981 -28.7451 -90.6165 656151.2420 -16.7020
-16.7164
[13136]ENERGY: 15960000 3139.0780 5256.8336 5296.9301
355.1664 -15756.1379 -1860.3666 0.0000 0.0000
7717.3704 4148.8740 308.7334 -3568.4964 4212.3768
310.1022 -2.3887 26.4917 656151.2420 -22.5327
-22.4966
[13186]ENERGY: 15970000 3223.3357 5206.2757 5309.7620
323.7395 -15858.8117 -1795.1874 0.0000 0.0000
7741.6802 4150.7940 309.7059 -3590.8862 4211.1299
310.0952 -77.5198 -103.7121 656151.2420 -17.5275
-17.5626
[13222]ENERGY: 15980000 3182.9397 5283.2721 5281.4045
328.3615 -15924.3452 -1793.5830 0.0000 0.0000
7789.1898 4147.2395 311.6065 -3641.9504 4211.1827
310.0526 -0.4010 -85.8878 656151.2420 -21.7959
-21.7892
[13272]ENERGY: 15990000 3159.6914 5320.3574 5263.2305
350.8320 -15859.4804 -1788.6709 0.0000 0.0000
7704.0841 4150.0441 308.2019 -3554.0400 4211.0950
310.1917 -31.9954 -31.7243 656151.2420 -23.5930
-23.5937
[13311]ENERGY: 16000000 3326.9996 5213.6788 5274.6319
327.9817 -15812.3518 -1883.2111 0.0000 0.0000
7705.7164 4153.4456 308.2672 -3552.2709 4211.7459
310.0182 -132.9502 -183.5554 656151.2420 -21.4491
-21.4488
[13361]ENERGY: 16010000 3259.2583 5207.4556 5301.0723
336.2511 -15861.9492 -1793.5630 0.0000 0.0000
7702.0194 4150.5446 308.1193 -3551.4748 4211.4174
309.9358 -12.9100 6.2862 656151.2420 -25.5962
-25.5840

[13397]ENERGY: 16020000 3208.9861 5203.0749 5268.4407
349.3763 -15794.1626 -1853.2760 0.0000 0.0000
7764.9490 4147.3884 310.6368 -3617.5607 4211.6027
310.1225 -68.3610 -107.7584 656151.2420 -14.5123
-14.5201
[13447]ENERGY: 16030000 3144.5403 5253.9820 5282.6416
346.9524 -15852.8306 -1757.3957 0.0000 0.0000
7729.7954 4147.6856 309.2305 -3582.1099 4210.7861
310.1118 11.1019 -47.2036 656151.2420 -22.0876
-22.1043
[13483]ENERGY: 16040000 3173.9066 5230.0384 5231.8467
336.0778 -15871.0904 -1800.6996 0.0000 0.0000
7847.8531 4147.9327 313.9534 -3699.9205 4211.7132
310.1770 107.9071 61.7320 656151.2420 -18.3773
-18.3763
[13533]ENERGY: 16050000 3143.7528 5269.7531 5295.6445
333.7699 -15924.9177 -1721.2217 0.0000 0.0000
7751.5172 4148.2981 310.0994 -3603.2191 4211.2993
310.0908 98.7981 38.3104 656151.2420 -14.0388
-14.0329
[13569]ENERGY: 16060000 3187.2184 5278.6076 5249.9989
341.1400 -15868.1154 -1775.1172 0.0000 0.0000
7733.6349 4147.3672 309.3841 -3586.2677 4210.0221
310.1311 48.1130 -5.0635 656151.2420 -16.1412
-16.1337
[13619]ENERGY: 16070000 3131.0753 5267.5027 5292.5542
367.6281 -15805.0782 -1835.2154 0.0000 0.0000
7733.5060 4151.9727 309.3789 -3581.5333 4212.5238
310.2723 -28.9048 -85.4466 656151.2420 -16.1489
-16.1405
[13655]ENERGY: 16080000 3170.2004 5261.6851 5290.0323
312.1626 -15915.2639 -1761.0666 0.0000 0.0000
7793.4056 4151.1556 311.7752 -3642.2500 4212.8512
310.1719 53.3761 57.0187 656151.2420 -18.2664
-18.2879
[13705]ENERGY: 16090000 3220.3680 5275.7716 5258.5428
336.1909 -15920.6205 -1761.6891 0.0000 0.0000
7741.0595 4149.6232 309.6811 -3591.4363 4213.9483
310.3668 61.7626 35.9863 656151.2420 -22.0593
-22.0578
[13744]ENERGY: 16100000 3233.3093 5261.9833 5262.0881
335.7412 -15926.4416 -1775.8183 0.0000 0.0000
7759.3992 4150.2612 310.4148 -3609.1380 4213.4648
310.3187 37.4046 16.9874 656151.2420 -16.5184
-16.5074
[13794]ENERGY: 16110000 3229.9388 5224.1359 5254.9514
331.2115 -15779.4471 -1831.4876 0.0000 0.0000
7719.8771 4149.1801 308.8337 -3570.6971 4213.6731
310.3584 -98.3403 -96.4379 656151.2420 -12.3452
-12.3575
[13830]ENERGY: 16120000 3205.1178 5270.4875 5243.3206
338.7323 -15899.6613 -1787.5767 0.0000 0.0000
7777.2529 4147.6729 311.1290 -3629.5799 4213.6604
310.3278 -117.9621 -16.4292 656151.2420 -17.3461

-17.3298
[13880]ENERGY: 16130000 3190.6545 5237.2909 5282.9477
348.5539 -15878.0012 -1733.6228 0.0000 0.0000
7706.8521 4154.6752 308.3126 -3552.1770 4215.2883
310.3015 14.7983 37.4683 656151.2420 -12.1012
-12.0958
[13916]ENERGY: 16140000 3204.7934 5176.9085 5261.0211
331.2118 -15787.5932 -1809.7111 0.0000 0.0000
7776.6392 4153.2698 311.1044 -3623.3694 4215.0254
310.3494 -70.3200 22.1151 656151.2420 -21.6596
-21.6637
[13966]ENERGY: 16150000 3217.5161 5138.5176 5310.7634
359.1545 -15764.5062 -1815.3244 0.0000 0.0000
7706.8975 4153.0185 308.3144 -3553.8790 4215.0322
310.5067 55.3203 20.9922 656151.2420 -9.8558
-9.8526
[14002]ENERGY: 16160000 3179.5736 5197.6405 5255.2586
348.0328 -15875.5939 -1752.4701 0.0000 0.0000
7799.7990 4152.2405 312.0310 -3647.5585 4214.1094
310.3548 89.6764 134.2696 656151.2420 -20.8323
-20.8425
[14052]ENERGY: 16170000 3093.1294 5283.5864 5232.5576
363.2840 -15839.5407 -1765.6970 0.0000 0.0000
7783.6448 4150.9643 311.3847 -3632.6805 4215.6793
310.2269 68.8306 79.2915 656151.2420 -22.7333
-22.7344
[14088]ENERGY: 16180000 3214.7873 5301.7827 5268.8628
341.9752 -15930.0597 -1732.5101 0.0000 0.0000
7690.8743 4155.7125 307.6734 -3535.1618 4214.6134
310.0362 60.1923 56.9560 656151.2420 -19.7771
-19.7640
[14138]ENERGY: 16190000 3176.5097 5279.8290 5310.4769
342.7753 -15820.5096 -1782.1786 0.0000 0.0000
7650.5174 4157.4201 306.0589 -3493.0973 4214.2203
310.3209 191.5206 66.9764 656151.2420 -17.3541
-17.3857
[14177]ENERGY: 16200000 3156.5079 5252.0015 5288.0534
337.0391 -15854.8076 -1814.2917 0.0000 0.0000
7785.5205 4150.0232 311.4597 -3635.4973 4215.0152
310.2683 -75.0564 -99.9981 656151.2420 -18.0731
-18.0507
[14227]ENERGY: 16210000 3230.0655 5307.9048 5260.2880
356.8659 -15957.2290 -1747.3620 0.0000 0.0000
7706.4529 4156.9861 308.2966 -3549.4668 4215.8368
310.0848 21.4747 -8.3462 656151.2420 -11.1570
-11.1533
[14263]ENERGY: 16220000 3227.2256 5193.1469 5307.6625
342.6698 -15809.7247 -1828.6239 0.0000 0.0000
7720.5772 4152.9334 308.8617 -3567.6439 4215.5142
310.3274 -69.9566 -64.6393 656151.2420 -15.4882
-15.4934
[14313]ENERGY: 16230000 3214.7654 5091.5759 5285.9363
338.8699 -15819.3770 -1776.6110 0.0000 0.0000
7817.0000 4152.1595 312.7191 -3664.8405 4215.0866

310.4652 -62.2755 -102.9838 656151.2420 -16.1583
-16.1715
[14349]ENERGY: 16240000 3184.4784 5141.3599 5297.4565
352.3387 -15856.1819 -1764.2980 0.0000 0.0000
7797.3091 4152.4628 311.9313 -3644.8463 4216.4632
310.4895 159.3525 134.0835 656151.2420 -15.1619
-15.1418
[14399]ENERGY: 16250000 3262.8548 5304.4159 5201.3398
348.4979 -15885.9889 -1788.1847 0.0000 0.0000
7712.6950 4155.6298 308.5464 -3557.0652 4216.7782
310.3535 -0.7921 27.8499 656151.2420 -13.6248
-13.6340
[14435]ENERGY: 16260000 3198.6403 5207.5777 5265.6356
322.8549 -15876.2133 -1717.8650 0.0000 0.0000
7754.5444 4155.1747 310.2205 -3599.3698 4217.0617
310.4848 174.2353 121.3323 656151.2420 -15.4948
-15.4871
[14485]ENERGY: 16270000 3143.7348 5262.9235 5258.6313
350.9489 -15833.3868 -1742.2181 0.0000 0.0000
7715.2022 4155.8358 308.6467 -3559.3664 4217.6669
310.3343 -100.7099 -60.1741 656151.2420 -15.0270
-15.0206
[14521]ENERGY: 16280000 3186.7800 5259.5366 5267.0371
374.7773 -15826.0207 -1818.0992 0.0000 0.0000
7714.0748 4158.0859 308.6016 -3555.9889 4218.8474
310.3574 -34.3348 -73.7867 656151.2420 -18.0109
-18.0302
[14571]ENERGY: 16290000 3145.0025 5199.7414 5271.1349
366.1929 -15769.8109 -1775.2945 0.0000 0.0000
7716.4958 4153.4622 308.6984 -3563.0335 4217.8939
310.4129 78.9065 39.7852 656151.2420 -12.9787
-12.9765
[14610]ENERGY: 16300000 3231.0951 5273.9224 5264.2542
346.1751 -15873.1363 -1790.9501 0.0000 0.0000
7705.6599 4157.0203 308.2649 -3548.6396 4218.3571
310.2637 -0.4762 11.1386 656151.2420 -21.0709
-21.0537
[14660]ENERGY: 16310000 3128.4274 5181.5292 5286.9142
340.6724 -15895.3218 -1722.6625 0.0000 0.0000
7833.0606 4152.6195 313.3616 -3680.4410 4218.2353
310.3006 -53.3581 -79.1721 656151.2420 -14.6387
-14.6524
[14696]ENERGY: 16320000 3200.2694 5148.0676 5236.4179
365.7270 -15898.1756 -1767.1567 0.0000 0.0000
7867.2166 4152.3663 314.7280 -3714.8503 4217.3623
310.4239 -119.7658 -43.4047 656151.2420 -19.1023
-19.0925
[14746]ENERGY: 16330000 3218.3326 5152.5271 5276.9423
334.8737 -15816.1666 -1811.0993 0.0000 0.0000
7798.3540 4153.7638 311.9732 -3644.5902 4219.2870
310.4408 62.5629 -30.9485 656151.2420 -12.8362
-12.8510
[14782]ENERGY: 16340000 3202.3630 5181.1587 5261.3146
336.3601 -15840.9662 -1701.2150 0.0000 0.0000

7717.5997 4156.6150 308.7426 -3560.9848 4218.5031
310.5832 -90.5804 -61.7963 656151.2420 -15.9182
-15.9217
[14832]ENERGY: 16350000 3140.2531 5388.5026 5244.2858
327.7194 -15892.5126 -1823.7482 0.0000 0.0000
7773.9583 4158.4584 310.9972 -3615.4999 4219.5611
310.6573 -32.0144 -124.1003 656151.2420 -16.1604
-16.1620
[14868]ENERGY: 16360000 3225.4955 5238.3774 5194.1315
341.8582 -15830.9887 -1825.3079 0.0000 0.0000
7814.0218 4157.5878 312.5999 -3656.4340 4219.2539
310.4601 -118.2843 -79.2974 656151.2420 -13.1382
-13.1241
[14918]ENERGY: 16370000 3174.3613 5202.2183 5269.0313
341.2484 -15771.9910 -1849.7450 0.0000 0.0000
7791.9093 4157.0326 311.7153 -3634.8767 4219.3875
310.2870 204.6939 96.9699 656151.2420 -14.6021
-14.5968
[14954]ENERGY: 16380000 3197.3396 5163.4614 5315.6702
347.7947 -15877.0824 -1778.0830 0.0000 0.0000
7788.0900 4157.1906 311.5625 -3630.8994 4220.2409
310.4189 149.4232 101.9557 656151.2420 -16.2772
-16.2955
[15004]ENERGY: 16390000 3229.9035 5223.0666 5293.9156
337.8419 -15877.7453 -1794.4580 0.0000 0.0000
7744.7495 4157.2738 309.8287 -3587.4757 4221.2600
310.3255 40.6939 42.8174 656151.2420 -17.8638
-17.8610
[15043]ENERGY: 16400000 3245.2016 5210.5828 5232.5423
344.0595 -15820.6685 -1842.9057 0.0000 0.0000
7785.8996 4154.7116 311.4749 -3631.1880 4220.5404
310.5031 -41.9954 -84.5608 656151.2420 -15.5632
-15.5457
[15093]ENERGY: 16410000 3154.3444 5244.5517 5292.9481
360.9805 -15815.3381 -1790.4141 0.0000 0.0000
7714.6295 4161.7019 308.6238 -3552.9276 4220.6646
310.1885 25.5295 69.3287 656151.2420 -19.4041
-19.4143
[15129]ENERGY: 16420000 3196.1788 5238.9900 5268.3014
342.8252 -15871.1026 -1767.1099 0.0000 0.0000
7747.4759 4155.5588 309.9378 -3591.9170 4219.7151
310.1835 -31.9746 -38.1214 656151.2420 -20.9850
-20.9829
[15179]ENERGY: 16430000 3251.2065 5285.6006 5274.0654
340.6769 -15842.6580 -1837.7906 0.0000 0.0000
7684.8848 4155.9855 307.4338 -3528.8993 4220.5860
310.3832 77.2970 69.1814 656151.2420 -18.7874
-18.7848
[15215]ENERGY: 16440000 3286.1452 5183.8455 5286.4887
350.9548 -15830.8025 -1770.9673 0.0000 0.0000
7658.1309 4163.7952 306.3635 -3494.3357 4220.7456
310.3560 -56.1281 -130.0966 656151.2420 -21.7260
-21.7332
[15265]ENERGY: 16450000 3182.3947 5275.2843 5316.3735

349.4985 -15867.3132 -1838.8438 0.0000 0.0000
7742.8386 4160.2326 309.7523 -3582.6060 4220.9829
310.4045 33.2260 -11.6762 656151.2420 -22.3913
-22.3885
[15301]ENERGY: 16460000 3165.6392 5338.1482 5241.7842
346.1948 -15907.9410 -1748.7298 0.0000 0.0000
7724.8470 4159.9425 309.0325 -3564.9045 4221.7217
310.5124 -120.7276 -69.5143 656151.2420 -21.6841
-21.6818
[15351]ENERGY: 16470000 3205.4824 5218.4656 5270.9606
333.7013 -15875.1377 -1767.0338 0.0000 0.0000
7773.0013 4159.4397 310.9589 -3613.5616 4221.5448
310.5307 63.5562 70.0857 656151.2420 -17.8683
-17.8673
[15387]ENERGY: 16480000 3161.8495 5180.7294 5276.4459
342.8066 -15850.8556 -1768.6744 0.0000 0.0000
7814.0130 4156.3144 312.5996 -3657.6986 4219.7931
310.6526 22.7162 16.5744 656151.2420 -21.5031
-21.5079
[15437]ENERGY: 16490000 3205.5077 5234.4807 5319.2329
324.1975 -15904.0299 -1780.9495 0.0000 0.0000
7760.0495 4158.4890 310.4408 -3601.5605 4221.8227
310.4764 -94.6463 -98.3990 656151.2420 -19.6710
-19.6863
[15476]ENERGY: 16500000 3156.4028 5244.3332 5251.4683
335.2423 -15875.4463 -1760.8979 0.0000 0.0000
7806.6924 4157.7948 312.3067 -3648.8976 4222.5003
310.4230 26.0006 -60.4537 656151.2420 -22.0500
-22.0335
[15526]ENERGY: 16510000 3196.9319 5244.8262 5264.7211
339.3514 -15808.5376 -1784.3930 0.0000 0.0000
7709.4446 4162.3446 308.4163 -3547.1000 4222.2657
310.1587 100.2868 60.7226 656151.2420 -26.5104
-26.5039
[15562]ENERGY: 16520000 3166.2292 5239.5608 5256.4132
361.5000 -15877.3287 -1743.8894 0.0000 0.0000
7754.6016 4157.0866 310.2228 -3597.5150 4223.0617
310.3232 79.1045 -18.3252 656151.2420 -18.6089
-18.6182
[15612]ENERGY: 16530000 3157.0596 5201.2231 5203.5743
340.1068 -15819.4603 -1759.4766 0.0000 0.0000
7835.0816 4158.1084 313.4424 -3676.9732 4221.9225
310.4275 53.1811 78.6475 656151.2420 -20.6357
-20.6233
[15648]ENERGY: 16540000 3230.3587 5242.4181 5227.7577
347.0632 -15881.1436 -1757.9999 0.0000 0.0000
7752.6434 4161.0977 310.1445 -3591.5457 4223.0691
310.4667 -141.7580 -141.4836 656151.2420 -21.2323
-21.2361
[15698]ENERGY: 16550000 3171.1706 5195.9573 5242.1144
337.7137 -15835.8216 -1764.4039 0.0000 0.0000
7812.8940 4159.6245 312.5548 -3653.2695 4223.1274
310.5661 8.3211 -54.3861 656151.2420 -17.9851
-17.9817

[15734]ENERGY: 16560000 3271.5096 5247.2600 5273.3081
309.0882 -15883.6418 -1769.8079 0.0000 0.0000
7713.3500 4161.0663 308.5726 -3552.2838 4223.4662
310.3705 -91.4405 -155.2148 656151.2420 -19.1461
-19.1482
[15784]ENERGY: 16570000 3182.7791 5235.3346 5241.4971
346.4686 -15803.5440 -1753.2091 0.0000 0.0000
7713.0354 4162.3616 308.5600 -3550.6738 4223.2019
310.4905 -40.2789 -24.0388 656151.2420 -22.7821
-22.7783
[15820]ENERGY: 16580000 3302.0108 5194.3185 5241.6333
347.3326 -15847.8198 -1764.6435 0.0000 0.0000
7692.9548 4165.7867 307.7567 -3527.1682 4224.8513
310.3865 -10.6361 -74.7410 656151.2420 -23.4615
-23.4715
[15870]ENERGY: 16590000 3203.6770 5281.6999 5199.7279
341.3282 -15846.6235 -1800.6058 0.0000 0.0000
7780.1386 4159.3423 311.2444 -3620.7963 4224.7241
310.4691 100.6743 45.4518 656151.2420 -16.9302
-16.9422
[15909]ENERGY: 16600000 3220.6194 5208.0959 5281.4395
325.9022 -15803.0312 -1823.3924 0.0000 0.0000
7753.2110 4162.8446 310.1672 -3590.3664 4224.7200
310.5114 -113.5827 -136.9375 656151.2420 -19.1165
-19.1248
[15959]ENERGY: 16610000 3218.6578 5234.9730 5260.2229
347.4384 -15790.4363 -1779.6049 0.0000 0.0000
7671.4440 4162.6949 306.8961 -3508.7492 4224.8817
310.4255 -6.5950 -60.4529 656151.2420 -23.3976
-23.3826
[15995]ENERGY: 16620000 3294.6717 5159.4218 5247.2743
330.8627 -15852.3905 -1765.4309 0.0000 0.0000
7746.4392 4160.8484 309.8963 -3585.5908 4225.0834
310.4349 -150.8201 -159.8927 656151.2420 -18.3827
-18.3805
[16045]ENERGY: 16630000 3178.4923 5176.7842 5251.6283
353.7687 -15863.1465 -1771.4328 0.0000 0.0000
7835.2682 4161.3624 313.4499 -3673.9058 4225.0698
310.5965 84.2736 15.0269 656151.2420 -17.4056
-17.3989
[16081]ENERGY: 16640000 3239.3638 5193.5237 5274.3163
331.1894 -15799.8422 -1875.0209 0.0000 0.0000
7797.7223 4161.2525 311.9479 -3636.4699 4224.8575
310.5857 -99.7458 -122.5602 656151.2420 -16.2050
-16.2115
[16131]ENERGY: 16650000 3129.3938 5186.8104 5298.4764
364.1993 -15910.9728 -1734.0124 0.0000 0.0000
7825.1115 4159.0062 313.0436 -3666.1054 4225.6124
310.5425 181.6130 146.2044 656151.2420 -19.0656
-19.0612
[16167]ENERGY: 16660000 3242.1562 5215.8849 5308.0329
339.2489 -15742.8805 -1914.6001 0.0000 0.0000
7716.8044 4164.6468 308.7108 -3552.1577 4226.6512
310.6137 -85.8209 -55.7851 656151.2420 -16.4947

-16.5076
[16217]ENERGY: 16670000 3174.6290 5271.1529 5300.8562
338.5341 -15889.3865 -1763.9442 0.0000 0.0000
7728.7344 4160.5760 309.1880 -3568.1584 4224.6092
310.6743 -111.7052 -53.3012 656151.2420 -20.7589
-20.7408
[16253]ENERGY: 16680000 3229.4553 5173.4053 5237.4482
361.2525 -15860.4214 -1778.2260 0.0000 0.0000
7801.7603 4164.6743 312.1094 -3637.0860 4226.3048
310.5137 63.2601 -60.4873 656151.2420 -19.3939
-19.4002
[16303]ENERGY: 16690000 3209.2247 5255.1913 5273.8309
333.9383 -15896.4774 -1747.0510 0.0000 0.0000
7737.0825 4165.7394 309.5220 -3571.3431 4226.2576
310.5541 -18.6963 -23.0456 656151.2420 -17.9394
-17.9516
[16342]ENERGY: 16700000 3220.4428 5256.2049 5262.3605
317.9402 -15810.7641 -1811.8610 0.0000 0.0000
7731.7205 4166.0437 309.3075 -3565.6768 4225.9786
310.7592 -116.9409 -147.3085 656151.2420 -22.8071
-22.8015
[16392]ENERGY: 16710000 3220.2378 5324.8138 5273.6908
340.8417 -15945.9011 -1803.9701 0.0000 0.0000
7753.1634 4162.8763 310.1653 -3590.2871 4224.9129
310.7047 -271.2699 -211.6604 656151.2420 -16.2685
-16.2418
[16428]ENERGY: 16720000 3241.4230 5238.6574 5322.3456
327.2841 -15876.1982 -1772.2753 0.0000 0.0000
7682.2868 4163.5235 307.3299 -3518.7633 4225.7479
310.6269 -15.9709 -38.7828 656151.2420 -22.4568
-22.4728
[16478]ENERGY: 16730000 3161.6212 5204.9393 5310.9328
357.0058 -15803.2022 -1821.6595 0.0000 0.0000
7756.9118 4166.5492 310.3153 -3590.3626 4228.1503
310.5224 4.4132 -79.9542 656151.2420 -20.4444
-20.4623
[16514]ENERGY: 16740000 3229.2766 5283.6661 5264.5884
351.4881 -15908.0533 -1765.9955 0.0000 0.0000
7714.4665 4169.4370 308.6172 -3545.0295 4229.5697
310.5575 109.2111 51.3602 656151.2420 -19.4943
-19.4819
[16564]ENERGY: 16750000 3209.4189 5298.8648 5233.0340
352.8621 -15882.0334 -1786.1957 0.0000 0.0000
7739.3317 4165.2823 309.6120 -3574.0493 4227.8167
310.5852 95.1167 -26.2909 656151.2420 -18.5153
-18.5183
[16600]ENERGY: 16760000 3201.6175 5257.9799 5306.4531
341.6959 -15944.7433 -1777.7757 0.0000 0.0000
7779.5505 4164.7779 311.2209 -3614.7726 4227.9335
310.5473 18.7752 48.8662 656151.2420 -19.9032
-19.9129
[16650]ENERGY: 16770000 3175.7996 5180.5107 5332.4951
329.8109 -15917.8500 -1749.4609 0.0000 0.0000
7811.3632 4162.6687 312.4936 -3648.6945 4227.7606

310.8260 169.1638 156.7680 656151.2420 -18.3488
-18.3407
[16686]ENERGY: 16780000 3232.5844 5221.1536 5262.3397
360.8385 -15909.8258 -1739.7380 0.0000 0.0000
7739.5298 4166.8822 309.6199 -3572.6476 4228.2338
310.9493 10.6872 -67.1124 656151.2420 -17.3682
-17.3673
[16736]ENERGY: 16790000 3208.5744 5211.1910 5258.0599
339.1337 -15891.7951 -1774.9915 0.0000 0.0000
7812.4531 4162.6254 312.5372 -3649.8277 4227.9294
310.7859 -44.1116 -90.0074 656151.2420 -22.8117
-22.8208
[16775]ENERGY: 16800000 3261.3311 5149.2541 5282.2388
368.9814 -15903.1643 -1795.1526 0.0000 0.0000
7800.9798 4164.4683 312.0782 -3636.5115 4228.6278
310.7691 -45.5493 -84.5234 656151.2420 -18.7740
-18.7661
[16825]ENERGY: 16810000 3255.2896 5154.7713 5263.0586
330.5245 -15872.8989 -1767.4866 0.0000 0.0000
7802.3903 4165.6489 312.1346 -3636.7414 4228.2107
310.7692 83.8872 -6.2723 656151.2420 -17.4561
-17.4567
[16861]ENERGY: 16820000 3233.1804 5212.9792 5249.8806
318.4334 -15803.8432 -1827.8565 0.0000 0.0000
7784.6524 4167.4262 311.4250 -3617.2261 4228.1592
310.5047 -36.8846 -52.7565 656151.2420 -24.0562
-24.0449
[16911]ENERGY: 16830000 3249.1573 5208.7761 5262.0737
330.3507 -15941.9101 -1714.6831 0.0000 0.0000
7773.2783 4167.0427 310.9700 -3606.2355 4228.5993
310.8415 52.5508 6.6877 656151.2420 -16.7042
-16.7054
[16947]ENERGY: 16840000 3151.0709 5254.1797 5266.2714
335.5174 -15762.8553 -1864.3516 0.0000 0.0000
7784.2705 4164.1030 311.4097 -3620.1675 4229.0505
310.7587 10.8060 30.7748 656151.2420 -21.1857
-21.1822
[16997]ENERGY: 16850000 3203.8600 5162.3084 5279.8405
340.2596 -15873.4043 -1768.0009 0.0000 0.0000
7820.8684 4165.7318 312.8738 -3655.1366 4228.6111
310.6377 -196.0746 -224.8897 656151.2420 -20.3726
-20.3866
[17033]ENERGY: 16860000 3198.7843 5239.3127 5232.9571
325.8623 -15864.1820 -1816.7379 0.0000 0.0000
7844.8512 4160.8476 313.8333 -3684.0036 4227.5337
310.5355 -148.5257 -114.0576 656151.2420 -20.7407
-20.7380
[17083]ENERGY: 16870000 3222.0999 5400.0605 5251.3491
346.9871 -15939.2006 -1849.5755 0.0000 0.0000
7736.4573 4168.1777 309.4970 -3568.2796 4229.5228
310.6453 -52.9540 -70.5804 656151.2420 -19.7703
-19.7691
[17119]ENERGY: 16880000 3187.7201 5266.4743 5253.5221
331.1158 -15830.7805 -1833.3284 0.0000 0.0000

7788.8706 4163.5940 311.5938 -3625.2766 4229.4891
310.6950 150.5038 82.7154 656151.2420 -17.9801
-17.9801
[17169]ENERGY: 16890000 3227.9365 5179.0915 5264.3387
363.0214 -15894.9205 -1727.5230 0.0000 0.0000
7752.8341 4164.7787 310.1521 -3588.0554 4229.7479
310.6407 129.3704 76.1916 656151.2420 -19.1679
-19.1697
[17208]ENERGY: 16900000 3194.0735 5244.1395 5209.5919
347.7232 -15871.0938 -1805.8767 0.0000 0.0000
7846.7051 4165.2627 313.9074 -3681.4425 4228.6634
310.4226 -256.1559 -201.4197 656151.2420 -23.0831
-23.0671
[17258]ENERGY: 16910000 3173.8895 5295.9522 5204.7084
330.5203 -15903.6952 -1767.3508 0.0000 0.0000
7829.0157 4163.0401 313.1998 -3665.9757 4228.9130
310.6267 -1.3286 -79.2026 656151.2420 -21.3730
-21.3918
[17294]ENERGY: 16920000 3171.1495 5346.7759 5299.6455
354.6964 -15954.1822 -1825.2007 0.0000 0.0000
7771.3842 4164.2687 310.8942 -3607.1156 4229.8617
310.5489 84.3176 -0.7975 656151.2420 -19.1766
-19.1551
[17344]ENERGY: 16930000 3252.8902 5209.0904 5261.8251
335.7893 -15844.7110 -1699.1402 0.0000 0.0000
7656.2095 4171.9535 306.2867 -3484.2560 4229.1474
310.7337 18.1980 -21.9993 656151.2420 -19.7816
-19.8003
[17380]ENERGY: 16940000 3242.3538 5221.0602 5292.6665
340.4705 -15855.7659 -1756.7689 0.0000 0.0000
7687.8621 4171.8781 307.5529 -3515.9840 4229.0596
310.7720 -93.5367 -31.0334 656151.2420 -15.3892
-15.3805
[17430]ENERGY: 16950000 3202.7278 5240.9907 5263.6468
337.6611 -15832.1951 -1822.5021 0.0000 0.0000
7774.9444 4165.2735 311.0366 -3609.6709 4228.7221
310.7261 -59.8712 -95.9210 656151.2420 -21.3532
-21.3555
[17466]ENERGY: 16960000 3227.3101 5220.1912 5358.2012
349.5731 -15874.0223 -1800.2546 0.0000 0.0000
7687.0514 4168.0502 307.5205 -3519.0013 4230.1991
310.8848 -77.8118 -138.3258 656151.2420 -15.8714
-15.8907
[17516]ENERGY: 16970000 3183.0416 5178.1422 5265.1612
349.9370 -15911.3679 -1731.1204 0.0000 0.0000
7832.2806 4166.0743 313.3304 -3666.2063 4229.4578
310.8219 41.7633 32.8118 656151.2420 -22.6048
-22.5905
[17552]ENERGY: 16980000 3181.0721 5314.7540 5341.8679
352.1698 -15883.6848 -1865.6677 0.0000 0.0000
7725.6661 4166.1775 309.0653 -3559.4886 4230.8351
310.9704 -97.7380 -139.5612 656151.2420 -21.2695
-21.2973
[17602]ENERGY: 16990000 3179.8175 5237.1839 5249.2372

342.1354 -15840.9887 -1813.2716 0.0000 0.0000
7813.1528 4167.2664 312.5652 -3645.8864 4229.8166
310.7742 251.2639 156.3589 656151.2420 -20.2059
-20.1675
[17641]ENERGY: 17000000 3095.9415 5290.4952 5268.6509
329.9174 -15877.0111 -1781.2642 0.0000 0.0000
7836.1540 4162.8836 313.4853 -3673.2704 4229.9426
310.6602 55.5550 67.8060 656151.2420 -15.8921
-15.8959
[17691]ENERGY: 17010000 3221.8722 5199.4650 5279.4107
345.5701 -15841.1509 -1759.8570 0.0000 0.0000
7725.9049 4171.2149 309.0748 -3554.6900 4230.2736
310.6252 16.1869 31.2089 656151.2420 -18.7989
-18.7979
[17727]ENERGY: 17020000 3262.0532 5283.3560 5259.5525
350.4422 -15907.6368 -1757.3283 0.0000 0.0000
7677.8700 4168.3089 307.1532 -3509.5611 4231.6207
310.6304 111.6998 80.5177 656151.2420 -16.4554
-16.4726
[17777]ENERGY: 17030000 3244.9678 5303.1950 5251.5225
334.3406 -15933.1582 -1775.8103 0.0000 0.0000
7747.3276 4172.3850 309.9318 -3574.9426 4232.1741
310.8560 30.4758 -31.2448 656151.2420 -21.8067
-21.8037
[17813]ENERGY: 17040000 3172.3020 5282.4510 5269.8668
335.2552 -15948.1550 -1727.2651 0.0000 0.0000
7782.2769 4166.7318 311.3300 -3615.5450 4231.2368
310.8821 30.9302 -26.2343 656151.2420 -23.3558
-23.3576
[17863]ENERGY: 17050000 3145.2985 5338.0207 5308.1590
339.7222 -15944.8048 -1790.8635 0.0000 0.0000
7769.9903 4165.5225 310.8385 -3604.4678 4230.4931
310.9621 23.9650 36.4140 656151.2420 -21.2036
-21.1976
[17899]ENERGY: 17060000 3181.9734 5229.3276 5226.6991
322.8734 -15900.3571 -1677.3432 0.0000 0.0000
7780.6575 4163.8307 311.2652 -3616.8268 4231.1473
310.8260 44.2892 -19.7065 656151.2420 -21.4019
-21.3969
[17949]ENERGY: 17070000 3212.3844 5179.2065 5250.0445
358.0362 -15813.5249 -1832.3130 0.0000 0.0000
7812.6394 4166.4732 312.5446 -3646.1662 4230.7902
310.8718 -145.5825 -139.9315 656151.2420 -18.0342
-18.0264
[17985]ENERGY: 17080000 3263.3402 5234.1589 5241.7074
322.1241 -15821.7056 -1786.7444 0.0000 0.0000
7718.0976 4170.9781 308.7625 -3547.1194 4230.9544
310.9060 -40.7942 -3.9509 656151.2420 -21.5872
-21.5959
[18035]ENERGY: 17090000 3271.2357 5237.6639 5289.7829
343.1240 -15951.8842 -1799.3133 0.0000 0.0000
7778.1906 4168.7996 311.1665 -3609.3911 4231.0676
310.9715 -218.3630 -153.1789 656151.2420 -22.5557
-22.5647

[18074]ENERGY: 17100000 3231.2345 5210.4221 5238.2410
337.2585 -15843.2132 -1775.3438 0.0000 0.0000
7769.9044 4168.5036 310.8350 -3601.4008 4231.5750
310.9309 -71.2448 -93.6807 656151.2420 -20.9034
-20.8967
[18124]ENERGY: 17110000 3105.0548 5226.7797 5287.9854
343.0885 -15826.7228 -1786.4951 0.0000 0.0000
7816.7990 4166.4895 312.7110 -3650.3095 4230.9326
310.9366 70.9439 64.5003 656151.2420 -22.1733
-22.1586
[18160]ENERGY: 17120000 3206.8884 5155.5963 5265.4818
359.3482 -15778.0905 -1773.2578 0.0000 0.0000
7732.7996 4168.7660 309.3506 -3564.0336 4232.0548
310.8877 -12.5325 -80.6687 656151.2420 -20.1728
-20.1864
[18210]ENERGY: 17130000 3178.3118 5215.0708 5216.9504
320.8887 -15893.5487 -1741.9050 0.0000 0.0000
7873.0705 4168.8384 314.9622 -3704.2321 4232.1577
310.8497 77.8992 75.3055 656151.2420 -15.9404
-15.9217
[18246]ENERGY: 17140000 3179.0089 5259.6061 5270.2478
334.2099 -15830.4586 -1845.3335 0.0000 0.0000
7800.8117 4168.0922 312.0715 -3632.7195 4233.7165
310.9568 11.3903 -49.4962 656151.2420 -20.3071
-20.3204
[18296]ENERGY: 17150000 3205.0325 5226.6838 5212.8303
332.2264 -15946.0651 -1693.0463 0.0000 0.0000
7830.9935 4168.6551 313.2789 -3662.3384 4232.6594
310.8995 41.9867 4.5973 656151.2420 -19.0378
-19.0326
[18332]ENERGY: 17160000 3132.0849 5230.0000 5250.6812
356.7755 -15817.1134 -1732.1721 0.0000 0.0000
7752.6632 4172.9192 310.1453 -3579.7439 4233.5617
310.7580 144.0405 133.5636 656151.2420 -16.7316
-16.7374
[18382]ENERGY: 17170000 3247.8879 5196.8168 5238.6703
348.0193 -15819.6869 -1775.7656 0.0000 0.0000
7737.1497 4173.0915 309.5247 -3564.0581 4234.6321
310.8174 -115.2113 -51.2108 656151.2420 -19.6220
-19.6131
[18418]ENERGY: 17180000 3156.1170 5293.4920 5233.7746
368.5522 -15811.8194 -1829.2250 0.0000 0.0000
7759.9181 4170.8095 310.4355 -3589.1085 4234.7199
310.8179 -1.1756 12.5909 656151.2420 -16.7137
-16.7295
[18468]ENERGY: 17190000 3231.4362 5287.3528 5264.6552
346.8367 -15889.3393 -1778.1311 0.0000 0.0000
7712.7763 4175.5868 308.5496 -3537.1895 4235.7081
310.8559 -45.4597 -138.9153 656151.2420 -19.1535
-19.1433
[18507]ENERGY: 17200000 3202.2079 5257.0025 5272.1459
354.1787 -15917.3220 -1791.1879 0.0000 0.0000
7796.6023 4173.6274 311.9031 -3622.9749 4235.0624
310.9220 -20.7260 64.0811 656151.2420 -22.2571

-22.2672
[18557]ENERGY: 17210000 3106.3191 5284.7818 5291.8837
323.7770 -15914.1831 -1738.0796 0.0000 0.0000
7816.1534 4170.6522 312.6852 -3645.5012 4235.9695
310.9152 125.0638 31.0243 656151.2420 -19.4642
-19.4569
[18593]ENERGY: 17220000 3244.8441 5198.6377 5296.4516
325.3573 -15910.1653 -1741.2985 0.0000 0.0000
7759.8922 4173.7191 310.4345 -3586.1731 4235.3819
310.8679 28.0124 -8.4349 656151.2420 -21.4151
-21.4313
[18643]ENERGY: 17230000 3231.0314 5249.0846 5244.1776
324.7268 -15946.9056 -1745.7194 0.0000 0.0000
7812.9119 4169.3074 312.5555 -3643.6046 4235.7893
310.8981 -58.9549 -84.2018 656151.2420 -19.7711
-19.7536
[18679]ENERGY: 17240000 3166.7783 5195.2036 5286.9177
365.2139 -15871.3974 -1813.0971 0.0000 0.0000
7843.9327 4173.5516 313.7965 -3670.3811 4236.4923
310.6488 -32.2767 -99.6479 656151.2420 -21.7603
-21.7510
[18729]ENERGY: 17250000 3184.5475 5192.2330 5291.2341
374.7619 -15860.9538 -1796.1551 0.0000 0.0000
7786.8126 4172.4802 311.5114 -3614.3324 4237.6306
310.6462 69.7607 29.8535 656151.2420 -18.8461
-18.8762
[18765]ENERGY: 17260000 3243.9949 5169.5400 5300.4228
361.2844 -15826.4430 -1808.4956 0.0000 0.0000
7738.4211 4178.7245 309.5755 -3559.6966 4237.1473
310.4866 12.3922 24.0185 656151.2420 -23.7361
-23.7190
[18815]ENERGY: 17270000 3186.0145 5336.9087 5304.5895
346.4051 -15983.9294 -1831.0881 0.0000 0.0000
7813.7154 4172.6158 312.5877 -3641.0996 4237.9715
310.5156 -6.5507 -46.7421 656151.2420 -23.3897
-23.3987
[18851]ENERGY: 17280000 3265.8072 5233.6773 5219.9537
294.7215 -15799.7140 -1838.5503 0.0000 0.0000
7797.7382 4173.6335 311.9485 -3624.1046 4237.0917
310.7208 -61.7006 -78.8111 656151.2420 -17.6252
-17.6240
[18901]ENERGY: 17290000 3143.8329 5275.0335 5277.8132
348.9199 -15880.0202 -1800.4061 0.0000 0.0000
7809.8902 4175.0634 312.4347 -3634.8268 4238.6010
310.5577 5.4595 -52.4708 656151.2420 -22.5170
-22.5094
[18940]ENERGY: 17300000 3263.2125 5263.7773 5257.8037
343.8094 -15886.6551 -1847.8012 0.0000 0.0000
7782.3025 4176.4491 311.3310 -3605.8534 4237.3749
310.7148 -18.3098 -99.4647 656151.2420 -28.3639
-28.3617
[18990]ENERGY: 17310000 3287.0413 5281.5059 5240.4350
347.3655 -15870.4864 -1839.3500 0.0000 0.0000
7728.0143 4174.5256 309.1592 -3553.4887 4238.6139

310.6164 -127.5799 -150.6237 656151.2420 -21.5228
-21.5356
[19026]ENERGY: 17320000 3244.6999 5284.0735 5293.4103
332.4988 -15908.3470 -1782.1063 0.0000 0.0000
7713.4994 4177.7286 308.5785 -3535.7708 4238.1875
310.7477 -164.6814 -54.4673 656151.2420 -23.5101
-23.5024
[19076]ENERGY: 17330000 3213.6993 5222.2343 5270.1949
333.2119 -15905.4714 -1779.8222 0.0000 0.0000
7819.7849 4173.8316 312.8305 -3645.9533 4238.6268
310.7092 -86.7719 -81.9043 656151.2420 -24.3320
-24.3269
[19112]ENERGY: 17340000 3258.5943 5274.2887 5290.8736
339.6854 -15884.7067 -1831.3421 0.0000 0.0000
7731.1144 4178.5077 309.2832 -3552.6068 4239.0459
310.5053 -45.8642 -80.0634 656151.2420 -23.2161
-23.2253
[19162]ENERGY: 17350000 3248.5406 5227.6367 5305.3187
344.3522 -15989.1768 -1730.0106 0.0000 0.0000
7768.2689 4174.9297 310.7696 -3593.3392 4239.6308
310.6559 -16.9291 -23.7952 656151.2420 -25.0886
-25.0657
[19198]ENERGY: 17360000 3207.2914 5416.4688 5248.0268
349.7514 -15811.9966 -1853.3385 0.0000 0.0000
7623.8486 4180.0519 304.9921 -3443.7968 4240.1990
310.6255 65.0302 80.2974 656151.2420 -20.8649
-20.8685
[19248]ENERGY: 17370000 3211.9992 5241.2689 5251.1755
343.5690 -15831.3321 -1863.3509 0.0000 0.0000
7819.9644 4173.2940 312.8377 -3646.6704 4239.1335
310.6104 -130.8900 -107.6209 656151.2420 -22.2620
-22.2658
[19284]ENERGY: 17380000 3189.0539 5262.9685 5268.1777
339.3454 -15882.7828 -1803.3171 0.0000 0.0000
7803.3836 4176.8291 312.1744 -3626.5545 4239.3781
310.6482 -16.3247 -77.4114 656151.2420 -22.9850
-22.9933
[19334]ENERGY: 17390000 3277.4484 5235.5139 5288.3052
336.0903 -15857.9317 -1816.3974 0.0000 0.0000
7716.5165 4179.5452 308.6992 -3536.9713 4240.2273
310.6354 -12.1783 -17.3829 656151.2420 -23.3532
-23.3476
[19373]ENERGY: 17400000 3193.3221 5289.6962 5261.0743
314.3594 -15863.5435 -1762.4941 0.0000 0.0000
7746.0539 4178.4682 309.8809 -3567.5857 4239.4413
310.6794 -40.6807 -48.4083 656151.2420 -23.8032
-23.8035
[19423]ENERGY: 17410000 3182.2423 5145.6360 5304.4207
353.7011 -15873.1648 -1720.3084 0.0000 0.0000
7783.7155 4176.2425 311.3875 -3607.4730 4239.5919
310.5028 36.3508 -30.9825 656151.2420 -23.6208
-23.6355
[19459]ENERGY: 17420000 3179.4746 5272.9171 5244.4930
353.1825 -15797.1572 -1836.3058 0.0000 0.0000

7765.0349 4181.6391 310.6402 -3583.3958 4240.8557
310.6268 -9.9350 -4.8778 656151.2420 -19.7621
-19.7476
[19509]ENERGY: 17430000 3232.4295 5277.3642 5253.8269
359.7248 -15925.2672 -1772.8068 0.0000 0.0000
7749.3942 4174.6655 310.0145 -3574.7287 4240.4866
310.8319 45.7423 52.7940 656151.2420 -20.8540
-20.8541
[19545]ENERGY: 17440000 3189.5779 5292.7635 5273.3497
342.7111 -15869.1471 -1800.5753 0.0000 0.0000
7747.6836 4176.3635 309.9461 -3571.3201 4240.3037
310.8651 -8.4390 -43.6005 656151.2420 -22.4987
-22.4925
[19595]ENERGY: 17450000 3182.4858 5295.6752 5264.8427
346.6083 -15859.4168 -1825.3184 0.0000 0.0000
7772.4000 4177.2769 310.9349 -3595.1231 4241.0893
310.7136 88.4840 78.1827 656151.2420 -22.0097
-22.0233
[19631]ENERGY: 17460000 3175.6760 5272.9622 5290.3718
351.1267 -15855.6895 -1747.1809 0.0000 0.0000
7693.1901 4180.4564 307.7661 -3512.7337 4239.1371
310.6022 10.3614 -34.1788 656151.2420 -20.9775
-20.9973
[19681]ENERGY: 17470000 3258.1608 5194.8341 5278.7259
322.9558 -15855.8861 -1843.3881 0.0000 0.0000
7819.6216 4175.0240 312.8240 -3644.5976 4239.9668
310.6022 -100.9758 -178.6599 656151.2420 -22.4071
-22.3807
[19717]ENERGY: 17480000 3192.4623 5245.0331 5261.4401
317.4537 -15861.7508 -1784.1414 0.0000 0.0000
7809.5615 4180.0586 312.4215 -3629.5029 4241.2205
310.6551 -78.0648 -96.7393 656151.2420 -21.5749
-21.5724
[19767]ENERGY: 17490000 3332.7069 5239.6071 5292.3933
330.1781 -15866.5562 -1734.4066 0.0000 0.0000
7589.1554 4183.0781 303.6042 -3406.0773 4240.5063
310.7880 -32.1864 -13.6090 656151.2420 -19.3028
-19.3008
[19806]ENERGY: 17500000 3209.7798 5223.8686 5283.6218
333.1606 -15914.8203 -1725.6379 0.0000 0.0000
7767.8780 4177.8507 310.7540 -3590.0273 4240.2013
310.7796 -55.1818 -39.3264 656151.2420 -16.5093
-16.5250
[19856]ENERGY: 17510000 3229.1297 5296.2333 5294.4794
327.4190 -15916.1602 -1770.1247 0.0000 0.0000
7718.1710 4179.1475 308.7654 -3539.0235 4240.9638
310.8131 41.3898 -12.1665 656151.2420 -18.8128
-18.8197
[19892]ENERGY: 17520000 3208.6984 5198.1097 5271.1958
356.8377 -15900.3240 -1748.1760 0.0000 0.0000
7794.0849 4180.4266 311.8024 -3613.6583 4240.5445
310.7545 8.1154 5.0016 656151.2420 -16.9184
-16.9123
[19942]ENERGY: 17530000 3237.1440 5299.7491 5224.8586

```
323.8275          -15758.6876     -1839.6886          0.0000          0.0000
7696.6999            4183.9029       307.9065     -3512.7970       4241.5701
310.8727             -10.3150        -46.1307    656151.2420        -14.9824
-14.9765
[19978]ENERGY: 17540000      3264.4020      5276.7898      5285.5543
334.7238          -15902.7113     -1772.3301          0.0000          0.0000
7692.4794            4178.9079       307.7376     -3513.5715       4241.9751
310.8136             -59.6382        -49.2045    656151.2420        -18.7248
-18.7147
[20028]ENERGY: 17550000      3241.7200      5282.2564      5275.6828
324.3763          -15847.7094     -1848.5410          0.0000          0.0000
7751.0470            4178.8320       310.0806     -3572.2150       4243.0984
310.8499             -81.6332        -53.7820    656151.2420        -15.9696
-15.9675
[20064]ENERGY: 17560000      3178.5913      5214.1162      5326.9956
338.0349          -15934.7531     -1733.5624          0.0000          0.0000
7789.6104            4179.0329       311.6234     -3610.5776       4240.5459
310.7514            -105.7306        -41.1581    656151.2420        -15.5109
-15.5271
[20114]ENERGY: 17570000      3187.4284      5204.2310      5278.0065
358.3039          -15840.6989     -1770.3849          0.0000          0.0000
7760.7839            4177.6699       310.4702     -3583.1140       4241.4158
310.7713             108.1882         69.0206    656151.2420        -19.4872
-19.4817
[20150]ENERGY: 17580000      3222.6410      5236.7892      5244.2887
323.6745          -15883.1057     -1700.4483          0.0000          0.0000
7734.9906            4178.8300       309.4383     -3556.1606       4240.4913
310.8969            -100.8253          1.3634    656151.2420        -14.6346
-14.6294
[20200]ENERGY: 17590000      3145.7480      5140.7910      5299.2596
315.1333          -15832.9949     -1759.8582          0.0000          0.0000
7870.7365            4178.8153       314.8688     -3691.9212       4241.0032
310.7651              88.7871         52.8540    656151.2420        -18.8127
-18.8218
[20239]ENERGY: 17600000      3193.4759      5197.3762      5245.4044
330.3635          -15869.8103     -1760.1552          0.0000          0.0000
7841.5873            4178.2419       313.7027     -3663.3455       4240.5537
310.7927             115.4427         81.6051    656151.2420        -19.9729
-19.9542
[20289]ENERGY: 17610000      3263.4850      5161.1565      5272.5320
357.3027          -15854.6489     -1763.7975          0.0000          0.0000
7745.4927            4181.5224       309.8584     -3563.9703       4243.0951
310.9745            -132.0007       -126.8661    656151.2420        -16.6762
-16.6896
[20325]ENERGY: 17620000      3242.5186      5201.7958      5278.9699
343.6016          -15924.2122     -1776.2783          0.0000          0.0000
7808.8545            4175.2499       312.3932     -3633.6045       4241.1779
311.0248             -55.0942         28.0064    656151.2420        -19.6899
-19.6898
[20375]ENERGY: 17630000      3233.6344      5241.9683      5232.4803
333.9554          -15878.5659     -1766.1134          0.0000          0.0000
7783.0395            4180.3985       311.3605     -3602.6410       4242.1610
310.9802              65.9076         23.2590    656151.2420        -17.5698
-17.5759
```

[20411]ENERGY: 17640000 3231.6795 5192.0811 5272.1437
341.8461 -15823.8492 -1775.7024 0.0000 0.0000
7741.9085 4180.1074 309.7150 -3561.8012 4241.8556
310.7992 15.4223 -9.5329 656151.2420 -19.9560
-19.9445
[20461]ENERGY: 17650000 3210.8851 5301.2722 5256.7221
334.2693 -15815.6549 -1841.6553 0.0000 0.0000
7737.0037 4182.8421 309.5188 -3554.1616 4242.3228
310.9801 -17.1727 -33.9625 656151.2420 -18.3417
-18.3556
[20497]ENERGY: 17660000 3192.7784 5213.4501 5285.0636
329.6647 -15877.4924 -1796.5756 0.0000 0.0000
7831.8434 4178.7321 313.3129 -3653.1113 4241.7812
310.9011 -48.1232 -18.5134 656151.2420 -17.5403
-17.5209
[20547]ENERGY: 17670000 3191.5031 5236.5144 5277.1338
344.7781 -15940.8126 -1655.9447 0.0000 0.0000
7726.4217 4179.5938 309.0955 -3546.8279 4241.7972
310.9374 287.1680 196.8269 656151.2420 -20.0695
-20.0859
[20583]ENERGY: 17680000 3201.2359 5270.6076 5197.3002
323.2811 -15843.2325 -1831.8402 0.0000 0.0000
7865.8919 4183.2441 314.6750 -3682.6478 4243.9685
310.9719 -136.7449 -201.7181 656151.2420 -18.3011
-18.3060
[20633]ENERGY: 17690000 3225.6185 5263.8303 5311.9353
326.3551 -15795.4615 -1857.5157 0.0000 0.0000
7706.4284 4181.1904 308.2957 -3525.2381 4244.0161
310.9876 -59.1302 -46.6961 656151.2420 -18.5968
-18.5782
[20672]ENERGY: 17700000 3195.8320 5245.6866 5300.1834
336.6968 -15885.1554 -1748.5406 0.0000 0.0000
7739.1252 4183.8279 309.6037 -3555.2973 4243.8175
310.9139 -14.6870 68.0764 656151.2420 -24.2300
-24.2378
[20722]ENERGY: 17710000 3138.2127 5200.3225 5294.1102
324.3665 -15900.0838 -1732.7346 0.0000 0.0000
7850.5973 4174.7907 314.0631 -3675.8066 4242.3966
311.1291 124.6545 46.8002 656151.2420 -22.6286
-22.6106
[20758]ENERGY: 17720000 3223.4844 5177.0092 5288.3031
330.9362 -15829.9934 -1765.1980 0.0000 0.0000
7756.2811 4180.8227 310.2900 -3575.4584 4242.7159
311.0985 29.1774 -50.8067 656151.2420 -19.1896
-19.2114
[20808]ENERGY: 17730000 3192.6910 5194.7496 5270.0095
350.9720 -15799.7355 -1814.0573 0.0000 0.0000
7783.0987 4177.7281 311.3629 -3605.3706 4243.3083
310.9496 -80.3490 -110.2865 656151.2420 -21.8077
-21.8179
[20844]ENERGY: 17740000 3154.0204 5275.7171 5257.8003
339.9182 -15826.0730 -1740.7996 0.0000 0.0000
7721.7975 4182.3810 308.9105 -3539.4165 4243.5327
310.8505 115.3638 81.9444 656151.2420 -17.7341

-17.7275
[20894]ENERGY: 17750000 3158.1787 5236.9970 5259.8954
329.1596 -15857.8410 -1751.2917 0.0000 0.0000
7801.2024 4176.3005 312.0871 -3624.9019 4242.9992
310.8319 145.7346 109.8133 656151.2420 -21.0923
-21.0792
[20930]ENERGY: 17760000 3182.4929 5248.9786 5240.3909
349.7062 -15890.5214 -1781.1114 0.0000 0.0000
7827.8858 4177.8216 313.1546 -3650.0642 4242.7682
310.8831 36.8405 55.2305 656151.2420 -19.2314
-19.2217
[20980]ENERGY: 17770000 3287.9294 5214.1578 5239.0212
344.0138 -15795.2037 -1833.4166 0.0000 0.0000
7723.2808 4179.7827 308.9698 -3543.4981 4243.8352
311.2330 -290.4132 -232.9177 656151.2420 -21.6296
-21.6484
[21016]ENERGY: 17780000 3227.4899 5306.1321 5249.0980
376.7406 -15867.7457 -1756.1212 0.0000 0.0000
7650.7431 4186.3367 306.0680 -3464.4064 4245.4077
311.1393 74.8768 -6.3483 656151.2420 -20.8719
-20.8615
[21066]ENERGY: 17790000 3165.6908 5251.9628 5220.4244
310.9629 -15850.6254 -1724.8452 0.0000 0.0000
7808.8831 4182.4534 312.3944 -3626.4297 4244.6571
311.1439 163.1393 85.2768 656151.2420 -19.8031
-19.8135
[21105]ENERGY: 17800000 3169.2823 5290.0427 5279.2421
332.8645 -15880.6140 -1759.8954 0.0000 0.0000
7754.7315 4185.6538 310.2280 -3569.0777 4245.5752
311.0244 245.3996 81.3957 656151.2420 -22.2307
-22.2470
[21155]ENERGY: 17810000 3217.6255 5267.1320 5230.6300
328.9286 -15850.3303 -1788.1579 0.0000 0.0000
7774.3894 4180.2173 311.0144 -3594.1721 4244.8300
310.9769 -27.0907 -103.6082 656151.2420 -20.8824
-20.8616
[21191]ENERGY: 17820000 3108.4892 5256.1989 5205.6862
317.2654 -15821.8039 -1763.2464 0.0000 0.0000
7878.4283 4181.0177 315.1765 -3697.4106 4246.3404
311.0305 91.3505 22.1578 656151.2420 -18.7466
-18.7454
[21241]ENERGY: 17830000 3187.7513 5256.5203 5278.0514
330.9397 -15897.8682 -1665.7975 0.0000 0.0000
7697.7637 4187.3608 307.9490 -3510.4030 4246.1467
311.0736 184.2457 84.6970 656151.2420 -21.2060
-21.2134
[21277]ENERGY: 17840000 3283.0552 5141.6295 5260.6993
342.9047 -15917.8439 -1712.8169 0.0000 0.0000
7788.2963 4185.9242 311.5708 -3602.3721 4246.5461
311.2035 -54.2085 -107.5457 656151.2420 -17.0356
-17.0361
[21327]ENERGY: 17850000 3136.9381 5287.5112 5238.1241
371.5034 -15806.7945 -1806.3390 0.0000 0.0000
7761.2081 4182.1515 310.4871 -3579.0566 4246.1172

311.1317 25.1526 13.3595 656151.2420 -15.8285
-15.8252
[21363]ENERGY: 17860000 3195.5101 5200.1692 5231.3318
332.7003 -15873.4912 -1745.1052 0.0000 0.0000
7842.0687 4183.1836 313.7220 -3658.8851 4245.9436
310.8472 116.9207 81.2338 656151.2420 -21.7684
-21.7706
[21413]ENERGY: 17870000 3144.7203 5248.9967 5272.8175
350.1388 -15781.2947 -1793.8626 0.0000 0.0000
7741.9954 4183.5114 309.7185 -3558.4840 4246.1382
310.9925 29.0703 -12.7122 656151.2420 -21.2467
-21.2611
[21449]ENERGY: 17880000 3157.4625 5230.3974 5334.8184
344.6100 -15843.4945 -1782.2131 0.0000 0.0000
7742.4844 4184.0651 309.7381 -3558.4193 4246.9056
311.0673 124.3004 2.4444 656151.2420 -20.6490
-20.6262
[21499]ENERGY: 17890000 3235.6304 5161.6801 5275.3094
351.6041 -15815.1361 -1819.8652 0.0000 0.0000
7798.1503 4187.3731 311.9650 -3610.7772 4247.6054
311.2319 210.1370 109.3306 656151.2420 -17.1164
-17.1269
[21538]ENERGY: 17900000 3202.6299 5262.4204 5254.2185
332.8490 -15886.1167 -1755.8978 0.0000 0.0000
7771.1170 4181.2204 310.8835 -3589.8966 4245.6908
311.1732 36.8919 98.0944 656151.2420 -19.8241
-19.8152
[21588]ENERGY: 17910000 3208.7156 5186.7783 5302.0525
332.3617 -15885.7004 -1748.7330 0.0000 0.0000
7785.4692 4180.9441 311.4577 -3604.5251 4245.3958
311.1602 6.3515 -22.7988 656151.2420 -17.6267
-17.6278
[21624]ENERGY: 17920000 3165.8669 5289.6236 5259.0609
352.1918 -15857.5785 -1805.4625 0.0000 0.0000
7778.5402 4182.2424 311.1805 -3596.2979 4247.2090
311.0775 -42.5836 -102.6461 656151.2420 -17.8617
-17.8676
[21674]ENERGY: 17930000 3198.3246 5249.9392 5248.9862
369.8704 -15863.0552 -1814.9448 0.0000 0.0000
7795.3817 4184.5021 311.8542 -3610.8796 4246.7015
311.2768 -38.2437 -5.2913 656151.2420 -18.0594
-18.0563
[21710]ENERGY: 17940000 3223.2343 5291.4491 5268.9434
327.7165 -15893.9321 -1758.8417 0.0000 0.0000
7723.4591 4182.0285 308.9770 -3541.4306 4246.2900
311.2838 -62.2342 -63.9690 656151.2420 -21.2317
-21.2323
[21760]ENERGY: 17950000 3226.1834 5309.7378 5247.4138
324.2704 -15910.7958 -1763.5228 0.0000 0.0000
7752.7359 4186.0227 310.1482 -3566.7132 4246.4522
311.2923 97.7199 40.4653 656151.2420 -22.3253
-22.3463
[21796]ENERGY: 17960000 3182.1951 5171.7164 5295.7460
355.7370 -15905.8848 -1754.9758 0.0000 0.0000

7833.5164 4178.0503 313.3798 -3655.4661 4246.8155
311.2082 119.5378 49.4677 656151.2420 -20.4237
-20.3984
[21846]ENERGY: 17970000 3253.8324 5229.8277 5283.8735
323.8073 -15980.2427 -1737.4122 0.0000 0.0000
7809.4441 4183.1301 312.4168 -3626.3140 4246.4516
311.0905 4.9211 14.3944 656151.2420 -20.2088
-20.2180
[21882]ENERGY: 17980000 3236.1600 5156.2709 5280.9513
335.0934 -15837.1782 -1780.1875 0.0000 0.0000
7790.6965 4181.8063 311.6668 -3608.8902 4246.2836
311.0758 -4.9536 74.1217 656151.2420 -21.8446
-21.8368
[21932]ENERGY: 17990000 3211.7358 5333.9772 5265.6732
308.2340 -15833.7123 -1858.3520 0.0000 0.0000
7757.3809 4184.9368 310.3340 -3572.4441 4246.8796
311.0697 -12.4917 -105.7053 656151.2420 -23.0281
-23.0335
[21971]ENERGY: 18000000 3220.4241 5302.3782 5300.2244
348.0062 -15870.1012 -1774.4717 0.0000 0.0000
7663.5403 4190.0003 306.5799 -3473.5401 4249.4616
311.1959 30.0186 -10.0013 656151.2420 -20.4985
-20.5057
[22021]ENERGY: 18010000 3113.9640 5231.6759 5280.7696
330.3778 -15896.0900 -1702.4192 0.0000 0.0000
7825.3245 4183.6025 313.0521 -3641.7219 4248.1499
310.9694 -35.4366 -72.9843 656151.2420 -20.5133
-20.4982
[22057]ENERGY: 18020000 3217.2947 5158.9525 5251.8344
308.6986 -15867.9477 -1732.5553 0.0000 0.0000
7847.9805 4184.2577 313.9585 -3663.7228 4248.1774
310.9543 -34.9822 -8.3711 656151.2420 -22.0258
-22.0528
[22107]ENERGY: 18030000 3237.3085 5308.1106 5269.4668
341.9738 -15940.7942 -1733.5211 0.0000 0.0000
7706.0258 4188.5702 308.2796 -3517.4556 4250.1011
311.1188 133.7147 61.6953 656151.2420 -22.7813
-22.7616
[22143]ENERGY: 18040000 3169.7267 5260.5343 5276.4319
352.7052 -15850.2164 -1782.9236 0.0000 0.0000
7759.6096 4185.8677 310.4232 -3573.7419 4249.4376
311.1248 2.2804 55.3282 656151.2420 -22.4899
-22.4812
[22193]ENERGY: 18050000 3301.4224 5244.0831 5275.1065
354.9551 -15987.7042 -1725.2352 0.0000 0.0000
7727.7068 4190.3345 309.1469 -3537.3723 4249.5853
311.1098 -80.3874 -116.6872 656151.2420 -23.8751
-23.8857
[22229]ENERGY: 18060000 3206.3524 5204.1120 5280.0464
344.2639 -15871.1507 -1765.1457 0.0000 0.0000
7789.5974 4188.0757 311.6228 -3601.5216 4250.8157
311.0709 73.4543 65.9589 656151.2420 -22.0295
-22.0190
[22279]ENERGY: 18070000 3213.4943 5322.2640 5256.0588

335.6169 -16021.5793 -1790.7928 0.0000 0.0000
7867.7283 4182.7902 314.7485 -3684.9381 4249.8899
311.2385 38.6831 -16.0509 656151.2420 -20.1967
-20.2021
[22315]ENERGY: 18080000 3192.6063 5194.4338 5289.4528
357.0377 -15867.6293 -1754.3421 0.0000 0.0000
7776.0778 4187.6370 311.0820 -3588.4408 4250.6372
311.2322 28.1411 -0.2035 656151.2420 -21.9057
-21.8969
[22365]ENERGY: 18090000 3154.6972 5290.7894 5229.5882
351.3297 -15885.7997 -1745.1077 0.0000 0.0000
7792.1470 4187.6440 311.7248 -3604.5029 4251.0024
311.1770 -16.0960 4.2351 656151.2420 -21.9647
-21.9720
[22404]ENERGY: 18100000 3216.8992 5276.7256 5226.7803
356.4559 -15850.7389 -1798.1400 0.0000 0.0000
7757.8906 4185.8728 310.3544 -3572.0178 4250.6218
311.3728 -72.5177 -75.4348 656151.2420 -20.6411
-20.6429
[22454]ENERGY: 18110000 3206.2218 5297.1529 5247.0174
340.3239 -15831.3209 -1832.5318 0.0000 0.0000
7763.2711 4190.1344 310.5697 -3573.1367 4251.6263
311.2245 151.3996 95.8288 656151.2420 -22.6843
-22.6734
[22490]ENERGY: 18120000 3243.0333 5194.5047 5277.9486
346.4545 -15817.3253 -1826.2587 0.0000 0.0000
7770.3799 4188.7370 310.8540 -3581.6429 4251.7897
311.1803 -52.5735 -104.3692 656151.2420 -19.5320
-19.5429
[22540]ENERGY: 18130000 3123.8172 5257.4332 5243.8611
335.7110 -15732.1552 -1844.4006 0.0000 0.0000
7801.1225 4185.3893 312.0839 -3615.7332 4251.9207
311.3056 90.0427 5.9175 656151.2420 -20.9318
-20.9481
[22576]ENERGY: 18140000 3250.6952 5275.8757 5292.2124
345.0978 -16013.5844 -1695.9086 0.0000 0.0000
7732.0434 4186.4315 309.3204 -3545.6119 4250.4732
311.2937 5.9618 9.2699 656151.2420 -21.4476
-21.4325
[22626]ENERGY: 18150000 3290.1789 5222.3392 5296.0009
333.8087 -15959.1368 -1733.8710 0.0000 0.0000
7738.8714 4188.1912 309.5935 -3550.6801 4250.3399
311.2653 -108.1687 -34.1884 656151.2420 -24.1210
-24.1203
[22662]ENERGY: 18160000 3252.6201 5232.2910 5197.4991
358.5563 -15810.7329 -1760.0608 0.0000 0.0000
7721.2879 4191.4607 308.8901 -3529.8273 4251.7574
311.1292 -14.7194 -69.1671 656151.2420 -23.9685
-23.9695
[22712]ENERGY: 18170000 3211.0855 5242.1224 5313.4381
338.6192 -15808.8440 -1792.6114 0.0000 0.0000
7686.2873 4190.0972 307.4899 -3496.1901 4252.1563
311.2756 -82.5729 -47.7544 656151.2420 -20.9812
-20.9834

[22748]ENERGY: 18180000 3135.4693 5293.6655 5280.1729
335.2225 -15827.9360 -1727.5192 0.0000 0.0000
7704.0501 4193.1252 308.2005 -3510.9249 4252.1010
311.2096 118.3131 16.8747 656151.2420 -25.5318
-25.5322
[22798]ENERGY: 18190000 3174.2237 5273.2053 5293.7020
340.3348 -15908.1870 -1784.4674 0.0000 0.0000
7801.1888 4190.0001 312.0866 -3611.1887 4252.4868
311.0553 35.2077 57.4034 656151.2420 -23.5194
-23.5066
[22837]ENERGY: 18200000 3150.7507 5254.8212 5279.8162
358.2660 -15922.4413 -1769.8438 0.0000 0.0000
7837.6337 4189.0027 313.5445 -3648.6310 4251.1897
311.3107 3.2577 -41.9341 656151.2420 -21.5995
-21.6062
[22887]ENERGY: 18210000 3236.1725 5291.0575 5198.4203
333.8263 -15883.2317 -1716.1045 0.0000 0.0000
7728.4658 4188.6062 309.1773 -3539.8596 4252.6204
311.2036 84.9223 -28.6298 656151.2420 -24.2331
-24.2450
[22923]ENERGY: 18220000 3220.3523 5293.3704 5264.8218
366.8170 -15904.2764 -1803.3673 0.0000 0.0000
7752.1702 4189.8880 310.1256 -3562.2822 4252.9326
311.2393 -7.9440 -105.8202 656151.2420 -24.5701
-24.5739
[22973]ENERGY: 18230000 3173.1195 5185.8815 5286.1290
328.8427 -15773.5684 -1788.6742 0.0000 0.0000
7779.1681 4190.8983 311.2056 -3588.2699 4252.6945
311.0717 -48.7586 -64.8092 656151.2420 -24.3578
-24.3398
[23009]ENERGY: 18240000 3215.0399 5231.6024 5247.9098
353.5853 -15841.2126 -1832.6927 0.0000 0.0000
7815.7012 4189.9333 312.6671 -3625.7679 4254.4597
311.0761 -55.0537 -4.1185 656151.2420 -27.3854
-27.3950
[23059]ENERGY: 18250000 3188.4741 5331.7899 5248.5402
339.7751 -15805.8121 -1793.2143 0.0000 0.0000
7686.6573 4196.2103 307.5047 -3490.4471 4255.0165
311.3003 -26.0114 -29.1990 656151.2420 -23.4844
-23.4899
[23095]ENERGY: 18260000 3196.3799 5168.4361 5289.0132
358.4049 -15816.0092 -1804.6378 0.0000 0.0000
7800.9664 4192.5535 312.0777 -3608.4128 4255.4880
311.2848 26.0194 -5.0648 656151.2420 -24.0524
-24.0437
[23145]ENERGY: 18270000 3241.4982 5141.3020 5260.1375
340.2965 -15802.5536 -1751.2783 0.0000 0.0000
7763.4237 4192.8260 310.5758 -3570.5977 4255.4760
311.4200 -155.6518 -149.8233 656151.2420 -24.5386
-24.5285
[23181]ENERGY: 18280000 3181.3881 5237.4930 5289.1673
347.1472 -15856.6509 -1824.9130 0.0000 0.0000
7816.3129 4189.9447 312.6916 -3626.3682 4255.6368
311.0822 -136.8955 -129.8079 656151.2420 -21.7957

-21.8015
[23231]ENERGY: 18290000 3232.1602 5300.0905 5300.7907
336.8615 -15961.8668 -1726.6485 0.0000 0.0000
7712.7510 4194.1387 308.5486 -3518.6123 4255.1257
311.1676 8.3476 1.1730 656151.2420 -23.1647
-23.1689
[23270]ENERGY: 18300000 3179.5981 5162.4267 5283.5926
340.2140 -15749.2182 -1768.2168 0.0000 0.0000
7749.3791 4197.7755 310.0139 -3551.6035 4255.9474
311.0879 18.9374 -40.9810 656151.2420 -22.6639
-22.6521
[23320]ENERGY: 18310000 3269.7110 5299.8125 5238.5426
362.1019 -15857.6088 -1806.4639 0.0000 0.0000
7690.3151 4196.4104 307.6511 -3493.9047 4255.7713
311.3201 -226.4143 -216.5043 656151.2420 -23.8132
-23.8233
[23356]ENERGY: 18320000 3130.7890 5190.4101 5316.0482
339.5156 -15813.0794 -1774.6357 0.0000 0.0000
7801.6773 4190.7251 312.1061 -3610.9522 4256.6620
311.1749 -250.5538 -95.5805 656151.2420 -20.6933
-20.6902
[23406]ENERGY: 18330000 3139.0622 5179.4125 5247.3204
358.4065 -15805.5817 -1795.7106 0.0000 0.0000
7869.2976 4192.2068 314.8112 -3677.0908 4256.9810
311.1794 137.1346 91.4735 656151.2420 -18.0548
-18.0540
[23442]ENERGY: 18340000 3244.0703 5183.5332 5244.3716
372.4888 -15883.2453 -1838.8012 0.0000 0.0000
7869.7732 4192.1906 314.8303 -3677.5826 4256.2561
311.1884 -20.7152 -47.9848 656151.2420 -24.0908
-24.0933
[23492]ENERGY: 18350000 3169.1360 5208.3050 5278.3349
353.5082 -15842.4812 -1747.4957 0.0000 0.0000
7778.4394 4197.7466 311.1765 -3580.6928 4257.5256
311.1028 -47.1950 -132.5688 656151.2420 -22.0368
-22.0357
[23528]ENERGY: 18360000 3157.4360 5146.0545 5297.7703
344.5201 -15842.6693 -1755.3592 0.0000 0.0000
7845.5808 4193.3333 313.8625 -3652.2474 4257.1036
311.1888 6.7590 -96.2868 656151.2420 -26.3803
-26.3893
[23578]ENERGY: 18370000 3242.5277 5271.0829 5240.7898
358.7936 -15953.9707 -1693.5810 0.0000 0.0000
7729.6268 4195.2691 309.2237 -3534.3576 4257.7684
311.2623 -0.6119 25.9782 656151.2420 -24.5426
-24.5215
[23614]ENERGY: 18380000 3254.1080 5142.7631 5269.0738
320.6219 -15744.1779 -1833.5550 0.0000 0.0000
7787.5643 4196.3981 311.5415 -3591.1662 4257.8783
311.1251 -100.0887 -147.9576 656151.2420 -24.8935
-24.9201
[23664]ENERGY: 18390000 3216.4769 5294.6533 5273.9491
334.7293 -15840.0953 -1854.2192 0.0000 0.0000
7768.0504 4193.5445 310.7609 -3574.5059 4258.5785

311.3870 -70.1805 -59.4834 656151.2420 -26.2914
-26.2781
[23703]ENERGY: 18400000 3230.5467 5250.7926 5223.2116
334.5323 -15866.7630 -1735.4468 0.0000 0.0000
7756.7411 4193.6146 310.3084 -3563.1265 4258.5047
311.1232 119.2192 94.4343 656151.2420 -23.2996
-23.3037
[23753]ENERGY: 18410000 3199.4494 5276.4482 5265.8712
351.8315 -15933.1930 -1732.2084 0.0000 0.0000
7767.2625 4195.4612 310.7293 -3571.8012 4258.3886
311.2432 9.4176 9.0861 656151.2420 -24.5851
-24.5767
[23789]ENERGY: 18420000 3210.2574 5265.3844 5235.0722
363.3186 -15878.7932 -1761.5102 0.0000 0.0000
7762.9253 4196.6545 310.5558 -3566.2708 4259.4094
311.3634 143.0651 64.4394 656151.2420 -26.0392
-26.0379
[23839]ENERGY: 18430000 3236.0237 5288.1618 5210.4836
319.4482 -15824.0834 -1770.2308 0.0000 0.0000
7737.1318 4196.9348 309.5240 -3540.1970 4258.1743
311.4110 -110.0995 -179.7882 656151.2420 -24.8280
-24.8567
[23875]ENERGY: 18440000 3207.9693 5176.5562 5229.2290
331.9526 -15879.6377 -1718.1181 0.0000 0.0000
7845.6589 4193.6101 313.8656 -3652.0489 4257.9492
311.4385 -90.5194 -87.2569 656151.2420 -22.8386
-22.8078
[23925]ENERGY: 18450000 3148.7212 5322.5845 5234.9121
358.6297 -15893.5515 -1700.2025 0.0000 0.0000
7727.1746 4198.2681 309.1256 -3528.9065 4259.4736
311.3843 121.5574 92.3505 656151.2420 -26.2684
-26.2722
[23961]ENERGY: 18460000 3228.6592 5223.8496 5247.6768
330.3710 -15825.7906 -1837.7832 0.0000 0.0000
7827.4062 4194.3889 313.1354 -3633.0173 4260.5514
311.3913 -83.0887 -79.6934 656151.2420 -22.4120
-22.4222
[24011]ENERGY: 18470000 3242.3389 5181.1555 5272.1043
322.6440 -15801.7806 -1829.6938 0.0000 0.0000
7811.7102 4198.4785 312.5075 -3613.2317 4260.9022
311.1898 -139.7910 -215.0665 656151.2420 -24.3132
-24.3234
[24047]ENERGY: 18480000 3170.6032 5298.0976 5248.6609
346.9329 -15799.4970 -1845.9097 0.0000 0.0000
7779.9190 4198.8069 311.2357 -3581.1122 4260.8429
311.3288 -53.4726 24.1567 656151.2420 -20.7776
-20.7545
[24097]ENERGY: 18490000 3204.9495 5272.2302 5306.1512
342.8123 -15878.9629 -1776.5694 0.0000 0.0000
7730.1164 4200.7273 309.2433 -3529.3890 4262.3450
311.3258 4.1987 -39.6152 656151.2420 -14.3670
-14.3811
[24136]ENERGY: 18500000 3188.9240 5175.6698 5251.8961
336.2781 -15872.3694 -1672.5294 0.0000 0.0000

7794.9421 4202.8113 311.8367 -3592.1308 4261.9251
311.4158 13.9923 6.0747 656151.2420 -15.1565
-15.1690
[24186]ENERGY: 18510000 3258.2534 5193.8193 5290.7450
314.5467 -15831.1452 -1814.4586 0.0000 0.0000
7789.2260 4200.9866 311.6080 -3588.2394 4263.5856
311.3829 -27.4879 -66.5688 656151.2420 -18.8662
-18.8529
[24222]ENERGY: 18520000 3186.4449 5210.6786 5269.1525
372.4317 -15834.7527 -1832.0097 0.0000 0.0000
7826.3482 4198.2934 313.0931 -3628.0547 4262.3057
311.3485 2.0505 -8.9624 656151.2420 -16.3078
-16.3051
[24272]ENERGY: 18530000 3256.4429 5230.1089 5265.8253
332.8689 -15915.8275 -1770.5653 0.0000 0.0000
7799.4564 4198.3097 312.0173 -3601.1467 4262.7855
311.4634 -21.9600 -41.9894 656151.2420 -21.0793
-21.0775
[24308]ENERGY: 18540000 3261.5321 5239.9005 5289.2183
345.8885 -15883.1136 -1870.7346 0.0000 0.0000
7816.7459 4199.4371 312.7089 -3617.3088 4263.9505
311.4275 -170.8362 -147.5692 656151.2420 -24.5747
-24.5716
[24358]ENERGY: 18550000 3196.7637 5243.0360 5234.2907
341.5551 -15764.6519 -1819.4253 0.0000 0.0000
7771.8216 4203.3899 310.9117 -3568.4318 4263.3307
311.3402 22.2363 -21.6162 656151.2420 -20.7232
-20.7420
[24394]ENERGY: 18560000 3202.0902 5246.3685 5265.5910
355.5751 -15861.8311 -1829.0490 0.0000 0.0000
7820.9332 4199.6779 312.8764 -3621.2554 4264.0549
311.5421 168.4477 42.9485 656151.2420 -22.8448
-22.8263
[24444]ENERGY: 18570000 3150.9508 5171.1671 5239.7447
324.2117 -15839.6300 -1708.1853 0.0000 0.0000
7860.1876 4198.4464 314.4468 -3661.7412 4264.5771
311.4890 15.0685 15.8200 656151.2420 -19.6501
-19.6641
[24480]ENERGY: 18580000 3224.8324 5156.3105 5269.9865
346.0063 -15883.3928 -1722.1069 0.0000 0.0000
7808.0924 4199.7284 312.3627 -3608.3640 4263.8447
311.5625 7.2119 -32.8445 656151.2420 -19.8393
-19.8148
[24530]ENERGY: 18590000 3205.1308 5179.9768 5233.5194
341.4324 -15900.2593 -1717.9256 0.0000 0.0000
7857.9858 4199.8602 314.3587 -3658.1255 4264.3472
311.5174 81.1697 16.7574 656151.2420 -23.7404
-23.7764
[24569]ENERGY: 18600000 3269.1209 5329.9314 5232.2576
315.5085 -15884.9041 -1821.3423 0.0000 0.0000
7761.0505 4201.6226 310.4808 -3559.4279 4265.8260
311.5903 -48.1135 -37.2641 656151.2420 -19.1839
-19.1577
[24619]ENERGY: 18610000 3213.7232 5278.3893 5283.9160

343.0298 -15882.1823 -1802.0531 0.0000 0.0000
7765.9231 4200.7459 310.6758 -3565.1773 4265.0984
311.2966 -49.3131 -46.2455 656151.2420 -15.9917
-15.9847
[24655]ENERGY: 18620000 3211.2856 5289.2275 5239.8078
330.7408 -15828.7701 -1747.8322 0.0000 0.0000
7706.3163 4200.7759 308.2912 -3505.5404 4266.6246
311.3715 -67.5482 -3.7218 656151.2420 -19.4621
-19.4854
[24705]ENERGY: 18630000 3171.8878 5234.8658 5278.9321
339.4221 -15755.0542 -1775.7485 0.0000 0.0000
7714.4682 4208.7733 308.6173 -3505.6949 4267.5263
311.3947 88.2581 -6.3960 656151.2420 -19.3942
-19.3930
[24741]ENERGY: 18640000 3208.9579 5282.4290 5261.3386
344.6524 -15952.2568 -1708.2880 0.0000 0.0000
7770.1295 4206.9626 310.8440 -3563.1669 4267.3262
311.3761 145.7854 -18.6257 656151.2420 -21.3716
-21.3638
[24791]ENERGY: 18650000 3266.7481 5264.1732 5282.6584
327.1827 -15904.9553 -1789.4716 0.0000 0.0000
7761.2325 4207.5679 310.4881 -3553.6646 4267.8763
311.4534 -167.8865 -186.1061 656151.2420 -21.9257
-21.9325
[24827]ENERGY: 18660000 3284.4900 5244.2294 5275.1540
333.9417 -15906.1861 -1818.5542 0.0000 0.0000
7792.0620 4205.1368 311.7214 -3586.9251 4267.8498
311.4793 -11.8995 10.4141 656151.2420 -20.6446
-20.6374
[24877]ENERGY: 18670000 3191.5026 5300.7362 5255.7861
347.6851 -15853.3384 -1757.2914 0.0000 0.0000
7721.2914 4206.3717 308.8903 -3514.9198 4267.9038
311.5600 -0.2605 -20.3404 656151.2420 -23.1171
-23.1136
[24913]ENERGY: 18680000 3248.2068 5274.8718 5285.3799
343.6651 -15895.0868 -1795.7290 0.0000 0.0000
7744.3292 4205.6371 309.8119 -3538.6921 4268.0703
311.5068 2.6605 9.3098 656151.2420 -22.0519
-22.0543
[24963]ENERGY: 18690000 3251.5660 5335.7703 5244.7094
322.6511 -15954.2478 -1739.6169 0.0000 0.0000
7743.8861 4204.7182 309.7942 -3539.1680 4267.9693
311.6388 31.8715 68.0448 656151.2420 -18.8039
-18.7783
[25002]ENERGY: 18700000 3266.6647 5247.4954 5301.5357
331.9808 -15846.0245 -1783.0947 0.0000 0.0000
7688.7347 4207.2921 307.5878 -3481.4426 4267.1009
311.7135 -124.6301 -153.5718 656151.2420 -24.3873
-24.4225
[25052]ENERGY: 18710000 3189.6325 5236.2253 5318.7087
334.5655 -15868.7543 -1787.4883 0.0000 0.0000
7785.7627 4208.6521 311.4694 -3577.1106 4270.3368
311.5889 64.6183 35.0236 656151.2420 -22.6091
-22.6084

[25088]ENERGY: 18720000 3200.3864 5233.7494 5221.1766
360.9096 -15894.1478 -1705.3675 0.0000 0.0000
7793.4260 4210.1327 311.7760 -3583.2932 4270.5457
311.4408 48.2659 -3.2683 656151.2420 -23.9731
-23.9589
[25138]ENERGY: 18730000 3202.2772 5131.4210 5284.7424
336.7094 -15855.5838 -1767.3234 0.0000 0.0000
7873.9947 4206.2375 314.9992 -3667.7572 4270.5616
311.4521 -202.5402 -186.3382 656151.2420 -23.9088
-23.8999
[25174]ENERGY: 18740000 3192.9849 5177.2185 5251.4383
323.9456 -15855.5043 -1762.1572 0.0000 0.0000
7879.3842 4207.3100 315.2148 -3672.0742 4271.3753
311.4646 -69.2691 -148.2279 656151.2420 -22.0279
-22.0447
[25224]ENERGY: 18750000 3195.7670 5248.5526 5250.6723
335.0680 -15901.8320 -1753.9367 0.0000 0.0000
7833.4054 4207.6966 313.3754 -3625.7088 4271.7909
311.5947 -98.2360 -101.9703 656151.2420 -23.8983
-23.8763
[25260]ENERGY: 18760000 3248.6920 5144.5895 5238.3118
339.1119 -15856.6853 -1743.1355 0.0000 0.0000
7839.4001 4210.2846 313.6152 -3629.1156 4270.5093
311.5533 37.7413 17.8193 656151.2420 -18.3109
-18.3162
[25310]ENERGY: 18770000 3145.2523 5148.4521 5268.1190
394.0451 -15814.5546 -1731.8438 0.0000 0.0000
7798.6027 4208.0729 311.9831 -3590.5299 4271.6187
311.5573 -96.9767 -63.4084 656151.2420 -22.4987
-22.5066
[25346]ENERGY: 18780000 3166.9264 5206.8925 5275.1720
328.5833 -15793.6116 -1800.1408 0.0000 0.0000
7825.4715 4209.2933 313.0580 -3616.1782 4271.4236
311.6472 -51.8525 -95.5492 656151.2420 -24.4049
-24.4091
[25396]ENERGY: 18790000 3225.1947 5255.4523 5229.0709
310.0809 -15865.2964 -1749.0416 0.0000 0.0000
7803.1384 4208.5992 312.1645 -3594.5392 4271.7219
311.5269 18.4320 -19.9582 656151.2420 -25.3975
-25.4156
[25435]ENERGY: 18800000 3197.8927 5316.8302 5228.9011
323.3791 -15853.2084 -1728.9506 0.0000 0.0000
7727.1304 4211.9745 309.1238 -3515.1559 4273.5633
311.1949 101.4114 -21.4231 656151.2420 -25.4616
-25.4421
[25485]ENERGY: 18810000 3214.0973 5298.5369 5232.1436
325.5821 -15889.5606 -1711.4160 0.0000 0.0000
7740.1907 4209.5740 309.6463 -3530.6167 4274.2797
311.4646 51.3580 6.2279 656151.2420 -21.4194
-21.4255
[25521]ENERGY: 18820000 3171.2499 5229.3320 5245.7531
338.3921 -15925.9280 -1702.3287 0.0000 0.0000
7855.4104 4211.8807 314.2557 -3643.5297 4274.4655
311.4550 198.6687 135.9164 656151.2420 -23.3701

-23.3500
[25571]ENERGY: 18830000 3249.3731 5256.4895 5257.2276
351.9846 -15926.4738 -1796.4662 0.0000 0.0000
7816.5284 4208.6632 312.7002 -3607.8652 4274.0501
311.3120 -217.3022 -153.3817 656151.2420 -27.4467
-27.4424
[25607]ENERGY: 18840000 3243.7460 5247.7638 5227.2107
342.4585 -15888.6404 -1724.1972 0.0000 0.0000
7764.6294 4212.9708 310.6240 -3551.6586 4274.3657
311.3267 134.6981 32.8906 656151.2420 -23.4099
-23.4220
[25657]ENERGY: 18850000 3261.9875 5273.0315 5236.6384
326.1320 -15829.2669 -1800.5301 0.0000 0.0000
7744.5014 4212.4938 309.8188 -3532.0076 4274.0200
311.3694 -89.7018 -134.8475 656151.2420 -24.4592
-24.4640
[25693]ENERGY: 18860000 3170.6323 5243.6213 5260.5917
349.3229 -15807.9798 -1801.3942 0.0000 0.0000
7794.6662 4209.4604 311.8256 -3585.2058 4274.3976
311.4207 -69.1057 -142.1179 656151.2420 -24.3086
-24.3107
[25743]ENERGY: 18870000 3205.6698 5281.2523 5270.4354
308.2749 -15802.9702 -1821.1944 0.0000 0.0000
7768.2441 4209.7120 310.7686 -3558.5322 4274.8721
311.4927 -130.9347 -105.0386 656151.2420 -26.3222
-26.3251
[25779]ENERGY: 18880000 3228.2015 5207.6181 5241.8038
342.6997 -15805.7727 -1805.5292 0.0000 0.0000
7801.6086 4210.6299 312.1033 -3590.9787 4276.1066
311.4672 -31.1676 -116.8420 656151.2420 -21.0729
-21.0647
[25829]ENERGY: 18890000 3214.4753 5236.3238 5229.2577
335.7125 -15787.5703 -1727.0719 0.0000 0.0000
7714.6299 4215.7569 308.6238 -3498.8730 4275.1351
311.3772 -251.2220 -173.8620 656151.2420 -24.8280
-24.8290
[25868]ENERGY: 18900000 3270.8701 5243.6565 5244.7368
326.4965 -15934.6338 -1705.1269 0.0000 0.0000
7767.4380 4213.4371 310.7364 -3554.0008 4274.9252
311.3119 62.8014 43.9184 656151.2420 -25.7227
-25.7311
[25918]ENERGY: 18910000 3186.2923 5274.5862 5254.8708
334.0375 -15884.6443 -1770.8415 0.0000 0.0000
7820.1435 4214.4445 312.8448 -3605.6990 4275.6005
311.5136 7.4275 -7.6151 656151.2420 -27.2069
-27.2100
[25954]ENERGY: 18920000 3211.9576 5171.4808 5261.7004
348.1306 -15771.9896 -1825.3462 0.0000 0.0000
7816.3918 4212.3253 312.6948 -3604.0665 4275.9938
311.6131 -139.6379 -116.6782 656151.2420 -23.8122
-23.7966
[26004]ENERGY: 18930000 3229.8767 5212.1297 5227.7846
354.8447 -15871.6752 -1688.9263 0.0000 0.0000
7750.1069 4214.1412 310.0430 -3535.9657 4274.8700

311.5225 -1.5991 -0.8520 656151.2420 -17.8288
-17.8239
[26040]ENERGY: 18940000 3274.3048 5221.7301 5255.7245
328.3274 -15768.0672 -1752.4022 0.0000 0.0000
7656.8287 4216.4460 306.3114 -3440.3827 4274.7795
311.3706 37.9140 -23.3242 656151.2420 -19.1007
-19.1160
[26090]ENERGY: 18950000 3170.0297 5387.2014 5251.1461
337.6021 -15947.3424 -1760.7175 0.0000 0.0000
7772.7256 4210.6450 310.9479 -3562.0806 4273.7586
311.5085 12.0821 11.7159 656151.2420 -25.2121
-25.2031
[26126]ENERGY: 18960000 3294.8892 5244.8565 5237.1547
349.8718 -15858.8100 -1711.1032 0.0000 0.0000
7653.6144 4210.4733 306.1828 -3443.1411 4272.6254
311.6819 -132.6395 -43.9503 656151.2420 -21.5223
-21.5296
[26176]ENERGY: 18970000 3160.6619 5261.2421 5243.0414
334.9895 -15805.5977 -1776.1088 0.0000 0.0000
7789.6932 4207.9216 311.6267 -3581.7716 4273.0493
311.5832 11.1639 -5.8585 656151.2420 -24.4734
-24.4698
[26212]ENERGY: 18980000 3238.1065 5145.8058 5344.9909
356.1057 -15932.3947 -1740.1529 0.0000 0.0000
7793.7755 4206.2368 311.7900 -3587.5387 4272.6108
311.5511 -168.9727 -177.6174 656151.2420 -21.5334
-21.5468
[26262]ENERGY: 18990000 3182.9210 5268.0933 5243.3434
333.4008 -15838.7445 -1742.9050 0.0000 0.0000
7765.9817 4212.0907 310.6781 -3553.8910 4274.1258
311.6375 -27.0330 -60.1580 656151.2420 -19.2794
-19.2787
[26301]ENERGY: 19000000 3189.0796 5164.3158 5276.9657
341.3293 -15824.8158 -1731.9027 0.0000 0.0000
7793.5371 4208.5090 311.7805 -3585.0282 4274.6704
311.5290 90.2209 82.6468 656151.2420 -24.0524
-24.0473
[26351]ENERGY: 19010000 3224.2466 5160.3881 5227.4206
341.7625 -15784.4578 -1751.3502 0.0000 0.0000
7797.6570 4215.6668 311.9453 -3581.9902 4276.4295
311.6450 36.4705 -105.6456 656151.2420 -24.6516
-24.6571
[26387]ENERGY: 19020000 3183.1314 5302.8879 5283.4156
340.1460 -15906.1405 -1813.3864 0.0000 0.0000
7819.7838 4209.8378 312.8304 -3609.9460 4275.0890
311.5014 16.9885 32.0425 656151.2420 -19.4178
-19.3922
[26437]ENERGY: 19030000 3199.4658 5229.7022 5222.6807
346.6755 -15777.4650 -1835.2066 0.0000 0.0000
7827.0213 4212.8739 313.1200 -3614.1474 4275.9139
311.4647 -127.5479 -115.1918 656151.2420 -18.0224
-18.0497
[26473]ENERGY: 19040000 3194.5716 5287.8677 5243.8412
340.9909 -16020.9189 -1701.0132 0.0000 0.0000

7863.4955 4208.8348 314.5791 -3654.6607 4274.8999
311.6197 256.7021 187.2748 656151.2420 -21.4317
-21.4052
[26523]ENERGY: 19050000 3284.1013 5170.3669 5290.6477
341.3316 -15893.0757 -1679.8525 0.0000 0.0000
7701.0542 4214.5736 308.0807 -3486.4806 4274.5937
311.6010 -108.7429 -101.9125 656151.2420 -19.7767
-19.7803
[26559]ENERGY: 19060000 3206.0148 5274.4291 5236.7657
354.9142 -15864.5555 -1753.4356 0.0000 0.0000
7757.3867 4211.5194 310.3343 -3545.8673 4275.4412
311.4001 15.0917 27.0114 656151.2420 -24.5982
-24.6191
[26609]ENERGY: 19070000 3265.1346 5247.7343 5256.3639
345.3480 -15892.6742 -1724.4739 0.0000 0.0000
7716.3452 4213.7778 308.6924 -3502.5674 4276.1611
311.4778 -99.3241 -64.5508 656151.2420 -23.4916
-23.4689
[26645]ENERGY: 19080000 3200.1407 5268.6147 5242.1454
343.8944 -15854.4221 -1753.7125 0.0000 0.0000
7764.2059 4210.8664 310.6071 -3553.3395 4276.1113
311.2723 -120.1771 -51.4345 656151.2420 -25.1688
-25.1870
[26695]ENERGY: 19090000 3236.2474 5203.8582 5245.6263
337.0956 -15843.4117 -1712.8261 0.0000 0.0000
7747.8779 4214.4675 309.9539 -3533.4105 4276.1053
311.4253 83.4756 58.9030 656151.2420 -20.7452
-20.7416
[26734]ENERGY: 19100000 3205.4690 5184.8996 5295.4348
342.2954 -15863.0626 -1795.9012 0.0000 0.0000
7843.0988 4212.2338 313.7632 -3630.8650 4276.8101
311.2960 39.0614 24.0937 656151.2420 -28.0838
-28.0889
[26784]ENERGY: 19110000 3196.0461 5296.5292 5305.1510
314.5281 -15963.5995 -1780.9804 0.0000 0.0000
7849.2325 4216.9070 314.0085 -3632.3255 4277.8200
311.4080 -17.9314 19.7886 656151.2420 -24.2401
-24.2175
[26820]ENERGY: 19120000 3295.5793 5245.9626 5253.0520
349.5825 -15950.1451 -1716.7708 0.0000 0.0000
7739.9592 4217.2197 309.6371 -3522.7395 4278.7209
311.4490 -76.7263 -37.3451 656151.2420 -24.5475
-24.5677
[26870]ENERGY: 19130000 3193.8483 5203.0495 5268.4371
348.5596 -15882.7383 -1813.6709 0.0000 0.0000
7896.2027 4213.6879 315.8876 -3682.5148 4279.0109
311.6205 7.3125 -113.0690 656151.2420 -22.8630
-22.8864
[26906]ENERGY: 19140000 3272.0464 5235.5085 5241.1680
354.0783 -15870.0319 -1789.1319 0.0000 0.0000
7773.4058 4217.0432 310.9751 -3556.3626 4278.4021
311.3720 27.4638 16.9690 656151.2420 -24.0067
-23.9812
[26956]ENERGY: 19150000 3260.0710 5293.3313 5214.9341

351.9111 -15917.6856 -1745.0620 0.0000 0.0000
7760.6539 4218.1539 310.4650 -3542.5000 4280.1955
311.6747 -96.8709 -81.1634 656151.2420 -20.1828
-20.1931
[26992]ENERGY: 19160000 3263.1202 5140.0939 5219.9425
348.9760 -15819.0764 -1767.5388 0.0000 0.0000
7832.0037 4217.5212 313.3193 -3614.4826 4279.8573
311.6586 -145.7125 -139.2259 656151.2420 -18.2215
-18.2050
[27042]ENERGY: 19170000 3206.2302 5338.8910 5231.7267
327.4381 -15893.3389 -1730.8154 0.0000 0.0000
7734.5339 4214.6656 309.4200 -3519.8683 4280.1657
311.5332 219.2379 173.0345 656151.2420 -22.6252
-22.6316
[27078]ENERGY: 19180000 3214.0562 5241.5386 5218.8507
363.7949 -15890.5050 -1753.3207 0.0000 0.0000
7821.8980 4216.3128 312.9150 -3605.5853 4280.3467
311.5400 55.0408 36.8110 656151.2420 -25.9534
-25.9462
[27128]ENERGY: 19190000 3214.7398 5252.1349 5278.4612
332.2008 -15918.7850 -1778.1329 0.0000 0.0000
7835.8022 4216.4210 313.4713 -3619.3813 4279.8358
311.8432 22.9951 -107.8060 656151.2420 -21.2626
-21.2662
[27167]ENERGY: 19200000 3201.2542 5199.9208 5309.9979
345.6076 -15815.2956 -1800.5979 0.0000 0.0000
7772.0574 4212.9443 310.9212 -3559.1131 4280.3089
311.4727 38.4889 32.3253 656151.2420 -23.2313
-23.2398
[27217]ENERGY: 19210000 3160.3832 5224.3222 5267.2114
351.6191 -15803.4361 -1791.0824 0.0000 0.0000
7810.8096 4219.8270 312.4714 -3590.9826 4280.1784
311.4342 8.0982 -18.7026 656151.2420 -23.7788
-23.7844
[27253]ENERGY: 19220000 3229.5213 5210.4153 5234.2209
340.2770 -15865.2603 -1751.2078 0.0000 0.0000
7818.2702 4216.2366 312.7699 -3602.0336 4279.3776
311.7809 -63.5178 -55.7835 656151.2420 -21.5493
-21.5386
[27303]ENERGY: 19230000 3235.3855 5296.3837 5234.4577
350.1561 -15816.8993 -1841.1712 0.0000 0.0000
7760.1837 4218.4961 310.4461 -3541.6876 4280.0794
311.7419 106.1315 50.0173 656151.2420 -25.8421
-25.8429
[27339]ENERGY: 19240000 3287.3462 5208.6881 5210.2899
340.1385 -15849.6056 -1756.2016 0.0000 0.0000
7780.8555 4221.5109 311.2731 -3559.3445 4281.0672
311.8698 36.3616 -67.5661 656151.2420 -24.1373
-24.1446
[27389]ENERGY: 19250000 3195.4484 5299.8418 5229.6295
313.0798 -15796.0463 -1764.5901 0.0000 0.0000
7739.3990 4216.7621 309.6147 -3522.6369 4279.6537
311.8573 76.6070 32.7130 656151.2420 -25.3421
-25.3199

[27425]ENERGY: 19260000 3110.9221 5268.1949 5257.9015
350.1967 -15769.2982 -1794.5075 0.0000 0.0000
7789.7946 4213.2042 311.6307 -3576.5904 4279.0996
311.6738 4.9748 -58.9518 656151.2420 -27.7709
-27.7902
[27475]ENERGY: 19270000 3133.5651 5307.2050 5229.1794
327.1923 -15781.5229 -1801.6092 0.0000 0.0000
7800.1605 4214.1701 312.0454 -3585.9904 4279.6069
311.7744 42.3324 41.5807 656151.2420 -23.2692
-23.2642
[27511]ENERGY: 19280000 3293.5029 5190.0008 5222.1453
353.8404 -15915.9177 -1732.5233 0.0000 0.0000
7809.8950 4220.9434 312.4348 -3588.9516 4279.4989
311.7851 29.0233 -110.5827 656151.2420 -26.9406
-26.9268
[27561]ENERGY: 19290000 3257.6422 5181.5049 5243.2702
326.7295 -15858.9905 -1751.9547 0.0000 0.0000
7823.3081 4221.5097 312.9714 -3601.7984 4280.0789
311.8250 7.6231 -144.6620 656151.2420 -26.6209
-26.6416
[27600]ENERGY: 19300000 3254.7353 5261.3226 5202.1242
340.7223 -15884.2798 -1703.9094 0.0000 0.0000
7748.6362 4219.3515 309.9842 -3529.2847 4279.9490
311.9230 76.9126 111.0202 656151.2420 -28.3111
-28.3016
[27650]ENERGY: 19310000 3279.5156 5144.9839 5243.6457
322.7341 -15787.1510 -1718.8032 0.0000 0.0000
7735.9308 4220.8560 309.4759 -3515.0749 4281.7964
311.8664 -59.6674 -16.3214 656151.2420 -25.1921
-25.1773
[27686]ENERGY: 19320000 3166.1788 5308.5373 5253.2590
346.9180 -15851.3060 -1785.2153 0.0000 0.0000
7778.5807 4216.9524 311.1821 -3561.6283 4281.8275
311.5602 -52.0830 -92.5625 656151.2420 -26.3212
-26.3520
[27736]ENERGY: 19330000 3238.7644 5220.1907 5244.5167
310.2946 -15874.7775 -1750.7196 0.0000 0.0000
7826.0206 4214.2899 313.0800 -3611.7307 4280.7156
311.8109 69.7951 92.4710 656151.2420 -27.0674
-27.0618
[27772]ENERGY: 19340000 3292.6222 5307.4435 5174.9287
360.8677 -15816.9549 -1790.4159 0.0000 0.0000
7693.6095 4222.1009 307.7828 -3471.5086 4281.9829
311.9615 -81.8465 -25.4011 656151.2420 -23.8456
-23.8332
[27822]ENERGY: 19350000 3194.2976 5266.7123 5210.6805
328.1151 -15849.3788 -1765.0545 0.0000 0.0000
7832.0480 4217.4201 313.3211 -3614.6279 4281.1022
311.7458 -32.9101 -82.0927 656151.2420 -27.8274
-27.8225
[27858]ENERGY: 19360000 3214.1766 5190.8648 5281.5670
346.3248 -15831.7926 -1754.0909 0.0000 0.0000
7773.8367 4220.8864 310.9923 -3552.9503 4281.6349
311.8495 50.5319 -9.0741 656151.2420 -23.4606

-23.4804
[27908]ENERGY: 19370000 3188.3874 5311.2695 5265.6926
370.4713 -15883.7159 -1802.7619 0.0000 0.0000
7771.8060 4221.1489 310.9111 -3550.6571 4282.6785
311.8783 64.4285 -26.9987 656151.2420 -24.9406
-24.9239
[27944]ENERGY: 19380000 3145.6498 5225.3348 5256.2208
375.1961 -15825.1135 -1742.2023 0.0000 0.0000
7785.1515 4220.2371 311.4450 -3564.9144 4281.7579
311.5879 -51.3396 -23.2891 656151.2420 -24.3420
-24.3346
[27994]ENERGY: 19390000 3170.2477 5282.9546 5271.9495
324.3537 -15866.1767 -1796.5165 0.0000 0.0000
7832.7622 4219.5744 313.3496 -3613.1878 4283.6629
312.0267 197.5447 151.3329 656151.2420 -26.1080
-26.1142
[28033]ENERGY: 19400000 3222.6288 5189.1262 5247.5181
351.5285 -15811.1239 -1863.0367 0.0000 0.0000
7876.5687 4213.2096 315.1021 -3663.3591 4282.4601
311.8589 -41.5828 -20.0974 656151.2420 -28.5249
-28.5265
[28083]ENERGY: 19410000 3278.8128 5259.6941 5266.2396
342.0083 -15922.1196 -1803.4685 0.0000 0.0000
7797.7805 4218.9473 311.9502 -3578.8332 4283.1432
311.9706 -131.8596 -86.6573 656151.2420 -23.4964
-23.4978
[28119]ENERGY: 19420000 3233.1141 5158.4197 5244.3559
341.1492 -15843.5820 -1769.5482 0.0000 0.0000
7853.2916 4217.2003 314.1709 -3636.0913 4283.1023
311.6457 -246.8226 -251.3678 656151.2420 -24.7611
-24.7600
[28169]ENERGY: 19430000 3262.5316 5220.6243 5212.7341
348.4960 -15815.1533 -1748.1611 0.0000 0.0000
7737.5134 4218.5850 309.5392 -3518.9284 4282.8126
311.6688 90.9554 73.1617 656151.2420 -20.1331
-20.1278
[28205]ENERGY: 19440000 3257.9766 5157.4112 5308.3344
346.0990 -15873.5311 -1785.9883 0.0000 0.0000
7812.1009 4222.4026 312.5231 -3589.6983 4283.6851
311.8052 -90.6011 -140.2652 656151.2420 -21.0137
-21.0222
[28255]ENERGY: 19450000 3231.8418 5208.5398 5297.2972
328.2108 -15829.6030 -1811.3494 0.0000 0.0000
7795.6542 4220.5913 311.8651 -3575.0629 4283.7801
311.9222 9.9158 -54.2803 656151.2420 -15.2595
-15.2676
[28291]ENERGY: 19460000 3233.8918 5262.8480 5260.1037
333.2257 -15857.7148 -1828.8857 0.0000 0.0000
7814.1816 4217.6503 312.6063 -3596.5313 4283.8860
311.9551 45.1169 48.2532 656151.2420 -15.6025
-15.5847
[28341]ENERGY: 19470000 3265.9894 5216.2040 5274.1312
330.8982 -15798.5673 -1858.1606 0.0000 0.0000
7791.1429 4221.6378 311.6847 -3569.5050 4282.6061

312.0912 -3.8650 -37.2810 656151.2420 -16.1510
-16.1582
[28377]ENERGY: 19480000 3173.5528 5378.3296 5253.7648
311.6267 -15894.9596 -1729.1848 0.0000 0.0000
7727.6160 4220.7455 309.1433 -3506.8705 4283.1954
312.2121 118.4221 72.5537 656151.2420 -18.5347
-18.5497
[28427]ENERGY: 19490000 3215.9716 5256.3289 5280.9394
332.7349 -15891.1555 -1762.6164 0.0000 0.0000
7788.0143 4220.2171 311.5595 -3567.7972 4283.7074
312.1972 -26.3525 35.8979 656151.2420 -17.6873
-17.6640
[28466]ENERGY: 19500000 3147.5495 5212.2463 5262.1458
336.6796 -15779.4938 -1832.6671 0.0000 0.0000
7872.9580 4219.4183 314.9577 -3653.5397 4283.3057
312.1468 -164.0291 -143.3503 656151.2420 -14.4446
-14.4634
[28516]ENERGY: 19510000 3165.2758 5195.0144 5269.6395
331.1165 -15797.4071 -1776.0307 0.0000 0.0000
7831.5236 4219.1319 313.3001 -3612.3917 4284.4802
312.2430 261.2361 49.2591 656151.2420 -13.9492
-13.9627
[28552]ENERGY: 19520000 3211.2565 5246.5674 5274.8613
345.1939 -15831.3943 -1767.5949 0.0000 0.0000
7743.5112 4222.4011 309.7792 -3521.1101 4285.0824
312.2713 125.3531 163.0991 656151.2420 -18.4889
-18.4731
[28602]ENERGY: 19530000 3267.8623 5305.1290 5206.2687
342.1396 -15884.6804 -1786.6180 0.0000 0.0000
7773.2708 4223.3720 310.9697 -3549.8989 4285.0348
312.3409 -38.7898 -89.3084 656151.2420 -15.4597
-15.4632
[28638]ENERGY: 19540000 3163.1558 5253.1806 5331.0115
331.7991 -15865.6363 -1781.0286 0.0000 0.0000
7790.0528 4222.5349 311.6411 -3567.5179 4283.6576
312.2943 112.7261 76.0017 656151.2420 -16.1693
-16.1645
[28688]ENERGY: 19550000 3233.4738 5315.5959 5307.0291
368.8957 -15934.3409 -1872.7115 0.0000 0.0000
7801.5023 4219.4444 312.0991 -3582.0579 4283.7251
312.3808 -214.8423 -71.2398 656151.2420 -14.9411
-14.9277
[28724]ENERGY: 19560000 3279.8392 5241.2549 5310.6243
332.5991 -15841.5746 -1830.6280 0.0000 0.0000
7728.3809 4220.4958 309.1739 -3507.8850 4283.9591
312.3472 -77.7328 -126.2182 656151.2420 -21.4768
-21.4802
[28774]ENERGY: 19570000 3171.2568 5280.3371 5315.7223
332.4800 -15813.3829 -1776.7804 0.0000 0.0000
7709.6227 4219.2555 308.4235 -3490.3672 4284.7286
312.1665 34.9828 53.7508 656151.2420 -11.7786
-11.7737
[28810]ENERGY: 19580000 3205.1312 5326.5313 5313.8146
348.7038 -15942.3570 -1779.6884 0.0000 0.0000

7748.9025 4221.0379 309.9948 -3527.8646 4285.6431
312.1774 129.4010 93.1829 656151.2420 -16.0106
-16.0232
[28860]ENERGY: 19590000 3263.6903 5153.8980 5255.2410
324.4805 -15836.9673 -1749.8931 0.0000 0.0000
7812.5903 4223.0398 312.5427 -3589.5505 4286.3877
312.3453 33.4558 -80.1577 656151.2420 -15.3820
-15.3852
[28899]ENERGY: 19600000 3274.5723 5329.1147 5268.7974
326.9829 -15923.5613 -1773.4543 0.0000 0.0000
7721.3465 4223.7981 308.8925 -3497.5484 4285.1801
312.3370 -90.9927 -98.3212 656151.2420 -15.1803
-15.1876
[28949]ENERGY: 19610000 3212.3778 5312.7599 5257.0592
340.4838 -15909.8653 -1787.4433 0.0000 0.0000
7797.5545 4222.9266 311.9412 -3574.6279 4286.0698
312.2311 28.1252 -0.4895 656151.2420 -20.4566
-20.4542
[28985]ENERGY: 19620000 3151.0706 5279.9167 5261.5416
341.9950 -15794.4827 -1778.1427 0.0000 0.0000
7761.5000 4223.3984 310.4988 -3538.1016 4285.7693
312.3371 76.0211 19.9925 656151.2420 -14.4108
-14.4237
[29035]ENERGY: 19630000 3149.8316 5227.6012 5243.8892
342.7893 -15865.6331 -1712.3586 0.0000 0.0000
7835.8606 4221.9803 313.4736 -3613.8804 4285.3833
312.3813 299.1514 156.0523 656151.2420 -18.3031
-18.2853
[29071]ENERGY: 19640000 3217.2791 5244.4886 5286.2157
335.5956 -15868.6486 -1801.8323 0.0000 0.0000
7809.8995 4222.9976 312.4350 -3586.9019 4287.0837
312.3036 72.8109 -36.8190 656151.2420 -16.9306
-16.9331
[29121]ENERGY: 19650000 3234.2109 5300.5627 5290.7583
333.7280 -15995.5922 -1797.9813 0.0000 0.0000
7859.8954 4225.5818 314.4351 -3634.3136 4286.1802
312.3873 150.7687 92.6181 656151.2420 -16.7155
-16.7087
[29157]ENERGY: 19660000 3239.7580 5236.9224 5227.4853
338.6991 -15789.6905 -1816.1046 0.0000 0.0000
7792.2671 4229.3369 311.7296 -3562.9303 4288.5137
312.2653 83.5106 -2.5234 656151.2420 -20.5773
-20.5879
[29207]ENERGY: 19670000 3233.0365 5282.9742 5272.7878
352.2081 -15930.1612 -1728.3276 0.0000 0.0000
7740.1841 4222.7020 309.6461 -3517.4822 4286.0767
312.2968 -42.3307 30.5869 656151.2420 -14.4765
-14.4694
[29243]ENERGY: 19680000 3242.4439 5331.4659 5271.8453
336.2076 -15883.5479 -1881.5227 0.0000 0.0000
7809.7995 4226.6916 312.4310 -3583.1079 4287.3778
312.3286 -172.0435 -182.0212 656151.2420 -23.5509
-23.5451
[29293]ENERGY: 19690000 3199.1256 5239.1129 5276.4070

346.9068 -15854.6631 -1790.8501 0.0000 0.0000
7809.4564 4225.4955 312.4173 -3583.9609 4287.0914
312.1707 0.8634 16.4612 656151.2420 -18.4196
-18.4145
[29332]ENERGY: 19700000 3272.6397 5220.7889 5250.0633
341.8325 -15852.6678 -1798.5201 0.0000 0.0000
7793.3333 4227.4699 311.7723 -3565.8635 4287.6164
312.1857 -63.1285 -23.8646 656151.2420 -18.0461
-18.0452
[29382]ENERGY: 19710000 3238.0622 5215.1324 5240.7061
365.4446 -15833.5828 -1791.8731 0.0000 0.0000
7788.5801 4222.4696 311.5821 -3566.1106 4287.1126
312.0902 -171.8146 -148.5688 656151.2420 -18.9035
-18.9100
[29418]ENERGY: 19720000 3176.5707 5205.2084 5276.7190
330.3560 -15857.6582 -1769.1782 0.0000 0.0000
7859.4327 4221.4503 314.4166 -3637.9824 4286.9593
312.2061 179.2933 85.4315 656151.2420 -14.9486
-14.9432
[29468]ENERGY: 19730000 3320.2078 5294.1633 5239.8555
341.1001 -15957.1686 -1778.6794 0.0000 0.0000
7762.4952 4221.9739 310.5386 -3540.5213 4287.5880
312.3880 38.7299 18.2027 656151.2420 -22.4400
-22.4365
[29504]ENERGY: 19740000 3298.2102 5191.7687 5262.7229
339.1909 -15922.9017 -1747.8106 0.0000 0.0000
7805.9615 4227.1418 312.2775 -3578.8197 4288.7121
312.4479 -66.5527 -62.9010 656151.2420 -16.3663
-16.3753
[29554]ENERGY: 19750000 3294.1965 5207.8588 5267.3816
342.0765 -15901.3012 -1791.3487 0.0000 0.0000
7808.2253 4227.0889 312.3681 -3581.1364 4288.8460
312.3633 -42.3701 -19.5549 656151.2420 -19.8934
-19.8862
[29590]ENERGY: 19760000 3164.0854 5302.0396 5306.6861
350.1564 -15926.9753 -1793.9654 0.0000 0.0000
7821.9754 4224.0022 312.9181 -3597.9732 4289.1143
312.4589 -96.2725 -51.2494 656151.2420 -19.7763
-19.7870
[29640]ENERGY: 19770000 3127.8609 5283.3227 5229.2273
343.7230 -15783.4232 -1829.5162 0.0000 0.0000
7853.6735 4224.8680 314.1862 -3628.8055 4289.6529
312.4064 -26.7125 -49.8428 656151.2420 -16.0161
-16.0146
[29676]ENERGY: 19780000 3217.9017 5252.7956 5318.4678
342.7554 -15822.0685 -1776.7087 0.0000 0.0000
7694.6062 4227.7495 307.8227 -3466.8566 4289.7817
312.6569 -86.8294 -62.8510 656151.2420 -16.4725
-16.4736
[29726]ENERGY: 19790000 3231.7435 5188.4530 5287.2435
360.1132 -15941.7351 -1748.5361 0.0000 0.0000
7848.5947 4225.8768 313.9830 -3622.7179 4289.2539
312.3594 -111.4778 -75.4965 656151.2420 -18.9917
-19.0032

[29765]ENERGY: 19800000 3197.6503 5273.3607 5259.8490
326.7265 -15871.7482 -1748.8457 0.0000 0.0000
7788.5320 4225.5245 311.5802 -3563.0075 4288.8099
312.2376 137.6705 141.8958 656151.2420 -17.8972
-17.8887
[29815]ENERGY: 19810000 3243.5788 5284.1572 5239.7033
365.4578 -15906.7271 -1767.9423 0.0000 0.0000
7767.2278 4225.4555 310.7279 -3541.7723 4289.5215
312.2864 19.8975 20.3660 656151.2420 -17.7940
-17.7854
[29851]ENERGY: 19820000 3228.0803 5318.0387 5285.8924
362.6870 -15888.2941 -1776.7611 0.0000 0.0000
7698.8486 4228.4919 307.9924 -3470.3567 4289.7711
312.1692 9.3482 -46.8143 656151.2420 -20.7139
-20.7187
[29901]ENERGY: 19830000 3277.1478 5280.7334 5263.0033
347.8879 -15892.0713 -1871.8105 0.0000 0.0000
7817.8582 4222.7488 312.7534 -3595.1094 4289.6599
312.3676 -14.1479 -52.5221 656151.2420 -15.3749
-15.3724
[29937]ENERGY: 19840000 3188.4166 5280.9397 5293.6097
336.1471 -15803.9833 -1840.7675 0.0000 0.0000
7771.0666 4225.4288 310.8815 -3545.6377 4290.5042
312.3119 -1.9495 -45.4848 656151.2420 -18.8935
-18.9103
[29987]ENERGY: 19850000 3219.1801 5355.9837 5288.0857
353.6447 -15915.8616 -1819.8323 0.0000 0.0000
7746.8684 4228.0688 309.9135 -3518.7996 4288.7824
312.5257 22.5320 -26.8351 656151.2420 -17.4288
-17.4056
[30023]ENERGY: 19860000 3233.3057 5313.7255 5264.6940
338.3155 -15889.3958 -1767.2131 0.0000 0.0000
7735.9743 4229.4061 309.4776 -3506.5682 4287.6003
312.4948 132.3026 191.4573 656151.2420 -20.5992
-20.5973
[30073]ENERGY: 19870000 3220.3135 5189.2123 5310.7647
332.6122 -15904.8733 -1666.3065 0.0000 0.0000
7746.6298 4228.3528 309.9039 -3518.2770 4288.6067
312.2832 200.7948 156.8744 656151.2420 -16.0546
-16.0541
[30109]ENERGY: 19880000 3221.9377 5252.8985 5299.3492
351.5108 -15904.2515 -1760.0848 0.0000 0.0000
7765.1868 4226.5466 310.6463 -3538.6402 4288.2095
312.2430 152.7555 -64.5070 656151.2420 -18.9011
-18.9383
[30159]ENERGY: 19890000 3233.9653 5216.5914 5257.0063
352.7545 -15786.2598 -1780.8260 0.0000 0.0000
7734.6613 4227.8931 309.4251 -3506.7683 4288.6749
312.3463 50.8713 78.1651 656151.2420 -17.8112
-17.7650
[30198]ENERGY: 19900000 3232.9015 5276.0677 5229.5685
333.6224 -15879.3839 -1765.3645 0.0000 0.0000
7800.9828 4228.3946 312.0783 -3572.5883 4288.6470
312.4223 56.5940 47.9220 656151.2420 -15.1066

-15.1284
[30248]ENERGY: 19910000 3238.0490 5285.2707 5229.1813
354.0407 -15919.5231 -1819.9813 0.0000 0.0000
7858.0730 4225.1104 314.3622 -3632.9627 4289.2293
312.3712 28.7156 -66.5686 656151.2420 -21.3035
-21.3067
[30284]ENERGY: 19920000 3165.1953 5234.6274 5296.1968
357.9667 -15864.6222 -1801.5468 0.0000 0.0000
7836.2688 4224.0860 313.4899 -3612.1828 4288.8531
312.4620 -64.3921 -73.2207 656151.2420 -12.4777
-12.4676
[30334]ENERGY: 19930000 3281.8117 5245.7775 5252.0760
358.8082 -15915.2632 -1807.6774 0.0000 0.0000
7814.6334 4230.1661 312.6244 -3584.4673 4290.7430
312.3421 126.7584 44.3698 656151.2420 -17.4089
-17.4152
[30370]ENERGY: 19940000 3224.4660 5203.4118 5250.0100
334.0202 -15824.7266 -1726.1572 0.0000 0.0000
7766.8956 4227.9197 310.7147 -3538.9758 4288.9067
312.4898 100.1055 95.7456 656151.2420 -12.0720
-12.0621
[30420]ENERGY: 19950000 3249.4184 5209.2065 5248.6792
354.7241 -15852.3370 -1809.6506 0.0000 0.0000
7827.4767 4227.5172 313.1382 -3599.9595 4290.5575
312.4134 -43.4568 -67.5047 656151.2420 -13.8916
-13.8994
[30456]ENERGY: 19960000 3163.6552 5264.9813 5250.1333
316.6500 -15797.5973 -1778.6156 0.0000 0.0000
7806.6432 4225.8502 312.3048 -3580.7930 4290.3958
312.5051 -102.5682 -133.8402 656151.2420 -14.3776
-14.3907
[30506]ENERGY: 19970000 3193.8147 5235.0979 5266.5453
350.8349 -15896.7484 -1729.0964 0.0000 0.0000
7807.2148 4227.6628 312.3276 -3579.5520 4290.6163
312.5562 191.1727 85.5751 656151.2420 -17.1206
-17.1240
[30542]ENERGY: 19980000 3267.4370 5213.8100 5323.3868
335.0350 -15981.6173 -1741.3324 0.0000 0.0000
7811.5083 4228.2275 312.4994 -3583.2808 4290.6674
312.3913 1.8106 0.3797 656151.2420 -13.4739
-13.4564
[30592]ENERGY: 19990000 3244.5913 5197.9735 5274.2000
332.2703 -15888.4338 -1768.8485 0.0000 0.0000
7836.1142 4227.8672 313.4837 -3608.2471 4290.9975
312.3670 -0.1967 -57.7561 656151.2420 -12.2882
-12.2988
[30631]ENERGY: 20000000 3240.3107 5209.5283 5248.1129
332.7802 -15858.3173 -1741.5292 0.0000 0.0000
7798.1638 4229.0494 311.9655 -3569.1144 4291.8761
312.3900 -15.7796 -41.2510 656151.2420 -15.5698
-15.5580

40.1ns Q9ZSP9 SIMULATION DATA

```
                 TS            BOND           ANGLE           DIHED           IMPRP
              ELECT             VDW        BOUNDARY            MISC         KINETIC
              TOTAL            TEMP       POTENTIAL          TOTAL3         TEMPAVG
           PRESSURE       GPRESSURE          VOLUME        PRESSAVG      GPRESSAVG
[191]ENERGY:      0       5073.0363       5427.9798       5983.4735        309.4550
         -9980.3682      -1816.1227          0.0000          0.0000       7946.2271
         12943.6807        296.3993       4997.4536      13055.9770        296.3993
          2833.7005        382.3025     723302.9074       2833.7005        382.3025
[279]ENERGY:  10000       3342.0155       5587.7607       5892.8456        317.6694
        -14480.9974      -1705.4155          0.0000          0.0000       8076.4840
          7030.3623        301.2580      -1046.1217       7096.5297        321.6125
            43.7578         40.2904     664265.7895          0.4459          0.5699
[235]ENERGY:  20000       3418.9013       5580.8599       5842.2846        352.7421
        -14424.5274      -1764.7867          0.0000          0.0000       8027.2712
          7032.7449        299.4556       -994.5263       7092.9904        303.3488
           -20.5344        -51.7714     664265.7895        -16.5787        -16.5267
[275]ENERGY:  30000       3382.5313       5566.4984       5965.5370        343.0036
        -14656.2553      -1775.4386          0.0000          0.0000       8203.1826
          7029.0590        306.0180      -1174.1236       7092.7689        305.2949
            90.4211        106.5638     664265.7895          1.0384          1.0323
[327]ENERGY:  40000       3472.1499       5600.3683       5934.7610        333.3618
        -14749.3830      -1839.6015          0.0000          0.0000       8275.5335
          7027.1901        308.7170      -1248.3434       7093.3753        307.6098
          -124.3150       -180.6324     664265.7895         -0.0262         -0.0386
[365]ENERGY:  50000       3462.3405       5503.0111       5959.5111        333.4270
        -14737.1868      -1732.6371          0.0000          0.0000       8243.1006
          7031.5665        307.5071      -1211.5341       7093.5946        308.5614
           105.6737         46.6620     664265.7895         -0.3793         -0.3797
[417]ENERGY:  60000       3453.9569       5521.6268       5958.9751        353.2740
        -14768.3274      -1772.5940          0.0000          0.0000       8283.2603
          7030.1718        309.0052      -1253.0885       7094.0431        309.4929
          -117.9982        -36.7381     664265.7895         -0.0619         -0.0524
[455]ENERGY:  70000       3398.4719       5643.5556       5983.7572        366.4920
        -14774.1420      -1841.1488          0.0000          0.0000       8252.5886
          7029.5744        307.8610      -1223.0142       7093.6368        309.8919
             9.9800        -40.3286     664265.7895          1.1050          1.1160
[506]ENERGY:  80000       3425.9042       5656.4920       5944.0189        369.4807
        -14892.8200      -1831.1339          0.0000          0.0000       8355.0148
          7026.9568        311.6820      -1328.0580       7093.6973        310.1831
          -214.7604       -156.5696     664265.7895         -0.1272         -0.1416
[543]ENERGY:  90000       3458.3705       5559.6670       5912.0512        355.6389
        -14883.2247      -1676.0441          0.0000          0.0000       8301.5055
          7027.9642        309.6859      -1273.5413       7092.7699        310.5325
           124.9259         82.1909     664265.7895         -0.3062         -0.2982
[596]ENERGY: 100000       3498.9873       5536.9068       5984.7780        346.9289
        -14778.1562      -1869.2356          0.0000          0.0000       8308.9444
          7029.1536        309.9634      -1279.7908       7095.0474        311.3199
            -0.8254        -64.5480     664265.7895          0.5862          0.5672
[633]ENERGY: 110000       3354.5402       5580.0216       6010.4190        339.9427
        -14856.3055      -1770.8349          0.0000          0.0000       8368.5542
          7026.3373        312.1871      -1342.2169       7094.1564        311.4881
           148.5714         61.7723     664265.7895         -0.6868         -0.6864
[684]ENERGY: 120000       3362.2428       5540.6680       5955.2676        362.7671
        -14711.5950      -1844.6917          0.0000          0.0000       8364.1928
```

```
        7028.8516       312.0244     -1335.3412      7094.2070       311.6584
        133.4142        74.0909    664265.7895       -1.4073        -1.3979
[720]ENERGY:  130000      3378.5010      5674.1238      5923.9499      354.4580
      -14826.1319     -1791.4470         0.0000         0.0000      8311.8927
        7025.3464       310.0734     -1286.5463      7094.7611       312.0327
        -47.7801       -31.4960    664265.7895        1.1020         1.1170
[770]ENERGY:  140000      3418.7666      5585.6806      5917.0779      349.1934
      -14786.4274     -1773.4511         0.0000         0.0000      8317.4428
        7028.2828       310.2804     -1289.1599      7093.2355       312.2614
        154.8482        21.1624    664265.7895        0.5689         0.5536
[806]ENERGY:  150000      3453.5642      5657.1076      5945.6558      347.7770
      -14931.2233     -1800.4422         0.0000         0.0000      8353.4800
        7025.9191       311.6248     -1327.5609      7093.0133       312.4295
        -19.6620        39.7204    664265.7895       -1.6620        -1.6466
[856]ENERGY:  160000      3356.3727      5593.5944      5904.1684      367.9491
      -14866.0838     -1748.1921         0.0000         0.0000      8417.5876
        7025.3962       314.0163     -1392.1914      7093.1953       312.2536
        134.9477       177.1515    664265.7895        0.8241         0.8182
[892]ENERGY:  170000      3423.0840      5593.6260      5940.7059      353.2931
      -14955.9119     -1730.3597         0.0000         0.0000      8399.5915
        7024.0288       313.3450     -1375.5626      7092.3347       312.4072
         -6.3173        35.6338    664265.7895        0.7369         0.7547
[942]ENERGY:  180000      3479.3787      5627.0509      5922.4013      360.6234
      -14990.8356     -1766.4699         0.0000         0.0000      8388.1563
        7020.3050       312.9184     -1367.8512      7091.6054       312.4546
        -85.0531      -111.7388    664265.7895        0.7807         0.7800
[978]ENERGY:  190000      3451.5107      5626.9474      5905.8200      330.9721
      -14809.1390     -1784.3500         0.0000         0.0000      8308.4136
        7030.1749       309.9436     -1278.2387      7093.2817       312.6460
        184.2557        68.2342    664265.7895        0.1553         0.1282
[1031]ENERGY:  200000      3454.5656      5625.4577      5912.9153
347.2812         -14931.4533     -1781.5473         0.0000         0.0000
8396.0307           7023.2499       313.2121     -1372.7808      7092.9323
312.4531           -133.4435       -50.0877    664265.7895        -2.7826
-2.7618
[1067]ENERGY:  210000      3417.4345      5622.5866      5911.9118
339.3518         -14846.5477     -1804.9872         0.0000         0.0000
8387.8253           7027.5752       312.9060     -1360.2501      7094.4733
312.6080            150.5638        52.8787    664265.7895         2.4164
 2.4103
[1117]ENERGY:  220000      3442.2155      5644.7992      5952.5391
363.6215         -14899.7258     -1771.2540         0.0000         0.0000
8295.3299           7027.5253       309.4555     -1267.8046      7092.8979
312.7279            -24.0479       -17.5427    664265.7895        -1.0636
-1.0676
[1153]ENERGY:  230000      3478.2782      5589.3416      5948.4686
363.0481         -15000.9260     -1761.3792         0.0000         0.0000
8408.6824           7025.5137       313.6841     -1383.1687      7094.0783
312.8479            159.4751        66.6615    664265.7895         2.0973
 2.0837
[1203]ENERGY:  240000      3396.6622      5663.4599      5870.4773
374.2320         -14957.4475     -1671.9988         0.0000         0.0000
8350.0189           7025.4041       311.4957     -1324.6148      7092.7515
312.7832             17.5592         7.1169    664265.7895        -0.2247
```

```
-0.2100
[1239]ENERGY:  250000      3477.3138      5467.6613      5959.4749
373.8198         -14899.7554     -1805.8269         0.0000         0.0000
8449.2018           7021.8893       315.1957     -1427.3125      7092.5918
312.6537           -130.7939      -174.6160    664265.7895        -2.5012
-2.5049
[1289]ENERGY:  260000      3415.0112      5673.4153      5928.1314
350.0087         -14995.8932     -1694.1107         0.0000         0.0000
8346.2748           7022.8374       311.3560     -1323.4373      7092.3157
312.8114             35.2435        80.2719    664265.7895         3.4384
 3.4426
[1325]ENERGY:  270000      3413.9162      5598.4195      5950.8047
352.5311         -14952.3604     -1750.6401         0.0000         0.0000
8415.5757           7028.2468       313.9412     -1387.3290      7093.4714
313.1717             -1.8647        99.3010    664265.7895        -0.6749
-0.6687
[1375]ENERGY:  280000      3416.5381      5631.0102      5999.6039
352.0292         -14970.0983     -1785.2447         0.0000         0.0000
8382.4988           7026.3373       312.7073     -1356.1615      7093.1153
313.3336            -34.1182       -88.7563    664265.7895         1.5623
 1.5427
[1411]ENERGY:  290000      3404.5179      5580.8342      5921.6985
367.9392         -14996.1412     -1731.2741         0.0000         0.0000
8477.6536           7025.2280       316.2570     -1452.4256      7093.6795
313.2834            -29.6551       -19.8791    664265.7895        -3.0673
-3.0530
[1464]ENERGY:  300000      3525.0202      5638.2335      5923.6474
329.4026         -14937.0556     -1829.2907         0.0000         0.0000
8375.0880           7025.0453       312.4309     -1350.0427      7093.6043
313.4812           -162.6649      -232.9050    664265.7895        -0.2224
-0.2412
[1500]ENERGY:  310000      3515.3765      5635.5826      5873.6993
354.1562         -14974.8726     -1813.3861         0.0000         0.0000
8433.8424           7024.3983       314.6227     -1409.4441      7093.4288
313.5080             55.2201       116.9883    664265.7895         0.8410
 0.8491
[1550]ENERGY:  320000      3495.8964      5644.1755      5917.4150
346.5828         -14955.4561     -1779.1726         0.0000         0.0000
8357.0568           7026.4978       311.7582     -1330.5591      7094.6134
313.6912            -21.6245       -39.0512    664265.7895        -0.1438
-0.1366
[1586]ENERGY:  330000      3509.8707      5704.1406      5879.2907
372.3326         -15135.9314     -1698.5280         0.0000         0.0000
8396.1721           7027.3472       313.2174     -1368.8249      7094.9145
313.9618             99.1836        63.6825    664265.7895         0.3853
 0.3890
[1636]ENERGY:  340000      3429.4163      5593.7688      5912.8179
374.3610         -14962.7986     -1786.8183         0.0000         0.0000
8465.9375           7026.6846       315.8200     -1439.2529      7095.4181
314.1327             30.9340       -40.2014    664265.7895        -0.2713
-0.2946
[1672]ENERGY:  350000      3458.1400      5656.1178      5972.9692
340.3582         -15008.7841     -1791.6956         0.0000         0.0000
8403.3654           7030.4709       313.4857     -1372.8945      7094.4157
```

314.3730 142.0180 77.8973 664265.7895 -0.3699
-0.3674
[1722]ENERGY: 360000 3385.6095 5663.2529 5919.4145
340.3764 -14972.3407 -1767.4069 0.0000 0.0000
8458.1830 7027.0888 315.5307 -1431.0942 7094.4813
314.2972 149.2979 120.3622 664265.7895 0.1659
0.1898
[1758]ENERGY: 370000 3490.4504 5686.4463 5972.1755
365.3797 -15124.1112 -1736.4893 0.0000 0.0000
8377.7173 7031.5687 312.5289 -1346.1486 7095.8687
314.1990 11.2771 -15.4018 664265.7895 0.5514
0.5404
[1808]ENERGY: 380000 3434.8474 5732.4348 5919.4548
348.9513 -15155.7993 -1727.0716 0.0000 0.0000
8470.8384 7023.6559 316.0028 -1447.1825 7094.3868
314.3058 45.9010 87.8050 664265.7895 -0.8854
-0.8831
[1844]ENERGY: 390000 3428.1535 5693.6210 5902.7471
371.7121 -15139.8955 -1732.2227 0.0000 0.0000
8500.6012 7024.7168 317.1131 -1475.8844 7095.1500
314.4479 -122.0283 -126.3984 664265.7895 1.7559
1.7708
[1897]ENERGY: 400000 3479.2372 5574.1573 5861.8107
366.3262 -14952.7250 -1719.7782 0.0000 0.0000
8424.4422 7033.4703 314.2720 -1390.9718 7096.8301
314.2233 3.7254 10.1544 664265.7895 -0.4491
-0.4572
[1933]ENERGY: 410000 3384.5830 5677.8802 5928.2009
368.1859 -15000.1269 -1713.6697 0.0000 0.0000
8386.9323 7031.9856 312.8727 -1354.9466 7096.6445
314.2037 122.2791 36.7316 664265.7895 -1.8312
-1.8296
[1983]ENERGY: 420000 3467.4521 5621.9570 5925.9536
356.2904 -15019.3828 -1752.0229 0.0000 0.0000
8427.8364 7028.0837 314.3986 -1399.7527 7095.9638
314.5345 133.5841 31.1702 664265.7895 1.0517
1.0284
[2019]ENERGY: 430000 3490.4044 5687.6761 5902.4849
337.6334 -15120.2324 -1772.9044 0.0000 0.0000
8501.2590 7026.3211 317.1376 -1474.9380 7096.0183
314.7168 51.7702 41.3323 664265.7895 -0.1746
-0.1587
[2069]ENERGY: 440000 3458.6501 5621.9201 5888.5541
359.4718 -14906.5982 -1791.7014 0.0000 0.0000
8398.6210 7028.9175 313.3087 -1369.7035 7096.8992
314.7156 9.5139 44.0388 664265.7895 1.3889
1.3898
[2105]ENERGY: 450000 3504.6352 5606.2943 5911.1319
339.7664 -15030.2437 -1742.8733 0.0000 0.0000
8438.9375 7027.6483 314.8127 -1411.2892 7096.6519
314.5703 -40.6374 -15.2886 664265.7895 -1.2900
-1.2799
[2155]ENERGY: 460000 3504.2789 5603.9684 5870.1601
355.7414 -14937.3076 -1796.7311 0.0000 0.0000

8428.6502 7028.7604 314.4290 -1399.8898 7097.5014
314.6200 83.7596 33.5908 664265.7895 -0.4930
-0.5105
[2191]ENERGY: 470000 3465.2708 5495.8180 5921.1250
354.3204 -14891.9588 -1760.1125 0.0000 0.0000
8444.0816 7028.5445 315.0046 -1415.5370 7096.5948
314.6419 -3.7176 -25.6913 664265.7895 -0.2606
-0.2661
[2241]ENERGY: 480000 3424.1069 5661.1575 5921.9167
345.6306 -15043.5106 -1785.6422 0.0000 0.0000
8505.7173 7029.3762 317.3040 -1476.3411 7098.1577
314.7609 101.8586 75.9857 664265.7895 1.6593
1.6707
[2277]ENERGY: 490000 3444.5570 5578.8679 5900.4965
350.4550 -14941.0392 -1843.1926 0.0000 0.0000
8533.3858 7023.5303 318.3361 -1509.8555 7096.6064
314.8496 -145.3379 -107.8231 664265.7895 -1.2928
-1.2782
[2330]ENERGY: 500000 3487.2389 5667.3582 5858.0840
357.7218 -15045.1850 -1749.1863 0.0000 0.0000
8453.3109 7029.3426 315.3489 -1423.9683 7097.5420
315.0739 119.5225 46.5591 664265.7895 -0.0877
-0.0914
[2366]ENERGY: 510000 3476.0016 5697.6239 5905.7599
349.1858 -15050.6183 -1827.8371 0.0000 0.0000
8477.4749 7027.5907 316.2504 -1449.8842 7097.4391
315.5300 -35.4453 48.6519 664265.7895 0.4028
0.3862
[2416]ENERGY: 520000 3507.8379 5667.1834 5880.6571
366.0487 -15053.6747 -1833.1861 0.0000 0.0000
8495.0894 7029.9557 316.9075 -1465.1337 7096.1354
315.6829 -83.8960 -55.0878 664265.7895 -1.1281
-1.1117
[2452]ENERGY: 530000 3488.1047 5646.7752 5890.9418
343.7699 -15150.4333 -1725.9326 0.0000 0.0000
8534.5927 7027.8183 318.3811 -1506.7743 7097.9110
315.5950 63.1087 127.0445 664265.7895 -0.0939
-0.1146
[2502]ENERGY: 540000 3530.9746 5712.4240 5870.7602
331.5287 -15046.5461 -1838.4459 0.0000 0.0000
8467.1340 7027.8294 315.8646 -1439.3046 7098.2868
315.3944 -102.5668 -63.4853 664265.7895 0.5915
0.6029
[2538]ENERGY: 550000 3519.5807 5581.3255 5915.8518
344.0924 -15071.9012 -1821.4751 0.0000 0.0000
8558.4895 7025.9637 319.2726 -1532.5258 7098.9491
315.7802 -10.5139 -31.7730 664265.7895 1.6705
1.6761
[2588]ENERGY: 560000 3559.0511 5610.9117 5915.7312
345.8857 -14930.0688 -1825.2098 0.0000 0.0000
8353.8274 7030.1285 311.6377 -1323.6989 7099.0122
315.6052 7.8680 -51.4340 664265.7895 -0.9586
-0.9595
[2624]ENERGY: 570000 3549.5997 5613.3541 5849.5486

377.5072 -15055.0877 -1792.1509 0.0000 0.0000
8488.0187 7030.7896 316.6437 -1457.2291 7098.4799
315.6000 -46.5827 -9.5565 664265.7895 -1.0327
-1.0465
[2674]ENERGY: 580000 3497.3842 5660.5698 5938.8816
358.7590 -15130.2138 -1684.1869 0.0000 0.0000
8389.9064 7031.1003 312.9837 -1358.8061 7098.5577
315.7796 26.6157 37.4878 664265.7895 1.1548
1.1733
[2710]ENERGY: 590000 3438.1716 5694.2998 5857.1450
336.8451 -15010.4626 -1794.0046 0.0000 0.0000
8506.6402 7028.6345 317.3384 -1478.0057 7099.3781
315.7765 -5.0254 -92.1295 664265.7895 -0.4974
-0.5086
[2763]ENERGY: 600000 3526.6934 5722.3610 5864.9842
349.5862 -15185.4442 -1747.2604 0.0000 0.0000
8498.2847 7029.2049 317.0267 -1469.0798 7100.1043
315.6800 -71.1317 16.6351 664265.7895 0.7726
0.7780
[2799]ENERGY: 610000 3502.8917 5668.8584 5829.6393
336.9871 -15097.4033 -1606.8863 0.0000 0.0000
8400.7419 7034.8288 313.3879 -1365.9131 7099.9107
315.6279 61.9704 55.8452 664265.7895 -0.5782
-0.5879
[2849]ENERGY: 620000 3517.1397 5633.9086 5902.0187
326.2668 -15118.3496 -1716.4203 0.0000 0.0000
8485.7442 7030.3080 316.5589 -1455.4362 7099.3772
315.6203 22.8445 30.7259 664265.7895 0.2709
0.2700
[2885]ENERGY: 630000 3523.6628 5622.9389 5866.4028
373.7067 -14988.1503 -1824.9466 0.0000 0.0000
8459.2920 7032.9063 315.5721 -1426.3857 7099.4792
315.5317 -41.6063 -36.3369 664265.7895 0.2673
0.2701
[2935]ENERGY: 640000 3444.3025 5686.6034 5884.3779
347.4349 -15023.5751 -1749.8577 0.0000 0.0000
8443.4891 7032.7751 314.9825 -1410.7141 7099.5514
315.3725 57.8553 81.4800 664265.7895 -1.2318
-1.2373
[2971]ENERGY: 650000 3471.6304 5643.6510 5905.8847
348.6054 -15006.9213 -1811.9286 0.0000 0.0000
8480.4891 7031.4107 316.3628 -1449.0783 7100.8517
315.3990 -183.9839 -132.4794 664265.7895 2.1196
2.1191
[3021]ENERGY: 660000 3405.2740 5600.0344 5891.7160
342.6803 -15007.1157 -1708.2374 0.0000 0.0000
8504.6670 7029.0186 317.2648 -1475.6484 7100.2784
315.7165 48.2915 41.2437 664265.7895 -1.8848
-1.8759
[3057]ENERGY: 670000 3441.5492 5677.1271 5917.6022
356.8931 -15051.6053 -1808.3340 0.0000 0.0000
8495.5053 7028.7377 316.9230 -1466.7676 7100.9463
315.7515 -19.6828 -54.1698 664265.7895 1.2326
1.2243

[3107]ENERGY: 680000 3576.0727 5597.7818 5885.3415
341.7638 -15093.1127 -1772.3836 0.0000 0.0000
8497.7555 7033.2191 317.0069 -1464.5364 7101.8095
315.8756 -8.5349 -116.7475 664265.7895 1.4003
1.3964
[3143]ENERGY: 690000 3415.9382 5722.3415 5875.8222
366.7277 -15017.3217 -1741.6022 0.0000 0.0000
8411.8584 7033.7640 313.8026 -1378.0943 7101.9977
315.9023 189.7015 89.6214 664265.7895 -2.0165
-1.9995
[3196]ENERGY: 700000 3448.5534 5655.0110 5930.3891
357.4769 -14950.3290 -1838.6841 0.0000 0.0000
8430.7469 7033.1642 314.5072 -1397.5827 7101.5855
315.9360 -99.6142 -50.8892 664265.7895 0.3004
0.3122
[3232]ENERGY: 710000 3444.1711 5700.1376 5893.8210
362.7736 -15028.1184 -1754.8687 0.0000 0.0000
8416.9189 7034.8352 313.9913 -1382.0837 7103.6086
315.9506 165.0241 120.2987 664265.7895 -2.6409
-2.6749
[3282]ENERGY: 720000 3544.7315 5720.1996 5918.4480
350.7104 -15084.6187 -1817.0976 0.0000 0.0000
8404.1770 7036.5502 313.5160 -1367.6269 7103.4173
316.2871 148.3087 76.0740 664265.7895 -2.1091
-2.0781
[3318]ENERGY: 730000 3453.3476 5743.4209 5882.2231
368.1552 -15202.8537 -1788.0633 0.0000 0.0000
8574.1401 7030.3699 319.8564 -1543.7702 7101.5928
317.0615 -83.2123 22.6712 664265.7895 -5.6970
-5.6926
[3368]ENERGY: 740000 3556.2968 5629.8658 5952.1065
341.7107 -15206.6296 -1781.6841 0.0000 0.0000
8543.7856 7035.4516 318.7241 -1508.3340 7100.3995
318.0858 41.3474 68.0264 664265.7895 -6.4541
-6.4810
[3404]ENERGY: 750000 3559.3608 5779.5642 5906.5039
352.5989 -15187.1281 -1865.4322 0.0000 0.0000
8489.9830 7035.4506 316.7170 -1454.5324 7102.1589
318.6641 21.0793 39.0401 664265.7895 -8.6660
-8.6388
[3454]ENERGY: 760000 3481.1441 5640.8714 5872.0603
365.1531 -15066.0515 -1836.4745 0.0000 0.0000
8575.7931 7032.4961 319.9181 -1543.2970 7101.2487
318.7074 -29.8927 -88.4980 664265.7895 -8.5268
-8.5466
[3490]ENERGY: 770000 3581.9971 5647.3814 5923.8627
356.5404 -15163.1157 -1871.0973 0.0000 0.0000
8559.2402 7034.8088 319.3006 -1524.4314 7101.6359
318.8478 34.4097 5.4662 664265.7895 -5.7521
-5.7545
[3540]ENERGY: 780000 3514.7610 5650.1971 5875.3809
332.2029 -15113.3349 -1801.8097 0.0000 0.0000
8579.1822 7036.5795 320.0445 -1542.6027 7101.0198
318.8377 -58.5613 -145.8306 664265.7895 -20.9937

-20.9911
[3576]ENERGY: 790000   3537.8558   5810.7267   5863.7652
335.9187   -15155.8188   -1867.3949   0.0000   0.0000
8508.7799   7033.8326   317.4182   -1474.9474   7100.2804
319.0549   -4.1505   -50.5991   664265.7895   -16.5709
-16.5708
[3629]ENERGY: 800000   3616.8274   5766.3376   5909.0216
342.6012   -15232.8778   -1865.7372   0.0000   0.0000
8499.6549   7035.8278   317.0778   -1463.8272   7100.8748
319.1513   -41.1238   -41.0475   664265.7895   -12.2574
-12.2567
[3665]ENERGY: 810000   3507.5212   5782.6241   5945.0767
343.4092   -15193.1336   -1849.4133   0.0000   0.0000
8495.7638   7031.8482   316.9326   -1463.9157   7101.3661
319.0911   11.8848   -91.9358   664265.7895   -10.6035
-10.6057
[3715]ENERGY: 820000   3505.0028   5652.1712   5912.8391
356.2163   -15231.1848   -1777.1719   0.0000   0.0000
8612.3701   7030.2429   321.2826   -1582.1273   7100.9467
319.1255   71.6419   22.5304   664265.7895   -12.0511
-12.0502
[3751]ENERGY: 830000   3514.5419   5796.3343   5890.9073
365.9597   -15243.9558   -1868.4503   0.0000   0.0000
8578.9529   7034.2900   320.0360   -1544.6629   7103.3504
319.0159   -67.9961   -94.3753   664265.7895   -18.5286
-18.5140
[3801]ENERGY: 840000   3461.8941   5753.4333   5896.9793
355.2193   -15199.6908   -1803.0320   0.0000   0.0000
8569.4875   7034.2907   319.6829   -1535.1968   7103.6506
319.3140   14.5487   20.9285   664265.7895   -12.6724
-12.6923
[3837]ENERGY: 850000   3509.2203   5727.7650   5867.5497
356.0214   -15220.3322   -1816.7348   0.0000   0.0000
8610.3157   7033.8051   321.2060   -1576.5106   7104.3608
319.4057   -68.6115   -94.6259   664265.7895   -8.7787
-8.7689
[3887]ENERGY: 860000   3491.3698   5790.8622   5904.0445
362.0689   -15171.9917   -1904.9002   0.0000   0.0000
8560.7667   7032.2203   319.3576   -1528.5464   7104.0561
319.3661   -28.4422   35.9736   664265.7895   -9.5496
-9.5433
[3923]ENERGY: 870000   3562.5579   5702.8449   5917.8733
347.9722   -15208.2889   -1756.0968   0.0000   0.0000
8470.0203   7036.8829   315.9723   -1433.1374   7104.1437
319.5286   -173.3270   -147.7073   664265.7895   -15.9202
-15.9148
[3973]ENERGY: 880000   3518.4842   5728.9699   5834.5221
343.0513   -15211.6274   -1787.2288   0.0000   0.0000
8605.9825   7032.1537   321.0443   -1573.8287   7103.5142
319.4771   74.6796   2.1260   664265.7895   -13.0934
-13.0989
[4009]ENERGY: 890000   3599.4617   5712.1056   5938.2489
362.5761   -15182.4169   -1854.1161   0.0000   0.0000
8462.8229   7038.6822   315.7038   -1424.1406   7104.7008

319.3378 46.8203 -7.9414 664265.7895 -19.5268
-19.5281
[4062]ENERGY: 900000 3626.5064 5735.7517 5892.3665
320.9385 -15246.5343 -1842.8171 0.0000 0.0000
8548.5021 7034.7137 318.9000 -1513.7884 7104.9435
319.4514 -292.8328 -201.0221 664265.7895 -12.0086
-12.0068
[4098]ENERGY: 910000 3413.9841 5668.8063 5848.1856
359.5440 -15080.2927 -1882.1202 0.0000 0.0000
8702.8277 7030.9349 324.6571 -1671.8928 7105.4797
319.2143 83.1398 -131.2131 664265.7895 -18.2312
-18.2406
[4148]ENERGY: 920000 3568.9714 5817.9294 5822.2992
348.9902 -15141.0064 -1882.9597 0.0000 0.0000
8501.8282 7036.0524 317.1589 -1465.7759 7104.8168
319.4367 -123.1863 -79.4961 664265.7895 -9.6287
-9.6296
[4184]ENERGY: 930000 3516.7154 5722.1642 5915.7171
340.7575 -15120.6515 -1801.2659 0.0000 0.0000
8463.7262 7037.1629 315.7375 -1426.5633 7105.8488
319.6707 -186.6561 -154.4953 664265.7895 -14.2192
-14.2042
[4234]ENERGY: 940000 3543.7145 5741.8177 5881.0229
344.2853 -15191.9263 -1827.0940 0.0000 0.0000
8543.7856 7035.6057 318.7241 -1508.1799 7104.9774
319.5157 -82.3702 -79.1339 664265.7895 -11.5187
-11.5367
[4270]ENERGY: 950000 3446.6829 5666.2125 5920.3264
343.3813 -15159.9937 -1760.4399 0.0000 0.0000
8580.5939 7036.7635 320.0972 -1543.8304 7106.7193
319.5164 -10.5715 -29.3883 664265.7895 -13.3131
-13.3155
[4320]ENERGY: 960000 3499.0998 5756.6720 5931.2613
374.6202 -15139.2273 -1867.2720 0.0000 0.0000
8485.4773 7040.6313 316.5489 -1444.8460 7107.9948
319.6811 -91.7567 -64.6145 664265.7895 -11.0963
-11.0933
[4356]ENERGY: 970000 3497.1715 5719.8019 5889.4242
366.1482 -15129.4946 -1797.4611 0.0000 0.0000
8492.7054 7038.2956 316.8185 -1454.4099 7105.6118
319.6248 -105.8279 -69.0651 664265.7895 -17.0783
-17.0673
[4406]ENERGY: 980000 3471.9856 5824.7955 5885.0843
337.1295 -15097.7732 -1932.9456 0.0000 0.0000
8549.3867 7037.6628 318.9330 -1511.7239 7107.1958
319.6647 50.5128 38.4516 664265.7895 -13.9892
-13.9948
[4442]ENERGY: 990000 3536.1770 5769.1191 5813.0235
338.7831 -15154.3641 -1855.1158 0.0000 0.0000
8594.1497 7041.7724 320.6029 -1552.3773 7107.7315
319.6894 26.0554 -5.9328 664265.7895 -18.4870
-18.5019
[4495]ENERGY: 1000000 3499.6059 5702.5003 5890.1385
338.9883 -15213.0103 -1736.1932 0.0000 0.0000

8557.7974 7039.8270 319.2468 -1517.9704 7105.7492
319.6962 148.2703 132.3973 664265.7895 -19.5908
-19.5890
[268]ENERGY: 1010000 3564.6794 5636.7333 5883.2314 364.5394
-15195.6911 -1812.9826 0.0000 0.0000 8597.2648
7037.7746 320.7191 -1559.4902 7107.4515 319.8131
-25.1495 -1.5146 664265.7895 -18.5601 -18.5383
[307]ENERGY: 1020000 3539.1870 5733.9983 5908.9809 364.2276
-15152.0874 -1861.4279 0.0000 0.0000 8506.7057
7039.5843 317.3408 -1467.1214 7107.3758 319.8780
-15.0692 -3.0424 664265.7895 -22.6391 -22.6636
[359]ENERGY: 1030000 3561.3961 5718.2051 5949.9086 340.2536
-15294.1332 -1860.2648 0.0000 0.0000 8620.1959
7035.5612 321.5746 -1584.6347 7108.1381 319.8159
-263.7548 -231.4252 664265.7895 -17.1114 -17.0863
[397]ENERGY: 1040000 3498.8255 5680.5072 5917.6178 349.7783
-15175.7446 -1805.5473 0.0000 0.0000 8571.3137
7036.7507 319.7510 -1534.5630 7106.9737 319.9228
54.6470 -11.2181 664265.7895 -19.9948 -20.0108
[449]ENERGY: 1050000 3579.7145 5603.8946 5911.4611 361.1449
-15179.7602 -1787.5962 0.0000 0.0000 8551.1605
7040.0193 318.9992 -1511.1412 7108.0197 319.9869
-5.3298 -38.3186 664265.7895 -19.4509 -19.4521
[487]ENERGY: 1060000 3601.5803 5719.2849 5894.6315 354.3160
-15223.6474 -1852.6753 0.0000 0.0000 8545.3564
7038.8463 318.7827 -1506.5101 7108.0574 320.0449
-109.6301 -162.3144 664265.7895 -19.4080 -19.3994
[538]ENERGY: 1070000 3476.3177 5841.3469 5894.3097 364.8748
-15258.7830 -1828.7240 0.0000 0.0000 8547.3857
7036.7278 318.8584 -1510.6579 7108.2686 319.7146
-17.1275 -163.4269 664265.7895 -16.4862 -16.4929
[575]ENERGY: 1080000 3497.5837 5740.4164 5905.0440 359.5358
-15157.9047 -1829.8781 0.0000 0.0000 8525.7851
7040.5821 318.0526 -1485.2030 7108.5263 319.7312
-106.1282 -148.0595 664265.7895 -18.6974 -18.7102
[626]ENERGY: 1090000 3518.9351 5819.4921 5895.6763 360.8016
-15164.6724 -1876.7929 0.0000 0.0000 8491.9418
7045.3817 316.7901 -1446.5602 7109.5680 319.8603
19.1135 -33.8778 664265.7895 -18.9984 -18.9878
[666]ENERGY: 1100000 3584.1196 5700.6215 5874.8279 341.9818
-15221.6115 -1734.7433 0.0000 0.0000 8495.3769
7040.5727 316.9182 -1454.8042 7107.7743 320.0457
42.6461 -5.1171 664265.7895 -6.6738 -6.6917
[717]ENERGY: 1110000 3518.9188 5659.7129 5870.5463 354.4174
-15195.7767 -1750.3719 0.0000 0.0000 8586.3020
7043.7488 320.3101 -1542.5532 7110.7501 320.1200
124.1860 37.4778 664265.7895 -10.4170 -10.3876
[753]ENERGY: 1120000 3471.0286 5768.0088 5889.7671 355.3761
-15152.5339 -1885.2228 0.0000 0.0000 8592.9864
7039.4103 320.5595 -1553.5761 7110.9557 320.0420
80.9906 -97.2689 664265.7895 -12.5648 -12.5733
[803]ENERGY: 1130000 3435.9600 5699.8953 5910.9633 381.3902
-15159.0212 -1795.2813 0.0000 0.0000 8569.5647
7043.4711 319.6858 -1526.0936 7111.0585 320.0710

68.9083 50.7349 664265.7895 -10.9169 -10.9370
[839]ENERGY: 1140000 3502.5752 5807.6131 5885.2333 318.8968
-15161.6131 -1870.6254 0.0000 0.0000 8559.3601
7041.4400 319.3051 -1517.9201 7110.9308 320.1090
-42.7024 -8.6703 664265.7895 -19.2689 -19.2472
[889]ENERGY: 1150000 3491.5365 5662.8597 5839.3721 338.1243
-15069.3814 -1799.4754 0.0000 0.0000 8579.6444
7042.6802 320.0618 -1536.9641 7112.6492 319.8924
63.1068 1.1657 664265.7895 -20.3419 -20.3351
[925]ENERGY: 1160000 3591.6218 5792.0661 5833.8578 361.7915
-15251.9507 -1860.8104 0.0000 0.0000 8578.4561
7045.0323 320.0175 -1533.4239 7113.7996 320.1325
-31.0692 -32.7593 664265.7895 -15.5410 -15.5515
[975]ENERGY: 1170000 3542.5982 5710.3730 5880.3072 371.4318
-15222.4932 -1838.5349 0.0000 0.0000 8600.1077
7043.7898 320.8252 -1556.3179 7112.6522 319.9752
-40.9827 -58.5018 664265.7895 -9.1255 -9.1144
[1011]ENERGY: 1180000 3508.9449 5774.1153 5858.8860
358.8004 -15255.0269 -1805.5606 0.0000 0.0000
8602.7481 7042.9073 320.9237 -1559.8408 7112.1472
319.8381 73.5895 83.6402 664265.7895 -19.5487
-19.5517
[1061]ENERGY: 1190000 3504.9802 5715.2261 5909.5015
349.4420 -15158.6095 -1798.5787 0.0000 0.0000
8522.3095 7044.2711 317.9229 -1478.0384 7112.7955
320.1978 -31.2041 -6.1329 664265.7895 -20.3718
-20.3595
[1100]ENERGY: 1200000 3475.6924 5813.2420 5867.2523
358.1195 -15193.3903 -1869.2116 0.0000 0.0000
8589.6660 7041.3703 320.4356 -1548.2957 7113.8952
320.0507 7.1894 -94.9941 664265.7895 -27.9058
-27.9289
[1150]ENERGY: 1210000 3577.6200 5876.3549 5873.2994
347.3084 -15232.5877 -1908.4490 0.0000 0.0000
8511.2319 7044.7779 317.5097 -1466.4540 7114.3645
320.0200 -39.8836 -114.1344 664265.7895 -22.9513
-22.9408
[1186]ENERGY: 1220000 3487.8093 5759.4313 5945.9985
351.6881 -15249.5186 -1789.3095 0.0000 0.0000
8542.4275 7048.5266 318.6734 -1493.9009 7114.8257
319.9593 247.8329 133.8605 664265.7895 -25.5673
-25.5667
[1236]ENERGY: 1230000 3517.1222 5802.6077 5825.5201
381.8810 -15221.9951 -1843.8655 0.0000 0.0000
8586.7335 7048.0039 320.3262 -1538.7296 7115.8143
319.9594 41.8841 -66.7678 664265.7895 -20.7481
-20.7364
[1272]ENERGY: 1240000 3501.2589 5763.6245 5861.4110
365.9635 -15202.4031 -1846.3198 0.0000 0.0000
8600.9263 7044.4613 320.8557 -1556.4650 7116.6493
319.8745 226.7908 93.5937 664265.7895 -23.1520
-23.1683
[1322]ENERGY: 1250000 3459.1379 5810.8587 5819.6034
348.3431 -15263.1979 -1780.0072 0.0000 0.0000

8650.5987 7045.3367 322.7087 -1605.2620 7116.4573
320.0514 116.1518 146.7744 664265.7895 -21.2440
-21.2478
[1358]ENERGY: 1260000 3530.5558 5722.9480 5910.5475
343.7053 -15171.3021 -1871.2751 0.0000 0.0000
8584.6458 7049.8253 320.2484 -1534.8206 7116.8825
320.0977 -22.2522 -9.8360 664265.7895 -21.3673
-21.3526
[1408]ENERGY: 1270000 3566.0445 5677.4739 5935.5859
364.1915 -15219.4859 -1838.9355 0.0000 0.0000
8564.3551 7049.2296 319.4914 -1515.1256 7118.5424
320.0941 -13.5412 -42.9238 664265.7895 -19.0537
-19.0639
[1444]ENERGY: 1280000 3436.6314 5743.6115 5856.7717
355.2969 -15209.8004 -1806.1872 0.0000 0.0000
8667.6661 7043.9899 323.3454 -1623.6761 7118.4317
320.1054 64.9006 44.8133 664265.7895 -23.3793
-23.3808
[1494]ENERGY: 1290000 3563.5740 5725.2614 5941.0141
352.4272 -15275.7057 -1785.4317 0.0000 0.0000
8531.7639 7052.9032 318.2756 -1478.8608 7120.7389
320.2454 -17.5710 -47.9249 664265.7895 -19.6110
-19.6147
[1533]ENERGY: 1300000 3532.4835 5768.8007 5883.5067
381.5877 -15239.3193 -1822.1533 0.0000 0.0000
8546.8944 7051.8004 318.8401 -1495.0940 7120.0624
320.0627 89.7438 67.2490 664265.7895 -10.1712
-10.1695
[1583]ENERGY: 1310000 3517.7578 5697.9903 5917.9903
368.5535 -15100.4036 -1905.7042 0.0000 0.0000
8552.0664 7048.2505 319.0330 -1503.8159 7121.0588
320.1531 -162.8877 -192.3068 664265.7895 -18.3914
-18.3890
[1619]ENERGY: 1320000 3488.0742 5747.7218 5819.2086
344.7252 -15264.4300 -1775.9468 0.0000 0.0000
8686.5690 7045.9220 324.0506 -1640.6469 7118.2550
320.5028 30.7346 101.8870 664265.7895 -24.8245
-24.8134
[1669]ENERGY: 1330000 3594.6184 5803.3080 5878.4078
356.3332 -15275.2269 -1821.0281 0.0000 0.0000
8516.2588 7052.6712 317.6972 -1463.5876 7121.8025
320.4080 -25.8492 -50.0525 664265.7895 -23.1547
-23.1575
[1705]ENERGY: 1340000 3538.1865 5698.0571 5911.4366
358.3058 -15204.9775 -1828.0174 0.0000 0.0000
8577.1426 7050.1337 319.9685 -1527.0088 7120.1359
320.6866 -207.5441 -140.7946 664265.7895 -23.6114
-23.6306
[1755]ENERGY: 1350000 3591.2585 5647.7553 5912.9134
363.7485 -15235.9268 -1804.2950 0.0000 0.0000
8580.6073 7056.0611 320.0977 -1524.5462 7121.7178
320.6344 -85.3739 -47.9436 664265.7895 -19.1986
-19.1970
[1791]ENERGY: 1360000 3557.8690 5664.0475 5932.5978

361.4980 -15180.2751 -1875.8412 0.0000 0.0000
8593.2935 7053.1896 320.5710 -1540.1039 7121.8662
320.5870 -87.8386 -37.4284 664265.7895 -17.9577
-17.9418
[1841]ENERGY: 1370000 3526.6662 5809.6324 5852.3374
342.5855 -15279.4332 -1819.9555 0.0000 0.0000
8619.4579 7051.2907 321.5470 -1568.1673 7120.9808
320.5687 -106.2685 -101.4249 664265.7895 -17.3634
-17.3644
[1877]ENERGY: 1380000 3501.3534 5738.6762 5892.9504
350.1227 -15219.8459 -1741.8603 0.0000 0.0000
8533.8842 7055.2807 318.3547 -1478.6035 7122.3028
320.5362 37.6860 59.9820 664265.7895 -21.1874
-21.1869
[1927]ENERGY: 1390000 3577.5940 5718.6635 5865.3023
321.9701 -15131.5939 -1884.1892 0.0000 0.0000
8589.3369 7057.0837 320.4234 -1532.2532 7124.9600
320.5245 69.0103 -99.1834 664265.7895 -19.3684
-19.3788
[1966]ENERGY: 1400000 3522.6388 5683.2454 5919.6151
356.3209 -15232.7997 -1788.5378 0.0000 0.0000
8592.5576 7053.0403 320.5435 -1539.5173 7123.3037
320.4654 193.4962 170.5582 664265.7895 -16.5867
-16.5771
[2016]ENERGY: 1410000 3610.8563 5716.8469 5923.5734
359.3075 -15243.1856 -1845.2000 0.0000 0.0000
8534.0485 7056.2470 318.3608 -1477.8015 7124.4065
320.6297 -18.5118 -39.5433 664265.7895 -19.1129
-19.1149
[2052]ENERGY: 1420000 3540.1689 5854.3447 5882.1188
327.2890 -15273.7161 -1881.1867 0.0000 0.0000
8603.7621 7052.7808 320.9615 -1550.9813 7124.0079
320.9556 -183.2567 -219.4508 664265.7895 -16.2450
-16.2412
[2102]ENERGY: 1430000 3572.6490 5735.0638 5857.5870
366.2123 -15217.6753 -1833.9352 0.0000 0.0000
8575.8890 7055.7906 319.9217 -1520.0984 7125.0419
320.6456 49.5867 6.3094 664265.7895 -20.5994
-20.6104
[2138]ENERGY: 1440000 3533.9785 5702.3843 5867.9825
360.4956 -15271.8578 -1802.7951 0.0000 0.0000
8663.8794 7054.0672 323.2042 -1609.8121 7124.8956
320.6471 -134.7325 -57.0019 664265.7895 -11.9038
-11.8931
[2188]ENERGY: 1450000 3384.9111 5691.1033 5934.8058
362.1186 -15175.1667 -1813.1426 0.0000 0.0000
8670.0800 7054.7096 323.4355 -1615.3705 7124.4236
320.6569 128.9118 136.2106 664265.7895 -10.5710
-10.5578
[2224]ENERGY: 1460000 3569.3994 5706.4514 5885.9650
346.0225 -15258.0911 -1787.2905 0.0000 0.0000
8591.7325 7054.1892 320.5127 -1537.5433 7124.7868
320.7066 -26.2689 8.9060 664265.7895 -16.1120
-16.1270

[2274]ENERGY: 1470000 3538.8208 5699.9593 5909.4533
377.4529 -15273.6499 -1814.1781 0.0000 0.0000
8615.0909 7052.9492 321.3841 -1562.1418 7123.3740
320.6406 -33.1178 -50.6229 664265.7895 -17.9998
-18.0119
[2310]ENERGY: 1480000 3603.0117 5770.9225 5835.3733
342.1215 -15196.9649 -1767.7784 0.0000 0.0000
8471.9819 7058.6676 316.0455 -1413.3142 7123.3067
320.4948 -42.1490 -17.7973 664265.7895 -22.2224
-22.2062
[2360]ENERGY: 1490000 3539.1244 5727.2972 5901.3946
360.8847 -15235.8757 -1863.2877 0.0000 0.0000
8624.9027 7054.4402 321.7501 -1570.4625 7123.7768
320.9066 -50.5853 -86.9255 664265.7895 -17.3216
-17.3509
[2399]ENERGY: 1500000 3467.0605 5678.8655 5917.8883
374.5596 -15224.5719 -1755.6627 0.0000 0.0000
8595.0954 7053.2346 320.6382 -1541.8608 7122.9940
320.9812 168.4652 142.5561 664265.7895 -20.0361
-20.0281
[2449]ENERGY: 1510000 3572.3070 5744.5193 5835.0554
344.8028 -15237.8430 -1874.0244 0.0000 0.0000
8673.8873 7058.7044 323.5775 -1615.1830 7125.3837
320.8387 -26.1315 -61.7735 664265.7895 -22.2211
-22.2124
[2485]ENERGY: 1520000 3484.8290 5725.1753 5871.2173
364.4997 -15139.3312 -1792.9754 0.0000 0.0000
8547.3505 7060.7653 318.8571 -1486.5852 7125.8791
321.0702 39.8936 -49.5388 664265.7895 -16.4296
-16.4336
[2535]ENERGY: 1530000 3510.9318 5779.7883 6003.8147
363.9789 -15339.2115 -1780.0314 0.0000 0.0000
8515.5289 7054.7997 317.6700 -1460.7292 7125.6287
320.8877 73.2589 -8.1432 664265.7895 -13.8150
-13.8220
[2571]ENERGY: 1540000 3511.5519 5733.3232 5855.5768
366.9196 -15263.3373 -1756.8107 0.0000 0.0000
8606.6633 7053.8869 321.0697 -1552.7764 7123.9293
320.9415 44.1092 6.1685 664265.7895 -7.7698
-7.7494
[2621]ENERGY: 1550000 3586.4640 5768.8501 5885.6667
363.5462 -15220.4880 -1852.1192 0.0000 0.0000
8527.8603 7059.7801 318.1300 -1468.0802 7123.1574
321.0507 -98.6470 -110.8486 664265.7895 -11.0575
-11.0551
[2657]ENERGY: 1560000 3497.9171 5679.5647 5796.3758
341.5850 -15190.0210 -1800.9655 0.0000 0.0000
8728.0484 7052.5044 325.5980 -1675.5439 7123.8818
320.9293 -77.9328 -46.4855 664265.7895 -12.5744
-12.5803
[2707]ENERGY: 1570000 3556.7884 5690.7268 5874.3789
374.6618 -15218.1014 -1801.9667 0.0000 0.0000
8578.1273 7054.6151 320.0052 -1523.5122 7123.3400
320.7110 44.5977 73.4039 664265.7895 -17.9183

-17.9303
[2743]ENERGY: 1580000 3569.9994 5786.2069 5884.2976
336.9465 -15204.8864 -1862.1868 0.0000 0.0000
8546.3255 7056.7027 318.8188 -1489.6228 7124.8890
320.9150 -56.6500 -80.8053 664265.7895 -15.1429
-15.1321
[2793]ENERGY: 1590000 3505.1245 5688.6357 5933.5425
358.0735 -15147.4637 -1884.4668 0.0000 0.0000
8597.6487 7051.0943 320.7334 -1546.5543 7124.2144
320.6200 -28.9193 -74.1860 664265.7895 -28.4350
-28.4465
[2832]ENERGY: 1600000 3534.2014 5632.3973 5924.2474
367.6179 -15262.4557 -1812.2808 0.0000 0.0000
8667.7782 7051.5057 323.3496 -1616.2726 7123.7313
320.9676 0.1533 45.8452 664265.7895 -19.4354
-19.4172
[2882]ENERGY: 1610000 3563.0825 5796.3932 5844.4506
351.8791 -15287.3288 -1769.4588 0.0000 0.0000
8554.8076 7053.8254 319.1353 -1500.9821 7124.0220
320.6292 105.9901 117.6842 664265.7895 -25.0123
-25.0205
[2918]ENERGY: 1620000 3555.6325 5706.4035 5856.0266
352.5595 -15152.8549 -1843.2698 0.0000 0.0000
8583.5486 7058.0460 320.2074 -1525.5026 7124.7308
320.7556 -187.1543 -104.2606 664265.7895 -24.0846
-24.0710
[2968]ENERGY: 1630000 3546.6443 5767.6694 5902.3954
369.5883 -15240.5915 -1856.2801 0.0000 0.0000
8568.5649 7057.9907 319.6485 -1510.5742 7125.2576
320.8774 203.4181 192.7973 664265.7895 -15.4639
-15.4761
[3004]ENERGY: 1640000 3553.0686 5709.5954 5846.3004
360.6043 -15199.7129 -1783.8971 0.0000 0.0000
8575.2929 7061.2517 319.8995 -1514.0412 7125.7847
320.8750 -63.4268 -62.2007 664265.7895 -23.5489
-23.5409
[3054]ENERGY: 1650000 3580.8847 5691.2822 5916.5993
354.7852 -15206.3225 -1808.9720 0.0000 0.0000
8530.1436 7058.4004 318.2152 -1471.7433 7125.3031
320.7165 -101.8751 -25.9588 664265.7895 -23.7836
-23.7863
[3090]ENERGY: 1660000 3517.0288 5772.2394 5918.1499
366.7265 -15292.5417 -1810.1154 0.0000 0.0000
8583.5823 7055.0698 320.2087 -1528.5125 7126.2213
320.9257 21.0045 -13.1698 664265.7895 -24.4310
-24.4461
[3140]ENERGY: 1670000 3596.1439 5728.2310 5888.5654
366.9125 -15262.3765 -1850.5683 0.0000 0.0000
8594.6236 7061.5316 320.6206 -1533.0920 7126.3182
320.7597 -30.4886 -105.4642 664265.7895 -22.8140
-22.8008
[3176]ENERGY: 1680000 3510.4375 5755.6320 5942.6844
364.0425 -15199.2449 -1832.4458 0.0000 0.0000
8520.0559 7061.1616 317.8388 -1458.8942 7128.0043

320.6904 187.8388 162.6133 664265.7895 -18.1712
-18.1708
[3226]ENERGY: 1690000 3517.7988 5715.8724 5903.7202
365.7676 -15133.5572 -1908.5361 0.0000 0.0000
8597.1106 7058.1763 320.7134 -1538.9343 7127.7922
320.7427 -115.8712 -50.8329 664265.7895 -20.0808
-20.0901
[3265]ENERGY: 1700000 3580.4542 5832.4632 5868.7378
345.6558 -15257.7884 -1798.2833 0.0000 0.0000
8487.0599 7058.2992 316.6079 -1428.7607 7126.8085
320.6998 -41.3140 -90.3796 664265.7895 -20.1754
-20.1692
[3315]ENERGY: 1710000 3539.8063 5727.4966 5904.3024
371.3663 -15287.1054 -1847.9538 0.0000 0.0000
8648.0623 7055.9746 322.6141 -1592.0876 7127.4371
320.8329 -39.2390 -94.2939 664265.7895 -21.9906
-21.9946
[3351]ENERGY: 1720000 3567.4332 5802.8814 5849.1987
342.6554 -15385.7960 -1763.5254 0.0000 0.0000
8644.2238 7057.0711 322.4709 -1587.1527 7126.5909
321.0492 -187.5685 -147.7950 664265.7895 -19.0296
-19.0242
[3401]ENERGY: 1730000 3542.8514 5813.1807 5868.6133
346.5503 -15234.5358 -1839.9186 0.0000 0.0000
8563.5270 7060.2684 319.4605 -1503.2587 7125.8359
320.9475 -115.1168 -114.0398 664265.7895 -17.8281
-17.8305
[3437]ENERGY: 1740000 3478.1048 5807.6352 5865.3370
344.7425 -15250.8674 -1755.4485 0.0000 0.0000
8568.3516 7057.8553 319.6405 -1510.4963 7127.0550
320.7319 276.0430 181.2160 664265.7895 -6.9634
-6.9764
[3487]ENERGY: 1750000 3546.9946 5657.7823 5953.0884
365.2126 -15284.7003 -1811.7159 0.0000 0.0000
8629.4976 7056.1592 321.9215 -1573.3383 7126.7558
320.8121 -74.4845 -50.5367 664265.7895 -6.0079
-6.0025
[3523]ENERGY: 1760000 3560.8836 5810.8139 5855.0471
324.4383 -15310.3851 -1748.8371 0.0000 0.0000
8567.5308 7059.4914 319.6099 -1508.0394 7127.2732
320.5860 -16.7125 56.3957 664265.7895 -8.9510
-8.9372
[3573]ENERGY: 1770000 3532.0681 5691.2302 5803.7424
365.7019 -15103.6959 -1807.3008 0.0000 0.0000
8576.9192 7058.6651 319.9601 -1518.2541 7127.2157
320.8777 146.8273 76.7069 664265.7895 -14.0124
-14.0240
[3609]ENERGY: 1780000 3534.9116 5650.1121 5877.8978
378.3965 -15206.4330 -1823.9568 0.0000 0.0000
8649.6877 7060.6159 322.6747 -1589.0717 7128.5947
320.9126 84.6182 25.8069 664265.7895 -11.7932
-11.7861
[3659]ENERGY: 1790000 3518.1780 5710.3934 5865.6433
352.2544 -15129.2754 -1806.7327 0.0000 0.0000

8553.2904 7063.7514 319.0787 -1489.5390 7129.8817
320.7528 22.5024 -29.0172 664265.7895 -10.8239
-10.8237
[3698]ENERGY: 1800000 3504.0011 5673.6773 5874.6380
346.2247 -15208.8084 -1766.1373 0.0000 0.0000
8638.1016 7061.6971 322.2425 -1576.4045 7128.8192
320.7407 -12.1221 50.7539 664265.7895 -22.2380
-22.2462
[3748]ENERGY: 1810000 3525.1463 5830.9255 5907.8384
359.9465 -15217.0247 -1864.7758 0.0000 0.0000
8520.8656 7062.9217 317.8691 -1457.9439 7128.3576
320.6090 -151.3520 -66.7500 664265.7895 -23.2907
-23.2775
[3784]ENERGY: 1820000 3461.9605 5769.5502 5905.5114
336.6158 -15208.9008 -1792.7136 0.0000 0.0000
8590.2652 7062.2887 320.4580 -1527.9765 7128.3754
320.5949 -25.1123 -21.1450 664265.7895 -18.6792
-18.6774
[3834]ENERGY: 1830000 3577.6788 5735.0053 5909.5368
348.3919 -15324.3669 -1841.1401 0.0000 0.0000
8653.3291 7058.4349 322.8106 -1594.8942 7129.3411
320.7723 -94.7614 -127.3462 664265.7895 -15.3844
-15.3842
[3870]ENERGY: 1840000 3553.5626 5729.5311 5847.2998
356.4731 -15170.2208 -1778.7545 0.0000 0.0000
8523.0346 7060.9259 317.9500 -1462.1087 7129.7056
320.7840 102.5097 17.3514 664265.7895 -15.7745
-15.7910
[3920]ENERGY: 1850000 3515.3080 5704.9909 5900.2751
342.2101 -15216.5556 -1773.7173 0.0000 0.0000
8590.1942 7062.7054 320.4553 -1527.4888 7129.7077
321.0174 157.5389 96.8668 664265.7895 -17.4378
-17.4450
[3956]ENERGY: 1860000 3642.9791 5740.6116 5890.0811
356.7854 -15279.9234 -1786.7575 0.0000 0.0000
8506.2490 7070.0252 317.3238 -1436.2238 7137.3528
320.7976 45.8658 -35.3535 664265.7895 -20.6162
-20.5971
[4006]ENERGY: 1870000 3644.7890 5791.7224 5864.7634
355.2268 -15383.3134 -1764.6827 0.0000 0.0000
8560.6777 7069.1832 319.3542 -1491.4945 7135.8695
321.0046 29.2069 -29.7735 664265.7895 -20.2030
-20.2174
[4042]ENERGY: 1880000 3554.7070 5724.2863 5917.8285
351.3205 -15242.0185 -1840.2856 0.0000 0.0000
8600.6016 7066.4399 320.8436 -1534.1618 7138.1031
320.8902 -113.7618 -131.6534 664265.7895 -20.7617
-20.7445
[4092]ENERGY: 1890000 3540.9236 5772.1898 5852.1863
342.0886 -15266.9948 -1784.8423 0.0000 0.0000
8610.4511 7066.0022 321.2110 -1544.4489 7137.7338
320.9587 12.8391 -23.5590 664265.7895 -20.3234
-20.3235
[4131]ENERGY: 1900000 3579.7381 5804.3430 5857.2177

370.3299 -15336.8533 -1761.5790 0.0000 0.0000
8557.7530 7070.9492 319.2451 -1486.8037 7136.1428
321.3285 -86.0027 -20.5398 664265.7895 -24.1835
-24.1820
[4181]ENERGY: 1910000 3544.9520 5667.7862 5915.7637
390.8187 -15302.3953 -1815.2156 0.0000 0.0000
8667.3127 7069.0223 323.3322 -1598.2904 7137.2686
321.7332 79.1529 12.9575 664265.7895 -19.6344
-19.6342
[4217]ENERGY: 1920000 3536.9547 5699.7059 5853.8143
368.6976 -15303.4930 -1802.9679 0.0000 0.0000
8712.9974 7065.7090 325.0365 -1647.2884 7136.8581
321.6571 123.5753 94.3713 664265.7895 -18.8664
-18.8778
[4267]ENERGY: 1930000 3514.6106 5714.6918 5904.2631
383.7208 -15255.8171 -1763.4939 0.0000 0.0000
8570.3581 7068.3334 319.7154 -1502.0247 7138.7888
321.7733 15.4487 11.1828 664265.7895 -17.7119
-17.7137
[4303]ENERGY: 1940000 3575.1932 5747.9427 5933.0975
347.7090 -15301.8469 -1840.4962 0.0000 0.0000
8608.2010 7069.8004 321.1271 -1538.4006 7138.3295
321.7670 97.6841 -2.2737 664265.7895 -17.6806
-17.6710
[4353]ENERGY: 1950000 3548.9027 5751.7761 5860.0521
351.7461 -15168.2519 -1884.3374 0.0000 0.0000
8609.3120 7069.1998 321.1685 -1540.1122 7138.6095
321.7738 -48.2513 -121.3822 664265.7895 -19.7105
-19.7196
[4389]ENERGY: 1960000 3614.5183 5729.2590 5855.2577
357.1529 -15244.5695 -1828.6512 0.0000 0.0000
8585.0535 7068.0207 320.2636 -1517.0328 7138.7571
321.9434 -14.2616 -55.4968 664265.7895 -18.5720
-18.5694
[4439]ENERGY: 1970000 3571.4645 5768.4593 5860.5260
337.2951 -15378.3267 -1773.4443 0.0000 0.0000
8682.9699 7068.9439 323.9163 -1614.0260 7138.7150
322.1725 17.5115 54.5618 664265.7895 -21.5969
-21.5945
[4475]ENERGY: 1980000 3552.7870 5804.0497 5926.6925
355.6712 -15399.4172 -1745.2817 0.0000 0.0000
8575.6679 7070.1695 319.9134 -1505.4984 7138.5889
321.9278 101.1926 105.7105 664265.7895 -19.6178
-19.6066
[4525]ENERGY: 1990000 3553.2714 5667.3171 5890.0727
361.5728 -15266.5307 -1772.3105 0.0000 0.0000
8636.8400 7070.2330 322.1955 -1566.6071 7137.6714
321.9946 -82.5980 -75.7442 664265.7895 -23.9068
-23.9277
[4564]ENERGY: 2000000 3491.0399 5779.1320 5898.5485
375.2215 -15246.1816 -1906.2353 0.0000 0.0000
8677.9368 7069.4619 323.7286 -1608.4749 7139.2229
322.1122 -20.7182 -19.5589 664265.7895 -17.2602
-17.2412

[4614]ENERGY: 2010000 3535.5320 5738.4869 5924.3877
390.7110 -15301.1927 -1876.4113 0.0000 0.0000
8660.2508 7071.7643 323.0688 -1588.4864 7140.0552
321.7860 4.2832 -43.5940 664265.7895 -19.2526
-19.2466
[4650]ENERGY: 2020000 3479.4844 5715.6702 5908.1341
351.9176 -15252.6863 -1943.2581 0.0000 0.0000
8806.8831 7066.1449 328.5389 -1740.7381 7141.5186
321.9165 -13.5204 -86.1575 664265.7895 -19.0974
-19.1224
[4700]ENERGY: 2030000 3524.9122 5774.0348 5925.2775
356.9382 -15308.3201 -1887.1825 0.0000 0.0000
8683.6191 7069.2793 323.9405 -1614.3399 7141.2017
322.0708 -70.4117 -79.7124 664265.7895 -20.6885
-20.6743
[4736]ENERGY: 2040000 3549.4555 5760.7878 5895.9742
365.4635 -15195.9830 -1785.4497 0.0000 0.0000
8484.6509 7074.8992 316.5181 -1409.7517 7142.2709
321.7754 -23.2608 14.4460 664265.7895 -17.6311
-17.6453
[4786]ENERGY: 2050000 3550.9264 5810.8036 5982.6864
343.1244 -15327.7549 -1856.0238 0.0000 0.0000
8569.5054 7073.2676 319.6836 -1496.2378 7140.8783
322.0249 63.7656 -8.1664 664265.7895 -14.9185
-14.9138
[4822]ENERGY: 2060000 3569.4139 5816.6203 5994.9509
379.8467 -15365.9028 -1803.9836 0.0000 0.0000
8485.9683 7076.9137 316.5672 -1409.0546 7142.0284
321.9530 130.1527 70.3092 664265.7895 -12.3667
-12.3719
[4872]ENERGY: 2070000 3484.2434 5786.6766 5892.1601
359.1687 -15317.5261 -1841.8265 0.0000 0.0000
8709.2138 7072.1099 324.8953 -1637.1038 7141.7236
322.1464 -24.2822 -54.9627 664265.7895 -9.7433
-9.7366
[4908]ENERGY: 2080000 3510.9463 5672.2698 5899.6486
360.7632 -15198.9237 -1813.5085 0.0000 0.0000
8639.2852 7070.4808 322.2867 -1568.8043 7142.2886
322.3692 122.2162 42.4251 664265.7895 -15.8579
-15.8556
[4958]ENERGY: 2090000 3546.2478 5766.3004 5930.9156
379.4140 -15316.0220 -1831.7172 0.0000 0.0000
8600.2517 7075.3902 320.8305 -1524.8615 7142.5464
322.1362 -62.3721 -50.0295 664265.7895 -9.4167
-9.4036
[4997]ENERGY: 2100000 3514.7100 5769.7202 5867.6422
337.9216 -15231.9614 -1875.8224 0.0000 0.0000
8688.3708 7070.5811 324.1178 -1617.7898 7142.0126
322.0192 51.4719 -50.4641 664265.7895 -11.7831
-11.7969
[5047]ENERGY: 2110000 3531.1515 5801.9748 5843.6574
343.6973 -15302.2628 -1828.2457 0.0000 0.0000
8679.7906 7069.7632 323.7977 -1610.0275 7141.6300
322.3093 103.8527 53.6366 664265.7895 -12.7766

-12.7791
[5083]ENERGY: 2120000 3559.6266 5742.1567 5883.9183
354.0508 -15357.6479 -1831.2564 0.0000 0.0000
8719.7813 7070.6294 325.2896 -1649.1519 7141.9752
322.5334 -27.3822 -85.7975 664265.7895 -7.3739
-7.3678
[5133]ENERGY: 2130000 3603.2348 5770.3651 5869.1604
376.5730 -15336.4893 -1794.3683 0.0000 0.0000
8582.1324 7070.6081 320.1546 -1511.5243 7140.0234
322.5065 50.9411 64.6332 664265.7895 -9.2739
-9.2855
[5169]ENERGY: 2140000 3514.1505 5792.6904 5865.8883
326.6960 -15251.7867 -1833.1277 0.0000 0.0000
8658.5258 7073.0366 323.0044 -1585.4891 7142.2533
322.5625 168.1621 151.0764 664265.7895 -5.8980
-5.8945
[5219]ENERGY: 2150000 3535.8962 5844.2683 5930.6256
359.1495 -15366.5638 -1836.8236 0.0000 0.0000
8603.8729 7070.4252 320.9656 -1533.4477 7141.8073
322.6224 41.1765 12.8018 664265.7895 -11.9775
-11.9772
[5255]ENERGY: 2160000 3585.4177 5798.2838 5922.7715
355.9203 -15362.3728 -1830.3522 0.0000 0.0000
8602.7734 7072.4417 320.9246 -1530.3317 7142.2556
322.7256 44.8106 115.3353 664265.7895 -11.4883
-11.4698
[5305]ENERGY: 2170000 3564.9158 5792.0942 5911.5633
361.2896 -15352.0898 -1781.4447 0.0000 0.0000
8579.4141 7075.7426 320.0532 -1503.6716 7141.7870
322.6563 -8.3410 -25.0713 664265.7895 -12.0351
-12.0413
[5341]ENERGY: 2180000 3501.2643 5781.0418 5865.8597
376.3443 -15304.9279 -1802.8037 0.0000 0.0000
8654.0835 7070.8620 322.8387 -1583.2216 7141.8360
322.7339 150.9758 34.4981 664265.7895 -10.0360
-10.0471
[5391]ENERGY: 2190000 3512.0948 5904.7039 5880.3484
373.4913 -15352.5590 -1833.6505 0.0000 0.0000
8587.4106 7071.8395 320.3515 -1515.5711 7142.4886
322.7051 -126.3361 -117.2109 664265.7895 -10.7149
-10.7127
[5430]ENERGY: 2200000 3597.9321 5719.1059 5910.5386
355.2131 -15237.2851 -1881.1541 0.0000 0.0000
8610.9413 7075.2917 321.2293 -1535.6496 7143.3649
322.5422 -204.6709 -123.8520 664265.7895 -13.0212
-13.0106
[5480]ENERGY: 2210000 3595.5159 5744.2006 5918.5423
350.6837 -15316.6657 -1803.7736 0.0000 0.0000
8587.7366 7076.2397 320.3637 -1511.4969 7141.7411
322.5588 39.8617 52.8217 664265.7895 -13.7870
-13.8081
[5516]ENERGY: 2220000 3581.7055 5681.3398 5932.7085
357.0865 -15241.4017 -1803.2189 0.0000 0.0000
8567.0375 7075.2573 319.5915 -1491.7803 7141.6350

322.5047 -20.1337 -35.4608 664265.7895 -13.7064
-13.6965
[5566]ENERGY: 2230000 3457.7666 5899.4150 5885.6214
360.7392 -15233.8363 -1872.5368 0.0000 0.0000
8575.6012 7072.7703 319.9110 -1502.8309 7143.0851
322.3400 -135.3248 -82.2538 664265.7895 -15.8307
-15.8177
[5602]ENERGY: 2240000 3446.3232 5851.4476 5928.8868
387.3443 -15284.2802 -1867.3085 0.0000 0.0000
8612.7011 7075.1142 321.2950 -1537.5869 7143.5878
322.3895 44.7967 89.6688 664265.7895 -12.6047
-12.6107
[5652]ENERGY: 2250000 3534.5439 5704.5087 5941.9054
348.8570 -15337.2183 -1791.2575 0.0000 0.0000
8671.7059 7073.0450 323.4961 -1598.6608 7142.3756
322.5530 111.6971 62.2636 664265.7895 -15.3640
-15.3744
[5688]ENERGY: 2260000 3536.1869 5768.2512 5922.6429
358.1238 -15279.9173 -1882.7462 0.0000 0.0000
8653.5679 7076.1094 322.8195 -1577.4585 7143.2110
322.5874 -145.3270 -126.0847 664265.7895 -11.2491
-11.2439
[5738]ENERGY: 2270000 3556.0374 5857.8220 5930.4400
361.8937 -15425.6381 -1767.1402 0.0000 0.0000
8562.0329 7075.4478 319.4048 -1486.5852 7144.0103
322.5849 104.0690 20.2120 664265.7895 -15.5956
-15.5928
[5774]ENERGY: 2280000 3526.6858 5803.8885 5919.0113
382.2429 -15327.9998 -1810.4190 0.0000 0.0000
8585.0640 7078.4737 320.2640 -1506.5903 7145.1517
322.5588 -15.6975 -58.2367 664265.7895 -12.9643
-12.9655
[5824]ENERGY: 2290000 3476.6880 5787.2658 5877.2625
385.9672 -15297.0865 -1786.4877 0.0000 0.0000
8631.1793 7074.7885 321.9843 -1556.3908 7145.5123
322.4567 195.7629 164.9340 664265.7895 -15.6962
-15.6806
[5863]ENERGY: 2300000 3474.0434 5748.0276 5904.5171
376.0372 -15254.4891 -1859.0079 0.0000 0.0000
8688.1785 7077.3069 324.1106 -1610.8717 7146.3950
322.4145 -23.0258 -34.4106 664265.7895 -10.9751
-11.0003
[5913]ENERGY: 2310000 3549.6984 5690.9150 5940.3414
377.7863 -15350.3854 -1805.9312 0.0000 0.0000
8674.7356 7077.1601 323.6091 -1597.5754 7147.0799
322.4319 165.6932 101.2325 664265.7895 -9.8493
-9.8511
[5949]ENERGY: 2320000 3463.8906 5883.7617 5882.3792
342.1725 -15310.8870 -1878.5664 0.0000 0.0000
8693.1132 7075.8639 324.2947 -1617.2493 7146.9632
322.3756 -30.9121 -55.0295 664265.7895 -11.5764
-11.5814
[5999]ENERGY: 2330000 3595.6495 5860.8236 5941.4837
361.8429 -15375.3642 -1784.2816 0.0000 0.0000

8478.8205 7078.9743 316.3006 -1399.8462 7145.5897
322.4518 67.2164 17.0546 664265.7895 -14.4726
-14.4621
[6035]ENERGY: 2340000 3480.4426 5737.0114 5898.9237
370.6019 -15242.0978 -1857.0644 0.0000 0.0000
8686.0186 7073.8361 324.0301 -1612.1826 7146.5422
322.4604 -49.2022 -27.9823 664265.7895 -15.4813
-15.4624
[6085]ENERGY: 2350000 3469.7188 5697.4864 5855.2164
328.8566 -15243.1410 -1737.2333 0.0000 0.0000
8705.1858 7076.0896 324.7451 -1629.0962 7146.7318
322.6726 90.4328 53.8621 664265.7895 -20.6148
-20.6439
[6121]ENERGY: 2360000 3503.6429 5756.7516 5970.6691
375.3012 -15304.9062 -1851.9967 0.0000 0.0000
8628.8885 7078.3504 321.8988 -1550.5381 7146.7121
322.5610 114.5278 160.5414 664265.7895 -18.7316
-18.7202
[6171]ENERGY: 2370000 3559.9238 5710.6363 5946.4017
374.1081 -15188.4520 -1881.2464 0.0000 0.0000
8561.3470 7082.7185 319.3792 -1478.6285 7147.3257
322.4706 -195.5355 -176.9319 664265.7895 -17.4291
-17.4240
[6207]ENERGY: 2380000 3518.9092 5607.0707 5951.2599
339.1406 -15255.7047 -1860.2161 0.0000 0.0000
8774.8246 7075.2843 327.3429 -1699.5404 7148.2371
322.6236 -87.7240 -77.9178 664265.7895 -14.8343
-14.8140
[6257]ENERGY: 2390000 3531.2487 5819.4623 5892.4492
364.1224 -15382.5920 -1859.0964 0.0000 0.0000
8711.8332 7077.4273 324.9931 -1634.4058 7148.1886
322.5885 38.6202 7.6854 664265.7895 -8.7536
-8.7815
[6296]ENERGY: 2400000 3633.4618 5734.2903 5930.6116
355.6751 -15433.7562 -1793.2365 0.0000 0.0000
8655.0989 7082.1449 322.8766 -1572.9539 7147.5471
322.6678 -79.9643 -112.4902 664265.7895 -13.0246
-13.0110
[6346]ENERGY: 2410000 3493.5693 5699.3607 5898.6536
352.3204 -15321.5153 -1720.1157 0.0000 0.0000
8678.2651 7080.5382 323.7408 -1597.7270 7147.5116
322.5567 30.3805 43.5002 664265.7895 -3.2945
-3.3047
[6382]ENERGY: 2420000 3444.2583 5688.2321 5913.1838
364.1471 -15189.0802 -1824.3571 0.0000 0.0000
8680.6919 7077.0759 323.8313 -1603.6161 7147.8786
322.6218 264.9288 184.7593 664265.7895 -6.4107
-6.4124
[6432]ENERGY: 2430000 3595.5593 5794.2278 5936.2096
360.1667 -15396.4632 -1831.2045 0.0000 0.0000
8619.2429 7077.7385 321.5390 -1541.5044 7147.2357
322.9440 -34.0515 117.7727 664265.7895 -0.6410
-0.6146
[6468]ENERGY: 2440000 3539.1152 5757.2410 5858.9427

350.9174 -15418.3227 -1759.8075 0.0000 0.0000
8750.4634 7078.5496 326.4342 -1671.9138 7147.8216
323.0687 -5.2957 52.6081 664265.7895 -3.4082
-3.4151
[6518]ENERGY: 2450000 3592.7935 5908.6566 5883.6811
357.4330 -15345.9709 -1872.8831 0.0000 0.0000
8558.6347 7082.3448 319.2780 -1476.2899 7149.3956
323.0382 72.7014 9.6706 664265.7895 1.6616
1.6477
[6554]ENERGY: 2460000 3495.8189 5834.9847 5873.2460
383.5204 -15321.8445 -1894.7946 0.0000 0.0000
8711.1291 7082.0600 324.9668 -1629.0691 7149.5377
322.9277 -136.8502 -100.7458 664265.7895 -2.4721
-2.4648
[6604]ENERGY: 2470000 3494.7656 5848.6591 5919.0952
375.9658 -15346.2421 -1861.5492 0.0000 0.0000
8649.6348 7080.3293 322.6728 -1569.3055 7149.9134
323.0305 82.6289 70.3645 664265.7895 -1.7212
-1.7342
[6640]ENERGY: 2480000 3511.1017 5787.7003 5927.8296
356.2559 -15398.6159 -1779.8592 0.0000 0.0000
8672.6371 7077.0494 323.5309 -1595.5877 7150.3216
323.1217 172.1248 171.6337 664265.7895 2.8200
2.8164
[6690]ENERGY: 2490000 3514.6078 5688.5326 5941.6849
367.0679 -15284.9135 -1794.1618 0.0000 0.0000
8645.5971 7078.4151 322.5221 -1567.1821 7151.1799
322.9407 84.1556 147.3420 664265.7895 -1.2225
-1.2164
[6729]ENERGY: 2500000 3560.4506 5847.4570 5932.5751
389.4061 -15381.4995 -1886.4686 0.0000 0.0000
8618.7014 7080.6222 321.5188 -1538.0792 7151.5221
322.9367 38.7263 -10.2217 664265.7895 -3.8841
-3.8778
[6779]ENERGY: 2510000 3551.9940 5734.9667 5909.9396
362.4040 -15380.5774 -1755.6907 0.0000 0.0000
8657.9240 7080.9602 322.9820 -1576.9638 7152.4895
323.0189 27.2915 7.6058 664265.7895 -1.5648
-1.5722
[6815]ENERGY: 2520000 3506.8105 5847.1780 5900.3521
323.2676 -15319.0682 -1862.5813 0.0000 0.0000
8683.0127 7078.9713 323.9179 -1604.0414 7151.3754
322.9974 12.1805 59.8880 664265.7895 0.8511
0.8614
[6865]ENERGY: 2530000 3552.8940 5834.0823 5905.9122
372.9474 -15315.6323 -1854.5856 0.0000 0.0000
8590.0106 7085.6287 320.4485 -1504.3819 7153.2643
323.0638 53.7666 29.2711 664265.7895 -2.0497
-2.0598
[6901]ENERGY: 2540000 3485.9250 5856.0714 5882.7700
349.6692 -15369.1176 -1762.6472 0.0000 0.0000
8640.8709 7083.5418 322.3458 -1557.3291 7153.0501
323.0392 10.4604 38.8528 664265.7895 -2.7421
-2.7352

[6951]ENERGY: 2550000 3603.0643 5786.9405 5906.8061
362.4698 -15344.6611 -1866.5413 0.0000 0.0000
8637.1663 7085.2446 322.2076 -1551.9217 7152.9467
323.1047 -138.3121 -134.2258 664265.7895 -1.8495
-1.8487
[6987]ENERGY: 2560000 3526.5826 5728.3898 5915.3937
365.4975 -15339.3569 -1846.8253 0.0000 0.0000
8729.3291 7079.0104 325.6457 -1650.3187 7152.6774
323.0120 3.6731 -13.8023 664265.7895 -5.7326
-5.7328
[7037]ENERGY: 2570000 3554.4398 5734.6995 5880.2795
346.2919 -15293.6516 -1838.6576 0.0000 0.0000
8696.6381 7080.0395 324.4262 -1616.5986 7152.0781
323.0839 -56.3132 -6.5146 664265.7895 -8.3351
-8.3307
[7073]ENERGY: 2580000 3603.7969 5793.9448 5889.3313
363.7158 -15295.0359 -1861.2486 0.0000 0.0000
8593.4948 7087.9992 320.5785 -1505.4957 7153.1517
323.1285 -11.2577 -12.1696 664265.7895 -1.0160
-1.0334
[7123]ENERGY: 2590000 3520.2874 5761.0121 5887.6481
367.7160 -15329.4430 -1774.5904 0.0000 0.0000
8654.6383 7087.2684 322.8594 -1567.3699 7153.2924
323.3006 201.5289 211.8551 664265.7895 -3.2604
-3.2537
[7162]ENERGY: 2600000 3596.3854 5803.4705 5945.3572
376.5700 -15302.8122 -1922.5624 0.0000 0.0000
8591.5838 7087.9922 320.5072 -1503.5916 7154.2563
323.3791 -152.6569 -207.3993 664265.7895 -4.8590
-4.8589
[7212]ENERGY: 2610000 3525.3549 5772.7696 5945.8981
370.7785 -15299.5266 -1866.2368 0.0000 0.0000
8634.6806 7083.7183 322.1149 -1550.9623 7153.8731
323.2216 -21.7304 -16.0251 664265.7895 0.7960
0.8036
[7248]ENERGY: 2620000 3565.1642 5726.7393 5945.2430
365.1905 -15345.5070 -1856.8037 0.0000 0.0000
8681.5624 7081.5887 323.8638 -1599.9737 7156.7032
323.2679 2.3533 -2.7279 664265.7895 1.6516
1.6462
[7298]ENERGY: 2630000 3608.0616 5839.4420 5827.7512
368.3787 -15461.2312 -1750.9914 0.0000 0.0000
8652.9655 7084.3763 322.7970 -1568.5891 7155.2932
323.4108 -19.3593 8.3873 664265.7895 -0.0449
-0.0394
[7334]ENERGY: 2640000 3465.0268 5717.5926 5936.4033
359.1940 -15292.7856 -1819.6771 0.0000 0.0000
8720.4033 7086.1573 325.3128 -1634.2460 7156.2951
323.3916 90.5704 30.7020 664265.7895 1.2293
1.2198
[7384]ENERGY: 2650000 3599.6444 5732.4216 5918.2016
366.4366 -15336.3079 -1874.5313 0.0000 0.0000
8679.0656 7084.9306 323.7707 -1594.1349 7157.5073
323.5966 88.0228 -83.9018 664265.7895 -0.3812

```
-0.3770
[7420]ENERGY: 2660000      3477.0477      5820.8976      5936.8264
366.2958         -15279.3754     -1816.0732         0.0000         0.0000
8581.6296           7087.2484       320.1358     -1494.3812      7158.2007
323.4809            -78.7321       -33.4208    664265.7895        -2.0219
-2.0101
[7470]ENERGY: 2670000      3532.7579      5710.5652      5891.2463
353.6004         -15311.1399     -1789.2658         0.0000         0.0000
8698.6139           7086.3780       324.4999     -1612.2359      7157.6916
323.4335             -8.7520        42.0295    664265.7895         3.3379
 3.3274
[7506]ENERGY: 2680000      3499.8892      5770.5605      5937.3603
350.0922         -15303.2808     -1829.5772         0.0000         0.0000
8663.5344           7088.5787       323.1913     -1574.9558      7159.3805
323.3243            174.9013        79.4573    664265.7895         1.3113
 1.3102
[7556]ENERGY: 2690000      3463.4402      5766.8418      5926.2306
380.3825         -15407.3989     -1712.1257         0.0000         0.0000
8668.7665           7086.1370       323.3865     -1582.6295      7158.2559
323.5614            290.5287       197.5396    664265.7895         3.9877
 3.9770
[7595]ENERGY: 2700000      3638.7077      5808.7312      5878.6213
378.5793         -15444.9315     -1829.7320         0.0000         0.0000
8660.2115           7090.1875       323.0673     -1570.0240      7159.4300
323.7622            221.0284       186.7824    664265.7895         1.4252
 1.4220
[7645]ENERGY: 2710000      3664.3235      5719.8784      5949.8340
382.0614         -15480.0610     -1785.5184         0.0000         0.0000
8638.9605           7089.4783       322.2746     -1549.4822      7159.2522
323.4992             22.0890       -40.8203    664265.7895         3.3175
 3.3341
[7681]ENERGY: 2720000      3577.0278      5797.4675      5896.5259
367.1002         -15356.1269     -1885.4287         0.0000         0.0000
8692.6546           7089.2203       324.2776     -1603.4342      7160.3965
323.5683            -55.3861      -119.3545    664265.7895        -2.2397
-2.2421
[7731]ENERGY: 2730000      3548.6411      5756.5025      5928.5041
389.4361         -15368.5230     -1838.9849         0.0000         0.0000
8674.7101           7090.2858       323.6082     -1584.4242      7160.4236
323.5938            129.9785        94.7709    664265.7895        -0.4097
-0.4062
[7767]ENERGY: 2740000      3537.4673      5753.2114      5945.7807
376.2916         -15407.8138     -1789.6469         0.0000         0.0000
8677.3993           7092.6895       323.7085     -1584.7098      7161.0002
323.5169            170.7761       105.5756    664265.7895        -0.1606
-0.1609
[7817]ENERGY: 2750000      3553.2221      5734.6051      5969.7125
388.5370         -15383.8024     -1864.6693         0.0000         0.0000
8693.2389           7090.8439       324.2994     -1602.3950      7161.5508
323.5479             72.0111       -13.0146    664265.7895         0.0846
 0.0815
[7853]ENERGY: 2760000      3582.0189      5693.4341      5924.0564
355.9392         -15345.4034     -1740.7917         0.0000         0.0000
8624.8704           7094.1241       321.7489     -1530.7464      7161.7365
```

323.4910 55.3591 99.9185 664265.7895 1.2142
1.2268
[7903]ENERGY: 2770000 3523.7877 5776.9999 5901.8521
345.7854 -15356.2597 -1818.9978 0.0000 0.0000
8719.4810 7092.6486 325.2784 -1626.8324 7160.6313
323.4236 54.6446 10.9887 664265.7895 -2.4038
-2.4025
[7939]ENERGY: 2780000 3531.0850 5821.7628 5925.6032
358.0782 -15322.1045 -1828.1636 0.0000 0.0000
8607.0174 7093.2786 321.0829 -1513.7388 7160.6882
323.4116 57.5346 -5.0731 664265.7895 0.7199
0.6819
[7989]ENERGY: 2790000 3602.5334 5859.0840 5942.1661
368.3792 -15435.6916 -1824.3044 0.0000 0.0000
8582.1460 7094.3128 320.1551 -1487.8332 7161.2508
323.4221 -6.9662 -11.5963 664265.7895 -2.4861
-2.4574
[8028]ENERGY: 2800000 3609.6996 5763.0647 5944.5700
360.3837 -15343.4601 -1874.9283 0.0000 0.0000
8634.5578 7093.8873 322.1103 -1540.6704 7160.6478
323.6376 -32.1619 -14.1151 664265.7895 -1.3699
-1.3799
[8078]ENERGY: 2810000 3619.2238 5762.2529 5927.2091
360.4306 -15403.6417 -1798.7319 0.0000 0.0000
8628.5316 7095.2745 321.8855 -1533.2572 7161.0126
323.7695 76.9355 144.1306 664265.7895 -0.1585
-0.1438
[8114]ENERGY: 2820000 3562.1568 5795.7347 5949.5453
352.6038 -15318.5531 -1888.9827 0.0000 0.0000
8643.6117 7096.1165 322.4481 -1547.4953 7161.7046
323.7051 -110.0422 -49.2501 664265.7895 3.1422
3.1452
[8164]ENERGY: 2830000 3536.3926 5818.5344 5876.6320
355.3154 -15322.9015 -1797.3907 0.0000 0.0000
8624.0802 7090.6623 321.7195 -1533.4179 7160.3026
323.7648 142.9877 113.8901 664265.7895 0.5268
0.5040
[8200]ENERGY: 2840000 3571.3825 5688.6481 5919.7258
361.5537 -15317.2484 -1880.1662 0.0000 0.0000
8744.4761 7088.3718 326.2108 -1656.1043 7162.7417
323.5923 -32.5954 -141.7161 664265.7895 -0.5523
-0.5393
[8250]ENERGY: 2850000 3625.8224 5730.5363 5932.2116
343.3048 -15372.3433 -1836.3519 0.0000 0.0000
8668.8917 7092.0714 323.3911 -1576.8202 7164.3537
323.8292 -50.3291 -132.9972 664265.7895 0.6918
0.7010
[8286]ENERGY: 2860000 3626.7479 5715.9766 5906.7210
354.2179 -15412.1249 -1798.3723 0.0000 0.0000
8700.8549 7094.0211 324.5835 -1606.8339 7162.8533
323.8677 -103.9636 -83.1442 664265.7895 -2.4970
-2.5050
[8336]ENERGY: 2870000 3559.8243 5855.3903 5911.4747
358.4798 -15421.3447 -1892.1573 0.0000 0.0000

8720.2261 7091.8931 325.3062 -1628.3330 7163.2577
323.9839 -45.2007 -31.8817 664265.7895 -0.7922
-0.7970
[8372]ENERGY: 2880000 3541.3801 5760.5409 5933.0432
359.4172 -15351.0598 -1834.9740 0.0000 0.0000
8685.1639 7093.5115 323.9982 -1591.6524 7163.7883
324.0424 -167.4098 -176.6238 664265.7895 0.4040
0.4021
[8422]ENERGY: 2890000 3493.0392 5814.9511 5939.6987
399.4358 -15401.3263 -1827.7162 0.0000 0.0000
8674.8202 7092.9025 323.6123 -1581.9177 7165.3867
323.9254 81.5249 -10.9873 664265.7895 0.7864
0.8099
[8461]ENERGY: 2900000 3600.4431 5817.5567 5923.5561
354.3877 -15365.5889 -1850.9030 0.0000 0.0000
8615.3372 7094.7889 321.3933 -1520.5483 7164.6172
323.8293 57.5855 4.3486 664265.7895 -5.0255
-5.0634
[8511]ENERGY: 2910000 3596.3626 5754.9238 5881.8379
360.2709 -15305.5848 -1904.8960 0.0000 0.0000
8709.5074 7092.4218 324.9063 -1617.0856 7163.9635
323.8333 -60.8633 -53.6727 664265.7895 -3.3371
-3.3052
[8547]ENERGY: 2920000 3503.7517 5829.4830 5872.8270
372.6395 -15299.0802 -1886.8120 0.0000 0.0000
8698.5167 7091.3256 324.4963 -1607.1911 7164.6990
323.9091 -119.3248 -68.4581 664265.7895 -4.5486
-4.5588
[8597]ENERGY: 2930000 3530.2981 5709.9370 5967.7388
340.7750 -15420.1136 -1830.1488 0.0000 0.0000
8795.9414 7094.4279 328.1307 -1701.5135 7165.5410
323.9974 0.5886 -8.4291 664265.7895 -2.8777
-2.8761
[8633]ENERGY: 2940000 3540.3831 5896.3834 5845.1430
354.7881 -15394.3113 -1885.8314 0.0000 0.0000
8741.6813 7098.2362 326.1065 -1643.4451 7166.4607
323.9515 24.8565 -74.1135 664265.7895 -6.9305
-6.9554
[8683]ENERGY: 2950000 3519.3334 5789.9646 5970.5100
361.3959 -15383.8279 -1812.6098 0.0000 0.0000
8649.8300 7094.5961 322.6800 -1555.2339 7165.3727
323.8164 41.6769 79.0932 664265.7895 -2.6029
-2.5809
[8719]ENERGY: 2960000 3580.1099 5762.2554 5864.4535
335.1529 -15318.8924 -1865.6495 0.0000 0.0000
8736.8547 7094.2844 325.9265 -1642.5702 7167.0620
323.9298 100.4491 47.3856 664265.7895 -2.1675
-2.1724
[8769]ENERGY: 2970000 3563.4940 5731.2325 5917.8927
378.3646 -15327.2938 -1852.9315 0.0000 0.0000
8685.5906 7096.3491 324.0141 -1589.2414 7168.1581
324.2116 35.6230 -58.7199 664265.7895 -7.3258
-7.3305
[8805]ENERGY: 2980000 3555.0899 5758.1951 5836.9797

346.8091 -15290.7558 -1834.7264 0.0000 0.0000
8724.6961 7096.2877 325.4729 -1628.4084 7166.2721
323.9630 140.9574 146.7094 664265.7895 -6.3664
-6.3427
[8855]ENERGY: 2990000 3586.5845 5748.1214 5879.5214
370.1452 -15329.8139 -1861.9415 0.0000 0.0000
8704.5903 7097.2075 324.7229 -1607.3829 7167.6097
324.1885 62.4264 44.5746 664265.7895 -5.0523
-5.0730
[8894]ENERGY: 3000000 3559.8746 5707.5592 5933.2417
369.8574 -15354.5397 -1834.9415 0.0000 0.0000
8718.5662 7099.6180 325.2442 -1618.9482 7168.6382
323.9552 122.9405 66.9559 664265.7895 -5.1286
-5.1306
[8944]ENERGY: 3010000 3533.1912 5833.6316 5906.9072
353.6618 -15350.9364 -1878.6037 0.0000 0.0000
8701.6700 7099.5217 324.6139 -1602.1483 7170.1169
324.1169 59.7764 33.0439 664265.7895 -4.0981
-4.0937
[8980]ENERGY: 3020000 3587.5434 5690.3261 5898.0165
366.6942 -15403.6583 -1791.9580 0.0000 0.0000
8752.3143 7099.2781 326.5032 -1653.0362 7168.9757
323.9127 100.5827 129.5669 664265.7895 -4.4444
-4.4413
[9030]ENERGY: 3030000 3553.9919 5935.9710 5820.5735
373.4014 -15340.9737 -1836.3311 0.0000 0.0000
8593.2236 7099.8565 320.5684 -1493.3671 7169.0034
323.9344 124.4710 46.5756 664265.7895 -2.4811
-2.5023
[9066]ENERGY: 3040000 3571.3237 5815.7075 5909.2209
351.6808 -15340.6526 -1847.3059 0.0000 0.0000
8637.9177 7097.8919 322.2357 -1540.0258 7169.7439
323.8765 8.7609 -20.7594 664265.7895 -6.2221
-6.1858
[9116]ENERGY: 3050000 3624.2760 5909.2421 5909.3514
364.1650 -15303.9178 -1857.2551 0.0000 0.0000
8455.8508 7101.7124 315.4437 -1354.1384 7170.1468
323.9047 -38.9711 31.2060 664265.7895 -1.7212
-1.7335
[9152]ENERGY: 3060000 3603.9853 5722.5315 5901.8200
374.1998 -15445.0767 -1792.0106 0.0000 0.0000
8735.1602 7100.6094 325.8633 -1634.5508 7168.7383
323.9469 23.5899 37.8376 664265.7895 -5.7404
-5.7444
[9202]ENERGY: 3070000 3500.3967 5737.6998 5893.7794
376.6969 -15271.8087 -1836.4175 0.0000 0.0000
8699.0808 7099.4273 324.5173 -1599.6535 7168.9881
323.8874 98.2267 106.8052 664265.7895 -5.2682
-5.2705
[9238]ENERGY: 3080000 3585.1882 5831.2960 5908.3728
358.3367 -15407.3528 -1823.5174 0.0000 0.0000
8646.9521 7099.2755 322.5727 -1547.6765 7170.8063
323.9812 77.4261 -21.6600 664265.7895 -5.3830
-5.3747

[9288]ENERGY: 3090000 3596.0515 5715.8132 5963.0263
375.7553 -15365.2369 -1778.5799 0.0000 0.0000
8598.6575 7105.4869 320.7711 -1493.1705 7170.8451
324.0409 115.5157 76.3053 664265.7895 -6.1200
-6.1380
[9327]ENERGY: 3100000 3545.3055 5805.7027 5865.4073
311.5498 -15307.8662 -1820.7134 0.0000 0.0000
8703.3403 7102.7261 324.6762 -1600.6142 7171.2395
323.9665 -154.6844 -57.9982 664265.7895 -5.7875
-5.7676
[9377]ENERGY: 3110000 3549.7221 5832.7788 5928.5372
323.3785 -15386.4179 -1878.1169 0.0000 0.0000
8727.6176 7097.4994 325.5819 -1630.1182 7169.3037
324.1235 58.3974 41.0133 664265.7895 -5.9169
-5.9219
[9413]ENERGY: 3120000 3610.5324 5836.4326 5873.6218
352.1413 -15312.3572 -1872.8955 0.0000 0.0000
8616.5983 7104.0738 321.4403 -1512.5245 7171.6886
324.0251 -142.2922 -84.2917 664265.7895 -8.7155
-8.7290
[9463]ENERGY: 3130000 3533.4294 5777.9723 5886.8704
362.9895 -15406.9547 -1807.3470 0.0000 0.0000
8752.7410 7099.7009 326.5191 -1653.0402 7171.5427
323.9471 3.0780 55.2398 664265.7895 -8.7326
-8.7287
[9499]ENERGY: 3140000 3562.1156 5814.4942 5876.4346
368.1227 -15379.0583 -1863.1790 0.0000 0.0000
8718.6020 7097.5318 325.2456 -1621.0702 7171.1342
324.0418 -48.0055 24.1897 664265.7895 -6.8527
-6.8543
[9549]ENERGY: 3150000 3606.3467 5764.2882 5964.6078
366.1866 -15365.7378 -1879.4163 0.0000 0.0000
8646.5700 7102.8451 322.5584 -1543.7249 7171.4889
323.8287 -16.8318 26.5480 664265.7895 -7.8246
-7.8187
[9585]ENERGY: 3160000 3629.7788 5819.7522 5887.2088
331.6974 -15368.3196 -1858.2181 0.0000 0.0000
8661.3770 7103.2764 323.1108 -1558.1006 7171.9401
323.7865 -60.8029 -94.8758 664265.7895 -6.4716
-6.4737
[9635]ENERGY: 3170000 3510.6505 5771.3388 5905.1890
348.4075 -15281.0613 -1811.7366 0.0000 0.0000
8658.3035 7101.0914 322.9961 -1557.2121 7171.6696
323.9745 28.2265 -34.6646 664265.7895 -7.8298
-7.8402
[9671]ENERGY: 3180000 3469.2843 5825.9999 5941.5124
368.5248 -15327.5968 -1779.5616 0.0000 0.0000
8602.7204 7100.8834 320.9226 -1501.8371 7172.5826
324.0356 111.1925 74.5620 664265.7895 -9.4553
-9.4402
[9721]ENERGY: 3190000 3534.3567 5868.2984 5865.0219
384.8781 -15336.8516 -1869.5011 0.0000 0.0000
8655.6477 7101.8501 322.8971 -1553.7975 7171.5665
324.0987 182.9780 87.6156 664265.7895 -7.6070

-7.6160
[9760]ENERGY: 3200000 3525.1122 5727.0876 5917.9218
373.8217 -15299.2336 -1857.3738 0.0000 0.0000
8715.9416 7103.2776 325.1463 -1612.6640 7172.1396
324.1776 66.4292 89.0490 664265.7895 -8.2522
-8.2367
[9810]ENERGY: 3210000 3610.4521 5870.4353 5870.7161
364.2747 -15360.8724 -1880.4679 0.0000 0.0000
8635.3190 7109.8569 322.1387 -1525.4621 7174.7543
324.0938 -55.7546 -115.5975 664265.7895 -10.0841
-10.0956
[9846]ENERGY: 3220000 3594.0332 5878.3785 5886.3908
368.4568 -15399.8298 -1847.1995 0.0000 0.0000
8624.5914 7104.8214 321.7385 -1519.7700 7173.6326
324.3085 -17.0295 5.1774 664265.7895 -7.8533
-7.8592
[9896]ENERGY: 3230000 3598.2447 5921.6463 5875.9397
368.4215 -15458.9316 -1880.1969 0.0000 0.0000
8680.9467 7106.0703 323.8408 -1574.8764 7174.8633
324.1026 -103.5887 -90.1318 664265.7895 -11.7948
-11.7864
[9932]ENERGY: 3240000 3598.2367 5815.6708 5920.6234
350.2289 -15376.9612 -1794.8275 0.0000 0.0000
8595.2628 7108.2339 320.6444 -1487.0289 7175.4124
323.8462 -26.7650 -70.5281 664265.7895 -10.4111
-10.4286
[9982]ENERGY: 3250000 3593.2742 5895.4498 5879.4569
362.4441 -15377.1423 -1951.5589 0.0000 0.0000
8705.2060 7107.1298 324.7458 -1598.0762 7177.1358
324.0802 -251.4468 -243.1929 664265.7895 -8.1824
-8.1638
[10018]ENERGY: 3260000 3484.2681 5806.3076 5932.0512
363.8279 -15298.7084 -1910.5696 0.0000 0.0000
8727.0723 7104.2490 325.5616 -1622.8233 7177.8698
323.9825 -89.7861 -108.9202 664265.7895 -10.8561
-10.8486
[10068]ENERGY: 3270000 3542.5733 5720.4089 5926.0712
360.8829 -15306.8419 -1873.1441 0.0000 0.0000
8734.6312 7104.5815 325.8435 -1630.0497 7176.3102
323.9908 138.6551 110.9576 664265.7895 -7.3024
-7.3018
[10104]ENERGY: 3280000 3580.0140 5730.3955 5953.3971
359.8583 -15329.8092 -1870.7254 0.0000 0.0000
8683.4236 7106.5538 323.9332 -1576.8698 7177.8351
323.8568 203.1066 90.3925 664265.7895 -9.6495
-9.6628
[10154]ENERGY: 3290000 3647.6719 5836.8852 5882.7730
353.6801 -15381.2405 -1904.3949 0.0000 0.0000
8671.1796 7106.5544 323.4765 -1564.6252 7177.9020
324.0034 -148.8553 -155.3564 664265.7895 -8.6830
-8.6871
[10193]ENERGY: 3300000 3547.3270 5769.8822 5891.9912
377.2372 -15301.8778 -1826.9489 0.0000 0.0000
8649.8158 7107.4267 322.6795 -1542.3891 7177.8830

323.9942 -23.0030 -33.6385 664265.7895 -10.2752
-10.2639
[10243]ENERGY: 3310000 3537.0764 5803.5694 5906.6107
363.8079 -15331.1940 -1891.4723 0.0000 0.0000
8717.2063 7105.6045 325.1935 -1611.6019 7176.7974
323.8962 113.2110 134.0169 664265.7895 -10.2040
-10.2038
[10279]ENERGY: 3320000 3600.5373 5910.1381 5923.4849
373.9519 -15391.6178 -1896.3527 0.0000 0.0000
8591.3195 7111.4613 320.4973 -1479.8583 7178.1580
324.0055 88.0769 -1.0565 664265.7895 -10.0986
-10.0905
[10329]ENERGY: 3330000 3556.6650 5864.5673 5982.6044
380.3738 -15441.0206 -1890.3831 0.0000 0.0000
8653.6099 7106.4166 322.8211 -1547.1932 7177.5247
323.9607 63.1438 60.6002 664265.7895 -9.3666
-9.3719
[10365]ENERGY: 3340000 3613.4875 5855.2419 5833.3045
376.1759 -15312.7838 -1842.9399 0.0000 0.0000
8585.3843 7107.8705 320.2759 -1477.5138 7177.3680
323.9752 -53.5670 -14.0779 664265.7895 -7.6081
-7.6180
[10415]ENERGY: 3350000 3630.7587 5794.6144 5860.4689
331.8215 -15294.8949 -1900.4997 0.0000 0.0000
8687.4749 7109.7439 324.0844 -1577.7310 7177.9607
324.1527 33.2605 -51.9801 664265.7895 -6.1889
-6.1884
[10451]ENERGY: 3360000 3630.4933 5861.7975 5936.0277
371.0993 -15379.6204 -1881.3909 0.0000 0.0000
8573.2331 7111.6396 319.8226 -1461.5935 7179.8344
324.3155 45.6595 -43.3024 664265.7895 6.0052
5.9907
[10501]ENERGY: 3370000 3540.0689 5743.0377 5899.7252
369.6504 -15291.2662 -1822.0714 0.0000 0.0000
8669.9161 7109.0606 323.4294 -1560.8555 7181.2222
324.0624 231.4141 135.1986 664265.7895 3.4011
3.4210
[10537]ENERGY: 3380000 3526.6953 5759.4384 5936.8309
349.8902 -15270.9983 -1908.8228 0.0000 0.0000
8718.7880 7111.8217 325.2525 -1606.9663 7179.9536
324.0184 -17.6279 -82.3823 664265.7895 -0.3397
-0.3340
[10587]ENERGY: 3390000 3489.4930 5836.4362 5912.2455
373.6011 -15382.8895 -1792.8340 0.0000 0.0000
8675.0657 7111.1180 323.6215 -1563.9477 7179.2657
324.1097 146.7154 93.9824 664265.7895 0.1222
0.1196
[10626]ENERGY: 3400000 3539.2337 5726.1659 5934.6617
356.3228 -15287.9620 -1845.3149 0.0000 0.0000
8687.5456 7110.6529 324.0870 -1576.8927 7180.0492
324.2808 151.9962 66.3483 664265.7895 0.0753
0.0482
[10676]ENERGY: 3410000 3576.2905 5788.1402 5927.7454
359.5151 -15320.6320 -1871.9127 0.0000 0.0000

8650.4281 7109.5746 322.7024 -1540.8535 7180.7145
324.1457 -16.5872 -156.3033 664265.7895 -10.7308
-10.7124
[10712]ENERGY: 3420000 3618.1166 5759.1282 5920.4829
351.1744 -15382.9655 -1876.0868 0.0000 0.0000
8718.3706 7108.2205 325.2369 -1610.1501 7180.0823
324.1704 -117.3758 -124.1277 664265.7895 -11.1936
-11.1965
[10762]ENERGY: 3430000 3469.0119 5755.1592 5908.1920
352.2612 -15251.4812 -1849.8014 0.0000 0.0000
8723.1948 7106.5365 325.4169 -1616.6583 7179.5405
323.8767 5.2069 62.1305 664265.7895 -9.0377
-9.0216
[10798]ENERGY: 3440000 3567.4596 5878.3832 5859.3203
376.1948 -15344.4755 -1935.0535 0.0000 0.0000
8705.9161 7107.7450 324.7723 -1598.1712 7180.7176
324.1254 -205.4314 -158.8073 664265.7895 -7.4583
-7.4635
[10848]ENERGY: 3450000 3503.5821 5812.8788 5918.8527
356.8412 -15343.4995 -1839.5484 0.0000 0.0000
8700.1673 7109.2742 324.5579 -1590.8931 7179.3199
324.0140 55.9158 -3.2983 664265.7895 -10.4960
-10.5077
[10884]ENERGY: 3460000 3520.8606 5820.6912 5905.0971
385.2524 -15378.2859 -1854.4947 0.0000 0.0000
8708.5343 7107.6549 324.8700 -1600.8794 7180.1125
324.0055 116.0702 66.8342 664265.7895 -5.1749
-5.1591
[10934]ENERGY: 3470000 3639.6734 5700.9445 5924.3863
338.0197 -15310.6484 -1866.2328 0.0000 0.0000
8687.3293 7113.4720 324.0789 -1573.8573 7180.9123
323.9207 143.3583 77.4880 664265.7895 -9.3792
-9.3999
[10970]ENERGY: 3480000 3467.4013 5743.5047 5919.6659
379.9986 -15289.0746 -1826.6030 0.0000 0.0000
8713.8689 7108.7618 325.0690 -1605.1070 7179.9611
324.1493 245.3356 162.0192 664265.7895 -5.2539
-5.2290
[11020]ENERGY: 3490000 3601.3917 5753.3583 5934.3072
371.5158 -15383.5416 -1827.4793 0.0000 0.0000
8664.7967 7114.3488 323.2384 -1550.4479 7179.7850
324.2648 123.3944 159.3582 664265.7895 0.9956
0.9857
[11059]ENERGY: 3500000 3561.5810 5810.5141 5900.7055
386.8848 -15328.6342 -1848.3492 0.0000 0.0000
8628.3660 7111.0679 321.8793 -1517.2981 7180.0574
323.9733 37.8288 1.8707 664265.7895 -4.2652
-4.2647
[11109]ENERGY: 3510000 3491.0622 5885.4942 5975.4115
345.8831 -15376.1303 -1857.1917 0.0000 0.0000
8647.5702 7112.0992 322.5957 -1535.4709 7182.3161
324.2008 70.9185 60.6540 664265.7895 -1.4199
-1.4200
[11145]ENERGY: 3520000 3608.0139 5685.2125 5914.0076

350.9063 -15370.8115 -1819.6596 0.0000 0.0000
8742.5438 7110.2130 326.1387 -1632.3308 7180.3423
324.2271 182.1383 106.3440 664265.7895 1.6749
1.6525
[11195]ENERGY: 3530000 3619.6596 5789.7221 5956.3611
359.7620 -15343.7904 -1868.1752 0.0000 0.0000
8598.0232 7111.5624 320.7474 -1486.4608 7181.0453
324.0984 60.2910 47.1304 664265.7895 0.6839
0.7022
[11231]ENERGY: 3540000 3520.7640 5820.2173 5893.1493
389.9813 -15292.2049 -1893.4756 0.0000 0.0000
8678.0674 7116.4988 323.7334 -1561.5686 7183.1224
324.0527 -64.4737 15.6391 664265.7895 -11.7354
-11.7297
[11281]ENERGY: 3550000 3499.0475 5855.1869 5896.2043
352.6331 -15314.5866 -1920.5370 0.0000 0.0000
8740.4454 7108.3936 326.0604 -1632.0519 7182.8467
324.2004 146.2766 90.3361 664265.7895 -12.3673
-12.3694
[11317]ENERGY: 3560000 3517.5928 5770.9834 5886.4095
341.9241 -15289.7274 -1811.5524 0.0000 0.0000
8695.4167 7111.0467 324.3806 -1584.3700 7184.0057
324.2372 49.8421 8.9233 664265.7895 -3.2048
-3.2074
[11367]ENERGY: 3570000 3614.4351 5809.8765 5897.2071
341.9312 -15329.1155 -1875.6573 0.0000 0.0000
8656.9164 7115.5934 322.9444 -1541.3229 7185.7008
324.2332 88.3512 82.1022 664265.7895 -4.7919
-4.8009
[11403]ENERGY: 3580000 3535.2910 5759.6235 5924.9709
367.4640 -15398.5867 -1761.3597 0.0000 0.0000
8687.9473 7115.3503 324.1020 -1572.5970 7186.4231
324.2113 147.3850 61.0796 664265.7895 -0.4764
-0.4693
[11453]ENERGY: 3590000 3583.5437 5769.8789 5877.6020
366.2376 -15266.4871 -1858.8096 0.0000 0.0000
8645.1001 7117.0656 322.5036 -1528.0345 7186.2311
324.5003 154.5418 116.6571 664265.7895 -2.6761
-2.6680
[11492]ENERGY: 3600000 3584.7974 5713.1174 5913.8842
358.4321 -15336.3666 -1822.4716 0.0000 0.0000
8701.7783 7113.1711 324.6180 -1588.6072 7185.9308
324.4579 142.8619 62.4117 664265.7895 -3.2114
-3.2159
[11542]ENERGY: 3610000 3634.3989 5764.9041 5947.9111
367.9523 -15381.5495 -1847.9849 0.0000 0.0000
8629.4597 7115.0918 321.9201 -1514.3679 7185.7526
324.4093 -105.7577 -82.9886 664265.7895 -3.1032
-3.1100
[11578]ENERGY: 3620000 3517.5960 5817.3474 5918.9118
362.7082 -15339.4531 -1812.3051 0.0000 0.0000
8650.5048 7115.3099 322.7052 -1535.1949 7184.7095
324.3165 112.1368 101.7819 664265.7895 -0.8532
-0.8313

[11628]ENERGY: 3630000 3512.4964 5782.4967 5902.2989
358.5518 -15320.2014 -1818.9758 0.0000 0.0000
8697.5953 7114.2618 324.4619 -1583.3334 7186.1735
324.3577 195.4522 125.7390 664265.7895 -7.9876
-8.0045
[11664]ENERGY: 3640000 3553.8954 5949.0449 5875.4244
378.8376 -15378.2649 -1871.3821 0.0000 0.0000
8607.5367 7115.0918 321.1023 -1492.4449 7185.3475
324.2323 -34.5904 -35.4880 664265.7895 -14.1315
-14.1248
[11714]ENERGY: 3650000 3553.0482 5736.7122 5874.3493
349.9871 -15309.1736 -1881.7504 0.0000 0.0000
8790.1019 7113.2747 327.9129 -1676.8272 7186.0421
324.2988 95.5110 30.2777 664265.7895 -11.0705
-11.0867
[11750]ENERGY: 3660000 3524.3122 5823.5554 5874.7271
378.5543 -15346.2327 -1852.3100 0.0000 0.0000
8710.0776 7112.6839 324.9276 -1597.3937 7185.2859
324.2383 20.7834 -74.7821 664265.7895 -13.0902
-13.0854
[11800]ENERGY: 3670000 3544.6155 5833.1113 5855.6008
387.1906 -15399.3831 -1741.6238 0.0000 0.0000
8633.6956 7113.2068 322.0782 -1520.4888 7184.4888
324.4589 42.7875 22.1174 664265.7895 -1.4211
-1.4154
[11836]ENERGY: 3680000 3572.8208 5782.8675 5934.3147
365.5167 -15387.2096 -1912.1315 0.0000 0.0000
8760.0765 7116.2552 326.7928 -1643.8213 7185.7250
324.4837 99.6883 14.3048 664265.7895 -1.1601
-1.1693
[11886]ENERGY: 3690000 3583.3476 5882.4235 5884.2415
356.5154 -15350.0571 -1871.1364 0.0000 0.0000
8629.7806 7115.1151 321.9321 -1514.6655 7184.7303
324.4353 -111.7222 -13.1143 664265.7895 -11.0933
-11.0877
[11925]ENERGY: 3700000 3563.5341 5823.6698 5941.2154
363.8295 -15376.5454 -1815.9555 0.0000 0.0000
8614.3870 7114.1350 321.3579 -1500.2521 7184.1647
324.4478 -31.1143 -53.0512 664265.7895 0.2681
0.2646
[11975]ENERGY: 3710000 3512.4388 5772.9399 5862.3026
355.0963 -15344.9432 -1825.7193 0.0000 0.0000
8779.9514 7112.0664 327.5342 -1667.8850 7184.5597
324.4206 106.9480 29.9070 664265.7895 -4.4039
-4.3928
[12011]ENERGY: 3720000 3591.0131 5832.6465 5861.0083
397.2868 -15358.8498 -1883.8948 0.0000 0.0000
8676.5258 7115.7358 323.6759 -1560.7899 7184.9276
324.5712 22.4409 -69.9810 664265.7895 -2.0569
-2.0642
[12061]ENERGY: 3730000 3544.6725 5751.9301 5910.9050
353.5191 -15259.4768 -1911.3569 0.0000 0.0000
8726.9551 7117.1481 325.5572 -1609.8069 7186.5110
324.5254 316.5728 131.1819 664265.7895 0.3057

0.3110
[12097]ENERGY: 3740000 3493.7585 5780.6584 5889.6691
377.8054 -15312.0897 -1828.5198 0.0000 0.0000
8714.6374 7115.9194 325.0977 -1598.7181 7186.1498
324.4021 196.2377 107.7543 664265.7895 -1.2529
-1.2447
[12147]ENERGY: 3750000 3498.9388 5870.6230 5956.3335
355.6414 -15423.1999 -1859.9027 0.0000 0.0000
8716.0249 7114.4589 325.1494 -1601.5660 7185.1982
324.4225 37.5650 30.6391 664265.7895 2.7792
2.7833
[12183]ENERGY: 3760000 3571.2179 5773.9961 5897.3411
360.0837 -15319.1718 -1905.4027 0.0000 0.0000
8736.7452 7114.8095 325.9224 -1621.9358 7186.8283
324.3435 -48.9263 -144.1770 664265.7895 -1.3192
-1.3230
[12233]ENERGY: 3770000 3487.6817 5826.9182 5849.8547
346.5271 -15338.1800 -1826.8991 0.0000 0.0000
8770.5552 7116.4578 327.1837 -1654.0974 7185.4706
324.2831 29.4842 15.7062 664265.7895 -2.3914
-2.4109
[12269]ENERGY: 3780000 3521.5160 5815.5744 5914.9435
344.5016 -15358.0689 -1846.6770 0.0000 0.0000
8723.1622 7114.9518 325.4157 -1608.2104 7185.7331
324.5907 22.0961 -13.7140 664265.7895 -6.9911
-6.9590
[12319]ENERGY: 3790000 3551.9950 5864.7387 5893.5693
352.9068 -15390.0933 -1852.9226 0.0000 0.0000
8696.4232 7116.6171 324.4182 -1579.8061 7186.6209
324.3540 -117.9564 -39.2110 664265.7895 -0.7139
-0.7524
[12358]ENERGY: 3800000 3549.5719 5738.3008 5919.3750
340.1578 -15300.8332 -1957.4500 0.0000 0.0000
8823.9262 7113.0486 329.1747 -1710.8776 7187.8118
324.5581 -150.9440 -168.1618 664265.7895 -4.0373
-4.0170
[12408]ENERGY: 3810000 3618.6711 5875.3270 5832.2275
359.9954 -15349.5224 -1839.8152 0.0000 0.0000
8620.9984 7117.8816 321.6045 -1503.1167 7186.2140
324.4146 -44.7228 -117.4982 664265.7895 -2.9239
-2.9318
[12444]ENERGY: 3820000 3556.4318 5875.1254 5874.0115
362.4433 -15415.8739 -1843.1166 0.0000 0.0000
8704.3257 7113.3472 324.7130 -1590.9785 7187.3755
324.3247 -49.6819 -39.0510 664265.7895 -0.1404
-0.1318
[12494]ENERGY: 3830000 3548.5131 5745.1909 5862.5668
364.0667 -15285.2036 -1801.0945 0.0000 0.0000
8681.9541 7115.9935 323.8784 -1565.9606 7186.6772
324.2858 -24.0464 23.6838 664265.7895 -3.7567
-3.7565
[12530]ENERGY: 3840000 3649.2009 5837.9976 5870.1718
378.2902 -15448.7475 -1836.6601 0.0000 0.0000
8666.9976 7117.2506 323.3205 -1549.7471 7186.8652

324.4358 77.5475 65.8000 664265.7895 -3.9550
-3.9593
[12580]ENERGY: 3850000 3569.2255 5872.2580 5934.4597
372.2919 -15373.5256 -1876.3659 0.0000 0.0000
8621.9478 7120.2914 321.6399 -1501.6564 7188.1754
324.4982 157.4442 73.6708 664265.7895 -3.7908
-3.7920
[12616]ENERGY: 3860000 3589.1299 5838.2909 5867.3707
355.7678 -15324.1504 -1946.5288 0.0000 0.0000
8736.9941 7116.8743 325.9317 -1620.1198 7188.1313
324.6729 -89.5423 -128.4527 664265.7895 -1.8858
-1.8852
[12666]ENERGY: 3870000 3603.5495 5829.5496 5943.8789
357.3004 -15413.1011 -1881.8196 0.0000 0.0000
8677.8445 7117.2022 323.7251 -1560.6423 7187.6127
324.6329 4.2339 32.9665 664265.7895 -6.7550
-6.7383
[12702]ENERGY: 3880000 3534.9821 5796.1474 5849.9920
360.1050 -15351.1430 -1818.7902 0.0000 0.0000
8747.5598 7118.8530 326.3258 -1628.7068 7188.9199
324.5913 116.0781 43.3182 664265.7895 -4.0333
-4.0451
[12752]ENERGY: 3890000 3522.7493 5738.9610 5912.0593
365.8693 -15405.5097 -1816.4238 0.0000 0.0000
8799.4451 7117.1504 328.2614 -1682.2947 7189.9981
324.5796 60.7505 74.3220 664265.7895 -3.5242
-3.5244
[12791]ENERGY: 3900000 3524.0229 5839.9693 5855.4040
372.2766 -15366.7970 -1758.6982 0.0000 0.0000
8654.1874 7120.3651 322.8426 -1533.8223 7190.7289
324.5359 220.6251 146.3226 664265.7895 -7.1637
-7.1622
[12841]ENERGY: 3910000 3663.9587 5939.5338 5880.5908
353.6843 -15383.7398 -1949.7645 0.0000 0.0000
8617.1273 7121.3906 321.4601 -1495.7368 7189.9122
324.6264 -35.9411 -71.7389 664265.7895 -6.6196
-6.6323
[12877]ENERGY: 3920000 3519.0785 5785.4172 5993.8274
336.5727 -15345.8888 -1851.4841 0.0000 0.0000
8680.0253 7117.5482 323.8065 -1562.4771 7189.6445
324.6649 107.3355 66.3579 664265.7895 -1.8199
-1.8165
[12927]ENERGY: 3930000 3566.3263 5744.5379 5895.9819
348.9704 -15266.6890 -1865.2441 0.0000 0.0000
8694.8028 7118.6861 324.3577 -1576.1167 7189.7402
324.4323 65.2239 -42.7942 664265.7895 -3.5196
-3.5271
[12963]ENERGY: 3940000 3557.4340 5843.8849 5936.8169
335.6020 -15406.2445 -1927.2867 0.0000 0.0000
8779.9686 7120.1751 327.5348 -1659.7936 7190.7717
324.6251 -167.5057 -145.6986 664265.7895 -3.6670
-3.6629
[13013]ENERGY: 3950000 3572.4653 5739.8713 5964.2215
357.4595 -15334.1367 -1839.5384 0.0000 0.0000

8661.3034 7121.6458 323.1081 -1539.6575 7190.7149
324.6325 83.3575 70.4330 664265.7895 -4.6685
-4.6754
[13049]ENERGY: 3960000 3632.1243 5687.7377 5883.0483
355.2230 -15232.9819 -1829.7675 0.0000 0.0000
8628.7453 7124.1292 321.8935 -1504.6161 7191.0765
324.6889 -53.6739 -43.9702 664265.7895 -3.5710
-3.5656
[13099]ENERGY: 3970000 3487.1243 5835.2766 5921.9284
368.9409 -15310.7206 -1886.5869 0.0000 0.0000
8704.2699 7120.2326 324.7109 -1584.0373 7190.2525
324.5136 41.3086 53.7475 664265.7895 -3.9934
-3.9733
[13135]ENERGY: 3980000 3545.7770 5921.6074 5830.1429
371.8737 -15375.6743 -1866.5119 0.0000 0.0000
8695.8562 7123.0709 324.3970 -1572.7853 7193.0844
324.5940 129.2765 58.7125 664265.7895 -7.4915
-7.5046
[13185]ENERGY: 3990000 3598.1044 5825.8299 5962.8429
328.2268 -15335.9659 -1888.3978 0.0000 0.0000
8633.3485 7123.9887 322.0652 -1509.3597 7193.5958
324.5357 -36.6719 -106.4061 664265.7895 -3.9917
-4.0042
[13224]ENERGY: 4000000 3538.4315 5780.6697 5866.1864
373.6898 -15234.3391 -1885.9441 0.0000 0.0000
8683.1214 7121.8155 323.9220 -1561.3060 7193.4720
324.4364 4.7207 -17.6997 664265.7895 -4.0489
-4.0187
[13274]ENERGY: 4010000 3515.3490 5775.0437 5893.3769
358.8477 -15370.4910 -1837.4520 0.0000 0.0000
8788.6043 7123.2786 327.8570 -1665.3258 7192.1293
324.3638 -12.8926 42.3276 664265.7895 -5.5111
-5.5303
[13310]ENERGY: 4020000 3539.1025 5708.6374 5940.3199
366.9614 -15237.5505 -1861.1447 0.0000 0.0000
8669.3746 7125.7007 323.4091 -1543.6739 7194.1655
324.4686 37.2294 7.4337 664265.7895 -5.2666
-5.2632
[13360]ENERGY: 4030000 3602.4533 5846.2213 5907.3425
361.7065 -15326.8881 -1849.0353 0.0000 0.0000
8586.0457 7127.8460 320.3006 -1458.1997 7193.8762
324.6113 -14.1419 -71.7809 664265.7895 -2.0630
-2.0817
[13396]ENERGY: 4040000 3519.4142 5871.6486 5897.4332
360.2442 -15355.8139 -1867.7498 0.0000 0.0000
8694.6370 7119.8136 324.3516 -1574.8234 7193.7926
324.6979 -87.8605 -77.7480 664265.7895 -3.9963
-3.9811
[13446]ENERGY: 4050000 3560.3382 5721.8374 5912.2134
363.5739 -15275.3788 -1827.4438 0.0000 0.0000
8669.7716 7124.9119 323.4240 -1544.8597 7193.3899
324.6948 4.9410 14.8554 664265.7895 -3.9283
-3.9303
[13482]ENERGY: 4060000 3520.3882 5805.9876 5896.8012

346.5742 -15341.8047 -1821.4687 0.0000 0.0000
8715.7960 7122.2736 325.1409 -1593.5223 7193.9774
324.5057 58.2086 -15.1888 664265.7895 -5.1093
-5.1008
[13532]ENERGY: 4070000 3505.2921 5882.5200 5889.6148
359.8902 -15279.5300 -1898.6395 0.0000 0.0000
8661.3538 7120.5014 323.1099 -1540.8524 7194.7745
324.2422 91.8267 57.8456 664265.7895 -1.2073
-1.2204
[13568]ENERGY: 4080000 3559.7413 5844.8408 5949.7765
353.9149 -15270.2085 -1930.8714 0.0000 0.0000
8621.9530 7129.1467 321.6401 -1492.8063 7196.3959
324.2901 -29.8871 -92.2779 664265.7895 -2.3969
-2.3852
[13618]ENERGY: 4090000 3548.5579 5803.5408 5851.6926
399.0557 -15292.7562 -1887.0363 0.0000 0.0000
8703.8447 7126.8992 324.6951 -1576.9455 7195.2738
324.1638 -106.8674 -102.9238 664265.7895 -4.2607
-4.2515
[13657]ENERGY: 4100000 3494.4054 5815.0023 5851.7537
357.7518 -15360.8302 -1786.6165 0.0000 0.0000
8755.7928 7127.2594 326.6330 -1628.5334 7195.2879
324.3495 64.5252 -11.0418 664265.7895 -8.3428
-8.3642
[13707]ENERGY: 4110000 3502.4853 5742.4608 5916.4661
357.4709 -15227.5269 -1919.1278 0.0000 0.0000
8748.9703 7121.1987 326.3785 -1627.7716 7196.8077
324.2521 -24.3851 -77.4099 664265.7895 -12.6671
-12.6521
[13743]ENERGY: 4120000 3578.4285 5677.6598 5930.3607
366.8123 -15299.3678 -1847.4256 0.0000 0.0000
8718.3124 7124.7805 325.2348 -1593.5320 7196.5983
324.3115 49.7438 -6.4022 664265.7895 -6.1913
-6.2077
[13793]ENERGY: 4130000 3560.8496 5802.5513 5856.8278
365.2955 -15261.8696 -1885.7300 0.0000 0.0000
8686.4039 7124.3285 324.0444 -1562.0754 7196.8077
324.1467 -78.5148 -61.4537 664265.7895 -7.6397
-7.6320
[13829]ENERGY: 4140000 3521.3722 5685.5096 5938.4382
347.6521 -15283.4748 -1821.2509 0.0000 0.0000
8733.0996 7121.3460 325.7864 -1611.7535 7195.2551
324.1308 53.5845 37.1900 664265.7895 0.4196
0.4015
[13879]ENERGY: 4150000 3624.2458 5774.1320 5889.4958
343.1551 -15304.5801 -1927.5890 0.0000 0.0000
8727.1535 7126.0131 325.5646 -1601.1404 7197.2421
324.1515 -166.9250 -148.3173 664265.7895 -2.2426
-2.2081
[13915]ENERGY: 4160000 3561.7952 5764.6352 5850.0251
366.4672 -15296.5238 -1900.1787 0.0000 0.0000
8775.7400 7121.9600 327.3771 -1653.7799 7196.9518
324.1963 -121.7572 -104.7966 664265.7895 -7.0192
-7.0162

[13965]ENERGY: 4170000 3539.1420 5734.4967 5961.0525
358.1418 -15278.6558 -1782.6285 0.0000 0.0000
8599.5271 7131.0757 320.8035 -1468.4513 7198.2044
324.1639 -32.4893 -57.5093 664265.7895 -10.2287
-10.2270
[14001]ENERGY: 4180000 3639.9835 5780.0528 5877.4391
354.9131 -15349.8854 -1819.1848 0.0000 0.0000
8645.2543 7128.5727 322.5094 -1516.6816 7198.6083
324.1226 -20.7938 -87.6297 664265.7895 -7.9558
-7.9924
[14051]ENERGY: 4190000 3638.5648 5786.1826 5879.7054
360.9144 -15292.6434 -1883.8341 0.0000 0.0000
8639.6417 7128.5314 322.3000 -1511.1103 7198.7788
324.2541 69.5663 68.3852 664265.7895 -9.4550
-9.4217
[14090]ENERGY: 4200000 3606.7468 5805.6896 5903.8944
369.7508 -15400.8199 -1829.1063 0.0000 0.0000
8674.0960 7130.2513 323.5853 -1543.8447 7197.6890
324.3237 -21.0507 -13.8459 664265.7895 -10.1666
-10.1656
[14140]ENERGY: 4210000 3544.0225 5752.8356 5919.0343
336.5917 -15309.3638 -1845.8523 0.0000 0.0000
8730.9295 7128.1975 325.7054 -1602.7320 7199.6170
324.4233 145.3296 79.3193 664265.7895 -3.4475
-3.4670
[14176]ENERGY: 4220000 3513.1439 5805.2785 5882.2468
351.1372 -15359.9078 -1838.5181 0.0000 0.0000
8770.3449 7123.7253 327.1758 -1646.6196 7199.9222
324.5219 147.0694 125.2313 664265.7895 -5.6114
-5.6129
[14226]ENERGY: 4230000 3542.1998 5787.0693 5873.2704
351.1373 -15392.9215 -1763.3328 0.0000 0.0000
8731.7422 7129.1647 325.7358 -1602.5775 7198.8758
324.5057 -104.2529 -70.8597 664265.7895 -2.4211
-2.4240
[14262]ENERGY: 4240000 3500.7037 5765.1862 5879.0244
374.4382 -15340.4592 -1835.1631 0.0000 0.0000
8783.8255 7127.5557 327.6787 -1656.2698 7197.8491
324.2761 -59.5924 -44.7159 664265.7895 -4.1223
-4.1139
[14312]ENERGY: 4250000 3554.4995 5859.3244 5923.9348
335.6581 -15316.2682 -1890.7763 0.0000 0.0000
8664.7059 7131.0784 323.2350 -1533.6275 7198.6648
324.3818 -38.7291 -46.0001 664265.7895 -1.2859
-1.2838
[14348]ENERGY: 4260000 3598.6475 5794.8806 5914.5721
359.7216 -15332.3517 -1896.0826 0.0000 0.0000
8689.9215 7129.3090 324.1756 -1560.6125 7198.8723
324.3837 -328.8106 -288.7415 664265.7895 -3.0892
-3.0758
[14398]ENERGY: 4270000 3491.7996 5792.9352 5975.1006
363.0948 -15340.7786 -1775.1089 0.0000 0.0000
8625.6808 7132.7235 321.7792 -1492.9573 7200.1452
324.4720 29.1149 61.0472 664265.7895 -5.6988

-5.7052
[14434]ENERGY: 4280000 3555.2196 5815.2635 5896.2373
343.3976 -15285.3044 -1856.3457 0.0000 0.0000
8664.9021 7133.3701 323.2423 -1531.5321 7201.1645
324.3440 35.3638 -75.5217 664265.7895 -6.1328
-6.1395
[14484]ENERGY: 4290000 3495.6512 5789.4957 5905.1038
331.7303 -15351.0687 -1804.3585 0.0000 0.0000
8760.3419 7126.8957 326.8027 -1633.4462 7200.8703
324.2630 169.7318 116.3388 664265.7895 -1.6172
-1.6186
[14523]ENERGY: 4300000 3591.2231 5794.3343 5887.0402
347.9219 -15354.9469 -1817.4840 0.0000 0.0000
8684.9700 7133.0585 323.9909 -1551.9115 7199.2878
324.3364 -48.3450 -40.7608 664265.7895 -5.2163
-5.2015
[14573]ENERGY: 4310000 3518.8929 5866.0601 5806.9819
352.6855 -15341.9683 -1857.4887 0.0000 0.0000
8784.4264 7129.5898 327.7011 -1654.8366 7200.4323
324.6256 169.1745 78.0889 664265.7895 -3.1810
-3.1966
[14609]ENERGY: 4320000 3549.7762 5752.6338 5870.0786
313.2983 -15331.0581 -1856.4553 0.0000 0.0000
8827.1862 7125.4597 329.2963 -1701.7265 7199.1793
324.3956 -31.2489 -75.9543 664265.7895 -4.1074
-4.1145
[14659]ENERGY: 4330000 3601.9722 5878.6497 5888.7810
347.8142 -15404.5894 -1826.9746 0.0000 0.0000
8647.8302 7133.4833 322.6054 -1514.3469 7200.8935
324.4019 134.4470 161.6235 664265.7895 -4.2408
-4.2263
[14695]ENERGY: 4340000 3616.2680 5836.2125 5856.1133
354.6700 -15366.4006 -1853.0754 0.0000 0.0000
8686.9258 7130.7137 324.0639 -1556.2121 7202.6526
324.2116 93.0799 12.5087 664265.7895 -1.5088
-1.4922
[14745]ENERGY: 4350000 3613.1157 5786.2640 5917.4739
348.5350 -15401.7845 -1826.0492 0.0000 0.0000
8695.0913 7132.6462 324.3685 -1562.4450 7202.3529
324.3030 -29.5856 17.4837 664265.7895 0.7350
0.7088
[14781]ENERGY: 4360000 3548.4285 5991.9637 5888.5449
337.5427 -15515.3551 -1819.9171 0.0000 0.0000
8701.1161 7132.3236 324.5933 -1568.7925 7203.5251
324.5275 -23.1518 -42.6686 664265.7895 2.1690
2.1815
[14831]ENERGY: 4370000 3492.9641 5726.2909 5908.1030
366.2652 -15311.0092 -1824.0599 0.0000 0.0000
8772.1032 7130.6574 327.2414 -1641.4458 7204.6224
324.4604 150.3517 111.7589 664265.7895 -1.3237
-1.3331
[14867]ENERGY: 4380000 3620.6401 5825.8771 5891.4482
355.3346 -15326.5137 -1868.1912 0.0000 0.0000
8638.3970 7136.9922 322.2535 -1501.4048 7204.7469

324.4802 -62.5475 -179.7568 664265.7895 0.0142
0.0167
[14917]ENERGY: 4390000 3579.6912 5782.5421 5869.1859
360.4449 -15389.9123 -1783.2065 0.0000 0.0000
8716.1494 7134.8948 325.1541 -1581.2546 7204.2977
324.4801 39.1114 96.7703 664265.7895 7.2166
7.2082
[14956]ENERGY: 4400000 3572.5061 5821.7227 5878.8142
373.2556 -15358.3855 -1904.1338 0.0000 0.0000
8750.0225 7133.8018 326.4177 -1616.2208 7205.1321
324.4394 -86.3726 -67.9674 664265.7895 2.2479
2.2258
[15006]ENERGY: 4410000 3630.6230 5848.5748 5878.9763
386.3063 -15375.6796 -1846.0735 0.0000 0.0000
8614.0970 7136.8243 321.3470 -1477.2727 7205.5897
324.5647 -115.7350 -144.6111 664265.7895 5.6993
5.7149
[15042]ENERGY: 4420000 3516.5079 5864.9428 5899.5241
351.9056 -15352.5579 -1857.1450 0.0000 0.0000
8714.4341 7137.6116 325.0901 -1576.8225 7206.2639
324.3518 -64.1675 -83.7518 664265.7895 4.7744
4.7851
[15092]ENERGY: 4430000 3494.7449 5804.2980 5925.2921
354.2994 -15351.9011 -1837.5205 0.0000 0.0000
8746.0408 7135.2537 326.2692 -1610.7871 7204.5644
324.4356 128.0426 84.0510 664265.7895 -0.4826
-0.4971
[15128]ENERGY: 4440000 3558.4920 5719.5939 5878.4285
343.7676 -15323.4775 -1821.8675 0.0000 0.0000
8781.1026 7136.0394 327.5771 -1645.0631 7205.2390
324.7783 133.3112 109.7472 664265.7895 0.8620
0.8741
[15178]ENERGY: 4450000 3654.8530 5846.1286 5943.7663
359.9060 -15422.0181 -1841.8376 0.0000 0.0000
8598.2820 7139.0802 320.7571 -1459.2018 7206.2273
324.7043 -33.3974 -27.9169 664265.7895 4.9449
4.9366
[15214]ENERGY: 4460000 3658.8873 5844.8193 5929.5034
327.9396 -15336.4355 -1907.8206 0.0000 0.0000
8619.3896 7136.2832 321.5445 -1483.1064 7205.4455
324.8990 -64.0157 -154.4767 664265.7895 0.4938
0.4940
[15264]ENERGY: 4470000 3549.8023 5780.3541 5919.9163
342.0773 -15245.6161 -1922.8400 0.0000 0.0000
8713.0975 7136.7914 325.0402 -1576.3062 7205.1440
324.8812 44.9551 13.6383 664265.7895 1.6307
1.6283
[15300]ENERGY: 4480000 3481.9067 5855.5689 5905.6759
367.0816 -15284.9669 -1866.1601 0.0000 0.0000
8677.1483 7136.2543 323.6991 -1540.8940 7205.1452
324.7310 -43.2312 -31.9284 664265.7895 2.5373
2.5542
[15350]ENERGY: 4490000 3535.2952 5789.1444 5924.6056
349.6779 -15294.9234 -1894.4020 0.0000 0.0000

8724.8976 7134.2954 325.4804 -1590.6023 7205.9258
324.8039 133.0136 101.6882 664265.7895 2.9458
2.9381
[15389]ENERGY: 4500000 3623.1912 5733.1270 5933.8125
357.3000 -15365.6154 -1929.7930 0.0000 0.0000
8784.4889 7136.5111 327.7035 -1647.9778 7206.7418
325.0819 -55.8410 -128.5019 664265.7895 0.1149
0.1209
[15439]ENERGY: 4510000 3637.1362 5671.7559 5916.4437
361.8849 -15375.5767 -1821.2861 0.0000 0.0000
8746.5040 7136.8620 326.2864 -1609.6421 7207.0738
325.2487 -31.2945 -6.6463 664265.7895 0.9998
0.9945
[15475]ENERGY: 4520000 3580.7192 5734.6696 5894.6503
367.0834 -15285.3078 -1826.5222 0.0000 0.0000
8671.6044 7136.8969 323.4923 -1534.7075 7207.4422
325.2800 58.6930 6.1764 664265.7895 3.0496
3.0407
[15525]ENERGY: 4530000 3634.7127 5810.9502 5887.3970
343.7359 -15396.6174 -1794.4982 0.0000 0.0000
8656.3418 7142.0220 322.9230 -1514.3198 7208.0636
325.1602 -84.9806 -81.5641 664265.7895 -2.1191
-2.1136
[15561]ENERGY: 4540000 3552.2232 5892.1915 5921.7053
363.1032 -15390.2802 -1894.2454 0.0000 0.0000
8695.6037 7140.3012 324.3876 -1555.3025 7209.7520
325.3134 3.7544 -17.6213 664265.7895 3.4444
3.4381
[15611]ENERGY: 4550000 3550.9623 5843.0967 5879.7965
372.1268 -15358.8975 -1814.2054 0.0000 0.0000
8669.6036 7142.4830 323.4177 -1527.1206 7210.1755
325.3010 78.3915 104.6143 664265.7895 0.1726
0.1769
[15647]ENERGY: 4560000 3509.9204 5835.7054 5850.2881
353.3244 -15269.6437 -1845.0444 0.0000 0.0000
8709.6770 7144.2272 324.9126 -1565.4498 7211.1404
325.2043 98.7465 28.3995 664265.7895 -2.7355
-2.7336
[15697]ENERGY: 4570000 3521.5817 5826.8144 5901.7679
358.1749 -15432.4204 -1821.3882 0.0000 0.0000
8786.1491 7140.6794 327.7654 -1645.4697 7211.7992
325.2450 98.4320 42.7134 664265.7895 3.2972
3.3009
[15733]ENERGY: 4580000 3560.6526 5851.9458 5849.0131
329.6232 -15392.8802 -1785.6706 0.0000 0.0000
8730.2450 7142.9291 325.6799 -1587.3160 7211.0689
325.3819 131.2670 196.8507 664265.7895 2.9653
2.9672
[15783]ENERGY: 4590000 3572.1145 5889.5702 5848.7773
366.8831 -15417.8091 -1855.8713 0.0000 0.0000
8738.1685 7141.8331 325.9755 -1596.3354 7212.8084
325.3052 45.4559 125.1921 664265.7895 3.2191
3.2197
[15822]ENERGY: 4600000 3632.5119 5873.3700 5938.0283

363.1742 -15434.7200 -1943.7762 0.0000 0.0000
8711.5647 7140.1530 324.9830 -1571.4117 7211.8994
325.3425 -92.3675 -49.9434 664265.7895 -4.3651
-4.3471
[15872]ENERGY: 4610000 3510.7213 5830.5180 5878.9142
354.8244 -15370.9695 -1837.9750 0.0000 0.0000
8775.6642 7141.6976 327.3743 -1633.9666 7213.9071
325.3875 140.7721 120.1191 664265.7895 0.2490
0.2461
[15908]ENERGY: 4620000 3664.1266 5840.0874 5871.7884
367.1577 -15369.2178 -1890.0015 0.0000 0.0000
8661.0158 7144.9566 323.0973 -1516.0592 7215.2233
325.3668 -134.5958 -123.0243 664265.7895 -1.3672
-1.3670
[15958]ENERGY: 4630000 3563.1877 5881.6920 5884.6758
358.7816 -15343.1826 -1849.7764 0.0000 0.0000
8651.9169 7147.2950 322.7579 -1504.6219 7215.6574
325.4247 -88.4961 13.8113 664265.7895 -0.0501
-0.0688
[15994]ENERGY: 4640000 3567.9812 5691.7022 5919.8409
385.7403 -15360.5236 -1819.9666 0.0000 0.0000
8765.0091 7149.7835 326.9768 -1615.2256 7215.7251
325.3982 155.8447 111.1720 664265.7895 3.4643
3.4641
[16044]ENERGY: 4650000 3501.7708 5813.2325 5886.8743
348.6991 -15357.4649 -1882.6271 0.0000 0.0000
8832.2645 7142.7491 329.4857 -1689.5154 7215.1713
324.9952 5.8629 -14.7444 664265.7895 2.1391
2.1364
[16080]ENERGY: 4660000 3579.6132 5774.1666 5907.5681
345.9664 -15285.9789 -1904.8324 0.0000 0.0000
8728.2669 7144.7700 325.6061 -1583.4969 7216.4014
325.3173 -172.9402 -172.3828 664265.7895 3.8064
3.8265
[16130]ENERGY: 4670000 3554.6016 5836.4200 5871.5629
380.4518 -15392.9175 -1875.8201 0.0000 0.0000
8771.9272 7146.2259 327.2349 -1625.7013 7218.3345
325.2329 -18.4544 -206.4823 664265.7895 7.3868
7.3720
[16166]ENERGY: 4680000 3593.8693 5821.6716 5862.9004
372.9955 -15353.8263 -1917.8266 0.0000 0.0000
8764.7150 7144.4990 326.9658 -1620.2161 7218.1217
325.3727 107.9638 57.6981 664265.7895 -1.7386
-1.7344
[16216]ENERGY: 4690000 3498.8230 5872.2866 5880.6574
365.2779 -15331.4133 -1852.3850 0.0000 0.0000
8719.0376 7152.2842 325.2618 -1566.7534 7218.2330
325.3900 119.6532 -10.5751 664265.7895 1.7614
1.7664
[16255]ENERGY: 4700000 3674.6757 5833.0849 5915.9268
332.6950 -15433.1379 -1773.4438 0.0000 0.0000
8598.8367 7148.6375 320.7778 -1450.1992 7217.2626
325.2167 -175.7204 -55.9543 664265.7895 0.3939
0.3864

[16305]ENERGY: 4710000 3669.7337 5842.6078 5958.8435
364.6703 -15425.5701 -1877.7738 0.0000 0.0000
8616.1795 7148.6909 321.4247 -1467.4886 7219.6510
325.2603 -78.1050 -111.9459 664265.7895 1.8050
1.8081
[16341]ENERGY: 4720000 3574.2354 5786.5469 5941.3564
377.3238 -15306.7291 -1897.3015 0.0000 0.0000
8673.5846 7149.0166 323.5662 -1524.5681 7220.5994
325.2986 117.5766 109.8509 664265.7895 -1.4507
-1.4483
[16391]ENERGY: 4730000 3566.4381 5762.0214 5938.0959
362.4394 -15365.1235 -1850.9441 0.0000 0.0000
8736.2620 7149.1894 325.9044 -1587.0727 7219.4891
325.3628 25.7897 36.0569 664265.7895 1.0161
1.0245
[16427]ENERGY: 4740000 3500.5751 5843.6665 5934.2892
377.6441 -15390.8303 -1837.2289 0.0000 0.0000
8720.3698 7148.4854 325.3115 -1571.8844 7221.7718
325.2424 79.6736 33.1785 664265.7895 2.4125
2.4108
[16477]ENERGY: 4750000 3606.7618 5824.5672 5919.2397
350.7917 -15289.0708 -1888.8883 0.0000 0.0000
8630.6446 7154.0459 321.9643 -1476.5987 7223.1741
325.2627 -156.4654 -202.5637 664265.7895 -0.6993
-0.7169
[16513]ENERGY: 4760000 3631.1309 5900.3303 5911.7453
367.6950 -15373.7929 -1917.7374 0.0000 0.0000
8634.0407 7153.4119 322.0910 -1480.6288 7223.4673
325.4676 -131.5374 -99.4231 664265.7895 5.9509
5.9671
[16563]ENERGY: 4770000 3545.2967 5877.6883 5901.2695
364.3491 -15349.0851 -1902.8301 0.0000 0.0000
8715.8625 7152.5508 325.1434 -1563.3117 7222.1692
325.6828 64.5911 75.8363 664265.7895 2.3101
2.2863
[16599]ENERGY: 4780000 3550.8810 5804.4613 5947.9790
354.2607 -15339.3092 -1891.7231 0.0000 0.0000
8723.1576 7149.7073 325.4155 -1573.4504 7222.0396
325.2294 -69.9145 -96.0611 664265.7895 6.4223
6.4276
[16649]ENERGY: 4790000 3553.2805 5825.9630 5892.3647
367.2461 -15394.0924 -1827.7825 0.0000 0.0000
8732.2959 7149.2753 325.7564 -1583.0206 7219.9185
325.0341 -39.9719 -127.4632 664265.7895 4.1638
4.1738
[16688]ENERGY: 4800000 3545.6241 5785.9262 5908.3131
350.2529 -15315.2404 -1870.0188 0.0000 0.0000
8743.5234 7148.3805 326.1753 -1595.1429 7219.5821
325.4660 8.3265 80.5619 664265.7895 -1.5605
-1.5691
[16738]ENERGY: 4810000 3586.2725 5827.3868 5938.9677
371.4616 -15313.1386 -1951.4208 0.0000 0.0000
8694.6311 7154.1603 324.3513 -1540.4708 7221.2525
325.5578 110.0088 0.1888 664265.7895 5.4883

5.4961
[16774]ENERGY: 4820000 3523.0029 5800.1277 5889.3304
338.8424 -15296.8805 -1880.0696 0.0000 0.0000
8773.6311 7147.9843 327.2984 -1625.6467 7220.1798
325.5196 145.6794 38.4857 664265.7895 -3.5050
-3.5139
[16824]ENERGY: 4830000 3545.2815 5849.6338 5920.9170
361.8880 -15385.8764 -1902.2666 0.0000 0.0000
8760.1199 7149.6971 326.7944 -1610.4228 7220.3004
325.5341 113.8702 36.5727 664265.7895 -3.4930
-3.4869
[16860]ENERGY: 4840000 3585.1081 5762.7664 5946.2891
372.9907 -15380.6042 -1819.9665 0.0000 0.0000
8683.9982 7150.5819 323.9547 -1533.4163 7219.3184
325.5367 182.6570 170.4175 664265.7895 -8.5970
-8.5868
[16910]ENERGY: 4850000 3579.5807 5901.1382 5862.3723
350.8024 -15403.5832 -1808.8754 0.0000 0.0000
8669.2771 7150.7120 323.4055 -1518.5651 7219.9197
325.5784 42.1689 -20.7055 664265.7895 -5.7761
-5.7924
[16946]ENERGY: 4860000 3562.4498 5876.6689 5928.6409
371.1602 -15292.1776 -1949.6512 0.0000 0.0000
8655.4695 7152.5604 322.8904 -1502.9091 7220.0666
325.3863 21.8882 -34.2694 664265.7895 -5.8093
-5.7837
[16996]ENERGY: 4870000 3597.6715 5854.6409 5872.9984
372.5629 -15400.3971 -1808.4059 0.0000 0.0000
8662.2325 7151.3035 323.1427 -1510.9291 7219.5433
325.3986 63.8063 53.7480 664265.7895 -5.9365
-5.9487
[17032]ENERGY: 4880000 3608.4066 5902.5546 5869.8841
398.6309 -15423.9008 -1901.4542 0.0000 0.0000
8696.3796 7150.5009 324.4166 -1545.8788 7220.7181
325.3717 -1.0153 -57.6000 664265.7895 -5.2730
-5.2709
[17082]ENERGY: 4890000 3604.5569 5827.7458 5925.2903
349.5487 -15353.4759 -1894.4527 0.0000 0.0000
8692.6492 7151.8622 324.2774 -1540.7870 7219.4111
325.1977 -112.3644 -55.2162 664265.7895 -8.6009
-8.6130
[17121]ENERGY: 4900000 3540.6317 5797.4625 5914.2675
359.5564 -15303.1436 -1853.3269 0.0000 0.0000
8694.1386 7149.5861 324.3330 -1544.5524 7219.3443
325.0875 -174.3050 -172.3587 664265.7895 -4.5147
-4.5088
[17171]ENERGY: 4910000 3551.0258 5835.5825 5877.7193
355.1205 -15269.3047 -1879.7221 0.0000 0.0000
8680.7039 7151.1252 323.8318 -1529.5787 7221.0009
325.3882 63.3047 0.0557 664265.7895 -7.6435
-7.6271
[17207]ENERGY: 4920000 3593.1312 5730.6083 5859.3136
352.6838 -15325.0472 -1801.3832 0.0000 0.0000
8743.5205 7152.8269 326.1751 -1590.6936 7219.9150

325.6288 44.3795 22.4948 664265.7895 -6.0877
-6.1028
[17257]ENERGY: 4930000 3527.8462 5825.0938 5865.1057
367.8140 -15388.1485 -1838.1541 0.0000 0.0000
8787.6071 7147.1641 327.8198 -1640.4429 7220.4936
325.6120 18.1659 41.7459 664265.7895 -4.8388
-4.8414
[17293]ENERGY: 4940000 3523.1400 5765.6867 5888.0046
364.5388 -15312.1147 -1876.7836 0.0000 0.0000
8797.7724 7150.2442 328.1990 -1647.5282 7221.5815
325.5262 72.1095 -19.9579 664265.7895 -6.4418
-6.4555
[17343]ENERGY: 4950000 3541.4163 5768.9054 5931.3782
374.8524 -15365.2395 -1823.8757 0.0000 0.0000
8724.5209 7151.9580 325.4664 -1572.5629 7219.7412
325.2767 47.1668 67.9101 664265.7895 -2.1530
-2.0958
[17379]ENERGY: 4960000 3596.8323 5791.7119 5841.4020
348.6549 -15442.8638 -1765.8544 0.0000 0.0000
8779.6459 7149.5289 327.5228 -1630.1171 7221.0890
325.4141 14.3528 54.2734 664265.7895 -1.3726
-1.4190
[17429]ENERGY: 4970000 3564.0597 5839.2755 5906.5006
345.6006 -15352.6815 -1847.4281 0.0000 0.0000
8696.0358 7151.3625 324.4037 -1544.6733 7221.0222
325.3946 -161.0456 -107.6510 664265.7895 -5.2313
-5.2360
[17465]ENERGY: 4980000 3575.1688 5892.3186 5856.8577
349.8371 -15373.8912 -1852.7188 0.0000 0.0000
8705.3985 7152.9706 324.7530 -1552.4279 7221.0401
325.5431 5.8616 -51.9821 664265.7895 -2.0269
-2.0424
[17515]ENERGY: 4990000 3586.2962 5827.8222 5875.9667
367.3646 -15346.2398 -1794.4553 0.0000 0.0000
8635.4241 7152.1787 322.1426 -1483.2454 7222.8874
325.5497 140.3181 43.2201 664265.7895 -0.6358
-0.6179
[17554]ENERGY: 5000000 3584.5100 5857.5275 5798.9922
353.9776 -15403.8172 -1781.8114 0.0000 0.0000
8742.4577 7151.8364 326.1355 -1590.6213 7222.6743
325.6278 -36.5670 -49.9545 664265.7895 -9.6049
-9.5996
[17604]ENERGY: 5010000 3535.7806 5880.6962 5877.4896
350.1832 -15480.2317 -1775.2016 0.0000 0.0000
8761.9766 7150.6929 326.8636 -1611.2837 7223.7638
325.6858 70.2552 -16.7862 664265.7895 -1.4385
-1.4486
[17640]ENERGY: 5020000 3632.6518 5778.8087 5859.0296
356.8833 -15392.3966 -1819.6524 0.0000 0.0000
8740.2642 7155.5884 326.0537 -1584.6757 7222.6080
325.7071 112.7121 48.7550 664265.7895 -4.4379
-4.4173
[17690]ENERGY: 5030000 3520.9160 5882.8502 5878.5480
325.2511 -15416.0907 -1894.4824 0.0000 0.0000

8855.2872 7152.2794 330.3446 -1703.0078 7224.0353
325.7000 -54.2144 -67.2177 664265.7895 -1.8757
-1.8914
[17726]ENERGY: 5040000 3606.3149 5838.4676 5945.5256
359.4995 -15414.9391 -1787.2874 0.0000 0.0000
8603.6597 7151.2407 320.9577 -1452.4190 7224.7729
325.5981 38.9623 4.4774 664265.7895 -0.2994
-0.2904
[17776]ENERGY: 5050000 3431.0623 5778.8496 5921.1241
368.5432 -15286.4740 -1827.8442 0.0000 0.0000
8769.6920 7154.9529 327.1515 -1614.7390 7225.1905
325.3003 87.3478 20.9883 664265.7895 -1.5699
-1.5697
[17812]ENERGY: 5060000 3594.0193 5711.0695 5930.6014
366.3337 -15350.5720 -1870.4295 0.0000 0.0000
8775.3995 7156.4220 327.3644 -1618.9775 7226.6591
325.5093 -98.5946 -129.4195 664265.7895 0.4059
0.4104
[17862]ENERGY: 5070000 3421.3748 5849.5440 5844.6690
369.1085 -15304.5886 -1810.5798 0.0000 0.0000
8783.5897 7153.1175 327.6699 -1630.4722 7224.7149
325.7573 76.9816 45.4978 664265.7895 -3.7090
-3.7112
[17898]ENERGY: 5080000 3556.5440 5811.2949 5942.6142
383.4370 -15377.8675 -1886.0233 0.0000 0.0000
8728.5904 7158.5897 325.6182 -1570.0006 7228.1841
325.7134 64.2490 -45.0436 664265.7895 -3.9763
-3.9907
[17948]ENERGY: 5090000 3484.6293 5759.2343 5885.9685
360.7443 -15319.8216 -1817.9833 0.0000 0.0000
8802.6114 7155.3829 328.3795 -1647.2285 7226.3902
325.6823 -42.6814 -26.0989 664265.7895 -1.4122
-1.3881
[17987]ENERGY: 5100000 3572.6411 5774.9003 5912.6094
365.2444 -15440.5304 -1771.6272 0.0000 0.0000
8744.3106 7157.5482 326.2046 -1586.7624 7227.1373
325.8616 27.9792 47.1570 664265.7895 0.4225
0.4077
[18037]ENERGY: 5110000 3561.0995 5918.5762 5891.2221
343.5620 -15368.3315 -1878.9005 0.0000 0.0000
8688.1782 7155.4061 324.1106 -1532.7721 7228.1567
325.8572 -23.5489 -79.8572 664265.7895 0.3544
0.3574
[18073]ENERGY: 5120000 3605.8797 5803.6826 5871.4593
359.9897 -15362.5654 -1857.3075 0.0000 0.0000
8734.2882 7155.4267 325.8307 -1578.8615 7228.2411
325.8457 -145.2894 -35.9133 664265.7895 1.2596
1.2544
[18123]ENERGY: 5130000 3574.7445 5780.8257 5907.5075
338.9331 -15388.3512 -1867.1785 0.0000 0.0000
8813.3129 7159.7940 328.7787 -1653.5189 7228.1867
325.6725 0.0814 34.8352 664265.7895 -3.1694
-3.1659
[18159]ENERGY: 5140000 3532.1929 5913.3631 5853.6048

366.3946 -15458.0395 -1766.5142 0.0000 0.0000
8719.2012 7160.2029 325.2679 -1558.9983 7228.8432
325.6126 54.4405 22.8892 664265.7895 -2.4236
-2.4310
[18209]ENERGY: 5150000 3591.7217 5756.1877 5853.7822
367.1807 -15320.6487 -1830.2251 0.0000 0.0000
8738.4292 7156.4277 325.9852 -1582.0014 7228.7642
325.7718 34.2132 -58.8502 664265.7895 0.2528
0.2474
[18245]ENERGY: 5160000 3616.2296 5885.4494 5884.6857
358.3949 -15415.5808 -1829.9918 0.0000 0.0000
8658.5143 7157.7011 323.0040 -1500.8131 7226.8059
325.6343 9.5565 -37.8399 664265.7895 -0.5946
-0.5650
[18295]ENERGY: 5170000 3607.5074 5814.0829 5903.2682
365.3298 -15392.4167 -1840.7738 0.0000 0.0000
8701.7175 7158.7152 324.6157 -1543.0023 7228.5308
325.5233 83.4770 55.4774 664265.7895 2.7499
2.7457
[18331]ENERGY: 5180000 3623.7982 5810.2175 5868.3705
361.9740 -15372.8696 -1826.5828 0.0000 0.0000
8696.3061 7161.2138 324.4138 -1535.0923 7227.4756
325.4867 99.4158 68.0113 664265.7895 2.5069
2.5140
[18381]ENERGY: 5190000 3618.4204 5789.1598 5858.4761
372.0897 -15402.1128 -1865.1833 0.0000 0.0000
8786.5684 7157.4184 327.7810 -1629.1501 7228.0339
325.6125 88.6907 5.2505 664265.7895 0.8468
0.8203
[18420]ENERGY: 5200000 3611.0965 5789.5797 5875.9002
363.5753 -15380.2988 -1775.6356 0.0000 0.0000
8673.0609 7157.2782 323.5467 -1515.7827 7227.9773
325.3849 187.6317 141.9674 664265.7895 0.0930
0.1133
[18470]ENERGY: 5210000 3564.4016 5798.8101 5969.5128
363.6261 -15312.5559 -1849.9486 0.0000 0.0000
8626.4561 7160.3021 321.8081 -1466.1540 7229.8596
325.3666 57.9648 20.3661 664265.7895 -0.2418
-0.2669
[18506]ENERGY: 5220000 3565.0792 5744.9135 5925.2257
363.8309 -15303.8438 -1844.9814 0.0000 0.0000
8711.8065 7162.0306 324.9921 -1549.7760 7230.9116
325.3179 140.9766 67.0671 664265.7895 -5.0694
-5.0576
[18556]ENERGY: 5230000 3593.7026 5766.0438 5885.8360
353.5870 -15398.3196 -1819.7942 0.0000 0.0000
8778.4395 7159.4952 327.4778 -1618.9443 7229.5659
325.4209 186.6137 110.9558 664265.7895 -0.7288
-0.7442
[18592]ENERGY: 5240000 3548.3511 5853.6519 5841.1902
355.3544 -15386.0608 -1829.5659 0.0000 0.0000
8774.9981 7157.9189 327.3494 -1617.0792 7230.3785
325.5734 -46.4798 -80.4217 664265.7895 -1.5932
-1.5585

[18642]ENERGY: 5250000 3543.3636 5755.5802 5930.1702
349.9488 -15271.9287 -1918.9418 0.0000 0.0000
8771.5756 7159.7678 327.2217 -1611.8078 7230.0551
325.7503 -8.6089 -54.0454 664265.7895 3.6351
3.5912
[18678]ENERGY: 5260000 3578.3525 5809.4118 5917.4311
367.2958 -15358.3817 -1867.1512 0.0000 0.0000
8709.9880 7156.9463 324.9242 -1553.0417 7230.9435
325.7378 -78.2879 -82.3975 664265.7895 0.6778
0.6955
[18728]ENERGY: 5270000 3584.6716 5786.7018 5896.5068
350.0599 -15317.7683 -1885.9255 0.0000 0.0000
8746.7645 7161.0108 326.2962 -1585.7537 7231.0113
325.4122 65.0836 18.1452 664265.7895 6.6464
6.6576
[18764]ENERGY: 5280000 3549.5592 5820.4396 5930.6979
372.1784 -15382.3324 -1855.9671 0.0000 0.0000
8723.5791 7158.1548 325.4312 -1565.4243 7230.1003
325.3173 -63.8007 -58.8425 664265.7895 -3.1627
-3.1630
[18814]ENERGY: 5290000 3548.1087 5864.2572 5860.6154
379.2601 -15291.0345 -1844.2362 0.0000 0.0000
8644.2631 7161.2338 322.4724 -1483.0293 7230.6531
325.4817 15.1126 31.6516 664265.7895 -2.0611
-2.0597
[18853]ENERGY: 5300000 3592.8248 5826.4367 5857.1887
333.3376 -15308.9132 -1821.0784 0.0000 0.0000
8681.7625 7161.5587 323.8713 -1520.2038 7230.6694
325.4642 199.5536 59.3776 664265.7895 1.4855
1.4783
[18903]ENERGY: 5310000 3600.1231 5771.5445 5935.4506
341.1670 -15323.8948 -1812.7288 0.0000 0.0000
8647.9938 7159.6553 322.6115 -1488.3385 7230.9983
325.5675 -160.3856 -86.7192 664265.7895 -7.2191
-7.2074
[18939]ENERGY: 5320000 3525.9312 5829.6678 5907.8908
362.8510 -15280.7383 -1872.3958 0.0000 0.0000
8687.6641 7160.8709 324.0914 -1526.7932 7231.0491
325.7592 133.2190 3.4060 664265.7895 -3.9513
-3.9807
[18989]ENERGY: 5330000 3567.0904 5801.1694 5920.1368
343.0455 -15311.3343 -1875.1775 0.0000 0.0000
8714.1864 7159.1168 325.0808 -1555.0696 7231.0006
325.8038 -170.0510 -213.6307 664265.7895 0.5378
0.5786
[19025]ENERGY: 5340000 3562.3154 5815.0796 5903.3968
382.8837 -15336.9123 -1867.4881 0.0000 0.0000
8701.3897 7160.6649 324.6035 -1540.7249 7231.4352
325.9743 -64.8569 -178.7269 664265.7895 0.1409
0.1201
[19075]ENERGY: 5350000 3647.3305 5837.1101 5888.0285
357.5299 -15416.1758 -1828.0168 0.0000 0.0000
8677.2424 7163.0489 323.7027 -1514.1935 7230.4329
325.6170 -135.6536 -107.0156 664265.7895 -2.2823

-2.2863
[19111]ENERGY: 5360000 3551.2117 5887.4160 5906.1668
364.5655 -15411.6650 -1892.9262 0.0000 0.0000
8755.7712 7160.5401 326.6322 -1595.2311 7232.1167
325.6687 96.1681 123.9339 664265.7895 -3.0543
-3.0459
[19161]ENERGY: 5370000 3506.6054 5893.6776 5888.3116
382.8131 -15370.8392 -1818.9223 0.0000 0.0000
8679.8382 7161.4844 323.7995 -1518.3537 7232.0702
325.8609 118.9113 71.4452 664265.7895 -6.3241
-6.3411
[19197]ENERGY: 5380000 3617.2614 5738.2055 5955.5970
377.4131 -15381.3453 -1842.4488 0.0000 0.0000
8693.6075 7158.2905 324.3132 -1535.3170 7232.3540
325.7797 -103.1985 -54.3226 664265.7895 -12.1703
-12.1424
[19247]ENERGY: 5390000 3610.3324 5771.0234 5898.3320
360.3654 -15414.6086 -1848.6427 0.0000 0.0000
8786.2946 7163.0966 327.7708 -1623.1980 7234.1830
325.8075 -8.9748 -82.7350 664265.7895 -14.8391
-14.8685
[19286]ENERGY: 5400000 3580.7751 5863.8546 5847.6758
359.3932 -15457.9541 -1778.5738 0.0000 0.0000
8748.2886 7163.4594 326.3530 -1584.8292 7234.4524
325.6304 107.6282 9.4184 664265.7895 -14.9015
-14.8922
[19336]ENERGY: 5410000 3577.8226 5770.0119 5884.6469
358.4886 -15378.7812 -1839.0732 0.0000 0.0000
8790.0460 7163.1616 327.9108 -1626.8844 7232.4616
325.6864 -89.9854 -37.8462 664265.7895 -12.1484
-12.1427
[19372]ENERGY: 5420000 3672.8739 5731.9352 5888.3442
359.0961 -15353.7339 -1904.8946 0.0000 0.0000
8772.6400 7166.2609 327.2614 -1606.3791 7235.9016
325.9934 -190.0364 -196.4016 664265.7895 -12.7006
-12.7017
[19422]ENERGY: 5430000 3588.7123 5778.9367 5919.0472
354.1246 -15350.8593 -1820.8105 0.0000 0.0000
8698.2869 7167.4379 324.4877 -1530.8490 7234.2681
325.9703 64.7898 -20.0479 664265.7895 -10.5489
-10.5669
[19458]ENERGY: 5440000 3554.7079 5825.6917 5890.4085
365.2444 -15344.5146 -1858.0553 0.0000 0.0000
8733.0480 7166.5305 325.7845 -1566.5175 7235.0083
326.1491 175.6201 126.6755 664265.7895 -12.5522
-12.5384
[19508]ENERGY: 5450000 3655.7394 5691.5108 5895.7272
373.3275 -15465.7763 -1786.3056 0.0000 0.0000
8800.0805 7164.3036 328.2851 -1635.7769 7233.7237
326.0305 -50.8123 -115.4322 664265.7895 -9.8011
-9.7939
[19544]ENERGY: 5460000 3575.2781 5816.7572 5927.6434
355.2632 -15351.8392 -1872.9793 0.0000 0.0000
8715.1120 7165.2354 325.1154 -1549.8765 7235.3063

326.1792 -147.3131 -82.7474 664265.7895 -12.7921
-12.7959
[19594]ENERGY: 5470000 3518.2503 5831.0100 5885.4221
356.0420 -15304.3493 -1877.0714 0.0000 0.0000
8754.5500 7163.8537 326.5866 -1590.6963 7235.8754
326.0341 113.7524 46.6619 664265.7895 -14.8379
-14.8489
[19630]ENERGY: 5480000 3585.7184 5890.3649 5946.2701
393.4021 -15434.8199 -1870.7973 0.0000 0.0000
8656.8653 7167.0035 322.9425 -1489.8617 7236.3010
326.1461 96.3135 24.1003 664265.7895 -15.3946
-15.3999
[19680]ENERGY: 5490000 3617.3232 5822.9209 5933.0312
339.7321 -15354.0568 -1898.1446 0.0000 0.0000
8705.8621 7166.6681 324.7703 -1539.1940 7235.1693
326.3633 -148.7788 -87.1065 664265.7895 -18.3158
-18.3101
[19719]ENERGY: 5500000 3592.7573 5772.2975 5889.2019
367.0539 -15345.1786 -1935.2043 0.0000 0.0000
8821.3935 7162.3212 329.0802 -1659.0723 7236.6279
326.4640 -2.8029 -10.5912 664265.7895 -13.2200
-13.2213
[19769]ENERGY: 5510000 3624.6953 5780.0768 5876.0604
365.8983 -15312.8458 -1873.8478 0.0000 0.0000
8708.1386 7168.1759 324.8552 -1539.9627 7236.4940
326.2453 -36.0338 -58.9736 664265.7895 -16.1793
-16.1572
[19805]ENERGY: 5520000 3619.3654 5833.2368 5953.2688
349.9371 -15476.6894 -1845.2649 0.0000 0.0000
8729.0570 7162.9108 325.6356 -1566.1463 7236.7865
326.3008 -24.6586 7.5915 664265.7895 -13.4272
-13.4444
[19855]ENERGY: 5530000 3510.1681 5855.1048 5912.7009
350.6427 -15345.8741 -1862.7559 0.0000 0.0000
8744.8808 7164.8673 326.2259 -1580.0135 7237.6008
326.3220 -124.6873 -33.6117 664265.7895 -14.1909
-14.1873
[19891]ENERGY: 5540000 3559.5490 5795.0302 5873.4219
378.6508 -15407.9002 -1831.4739 0.0000 0.0000
8798.7628 7166.0407 328.2360 -1632.7221 7235.8741
326.0740 47.8385 14.7924 664265.7895 -16.3974
-16.4062
[19941]ENERGY: 5550000 3639.2222 5845.1649 5936.3230
327.7259 -15384.2659 -1887.6469 0.0000 0.0000
8691.6486 7168.1719 324.2401 -1523.4767 7236.5605
326.3720 107.0237 49.4331 664265.7895 -16.6042
-16.6060
[19977]ENERGY: 5560000 3552.4341 5836.0479 5864.3426
344.0768 -15390.6032 -1830.3256 0.0000 0.0000
8790.9641 7166.9366 327.9450 -1624.0275 7235.6967
326.2725 29.6520 19.2507 664265.7895 -16.9560
-16.9304
[20027]ENERGY: 5570000 3488.3171 5901.5877 5850.6429
363.6602 -15346.6149 -1803.8638 0.0000 0.0000

8714.6864 7168.4155 325.0995 -1546.2709 7234.9642
326.1844 71.8592 87.5502 664265.7895 -11.2870
-11.2854
[20063]ENERGY: 5580000 3624.2787 5852.5413 5903.7161
345.2156 -15263.2834 -1934.1752 0.0000 0.0000
8636.3295 7164.6225 322.1764 -1471.7070 7236.1111
326.0647 -73.1694 -116.3984 664265.7895 -14.8329
-14.8568
[20113]ENERGY: 5590000 3622.2294 5899.8835 5865.3558
375.6746 -15341.6469 -1936.6973 0.0000 0.0000
8681.9260 7166.7251 323.8774 -1515.2009 7237.4243
326.4236 64.5622 -26.7582 664265.7895 -13.3332
-13.3206
[20152]ENERGY: 5600000 3593.4884 5823.8023 5936.8764
362.0400 -15422.4275 -1914.8046 0.0000 0.0000
8784.5513 7163.5264 327.7058 -1621.0249 7236.3361
326.5321 64.8513 -41.6088 664265.7895 -17.6176
-17.6097
[20202]ENERGY: 5610000 3622.5105 5831.3519 5898.6630
361.7395 -15360.4963 -1837.2645 0.0000 0.0000
8651.5858 7168.0899 322.7455 -1483.4959 7236.7423
326.3862 38.8348 0.1719 664265.7895 -18.5290
-18.5340
[20238]ENERGY: 5620000 3544.8837 5899.5678 5853.7546
381.0810 -15407.1293 -1828.6734 0.0000 0.0000
8722.6536 7166.1380 325.3967 -1556.5157 7236.3265
326.5129 -7.5868 -44.5211 664265.7895 -15.6293
-15.6532
[20288]ENERGY: 5630000 3568.0720 5861.2211 5897.7159
336.1713 -15367.7880 -1861.7139 0.0000 0.0000
8734.4280 7168.1063 325.8360 -1566.3216 7236.1859
326.2811 14.9066 4.9769 664265.7895 -17.4415
-17.4192
[20324]ENERGY: 5640000 3568.3515 5757.2225 5873.8651
357.3943 -15357.2886 -1887.8896 0.0000 0.0000
8853.5715 7165.2266 330.2806 -1688.3449 7237.5178
326.2662 27.0418 -15.3284 664265.7895 -14.5057
-14.5144
[20374]ENERGY: 5650000 3607.7241 5819.1544 5919.4929
365.5878 -15317.2199 -1893.5722 0.0000 0.0000
8666.6303 7167.7973 323.3068 -1498.8330 7236.4376
326.1185 12.5751 -6.4243 664265.7895 -20.3025
-20.2968
[20410]ENERGY: 5660000 3660.6503 5706.3888 5898.6331
345.5212 -15253.6672 -1896.3219 0.0000 0.0000
8703.8472 7165.0514 324.6951 -1538.7958 7236.3438
326.1709 -29.8070 -76.2136 664265.7895 -16.9866
-16.9974
[20460]ENERGY: 5670000 3628.5666 5775.8887 5855.3765
366.2056 -15326.0765 -1860.6894 0.0000 0.0000
8727.4519 7166.7234 325.5757 -1560.7284 7238.2022
326.3537 50.9756 43.8091 664265.7895 -19.6577
-19.6574
[20496]ENERGY: 5680000 3585.0765 5741.1299 5905.2128

354.8109 -15334.8326 -1861.7315 0.0000 0.0000
8775.8513 7165.5172 327.3812 -1610.3341 7237.0740
326.4603 -51.6437 9.8431 664265.7895 -17.5200
-17.5141
[20546]ENERGY: 5690000 3649.5248 5856.3524 5887.3526
354.7135 -15397.3126 -1853.5438 0.0000 0.0000
8671.0805 7168.1674 323.4728 -1502.9131 7237.0273
326.5646 -75.8457 -109.2546 664265.7895 -14.6282
-14.6264
[20585]ENERGY: 5700000 3536.0360 5846.0767 5899.1413
370.1300 -15362.3829 -1828.4590 0.0000 0.0000
8707.5105 7168.0525 324.8318 -1539.4579 7236.3303
326.2996 -24.3890 -85.1813 664265.7895 -15.8288
-15.8463
[20635]ENERGY: 5710000 3668.6200 5790.1505 5896.0965
360.8688 -15407.3130 -1861.2914 0.0000 0.0000
8715.6603 7162.7916 325.1358 -1552.8687 7235.4438
326.3087 -237.2530 -90.6011 664265.7895 -20.7227
-20.7043
[20671]ENERGY: 5720000 3664.5580 5864.0347 5867.1113
372.2939 -15395.8047 -1871.1077 0.0000 0.0000
8668.7292 7169.8146 323.3851 -1498.9146 7236.7721
326.5114 -103.0071 -85.1723 664265.7895 -11.5468
-11.5443
[20721]ENERGY: 5730000 3589.4507 5864.8905 5898.7601
351.1804 -15441.6070 -1840.5053 0.0000 0.0000
8742.2680 7164.4375 326.1284 -1577.8306 7236.1320
326.3288 114.4749 109.0459 664265.7895 -9.5963
-9.6003
[20757]ENERGY: 5740000 3572.4753 5740.7765 5914.7888
359.9416 -15277.0310 -1890.3571 0.0000 0.0000
8749.2835 7169.8775 326.3901 -1579.4060 7238.6527
326.1091 -108.5115 -111.7754 664265.7895 -10.7451
-10.7505
[20807]ENERGY: 5750000 3623.7955 5881.7529 5859.8198
339.6629 -15316.7845 -1913.1700 0.0000 0.0000
8691.8785 7166.9552 324.2487 -1524.9233 7238.1912
326.1713 -129.8622 -35.1523 664265.7895 -13.0172
-12.9966
[20843]ENERGY: 5760000 3603.4385 5834.4838 5899.0148
347.9558 -15325.7447 -1850.5381 0.0000 0.0000
8661.1902 7169.8003 323.1038 -1491.3899 7238.6522
326.1150 11.9391 -18.8326 664265.7895 -11.2538
-11.2834
[20893]ENERGY: 5770000 3543.9961 5934.0074 5893.2660
362.6309 -15338.7061 -1879.6084 0.0000 0.0000
8655.0111 7170.5970 322.8733 -1484.4141 7238.1598
326.4459 183.6111 219.9544 664265.7895 -13.4581
-13.4567
[20929]ENERGY: 5780000 3635.1148 5823.2994 5877.4381
374.4834 -15340.5719 -1950.5288 0.0000 0.0000
8751.1065 7170.3416 326.4581 -1580.7649 7240.0211
326.2946 -48.2132 -137.3763 664265.7895 -15.7719
-15.7585

[20979]ENERGY: 5790000 3623.2008 5786.1473 5848.6192
345.3551 -15351.2619 -1827.8139 0.0000 0.0000
8746.4635 7170.7100 326.2849 -1575.7535 7238.8801
326.5269 -44.2260 -5.7679 664265.7895 -7.0967
-7.0921
[21018]ENERGY: 5800000 3574.4895 5890.1538 5899.7674
354.1422 -15455.6217 -1824.2795 0.0000 0.0000
8731.5038 7170.1555 325.7269 -1561.3483 7239.7208
326.4495 -55.8027 -41.1664 664265.7895 -12.2876
-12.2985
[21068]ENERGY: 5810000 3547.9120 5831.0263 5898.0179
360.1454 -15344.1697 -1844.0491 0.0000 0.0000
8723.5257 7172.4084 325.4292 -1551.1173 7239.6371
326.4726 77.3525 -0.5090 664265.7895 -6.5870
-6.5841
[21104]ENERGY: 5820000 3596.9742 5932.7324 5917.7185
348.0485 -15447.4635 -1913.9965 0.0000 0.0000
8735.5990 7169.6127 325.8796 -1565.9864 7238.9329
326.3950 -91.0028 -104.6949 664265.7895 -11.2976
-11.3063
[21154]ENERGY: 5830000 3543.1216 5855.8911 5919.0215
373.8936 -15375.5600 -1883.9558 0.0000 0.0000
8737.2597 7169.6716 325.9416 -1567.5882 7239.8741
326.4396 124.4476 53.4443 664265.7895 -9.0347
-9.0365
[21190]ENERGY: 5840000 3533.3585 5908.9966 5898.3885
361.0825 -15404.0574 -1916.6035 0.0000 0.0000
8783.6680 7164.8331 327.6728 -1618.8349 7238.3056
326.1401 34.5688 53.8548 664265.7895 -10.6529
-10.6346
[21240]ENERGY: 5850000 3566.4641 5897.1869 5911.5619
353.7816 -15369.5973 -1893.7695 0.0000 0.0000
8704.6893 7170.3171 324.7266 -1534.3722 7240.4271
326.3480 209.7094 106.7783 664265.7895 -14.3938
-14.4046
[21276]ENERGY: 5860000 3601.0734 5896.1331 5878.8792
351.8230 -15413.7741 -1812.3363 0.0000 0.0000
8668.6554 7170.4536 323.3823 -1498.2018 7238.8906
326.2054 8.9587 31.2591 664265.7895 -10.4846
-10.5049
[21326]ENERGY: 5870000 3708.2391 5878.8313 5885.1093
350.2601 -15440.4417 -1902.6588 0.0000 0.0000
8690.5223 7169.8615 324.1981 -1520.6608 7239.9781
326.2962 -83.3184 -151.2258 664265.7895 -11.0400
-11.0162
[21362]ENERGY: 5880000 3542.5040 5770.3417 5910.3036
369.8785 -15330.5660 -1822.4857 0.0000 0.0000
8730.4158 7170.3919 325.6863 -1560.0239 7240.7738
326.1251 175.3260 142.3370 664265.7895 -12.7719
-12.7819
[21412]ENERGY: 5890000 3576.6019 5821.2847 5935.8141
362.1046 -15435.5908 -1862.1516 0.0000 0.0000
8774.6036 7172.6665 327.3347 -1601.9371 7240.2089
326.2489 22.0348 22.1143 664265.7895 -10.4457

-10.4355
[21451]ENERGY: 5900000   3541.5831   5853.9531   5874.1668
346.6521   -15323.4648   -1901.2834   0.0000   0.0000
8777.8615   7169.4684   327.4562   -1608.3932   7240.4572
326.1569   45.6491   41.3628   664265.7895   -14.3923
-14.3822
[21501]ENERGY: 5910000   3551.9715   5768.6238   5966.3560
355.7759   -15366.0288   -1928.1288   0.0000   0.0000
8819.8666   7168.4362   329.0232   -1651.4303   7239.6585
326.1812   -65.8963   3.5432   664265.7895   -10.6738
-10.6702
[21537]ENERGY: 5920000   3610.0686   5832.4958   5935.1482
358.4227   -15459.6895   -1859.2931   0.0000   0.0000
8755.7833   7172.9362   326.6326   -1582.8471   7241.3895
326.2055   58.3211   -105.6066   664265.7895   -15.1961
-15.2172
[21587]ENERGY: 5930000   3626.6797   5861.9205   5938.8084
343.8723   -15469.5659   -1883.9402   0.0000   0.0000
8751.7223   7169.4972   326.4811   -1582.2251   7241.1052
326.3519   35.0930   -36.9111   664265.7895   -14.6753
-14.6672
[21623]ENERGY: 5940000   3542.1456   5760.5924   5930.4742
359.7742   -15369.7919   -1866.6949   0.0000   0.0000
8814.5513   7171.0509   328.8249   -1643.5003   7241.6058
326.2071   52.0373   -100.9469   664265.7895   -14.1949
-14.2067
[21673]ENERGY: 5950000   3665.6348   5842.6217   5994.4031
375.5518   -15426.6386   -1930.5746   0.0000   0.0000
8654.4842   7175.4825   322.8537   -1479.0017   7241.3612
326.0799   -103.5676   -122.7361   664265.7895   -14.9410
-14.9336
[21709]ENERGY: 5960000   3640.6838   5894.8447   5896.7473
371.8779   -15440.9905   -1906.8963   0.0000   0.0000
8716.0450   7172.3117   325.1502   -1543.7332   7240.1735
326.0953   -53.7475   -39.6346   664265.7895   -13.3298
-13.3269
[21759]ENERGY: 5970000   3611.0142   5796.2842   5855.9794
362.0622   -15361.9297   -1871.7388   0.0000   0.0000
8781.1423   7172.8138   327.5786   -1608.3285   7241.2555
326.4665   -188.1163   -223.3853   664265.7895   -13.9296
-13.9289
[21795]ENERGY: 5980000   3612.6498   5733.1341   5930.0674
354.7023   -15365.5497   -1841.5265   0.0000   0.0000
8742.7998   7166.2773   326.1483   -1576.5225   7240.5008
326.6319   169.1684   61.6283   664265.7895   -14.3627
-14.3480
[21845]ENERGY: 5990000   3552.8702   5902.6131   5921.6453
356.7242   -15418.5296   -1867.8597   0.0000   0.0000
8721.6446   7169.1081   325.3591   -1552.5366   7240.3212
326.4472   99.7438   64.1511   664265.7895   -13.7533
-13.7721
[21884]ENERGY: 6000000   3520.5340   5874.9767   5960.5393
331.5135   -15378.5630   -1892.6602   0.0000   0.0000
8756.4666   7172.8069   326.6581   -1583.6597   7240.9359

326.4237 -127.6414 -104.8939 664265.7895 -9.2332
-9.2356
[21934]ENERGY: 6010000 3654.6907 5901.0032 5867.9965
364.2313 -15391.9086 -1909.1439 0.0000 0.0000
8684.0759 7170.9451 323.9576 -1513.1308 7242.1113
326.4967 -196.4010 -214.6720 664265.7895 -9.3547
-9.3485
[21970]ENERGY: 6020000 3612.2060 5773.4832 5873.4905
350.5095 -15391.2155 -1872.7061 0.0000 0.0000
8824.5550 7170.3226 329.1981 -1654.2323 7241.2448
326.4265 6.5086 -40.2516 664265.7895 -11.8721
-11.8680
[22020]ENERGY: 6030000 3575.8111 5834.8565 5949.2084
361.9587 -15386.8213 -1872.1021 0.0000 0.0000
8709.3996 7172.3108 324.9023 -1537.0888 7240.7170
326.6285 14.3134 29.5183 664265.7895 -13.2082
-13.1870
[22056]ENERGY: 6040000 3646.5965 5806.1815 5918.2030
365.9285 -15376.7906 -1906.8325 0.0000 0.0000
8719.4472 7172.7336 325.2771 -1546.7136 7242.6152
326.6459 -200.9105 -153.4000 664265.7895 -12.0148
-12.0357
[22106]ENERGY: 6050000 3539.0283 5752.3042 5946.9201
360.4810 -15380.8083 -1824.3583 0.0000 0.0000
8778.2343 7171.8011 327.4701 -1606.4331 7242.1431
326.5609 -28.3720 -9.5149 664265.7895 -13.2248
-13.2223
[22142]ENERGY: 6060000 3513.2175 5857.9508 5868.1972
327.4122 -15262.8975 -1893.9154 0.0000 0.0000
8758.6992 7168.6641 326.7414 -1590.0351 7243.4010
326.4650 27.8414 44.1520 664265.7895 -14.0586
-14.0701
[22192]ENERGY: 6070000 3653.4447 5823.3549 5876.8342
367.9315 -15319.6638 -1884.8878 0.0000 0.0000
8664.6819 7181.6956 323.2341 -1482.9863 7245.3127
326.4655 -121.9374 -137.2991 664265.7895 -16.8491
-16.8541
[22228]ENERGY: 6080000 3592.1408 5833.6532 5931.5741
364.7211 -15374.9041 -1865.3100 0.0000 0.0000
8693.9443 7175.8194 324.3257 -1518.1250 7245.6182
326.5088 -110.3125 -112.5043 664265.7895 -19.4266
-19.4035
[22278]ENERGY: 6090000 3590.6674 5824.2300 5871.6294
360.5730 -15445.7798 -1846.0965 0.0000 0.0000
8816.1555 7171.3790 328.8848 -1644.7765 7245.0815
326.6061 28.6843 -17.3728 664265.7895 -17.1732
-17.1641
[22317]ENERGY: 6100000 3610.5157 5834.4363 5931.6965
367.9487 -15334.4296 -1876.3418 0.0000 0.0000
8643.7743 7177.6000 322.4541 -1466.1743 7246.5325
326.3226 -5.4495 -96.5363 664265.7895 -17.3603
-17.3852
[22367]ENERGY: 6110000 3660.7331 5803.2640 5968.3130
344.5008 -15430.4638 -1880.5240 0.0000 0.0000

8708.7394 7174.5626 324.8776 -1534.1768 7245.1074
326.5380 -129.8404 -70.0938 664265.7895 -19.5294
-19.5049
[22403]ENERGY: 6120000 3623.2793 5892.4139 5841.9718
373.7003 -15463.5453 -1791.6704 0.0000 0.0000
8696.0337 7172.1833 324.4037 -1523.8504 7246.4514
326.2819 97.9126 97.6003 664265.7895 -18.0945
-18.1045
[22453]ENERGY: 6130000 3569.9765 5912.5154 5919.6829
365.0673 -15358.8491 -1975.2512 0.0000 0.0000
8743.6574 7176.7992 326.1803 -1566.8582 7247.1932
326.4163 -136.0285 -110.4236 664265.7895 -16.4293
-16.4474
[22489]ENERGY: 6140000 3588.7049 5811.3976 5832.0788
377.0392 -15365.8282 -1813.2754 0.0000 0.0000
8747.8834 7178.0004 326.3379 -1569.8831 7246.6999
326.5755 224.5651 87.7273 664265.7895 -14.6941
-14.6829
[22539]ENERGY: 6150000 3562.4170 5805.0104 5871.3869
369.1799 -15386.8497 -1825.6784 0.0000 0.0000
8780.6748 7176.1410 327.5612 -1604.5339 7246.5921
326.5925 -90.9171 5.4664 664265.7895 -15.8965
-15.8952
[22575]ENERGY: 6160000 3655.6360 5877.6687 5880.0387
349.9773 -15376.7601 -1867.5991 0.0000 0.0000
8661.1681 7180.1296 323.1030 -1481.0385 7248.5445
326.5581 42.4489 -17.6680 664265.7895 -15.9993
-16.0208
[22625]ENERGY: 6170000 3645.1899 5838.2528 5908.9374
357.6264 -15444.3939 -1833.9597 0.0000 0.0000
8708.3325 7179.9854 324.8625 -1528.3471 7248.0916
326.5866 -19.3357 36.1061 664265.7895 -16.1315
-16.1090
[22661]ENERGY: 6180000 3625.7070 5767.6482 5840.6444
390.0178 -15394.2370 -1832.8075 0.0000 0.0000
8776.7817 7173.7546 327.4160 -1603.0272 7246.5939
326.5929 -46.8250 -44.4129 664265.7895 -16.8130
-16.8227
[22711]ENERGY: 6190000 3587.2098 5799.5495 5889.0564
332.3972 -15321.4552 -1876.3614 0.0000 0.0000
8764.4308 7174.8270 326.9552 -1589.6038 7249.0935
326.7397 -9.7691 -0.1450 664265.7895 -18.4058
-18.3984
[22750]ENERGY: 6200000 3480.8759 5782.3159 5890.8371
346.2616 -15365.2535 -1821.9726 0.0000 0.0000
8862.1749 7175.2393 330.6015 -1686.9356 7248.5912
326.6197 168.6081 191.6698 664265.7895 -18.9181
-18.9207
[22800]ENERGY: 6210000 3627.4267 5733.2004 5923.7992
361.4087 -15382.5831 -1813.8441 0.0000 0.0000
8727.9946 7177.4024 325.5960 -1550.5921 7247.6243
326.4381 2.1869 -13.9963 664265.7895 -19.4432
-19.4243
[22836]ENERGY: 6220000 3581.2635 5846.0878 5860.6966

357.3991 -15415.0183 -1845.8873 0.0000 0.0000
8791.0620 7175.6035 327.9487 -1615.4585 7248.1767
326.4154 -45.2329 -62.5518 664265.7895 -15.7853
-15.7958
[22886]ENERGY: 6230000 3653.7652 5795.6644 5932.1086
343.3788 -15391.4801 -1841.1647 0.0000 0.0000
8687.5116 7179.7839 324.0857 -1507.7277 7248.8958
326.5047 -75.3848 -99.7991 664265.7895 -19.2489
-19.2411
[22922]ENERGY: 6240000 3560.3912 5821.3892 5909.0956
364.8443 -15449.3655 -1776.2170 0.0000 0.0000
8747.4252 7177.5629 326.3208 -1569.8623 7249.4685
326.2827 182.4400 118.4571 664265.7895 -18.8148
-18.8100
[22972]ENERGY: 6250000 3511.9176 5917.6834 5879.8992
358.0034 -15374.7698 -1832.5681 0.0000 0.0000
8715.4826 7175.6482 325.1292 -1539.8344 7248.2545
326.5235 161.0653 130.7545 664265.7895 -17.6472
-17.6519
[23008]ENERGY: 6260000 3655.6755 5765.3158 5917.1705
354.0939 -15391.4059 -1915.2948 0.0000 0.0000
8791.4013 7176.9563 327.9613 -1614.4450 7248.4079
326.7042 -69.4213 28.0214 664265.7895 -17.8173
-17.8202
[23058]ENERGY: 6270000 3521.2068 5894.6989 5895.3284
359.5857 -15453.6047 -1769.2917 0.0000 0.0000
8729.3568 7177.2803 325.6468 -1552.0765 7248.4339
326.6740 159.1959 66.6074 664265.7895 -17.1906
-17.2082
[23094]ENERGY: 6280000 3624.2409 5852.0825 5908.1262
352.8762 -15416.6950 -1878.5309 0.0000 0.0000
8737.0020 7179.1020 325.9320 -1557.9000 7248.4312
326.7366 108.9928 -33.5377 664265.7895 -11.6247
-11.6147
[23144]ENERGY: 6290000 3570.9336 5829.7671 5908.0879
353.4882 -15363.2810 -1914.9334 0.0000 0.0000
8794.1304 7178.1929 328.0631 -1615.9375 7248.4720
326.6895 33.7154 25.3657 664265.7895 -18.8871
-18.8875
[23183]ENERGY: 6300000 3595.6506 5908.4864 5905.4288
353.9985 -15412.4603 -1856.4002 0.0000 0.0000
8684.7829 7179.4868 323.9840 -1505.2961 7248.5873
326.6475 65.5276 87.4023 664265.7895 -14.9744
-14.9728
[23233]ENERGY: 6310000 3692.2128 5824.7811 5907.7035
368.2977 -15446.8738 -1847.1906 0.0000 0.0000
8681.7047 7180.6353 323.8691 -1501.0693 7248.5303
326.8555 39.3677 47.2622 664265.7895 -18.3350
-18.3474
[23269]ENERGY: 6320000 3760.4163 5656.3067 5857.2137
336.1740 -15406.5680 -1826.6284 0.0000 0.0000
8801.8032 7178.7175 328.3494 -1623.0857 7248.8230
326.8245 -161.3310 -165.0525 664265.7895 -19.4019
-19.3947

[23319]ENERGY: 6330000 3598.1229 5743.8320 5974.5900
362.7978 -15452.8727 -1852.8391 0.0000 0.0000
8803.8200 7177.4509 328.4246 -1626.3691 7250.4921
326.7227 -70.0389 -78.4974 664265.7895 -17.4010
-17.3981
[23355]ENERGY: 6340000 3545.3331 5840.6666 5947.7802
345.0837 -15399.2050 -1868.5494 0.0000 0.0000
8768.3334 7179.4427 327.1008 -1588.8907 7251.3324
326.7619 44.0492 124.4586 664265.7895 -15.4472
-15.4487
[23405]ENERGY: 6350000 3508.2761 5873.3539 5941.0739
372.3324 -15440.3306 -1889.3295 0.0000 0.0000
8813.8442 7179.2203 328.7986 -1634.6239 7251.1946
326.6550 79.6594 14.6624 664265.7895 -16.0051
-16.0312
[23441]ENERGY: 6360000 3554.3595 5792.0219 5872.8878
375.0159 -15362.6272 -1856.7246 0.0000 0.0000
8805.1553 7180.0888 328.4744 -1625.0666 7251.4326
326.6303 -102.0561 -110.3846 664265.7895 -10.6657
-10.6392
[23491]ENERGY: 6370000 3602.6549 5734.1072 5872.4151
329.9573 -15284.5251 -1893.4999 0.0000 0.0000
8819.0219 7180.1314 328.9917 -1638.8905 7251.7558
326.3442 49.1459 -64.2689 664265.7895 -15.2968
-15.2962
[23527]ENERGY: 6380000 3634.2997 5772.9318 5889.8937
344.5918 -15493.3971 -1799.4743 0.0000 0.0000
8828.9510 7177.7966 329.3621 -1651.1544 7249.6338
326.4421 30.0378 59.0669 664265.7895 -18.1644
-18.1643
[23577]ENERGY: 6390000 3493.1713 5777.8183 5919.0901
363.9893 -15280.9690 -1837.5452 0.0000 0.0000
8741.1752 7176.7300 326.0877 -1564.4453 7248.5589
326.4732 -30.3603 -0.5966 664265.7895 -18.8989
-18.8831
[23616]ENERGY: 6400000 3583.6165 5830.7456 5850.3855
354.5441 -15342.7902 -1852.2155 0.0000 0.0000
8756.4257 7180.7116 326.6566 -1575.7141 7249.3982
326.5650 139.8924 60.9741 664265.7895 -19.2059
-19.2302
[23666]ENERGY: 6410000 3552.4939 5866.3957 5834.7017
361.8856 -15351.8333 -1904.2841 0.0000 0.0000
8820.0750 7179.4343 329.0310 -1640.6406 7250.1520
326.9749 -31.4529 12.2521 664265.7895 -21.8852
-21.8777
[23702]ENERGY: 6420000 3674.4665 5801.4727 5879.8163
385.5282 -15366.6012 -1868.2121 0.0000 0.0000
8674.6367 7181.1071 323.6055 -1493.5296 7249.2874
327.0322 -80.4083 -87.8043 664265.7895 -22.7161
-22.7052
[23752]ENERGY: 6430000 3685.6162 5854.5033 5869.8219
356.1447 -15428.6263 -1883.2397 0.0000 0.0000
8722.9140 7177.1341 325.4064 -1545.7799 7249.3526
326.7977 -40.7576 -17.8137 664265.7895 -25.0900

-25.1118
[23788]ENERGY: 6440000 3589.4336 5828.0652 5914.2000
333.1509 -15307.0242 -1893.3194 0.0000 0.0000
8711.7229 7176.2290 324.9889 -1535.4939 7249.5544
326.7838 -94.6012 -161.4884 664265.7895 -20.1541
-20.1401
[23838]ENERGY: 6450000 3619.4567 5803.2815 5919.0188
349.4177 -15416.2955 -1842.8556 0.0000 0.0000
8745.0965 7177.1199 326.2339 -1567.9765 7249.2867
326.6976 -157.2185 -128.6319 664265.7895 -20.3156
-20.3235
[23874]ENERGY: 6460000 3596.5634 5873.1299 5868.2292
345.5715 -15445.3375 -1861.3169 0.0000 0.0000
8799.7058 7176.5455 328.2711 -1623.1603 7250.2022
326.8639 81.7548 55.7118 664265.7895 -16.0265
-16.0333
[23924]ENERGY: 6470000 3552.3610 5819.2340 5871.2994
383.4457 -15345.7342 -1909.9165 0.0000 0.0000
8803.0948 7173.7842 328.3976 -1629.3106 7250.6217
326.7941 -63.6923 -96.9363 664265.7895 -20.2605
-20.2466
[23960]ENERGY: 6480000 3608.5919 5875.3550 5903.4336
345.3016 -15493.5387 -1835.2503 0.0000 0.0000
8774.0937 7177.9868 327.3157 -1596.1069 7251.3266
326.6003 -199.6881 -155.6529 664265.7895 -16.7532
-16.7422
[24010]ENERGY: 6490000 3551.0791 5826.9430 5938.5999
370.4839 -15382.4381 -1845.1799 0.0000 0.0000
8722.5383 7182.0262 325.3924 -1540.5121 7252.4920
326.7084 -81.5194 -53.7840 664265.7895 -17.4311
-17.4368
[24049]ENERGY: 6500000 3560.7369 5863.9946 5890.1037
366.0784 -15383.5760 -1802.7183 0.0000 0.0000
8687.9548 7182.5741 324.1023 -1505.3807 7251.3754
326.6085 -68.0470 -89.0805 664265.7895 -18.2757
-18.2749
[24099]ENERGY: 6510000 3629.0721 5868.6113 5862.7306
368.1782 -15408.6355 -1887.2558 0.0000 0.0000
8749.4078 7182.1087 326.3948 -1567.2991 7251.8308
326.7384 182.8134 58.9448 664265.7895 -16.4786
-16.4902
[24135]ENERGY: 6520000 3566.9393 5880.1390 5940.6555
386.4662 -15517.9536 -1854.8577 0.0000 0.0000
8779.2534 7180.6421 327.5082 -1598.6113 7250.1942
327.0003 -23.3345 34.7980 664265.7895 -10.2185
-10.1870
[24185]ENERGY: 6530000 3579.3321 5782.3326 5925.1189
383.1088 -15399.6879 -1869.4926 0.0000 0.0000
8779.2520 7179.9639 327.5081 -1599.2881 7251.3443
326.8907 87.4024 66.7488 664265.7895 -11.8304
-11.8604
[24221]ENERGY: 6540000 3614.1458 5824.3209 5952.3923
358.9562 -15393.7341 -1915.3882 0.0000 0.0000
8741.9875 7182.6803 326.1180 -1559.3072 7252.7941

326.7566 -34.8757 -42.5580 664265.7895 -15.4703
-15.4669
[24271]ENERGY: 6550000 3654.2888 5840.6943 5939.3914
375.2493 -15349.7040 -1931.4773 0.0000 0.0000
8659.9643 7188.4069 323.0581 -1471.5574 7253.4643
326.7843 31.6123 -30.3568 664265.7895 -18.0503
-18.0506
[24307]ENERGY: 6560000 3609.7562 5819.8477 5925.8118
355.3011 -15332.0963 -1861.7535 0.0000 0.0000
8669.0704 7185.9376 323.3978 -1483.1328 7253.3608
326.6756 132.9912 62.3297 664265.7895 -17.8223
-17.8368
[24357]ENERGY: 6570000 3588.0015 5787.8798 5905.9738
354.2337 -15367.8532 -1878.8464 0.0000 0.0000
8793.0634 7182.4525 328.0233 -1610.6109 7252.8120
326.6628 54.1639 -48.5607 664265.7895 -16.9411
-16.9268
[24393]ENERGY: 6580000 3631.8554 5857.6220 5961.8947
387.3147 -15471.9019 -1839.3152 0.0000 0.0000
8657.7735 7185.2432 322.9764 -1472.5303 7253.7309
326.5771 163.4038 168.6800 664265.7895 -15.8738
-15.8655
[24443]ENERGY: 6590000 3590.8269 5748.3252 5913.2755
335.1365 -15352.3317 -1858.1188 0.0000 0.0000
8806.8544 7183.9682 328.5378 -1622.8863 7253.9593
326.6395 -96.0983 -98.8028 664265.7895 -18.3041
-18.3132
[24482]ENERGY: 6600000 3603.8132 5761.0494 5923.6268
357.1663 -15373.6095 -1821.0608 0.0000 0.0000
8735.4234 7186.4089 325.8731 -1549.0145 7253.9933
326.7786 36.5368 105.3964 664265.7895 -11.7298
-11.7372
[24532]ENERGY: 6610000 3593.8346 5785.9719 5885.1509
343.8818 -15367.2123 -1847.0824 0.0000 0.0000
8791.9568 7186.5013 327.9821 -1605.4555 7254.0284
326.8704 106.1304 78.9613 664265.7895 -11.1648
-11.1636
[24568]ENERGY: 6620000 3619.1854 5832.2000 5963.1991
346.2042 -15433.9079 -1896.7689 0.0000 0.0000
8753.1098 7183.2217 326.5329 -1569.8881 7254.0656
326.8566 16.9411 -50.1216 664265.7895 -12.4587
-12.4759
[24618]ENERGY: 6630000 3591.6409 5788.0494 5921.2962
370.8275 -15389.4318 -1833.6051 0.0000 0.0000
8734.5719 7183.3490 325.8413 -1551.2229 7255.2419
327.0881 6.5754 -38.3226 664265.7895 -12.2096
-12.1895
[24654]ENERGY: 6640000 3609.9116 5799.7581 5982.8195
355.2289 -15504.1347 -1846.3335 0.0000 0.0000
8781.9495 7179.1994 327.6087 -1602.7501 7254.5432
326.6923 13.5942 -33.1326 664265.7895 -13.0495
-13.0572
[24704]ENERGY: 6650000 3545.6914 5809.5549 5874.6068
359.2223 -15375.9918 -1815.5474 0.0000 0.0000

8786.4394 7183.9756 327.7762 -1602.4637 7253.9218
326.8215 11.9179 -57.1084 664265.7895 -13.2763
-13.2759
[24740]ENERGY: 6660000 3644.0939 5861.7475 5925.1325
336.3792 -15420.5391 -1883.0939 0.0000 0.0000
8717.6747 7181.3947 325.2110 -1536.2800 7252.5324
326.8341 -139.9287 -164.0442 664265.7895 -5.5975
-5.5929
[24790]ENERGY: 6670000 3602.4796 5832.2300 5897.4211
342.2975 -15353.1828 -1892.3822 0.0000 0.0000
8755.9020 7184.7653 326.6370 -1571.1368 7255.3479
326.8837 134.1911 98.9722 664265.7895 -8.1110
-8.1107
[24826]ENERGY: 6680000 3558.4082 5806.8083 5896.7934
345.8722 -15326.4090 -1838.7948 0.0000 0.0000
8743.7527 7186.4310 326.1838 -1557.3217 7257.2754
326.8006 140.2935 83.9341 664265.7895 -9.8547
-9.8550
[24876]ENERGY: 6690000 3615.1797 5952.6949 5905.6315
352.3475 -15411.4812 -1911.2324 0.0000 0.0000
8682.9227 7186.0628 323.9146 -1496.8599 7255.7434
326.7959 -39.2072 -40.9212 664265.7895 -13.0716
-13.0601
[24915]ENERGY: 6700000 3581.9732 5874.0512 5911.6951
345.2066 -15353.7818 -1850.3393 0.0000 0.0000
8677.8109 7186.6158 323.7239 -1491.1950 7256.9064
326.6762 -38.0279 -103.8978 664265.7895 -15.9417
-15.9590
[24965]ENERGY: 6710000 3637.7423 5843.7273 5915.5795
341.9452 -15451.3777 -1858.9823 0.0000 0.0000
8759.5195 7188.1538 326.7720 -1571.3657 7256.9080
326.7330 -81.3966 -135.3173 664265.7895 -17.1303
-17.1247
[25001]ENERGY: 6720000 3620.3714 5734.6084 5894.0189
371.8677 -15311.3331 -1896.6541 0.0000 0.0000
8773.4134 7186.2926 327.2903 -1587.1208 7256.6727
326.8851 -51.4441 -37.9988 664265.7895 -14.9692
-14.9711
[25051]ENERGY: 6730000 3757.0667 5835.1650 5940.9691
351.2056 -15415.9459 -1962.9585 0.0000 0.0000
8681.4705 7186.9724 323.8604 -1494.4981 7257.0229
326.9687 -273.8605 -234.8621 664265.7895 -16.3011
-16.3050
[25087]ENERGY: 6740000 3535.2956 5921.2074 5896.9623
353.4472 -15368.7063 -1848.3093 0.0000 0.0000
8697.7959 7187.6928 324.4694 -1510.1032 7259.2598
327.1474 -8.5877 17.1834 664265.7895 -17.9479
-17.9467
[25137]ENERGY: 6750000 3549.9163 5905.5861 5915.6177
350.2539 -15503.9403 -1813.2503 0.0000 0.0000
8780.4166 7184.6000 327.5516 -1595.8166 7259.4615
326.8172 102.4229 73.8261 664265.7895 -15.7322
-15.7271
[25173]ENERGY: 6760000 3570.3282 5830.5859 5923.2389

371.2859 -15388.7657 -1873.9011 0.0000 0.0000
8755.4319 7188.2041 326.6195 -1567.2279 7258.9337
326.7960 -2.7473 -15.9381 664265.7895 -17.7213
-17.6969
[25223]ENERGY: 6770000 3603.2876 5817.9952 5933.4926
379.1882 -15374.6617 -1878.6095 0.0000 0.0000
8710.9559 7191.6483 324.9603 -1519.3076 7258.7832
327.1665 -24.0081 18.3526 664265.7895 -13.9413
-13.9508
[25259]ENERGY: 6780000 3508.2221 5819.4808 5894.6285
341.3796 -15446.9151 -1800.2932 0.0000 0.0000
8866.3364 7182.8390 330.7568 -1683.4974 7258.8182
327.1256 66.2976 33.8283 664265.7895 -19.8722
-19.8842
[25309]ENERGY: 6790000 3585.8908 5724.2229 5944.3132
344.2387 -15407.1121 -1918.1422 0.0000 0.0000
8909.5915 7183.0029 332.3704 -1726.5886 7258.9155
326.9019 -38.3039 -52.9671 664265.7895 -16.2987
-16.2984
[25348]ENERGY: 6800000 3666.2414 5771.0005 5940.6382
355.7174 -15358.7458 -1904.2388 0.0000 0.0000
8719.8788 7190.4917 325.2932 -1529.3871 7259.7955
326.9361 -134.8973 -100.1877 664265.7895 -14.3176
-14.3083
[25398]ENERGY: 6810000 3651.4491 5810.6068 5957.1965
341.2226 -15460.5818 -1820.2147 0.0000 0.0000
8711.0521 7190.7305 324.9639 -1520.3216 7258.6071
327.2004 -78.2519 18.5473 664265.7895 -16.8419
-16.8336
[25434]ENERGY: 6820000 3578.7770 5838.0034 5895.6130
364.6988 -15460.2020 -1881.7281 0.0000 0.0000
8850.0999 7185.2621 330.1511 -1664.8378 7259.1666
327.3037 -7.5834 -70.5101 664265.7895 -12.5513
-12.5688
[25484]ENERGY: 6830000 3555.6387 5786.4891 5876.0044
386.4541 -15348.7705 -1800.0186 0.0000 0.0000
8732.2095 7188.0066 325.7532 -1544.2028 7259.4293
326.8525 43.0934 -14.5452 664265.7895 -13.5079
-13.5074
[25520]ENERGY: 6840000 3612.3838 5741.4500 5919.7571
343.3059 -15337.8117 -1877.1615 0.0000 0.0000
8789.1292 7191.0528 327.8766 -1598.0764 7260.1427
326.9409 -152.9587 -155.2226 664265.7895 -18.6644
-18.6712
[25570]ENERGY: 6850000 3583.1077 5772.7511 5860.2704
353.1318 -15331.5229 -1865.2298 0.0000 0.0000
8816.2323 7188.7407 328.8876 -1627.4916 7262.7005
326.9498 -70.0170 -77.1376 664265.7895 -15.9563
-15.9542
[25606]ENERGY: 6860000 3562.1780 5800.2130 5849.2403
376.0448 -15293.5750 -1899.5878 0.0000 0.0000
8798.4981 7193.0114 328.2261 -1605.4867 7265.4008
327.1044 -54.6051 -115.2960 664265.7895 -15.2216
-15.2225

[25656]ENERGY: 6870000 3514.4365 5792.6364 5873.7426
358.5723 -15342.8403 -1880.3016 0.0000 0.0000
8874.2420 7190.4879 331.0517 -1683.7541 7263.4749
327.2929 7.3854 5.6313 664265.7895 -14.5057
-14.4899
[25692]ENERGY: 6880000 3521.0652 5769.9873 5899.2937
349.0331 -15330.9802 -1875.2292 0.0000 0.0000
8860.2149 7193.3848 330.5284 -1666.8300 7264.0624
327.0665 16.3694 79.3877 664265.7895 -19.4982
-19.5015
[25742]ENERGY: 6890000 3643.0395 5758.2507 5877.3322
361.6202 -15343.2754 -1884.2758 0.0000 0.0000
8780.5988 7193.2901 327.5583 -1587.3086 7266.7175
327.1118 30.1653 -14.7519 664265.7895 -16.9306
-16.9535
[25781]ENERGY: 6900000 3593.3200 5730.2732 5831.1247
357.2706 -15316.3114 -1897.9768 0.0000 0.0000
8894.3430 7192.0433 331.8015 -1702.2996 7266.9689
327.1343 -28.0329 -42.8644 664265.7895 -16.4231
-16.4151
[25831]ENERGY: 6910000 3553.2708 5871.6620 5952.4973
329.8837 -15469.8080 -1839.6758 0.0000 0.0000
8797.7338 7195.5637 328.1976 -1602.1700 7268.1285
327.1140 155.1506 69.7836 664265.7895 -14.5773
-14.5765
[25867]ENERGY: 6920000 3621.5064 5767.1888 5974.6516
374.0080 -15441.2331 -1828.5098 0.0000 0.0000
8730.1182 7197.7302 325.6752 -1532.3880 7267.8389
327.1371 -66.1245 -145.8273 664265.7895 -15.7375
-15.7240
[25917]ENERGY: 6930000 3616.3720 5803.4823 5921.4158
350.6259 -15330.9708 -1873.7661 0.0000 0.0000
8713.1914 7200.3505 325.0437 -1512.8410 7268.5638
326.9558 -24.8419 50.9971 664265.7895 -18.5414
-18.5219
[25953]ENERGY: 6940000 3577.6393 5860.4032 5938.1126
347.8833 -15406.2825 -1865.3058 0.0000 0.0000
8746.4233 7198.8736 326.2834 -1547.5497 7269.0656
327.0054 49.8200 3.7549 664265.7895 -16.3973
-16.4418
[26003]ENERGY: 6950000 3645.2597 5776.7519 5911.7029
342.1397 -15322.2778 -1833.7480 0.0000 0.0000
8681.5061 7201.3346 323.8617 -1480.1715 7268.9745
326.9256 1.9522 -37.4468 664265.7895 -17.1736
-17.1578
[26039]ENERGY: 6960000 3578.6188 5770.1612 5849.6619
373.2821 -15252.5245 -1808.8177 0.0000 0.0000
8690.5672 7200.9489 324.1997 -1489.6183 7267.7654
326.8582 72.0083 -35.8862 664265.7895 -18.8663
-18.8804
[26089]ENERGY: 6970000 3619.3528 5906.5545 5937.9673
351.9834 -15470.4730 -1895.4923 0.0000 0.0000
8749.1454 7199.0379 326.3850 -1550.1074 7270.6723
327.0311 -200.2502 -124.8708 664265.7895 -18.0994

-18.0839
[26125]ENERGY: 6980000 3555.7928 5868.0424 5933.8610
348.5648 -15464.0294 -1838.6687 0.0000 0.0000
8795.3451 7198.9080 328.1085 -1596.4371 7270.3623
327.0584 -102.5366 -98.4743 664265.7895 -16.3064
-16.2978
[26175]ENERGY: 6990000 3540.4747 5818.4280 5896.8781
351.5640 -15365.8246 -1873.4022 0.0000 0.0000
8828.5864 7196.7045 329.3485 -1631.8820 7270.3434
326.9516 26.3120 11.6385 664265.7895 -21.5265
-21.5186
[26214]ENERGY: 7000000 3582.8810 5906.3623 5935.9308
380.6244 -15407.3326 -1839.4348 0.0000 0.0000
8639.1779 7198.2089 322.2827 -1440.9690 7270.7442
326.9557 -52.5796 -69.8131 664265.7895 -19.9129
-19.9418
[26264]ENERGY: 7010000 3610.9637 5867.3276 5890.7360
373.1837 -15396.4527 -1829.7076 0.0000 0.0000
8681.8821 7197.9327 323.8757 -1483.9494 7269.9410
327.0432 12.3125 16.2573 664265.7895 -8.2505
-8.2572
[26300]ENERGY: 7020000 3573.2955 5830.4228 5896.9325
365.9056 -15294.2471 -1809.8907 0.0000 0.0000
8640.1474 7202.5660 322.3188 -1437.5814 7271.6935
326.9326 -9.6104 -6.4851 664265.7895 -15.5761
-15.5671
[26350]ENERGY: 7030000 3483.7422 5713.2787 5885.7855
346.1206 -15161.8893 -1901.2433 0.0000 0.0000
8834.1614 7199.9557 329.5565 -1634.2057 7271.9601
326.7149 84.4723 12.6125 664265.7895 -15.0598
-15.0613
[26386]ENERGY: 7040000 3613.2228 5708.1508 5885.6084
363.3407 -15268.5143 -1857.8670 0.0000 0.0000
8756.8636 7200.8050 326.6729 -1556.0587 7271.4129
326.6442 -144.3705 -83.5515 664265.7895 -20.0621
-20.0493
[26436]ENERGY: 7050000 3636.0751 5862.4985 5892.1288
345.1575 -15324.1389 -1894.8546 0.0000 0.0000
8686.9655 7203.8319 324.0654 -1483.1336 7272.9787
326.7718 -179.7306 -34.4568 664265.7895 -17.1178
-17.0943
[26472]ENERGY: 7060000 3600.7573 5707.3054 5882.8654
347.0974 -15276.7019 -1768.8761 0.0000 0.0000
8712.2586 7204.7061 325.0089 -1507.5525 7272.3530
326.7070 258.4100 227.4650 664265.7895 -18.7814
-18.8112
[26522]ENERGY: 7070000 3563.3645 5805.9809 5886.8011
376.1101 -15319.4936 -1874.6199 0.0000 0.0000
8766.8872 7205.0303 327.0468 -1561.8569 7273.7314
326.9271 22.0757 -3.3624 664265.7895 -18.3857
-18.3822
[26558]ENERGY: 7080000 3635.6741 5735.7575 5927.5127
373.5332 -15366.8996 -1865.8787 0.0000 0.0000
8761.3928 7201.0921 326.8419 -1560.3007 7272.3983

326.7938 20.2359 -13.7547 664265.7895 -16.3109
-16.3041
[26608]ENERGY: 7090000 3638.6585 5825.2811 5866.6354
349.4560 -15404.9881 -1837.6184 0.0000 0.0000
8764.1299 7201.5543 326.9440 -1562.5755 7273.2840
327.0828 12.4162 -49.8009 664265.7895 -14.5629
-14.5598
[26647]ENERGY: 7100000 3607.5189 5742.4519 5926.3605
372.0260 -15340.3490 -1885.5342 0.0000 0.0000
8778.1810 7200.6550 327.4681 -1577.5259 7272.5990
327.0659 -87.3077 -15.6162 664265.7895 -17.2573
-17.2484
[26697]ENERGY: 7110000 3635.6233 5819.8995 5900.2288
348.8217 -15300.0138 -1866.3356 0.0000 0.0000
8670.4686 7208.6924 323.4500 -1461.7762 7274.5660
327.1710 152.5674 -41.4010 664265.7895 -20.4930
-20.5026
[26733]ENERGY: 7120000 3612.8994 5986.6852 5866.9117
337.3792 -15403.8222 -1895.3854 0.0000 0.0000
8700.2797 7204.9476 324.5621 -1495.3321 7274.6369
326.9242 -10.8395 46.9065 664265.7895 -17.9653
-17.9623
[26783]ENERGY: 7130000 3506.7478 5843.5557 5910.0085
372.1280 -15301.1028 -1799.0732 0.0000 0.0000
8674.4597 7206.7236 323.5989 -1467.7361 7273.6501
326.7572 117.2652 104.1046 664265.7895 -17.7427
-17.7500
[26819]ENERGY: 7140000 3572.3408 5867.9442 5928.7372
360.9351 -15399.1924 -1844.7091 0.0000 0.0000
8719.0534 7205.1091 325.2624 -1513.9443 7272.3063
326.9556 7.1149 36.5708 664265.7895 -19.3412
-19.3493
[26869]ENERGY: 7150000 3581.3928 5709.5733 5890.2340
346.2629 -15389.6250 -1786.5664 0.0000 0.0000
8851.0485 7202.3201 330.1865 -1648.7284 7272.6989
326.7867 104.6474 2.3866 664265.7895 -18.1575
-18.1533
[26905]ENERGY: 7160000 3542.7836 5822.3109 5908.9097
359.0312 -15416.1422 -1814.0178 0.0000 0.0000
8798.6956 7201.5710 328.2334 -1597.1246 7273.8732
326.9855 6.6784 23.8537 664265.7895 -18.1134
-18.1094
[26955]ENERGY: 7170000 3606.7220 5834.1512 5890.5071
339.0142 -15305.1082 -1872.8722 0.0000 0.0000
8711.4068 7203.8207 324.9772 -1507.5860 7272.5728
327.2861 22.9448 56.7322 664265.7895 -19.8568
-19.8608
[26991]ENERGY: 7180000 3562.4540 5828.6761 5962.7834
361.1803 -15327.9401 -1873.9248 0.0000 0.0000
8693.0808 7206.3096 324.2935 -1486.7712 7272.7281
327.2525 -81.0717 -73.2078 664265.7895 -15.0259
-15.0229
[27041]ENERGY: 7190000 3519.4473 5758.6227 5944.6035
357.6569 -15250.8854 -1874.8818 0.0000 0.0000

8752.6824 7207.2456 326.5169 -1545.4368 7274.6957
327.1576 113.5774 64.0524 664265.7895 -16.7267
-16.7458
[27080]ENERGY: 7200000 3501.8066 5780.0172 5946.6639
345.4985 -15301.9492 -1863.9057 0.0000 0.0000
8797.4048 7205.5361 328.1853 -1591.8687 7275.0856
327.1158 92.9705 58.7524 664265.7895 -17.2338
-17.2043
[27130]ENERGY: 7210000 3579.8067 5871.1762 5903.7070
373.4485 -15436.3648 -1899.7256 0.0000 0.0000
8808.9399 7200.9879 328.6156 -1607.9520 7275.0091
327.1999 148.6327 65.3302 664265.7895 -18.9468
-18.9646
[27166]ENERGY: 7220000 3534.6374 5849.5830 5902.0016
381.3115 -15389.7587 -1868.6718 0.0000 0.0000
8790.4322 7199.5352 327.9252 -1590.8971 7272.9665
327.2539 -92.8601 -42.2091 664265.7895 -15.8045
-15.7997
[27216]ENERGY: 7230000 3558.4316 5854.9789 5926.3104
360.9498 -15348.9050 -1876.5449 0.0000 0.0000
8728.9233 7204.1441 325.6306 -1524.7792 7274.6178
327.2295 -29.2645 27.0555 664265.7895 -16.1751
-16.1701
[27252]ENERGY: 7240000 3602.1155 5853.5669 5831.1224
345.8624 -15320.5786 -1849.2400 0.0000 0.0000
8742.2755 7205.1241 326.1287 -1537.1514 7275.1723
327.1115 107.8990 38.3950 664265.7895 -14.1447
-14.1435
[27302]ENERGY: 7250000 3596.8467 5812.6637 5858.9996
357.5417 -15324.4819 -1883.4066 0.0000 0.0000
8786.6158 7204.7790 327.7828 -1581.8368 7273.2736
327.2205 -113.3623 -77.5744 664265.7895 -19.7593
-19.7462
[27338]ENERGY: 7260000 3609.2207 5710.2446 5890.5917
376.9554 -15373.1382 -1859.2562 0.0000 0.0000
8844.2099 7198.8277 329.9313 -1645.3821 7272.4763
327.3728 56.5513 22.7752 664265.7895 -22.4662
-22.4891
[27388]ENERGY: 7270000 3550.7954 5856.3487 5902.3828
368.2155 -15416.7494 -1825.8847 0.0000 0.0000
8771.5234 7206.6316 327.2198 -1564.8918 7273.3371
327.2003 68.3206 46.7888 664265.7895 -23.2119
-23.1933
[27424]ENERGY: 7280000 3572.6765 5855.5230 5918.4192
360.6484 -15454.2594 -1851.8926 0.0000 0.0000
8795.7848 7196.8999 328.1249 -1598.8849 7273.6347
327.2055 -85.9055 -100.4640 664265.7895 -17.2887
-17.3026
[27474]ENERGY: 7290000 3534.5454 5758.4854 5882.8113
377.1628 -15329.5287 -1857.1961 0.0000 0.0000
8835.7577 7202.0378 329.6160 -1633.7198 7275.1966
327.0397 4.8483 -55.4180 664265.7895 -18.6027
-18.6126
[27513]ENERGY: 7300000 3644.8470 5826.4860 5836.6950

360.3363 -15388.0732 -1866.0408 0.0000 0.0000
8789.4706 7203.7210 327.8893 -1585.7495 7275.1372
327.2193 152.2672 45.7823 664265.7895 -17.7571
-17.7488
[27563]ENERGY: 7310000 3615.5280 5760.4134 5910.1749
360.1259 -15395.9207 -1811.1390 0.0000 0.0000
8769.5386 7208.7211 327.1457 -1560.8175 7275.9485
327.1615 27.6611 -51.0062 664265.7895 -22.1332
-22.1300
[27599]ENERGY: 7320000 3695.1898 5793.5632 5910.5944
360.6049 -15433.8407 -1858.7369 0.0000 0.0000
8739.8754 7207.2501 326.0392 -1532.6253 7273.7201
327.1791 -202.8772 -116.4720 664265.7895 -17.7546
-17.7459
[27649]ENERGY: 7330000 3586.2409 5821.1992 5907.4671
353.3239 -15314.1449 -1853.0244 0.0000 0.0000
8706.1302 7207.1920 324.7803 -1498.9382 7276.2132
327.1311 -112.8649 -56.6777 664265.7895 -15.4103
-15.4137
[27685]ENERGY: 7340000 3669.3719 5747.8097 5857.1240
382.4297 -15343.0827 -1825.7031 0.0000 0.0000
8715.5603 7203.5098 325.1321 -1512.0505 7274.4953
327.0846 80.4699 85.2082 664265.7895 -15.3766
-15.3907
[27735]ENERGY: 7350000 3557.1785 5739.9995 5891.0646
333.9991 -15291.8130 -1864.4345 0.0000 0.0000
8834.4315 7200.4258 329.5666 -1634.0057 7275.1413
327.1591 98.2845 -60.1441 664265.7895 -19.4144
-19.4049
[27771]ENERGY: 7360000 3575.7535 5654.7095 5869.0082
365.4469 -15279.6331 -1854.5129 0.0000 0.0000
8870.8593 7201.6313 330.9255 -1669.2280 7276.2545
327.0646 -51.9830 -70.6837 664265.7895 -14.6722
-14.6730
[27821]ENERGY: 7370000 3560.7434 5846.8696 5901.8599
355.0400 -15266.1502 -1901.7364 0.0000 0.0000
8712.9952 7209.6215 325.0364 -1503.3737 7276.3599
326.9210 73.2836 -16.9477 664265.7895 -15.8032
-15.8031
[27857]ENERGY: 7380000 3566.8073 5791.9383 5872.6772
370.8138 -15291.0166 -1808.9082 0.0000 0.0000
8703.5986 7205.9105 324.6859 -1497.6881 7274.4620
326.9373 40.6839 50.5339 664265.7895 -17.9223
-17.9186
[27907]ENERGY: 7390000 3572.5091 5792.5327 5894.1068
360.5976 -15386.1980 -1886.6387 0.0000 0.0000
8854.9450 7201.8545 330.3318 -1653.0905 7274.5094
326.9484 -179.4778 -156.6848 664265.7895 -16.1797
-16.1730
[27946]ENERGY: 7400000 3615.8891 5831.7279 5866.8396
340.4173 -15288.9504 -1906.3436 0.0000 0.0000
8747.2947 7206.8747 326.3159 -1540.4200 7275.4584
327.1554 -8.3443 -74.3658 664265.7895 -11.5195
-11.5304

[27996]ENERGY: 7410000 3523.4083 5899.9358 5908.2337
364.5251 -15401.8764 -1985.7615 0.0000 0.0000
8894.0198 7202.4848 331.7895 -1691.5350 7276.7885
327.2306 151.5572 53.8283 664265.7895 -11.4919
-11.4879
[28032]ENERGY: 7420000 3581.3264 5868.1866 5893.9608
324.2618 -15351.0619 -1857.1493 0.0000 0.0000
8745.6375 7205.1620 326.2541 -1540.4755 7275.9027
327.2275 -232.2428 -139.4893 664265.7895 -9.9804
-9.9910
[28082]ENERGY: 7430000 3626.2763 5832.2689 5894.5593
333.1025 -15272.4992 -1958.6993 0.0000 0.0000
8753.0562 7208.0646 326.5309 -1544.9916 7275.9602
327.0344 -179.2164 -153.4693 664265.7895 -11.6103
-11.6028
[28118]ENERGY: 7440000 3594.9581 5793.2706 5937.7797
370.3395 -15375.9598 -1907.2309 0.0000 0.0000
8791.0838 7204.2410 327.9495 -1586.8428 7275.7776
327.5462 -120.7598 -205.0618 664265.7895 -9.8311
-9.8309
[28168]ENERGY: 7450000 3670.2354 5772.6751 5879.7123
350.3450 -15317.3533 -1881.6296 0.0000 0.0000
8731.2240 7205.2088 325.7164 -1526.0152 7276.5865
327.5851 -147.7973 -144.8280 664265.7895 -6.1954
-6.2095
[28204]ENERGY: 7460000 3613.0923 5857.1771 5849.0679
353.4916 -15420.7371 -1875.8936 0.0000 0.0000
8829.9329 7206.1311 329.3987 -1623.8018 7276.8991
327.5183 -27.3231 -81.7017 664265.7895 -8.4331
-8.4308
[28254]ENERGY: 7470000 3579.3339 5861.9201 5969.1294
344.2230 -15370.8875 -1920.5713 0.0000 0.0000
8740.1176 7203.2651 326.0482 -1536.8525 7276.9586
327.4346 14.5709 -60.4700 664265.7895 -9.9127
-9.9023
[28290]ENERGY: 7480000 3574.8931 5859.0016 5841.5303
346.0696 -15352.6299 -1890.0354 0.0000 0.0000
8826.8101 7205.6393 329.2822 -1621.1708 7278.0181
327.5922 2.4778 -87.4920 664265.7895 -11.3221
-11.3406
[28340]ENERGY: 7490000 3644.7182 5792.7611 5875.5080
355.3584 -15373.2662 -1887.9675 0.0000 0.0000
8799.1150 7206.2270 328.2491 -1592.8879 7277.8069
327.5271 -91.7746 -76.6787 664265.7895 -8.2655
-8.2319
[28379]ENERGY: 7500000 3604.2400 5813.7046 5907.5042
363.8957 -15414.9170 -1792.8807 0.0000 0.0000
8728.7603 7210.3071 325.6245 -1518.4532 7278.5629
327.3362 -19.4998 20.9233 664265.7895 -8.7893
-8.7984
[28429]ENERGY: 7510000 3595.8576 5866.7397 5861.4346
376.0393 -15419.0784 -1832.3527 0.0000 0.0000
8758.6428 7207.2829 326.7393 -1551.3599 7276.4555
327.3305 -47.6766 7.5681 664265.7895 -3.9839

-4.0070
[28465]ENERGY: 7520000 3614.7904 5905.5990 5858.1586
353.2042 -15379.3987 -1894.6773 0.0000 0.0000
8747.0475 7204.7236 326.3067 -1542.3238 7277.7422
327.4365 10.5069 66.4535 664265.7895 -6.3297
-6.2967
[28515]ENERGY: 7530000 3606.6904 6023.3746 5852.7992
370.1458 -15312.1089 -1981.4315 0.0000 0.0000
8649.5262 7208.9957 322.6687 -1440.5305 7279.2089
327.3437 -77.5349 -77.2767 664265.7895 -9.6506
-9.6594
[28551]ENERGY: 7540000 3637.3052 5815.5466 5905.5407
362.9994 -15350.2820 -1924.3488 0.0000 0.0000
8762.9198 7209.6809 326.8988 -1553.2389 7278.7638
327.2066 131.0296 91.7614 664265.7895 -7.2221
-7.2397
[28601]ENERGY: 7550000 3596.8756 5772.7636 5887.6486
382.4618 -15353.9912 -1846.0100 0.0000 0.0000
8771.9184 7211.6668 327.2345 -1560.2516 7279.6923
327.3509 -73.6334 -76.7826 664265.7895 -9.1408
-9.1583
[28637]ENERGY: 7560000 3544.0272 5942.7223 5859.5671
385.8821 -15407.6982 -1853.6738 0.0000 0.0000
8738.9307 7209.7573 326.0039 -1529.1733 7279.8627
327.5583 145.6901 21.8938 664265.7895 -8.7845
-8.7886
[28687]ENERGY: 7570000 3591.6374 5909.1905 5885.3311
375.5956 -15368.8723 -1947.1348 0.0000 0.0000
8765.0898 7210.8373 326.9798 -1554.2526 7281.2083
327.5878 -115.4321 -19.1397 664265.7895 -7.9096
-7.8808
[28723]ENERGY: 7580000 3564.0638 5839.0933 5888.0768
372.7734 -15420.8531 -1785.5020 0.0000 0.0000
8757.6160 7215.2682 326.7010 -1542.3478 7282.3769
327.5671 175.2596 92.5184 664265.7895 -11.8532
-11.8607
[28773]ENERGY: 7590000 3537.8391 5799.1905 5879.4893
353.3207 -15352.2830 -1850.0612 0.0000 0.0000
8845.5550 7213.0504 329.9815 -1632.5046 7283.3170
327.5735 127.8433 10.5099 664265.7895 -8.3628
-8.3537
[28812]ENERGY: 7600000 3513.7092 5804.0013 5924.9304
356.3851 -15376.7921 -1842.4072 0.0000 0.0000
8831.4102 7211.2369 329.4539 -1620.1733 7282.3741
327.6420 160.2501 204.7227 664265.7895 -9.6323
-9.6322
[28862]ENERGY: 7610000 3620.5555 5758.8521 5848.0160
357.1828 -15264.8244 -1896.9685 0.0000 0.0000
8790.3258 7213.1393 327.9212 -1577.1865 7281.4856
327.4910 -19.1201 -24.5453 664265.7895 -4.3876
-4.3854
[28898]ENERGY: 7620000 3578.5050 5717.4136 5912.7078
402.9215 -15373.8918 -1797.0225 0.0000 0.0000
8767.6714 7208.3051 327.0761 -1559.3663 7282.0251

327.4509 133.6339 98.5376 664265.7895 -11.9278
-11.9264
[28948]ENERGY: 7630000 3573.1040 5843.5421 5936.2082
348.6305 -15406.2102 -1873.4933 0.0000 0.0000
8783.8310 7205.6123 327.6789 -1578.2187 7281.0403
327.5834 -71.2013 -73.1622 664265.7895 -9.5443
-9.5430
[28984]ENERGY: 7640000 3547.1225 5783.0265 5907.7853
362.0802 -15357.8104 -1850.6840 0.0000 0.0000
8820.0514 7211.5716 329.0301 -1608.4798 7280.7249
327.6098 134.7311 59.2129 664265.7895 -9.6485
-9.6672
[29034]ENERGY: 7650000 3613.2620 5820.7461 5921.3600
353.3521 -15339.9581 -1955.0520 0.0000 0.0000
8796.3161 7210.0261 328.1447 -1586.2900 7282.5705
327.6070 -189.5837 -103.1513 664265.7895 -6.6283
-6.6131
[29070]ENERGY: 7660000 3636.9106 5777.7713 5904.3096
367.7333 -15367.9071 -1875.1287 0.0000 0.0000
8769.5657 7213.2547 327.1468 -1556.3110 7282.8307
327.5701 -43.4520 -43.8171 664265.7895 -10.8336
-10.8303
[29120]ENERGY: 7670000 3480.0744 5753.3293 5869.0881
348.8094 -15396.3006 -1824.0036 0.0000 0.0000
8976.1506 7207.1476 334.8534 -1769.0030 7282.2751
327.5194 180.6381 212.2698 664265.7895 -8.8465
-8.8395
[29156]ENERGY: 7680000 3603.9942 5827.9973 5917.7309
353.1366 -15440.0348 -1852.0145 0.0000 0.0000
8804.3266 7215.1364 328.4435 -1589.1902 7281.4010
327.5867 -89.3718 -63.3162 664265.7895 -10.9855
-10.9928
[29206]ENERGY: 7690000 3573.1686 5866.9561 5904.7451
349.6621 -15360.4262 -1877.1656 0.0000 0.0000
8751.9516 7208.8917 326.4897 -1543.0599 7282.8417
327.6936 -21.5026 11.9673 664265.7895 -8.5776
-8.5784
[29245]ENERGY: 7700000 3535.6534 5814.2715 5874.7656
376.1295 -15349.8499 -1840.9335 0.0000 0.0000
8804.3858 7214.4223 328.4457 -1589.9634 7283.5967
327.7249 350.3178 201.7953 664265.7895 -12.7536
-12.7637
[29295]ENERGY: 7710000 3621.4515 5956.6356 5848.1729
339.3037 -15437.6068 -1881.1363 0.0000 0.0000
8766.9595 7213.7801 327.0495 -1553.1794 7283.1853
327.7539 100.8197 20.5018 664265.7895 -8.1705
-8.1472
[29331]ENERGY: 7720000 3621.9120 5706.4128 5932.2142
335.5525 -15339.7588 -1855.6157 0.0000 0.0000
8814.5802 7215.2972 328.8260 -1599.2830 7282.3849
327.7189 0.3933 -33.5225 664265.7895 -10.4103
-10.4320
[29381]ENERGY: 7730000 3581.1898 5758.2251 5896.8334
367.3868 -15344.8336 -1890.6347 0.0000 0.0000

8844.7743 7212.9412 329.9524 -1631.8331 7284.1876
327.7036 -115.7150 -79.4409 664265.7895 -10.3740
-10.3592
[29417]ENERGY: 7740000 3680.8046 5810.2354 5954.5268
356.6133 -15402.3601 -1893.5170 0.0000 0.0000
8706.2451 7212.5481 324.7846 -1493.6970 7284.6214
327.7822 -45.7690 -37.7736 664265.7895 -10.5603
-10.5869
[29467]ENERGY: 7750000 3618.2680 5777.2862 5915.3314
366.4058 -15355.3262 -1885.3084 0.0000 0.0000
8779.8304 7216.4872 327.5297 -1563.3432 7284.6415
327.4267 31.6010 17.5935 664265.7895 -5.8021
-5.7787
[29503]ENERGY: 7760000 3573.8803 5878.0577 5913.3241
374.8582 -15405.3695 -1914.6394 0.0000 0.0000
8793.6844 7213.7959 328.0465 -1579.8885 7285.2931
327.6657 31.0922 -14.6893 664265.7895 -12.4158
-12.4033
[29553]ENERGY: 7770000 3548.5097 5800.9952 5864.7192
337.8805 -15316.6887 -1830.2643 0.0000 0.0000
8806.7341 7211.8857 328.5333 -1594.8484 7285.8264
327.6730 -20.4886 -56.4846 664265.7895 -8.2307
-8.2404
[29589]ENERGY: 7780000 3591.1891 5907.6004 5837.9176
357.6083 -15422.0292 -1838.1983 0.0000 0.0000
8780.1689 7214.2568 327.5423 -1565.9121 7285.7778
327.5367 -11.4257 -31.2679 664265.7895 -5.9912
-6.0006
[29639]ENERGY: 7790000 3528.6497 5842.8619 5870.8287
365.0649 -15293.2246 -1861.4062 0.0000 0.0000
8757.6854 7210.4598 326.7036 -1547.2256 7285.1788
327.5726 92.1928 33.1615 664265.7895 -11.7210
-11.7173
[29678]ENERGY: 7800000 3537.0179 5902.4532 5850.5378
365.4453 -15399.8102 -1813.5723 0.0000 0.0000
8772.2160 7214.2878 327.2456 -1557.9282 7284.2632
327.7357 87.5329 29.4138 664265.7895 -13.1187
-13.1247
[29728]ENERGY: 7810000 3603.6905 5872.9757 5872.9203
366.0520 -15304.3860 -1934.4275 0.0000 0.0000
8737.1226 7213.9476 325.9365 -1523.1750 7285.7179
327.9588 -71.7163 -86.6514 664265.7895 -10.1108
-10.1041
[29764]ENERGY: 7820000 3514.8118 5888.4301 5854.1269
347.7471 -15365.3462 -1871.1475 0.0000 0.0000
8843.4164 7212.0385 329.9017 -1631.3779 7285.0381
327.8358 171.9164 145.2045 664265.7895 -11.8778
-11.8793
[29814]ENERGY: 7830000 3514.2177 5837.8566 5960.3134
373.9062 -15392.8979 -1883.9161 0.0000 0.0000
8805.0993 7214.5791 328.4723 -1590.5202 7286.5378
327.7677 26.2825 41.0694 664265.7895 -7.9645
-7.9644
[29850]ENERGY: 7840000 3588.5907 5663.9069 5897.0549

354.9266 -15323.6614 -1792.2746 0.0000 0.0000
8830.1886 7218.7318 329.4083 -1611.4569 7285.3800
327.6250 219.8547 62.2947 664265.7895 -14.8522
-14.8675
[29900]ENERGY: 7850000 3626.2140 5903.4652 5907.0280
334.8769 -15264.0787 -1972.9850 0.0000 0.0000
8682.1825 7216.7028 323.8869 -1465.4797 7285.8030
327.7111 22.5802 -80.4732 664265.7895 -10.5914
-10.5789
[29936]ENERGY: 7860000 3567.7166 5869.8340 5853.2796
344.4737 -15390.9128 -1827.3556 0.0000 0.0000
8799.7211 7216.7567 328.2717 -1582.9644 7285.8202
327.9342 47.3737 73.4024 664265.7895 -11.3392
-11.3577
[29986]ENERGY: 7870000 3565.3564 5836.9378 5944.5214
381.0583 -15428.6959 -1958.1608 0.0000 0.0000
8872.6314 7213.6486 330.9916 -1658.9829 7285.5400
327.8767 45.1137 -31.9469 664265.7895 -9.6298
-9.6173
[30022]ENERGY: 7880000 3572.9718 5874.5374 5924.5111
368.9329 -15423.4991 -1845.0775 0.0000 0.0000
8743.7736 7216.1501 326.1846 -1527.6235 7286.8643
327.8206 -153.7273 -125.6270 664265.7895 -13.2235
-13.2149
[30072]ENERGY: 7890000 3639.5631 5878.1105 5890.4868
355.7126 -15451.2601 -1872.4071 0.0000 0.0000
8779.2690 7219.4746 327.5087 -1559.7944 7286.9156
327.8770 123.9764 28.9607 664265.7895 -11.7773
-11.7954
[30111]ENERGY: 7900000 3576.6543 5875.7188 5907.6441
370.2701 -15370.9932 -1884.7400 0.0000 0.0000
8739.8715 7214.4257 326.0390 -1525.4458 7286.8349
327.7340 135.7760 54.3195 664265.7895 -13.9194
-13.8999
[30161]ENERGY: 7910000 3621.6387 5749.3113 5928.1012
349.0264 -15377.7489 -1875.9256 0.0000 0.0000
8822.6448 7217.0479 329.1269 -1605.5969 7287.8448
327.6102 -49.3696 9.4976 664265.7895 -15.5308
-15.5268
[30197]ENERGY: 7920000 3611.2645 5786.3555 5932.3788
362.9740 -15403.9201 -1873.4409 0.0000 0.0000
8800.4542 7216.0661 328.2990 -1584.3881 7287.5250
327.8268 -102.9874 -56.0012 664265.7895 -16.1995
-16.2054
[30247]ENERGY: 7930000 3609.0628 5824.3572 5915.3530
380.2282 -15399.7317 -1822.7330 0.0000 0.0000
8715.4704 7222.0068 325.1287 -1493.4636 7287.7854
327.9199 88.4644 71.7535 664265.7895 -13.3957
-13.4050
[30283]ENERGY: 7940000 3550.6904 5856.2304 5907.9152
338.1280 -15451.2335 -1862.4214 0.0000 0.0000
8873.6256 7212.9347 331.0287 -1660.6909 7287.0757
327.7155 48.5390 67.5737 664265.7895 -10.0756
-10.0682

[30333]ENERGY: 7950000 3583.9614 5908.3559 5920.0511
349.0915 -15446.9131 -1901.1012 0.0000 0.0000
8804.1212 7217.5668 328.4358 -1586.5545 7287.5067
327.6216 -23.0856 -52.5301 664265.7895 -10.1736
-10.1594
[30369]ENERGY: 7960000 3579.6736 5869.8835 5936.0578
339.0674 -15326.2843 -1892.4593 0.0000 0.0000
8708.6033 7214.5419 324.8726 -1494.0614 7286.8321
327.7293 -82.7354 -8.1776 664265.7895 -14.5878
-14.5884
[30419]ENERGY: 7970000 3629.3900 5910.3327 5919.8590
363.2847 -15451.0131 -1885.6619 0.0000 0.0000
8728.4792 7214.6706 325.6140 -1513.8086 7287.1199
327.5613 -120.2859 -135.9064 664265.7895 -11.9540
-11.9827
[30455]ENERGY: 7980000 3600.8922 5825.9355 5885.6171
336.5183 -15458.1982 -1845.8494 0.0000 0.0000
8871.9880 7216.9034 330.9676 -1655.0846 7286.4832
327.7052 60.5013 21.4036 664265.7895 -10.1203
-10.1062
[30505]ENERGY: 7990000 3641.2136 5796.6314 5910.8562
385.5789 -15385.4957 -1893.8409 0.0000 0.0000
8762.2771 7217.2207 326.8749 -1545.0564 7288.4306
327.6082 -198.6256 -231.9724 664265.7895 -13.5117
-13.5127
[30544]ENERGY: 8000000 3548.2834 5811.6266 5906.7873
380.7556 -15344.6134 -1896.8298 0.0000 0.0000
8812.7200 7218.7297 328.7566 -1593.9903 7289.1066
327.7864 51.9051 34.5002 664265.7895 -3.4598
-3.4565
[30594]ENERGY: 8010000 3582.5707 5829.2576 5929.7686
353.4891 -15340.8607 -1836.4209 0.0000 0.0000
8704.8862 7222.6906 324.7339 -1482.1956 7288.9030
327.8553 147.3849 85.1579 664265.7895 -9.3003
-9.3014
[30630]ENERGY: 8020000 3563.1926 5766.9617 5852.1519
355.8948 -15381.8271 -1823.9312 0.0000 0.0000
8887.1233 7219.5661 331.5322 -1667.5572 7289.6407
328.0088 47.9718 40.6999 664265.7895 -7.8808
-7.8825
[30680]ENERGY: 8030000 3564.4537 5881.2653 5945.0785
350.6197 -15428.6006 -1862.1371 0.0000 0.0000
8769.4884 7220.1680 327.1439 -1549.3204 7290.5285
328.1066 41.2620 19.1249 664265.7895 -10.7498
-10.7450
[30716]ENERGY: 8040000 3627.0350 5803.8444 5946.2587
370.0879 -15411.5994 -1893.7888 0.0000 0.0000
8778.1605 7219.9983 327.4674 -1558.1622 7290.0891
328.2555 23.7747 -66.0187 664265.7895 -9.4968
-9.5161
[30766]ENERGY: 8050000 3612.5453 5841.2863 5883.8751
329.0901 -15407.5723 -1865.3707 0.0000 0.0000
8826.1186 7219.9725 329.2565 -1606.1461 7291.4339
327.9979 8.5375 -47.1953 664265.7895 -10.6899

-10.6828
[30802]ENERGY: 8060000 3553.8929 5921.9287 5831.1254
355.1137 -15382.8866 -1862.8449 0.0000 0.0000
8809.0346 7225.3638 328.6191 -1583.6708 7292.8749
327.9695 113.4335 76.6886 664265.7895 -9.7591
-9.7465
[30852]ENERGY: 8070000 3571.2055 5860.1852 5853.8911
370.3921 -15401.4553 -1852.4802 0.0000 0.0000
8820.1298 7221.8681 329.0330 -1598.2617 7294.0731
328.1193 211.9102 162.4915 664265.7895 -10.9275
-10.9319
[30888]ENERGY: 8080000 3672.0309 5887.1490 5860.3598
358.2664 -15397.6451 -1866.3845 0.0000 0.0000
8710.5000 7224.2765 324.9433 -1486.2235 7293.9186
328.0945 -143.4696 -175.9964 664265.7895 -9.1279
-9.1304
[30938]ENERGY: 8090000 3636.0595 5868.1732 5888.2609
346.1788 -15419.5599 -1938.9227 0.0000 0.0000
8844.5087 7224.6985 329.9425 -1619.8102 7294.7724
328.1045 -27.4855 -75.6548 664265.7895 -11.4237
-11.4233
[30977]ENERGY: 8100000 3551.3100 5822.4334 5840.5075
363.3074 -15358.3711 -1930.2691 0.0000 0.0000
8932.1622 7221.0803 333.2124 -1711.0819 7295.5794
328.3184 83.0088 -48.7486 664265.7895 -8.7178
-8.7215
[31027]ENERGY: 8110000 3660.5515 5769.2549 5912.8443
363.9380 -15412.8322 -1911.2774 0.0000 0.0000
8842.2343 7224.7135 329.8576 -1617.5208 7295.5780
328.2562 -14.5294 -50.8967 664265.7895 -14.1453
-14.1445
[31063]ENERGY: 8120000 3597.8286 5886.6427 5916.0296
357.6340 -15410.8409 -1898.5753 0.0000 0.0000
8775.8531 7224.5719 327.3813 -1551.2812 7295.2245
328.4325 171.1688 95.4318 664265.7895 -5.0140
-5.0026
[31113]ENERGY: 8130000 3613.6393 5923.6919 5955.7693
379.2256 -15503.6735 -1862.5330 0.0000 0.0000
8718.6813 7224.8011 325.2485 -1493.8803 7295.6734
328.2125 66.2283 -46.8902 664265.7895 -10.6346
-10.6514
[31149]ENERGY: 8140000 3579.1676 5854.7789 5872.8779
337.9966 -15305.3374 -1935.5531 0.0000 0.0000
8820.6248 7224.5553 329.0515 -1596.0695 7296.2962
328.1459 10.8451 -14.9073 664265.7895 -6.9636
-6.9533
[31199]ENERGY: 8150000 3706.6565 5751.1740 6014.3177
364.1036 -15340.1377 -1960.6633 0.0000 0.0000
8694.5804 7230.0311 324.3494 -1464.5493 7296.2732
328.2324 -191.9093 -114.1487 664265.7895 -10.0517
-10.0504
[31235]ENERGY: 8160000 3501.8120 5839.5442 5925.6375
376.4965 -15381.5493 -1876.9040 0.0000 0.0000
8840.8567 7225.8937 329.8063 -1614.9630 7296.0335

328.0641 125.9490 40.0344 664265.7895 -8.8673
-8.8622
[31285]ENERGY: 8170000 3657.6095 5884.4253 5912.0140
338.9949 -15373.9827 -1836.6217 0.0000 0.0000
8647.4393 7229.8786 322.5909 -1417.5606 7296.3391
328.3826 -59.7231 -40.2727 664265.7895 -8.0633
-8.0731
[31321]ENERGY: 8180000 3572.4835 5803.1223 5914.5056
384.1521 -15369.1437 -1845.9325 0.0000 0.0000
8767.3730 7226.5603 327.0650 -1540.8127 7295.4349
328.2089 126.5255 72.4570 664265.7895 -7.0490
-7.0266
[31371]ENERGY: 8190000 3649.1763 5842.8002 5869.6743
378.7561 -15428.8740 -1794.0506 0.0000 0.0000
8709.7113 7227.1938 324.9139 -1482.5175 7296.3026
328.1062 -61.1716 -55.5430 664265.7895 -9.3323
-9.3466
[31410]ENERGY: 8200000 3707.8046 5809.1251 5845.1311
359.9675 -15409.3028 -1882.1198 0.0000 0.0000
8796.0360 7226.6417 328.1342 -1569.3943 7295.5972
328.1223 -10.0062 -47.4248 664265.7895 -9.2920
-9.2829
[31460]ENERGY: 8210000 3627.3767 5780.0452 5867.2140
355.9569 -15316.4652 -1886.0183 0.0000 0.0000
8799.9019 7228.0112 328.2784 -1571.8906 7297.3273
328.2232 -17.2308 -63.7546 664265.7895 -6.7697
-6.7891
[31496]ENERGY: 8220000 3619.8675 5840.7223 5881.7959
363.3179 -15380.2522 -1837.9327 0.0000 0.0000
8736.2071 7223.7258 325.9023 -1512.4813 7296.9405
328.1216 -6.4969 -38.4922 664265.7895 -10.6222
-10.6290
[31546]ENERGY: 8230000 3583.4141 5996.6852 5879.8378
341.7708 -15488.0712 -1807.4642 0.0000 0.0000
8718.5426 7224.7152 325.2434 -1493.8274 7296.1356
327.9834 -20.6346 7.6139 664265.7895 -9.4374
-9.4237
[31582]ENERGY: 8240000 3528.8623 5725.9656 5955.1205
351.0090 -15358.7970 -1800.9008 0.0000 0.0000
8823.1016 7224.3611 329.1439 -1598.7405 7297.6649
328.0211 15.8356 62.3029 664265.7895 -8.9289
-8.9285
[31632]ENERGY: 8250000 3668.2044 5747.0000 5902.4615
337.5755 -15384.2603 -1820.2219 0.0000 0.0000
8776.2595 7227.0188 327.3965 -1549.2408 7298.8247
328.0838 25.6018 -4.9612 664265.7895 -13.8190
-13.8266
[31668]ENERGY: 8260000 3557.3629 5781.5364 5937.2585
363.6181 -15360.4398 -1802.1846 0.0000 0.0000
8749.1914 7226.3429 326.3867 -1522.8485 7298.6040
328.0864 187.6676 174.5557 664265.7895 -10.7192
-10.7187
[31718]ENERGY: 8270000 3635.4149 5781.2613 5909.2745
344.5212 -15367.0657 -1851.2754 0.0000 0.0000

8775.8777 7228.0086 327.3822 -1547.8691 7299.0565
328.0882 119.1968 26.0043 664265.7895 -9.2128
-9.2155
[31754]ENERGY: 8280000 3654.0108 5776.4103 6006.0660
370.1630 -15403.2626 -1894.3152 0.0000 0.0000
8721.9074 7230.9798 325.3689 -1490.9276 7300.3517
328.0258 -97.9295 -23.3257 664265.7895 -8.4211
-8.4038
[31804]ENERGY: 8290000 3586.1199 5818.7656 5898.8502
381.9584 -15420.8093 -1864.5363 0.0000 0.0000
8827.8460 7228.1944 329.3209 -1599.6516 7300.4934
328.1713 51.3966 59.4902 664265.7895 -11.6838
-11.6924
[31843]ENERGY: 8300000 3532.3505 5881.2664 5945.9341
357.9694 -15435.1722 -1839.4113 0.0000 0.0000
8788.1793 7231.1162 327.8411 -1557.0631 7300.7650
328.2852 125.6401 100.5897 664265.7895 -9.3552
-9.3680
[31893]ENERGY: 8310000 3628.0186 5792.3364 5909.8665
351.6794 -15414.9796 -1911.9872 0.0000 0.0000
8870.5167 7225.4509 330.9127 -1645.0658 7300.4510
328.2783 -69.3872 -84.0158 664265.7895 -10.6545
-10.6449
[31929]ENERGY: 8320000 3518.5233 5779.5570 5909.0543
384.4691 -15328.9046 -1891.0682 0.0000 0.0000
8859.9291 7231.5599 330.5177 -1628.3691 7302.2584
328.3817 -11.6280 -59.8589 664265.7895 -12.7260
-12.7378
[31979]ENERGY: 8330000 3590.1955 5887.0914 5934.0608
336.1069 -15412.2151 -1973.0631 0.0000 0.0000
8863.9924 7226.1689 330.6693 -1637.8235 7302.3786
328.1566 100.9272 -2.6811 664265.7895 -14.7067
-14.6851
[32015]ENERGY: 8340000 3580.3642 5775.5220 5928.0234
362.4929 -15457.4662 -1880.1741 0.0000 0.0000
8924.5700 7233.3323 332.9292 -1691.2377 7302.4577
328.1792 -94.8584 -113.9387 664265.7895 -11.1841
-11.1885
[32065]ENERGY: 8350000 3611.0711 5873.0854 5886.9322
313.6304 -15427.0893 -1853.5045 0.0000 0.0000
8826.4784 7230.6037 329.2699 -1595.8746 7304.2239
328.1566 39.0157 -45.2401 664265.7895 -14.8197
-14.8230
[32101]ENERGY: 8360000 3579.9561 5861.1114 5869.8872
330.5375 -15500.2348 -1756.7826 0.0000 0.0000
8849.3147 7233.7896 330.1218 -1615.5251 7303.5598
328.2271 -80.7201 -91.6333 664265.7895 -6.4155
-6.4099
[32151]ENERGY: 8370000 3588.5921 5947.8084 5845.4310
368.7501 -15438.3092 -1894.6959 0.0000 0.0000
8814.8369 7232.4134 328.8356 -1582.4236 7304.3442
328.2216 -165.5083 -72.2827 664265.7895 -11.8745
-11.8775
[32187]ENERGY: 8380000 3516.8618 5867.6713 5862.4788

369.8694 -15390.2821 -1761.1942 0.0000 0.0000
8769.3576 7234.7626 327.1390 -1534.5950 7305.2765
328.3118 136.0811 212.1393 664265.7895 -9.4145
-9.4129
[32237]ENERGY: 8390000 3614.0572 5852.9686 5888.0715
355.8811 -15459.4207 -1872.7544 0.0000 0.0000
8852.2716 7231.0749 330.2321 -1621.1967 7305.3180
328.2176 49.9706 78.9687 664265.7895 -12.8705
-12.8654
[32276]ENERGY: 8400000 3556.5551 5851.3795 5987.8061
338.0276 -15488.7069 -1812.0590 0.0000 0.0000
8798.8258 7231.8283 328.2383 -1566.9975 7304.8911
328.5595 20.2896 50.7349 664265.7895 -10.1122
-10.1034
[32326]ENERGY: 8410000 3524.8118 5808.6439 5835.9162
375.7066 -15352.8441 -1762.1271 0.0000 0.0000
8806.8723 7236.9796 328.5385 -1569.8927 7306.7915
328.3632 142.6830 62.4929 664265.7895 -10.5463
-10.5564
[32362]ENERGY: 8420000 3644.0369 5822.9931 5882.6058
384.9172 -15421.3644 -1843.1966 0.0000 0.0000
8763.7451 7233.7372 326.9296 -1530.0079 7305.4539
328.2630 -88.5082 -49.8369 664265.7895 -15.0314
-15.0446
[32412]ENERGY: 8430000 3592.2607 5817.9972 5922.3754
325.1819 -15430.4259 -1830.8125 0.0000 0.0000
8837.6103 7234.1871 329.6851 -1603.4232 7306.1926
328.2706 -136.0712 -59.6747 664265.7895 -14.7235
-14.7076
[32448]ENERGY: 8440000 3628.6391 5945.9216 5889.7797
384.3397 -15443.6509 -1856.5376 0.0000 0.0000
8687.2006 7235.6922 324.0741 -1451.5083 7306.2038
328.2111 105.0396 -14.3744 664265.7895 -14.9748
-14.9824
[32498]ENERGY: 8450000 3597.2022 5814.0931 5848.1430
346.2539 -15359.8751 -1793.1790 0.0000 0.0000
8786.4710 7239.1092 327.7774 -1547.3618 7306.8534
328.0094 156.2132 90.3381 664265.7895 -16.7854
-16.7799
[32534]ENERGY: 8460000 3638.6954 5878.5435 5952.3149
348.5154 -15440.0021 -1777.9774 0.0000 0.0000
8642.3455 7242.4351 322.4008 -1399.9103 7306.6746
328.2902 32.2804 1.9578 664265.7895 -12.1929
-12.1966
[32584]ENERGY: 8470000 3572.9972 5884.4171 5943.1740
360.2224 -15321.0822 -1938.7285 0.0000 0.0000
8735.2723 7236.2723 325.8674 -1499.0000 7307.3943
328.0200 -29.5354 23.1443 664265.7895 -9.8820
-9.8716
[32620]ENERGY: 8480000 3675.8198 5864.9940 5921.3001
344.5524 -15452.3830 -1852.1485 0.0000 0.0000
8735.8995 7238.0344 325.8909 -1497.8651 7306.5999
328.1119 -95.3509 -36.8142 664265.7895 -16.4264
-16.4466

[32670]ENERGY: 8490000 3626.1493 5897.7315 5910.0385
370.1265 -15403.6674 -1891.7663 0.0000 0.0000
8729.7681 7238.3803 325.6621 -1491.3878 7307.4285
328.1214 21.0335 -81.8061 664265.7895 -11.6635
-11.6532
[32709]ENERGY: 8500000 3666.5824 5879.3110 5851.0685
367.8853 -15346.2847 -1947.1747 0.0000 0.0000
8765.7126 7237.1004 327.0030 -1528.6122 7308.2822
328.1197 -84.5318 -154.9884 664265.7895 -10.4878
-10.5116
[32759]ENERGY: 8510000 3616.2314 5881.1221 5855.0438
361.1680 -15397.3052 -1847.6233 0.0000 0.0000
8769.3140 7237.9509 327.1374 -1531.3631 7308.9240
328.0802 135.5994 12.6984 664265.7895 -14.3835
-14.3694
[32795]ENERGY: 8520000 3697.0938 5833.5675 5890.9451
340.7712 -15359.8974 -1840.7378 0.0000 0.0000
8680.2311 7241.9734 323.8142 -1438.2577 7309.6278
327.9994 -8.4410 -91.2142 664265.7895 -12.2137
-12.2060
[32845]ENERGY: 8530000 3658.5439 5891.6283 5855.9416
361.9614 -15379.2966 -1851.1041 0.0000 0.0000
8704.8201 7242.4945 324.7314 -1462.3256 7310.4197
328.0323 67.2480 20.9661 664265.7895 -11.0912
-11.0986
[32881]ENERGY: 8540000 3656.4940 5832.9596 5868.6311
347.9531 -15458.7216 -1823.6726 0.0000 0.0000
8817.0036 7240.6471 328.9164 -1576.3565 7309.2871
328.3363 -104.8184 -41.3922 664265.7895 -9.4600
-9.4590
[32931]ENERGY: 8550000 3603.1622 5804.3758 5892.9054
383.1746 -15338.2032 -1847.4244 0.0000 0.0000
8744.0742 7242.0647 326.1958 -1502.0095 7310.7255
328.0609 178.4179 160.1831 664265.7895 -8.3884
-8.3716
[32967]ENERGY: 8560000 3592.4087 5775.9893 5866.3149
390.2804 -15408.9602 -1837.8030 0.0000 0.0000
8863.4789 7241.7090 330.6502 -1621.7699 7311.3000
328.0274 -1.8521 -25.0044 664265.7895 -7.7187
-7.7320
[33017]ENERGY: 8570000 3572.6812 5753.9287 5917.0201
343.9448 -15322.3458 -1801.0011 0.0000 0.0000
8778.2956 7242.5234 327.4724 -1535.7722 7311.4700
328.1117 -36.1359 -114.4222 664265.7895 -12.3539
-12.3547
[33053]ENERGY: 8580000 3568.4762 5782.5069 5901.1402
370.1879 -15375.4922 -1870.6375 0.0000 0.0000
8863.8559 7240.0373 330.6642 -1623.8185 7312.5883
328.0751 62.2266 24.4008 664265.7895 -14.3396
-14.3231
[33103]ENERGY: 8590000 3577.3134 5966.4082 5885.5040
350.6672 -15313.7576 -1981.0328 0.0000 0.0000
8755.3520 7240.4544 326.6165 -1514.8976 7312.8701
328.0639 8.1533 -68.5466 664265.7895 -15.3763

-15.4013
[33142]ENERGY: 8600000 3620.4362 5773.4830 5902.1923
351.4837 -15318.8084 -1866.8646 0.0000 0.0000
8781.1589 7243.0810 327.5792 -1538.0779 7312.8154
327.9928 13.1161 23.2209 664265.7895 -9.5625
-9.5572
[33192]ENERGY: 8610000 3723.4392 5731.9476 5944.7396
368.2780 -15505.1130 -1854.3280 0.0000 0.0000
8832.1399 7241.1033 329.4811 -1591.0366 7311.2707
328.2192 -47.2875 -69.4363 664265.7895 -10.2843
-10.2713
[33228]ENERGY: 8620000 3555.2280 5762.5090 5898.5502
371.6085 -15378.9762 -1846.2464 0.0000 0.0000
8876.9188 7239.5920 331.1515 -1637.3269 7311.5999
328.4898 -23.2059 -37.0622 664265.7895 -14.1104
-14.1281
[33278]ENERGY: 8630000 3556.1324 5822.3049 5882.4026
363.8890 -15316.0335 -1883.3298 0.0000 0.0000
8816.7267 7242.0923 328.9061 -1574.6344 7312.8301
328.2651 -204.2409 -113.6710 664265.7895 -9.8960
-9.8611
[33314]ENERGY: 8640000 3569.7415 5862.5473 5910.5242
360.7094 -15360.3113 -1881.5115 0.0000 0.0000
8782.1339 7243.8335 327.6156 -1538.3003 7312.1834
328.2054 114.4907 77.7588 664265.7895 -12.0036
-12.0312
[33364]ENERGY: 8650000 3538.5380 5857.7516 5929.7230
369.9574 -15503.0477 -1773.1740 0.0000 0.0000
8824.2939 7244.0422 329.1884 -1580.2517 7313.2044
328.3030 29.7227 38.2791 664265.7895 -10.4079
-10.4180
[33400]ENERGY: 8660000 3554.6787 5755.6532 5907.8877
334.2268 -15321.3223 -1792.9426 0.0000 0.0000
8802.6675 7240.8490 328.3816 -1561.8185 7311.4026
328.1226 5.9274 -1.0804 664265.7895 -11.3977
-11.3897
[33450]ENERGY: 8670000 3633.1779 5826.3660 5940.6544
353.4353 -15330.4376 -1900.2730 0.0000 0.0000
8725.9676 7248.8906 325.5203 -1477.0770 7314.4976
328.3562 15.9914 -56.6795 664265.7895 -9.9292
-9.9086
[33486]ENERGY: 8680000 3538.7365 5780.3618 5893.0940
362.3837 -15224.9082 -1877.3310 0.0000 0.0000
8772.5340 7244.8708 327.2575 -1527.6632 7316.2014
328.1447 139.9868 50.4172 664265.7895 -7.1747
-7.1879
[33536]ENERGY: 8690000 3574.0078 5801.0426 5937.1239
388.6076 -15345.1541 -1935.6570 0.0000 0.0000
8825.0891 7245.0599 329.2180 -1580.0292 7315.4728
328.2855 -14.7095 -25.4917 664265.7895 -9.5504
-9.5681
[33575]ENERGY: 8700000 3616.9086 5743.9638 5965.6697
368.0460 -15372.7026 -1864.7019 0.0000 0.0000
8784.6028 7241.7865 327.7077 -1542.8163 7315.3088

328.4113 -226.3811 -204.5154 664265.7895 -7.2508
-7.2450
[33625]ENERGY: 8710000 3616.0479 5734.6472 5939.6565
377.1139 -15391.3873 -1857.8641 0.0000 0.0000
8827.5198 7245.7340 329.3087 -1581.7858 7315.4858
328.1996 50.4483 -54.0839 664265.7895 -11.5576
-11.5528
[33661]ENERGY: 8720000 3659.4737 5865.2970 5912.2196
343.3784 -15424.2429 -1850.5258 0.0000 0.0000
8743.9886 7249.5887 326.1926 -1494.3999 7314.6042
328.3426 -94.4309 -4.8698 664265.7895 -9.4539
-9.4405
[33711]ENERGY: 8730000 3536.5091 5854.1156 5935.7485
367.2035 -15396.9217 -1844.8259 0.0000 0.0000
8793.3201 7245.1493 328.0329 -1548.1708 7315.6496
328.4817 184.2966 17.3353 664265.7895 -10.3955
-10.4065
[33747]ENERGY: 8740000 3566.9755 5707.1737 5902.4815
363.3604 -15283.3144 -1846.5641 0.0000 0.0000
8832.5141 7242.6267 329.4950 -1589.8874 7314.8897
328.2018 207.2703 63.5861 664265.7895 -9.4797
-9.4897
[33797]ENERGY: 8750000 3557.5431 5744.8838 5943.3681
358.1430 -15360.7645 -1812.6152 0.0000 0.0000
8812.6316 7243.1900 328.7533 -1569.4416 7315.4712
327.9873 94.0938 10.5920 664265.7895 -9.6200
-9.6321
[33833]ENERGY: 8760000 3604.8287 5797.4891 5938.2644
368.2517 -15326.6468 -1948.1203 0.0000 0.0000
8809.1647 7243.2315 328.6240 -1565.9332 7316.7929
327.9656 -19.0449 -7.7353 664265.7895 -13.5850
-13.5630
[33883]ENERGY: 8770000 3627.3005 5819.8072 5974.4876
374.1714 -15404.6037 -1840.0698 0.0000 0.0000
8691.8081 7242.9013 324.2460 -1448.9068 7315.6614
328.0299 -2.5135 -27.2889 664265.7895 -11.8769
-11.8666
[33919]ENERGY: 8780000 3653.2169 5754.9538 5920.9430
380.0990 -15393.6554 -1863.9701 0.0000 0.0000
8797.7588 7249.3460 328.1985 -1548.4128 7316.6278
328.3097 8.6773 -19.2642 664265.7895 -8.1959
-8.2094
[33969]ENERGY: 8790000 3511.3457 5834.1011 5959.3942
365.3750 -15396.3442 -1846.9153 0.0000 0.0000
8819.5135 7246.4702 329.0101 -1573.0434 7316.6248
328.3383 225.1601 147.0156 664265.7895 -8.5613
-8.5595
[34008]ENERGY: 8800000 3678.6103 5898.3590 5920.3832
358.9929 -15538.2310 -1819.5746 0.0000 0.0000
8751.6235 7250.1634 326.4774 -1501.4601 7316.1069
328.6407 -78.6849 -118.1746 664265.7895 -7.5919
-7.5854
[34058]ENERGY: 8810000 3610.5677 5794.1315 5964.7559
363.6703 -15469.1453 -1775.4238 0.0000 0.0000

8756.5357 7245.0921 326.6607 -1511.4436 7315.6034
328.6695 121.3090 78.8193 664265.7895 -7.5918
-7.5978
[34094]ENERGY: 8820000 3597.8374 5775.7896 5896.0155
367.8138 -15418.4998 -1876.8701 0.0000 0.0000
8900.4924 7242.5789 332.0310 -1657.9135 7317.1405
328.6119 -46.8430 -64.1583 664265.7895 -6.1523
-6.1441
[34144]ENERGY: 8830000 3580.3970 5927.6092 5873.1408
355.3412 -15478.6754 -1840.1327 0.0000 0.0000
8828.0856 7245.7656 329.3298 -1582.3199 7317.0893
328.5252 136.1860 77.6190 664265.7895 -8.7751
-8.7634
[34180]ENERGY: 8840000 3529.3145 5846.1372 5893.8639
349.9313 -15400.4339 -1834.6771 0.0000 0.0000
8858.0138 7242.1496 330.4463 -1615.8642 7316.9439
328.9732 59.0468 57.9218 664265.7895 -6.5364
-6.5535
[34230]ENERGY: 8850000 3593.6757 5782.5132 5882.4877
357.4779 -15397.4289 -1831.1157 0.0000 0.0000
8854.4542 7242.0643 330.3135 -1612.3899 7316.8186
328.5512 63.1943 53.0044 664265.7895 -10.0840
-10.0745
[34266]ENERGY: 8860000 3583.1244 5780.7397 5887.7245
353.0493 -15318.6508 -1885.8020 0.0000 0.0000
8848.5478 7248.7329 330.0932 -1599.8149 7317.4175
328.5964 36.9236 -46.7976 664265.7895 -8.4086
-8.4190
[34316]ENERGY: 8870000 3584.8679 5774.2309 5891.2658
369.6679 -15350.2556 -1879.8704 0.0000 0.0000
8854.6843 7244.5908 330.3221 -1610.0935 7318.1919
328.4741 56.7184 34.0378 664265.7895 -5.3940
-5.3937
[34352]ENERGY: 8880000 3574.1878 5934.7086 5911.0005
350.8774 -15509.1696 -1800.6639 0.0000 0.0000
8785.4950 7246.4358 327.7410 -1539.0591 7316.9330
328.6614 36.0508 101.0941 664265.7895 -6.5700
-6.5540
[34402]ENERGY: 8890000 3613.5925 5794.7600 5870.3703
366.5157 -15373.4608 -1784.9490 0.0000 0.0000
8760.8595 7247.6881 326.8220 -1513.1713 7317.0241
328.6854 14.1292 -52.3071 664265.7895 -4.5021
-4.5212
[34441]ENERGY: 8900000 3604.2627 5855.1281 5882.6487
365.5714 -15362.1805 -1944.8283 0.0000 0.0000
8848.0831 7248.6853 330.0758 -1599.3978 7317.3364
328.6838 -119.6380 -56.8399 664265.7895 -2.2979
-2.2914
[34491]ENERGY: 8910000 3638.9551 5794.8687 5962.8280
349.6043 -15520.7339 -1781.4359 0.0000 0.0000
8803.1046 7247.1909 328.3979 -1555.9137 7316.0090
328.7197 84.7347 71.1157 664265.7895 -3.3328
-3.3245
[34527]ENERGY: 8920000 3588.2772 5868.7937 5882.5123

386.1237 -15391.5355 -1878.7693 0.0000 0.0000
8789.7197 7245.1218 327.8986 -1544.5978 7316.6520
328.6984 133.4976 75.4413 664265.7895 -8.2554
-8.2677
[34577]ENERGY: 8930000 3592.0715 5954.6622 5877.8838
351.8334 -15532.3093 -1844.5539 0.0000 0.0000
8844.7617 7244.3494 329.9519 -1600.4123 7316.3427
328.7635 -137.7834 -82.7726 664265.7895 -9.5439
-9.5442
[34613]ENERGY: 8940000 3622.6003 5962.4431 5872.8890
344.2272 -15450.0628 -1905.7684 0.0000 0.0000
8797.8891 7244.2175 328.2034 -1553.6716 7316.9151
328.8864 -66.4109 -17.5834 664265.7895 -2.2403
-2.2534
[34663]ENERGY: 8950000 3701.6733 5779.0552 5903.3354
369.0657 -15527.3945 -1794.6499 0.0000 0.0000
8815.1335 7246.2187 328.8467 -1568.9148 7317.7733
328.8978 26.9245 -89.7754 664265.7895 -6.4217
-6.4124
[34699]ENERGY: 8960000 3566.6112 5929.2281 5884.0202
358.7275 -15530.3513 -1735.2972 0.0000 0.0000
8774.2309 7247.1695 327.3208 -1527.0615 7317.6073
328.8248 151.4959 49.9941 664265.7895 -8.1955
-8.1851
[34749]ENERGY: 8970000 3546.5575 5789.5531 5959.9230
377.4493 -15426.6224 -1842.8010 0.0000 0.0000
8841.8780 7245.9376 329.8444 -1595.9404 7317.7020
328.7778 167.7880 143.8390 664265.7895 -2.5782
-2.5986
[34785]ENERGY: 8980000 3609.6035 5949.6033 5897.9588
372.5688 -15491.8452 -1820.8379 0.0000 0.0000
8726.5792 7243.6305 325.5432 -1482.9487 7318.1559
328.9000 25.4762 103.4745 664265.7895 -3.0037
-2.9858
[34835]ENERGY: 8990000 3617.7931 5937.0140 5931.2068
370.0608 -15521.4513 -1794.6699 0.0000 0.0000
8706.4835 7246.4369 324.7935 -1460.0466 7318.2343
328.8250 -51.6660 -45.1870 664265.7895 -5.7958
-5.7992
[34874]ENERGY: 9000000 3613.9099 5885.3924 5898.3741
372.5520 -15359.2395 -1863.1242 0.0000 0.0000
8701.6281 7249.4927 324.6124 -1452.1353 7319.1418
328.6053 -23.8557 -107.8771 664265.7895 -4.9710
-4.9808
[34924]ENERGY: 9010000 3554.3552 5861.8770 5895.8748
368.8678 -15344.9587 -1810.2212 0.0000 0.0000
8725.2785 7251.0734 325.4946 -1474.2051 7319.4925
328.5961 176.8213 104.7038 664265.7895 -7.1626
-7.1519
[34960]ENERGY: 9020000 3587.8952 5906.7079 5913.7863
360.5926 -15477.4925 -1791.2911 0.0000 0.0000
8747.4233 7247.6217 326.3207 -1499.8016 7319.3401
328.6808 74.2230 12.5087 664265.7895 -10.2229
-10.2286

[35010]ENERGY: 9030000 3550.2672 5821.8748 5897.4861
373.6587 -15417.1595 -1780.4686 0.0000 0.0000
8802.2940 7247.9527 328.3677 -1554.3413 7318.2666
328.4631 40.6082 3.4041 664265.7895 -4.4532
-4.4588
[35046]ENERGY: 9040000 3620.7776 5815.3067 5926.1843
359.2307 -15476.0826 -1866.3460 0.0000 0.0000
8867.3574 7246.4282 330.7949 -1620.9292 7317.8678
328.5478 -149.8174 -83.2460 664265.7895 -9.0418
-9.0116
[35096]ENERGY: 9050000 3569.7229 5929.7347 5921.2222
343.6409 -15462.7139 -1834.8925 0.0000 0.0000
8777.1428 7243.8573 327.4294 -1533.2856 7317.2879
328.7007 -58.2129 -34.0269 664265.7895 -9.6966
-9.7009
[35132]ENERGY: 9060000 3637.4620 5798.6293 5911.4487
362.7722 -15418.8716 -1865.8033 0.0000 0.0000
8821.5409 7247.1782 329.0857 -1574.3627 7319.2008
328.6101 52.9630 20.8124 664265.7895 -8.7839
-8.7975
[35182]ENERGY: 9070000 3606.5942 5878.8383 5858.3354
341.9912 -15432.1331 -1806.3310 0.0000 0.0000
8801.7629 7249.0578 328.3479 -1552.7051 7317.6910
328.6742 107.5625 161.9403 664265.7895 -8.0293
-8.0117
[35218]ENERGY: 9080000 3565.7109 5947.7318 5893.8300
360.8201 -15382.8291 -1917.6662 0.0000 0.0000
8779.2323 7246.8298 327.5074 -1532.4025 7318.1110
328.2993 -238.6264 -170.1273 664265.7895 -8.8457
-8.8697
[35268]ENERGY: 9090000 3553.4907 5866.7542 5895.4073
343.3353 -15468.8600 -1751.9911 0.0000 0.0000
8811.4709 7249.6074 328.7100 -1561.8635 7318.9738
328.4117 165.6351 93.6137 664265.7895 -8.4894
-8.4794
[35307]ENERGY: 9100000 3545.6060 5837.1854 5946.7777
361.5630 -15444.2662 -1828.8571 0.0000 0.0000
8833.1099 7251.1186 329.5173 -1581.9913 7319.8506
328.4582 125.6973 33.2564 664265.7895 -5.7547
-5.7742
[35357]ENERGY: 9110000 3527.5569 5789.0472 5918.6936
378.3711 -15336.3669 -1818.6275 0.0000 0.0000
8791.1989 7249.8732 327.9538 -1541.3256 7320.6563
328.4986 179.6871 74.8324 664265.7895 -3.6591
-3.6482
[35393]ENERGY: 9120000 3558.4055 5814.9034 5916.9348
368.1281 -15481.1049 -1804.7227 0.0000 0.0000
8872.9371 7245.4813 331.0030 -1627.4558 7319.3477
328.6908 68.5057 157.2270 664265.7895 -7.8940
-7.8719
[35443]ENERGY: 9130000 3637.9098 5756.2151 5922.3820
379.4736 -15417.0911 -1860.3191 0.0000 0.0000
8831.2259 7249.7962 329.4470 -1581.4298 7320.0816
328.5422 -82.7587 -54.1961 664265.7895 -3.1838

-3.2032
[35479]ENERGY: 9140000 3665.4421 5839.8782 5945.4952
337.2329 -15413.4622 -1819.4822 0.0000 0.0000
8696.2321 7251.3362 324.4111 -1444.8959 7319.7048
328.5184 30.8164 -13.5574 664265.7895 -9.4558
-9.4629
[35529]ENERGY: 9150000 3666.2492 5698.9259 5890.8337
345.7108 -15331.9327 -1827.2628 0.0000 0.0000
8806.1109 7248.6352 328.5101 -1557.4757 7319.9836
328.4046 -130.9152 -70.6067 664265.7895 -6.1105
-6.0847
[35565]ENERGY: 9160000 3608.0826 5758.4547 5874.7892
379.2714 -15383.7904 -1792.2519 0.0000 0.0000
8802.8970 7247.4527 328.3902 -1555.4442 7321.1801
328.5609 128.2760 173.5899 664265.7895 -4.3102
-4.3106
[35615]ENERGY: 9170000 3603.6798 5815.0678 5875.7499
356.3267 -15380.9217 -1885.1480 0.0000 0.0000
8862.2104 7246.9649 330.6028 -1615.2455 7321.5036
328.6542 -54.4944 -85.8197 664265.7895 -3.5102
-3.5261
[35651]ENERGY: 9180000 3530.7181 5817.3646 5975.1512
331.2516 -15336.8663 -1854.2915 0.0000 0.0000
8791.0297 7254.3575 327.9475 -1536.6723 7320.8709
328.5516 78.5202 40.1708 664265.7895 -3.5318
-3.5182
[35701]ENERGY: 9190000 3649.3547 5825.0013 5919.0442
356.2751 -15368.3650 -1895.9997 0.0000 0.0000
8769.2457 7254.5563 327.1348 -1514.6894 7322.2628
328.5889 -28.1516 -29.7503 664265.7895 -8.5988
-8.6171
[35740]ENERGY: 9200000 3569.7126 5857.2347 5874.2922
354.9587 -15292.8939 -1878.4924 0.0000 0.0000
8769.0080 7253.8199 327.1260 -1515.1882 7321.5607
328.7331 -141.9184 -76.3154 664265.7895 -5.3796
-5.3691
[35790]ENERGY: 9210000 3575.7598 5871.6431 5982.7768
341.1116 -15492.0941 -1851.2186 0.0000 0.0000
8820.7801 7248.7587 329.0573 -1572.0214 7321.5854
328.7259 215.3618 158.2466 664265.7895 -3.1336
-3.1445
[35826]ENERGY: 9220000 3712.0860 5901.7868 5893.2053
346.2967 -15504.2910 -1859.7942 0.0000 0.0000
8763.8858 7253.1754 326.9349 -1510.7104 7322.2080
328.5654 22.2890 25.6179 664265.7895 -7.4138
-7.4011
[35876]ENERGY: 9230000 3618.8629 5849.4644 5951.7865
341.0377 -15412.6279 -1908.2931 0.0000 0.0000
8811.0826 7251.3131 328.6955 -1559.7695 7323.2046
328.7006 -44.1234 -61.9097 664265.7895 -9.4987
-9.5111
[35912]ENERGY: 9240000 3548.8697 5821.7856 5938.3215
372.8291 -15401.0314 -1837.7224 0.0000 0.0000
8808.3176 7251.3698 328.5924 -1556.9478 7323.4626

328.8285 -106.5804 -17.1837 664265.7895 -3.1191
-3.1158
[35962]ENERGY: 9250000 3552.7606 5911.2909 5902.0080
335.1632 -15434.9495 -1827.9989 0.0000 0.0000
8816.7815 7255.0559 328.9081 -1561.7257 7322.1923
328.9074 30.7872 39.6565 664265.7895 -3.8596
-3.8428
[35998]ENERGY: 9260000 3652.7773 5895.9347 5849.9361
324.6543 -15515.3445 -1809.2372 0.0000 0.0000
8851.9078 7250.6285 330.2185 -1601.2793 7323.3633
328.8463 -74.6257 -146.3610 664265.7895 -3.8375
-3.8466
[36048]ENERGY: 9270000 3578.1664 5840.1809 5874.7229
362.2523 -15347.4735 -1908.9614 0.0000 0.0000
8851.8449 7250.7325 330.2162 -1601.1124 7323.3579
328.8270 126.0062 18.2363 664265.7895 1.0329
1.0196
[36084]ENERGY: 9280000 3645.0594 5652.7338 5925.5128
352.1140 -15362.6672 -1864.4186 0.0000 0.0000
8900.4918 7248.8262 332.0309 -1651.6656 7322.1461
328.7812 -12.6398 23.3289 664265.7895 1.3998
1.4056
[36134]ENERGY: 9290000 3633.8221 5788.1257 5969.8971
378.6597 -15373.0500 -1913.8582 0.0000 0.0000
8771.0917 7254.6882 327.2037 -1516.4035 7321.7661
328.8640 -63.6861 -25.0462 664265.7895 -2.9679
-2.9682
[36173]ENERGY: 9300000 3626.3897 5866.2475 5868.5439
364.0754 -15430.4725 -1840.1441 0.0000 0.0000
8800.5002 7255.1401 328.3008 -1545.3602 7323.5151
328.7334 28.2199 19.2288 664265.7895 -1.6039
-1.6104
[36223]ENERGY: 9310000 3606.1609 5878.9512 5916.6258
376.8939 -15410.8007 -1922.6534 0.0000 0.0000
8809.6683 7254.8459 328.6428 -1554.8224 7323.6152
328.9035 -145.7781 -191.0218 664265.7895 -3.0348
-3.0396
[36259]ENERGY: 9320000 3574.9729 5851.9134 5919.5786
354.6311 -15424.8370 -1837.4932 0.0000 0.0000
8811.7014 7250.4671 328.7186 -1561.2343 7324.7529
328.7516 82.5789 -75.5073 664265.7895 -8.4391
-8.4361
[36309]ENERGY: 9330000 3603.0858 5793.4604 5947.2708
362.1854 -15333.3101 -1867.3068 0.0000 0.0000
8750.1730 7255.5585 326.4233 -1494.6144 7324.2943
328.7842 5.4064 29.5414 664265.7895 1.5344
1.5368
[36345]ENERGY: 9340000 3553.6857 5910.4552 5931.4801
369.7200 -15420.8703 -1904.5646 0.0000 0.0000
8817.6129 7257.5190 328.9391 -1560.0939 7325.0943
328.7763 -6.9141 -82.1790 664265.7895 -4.9906
-4.9883
[36395]ENERGY: 9350000 3632.2043 5879.1860 5880.0429
355.1428 -15440.0241 -1870.1361 0.0000 0.0000

8816.2340 7252.6498 328.8877 -1563.5842 7324.8961
328.7166 13.3154 -19.6103 664265.7895 -7.0522
-7.0479
[36431]ENERGY: 9360000 3586.5100 5804.2915 5973.5264
351.4555 -15363.8099 -1889.7094 0.0000 0.0000
8794.2830 7256.5472 328.0688 -1537.7358 7324.6842
328.7154 9.1422 -22.6288 664265.7895 -5.5949
-5.6031
[36481]ENERGY: 9370000 3538.4121 5831.8822 5925.5196
338.9021 -15370.2998 -1894.8214 0.0000 0.0000
8881.9044 7251.4992 331.3375 -1630.4051 7325.4804
328.8706 63.0528 81.8452 664265.7895 -6.0384
-6.0330
[36517]ENERGY: 9380000 3665.6338 5850.0590 5907.1915
356.6778 -15403.9009 -1874.3134 0.0000 0.0000
8756.7390 7258.0867 326.6683 -1498.6522 7326.4537
328.5303 -27.7938 14.2555 664265.7895 -4.7087
-4.7051
[36567]ENERGY: 9390000 3579.2754 5777.7801 5877.7783
351.9148 -15384.6347 -1764.8139 0.0000 0.0000
8817.6930 7254.9931 328.9421 -1562.6999 7325.8120
328.2976 46.6703 40.3477 664265.7895 -12.9571
-12.9687
[36606]ENERGY: 9400000 3601.9625 5802.5664 5926.0824
354.3377 -15409.2534 -1876.6016 0.0000 0.0000
8855.3180 7254.4119 330.3457 -1600.9061 7327.5038
328.3144 39.3385 31.5500 664265.7895 -7.8780
-7.8548
[36656]ENERGY: 9410000 3570.8318 5828.9761 5934.6290
365.7119 -15360.0905 -1862.7140 0.0000 0.0000
8779.8007 7257.1450 327.5286 -1522.6557 7326.8501
328.5225 96.9602 40.0015 664265.7895 -7.2793
-7.2841
[36692]ENERGY: 9420000 3608.5706 5756.0118 5911.1689
354.5546 -15399.4149 -1842.6935 0.0000 0.0000
8866.5776 7254.7752 330.7658 -1611.8024 7326.4994
328.4507 -2.1210 42.9119 664265.7895 -10.1937
-10.1882
[36742]ENERGY: 9430000 3557.4944 5863.5185 5874.6967
363.5329 -15404.1830 -1843.8172 0.0000 0.0000
8847.9898 7259.2322 330.0724 -1588.7576 7327.5072
328.5160 -11.4856 11.9141 664265.7895 -9.9626
-9.9820
[36778]ENERGY: 9440000 3637.7014 5803.8874 5948.2655
376.2664 -15367.0700 -1888.9485 0.0000 0.0000
8747.8134 7257.9156 326.3353 -1489.8978 7325.1021
328.6781 -15.4315 9.5660 664265.7895 -10.8800
-10.8701
[36828]ENERGY: 9450000 3674.2163 5792.8207 5912.8806
375.8607 -15457.7536 -1828.5115 0.0000 0.0000
8785.4473 7254.9605 327.7392 -1530.4868 7324.3839
328.6950 29.1904 63.9595 664265.7895 -6.9003
-6.9031
[36864]ENERGY: 9460000 3605.6802 5801.5986 5937.9741

360.3929 -15414.2696 -1860.0778 0.0000 0.0000
8822.7511 7254.0494 329.1308 -1568.7017 7327.6482
328.8531 104.6778 -20.8571 664265.7895 -2.4980
-2.5079
[36914]ENERGY: 9470000 3604.9868 5826.3743 5890.4119
358.2880 -15383.9715 -1828.4467 0.0000 0.0000
8791.6932 7259.3360 327.9722 -1532.3572 7328.6014
328.9409 145.1218 77.1097 664265.7895 -0.6934
-0.6937
[36950]ENERGY: 9480000 3564.2462 5735.4486 5908.4541
347.8429 -15367.1555 -1841.6443 0.0000 0.0000
8908.9039 7256.0960 332.3447 -1652.8079 7330.5428
329.0417 82.3686 10.1899 664265.7895 -8.1131
-8.1003
[37000]ENERGY: 9490000 3559.7375 5814.9578 5842.1365
354.8899 -15349.6256 -1854.4630 0.0000 0.0000
8891.1147 7258.7479 331.6811 -1632.3669 7330.9347
329.1730 187.8715 97.2003 664265.7895 -1.4466
-1.4610
[37039]ENERGY: 9500000 3581.8575 5952.3288 5868.1255
345.3314 -15411.3316 -1885.4597 0.0000 0.0000
8810.5440 7261.3958 328.6754 -1549.1482 7332.1215
329.1574 -158.1871 -153.1440 664265.7895 -7.2325
-7.2150
[37089]ENERGY: 9510000 3617.5893 5806.8168 5902.4752
357.4259 -15359.7249 -1931.2228 0.0000 0.0000
8865.2248 7258.5843 330.7153 -1606.6406 7332.6597
329.0745 -186.7239 -78.7561 664265.7895 -5.7062
-5.7155
[37125]ENERGY: 9520000 3610.9783 5965.0299 5844.3326
356.7948 -15342.7202 -1935.8523 0.0000 0.0000
8762.2267 7260.7898 326.8730 -1501.4369 7333.0349
329.0459 -52.2486 -10.2968 664265.7895 -8.5659
-8.5676
[37175]ENERGY: 9530000 3676.6114 5784.2832 5889.9422
343.7850 -15390.8581 -1809.0298 0.0000 0.0000
8767.0010 7261.7349 327.0511 -1505.2660 7332.6903
329.0079 8.7999 4.0164 664265.7895 -5.8450
-5.8332
[37211]ENERGY: 9540000 3634.4815 5837.6715 5887.7588
361.5861 -15269.0091 -1915.6028 0.0000 0.0000
8725.9549 7262.8409 325.5199 -1463.1140 7334.4045
329.0638 -28.2204 -50.4313 664265.7895 -1.0341
-1.0553
[37261]ENERGY: 9550000 3637.8525 5828.8135 5833.8006
363.5637 -15367.2498 -1862.5662 0.0000 0.0000
8828.5548 7262.7691 329.3473 -1565.7857 7332.9165
329.0840 3.8620 61.3373 664265.7895 -5.9467
-5.9357
[37297]ENERGY: 9560000 3685.2969 5822.2519 5913.6985
374.1112 -15382.6654 -1917.5126 0.0000 0.0000
8769.2886 7264.4690 327.1364 -1504.8195 7333.7414
329.1626 26.7331 -30.1731 664265.7895 -8.1170
-8.1178

[37347]ENERGY: 9570000 3559.8824 5816.7276 5941.8113
371.1256 -15551.3496 -1778.3744 0.0000 0.0000
8899.2397 7259.0626 331.9842 -1640.1771 7332.9509
329.3096 243.8684 179.8359 664265.7895 -2.4109
-2.4114
[37383]ENERGY: 9580000 3651.9365 5763.4311 5955.0745
356.3848 -15398.8996 -1866.9398 0.0000 0.0000
8802.3842 7263.3716 328.3710 -1539.0125 7332.3674
329.2401 -114.2873 -95.6796 664265.7895 -4.9853
-4.9801
[37433]ENERGY: 9590000 3631.5657 5788.1204 5867.8264
359.8016 -15274.1408 -1925.0508 0.0000 0.0000
8813.3908 7261.5133 328.7816 -1551.8775 7333.3002
329.0290 -190.3902 -166.4408 664265.7895 -2.3348
-2.3480
[37472]ENERGY: 9600000 3604.1363 5860.3056 5890.7476
351.6522 -15401.0605 -1865.8232 0.0000 0.0000
8818.9547 7258.9128 328.9892 -1560.0420 7331.2280
329.1356 -151.3534 -93.2326 664265.7895 -4.4866
-4.4643
[37522]ENERGY: 9610000 3696.7730 5777.6505 5974.3976
355.9348 -15353.6384 -1907.9660 0.0000 0.0000
8722.0113 7265.1629 325.3728 -1456.8484 7331.6600
329.1846 -155.5403 -109.0970 664265.7895 -5.0079
-5.0112
[37558]ENERGY: 9620000 3619.9980 5839.8869 5947.5653
366.4103 -15328.1812 -1907.2151 0.0000 0.0000
8724.7238 7263.1880 325.4739 -1461.5358 7332.9981
328.8616 -93.8370 -103.0886 664265.7895 -6.3370
-6.3301
[37608]ENERGY: 9630000 3578.5816 5762.7383 5920.6882
344.1101 -15273.4310 -1890.2972 0.0000 0.0000
8820.0735 7262.4634 329.0309 -1557.6100 7333.8390
328.7762 3.4496 -1.3485 664265.7895 -10.1624
-10.1861
[37644]ENERGY: 9640000 3598.6426 5803.0986 5835.8679
360.4553 -15293.1428 -1843.9088 0.0000 0.0000
8803.5290 7264.5418 328.4138 -1538.9872 7333.8527
328.9728 92.7898 129.0240 664265.7895 -12.4083
-12.3982
[37694]ENERGY: 9650000 3591.7876 5835.6037 5895.3865
359.0487 -15364.3977 -1873.1910 0.0000 0.0000
8822.2396 7266.4772 329.1117 -1555.7624 7337.0261
328.9347 33.3733 -71.1554 664265.7895 -12.1471
-12.1542
[37730]ENERGY: 9660000 3667.2033 5848.9430 5858.7471
375.6912 -15363.7537 -1878.1760 0.0000 0.0000
8757.0373 7265.6922 326.6794 -1491.3451 7336.9944
329.0471 -116.5049 -129.9311 664265.7895 -6.7620
-6.7477
[37780]ENERGY: 9670000 3561.9889 5789.0593 5910.4352
380.6795 -15324.9324 -1853.0830 0.0000 0.0000
8802.4689 7266.6164 328.3742 -1535.8525 7335.8508
329.0874 -101.0260 -93.2143 664265.7895 -9.0137

-9.0200
[37816]ENERGY: 9680000 3548.8366 5794.3437 5891.3062
393.2347 -15343.7011 -1849.2562 0.0000 0.0000
8828.8270 7263.5910 329.3575 -1565.2360 7335.8630
329.0378 117.8611 125.3118 664265.7895 -7.8551
-7.8632
[37866]ENERGY: 9690000 3557.8811 5878.9018 5884.9199
366.6619 -15299.3551 -1934.9350 0.0000 0.0000
8814.1837 7268.2582 328.8112 -1545.9255 7337.4257
329.1046 84.5128 -30.2647 664265.7895 -8.4323
-8.4479
[37905]ENERGY: 9700000 3588.8177 5836.4643 5867.2850
337.1590 -15400.3902 -1817.7575 0.0000 0.0000
8853.4134 7264.9916 330.2747 -1588.4218 7336.7864
328.9658 -107.3020 -123.3977 664265.7895 -8.7044
-8.6750
[37955]ENERGY: 9710000 3644.6272 5824.3207 5856.8245
339.5961 -15371.1268 -1846.8202 0.0000 0.0000
8819.3855 7266.8069 329.0053 -1552.5786 7338.1975
328.9359 26.7696 -88.0743 664265.7895 -4.6068
-4.6194
[37991]ENERGY: 9720000 3636.4319 5828.0107 5868.4841
357.1121 -15432.4358 -1915.2687 0.0000 0.0000
8922.4935 7264.8277 332.8517 -1657.6657 7339.9855
329.0787 -27.2019 -83.7825 664265.7895 -6.5809
-6.6014
[38041]ENERGY: 9730000 3626.3627 5894.6984 5887.4351
358.7349 -15374.6264 -1914.2952 0.0000 0.0000
8793.4605 7271.7700 328.0381 -1521.6905 7342.1372
329.1885 -161.7784 -138.9589 664265.7895 -4.4392
-4.4191
[38077]ENERGY: 9740000 3640.7994 5833.0565 5891.0207
348.9404 -15370.7171 -1897.1683 0.0000 0.0000
8822.9342 7268.8658 329.1377 -1554.0684 7341.0462
329.2166 4.8393 -101.1728 664265.7895 -4.7486
-4.7534
[38127]ENERGY: 9750000 3729.7048 5746.7516 5867.7244
341.9675 -15308.9177 -1943.2385 0.0000 0.0000
8838.8678 7272.8600 329.7321 -1566.0079 7340.9823
329.1861 -134.5732 -97.2265 664265.7895 -8.2367
-8.2373
[38163]ENERGY: 9760000 3543.8147 5927.2356 5881.3943
355.2232 -15338.0544 -1829.2392 0.0000 0.0000
8729.2578 7269.6320 325.6431 -1459.6258 7340.3059
329.1925 -30.9331 -5.4186 664265.7895 -8.0883
-8.0896
[38213]ENERGY: 9770000 3594.3833 5814.9657 5988.5244
361.6307 -15415.6381 -1839.2483 0.0000 0.0000
8763.9328 7268.5505 326.9366 -1495.3823 7342.2915
329.2260 38.0050 -7.4103 664265.7895 -5.2483
-5.2292
[38249]ENERGY: 9780000 3664.7723 5849.7612 5906.7833
353.3825 -15433.9612 -1872.8203 0.0000 0.0000
8804.4097 7272.3276 328.4466 -1532.0821 7341.8059

329.2884 -206.3797 -209.5218 664265.7895 -11.0453
-11.0685
[38299]ENERGY: 9790000 3589.8180 5799.8113 5952.1610
357.7111 -15496.1334 -1795.2816 0.0000 0.0000
8862.9698 7271.0562 330.6312 -1591.9136 7342.5004
329.2503 -25.0952 74.0238 664265.7895 -10.7591
-10.7338
[38338]ENERGY: 9800000 3700.5499 5786.1129 5887.8801
350.8623 -15398.7999 -1827.5633 0.0000 0.0000
8774.5365 7273.5786 327.3322 -1500.9579 7344.5411
329.2779 238.2948 176.1927 664265.7895 -9.2247
-9.2520
[38388]ENERGY: 9810000 3644.1149 5819.4357 5910.7936
351.5391 -15393.0453 -1897.9122 0.0000 0.0000
8836.7552 7271.6810 329.6532 -1565.0742 7345.0070
329.3062 124.9722 -64.9169 664265.7895 -9.4774
-9.4560
[38424]ENERGY: 9820000 3620.5836 5882.4914 5933.3428
342.8078 -15403.5088 -1885.8149 0.0000 0.0000
8787.4745 7277.3765 327.8148 -1510.0980 7343.9982
329.3687 4.1422 38.8777 664265.7895 -11.2716
-11.2655
[38474]ENERGY: 9830000 3614.3299 5804.8165 5867.7273
376.4951 -15265.9064 -1877.9399 0.0000 0.0000
8756.0321 7275.5545 326.6419 -1480.4776 7344.2566
328.9845 58.6420 -25.5632 664265.7895 -8.8606
-8.8653
[38510]ENERGY: 9840000 3539.4261 5867.3199 5875.0287
340.7472 -15347.4123 -1867.6320 0.0000 0.0000
8862.1180 7269.5955 330.5994 -1592.5225 7342.9072
328.9924 -2.2600 -71.1493 664265.7895 -3.9827
-3.9897
[38560]ENERGY: 9850000 3613.7864 5926.1560 5851.9413
385.1458 -15471.9372 -1801.7311 0.0000 0.0000
8771.0383 7274.3995 327.2017 -1496.6388 7344.7046
328.9366 82.1040 10.3688 664265.7895 -3.3010
-3.2903
[38596]ENERGY: 9860000 3632.0199 5816.5133 5884.9413
362.1773 -15282.0810 -1930.2008 0.0000 0.0000
8791.2825 7274.6526 327.9569 -1516.6299 7345.7397
329.1548 -142.9052 -97.0231 664265.7895 -6.4306
-6.4467
[38646]ENERGY: 9870000 3607.1770 5850.5639 5943.9968
358.9517 -15413.2045 -1906.9002 0.0000 0.0000
8834.0385 7274.6232 329.5519 -1559.4153 7344.9744
329.0214 144.5026 90.2652 664265.7895 -7.2462
-7.2342
[38682]ENERGY: 9880000 3639.7495 5823.2347 5925.5101
373.2806 -15377.3632 -1889.5225 0.0000 0.0000
8778.4449 7273.3341 327.4780 -1505.1108 7344.5816
329.0182 -105.0440 -171.2152 664265.7895 -6.0805
-6.0701
[38732]ENERGY: 9890000 3610.8728 5831.1813 6003.0117
366.0623 -15424.7752 -1896.4219 0.0000 0.0000

8782.9307 7272.8617 327.6453 -1510.0690 7344.7658
329.0030 88.9251 42.7236 664265.7895 -2.0010
-2.0153
[38771]ENERGY: 9900000 3681.2392 5902.9066 5885.6319
355.5067 -15404.5295 -1939.9810 0.0000 0.0000
8792.6938 7273.4677 328.0095 -1519.2261 7344.4596
329.0784 -27.3408 -2.3009 664265.7895 -1.8351
-1.8353
[38821]ENERGY: 9910000 3617.5658 5890.7469 5884.1045
337.8555 -15454.3130 -1831.1170 0.0000 0.0000
8826.0897 7270.9324 329.2554 -1555.1574 7346.2154
329.0965 -35.5927 -90.6758 664265.7895 -6.6559
-6.6589
[38857]ENERGY: 9920000 3596.3797 5810.4229 5879.7318
364.8810 -15361.1391 -1892.6629 0.0000 0.0000
8876.3389 7273.9524 331.1299 -1602.3865 7345.1009
329.0832 -29.9141 -53.9405 664265.7895 -3.5354
-3.5350
[38907]ENERGY: 9930000 3663.0686 5790.4429 5898.7664
369.1321 -15399.7949 -1836.3316 0.0000 0.0000
8786.6892 7271.9728 327.7855 -1514.7165 7345.2612
329.1937 -50.1233 -118.3582 664265.7895 -0.8580
-0.8612
[38943]ENERGY: 9940000 3642.8695 5811.7795 5920.8015
368.1680 -15480.5101 -1895.6645 0.0000 0.0000
8898.3041 7265.7481 331.9493 -1632.5560 7344.8793
329.1394 -189.2682 -111.1777 664265.7895 -5.4206
-5.4151
[38993]ENERGY: 9950000 3617.0011 5852.7546 5903.9836
375.3761 -15400.3760 -1860.3606 0.0000 0.0000
8785.4315 7273.8103 327.7386 -1511.6212 7344.1630
329.2113 51.7182 17.5269 664265.7895 -6.6650
-6.6813
[39029]ENERGY: 9960000 3654.1351 5834.1038 5927.1562
354.8104 -15489.9821 -1803.1279 0.0000 0.0000
8795.9728 7273.0682 328.1319 -1522.9046 7344.5764
329.2656 135.9224 69.1002 664265.7895 -4.1060
-4.1040
[39079]ENERGY: 9970000 3615.8092 5809.6055 5896.3141
357.2878 -15431.7220 -1839.7023 0.0000 0.0000
8867.7665 7275.3589 330.8101 -1592.4077 7346.6329
329.1407 -37.9628 -82.4864 664265.7895 -4.1056
-4.1039
[39115]ENERGY: 9980000 3599.4796 5929.3339 5854.3859
353.2427 -15497.9397 -1838.6026 0.0000 0.0000
8875.4145 7275.3143 331.0954 -1600.1002 7347.9212
328.9660 133.8679 42.7181 664265.7895 -2.6738
-2.6520
[39165]ENERGY: 9990000 3667.2067 5926.5284 5920.2857
392.7777 -15486.3535 -1854.9725 0.0000 0.0000
8712.5738 7278.0464 325.0207 -1434.5274 7346.8803
329.1598 30.6852 -35.0391 664265.7895 1.6553
1.6533
[39204]ENERGY: 10000000 3636.6527 5991.4960 5869.2314

363.4262 -15435.8825 -1845.5639 0.0000 0.0000
8698.6882 7278.0480 324.5027 -1420.6402 7347.4669
329.1957 176.1773 123.7576 664265.7895 1.6311
1.6337
[39254]ENERGY: 10010000 3618.9040 5914.8687 5917.4605
389.5232 -15478.3792 -1870.5762 0.0000 0.0000
8782.8598 7274.6608 327.6427 -1508.1990 7347.4488
328.9374 18.5427 21.0555 664265.7895 1.8047
1.7912
[39290]ENERGY: 10020000 3545.6245 5811.9756 5901.9987
365.0308 -15386.9303 -1862.3394 0.0000 0.0000
8898.2851 7273.6449 331.9486 -1624.6402 7347.7194
328.9717 74.4384 46.0695 664265.7895 0.6251
0.6207
[39340]ENERGY: 10030000 3525.9154 5860.7384 5925.7225
370.8158 -15399.9697 -1832.0565 0.0000 0.0000
8823.5821 7274.7481 329.1618 -1548.8340 7348.0992
328.9098 98.2582 19.5044 664265.7895 -2.4992
-2.4835
[39376]ENERGY: 10040000 3538.6734 5838.3685 5894.9368
364.5077 -15341.2212 -1866.2587 0.0000 0.0000
8846.6351 7275.6416 330.0218 -1570.9935 7348.8059
329.2371 42.3046 88.4258 664265.7895 -0.1457
-0.1608
[39426]ENERGY: 10050000 3577.4257 5873.7794 5909.8397
365.2668 -15344.5434 -1880.9737 0.0000 0.0000
8777.7149 7278.5093 327.4508 -1499.2056 7348.7788
329.0228 -72.7544 -63.9317 664265.7895 -4.1385
-4.1211
[39462]ENERGY: 10060000 3650.2485 5919.2136 5884.2941
378.9419 -15333.0235 -1966.9442 0.0000 0.0000
8747.8072 7280.5377 326.3351 -1467.2696 7349.1484
328.8587 -132.9913 -121.4092 664265.7895 -5.8102
-5.8178
[39512]ENERGY: 10070000 3629.5102 5820.5027 5863.9026
392.1130 -15420.1009 -1816.9130 0.0000 0.0000
8808.4734 7277.4880 328.5982 -1530.9854 7348.7823
329.1651 48.7999 0.3525 664265.7895 -4.3339
-4.3353
[39548]ENERGY: 10080000 3562.7603 5780.3557 5963.1478
388.4581 -15441.1217 -1811.3843 0.0000 0.0000
8834.3184 7276.5342 329.5623 -1557.7842 7349.6776
329.0060 122.8408 72.7029 664265.7895 -7.3626
-7.3727
[39598]ENERGY: 10090000 3603.9344 5854.1016 5930.7232
356.2032 -15435.9500 -1808.9393 0.0000 0.0000
8776.9043 7276.9773 327.4205 -1499.9269 7349.0516
329.0482 -98.0100 -138.9246 664265.7895 -4.9405
-4.9301
[39637]ENERGY: 10100000 3629.2717 5895.8270 5943.7285
361.0588 -15373.3354 -1917.4407 0.0000 0.0000
8741.1026 7280.2127 326.0849 -1460.8899 7349.7210
329.2813 -110.5585 -172.6302 664265.7895 -8.9686
-8.9895

[39687]ENERGY: 10110000 3607.4132 5836.5762 5891.8785
372.4896 -15388.0025 -1807.0408 0.0000 0.0000
8764.2942 7277.6084 326.9501 -1486.6858 7350.0586
329.1922 68.4373 53.6838 664265.7895 -11.5351
-11.4979
[39723]ENERGY: 10120000 3631.3975 5792.7681 5891.4794
368.2154 -15412.1944 -1816.0344 0.0000 0.0000
8825.8494 7281.4809 329.2464 -1544.3685 7350.9467
329.2362 -36.7834 -9.8734 664265.7895 -10.5748
-10.6071
[39773]ENERGY: 10130000 3608.9343 5818.2840 5983.2639
334.8646 -15455.0624 -1839.2079 0.0000 0.0000
8827.9508 7279.0273 329.3248 -1548.9235 7352.8823
329.2935 18.4590 -91.8198 664265.7895 -7.3354
-7.3067
[39809]ENERGY: 10140000 3605.1649 5832.7418 5867.3737
350.7670 -15380.1778 -1870.0089 0.0000 0.0000
8874.5277 7280.3885 331.0623 -1594.1392 7352.8387
329.0710 102.6022 44.1055 664265.7895 -12.3205
-12.3316
[39859]ENERGY: 10150000 3608.8595 5832.8787 5922.1392
360.5365 -15354.2551 -1884.7576 0.0000 0.0000
8800.5289 7285.9300 328.3018 -1514.5989 7352.9995
329.2134 -88.4740 -75.8628 664265.7895 -8.4595
-8.4507
[39895]ENERGY: 10160000 3625.9788 5845.5838 5956.4537
381.4394 -15486.7407 -1828.5731 0.0000 0.0000
8787.6567 7281.7986 327.8216 -1505.8581 7353.1151
328.8521 -2.1755 -43.6327 664265.7895 -11.5499
-11.5697
[39945]ENERGY: 10170000 3671.4528 5788.5048 5881.7697
365.6399 -15348.9693 -1902.0567 0.0000 0.0000
8825.4871 7281.8284 329.2329 -1543.6586 7354.3569
328.9584 67.1278 -32.9899 664265.7895 -6.7817
-6.7696
[39981]ENERGY: 10180000 3658.1208 5769.4566 5920.3145
353.6723 -15504.7248 -1790.3447 0.0000 0.0000
8873.9365 7280.4312 331.0403 -1593.5053 7354.5757
328.9413 -19.0902 -69.6759 664265.7895 -9.1819
-9.1942
[40031]ENERGY: 10190000 3594.4066 5827.4199 5921.6789
369.1893 -15351.7852 -1865.2222 0.0000 0.0000
8785.1613 7280.8486 327.7286 -1504.3128 7354.5646
329.0613 -78.2681 -68.8032 664265.7895 -3.8567
-3.8313
[40070]ENERGY: 10200000 3558.2946 5942.3062 5935.3855
335.4420 -15443.6605 -1861.6758 0.0000 0.0000
8816.9383 7283.0303 328.9140 -1533.9080 7356.0237
329.1931 -45.2572 -95.2231 664265.7895 -6.4076
-6.4284
[40120]ENERGY: 10210000 3657.5452 5795.6874 5984.8559
362.2559 -15403.6152 -1920.9423 0.0000 0.0000
8810.6858 7286.4726 328.6807 -1524.2132 7356.8857
329.0320 12.4086 -171.7866 664265.7895 -8.0024

-8.0026
[40156]ENERGY: 10220000 3612.2151 5890.1082 5915.8520
389.6435 -15451.2395 -1871.1157 0.0000 0.0000
8798.8109 7284.2747 328.2377 -1514.5362 7356.2880
329.3711 -24.5543 -33.1780 664265.7895 -6.2659
-6.2617
[40206]ENERGY: 10230000 3592.5714 5882.3104 5956.4701
350.5992 -15527.0932 -1818.2692 0.0000 0.0000
8846.5055 7283.0941 330.0170 -1563.4113 7355.3374
329.4206 145.0382 80.9443 664265.7895 -7.1280
-7.1364
[40242]ENERGY: 10240000 3582.3093 5931.3677 5889.4088
353.0816 -15514.2183 -1793.5335 0.0000 0.0000
8831.4853 7279.9011 329.4567 -1551.5842 7354.7506
329.1505 169.8826 156.1163 664265.7895 -3.4851
-3.4881
[40292]ENERGY: 10250000 3551.3465 5837.5840 5949.8343
380.2112 -15311.7551 -2018.9222 0.0000 0.0000
8895.6345 7283.9332 331.8497 -1611.7013 7355.7493
329.4476 -147.0395 -217.6751 664265.7895 -5.2323
-5.2148
[40328]ENERGY: 10260000 3599.0012 5901.9481 5886.7725
347.5125 -15372.6926 -1887.5649 0.0000 0.0000
8810.4465 7285.4231 328.6718 -1525.0233 7357.0610
329.3781 193.5918 94.2024 664265.7895 -6.0118
-6.0193
[40378]ENERGY: 10270000 3569.3694 5851.6341 5901.0107
394.2537 -15349.2469 -1908.1155 0.0000 0.0000
8827.3606 7286.2661 329.3028 -1541.0945 7356.8069
329.1205 -27.2542 -14.1011 664265.7895 -8.4029
-8.4012
[40414]ENERGY: 10280000 3629.0840 5840.4226 5910.0366
365.5570 -15419.2773 -1816.4297 0.0000 0.0000
8772.1968 7281.5900 327.2449 -1490.6068 7355.8982
329.1196 -53.8604 55.5400 664265.7895 -5.8045
-5.8001
[40464]ENERGY: 10290000 3599.6236 5834.2458 5856.1343
345.7384 -15320.3370 -1889.2652 0.0000 0.0000
8860.9501 7287.0899 330.5558 -1573.8601 7358.0199
329.0433 40.9827 69.8706 664265.7895 -7.8731
-7.8812
[40503]ENERGY: 10300000 3619.4718 5920.6257 5899.1462
347.9103 -15348.6273 -1919.8318 0.0000 0.0000
8769.7431 7288.4380 327.1534 -1481.3051 7357.4997
329.2177 125.8191 67.0594 664265.7895 -7.6581
-7.6494
[40553]ENERGY: 10310000 3584.5138 5845.1097 5877.2839
351.3098 -15430.1886 -1829.3934 0.0000 0.0000
8888.4367 7287.0720 331.5812 -1601.3648 7357.0530
329.2231 -44.6087 -40.5489 664265.7895 -4.4224
-4.4202
[40589]ENERGY: 10320000 3571.6126 5753.8341 5920.2391
365.7954 -15347.5235 -1857.9640 0.0000 0.0000
8879.9141 7285.9078 331.2633 -1594.0063 7356.8684

329.2480 -1.9837 -29.5516 664265.7895 -1.7120
-1.7137
[40639]ENERGY: 10330000 3532.8123 5874.7159 5917.9402
352.4265 -15432.5565 -1855.8271 0.0000 0.0000
8896.5781 7286.0893 331.8849 -1610.4888 7358.5536
329.2694 17.5375 39.0880 664265.7895 -0.6943
-0.7095
[40675]ENERGY: 10340000 3506.9369 5822.2236 5913.3743
349.9176 -15305.1001 -1865.7559 0.0000 0.0000
8864.8341 7286.4304 330.7007 -1578.4037 7358.2261
329.1235 39.6727 -7.5423 664265.7895 -13.8117
-13.8094
[40725]ENERGY: 10350000 3588.1418 5847.8223 5954.1533
364.7747 -15434.4898 -1851.4333 0.0000 0.0000
8821.4480 7290.4170 329.0822 -1531.0310 7360.2255
329.2271 78.5141 23.0395 664265.7895 -6.2894
-6.2739
[40761]ENERGY: 10360000 3613.9866 5888.2150 5895.5703
360.2456 -15419.8493 -1860.6787 0.0000 0.0000
8813.9804 7291.4700 328.8036 -1522.5105 7359.4164
329.0921 -108.2218 -122.6184 664265.7895 -5.2924
-5.2961
[40811]ENERGY: 10370000 3675.7392 5837.0123 5922.3667
350.9420 -15408.9751 -1910.6014 0.0000 0.0000
8823.0250 7289.5086 329.1410 -1533.5164 7359.7333
329.0653 -119.8345 -202.9010 664265.7895 -2.2506
-2.2558
[40847]ENERGY: 10380000 3582.5387 5852.1119 5913.6638
372.0473 -15339.0604 -1898.8230 0.0000 0.0000
8809.0254 7291.5038 328.6188 -1517.5217 7359.6824
329.3029 27.7481 62.9595 664265.7895 -4.1771
-4.1719
[40897]ENERGY: 10390000 3514.3075 5939.2032 5867.5309
352.2444 -15269.1793 -1948.8038 0.0000 0.0000
8830.5717 7285.8745 329.4226 -1544.6972 7358.9140
329.3733 -109.8117 -41.4587 664265.7895 -6.0869
-6.0846
[40936]ENERGY: 10400000 3581.2307 5790.6833 5978.0370
352.4147 -15456.6471 -1844.8511 0.0000 0.0000
8886.0114 7286.8789 331.4907 -1599.1325 7357.6625
329.3850 97.3475 68.7742 664265.7895 -2.0609
-2.0704
[40986]ENERGY: 10410000 3644.4379 5877.2148 5867.0102
354.0043 -15441.7948 -1861.1292 0.0000 0.0000
8844.0184 7283.7616 329.9242 -1560.2568 7358.2080
329.5214 182.5928 48.0789 664265.7895 -5.2841
-5.2676
[41022]ENERGY: 10420000 3680.5549 5842.3082 5945.9680
367.5147 -15436.1459 -1875.2743 0.0000 0.0000
8764.9691 7289.8946 326.9753 -1475.0745 7359.1208
329.6472 -175.6676 -144.8780 664265.7895 -0.6652
-0.6753
[41072]ENERGY: 10430000 3680.2660 5858.5761 5897.4738
360.1179 -15424.1357 -1884.0145 0.0000 0.0000

8800.2803 7288.5640 328.2926 -1511.7163 7360.3649
329.4009 2.2635 -84.1045 664265.7895 -6.2303
-6.2440
[41108]ENERGY: 10440000 3523.6241 5890.7464 5940.5850
346.6638 -15321.0914 -1945.1081 0.0000 0.0000
8852.3022 7287.7220 330.2332 -1564.5802 7360.5605
329.6311 54.9962 -5.4061 664265.7895 -2.1626
-2.1520
[41158]ENERGY: 10450000 3660.4480 5954.4618 5833.6366
362.7875 -15416.0766 -1863.0610 0.0000 0.0000
8759.8235 7292.0197 326.7833 -1467.8038 7359.5548
329.5084 -30.3172 -55.3011 664265.7895 -2.1086
-2.0982
[41194]ENERGY: 10460000 3644.1553 5776.2819 5924.3520
359.5179 -15399.9270 -1860.3505 0.0000 0.0000
8845.8662 7289.8957 329.9931 -1555.9704 7361.4557
329.5578 -88.8842 -139.2301 664265.7895 -2.7133
-2.7183
[41244]ENERGY: 10470000 3541.0021 5844.5586 5876.7215
356.1163 -15361.2839 -1887.2800 0.0000 0.0000
8920.3339 7290.1683 332.7711 -1630.1656 7362.0709
329.6960 -46.2388 -76.4312 664265.7895 -4.7925
-4.7923
[41280]ENERGY: 10480000 3642.9915 5865.3485 5867.6753
353.0509 -15295.1067 -1913.7727 0.0000 0.0000
8770.4623 7290.6492 327.1802 -1479.8131 7363.0097
329.6477 13.2987 -59.2738 664265.7895 -6.2734
-6.2833
[41330]ENERGY: 10490000 3582.5295 5771.4730 5932.7172
352.3486 -15305.0009 -1852.0138 0.0000 0.0000
8811.0679 7293.1215 328.6950 -1517.9464 7362.0918
329.6865 71.6319 84.3017 664265.7895 -6.8996
-6.9034
[41369]ENERGY: 10500000 3545.9934 5790.2484 5933.0173
366.1748 -15385.4184 -1799.5047 0.0000 0.0000
8843.8848 7294.3956 329.9192 -1549.4892 7363.1949
329.4572 0.8138 66.8101 664265.7895 -7.9676
-7.9531
[41419]ENERGY: 10510000 3623.6417 5790.8821 5949.3212
347.4375 -15426.0247 -1796.9410 0.0000 0.0000
8807.2353 7295.5521 328.5520 -1511.6832 7365.5831
329.3397 185.4142 99.9283 664265.7895 -12.3187
-12.3376
[41455]ENERGY: 10520000 3602.2885 5771.2590 5941.4603
351.8183 -15428.3810 -1885.6137 0.0000 0.0000
8937.1292 7289.9606 333.3977 -1647.1686 7364.0770
329.7618 100.0381 50.1485 664265.7895 -8.0437
-8.0132
[41505]ENERGY: 10530000 3638.1245 5847.3259 5924.0033
385.1675 -15396.3178 -1879.2227 0.0000 0.0000
8776.2053 7295.2861 327.3944 -1480.9192 7363.5555
329.7396 -31.7052 18.6501 664265.7895 -10.9269
-10.9358
[41541]ENERGY: 10540000 3679.0831 5810.2999 5891.0637

338.3303 -15421.1404 -1821.1539 0.0000 0.0000
8816.5893 7293.0720 328.9010 -1523.5173 7365.8158
329.6293 56.4692 -1.2787 664265.7895 -7.5049
-7.5105
[41591]ENERGY: 10550000 3546.2750 5910.3231 5924.2552
355.2305 -15468.1625 -1853.8865 0.0000 0.0000
8876.0622 7290.0970 331.1196 -1585.9652 7364.9038
329.9039 89.5062 64.8675 664265.7895 -5.5762
-5.5741
[41627]ENERGY: 10560000 3629.3915 5906.2718 5879.2253
371.3267 -15358.1880 -1865.6468 0.0000 0.0000
8735.6383 7298.0187 325.8811 -1437.6196 7365.9674
329.7412 13.9203 53.2925 664265.7895 -2.5670
-2.5764
[41677]ENERGY: 10570000 3549.3253 5930.6871 5878.6022
365.7435 -15426.9597 -1861.1193 0.0000 0.0000
8856.1960 7292.4752 330.3785 -1563.7209 7365.7822
329.6947 69.3850 10.5111 664265.7895 -2.5642
-2.5489
[41713]ENERGY: 10580000 3574.7874 5840.3701 5898.5128
360.4747 -15428.4327 -1791.9861 0.0000 0.0000
8840.1931 7293.9192 329.7815 -1546.2739 7365.1173
329.7551 -5.1096 -50.7456 664265.7895 -7.7974
-7.8240
[41763]ENERGY: 10590000 3679.7425 5751.2658 5864.4163
397.2983 -15372.1849 -1876.4753 0.0000 0.0000
8850.1938 7294.2565 330.1546 -1555.9373 7366.9635
329.9587 -133.5154 -151.2790 664265.7895 -5.7088
-5.6978
[41802]ENERGY: 10600000 3585.0063 5884.0670 5915.3863
362.4521 -15372.8037 -1941.1786 0.0000 0.0000
8863.4275 7296.3568 330.6483 -1567.0707 7367.5729
329.9503 23.2476 -45.5446 664265.7895 -5.0271
-5.0186
[41852]ENERGY: 10610000 3564.4593 5778.4334 5853.3970
347.0220 -15388.3291 -1791.4370 0.0000 0.0000
8931.1052 7294.6509 333.1730 -1636.4544 7366.2848
329.8714 -10.8376 -26.6711 664265.7895 -5.4044
-5.4194
[41888]ENERGY: 10620000 3552.9026 5918.4869 5874.3286
373.6652 -15494.8407 -1796.9758 0.0000 0.0000
8865.3400 7292.9068 330.7196 -1572.4332 7366.8922
329.9557 160.5301 71.1977 664265.7895 -5.7225
-5.7227
[41938]ENERGY: 10630000 3584.0573 5926.3113 5883.1374
333.7166 -15392.0698 -1891.4872 0.0000 0.0000
8852.9324 7296.5980 330.2567 -1556.3345 7367.4889
330.0123 127.1563 37.1711 664265.7895 -10.3565
-10.3581
[41974]ENERGY: 10640000 3651.5946 5805.0689 5885.4894
396.5919 -15495.3025 -1846.3287 0.0000 0.0000
8896.8278 7293.9415 331.8942 -1602.8863 7366.7172
330.3240 78.3436 84.5740 664265.7895 -4.1619
-4.1467

[42024]ENERGY: 10650000 3563.0146 5876.4029 5907.7833
365.3230 -15408.0583 -1849.1097 0.0000 0.0000
8839.8030 7295.1587 329.7669 -1544.6443 7368.1190
330.1584 61.7115 8.5186 664265.7895 -5.8645
-5.8710
[42060]ENERGY: 10660000 3580.5449 5815.5542 5916.8000
357.3867 -15399.0665 -1875.2140 0.0000 0.0000
8900.4400 7296.4453 332.0290 -1603.9947 7368.2837
330.2970 45.8429 64.7726 664265.7895 -5.3798
-5.3876
[42110]ENERGY: 10670000 3638.6289 5899.0030 5889.9602
350.3209 -15521.0543 -1847.5519 0.0000 0.0000
8885.3244 7294.6313 331.4651 -1590.6931 7369.0558
330.0846 85.8004 16.7516 664265.7895 -2.3144
-2.2825
[42146]ENERGY: 10680000 3681.3935 5890.4481 5917.0820
335.9264 -15512.1783 -1872.1852 0.0000 0.0000
8855.5395 7296.0260 330.3540 -1559.5135 7370.3098
330.0435 47.5899 -16.4773 664265.7895 -8.1289
-8.1442
[42196]ENERGY: 10690000 3560.6833 5900.6005 5884.1663
363.3609 -15429.2577 -1898.5763 0.0000 0.0000
8914.8025 7295.7796 332.5648 -1619.0229 7370.2027
329.9877 86.6175 56.6102 664265.7895 -9.0388
-9.0509
[42235]ENERGY: 10700000 3631.8200 5903.0200 5967.0983
348.4864 -15469.1097 -1852.1316 0.0000 0.0000
8770.4531 7299.6366 327.1799 -1470.8165 7370.9005
330.0015 122.3380 -1.5860 664265.7895 -4.9386
-4.9326
[42285]ENERGY: 10710000 3640.6077 5858.4068 5900.8416
364.1172 -15291.4567 -1945.0639 0.0000 0.0000
8776.9866 7304.4394 327.4236 -1472.5473 7371.3181
329.7869 -52.2920 -32.4162 664265.7895 -6.2252
-6.2105
[42321]ENERGY: 10720000 3650.5649 5835.4176 5868.5892
378.9794 -15328.3413 -1911.9923 0.0000 0.0000
8809.6703 7302.8878 328.6429 -1506.7825 7372.0248
330.0487 -27.0676 17.5955 664265.7895 -2.8165
-2.8431
[42371]ENERGY: 10730000 3563.1469 5841.0565 5913.2388
343.9763 -15352.0919 -1856.5620 0.0000 0.0000
8848.5887 7301.3534 330.0947 -1547.2352 7372.6030
330.0472 85.0039 14.8300 664265.7895 -1.1849
-1.1607
[42407]ENERGY: 10740000 3631.8767 5834.5960 5870.7979
369.5831 -15345.1139 -1840.7432 0.0000 0.0000
8781.7448 7302.7414 327.6011 -1479.0034 7372.2542
329.9245 27.8737 37.8528 664265.7895 -2.3961
-2.4035
[42457]ENERGY: 10750000 3656.2165 5855.4459 5907.6878
380.5903 -15445.8075 -1879.9092 0.0000 0.0000
8827.0436 7301.2674 329.2910 -1525.7763 7373.6038
329.8566 -18.6083 -1.9483 664265.7895 -3.0084

-2.9982
[42493]ENERGY: 10760000 3649.1331 5739.3211 5972.2563
343.8264 -15290.6458 -1953.5704 0.0000 0.0000
8843.5131 7303.8338 329.9053 -1539.6793 7374.2317
329.8857 44.0078 -67.1840 664265.7895 -2.6408
-2.6481
[42543]ENERGY: 10770000 3656.0392 5818.1420 5879.4908
364.2849 -15386.0411 -1810.9362 0.0000 0.0000
8781.4145 7302.3942 327.5888 -1479.0203 7372.5472
330.0367 48.2456 77.7635 664265.7895 -5.5512
-5.5419
[42579]ENERGY: 10780000 3538.4752 5972.7111 5955.2774
355.0487 -15399.6575 -1902.9257 0.0000 0.0000
8781.4381 7300.3673 327.5897 -1481.0708 7373.8114
330.0295 249.9427 124.1673 664265.7895 -9.5275
-9.5402
[42629]ENERGY: 10790000 3554.9481 5822.3075 5893.7822
360.7167 -15478.2210 -1822.7483 0.0000 0.0000
8969.6108 7300.3960 334.6094 -1669.2148 7372.2455
330.1016 -4.1417 19.9016 664265.7895 -5.4478
-5.4379
[42668]ENERGY: 10800000 3582.0985 5856.9550 5864.1581
368.6556 -15489.2157 -1873.3734 0.0000 0.0000
8992.5188 7301.7970 335.4640 -1690.7218 7373.1273
330.3218 86.4736 33.2869 664265.7895 -4.1093
-4.1368
[42718]ENERGY: 10810000 3620.2996 5880.4036 5944.3645
377.4530 -15401.6550 -1876.2112 0.0000 0.0000
8758.7455 7303.4000 326.7431 -1455.3455 7373.6631
330.2448 112.4160 16.1826 664265.7895 -3.8552
-3.8531
[42754]ENERGY: 10820000 3634.7246 5830.0169 5891.5810
359.5845 -15450.2966 -1815.5190 0.0000 0.0000
8854.5044 7304.5957 330.3154 -1549.9087 7375.1625
330.1550 51.3668 38.3595 664265.7895 -3.3892
-3.3864
[42804]ENERGY: 10830000 3606.0910 5896.2296 5904.3642
327.4462 -15451.2765 -1877.2652 0.0000 0.0000
8895.8772 7301.4666 331.8588 -1594.4106 7375.1164
330.0818 -62.0870 -67.1564 664265.7895 -1.2310
-1.2223
[42840]ENERGY: 10840000 3585.0046 5941.1742 5915.8362
369.6264 -15391.3664 -1880.2783 0.0000 0.0000
8765.8047 7305.8013 327.0065 -1460.0034 7374.8625
329.9608 42.6980 24.9933 664265.7895 -5.3960
-5.3910
[42890]ENERGY: 10850000 3587.5250 5890.7964 5893.2969
369.4292 -15423.2987 -1932.9969 0.0000 0.0000
8919.0100 7303.7619 332.7217 -1615.2481 7375.3000
330.1528 75.3299 67.9942 664265.7895 -3.5079
-3.5237
[42926]ENERGY: 10860000 3639.0561 5869.5493 5839.9868
377.0277 -15287.8590 -1939.1538 0.0000 0.0000
8811.3864 7309.9935 328.7069 -1501.3929 7376.6985

330.1474 -16.1224 -42.2424 664265.7895 -3.8980
-3.8943
[42976]ENERGY: 10870000 3576.4028 5808.2701 5891.4981
350.9067 -15314.2287 -1844.7006 0.0000 0.0000
8839.8490 7307.9973 329.7687 -1531.8517 7376.2894
330.2708 223.0073 145.4542 664265.7895 -2.4765
-2.4669
[43012]ENERGY: 10880000 3553.4105 5830.7858 5915.4449
365.4311 -15434.1912 -1901.9812 0.0000 0.0000
8971.9783 7300.8782 334.6977 -1671.1001 7374.8675
330.1098 40.7983 31.9218 664265.7895 -5.9821
-5.9907
[43062]ENERGY: 10890000 3688.4461 5792.6765 5932.6029
335.4334 -15410.6120 -1852.4599 0.0000 0.0000
8823.0807 7309.1676 329.1431 -1513.9131 7376.7160
330.2603 -24.2302 -67.0352 664265.7895 -3.4494
-3.4557
[43101]ENERGY: 10900000 3607.8345 5946.7076 5856.4398
360.6205 -15449.0710 -1860.6238 0.0000 0.0000
8844.5106 7306.4182 329.9426 -1538.0925 7377.4584
330.4202 27.2053 17.9513 664265.7895 -3.1482
-3.1398
[43151]ENERGY: 10910000 3473.8628 5940.2580 5924.4023
379.6134 -15370.7488 -1949.5511 0.0000 0.0000
8907.7505 7305.5872 332.3017 -1602.1633 7378.2427
330.5110 123.1283 15.4836 664265.7895 -6.2800
-6.2814
[43187]ENERGY: 10920000 3576.5225 5794.3445 5895.5659
363.0349 -15359.5183 -1884.7878 0.0000 0.0000
8921.2934 7306.4550 332.8069 -1614.8383 7377.8516
330.0784 61.2178 96.9288 664265.7895 -7.4468
-7.4342
[43237]ENERGY: 10930000 3577.9287 5976.3288 5911.2080
337.7659 -15363.4825 -1937.3437 0.0000 0.0000
8807.8380 7310.2433 328.5745 -1497.5947 7380.8032
330.1055 107.0402 -17.3321 664265.7895 -6.7908
-6.8083
[43273]ENERGY: 10940000 3601.8488 5881.4081 5918.7619
360.6033 -15423.9757 -1839.1665 0.0000 0.0000
8813.1759 7312.6559 328.7736 -1500.5201 7380.8969
330.2073 74.9639 56.7687 664265.7895 -4.5501
-4.5483
[43323]ENERGY: 10950000 3607.1662 5889.8056 5949.1938
365.1320 -15432.3884 -1930.2229 0.0000 0.0000
8859.8745 7308.5608 330.5157 -1551.3137 7382.0156
330.3337 -102.0873 -104.8804 664265.7895 -5.4118
-5.4090
[43359]ENERGY: 10960000 3654.7915 5824.8710 5898.0716
351.4991 -15370.8903 -1941.4910 0.0000 0.0000
8893.3502 7310.2021 331.7645 -1583.1481 7381.9812
330.2502 -78.2024 -83.7884 664265.7895 -15.7303
-15.7357
[43409]ENERGY: 10970000 3657.6554 5904.1437 5896.9282
344.1647 -15297.0986 -1882.9877 0.0000 0.0000

8691.9073 7314.7131 324.2497 -1377.1941 7383.7216
330.1655 86.7748 -31.9817 664265.7895 -10.4498
-10.4416
[43445]ENERGY: 10980000 3539.7864 5947.9558 5931.6768
363.2712 -15509.4706 -1852.7171 0.0000 0.0000
8891.2405 7311.7431 331.6858 -1579.4975 7382.5281
330.5193 45.3314 78.6856 664265.7895 -12.6341
-12.6469
[43495]ENERGY: 10990000 3667.5285 5953.0983 5956.4122
365.3643 -15451.6885 -1912.8701 0.0000 0.0000
8737.5172 7315.3619 325.9512 -1422.1553 7383.1917
330.3797 63.9637 102.7108 664265.7895 -6.5365
-6.5334
[43534]ENERGY: 11000000 3633.0093 5799.6111 5944.5064
356.9687 -15395.1721 -1898.6967 0.0000 0.0000
8872.5188 7312.7456 330.9874 -1559.7732 7382.1513
330.3903 -75.0535 -81.9041 664265.7895 -5.4714
-5.4470
[43584]ENERGY: 11010000 3576.5464 5878.0085 5962.5943
354.7015 -15404.1674 -1927.5150 0.0000 0.0000
8870.6911 7310.8594 330.9192 -1559.8317 7382.9280
330.3063 -95.8740 -140.2191 664265.7895 -9.7979
-9.8019
[43620]ENERGY: 11020000 3569.1926 5859.0619 5936.1205
347.5364 -15314.0911 -1937.3452 0.0000 0.0000
8851.3066 7311.7818 330.1961 -1539.5248 7383.2703
330.3114 20.4885 -57.9163 664265.7895 -7.8492
-7.8616
[43670]ENERGY: 11030000 3628.1850 5865.3828 5890.0345
353.1424 -15356.5153 -1934.3196 0.0000 0.0000
8864.1452 7310.0549 330.6750 -1554.0902 7384.4339
330.0913 15.8504 36.1635 664265.7895 -4.4028
-4.3875
[43706]ENERGY: 11040000 3621.4000 5880.8807 5907.1687
367.3904 -15278.0009 -1898.2162 0.0000 0.0000
8716.4749 7317.0976 325.1662 -1399.3773 7384.5114
330.2557 -56.9231 -68.0828 664265.7895 -4.2946
-4.2957
[43756]ENERGY: 11050000 3589.7546 5722.9549 5870.5472
339.0456 -15270.5220 -1821.0662 0.0000 0.0000
8886.8753 7317.5895 331.5230 -1569.2858 7384.7778
330.2884 65.7937 102.5633 664265.7895 -5.9906
-5.9936
[43792]ENERGY: 11060000 3636.0780 5918.2818 5933.2282
379.0865 -15400.0690 -1866.6256 0.0000 0.0000
8719.7140 7319.6938 325.2871 -1400.0202 7386.8377
330.1716 -45.4297 -67.9200 664265.7895 -7.2425
-7.2652
[43842]ENERGY: 11070000 3659.0114 5860.9410 5894.0714
382.0646 -15391.3024 -1878.4849 0.0000 0.0000
8789.9943 7316.2954 327.9088 -1473.6989 7386.7409
330.2317 -149.8291 -190.0117 664265.7895 -3.5338
-3.5166
[43878]ENERGY: 11080000 3560.4636 5907.7919 5887.7329

388.1062 -15470.5811 -1872.1131 0.0000 0.0000
8910.8461 7312.2466 332.4172 -1598.5995 7385.1917
330.3260 82.3625 3.9930 664265.7895 -5.8002
-5.7963
[43928]ENERGY: 11090000 3635.1390 5911.7857 5947.4041
341.9235 -15349.7757 -1962.2088 0.0000 0.0000
8787.9561 7312.2239 327.8328 -1475.7322 7386.3442
330.0820 20.1115 -33.8349 664265.7895 -7.9343
-7.9479
[43967]ENERGY: 11100000 3652.9666 5810.0633 5891.5085
344.2946 -15370.8175 -1874.4551 0.0000 0.0000
8856.8615 7310.4219 330.4033 -1546.4396 7384.6182
330.3727 -73.5055 -102.6010 664265.7895 -9.6896
-9.6737
[44017]ENERGY: 11110000 3676.0815 5929.6591 5863.2102
360.1392 -15404.6354 -1920.3540 0.0000 0.0000
8814.0088 7318.1094 328.8047 -1495.8994 7385.1810
330.4381 -23.6613 -105.9574 664265.7895 -10.0555
-10.0592
[44053]ENERGY: 11120000 3677.5889 5832.4228 5908.2623
360.7380 -15374.3242 -1941.3329 0.0000 0.0000
8851.2897 7314.6446 330.1955 -1536.6451 7386.1195
330.3032 -58.0481 -149.9137 664265.7895 -6.1694
-6.1692
[44103]ENERGY: 11130000 3614.4743 5844.3299 5885.5833
361.0958 -15323.3379 -1870.6633 0.0000 0.0000
8803.8061 7315.2881 328.4241 -1488.5180 7385.4862
330.2935 195.3095 148.1399 664265.7895 -5.8993
-5.9085
[44139]ENERGY: 11140000 3607.1784 5805.7550 5875.8661
347.5006 -15295.0937 -1930.8104 0.0000 0.0000
8901.8396 7312.2357 332.0812 -1589.6039 7385.3770
330.5028 -18.2627 -55.0454 664265.7895 -6.4475
-6.4394
[44189]ENERGY: 11150000 3650.9334 5825.0267 5904.5061
370.3131 -15354.9765 -1839.8890 0.0000 0.0000
8754.8621 7310.7760 326.5982 -1444.0861 7384.3056
330.3464 -53.8694 -24.8535 664265.7895 -5.6946
-5.6866
[44225]ENERGY: 11160000 3641.9208 5793.4834 5943.0879
388.5885 -15277.7437 -1934.3543 0.0000 0.0000
8760.9427 7315.9251 326.8251 -1445.0176 7385.0830
330.1212 87.2742 -16.7580 664265.7895 -10.5002
-10.5152
[44275]ENERGY: 11170000 3597.3155 5960.4131 5902.7693
367.9108 -15491.4418 -1873.7136 0.0000 0.0000
8847.5962 7310.8497 330.0577 -1536.7466 7385.9961
330.1990 54.3920 -24.7655 664265.7895 -8.0221
-8.0173
[44311]ENERGY: 11180000 3594.7959 5893.3528 5931.3985
375.6002 -15316.2079 -2000.5075 0.0000 0.0000
8836.8726 7315.3046 329.6576 -1521.5680 7386.8639
330.1297 -64.7801 -76.3719 664265.7895 -5.1936
-5.2106

[44361]ENERGY: 11190000 3525.4756 5919.3538 5929.1302
376.8778 -15425.1462 -1834.7471 0.0000 0.0000
8822.5806 7313.5247 329.1245 -1509.0559 7386.3551
330.1110 61.7377 51.3083 664265.7895 -7.0757
-7.0704
[44400]ENERGY: 11200000 3646.6189 5875.1766 5891.1837
357.2801 -15359.5363 -1940.9221 0.0000 0.0000
8846.4310 7316.2320 330.0142 -1530.1991 7387.3787
329.9924 -131.3481 -155.2622 664265.7895 -8.4589
-8.4645
[44450]ENERGY: 11210000 3520.2181 5776.8793 5932.3696
350.8954 -15326.3935 -1823.7630 0.0000 0.0000
8886.4669 7316.6728 331.5077 -1569.7942 7388.3237
329.8468 49.0616 22.7859 664265.7895 -5.9248
-5.8944
[44486]ENERGY: 11220000 3569.0501 5840.5944 5934.8820
378.7983 -15339.9606 -1923.3480 0.0000 0.0000
8853.8124 7313.8286 330.2896 -1539.9838 7388.4401
329.9364 65.8542 -17.1883 664265.7895 -11.8951
-11.9127
[44536]ENERGY: 11230000 3636.1954 5809.3724 5913.5942
354.0301 -15319.0429 -1922.0988 0.0000 0.0000
8849.6306 7321.6810 330.1336 -1527.9496 7389.8383
329.9624 18.6637 -41.6274 664265.7895 -11.0144
-11.0189
[44572]ENERGY: 11240000 3570.2311 5879.1745 5916.4552
366.3620 -15364.4588 -1926.5447 0.0000 0.0000
8871.8992 7313.1184 330.9643 -1558.7808 7387.5522
330.2453 -99.1979 -61.9460 664265.7895 -12.5254
-12.5164
[44622]ENERGY: 11250000 3586.1319 5892.9096 5902.8954
359.8037 -15474.1909 -1833.6159 0.0000 0.0000
8882.8666 7316.8003 331.3734 -1566.0663 7389.1219
330.1208 182.0542 -17.1645 664265.7895 -7.0262
-7.0435
[44658]ENERGY: 11260000 3738.3659 5863.4078 5940.2941
368.4729 -15451.5662 -1866.2249 0.0000 0.0000
8728.6766 7321.4262 325.6214 -1407.2504 7388.8902
330.1473 -50.9980 -27.7489 664265.7895 -6.4467
-6.4270
[44708]ENERGY: 11270000 3695.1430 5780.5800 5946.4155
356.2991 -15445.7921 -1907.1298 0.0000 0.0000
8889.2740 7314.7898 331.6125 -1574.4842 7388.6831
330.1847 -186.8076 -144.0546 664265.7895 -6.0529
-6.0451
[44744]ENERGY: 11280000 3621.7192 5867.5272 5921.1953
352.9158 -15355.5204 -1879.4699 0.0000 0.0000
8792.6266 7320.9936 328.0070 -1471.6329 7390.0269
330.2513 77.2651 52.1297 664265.7895 -7.4731
-7.4744
[44794]ENERGY: 11290000 3546.0235 5791.6429 5858.4824
373.0653 -15347.6180 -1810.8848 0.0000 0.0000
8909.1252 7319.8365 332.3530 -1589.2887 7390.6940
330.1315 255.5281 168.6246 664265.7895 -5.4158

-5.4147
[44833]ENERGY: 11300000 3632.9922 5865.4971 5945.9655
357.4869 -15529.6545 -1812.1945 0.0000 0.0000
8862.1782 7322.2710 330.6016 -1539.9072 7393.2115
330.3508 96.4542 66.1377 664265.7895 -7.8806
-7.9026
[44883]ENERGY: 11310000 3634.6992 5753.2386 5904.1763
359.9013 -15361.6040 -1904.4185 0.0000 0.0000
8934.1455 7320.1384 333.2864 -1614.0070 7392.8314
330.3684 87.9602 17.0193 664265.7895 -6.0174
-6.0034
[44919]ENERGY: 11320000 3607.8325 5850.9463 5938.9272
350.0135 -15354.5280 -1864.4697 0.0000 0.0000
8796.0161 7324.7379 328.1335 -1471.2782 7392.6763
330.1635 -57.7151 -118.7814 664265.7895 -7.0146
-7.0101
[44969]ENERGY: 11330000 3588.0823 5788.1848 5842.2098
360.9759 -15347.8009 -1827.7287 0.0000 0.0000
8917.5854 7321.5085 332.6686 -1596.0769 7392.5593
330.2179 57.3882 -18.2735 664265.7895 -2.3647
-2.4002
[45005]ENERGY: 11340000 3620.7236 5900.1555 5914.5432
370.6881 -15403.3526 -1860.3534 0.0000 0.0000
8782.6666 7325.0710 327.6355 -1457.5956 7395.1263
330.1936 69.8911 60.7335 664265.7895 -8.2820
-8.2513
[45055]ENERGY: 11350000 3614.0664 5911.9773 5843.0924
354.6826 -15441.4927 -1852.2865 0.0000 0.0000
8892.5269 7322.5663 331.7338 -1569.9606 7395.2984
330.3114 163.7200 148.7944 664265.7895 -7.2847
-7.2969
[45091]ENERGY: 11360000 3501.6068 5789.6556 5889.0407
370.5225 -15278.0620 -1847.2362 0.0000 0.0000
8899.0290 7324.5564 331.9764 -1574.4726 7395.8067
330.4460 102.5369 19.5675 664265.7895 -6.1050
-6.1012
[45141]ENERGY: 11370000 3679.5509 5776.1426 5925.4489
362.2165 -15318.6446 -1895.3518 0.0000 0.0000
8795.8803 7325.2429 328.1284 -1470.6375 7396.3080
330.0633 -169.3058 -131.0730 664265.7895 -7.7727
-7.7640
[45177]ENERGY: 11380000 3607.5596 5946.7338 5870.9625
362.4064 -15335.8562 -1944.1645 0.0000 0.0000
8819.7835 7327.4250 329.0201 -1492.3585 7397.2106
330.1372 -89.4983 -77.6041 664265.7895 -12.8244
-12.8114
[45227]ENERGY: 11390000 3598.4806 5875.7239 5859.5434
372.5481 -15358.5201 -1888.8396 0.0000 0.0000
8860.6696 7319.6059 330.5454 -1541.0637 7395.9895
330.1854 45.8984 25.3651 664265.7895 -8.7489
-8.7452
[45266]ENERGY: 11400000 3524.9096 5812.8508 5860.0907
381.8968 -15329.7899 -1850.2382 0.0000 0.0000
8920.6580 7320.3778 332.7832 -1600.2803 7395.6983

330.2206 209.2082 86.8003 664265.7895 -10.9788
-11.0057
[45316]ENERGY: 11410000 3692.1768 5797.6432 5932.5836
372.0593 -15375.2484 -1917.5644 0.0000 0.0000
8824.0114 7325.6616 329.1778 -1498.3499 7397.3340
330.4726 -55.0894 -126.8007 664265.7895 -9.9220
-9.9125
[45352]ENERGY: 11420000 3580.7781 6020.2297 5916.1780
375.0579 -15361.6940 -1938.4051 0.0000 0.0000
8732.1463 7324.2910 325.7508 -1407.8553 7396.1778
330.4179 -69.0062 -98.8524 664265.7895 -11.4015
-11.4112
[45402]ENERGY: 11430000 3610.6176 5879.5323 5862.3690
352.9817 -15414.8047 -1881.9762 0.0000 0.0000
8912.6334 7321.3531 332.4839 -1591.2803 7396.7320
330.4183 -110.2959 -109.7920 664265.7895 -10.7429
-10.7243
[45438]ENERGY: 11440000 3679.3441 5808.0098 5840.7516
337.6496 -15329.6853 -1907.9084 0.0000 0.0000
8898.2775 7326.4389 331.9483 -1571.8386 7396.5850
330.3861 -9.9234 -88.6642 664265.7895 -12.2685
-12.2789
[45488]ENERGY: 11450000 3648.3814 5759.2136 5920.6987
371.8806 -15369.4158 -1886.3188 0.0000 0.0000
8879.8775 7324.3173 331.2619 -1555.5602 7395.3549
330.3616 -83.2021 -137.0821 664265.7895 -11.3816
-11.3759
[45524]ENERGY: 11460000 3649.8363 5837.5983 5928.8186
357.4415 -15369.6883 -1910.9290 0.0000 0.0000
8833.7192 7326.7965 329.5400 -1506.9227 7397.7945
330.1169 -26.7349 -79.6881 664265.7895 -9.5812
-9.5755
[45574]ENERGY: 11470000 3580.9457 5838.5697 5907.4742
348.1867 -15360.3242 -1940.1048 0.0000 0.0000
8946.9070 7321.6542 333.7624 -1625.2528 7397.1706
330.2582 141.3291 48.0343 664265.7895 -14.0440
-14.0674
[45610]ENERGY: 11480000 3530.8986 5841.5580 5953.3704
352.2468 -15384.2558 -1870.6501 0.0000 0.0000
8897.3945 7320.5623 331.9154 -1576.8322 7396.3787
330.3356 -103.0056 -53.4826 664265.7895 -11.3204
-11.3071
[45660]ENERGY: 11490000 3573.8431 5956.3746 5905.0118
335.7507 -15354.7212 -1927.4247 0.0000 0.0000
8837.9911 7326.8255 329.6994 -1511.1656 7396.0415
330.2707 157.7348 112.7979 664265.7895 -9.3915
-9.3780
[45699]ENERGY: 11500000 3660.5520 5837.8407 5941.0796
394.3794 -15477.6507 -1853.3958 0.0000 0.0000
8822.9833 7325.7885 329.1395 -1497.1947 7397.7257
330.3403 186.3234 156.5836 664265.7895 -11.1740
-11.1788
[45749]ENERGY: 11510000 3544.0673 5852.4794 5900.5321
366.0767 -15370.4444 -1853.7708 0.0000 0.0000

8888.0835 7327.0239 331.5680 -1561.0597 7396.0112
330.5109 -26.0554 -33.7674 664265.7895 -14.3421
-14.3613
[45785]ENERGY: 11520000 3583.6990 5832.1158 5878.8095
369.8923 -15426.9654 -1927.0515 0.0000 0.0000
9012.7675 7323.2673 336.2193 -1689.5002 7396.4718
330.7545 -19.3028 -127.6212 664265.7895 -11.1556
-11.1565
[45835]ENERGY: 11530000 3509.7194 5898.5985 5931.3056
376.4260 -15354.7158 -1860.7613 0.0000 0.0000
8818.4208 7318.9931 328.9693 -1499.4277 7395.3259
330.5795 52.5943 -3.5206 664265.7895 -11.3505
-11.3425
[45871]ENERGY: 11540000 3529.5926 5858.7189 5888.8282
353.0637 -15324.0429 -1895.1073 0.0000 0.0000
8910.3861 7321.4393 332.4000 -1588.9467 7394.3648
330.3287 -58.1355 -3.3080 664265.7895 -13.3428
-13.3369
[45921]ENERGY: 11550000 3709.1033 5889.8306 5838.4562
365.9915 -15362.2946 -1934.1787 0.0000 0.0000
8817.3821 7324.2903 328.9305 -1493.0917 7397.2497
330.4392 -8.0988 18.5721 664265.7895 -10.6535
-10.6741
[45957]ENERGY: 11560000 3642.1472 5911.6030 5863.9110
380.2613 -15443.7974 -1960.2611 0.0000 0.0000
8928.7269 7322.5907 333.0842 -1606.1361 7395.3892
330.5146 101.9708 6.8881 664265.7895 -7.3587
-7.3497
[46007]ENERGY: 11570000 3627.3554 5819.3620 5942.8091
365.8416 -15456.9074 -1831.2295 0.0000 0.0000
8854.5294 7321.7606 330.3163 -1532.7688 7395.5706
330.4159 215.4345 178.4081 664265.7895 -10.5386
-10.5402
[46043]ENERGY: 11580000 3634.4262 5881.9108 5872.2036
347.7369 -15288.6981 -1938.1934 0.0000 0.0000
8815.5586 7324.9445 328.8625 -1490.6141 7396.7745
330.5225 -17.3509 -62.6021 664265.7895 -12.0385
-12.0497
[46093]ENERGY: 11590000 3553.0612 5930.3587 5971.5103
376.9394 -15423.4397 -1992.3009 0.0000 0.0000
8902.0647 7318.1937 332.0896 -1583.8710 7393.9816
330.4275 -68.7631 -71.1966 664265.7895 -10.6888
-10.6719
[46132]ENERGY: 11600000 3647.7251 5904.9134 5890.6037
363.1352 -15406.9985 -1880.7589 0.0000 0.0000
8806.6459 7325.2660 328.5300 -1481.3800 7394.3540
330.1806 13.4468 30.2921 664265.7895 -11.5473
-11.5496
[46182]ENERGY: 11610000 3620.6430 5770.6242 5910.6802
362.1168 -15401.9103 -1902.2230 0.0000 0.0000
8963.8962 7323.8270 334.3962 -1640.0692 7394.0359
330.3104 -145.3247 -142.8894 664265.7895 -12.0953
-12.0885
[46218]ENERGY: 11620000 3631.9062 5885.2599 5965.2168

365.2396 -15435.0645 -1828.1924 0.0000 0.0000
8742.2476 7326.6132 326.1277 -1415.6345 7394.5088
330.2449 -77.1195 -151.2059 664265.7895 -10.6871
-10.7045
[46268]ENERGY: 11630000 3638.8237 5859.7984 5942.2142
357.8192 -15384.3359 -1893.7711 0.0000 0.0000
8799.4933 7320.0418 328.2632 -1479.4515 7394.4714
330.3390 -71.6190 -56.0596 664265.7895 -5.1685
-5.1481
[46304]ENERGY: 11640000 3539.6106 5876.9600 5892.3941
376.9748 -15370.8756 -1859.5344 0.0000 0.0000
8866.3922 7321.9217 330.7588 -1544.4704 7394.7499
330.6248 77.7376 117.9473 664265.7895 -11.1151
-11.1101
[46354]ENERGY: 11650000 3555.5492 5914.3668 5894.5518
358.6964 -15348.1157 -1909.6254 0.0000 0.0000
8860.1054 7325.5285 330.5243 -1534.5769 7397.5269
330.3313 64.1032 -50.2765 664265.7895 -14.8674
-14.8781
[46390]ENERGY: 11660000 3562.1071 5926.7715 5900.5334
392.8529 -15490.7754 -1800.0130 0.0000 0.0000
8830.4755 7321.9520 329.4190 -1508.5235 7395.8587
330.3225 63.0732 -11.0514 664265.7895 -11.2973
-11.3063
[46440]ENERGY: 11670000 3670.0853 5772.3939 5905.5771
347.6596 -15293.8015 -1897.9675 0.0000 0.0000
8821.7030 7325.6500 329.0917 -1496.0530 7397.1718
330.3158 -34.1444 -45.7759 664265.7895 -8.1887
-8.1780
[46476]ENERGY: 11680000 3668.5307 5910.7433 5907.4223
358.3623 -15519.4996 -1794.6811 0.0000 0.0000
8794.3160 7325.1939 328.0701 -1469.1221 7398.2702
330.4341 -65.2947 -129.8452 664265.7895 -11.4942
-11.4850
[46526]ENERGY: 11690000 3574.1052 5863.7864 5924.1461
363.7149 -15366.0497 -1873.5807 0.0000 0.0000
8842.4828 7328.6050 329.8669 -1513.8778 7398.5051
330.5236 140.0056 41.7971 664265.7895 -12.5471
-12.5643
[46565]ENERGY: 11700000 3789.8423 5841.9323 5888.1829
373.2766 -15439.0819 -1938.4587 0.0000 0.0000
8810.9345 7326.6281 328.6900 -1484.3065 7397.4127
330.4696 -160.7875 -199.4730 664265.7895 -8.4497
-8.4291
[46615]ENERGY: 11710000 3615.2122 5766.4939 5952.9323
344.4314 -15301.5214 -1859.0844 0.0000 0.0000
8807.8143 7326.2782 328.5736 -1481.5361 7397.5567
330.3159 -28.4040 -58.1846 664265.7895 -6.2550
-6.2701
[46651]ENERGY: 11720000 3671.9440 5872.6015 5855.1393
345.4874 -15303.1928 -1880.9911 0.0000 0.0000
8767.2532 7328.2415 327.0605 -1439.0118 7398.6563
330.4072 -159.9211 -171.0488 664265.7895 -10.2063
-10.2245

[46701]ENERGY: 11730000 3608.3774 5830.6074 5945.8621
366.4356 -15428.2967 -1828.8114 0.0000 0.0000
8833.0198 7327.1941 329.5139 -1505.8257 7398.7271
330.1065 61.1757 82.6778 664265.7895 -12.5183
-12.4858
[46737]ENERGY: 11740000 3594.5921 5826.2013 5922.7439
341.0871 -15300.4208 -1895.0721 0.0000 0.0000
8839.1662 7328.2978 329.7432 -1510.8685 7398.7283
330.1715 112.2995 63.1368 664265.7895 -10.8141
-10.8197
[46787]ENERGY: 11750000 3700.7855 5915.6053 5904.1785
370.3564 -15537.7258 -1836.1396 0.0000 0.0000
8812.2086 7329.2688 328.7375 -1482.9397 7398.6059
330.2491 82.0270 8.1293 664265.7895 -7.4550
-7.4416
[46823]ENERGY: 11760000 3703.8323 5744.6324 5879.4390
354.0220 -15336.6744 -1850.0345 0.0000 0.0000
8827.7447 7322.9615 329.3171 -1504.7831 7398.5834
330.3710 -80.9399 -110.6764 664265.7895 -12.4946
-12.5194
[46873]ENERGY: 11770000 3612.0655 5937.7518 5902.6749
353.0751 -15342.9749 -1904.3051 0.0000 0.0000
8768.0101 7326.2974 327.0887 -1441.7127 7398.8615
330.2478 5.6629 67.2097 664265.7895 -12.7750
-12.7670
[46909]ENERGY: 11780000 3555.8004 5940.8444 5957.4225
348.8251 -15396.8112 -1874.8957 0.0000 0.0000
8793.9902 7325.1757 328.0579 -1468.8146 7399.9813
330.4372 -115.3020 -46.8071 664265.7895 -11.8154
-11.8227
[46959]ENERGY: 11790000 3644.3798 5882.8939 5856.1017
374.2146 -15348.5492 -1904.2747 0.0000 0.0000
8824.4176 7329.1836 329.1930 -1495.2339 7400.1343
330.4696 15.2782 -11.9661 664265.7895 -11.7275
-11.7195
[46998]ENERGY: 11800000 3601.5437 5858.7794 5937.5247
360.6243 -15393.2098 -1820.7538 0.0000 0.0000
8783.2577 7327.7662 327.6575 -1455.4916 7400.3313
330.5682 107.5763 119.2208 664265.7895 -11.8617
-11.8521
[47048]ENERGY: 11810000 3558.3048 5804.1043 5930.9395
373.5315 -15339.0381 -1865.1430 0.0000 0.0000
8865.3587 7328.0576 330.7203 -1537.3010 7400.1877
330.4765 50.9267 77.3389 664265.7895 -11.5523
-11.5658
[47084]ENERGY: 11820000 3591.7188 5850.4506 5893.6564
345.0045 -15402.9444 -1857.3564 0.0000 0.0000
8907.5409 7328.0702 332.2939 -1579.4706 7400.4015
330.3739 136.6009 115.0885 664265.7895 -12.0630
-12.0696
[47134]ENERGY: 11830000 3588.8861 5793.5406 5890.0459
373.1925 -15375.3781 -1849.5480 0.0000 0.0000
8905.3582 7326.0972 332.2125 -1579.2610 7399.2957
330.3889 48.6787 19.5891 664265.7895 -10.4760

-10.4732
[47170]ENERGY: 11840000 3639.1274 5745.4635 5970.2617
359.6492 -15468.6670 -1843.8447 0.0000 0.0000
8921.9654 7323.9555 332.8320 -1598.0099 7396.4863
330.2135 29.8187 -28.5938 664265.7895 -10.4503
-10.4441
[47220]ENERGY: 11850000 3679.1469 5777.3009 5950.9414
376.1635 -15422.6462 -1910.5815 0.0000 0.0000
8873.8248 7324.1498 331.0361 -1549.6750 7399.4943
330.5539 -12.4477 -55.1122 664265.7895 -12.1733
-12.1701
[47256]ENERGY: 11860000 3644.2599 5931.4327 5879.1776
369.2754 -15397.5867 -1900.4524 0.0000 0.0000
8806.6065 7332.7130 328.5286 -1473.8935 7400.1648
330.6758 174.8020 104.9477 664265.7895 -11.0869
-11.0916
[47306]ENERGY: 11870000 3676.0656 5845.4162 5920.7689
375.2169 -15524.6401 -1802.1305 0.0000 0.0000
8833.9209 7324.6178 329.5475 -1509.3031 7397.9220
330.5321 27.9641 87.9129 664265.7895 -11.3818
-11.3619
[47342]ENERGY: 11880000 3682.8681 5825.8396 5855.0146
378.4157 -15370.5239 -1851.8567 0.0000 0.0000
8809.6671 7329.4245 328.6427 -1480.2425 7400.3430
330.6523 -17.1405 -65.9518 664265.7895 -6.2391
-6.2722
[47392]ENERGY: 11890000 3666.8617 5803.4413 5915.8883
379.5300 -15413.8972 -1885.4252 0.0000 0.0000
8862.7290 7329.1279 330.6222 -1533.6011 7399.2447
330.4677 -200.1877 -145.0723 664265.7895 -8.9991
-8.9838
[47431]ENERGY: 11900000 3652.7503 5814.2233 5920.3364
390.0510 -15428.0984 -1845.7367 0.0000 0.0000
8827.9699 7331.4958 329.3255 -1496.4741 7399.0793
330.5635 -71.6910 -68.2877 664265.7895 -12.0462
-12.0426
[47481]ENERGY: 11910000 3620.9227 5800.2496 5955.4916
337.3079 -15445.3529 -1835.6197 0.0000 0.0000
8893.6568 7326.6559 331.7759 -1567.0009 7400.5913
330.4388 119.4976 -19.1003 664265.7895 -7.6296
-7.6421
[47517]ENERGY: 11920000 3656.6891 5808.9609 5936.6271
367.0229 -15391.8322 -1875.4435 0.0000 0.0000
8828.3687 7330.3931 329.3404 -1497.9757 7400.4748
330.5152 -20.5055 -46.6544 664265.7895 -6.0211
-6.0057
[47567]ENERGY: 11930000 3528.9001 5926.2150 5947.0238
354.3364 -15345.0807 -1913.3603 0.0000 0.0000
8833.2412 7331.2755 329.5222 -1501.9657 7401.8001
330.7167 -80.6892 -143.3974 664265.7895 -6.5228
-6.5303
[47603]ENERGY: 11940000 3631.2391 5846.0555 5903.0479
360.8810 -15322.4166 -1943.5111 0.0000 0.0000
8858.7786 7334.0744 330.4748 -1524.7041 7402.1093

330.5475 -45.9310 -96.7224 664265.7895 -8.0013
-8.0090
[47653]ENERGY: 11950000 3575.3076 5742.5236 5868.1949
369.6967 -15230.9723 -1858.5671 0.0000 0.0000
8866.6604 7332.8437 330.7689 -1533.8167 7402.4805
330.4530 39.5987 22.6662 664265.7895 -10.3362
-10.3372
[47689]ENERGY: 11960000 3620.9394 5840.4108 5869.8787
369.3337 -15392.8078 -1877.8479 0.0000 0.0000
8899.8554 7329.7623 332.0072 -1570.0931 7402.5041
330.4789 -45.6518 -146.2654 664265.7895 -8.1692
-8.1550
[47739]ENERGY: 11970000 3644.0304 5828.9961 5958.9749
364.8314 -15424.4700 -1859.9909 0.0000 0.0000
8819.4970 7331.8688 329.0094 -1487.6282 7404.4321
330.5479 72.3865 65.7196 664265.7895 -5.8634
-5.8619
[47775]ENERGY: 11980000 3660.9527 5794.6151 5870.6637
360.5586 -15373.8363 -1811.1155 0.0000 0.0000
8830.2569 7332.0951 329.4108 -1498.1618 7402.8926
330.1535 36.3211 -3.0815 664265.7895 -7.3885
-7.3912
[47825]ENERGY: 11990000 3623.9655 5871.0356 5921.0799
363.5432 -15316.5759 -1899.8749 0.0000 0.0000
8771.5091 7334.6824 327.2193 -1436.8267 7404.1752
330.2689 111.8817 10.0583 664265.7895 -9.6589
-9.6688
[47864]ENERGY: 12000000 3548.0086 5916.2641 5903.8213
368.6273 -15432.0776 -1813.4903 0.0000 0.0000
8841.0370 7332.1904 329.8130 -1508.8466 7404.6467
330.2136 69.0215 53.2707 664265.7895 -7.9479
-7.9412
[47914]ENERGY: 12010000 3699.4965 5859.6216 5860.7728
382.4455 -15289.4698 -1872.6635 0.0000 0.0000
8696.6713 7336.8745 324.4274 -1359.7968 7404.8383
330.3956 87.6851 -55.9706 664265.7895 -11.3537
-11.3742
[47950]ENERGY: 12020000 3788.5164 5816.5522 5920.8498
341.2750 -15437.8903 -1915.6563 0.0000 0.0000
8817.4936 7331.1404 328.9347 -1486.3532 7402.9461
330.3307 -107.4525 -143.0283 664265.7895 -11.1178
-11.0986
[48000]ENERGY: 12030000 3614.0195 5881.5049 5933.1578
362.6371 -15429.8765 -1900.7533 0.0000 0.0000
8866.4828 7327.1722 330.7622 -1539.3106 7402.5677
330.5529 -182.4118 -196.9065 664265.7895 -12.0486
-12.0503
[48036]ENERGY: 12040000 3561.3898 5808.8604 5929.6736
325.0204 -15326.1740 -1892.5542 0.0000 0.0000
8923.0659 7329.2819 332.8731 -1593.7840 7403.6504
330.4432 27.0782 -55.2101 664265.7895 -11.4717
-11.4576
[48086]ENERGY: 12050000 3620.7953 5917.9684 5875.4535
348.6626 -15457.5146 -1792.0690 0.0000 0.0000

8824.6682 7337.9645 329.2023 -1486.7038 7404.0202
330.3708 218.7296 162.5082 664265.7895 -12.5569
-12.5684
[48122]ENERGY: 12060000 3571.6817 5803.6513 5918.0851
352.6292 -15390.6606 -1825.4119 0.0000 0.0000
8897.1861 7327.1608 331.9076 -1570.0253 7402.0584
330.5119 248.9341 152.4438 664265.7895 -8.6831
-8.6989
[48172]ENERGY: 12070000 3686.6826 5825.8905 5911.5412
371.0201 -15444.4796 -1894.7916 0.0000 0.0000
8878.1245 7333.9876 331.1965 -1544.1369 7404.8911
330.1530 -35.3647 -83.9798 664265.7895 -7.4376
-7.4434
[48208]ENERGY: 12080000 3655.7054 5872.2947 5846.8680
388.9714 -15380.6674 -1770.8788 0.0000 0.0000
8722.6534 7334.9467 325.3967 -1387.7067 7404.4445
330.1410 103.2061 69.0235 664265.7895 -11.0757
-11.0478
[48258]ENERGY: 12090000 3630.9236 5803.8876 5941.6342
388.4584 -15343.5006 -1904.5708 0.0000 0.0000
8818.5196 7335.3519 328.9730 -1483.1676 7404.6080
330.0632 -158.2782 -152.2107 664265.7895 -10.9939
-11.0110
[48297]ENERGY: 12100000 3630.8288 5724.0154 5924.9201
380.9913 -15379.9108 -1835.6877 0.0000 0.0000
8889.7994 7334.9565 331.6321 -1554.8430 7405.3497
330.2858 -15.3962 9.3986 664265.7895 -2.0586
-2.0514
[48347]ENERGY: 12110000 3606.2909 5868.1358 5899.1787
343.5354 -15330.0314 -1880.7612 0.0000 0.0000
8824.0784 7330.4268 329.1803 -1493.6517 7404.3014
330.3773 140.6483 93.3976 664265.7895 -4.4450
-4.4413
[48383]ENERGY: 12120000 3632.6800 5927.7907 5924.9415
377.9656 -15452.8588 -1884.2695 0.0000 0.0000
8809.6286 7335.8781 328.6413 -1473.7505 7407.1305
330.4849 -6.2219 35.6019 664265.7895 -5.6284
-5.6200
[48433]ENERGY: 12130000 3553.3056 5905.0767 5905.2646
360.6662 -15416.2528 -1860.5013 0.0000 0.0000
8885.8020 7333.3609 331.4829 -1552.4411 7406.9423
330.4740 53.3002 49.8213 664265.7895 -8.0779
-8.0754
[48469]ENERGY: 12140000 3708.1974 5856.9342 5936.1871
354.0004 -15485.8266 -1847.1907 0.0000 0.0000
8815.8560 7338.1578 328.8736 -1477.6982 7409.0138
330.5303 -10.5460 -83.6587 664265.7895 -6.3390
-6.3336
[48519]ENERGY: 12150000 3729.1656 5916.1812 5912.0695
374.5122 -15480.4834 -1838.6715 0.0000 0.0000
8728.0314 7340.8049 325.5973 -1387.2265 7409.1300
330.7406 94.8392 54.3788 664265.7895 -5.6024
-5.6306
[48555]ENERGY: 12160000 3572.9216 5943.5239 5910.3675

351.4222 -15457.7841 -1888.1329 0.0000 0.0000
8906.5982 7338.9164 332.2587 -1567.6818 7409.9321
330.5556 -15.5592 -25.1743 664265.7895 -7.3291
-7.3116
[48605]ENERGY: 12170000 3577.0585 5894.0811 5877.7523
356.0001 -15417.6009 -1811.3794 0.0000 0.0000
8861.3497 7337.2614 330.5707 -1524.0883 7409.3312
330.4205 31.9054 37.2834 664265.7895 -9.2483
-9.2450
[48641]ENERGY: 12180000 3676.3853 5872.5239 5899.7115
365.2637 -15437.5012 -1811.8619 0.0000 0.0000
8774.8522 7339.3733 327.3440 -1435.4789 7409.3552
330.3169 -61.6527 -23.3232 664265.7895 -9.8601
-9.8504
[48691]ENERGY: 12190000 3644.7707 5812.0507 5900.3341
346.5386 -15363.0141 -1866.0283 0.0000 0.0000
8860.7212 7335.3729 330.5473 -1525.3483 7408.6762
330.4213 8.9735 -50.9880 664265.7895 -7.4512
-7.4795
[48730]ENERGY: 12200000 3572.7338 5912.1381 5865.4037
338.0113 -15348.7959 -1869.4584 0.0000 0.0000
8870.1196 7340.1522 330.8979 -1529.9674 7408.6759
330.3573 152.2301 168.8609 664265.7895 -8.9437
-8.9206
[48780]ENERGY: 12210000 3567.5383 5976.6816 5844.7774
377.2343 -15379.5853 -1856.2588 0.0000 0.0000
8807.6796 7338.0672 328.5686 -1469.6124 7410.6926
330.3938 39.5072 57.8435 664265.7895 -11.0238
-11.0423
[48816]ENERGY: 12220000 3620.8631 5814.2910 5919.8409
366.1167 -15389.4774 -1857.0598 0.0000 0.0000
8862.9825 7337.5571 330.6317 -1525.4254 7411.2877
330.3915 -90.6956 -117.2663 664265.7895 -4.9193
-4.9160
[48866]ENERGY: 12230000 3699.7230 5818.2696 5856.5459
363.5733 -15372.3797 -1851.4657 0.0000 0.0000
8826.6780 7340.9444 329.2773 -1485.7336 7411.8689
330.4439 -17.6236 -14.5835 664265.7895 -6.8974
-6.9070
[48902]ENERGY: 12240000 3554.8914 5857.4314 5957.8295
368.4339 -15479.3885 -1820.8961 0.0000 0.0000
8897.9000 7336.2016 331.9342 -1561.6983 7410.9764
330.5210 98.6850 32.7683 664265.7895 -8.1609
-8.1635
[48952]ENERGY: 12250000 3663.2094 5862.0663 5864.8040
356.3420 -15372.9770 -1828.7639 0.0000 0.0000
8794.6287 7339.3094 328.0817 -1455.3192 7410.3916
330.6739 14.6249 27.9803 664265.7895 -10.1437
-10.1185
[48988]ENERGY: 12260000 3579.8701 5819.0166 6026.9455
398.0424 -15467.0605 -1818.3632 0.0000 0.0000
8802.6070 7341.0579 328.3794 -1461.5492 7412.1518
330.5233 50.5462 -79.0924 664265.7895 -7.8637
-7.8796

[49038]ENERGY: 12270000 3626.0370 5888.8486 5927.6114
360.3764 -15353.0912 -1891.1557 0.0000 0.0000
8785.2269 7343.8534 327.7310 -1441.3735 7413.4401
330.5889 -16.4888 -29.3747 664265.7895 -4.8061
-4.8020
[49074]ENERGY: 12280000 3649.7225 5857.0438 5903.6671
351.2892 -15395.7329 -1836.2188 0.0000 0.0000
8812.6358 7342.4068 328.7535 -1470.2290 7413.3486
330.7498 -8.6985 0.5651 664265.7895 -3.6454
-3.6638
[49124]ENERGY: 12290000 3623.9037 5826.5276 5911.5907
373.4858 -15406.6327 -1788.2200 0.0000 0.0000
8803.3350 7343.9902 328.4065 -1459.3448 7413.8722
330.7361 162.0466 110.6125 664265.7895 -0.8969
-0.8988
[49163]ENERGY: 12300000 3633.5779 5809.9880 5930.3537
356.2356 -15485.2526 -1808.4472 0.0000 0.0000
8903.8356 7340.2908 332.1557 -1563.5448 7413.7588
330.6052 41.1115 42.9913 664265.7895 -3.3392
-3.3103
[49213]ENERGY: 12310000 3592.2678 5910.8591 5874.1351
364.4450 -15385.9403 -1941.2279 0.0000 0.0000
8930.8641 7345.4029 333.1640 -1585.4612 7416.3116
330.8159 -50.2720 -72.2206 664265.7895 -5.2415
-5.2478
[49249]ENERGY: 12320000 3633.0368 5867.7194 5906.7801
348.8586 -15363.5066 -1904.8221 0.0000 0.0000
8856.8722 7344.9384 330.4037 -1511.9338 7416.4069
330.6115 -67.6455 -166.0245 664265.7895 -6.8158
-6.8300
[49299]ENERGY: 12330000 3624.0562 5863.1819 5916.6762
387.4263 -15519.3378 -1789.5156 0.0000 0.0000
8862.2830 7344.7703 330.6056 -1517.5127 7415.1577
330.4245 -88.2504 -74.0412 664265.7895 -8.5776
-8.5730
[49335]ENERGY: 12340000 3598.9667 5893.4736 5959.2026
346.7403 -15410.6169 -1804.1371 0.0000 0.0000
8766.6097 7350.2389 327.0365 -1416.3709 7417.2026
330.6779 -40.1814 -58.7387 664265.7895 -4.6401
-4.6480
[49385]ENERGY: 12350000 3741.4033 5818.8646 5894.4053
361.9240 -15406.7904 -1839.6843 0.0000 0.0000
8774.8064 7344.9290 327.3423 -1429.8774 7415.2853
330.6600 -7.2152 -37.2453 664265.7895 -7.1555
-7.1367
[49421]ENERGY: 12360000 3674.8781 5775.6557 5911.5656
350.7999 -15335.5486 -1929.6031 0.0000 0.0000
8896.7674 7344.5151 331.8920 -1552.2524 7417.5606
330.8180 -229.3214 -186.7451 664265.7895 -9.2886
-9.2770
[49471]ENERGY: 12370000 3673.4131 5858.3605 5876.4707
348.8401 -15333.2620 -1920.1405 0.0000 0.0000
8840.5914 7344.2732 329.7964 -1496.3182 7418.0273
330.5349 -139.4003 -159.3969 664265.7895 -5.2702

-5.2727
[49507]ENERGY: 12380000 3623.4675 5848.2696 5960.9006
373.6940 -15335.1499 -1897.4939 0.0000 0.0000
8774.5762 7348.2640 327.3337 -1426.3122 7418.6339
330.5622 -36.5267 -28.8080 664265.7895 -5.8974
-5.9094
[49557]ENERGY: 12390000 3586.8735 5813.9085 5908.7613
376.8414 -15400.5015 -1787.7503 0.0000 0.0000
8846.4025 7344.5353 330.0131 -1501.8672 7419.1734
330.5721 264.7815 225.0194 664265.7895 -6.7375
-6.7476
[49596]ENERGY: 12400000 3699.0365 5770.3128 5988.5955
383.3714 -15488.3976 -1818.2749 0.0000 0.0000
8810.1820 7344.8256 328.6619 -1465.3564 7418.4227
330.5270 -68.7928 -144.7537 664265.7895 -5.9127
-5.9046
[49646]ENERGY: 12410000 3617.9205 5847.5319 5929.1464
353.3469 -15482.8721 -1833.7489 0.0000 0.0000
8911.2686 7342.5932 332.4330 -1568.6754 7419.7534
330.5731 25.6170 -54.6435 664265.7895 -6.0395
-6.0242
[49682]ENERGY: 12420000 3608.3408 5831.7160 5884.7265
370.3370 -15369.1033 -1834.3194 0.0000 0.0000
8857.5162 7349.2137 330.4277 -1508.3025 7419.9655
330.6679 132.8428 81.2357 664265.7895 -9.3299
-9.3533
[49732]ENERGY: 12430000 3606.2506 6030.9653 5931.8433
356.4877 -15508.6305 -1880.6776 0.0000 0.0000
8813.4031 7349.6419 328.7821 -1463.7612 7419.9280
330.7573 127.2122 108.9163 664265.7895 -8.0135
-8.0035
[49768]ENERGY: 12440000 3648.7265 5839.1433 5906.8010
367.9198 -15420.2902 -1901.1320 0.0000 0.0000
8909.3545 7350.5231 332.3616 -1558.8314 7421.4316
330.5538 -40.8521 -24.9446 664265.7895 -5.9467
-5.9419
[49818]ENERGY: 12450000 3738.3080 5855.8537 5913.1513
382.0477 -15458.8989 -1839.1333 0.0000 0.0000
8765.1692 7356.4977 326.9827 -1408.6715 7422.6984
330.5932 94.8574 -24.4365 664265.7895 -9.9682
-9.9586
[49854]ENERGY: 12460000 3614.7588 5864.4514 5892.4440
356.6355 -15473.5647 -1816.6385 0.0000 0.0000
8905.5878 7343.6742 332.2210 -1561.9135 7421.2267
330.8289 110.2712 100.1138 664265.7895 -11.0754
-11.0980
[49904]ENERGY: 12470000 3610.9572 5822.5858 5906.1059
350.4752 -15318.7716 -1922.4133 0.0000 0.0000
8902.6371 7351.5763 332.1110 -1551.0608 7422.7772
330.7525 -101.5688 -160.4230 664265.7895 -7.1434
-7.1246
[49940]ENERGY: 12480000 3602.9703 5868.2591 5949.6019
374.4318 -15500.3826 -1869.6192 0.0000 0.0000
8921.6237 7346.8850 332.8193 -1574.7387 7422.6435

330.6678 -27.4895 -139.1732 664265.7895 -11.8698
-11.8840
[49990]ENERGY: 12490000 3588.1069 5886.3209 5882.0758
367.9216 -15391.1799 -1789.3425 0.0000 0.0000
8811.2817 7355.1846 328.7030 -1456.0972 7422.6757
330.5300 182.7560 137.0779 664265.7895 -13.1347
-13.1581
[50029]ENERGY: 12500000 3626.3172 5836.1616 5918.3549
343.6223 -15429.3395 -1841.3482 0.0000 0.0000
8894.9733 7348.7416 331.8251 -1546.2317 7420.9868
330.7397 -1.2974 -24.9367 664265.7895 -7.5326
-7.4991
[50079]ENERGY: 12510000 3624.9209 5798.3826 5907.4083
345.9846 -15370.0838 -1885.7027 0.0000 0.0000
8927.5422 7348.4521 333.0400 -1579.0902 7421.4716
330.8881 -165.2324 -160.9375 664265.7895 -8.8762
-8.8848
[50115]ENERGY: 12520000 3601.1586 5772.9636 5935.5792
371.1670 -15370.4654 -1819.5376 0.0000 0.0000
8861.6526 7352.5180 330.5820 -1509.1346 7422.2384
330.6747 -3.2416 54.1483 664265.7895 -8.7877
-8.7885
[50165]ENERGY: 12530000 3612.3562 5840.8668 5917.5810
325.9451 -15267.8210 -1954.3078 0.0000 0.0000
8875.7386 7350.3588 331.1075 -1525.3798 7422.9192
330.7301 50.4785 -1.5099 664265.7895 -8.4701
-8.5005
[50201]ENERGY: 12540000 3681.8368 5821.9372 5904.2885
391.8739 -15339.5226 -1985.0259 0.0000 0.0000
8869.9653 7345.3533 330.8921 -1524.6121 7423.2581
330.7771 -189.4283 -207.7133 664265.7895 -11.7878
-11.7411
[50251]ENERGY: 12550000 3616.2659 5780.3059 5901.8957
359.4276 -15331.3087 -1901.4970 0.0000 0.0000
8922.7644 7347.8538 332.8618 -1574.9106 7421.4062
330.9215 -73.4045 -154.2179 664265.7895 -8.8104
-8.8287
[50287]ENERGY: 12560000 3688.4838 5858.8143 5935.3282
375.7789 -15452.8926 -1901.3851 0.0000 0.0000
8844.9556 7349.0832 329.9592 -1495.8724 7421.0399
330.7310 -8.1619 -53.6042 664265.7895 -12.1996
-12.1997
[50337]ENERGY: 12570000 3680.2667 5849.6034 5880.4732
366.3131 -15306.3334 -1930.1808 0.0000 0.0000
8813.3004 7353.4426 328.7783 -1459.8577 7421.4104
330.6309 22.5341 -36.8192 664265.7895 -7.2127
-7.2042
[50373]ENERGY: 12580000 3613.6423 5803.2142 5883.9524
361.3776 -15370.9042 -1883.1221 0.0000 0.0000
8943.8044 7351.9645 333.6467 -1591.8399 7422.1641
330.6488 10.9745 18.3061 664265.7895 -11.1840
-11.1947
[50423]ENERGY: 12590000 3605.7517 5813.8262 5957.0895
373.6354 -15402.5176 -1842.0146 0.0000 0.0000

8848.2714 7354.0420 330.0829 -1494.2294 7421.3352
330.5551 -16.3795 -16.9170 664265.7895 -13.0151
-13.0026
[50462]ENERGY: 12600000 3645.0059 5856.3893 5903.1906
366.7873 -15462.0737 -1830.6026 0.0000 0.0000
8873.3720 7352.0687 331.0192 -1521.3033 7422.1776
330.9712 -85.3370 -49.6986 664265.7895 -8.9292
-8.9298
[50512]ENERGY: 12610000 3645.2734 5810.5387 5915.7625
354.7428 -15366.7823 -1907.0448 0.0000 0.0000
8900.5003 7352.9906 332.0312 -1547.5097 7424.2319
331.0284 -134.5821 -117.9702 664265.7895 -9.8155
-9.8158
[50548]ENERGY: 12620000 3649.9214 5887.9369 5890.7854
343.6690 -15363.4673 -1922.6560 0.0000 0.0000
8865.8392 7352.0287 330.7382 -1513.8106 7423.8368
330.8540 -96.1743 -101.5591 664265.7895 -8.1915
-8.2017
[50598]ENERGY: 12630000 3627.9136 5855.9475 5954.4426
349.0246 -15399.9067 -1885.2148 0.0000 0.0000
8850.8112 7353.0180 330.1776 -1497.7932 7423.7254
331.0364 -54.8389 -50.4368 664265.7895 -9.6858
-9.7033
[50634]ENERGY: 12640000 3612.4177 5774.5868 5924.5989
362.2840 -15448.4126 -1731.8162 0.0000 0.0000
8856.0292 7349.6880 330.3723 -1506.3412 7423.2616
331.0388 185.1488 163.2334 664265.7895 -7.1388
-7.1113
[50684]ENERGY: 12650000 3700.8732 5779.9133 5917.3496
355.7888 -15390.1438 -1855.1474 0.0000 0.0000
8838.7364 7347.3702 329.7272 -1491.3662 7423.1553
330.9184 -61.9895 -36.0264 664265.7895 -9.2215
-9.2296
[50720]ENERGY: 12660000 3665.7138 5829.9584 5964.0150
373.0500 -15540.8100 -1817.4437 0.0000 0.0000
8876.5013 7350.9848 331.1360 -1525.5165 7424.5905
330.8757 -18.0597 60.2296 664265.7895 -9.6151
-9.6111
[50770]ENERGY: 12670000 3636.2651 5840.0036 5884.9717
349.6456 -15435.4781 -1848.8133 0.0000 0.0000
8923.3425 7349.9371 332.8834 -1573.4054 7422.8544
331.0436 162.9238 138.7778 664265.7895 -9.0843
-9.0940
[50806]ENERGY: 12680000 3721.8278 5852.4865 5871.1440
376.4506 -15452.9578 -1818.0549 0.0000 0.0000
8802.5538 7353.4499 328.3774 -1449.1039 7424.2684
330.9330 67.4667 14.1303 664265.7895 -4.1360
-4.1479
[50856]ENERGY: 12690000 3609.6153 5802.6698 5982.8934
357.2442 -15492.1160 -1889.3024 0.0000 0.0000
8980.9575 7351.9619 335.0327 -1628.9957 7423.9796
331.0771 64.8367 1.1278 664265.7895 -9.8942
-9.8685
[50895]ENERGY: 12700000 3598.3648 5790.5489 5917.1952

339.2782 -15376.8637 -1856.5056 0.0000 0.0000
8940.8384 7352.8562 333.5361 -1587.9823 7424.5705
330.9632 132.0094 13.0327 664265.7895 -3.4580
-3.4787
[50945]ENERGY: 12710000 3660.3142 5778.7495 5954.9150
350.4166 -15515.0057 -1777.2131 0.0000 0.0000
8902.1360 7354.3126 332.0923 -1547.8233 7423.9269
331.0352 36.0606 -7.4625 664265.7895 -8.8051
-8.8069
[50981]ENERGY: 12720000 3676.6841 5921.4478 5941.8538
382.0027 -15472.0549 -1824.9861 0.0000 0.0000
8731.1044 7356.0519 325.7120 -1375.0526 7424.5238
330.9792 43.3886 30.6574 664265.7895 -7.7852
-7.7774
[51031]ENERGY: 12730000 3560.5290 5891.6218 5924.4416
401.6608 -15342.7681 -1894.5799 0.0000 0.0000
8814.6973 7355.6024 328.8304 -1459.0949 7425.3830
331.1151 101.8481 102.9550 664265.7895 -11.1382
-11.1287
[51067]ENERGY: 12740000 3540.4333 5876.8345 5897.7127
359.4271 -15443.0550 -1834.1932 0.0000 0.0000
8957.0542 7354.2136 334.1410 -1602.8406 7426.2906
331.1036 23.3810 61.7834 664265.7895 -4.5765
-4.5803
[51117]ENERGY: 12750000 3659.5098 5880.7154 5906.3455
378.8921 -15385.2708 -1946.9281 0.0000 0.0000
8858.6992 7351.9630 330.4719 -1506.7362 7427.6240
331.0853 -50.2887 -49.1844 664265.7895 -4.1707
-4.1716
[51153]ENERGY: 12760000 3597.3528 5888.6770 5906.7885
383.0311 -15354.3379 -1855.0051 0.0000 0.0000
8791.7609 7358.2673 327.9747 -1433.4936 7426.9607
331.1758 138.6872 127.7339 664265.7895 -8.0290
-8.0385
[51203]ENERGY: 12770000 3631.7751 5927.7137 5891.6659
356.0597 -15441.7309 -1893.3002 0.0000 0.0000
8883.5253 7355.7086 331.3980 -1527.8167 7426.7845
330.9862 -116.9235 46.1514 664265.7895 -7.6172
-7.5979
[51239]ENERGY: 12780000 3603.9708 5775.5456 5923.0013
380.0812 -15352.7030 -1853.7488 0.0000 0.0000
8881.7292 7357.8763 331.3310 -1523.8529 7427.6784
331.0196 26.0231 -43.0863 664265.7895 -6.8138
-6.8153
[51289]ENERGY: 12790000 3588.9886 5964.7558 5924.9215
351.4961 -15376.0149 -1938.2353 0.0000 0.0000
8839.4163 7355.3280 329.7525 -1484.0883 7428.3943
330.9662 -33.3211 -117.6609 664265.7895 -5.7584
-5.7664
[51328]ENERGY: 12800000 3629.2016 5804.6089 5901.2610
370.9878 -15383.2195 -1855.3915 0.0000 0.0000
8888.5390 7355.9874 331.5850 -1532.5516 7429.0222
331.0560 -44.9425 -67.2935 664265.7895 -8.5835
-8.5827

[51378]ENERGY: 12810000 3638.8285 5894.3558 5920.6972
345.6047 -15349.2953 -1929.5273 0.0000 0.0000
8836.8250 7357.4887 329.6559 -1479.3363 7429.1883
331.0112 -13.0353 -57.1917 664265.7895 -5.6328
-5.6458
[51414]ENERGY: 12820000 3626.6914 5826.6910 5915.3714
383.8900 -15359.0605 -1844.2832 0.0000 0.0000
8808.3021 7357.6021 328.5918 -1450.7000 7427.0119
330.9150 -35.8236 -22.1086 664265.7895 -8.6677
-8.6513
[51464]ENERGY: 12830000 3709.5413 5795.7591 5943.6883
348.1683 -15316.5457 -1971.0808 0.0000 0.0000
8849.1369 7358.6675 330.1151 -1490.4694 7428.7323
331.0386 -257.6080 -146.8523 664265.7895 -4.1053
-4.1111
[51500]ENERGY: 12840000 3615.7104 5767.2396 5893.6691
389.3171 -15419.7899 -1824.6092 0.0000 0.0000
8938.1997 7359.7369 333.4376 -1578.4628 7429.7002
331.0102 41.5565 -92.1611 664265.7895 -7.3497
-7.3615
[51550]ENERGY: 12850000 3614.9480 5912.5335 5946.6285
372.7232 -15360.4812 -1883.7575 0.0000 0.0000
8754.8470 7357.4415 326.5977 -1397.4055 7429.4502
330.9315 -39.5040 46.5240 664265.7895 -5.5327
-5.5216
[51586]ENERGY: 12860000 3572.4806 5892.2674 5889.5252
383.8040 -15388.5158 -1842.6341 0.0000 0.0000
8850.9612 7357.8885 330.1832 -1493.0728 7430.7388
331.0557 53.0339 13.7126 664265.7895 -2.7723
-2.7818
[51636]ENERGY: 12870000 3575.5369 5859.1919 5959.4238
367.5656 -15382.2327 -1853.5454 0.0000 0.0000
8830.2126 7356.1528 329.4092 -1474.0598 7429.2077
331.0388 -13.8096 -43.4393 664265.7895 -3.5054
-3.4858
[51672]ENERGY: 12880000 3561.3092 5928.8217 5934.7970
376.4497 -15418.4338 -1839.6973 0.0000 0.0000
8818.3801 7361.6266 328.9678 -1456.7534 7430.1187
331.1070 68.8917 5.8531 664265.7895 -7.4580
-7.4734
[51722]ENERGY: 12890000 3646.7807 5929.5006 5931.7271
365.5417 -15439.6967 -1913.4181 0.0000 0.0000
8837.8014 7358.2367 329.6923 -1479.5647 7431.0579
331.3465 -63.2264 59.3011 664265.7895 -8.7777
-8.7685
[51761]ENERGY: 12900000 3677.2670 5814.4558 5904.4857
361.2455 -15370.3517 -1851.5031 0.0000 0.0000
8826.6527 7362.2519 329.2764 -1464.4008 7431.6308
331.3321 121.2449 47.6317 664265.7895 -8.5051
-8.5079
[51811]ENERGY: 12910000 3590.4388 5906.1718 5914.4947
371.3154 -15443.9415 -1899.2146 0.0000 0.0000
8916.5003 7355.7648 332.6281 -1560.7355 7429.7753
331.3060 -91.9378 -149.5810 664265.7895 -8.6742

```
-8.6743
[51847]ENERGY: 12920000      3630.0780      5825.8003      5926.7793
360.5494         -15452.0585     -1852.7363         0.0000         0.0000
8920.1438           7358.5560       332.7640     -1561.5878      7429.4368
331.1002             67.3652        91.2177    664265.7895        -7.9093
-7.9011
[51897]ENERGY: 12930000      3550.2343      5895.6270      5846.1634
352.8224         -15306.7679     -1898.1521         0.0000         0.0000
8920.0437           7359.9707       332.7603     -1560.0730      7430.1635
330.9423             86.3506       102.4773    664265.7895       -10.1177
-10.1328
[51933]ENERGY: 12940000      3603.7034      5896.0460      5943.0360
363.8203         -15326.9148     -1905.0812         0.0000         0.0000
8785.7310           7360.3406       327.7498     -1425.3904      7431.5114
330.8557            -70.7046      -138.0823    664265.7895        -4.0970
-4.0901
[51983]ENERGY: 12950000      3660.3658      5882.9433      5917.8338
368.6380         -15427.9706     -1858.4238         0.0000         0.0000
8819.5106           7362.8971       329.0099     -1456.6135      7432.3503
331.0315             52.1208       -44.0985    664265.7895        -4.8520
-4.8618
[52019]ENERGY: 12960000      3627.9964      5837.6136      5906.9342
376.4305         -15436.3783     -1766.0979         0.0000         0.0000
8814.3829           7360.8813       328.8187     -1453.5016      7430.2302
330.9695             -2.1792        10.3915    664265.7895         0.5672
 0.5772
[52069]ENERGY: 12970000      3636.1684      5844.6319      5904.1018
361.9412         -15388.2793     -1880.8121         0.0000         0.0000
8878.6483           7356.4002       331.2161     -1522.2481      7430.6255
330.9564             73.9455        -5.2123    664265.7895        -3.3413
-3.3589
[52105]ENERGY: 12980000      3648.3847      5839.4502      5894.6101
353.9069         -15329.0604     -1924.8168         0.0000         0.0000
8880.4757           7362.9502       331.2842     -1517.5254      7430.3662
330.9420           -100.0074      -123.1930    664265.7895        -0.8727
-0.8597
[52155]ENERGY: 12990000      3662.9759      5917.2911      5955.8112
372.6476         -15474.4032     -1880.6834         0.0000         0.0000
8805.8165           7359.4557       328.4991     -1446.3608      7429.9239
331.0855             78.3010        26.0786    664265.7895        -4.1796
-4.1744
[52194]ENERGY: 13000000      3707.5367      5899.1344      5940.7239
375.4513         -15547.4708     -1840.7034         0.0000         0.0000
8827.3160           7361.9881       329.3011     -1465.3279      7431.6083
331.1828             22.4916       -14.6614    664265.7895        -0.9246
-0.9302
[52244]ENERGY: 13010000      3683.1825      5830.4428      5945.6905
360.8825         -15470.1653     -1902.7466         0.0000         0.0000
8912.8456           7360.1321       332.4918     -1552.7135      7431.8114
331.0126             -3.4261         2.8746    664265.7895        -1.4344
-1.4262
[52280]ENERGY: 13020000      3566.6624      5987.3492      5903.8256
356.4849         -15379.4653     -1874.0280         0.0000         0.0000
8798.3426           7359.1713       328.2203     -1439.1712      7431.5351
```

330.8416 105.9416 75.9392 664265.7895 -7.1578
-7.1588
[52330]ENERGY: 13030000 3732.3171 5818.0209 5932.2056
353.3266 -15414.8296 -1843.5963 0.0000 0.0000
8783.5236 7360.9680 327.6675 -1422.5556 7432.6149
330.7853 -47.9417 -132.7547 664265.7895 -3.3421
-3.3354
[52366]ENERGY: 13040000 3642.3285 5768.6869 5851.5578
343.2742 -15438.1741 -1809.2563 0.0000 0.0000
9002.1190 7360.5360 335.8221 -1641.5831 7431.5105
330.7498 11.2133 16.6843 664265.7895 -5.3888
-5.3866
[52416]ENERGY: 13050000 3649.6667 5844.6832 5892.6753
335.1129 -15385.7890 -1809.3018 0.0000 0.0000
8834.4755 7361.5228 329.5682 -1472.9527 7432.6904
331.0186 155.0131 40.6197 664265.7895 -2.7136
-2.7398
[52452]ENERGY: 13060000 3717.8095 5842.3862 5905.6094
348.0279 -15496.9946 -1785.0112 0.0000 0.0000
8828.3420 7360.1692 329.3394 -1468.1728 7434.2178
330.8184 99.4945 54.2811 664265.7895 -4.9133
-4.8833
[52502]ENERGY: 13070000 3673.2794 5897.8015 5956.2482
359.2467 -15443.2947 -1920.7199 0.0000 0.0000
8841.4928 7364.0539 329.8300 -1477.4389 7436.4369
331.0436 -63.6527 -94.4725 664265.7895 -2.7652
-2.7725
[52538]ENERGY: 13080000 3613.5311 5861.5401 5912.8477
362.8605 -15404.6264 -1892.8716 0.0000 0.0000
8908.4726 7361.7540 332.3286 -1546.7186 7436.2229
330.9157 111.4093 20.7964 664265.7895 -5.7522
-5.7546
[52588]ENERGY: 13090000 3566.7373 5788.0582 5893.7917
363.8817 -15394.6067 -1840.8730 0.0000 0.0000
8987.6212 7364.6105 335.2813 -1623.0107 7436.0933
331.0126 44.7538 -39.7838 664265.7895 -8.2791
-8.2808
[52627]ENERGY: 13100000 3624.1711 5944.0551 5941.2905
363.8082 -15426.6778 -1867.8246 0.0000 0.0000
8786.4024 7365.2249 327.7748 -1421.1775 7435.3383
331.0370 -58.0953 -4.4381 664265.7895 -7.4981
-7.4985
[52677]ENERGY: 13110000 3693.2447 5777.5785 5919.5198
381.6368 -15417.3263 -1870.5385 0.0000 0.0000
8881.4110 7365.5261 331.3191 -1515.8850 7435.7331
331.1790 -114.6381 -122.1185 664265.7895 -10.7674
-10.7695
[52713]ENERGY: 13120000 3643.0218 5889.4578 5865.2292
353.3660 -15403.8515 -1881.2383 0.0000 0.0000
8896.5695 7362.5545 331.8846 -1534.0150 7435.9022
331.2161 69.3751 -4.0121 664265.7895 -3.5364
-3.5195
[52763]ENERGY: 13130000 3649.4883 5908.8469 5867.1289
371.9391 -15348.0913 -1887.7242 0.0000 0.0000

8808.1686 7369.7564 328.5868 -1438.4122 7436.0029
331.2160 -101.1326 -163.2321 664265.7895 -9.4174
-9.4242
[52799]ENERGY: 13140000 3625.2816 5888.3589 5968.1423
386.0619 -15385.3739 -1891.6757 0.0000 0.0000
8778.3652 7369.1602 327.4750 -1409.2049 7438.2517
331.2748 107.7235 89.0692 664265.7895 -1.7853
-1.7805
[52849]ENERGY: 13150000 3639.9102 5839.7319 5961.0953
363.8247 -15424.3028 -1889.3488 0.0000 0.0000
8872.6310 7363.5416 330.9916 -1509.0894 7437.8003
331.1605 -15.2472 -36.8404 664265.7895 -2.2854
-2.2960
[52885]ENERGY: 13160000 3581.7199 5890.6878 5917.0670
357.6475 -15408.0625 -1863.3590 0.0000 0.0000
8890.4095 7366.1101 331.6548 -1524.2994 7438.0920
331.4720 95.4893 137.1541 664265.7895 -7.1311
-7.1313
[52935]ENERGY: 13170000 3701.7921 5817.7131 5951.3661
379.4414 -15439.0073 -1898.6856 0.0000 0.0000
8852.2052 7364.8250 330.2296 -1487.3802 7436.4199
331.5606 -19.2089 -99.3648 664265.7895 -5.7284
-5.7519
[52971]ENERGY: 13180000 3596.1040 5872.4817 5931.6020
360.5630 -15426.4802 -1834.4347 0.0000 0.0000
8864.8873 7364.7231 330.7027 -1500.1642 7437.7488
331.4793 148.1166 119.0316 664265.7895 -7.8349
-7.8232
[53021]ENERGY: 13190000 3535.7246 5942.5714 5868.5640
367.6318 -15407.1494 -1844.2953 0.0000 0.0000
8902.4851 7365.5322 332.1053 -1536.9529 7438.2103
331.3837 85.1450 67.5331 664265.7895 -8.5510
-8.5462
[53060]ENERGY: 13200000 3645.7459 5856.0287 5927.3496
369.5900 -15316.1601 -1944.7391 0.0000 0.0000
8830.3793 7368.1943 329.4154 -1462.1849 7438.8927
331.3957 -99.8766 -37.5264 664265.7895 -10.6080
-10.5932
[53110]ENERGY: 13210000 3643.4235 5918.0888 5969.7292
357.8913 -15415.8407 -1891.0258 0.0000 0.0000
8784.8773 7367.1436 327.7180 -1417.7337 7439.7327
331.1768 -86.7908 -105.2486 664265.7895 -2.3250
-2.3103
[53146]ENERGY: 13220000 3665.2432 5864.4529 5883.9157
371.1851 -15417.8446 -1875.6275 0.0000 0.0000
8877.8215 7369.1462 331.1852 -1508.6753 7439.0649
331.2167 -114.1828 -105.9803 664265.7895 -4.1497
-4.1587
[53196]ENERGY: 13230000 3607.4209 5951.1575 5951.8889
362.0414 -15417.1436 -1915.1973 0.0000 0.0000
8830.4414 7370.6094 329.4177 -1459.8321 7439.7649
331.3745 2.2878 -79.9330 664265.7895 -7.6743
-7.6992
[53232]ENERGY: 13240000 3552.3222 5802.4319 5894.3550

372.8873 -15343.4370 -1820.0922 0.0000 0.0000
8908.7854 7367.2526 332.3403 -1541.5328 7439.1268
331.3344 203.7696 25.2240 664265.7895 -4.1538
-4.1529
[53282]ENERGY: 13250000 3635.2115 5838.7375 5912.8731
350.9558 -15439.7403 -1827.3962 0.0000 0.0000
8900.8378 7371.4792 332.0438 -1529.3586 7438.9862
331.0423 -6.9650 -48.6683 664265.7895 -9.1880
-9.1819
[53318]ENERGY: 13260000 3658.7440 5914.0434 5947.3563
381.8192 -15476.3117 -1823.1027 0.0000 0.0000
8769.7751 7372.3236 327.1546 -1397.4515 7439.8241
331.0489 -53.9883 -122.5877 664265.7895 -6.3890
-6.3801
[53368]ENERGY: 13270000 3621.0950 5832.1392 5909.6337
374.4756 -15398.9754 -1856.8139 0.0000 0.0000
8889.3819 7370.9360 331.6165 -1518.4458 7438.7637
331.2947 104.6807 61.2746 664265.7895 -4.2576
-4.2750
[53404]ENERGY: 13280000 3588.7326 5875.0678 5949.4315
371.9523 -15479.6129 -1857.7424 0.0000 0.0000
8922.5112 7370.3401 332.8524 -1552.1711 7441.3264
331.2100 75.3835 -34.4999 664265.7895 -9.5667
-9.5507
[53454]ENERGY: 13290000 3671.0397 5878.0468 5948.8426
344.6567 -15513.2704 -1821.3925 0.0000 0.0000
8860.3607 7368.2835 330.5338 -1492.0771 7439.5754
331.2538 -12.9763 -25.0280 664265.7895 -6.4535
-6.4674
[53493]ENERGY: 13300000 3567.0191 5892.8412 5920.3419
370.7178 -15476.7230 -1851.0615 0.0000 0.0000
8940.7763 7363.9118 333.5337 -1576.8645 7440.6030
331.2917 -8.4162 16.3081 664265.7895 -4.1179
-4.0972
[53543]ENERGY: 13310000 3630.9518 5900.3656 5890.8143
360.5550 -15416.3256 -1852.2408 0.0000 0.0000
8856.1462 7370.2666 330.3766 -1485.8796 7441.7116
331.3959 51.9100 1.8542 664265.7895 -3.2989
-3.3000
[53579]ENERGY: 13320000 3583.6063 5880.3075 5906.1665
357.9080 -15439.1360 -1819.9900 0.0000 0.0000
8899.5963 7368.4586 331.9975 -1531.1377 7440.5437
331.1954 123.6535 64.8057 664265.7895 -7.7427
-7.7460
[53629]ENERGY: 13330000 3616.0495 5880.4562 5907.9816
378.1738 -15407.2583 -1816.4307 0.0000 0.0000
8811.5968 7370.5690 328.7147 -1441.0279 7440.3743
331.2163 10.4443 68.5292 664265.7895 -5.4758
-5.4861
[53665]ENERGY: 13340000 3580.1135 5835.3255 5980.5437
375.1164 -15427.9614 -1929.1465 0.0000 0.0000
8954.3455 7368.3366 334.0399 -1586.0088 7441.8853
330.9694 -2.2858 -67.3623 664265.7895 -3.4992
-3.5026

[53715]ENERGY: 13350000 3671.4196 5937.2352 5921.0784
353.9248 -15473.0799 -1838.9676 0.0000 0.0000
8798.9044 7370.5149 328.2412 -1428.3895 7442.6114
331.2541 -101.1083 -77.8125 664265.7895 -1.6481
-1.6409
[53751]ENERGY: 13360000 3692.9585 5798.8062 5888.0195
358.0349 -15485.9778 -1854.4052 0.0000 0.0000
8972.1082 7369.5442 334.7026 -1602.5640 7444.3619
331.1544 -39.3397 -57.9138 664265.7895 -5.8236
-5.8062
[53801]ENERGY: 13370000 3668.1436 5812.0508 5916.8894
371.4837 -15370.3899 -1923.3540 0.0000 0.0000
8899.9214 7374.7449 332.0096 -1525.1764 7445.8029
331.4191 -5.3975 -45.1918 664265.7895 -5.2495
-5.2633
[53837]ENERGY: 13380000 3623.0063 5824.3103 5911.4372
367.4579 -15270.7260 -1934.5210 0.0000 0.0000
8855.8159 7376.7807 330.3643 -1479.0352 7444.9659
331.5809 47.8789 48.8411 664265.7895 -2.7086
-2.7031
[53887]ENERGY: 13390000 3619.9238 5938.7894 5906.0074
359.2615 -15471.5284 -1863.1792 0.0000 0.0000
8880.8557 7370.1302 331.2984 -1510.7255 7444.9426
331.5804 7.7179 -30.4243 664265.7895 -4.8967
-4.9085
[53926]ENERGY: 13400000 3578.9006 5940.9254 5928.0481
389.0278 -15471.6032 -1795.9169 0.0000 0.0000
8807.3489 7376.7307 328.5563 -1430.6182 7444.6519
331.5357 144.1237 44.3494 664265.7895 -6.7526
-6.7554
[53976]ENERGY: 13410000 3614.3383 5873.9407 5896.7013
362.6032 -15445.1629 -1875.3105 0.0000 0.0000
8947.3362 7374.4463 333.7784 -1572.8899 7446.9974
331.6266 126.1121 43.9348 664265.7895 -4.2403
-4.2476
[54012]ENERGY: 13420000 3621.1602 5893.7460 5953.2057
358.2349 -15404.5678 -1871.3458 0.0000 0.0000
8825.3677 7375.8009 329.2284 -1449.5668 7446.6438
331.5090 173.4864 110.2858 664265.7895 -7.9975
-7.9994
[54062]ENERGY: 13430000 3609.4709 5903.6399 5918.8330
363.0333 -15443.5904 -1854.4175 0.0000 0.0000
8880.2806 7377.2497 331.2770 -1503.0309 7446.3159
331.5075 -51.7973 -5.5638 664265.7895 -4.6778
-4.6708
[54098]ENERGY: 13440000 3580.3945 5961.1297 5908.9319
356.7596 -15417.8305 -1888.1307 0.0000 0.0000
8872.5616 7373.8161 330.9890 -1498.7455 7446.7161
331.4679 43.7735 52.6521 664265.7895 -1.6578
-1.6478
[54148]ENERGY: 13450000 3652.6296 5803.1053 5851.2122
366.0202 -15398.6954 -1808.4328 0.0000 0.0000
8909.6990 7375.5381 332.3744 -1534.1609 7446.3935
331.6069 47.6897 85.2991 664265.7895 -3.9666

-3.9768
[54184]ENERGY: 13460000 3669.9561 5860.5225 5878.8988
383.9409 -15430.3072 -1861.4636 0.0000 0.0000
8872.3836 7373.9311 330.9824 -1498.4525 7447.3265
331.4738 174.1085 50.6301 664265.7895 -1.8376
-1.8288
[54234]ENERGY: 13470000 3549.5673 5936.2377 5889.1557
358.7998 -15484.3137 -1807.3891 0.0000 0.0000
8933.1327 7375.1904 333.2486 -1557.9423 7447.2270
331.5225 -45.3990 -33.1669 664265.7895 -5.9030
-5.9109
[54270]ENERGY: 13480000 3645.5879 5814.9179 5961.7729
356.7485 -15479.0123 -1820.6227 0.0000 0.0000
8897.6876 7377.0797 331.9263 -1520.6079 7449.2209
331.6983 65.1526 89.3830 664265.7895 -1.2903
-1.2893
[54320]ENERGY: 13490000 3656.7199 5790.3108 5925.8294
369.7784 -15425.8687 -1853.3444 0.0000 0.0000
8913.3731 7376.7985 332.5115 -1536.5746 7448.1534
331.6560 75.5227 58.5344 664265.7895 -6.3451
-6.3247
[54359]ENERGY: 13500000 3683.2010 5918.6090 5949.1347
365.7858 -15494.5790 -1799.7719 0.0000 0.0000
8764.1100 7386.4895 326.9432 -1377.6204 7450.0872
331.6000 44.3212 65.7922 664265.7895 -5.1311
-5.1488
[54409]ENERGY: 13510000 3699.8061 5900.6128 5904.5732
356.8167 -15478.2223 -1854.4985 0.0000 0.0000
8847.1254 7376.2134 330.0401 -1470.9121 7450.3927
331.6423 -15.3081 9.4209 664265.7895 -5.5867
-5.5714
[54445]ENERGY: 13520000 3650.8970 5888.7375 5943.9013
358.6922 -15403.1315 -1908.4076 0.0000 0.0000
8849.8632 7380.5522 330.1422 -1469.3110 7450.1825
331.5649 -149.3722 -100.9899 664265.7895 -4.5338
-4.5363
[54495]ENERGY: 13530000 3627.3673 5923.8861 5949.4532
364.3568 -15507.0797 -1799.9492 0.0000 0.0000
8817.2711 7375.3055 328.9264 -1441.9656 7448.8608
331.3697 0.7720 97.8126 664265.7895 -5.8345
-5.8359
[54531]ENERGY: 13540000 3727.7046 5853.2664 5908.3774
353.2732 -15521.4113 -1865.3833 0.0000 0.0000
8920.7566 7376.5838 332.7869 -1544.1729 7449.3640
331.5791 11.8076 -92.5963 664265.7895 -6.9993
-7.0065
[54581]ENERGY: 13550000 3629.1961 5886.4570 5909.6620
402.4778 -15446.2272 -1795.5635 0.0000 0.0000
8792.3465 7378.3486 327.9966 -1413.9979 7450.0132
331.5027 254.1786 167.1628 664265.7895 -7.8609
-7.8611
[54617]ENERGY: 13560000 3602.4060 5848.1683 5912.3438
371.8097 -15350.2293 -1825.0877 0.0000 0.0000
8820.4429 7379.8537 329.0447 -1440.5891 7449.1832

331.4487 -88.0259 -57.2557 664265.7895 -6.6004
-6.6094
[54667]ENERGY: 13570000 3620.3847 5854.3181 5966.4505
372.1576 -15466.5932 -1874.6701 0.0000 0.0000
8904.2981 7376.3457 332.1729 -1527.9524 7451.2672
331.3780 8.8552 -20.0887 664265.7895 -8.7700
-8.7640
[54703]ENERGY: 13580000 3647.9842 5792.4565 5927.0761
365.4329 -15362.2849 -1922.9673 0.0000 0.0000
8935.7708 7383.4683 333.3470 -1552.3025 7452.8425
331.5972 22.7596 -56.4606 664265.7895 -1.9163
-1.9020
[54753]ENERGY: 13590000 3689.2285 5953.8977 5914.8749
365.5598 -15430.7761 -1918.5182 0.0000 0.0000
8807.0905 7381.3570 328.5466 -1425.7335 7452.4949
331.3607 -183.1992 -157.5000 664265.7895 1.7253
1.7252
[54792]ENERGY: 13600000 3652.1279 5899.1093 5906.9883
369.1696 -15467.2394 -1898.5400 0.0000 0.0000
8920.6179 7382.2336 332.7817 -1538.3843 7453.8552
331.3485 63.1363 -13.8786 664265.7895 -2.9955
-3.0281
[54842]ENERGY: 13610000 3577.6174 5872.8546 5928.3540
350.1261 -15421.2053 -1813.6316 0.0000 0.0000
8888.7182 7382.8334 331.5917 -1505.8848 7452.6494
331.3693 34.8767 -22.3689 664265.7895 0.9919
0.9988
[54878]ENERGY: 13620000 3668.6406 5892.3948 5903.4480
364.7983 -15393.8735 -1834.8044 0.0000 0.0000
8783.8488 7384.4526 327.6796 -1399.3962 7452.2806
331.1100 48.9076 55.5161 664265.7895 -4.8774
-4.8563
[54928]ENERGY: 13630000 3695.4570 5962.6067 5908.9630
359.6478 -15473.8722 -1895.5902 0.0000 0.0000
8828.7224 7385.9345 329.3536 -1442.7879 7455.0204
331.2385 72.7646 114.2403 664265.7895 -1.6691
-1.6772
[54964]ENERGY: 13640000 3677.8922 5882.3643 5872.0978
360.0646 -15401.6394 -1817.8344 0.0000 0.0000
8811.8087 7384.7537 328.7226 -1427.0550 7454.7818
331.0947 -1.4267 -21.2451 664265.7895 -2.8206
-2.8213
[55014]ENERGY: 13650000 3647.5852 5886.9770 5963.1890
358.2380 -15463.7026 -1883.6182 0.0000 0.0000
8875.9389 7384.6073 331.1150 -1491.3316 7455.4273
331.3694 -162.9324 -115.6144 664265.7895 -2.3673
-2.3540
[55050]ENERGY: 13660000 3674.6224 5914.4793 5940.9298
351.3621 -15408.1623 -1872.7322 0.0000 0.0000
8787.0762 7387.5753 327.8000 -1399.5010 7455.8196
331.1880 0.4942 -58.9957 664265.7895 -2.5854
-2.5945
[55100]ENERGY: 13670000 3592.9958 5888.1716 6015.9677
364.1624 -15497.6689 -1814.8726 0.0000 0.0000

8835.7736 7384.5297 329.6166 -1451.2440 7455.6886
330.9506 89.1744 -27.3894 664265.7895 -2.0023
-2.0200
[55136]ENERGY: 13680000 3659.7743 5931.9555 5909.3998
342.8608 -15549.4147 -1770.3603 0.0000 0.0000
8860.6978 7384.9132 330.5464 -1475.7846 7455.7507
331.0742 148.1315 147.7026 664265.7895 0.6815
0.7027
[55186]ENERGY: 13690000 3634.4173 5859.6888 5902.8562
348.9983 -15498.2921 -1808.0388 0.0000 0.0000
8945.1598 7384.7894 333.6973 -1560.3704 7455.7291
331.1368 -47.4380 -24.8688 664265.7895 -3.6020
-3.6154
[55225]ENERGY: 13700000 3591.8441 5876.7951 5927.6678
360.9759 -15412.3138 -1771.0304 0.0000 0.0000
8811.0858 7385.0247 328.6957 -1426.0611 7455.7130
331.3191 293.9740 265.5066 664265.7895 -2.2940
-2.2950
[55275]ENERGY: 13710000 3687.1023 5957.5417 5929.4078
371.0764 -15459.0109 -1850.7457 0.0000 0.0000
8749.4275 7384.7993 326.3955 -1364.6283 7454.8452
331.0402 -146.1525 -91.6511 664265.7895 -3.4501
-3.4226
[55311]ENERGY: 13720000 3704.2235 5879.7980 5933.2108
370.4479 -15460.8900 -1856.4548 0.0000 0.0000
8813.8008 7384.1363 328.7969 -1429.6645 7455.4206
331.0758 46.8200 60.9648 664265.7895 -3.1933
-3.2004
[55361]ENERGY: 13730000 3551.5846 5890.3658 5917.3357
366.6017 -15489.5850 -1763.8204 0.0000 0.0000
8909.2997 7381.7821 332.3595 -1527.5176 7454.8442
331.4171 80.3178 92.9980 664265.7895 -2.9033
-2.9032
[55397]ENERGY: 13740000 3749.1100 5851.7974 5905.8123
352.7003 -15442.3027 -1837.8400 0.0000 0.0000
8805.7706 7385.0479 328.4974 -1420.7228 7455.2196
331.5108 -111.8089 -104.5844 664265.7895 -5.2010
-5.2158
[55447]ENERGY: 13750000 3563.2687 5927.8938 5967.9937
352.3857 -15430.6444 -1854.6417 0.0000 0.0000
8862.5698 7388.8256 330.6163 -1473.7441 7458.6176
331.2518 38.0897 -5.1219 664265.7895 -0.8974
-0.8969
[55483]ENERGY: 13760000 3599.5626 5858.5007 5871.7832
375.1154 -15462.3446 -1723.2781 0.0000 0.0000
8864.3632 7383.7023 330.6832 -1480.6609 7456.9620
331.4504 62.8152 22.9571 664265.7895 -2.5252
-2.5227
[55533]ENERGY: 13770000 3653.9189 5961.0400 5906.4730
344.4111 -15507.8223 -1884.0316 0.0000 0.0000
8911.7539 7385.7431 332.4511 -1526.0108 7457.0061
331.6539 -6.9286 11.7890 664265.7895 -5.9326
-5.9322
[55569]ENERGY: 13780000 3723.3483 5980.4765 5937.8137

386.7978   -15551.9801   -1843.9674   0.0000   0.0000
8755.2004   7387.6892   326.6109   -1367.5112   7457.7850
331.6270   32.6727   -24.6388   664265.7895   -9.5106
-9.5219
[55619]ENERGY: 13790000   3616.0449   5941.9682   5897.2851
318.7233   -15400.2447   -1860.9349   0.0000   0.0000
8875.4909   7388.3329   331.0983   -1487.1581   7457.0765
331.5307   -146.1280   -46.2892   664265.7895   -9.1990
-9.1751
[55658]ENERGY: 13800000   3618.3845   5861.7557   5952.1634
394.4172   -15403.2057   -1813.0709   0.0000   0.0000
8782.6200   7393.0642   327.6337   -1389.5558   7458.4889
331.6605   -54.0810   -58.6133   664265.7895   -4.8618
-4.8737
[55708]ENERGY: 13810000   3675.3759   5869.3277   5915.8266
358.4047   -15340.2289   -1965.6210   0.0000   0.0000
8874.3778   7387.4628   331.0568   -1486.9150   7460.1162
331.7026   -239.5555   -251.8632   664265.7895   -8.5772
-8.5837
[55744]ENERGY: 13820000   3697.4445   5916.0029   5919.2129
395.8349   -15463.5207   -1908.5822   0.0000   0.0000
8834.8137   7391.2060   329.5808   -1443.6078   7461.0607
331.2923   13.6677   12.1857   664265.7895   -5.7396
-5.7287
[55794]ENERGY: 13830000   3708.2966   5790.8960   5915.2627
367.3561   -15476.1067   -1855.5832   0.0000   0.0000
8937.9875   7388.1089   333.4297   -1549.8785   7459.4838
331.5973   -65.1035   -24.4510   664265.7895   -9.7466
-9.7564
[55830]ENERGY: 13840000   3682.4496   5812.1138   5983.4242
354.3983   -15503.6876   -1796.0123   0.0000   0.0000
8855.7872   7388.4732   330.3632   -1467.3140   7460.7509
331.6241   82.7955   74.8596   664265.7895   -5.3934
-5.3831
[55880]ENERGY: 13850000   3626.5222   5834.3803   5937.5695
356.5289   -15462.8930   -1825.6316   0.0000   0.0000
8924.0009   7390.4771   332.9079   -1533.5238   7462.4979
331.5981   -149.1441   -180.9712   664265.7895   -4.9703
-4.9920
[55916]ENERGY: 13860000   3764.6341   5824.5331   5905.4110
356.2723   -15392.3001   -1895.0422   0.0000   0.0000
8825.5857   7389.0938   329.2366   -1436.4918   7460.6167
331.4918   -121.6304   -122.4107   664265.7895   -5.2349
-5.2035
[55966]ENERGY: 13870000   3604.8133   5874.1008   5914.3700
370.3691   -15489.2217   -1836.2233   0.0000   0.0000
8951.1702   7389.3783   333.9215   -1561.7919   7461.5993
331.5693   164.7165   63.3532   664265.7895   -2.8049
-2.8186
[56002]ENERGY: 13880000   3699.6006   5840.5458   5890.2744
361.2571   -15504.8900   -1848.9356   0.0000   0.0000
8951.4561   7389.3085   333.9321   -1562.1476   7462.1569
331.8286   -92.3207   -63.0958   664265.7895   -4.8231
-4.8417

[56052]ENERGY: 13890000 3628.1295 5837.8869 5895.3510
352.1924 -15447.3009 -1764.5365 0.0000 0.0000
8888.6922 7390.4146 331.5907 -1498.2775 7462.2199
331.6756 7.0468 81.4687 664265.7895 -1.1787
-1.1592
[56091]ENERGY: 13900000 3575.1482 5867.1248 5905.5210
368.4160 -15431.1493 -1873.5857 0.0000 0.0000
8981.5049 7392.9799 335.0531 -1588.5250 7463.7568
331.5235 -61.8770 -145.9253 664265.7895 -5.9181
-5.9271
[56141]ENERGY: 13910000 3666.8279 5888.1995 5908.7364
363.3815 -15435.1161 -1862.6367 0.0000 0.0000
8864.3118 7393.7043 330.6812 -1470.6075 7464.3789
331.8253 -37.4051 -53.9593 664265.7895 -4.5912
-4.5799
[56177]ENERGY: 13920000 3634.4637 5953.4782 5844.5931
372.6662 -15436.8819 -1914.6182 0.0000 0.0000
8941.2353 7394.9365 333.5509 -1546.2988 7465.8480
331.7958 41.9587 -94.8538 664265.7895 -8.8838
-8.9005
[56227]ENERGY: 13930000 3664.9039 5833.6943 5935.1588
386.9402 -15491.4816 -1835.0526 0.0000 0.0000
8901.6970 7395.8600 332.0759 -1505.8370 7466.7177
331.9985 40.7967 -6.4492 664265.7895 -0.5831
-0.5600
[56263]ENERGY: 13940000 3653.8785 5797.8799 5953.6763
370.6286 -15360.2336 -1902.0847 0.0000 0.0000
8887.2584 7401.0034 331.5373 -1486.2550 7467.1051
331.9872 -84.3804 -62.5728 664265.7895 -1.2393
-1.2367
[56313]ENERGY: 13950000 3599.2610 5935.8867 5924.2452
367.9563 -15449.6715 -1833.4016 0.0000 0.0000
8853.9237 7398.1999 330.2937 -1455.7238 7468.2803
331.8259 185.1933 176.6059 664265.7895 -1.5428
-1.5699
[56349]ENERGY: 13960000 3572.1109 5796.2248 5993.2728
361.4702 -15407.7322 -1854.2863 0.0000 0.0000
8936.6163 7397.6765 333.3785 -1538.9398 7469.8212
331.7444 86.8982 3.6122 664265.7895 -2.3267
-2.3205
[56399]ENERGY: 13970000 3567.8585 5891.9246 5973.5521
354.4915 -15531.1536 -1785.3644 0.0000 0.0000
8922.4993 7393.8081 332.8519 -1528.6913 7468.0342
331.7847 70.0220 24.4312 664265.7895 -2.9539
-2.9400
[56435]ENERGY: 13980000 3572.2058 6003.8457 5943.6914
360.6688 -15413.2155 -1855.7061 0.0000 0.0000
8788.4727 7399.9628 327.8521 -1388.5100 7468.8639
331.8729 -45.9674 -74.7075 664265.7895 -2.8640
-2.8635
[56485]ENERGY: 13990000 3635.6550 5892.9624 5888.6700
346.7461 -15455.7546 -1858.5674 0.0000 0.0000
8948.2011 7397.9127 333.8107 -1550.2884 7468.4362
331.8023 62.2493 81.4838 664265.7895 -2.0209

-2.0283
[56524]ENERGY: 14000000 3689.3364 5919.1814 5899.7786
341.6367 -15423.8189 -1858.4800 0.0000 0.0000
8834.9036 7402.5378 329.5842 -1432.3658 7467.9069
331.8619 30.3538 67.4973 664265.7895 -7.4926
-7.5006
[56574]ENERGY: 14010000 3584.4712 5950.5960 5913.9632
394.6366 -15452.3669 -1889.7976 0.0000 0.0000
8894.0193 7395.5217 331.7895 -1498.4976 7468.5843
331.8299 70.2794 18.4152 664265.7895 -2.9963
-2.9908
[56610]ENERGY: 14020000 3709.9385 5893.2846 5924.9807
348.0096 -15469.8087 -1899.1470 0.0000 0.0000
8889.4454 7396.7031 331.6188 -1492.7423 7469.6675
331.8375 -57.7782 -96.0900 664265.7895 -5.9316
-5.9221
[56660]ENERGY: 14030000 3610.1002 5924.4981 5893.0676
346.9315 -15405.6182 -1835.4242 0.0000 0.0000
8868.3714 7401.9263 330.8327 -1466.4451 7470.0284
331.8077 -7.0722 -102.1278 664265.7895 -5.8890
-5.9188
[56696]ENERGY: 14040000 3602.1445 5831.5839 5892.8899
357.5404 -15457.6766 -1826.9054 0.0000 0.0000
8996.8571 7396.4336 335.6258 -1600.4234 7471.3614
331.7974 146.4401 112.1908 664265.7895 -5.4621
-5.4452
[56746]ENERGY: 14050000 3679.4415 5950.5346 5854.0652
368.8451 -15437.1679 -1873.6405 0.0000 0.0000
8856.8469 7398.9248 330.4028 -1457.9220 7470.2735
332.2054 -71.1236 -58.8376 664265.7895 -4.9805
-4.9802
[56782]ENERGY: 14060000 3606.7357 5868.9396 5794.7660
338.8229 -15396.4589 -1796.2219 0.0000 0.0000
8982.0375 7398.6209 335.0730 -1583.4166 7470.6691
332.0772 60.5232 10.5381 664265.7895 -3.7929
-3.7806
[56832]ENERGY: 14070000 3584.5948 5822.1923 5887.8811
359.7721 -15403.3292 -1797.9518 0.0000 0.0000
8947.3410 7400.5002 333.7786 -1546.8407 7470.1189
332.0286 -102.6656 -116.6707 664265.7895 -4.4080
-4.4130
[56868]ENERGY: 14080000 3576.7198 5858.2839 5964.9818
365.6938 -15414.3444 -1844.8573 0.0000 0.0000
8893.0551 7399.5327 331.7535 -1493.5224 7471.4737
332.2462 -86.7081 -41.9405 664265.7895 -5.5610
-5.5650
[56918]ENERGY: 14090000 3623.4525 5897.9555 5878.1389
372.8336 -15489.2219 -1841.2601 0.0000 0.0000
8957.9059 7399.8045 334.1727 -1558.1014 7471.3615
332.0889 -68.3632 -86.8787 664265.7895 -5.2054
-5.2047
[56957]ENERGY: 14100000 3669.5510 5877.2812 5913.6092
335.2411 -15440.1538 -1843.6251 0.0000 0.0000
8890.3483 7402.2518 331.6525 -1488.0965 7473.4637

331.9578 -14.8848 39.2149 664265.7895 -6.1878
-6.1779
[57007]ENERGY: 14110000 3589.6383 5879.9031 5931.2197
367.8449 -15454.5454 -1867.8027 0.0000 0.0000
8955.4037 7401.6615 334.0794 -1553.7421 7474.2207
332.2042 -136.6469 -154.3175 664265.7895 -4.9493
-4.9455
[57043]ENERGY: 14120000 3693.3887 5893.2067 5923.7585
359.6303 -15546.5500 -1818.6129 0.0000 0.0000
8898.0395 7402.8608 331.9394 -1495.1788 7473.2022
332.2060 -67.4848 -0.5772 664265.7895 -9.4514
-9.4719
[57093]ENERGY: 14130000 3609.9684 5866.8255 5931.8462
374.0738 -15397.3941 -1842.0287 0.0000 0.0000
8861.6182 7404.9092 330.5808 -1456.7089 7474.9512
332.0150 49.0893 11.7457 664265.7895 -2.6640
-2.6537
[57129]ENERGY: 14140000 3647.2327 5847.2024 5902.6722
368.7783 -15431.8600 -1855.6542 0.0000 0.0000
8923.8733 7402.2447 332.9032 -1521.6286 7474.2016
332.0237 -114.8157 -46.0218 664265.7895 -7.6260
-7.6270
[57179]ENERGY: 14150000 3564.8270 5815.0130 5900.4326
340.1562 -15334.2719 -1852.1380 0.0000 0.0000
8971.0409 7405.0598 334.6627 -1565.9811 7475.4761
332.0709 -30.7294 -64.4073 664265.7895 -1.1180
-1.1055
[57215]ENERGY: 14160000 3668.2910 5877.2443 5880.4336
360.5826 -15479.1479 -1816.4179 0.0000 0.0000
8913.0285 7404.0143 332.4986 -1509.0143 7474.3418
332.1112 -59.6765 -122.6102 664265.7895 -2.2441
-2.2686
[57265]ENERGY: 14170000 3556.2603 5937.9214 5969.8298
363.0564 -15460.8224 -1834.6290 0.0000 0.0000
8872.4222 7404.0386 330.9838 -1468.3836 7475.7106
332.0388 225.8222 243.4134 664265.7895 -3.8227
-3.8139
[57301]ENERGY: 14180000 3593.9728 5861.5599 5885.3761
353.3179 -15370.9947 -1853.7109 0.0000 0.0000
8936.9369 7406.4579 333.3905 -1530.4790 7476.7341
332.0425 149.0535 66.4702 664265.7895 -2.4546
-2.4518
[57351]ENERGY: 14190000 3619.9911 5813.1188 5900.9779
378.5113 -15464.1046 -1749.4406 0.0000 0.0000
8904.4375 7403.4913 332.1781 -1500.9461 7478.1068
332.0332 32.0311 -7.4674 664265.7895 -3.0402
-3.0401
[57390]ENERGY: 14200000 3651.3207 5944.1872 5880.6259
366.9418 -15394.8816 -1923.9367 0.0000 0.0000
8883.5487 7407.8061 331.3989 -1475.7426 7480.4466
331.9678 -146.4541 -143.7793 664265.7895 -4.6584
-4.6511
[57440]ENERGY: 14210000 3612.2890 5945.1500 5883.6448
402.8512 -15487.0377 -1780.2065 0.0000 0.0000

8827.2083 7403.8991 329.2971 -1423.3091 7477.2621
332.1384 -7.6313 27.8693 664265.7895 -5.7841
-5.8114
[57476]ENERGY: 14220000 3604.8403 5948.8330 5902.0729
355.5503 -15441.4175 -1868.2679 0.0000 0.0000
8906.2823 7407.8934 332.2469 -1498.3889 7478.6655
332.1639 -56.6230 -57.8906 664265.7895 -5.0956
-5.0790
[57526]ENERGY: 14230000 3594.8306 5882.4403 5925.5463
378.0153 -15425.9290 -1865.8021 0.0000 0.0000
8916.1423 7405.2436 332.6148 -1510.8987 7480.7148
332.1761 151.8772 91.8567 664265.7895 -4.1209
-4.1223
[57562]ENERGY: 14240000 3695.7386 5835.7904 5974.3160
340.3314 -15518.6259 -1785.7239 0.0000 0.0000
8867.3240 7409.1508 330.7936 -1458.1732 7479.9073
331.9884 5.3928 59.7325 664265.7895 -3.5252
-3.5029
[57612]ENERGY: 14250000 3616.2046 5965.4179 5937.7368
382.0158 -15486.9418 -1864.0759 0.0000 0.0000
8856.7200 7407.0775 330.3980 -1449.6425 7481.6740
331.9350 43.1934 50.7603 664265.7895 -3.4294
-3.4569
[57648]ENERGY: 14260000 3642.7765 5871.8785 5896.8891
356.9062 -15370.4134 -1894.2997 0.0000 0.0000
8907.1384 7410.8756 332.2789 -1496.2629 7482.9277
331.9917 -45.9341 -76.0318 664265.7895 -4.8496
-4.8334
[57698]ENERGY: 14270000 3639.3477 5868.5405 5876.5670
377.0273 -15477.2408 -1862.1615 0.0000 0.0000
8989.4447 7411.5250 335.3493 -1577.9197 7481.7321
332.0915 -3.4593 -68.5846 664265.7895 -7.5475
-7.5602
[57734]ENERGY: 14280000 3623.6832 5862.3415 5920.2644
348.2279 -15396.3467 -1826.7607 0.0000 0.0000
8876.1058 7407.5153 331.1212 -1468.5904 7481.5237
332.0055 28.4103 9.3534 664265.7895 -6.1787
-6.1551
[57784]ENERGY: 14290000 3664.9620 5898.8691 5922.3875
365.2139 -15416.9974 -1936.5861 0.0000 0.0000
8912.2287 7410.0776 332.4688 -1502.1510 7482.1656
332.0265 -121.4535 -147.7496 664265.7895 -6.6707
-6.6748
[57823]ENERGY: 14300000 3691.2801 5885.3251 5903.3794
380.6768 -15395.6228 -1839.0533 0.0000 0.0000
8786.3436 7412.3289 327.7727 -1374.0146 7482.2394
332.0240 -24.2124 -104.0313 664265.7895 -4.0125
-4.0486
[57873]ENERGY: 14310000 3658.5867 5931.0464 5975.9320
371.9783 -15513.7302 -1830.2745 0.0000 0.0000
8817.7917 7411.3306 328.9458 -1406.4611 7483.1017
332.3097 -63.4783 -29.6625 664265.7895 -1.7601
-1.7273
[57909]ENERGY: 14320000 3657.9201 5830.0867 5937.4941

361.6681 -15452.6635 -1842.1112 0.0000 0.0000
8918.5542 7410.9486 332.7047 -1507.6056 7483.8473
332.2524 -5.9421 -74.3525 664265.7895 -0.2150
-0.2196
[57959]ENERGY: 14330000 3656.3062 5893.3593 5910.9039
374.3469 -15508.2386 -1816.8182 0.0000 0.0000
8900.7596 7410.6191 332.0409 -1490.1404 7482.2167
332.0547 11.4510 -56.7053 664265.7895 -0.5838
-0.5894
[57995]ENERGY: 14340000 3636.2815 6029.0803 5932.3431
364.3351 -15504.0732 -1896.6763 0.0000 0.0000
8851.7855 7413.0761 330.2140 -1438.7094 7481.8094
332.0598 153.3554 36.5934 664265.7895 -6.7532
-6.7617
[58045]ENERGY: 14350000 3688.3854 5840.5171 5907.5365
352.6211 -15406.2496 -1855.9452 0.0000 0.0000
8888.6496 7415.5151 331.5892 -1473.1345 7482.5629
332.1261 -29.5376 -42.4757 664265.7895 -3.8171
-3.8158
[58081]ENERGY: 14360000 3713.1438 5868.6024 5964.6178
323.5900 -15491.1380 -1857.4626 0.0000 0.0000
8890.1442 7411.4977 331.6449 -1478.6465 7483.2796
332.3072 -33.5243 -123.1800 664265.7895 -4.7840
-4.8001
[58131]ENERGY: 14370000 3626.1089 5932.4551 5948.1306
367.8540 -15533.7348 -1810.4022 0.0000 0.0000
8883.1529 7413.5646 331.3841 -1469.5883 7483.4108
332.2149 5.9588 -45.0189 664265.7895 -9.3606
-9.3379
[58167]ENERGY: 14380000 3584.9042 5860.3258 5908.2647
344.3782 -15444.9249 -1849.6369 0.0000 0.0000
9005.1572 7408.4683 335.9354 -1596.6889 7483.4976
332.4000 125.7962 -13.6701 664265.7895 -2.0771
-2.0944
[58217]ENERGY: 14390000 3629.6989 5855.8028 5889.5346
351.9480 -15381.9165 -1870.3952 0.0000 0.0000
8935.4903 7410.1630 333.3365 -1525.3274 7484.1137
332.3916 116.7398 -49.2294 664265.7895 -5.5592
-5.5532
[58256]ENERGY: 14400000 3650.3207 5859.4168 5898.7330
379.6401 -15406.2097 -1900.7853 0.0000 0.0000
8933.3776 7414.4933 333.2577 -1518.8844 7484.4652
332.0281 22.2290 -26.9255 664265.7895 -4.0901
-4.0783
[58306]ENERGY: 14410000 3635.7851 5923.5490 5884.6345
373.6349 -15389.2153 -1846.3289 0.0000 0.0000
8825.3628 7407.4221 329.2283 -1417.9407 7483.4763
332.0783 -76.1253 -64.3974 664265.7895 -2.9946
-3.0071
[58342]ENERGY: 14420000 3581.5691 5915.1434 5948.7200
356.8379 -15398.2910 -1866.1589 0.0000 0.0000
8873.5024 7411.3230 331.0241 -1462.1794 7483.4711
331.8581 63.5766 8.1620 664265.7895 -0.1436
-0.1225

[58392]ENERGY: 14430000 3677.3468 5813.4105 5922.0250
349.7021 -15449.0758 -1807.7023 0.0000 0.0000
8906.6653 7412.3715 332.2612 -1494.2937 7484.0738
331.7944 -27.7721 19.4269 664265.7895 -0.3537
-0.3657
[58428]ENERGY: 14440000 3609.0907 5856.2442 5971.2647
361.7256 -15485.4976 -1850.2372 0.0000 0.0000
8954.4338 7417.0242 334.0432 -1537.4096 7487.0859
332.0067 29.0515 -6.5178 664265.7895 -5.2772
-5.2730
[58478]ENERGY: 14450000 3610.6578 5945.3878 5926.4353
359.0300 -15393.5224 -1878.4562 0.0000 0.0000
8845.5158 7415.0481 329.9801 -1430.4677 7485.3347
331.8673 103.7887 108.1287 664265.7895 -3.6070
-3.6046
[58514]ENERGY: 14460000 3702.8271 5895.7865 5867.6811
366.4021 -15393.7952 -1851.3021 0.0000 0.0000
8829.1105 7416.7101 329.3681 -1412.4005 7486.1407
331.8642 4.9249 -25.2039 664265.7895 -3.4945
-3.4912
[58564]ENERGY: 14470000 3618.7790 5827.3232 5927.6265
367.0874 -15468.9923 -1813.9805 0.0000 0.0000
8955.4280 7413.2714 334.0803 -1542.1567 7484.7981
331.8993 210.3503 174.3718 664265.7895 -5.1872
-5.1979
[58600]ENERGY: 14480000 3801.0030 5703.7419 5937.5182
344.6225 -15408.2195 -1897.5561 0.0000 0.0000
8933.7947 7414.9047 333.2733 -1518.8900 7484.9742
332.1745 -4.0515 -52.4981 664265.7895 -4.2048
-4.2117
[58650]ENERGY: 14490000 3660.7424 5962.0163 5989.8720
340.5943 -15471.7423 -1880.9241 0.0000 0.0000
8815.8172 7416.3757 328.8722 -1399.4415 7485.9406
332.1278 -10.4776 -115.0630 664265.7895 -4.2620
-4.2616
[58689]ENERGY: 14500000 3675.8426 5885.9136 5896.1810
361.6076 -15393.4138 -1914.4494 0.0000 0.0000
8901.0407 7412.7223 332.0514 -1488.3184 7486.0654
332.1696 -35.7280 -124.3263 664265.7895 -5.3454
-5.3447
[58739]ENERGY: 14510000 3581.6267 5992.9859 5949.3265
375.5905 -15507.1037 -1834.8971 0.0000 0.0000
8856.6316 7414.1604 330.3947 -1442.4711 7486.9467
332.1229 155.8683 116.5468 664265.7895 -4.4943
-4.4825
[58775]ENERGY: 14520000 3652.2594 5852.4970 5936.1066
363.3110 -15446.5315 -1793.7853 0.0000 0.0000
8854.6567 7418.5138 330.3211 -1436.1429 7487.8771
332.2148 34.5715 14.3502 664265.7895 -6.9301
-6.9467
[58825]ENERGY: 14530000 3670.1131 5788.2864 5952.6867
370.5506 -15352.7759 -1902.7423 0.0000 0.0000
8891.4868 7417.6055 331.6950 -1473.8812 7488.7623
332.3321 -48.8184 -142.6854 664265.7895 -2.5278

-2.5198
[58861]ENERGY: 14540000 3615.1666 5817.6080 6003.4487
357.2778 -15495.0363 -1847.5834 0.0000 0.0000
8965.3100 7416.1916 334.4490 -1549.1184 7489.0390
332.2837 -58.3496 -35.0833 664265.7895 -2.4135
-2.4128
[58911]ENERGY: 14550000 3593.4419 5803.0716 5964.9959
371.6347 -15415.1389 -1890.0281 0.0000 0.0000
8986.0888 7414.0658 335.2241 -1572.0230 7488.2007
332.3697 87.7588 -1.1730 664265.7895 -1.7794
-1.7640
[58947]ENERGY: 14560000 3575.9792 5958.7393 5952.1943
383.8892 -15509.7212 -1840.1300 0.0000 0.0000
8894.3932 7415.3440 331.8034 -1479.0492 7488.1571
332.4633 -88.6291 -128.7898 664265.7895 -3.9900
-3.9951
[58997]ENERGY: 14570000 3574.0033 5885.2468 5941.1601
362.9325 -15362.9296 -1880.0032 0.0000 0.0000
8893.0963 7413.5062 331.7550 -1479.5901 7488.2120
332.3831 137.1353 63.6733 664265.7895 -3.0667
-3.0852
[59033]ENERGY: 14580000 3625.8368 5755.6355 5963.8194
350.8565 -15364.8565 -1833.4365 0.0000 0.0000
8917.0852 7414.9403 332.6499 -1502.1449 7488.2254
332.1893 -14.2947 -1.6332 664265.7895 -5.5319
-5.5060
[59083]ENERGY: 14590000 3623.7369 5924.6497 5931.9236
360.9673 -15392.5757 -1810.7836 0.0000 0.0000
8787.2637 7425.1819 327.8070 -1362.0818 7490.8893
332.4182 3.6754 -38.7575 664265.7895 -2.5110
-2.5091
[59122]ENERGY: 14600000 3563.7481 5872.6356 5904.8843
346.4157 -15425.7623 -1839.7367 0.0000 0.0000
8995.9777 7418.1624 335.5930 -1577.8153 7490.9702
332.5866 120.5344 10.3669 664265.7895 0.0370
0.0177
[59172]ENERGY: 14610000 3627.4973 5880.8166 5948.7782
337.5586 -15443.4946 -1883.6972 0.0000 0.0000
8951.0925 7418.5514 333.9186 -1532.5411 7489.0799
332.5947 -14.6241 3.7475 664265.7895 -1.7985
-1.8012
[59208]ENERGY: 14620000 3648.7296 5962.6710 5967.5959
370.6478 -15519.1437 -1911.0555 0.0000 0.0000
8898.8271 7418.2721 331.9688 -1480.5550 7491.2708
332.5800 -88.6830 -77.1643 664265.7895 -0.2109
-0.2040
[59258]ENERGY: 14630000 3608.4637 5960.1805 5914.5580
344.3536 -15410.1379 -1813.5802 0.0000 0.0000
8819.9153 7423.7530 329.0250 -1396.1623 7491.7571
332.4907 117.9995 47.2692 664265.7895 1.6588
1.6647
[59294]ENERGY: 14640000 3625.9162 5890.4532 5932.6023
376.6233 -15539.7547 -1790.9743 0.0000 0.0000
8922.9868 7417.8527 332.8701 -1505.1341 7490.4553

332.4764 -65.2923 -15.5578 664265.7895 -2.7032
-2.6935
[59344]ENERGY: 14650000 3652.7136 5899.0650 5881.1441
336.0292 -15472.3615 -1836.3147 0.0000 0.0000
8960.0032 7420.2789 334.2510 -1539.7243 7492.0641
332.5772 25.4897 19.9100 664265.7895 -0.3453
-0.3546
[59380]ENERGY: 14660000 3566.9444 5957.0621 5857.1488
363.4951 -15418.4487 -1801.2171 0.0000 0.0000
8893.2596 7418.2442 331.7611 -1475.0154 7491.4494
332.6769 -15.2736 -3.2490 664265.7895 -7.9772
-7.9951
[59430]ENERGY: 14670000 3599.6310 5827.3802 5927.5788
339.2783 -15360.3768 -1818.4094 0.0000 0.0000
8904.3707 7419.4529 332.1756 -1484.9178 7492.0562
332.5391 -35.6599 -94.0652 664265.7895 -3.6627
-3.6490
[59466]ENERGY: 14680000 3630.0146 5944.2550 5921.6600
375.0673 -15497.7956 -1872.3135 0.0000 0.0000
8919.1803 7420.0681 332.7281 -1499.1122 7492.3640
332.6462 29.6304 -62.1446 664265.7895 -4.3470
-4.3484
[59516]ENERGY: 14690000 3634.2550 5844.2771 5877.5833
362.6788 -15385.1027 -1839.4410 0.0000 0.0000
8926.3511 7420.6016 332.9956 -1505.7495 7492.6136
332.6427 211.0039 124.8456 664265.7895 -3.0184
-3.0332
[59555]ENERGY: 14700000 3640.2548 5835.1032 5893.8839
351.8339 -15471.7114 -1831.0660 0.0000 0.0000
9001.7955 7420.0939 335.8100 -1581.7016 7492.1518
332.5776 -106.7198 -67.0014 664265.7895 -3.3369
-3.3272
[59605]ENERGY: 14710000 3604.3174 5924.3227 5875.6778
354.1386 -15372.1034 -1874.9387 0.0000 0.0000
8912.2265 7423.6409 332.4687 -1488.5856 7493.0910
332.6181 1.7229 32.9771 664265.7895 -4.5854
-4.5803
[59641]ENERGY: 14720000 3604.5532 5912.4491 5950.0106
359.8858 -15507.5485 -1840.9237 0.0000 0.0000
8941.1735 7419.5999 333.5486 -1521.5736 7493.2926
332.7986 -23.1147 -104.9516 664265.7895 0.8215
0.8167
[59691]ENERGY: 14730000 3644.3551 5902.0703 5931.8638
392.7918 -15562.3468 -1806.6311 0.0000 0.0000
8915.1012 7417.2043 332.5759 -1497.8969 7493.1079
332.7041 -25.4233 18.5729 664265.7895 -4.9683
-4.9494
[59727]ENERGY: 14740000 3628.0801 5843.2338 5919.4700
365.0679 -15351.1648 -1934.3781 0.0000 0.0000
8952.7169 7423.0258 333.9792 -1529.6911 7494.6536
332.5957 34.6132 -50.8368 664265.7895 -6.9886
-7.0112
[59777]ENERGY: 14750000 3654.8610 5852.5115 5958.9419
339.6935 -15519.6257 -1801.7411 0.0000 0.0000

8936.1947 7420.8357 333.3628 -1515.3590 7494.0145
332.7260 102.1260 14.5091 664265.7895 -2.3770
-2.3853
[59813]ENERGY: 14760000 3601.7636 5872.1147 5914.0196
357.5844 -15385.4988 -1850.7593 0.0000 0.0000
8912.4108 7421.6350 332.4756 -1490.7758 7494.2272
332.7962 57.4680 47.2278 664265.7895 -1.7054
-1.6782
[59863]ENERGY: 14770000 3577.4344 5877.3151 5912.0313
359.0076 -15359.6598 -1907.6865 0.0000 0.0000
8964.7588 7423.2010 334.4284 -1541.5578 7495.9185
332.5239 71.7202 6.3106 664265.7895 -5.2346
-5.2449
[59899]ENERGY: 14780000 3610.4109 5861.4715 5913.9198
340.4489 -15390.9940 -1865.3268 0.0000 0.0000
8954.5800 7424.5103 334.0487 -1530.0697 7495.3708
332.5591 -116.1977 -68.1704 664265.7895 -7.5251
-7.5121
[59949]ENERGY: 14790000 3637.5769 5995.2295 5913.4205
344.2985 -15452.6237 -1877.2867 0.0000 0.0000
8865.0723 7425.6874 330.7096 -1439.3849 7496.5692
332.6405 57.2967 25.9371 664265.7895 -1.7200
-1.7307
[59988]ENERGY: 14800000 3633.3657 5882.9050 5918.6359
359.0484 -15399.4579 -1842.7988 0.0000 0.0000
8875.3473 7427.0456 331.0929 -1448.3017 7498.0807
332.4745 105.9246 15.9859 664265.7895 -9.1073
-9.1234
[60038]ENERGY: 14810000 3664.5908 5871.7164 5945.1379
348.4265 -15549.5443 -1792.8963 0.0000 0.0000
8938.9501 7426.3811 333.4656 -1512.5690 7496.7346
332.8208 76.3374 56.1120 664265.7895 -5.7474
-5.7226
[60074]ENERGY: 14820000 3599.8279 5951.3137 5918.3513
361.3354 -15485.9546 -1908.8652 0.0000 0.0000
8987.4337 7423.4422 335.2743 -1563.9914 7497.0097
332.9148 36.9631 35.4775 664265.7895 -6.7683
-6.7795
[60124]ENERGY: 14830000 3627.1968 5876.4189 5943.7009
362.5915 -15457.8565 -1820.1466 0.0000 0.0000
8895.9671 7427.8721 331.8621 -1468.0950 7497.3790
332.8754 -74.7058 -13.2280 664265.7895 -4.9868
-4.9864
[60160]ENERGY: 14840000 3656.5210 5935.9603 5875.4322
343.0046 -15479.2157 -1846.5408 0.0000 0.0000
8939.4817 7424.6433 333.4854 -1514.8384 7498.2476
332.8692 69.7381 -0.5253 664265.7895 -8.1440
-8.1579
[60210]ENERGY: 14850000 3631.1100 6009.1267 5905.9570
367.2719 -15563.3982 -1883.8516 0.0000 0.0000
8956.3786 7422.5945 334.1158 -1533.7841 7498.5500
332.7649 -54.4422 -28.2332 664265.7895 -7.7982
-7.7686
[60246]ENERGY: 14860000 3709.9472 5916.8646 5927.8957

383.2021 -15547.0179 -1867.5223 0.0000 0.0000
8901.6968 7425.0663 332.0759 -1476.6305 7498.6184
332.8865 60.7489 79.5289 664265.7895 -5.7442
-5.7594
[60296]ENERGY: 14870000 3647.9911 5921.7373 5960.1783
372.5135 -15430.9227 -1831.2326 0.0000 0.0000
8791.0109 7431.2758 327.9468 -1359.7352 7499.4672
332.6123 279.3686 93.4325 664265.7895 -8.1985
-8.2205
[60332]ENERGY: 14880000 3675.6731 5855.5907 5973.2693
350.8205 -15477.9210 -1861.8275 0.0000 0.0000
8912.1500 7427.7551 332.4658 -1484.3949 7497.5793
332.6086 29.8163 -37.8754 664265.7895 -3.2766
-3.2508
[60382]ENERGY: 14890000 3661.6292 5926.7867 5914.6688
353.9174 -15412.2862 -1868.3161 0.0000 0.0000
8850.2032 7426.6029 330.1549 -1423.6003 7496.3180
332.8132 6.1394 20.2411 664265.7895 -9.2841
-9.2763
[60421]ENERGY: 14900000 3631.7793 5984.6174 5948.0197
358.8306 -15450.3552 -1857.2580 0.0000 0.0000
8813.0347 7428.6685 328.7684 -1384.3662 7498.8659
332.7051 -42.6556 1.8832 664265.7895 -6.4220
-6.4227
[60471]ENERGY: 14910000 3685.7583 5857.1957 5930.6108
347.3923 -15433.7123 -1845.3062 0.0000 0.0000
8883.5748 7425.5135 331.3998 -1458.0614 7497.5918
332.4886 66.5124 2.6873 664265.7895 -10.3190
-10.3197
[60507]ENERGY: 14920000 3706.4122 5952.0230 5903.4309
352.7657 -15426.6394 -1924.7636 0.0000 0.0000
8860.7091 7423.9379 330.5468 -1436.7712 7498.1939
332.3818 -218.3378 -142.1077 664265.7895 -8.3053
-8.3179
[60557]ENERGY: 14930000 3657.8370 5916.1116 5931.6975
374.0043 -15526.2039 -1790.8875 0.0000 0.0000
8863.7969 7426.3558 330.6620 -1437.4410 7498.6556
332.4278 22.1784 134.2302 664265.7895 -7.3847
-7.3847
[60593]ENERGY: 14940000 3658.1783 5842.6363 5922.0795
344.4695 -15431.2796 -1828.1877 0.0000 0.0000
8920.2706 7428.1669 332.7688 -1492.1037 7500.5262
332.7337 93.2734 54.7151 664265.7895 -6.7325
-6.7319
[60643]ENERGY: 14950000 3579.8088 5764.7759 5973.3106
345.6111 -15361.9071 -1758.6109 0.0000 0.0000
8888.7746 7431.7629 331.5938 -1457.0117 7500.8266
332.6612 108.6082 39.3496 664265.7895 -7.6120
-7.6182
[60679]ENERGY: 14960000 3707.8390 5923.8559 5961.0392
356.5699 -15431.3820 -1891.6869 0.0000 0.0000
8806.6054 7432.8405 328.5285 -1373.7649 7503.3665
332.8801 -188.2572 -200.5972 664265.7895 -7.3415
-7.3333

[60729]ENERGY: 14970000 3653.3089 5877.1048 5909.2002
337.0294 -15527.9385 -1806.9321 0.0000 0.0000
8990.1962 7431.9690 335.3773 -1558.2272 7503.2244
332.9243 56.8791 62.3913 664265.7895 -0.5996
-0.6051
[60765]ENERGY: 14980000 3599.2865 5864.1644 5910.1379
383.0584 -15433.5443 -1846.0779 0.0000 0.0000
8956.9813 7434.0063 334.1383 -1522.9750 7503.5297
332.9528 126.2506 35.4428 664265.7895 -4.6612
-4.6588
[60815]ENERGY: 14990000 3703.9875 5846.5948 5923.6897
382.9098 -15546.4637 -1797.2500 0.0000 0.0000
8916.2895 7429.7576 332.6203 -1486.5319 7502.1799
332.8429 228.3451 83.7771 664265.7895 -8.9405
-8.9231
[60854]ENERGY: 15000000 3579.3094 5826.6884 5898.5987
344.8933 -15388.9867 -1825.6618 0.0000 0.0000
8993.2782 7428.1194 335.4923 -1565.1588 7503.9689
332.9609 123.8898 17.0744 664265.7895 1.4008
1.3843
[60904]ENERGY: 15010000 3665.3753 5905.5143 5905.4174
361.3185 -15535.4593 -1823.1984 0.0000 0.0000
8955.1957 7434.1635 334.0716 -1521.0322 7503.4154
332.8802 -26.0312 -82.6003 664265.7895 -2.9930
-2.9809
[60940]ENERGY: 15020000 3625.0751 5844.4902 5892.4715
345.1185 -15399.2124 -1853.4528 0.0000 0.0000
8975.4653 7429.9555 334.8278 -1545.5099 7505.9471
332.7641 90.3407 54.5240 664265.7895 -2.3700
-2.3929
[60990]ENERGY: 15030000 3638.9971 5924.4745 5886.7043
395.8191 -15385.3543 -1818.0108 0.0000 0.0000
8794.1063 7436.7361 328.0622 -1357.3702 7505.5270
332.6273 16.6486 36.6710 664265.7895 0.2304
0.2483
[61026]ENERGY: 15040000 3628.4173 5827.1680 5887.3797
342.3372 -15407.4496 -1810.7610 0.0000 0.0000
8964.4519 7431.5435 334.4169 -1532.9084 7505.6666
332.5348 -33.9350 13.7869 664265.7895 -8.5727
-8.5799
[61076]ENERGY: 15050000 3672.7952 5853.5125 5905.7352
353.6609 -15380.8224 -1883.2836 0.0000 0.0000
8910.6670 7432.2649 332.4105 -1478.4022 7504.9942
332.7253 -110.9481 -130.6888 664265.7895 -1.9656
-1.9744
[61112]ENERGY: 15060000 3628.4397 5998.7340 5898.7613
360.8084 -15429.7298 -1801.0368 0.0000 0.0000
8785.1905 7441.1673 327.7296 -1344.0233 7507.2730
332.7345 15.3538 66.8162 664265.7895 -4.8177
-4.7923
[61162]ENERGY: 15070000 3653.2489 5869.4294 5898.3330
362.9002 -15462.7694 -1892.1922 0.0000 0.0000
9003.7021 7432.6521 335.8812 -1571.0500 7506.9270
332.8117 40.5290 -7.4400 664265.7895 -5.6596

-5.6741
[61198]ENERGY: 15080000 3609.1090 5929.2592 5895.2676
350.2768 -15378.8953 -1914.2545 0.0000 0.0000
8941.1189 7431.8817 333.5465 -1509.2372 7507.7532
332.8567 -59.9946 -52.4584 664265.7895 -10.8466
-10.8437
[61248]ENERGY: 15090000 3640.2960 5879.8121 5930.8512
364.5932 -15514.5771 -1833.5852 0.0000 0.0000
8967.6088 7434.9990 334.5347 -1532.6098 7505.8082
332.6451 65.3640 75.5855 664265.7895 -8.1313
-8.1311
[61287]ENERGY: 15100000 3551.1906 5931.1006 5941.0403
356.9475 -15394.9613 -1833.0677 0.0000 0.0000
8883.0017 7435.2517 331.3785 -1447.7500 7505.4751
332.7576 280.8260 269.4109 664265.7895 -9.3423
-9.3343
[61337]ENERGY: 15110000 3731.5972 5814.7046 5953.6711
362.4280 -15423.4020 -1961.9561 0.0000 0.0000
8957.0885 7434.1312 334.1423 -1522.9573 7506.6590
332.6985 -239.0608 -193.6685 664265.7895 -3.6764
-3.6979
[61373]ENERGY: 15120000 3681.7122 5897.3775 5919.8385
357.7177 -15479.1792 -1851.2805 0.0000 0.0000
8912.4163 7438.6025 332.4758 -1473.8138 7508.1167
332.7131 -64.5693 -64.3407 664265.7895 -5.4481
-5.4381
[61423]ENERGY: 15130000 3714.0078 5847.4099 5934.9544
371.2214 -15496.6894 -1859.7679 0.0000 0.0000
8925.1845 7436.3208 332.9521 -1488.8637 7508.1622
332.7733 123.9662 19.6818 664265.7895 -5.1837
-5.1917
[61459]ENERGY: 15140000 3727.8242 5983.2776 5882.8660
376.6234 -15535.6764 -1833.4409 0.0000 0.0000
8837.2062 7438.6801 329.6701 -1398.5261 7508.6125
332.9151 -235.7597 -243.6726 664265.7895 -7.6325
-7.6366
[61509]ENERGY: 15150000 3671.4202 5859.0866 5956.2158
376.7650 -15467.7434 -1906.1389 0.0000 0.0000
8945.6811 7435.2864 333.7167 -1510.3947 7508.2447
333.0725 -200.7712 -107.2320 664265.7895 -5.2998
-5.3066
[61545]ENERGY: 15160000 3642.2563 5945.7703 5966.5320
370.4030 -15433.3260 -1927.3907 0.0000 0.0000
8871.9768 7436.2216 330.9672 -1435.7552 7509.5935
332.7588 -84.0335 -133.9969 664265.7895 -5.5286
-5.5120
[61595]ENERGY: 15170000 3640.9993 5915.0853 5952.1545
350.5507 -15459.8030 -1882.4140 0.0000 0.0000
8916.4552 7433.0281 332.6264 -1483.4271 7509.2865
333.0176 58.6823 34.3089 664265.7895 -4.9187
-4.8880
[61631]ENERGY: 15180000 3728.6729 5962.0760 5870.7079
360.4781 -15576.8671 -1851.3374 0.0000 0.0000
8942.6901 7436.4206 333.6051 -1506.2695 7510.1869

333.0281 22.8782 -23.2376 664265.7895 -0.1648
-0.1800
[61681]ENERGY: 15190000 3617.8183 5839.1662 5850.4720
370.4108 -15443.5886 -1815.9162 0.0000 0.0000
9020.9255 7439.2881 336.5237 -1581.6374 7510.3696
333.0514 79.9076 51.3157 664265.7895 -4.7174
-4.7408
[61720]ENERGY: 15200000 3644.4772 5821.4593 5935.8251
353.7824 -15438.8086 -1799.9262 0.0000 0.0000
8921.9589 7438.7680 332.8318 -1483.1909 7510.2712
333.0346 70.5489 44.9931 664265.7895 -3.3895
-3.3844
[61770]ENERGY: 15210000 3588.1344 5793.2432 5946.6006
337.6076 -15423.2262 -1843.7270 0.0000 0.0000
9038.9446 7437.5773 337.1959 -1601.3673 7510.5249
332.8254 -47.1161 -36.7262 664265.7895 -2.2968
-2.2883
[61806]ENERGY: 15220000 3578.5461 5957.5811 5959.9859
347.1458 -15494.0513 -1872.4179 0.0000 0.0000
8960.3962 7437.1859 334.2657 -1523.2103 7510.1758
333.0191 269.7543 128.9136 664265.7895 -1.2756
-1.2718
[61856]ENERGY: 15230000 3736.1078 5814.0889 5954.1751
369.4325 -15500.3215 -1856.9886 0.0000 0.0000
8920.1193 7436.6136 332.7631 -1483.5057 7509.5376
333.0425 -21.3732 -47.2001 664265.7895 -3.8917
-3.8979
[61892]ENERGY: 15240000 3602.1232 5964.6139 5936.3896
368.5604 -15514.9824 -1854.6259 0.0000 0.0000
8935.1208 7437.1995 333.3228 -1497.9213 7509.0654
332.9971 87.1982 39.9763 664265.7895 -4.0471
-4.0586
[61942]ENERGY: 15250000 3703.1889 5848.0191 5894.0660
367.2688 -15422.7751 -1815.0801 0.0000 0.0000
8866.7410 7441.4287 330.7719 -1425.3123 7510.7358
333.2108 -13.2736 -36.2154 664265.7895 -4.5005
-4.4673
[61978]ENERGY: 15260000 3666.0237 5903.7703 5921.2867
368.8242 -15497.6428 -1914.3830 0.0000 0.0000
8987.6239 7435.5031 335.2814 -1552.1208 7510.1904
333.2460 -174.8391 -129.7677 664265.7895 -3.5392
-3.5593
[62028]ENERGY: 15270000 3657.2912 5932.5311 5937.4647
359.3681 -15422.7816 -1936.4195 0.0000 0.0000
8909.6811 7437.1351 332.3737 -1472.5460 7510.5254
333.1819 -39.9908 -50.3708 664265.7895 -3.6561
-3.6593
[62064]ENERGY: 15280000 3612.0798 5961.4344 5967.3088
350.4363 -15527.5053 -1849.1056 0.0000 0.0000
8928.5293 7443.1778 333.0769 -1485.3515 7511.0139
333.2064 161.1685 59.5544 664265.7895 -3.9260
-3.9358
[62114]ENERGY: 15290000 3622.7362 5870.4943 5954.3526
339.0913 -15421.6510 -1893.6374 0.0000 0.0000

8967.2420 7438.6279 334.5210 -1528.6141 7513.6006
333.1721 -71.7708 -121.3850 664265.7895 -0.0482
-0.0266
[62153]ENERGY: 15300000 3643.9506 5919.4264 5954.1178
385.8401 -15388.5666 -1970.5992 0.0000 0.0000
8896.1524 7440.3215 331.8690 -1455.8309 7513.3909
332.7940 -224.0934 -206.6769 664265.7895 -8.3264
-8.3352
[62203]ENERGY: 15310000 3610.8891 5765.1740 6005.4172
372.3471 -15359.4692 -1928.8169 0.0000 0.0000
8968.4821 7434.0233 334.5673 -1534.4588 7511.1897
332.6685 -194.7453 -105.8080 664265.7895 -8.9889
-8.9903
[62239]ENERGY: 15320000 3638.0302 5850.2944 5976.8866
366.1704 -15443.5907 -1885.6997 0.0000 0.0000
8940.3103 7442.4014 333.5163 -1497.9089 7511.6652
332.6985 -130.6132 -109.4648 664265.7895 -5.8576
-5.8696
[62289]ENERGY: 15330000 3622.9163 5949.1564 5927.7640
389.7353 -15497.0878 -1808.8935 0.0000 0.0000
8859.4993 7443.0899 330.5017 -1416.4094 7512.0108
332.8302 -97.7723 -94.4341 664265.7895 -9.1328
-9.1254
[62325]ENERGY: 15340000 3648.7415 5949.8183 5967.3983
360.2351 -15522.4683 -1898.3177 0.0000 0.0000
8931.9127 7437.3199 333.2031 -1494.5928 7511.3398
333.0518 206.6529 148.9852 664265.7895 -10.0877
-10.0894
[62375]ENERGY: 15350000 3705.6463 5801.5516 5937.4173
359.4178 -15368.6708 -1911.0506 0.0000 0.0000
8916.6169 7440.9285 332.6325 -1475.6884 7510.5364
332.8922 2.3315 -76.1875 664265.7895 -8.0189
-8.0201
[62411]ENERGY: 15360000 3711.2579 5767.8115 5914.9326
353.3671 -15466.2767 -1796.6649 0.0000 0.0000
8953.4790 7437.9065 334.0076 -1515.5725 7511.5823
332.7085 -13.3442 -47.7573 664265.7895 -8.0803
-8.0675
[62461]ENERGY: 15370000 3580.7730 5949.9589 5907.3620
374.7255 -15372.8158 -1920.1982 0.0000 0.0000
8917.8805 7437.6859 332.6796 -1480.1946 7511.8112
332.8921 194.1353 79.7179 664265.7895 -10.5693
-10.6029
[62497]ENERGY: 15380000 3649.4060 5925.5797 5943.7295
372.8722 -15390.9191 -1913.1065 0.0000 0.0000
8848.1676 7435.7294 330.0790 -1412.4382 7510.9935
332.9847 8.9658 -32.3343 664265.7895 -10.9115
-10.8921
[62547]ENERGY: 15390000 3583.5313 5836.0638 5894.0109
348.7040 -15358.8467 -1846.2103 0.0000 0.0000
8981.1166 7438.3697 335.0386 -1542.7469 7511.0890
332.9050 47.6878 6.5296 664265.7895 -12.0488
-12.0407
[62586]ENERGY: 15400000 3637.4476 5971.7885 5936.7481

365.6173 -15435.0001 -1961.8514 0.0000 0.0000
8925.8717 7440.6216 332.9777 -1485.2501 7511.5026
332.8652 27.5688 -60.6284 664265.7895 -8.9552
-8.9443
[62636]ENERGY: 15410000 3647.5067 5871.6841 5981.2603
372.8129 -15568.8442 -1846.9335 0.0000 0.0000
8979.9841 7437.4704 334.9964 -1542.5137 7510.2932
332.9719 -97.4297 -23.3425 664265.7895 -6.1321
-6.1268
[62672]ENERGY: 15420000 3605.7090 5889.4316 5901.5264
367.2752 -15421.0031 -1876.9989 0.0000 0.0000
8972.9190 7438.8592 334.7328 -1534.0597 7511.1948
332.9939 -44.3406 -67.3029 664265.7895 -7.8859
-7.9003
[62722]ENERGY: 15430000 3650.2359 5917.3167 5899.9013
333.2325 -15417.2091 -1830.4064 0.0000 0.0000
8887.5668 7440.6377 331.5488 -1446.9291 7511.0932
333.0190 220.7487 143.8536 664265.7895 -7.7598
-7.7588
[62758]ENERGY: 15440000 3593.8846 5908.7963 5897.9523
363.2696 -15334.9472 -1822.2039 0.0000 0.0000
8835.3367 7442.0883 329.6003 -1393.2484 7510.5488
332.7809 -43.7760 36.5376 664265.7895 -7.4602
-7.4455
[62808]ENERGY: 15450000 3556.6473 5809.3319 5948.2605
376.4349 -15466.6659 -1839.9568 0.0000 0.0000
9053.6537 7437.7055 337.7446 -1615.9482 7512.0702
332.7033 43.7323 -44.3706 664265.7895 -12.0501
-12.0602
[62844]ENERGY: 15460000 3660.9649 5951.3507 5931.0106
380.1896 -15490.7543 -1899.5064 0.0000 0.0000
8908.3250 7441.5801 332.3231 -1466.7449 7512.3052
332.9424 71.7186 -19.3258 664265.7895 -13.3691
-13.3638
[62894]ENERGY: 15470000 3693.9789 5740.2033 5959.4745
363.9530 -15425.0368 -1866.6336 0.0000 0.0000
8974.0992 7440.0384 334.7768 -1534.0608 7512.2972
333.0441 22.8454 57.5977 664265.7895 -9.2323
-9.2258
[62930]ENERGY: 15480000 3621.2742 5917.0144 5903.2945
382.7607 -15473.7708 -1747.9689 0.0000 0.0000
8841.2323 7443.8363 329.8203 -1397.3960 7512.4983
333.0376 4.9074 -2.3758 664265.7895 -9.6967
-9.7154
[62980]ENERGY: 15490000 3628.8409 5923.0305 5946.6354
346.5010 -15448.1531 -1878.9477 0.0000 0.0000
8922.4468 7440.3538 332.8500 -1482.0930 7513.3556
333.0289 25.6213 50.5152 664265.7895 -6.6570
-6.6466
[63019]ENERGY: 15500000 3666.8998 5867.2240 5887.6010
351.8047 -15491.7325 -1823.3540 0.0000 0.0000
8981.8961 7440.3391 335.0677 -1541.5571 7513.0543
332.9847 0.7330 37.3484 664265.7895 -7.1050
-7.1080

[63069]ENERGY: 15510000 3679.9231 5833.6998 5935.7491
368.4954 -15358.0974 -1983.4226 0.0000 0.0000
8962.5729 7438.9201 334.3468 -1523.6527 7512.8458
333.2681 -235.9473 -225.4458 664265.7895 -6.7050
-6.7049
[63105]ENERGY: 15520000 3629.7794 5839.7871 5965.4298
367.8300 -15468.9822 -1815.2625 0.0000 0.0000
8926.8806 7445.4623 333.0154 -1481.4183 7514.8210
333.0646 143.4530 120.3721 664265.7895 -7.7590
-7.7673
[63155]ENERGY: 15530000 3634.6468 5893.0712 5942.1667
355.9855 -15496.8010 -1893.0944 0.0000 0.0000
9003.2359 7439.2108 335.8638 -1564.0252 7514.4239
333.1245 147.2982 77.1738 664265.7895 -4.2328
-4.2121
[63191]ENERGY: 15540000 3669.9581 5904.8830 5926.4283
364.1926 -15505.2519 -1827.6636 0.0000 0.0000
8912.2567 7444.8031 332.4698 -1467.4536 7514.3489
333.0443 -7.3435 1.7662 664265.7895 -7.0407
-7.0560
[63241]ENERGY: 15550000 3599.6060 5900.5438 5962.9171
358.1340 -15374.3034 -1887.2381 0.0000 0.0000
8887.5597 7447.2191 331.5485 -1440.3406 7515.5211
332.8460 -13.4173 -30.5835 664265.7895 -10.9526
-10.9402
[63277]ENERGY: 15560000 3660.5021 5880.6871 5950.2474
364.0011 -15418.7213 -1887.6365 0.0000 0.0000
8898.1582 7447.2380 331.9439 -1450.9202 7516.8802
332.9794 -64.4739 -65.0724 664265.7895 -4.6507
-4.6772
[63327]ENERGY: 15570000 3630.7549 6007.9696 5926.3695
353.8541 -15527.9315 -1842.0360 0.0000 0.0000
8899.5079 7448.4885 331.9942 -1451.0194 7517.2955
333.0631 244.2767 131.3167 664265.7895 -2.9201
-2.9343
[63363]ENERGY: 15580000 3693.4091 5872.6065 5932.2387
354.9217 -15430.7948 -1872.8025 0.0000 0.0000
8900.2622 7449.8410 332.0224 -1450.4211 7516.8164
332.9813 57.7922 26.3131 664265.7895 -5.0267
-5.0204
[63413]ENERGY: 15590000 3653.6087 5890.3336 5895.2481
354.1571 -15419.9748 -1811.2081 0.0000 0.0000
8883.2268 7445.3915 331.3869 -1437.8354 7517.7528
332.7497 155.9857 130.8498 664265.7895 -6.5035
-6.4843
[63452]ENERGY: 15600000 3617.8980 5923.2969 5895.9774
366.7753 -15455.5585 -1875.7074 0.0000 0.0000
8970.1747 7442.8565 334.6304 -1527.3182 7516.1426
332.9373 -126.2962 -141.8031 664265.7895 -5.5078
-5.5333
[63502]ENERGY: 15610000 3562.3626 5938.7000 5985.8826
381.1757 -15564.2404 -1794.4369 0.0000 0.0000
8933.0754 7442.5190 333.2465 -1490.5563 7515.7788
332.8756 81.4851 51.0855 664265.7895 -3.3967

-3.3765
[63538]ENERGY: 15620000 3693.3654 5760.5145 5878.1213
353.1095 -15415.0938 -1863.0260 0.0000 0.0000
9037.1048 7444.0957 337.1272 -1593.0091 7518.0532
332.8918 30.1803 56.2442 664265.7895 -5.3992
-5.3860
[63588]ENERGY: 15630000 3582.0096 5837.4454 5907.3600
364.4975 -15351.9874 -1867.2747 0.0000 0.0000
8973.9858 7446.0362 334.7726 -1527.9496 7518.4726
332.7061 -12.3516 -58.8957 664265.7895 -6.5926
-6.6136
[63624]ENERGY: 15640000 3637.8145 5869.2817 5983.7419
356.6649 -15480.1652 -1853.3642 0.0000 0.0000
8931.6401 7445.6137 333.1929 -1486.0264 7519.4088
332.9143 44.2056 101.7422 664265.7895 -5.6699
-5.6550
[63674]ENERGY: 15650000 3712.8472 5917.8392 5876.1376
350.2805 -15469.5075 -1916.7114 0.0000 0.0000
8974.9698 7445.8555 334.8093 -1529.1142 7518.8462
332.8552 -76.2375 -1.4368 664265.7895 -7.1610
-7.1631
[63710]ENERGY: 15660000 3645.3857 5940.4181 5908.4897
373.2785 -15448.9985 -1840.3125 0.0000 0.0000
8870.6051 7448.8662 330.9160 -1421.7388 7520.2044
332.8717 -91.3323 -167.9048 664265.7895 -3.6781
-3.6886
[63760]ENERGY: 15670000 3728.1424 5799.6118 5930.3176
334.8939 -15383.8416 -1833.8859 0.0000 0.0000
8873.9116 7449.1498 331.0394 -1424.7618 7519.8358
332.6561 -150.9629 -162.7995 664265.7895 -0.2917
-0.2784
[63796]ENERGY: 15680000 3599.5108 5912.5888 5825.5234
348.1373 -15387.6036 -1936.9168 0.0000 0.0000
9085.2063 7446.4462 338.9217 -1638.7601 7520.3196
332.6682 -33.2376 -58.9647 664265.7895 -6.1238
-6.1348
[63846]ENERGY: 15690000 3627.1649 5743.8460 5935.0061
358.5029 -15484.1254 -1764.1905 0.0000 0.0000
9027.3243 7443.5283 336.7624 -1583.7960 7519.4320
332.5011 178.3470 116.2660 664265.7895 -3.5739
-3.5710
[63885]ENERGY: 15700000 3649.1261 5919.8434 5876.5930
367.2613 -15458.3231 -1773.0912 0.0000 0.0000
8864.8588 7446.2683 330.7016 -1418.5905 7519.6611
332.6342 103.2678 46.0631 664265.7895 -8.5648
-8.5554
[63935]ENERGY: 15710000 3586.1954 5877.4642 5903.5264
380.6380 -15385.3621 -1915.1489 0.0000 0.0000
9000.0953 7447.4084 335.7466 -1552.6869 7520.9054
332.8439 92.5215 33.2523 664265.7895 -5.0887
-5.1113
[63971]ENERGY: 15720000 3572.1528 6025.6334 5896.3147
366.2646 -15462.8748 -1842.4766 0.0000 0.0000
8893.9000 7448.9141 331.7850 -1444.9859 7519.3396

333.0000 -44.5240 -24.1636 664265.7895 -8.5919
-8.5690
[64021]ENERGY: 15730000 3698.0913 5794.4517 5977.7251
348.5598 -15467.3815 -1802.3237 0.0000 0.0000
8899.4814 7448.6042 331.9932 -1450.8772 7519.5402
332.9490 -129.8097 -42.0717 664265.7895 -4.2632
-4.2528
[64057]ENERGY: 15740000 3713.5032 5854.1828 5935.7900
374.0900 -15484.8662 -1815.3530 0.0000 0.0000
8875.4602 7452.8070 331.0971 -1422.6532 7519.7224
333.0909 -80.4266 -166.8890 664265.7895 -6.4489
-6.4728
[64107]ENERGY: 15750000 3604.0915 5890.2730 5932.1947
362.7615 -15381.3970 -1889.7763 0.0000 0.0000
8929.9840 7448.1314 333.1311 -1481.8526 7519.9713
333.1639 28.5057 -4.1746 664265.7895 -6.4904
-6.4839
[64143]ENERGY: 15760000 3664.6845 5887.1637 5954.3125
347.8200 -15459.3929 -1832.0076 0.0000 0.0000
8886.0433 7448.6234 331.4919 -1437.4198 7519.3407
333.0585 -23.5150 -22.6742 664265.7895 -3.4199
-3.4076
[64193]ENERGY: 15770000 3634.4046 5906.4804 5950.0365
347.7273 -15381.4472 -1864.1581 0.0000 0.0000
8857.0079 7450.0514 330.4088 -1406.9565 7520.5245
333.2548 -89.9362 -71.0519 664265.7895 -7.1162
-7.1205
[64229]ENERGY: 15780000 3599.3141 5940.2668 5977.1693
346.3429 -15446.2842 -1883.6087 0.0000 0.0000
8914.0338 7447.2340 332.5361 -1466.7998 7520.2118
333.2574 10.8233 124.3234 664265.7895 -6.5073
-6.5083
[64279]ENERGY: 15790000 3660.1721 5932.1815 5960.6099
374.8318 -15477.4790 -1838.1937 0.0000 0.0000
8837.0184 7449.1410 329.6631 -1387.8774 7519.8462
333.3905 -121.2841 -65.1886 664265.7895 -5.2780
-5.2694
[64318]ENERGY: 15800000 3564.3325 5917.6846 5985.3443
371.8477 -15389.3285 -1924.0684 0.0000 0.0000
8920.5229 7446.3350 332.7782 -1474.1878 7521.3393
333.1218 -54.3730 -62.4441 664265.7895 -3.5875
-3.6018
[64368]ENERGY: 15810000 3645.5511 5946.2866 5954.2973
363.0974 -15448.9589 -1904.3304 0.0000 0.0000
8893.9295 7449.8726 331.7861 -1444.0569 7520.4120
333.0675 110.9785 85.4082 664265.7895 -3.9953
-3.9935
[64404]ENERGY: 15820000 3661.2871 5994.2288 5874.7468
382.4247 -15407.8356 -1855.7552 0.0000 0.0000
8803.0835 7452.1800 328.3971 -1350.9034 7521.2393
333.1437 -60.1367 -29.3940 664265.7895 -2.0273
-2.0302
[64454]ENERGY: 15830000 3590.5576 5879.9737 5929.1146
365.3373 -15415.4597 -1885.6604 0.0000 0.0000

8984.0954 7447.9586 335.1497 -1536.1368 7519.8512
333.1529 -144.7217 -49.0589 664265.7895 -6.8108
-6.7916
[64490]ENERGY: 15840000 3660.6783 5842.6709 5928.2073
350.6279 -15507.7002 -1822.1528 0.0000 0.0000
8995.9164 7448.2479 335.5907 -1547.6685 7521.9482
333.1837 35.8768 -49.9185 664265.7895 -4.5874
-4.6196
[64540]ENERGY: 15850000 3698.5421 5931.6414 5919.4169
368.4598 -15505.4164 -1825.8163 0.0000 0.0000
8865.5094 7452.3368 330.7259 -1413.1725 7521.6871
332.9401 -9.3284 -24.4751 664265.7895 -6.7202
-6.7102
[64576]ENERGY: 15860000 3574.8563 5887.9288 5894.4754
369.9839 -15455.3519 -1805.3563 0.0000 0.0000
8982.9418 7449.4780 335.1067 -1533.4638 7521.7479
333.0470 16.4702 -28.5947 664265.7895 -5.5691
-5.5711
[64626]ENERGY: 15870000 3657.5990 5824.3244 5985.5087
359.4413 -15401.7705 -1887.2011 0.0000 0.0000
8912.6036 7450.5054 332.4828 -1462.0982 7521.6295
332.9169 64.2917 -23.1492 664265.7895 -9.4217
-9.4165
[64662]ENERGY: 15880000 3678.3197 5837.0460 5947.3495
364.2363 -15468.5862 -1818.8495 0.0000 0.0000
8909.1307 7448.6464 332.3532 -1460.4843 7521.8642
332.8330 50.3786 -10.2500 664265.7895 -7.6310
-7.6345
[64712]ENERGY: 15890000 3655.7551 6031.8373 5904.0964
357.0183 -15407.2046 -1908.4651 0.0000 0.0000
8820.1198 7453.1572 329.0327 -1366.9626 7522.7677
332.8140 79.2979 -12.1261 664265.7895 -5.5904
-5.5936
[64751]ENERGY: 15900000 3564.5792 5921.4084 6003.5943
370.9568 -15487.0559 -1855.9461 0.0000 0.0000
8934.2967 7451.8335 333.2920 -1482.4632 7523.8747
332.8147 -8.8147 40.4657 664265.7895 -6.2972
-6.2712
[64801]ENERGY: 15910000 3576.5322 5900.6289 5962.8565
363.3823 -15390.8215 -1894.7723 0.0000 0.0000
8933.0744 7450.8805 333.2464 -1482.1939 7523.8977
333.0776 111.1002 74.0687 664265.7895 -2.6612
-2.6863
[64837]ENERGY: 15920000 3614.1742 5820.3977 5913.6919
364.2631 -15375.5236 -1887.2732 0.0000 0.0000
8999.4732 7449.2032 335.7234 -1550.2700 7524.7833
333.2117 -88.6005 -49.2232 664265.7895 -6.0121
-6.0089
[64887]ENERGY: 15930000 3775.7032 5888.0436 5938.1765
370.1027 -15568.7283 -1824.2456 0.0000 0.0000
8875.7465 7454.7986 331.1078 -1420.9479 7525.4805
333.1455 43.9309 58.7251 664265.7895 -9.6042
-9.5898
[64923]ENERGY: 15940000 3582.3410 5902.7343 5950.2543

384.8389 -15387.1551 -1896.6357 0.0000 0.0000
8919.1238 7455.5015 332.7260 -1463.6223 7524.8507
333.2341 207.2201 61.6280 664265.7895 -3.7805
-3.7933
[64973]ENERGY: 15950000 3595.7121 5899.8059 5952.1807
366.1887 -15352.3283 -1902.6613 0.0000 0.0000
8895.3906 7454.2885 331.8406 -1441.1021 7524.2914
333.2039 52.6098 1.7320 664265.7895 -6.6541
-6.6596
[65009]ENERGY: 15960000 3623.4606 5820.6754 5947.4504
368.3391 -15493.4694 -1781.3793 0.0000 0.0000
8968.3817 7453.4583 334.5635 -1514.9233 7525.2013
333.2761 151.1415 97.5608 664265.7895 -4.7580
-4.7469
[65059]ENERGY: 15970000 3582.5819 5848.4115 5951.5989
360.1150 -15339.5949 -1913.9341 0.0000 0.0000
8963.7531 7452.9314 334.3909 -1510.8217 7526.4802
333.3625 31.5163 -22.7075 664265.7895 -7.5168
-7.5238
[65095]ENERGY: 15980000 3646.8313 5844.5252 5970.6754
371.3356 -15427.4861 -1878.7066 0.0000 0.0000
8927.3066 7454.4815 333.0313 -1472.8251 7524.9489
332.9164 76.2199 14.4356 664265.7895 -7.2811
-7.2722
[65145]ENERGY: 15990000 3583.6021 5869.8391 5958.5464
361.5673 -15302.8759 -1856.5885 0.0000 0.0000
8838.5167 7452.6072 329.7190 -1385.9095 7524.9724
332.9958 11.8001 -81.5972 664265.7895 -4.0045
-4.0073
[65184]ENERGY: 16000000 3639.9152 5933.1749 5935.5676
383.1646 -15366.9581 -1953.0877 0.0000 0.0000
8880.3636 7452.1399 331.2801 -1428.2236 7524.4069
333.1156 26.2003 62.6937 664265.7895 -6.8910
-6.8819
[65234]ENERGY: 16010000 3639.1169 5910.9713 5963.5354
370.8786 -15443.3463 -1925.3145 0.0000 0.0000
8938.2829 7454.1242 333.4407 -1484.1587 7525.7391
333.3839 -44.4023 -111.2903 664265.7895 -5.0642
-5.0745
[65270]ENERGY: 16020000 3623.2451 5848.3939 5872.3282
361.7488 -15350.4843 -1867.9382 0.0000 0.0000
8962.9073 7450.2008 334.3593 -1512.7065 7526.0120
333.0329 108.6205 51.6093 664265.7895 -7.9618
-7.9723
[65320]ENERGY: 16030000 3637.8033 5897.1947 5965.7818
361.4294 -15471.2846 -1802.9028 0.0000 0.0000
8870.9824 7459.0040 330.9301 -1411.9784 7527.5637
332.8398 -30.3167 -0.8619 664265.7895 -3.7692
-3.7519
[65356]ENERGY: 16040000 3651.3784 5921.4182 5966.9060
367.5195 -15473.1131 -1829.4012 0.0000 0.0000
8852.0930 7456.8008 330.2254 -1395.2923 7526.8462
332.9708 -15.1219 26.0257 664265.7895 -9.9867
-9.9978

[65406]ENERGY: 16050000 3609.8844 5932.6081 5956.0054
390.4379 -15482.1731 -1888.5607 0.0000 0.0000
8935.8859 7454.0879 333.3513 -1481.7980 7526.2332
333.0666 19.8801 7.7505 664265.7895 -9.3361
-9.3186
[65442]ENERGY: 16060000 3675.1562 5894.4940 5951.9086
377.4238 -15508.2525 -1819.9376 0.0000 0.0000
8886.3353 7457.1277 331.5028 -1429.2076 7526.7136
333.1705 84.4461 30.8216 664265.7895 -4.6795
-4.6815
[65492]ENERGY: 16070000 3694.3871 5868.1430 5927.1916
351.9629 -15452.5548 -1881.5564 0.0000 0.0000
8946.9828 7454.5562 333.7653 -1492.4266 7527.3900
332.8750 -51.1254 -46.6846 664265.7895 -6.5457
-6.5338
[65528]ENERGY: 16080000 3632.0868 5918.3978 5942.5902
384.7803 -15441.4034 -1910.2797 0.0000 0.0000
8929.2829 7455.4550 333.1050 -1473.8279 7529.7895
333.0418 54.1016 103.9005 664265.7895 -4.8909
-4.9239
[65578]ENERGY: 16090000 3582.6543 5897.0631 5924.6253
365.6468 -15417.1522 -1915.2604 0.0000 0.0000
9020.9107 7458.4877 336.5231 -1562.4231 7527.4587
332.9667 29.8361 -28.5020 664265.7895 -5.4789
-5.4756
[65617]ENERGY: 16100000 3581.0121 6015.9552 5966.6049
390.3584 -15447.2650 -1855.5578 0.0000 0.0000
8809.4876 7460.5955 328.6360 -1348.8921 7531.3896
333.1366 98.1527 -2.0147 664265.7895 -4.2286
-4.2061
[65667]ENERGY: 16110000 3598.3501 5908.3770 5989.4995
356.1767 -15561.0986 -1763.3399 0.0000 0.0000
8929.4726 7457.4374 333.1121 -1472.0352 7529.5078
333.1409 158.0232 35.6399 664265.7895 -4.5545
-4.5790
[65703]ENERGY: 16120000 3602.2083 5809.8453 5978.6904
378.2495 -15378.6020 -1876.8923 0.0000 0.0000
8947.9872 7461.4863 333.8027 -1486.5009 7531.9981
333.2987 85.0288 83.1857 664265.7895 -6.1816
-6.1939
[65753]ENERGY: 16130000 3598.1272 5932.2686 5912.7492
366.9023 -15513.3933 -1771.6674 0.0000 0.0000
8934.7813 7459.7678 333.3101 -1475.0135 7529.3894
333.1112 94.7021 133.4621 664265.7895 -3.2053
-3.1704
[65789]ENERGY: 16140000 3592.3162 5779.7606 5905.3423
359.3501 -15455.0059 -1807.7831 0.0000 0.0000
9080.5969 7454.5771 338.7497 -1626.0198 7529.7375
333.0826 101.9279 29.6803 664265.7895 -6.0178
-6.0366
[65839]ENERGY: 16150000 3635.6891 5986.8354 5930.9958
343.0270 -15459.3598 -1836.5872 0.0000 0.0000
8856.6581 7457.2584 330.3957 -1399.3997 7530.4997
333.2550 55.8934 16.7839 664265.7895 -0.2455

-0.2478
[65875]ENERGY: 16160000 3769.2734 5915.8565 5985.2834
335.8512 -15575.7888 -1897.9328 0.0000 0.0000
8927.9803 7460.5232 333.0564 -1467.4570 7530.1192
333.3030 69.0754 64.8242 664265.7895 -6.2536
-6.2327
[65925]ENERGY: 16170000 3642.7029 5838.7217 5979.2362
359.4478 -15496.1208 -1861.9223 0.0000 0.0000
8995.9347 7458.0002 335.5914 -1537.9345 7532.2441
333.2101 39.7416 -13.1049 664265.7895 -5.0984
-5.1090
[65961]ENERGY: 16180000 3595.0549 5905.4356 5996.1367
391.9606 -15427.6450 -1902.0819 0.0000 0.0000
8900.5540 7459.4148 332.0332 -1441.1392 7532.5655
333.2541 59.6404 41.9138 664265.7895 -4.8501
-4.8375
[66011]ENERGY: 16190000 3673.5203 6069.6118 5864.6677
350.9770 -15432.4556 -1941.8374 0.0000 0.0000
8875.4104 7459.8941 331.0953 -1415.5163 7532.5498
333.4044 -11.1134 -63.9561 664265.7895 -0.5825
-0.5940
[66050]ENERGY: 16200000 3604.3920 5971.8122 5989.7504
381.2310 -15544.6770 -1879.5210 0.0000 0.0000
8934.5503 7457.5379 333.3015 -1477.0124 7532.6813
333.4519 183.2666 58.4321 664265.7895 -3.8540
-3.8551
[66100]ENERGY: 16210000 3678.1357 5936.2321 5907.0441
349.4476 -15503.0737 -1885.3521 0.0000 0.0000
8974.4313 7456.8649 334.7892 -1517.5663 7532.1123
333.2811 14.7757 -41.1917 664265.7895 -6.1379
-6.1441
[66136]ENERGY: 16220000 3603.0022 5869.0886 5954.2041
354.7816 -15404.5611 -1887.9797 0.0000 0.0000
8971.8391 7460.3747 334.6925 -1511.4644 7532.5831
333.3729 29.7593 -23.3190 664265.7895 -4.2130
-4.2007
[66186]ENERGY: 16230000 3699.0766 5833.3110 5918.0120
361.2440 -15492.0354 -1847.9765 0.0000 0.0000
8988.0610 7459.6928 335.2977 -1528.3682 7531.7532
333.2111 18.1705 -53.7942 664265.7895 -6.1940
-6.2123
[66222]ENERGY: 16240000 3612.2870 5890.4560 5984.2062
354.0931 -15361.1275 -1946.0289 0.0000 0.0000
8927.9869 7461.8727 333.0566 -1466.1142 7533.1617
333.2328 -29.6361 -114.8464 664265.7895 -5.2686
-5.2687
[66272]ENERGY: 16250000 3670.3526 5904.3254 5890.1472
393.1665 -15457.9409 -1804.5728 0.0000 0.0000
8865.3872 7460.8652 330.7214 -1404.5220 7532.1898
333.3992 -15.0838 37.8346 664265.7895 -4.2407
-4.2433
[66308]ENERGY: 16260000 3561.3563 6022.2154 5923.5961
352.2250 -15531.3262 -1866.5604 0.0000 0.0000
8993.7487 7455.2548 335.5099 -1538.4939 7531.5652

333.3559 94.5035 114.7448 664265.7895 -4.5940
-4.5853
[66358]ENERGY: 16270000 3622.4098 5865.0904 5958.8003
370.3297 -15338.3164 -1898.7588 0.0000 0.0000
8882.7948 7462.3498 331.3707 -1420.4450 7532.1334
333.4155 -214.7098 -126.6623 664265.7895 -3.0963
-3.1060
[66394]ENERGY: 16280000 3619.6951 5853.0479 5955.1325
341.9997 -15475.4310 -1832.5085 0.0000 0.0000
9000.6912 7462.6270 335.7688 -1538.0641 7533.6182
333.2268 84.8425 70.9943 664265.7895 -4.7927
-4.7978
[66444]ENERGY: 16290000 3574.2543 5872.9336 5923.3717
393.7111 -15325.7454 -1835.5270 0.0000 0.0000
8865.8184 7468.8166 330.7374 -1397.0018 7535.4674
333.0836 38.5119 -21.3021 664265.7895 -5.9552
-5.9577
[66483]ENERGY: 16300000 3586.6809 5977.9092 5856.9921
353.1033 -15498.2832 -1818.8824 0.0000 0.0000
9004.1677 7461.6876 335.8985 -1542.4800 7534.5946
333.2535 135.6252 60.8235 664265.7895 -6.6232
-6.5912
[66533]ENERGY: 16310000 3612.5148 5856.1974 5955.1946
350.3698 -15438.5661 -1814.8397 0.0000 0.0000
8938.7901 7459.6608 333.4596 -1479.1293 7533.3799
333.4351 -143.0119 -51.4924 664265.7895 -9.3065
-9.3329
[66569]ENERGY: 16320000 3654.2712 5886.2152 5965.5393
361.1431 -15399.3313 -1888.8208 0.0000 0.0000
8883.5707 7462.5875 331.3997 -1420.9832 7534.8199
333.5195 -51.1579 -81.2097 664265.7895 -5.1643
-5.1591
[66619]ENERGY: 16330000 3607.3502 5976.4921 5910.8190
367.1634 -15446.7192 -1852.5802 0.0000 0.0000
8899.9453 7462.4707 332.0105 -1437.4746 7534.7110
333.3892 9.6124 -58.1579 664265.7895 -4.3864
-4.3978
[66655]ENERGY: 16340000 3649.3691 5851.7207 6001.9016
373.3857 -15456.2705 -1846.2392 0.0000 0.0000
8889.3834 7463.2507 331.6165 -1426.1327 7536.5638
333.4348 105.2917 16.7633 664265.7895 -6.9686
-6.9640
[66705]ENERGY: 16350000 3691.5363 5835.3878 5901.4643
353.2577 -15426.0934 -1863.3557 0.0000 0.0000
8970.0350 7462.2320 334.6252 -1507.8030 7536.8053
333.4498 69.1104 -12.0277 664265.7895 -5.9717
-5.9699
[66741]ENERGY: 16360000 3708.9241 5852.5757 5934.5935
377.9096 -15478.4621 -1840.2673 0.0000 0.0000
8908.7049 7463.9784 332.3373 -1444.7265 7536.1195
333.5784 4.3031 25.1394 664265.7895 -6.8049
-6.7881
[66791]ENERGY: 16370000 3682.8926 5809.3179 5917.9717
340.9148 -15410.8709 -1851.0554 0.0000 0.0000

8972.9731 7462.1438 334.7348 -1510.8292 7535.5420
333.5015 0.6115 13.5455 664265.7895 -3.5437
-3.5446
[66827]ENERGY: 16380000 3756.9611 5956.1674 5924.5277
368.2394 -15558.8988 -1844.6433 0.0000 0.0000
8863.4777 7465.8312 330.6501 -1397.6465 7536.1568
333.4728 139.9471 57.3485 664265.7895 -6.1592
-6.1760
[66877]ENERGY: 16390000 3609.3041 5934.4550 5905.4731
349.4013 -15431.7492 -1823.8756 0.0000 0.0000
8917.7787 7460.7873 332.6758 -1456.9914 7537.0087
333.4916 109.0483 44.7354 664265.7895 -7.9736
-7.9728
[66916]ENERGY: 16400000 3713.8288 5864.8263 6003.6974
369.9519 -15534.2231 -1867.9533 0.0000 0.0000
8913.6991 7463.8269 332.5236 -1449.8721 7536.3075
333.4640 -71.9040 -123.3734 664265.7895 -8.0576
-8.0514
[66966]ENERGY: 16410000 3718.1486 5965.5615 5950.2104
369.4699 -15529.7271 -1881.8890 0.0000 0.0000
8870.7797 7462.5540 330.9225 -1408.2257 7535.7142
333.1813 -11.7613 -9.3449 664265.7895 -6.5544
-6.5491
[67002]ENERGY: 16420000 3689.9230 5839.3650 5963.2084
370.7697 -15414.3932 -1856.1721 0.0000 0.0000
8871.8136 7464.5145 330.9611 -1407.2992 7536.5453
333.5413 -31.7863 -48.5313 664265.7895 -6.0539
-6.0577
[67052]ENERGY: 16430000 3670.7023 5919.1651 5893.9222
367.0682 -15515.5315 -1790.1586 0.0000 0.0000
8921.9070 7467.0747 332.8298 -1454.8323 7539.2042
333.5171 43.4257 12.0269 664265.7895 -6.2264
-6.2091
[67088]ENERGY: 16440000 3641.9972 5915.7678 5964.2212
386.8100 -15492.3845 -1848.5899 0.0000 0.0000
8900.2057 7468.0274 332.0203 -1432.1783 7539.2891
333.5535 25.4332 -59.7066 664265.7895 -1.7412
-1.7666
[67138]ENERGY: 16450000 3631.2378 5972.3052 5929.9763
373.5360 -15484.3313 -1888.2017 0.0000 0.0000
8932.0195 7466.5418 333.2071 -1465.4777 7539.2331
333.5103 54.2993 -20.1863 664265.7895 -3.7042
-3.6981
[67174]ENERGY: 16460000 3598.3792 5869.9882 5927.2464
391.9621 -15515.5782 -1837.8915 0.0000 0.0000
9030.6469 7464.7530 336.8863 -1565.8939 7538.7333
333.7045 -5.4847 0.2177 664265.7895 -8.4932
-8.5012
[67224]ENERGY: 16470000 3652.8512 5847.3629 5879.4949
346.1498 -15425.0508 -1823.0192 0.0000 0.0000
8989.6188 7467.4075 335.3558 -1522.2112 7538.1851
333.5177 22.4488 105.1990 664265.7895 -12.7060
-12.6891
[67260]ENERGY: 16480000 3655.3045 5897.6997 5933.4972

340.7995 -15506.1420 -1795.3261 0.0000 0.0000
8939.9774 7465.8102 333.5039 -1474.1672 7540.5108
333.5375 70.0070 61.8133 664265.7895 -7.9602
-7.9651
[67310]ENERGY: 16490000 3589.6442 5907.5845 6012.9098
377.6066 -15461.4464 -1885.5527 0.0000 0.0000
8926.9620 7467.7079 333.0184 -1459.2541 7539.7771
333.5528 -93.2964 -38.4659 664265.7895 -10.4062
-10.3970
[67349]ENERGY: 16500000 3576.1332 5866.4304 5921.2981
385.9984 -15458.1329 -1833.0110 0.0000 0.0000
9006.1936 7464.9098 335.9741 -1541.2838 7540.9896
333.5620 0.6460 -9.4422 664265.7895 -9.7593
-9.7577
[67399]ENERGY: 16510000 3573.3526 5913.0366 5931.1293
353.9587 -15434.7035 -1840.9357 0.0000 0.0000
8971.4052 7467.2431 334.6763 -1504.1620 7539.5422
333.3950 -19.3110 27.9786 664265.7895 -10.9796
-10.9815
[67435]ENERGY: 16520000 3616.6672 5885.9905 5944.5172
399.8282 -15354.9908 -1868.7699 0.0000 0.0000
8851.8040 7475.0464 330.2146 -1376.7576 7542.3466
333.2887 42.3033 -53.2854 664265.7895 -8.3109
-8.3311
[67485]ENERGY: 16530000 3571.7333 5890.5407 5940.9743
381.8016 -15383.8694 -1800.6333 0.0000 0.0000
8870.7623 7471.3096 330.9219 -1399.4527 7541.2883
333.2160 -123.9942 -98.2630 664265.7895 -8.6427
-8.6260
[67521]ENERGY: 16540000 3602.9861 5961.6090 5933.8673
346.2274 -15394.9636 -1868.7103 0.0000 0.0000
8889.6117 7470.6275 331.6251 -1418.9842 7543.0003
333.0617 -2.2968 -24.9602 664265.7895 -11.4455
-11.4534
[67571]ENERGY: 16550000 3753.7455 5875.5492 6019.7006
386.3583 -15547.0401 -1882.4603 0.0000 0.0000
8861.9937 7467.8469 330.5948 -1394.1468 7541.7780
333.2495 -304.2706 -231.4032 664265.7895 -11.3551
-11.3522
[67607]ENERGY: 16560000 3576.5861 5864.5348 5970.1108
402.8923 -15385.4936 -1861.3927 0.0000 0.0000
8902.8465 7470.0842 332.1188 -1432.7622 7543.3462
333.2535 53.2003 35.4829 664265.7895 -11.1410
-11.1420
[67657]ENERGY: 16570000 3698.3990 5896.9970 6019.8317
350.7699 -15528.3297 -1820.0645 0.0000 0.0000
8854.4163 7472.0197 330.3121 -1382.3966 7542.6795
333.2922 128.5167 68.5910 664265.7895 -7.2243
-7.2292
[67693]ENERGY: 16580000 3625.3753 5935.5643 5909.8329
358.0818 -15389.3890 -1853.2687 0.0000 0.0000
8883.3231 7469.5198 331.3905 -1413.8033 7541.8676
333.0262 9.0863 -35.9459 664265.7895 0.1141
0.1255

[67743]ENERGY: 16590000 3651.8425 6001.6176 5933.3942
356.9272 -15463.1190 -1803.5610 0.0000 0.0000
8797.9375 7475.0389 328.2052 -1322.8986 7544.7786
333.0804 95.7993 140.1743 664265.7895 -0.8438
-0.8557
[67782]ENERGY: 16600000 3647.2316 5966.6175 5907.2113
381.2310 -15493.7755 -1848.2769 0.0000 0.0000
8908.0898 7468.3288 332.3144 -1439.7610 7543.8446
333.4238 72.3909 85.1804 664265.7895 -4.4741
-4.4597
[67832]ENERGY: 16610000 3628.4747 6007.6789 5941.2161
386.0975 -15490.2707 -1829.2655 0.0000 0.0000
8830.9035 7474.8344 329.4350 -1356.0691 7544.2319
333.7835 -91.2198 -34.3343 664265.7895 -8.0019
-8.0027
[67868]ENERGY: 16620000 3690.6431 5773.6579 5896.0314
359.6726 -15389.7422 -1877.6532 0.0000 0.0000
9024.7517 7477.3614 336.6664 -1547.3903 7545.6046
333.6255 -185.2227 -215.0699 664265.7895 -7.5080
-7.5055
[67918]ENERGY: 16630000 3638.7144 5873.2146 5925.3866
325.1644 -15367.8148 -1877.6713 0.0000 0.0000
8958.8291 7475.8230 334.2072 -1483.0061 7548.0243
333.6982 -13.5008 -158.3248 664265.7895 -10.1954
-10.1990
[67954]ENERGY: 16640000 3660.7474 5966.0544 5922.9312
361.7555 -15489.0507 -1889.2220 0.0000 0.0000
8943.0658 7476.2815 333.6191 -1466.7843 7546.4478
333.3859 -57.3225 -14.3089 664265.7895 -7.9809
-7.9807
[68004]ENERGY: 16650000 3657.6481 5860.5176 5931.0474
391.7427 -15402.7758 -1879.0882 0.0000 0.0000
8917.9715 7477.0634 332.6830 -1440.9081 7548.1064
333.4182 -13.4097 -44.8501 664265.7895 -12.5295
-12.5486
[68040]ENERGY: 16660000 3616.9639 5849.9463 5927.5028
375.3101 -15367.9447 -1909.5857 0.0000 0.0000
8984.8841 7477.0768 335.1792 -1507.8072 7549.0706
333.6781 279.8286 122.0910 664265.7895 -6.3840
-6.3785
[68090]ENERGY: 16670000 3608.6183 5972.2847 5961.3150
366.4037 -15467.2039 -1882.6424 0.0000 0.0000
8916.4336 7475.2089 332.6256 -1441.2246 7547.8081
333.6230 57.6738 68.7272 664265.7895 -10.1779
-10.1646
[68126]ENERGY: 16680000 3587.7034 5973.0006 5947.6205
364.3294 -15475.8910 -1887.6419 0.0000 0.0000
8963.2650 7472.3861 334.3727 -1490.8789 7547.1380
333.5816 96.3292 32.5973 664265.7895 -5.1517
-5.1625
[68176]ENERGY: 16690000 3693.3180 5928.0188 5940.7011
360.2953 -15449.1499 -1866.5595 0.0000 0.0000
8872.2157 7478.8396 330.9761 -1393.3761 7548.3411
333.6818 -1.3476 15.7132 664265.7895 -7.1457

-7.1409
[68215]ENERGY: 16700000 3659.8438 5899.5319 5932.8871
377.8381 -15481.9247 -1811.7116 0.0000 0.0000
8897.6597 7474.1245 331.9253 -1423.5353 7546.7211
333.6492 55.4637 6.5893 664265.7895 -7.5055
-7.5108
[68265]ENERGY: 16710000 3712.7510 5961.0605 5958.3894
358.5986 -15490.6408 -1863.6311 0.0000 0.0000
8846.1157 7482.6433 330.0024 -1363.4724 7548.0362
333.6186 64.5310 -1.6879 664265.7895 -6.3046
-6.3008
[68301]ENERGY: 16720000 3727.4973 5972.3591 5919.1499
358.5837 -15489.0659 -1859.2885 0.0000 0.0000
8848.7188 7477.9545 330.0995 -1370.7643 7547.7500
333.5243 -139.2609 -123.8196 664265.7895 -5.7793
-5.7864
[68351]ENERGY: 16730000 3623.6607 5926.7993 5919.0630
356.8188 -15417.8386 -1938.1604 0.0000 0.0000
9002.3160 7472.6587 335.8295 -1529.6573 7548.5024
333.4830 70.3964 31.0464 664265.7895 -5.5635
-5.5513
[68387]ENERGY: 16740000 3647.4226 5881.1818 5936.3059
374.6390 -15448.6480 -1883.4209 0.0000 0.0000
8965.3825 7472.8629 334.4517 -1492.5196 7546.7870
333.6699 -113.6873 -28.2116 664265.7895 -3.7283
-3.7186
[68437]ENERGY: 16750000 3601.6215 5928.3432 5941.6964
389.9721 -15359.0164 -1940.7031 0.0000 0.0000
8911.1079 7473.0215 332.4270 -1438.0864 7547.4564
333.6021 -65.4904 -67.9310 664265.7895 -5.2653
-5.2831
[68473]ENERGY: 16760000 3641.7935 5905.6792 5911.0899
370.1650 -15386.1317 -1921.3043 0.0000 0.0000
8953.5798 7474.8715 334.0114 -1478.7084 7546.7625
333.5415 -161.9852 -92.4309 664265.7895 -4.6211
-4.6138
[68523]ENERGY: 16770000 3620.4586 5802.7150 5910.7690
374.6756 -15439.7265 -1852.0181 0.0000 0.0000
9054.7838 7471.6573 337.7868 -1583.1265 7546.7465
333.4744 92.1545 28.8219 664265.7895 -2.8058
-2.8021
[68559]ENERGY: 16780000 3626.9070 5974.8459 5971.0955
360.9104 -15483.4956 -1852.1126 0.0000 0.0000
8879.9666 7478.1173 331.2652 -1401.8494 7548.2374
333.2621 1.5912 112.0218 664265.7895 -6.2179
-6.2262
[68609]ENERGY: 16790000 3649.1948 5841.6942 5934.0369
383.9159 -15384.2878 -1821.0834 0.0000 0.0000
8877.8926 7481.3631 331.1879 -1396.5295 7549.1035
333.5927 -16.1764 -36.2444 664265.7895 -3.7689
-3.7562
[68648]ENERGY: 16800000 3690.2232 5946.0171 5958.7651
383.5744 -15357.7685 -1928.1793 0.0000 0.0000
8787.4963 7480.1282 327.8157 -1307.3681 7547.9650

333.6687 87.4020 46.1026 664265.7895 -2.3922
-2.3992
[68698]ENERGY: 16810000 3662.9683 5948.5297 5948.9757
380.8265 -15399.2197 -1927.2299 0.0000 0.0000
8861.9993 7476.8500 330.5950 -1385.1493 7547.6815
333.5587 -145.3205 -128.5026 664265.7895 -4.8282
-4.8249
[68734]ENERGY: 16820000 3655.3790 5910.2008 5911.5024
377.6337 -15441.8465 -1882.2457 0.0000 0.0000
8939.9244 7470.5481 333.5020 -1469.3763 7547.7106
333.6421 -4.7417 -79.8146 664265.7895 -5.1440
-5.1549
[68784]ENERGY: 16830000 3686.3496 6006.7573 5912.9279
340.8303 -15488.7523 -1837.3945 0.0000 0.0000
8859.6119 7480.3303 330.5059 -1379.2816 7548.7677
333.6181 97.2122 131.8134 664265.7895 -4.2782
-4.2726
[68820]ENERGY: 16840000 3591.0296 5893.3815 5967.1559
368.4675 -15475.0118 -1799.4204 0.0000 0.0000
8930.9789 7476.5812 333.1682 -1454.3977 7548.8688
333.8048 159.0979 83.6659 664265.7895 -1.9401
-1.9540
[68870]ENERGY: 16850000 3673.1817 5919.4183 5951.3682
361.3788 -15523.3770 -1869.7043 0.0000 0.0000
8965.7579 7478.0235 334.4657 -1487.7343 7548.7049
333.9467 86.2338 9.2976 664265.7895 -3.3987
-3.3993
[68906]ENERGY: 16860000 3629.2077 5888.8316 5882.2964
342.2344 -15441.8464 -1881.7775 0.0000 0.0000
9056.5326 7475.4787 337.8520 -1581.0538 7548.1139
333.9171 8.9462 -73.7329 664265.7895 -3.1919
-3.1752
[68956]ENERGY: 16870000 3672.9107 5840.4927 5997.0250
372.7999 -15447.2370 -1906.7111 0.0000 0.0000
8945.2765 7474.5568 333.7016 -1470.7197 7549.0892
334.0947 -14.2134 78.8549 664265.7895 -4.2277
-4.2173
[68992]ENERGY: 16880000 3641.4603 5902.0207 5942.4742
368.7035 -15491.6253 -1916.9296 0.0000 0.0000
9027.9505 7474.0543 336.7857 -1553.8962 7549.5865
333.9633 -88.2932 -33.3030 664265.7895 -5.9182
-5.9076
[69042]ENERGY: 16890000 3693.0076 5871.4108 5911.6816
373.0035 -15469.6769 -1762.7603 0.0000 0.0000
8858.8421 7475.5084 330.4772 -1383.3337 7547.6615
333.8365 -43.8160 3.4682 664265.7895 -5.5565
-5.5792
[69081]ENERGY: 16900000 3591.4090 5902.6739 5910.1456
374.8294 -15385.1808 -1896.4912 0.0000 0.0000
8980.5333 7477.9192 335.0169 -1502.6141 7548.9061
333.8470 41.6768 -20.4316 664265.7895 -6.3213
-6.3206
[69131]ENERGY: 16910000 3563.3633 5891.1478 5963.3690
371.4640 -15476.7020 -1888.4278 0.0000 0.0000

9048.1394 7472.3538 337.5389 -1575.7856 7548.3385
334.0861 91.0756 -30.7714 664265.7895 -4.3803
-4.3809
[69167]ENERGY: 16920000 3697.0885 5833.7449 5945.6014
364.3154 -15425.5614 -1846.6717 0.0000 0.0000
8908.1015 7476.6186 332.3148 -1431.4829 7549.1305
333.8193 -94.9829 -7.2641 664265.7895 0.0483
0.0539
[69217]ENERGY: 16930000 3608.5512 6019.0651 5947.1797
379.4126 -15412.2733 -1948.9104 0.0000 0.0000
8882.0783 7475.1032 331.3440 -1406.9751 7548.1455
333.9626 -81.2675 -90.3327 664265.7895 -4.5191
-4.5322
[69253]ENERGY: 16940000 3698.2817 5934.5219 5964.2666
349.5084 -15445.0759 -1890.1067 0.0000 0.0000
8867.7298 7479.1258 330.8088 -1388.6041 7550.1520
333.9865 -16.5886 14.9235 664265.7895 -4.5816
-4.5863
[69303]ENERGY: 16950000 3587.4264 5988.9644 5900.5632
373.3937 -15527.3540 -1850.5915 0.0000 0.0000
9003.8121 7476.2143 335.8853 -1527.5978 7548.8311
334.0112 75.4819 37.3714 664265.7895 -5.0202
-5.0288
[69339]ENERGY: 16960000 3644.7283 5926.7943 5970.5791
356.1778 -15439.4554 -1881.2359 0.0000 0.0000
8900.6263 7478.2144 332.0359 -1422.4119 7549.3057
334.0309 14.3481 -43.8601 664265.7895 -1.9786
-1.9447
[69389]ENERGY: 16970000 3712.1571 5891.7631 5905.4299
357.8284 -15482.4425 -1873.3734 0.0000 0.0000
8966.7466 7478.1092 334.5025 -1488.6374 7549.0428
334.0583 -13.1521 7.8015 664265.7895 -1.2954
-1.2986
[69425]ENERGY: 16980000 3671.9365 5999.8048 5928.3600
351.8315 -15471.9522 -1942.5378 0.0000 0.0000
8943.4168 7480.8597 333.6322 -1462.5571 7550.9744
333.8117 4.7101 -113.5832 664265.7895 0.7654
0.7484
[69475]ENERGY: 16990000 3600.7307 5975.2307 5891.3903
380.7341 -15447.1864 -1906.9326 0.0000 0.0000
8979.5736 7473.5404 334.9811 -1506.0331 7548.8338
333.9635 -43.0370 5.4616 664265.7895 -0.6199
-0.6052
[69514]ENERGY: 17000000 3652.4300 5825.1813 5853.8471
359.2299 -15403.8562 -1789.4942 0.0000 0.0000
8983.6853 7481.0233 335.1344 -1502.6620 7549.3610
333.8659 117.8492 77.3944 664265.7895 -2.7066
-2.7188
[69564]ENERGY: 17010000 3661.3814 5926.6553 5958.5581
350.7568 -15502.0651 -1860.7047 0.0000 0.0000
8944.7429 7479.3247 333.6817 -1465.4182 7550.6623
333.9250 -46.8439 -108.9553 664265.7895 0.7196
0.7110
[69600]ENERGY: 17020000 3752.1385 5992.0528 5905.2182

348.6903 -15513.8543 -1829.9453 0.0000 0.0000
8823.9048 7478.2049 329.1739 -1345.6999 7549.9608
333.8792 100.0057 54.2490 664265.7895 -0.4242
-0.4176
[69650]ENERGY: 17030000 3621.9600 5883.0495 5908.3743
370.9145 -15478.8326 -1832.4178 0.0000 0.0000
9005.6526 7478.7005 335.9539 -1526.9521 7552.1227
333.8675 -7.2305 -45.7529 664265.7895 3.8969
3.9007
[69686]ENERGY: 17040000 3728.8334 5898.4745 5924.9060
357.3733 -15509.4067 -1908.9186 0.0000 0.0000
8988.7108 7479.9727 335.3219 -1508.7380 7550.8853
333.6754 -175.6268 -264.2464 664265.7895 0.9725
0.9709
[69736]ENERGY: 17050000 3685.4529 5975.7099 5892.6005
347.8622 -15462.6312 -1830.6275 0.0000 0.0000
8870.5969 7478.9636 330.9157 -1391.6333 7551.6764
333.7608 27.6438 37.4210 664265.7895 -0.1083
-0.1172
[69772]ENERGY: 17060000 3653.2200 5945.6469 5916.5830
356.3754 -15484.9656 -1854.0588 0.0000 0.0000
8948.7311 7481.5319 333.8305 -1467.1991 7552.3337
334.0327 9.4510 0.9659 664265.7895 2.1525
2.1793
[69822]ENERGY: 17070000 3691.9742 5908.5695 5897.1032
360.0580 -15537.7668 -1817.3691 0.0000 0.0000
8981.8217 7484.3907 335.0649 -1497.4310 7553.0610
333.8425 91.9653 52.1309 664265.7895 -1.6456
-1.6732
[69858]ENERGY: 17080000 3606.5295 5956.3123 5894.6724
345.5006 -15478.5529 -1828.3409 0.0000 0.0000
8983.1257 7479.2466 335.1136 -1503.8791 7551.9139
333.8246 270.1041 81.5215 664265.7895 2.2385
2.2423
[69908]ENERGY: 17090000 3719.4347 5959.9391 5881.8310
371.6423 -15513.8654 -1819.2017 0.0000 0.0000
8883.5937 7483.3738 331.4006 -1400.2199 7553.3079
333.8599 -1.3161 16.6715 664265.7895 -1.0472
-1.0341
[69947]ENERGY: 17100000 3663.0908 5942.6651 5863.6271
372.0457 -15544.2345 -1815.0339 0.0000 0.0000
8995.1688 7477.3290 335.5628 -1517.8398 7550.7817
333.8310 -65.8339 -8.9257 664265.7895 -2.0486
-2.0679
[69997]ENERGY: 17110000 3681.0023 5978.4234 5873.8841
339.7018 -15509.2586 -1861.4622 0.0000 0.0000
8973.4894 7475.7801 334.7541 -1497.7093 7551.4869
334.0960 -34.9261 -17.9617 664265.7895 -4.0197
-3.9847
[70033]ENERGY: 17120000 3701.2896 5961.0217 5936.4384
370.2085 -15524.4129 -1851.7447 0.0000 0.0000
8889.2074 7482.0081 331.6100 -1407.1993 7551.8551
333.8940 -35.9698 -43.7787 664265.7895 -1.9102
-1.9450

[70083]ENERGY: 17130000 3660.1489 5964.9109 5882.4471
371.2078 -15436.5429 -1916.4310 0.0000 0.0000
8956.4692 7482.2100 334.1192 -1474.2592 7552.7396
333.9220 30.2743 -81.0908 664265.7895 -5.7013
-5.6773
[70119]ENERGY: 17140000 3631.2320 5831.9423 5905.1101
352.8845 -15514.5437 -1785.8686 0.0000 0.0000
9060.5925 7481.3491 338.0035 -1579.2434 7552.8424
333.8846 167.9099 128.2566 664265.7895 -3.0169
-3.0294
[70169]ENERGY: 17150000 3712.4614 5952.0456 5938.1771
386.4420 -15469.3797 -1944.7785 0.0000 0.0000
8905.2309 7480.1988 332.2077 -1425.0321 7551.3562
334.0003 -229.5148 -210.9640 664265.7895 -7.1992
-7.1911
[70205]ENERGY: 17160000 3622.9822 5966.3560 5919.4300
350.7709 -15436.5552 -1867.0783 0.0000 0.0000
8924.7223 7480.6279 332.9348 -1444.0944 7553.0025
333.7588 -2.6509 -97.9022 664265.7895 -6.1003
-6.1077
[70255]ENERGY: 17170000 3715.6077 5824.3818 5885.7490
370.0245 -15537.9110 -1801.7801 0.0000 0.0000
9019.4965 7475.5682 336.4704 -1543.9282 7549.9362
333.6181 -65.3583 -71.1593 664265.7895 -5.9982
-5.9830
[70291]ENERGY: 17180000 3582.6524 5829.4279 5971.7766
359.4798 -15329.2979 -1874.6888 0.0000 0.0000
8943.1100 7482.4600 333.6208 -1460.6500 7553.1802
333.7596 175.9729 37.6891 664265.7895 -6.7236
-6.7283
[70341]ENERGY: 17190000 3651.8094 5903.1964 5892.4222
356.2513 -15481.9503 -1778.5386 0.0000 0.0000
8939.2818 7482.4722 333.4780 -1456.8097 7552.2668
333.7303 -8.8267 -73.5498 664265.7895 -9.8141
-9.8418
[70380]ENERGY: 17200000 3720.8870 5844.8903 5888.8329
347.4819 -15423.6564 -1886.8497 0.0000 0.0000
8989.1517 7480.7377 335.3384 -1508.4140 7552.5832
333.9294 58.1696 28.2380 664265.7895 -6.4211
-6.3783
[70430]ENERGY: 17210000 3593.8403 5886.0156 5939.4308
349.5246 -15526.7700 -1705.3787 0.0000 0.0000
8946.3735 7483.0360 333.7425 -1463.3374 7553.5199
333.7914 153.0056 94.9800 664265.7895 -10.3870
-10.4077
[70466]ENERGY: 17220000 3650.1366 5887.3260 5893.1961
374.1656 -15480.0428 -1771.6431 0.0000 0.0000
8932.1280 7485.2665 333.2111 -1446.8616 7553.4369
333.9212 -13.5415 15.7983 664265.7895 -8.0600
-8.0533
[70516]ENERGY: 17230000 3636.5029 5952.2556 5907.5172
375.2587 -15500.3319 -1837.6219 0.0000 0.0000
8942.5164 7476.0971 333.5986 -1466.4193 7553.3679
333.7630 -24.8691 -22.2334 664265.7895 -10.3687

```
-10.3709
[70552]ENERGY: 17240000      3676.0632      5880.1442      5919.9269
352.0597         -15461.6037     -1871.8170         0.0000         0.0000
8983.7830          7478.5562       335.1381     -1505.2268      7553.9177
333.6109           -29.6709       -74.1930    664265.7895        -7.6502
-7.6619
[70602]ENERGY: 17250000      3681.9714      5882.5162      5933.5832
379.9470         -15521.8065     -1847.5375         0.0000         0.0000
8973.4300          7482.1039       334.7519     -1491.3261      7555.6850
333.7108            64.9516        62.3050    664265.7895        -6.9067
-6.8824
[70638]ENERGY: 17260000      3636.1509      5960.3492      5928.0874
362.3602         -15478.3572     -1824.0519         0.0000         0.0000
8899.9580          7484.4966       332.0110     -1415.4614      7556.5758
333.8048            -5.9963      -122.0563    664265.7895        -5.2248
-5.2509
[70688]ENERGY: 17270000      3640.1145      5778.7033      5930.0907
370.7162         -15405.2055     -1884.2466         0.0000         0.0000
9052.7344          7482.9070       337.7103     -1569.8274      7555.3685
333.8657           190.1601       144.6188    664265.7895        -3.7457
-3.7258
[70724]ENERGY: 17280000      3569.3086      5906.9304      5926.3387
373.2699         -15439.4560     -1878.9407         0.0000         0.0000
9022.8533          7480.3042       336.5956     -1542.5491      7555.3512
334.1398           -69.1747       -99.7412    664265.7895        -2.1839
-2.1914
[70774]ENERGY: 17290000      3585.4140      5900.2016      5953.1733
370.1581         -15565.8220     -1751.7587         0.0000         0.0000
8990.5826          7481.9489       335.3917     -1508.6338      7555.7546
333.7876            55.6547        16.4900    664265.7895        -9.3255
-9.3329
[70813]ENERGY: 17300000      3550.4427      5893.1112      5945.2203
327.2260         -15440.5929     -1758.0210         0.0000         0.0000
8964.9207          7482.3071       334.4344     -1482.6136      7557.1388
333.5894           214.6049       156.6776    664265.7895        -2.6696
-2.6758
[70863]ENERGY: 17310000      3663.7692      5860.0967      5910.7001
347.4206         -15412.1209     -1889.3633         0.0000         0.0000
9002.6337          7483.1362       335.8413     -1519.4975      7556.4625
333.6620          -106.4495       -49.7107    664265.7895        -7.2034
-7.1950
[70899]ENERGY: 17320000      3710.3202      5877.4200      5991.6034
355.7637         -15411.2590     -1911.7359         0.0000         0.0000
8875.9943          7488.1067       331.1171     -1387.8876      7558.1056
333.8782           -50.3738       -73.0828    664265.7895        -5.5761
-5.5641
[70949]ENERGY: 17330000      3646.4859      5966.7324      5899.4163
352.7786         -15410.3083     -1886.9988         0.0000         0.0000
8918.0702          7486.1763       332.6867     -1431.8939      7558.2447
334.1262            34.1263        20.8494    664265.7895        -3.6407
-3.6471
[70985]ENERGY: 17340000      3718.1406      5865.2988      5906.9265
368.8925         -15454.0762     -1865.0491         0.0000         0.0000
8949.9805          7490.1136       333.8771     -1459.8668      7558.6607
```

334.0994 -57.6607 -0.4167 664265.7895 -4.4002
-4.4035
[71035]ENERGY: 17350000 3641.5069 5887.4014 5920.4050
371.2516 -15355.1237 -1862.6320 0.0000 0.0000
8887.1002 7489.9093 331.5314 -1397.1909 7557.8868
333.9766 -55.9420 -61.0168 664265.7895 -7.2948
-7.2906
[71071]ENERGY: 17360000 3710.2439 5936.7579 5928.0530
356.5927 -15456.8101 -1922.2715 0.0000 0.0000
8934.6274 7487.1933 333.3043 -1447.4341 7559.4998
333.8671 -146.5681 -194.2911 664265.7895 -4.5658
-4.5688
[71121]ENERGY: 17370000 3662.4649 5941.0643 5891.4544
339.8073 -15300.7200 -1884.8382 0.0000 0.0000
8840.4917 7489.7243 329.7926 -1350.7674 7559.9547
333.8640 98.4397 102.7580 664265.7895 -8.5318
-8.5370
[71157]ENERGY: 17380000 3722.0916 5908.8896 5917.5705
356.9012 -15518.3466 -1793.7961 0.0000 0.0000
8896.4333 7489.7435 331.8795 -1406.6898 7559.7949
333.9707 52.2131 -33.5884 664265.7895 -5.6750
-5.6645
[71207]ENERGY: 17390000 3698.8007 5827.5896 5868.3348
378.0310 -15432.9614 -1802.4496 0.0000 0.0000
8949.6620 7487.0070 333.8652 -1462.6550 7559.2002
333.9870 38.5120 42.5980 664265.7895 -7.4381
-7.4359
[71246]ENERGY: 17400000 3605.3491 5866.6453 5940.9208
343.4860 -15498.1975 -1797.4427 0.0000 0.0000
9023.6392 7484.4002 336.6249 -1539.2390 7558.5097
333.7284 -220.3723 -190.5810 664265.7895 -9.7817
-9.7983
[71296]ENERGY: 17410000 3756.7852 5889.3433 5961.2657
380.4563 -15464.5781 -1875.1591 0.0000 0.0000
8842.2083 7490.3216 329.8567 -1351.8867 7558.9142
333.6435 53.6541 -43.2603 664265.7895 -8.2472
-8.2430
[71332]ENERGY: 17420000 3601.3101 5946.6043 5920.5425
341.6460 -15409.9016 -1836.6565 0.0000 0.0000
8920.7937 7484.3385 332.7883 -1436.4552 7558.7337
333.6420 -62.7336 -43.3796 664265.7895 -7.1035
-7.0978
[71382]ENERGY: 17430000 3612.7496 6038.7345 5947.2483
338.3513 -15522.3161 -1839.3499 0.0000 0.0000
8914.6481 7490.0657 332.5590 -1424.5823 7558.6127
333.4705 -80.3660 -83.0052 664265.7895 -7.4789
-7.4748
[71418]ENERGY: 17440000 3561.9451 5961.7546 5910.9594
384.4053 -15416.0675 -1838.7843 0.0000 0.0000
8927.5090 7491.7216 333.0388 -1435.7874 7558.3475
333.6082 35.6148 25.2186 664265.7895 -8.4459
-8.4562
[71468]ENERGY: 17450000 3580.1457 5877.2702 5891.2472
363.9359 -15369.9866 -1904.9051 0.0000 0.0000

9046.5960 7484.3033 337.4813 -1562.2927 7558.4750
333.8378 92.0651 30.1526 664265.7895 -4.9557
-4.9721
[71504]ENERGY: 17460000 3621.8513 5872.0295 5853.1778
380.0427 -15400.5040 -1835.4909 0.0000 0.0000
8994.2002 7485.3066 335.5267 -1508.8936 7556.8079
333.7193 37.3825 -6.3840 664265.7895 -4.1176
-4.0995
[71554]ENERGY: 17470000 3710.0888 5940.2240 5895.4099
344.1173 -15518.9664 -1832.6370 0.0000 0.0000
8949.6370 7487.8735 333.8643 -1461.7635 7558.2424
333.9066 69.6776 3.7660 664265.7895 -8.5905
-8.5986
[71590]ENERGY: 17480000 3649.4532 5934.9396 5909.3093
367.5174 -15508.2441 -1828.2435 0.0000 0.0000
8959.0780 7483.8100 334.2165 -1475.2680 7558.7795
333.6995 -161.4724 -107.5056 664265.7895 -7.7468
-7.7232
[71640]ENERGY: 17490000 3636.6162 5912.6532 5894.4519
361.3933 -15405.5461 -1902.2613 0.0000 0.0000
8988.4548 7485.7619 335.3124 -1502.6929 7559.1018
333.8939 -175.7725 -136.0833 664265.7895 -6.1998
-6.2058
[71679]ENERGY: 17500000 3623.8626 5809.1964 5884.1868
368.4251 -15424.0725 -1841.5116 0.0000 0.0000
9063.8487 7483.9356 338.1249 -1579.9131 7558.9336
334.0496 -4.5007 -15.6308 664265.7895 -9.2860
-9.2958
[71729]ENERGY: 17510000 3691.9316 5815.2722 5888.4202
365.3963 -15394.7570 -1835.4747 0.0000 0.0000
8955.5603 7486.3488 334.0852 -1469.2115 7559.3052
333.9856 34.1866 74.3805 664265.7895 -5.7052
-5.6990
[71765]ENERGY: 17520000 3681.5653 5943.9183 5957.7515
341.7022 -15472.6338 -1908.6434 0.0000 0.0000
8943.7873 7487.4473 333.6461 -1456.3399 7560.4387
333.8588 -38.6019 22.2372 664265.7895 -9.1573
-9.1643
[71815]ENERGY: 17530000 3622.2219 6019.3476 5904.9986
366.7779 -15543.2173 -1817.0202 0.0000 0.0000
8935.8758 7488.9843 333.3509 -1446.8915 7560.0439
333.6202 165.3166 96.0194 664265.7895 -6.4873
-6.4966
[71851]ENERGY: 17540000 3622.8991 5843.2343 5909.8626
354.6805 -15420.8912 -1768.5255 0.0000 0.0000
8945.3506 7486.6104 333.7044 -1458.7402 7559.5162
333.7426 -0.3627 -20.0972 664265.7895 -7.3161
-7.3124
[71901]ENERGY: 17550000 3640.0582 5963.3141 5929.6137
350.6712 -15452.6463 -1798.9975 0.0000 0.0000
8858.6593 7490.6726 330.4704 -1367.9867 7556.8679
333.4161 5.5744 -25.6445 664265.7895 -8.3752
-8.3715
[71937]ENERGY: 17560000 3647.1603 5801.1770 5902.0204

378.7343 -15428.7606 -1814.5818 0.0000 0.0000
8999.4395 7485.1890 335.7222 -1514.2505 7558.6552
333.3937 244.2650 204.6607 664265.7895 -12.9767
-12.9706
[71987]ENERGY: 17570000 3618.2497 5865.4463 5885.9190
378.9372 -15433.5719 -1812.8316 0.0000 0.0000
8986.8785 7489.0273 335.2536 -1497.8512 7558.4284
333.4233 150.5470 121.1176 664265.7895 -4.8423
-4.8255
[72023]ENERGY: 17580000 3686.9265 5888.3202 5904.6748
331.8308 -15420.5805 -1785.7416 0.0000 0.0000
8883.5808 7489.0111 331.4001 -1394.5697 7559.2992
333.2419 102.0017 114.0718 664265.7895 -6.1130
-6.1260
[72073]ENERGY: 17590000 3657.7755 5966.2743 5896.3845
363.4387 -15415.1733 -1901.4297 0.0000 0.0000
8917.1509 7484.4209 332.6524 -1432.7300 7558.9340
333.6450 -209.4836 -152.9174 664265.7895 -7.1698
-7.1601
[72112]ENERGY: 17600000 3672.0924 5927.8246 5916.7253
353.0313 -15535.1187 -1822.1167 0.0000 0.0000
8973.4067 7485.8450 334.7510 -1487.5617 7561.2947
333.8799 165.7273 182.4418 664265.7895 -5.1907
-5.2117
[72162]ENERGY: 17610000 3645.0124 5964.1436 5929.8099
349.6256 -15497.4126 -1916.9164 0.0000 0.0000
9013.9600 7488.2226 336.2638 -1525.7375 7562.7304
333.9390 -28.6378 -35.6276 664265.7895 -9.1683
-9.1618
[72198]ENERGY: 17620000 3643.3977 5983.0129 5931.2508
373.6743 -15427.5535 -1876.7097 0.0000 0.0000
8865.3840 7492.4564 330.7212 -1372.9275 7562.8787
333.9059 143.0336 45.7689 664265.7895 -4.4052
-4.4069
[72248]ENERGY: 17630000 3557.1410 5906.3929 5976.3190
377.1909 -15412.8952 -1905.2773 0.0000 0.0000
8990.8889 7489.7602 335.4032 -1501.1287 7561.1023
334.0574 -40.5813 -62.7910 664265.7895 -4.4385
-4.4353
[72284]ENERGY: 17640000 3557.4174 5854.1819 5964.4518
335.8129 -15431.3050 -1813.3467 0.0000 0.0000
9021.1150 7488.3272 336.5308 -1532.7878 7563.5781
333.9522 87.5517 63.3667 664265.7895 -9.3184
-9.3231
[72334]ENERGY: 17650000 3551.2770 5940.6151 5911.8401
377.0854 -15452.4143 -1749.2902 0.0000 0.0000
8913.8520 7492.9651 332.5293 -1420.8869 7561.9956
333.9627 166.0714 109.2931 664265.7895 -4.9652
-4.9723
[72370]ENERGY: 17660000 3672.4590 5957.5333 5940.0127
371.8320 -15379.4026 -1903.4100 0.0000 0.0000
8837.5860 7496.6105 329.6842 -1340.9755 7563.5164
333.7814 -102.0949 -136.0290 664265.7895 -6.3858
-6.3850

[72420]ENERGY: 17670000 3635.1737 6007.2950 5980.5332
381.2027 -15524.7044 -1830.8717 0.0000 0.0000
8841.3711 7489.9995 329.8254 -1351.3715 7562.5491
333.7710 -13.5971 -39.2845 664265.7895 -7.3343
-7.3399
[72456]ENERGY: 17680000 3652.7415 5927.9596 5923.1901
356.3901 -15475.9893 -1870.9309 0.0000 0.0000
8978.5263 7491.8873 334.9420 -1486.6390 7564.3393
333.6367 -101.1968 -142.8135 664265.7895 -6.7123
-6.6973
[72506]ENERGY: 17690000 3615.9263 5798.8741 5950.1812
372.9252 -15455.7418 -1884.2467 0.0000 0.0000
9093.3428 7491.2611 339.2252 -1602.0817 7565.2680
333.7244 49.1968 59.9114 664265.7895 -12.9325
-12.9383
[72545]ENERGY: 17700000 3596.2690 5899.7274 5919.9975
353.7069 -15455.5223 -1782.5407 0.0000 0.0000
8959.2627 7490.9005 334.2234 -1468.3622 7565.6979
333.5886 174.3536 158.9905 664265.7895 -8.3032
-8.2821
[72595]ENERGY: 17710000 3666.2587 5985.0859 5904.2200
362.5886 -15452.0831 -1837.0759 0.0000 0.0000
8866.8062 7495.8004 330.7743 -1371.0058 7566.5700
333.7553 85.2465 -12.0384 664265.7895 -10.7341
-10.7618
[72631]ENERGY: 17720000 3671.1395 6010.6581 5888.3313
353.7137 -15461.7673 -1901.2681 0.0000 0.0000
8936.4725 7497.2798 333.3732 -1439.1927 7566.3324
333.8897 96.3764 0.1962 664265.7895 -10.7919
-10.7797
[72681]ENERGY: 17730000 3645.4294 5836.8978 5900.0646
346.0641 -15455.2674 -1827.2487 0.0000 0.0000
9047.8341 7493.7739 337.5275 -1554.0603 7567.4711
333.8595 -34.5848 0.6906 664265.7895 -11.8577
-11.8647
[72717]ENERGY: 17740000 3712.0471 5865.4200 5950.5652
378.5991 -15454.9290 -1871.0111 0.0000 0.0000
8920.4051 7501.0964 332.7738 -1419.3086 7569.1517
333.7882 -19.6615 -104.6253 664265.7895 -13.1149
-13.1188
[72767]ENERGY: 17750000 3670.8801 5915.8468 5915.5396
348.9379 -15426.9121 -1903.4195 0.0000 0.0000
8975.3907 7496.2634 334.8250 -1479.1273 7568.4810
333.6686 -11.8492 -112.0530 664265.7895 -12.1076
-12.0955
[72803]ENERGY: 17760000 3643.8264 5855.5153 5982.9653
366.6466 -15444.9948 -1890.3724 0.0000 0.0000
8981.7708 7495.3572 335.0630 -1486.4136 7570.4432
333.6114 -42.2844 -52.8034 664265.7895 -12.6416
-12.6330
[72853]ENERGY: 17770000 3616.8245 5980.9723 5941.0985
356.5277 -15457.1087 -1834.0478 0.0000 0.0000
8896.3561 7500.6228 331.8766 -1395.7333 7569.5044
333.7767 9.3338 28.3344 664265.7895 -12.3486

-12.3403
[72889]ENERGY: 17780000 3664.5010 5906.0943 5908.8464
359.0185 -15395.3014 -1811.2839 0.0000 0.0000
8871.3889 7503.2639 330.9453 -1368.1250 7570.3018
333.9449 32.9864 -56.1560 664265.7895 -8.9644
-8.9842
[72939]ENERGY: 17790000 3590.9693 5900.5357 5899.2301
352.5813 -15409.8904 -1849.0716 0.0000 0.0000
9015.8975 7500.2520 336.3361 -1515.6455 7569.3884
334.1208 45.3223 11.3568 664265.7895 -9.6638
-9.6646
[72978]ENERGY: 17800000 3630.1702 5925.6410 5954.3724
370.9966 -15483.4401 -1877.2850 0.0000 0.0000
8977.1208 7497.5760 334.8896 -1479.5449 7569.7313
333.7425 -1.2303 -54.1194 664265.7895 -10.2872
-10.2901
[73028]ENERGY: 17810000 3642.7256 5789.3029 6001.2924
347.8828 -15460.5998 -1820.7719 0.0000 0.0000
8998.0200 7497.8520 335.6692 -1500.1680 7569.2472
333.7414 149.5847 144.8762 664265.7895 -13.0522
-13.0340
[73064]ENERGY: 17820000 3660.5396 5891.4129 5852.7892
370.0443 -15409.7752 -1816.0375 0.0000 0.0000
8948.7420 7497.7153 333.8309 -1451.0267 7570.5857
333.8373 24.4830 53.7395 664265.7895 -12.2672
-12.2842
[73114]ENERGY: 17830000 3655.4908 5855.1245 5940.7217
379.6132 -15514.0852 -1879.9554 0.0000 0.0000
9060.1553 7497.0650 337.9871 -1563.0903 7570.2686
333.8170 -235.4649 -203.0928 664265.7895 -11.9046
-11.9008
[73150]ENERGY: 17840000 3611.2754 5967.5979 5910.1564
362.6083 -15451.0676 -1826.7450 0.0000 0.0000
8926.1292 7499.9546 332.9873 -1426.1745 7571.4322
333.7581 155.1920 81.7653 664265.7895 -18.4056
-18.4250
[73200]ENERGY: 17850000 3700.3000 5899.8889 5914.6932
386.1992 -15420.9736 -1870.7914 0.0000 0.0000
8891.5679 7500.8842 331.6980 -1390.6837 7573.5787
333.8503 82.4586 103.7151 664265.7895 -15.0023
-14.9876
[73236]ENERGY: 17860000 3673.7014 6031.9848 5846.9295
363.0576 -15543.4193 -1847.1819 0.0000 0.0000
8975.8396 7500.9118 334.8418 -1474.9279 7572.1636
333.7485 0.7154 20.4110 664265.7895 -15.4784
-15.4846
[73286]ENERGY: 17870000 3660.0775 6029.9604 5875.4233
345.0677 -15437.3423 -1863.6830 0.0000 0.0000
8891.0790 7500.5826 331.6798 -1390.4964 7572.5132
333.8687 79.6938 67.0485 664265.7895 -14.5221
-14.5063
[73322]ENERGY: 17880000 3630.4269 5935.5543 5899.7908
372.7372 -15483.5204 -1824.9231 0.0000 0.0000
8967.1966 7497.2623 334.5193 -1469.9343 7571.9375

334.0157 -149.7019 -75.8135 664265.7895 -18.4849
-18.5079
[73372]ENERGY: 17890000 3612.5685 5868.1595 5953.9434
367.3415 -15425.1390 -1822.8010 0.0000 0.0000
8948.1327 7502.2055 333.8082 -1445.9272 7573.7612
334.0778 -28.5336 -149.2800 664265.7895 -18.8145
-18.8133
[73411]ENERGY: 17900000 3715.2683 5927.0727 5853.1971
363.9098 -15422.0042 -1890.8235 0.0000 0.0000
8955.8287 7502.4490 334.0953 -1453.3797 7573.8006
334.0589 -206.9220 -203.6333 664265.7895 -14.3298
-14.3139
[73461]ENERGY: 17910000 3623.5510 5873.7519 5918.5470
366.8552 -15402.1519 -1861.0385 0.0000 0.0000
8988.9160 7508.4306 335.3296 -1480.4854 7573.6773
334.0440 132.6435 67.9233 664265.7895 -17.5608
-17.5621
[73497]ENERGY: 17920000 3604.4554 5871.8039 5851.7524
383.1410 -15411.6682 -1846.7429 0.0000 0.0000
9046.3582 7499.0997 337.4724 -1547.2584 7574.2927
334.0111 -78.4561 -82.2623 664265.7895 -12.3236
-12.3090
[73547]ENERGY: 17930000 3692.4060 5908.4678 5938.4076
377.5711 -15449.5960 -1855.5900 0.0000 0.0000
8890.3451 7502.0116 331.6524 -1388.3335 7576.3863
334.1721 -14.0184 6.4809 664265.7895 -11.0012
-11.0295
[73583]ENERGY: 17940000 3675.7074 5943.6302 5921.8673
372.3176 -15409.8068 -1855.2080 0.0000 0.0000
8857.8644 7506.3720 330.4407 -1351.4924 7575.4301
334.1015 -26.7522 21.6659 664265.7895 -16.1924
-16.1701
[73633]ENERGY: 17950000 3764.8329 5883.3640 5939.3446
365.7973 -15512.9359 -1874.3055 0.0000 0.0000
8936.0478 7502.1452 333.3573 -1433.9026 7575.0833
334.1255 -101.1220 -74.8732 664265.7895 -16.1720
-16.1823
[73669]ENERGY: 17960000 3733.9932 5915.4857 5913.4754
366.7298 -15422.5481 -1900.8823 0.0000 0.0000
8898.2415 7504.4953 331.9470 -1393.7462 7575.5754
334.3165 0.2797 -37.7236 664265.7895 -19.1562
-19.1760
[73719]ENERGY: 17970000 3688.0709 5969.6321 5905.6740
369.3816 -15448.1112 -1793.7805 0.0000 0.0000
8817.3427 7508.2096 328.9291 -1309.1331 7576.5711
334.2465 -21.2108 -34.8478 664265.7895 -16.0954
-16.0784
[73755]ENERGY: 17980000 3707.0768 5989.6985 5845.0502
375.0364 -15443.9065 -1875.5870 0.0000 0.0000
8908.1362 7505.5047 332.3161 -1402.6315 7575.2777
334.2949 -178.2988 -102.6863 664265.7895 -16.9075
-16.8980
[73805]ENERGY: 17990000 3660.3123 5866.9823 5863.6986
354.2563 -15471.4406 -1801.7442 0.0000 0.0000

9029.4823 7501.5471 336.8429 -1527.9352 7576.8465
334.3944 40.0847 -62.2651 664265.7895 -16.4579
-16.4750
[73844]ENERGY: 18000000 3534.8349 5967.2269 5852.4137
366.6799 -15465.7910 -1806.5178 0.0000 0.0000
9051.2668 7500.1133 337.6556 -1551.1535 7577.2322
334.2279 82.1217 96.1260 664265.7895 -15.0382
-15.0310
[73894]ENERGY: 18010000 3651.1171 5892.5563 5969.6610
362.1486 -15462.8636 -1831.7576 0.0000 0.0000
8922.5561 7503.4179 332.8540 -1419.1382 7576.3016
334.2282 -48.7470 29.2845 664265.7895 -12.2716
-12.2688
[73930]ENERGY: 18020000 3709.0373 5873.4694 5907.8280
335.4036 -15404.1036 -1819.9283 0.0000 0.0000
8902.3278 7504.0342 332.0994 -1398.2937 7574.6824
334.2475 -72.8009 -54.9496 664265.7895 -12.0725
-12.0479
[73980]ENERGY: 18030000 3662.8955 5935.9855 5937.8392
354.3664 -15436.6415 -1893.3531 0.0000 0.0000
8940.5830 7501.6750 333.5265 -1438.9080 7576.6452
334.2904 -112.2093 -141.1696 664265.7895 -9.1414
-9.1632
[74016]ENERGY: 18040000 3688.9135 5842.5243 5881.6482
379.0105 -15365.7680 -1900.9821 0.0000 0.0000
8978.0266 7503.3729 334.9233 -1474.6537 7575.6971
334.4266 -167.3533 -159.6106 664265.7895 -13.9003
-13.9123
[74066]ENERGY: 18050000 3745.3761 5827.2766 5903.6725
367.1774 -15398.7758 -1883.9095 0.0000 0.0000
8941.5329 7502.3503 333.5620 -1439.1826 7574.8168
334.3290 22.4049 -5.3190 664265.7895 -12.6979
-12.6800
[74102]ENERGY: 18060000 3714.7828 5966.8785 5869.8992
371.4519 -15349.0509 -1923.4091 0.0000 0.0000
8856.8130 7507.3654 330.4015 -1349.4477 7575.6410
334.1054 -38.4657 -57.7095 664265.7895 -12.5595
-12.5675
[74152]ENERGY: 18070000 3625.4180 5953.5166 5840.1747
361.8199 -15391.0998 -1805.9477 0.0000 0.0000
8923.1162 7506.9978 332.8749 -1416.1184 7576.0630
334.3932 104.7897 -19.7561 664265.7895 -11.8190
-11.8209
[74188]ENERGY: 18080000 3624.4886 5909.7420 5850.5692
341.7505 -15404.3480 -1884.4629 0.0000 0.0000
9061.6403 7499.3797 338.0425 -1562.2606 7575.9136
334.0450 -21.9409 -61.9316 664265.7895 -17.2290
-17.2228
[74238]ENERGY: 18090000 3639.4902 5892.4491 5899.3188
338.0878 -15408.3362 -1877.0880 0.0000 0.0000
9018.9944 7502.9161 336.4516 -1516.0783 7576.0138
334.1700 -52.8509 -84.2445 664265.7895 -13.9964
-13.9938
[74277]ENERGY: 18100000 3574.8895 5868.5270 5900.2098

360.6218 -15441.8632 -1880.4086 0.0000 0.0000
9120.2996 7502.2759 340.2308 -1618.0238 7575.5931
334.0486 23.8998 -24.2931 664265.7895 -10.7602
-10.7501
[74327]ENERGY: 18110000 3676.3741 5926.7956 5894.5121
340.0872 -15426.5595 -1903.3007 0.0000 0.0000
8993.1155 7501.0242 335.4862 -1492.0913 7575.0533
334.1858 32.2625 -26.6151 664265.7895 -11.9313
-11.9493
[74363]ENERGY: 18120000 3691.6673 5907.7462 5922.5812
384.6411 -15426.4609 -1860.6631 0.0000 0.0000
8883.2610 7502.7729 331.3881 -1380.4882 7576.3004
334.4090 16.1163 -9.1688 664265.7895 -14.4474
-14.4305
[74413]ENERGY: 18130000 3681.5889 5843.4587 5895.9708
341.6396 -15403.5223 -1848.3278 0.0000 0.0000
8993.1998 7504.0077 335.4894 -1489.1921 7576.2359
334.1107 -30.0404 -127.7970 664265.7895 -11.0184
-11.0298
[74449]ENERGY: 18140000 3671.4058 5868.0615 5888.6182
358.3799 -15356.6804 -1833.4066 0.0000 0.0000
8908.9431 7505.3216 332.3462 -1403.6215 7576.0430
334.4810 98.3799 -123.3100 664265.7895 -10.4846
-10.4967
[74499]ENERGY: 18150000 3631.4163 5958.9092 5906.1129
370.2537 -15389.0177 -1933.8320 0.0000 0.0000
8958.9423 7502.7847 334.2114 -1456.1576 7575.7155
334.3497 -19.9378 -36.7521 664265.7895 -11.0336
-11.0220
[74535]ENERGY: 18160000 3609.5318 5949.2674 5902.5499
374.7338 -15380.0020 -1859.8645 0.0000 0.0000
8910.2928 7506.5093 332.3966 -1403.7835 7575.3260
334.1809 40.3289 -11.5424 664265.7895 -12.2106
-12.2133
[74585]ENERGY: 18170000 3661.4652 5992.1807 5910.3225
365.3543 -15539.5206 -1825.2001 0.0000 0.0000
8939.5896 7504.1917 333.4895 -1435.3980 7575.2761
334.4806 -99.3722 -63.6099 664265.7895 -12.2354
-12.2260
[74621]ENERGY: 18180000 3631.0857 5864.5345 5918.6386
357.8035 -15486.1333 -1866.1167 0.0000 0.0000
9080.4620 7500.2744 338.7447 -1580.1876 7577.1945
334.5625 138.7562 72.3007 664265.7895 -15.9117
-15.9385
[74671]ENERGY: 18190000 3618.6932 6004.6672 5913.7269
379.5804 -15499.1923 -1825.7418 0.0000 0.0000
8912.3336 7504.0673 332.4727 -1408.2663 7575.7358
334.4190 95.9975 -6.8229 664265.7895 -7.2130
-7.2112
[74710]ENERGY: 18200000 3631.6475 5869.1339 5919.1867
359.2972 -15377.7095 -1926.7676 0.0000 0.0000
9026.6668 7501.4551 336.7379 -1525.2118 7575.3460
334.2284 -61.2481 -55.3486 664265.7895 -12.6926
-12.6779

[74760]ENERGY: 18210000 3675.8446 5852.2859 5862.0590
357.3040 -15421.5660 -1838.8139 0.0000 0.0000
9013.1723 7500.2858 336.2344 -1512.8865 7575.8847
334.2887 -59.4364 16.7128 664265.7895 -13.3372
-13.3395
[74796]ENERGY: 18220000 3711.0756 5950.4116 5875.0047
389.4520 -15577.0829 -1848.5295 0.0000 0.0000
9000.2343 7500.5658 335.7518 -1499.6685 7575.0231
334.3213 -30.2082 -82.3665 664265.7895 -10.9752
-10.9785
[74846]ENERGY: 18230000 3621.6085 5928.5077 5925.0429
365.7929 -15442.7395 -1812.8612 0.0000 0.0000
8920.5952 7505.9465 332.7809 -1414.6488 7575.5201
334.2792 74.8731 98.7617 664265.7895 -7.0669
-7.0492
[74882]ENERGY: 18240000 3699.7540 5968.1362 5921.9006
372.1709 -15445.2467 -1924.7505 0.0000 0.0000
8908.8128 7500.7774 332.3413 -1408.0355 7574.6496
334.1081 -269.7831 -239.5329 664265.7895 -9.2988
-9.3161
[74932]ENERGY: 18250000 3682.2459 5929.2605 5913.4550
381.5009 -15436.6375 -1883.8925 0.0000 0.0000
8918.8879 7504.8203 332.7172 -1414.0676 7576.7749
334.2950 -95.0826 -103.9235 664265.7895 -10.0015
-9.9751
[74968]ENERGY: 18260000 3641.2496 5872.1183 5936.7822
388.7470 -15483.2398 -1869.2975 0.0000 0.0000
9019.8038 7506.1636 336.4818 -1513.6401 7576.0761
334.4383 49.2457 1.1163 664265.7895 -5.7207
-5.7385
[75018]ENERGY: 18270000 3596.8116 5975.4207 5913.9372
344.5648 -15374.1501 -1882.0292 0.0000 0.0000
8928.9538 7503.5089 333.0927 -1425.4450 7575.6805
334.6485 334.3369 152.3933 664265.7895 -9.3640
-9.3797
[75054]ENERGY: 18280000 3672.3339 5967.2986 5922.8679
388.3532 -15554.0530 -1821.1509 0.0000 0.0000
8927.2067 7502.8564 333.0275 -1424.3503 7574.0269
334.3225 77.4238 30.8738 664265.7895 -11.8463
-11.8544
[75104]ENERGY: 18290000 3641.4754 5863.5597 5895.5097
365.0645 -15389.8101 -1826.4655 0.0000 0.0000
8955.5473 7504.8810 334.0848 -1450.6664 7575.0002
334.3124 111.9532 92.4552 664265.7895 -9.3901
-9.3632
[75143]ENERGY: 18300000 3770.0356 5901.6765 5980.4604
362.1340 -15482.1587 -1906.9982 0.0000 0.0000
8882.8139 7507.9636 331.3715 -1374.8504 7577.5622
334.2472 -34.0661 -68.2011 664265.7895 -9.9001
-9.9143
[75193]ENERGY: 18310000 3569.2241 5866.1945 5876.5862
355.9332 -15422.1314 -1817.1063 0.0000 0.0000
9075.6542 7504.3545 338.5653 -1571.2997 7577.5027
334.2738 34.2400 -1.3988 664265.7895 -8.3968

-8.3914
[75229]ENERGY: 18320000 3717.7079 5896.7061 5899.8079
347.6288 -15445.8188 -1891.4981 0.0000 0.0000
8979.9674 7504.5012 334.9957 -1475.4662 7576.8111
334.4089 -71.1917 -158.4241 664265.7895 -11.5499
-11.5437
[75279]ENERGY: 18330000 3641.4633 5949.4390 5881.4594
352.7758 -15427.5515 -1856.7303 0.0000 0.0000
8962.0423 7502.8980 334.3271 -1459.1443 7576.7914
334.1771 -48.2547 -112.0966 664265.7895 -11.1611
-11.1509
[75315]ENERGY: 18340000 3656.5168 5877.4892 5905.4883
356.6768 -15415.1915 -1834.4591 0.0000 0.0000
8958.7500 7505.2704 334.2042 -1453.4795 7576.5126
334.5033 -93.5498 -93.7817 664265.7895 -14.6268
-14.6449
[75365]ENERGY: 18350000 3764.6369 5752.0338 5983.9536
364.2867 -15405.0558 -1881.4238 0.0000 0.0000
8929.0708 7507.5022 333.0971 -1421.5686 7578.3064
334.5659 -80.9374 -76.9903 664265.7895 -9.7792
-9.7680
[75401]ENERGY: 18360000 3689.2939 5884.9175 5940.6492
386.1517 -15467.4820 -1878.0438 0.0000 0.0000
8951.2425 7506.7291 333.9242 -1444.5135 7576.1522
334.3483 71.4849 59.4187 664265.7895 -13.1370
-13.1470
[75451]ENERGY: 18370000 3492.8808 5932.3142 5955.8684
367.1576 -15426.0944 -1824.0694 0.0000 0.0000
9006.1940 7504.2513 335.9741 -1501.9427 7578.0766
334.3962 156.5157 170.8348 664265.7895 -10.5871
-10.5766
[75487]ENERGY: 18380000 3583.9533 5881.8888 5894.7039
376.3374 -15388.2312 -1891.3603 0.0000 0.0000
9047.1556 7504.4474 337.5022 -1542.7082 7576.8254
334.2926 -2.7142 -88.4703 664265.7895 -9.1763
-9.1967
[75537]ENERGY: 18390000 3644.4247 5870.6407 5891.8561
358.3442 -15408.7450 -1826.3970 0.0000 0.0000
8974.8713 7504.9951 334.8056 -1469.8762 7577.9261
334.3577 -156.5484 -69.3602 664265.7895 -6.3070
-6.3152
[75576]ENERGY: 18400000 3635.2776 5849.8246 5935.2925
361.1066 -15448.3991 -1830.8374 0.0000 0.0000
9006.9760 7509.2409 336.0033 -1497.7351 7580.3135
334.2678 67.3914 17.8482 664265.7895 -8.6686
-8.6495
[75626]ENERGY: 18410000 3587.1193 5846.6304 5937.4218
369.1937 -15359.9444 -1843.3543 0.0000 0.0000
8974.8667 7511.9332 334.8055 -1462.9335 7578.6476
334.2534 8.2821 55.4122 664265.7895 -8.3996
-8.3979
[75662]ENERGY: 18420000 3660.2965 5826.7415 5948.3257
369.6515 -15307.9826 -1977.2607 0.0000 0.0000
8988.4409 7508.2128 335.3118 -1480.2281 7580.3706

334.3705 -45.8971 -133.1336 664265.7895 -9.0785
-9.0625
[75712]ENERGY: 18430000 3678.5096 5948.3613 5902.5755
364.2413 -15485.2321 -1852.1134 0.0000 0.0000
8949.5974 7505.9394 333.8628 -1443.6580 7580.6778
334.4612 23.5217 -49.6313 664265.7895 -7.6895
-7.6997
[75748]ENERGY: 18440000 3566.9399 5916.4071 5937.9858
353.1090 -15422.4884 -1833.6586 0.0000 0.0000
8990.4497 7508.7445 335.3868 -1481.7052 7582.5943
334.2376 151.3524 173.4837 664265.7895 -13.4030
-13.4074
[75798]ENERGY: 18450000 3660.6922 5876.4874 5955.4317
355.9632 -15454.2854 -1850.0781 0.0000 0.0000
8962.7847 7506.9957 334.3548 -1455.7890 7580.9515
334.4934 72.0073 97.6878 664265.7895 -12.5571
-12.5420
[75834]ENERGY: 18460000 3665.6724 5898.0535 5923.7134
335.6005 -15379.7982 -1866.9028 0.0000 0.0000
8932.6530 7508.9918 333.2307 -1423.6612 7583.1074
334.5135 150.4880 46.7010 664265.7895 -9.3627
-9.3880
[75884]ENERGY: 18470000 3664.6937 5891.0602 5923.0651
373.4722 -15370.7825 -1895.9484 0.0000 0.0000
8923.6003 7509.1604 332.8930 -1414.4398 7582.3037
334.5354 -6.3206 26.0445 664265.7895 -10.5199
-10.5015
[75920]ENERGY: 18480000 3643.0650 5822.3070 5948.3058
373.6387 -15368.9211 -1906.8823 0.0000 0.0000
8999.6332 7511.1462 335.7294 -1488.4869 7582.7868
334.5567 127.8486 29.6573 664265.7895 -9.4468
-9.4312
[75970]ENERGY: 18490000 3639.7515 5969.0164 5940.4767
372.4310 -15421.1057 -1876.0938 0.0000 0.0000
8888.2331 7512.7093 331.5736 -1375.5238 7581.4662
334.3539 -8.0745 -46.8719 664265.7895 -5.9079
-5.9394
[76009]ENERGY: 18500000 3631.1989 5910.0685 5939.9101
362.4340 -15470.9209 -1850.5084 0.0000 0.0000
8990.0975 7512.2798 335.3737 -1477.8177 7580.8032
334.1239 -20.5099 -46.4704 664265.7895 -6.7993
-6.7822
[76059]ENERGY: 18510000 3730.8635 5778.5831 5909.9582
369.6166 -15470.1597 -1807.6378 0.0000 0.0000
8997.3670 7508.5910 335.6448 -1488.7760 7582.4470
334.1770 41.7142 11.2919 664265.7895 -4.0131
-4.0281
[76095]ENERGY: 18520000 3653.1590 5883.4099 5831.4009
363.5451 -15355.0331 -1911.4382 0.0000 0.0000
9042.7250 7507.7686 337.3369 -1534.9564 7582.6640
334.2362 33.8769 45.8410 664265.7895 -6.1622
-6.1570
[76145]ENERGY: 18530000 3606.0457 6003.9346 5847.8000
358.9549 -15437.6090 -1838.6741 0.0000 0.0000

8971.4625 7511.9145 334.6785 -1459.5480 7582.5739
334.6306 22.7785 -13.4794 664265.7895 -7.7266
-7.7322
[76181]ENERGY: 18540000 3697.8950 5903.0565 5879.9612
352.4086 -15425.2586 -1850.7725 0.0000 0.0000
8952.8872 7510.1773 333.9855 -1442.7099 7582.3410
334.6314 -74.4615 -26.2954 664265.7895 -11.2238
-11.2136
[76231]ENERGY: 18550000 3703.4707 5871.5553 5891.6673
352.6647 -15428.1660 -1872.5895 0.0000 0.0000
8992.6619 7511.2645 335.4693 -1481.3973 7583.4890
334.4931 -88.2459 -148.4470 664265.7895 -7.3700
-7.3646
[76267]ENERGY: 18560000 3679.9375 5923.5806 5925.3289
364.5565 -15402.6235 -1911.1003 0.0000 0.0000
8931.1669 7510.8465 333.1753 -1420.3204 7582.6620
334.3897 46.2426 73.2361 664265.7895 -6.0933
-6.1113
[76317]ENERGY: 18570000 3705.2993 5935.3365 5950.3698
364.1436 -15476.8468 -1838.2139 0.0000 0.0000
8870.9992 7511.0877 330.9307 -1359.9115 7583.9728
334.4445 22.1123 -17.1124 664265.7895 -7.5027
-7.4942
[76353]ENERGY: 18580000 3659.9169 5901.8049 5905.3484
377.8323 -15380.2795 -1947.5148 0.0000 0.0000
8991.3669 7508.4751 335.4210 -1482.8917 7584.9000
334.5746 18.0323 -62.4201 664265.7895 -8.9881
-8.9716
[76403]ENERGY: 18590000 3676.2619 5908.1863 5874.5680
358.6047 -15402.3378 -1849.6290 0.0000 0.0000
8946.6719 7512.3260 333.7537 -1434.3459 7582.8274
334.6938 235.3060 118.6703 664265.7895 -8.4578
-8.4879
[76442]ENERGY: 18600000 3683.4268 5815.9188 5953.4701
354.2432 -15457.5147 -1810.7384 0.0000 0.0000
8976.2926 7515.0984 334.8587 -1461.1942 7583.5371
334.6835 6.1009 28.8051 664265.7895 -12.6065
-12.5732
[76492]ENERGY: 18610000 3617.9686 5927.2288 5982.7869
351.0069 -15393.8818 -1925.1977 0.0000 0.0000
8949.2960 7509.2075 333.8516 -1440.0884 7583.9726
334.4865 -31.3299 -15.4280 664265.7895 -10.1486
-10.1688
[76528]ENERGY: 18620000 3661.1408 5913.1658 5977.2833
346.2692 -15449.8374 -1835.4904 0.0000 0.0000
8900.9580 7513.4893 332.0483 -1387.4687 7582.8884
334.6502 151.2468 110.8957 664265.7895 -3.9472
-3.9485
[76578]ENERGY: 18630000 3632.3394 5871.4774 5924.0826
359.8570 -15309.5753 -1914.2364 0.0000 0.0000
8948.2326 7512.1773 333.8119 -1436.0553 7583.1971
334.6034 -49.0794 -17.4197 664265.7895 -10.2519
-10.2511
[76614]ENERGY: 18640000 3737.5371 5846.8573 5955.2723

370.1412 -15408.8074 -1952.8779 0.0000 0.0000
8964.1936 7512.3162 334.4073 -1451.8774 7583.6773
334.5303 17.7223 -82.5436 664265.7895 -4.7236
-4.7343
[76664]ENERGY: 18650000 3734.0397 5890.7319 5864.3562
371.4014 -15411.5512 -1881.2270 0.0000 0.0000
8942.8311 7510.5821 333.6104 -1432.2491 7584.3219
334.3974 -4.3962 -12.5516 664265.7895 -10.1446
-10.1403
[76700]ENERGY: 18660000 3747.2154 5832.4851 5906.6519
359.9790 -15419.0209 -1821.7531 0.0000 0.0000
8907.0669 7512.6242 332.2762 -1394.4427 7584.1632
334.4050 -129.7582 13.4451 664265.7895 -8.1067
-8.1033
[76750]ENERGY: 18670000 3718.7652 5841.9361 5974.4115
364.4294 -15393.6060 -1911.1119 0.0000 0.0000
8916.6477 7511.4721 332.6336 -1405.1757 7583.4744
334.3010 -53.5239 -55.8477 664265.7895 -6.5633
-6.5611
[76786]ENERGY: 18680000 3603.9953 5982.7711 5857.9076
335.9257 -15323.3362 -1877.4223 0.0000 0.0000
8935.8508 7515.6921 333.3500 -1420.1587 7585.2272
334.2922 86.8520 49.1398 664265.7895 -7.5776
-7.5809
[76836]ENERGY: 18690000 3617.4956 5918.7975 5918.3802
363.5677 -15490.0721 -1857.5125 0.0000 0.0000
9037.9075 7508.5639 337.1572 -1529.3436 7584.4860
334.4575 45.1489 43.5291 664265.7895 -7.3929
-7.3786
[76875]ENERGY: 18700000 3624.7738 5890.1335 5938.4136
357.0688 -15445.4151 -1845.8048 0.0000 0.0000
8991.7650 7510.9348 335.4359 -1480.8302 7585.8651
334.5195 10.3057 -19.5878 664265.7895 -9.3334
-9.3373
[76925]ENERGY: 18710000 3705.8871 5931.6152 5950.7045
369.1690 -15495.5818 -1882.8375 0.0000 0.0000
8936.8026 7515.7591 333.3855 -1421.0435 7585.4012
334.4641 23.4884 -52.5231 664265.7895 -10.1936
-10.2037
[76961]ENERGY: 18720000 3648.8860 6014.6874 5898.8598
368.8614 -15446.9747 -1886.6969 0.0000 0.0000
8915.1262 7512.7492 332.5769 -1402.3770 7585.2269
334.5364 59.7741 29.5619 664265.7895 -11.9624
-11.9496
[77011]ENERGY: 18730000 3609.7540 5957.4131 5940.1419
382.0410 -15419.5671 -1888.0934 0.0000 0.0000
8930.3721 7512.0616 333.1456 -1418.3106 7583.7508
334.4650 -13.8704 28.8382 664265.7895 -7.2625
-7.2608
[77047]ENERGY: 18740000 3643.3813 5941.0501 5962.0883
352.0071 -15393.6643 -1877.1423 0.0000 0.0000
8885.2827 7513.0029 331.4636 -1372.2798 7583.3173
334.3587 44.8039 47.7849 664265.7895 -7.4804
-7.4818

[77097]ENERGY: 18750000 3644.8780 5898.7734 5882.1104
338.1492 -15435.2574 -1871.2678 0.0000 0.0000
9055.5165 7512.9024 337.8141 -1542.6142 7586.4084
334.5909 35.0159 46.5440 664265.7895 -5.2594
-5.2621
[77133]ENERGY: 18760000 3680.5402 5826.5195 5899.1284
349.2623 -15319.8544 -1936.1238 0.0000 0.0000
9012.7235 7512.1957 336.2177 -1500.5277 7586.3398
334.6917 -95.0498 -59.3234 664265.7895 -9.6276
-9.6327
[77183]ENERGY: 18770000 3626.1435 5967.7900 5972.8206
387.0623 -15479.7805 -1846.7954 0.0000 0.0000
8889.1879 7516.4284 331.6092 -1372.7595 7587.1119
334.6863 128.6071 133.1306 664265.7895 -6.8993
-6.8953
[77219]ENERGY: 18780000 3637.2849 5857.3053 5920.3867
391.7212 -15447.5999 -1856.1301 0.0000 0.0000
9010.1814 7513.1495 336.1229 -1497.0319 7586.9896
334.7069 -17.5739 -22.1745 664265.7895 -6.3341
-6.3304
[77269]ENERGY: 18790000 3647.1385 5881.3700 5923.9940
360.9865 -15445.0159 -1838.3020 0.0000 0.0000
8983.2505 7513.4215 335.1182 -1469.8290 7587.4057
334.6182 -51.7871 -43.1753 664265.7895 -6.1066
-6.1200
[77308]ENERGY: 18800000 3651.8059 5874.8262 5958.3740
336.4198 -15333.9253 -1937.6649 0.0000 0.0000
8966.6552 7516.4909 334.4991 -1450.1643 7588.4829
334.6802 -15.2933 -79.8219 664265.7895 -3.8272
-3.8143
[77358]ENERGY: 18810000 3776.0912 5766.3662 5952.0006
345.2388 -15324.4675 -1947.4522 0.0000 0.0000
8949.2422 7517.0194 333.8496 -1432.2229 7588.5814
334.6073 -60.3666 -109.0145 664265.7895 -6.0977
-6.0932
[77394]ENERGY: 18820000 3627.4881 5878.4249 5916.6609
355.7284 -15398.8171 -1924.3856 0.0000 0.0000
9061.1660 7516.2656 338.0248 -1544.9004 7589.4895
334.6947 -34.1123 -173.1244 664265.7895 -6.5255
-6.5658
[77444]ENERGY: 18830000 3737.2392 5961.2018 5942.5322
349.4696 -15504.2454 -1842.2625 0.0000 0.0000
8876.3310 7520.2660 331.1296 -1356.0650 7588.2252
334.9001 43.1883 151.1841 664265.7895 -2.8340
-2.8012
[77480]ENERGY: 18840000 3689.5339 5897.1820 5909.2261
383.8705 -15306.9295 -1938.2481 0.0000 0.0000
8889.5144 7524.1493 331.6214 -1365.3651 7590.3177
334.6887 251.3782 115.0469 664265.7895 -6.3209
-6.3341
[77530]ENERGY: 18850000 3741.8260 5907.3449 5948.0591
342.4624 -15443.6609 -1933.8607 0.0000 0.0000
8955.8001 7517.9708 334.0942 -1437.8292 7587.9686
334.7613 -59.9588 -138.0752 664265.7895 -3.9006

-3.9113
[77566]ENERGY: 18860000 3627.3396 5905.7439 5912.6198
367.6933 -15491.7771 -1815.6834 0.0000 0.0000
9003.0885 7509.0245 335.8583 -1494.0640 7588.9496
334.8229 -24.3384 -34.6436 664265.7895 -0.4629
-0.4518
[77616]ENERGY: 18870000 3626.0480 5926.7352 5939.2736
375.3363 -15437.8999 -1841.0073 0.0000 0.0000
8927.1239 7515.6097 333.0244 -1411.5142 7589.5148
334.6872 121.5459 8.8198 664265.7895 -4.8125
-4.8097
[77652]ENERGY: 18880000 3660.6016 5951.9481 5860.7869
353.4555 -15474.9347 -1801.9965 0.0000 0.0000
8964.8814 7514.7421 334.4330 -1450.1393 7588.1431
334.5808 130.4816 128.0070 664265.7895 -5.8027
-5.8022
[77702]ENERGY: 18890000 3734.7150 5959.8606 5922.7116
357.6206 -15448.0019 -1888.8265 0.0000 0.0000
8880.5042 7518.5836 331.2853 -1361.9206 7589.9368
334.4619 51.5663 28.0286 664265.7895 -7.9386
-7.9229
[77741]ENERGY: 18900000 3672.9735 5853.7093 5944.9467
354.4087 -15394.2465 -1926.0499 0.0000 0.0000
9015.2825 7521.0244 336.3132 -1494.2582 7591.7955
334.5609 -106.8197 -6.4404 664265.7895 -8.3051
-8.3056
[77791]ENERGY: 18910000 3640.1956 5957.1104 5883.1530
351.9550 -15486.1641 -1873.6414 0.0000 0.0000
9043.3920 7516.0005 337.3618 -1527.3915 7591.4161
334.6231 91.6804 10.0754 664265.7895 -9.2988
-9.3067
[77827]ENERGY: 18920000 3666.8933 5823.7950 5888.3901
400.7614 -15358.4291 -1906.0241 0.0000 0.0000
9002.2742 7517.6608 335.8279 -1484.6133 7590.5049
334.6397 -136.4018 -30.5157 664265.7895 -7.5506
-7.5460
[77877]ENERGY: 18930000 3661.6387 5958.7786 5942.7523
351.6778 -15550.7068 -1820.5342 0.0000 0.0000
8974.6309 7518.2373 334.7967 -1456.3936 7591.5412
334.8819 61.4221 61.1113 664265.7895 -6.4625
-6.4701
[77913]ENERGY: 18940000 3683.2105 5922.9703 5915.9205
360.4057 -15377.7614 -1960.5368 0.0000 0.0000
8975.1769 7519.3855 334.8170 -1455.7913 7592.7004
334.6411 -149.2106 -174.1396 664265.7895 -7.6537
-7.6475
[77963]ENERGY: 18950000 3710.2044 5889.7555 5904.7491
390.3181 -15433.6001 -1830.9958 0.0000 0.0000
8887.0857 7517.5169 331.5308 -1369.5688 7591.0389
334.5077 47.7631 82.4989 664265.7895 -7.4386
-7.4423
[77999]ENERGY: 18960000 3608.1092 5957.6064 5866.2483
375.9028 -15387.4496 -1880.1500 0.0000 0.0000
8978.8224 7519.0895 334.9530 -1459.7328 7591.7514

334.7890 14.8747 -74.1098 664265.7895 -7.4680
-7.4814
[78049]ENERGY: 18970000 3651.6382 5925.3547 5935.1052
347.3201 -15431.1903 -1863.6486 0.0000 0.0000
8952.9300 7517.5093 333.9871 -1435.4207 7591.9627
334.8586 -25.4872 -56.3483 664265.7895 -9.7577
-9.7257
[78085]ENERGY: 18980000 3710.5548 5946.8041 5925.3122
367.8674 -15509.1124 -1918.5288 0.0000 0.0000
8996.7718 7519.6691 335.6226 -1477.1027 7593.0458
334.8568 24.3818 -25.0716 664265.7895 -6.7362
-6.7721
[78135]ENERGY: 18990000 3672.7993 5850.0900 5920.9153
387.0086 -15290.2428 -1963.0678 0.0000 0.0000
8942.3887 7519.8913 333.5939 -1422.4974 7593.0188
334.6725 -101.7812 -150.6632 664265.7895 -8.7283
-8.7204
[78174]ENERGY: 19000000 3632.6170 5895.8338 5910.3329
359.2858 -15505.9165 -1759.7490 0.0000 0.0000
8986.8813 7519.2854 335.2537 -1467.5960 7593.9414
334.7018 -22.5958 8.1846 664265.7895 -8.2373
-8.2262
[78224]ENERGY: 19010000 3650.6226 5877.0817 5859.7641
361.0764 -15375.7433 -1874.2963 0.0000 0.0000
9025.5733 7524.0785 336.6971 -1501.4948 7596.3420
334.8403 -22.7338 -54.9468 664265.7895 -6.4197
-6.4333
[78260]ENERGY: 19020000 3655.4529 5971.6728 5925.0541
362.1666 -15412.7107 -1875.4307 0.0000 0.0000
8896.8302 7523.0352 331.8943 -1373.7950 7596.2802
334.9688 27.2526 71.9608 664265.7895 -5.8419
-5.8168
[78310]ENERGY: 19030000 3623.5920 5981.0207 5960.8523
347.4076 -15379.9681 -1915.6036 0.0000 0.0000
8909.3438 7526.6446 332.3612 -1382.6992 7597.7793
334.9219 -180.6013 -98.6154 664265.7895 -4.2086
-4.2108
[78346]ENERGY: 19040000 3544.4697 5971.9049 5957.3541
382.7553 -15435.9040 -1876.2242 0.0000 0.0000
8977.6540 7522.0097 334.9094 -1455.6443 7597.1923
334.7662 -36.7589 30.9970 664265.7895 -9.0430
-9.0404
[78396]ENERGY: 19050000 3593.0909 5928.7272 5937.1732
339.2933 -15411.8113 -1897.7790 0.0000 0.0000
9037.2187 7525.9131 337.1315 -1511.3057 7597.6288
334.8424 178.3161 161.7921 664265.7895 -6.1644
-6.1924
[78432]ENERGY: 19060000 3599.8885 5901.4854 5949.6517
362.7526 -15308.0452 -1903.4064 0.0000 0.0000
8924.2705 7526.5972 332.9180 -1397.6733 7597.3406
334.9038 -122.2220 -101.6943 664265.7895 -8.6008
-8.5963
[78482]ENERGY: 19070000 3656.3222 5936.0039 5886.1011
378.6322 -15365.8528 -1904.6035 0.0000 0.0000

8941.6516 7528.2547 333.5664 -1413.3969 7599.0841
334.8906 -74.5783 -103.3770 664265.7895 -7.9910
-7.9776
[78518]ENERGY: 19080000 3664.2613 5827.8975 5906.7733
328.6587 -15385.3902 -1830.2235 0.0000 0.0000
9013.4459 7525.4231 336.2447 -1488.0228 7598.5177
334.9588 165.0184 75.0059 664265.7895 -7.8637
-7.8666
[78568]ENERGY: 19090000 3784.1322 5937.1680 5938.9886
365.0144 -15448.3206 -1920.0827 0.0000 0.0000
8869.9022 7526.8021 330.8898 -1343.1001 7597.7788
334.5131 27.3270 -3.2503 664265.7895 -9.0126
-9.0234
[78607]ENERGY: 19100000 3683.2206 5922.7453 5893.4718
360.1409 -15408.1318 -1877.5697 0.0000 0.0000
8953.0876 7526.9646 333.9930 -1426.1230 7596.8013
334.8230 -71.7566 -78.9422 664265.7895 -9.7711
-9.7551
[78657]ENERGY: 19110000 3634.3052 5881.9284 5973.9402
347.7513 -15462.2766 -1847.7976 0.0000 0.0000
8995.7907 7523.6416 335.5860 -1472.1491 7596.3807
334.9064 13.0272 120.0838 664265.7895 -11.1996
-11.1737
[78693]ENERGY: 19120000 3725.5624 5882.3490 5869.1506
349.6029 -15506.2132 -1799.6731 0.0000 0.0000
8999.5222 7520.3008 335.7252 -1479.2213 7596.5630
334.7021 39.6863 -5.0767 664265.7895 -9.5553
-9.5946
[78743]ENERGY: 19130000 3679.1127 5897.0447 5934.5063
369.4193 -15397.5937 -1879.3971 0.0000 0.0000
8923.3605 7526.4527 332.8840 -1396.9077 7596.0964
334.7245 59.6588 44.5242 664265.7895 -5.0018
-5.0091
[78779]ENERGY: 19140000 3710.2968 5916.5048 5951.0842
340.6173 -15459.3946 -1940.7787 0.0000 0.0000
9001.4822 7519.8120 335.7984 -1481.6702 7595.0803
334.9701 78.3310 14.4733 664265.7895 -11.7336
-11.7038
[78829]ENERGY: 19150000 3601.6648 6000.7992 5927.9895
365.5792 -15459.4866 -1926.7047 0.0000 0.0000
9016.8038 7526.6452 336.3699 -1490.1587 7598.5142
334.8922 171.8610 54.1206 664265.7895 -11.1291
-11.1491
[78865]ENERGY: 19160000 3650.9517 5869.6592 5919.4344
370.2620 -15322.8948 -1924.5243 0.0000 0.0000
8966.5954 7529.4835 334.4969 -1437.1119 7598.9513
334.6669 -4.7887 -46.3201 664265.7895 -9.7286
-9.7299
[78915]ENERGY: 19170000 3595.8145 5905.1702 5890.1699
355.9889 -15424.8233 -1844.7647 0.0000 0.0000
9045.7100 7523.2655 337.4483 -1522.4445 7597.2890
334.8401 146.5785 -75.7889 664265.7895 -10.5048
-10.5114
[78951]ENERGY: 19180000 3692.3392 5982.6006 5935.2002

358.1677 -15549.6697 -1787.1552 0.0000 0.0000
8893.5435 7525.0263 331.7717 -1368.5172 7597.2510
334.7983 -98.8178 -15.8037 664265.7895 -9.2642
-9.2586
[79001]ENERGY: 19190000 3644.9202 5849.2806 5921.1220
359.9909 -15372.1797 -1922.7529 0.0000 0.0000
9045.4568 7525.8381 337.4388 -1519.6187 7597.7948
335.0385 -73.0902 -85.6369 664265.7895 -7.2894
-7.2801
[79040]ENERGY: 19200000 3682.4529 5898.9013 5901.3642
359.2912 -15445.5590 -1864.0269 0.0000 0.0000
8993.1594 7525.5832 335.4879 -1467.5762 7598.6888
334.7120 -115.7498 -85.9858 664265.7895 -7.7824
-7.7723
[79090]ENERGY: 19210000 3594.7387 5931.7935 5880.5119
351.0152 -15453.9642 -1834.0065 0.0000 0.0000
9052.2041 7522.2926 337.6905 -1529.9115 7596.7220
334.9805 136.3883 46.8755 664265.7895 -8.3756
-8.4149
[79126]ENERGY: 19220000 3570.3355 5891.9432 5898.4217
367.1969 -15412.9732 -1780.1591 0.0000 0.0000
8990.4318 7525.1967 335.3861 -1465.2351 7596.5075
334.9072 88.1229 99.9397 664265.7895 -10.0923
-10.0652
[79176]ENERGY: 19230000 3731.3105 5939.1541 5961.2523
379.5083 -15463.4200 -1913.3049 0.0000 0.0000
8891.5326 7526.0328 331.6967 -1365.4998 7596.6726
334.9699 -142.5089 -119.0465 664265.7895 -7.7193
-7.7127
[79212]ENERGY: 19240000 3573.8118 5917.9204 5884.1412
353.4706 -15326.2549 -1841.0312 0.0000 0.0000
8962.8790 7524.9368 334.3583 -1437.9422 7596.3948
334.7776 -50.2356 16.6142 664265.7895 -9.3919
-9.4167
[79262]ENERGY: 19250000 3606.8092 5928.4749 5911.0711
369.6342 -15367.6262 -1906.2416 0.0000 0.0000
8985.7530 7527.8745 335.2116 -1457.8785 7598.6656
334.8918 150.8017 -12.8990 664265.7895 -14.9163
-14.9050
[79298]ENERGY: 19260000 3697.3645 6037.4380 5922.9221
401.8327 -15571.6186 -1858.6254 0.0000 0.0000
8899.1184 7528.4317 331.9797 -1370.6867 7597.1050
334.8473 -86.7929 -97.8092 664265.7895 -10.4726
-10.4618
[79348]ENERGY: 19270000 3602.3323 5939.1030 5914.8259
348.1281 -15429.5351 -1845.5462 0.0000 0.0000
8997.9040 7527.2122 335.6649 -1470.6919 7599.3383
334.9074 143.0822 40.8093 664265.7895 -4.5129
-4.5022
[79384]ENERGY: 19280000 3726.0799 5924.9805 5906.5047
365.7838 -15467.1372 -1846.7548 0.0000 0.0000
8919.8467 7529.3037 332.7530 -1390.5430 7598.1597
335.0245 105.9046 84.9854 664265.7895 -7.0429
-7.0567

[79434]ENERGY: 19290000 3776.1310 5806.9834 5948.0974
366.1452 -15444.6382 -1834.7650 0.0000 0.0000
8910.7978 7528.7516 332.4154 -1382.0462 7598.1262
334.6864 41.0023 66.3738 664265.7895 -11.6754
-11.6809
[79473]ENERGY: 19300000 3748.5656 5878.7459 5895.0455
387.6582 -15416.1274 -1938.8691 0.0000 0.0000
8972.1141 7527.1327 334.7028 -1444.9814 7599.3691
334.7355 40.9993 -28.1642 664265.7895 -7.4517
-7.4586
[79523]ENERGY: 19310000 3709.8632 5900.3818 5904.8782
359.8910 -15508.2877 -1860.5436 0.0000 0.0000
9015.1645 7521.3476 336.3088 -1493.8170 7598.8884
334.7525 -201.2923 -92.5396 664265.7895 -5.6636
-5.6458
[79559]ENERGY: 19320000 3624.0290 5931.9031 5888.5407
353.4863 -15401.0732 -1876.3059 0.0000 0.0000
9005.7619 7526.3419 335.9580 -1479.4200 7600.5306
334.8428 48.3397 56.2937 664265.7895 -7.9128
-7.9336
[79609]ENERGY: 19330000 3661.6130 5988.0434 5925.1635
369.5150 -15399.4762 -1896.6614 0.0000 0.0000
8881.1080 7529.3054 331.3078 -1351.8026 7601.0770
335.0288 -136.0662 -103.4901 664265.7895 -7.1122
-7.1111
[79645]ENERGY: 19340000 3727.1620 5945.5036 5874.2524
347.9457 -15402.5185 -1897.5315 0.0000 0.0000
8940.2656 7535.0793 333.5147 -1405.1863 7604.0005
335.2322 -49.5177 -62.5349 664265.7895 -8.4665
-8.4521
[79695]ENERGY: 19350000 3761.9833 5884.9011 5896.1924
361.1685 -15488.9261 -1816.0148 0.0000 0.0000
8931.4260 7530.7303 333.1849 -1400.6957 7603.2779
335.0361 -129.3902 -114.9747 664265.7895 -5.8676
-5.8690
[79731]ENERGY: 19360000 3656.9623 6038.6639 5871.9846
368.3876 -15456.4778 -1893.7242 0.0000 0.0000
8944.5148 7530.3112 333.6732 -1414.2036 7603.7677
335.1140 28.4433 -26.8885 664265.7895 -7.4961
-7.4905
[79781]ENERGY: 19370000 3724.1629 5842.5579 5928.0017
361.0097 -15328.7688 -1881.5791 0.0000 0.0000
8891.0568 7536.4411 331.6790 -1354.6157 7604.2516
334.9852 -89.1014 -113.4675 664265.7895 -12.4749
-12.4808
[79817]ENERGY: 19380000 3707.0135 6065.6357 5892.7722
360.3519 -15549.3430 -1837.0099 0.0000 0.0000
8888.0756 7527.4960 331.5677 -1360.5796 7602.8402
334.8823 69.3545 66.7070 664265.7895 -13.7361
-13.7343
[79867]ENERGY: 19390000 3676.7823 5875.8485 5899.6220
372.0597 -15421.7072 -1892.8360 0.0000 0.0000
9017.5266 7527.2961 336.3969 -1490.2305 7603.8667
335.1302 90.6146 162.7137 664265.7895 -9.3255

-9.3031
[79906]ENERGY: 19400000 3633.8232 6005.6604 5935.5722
358.9275 -15449.2368 -1904.9270 0.0000 0.0000
8950.5505 7530.3700 333.8984 -1420.1805 7604.8913
335.0868 -74.2800 -35.4427 664265.7895 -9.4504
-9.4655
[79956]ENERGY: 19410000 3667.5690 5954.8939 5945.0830
354.7303 -15433.4287 -1873.1627 0.0000 0.0000
8917.2447 7532.9296 332.6559 -1384.3151 7605.7377
334.8955 -60.5617 -71.3963 664265.7895 -8.5713
-8.5739
[79992]ENERGY: 19420000 3728.7063 5909.4183 5935.4354
345.3112 -15571.4398 -1788.5181 0.0000 0.0000
8973.0981 7532.0114 334.7395 -1441.0867 7604.1378
335.0337 -3.7367 45.8130 664265.7895 -8.2185
-8.2256
[80042]ENERGY: 19430000 3641.3268 6011.0675 5896.5114
339.6332 -15449.2812 -1899.7065 0.0000 0.0000
8994.8372 7534.3885 335.5505 -1460.4487 7606.3144
334.8915 15.7814 76.5068 664265.7895 -10.2973
-10.3025
[80078]ENERGY: 19440000 3679.9900 5846.8801 5847.2092
337.5161 -15417.0367 -1840.5689 0.0000 0.0000
9076.9121 7530.9019 338.6123 -1546.0102 7605.5653
334.8626 -27.3258 -96.0049 664265.7895 -8.9352
-8.9331
[80128]ENERGY: 19450000 3706.1073 5961.9217 5854.2165
355.6870 -15530.8544 -1843.4176 0.0000 0.0000
9027.1785 7530.8390 336.7569 -1496.3395 7605.6025
334.9908 -49.1992 53.9636 664265.7895 -8.8589
-8.8615
[80164]ENERGY: 19460000 3548.2646 5907.5163 5920.3484
352.4340 -15383.0945 -1839.2598 0.0000 0.0000
9027.2681 7533.4770 336.7603 -1493.7910 7607.1512
335.0670 49.2101 110.5970 664265.7895 -8.0430
-8.0393
[80214]ENERGY: 19470000 3606.6174 5906.6161 5911.0073
369.9435 -15304.5473 -1897.0704 0.0000 0.0000
8943.2313 7535.7980 333.6253 -1407.4333 7606.1299
335.0629 21.6756 -15.2425 664265.7895 -6.5099
-6.5000
[80250]ENERGY: 19480000 3684.3978 5918.1793 5891.8020
355.7886 -15390.5412 -1849.3149 0.0000 0.0000
8924.1453 7534.4568 332.9133 -1389.6885 7607.9536
335.0973 117.4060 58.1629 664265.7895 -6.4213
-6.4124
[80300]ENERGY: 19490000 3613.9842 6031.3666 5846.3536
346.0330 -15304.7068 -1927.1974 0.0000 0.0000
8930.3158 7536.1491 333.1435 -1394.1667 7606.8380
334.8957 -1.2463 0.9869 664265.7895 -6.6665
-6.6882
[80339]ENERGY: 19500000 3620.9855 5946.3447 5888.7613
385.6034 -15387.8810 -1846.3504 0.0000 0.0000
8929.4451 7536.9085 333.1110 -1392.5366 7608.4205

334.9493 74.5793 7.1173 664265.7895 -8.3697
-8.3773
[80389]ENERGY: 19510000 3681.6714 5974.4403 5917.8310
356.0930 -15591.5901 -1789.7245 0.0000 0.0000
8986.0599 7534.7809 335.2230 -1451.2789 7607.3929
334.8607 -16.2960 -17.8915 664265.7895 -6.5945
-6.5910
[80425]ENERGY: 19520000 3668.4912 5902.7516 5894.9997
378.4702 -15454.9433 -1800.4250 0.0000 0.0000
8947.3535 7536.6978 333.7791 -1410.6557 7607.1898
334.7287 208.8356 101.0103 664265.7895 -6.2887
-6.2668
[80475]ENERGY: 19530000 3627.9831 6020.1805 5909.7713
362.3903 -15443.8244 -1827.2748 0.0000 0.0000
8886.2913 7535.5173 331.5012 -1350.7740 7607.1538
334.7947 -145.1795 -150.8671 664265.7895 -5.8666
-5.8838
[80511]ENERGY: 19540000 3711.3042 5924.7183 5892.3103
351.3063 -15466.9082 -1879.7079 0.0000 0.0000
9001.8581 7534.8812 335.8124 -1466.9769 7606.7369
334.9156 -145.8511 -72.2167 664265.7895 -5.3283
-5.3110
[80561]ENERGY: 19550000 3609.7550 6014.7052 5911.4549
346.6070 -15424.6397 -1874.8336 0.0000 0.0000
8952.2113 7535.2602 333.9603 -1416.9512 7607.8077
334.8652 -47.8565 48.1384 664265.7895 -7.3397
-7.3382
[80597]ENERGY: 19560000 3763.6566 5884.7726 5967.1372
334.8723 -15521.5483 -1903.8980 0.0000 0.0000
9011.6838 7536.6762 336.1789 -1475.0075 7609.0213
334.7915 -80.4723 -66.2082 664265.7895 -7.4346
-7.4452
[80647]ENERGY: 19570000 3569.6171 5857.6895 5919.6829
344.0345 -15352.1978 -1815.6126 0.0000 0.0000
9013.9682 7537.1817 336.2641 -1476.7864 7608.3018
334.8329 148.8835 115.6850 664265.7895 -6.1929
-6.2096
[80683]ENERGY: 19580000 3596.5764 5949.6948 5899.8775
368.1810 -15416.8762 -1785.4470 0.0000 0.0000
8926.2486 7538.2551 332.9918 -1387.9935 7608.4431
334.6787 30.1385 98.2424 664265.7895 -8.7149
-8.6896
[80733]ENERGY: 19590000 3618.2351 5927.7672 5864.4286
398.2887 -15368.0104 -1849.7076 0.0000 0.0000
8948.7328 7539.7344 333.8306 -1408.9984 7609.6424
334.8198 99.7526 37.4146 664265.7895 -5.4406
-5.4499
[80772]ENERGY: 19600000 3685.6355 5929.6623 5942.0296
356.4287 -15358.5620 -1866.0233 0.0000 0.0000
8854.5679 7543.7387 330.3177 -1310.8292 7609.6160
334.8128 118.6596 23.2508 664265.7895 -9.6130
-9.6166
[80822]ENERGY: 19610000 3654.0691 5916.5639 5865.5122
357.0978 -15410.0321 -1811.8769 0.0000 0.0000

8970.9303 7542.2643 334.6586 -1428.6660 7610.0894
334.8867 -7.3603 11.4743 664265.7895 -9.6708
-9.6763
[80858]ENERGY: 19620000 3672.6706 5916.2111 5862.7034
356.9314 -15325.8488 -1854.8396 0.0000 0.0000
8912.4243 7540.2523 332.4761 -1372.1720 7609.2305
334.8945 79.0994 -9.3458 664265.7895 -8.8434
-8.8614
[80908]ENERGY: 19630000 3696.3084 5903.1037 5912.3768
354.6926 -15434.1264 -1818.9820 0.0000 0.0000
8926.6537 7540.0267 333.0069 -1386.6270 7610.1906
334.9484 25.3268 -72.1813 664265.7895 -11.4704
-11.4466
[80944]ENERGY: 19640000 3640.7850 5855.0240 5908.4130
358.0608 -15438.1641 -1897.2779 0.0000 0.0000
9107.3239 7534.1648 339.7468 -1573.1591 7610.4141
334.6406 -60.8408 -48.0186 664265.7895 -9.4442
-9.4648
[80994]ENERGY: 19650000 3652.0246 5971.9828 5940.9239
383.8734 -15461.5092 -1884.4024 0.0000 0.0000
8933.9648 7536.8580 333.2796 -1397.1068 7611.6000
334.9068 -49.9086 -44.8017 664265.7895 -7.8700
-7.8542
[81030]ENERGY: 19660000 3609.8963 5745.2055 5929.2262
344.6939 -15352.5756 -1846.2574 0.0000 0.0000
9109.0429 7539.2317 339.8109 -1569.8112 7610.7011
334.7716 63.5829 53.1667 664265.7895 -9.1844
-9.1729
[81080]ENERGY: 19670000 3639.3696 5918.8002 5923.7976
353.7975 -15371.2996 -1800.2749 0.0000 0.0000
8876.2490 7540.4395 331.1266 -1335.8096 7612.4856
335.0191 35.3089 84.4960 664265.7895 -5.8822
-5.8905
[81116]ENERGY: 19680000 3667.0392 5781.1169 5921.9011
352.8783 -15483.7104 -1810.8559 0.0000 0.0000
9105.2662 7533.6355 339.6700 -1571.6308 7610.1147
335.0426 36.1148 80.6549 664265.7895 -6.7148
-6.7172
[81166]ENERGY: 19690000 3626.3759 5932.1575 5945.1072
378.6000 -15474.4046 -1876.1076 0.0000 0.0000
9003.0829 7534.8113 335.8581 -1468.2715 7610.2216
335.0593 34.7882 -68.0485 664265.7895 -8.2366
-8.2403
[81205]ENERGY: 19700000 3683.1025 5864.6279 5922.5545
368.8042 -15517.4286 -1881.0861 0.0000 0.0000
9093.9837 7534.5582 339.2491 -1559.4255 7610.4363
335.0165 -207.9326 -175.1551 664265.7895 -9.8219
-9.7997
[81255]ENERGY: 19710000 3687.8085 5816.6698 5945.8229
355.1399 -15317.4509 -1934.8234 0.0000 0.0000
8985.4815 7538.6482 335.2015 -1446.8333 7611.2597
334.7209 -93.0830 -52.5125 664265.7895 -12.4644
-12.4605
[81291]ENERGY: 19720000 3600.5518 5937.8290 5924.2603

359.6775 -15395.3370 -1828.4560 0.0000 0.0000
8940.6981 7539.2236 333.5308 -1401.4745 7610.8369
334.7655 49.5920 126.7976 664265.7895 -11.3808
-11.4011
[81341]ENERGY: 19730000 3748.4942 5869.8039 5915.4239
353.1687 -15398.8735 -1872.1755 0.0000 0.0000
8921.9636 7537.8053 332.8319 -1384.1582 7609.3561
334.9369 -44.0608 -23.1914 664265.7895 -10.0295
-10.0241
[81377]ENERGY: 19740000 3690.2651 5831.8026 5894.1720
354.4555 -15387.5229 -1863.3620 0.0000 0.0000
9018.9440 7538.7541 336.4498 -1480.1898 7611.5076
334.8728 -93.3907 -166.7281 664265.7895 -12.1600
-12.1794
[81427]ENERGY: 19750000 3559.9028 5919.5978 5907.6025
357.2007 -15374.4479 -1887.6982 0.0000 0.0000
9054.8917 7537.0494 337.7908 -1517.8423 7610.6576
334.9475 12.9141 48.5685 664265.7895 -7.4737
-7.4619
[81463]ENERGY: 19760000 3725.9071 5907.5266 5869.4622
371.4556 -15476.1083 -1889.1379 0.0000 0.0000
9031.5661 7540.6712 336.9206 -1490.8949 7611.9054
334.9840 -64.1186 -117.9134 664265.7895 -10.4619
-10.4718
[81513]ENERGY: 19770000 3620.6053 5934.9012 5882.6861
342.4645 -15362.3555 -1893.7094 0.0000 0.0000
9015.1948 7539.7870 336.3099 -1475.4079 7613.5499
335.0149 60.7082 100.5179 664265.7895 -11.7539
-11.7393
[81549]ENERGY: 19780000 3579.6526 5855.9159 5940.7285
361.6494 -15399.2919 -1863.0286 0.0000 0.0000
9062.6005 7538.2265 338.0784 -1524.3741 7613.7698
334.9210 122.8220 31.9004 664265.7895 -9.5190
-9.5312
[81599]ENERGY: 19790000 3593.1517 5869.4754 5921.6462
354.7189 -15371.4524 -1843.2919 0.0000 0.0000
9015.3179 7539.5658 336.3145 -1475.7521 7612.4344
334.8437 73.0914 79.0541 664265.7895 -9.6087
-9.6047
[81638]ENERGY: 19800000 3673.7143 5838.2163 5946.4266
347.6658 -15457.0986 -1878.9417 0.0000 0.0000
9070.6141 7540.5968 338.3773 -1530.0173 7613.7127
335.0224 118.4894 28.0706 664265.7895 -10.2764
-10.2769
[81688]ENERGY: 19810000 3653.7010 5866.2176 5968.3259
370.5145 -15393.0814 -1923.0786 0.0000 0.0000
8998.7385 7541.3375 335.6960 -1457.4010 7617.1734
335.1145 124.9705 57.8508 664265.7895 -8.2680
-8.2721
[81724]ENERGY: 19820000 3663.3256 5986.3020 5844.2451
342.7528 -15458.3855 -1825.0408 0.0000 0.0000
8990.8793 7544.0785 335.4028 -1446.8008 7615.4450
334.9868 -47.2650 -24.9880 664265.7895 -8.3551
-8.3384

[81774]ENERGY: 19830000 3606.6820 5999.3987 5893.7790
356.3058 -15414.2567 -1872.4937 0.0000 0.0000
8974.6414 7544.0565 334.7971 -1430.5849 7615.4568
334.8889 17.1359 -8.5380 664265.7895 -8.7488
-8.7365
[81810]ENERGY: 19840000 3681.1097 6009.3621 5859.7201
354.9645 -15461.7038 -1871.2772 0.0000 0.0000
8970.6017 7542.7771 334.6464 -1427.8246 7617.4249
335.0751 61.7126 -69.3298 664265.7895 -7.3106
-7.3410
[81860]ENERGY: 19850000 3640.3434 5883.7024 5926.7158
365.0565 -15370.2766 -1880.1162 0.0000 0.0000
8978.6551 7544.0802 334.9468 -1434.5748 7616.3141
335.0348 66.8633 -66.5428 664265.7895 -9.7198
-9.7155
[81896]ENERGY: 19860000 3683.3255 5903.4426 5914.8916
352.6396 -15431.8890 -1800.7935 0.0000 0.0000
8924.5065 7546.1233 332.9268 -1378.3832 7617.2875
335.1501 27.5341 26.6772 664265.7895 -4.8686
-4.8609
[81946]ENERGY: 19870000 3670.0106 5942.2603 5910.3937
363.5375 -15441.6755 -1835.9320 0.0000 0.0000
8940.0457 7548.6403 333.5065 -1391.4054 7617.6970
335.0312 -10.1726 -21.7286 664265.7895 -7.2188
-7.2075
[81982]ENERGY: 19880000 3683.9541 5970.5241 5870.3836
370.3940 -15455.7838 -1838.2038 0.0000 0.0000
8944.0294 7545.2977 333.6551 -1398.7317 7617.2287
335.0395 89.3374 76.4723 664265.7895 -8.3211
-8.3269
[82032]ENERGY: 19890000 3624.0955 5917.7220 5865.3720
334.0565 -15422.0638 -1809.3489 0.0000 0.0000
9037.9000 7547.7332 337.1569 -1490.1667 7617.9075
335.1868 130.9413 17.7935 664265.7895 -12.4762
-12.4616
[82071]ENERGY: 19900000 3645.8248 5989.6009 5909.2283
362.9527 -15503.3663 -1835.3756 0.0000 0.0000
8974.8844 7543.7492 334.8061 -1431.1352 7617.7232
335.2392 -8.3196 -79.1421 664265.7895 -11.8494
-11.8633
[82121]ENERGY: 19910000 3644.4354 5990.1747 5868.9478
376.8259 -15528.7459 -1784.5449 0.0000 0.0000
8978.5999 7545.6929 334.9447 -1432.9070 7617.3549
335.2354 -7.1650 3.7928 664265.7895 -8.1253
-8.1353
[82157]ENERGY: 19920000 3696.7217 6032.7140 5864.4661
353.7606 -15586.2617 -1806.6178 0.0000 0.0000
8991.0074 7545.7902 335.4076 -1445.2172 7618.2978
335.2579 73.7068 22.8379 664265.7895 -10.7409
-10.7575
[82207]ENERGY: 19930000 3628.1049 5875.3370 5980.1045
379.4657 -15492.1588 -1872.5545 0.0000 0.0000
9047.9359 7546.2348 337.5313 -1501.7011 7618.3226
335.1571 -9.4016 -15.0346 664265.7895 -12.2481

```
-12.2294
[82243]ENERGY: 19940000      3730.7512      5876.0600      5889.7032
355.1020         -15408.5337     -1885.2163         0.0000         0.0000
8987.6588           7545.5252       335.2827     -1442.1336      7618.3223
335.1870           -109.4774      -174.5870    664265.7895        -8.8332
-8.8405
[82293]ENERGY: 19950000      3703.7563      5847.4328      5873.8903
335.8592         -15370.4397     -1862.6807         0.0000         0.0000
9019.9436           7547.7619       336.4871     -1472.1817      7618.9998
335.0428             16.6848        58.1485    664265.7895       -10.1633
-10.1352
[82329]ENERGY: 19960000      3577.9693      5884.2922      5921.9310
388.6639         -15395.0622     -1836.6339         0.0000         0.0000
9006.6605           7547.8209       335.9915     -1458.8397      7617.2164
334.8656             37.4979       100.6687    664265.7895        -8.5862
-8.5934
[82379]ENERGY: 19970000      3668.1476      5999.0197      5927.6697
356.2152         -15489.4931     -1880.2959         0.0000         0.0000
8962.1177           7543.3809       334.3299     -1418.7368      7616.6982
335.0338            -38.1313       -36.0085    664265.7895        -5.7119
-5.7287
[82415]ENERGY: 19980000      3662.6598      5882.5886      5912.6364
369.6424         -15453.8194     -1840.7259         0.0000         0.0000
9011.7375           7544.7195       336.1809     -1467.0181      7618.6432
335.2995            -16.4432       -39.0769    664265.7895       -15.5055
-15.4924
[82465]ENERGY: 19990000      3622.9186      5946.4593      5847.0585
352.4763         -15356.6894     -1886.1192         0.0000         0.0000
9018.0070           7544.1111       336.4148     -1473.8959      7618.0728
335.1493           -120.9222       -64.7377    664265.7895        -8.2970
-8.3024
[82504]ENERGY: 20000000      3670.6430      5934.7480      5899.1671
369.0531         -15453.2987     -1875.9043         0.0000         0.0000
9000.0801           7544.4884       335.7460     -1455.5917      7619.7670
335.0234             81.6217        69.9675    664265.7895        -9.7996
-9.8123
```
