# Supplementary material for: Insights into the mechanism(s) of digestion of crystalline cellulose by plant class C GH9 endoglucanases
Source: J Mol Model. 2019 Jul 23;25(8):240. doi: 10.1007/s00894-019-4133-1 (PMC7385011; doi:10.1007/s00894-019-4133-1)
Supplement: Supplementary file 9 — (PDF 123 kb) [file 894_2019_4133_MOESM9_ESM.pdf]

Supplementary Text 7

ENSEMBLE FREQUENCY DATA (40.1ns)

```
        [,1]     [,2]     [,3]     [,4]     [,5]     [,6]     [,7]     [,8]
[,9]    [,10]    [,11]    [,12]    [,13]    [,14]    [,15]    [,16]
[1,] 0.000341 0.000681 0.001372 0.004374 0.007478 0.011097 0.011649 0.016902
0.021270 0.022669 0.024547 0.028478 0.030438 0.032352 0.033174 0.035256
[2,] 0.001501 0.002125 0.002679 0.008596 0.009766 0.012013 0.012935 0.015446
0.016413 0.018647 0.020581 0.024892 0.025868 0.027625 0.028705 0.030541
[3,] 0.001585 0.001792 0.003272 0.009507 0.012084 0.013286 0.014810 0.019636
0.022368 0.025337 0.026735 0.029003 0.029923 0.033015 0.034426 0.037445
[4,] 0.001387 0.001651 0.004025 0.009480 0.010541 0.011355 0.015374 0.018253
0.018891 0.022076 0.022805 0.027093 0.029739 0.031318 0.033748 0.035132
        [,17]    [,18]    [,19]    [,20]    [,21]    [,22]    [,23]    [,24]
[,25]    [,26]    [,27]    [,28]    [,29]    [,30]    [,31]    [,32]
[1,] 0.037530 0.039922 0.040963 0.043274 0.044135 0.046726 0.048099 0.050465
0.051716 0.054081 0.054390 0.055265 0.057803 0.059187 0.061302 0.062519
[2,] 0.032226 0.035590 0.036571 0.038129 0.039119 0.040685 0.042443 0.043248
0.045578 0.045956 0.048950 0.051083 0.053763 0.054131 0.055626 0.056344
[3,] 0.037906 0.039151 0.041693 0.042667 0.046999 0.049171 0.051067 0.051649
0.053072 0.053916 0.056735 0.059112 0.060773 0.061707 0.061975 0.062571
[4,] 0.036780 0.037600 0.038662 0.038816 0.042719 0.043474 0.044808 0.046258
0.048511 0.049747 0.051358 0.052651 0.054778 0.057877 0.058768 0.059049
        [,33]    [,34]    [,35]    [,36]    [,37]    [,38]    [,39]    [,40]
[,41]    [,42]    [,43]    [,44]    [,45]    [,46]    [,47]    [,48]
[1,] 0.064745 0.066898 0.068881 0.070308 0.070985 0.073428 0.073974 0.074767
0.076034 0.078554 0.079326 0.080331 0.081504 0.083287 0.083887 0.085269
[2,] 0.057225 0.058331 0.059857 0.061666 0.063016 0.063871 0.065411 0.066169
0.066706 0.067219 0.068733 0.071206 0.071567 0.072386 0.075078 0.076441
[3,] 0.064491 0.065628 0.067570 0.070635 0.070962 0.072967 0.073747 0.075273
0.075654 0.077114 0.078649 0.080382 0.080782 0.084173 0.084798 0.085673
[4,] 0.059781 0.062854 0.063139 0.066202 0.066675 0.068122 0.069734 0.071652
0.072872 0.074585 0.077709 0.079060 0.080397 0.081275 0.083024 0.083337
        [,49]    [,50]    [,51]    [,52]    [,53]    [,54]    [,55]    [,56]
[,57]    [,58]    [,59]    [,60]    [,61]    [,62]    [,63]    [,64]
[1,] 0.087385 0.088450 0.090890 0.091083 0.092721 0.094275 0.097329 0.100298
0.102193 0.102997 0.103523 0.105597 0.105928 0.107102 0.108286 0.108594
[2,] 0.077430 0.078203 0.079879 0.080791 0.081588 0.082679 0.084311 0.085167
0.086891 0.087736 0.089185 0.089857 0.091033 0.091439 0.093802 0.095650
[3,] 0.086254 0.087331 0.088249 0.089070 0.090323 0.090962 0.091971 0.093361
0.094457 0.095345 0.097405 0.098491 0.099418 0.100971 0.103018 0.104528
[4,] 0.084593 0.086059 0.087966 0.088914 0.089976 0.092462 0.092963 0.093982
0.094519 0.095340 0.096247 0.098554 0.100192 0.101020 0.101554 0.103005
        [,65]    [,66]    [,67]    [,68]    [,69]    [,70]    [,71]    [,72]
[,73]    [,74]    [,75]    [,76]    [,77]    [,78]    [,79]    [,80]
[1,] 0.109182 0.109782 0.110971 0.112000 0.113009 0.114690 0.116870 0.117993
0.119233 0.119844 0.120873 0.121499 0.124018 0.126402 0.127493 0.128104
[2,] 0.096371 0.096786 0.097221 0.099166 0.099742 0.101045 0.101503 0.102914
0.104773 0.106999 0.107741 0.108170 0.109937 0.110368 0.111467 0.113059
[3,] 0.104754 0.106659 0.107035 0.109273 0.110406 0.111654 0.112920 0.114082
0.115569 0.116876 0.117210 0.119445 0.119584 0.120988 0.122137 0.122487
[4,] 0.103290 0.104435 0.105744 0.105879 0.108150 0.109043 0.110084 0.111023
0.111093 0.112478 0.114057 0.114613 0.116280 0.116937 0.118347 0.119479
        [,81]    [,82]    [,83]    [,84]    [,85]    [,86]    [,87]    [,88]
[,89]    [,90]    [,91]    [,92]    [,93]    [,94]    [,95]    [,96]
```

[1,] 0.128898 0.130397 0.131375 0.133177 0.134631 0.135685 0.137903 0.139579
0.140085 0.142532 0.143447 0.144222 0.147485 0.149057 0.150153 0.151243
[2,] 0.114192 0.114618 0.116219 0.117446 0.118149 0.119355 0.120529 0.121979
0.123415 0.124753 0.125920 0.127840 0.128287 0.129049 0.129444 0.131188
[3,] 0.123228 0.123879 0.125559 0.126281 0.128484 0.129154 0.130763 0.131460
0.133421 0.134557 0.135933 0.136273 0.136477 0.138221 0.138698 0.140453
[4,] 0.120327 0.121293 0.122687 0.123348 0.124395 0.125306 0.126108 0.127562
0.129108 0.130687 0.130913 0.132846 0.134378 0.135987 0.137150 0.138033
[,97] [,98] [,99] [,100] [,101] [,102] [,103] [,104]
[,105] [,106] [,107] [,108] [,109] [,110] [,111] [,112]
[1,] 0.152356 0.153639 0.154582 0.154953 0.156261 0.157014 0.158693 0.158706
0.160897 0.161853 0.164534 0.165643 0.167294 0.167649 0.169080 0.170770
[2,] 0.131412 0.132764 0.133437 0.136405 0.137110 0.138806 0.139317 0.140341
0.140893 0.141506 0.142467 0.143373 0.144218 0.145084 0.147516 0.147988
[3,] 0.141758 0.142316 0.142903 0.143434 0.145647 0.146568 0.147265 0.148290
0.149246 0.149960 0.151795 0.152863 0.153657 0.154194 0.155001 0.157122
[4,] 0.138751 0.139226 0.141501 0.142019 0.143973 0.144298 0.144964 0.145315
0.147365 0.149182 0.149303 0.149752 0.150615 0.152293 0.153742 0.154773
[,113] [,114] [,115] [,116] [,117] [,118] [,119] [,120]
[,121] [,122] [,123] [,124] [,125] [,126] [,127] [,128]
[1,] 0.171665 0.171777 0.173363 0.174774 0.177217 0.178412 0.179749 0.180571
0.180779 0.182737 0.183700 0.184263 0.185588 0.185869 0.186330 0.188384
[2,] 0.149467 0.150009 0.151500 0.151974 0.152367 0.153539 0.154042 0.156381
0.157297 0.158066 0.158819 0.159968 0.160784 0.162017 0.162862 0.164652
[3,] 0.157299 0.158337 0.159327 0.160053 0.160549 0.162105 0.163455 0.165621
0.166028 0.167537 0.167912 0.170582 0.171480 0.172214 0.173054 0.174898
[4,] 0.155016 0.155678 0.156488 0.157475 0.158665 0.160518 0.161821 0.162505
0.163354 0.165066 0.166338 0.167381 0.167930 0.168358 0.169394 0.171010
[,129] [,130] [,131] [,132] [,133] [,134] [,135] [,136]
[,137] [,138] [,139] [,140] [,141] [,142] [,143] [,144]
[1,] 0.189147 0.190340 0.190911 0.192505 0.194101 0.194330 0.195302 0.196795
0.198806 0.198943 0.201017 0.201896 0.202077 0.203579 0.204937 0.206280
[2,] 0.165134 0.165623 0.166996 0.167875 0.168179 0.168351 0.169610 0.171331
0.171490 0.172832 0.173460 0.175013 0.176900 0.177425 0.177719 0.180004
[3,] 0.175798 0.176769 0.178122 0.178798 0.180105 0.181425 0.181836 0.182164
0.183748 0.184616 0.184923 0.186080 0.186899 0.187908 0.188497 0.190980
[4,] 0.171844 0.173515 0.174875 0.175827 0.176660 0.177798 0.179331 0.180244
0.181357 0.183268 0.183945 0.184917 0.185986 0.187894 0.189126 0.189475
[,145] [,146] [,147] [,148] [,149] [,150] [,151] [,152]
[,153] [,154] [,155] [,156] [,157] [,158] [,159] [,160]
[1,] 0.207377 0.208574 0.209220 0.211358 0.212190 0.212548 0.215194 0.215567
0.217418 0.217804 0.218376 0.219032 0.220161 0.221624 0.222228 0.223727
[2,] 0.180477 0.180602 0.181312 0.182400 0.183605 0.184005 0.185243 0.186499
0.186617 0.188949 0.189288 0.190762 0.191878 0.192402 0.194118 0.194565
[3,] 0.191493 0.192258 0.194242 0.195945 0.196975 0.197867 0.198736 0.200068
0.200852 0.201860 0.203022 0.204081 0.205406 0.205822 0.206006 0.206669
[4,] 0.191090 0.191605 0.192537 0.194520 0.195609 0.196912 0.197799 0.198324
0.198590 0.200628 0.201004 0.201712 0.203183 0.204166 0.204649 0.205400
[,161] [,162] [,163] [,164] [,165] [,166] [,167] [,168]
[,169] [,170] [,171] [,172] [,173] [,174] [,175] [,176]
[1,] 0.224407 0.224817 0.226372 0.227112 0.228386 0.229842 0.231393 0.233959
0.235578 0.237340 0.237753 0.238574 0.240540 0.242642 0.243037 0.244057
[2,] 0.196041 0.196731 0.197995 0.198730 0.199427 0.201565 0.202469 0.203058
0.204275 0.204371 0.205255 0.206395 0.207249 0.209082 0.210390 0.210476

[3,] 0.207170 0.209026 0.209536 0.212585 0.213392 0.213877 0.214785 0.215958
0.218572 0.219690 0.220595 0.222662 0.223441 0.224133 0.225550 0.226006
[4,] 0.206632 0.207528 0.210197 0.210865 0.212205 0.213181 0.214101 0.214902
0.215246 0.216205 0.216623 0.218254 0.219394 0.220451 0.221793 0.222574
[,177] [,178] [,179] [,180] [,181] [,182] [,183] [,184]
[,185] [,186] [,187] [,188] [,189] [,190] [,191] [,192]
[1,] 0.246163 0.247274 0.249405 0.250091 0.250370 0.250571 0.251974 0.253454
0.255138 0.255212 0.256662 0.257083 0.258132 0.259317 0.259687 0.261000
[2,] 0.211857 0.212564 0.213552 0.214448 0.216163 0.217743 0.218869 0.219845
0.221635 0.221968 0.222320 0.223728 0.225021 0.226436 0.226889 0.228780
[3,] 0.227379 0.227961 0.228981 0.229250 0.230666 0.231157 0.231417 0.233806
0.234967 0.235472 0.236899 0.238298 0.238705 0.239857 0.241163 0.243014
[4,] 0.222612 0.224012 0.224883 0.226495 0.227609 0.228799 0.230410 0.230624
0.232824 0.234059 0.234397 0.235659 0.236995 0.237609 0.238291 0.240527
[,193] [,194] [,195] [,196] [,197] [,198] [,199] [,200]
[,201] [,202] [,203] [,204] [,205] [,206] [,207] [,208]
[1,] 0.261894 0.262343 0.263348 0.265257 0.265882 0.266814 0.268826 0.269437
0.271533 0.272328 0.273240 0.274432 0.275458 0.276583 0.278460 0.279066
[2,] 0.230948 0.231467 0.232241 0.233194 0.233316 0.234550 0.235124 0.236395
0.237779 0.239133 0.240298 0.241937 0.242774 0.243429 0.244096 0.244426
[3,] 0.244671 0.246587 0.249073 0.249753 0.250638 0.252279 0.253693 0.254394
0.255855 0.258522 0.258962 0.259319 0.260451 0.260898 0.262773 0.264672
[4,] 0.241166 0.242539 0.243051 0.244219 0.245257 0.246987 0.247789 0.248588
0.250638 0.251667 0.252325 0.253762 0.254168 0.255246 0.256524 0.257336
[,209] [,210] [,211] [,212] [,213] [,214] [,215] [,216]
[,217] [,218] [,219] [,220] [,221] [,222] [,223] [,224]
[1,] 0.281016 0.282259 0.284745 0.286112 0.287974 0.289215 0.290539 0.291744
0.292097 0.293173 0.294369 0.295309 0.296727 0.297300 0.298842 0.301256
[2,] 0.246528 0.248066 0.250380 0.250778 0.251760 0.252606 0.253728 0.256127
0.256823 0.258843 0.259067 0.260432 0.261371 0.261835 0.263857 0.264710
[3,] 0.265437 0.267118 0.268909 0.269415 0.270467 0.271510 0.271787 0.272995
0.273739 0.275276 0.277824 0.278115 0.280069 0.280585 0.282145 0.283399
[4,] 0.258615 0.260184 0.261184 0.262168 0.263952 0.264434 0.266094 0.267052
0.268603 0.269137 0.269685 0.270210 0.272278 0.273531 0.277419 0.279142
[,225] [,226] [,227] [,228] [,229] [,230] [,231] [,232]
[,233] [,234] [,235] [,236] [,237] [,238] [,239] [,240]
[1,] 0.302221 0.303184 0.304868 0.306112 0.307225 0.308514 0.309759 0.311836
0.312528 0.313034 0.314551 0.316581 0.317200 0.318463 0.319599 0.322623
[2,] 0.265833 0.266797 0.267637 0.268566 0.270341 0.270749 0.271497 0.272551
0.274466 0.275716 0.277610 0.278646 0.279787 0.280736 0.281490 0.282347
[3,] 0.283584 0.284415 0.285214 0.285673 0.286170 0.288408 0.289956 0.291278
0.291912 0.292859 0.294093 0.295770 0.297428 0.297812 0.299009 0.300088
[4,] 0.279566 0.280567 0.280759 0.282639 0.283810 0.284889 0.286955 0.287688
0.288013 0.288460 0.289982 0.291882 0.292401 0.293023 0.294361 0.295859
[,241] [,242] [,243] [,244] [,245] [,246] [,247] [,248]
[,249] [,250] [,251] [,252] [,253] [,254] [,255] [,256]
[1,] 0.322873 0.324240 0.326091 0.326923 0.327523 0.328921 0.329579 0.331154
0.331527 0.332681 0.333660 0.334541 0.337965 0.338290 0.338670 0.341339
[2,] 0.283353 0.285129 0.286129 0.286496 0.287709 0.289910 0.290654 0.291115
0.291832 0.292529 0.294439 0.295475 0.296313 0.297287 0.298225 0.299456
[3,] 0.301125 0.303266 0.303639 0.304006 0.304972 0.306170 0.307040 0.308537
0.309722 0.311051 0.312290 0.313409 0.313542 0.315771 0.316866 0.318191
[4,] 0.297396 0.297988 0.299228 0.300213 0.301277 0.301784 0.303786 0.305316
0.306909 0.307934 0.309535 0.310871 0.311701 0.312879 0.314209 0.315264

```
       [,257]   [,258]   [,259]   [,260]   [,261]   [,262]   [,263]   [,264]
[,265]   [,266]   [,267]   [,268]   [,269]   [,270]   [,271]   [,272]
[1,] 0.342717 0.342842 0.344087 0.344920 0.348215 0.349255 0.349911 0.351131
0.353443 0.355932 0.356949 0.357493 0.359418 0.362312 0.362702 0.363173
[2,] 0.302610 0.303144 0.303406 0.304435 0.306398 0.307166 0.308641 0.309790
0.310961 0.311598 0.312521 0.314565 0.315440 0.315735 0.318629 0.319850
[3,] 0.319391 0.320018 0.320940 0.322168 0.324183 0.325761 0.327197 0.328212
0.330335 0.332128 0.332877 0.333618 0.335982 0.337099 0.337492 0.338827
[4,] 0.316648 0.317005 0.317988 0.319126 0.321582 0.322401 0.322927 0.323727
0.325268 0.325775 0.327284 0.328441 0.330300 0.331807 0.332991 0.334799
       [,273]   [,274]   [,275]   [,276]   [,277]   [,278]   [,279]   [,280]
[,281]   [,282]   [,283]   [,284]   [,285]   [,286]   [,287]   [,288]
[1,] 0.363975 0.364808 0.366734 0.367385 0.369021 0.369975 0.371862 0.372507
0.374076 0.375540 0.377132 0.377822 0.378583 0.379722 0.381091 0.381470
[2,] 0.320452 0.321797 0.322330 0.323491 0.325260 0.325856 0.326826 0.329707
0.330182 0.331671 0.332358 0.333385 0.334649 0.335280 0.336330 0.337932
[3,] 0.339681 0.340782 0.341792 0.344092 0.344423 0.346790 0.347600 0.348014
0.348433 0.349177 0.350833 0.351747 0.353463 0.355648 0.357085 0.357406
[4,] 0.335990 0.337151 0.338267 0.339440 0.339971 0.340986 0.342432 0.342630
0.345890 0.346299 0.347709 0.349615 0.349881 0.350788 0.352195 0.353160
       [,289]   [,290]   [,291]   [,292]   [,293]   [,294]   [,295]   [,296]
[,297]   [,298]   [,299]   [,300]   [,301]   [,302]   [,303]   [,304]
[1,] 0.384211 0.385663 0.386247 0.386955 0.388641 0.390287 0.390837 0.392929
0.393101 0.394557 0.394849 0.395659 0.397545 0.398249 0.399832 0.401267
[2,] 0.338792 0.339215 0.340903 0.342674 0.342918 0.345431 0.345837 0.347581
0.347776 0.350269 0.350805 0.352759 0.354499 0.354933 0.356379 0.357195
[3,] 0.359082 0.359498 0.360596 0.361894 0.362903 0.364098 0.364745 0.365268
0.366417 0.367066 0.368357 0.370767 0.372147 0.372974 0.373931 0.375205
[4,] 0.355106 0.356956 0.358550 0.359680 0.360212 0.361726 0.363265 0.363930
0.365009 0.366617 0.368054 0.369356 0.370222 0.371114 0.371930 0.373225
       [,305]   [,306]   [,307]   [,308]   [,309]   [,310]   [,311]   [,312]
[,313]   [,314]   [,315]   [,316]   [,317]   [,318]   [,319]   [,320]
[1,] 0.403138 0.404211 0.405033 0.406658 0.408674 0.409145 0.409762 0.411056
0.412772 0.413812 0.414613 0.416594 0.418924 0.420722 0.422082 0.422923
[2,] 0.357997 0.359144 0.360333 0.363153 0.363841 0.365971 0.367590 0.368102
0.368921 0.370170 0.372147 0.374255 0.375111 0.376164 0.377181 0.377956
[3,] 0.376422 0.377108 0.377688 0.378986 0.379221 0.381822 0.383146 0.384480
0.385101 0.386139 0.388582 0.389066 0.390990 0.392165 0.392791 0.394430
[4,] 0.374077 0.374990 0.376954 0.377936 0.379412 0.380818 0.381699 0.383165
0.384167 0.384628 0.386526 0.388284 0.388583 0.390214 0.391337 0.392620
       [,321]   [,322]   [,323]   [,324]   [,325]   [,326]   [,327]   [,328]
[,329]   [,330]   [,331]   [,332]   [,333]   [,334]   [,335]   [,336]
[1,] 0.423589 0.425888 0.426559 0.428097 0.429076 0.429456 0.431092 0.432191
0.433288 0.434042 0.435926 0.437891 0.438476 0.439938 0.441443 0.443266
[2,] 0.379665 0.380963 0.381663 0.384000 0.384214 0.385581 0.388200 0.389099
0.390343 0.391694 0.392553 0.392801 0.393707 0.395200 0.397082 0.398010
[3,] 0.395266 0.397215 0.398433 0.399229 0.400091 0.401405 0.402992 0.403561
0.405310 0.405800 0.406509 0.407198 0.409659 0.410607 0.411232 0.413862
[4,] 0.393187 0.394868 0.396610 0.399203 0.399848 0.400850 0.401851 0.403942
0.407256 0.407766 0.408623 0.409352 0.410164 0.411467 0.412830 0.413765
       [,337]   [,338]   [,339]   [,340]   [,341]   [,342]   [,343]   [,344]
[,345]   [,346]   [,347]   [,348]   [,349]   [,350]   [,351]   [,352]
[1,] 0.444247 0.444928 0.446586 0.447579 0.448852 0.451042 0.452584 0.455736
0.457050 0.458955 0.459956 0.461176 0.462152 0.462804 0.463684 0.463778
```

```
[2,] 0.399533 0.401866 0.403226 0.403977 0.405396 0.405770 0.407890 0.409067
0.410013 0.411837 0.412575 0.413458 0.415382 0.416927 0.417418 0.419235
[3,] 0.415152 0.417003 0.417681 0.419469 0.420788 0.422655 0.423410 0.425415
0.427938 0.428720 0.430141 0.430929 0.433225 0.435219 0.435789 0.436105
[4,] 0.414781 0.416910 0.418062 0.420119 0.420743 0.421150 0.423475 0.425144
0.426721 0.426980 0.429339 0.429598 0.430593 0.432062 0.433636 0.436517
       [,353]   [,354]   [,355]   [,356]   [,357]   [,358]   [,359]   [,360]
[,361]   [,362]   [,363]   [,364]   [,365]   [,366]   [,367]   [,368]
[1,] 0.467604 0.467953 0.470086 0.471363 0.472482 0.473559 0.476121 0.477021
0.477702 0.479433 0.482142 0.483816 0.485267 0.487702 0.488621 0.489194
[2,] 0.420281 0.421711 0.423515 0.423821 0.425122 0.428168 0.428231 0.429135
0.429740 0.431607 0.433003 0.433157 0.433940 0.436008 0.436661 0.439732
[3,] 0.437129 0.438500 0.441287 0.441977 0.442186 0.443719 0.445036 0.445485
0.446999 0.449011 0.451182 0.453060 0.453847 0.455374 0.456803 0.456951
[4,] 0.436702 0.437709 0.438812 0.440470 0.442362 0.443583 0.445143 0.445947
0.447977 0.450344 0.451000 0.452630 0.453509 0.454744 0.456278 0.456699
       [,369]   [,370]   [,371]   [,372]   [,373]   [,374]   [,375]   [,376]
[,377]   [,378]   [,379]   [,380]   [,381]   [,382]   [,383]   [,384]
[1,] 0.491194 0.492942 0.493488 0.494252 0.497352 0.498191 0.498973 0.499957
0.500716 0.502141 0.502286 0.503487 0.505207 0.507420 0.507728 0.509778
[2,] 0.441183 0.441577 0.442672 0.443185 0.444550 0.445616 0.446780 0.448222
0.449537 0.450118 0.452889 0.454320 0.456091 0.457017 0.458760 0.460581
[3,] 0.457650 0.459869 0.462032 0.463461 0.464896 0.466168 0.468124 0.469232
0.472420 0.472972 0.474310 0.474812 0.476302 0.478170 0.480515 0.481576
[4,] 0.457593 0.458999 0.460254 0.462185 0.463235 0.464400 0.465015 0.468030
0.468684 0.470261 0.471948 0.473721 0.474435 0.475841 0.476191 0.479164
       [,385]   [,386]   [,387]   [,388]   [,389]   [,390]   [,391]   [,392]
[,393]   [,394]   [,395]   [,396]   [,397]   [,398]   [,399]   [,400]
[1,] 0.512064 0.512721 0.514379 0.514917 0.516191 0.516616 0.518831 0.522161
0.522567 0.524345 0.526165 0.528304 0.528620 0.530953 0.533518 0.534214
[2,] 0.462767 0.464373 0.465501 0.466700 0.469462 0.471061 0.472380 0.474349
0.475673 0.476572 0.478037 0.481009 0.481410 0.483178 0.484365 0.487238
[3,] 0.482973 0.483917 0.484993 0.486036 0.487079 0.488097 0.488763 0.490995
0.491339 0.492768 0.494042 0.494623 0.496098 0.497801 0.499520 0.500880
[4,] 0.480099 0.480886 0.482172 0.483771 0.486368 0.487200 0.487897 0.489755
0.491791 0.492933 0.494655 0.495392 0.495841 0.497421 0.499691 0.500287
       [,401]   [,402]   [,403]   [,404]   [,405]   [,406]   [,407]   [,408]
[,409]   [,410]   [,411]   [,412]   [,413]   [,414]   [,415]   [,416]
[1,] 0.535168 0.537226 0.538448 0.539421 0.540741 0.542429 0.543521 0.545442
0.546931 0.547397 0.549326 0.550360 0.552647 0.553415 0.553827 0.555719
[2,] 0.488432 0.489015 0.490279 0.493862 0.495836 0.498515 0.499905 0.500562
0.501569 0.503283 0.504898 0.505877 0.506769 0.507907 0.509234 0.510993
[3,] 0.501404 0.502391 0.504409 0.505249 0.506281 0.508678 0.509058 0.510215
0.513089 0.514305 0.515583 0.517513 0.519167 0.520340 0.521247 0.522175
[4,] 0.504307 0.504420 0.505532 0.507030 0.507784 0.508844 0.510494 0.512709
0.512889 0.514340 0.515242 0.517640 0.518541 0.518935 0.520762 0.521921
       [,417]   [,418]   [,419]   [,420]   [,421]   [,422]   [,423]   [,424]
[,425]   [,426]   [,427]   [,428]   [,429]   [,430]   [,431]   [,432]
[1,] 0.557942 0.558143 0.558615 0.562503 0.563829 0.564578 0.565652 0.566682
0.570564 0.571679 0.574142 0.575724 0.578356 0.579821 0.580599 0.582001
[2,] 0.511761 0.512448 0.514888 0.516431 0.517574 0.519350 0.519784 0.521119
0.523309 0.524850 0.525339 0.527740 0.528626 0.530184 0.533772 0.535391
[3,] 0.522999 0.525138 0.527124 0.527967 0.529280 0.530673 0.531162 0.532233
0.534352 0.535378 0.536722 0.539995 0.541171 0.542315 0.542712 0.543232
```

[4,] 0.523340 0.525378 0.526428 0.527696 0.530375 0.531335 0.533268 0.534986
0.537491 0.539236 0.540977 0.541397 0.543178 0.544211 0.547292 0.547733
[,433] [,434] [,435] [,436] [,437] [,438] [,439] [,440]
[,441] [,442] [,443] [,444] [,445] [,446] [,447] [,448]
[1,] 0.585319 0.586083 0.587792 0.588292 0.589122 0.590319 0.591722 0.593242
0.595977 0.597835 0.600594 0.605437 0.606481 0.607505 0.609636 0.610374
[2,] 0.539706 0.540846 0.542657 0.543472 0.545668 0.545904 0.547648 0.547938
0.550998 0.554473 0.555272 0.556503 0.559802 0.561109 0.562080 0.563696
[3,] 0.545281 0.546546 0.548434 0.550402 0.551869 0.552958 0.554290 0.556927
0.559064 0.560592 0.561055 0.563439 0.565821 0.567557 0.569161 0.569896
[4,] 0.548568 0.550821 0.552414 0.553275 0.555879 0.556013 0.557367 0.558849
0.560093 0.562043 0.563256 0.564838 0.566726 0.568348 0.569573 0.571078
[,449] [,450] [,451] [,452] [,453] [,454] [,455] [,456]
[,457] [,458] [,459] [,460] [,461] [,462] [,463] [,464]
[1,] 0.613857 0.614938 0.615672 0.618126 0.619543 0.620709 0.623027 0.624728
0.626500 0.629247 0.630104 0.632414 0.636077 0.636807 0.637681 0.641739
[2,] 0.566293 0.567339 0.569155 0.573522 0.574614 0.575170 0.576464 0.576571
0.578633 0.582015 0.583849 0.583882 0.585015 0.586333 0.587859 0.590778
[3,] 0.570831 0.571699 0.574074 0.577410 0.581724 0.583278 0.583873 0.584767
0.587046 0.587590 0.589933 0.591454 0.594339 0.595044 0.595339 0.596535
[4,] 0.572256 0.573325 0.575444 0.576757 0.577618 0.578952 0.581544 0.581774
0.582442 0.583455 0.584683 0.586203 0.588839 0.589304 0.592647 0.595111
[,465] [,466] [,467] [,468] [,469] [,470] [,471] [,472]
[,473] [,474] [,475] [,476] [,477] [,478] [,479] [,480]
[1,] 0.642590 0.643478 0.646803 0.647470 0.649259 0.649922 0.653282 0.655680
0.657056 0.658884 0.661918 0.663878 0.664431 0.667630 0.669205 0.669589
[2,] 0.591592 0.592921 0.594067 0.594697 0.595689 0.596751 0.598933 0.600054
0.602398 0.603546 0.606034 0.606352 0.608702 0.610343 0.613060 0.617339
[3,] 0.597848 0.599931 0.600600 0.602555 0.604769 0.605600 0.606332 0.608485
0.610271 0.611546 0.612203 0.614993 0.616729 0.618734 0.620710 0.622346
[4,] 0.597277 0.597978 0.598583 0.599630 0.601736 0.604092 0.604873 0.605874
0.606196 0.611261 0.612125 0.615310 0.616707 0.617513 0.619239 0.621772
[,481] [,482] [,483] [,484] [,485] [,486] [,487] [,488]
[,489] [,490] [,491] [,492] [,493] [,494] [,495] [,496]
[1,] 0.672450 0.673878 0.676312 0.678650 0.681559 0.684910 0.687840 0.689507
0.690058 0.692170 0.694833 0.696925 0.699798 0.700742 0.702814 0.705303
[2,] 0.618571 0.620315 0.621185 0.622727 0.624249 0.624592 0.626921 0.628631
0.630147 0.633245 0.634235 0.635188 0.636517 0.637335 0.639347 0.640229
[3,] 0.623223 0.624779 0.626909 0.628869 0.630617 0.632378 0.633733 0.634966
0.635375 0.637198 0.637626 0.639228 0.639931 0.642817 0.643552 0.644291
[4,] 0.621930 0.626103 0.627924 0.630626 0.632445 0.635337 0.636774 0.639415
0.640488 0.642656 0.643350 0.644870 0.646360 0.646876 0.649637 0.651132
[,497] [,498] [,499] [,500] [,501] [,502] [,503] [,504]
[,505] [,506] [,507] [,508] [,509] [,510] [,511] [,512]
[1,] 0.706998 0.709079 0.711725 0.712131 0.715865 0.715955 0.721319 0.722733
0.723883 0.725645 0.725823 0.727420 0.728816 0.730199 0.733334 0.734902
[2,] 0.642107 0.644621 0.646118 0.647093 0.648742 0.649639 0.652661 0.654546
0.655377 0.657068 0.658550 0.663351 0.666232 0.668363 0.669027 0.669567
[3,] 0.646087 0.646864 0.648147 0.650560 0.651085 0.653291 0.654553 0.656663
0.657514 0.659566 0.661039 0.662558 0.663693 0.665931 0.669091 0.670294
[4,] 0.656324 0.657402 0.660251 0.663971 0.664586 0.667172 0.669637 0.674250
0.674402 0.675127 0.678581 0.680089 0.681465 0.682874 0.684863 0.685665
[,513] [,514] [,515] [,516] [,517] [,518] [,519] [,520]
[,521] [,522] [,523] [,524] [,525] [,526] [,527] [,528]

[1,] 0.737509 0.741840 0.742131 0.744117 0.745271 0.749768 0.751526 0.754016
0.754806 0.760421 0.761747 0.762757 0.766070 0.769045 0.771021 0.772440
[2,] 0.672596 0.674264 0.675998 0.677749 0.681224 0.683069 0.684508 0.687338
0.689950 0.692747 0.694925 0.696232 0.697566 0.702143 0.703189 0.703974
[3,] 0.672835 0.676008 0.678669 0.679541 0.679607 0.681802 0.683897 0.684838
0.688864 0.689904 0.690460 0.693318 0.695796 0.697243 0.697789 0.700130
[4,] 0.686981 0.688740 0.689279 0.690585 0.693601 0.696754 0.697924 0.702769
0.705270 0.707085 0.708523 0.711159 0.714068 0.715101 0.715601 0.716227
[,529] [,530] [,531] [,532] [,533] [,534] [,535] [,536]
[,537] [,538] [,539] [,540] [,541] [,542] [,543] [,544]
[1,] 0.777031 0.778965 0.781912 0.783911 0.786400 0.787225 0.787829 0.790594
0.793138 0.794050 0.795358 0.798076 0.799123 0.801565 0.804707 0.805228
[2,] 0.705399 0.710001 0.710937 0.711187 0.713368 0.714488 0.718234 0.725250
0.726399 0.729271 0.729983 0.731136 0.733560 0.735549 0.735957 0.740436
[3,] 0.701221 0.703083 0.703883 0.706082 0.706560 0.708830 0.709355 0.710507
0.712189 0.713675 0.715538 0.719683 0.721856 0.724111 0.726429 0.728022
[4,] 0.717184 0.718647 0.719172 0.720105 0.720909 0.723001 0.723962 0.725132
0.728000 0.729895 0.731969 0.734903 0.737296 0.739874 0.741091 0.743309
[,545] [,546] [,547] [,548] [,549] [,550] [,551] [,552]
[,553] [,554] [,555] [,556] [,557] [,558] [,559] [,560]
[1,] 0.806090 0.807252 0.810031 0.811694 0.814738 0.817564 0.819100 0.820694
0.823485 0.823558 0.824609 0.827630 0.830009 0.831735 0.837108 0.837577
[2,] 0.742765 0.743599 0.743942 0.744710 0.748877 0.749871 0.751716 0.753546
0.756230 0.757872 0.759457 0.763039 0.765420 0.768036 0.769734 0.772081
[3,] 0.728934 0.731268 0.732771 0.735214 0.739686 0.743389 0.744177 0.744872
0.748708 0.751302 0.753211 0.755344 0.757287 0.758534 0.761319 0.763415
[4,] 0.745324 0.746741 0.748229 0.749286 0.750025 0.751611 0.753657 0.755872
0.757135 0.759006 0.762569 0.763857 0.766008 0.768134 0.769914 0.772788
[,561] [,562] [,563] [,564] [,565] [,566] [,567] [,568]
[,569] [,570] [,571] [,572] [,573] [,574] [,575] [,576]
[1,] 0.841390 0.843278 0.850064 0.851576 0.858025 0.860534 0.863457 0.865692
0.866833 0.867600 0.873034 0.876625 0.876996 0.879298 0.881612 0.883226
[2,] 0.773526 0.774950 0.775903 0.780304 0.782531 0.786339 0.787623 0.789151
0.794665 0.795547 0.796906 0.798658 0.800588 0.801293 0.802434 0.804403
[3,] 0.764632 0.766716 0.767808 0.773109 0.774572 0.777555 0.781218 0.783635
0.783889 0.786014 0.790423 0.794392 0.796975 0.798796 0.801370 0.804203
[4,] 0.774540 0.776100 0.777173 0.779384 0.781428 0.783785 0.786319 0.788076
0.790997 0.792207 0.793235 0.795565 0.797105 0.797679 0.799248 0.805005
[,577] [,578] [,579] [,580] [,581] [,582] [,583] [,584]
[,585] [,586] [,587] [,588] [,589] [,590] [,591] [,592]
[1,] 0.886236 0.887402 0.890006 0.894088 0.896663 0.899990 0.902254 0.905069
0.907259 0.910580 0.911959 0.912820 0.914281 0.919581 0.920832 0.923449
[2,] 0.805608 0.807629 0.808591 0.813148 0.816021 0.817573 0.819547 0.821020
0.822824 0.825737 0.828382 0.828997 0.832385 0.834967 0.837317 0.838216
[3,] 0.806461 0.807006 0.807470 0.809373 0.810626 0.814618 0.815886 0.818696
0.819386 0.819966 0.823282 0.824455 0.827319 0.830405 0.831201 0.832889
[4,] 0.805135 0.809294 0.810973 0.811540 0.812784 0.816478 0.817818 0.822401
0.822724 0.826909 0.831415 0.832759 0.834498 0.837060 0.839833 0.843985
[,593] [,594] [,595] [,596] [,597] [,598] [,599] [,600]
[,601] [,602] [,603] [,604] [,605] [,606] [,607] [,608]
[1,] 0.926884 0.927552 0.929808 0.931899 0.935743 0.939454 0.941201 0.942846
0.943808 0.950107 0.952189 0.952488 0.955652 0.956738 0.958942 0.960933
[2,] 0.839458 0.841786 0.843252 0.846589 0.847633 0.848731 0.855024 0.859663
0.860706 0.862856 0.866012 0.867795 0.872609 0.875647 0.877175 0.880556

```
[3,] 0.834775 0.839445 0.840718 0.841696 0.844581 0.848120 0.852682 0.853850
0.856693 0.859275 0.860612 0.861515 0.864720 0.867215 0.868821 0.870707
[4,] 0.844350 0.846880 0.848502 0.850112 0.852126 0.855338 0.858239 0.858481
0.860939 0.862483 0.864339 0.871477 0.872243 0.876548 0.880914 0.882495
       [,609]   [,610]   [,611]   [,612]   [,613]   [,614]   [,615]   [,616]
[,617]   [,618]   [,619]   [,620]   [,621]   [,622]   [,623]   [,624]
[1,] 0.964113 0.966055 0.968179 0.969254 0.970284 0.972686 0.976725 0.977544
0.979188 0.983218 0.984662 0.985774 0.988467 0.991987 0.994949 0.996709
[2,] 0.882607 0.883442 0.885732 0.886869 0.891012 0.896154 0.898744 0.900851
0.902077 0.905049 0.906937 0.909126 0.910409 0.913550 0.915710 0.919209
[3,] 0.872698 0.875295 0.876149 0.878355 0.879159 0.880441 0.882981 0.884784
0.887155 0.889579 0.893694 0.896922 0.900083 0.904707 0.907095 0.910268
[4,] 0.884329 0.890099 0.893203 0.894709 0.898483 0.899701 0.903159 0.904468
0.906147 0.908775 0.912556 0.914100 0.915202 0.917319 0.919449 0.920997
       [,625]   [,626]   [,627]   [,628]   [,629]   [,630]   [,631]   [,632]
[,633]   [,634]   [,635]   [,636]   [,637]   [,638]   [,639]   [,640]
[1,] 0.997402 1.000299 1.002822 1.003458 1.005211 1.007667 1.012443 1.013277
1.019041 1.020338 1.021866 1.024910 1.027601 1.030579 1.032590 1.037089
[2,] 0.923449 0.925425 0.927972 0.929562 0.931118 0.933406 0.937918 0.938645
0.941172 0.943313 0.945100 0.948833 0.950804 0.952744 0.954355 0.959180
[3,] 0.910915 0.911798 0.913694 0.918466 0.921402 0.922523 0.923488 0.928695
0.929498 0.932411 0.934678 0.938569 0.941218 0.943061 0.944267 0.948933
[4,] 0.924756 0.925540 0.929387 0.930608 0.934510 0.936399 0.939626 0.940956
0.946292 0.947695 0.948207 0.950457 0.952013 0.953530 0.955844 0.957736
       [,641]   [,642]   [,643]   [,644]   [,645]   [,646]   [,647]   [,648]
[,649]   [,650]   [,651]   [,652]   [,653]   [,654]   [,655]   [,656]
[1,] 1.038794 1.042261 1.045931 1.046849 1.048663 1.052664 1.056583 1.061057
1.062598 1.063278 1.064126 1.065953 1.070881 1.073870 1.074740 1.076601
[2,] 0.960113 0.967761 0.969995 0.971695 0.976194 0.979164 0.979538 0.980520
0.982086 0.982946 0.989151 0.992666 0.995570 0.996598 1.000951 1.001910
[3,] 0.949838 0.953743 0.954111 0.957491 0.958599 0.959913 0.961265 0.962203
0.964032 0.966128 0.968504 0.971899 0.973139 0.973324 0.979168 0.981979
[4,] 0.960199 0.963317 0.967146 0.968450 0.968980 0.969945 0.973911 0.974706
0.979855 0.982167 0.986634 0.988646 0.991723 0.993078 0.994626 0.997806
       [,657]   [,658]   [,659]   [,660]   [,661]   [,662]   [,663]   [,664]
[,665]   [,666]   [,667]   [,668]   [,669]   [,670]   [,671]   [,672]
[1,] 1.079940 1.082384 1.083515 1.089455 1.092492 1.099376 1.099606 1.101198
1.102255 1.103946 1.109240 1.110432 1.111990 1.115577 1.117613 1.120523
[2,] 1.005819 1.006815 1.012160 1.014587 1.016858 1.019766 1.020776 1.024567
1.026599 1.032132 1.035374 1.037510 1.048403 1.049349 1.050372 1.053698
[3,] 0.982647 0.984223 0.987609 0.988149 0.992586 0.993978 0.994741 1.000813
1.001290 1.005789 1.008691 1.013040 1.016472 1.020535 1.021713 1.021987
[4,] 1.001749 1.003808 1.006024 1.012006 1.015236 1.015739 1.018005 1.018847
1.021944 1.026088 1.028591 1.032877 1.034607 1.036691 1.038758 1.041816
       [,673]   [,674]   [,675]   [,676]   [,677]   [,678]   [,679]   [,680]
[,681]   [,682]   [,683]   [,684]   [,685]   [,686]   [,687]   [,688]
[1,] 1.121279 1.122707 1.124952 1.125704 1.128209 1.135868 1.137564 1.139473
1.141487 1.143616 1.147446 1.150658 1.156280 1.159346 1.163172 1.166635
[2,] 1.054672 1.061317 1.063391 1.064049 1.069852 1.074338 1.079913 1.081993
1.085986 1.092034 1.092545 1.093745 1.097970 1.098955 1.101143 1.107213
[3,] 1.025598 1.027447 1.029569 1.033373 1.038548 1.043304 1.044463 1.045554
1.048896 1.051195 1.052768 1.057359 1.058307 1.061215 1.061902 1.066769
[4,] 1.044917 1.047807 1.050681 1.054402 1.055454 1.060520 1.061202 1.062431
1.068274 1.071385 1.076086 1.076429 1.080571 1.083306 1.086889 1.089790
```

```
      [,689]   [,690]   [,691]   [,692]   [,693]   [,694]   [,695]   [,696]
[,697]   [,698]   [,699]   [,700]   [,701]   [,702]   [,703]   [,704]
[1,] 1.168781 1.171326 1.174439 1.175997 1.178714 1.179582 1.182271 1.183685
1.186558 1.192195 1.193581 1.194701 1.201727 1.204405 1.206147 1.210011
[2,] 1.111338 1.113745 1.117908 1.119397 1.122205 1.126871 1.129365 1.132297
1.133693 1.134914 1.135270 1.138463 1.143631 1.148127 1.149793 1.155835
[3,] 1.067700 1.074782 1.077662 1.083719 1.086189 1.090432 1.091379 1.094204
1.096408 1.097245 1.098355 1.103586 1.105409 1.107137 1.108209 1.111030
[4,] 1.091347 1.095589 1.102560 1.102662 1.106637 1.109924 1.112152 1.114081
1.115214 1.116792 1.122298 1.123858 1.128691 1.130688 1.131947 1.132578
      [,705]   [,706]   [,707]   [,708]   [,709]   [,710]   [,711]   [,712]
[,713]   [,714]   [,715]   [,716]   [,717]   [,718]   [,719]   [,720]
[1,] 1.212339 1.213999 1.216421 1.217894 1.222178 1.222479 1.230127 1.232758
1.235908 1.241502 1.246513 1.251659 1.259216 1.262658 1.263846 1.264202
[2,] 1.164075 1.168300 1.175503 1.177594 1.185680 1.189411 1.190544 1.192796
1.199148 1.205327 1.206751 1.212901 1.214824 1.217295 1.219501 1.220609
[3,] 1.114367 1.117013 1.120865 1.122197 1.123762 1.129439 1.132304 1.135072
1.136545 1.141922 1.143469 1.145679 1.146935 1.148816 1.149872 1.153420
[4,] 1.135078 1.137835 1.140215 1.144923 1.148715 1.154266 1.156951 1.159038
1.161358 1.163279 1.168538 1.168897 1.173103 1.173814 1.178102 1.180267
      [,721]   [,722]   [,723]   [,724]   [,725]   [,726]   [,727]   [,728]
[,729]   [,730]   [,731]   [,732]   [,733]   [,734]   [,735]   [,736]
[1,] 1.264225 1.268854 1.272612 1.277006 1.278033 1.280524 1.283200 1.290387
1.293172 1.296234 1.300693 1.304300 1.304901 1.308872 1.312031 1.315882
[2,] 1.220945 1.222970 1.228974 1.233391 1.234842 1.242753 1.242933 1.247018
1.248551 1.251432 1.253468 1.257530 1.259052 1.259175 1.261735 1.265762
[3,] 1.154763 1.157591 1.162683 1.165111 1.168243 1.172724 1.175470 1.179510
1.181192 1.181650 1.185370 1.187431 1.190111 1.190848 1.193943 1.197005
[4,] 1.186191 1.188776 1.191151 1.193553 1.198431 1.201193 1.203329 1.205769
1.209379 1.212446 1.213027 1.215375 1.217953 1.220470 1.222351 1.226921
      [,737]   [,738]   [,739]   [,740]   [,741]   [,742]   [,743]   [,744]
[,745]   [,746]   [,747]   [,748]   [,749]   [,750]   [,751]   [,752]
[1,] 1.317069 1.317980 1.320406 1.323852 1.325682 1.327086 1.331667 1.335143
1.337568 1.340108 1.347231 1.351551 1.355262 1.360818 1.362801 1.364260
[2,] 1.269650 1.272200 1.275220 1.277977 1.279384 1.281185 1.284804 1.292864
1.293507 1.298795 1.301319 1.303558 1.306701 1.308804 1.314292 1.315846
[3,] 1.199168 1.201030 1.204778 1.207482 1.216843 1.219074 1.222014 1.223124
1.223487 1.228068 1.231739 1.238617 1.239365 1.241014 1.246278 1.252628
[4,] 1.229242 1.231985 1.234963 1.237084 1.240978 1.244587 1.252816 1.254574
1.255404 1.259199 1.263240 1.265968 1.268436 1.272368 1.276613 1.281086
      [,753]   [,754]   [,755]   [,756]   [,757]   [,758]   [,759]   [,760]
[,761]   [,762]   [,763]   [,764]   [,765]   [,766]   [,767]   [,768]
[1,] 1.368893 1.373510 1.376980 1.379409 1.379936 1.387344 1.388629 1.391094
1.392748 1.394829 1.403657 1.407537 1.410589 1.414733 1.419695 1.424354
[2,] 1.319790 1.323610 1.329594 1.330169 1.334479 1.335173 1.341588 1.343416
1.348099 1.350796 1.360751 1.362163 1.367968 1.369894 1.374516 1.375746
[3,] 1.255151 1.256439 1.259215 1.260951 1.262089 1.265299 1.272730 1.273185
1.277635 1.282712 1.286273 1.286643 1.291252 1.292677 1.299593 1.301804
[4,] 1.283948 1.288854 1.291317 1.298387 1.303280 1.305811 1.309028 1.310769
1.313795 1.315850 1.317257 1.326534 1.329072 1.329718 1.332449 1.335414
      [,769]   [,770]   [,771]   [,772]   [,773]   [,774]   [,775]   [,776]
[,777]   [,778]   [,779]   [,780]   [,781]   [,782]   [,783]   [,784]
[1,] 1.426486 1.430051 1.434497 1.436424 1.439726 1.444274 1.451421 1.452979
1.454972 1.457557 1.460280 1.472083 1.475484 1.478274 1.479409 1.482000
```

```
[2,] 1.376658 1.379156 1.381143 1.388634 1.390356 1.393731 1.396884 1.397883
1.401889 1.402062 1.407339 1.411089 1.412722 1.413384 1.416627 1.420346
[3,] 1.303420 1.307876 1.310027 1.310669 1.312038 1.317764 1.321745 1.329623
1.332736 1.334471 1.335592 1.337713 1.338094 1.342238 1.343144 1.354409
[4,] 1.335689 1.338789 1.343954 1.346743 1.349358 1.350673 1.357353 1.360137
1.366101 1.370486 1.374125 1.377200 1.379639 1.382990 1.384103 1.387954
       [,785]   [,786]   [,787]   [,788]   [,789]   [,790]   [,791]   [,792]
[,793]   [,794]   [,795]   [,796]   [,797]   [,798]   [,799]   [,800]
[1,] 1.487292 1.490259 1.498111 1.498549 1.500625 1.503686 1.504835 1.514975
1.523064 1.532826 1.537066 1.542906 1.543983 1.548743 1.553771 1.557031
[2,] 1.427783 1.428736 1.432532 1.434155 1.439420 1.441323 1.442903 1.448918
1.453057 1.460098 1.462385 1.464937 1.469812 1.473741 1.475454 1.478969
[3,] 1.355878 1.358669 1.359938 1.371386 1.372959 1.376850 1.379599 1.384151
1.386135 1.392360 1.394245 1.400239 1.403863 1.407937 1.410324 1.419513
[4,] 1.389735 1.391301 1.395694 1.397185 1.400407 1.405565 1.407393 1.408790
1.418149 1.420780 1.429591 1.432229 1.436230 1.437412 1.442296 1.442966
       [,801]   [,802]   [,803]   [,804]   [,805]   [,806]   [,807]   [,808]
[,809]   [,810]   [,811]   [,812]   [,813]   [,814]   [,815]   [,816]
[1,] 1.564014 1.569333 1.576105 1.578033 1.579326 1.580277 1.582990 1.584922
1.587982 1.591308 1.593624 1.600388 1.602035 1.608925 1.610818 1.613471
[2,] 1.485796 1.486633 1.495599 1.498302 1.501453 1.508216 1.509304 1.512077
1.515533 1.517619 1.523661 1.529123 1.531795 1.539366 1.545442 1.546787
[3,] 1.426927 1.429017 1.429132 1.435531 1.438517 1.440917 1.442513 1.445341
1.447865 1.454839 1.458335 1.462744 1.465017 1.467283 1.471777 1.476683
[4,] 1.450769 1.453793 1.457621 1.461460 1.462312 1.465534 1.469069 1.475398
1.476687 1.479122 1.482074 1.485485 1.489651 1.501669 1.503750 1.505780
       [,817]   [,818]   [,819]   [,820]   [,821]   [,822]   [,823]   [,824]
[,825]   [,826]   [,827]   [,828]   [,829]   [,830]   [,831]   [,832]
[1,] 1.615567 1.615859 1.624049 1.627969 1.634312 1.639121 1.641390 1.644427
1.647572 1.649824 1.655549 1.660197 1.667318 1.668790 1.673871 1.675409
[2,] 1.559413 1.560081 1.565216 1.569639 1.570316 1.577172 1.580938 1.588108
1.591545 1.596682 1.608148 1.613073 1.615390 1.624619 1.630462 1.634754
[3,] 1.480334 1.480862 1.487143 1.490986 1.491828 1.495202 1.500605 1.505731
1.509765 1.511855 1.512920 1.516443 1.522640 1.525056 1.527196 1.531210
[4,] 1.507036 1.510969 1.514932 1.515788 1.521948 1.524092 1.533281 1.542806
1.546836 1.554949 1.555428 1.557085 1.562715 1.574247 1.582982 1.584692
       [,833]   [,834]   [,835]   [,836]   [,837]   [,838]   [,839]   [,840]
[,841]   [,842]   [,843]   [,844]   [,845]   [,846]   [,847]   [,848]
[1,] 1.679960 1.681241 1.683945 1.686297 1.690093 1.694895 1.707906 1.716737
1.721910 1.725407 1.737414 1.740753 1.745670 1.746882 1.747020 1.750239
[2,] 1.636796 1.638791 1.642114 1.646013 1.647354 1.649542 1.655145 1.663182
1.664979 1.671523 1.673268 1.675605 1.682109 1.684232 1.686882 1.693682
[3,] 1.532847 1.535398 1.537215 1.547227 1.550725 1.555068 1.558919 1.560777
1.570867 1.582695 1.585381 1.589624 1.596893 1.598608 1.601193 1.614211
[4,] 1.587797 1.590366 1.595645 1.602377 1.604437 1.607889 1.614671 1.616154
1.621633 1.621900 1.625235 1.628189 1.630487 1.632437 1.634673 1.647662
       [,849]   [,850]   [,851]   [,852]   [,853]   [,854]   [,855]   [,856]
[,857]   [,858]   [,859]   [,860]   [,861]   [,862]   [,863]   [,864]
[1,] 1.754886 1.771705 1.776002 1.780959 1.787415 1.789285 1.795895 1.800000
1.803712 1.807283 1.811615 1.814832 1.822601 1.827364 1.828156 1.835424
[2,] 1.697078 1.700090 1.700282 1.703906 1.711026 1.715612 1.723580 1.726017
1.729930 1.731190 1.734266 1.740458 1.745361 1.755947 1.761332 1.767370
[3,] 1.617728 1.620010 1.624078 1.629758 1.631585 1.632826 1.636855 1.642422
1.645373 1.650324 1.658111 1.672540 1.684189 1.684803 1.688499 1.691656
```

```
[4,] 1.654525 1.656202 1.658814 1.673175 1.674475 1.679020 1.683837 1.684891
1.699079 1.705011 1.712070 1.713177 1.719518 1.720692 1.730690 1.738439
       [,865]   [,866]   [,867]   [,868]   [,869]   [,870]   [,871]   [,872]
[,873]   [,874]   [,875]   [,876]   [,877]   [,878]   [,879]   [,880]
[1,] 1.837924 1.847070 1.848526 1.858362 1.864690 1.866443 1.871880 1.881059
1.882548 1.889704 1.893269 1.901145 1.901977 1.907519 1.912184 1.921194
[2,] 1.768729 1.775000 1.781134 1.792047 1.803087 1.805800 1.807103 1.811108
1.815499 1.815618 1.818911 1.836114 1.838905 1.849492 1.851418 1.853703
[3,] 1.704985 1.707766 1.709673 1.714904 1.720792 1.725020 1.727383 1.737390
1.738760 1.744140 1.752389 1.763355 1.766175 1.767310 1.773083 1.780141
[4,] 1.745875 1.750287 1.755643 1.756074 1.761078 1.765504 1.767487 1.775969
1.785100 1.786438 1.794964 1.795783 1.798551 1.803179 1.806368 1.811829
       [,881]   [,882]   [,883]   [,884]   [,885]   [,886]   [,887]   [,888]
[,889]   [,890]   [,891]   [,892]   [,893]   [,894]   [,895]   [,896]
[1,] 1.923994 1.929889 1.935604 1.947266 1.951285 1.956309 1.962835 1.963386
1.964087 1.972141 1.974908 1.981969 1.986831 1.990724 1.999097 2.017063
[2,] 1.858960 1.863301 1.867672 1.878813 1.879698 1.896640 1.899216 1.900221
1.905759 1.915078 1.923157 1.936442 1.940985 1.942913 1.951957 1.966145
[3,] 1.784939 1.791113 1.796326 1.800198 1.803139 1.810575 1.816038 1.818074
1.819989 1.828545 1.832181 1.844820 1.855699 1.863601 1.864919 1.874715
[4,] 1.812751 1.816093 1.821269 1.835422 1.839858 1.843125 1.846220 1.848403
1.865173 1.880856 1.883363 1.891498 1.897981 1.902324 1.920581 1.923995
       [,897]   [,898]   [,899]   [,900]   [,901]   [,902]   [,903]   [,904]
[,905]   [,906]   [,907]   [,908]   [,909]   [,910]   [,911]   [,912]
[1,] 2.032293 2.034843 2.042715 2.048220 2.056246 2.059064 2.064475 2.072462
2.105790 2.111305 2.121956 2.128406 2.130855 2.152285 2.157839 2.185583
[2,] 1.974339 1.982348 1.984242 1.988765 1.992741 2.000620 2.007409 2.009522
2.013796 2.022290 2.026404 2.035269 2.043624 2.051905 2.060822 2.063386
[3,] 1.877036 1.878941 1.881193 1.881698 1.885057 1.889834 1.896088 1.901491
1.909511 1.924253 1.928612 1.930615 1.952287 1.956885 1.963820 1.989498
[4,] 1.927568 1.936751 1.944565 1.944776 1.949942 1.965052 1.965546 1.980764
1.981763 1.990105 2.004984 2.012317 2.018163 2.026415 2.029316 2.036879
       [,913]   [,914]   [,915]   [,916]   [,917]   [,918]   [,919]   [,920]
[,921]   [,922]   [,923]   [,924]   [,925]   [,926]   [,927]   [,928]
[1,] 2.197149 2.200461 2.208806 2.216388 2.220402 2.221169 2.240313 2.245863
2.246890 2.254692 2.277293 2.281108 2.286476 2.287394 2.302789 2.323058
[2,] 2.070010 2.078948 2.100785 2.117409 2.120502 2.125223 2.130451 2.135367
2.141255 2.141806 2.161863 2.165044 2.178751 2.181824 2.188993 2.190655
[3,] 1.989824 1.991783 1.996538 1.999698 2.006880 2.016659 2.022976 2.028550
2.038180 2.040702 2.042116 2.047763 2.056979 2.066332 2.077916 2.080960
[4,] 2.048873 2.056900 2.064675 2.078006 2.089813 2.095092 2.097279 2.098010
2.110133 2.119818 2.133816 2.135206 2.135837 2.142948 2.143850 2.158908
       [,929]   [,930]   [,931]   [,932]   [,933]   [,934]   [,935]   [,936]
[,937]   [,938]   [,939]   [,940]   [,941]   [,942]   [,943]   [,944]
[1,] 2.328445 2.331247 2.350536 2.350672 2.364186 2.374046 2.386677 2.390814
2.406057 2.407789 2.413227 2.426824 2.432251 2.436264 2.446429 2.454315
[2,] 2.203945 2.207811 2.216443 2.225324 2.246240 2.249786 2.257095 2.272192
2.278845 2.288886 2.292473 2.294476 2.296242 2.307507 2.313786 2.317753
[3,] 2.082514 2.087896 2.102769 2.105457 2.113414 2.122168 2.126246 2.129085
2.139913 2.149540 2.152232 2.161686 2.175209 2.192540 2.193939 2.198057
[4,] 2.172858 2.187274 2.198294 2.202227 2.207671 2.215981 2.219976 2.229914
2.232502 2.233422 2.234824 2.237277 2.256127 2.265996 2.271878 2.278281
       [,945]   [,946]   [,947]   [,948]   [,949]   [,950]   [,951]   [,952]
[,953]   [,954]   [,955]   [,956]   [,957]   [,958]   [,959]   [,960]
```

```
[1,] 2.474514 2.509291 2.521437 2.527344 2.537108 2.547981 2.558778 2.561988
2.572043 2.590976 2.597448 2.598216 2.610169 2.613818 2.632575 2.643769
[2,] 2.332344 2.351448 2.359766 2.365571 2.371992 2.377965 2.390455 2.399442
2.416172 2.423432 2.433768 2.456146 2.458989 2.464719 2.473987 2.479790
[3,] 2.201422 2.215230 2.225998 2.234227 2.251085 2.262863 2.266564 2.271065
2.288135 2.290402 2.313708 2.319714 2.323577 2.324621 2.343763 2.352341
[4,] 2.296310 2.296478 2.310637 2.316032 2.324079 2.329430 2.338012 2.341869
2.359139 2.366009 2.368324 2.376029 2.381934 2.388657 2.396146 2.405033
       [,961]   [,962]   [,963]   [,964]   [,965]   [,966]   [,967]   [,968]
[,969]   [,970]   [,971]   [,972]   [,973]   [,974]   [,975]   [,976]
[1,] 2.647071 2.661206 2.715787 2.730040 2.733944 2.753298 2.760804 2.766135
2.808936 2.811130 2.845699 2.862934 2.885316 2.894373 2.895691 2.905883
[2,] 2.500962 2.512373 2.544376 2.556951 2.561952 2.579751 2.584249 2.591191
2.599618 2.616087 2.624142 2.628669 2.633804 2.649726 2.671985 2.683591
[3,] 2.352642 2.364089 2.369665 2.372280 2.382581 2.392219 2.398697 2.400927
2.406814 2.414049 2.419127 2.428318 2.457953 2.462259 2.473330 2.515339
[4,] 2.426759 2.442823 2.444447 2.456566 2.471261 2.474985 2.497065 2.497414
2.504004 2.524728 2.525289 2.539640 2.540471 2.555023 2.571791 2.596205
       [,977]   [,978]   [,979]   [,980]   [,981]   [,982]   [,983]   [,984]
[,985]   [,986]   [,987]   [,988]   [,989]   [,990]   [,991]   [,992]
[1,] 2.933356 2.936525 2.942132 2.947038 2.953302 2.985455 2.998302 3.002716
3.015747 3.021686 3.035334 3.036636 3.041501 3.046304 3.047668 3.089330
[2,] 2.692780 2.699994 2.720907 2.726395 2.739195 2.752869 2.807276 2.809779
2.827581 2.840908 2.843245 2.846128 2.873075 2.877078 2.905634 2.909191
[3,] 2.520418 2.526407 2.532784 2.543086 2.550366 2.554991 2.564611 2.586427
2.589562 2.593426 2.634850 2.637642 2.647121 2.696237 2.699770 2.736373
[4,] 2.611435 2.616491 2.623497 2.639700 2.646649 2.659577 2.665204 2.708481
2.711971 2.726095 2.726543 2.728333 2.740636 2.749695 2.751382 2.767101
       [,993]   [,994]   [,995]   [,996]   [,997]   [,998]   [,999]  [,1000]
[,1001]  [,1002]  [,1003]  [,1004]  [,1005]  [,1006]  [,1007]  [,1008]
[1,] 3.091928 3.103883 3.131883 3.144838 3.148517 3.160920 3.164338 3.171989
3.175808 3.186890 3.189950 3.209824 3.236837 3.256063 3.291775 3.299046
[2,] 2.924095 2.938035 2.990118 3.006046 3.028255 3.062335 3.077358 3.080912
3.108435 3.109684 3.145415 3.157270 3.163539 3.167450 3.188868 3.202408
[3,] 2.742635 2.767091 2.771612 2.782576 2.795843 2.799593 2.830985 2.868446
2.895987 2.897396 2.915529 2.924809 2.948471 2.970647 3.011656 3.017247
[4,] 2.771491 2.806178 2.817790 2.847013 2.864684 2.872781 2.921662 2.926642
2.944604 2.979388 2.980317 2.998444 3.006975 3.008711 3.035530 3.041158
      [,1009]  [,1010]  [,1011]  [,1012]  [,1013]  [,1014]  [,1015]  [,1016]
[,1017]  [,1018]  [,1019]  [,1020]  [,1021]  [,1022]  [,1023]  [,1024]
[1,] 3.307180 3.363044 3.366812 3.395537 3.397823 3.426703 3.443193 3.447738
3.462433 3.488152 3.490725 3.535562 3.552923 3.555891 3.557704 3.590257
[2,] 3.221264 3.223689 3.283177 3.306472 3.309727 3.310388 3.312849 3.352869
3.357066 3.359401 3.361686 3.391627 3.460020 3.520470 3.535030 3.567385
[3,] 3.036177 3.038559 3.062240 3.066192 3.069376 3.071585 3.101018 3.120066
3.170574 3.178010 3.199774 3.209990 3.233980 3.244497 3.259891 3.278186
[4,] 3.058164 3.065006 3.091309 3.111752 3.143073 3.148023 3.148854 3.159601
3.184038 3.231289 3.248966 3.302784 3.312642 3.331861 3.360267 3.366556
      [,1025]  [,1026]  [,1027]  [,1028]  [,1029]  [,1030]  [,1031]  [,1032]
[,1033]  [,1034]  [,1035]  [,1036]  [,1037]  [,1038]  [,1039]  [,1040]
[1,] 3.594233 3.609783 3.611532 3.626981 3.629178 3.641583 3.668128 3.683517
3.691200 3.700933 3.710749 3.713068 3.714640 3.715855 3.774646 3.786616
[2,] 3.572462 3.590294 3.605984 3.661839 3.664831 3.671004 3.727324 3.829924
3.838676 3.849196 3.855963 3.968715 3.981663 3.999323 4.022891 4.091331
```

[3,] 3.285013 3.297232 3.311627 3.334518 3.356514 3.375354 3.416421 3.464031
3.482240 3.511301 3.515213 3.545893 3.576077 3.590550 3.655943 3.668537
[4,] 3.385316 3.459886 3.471731 3.479602 3.483020 3.488138 3.615415 3.638264
3.639313 3.640479 3.662338 3.706677 3.731837 3.801454 3.821144 3.860602
[,1041] [,1042] [,1043] [,1044] [,1045] [,1046] [,1047] [,1048]
[,1049] [,1050] [,1051] [,1052] [,1053] [,1054] [,1055] [,1056]
[1,] 3.788936 3.796039 3.810314 3.884160 3.897168 3.968883 3.985277 3.994489
4.026790 4.039747 4.125728 4.142024 4.153973 4.182223 4.207327 4.242485
[2,] 4.152080 4.187964 4.214050 4.270669 4.290374 4.329122 4.418281 4.440118
4.450828 4.457247 4.626850 4.691063 4.739000 4.748957 4.770807 4.792276
[3,] 3.676437 3.697376 3.735159 3.745106 3.775379 3.853819 3.868832 3.956384
3.972218 3.975946 4.013334 4.084877 4.096543 4.140540 4.192410 4.216010
[4,] 3.871281 3.872884 3.964118 4.037217 4.041586 4.070889 4.076193 4.083993
4.089350 4.102306 4.118657 4.246493 4.293473 4.328267 4.366532 4.412344
[,1057] [,1058] [,1059] [,1060] [,1061] [,1062] [,1063] [,1064]
[,1065] [,1066] [,1067] [,1068] [,1069] [,1070] [,1071] [,1072]
[1,] 4.309499 4.391903 4.450638 4.522323 4.534335 4.587375 4.676195 4.710539
4.712829 4.837881 4.840807 4.916083 5.029609 5.058520 5.094966 5.127141
[2,] 4.794684 4.832573 4.976395 5.069431 5.105509 5.152441 5.193311 5.281123
5.314034 5.431704 5.446599 5.479390 5.489572 5.531433 5.643898 5.702819
[3,] 4.223498 4.261899 4.286573 4.342234 4.421135 4.441466 4.477325 4.483374
4.512226 4.578994 4.589652 4.790686 4.809058 4.878752 4.883859 4.911377
[4,] 4.432620 4.447647 4.456050 4.504327 4.515021 4.519843 4.551666 4.612478
4.617959 4.618972 4.633077 4.645212 4.695019 4.708731 4.766851 4.813182
[,1073] [,1074] [,1075] [,1076] [,1077] [,1078] [,1079] [,1080]
[,1081] [,1082] [,1083] [,1084] [,1085] [,1086] [,1087] [,1088]
[1,] 5.128941 5.140489 5.195847 5.210703 5.275156 5.366016 5.429779 5.505288
5.551721 5.676272 5.711754 5.715120 5.747152 5.978317 6.077793 6.312579
[2,] 5.704530 5.769814 5.876373 5.881759 6.061185 6.083402 6.093946 6.214718
6.238841 6.427320 6.505008 6.534509 6.656681 6.691194 6.767705 6.771871
[3,] 4.983301 5.065126 5.071296 5.076553 5.135154 5.239758 5.294629 5.349456
5.398783 5.422090 5.466892 5.555116 5.604680 5.655394 5.708851 5.811660
[4,] 4.968351 4.975678 4.998956 5.035930 5.154867 5.247719 5.256196 5.308223
5.341315 5.434942 5.643954 5.906225 5.960616 6.019948 6.143725 6.192630
[,1089] [,1090] [,1091] [,1092] [,1093] [,1094] [,1095] [,1096]
[,1097] [,1098] [,1099] [,1100] [,1101] [,1102] [,1103] [,1104]
[1,] 6.368805 6.571286 6.609050 6.701800 6.824083 6.853112 7.124230 7.141621
7.185217 7.268873 7.318555 7.645183 7.682744 7.769045 7.931278 8.051296
[2,] 6.840251 7.219294 7.248150 7.255217 7.507908 7.958860 8.001750 8.089375
8.137326 8.138831 8.340229 8.511189 8.601207 8.630126 8.666355 9.015444
[3,] 5.927496 5.970143 6.003451 6.307972 6.361107 6.373454 6.595998 6.728483
6.988837 7.056284 7.115802 7.251656 7.562710 7.703251 7.769677 8.044694
[4,] 6.353640 6.357688 6.452763 6.585820 6.652958 6.738501 6.782910 6.888436
7.015710 7.109123 7.410997 7.462464 7.494469 7.812100 7.841033 7.878033
[,1105] [,1106] [,1107] [,1108] [,1109] [,1110] [,1111] [,1112]
[,1113] [,1114] [,1115] [,1116] [,1117] [,1118] [,1119] [,1120]
[1,] 8.052302 8.188693 8.380909 8.404470 8.700907 8.775307 9.227867 9.230365
9.298963 9.404558 9.475492 9.530215 9.571570 9.633796 9.674709 9.767860
[2,] 9.032474 9.165109 9.190018 9.253088 9.297469 9.465050 9.538323 9.560090
9.844394 9.924775 10.037576 10.046619 10.073235 10.100077 10.225229 10.234968
[3,] 8.056667 8.185948 8.314648 8.342765 8.446604 8.522491 8.528979 8.551846
8.876551 8.906558 9.075124 9.188014 9.239436 9.300324 9.409326 9.542245
[4,] 7.909190 7.948062 8.227223 8.351373 8.382749 8.587205 8.601011 8.710496
8.752242 8.834997 8.836452 8.955752 9.327543 9.377757 9.401344 9.509080

```
      [,1121]   [,1122]   [,1123]   [,1124]   [,1125]   [,1126]   [,1127]
[,1128]   [,1129]  [,1130]  [,1131]  [,1132]  [,1133]  [,1134]  [,1135]
[1,]  9.919116  9.981594  9.992363 10.080782 10.141184 10.195181 10.297841
10.328771 10.459200 10.60301 10.69456 10.72242 10.87386 10.93336 10.96062
[2,] 10.255994 10.269347 10.345144 10.358492 10.370722 10.409125 10.599349
10.624698 10.677852 10.70972 10.85021 10.90134 10.94407 10.95450 10.96015
[3,]  9.547902  9.604181  9.620001  9.638774  9.757796  9.851363  9.883684
9.892701  9.899603  9.90238 10.12907 10.15817 10.20558 10.22339 10.24776
[4,]  9.538769  9.761283  9.837985  9.867330  9.879445 10.014518 10.041885
10.245344 10.253515 10.30085 10.57675 10.69399 10.86168 11.01500 11.05148
      [,1136]  [,1137]  [,1138]  [,1139]  [,1140]  [,1141]  [,1142]  [,1143]
[,1144]  [,1145]  [,1146]  [,1147]  [,1148]  [,1149]  [,1150]  [,1151]
[1,] 10.97712 11.03637 11.05574 11.05813 11.09932 11.13181 11.13908 11.20597
11.39690 11.41496 11.41843 11.43039 11.43729 11.55051 11.72769 11.76520
[2,] 10.96859 11.00310 11.21485 11.24863 11.31478 11.34258 11.35448 11.48270
11.48611 11.50568 11.54523 11.64715 11.70926 11.72499 11.77056 11.78930
[3,] 10.30002 10.32707 10.34750 10.47973 10.48638 10.49533 10.50075 10.80189
10.88440 10.93949 10.96955 11.00841 11.02286 11.08384 11.10777 11.12442
[4,] 11.05861 11.06490 11.13664 11.24633 11.25018 11.27083 11.44427 11.50728
11.50911 11.52573 11.54638 11.60155 11.65567 11.67169 11.68436 11.73063
      [,1152]  [,1153]  [,1154]  [,1155]  [,1156]  [,1157]  [,1158]  [,1159]
[,1160]  [,1161]  [,1162]  [,1163]  [,1164]  [,1165]  [,1166]  [,1167]
[1,] 11.84797 12.22878 12.23755 12.39199 12.41224 12.60086 12.62965 12.65424
12.65877 12.68383 12.69971 12.75144 12.84811 12.85862 12.90896 12.99960
[2,] 11.81450 11.85277 11.90944 12.08828 12.09117 12.12990 12.14151 12.15918
12.16531 12.30390 12.31452 12.32316 12.38399 12.44860 12.45766 12.48461
[3,] 11.12957 11.14657 11.15027 11.26726 11.26911 11.40097 11.41593 11.41912
11.42791 11.43418 11.47052 11.62282 11.66598 11.72635 11.83496 11.88456
[4,] 11.75365 11.83437 11.94940 12.01161 12.05638 12.12913 12.16167 12.16334
12.19115 12.21824 12.31107 12.31245 12.36847 12.38110 12.40183 12.40608
      [,1168]  [,1169]  [,1170]  [,1171]  [,1172]  [,1173]  [,1174]  [,1175]
[,1176]  [,1177]  [,1178]  [,1179]  [,1180]  [,1181]  [,1182]  [,1183]
[1,] 13.05309 13.09388 13.10305 13.20360 13.21313 13.24323 13.25524 13.27712
13.28433 13.32251 13.39223 13.40424 13.40917 13.44177 13.44547 13.47573
[2,] 12.52403 12.55035 12.66432 12.67478 12.70512 12.77062 12.81206 12.85696
12.90852 12.98249 13.02892 13.10433 13.12015 13.13975 13.17593 13.19457
[3,] 11.96346 12.02792 12.11815 12.12709 12.17863 12.18881 12.25534 12.27506
12.28257 12.28777 12.29642 12.34784 12.42300 12.42420 12.46410 12.50694
[4,] 12.42749 12.44814 12.49421 12.55115 12.55973 12.57130 12.60384 12.60580
12.73017 12.79079 12.79841 12.83094 12.88540 12.92839 12.93797 12.98342
      [,1184]  [,1185]  [,1186]  [,1187]  [,1188]  [,1189]  [,1190]  [,1191]
[,1192]  [,1193]  [,1194]  [,1195]  [,1196]  [,1197]  [,1198]  [,1199]
[1,] 13.50820 13.52589 13.53686 13.54444 13.57616 13.58737 13.59058 13.63668
13.64768 13.66741 13.74054 13.81202 13.82294 13.82863 13.85056 13.85463
[2,] 13.21988 13.22957 13.23999 13.27141 13.31668 13.35350 13.38493 13.46408
13.51940 13.52523 13.54315 13.59054 13.60260 13.67106 13.70377 13.83874
[3,] 12.51150 12.53092 12.57526 12.57667 12.59754 12.61496 12.67757 12.70185
12.76336 12.84140 12.84555 12.90541 12.94973 12.96319 12.97771 12.98716
[4,] 13.01088 13.04708 13.06662 13.17898 13.19701 13.21553 13.22428 13.24188
13.32416 13.32819 13.33914 13.42783 13.48441 13.49619 13.58176 13.58270
      [,1200]  [,1201]  [,1202]  [,1203]  [,1204]  [,1205]  [,1206]  [,1207]
[,1208]  [,1209]  [,1210]  [,1211]  [,1212]  [,1213]  [,1214]  [,1215]
[1,] 13.90367 13.90911 13.94003 13.96384 14.03653 14.06533 14.08958 14.19286
14.26414 14.30106 14.39893 14.41918 14.48357 14.48438 14.53397 14.57277
```

[2,] 13.87772 13.96644 14.01674 14.13429 14.16779 14.19898 14.20300 14.21524
14.23719 14.24688 14.36279 14.36457 14.36907 14.39531 14.39955 14.40475
[3,] 13.01215 13.02426 13.10283 13.13633 13.15024 13.16899 13.20538 13.21291
13.21416 13.30478 13.32384 13.38624 13.41583 13.43338 13.46108 13.54034
[4,] 13.67444 13.72800 13.75063 13.75180 13.76643 13.78923 13.79687 13.80791
13.81882 13.82174 13.84594 13.87201 13.88715 13.89745 13.93820 13.96776
[,1216] [,1217] [,1218] [,1219] [,1220] [,1221] [,1222] [,1223]
[,1224] [,1225] [,1226] [,1227] [,1228] [,1229] [,1230] [,1231]
[1,] 14.59396 14.60441 14.62697 14.64782 14.67437 14.67598 14.69738 14.70670
14.72443 14.73986 14.78107 14.78943 14.82411 14.86963 14.88403 14.88765
[2,] 14.41051 14.41828 14.46329 14.46926 14.47544 14.49852 14.51123 14.51202
14.60712 14.64198 14.66338 14.67106 14.67486 14.68029 14.68326 14.73210
[3,] 13.54724 13.60054 13.61283 13.65836 13.76541 13.80296 13.83366 13.90656
13.93498 13.96423 13.96783 13.99193 14.06245 14.08718 14.11419 14.13289
[4,] 13.98024 14.00325 14.03447 14.05955 14.06523 14.07328 14.11753 14.14516
14.17571 14.31544 14.39316 14.39720 14.41028 14.42478 14.43097 14.44898
[,1232] [,1233] [,1234] [,1235] [,1236] [,1237] [,1238] [,1239]
[,1240] [,1241] [,1242] [,1243] [,1244] [,1245] [,1246] [,1247]
[1,] 14.90206 14.91082 14.93188 14.94381 14.96992 15.04051 15.08745 15.15135
15.19976 15.22302 15.31473 15.32958 15.35435 15.37824 15.40108 15.49183
[2,] 14.75313 14.77688 14.79418 14.80324 14.85091 14.86392 14.88652 14.95630
14.97248 14.97648 14.98142 15.00286 15.09238 15.10044 15.10093 15.11167
[3,] 14.18314 14.20225 14.21912 14.22368 14.26403 14.28061 14.40533 14.43425
14.46051 14.50469 14.51369 14.51911 14.52160 14.55462 14.56454 14.57312
[4,] 14.52488 14.52839 14.54755 14.59423 14.62625 14.67599 14.72110 14.73827
14.73891 14.75166 14.76276 14.78126 14.78605 14.80560 14.82794 14.83806
[,1248] [,1249] [,1250] [,1251] [,1252] [,1253] [,1254] [,1255]
[,1256] [,1257] [,1258] [,1259] [,1260] [,1261] [,1262] [,1263]
[1,] 15.55613 15.56250 15.56805 15.57909 15.61384 15.62656 15.65766 15.66444
15.67910 15.69226 15.72517 15.75207 15.79181 15.79578 15.80512 15.83962
[2,] 15.15988 15.18385 15.21462 15.24315 15.26654 15.36441 15.36934 15.37859
15.38557 15.39278 15.39685 15.42500 15.45539 15.45859 15.46959 15.51288
[3,] 14.58371 14.60378 14.62533 14.71078 14.77988 14.78129 14.80388 14.83398
14.84013 14.86685 14.91736 14.95537 14.97587 14.98628 15.02728 15.06949
[4,] 14.84835 14.88889 14.90791 14.90977 14.93517 14.96632 15.04865 15.05500
15.05671 15.08329 15.09315 15.11485 15.13265 15.13986 15.14300 15.15140
[,1264] [,1265] [,1266] [,1267] [,1268] [,1269] [,1270] [,1271]
[,1272] [,1273] [,1274] [,1275] [,1276] [,1277] [,1278] [,1279]
[1,] 15.90411 15.92541 15.92571 15.93276 15.93897 15.94204 15.99271 16.00346
16.05002 16.05440 16.05889 16.06843 16.12120 16.12122 16.17721 16.20942
[2,] 15.52543 15.55206 15.55549 15.56179 15.58637 15.61981 15.66723 15.70579
15.71564 15.74517 15.75642 15.84195 15.89545 15.91950 15.96646 15.97199
[3,] 15.17812 15.20279 15.21233 15.22226 15.22637 15.25242 15.27972 15.29776
15.30272 15.32383 15.33086 15.41368 15.43884 15.44545 15.44917 15.51222
[4,] 15.24706 15.25929 15.28158 15.29133 15.35839 15.38328 15.39344 15.39475
15.39686 15.39815 15.41404 15.41660 15.44347 15.48725 15.51166 15.57773
[,1280] [,1281] [,1282] [,1283] [,1284] [,1285] [,1286] [,1287]
[,1288] [,1289] [,1290] [,1291] [,1292] [,1293] [,1294] [,1295]
[1,] 16.21000 16.26609 16.30602 16.35950 16.36732 16.37691 16.39634 16.49293
16.54046 16.54970 16.55730 16.60338 16.63421 16.71780 16.72075 16.74896
[2,] 15.98885 16.01022 16.07014 16.07786 16.08727 16.11639 16.14271 16.16011
16.16336 16.19436 16.19466 16.20036 16.22137 16.27687 16.29680 16.30451
[3,] 15.51895 15.52624 15.61176 15.66170 15.67087 15.67511 15.71873 15.73248
15.73784 15.82923 15.86100 15.86596 15.87251 15.88596 15.89979 15.91219

```
[4,] 15.61872 15.62486 15.64258 15.65827 15.66681 15.72688 15.73751 15.78784
15.79710 15.86385 15.90116 15.91098 15.92184 15.98108 15.98164 15.98519
      [,1296]  [,1297]  [,1298]  [,1299]  [,1300]  [,1301]  [,1302]  [,1303]
[,1304]  [,1305]  [,1306]  [,1307]  [,1308]  [,1309]  [,1310]  [,1311]
[1,] 16.77621 16.79800 16.80639 16.81197 16.81263 16.87825 16.94941 16.98202
16.99843 17.01574 17.04114 17.05086 17.06246 17.13108 17.14488 17.18299
[2,] 16.31140 16.31880 16.32160 16.34698 16.35109 16.36888 16.38955 16.41134
16.44030 16.53020 16.60367 16.64069 16.66195 16.69860 16.74284 16.75615
[3,] 15.92518 16.00387 16.01565 16.10999 16.12347 16.15678 16.16532 16.17246
16.23139 16.24831 16.28165 16.32794 16.33385 16.35197 16.42276 16.44186
[4,] 16.05354 16.09760 16.19444 16.23438 16.24713 16.24867 16.26687 16.32688
16.34570 16.35076 16.35085 16.38463 16.38533 16.38807 16.41941 16.52477
      [,1312]  [,1313]  [,1314]  [,1315]  [,1316]  [,1317]  [,1318]  [,1319]
[,1320]  [,1321]  [,1322]  [,1323]  [,1324]  [,1325]  [,1326]  [,1327]
[1,] 17.18602 17.18834 17.20830 17.23218 17.33075 17.33135 17.48693 17.53096
17.55574 17.57196 17.61290 17.65558 17.67017 17.68276 17.79078 17.86240
[2,] 16.75771 16.76004 16.79306 16.88866 16.91444 16.91512 16.93397 17.01627
17.04832 17.06771 17.06803 17.08426 17.13687 17.17916 17.21830 17.24619
[3,] 16.45867 16.47121 16.48224 16.48971 16.50592 16.54096 16.54594 16.54876
16.55347 16.57127 16.57648 16.58632 16.69046 16.70109 16.72408 16.75281
[4,] 16.61399 16.67385 16.73169 16.75404 16.79405 16.83866 16.85852 16.86719
16.87194 16.91022 16.91708 16.93349 16.96015 17.01491 17.03804 17.04038
      [,1328]  [,1329]  [,1330]  [,1331]  [,1332]  [,1333]  [,1334]  [,1335]
[,1336]  [,1337]  [,1338]  [,1339]  [,1340]  [,1341]  [,1342]  [,1343]
[1,] 17.88026 17.89311 17.95653 17.96784 18.00796 18.02864 18.04483 18.10130
18.15607 18.16327 18.17578 18.18532 18.24765 18.26844 18.28010 18.28350
[2,] 17.31101 17.35834 17.41611 17.43996 17.44835 17.50767 17.52553 17.53638
17.54801 17.58302 17.60982 17.60987 17.61450 17.67543 17.68129 17.72631
[3,] 16.75581 16.76292 16.80336 16.84858 16.88331 16.92610 16.92912 16.96615
17.05759 17.09094 17.12664 17.14144 17.16618 17.19966 17.20845 17.22824
[4,] 17.06666 17.06687 17.11529 17.14948 17.15651 17.18039 17.20549 17.21740
17.22408 17.22999 17.25714 17.26278 17.39712 17.43152 17.45295 17.47026
      [,1344]  [,1345]  [,1346]  [,1347]  [,1348]  [,1349]  [,1350]  [,1351]
[,1352]  [,1353]  [,1354]  [,1355]  [,1356]  [,1357]  [,1358]  [,1359]
[1,] 18.32815 18.34376 18.37929 18.38618 18.40042 18.42411 18.45229 18.51059
18.53400 18.53984 18.56373 18.56630 18.57004 18.59671 18.60943 18.66330
[2,] 17.74754 17.78161 17.79603 17.81129 17.93312 17.94667 18.01740 18.03583
18.04862 18.07649 18.10230 18.17608 18.20047 18.21577 18.22045 18.25681
[3,] 17.28615 17.31820 17.35963 17.39463 17.39846 17.41920 17.53333 17.59249
17.61374 17.66482 17.67860 17.71297 17.72721 17.75410 17.77039 17.77375
[4,] 17.48106 17.48733 17.67773 17.71671 17.74948 17.77470 17.79419 17.80280
17.81862 17.85846 17.86282 17.89417 17.90843 17.91411 17.92092 17.95426
      [,1360]  [,1361]  [,1362]  [,1363]  [,1364]  [,1365]  [,1366]  [,1367]
[,1368]  [,1369]  [,1370]  [,1371]  [,1372]  [,1373]  [,1374]  [,1375]
[1,] 18.68420 18.70974 18.73390 18.74939 18.78180 18.80700 18.86344 18.94144
18.96747 19.00026 19.06398 19.09666 19.10366 19.14607 19.17088 19.17513
[2,] 18.27150 18.32343 18.34650 18.36805 18.38077 18.40882 18.45303 18.45551
18.48936 18.53192 18.57573 18.59205 18.61059 18.65221 18.68788 18.70655
[3,] 17.89021 17.92057 17.92340 17.96209 17.96376 17.99208 17.99544 17.99868
18.06794 18.08029 18.09938 18.14259 18.15429 18.20485 18.22104 18.26414
[4,] 18.01335 18.05425 18.05788 18.08451 18.09498 18.09725 18.10743 18.11565
18.16418 18.16997 18.18886 18.19656 18.22180 18.24401 18.29215 18.39205
      [,1376]  [,1377]  [,1378]  [,1379]  [,1380]  [,1381]  [,1382]  [,1383]
[,1384]  [,1385]  [,1386]  [,1387]  [,1388]  [,1389]  [,1390]  [,1391]
```

[1,] 19.17553 19.17887 19.19219 19.19458 19.26959 19.27212 19.37988 19.50220
19.54880 19.61732 19.62953 19.64981 19.73056 19.75419 19.81347 19.84680
[2,] 18.73715 18.76266 18.78451 18.79199 18.81771 18.82776 18.84454 18.94518
19.00312 19.04905 19.05563 19.06666 19.09645 19.12484 19.12886 19.16783
[3,] 18.27710 18.29862 18.35178 18.36787 18.42268 18.45541 18.46095 18.47207
18.50415 18.51882 18.52668 18.54698 18.64374 18.66761 18.75577 18.76498
[4,] 18.39230 18.39828 18.40010 18.43738 18.46737 18.49717 18.51281 18.53908
18.55193 18.56183 18.62158 18.66365 18.69664 18.79521 18.79857 18.83708
[,1392] [,1393] [,1394] [,1395] [,1396] [,1397] [,1398] [,1399]
[,1400] [,1401] [,1402] [,1403] [,1404] [,1405] [,1406] [,1407]
[1,] 19.85013 19.85920 19.87187 19.89279 19.90560 19.91048 19.91134 19.92466
19.92951 19.94919 19.95984 19.97639 19.98229 20.03962 20.08249 20.08765
[2,] 19.17588 19.21286 19.31538 19.34041 19.35031 19.41357 19.46341 19.47496
19.47888 19.49679 19.51471 19.52004 19.55407 19.56065 19.57665 19.59498
[3,] 18.84028 18.87089 18.92663 18.94131 18.94939 18.98193 19.06631 19.14620
19.16376 19.18563 19.20746 19.21710 19.22947 19.23592 19.27530 19.30375
[4,] 18.85828 18.88470 18.98220 18.98790 18.99057 18.99201 19.04340 19.04945
19.05045 19.08644 19.13158 19.20638 19.25287 19.27581 19.28748 19.29363
[,1408] [,1409] [,1410] [,1411] [,1412] [,1413] [,1414] [,1415]
[,1416] [,1417] [,1418] [,1419] [,1420] [,1421] [,1422] [,1423]
[1,] 20.09543 20.11233 20.20192 20.22059 20.22350 20.33162 20.36045 20.38022
20.39811 20.41725 20.49463 20.49643 20.58180 20.59470 20.62124 20.69525
[2,] 19.65616 19.68946 19.70004 19.70242 19.76384 19.77477 19.78609 19.82169
19.83366 19.85035 19.87514 19.94021 19.96897 19.99598 20.01835 20.05416
[3,] 19.35032 19.47699 19.50320 19.52844 19.54533 19.54666 19.54842 19.56302
19.61403 19.67123 19.69245 19.70562 19.71710 19.73575 19.75824 19.78712
[4,] 19.32266 19.39946 19.42953 19.50258 19.50420 19.60437 19.61926 19.65673
19.74937 19.77731 19.78179 19.78650 19.80900 19.85722 19.88990 20.00781
[,1424] [,1425] [,1426] [,1427] [,1428] [,1429] [,1430] [,1431]
[,1432] [,1433] [,1434] [,1435] [,1436] [,1437] [,1438] [,1439]
[1,] 20.70728 20.71609 20.72446 20.84797 20.85144 20.92059 20.96106 21.06902
21.18205 21.21914 21.23657 21.30574 21.31673 21.32033 21.33952 21.36159
[2,] 20.06133 20.16249 20.20163 20.23197 20.27551 20.35726 20.36391 20.36514
20.38668 20.39907 20.42245 20.47270 20.49586 20.49923 20.53047 20.56954
[3,] 19.85324 19.85787 19.86196 19.88791 19.89753 19.90572 20.00770 20.05667
20.06549 20.08658 20.20064 20.23292 20.23367 20.26897 20.27435 20.32421
[4,] 20.01204 20.03213 20.05188 20.07743 20.09691 20.12760 20.14059 20.16131
20.16892 20.21125 20.22396 20.22448 20.23273 20.29122 20.36526 20.51680
[,1440] [,1441] [,1442] [,1443] [,1444] [,1445] [,1446] [,1447]
[,1448] [,1449] [,1450] [,1451] [,1452] [,1453] [,1454] [,1455]
[1,] 21.39526 21.40364 21.44087 21.44962 21.45368 21.50557 21.54180 21.56837
21.59142 21.61202 21.65474 21.65839 21.68001 21.69207 21.71399 21.73602
[2,] 20.59627 20.60738 20.62758 20.67055 20.68640 20.69194 20.74769 20.86535
20.86603 20.95014 20.95865 21.02812 21.08514 21.09606 21.18301 21.20298
[3,] 20.36157 20.37684 20.41281 20.41948 20.45802 20.46391 20.52974 20.54158
20.54507 20.58016 20.62788 20.63613 20.64241 20.69185 20.69786 20.73069
[4,] 20.52943 20.56200 20.56367 20.58419 20.59527 20.62899 20.63115 20.68396
20.72302 20.74187 20.74454 20.78413 20.85822 20.97544 20.97572 20.97737
[,1456] [,1457] [,1458] [,1459] [,1460] [,1461] [,1462] [,1463]
[,1464] [,1465] [,1466] [,1467] [,1468] [,1469] [,1470] [,1471]
[1,] 21.75009 21.79095 21.79424 21.80585 21.88667 21.90226 21.90260 21.90746
21.91529 21.96580 22.08809 22.13965 22.15689 22.17073 22.17484 22.18675
[2,] 21.22295 21.30054 21.33274 21.34183 21.35922 21.41465 21.42047 21.43254
21.43713 21.46440 21.50451 21.58299 21.68503 21.69428 21.72317 21.74405

[3,] 20.73853 20.76821 20.83162 20.88515 20.95540 21.03510 21.08983 21.10890
21.11722 21.15426 21.16498 21.18579 21.19161 21.21070 21.28224 21.31231
[4,] 20.99609 21.01319 21.03548 21.05811 21.07419 21.09103 21.11040 21.21915
21.24430 21.24778 21.30305 21.33038 21.37763 21.39213 21.40810 21.41126
[,1472] [,1473] [,1474] [,1475] [,1476] [,1477] [,1478] [,1479]
[,1480] [,1481] [,1482] [,1483] [,1484] [,1485] [,1486] [,1487]
[1,] 22.29131 22.30304 22.30946 22.31020 22.34136 22.39635 22.40854 22.42249
22.59509 22.61440 22.67382 22.69630 22.72360 22.75469 22.76644 22.79482
[2,] 21.83794 22.02922 22.10936 22.17432 22.18904 22.25563 22.27334 22.36869
22.44128 22.52737 22.53139 22.58953 22.69105 22.71206 22.72062 22.74945
[3,] 21.31887 21.35178 21.40747 21.41266 21.48759 21.59085 21.69862 21.73467
21.74660 21.77404 21.80587 21.85259 21.88450 21.98085 22.06150 22.06364
[4,] 21.45968 21.51466 21.52943 21.61137 21.68451 21.68825 21.70502 21.71189
21.75584 21.86519 21.88872 21.93922 21.99927 22.02517 22.05499 22.07040
[,1488] [,1489] [,1490] [,1491] [,1492] [,1493] [,1494] [,1495]
[,1496] [,1497] [,1498] [,1499] [,1500] [,1501] [,1502] [,1503]
[1,] 22.80411 22.81222 22.83306 22.86527 22.87066 23.02894 23.05118 23.06688
23.13550 23.15740 23.22969 23.27486 23.39927 23.43963 23.44958 23.49949
[2,] 22.78395 22.81599 22.86265 22.88315 22.91531 22.92556 22.92818 22.94366
22.98221 23.03188 23.07112 23.07325 23.12962 23.16538 23.17781 23.23152
[3,] 22.12802 22.17612 22.21331 22.24465 22.27524 22.29392 22.37302 22.45908
22.50119 22.50824 22.56517 22.67782 22.67969 22.75657 22.82931 22.83812
[4,] 22.07723 22.09525 22.14727 22.15936 22.16050 22.23480 22.34479 22.34989
22.39049 22.40991 22.41293 22.41770 22.45127 22.57229 22.60117 22.63573
[,1504] [,1505] [,1506] [,1507] [,1508] [,1509] [,1510] [,1511]
[,1512] [,1513] [,1514] [,1515] [,1516] [,1517] [,1518] [,1519]
[1,] 23.50505 23.50746 23.64158 23.82212 23.84924 23.88083 23.88692 23.94344
23.95696 23.97741 24.01479 24.02754 24.05616 24.08419 24.09087 24.15419
[2,] 23.24659 23.26122 23.28738 23.30189 23.33210 23.33825 23.35874 23.36347
23.38422 23.41004 23.57140 23.59070 23.62659 23.66400 23.70549 23.84520
[3,] 22.90327 23.00634 23.03634 23.05444 23.10897 23.12118 23.12227 23.15137
23.17014 23.18091 23.21369 23.23819 23.26667 23.28277 23.31069 23.37773
[4,] 22.67461 22.70758 22.74298 22.75910 22.76375 22.78449 22.86259 22.95041
23.00697 23.02534 23.05085 23.06584 23.20661 23.25915 23.28898 23.33345
[,1520] [,1521] [,1522] [,1523] [,1524] [,1525] [,1526] [,1527]
[,1528] [,1529] [,1530] [,1531] [,1532] [,1533] [,1534] [,1535]
[1,] 24.24364 24.26848 24.41362 24.46976 24.50970 24.57903 24.58189 24.64084
24.67947 24.77400 24.84994 24.86465 24.94509 25.08790 25.09037 25.10656
[2,] 23.86822 23.96653 23.99846 24.06930 24.15135 24.17684 24.18171 24.18367
24.20033 24.22879 24.27651 24.29873 24.34833 24.37896 24.39153 24.40342
[3,] 23.44811 23.50696 23.57906 23.59206 23.62054 23.68122 23.78642 23.82106
23.89667 23.95039 23.98302 24.04556 24.06963 24.09685 24.34430 24.37813
[4,] 23.39082 23.39977 23.45923 23.47331 23.53426 23.59774 23.60399 23.81942
23.88894 23.90334 23.95302 24.00769 24.01601 24.02964 24.04702 24.05249
[,1536] [,1537] [,1538] [,1539] [,1540] [,1541] [,1542] [,1543]
[,1544] [,1545] [,1546] [,1547] [,1548] [,1549] [,1550] [,1551]
[1,] 25.23300 25.24204 25.27480 25.28206 25.32179 25.37960 25.43096 25.43614
25.48170 25.75426 25.83516 25.83803 25.89035 25.91953 25.96333 26.08478
[2,] 24.40892 24.40926 24.59690 24.67439 24.79347 24.86582 25.01824 25.06871
25.07509 25.14982 25.20811 25.33310 25.37201 25.41433 25.44066 25.61809
[3,] 24.45085 24.59029 24.65016 24.75042 24.85789 25.03899 25.16374 25.18767
25.23059 25.25982 25.33840 25.47686 25.49347 25.70307 25.73207 25.82100
[4,] 24.06873 24.32003 24.33361 24.47117 24.50198 24.59463 24.64929 24.65315
24.65847 24.69038 24.69870 24.72444 24.75559 24.78964 24.83574 24.93829

```
      [,1552]  [,1553]  [,1554]  [,1555]  [,1556]  [,1557]  [,1558]  [,1559]
[,1560]  [,1561]  [,1562]  [,1563]  [,1564]  [,1565]  [,1566]  [,1567]
[1,] 26.08956 26.19575 26.36778 26.39646 26.48340 26.62057 26.71200 26.72347
26.96127 26.99025 27.07200 27.12741 27.26452 27.35319 27.42122 27.43112
[2,] 25.67090 25.71401 25.99565 26.08222 26.10362 26.13190 26.13948 26.20936
26.24826 26.35056 26.43535 26.47971 26.55287 26.57613 26.69585 26.74933
[3,] 25.83589 25.88920 25.90848 26.00438 26.07499 26.08343 26.11656 26.13055
26.16858 26.30745 26.39153 26.41396 26.43121 26.44484 26.51371 26.53059
[4,] 24.98991 25.13760 25.20141 25.25787 25.57552 25.60377 25.95500 26.01762
26.25086 26.40522 26.48268 26.67708 26.70244 26.75130 26.75640 26.84929
      [,1568]  [,1569]  [,1570]  [,1571]  [,1572]  [,1573]  [,1574]  [,1575]
[,1576]  [,1577]  [,1578]  [,1579]  [,1580]  [,1581]  [,1582]  [,1583]
[1,] 27.48566 27.62873 27.65430 27.68209 27.85098 27.94708 27.95272 28.04755
28.06187 28.25541 28.30442 28.45804 28.59823 28.79963 28.91658 29.40914
[2,] 26.92207 26.99032 26.99843 27.00222 27.08533 27.27941 27.33528 27.36111
27.41612 27.45852 27.46254 27.64197 27.65461 27.65700 27.69839 27.73895
[3,] 26.58906 26.60972 26.69347 27.00479 27.09796 27.11355 27.19678 27.24468
27.26044 27.49054 27.61350 27.76154 27.79248 27.83363 27.86161 28.11394
[4,] 26.95899 27.12756 27.38475 27.50144 27.52520 27.55489 27.61621 27.85065
27.85072 27.99153 28.19773 28.20027 28.22027 28.24161 28.30778 28.44339
      [,1584]  [,1585]  [,1586]  [,1587]  [,1588]  [,1589]  [,1590]  [,1591]
[,1592]  [,1593]  [,1594]  [,1595]  [,1596]  [,1597]  [,1598]  [,1599]
[1,] 29.49277 29.57565 29.58909 30.04877 30.06091 30.10348 30.20533 30.51143
30.51482 30.52115 30.55766 30.61987 30.72796 30.78083 30.89636 30.99805
[2,] 27.88511 27.88868 28.00436 28.03989 28.21030 28.33097 28.52442 28.84168
28.85281 29.01996 29.03050 29.26330 29.64435 29.78267 30.45889 30.58097
[3,] 28.19900 28.24216 28.39231 28.54965 28.77146 28.86474 28.89614 29.09418
29.17708 29.43516 29.44261 29.68886 29.73518 29.74146 29.77952 29.91484
[4,] 28.56426 28.56720 28.60846 28.73716 28.75409 28.84885 29.07392 29.09956
29.14194 29.17146 29.22423 29.27072 29.42273 29.92496 29.99964 30.06488
      [,1600]  [,1601]  [,1602]  [,1603]  [,1604]  [,1605]  [,1606]  [,1607]
[,1608]  [,1609]  [,1610]  [,1611]  [,1612]  [,1613]  [,1614]  [,1615]
[1,] 31.04158 31.58326 31.67215 31.88746 31.96294 32.08513 32.29789 32.50146
32.55829 32.74462 32.75651 33.21823 33.23161 33.45067 33.72803 33.87995
[2,] 31.23578 31.53348 31.87202 32.01760 32.10798 32.32222 32.62268 32.63856
33.04455 33.27935 33.58537 33.67262 33.84781 33.88938 34.05436 34.06907
[3,] 29.94339 29.97682 30.20342 30.23845 30.24975 30.38670 30.52073 30.58442
30.60596 30.68141 30.85179 31.52934 32.21052 33.13204 33.45326 33.55628
[4,] 30.07849 30.44987 30.55826 30.59966 30.63312 30.67170 31.17584 31.42891
31.57121 31.61737 32.04804 32.09698 32.31901 32.64975 32.76169 32.90113
      [,1616]  [,1617]  [,1618]  [,1619]  [,1620]  [,1621]  [,1622]  [,1623]
[,1624]  [,1625]  [,1626]  [,1627]  [,1628]  [,1629]  [,1630]  [,1631]
[1,] 34.02591 34.43461 34.44767 34.74477 35.15327 35.64716 35.67856 36.00984
36.43702 36.72741 37.11649 37.60469 37.66099 38.72832 39.34265 39.67507
[2,] 34.18783 34.32338 34.33451 35.62930 35.73990 36.81259 37.11395 37.33202
37.60243 37.73903 38.36437 38.57789 39.87714 40.09287 40.16423 40.37645
[3,] 33.85826 33.88623 33.95320 34.10548 34.82406 34.95019 35.18094 35.19489
35.46754 35.49868 35.89120 35.96439 36.51135 36.81825 37.22929 37.36006
[4,] 32.92949 32.96881 33.04179 33.10242 33.10366 33.65626 34.00607 34.10929
34.22943 35.28963 35.62492 35.85952 36.17451 36.29894 37.18108 37.59223
      [,1632]  [,1633]  [,1634]  [,1635]  [,1636]  [,1637]  [,1638]  [,1639]
[,1640]  [,1641]
[1,] 39.84957 40.82208 41.65912 43.85342 44.44576 44.67558 44.70061 45.78391
47.38233 48.31554
```

```
[2,] 40.80581 40.93392 41.03700 41.11016 41.96606 42.12898 42.73162 43.26662
45.54006 60.61139
[3,] 37.81904 38.21428 38.36271 38.66483 39.34870 39.68275 40.32949 40.53014
40.81917 44.62958
[4,] 37.95060 38.47463 38.47947 38.99362 39.04902 42.77472 43.01716 43.23914
43.94874 46.37200
```

ENSEMBLE FLUCTUATION DATA (40.1ns)

```
              [,1]      [,2]      [,3]       [,4]      [,5]       [,6]
[,7]       [,8]       [,9]      [,10]      [,11]      [,12]      [,13]
NAT0.pdb 0.2365321 0.1813386 0.1256330 0.12299297 0.1534674 0.13454416
0.10663230 0.12113080 0.12505568 0.10195806 0.11437727 0.13228003 0.12880047
LJP6.pdb 0.2153278 0.1404102 0.1009381 0.09556295 0.1080003 0.09760085
0.09089987 0.09488369 0.08728362 0.08894002 0.10330932 0.09658444 0.09352418
3WY9.pdb 0.1780933 0.1985338 0.1312768 0.10301962 0.1441068 0.14620944
0.12197626 0.10887856 0.09211886 0.08725563 0.10088206 0.10740163 0.09403384
ZSP9.pdb 0.1930934 0.1387591 0.1085518 0.12322295 0.1208260 0.09935632
0.09993325 0.09230788 0.08356642 0.07879098 0.09186658 0.09305737 0.09273552
              [,14]     [,15]     [,16]     [,17]     [,18]     [,19]     [,20]
   [,21]     [,22]     [,23]     [,24]     [,25]     [,26]     [,27]
NAT0.pdb 0.11991335 0.1490893 0.1670129 0.1654714 0.1688336 0.1848529 0.2364275
0.2901803 0.3439253 0.3328937 0.4476543 0.3745364 0.3392365 0.3178798
LJP6.pdb 0.09263718 0.1056468 0.1114841 0.1169246 0.1218900 0.1109509 0.1163201
0.1692593 0.2754754 0.5033790 0.3648346 0.2629286 0.1794067 0.1934798
3WY9.pdb 0.09611119 0.1114621 0.1117091 0.1135157 0.1240452 0.1171931 0.1341648
0.1953487 0.2759119 0.2426469 0.1975013 0.2264809 0.3467945 0.2231925
ZSP9.pdb 0.09266690 0.0996687 0.1055954 0.1169712 0.1164653 0.1040672 0.1098896
0.1656103 0.1970906 0.2091108 0.2340340 0.2584751 0.1718548 0.1787996
             [,28]     [,29]     [,30]     [,31]     [,32]     [,33]     [,34]
  [,35]     [,36]     [,37]     [,38]     [,39]     [,40]     [,41]
NAT0.pdb 0.2345952 0.2854813 0.3020336 0.3002602 0.3346322 0.3611532 0.2805123
0.2932278 0.3104499 0.2967808 0.2515676 0.2412928 0.3208929 0.3893564
LJP6.pdb 0.1494687 0.1804392 0.1854315 0.2236951 0.2231396 0.1844816 0.1421461
0.1411833 0.1773388 0.1845922 0.1333120 0.1254938 0.1770548 0.2530750
3WY9.pdb 0.1470048 0.1602110 0.1713096 0.1404325 0.1450080 0.1564317 0.1256434
0.1277580 0.1634383 0.1618605 0.1350285 0.1229455 0.1690930 0.2204120
ZSP9.pdb 0.1436685 0.1594765 0.2141350 0.2098407 0.1926500 0.1516984 0.1221788
0.1393917 0.1621895 0.1657069 0.1330047 0.1343561 0.1912092 0.2846198
             [,42]     [,43]     [,44]     [,45]     [,46]     [,47]      [,48]
    [,49]      [,50]      [,51]      [,52]      [,53]      [,54]     [,55]
NAT0.pdb 0.3749767 0.3852347 0.3374722 0.2956296 0.2145461 0.2410066 0.17472685
0.14824932 0.13877025 0.14151725 0.11547258 0.13193420 0.19525064 0.1980038
LJP6.pdb 0.2177436 0.2531954 0.1869011 0.1646925 0.1268506 0.1462922 0.10409085
0.11156501 0.09419868 0.08904401 0.08817435 0.07954794 0.09715711 0.1170075
3WY9.pdb 0.2141431 0.2229097 0.2110046 0.1713298 0.1264578 0.1517776 0.09869125
0.10645690 0.09816176 0.09365623 0.09014560 0.09261042 0.10469173 0.1383260
ZSP9.pdb 0.2758463 0.2735440 0.2649149 0.2088917 0.1420228 0.1605814 0.11979313
0.09957599 0.09147657 0.10472935 0.08956794 0.08976158 0.10536603 0.1297989
             [,56]     [,57]      [,58]      [,59]      [,60]      [,61]
[,62]      [,63]      [,64]      [,65]      [,66]      [,67]      [,68]
NAT0.pdb 0.2108143 0.1909457 0.16605553 0.14791785 0.13770364 0.09895471
0.09642133 0.10104908 0.08140190 0.08377417 0.09226780 0.08067959 0.07458826
```

LJP6.pdb 0.1162650 0.1228450 0.10039409 0.10425679 0.10093572 0.07985173
0.08819917 0.07611161 0.07189573 0.07425300 0.06983824 0.06971338 0.06234776
3WY9.pdb 0.1179104 0.1105675 0.09221628 0.08178287 0.08742477 0.07928339
0.07580254 0.07362024 0.06411196 0.06561387 0.07451222 0.07168718 0.06146599
ZSP9.pdb 0.1189712 0.1161110 0.09973289 0.09216002 0.08596137 0.07670504
0.08362430 0.07669041 0.06447518 0.07249380 0.07329775 0.06569326 0.06106924
[,69] [,70] [,71] [,72] [,73] [,74]
[,75] [,76] [,77] [,78] [,79] [,80] [,81] [,82]
NAT0.pdb 0.08764426 0.09602528 0.10093654 0.11230518 0.10966293 0.1374745
0.2017716 0.1794159 0.1383749 0.1343416 0.1720610 0.1393077 0.1210884 0.1483813
LJP6.pdb 0.06990181 0.07074768 0.07093554 0.08691805 0.08935927 0.1116247
0.1226342 0.1256043 0.1155785 0.1605451 0.2213386 0.1644613 0.1550200 0.2290651
3WY9.pdb 0.07267770 0.07985128 0.08088749 0.09140286 0.09705523 0.1116687
0.1443594 0.2458956 0.1942574 0.1905314 0.2180897 0.1532145 0.1340811 0.1419542
ZSP9.pdb 0.06822085 0.07214053 0.07473843 0.08521171 0.08540279 0.1111316
0.1232993 0.1549835 0.1055578 0.1677348 0.1882429 0.2536072 0.3422077 0.2332851
[,83] [,84] [,85] [,86] [,87] [,88] [,89]
[,90] [,91] [,92] [,93] [,94] [,95] [,96]
NAT0.pdb 0.1823971 0.1654512 0.1678275 0.1470042 0.1381088 0.1857013 0.1662051
0.1321363 0.1490697 0.1714635 0.1444409 0.1242692 0.1475839 0.15224678
LJP6.pdb 0.2172220 0.1716193 0.2123208 0.1898934 0.1816828 0.1944632 0.1361120
0.1161259 0.1311943 0.1252441 0.1058200 0.1014700 0.1025203 0.09649121
3WY9.pdb 0.1403181 0.1046233 0.1266232 0.1706983 0.1662117 0.1876659 0.1577979
0.1264951 0.1422918 0.1386647 0.1191805 0.1124156 0.1115585 0.10896926
ZSP9.pdb 0.2306041 0.2758403 0.2046263 0.1342230 0.1588299 0.1507280 0.1158342
0.1048824 0.1235041 0.1287004 0.1029557 0.1003066 0.1074085 0.10091746
[,97] [,98] [,99] [,100] [,101] [,102]
[,103] [,104] [,105] [,106] [,107] [,108] [,109] [,110]
NAT0.pdb 0.12168097 0.14380524 0.1720403 0.15384022 0.15315575 0.1957756
0.2206992 0.1889674 0.2129514 0.4449146 0.3635885 0.3959843 0.2453483 0.2172837
LJP6.pdb 0.08498856 0.09249293 0.1016562 0.09485893 0.09766341 0.1331508
0.1289261 0.1143556 0.1316223 0.2186762 0.2028222 0.1439049 0.1751721 0.1382241
3WY9.pdb 0.09027171 0.10289270 0.1122245 0.10168545 0.10718627 0.1501020
0.1458097 0.1261170 0.1631140 0.3742038 0.3121903 0.4018671 0.1707627 0.1443879
ZSP9.pdb 0.08133203 0.09543515 0.1049544 0.09855310 0.10551732 0.1194637
0.1173867 0.1267921 0.1498121 0.3501359 0.3257955 0.4362244 0.2358637 0.1669205
[,111] [,112] [,113] [,114] [,115] [,116]
[,117] [,118] [,119] [,120] [,121] [,122] [,123] [,124]
NAT0.pdb 0.1751123 0.1750447 0.17474943 0.16578089 0.20578533 0.2539220
0.2770776 0.4229888 0.3628190 0.3621009 0.2792837 0.2841292 0.3218561 0.3012484
LJP6.pdb 0.1197948 0.1093159 0.11823611 0.10555004 0.11294525 0.1372884
0.1540972 0.2123744 0.2870840 0.1870081 0.1434769 0.1713436 0.2217779 0.2229970
3WY9.pdb 0.1182362 0.1122184 0.11168806 0.08044478 0.09791467 0.1275395
0.1414933 0.2070516 0.3066941 0.2222248 0.1461972 0.1753870 0.2199832 0.1875701
ZSP9.pdb 0.1161748 0.1020429 0.08457319 0.09724340 0.10356198 0.1141457
0.1422696 0.1483010 0.2694158 0.2120844 0.1541372 0.1596533 0.2091523 0.1670599
[,125] [,126] [,127] [,128] [,129] [,130] [,131]
[,132] [,133] [,134] [,135] [,136] [,137] [,138]
NAT0.pdb 0.2597133 0.2640444 0.2336661 0.2202934 0.2418285 0.2669629 0.2565560
0.3207530 0.3403668 0.3412946 0.2755458 0.2708739 0.2384518 0.2139925
LJP6.pdb 0.1632289 0.1407704 0.1382648 0.1405146 0.1327044 0.1421743 0.2009411
0.2767488 0.2179987 0.2106183 0.1486031 0.1467121 0.1228349 0.1338074
3WY9.pdb 0.1546346 0.1485635 0.1269883 0.1277561 0.1227658 0.1382294 0.1327908
0.1839701 0.2021832 0.1733736 0.1352706 0.1479797 0.1307152 0.1338484

ZSP9.pdb 0.1383369 0.1294147 0.1176088 0.1260994 0.1216397 0.1342139 0.1516485 0.1793019 0.2152383 0.2188698 0.1373343 0.1536466 0.1481253 0.1449251

[,139] [,140] [,141] [,142] [,143] [,144] [,145] [,146] [,147] [,148] [,149] [,150] [,151] [,152]
NAT0.pdb 0.2205671 0.2186980 0.2235344 0.2277771 0.3480395 0.2413091 0.1594568 0.12648444 0.11213608 0.10725804 0.09843641 0.10170905 0.08040306 0.07790787
LJP6.pdb 0.1373078 0.1091416 0.1463484 0.2138406 0.2875955 0.1418989 0.1154331 0.09253935 0.08680029 0.07722184 0.07689372 0.06917195 0.05745224 0.06382149
3WY9.pdb 0.1379316 0.1376091 0.1615948 0.1470590 0.2011992 0.1609048 0.1437261 0.10299130 0.09440009 0.08275693 0.09615145 0.08086967 0.06714201 0.06675885
ZSP9.pdb 0.1321511 0.1352802 0.1885245 0.3025930 0.3775615 0.1713422 0.1314175 0.09103506 0.07851813 0.06993610 0.07299142 0.06467479 0.05525384 0.06042331

[,153] [,154] [,155] [,156] [,157] [,158] [,159] [,160] [,161] [,162] [,163] [,164] [,165]
NAT0.pdb 0.09324579 0.08893558 0.07071468 0.07170809 0.09460454 0.08511111 0.08280719 0.10581840 0.11414231 0.1269360 0.1457686 0.1601197 0.2193785
LJP6.pdb 0.06785655 0.06014627 0.05756084 0.05631861 0.06268247 0.05996251 0.06876905 0.07942991 0.08503033 0.1106666 0.1207223 0.1539323 0.1677481
3WY9.pdb 0.07014397 0.07323912 0.05992488 0.06063842 0.06951454 0.06326584 0.06876197 0.08129450 0.08641431 0.1127995 0.1235042 0.1386829 0.1705745
ZSP9.pdb 0.06455611 0.05900623 0.05753150 0.05477336 0.06524619 0.06670020 0.07131896 0.08153713 0.10167841 0.1244375 0.1185325 0.1582018 0.1865836

[,166] [,167] [,168] [,169] [,170] [,171] [,172] [,173] [,174] [,175] [,176] [,177] [,178] [,179]
NAT0.pdb 0.3329886 0.3641181 0.3279881 0.2533561 0.3540609 0.2957123 0.1770549 0.1906597 0.2616877 0.2088737 0.1552312 0.1895203 0.1818305 0.13593050
LJP6.pdb 0.4298484 0.3821662 0.3492724 0.2830461 0.2596728 0.1779051 0.1364179 0.1627108 0.1688392 0.1044792 0.1015684 0.1448051 0.1134460 0.08085466
3WY9.pdb 0.2764953 0.3416126 0.3848292 0.5851545 0.8634089 0.4436132 0.3425601 0.2038456 0.1892313 0.1128224 0.1108526 0.1451339 0.1405664 0.10373435
ZSP9.pdb 0.3255416 0.4773707 0.5944080 0.5017671 0.6906309 0.3875026 0.2106723 0.2019374 0.1989529 0.1192442 0.1074462 0.1442720 0.1261895 0.08504497

[,180] [,181] [,182] [,183] [,184] [,185] [,186] [,187] [,188] [,189] [,190] [,191] [,192] [,193]
NAT0.pdb 0.1555509 0.1876112 0.15801146 0.13506245 0.1871945 0.2007623 0.15559756 0.1933866 0.2868852 0.2262385 0.1759581 0.1968497 0.1604526 0.2633498
LJP6.pdb 0.1022278 0.1035529 0.07979340 0.08094945 0.1125624 0.1075496 0.07930322 0.1355459 0.1902910 0.1617049 0.1408325 0.1331218 0.1531229 0.1374897
3WY9.pdb 0.1165849 0.1326454 0.11569218 0.10214136 0.1354188 0.1356538 0.11191458 0.1180542 0.1919287 0.1997963 0.1826314 0.1710325 0.1327868 0.1561063
ZSP9.pdb 0.1107073 0.1198802 0.08542443 0.08029089 0.1269505 0.1119159 0.07547936 0.1154287 0.1635664 0.1641673 0.1376829 0.2019489 0.1603026 0.1583894

[,194] [,195] [,196] [,197] [,198] [,199] [,200] [,201] [,202] [,203] [,204] [,205] [,206] [,207]
NAT0.pdb 0.2863335 0.1913605 0.2367376 0.2295486 0.18056838 0.204801 0.3134693 0.3446119 0.2408780 0.2331650 0.1988488 0.2222486 0.2206891 0.1557748
LJP6.pdb 0.1298818 0.2113133 0.2849050 0.1911275 0.23470705 0.200577 0.1249653 0.1691588 0.2379529 0.1827396 0.1670275 0.1356089 0.1588665 0.1055928
3WY9.pdb 0.1555174 0.1376367 0.1985492 0.2423422 0.14877757 0.183041 0.2448804 0.1807611 0.1962255 0.1449474 0.1444057 0.1353799 0.1485744 0.1664152
ZSP9.pdb 0.1122646 0.1017227 0.1128618 0.1051318 0.08473146 0.114422 0.1265379 0.1829123 0.2510354 0.2570325 0.1714453 0.2061842 0.1279590 0.1139263

[,208] [,209] [,210] [,211] [,212] [,213] [,214] [,215] [,216] [,217] [,218] [,219] [,220]

```
NAT0.pdb 0.22033232 0.19432140 0.1734512 0.15428501 0.11698753 0.09920997
0.11541808 0.08950451 0.07981323 0.10679196 0.10542053 0.08988321 0.11211461
LJP6.pdb 0.08927039 0.14316163 0.1502700 0.09463102 0.08154333 0.09406953
0.09166820 0.07962314 0.06573319 0.07940184 0.09197217 0.08319939 0.08649728
3WY9.pdb 0.11624469 0.12872689 0.1054290 0.08310896 0.07334663 0.07800803
0.08035792 0.07283013 0.06605270 0.07545716 0.07802222 0.07506071 0.08015334
ZSP9.pdb 0.10879844 0.09853531 0.1027562 0.09626152 0.08147981 0.07777099
0.08404718 0.07799207 0.06308325 0.08443651 0.08896792 0.08554513 0.10179984
            [,221]    [,222]    [,223]    [,224]    [,225]    [,226]    [,227]
 [,228]    [,229]    [,230]    [,231]    [,232]    [,233]    [,234]
NAT0.pdb 0.1337119 0.1382596 0.1368390 0.1930910 0.2206150 0.3136998 0.2698404
0.2741737 0.2355581 0.1735600 0.2316214 0.2236218 0.1921533 0.2225755
LJP6.pdb 0.1235444 0.1325850 0.1154239 0.1713558 0.2536034 0.3244578 0.2736994
0.3134108 0.2579695 0.2566143 0.2080788 0.1638583 0.1558872 0.1938291
3WY9.pdb 0.1036171 0.1436126 0.1187242 0.1691899 0.1998559 0.2545307 0.3590909
0.3278058 0.1637995 0.1570673 0.1634906 0.1616509 0.1375176 0.1405405
ZSP9.pdb 0.1138400 0.1443245 0.1798205 0.2666033 0.2296081 0.2775710 0.2688832
0.2763118 0.1700632 0.1683622 0.1977237 0.1791511 0.1351374 0.1671138
            [,235]    [,236]    [,237]    [,238]    [,239]    [,240]    [,241]
 [,242]    [,243]    [,244]    [,245]    [,246]    [,247]    [,248]
NAT0.pdb 0.2983852 0.3195009 0.3492565 0.3924252 0.3328521 0.3605473 0.3923061
0.4759413 0.2665227 0.1749805 0.1770899 0.1448934 0.1457123 0.1363732
LJP6.pdb 0.2411667 0.2057732 0.2758658 0.2940714 0.2022643 0.1795453 0.1936163
0.2402546 0.2399068 0.1930181 0.2731325 0.2339469 0.1815400 0.1858760
3WY9.pdb 0.1970678 0.1849851 0.1533737 0.1985958 0.2031406 0.1626635 0.1887691
0.2360869 0.2665613 0.3063519 0.2091925 0.1764619 0.2034356 0.1652145
ZSP9.pdb 0.2002879 0.1720401 0.1519098 0.2267023 0.1800932 0.1264696 0.1164994
0.1673756 0.1761469 0.1556049 0.2139163 0.1543714 0.1447687 0.1423243
            [,249]     [,250]     [,251]    [,252]     [,253]     [,254]
[,255]     [,256]     [,257]     [,258]     [,259]     [,260]     [,261]
NAT0.pdb 0.1210757 0.09655992 0.11947573 0.1186437 0.10387164 0.10980652
0.10531925 0.11831278 0.11575767 0.10451082 0.10650973 0.11517710 0.11581565
LJP6.pdb 0.1829407 0.12440564 0.14867780 0.1552034 0.13715699 0.10019022
0.10165779 0.10193435 0.10635497 0.10096178 0.09722489 0.10115378 0.10264165
3WY9.pdb 0.1512245 0.08821586 0.11729266 0.1292808 0.11130725 0.08052271
0.08315728 0.09945880 0.10089854 0.08429797 0.08252232 0.09625502 0.09207652
ZSP9.pdb 0.1224388 0.08474760 0.09283702 0.1258288 0.08065931 0.08126128
0.08714973 0.09211557 0.09055274 0.09018325 0.08682929 0.08449583 0.09179697
             [,262]    [,263]    [,264]    [,265]    [,266]    [,267]    [,268]
  [,269]    [,270]    [,271]    [,272]    [,273]    [,274]    [,275]
NAT0.pdb 0.11712269 0.1370915 0.1582253 0.1726857 0.1842126 0.2373062 0.2922870
0.2767916 0.3154379 0.2914192 0.2578779 0.2600696 0.2916868 0.2974375
LJP6.pdb 0.10843348 0.1107354 0.1289283 0.1498479 0.1466117 0.1736477 0.2188442
0.2431811 0.3225806 0.2644990 0.4450073 0.8263870 0.4104998 0.3815033
3WY9.pdb 0.09336009 0.1105985 0.1114216 0.1219946 0.1867323 0.2255975 0.3186937
0.2414302 0.3230517 0.4695927 0.5720662 0.5936136 0.3458826 0.2004640
ZSP9.pdb 0.10593160 0.1118736 0.1186046 0.1391416 0.1550122 0.1777790 0.2369083
0.2908168 0.3873032 0.2881978 0.3404312 0.3320169 0.2582755 0.3896286
            [,276]    [,277]    [,278]    [,279]    [,280]    [,281]    [,282]
 [,283]    [,284]    [,285]    [,286]    [,287]    [,288]    [,289]
NAT0.pdb 0.3064037 0.3298440 0.2554401 0.2036947 0.2574322 0.2545359 0.1778866
0.1880339 0.2211002 0.1767859 0.1436231 0.1698031 0.1712525 0.1393520
LJP6.pdb 0.3364450 0.3527404 0.2671459 0.1873195 0.2049644 0.1997435 0.1505117
0.1419926 0.1580612 0.1503472 0.1196237 0.1363204 0.1539882 0.1277310
```

3WY9.pdb 0.1752763 0.1439253 0.1305932 0.1567095 0.2148508 0.2194160 0.1464736 0.1273313 0.1564962 0.1485624 0.1205640 0.1324174 0.1576505 0.1374110
ZSP9.pdb 0.5637868 0.3707534 0.4011721 0.2801806 0.2362466 0.2324031 0.1610781 0.1299898 0.1440777 0.1439688 0.1204145 0.1202990 0.1395985 0.1176308

[,290] [,291] [,292] [,293] [,294] [,295] [,296] [,297] [,298] [,299] [,300] [,301] [,302] [,303]
NAT0.pdb 0.1541326 0.1865555 0.1594475 0.1996766 0.2750987 0.3085150 0.2241715 0.3358709 0.3168332 0.2385565 0.1873699 0.1510162 0.1753417 0.1696843
LJP6.pdb 0.1322246 0.1606873 0.1431143 0.1507708 0.2115843 0.2432692 0.3099587 0.5126453 0.4643749 0.4560973 0.2039252 0.1811379 0.1687522 0.1669356
3WY9.pdb 0.1282607 0.1682373 0.1566423 0.1485783 0.1864188 0.1927704 0.1822375 0.2309986 0.2794400 0.4535589 0.2121046 0.1338585 0.1311584 0.1229267
ZSP9.pdb 0.1199312 0.1333773 0.1183623 0.1276523 0.1644894 0.1928941 0.1869202 0.3489318 0.5726479 0.2509083 0.1366967 0.1224170 0.1553276 0.1530467

[,304] [,305] [,306] [,307] [,308] [,309] [,310] [,311] [,312] [,313] [,314] [,315] [,316] [,317]
NAT0.pdb 0.1953252 0.3495177 0.3188123 0.2312266 0.1657490 0.1257536 0.1504597 0.1585362 0.2035942 0.1656128 0.1440976 0.10754993 0.10273831 0.08803621
LJP6.pdb 0.1645966 0.2786674 0.3078658 0.2391334 0.1678348 0.1375258 0.1486165 0.2131750 0.2307928 0.1784817 0.1181145 0.08559416 0.08767864 0.07491796
3WY9.pdb 0.1328945 0.2566161 0.2294252 0.1668393 0.1111584 0.1057016 0.1332338 0.1829433 0.1716634 0.1511138 0.1247158 0.10214676 0.08939615 0.07600803
ZSP9.pdb 0.1646804 0.2821554 0.2878185 0.2283916 0.1740865 0.1165151 0.1232995 0.1617007 0.1555029 0.1260269 0.1032258 0.08343299 0.08973657 0.07769882

[,318] [,319] [,320] [,321] [,322] [,323] [,324] [,325] [,326] [,327] [,328] [,329] [,330]
NAT0.pdb 0.07851627 0.07850605 0.08177509 0.07142857 0.07376244 0.08048762 0.08175405 0.07738259 0.08931332 0.10702719 0.10505287 0.12560151 0.12917194
LJP6.pdb 0.07583045 0.08023750 0.07923515 0.07112116 0.07320119 0.08699787 0.09073145 0.08441104 0.09505058 0.10330669 0.11242739 0.12966533 0.14618735
3WY9.pdb 0.07708583 0.07409934 0.07910411 0.06865353 0.06424496 0.07055305 0.07439339 0.07478512 0.07789739 0.07594838 0.07765020 0.09137302 0.09686538
ZSP9.pdb 0.07594056 0.06861424 0.07439499 0.06379371 0.06346399 0.07943027 0.07234272 0.07107836 0.08312469 0.08126412 0.09074188 0.10991553 0.10632067

[,331] [,332] [,333] [,334] [,335] [,336] [,337] [,338] [,339] [,340] [,341] [,342] [,343] [,344]
NAT0.pdb 0.1494538 0.2201149 0.2075418 0.3039416 0.2396661 0.1751644 0.1829043 0.2333876 0.2980291 0.3005466 0.4471380 0.4268013 0.2653323 0.2095565
LJP6.pdb 0.1727613 0.2440908 0.3492976 0.4132118 0.5091124 0.3547377 0.3410902 0.5083981 0.6636304 0.6116508 0.7022662 0.7138513 0.5967402 0.6259376
3WY9.pdb 0.1070082 0.1401511 0.1400753 0.1438406 0.1288149 0.2018099 0.2591445 0.3855517 0.4113570 0.1989328 0.1925486 0.2630516 0.1977881 0.1554875
ZSP9.pdb 0.1195803 0.1767777 0.1886145 0.1945133 0.1884255 0.1905536 0.2843165 0.4067012 0.7765975 0.4711978 0.3066807 0.2778120 0.4856854 0.5262097

[,345] [,346] [,347] [,348] [,349] [,350] [,351] [,352] [,353] [,354] [,355] [,356] [,357] [,358]
NAT0.pdb 0.5770420 0.3895089 0.2411226 0.2005375 0.2392629 0.2280160 0.1644300 0.1502209 0.1878697 0.1773958 0.12827345 0.1411641 0.1612093 0.14066763
LJP6.pdb 0.4051094 0.3627019 0.4657778 0.2862364 0.2622883 0.2354292 0.1460136 0.1361598 0.1564896 0.1394115 0.10981807 0.1182928 0.1275887 0.11047083
3WY9.pdb 0.1495198 0.1964950 0.2581269 0.2004302 0.2545696 0.1796184 0.1296573 0.1358641 0.1466058 0.1067865 0.09369931 0.1258795 0.1225512 0.09960842
ZSP9.pdb 0.4091533 0.2734572 0.2038928 0.1919688 0.2326506 0.1804855 0.1281788 0.1348247 0.1473188 0.1196701 0.09519888 0.1064106 0.1238329 0.10384346

[,359] [,360] [,361] [,362] [,363] [,364] [,365]
[,366] [,367] [,368] [,369] [,370] [,371] [,372]
NAT0.pdb 0.1242821 0.14300227 0.1593902 0.1528950 0.14574559 0.1801450 0.1993854
0.2241166 0.2857188 0.3078298 0.2967247 0.2338494 0.18094826 0.15837994
LJP6.pdb 0.1007726 0.09230259 0.1025321 0.1028041 0.09577218 0.1033351 0.1378982
0.1367792 0.2709692 0.3272366 0.2221389 0.1666048 0.10242890 0.09522115
3WY9.pdb 0.1155678 0.11209056 0.1048258 0.1162369 0.10042184 0.1016760 0.1263195
0.1656860 0.2965616 0.2519998 0.1883481 0.1235769 0.09974689 0.10686885
ZSP9.pdb 0.1027626 0.11029982 0.1156449 0.1131746 0.10492582 0.1148862 0.1572911
0.2467501 0.5315780 0.3044056 0.2497513 0.1859014 0.11925719 0.10707154
[,373] [,374] [,375] [,376] [,377] [,378] [,379]
[,380] [,381] [,382] [,383] [,384] [,385] [,386]
NAT0.pdb 0.16103669 0.1906149 0.2509899 0.2499770 0.3458007 0.4717073 0.4935499
0.2787691 0.2480746 0.2807484 0.2465364 0.1996619 0.1875632 0.1827617
LJP6.pdb 0.09676326 0.1064221 0.1317414 0.1634548 0.1597734 0.1760777 0.1854248
0.1242929 0.1507968 0.1765119 0.1477960 0.1321331 0.1100806 0.1090082
3WY9.pdb 0.10479716 0.1158293 0.1609118 0.1763207 0.1674864 0.2043666 0.2031244
0.1523774 0.1961278 0.2357430 0.1845204 0.1550089 0.1515704 0.1663751
ZSP9.pdb 0.09728027 0.1015804 0.1379976 0.1411201 0.2175720 0.2178031 0.1774410
0.1285652 0.1615458 0.2014758 0.1617374 0.1290670 0.1193349 0.1080971
[,387] [,388] [,389] [,390] [,391] [,392] [,393]
[,394] [,395] [,396] [,397] [,398] [,399] [,400]
NAT0.pdb 0.1996380 0.2385848 0.2226487 0.2275992 0.2867850 0.3160934 0.3421839
0.4709449 0.5204111 0.5275549 0.4652498 0.4694172 0.4266780 0.3820241
LJP6.pdb 0.1203140 0.1279665 0.1390261 0.1314919 0.1688749 0.1807748 0.1618297
0.1774845 0.2333208 0.2470716 0.2431062 0.2854526 0.3116346 0.3396889
3WY9.pdb 0.1434280 0.1289568 0.1251348 0.1256745 0.1146972 0.1258899 0.1491786
0.2728797 0.2177836 0.2822204 0.2698497 0.2307020 0.1869522 0.1807905
ZSP9.pdb 0.1215601 0.1177108 0.1257336 0.1317255 0.1124514 0.1274963 0.1390658
0.1678675 0.1595745 0.1795396 0.2141297 0.2483611 0.2750796 0.2948160
[,401] [,402] [,403] [,404] [,405] [,406] [,407]
[,408] [,409] [,410] [,411] [,412] [,413] [,414]
NAT0.pdb 0.3692045 0.3117658 0.2705034 0.3487298 0.2747648 0.2419917 0.2460691
0.1884653 0.14311969 0.1523914 0.12420767 0.12206388 0.10077745 0.12463371
LJP6.pdb 0.3273908 0.2055821 0.1605977 0.2256814 0.1579532 0.1644187 0.1442653
0.1190383 0.10350388 0.1020479 0.09140778 0.08123802 0.06997326 0.09513239
3WY9.pdb 0.1746887 0.2015528 0.1675691 0.2068899 0.1818558 0.1849056 0.1641288
0.1218565 0.09935158 0.1020921 0.08819457 0.09082390 0.07954992 0.09123519
ZSP9.pdb 0.3037557 0.3108450 0.1835692 0.1795231 0.1467417 0.2007188 0.2068533
0.1188738 0.09534661 0.1006778 0.08092787 0.07699300 0.07527091 0.10139895
[,415] [,416] [,417] [,418] [,419] [,420] [,421]
[,422] [,423] [,424] [,425] [,426] [,427] [,428]
NAT0.pdb 0.1488694 0.1717240 0.2465182 0.2947057 0.2056537 0.2473887 0.2349032
0.2695409 0.2299007 0.2882762 0.3070730 0.3978018 0.3192726 0.2334052
LJP6.pdb 0.1460826 0.1713449 0.1905477 0.2203179 0.1830979 0.2344974 0.2061888
0.2403655 0.1882660 0.1961958 0.1404949 0.2088016 0.2101068 0.1517081
3WY9.pdb 0.1136763 0.1274614 0.1570074 0.1813731 0.1498674 0.1581766 0.1985360
0.2057770 0.1917936 0.2141512 0.2164907 0.1981845 0.1856328 0.1410228
ZSP9.pdb 0.1294105 0.1529899 0.1727889 0.2202393 0.1452409 0.2048602 0.2075729
0.2623477 0.2367903 0.2429034 0.2089887 0.2071990 0.1812465 0.1371221
[,429] [,430] [,431] [,432] [,433] [,434]
[,435] [,436] [,437] [,438] [,439] [,440] [,441]
NAT0.pdb 0.1868634 0.13867225 0.15428791 0.13756003 0.12833644 0.13053574
0.10955194 0.09815810 0.08300441 0.08785982 0.09278086 0.07580965 0.08194394

LJP6.pdb 0.1598458 0.08827266 0.08890139 0.08455605 0.08165758 0.08766826
0.08794052 0.08009313 0.07090474 0.07477115 0.07276580 0.07179110 0.07632622
3WY9.pdb 0.1507720 0.10953491 0.10529537 0.10564289 0.09541446 0.08622984
0.09512202 0.08546113 0.07478907 0.08660729 0.08191600 0.07684370 0.08951027
ZSP9.pdb 0.1224171 0.10283181 0.09987830 0.08707706 0.08203986 0.09262701
0.09629352 0.08725654 0.06701178 0.08356580 0.07388176 0.07240913 0.07656413
[,442] [,443] [,444] [,445] [,446] [,447]
[,448] [,449] [,450] [,451] [,452] [,453] [,454] [,455]
NAT0.pdb 0.09345287 0.08091969 0.09377309 0.09971285 0.11882307 0.1658506
0.1387155 0.1286080 0.1974216 0.2323109 0.2672856 0.2517521 0.1792075 0.1912925
LJP6.pdb 0.08119977 0.07677457 0.08271319 0.08469732 0.08809932 0.1136755
0.1539144 0.1043170 0.1362320 0.1857139 0.1655989 0.1543665 0.1526124 0.2706269
3WY9.pdb 0.09983868 0.08574980 0.09860449 0.13187198 0.17269455 0.1635609
0.1498769 0.2480927 0.2427851 0.1762952 0.1580195 0.1510126 0.4225018 0.2347538
ZSP9.pdb 0.08050414 0.07721212 0.08143502 0.08483202 0.09230755 0.1249927
0.2104453 0.1478785 0.1597058 0.2625695 0.3166760 0.2886178 0.4404197 0.2049784
[,456] [,457] [,458] [,459] [,460] [,461] [,462]
[,463] [,464] [,465] [,466] [,467] [,468] [,469]
NAT0.pdb 0.1635406 0.2182355 0.1997568 0.2402447 0.2625996 0.2656420 0.2417605
0.2840994 0.3066952 0.3176420 0.3753989 0.4025570 0.4251428 0.4597450
LJP6.pdb 0.4350492 0.5033801 0.3323444 0.3918813 0.2653815 0.3115269 0.5328169
0.6791606 0.4520419 0.6336833 0.5330236 0.5465563 0.5248486 0.8120374
3WY9.pdb 0.2001057 0.2274800 0.2947547 0.3648345 0.3290661 0.2985532 0.3850860
0.4302416 0.3970059 0.3658440 0.3504789 0.6792074 0.6811875 0.8005201
ZSP9.pdb 0.2178641 0.2644349 0.1753793 0.2170425 0.2856544 0.3778317 0.2703843
0.1851134 0.2186441 0.2694358 0.2357109 0.1763481 0.2158235 0.1966501
[,470] [,471] [,472] [,473] [,474] [,475] [,476]
[,477] [,478] [,479] [,480] [,481] [,482] [,483]
NAT0.pdb 0.9984210 1.3124799 2.1777433 1.9649198 1.8379648 1.0470552 0.7010458
0.4794295 0.6275800 0.5298579 0.4109201 0.3564632 0.3774793 0.3409679
LJP6.pdb 0.8053116 0.7882479 0.6615047 0.7175290 0.5749587 0.4677658 0.4843564
0.4158436 0.5212498 0.5249535 0.4298585 0.2550002 0.1589632 0.1690918
3WY9.pdb 1.0653723 1.1009108 0.7579099 0.9411225 0.6872933 0.5426080 0.3732866
0.2841027 0.1987759 0.1647627 0.1840423 0.1996722 0.1560522 0.1273601
ZSP9.pdb 0.9545836 1.1325354 1.1502899 1.1297625 0.8633968 0.6785148 0.5500422
0.3936349 0.3057945 0.2001729 0.1936015 0.1460044 0.1492540 0.1276407
[,484] [,485] [,486] [,487] [,488] [,489] [,490]
[,491] [,492] [,493] [,494] [,495] [,496] [,497]
NAT0.pdb 0.3239282 0.3205175 0.3387738 0.3977448 0.3786658 0.3539763 0.3904919
0.4539133 0.5512050 0.8900062 0.8476848 0.9529706 0.8087526 0.7058663
LJP6.pdb 0.1287853 0.1505818 0.1578488 0.1860186 0.2223193 0.2046314 0.2847324
0.1837994 0.2935116 0.2956744 0.4251257 0.3393510 0.2681686 0.2066172
3WY9.pdb 0.1329087 0.1142872 0.1419095 0.1442018 0.1724742 0.1851030 0.3300464
0.3126014 0.2708396 0.3106521 0.2717087 0.2518335 0.3415010 0.3570969
ZSP9.pdb 0.1239379 0.1209615 0.1368727 0.1601213 0.1672153 0.1483410 0.1628530
0.1527222 0.1932896 0.2079068 0.2486515 0.2967171 0.3738243 0.4538956
[,498] [,499] [,500] [,501] [,502] [,503] [,504]
[,505] [,506] [,507] [,508] [,509] [,510] [,511]
NAT0.pdb 1.2779686 1.5712243 2.2817021 1.9359965 2.9252985 3.4083935 3.1755399
3.4750826 4.4748631 3.5047970 2.5923564 1.7352973 2.0325981 2.9422522
LJP6.pdb 0.2332361 0.2679077 0.3530099 0.3752538 0.5254895 0.5025527 0.7736171
0.9596243 1.1777158 0.9449434 0.5945883 0.7494084 0.7327547 0.6782447
3WY9.pdb 0.4208362 0.4834495 0.6347275 0.7038189 0.8487456 0.7326189 0.5723559
0.7768791 0.8622096 0.9120707 0.8451898 0.6179218 0.8069740 0.7128465

```
ZSP9.pdb 0.5173512 0.6593893 0.7775792 0.6576519 0.7912923 0.8508240 1.1697394
1.3839332 1.3528536 1.1412301 0.9464959 0.7205225 0.8430137 0.8801162
            [,512]    [,513]    [,514]    [,515]    [,516]    [,517]    [,518]
 [,519]    [,520]    [,521]    [,522]    [,523]    [,524]    [,525]
NAT0.pdb 2.5617020 1.8239816 1.7919224 1.1873080 1.0830486 0.7706633 0.5054479
0.4975874 0.7530864 1.1001559 1.0880794 1.9282760 2.3267321 2.3962984
LJP6.pdb 0.4196323 0.4073230 0.3491335 0.5021969 0.6581652 0.7292921 0.8241114
0.5446555 0.4293314 0.2814568 0.2151371 0.3873922 0.5289564 0.3993622
3WY9.pdb 0.5527492 0.6271469 0.6259316 0.5458128 0.3988011 0.3964793 0.2386901
0.2420049 0.1831392 0.1326469 0.2128595 0.2737468 0.3900712 0.4304750
ZSP9.pdb 0.7341492 0.6335033 0.4950139 0.4255940 0.3000880 0.3971056 0.3940056
0.3630008 0.2858598 0.2697060 0.2971977 0.3363237 0.3504466 0.2935720
            [,526]    [,527]    [,528]    [,529]    [,530]    [,531]    [,532]
 [,533]    [,534]    [,535]    [,536]    [,537]    [,538]    [,539]
NAT0.pdb 2.9368933 2.7505444 3.3289491 3.4918263 2.2970553 2.1984161 1.0088046
0.6138812 0.6105478 0.3963425 0.4702717 0.4291137 0.3877986 0.5321231
LJP6.pdb 0.4855117 0.8303029 1.0261676 1.0493227 0.5430757 0.3999963 0.2945072
0.2773989 0.2442172 0.2653891 0.3100122 0.4259636 0.5581686 0.7141402
3WY9.pdb 0.3968123 0.5318870 0.7381205 0.8053084 1.0143902 0.8862299 0.5285434
0.3771889 0.2938405 0.2839577 0.2169457 0.1858694 0.1902420 0.2942245
ZSP9.pdb 0.3954087 0.5364779 0.8778753 0.9595990 0.7081473 0.6936895 0.4576468
0.3708126 0.2936262 0.2885947 0.2052512 0.2248406 0.1784782 0.2812839
            [,540]    [,541]    [,542]    [,543]    [,544]    [,545]    [,546]
 [,547]    [,548]    [,549]
NAT0.pdb 0.4321890 0.4137139 0.3760984 0.3259450 0.3119003 0.3023287 0.3606906
0.5126954 0.6264526 0.4321185
LJP6.pdb 0.3492234 0.2834359 0.1864827 0.1709131 0.1375596 0.1154110 0.1486692
0.1984214 0.2728965 0.2737104
3WY9.pdb 0.4081412 0.2616399 0.2590172 0.2013022 0.1798980 0.1483841 0.1234284
0.1160558 0.1097189 0.1314274
ZSP9.pdb 0.3969269 0.3196120 0.3456172 0.1794254 0.1745754 0.1746772 0.1494475
0.1930186 0.2044315 0.2392069
```
