# Supplementary material for: Insights into the mechanism(s) of digestion of crystalline cellulose by plant class C GH9 endoglucanases
Source: J Mol Model. 2019 Jul 23;25(8):240. doi: 10.1007/s00894-019-4133-1 (PMC7385011; doi:10.1007/s00894-019-4133-1)
Supplement: Supplementary file 10 — (PDF 155 kb) [file 894_2019_4133_MOESM10_ESM.pdf]

Supplementary Text 8

```
 modes40.1_Q5NAT0

Call:
  nma.pdb(pdb = pdb40.1_NAT0)

Class:
  VibrationalModes (nma)

Number of modes:
  1704 (6 trivial)

Frequencies:
  Mode 7:       0.003
  Mode 8:       0.004
  Mode 9:       0.005
  Mode 10:      0.01
  Mode 11:      0.013
  Mode 12:      0.015

+ attr: modes, frequencies, force.constants, fluctuations,
        U, L, xyz, mass, temp, triv.modes, natoms, call

 modes40.1_Q5NAT0 FREQUENCIES

   [1] 0.000000000 0.000000000 0.000000000 0.000000000 0.000000000 0.000000000
0.002756644 0.003862583 0.005280971 0.009949412 0.012970884 0.015485539
  [13] 0.016846383 0.018986858 0.020760419 0.022085293 0.024375139 0.024730036
0.026728555 0.027382977 0.028333156 0.028658056 0.029752611 0.031163903
  [25] 0.031422523 0.031677032 0.032036036 0.032806772 0.034360148 0.034877817
0.035223617 0.035354973 0.036132729 0.036669934 0.036912969 0.037802566
  [37] 0.038198844 0.038558878 0.039215145 0.039391733 0.039879668 0.040460430
0.040904988 0.041172864 0.041420666 0.041977399 0.042550663 0.042985390
  [49] 0.043051339 0.043622102 0.043941755 0.044408792 0.044621040 0.044797805
0.045324802 0.045676914 0.046235538 0.046550848 0.047208962 0.047323111
  [61] 0.047646641 0.047943429 0.048150618 0.048339630 0.048615779 0.049388064
0.049754951 0.050098427 0.050407916 0.050726503 0.051108317 0.051892322
  [73] 0.052173457 0.052224167 0.052323262 0.052535839 0.052747079 0.052804914
0.053186810 0.053307403 0.053652215 0.053914310 0.054192901 0.054535133
  [85] 0.054677078 0.054902458 0.055098880 0.055849100 0.056135688 0.056329609
0.056586465 0.056837704 0.056888264 0.057204414 0.057394944 0.057541721
  [97] 0.058052734 0.058471629 0.058586748 0.059055492 0.059437926 0.059873362
0.060239883 0.060490186 0.060810311 0.060954681 0.061235782 0.061469669
 [109] 0.061573837 0.062015311 0.062028176 0.062369652 0.062637530 0.062849680
0.063070757 0.063270646 0.063691812 0.063845338 0.064049534 0.064461289
 [121] 0.064723054 0.064863987 0.065067516 0.065525464 0.065539959 0.065974091
0.066165784 0.066697316 0.066885610 0.066980788 0.067098108 0.067339470
 [133] 0.067451660 0.067604329 0.067888865 0.068078515 0.068302508 0.068417191
0.068493232 0.068694489 0.069120719 0.069331664 0.069517378 0.069734388
 [145] 0.069870108 0.070016418 0.070183901 0.070349725 0.070507616 0.070872395
0.071051587 0.071207921 0.071239573 0.071379351 0.071720452 0.071941731
 [157] 0.071974293 0.072379287 0.072444351 0.072686256 0.073040675 0.073107403
0.073231684 0.073374054 0.073468928 0.073595524 0.073880986 0.074036136
 [169] 0.074113930 0.074203763 0.074388891 0.074567445 0.074829739 0.075047758
0.075125854 0.075174391 0.075357471 0.075693194 0.075881699 0.075985110
```

 [181] 0.076291516 0.076341303 0.076620833 0.076951374 0.077230008 0.077332599
0.077472010 0.077896874 0.078095143 0.078299380 0.078568075 0.078718814
 [193] 0.078883233 0.079170106 0.079431552 0.079457856 0.079617728 0.079771245
0.079922567 0.080011101 0.080271859 0.080411056 0.080539635 0.080742394
 [205] 0.080810756 0.080982189 0.081149670 0.081245598 0.081396823 0.081452664
0.081548079 0.081565938 0.081955369 0.082211655 0.082296650 0.082337422
 [217] 0.082636367 0.082696578 0.083003842 0.083101287 0.083309214 0.083472026
0.083630130 0.083911491 0.083984059 0.084266042 0.084516220 0.084725458
 [229] 0.085076319 0.085256111 0.085434041 0.085653902 0.085752766 0.085838828
0.085924215 0.086231288 0.086474462 0.086509899 0.086665676 0.086985275
 [241] 0.087143981 0.087336338 0.087409541 0.087712714 0.087784015 0.087954096
0.088140232 0.088449205 0.088656161 0.088948962 0.089184017 0.089405280
 [253] 0.089535371 0.089625291 0.089825871 0.089871824 0.090116840 0.090264568
0.090407713 0.090623892 0.090791165 0.091024523 0.091078911 0.091126178
 [265] 0.091486409 0.091599857 0.091861084 0.091920764 0.092220917 0.092581266
0.092630638 0.092780780 0.092965834 0.093064824 0.093193611 0.093284349
 [277] 0.093319100 0.093596504 0.093767928 0.093908834 0.094219592 0.094515259
0.094657862 0.094962034 0.094977104 0.095099973 0.095247419 0.095441226
 [289] 0.095707584 0.095906800 0.096041140 0.096094006 0.096221767 0.096502770
0.096691835 0.096878177 0.096903405 0.097066380 0.097113081 0.097390472
 [301] 0.097465349 0.097521079 0.097914444 0.097974961 0.098078709 0.098490037
0.098727648 0.098799589 0.098974414 0.099144716 0.099198481 0.099291385
 [313] 0.099487499 0.099719939 0.099809947 0.099900760 0.099962609 0.100105170
0.100177386 0.100403057 0.100528491 0.100629103 0.100745582 0.100973119
 [325] 0.101249692 0.101463242 0.101594723 0.101665880 0.101817501 0.101960947
0.102024898 0.102122672 0.102327098 0.102366450 0.102449682 0.102608662
 [337] 0.102819636 0.103117911 0.103269117 0.103431490 0.103551788 0.103925131
0.103956568 0.104183414 0.104306123 0.104410495 0.104457185 0.104600886
 [349] 0.104709197 0.105064325 0.105109641 0.105314042 0.105552969 0.105602153
0.105713991 0.105792315 0.105998387 0.106052858 0.106320267 0.106349805
 [361] 0.106509385 0.106958990 0.107053204 0.107140242 0.107371332 0.107734143
0.107888036 0.107962436 0.108017910 0.108104523 0.108374934 0.108512629
 [373] 0.108902809 0.109062717 0.109154303 0.109346859 0.109504268 0.109593983
0.109783575 0.109935521 0.110130966 0.110180750 0.110352235 0.110512796
 [385] 0.110838021 0.110940585 0.110985898 0.111164345 0.111300979 0.111592700
0.111695819 0.111956764 0.112005850 0.112226469 0.112369138 0.112405425
 [397] 0.112639318 0.112756307 0.112881814 0.113158493 0.113270138 0.113347040
0.113680088 0.113791778 0.113872331 0.113950716 0.114009719 0.114259616
 [409] 0.114364760 0.114447787 0.114835337 0.114876688 0.115062640 0.115105890
0.115286418 0.115380528 0.115726439 0.115914524 0.116018059 0.116119647
 [421] 0.116284444 0.116390914 0.116523702 0.116733289 0.117212402 0.117356672
0.117471659 0.117620779 0.117634884 0.117791755 0.117932095 0.118089429
 [433] 0.118276754 0.118359712 0.118657989 0.118894388 0.119113414 0.119179638
0.119375438 0.119418293 0.119601207 0.119768827 0.119852760 0.120029631
 [445] 0.120139003 0.120347449 0.120551754 0.120632098 0.121129878 0.121173680
0.121318459 0.121619685 0.121849922 0.121903752 0.122123291 0.122214106
 [457] 0.122415398 0.122729205 0.122919763 0.123206487 0.123258285 0.123468646
0.123634711 0.123962801 0.124045122 0.124361334 0.124440236 0.124566376
 [469] 0.124690966 0.124865447 0.125031580 0.125142956 0.125287293 0.125480022
0.125745600 0.125879386 0.126015141 0.126109580 0.126271971 0.126319505
 [481] 0.126585823 0.126776578 0.126885623 0.127113298 0.127449724 0.127601080
0.127645439 0.127867205 0.128088982 0.128402831 0.128479350 0.128701154
 [493] 0.129059940 0.129220681 0.129455304 0.129639981 0.129821566 0.130124813
0.130221426 0.130343040 0.130388021 0.130537328 0.130878694 0.130955217

[505] 0.131059336 0.131427007 0.131634801 0.131889330 0.132096398 0.132288685
0.132395678 0.132707644 0.132914199 0.133047059 0.133193196 0.133431085
[517] 0.133606759 0.133845333 0.133991544 0.134080932 0.134288203 0.134369288
0.134580257 0.134740522 0.134857215 0.135170903 0.135500690 0.135570867
[529] 0.135723058 0.135884679 0.135979901 0.136143077 0.136374799 0.136816517
0.137113623 0.137306079 0.137603324 0.137654489 0.137721178 0.137831581
[541] 0.138038817 0.138284948 0.138581831 0.138682872 0.138931965 0.139269488
0.139363124 0.139536232 0.139650369 0.139902084 0.140347861 0.140553190
[553] 0.140577427 0.140758669 0.140824427 0.140888717 0.140979930 0.141441652
0.141510405 0.141772039 0.141876076 0.142002692 0.142303300 0.142323412
[565] 0.142584621 0.142632312 0.142865743 0.142915556 0.143156841 0.143199035
0.143345424 0.143703959 0.143871491 0.144107045 0.144226346 0.144455535
[577] 0.144639361 0.144844115 0.144952325 0.145435400 0.145700592 0.145724842
0.145917571 0.146366400 0.146457142 0.146530629 0.146780986 0.147024197
[589] 0.147354874 0.147544015 0.147801224 0.147916176 0.148046181 0.148288940
0.148513310 0.148807489 0.148973023 0.149187025 0.149399450 0.149530368
[601] 0.149754484 0.149791607 0.150093069 0.150111632 0.150181644 0.150472729
0.150716874 0.151191500 0.151243679 0.151362878 0.151850081 0.151915206
[613] 0.152153957 0.152314108 0.152580874 0.152755916 0.153019512 0.153222159
0.153385241 0.153509460 0.153680725 0.154064451 0.154238549 0.154281653
[625] 0.154408596 0.154472643 0.154992402 0.155221769 0.155292250 0.155389598
0.155478414 0.155531679 0.155853245 0.155906788 0.156115829 0.156218745
[637] 0.156504510 0.156881248 0.157098828 0.157154365 0.157396112 0.157505187
0.157728009 0.157859080 0.157976013 0.158117372 0.158473095 0.158610737
[649] 0.158842773 0.158901845 0.159100901 0.159241182 0.159465152 0.159495409
0.159727428 0.160068022 0.160460153 0.160575349 0.160887385 0.161272105
[661] 0.161462356 0.161504709 0.161540150 0.161952022 0.162057015 0.162187165
0.162442473 0.162611574 0.162804149 0.163232075 0.163448252 0.163665305
[673] 0.163730914 0.163862207 0.163937240 0.164108427 0.164410446 0.164517488
0.164727903 0.164812532 0.165060788 0.165406785 0.165430826 0.165463590
[685] 0.165812414 0.165904887 0.166295897 0.166392516 0.166838719 0.166963700
0.167040297 0.167172098 0.167411180 0.167497780 0.167600886 0.167996694
[697] 0.168249660 0.168452106 0.168458948 0.168512770 0.168746425 0.168782673
0.168980058 0.169102932 0.169486863 0.169750219 0.169822650 0.170085262
[709] 0.170316761 0.170552920 0.170789273 0.170797800 0.171139008 0.171406401
0.171598775 0.171887044 0.171970506 0.172222343 0.172412778 0.172584438
[721] 0.172719341 0.172885935 0.172959470 0.173145949 0.173276614 0.173634250
0.173934728 0.174346013 0.174602258 0.174610744 0.174794593 0.174803287
[733] 0.175108992 0.175373149 0.175735527 0.175833802 0.175956065 0.176360123
0.176919530 0.177106774 0.177228087 0.177352888 0.177738095 0.178028801
[745] 0.178543900 0.178660551 0.178768908 0.178859639 0.179011605 0.179231928
0.179285271 0.179731811 0.179918803 0.180154257 0.180481635 0.180932363
[757] 0.181307800 0.181409549 0.181585607 0.181799954 0.182051899 0.182377746
0.182480356 0.182603024 0.182735729 0.182804885 0.182896661 0.182971295
[769] 0.183356718 0.183435514 0.183682250 0.183778688 0.183931685 0.184176587
0.184524285 0.184966188 0.185121077 0.185390097 0.185491382 0.185659818
[781] 0.185732318 0.185955984 0.186060229 0.186155775 0.186356914 0.186838346
0.186852987 0.186971431 0.187267823 0.187374312 0.187639898 0.187721214
[793] 0.188200501 0.188393340 0.188664340 0.188952312 0.189030049 0.189646188
0.189948541 0.189955342 0.190071520 0.190427077 0.190779914 0.191238225
[805] 0.191822646 0.192035130 0.192192626 0.192280382 0.192849365 0.192992612
0.193346597 0.193499687 0.193761780 0.193905921 0.194155724 0.194471646
[817] 0.194803765 0.194859605 0.195010208 0.195157320 0.195207869 0.195860770
0.196441175 0.196828276 0.196976281 0.197229324 0.197763258 0.197875492

[829] 0.198136143 0.198359933 0.198481912 0.199013567 0.199319503 0.199842149
0.199958599 0.200246015 0.200365075 0.200483433 0.200600332 0.200647805
[841] 0.201021073 0.201524098 0.201655531 0.201849759 0.202155662 0.202162240
0.202283366 0.202444775 0.202559354 0.202766833 0.203578210 0.203863318
[853] 0.203964992 0.204173152 0.204346642 0.204627027 0.204876617 0.205460402
0.205640689 0.205813373 0.206014931 0.206192646 0.206420157 0.206549946
[865] 0.206627069 0.206843877 0.207110303 0.207193514 0.207728602 0.208121790
0.208495588 0.208833059 0.208980986 0.209778351 0.210130940 0.210333057
[877] 0.210464886 0.210705998 0.211156928 0.211853703 0.211962539 0.212087682
0.212882989 0.213207873 0.213551242 0.213616410 0.213821512 0.214148461
[889] 0.214322741 0.214351990 0.214481703 0.214870550 0.215255281 0.215269107
0.215713493 0.216313115 0.216397060 0.217016855 0.217275645 0.217440486
[901] 0.217742343 0.218232014 0.218694297 0.218994310 0.219306387 0.219467049
0.220023118 0.220197867 0.220559755 0.220740849 0.221108779 0.221363243
[913] 0.221543052 0.221917414 0.222022229 0.222600023 0.222711171 0.223002316
0.223297675 0.223370546 0.223645033 0.223726397 0.224094625 0.224446224
[925] 0.224983062 0.225371211 0.225655556 0.226623897 0.226971801 0.227041485
0.227684483 0.227926438 0.228220530 0.228337928 0.229116131 0.230847319
[937] 0.230882758 0.231140324 0.231749381 0.232467901 0.233471676 0.233810908
0.234500105 0.235300720 0.235733079 0.235908968 0.236107417 0.236568017
[949] 0.236904365 0.237150051 0.238210825 0.238469717 0.238515494 0.238979242
0.239132195 0.240176305 0.240655537 0.240714051 0.241354260 0.241508908
[961] 0.241521966 0.242575995 0.242908195 0.243004426 0.244001502 0.244006796
0.244321034 0.244733265 0.245228950 0.246121940 0.246872814 0.246963962
[973] 0.247259536 0.247546009 0.247982708 0.248936088 0.249334340 0.250339899
0.251609368 0.251781510 0.252242460 0.252757643 0.253383217 0.254047884
[985] 0.254506670 0.254735230 0.254792599 0.256182067 0.256491017 0.256530566
0.257241116 0.258236534 0.258784469 0.258978447 0.259622059 0.260546850
[997] 0.262275612 0.262963415 0.263155130 0.263590954 0.264224433 0.264644809
0.265976291 0.266819595 0.266913896 0.268482266 0.269294961 0.269923640
[1009] 0.270822795 0.271308953 0.271966042 0.272582706 0.272730188 0.272977871
0.273486688 0.274409108 0.274981194 0.275586791 0.275796276 0.276488326
[1021] 0.276535091 0.276878328 0.277464312 0.277579316 0.278966701 0.279738934
0.279854089 0.280587863 0.281584633 0.281658522 0.282240550 0.282404694
[1033] 0.282959272 0.283112398 0.283457098 0.283628979 0.284111188 0.284254293
0.285141705 0.287188160 0.288245508 0.288629277 0.288771020 0.289078131
[1045] 0.290358293 0.291869902 0.292031757 0.293365778 0.295324209 0.295511174
0.295638350 0.296148777 0.297307014 0.297405531 0.298724713 0.299969282
[1057] 0.300001284 0.300115712 0.300176476 0.301571698 0.302914674 0.303114172
0.305456940 0.305673383 0.306165508 0.306552624 0.306619547 0.306660024
[1069] 0.306748228 0.307667335 0.308304775 0.308403967 0.309214872 0.309706850
0.311104235 0.312373679 0.313864766 0.316573505 0.317789342 0.317907885
[1081] 0.318010693 0.319374008 0.319660197 0.320532840 0.323303044 0.324375813
0.325369462 0.327781064 0.327814755 0.327857097 0.332848621 0.333550261
[1093] 0.335762466 0.338454914 0.338917565 0.340683786 0.344168176 0.345406343
0.345510243 0.345871632 0.350064219 0.352882975 0.356923474 0.357263499
[1105] 0.357957654 0.358164400 0.360377578 0.360391214 0.360846377 0.362783979
0.363428492 0.365539962 0.366188236 0.368677092 0.370861071 0.373469274
[1117] 0.374168532 0.375002348 0.379229227 0.381545588 0.389143339 0.392367551
0.399874348 0.401021855 0.401651236 0.407986052 0.412017690 0.414441611
[1129] 0.415759596 0.416642959 0.424804489 0.425322670 0.426618885 0.429095216
0.430559129 0.440162555 0.441141875 0.443612679 0.451261943 0.451627159
[1141] 0.455053068 0.457740684 0.457862936 0.461397564 0.466612518 0.469353000
0.474066475 0.474130055 0.483421396 0.483538072 0.485336469 0.487615501

[1153] 0.488077483 0.489915186 0.491535065 0.492392831 0.493990793 0.495051956
0.497416108 0.501252575 0.502828728 0.503641637 0.505306367 0.506831384
[1165] 0.508180039 0.510732230 0.511498534 0.514718011 0.518244567 0.520477132
0.521154508 0.524822058 0.526255897 0.526932934 0.527307900 0.527894130
[1177] 0.528729249 0.529324218 0.529564558 0.530235030 0.531010492 0.531182192
0.532776325 0.535216469 0.536873692 0.537803110 0.538084739 0.538247170
[1189] 0.539296260 0.540904599 0.545037553 0.547799038 0.547888759 0.556561809
0.556759092 0.560183055 0.560718970 0.563624620 0.564963262 0.565041427
[1201] 0.565608298 0.566158651 0.566311711 0.566820269 0.567203008 0.568328909
0.569477048 0.570478944 0.570704017 0.571829425 0.573869845 0.575011942
[1213] 0.575841895 0.575909611 0.579185393 0.579202012 0.579447194 0.579925387
0.580082761 0.581064257 0.582433837 0.582695025 0.582799802 0.583509840
[1225] 0.583590469 0.583811788 0.584950085 0.585333108 0.585587769 0.585734367
0.586421285 0.586661441 0.586731060 0.587725395 0.587962434 0.588784156
[1237] 0.589959316 0.590961292 0.591725493 0.591847717 0.592316605 0.592403433
0.593450881 0.593567053 0.594226587 0.594737405 0.596279469 0.596890833
[1249] 0.597405373 0.599591065 0.601094762 0.601873115 0.603928023 0.604352647
0.605409023 0.605700118 0.605717431 0.606753439 0.607562879 0.608013024
[1261] 0.608222000 0.608691633 0.609125349 0.609677012 0.610154969 0.610373960
0.610716070 0.611035956 0.611065616 0.612077508 0.612779808 0.612993936
[1273] 0.613719951 0.614091629 0.614388752 0.614569286 0.615003141 0.615710455
0.615786168 0.617236973 0.617391339 0.618200106 0.619509405 0.620494056
[1285] 0.620969949 0.621127690 0.622837533 0.623136420 0.623642770 0.624127687
0.624509091 0.626001186 0.626428700 0.627741658 0.628019920 0.628190165
[1297] 0.628356897 0.628890562 0.629148166 0.629686531 0.629778322 0.630240231
0.631103636 0.631320717 0.631668044 0.632464462 0.632544618 0.632730920
[1309] 0.633400263 0.633421198 0.634709235 0.635134138 0.635140080 0.635404579
0.635465708 0.636474786 0.636915095 0.637614350 0.637701088 0.637785093
[1321] 0.639026421 0.639026817 0.639669120 0.640135486 0.640783872 0.640923951
0.641065936 0.641891636 0.642681475 0.643732108 0.644074394 0.644456472
[1333] 0.646351914 0.647283310 0.647322481 0.647465450 0.647773542 0.648513215
0.649320629 0.650519970 0.650800637 0.651354414 0.652302296 0.652474552
[1345] 0.652586311 0.653858579 0.655439148 0.655909304 0.656182435 0.656516687
0.657006140 0.657283055 0.657417114 0.658737753 0.659002908 0.659734886
[1357] 0.659793186 0.659837622 0.660220630 0.660678528 0.661595613 0.662565507
0.662576900 0.665544203 0.666381500 0.666852450 0.667160398 0.667963475
[1369] 0.668746591 0.669027032 0.669260521 0.671301570 0.672611932 0.672987406
0.673234910 0.674421489 0.674633662 0.675386598 0.675774250 0.676065227
[1381] 0.676278475 0.676881763 0.677135156 0.678158279 0.678247076 0.678295793
0.678677062 0.678707853 0.680238262 0.680253734 0.680483259 0.680534403
[1393] 0.681655475 0.682193821 0.682314393 0.683064685 0.683668005 0.684106154
0.684747140 0.684788681 0.685180126 0.685287919 0.685776765 0.685845926
[1405] 0.686338231 0.686572953 0.687920339 0.687983596 0.688230307 0.688864918
0.689149872 0.689745225 0.690207996 0.691242716 0.692670343 0.693146199
[1417] 0.693554896 0.695166419 0.695502731 0.695630262 0.695870204 0.696402163
0.696852953 0.696930228 0.696998245 0.697126775 0.697240222 0.697283544
[1429] 0.698679415 0.698690091 0.700641149 0.702858070 0.703688062 0.704504888
0.705503704 0.706951943 0.707718511 0.708435624 0.709031288 0.709090625
[1441] 0.709206052 0.709252964 0.709478945 0.710081707 0.710168296 0.710291590
0.710423205 0.710507168 0.710857941 0.711156914 0.711342355 0.711447394
[1453] 0.712513964 0.713228901 0.713331709 0.713758410 0.713772907 0.715250778
0.715676883 0.715728485 0.716005755 0.717639786 0.718147843 0.718561937
[1465] 0.718811597 0.719149425 0.720510230 0.720554578 0.722040969 0.722267156
0.722732463 0.724028286 0.724238621 0.724392670 0.724541215 0.726694725

[1477] 0.726755287 0.727887994 0.728714418 0.730556868 0.733135097 0.733653967
0.734298841 0.734629720 0.734819096 0.734881039 0.735197289 0.735413006
[1489] 0.735591833 0.736170342 0.736421462 0.736954706 0.737054154 0.737175425
0.738066630 0.738688072 0.738803520 0.739446643 0.739538460 0.739891098
[1501] 0.740628601 0.740684363 0.741054008 0.741639681 0.741842394 0.742010676
0.742287922 0.742947906 0.743003342 0.743201678 0.743519993 0.744578552
[1513] 0.744848551 0.745064331 0.745754839 0.746913927 0.748003563 0.748868353
0.749159866 0.749393873 0.749589403 0.749754832 0.751424643 0.751428873
[1525] 0.751626621 0.751734843 0.751747277 0.752272015 0.753197250 0.753402266
0.755839057 0.756225244 0.756531785 0.756765270 0.757848562 0.758680045
[1537] 0.759198940 0.759398334 0.759868254 0.760022980 0.760158014 0.760456142
0.760505055 0.761041728 0.761131721 0.763760328 0.764129103 0.764389231
[1549] 0.766312673 0.767474824 0.767827497 0.768915349 0.770540599 0.770703617
0.771054913 0.771523922 0.771654695 0.773852889 0.776802083 0.777244094
[1561] 0.777800221 0.777857780 0.778777549 0.778997408 0.779329705 0.779937057
0.780608530 0.781063208 0.781171438 0.782197729 0.782852961 0.783644451
[1573] 0.784045760 0.786386893 0.787498980 0.787932677 0.788257720 0.789046373
0.789092166 0.790656785 0.790933264 0.792169600 0.793383608 0.793617662
[1585] 0.794900252 0.797177290 0.797467517 0.799470497 0.799617654 0.799873413
0.800136324 0.800992881 0.801491905 0.801793492 0.802686086 0.803404615
[1597] 0.804181280 0.804333749 0.807689885 0.808957522 0.809002407 0.809108340
0.809821155 0.810277307 0.811952976 0.812856119 0.812930655 0.814583374
[1609] 0.816962756 0.817328941 0.817698138 0.818659921 0.819043517 0.821275747
0.822570841 0.822747411 0.826399977 0.826844011 0.829013609 0.831086113
[1621] 0.832379287 0.832687635 0.833419156 0.833569604 0.834397958 0.836953688
0.837374203 0.838088985 0.841311170 0.841457527 0.842883572 0.843098942
[1633] 0.846001151 0.846734497 0.849029404 0.851117900 0.854109625 0.855859624
0.863100309 0.864326713 0.865540421 0.865736959 0.867261353 0.872435864
[1645] 0.872612039 0.874127820 0.874705663 0.875341813 0.877091845 0.879126570
0.879266647 0.879792388 0.880687426 0.882674534 0.882999183 0.884654716
[1657] 0.886109292 0.886730019 0.887101271 0.894434652 0.895692403 0.898731758
0.899794840 0.904497173 0.907360895 0.908136125 0.909023444 0.910730992
[1669] 0.910896674 0.917293667 0.917868855 0.920497321 0.926385009 0.927970335
0.933315879 0.933968656 0.934281635 0.937436571 0.938133935 0.946511681
[1681] 0.950238463 0.950246180 0.950656936 0.955060210 0.960708398 0.964529642
0.969624541 0.970646541 0.975985789 0.976710817 0.990454381 0.998129653
[1693] 0.998279002 1.002487525 1.004689678 1.016875245 1.027247725 1.061048633
1.063788413 1.064086335 1.076902185 1.095543372 1.106276164 1.137566199

modes40.1_Q5NAT0 FLUCTUATIONS

[1] 0.25030123 0.19443150 0.13472431 0.13225356 0.16508121 0.14378366
0.11313110 0.12957475 0.13494185 0.10851510 0.12020922 0.14158698 0.13787122
[14] 0.12624721 0.15803666 0.17919036 0.17613798 0.17930226 0.19772209
0.25328327 0.31020710 0.36294852 0.34792850 0.46385028 0.38835056 0.35665460
[27] 0.33460526 0.25155562 0.30752557 0.32455812 0.32257282 0.36100804
0.39030080 0.30497603 0.31863205 0.33493889 0.32132544 0.27028178 0.26036874
[40] 0.34623016 0.41789674 0.39971106 0.41136736 0.35778368 0.31443213
0.22869997 0.25624183 0.18618418 0.15577318 0.14602321 0.14964570 0.12059789
[53] 0.13941716 0.20617728 0.21172070 0.22591722 0.20256168 0.17469916
0.15371853 0.14194539 0.10124663 0.09842722 0.10394507 0.08318810 0.08445922
[66] 0.09338609 0.08209778 0.07547598 0.08834423 0.09755697 0.10309022
0.11457816 0.11241040 0.14141078 0.20724543 0.18477872 0.14299003 0.13954505

```
 [79] 0.17942922 0.14653007 0.12662928 0.15552276 0.19178928 0.17436186
0.17642701 0.15338881 0.14333449 0.19297539 0.17327684 0.13649263 0.15444663
 [92] 0.17894156 0.15019187 0.12860754 0.15471938 0.16048489 0.12721379
0.15048030 0.18211572 0.16269486 0.16092080 0.20659244 0.23461060 0.20074622
[105] 0.22527183 0.46212184 0.38124379 0.41220162 0.25789530 0.22926614
0.18433417 0.18611428 0.18250447 0.17419480 0.21805270 0.26906175 0.29671141
[118] 0.44594716 0.38898554 0.39056032 0.30229353 0.30411715 0.34627943
0.32989958 0.28497035 0.29029079 0.25677590 0.23868486 0.26312101 0.29356721
[131] 0.28004845 0.34999688 0.36745755 0.36922568 0.29722922 0.29063612
0.25389265 0.22630392 0.23193999 0.22756043 0.23418979 0.24035759 0.36202806
[144] 0.25188984 0.16776074 0.13302922 0.11795682 0.11069509 0.10211131
0.10675949 0.08424829 0.08003815 0.09655971 0.09333513 0.07357092 0.07405999
[157] 0.09896354 0.08969924 0.08567845 0.10987106 0.12025943 0.13286882
0.15147053 0.16790072 0.22913441 0.34304546 0.37491145 0.33994161 0.26526746
[170] 0.36811957 0.30679733 0.18567981 0.20085751 0.27442861 0.21882709
0.16361741 0.20110513 0.19314245 0.14389947 0.16503787 0.20009050 0.16784096
[183] 0.14362312 0.19972092 0.21390406 0.16552299 0.20516474 0.30239621
0.23926005 0.18643749 0.20669324 0.16771011 0.27173894 0.29651267 0.19912191
[196] 0.24593436 0.23628773 0.18551795 0.21197264 0.32366257 0.35431275
0.27919463 0.24535201 0.23772683 0.20245485 0.22753555 0.22664624 0.16051135
[209] 0.22776980 0.20362415 0.18311291 0.16320645 0.12245491 0.10492895
0.12220171 0.09345403 0.08446019 0.11434672 0.11163115 0.09435081 0.11938064
[222] 0.14289727 0.14500002 0.14387633 0.20407162 0.23217584 0.32958035
0.28824786 0.29299853 0.24894974 0.18689314 0.25038437 0.23940741 0.20551277
[235] 0.24037789 0.32155918 0.34187851 0.36754597 0.40914338 0.34756675
0.37471292 0.41137705 0.49692611 0.28186523 0.18579867 0.18616014 0.15253312
[248] 0.15372924 0.14174162 0.12496565 0.09892737 0.12083932 0.12056030
0.10711979 0.11327192 0.10812254 0.12355709 0.12105963 0.10786968 0.11204326
[261] 0.12342421 0.12260905 0.12342690 0.14741961 0.17034380 0.18400821
0.19742764 0.25565709 0.31131292 0.29238760 0.33568898 0.31203178 0.27283239
[274] 0.27696298 0.31228006 0.31912685 0.33059628 0.35657685 0.27685760
0.22113275 0.27917066 0.27558076 0.19252666 0.20382497 0.23950865 0.19039310
[287] 0.15435032 0.18378836 0.18475759 0.14840870 0.16482234 0.20084196
0.17074304 0.20976968 0.28923695 0.32528605 0.23950288 0.23581561 0.35415572
[300] 0.33174270 0.25204242 0.19874778 0.15893766 0.18347346 0.17869214
0.20657485 0.36458152 0.33284243 0.24222415 0.17382424 0.13164961 0.15646674
[313] 0.16350397 0.21049506 0.17473228 0.15213196 0.11362046 0.10800067
0.09278727 0.08122137 0.08086805 0.08519783 0.07395346 0.07496932 0.08290387
[326] 0.08518238 0.07980995 0.09217373 0.11204539 0.11040899 0.13056847
0.13649401 0.15870924 0.23120695 0.21792388 0.31882388 0.25547176 0.18753347
[339] 0.19664805 0.24999560 0.31885235 0.32123725 0.47185837 0.45259410
0.28514071 0.22590585 0.28209403 0.37133996 0.59563903 0.59907707 0.41019339
[352] 0.25807102 0.21377101 0.25360993 0.24179122 0.17449308 0.15928393
0.19904536 0.18714098 0.13497273 0.14938933 0.17139563 0.14814548 0.13125869
[365] 0.15342718 0.17020031 0.16187261 0.15621580 0.19489077 0.21542730
0.24078814 0.30713129 0.32643548 0.31508655 0.25322231 0.19738962 0.17128858
[378] 0.17402462 0.20730848 0.26921357 0.26725344 0.36736190 0.49787810
0.52157840 0.30137540 0.26812717 0.30569613 0.27175829 0.21866822 0.20505651
[391] 0.19814807 0.21903580 0.25929927 0.24461660 0.25149563 0.31573485
0.34523139 0.37434946 0.50903437 0.55418400 0.56285107 0.49748907 0.50802026
[404] 0.46557592 0.41769204 0.40401153 0.34368672 0.29887339 0.38168742
0.30327369 0.26589031 0.26655027 0.20530940 0.15503856 0.16351277 0.13328057
[417] 0.13212089 0.10874943 0.13437533 0.15908334 0.18159875 0.25880173
0.31050550 0.21936443 0.26371980 0.25130955 0.28939560 0.25002664 0.31254687
```

[430] 0.33305066 0.42386858 0.33954981 0.24989056 0.19892260 0.15040704
0.16888775 0.14952080 0.13931572 0.13858099 0.11545469 0.10185912 0.08723942
[443] 0.09216956 0.09514634 0.07722244 0.08499764 0.09677014 0.08339088
0.09757695 0.10509117 0.12405694 0.17323775 0.14753328 0.13798337 0.20911757
[456] 0.24423967 0.28064731 0.26662921 0.19223416 0.20479128 0.17456411
0.23179057 0.21196742 0.25279783 0.27721087 0.28261836 0.25998509 0.30700350
[469] 0.33281201 0.34793170 0.41022334 0.44155439 0.46492178 0.50093544
0.39509699 0.41657782 0.53084013 0.52237813 0.60414447 0.91372723 0.99902666
[482] 1.28172105 2.08995470 1.85962542 1.69264001 1.80690198 2.30200355
1.59774704 1.49672827 0.94242908 0.62273549 0.44379583 0.61119589 0.53970216
[495] 0.44056262 0.39257082 0.41673266 0.38071069 0.36070741 0.35590382
0.37260737 0.42922037 0.41296129 0.38943659 0.43154980 0.49848592 0.59899767
[508] 0.93652002 0.88772069 0.95864486 0.77631679 0.64542596 1.17556865
1.42535129 2.07305306 1.72556724 2.65305267 3.09196684 2.87353146 3.16918296
[521] 4.53578227 4.39389505 4.99970576 4.13896441 3.23442743 2.38863172
1.57147010 1.89996849 2.75689214 2.35866376 1.67272472 1.61751986 1.06072015
[534] 0.99363353 0.72364116 0.48260972 0.44158213 0.70312840 1.01990272
1.02396058 1.86131977 2.22658849 2.27903919 2.76412910 2.54974847 3.06688502
[547] 3.24933666 2.10441037 2.05307323 1.49997201 0.90106776 0.54415927
0.58767181 0.39146851 0.49336707 0.46014335 0.42766251 0.57391339 0.47343700
[560] 0.45481463 0.41353763 0.36139582 0.34507606 0.33453926 0.39715070
0.55198997 0.66890453 0.47277707

modes40.1_Q5NAT0 MASSES

[1] 138.14694 114.07900 163.17300 57.05100 131.19600 71.07800 113.15800
87.07700 129.18000 87.07700 113.15800 113.15800 163.17300 147.17400 128.10600
[16] 71.07800 117.12600 157.19400 87.07700 57.05100 99.13100 113.15800
97.11500 57.05100 87.07700 117.12600 157.19400 113.15800 71.07800 186.21000
[31] 157.19400 71.07800 114.10300 87.07700 57.05100 113.15800 71.07800
114.07900 57.05100 129.18000 71.07800 114.10300 57.05100 99.13100 114.07900
[46] 113.15800 99.13100 57.05100 57.05100 163.17300 163.17300 114.07900
71.07800 57.05100 114.07900 114.10300 99.13100 129.18000 147.17400 57.05100
[61] 113.15800 97.11500 131.19600 71.07800 147.17400 101.10400 99.13100
101.10400 131.19600 131.19600 71.07800 186.21000 87.07700 99.13100 113.15800
[76] 128.10600 163.17300 57.05100 128.10600 128.10600 131.19600 71.07800
71.07800 71.07800 57.05100 128.10600 113.15800 57.05100 137.13900 71.07800
[91] 99.13100 128.10600 71.07800 113.15800 129.18000 186.21000 57.05100
101.10400 114.07900 163.17300 147.17400 71.07800 129.18000 71.07800 137.13900
[106] 97.11500 128.10600 97.11500 114.10300 99.13100 113.15800 163.17300
71.07800 128.10600 99.13100 57.05100 114.07900 57.05100 114.07900 87.07700
[121] 114.07900 137.13900 114.10300 103.14300 186.21000 117.12600 157.19400
97.11500 128.10600 114.07900 131.19600 101.10400 101.10400 87.07700 157.19400
[136] 117.12600 71.07800 163.17300 157.19400 113.15800 114.07900 97.11500
117.12600 114.10300 97.11500 57.05100 87.07700 114.07900 113.15800 71.07800
[151] 57.05100 128.10600 101.10400 71.07800 71.07800 71.07800 131.19600
71.07800 71.07800 71.07800 87.07700 113.15800 99.13100 147.17400 157.19400
[166] 87.07700 87.07700 114.10300 97.11500 57.05100 163.17300 71.07800
114.07900 117.12600 113.15800 113.15800 117.12600 137.13900 87.07700 129.18000
[181] 117.12600 113.15800 147.17400 114.07900 147.17400 71.07800 114.07900
129.18000 163.17300 157.19400 57.05100 157.19400 163.17300 114.07900 114.10300
[196] 87.07700 113.15800 101.10400 99.13100 71.07800 157.19400 114.10300

```
163.17300 163.17300  57.05100  87.07700 147.17400  87.07700  57.05100 163.17300
[211]  57.05100 114.07900 128.10600 113.15800 113.15800 186.21000  71.07800
87.07700  71.07800 186.21000 113.15800 163.17300 117.12600  71.07800  87.07700
[226] 114.07900 114.07900 157.19400 157.19400 163.17300 113.15800 114.07900
163.17300 113.15800  71.07800 114.10300 114.10300  71.07800 114.07900  71.07800
[241] 113.15800  57.05100  57.05100 101.10400  57.05100 186.21000  87.07700
113.15800 114.10300 117.12600 147.17400  57.05100 186.21000 114.07900  99.13100
[256] 129.18000 163.17300  97.11500  57.05100  99.13100 117.12600 113.15800
113.15800  71.07800  71.07800 129.18000 147.17400 113.15800 113.15800 117.12600
[271]  57.05100 129.18000  71.07800  57.05100 128.10600 137.13900  71.07800
57.05100  99.13100 113.15800 117.12600  57.05100 163.17300 157.19400 157.19400
[286] 129.18000  71.07800 114.07900 147.17400 147.17400  71.07800 103.14300
87.07700 103.14300 113.15800  57.05100 129.18000 114.07900  71.07800  71.07800
[301] 114.07900 114.10300  99.13100  57.05100 157.19400 101.10400  97.11500
57.05100  57.05100 131.19600 113.15800 163.17300 137.13900 117.12600 157.19400
[316] 186.21000 114.10300 114.10300 113.15800 117.12600 147.17400  99.13100
101.10400  87.07700  71.07800  87.07700 147.17400 113.15800 113.15800  71.07800
[331]  99.13100 163.17300  87.07700 114.07900 137.13900 113.15800  71.07800
57.05100  57.05100  71.07800  99.13100 157.19400 103.14300  87.07700  57.05100
[346]  57.05100  57.05100  57.05100  71.07800  99.13100  71.07800  57.05100
71.07800  71.07800 128.10600 113.15800 113.15800  71.07800 147.17400  71.07800
[361] 129.18000  87.07700 117.12600  99.13100 114.07900 163.17300 113.15800
113.15800  57.05100  87.07700 114.10300  97.11500 157.19400  57.05100 101.10400
[376]  87.07700 163.17300 131.19600  99.13100  57.05100 163.17300  57.05100
71.07800  99.13100 163.17300  97.11500 157.19400 117.12600  71.07800 137.13900
[391] 137.13900 157.19400  57.05100  87.07700  87.07700 113.15800  71.07800
87.07700 113.15800 157.19400  71.07800  87.07700  97.11500  87.07700 147.17400
[406]  57.05100  57.05100 114.10300  97.11500 114.10300 113.15800 113.15800
114.07900  57.05100  71.07800  99.13100  99.13100  57.05100  57.05100  97.11500
[421] 114.07900 128.10600 137.13900 114.07900 114.07900 147.17400  71.07800
114.07900 128.10600 157.19400 114.10300 114.10300 163.17300 128.10600 117.12600
[436] 101.10400 128.10600  71.07800  71.07800 101.10400 163.17300 114.10300
114.10300  71.07800  97.11500 113.15800 131.19600  57.05100 113.15800 113.15800
[451]  71.07800 157.19400 113.15800  71.07800  71.07800  57.05100 137.13900
57.05100  71.07800 157.19400  71.07800 157.19400  57.05100 157.19400 113.15800
[466]  57.05100 117.12600  87.07700 113.15800 117.12600 137.13900  57.05100
113.15800  71.07800  71.07800 114.10300 137.13900 101.10400  87.07700 113.15800
[481]  97.11500 137.13900  57.05100  71.07800 114.10300 137.13900 117.12600
137.13900  71.07800  87.07700  97.11500  99.13100 128.10600 113.15800 128.10600
[496] 117.12600 129.18000  71.07800 101.10400  71.07800  87.07700 186.21000
128.10600 129.18000 114.07900  57.05100 157.19400 101.10400 163.17300 137.13900
[511] 157.19400 163.17300  71.07800  99.13100 101.10400  99.13100  87.07700
114.10300 157.19400  87.07700  97.11500  71.07800  57.05100  57.05100 129.18000
[526] 101.10400  99.13100 128.10600 128.10600 113.15800 137.13900 113.15800
57.05100 113.15800  57.05100 129.18000 113.15800 163.17300  57.05100  97.11500
[541]  99.13100 186.21000  57.05100 113.15800 128.10600 129.18000  71.07800
71.07800 157.19400 163.17300  57.05100 163.17300  99.13100 113.15800  97.11500
[556]  87.07700 186.21000 101.10400  97.11500  87.07700 113.15800  97.11500
71.07800  57.05100 128.10600  87.07700  71.07800  88.08534


 modes40.1_Q8LJP6

Call:
```

```
  nma.pdb(pdb = pdb40.1_LJP6)

Class:
  VibrationalModes (nma)

Number of modes:
  1680 (6 trivial)

Frequencies:
  Mode 7:       0.006
  Mode 8:       0.007
  Mode 9:       0.008
  Mode 10:      0.014
  Mode 11:      0.016
  Mode 12:      0.017

+ attr: modes, frequencies, force.constants, fluctuations,
        U, L, xyz, mass, temp, triv.modes, natoms, call


 modes40.1_Q8LJP6 FREQUENCIES


   [1] 0.000000000 0.000000000 0.000000000 0.000000000 0.000000000 0.000000000
0.006085466 0.007235864 0.008126259 0.014299166 0.015515770 0.017312078
  [13] 0.017909705 0.019293229 0.020200142 0.021508894 0.022647586 0.024823069
0.025260552 0.026099470 0.026800483 0.027456419 0.028383623 0.029224765
  [25] 0.030285057 0.030461025 0.030903530 0.031935065 0.032453953 0.032789781
0.033604691 0.034024930 0.034790193 0.035748624 0.036275109 0.036500652
  [37] 0.037002412 0.037541679 0.037863158 0.038051015 0.038333221 0.039277750
0.039464008 0.039997002 0.040264313 0.040555478 0.040821614 0.040924489
  [49] 0.041423418 0.041861078 0.042387835 0.042702787 0.043096620 0.043394464
0.044042806 0.044188647 0.044464086 0.045055750 0.045267762 0.045344916
  [61] 0.045852923 0.045862589 0.046365471 0.046499943 0.046736033 0.047330336
0.047566032 0.047943165 0.048365561 0.048643906 0.049038580 0.049279727
  [73] 0.049449315 0.049745532 0.049979721 0.050112835 0.050467679 0.050827770
0.050926846 0.051400641 0.051880117 0.051979868 0.052192145 0.052450187
  [85] 0.052703361 0.053120808 0.053364868 0.053728644 0.053761871 0.053797196
0.054089270 0.054331311 0.054383968 0.054765029 0.055089915 0.055600683
  [97] 0.055671480 0.056076319 0.056265493 0.056494625 0.056758098 0.057089834
0.057250004 0.057570107 0.057649031 0.057995983 0.058212212 0.058744346
 [109] 0.058896791 0.059013442 0.059221253 0.059305027 0.059643408 0.059789113
0.059867862 0.059947775 0.060161832 0.060484532 0.060759679 0.061147818
 [121] 0.061377707 0.061597898 0.061687068 0.061791484 0.061823041 0.062020008
0.062320491 0.062372089 0.062666438 0.062873052 0.063091637 0.063204153
 [133] 0.063488261 0.063650835 0.063786593 0.064269832 0.064439279 0.064532767
0.064615143 0.064838989 0.064959593 0.065107990 0.065166516 0.065280306
 [145] 0.065673547 0.065757769 0.065939720 0.065963916 0.066058888 0.066474201
0.066634812 0.067002719 0.067144715 0.067358840 0.067576034 0.067627743
 [157] 0.067813267 0.067961770 0.068046323 0.068279326 0.068445323 0.068556812
0.068754567 0.069198732 0.069388270 0.069641155 0.069702052 0.069929719
 [169] 0.070088194 0.070202305 0.070423140 0.070712633 0.070738240 0.070893121
0.071019316 0.071433625 0.071641472 0.071724160 0.071852949 0.071995934
 [181] 0.072105445 0.072285610 0.072467599 0.072525250 0.072837343 0.072935520
```

```
0.073299102 0.073335033 0.073398043 0.073652120 0.073811869 0.074259554
 [193] 0.074457813 0.074513074 0.074920742 0.075101742 0.075127202 0.075437764
0.075590388 0.075739194 0.075783160 0.075833948 0.076104858 0.076343791
 [205] 0.076439785 0.076668423 0.076790075 0.076847779 0.077114799 0.077240011
0.077544888 0.077727272 0.077781514 0.078064810 0.078151236 0.078331238
 [217] 0.078576135 0.078755488 0.078938606 0.079370939 0.079449089 0.079719470
0.079790930 0.079942848 0.080178874 0.080465692 0.080722628 0.080794611
 [229] 0.080963889 0.081054880 0.081210048 0.081367877 0.081405848 0.081568578
0.081661531 0.081865844 0.081985344 0.082215506 0.082358646 0.082467298
 [241] 0.082725212 0.082761948 0.082890700 0.083146082 0.083339917 0.083818162
0.083946501 0.084158359 0.084269349 0.084414408 0.084506479 0.084728448
 [253] 0.085007514 0.085213316 0.085290865 0.085479985 0.085646508 0.085735632
0.085844730 0.085909326 0.086099882 0.086225119 0.086345627 0.086533173
 [265] 0.086634397 0.086848739 0.087125957 0.087203403 0.087357218 0.087550120
0.087689896 0.087879042 0.087962304 0.088086474 0.088264726 0.088536938
 [277] 0.088729273 0.088847098 0.088966047 0.089104171 0.089300816 0.089429501
0.089642953 0.089778061 0.089885634 0.090014186 0.090103769 0.090185117
 [289] 0.090436147 0.090652817 0.090966762 0.091083221 0.091105606 0.091496238
0.091683332 0.091829920 0.091907949 0.091964431 0.092091594 0.092273227
 [301] 0.092369668 0.092643626 0.092689685 0.092854191 0.092982181 0.093208559
0.093352209 0.093639660 0.093723615 0.093813165 0.093980017 0.094204939
 [313] 0.094300479 0.094523566 0.094732627 0.095000304 0.095108762 0.095257258
0.095522405 0.095703084 0.095755608 0.095992071 0.096053799 0.096234665
 [325] 0.096428721 0.096586334 0.096610459 0.096750629 0.096959981 0.097074731
0.097193384 0.097408806 0.097532767 0.097736686 0.097835509 0.097912504
 [337] 0.098175253 0.098341271 0.098463029 0.098480650 0.098752660 0.098840730
0.099089515 0.099210865 0.099403570 0.099489409 0.099619553 0.099732258
 [349] 0.099775680 0.099938533 0.100222510 0.100488167 0.100538317 0.100658173
0.100781027 0.100963711 0.101225797 0.101339215 0.101467486 0.101678088
 [361] 0.101811655 0.102067593 0.102294170 0.102327717 0.102557302 0.102659873
0.102730293 0.102952829 0.103074914 0.103314485 0.103389849 0.103503833
 [373] 0.103792210 0.103840886 0.104056665 0.104130398 0.104267139 0.104414013
0.104511732 0.104670242 0.104849895 0.105018386 0.105150239 0.105249081
 [385] 0.105533409 0.105613786 0.105862805 0.106029687 0.106139762 0.106233630
0.106473943 0.106668844 0.106717039 0.106815853 0.107097087 0.107216579
 [397] 0.107427229 0.107708512 0.107737905 0.107931814 0.108269939 0.108519749
0.108604796 0.108722981 0.109132372 0.109290553 0.109505309 0.109547169
 [409] 0.109655792 0.109840551 0.110053658 0.110090133 0.110459149 0.110575352
0.110655157 0.110814022 0.110920491 0.111141784 0.111331471 0.111488236
 [421] 0.111605751 0.112057965 0.112127001 0.112381761 0.112527610 0.112619302
0.112840181 0.112931507 0.113167447 0.113218692 0.113293952 0.113501245
 [433] 0.113610435 0.113833508 0.114037043 0.114205954 0.114404288 0.114526663
0.114848902 0.114950422 0.115082231 0.115208722 0.115332989 0.115500333
 [445] 0.115624067 0.116050804 0.116110267 0.116302522 0.116477717 0.116794573
0.116971845 0.117269008 0.117344045 0.117498609 0.117673745 0.117734755
 [457] 0.118109697 0.118235949 0.118424861 0.118587415 0.118780246 0.118982451
0.119081511 0.119289045 0.119530342 0.119723876 0.119788177 0.119856881
 [469] 0.120313453 0.120458530 0.120643121 0.120678389 0.120721096 0.120902040
0.121100284 0.121400695 0.121428755 0.121536555 0.121786606 0.121888374
 [481] 0.122140609 0.122523777 0.122598800 0.122716821 0.122775841 0.122888849
0.123077616 0.123156106 0.123247290 0.123359969 0.123603873 0.123794414
 [493] 0.124045530 0.124155546 0.124194712 0.124484603 0.124675831 0.124933082
0.125051230 0.125103682 0.125139110 0.125443984 0.125750636 0.125809340
 [505] 0.125923850 0.125982975 0.126148240 0.126402495 0.126573616 0.126772182
```

0.126868154 0.126962458 0.127046125 0.127274607 0.127383922 0.127520657
[517] 0.127873643 0.127959584 0.128067326 0.128174581 0.128394742 0.128479350
0.128761365 0.129143228 0.129182941 0.129523477 0.129621711 0.129760968
[529] 0.129963436 0.130098823 0.130217925 0.130503657 0.130717957 0.131118272
0.131325686 0.131384117 0.131660776 0.131871180 0.131902198 0.132310224
[541] 0.132467404 0.132566419 0.132676242 0.132883417 0.133213068 0.133489827
0.133524642 0.133781351 0.133941249 0.134064022 0.134152135 0.134474249
[553] 0.134641037 0.135108955 0.135425333 0.135615889 0.135684233 0.135810467
0.136212737 0.136381857 0.136434223 0.136544646 0.136624484 0.137036288
[565] 0.137049226 0.137196177 0.137310414 0.137372937 0.137546891 0.137582982
0.137830386 0.138210834 0.138340714 0.138457300 0.138620849 0.138704697
[577] 0.138856098 0.139152035 0.139325677 0.139717102 0.139953313 0.140105535
0.140242058 0.140286664 0.140594634 0.140760828 0.141058515 0.141345450
[589] 0.141639673 0.141677850 0.141836524 0.141953541 0.142000017 0.142183988
0.142443585 0.142596345 0.142684870 0.142855902 0.142963403 0.143061084
[601] 0.143480278 0.143629731 0.143953513 0.144156078 0.144254795 0.144420987
0.144524607 0.144813333 0.144844202 0.144972332 0.145180717 0.145366326
[613] 0.145571798 0.145732316 0.145903770 0.146168544 0.146352814 0.146479452
0.146594490 0.146711596 0.147330548 0.147672890 0.147841836 0.148161969
[625] 0.148329504 0.148656851 0.148906440 0.148973278 0.149135655 0.149429117
0.149614789 0.149739006 0.149898527 0.150175234 0.150635004 0.150719227
[637] 0.150791057 0.150998373 0.151313920 0.151440424 0.151605422 0.151800029
0.152093846 0.152554558 0.152724904 0.152954608 0.153169001 0.153351135
[649] 0.153561429 0.153646768 0.153790953 0.154218348 0.154311941 0.154479202
0.154572965 0.154726929 0.154806226 0.154990523 0.155247958 0.155436213
[661] 0.155738703 0.156196124 0.156519966 0.156812935 0.156849841 0.157297992
0.157465941 0.157562509 0.157671871 0.157953884 0.158322774 0.158604110
[673] 0.158886302 0.159087367 0.159189237 0.159426786 0.159657239 0.159690077
0.159706811 0.160293286 0.160317778 0.160505531 0.160785174 0.160991971
[685] 0.161158585 0.161863550 0.162024344 0.162416742 0.162568497 0.162926317
0.163116580 0.163215470 0.163313757 0.163650833 0.164124479 0.164127797
[697] 0.164180570 0.164678997 0.164917009 0.165145399 0.165254648 0.165636411
0.165927482 0.166360849 0.166495926 0.166537986 0.166915297 0.167467003
[709] 0.167834151 0.168059406 0.168377355 0.168406312 0.168679689 0.169185897
0.169279969 0.169381466 0.169545513 0.169658124 0.169947002 0.170025830
[721] 0.170110503 0.170652770 0.170752561 0.171131533 0.171402115 0.171971758
0.172122741 0.172250065 0.172342244 0.173138561 0.173267843 0.173654089
[733] 0.173967419 0.174240794 0.174494362 0.174539429 0.174749446 0.175412359
0.175577047 0.175852457 0.175870965 0.176004212 0.176266812 0.176784035
[745] 0.177108848 0.177335034 0.177444129 0.177680367 0.177968535 0.178049075
0.178182684 0.178506300 0.178562058 0.178648712 0.178946927 0.179102779
[757] 0.179201328 0.179593713 0.179633482 0.179736884 0.179903386 0.180214706
0.180306606 0.180518613 0.180983245 0.181073566 0.181317788 0.181545498
[769] 0.181730833 0.182147670 0.182459256 0.182508671 0.182696981 0.183117912
0.183163278 0.183461611 0.183554093 0.183852068 0.183953511 0.184198453
[781] 0.184407703 0.184478430 0.184827273 0.185083924 0.185701971 0.185774387
0.185870284 0.186218493 0.186566391 0.186681150 0.186738130 0.186984030
[793] 0.187131429 0.187347408 0.187684171 0.187898032 0.187972364 0.188252110
0.188450139 0.188688707 0.188837927 0.189028909 0.189109696 0.189292844
[805] 0.189514179 0.189628289 0.190119556 0.190233171 0.190410582 0.190580385
0.190640983 0.190951445 0.191072983 0.191182321 0.191700857 0.191848989
[817] 0.192156510 0.192463211 0.192582940 0.192772774 0.192925663 0.193035198
0.193256245 0.193787467 0.193906247 0.194226944 0.194362464 0.194557723
[829] 0.194803960 0.194968248 0.195478038 0.195554670 0.195712763 0.195933892

0.196091420 0.196916733 0.197075211 0.197476206 0.197823576 0.197960729
 [841] 0.198737817 0.198870836 0.199194540 0.199573064 0.199881311 0.200087199
0.200279723 0.200535986 0.200948164 0.201160075 0.201767231 0.202237467
 [853] 0.202877048 0.203169872 0.203344343 0.203437374 0.203760350 0.203945183
0.204105775 0.204161738 0.204358851 0.204441076 0.204918649 0.205446840
 [865] 0.205473593 0.205812696 0.205831157 0.205920358 0.206050155 0.206403038
0.206558040 0.206906813 0.207253654 0.207385548 0.207555865 0.207783102
 [877] 0.207932751 0.208402687 0.208972562 0.209168411 0.209445184 0.209527165
0.209678710 0.209967176 0.210078436 0.210270304 0.211161786 0.211575532
 [889] 0.211721602 0.212084398 0.212662154 0.213067755 0.213224089 0.213716941
0.213981379 0.214220839 0.214570791 0.214714588 0.215514188 0.216066772
 [901] 0.216182568 0.216357140 0.216690434 0.216762021 0.216916919 0.217220728
0.217510720 0.218504435 0.218653291 0.219361821 0.219389533 0.219599335
 [913] 0.220739529 0.220901098 0.221451107 0.221838641 0.222226355 0.222372321
0.223121607 0.223767492 0.223818369 0.224407059 0.224669851 0.224844537
 [925] 0.225112276 0.225427288 0.225757570 0.225860099 0.225937862 0.226395935
0.226937593 0.227071439 0.227560348 0.227927716 0.228463913 0.228600467
 [937] 0.229475158 0.229954881 0.231091333 0.231619934 0.231787032 0.232409163
0.232670266 0.232908510 0.233973086 0.234171824 0.234613497 0.234810722
 [949] 0.235123245 0.235503124 0.235655216 0.236281956 0.236483520 0.236580544
0.237233990 0.237625739 0.238728436 0.238918553 0.239201090 0.239982170
 [961] 0.240371495 0.240858804 0.240983552 0.241167218 0.241667702 0.241828643
0.242307220 0.243263163 0.243901562 0.244338139 0.244588424 0.245093032
 [973] 0.245292932 0.245426469 0.246183735 0.246641794 0.247400357 0.247754665
0.248126538 0.248297014 0.249426112 0.249512266 0.249867026 0.250394330
 [985] 0.250630178 0.251696284 0.252273437 0.254413196 0.254744826 0.255232629
0.255628207 0.255857944 0.256199765 0.256612460 0.257417709 0.257818334
 [997] 0.257950297 0.258292684 0.259073109 0.260176861 0.261164290 0.261512688
0.262528961 0.262731503 0.262793488 0.263397825 0.263476574 0.264070187
[1009] 0.266663288 0.266781239 0.267652908 0.268255645 0.268365959 0.268502361
0.269784529 0.271294015 0.271460206 0.272154720 0.272844356 0.275210008
[1021] 0.275942178 0.277135785 0.278512601 0.279356960 0.280617110 0.280658495
0.282239293 0.282793837 0.283070881 0.283251625 0.284160309 0.284811940
[1033] 0.285082713 0.285649428 0.285756883 0.288380720 0.289379840 0.289545230
0.289574054 0.291420741 0.291608124 0.291709954 0.291807235 0.292106862
[1045] 0.293105596 0.296046163 0.298621118 0.299238043 0.300604465 0.300821199
0.301569304 0.302226311 0.304597115 0.304873081 0.305704250 0.307268970
[1057] 0.311469273 0.311827386 0.312262485 0.312526293 0.317063930 0.317691406
0.319218796 0.319780621 0.321923321 0.325152224 0.325702882 0.326715677
[1069] 0.328903192 0.329654803 0.331144837 0.334539034 0.335364731 0.335768954
0.336010990 0.337454383 0.342344095 0.344711457 0.346468285 0.346832072
[1081] 0.347629045 0.348414817 0.348497869 0.349905282 0.355010718 0.358343672
0.359616537 0.361265630 0.362695608 0.365749106 0.366886977 0.370322739
[1093] 0.370927386 0.371453317 0.372551739 0.374309757 0.378090045 0.380071168
0.380128180 0.382296932 0.385619980 0.385815645 0.391831131 0.392548561
[1105] 0.392896728 0.396762707 0.397532061 0.403492153 0.405925111 0.406842779
0.410627742 0.411691540 0.414038701 0.414169328 0.416251825 0.427629459
[1117] 0.428479544 0.428691927 0.434393876 0.436093489 0.449014683 0.450950218
0.452665729 0.454047352 0.458447326 0.459630796 0.462888301 0.463017154
[1129] 0.464317710 0.466827602 0.467550742 0.468531041 0.477874372 0.478325718
0.481824577 0.482478728 0.484131627 0.485291295 0.489645323 0.491536920
[1141] 0.492097458 0.499361005 0.501395540 0.504236870 0.505131689 0.505804250
0.507506908 0.508952798 0.509200490 0.509694171 0.510025096 0.511903882
[1153] 0.512536298 0.513484437 0.518159343 0.518774271 0.520070333 0.520795345

0.521069881 0.524250850 0.525484072 0.526513539 0.526764418 0.526902721
[1165] 0.527102983 0.528098262 0.532987592 0.533789474 0.535356776 0.536013883
0.536294940 0.539314461 0.539396131 0.539854036 0.540781096 0.543184186
[1177] 0.544609007 0.544975343 0.546032777 0.546467229 0.547853529 0.549218343
0.549684079 0.553418813 0.553990672 0.554340002 0.554973538 0.555113505
[1189] 0.558264226 0.558507374 0.558703289 0.559566916 0.560099802 0.561542937
0.561743814 0.562353244 0.563829989 0.566384010 0.566617392 0.567295242
[1201] 0.567690735 0.568756106 0.569675638 0.570675854 0.571380432 0.571829558
0.573464590 0.574479175 0.576139421 0.576486952 0.576917526 0.577711113
[1213] 0.578119700 0.578673891 0.578885889 0.579113861 0.579800671 0.580837813
0.581591078 0.582275053 0.583994032 0.585192533 0.585317788 0.585706344
[1225] 0.586729420 0.586990574 0.588463129 0.589169458 0.592063747 0.592893713
0.594146187 0.594849643 0.598351893 0.598388638 0.599063688 0.599720239
[1237] 0.599805171 0.600063569 0.600526632 0.600731044 0.603169791 0.603235888
0.603302377 0.603852311 0.603941465 0.604050105 0.604333785 0.605124821
[1249] 0.605276418 0.605530431 0.606013518 0.606278754 0.606295131 0.608278366
0.609003868 0.609491122 0.609627527 0.609687398 0.609800165 0.609861743
[1261] 0.610875422 0.611307051 0.611802780 0.612161016 0.612348307 0.613363225
0.613621733 0.613824426 0.614068984 0.615506017 0.615840299 0.615925846
[1273] 0.616022566 0.616465547 0.618343110 0.618465378 0.618485406 0.618694611
0.619680823 0.620171431 0.620798580 0.621380298 0.621859484 0.623856621
[1285] 0.623947280 0.624144246 0.624276475 0.624422712 0.624504589 0.625075827
0.625691239 0.625756171 0.625978991 0.626854408 0.627107375 0.627645029
[1297] 0.627716215 0.628003887 0.629006974 0.629111346 0.629965302 0.631014065
0.631529682 0.632362006 0.633468104 0.634140463 0.634536347 0.635019727
[1309] 0.635952305 0.636062367 0.636823140 0.638013578 0.638166888 0.638353573
0.638935602 0.639452575 0.639861522 0.639889886 0.640480425 0.640497016
[1321] 0.640670825 0.641012789 0.642104222 0.642497368 0.642636364 0.642785025
0.642930973 0.642985261 0.643537753 0.643566978 0.643916668 0.644375951
[1333] 0.644751132 0.645320384 0.647081742 0.648518312 0.649238760 0.649659015
0.650369455 0.651230444 0.651489168 0.651519611 0.651556584 0.652222860
[1345] 0.652985423 0.654060208 0.654559257 0.654573188 0.654936901 0.656526622
0.657144612 0.657518132 0.657524392 0.657836938 0.658848968 0.659661471
[1357] 0.660412511 0.660947063 0.662178213 0.663092765 0.664195014 0.664650231
0.664809648 0.665938877 0.666278442 0.666484674 0.666705623 0.667364688
[1369] 0.667700436 0.667879814 0.667959588 0.669121736 0.669232683 0.670084026
0.670485322 0.671139902 0.671400612 0.671688355 0.673970128 0.673982700
[1381] 0.674236338 0.675563467 0.676145571 0.676670408 0.677048607 0.678878689
0.678969108 0.679272421 0.679359488 0.680037132 0.680310667 0.681269533
[1393] 0.681705472 0.682105649 0.682341901 0.682861865 0.683681713 0.683822321
0.684354374 0.684879818 0.685198407 0.685950918 0.686252326 0.686594277
[1405] 0.687361235 0.688019253 0.688362847 0.688924963 0.689782040 0.689795150
0.689937358 0.690404439 0.690589345 0.690896942 0.692738870 0.693801323
[1417] 0.694710470 0.694755280 0.694956178 0.695545086 0.696016538 0.696102767
0.696797681 0.696952380 0.697615513 0.699474513 0.699927396 0.700106531
[1429] 0.701249936 0.702357544 0.702427362 0.702623002 0.702751422 0.703074180
0.703170242 0.703790411 0.703901435 0.704192300 0.704518605 0.705617779
[1441] 0.706215085 0.706447649 0.706569262 0.706751821 0.707637868 0.707743457
0.707946037 0.708583367 0.709094573 0.709149136 0.709833740 0.710702188
[1453] 0.711210126 0.711933791 0.712156822 0.712760376 0.712868297 0.714662584
0.715341345 0.715878297 0.716648306 0.718091195 0.718209108 0.718264089
[1465] 0.718610265 0.718791070 0.719240329 0.720124623 0.720556635 0.720591225
0.721140057 0.721826957 0.722294739 0.722843451 0.723595967 0.723873267
[1477] 0.723970348 0.724945035 0.727000251 0.727424140 0.728473316 0.728605350

0.728730842 0.729684244 0.730816623 0.732334120 0.732511226 0.732884302
[1489] 0.733200947 0.733740468 0.734541100 0.735094988 0.735551078 0.736511867
0.736604927 0.736809764 0.736891546 0.737399289 0.737823589 0.738048698
[1501] 0.738888218 0.741139850 0.741298058 0.741791363 0.742147742 0.743748265
0.746998401 0.748355885 0.749454510 0.749703156 0.750827390 0.751136977
[1513] 0.752732116 0.753952411 0.755397296 0.755464693 0.756438715 0.758136555
0.758487426 0.758630363 0.759111837 0.759690824 0.760220941 0.760997825
[1525] 0.761345664 0.761873798 0.762044189 0.762087433 0.762344934 0.762990341
0.763809163 0.764459265 0.764494718 0.765428460 0.766217800 0.766225287
[1537] 0.767015228 0.767112346 0.767602492 0.768034644 0.768079727 0.768771919
0.768858620 0.768889818 0.769209868 0.769287745 0.769629269 0.770054034
[1549] 0.772641616 0.773019671 0.773607485 0.774220471 0.774898074 0.777239679
0.777553392 0.779153017 0.779672127 0.781259291 0.781442440 0.782151451
[1561] 0.782564111 0.782674689 0.782695111 0.782944184 0.783404362 0.784175479
0.784534303 0.785334613 0.785828398 0.786030978 0.786225483 0.786311049
[1573] 0.786316589 0.789333124 0.790575503 0.792480888 0.793636285 0.796064961
0.796867566 0.796968962 0.798155629 0.799080579 0.801058656 0.801077707
[1585] 0.801673607 0.802757370 0.805508607 0.806381776 0.807058569 0.811466348
0.812816230 0.813149628 0.813593624 0.813708002 0.814795002 0.815399376
[1597] 0.816982723 0.818300259 0.818984445 0.820117160 0.820476149 0.822347446
0.823145504 0.824980841 0.825032837 0.825798991 0.826845084 0.827027341
[1609] 0.827049347 0.828582721 0.831261331 0.832119623 0.832505248 0.832662797
0.833341680 0.833985904 0.834046905 0.836771201 0.836994665 0.836996935
[1621] 0.837620702 0.838388889 0.840447858 0.840493050 0.842234455 0.842768540
0.847131529 0.850018838 0.850566797 0.854732831 0.854897706 0.857370441
[1633] 0.857526169 0.860957620 0.866546196 0.868564199 0.878369311 0.880127857
0.889500859 0.893729634 0.898817561 0.900563813 0.901834067 0.904837718
[1645] 0.909033642 0.909260715 0.914892469 0.918143513 0.922348869 0.923546157
0.926057667 0.926513950 0.928766422 0.928966994 0.930585145 0.932427677
[1657] 0.932578884 0.950012573 0.951474134 0.965646816 0.969591363 0.972435673
0.975951179 0.977722250 0.985789465 0.988656159 1.007752058 1.008648492
[1669] 1.011451239 1.016672658 1.018267306 1.019548640 1.020457064 1.031025057
1.033024494 1.040386794 1.046879249 1.074031318 1.075253976 1.239074007

modes40.1_Q8LJP6 FLUCTUATIONS

[1] 0.21412178 0.13859720 0.09974688 0.09373233 0.10539406 0.09610681
0.08974582 0.09299112 0.08574024 0.08799903 0.10218392 0.09518221 0.09257520
[14] 0.09184709 0.10451760 0.11046836 0.11630196 0.12126641 0.11048564
0.11586272 0.16852383 0.27452459 0.50225947 0.36339720 0.26131274 0.17752811
[27] 0.19116115 0.14724933 0.17795970 0.18350121 0.22226960 0.22207782
0.18359373 0.14167353 0.14086502 0.17701434 0.18445532 0.13328211 0.12560172
[40] 0.17715780 0.25327565 0.21801732 0.25346537 0.18708061 0.16474256
0.12683086 0.14612391 0.10389118 0.11136922 0.09389908 0.08881470 0.08807484
[53] 0.07950005 0.09693385 0.11690145 0.11615651 0.12284151 0.10035375
0.10435481 0.10111016 0.08006919 0.08836047 0.07618977 0.07204592 0.07441355
[66] 0.06991170 0.06979965 0.06251324 0.07002034 0.07077604 0.07104891
0.08705205 0.08939526 0.11158508 0.12267835 0.12564394 0.11551039 0.16033805
[79] 0.22124370 0.16425703 0.15447097 0.22806550 0.21584457 0.17004270
0.21020900 0.18846694 0.18044872 0.19331005 0.13515522 0.11548244 0.13061440
[92] 0.12467114 0.10541783 0.10126444 0.10228810 0.09624614 0.08487787
0.09246590 0.10159653 0.09483276 0.09775917 0.13332977 0.12912887 0.11458083

[105] 0.13201548 0.21918611 0.20348093 0.14450618 0.17585078 0.13880794
0.12022532 0.10963000 0.11845107 0.10572301 0.11302576 0.13740261 0.15434131
[118] 0.21271577 0.28749893 0.18724340 0.14356603 0.17149389 0.22197153
0.22298238 0.16308276 0.14053962 0.13792134 0.14024556 0.13231152 0.14198469
[131] 0.20078052 0.27672412 0.21809491 0.21085480 0.14869895 0.14693853
0.12302411 0.13414203 0.13768789 0.10959506 0.14691998 0.21455377 0.28828191
[144] 0.14246736 0.11601760 0.09305470 0.08725370 0.07759356 0.07722286
0.06955383 0.05781350 0.06411569 0.06815215 0.06052347 0.05789450 0.05658148
[157] 0.06302442 0.06036826 0.06909761 0.07972843 0.08551403 0.11117957
0.12111061 0.15443375 0.16847463 0.43080736 0.38295464 0.35008178 0.28397085
[170] 0.26038745 0.17844231 0.13711444 0.16353638 0.16960205 0.10503955
0.10222596 0.14558078 0.11406635 0.08137269 0.10285603 0.10422323 0.08031427
[183] 0.08146297 0.11319312 0.10816002 0.07980273 0.13608314 0.19093596
0.16232237 0.14138798 0.13368794 0.15361943 0.13791660 0.13035614 0.21190498
[196] 0.28549481 0.19157339 0.23510266 0.20092930 0.12536252 0.16968973
0.23848082 0.18315223 0.16747077 0.13600775 0.15930707 0.10597107 0.08966280
[209] 0.14358514 0.15065578 0.09498265 0.08190222 0.09446754 0.09205145
0.07998495 0.06612047 0.07984842 0.09241799 0.08365292 0.08703184 0.12418893
[222] 0.13325471 0.11605018 0.17216079 0.25459896 0.32554294 0.27471524
0.31437331 0.25875915 0.25726134 0.20873543 0.16445832 0.15636618 0.19430447
[235] 0.24166978 0.20614548 0.27618294 0.29421172 0.20244944 0.17977206
0.19380192 0.24033253 0.23991154 0.19307237 0.27315120 0.23411964 0.18182791
[248] 0.18632697 0.18337446 0.12479156 0.14903943 0.15552840 0.13753052
0.10054919 0.10197877 0.10222700 0.10665222 0.10125858 0.09750419 0.10136866
[261] 0.10288824 0.10872967 0.11100074 0.12913316 0.15016055 0.14703012
0.17400376 0.21929969 0.24384381 0.32346881 0.26527111 0.44592633 0.82710865
[274] 0.41088108 0.38141936 0.33612087 0.35218377 0.26682248 0.18714428
0.20459554 0.19939402 0.15047355 0.14200863 0.15807118 0.15047790 0.11986067
[287] 0.13657215 0.15433233 0.12815213 0.13269401 0.16130363 0.14378576
0.15140393 0.21237561 0.24428228 0.31115253 0.51402005 0.46568633 0.45716679
[300] 0.20484664 0.18183633 0.16945143 0.16766398 0.16523592 0.27935526
0.30842854 0.23960897 0.16832051 0.13808637 0.14917121 0.21371897 0.23128568
[313] 0.17892795 0.11841708 0.08578320 0.08796102 0.07508420 0.07599200
0.08053608 0.07955755 0.07131013 0.07341701 0.08733201 0.09104751 0.08461140
[326] 0.09528408 0.10352698 0.11256051 0.12973629 0.14613317 0.17256029
0.24376479 0.34816142 0.41027302 0.44321685 0.43320637 0.50393164 0.35118385
[339] 0.33785661 0.50443894 0.66047776 0.60994586 0.70087097 0.71105051
0.59369343 0.62127952 0.40139878 0.36065553 0.46501398 0.28622836 0.26250424
[352] 0.23595317 0.14640891 0.13646943 0.15693068 0.13990431 0.11012171
0.11845532 0.12782162 0.11068670 0.10077773 0.09204503 0.10239025 0.10255633
[365] 0.09521143 0.10263161 0.13727936 0.13642327 0.27092459 0.32711562
0.22167819 0.16599089 0.10177988 0.09472830 0.09601887 0.10536681 0.13007592
[378] 0.16195820 0.15851338 0.17491330 0.18436901 0.12326852 0.15008348
0.17553331 0.14706099 0.13168770 0.10985179 0.10860291 0.11998575 0.12747831
[391] 0.13827622 0.13089583 0.16836790 0.18034254 0.16157203 0.17722489
0.23298034 0.24683050 0.24274450 0.28516760 0.31114871 0.33904759 0.32645148
[404] 0.20446006 0.15948695 0.22424571 0.15638665 0.16300112 0.14242914
0.11704267 0.10209574 0.10115659 0.09091129 0.08099781 0.06998748 0.09518988
[417] 0.14631991 0.17171063 0.19110201 0.22089664 0.18354261 0.23473388
0.20604446 0.23999917 0.18775076 0.19595572 0.14031194 0.20875283 0.21023838
[430] 0.15182380 0.16007328 0.08843408 0.08888961 0.08447802 0.08142100
0.08755778 0.08777127 0.08009635 0.07088719 0.07460243 0.07269461 0.07180943
[443] 0.07623834 0.08092901 0.07667981 0.08265246 0.08434481 0.08771775
0.11358863 0.15356080 0.10342066 0.13461397 0.18465777 0.16436835 0.15215208

[456] 0.14958810 0.23715984 0.18959626 0.27524849 0.23497475 0.19443309
0.17948874 0.18935790 0.17638830 0.26849631 0.43371891 0.50276189 0.33225779
[469] 0.39265086 0.26609632 0.31301626 0.53464475 0.68085907 0.45308295
0.63343729 0.53219465 0.54533292 0.52286758 0.80798405 1.01522135 0.80203929
[482] 0.78622171 0.66176672 0.71784888 0.57434300 0.46687638 0.48236185
0.41429751 0.51886124 0.52301958 0.42907217 0.25526254 0.15980104 0.16966578
[495] 0.12884202 0.15055083 0.15758432 0.18549835 0.22161396 0.20428797
0.28466084 0.18382573 0.29388000 0.29642752 0.42693946 0.34164703 0.27083724
[508] 0.20867618 0.23514407 0.26918548 0.35439769 0.37623236 0.52658748
0.50462684 0.77660407 0.96328986 1.18291075 0.95009274 0.59833265 0.75313232
[521] 0.73492759 0.67899750 0.42018073 0.40657653 0.34823841 0.50009795
0.65514229 0.72685725 0.82204563 0.54388534 0.42961469 0.28221322 0.21705443
[534] 0.39032767 0.53323306 0.40354448 0.48992419 0.83585346 1.03166767
1.05384661 0.54602200 0.40301940 0.29755738 0.28077381 0.24725341 0.26780809
[547] 0.31140558 0.42707132 0.55883510 0.71485214 0.34969051 0.28410498
0.18688150 0.17102467 0.13749167 0.11524844 0.14850031 0.19828906 0.27292526
[560] 0.27388242

modes40.1_Q8LJP6 MASSES

[1] 138.1469 114.0790 163.1730 114.1030 117.1260 71.0780 113.1580 87.0770
129.1800 87.0770 113.1580 113.1580 147.1740 147.1740 128.1060 71.0780
[17] 117.1260 157.1940 87.0770 57.0510 163.1730 113.1580 97.1150 137.1390
114.1030 117.1260 157.1940 99.1310 101.1040 186.2100 157.1940 71.0780
[33] 114.1030 87.0770 57.0510 113.1580 114.1030 114.0790 57.0510 129.1800
71.0780 87.0770 57.0510 99.1310 114.0790 113.1580 99.1310 57.0510
[49] 57.0510 163.1730 163.1730 114.0790 71.0780 57.0510 114.0790 114.1030
99.1310 129.1800 147.1740 57.0510 113.1580 97.1150 131.1960 71.0780
[65] 147.1740 101.1040 113.1580 101.1040 131.1960 131.1960 87.0770 186.2100
87.0770 113.1580 113.1580 128.1060 163.1730 57.0510 129.1800 117.1260
[81] 131.1960 57.0510 71.0780 101.1040 57.0510 128.1060 113.1580 57.0510
137.1390 71.0780 131.1960 114.0790 71.0780 99.1310 129.1800 186.2100
[97] 57.0510 101.1040 114.0790 163.1730 113.1580 113.1580 129.1800 71.0780
137.1390 97.1150 128.1060 97.1150 163.1730 99.1310 113.1580 163.1730
[113] 57.0510 128.1060 99.1310 57.0510 114.0790 57.0510 114.1030 87.0770
114.0790 137.1390 163.1730 103.1430 186.2100 117.1260 157.1940 97.1150
[129] 128.1060 114.0790 113.1580 101.1040 101.1040 114.1030 157.1940 137.1390
71.0780 163.1730 129.1800 129.1800 114.0790 97.1150 87.0770 114.1030
[145] 97.1150 57.0510 87.0770 114.0790 113.1580 71.0780 57.0510 128.1060
101.1040 71.0780 71.0780 71.0780 131.1960 71.0780 71.0780 71.0780
[161] 87.0770 113.1580 99.1310 147.1740 157.1940 157.1940 87.0770 114.1030
97.1150 71.0780 163.1730 87.0770 101.1040 128.1060 113.1580 113.1580
[177] 157.1940 137.1390 71.0780 163.1730 117.1260 113.1580 147.1740 128.1060
147.1740 71.0780 114.0790 129.1800 163.1730 157.1940 57.0510 129.1800
[193] 163.1730 114.0790 87.0770 87.0770 113.1580 101.1040 99.1310 71.0780
117.1260 163.1730 163.1730 157.1940 87.0770 99.1310 87.0770 57.0510
[209] 163.1730 114.1030 114.0790 128.1060 113.1580 113.1580 186.2100 71.0780
71.0780 71.0780 186.2100 113.1580 163.1730 117.1260 71.0780 87.0770
[225] 114.1030 114.1030 117.1260 163.1730 163.1730 113.1580 114.1030 163.1730
113.1580 57.0510 129.1800 114.1030 57.0510 114.0790 87.0770 131.1960
[241] 57.0510 57.0510 101.1040 57.0510 186.2100 71.0780 131.1960 101.1040
128.1060 147.1740 57.0510 186.2100 114.0790 99.1310 129.1800 163.1730

```
[257] 101.1040  57.0510  99.1310 117.1260 101.1040 113.1580  99.1310  71.0780
129.1800 147.1740 113.1580 131.1960 117.1260  57.0510 129.1800  71.0780
[273]  57.0510 131.1960 137.1390  71.0780  97.1150  99.1310 147.1740 128.1060
157.1940 163.1730 137.1390 117.1260 129.1800  71.0780 128.1060 137.1390
[289] 147.1740 131.1960 103.1430  87.0770 113.1580 113.1580  57.0510 129.1800
57.0510 113.1580 157.1940 114.1030  99.1310 117.1260 129.1800 101.1040
[305]  97.1150  57.0510  57.0510 113.1580 131.1960 147.1740 157.1940 117.1260
129.1800 186.2100 114.1030 114.1030 131.1960 117.1260 147.1740  99.1310
[321] 101.1040  87.0770  71.0780  87.0770 147.1740 113.1580  71.0780 101.1040
99.1310 163.1730  87.0770 114.0790 163.1730 113.1580  99.1310  87.0770
[337]  87.0770 157.1940  57.0510 101.1040 113.1580 157.1940 103.1430  71.0780
71.0780  57.0510 114.1030  99.1310  71.0780  97.1150 101.1040 117.1260
[353] 113.1580 113.1580  87.0770 147.1740  71.0780 129.1800  87.0770 117.1260
99.1310 114.0790 163.1730 113.1580 113.1580  57.0510 114.0790 114.1030
[369]  97.1150 157.1940  57.0510 101.1040  87.0770 163.1730 131.1960  99.1310
57.0510 163.1730  57.0510 114.1030 114.1030 147.1740  97.1150 157.1940
[385] 117.1260  99.1310 137.1390 137.1390 157.1940  57.0510  87.0770 157.1940
157.1940  57.0510 163.1730  71.0780  71.0780 186.2100 163.1730 101.1040
[401] 157.1940 129.1800  71.0780  87.0770 114.0790  97.1150 114.1030  99.1310
113.1580 101.1040  57.0510  71.0780  99.1310  99.1310  57.0510  57.0510
[417]  97.1150 114.0790  71.0780 163.1730 114.0790 114.1030 147.1740  71.0780
114.0790 128.1060 157.1940 114.0790 114.1030 163.1730 128.1060 117.1260
[433] 101.1040 114.0790  97.1150  57.0510 101.1040 163.1730 114.1030 114.1030
71.0780  97.1150 113.1580 113.1580  57.0510 113.1580 113.1580  71.0780
[449] 157.1940 113.1580 114.1030  71.0780  57.0510 137.1390  57.0510  57.0510
163.1730 114.1030 117.1260 113.1580 113.1580  97.1150  99.1310  99.1310
[465] 101.1040 101.1040 117.1260  97.1150 129.1800  99.1310  71.0780  97.1150
113.1580  97.1150 129.1800  99.1310  71.0780  97.1150  71.0780  87.0770
[481]  97.1150  71.0780  97.1150  87.0770  87.0770  87.0770  97.1150 113.1580
71.0780 113.1580  87.0770 117.1260 157.1940 129.1800 101.1040  87.0770
[497]  87.0770 186.2100 113.1580  87.0770 129.1800  57.0510  99.1310 101.1040
163.1730 163.1730 157.1940 163.1730  87.0770  71.0780 113.1580  99.1310
[513] 101.1040 114.1030 129.1800  87.0770  71.0780 129.1800 128.1060 113.1580
101.1040 114.1030 113.1580 129.1800 113.1580  87.0770 113.1580  87.0770
[529] 129.1800 113.1580 163.1730  57.0510  97.1150 113.1580 186.2100  57.0510
113.1580 101.1040 129.1800  71.0780  57.0510 114.1030  87.0770 163.1730
[545]  57.0510 147.1740  97.1150  87.0770 186.2100 113.1580 114.1030  87.0770
113.1580  97.1150  99.1310  57.0510 129.1800  87.0770 131.1960 145.1133


 modes40.1_Q93WY9

Call:
  nma.pdb(pdb = pdb40.1_3WY9)

Class:
  VibrationalModes (nma)

Number of modes:
  1728 (6 trivial)

Frequencies:
  Mode 7:       0.006
  Mode 8:       0.006
```

```
  Mode 9:       0.008
  Mode 10:      0.015
  Mode 11:      0.017
  Mode 12:      0.018

+ attr: modes, frequencies, force.constants, fluctuations,
        U, L, xyz, mass, temp, triv.modes, natoms, call


 modes40.1_Q93WY9 FREQUENCIES


   [1] 0.000000000 0.000000000 0.000000000 0.000000000 0.000000000 0.000000000
0.005858545 0.006320275 0.008283705 0.014953791 0.016612452 0.018014765
  [13] 0.018227237 0.021712258 0.022981224 0.025080415 0.025530854 0.026576016
0.026679230 0.028262889 0.029213062 0.030438565 0.030637637 0.031033990
  [25] 0.032098044 0.032459025 0.033466845 0.034607327 0.035033253 0.035454421
0.035569973 0.036118355 0.036689616 0.037723415 0.038352048 0.038948437
  [37] 0.039054625 0.039629581 0.039818328 0.040081143 0.040332827 0.041284682
0.041649058 0.041970157 0.042162244 0.042337309 0.042953263 0.043418682
  [49] 0.043562252 0.044313149 0.044369989 0.044672953 0.045062496 0.045805666
0.046299593 0.046344979 0.046471336 0.046642718 0.047005975 0.047206279
  [61] 0.047515947 0.047604358 0.047867552 0.048028152 0.048362942 0.048642604
0.049771749 0.049849808 0.050057456 0.050392336 0.050663296 0.050854176
  [73] 0.051188297 0.051737598 0.051781153 0.051961591 0.052257866 0.052498941
0.052802756 0.053060454 0.053438390 0.053501870 0.053799550 0.054046169
  [85] 0.054301698 0.054527933 0.054599889 0.054869922 0.054888615 0.055363963
0.055589976 0.055855676 0.055913467 0.056123278 0.056493728 0.056656254
  [97] 0.057051664 0.057201093 0.057607274 0.057872248 0.058127299 0.058390563
0.058576803 0.058753400 0.058889265 0.059118510 0.059272557 0.059660818
 [109] 0.059825113 0.060120767 0.060347013 0.060539787 0.060638033 0.060740708
0.060902298 0.060997884 0.061099332 0.061446795 0.061482442 0.061875053
 [121] 0.061914341 0.062142821 0.062238944 0.062754493 0.062784961 0.063053686
0.063239010 0.063353664 0.063540904 0.063650636 0.063981871 0.064176554
 [133] 0.064310217 0.064680773 0.064883705 0.065274291 0.065350888 0.065469194
0.065919549 0.066017463 0.066203865 0.066304797 0.066546372 0.066696936
 [145] 0.066869703 0.066977385 0.067350566 0.067456729 0.067761327 0.067892036
0.068031990 0.068237392 0.068339583 0.068547943 0.068758619 0.069029959
 [157] 0.069311749 0.069398673 0.069545793 0.069808631 0.070017142 0.070101023
0.070233509 0.070497376 0.070595040 0.070786744 0.071018602 0.071058716
 [169] 0.071193157 0.071291822 0.071580455 0.071729281 0.072030405 0.072235658
0.072383486 0.072556677 0.072720226 0.072950105 0.073056280 0.073283723
 [181] 0.073298066 0.073649885 0.073904811 0.074149979 0.074233796 0.074482643
0.074714389 0.075157204 0.075188541 0.075339821 0.075449683 0.075719794
 [193] 0.075775138 0.075902059 0.076086550 0.076218437 0.076578671 0.076789250
0.076879416 0.076978526 0.077299509 0.077525123 0.077663535 0.077816514
 [205] 0.077887932 0.078044690 0.078463226 0.078673751 0.078906189 0.079016542
0.079202894 0.079274181 0.079344446 0.079401570 0.079713592 0.079978011
 [217] 0.080292525 0.080462387 0.080651522 0.080852904 0.080892525 0.081111112
0.081262743 0.081652070 0.081962941 0.082094181 0.082134283 0.082270791
 [229] 0.082306037 0.082358339 0.082606475 0.082723834 0.082886574 0.082908880
0.083229514 0.083344476 0.083726543 0.083830853 0.083908321 0.084235977
 [241] 0.084452808 0.084486993 0.084634223 0.084739508 0.084853619 0.085034924
0.085412691 0.085557588 0.085709776 0.085857712 0.086143412 0.086257574
```

 [253] 0.086317167 0.086448535 0.086542101 0.086676489 0.086796517 0.086974208
0.087156188 0.087245657 0.087408237 0.087621265 0.087685708 0.087925580
 [265] 0.088137645 0.088185196 0.088198408 0.088432307 0.088507608 0.088808173
0.088884438 0.088904954 0.089084838 0.089308474 0.089450600 0.089744056
 [277] 0.089867456 0.089983086 0.090117262 0.090363574 0.090625569 0.090731161
0.090883186 0.091032036 0.091222304 0.091371434 0.091552835 0.091581466
 [289] 0.091763556 0.091990869 0.092215011 0.092361166 0.092436692 0.092708950
0.092813672 0.093111627 0.093307020 0.093504850 0.093655618 0.093753340
 [301] 0.093883341 0.094147380 0.094257491 0.094479206 0.094649299 0.094753480
0.094971770 0.095115686 0.095164675 0.095249679 0.095443880 0.095526515
 [313] 0.095703746 0.095744762 0.095923834 0.095944165 0.096100860 0.096298474
0.096526522 0.096640606 0.096794734 0.097031536 0.097096516 0.097212668
 [325] 0.097390342 0.097444945 0.097595856 0.097720357 0.097940052 0.098059466
0.098167899 0.098288970 0.098360716 0.098450809 0.098552642 0.098908619
 [337] 0.098998596 0.099086192 0.099379487 0.099548841 0.099589163 0.099855364
0.100032522 0.100301208 0.100321535 0.100670125 0.100842712 0.100897832
 [349] 0.101002716 0.101165222 0.101254820 0.101290712 0.101468110 0.101635729
0.101855682 0.102135693 0.102261355 0.102311131 0.102531488 0.102620511
 [361] 0.103090395 0.103095187 0.103189123 0.103404303 0.103454263 0.103612435
0.103819052 0.103953401 0.104076138 0.104175147 0.104431357 0.104606334
 [373] 0.104699036 0.104731813 0.104871152 0.105041780 0.105176373 0.105321858
0.105596156 0.105770285 0.105819727 0.105916748 0.105949865 0.106077695
 [385] 0.106160881 0.106423496 0.107002083 0.107034510 0.107298056 0.107501241
0.107558719 0.107708394 0.107813936 0.107961146 0.108179595 0.108385918
 [397] 0.108440825 0.108725544 0.108824978 0.109233407 0.109356820 0.109526010
0.109568671 0.109705446 0.109760154 0.109843087 0.110015559 0.110214769
 [409] 0.110421534 0.110522766 0.110715916 0.110764980 0.110850818 0.110932708
0.111061303 0.111296086 0.111439946 0.111566253 0.111645236 0.111824895
 [421] 0.112024619 0.112301943 0.112303183 0.112466027 0.112628860 0.112742153
0.112881141 0.113081351 0.113230549 0.113360671 0.113437735 0.113497004
 [433] 0.113618238 0.113892349 0.114066249 0.114115426 0.114281783 0.114570336
0.114771462 0.114835006 0.115026421 0.115085753 0.115189813 0.115444944
 [445] 0.115580244 0.115620014 0.115783115 0.115899772 0.116014893 0.116153018
0.116294572 0.116345638 0.116579122 0.116680222 0.116947806 0.116999777
 [457] 0.117170470 0.117216616 0.117292766 0.117527062 0.117641667 0.117712055
0.117931235 0.118058322 0.118193523 0.118382181 0.118598842 0.118764678
 [469] 0.118815321 0.118947639 0.119076087 0.119310172 0.119357294 0.119466221
0.119872731 0.119968205 0.120173786 0.120258069 0.120329979 0.120669678
 [481] 0.121048922 0.121195941 0.121331403 0.121469738 0.121615207 0.121827885
0.122030645 0.122179384 0.122253272 0.122463394 0.122594047 0.122741279
 [493] 0.122873491 0.122975699 0.123300818 0.123467313 0.123600389 0.123770267
0.123822443 0.123909968 0.124187675 0.124272701 0.124458657 0.124547260
 [505] 0.124672783 0.124804370 0.125078674 0.125132228 0.125622056 0.125669432
0.125840040 0.125979859 0.126115606 0.126245088 0.126278089 0.126452984
 [517] 0.126533785 0.126657040 0.126858970 0.127027382 0.127121567 0.127255599
0.127332409 0.127523240 0.127653972 0.127782093 0.127907809 0.128033005
 [529] 0.128166874 0.128361101 0.128459830 0.128672219 0.128716111 0.128740610
0.128951161 0.129048065 0.129160880 0.129386019 0.129531201 0.129581840
 [541] 0.129652388 0.129891789 0.130338084 0.130439492 0.130761356 0.131028796
0.131132084 0.131150819 0.131344973 0.131502633 0.131697229 0.131959605
 [553] 0.132112888 0.132145191 0.132341235 0.132489584 0.132625639 0.132830224
0.133092935 0.133206602 0.133453104 0.133605906 0.133684468 0.133980201
 [565] 0.134017157 0.134069880 0.134157138 0.134352604 0.134618553 0.134665022
0.135014150 0.135234040 0.135429167 0.135561058 0.135569373 0.135647544

[577] 0.136080082 0.136201207 0.136617161 0.136762723 0.136988035 0.137171896
0.137327385 0.137364178 0.137621639 0.137874210 0.138069826 0.138128062
[589] 0.138305737 0.138457483 0.138620027 0.139034665 0.139114895 0.139289493
0.139462510 0.139596759 0.139657352 0.139944445 0.140182984 0.140261022
[601] 0.140406956 0.140631473 0.140838546 0.141166128 0.141335504 0.141396605
0.141495458 0.141890626 0.141998233 0.142333468 0.142516298 0.142754354
[613] 0.142940013 0.142995557 0.143359825 0.143422272 0.143474275 0.143630789
0.143657152 0.143878181 0.143989932 0.144195432 0.144361780 0.144519612
[625] 0.144629466 0.144945684 0.145233573 0.145353518 0.145534208 0.145565533
0.145946385 0.146121054 0.146400230 0.146638718 0.146728515 0.146842150
[637] 0.147083538 0.147335620 0.147452911 0.147634891 0.147841236 0.148096219
0.148324295 0.148483118 0.148581006 0.148807404 0.148964436 0.149104910
[649] 0.149209266 0.149380289 0.149488520 0.149757021 0.150015248 0.150090031
0.150297977 0.150723681 0.151056488 0.151172232 0.151268129 0.151306220
[661] 0.151707891 0.151785010 0.152031296 0.152216873 0.152472927 0.152569003
0.152696043 0.152813860 0.153110778 0.153301904 0.154071603 0.154242819
[673] 0.154455260 0.154527074 0.154763759 0.155000737 0.155140480 0.155247306
0.155447864 0.155699663 0.155783749 0.155954304 0.156024373 0.156251333
[685] 0.156307576 0.156400243 0.156611618 0.156722936 0.156824081 0.156920640
0.156950581 0.157032546 0.157129461 0.157307171 0.157504061 0.157871194
[697] 0.158284772 0.158504820 0.158739245 0.158886062 0.158973483 0.159235694
0.159952143 0.160034312 0.160139296 0.160245556 0.160546006 0.160692197
[709] 0.160739480 0.160868570 0.161426191 0.161677061 0.161933878 0.162195442
0.162563589 0.162894287 0.163026022 0.163246428 0.163547638 0.163685578
[721] 0.163732074 0.163927583 0.164011775 0.164251603 0.164638231 0.164715755
0.164947956 0.165358846 0.165515096 0.165546313 0.165854037 0.166247985
[733] 0.166357727 0.166588932 0.166643662 0.166688649 0.166901942 0.167020052
0.167113448 0.167326200 0.167699699 0.167836566 0.168108082 0.168394128
[745] 0.168505780 0.168580098 0.168997446 0.169120008 0.169405765 0.169629381
0.169857176 0.170298243 0.170404486 0.170569108 0.170942263 0.171086826
[757] 0.171335896 0.171511218 0.171719333 0.171954819 0.171989138 0.172080574
0.172279106 0.172500098 0.172650692 0.172880953 0.172936182 0.173109591
[769] 0.173417478 0.173617764 0.173678228 0.174172090 0.174224656 0.174411671
0.174451823 0.174602910 0.174980202 0.175125482 0.175302288 0.175792669
[781] 0.175903080 0.175989820 0.176088241 0.176229229 0.176668941 0.176814480
0.177360529 0.177446913 0.177544954 0.177762036 0.178066075 0.178326491
[793] 0.178509847 0.178591988 0.179077603 0.179230868 0.179452403 0.179511537
0.179930488 0.180156717 0.180223350 0.180419520 0.180623612 0.180768700
[805] 0.180964699 0.181424698 0.181546963 0.181646068 0.181855121 0.182094748
0.182164635 0.182446136 0.182683878 0.182868683 0.183553127 0.183720514
[817] 0.183805838 0.184048155 0.184141719 0.184327948 0.184379405 0.184434624
0.185002612 0.185074275 0.185262301 0.185486125 0.186286085 0.186411887
[829] 0.186668531 0.186739893 0.186934578 0.187204239 0.187286623 0.187704684
0.188054008 0.188267315 0.188428967 0.188690787 0.189089335 0.189423670
[841] 0.189641847 0.190145069 0.190157391 0.190270783 0.190710727 0.190770487
0.190978902 0.191066553 0.191403324 0.191600673 0.191796565 0.192040604
[853] 0.192311469 0.192484991 0.192588135 0.192935970 0.193055668 0.193298314
0.193419556 0.193629960 0.193687119 0.193830139 0.193987809 0.194339721
[865] 0.194494894 0.194697957 0.194874813 0.195007415 0.195662345 0.195828242
0.196243660 0.196426281 0.196458453 0.196615304 0.196764242 0.196944323
[877] 0.197218471 0.197368622 0.197766204 0.198221843 0.198436474 0.198723159
0.198881344 0.199555675 0.200076945 0.200396804 0.200460563 0.200653423
[889] 0.201234732 0.201456086 0.202123018 0.202288061 0.202406546 0.202473738
0.203130970 0.203264292 0.203480762 0.203696007 0.203989084 0.204125754

[901] 0.204465916 0.204916362 0.205213806 0.205976812 0.206148662 0.206563190 0.206606228 0.207225909 0.207326362 0.207906375 0.208064031 0.208322087
[913] 0.208423713 0.208956197 0.209108458 0.209135167 0.209658112 0.209869435 0.210147876 0.210790373 0.211099399 0.211438645 0.211532607 0.211643643
[925] 0.211684152 0.212095087 0.212390945 0.212436140 0.212783731 0.213011991 0.213347186 0.213673854 0.214085348 0.214423059 0.214609391 0.214827576
[937] 0.215074573 0.215601087 0.216308548 0.216865883 0.217246206 0.217340396 0.218126538 0.218216634 0.218289809 0.218611061 0.218757934 0.218883242
[949] 0.219413951 0.219663344 0.220021966 0.220778599 0.221690667 0.222360474 0.222509425 0.222647811 0.223115534 0.224478498 0.224595766 0.224680336
[961] 0.224739122 0.225446502 0.225921549 0.226260010 0.226452374 0.226775857 0.226821034 0.226837785 0.227599971 0.227764403 0.228117119 0.228271747
[973] 0.228497006 0.228610384 0.229532716 0.229598479 0.229690471 0.229973496 0.230835742 0.230938539 0.231630049 0.231884000 0.232112084 0.232265688
[985] 0.232813383 0.233452852 0.233529605 0.234385632 0.235369553 0.235705999 0.235820583 0.236108383 0.236704069 0.237452828 0.237748294 0.237972673
[997] 0.238797182 0.239455792 0.239673024 0.240708369 0.240861328 0.242047932 0.242097217 0.242403533 0.242620422 0.243096920 0.243637941 0.244118470
[1009] 0.244700091 0.245026052 0.245216813 0.245390240 0.245593250 0.246516981 0.246521348 0.246630189 0.246981603 0.247486397 0.247810839 0.248937207
[1021] 0.249514245 0.249733175 0.250277916 0.252281671 0.252543641 0.253031134 0.253296329 0.253797497 0.254622891 0.254863373 0.254884443 0.255959252
[1033] 0.256118581 0.256294415 0.258619878 0.258945582 0.261336633 0.261525280 0.262803464 0.263322948 0.263364068 0.264178269 0.264811252 0.264964732
[1045] 0.265416816 0.266119820 0.266322104 0.267802584 0.269555996 0.270486155 0.270990865 0.271719301 0.272101803 0.273681120 0.274343653 0.274724483
[1057] 0.276199453 0.277005098 0.277420625 0.278505917 0.278684577 0.278721613 0.278817476 0.279236429 0.280270138 0.281128275 0.283548769 0.283827708
[1069] 0.284540553 0.285153208 0.286050849 0.286660984 0.287357853 0.288462133 0.288997988 0.289626403 0.291559085 0.291583757 0.292398775 0.294174912
[1081] 0.296217622 0.296992591 0.298232328 0.298396631 0.299223145 0.299699281 0.300973107 0.301639180 0.304321731 0.304878357 0.305165399 0.305927638
[1093] 0.307747885 0.308171113 0.309212374 0.309648693 0.312439476 0.313047740 0.314401597 0.316569744 0.317462611 0.317587817 0.320900335 0.322132672
[1105] 0.323853404 0.325875722 0.327083815 0.327279067 0.328565194 0.329501589 0.331676954 0.334646837 0.335467475 0.336993633 0.337081265 0.337742989
[1117] 0.338047085 0.338314109 0.340965127 0.348352506 0.349019787 0.349374936 0.351726771 0.352946137 0.355286489 0.357449105 0.358191662 0.358404423
[1129] 0.358767438 0.360688469 0.364313904 0.365162002 0.368141794 0.369800817 0.371937713 0.372086932 0.375116958 0.376786681 0.378493076 0.380272087
[1141] 0.386467169 0.387494112 0.388613908 0.388889135 0.390135256 0.394175549 0.399728404 0.400173101 0.401408455 0.401797174 0.403236064 0.412837124
[1153] 0.415546760 0.420746885 0.422772901 0.424553172 0.428586738 0.433084932 0.437682257 0.441730265 0.443632064 0.451414653 0.451750702 0.455412352
[1165] 0.458927522 0.459700753 0.462552595 0.464625891 0.464988319 0.466574653 0.474178936 0.474985522 0.479453414 0.482426278 0.483774377 0.485365799
[1177] 0.488259637 0.491491286 0.491638584 0.491853668 0.493258529 0.493761583 0.497160023 0.498266190 0.499537726 0.500356513 0.500694748 0.500793490
[1189] 0.501433378 0.506530228 0.507256766 0.508566914 0.508883863 0.509485575 0.511462183 0.511960388 0.512769144 0.512782309 0.515229488 0.515428093
[1201] 0.515606259 0.515739254 0.519813177 0.523082279 0.525078883 0.526408457 0.527126241 0.528062838 0.528388347 0.530789872 0.530829623 0.531162068
[1213] 0.531360939 0.531450409 0.534231109 0.534313082 0.537429010 0.537744727 0.537843308 0.538026292 0.538384687 0.541054808 0.542958179 0.543001447

[1225] 0.543601787 0.545099616 0.547524395 0.548709789 0.550488926 0.551981535
0.554056693 0.554240677 0.555416571 0.555658982 0.557311640 0.557615237
[1237] 0.557782403 0.558077914 0.558552612 0.560964892 0.560989817 0.561267573
0.561889105 0.562859619 0.562956367 0.563634552 0.564420808 0.564952748
[1249] 0.565279755 0.565883452 0.566680400 0.567153994 0.568585751 0.568607134
0.570330201 0.570429256 0.571749929 0.572730711 0.573028305 0.573355721
[1261] 0.573557937 0.574109318 0.574377114 0.576106468 0.576847140 0.577147746
0.577559013 0.578358552 0.578521322 0.578548752 0.580529016 0.580944691
[1273] 0.582304656 0.583268081 0.583381809 0.583777316 0.583931418 0.585645535
0.585794778 0.587002182 0.587211173 0.587483517 0.588195437 0.590493023
[1285] 0.591297820 0.591955111 0.593512790 0.594123335 0.594826413 0.594907893
0.595371010 0.596830018 0.597358688 0.597926990 0.598307124 0.599387172
[1297] 0.599793347 0.600145477 0.601097796 0.601441729 0.604064845 0.604657299
0.604895094 0.605218245 0.606147028 0.606330016 0.606445182 0.606495636
[1309] 0.607184451 0.607249882 0.607544993 0.608208881 0.608657508 0.608742504
0.610424214 0.611868648 0.611894687 0.612361503 0.612984783 0.613135324
[1321] 0.613662785 0.614747747 0.615486777 0.615915729 0.616122642 0.617708691
0.617831064 0.618069958 0.619981845 0.620051195 0.620557267 0.620954529
[1333] 0.621038781 0.621569233 0.622132947 0.622495451 0.622594690 0.623165504
0.623368608 0.624846543 0.625478683 0.625497575 0.625749512 0.626838507
[1345] 0.626980389 0.627122583 0.629048512 0.629852626 0.630059283 0.630549750
0.630930564 0.630988635 0.631427796 0.631934076 0.633399903 0.633850042
[1357] 0.633948002 0.634078546 0.634351693 0.634623026 0.634877407 0.635129532
0.636607716 0.636696799 0.636932713 0.638804147 0.639081338 0.639731327
[1369] 0.639900811 0.639995491 0.640041579 0.641540902 0.642597085 0.642847088
0.643118323 0.643227238 0.643922097 0.645350490 0.645438248 0.645680404
[1381] 0.645926332 0.646142373 0.646289051 0.646606499 0.647203180 0.647292389
0.647389861 0.647445008 0.647547445 0.647785175 0.647885310 0.648179877
[1393] 0.648300797 0.650210900 0.650418547 0.650865555 0.651620844 0.651846170
0.652015710 0.652406358 0.653956700 0.654638545 0.654818677 0.654843473
[1405] 0.655559346 0.657340743 0.658652344 0.658936812 0.659412273 0.660054905
0.660223488 0.661712396 0.662331207 0.663117347 0.663786004 0.663858505
[1417] 0.664254029 0.664256126 0.664831976 0.666426694 0.667800922 0.668060433
0.668920899 0.669181792 0.669876841 0.670101169 0.670609263 0.670916695
[1429] 0.671729063 0.671901391 0.673550357 0.673746074 0.673812973 0.674525856
0.674557344 0.675148569 0.675151627 0.675212722 0.676510397 0.676741790
[1441] 0.677098683 0.677906407 0.678083403 0.679086354 0.679372035 0.680184787
0.680413311 0.681805584 0.682102307 0.682868337 0.683119325 0.683725801
[1453] 0.683828526 0.684034448 0.684689911 0.684900252 0.685391315 0.685513615
0.686821000 0.688679935 0.689267554 0.689436637 0.690818259 0.691446704
[1465] 0.692399588 0.692815836 0.693368716 0.693410307 0.694949872 0.696493799
0.696723791 0.697123813 0.697517560 0.697692413 0.697933170 0.698023316
[1477] 0.698061291 0.698748277 0.699263630 0.700109045 0.702400171 0.702972430
0.703467801 0.703471042 0.703625551 0.703649473 0.703947567 0.703954709
[1489] 0.704864326 0.705925520 0.706268831 0.706505536 0.706713488 0.707310800
0.707447535 0.707965518 0.709145921 0.709302409 0.709419265 0.709899522
[1501] 0.709936862 0.710095886 0.711899527 0.712770237 0.712927155 0.713616388
0.715323339 0.715895157 0.715912813 0.716532629 0.716628884 0.717697812
[1513] 0.718169500 0.718438353 0.719173588 0.719190617 0.719971966 0.720322384
0.721127289 0.721335082 0.721396374 0.721987380 0.722264859 0.722848742
[1525] 0.723103333 0.723973164 0.724086726 0.724306942 0.724659686 0.724775167
0.725303372 0.726409697 0.727342530 0.728566690 0.729948943 0.730711023
[1537] 0.731229207 0.731374787 0.732013450 0.732244012 0.732559153 0.732632005
0.732659888 0.732984976 0.734224636 0.734743048 0.734872335 0.735422943

[1549] 0.736380821 0.736470819 0.737759164 0.739069768 0.740633491 0.741371930
0.742010420 0.742191617 0.742659566 0.743201951 0.743997645 0.744540722
[1561] 0.745427607 0.745841057 0.746177867 0.747545617 0.747581805 0.748671653
0.749485739 0.750118268 0.750325679 0.750642221 0.750690795 0.751177476
[1573] 0.751473790 0.753782577 0.754251312 0.754959806 0.755076423 0.756030693
0.757946739 0.759230369 0.759230853 0.760442868 0.760589434 0.761673922
[1585] 0.763387427 0.763882980 0.764183052 0.765086218 0.765299138 0.765343538
0.765792164 0.766100101 0.766276312 0.766687059 0.766817914 0.767222478
[1597] 0.767692459 0.767958059 0.768424508 0.770034758 0.770679855 0.771646653
0.772828987 0.773042428 0.773508824 0.774735759 0.776219885 0.778016563
[1609] 0.778988352 0.779046409 0.779421162 0.780436448 0.780827176 0.781268353
0.785269618 0.785815005 0.787788561 0.789227105 0.790187287 0.792474431
[1621] 0.792924941 0.793509693 0.796394987 0.798376529 0.798755992 0.799436499
0.799899270 0.801144676 0.801409446 0.803328281 0.803590172 0.806886901
[1633] 0.807342012 0.808735801 0.808970141 0.809159931 0.809803279 0.810238401
0.811604558 0.812705171 0.812835255 0.813371516 0.813569013 0.814266631
[1645] 0.816406329 0.817621759 0.817969363 0.818236149 0.818449246 0.819521071
0.819773015 0.820675840 0.822288950 0.823371866 0.827281362 0.828492151
[1657] 0.828731098 0.830001573 0.830972344 0.831035411 0.834471966 0.836336178
0.838577790 0.839042218 0.839663033 0.840085071 0.843883273 0.845156267
[1669] 0.845802707 0.848048419 0.850394693 0.853691804 0.855076132 0.855539391
0.858466140 0.859688371 0.863591338 0.863826396 0.867012267 0.867195342
[1681] 0.867882689 0.868418662 0.868518309 0.870935215 0.870995111 0.871390682
0.874677950 0.875185203 0.875348541 0.877327776 0.879260698 0.880177430
[1693] 0.880487350 0.880533018 0.881572059 0.884016343 0.893670864 0.903272911
0.916121982 0.916430719 0.920532943 0.921949377 0.926162231 0.926470849
[1705] 0.927385911 0.929463260 0.939203742 0.940906081 0.944004023 0.944192196
0.947841421 0.948263155 0.953485681 0.954457276 0.961687748 0.965721113
[1717] 0.971096781 0.972800737 0.978944137 0.983859583 0.985768112 0.989642086
0.998355781 1.002584611 1.010721470 1.013232618 1.016985952 1.063240574

modes40.1_Q93WY9 FLUCTUATIONS

[1] 0.18114095 0.20160406 0.13320476 0.10458874 0.14589535 0.14794210
0.12291949 0.10978411 0.09355055 0.08811646 0.10184847 0.10940901 0.09624456
[14] 0.09789934 0.11432675 0.11569191 0.11720827 0.12824289 0.12284706
0.14121923 0.20392490 0.28358443 0.25081167 0.20445848 0.23437307 0.35281702
[27] 0.22683984 0.15128932 0.16682660 0.17958996 0.14750445 0.15272415
0.16635894 0.13475579 0.13664507 0.17469227 0.17402820 0.14443873 0.13197888
[40] 0.18118972 0.23390809 0.22568560 0.23528930 0.22094706 0.18039574
0.13321027 0.15835407 0.10338889 0.10971558 0.10124865 0.09777695 0.09350100
[53] 0.09685380 0.10964994 0.14550533 0.12567990 0.11671585 0.09705142
0.08578632 0.09041951 0.08122318 0.07731510 0.07558363 0.06544929 0.06620695
[66] 0.07518343 0.07253658 0.06194181 0.07289873 0.08041288 0.08182687
0.09231087 0.09804295 0.11354088 0.14676350 0.24924767 0.19710761 0.19315560
[79] 0.22118696 0.15536478 0.13531889 0.14365497 0.14236446 0.10579353
0.12764693 0.17213814 0.16771650 0.19024211 0.16012769 0.12782579 0.14415114
[92] 0.14135817 0.12097439 0.11384085 0.11410178 0.11204864 0.09256340
0.10573965 0.11665466 0.10615878 0.11087718 0.15525155 0.15261323 0.13209757
[105] 0.16979055 0.38340420 0.32187162 0.41080335 0.17741611 0.15084380
0.12323226 0.11815713 0.11622235 0.08524744 0.10404337 0.13528056 0.15147652
[118] 0.21688903 0.31890968 0.23432813 0.15592571 0.18387418 0.23027678

0.19928075 0.16753104 0.16062583 0.13767043 0.13633554 0.13296636 0.15035256
[131] 0.14379113 0.19780384 0.21538355 0.18635317 0.14560043 0.15769239
0.13825813 0.14056814 0.14496728 0.14305022 0.16814251 0.15367821 0.20882098
[144] 0.16723404 0.15017396 0.10800026 0.09853603 0.08575844 0.09942469
0.08395518 0.06922508 0.06802776 0.07212983 0.07551309 0.06142203 0.06185795
[157] 0.07170512 0.06559009 0.07034144 0.08323635 0.08952292 0.11610489
0.12657837 0.14280484 0.17581219 0.28238872 0.34745081 0.39060117 0.59287372
[170] 0.87162895 0.45027122 0.34873749 0.20988056 0.19452940 0.11670667
0.11510003 0.15069409 0.14584104 0.10749846 0.12106551 0.13827279 0.12059310
[183] 0.10609063 0.14057069 0.14165903 0.11699157 0.12260286 0.19800671
0.20632100 0.18817107 0.17651891 0.13773632 0.16267352 0.16195207 0.14257392
[196] 0.20488351 0.25023697 0.15490978 0.18885625 0.25160779 0.18541006
0.26432660 0.20298080 0.15041520 0.14812739 0.13890670 0.15111830 0.16972627
[209] 0.11904334 0.13229590 0.10919212 0.08597843 0.07550066 0.08089176
0.08350973 0.07482489 0.06808679 0.07866895 0.08115177 0.07738148 0.08344030
[222] 0.10826344 0.14808625 0.12289380 0.17480640 0.20698510 0.26245762
0.36968491 0.33787115 0.17119883 0.16534592 0.17313321 0.16918448 0.14364435
[235] 0.14879061 0.20640852 0.19217646 0.15988553 0.20697304 0.21014032
0.16844451 0.19668365 0.24336831 0.27328270 0.31275305 0.21400415 0.18076019
[248] 0.20799703 0.16839710 0.15336826 0.08949477 0.11803360 0.13013608
0.11279267 0.08238890 0.08480414 0.10228102 0.10426076 0.08641281 0.08537147
[261] 0.10074570 0.09586052 0.09624869 0.11507107 0.11724409 0.12677469
0.19363449 0.23375056 0.32989042 0.25445440 0.33773037 0.48733400 0.59046971
[274] 0.61013021 0.36059126 0.21254965 0.18535135 0.15263294 0.13915085
0.16740049 0.22795001 0.23127431 0.15515261 0.13577463 0.16639249 0.15612421
[287] 0.12655074 0.14017357 0.16532639 0.14278050 0.13376006 0.17575289
0.16296974 0.15316484 0.19246013 0.19955768 0.18895823 0.23826224 0.28659717
[300] 0.46174553 0.21854314 0.13841065 0.13573798 0.12733975 0.13813095
0.26333733 0.23487439 0.17061670 0.11451846 0.10908716 0.13759592 0.18690491
[313] 0.17604607 0.15639180 0.12946129 0.10504104 0.09222102 0.07800761
0.07827776 0.07540331 0.08059375 0.06947922 0.06482500 0.07185562 0.07582182
[326] 0.07570748 0.07938698 0.07851153 0.08008260 0.09387732 0.10066997
0.11172700 0.14491506 0.14521946 0.15135730 0.11898077 0.11703370 0.13614537
[339] 0.21070465 0.27113357 0.39920786 0.42427977 0.20997969 0.20363942
0.27756297 0.21054596 0.16619576 0.15825692 0.20756915 0.26894732 0.20861577
[352] 0.26320377 0.18635322 0.13460694 0.14149058 0.15226309 0.11056808
0.09683299 0.12965381 0.12622157 0.10196924 0.11741314 0.11472442 0.10792999
[365] 0.11851300 0.10256674 0.10490004 0.13038010 0.17024876 0.30291640
0.25835159 0.19372872 0.12820648 0.10312660 0.10988629 0.10724886 0.11943069
[378] 0.16487998 0.17957224 0.17149456 0.20978517 0.20941911 0.15728484
0.20209694 0.24335144 0.19267845 0.16219802 0.16002987 0.17506751 0.15439485
[391] 0.13975244 0.13551495 0.13717205 0.12706806 0.13829048 0.16161956
0.28355084 0.22978785 0.29460693 0.28118473 0.24269347 0.19968712 0.19402669
[404] 0.18711447 0.21274848 0.17725463 0.21670667 0.18992810 0.19058263
0.16850806 0.12525983 0.10144759 0.10452386 0.09035388 0.09412860 0.08254773
[417] 0.09537475 0.11765451 0.13196782 0.16287011 0.18779922 0.15481231
0.16407538 0.20459276 0.21247232 0.19928763 0.22363138 0.22750340 0.20912069
[430] 0.19504672 0.14926046 0.15786735 0.11551583 0.11152225 0.11092224
0.09961906 0.08925618 0.09727669 0.08652516 0.07602074 0.08741212 0.08228722
[443] 0.07699993 0.08994068 0.10033173 0.08631087 0.09974729 0.13362214
0.17583327 0.16625741 0.15209731 0.25148368 0.24609731 0.17914883 0.16039999
[456] 0.15275933 0.42437761 0.25518425 0.50842746 0.32267483 0.22688165
0.18367011 0.23597725 0.24773943 0.27173268 0.24389970 0.21031123 0.24066336
[469] 0.30571936 0.37551484 0.33773308 0.30778175 0.38815910 0.42609546

0.38683444 0.34185191 0.32966209 0.64247784 0.62444634 0.72736831 0.97486160
[482] 1.00002351 0.65789319 0.82856405 0.59416663 0.47170361 0.32451543
0.25149962 0.18211100 0.15816458 0.18402204 0.20674480 0.16478340 0.13988887
[495] 0.14546661 0.12719506 0.15374827 0.15622855 0.18539307 0.19944883
0.34555185 0.32767034 0.28404406 0.32070460 0.27750357 0.24973133 0.33316004
[508] 0.33712296 0.38613171 0.43182171 0.56374197 0.63580510 0.75976038
0.64012594 0.49953948 0.69588144 0.78953246 0.85636683 0.78776359 0.56231355
[521] 0.73248508 0.62762093 0.48320259 0.55408784 0.56549522 0.49289673
0.36850420 0.37313849 0.22964304 0.23623330 0.18713246 0.13561013 0.20927552
[534] 0.26188823 0.37867319 0.40651692 0.36506802 0.50680882 0.70427458
0.75789067 0.96659299 0.84927279 0.49147344 0.35225632 0.27594842 0.27567252
[547] 0.21677861 0.19097356 0.19963958 0.30447788 0.41931113 0.27230238
0.27255441 0.21518525 0.19404462 0.16141001 0.13609424 0.12824469 0.12019074
[560] 0.13586461 0.13115106 0.17448473 0.24497807 0.30976053 0.31523196
0.41874931 0.40299832 0.57109069 0.81522261 1.06652476 0.90825740 0.73552534
[573] 0.47608805 0.59049378 0.51727155 0.48813105

modes40.1_Q93WY9 MASSES

[1] 138.1469 114.1030 163.1730 57.0510 128.1060 71.0780 113.1580 87.0770
129.1800 87.0770 147.1740 113.1580 147.1740 163.1730 128.1060 71.0780
[17] 117.1260 157.1940 87.0770 57.0510 163.1730 113.1580 97.1150 137.1390
114.0790 117.1260 157.1940 99.1310 117.1260 186.2100 157.1940 57.0510
[33] 114.1030 87.0770 57.0510 113.1580 113.1580 114.0790 57.0510 129.1800
71.0780 87.0770 57.0510 99.1310 114.0790 113.1580 99.1310 57.0510
[49] 57.0510 163.1730 163.1730 114.0790 71.0780 57.0510 114.0790 114.1030
99.1310 129.1800 147.1740 57.0510 113.1580 97.1150 131.1960 71.0780
[65] 147.1740 101.1040 99.1310 101.1040 131.1960 131.1960 87.0770 186.2100
87.0770 113.1580 113.1580 128.1060 163.1730 57.0510 129.1800 117.1260
[81] 131.1960 57.0510 128.1060 87.0770 57.0510 128.1060 113.1580 87.0770
114.1030 71.0780 113.1580 114.0790 71.0780 99.1310 129.1800 186.2100
[97] 57.0510 101.1040 114.0790 163.1730 113.1580 113.1580 129.1800 71.0780
137.1390 97.1150 128.1060 97.1150 114.1030 99.1310 113.1580 163.1730
[113] 57.0510 128.1060 99.1310 57.0510 114.0790 57.0510 101.1040 101.1040
114.0790 137.1390 163.1730 103.1430 186.2100 117.1260 157.1940 97.1150
[129] 128.1060 114.0790 131.1960 101.1040 101.1040 87.0770 157.1940 71.0780
71.0780 163.1730 157.1940 113.1580 114.0790 97.1150 87.0770 157.1940
[145] 97.1150 57.0510 87.0770 114.0790 113.1580 71.0780 57.0510 128.1060
101.1040 71.0780 71.0780 71.0780 131.1960 71.0780 71.0780 71.0780
[161] 87.0770 113.1580 99.1310 147.1740 157.1940 114.1030 87.0770 114.1030
97.1150 71.0780 163.1730 71.0780 129.1800 128.1060 113.1580 113.1580
[177] 101.1040 137.1390 71.0780 163.1730 117.1260 113.1580 147.1740 128.1060
147.1740 71.0780 114.0790 129.1800 163.1730 157.1940 57.0510 129.1800
[193] 163.1730 114.0790 87.0770 87.0770 113.1580 101.1040 99.1310 71.0780
117.1260 129.1800 163.1730 163.1730 157.1940 87.0770 99.1310 87.0770
[209] 57.0510 163.1730 71.0780 114.0790 128.1060 113.1580 113.1580 186.2100
71.0780 71.0780 71.0780 186.2100 113.1580 163.1730 129.1800 71.0780
[225] 87.0770 114.1030 129.1800 128.1060 163.1730 163.1730 113.1580 114.1030
163.1730 113.1580 57.0510 128.1060 114.1030 57.0510 114.0790 71.0780
[241] 113.1580 57.0510 57.0510 101.1040 57.0510 186.2100 87.0770 131.1960
101.1040 128.1060 147.1740 57.0510 186.2100 114.0790 99.1310 129.1800
[257] 163.1730 71.0780 57.0510 99.1310 117.1260 101.1040 113.1580 71.0780

```
71.0780 129.1800 147.1740 113.1580 131.1960 117.1260  57.0510 114.1030
[273]  71.0780  57.0510 114.1030 137.1390  71.0780  97.1150  99.1310 147.1740
128.1060 129.1800 163.1730 117.1260 128.1060 129.1800  71.0780 128.1060
[289] 114.1030 147.1740 131.1960 103.1430  71.0780 103.1430 113.1580  57.0510
129.1800  57.0510 114.1030 117.1260 114.1030 113.1580 137.1390 129.1800
[305]  87.0770  97.1150  57.0510  57.0510 113.1580 113.1580 147.1740 157.1940
117.1260 157.1940 186.2100 114.1030 114.1030 131.1960 117.1260 147.1740
[321]  99.1310 101.1040  87.0770  71.0780  87.0770 147.1740 113.1580  71.0780
101.1040  99.1310 163.1730  87.0770 114.0790 163.1730 113.1580  71.0780
[337]  87.0770  71.0780 157.1940 129.1800  87.0770 113.1580 129.1800 103.1430
87.0770  87.0770  57.0510 101.1040  99.1310 113.1580  97.1150  87.0770
[353] 128.1060 113.1580 113.1580  87.0770 147.1740  71.0780 129.1800  87.0770
117.1260  99.1310 114.0790 163.1730 113.1580 113.1580  57.0510 114.0790
[369] 114.1030  97.1150 157.1940  71.0780 101.1040  87.0770 163.1730 131.1960
99.1310  57.0510 163.1730  57.0510 114.1030 114.1030 163.1730  97.1150
[385] 157.1940 117.1260  99.1310 137.1390 137.1390 157.1940  57.0510  87.0770
157.1940  57.0510  57.0510 163.1730  71.0780 101.1040 186.2100 147.1740
[401]  87.0770 157.1940 129.1800  71.0780  87.0770 114.0790  97.1150 114.1030
113.1580 113.1580  71.0780  57.0510  71.0780 113.1580  99.1310  57.0510
[417]  57.0510  97.1150 114.0790  71.0780 163.1730 114.0790 114.1030 147.1740
71.0780 114.0790 117.1260 157.1940 114.0790 114.1030 163.1730 128.1060
[433] 117.1260 101.1040 128.1060  97.1150  71.0780 101.1040 163.1730 114.1030
114.1030  71.0780  97.1150 113.1580 113.1580  57.0510  99.1310 113.1580
[449]  71.0780 157.1940 113.1580 137.1390  57.0510  57.0510 117.1260  87.0770
129.1800 163.1730  87.0770 117.1260 113.1580 113.1580  97.1150  99.1310
[465]  71.0780 113.1580  97.1150 117.1260  97.1150 129.1800  97.1150 114.0790
97.1150 128.1060 117.1260 129.1800  99.1310 101.1040  97.1150  71.0780
[481]  97.1150  71.0780  87.0770  87.0770 101.1040  71.0780 114.0790 113.1580
101.1040 113.1580 128.1060 117.1260 129.1800 128.1060 101.1040  71.0780
[497]  87.0770 186.2100  99.1310  97.1150 129.1800  57.0510 129.1800 101.1040
163.1730 163.1730 157.1940 163.1730  87.0770  99.1310 113.1580  99.1310
[513] 101.1040 114.1030 129.1800  87.0770  71.0780 131.1960 101.1040 131.1960
129.1800 114.1030 113.1580 129.1800 113.1580  87.0770 113.1580 163.1730
[529] 117.1260 113.1580 163.1730  57.0510  87.0770 113.1580 186.2100  57.0510
113.1580  87.0770 129.1800 163.1730  57.0510 114.0790  87.0770 163.1730
[545]  99.1310 147.1740  97.1150  71.0780 186.2100 113.1580 114.1030  87.0770
113.1580  97.1150  71.0780  57.0510 129.1800 101.1040 113.1580 128.1060
[561] 147.1740  99.1310 163.1730  99.1310 137.1390  87.0770  71.0780  87.0770
97.1150  71.0780 101.1040  99.1310  87.0770 113.1580  87.0770 104.0843


 modes40.1_Q9ZSP9

Call:
  nma.pdb(pdb = pdb40.1_ZSP9)

Class:
  VibrationalModes (nma)

Number of modes:
  1755 (6 trivial)

Frequencies:
  Mode 7:       0.005
```

```
  Mode 8:       0.006
  Mode 9:       0.009
  Mode 10:      0.014
  Mode 11:      0.016
  Mode 12:      0.016

+ attr: modes, frequencies, force.constants, fluctuations,
        U, L, xyz, mass, temp, triv.modes, natoms, call
```

modes40.1_Q9ZSP9 FREQUENCIES

```
   [1] 0.000000000 0.000000000 0.000000000 0.000000000 0.000000000 0.000000000
0.005273771 0.005689650 0.008582570 0.014111040 0.015510872 0.016058095
  [13] 0.018588453 0.020566120 0.021649168 0.022350364 0.022905598 0.025537798
0.026799065 0.027501127 0.027704848 0.028687209 0.029351468 0.029964273
  [25] 0.030256188 0.030668212 0.030970669 0.031523930 0.032141813 0.032737208
0.033438450 0.033964575 0.034626717 0.035064330 0.035144785 0.036183868
  [37] 0.036285233 0.037299345 0.038091933 0.038324960 0.038471407 0.038553622
0.039270656 0.039730761 0.040089042 0.040530174 0.041107907 0.041165481
  [49] 0.041493681 0.041907343 0.042060288 0.042564650 0.043400301 0.043967687
0.044238490 0.044570204 0.044850360 0.045431140 0.045621980 0.045869769
  [61] 0.046380492 0.046531527 0.047075170 0.047616063 0.047992539 0.048376296
0.048505717 0.048723512 0.049069820 0.049104910 0.049335979 0.049541690
  [73] 0.049930028 0.050136081 0.050324179 0.050685540 0.050857414 0.051137551
0.051222183 0.051393987 0.051639587 0.052022977 0.052352542 0.052540901
  [85] 0.052667302 0.052970868 0.053159895 0.053243692 0.053835086 0.053947657
0.054091612 0.054426104 0.054704867 0.054921832 0.054999951 0.055249235
  [97] 0.055414497 0.055575849 0.055910069 0.056584675 0.056763676 0.056900953
0.057066980 0.057453171 0.057623321 0.058031568 0.058041388 0.058520993
 [109] 0.058604904 0.058879801 0.058969860 0.059164980 0.059377380 0.059563512
0.059795256 0.060025894 0.060242406 0.060471758 0.060503584 0.060716307
 [121] 0.060924130 0.061141811 0.061267212 0.061455657 0.061545445 0.061710059
0.061995702 0.062252780 0.062363560 0.062640361 0.062736933 0.062988572
 [133] 0.063072363 0.063177897 0.063473298 0.063772693 0.063918892 0.064283428
0.064530215 0.064648848 0.064782709 0.065216639 0.065230037 0.065592114
 [145] 0.065636896 0.065802822 0.066166742 0.066196404 0.066231216 0.066433607
0.066721807 0.066887693 0.066994968 0.067335144 0.067403950 0.067820177
 [157] 0.067853783 0.067949842 0.068316228 0.068620148 0.068704628 0.068979853
0.069190495 0.069315404 0.069479473 0.069645883 0.069668974 0.069862675
 [169] 0.069998689 0.070356206 0.070434829 0.070623739 0.070821804 0.070883295
0.071215035 0.071423164 0.071516199 0.071676821 0.071788583 0.072041129
 [181] 0.072215492 0.072376837 0.072686604 0.072862551 0.073009457 0.073159703
0.073288389 0.073469963 0.073663813 0.073710563 0.073798313 0.073916120
 [193] 0.074145538 0.074268934 0.074546891 0.074654527 0.074791309 0.074921249
0.075205889 0.075477543 0.075605131 0.075816577 0.075893214 0.076033432
 [205] 0.076201818 0.076279728 0.076409292 0.076757411 0.076891935 0.076934255
0.076985107 0.077209670 0.077394481 0.077661578 0.077751221 0.078021643
 [217] 0.078251001 0.078522120 0.078533732 0.078734901 0.078886444 0.079003879
0.079082232 0.079227516 0.079554868 0.079615500 0.079781565 0.079860265
 [229] 0.080091632 0.080243927 0.080327220 0.080596227 0.080710389 0.080912877
0.081338609 0.081452819 0.081511418 0.081682931 0.081798674 0.082052053
 [241] 0.082190701 0.082356186 0.082538067 0.082630543 0.082736081 0.082771894
```

0.083165730 0.083530422 0.083674794 0.083812420 0.084082929 0.084125395
[253] 0.084354672 0.084434810 0.084581232 0.084623897 0.084873617 0.084945512
0.084981735 0.085107724 0.085312246 0.085599027 0.085767829 0.085898711
[265] 0.086100029 0.086353107 0.086649014 0.086662753 0.086914484 0.087074773
0.087233171 0.087426781 0.087575721 0.087743608 0.087854538 0.088030525
[277] 0.088178733 0.088249227 0.088313929 0.088504173 0.088676158 0.088737409
0.088894554 0.089114546 0.089253008 0.089268332 0.089566344 0.089733047
[289] 0.090018970 0.090063278 0.090238326 0.090613829 0.090678241 0.090704774
0.090885137 0.090970103 0.091101713 0.091356878 0.091581466 0.091677392
[301] 0.091871148 0.092080179 0.092182318 0.092385160 0.092521602 0.092593714
0.092732444 0.092863875 0.093128628 0.093273352 0.093316114 0.093484395
[313] 0.093691042 0.093828959 0.093961957 0.094124777 0.094318206 0.094441127
0.094731557 0.094771256 0.094868095 0.094992171 0.095339258 0.095461130
[325] 0.095594903 0.095795808 0.095850792 0.096065797 0.096204523 0.096483607
0.096644669 0.096713969 0.096785574 0.097052157 0.097283906 0.097309941
[337] 0.097523417 0.097637763 0.097874079 0.098093429 0.098159900 0.098263195
0.098485022 0.098500710 0.098641789 0.098723415 0.098839320 0.098901704
[349] 0.099116736 0.099282201 0.099563852 0.099675604 0.099747115 0.099851305
0.100023152 0.100175363 0.100337946 0.100483504 0.100601788 0.100808922
[361] 0.100839447 0.101075920 0.101347338 0.101365207 0.101551705 0.101702748
0.101916717 0.101967158 0.102134205 0.102249588 0.102335020 0.102432497
[373] 0.102680474 0.102830106 0.103060046 0.103151804 0.103335568 0.103472013
0.103539190 0.103620502 0.103820516 0.104040354 0.104118234 0.104192288
[385] 0.104286572 0.104419956 0.104585750 0.104812442 0.104910274 0.105185284
0.105334725 0.105543370 0.105586201 0.105700812 0.105844978 0.105990620
[397] 0.106237921 0.106328724 0.106469423 0.106840869 0.106924527 0.107085378
0.107401524 0.107597923 0.107671701 0.107776221 0.107835079 0.108020841
[409] 0.108249583 0.108267716 0.108355533 0.108600831 0.108734047 0.108833241
0.109030429 0.109127265 0.109265172 0.109398389 0.109476738 0.109525316
[421] 0.109710410 0.109845739 0.110022697 0.110212931 0.110387923 0.110541902
0.110704133 0.110958279 0.111078863 0.111193849 0.111298020 0.111554673
[433] 0.111640585 0.111709425 0.111902790 0.112030384 0.112123838 0.112266412
0.112406664 0.112453414 0.112889556 0.112951132 0.113131852 0.113202471
[445] 0.113334077 0.113551559 0.113615563 0.113707826 0.113861319 0.114079128
0.114281894 0.114324774 0.114539711 0.114632446 0.114784152 0.114936428
[457] 0.115068474 0.115282463 0.115466884 0.115556024 0.115747669 0.115982575
0.116035960 0.116278671 0.116416701 0.116653734 0.116713215 0.116930802
[469] 0.117004107 0.117161931 0.117296977 0.117333035 0.117484919 0.117663950
0.117770571 0.117896005 0.118048452 0.118369342 0.118417481 0.118625857
[481] 0.118779926 0.118993841 0.119199402 0.119347850 0.119502578 0.119575260
0.119785534 0.119928398 0.120092503 0.120186959 0.120232053 0.120623068
[493] 0.120641651 0.120833929 0.120930425 0.121178906 0.121298204 0.121529156
0.121546975 0.121581148 0.121687563 0.121815825 0.122123706 0.122274300
[505] 0.122379803 0.122551786 0.122728896 0.122878439 0.122956027 0.123088215
0.123370748 0.123476647 0.123547606 0.123716738 0.123916510 0.123976491
[517] 0.124217655 0.124443290 0.124599822 0.124790873 0.124984266 0.125164814
0.125249985 0.125638892 0.125804004 0.125998155 0.126349379 0.126525277
[529] 0.126573516 0.126900495 0.127016514 0.127360157 0.127398636 0.127526914
0.127719338 0.127770298 0.127832335 0.127912858 0.128403620 0.128578776
[541] 0.128885929 0.129007722 0.129409216 0.129590636 0.129780195 0.130033777
0.130084901 0.130186409 0.130329727 0.130435026 0.130645076 0.131003758
[553] 0.131105134 0.131312570 0.131332244 0.131463428 0.131568396 0.131914968
0.132059000 0.132294238 0.132431354 0.132499238 0.132667650 0.132774051
[565] 0.133050582 0.133084180 0.133273046 0.133464777 0.133665235 0.133806531

0.134146753 0.134189610 0.134352510 0.134545526 0.134666244 0.134720217
[577] 0.134760824 0.134834486 0.134859563 0.135145415 0.135306790 0.135599451
0.135653613 0.135754502 0.135881137 0.135998248 0.136277344 0.136583690
[589] 0.136777725 0.136963070 0.137179652 0.137221376 0.137449530 0.137548365
0.137737638 0.137865300 0.138114307 0.138244094 0.138279545 0.138410092
[601] 0.138679767 0.139066088 0.139132465 0.139157405 0.139295949 0.139428087
0.139635131 0.139721906 0.140136266 0.140534265 0.140563372 0.140723573
[613] 0.140778013 0.141107261 0.141370448 0.141530541 0.141645843 0.141679280
0.141876701 0.142093458 0.142205988 0.142373504 0.142439940 0.142601852
[625] 0.142697473 0.142906516 0.143112334 0.143307427 0.143344894 0.143570286
0.143690121 0.143981312 0.144072853 0.144430721 0.144857492 0.145209328
[637] 0.145411363 0.145475454 0.145823887 0.145957318 0.146009633 0.146191591
0.146473659 0.146637768 0.146903806 0.147029366 0.147075788 0.147221419
[649] 0.147566503 0.147605892 0.147782971 0.147962320 0.148361691 0.148563360
0.148604275 0.149048838 0.149158753 0.149266889 0.149458017 0.149757275
[661] 0.149957490 0.150191004 0.150356363 0.150697461 0.150880649 0.150982436
0.151172064 0.151272818 0.151459992 0.151550359 0.151653032 0.151846578
[673] 0.151940215 0.152064948 0.152193657 0.152566430 0.152632495 0.152727973
0.153128892 0.153329248 0.153397626 0.153695147 0.153764762 0.154019971
[685] 0.154261457 0.154378818 0.154871008 0.155076790 0.155137868 0.155417145
0.155520686 0.155589159 0.155756024 0.155971438 0.155982481 0.156046369
[697] 0.156427936 0.156596737 0.156775536 0.156980032 0.157233163 0.157287444
0.157521349 0.157783244 0.158145965 0.158496270 0.158590853 0.158652334
[709] 0.158792612 0.159121597 0.159394927 0.159987850 0.160119207 0.160187375
0.160395259 0.160692985 0.160993702 0.161336725 0.161483377 0.161546187
[721] 0.161633109 0.161811196 0.161976342 0.162182635 0.162598878 0.162763302
0.162952512 0.163159047 0.163297548 0.163530678 0.163658959 0.163981579
[733] 0.164102022 0.164163830 0.164574215 0.164835738 0.165192021 0.165315566
0.165466040 0.166045369 0.166130623 0.166262611 0.166748359 0.166956873
[745] 0.167334678 0.167436900 0.167536188 0.167688823 0.168147028 0.168204865
0.168358850 0.168415487 0.168630952 0.168746651 0.168867820 0.169204760
[757] 0.169215913 0.169322983 0.169462575 0.169664619 0.170095463 0.170241712
0.170646387 0.170673030 0.170757160 0.171186882 0.171224018 0.171368639
[769] 0.171543928 0.171844302 0.171909589 0.171946790 0.172327105 0.172378249
0.172666464 0.172905860 0.173170598 0.173221421 0.173478377 0.173638262
[781] 0.173814832 0.174030963 0.174291885 0.174414213 0.174568234 0.174728427
0.174825747 0.175047069 0.175216004 0.175295136 0.175681899 0.175759596
[793] 0.175891920 0.176293970 0.176467811 0.176604051 0.177138522 0.177384949
0.177838255 0.177921560 0.178090541 0.178326491 0.178510911 0.178823168
[805] 0.178890793 0.179217653 0.179565291 0.179703199 0.179934007 0.180070719
0.180172675 0.180663786 0.180697432 0.181005567 0.181466649 0.181730973
[817] 0.181831301 0.182033253 0.182050090 0.182213366 0.182436764 0.182832181
0.183247272 0.183515657 0.183607284 0.183607974 0.183748776 0.183831469
[829] 0.184011542 0.184649847 0.184808086 0.184915649 0.185257789 0.185460791
0.185619361 0.185987380 0.186144345 0.186231891 0.186487695 0.186597140
[841] 0.186775768 0.187216484 0.187259977 0.187445608 0.187482834 0.187613707
0.188056972 0.188139117 0.188760178 0.188924828 0.189089201 0.189208253
[853] 0.189784779 0.189860641 0.190282964 0.190583110 0.190829498 0.190904480
0.191263654 0.191431444 0.191896515 0.192289076 0.192400225 0.192479069
[865] 0.192809497 0.193054224 0.193159687 0.193343584 0.193694835 0.194244484
0.194918482 0.195126816 0.195310417 0.195449528 0.195533879 0.195883725
[877] 0.196108535 0.196246564 0.196298767 0.197542382 0.197957018 0.198194750
0.198505584 0.198759674 0.199064918 0.199134510 0.199378588 0.199595465
[889] 0.200139477 0.200414626 0.200468587 0.200717353 0.200927049 0.201404528

0.201537798 0.201739422 0.201876675 0.202251494 0.202637746 0.202733226
[901] 0.202997622 0.203057820 0.203108772 0.203262547 0.203425794 0.203949033
0.204116447 0.204721641 0.204760860 0.205173377 0.205950801 0.206104545
[913] 0.206356582 0.206420771 0.206550007 0.207124001 0.207253532 0.207531516
0.207858001 0.208315643 0.208365977 0.208756265 0.208910612 0.209111244
[925] 0.209606335 0.210094954 0.210395852 0.210561328 0.210783043 0.210942031
0.210988857 0.211287343 0.211384249 0.212064332 0.212170314 0.212507909
[937] 0.212742776 0.213014310 0.213303668 0.213636212 0.213812035 0.213940358
0.214567191 0.214844259 0.215615537 0.215843033 0.216070582 0.216133409
[949] 0.216314813 0.217388816 0.217463027 0.218407152 0.218865362 0.218898170
0.219183632 0.220656261 0.220823971 0.220951203 0.221025650 0.221561345
[961] 0.221941725 0.222218946 0.222857956 0.223114625 0.223569815 0.224033861
0.224304600 0.224428674 0.224531414 0.224954238 0.225412512 0.226058182
[973] 0.226440516 0.226716202 0.227145888 0.228191393 0.228348577 0.228689593
0.229405496 0.229510092 0.229777079 0.230458721 0.230503890 0.230849568
[985] 0.231287783 0.231841285 0.232013738 0.232318029 0.233021861 0.233613977
0.234590823 0.235101590 0.236176337 0.236182396 0.236291282 0.236501729
[997] 0.236526790 0.237121798 0.237202917 0.237672051 0.237968416 0.238023760
0.238214599 0.239162965 0.239170590 0.239633653 0.240099989 0.240311579
[1009] 0.241178614 0.241285823 0.242046885 0.242233405 0.242646104 0.242922846
0.243359045 0.243567545 0.244468210 0.244885004 0.244941267 0.245637648
[1021] 0.246164133 0.246436359 0.246812424 0.247439363 0.247454462 0.247982912
0.248757089 0.248892279 0.249451245 0.250202201 0.250390688 0.251495179
[1033] 0.251635441 0.251834674 0.252908422 0.253643001 0.253697572 0.254408318
0.255229900 0.255498861 0.256444548 0.257195767 0.257473004 0.257810425
[1045] 0.257885626 0.257943276 0.258603765 0.259542091 0.259610058 0.262082431
0.262483416 0.262765052 0.262824620 0.262887932 0.263669886 0.263938020
[1057] 0.263987824 0.264142023 0.264923622 0.266374172 0.266753086 0.268535142
0.269391028 0.269762089 0.272028484 0.272272804 0.273124582 0.274747118
[1069] 0.274900534 0.275592674 0.275993762 0.276063092 0.277292264 0.277627722
0.278247906 0.278603808 0.279833316 0.280762537 0.281475675 0.282164658
[1081] 0.282398460 0.282422991 0.283137091 0.283998740 0.286697873 0.286926128
0.287897039 0.289238527 0.289601170 0.290511704 0.291636788 0.292020264
[1093] 0.292832769 0.296043639 0.296070889 0.296900891 0.297028538 0.297246900
0.303576696 0.303603229 0.303765252 0.306144038 0.306416916 0.306924108
[1105] 0.307706687 0.308793362 0.310309491 0.311114086 0.312647480 0.312715326
0.313207466 0.316878651 0.319787117 0.319941618 0.321114139 0.321186111
[1117] 0.321328706 0.321844864 0.321929695 0.322369231 0.323523441 0.327055973
0.327971808 0.329780141 0.330573782 0.331162506 0.333038706 0.334314192
[1129] 0.335082619 0.335648983 0.337208350 0.337781199 0.338181859 0.338364607
0.338756576 0.339551243 0.341812021 0.342015345 0.342574388 0.343022695
[1141] 0.345360689 0.345729049 0.347484887 0.349169514 0.354753262 0.355014642
0.355848814 0.357157629 0.358974777 0.361350670 0.364592920 0.364885122
[1153] 0.366686323 0.367827527 0.370855812 0.372286410 0.372526683 0.378104516
0.388566779 0.391190839 0.394490015 0.396056972 0.401172757 0.405326771
[1165] 0.406401828 0.408436983 0.410509255 0.412313389 0.413143394 0.414695269
0.415084818 0.417714360 0.421556651 0.424353461 0.427295900 0.432347618
[1177] 0.433269978 0.434772446 0.435764810 0.444842991 0.445668797 0.446713422
0.447597777 0.448694707 0.456156392 0.456507206 0.460012330 0.460387936
[1189] 0.466748511 0.466761454 0.469722890 0.470856180 0.473067742 0.473106694
0.477000694 0.482209799 0.486075638 0.487536482 0.487999240 0.489556854
[1201] 0.490807137 0.491548567 0.497248617 0.497324946 0.499198429 0.499943247
0.500249431 0.501856648 0.503657781 0.504345089 0.509430758 0.509640123
[1213] 0.516783943 0.517604395 0.520491197 0.524565397 0.528299293 0.529150144

0.529261693 0.529400396 0.529412047 0.531158586 0.533182005 0.533826249
[1225] 0.533833794 0.534316045 0.538454574 0.539892697 0.542098675 0.543361456
0.543695765 0.543736483 0.544248474 0.545105727 0.545647072 0.547510816
[1237] 0.550215166 0.551595467 0.552622587 0.554287543 0.555030359 0.555703860
0.555717830 0.556319723 0.558429337 0.558462721 0.560013010 0.560066177
[1249] 0.560480012 0.560658206 0.561529292 0.562567204 0.563498252 0.563847712
0.564040448 0.564425992 0.565030062 0.565077222 0.567854711 0.569374579
[1261] 0.570120998 0.570891243 0.572242588 0.572470618 0.573206598 0.573475169
0.574081410 0.574879573 0.575321675 0.577764712 0.578173174 0.578440468
[1273] 0.578772700 0.579164904 0.580951667 0.581076485 0.581278193 0.583231470
0.583565597 0.584457714 0.584689991 0.586541419 0.586561133 0.587195255
[1285] 0.588538517 0.589770047 0.590175844 0.590209342 0.590515007 0.591003704
0.591167234 0.591637303 0.591699787 0.592149884 0.592235710 0.592775700
[1297] 0.593099560 0.593320940 0.594190097 0.594817428 0.595083227 0.595623293
0.596243508 0.596768772 0.596888987 0.597084061 0.597996314 0.598265675
[1309] 0.598583709 0.599228633 0.602236101 0.603483540 0.603807132 0.603994896
0.604173483 0.604470055 0.604599632 0.605104415 0.605528109 0.606563689
[1321] 0.606636953 0.608015190 0.608677400 0.609783508 0.610686891 0.611003082
0.611280614 0.611510366 0.611899386 0.612400013 0.612481263 0.612859025
[1333] 0.613068146 0.613280542 0.614117223 0.614509478 0.614549357 0.615070705
0.615684947 0.617403689 0.617526389 0.617535987 0.617569129 0.618113358
[1345] 0.618316094 0.618761609 0.618796895 0.619123898 0.619271352 0.619335955
0.619507503 0.621460681 0.621709364 0.622163218 0.622363536 0.623724750
[1357] 0.624312403 0.624436261 0.624521847 0.624744459 0.624858765 0.625029669
0.625572934 0.626128411 0.626251808 0.626359086 0.627157096 0.627950522
[1369] 0.628162745 0.628988772 0.629469067 0.629784496 0.630123625 0.630555555
0.631376225 0.632384838 0.632570326 0.633905427 0.634655316 0.634846266
[1381] 0.635062845 0.636220805 0.636254447 0.636325327 0.637684047 0.638571145
0.640491558 0.641247314 0.641536262 0.641548719 0.641907066 0.642415517
[1393] 0.643090870 0.643495222 0.643560070 0.643600786 0.644233732 0.644240160
0.644296265 0.645925312 0.646976824 0.648719726 0.649646791 0.649887379
[1405] 0.651452134 0.652225617 0.653131185 0.653478781 0.653698991 0.653779294
0.654477502 0.654610375 0.654927619 0.655443225 0.656500405 0.656980463
[1417] 0.657008974 0.657488757 0.657524835 0.658434194 0.659093422 0.659257621
0.659690750 0.660166704 0.660521738 0.660533415 0.661158713 0.661288731
[1429] 0.662797278 0.662857811 0.663911368 0.664495465 0.664898076 0.665134652
0.665226920 0.665551834 0.665672851 0.669165231 0.669909794 0.670521966
[1441] 0.670997995 0.671377107 0.671831568 0.672818689 0.673251709 0.673421390
0.673517581 0.673624307 0.673762296 0.674404682 0.674634225 0.675487610
[1453] 0.676254335 0.676676378 0.676820706 0.677062431 0.677249297 0.677404067
0.678020400 0.678289015 0.678496525 0.678814845 0.678922193 0.679384096
[1465] 0.679798607 0.680694975 0.682551240 0.682555731 0.682666702 0.682730111
0.683397686 0.684233422 0.684528001 0.684788293 0.685274002 0.685465097
[1477] 0.685694373 0.686140661 0.686801306 0.687572324 0.688179827 0.689991637
0.690056759 0.690759681 0.691148148 0.691632248 0.691640964 0.693416352
[1489] 0.693519340 0.693574544 0.693595617 0.694534356 0.694642538 0.694660825
0.695728790 0.696138263 0.697504196 0.698341622 0.698757883 0.698969173
[1501] 0.699080528 0.699818745 0.700994799 0.701538200 0.702317673 0.702855943
0.702885044 0.704687461 0.704957898 0.705627885 0.707288811 0.707364104
[1513] 0.707788999 0.707869374 0.707954212 0.708355849 0.709229660 0.709801034
0.711906536 0.712024655 0.712335780 0.712687003 0.713285385 0.713484935
[1525] 0.713778248 0.714034724 0.714260025 0.714655282 0.714762032 0.715517524
0.715748481 0.715784454 0.716833881 0.716926280 0.718232667 0.720902939
[1537] 0.721078444 0.721689822 0.721722902 0.721852030 0.722160341 0.722290267

0.722868839 0.723898829 0.723929901 0.724513788 0.724847753 0.724969564
[1549] 0.726883277 0.727040162 0.728901874 0.728918050 0.728937076 0.728949464
0.729294597 0.729569384 0.730279781 0.730282764 0.730626851 0.731372882
[1561] 0.731934411 0.732043087 0.733135200 0.733803502 0.734583325 0.735057359
0.735293322 0.736021150 0.736376246 0.736487036 0.737278931 0.737707970
[1573] 0.738222786 0.738285043 0.738508588 0.740002833 0.741109585 0.741132075
0.741195814 0.741328913 0.741481871 0.741656075 0.742008645 0.742348867
[1585] 0.744212156 0.744612537 0.745470914 0.745948810 0.746491079 0.747435281
0.747712938 0.748117235 0.748781283 0.748997332 0.749201706 0.749590992
[1597] 0.750209589 0.750475942 0.752329810 0.752415644 0.753426658 0.753556269
0.754166341 0.756564613 0.756692850 0.756943402 0.757211853 0.757861882
[1609] 0.758413785 0.759003800 0.759286851 0.759485371 0.761007278 0.761820966
0.762271466 0.762457183 0.763395938 0.763700712 0.764123617 0.764213579
[1621] 0.764376705 0.766700952 0.767623661 0.767916086 0.768061407 0.769573382
0.769737971 0.770267488 0.770862625 0.770913047 0.771093923 0.772094372
[1633] 0.773138249 0.773238137 0.774786713 0.776959875 0.777890816 0.778147336
0.778933250 0.779703152 0.779891360 0.780075788 0.780178489 0.780460222
[1645] 0.780549031 0.780812496 0.784940691 0.785097203 0.787313460 0.787808544
0.789296636 0.790173358 0.790235594 0.790320340 0.790831656 0.790964889
[1657] 0.791376890 0.791881733 0.793156511 0.794792027 0.795616134 0.797961746
0.799959925 0.803296575 0.804882173 0.806310326 0.807289489 0.810831848
[1669] 0.811809084 0.815439852 0.817833762 0.819033342 0.822033047 0.822423617
0.823254170 0.824566354 0.824682251 0.826365095 0.828988355 0.829159847
[1681] 0.832864812 0.834637809 0.834742445 0.835042640 0.835448064 0.836390814
0.839919882 0.839920832 0.842041426 0.845137550 0.845175224 0.845223774
[1693] 0.845488492 0.845794501 0.848810585 0.850612166 0.850655940 0.851189175
0.853182757 0.853434009 0.854846861 0.858167689 0.858545582 0.859170450
[1705] 0.859613690 0.860376578 0.860403443 0.863299764 0.870636631 0.871722338
0.872669644 0.872867159 0.878239343 0.880261171 0.880877925 0.881432463
[1717] 0.881879550 0.887872422 0.888646937 0.892246331 0.894263938 0.894917475
0.900993985 0.901681076 0.904799715 0.906765493 0.910335417 0.910968290
[1729] 0.912915986 0.913298240 0.913843402 0.914854261 0.915693176 0.915779285
0.923322050 0.928653518 0.929515161 0.931150735 0.945471925 0.949941993
[1741] 0.957241451 0.957365217 0.958886211 0.970469501 0.975818776 0.980459130
0.987205051 0.987267220 0.993841025 0.994546785 1.040911242 1.043856991
[1753] 1.046546755 1.055099339 1.083797240

modes40.1_Q9ZSP9 FLUCTUATIONS

[1] 0.19570552 0.14072293 0.10970653 0.12388738 0.12228982 0.10070675
0.10049360 0.09321722 0.08562947 0.07990409 0.09261085 0.09534385 0.09557260
[14] 0.09449533 0.10233131 0.11025961 0.12157938 0.12098968 0.11017306
0.11770908 0.17478079 0.20547080 0.21490344 0.23914506 0.26366267 0.17681274
[27] 0.18326514 0.14969188 0.16793368 0.22539205 0.21950075 0.20375854
0.16353649 0.13326230 0.15080697 0.17699569 0.18108331 0.14535675 0.14799433
[40] 0.20765833 0.30492816 0.29414285 0.29111202 0.27829927 0.22049579
0.15106140 0.16889070 0.12559196 0.10364322 0.09569010 0.11107123 0.09561391
[53] 0.09712839 0.11514153 0.14040390 0.13016668 0.12612596 0.10669762
0.09875379 0.09073441 0.08029603 0.08699633 0.07944704 0.06640631 0.07372626
[66] 0.07420495 0.06643288 0.06168144 0.06843995 0.07233946 0.07545849
0.08607228 0.08594958 0.11225346 0.12559449 0.15771793 0.10735013 0.16990044
[79] 0.19159605 0.25600702 0.34423350 0.23457184 0.23212122 0.27796580

0.20691442 0.13557913 0.16013736 0.15295155 0.11789229 0.10588297 0.12515224
[92] 0.13125487 0.10473633 0.10154869 0.10985298 0.10411542 0.08353848
0.09773608 0.10891540 0.10287604 0.10946377 0.12469076 0.12432837 0.13381724
[105] 0.15779953 0.36085866 0.33904362 0.44889486 0.24640501 0.17726192
0.12397610 0.11067316 0.09222809 0.10712876 0.11343212 0.12671577 0.15754126
[118] 0.16298593 0.28883998 0.23255372 0.17082290 0.17453728 0.22704978
0.18593068 0.15551040 0.14631071 0.13275110 0.13873949 0.13633114 0.15173627
[131] 0.16811486 0.19901864 0.23545924 0.23988824 0.15373637 0.17029036
0.16132341 0.15835830 0.14362199 0.14588792 0.20102420 0.31690589 0.39450354
[144] 0.18514897 0.14304437 0.09950317 0.08561461 0.07514103 0.07802959
0.06997184 0.05947156 0.06313290 0.06756264 0.06258971 0.05992681 0.05634093
[157] 0.06800439 0.07022096 0.07355524 0.08386817 0.10613783 0.12941980
0.12226351 0.16354164 0.19467372 0.33419597 0.48739324 0.60744563 0.51502114
[170] 0.70460984 0.39781606 0.21797462 0.21076634 0.20688038 0.12462298
0.11379368 0.15253100 0.13300899 0.09026906 0.11817520 0.12831531 0.09188398
[183] 0.08654808 0.13577638 0.12082519 0.08265553 0.12412024 0.17458456
0.17596563 0.14852647 0.21454229 0.17117104 0.17148224 0.12235226 0.10998525
[196] 0.12400436 0.11590608 0.09278163 0.12400737 0.13859544 0.19737095
0.32439866 0.26460748 0.26908094 0.17996573 0.21439338 0.13427852 0.12068084
[209] 0.11426272 0.10551829 0.10979037 0.10232446 0.08646857 0.08374434
0.09008500 0.08190562 0.06728439 0.09066785 0.09441875 0.08968566 0.10814203
[222] 0.12214845 0.15129307 0.18732309 0.27794318 0.24255744 0.29241480
0.28582955 0.29208722 0.18138413 0.18114524 0.21256517 0.19105858 0.14518374
[235] 0.18091668 0.21500400 0.18362917 0.16231966 0.23957637 0.19177272
0.13485823 0.12462024 0.17849650 0.18674536 0.16476459 0.22542505 0.16629484
[248] 0.15472705 0.14917833 0.12752359 0.08786956 0.09528349 0.12937063
0.08510912 0.08635894 0.09104441 0.09758281 0.09530665 0.09337919 0.09154444
[261] 0.09091961 0.09735254 0.11146686 0.12012170 0.12823674 0.14855484
0.16610074 0.19231277 0.25235295 0.30743384 0.40860109 0.30687652 0.36269240
[274] 0.35289075 0.27596458 0.40939682 0.58807640 0.39311845 0.42504521
0.29973118 0.25552449 0.24979310 0.17457335 0.14266858 0.15828539 0.15577512
[287] 0.12901113 0.13049175 0.15075905 0.12560210 0.12741456 0.14356197
0.12807275 0.13501476 0.17382662 0.20485925 0.20061610 0.36577749 0.59083518
[300] 0.26568158 0.14895029 0.13255937 0.16699636 0.16360399 0.17710661
0.29736239 0.30192149 0.23891415 0.18161516 0.12310798 0.13140552 0.16980451
[313] 0.16525193 0.13723236 0.11307142 0.08995672 0.09594003 0.08190188
0.07851025 0.07186691 0.07805617 0.06593871 0.06511842 0.08233268 0.07503340
[326] 0.07272046 0.08578821 0.08557062 0.09460955 0.11392036 0.11233864
0.12742285 0.18475600 0.19836982 0.20721557 0.17058383 0.18180511 0.20464701
[339] 0.20882846 0.30407515 0.42959833 0.80640031 0.49843769 0.33035963
0.29730264 0.50581611 0.54904830 0.42899568 0.29357012 0.21848011 0.20468251
[352] 0.24557261 0.19199541 0.13644582 0.14203696 0.15572175 0.12631831
0.09942904 0.11088817 0.12976121 0.10835386 0.10592256 0.11461669 0.12152714
[365] 0.11812587 0.10974446 0.12225663 0.16610634 0.25809454 0.54732296
0.32075486 0.26455205 0.19729056 0.12742238 0.11438307 0.10266281 0.10845357
[378] 0.14547759 0.14714918 0.22362764 0.22503726 0.18703842 0.13807206
0.17326127 0.21583783 0.17724823 0.14101370 0.13195738 0.11840704 0.13432800
[391] 0.13028967 0.13869218 0.14773240 0.12946190 0.14646218 0.15863617
0.18936331 0.18035591 0.19983051 0.23218526 0.26818695 0.29640557 0.31701538
[404] 0.32491842 0.33079369 0.20065538 0.19723428 0.16094780 0.21278025
0.21543731 0.12478312 0.09914456 0.10510694 0.08540470 0.08383544 0.08252799
[417] 0.11002315 0.13951758 0.16458168 0.18654870 0.23666006 0.15802780
0.21972692 0.22203459 0.27967274 0.25376373 0.26316829 0.22941158 0.22827502
[430] 0.19867736 0.15248489 0.13536977 0.11478632 0.11166229 0.09666383

0.08866992 0.09802859 0.09964477 0.08931787 0.06957857 0.08511219 0.07440662
[443] 0.07292390 0.07750703 0.08090555 0.07777372 0.08272477 0.08606580
0.09352154 0.12733834 0.21325040 0.14944767 0.16080616 0.26423441 0.31808141
[456] 0.28949155 0.44125536 0.39305379 0.28111854 0.20674095 0.28121258
0.39457226 0.20536554 0.21716727 0.18895354 0.18265905 0.23544556 0.22834800
[469] 0.21355317 0.22839588 0.27958266 0.19155183 0.23468811 0.30675759
0.40091953 0.29151792 0.20592099 0.24233654 0.29295552 0.25555449 0.19613845
[482] 0.22997610 0.20244082 0.35906365 0.34247137 0.55802620 0.61298820
1.13787906 0.87237779 0.80621514 0.95654590 0.95836095 0.93924346 0.71240964
[495] 0.54782050 0.45111833 0.32486237 0.26280204 0.17885116 0.19096316
0.16001801 0.16559970 0.14777783 0.14378722 0.13967214 0.15481542 0.17640109
[508] 0.18348883 0.16621678 0.18495897 0.17578340 0.21756966 0.23080319
0.26981386 0.31154071 0.37123286 0.43598340 0.46753490 0.58235633 0.66747298
[521] 0.54254573 0.66754188 0.70365451 1.00384640 1.18974996 1.15455832
0.97975890 0.80696013 0.61558537 0.75336744 0.77368077 0.62123141 0.54413915
[534] 0.43569177 0.38229920 0.26554561 0.35685022 0.37366555 0.35074987
0.28979756 0.27618338 0.29545886 0.31597499 0.31374969 0.25008222 0.33083577
[547] 0.48508535 0.81996786 0.87094736 0.62121060 0.60427388 0.38819132
0.32987810 0.27010902 0.27739214 0.19953336 0.23354734 0.19330807 0.30111737
[560] 0.41352681 0.33607335 0.36662921 0.20178070 0.19558583 0.19453503
0.16979780 0.20862261 0.21583516 0.23319986 0.22612292 0.25599177 0.27453321
[573] 0.39657117 0.45669339 0.63252770 0.79228024 1.07591810 1.11197766
0.85950822 0.97153290 0.87053775 0.66086461 0.86967335 0.78452476 0.84822753

modes40.1_Q9ZSP9 MASSES

[1] 138.1469 114.1030 163.1730 57.0510 128.1060 71.0780 113.1580 87.0770
129.1800 87.0770 147.1740 113.1580 147.1740 163.1730 128.1060 71.0780
[17] 117.1260 157.1940 87.0770 57.0510 163.1730 113.1580 97.1150 157.1940
114.1030 117.1260 157.1940 99.1310 117.1260 186.2100 157.1940 57.0510
[33] 114.1030 87.0770 57.0510 113.1580 114.1030 114.0790 57.0510 129.1800
71.0780 87.0770 57.0510 113.1580 114.0790 113.1580 99.1310 57.0510
[49] 57.0510 163.1730 163.1730 114.0790 71.0780 57.0510 114.0790 114.1030
99.1310 129.1800 147.1740 57.0510 113.1580 97.1150 131.1960 71.0780
[65] 147.1740 101.1040 99.1310 101.1040 131.1960 113.1580 87.0770 186.2100
87.0770 113.1580 113.1580 128.1060 163.1730 57.0510 157.1940 117.1260
[81] 131.1960 71.0780 71.0780 87.0770 57.0510 128.1060 113.1580 87.0770
137.1390 71.0780 131.1960 114.0790 71.0780 99.1310 129.1800 186.2100
[97] 57.0510 101.1040 114.0790 163.1730 113.1580 113.1580 129.1800 71.0780
137.1390 97.1150 128.1060 97.1150 163.1730 99.1310 113.1580 163.1730
[113] 57.0510 128.1060 99.1310 57.0510 114.0790 57.0510 114.1030 101.1040
114.0790 137.1390 163.1730 103.1430 186.2100 117.1260 157.1940 97.1150
[129] 128.1060 114.0790 131.1960 101.1040 101.1040 87.0770 157.1940 71.0780
71.0780 163.1730 157.1940 113.1580 114.0790 97.1150 114.1030 137.1390
[145] 97.1150 57.0510 87.0770 114.0790 113.1580 71.0780 57.0510 128.1060
101.1040 71.0780 71.0780 71.0780 131.1960 71.0780 71.0780 71.0780
[161] 87.0770 113.1580 99.1310 147.1740 157.1940 157.1940 163.1730 114.1030
97.1150 57.0510 163.1730 87.0770 114.1030 128.1060 113.1580 113.1580
[177] 114.1030 137.1390 71.0780 137.1390 117.1260 113.1580 147.1740 128.1060
147.1740 71.0780 114.0790 129.1800 163.1730 157.1940 57.0510 129.1800
[193] 163.1730 114.0790 87.0770 87.0770 113.1580 101.1040 99.1310 71.0780
117.1260 129.1800 163.1730 163.1730 157.1940 87.0770 99.1310 87.0770

```
[209]  57.0510 163.1730  71.0780 114.0790 128.1060 113.1580 113.1580 186.2100
57.0510  71.0780  71.0780 186.2100 113.1580 163.1730 129.1800  71.0780
[225]  87.0770 114.1030 114.1030 117.1260 147.1740 163.1730 113.1580 114.1030
163.1730 113.1580  57.0510 157.1940 114.1030  57.0510 114.0790  71.0780
[241] 113.1580  57.0510  57.0510 101.1040  57.0510 186.2100  87.0770 131.1960
101.1040 128.1060 147.1740  57.0510 186.2100 114.0790  99.1310 129.1800
[257] 163.1730  71.0780  57.0510  99.1310 117.1260 101.1040 113.1580  99.1310
71.0780 117.1260 147.1740 113.1580 131.1960  87.0770  57.0510 129.1800
[273]  71.0780  57.0510 137.1390 114.1030  71.0780  97.1150  99.1310 147.1740
128.1060 129.1800 163.1730 117.1260 117.1260 129.1800  71.0780 128.1060
[289] 114.1030 147.1740 131.1960 103.1430  87.0770 131.1960 113.1580  57.0510
129.1800  57.0510 114.1030 157.1940 114.1030 101.1040 117.1260 129.1800
[305] 101.1040  97.1150  57.0510  57.0510 113.1580 113.1580 163.1730 157.1940
117.1260 157.1940 186.2100 114.1030 114.1030 131.1960 117.1260 147.1740
[321]  99.1310 101.1040  87.0770  71.0780  71.0780 147.1740 113.1580  71.0780
101.1040 101.1040 163.1730  87.0770 114.0790 163.1730 113.1580  71.0780
[337]  87.0770  71.0780  57.0510 129.1800 163.1730 113.1580 129.1800 103.1430
87.0770  87.0770  57.0510 147.1740  99.1310  87.0770  97.1150 114.1030
[353] 128.1060 113.1580 113.1580  87.0770 147.1740  71.0780 129.1800  87.0770
117.1260  99.1310 114.0790 163.1730 113.1580 113.1580  57.0510 114.0790
[369] 114.1030  97.1150 157.1940  71.0780 101.1040  87.0770 163.1730 131.1960
99.1310  57.0510 163.1730  57.0510 114.1030 114.1030 163.1730  97.1150
[385] 157.1940 117.1260  99.1310 137.1390 137.1390 157.1940  71.0780  87.0770
157.1940  57.0510  57.0510 163.1730  71.0780 101.1040 186.2100 163.1730
[401]  87.0770 157.1940 129.1800  71.0780  87.0770 114.0790  97.1150 114.1030
113.1580 113.1580 101.1040  57.0510  71.0780 113.1580  99.1310  57.0510
[417]  57.0510  97.1150 114.0790  71.0780 163.1730 114.0790 114.1030 147.1740
71.0780 114.0790 117.1260 157.1940 114.0790 114.1030 163.1730 128.1060
[433] 117.1260 101.1040 128.1060  97.1150  71.0780 101.1040 163.1730 114.1030
114.1030  71.0780  97.1150 113.1580 113.1580  57.0510  99.1310 113.1580
[449]  71.0780 157.1940 113.1580 137.1390  71.0780  57.0510 137.1390  87.0770
57.0510 163.1730 114.1030 117.1260 113.1580 113.1580  97.1150  99.1310
[465]  99.1310  97.1150 114.0790  97.1150 129.1800  97.1150 101.1040  97.1150
129.1800  97.1150  71.0780  97.1150 157.1940 101.1040 129.1800  99.1310
[481] 101.1040  97.1150  71.0780  97.1150 157.1940  97.1150 157.1940  99.1310
113.1580  97.1150  99.1310  97.1150  71.0780 114.1030  71.0780 137.1390
[497]  99.1310 101.1040 113.1580 117.1260 117.1260 157.1940  71.0780 101.1040
87.0770  87.0770 186.2100  71.0780 113.1580 114.1030  57.0510 129.1800
[513] 101.1040 163.1730 163.1730 157.1940 163.1730  87.0770  71.0780  99.1310
99.1310 101.1040 114.1030 129.1800  87.0770  57.0510 129.1800 101.1040
[529]  99.1310 129.1800 114.1030 113.1580 129.1800 113.1580  87.0770 113.1580
99.1310 129.1800 113.1580 163.1730  57.0510  97.1150 113.1580 186.2100
[545]  57.0510 113.1580 101.1040 129.1800 163.1730  57.0510 114.1030  87.0770
147.1740 113.1580 147.1740  97.1150  71.0780 186.2100 113.1580 114.1030
[561]  87.0770 113.1580  97.1150  71.0780  57.0510 129.1800  87.0770 113.1580
128.1060 147.1740  99.1310 163.1730 113.1580 137.1390 101.1040  71.0780
[577]  87.0770  97.1150  71.0780 113.1580  99.1310  87.0770  99.1310  87.0770
104.0843
```
