# Supplementary material for: Insights into the mechanism(s) of digestion of crystalline cellulose by plant class C GH9 endoglucanases
Source: J Mol Model. 2019 Jul 23;25(8):240. doi: 10.1007/s00894-019-4133-1 (PMC7385011; doi:10.1007/s00894-019-4133-1)
Supplement: Supplementary file 11 — (PDF 129 kb) [file 894_2019_4133_MOESM11_ESM.pdf]

Supplementary Text 9

Q5NAT0_40.1ns (DCCM)

1 2 3 4 5
6 7 8 9 10 11
1.0000000000 0.6329574727 0.5487618611 0.5034660769 0.4982403161
0.4660173095 0.4174232619 0.3910167831 0.3888448086 0.3427715227
0.2654670674
12 13 14 15 16
17 18 19 20 21 22
0.2848478804 0.2583188158 0.1901067227 0.1692766791 0.1918914960
0.1379938618 0.0861545053 0.0421602413 0.0302260522 0.0583042947
0.0860376069
23 24 25 26 27
28 29 30 31 32 33
0.0708933861 0.0634811032 0.1012226187 0.1422060437 0.1858663745
0.2001954136 0.2061858446 0.1713842060 0.1369330807 0.1159130968
0.0821405269
34 35 36 37 38
39 40 41 42 43 44
0.0721436341 0.0403727890 -0.0044060569 -0.0252891255 -0.0398998052
-0.0898019199 -0.0859473839 -0.0943720391 -0.1276536912 -0.1192855976
-0.1293641073
45 46 47 48 49
50 51 52 53 54 55
-0.0986950113 -0.0907779985 -0.0375210347 -0.0238687495 -0.0156981231
0.0433018119 0.0556066513 0.0612227180 0.0820901813 0.1178339077
0.0839497620
56 57 58 59 60
61 62 63 64 65 66
0.0477047161 0.0016561867 -0.0250345960 -0.0652476962 -0.1191080873
-0.1351611153 -0.0449927530 -0.0474583777 -0.1231882577 -0.0697504621
0.0065356722
67 68 69 70 71
72 73 74 75 76 77
-0.0573620859 -0.0878844549 0.0155867421 0.0343491299 -0.0443748395
-0.0171270963 0.0715250005 0.0297316530 -0.0023182185 0.0389489205
0.1310750444
78 79 80 81 82
83 84 85 86 87 88
0.1391592846 0.1923047675 0.2872027943 0.2466928796 0.2056292888
0.2674699444 0.3109124700 0.2504470819 0.2230476613 0.1536830689
0.1242876453
89 90 91 92 93
94 95 96 97 98 99
0.1391678639 0.0984770326 0.0371273250 0.0382861904 0.0503530052
-0.0223284134 -0.0439739776 -0.0204812253 -0.0606014843 -0.1170949883
-0.0976797582
100 101 102 103 104
105 106 107 108 109 110
-0.1007226571 -0.1644890361 -0.1669826013 -0.1529185778 -0.1804734287
-0.2245998196 -0.1775985947 -0.2322883698 -0.2424611294 -0.2928858523
-0.2726210529
111 112 113 114 115
116 117 118 119 120 121
-0.2379306316 -0.2016620875 -0.1533703862 -0.1206460262 -0.0791618878

-0.0429608132 -0.0438828408 -0.0188109715 0.0184124899 0.0153720737
0.0293836269
122 123 124 125 126
127 128 129 130 131 132
0.0472008959 0.0667915685 0.0797875772 0.1064539429 0.1052504590
0.1201735248 0.0944608636 0.1029718330 0.0882785539 0.0483133869
0.0304089839
133 134 135 136 137
138 139 140 141 142 143
0.0092659014 -0.0417045315 -0.0606154975 -0.1100902057 -0.1346941014
-0.1634368670 -0.2171592928 -0.2333675749 -0.2849681936 -0.3296363521
-0.2666537967
144 145 146 147 148
149 150 151 152 153 154
-0.2802941654 -0.3277646549 -0.3281554862 -0.3347922302 -0.2626511698
-0.2817899118 -0.3406440935 -0.3427275512 -0.2581478284 -0.2603631213
-0.3204499692
155 156 157 158 159
160 161 162 163 164 165
-0.2865063991 -0.2148023151 -0.2442728128 -0.2650342081 -0.1711525360
-0.1481504267 -0.2059645139 -0.1654970302 -0.0974507764 -0.1369050959
-0.1524018493
166 167 168 169 170
171 172 173 174 175 176
-0.0878814593 -0.0725904729 -0.1101739066 -0.1718001099 -0.1547934725
-0.1378811903 -0.1960984515 -0.2412999122 -0.2414313545 -0.2433079100
-0.2821427185
177 178 179 180 181
182 183 184 185 186 187
-0.3166705294 -0.3269039645 -0.3328047796 -0.3486063777 -0.3674892940
-0.3627054506 -0.3763350947 -0.3794280849 -0.3804664788 -0.3797332087
-0.3627609882
188 189 190 191 192
193 194 195 196 197 198
-0.3429655826 -0.3531093558 -0.3518263693 -0.3129073153 -0.2797271139
-0.2072805235 -0.2286886816 -0.2463056225 -0.2025828331 -0.1625104576
-0.1565656669
199 200 201 202 203
204 205 206 207 208 209
-0.1638099927 -0.1423320332 -0.1020504119 -0.0855134921 -0.0744673386
-0.0902574377 -0.0738109340 -0.0766479442 -0.1238177689 -0.1784925605
-0.2074592608
210 211 212 213 214
215 216 217 218 219 220
-0.2780676696 -0.3195817230 -0.3106708907 -0.2833502078 -0.3509459614
-0.3240791875 -0.2844828436 -0.3464652919 -0.3437477065 -0.2773235000
-0.2640823789
221 222 223 224 225
226 227 228 229 230 231
-0.3056515031 -0.2746704913 -0.2014540208 -0.2220601025 -0.2326322669
-0.2608418643 -0.2679249258 -0.3087910038 -0.3355177817 -0.3186834111
-0.3382401497
232 233 234 235 236
237 238 239 240 241 242

-0.3389090325 -0.3294709823 -0.2980729187 -0.2931538398 -0.2925353892
-0.3051805203 -0.2657448907 -0.2192069265 -0.2312663046 -0.1855439330
-0.1915210281
243 244 245 246 247
248 249 250 251 252 253
-0.1522895764 -0.1556154172 -0.1497319767 -0.1356550001 -0.0786931777
-0.0444726570 -0.0267527844 0.0268301788 -0.0323227066 -0.0248073067
-0.0887845296
254 255 256 257 258
259 260 261 262 263 264
-0.0961504760 -0.1444986147 -0.1761553287 -0.2036775262 -0.1343302093
-0.1269398048 -0.2084030373 -0.1876063591 -0.1188956469 -0.1586740352
-0.2100111052
265 266 267 268 269
270 271 272 273 274 275
-0.1507775463 -0.1339613663 -0.1919755978 -0.1831730142 -0.1414337874
-0.1490340821 -0.1867922540 -0.2267167654 -0.2211725704 -0.2622351235
-0.2443012207
276 277 278 279 280
281 282 283 284 285 286
-0.2214826251 -0.2456660995 -0.2398733021 -0.2577563697 -0.2390929938
-0.2008624918 -0.2147458592 -0.2127804857 -0.1659497335 -0.1554089867
-0.1543464227
287 288 289 290 291
292 293 294 295 296 297
-0.1162959154 -0.0739437750 -0.0840225634 -0.0456008124 0.0114944419
0.0008765822 0.0032618303 0.0694339601 0.0739273432 0.0332334994
-0.0134003203
298 299 300 301 302
303 304 305 306 307 308
-0.0726823196 -0.1007486681 -0.0939163059 -0.0461810365 0.0060731953
0.0345110074 0.0762852314 0.1599092630 0.2150748974 0.2070250942
0.2524542699
309 310 311 312 313
314 315 316 317 318 319
0.2394879594 0.2420919383 0.1776631520 0.1279445759 0.0649260898
0.0710302664 0.1453628353 0.1280708628 0.1769718561 0.1966180278
0.2734024383
320 321 322 323 324
325 326 327 328 329 330
0.1905546335 0.1337442005 0.2209136477 0.2518215201 0.1272916654
0.1112645227 0.2041929721 0.1854140626 0.0767434100 0.1137684540
0.1712043199
331 332 333 334 335
336 337 338 339 340 341
0.0889848383 0.0364038965 0.0937540806 0.0754614273 0.0171874527
-0.0225873620 -0.0662620795 -0.0245019963 0.0059737049 0.0359882126
-0.0206710767
342 343 344 345 346
347 348 349 350 351 352
-0.0245399506 -0.0575105971 -0.0784637232 -0.0579155807 -0.0056271608
0.0525649610 0.0593295223 0.0799084094 0.1025692198 0.0929809783
0.0879487337
353 354 355 356 357

```
358           359           360           361           362           363
 0.1109399449  0.1646377339  0.1691921497  0.1621847137  0.2197961301
0.2644336672  0.2586170465  0.2892993157  0.3702618051  0.3973620484
0.3675564616
          364           365           366           367           368
369           370           371           372           373           374
 0.4594880713  0.5584789962  0.4625872368  0.4463832102  0.5834528282
0.5806619502  0.6022651105  0.4993424169  0.4461901935  0.3790837304
0.3551645184
          375           376           377           378           379
380           381           382           383           384           385
 0.4581096251  0.5128886308  0.4232815991  0.4091591148  0.3755112580
0.3628408384  0.4102160472  0.4114635697  0.3554112543  0.3303039187
0.3732906052
          386           387           388           389           390
391           392           393           394           395           396
 0.3271235186  0.3003851282  0.2864439177  0.2753357958  0.2203023391
0.1807797273  0.1527434424  0.1634337620  0.2153565862  0.2071945578
0.1751820789
          397           398           399           400           401
402           403           404           405           406           407
 0.1521061245  0.1246640313  0.1122912211  0.0937380393  0.1196048352
0.1502884484  0.1796075046  0.1712752361  0.1926993437  0.2236861292
0.2588691680
          408           409           410           411           412
413           414           415           416           417           418
 0.2477665183  0.2185315058  0.2203105013  0.2481591412  0.2563106612
0.3181156065  0.3277209599  0.3026131830  0.3400736218  0.3208825195
0.2739020477
          419           420           421           422           423
424           425           426           427           428           429
 0.3126220880  0.2945190023  0.2135101770  0.1650175826  0.2168567348
0.2754380868  0.3158796585  0.3178978610  0.3214673802  0.2984937786
0.2571528974
          430           431           432           433           434
435           436           437           438           439           440
 0.2165445994  0.1752380556  0.1766393272  0.1686018590  0.1646219588
0.2543056332  0.2733758680  0.2270606363  0.2504696180  0.1729201359
0.1738864495
          441           442           443           444           445
446           447           448           449           450           451
 0.1501322699  0.2528516913  0.2739915340  0.1729230814  0.1925851969
0.3008465608  0.2963220686  0.2515775861  0.3030333672  0.3904867784
0.3042671692
          452           453           454           455           456
457           458           459           460           461           462
 0.2839156830  0.4137788376  0.4639163536  0.3780422189  0.3328971967
0.3783477207  0.4199323458  0.5035578423  0.4662190741  0.4149744773
0.3786138140
          463           464           465           466           467
468           469           470           471           472           473
 0.3797601114  0.3029700528  0.3069623634  0.2733587561  0.2725444458
0.2804561833  0.2490631701  0.2424189088  0.2119031610  0.2151558765
0.1767403703
```

```
          474           475           476           477           478
479         480         481         482         483         484
 0.1364417661  0.1202762661  0.1035550744  0.0541819087  0.0075027533
-0.0106809976 -0.0663416064 -0.0362563839 -0.0861659367 -0.1105122051
-0.0499239458
          485           486           487           488           489
490         491         492         493         494         495
-0.1489382326 -0.1426036249 -0.2048682918 -0.2504635338 -0.2772157514
-0.2296491723 -0.0818045808 -0.1107585889  0.0221547738 -0.0288039886
0.0541908604
          496           497           498           499           500
501         502         503         504         505         506
 0.0721141390  0.0900868825  0.1057354724  0.1156701186  0.1206372622
0.1426074604  0.1384406426  0.1146996567  0.1010833120  0.0971202428
0.0906259595
          507           508           509           510           511
512         513         514         515         516         517
 0.0664430870  0.0222449587  0.0392431199 -0.1159869544 -0.1808489925
-0.2954090948 -0.3401883945 -0.3610031299 -0.3538298375 -0.2783841019
-0.2849419583
          518           519           520           521           522
523         524         525         526         527         528
-0.2502338562 -0.1654351417 -0.1664715005 -0.1738126819 -0.1106494529
-0.0500615565 -0.0781016089 -0.0206273257 -0.0125594328 -0.0069371962
0.1154733157
          529           530           531           532           533
534         535         536         537         538         539
 0.1159078363  0.0752803640  0.1116238033  0.0221671993  0.0341784434
0.1163018312  0.1517072078  0.1672120146  0.0571927312  0.1771054752
0.1146708827
          540           541           542           543           544
545         546         547         548         549         550
 0.0363417246  0.0829383602 -0.0188021525 -0.1332410026 -0.2103245178
-0.1783745655 -0.2440954818 -0.3029450060 -0.3004659060 -0.3009262267
-0.2073828180
          551           552           553           554           555
556         557         558         559         560         561
-0.2299325874 -0.0854479100  0.0549033382  0.0162237024  0.1762774654
0.1004254300  0.1419352329  0.1212391096  0.1127250155  0.1096963795
0.1109239927
          562           563           564           565           566
567         568
 0.1062661633  0.1190881896  0.1087333223  0.0969228878  0.0792567961
0.0757942502  0.1001905612
```

Q5NAT0_40.1ns (MATRIX)

```
          1            2            3            4            5            6
       7            8            9           10           11           12
 0.632957473  1.000000000  0.780791303  0.681673522  0.675510072  0.621155311
0.555590245  0.519326855  0.508837183  0.454248597  0.370478297  0.391840364
         13           14           15           16           17           18
      19           20           21           22           23           24
```

0.351565519 0.279935346 0.258712976 0.277253982 0.210776570 0.156529699
0.107157946 0.091278814 0.123281973 0.150038603 0.134542982 0.123313754
25 26 27 28 29 30
31 32 33 34 35 36
0.166595448 0.216149747 0.263832475 0.282453852 0.282252008 0.234916563
0.203292727 0.180663657 0.146383775 0.135083070 0.097281661 0.049321459
37 38 39 40 41 42
43 44 45 46 47 48
0.021650227 0.002297939 -0.053843984 -0.049147698 -0.064674922 -0.102226284
-0.087010132 -0.097511209 -0.058316302 -0.049894872 0.011126795 0.032376361
49 50 51 52 53 54
55 56 57 58 59 60
0.035389059 0.101223336 0.102024391 0.098969777 0.114758084 0.150885407
0.118819797 0.087050984 0.031847894 0.006915792 -0.047844030 -0.106336804
61 62 63 64 65 66
67 68 69 70 71 72
-0.133259428 -0.032499106 -0.019436068 -0.104475106 -0.058171513 0.036151609
-0.025173937 -0.070707395 0.040089690 0.074933277 -0.014907316 0.006486512
73 74 75 76 77 78
79 80 81 82 83 84
0.114680803 0.073854124 0.026270161 0.069719580 0.178113108 0.198726223
0.259751301 0.376485043 0.332417532 0.282221622 0.353624599 0.410969572
85 86 87 88 89 90
91 92 93 94 95 96
0.339354962 0.308317423 0.223988897 0.191332274 0.211170742 0.161573155
0.097879948 0.101363017 0.110820507 0.030799064 0.014331864 0.038581475
97 98 99 100 101 102
103 104 105 106 107 108
-0.011005326 -0.073394979 -0.048603646 -0.057108032 -0.132732512 -0.130564909
-0.114894397 -0.150974154 -0.203515325 -0.159538040 -0.221626987 -0.238040027
109 110 111 112 113 114
115 116 117 118 119 120
-0.294956528 -0.269378328 -0.226963843 -0.183934589 -0.135041967 -0.102950414
-0.053507300 -0.018449935 -0.018508956 0.002170976 0.045241796 0.047980719
121 122 123 124 125 126
127 128 129 130 131 132
0.065222177 0.078593126 0.098316033 0.119581266 0.149086487 0.153205439
0.170430461 0.146054228 0.160069469 0.144734298 0.100204793 0.079386591
133 134 135 136 137 138
139 140 141 142 143 144
0.049787880 -0.006298146 -0.029350969 -0.086928280 -0.113402332 -0.154268344
-0.213880719 -0.239846598 -0.295905821 -0.351599703 -0.286829904 -0.306370102
145 146 147 148 149 150
151 152 153 154 155 156
-0.357254064 -0.368099302 -0.384838154 -0.301864818 -0.307755326 -0.367512413
-0.374659542 -0.274206293 -0.262737164 -0.327542239 -0.293777108 -0.200439305
157 158 159 160 161 162
163 164 165 166 167 168
-0.227269809 -0.253645380 -0.150315513 -0.115185734 -0.179501368 -0.142258100
-0.061164110 -0.099254015 -0.125729823 -0.059875844 -0.038571668 -0.076101819
169 170 171 172 173 174
175 176 177 178 179 180
-0.143089896 -0.126362620 -0.106333231 -0.167721468 -0.223096534 -0.225972906
-0.227823293 -0.273507599 -0.316017574 -0.327226021 -0.337884589 -0.364063973

```
        181         182         183         184         185         186 
        187         188         189         190         191         192 
-0.384993576 -0.381915582 -0.406056847 -0.412735697 -0.414098239 -0.419741448 
-0.408652948 -0.385738724 -0.399646533 -0.405893295 -0.363558070 -0.329277273 
        193         194         195         196         197         198 
        199         200         201         202         203         204 
-0.249644667 -0.266817226 -0.280625415 -0.226300430 -0.177492181 -0.180253741 
-0.194738809 -0.164852997 -0.114067872 -0.101141940 -0.088233044 -0.115814848 
        205         206         207         208         209         210 
        211         212         213         214         215         216 
-0.107053426 -0.119374016 -0.172708521 -0.230258681 -0.261102068 -0.337780799 
-0.378103463 -0.368722863 -0.338121640 -0.400661853 -0.371032377 -0.322325299 
        217         218         219         220         221         222 
        223         224         225         226         227         228 
-0.375490149 -0.378811280 -0.308727810 -0.278430721 -0.319677218 -0.294290927 
-0.208443960 -0.219059230 -0.235386120 -0.267056730 -0.280494088 -0.333681968 
        229         230         231         232         233         234 
        235         236         237         238         239         240 
-0.365922955 -0.346870259 -0.374695938 -0.381148047 -0.376400140 -0.344643246 
-0.337777285 -0.340870387 -0.356121705 -0.315719881 -0.267931228 -0.286256914 
        241         242         243         244         245         246 
        247         248         249         250         251         252 
-0.239387435 -0.247778939 -0.206128790 -0.218130866 -0.213470830 -0.200170071 
-0.140321490 -0.110739827 -0.083167023 -0.015911113 -0.072323254 -0.052413795 
        253         254         255         256         257         258 
        259         260         261         262         263         264 
-0.123002812 -0.147527981 -0.197552556 -0.220763874 -0.256653483 -0.184145459 
-0.162271691 -0.251095715 -0.238176543 -0.156065599 -0.187918414 -0.249835960 
        265         266         267         268         269         270 
        271         272         273         274         275         276 
-0.187927307 -0.157958307 -0.219747965 -0.216230055 -0.164260523 -0.164910137 
-0.205800613 -0.243933468 -0.234211663 -0.284018344 -0.270565257 -0.252792778 
        277         278         279         280         281         282 
        283         284         285         286         287         288 
-0.285827802 -0.286743061 -0.310186334 -0.286884859 -0.249475969 -0.270107953 
-0.268060651 -0.215609478 -0.210908835 -0.213276195 -0.167515372 -0.126246925 
        289         290         291         292         293         294 
        295         296         297         298         299         300 
-0.144174196 -0.099633790 -0.035492037 -0.054239073 -0.057006483  0.025558350 
0.030663772 -0.017278552 -0.075885790 -0.137255519 -0.162451270 -0.155765292 
        301         302         303         304         305         306 
        307         308         309         310         311         312 
-0.111001803 -0.056219102 -0.021033824  0.020104285  0.114084857  0.173927964 
0.172366543  0.223732521  0.206222281  0.212645423  0.139783181  0.088893269 
        313         314         315         316         317         318 
        319         320         321         322         323         324 
 0.029115052  0.039172115  0.118369584  0.114810428  0.171707454  0.175722166 
0.265344906  0.191855068  0.112598593  0.199687606  0.254525415  0.124249488 
        325         326         327         328         329         330 
        331         332         333         334         335         336 
 0.086870997  0.193042722  0.206731242  0.072055286  0.093754466  0.171434407 
0.092317060  0.019458743  0.078554318  0.069833575  0.010185424 -0.034169886 
        337         338         339         340         341         342 
        343         344         345         346         347         348 
```

-0.090301325 -0.049791320 -0.028055620 0.009179381 -0.052103531 -0.062752691
-0.096877024 -0.125559517 -0.110738941 -0.053827211 0.010959756 0.020836450
349 350 351 352 353 354
355 356 357 358 359 360
0.048608922 0.077901760 0.066534748 0.053445845 0.087084061 0.147062569
0.140840061 0.130957881 0.208056066 0.250717532 0.228874439 0.268597319
361 362 363 364 365 366
367 368 369 370 371 372
0.364159589 0.373051764 0.337324626 0.424243119 0.514954658 0.440554218
0.417334734 0.523143003 0.537705826 0.560156069 0.481397925 0.430770398
373 374 375 376 377 378
379 380 381 382 383 384
0.363080462 0.349070921 0.448263549 0.492460387 0.426423496 0.444069376
0.429600456 0.429121981 0.466670994 0.444132322 0.385526540 0.351083960
385 386 387 388 389 390
391 392 393 394 395 396
0.398464988 0.353672413 0.328484847 0.311561225 0.302532407 0.249498345
0.218721681 0.195571068 0.212010102 0.263726233 0.247943734 0.215573213
397 398 399 400 401 402
403 404 405 406 407 408
0.187712979 0.162967996 0.144918542 0.120192850 0.138191542 0.167184040
0.201094483 0.200590247 0.224228693 0.250605748 0.291192103 0.289300565
409 410 411 412 413 414
415 416 417 418 419 420
0.265039607 0.276337060 0.303434390 0.320741410 0.394271103 0.418189637
0.363142301 0.376487019 0.342758494 0.280725342 0.308931166 0.275522599
421 422 423 424 425 426
427 428 429 430 431 432
0.185473122 0.135638631 0.193523364 0.246233963 0.294003346 0.305690171
0.317916412 0.308964917 0.267351920 0.231324306 0.181511015 0.178216500
433 434 435 436 437 438
439 440 441 442 443 444
0.173398353 0.159033190 0.249246675 0.285578874 0.247461423 0.285943300
0.209337848 0.222851162 0.181952428 0.285985280 0.332035772 0.224812511
445 446 447 448 449 450
451 452 453 454 455 456
0.238408462 0.354596131 0.384490219 0.319540613 0.361961433 0.499568525
0.387801714 0.352164328 0.533344421 0.616707845 0.495225876 0.424817864
457 458 459 460 461 462
463 464 465 466 467 468
0.497971342 0.583581848 0.715364133 0.636932065 0.549594598 0.494915068
0.501723589 0.402233661 0.404433977 0.367132593 0.356416912 0.354928645
469 470 471 472 473 474
475 476 477 478 479 480
0.316851447 0.301704736 0.270693565 0.268680938 0.226697770 0.184717587
0.171539492 0.152396256 0.096897321 0.049731128 0.038553283 -0.023202325
481 482 483 484 485 486
487 488 489 490 491 492
0.018280625 -0.045295552 -0.076496224 -0.013508792 -0.134546094 -0.141199463
-0.223258624 -0.283508379 -0.297979797 -0.232132355 -0.072076777 -0.096803390
493 494 495 496 497 498
499 500 501 502 503 504
0.070016007 0.015730741 0.103661879 0.117094786 0.140998573 0.156754824
0.168098982 0.172854010 0.194853349 0.192834143 0.168978458 0.149753737

505 506 507 508 509 510
511 512 513 514 515 516
0.144122369 0.127581934 0.094896835 0.027822344 0.021532486 -0.161342080
-0.251346315 -0.364889895 -0.419054485 -0.428536765 -0.423748094 -0.346706150
517 518 519 520 521 522
523 524 525 526 527 528
-0.356764683 -0.309141478 -0.221563878 -0.233922353 -0.236559952 -0.161621334
-0.099712732 -0.139429115 -0.074133433 -0.071223878 -0.061197561 0.074937582
529 530 531 532 533 534
535 536 537 538 539 540
0.079630886 0.043472631 0.089679777 0.003149579 0.026167712 0.135269075
0.188659666 0.188411900 0.043331622 0.160404081 0.075417906 -0.023873558
541 542 543 544 545 546
547 548 549 550 551 552
0.028712685 -0.088028521 -0.215172303 -0.297289118 -0.260724868 -0.325483334
-0.387257094 -0.384647887 -0.387405386 -0.292915470 -0.311897426 -0.151695333
553 554 555 556 557 558
559 560 561 562 563 564
0.006910397 -0.014072071 0.180755411 0.128586528 0.179990866 0.161407066
0.162602984 0.155460016 0.158936184 0.157074627 0.169184452 0.155502649
565 566 567 568
0.140709036 0.116334649 0.115547287 0.145995382

Q8LJP6_40.1ns (DCCM)

1 2 3 4 5
6 7 8 9 10 11
1.000000e+00 3.756877e-01 3.051341e-01 2.813176e-01 2.839402e-01
2.463647e-01 2.008131e-01 1.953260e-01 1.850028e-01 1.407454e-01
1.142384e-01
12 13 14 15 16
17 18 19 20 21 22
1.296846e-01 1.158644e-01 9.283592e-02 8.634024e-02 8.469080e-02
6.547516e-02 5.461470e-02 4.748325e-02 4.573840e-02 3.489448e-02
3.135658e-02
23 24 25 26 27
28 29 30 31 32 33
1.995156e-02 2.545453e-02 3.755150e-02 5.678868e-02 6.162320e-02
6.990018e-02 5.372849e-02 4.652209e-02 3.700942e-02 3.842223e-02
3.763453e-02
34 35 36 37 38
39 40 41 42 43 44
4.267094e-02 4.339204e-02 3.701674e-02 3.119059e-02 3.337605e-02
2.584997e-02 2.256396e-02 2.122571e-02 1.687683e-02 1.022014e-02
1.124243e-02
45 46 47 48 49
50 51 52 53 54 55
1.897250e-02 2.219259e-02 2.943837e-02 3.196596e-02 3.090297e-02
4.795140e-02 5.286128e-02 5.240120e-02 6.579153e-02 6.497299e-02
5.082470e-02
56 57 58 59 60
61 62 63 64 65 66
4.646677e-02 3.979756e-02 3.953575e-02 2.439309e-02 1.401875e-02
5.809453e-03 1.710016e-02 2.395691e-02 4.132287e-03 2.214499e-03

2.305351e-02
67 68 69 70 71
72 73 74 75 76 77
1.105043e-02 -1.005194e-02 6.920353e-03 2.564262e-02 1.864894e-03
-6.853321e-03 2.668691e-02 2.849843e-02 -2.201454e-03 5.157428e-03
4.500466e-02
78 79 80 81 82
83 84 85 86 87 88
3.961389e-02 2.655847e-02 6.381039e-02 9.064321e-02 9.270224e-02
1.371886e-01 1.441667e-01 1.143287e-01 9.219117e-02 7.619052e-02
6.375467e-02
89 90 91 92 93
94 95 96 97 98 99
7.147588e-02 6.263187e-02 4.252797e-02 4.272043e-02 4.444989e-02
2.285215e-02 2.007438e-02 2.979132e-02 1.891204e-02 4.197268e-03
1.420420e-02
100 101 102 103 104
105 106 107 108 109 110
1.379039e-02 -2.196925e-03 -5.363446e-03 5.995367e-03 2.925459e-03
-9.542362e-03 -5.825331e-03 -1.960382e-02 -3.018572e-02 -3.182264e-02
-2.140301e-02
111 112 113 114 115
116 117 118 119 120 121
-1.514398e-02 4.247048e-03 1.010819e-02 2.281212e-02 3.417266e-02
3.920471e-02 4.045248e-02 3.389890e-02 3.193764e-02 4.231474e-02
4.595643e-02
122 123 124 125 126
127 128 129 130 131 132
4.410349e-02 3.838215e-02 4.445907e-02 4.328313e-02 4.002155e-02
4.197530e-02 4.517043e-02 4.516489e-02 4.015177e-02 4.465571e-02
3.650440e-02
133 134 135 136 137
138 139 140 141 142 143
4.113151e-02 3.395648e-02 3.832102e-02 3.154238e-02 2.898179e-02
1.546762e-02 3.173858e-04 -1.676036e-02 -1.896651e-02 -2.166285e-02
-1.107008e-02
144 145 146 147 148
149 150 151 152 153 154
-1.957446e-02 -3.737745e-02 -4.773214e-02 -5.485226e-02 -4.498385e-02
-2.598304e-02 -4.173912e-02 -6.143511e-02 -3.893235e-02 -2.777644e-02
-5.372692e-02
155 156 157 158 159
160 161 162 163 164 165
-5.672413e-02 -2.817184e-02 -3.288183e-02 -5.577529e-02 -3.893827e-02
-2.163386e-02 -3.955605e-02 -4.111086e-02 -2.221457e-02 -1.864589e-02
-3.432060e-02
166 167 168 169 170
171 172 173 174 175 176
-2.235811e-02 -1.231566e-02 -1.124004e-02 -1.899180e-02 -1.077853e-02
-1.346349e-02 -3.091251e-02 -3.755674e-02 -2.809461e-02 -3.255694e-02
-5.344580e-02
177 178 179 180 181
182 183 184 185 186 187
-4.700609e-02 -4.027553e-02 -5.611991e-02 -6.784480e-02 -5.897240e-02

```
-5.420346e-02 -7.243510e-02 -6.965796e-02 -5.692029e-02 -6.593589e-02
-6.704017e-02
          188           189           190           191           192
193           194           195           196           197           198
-5.620094e-02 -5.076671e-02 -4.866351e-02 -3.607898e-02 -2.543899e-02
-1.287727e-02 -8.038187e-03 -8.512831e-03  8.243597e-04  6.517083e-03
1.690148e-02
          199           200           201           202           203
204           205           206           207           208           209
 1.817608e-02  6.212519e-03  7.331635e-03  7.855464e-03  5.704904e-03
-8.744125e-03 -1.984550e-02 -3.003743e-02 -4.563782e-02 -5.356778e-02
-5.454525e-02
          210           211           212           213           214
215           216           217           218           219           220
-6.394654e-02 -7.537551e-02 -6.871920e-02 -8.205446e-02 -9.623305e-02
-8.277904e-02 -8.438893e-02 -1.042993e-01 -1.027143e-01 -8.460202e-02
-8.776239e-02
          221           222           223           224           225
226           227           228           229           230           231
-9.694247e-02 -8.666315e-02 -7.154352e-02 -7.055312e-02 -7.079865e-02
-7.757101e-02 -9.861802e-02 -9.678600e-02 -9.422675e-02 -1.011535e-01
-1.053966e-01
          232           233           234           235           236
237           238           239           240           241           242
-9.888944e-02 -1.077153e-01 -1.072529e-01 -8.494805e-02 -8.627287e-02
-8.675406e-02 -8.665736e-02 -8.800010e-02 -8.007394e-02 -7.379456e-02
-8.144044e-02
          243           244           245           246           247
248           249           250           251           252           253
-9.547709e-02 -9.647851e-02 -8.543417e-02 -7.040294e-02 -6.672841e-02
-4.203281e-02 -3.734461e-02 -3.210719e-02 -1.091263e-02 -1.124838e-02
-1.826417e-02
          254           255           256           257           258
259           260           261           262           263           264
-4.200634e-02 -5.001815e-02 -7.267475e-02 -6.829950e-02 -5.567347e-02
-7.410493e-02 -9.592393e-02 -8.839830e-02 -8.251888e-02 -1.032754e-01
-1.105648e-01
          265           266           267           268           269
270           271           272           273           274           275
-9.844241e-02 -1.006867e-01 -1.122829e-01 -1.103605e-01 -9.515240e-02
-8.957223e-02 -1.049889e-01 -9.011017e-02 -7.356246e-02 -1.029350e-01
-1.058856e-01
          276           277           278           279           280
281           282           283           284           285           286
-1.153073e-01 -1.143950e-01 -1.154671e-01 -1.258869e-01 -1.239791e-01
-1.216942e-01 -1.154434e-01 -1.090043e-01 -1.068236e-01 -9.228566e-02
-8.055656e-02
          287           288           289           290           291
292           293           294           295           296           297
-7.702766e-02 -7.003494e-02 -5.331043e-02 -3.681465e-02 -3.527791e-02
-3.151117e-02 -7.978821e-03  4.521203e-03 -1.486403e-03  5.348265e-03
-3.887882e-03
          298           299           300           301           302
303           304           305           306           307           308
```

```
-1.689639e-02 -2.570619e-02 -1.650761e-02 -1.257935e-02  1.770281e-02
5.255806e-02  7.628967e-02  7.366350e-02  8.534712e-02  9.893609e-02
8.997747e-02
          309           310           311           312           313
314           315           316           317           318           319
 6.059722e-02  3.979560e-02  1.488959e-02  3.046133e-03  2.722923e-02
3.737814e-02  6.329930e-02  7.519584e-02  1.024042e-01  6.928949e-02
4.887768e-02
          320           321           322           323           324
325           326           327           328           329           330
 7.828961e-02  8.844304e-02  5.132316e-02  3.712183e-02  5.472511e-02
3.866185e-02  1.089919e-03  5.505733e-03  1.010025e-02 -1.700443e-02
-4.098225e-02
          331           332           333           334           335
336           337           338           339           340           341
-3.944685e-02 -3.644848e-02 -6.012777e-02 -7.757469e-02 -9.671350e-02
-1.147528e-01 -1.104425e-01 -1.127237e-01 -1.190783e-01 -1.165679e-01
-1.010264e-01
          342           343           344           345           346
347           348           349           350           351           352
-8.994159e-02 -8.621468e-02 -9.952154e-02 -9.857879e-02 -9.802249e-02
-1.015961e-01 -8.935691e-02 -5.362838e-02 -3.531915e-02 -8.894308e-03
7.676063e-03
          353           354           355           356           357
358           359           360           361           362           363
 1.761005e-02  4.284853e-02  6.711510e-02  8.332762e-02  1.179977e-01
1.682075e-01  1.707747e-01  1.731626e-01  2.493586e-01  3.545127e-01
2.487148e-01
          364           365           366           367           368
369           370           371           372           373           374
 2.671918e-01  4.071857e-01  3.998022e-01  3.713057e-01  2.579027e-01
1.581557e-01  1.165084e-01  1.793823e-01  1.923587e-01  2.820303e-01
2.126232e-01
          375           376           377           378           379
380           381           382           383           384           385
 2.020764e-01  1.782686e-01  1.908962e-01  2.117517e-01  3.171810e-01
2.619272e-01  1.998549e-01  2.007018e-01  1.370574e-01  1.160459e-01
1.077089e-01
          386           387           388           389           390
391           392           393           394           395           396
 8.722911e-02  7.139656e-02  6.560370e-02  5.317671e-02  4.686183e-02
5.327301e-02  3.886067e-02  1.519265e-02 -1.635619e-02 -3.193623e-02
-2.488663e-02
          397           398           399           400           401
402           403           404           405           406           407
-8.049898e-03  1.069118e-02  2.688963e-02  3.270168e-02  2.738873e-02
3.289341e-02  4.743014e-02  7.994259e-02  6.929158e-02  4.745067e-02
7.251404e-02
          408           409           410           411           412
413           414           415           416           417           418
 8.173474e-02  1.040725e-01  1.519927e-01  1.538353e-01  1.316222e-01
1.353050e-01  1.279672e-01  1.097424e-01  1.240339e-01  9.139154e-02
6.140114e-02
          419           420           421           422           423
```

424 425 426 427 428 429
4.522289e-02 5.962538e-02 8.854426e-02 8.066295e-02 1.062364e-01
1.153831e-01 1.121741e-01 8.464467e-02 7.713196e-02 4.911230e-02
4.240698e-02
430 431 432 433 434
435 436 437 438 439 440
4.111949e-02 4.157442e-02 7.607748e-02 9.709956e-02 8.437628e-02
9.662887e-02 7.059279e-02 6.348465e-02 4.796775e-02 7.828766e-02
8.816183e-02
441 442 443 444 445
446 447 448 449 450 451
6.078087e-02 6.466614e-02 1.028608e-01 1.054520e-01 8.106619e-02
1.034918e-01 1.613376e-01 1.358761e-01 9.961699e-02 1.340770e-01
2.041424e-01
452 453 454 455 456
457 458 459 460 461 462
2.328640e-01 2.022290e-01 2.548492e-01 3.063049e-01 3.180887e-01
2.193291e-01 1.930339e-01 1.324324e-01 9.959273e-02 9.216680e-02
8.391641e-02
463 464 465 466 467
468 469 470 471 472 473
7.524850e-02 5.324734e-02 2.905143e-02 6.645036e-05 -2.307607e-02
-3.679062e-02 -8.328504e-02 -9.165274e-02 -9.165938e-02 -1.111190e-01
-1.387374e-01
474 475 476 477 478
479 480 481 482 483 484
-1.654519e-01 -1.559327e-01 -1.668429e-01 -1.784567e-01 -1.737778e-01
-1.546103e-01 -1.453910e-01 -1.578642e-01 -1.350418e-01 -1.258502e-01
-8.995162e-02
485 486 487 488 489
490 491 492 493 494 495
-9.727712e-02 -1.391350e-01 -1.327844e-01 -1.529312e-01 -1.343760e-01
-1.004068e-01 -9.639531e-02 -6.167257e-02 -8.467834e-02 -1.257075e-02
-2.550058e-02
496 497 498 499 500
501 502 503 504 505 506
-2.538191e-02 -1.678797e-02 -7.079531e-03 9.788194e-03 1.388352e-02
1.451376e-02 3.121453e-02 3.627683e-02 6.561164e-02 3.602114e-02
3.575864e-02
507 508 509 510 511
512 513 514 515 516 517
-2.924876e-05 -6.359139e-02 -1.172628e-01 -1.563019e-01 -1.722818e-01
-1.551065e-01 -1.490317e-01 -1.075285e-01 -8.676127e-02 -5.155894e-02
-1.972103e-02
518 519 520 521 522
523 524 525 526 527 528
-3.917566e-02 -4.560306e-02 -1.082023e-02 -3.046762e-02 -3.371139e-02
-7.093562e-02 -8.484247e-02 -9.437093e-02 -9.410987e-02 -1.021625e-01
-6.892711e-02
529 530 531 532 533
534 535 536 537 538 539
-3.981689e-02 -3.037709e-02 -2.167015e-02 -6.226678e-02 -5.464463e-02
-1.084990e-02 6.903506e-03 -2.615637e-02 -6.399676e-02 -5.790475e-02
-8.559072e-02

          540   541   542   543   544
545   546   547   548   549   550
-1.057671e-01 -1.347286e-01 -1.350893e-01 -9.808617e-02 -5.673727e-02
-2.346654e-02 1.277309e-02 -8.018811e-03 2.037657e-02 1.372177e-02
3.119660e-02
          551   552   553   554   555
556   557   558   559   560
 4.165989e-02 5.001075e-02 2.865809e-02 3.459682e-02 1.426707e-02
-5.906036e-03 2.814051e-03 -1.773102e-03 1.323819e-02 3.584996e-02

Q8LJP6_40.1ns (MATRIX)

            1   2   3   4   5
 6   7   8   9   10   11
 3.756877e-01 1.000000e+00 6.750240e-01 5.049690e-01 5.378412e-01
4.723403e-01 3.589065e-01 3.260511e-01 3.178642e-01 2.484844e-01
1.970019e-01
           12   13   14   15   16
17   18   19   20   21   22
 2.135084e-01 1.851675e-01 1.560327e-01 1.460189e-01 1.343538e-01
1.041519e-01 8.811994e-02 7.283652e-02 6.528366e-02 5.310920e-02
4.876003e-02
           23   24   25   26   27
28   29   30   31   32   33
 3.265918e-02 4.258812e-02 6.147619e-02 9.124125e-02 1.013295e-01
1.079879e-01 8.388582e-02 6.516361e-02 5.729681e-02 5.587254e-02
5.389585e-02
           34   35   36   37   38
39   40   41   42   43   44
 5.611806e-02 5.542197e-02 4.912262e-02 3.755160e-02 3.896819e-02
2.768018e-02 2.579500e-02 2.141806e-02 1.477219e-02 9.665382e-03
1.328579e-02
           45   46   47   48   49
50   51   52   53   54   55
 2.518415e-02 2.807565e-02 3.954018e-02 4.602975e-02 4.421463e-02
6.721463e-02 6.438703e-02 5.806309e-02 6.600290e-02 6.846874e-02
5.225698e-02
           56   57   58   59   60
61   62   63   64   65   66
 4.827316e-02 4.126806e-02 4.393847e-02 2.285760e-02 1.156434e-02
7.742309e-05 1.465107e-02 2.780880e-02 5.202967e-03 3.841601e-03
3.254963e-02
           67   68   69   70   71
72   73   74   75   76   77
 2.080810e-02 -1.794989e-03 2.320620e-02 5.126610e-02 1.920557e-02
1.893094e-02 6.758772e-02 6.395544e-02 2.226110e-02 4.190120e-02
9.868913e-02
           78   79   80   81   82
83   84   85   86   87   88
 7.662634e-02 6.056320e-02 1.174644e-01 1.473673e-01 1.152414e-01
1.936955e-01 2.193803e-01 1.580268e-01 1.341275e-01 1.078235e-01
8.825538e-02
           89   90   91   92   93

```
94             95             96             97             98             99
 1.057232e-01  9.853053e-02  6.908333e-02  6.710339e-02  7.106735e-02
4.260924e-02  3.786353e-02  4.796476e-02  3.260089e-02  1.392633e-02
2.488016e-02
          100            101            102            103            104
105            106            107            108            109            110
 2.115684e-02  9.795270e-04 -6.923136e-04  1.186947e-02  5.824283e-03
-1.010505e-02 -6.428580e-03 -2.244326e-02 -3.620098e-02 -4.125533e-02
-3.049253e-02
          111            112            113            114            115
116            117            118            119            120            121
-2.161921e-02 -5.019138e-04  7.764394e-03  2.010148e-02  3.462515e-02
3.893138e-02  3.973421e-02  3.160202e-02  2.968263e-02  4.223738e-02
4.716861e-02
          122            123            124            125            126
127            128            129            130            131            132
 4.586672e-02  3.993068e-02  4.720427e-02  4.399050e-02  4.052909e-02
4.399664e-02  4.985022e-02  5.127168e-02  4.312444e-02  4.977898e-02
3.948213e-02
          133            134            135            136            137
138            139            140            141            142            143
 4.313614e-02  3.399334e-02  3.951983e-02  2.975858e-02  2.776793e-02
7.940505e-03 -7.786555e-03 -2.929654e-02 -3.005234e-02 -3.459199e-02
-2.274212e-02
          144            145            146            147            148
149            150            151            152            153            154
-3.605842e-02 -5.629442e-02 -6.567833e-02 -7.591019e-02 -6.418567e-02
-3.806458e-02 -5.403245e-02 -7.686517e-02 -4.911200e-02 -3.233384e-02
-6.118028e-02
          155            156            157            158            159
160            161            162            163            164            165
-6.412096e-02 -2.657462e-02 -3.046540e-02 -5.567414e-02 -3.538529e-02
-1.422559e-02 -3.626896e-02 -3.798748e-02 -1.487696e-02 -1.272451e-02
-3.216248e-02
          166            167            168            169            170
171            172            173            174            175            176
-2.125701e-02 -8.378740e-03 -6.851592e-03 -1.574125e-02 -5.850134e-03
-8.150034e-03 -2.765648e-02 -3.633817e-02 -2.572445e-02 -2.968120e-02
-5.496944e-02
          177            178            179            180            181
182            183            184            185            186            187
-4.878452e-02 -4.150943e-02 -6.078324e-02 -7.591829e-02 -6.672553e-02
-6.322447e-02 -8.650686e-02 -8.349620e-02 -7.107113e-02 -8.448161e-02
-8.574598e-02
          188            189            190            191            192
193            194            195            196            197            198
-7.275982e-02 -7.019264e-02 -7.101267e-02 -5.774331e-02 -4.310135e-02
-2.771044e-02 -2.589357e-02 -2.545931e-02 -1.177025e-02 -4.472606e-03
8.906241e-03
          199            200            201            202            203
204            205            206            207            208            209
 9.655456e-03 -8.235418e-03 -9.008108e-03 -5.894330e-03 -8.113307e-03
-2.623865e-02 -3.886737e-02 -5.087401e-02 -7.018475e-02 -7.859536e-02
-7.860676e-02
```

```
          210            211            212            213            214 
215            216            217            218            219            220 
-8.726153e-02 -1.016209e-01 -9.071406e-02 -1.042633e-01 -1.203439e-01 
-1.033204e-01 -1.012352e-01 -1.244889e-01 -1.221350e-01 -9.698819e-02 
-9.917254e-02 
          221            222            223            224            225 
226            227            228            229            230            231 
-1.113866e-01 -9.789411e-02 -7.698222e-02 -7.684302e-02 -7.793090e-02 
-8.775063e-02 -1.136230e-01 -1.127004e-01 -1.102183e-01 -1.201722e-01 
-1.254663e-01 
          232            233            234            235            236 
237            238            239            240            241            242 
-1.177651e-01 -1.299749e-01 -1.301452e-01 -1.039549e-01 -1.071897e-01 
-1.070013e-01 -1.073314e-01 -1.112483e-01 -1.028579e-01 -9.601065e-02 
-1.037663e-01 
          243            244            245            246            247 
248            249            250            251            252            253 
-1.194668e-01 -1.214013e-01 -1.078700e-01 -9.305723e-02 -8.928735e-02 
-6.262716e-02 -5.800605e-02 -5.218705e-02 -2.709255e-02 -2.538232e-02 
-3.639886e-02 
          254            255            256            257            258 
259            260            261            262            263            264 
-6.582952e-02 -7.120060e-02 -9.681732e-02 -8.915636e-02 -7.228710e-02 
-9.265239e-02 -1.168325e-01 -1.056425e-01 -9.710087e-02 -1.213802e-01 
-1.285682e-01 
          265            266            267            268            269 
270            271            272            273            274            275 
-1.128923e-01 -1.157323e-01 -1.298189e-01 -1.274895e-01 -1.092872e-01 
-1.026741e-01 -1.218345e-01 -1.047184e-01 -8.600636e-02 -1.196716e-01 
-1.232783e-01 
          276            277            278            279            280 
281            282            283            284            285            286 
-1.352356e-01 -1.338458e-01 -1.350661e-01 -1.481593e-01 -1.462470e-01 
-1.437605e-01 -1.375568e-01 -1.302884e-01 -1.281055e-01 -1.143956e-01 
-1.012505e-01 
          287            288            289            290            291 
292            293            294            295            296            297 
-9.480949e-02 -9.036317e-02 -7.417833e-02 -5.214805e-02 -5.074030e-02 
-5.250447e-02 -2.573108e-02 -1.139011e-02 -1.867486e-02 -1.227213e-02 
-1.908254e-02 
          298            299            300            301            302 
303            304            305            306            307            308 
-3.221407e-02 -4.168202e-02 -3.845684e-02 -3.299083e-02 -8.771964e-03 
3.055485e-02  5.540234e-02  5.935382e-02  7.429080e-02  8.279061e-02 
7.171248e-02 
          309            310            311            312            313 
314            315            316            317            318            319 
 3.777462e-02  2.058206e-02 -2.494991e-03 -9.669736e-03  1.220661e-02 
2.682465e-02  5.128291e-02  5.455787e-02  8.645620e-02  6.203900e-02 
3.339446e-02 
          320            321            322            323            324 
325            326            327            328            329            330 
 5.954068e-02  8.454249e-02  4.879118e-02  2.396345e-02  4.730204e-02 
5.451922e-02  6.324751e-03  9.306724e-03  3.037000e-02 -3.848348e-04 
```

```
-3.753904e-02
          331            332            333            334            335 
336            337            338            339            340            341 
-3.480360e-02 -2.858435e-02 -6.131112e-02 -8.697085e-02 -1.093061e-01 
-1.317182e-01 -1.276635e-01 -1.316074e-01 -1.411928e-01 -1.381310e-01 
-1.201301e-01 
          342            343            344            345            346 
347            348            349            350            351            352 
-1.083930e-01 -1.050446e-01 -1.199545e-01 -1.185214e-01 -1.162393e-01 
-1.201199e-01 -1.064570e-01 -6.443143e-02 -4.645140e-02 -2.412595e-02 
-1.444951e-02 
          353            354            355            356            357 
358            359            360            361            362            363 
-4.393717e-03  2.752647e-02  4.615556e-02  5.408216e-02  8.530666e-02 
1.373403e-01  1.383443e-01  1.299328e-01  1.748736e-01  2.662543e-01 
2.186675e-01 
          364            365            366            367            368 
369            370            371            372            373            374 
 2.016175e-01  2.794632e-01  3.106581e-01  3.062329e-01  2.531326e-01 
1.647564e-01  1.369267e-01  1.834116e-01  1.883512e-01  2.401762e-01 
1.821908e-01 
          375            376            377            378            379 
380            381            382            383            384            385 
 1.920283e-01  1.772503e-01  1.891244e-01  2.049237e-01  2.472494e-01 
2.227474e-01  1.860552e-01  1.839390e-01  1.283811e-01  1.093870e-01 
9.647940e-02 
          386            387            388            389            390 
391            392            393            394            395            396 
 8.420530e-02  6.773437e-02  6.759787e-02  5.424762e-02  5.140074e-02 
5.529707e-02  3.551396e-02  9.787710e-03 -2.639667e-02 -4.478817e-02 
-3.395778e-02 
          397            398            399            400            401 
402            403            404            405            406            407 
-1.440222e-02  5.832029e-03  2.338156e-02  2.707579e-02  2.159562e-02 
2.611945e-02  4.001804e-02  6.890758e-02  6.404713e-02  4.840883e-02 
8.041326e-02 
          408            409            410            411            412 
413            414            415            416            417            418 
 9.122680e-02  1.277849e-01  1.861141e-01  2.090619e-01  1.658845e-01 
1.472714e-01  1.236245e-01  9.820180e-02  1.049499e-01  7.344371e-02 
4.628263e-02 
          419            420            421            422            423 
424            425            426            427            428            429 
 3.071498e-02  4.709872e-02  7.179643e-02  6.866724e-02  9.351892e-02 
1.019550e-01  9.912406e-02  7.466188e-02  6.771660e-02  3.644108e-02 
3.443468e-02 
          430            431            432            433            434 
435            436            437            438            439            440 
 3.579675e-02  3.304489e-02  6.236806e-02  8.612057e-02  8.098066e-02 
9.971813e-02  7.614755e-02  8.165880e-02  6.151298e-02  9.290192e-02 
1.235838e-01 
          441            442            443            444            445 
446            447            448            449            450            451 
 9.331527e-02  1.046396e-01  1.553365e-01  1.856361e-01  1.581761e-01 
```

2.123518e-01 3.109545e-01 2.486253e-01 2.014124e-01 2.633675e-01
3.643655e-01
452 453 454 455 456
457 458 459 460 461 462
3.907793e-01 3.680605e-01 5.414448e-01 6.141419e-01 5.860379e-01
3.639714e-01 3.123278e-01 2.243738e-01 1.589507e-01 1.522220e-01
1.167991e-01
463 464 465 466 467
468 469 470 471 472 473
9.831725e-02 7.174771e-02 4.056257e-02 1.682308e-03 -3.039405e-02
-5.011827e-02 -1.104716e-01 -1.172855e-01 -1.229640e-01 -1.467274e-01
-1.743700e-01
474 475 476 477 478
479 480 481 482 483 484
-2.008428e-01 -1.824921e-01 -1.880036e-01 -1.994923e-01 -1.871279e-01
-1.599856e-01 -1.507815e-01 -1.707018e-01 -1.418774e-01 -1.377621e-01
-9.181259e-02
485 486 487 488 489
490 491 492 493 494 495
-9.450009e-02 -1.454116e-01 -1.309100e-01 -1.582962e-01 -1.335425e-01
-9.444179e-02 -1.011824e-01 -6.904878e-02 -1.055495e-01 -2.257515e-02
-3.609313e-02
496 497 498 499 500
501 502 503 504 505 506
-3.752540e-02 -2.788798e-02 -1.276369e-02 1.220533e-02 1.338606e-02
1.351073e-02 3.216463e-02 3.780232e-02 7.312801e-02 3.810220e-02
3.377265e-02
507 508 509 510 511
512 513 514 515 516 517
-1.548686e-02 -8.677333e-02 -1.527735e-01 -1.884584e-01 -2.059332e-01
-1.784607e-01 -1.717799e-01 -1.240107e-01 -1.019069e-01 -5.940862e-02
-2.622971e-02
518 519 520 521 522
523 524 525 526 527 528
-5.351665e-02 -5.700742e-02 -1.111377e-02 -2.644825e-02 -2.244859e-02
-6.591435e-02 -7.433951e-02 -8.456222e-02 -8.131046e-02 -8.996664e-02
-5.089539e-02
529 530 531 532 533
534 535 536 537 538 539
-1.747191e-02 -8.922237e-03 -3.193719e-03 -5.617689e-02 -6.030095e-02
-1.304084e-02 -1.992879e-03 -4.813257e-02 -9.151087e-02 -8.875768e-02
-1.196506e-01
540 541 542 543 544
545 546 547 548 549 550
-1.377065e-01 -1.677887e-01 -1.738834e-01 -1.289380e-01 -8.698261e-02
-3.814664e-02 1.371555e-02 8.833152e-04 3.830180e-02 2.998270e-02
4.729799e-02
551 552 553 554 555
556 557 558 559 560
5.474466e-02 5.908031e-02 3.170921e-02 3.630233e-02 1.036670e-02
-1.393153e-02 -1.891855e-03 -5.558325e-03 1.436363e-02 4.160027e-02

```
            1             2             3             4             5 
 6             7             8             9             10            11 
 1.000000e+00  4.145520e-01  3.255244e-01  3.042981e-01  2.744211e-01 
2.831764e-01  2.173967e-01  1.995772e-01  1.619080e-01  1.273567e-01 
1.024690e-01 
           12            13            14            15            16 
17            18            19            20            21            22 
 1.007860e-01  8.426675e-02  6.114644e-02  5.276657e-02  4.661500e-02 
2.652078e-02  1.612062e-02  6.225996e-03  2.725771e-03 -1.942248e-05 
4.169350e-03 
           23            24            25            26            27 
28            29            30            31            32            33 
 7.731004e-03  1.296736e-02  1.118890e-02  1.905428e-02  2.951308e-02 
2.265423e-02  7.361874e-03 -1.428519e-02  6.567365e-03  6.374527e-03 
2.304159e-03 
           34            35            36            37            38 
39            40            41            42            43            44 
 1.435856e-03 -3.593407e-03 -4.891243e-03 -1.499585e-02 -1.506309e-02 
-3.013135e-02 -2.872051e-02 -2.992896e-02 -3.661449e-02 -3.693708e-02 
-3.703737e-02 
           45            46            47            48            49 
50            51            52            53            54            55 
-2.889030e-02 -2.947007e-02 -1.236913e-02 -1.180108e-02 -1.135058e-02 
-2.045173e-03 -1.884095e-03 -4.558879e-03  9.668203e-03  1.690579e-02 
3.684234e-03 
           56            57            58            59            60 
61            62            63            64            65            66 
-2.964698e-03 -1.023790e-02 -1.989029e-02 -3.705560e-02 -4.615857e-02 
-4.813531e-02 -2.711750e-02 -2.337672e-02 -4.096177e-02 -2.786247e-02 
1.932323e-03 
           67            68            69            70            71 
72            73            74            75            76            77 
-9.209423e-03 -2.308118e-02  9.632225e-03  2.391557e-02 -4.689395e-03 
3.649154e-03  4.453452e-02  3.388845e-02 -4.131186e-03 -4.477981e-03 
2.963204e-02 
           78            79            80            81            82 
83            84            85            86            87            88 
 5.893776e-02  8.768916e-02  1.310381e-01  1.258671e-01  1.433258e-01 
1.939894e-01  2.168107e-01  1.706825e-01  1.304883e-01  1.134347e-01 
8.104214e-02 
           89            90            91            92            93 
94            95            96            97            98            99 
 7.202386e-02  7.571489e-02  4.930957e-02  3.364128e-02  3.417958e-02 
1.169343e-02 -7.268791e-05 -1.478644e-03 -1.900891e-02 -2.999449e-02 
-2.368397e-02 
          100           101           102           103           104 
105           106           107           108           109           110 
-3.103767e-02 -4.369817e-02 -4.125322e-02 -3.975056e-02 -4.868380e-02 
-5.977178e-02 -4.258543e-02 -5.614122e-02 -5.468373e-02 -8.365933e-02 
-8.334700e-02 
          111           112           113           114           115 
116           117           118           119           120           121 
-7.374733e-02 -6.189727e-02 -5.332885e-02 -5.252323e-02 -3.835204e-02 
```

-2.826358e-02 -2.878469e-02 -2.923048e-02 -2.157081e-02 -1.017489e-02
-9.934835e-03
122 123 124 125 126
127 128 129 130 131 132
-2.935499e-03 -6.958600e-04 -3.700578e-03 3.784574e-03 9.302779e-03
4.641853e-03 5.108159e-03 5.503208e-03 6.979542e-03 1.972616e-03
-4.155708e-03
133 134 135 136 137
138 139 140 141 142 143
-1.400593e-02 -2.781033e-02 -3.150000e-02 -3.993179e-02 -4.541422e-02
-5.554064e-02 -7.466031e-02 -8.045299e-02 -9.184183e-02 -1.076056e-01
-9.789273e-02
144 145 146 147 148
149 150 151 152 153 154
-1.007385e-01 -1.130642e-01 -1.173988e-01 -1.244879e-01 -1.151989e-01
-9.655385e-02 -1.078442e-01 -1.096955e-01 -8.015252e-02 -8.032774e-02
-9.347878e-02
155 156 157 158 159
160 161 162 163 164 165
-8.898061e-02 -5.752847e-02 -6.631594e-02 -8.386545e-02 -5.613508e-02
-3.713253e-02 -5.520059e-02 -4.728267e-02 -2.707021e-02 -3.046278e-02
-4.142490e-02
166 167 168 169 170
171 172 173 174 175 176
-3.054684e-02 -1.735537e-02 -9.356644e-03 -1.010424e-02 -5.957688e-03
-1.229866e-02 -2.468786e-02 -3.940336e-02 -4.894191e-02 -6.049229e-02
-7.704150e-02
177 178 179 180 181
182 183 184 185 186 187
-8.304375e-02 -8.045545e-02 -8.993376e-02 -1.015395e-01 -1.058402e-01
-1.061919e-01 -1.135663e-01 -1.143390e-01 -1.212134e-01 -1.252710e-01
-1.231964e-01
188 189 190 191 192
193 194 195 196 197 198
-1.107799e-01 -1.099933e-01 -1.089102e-01 -1.053038e-01 -1.048746e-01
-1.015994e-01 -9.035960e-02 -8.362129e-02 -7.414281e-02 -6.345914e-02
-6.511694e-02
199 200 201 202 203
204 205 206 207 208 209
-5.171831e-02 -3.191438e-02 -2.397102e-02 -1.290021e-02 -3.092404e-02
-4.506032e-02 -4.407712e-02 -5.987211e-02 -4.740191e-02 -5.575232e-02
-7.630043e-02
210 211 212 213 214
215 216 217 218 219 220
-9.691928e-02 -1.192610e-01 -1.110159e-01 -1.100392e-01 -1.298060e-01
-1.263580e-01 -1.051801e-01 -1.164868e-01 -1.307378e-01 -1.138442e-01
-9.621380e-02
221 222 223 224 225
226 227 228 229 230 231
-1.113608e-01 -1.111374e-01 -7.909033e-02 -8.153964e-02 -8.523465e-02
-8.913402e-02 -9.394421e-02 -9.301416e-02 -1.019502e-01 -1.259883e-01
-1.281128e-01
232 233 234 235 236
237 238 239 240 241 242

```
-1.401850e-01 -1.344318e-01 -1.278040e-01 -1.364923e-01 -1.297262e-01
-1.246197e-01 -1.206900e-01 -1.164497e-01 -1.115982e-01 -1.044789e-01
-1.048956e-01
          243           244           245           246           247
248           249           250           251           252           253
-7.941746e-02 -8.275007e-02 -6.932797e-02 -7.121895e-02 -6.859499e-02
-4.970570e-02 -3.734806e-02 -2.156131e-02 -1.937674e-02 -7.404236e-03
-2.101048e-02
          254           255           256           257           258
259           260           261           262           263           264
-4.118563e-02 -6.451831e-02 -7.260718e-02 -7.995161e-02 -6.397197e-02
-5.572337e-02 -8.632861e-02 -8.803004e-02 -6.219765e-02 -6.704972e-02
-8.630020e-02
          265           266           267           268           269
270           271           272           273           274           275
-7.706951e-02 -4.734126e-02 -3.970317e-02 -5.671438e-02 -6.438019e-02
-9.093997e-02 -9.327577e-02 -9.051948e-02 -8.919688e-02 -9.023098e-02
-1.063504e-01
          276           277           278           279           280
281           282           283           284           285           286
-1.221868e-01 -1.120374e-01 -1.171565e-01 -1.039415e-01 -1.011973e-01
-1.065146e-01 -1.076706e-01 -1.117231e-01 -9.992531e-02 -9.879740e-02
-9.823136e-02
          287           288           289           290           291
292           293           294           295           296           297
-8.038513e-02 -7.254268e-02 -7.721364e-02 -6.269818e-02 -4.058490e-02
-3.827701e-02 -3.784211e-02 -1.436434e-02  5.811705e-03 -4.965560e-03
1.359151e-02
          298           299           300           301           302
303           304           305           306           307           308
 9.007175e-03 -1.317783e-02 -2.855894e-02 -3.924755e-02 -2.674725e-02
2.004846e-03  3.761404e-02  7.951269e-02  7.742544e-02  1.046950e-01
1.235518e-01
          309           310           311           312           313
314           315           316           317           318           319
 1.050134e-01  5.415212e-02  2.259587e-02 -1.271443e-02 -9.974784e-03
1.378410e-03 -5.159348e-03  6.240208e-03  2.666301e-02  7.235706e-02
4.754242e-02
          320           321           322           323           324
325           326           327           328           329           330
 3.939443e-02  9.054675e-02  1.089120e-01  6.065751e-02  6.934832e-02
1.176112e-01  8.560580e-02  4.416218e-02  7.895377e-02  8.780888e-02
3.900701e-02
          331           332           333           334           335
336           337           338           339           340           341
 1.417854e-02  3.682394e-02  2.712227e-02 -5.884120e-03 -3.276142e-02
-4.628849e-02 -2.090507e-02 -1.285721e-02 -2.201826e-03 -2.230840e-02
-2.195716e-02
          342           343           344           345           346
347           348           349           350           351           352
-2.359293e-02 -4.279818e-02 -6.375148e-02 -6.995173e-02 -8.616905e-02
-7.670681e-02 -5.103556e-02 -3.874213e-02 -1.791733e-02  4.224504e-03
3.047642e-02
          353           354           355           356           357
```

```
358           359           360           361           362           363 
 6.472637e-02  7.333980e-02  8.323791e-02  1.250029e-01  1.530245e-01 
1.507563e-01  1.955851e-01  2.479219e-01  2.554858e-01  3.393081e-01 
4.701508e-01 
          364           365           366           367           368 
369           370           371           372           373           374 
 3.593094e-01  2.753006e-01  4.190955e-01  4.929664e-01  4.351854e-01 
2.753082e-01  1.930050e-01  1.821765e-01  2.292516e-01  3.561161e-01 
3.768127e-01 
          375           376           377           378           379 
380           381           382           383           384           385 
 2.539146e-01  2.142276e-01  1.788988e-01  1.681026e-01  2.132738e-01 
3.056259e-01  2.577513e-01  2.085597e-01  1.933139e-01  1.247019e-01 
1.037099e-01 
          386           387           388           389           390 
391           392           393           394           395           396 
 8.148094e-02  4.318202e-02  2.170444e-02 -3.504593e-03 -3.651717e-03 
-1.084786e-02 -2.662726e-02 -3.064877e-02 -3.071501e-02 -1.310923e-02 
-2.171369e-02 
          397           398           399           400           401 
402           403           404           405           406           407 
 4.272526e-03  6.027451e-03 -2.393852e-03 -6.012911e-03 -2.329918e-02 
-3.464360e-02 -4.244126e-02 -4.390396e-02 -3.367974e-02 -1.298181e-02 
-9.365742e-03 
          408           409           410           411           412 
413           414           415           416           417           418 
 7.799533e-03  3.467131e-02  6.701690e-02  1.161554e-01  1.232235e-01 
1.116125e-01  1.304629e-01  9.255672e-02  6.114862e-02  6.252053e-02 
1.015199e-01 
          419           420           421           422           423 
424           425           426           427           428           429 
 6.944524e-02  5.972889e-02  9.235919e-02  1.232765e-01  1.123546e-01 
1.232902e-01  7.679254e-02  8.562645e-02  6.435512e-02  4.222494e-02 
1.801107e-02 
          430           431           432           433           434 
435           436           437           438           439           440 
 1.703782e-02  6.154780e-03 -1.824964e-03  3.367129e-02  5.167166e-02 
3.195205e-02  4.995286e-02  2.860629e-02  3.102924e-02  3.586292e-02 
6.751725e-02 
          441           442           443           444           445 
446           447           448           449           450           451 
 8.616433e-02  6.254167e-02  7.074900e-02  1.140512e-01  1.003484e-01 
7.891977e-02  1.200996e-01  1.380214e-01  1.397348e-01  1.837039e-01 
2.190533e-01 
          452           453           454           455           456 
457           458           459           460           461           462 
 2.134257e-01  2.218629e-01  2.882083e-01  2.620503e-01  2.458346e-01 
1.412222e-01  1.352775e-01  7.828035e-02  6.876634e-02  5.637128e-02 
4.087733e-02 
          463           464           465           466           467 
468           469           470           471           472           473 
 1.640503e-02 -1.788048e-03 -9.366537e-03 -2.494835e-02 -5.038498e-02 
-6.258310e-02 -1.205878e-01 -1.288877e-01 -1.440287e-01 -1.299053e-01 
-1.250868e-01 
```

```
          474           475           476           477           478 
479           480           481           482           483           484 
-1.104749e-01 -1.554176e-01 -1.549515e-01 -1.784475e-01 -1.602977e-01 
-1.465678e-01 -1.480417e-01 -1.337955e-01 -1.139932e-01 -8.864285e-02 
-6.254132e-02 
          485           486           487           488           489 
490           491           492           493           494           495 
-3.706707e-02 -7.392721e-02 -7.342036e-02 -1.034788e-01 -1.346838e-01 
-1.206753e-01 -1.620750e-01 -1.572286e-01 -1.259552e-01 -8.685949e-02 
-7.870409e-02 
          496           497           498           499           500 
501           502           503           504           505           506 
-4.286929e-02 -4.553163e-02 -3.994228e-02 -2.491719e-02 -2.550908e-02 
-2.859290e-02 -6.582732e-02 -3.342839e-02 -6.990385e-02 -9.943620e-02 
-1.498838e-01 
          507           508           509           510           511 
512           513           514           515           516           517 
-1.073327e-01 -1.220312e-01 -1.030570e-01 -6.661283e-02 -5.835026e-02 
-2.498277e-02  4.374925e-03  9.131935e-03  3.546293e-02  6.811612e-02 
4.221065e-02 
          518           519           520           521           522 
523           524           525           526           527           528 
 5.644116e-02  8.946959e-02  9.305247e-02  1.147403e-01  9.028500e-02 
7.909871e-02  9.140191e-02  7.217791e-02  7.393844e-02  5.522920e-02 
1.511009e-02 
          529           530           531           532           533 
534           535           536           537           538           539 
 1.492788e-02  6.864886e-02  6.186933e-02  3.192480e-02  8.183405e-02 
7.319828e-02  9.856092e-02  8.153415e-02  4.478328e-02  3.065622e-02 
2.337172e-02 
          540           541           542           543           544 
545           546           547           548           549           550 
-8.827228e-04 -4.323504e-02 -6.124234e-02 -3.724039e-02 -5.176305e-02 
-2.822459e-02  1.374219e-02  2.147295e-02  5.268923e-02 -6.359497e-03 
2.127557e-02 
          551           552           553           554           555 
556           557           558           559           560           561 
 5.107039e-02  5.211906e-02  3.888067e-02  2.918566e-02  2.263912e-02 
4.816804e-03 -5.843647e-03 -3.423208e-02 -3.370983e-02 -6.427204e-02 
-3.154633e-02 
          562           563           564           565           566 
567           568           569           570           571           572 
-4.983678e-02 -1.205685e-04  1.615499e-03 -7.963427e-03  2.792324e-02 
2.112887e-02  2.043531e-02  5.748358e-02  4.413698e-02  3.786795e-02 
-7.538518e-03 
          573           574           575           576 
-3.217432e-02 -5.886295e-02 -8.714950e-02 -1.166533e-01 


 Q93WY9_40.1ns (MATRIX)

           1             2             3             4             5 
 6             7             8             9             10            11 
 0.4145520052  1.0000000000  0.5973829880  0.4444243934  0.3924631378 
```

```
0.4643729518  0.3201900504  0.2748038132  0.2306620698  0.1886092616
0.1563005057
           12            13            14            15            16
17            18            19            20            21            22
 0.1528730198  0.1299785222  0.1049547703  0.0947384513  0.0851878279
0.0635428544  0.0473887287  0.0396339291  0.0324897764  0.0256961589
0.0258171530
           23            24            25            26            27
28            29            30            31            32            33
 0.0310289264  0.0404280647  0.0368391745  0.0404837444  0.0548519638
0.0508276261  0.0325243790  0.0065293041  0.0363305460  0.0342709645
0.0304608977
           34            35            36            37            38
39            40            41            42            43            44
 0.0310249204  0.0241363271  0.0222135850  0.0096025904  0.0084412595
-0.0072563260 -0.0058792950 -0.0122765241 -0.0199816017 -0.0172121027
-0.0159561188
           45            46            47            48            49
50            51            52            53            54            55
-0.0042649278 -0.0053823706  0.0113715082  0.0175489301  0.0126690511
0.0221273115  0.0157841047  0.0086382494  0.0189298943  0.0287824310
0.0172904251
           56            57            58            59            60
61            62            63            64            65            66
 0.0125570269  0.0041218081 -0.0026557463 -0.0242245964 -0.0326937234
-0.0402080363 -0.0197232286 -0.0083008064 -0.0255620829 -0.0186857777
0.0150083580
           67            68            69            70            71
72            73            74            75            76            77
 0.0085456736 -0.0084192634  0.0218392164  0.0438822454  0.0139589030
0.0225771811  0.0700886450  0.0602301557  0.0178214945  0.0150028909
0.0533378873
           78            79            80            81            82
83            84            85            86            87            88
 0.0921803983  0.1156142542  0.1732891993  0.1693233376  0.1688134524
0.2341741539  0.2910316980  0.2114663188  0.1527040304  0.1296202924
0.0926843472
           89            90            91            92            93
94            95            96            97            98            99
 0.0904168142  0.0993625010  0.0720940262  0.0564122873  0.0598340136
0.0367749199  0.0293615052  0.0271020185  0.0081163596 -0.0029910448
0.0054323322
          100           101           102           103           104
105           106           107           108           109           110
-0.0040720547 -0.0198749559 -0.0167737240 -0.0140704114 -0.0248921710
-0.0388973524 -0.0267367390 -0.0414705671 -0.0430185640 -0.0679521621
-0.0673517908
          111           112           113           114           115
116           117           118           119           120           121
-0.0559795573 -0.0434196811 -0.0381514917 -0.0386127899 -0.0209032775
-0.0142584795 -0.0155045040 -0.0206936270 -0.0141983890  0.0001185827
0.0029014308
          122           123           124           125           126
127           128           129           130           131           132
```

```
 0.0063270860  0.0079345319  0.0084479964  0.0199781327  0.0279777083 
0.0248961978  0.0264929478  0.0299813480  0.0302614934  0.0232218429 
0.0144186050 
          133           134           135           136           137 
138           139           140           141           142           143 
 0.0014873737 -0.0128311531 -0.0139471883 -0.0252371507 -0.0299728007 
-0.0443478384 -0.0605113398 -0.0695281772 -0.0807111698 -0.0980308173 
-0.0923135170 
          144           145           146           147           148 
149           150           151           152           153           154 
-0.0960005045 -0.1115694306 -0.1162091973 -0.1231022108 -0.1153957256 
-0.0919681502 -0.0990888629 -0.1040350683 -0.0733207261 -0.0677252848 
-0.0790527946 
          155           156           157           158           159 
160           161           162           163           164           165 
-0.0755504744 -0.0397255096 -0.0445687507 -0.0629873386 -0.0375285459 
-0.0146068953 -0.0322434195 -0.0275770951 -0.0062084509 -0.0084001175 
-0.0214970014 
          166           167           168           169           170 
171           172           173           174           175           176 
-0.0148589210 -0.0019205683  0.0062624220  0.0035778749  0.0058591460 
0.0031140213 -0.0081264937 -0.0193741543 -0.0303587602 -0.0386319656 
-0.0571049572 
          177           178           179           180           181 
182           183           184           185           186           187 
-0.0647949454 -0.0621060179 -0.0730668321 -0.0878579706 -0.0923467227 
-0.0933091710 -0.1042948341 -0.1074606452 -0.1148380459 -0.1205786248 
-0.1221765939 
          188           189           190           191           192 
193           194           195           196           197           198 
-0.1109892666 -0.1105867100 -0.1122941140 -0.1092852337 -0.1087989173 
-0.1036984833 -0.0889389836 -0.0841283508 -0.0776087539 -0.0641530254 
-0.0642381198 
          199           200           201           202           203 
204           205           206           207           208           209 
-0.0549483694 -0.0344142866 -0.0255988127 -0.0125775456 -0.0268074031 
-0.0393031661 -0.0415962328 -0.0616071191 -0.0551445549 -0.0680223083 
-0.0904328473 
          210           211           212           213           214 
215           216           217           218           219           220 
-0.1090343721 -0.1305612493 -0.1233930240 -0.1174907690 -0.1338083287 
-0.1316767636 -0.1076903629 -0.1124875849 -0.1274603324 -0.1115638821 
-0.0869362784 
          221           222           223           224           225 
226           227           228           229           230           231 
-0.0992560515 -0.1028174184 -0.0701914837 -0.0668162391 -0.0719261623 
-0.0796916918 -0.0866889029 -0.0886382409 -0.0988830891 -0.1221794281 
-0.1270433854 
          232           233           234           235           236 
237           238           239           240           241           242 
-0.1424980991 -0.1360980246 -0.1309230537 -0.1425841704 -0.1369001812 
-0.1321288612 -0.1304261904 -0.1284985059 -0.1237552372 -0.1183465452 
-0.1212604186 
          243           244           245           246           247 
```

```
248           249           250           251           252           253 
-0.0962126846 -0.0973041662 -0.0845676472 -0.0872247687 -0.0856663611 
-0.0675543250 -0.0545631970 -0.0381822952 -0.0385651571 -0.0202971247 
-0.0290411343 
          254           255           256           257           258 
259           260           261           262           263           264 
-0.0541442811 -0.0829677518 -0.0850956842 -0.0955005637 -0.0806429270 
-0.0680882492 -0.0965049075 -0.0986543221 -0.0706369163 -0.0692983963 
-0.0875790831 
          265           266           267           268           269 
270           271           272           273           274           275 
-0.0785525608 -0.0474384189 -0.0374150715 -0.0542279865 -0.0637927890 
-0.0919175860 -0.0933034159 -0.0919111931 -0.0922005228 -0.0924299782 
-0.1068831794 
          276           277           278           279           280 
281           282           283           284           285           286 
-0.1242738242 -0.1120668828 -0.1198597029 -0.1076517657 -0.1084917990 
-0.1132378035 -0.1172706529 -0.1225377261 -0.1145243760 -0.1163477960 
-0.1162611918 
          287           288           289           290           291 
292           293           294           295           296           297 
-0.0987300542 -0.0944905170 -0.1002971422 -0.0858523967 -0.0642604574 
-0.0630829091 -0.0641506444 -0.0393314228 -0.0206327814 -0.0308545866 
-0.0154634999 
          298           299           300           301           302 
303           304           305           306           307           308 
-0.0164698815 -0.0335977967 -0.0439812058 -0.0596118605 -0.0503778033 
-0.0252814337  0.0073697777  0.0457387020  0.0488788316  0.0731493922 
0.0877083088 
          309           310           311           312           313 
314           315           316           317           318           319 
 0.0681160191  0.0235312064  0.0024300877 -0.0261456078 -0.0184373946 
-0.0075825027 -0.0085394734  0.0006034987  0.0108346511  0.0494587443 
0.0306232089 
          320           321           322           323           324 
325           326           327           328           329           330 
 0.0109649964  0.0543795358  0.0781237254  0.0321782736  0.0274683210 
0.0746345132  0.0597222112  0.0147922755  0.0393079642  0.0649524359 
0.0230188549 
          331           332           333           334           335 
336           337           338           339           340           341 
-0.0073019399  0.0173838220  0.0224444944 -0.0112624636 -0.0388251678 
-0.0582504948 -0.0376778895 -0.0344500406 -0.0179562286 -0.0374397945 
-0.0317394241 
          342           343           344           345           346 
347           348           349           350           351           352 
-0.0300076809 -0.0527540249 -0.0727575454 -0.0793242776 -0.0989195666 
-0.0942779510 -0.0688165601 -0.0602316944 -0.0456247270 -0.0265619692 
-0.0005123852 
          353           354           355           356           357 
358           359           360           361           362           363 
 0.0252591363  0.0271054702  0.0434874298  0.0802969161  0.0961051476 
0.1034650770  0.1470390873  0.1806525111  0.1850915945  0.2213873282 
0.3071446766 
```

```
          364           365           366           367           368 
369           370           371           372           373           374 
 0.2683461934  0.1975724238  0.2660014799  0.3269025711  0.3126374287 
0.2238769925  0.1521711090  0.1326054324  0.1631624309  0.2548214294 
0.2563916720 
          375           376           377           378           379 
380           381           382           383           384           385 
 0.1742109004  0.1734360539  0.1590242095  0.1761893120  0.2124162472 
0.2525098170  0.2042999872  0.1618936672  0.1503408468  0.1002891307 
0.0849834833 
          386           387           388           389           390 
391           392           393           394           395           396 
 0.0663809713  0.0444994229  0.0256036498  0.0095790759  0.0114603016 
0.0087840466 -0.0063232889 -0.0095202343 -0.0092013019  0.0099514554 
-0.0010222517 
          397           398           399           400           401 
402           403           404           405           406           407 
 0.0211578320  0.0271488880  0.0135574019  0.0109567682 -0.0058852565 
-0.0156235455 -0.0240698377 -0.0258918794 -0.0172367712  0.0023618706 
0.0058866168 
          408           409           410           411           412 
413           414           415           416           417           418 
 0.0241634048  0.0490624181  0.0829303699  0.1353667089  0.1565443119 
0.1248920499  0.1098653826  0.0798427389  0.0489246485  0.0450750449 
0.0733974874 
          419           420           421           422           423 
424           425           426           427           428           429 
 0.0434944585  0.0358006299  0.0629312318  0.0875116520  0.0817311765 
0.0928516751  0.0558134302  0.0665166349  0.0496770776  0.0349649730 
0.0168538421 
          430           431           432           433           434 
435           436           437           438           439           440 
 0.0136765954  0.0061601925 -0.0043673962  0.0237731543  0.0441678199 
0.0306847350  0.0506915966  0.0359170517  0.0437240216  0.0430271636 
0.0671898633 
          441           442           443           444           445 
446           447           448           449           450           451 
 0.0931085285  0.0711043901  0.0742145898  0.1232341777  0.1226803480 
0.0964052403  0.1330474392  0.2091930172  0.2041432972  0.2495299548 
0.3432205113 
          452           453           454           455           456 
457           458           459           460           461           462 
 0.3070868435  0.3143085633  0.4288928080  0.3285298900  0.3043996856 
0.1650233642  0.1596039291  0.0965727633  0.0929713699  0.0835259832 
0.0643563984 
          463           464           465           466           467 
468           469           470           471           472           473 
 0.0325710039  0.0144811515  0.0068166088 -0.0073919816 -0.0330675387 
-0.0469676636 -0.1061504107 -0.1215791401 -0.1350503703 -0.1146760667 
-0.1072429976 
          474           475           476           477           478 
479           480           481           482           483           484 
-0.0927571355 -0.1420863759 -0.1424196272 -0.1764351567 -0.1608338381 
-0.1470993735 -0.1542835809 -0.1406617939 -0.1146502570 -0.0914499109 
```

```
-0.0578300712
          485           486           487           488           489
490           491           492           493           494           495
-0.0314847705 -0.0671410488 -0.0624377143 -0.1002685961 -0.1271987673
-0.1240260302 -0.1634652407 -0.1612523061 -0.1186091892 -0.0754226915
-0.0592323106
          496           497           498           499           500
501           502           503           504           505           506
-0.0225995366 -0.0257522160 -0.0198635918 -0.0090122475 -0.0148795599
-0.0279569235 -0.0706587035 -0.0452036638 -0.0845053904 -0.1146106506
-0.1668584225
          507           508           509           510           511
512           513           514           515           516           517
-0.1345954109 -0.1441409527 -0.1337711788 -0.0957938300 -0.0892332199
-0.0622860823 -0.0311977165 -0.0205343028  0.0032827488  0.0343408935
0.0036767469
          518           519           520           521           522
523           524           525           526           527           528
 0.0161505709  0.0538400911  0.0612447925  0.0888521216  0.0674385823
0.0607435924  0.0822331871  0.0706725911  0.0812299065  0.0663070820
0.0345159744
          529           530           531           532           533
534           535           536           537           538           539
 0.0332936953  0.0796970105  0.0711158495  0.0314955783  0.0769203753
0.0580511376  0.0752519093  0.0543799807  0.0102345422 -0.0083787783
-0.0182994287
          540           541           542           543           544
545           546           547           548           549           550
-0.0435387973 -0.0822499362 -0.0977644851 -0.0779757709 -0.0884829871
-0.0577976529 -0.0178448703  0.0016621151  0.0385127044 -0.0193755800
0.0046468647
          551           552           553           554           555
556           557           558           559           560           561
 0.0400026472  0.0475643153  0.0426558773  0.0395804233  0.0391144390
0.0239005023  0.0141591421 -0.0143697491 -0.0242184412 -0.0544811279
-0.0249198716
          562           563           564           565           566
567           568           569           570           571           572
-0.0362887470  0.0066932943  0.0090696045 -0.0128565173  0.0199391567
0.0017267810  0.0010820511  0.0386682603  0.0339396128  0.0341458690
-0.0117881356
          573           574           575           576
-0.0415439173 -0.0740974098 -0.1047922843 -0.1251241471
```

Q9ZSP9_40.1ns (DCCM)

```
            1             2             3             4             5
 6             7             8             9             10            11
 1.0000000000  0.5838595796  0.4154414395  0.3473103534  0.3173494637
0.2997366306  0.2316186642  0.2042338606  0.2086222350  0.1634184522
0.1194887129
           12            13            14            15            16
17            18            19            20            21            22
```

0.1326549156 0.1113752103 0.0764163088 0.0770178208 0.0782427034
0.0515569880 0.0315948270 0.0180902039 0.0119858803 0.0057574819
0.0121384572
23 24 25 26 27
28 29 30 31 32 33
0.0167615398 0.0137926141 0.0258538555 0.0467747567 0.0656284495
0.0720290049 0.0565906703 0.0366107317 0.0261985597 0.0291856002
0.0187412903
34 35 36 37 38
39 40 41 42 43 44
0.0214983458 0.0101793474 0.0114902548 0.0004284099 -0.0046497287
-0.0208239826 -0.0200914769 -0.0160985443 -0.0230798496 -0.0290281647
-0.0305812510
45 46 47 48 49
50 51 52 53 54 55
-0.0259903557 -0.0251890528 -0.0141650872 -0.0117161497 -0.0059735898
0.0182971065 0.0243210653 0.0331685706 0.0550095426 0.0554182488
0.0350443263
56 57 58 59 60
61 62 63 64 65 66
0.0243989943 0.0073737960 -0.0005151268 -0.0114664373 -0.0321904872
-0.0331066861 -0.0034919968 -0.0105992158 -0.0396349803 -0.0214976736
0.0036988921
67 68 69 70 71
72 73 74 75 76 77
-0.0225164114 -0.0416893464 -0.0031630070 0.0096310756 -0.0264339372
-0.0193717614 0.0237378126 0.0149181265 -0.0190672633 -0.0015123208
0.0449638936
78 79 80 81 82
83 84 85 86 87 88
0.0412800348 0.0576116614 0.0783432133 0.0799439872 0.0901561106
0.1116916564 0.0820519073 0.0629772910 0.0781395262 0.0451572562
0.0297323122
89 90 91 92 93
94 95 96 97 98 99
0.0418892878 0.0360350417 0.0069276543 0.0023341148 0.0127834065
-0.0129765484 -0.0294873534 -0.0191398371 -0.0227459883 -0.0512354095
-0.0550842383
100 101 102 103 104
105 106 107 108 109 110
-0.0385856546 -0.0548935084 -0.0645187538 -0.0517371275 -0.0510490576
-0.0669817234 -0.0475278296 -0.0622049141 -0.0637330610 -0.0823322527
-0.0794009623
111 112 113 114 115
116 117 118 119 120 121
-0.0719578469 -0.0532641646 -0.0446833002 -0.0246180377 -0.0149208089
-0.0038253326 -0.0048537609 0.0007155160 0.0010628543 0.0093227988
0.0183385381
122 123 124 125 126
127 128 129 130 131 132
0.0259990694 0.0298831231 0.0321802029 0.0385570928 0.0366471657
0.0388140642 0.0335536594 0.0369971251 0.0381724822 0.0282600080
0.0241781326
133 134 135 136 137

```
138           139           140           141           142           143 
 0.0114677224 -0.0010944026 -0.0028580546 -0.0162796035 -0.0208341829 
-0.0375447978 -0.0539009846 -0.0671765543 -0.0753740675 -0.0734751368 
-0.0621910568 
          144           145           146           147           148 
149           150           151           152           153           154 
-0.0797299118 -0.0942376816 -0.1052580822 -0.1054226538 -0.0877239176 
-0.0751271611 -0.1103731259 -0.1239176275 -0.0875209992 -0.0865810233 
-0.1223924152 
          155           156           157           158           159 
160           161           162           163           164           165 
-0.1075099933 -0.0795486394 -0.0955720540 -0.1165852501 -0.0846674401 
-0.0681860140 -0.0885201160 -0.0812253405 -0.0588015552 -0.0598947430 
-0.0782331306 
          166           167           168           169           170 
171           172           173           174           175           176 
-0.0576051818 -0.0458828527 -0.0512110233 -0.0631202894 -0.0569737072 
-0.0622458625 -0.0732434519 -0.0912938064 -0.0823874629 -0.0919558216 
-0.1162389190 
          177           178           179           180           181 
182           183           184           185           186           187 
-0.1147621722 -0.1073749652 -0.1234708646 -0.1328472768 -0.1284927414 
-0.1266034873 -0.1405593697 -0.1314597647 -0.1261822188 -0.1321237264 
-0.1173317966 
          188           189           190           191           192 
193           194           195           196           197           198 
-0.1149558577 -0.1030613164 -0.0939093160 -0.0672736448 -0.0573347812 
-0.0533281090 -0.0691042547 -0.0524957855 -0.0388575919 -0.0421040014 
-0.0317997615 
          199           200           201           202           203 
204           205           206           207           208           209 
-0.0083738826 -0.0104542624 -0.0147955148  0.0016875703  0.0108908476 
0.0229408768  0.0173008776  0.0061463472 -0.0100929290 -0.0123139602 
-0.0356901454 
          210           211           212           213           214 
215           216           217           218           219           220 
-0.0649795075 -0.0780946530 -0.0983012820 -0.1041533276 -0.1344238784 
-0.1315816623 -0.1116246510 -0.1382729520 -0.1469201554 -0.1309927181 
-0.1201440042 
          221           222           223           224           225 
226           227           228           229           230           231 
-0.1346553705 -0.1421876190 -0.1117515332 -0.1021530302 -0.1040886005 
-0.1215290519 -0.1222286896 -0.1340173308 -0.1294486937 -0.1410427215 
-0.1484006727 
          232           233           234           235           236 
237           238           239           240           241           242 
-0.1474251809 -0.1368041870 -0.1419828910 -0.1475894254 -0.1378215955 
-0.1240761393 -0.1202578426 -0.1020278398 -0.1019758248 -0.0954416688 
-0.0752050347 
          243           244           245           246           247 
248           249           250           251           252           253 
-0.0733708220 -0.0694883598 -0.0459041666 -0.0347428377 -0.0147721919 
-0.0021238528 -0.0010664353 -0.0009608805  0.0011638162  0.0191384818 
0.0016340833 
```

```
          254           255           256           257           258 
259           260           261           262           263           264 
-0.0122056616 -0.0488698461 -0.0617699672 -0.0768384150 -0.0552058890 
-0.0547730813 -0.0938642348 -0.0996000550 -0.0776496040 -0.0916217906 
-0.1179451284 
          265           266           267           268           269 
270           271           272           273           274           275 
-0.1093163050 -0.1008153254 -0.1188900184 -0.1246939758 -0.1025305077 
-0.1021504772 -0.1053492194 -0.1210945693 -0.1239513881 -0.1284868938 
-0.1318902493 
          276           277           278           279           280 
281           282           283           284           285           286 
-0.1124158673 -0.0996288089 -0.1143882610 -0.1063046997 -0.1092198207 
-0.1048126143 -0.1035751109 -0.1123854780 -0.1033839172 -0.0896629449 
-0.0853902672 
          287           288           289           290           291 
292           293           294           295           296           297 
-0.0686537314 -0.0529230461 -0.0451628492 -0.0309526879 -0.0065475194 
0.0021879394  0.0016152884  0.0256433704  0.0390628087  0.0320749783 
0.0298430545 
          298           299           300           301           302 
303           304           305           306           307           308 
 0.0107428161 -0.0078514676 -0.0199668945 -0.0041873183  0.0059012745 
0.0315984108  0.0639427033  0.0961388638  0.0805581527  0.1013648021 
0.1147739240 
          309           310           311           312           313 
314           315           316           317           318           319 
 0.1128556045  0.0924940254  0.0771109186  0.0485466668  0.0539677034 
0.0859427364  0.0779504006  0.0939364402  0.0996022148  0.1282852611 
0.0892319494 
          320           321           322           323           324 
325           326           327           328           329           330 
 0.0847364023  0.1232057397  0.1319599587  0.0777218475  0.0724056949 
0.1016790738  0.0651302730  0.0213770715  0.0380034123  0.0388046981 
-0.0042456904 
          331           332           333           334           335 
336           337           338           339           340           341 
-0.0306517008 -0.0220720485 -0.0234620101 -0.0530068741 -0.0612412957 
-0.0755983593 -0.0631125748 -0.0782132985 -0.0639631673 -0.0576640308 
-0.0448697554 
          342           343           344           345           346 
347           348           349           350           351           352 
-0.0502835781 -0.0638857959 -0.0596289391 -0.0566802663 -0.0423770321 
-0.0305321297 -0.0232034588 -0.0355797210 -0.0201915368 -0.0125884005 
0.0171304187 
          353           354           355           356           357 
358           359           360           361           362           363 
 0.0354805445  0.0392806536  0.0665181080  0.0982502333  0.1223798885 
0.1313973094  0.1895833280  0.2067471898  0.2235274253  0.2744253671 
0.3541336753 
          364           365           366           367           368 
369           370           371           372           373           374 
 0.3033206564  0.2784161014  0.3665645229  0.3510874622  0.2804258534 
0.1962174440  0.1333561454  0.1679853935  0.1909607741  0.2422431378 
```

0.3010963584
375 376 377 378 379
380 381 382 383 384 385
0.2294128471 0.2199414616 0.2017280345 0.2041708994 0.2420291896
0.2764257127 0.3083574187 0.2786746375 0.2357148429 0.1723713997
0.1455911076
386 387 388 389 390
391 392 393 394 395 396
0.1243929293 0.1184755049 0.0907319231 0.0743086873 0.0496822355
0.0408635288 0.0490612082 0.0450285540 0.0334296008 0.0287704210
0.0096143021
397 398 399 400 401
402 403 404 405 406 407
0.0237790557 0.0311823459 0.0396275678 0.0407628222 0.0532931827
0.0369653789 0.0353216933 0.0449412272 0.0469547188 0.0482376628
0.0427281350
408 409 410 411 412
413 414 415 416 417 418
0.0532142018 0.0691295911 0.0868198991 0.1490542059 0.1602496842
0.1374528679 0.1653586828 0.1543578977 0.1374236244 0.1549254601
0.1473766705
419 420 421 422 423
424 425 426 427 428 429
0.1115737911 0.0913824382 0.1062053468 0.1408960507 0.1423540777
0.1471715531 0.1249627453 0.1281245911 0.1127742862 0.0976880394
0.0785924660
430 431 432 433 434
435 436 437 438 439 440
0.0902105236 0.0873764644 0.0888424663 0.1253629585 0.1290467237
0.1071651012 0.1164888930 0.0736511331 0.0565094879 0.0494659467
0.1004656539
441 442 443 444 445
446 447 448 449 450 451
0.1066890151 0.0672978730 0.0777901658 0.1277890656 0.1232233412
0.0908350829 0.1239185052 0.1873518820 0.1472852887 0.1146453373
0.1399439227
452 453 454 455 456
457 458 459 460 461 462
0.2258691083 0.2951956886 0.1902565382 0.2257599583 0.2904576522
0.2847367599 0.3676115543 0.5264337199 0.4648977822 0.3629173596
0.2540037613
463 464 465 466 467
468 469 470 471 472 473
0.2786022476 0.2373065980 0.1944058465 0.1542814700 0.1323027427
0.1029170765 0.0790875054 0.0559171016 0.0442120954 0.0288602341
0.0040258934
474 475 476 477 478
479 480 481 482 483 484
-0.0030085007 -0.0171245432 -0.0243835319 -0.0229866069 -0.0295333274
-0.0473380236 -0.0447849147 -0.0475728919 -0.0713650530 -0.0765628931
-0.1038750046
485 486 487 488 489
490 491 492 493 494 495
-0.1069483393 -0.0882681571 -0.0940877803 -0.0460292814 -0.0506709229

```
-0.0977582766 -0.0687643052 -0.0817449147 -0.0381298061 -0.0233743559 
-0.0712923049 
          496           497           498           499           500 
501           502           503           504           505           506 
-0.0370310081 -0.0753363919 -0.0316159906 -0.0660370215 -0.0299282366 
-0.0078176728 -0.0113854991  0.0146458298  0.0152310058  0.0263561175 
0.0254388629 
          507           508           509           510           511 
512           513           514           515           516           517 
 0.0294287738  0.0312649789  0.0329191720  0.0319549198  0.0282418927 
0.0096213839 -0.0312291443 -0.0602514882 -0.0961967832 -0.1428734815 
-0.1692727805 
          518           519           520           521           522 
523           524           525           526           527           528 
-0.2084003518 -0.2004444806 -0.2154607308 -0.1892847980 -0.1872600251 
-0.1526904701 -0.1479135345 -0.1163375981 -0.0773663013 -0.0558156912 
-0.0988394881 
          529           530           531           532           533 
534           535           536           537           538           539 
-0.1088564097 -0.0656973519 -0.0273549109 -0.0422506404 -0.0002808340 
0.0026775641  0.0525363929  0.0440388640  0.0853713077  0.1062262165 
0.0993399529 
          540           541           542           543           544 
545           546           547           548           549           550 
 0.0822409004  0.0514255968  0.0097343288  0.0070445291 -0.0470684675 
-0.1190477838 -0.1559166475 -0.1354582231 -0.1412622132 -0.1527890350 
-0.1880409327 
          551           552           553           554           555 
556           557           558           559           560           561 
-0.2158601447 -0.2090947103 -0.2042470085 -0.1457466978 -0.0985460738 
-0.0487012399 -0.0286796178  0.0234074785  0.0525779986  0.0884251563 
0.0790455915 
          562           563           564           565           566 
567           568           569           570           571           572 
 0.0584473299  0.0420902529  0.0405889967  0.0449287191  0.0366434254 
0.0252699968  0.0554153868  0.0329142951  0.0350958743  0.0232186823 
-0.0208903984 
          573           574           575           576           577 
578           579           580           581           582           583 
-0.0267187946 -0.0730675911 -0.0683060390 -0.0960556148 -0.0567493239 
-0.0460754465 -0.0909032726 -0.1226953661 -0.1588513265 -0.1501657523 
-0.1617400214 
          584           585 
-0.1735560919 -0.1643225355 


 Q9ZSP9_40.1ns (MATRIX)

            1             2             3             4             5 
 6             7             8             9            10            11 
 5.838596e-01  1.000000e+00  6.439711e-01  5.142996e-01  4.744012e-01 
4.397956e-01  3.386895e-01  2.996427e-01  3.039734e-01  2.452531e-01 
1.868322e-01 
           12            13            14            15            16 
```

```
17              18              19              20              21              22
 2.028305e-01  1.720435e-01  1.285601e-01  1.311149e-01  1.290546e-01
9.163617e-02  6.767542e-02  5.235785e-02  4.378968e-02  3.393820e-02
4.004868e-02
           23              24              25              26              27
28              29              30              31              32              33
 4.461825e-02  4.182322e-02  5.645656e-02  8.617418e-02  1.085908e-01
1.174677e-01  9.746123e-02  6.745548e-02  5.418860e-02  5.874272e-02
5.014209e-02
           34              35              36              37              38
39              40              41              42              43              44
 5.272511e-02  3.732288e-02  3.890553e-02  2.436504e-02  1.885194e-02
-7.592551e-04 -8.415642e-04 -2.469355e-04 -9.917674e-03 -1.511839e-02
-1.774802e-02
           45              46              47              48              49
50              51              52              53              54              55
-9.070653e-03 -6.700592e-03  6.518157e-03  1.264622e-02  1.905950e-02
4.898107e-02  5.098406e-02  5.971307e-02  8.682896e-02  8.426879e-02
5.975640e-02
           56              57              58              59              60
61              62              63              64              65              66
 5.019913e-02  2.812844e-02  1.922895e-02  4.935625e-05 -2.454427e-02
-3.122824e-02  5.052121e-03  3.428943e-03 -3.262074e-02 -1.675391e-02
1.819418e-02
           67              68              69              70              71
72              73              74              75              76              77
-8.041422e-03 -3.548828e-02  9.208128e-03  3.246448e-02 -9.748657e-03
-3.061742e-03  5.269637e-02  4.548063e-02  9.456546e-04  2.120897e-02
8.406575e-02
           78              79              80              81              82
83              84              85              86              87              88
 7.906323e-02  1.006613e-01  1.257403e-01  1.219102e-01  1.419759e-01
1.703936e-01  1.297496e-01  1.060199e-01  1.298848e-01  8.535536e-02
6.749577e-02
           89              90              91              92              93
94              95              96              97              98              99
 8.563549e-02  7.654227e-02  3.906847e-02  3.346414e-02  4.593197e-02
1.151946e-02 -6.089422e-03  7.605676e-03  4.398829e-04 -3.730990e-02
-3.979907e-02
          100             101             102             103             104
105             106             107             108             109             110
-2.077795e-02 -4.544980e-02 -5.464316e-02 -3.713880e-02 -4.108618e-02
-6.231432e-02 -4.306056e-02 -6.105732e-02 -6.678219e-02 -8.870928e-02
-8.189529e-02
          111             112             113             114             115
116             117             118             119             120             121
-7.128511e-02 -4.665321e-02 -3.912363e-02 -1.386401e-02  2.728092e-03
1.745056e-02  1.238413e-02  1.617574e-02  1.329567e-02  2.726165e-02
4.164428e-02
          122             123             124             125             126
127             128             129             130             131             132
 4.853996e-02  5.241187e-02  5.989076e-02  7.037334e-02  6.972105e-02
7.134504e-02  6.419971e-02  6.964236e-02  6.913186e-02  5.587527e-02
5.062545e-02
```

```
          133           134           135           136           137 
138           139           140           141           142           143 
 3.347713e-02  1.745704e-02  1.876934e-02  6.419130e-04 -6.624615e-03 
-3.012599e-02 -5.156632e-02 -7.137335e-02 -8.076844e-02 -8.189339e-02 
-7.004435e-02 
          144           145           146           147           148 
149           150           151           152           153           154 
-9.105753e-02 -1.118756e-01 -1.235453e-01 -1.293944e-01 -1.080653e-01 
-8.470572e-02 -1.245443e-01 -1.455461e-01 -9.930038e-02 -9.040626e-02 
-1.340809e-01 
          155           156           157           158           159 
160           161           162           163           164           165 
-1.199340e-01 -7.861581e-02 -9.412111e-02 -1.227960e-01 -8.578202e-02 
-5.989652e-02 -8.597158e-02 -8.044896e-02 -4.966689e-02 -5.090394e-02 
-7.621411e-02 
          166           167           168           169           170 
171           172           173           174           175           176 
-5.607894e-02 -4.220823e-02 -4.951393e-02 -6.460142e-02 -5.800217e-02 
-6.020700e-02 -7.015962e-02 -9.294976e-02 -8.097585e-02 -9.062493e-02 
-1.225480e-01 
          177           178           179           180           181 
182           183           184           185           186           187 
-1.225652e-01 -1.132074e-01 -1.340699e-01 -1.495078e-01 -1.435047e-01 
-1.410624e-01 -1.638916e-01 -1.550586e-01 -1.471676e-01 -1.581528e-01 
-1.463419e-01 
          188           189           190           191           192 
193           194           195           196           197           198 
-1.411299e-01 -1.259033e-01 -1.176565e-01 -8.654151e-02 -7.436337e-02 
-6.572195e-02 -8.199110e-02 -6.331594e-02 -4.585795e-02 -4.320970e-02 
-3.044463e-02 
          199           200           201           202           203 
204           205           206           207           208           209 
-6.952839e-03 -5.644757e-03 -1.465003e-02  3.496420e-03  1.452381e-02 
2.689209e-02  2.049051e-02  2.667056e-03 -1.948740e-02 -2.943241e-02 
-5.695245e-02 
          210           211           212           213           214 
215           216           217           218           219           220 
-9.275253e-02 -1.117874e-01 -1.343494e-01 -1.338687e-01 -1.675215e-01 
-1.690180e-01 -1.416816e-01 -1.661334e-01 -1.799071e-01 -1.631201e-01 
-1.418277e-01 
          221           222           223           224           225 
226           227           228           229           230           231 
-1.567427e-01 -1.711563e-01 -1.335328e-01 -1.165508e-01 -1.208487e-01 
-1.466198e-01 -1.466227e-01 -1.642190e-01 -1.575226e-01 -1.720767e-01 
-1.856092e-01 
          232           233           234           235           236 
237           238           239           240           241           242 
-1.855702e-01 -1.739880e-01 -1.832985e-01 -1.917563e-01 -1.801434e-01 
-1.652353e-01 -1.630405e-01 -1.416341e-01 -1.432769e-01 -1.366978e-01 
-1.144764e-01 
          243           244           245           246           247 
248           249           250           251           252           253 
-1.126911e-01 -1.084816e-01 -8.169143e-02 -6.688594e-02 -4.957989e-02 
-3.192743e-02 -2.432390e-02 -2.596985e-02 -2.201664e-02  2.879271e-03 
```

```
-9.557249e-03
          254            255            256            257            258 
259            260            261            262            263            264 
-3.435733e-02 -8.024868e-02 -9.234821e-02 -1.152050e-01 -9.056808e-02 
-8.413549e-02 -1.305415e-01 -1.429290e-01 -1.144359e-01 -1.249721e-01 
-1.584724e-01 
          265            266            267            268            269 
270            271            272            273            274            275 
-1.510718e-01 -1.365081e-01 -1.556118e-01 -1.648958e-01 -1.368453e-01 
-1.327603e-01 -1.360403e-01 -1.553859e-01 -1.602295e-01 -1.648586e-01 
-1.721366e-01 
          276            277            278            279            280 
281            282            283            284            285            286 
-1.485194e-01 -1.326290e-01 -1.528470e-01 -1.445643e-01 -1.506294e-01 
-1.497556e-01 -1.479149e-01 -1.595885e-01 -1.526529e-01 -1.389296e-01 
-1.317245e-01 
          287            288            289            290            291 
292            293            294            295            296            297 
-1.111576e-01 -9.636392e-02 -8.843512e-02 -7.118781e-02 -4.150490e-02 
-3.476423e-02 -3.752496e-02 -4.550283e-03  1.236398e-02  3.999855e-03 
1.376050e-03 
          298            299            300            301            302 
303            304            305            306            307            308 
-1.428585e-02 -3.071456e-02 -5.355818e-02 -4.294162e-02 -2.799299e-02 
5.272733e-03  4.416801e-02  8.072983e-02  6.488373e-02  9.050918e-02 
1.042629e-01 
          309            310            311            312            313 
314            315            316            317            318            319 
 1.006078e-01  7.307807e-02  6.475792e-02  3.776563e-02  5.236331e-02 
9.118622e-02  9.029951e-02  1.078559e-01  1.029281e-01  1.339769e-01 
9.100242e-02 
          320            321            322            323            324 
325            326            327            328            329            330 
 7.007594e-02  1.102044e-01  1.247020e-01  6.041397e-02  4.487274e-02 
8.253526e-02  5.714534e-02 -1.571598e-03  6.371767e-03  2.393036e-02 
-2.120148e-02 
          331            332            333            334            335 
336            337            338            339            340            341 
-6.176480e-02 -5.116887e-02 -4.252185e-02 -7.739585e-02 -9.207117e-02 
-1.117379e-01 -1.033549e-01 -1.237113e-01 -1.118408e-01 -9.731261e-02 
-7.987444e-02 
          342            343            344            345            346 
347            348            349            350            351            352 
-8.017759e-02 -1.013539e-01 -1.030351e-01 -9.838079e-02 -7.497545e-02 
-6.225718e-02 -5.554111e-02 -7.587227e-02 -6.065857e-02 -4.800505e-02 
-1.176949e-02 
          353            354            355            356            357 
358            359            360            361            362            363 
-1.097790e-03  7.632390e-04  4.191126e-02  7.108981e-02  8.561756e-02 
1.052607e-01  1.738111e-01  1.823601e-01  2.006466e-01  2.494216e-01 
3.300870e-01 
          364            365            366            367            368 
369            370            371            372            373            374 
 2.916857e-01  2.677389e-01  3.453737e-01  3.427513e-01  2.744381e-01 
```

```
1.954961e-01  1.340725e-01  1.693425e-01  1.954809e-01  2.474051e-01
3.034286e-01
          375           376           377           378           379
380           381           382           383           384           385
 2.490873e-01  2.618289e-01  2.594507e-01  2.775005e-01  3.149495e-01
3.690323e-01  3.508615e-01  3.305711e-01  2.890912e-01  2.121107e-01
1.812971e-01
          386           387           388           389           390
391           392           393           394           395           396
 1.611780e-01  1.561376e-01  1.259513e-01  1.114391e-01  8.342234e-02
7.529608e-02  8.462434e-02  7.892285e-02  6.763970e-02  6.230326e-02
4.059737e-02
          397           398           399           400           401
402           403           404           405           406           407
 5.345384e-02  6.152299e-02  6.864493e-02  6.755930e-02  7.784116e-02
5.930995e-02  5.795010e-02  6.795347e-02  7.102137e-02  7.831176e-02
7.456178e-02
          408           409           410           411           412
413           414           415           416           417           418
 9.011037e-02  1.060301e-01  1.304111e-01  2.165947e-01  2.296420e-01
1.899036e-01  2.051479e-01  1.877242e-01  1.583102e-01  1.722275e-01
1.518190e-01
          419           420           421           422           423
424           425           426           427           428           429
 1.111503e-01  8.382500e-02  1.000235e-01  1.372244e-01  1.461098e-01
1.592196e-01  1.418655e-01  1.556824e-01  1.386646e-01  1.271754e-01
1.020953e-01
          430           431           432           433           434
435           436           437           438           439           440
 1.129326e-01  1.120583e-01  1.072353e-01  1.467138e-01  1.608993e-01
1.383571e-01  1.517435e-01  1.028397e-01  8.563021e-02  7.120571e-02
1.282626e-01
          441           442           443           444           445
446           447           448           449           450           451
 1.427431e-01  9.630028e-02  9.989693e-02  1.657904e-01  1.845395e-01
1.420597e-01  1.743395e-01  2.733528e-01  2.281912e-01  1.750333e-01
2.095005e-01
          452           453           454           455           456
457           458           459           460           461           462
 3.444114e-01  4.189671e-01  2.707203e-01  2.528741e-01  3.454096e-01
2.913460e-01  3.065091e-01  4.472971e-01  6.006762e-01  5.357308e-01
3.785613e-01
          463           464           465           466           467
468           469           470           471           472           473
 4.319756e-01  3.641575e-01  2.927618e-01  2.335054e-01  1.985870e-01
1.528776e-01  1.255755e-01  9.340579e-02  7.505288e-02  5.861449e-02
2.269492e-02
          474           475           476           477           478
479           480           481           482           483           484
 1.030761e-02 -1.033160e-02 -1.489069e-02 -1.121157e-02 -2.301405e-02
-4.311174e-02 -3.517544e-02 -3.796919e-02 -6.633628e-02 -7.163065e-02
-9.854430e-02
          485           486           487           488           489
490           491           492           493           494           495
```

```
-1.020200e-01 -7.470737e-02 -8.330658e-02 -2.729880e-02 -3.420754e-02
-9.316875e-02 -6.258422e-02 -8.523198e-02 -3.928792e-02 -2.044682e-02
-7.176108e-02
          496           497           498           499           500
501           502           503           504           505           506
-2.873215e-02 -7.258866e-02 -1.576461e-02 -6.351118e-02 -1.341373e-02
1.039700e-02  1.379080e-02  4.118106e-02  4.327562e-02  5.535198e-02
5.520484e-02
          507           508           509           510           511
512           513           514           515           516           517
 5.999531e-02  5.858096e-02  5.915056e-02  5.500490e-02  4.938637e-02
1.317576e-02 -3.322280e-02 -7.712489e-02 -1.133782e-01 -1.764602e-01
-1.999948e-01
          518           519           520           521           522
523           524           525           526           527           528
-2.491698e-01 -2.337244e-01 -2.522749e-01 -2.281895e-01 -2.350864e-01
-1.962885e-01 -1.940362e-01 -1.574462e-01 -1.127796e-01 -9.197486e-02
-1.441374e-01
          529           530           531           532           533
534           535           536           537           538           539
-1.580788e-01 -1.135036e-01 -6.582795e-02 -7.490661e-02 -2.285550e-02
-2.662720e-02  3.476743e-02  3.411462e-02  9.370645e-02  1.181256e-01
9.943541e-02
          540           541           542           543           544
545           546           547           548           549           550
 8.207795e-02  3.394199e-02 -2.464683e-02 -2.665013e-02 -8.860866e-02
-1.647257e-01 -2.101026e-01 -1.944208e-01 -1.996602e-01 -2.112620e-01
-2.464155e-01
          551           552           553           554           555
556           557           558           559           560           561
-2.708843e-01 -2.613004e-01 -2.614069e-01 -1.934760e-01 -1.490610e-01
-8.111284e-02 -4.965397e-02  1.818505e-02  4.401125e-02  8.774206e-02
8.967682e-02
          562           563           564           565           566
567           568           569           570           571           572
 7.568937e-02  6.709869e-02  6.937443e-02  7.648835e-02  6.921764e-02
5.311304e-02  8.130731e-02  5.085948e-02  4.250460e-02  3.307234e-02
-2.919437e-02
          573           574           575           576           577
578           579           580           581           582           583
-3.267871e-02 -9.102012e-02 -8.406498e-02 -1.158298e-01 -7.101522e-02
-6.722696e-02 -1.228717e-01 -1.523422e-01 -1.928903e-01 -1.782528e-01
-1.827061e-01
          584           585
-1.939270e-01 -1.778137e-01
```
