# Supplementary material for: Insights into the mechanism(s) of digestion of crystalline cellulose by plant class C GH9 endoglucanases
Source: J Mol Model. 2019 Jul 23;25(8):240. doi: 10.1007/s00894-019-4133-1 (PMC7385011; doi:10.1007/s00894-019-4133-1)
Supplement: Supplementary file 3 — (PDF 2382 kb) [file 894_2019_4133_MOESM3_ESM.pdf]

Supplementary Text 10

```
Call:
  pca.xyz(xyz = xyz96, rm.gaps = TRUE)

Class:
  pca

Number of eigenvalues:
  1089

      Eigenvalue Variance Cumulative
   PC 1   1451.111   72.530     72.530
   PC 2    128.498    6.423     78.952
   PC 3     99.560    4.976     83.929
   PC 4     67.942    3.396     87.324
   PC 5     46.299    2.314     89.639
   PC 6     44.221    2.210     91.849

   (Obtained from 96 conformers with 1089 xyz input values).

+ attr: L, U, z, au, sdev, mean, call

[,1]         [,2]       [,3]        [,4]          [,5]       [,6]
E:\\R-3.1.2\\scripts\\40.1ns_data\\NAT0.pdb
-26.174766 -49.19484841 -13.655031  -8.4493930  1.648399e+00  -4.410633
E:\\R-3.1.2\\scripts\\40.1ns_data\\LJP6.pdb
-7.137154 -43.56926318 -12.301589  15.2993715  1.291078e+01  10.959699
E:\\R-3.1.2\\scripts\\40.1ns_data\\3WY9.pdb
-9.109342 -41.94685048   1.522348 -34.4764359 -1.993688e+01  11.397632
E:\\R-3.1.2\\scripts\\40.1ns_data\\ZSP9.pdb
-19.659492 -40.28741188 -26.246999  12.7487217  9.812384e+00 -12.332311
E:\\R-3.1.2\\scripts\\mPDBS\\summary_pdb_93\\013d855ad26c877c.final.pdb
52.364186  -7.89376335  10.028222   3.1244011 -2.053072e+00  -9.113038
E:\\R-3.1.2\\scripts\\mPDBS\\summary_pdb_93\\0a2a705342ca0d54.final.pdb
-24.106361   0.06297032 -15.802606   7.3011151  1.205262e+01  -2.752591
E:\\R-3.1.2\\scripts\\mPDBS\\summary_pdb_93\\0a59fceadb4194d9.final.pdb
51.814984   6.72738105  -4.898246  -3.8622086  1.459474e+00   3.485965
E:\\R-3.1.2\\scripts\\mPDBS\\summary_pdb_93\\0dba59f3306825e8.final.pdb
52.321256  -5.67851580  10.586261   3.2141411  1.090832e+00  -4.835424
E:\\R-3.1.2\\scripts\\mPDBS\\summary_pdb_93\\126f26a78b57339c.final.pdb
51.837928  -1.75043352  10.253150   2.5403043  6.869624e+00   2.897134
E:\\R-3.1.2\\scripts\\mPDBS\\summary_pdb_93\\12f06f3e342df706.final.pdb
-24.134824   1.31338096 -17.286996   7.0723233  1.152521e+01  -2.362576
E:\\R-3.1.2\\scripts\\mPDBS\\summary_pdb_93\\18618a2a251b867b.final.pdb
-40.739549   8.82544584  -2.865992  -4.7330800  1.223670e+00  -2.312585
E:\\R-3.1.2\\scripts\\mPDBS\\summary_pdb_93\\1f305f598641eeb2.final.pdb
-32.005064  -9.71362521  13.466948  -0.5339163 -5.491641e+00  -9.002533
E:\\R-3.1.2\\scripts\\mPDBS\\summary_pdb_93\\1f8a00b551be0a39.final.pdb
-40.739549   8.82544584  -2.865992  -4.7330800  1.223670e+00  -2.312585
E:\\R-3.1.2\\scripts\\mPDBS\\summary_pdb_93\\219f45328d47579a.final.pdb
52.299583   2.79894692  -4.531414  -3.1529005 -4.293366e+00  -4.249328
E:\\R-3.1.2\\scripts\\mPDBS\\summary_pdb_93\\238dd3918e54336d.final.pdb
52.342705   0.62283021  -5.059441  -3.2313690 -7.361823e+00  -8.448938
E:\\R-3.1.2\\scripts\\mPDBS\\summary_pdb_93\\2b3027889e2fdc42.final.pdb
```

```
52.532401   4.27560457  -4.147460  -2.9836572 -4.734571e+00  -5.132649
E:\\R-3.1.2\\scripts\\mPDBS\\summary_pdb_93\\2fcb3b46e33793b0.final.pdb
-32.505208  -3.41648142  13.458951  -1.0404743  3.628293e+00   3.215256
E:\\R-3.1.2\\scripts\\mPDBS\\summary_pdb_93\\34eb32825184200б.final.pdb
-28.902894  -6.40891637  10.824239  -1.2267218  4.192342e+00   6.727459
E:\\R-3.1.2\\scripts\\mPDBS\\summary_pdb_93\\354b32871797b7ab.final.pdb
-24.485242  -3.90256739  -5.573500  -5.6588793 -8.822040e+00  -5.565115
E:\\R-3.1.2\\scripts\\mPDBS\\summary_pdb_93\\397368adbc32d5de.final.pdb
51.837928  -1.75043352  10.253150   2.5403043  6.869624e+00   2.897134
E:\\R-3.1.2\\scripts\\mPDBS\\summary_pdb_93\\411ca13cd3df6edd.final.pdb
-33.307115   0.17342018  16.643395  14.9074755 -2.255632e+00   9.848612
E:\\R-3.1.2\\scripts\\mPDBS\\summary_pdb_93\\42357eee6642bf12.final.pdb
-3.018193  10.44977856  -6.042500  -2.3170632  5.685494e+00  -4.742247
E:\\R-3.1.2\\scripts\\mPDBS\\summary_pdb_93\\44342d1f75a7fcfc.final.pdb
-31.213544   3.19369724  -1.717117  -6.1904130 -7.007488e+00  -6.298543
E:\\R-3.1.2\\scripts\\mPDBS\\summary_pdb_93\\448ef21dffe8cdfb.final.pdb
-32.021879  -7.34456371  13.792061  -0.3666375 -2.150499e+00  -4.517302
E:\\R-3.1.2\\scripts\\mPDBS\\summary_pdb_93\\472a40b3e8f113c7.final.pdb
52.321256  -5.67851580  10.586261   3.2141411  1.090832e+00  -4.835424
E:\\R-3.1.2\\scripts\\mPDBS\\summary_pdb_93\\48a26225951ea153.final.pdb
51.409674   9.88838643  -4.823055   9.2992846 -8.643122e+00   9.160267
E:\\R-3.1.2\\scripts\\mPDBS\\summary_pdb_93\\4ab4a5c7c6682ce6.final.pdb
51.441478  -1.51202338  13.383862   5.4258168  1.114719e+01   3.728844
E:\\R-3.1.2\\scripts\\mPDBS\\summary_pdb_93\\4eaa45b62cb6a25f.final.pdb
-25.012963   2.20198344  -5.412306  -6.2897189 -7.419569e-04   6.369788
E:\\R-3.1.2\\scripts\\mPDBS\\summary_pdb_93\\58c371611d85ac96.final.pdb
51.837928  -1.75043352  10.253150   2.5403043  6.869624e+00   2.897134
E:\\R-3.1.2\\scripts\\mPDBS\\summary_pdb_93\\59a647f04822d30a.final.pdb
-31.698143   7.12213137  -2.083950  -6.8997210 -1.254647e+00   1.436750
E:\\R-3.1.2\\scripts\\mPDBS\\summary_pdb_93\\59b091fe385e05fc.final.pdb
51.814984   6.72738105  -4.898246  -3.8622086  1.459474e+00   3.485965
E:\\R-3.1.2\\scripts\\mPDBS\\summary_pdb_93\\5c35ac30f930d2db.final.pdb
-25.012963   2.20198344  -5.412306  -6.2897189 -7.419569e-04   6.369788
E:\\R-3.1.2\\scripts\\mPDBS\\summary_pdb_93\\5f60752431cf2dcb.final.pdb
-23.608251  -4.77404887 -17.578573   7.6593549  2.655689e+00 -14.304201
E:\\R-3.1.2\\scripts\\mPDBS\\summary_pdb_93\\626456dd6ff2868d.final.pdb
52.321256  -5.67851580  10.586261   3.2141411  1.090832e+00  -4.835424
E:\\R-3.1.2\\scripts\\mPDBS\\summary_pdb_93\\6583d53ff3c8f7ef.final.pdb
-31.698143   7.12213137  -2.083950  -6.8997210 -1.254647e+00   1.436750
E:\\R-3.1.2\\scripts\\mPDBS\\summary_pdb_93\\69760178986bef01.final.pdb
19.207839   5.30125613  -4.303739  -6.9120495  4.977779e-01   5.047911
E:\\R-3.1.2\\scripts\\mPDBS\\summary_pdb_93\\6a9d87d47926b1c8.final.pdb
52.002379  -2.78615454  10.701147  16.9283217 -9.597930e+00   1.091975
E:\\R-3.1.2\\scripts\\mPDBS\\summary_pdb_93\\6bb3547ea982ffc4.final.pdb
38.855495   2.15919718  -4.706115  -6.3852443 -5.273944e-01   8.868925
E:\\R-3.1.2\\scripts\\mPDBS\\summary_pdb_93\\716d9624611d1c42.final.pdb
-24.135972   1.33050197 -17.417379   7.0285153  1.147699e+01  -2.369298
E:\\R-3.1.2\\scripts\\mPDBS\\summary_pdb_93\\75f27e70ad0f1be4.final.pdb
-31.698143   7.12213137  -2.083950  -6.8997210 -1.254647e+00   1.436750
E:\\R-3.1.2\\scripts\\mPDBS\\summary_pdb_93\\769f910fffb22872.final.pdb
-41.207646  12.17629586  -3.820193   8.1660049 -9.483033e+00   3.317251
E:\\R-3.1.2\\scripts\\mPDBS\\summary_pdb_93\\791f8c80dd5c3135.final.pdb
51.814984   6.72738105  -4.898246  -3.8622086  1.459474e+00   3.485965
E:\\R-3.1.2\\scripts\\mPDBS\\summary_pdb_93\\7fad98e66a3f6099.final.pdb
```

```
-38.545747   8.28082718  -3.047010  -4.7473510  4.885690e-01  -1.313072
E:\\R-3.1.2\\scripts\\mPDBS\\summary_pdb_93\\804b89c94a633196.final.pdb
-32.505208  -3.41648142  13.458951  -1.0404743  3.628293e+00   3.215256
E:\\R-3.1.2\\scripts\\mPDBS\\summary_pdb_93\\813cc65a2298feba.final.pdb
-32.505208  -3.41648142  13.458951  -1.0404743  3.628293e+00   3.215256
E:\\R-3.1.2\\scripts\\mPDBS\\summary_pdb_93\\83c0eefcfe12d3f9.final.pdb
-33.307115   0.17342018  16.643395  14.9074755 -2.255632e+00   9.848612
E:\\R-3.1.2\\scripts\\mPDBS\\summary_pdb_93\\86d50469ef569760.final.pdb
52.321256  -5.67851580  10.586261   3.2141411  1.090832e+00  -4.835424
E:\\R-3.1.2\\scripts\\mPDBS\\summary_pdb_93\\89d0666e1402203d.final.pdb
-31.698143   7.12213137  -2.083950  -6.8997210 -1.254647e+00   1.436750
E:\\R-3.1.2\\scripts\\mPDBS\\summary_pdb_93\\8b34852716dadc62.final.pdb
-31.978950  -9.55981126  13.234022  -0.4563775 -5.294403e+00  -8.794916
E:\\R-3.1.2\\scripts\\mPDBS\\summary_pdb_93\\8b6ef9ac3fe74395.final.pdb
-21.124715   4.85636057  16.621713   4.0672685  1.291127e+01  -5.779951
E:\\R-3.1.2\\scripts\\mPDBS\\summary_pdb_93\\98bbe0d5986c138f.final.pdb
51.814984   6.72738105  -4.898246  -3.8622086  1.459474e+00   3.485965
E:\\R-3.1.2\\scripts\\mPDBS\\summary_pdb_93\\9c22f4909d67e2fa.final.pdb
-32.901658  -3.17807128  16.589663   1.8450382  7.905855e+00   4.046966
E:\\R-3.1.2\\scripts\\mPDBS\\summary_pdb_93\\9dda8ed0d81fb3ef.final.pdb
-31.149909   0.31681718  -1.220071  -6.2908585 -9.896429e+00 -10.844237
E:\\R-3.1.2\\scripts\\mPDBS\\summary_pdb_93\\9f752b3616e6033b.final.pdb
-4.055341  -8.07198586 -16.918572   1.4175543  5.514134e+00  12.329708
E:\\R-3.1.2\\scripts\\mPDBS\\summary_pdb_93\\a02c70083816d6ba.final.pdb
-25.012963   2.20198344  -5.412306  -6.2897189 -7.419569e-04   6.369788
E:\\R-3.1.2\\scripts\\mPDBS\\summary_pdb_93\\a0c997d891e4e082.final.pdb
-20.421520  14.14537411  -4.421288  -1.7576093  6.586276e+00  -9.719173
E:\\R-3.1.2\\scripts\\mPDBS\\summary_pdb_93\\ab8039a2d823c616.final.pdb
-20.714082  15.03520077  -3.202218  -0.1012829  9.474955e+00 -10.109546
E:\\R-3.1.2\\scripts\\mPDBS\\summary_pdb_93\\acf4d74b9c71d4dc.final.pdb
-32.505208  -3.41648142  13.458951  -1.0404743  3.628293e+00   3.215256
E:\\R-3.1.2\\scripts\\mPDBS\\summary_pdb_93\\ad8a823e4b4c668e.final.pdb
-24.715050  -1.42588958 -18.955711  44.6148030 -2.268616e+01  -2.718545
E:\\R-3.1.2\\scripts\\mPDBS\\summary_pdb_93\\b16ababd03b7b857.final.pdb
-32.901658  -3.17807128  16.589663   1.8450382  7.905855e+00   4.046966
E:\\R-3.1.2\\scripts\\mPDBS\\summary_pdb_93\\b226923e697d3c63.final.pdb
-32.386416  10.95778530  -0.502680   7.8452512 -8.625127e+00   6.931065
E:\\R-3.1.2\\scripts\\mPDBS\\summary_pdb_93\\b28e48f6fa27a165.final.pdb
51.814984   6.72738105  -4.898246  -3.8622086  1.459474e+00   3.485965
E:\\R-3.1.2\\scripts\\mPDBS\\summary_pdb_93\\b42c4924e3cd1aec.final.pdb
-31.677630   6.42136801  -1.058877  -6.9216980 -1.075131e+00   1.090666
E:\\R-3.1.2\\scripts\\mPDBS\\summary_pdb_93\\b4dd239fcabff1df.final.pdb
51.814984   6.72738105  -4.898246  -3.8622086  1.459474e+00   3.485965
E:\\R-3.1.2\\scripts\\mPDBS\\summary_pdb_93\\b669942ef97459a2.final.pdb
-31.978950  -9.55981126  13.234022  -0.4563775 -5.294403e+00  -8.794916
E:\\R-3.1.2\\scripts\\mPDBS\\summary_pdb_93\\b99f4d9d87c1017b.final.pdb
51.814984   6.72738105  -4.898246  -3.8622086  1.459474e+00   3.485965
E:\\R-3.1.2\\scripts\\mPDBS\\summary_pdb_93\\b9d2cbf3975403e7.final.pdb
51.814984   6.72738105  -4.898246  -3.8622086  1.459474e+00   3.485965
E:\\R-3.1.2\\scripts\\mPDBS\\summary_pdb_93\\bc88c8a505f3a288.final.pdb
52.494066  -3.82676802  -7.457262  -3.4716925 -9.234365e+00 -10.277409
E:\\R-3.1.2\\scripts\\mPDBS\\summary_pdb_93\\bdac0e64f4ef427e.final.pdb
-32.910665  -0.06498996  13.512683  12.0219630 -6.533194e+00   9.016902
E:\\R-3.1.2\\scripts\\mPDBS\\summary_pdb_93\\ca2a1f55fc9d0681.final.pdb
```

```
-31.978950  -9.55981126  13.234022  -0.4563775 -5.294403e+00  -8.794916
E:\\R-3.1.2\\scripts\\mPDBS\\summary_pdb_93\\ce34248de3e81a3c.final.pdb
8.504919  -7.45048531  -8.031510  -5.2420083 -5.277923e+00   4.798081
E:\\R-3.1.2\\scripts\\mPDBS\\summary_pdb_93\\d02e697772494a61.final.pdb
52.363219  -0.07793314  -4.034368  -3.2533461 -7.182307e+00  -8.795022
E:\\R-3.1.2\\scripts\\mPDBS\\summary_pdb_93\\d04221245d015047.final.pdb
-20.704482  14.82002266  -2.915211  -0.1741302  9.318393e+00  -9.899160
E:\\R-3.1.2\\scripts\\mPDBS\\summary_pdb_93\\d678141e4018054a.final.pdb
-28.610665   5.96220875  -1.723666  -7.6565781 -1.378050e+00   3.098357
E:\\R-3.1.2\\scripts\\mPDBS\\summary_pdb_93\\d71b40af0f0efe39.final.pdb
13.943274   6.69178313  -4.886790  -5.3947333  2.084602e+00   2.168631
E:\\R-3.1.2\\scripts\\mPDBS\\summary_pdb_93\\dc0809eff10197e2.final.pdb
49.546987  -1.75794058  -5.328683  -3.9312071 -7.809353e+00  -5.878839
E:\\R-3.1.2\\scripts\\mPDBS\\summary_pdb_93\\e1163918d3707839.final.pdb
-31.170422   1.01758054  -2.245144  -6.2688814 -1.007595e+01 -10.498153
E:\\R-3.1.2\\scripts\\mPDBS\\summary_pdb_93\\e4b48b06824ef848.final.pdb
-40.739549   8.82544584  -2.865992  -4.7330800  1.223670e+00  -2.312585
E:\\R-3.1.2\\scripts\\mPDBS\\summary_pdb_93\\e529e19ad281fea2.final.pdb
52.364186  -7.89376335  10.028222   3.1244011 -2.053072e+00  -9.113038
E:\\R-3.1.2\\scripts\\mPDBS\\summary_pdb_93\\e5602082718ec3a1.final.pdb
-32.505208  -3.41648142  13.458951  -1.0404743  3.628293e+00   3.215256
E:\\R-3.1.2\\scripts\\mPDBS\\summary_pdb_93\\e5d589d996d0a960.final.pdb
-33.131949   7.59861551  -1.733757  -6.8126959 -1.489380e+00   1.008771
E:\\R-3.1.2\\scripts\\mPDBS\\summary_pdb_93\\e9df9e72307a2fbd.final.pdb
-25.012963   2.20198344  -5.412306  -6.2897189 -7.419569e-04   6.369788
E:\\R-3.1.2\\scripts\\mPDBS\\summary_pdb_93\\ea00f8694090cae7.final.pdb
-32.505208  -3.41648142  13.458951  -1.0404743  3.628293e+00   3.215256
E:\\R-3.1.2\\scripts\\mPDBS\\summary_pdb_93\\eba517be32db050e.final.pdb
51.814984   6.72738105  -4.898246  -3.8622086  1.459474e+00   3.485965
E:\\R-3.1.2\\scripts\\mPDBS\\summary_pdb_93\\ec04b9545899c29d.final.pdb
-32.090016   9.27176564  -3.642397   2.9193257 -9.438335e+00   5.196032
E:\\R-3.1.2\\scripts\\mPDBS\\summary_pdb_93\\ee6862cbcad52078.final.pdb
51.922946   4.87625360  -3.779175  -4.1070775  2.059351e+00   3.214305
E:\\R-3.1.2\\scripts\\mPDBS\\summary_pdb_93\\ef6a42fc83b81e5d.final.pdb
-40.794879   8.90289420  -2.113708  -4.8426400  1.268972e+00  -2.592993
E:\\R-3.1.2\\scripts\\mPDBS\\summary_pdb_93\\ef8e88d4ad93a0ad.final.pdb
-20.704482  14.82002266  -2.915211  -0.1741302  9.318393e+00  -9.899160
E:\\R-3.1.2\\scripts\\mPDBS\\summary_pdb_93\\f14fcae8deec4fa3.final.pdb
-4.055341  -8.07198586 -16.918572   1.4175543  5.514134e+00  12.329708
E:\\R-3.1.2\\scripts\\mPDBS\\summary_pdb_93\\f83584e22003fb92.final.pdb
51.409674   9.88838643  -4.823055   9.2992846 -8.643122e+00   9.160267
E:\\R-3.1.2\\scripts\\mPDBS\\summary_pdb_93\\f8e2bf87e52caa29.final.pdb
52.364186  -7.89376335  10.028222   3.1244011 -2.053072e+00  -9.113038

[,7]        [,8]        [,9]        [,10]        [,11]        [,12]
E:\\R-3.1.2\\scripts\\40.1ns_data\\NAT0.pdb
17.06345632 -24.6291789  16.7251184  -1.12762224  15.97285860  -5.31095965
E:\\R-3.1.2\\scripts\\40.1ns_data\\LJP6.pdb
0.02495566 -22.5977242 -14.5192516  -7.59201751 -19.30663541   3.29050749
E:\\R-3.1.2\\scripts\\40.1ns_data\\3WY9.pdb
21.41368785  21.8273251 -12.9965379 -11.96713478   1.62777178   0.96118231
E:\\R-3.1.2\\scripts\\40.1ns_data\\ZSP9.pdb
13.66185423  21.2789629  13.0804015  17.81886777 -11.24871290   2.03112756
E:\\R-3.1.2\\scripts\\mPDBS\\summary_pdb_93\\013d855ad26c877c.final.pdb
```

-3.64246511 0.7569109 -0.6655648 -1.08306161 0.47400424 0.63188736
E:\\R-3.1.2\\scripts\\mPDBS\\summary_pdb_93\\0a2a705342ca0d54.final.pdb
-5.79142728 6.1537747 0.6867361 -6.72779820 5.18515905 3.77263176
E:\\R-3.1.2\\scripts\\mPDBS\\summary_pdb_93\\0a59fceadb4194d9.final.pdb
1.52248558 -0.5869192 1.5805519 -0.26987727 -0.50029384 1.40808310
E:\\R-3.1.2\\scripts\\mPDBS\\summary_pdb_93\\0dba59f3306825e8.final.pdb
-2.79492795 1.8929649 1.0360744 -1.41074777 -0.62236801 -2.16266062
E:\\R-3.1.2\\scripts\\mPDBS\\summary_pdb_93\\126f26a78b57339c.final.pdb
0.10539184 2.3491297 3.8360117 -3.52176292 -0.15041229 -0.35766305
E:\\R-3.1.2\\scripts\\mPDBS\\summary_pdb_93\\12f06f3e342df706.final.pdb
-5.95823681 5.7447785 -0.3022216 -6.10829374 4.03228687 2.69666386
E:\\R-3.1.2\\scripts\\mPDBS\\summary_pdb_93\\18618a2a251b867b.final.pdb
0.54604394 -0.5153787 2.5680971 -2.39608401 -1.63004817 1.72865288
E:\\R-3.1.2\\scripts\\mPDBS\\summary_pdb_93\\1f305f598641eeb2.final.pdb
-7.32041527 -0.2265019 -0.5807946 0.32138921 -0.29247973 0.12557839
E:\\R-3.1.2\\scripts\\mPDBS\\summary_pdb_93\\1f8a00b551be0a39.final.pdb
0.54604394 -0.5153787 2.5680971 -2.39608401 -1.63004817 1.72865288
E:\\R-3.1.2\\scripts\\mPDBS\\summary_pdb_93\\219f45328d47579a.final.pdb
-1.43229579 -1.1784035 -1.2383711 1.82325630 -0.96508965 -0.39903836
E:\\R-3.1.2\\scripts\\mPDBS\\summary_pdb_93\\238dd3918e54336d.final.pdb
-2.29268129 -2.3760145 -2.9196718 2.12441540 0.11256985 2.35147824
E:\\R-3.1.2\\scripts\\mPDBS\\summary_pdb_93\\2b3027889e2fdc42.final.pdb
-3.41807833 -1.9282377 -1.0798427 3.75297605 0.86481284 -1.71485176
E:\\R-3.1.2\\scripts\\mPDBS\\summary_pdb_93\\2fcb3b46e33793b0.final.pdb
-3.41004610 1.3064727 4.0089597 -1.97079482 -1.05945885 -0.83818845
E:\\R-3.1.2\\scripts\\mPDBS\\summary_pdb_93\\34eb328251842006.final.pdb
-5.77687187 1.7427085 0.9881965 -0.43115116 -0.07420188 -5.00261139
E:\\R-3.1.2\\scripts\\mPDBS\\summary_pdb_93\\354b32871797b7ab.final.pdb
-8.05779080 -2.4225945 -5.7155234 4.41676041 0.42688489 -1.61160523
E:\\R-3.1.2\\scripts\\mPDBS\\summary_pdb_93\\397368adbc32d5de.final.pdb
0.10539184 2.3491297 3.8360117 -3.52176292 -0.15041229 -0.35766305
E:\\R-3.1.2\\scripts\\mPDBS\\summary_pdb_93\\411ca13cd3df6edd.final.pdb
7.28573592 -0.9562578 -4.8017624 6.73218438 4.26913720 3.93774730
E:\\R-3.1.2\\scripts\\mPDBS\\summary_pdb_93\\42357eee6642bf12.final.pdb
4.21215568 0.7998298 -1.3459308 -2.13628745 -2.23733595 -6.47709626
E:\\R-3.1.2\\scripts\\mPDBS\\summary_pdb_93\\44342d1f75a7fcfc.final.pdb
-3.48501730 -2.0464649 -0.1984542 2.05975188 -2.08964349 -0.20725649
E:\\R-3.1.2\\scripts\\mPDBS\\summary_pdb_93\\448ef21dffe8cdfb.final.pdb
-6.31036588 0.8503078 1.2090224 0.14022033 -1.53141456 -2.64318602
E:\\R-3.1.2\\scripts\\mPDBS\\summary_pdb_93\\472a40b3e8f113c7.final.pdb
-2.79492795 1.8929649 1.0360744 -1.41074777 -0.62236801 -2.16266062
E:\\R-3.1.2\\scripts\\mPDBS\\summary_pdb_93\\48a26225951ea153.final.pdb
5.90438095 -0.8914895 2.4181353 0.20996334 -0.93579462 0.46350045
E:\\R-3.1.2\\scripts\\mPDBS\\summary_pdb_93\\4ab4a5c7c6682ce6.final.pdb
6.40440133 0.4158464 -5.8258318 4.45148379 5.62833963 5.13939702
E:\\R-3.1.2\\scripts\\mPDBS\\summary_pdb_93\\4eaa45b62cb6a25f.final.pdb
-4.24262393 -0.6334991 -1.2152996 2.02246774 -0.18597880 -2.55500038
E:\\R-3.1.2\\scripts\\mPDBS\\summary_pdb_93\\58c371611d85ac96.final.pdb
0.10539184 2.3491297 3.8360117 -3.52176292 -0.15041229 -0.35766305
E:\\R-3.1.2\\scripts\\mPDBS\\summary_pdb_93\\59a647f04822d30a.final.pdb
-0.53023594 -1.4549806 2.6204689 -0.03338169 -1.62484768 1.59986497
E:\\R-3.1.2\\scripts\\mPDBS\\summary_pdb_93\\59b091fe385e05fc.final.pdb
1.52248558 -0.5869192 1.5805519 -0.26987727 -0.50029384 1.40808310
E:\\R-3.1.2\\scripts\\mPDBS\\summary_pdb_93\\5c35ac30f930d2db.final.pdb

-4.24262393 -0.6334991 -1.2152996   2.02246774 -0.18597880 -2.55500038
E:\\R-3.1.2\\scripts\\mPDBS\\summary_pdb_93\\5f60752431cf2dcb.final.pdb
-9.69361496   3.8521675 -4.7374691 -3.55445347   4.56837638   3.60170154
E:\\R-3.1.2\\scripts\\mPDBS\\summary_pdb_93\\626456dd6ff2868d.final.pdb
-2.79492795   1.8929649   1.0360744 -1.41074777 -0.62236801 -2.16266062
E:\\R-3.1.2\\scripts\\mPDBS\\summary_pdb_93\\6583d53ff3c8f7ef.final.pdb
-0.53023594 -1.4549806   2.6204689 -0.03338169 -1.62484768   1.59986497
E:\\R-3.1.2\\scripts\\mPDBS\\summary_pdb_93\\69760178986bef01.final.pdb
0.76069826 -1.1874767 -2.1286717   3.29007087 -1.69246185 -7.35444915
E:\\R-3.1.2\\scripts\\mPDBS\\summary_pdb_93\\6a9d87d47926b1c8.final.pdb
2.17966073   1.2829176   2.2795388 -1.03736955 -1.73631501 -4.15891654
E:\\R-3.1.2\\scripts\\mPDBS\\summary_pdb_93\\6bb3547ea982ffc4.final.pdb
-1.55967708 -1.0474029 -0.6311491   4.54610223 -0.34240511 -3.60905005
E:\\R-3.1.2\\scripts\\mPDBS\\summary_pdb_93\\716d9624611d1c42.final.pdb
-5.87844810   5.6412629 -0.2372453 -5.94874614   3.95551269   2.65830640
E:\\R-3.1.2\\scripts\\mPDBS\\summary_pdb_93\\75f27e70ad0f1be4.final.pdb
-0.53023594 -1.4549806   2.6204689 -0.03338169 -1.62484768   1.59986497
E:\\R-3.1.2\\scripts\\mPDBS\\summary_pdb_93\\769f910fffb22872.final.pdb
5.78050428   0.2261952   4.2202842 -0.89136890 -2.59730555   1.09323264
E:\\R-3.1.2\\scripts\\mPDBS\\summary_pdb_93\\791f8c80dd5c3135.final.pdb
1.52248558 -0.5869192   1.5805519 -0.26987727 -0.50029384   1.40808310
E:\\R-3.1.2\\scripts\\mPDBS\\summary_pdb_93\\7fad98e66a3f6099.final.pdb
-0.45699710 -0.5797001   3.5701787 -2.60761972 -1.05227631   4.09156284
E:\\R-3.1.2\\scripts\\mPDBS\\summary_pdb_93\\804b89c94a633196.final.pdb
-3.41004610   1.3064727   4.0089597 -1.97079482 -1.05945885 -0.83818845
E:\\R-3.1.2\\scripts\\mPDBS\\summary_pdb_93\\813cc65a2298feba.final.pdb
-3.41004610   1.3064727   4.0089597 -1.97079482 -1.05945885 -0.83818845
E:\\R-3.1.2\\scripts\\mPDBS\\summary_pdb_93\\83c0eefcfe12d3f9.final.pdb
7.28573592 -0.9562578 -4.8017624   6.73218438   4.26913720   3.93774730
E:\\R-3.1.2\\scripts\\mPDBS\\summary_pdb_93\\86d50469ef569760.final.pdb
-2.79492795   1.8929649   1.0360744 -1.41074777 -0.62236801 -2.16266062
E:\\R-3.1.2\\scripts\\mPDBS\\summary_pdb_93\\89d0666e1402203d.final.pdb
-0.53023594 -1.4549806   2.6204689 -0.03338169 -1.62484768   1.59986497
E:\\R-3.1.2\\scripts\\mPDBS\\summary_pdb_93\\8b34852716dadc62.final.pdb
-7.15790305 -0.2857462 -0.4926168   0.46790648 -0.43504232   0.15136195
E:\\R-3.1.2\\scripts\\mPDBS\\summary_pdb_93\\8b6ef9ac3fe74395.final.pdb
10.79309532   0.1263406 -7.8534141   2.33288871   2.31732888 -2.11734005
E:\\R-3.1.2\\scripts\\mPDBS\\summary_pdb_93\\98bbe0d5986c138f.final.pdb
1.52248558 -0.5869192   1.5805519 -0.26987727 -0.50029384   1.40808310
E:\\R-3.1.2\\scripts\\mPDBS\\summary_pdb_93\\9c22f4909d67e2fa.final.pdb
2.88896339 -0.6268106 -5.6528838   6.00245188   4.71929308   4.65887161
E:\\R-3.1.2\\scripts\\mPDBS\\summary_pdb_93\\9dda8ed0d81fb3ef.final.pdb
-4.33975278 -2.7238182 -1.3095550   1.85638694   0.07884365   3.56050232
E:\\R-3.1.2\\scripts\\mPDBS\\summary_pdb_93\\9f752b3616e6033b.final.pdb
-12.57544097   3.5180732 -3.0000051   2.14175007   5.17018932 -0.19038377
E:\\R-3.1.2\\scripts\\mPDBS\\summary_pdb_93\\a02c70083816d6ba.final.pdb
-4.24262393 -0.6334991 -1.2152996   2.02246774 -0.18597880 -2.55500038
E:\\R-3.1.2\\scripts\\mPDBS\\summary_pdb_93\\a0c997d891e4e082.final.pdb
6.72952432   0.7321309 -0.1430557 -4.77544263 -3.23733538 -5.19857518
E:\\R-3.1.2\\scripts\\mPDBS\\summary_pdb_93\\ab8039a2d823c616.final.pdb
11.40040293 -1.1948752 -6.1655006   0.09511137   0.98115967 -3.65915620
E:\\R-3.1.2\\scripts\\mPDBS\\summary_pdb_93\\acf4d74b9c71d4dc.final.pdb
-3.41004610   1.3064727   4.0089597 -1.97079482 -1.05945885 -0.83818845
E:\\R-3.1.2\\scripts\\mPDBS\\summary_pdb_93\\ad8a823e4b4c668e.final.pdb

```
2.16051984   4.4608694  -3.3555533  -8.75761324   5.38358531  -5.99690469
E:\\R-3.1.2\\scripts\\mPDBS\\summary_pdb_93\\b16ababd03b7b857.final.pdb
2.88896339  -0.6268106  -5.6528838   6.00245188   4.71929308   4.65887161
E:\\R-3.1.2\\scripts\\mPDBS\\summary_pdb_93\\b226923e697d3c63.final.pdb
8.52080949  -3.0474569  -3.0058975   4.91367183   1.71153713   2.51021389
E:\\R-3.1.2\\scripts\\mPDBS\\summary_pdb_93\\b28e48f6fa27a165.final.pdb
1.52248558  -0.5869192   1.5805519  -0.26987727  -0.50029384   1.40808310
E:\\R-3.1.2\\scripts\\mPDBS\\summary_pdb_93\\b42c4924e3cd1aec.final.pdb
-0.52458592  -0.9347228   3.1906688  -0.53790574  -0.53402004   2.61710718
E:\\R-3.1.2\\scripts\\mPDBS\\summary_pdb_93\\b4dd239fcabff1df.final.pdb
1.52248558  -0.5869192   1.5805519  -0.26987727  -0.50029384   1.40808310
E:\\R-3.1.2\\scripts\\mPDBS\\summary_pdb_93\\b669942ef97459a2.final.pdb
-7.15790305  -0.2857462  -0.4926168   0.46790648  -0.43504232   0.15136195
E:\\R-3.1.2\\scripts\\mPDBS\\summary_pdb_93\\b99f4d9d87c1017b.final.pdb
1.52248558  -0.5869192   1.5805519  -0.26987727  -0.50029384   1.40808310
E:\\R-3.1.2\\scripts\\mPDBS\\summary_pdb_93\\b9d2cbf3975403e7.final.pdb
1.52248558  -0.5869192   1.5805519  -0.26987727  -0.50029384   1.40808310
E:\\R-3.1.2\\scripts\\mPDBS\\summary_pdb_93\\bc88c8a505f3a288.final.pdb
2.64993041  -8.1922644  -2.6784230   0.38869912   0.86608328   9.11871761
E:\\R-3.1.2\\scripts\\mPDBS\\summary_pdb_93\\bdac0e64f4ef427e.final.pdb
0.98672643   0.9770255   4.8600811  -1.24106233  -1.50961472  -1.55931277
E:\\R-3.1.2\\scripts\\mPDBS\\summary_pdb_93\\ca2a1f55fc9d0681.final.pdb
-7.15790305  -0.2857462  -0.4926168   0.46790648  -0.43504232   0.15136195
E:\\R-3.1.2\\scripts\\mPDBS\\summary_pdb_93\\ce34248de3e81a3c.final.pdb
-9.24141398  -0.5860707  -6.7141601   9.32564369   0.39525388 -10.25509356
E:\\R-3.1.2\\scripts\\mPDBS\\summary_pdb_93\\d02e697772494a61.final.pdb
-2.28703127  -1.8557567  -2.3494719   1.61989136   1.20339749   3.36872045
E:\\R-3.1.2\\scripts\\mPDBS\\summary_pdb_93\\d04221245d015047.final.pdb
11.39867438  -0.5557751  -6.6070054  -0.30822971   0.53455022  -3.34364361
E:\\R-3.1.2\\scripts\\mPDBS\\summary_pdb_93\\d678141e4018054a.final.pdb
-0.84135495  -1.8369154   0.9718182   1.27893215  -2.01749253  -0.66889886
E:\\R-3.1.2\\scripts\\mPDBS\\summary_pdb_93\\d71b40af0f0efe39.final.pdb
1.78267753  -0.4708000  -1.6989796   1.80290121  -1.72342810  -7.05818012
E:\\R-3.1.2\\scripts\\mPDBS\\summary_pdb_93\\dc0809eff10197e2.final.pdb
-3.86587992  -2.4155002  -4.2311126   4.22368270   0.27383581   0.07389532
E:\\R-3.1.2\\scripts\\mPDBS\\summary_pdb_93\\e1163918d3707839.final.pdb
-4.34540280  -3.2440760  -1.8797549   2.36091098  -1.01198399   2.54326012
E:\\R-3.1.2\\scripts\\mPDBS\\summary_pdb_93\\e4b48b06824ef848.final.pdb
0.54604394  -0.5153787   2.5680971  -2.39608401  -1.63004817   1.72865288
E:\\R-3.1.2\\scripts\\mPDBS\\summary_pdb_93\\e529e19ad281fea2.final.pdb
-3.64246511   0.7569109  -0.6655648  -1.08306161   0.47400424   0.63188736
E:\\R-3.1.2\\scripts\\mPDBS\\summary_pdb_93\\e5602082718ec3a1.final.pdb
-3.41004610   1.3064727   4.0089597  -1.97079482  -1.05945885  -0.83818845
E:\\R-3.1.2\\scripts\\mPDBS\\summary_pdb_93\\e5d589d996d0a960.final.pdb
-0.52759750  -1.5570297   3.3622866  -0.84389287  -1.59195654   3.45436691
E:\\R-3.1.2\\scripts\\mPDBS\\summary_pdb_93\\e9df9e72307a2fbd.final.pdb
-4.24262393  -0.6334991  -1.2152996   2.02246774  -0.18597880  -2.55500038
E:\\R-3.1.2\\scripts\\mPDBS\\summary_pdb_93\\ea00f8694090cae7.final.pdb
-3.41004610   1.3064727   4.0089597  -1.97079482  -1.05945885  -0.83818845
E:\\R-3.1.2\\scripts\\mPDBS\\summary_pdb_93\\eba517be32db050e.final.pdb
1.52248558  -0.5869192   1.5805519  -0.26987727  -0.50029384   1.40808310
E:\\R-3.1.2\\scripts\\mPDBS\\summary_pdb_93\\ec04b9545899c29d.final.pdb
3.49815039  -0.6893704   3.7680826   0.32431282  -2.82848079   0.38338835
E:\\R-3.1.2\\scripts\\mPDBS\\summary_pdb_93\\ee6862cbcad52078.final.pdb
```

```
2.86301556  -0.1152982   4.0142605  -1.10186286   2.05229672   3.19795907
E:\\R-3.1.2\\scripts\\mPDBS\\summary_pdb_93\\ef6a42fc83b81e5d.final.pdb
0.48171388   0.5745522   3.3279979  -2.73958005  -1.14381389   2.12598443
E:\\R-3.1.2\\scripts\\mPDBS\\summary_pdb_93\\ef8e88d4ad93a0ad.final.pdb
11.39867438  -0.5557751  -6.6070054  -0.30822971   0.53455022  -3.34364361
E:\\R-3.1.2\\scripts\\mPDBS\\summary_pdb_93\\f14fcae8deec4fa3.final.pdb
-12.57544097   3.5180732  -3.0000051   2.14175007   5.17018932  -0.19038377
E:\\R-3.1.2\\scripts\\mPDBS\\summary_pdb_93\\f83584e22003fb92.final.pdb
5.90438095  -0.8914895   2.4181353   0.20996334  -0.93579462   0.46350045
E:\\R-3.1.2\\scripts\\mPDBS\\summary_pdb_93\\f8e2bf87e52caa29.final.pdb
-3.64246511   0.7569109  -0.6655648  -1.08306161   0.47400424   0.63188736

[,13]       [,14]        [,15]        [,16]       [,17]       [,18]
E:\\R-3.1.2\\scripts\\40.1ns_data\\NAT0.pdb
-2.0618508 -0.66961634 -0.284497882 -1.464137687 -0.31560649 -0.02088582
E:\\R-3.1.2\\scripts\\40.1ns_data\\LJP6.pdb
-2.5094341 -0.54132010  1.415742905  0.835344856  0.48770179  0.35041078
E:\\R-3.1.2\\scripts\\40.1ns_data\\3WY9.pdb
-1.7682242  0.90677129 -0.321784873 -0.334349154  0.36374686 -0.01813355
E:\\R-3.1.2\\scripts\\40.1ns_data\\ZSP9.pdb
1.7702131 -0.15672331  0.848511742  0.097777499  0.44286312 -0.19023523
E:\\R-3.1.2\\scripts\\mPDBS\\summary_pdb_93\\013d855ad26c877c.final.pdb
0.6576695 -3.41063500 -0.369099020 -1.565882439  1.34214071 -0.59875158
E:\\R-3.1.2\\scripts\\mPDBS\\summary_pdb_93\\0a2a705342ca0d54.final.pdb
-3.3031546 -0.71044303 -2.758888849  5.123714725  0.60371819 -0.84082589
E:\\R-3.1.2\\scripts\\mPDBS\\summary_pdb_93\\0a59fceadb4194d9.final.pdb
0.4425926 -0.71789746  1.360764591 -1.433499856 -0.35551152  0.14573774
E:\\R-3.1.2\\scripts\\mPDBS\\summary_pdb_93\\0dba59f3306825e8.final.pdb
-2.4975974  2.85560085  0.180057501  0.536084370 -3.21647480 -0.31373380
E:\\R-3.1.2\\scripts\\mPDBS\\summary_pdb_93\\126f26a78b57339c.final.pdb
1.6476207 -0.62049395  0.398156112 -0.678465231  0.60214758 -0.91115104
E:\\R-3.1.2\\scripts\\mPDBS\\summary_pdb_93\\12f06f3e342df706.final.pdb
-2.1767387  1.29833346 -2.426742309 -1.316704205 -0.13920930 -0.22705330
E:\\R-3.1.2\\scripts\\mPDBS\\summary_pdb_93\\18618a2a251b867b.final.pdb
-0.4059019  0.91323015  0.703738994 -0.808599124 -0.36776642  0.67357207
E:\\R-3.1.2\\scripts\\mPDBS\\summary_pdb_93\\1f305f598641eeb2.final.pdb
1.0157226 -2.67970634 -0.504859899 -0.957472293  2.27213107  0.81642969
E:\\R-3.1.2\\scripts\\mPDBS\\summary_pdb_93\\1f8a00b551be0a39.final.pdb
-0.4059019  0.91323015  0.703738994 -0.808599124 -0.36776642  0.67357207
E:\\R-3.1.2\\scripts\\mPDBS\\summary_pdb_93\\219f45328d47579a.final.pdb
-3.7069366  2.76650438  1.149872993 -0.206315868 -4.16300249  0.74808556
E:\\R-3.1.2\\scripts\\mPDBS\\summary_pdb_93\\238dd3918e54336d.final.pdb
-0.6247266 -3.39646076  0.581556521 -2.270407958  0.31415049  0.46654723
E:\\R-3.1.2\\scripts\\mPDBS\\summary_pdb_93\\2b3027889e2fdc42.final.pdb
-9.1631438 14.02244324  6.272260214  0.522859041 12.52115358 -0.66134432
E:\\R-3.1.2\\scripts\\mPDBS\\summary_pdb_93\\2fcb3b46e33793b0.final.pdb
1.8917372  0.57254799  0.393349573  0.235028651  0.83870835  0.14695232
E:\\R-3.1.2\\scripts\\mPDBS\\summary_pdb_93\\34eb328251842006.final.pdb
3.1548670 -0.08018220 -1.036419490  1.021130240  1.32557241 -1.47232063
E:\\R-3.1.2\\scripts\\mPDBS\\summary_pdb_93\\354b32871797b7ab.final.pdb
0.6371079 -2.88999343 -1.017664788 -0.836412791  1.40219208 -0.33605210
E:\\R-3.1.2\\scripts\\mPDBS\\summary_pdb_93\\397368adbc32d5de.final.pdb
1.6476207 -0.62049395  0.398156112 -0.678465231  0.60214758 -0.91115104
E:\\R-3.1.2\\scripts\\mPDBS\\summary_pdb_93\\411ca13cd3df6edd.final.pdb
```

-1.7113667 -0.04310146 -1.699678431 0.022304714 0.70409702 0.88433289
E:\\R-3.1.2\\scripts\\mPDBS\\summary_pdb_93\\42357eee6642bf12.final.pdb
1.7575149 -0.99843511 -1.825931250 0.698886646 1.16550145 -0.60874107
E:\\R-3.1.2\\scripts\\mPDBS\\summary_pdb_93\\44342d1f75a7fcfc.final.pdb
-4.2034010 4.08326522 1.540710216 0.935455517 -5.32509996 -0.83203903
E:\\R-3.1.2\\scripts\\mPDBS\\summary_pdb_93\\448ef21dffe8cdfb.final.pdb
-2.2534810 4.04864279 0.175250962 1.449578253 -2.97991402 0.74436957
E:\\R-3.1.2\\scripts\\mPDBS\\summary_pdb_93\\472a40b3e8f113c7.final.pdb
-2.4975974 2.85560085 0.180057501 0.536084370 -3.21647480 -0.31373380
E:\\R-3.1.2\\scripts\\mPDBS\\summary_pdb_93\\48a26225951ea153.final.pdb
-1.2547374 -1.90468423 -2.933221911 -1.360534914 1.54973768 0.42955912
E:\\R-3.1.2\\scripts\\mPDBS\\summary_pdb_93\\4ab4a5c7c6682ce6.final.pdb
0.1611864 -0.01589471 3.446008618 -0.927412928 -1.58343788 -0.45714883
E:\\R-3.1.2\\scripts\\mPDBS\\summary_pdb_93\\4eaa45b62cb6a25f.final.pdb
1.7044270 -0.21143014 -0.238456719 0.000495311 0.73253008 -0.65686159
E:\\R-3.1.2\\scripts\\mPDBS\\summary_pdb_93\\58c371611d85ac96.final.pdb
1.6476207 -0.62049395 0.398156112 -0.678465231 0.60214758 -0.91115104
E:\\R-3.1.2\\scripts\\mPDBS\\summary_pdb_93\\59a647f04822d30a.final.pdb
-0.0538718 0.59886337 1.751601814 -0.291728471 -1.51760899 -1.43438685
E:\\R-3.1.2\\scripts\\mPDBS\\summary_pdb_93\\59b091fe385e05fc.final.pdb
0.4425926 -0.71789746 1.360764591 -1.433499856 -0.35551152 0.14573774
E:\\R-3.1.2\\scripts\\mPDBS\\summary_pdb_93\\5c35ac30f930d2db.final.pdb
1.7044270 -0.21143014 -0.238456719 0.000495311 0.73253008 -0.65686159
E:\\R-3.1.2\\scripts\\mPDBS\\summary_pdb_93\\5f60752431cf2dcb.final.pdb
-3.2212585 -1.42661139 -3.111025017 -2.096838276 0.53194543 0.12659123
E:\\R-3.1.2\\scripts\\mPDBS\\summary_pdb_93\\626456dd6ff2868d.final.pdb
-2.4975974 2.85560085 0.180057501 0.536084370 -3.21647480 -0.31373380
E:\\R-3.1.2\\scripts\\mPDBS\\summary_pdb_93\\6583d53ff3c8f7ef.final.pdb
-0.0538718 0.59886337 1.751601814 -0.291728471 -1.51760899 -1.43438685
E:\\R-3.1.2\\scripts\\mPDBS\\summary_pdb_93\\69760178986bef01.final.pdb
3.0263693 -1.56254983 -0.454270535 1.013591084 0.25031099 -0.61887825
E:\\R-3.1.2\\scripts\\mPDBS\\summary_pdb_93\\6a9d87d47926b1c8.final.pdb
-7.5242050 2.77374502 -9.045856795 1.483040877 -4.07070545 -0.20481776
E:\\R-3.1.2\\scripts\\mPDBS\\summary_pdb_93\\6bb3547ea982ffc4.final.pdb
2.4562157 -1.26681679 0.585600837 -0.197936353 0.15645567 2.33969061
E:\\R-3.1.2\\scripts\\mPDBS\\summary_pdb_93\\716d9624611d1c42.final.pdb
-2.1539393 1.25195191 -2.331816947 -1.259930174 -0.13771658 -0.19421827
E:\\R-3.1.2\\scripts\\mPDBS\\summary_pdb_93\\75f27e70ad0f1be4.final.pdb
-0.0538718 0.59886337 1.751601814 -0.291728471 -1.51760899 -1.43438685
E:\\R-3.1.2\\scripts\\mPDBS\\summary_pdb_93\\769f910fffb22872.final.pdb
-2.1496529 -0.29528479 -3.468652298 -0.769422718 1.71008436 0.34440140
E:\\R-3.1.2\\scripts\\mPDBS\\summary_pdb_93\\791f8c80dd5c3135.final.pdb
0.4425926 -0.71789746 1.360764591 -1.433499856 -0.35551152 0.14573774
E:\\R-3.1.2\\scripts\\mPDBS\\summary_pdb_93\\7fad98e66a3f6099.final.pdb
-1.0696108 1.23249244 1.440905467 -1.317878692 -1.10837899 0.22444351
E:\\R-3.1.2\\scripts\\mPDBS\\summary_pdb_93\\804b89c94a633196.final.pdb
1.8917372 0.57254799 0.393349573 0.235028651 0.83870835 0.14695232
E:\\R-3.1.2\\scripts\\mPDBS\\summary_pdb_93\\813cc65a2298feba.final.pdb
1.8917372 0.57254799 0.393349573 0.235028651 0.83870835 0.14695232
E:\\R-3.1.2\\scripts\\mPDBS\\summary_pdb_93\\83c0eefcfe12d3f9.final.pdb
-1.7113667 -0.04310146 -1.699678431 0.022304714 0.70409702 0.88433289
E:\\R-3.1.2\\scripts\\mPDBS\\summary_pdb_93\\86d50469ef569760.final.pdb
-2.4975974 2.85560085 0.180057501 0.536084370 -3.21647480 -0.31373380
E:\\R-3.1.2\\scripts\\mPDBS\\summary_pdb_93\\89d0666e1402203d.final.pdb

```
-0.0538718  0.59886337  1.751601814 -0.291728471 -1.51760899 -1.43438685
E:\\R-3.1.2\\scripts\\mPDBS\\summary_pdb_93\\8b34852716dadc62.final.pdb
0.9017860 -2.21759306 -0.373905559 -0.652388556  1.57870148  0.45935179
E:\\R-3.1.2\\scripts\\mPDBS\\summary_pdb_93\\8b6ef9ac3fe74395.final.pdb
1.6081771  0.03791940  0.909388952  1.027470898  0.37801056 -0.09345033
E:\\R-3.1.2\\scripts\\mPDBS\\summary_pdb_93\\98bbe0d5986c138f.final.pdb
0.4425926 -0.71789746  1.360764591 -1.433499856 -0.35551152  0.14573774
E:\\R-3.1.2\\scripts\\mPDBS\\summary_pdb_93\\9c22f4909d67e2fa.final.pdb
0.4053029  1.17714723  3.441202079 -0.013919045 -1.34687711  0.60095454
E:\\R-3.1.2\\scripts\\mPDBS\\summary_pdb_93\\9dda8ed0d81fb3ef.final.pdb
-2.0448768 -3.74324546  0.669875112  3.862799893 -0.38660025 -1.96071687
E:\\R-3.1.2\\scripts\\mPDBS\\summary_pdb_93\\9f752b3616e6033b.final.pdb
0.1501291  0.29238544 -1.610076459 -0.720786616 -0.14810426 -0.35998627
E:\\R-3.1.2\\scripts\\mPDBS\\summary_pdb_93\\a02c70083816d6ba.final.pdb
1.7044270 -0.21143014 -0.238456719  0.000495311  0.73253008 -0.65686159
E:\\R-3.1.2\\scripts\\mPDBS\\summary_pdb_93\\a0c997d891e4e082.final.pdb
0.9773281 -0.39219987 -1.945318735  0.251560120  1.47139456  0.57910945
E:\\R-3.1.2\\scripts\\mPDBS\\summary_pdb_93\\ab8039a2d823c616.final.pdb
-0.1731402 -0.37462495 -1.070814252 -0.187387876  0.08736094 -0.47586694
E:\\R-3.1.2\\scripts\\mPDBS\\summary_pdb_93\\acf4d74b9c71d4dc.final.pdb
1.8917372  0.57254799  0.393349573  0.235028651  0.83870835  0.14695232
E:\\R-3.1.2\\scripts\\mPDBS\\summary_pdb_93\\ad8a823e4b4c668e.final.pdb
8.2369323  0.79333924 11.964810173  1.055735656 -1.63267827  0.45836228
E:\\R-3.1.2\\scripts\\mPDBS\\summary_pdb_93\\b16ababd03b7b857.final.pdb
0.4053029  1.17714723  3.441202079 -0.013919045 -1.34687711  0.60095454
E:\\R-3.1.2\\scripts\\mPDBS\\summary_pdb_93\\b226923e697d3c63.final.pdb
-2.9125488 -0.46418508 -1.646168726 -0.610289853 -0.91568468 -2.18157582
E:\\R-3.1.2\\scripts\\mPDBS\\summary_pdb_93\\b28e48f6fa27a165.final.pdb
0.4425926 -0.71789746  1.360764591 -1.433499856 -0.35551152  0.14573774
E:\\R-3.1.2\\scripts\\mPDBS\\summary_pdb_93\\b42c4924e3cd1aec.final.pdb
-0.9775576 -1.06468217  1.449083181  4.699707995 -1.05626226 -2.28152637
E:\\R-3.1.2\\scripts\\mPDBS\\summary_pdb_93\\b4dd239fcabff1df.final.pdb
0.4425926 -0.71789746  1.360764591 -1.433499856 -0.35551152  0.14573774
E:\\R-3.1.2\\scripts\\mPDBS\\summary_pdb_93\\b669942ef97459a2.final.pdb
0.9017860 -2.21759306 -0.373905559 -0.652388556  1.57870148  0.45935179
E:\\R-3.1.2\\scripts\\mPDBS\\summary_pdb_93\\b99f4d9d87c1017b.final.pdb
0.4425926 -0.71789746  1.360764591 -1.433499856 -0.35551152  0.14573774
E:\\R-3.1.2\\scripts\\mPDBS\\summary_pdb_93\\b9d2cbf3975403e7.final.pdb
0.4425926 -0.71789746  1.360764591 -1.433499856 -0.35551152  0.14573774
E:\\R-3.1.2\\scripts\\mPDBS\\summary_pdb_93\\bc88c8a505f3a288.final.pdb
17.5223677 11.74057704 -8.981473910  3.056205991 -0.19619160 -0.38008158
E:\\R-3.1.2\\scripts\\mPDBS\\summary_pdb_93\\bdac0e64f4ef427e.final.pdb
-0.2249324 -0.64770070 -4.747530938  0.271252410  2.88968249  0.43033067
E:\\R-3.1.2\\scripts\\mPDBS\\summary_pdb_93\\ca2a1f55fc9d0681.final.pdb
0.9017860 -2.21759306 -0.373905559 -0.652388556  1.57870148  0.45935179
E:\\R-3.1.2\\scripts\\mPDBS\\summary_pdb_93\\ce34248de3e81a3c.final.pdb
-0.5994534  2.14345742 -1.416272320  2.504055925 -3.42873022  2.82684289
E:\\R-3.1.2\\scripts\\mPDBS\\summary_pdb_93\\d02e697772494a61.final.pdb
-1.5484124 -5.06000630  0.279037889  2.721028508  0.77549722 -0.38059228
E:\\R-3.1.2\\scripts\\mPDBS\\summary_pdb_93\\d04221245d015047.final.pdb
-0.1840189 -0.26846156 -1.049102773 -0.139966204  0.16806966 -0.45190089
E:\\R-3.1.2\\scripts\\mPDBS\\summary_pdb_93\\d678141e4018054a.final.pdb
0.9942015  0.12351613  1.075611557 -0.105921821 -0.16007934 -0.15082563
E:\\R-3.1.2\\scripts\\mPDBS\\summary_pdb_93\\d71b40af0f0efe39.final.pdb
```

```
2.6584788 -1.37690172 -0.850635548  0.905999631  0.51461139 -0.19327067
E:\\R-3.1.2\\scripts\\mPDBS\\summary_pdb_93\\dc0809eff10197e2.final.pdb
0.3752222 -3.72182837  0.005374973 -1.906764165  1.03550393  2.14294215
E:\\R-3.1.2\\scripts\\mPDBS\\summary_pdb_93\\e1163918d3707839.final.pdb
-1.1211910 -2.07969992  0.972393744 -1.128636573 -0.84794698 -1.11357735
E:\\R-3.1.2\\scripts\\mPDBS\\summary_pdb_93\\e4b48b06824ef848.final.pdb
-0.4059019  0.91323015  0.703738994 -0.808599124 -0.36776642  0.67357207
E:\\R-3.1.2\\scripts\\mPDBS\\summary_pdb_93\\e529e19ad281fea2.final.pdb
0.6576695 -3.41063500 -0.369099020 -1.565882439  1.34214071 -0.59875158
E:\\R-3.1.2\\scripts\\mPDBS\\summary_pdb_93\\e5602082718ec3a1.final.pdb
1.8917372  0.57254799  0.393349573  0.235028651  0.83870835  0.14695232
E:\\R-3.1.2\\scripts\\mPDBS\\summary_pdb_93\\e5d589d996d0a960.final.pdb
-0.6336438  0.87215332  2.195751805 -0.643131760 -1.76416005 -1.97695532
E:\\R-3.1.2\\scripts\\mPDBS\\summary_pdb_93\\e9df9e72307a2fbd.final.pdb
1.7044270 -0.21143014 -0.238456719  0.000495311  0.73253008 -0.65686159
E:\\R-3.1.2\\scripts\\mPDBS\\summary_pdb_93\\ea00f8694090cae7.final.pdb
1.8917372  0.57254799  0.393349573  0.235028651  0.83870835  0.14695232
E:\\R-3.1.2\\scripts\\mPDBS\\summary_pdb_93\\eba517be32db050e.final.pdb
0.4425926 -0.71789746  1.360764591 -1.433499856 -0.35551152  0.14573774
E:\\R-3.1.2\\scripts\\mPDBS\\summary_pdb_93\\ec04b9545899c29d.final.pdb
0.5876904 -0.07076371  3.241113224  0.338805040 -1.04662627 -2.53697015
E:\\R-3.1.2\\scripts\\mPDBS\\summary_pdb_93\\ee6862cbcad52078.final.pdb
-1.5691579 -5.06723168  1.906218141 15.577635944  1.91934218  3.31520257
E:\\R-3.1.2\\scripts\\mPDBS\\summary_pdb_93\\ef6a42fc83b81e5d.final.pdb
-0.1639485  1.66960349  0.760495727 -2.368937705 -1.05408334 12.76214684
E:\\R-3.1.2\\scripts\\mPDBS\\summary_pdb_93\\ef8e88d4ad93a0ad.final.pdb
-0.1840189 -0.26846156 -1.049102773 -0.139966204  0.16806966 -0.45190089
E:\\R-3.1.2\\scripts\\mPDBS\\summary_pdb_93\\f14fcae8deec4fa3.final.pdb
0.1501291  0.29238544 -1.610076459 -0.720786616 -0.14810426 -0.35998627
E:\\R-3.1.2\\scripts\\mPDBS\\summary_pdb_93\\f83584e22003fb92.final.pdb
-1.2547374 -1.90468423 -2.933221911 -1.360534914  1.54973768  0.42955912
E:\\R-3.1.2\\scripts\\mPDBS\\summary_pdb_93\\f8e2bf87e52caa29.final.pdb
0.6576695 -3.41063500 -0.369099020 -1.565882439  1.34214071 -0.59875158

[,19]        [,20]       [,21]       [,22]       [,23]       [,24]
E:\\R-3.1.2\\scripts\\40.1ns_data\\NAT0.pdb
-0.02225261  0.339605924 -0.30942946 -0.23396654 -0.03635356  0.08262730
E:\\R-3.1.2\\scripts\\40.1ns_data\\LJP6.pdb
0.17067576  0.041202342  0.33551692 -0.09081401 -0.04602278 -0.00277417
E:\\R-3.1.2\\scripts\\40.1ns_data\\3WY9.pdb
0.31849616  0.159649238 -0.04646014  0.08202995 -0.01167704 -0.11200456
E:\\R-3.1.2\\scripts\\40.1ns_data\\ZSP9.pdb
-0.58333227 -0.503918047  0.12981843  0.17153943  0.55793907  0.08779495
E:\\R-3.1.2\\scripts\\mPDBS\\summary_pdb_93\\013d855ad26c877c.final.pdb
-0.45857575  0.013212917  0.76244480 -0.96786909 -0.32638089 -1.06767521
E:\\R-3.1.2\\scripts\\mPDBS\\summary_pdb_93\\0a2a705342ca0d54.final.pdb
1.21137614  2.657636917  1.75310085 -0.20076461  0.88579783  2.93888498
E:\\R-3.1.2\\scripts\\mPDBS\\summary_pdb_93\\0a59fceadb4194d9.final.pdb
0.09546248 -0.217308714 -0.43966791  0.72492675  0.02042816  0.46649639
E:\\R-3.1.2\\scripts\\mPDBS\\summary_pdb_93\\0dba59f3306825e8.final.pdb
0.49933552 -0.500980956 -0.65639268 -2.34158965  0.67063515  0.20833115
E:\\R-3.1.2\\scripts\\mPDBS\\summary_pdb_93\\126f26a78b57339c.final.pdb
-0.04990504 -0.015444547  1.14608230 -0.57045518 -0.05659587 -0.35809338
E:\\R-3.1.2\\scripts\\mPDBS\\summary_pdb_93\\12f06f3e342df706.final.pdb
```

```
1.17927789  1.134666468 -0.88756796  0.46089729 -0.27869706 -1.38272748
E:\\R-3.1.2\\scripts\\mPDBS\\summary_pdb_93\\18618a2a251b867b.final.pdb
0.26565218 -0.043698753 -1.65095603  1.25306725  0.18226422  0.59200913
E:\\R-3.1.2\\scripts\\mPDBS\\summary_pdb_93\\1f305f598641eeb2.final.pdb
1.27618713 -1.394790060 -0.56114201  1.74926814 -1.63033525 -1.02839256
E:\\R-3.1.2\\scripts\\mPDBS\\summary_pdb_93\\1f8a00b551be0a39.final.pdb
0.26565218 -0.043698753 -1.65095603  1.25306725  0.18226422  0.59200913
E:\\R-3.1.2\\scripts\\mPDBS\\summary_pdb_93\\219f45328d47579a.final.pdb
0.66714971 -0.654294103 -2.23705915 -1.05693809  0.74723495  1.05279251
E:\\R-3.1.2\\scripts\\mPDBS\\summary_pdb_93\\238dd3918e54336d.final.pdb
-0.26952708 -0.129419558 -0.84304595  0.27282339 -0.24840276 -0.19175687
E:\\R-3.1.2\\scripts\\mPDBS\\summary_pdb_93\\2b3027889e2fdc42.final.pdb
-0.53845283  0.459128994  1.29450413  0.55999388 -0.18555675 -0.33456731
E:\\R-3.1.2\\scripts\\mPDBS\\summary_pdb_93\\2fcb3b46e33793b0.final.pdb
1.02237924 -0.690079626  0.19667356  1.00416199 -0.11894334  0.64784366
E:\\R-3.1.2\\scripts\\mPDBS\\summary_pdb_93\\34eb328251842006.final.pdb
-2.83206036 -0.308684934  0.88248263 -2.23735474  0.40637509 -0.29341507
E:\\R-3.1.2\\scripts\\mPDBS\\summary_pdb_93\\354b32871797b7ab.final.pdb
-3.77287240 -0.897365137 -1.99071058 -1.47170929  0.54212355  0.45848823
E:\\R-3.1.2\\scripts\\mPDBS\\summary_pdb_93\\397368adbc32d5de.final.pdb
-0.04990504 -0.015444547  1.14608230 -0.57045518 -0.05659587 -0.35809338
E:\\R-3.1.2\\scripts\\mPDBS\\summary_pdb_93\\411ca13cd3df6edd.final.pdb
0.59601451  1.411137482 -0.95447179 -0.40572712  0.99495712 -0.15671802
E:\\R-3.1.2\\scripts\\mPDBS\\summary_pdb_93\\42357eee6642bf12.final.pdb
-0.47792357  3.432412219 -0.63866972 -0.11295133  0.90881355 -1.51682845
E:\\R-3.1.2\\scripts\\mPDBS\\summary_pdb_93\\44342d1f75a7fcfc.final.pdb
0.46683305 -0.007881935 -0.89767377 -1.10912872  1.05248071 -1.21283620
E:\\R-3.1.2\\scripts\\mPDBS\\summary_pdb_93\\448ef21dffe8cdfb.final.pdb
1.57161979 -1.175616035 -1.60580142 -0.76697249  0.60828768  1.21426819
E:\\R-3.1.2\\scripts\\mPDBS\\summary_pdb_93\\472a40b3e8f113c7.final.pdb
0.49933552 -0.500980956 -0.65639268 -2.34158965  0.67063515  0.20833115
E:\\R-3.1.2\\scripts\\mPDBS\\summary_pdb_93\\48a26225951ea153.final.pdb
0.89757471 -0.004888646 -0.50582081 -0.85853422  1.66707110  0.05045166
E:\\R-3.1.2\\scripts\\mPDBS\\summary_pdb_93\\4ab4a5c7c6682ce6.final.pdb
-1.34508556  1.718760442  0.09916984 -0.25908301 -0.41902634 -0.67037029
E:\\R-3.1.2\\scripts\\mPDBS\\summary_pdb_93\\4eaa45b62cb6a25f.final.pdb
-3.40788283 -0.985254293 -1.58733255 -1.01960593  0.81095448  1.11674149
E:\\R-3.1.2\\scripts\\mPDBS\\summary_pdb_93\\58c371611d85ac96.final.pdb
-0.04990504 -0.015444547  1.14608230 -0.57045518 -0.05659587 -0.35809338
E:\\R-3.1.2\\scripts\\mPDBS\\summary_pdb_93\\59a647f04822d30a.final.pdb
-0.10485417  0.429103454  0.89971747  0.67273612  0.32567392 -1.79913233
E:\\R-3.1.2\\scripts\\mPDBS\\summary_pdb_93\\59b091fe385e05fc.final.pdb
0.09546248 -0.217308714 -0.43966791  0.72492675  0.02042816  0.46649639
E:\\R-3.1.2\\scripts\\mPDBS\\summary_pdb_93\\5c35ac30f930d2db.final.pdb
-3.40788283 -0.985254293 -1.58733255 -1.01960593  0.81095448  1.11674149
E:\\R-3.1.2\\scripts\\mPDBS\\summary_pdb_93\\5f60752431cf2dcb.final.pdb
0.72346592  1.140894049 -1.21694495 -0.01661284 -0.47589134 -1.84671443
E:\\R-3.1.2\\scripts\\mPDBS\\summary_pdb_93\\626456dd6ff2868d.final.pdb
0.49933552 -0.500980956 -0.65639268 -2.34158965  0.67063515  0.20833115
E:\\R-3.1.2\\scripts\\mPDBS\\summary_pdb_93\\6583d53ff3c8f7ef.final.pdb
-0.10485417  0.429103454  0.89971747  0.67273612  0.32567392 -1.79913233
E:\\R-3.1.2\\scripts\\mPDBS\\summary_pdb_93\\69760178986bef01.final.pdb
0.03153472  2.732829689  1.61787709 -0.14698352  0.24279792 -1.92706551
E:\\R-3.1.2\\scripts\\mPDBS\\summary_pdb_93\\6a9d87d47926b1c8.final.pdb
```

-7.11900033 0.307371788 1.46695559 7.07597115 -2.84513825 0.20660696
E:\\R-3.1.2\\scripts\\mPDBS\\summary_pdb_93\\6bb3547ea982ffc4.final.pdb
3.69643515 1.144476433 0.65168605 2.77426989 -0.83874668 0.75940968
E:\\R-3.1.2\\scripts\\mPDBS\\summary_pdb_93\\716d9624611d1c42.final.pdb
1.08845549 1.053004894 -0.81356692 0.43549052 -0.20706041 -1.18846117
E:\\R-3.1.2\\scripts\\mPDBS\\summary_pdb_93\\75f27e70ad0f1be4.final.pdb
-0.10485417 0.429103454 0.89971747 0.67273612 0.32567392 -1.79913233
E:\\R-3.1.2\\scripts\\mPDBS\\summary_pdb_93\\769f910fffb22872.final.pdb
1.34288125 1.462748398 -2.59682141 -2.94318225 -3.82469759 1.88178154
E:\\R-3.1.2\\scripts\\mPDBS\\summary_pdb_93\\791f8c80dd5c3135.final.pdb
0.09546248 -0.217308714 -0.43966791 0.72492675 0.02042816 0.46649639
E:\\R-3.1.2\\scripts\\mPDBS\\summary_pdb_93\\7fad98e66a3f6099.final.pdb
-0.48889315 -0.652233718 -1.49174096 0.82085212 0.19112806 0.47074356
E:\\R-3.1.2\\scripts\\mPDBS\\summary_pdb_93\\804b89c94a633196.final.pdb
1.02237924 -0.690079626 0.19667356 1.00416199 -0.11894334 0.64784366
E:\\R-3.1.2\\scripts\\mPDBS\\summary_pdb_93\\813cc65a2298feba.final.pdb
1.02237924 -0.690079626 0.19667356 1.00416199 -0.11894334 0.64784366
E:\\R-3.1.2\\scripts\\mPDBS\\summary_pdb_93\\83c0eefcfe12d3f9.final.pdb
0.59601451 1.411137482 -0.95447179 -0.40572712 0.99495712 -0.15671802
E:\\R-3.1.2\\scripts\\mPDBS\\summary_pdb_93\\86d50469ef569760.final.pdb
0.49933552 -0.500980956 -0.65639268 -2.34158965 0.67063515 0.20833115
E:\\R-3.1.2\\scripts\\mPDBS\\summary_pdb_93\\89d0666e1402203d.final.pdb
-0.10485417 0.429103454 0.89971747 0.67273612 0.32567392 -1.79913233
E:\\R-3.1.2\\scripts\\mPDBS\\summary_pdb_93\\8b34852716dadc62.final.pdb
0.61370852 -0.661422161 -0.18696394 0.60674807 -0.38872836 -0.06173817
E:\\R-3.1.2\\scripts\\mPDBS\\summary_pdb_93\\8b6ef9ac3fe74395.final.pdb
-2.54669659 4.122186026 -1.86415527 -0.78381971 0.54479717 -0.63875238
E:\\R-3.1.2\\scripts\\mPDBS\\summary_pdb_93\\98bbe0d5986c138f.final.pdb
0.09546248 -0.217308714 -0.43966791 0.72492675 0.02042816 0.46649639
E:\\R-3.1.2\\scripts\\mPDBS\\summary_pdb_93\\9c22f4909d67e2fa.final.pdb
-0.27280129 1.044125363 -0.85023890 1.31553416 -0.48137381 0.33556675
E:\\R-3.1.2\\scripts\\mPDBS\\summary_pdb_93\\9dda8ed0d81fb3ef.final.pdb
-0.42210138 2.275328296 3.32999375 -0.23746844 1.37250843 1.89297777
E:\\R-3.1.2\\scripts\\mPDBS\\summary_pdb_93\\9f752b3616e6033b.final.pdb
-0.31732413 -1.422839940 0.85913331 -0.49557327 -0.78461114 -0.18761139
E:\\R-3.1.2\\scripts\\mPDBS\\summary_pdb_93\\a02c70083816d6ba.final.pdb
-3.40788283 -0.985254293 -1.58733255 -1.01960593 0.81095448 1.11674149
E:\\R-3.1.2\\scripts\\mPDBS\\summary_pdb_93\\a0c997d891e4e082.final.pdb
-0.14985364 2.944612131 -2.19294303 0.34119146 0.77431403 -0.22372798
E:\\R-3.1.2\\scripts\\mPDBS\\summary_pdb_93\\ab8039a2d823c616.final.pdb
0.72414439 -3.474647051 1.25053321 0.01954615 -0.82510511 0.96456051
E:\\R-3.1.2\\scripts\\mPDBS\\summary_pdb_93\\acf4d74b9c71d4dc.final.pdb
1.02237924 -0.690079626 0.19667356 1.00416199 -0.11894334 0.64784366
E:\\R-3.1.2\\scripts\\mPDBS\\summary_pdb_93\\ad8a823e4b4c668e.final.pdb
-0.84792384 -0.581062732 0.11016850 1.52513637 1.11094599 0.33094372
E:\\R-3.1.2\\scripts\\mPDBS\\summary_pdb_93\\b16ababd03b7b857.final.pdb
-0.27280129 1.044125363 -0.85023890 1.31553416 -0.48137381 0.33556675
E:\\R-3.1.2\\scripts\\mPDBS\\summary_pdb_93\\b226923e697d3c63.final.pdb
1.53850143 -5.312016720 4.11956274 -1.21579508 0.48783465 -1.34898961
E:\\R-3.1.2\\scripts\\mPDBS\\summary_pdb_93\\b28e48f6fa27a165.final.pdb
0.09546248 -0.217308714 -0.43966791 0.72492675 0.02042816 0.46649639
E:\\R-3.1.2\\scripts\\mPDBS\\summary_pdb_93\\b42c4924e3cd1aec.final.pdb
-0.05711181 2.187439140 3.73337178 0.21463492 1.64133936 2.55123102
E:\\R-3.1.2\\scripts\\mPDBS\\summary_pdb_93\\b4dd239fcabff1df.final.pdb

0.09546248 -0.217308714 -0.43966791 0.72492675 0.02042816 0.46649639
E:\\R-3.1.2\\scripts\\mPDBS\\summary_pdb_93\\b669942ef97459a2.final.pdb
0.61370852 -0.661422161 -0.18696394 0.60674807 -0.38872836 -0.06173817
E:\\R-3.1.2\\scripts\\mPDBS\\summary_pdb_93\\b99f4d9d87c1017b.final.pdb
0.09546248 -0.217308714 -0.43966791 0.72492675 0.02042816 0.46649639
E:\\R-3.1.2\\scripts\\mPDBS\\summary_pdb_93\\b9d2cbf3975403e7.final.pdb
0.09546248 -0.217308714 -0.43966791 0.72492675 0.02042816 0.46649639
E:\\R-3.1.2\\scripts\\mPDBS\\summary_pdb_93\\bc88c8a505f3a288.final.pdb
0.29551654 0.012091159 0.24869827 -0.24039542 -0.26757637 -0.06451473
E:\\R-3.1.2\\scripts\\mPDBS\\summary_pdb_93\\bdac0e64f4ef427e.final.pdb
1.89119503 -0.323067507 0.09244066 -0.71709930 1.35738759 0.15555889
E:\\R-3.1.2\\scripts\\mPDBS\\summary_pdb_93\\ca2a1f55fc9d0681.final.pdb
0.61370852 -0.661422161 -0.18696394 0.60674807 -0.38872836 -0.06173817
E:\\R-3.1.2\\scripts\\mPDBS\\summary_pdb_93\\ce34248de3e81a3c.final.pdb
5.59356165 1.957936244 0.83732807 0.93149314 -0.65362281 -0.28130261
E:\\R-3.1.2\\scripts\\mPDBS\\summary_pdb_93\\d02e697772494a61.final.pdb
-0.22178472 1.628916128 1.99060837 -0.18527780 1.06726267 4.15860648
E:\\R-3.1.2\\scripts\\mPDBS\\summary_pdb_93\\d04221245d015047.final.pdb
0.69138973 -3.008928110 1.09305515 0.03612124 -0.71016817 0.64245947
E:\\R-3.1.2\\scripts\\mPDBS\\summary_pdb_93\\d678141e4018054a.final.pdb
-0.03153379 -0.568935309 -0.55554813 1.26704069 0.19618006 0.27217207
E:\\R-3.1.2\\scripts\\mPDBS\\summary_pdb_93\\d71b40af0f0efe39.final.pdb
0.59667812 3.195932941 0.99142470 0.24908965 0.34058015 -1.69788064
E:\\R-3.1.2\\scripts\\mPDBS\\summary_pdb_93\\dc0809eff10197e2.final.pdb
1.49389568 -0.001153945 -1.25034439 1.60169880 -0.69724678 0.96288203
E:\\R-3.1.2\\scripts\\mPDBS\\summary_pdb_93\\e1163918d3707839.final.pdb
-0.46984374 0.516992610 0.49633944 0.22063276 0.05684299 -2.45738559
E:\\R-3.1.2\\scripts\\mPDBS\\summary_pdb_93\\e4b48b06824ef848.final.pdb
0.26565218 -0.043698753 -1.65095603 1.25306725 0.18226422 0.59200913
E:\\R-3.1.2\\scripts\\mPDBS\\summary_pdb_93\\e529e19ad281fea2.final.pdb
-0.45857575 0.013212917 0.76244480 -0.96786909 -0.32638089 -1.06767521
E:\\R-3.1.2\\scripts\\mPDBS\\summary_pdb_93\\e5602082718ec3a1.final.pdb
1.02237924 -0.690079626 0.19667356 1.00416199 -0.11894334 0.64784366
E:\\R-3.1.2\\scripts\\mPDBS\\summary_pdb_93\\e5d589d996d0a960.final.pdb
-0.78492018 -0.279445712 0.72638510 0.42075210 0.44096868 -1.81361532
E:\\R-3.1.2\\scripts\\mPDBS\\summary_pdb_93\\e9df9e72307a2fbd.final.pdb
-3.40788283 -0.985254293 -1.58733255 -1.01960593 0.81095448 1.11674149
E:\\R-3.1.2\\scripts\\mPDBS\\summary_pdb_93\\ea00f8694090cae7.final.pdb
1.02237924 -0.690079626 0.19667356 1.00416199 -0.11894334 0.64784366
E:\\R-3.1.2\\scripts\\mPDBS\\summary_pdb_93\\eba517be32db050e.final.pdb
0.09546248 -0.217308714 -0.43966791 0.72492675 0.02042816 0.46649639
E:\\R-3.1.2\\scripts\\mPDBS\\summary_pdb_93\\ec04b9545899c29d.final.pdb
0.07820525 2.665521348 0.41705645 -3.74721952 -9.04079369 0.75434148
E:\\R-3.1.2\\scripts\\mPDBS\\summary_pdb_93\\ee6862cbcad52078.final.pdb
-0.50920743 -3.621222184 -3.20495833 -0.07572020 -1.43350257 -3.41786470
E:\\R-3.1.2\\scripts\\mPDBS\\summary_pdb_93\\ef6a42fc83b81e5d.final.pdb
-3.79707069 0.641007319 5.07270015 -3.01765866 -0.12101101 -0.47336105
E:\\R-3.1.2\\scripts\\mPDBS\\summary_pdb_93\\ef8e88d4ad93a0ad.final.pdb
0.69138973 -3.008928110 1.09305515 0.03612124 -0.71016817 0.64245947
E:\\R-3.1.2\\scripts\\mPDBS\\summary_pdb_93\\f14fcae8deec4fa3.final.pdb
-0.31732413 -1.422839940 0.85913331 -0.49557327 -0.78461114 -0.18761139
E:\\R-3.1.2\\scripts\\mPDBS\\summary_pdb_93\\f83584e22003fb92.final.pdb
0.89757471 -0.004888646 -0.50582081 -0.85853422 1.66707110 0.05045166
E:\\R-3.1.2\\scripts\\mPDBS\\summary_pdb_93\\f8e2bf87e52caa29.final.pdb

```
-0.45857575  0.013212917  0.76244480 -0.96786909 -0.32638089 -1.06767521

[,25]       [,26]         [,27]         [,28]         [,29]         [,30]
E:\\R-3.1.2\\scripts\\40.1ns_data\\NAT0.pdb
0.0284692772  0.07340220 -0.0009420407  0.0075243535 -0.0712687775 -0.0113249313
E:\\R-3.1.2\\scripts\\40.1ns_data\\LJP6.pdb
0.0172069677 -0.10253460  0.0335688167  0.0484464084  0.0473167551 -0.0097899560
E:\\R-3.1.2\\scripts\\40.1ns_data\\3WY9.pdb
-0.0560896842  0.01144485  0.0018995517 -0.0217366604  0.0599632351
0.0104962755
E:\\R-3.1.2\\scripts\\40.1ns_data\\ZSP9.pdb
0.0590745982 -0.03797804  0.0161760346 -0.0608470022 -0.0104151174 -0.0054482677
E:\\R-3.1.2\\scripts\\mPDBS\\summary_pdb_93\\013d855ad26c877c.final.pdb
-0.8044053354 -0.45924817 -0.2955269396 -0.0724298678 -0.0155896011
0.2267311863
E:\\R-3.1.2\\scripts\\mPDBS\\summary_pdb_93\\0a2a705342ca0d54.final.pdb
0.8771627591  0.28590972  1.2520360950  0.0663914283 -0.2202903377  2.9545834396
E:\\R-3.1.2\\scripts\\mPDBS\\summary_pdb_93\\0a59fceadb4194d9.final.pdb
0.2100304121  0.28610420  0.1078257329  0.0688130529  0.0122171194  0.0191132209
E:\\R-3.1.2\\scripts\\mPDBS\\summary_pdb_93\\0dba59f3306825e8.final.pdb
0.3356656311 -0.05567181 -0.0146939269 -0.0559181756 -0.0249772828 -0.0181019025
E:\\R-3.1.2\\scripts\\mPDBS\\summary_pdb_93\\126f26a78b57339c.final.pdb
-0.0335302674 -0.33115484 -0.1431781035  0.0399135696 -0.0098602153
-0.0413593520
E:\\R-3.1.2\\scripts\\mPDBS\\summary_pdb_93\\12f06f3e342df706.final.pdb
-0.0345908893  0.45582903  1.3330039444  0.2311423255  0.2443640767
-2.0116652843
E:\\R-3.1.2\\scripts\\mPDBS\\summary_pdb_93\\18618a2a251b867b.final.pdb
-0.3604235999 -1.46639864 -0.5675781316 -0.6160786620 -0.1684080332
0.0113770455
E:\\R-3.1.2\\scripts\\mPDBS\\summary_pdb_93\\1f305f598641eeb2.final.pdb
8.9206139740 -0.52666376 -0.0393603444 -0.0160811105 -0.0063295997 -0.0299609452
E:\\R-3.1.2\\scripts\\mPDBS\\summary_pdb_93\\1f8a00b551be0a39.final.pdb
-0.3604235999 -1.46639864 -0.5675781316 -0.6160786620 -0.1684080332
0.0113770455
E:\\R-3.1.2\\scripts\\mPDBS\\summary_pdb_93\\219f45328d47579a.final.pdb
0.5330225918  0.56536161  0.2505481623 -0.0042453698 -0.0028927413 -0.0386094158
E:\\R-3.1.2\\scripts\\mPDBS\\summary_pdb_93\\238dd3918e54336d.final.pdb
-0.6036455022  0.17538640 -0.0139297203 -0.0196763832  0.0007057119
0.1666714728
E:\\R-3.1.2\\scripts\\mPDBS\\summary_pdb_93\\2b3027889e2fdc42.final.pdb
-0.1218886499 -0.09656743 -0.0132292119 -0.0063416941  0.0136480798
0.0002824348
E:\\R-3.1.2\\scripts\\mPDBS\\summary_pdb_93\\2fcb3b46e33793b0.final.pdb
-0.5586318059  0.53813062  0.2166606142  0.1769957731  0.0107913765
-0.1125329875
E:\\R-3.1.2\\scripts\\mPDBS\\summary_pdb_93\\34eb328251842006.final.pdb
-0.1930194634 -2.79382895 -0.1092972403 -0.2727722119  0.0662732004
-0.0223752220
E:\\R-3.1.2\\scripts\\mPDBS\\summary_pdb_93\\354b32871797b7ab.final.pdb
-0.4850000472  0.07802118  0.8891338117  0.0250090729  0.0020030553
0.1197413387
E:\\R-3.1.2\\scripts\\mPDBS\\summary_pdb_93\\397368adbc32d5de.final.pdb
-0.0335302674 -0.33115484 -0.1431781035  0.0399135696 -0.0098602153
```

-0.0413593520
E:\\R-3.1.2\\scripts\\mPDBS\\summary_pdb_93\\411ca13cd3df6edd.final.pdb
0.0387168651 0.47059359 0.2195279764 -0.5474152340 0.0501681950 -0.0737620836
E:\\R-3.1.2\\scripts\\mPDBS\\summary_pdb_93\\42357eee6642bf12.final.pdb
0.3873865031 1.81043870 0.8807343884 0.4220617837 -0.0327822341 -0.0406284794
E:\\R-3.1.2\\scripts\\mPDBS\\summary_pdb_93\\44342d1f75a7fcfc.final.pdb
0.2889891557 0.26700895 0.1699138684 0.1283332889 0.1075641577 0.1195202585
E:\\R-3.1.2\\scripts\\mPDBS\\summary_pdb_93\\448ef21dffe8cdfb.final.pdb
-0.1894359074 0.81361364 0.3451447908 0.0811640279 -0.0043256909
-0.0892755380
E:\\R-3.1.2\\scripts\\mPDBS\\summary_pdb_93\\472a40b3e8f113c7.final.pdb
0.3356656311 -0.05567181 -0.0146939269 -0.0559181756 -0.0249772828 -0.0181019025
E:\\R-3.1.2\\scripts\\mPDBS\\summary_pdb_93\\48a26225951ea153.final.pdb
0.4514330076 0.48172114 -0.0620891319 -0.2578746220 0.0459748781 -0.0042638971
E:\\R-3.1.2\\scripts\\mPDBS\\summary_pdb_93\\4ab4a5c7c6682ce6.final.pdb
0.3416526652 -0.80895862 -0.1228711922 0.2826554362 0.0636127901 0.0843708277
E:\\R-3.1.2\\scripts\\mPDBS\\summary_pdb_93\\4eaa45b62cb6a25f.final.pdb
0.3286758671 0.18873898 1.0108892650 0.1134985089 0.0135144628 -0.0278169132
E:\\R-3.1.2\\scripts\\mPDBS\\summary_pdb_93\\58c371611d85ac96.final.pdb
-0.0335302674 -0.33115484 -0.1431781035 0.0399135696 -0.0098602153
-0.0413593520
E:\\R-3.1.2\\scripts\\mPDBS\\summary_pdb_93\\59a647f04822d30a.final.pdb
-0.0340030239 -0.01224846 0.0271914390 0.2013917115 0.1226740184
0.1772428951
E:\\R-3.1.2\\scripts\\mPDBS\\summary_pdb_93\\59b091fe385e05fc.final.pdb
0.2100304121 0.28610420 0.1078257329 0.0688130529 0.0122171194 0.0191132209
E:\\R-3.1.2\\scripts\\mPDBS\\summary_pdb_93\\5c35ac30f930d2db.final.pdb
0.3286758671 0.18873898 1.0108892650 0.1134985089 0.0135144628 -0.0278169132
E:\\R-3.1.2\\scripts\\mPDBS\\summary_pdb_93\\5f60752431cf2dcb.final.pdb
-0.8283045190 0.24084428 0.7909640693 -0.0023736831 0.0366166643
-0.2467727937
E:\\R-3.1.2\\scripts\\mPDBS\\summary_pdb_93\\626456dd6ff2868d.final.pdb
0.3356656311 -0.05567181 -0.0146939269 -0.0559181756 -0.0249772828 -0.0181019025
E:\\R-3.1.2\\scripts\\mPDBS\\summary_pdb_93\\6583d53ff3c8f7ef.final.pdb
-0.0340030239 -0.01224846 0.0271914390 0.2013917115 0.1226740184
0.1772428951
E:\\R-3.1.2\\scripts\\mPDBS\\summary_pdb_93\\69760178986bef01.final.pdb
0.1439152736 -0.60381025 -0.4723439873 0.1330050597 -0.0471003679 0.4997306111
E:\\R-3.1.2\\scripts\\mPDBS\\summary_pdb_93\\6a9d87d47926b1c8.final.pdb
-0.3320651483 -0.09967046 -0.0014206692 0.0975852077 0.0058143588
0.0098053506
E:\\R-3.1.2\\scripts\\mPDBS\\summary_pdb_93\\6bb3547ea982ffc4.final.pdb
-0.5619807990 -1.69253431 1.9104633923 0.0051325327 0.0805395329
-0.1248681552
E:\\R-3.1.2\\scripts\\mPDBS\\summary_pdb_93\\716d9624611d1c42.final.pdb
-0.0146286047 0.35156208 0.9127195226 0.0861157529 0.0481280718
-0.3943310456
E:\\R-3.1.2\\scripts\\mPDBS\\summary_pdb_93\\75f27e70ad0f1be4.final.pdb
-0.0340030239 -0.01224846 0.0271914390 0.2013917115 0.1226740184
0.1772428951
E:\\R-3.1.2\\scripts\\mPDBS\\summary_pdb_93\\769f910fffb22872.final.pdb
-0.2771955309 -1.68802609 -1.3165735772 3.5173269571 0.5968740983
0.0481246002
E:\\R-3.1.2\\scripts\\mPDBS\\summary_pdb_93\\791f8c80dd5c3135.final.pdb

0.2100304121 0.28610420 0.1078257329 0.0688130529 0.0122171194 0.0191132209
E:\\R-3.1.2\\scripts\\mPDBS\\summary_pdb_93\\7fad98e66a3f6099.final.pdb
-0.4684668206 -3.49100911 -0.0768376542 -1.0524785626 -0.3170248623
0.3900107204
E:\\R-3.1.2\\scripts\\mPDBS\\summary_pdb_93\\804b89c94a633196.final.pdb
-0.5586318059 0.53813062 0.2166606142 0.1769957731 0.0107913765
-0.1125329875
E:\\R-3.1.2\\scripts\\mPDBS\\summary_pdb_93\\813cc65a2298feba.final.pdb
-0.5586318059 0.53813062 0.2166606142 0.1769957731 0.0107913765
-0.1125329875
E:\\R-3.1.2\\scripts\\mPDBS\\summary_pdb_93\\83c0eefcfe12d3f9.final.pdb
0.0387168651 0.47059359 0.2195279764 -0.5474152340 0.0501681950 -0.0737620836
E:\\R-3.1.2\\scripts\\mPDBS\\summary_pdb_93\\86d50469ef569760.final.pdb
0.3356656311 -0.05567181 -0.0146939269 -0.0559181756 -0.0249772828 -0.0181019025
E:\\R-3.1.2\\scripts\\mPDBS\\summary_pdb_93\\89d0666e1402203d.final.pdb
-0.0340030239 -0.01224846 0.0271914390 0.2013917115 0.1226740184
0.1772428951
E:\\R-3.1.2\\scripts\\mPDBS\\summary_pdb_93\\8b34852716dadc62.final.pdb
-1.3295068739 0.41003729 0.0643117781 0.0646523358 0.0050619907
0.1555575508
E:\\R-3.1.2\\scripts\\mPDBS\\summary_pdb_93\\8b6ef9ac3fe74395.final.pdb
0.3526315493 -0.13989674 -1.2536134037 -0.1144560629 -0.2229048965 0.0577756267
E:\\R-3.1.2\\scripts\\mPDBS\\summary_pdb_93\\98bbe0d5986c138f.final.pdb
0.2100304121 0.28610420 0.1078257329 0.0688130529 0.0122171194 0.0191132209
E:\\R-3.1.2\\scripts\\mPDBS\\summary_pdb_93\\9c22f4909d67e2fa.final.pdb
-0.1834488733 0.06032683 0.2369675255 0.4197376397 0.0842643819
0.0131971922
E:\\R-3.1.2\\scripts\\mPDBS\\summary_pdb_93\\9dda8ed0d81fb3ef.final.pdb
-0.0174493194 -0.26363017 -0.5509077881 0.0002147833 0.0882290710
-0.9035259844
E:\\R-3.1.2\\scripts\\mPDBS\\summary_pdb_93\\9f752b3616e6033b.final.pdb
0.0270221234 0.03925750 -1.7723855810 -0.2180312611 -0.0560332513 -0.1032216402
E:\\R-3.1.2\\scripts\\mPDBS\\summary_pdb_93\\a02c70083816d6ba.final.pdb
0.3286758671 0.18873898 1.0108892650 0.1134985089 0.0135144628 -0.0278169132
E:\\R-3.1.2\\scripts\\mPDBS\\summary_pdb_93\\a0c997d891e4e082.final.pdb
0.1208643070 0.63041885 -1.5817285593 -0.5573368708 -0.3328629279 -0.1841924858
E:\\R-3.1.2\\scripts\\mPDBS\\summary_pdb_93\\ab8039a2d823c616.final.pdb
-0.1208660187 0.10134047 -0.2423531892 -0.8311641952 3.9386906424
0.2692375462
E:\\R-3.1.2\\scripts\\mPDBS\\summary_pdb_93\\acf4d74b9c71d4dc.final.pdb
-0.5586318059 0.53813062 0.2166606142 0.1769957731 0.0107913765
-0.1125329875
E:\\R-3.1.2\\scripts\\mPDBS\\summary_pdb_93\\ad8a823e4b4c668e.final.pdb
0.0002851169 -0.16357957 -0.1484207523 0.2619993023 0.0528828164 0.0080263675
E:\\R-3.1.2\\scripts\\mPDBS\\summary_pdb_93\\b16ababd03b7b857.final.pdb
-0.1834488733 0.06032683 0.2369675255 0.4197376397 0.0842643819
0.0131971922
E:\\R-3.1.2\\scripts\\mPDBS\\summary_pdb_93\\b226923e697d3c63.final.pdb
-0.0432387783 -0.43482954 1.3513409780 0.5703215358 -0.6733776489
0.1829526014
E:\\R-3.1.2\\scripts\\mPDBS\\summary_pdb_93\\b28e48f6fa27a165.final.pdb
0.2100304121 0.28610420 0.1078257329 0.0688130529 0.0122171194 0.0191132209
E:\\R-3.1.2\\scripts\\mPDBS\\summary_pdb_93\\b42c4924e3cd1aec.final.pdb
0.7962265949 -0.15291237 -0.4291523348 0.0887042194 0.0997404785 -1.0510842363

E:\\R-3.1.2\\scripts\\mPDBS\\summary_pdb_93\\b4dd239fcabff1df.final.pdb
0.2100304121 0.28610420 0.1078257329 0.0688130529 0.0122171194 0.0191132209
E:\\R-3.1.2\\scripts\\mPDBS\\summary_pdb_93\\b669942ef97459a2.final.pdb
-1.3295068739 0.41003729 0.0643117781 0.0646523358 0.0050619907
0.1555575508
E:\\R-3.1.2\\scripts\\mPDBS\\summary_pdb_93\\b99f4d9d87c1017b.final.pdb
0.2100304121 0.28610420 0.1078257329 0.0688130529 0.0122171194 0.0191132209
E:\\R-3.1.2\\scripts\\mPDBS\\summary_pdb_93\\b9d2cbf3975403e7.final.pdb
0.2100304121 0.28610420 0.1078257329 0.0688130529 0.0122171194 0.0191132209
E:\\R-3.1.2\\scripts\\mPDBS\\summary_pdb_93\\bc88c8a505f3a288.final.pdb
0.0577981562 0.01492905 0.0075411211 -0.0388809653 0.0004043916 0.0032030255
E:\\R-3.1.2\\scripts\\mPDBS\\summary_pdb_93\\bdac0e64f4ef427e.final.pdb
-0.3364660675 0.94839737 0.1992210651 -0.7901571006 -0.0233048104
-0.1994922633
E:\\R-3.1.2\\scripts\\mPDBS\\summary_pdb_93\\ca2a1f55fc9d0681.final.pdb
-1.3295068739 0.41003729 0.0643117781 0.0646523358 0.0050619907
0.1555575508
E:\\R-3.1.2\\scripts\\mPDBS\\summary_pdb_93\\ce34248de3e81a3c.final.pdb
-0.4203473164 -0.89240940 -0.5509081212 -0.1168799632 -0.0538791672
0.0593004412
E:\\R-3.1.2\\scripts\\mPDBS\\summary_pdb_93\\d02e697772494a61.final.pdb
0.2265841167 0.03472249 -0.4702734941 -0.1323638753 -0.0222278280 -1.0616556587
E:\\R-3.1.2\\scripts\\mPDBS\\summary_pdb_93\\d04221245d015047.final.pdb
-0.1297740428 0.01222084 -0.0876641555 0.1382806284 -1.1626723538
-0.1551056616
E:\\R-3.1.2\\scripts\\mPDBS\\summary_pdb_93\\d678141e4018054a.final.pdb
0.0494360741 3.33510391 -2.6253473321 0.1387027026 -0.2678161716 0.5848568271
E:\\R-3.1.2\\scripts\\mPDBS\\summary_pdb_93\\d71b40af0f0efe39.final.pdb
0.1142101297 -0.74330690 1.3422338606 0.3438718556 0.1776023577 -0.1901248131
E:\\R-3.1.2\\scripts\\mPDBS\\summary_pdb_93\\dc0809eff10197e2.final.pdb
-0.9868142698 0.46353267 0.1772193606 -0.0947542998 -0.2061751280
0.4957407545
E:\\R-3.1.2\\scripts\\mPDBS\\summary_pdb_93\\e1163918d3707839.final.pdb
-0.8476789382 -0.12296627 -0.0945640143 0.1129022754 0.1111626109
0.3248011471
E:\\R-3.1.2\\scripts\\mPDBS\\summary_pdb_93\\e4b48b06824ef848.final.pdb
-0.3604235999 -1.46639864 -0.5675781316 -0.6160786620 -0.1684080332
0.0113770455
E:\\R-3.1.2\\scripts\\mPDBS\\summary_pdb_93\\e529e19ad281fea2.final.pdb
-0.8044053354 -0.45924817 -0.2955269396 -0.0724298678 -0.0155896011
0.2267311863
E:\\R-3.1.2\\scripts\\mPDBS\\summary_pdb_93\\e5602082718ec3a1.final.pdb
-0.5586318059 0.53813062 0.2166606142 0.1769957731 0.0107913765
-0.1125329875
E:\\R-3.1.2\\scripts\\mPDBS\\summary_pdb_93\\e5d589d996d0a960.final.pdb
0.1146919898 0.98121299 -0.4622555753 0.4004748543 0.2062963337 0.2310491002
E:\\R-3.1.2\\scripts\\mPDBS\\summary_pdb_93\\e9df9e72307a2fbd.final.pdb
0.3286758671 0.18873898 1.0108892650 0.1134985089 0.0135144628 -0.0278169132
E:\\R-3.1.2\\scripts\\mPDBS\\summary_pdb_93\\ea00f8694090cae7.final.pdb
-0.5586318059 0.53813062 0.2166606142 0.1769957731 0.0107913765
-0.1125329875
E:\\R-3.1.2\\scripts\\mPDBS\\summary_pdb_93\\eba517be32db050e.final.pdb
0.2100304121 0.28610420 0.1078257329 0.0688130529 0.0122171194 0.0191132209
E:\\R-3.1.2\\scripts\\mPDBS\\summary_pdb_93\\ec04b9545899c29d.final.pdb

-0.0420658372 1.29880465 0.9289769013 -1.9379581838 -0.3531834654
-0.0590305111
E:\\R-3.1.2\\scripts\\mPDBS\\summary_pdb_93\\ee6862cbcad52078.final.pdb
-0.5804563510 -0.08489528 -0.0869741715 0.0095796295 0.0362807268
-0.2229452700
E:\\R-3.1.2\\scripts\\mPDBS\\summary_pdb_93\\ef6a42fc83b81e5d.final.pdb
0.2952395809 0.49486939 0.1471674344 0.0849920422 0.0136696230 0.0019169467
E:\\R-3.1.2\\scripts\\mPDBS\\summary_pdb_93\\ef8e88d4ad93a0ad.final.pdb
-0.1297740428 0.01222084 -0.0876641555 0.1382806284 -1.1626723538
-0.1551056616
E:\\R-3.1.2\\scripts\\mPDBS\\summary_pdb_93\\f14fcae8deec4fa3.final.pdb
0.0270221234 0.03925750 -1.7723855810 -0.2180312611 -0.0560332513 -0.1032216402
E:\\R-3.1.2\\scripts\\mPDBS\\summary_pdb_93\\f83584e22003fb92.final.pdb
0.4514330076 0.48172114 -0.0620891319 -0.2578746220 0.0459748781 -0.0042638971
E:\\R-3.1.2\\scripts\\mPDBS\\summary_pdb_93\\f8e2bf87e52caa29.final.pdb
-0.8044053354 -0.45924817 -0.2955269396 -0.0724298678 -0.0155896011
0.2267311863

[,31]   [,32]   [,33]   [,34]   [,35]   [,36]
E:\\R-3.1.2\\scripts\\40.1ns_data\\NAT0.pdb
-3.118351e-06 0.0035186507 0.000743965 0.0029183115 0.0050915813
-0.0016777078
E:\\R-3.1.2\\scripts\\40.1ns_data\\LJP6.pdb
6.598122e-03 -0.0102173352 0.005561929 0.0025198041 0.0019553538
-0.0086058287
E:\\R-3.1.2\\scripts\\40.1ns_data\\3WY9.pdb
4.996411e-03 -0.0144334521 -0.002014190 -0.0031484732 -0.0051516271
0.0036707959
E:\\R-3.1.2\\scripts\\40.1ns_data\\ZSP9.pdb
-4.415123e-03 0.0307954296 -0.012617964 -0.0026725418 -0.0052894977
0.0053014931
E:\\R-3.1.2\\scripts\\mPDBS\\summary_pdb_93\\013d855ad26c877c.final.pdb
6.643049e-02 -0.0651772559 0.345656478 0.1290851889 0.1724534229
-0.1878806762
E:\\R-3.1.2\\scripts\\mPDBS\\summary_pdb_93\\0a2a705342ca0d54.final.pdb
-1.814413e-01 0.4864645942 0.377127337 0.2524572968 -0.1680940502
0.0514056079
E:\\R-3.1.2\\scripts\\mPDBS\\summary_pdb_93\\0a59fceadb4194d9.final.pdb
-2.695136e-01 0.1606866285 0.012741142 -0.0898484036 0.0312454697
-0.1116528737
E:\\R-3.1.2\\scripts\\mPDBS\\summary_pdb_93\\0dba59f3306825e8.final.pdb
1.716963e-01 -0.1423533127 0.046398420 0.1012552430 0.1408583742
-0.0911145381
E:\\R-3.1.2\\scripts\\mPDBS\\summary_pdb_93\\126f26a78b57339c.final.pdb
2.592581e-01 -0.1932705827 -0.064109958 -0.1303596181 -0.3293296546
0.1229777036
E:\\R-3.1.2\\scripts\\mPDBS\\summary_pdb_93\\12f06f3e342df706.final.pdb
9.200005e-01 1.7301354076 0.855396138 0.6720751838 -0.2171371594
0.0574321553
E:\\R-3.1.2\\scripts\\mPDBS\\summary_pdb_93\\18618a2a251b867b.final.pdb
-2.775994e-01 0.0476851836 0.024401769 0.0663040773 0.0231669889
-0.2261852614
E:\\R-3.1.2\\scripts\\mPDBS\\summary_pdb_93\\1f305f598641eeb2.final.pdb
3.056070e-02 -0.0107851303 -0.021762472 -0.0043516722 -0.0149708196

0.0036283210
E:\\R-3.1.2\\scripts\\mPDBS\\summary_pdb_93\\1f8a00b551be0a39.final.pdb
-2.775994e-01 0.0476851836 0.024401769 0.0663040773 0.0231669889
-0.2261852614
E:\\R-3.1.2\\scripts\\mPDBS\\summary_pdb_93\\219f45328d47579a.final.pdb
-3.596739e-01 0.2380306139 -0.256905209 -0.0631080748 -0.3095890437
0.1343323943
E:\\R-3.1.2\\scripts\\mPDBS\\summary_pdb_93\\238dd3918e54336d.final.pdb
-4.432551e-01 0.2989878653 -0.085435587 -0.0811188132 -0.4501844166
0.1739432325
E:\\R-3.1.2\\scripts\\mPDBS\\summary_pdb_93\\2b3027889e2fdc42.final.pdb
1.120095e-02 -0.0045575621 0.002264678 0.0005893378 0.0012649480
-0.0009000797
E:\\R-3.1.2\\scripts\\mPDBS\\summary_pdb_93\\2fcb3b46e33793b0.final.pdb
-4.891594e-02 0.0016807755 -0.098870765 -0.1844624296 -0.1110341283
0.1951618187
E:\\R-3.1.2\\scripts\\mPDBS\\summary_pdb_93\\34eb328251842006.final.pdb
-7.621378e-01 1.1492750791 -1.116919647 0.6099348978 0.0227824348
0.1148830481
E:\\R-3.1.2\\scripts\\mPDBS\\summary_pdb_93\\354b32871797b7ab.final.pdb
4.814485e-03 0.0059751644 0.073232390 -0.0892305923 -0.4195897121
0.2252196079
E:\\R-3.1.2\\scripts\\mPDBS\\summary_pdb_93\\397368adbc32d5de.final.pdb
2.592581e-01 -0.1932705827 -0.064109958 -0.1303596181 -0.3293296546
0.1229777036
E:\\R-3.1.2\\scripts\\mPDBS\\summary_pdb_93\\411ca13cd3df6edd.final.pdb
-2.191472e-02 -0.1137491537 -0.097550578 0.1484486488 -0.2975417754
-0.5339661826
E:\\R-3.1.2\\scripts\\mPDBS\\summary_pdb_93\\42357eee6642bf12.final.pdb
7.528551e-01 -1.1510428549 -0.344818892 0.7308066564 0.1291457432
-0.0632712074
E:\\R-3.1.2\\scripts\\mPDBS\\summary_pdb_93\\44342d1f75a7fcfc.final.pdb
-1.244949e-01 -0.0853088981 -0.039639547 -0.0318865727 -0.4196857682
0.1701517329
E:\\R-3.1.2\\scripts\\mPDBS\\summary_pdb_93\\448ef21dffe8cdfb.final.pdb
-1.364778e-01 0.0525980454 0.011637613 0.0471524315 0.3591539005
-0.0189304229
E:\\R-3.1.2\\scripts\\mPDBS\\summary_pdb_93\\472a40b3e8f113c7.final.pdb
1.716963e-01 -0.1423533127 0.046398420 0.1012552430 0.1408583742
-0.0911145381
E:\\R-3.1.2\\scripts\\mPDBS\\summary_pdb_93\\48a26225951ea153.final.pdb
-1.666599e-01 0.1490016192 0.064800598 -0.0479441841 0.3177719510
0.4225144482
E:\\R-3.1.2\\scripts\\mPDBS\\summary_pdb_93\\4ab4a5c7c6682ce6.final.pdb
2.136364e-01 -0.2319125719 0.056312081 0.0950039225 0.0119706188
0.1045795936
E:\\R-3.1.2\\scripts\\mPDBS\\summary_pdb_93\\4eaa45b62cb6a25f.final.pdb
1.785559e-01 -0.1323260724 0.171409119 -0.0979601827 0.0618401742
-0.0603764984
E:\\R-3.1.2\\scripts\\mPDBS\\summary_pdb_93\\58c371611d85ac96.final.pdb
2.592581e-01 -0.1932705827 -0.064109958 -0.1303596181 -0.3293296546
0.1229777036
E:\\R-3.1.2\\scripts\\mPDBS\\summary_pdb_93\\59a647f04822d30a.final.pdb
-3.433465e-02 -0.1626528834 0.230006804 -0.0586269015 -0.0788512547

-0.0758335351
E:\\R-3.1.2\\scripts\\mPDBS\\summary_pdb_93\\59b091fe385e05fc.final.pdb
-2.695136e-01 0.1606866285 0.012741142 -0.0898484036 0.0312454697
-0.1116528737
E:\\R-3.1.2\\scripts\\mPDBS\\summary_pdb_93\\5c35ac30f930d2db.final.pdb
1.785559e-01 -0.1323260724 0.171409119 -0.0979601827 0.0618401742
-0.0603764984
E:\\R-3.1.2\\scripts\\mPDBS\\summary_pdb_93\\5f60752431cf2dcb.final.pdb
-2.892377e-01 -0.3513756490 -0.923545785 -0.7326989851 0.0508095156
0.1208804677
E:\\R-3.1.2\\scripts\\mPDBS\\summary_pdb_93\\626456dd6ff2868d.final.pdb
1.716963e-01 -0.1423533127 0.046398420 0.1012552430 0.1408583742
-0.0911145381
E:\\R-3.1.2\\scripts\\mPDBS\\summary_pdb_93\\6583d53ff3c8f7ef.final.pdb
-3.433465e-02 -0.1626528834 0.230006804 -0.0586269015 -0.0788512547
-0.0758335351
E:\\R-3.1.2\\scripts\\mPDBS\\summary_pdb_93\\69760178986bef01.final.pdb
7.790807e-01 1.1054343466 -0.501478112 -0.4999231693 0.3032453083
-0.4361747859
E:\\R-3.1.2\\scripts\\mPDBS\\summary_pdb_93\\6a9d87d47926b1c8.final.pdb
-1.323767e-02 0.0087161858 0.002124344 -0.0059693758 0.0059129433
0.0116883867
E:\\R-3.1.2\\scripts\\mPDBS\\summary_pdb_93\\6bb3547ea982ffc4.final.pdb
9.126906e-02 -0.7853114215 0.087609374 0.8369340770 0.1230997540
0.3687395491
E:\\R-3.1.2\\scripts\\mPDBS\\summary_pdb_93\\716d9624611d1c42.final.pdb
-1.154963e-01 -0.4896768858 -0.825369056 -0.7414285754 0.5322394019
-0.1647156386
E:\\R-3.1.2\\scripts\\mPDBS\\summary_pdb_93\\75f27e70ad0f1be4.final.pdb
-3.433465e-02 -0.1626528834 0.230006804 -0.0586269015 -0.0788512547
-0.0758335351
E:\\R-3.1.2\\scripts\\mPDBS\\summary_pdb_93\\769f910fffb22872.final.pdb
1.255814e-01 -0.1350966146 0.001709947 0.0102119570 -0.1195226667
-0.1477510215
E:\\R-3.1.2\\scripts\\mPDBS\\summary_pdb_93\\791f8c80dd5c3135.final.pdb
-2.695136e-01 0.1606866285 0.012741142 -0.0898484036 0.0312454697
-0.1116528737
E:\\R-3.1.2\\scripts\\mPDBS\\summary_pdb_93\\7fad98e66a3f6099.final.pdb
1.730166e+00 -0.3014022810 0.025683695 -0.3065345825 0.0008973886
-0.0062538520
E:\\R-3.1.2\\scripts\\mPDBS\\summary_pdb_93\\804b89c94a633196.final.pdb
-4.891594e-02 0.0016807755 -0.098870765 -0.1844624296 -0.1110341283
0.1951618187
E:\\R-3.1.2\\scripts\\mPDBS\\summary_pdb_93\\813cc65a2298feba.final.pdb
-4.891594e-02 0.0016807755 -0.098870765 -0.1844624296 -0.1110341283
0.1951618187
E:\\R-3.1.2\\scripts\\mPDBS\\summary_pdb_93\\83c0eefcfe12d3f9.final.pdb
-2.191472e-02 -0.1137491537 -0.097550578 0.1484486488 -0.2975417754
-0.5339661826
E:\\R-3.1.2\\scripts\\mPDBS\\summary_pdb_93\\86d50469ef569760.final.pdb
1.716963e-01 -0.1423533127 0.046398420 0.1012552430 0.1408583742
-0.0911145381
E:\\R-3.1.2\\scripts\\mPDBS\\summary_pdb_93\\89d0666e1402203d.final.pdb
-3.433465e-02 -0.1626528834 0.230006804 -0.0586269015 -0.0788512547

-0.0758335351
E:\\R-3.1.2\\scripts\\mPDBS\\summary_pdb_93\\8b34852716dadc62.final.pdb
-2.417435e-01 0.1297741022 0.310895671 0.0749823774 0.3907489493
-0.1156965610
E:\\R-3.1.2\\scripts\\mPDBS\\summary_pdb_93\\8b6ef9ac3fe74395.final.pdb
-1.506530e-02 0.5003056237 0.117893468 -0.4529439127 0.1295307191
0.6194193691
E:\\R-3.1.2\\scripts\\mPDBS\\summary_pdb_93\\98bbe0d5986c138f.final.pdb
-2.695136e-01 0.1606866285 0.012741142 -0.0898484036 0.0312454697
-0.1116528737
E:\\R-3.1.2\\scripts\\mPDBS\\summary_pdb_93\\9c22f4909d67e2fa.final.pdb
-9.453762e-02 -0.0369612138 0.021551274 0.0409011110 0.2302661451
0.1767637088
E:\\R-3.1.2\\scripts\\mPDBS\\summary_pdb_93\\9dda8ed0d81fb3ef.final.pdb
9.193628e-02 -0.2107217759 -0.080578760 -0.0595518550 -0.1398333123
0.0856985198
E:\\R-3.1.2\\scripts\\mPDBS\\summary_pdb_93\\9f752b3616e6033b.final.pdb
-1.676859e-01 -0.4415254570 0.179894884 0.1618788457 -0.0153818365
0.0277065584
E:\\R-3.1.2\\scripts\\mPDBS\\summary_pdb_93\\a02c70083816d6ba.final.pdb
1.785559e-01 -0.1323260724 0.171409119 -0.0979601827 0.0618401742
-0.0603764984
E:\\R-3.1.2\\scripts\\mPDBS\\summary_pdb_93\\a0c997d891e4e082.final.pdb
-4.716573e-01 -0.4897628702 0.343915277 0.3108187023 -0.1440187533
0.1914318604
E:\\R-3.1.2\\scripts\\mPDBS\\summary_pdb_93\\ab8039a2d823c616.final.pdb
1.511528e-01 -0.0198962064 -0.011792279 -0.0402937675 0.0040998906
0.0133468579
E:\\R-3.1.2\\scripts\\mPDBS\\summary_pdb_93\\acf4d74b9c71d4dc.final.pdb
-4.891594e-02 0.0016807755 -0.098870765 -0.1844624296 -0.1110341283
0.1951618187
E:\\R-3.1.2\\scripts\\mPDBS\\summary_pdb_93\\ad8a823e4b4c668e.final.pdb
5.946589e-03 -0.0148990142 -0.011169528 0.0161472824 -0.0567589519
-0.0629321178
E:\\R-3.1.2\\scripts\\mPDBS\\summary_pdb_93\\b16ababd03b7b857.final.pdb
-9.453762e-02 -0.0369612138 0.021551274 0.0409011110 0.2302661451
0.1767637088
E:\\R-3.1.2\\scripts\\mPDBS\\summary_pdb_93\\b226923e697d3c63.final.pdb
3.811116e-01 0.2576068703 -0.052816347 -0.2434187375 0.2616750642
0.1945687981
E:\\R-3.1.2\\scripts\\mPDBS\\summary_pdb_93\\b28e48f6fa27a165.final.pdb
-2.695136e-01 0.1606866285 0.012741142 -0.0898484036 0.0312454697
-0.1116528737
E:\\R-3.1.2\\scripts\\mPDBS\\summary_pdb_93\\b42c4924e3cd1aec.final.pdb
2.656777e-01 -0.3490230128 0.017597969 -0.0682814453 0.3415965741
-0.1998975864
E:\\R-3.1.2\\scripts\\mPDBS\\summary_pdb_93\\b4dd239fcabff1df.final.pdb
-2.695136e-01 0.1606866285 0.012741142 -0.0898484036 0.0312454697
-0.1116528737
E:\\R-3.1.2\\scripts\\mPDBS\\summary_pdb_93\\b669942ef97459a2.final.pdb
-2.417435e-01 0.1297741022 0.310895671 0.0749823774 0.3907489493
-0.1156965610
E:\\R-3.1.2\\scripts\\mPDBS\\summary_pdb_93\\b99f4d9d87c1017b.final.pdb
-2.695136e-01 0.1606866285 0.012741142 -0.0898484036 0.0312454697

-0.1116528737
E:\\R-3.1.2\\scripts\\mPDBS\\summary_pdb_93\\b9d2cbf3975403e7.final.pdb
-2.695136e-01 0.1606866285 0.012741142 -0.0898484036 0.0312454697
-0.1116528737
E:\\R-3.1.2\\scripts\\mPDBS\\summary_pdb_93\\bc88c8a505f3a288.final.pdb
3.692449e-03 -0.0041387704 0.003830339 -0.0026259582 0.0100056887
0.0101097868
E:\\R-3.1.2\\scripts\\mPDBS\\summary_pdb_93\\bdac0e64f4ef427e.final.pdb
2.370696e-02 -0.0751071645 -0.217972617 -0.0769148918 -0.6388420487
-0.5155680726
E:\\R-3.1.2\\scripts\\mPDBS\\summary_pdb_93\\ca2a1f55fc9d0681.final.pdb
-2.417435e-01 0.1297741022 0.310895671 0.0749823774 0.3907489493
-0.1156965610
E:\\R-3.1.2\\scripts\\mPDBS\\summary_pdb_93\\ce34248de3e81a3c.final.pdb
-7.093445e-02 0.3607932575 0.119741102 -0.3368103602 -0.1532568368
0.0637487026
E:\\R-3.1.2\\scripts\\mPDBS\\summary_pdb_93\\d02e697772494a61.final.pdb
-1.432427e-01 0.1126177360 -0.297844421 -0.0907733571 -0.0297365878
0.0498791813
E:\\R-3.1.2\\scripts\\mPDBS\\summary_pdb_93\\d04221245d015047.final.pdb
-1.590648e-01 -0.0578181071 0.009032671 0.0841226469 -0.0900189157
-0.0723331282
E:\\R-3.1.2\\scripts\\mPDBS\\summary_pdb_93\\d678141e4018054a.final.pdb
1.114307e+00 0.6390368824 -0.097253818 -0.1871793500 0.1414339952
-0.0795218894
E:\\R-3.1.2\\scripts\\mPDBS\\summary_pdb_93\\d71b40af0f0efe39.final.pdb
-8.256821e-01 -0.0814725073 0.409615666 -0.4011402378 0.0101951168
-0.3668247598
E:\\R-3.1.2\\scripts\\mPDBS\\summary_pdb_93\\dc0809eff10197e2.final.pdb
1.469336e+00 -0.0070538254 -0.774240755 0.4734259553 -0.1183572769
0.0971535564
E:\\R-3.1.2\\scripts\\mPDBS\\summary_pdb_93\\e1163918d3707839.final.pdb
-2.080761e-01 -0.0243516466 0.131830075 -0.0498973112 -0.5602811411
0.2097625711
E:\\R-3.1.2\\scripts\\mPDBS\\summary_pdb_93\\e4b48b06824ef848.final.pdb
-2.775994e-01 0.0476851836 0.024401769 0.0663040773 0.0231669889
-0.2261852614
E:\\R-3.1.2\\scripts\\mPDBS\\summary_pdb_93\\e529e19ad281fea2.final.pdb
6.643049e-02 -0.0651772559 0.345656478 0.1290851889 0.1724534229
-0.1878806762
E:\\R-3.1.2\\scripts\\mPDBS\\summary_pdb_93\\e5602082718ec3a1.final.pdb
-4.891594e-02 0.0016807755 -0.098870765 -0.1844624296 -0.1110341283
0.1951618187
E:\\R-3.1.2\\scripts\\mPDBS\\summary_pdb_93\\e5d589d996d0a960.final.pdb
-5.537132e-01 0.2238103797 -0.947669935 1.2866306122 0.1537974105
-0.0401570046
E:\\R-3.1.2\\scripts\\mPDBS\\summary_pdb_93\\e9df9e72307a2fbd.final.pdb
1.785559e-01 -0.1323260724 0.171409119 -0.0979601827 0.0618401742
-0.0603764984
E:\\R-3.1.2\\scripts\\mPDBS\\summary_pdb_93\\ea00f8694090cae7.final.pdb
-4.891594e-02 0.0016807755 -0.098870765 -0.1844624296 -0.1110341283
0.1951618187
E:\\R-3.1.2\\scripts\\mPDBS\\summary_pdb_93\\eba517be32db050e.final.pdb
-2.695136e-01 0.1606866285 0.012741142 -0.0898484036 0.0312454697

```
-0.1116528737
E:\\R-3.1.2\\scripts\\mPDBS\\summary_pdb_93\\ec04b9545899c29d.final.pdb
-6.121254e-02  0.0633397136  0.003088716 -0.0197214713  0.1057846057
0.1105712286
E:\\R-3.1.2\\scripts\\mPDBS\\summary_pdb_93\\ee6862cbcad52078.final.pdb
1.578975e-02 -0.0504401714 -0.029370893 -0.0219330951  0.0101604751
-0.0025382963
E:\\R-3.1.2\\scripts\\mPDBS\\summary_pdb_93\\ef6a42fc83b81e5d.final.pdb
1.589370e-02 -0.0003673116  0.002261990  0.0006906221  0.0001090582
0.0016932849
E:\\R-3.1.2\\scripts\\mPDBS\\summary_pdb_93\\ef8e88d4ad93a0ad.final.pdb
-1.590648e-01 -0.0578181071  0.009032671  0.0841226469 -0.0900189157
-0.0723331282
E:\\R-3.1.2\\scripts\\mPDBS\\summary_pdb_93\\f14fcae8deec4fa3.final.pdb
-1.676859e-01 -0.4415254570  0.179894884  0.1618788457 -0.0153818365
0.0277065584
E:\\R-3.1.2\\scripts\\mPDBS\\summary_pdb_93\\f83584e22003fb92.final.pdb
-1.666599e-01  0.1490016192  0.064800598 -0.0479441841  0.3177719510
0.4225144482
E:\\R-3.1.2\\scripts\\mPDBS\\summary_pdb_93\\f8e2bf87e52caa29.final.pdb
6.643049e-02 -0.0651772559  0.345656478  0.1290851889  0.1724534229
-0.1878806762

[,37]         [,38]         [,39]         [,40]         [,41]         [,42]
E:\\R-3.1.2\\scripts\\40.1ns_data\\NAT0.pdb
-0.0005236504  0.0002630758  9.581330e-15 -2.347540e-14  5.518401e-14
-8.613355e-15
E:\\R-3.1.2\\scripts\\40.1ns_data\\LJP6.pdb
-0.0026782897  0.0003108532 -8.206233e-16 -4.141711e-14 -1.011965e-15
5.656571e-15
E:\\R-3.1.2\\scripts\\40.1ns_data\\3WY9.pdb
0.0014139494 -0.0001790228 -1.375784e-14 -6.122318e-14  2.867313e-14
1.971429e-14
E:\\R-3.1.2\\scripts\\40.1ns_data\\ZSP9.pdb
0.0008844115 -0.0004823669  4.083097e-14  4.038695e-14 -4.564094e-14
-2.682444e-14
E:\\R-3.1.2\\scripts\\mPDBS\\summary_pdb_93\\013d855ad26c877c.final.pdb
-0.0347886459  0.1079362579 -4.075853e-13 -4.552624e-13  3.521304e-13
1.803434e-13
E:\\R-3.1.2\\scripts\\mPDBS\\summary_pdb_93\\0a2a705342ca0d54.final.pdb
0.0218932863 -0.0027443233  1.168407e-14  1.015838e-13 -9.806263e-14
6.146168e-14
E:\\R-3.1.2\\scripts\\mPDBS\\summary_pdb_93\\0a59fceadb4194d9.final.pdb
-0.0940224196 -0.0071517950 -5.219251e-15 -5.205102e-14  1.050819e-14
7.088145e-15
E:\\R-3.1.2\\scripts\\mPDBS\\summary_pdb_93\\0dba59f3306825e8.final.pdb
-0.0023513075 -0.1237308648  2.751900e-13 -2.904999e-14 -5.971546e-14
-6.798229e-14
E:\\R-3.1.2\\scripts\\mPDBS\\summary_pdb_93\\126f26a78b57339c.final.pdb
0.1309313201  0.0360155893 -1.000580e-14  2.324522e-13 -1.117701e-13
-1.410395e-14
E:\\R-3.1.2\\scripts\\mPDBS\\summary_pdb_93\\12f06f3e342df706.final.pdb
0.0471226534 -0.0018637800 -2.248668e-14 -3.620984e-13  2.473681e-13
-2.527449e-14
```

E:\\R-3.1.2\\scripts\\mPDBS\\summary_pdb_93\\18618a2a251b867b.final.pdb
0.3174463829 -0.0135751436 -1.148705e-13 -4.723144e-13 3.063538e-13
-9.448766e-14
E:\\R-3.1.2\\scripts\\mPDBS\\summary_pdb_93\\1f305f598641eeb2.final.pdb
0.0025169308 -0.0002519726 -8.780551e-14 -2.221505e-13 3.338616e-14
-6.225671e-14
E:\\R-3.1.2\\scripts\\mPDBS\\summary_pdb_93\\1f8a00b551be0a39.final.pdb
0.3174463829 -0.0135751436 -1.148705e-13 -4.723144e-13 3.063538e-13
-9.448766e-14
E:\\R-3.1.2\\scripts\\mPDBS\\summary_pdb_93\\219f45328d47579a.final.pdb
-0.0215768608 0.2891958917 -6.832664e-13 3.044257e-14 1.010798e-13
2.560201e-13
E:\\R-3.1.2\\scripts\\mPDBS\\summary_pdb_93\\238dd3918e54336d.final.pdb
0.0195621018 -0.1219446210 5.801276e-13 9.082818e-13 -5.517179e-13
-2.078244e-13
E:\\R-3.1.2\\scripts\\mPDBS\\summary_pdb_93\\2b3027889e2fdc42.final.pdb
0.0000833949 -0.0002764205 -4.500936e-14 -1.100679e-13 -8.929165e-15
1.301006e-14
E:\\R-3.1.2\\scripts\\mPDBS\\summary_pdb_93\\2fcb3b46e33793b0.final.pdb
0.0683769531 -0.0043970162 9.880194e-14 3.357930e-13 -2.039388e-13
-5.764234e-14
E:\\R-3.1.2\\scripts\\mPDBS\\summary_pdb_93\\34eb328251842006.final.pdb
-0.1158449284 0.0180362168 9.458520e-14 -2.887498e-13 1.115067e-13
-2.357195e-14
E:\\R-3.1.2\\scripts\\mPDBS\\summary_pdb_93\\354b32871797b7ab.final.pdb
0.1107645538 -0.0838047286 3.595454e-13 5.109628e-13 -3.399880e-13
-1.558259e-13
E:\\R-3.1.2\\scripts\\mPDBS\\summary_pdb_93\\397368adbc32d5de.final.pdb
0.1309313201 0.0360155893 -1.000580e-14 2.324522e-13 -1.117701e-13
-1.410395e-14
E:\\R-3.1.2\\scripts\\mPDBS\\summary_pdb_93\\411ca13cd3df6edd.final.pdb
-0.1266182179 -0.0038730500 -2.126857e-14 -8.024727e-14 8.772060e-15
-7.898173e-16
E:\\R-3.1.2\\scripts\\mPDBS\\summary_pdb_93\\42357eee6642bf12.final.pdb
0.3410965722 0.0750966992 -1.743952e-13 4.175584e-13 -2.784901e-13
5.430012e-14
E:\\R-3.1.2\\scripts\\mPDBS\\summary_pdb_93\\44342d1f75a7fcfc.final.pdb
-0.0442175949 0.2590987450 -5.944034e-13 1.688728e-13 2.897300e-14
2.368452e-13
E:\\R-3.1.2\\scripts\\mPDBS\\summary_pdb_93\\448ef21dffe8cdfb.final.pdb
-0.0649056746 -0.1641434703 3.879372e-13 6.714135e-14 -1.622612e-13
-1.208349e-13
E:\\R-3.1.2\\scripts\\mPDBS\\summary_pdb_93\\472a40b3e8f113c7.final.pdb
-0.0023513075 -0.1237308648 2.751900e-13 -2.904999e-14 -5.971546e-14
-6.798229e-14
E:\\R-3.1.2\\scripts\\mPDBS\\summary_pdb_93\\48a26225951ea153.final.pdb
0.0601328410 0.0129200188 -6.010525e-14 1.843958e-14 -3.001256e-14
5.472062e-14
E:\\R-3.1.2\\scripts\\mPDBS\\summary_pdb_93\\4ab4a5c7c6682ce6.final.pdb
0.3668996323 0.0345039946 -1.637627e-13 -1.690283e-13 1.663555e-13
-2.042528e-14
E:\\R-3.1.2\\scripts\\mPDBS\\summary_pdb_93\\4eaa45b62cb6a25f.final.pdb
-0.0028199676 0.0309880974 -2.315823e-13 -4.441127e-13 2.368661e-13
6.147100e-14

E:\\R-3.1.2\\scripts\\mPDBS\\summary_pdb_93\\58c371611d85ac96.final.pdb
0.1309313201 0.0360155893 -1.000580e-14 2.324522e-13 -1.117701e-13
-1.410395e-14
E:\\R-3.1.2\\scripts\\mPDBS\\summary_pdb_93\\59a647f04822d30a.final.pdb
-0.1166631537 -0.0372489416 9.454331e-14 9.761287e-14 -4.268322e-14
-3.029098e-14
E:\\R-3.1.2\\scripts\\mPDBS\\summary_pdb_93\\59b091fe385e05fc.final.pdb
-0.0940224196 -0.0071517950 -5.219251e-15 -5.205102e-14 1.050819e-14
7.088145e-15
E:\\R-3.1.2\\scripts\\mPDBS\\summary_pdb_93\\5c35ac30f930d2db.final.pdb
-0.0028199676 0.0309880974 -2.315823e-13 -4.441127e-13 2.368661e-13
6.147100e-14
E:\\R-3.1.2\\scripts\\mPDBS\\summary_pdb_93\\5f60752431cf2dcb.final.pdb
-0.0673277395 -0.0543939719 2.843431e-13 3.352994e-13 -8.222071e-14
-7.622115e-14
E:\\R-3.1.2\\scripts\\mPDBS\\summary_pdb_93\\626456dd6ff2868d.final.pdb
-0.0023513075 -0.1237308648 2.751900e-13 -2.904999e-14 -5.971546e-14
-6.798229e-14
E:\\R-3.1.2\\scripts\\mPDBS\\summary_pdb_93\\6583d53ff3c8f7ef.final.pdb
-0.1166631537 -0.0372489416 9.454331e-14 9.761287e-14 -4.268322e-14
-3.029098e-14
E:\\R-3.1.2\\scripts\\mPDBS\\summary_pdb_93\\69760178986bef01.final.pdb
0.0847796710 -0.0520722053 -3.483770e-15 -9.841789e-13 7.999021e-13
-5.262933e-15
E:\\R-3.1.2\\scripts\\mPDBS\\summary_pdb_93\\6a9d87d47926b1c8.final.pdb
0.0050990526 0.0001531810 -5.075782e-14 -8.267121e-15 -3.029965e-14
-6.242181e-15
E:\\R-3.1.2\\scripts\\mPDBS\\summary_pdb_93\\6bb3547ea982ffc4.final.pdb
-0.7053714647 -0.0602332355 1.510392e-13 1.415007e-13 -5.815425e-13
1.612855e-13
E:\\R-3.1.2\\scripts\\mPDBS\\summary_pdb_93\\716d9624611d1c42.final.pdb
-0.1809122608 0.0603988541 -2.966361e-13 -6.243545e-13 4.918782e-13
1.274081e-13
E:\\R-3.1.2\\scripts\\mPDBS\\summary_pdb_93\\75f27e70ad0f1be4.final.pdb
-0.1166631537 -0.0372489416 9.454331e-14 9.761287e-14 -4.268322e-14
-3.029098e-14
E:\\R-3.1.2\\scripts\\mPDBS\\summary_pdb_93\\769f910fffb22872.final.pdb
-0.0853715736 -0.0011515745 -3.616283e-14 -2.157058e-13 2.597674e-14
-6.203944e-14
E:\\R-3.1.2\\scripts\\mPDBS\\summary_pdb_93\\791f8c80dd5c3135.final.pdb
-0.0940224196 -0.0071517950 -5.219251e-15 -5.205102e-14 1.050819e-14
7.088145e-15
E:\\R-3.1.2\\scripts\\mPDBS\\summary_pdb_93\\7fad98e66a3f6099.final.pdb
-0.3688776182 0.0401555682 -3.322658e-13 -1.254784e-12 6.133045e-13
2.811805e-13
E:\\R-3.1.2\\scripts\\mPDBS\\summary_pdb_93\\804b89c94a633196.final.pdb
0.0683769531 -0.0043970162 9.880194e-14 3.357930e-13 -2.039388e-13
-5.764234e-14
E:\\R-3.1.2\\scripts\\mPDBS\\summary_pdb_93\\813cc65a2298feba.final.pdb
0.0683769531 -0.0043970162 9.880194e-14 3.357930e-13 -2.039388e-13
-5.764234e-14
E:\\R-3.1.2\\scripts\\mPDBS\\summary_pdb_93\\83c0eefcfe12d3f9.final.pdb
-0.1266182179 -0.0038730500 -2.126857e-14 -8.024727e-14 8.772060e-15
-7.898173e-16

E:\\R-3.1.2\\scripts\\mPDBS\\summary_pdb_93\\86d50469ef569760.final.pdb
-0.0023513075 -0.1237308648 2.751900e-13 -2.904999e-14 -5.971546e-14
-6.798229e-14
E:\\R-3.1.2\\scripts\\mPDBS\\summary_pdb_93\\89d0666e1402203d.final.pdb
-0.1166631537 -0.0372489416 9.454331e-14 9.761287e-14 -4.268322e-14
-3.029098e-14
E:\\R-3.1.2\\scripts\\mPDBS\\summary_pdb_93\\8b34852716dadc62.final.pdb
-0.0973430129 0.0675236524 -3.084485e-13 -3.523900e-13 2.760462e-13
1.348183e-13
E:\\R-3.1.2\\scripts\\mPDBS\\summary_pdb_93\\8b6ef9ac3fe74395.final.pdb
-0.7174832536 -0.0151005393 1.781637e-13 1.149937e-13 -1.735212e-13
1.757265e-13
E:\\R-3.1.2\\scripts\\mPDBS\\summary_pdb_93\\98bbe0d5986c138f.final.pdb
-0.0940224196 -0.0071517950 -5.219251e-15 -5.205102e-14 1.050819e-14
7.088145e-15
E:\\R-3.1.2\\scripts\\mPDBS\\summary_pdb_93\\9c22f4909d67e2fa.final.pdb
0.3043452652 -0.0059086109 -5.002085e-14 -5.020077e-14 7.452589e-14
-6.878339e-14
E:\\R-3.1.2\\scripts\\mPDBS\\summary_pdb_93\\9dda8ed0d81fb3ef.final.pdb
0.0227318059 -0.0473145862 1.718109e-13 2.970443e-13 -2.191270e-13
-6.980841e-14
E:\\R-3.1.2\\scripts\\mPDBS\\summary_pdb_93\\9f752b3616e6033b.final.pdb
-0.0092396663 -0.0199214466 1.066423e-13 3.494259e-13 -2.032586e-13
-4.022787e-14
E:\\R-3.1.2\\scripts\\mPDBS\\summary_pdb_93\\a02c70083816d6ba.final.pdb
-0.0028199676 0.0309880974 -2.315823e-13 -4.441127e-13 2.368661e-13
6.147100e-14
E:\\R-3.1.2\\scripts\\mPDBS\\summary_pdb_93\\a0c997d891e4e082.final.pdb
-0.0857684479 -0.0133207732 1.521238e-13 6.926356e-13 -5.218926e-13
-2.184331e-14
E:\\R-3.1.2\\scripts\\mPDBS\\summary_pdb_93\\ab8039a2d823c616.final.pdb
0.0048388116 -0.0003532589 -5.641534e-15 -3.230749e-14 9.887992e-14
2.195217e-14
E:\\R-3.1.2\\scripts\\mPDBS\\summary_pdb_93\\acf4d74b9c71d4dc.final.pdb
0.0683769531 -0.0043970162 9.880194e-14 3.357930e-13 -2.039388e-13
-5.764234e-14
E:\\R-3.1.2\\scripts\\mPDBS\\summary_pdb_93\\ad8a823e4b4c668e.final.pdb
-0.0251991338 -0.0011683232 2.523244e-14 5.408712e-14 -7.682754e-15
-1.600140e-14
E:\\R-3.1.2\\scripts\\mPDBS\\summary_pdb_93\\b16ababd03b7b857.final.pdb
0.3043452652 -0.0059086109 -5.002085e-14 -5.020077e-14 7.452589e-14
-6.878339e-14
E:\\R-3.1.2\\scripts\\mPDBS\\summary_pdb_93\\b226923e697d3c63.final.pdb
0.0907770372 -0.0027741524 -6.440609e-14 -3.459411e-13 2.631260e-13
1.947715e-14
E:\\R-3.1.2\\scripts\\mPDBS\\summary_pdb_93\\b28e48f6fa27a165.final.pdb
-0.0940224196 -0.0071517950 -5.219251e-15 -5.205102e-14 1.050819e-14
7.088145e-15
E:\\R-3.1.2\\scripts\\mPDBS\\summary_pdb_93\\b42c4924e3cd1aec.final.pdb
-0.0908527155 0.0674782399 -3.946421e-13 -6.421395e-13 3.560443e-13
1.342582e-13
E:\\R-3.1.2\\scripts\\mPDBS\\summary_pdb_93\\b4dd239fcabff1df.final.pdb
-0.0940224196 -0.0071517950 -5.219251e-15 -5.205102e-14 1.050819e-14
7.088145e-15

E:\\R-3.1.2\\scripts\\mPDBS\\summary_pdb_93\\b669942ef97459a2.final.pdb
-0.0973430129 0.0675236524 -3.084485e-13 -3.523900e-13 2.760462e-13
1.348183e-13
E:\\R-3.1.2\\scripts\\mPDBS\\summary_pdb_93\\b99f4d9d87c1017b.final.pdb
-0.0940224196 -0.0071517950 -5.219251e-15 -5.205102e-14 1.050819e-14
7.088145e-15
E:\\R-3.1.2\\scripts\\mPDBS\\summary_pdb_93\\b9d2cbf3975403e7.final.pdb
-0.0940224196 -0.0071517950 -5.219251e-15 -5.205102e-14 1.050819e-14
7.088145e-15
E:\\R-3.1.2\\scripts\\mPDBS\\summary_pdb_93\\bc88c8a505f3a288.final.pdb
0.0042641023 0.0001537794 2.466304e-14 1.013460e-13 8.294579e-15
1.279929e-14
E:\\R-3.1.2\\scripts\\mPDBS\\summary_pdb_93\\bdac0e64f4ef427e.final.pdb
-0.3625865300 -0.0023614554 1.429509e-13 3.350887e-13 -2.576547e-13
4.356457e-15
E:\\R-3.1.2\\scripts\\mPDBS\\summary_pdb_93\\ca2a1f55fc9d0681.final.pdb
-0.0973430129 0.0675236524 -3.084485e-13 -3.523900e-13 2.760462e-13
1.348183e-13
E:\\R-3.1.2\\scripts\\mPDBS\\summary_pdb_93\\ce34248de3e81a3c.final.pdb
0.1319231979 0.1206745894 -3.967284e-13 -3.650695e-13 3.587508e-13
8.676720e-14
E:\\R-3.1.2\\scripts\\mPDBS\\summary_pdb_93\\d02e697772494a61.final.pdb
0.0453725400 -0.0172174395 8.402143e-14 1.664395e-13 -1.628779e-13
-5.350857e-14
E:\\R-3.1.2\\scripts\\mPDBS\\summary_pdb_93\\d04221245d015047.final.pdb
-0.0324835176 0.0010822023 3.280259e-14 1.685410e-13 -1.729096e-13
-2.782637e-14
E:\\R-3.1.2\\scripts\\mPDBS\\summary_pdb_93\\d678141e4018054a.final.pdb
-0.0478839342 -0.0169784857 2.465815e-13 6.007023e-13 -1.973010e-13
1.578582e-14
E:\\R-3.1.2\\scripts\\mPDBS\\summary_pdb_93\\d71b40af0f0efe39.final.pdb
0.4376062011 -0.0803564122 -6.790551e-14 -6.408026e-13 4.747695e-13
-2.376695e-13
E:\\R-3.1.2\\scripts\\mPDBS\\summary_pdb_93\\dc0809eff10197e2.final.pdb
0.2891731920 -0.0579377341 3.686774e-13 6.382116e-13 -3.937086e-13
-3.808325e-14
E:\\R-3.1.2\\scripts\\mPDBS\\summary_pdb_93\\e1163918d3707839.final.pdb
-0.0030786323 -0.1520417677 6.932981e-13 1.053850e-12 -6.215392e-13
-2.456003e-13
E:\\R-3.1.2\\scripts\\mPDBS\\summary_pdb_93\\e4b48b06824ef848.final.pdb
0.3174463829 -0.0135751436 -1.148705e-13 -4.723144e-13 3.063538e-13
-9.448766e-14
E:\\R-3.1.2\\scripts\\mPDBS\\summary_pdb_93\\e529e19ad281fea2.final.pdb
-0.0347886459 0.1079362579 -4.075853e-13 -4.552624e-13 3.521304e-13
1.803434e-13
E:\\R-3.1.2\\scripts\\mPDBS\\summary_pdb_93\\e5602082718ec3a1.final.pdb
0.0683769531 -0.0043970162 9.880194e-14 3.357930e-13 -2.039388e-13
-5.764234e-14
E:\\R-3.1.2\\scripts\\mPDBS\\summary_pdb_93\\e5d589d996d0a960.final.pdb
-0.1069164598 0.0246613530 2.476673e-13 9.346372e-13 -5.875053e-13
-1.754118e-14
E:\\R-3.1.2\\scripts\\mPDBS\\summary_pdb_93\\e9df9e72307a2fbd.final.pdb
-0.0028199676 0.0309880974 -2.315823e-13 -4.441127e-13 2.368661e-13
6.147100e-14

E:\\R-3.1.2\\scripts\\mPDBS\\summary_pdb_93\\ea00f8694090cae7.final.pdb
0.0683769531 -0.0043970162 9.880194e-14 3.357930e-13 -2.039388e-13
-5.764234e-14
E:\\R-3.1.2\\scripts\\mPDBS\\summary_pdb_93\\eba517be32db050e.final.pdb
-0.0940224196 -0.0071517950 -5.219251e-15 -5.205102e-14 1.050819e-14
7.088145e-15
E:\\R-3.1.2\\scripts\\mPDBS\\summary_pdb_93\\ec04b9545899c29d.final.pdb
0.0584448151 0.0019652672 7.792407e-14 1.718132e-13 -8.779991e-14
2.288090e-14
E:\\R-3.1.2\\scripts\\mPDBS\\summary_pdb_93\\ee6862cbcad52078.final.pdb
-0.0014141710 0.0001402097 -4.009619e-14 -1.281408e-13 1.043359e-13
3.257730e-14
E:\\R-3.1.2\\scripts\\mPDBS\\summary_pdb_93\\ef6a42fc83b81e5d.final.pdb
-0.0044926343 0.0001000091 4.541107e-14 1.272492e-13 -2.696572e-14
5.661846e-14
E:\\R-3.1.2\\scripts\\mPDBS\\summary_pdb_93\\ef8e88d4ad93a0ad.final.pdb
-0.0324835176 0.0010822023 3.280259e-14 1.685410e-13 -1.729096e-13
-2.782637e-14
E:\\R-3.1.2\\scripts\\mPDBS\\summary_pdb_93\\f14fcae8deec4fa3.final.pdb
-0.0092396663 -0.0199214466 1.066423e-13 3.494259e-13 -2.032586e-13
-4.022787e-14
E:\\R-3.1.2\\scripts\\mPDBS\\summary_pdb_93\\f83584e22003fb92.final.pdb
0.0601328410 0.0129200188 -6.010525e-14 1.843958e-14 -3.001256e-14
5.472062e-14
E:\\R-3.1.2\\scripts\\mPDBS\\summary_pdb_93\\f8e2bf87e52caa29.final.pdb
-0.0347886459 0.1079362579 -4.075853e-13 -4.552624e-13 3.521304e-13
1.803434e-13

[,43]   [,44]   [,45]   [,46]   [,47]   [,48]
E:\\R-3.1.2\\scripts\\40.1ns_data\\NAT0.pdb
-4.299504e-15 6.682514e-15 5.846438e-15 -1.000002e-14 1.502840e-14
3.228776e-14
E:\\R-3.1.2\\scripts\\40.1ns_data\\LJP6.pdb
3.929081e-15 -2.700482e-15 1.436208e-14 -7.185894e-15 1.849309e-14
-3.185052e-14
E:\\R-3.1.2\\scripts\\40.1ns_data\\3WY9.pdb
1.082499e-14 -8.850340e-15 -4.960439e-15 8.347176e-15 -1.012065e-14
-2.446816e-14
E:\\R-3.1.2\\scripts\\40.1ns_data\\ZSP9.pdb
4.073680e-15 -7.013893e-15 -2.627609e-15 6.088045e-15 -1.369197e-14
-4.926481e-14
E:\\R-3.1.2\\scripts\\mPDBS\\summary_pdb_93\\013d855ad26c877c.final.pdb
-2.431776e-14 4.475164e-14 6.436609e-14 9.099464e-15 6.858779e-15
-2.460359e-14
E:\\R-3.1.2\\scripts\\mPDBS\\summary_pdb_93\\0a2a705342ca0d54.final.pdb
-2.036519e-14 -1.247873e-14 -8.543252e-15 7.998233e-15 -7.288705e-15
1.797631e-13
E:\\R-3.1.2\\scripts\\mPDBS\\summary_pdb_93\\0a59fceadb4194d9.final.pdb
-1.577107e-16 2.920932e-14 6.426787e-15 -1.787546e-14 -1.197798e-14
1.488272e-14
E:\\R-3.1.2\\scripts\\mPDBS\\summary_pdb_93\\0dba59f3306825e8.final.pdb
9.509933e-14 -4.561184e-14 -3.717469e-15 -1.515696e-14 -3.137236e-14
-1.813879e-15
E:\\R-3.1.2\\scripts\\mPDBS\\summary_pdb_93\\126f26a78b57339c.final.pdb

-4.271923e-14 -1.479958e-14 -1.501207e-15 1.793154e-14 4.124056e-14
-1.018847e-14
E:\\R-3.1.2\\scripts\\mPDBS\\summary_pdb_93\\12f06f3e342df706.final.pdb
-1.771205e-14 1.288705e-14 -4.441963e-15 9.524298e-15 -1.104178e-14
-1.649350e-14
E:\\R-3.1.2\\scripts\\mPDBS\\summary_pdb_93\\18618a2a251b867b.final.pdb
7.895674e-14 1.522316e-14 2.659947e-14 -4.316257e-14 6.300872e-14
-7.787252e-14
E:\\R-3.1.2\\scripts\\mPDBS\\summary_pdb_93\\1f305f598641eeb2.final.pdb
-2.850370e-14 6.514464e-14 2.065039e-14 -5.135556e-15 -6.120003e-15
7.944392e-14
E:\\R-3.1.2\\scripts\\mPDBS\\summary_pdb_93\\1f8a00b551be0a39.final.pdb
7.895674e-14 1.522316e-14 2.659947e-14 -4.316257e-14 6.300872e-14
-7.787252e-14
E:\\R-3.1.2\\scripts\\mPDBS\\summary_pdb_93\\219f45328d47579a.final.pdb
-1.615001e-13 7.686131e-14 4.783811e-14 5.974908e-14 6.826556e-14
1.026437e-13
E:\\R-3.1.2\\scripts\\mPDBS\\summary_pdb_93\\238dd3918e54336d.final.pdb
-2.304718e-14 -6.401602e-14 -8.480235e-14 -1.264757e-14 9.071136e-16
4.886162e-14
E:\\R-3.1.2\\scripts\\mPDBS\\summary_pdb_93\\2b3027889e2fdc42.final.pdb
-4.229251e-14 1.096876e-14 -3.290334e-15 -8.558087e-15 5.774314e-15
-8.327003e-15
E:\\R-3.1.2\\scripts\\mPDBS\\summary_pdb_93\\2fcb3b46e33793b0.final.pdb
-3.201416e-14 -9.805824e-15 -1.989747e-14 -3.438780e-15 1.561131e-14
-4.005168e-15
E:\\R-3.1.2\\scripts\\mPDBS\\summary_pdb_93\\34eb328251842006.final.pdb
6.633128e-14 1.688604e-14 -4.261925e-14 8.739853e-14 -2.706957e-14
3.797077e-14
E:\\R-3.1.2\\scripts\\mPDBS\\summary_pdb_93\\354b32871797b7ab.final.pdb
-3.743180e-15 -6.699047e-14 -6.599817e-14 -1.350068e-14 3.652857e-14
5.313352e-14
E:\\R-3.1.2\\scripts\\mPDBS\\summary_pdb_93\\397368adbc32d5de.final.pdb
-4.271923e-14 -1.479958e-14 -1.501207e-15 1.793154e-14 4.124056e-14
-1.018847e-14
E:\\R-3.1.2\\scripts\\mPDBS\\summary_pdb_93\\411ca13cd3df6edd.final.pdb
4.335775e-14 -3.962756e-14 2.168716e-14 -3.755424e-14 1.772811e-14
5.104325e-14
E:\\R-3.1.2\\scripts\\mPDBS\\summary_pdb_93\\42357eee6642bf12.final.pdb
-9.803702e-14 -5.406805e-14 6.025758e-14 -1.763350e-14 6.511232e-14
2.228970e-13
E:\\R-3.1.2\\scripts\\mPDBS\\summary_pdb_93\\44342d1f75a7fcfc.final.pdb
-1.851640e-13 6.125322e-14 5.991790e-14 7.937130e-14 7.625044e-14
4.286403e-14
E:\\R-3.1.2\\scripts\\mPDBS\\summary_pdb_93\\448ef21dffe8cdfb.final.pdb
8.946302e-14 -2.458393e-14 -5.040475e-14 -4.914723e-14 -6.710637e-14
7.139129e-15
E:\\R-3.1.2\\scripts\\mPDBS\\summary_pdb_93\\472a40b3e8f113c7.final.pdb
9.509933e-14 -4.561184e-14 -3.717469e-15 -1.515696e-14 -3.137236e-14
-1.813879e-15
E:\\R-3.1.2\\scripts\\mPDBS\\summary_pdb_93\\48a26225951ea153.final.pdb
-1.451809e-14 1.259636e-14 -8.560749e-15 1.209130e-14 -2.289676e-14
-1.956758e-14
E:\\R-3.1.2\\scripts\\mPDBS\\summary_pdb_93\\4ab4a5c7c6682ce6.final.pdb

-2.900109e-14 3.655518e-14 1.481083e-14 -2.280735e-14 6.134948e-14
-1.685697e-14
E:\\R-3.1.2\\scripts\\mPDBS\\summary_pdb_93\\4eaa45b62cb6a25f.final.pdb
2.624486e-14 2.229334e-15 2.581785e-14 -7.741597e-15 1.308639e-14
1.471926e-14
E:\\R-3.1.2\\scripts\\mPDBS\\summary_pdb_93\\58c371611d85ac96.final.pdb
-4.271923e-14 -1.479958e-14 -1.501207e-15 1.793154e-14 4.124056e-14
-1.018847e-14
E:\\R-3.1.2\\scripts\\mPDBS\\summary_pdb_93\\59a647f04822d30a.final.pdb
-3.074082e-14 -2.623631e-15 -2.623083e-17 -1.517406e-14 -1.964551e-14
-1.592391e-14
E:\\R-3.1.2\\scripts\\mPDBS\\summary_pdb_93\\59b091fe385e05fc.final.pdb
-1.577107e-16 2.920932e-14 6.426787e-15 -1.787546e-14 -1.197798e-14
1.488272e-14
E:\\R-3.1.2\\scripts\\mPDBS\\summary_pdb_93\\5c35ac30f930d2db.final.pdb
2.624486e-14 2.229334e-15 2.581785e-14 -7.741597e-15 1.308639e-14
1.471926e-14
E:\\R-3.1.2\\scripts\\mPDBS\\summary_pdb_93\\5f60752431cf2dcb.final.pdb
9.352726e-14 4.084586e-14 -2.945205e-14 -5.940097e-15 5.824937e-15
-1.770135e-13
E:\\R-3.1.2\\scripts\\mPDBS\\summary_pdb_93\\626456dd6ff2868d.final.pdb
9.509933e-14 -4.561184e-14 -3.717469e-15 -1.515696e-14 -3.137236e-14
-1.813879e-15
E:\\R-3.1.2\\scripts\\mPDBS\\summary_pdb_93\\6583d53ff3c8f7ef.final.pdb
-3.074082e-14 -2.623631e-15 -2.623083e-17 -1.517406e-14 -1.964551e-14
-1.592391e-14
E:\\R-3.1.2\\scripts\\mPDBS\\summary_pdb_93\\69760178986bef01.final.pdb
2.555827e-13 1.020852e-13 3.147451e-14 -1.733731e-14 1.513945e-14
-3.727172e-13
E:\\R-3.1.2\\scripts\\mPDBS\\summary_pdb_93\\6a9d87d47926b1c8.final.pdb
7.762034e-15 -3.856939e-15 3.242394e-15 -2.813179e-16 1.746115e-15
3.044764e-14
E:\\R-3.1.2\\scripts\\mPDBS\\summary_pdb_93\\6bb3547ea982ffc4.final.pdb
-5.482566e-15 -1.931166e-13 -5.615668e-14 1.685899e-13 -1.522146e-13
3.939441e-13
E:\\R-3.1.2\\scripts\\mPDBS\\summary_pdb_93\\716d9624611d1c42.final.pdb
9.138930e-14 1.233965e-13 4.128711e-14 -9.403103e-15 -1.068486e-14
-2.046094e-13
E:\\R-3.1.2\\scripts\\mPDBS\\summary_pdb_93\\75f27e70ad0f1be4.final.pdb
-3.074082e-14 -2.623631e-15 -2.623083e-17 -1.517406e-14 -1.964551e-14
-1.592391e-14
E:\\R-3.1.2\\scripts\\mPDBS\\summary_pdb_93\\769f910fffb22872.final.pdb
-1.535093e-13 1.523443e-13 4.210175e-14 7.193408e-14 1.319873e-14
6.149613e-14
E:\\R-3.1.2\\scripts\\mPDBS\\summary_pdb_93\\791f8c80dd5c3135.final.pdb
-1.577107e-16 2.920932e-14 6.426787e-15 -1.787546e-14 -1.197798e-14
1.488272e-14
E:\\R-3.1.2\\scripts\\mPDBS\\summary_pdb_93\\7fad98e66a3f6099.final.pdb
2.211832e-13 -1.231311e-13 3.270179e-14 1.519072e-13 -1.071978e-14
-2.131536e-13
E:\\R-3.1.2\\scripts\\mPDBS\\summary_pdb_93\\804b89c94a633196.final.pdb
-3.201416e-14 -9.805824e-15 -1.989747e-14 -3.438780e-15 1.561131e-14
-4.005168e-15
E:\\R-3.1.2\\scripts\\mPDBS\\summary_pdb_93\\813cc65a2298feba.final.pdb

-3.201416e-14 -9.805824e-15 -1.989747e-14 -3.438780e-15 1.561131e-14
-4.005168e-15
E:\\R-3.1.2\\scripts\\mPDBS\\summary_pdb_93\\83c0eefcfe12d3f9.final.pdb
4.335775e-14 -3.962756e-14 2.168716e-14 -3.755424e-14 1.772811e-14
5.104325e-14
E:\\R-3.1.2\\scripts\\mPDBS\\summary_pdb_93\\86d50469ef569760.final.pdb
9.509933e-14 -4.561184e-14 -3.717469e-15 -1.515696e-14 -3.137236e-14
-1.813879e-15
E:\\R-3.1.2\\scripts\\mPDBS\\summary_pdb_93\\89d0666e1402203d.final.pdb
-3.074082e-14 -2.623631e-15 -2.623083e-17 -1.517406e-14 -1.964551e-14
-1.592391e-14
E:\\R-3.1.2\\scripts\\mPDBS\\summary_pdb_93\\8b34852716dadc62.final.pdb
-2.028111e-14 5.153400e-14 4.178973e-14 -6.689869e-15 -3.606480e-14
-2.865364e-14
E:\\R-3.1.2\\scripts\\mPDBS\\summary_pdb_93\\8b6ef9ac3fe74395.final.pdb
-1.775165e-14 -1.180981e-14 -5.583617e-14 1.406991e-13 -1.454527e-13
-7.121626e-14
E:\\R-3.1.2\\scripts\\mPDBS\\summary_pdb_93\\98bbe0d5986c138f.final.pdb
-1.577107e-16 2.920932e-14 6.426787e-15 -1.787546e-14 -1.197798e-14
1.488272e-14
E:\\R-3.1.2\\scripts\\mPDBS\\summary_pdb_93\\9c22f4909d67e2fa.final.pdb
-6.801229e-16 4.274583e-14 8.664453e-15 -4.657916e-14 2.648846e-14
-1.892834e-14
E:\\R-3.1.2\\scripts\\mPDBS\\summary_pdb_93\\9dda8ed0d81fb3ef.final.pdb
1.348875e-15 -5.054396e-14 -1.196149e-15 1.746700e-14 -6.459876e-15
-2.735628e-14
E:\\R-3.1.2\\scripts\\mPDBS\\summary_pdb_93\\9f752b3616e6033b.final.pdb
-8.862383e-14 -3.220920e-14 3.951244e-15 3.302737e-15 -2.620916e-14
6.805999e-14
E:\\R-3.1.2\\scripts\\mPDBS\\summary_pdb_93\\a02c70083816d6ba.final.pdb
2.624486e-14 2.229334e-15 2.581785e-14 -7.741597e-15 1.308639e-14
1.471926e-14
E:\\R-3.1.2\\scripts\\mPDBS\\summary_pdb_93\\a0c997d891e4e082.final.pdb
-1.476225e-13 -6.626498e-14 -1.676172e-14 2.326750e-14 -1.976720e-14
1.728077e-13
E:\\R-3.1.2\\scripts\\mPDBS\\summary_pdb_93\\ab8039a2d823c616.final.pdb
5.658107e-14 2.397982e-15 -4.962489e-15 -4.429458e-14 2.458678e-16
-2.391727e-14
E:\\R-3.1.2\\scripts\\mPDBS\\summary_pdb_93\\acf4d74b9c71d4dc.final.pdb
-3.201416e-14 -9.805824e-15 -1.989747e-14 -3.438780e-15 1.561131e-14
-4.005168e-15
E:\\R-3.1.2\\scripts\\mPDBS\\summary_pdb_93\\ad8a823e4b4c668e.final.pdb
-2.047043e-14 -3.218896e-14 -6.420380e-15 1.433466e-15 -1.313140e-14
5.939357e-14
E:\\R-3.1.2\\scripts\\mPDBS\\summary_pdb_93\\b16ababd03b7b857.final.pdb
-6.801229e-16 4.274583e-14 8.664453e-15 -4.657916e-14 2.648846e-14
-1.892834e-14
E:\\R-3.1.2\\scripts\\mPDBS\\summary_pdb_93\\b226923e697d3c63.final.pdb
6.365762e-14 3.898767e-14 5.941835e-15 1.211323e-14 -1.035216e-14
-1.319349e-13
E:\\R-3.1.2\\scripts\\mPDBS\\summary_pdb_93\\b28e48f6fa27a165.final.pdb
-1.577107e-16 2.920932e-14 6.426787e-15 -1.787546e-14 -1.197798e-14
1.488272e-14
E:\\R-3.1.2\\scripts\\mPDBS\\summary_pdb_93\\b42c4924e3cd1aec.final.pdb

1.497118e-14 1.041455e-14 7.288618e-14 -6.073787e-15 -1.683482e-14
-1.917175e-14
E:\\R-3.1.2\\scripts\\mPDBS\\summary_pdb_93\\b4dd239fcabff1df.final.pdb
-1.577107e-16 2.920932e-14 6.426787e-15 -1.787546e-14 -1.197798e-14
1.488272e-14
E:\\R-3.1.2\\scripts\\mPDBS\\summary_pdb_93\\b669942ef97459a2.final.pdb
-2.028111e-14 5.153400e-14 4.178973e-14 -6.689869e-15 -3.606480e-14
-2.865364e-14
E:\\R-3.1.2\\scripts\\mPDBS\\summary_pdb_93\\b99f4d9d87c1017b.final.pdb
-1.577107e-16 2.920932e-14 6.426787e-15 -1.787546e-14 -1.197798e-14
1.488272e-14
E:\\R-3.1.2\\scripts\\mPDBS\\summary_pdb_93\\b9d2cbf3975403e7.final.pdb
-1.577107e-16 2.920932e-14 6.426787e-15 -1.787546e-14 -1.197798e-14
1.488272e-14
E:\\R-3.1.2\\scripts\\mPDBS\\summary_pdb_93\\bc88c8a505f3a288.final.pdb
2.316443e-14 -2.436767e-14 9.103283e-15 3.475974e-15 3.489683e-15
6.115994e-14
E:\\R-3.1.2\\scripts\\mPDBS\\summary_pdb_93\\bdac0e64f4ef427e.final.pdb
1.465810e-14 -9.012715e-14 -1.320882e-14 -1.830096e-14 1.284231e-15
5.942889e-14
E:\\R-3.1.2\\scripts\\mPDBS\\summary_pdb_93\\ca2a1f55fc9d0681.final.pdb
-2.028111e-14 5.153400e-14 4.178973e-14 -6.689869e-15 -3.606480e-14
-2.865364e-14
E:\\R-3.1.2\\scripts\\mPDBS\\summary_pdb_93\\ce34248de3e81a3c.final.pdb
-3.065609e-14 8.113308e-14 1.882598e-14 6.287730e-15 5.516355e-14
-9.421000e-14
E:\\R-3.1.2\\scripts\\mPDBS\\summary_pdb_93\\d02e697772494a61.final.pdb
1.021105e-14 -3.395110e-14 -2.103522e-14 -8.769591e-15 -1.255219e-15
4.565781e-14
E:\\R-3.1.2\\scripts\\mPDBS\\summary_pdb_93\\d04221245d015047.final.pdb
-4.195704e-14 -2.205107e-15 5.621418e-15 5.405055e-15 3.544011e-15
7.844746e-14
E:\\R-3.1.2\\scripts\\mPDBS\\summary_pdb_93\\d678141e4018054a.final.pdb
-4.486602e-14 -3.630757e-14 1.955671e-14 9.501623e-15 -4.500988e-14
-1.736126e-13
E:\\R-3.1.2\\scripts\\mPDBS\\summary_pdb_93\\d71b40af0f0efe39.final.pdb
7.781191e-14 1.332360e-13 2.536439e-14 -1.666957e-13 9.233413e-14
-6.559943e-14
E:\\R-3.1.2\\scripts\\mPDBS\\summary_pdb_93\\dc0809eff10197e2.final.pdb
8.542116e-14 -1.502056e-13 -2.416401e-14 4.150029e-14 3.834351e-14
1.227014e-14
E:\\R-3.1.2\\scripts\\mPDBS\\summary_pdb_93\\e1163918d3707839.final.pdb
-4.802142e-14 -8.997346e-14 -7.113587e-14 -1.160029e-14 -4.543874e-15
1.131417e-14
E:\\R-3.1.2\\scripts\\mPDBS\\summary_pdb_93\\e4b48b06824ef848.final.pdb
7.895674e-14 1.522316e-14 2.659947e-14 -4.316257e-14 6.300872e-14
-7.787252e-14
E:\\R-3.1.2\\scripts\\mPDBS\\summary_pdb_93\\e529e19ad281fea2.final.pdb
-2.431776e-14 4.475164e-14 6.436609e-14 9.099464e-15 6.858779e-15
-2.460359e-14
E:\\R-3.1.2\\scripts\\mPDBS\\summary_pdb_93\\e5602082718ec3a1.final.pdb
-3.201416e-14 -9.805824e-15 -1.989747e-14 -3.438780e-15 1.561131e-14
-4.005168e-15
E:\\R-3.1.2\\scripts\\mPDBS\\summary_pdb_93\\e5d589d996d0a960.final.pdb

-1.335155e-13 -5.104514e-14 -3.112507e-15 6.767784e-14 -7.261192e-14
2.525500e-13
E:\\R-3.1.2\\scripts\\mPDBS\\summary_pdb_93\\e9df9e72307a2fbd.final.pdb
2.624486e-14 2.229334e-15 2.581785e-14 -7.741597e-15 1.308639e-14
1.471926e-14
E:\\R-3.1.2\\scripts\\mPDBS\\summary_pdb_93\\ea00f8694090cae7.final.pdb
-3.201416e-14 -9.805824e-15 -1.989747e-14 -3.438780e-15 1.561131e-14
-4.005168e-15
E:\\R-3.1.2\\scripts\\mPDBS\\summary_pdb_93\\eba517be32db050e.final.pdb
-1.577107e-16 2.920932e-14 6.426787e-15 -1.787546e-14 -1.197798e-14
1.488272e-14
E:\\R-3.1.2\\scripts\\mPDBS\\summary_pdb_93\\ec04b9545899c29d.final.pdb
7.669735e-14 -2.324602e-14 -4.415705e-14 -2.923247e-14 9.061931e-16
-4.197870e-14
E:\\R-3.1.2\\scripts\\mPDBS\\summary_pdb_93\\ee6862cbcad52078.final.pdb
2.185115e-14 -1.423683e-14 -6.453643e-15 3.351051e-15 -1.200010e-14
-3.143223e-14
E:\\R-3.1.2\\scripts\\mPDBS\\summary_pdb_93\\ef6a42fc83b81e5d.final.pdb
2.236219e-14 -3.852779e-15 1.296331e-14 4.544109e-15 7.154524e-15
-5.530855e-14
E:\\R-3.1.2\\scripts\\mPDBS\\summary_pdb_93\\ef8e88d4ad93a0ad.final.pdb
-4.195704e-14 -2.205107e-15 5.621418e-15 5.405055e-15 3.544011e-15
7.844746e-14
E:\\R-3.1.2\\scripts\\mPDBS\\summary_pdb_93\\f14fcae8deec4fa3.final.pdb
-8.862383e-14 -3.220920e-14 3.951244e-15 3.302737e-15 -2.620916e-14
6.805999e-14
E:\\R-3.1.2\\scripts\\mPDBS\\summary_pdb_93\\f83584e22003fb92.final.pdb
-1.451809e-14 1.259636e-14 -8.560749e-15 1.209130e-14 -2.289676e-14
-1.956758e-14
E:\\R-3.1.2\\scripts\\mPDBS\\summary_pdb_93\\f8e2bf87e52caa29.final.pdb
-2.431776e-14 4.475164e-14 6.436609e-14 9.099464e-15 6.858779e-15
-2.460359e-14

[,49]   [,50]   [,51]   [,52]   [,53]   [,54]
E:\\R-3.1.2\\scripts\\40.1ns_data\\NAT0.pdb
-2.770907e-15 -5.880075e-16 1.745740e-14 -3.184710e-15 6.415690e-15
-5.087488e-15
E:\\R-3.1.2\\scripts\\40.1ns_data\\LJP6.pdb
-1.329317e-15 2.257375e-15 1.224097e-14 -1.003331e-14 -1.132679e-15
1.525927e-16
E:\\R-3.1.2\\scripts\\40.1ns_data\\3WY9.pdb
-1.494349e-14 5.227116e-15 3.760672e-15 5.183850e-15 2.950899e-15
1.779989e-15
E:\\R-3.1.2\\scripts\\40.1ns_data\\ZSP9.pdb
7.532164e-15 7.826037e-15 -3.278179e-14 -6.133335e-15 3.853632e-15
-6.647398e-15
E:\\R-3.1.2\\scripts\\mPDBS\\summary_pdb_93\\013d855ad26c877c.final.pdb
-3.243025e-15 2.602751e-14 6.256328e-14 3.038498e-14 3.906482e-14
5.405070e-14
E:\\R-3.1.2\\scripts\\mPDBS\\summary_pdb_93\\0a2a705342ca0d54.final.pdb
-1.013345e-14 -3.171548e-15 1.991855e-14 -1.778422e-14 1.263159e-14
-4.407448e-15
E:\\R-3.1.2\\scripts\\mPDBS\\summary_pdb_93\\0a59fceadb4194d9.final.pdb
1.058747e-14 -1.010719e-14 1.050171e-14 -2.504137e-15 1.300640e-14

5.609272e-15
E:\\R-3.1.2\\scripts\\mPDBS\\summary_pdb_93\\0dba59f3306825e8.final.pdb
-9.254342e-16 -6.703257e-15 -6.046808e-14 -4.399996e-14 -1.441844e-14
-2.545167e-14
E:\\R-3.1.2\\scripts\\mPDBS\\summary_pdb_93\\126f26a78b57339c.final.pdb
-2.647943e-14 8.848539e-15 -2.775814e-14 6.160164e-15 -2.348194e-14
-1.274396e-14
E:\\R-3.1.2\\scripts\\mPDBS\\summary_pdb_93\\12f06f3e342df706.final.pdb
4.464782e-14 -1.956165e-14 7.292530e-14 4.366196e-14 -3.899787e-15
-9.880935e-15
E:\\R-3.1.2\\scripts\\mPDBS\\summary_pdb_93\\18618a2a251b867b.final.pdb
2.506120e-14 5.996521e-15 -8.805644e-14 -1.019886e-14 -2.067039e-14
-5.010631e-14
E:\\R-3.1.2\\scripts\\mPDBS\\summary_pdb_93\\1f305f598641eeb2.final.pdb
-7.695405e-15 -2.126398e-16 -1.375547e-14 -1.205266e-14 -8.331834e-15
1.115352e-14
E:\\R-3.1.2\\scripts\\mPDBS\\summary_pdb_93\\1f8a00b551be0a39.final.pdb
2.506120e-14 5.996521e-15 -8.805644e-14 -1.019886e-14 -2.067039e-14
-5.010631e-14
E:\\R-3.1.2\\scripts\\mPDBS\\summary_pdb_93\\219f45328d47579a.final.pdb
2.287684e-15 3.150536e-14 1.498848e-13 1.071568e-13 5.496698e-14
6.642391e-14
E:\\R-3.1.2\\scripts\\mPDBS\\summary_pdb_93\\238dd3918e54336d.final.pdb
-1.906318e-14 -2.285502e-14 -6.262751e-14 -3.617485e-14 -4.215586e-14
-9.672077e-14
E:\\R-3.1.2\\scripts\\mPDBS\\summary_pdb_93\\2b3027889e2fdc42.final.pdb
-2.980454e-15 -1.407125e-14 3.169092e-14 7.026163e-15 -2.952623e-15
2.173218e-15
E:\\R-3.1.2\\scripts\\mPDBS\\summary_pdb_93\\2fcb3b46e33793b0.final.pdb
7.764450e-15 -1.067649e-14 -2.968543e-14 -9.470528e-15 -6.807880e-15
-9.297136e-15
E:\\R-3.1.2\\scripts\\mPDBS\\summary_pdb_93\\34eb328251842006.final.pdb
-9.352133e-15 -3.371810e-14 7.259466e-14 4.653460e-14 -2.738892e-14
-1.414645e-14
E:\\R-3.1.2\\scripts\\mPDBS\\summary_pdb_93\\354b32871797b7ab.final.pdb
1.297834e-14 -4.943922e-15 -4.663655e-14 -3.026418e-14 -2.809121e-14
-4.720260e-14
E:\\R-3.1.2\\scripts\\mPDBS\\summary_pdb_93\\397368adbc32d5de.final.pdb
-2.647943e-14 8.848539e-15 -2.775814e-14 6.160164e-15 -2.348194e-14
-1.274396e-14
E:\\R-3.1.2\\scripts\\mPDBS\\summary_pdb_93\\411ca13cd3df6edd.final.pdb
1.802859e-14 1.499915e-14 7.608084e-15 -3.751741e-15 -4.236152e-15
-2.215127e-15
E:\\R-3.1.2\\scripts\\mPDBS\\summary_pdb_93\\42357eee6642bf12.final.pdb
1.067258e-14 1.191476e-14 1.944816e-14 -4.656874e-15 -1.340055e-14
3.037927e-14
E:\\R-3.1.2\\scripts\\mPDBS\\summary_pdb_93\\44342d1f75a7fcfc.final.pdb
-4.423259e-14 3.234497e-14 1.422936e-13 8.055188e-14 2.185603e-14
7.603450e-14
E:\\R-3.1.2\\scripts\\mPDBS\\summary_pdb_93\\448ef21dffe8cdfb.final.pdb
1.111594e-14 -2.499143e-14 -3.827101e-14 -3.921149e-14 -1.785807e-14
-4.757381e-14
E:\\R-3.1.2\\scripts\\mPDBS\\summary_pdb_93\\472a40b3e8f113c7.final.pdb
-9.254342e-16 -6.703257e-15 -6.046808e-14 -4.399996e-14 -1.441844e-14

-2.545167e-14
E:\\R-3.1.2\\scripts\\mPDBS\\summary_pdb_93\\48a26225951ea153.final.pdb
5.007080e-15 -3.065867e-14 1.946134e-14 -3.008755e-16 7.900675e-15
7.708287e-15
E:\\R-3.1.2\\scripts\\mPDBS\\summary_pdb_93\\4ab4a5c7c6682ce6.final.pdb
6.934815e-15 1.720991e-14 -5.384404e-14 -2.560291e-15 -2.207291e-14
-2.581948e-15
E:\\R-3.1.2\\scripts\\mPDBS\\summary_pdb_93\\4eaa45b62cb6a25f.final.pdb
1.153147e-14 8.313919e-15 1.593666e-14 2.311949e-14 1.999381e-14
2.977142e-14
E:\\R-3.1.2\\scripts\\mPDBS\\summary_pdb_93\\58c371611d85ac96.final.pdb
-2.647943e-14 8.848539e-15 -2.775814e-14 6.160164e-15 -2.348194e-14
-1.274396e-14
E:\\R-3.1.2\\scripts\\mPDBS\\summary_pdb_93\\59a647f04822d30a.final.pdb
-1.826378e-14 6.410856e-15 -1.015893e-14 -2.418682e-14 6.879509e-15
-1.133485e-14
E:\\R-3.1.2\\scripts\\mPDBS\\summary_pdb_93\\59b091fe385e05fc.final.pdb
1.058747e-14 -1.010719e-14 1.050171e-14 -2.504137e-15 1.300640e-14
5.609272e-15
E:\\R-3.1.2\\scripts\\mPDBS\\summary_pdb_93\\5c35ac30f930d2db.final.pdb
1.153147e-14 8.313919e-15 1.593666e-14 2.311949e-14 1.999381e-14
2.977142e-14
E:\\R-3.1.2\\scripts\\mPDBS\\summary_pdb_93\\5f60752431cf2dcb.final.pdb
-6.538018e-15 -1.689516e-14 -7.209205e-14 -5.644027e-15 -2.200781e-14
-1.362750e-14
E:\\R-3.1.2\\scripts\\mPDBS\\summary_pdb_93\\626456dd6ff2868d.final.pdb
-9.254342e-16 -6.703257e-15 -6.046808e-14 -4.399996e-14 -1.441844e-14
-2.545167e-14
E:\\R-3.1.2\\scripts\\mPDBS\\summary_pdb_93\\6583d53ff3c8f7ef.final.pdb
-1.826378e-14 6.410856e-15 -1.015893e-14 -2.418682e-14 6.879509e-15
-1.133485e-14
E:\\R-3.1.2\\scripts\\mPDBS\\summary_pdb_93\\69760178986bef01.final.pdb
-9.997994e-15 -2.480296e-15 -1.477870e-13 -9.912941e-15 -9.838340e-15
-5.865719e-15
E:\\R-3.1.2\\scripts\\mPDBS\\summary_pdb_93\\6a9d87d47926b1c8.final.pdb
-6.682765e-15 -1.213468e-14 1.335047e-14 -1.144554e-14 1.138995e-15
-2.554192e-15
E:\\R-3.1.2\\scripts\\mPDBS\\summary_pdb_93\\6bb3547ea982ffc4.final.pdb
-8.043346e-14 -1.289764e-14 2.847986e-13 6.324457e-14 5.318854e-15
4.581066e-14
E:\\R-3.1.2\\scripts\\mPDBS\\summary_pdb_93\\716d9624611d1c42.final.pdb
1.007434e-14 8.825799e-15 1.576665e-15 4.100513e-14 4.670567e-14
6.802550e-14
E:\\R-3.1.2\\scripts\\mPDBS\\summary_pdb_93\\75f27e70ad0f1be4.final.pdb
-1.826378e-14 6.410856e-15 -1.015893e-14 -2.418682e-14 6.879509e-15
-1.133485e-14
E:\\R-3.1.2\\scripts\\mPDBS\\summary_pdb_93\\769f910fffb22872.final.pdb
-8.429481e-15 2.991112e-15 5.116683e-14 -2.193589e-14 -2.412943e-14
6.114341e-15
E:\\R-3.1.2\\scripts\\mPDBS\\summary_pdb_93\\791f8c80dd5c3135.final.pdb
1.058747e-14 -1.010719e-14 1.050171e-14 -2.504137e-15 1.300640e-14
5.609272e-15
E:\\R-3.1.2\\scripts\\mPDBS\\summary_pdb_93\\7fad98e66a3f6099.final.pdb
-1.057554e-13 5.861954e-14 7.787324e-14 7.381019e-14 1.487625e-14

9.609178e-14
E:\\R-3.1.2\\scripts\\mPDBS\\summary_pdb_93\\804b89c94a633196.final.pdb
7.764450e-15 -1.067649e-14 -2.968543e-14 -9.470528e-15 -6.807880e-15
-9.297136e-15
E:\\R-3.1.2\\scripts\\mPDBS\\summary_pdb_93\\813cc65a2298feba.final.pdb
7.764450e-15 -1.067649e-14 -2.968543e-14 -9.470528e-15 -6.807880e-15
-9.297136e-15
E:\\R-3.1.2\\scripts\\mPDBS\\summary_pdb_93\\83c0eefcfe12d3f9.final.pdb
1.802859e-14 1.499915e-14 7.608084e-15 -3.751741e-15 -4.236152e-15
-2.215127e-15
E:\\R-3.1.2\\scripts\\mPDBS\\summary_pdb_93\\86d50469ef569760.final.pdb
-9.254342e-16 -6.703257e-15 -6.046808e-14 -4.399996e-14 -1.441844e-14
-2.545167e-14
E:\\R-3.1.2\\scripts\\mPDBS\\summary_pdb_93\\89d0666e1402203d.final.pdb
-1.826378e-14 6.410856e-15 -1.015893e-14 -2.418682e-14 6.879509e-15
-1.133485e-14
E:\\R-3.1.2\\scripts\\mPDBS\\summary_pdb_93\\8b34852716dadc62.final.pdb
1.510580e-14 -1.129405e-14 7.046536e-14 1.864500e-14 5.153000e-14
4.196221e-14
E:\\R-3.1.2\\scripts\\mPDBS\\summary_pdb_93\\8b6ef9ac3fe74395.final.pdb
-9.432072e-14 -3.043460e-16 1.677766e-13 1.543381e-14 4.900237e-14
4.150357e-14
E:\\R-3.1.2\\scripts\\mPDBS\\summary_pdb_93\\98bbe0d5986c138f.final.pdb
1.058747e-14 -1.010719e-14 1.050171e-14 -2.504137e-15 1.300640e-14
5.609272e-15
E:\\R-3.1.2\\scripts\\mPDBS\\summary_pdb_93\\9c22f4909d67e2fa.final.pdb
2.367956e-14 -4.156534e-15 -5.608749e-14 -2.689566e-14 -4.538428e-15
-1.879518e-14
E:\\R-3.1.2\\scripts\\mPDBS\\summary_pdb_93\\9dda8ed0d81fb3ef.final.pdb
-1.907460e-14 -9.515857e-15 -2.426245e-14 -1.574289e-14 -4.615532e-14
-1.352363e-14
E:\\R-3.1.2\\scripts\\mPDBS\\summary_pdb_93\\9f752b3616e6033b.final.pdb
-2.010086e-14 -1.052585e-15 1.247396e-14 -3.416156e-14 -2.055746e-15
-2.059825e-14
E:\\R-3.1.2\\scripts\\mPDBS\\summary_pdb_93\\a02c70083816d6ba.final.pdb
1.153147e-14 8.313919e-15 1.593666e-14 2.311949e-14 1.999381e-14
2.977142e-14
E:\\R-3.1.2\\scripts\\mPDBS\\summary_pdb_93\\a0c997d891e4e082.final.pdb
-1.700460e-14 1.319531e-15 8.432690e-14 -2.691454e-14 6.057612e-15
-2.115118e-14
E:\\R-3.1.2\\scripts\\mPDBS\\summary_pdb_93\\ab8039a2d823c616.final.pdb
2.449703e-14 -1.843311e-14 -2.266456e-14 2.022858e-14 2.188745e-15
-8.241805e-15
E:\\R-3.1.2\\scripts\\mPDBS\\summary_pdb_93\\acf4d74b9c71d4dc.final.pdb
7.764450e-15 -1.067649e-14 -2.968543e-14 -9.470528e-15 -6.807880e-15
-9.297136e-15
E:\\R-3.1.2\\scripts\\mPDBS\\summary_pdb_93\\ad8a823e4b4c668e.final.pdb
6.456849e-15 4.489943e-15 3.194193e-15 -5.433404e-16 7.199838e-15
-3.566112e-15
E:\\R-3.1.2\\scripts\\mPDBS\\summary_pdb_93\\b16ababd03b7b857.final.pdb
2.367956e-14 -4.156534e-15 -5.608749e-14 -2.689566e-14 -4.538428e-15
-1.879518e-14
E:\\R-3.1.2\\scripts\\mPDBS\\summary_pdb_93\\b226923e697d3c63.final.pdb
-1.140804e-14 -3.767634e-15 -5.042295e-14 2.257124e-14 -4.052457e-16

2.473526e-14
E:\\R-3.1.2\\scripts\\mPDBS\\summary_pdb_93\\b28e48f6fa27a165.final.pdb
1.058747e-14 -1.010719e-14 1.050171e-14 -2.504137e-15 1.300640e-14
5.609272e-15
E:\\R-3.1.2\\scripts\\mPDBS\\summary_pdb_93\\b42c4924e3cd1aec.final.pdb
1.049783e-14 1.608823e-14 4.883836e-14 1.940651e-14 2.862405e-14
7.270340e-14
E:\\R-3.1.2\\scripts\\mPDBS\\summary_pdb_93\\b4dd239fcabff1df.final.pdb
1.058747e-14 -1.010719e-14 1.050171e-14 -2.504137e-15 1.300640e-14
5.609272e-15
E:\\R-3.1.2\\scripts\\mPDBS\\summary_pdb_93\\b669942ef97459a2.final.pdb
1.510580e-14 -1.129405e-14 7.046536e-14 1.864500e-14 5.153000e-14
4.196221e-14
E:\\R-3.1.2\\scripts\\mPDBS\\summary_pdb_93\\b99f4d9d87c1017b.final.pdb
1.058747e-14 -1.010719e-14 1.050171e-14 -2.504137e-15 1.300640e-14
5.609272e-15
E:\\R-3.1.2\\scripts\\mPDBS\\summary_pdb_93\\b9d2cbf3975403e7.final.pdb
1.058747e-14 -1.010719e-14 1.050171e-14 -2.504137e-15 1.300640e-14
5.609272e-15
E:\\R-3.1.2\\scripts\\mPDBS\\summary_pdb_93\\bc88c8a505f3a288.final.pdb
1.155669e-15 -4.513573e-15 -3.102605e-14 5.382512e-15 1.082325e-14
1.303736e-14
E:\\R-3.1.2\\scripts\\mPDBS\\summary_pdb_93\\bdac0e64f4ef427e.final.pdb
-1.609255e-14 1.166241e-14 3.850351e-14 2.016332e-14 1.960372e-14
-1.347435e-14
E:\\R-3.1.2\\scripts\\mPDBS\\summary_pdb_93\\ca2a1f55fc9d0681.final.pdb
1.510580e-14 -1.129405e-14 7.046536e-14 1.864500e-14 5.153000e-14
4.196221e-14
E:\\R-3.1.2\\scripts\\mPDBS\\summary_pdb_93\\ce34248de3e81a3c.final.pdb
8.435100e-16 1.033860e-14 7.518383e-15 3.388365e-14 1.045985e-14
2.640258e-14
E:\\R-3.1.2\\scripts\\mPDBS\\summary_pdb_93\\d02e697772494a61.final.pdb
2.060561e-14 -1.113523e-14 -7.283326e-15 3.065464e-15 -7.986574e-15
-2.438019e-14
E:\\R-3.1.2\\scripts\\mPDBS\\summary_pdb_93\\d04221245d015047.final.pdb
3.116457e-15 -4.155256e-15 2.831961e-14 -3.958941e-15 2.931640e-15
-5.469283e-15
E:\\R-3.1.2\\scripts\\mPDBS\\summary_pdb_93\\d678141e4018054a.final.pdb
-3.822997e-14 -2.899391e-15 -3.685462e-14 -6.065154e-14 4.071122e-15
-7.741434e-15
E:\\R-3.1.2\\scripts\\mPDBS\\summary_pdb_93\\d71b40af0f0efe39.final.pdb
9.132135e-14 1.362776e-14 -2.041706e-13 -3.870356e-14 -1.633482e-14
-4.983763e-14
E:\\R-3.1.2\\scripts\\mPDBS\\summary_pdb_93\\dc0809eff10197e2.final.pdb
-2.160573e-14 -9.205018e-15 -3.966225e-14 -1.153086e-14 -4.231709e-14
-3.306382e-14
E:\\R-3.1.2\\scripts\\mPDBS\\summary_pdb_93\\e1163918d3707839.final.pdb
-2.922473e-14 8.632386e-15 -8.697075e-14 -7.657791e-14 -5.862235e-14
-8.703081e-14
E:\\R-3.1.2\\scripts\\mPDBS\\summary_pdb_93\\e4b48b06824ef848.final.pdb
2.506120e-14 5.996521e-15 -8.805644e-14 -1.019886e-14 -2.067039e-14
-5.010631e-14
E:\\R-3.1.2\\scripts\\mPDBS\\summary_pdb_93\\e529e19ad281fea2.final.pdb
-3.243025e-15 2.602751e-14 6.256328e-14 3.038498e-14 3.906482e-14

5.405070e-14
E:\\R-3.1.2\\scripts\\mPDBS\\summary_pdb_93\\e5602082718ec3a1.final.pdb
7.764450e-15 -1.067649e-14 -2.968543e-14 -9.470528e-15 -6.807880e-15
-9.297136e-15
E:\\R-3.1.2\\scripts\\mPDBS\\summary_pdb_93\\e5d589d996d0a960.final.pdb
-2.112505e-14 -3.741017e-14 1.374997e-13 3.489597e-16 -2.311816e-14
-1.550385e-14
E:\\R-3.1.2\\scripts\\mPDBS\\summary_pdb_93\\e9df9e72307a2fbd.final.pdb
1.153147e-14 8.313919e-15 1.593666e-14 2.311949e-14 1.999381e-14
2.977142e-14
E:\\R-3.1.2\\scripts\\mPDBS\\summary_pdb_93\\ea00f8694090cae7.final.pdb
7.764450e-15 -1.067649e-14 -2.968543e-14 -9.470528e-15 -6.807880e-15
-9.297136e-15
E:\\R-3.1.2\\scripts\\mPDBS\\summary_pdb_93\\eba517be32db050e.final.pdb
1.058747e-14 -1.010719e-14 1.050171e-14 -2.504137e-15 1.300640e-14
5.609272e-15
E:\\R-3.1.2\\scripts\\mPDBS\\summary_pdb_93\\ec04b9545899c29d.final.pdb
1.961304e-14 -2.076185e-14 -5.943961e-14 1.259149e-14 2.394598e-15
-3.599047e-14
E:\\R-3.1.2\\scripts\\mPDBS\\summary_pdb_93\\ee6862cbcad52078.final.pdb
-5.589970e-16 -6.172836e-15 1.616736e-16 -6.768521e-15 -4.587383e-15
4.262259e-15
E:\\R-3.1.2\\scripts\\mPDBS\\summary_pdb_93\\ef6a42fc83b81e5d.final.pdb
-8.353261e-15 3.920274e-15 1.088937e-14 -1.144196e-14 2.705221e-15
-5.192418e-16
E:\\R-3.1.2\\scripts\\mPDBS\\summary_pdb_93\\ef8e88d4ad93a0ad.final.pdb
3.116457e-15 -4.155256e-15 2.831961e-14 -3.958941e-15 2.931640e-15
-5.469283e-15
E:\\R-3.1.2\\scripts\\mPDBS\\summary_pdb_93\\f14fcae8deec4fa3.final.pdb
-2.010086e-14 -1.052585e-15 1.247396e-14 -3.416156e-14 -2.055746e-15
-2.059825e-14
E:\\R-3.1.2\\scripts\\mPDBS\\summary_pdb_93\\f83584e22003fb92.final.pdb
5.007080e-15 -3.065867e-14 1.946134e-14 -3.008755e-16 7.900675e-15
7.708287e-15
E:\\R-3.1.2\\scripts\\mPDBS\\summary_pdb_93\\f8e2bf87e52caa29.final.pdb
-3.243025e-15 2.602751e-14 6.256328e-14 3.038498e-14 3.906482e-14
5.405070e-14

[,55]   [,56]   [,57]   [,58]   [,59]   [,60]
E:\\R-3.1.2\\scripts\\40.1ns_data\\NAT0.pdb
5.711352e-15 4.140069e-15 3.926359e-15 -2.440733e-14 1.148276e-15
-3.095543e-17
E:\\R-3.1.2\\scripts\\40.1ns_data\\LJP6.pdb
-4.293719e-15 -1.525319e-15 -9.027550e-16 2.756040e-14 -9.371331e-16
1.927776e-15
E:\\R-3.1.2\\scripts\\40.1ns_data\\3WY9.pdb
2.437993e-14 1.045955e-16 1.375426e-15 2.285122e-14 -1.083461e-14
-1.660034e-16
E:\\R-3.1.2\\scripts\\40.1ns_data\\ZSP9.pdb
-2.621138e-14 -3.481653e-15 7.600145e-15 1.081127e-14 -1.448294e-15
1.982001e-15
E:\\R-3.1.2\\scripts\\mPDBS\\summary_pdb_93\\013d855ad26c877c.final.pdb
1.486777e-14 -6.538121e-15 -1.014233e-14 -9.592550e-14 2.899925e-14
-4.004864e-16

E:\\R-3.1.2\\scripts\\mPDBS\\summary_pdb_93\\0a2a705342ca0d54.final.pdb
-8.780067e-14 1.255154e-16 5.933320e-15 -9.873103e-14 -4.036499e-15
-4.273988e-15
E:\\R-3.1.2\\scripts\\mPDBS\\summary_pdb_93\\0a59fceadb4194d9.final.pdb
-1.436763e-14 9.552259e-16 -3.457347e-15 3.255239e-15 -3.825799e-15
1.005832e-14
E:\\R-3.1.2\\scripts\\mPDBS\\summary_pdb_93\\0dba59f3306825e8.final.pdb
-3.083613e-14 -1.728950e-14 4.727718e-15 5.063435e-14 -4.734983e-14
-3.202103e-14
E:\\R-3.1.2\\scripts\\mPDBS\\summary_pdb_93\\126f26a78b57339c.final.pdb
1.521346e-15 -2.045775e-16 4.163499e-15 3.764726e-15 -3.379143e-15
1.294509e-14
E:\\R-3.1.2\\scripts\\mPDBS\\summary_pdb_93\\12f06f3e342df706.final.pdb
3.834018e-14 1.161813e-14 6.044342e-15 3.532103e-14 6.676069e-15
2.400282e-14
E:\\R-3.1.2\\scripts\\mPDBS\\summary_pdb_93\\18618a2a251b867b.final.pdb
-4.119864e-14 -4.117746e-14 1.494936e-14 2.184774e-13 -1.200074e-14
-2.542919e-14
E:\\R-3.1.2\\scripts\\mPDBS\\summary_pdb_93\\1f305f598641eeb2.final.pdb
6.640602e-14 1.190608e-14 -2.170441e-15 -9.730255e-14 -3.814948e-15
9.691032e-15
E:\\R-3.1.2\\scripts\\mPDBS\\summary_pdb_93\\1f8a00b551be0a39.final.pdb
-4.119864e-14 -4.117746e-14 1.494936e-14 2.184774e-13 -1.200074e-14
-2.542919e-14
E:\\R-3.1.2\\scripts\\mPDBS\\summary_pdb_93\\219f45328d47579a.final.pdb
1.187440e-13 3.273775e-14 5.686815e-15 -1.343677e-13 9.418825e-14
7.640456e-14
E:\\R-3.1.2\\scripts\\mPDBS\\summary_pdb_93\\238dd3918e54336d.final.pdb
-2.812952e-14 -2.486005e-15 9.364428e-15 1.035515e-13 -4.471930e-14
4.361668e-15
E:\\R-3.1.2\\scripts\\mPDBS\\summary_pdb_93\\2b3027889e2fdc42.final.pdb
-3.079786e-15 -3.816294e-15 -4.680977e-15 1.369329e-14 -4.319545e-15
5.801474e-16
E:\\R-3.1.2\\scripts\\mPDBS\\summary_pdb_93\\2fcb3b46e33793b0.final.pdb
-1.810623e-14 1.065708e-14 1.066478e-14 4.780707e-14 5.543885e-15
2.756389e-14
E:\\R-3.1.2\\scripts\\mPDBS\\summary_pdb_93\\34eb328251842006.final.pdb
1.431852e-13 -7.929371e-15 -5.477526e-14 -1.296228e-14 -1.991487e-14
-1.698484e-14
E:\\R-3.1.2\\scripts\\mPDBS\\summary_pdb_93\\354b32871797b7ab.final.pdb
-6.247324e-14 9.733297e-15 2.366074e-14 1.319298e-13 -2.204543e-14
-4.965871e-15
E:\\R-3.1.2\\scripts\\mPDBS\\summary_pdb_93\\397368adbc32d5de.final.pdb
1.521346e-15 -2.045775e-16 4.163499e-15 3.764726e-15 -3.379143e-15
1.294509e-14
E:\\R-3.1.2\\scripts\\mPDBS\\summary_pdb_93\\411ca13cd3df6edd.final.pdb
-1.056593e-14 -2.102511e-14 1.374781e-14 1.700792e-14 -6.904945e-16
-9.438117e-15
E:\\R-3.1.2\\scripts\\mPDBS\\summary_pdb_93\\42357eee6642bf12.final.pdb
1.552072e-14 -3.120624e-14 6.939236e-14 -8.582388e-14 4.607728e-14
3.422306e-14
E:\\R-3.1.2\\scripts\\mPDBS\\summary_pdb_93\\44342d1f75a7fcfc.final.pdb
1.020523e-13 -4.583977e-16 -1.957613e-15 -1.936749e-13 8.788637e-14
6.754444e-14

E:\\R-3.1.2\\scripts\\mPDBS\\summary_pdb_93\\448ef21dffe8cdfb.final.pdb
-4.292752e-14 2.785336e-15 1.847277e-14 7.086328e-14 -4.543704e-14
-1.224975e-14
E:\\R-3.1.2\\scripts\\mPDBS\\summary_pdb_93\\472a40b3e8f113c7.final.pdb
-3.083613e-14 -1.728950e-14 4.727718e-15 5.063435e-14 -4.734983e-14
-3.202103e-14
E:\\R-3.1.2\\scripts\\mPDBS\\summary_pdb_93\\48a26225951ea153.final.pdb
3.659442e-14 2.589823e-15 -8.223716e-15 -1.099400e-14 8.523480e-15
3.474900e-14
E:\\R-3.1.2\\scripts\\mPDBS\\summary_pdb_93\\4ab4a5c7c6682ce6.final.pdb
-3.472744e-14 -2.965801e-14 3.189826e-14 5.557571e-14 5.334806e-15
1.113057e-14
E:\\R-3.1.2\\scripts\\mPDBS\\summary_pdb_93\\4eaa45b62cb6a25f.final.pdb
-3.421557e-14 1.100807e-14 9.347539e-15 4.782763e-14 4.288101e-15
-1.378972e-14
E:\\R-3.1.2\\scripts\\mPDBS\\summary_pdb_93\\58c371611d85ac96.final.pdb
1.521346e-15 -2.045775e-16 4.163499e-15 3.764726e-15 -3.379143e-15
1.294509e-14
E:\\R-3.1.2\\scripts\\mPDBS\\summary_pdb_93\\59a647f04822d30a.final.pdb
-1.907459e-14 2.112056e-16 8.565219e-15 -5.504885e-14 -2.127674e-14
-1.132130e-14
E:\\R-3.1.2\\scripts\\mPDBS\\summary_pdb_93\\59b091fe385e05fc.final.pdb
-1.436763e-14 9.552259e-16 -3.457347e-15 3.255239e-15 -3.825799e-15
1.005832e-14
E:\\R-3.1.2\\scripts\\mPDBS\\summary_pdb_93\\5c35ac30f930d2db.final.pdb
-3.421557e-14 1.100807e-14 9.347539e-15 4.782763e-14 4.288101e-15
-1.378972e-14
E:\\R-3.1.2\\scripts\\mPDBS\\summary_pdb_93\\5f60752431cf2dcb.final.pdb
-6.089966e-14 2.781306e-14 -9.624228e-16 9.438078e-14 -3.759841e-14
-6.191373e-15
E:\\R-3.1.2\\scripts\\mPDBS\\summary_pdb_93\\626456dd6ff2868d.final.pdb
-3.083613e-14 -1.728950e-14 4.727718e-15 5.063435e-14 -4.734983e-14
-3.202103e-14
E:\\R-3.1.2\\scripts\\mPDBS\\summary_pdb_93\\6583d53ff3c8f7ef.final.pdb
-1.907459e-14 2.112056e-16 8.565219e-15 -5.504885e-14 -2.127674e-14
-1.132130e-14
E:\\R-3.1.2\\scripts\\mPDBS\\summary_pdb_93\\69760178986bef01.final.pdb
-1.619974e-13 -4.916267e-15 -4.190174e-14 2.172322e-13 -6.691360e-14
-6.338208e-14
E:\\R-3.1.2\\scripts\\mPDBS\\summary_pdb_93\\6a9d87d47926b1c8.final.pdb
1.357334e-14 6.832328e-15 2.057545e-15 -7.507507e-15 4.792272e-15
4.838731e-15
E:\\R-3.1.2\\scripts\\mPDBS\\summary_pdb_93\\6bb3547ea982ffc4.final.pdb
2.482117e-13 3.297788e-14 -5.173501e-14 -4.156296e-13 -1.990104e-14
-6.628858e-14
E:\\R-3.1.2\\scripts\\mPDBS\\summary_pdb_93\\716d9624611d1c42.final.pdb
-4.236414e-14 3.913554e-14 -1.365214e-14 -1.229713e-14 3.639652e-15
-1.594261e-14
E:\\R-3.1.2\\scripts\\mPDBS\\summary_pdb_93\\75f27e70ad0f1be4.final.pdb
-1.907459e-14 2.112056e-16 8.565219e-15 -5.504885e-14 -2.127674e-14
-1.132130e-14
E:\\R-3.1.2\\scripts\\mPDBS\\summary_pdb_93\\769f910fffb22872.final.pdb
2.595630e-14 -2.342737e-14 3.760608e-15 -2.163627e-13 -5.766795e-15
-2.003264e-14

E:\\R-3.1.2\\scripts\\mPDBS\\summary_pdb_93\\791f8c80dd5c3135.final.pdb
-1.436763e-14 9.552259e-16 -3.457347e-15 3.255239e-15 -3.825799e-15
1.005832e-14
E:\\R-3.1.2\\scripts\\mPDBS\\summary_pdb_93\\7fad98e66a3f6099.final.pdb
7.653547e-14 5.674239e-14 -7.880261e-14 -8.423990e-14 -3.232403e-14
-1.111279e-13
E:\\R-3.1.2\\scripts\\mPDBS\\summary_pdb_93\\804b89c94a633196.final.pdb
-1.810623e-14 1.065708e-14 1.066478e-14 4.780707e-14 5.543885e-15
2.756389e-14
E:\\R-3.1.2\\scripts\\mPDBS\\summary_pdb_93\\813cc65a2298feba.final.pdb
-1.810623e-14 1.065708e-14 1.066478e-14 4.780707e-14 5.543885e-15
2.756389e-14
E:\\R-3.1.2\\scripts\\mPDBS\\summary_pdb_93\\83c0eefcfe12d3f9.final.pdb
-1.056593e-14 -2.102511e-14 1.374781e-14 1.700792e-14 -6.904945e-16
-9.438117e-15
E:\\R-3.1.2\\scripts\\mPDBS\\summary_pdb_93\\86d50469ef569760.final.pdb
-3.083613e-14 -1.728950e-14 4.727718e-15 5.063435e-14 -4.734983e-14
-3.202103e-14
E:\\R-3.1.2\\scripts\\mPDBS\\summary_pdb_93\\89d0666e1402203d.final.pdb
-1.907459e-14 2.112056e-16 8.565219e-15 -5.504885e-14 -2.127674e-14
-1.132130e-14
E:\\R-3.1.2\\scripts\\mPDBS\\summary_pdb_93\\8b34852716dadc62.final.pdb
1.150932e-15 2.791841e-15 9.455675e-15 -6.594568e-14 2.755232e-14
6.360363e-15
E:\\R-3.1.2\\scripts\\mPDBS\\summary_pdb_93\\8b6ef9ac3fe74395.final.pdb
1.627820e-13 6.468803e-14 -7.939405e-14 -3.233119e-13 -2.560266e-14
-8.900300e-15
E:\\R-3.1.2\\scripts\\mPDBS\\summary_pdb_93\\98bbe0d5986c138f.final.pdb
-1.436763e-14 9.552259e-16 -3.457347e-15 3.255239e-15 -3.825799e-15
1.005832e-14
E:\\R-3.1.2\\scripts\\mPDBS\\summary_pdb_93\\9c22f4909d67e2fa.final.pdb
-3.258445e-14 -1.439259e-14 3.073423e-14 9.725927e-14 3.547435e-15
1.911648e-14
E:\\R-3.1.2\\scripts\\mPDBS\\summary_pdb_93\\9dda8ed0d81fb3ef.final.pdb
1.174245e-14 -1.721489e-14 -1.453761e-14 4.478102e-14 -2.557213e-14
4.467640e-15
E:\\R-3.1.2\\scripts\\mPDBS\\summary_pdb_93\\9f752b3616e6033b.final.pdb
6.434396e-14 -1.776356e-14 1.775325e-14 -8.935339e-14 1.666910e-15
1.872146e-14
E:\\R-3.1.2\\scripts\\mPDBS\\summary_pdb_93\\a02c70083816d6ba.final.pdb
-3.421557e-14 1.100807e-14 9.347539e-15 4.782763e-14 4.288101e-15
-1.378972e-14
E:\\R-3.1.2\\scripts\\mPDBS\\summary_pdb_93\\a0c997d891e4e082.final.pdb
1.233362e-13 -3.345026e-14 1.728841e-14 -1.444297e-13 1.451897e-14
4.415245e-14
E:\\R-3.1.2\\scripts\\mPDBS\\summary_pdb_93\\ab8039a2d823c616.final.pdb
3.140806e-14 7.482089e-15 7.540564e-15 3.319017e-14 6.482186e-17
1.569102e-14
E:\\R-3.1.2\\scripts\\mPDBS\\summary_pdb_93\\acf4d74b9c71d4dc.final.pdb
-1.810623e-14 1.065708e-14 1.066478e-14 4.780707e-14 5.543885e-15
2.756389e-14
E:\\R-3.1.2\\scripts\\mPDBS\\summary_pdb_93\\ad8a823e4b4c668e.final.pdb
5.327093e-15 -2.343242e-15 -1.690844e-15 -4.548049e-14 -7.080176e-15
1.029382e-17

E:\\R-3.1.2\\scripts\\mPDBS\\summary_pdb_93\\b16ababd03b7b857.final.pdb
-3.258445e-14 -1.439259e-14 3.073423e-14 9.725927e-14 3.547435e-15
1.911648e-14
E:\\R-3.1.2\\scripts\\mPDBS\\summary_pdb_93\\b226923e697d3c63.final.pdb
-7.970393e-14 3.129379e-14 -1.116661e-14 2.240265e-14 -2.144392e-14
-2.321525e-14
E:\\R-3.1.2\\scripts\\mPDBS\\summary_pdb_93\\b28e48f6fa27a165.final.pdb
-1.436763e-14 9.552259e-16 -3.457347e-15 3.255239e-15 -3.825799e-15
1.005832e-14
E:\\R-3.1.2\\scripts\\mPDBS\\summary_pdb_93\\b42c4924e3cd1aec.final.pdb
6.458755e-14 5.367644e-15 -4.939169e-15 -5.863171e-14 2.886414e-14
-3.457973e-15
E:\\R-3.1.2\\scripts\\mPDBS\\summary_pdb_93\\b4dd239fcabff1df.final.pdb
-1.436763e-14 9.552259e-16 -3.457347e-15 3.255239e-15 -3.825799e-15
1.005832e-14
E:\\R-3.1.2\\scripts\\mPDBS\\summary_pdb_93\\b669942ef97459a2.final.pdb
1.150932e-15 2.791841e-15 9.455675e-15 -6.594568e-14 2.755232e-14
6.360363e-15
E:\\R-3.1.2\\scripts\\mPDBS\\summary_pdb_93\\b99f4d9d87c1017b.final.pdb
-1.436763e-14 9.552259e-16 -3.457347e-15 3.255239e-15 -3.825799e-15
1.005832e-14
E:\\R-3.1.2\\scripts\\mPDBS\\summary_pdb_93\\b9d2cbf3975403e7.final.pdb
-1.436763e-14 9.552259e-16 -3.457347e-15 3.255239e-15 -3.825799e-15
1.005832e-14
E:\\R-3.1.2\\scripts\\mPDBS\\summary_pdb_93\\bc88c8a505f3a288.final.pdb
-1.258163e-14 -2.837340e-15 1.636718e-16 -1.427761e-14 -4.398041e-15
4.009167e-15
E:\\R-3.1.2\\scripts\\mPDBS\\summary_pdb_93\\bdac0e64f4ef427e.final.pdb
1.789362e-14 9.766419e-15 2.165500e-15 -2.807190e-14 -2.270912e-14
1.424675e-14
E:\\R-3.1.2\\scripts\\mPDBS\\summary_pdb_93\\ca2a1f55fc9d0681.final.pdb
1.150932e-15 2.791841e-15 9.455675e-15 -6.594568e-14 2.755232e-14
6.360363e-15
E:\\R-3.1.2\\scripts\\mPDBS\\summary_pdb_93\\ce34248de3e81a3c.final.pdb
2.147405e-14 5.990998e-16 -2.225915e-15 3.788944e-15 2.560187e-14
3.585709e-14
E:\\R-3.1.2\\scripts\\mPDBS\\summary_pdb_93\\d02e697772494a61.final.pdb
3.880739e-14 1.417992e-14 1.633195e-14 9.167648e-14 -7.193331e-15
8.780009e-15
E:\\R-3.1.2\\scripts\\mPDBS\\summary_pdb_93\\d04221245d015047.final.pdb
9.592937e-15 -2.527943e-15 9.685983e-15 -5.301496e-14 6.932254e-16
5.652002e-15
E:\\R-3.1.2\\scripts\\mPDBS\\summary_pdb_93\\d678141e4018054a.final.pdb
-3.962357e-14 6.141813e-15 -5.331948e-15 -5.243256e-14 -2.245803e-14
4.301994e-14
E:\\R-3.1.2\\scripts\\mPDBS\\summary_pdb_93\\d71b40af0f0efe39.final.pdb
-2.069089e-13 -3.691945e-14 4.877432e-14 3.151011e-13 -4.400508e-15
-2.026129e-14
E:\\R-3.1.2\\scripts\\mPDBS\\summary_pdb_93\\dc0809eff10197e2.final.pdb
-2.555274e-14 -2.956743e-14 1.546996e-14 1.197259e-13 -2.725759e-14
-1.341384e-15
E:\\R-3.1.2\\scripts\\mPDBS\\summary_pdb_93\\e1163918d3707839.final.pdb
-5.188092e-14 -4.020029e-15 1.261710e-14 5.740915e-14 -3.330661e-14
-9.052456e-15

E:\\R-3.1.2\\scripts\\mPDBS\\summary_pdb_93\\e4b48b06824ef848.final.pdb
-4.119864e-14 -4.117746e-14 1.494936e-14 2.184774e-13 -1.200074e-14
-2.542919e-14
E:\\R-3.1.2\\scripts\\mPDBS\\summary_pdb_93\\e529e19ad281fea2.final.pdb
1.486777e-14 -6.538121e-15 -1.014233e-14 -9.592550e-14 2.899925e-14
-4.004864e-16
E:\\R-3.1.2\\scripts\\mPDBS\\summary_pdb_93\\e5602082718ec3a1.final.pdb
-1.810623e-14 1.065708e-14 1.066478e-14 4.780707e-14 5.543885e-15
2.756389e-14
E:\\R-3.1.2\\scripts\\mPDBS\\summary_pdb_93\\e5d589d996d0a960.final.pdb
1.592634e-13 -5.176787e-14 8.062719e-15 -2.184736e-13 -1.920306e-15
4.897938e-14
E:\\R-3.1.2\\scripts\\mPDBS\\summary_pdb_93\\e9df9e72307a2fbd.final.pdb
-3.421557e-14 1.100807e-14 9.347539e-15 4.782763e-14 4.288101e-15
-1.378972e-14
E:\\R-3.1.2\\scripts\\mPDBS\\summary_pdb_93\\ea00f8694090cae7.final.pdb
-1.810623e-14 1.065708e-14 1.066478e-14 4.780707e-14 5.543885e-15
2.756389e-14
E:\\R-3.1.2\\scripts\\mPDBS\\summary_pdb_93\\eba517be32db050e.final.pdb
-1.436763e-14 9.552259e-16 -3.457347e-15 3.255239e-15 -3.825799e-15
1.005832e-14
E:\\R-3.1.2\\scripts\\mPDBS\\summary_pdb_93\\ec04b9545899c29d.final.pdb
-3.622515e-14 1.829830e-14 2.072129e-14 1.819291e-14 2.191035e-15
2.447385e-14
E:\\R-3.1.2\\scripts\\mPDBS\\summary_pdb_93\\ee6862cbcad52078.final.pdb
-7.661407e-15 -5.133003e-15 -3.689800e-15 3.912325e-14 -1.642857e-14
1.387298e-14
E:\\R-3.1.2\\scripts\\mPDBS\\summary_pdb_93\\ef6a42fc83b81e5d.final.pdb
-5.174966e-14 3.171990e-15 -2.192963e-15 5.030398e-14 -1.220518e-15
1.060934e-14
E:\\R-3.1.2\\scripts\\mPDBS\\summary_pdb_93\\ef8e88d4ad93a0ad.final.pdb
9.592937e-15 -2.527943e-15 9.685983e-15 -5.301496e-14 6.932254e-16
5.652002e-15
E:\\R-3.1.2\\scripts\\mPDBS\\summary_pdb_93\\f14fcae8deec4fa3.final.pdb
6.434396e-14 -1.776356e-14 1.775325e-14 -8.935339e-14 1.666910e-15
1.872146e-14
E:\\R-3.1.2\\scripts\\mPDBS\\summary_pdb_93\\f83584e22003fb92.final.pdb
3.659442e-14 2.589823e-15 -8.223716e-15 -1.099400e-14 8.523480e-15
3.474900e-14
E:\\R-3.1.2\\scripts\\mPDBS\\summary_pdb_93\\f8e2bf87e52caa29.final.pdb
1.486777e-14 -6.538121e-15 -1.014233e-14 -9.592550e-14 2.899925e-14
-4.004864e-16

[,61]   [,62]   [,63]   [,64]   [,65]   [,66]
E:\\R-3.1.2\\scripts\\40.1ns_data\\NAT0.pdb
6.718333e-15 1.999317e-15 6.335994e-15 -9.875608e-16 9.897662e-15
-4.343967e-15
E:\\R-3.1.2\\scripts\\40.1ns_data\\LJP6.pdb
-2.774314e-15 4.031521e-15 6.173417e-15 3.056584e-15 1.878851e-14
-6.762647e-15
E:\\R-3.1.2\\scripts\\40.1ns_data\\3WY9.pdb
-1.034586e-14 5.123330e-16 2.051530e-15 -3.286997e-15 -1.311189e-15
4.064722e-15
E:\\R-3.1.2\\scripts\\40.1ns_data\\ZSP9.pdb

-7.701131e-15 5.048490e-15 3.296685e-15 8.024277e-16 -4.574498e-15
2.958279e-15
E:\\R-3.1.2\\scripts\\mPDBS\\summary_pdb_93\\013d855ad26c877c.final.pdb
-6.427032e-15 2.839955e-14 4.515434e-15 5.322048e-16 -5.066744e-14
9.427599e-15
E:\\R-3.1.2\\scripts\\mPDBS\\summary_pdb_93\\0a2a705342ca0d54.final.pdb
-3.762464e-15 1.796162e-15 -1.532208e-15 7.982925e-15 2.732218e-14
6.882928e-15
E:\\R-3.1.2\\scripts\\mPDBS\\summary_pdb_93\\0a59fceadb4194d9.final.pdb
-4.337579e-15 5.338666e-15 3.351783e-15 5.953267e-15 3.014653e-15
-8.695682e-15
E:\\R-3.1.2\\scripts\\mPDBS\\summary_pdb_93\\0dba59f3306825e8.final.pdb
-3.931110e-14 -1.783083e-14 2.620230e-14 -4.718803e-15 4.928841e-14
-2.715598e-14
E:\\R-3.1.2\\scripts\\mPDBS\\summary_pdb_93\\126f26a78b57339c.final.pdb
2.362206e-14 1.897311e-15 -5.058071e-15 -1.330482e-14 2.930856e-15
3.028505e-14
E:\\R-3.1.2\\scripts\\mPDBS\\summary_pdb_93\\12f06f3e342df706.final.pdb
-5.796800e-16 4.027450e-16 7.489439e-15 1.986643e-14 -2.200999e-14
-1.898353e-14
E:\\R-3.1.2\\scripts\\mPDBS\\summary_pdb_93\\18618a2a251b867b.final.pdb
-4.121028e-14 4.425473e-15 4.022317e-14 -1.832156e-14 -4.896889e-15
-1.646924e-14
E:\\R-3.1.2\\scripts\\mPDBS\\summary_pdb_93\\1f305f598641eeb2.final.pdb
-1.471476e-14 -2.098111e-16 1.376079e-14 -3.103492e-15 -4.537398e-15
8.012838e-15
E:\\R-3.1.2\\scripts\\mPDBS\\summary_pdb_93\\1f8a00b551be0a39.final.pdb
-4.121028e-14 4.425473e-15 4.022317e-14 -1.832156e-14 -4.896889e-15
-1.646924e-14
E:\\R-3.1.2\\scripts\\mPDBS\\summary_pdb_93\\219f45328d47579a.final.pdb
7.468038e-14 3.772248e-14 -6.228083e-14 6.432783e-16 -8.773426e-14
5.380771e-14
E:\\R-3.1.2\\scripts\\mPDBS\\summary_pdb_93\\238dd3918e54336d.final.pdb
1.311005e-14 -3.561425e-14 -2.070019e-16 -2.793643e-15 6.563763e-14
-2.158077e-14
E:\\R-3.1.2\\scripts\\mPDBS\\summary_pdb_93\\2b3027889e2fdc42.final.pdb
5.336757e-15 4.243188e-15 2.422622e-15 -3.153164e-15 2.332473e-15
-5.744483e-15
E:\\R-3.1.2\\scripts\\mPDBS\\summary_pdb_93\\2fcb3b46e33793b0.final.pdb
6.551002e-15 -4.352331e-15 2.508944e-15 3.308046e-15 1.773428e-14
3.002570e-16
E:\\R-3.1.2\\scripts\\mPDBS\\summary_pdb_93\\34eb328251842006.final.pdb
5.098541e-14 2.060134e-14 -6.740450e-14 -3.557541e-14 -2.481504e-14
9.469606e-15
E:\\R-3.1.2\\scripts\\mPDBS\\summary_pdb_93\\354b32871797b7ab.final.pdb
2.492073e-15 -1.695724e-14 1.443239e-14 -2.189826e-14 3.090458e-14
1.167917e-14
E:\\R-3.1.2\\scripts\\mPDBS\\summary_pdb_93\\397368adbc32d5de.final.pdb
2.362206e-14 1.897311e-15 -5.058071e-15 -1.330482e-14 2.930856e-15
3.028505e-14
E:\\R-3.1.2\\scripts\\mPDBS\\summary_pdb_93\\411ca13cd3df6edd.final.pdb
-1.157708e-14 6.311882e-15 2.375540e-14 -1.222380e-14 5.369500e-15
-1.061376e-14
E:\\R-3.1.2\\scripts\\mPDBS\\summary_pdb_93\\42357eee6642bf12.final.pdb

-3.672968e-14 -1.570846e-14 4.524618e-14 -2.434893e-14 3.858961e-14
-2.341390e-14
E:\\R-3.1.2\\scripts\\mPDBS\\summary_pdb_93\\44342d1f75a7fcfc.final.pdb
8.185714e-14 3.927906e-14 -5.655800e-14 1.306742e-14 -7.326297e-14
7.074278e-14
E:\\R-3.1.2\\scripts\\mPDBS\\summary_pdb_93\\448ef21dffe8cdfb.final.pdb
-2.808535e-14 -1.802889e-14 3.599366e-14 -8.434297e-15 6.049088e-14
-4.839864e-14
E:\\R-3.1.2\\scripts\\mPDBS\\summary_pdb_93\\472a40b3e8f113c7.final.pdb
-3.931110e-14 -1.783083e-14 2.620230e-14 -4.718803e-15 4.928841e-14
-2.715598e-14
E:\\R-3.1.2\\scripts\\mPDBS\\summary_pdb_93\\48a26225951ea153.final.pdb
9.378663e-15 9.744863e-15 -9.796553e-15 1.573364e-14 1.110252e-15
-1.195219e-14
E:\\R-3.1.2\\scripts\\mPDBS\\summary_pdb_93\\4ab4a5c7c6682ce6.final.pdb
-2.665304e-14 5.051038e-15 3.351936e-14 -1.930869e-14 -1.948524e-14
8.329113e-16
E:\\R-3.1.2\\scripts\\mPDBS\\summary_pdb_93\\4eaa45b62cb6a25f.final.pdb
-8.514097e-15 1.586069e-14 1.815100e-15 3.478571e-15 -1.280503e-15
1.599517e-14
E:\\R-3.1.2\\scripts\\mPDBS\\summary_pdb_93\\58c371611d85ac96.final.pdb
2.362206e-14 1.897311e-15 -5.058071e-15 -1.330482e-14 2.930856e-15
3.028505e-14
E:\\R-3.1.2\\scripts\\mPDBS\\summary_pdb_93\\59a647f04822d30a.final.pdb
-1.002466e-14 -4.751689e-15 1.866112e-14 3.766703e-15 2.077368e-14
-1.099392e-14
E:\\R-3.1.2\\scripts\\mPDBS\\summary_pdb_93\\59b091fe385e05fc.final.pdb
-4.337579e-15 5.338666e-15 3.351783e-15 5.953267e-15 3.014653e-15
-8.695682e-15
E:\\R-3.1.2\\scripts\\mPDBS\\summary_pdb_93\\5c35ac30f930d2db.final.pdb
-8.514097e-15 1.586069e-14 1.815100e-15 3.478571e-15 -1.280503e-15
1.599517e-14
E:\\R-3.1.2\\scripts\\mPDBS\\summary_pdb_93\\5f60752431cf2dcb.final.pdb
4.117707e-14 1.244389e-14 -1.554205e-14 -1.733016e-14 2.757795e-14
2.382554e-14
E:\\R-3.1.2\\scripts\\mPDBS\\summary_pdb_93\\626456dd6ff2868d.final.pdb
-3.931110e-14 -1.783083e-14 2.620230e-14 -4.718803e-15 4.928841e-14
-2.715598e-14
E:\\R-3.1.2\\scripts\\mPDBS\\summary_pdb_93\\6583d53ff3c8f7ef.final.pdb
-1.002466e-14 -4.751689e-15 1.866112e-14 3.766703e-15 2.077368e-14
-1.099392e-14
E:\\R-3.1.2\\scripts\\mPDBS\\summary_pdb_93\\69760178986bef01.final.pdb
-2.868290e-14 4.268395e-14 3.252322e-14 -8.979540e-15 -1.336132e-14
3.085365e-14
E:\\R-3.1.2\\scripts\\mPDBS\\summary_pdb_93\\6a9d87d47926b1c8.final.pdb
-9.197189e-16 3.416929e-15 1.096015e-14 -6.228880e-15 6.832249e-15
-3.139605e-15
E:\\R-3.1.2\\scripts\\mPDBS\\summary_pdb_93\\6bb3547ea982ffc4.final.pdb
6.446348e-14 7.165917e-15 -1.033996e-13 -1.494168e-15 2.792562e-14
1.331169e-18
E:\\R-3.1.2\\scripts\\mPDBS\\summary_pdb_93\\716d9624611d1c42.final.pdb
1.606152e-14 4.077106e-14 -3.270617e-15 -2.533837e-15 -3.450846e-14
1.200166e-14
E:\\R-3.1.2\\scripts\\mPDBS\\summary_pdb_93\\75f27e70ad0f1be4.final.pdb

-1.002466e-14 -4.751689e-15 1.866112e-14 3.766703e-15 2.077368e-14
-1.099392e-14
E:\\R-3.1.2\\scripts\\mPDBS\\summary_pdb_93\\769f910fffb22872.final.pdb
-4.006818e-14 4.423304e-15 1.022322e-15 4.978813e-15 2.983520e-14
3.771615e-14
E:\\R-3.1.2\\scripts\\mPDBS\\summary_pdb_93\\791f8c80dd5c3135.final.pdb
-4.337579e-15 5.338666e-15 3.351783e-15 5.953267e-15 3.014653e-15
-8.695682e-15
E:\\R-3.1.2\\scripts\\mPDBS\\summary_pdb_93\\7fad98e66a3f6099.final.pdb
4.310837e-14 5.950347e-14 -5.498989e-14 -2.630965e-14 -1.147134e-13
8.941137e-14
E:\\R-3.1.2\\scripts\\mPDBS\\summary_pdb_93\\804b89c94a633196.final.pdb
6.551002e-15 -4.352331e-15 2.508944e-15 3.308046e-15 1.773428e-14
3.002570e-16
E:\\R-3.1.2\\scripts\\mPDBS\\summary_pdb_93\\813cc65a2298feba.final.pdb
6.551002e-15 -4.352331e-15 2.508944e-15 3.308046e-15 1.773428e-14
3.002570e-16
E:\\R-3.1.2\\scripts\\mPDBS\\summary_pdb_93\\83c0eefcfe12d3f9.final.pdb
-1.157708e-14 6.311882e-15 2.375540e-14 -1.222380e-14 5.369500e-15
-1.061376e-14
E:\\R-3.1.2\\scripts\\mPDBS\\summary_pdb_93\\86d50469ef569760.final.pdb
-3.931110e-14 -1.783083e-14 2.620230e-14 -4.718803e-15 4.928841e-14
-2.715598e-14
E:\\R-3.1.2\\scripts\\mPDBS\\summary_pdb_93\\89d0666e1402203d.final.pdb
-1.002466e-14 -4.751689e-15 1.866112e-14 3.766703e-15 2.077368e-14
-1.099392e-14
E:\\R-3.1.2\\scripts\\mPDBS\\summary_pdb_93\\8b34852716dadc62.final.pdb
-1.346576e-14 1.352920e-14 8.535807e-15 2.630354e-14 -2.506265e-14
-1.852843e-14
E:\\R-3.1.2\\scripts\\mPDBS\\summary_pdb_93\\8b6ef9ac3fe74395.final.pdb
1.055979e-13 2.121516e-14 -1.028893e-13 3.985812e-14 -2.169405e-14
9.065320e-15
E:\\R-3.1.2\\scripts\\mPDBS\\summary_pdb_93\\98bbe0d5986c138f.final.pdb
-4.337579e-15 5.338666e-15 3.351783e-15 5.953267e-15 3.014653e-15
-8.695682e-15
E:\\R-3.1.2\\scripts\\mPDBS\\summary_pdb_93\\9c22f4909d67e2fa.final.pdb
-3.593715e-14 -6.441372e-15 3.204998e-14 -2.591315e-15 3.327144e-14
-1.195166e-14
E:\\R-3.1.2\\scripts\\mPDBS\\summary_pdb_93\\9dda8ed0d81fb3ef.final.pdb
8.911586e-15 1.838959e-15 1.584722e-15 6.723323e-15 8.774813e-15
1.908957e-15
E:\\R-3.1.2\\scripts\\mPDBS\\summary_pdb_93\\9f752b3616e6033b.final.pdb
-1.727354e-14 -1.615089e-14 1.296924e-14 6.562648e-15 1.715204e-14
-3.385668e-14
E:\\R-3.1.2\\scripts\\mPDBS\\summary_pdb_93\\a02c70083816d6ba.final.pdb
-8.514097e-15 1.586069e-14 1.815100e-15 3.478571e-15 -1.280503e-15
1.599517e-14
E:\\R-3.1.2\\scripts\\mPDBS\\summary_pdb_93\\a0c997d891e4e082.final.pdb
3.927762e-15 -1.949052e-14 -1.046983e-14 1.028087e-15 3.957735e-14
-3.605557e-14
E:\\R-3.1.2\\scripts\\mPDBS\\summary_pdb_93\\ab8039a2d823c616.final.pdb
7.903531e-15 9.154677e-16 3.852478e-15 -9.369210e-16 -2.103608e-15
-1.704257e-14
E:\\R-3.1.2\\scripts\\mPDBS\\summary_pdb_93\\acf4d74b9c71d4dc.final.pdb

6.551002e-15 -4.352331e-15 2.508944e-15 3.308046e-15 1.773428e-14
3.002570e-16
E:\\R-3.1.2\\scripts\\mPDBS\\summary_pdb_93\\ad8a823e4b4c668e.final.pdb
-2.719230e-15 -9.235546e-16 3.899979e-15 5.928321e-16 -1.300845e-14
-2.955830e-16
E:\\R-3.1.2\\scripts\\mPDBS\\summary_pdb_93\\b16ababd03b7b857.final.pdb
-3.593715e-14 -6.441372e-15 3.204998e-14 -2.591315e-15 3.327144e-14
-1.195166e-14
E:\\R-3.1.2\\scripts\\mPDBS\\summary_pdb_93\\b226923e697d3c63.final.pdb
2.935030e-15 1.328987e-14 5.882500e-15 1.545824e-15 -1.378700e-14
2.944119e-14
E:\\R-3.1.2\\scripts\\mPDBS\\summary_pdb_93\\b28e48f6fa27a165.final.pdb
-4.337579e-15 5.338666e-15 3.351783e-15 5.953267e-15 3.014653e-15
-8.695682e-15
E:\\R-3.1.2\\scripts\\mPDBS\\summary_pdb_93\\b42c4924e3cd1aec.final.pdb
-2.271090e-14 2.576860e-14 1.590465e-14 7.303371e-15 -3.215826e-14
-9.638663e-15
E:\\R-3.1.2\\scripts\\mPDBS\\summary_pdb_93\\b4dd239fcabff1df.final.pdb
-4.337579e-15 5.338666e-15 3.351783e-15 5.953267e-15 3.014653e-15
-8.695682e-15
E:\\R-3.1.2\\scripts\\mPDBS\\summary_pdb_93\\b669942ef97459a2.final.pdb
-1.346576e-14 1.352920e-14 8.535807e-15 2.630354e-14 -2.506265e-14
-1.852843e-14
E:\\R-3.1.2\\scripts\\mPDBS\\summary_pdb_93\\b99f4d9d87c1017b.final.pdb
-4.337579e-15 5.338666e-15 3.351783e-15 5.953267e-15 3.014653e-15
-8.695682e-15
E:\\R-3.1.2\\scripts\\mPDBS\\summary_pdb_93\\b9d2cbf3975403e7.final.pdb
-4.337579e-15 5.338666e-15 3.351783e-15 5.953267e-15 3.014653e-15
-8.695682e-15
E:\\R-3.1.2\\scripts\\mPDBS\\summary_pdb_93\\bc88c8a505f3a288.final.pdb
-1.417433e-14 -4.587803e-16 -1.131826e-15 -4.641123e-15 1.251823e-14
1.993684e-15
E:\\R-3.1.2\\scripts\\mPDBS\\summary_pdb_93\\bdac0e64f4ef427e.final.pdb
3.469039e-14 1.278544e-14 -1.495777e-14 1.729671e-16 1.354883e-14
2.693130e-14
E:\\R-3.1.2\\scripts\\mPDBS\\summary_pdb_93\\ca2a1f55fc9d0681.final.pdb
-1.346576e-14 1.352920e-14 8.535807e-15 2.630354e-14 -2.506265e-14
-1.852843e-14
E:\\R-3.1.2\\scripts\\mPDBS\\summary_pdb_93\\ce34248de3e81a3c.final.pdb
3.258363e-14 2.924935e-14 -7.338674e-16 6.388318e-15 -5.822977e-14
3.558096e-14
E:\\R-3.1.2\\scripts\\mPDBS\\summary_pdb_93\\d02e697772494a61.final.pdb
3.520936e-15 -5.391900e-15 -1.651593e-15 -1.501130e-14 -5.917764e-15
-1.795260e-14
E:\\R-3.1.2\\scripts\\mPDBS\\summary_pdb_93\\d04221245d015047.final.pdb
-9.790431e-15 -1.598580e-15 4.445320e-15 2.943222e-15 5.804021e-15
-1.760088e-15
E:\\R-3.1.2\\scripts\\mPDBS\\summary_pdb_93\\d678141e4018054a.final.pdb
-9.567968e-15 -5.449815e-15 1.314746e-14 2.839292e-14 -3.628236e-15
-2.990743e-14
E:\\R-3.1.2\\scripts\\mPDBS\\summary_pdb_93\\d71b40af0f0efe39.final.pdb
-8.927497e-14 -2.629612e-14 1.037035e-13 -2.159206e-14 6.386475e-14
-3.561653e-14
E:\\R-3.1.2\\scripts\\mPDBS\\summary_pdb_93\\dc0809eff10197e2.final.pdb

-8.860636e-15 -5.615253e-15 2.467642e-15 -3.293960e-14 3.169112e-14
1.916594e-14
E:\\R-3.1.2\\scripts\\mPDBS\\summary_pdb_93\\e1163918d3707839.final.pdb
7.357057e-15 -4.041110e-14 7.619392e-15 -1.777726e-14 8.646495e-14
-1.338046e-14
E:\\R-3.1.2\\scripts\\mPDBS\\summary_pdb_93\\e4b48b06824ef848.final.pdb
-4.121028e-14 4.425473e-15 4.022317e-14 -1.832156e-14 -4.896889e-15
-1.646924e-14
E:\\R-3.1.2\\scripts\\mPDBS\\summary_pdb_93\\e529e19ad281fea2.final.pdb
-6.427032e-15 2.839955e-14 4.515434e-15 5.322048e-16 -5.066744e-14
9.427599e-15
E:\\R-3.1.2\\scripts\\mPDBS\\summary_pdb_93\\e5602082718ec3a1.final.pdb
6.551002e-15 -4.352331e-15 2.508944e-15 3.308046e-15 1.773428e-14
3.002570e-16
E:\\R-3.1.2\\scripts\\mPDBS\\summary_pdb_93\\e5d589d996d0a960.final.pdb
1.108713e-14 1.928012e-14 -4.418929e-14 -1.324305e-14 4.005829e-14
-4.498079e-14
E:\\R-3.1.2\\scripts\\mPDBS\\summary_pdb_93\\e9df9e72307a2fbd.final.pdb
-8.514097e-15 1.586069e-14 1.815100e-15 3.478571e-15 -1.280503e-15
1.599517e-14
E:\\R-3.1.2\\scripts\\mPDBS\\summary_pdb_93\\ea00f8694090cae7.final.pdb
6.551002e-15 -4.352331e-15 2.508944e-15 3.308046e-15 1.773428e-14
3.002570e-16
E:\\R-3.1.2\\scripts\\mPDBS\\summary_pdb_93\\eba517be32db050e.final.pdb
-4.337579e-15 5.338666e-15 3.351783e-15 5.953267e-15 3.014653e-15
-8.695682e-15
E:\\R-3.1.2\\scripts\\mPDBS\\summary_pdb_93\\ec04b9545899c29d.final.pdb
5.459920e-15 -9.497893e-15 1.099733e-14 -4.286750e-15 -3.481716e-14
-2.834766e-14
E:\\R-3.1.2\\scripts\\mPDBS\\summary_pdb_93\\ee6862cbcad52078.final.pdb
4.042871e-15 3.116485e-15 4.628323e-15 5.478685e-16 -1.317324e-14
1.184171e-14
E:\\R-3.1.2\\scripts\\mPDBS\\summary_pdb_93\\ef6a42fc83b81e5d.final.pdb
-1.559643e-14 7.641650e-15 7.810729e-15 -4.840156e-15 1.600610e-14
-6.916349e-17
E:\\R-3.1.2\\scripts\\mPDBS\\summary_pdb_93\\ef8e88d4ad93a0ad.final.pdb
-9.790431e-15 -1.598580e-15 4.445320e-15 2.943222e-15 5.804021e-15
-1.760088e-15
E:\\R-3.1.2\\scripts\\mPDBS\\summary_pdb_93\\f14fcae8deec4fa3.final.pdb
-1.727354e-14 -1.615089e-14 1.296924e-14 6.562648e-15 1.715204e-14
-3.385668e-14
E:\\R-3.1.2\\scripts\\mPDBS\\summary_pdb_93\\f83584e22003fb92.final.pdb
9.378663e-15 9.744863e-15 -9.796553e-15 1.573364e-14 1.110252e-15
-1.195219e-14
E:\\R-3.1.2\\scripts\\mPDBS\\summary_pdb_93\\f8e2bf87e52caa29.final.pdb
-6.427032e-15 2.839955e-14 4.515434e-15 5.322048e-16 -5.066744e-14
9.427599e-15

[,67]   [,68]   [,69]   [,70]   [,71]   [,72]
E:\\R-3.1.2\\scripts\\40.1ns_data\\NAT0.pdb
-6.140993e-15 1.053464e-15 -1.296211e-14 -3.810666e-16 -4.030663e-15
8.010186e-16
E:\\R-3.1.2\\scripts\\40.1ns_data\\LJP6.pdb
-6.994410e-15 -1.199411e-14 4.670897e-16 3.480797e-15 1.624169e-17

-2.872677e-15
E:\\R-3.1.2\\scripts\\40.1ns_data\\3WY9.pdb
5.010939e-15 5.578719e-15 6.266470e-15 -5.466149e-16 4.739741e-16
-8.241478e-15
E:\\R-3.1.2\\scripts\\40.1ns_data\\ZSP9.pdb
-5.194232e-15 1.070384e-14 -9.438986e-15 -3.469998e-16 -1.399115e-15
-3.542286e-15
E:\\R-3.1.2\\scripts\\mPDBS\\summary_pdb_93\\013d855ad26c877c.final.pdb
1.115698e-16 -1.184107e-13 2.679886e-14 1.581199e-14 -1.978563e-15
-7.018334e-15
E:\\R-3.1.2\\scripts\\mPDBS\\summary_pdb_93\\0a2a705342ca0d54.final.pdb
-5.388707e-15 1.452629e-14 -1.066367e-15 1.504830e-15 -1.014765e-14
-3.905033e-15
E:\\R-3.1.2\\scripts\\mPDBS\\summary_pdb_93\\0a59fceadb4194d9.final.pdb
1.177826e-15 -2.021332e-15 -8.125806e-15 3.706923e-15 8.043401e-15
-5.388677e-15
E:\\R-3.1.2\\scripts\\mPDBS\\summary_pdb_93\\0dba59f3306825e8.final.pdb
-7.609961e-16 7.236383e-14 -5.336271e-14 -8.685774e-15 1.140483e-14
-4.849930e-15
E:\\R-3.1.2\\scripts\\mPDBS\\summary_pdb_93\\126f26a78b57339c.final.pdb
2.730569e-15 1.506591e-14 1.443028e-14 3.279481e-15 -6.256825e-15
1.742999e-14
E:\\R-3.1.2\\scripts\\mPDBS\\summary_pdb_93\\12f06f3e342df706.final.pdb
1.522566e-14 1.070259e-15 -1.050917e-14 5.943987e-15 -2.180063e-15
-6.717887e-15
E:\\R-3.1.2\\scripts\\mPDBS\\summary_pdb_93\\18618a2a251b867b.final.pdb
1.893073e-14 2.342477e-14 -1.523674e-14 -4.707346e-15 3.495846e-14
1.391068e-14
E:\\R-3.1.2\\scripts\\mPDBS\\summary_pdb_93\\1f305f598641eeb2.final.pdb
2.171969e-15 -4.634124e-15 -1.858697e-15 7.646132e-16 -3.068569e-16
-4.445815e-15
E:\\R-3.1.2\\scripts\\mPDBS\\summary_pdb_93\\1f8a00b551be0a39.final.pdb
1.893073e-14 2.342477e-14 -1.523674e-14 -4.707346e-15 3.495846e-14
1.391068e-14
E:\\R-3.1.2\\scripts\\mPDBS\\summary_pdb_93\\219f45328d47579a.final.pdb
2.625542e-14 -1.816424e-13 1.143691e-13 2.095962e-14 -2.708692e-14
5.200942e-15
E:\\R-3.1.2\\scripts\\mPDBS\\summary_pdb_93\\238dd3918e54336d.final.pdb
-1.357255e-15 1.239959e-13 -5.154680e-14 -1.579701e-14 -9.399244e-15
1.684441e-14
E:\\R-3.1.2\\scripts\\mPDBS\\summary_pdb_93\\2b3027889e2fdc42.final.pdb
-1.028091e-15 -6.797026e-15 -2.627600e-15 2.905914e-15 2.310573e-15
3.171316e-15
E:\\R-3.1.2\\scripts\\mPDBS\\summary_pdb_93\\2fcb3b46e33793b0.final.pdb
-1.154449e-14 3.385419e-14 -3.260354e-15 -6.205506e-15 2.090531e-15
7.679902e-15
E:\\R-3.1.2\\scripts\\mPDBS\\summary_pdb_93\\34eb328251842006.final.pdb
-2.402289e-14 1.745721e-14 -5.198395e-14 5.826612e-15 -2.754944e-14
-3.685986e-14
E:\\R-3.1.2\\scripts\\mPDBS\\summary_pdb_93\\354b32871797b7ab.final.pdb
-1.068197e-14 8.479123e-14 -1.234592e-14 -1.451930e-14 -3.603793e-15
1.974087e-14
E:\\R-3.1.2\\scripts\\mPDBS\\summary_pdb_93\\397368adbc32d5de.final.pdb
2.730569e-15 1.506591e-14 1.443028e-14 3.279481e-15 -6.256825e-15

1.742999e-14
E:\\R-3.1.2\\scripts\\mPDBS\\summary_pdb_93\\411ca13cd3df6edd.final.pdb
5.719912e-15 2.761612e-14 -1.041609e-14 -5.687691e-15 1.587855e-14
1.824343e-15
E:\\R-3.1.2\\scripts\\mPDBS\\summary_pdb_93\\42357eee6642bf12.final.pdb
2.912148e-14 4.602084e-15 1.982758e-14 3.444584e-15 2.069250e-14
7.760066e-15
E:\\R-3.1.2\\scripts\\mPDBS\\summary_pdb_93\\44342d1f75a7fcfc.final.pdb
-8.220568e-15 -1.486360e-13 8.833811e-14 3.092697e-14 -2.672351e-14
8.741512e-15
E:\\R-3.1.2\\scripts\\mPDBS\\summary_pdb_93\\448ef21dffe8cdfb.final.pdb
-1.990412e-14 7.492723e-14 -5.250221e-14 -7.069398e-15 5.086398e-15
-4.078053e-15
E:\\R-3.1.2\\scripts\\mPDBS\\summary_pdb_93\\472a40b3e8f113c7.final.pdb
-7.609961e-16 7.236383e-14 -5.336271e-14 -8.685774e-15 1.140483e-14
-4.849930e-15
E:\\R-3.1.2\\scripts\\mPDBS\\summary_pdb_93\\48a26225951ea153.final.pdb
-2.068685e-14 -4.881897e-14 -1.966670e-15 2.704686e-15 -1.984141e-14
-3.718138e-15
E:\\R-3.1.2\\scripts\\mPDBS\\summary_pdb_93\\4ab4a5c7c6682ce6.final.pdb
5.032547e-15 4.040288e-16 2.011397e-15 -8.699774e-16 1.567181e-14
1.910573e-14
E:\\R-3.1.2\\scripts\\mPDBS\\summary_pdb_93\\4eaa45b62cb6a25f.final.pdb
7.049290e-15 -2.974996e-14 3.165794e-14 3.131081e-15 4.265346e-15
-1.040250e-15
E:\\R-3.1.2\\scripts\\mPDBS\\summary_pdb_93\\58c371611d85ac96.final.pdb
2.730569e-15 1.506591e-14 1.443028e-14 3.279481e-15 -6.256825e-15
1.742999e-14
E:\\R-3.1.2\\scripts\\mPDBS\\summary_pdb_93\\59a647f04822d30a.final.pdb
-1.053035e-14 8.807460e-15 -2.341364e-14 -2.321354e-16 -4.464407e-15
1.957876e-15
E:\\R-3.1.2\\scripts\\mPDBS\\summary_pdb_93\\59b091fe385e05fc.final.pdb
1.177826e-15 -2.021332e-15 -8.125806e-15 3.706923e-15 8.043401e-15
-5.388677e-15
E:\\R-3.1.2\\scripts\\mPDBS\\summary_pdb_93\\5c35ac30f930d2db.final.pdb
7.049290e-15 -2.974996e-14 3.165794e-14 3.131081e-15 4.265346e-15
-1.040250e-15
E:\\R-3.1.2\\scripts\\mPDBS\\summary_pdb_93\\5f60752431cf2dcb.final.pdb
-1.985218e-14 1.092110e-13 -1.844569e-14 6.717783e-17 1.309418e-14
-8.165514e-15
E:\\R-3.1.2\\scripts\\mPDBS\\summary_pdb_93\\626456dd6ff2868d.final.pdb
-7.609961e-16 7.236383e-14 -5.336271e-14 -8.685774e-15 1.140483e-14
-4.849930e-15
E:\\R-3.1.2\\scripts\\mPDBS\\summary_pdb_93\\6583d53ff3c8f7ef.final.pdb
-1.053035e-14 8.807460e-15 -2.341364e-14 -2.321354e-16 -4.464407e-15
1.957876e-15
E:\\R-3.1.2\\scripts\\mPDBS\\summary_pdb_93\\69760178986bef01.final.pdb
-6.073230e-15 1.053908e-13 -1.028024e-14 7.318691e-15 6.359287e-14
-3.525135e-14
E:\\R-3.1.2\\scripts\\mPDBS\\summary_pdb_93\\6a9d87d47926b1c8.final.pdb
-5.349773e-15 -6.614662e-15 5.064511e-15 2.200483e-15 -3.951377e-15
6.170510e-16
E:\\R-3.1.2\\scripts\\mPDBS\\summary_pdb_93\\6bb3547ea982ffc4.final.pdb
-3.099325e-14 -1.159271e-13 -1.309079e-14 -1.872049e-15 -1.074054e-13

-5.092567e-14
E:\\R-3.1.2\\scripts\\mPDBS\\summary_pdb_93\\716d9624611d1c42.final.pdb
-7.262643e-15 -3.134240e-14 4.654832e-14 1.424030e-14 1.161836e-14
-2.256806e-14
E:\\R-3.1.2\\scripts\\mPDBS\\summary_pdb_93\\75f27e70ad0f1be4.final.pdb
-1.053035e-14 8.807460e-15 -2.341364e-14 -2.321354e-16 -4.464407e-15
1.957876e-15
E:\\R-3.1.2\\scripts\\mPDBS\\summary_pdb_93\\769f910fffb22872.final.pdb
-1.160084e-14 1.678251e-14 -9.429758e-15 1.669497e-15 -1.622951e-15
-2.576995e-15
E:\\R-3.1.2\\scripts\\mPDBS\\summary_pdb_93\\791f8c80dd5c3135.final.pdb
1.177826e-15 -2.021332e-15 -8.125806e-15 3.706923e-15 8.043401e-15
-5.388677e-15
E:\\R-3.1.2\\scripts\\mPDBS\\summary_pdb_93\\7fad98e66a3f6099.final.pdb
-1.940082e-14 -1.258334e-13 6.304913e-14 6.329974e-15 -3.589974e-14
-3.207535e-14
E:\\R-3.1.2\\scripts\\mPDBS\\summary_pdb_93\\804b89c94a633196.final.pdb
-1.154449e-14 3.385419e-14 -3.260354e-15 -6.205506e-15 2.090531e-15
7.679902e-15
E:\\R-3.1.2\\scripts\\mPDBS\\summary_pdb_93\\813cc65a2298feba.final.pdb
-1.154449e-14 3.385419e-14 -3.260354e-15 -6.205506e-15 2.090531e-15
7.679902e-15
E:\\R-3.1.2\\scripts\\mPDBS\\summary_pdb_93\\83c0eefcfe12d3f9.final.pdb
5.719912e-15 2.761612e-14 -1.041609e-14 -5.687691e-15 1.587855e-14
1.824343e-15
E:\\R-3.1.2\\scripts\\mPDBS\\summary_pdb_93\\86d50469ef569760.final.pdb
-7.609961e-16 7.236383e-14 -5.336271e-14 -8.685774e-15 1.140483e-14
-4.849930e-15
E:\\R-3.1.2\\scripts\\mPDBS\\summary_pdb_93\\89d0666e1402203d.final.pdb
-1.053035e-14 8.807460e-15 -2.341364e-14 -2.321354e-16 -4.464407e-15
1.957876e-15
E:\\R-3.1.2\\scripts\\mPDBS\\summary_pdb_93\\8b34852716dadc62.final.pdb
5.232021e-15 -1.001411e-13 2.576504e-14 8.855364e-15 -2.208114e-15
-1.342821e-14
E:\\R-3.1.2\\scripts\\mPDBS\\summary_pdb_93\\8b6ef9ac3fe74395.final.pdb
-8.608335e-14 -1.136764e-13 1.582210e-15 1.022048e-14 -8.375997e-14
-3.857879e-14
E:\\R-3.1.2\\scripts\\mPDBS\\summary_pdb_93\\98bbe0d5986c138f.final.pdb
1.177826e-15 -2.021332e-15 -8.125806e-15 3.706923e-15 8.043401e-15
-5.388677e-15
E:\\R-3.1.2\\scripts\\mPDBS\\summary_pdb_93\\9c22f4909d67e2fa.final.pdb
1.978223e-14 3.353066e-14 -8.588557e-15 4.493272e-16 1.632741e-14
9.615854e-15
E:\\R-3.1.2\\scripts\\mPDBS\\summary_pdb_93\\9dda8ed0d81fb3ef.final.pdb
-2.196023e-14 6.909685e-14 -3.902962e-14 -6.601784e-16 1.584140e-15
-2.197655e-15
E:\\R-3.1.2\\scripts\\mPDBS\\summary_pdb_93\\9f752b3616e6033b.final.pdb
-5.131730e-15 -1.625208e-14 -3.591077e-14 1.774606e-15 -1.159285e-14
8.227525e-15
E:\\R-3.1.2\\scripts\\mPDBS\\summary_pdb_93\\a02c70083816d6ba.final.pdb
7.049290e-15 -2.974996e-14 3.165794e-14 3.131081e-15 4.265346e-15
-1.040250e-15
E:\\R-3.1.2\\scripts\\mPDBS\\summary_pdb_93\\a0c997d891e4e082.final.pdb
-1.572623e-14 -5.153481e-14 -2.144056e-14 -3.377492e-17 -3.152746e-14

6.441698e-15
E:\\R-3.1.2\\scripts\\mPDBS\\summary_pdb_93\\ab8039a2d823c616.final.pdb
1.015996e-14 -1.561563e-14 -1.705258e-14 4.649545e-15 3.276301e-15
1.854222e-15
E:\\R-3.1.2\\scripts\\mPDBS\\summary_pdb_93\\acf4d74b9c71d4dc.final.pdb
-1.154449e-14 3.385419e-14 -3.260354e-15 -6.205506e-15 2.090531e-15
7.679902e-15
E:\\R-3.1.2\\scripts\\mPDBS\\summary_pdb_93\\ad8a823e4b4c668e.final.pdb
-1.458226e-14 6.765370e-15 -3.782143e-15 3.706765e-15 6.489546e-15
-4.115773e-16
E:\\R-3.1.2\\scripts\\mPDBS\\summary_pdb_93\\b16ababd03b7b857.final.pdb
1.978223e-14 3.353066e-14 -8.588557e-15 4.493272e-16 1.632741e-14
9.615854e-15
E:\\R-3.1.2\\scripts\\mPDBS\\summary_pdb_93\\b226923e697d3c63.final.pdb
1.056299e-15 8.569803e-15 3.961161e-15 -2.692841e-15 1.649409e-14
-8.891952e-15
E:\\R-3.1.2\\scripts\\mPDBS\\summary_pdb_93\\b28e48f6fa27a165.final.pdb
1.177826e-15 -2.021332e-15 -8.125806e-15 3.706923e-15 8.043401e-15
-5.388677e-15
E:\\R-3.1.2\\scripts\\mPDBS\\summary_pdb_93\\b42c4924e3cd1aec.final.pdb
8.577410e-15 -6.069163e-14 2.216882e-14 1.785279e-14 8.210350e-15
-6.751304e-15
E:\\R-3.1.2\\scripts\\mPDBS\\summary_pdb_93\\b4dd239fcabff1df.final.pdb
1.177826e-15 -2.021332e-15 -8.125806e-15 3.706923e-15 8.043401e-15
-5.388677e-15
E:\\R-3.1.2\\scripts\\mPDBS\\summary_pdb_93\\b669942ef97459a2.final.pdb
5.232021e-15 -1.001411e-13 2.576504e-14 8.855364e-15 -2.208114e-15
-1.342821e-14
E:\\R-3.1.2\\scripts\\mPDBS\\summary_pdb_93\\b99f4d9d87c1017b.final.pdb
1.177826e-15 -2.021332e-15 -8.125806e-15 3.706923e-15 8.043401e-15
-5.388677e-15
E:\\R-3.1.2\\scripts\\mPDBS\\summary_pdb_93\\b9d2cbf3975403e7.final.pdb
1.177826e-15 -2.021332e-15 -8.125806e-15 3.706923e-15 8.043401e-15
-5.388677e-15
E:\\R-3.1.2\\scripts\\mPDBS\\summary_pdb_93\\bc88c8a505f3a288.final.pdb
2.640849e-15 1.196406e-14 -3.098523e-15 1.324714e-15 -1.743042e-15
-4.958896e-16
E:\\R-3.1.2\\scripts\\mPDBS\\summary_pdb_93\\bdac0e64f4ef427e.final.pdb
2.893614e-15 4.697824e-14 2.655261e-16 1.777965e-14 3.456192e-15
6.539322e-15
E:\\R-3.1.2\\scripts\\mPDBS\\summary_pdb_93\\ca2a1f55fc9d0681.final.pdb
5.232021e-15 -1.001411e-13 2.576504e-14 8.855364e-15 -2.208114e-15
-1.342821e-14
E:\\R-3.1.2\\scripts\\mPDBS\\summary_pdb_93\\ce34248de3e81a3c.final.pdb
5.026166e-15 -5.679872e-14 4.843694e-14 1.481221e-14 1.323353e-15
1.053126e-14
E:\\R-3.1.2\\scripts\\mPDBS\\summary_pdb_93\\d02e697772494a61.final.pdb
5.103723e-15 3.825548e-14 -4.473345e-15 -6.701852e-15 1.474870e-15
1.667748e-16
E:\\R-3.1.2\\scripts\\mPDBS\\summary_pdb_93\\d04221245d015047.final.pdb
9.756861e-16 7.714666e-15 -1.090472e-14 2.299428e-15 -6.223045e-15
8.030705e-15
E:\\R-3.1.2\\scripts\\mPDBS\\summary_pdb_93\\d678141e4018054a.final.pdb
-1.112458e-14 2.286573e-14 -2.010996e-14 1.586057e-14 3.559807e-15

-5.174177e-15
E:\\R-3.1.2\\scripts\\mPDBS\\summary_pdb_93\\d71b40af0f0efe39.final.pdb
4.018363e-14 1.135122e-13 -1.152496e-14 -2.418320e-14 7.477485e-14
2.577416e-14
E:\\R-3.1.2\\scripts\\mPDBS\\summary_pdb_93\\dc0809eff10197e2.final.pdb
1.229522e-14 1.341776e-13 -3.669049e-14 5.583046e-16 1.431047e-14
-1.454943e-14
E:\\R-3.1.2\\scripts\\mPDBS\\summary_pdb_93\\e1163918d3707839.final.pdb
-2.124205e-14 1.254954e-13 -6.680255e-14 -1.306172e-14 -5.452332e-15
2.163571e-14
E:\\R-3.1.2\\scripts\\mPDBS\\summary_pdb_93\\e4b48b06824ef848.final.pdb
1.893073e-14 2.342477e-14 -1.523674e-14 -4.707346e-15 3.495846e-14
1.391068e-14
E:\\R-3.1.2\\scripts\\mPDBS\\summary_pdb_93\\e529e19ad281fea2.final.pdb
1.115698e-16 -1.184107e-13 2.679886e-14 1.581199e-14 -1.978563e-15
-7.018334e-15
E:\\R-3.1.2\\scripts\\mPDBS\\summary_pdb_93\\e5602082718ec3a1.final.pdb
-1.154449e-14 3.385419e-14 -3.260354e-15 -6.205506e-15 2.090531e-15
7.679902e-15
E:\\R-3.1.2\\scripts\\mPDBS\\summary_pdb_93\\e5d589d996d0a960.final.pdb
-2.606708e-14 1.255998e-14 -8.883192e-14 1.641627e-14 -3.511936e-14
-3.082985e-14
E:\\R-3.1.2\\scripts\\mPDBS\\summary_pdb_93\\e9df9e72307a2fbd.final.pdb
7.049290e-15 -2.974996e-14 3.165794e-14 3.131081e-15 4.265346e-15
-1.040250e-15
E:\\R-3.1.2\\scripts\\mPDBS\\summary_pdb_93\\ea00f8694090cae7.final.pdb
-1.154449e-14 3.385419e-14 -3.260354e-15 -6.205506e-15 2.090531e-15
7.679902e-15
E:\\R-3.1.2\\scripts\\mPDBS\\summary_pdb_93\\eba517be32db050e.final.pdb
1.177826e-15 -2.021332e-15 -8.125806e-15 3.706923e-15 8.043401e-15
-5.388677e-15
E:\\R-3.1.2\\scripts\\mPDBS\\summary_pdb_93\\ec04b9545899c29d.final.pdb
3.517438e-15 -3.961214e-14 1.027035e-14 1.238043e-15 -2.550139e-15
1.603125e-15
E:\\R-3.1.2\\scripts\\mPDBS\\summary_pdb_93\\ee6862cbcad52078.final.pdb
-4.337293e-15 8.115524e-15 4.289831e-16 1.528110e-15 5.467931e-16
-3.181242e-15
E:\\R-3.1.2\\scripts\\mPDBS\\summary_pdb_93\\ef6a42fc83b81e5d.final.pdb
1.030677e-14 5.663192e-16 -5.727858e-15 3.059444e-15 -5.391824e-16
-4.054979e-15
E:\\R-3.1.2\\scripts\\mPDBS\\summary_pdb_93\\ef8e88d4ad93a0ad.final.pdb
9.756861e-16 7.714666e-15 -1.090472e-14 2.299428e-15 -6.223045e-15
8.030705e-15
E:\\R-3.1.2\\scripts\\mPDBS\\summary_pdb_93\\f14fcae8deec4fa3.final.pdb
-5.131730e-15 -1.625208e-14 -3.591077e-14 1.774606e-15 -1.159285e-14
8.227525e-15
E:\\R-3.1.2\\scripts\\mPDBS\\summary_pdb_93\\f83584e22003fb92.final.pdb
-2.068685e-14 -4.881897e-14 -1.966670e-15 2.704686e-15 -1.984141e-14
-3.718138e-15
E:\\R-3.1.2\\scripts\\mPDBS\\summary_pdb_93\\f8e2bf87e52caa29.final.pdb
1.115698e-16 -1.184107e-13 2.679886e-14 1.581199e-14 -1.978563e-15
-7.018334e-15

[,73]   [,74]   [,75]   [,76]   [,77]   [,78]

E:\\R-3.1.2\\scripts\\40.1ns_data\\NAT0.pdb
2.701349e-15 3.855545e-15 -2.171659e-16 3.927187e-15 3.036002e-15
-7.871154e-16
E:\\R-3.1.2\\scripts\\40.1ns_data\\LJP6.pdb
-4.577579e-17 2.075423e-15 2.651237e-15 7.558428e-15 2.846456e-15
1.393274e-15
E:\\R-3.1.2\\scripts\\40.1ns_data\\3WY9.pdb
-4.320799e-17 3.827699e-15 9.725520e-16 2.360838e-15 2.300272e-15
5.542772e-15
E:\\R-3.1.2\\scripts\\40.1ns_data\\ZSP9.pdb
-3.325268e-15 2.507850e-15 4.244017e-16 3.163232e-15 7.262925e-16
1.982364e-15
E:\\R-3.1.2\\scripts\\mPDBS\\summary_pdb_93\\013d855ad26c877c.final.pdb
3.598533e-14 -1.298896e-14 8.245461e-15 -1.544399e-14 4.885447e-15
4.825847e-15
E:\\R-3.1.2\\scripts\\mPDBS\\summary_pdb_93\\0a2a705342ca0d54.final.pdb
-1.803249e-15 3.998253e-15 1.889853e-15 1.183164e-14 1.252936e-15
3.794861e-16
E:\\R-3.1.2\\scripts\\mPDBS\\summary_pdb_93\\0a59fceadb4194d9.final.pdb
2.114877e-15 3.677278e-15 6.102134e-15 5.860816e-15 -3.249562e-15
-2.833522e-16
E:\\R-3.1.2\\scripts\\mPDBS\\summary_pdb_93\\0dba59f3306825e8.final.pdb
-6.849359e-14 1.811463e-14 -6.589895e-15 1.817322e-14 1.754271e-15
-4.390305e-15
E:\\R-3.1.2\\scripts\\mPDBS\\summary_pdb_93\\126f26a78b57339c.final.pdb
1.698561e-14 -7.730403e-16 -4.644911e-16 -7.473798e-15 3.207661e-15
1.266411e-15
E:\\R-3.1.2\\scripts\\mPDBS\\summary_pdb_93\\12f06f3e342df706.final.pdb
2.716772e-14 3.945344e-15 4.755874e-15 4.511109e-15 6.515600e-16
2.248651e-15
E:\\R-3.1.2\\scripts\\mPDBS\\summary_pdb_93\\18618a2a251b867b.final.pdb
-1.670336e-14 1.928467e-14 -1.027857e-14 7.692112e-15 7.323001e-15
3.602392e-15
E:\\R-3.1.2\\scripts\\mPDBS\\summary_pdb_93\\1f305f598641eeb2.final.pdb
8.847200e-15 4.075264e-15 6.325324e-16 -2.538737e-15 2.264642e-15
1.452041e-15
E:\\R-3.1.2\\scripts\\mPDBS\\summary_pdb_93\\1f8a00b551be0a39.final.pdb
-1.670336e-14 1.928467e-14 -1.027857e-14 7.692112e-15 7.323001e-15
3.602392e-15
E:\\R-3.1.2\\scripts\\mPDBS\\summary_pdb_93\\219f45328d47579a.final.pdb
1.500673e-13 -3.042045e-14 7.901909e-15 -4.418682e-14 6.281876e-15
1.395439e-14
E:\\R-3.1.2\\scripts\\mPDBS\\summary_pdb_93\\238dd3918e54336d.final.pdb
-4.188037e-14 1.653331e-14 -9.676857e-15 1.626352e-14 2.421964e-15
-2.740696e-15
E:\\R-3.1.2\\scripts\\mPDBS\\summary_pdb_93\\2b3027889e2fdc42.final.pdb
-6.090094e-15 2.545371e-15 2.487065e-15 -4.024026e-16 6.116245e-16
4.563990e-16
E:\\R-3.1.2\\scripts\\mPDBS\\summary_pdb_93\\2fcb3b46e33793b0.final.pdb
1.204863e-14 8.850958e-15 -1.029904e-16 9.311159e-15 3.690585e-15
5.269998e-16
E:\\R-3.1.2\\scripts\\mPDBS\\summary_pdb_93\\34eb328251842006.final.pdb
5.567936e-15 -1.346625e-14 9.755151e-15 -6.494386e-14 -1.724208e-14
-4.688821e-15

E:\\R-3.1.2\\scripts\\mPDBS\\summary_pdb_93\\354b32871797b7ab.final.pdb
-3.033997e-14 1.398933e-14 3.008125e-15 2.344229e-14 -4.025692e-15
-8.388192e-16
E:\\R-3.1.2\\scripts\\mPDBS\\summary_pdb_93\\397368adbc32d5de.final.pdb
1.698561e-14 -7.730403e-16 -4.644911e-16 -7.473798e-15 3.207661e-15
1.266411e-15
E:\\R-3.1.2\\scripts\\mPDBS\\summary_pdb_93\\411ca13cd3df6edd.final.pdb
-6.334070e-15 7.263686e-15 -4.802451e-15 -1.051413e-14 5.989402e-15
-5.720824e-15
E:\\R-3.1.2\\scripts\\mPDBS\\summary_pdb_93\\42357eee6642bf12.final.pdb
-1.282145e-14 -9.208147e-15 -5.067583e-15 2.214751e-14 -3.502624e-15
-1.736524e-15
E:\\R-3.1.2\\scripts\\mPDBS\\summary_pdb_93\\44342d1f75a7fcfc.final.pdb
1.287524e-13 -2.678806e-14 1.892110e-14 -3.645522e-14 1.345797e-14
2.014345e-15
E:\\R-3.1.2\\scripts\\mPDBS\\summary_pdb_93\\448ef21dffe8cdfb.final.pdb
-6.912949e-14 -3.999874e-15 -8.755791e-15 4.922541e-14 -5.564165e-15
6.679197e-15
E:\\R-3.1.2\\scripts\\mPDBS\\summary_pdb_93\\472a40b3e8f113c7.final.pdb
-6.849359e-14 1.811463e-14 -6.589895e-15 1.817322e-14 1.754271e-15
-4.390305e-15
E:\\R-3.1.2\\scripts\\mPDBS\\summary_pdb_93\\48a26225951ea153.final.pdb
8.972944e-15 -2.431972e-16 6.193302e-15 7.266810e-15 1.754339e-14
-1.350966e-15
E:\\R-3.1.2\\scripts\\mPDBS\\summary_pdb_93\\4ab4a5c7c6682ce6.final.pdb
9.126446e-15 2.017201e-14 2.800258e-15 2.568516e-15 -4.836251e-15
-2.295844e-15
E:\\R-3.1.2\\scripts\\mPDBS\\summary_pdb_93\\4eaa45b62cb6a25f.final.pdb
6.552014e-15 9.250064e-15 -5.073467e-16 1.037037e-15 1.052272e-14
6.553488e-15
E:\\R-3.1.2\\scripts\\mPDBS\\summary_pdb_93\\58c371611d85ac96.final.pdb
1.698561e-14 -7.730403e-16 -4.644911e-16 -7.473798e-15 3.207661e-15
1.266411e-15
E:\\R-3.1.2\\scripts\\mPDBS\\summary_pdb_93\\59a647f04822d30a.final.pdb
-1.660053e-14 2.089012e-15 1.261552e-17 1.118375e-14 4.108682e-15
5.946963e-15
E:\\R-3.1.2\\scripts\\mPDBS\\summary_pdb_93\\59b091fe385e05fc.final.pdb
2.114877e-15 3.677278e-15 6.102134e-15 5.860816e-15 -3.249562e-15
-2.833522e-16
E:\\R-3.1.2\\scripts\\mPDBS\\summary_pdb_93\\5c35ac30f930d2db.final.pdb
6.552014e-15 9.250064e-15 -5.073467e-16 1.037037e-15 1.052272e-14
6.553488e-15
E:\\R-3.1.2\\scripts\\mPDBS\\summary_pdb_93\\5f60752431cf2dcb.final.pdb
-5.253539e-16 6.120687e-15 -4.070562e-15 1.804940e-15 3.609250e-15
2.495415e-15
E:\\R-3.1.2\\scripts\\mPDBS\\summary_pdb_93\\626456dd6ff2868d.final.pdb
-6.849359e-14 1.811463e-14 -6.589895e-15 1.817322e-14 1.754271e-15
-4.390305e-15
E:\\R-3.1.2\\scripts\\mPDBS\\summary_pdb_93\\6583d53ff3c8f7ef.final.pdb
-1.660053e-14 2.089012e-15 1.261552e-17 1.118375e-14 4.108682e-15
5.946963e-15
E:\\R-3.1.2\\scripts\\mPDBS\\summary_pdb_93\\69760178986bef01.final.pdb
-8.813981e-16 2.010160e-14 3.633071e-15 -8.542088e-15 -7.728441e-15
5.677983e-16

E:\\R-3.1.2\\scripts\\mPDBS\\summary_pdb_93\\6a9d87d47926b1c8.final.pdb
-8.543447e-15 1.515060e-15 2.814136e-15 -3.508220e-15 3.287459e-15
6.974218e-16
E:\\R-3.1.2\\scripts\\mPDBS\\summary_pdb_93\\6bb3547ea982ffc4.final.pdb
-1.043492e-13 -3.171887e-14 -4.092164e-15 -5.831417e-14 1.638508e-14
-3.292407e-15
E:\\R-3.1.2\\scripts\\mPDBS\\summary_pdb_93\\716d9624611d1c42.final.pdb
4.611675e-14 9.919774e-16 1.270846e-14 -7.088120e-15 -2.037834e-15
8.730228e-15
E:\\R-3.1.2\\scripts\\mPDBS\\summary_pdb_93\\75f27e70ad0f1be4.final.pdb
-1.660053e-14 2.089012e-15 1.261552e-17 1.118375e-14 4.108682e-15
5.946963e-15
E:\\R-3.1.2\\scripts\\mPDBS\\summary_pdb_93\\769f910fffb22872.final.pdb
-2.236539e-14 7.673607e-16 1.192244e-15 4.257794e-15 2.781650e-15
6.856716e-16
E:\\R-3.1.2\\scripts\\mPDBS\\summary_pdb_93\\791f8c80dd5c3135.final.pdb
2.114877e-15 3.677278e-15 6.102134e-15 5.860816e-15 -3.249562e-15
-2.833522e-16
E:\\R-3.1.2\\scripts\\mPDBS\\summary_pdb_93\\7fad98e66a3f6099.final.pdb
-5.527990e-15 -9.629429e-15 1.522689e-14 -9.286086e-14 -1.061062e-14
-2.189556e-16
E:\\R-3.1.2\\scripts\\mPDBS\\summary_pdb_93\\804b89c94a633196.final.pdb
1.204863e-14 8.850958e-15 -1.029904e-16 9.311159e-15 3.690585e-15
5.269998e-16
E:\\R-3.1.2\\scripts\\mPDBS\\summary_pdb_93\\813cc65a2298feba.final.pdb
1.204863e-14 8.850958e-15 -1.029904e-16 9.311159e-15 3.690585e-15
5.269998e-16
E:\\R-3.1.2\\scripts\\mPDBS\\summary_pdb_93\\83c0eefcfe12d3f9.final.pdb
-6.334070e-15 7.263686e-15 -4.802451e-15 -1.051413e-14 5.989402e-15
-5.720824e-15
E:\\R-3.1.2\\scripts\\mPDBS\\summary_pdb_93\\86d50469ef569760.final.pdb
-6.849359e-14 1.811463e-14 -6.589895e-15 1.817322e-14 1.754271e-15
-4.390305e-15
E:\\R-3.1.2\\scripts\\mPDBS\\summary_pdb_93\\89d0666e1402203d.final.pdb
-1.660053e-14 2.089012e-15 1.261552e-17 1.118375e-14 4.108682e-15
5.946963e-15
E:\\R-3.1.2\\scripts\\mPDBS\\summary_pdb_93\\8b34852716dadc62.final.pdb
2.129904e-14 6.682673e-16 2.006433e-15 9.545347e-15 7.486160e-15
3.367176e-15
E:\\R-3.1.2\\scripts\\mPDBS\\summary_pdb_93\\8b6ef9ac3fe74395.final.pdb
-3.403578e-15 -3.239093e-14 1.599725e-14 -4.935397e-14 -2.159702e-15
2.898469e-15
E:\\R-3.1.2\\scripts\\mPDBS\\summary_pdb_93\\98bbe0d5986c138f.final.pdb
2.114877e-15 3.677278e-15 6.102134e-15 5.860816e-15 -3.249562e-15
-2.833522e-16
E:\\R-3.1.2\\scripts\\mPDBS\\summary_pdb_93\\9c22f4909d67e2fa.final.pdb
1.598131e-16 9.625512e-15 -5.167626e-16 3.596865e-14 8.705823e-15
1.077781e-14
E:\\R-3.1.2\\scripts\\mPDBS\\summary_pdb_93\\9dda8ed0d81fb3ef.final.pdb
-2.452708e-14 5.132584e-15 4.071096e-15 1.959794e-15 4.806600e-15
-1.726028e-14
E:\\R-3.1.2\\scripts\\mPDBS\\summary_pdb_93\\9f752b3616e6033b.final.pdb
-2.748703e-14 -4.444914e-15 1.980634e-16 1.427485e-14 -7.473994e-17
-5.765684e-16

E:\\R-3.1.2\\scripts\\mPDBS\\summary_pdb_93\\a02c70083816d6ba.final.pdb
6.552014e-15 9.250064e-15 -5.073467e-16 1.037037e-15 1.052272e-14
6.553488e-15
E:\\R-3.1.2\\scripts\\mPDBS\\summary_pdb_93\\a0c997d891e4e082.final.pdb
-4.203854e-14 2.139071e-16 -5.468337e-15 9.599140e-15 6.723179e-15
2.198499e-15
E:\\R-3.1.2\\scripts\\mPDBS\\summary_pdb_93\\ab8039a2d823c616.final.pdb
1.124777e-14 1.178413e-15 4.413570e-15 5.781439e-16 8.345080e-16
1.648917e-15
E:\\R-3.1.2\\scripts\\mPDBS\\summary_pdb_93\\acf4d74b9c71d4dc.final.pdb
1.204863e-14 8.850958e-15 -1.029904e-16 9.311159e-15 3.690585e-15
5.269998e-16
E:\\R-3.1.2\\scripts\\mPDBS\\summary_pdb_93\\ad8a823e4b4c668e.final.pdb
-4.921147e-16 2.713316e-15 1.876570e-15 8.849566e-15 1.355241e-15
-4.433468e-16
E:\\R-3.1.2\\scripts\\mPDBS\\summary_pdb_93\\b16ababd03b7b857.final.pdb
1.598131e-16 9.625512e-15 -5.167626e-16 3.596865e-14 8.705823e-15
1.077781e-14
E:\\R-3.1.2\\scripts\\mPDBS\\summary_pdb_93\\b226923e697d3c63.final.pdb
1.506283e-14 9.007263e-16 -5.639073e-15 8.739094e-15 -1.083091e-14
1.129826e-14
E:\\R-3.1.2\\scripts\\mPDBS\\summary_pdb_93\\b28e48f6fa27a165.final.pdb
2.114877e-15 3.677278e-15 6.102134e-15 5.860816e-15 -3.249562e-15
-2.833522e-16
E:\\R-3.1.2\\scripts\\mPDBS\\summary_pdb_93\\b42c4924e3cd1aec.final.pdb
3.050247e-14 -6.065923e-15 9.875332e-15 -7.070309e-15 -3.132769e-15
8.130654e-15
E:\\R-3.1.2\\scripts\\mPDBS\\summary_pdb_93\\b4dd239fcabff1df.final.pdb
2.114877e-15 3.677278e-15 6.102134e-15 5.860816e-15 -3.249562e-15
-2.833522e-16
E:\\R-3.1.2\\scripts\\mPDBS\\summary_pdb_93\\b669942ef97459a2.final.pdb
2.129904e-14 6.682673e-16 2.006433e-15 9.545347e-15 7.486160e-15
3.367176e-15
E:\\R-3.1.2\\scripts\\mPDBS\\summary_pdb_93\\b99f4d9d87c1017b.final.pdb
2.114877e-15 3.677278e-15 6.102134e-15 5.860816e-15 -3.249562e-15
-2.833522e-16
E:\\R-3.1.2\\scripts\\mPDBS\\summary_pdb_93\\b9d2cbf3975403e7.final.pdb
2.114877e-15 3.677278e-15 6.102134e-15 5.860816e-15 -3.249562e-15
-2.833522e-16
E:\\R-3.1.2\\scripts\\mPDBS\\summary_pdb_93\\bc88c8a505f3a288.final.pdb
8.654966e-16 5.784100e-15 2.296110e-15 -2.107636e-15 -6.768109e-16
1.152132e-15
E:\\R-3.1.2\\scripts\\mPDBS\\summary_pdb_93\\bdac0e64f4ef427e.final.pdb
3.296302e-15 -7.024364e-15 1.220040e-14 1.412963e-15 -6.118815e-15
1.021901e-14
E:\\R-3.1.2\\scripts\\mPDBS\\summary_pdb_93\\ca2a1f55fc9d0681.final.pdb
2.129904e-14 6.682673e-16 2.006433e-15 9.545347e-15 7.486160e-15
3.367176e-15
E:\\R-3.1.2\\scripts\\mPDBS\\summary_pdb_93\\ce34248de3e81a3c.final.pdb
7.899636e-14 4.707579e-15 1.335268e-14 -1.696943e-14 6.178915e-16
1.810452e-15
E:\\R-3.1.2\\scripts\\mPDBS\\summary_pdb_93\\d02e697772494a61.final.pdb
-2.107494e-15 4.027692e-15 -3.797012e-15 3.603508e-15 1.151279e-15
1.662966e-14

E:\\R-3.1.2\\scripts\\mPDBS\\summary_pdb_93\\d04221245d015047.final.pdb
-8.464706e-15 3.613965e-15 4.933065e-15 1.154064e-16 6.202762e-15
-9.101250e-16
E:\\R-3.1.2\\scripts\\mPDBS\\summary_pdb_93\\d678141e4018054a.final.pdb
3.365512e-14 -1.551358e-14 4.271457e-15 2.041510e-14 1.229691e-15
-8.807341e-15
E:\\R-3.1.2\\scripts\\mPDBS\\summary_pdb_93\\d71b40af0f0efe39.final.pdb
-1.808264e-14 4.280667e-14 -1.657485e-14 7.120326e-14 4.308363e-15
1.645658e-14
E:\\R-3.1.2\\scripts\\mPDBS\\summary_pdb_93\\dc0809eff10197e2.final.pdb
-3.558244e-14 1.183038e-14 1.446086e-15 -1.955381e-14 -1.587872e-14
1.131451e-15
E:\\R-3.1.2\\scripts\\mPDBS\\summary_pdb_93\\e1163918d3707839.final.pdb
-6.394151e-14 1.928966e-14 2.516635e-15 2.287015e-14 -1.279518e-14
-8.388358e-15
E:\\R-3.1.2\\scripts\\mPDBS\\summary_pdb_93\\e4b48b06824ef848.final.pdb
-1.670336e-14 1.928467e-14 -1.027857e-14 7.692112e-15 7.323001e-15
3.602392e-15
E:\\R-3.1.2\\scripts\\mPDBS\\summary_pdb_93\\e529e19ad281fea2.final.pdb
3.598533e-14 -1.298896e-14 8.245461e-15 -1.544399e-14 4.885447e-15
4.825847e-15
E:\\R-3.1.2\\scripts\\mPDBS\\summary_pdb_93\\e5602082718ec3a1.final.pdb
1.204863e-14 8.850958e-15 -1.029904e-16 9.311159e-15 3.690585e-15
5.269998e-16
E:\\R-3.1.2\\scripts\\mPDBS\\summary_pdb_93\\e5d589d996d0a960.final.pdb
-2.406807e-14 -2.440343e-14 6.640880e-15 -2.782840e-14 -9.005447e-15
-8.196619e-15
E:\\R-3.1.2\\scripts\\mPDBS\\summary_pdb_93\\e9df9e72307a2fbd.final.pdb
6.552014e-15 9.250064e-15 -5.073467e-16 1.037037e-15 1.052272e-14
6.553488e-15
E:\\R-3.1.2\\scripts\\mPDBS\\summary_pdb_93\\ea00f8694090cae7.final.pdb
1.204863e-14 8.850958e-15 -1.029904e-16 9.311159e-15 3.690585e-15
5.269998e-16
E:\\R-3.1.2\\scripts\\mPDBS\\summary_pdb_93\\eba517be32db050e.final.pdb
2.114877e-15 3.677278e-15 6.102134e-15 5.860816e-15 -3.249562e-15
-2.833522e-16
E:\\R-3.1.2\\scripts\\mPDBS\\summary_pdb_93\\ec04b9545899c29d.final.pdb
1.186890e-15 2.661471e-15 2.290307e-15 1.027042e-14 1.289757e-15
1.806178e-15
E:\\R-3.1.2\\scripts\\mPDBS\\summary_pdb_93\\ee6862cbcad52078.final.pdb
-2.247953e-15 2.054444e-15 1.721090e-15 2.852806e-15 5.590238e-16
1.367237e-15
E:\\R-3.1.2\\scripts\\mPDBS\\summary_pdb_93\\ef6a42fc83b81e5d.final.pdb
2.272738e-16 2.208916e-15 4.140352e-15 -6.951556e-15 1.274328e-15
1.560189e-15
E:\\R-3.1.2\\scripts\\mPDBS\\summary_pdb_93\\ef8e88d4ad93a0ad.final.pdb
-8.464706e-15 3.613965e-15 4.933065e-15 1.154064e-16 6.202762e-15
-9.101250e-16
E:\\R-3.1.2\\scripts\\mPDBS\\summary_pdb_93\\f14fcae8deec4fa3.final.pdb
-2.748703e-14 -4.444914e-15 1.980634e-16 1.427485e-14 -7.473994e-17
-5.765684e-16
E:\\R-3.1.2\\scripts\\mPDBS\\summary_pdb_93\\f83584e22003fb92.final.pdb
8.972944e-15 -2.431972e-16 6.193302e-15 7.266810e-15 1.754339e-14
-1.350966e-15

E:\\R-3.1.2\\scripts\\mPDBS\\summary_pdb_93\\f8e2bf87e52caa29.final.pdb
3.598533e-14 -1.298896e-14 8.245461e-15 -1.544399e-14 4.885447e-15
4.825847e-15

[,79]   [,80]   [,81]   [,82]   [,83]   [,84]
E:\\R-3.1.2\\scripts\\40.1ns_data\\NAT0.pdb
-1.126949e-15 -4.822188e-15 5.576255e-15 1.000234e-15 -4.561263e-15
-3.177650e-17
E:\\R-3.1.2\\scripts\\40.1ns_data\\LJP6.pdb
-7.050674e-16 -5.794757e-15 2.044120e-15 -1.113844e-15 -2.257105e-15
-2.054543e-15
E:\\R-3.1.2\\scripts\\40.1ns_data\\3WY9.pdb
-2.091771e-15 -2.050630e-15 1.499322e-15 -2.750874e-15 -5.935525e-15
1.647838e-16
E:\\R-3.1.2\\scripts\\40.1ns_data\\ZSP9.pdb
1.815282e-15 -3.713428e-15 5.288771e-15 5.466345e-16 -4.844259e-15
-1.798678e-15
E:\\R-3.1.2\\scripts\\mPDBS\\summary_pdb_93\\013d855ad26c877c.final.pdb
8.015421e-15 -1.813374e-15 -3.148146e-14 3.807936e-15 -6.222692e-15
6.678067e-15
E:\\R-3.1.2\\scripts\\mPDBS\\summary_pdb_93\\0a2a705342ca0d54.final.pdb
3.359766e-15 -5.339126e-15 -1.429166e-15 9.166806e-16 -2.170665e-15
3.179103e-16
E:\\R-3.1.2\\scripts\\mPDBS\\summary_pdb_93\\0a59fceadb4194d9.final.pdb
7.713418e-15 -4.953917e-15 -2.915616e-17 4.000930e-16 -8.864234e-16
-2.030386e-15
E:\\R-3.1.2\\scripts\\mPDBS\\summary_pdb_93\\0dba59f3306825e8.final.pdb
4.729855e-15 -4.773679e-15 2.311029e-14 -6.284685e-15 1.776118e-15
-8.745359e-15
E:\\R-3.1.2\\scripts\\mPDBS\\summary_pdb_93\\126f26a78b57339c.final.pdb
-1.735459e-15 -6.565106e-15 5.106139e-15 3.696480e-15 -4.736034e-15
2.517550e-15
E:\\R-3.1.2\\scripts\\mPDBS\\summary_pdb_93\\12f06f3e342df706.final.pdb
3.305990e-15 -5.793298e-15 2.113139e-15 2.388810e-15 -1.553754e-15
3.144408e-16
E:\\R-3.1.2\\scripts\\mPDBS\\summary_pdb_93\\18618a2a251b867b.final.pdb
-1.431746e-14 -1.139136e-14 -7.009297e-15 -4.371554e-15 -3.220514e-15
-2.083160e-15
E:\\R-3.1.2\\scripts\\mPDBS\\summary_pdb_93\\1f305f598641eeb2.final.pdb
2.010850e-15 -5.839590e-15 -6.006774e-16 -6.673518e-16 -2.539473e-15
-1.280866e-15
E:\\R-3.1.2\\scripts\\mPDBS\\summary_pdb_93\\1f8a00b551be0a39.final.pdb
-1.431746e-14 -1.139136e-14 -7.009297e-15 -4.371554e-15 -3.220514e-15
-2.083160e-15
E:\\R-3.1.2\\scripts\\mPDBS\\summary_pdb_93\\219f45328d47579a.final.pdb
4.840716e-15 -9.361416e-15 -5.126855e-14 1.530137e-14 -1.008826e-14
1.614691e-14
E:\\R-3.1.2\\scripts\\mPDBS\\summary_pdb_93\\238dd3918e54336d.final.pdb
-1.207934e-14 -1.357961e-14 4.024093e-14 -6.268401e-15 -9.267306e-15
-6.348384e-15
E:\\R-3.1.2\\scripts\\mPDBS\\summary_pdb_93\\2b3027889e2fdc42.final.pdb
4.371907e-15 -4.457678e-15 -4.048077e-15 -1.304056e-15 -2.380454e-15
-1.403446e-15
E:\\R-3.1.2\\scripts\\mPDBS\\summary_pdb_93\\2fcb3b46e33793b0.final.pdb

9.265464e-15 -3.127350e-15 5.267892e-15 -4.292924e-15 -5.153701e-15
-3.130082e-15
E:\\R-3.1.2\\scripts\\mPDBS\\summary_pdb_93\\34eb328251842006.final.pdb
1.864987e-14 -8.591661e-15 -9.382392e-15 1.115121e-14 9.290391e-15
5.097986e-15
E:\\R-3.1.2\\scripts\\mPDBS\\summary_pdb_93\\354b32871797b7ab.final.pdb
3.886695e-15 4.240922e-16 1.207841e-14 -1.325442e-14 -8.510770e-15
-1.771731e-15
E:\\R-3.1.2\\scripts\\mPDBS\\summary_pdb_93\\397368adbc32d5de.final.pdb
-1.735459e-15 -6.565106e-15 5.106139e-15 3.696480e-15 -4.736034e-15
2.517550e-15
E:\\R-3.1.2\\scripts\\mPDBS\\summary_pdb_93\\411ca13cd3df6edd.final.pdb
7.393697e-15 -6.327589e-15 1.081462e-15 8.769858e-16 -1.420576e-15
-2.119822e-15
E:\\R-3.1.2\\scripts\\mPDBS\\summary_pdb_93\\42357eee6642bf12.final.pdb
6.449297e-15 -2.325134e-15 -2.949855e-14 1.510460e-15 -2.190590e-14
1.090955e-14
E:\\R-3.1.2\\scripts\\mPDBS\\summary_pdb_93\\44342d1f75a7fcfc.final.pdb
1.742000e-14 -6.202168e-15 -2.667454e-14 1.472121e-14 -9.370929e-15
8.737820e-15
E:\\R-3.1.2\\scripts\\mPDBS\\summary_pdb_93\\448ef21dffe8cdfb.final.pdb
-2.131077e-14 -4.518598e-15 1.881608e-14 -1.173836e-14 -4.305421e-15
-1.815161e-15
E:\\R-3.1.2\\scripts\\mPDBS\\summary_pdb_93\\472a40b3e8f113c7.final.pdb
4.729855e-15 -4.773679e-15 2.311029e-14 -6.284685e-15 1.776118e-15
-8.745359e-15
E:\\R-3.1.2\\scripts\\mPDBS\\summary_pdb_93\\48a26225951ea153.final.pdb
7.560329e-15 -1.245404e-15 -1.773854e-15 -1.416813e-15 6.676971e-15
-4.249694e-15
E:\\R-3.1.2\\scripts\\mPDBS\\summary_pdb_93\\4ab4a5c7c6682ce6.final.pdb
-2.765885e-15 -1.322772e-15 -2.611715e-14 -1.120479e-14 -1.452203e-15
-1.073328e-15
E:\\R-3.1.2\\scripts\\mPDBS\\summary_pdb_93\\4eaa45b62cb6a25f.final.pdb
-2.362213e-15 -6.815559e-15 -2.537720e-15 -1.638490e-15 -2.050226e-15
-5.839387e-15
E:\\R-3.1.2\\scripts\\mPDBS\\summary_pdb_93\\58c371611d85ac96.final.pdb
-1.735459e-15 -6.565106e-15 5.106139e-15 3.696480e-15 -4.736034e-15
2.517550e-15
E:\\R-3.1.2\\scripts\\mPDBS\\summary_pdb_93\\59a647f04822d30a.final.pdb
-3.405353e-15 -4.885512e-15 2.054220e-15 -3.614815e-15 4.380647e-16
-4.522840e-15
E:\\R-3.1.2\\scripts\\mPDBS\\summary_pdb_93\\59b091fe385e05fc.final.pdb
7.713418e-15 -4.953917e-15 -2.915616e-17 4.000930e-16 -8.864234e-16
-2.030386e-15
E:\\R-3.1.2\\scripts\\mPDBS\\summary_pdb_93\\5c35ac30f930d2db.final.pdb
-2.362213e-15 -6.815559e-15 -2.537720e-15 -1.638490e-15 -2.050226e-15
-5.839387e-15
E:\\R-3.1.2\\scripts\\mPDBS\\summary_pdb_93\\5f60752431cf2dcb.final.pdb
-2.072590e-16 -9.271636e-15 1.852666e-14 -1.984428e-15 1.159437e-14
-1.768391e-14
E:\\R-3.1.2\\scripts\\mPDBS\\summary_pdb_93\\626456dd6ff2868d.final.pdb
4.729855e-15 -4.773679e-15 2.311029e-14 -6.284685e-15 1.776118e-15
-8.745359e-15
E:\\R-3.1.2\\scripts\\mPDBS\\summary_pdb_93\\6583d53ff3c8f7ef.final.pdb

-3.405353e-15 -4.885512e-15 2.054220e-15 -3.614815e-15 4.380647e-16
-4.522840e-15
E:\\R-3.1.2\\scripts\\mPDBS\\summary_pdb_93\\69760178986bef01.final.pdb
-1.541249e-14 -6.513208e-15 -1.755826e-14 -9.993966e-15 1.364334e-14
-1.939012e-14
E:\\R-3.1.2\\scripts\\mPDBS\\summary_pdb_93\\6a9d87d47926b1c8.final.pdb
4.776692e-15 -5.323912e-15 -1.987145e-15 -9.838039e-16 -3.478360e-15
-1.184350e-15
E:\\R-3.1.2\\scripts\\mPDBS\\summary_pdb_93\\6bb3547ea982ffc4.final.pdb
2.471472e-14 -4.666496e-15 5.565781e-14 7.037930e-15 -2.870939e-15
-3.949483e-15
E:\\R-3.1.2\\scripts\\mPDBS\\summary_pdb_93\\716d9624611d1c42.final.pdb
-5.806513e-15 -3.258759e-15 -2.863137e-14 4.072143e-15 3.247350e-16
-7.675930e-16
E:\\R-3.1.2\\scripts\\mPDBS\\summary_pdb_93\\75f27e70ad0f1be4.final.pdb
-3.405353e-15 -4.885512e-15 2.054220e-15 -3.614815e-15 4.380647e-16
-4.522840e-15
E:\\R-3.1.2\\scripts\\mPDBS\\summary_pdb_93\\769f910fffb22872.final.pdb
3.743609e-15 -5.083362e-15 7.368939e-16 -4.698983e-15 -3.210105e-15
-1.836938e-15
E:\\R-3.1.2\\scripts\\mPDBS\\summary_pdb_93\\791f8c80dd5c3135.final.pdb
7.713418e-15 -4.953917e-15 -2.915616e-17 4.000930e-16 -8.864234e-16
-2.030386e-15
E:\\R-3.1.2\\scripts\\mPDBS\\summary_pdb_93\\7fad98e66a3f6099.final.pdb
2.905939e-14 4.656210e-15 -1.903832e-14 2.164915e-15 6.955403e-15
1.149215e-15
E:\\R-3.1.2\\scripts\\mPDBS\\summary_pdb_93\\804b89c94a633196.final.pdb
9.265464e-15 -3.127350e-15 5.267892e-15 -4.292924e-15 -5.153701e-15
-3.130082e-15
E:\\R-3.1.2\\scripts\\mPDBS\\summary_pdb_93\\813cc65a2298feba.final.pdb
9.265464e-15 -3.127350e-15 5.267892e-15 -4.292924e-15 -5.153701e-15
-3.130082e-15
E:\\R-3.1.2\\scripts\\mPDBS\\summary_pdb_93\\83c0eefcfe12d3f9.final.pdb
7.393697e-15 -6.327589e-15 1.081462e-15 8.769858e-16 -1.420576e-15
-2.119822e-15
E:\\R-3.1.2\\scripts\\mPDBS\\summary_pdb_93\\86d50469ef569760.final.pdb
4.729855e-15 -4.773679e-15 2.311029e-14 -6.284685e-15 1.776118e-15
-8.745359e-15
E:\\R-3.1.2\\scripts\\mPDBS\\summary_pdb_93\\89d0666e1402203d.final.pdb
-3.405353e-15 -4.885512e-15 2.054220e-15 -3.614815e-15 4.380647e-16
-4.522840e-15
E:\\R-3.1.2\\scripts\\mPDBS\\summary_pdb_93\\8b34852716dadc62.final.pdb
1.282267e-15 -1.002721e-14 -1.367355e-14 2.246984e-15 -2.603657e-15
7.730463e-16
E:\\R-3.1.2\\scripts\\mPDBS\\summary_pdb_93\\8b6ef9ac3fe74395.final.pdb
2.915253e-14 -5.145933e-15 2.953836e-14 1.205167e-14 2.663438e-15
-6.262158e-15
E:\\R-3.1.2\\scripts\\mPDBS\\summary_pdb_93\\98bbe0d5986c138f.final.pdb
7.713418e-15 -4.953917e-15 -2.915616e-17 4.000930e-16 -8.864234e-16
-2.030386e-15
E:\\R-3.1.2\\scripts\\mPDBS\\summary_pdb_93\\9c22f4909d67e2fa.final.pdb
-1.135433e-14 -6.577498e-15 -5.230871e-15 -2.624120e-15 -8.771900e-15
2.996420e-15
E:\\R-3.1.2\\scripts\\mPDBS\\summary_pdb_93\\9dda8ed0d81fb3ef.final.pdb

9.650176e-15 2.573040e-15 3.685245e-14 1.309414e-15 1.049686e-14
-9.233699e-15
E:\\R-3.1.2\\scripts\\mPDBS\\summary_pdb_93\\9f752b3616e6033b.final.pdb
7.012994e-15 -1.837210e-15 1.310514e-14 1.757242e-16 -5.677499e-15
4.514904e-15
E:\\R-3.1.2\\scripts\\mPDBS\\summary_pdb_93\\a02c70083816d6ba.final.pdb
-2.362213e-15 -6.815559e-15 -2.537720e-15 -1.638490e-15 -2.050226e-15
-5.839387e-15
E:\\R-3.1.2\\scripts\\mPDBS\\summary_pdb_93\\a0c997d891e4e082.final.pdb
9.898528e-15 -7.261876e-16 2.860883e-14 -5.419290e-15 -1.011080e-14
3.288485e-15
E:\\R-3.1.2\\scripts\\mPDBS\\summary_pdb_93\\ab8039a2d823c616.final.pdb
2.491692e-15 -4.818183e-15 -2.970726e-15 -9.092256e-16 4.441293e-16
-1.896440e-15
E:\\R-3.1.2\\scripts\\mPDBS\\summary_pdb_93\\acf4d74b9c71d4dc.final.pdb
9.265464e-15 -3.127350e-15 5.267892e-15 -4.292924e-15 -5.153701e-15
-3.130082e-15
E:\\R-3.1.2\\scripts\\mPDBS\\summary_pdb_93\\ad8a823e4b4c668e.final.pdb
1.464396e-15 -7.809349e-15 -3.161976e-15 -1.537983e-15 -2.285352e-15
-1.391202e-15
E:\\R-3.1.2\\scripts\\mPDBS\\summary_pdb_93\\b16ababd03b7b857.final.pdb
-1.135433e-14 -6.577498e-15 -5.230871e-15 -2.624120e-15 -8.771900e-15
2.996420e-15
E:\\R-3.1.2\\scripts\\mPDBS\\summary_pdb_93\\b226923e697d3c63.final.pdb
-2.420898e-15 -1.257392e-14 -1.472468e-14 1.427592e-15 -5.489052e-15
-4.449006e-15
E:\\R-3.1.2\\scripts\\mPDBS\\summary_pdb_93\\b28e48f6fa27a165.final.pdb
7.713418e-15 -4.953917e-15 -2.915616e-17 4.000930e-16 -8.864234e-16
-2.030386e-15
E:\\R-3.1.2\\scripts\\mPDBS\\summary_pdb_93\\b42c4924e3cd1aec.final.pdb
5.220993e-15 -4.296791e-15 -2.786911e-14 1.823760e-15 -9.817187e-15
5.260404e-15
E:\\R-3.1.2\\scripts\\mPDBS\\summary_pdb_93\\b4dd239fcabff1df.final.pdb
7.713418e-15 -4.953917e-15 -2.915616e-17 4.000930e-16 -8.864234e-16
-2.030386e-15
E:\\R-3.1.2\\scripts\\mPDBS\\summary_pdb_93\\b669942ef97459a2.final.pdb
1.282267e-15 -1.002721e-14 -1.367355e-14 2.246984e-15 -2.603657e-15
7.730463e-16
E:\\R-3.1.2\\scripts\\mPDBS\\summary_pdb_93\\b99f4d9d87c1017b.final.pdb
7.713418e-15 -4.953917e-15 -2.915616e-17 4.000930e-16 -8.864234e-16
-2.030386e-15
E:\\R-3.1.2\\scripts\\mPDBS\\summary_pdb_93\\b9d2cbf3975403e7.final.pdb
7.713418e-15 -4.953917e-15 -2.915616e-17 4.000930e-16 -8.864234e-16
-2.030386e-15
E:\\R-3.1.2\\scripts\\mPDBS\\summary_pdb_93\\bc88c8a505f3a288.final.pdb
2.430318e-15 -4.900032e-15 7.384304e-17 -4.115408e-16 -2.837554e-15
-5.702911e-16
E:\\R-3.1.2\\scripts\\mPDBS\\summary_pdb_93\\bdac0e64f4ef427e.final.pdb
-1.099481e-14 -4.923114e-15 9.612616e-15 -2.772872e-15 -4.554788e-15
3.069305e-17
E:\\R-3.1.2\\scripts\\mPDBS\\summary_pdb_93\\ca2a1f55fc9d0681.final.pdb
1.282267e-15 -1.002721e-14 -1.367355e-14 2.246984e-15 -2.603657e-15
7.730463e-16
E:\\R-3.1.2\\scripts\\mPDBS\\summary_pdb_93\\ce34248de3e81a3c.final.pdb

8.326906e-15 -4.809014e-15 -2.660347e-14 4.762006e-15 -5.323154e-15
1.609705e-15
E:\\R-3.1.2\\scripts\\mPDBS\\summary_pdb_93\\d02e697772494a61.final.pdb
-8.929085e-15 -1.358655e-14 -6.469100e-15 -4.268265e-15 -3.447309e-15
6.078572e-16
E:\\R-3.1.2\\scripts\\mPDBS\\summary_pdb_93\\d04221245d015047.final.pdb
4.510477e-15 -3.321334e-15 7.525002e-15 7.112230e-16 -1.589400e-15
3.984361e-16
E:\\R-3.1.2\\scripts\\mPDBS\\summary_pdb_93\\d678141e4018054a.final.pdb
2.967792e-15 -2.046914e-15 5.324249e-15 1.602338e-14 8.409176e-15
6.982378e-15
E:\\R-3.1.2\\scripts\\mPDBS\\summary_pdb_93\\d71b40af0f0efe39.final.pdb
-3.150485e-14 -1.577973e-14 -2.509634e-15 -1.037475e-14 -6.720895e-15
1.847160e-15
E:\\R-3.1.2\\scripts\\mPDBS\\summary_pdb_93\\dc0809eff10197e2.final.pdb
3.852155e-15 2.167662e-16 7.044260e-15 -8.595260e-15 -5.334035e-15
-6.841268e-16
E:\\R-3.1.2\\scripts\\mPDBS\\summary_pdb_93\\e1163918d3707839.final.pdb
1.511412e-14 4.875993e-15 4.496065e-14 5.163103e-15 1.851431e-15
5.306374e-15
E:\\R-3.1.2\\scripts\\mPDBS\\summary_pdb_93\\e4b48b06824ef848.final.pdb
-1.431746e-14 -1.139136e-14 -7.009297e-15 -4.371554e-15 -3.220514e-15
-2.083160e-15
E:\\R-3.1.2\\scripts\\mPDBS\\summary_pdb_93\\e529e19ad281fea2.final.pdb
8.015421e-15 -1.813374e-15 -3.148146e-14 3.807936e-15 -6.222692e-15
6.678067e-15
E:\\R-3.1.2\\scripts\\mPDBS\\summary_pdb_93\\e5602082718ec3a1.final.pdb
9.265464e-15 -3.127350e-15 5.267892e-15 -4.292924e-15 -5.153701e-15
-3.130082e-15
E:\\R-3.1.2\\scripts\\mPDBS\\summary_pdb_93\\e5d589d996d0a960.final.pdb
1.209142e-14 -3.582418e-15 1.113551e-14 7.310638e-15 -3.348522e-15
9.900159e-16
E:\\R-3.1.2\\scripts\\mPDBS\\summary_pdb_93\\e9df9e72307a2fbd.final.pdb
-2.362213e-15 -6.815559e-15 -2.537720e-15 -1.638490e-15 -2.050226e-15
-5.839387e-15
E:\\R-3.1.2\\scripts\\mPDBS\\summary_pdb_93\\ea00f8694090cae7.final.pdb
9.265464e-15 -3.127350e-15 5.267892e-15 -4.292924e-15 -5.153701e-15
-3.130082e-15
E:\\R-3.1.2\\scripts\\mPDBS\\summary_pdb_93\\eba517be32db050e.final.pdb
7.713418e-15 -4.953917e-15 -2.915616e-17 4.000930e-16 -8.864234e-16
-2.030386e-15
E:\\R-3.1.2\\scripts\\mPDBS\\summary_pdb_93\\ec04b9545899c29d.final.pdb
2.147497e-15 -6.242934e-15 3.074238e-15 3.230073e-16 -2.668825e-15
-1.673991e-15
E:\\R-3.1.2\\scripts\\mPDBS\\summary_pdb_93\\ee6862cbcad52078.final.pdb
3.331507e-15 -4.595263e-15 3.619835e-15 1.111330e-15 -2.979367e-15
-1.224607e-15
E:\\R-3.1.2\\scripts\\mPDBS\\summary_pdb_93\\ef6a42fc83b81e5d.final.pdb
3.226227e-15 -5.653869e-15 2.566810e-15 -9.587024e-16 -2.513180e-15
-1.234718e-15
E:\\R-3.1.2\\scripts\\mPDBS\\summary_pdb_93\\ef8e88d4ad93a0ad.final.pdb
4.510477e-15 -3.321334e-15 7.525002e-15 7.112230e-16 -1.589400e-15
3.984361e-16
E:\\R-3.1.2\\scripts\\mPDBS\\summary_pdb_93\\f14fcae8deec4fa3.final.pdb

7.012994e-15 -1.837210e-15 1.310514e-14 1.757242e-16 -5.677499e-15
4.514904e-15
E:\\R-3.1.2\\scripts\\mPDBS\\summary_pdb_93\\f83584e22003fb92.final.pdb
7.560329e-15 -1.245404e-15 -1.773854e-15 -1.416813e-15 6.676971e-15
-4.249694e-15
E:\\R-3.1.2\\scripts\\mPDBS\\summary_pdb_93\\f8e2bf87e52caa29.final.pdb
8.015421e-15 -1.813374e-15 -3.148146e-14 3.807936e-15 -6.222692e-15
6.678067e-15

[,85]   [,86]   [,87]   [,88]   [,89]   [,90]
E:\\R-3.1.2\\scripts\\40.1ns_data\\NAT0.pdb
2.891103e-15 4.378681e-15 1.676542e-15 1.312444e-15 3.425034e-15
1.992909e-15
E:\\R-3.1.2\\scripts\\40.1ns_data\\LJP6.pdb
1.443981e-15 3.612021e-15 1.859945e-15 -1.294646e-15 4.626648e-15
3.335652e-16
E:\\R-3.1.2\\scripts\\40.1ns_data\\3WY9.pdb
5.416414e-15 7.051135e-15 5.668438e-15 2.467777e-16 3.234264e-15
-1.032879e-15
E:\\R-3.1.2\\scripts\\40.1ns_data\\ZSP9.pdb
-2.073185e-16 5.118219e-15 4.203942e-15 6.420404e-17 5.685833e-15
-2.234870e-15
E:\\R-3.1.2\\scripts\\mPDBS\\summary_pdb_93\\013d855ad26c877c.final.pdb
-3.028361e-15 1.624442e-15 4.997963e-15 4.561950e-16 -1.518366e-16
5.153908e-16
E:\\R-3.1.2\\scripts\\mPDBS\\summary_pdb_93\\0a2a705342ca0d54.final.pdb
9.236635e-16 2.986249e-15 7.221863e-16 1.578924e-15 6.136201e-15
-9.431556e-16
E:\\R-3.1.2\\scripts\\mPDBS\\summary_pdb_93\\0a59fceadb4194d9.final.pdb
1.125166e-15 7.154897e-15 5.634003e-15 1.570263e-15 4.923059e-15
-4.713177e-16
E:\\R-3.1.2\\scripts\\mPDBS\\summary_pdb_93\\0dba59f3306825e8.final.pdb
3.993799e-15 1.409016e-14 1.462596e-15 4.154625e-15 5.461729e-15
-2.943648e-15
E:\\R-3.1.2\\scripts\\mPDBS\\summary_pdb_93\\126f26a78b57339c.final.pdb
-2.920808e-15 -3.906724e-15 1.628696e-15 -3.508082e-15 7.390741e-15
1.165045e-15
E:\\R-3.1.2\\scripts\\mPDBS\\summary_pdb_93\\12f06f3e342df706.final.pdb
6.886085e-16 7.249766e-15 4.073672e-15 -4.684833e-16 6.616286e-15
-1.707301e-15
E:\\R-3.1.2\\scripts\\mPDBS\\summary_pdb_93\\18618a2a251b867b.final.pdb
-2.263026e-17 3.998751e-16 4.765847e-16 1.491020e-15 1.112383e-14
6.241926e-15
E:\\R-3.1.2\\scripts\\mPDBS\\summary_pdb_93\\1f305f598641eeb2.final.pdb
1.685250e-15 6.294386e-15 1.318543e-16 1.517920e-15 5.790520e-15
-2.053376e-15
E:\\R-3.1.2\\scripts\\mPDBS\\summary_pdb_93\\1f8a00b551be0a39.final.pdb
-2.263026e-17 3.998751e-16 4.765847e-16 1.491020e-15 1.112383e-14
6.241926e-15
E:\\R-3.1.2\\scripts\\mPDBS\\summary_pdb_93\\219f45328d47579a.final.pdb
1.347211e-15 4.321226e-15 1.675364e-15 -2.750933e-15 1.013590e-14
-1.367907e-14
E:\\R-3.1.2\\scripts\\mPDBS\\summary_pdb_93\\238dd3918e54336d.final.pdb
-1.794374e-15 1.011780e-14 4.082122e-17 2.219483e-15 9.766407e-15

-5.986004e-15
E:\\R-3.1.2\\scripts\\mPDBS\\summary_pdb_93\\2b3027889e2fdc42.final.pdb
-8.563218e-16 5.499103e-15 3.475574e-15 2.008714e-15 6.617883e-15
-1.031200e-15
E:\\R-3.1.2\\scripts\\mPDBS\\summary_pdb_93\\2fcb3b46e33793b0.final.pdb
5.765319e-15 7.642266e-15 -1.415519e-15 3.370605e-15 4.038449e-15
-2.056845e-15
E:\\R-3.1.2\\scripts\\mPDBS\\summary_pdb_93\\34eb328251842006.final.pdb
1.837992e-16 1.204174e-14 1.220464e-14 1.482057e-14 6.569847e-15
-1.391032e-14
E:\\R-3.1.2\\scripts\\mPDBS\\summary_pdb_93\\354b32871797b7ab.final.pdb
7.333201e-15 5.975107e-17 7.223804e-15 2.163164e-14 1.145289e-14
1.196944e-14
E:\\R-3.1.2\\scripts\\mPDBS\\summary_pdb_93\\397368adbc32d5de.final.pdb
-2.920808e-15 -3.906724e-15 1.628696e-15 -3.508082e-15 7.390741e-15
1.165045e-15
E:\\R-3.1.2\\scripts\\mPDBS\\summary_pdb_93\\411ca13cd3df6edd.final.pdb
3.564515e-16 7.798391e-15 -4.426565e-15 4.871575e-15 6.814007e-15
-7.965684e-16
E:\\R-3.1.2\\scripts\\mPDBS\\summary_pdb_93\\42357eee6642bf12.final.pdb
5.446046e-15 1.099483e-14 4.984744e-15 7.864190e-15 1.223002e-14
2.041357e-15
E:\\R-3.1.2\\scripts\\mPDBS\\summary_pdb_93\\44342d1f75a7fcfc.final.pdb
-1.083801e-15 -4.610510e-15 -5.038057e-15 -3.775751e-15 -2.226567e-15
4.275918e-16
E:\\R-3.1.2\\scripts\\mPDBS\\summary_pdb_93\\448ef21dffe8cdfb.final.pdb
-2.067881e-14 -7.137578e-15 1.577906e-14 4.904101e-15 1.200430e-14
5.787575e-15
E:\\R-3.1.2\\scripts\\mPDBS\\summary_pdb_93\\472a40b3e8f113c7.final.pdb
3.993799e-15 1.409016e-14 1.462596e-15 4.154625e-15 5.461729e-15
-2.943648e-15
E:\\R-3.1.2\\scripts\\mPDBS\\summary_pdb_93\\48a26225951ea153.final.pdb
-7.805624e-15 -3.318062e-15 3.184140e-15 5.654245e-16 -2.090326e-17
6.749008e-16
E:\\R-3.1.2\\scripts\\mPDBS\\summary_pdb_93\\4ab4a5c7c6682ce6.final.pdb
5.766687e-15 2.668745e-15 4.664462e-15 8.564824e-16 5.978676e-15
3.372480e-15
E:\\R-3.1.2\\scripts\\mPDBS\\summary_pdb_93\\4eaa45b62cb6a25f.final.pdb
-1.137453e-15 3.804585e-15 -2.202250e-15 -8.408998e-15 3.818371e-15
4.364785e-17
E:\\R-3.1.2\\scripts\\mPDBS\\summary_pdb_93\\58c371611d85ac96.final.pdb
-2.920808e-15 -3.906724e-15 1.628696e-15 -3.508082e-15 7.390741e-15
1.165045e-15
E:\\R-3.1.2\\scripts\\mPDBS\\summary_pdb_93\\59a647f04822d30a.final.pdb
-6.015478e-16 4.743119e-15 2.121147e-15 5.248281e-15 6.271844e-15
-1.356138e-15
E:\\R-3.1.2\\scripts\\mPDBS\\summary_pdb_93\\59b091fe385e05fc.final.pdb
1.125166e-15 7.154897e-15 5.634003e-15 1.570263e-15 4.923059e-15
-4.713177e-16
E:\\R-3.1.2\\scripts\\mPDBS\\summary_pdb_93\\5c35ac30f930d2db.final.pdb
-1.137453e-15 3.804585e-15 -2.202250e-15 -8.408998e-15 3.818371e-15
4.364785e-17
E:\\R-3.1.2\\scripts\\mPDBS\\summary_pdb_93\\5f60752431cf2dcb.final.pdb
2.009167e-15 -3.556958e-16 -2.621060e-15 -2.624745e-15 3.036250e-15

8.019762e-16
E:\\R-3.1.2\\scripts\\mPDBS\\summary_pdb_93\\626456dd6ff2868d.final.pdb
3.993799e-15 1.409016e-14 1.462596e-15 4.154625e-15 5.461729e-15
-2.943648e-15
E:\\R-3.1.2\\scripts\\mPDBS\\summary_pdb_93\\6583d53ff3c8f7ef.final.pdb
-6.015478e-16 4.743119e-15 2.121147e-15 5.248281e-15 6.271844e-15
-1.356138e-15
E:\\R-3.1.2\\scripts\\mPDBS\\summary_pdb_93\\69760178986bef01.final.pdb
-3.256581e-15 -2.366574e-15 -1.491010e-15 -6.893642e-15 -2.583302e-15
-5.539442e-15
E:\\R-3.1.2\\scripts\\mPDBS\\summary_pdb_93\\6a9d87d47926b1c8.final.pdb
-9.709793e-16 5.094756e-15 3.724242e-15 1.807111e-15 4.250892e-15
-1.842098e-15
E:\\R-3.1.2\\scripts\\mPDBS\\summary_pdb_93\\6bb3547ea982ffc4.final.pdb
-2.105614e-14 1.062806e-14 7.467225e-15 1.415797e-14 1.412917e-14
-8.684376e-15
E:\\R-3.1.2\\scripts\\mPDBS\\summary_pdb_93\\716d9624611d1c42.final.pdb
3.477353e-16 7.162596e-15 8.918755e-15 -1.108163e-15 3.322480e-15
-7.370740e-15
E:\\R-3.1.2\\scripts\\mPDBS\\summary_pdb_93\\75f27e70ad0f1be4.final.pdb
-6.015478e-16 4.743119e-15 2.121147e-15 5.248281e-15 6.271844e-15
-1.356138e-15
E:\\R-3.1.2\\scripts\\mPDBS\\summary_pdb_93\\769f910fffb22872.final.pdb
5.068941e-16 3.590032e-15 2.049112e-15 3.509371e-15 5.926594e-15
-2.497611e-15
E:\\R-3.1.2\\scripts\\mPDBS\\summary_pdb_93\\791f8c80dd5c3135.final.pdb
1.125166e-15 7.154897e-15 5.634003e-15 1.570263e-15 4.923059e-15
-4.713177e-16
E:\\R-3.1.2\\scripts\\mPDBS\\summary_pdb_93\\7fad98e66a3f6099.final.pdb
3.162654e-15 1.654022e-14 -3.013494e-15 8.313600e-15 9.624919e-16
-1.293488e-15
E:\\R-3.1.2\\scripts\\mPDBS\\summary_pdb_93\\804b89c94a633196.final.pdb
5.765319e-15 7.642266e-15 -1.415519e-15 3.370605e-15 4.038449e-15
-2.056845e-15
E:\\R-3.1.2\\scripts\\mPDBS\\summary_pdb_93\\813cc65a2298feba.final.pdb
5.765319e-15 7.642266e-15 -1.415519e-15 3.370605e-15 4.038449e-15
-2.056845e-15
E:\\R-3.1.2\\scripts\\mPDBS\\summary_pdb_93\\83c0eefcfe12d3f9.final.pdb
3.564515e-16 7.798391e-15 -4.426565e-15 4.871575e-15 6.814007e-15
-7.965684e-16
E:\\R-3.1.2\\scripts\\mPDBS\\summary_pdb_93\\86d50469ef569760.final.pdb
3.993799e-15 1.409016e-14 1.462596e-15 4.154625e-15 5.461729e-15
-2.943648e-15
E:\\R-3.1.2\\scripts\\mPDBS\\summary_pdb_93\\89d0666e1402203d.final.pdb
-6.015478e-16 4.743119e-15 2.121147e-15 5.248281e-15 6.271844e-15
-1.356138e-15
E:\\R-3.1.2\\scripts\\mPDBS\\summary_pdb_93\\8b34852716dadc62.final.pdb
7.138046e-16 6.283977e-15 -3.122349e-16 -3.228283e-15 8.732611e-15
-3.455032e-15
E:\\R-3.1.2\\scripts\\mPDBS\\summary_pdb_93\\8b6ef9ac3fe74395.final.pdb
-5.635174e-15 1.120782e-14 7.513573e-15 4.976519e-15 3.708031e-15
-1.357670e-14
E:\\R-3.1.2\\scripts\\mPDBS\\summary_pdb_93\\98bbe0d5986c138f.final.pdb
1.125166e-15 7.154897e-15 5.634003e-15 1.570263e-15 4.923059e-15

-4.713177e-16
E:\\R-3.1.2\\scripts\\mPDBS\\summary_pdb_93\\9c22f4909d67e2fa.final.pdb
3.452933e-15 1.758951e-15 1.989743e-15 -4.469478e-15 5.016833e-15
2.705459e-18
E:\\R-3.1.2\\scripts\\mPDBS\\summary_pdb_93\\9dda8ed0d81fb3ef.final.pdb
1.904948e-17 -7.871790e-15 -5.296965e-15 -8.879741e-15 -4.436171e-15
5.987381e-15
E:\\R-3.1.2\\scripts\\mPDBS\\summary_pdb_93\\9f752b3616e6033b.final.pdb
1.178640e-15 8.077711e-15 3.342728e-15 4.845163e-15 7.966353e-15
-3.509495e-15
E:\\R-3.1.2\\scripts\\mPDBS\\summary_pdb_93\\a02c70083816d6ba.final.pdb
-1.137453e-15 3.804585e-15 -2.202250e-15 -8.408998e-15 3.818371e-15
4.364785e-17
E:\\R-3.1.2\\scripts\\mPDBS\\summary_pdb_93\\a0c997d891e4e082.final.pdb
3.231642e-15 8.727170e-16 2.722699e-15 2.444461e-15 7.263066e-15
-4.050464e-15
E:\\R-3.1.2\\scripts\\mPDBS\\summary_pdb_93\\ab8039a2d823c616.final.pdb
2.189073e-15 6.746057e-15 5.027713e-15 -1.267414e-15 5.859674e-15
-2.738579e-15
E:\\R-3.1.2\\scripts\\mPDBS\\summary_pdb_93\\acf4d74b9c71d4dc.final.pdb
5.765319e-15 7.642266e-15 -1.415519e-15 3.370605e-15 4.038449e-15
-2.056845e-15
E:\\R-3.1.2\\scripts\\mPDBS\\summary_pdb_93\\ad8a823e4b4c668e.final.pdb
-9.001433e-16 6.227297e-15 1.165040e-15 1.298896e-15 5.415635e-15
-1.271191e-15
E:\\R-3.1.2\\scripts\\mPDBS\\summary_pdb_93\\b16ababd03b7b857.final.pdb
3.452933e-15 1.758951e-15 1.989743e-15 -4.469478e-15 5.016833e-15
2.705459e-18
E:\\R-3.1.2\\scripts\\mPDBS\\summary_pdb_93\\b226923e697d3c63.final.pdb
7.997043e-15 7.145711e-15 2.289415e-15 3.988004e-15 1.346574e-14
3.490955e-16
E:\\R-3.1.2\\scripts\\mPDBS\\summary_pdb_93\\b28e48f6fa27a165.final.pdb
1.125166e-15 7.154897e-15 5.634003e-15 1.570263e-15 4.923059e-15
-4.713177e-16
E:\\R-3.1.2\\scripts\\mPDBS\\summary_pdb_93\\b42c4924e3cd1aec.final.pdb
4.913138e-15 1.285816e-14 8.066478e-15 2.986808e-16 3.926498e-15
-5.015537e-15
E:\\R-3.1.2\\scripts\\mPDBS\\summary_pdb_93\\b4dd239fcabff1df.final.pdb
1.125166e-15 7.154897e-15 5.634003e-15 1.570263e-15 4.923059e-15
-4.713177e-16
E:\\R-3.1.2\\scripts\\mPDBS\\summary_pdb_93\\b669942ef97459a2.final.pdb
7.138046e-16 6.283977e-15 -3.122349e-16 -3.228283e-15 8.732611e-15
-3.455032e-15
E:\\R-3.1.2\\scripts\\mPDBS\\summary_pdb_93\\b99f4d9d87c1017b.final.pdb
1.125166e-15 7.154897e-15 5.634003e-15 1.570263e-15 4.923059e-15
-4.713177e-16
E:\\R-3.1.2\\scripts\\mPDBS\\summary_pdb_93\\b9d2cbf3975403e7.final.pdb
1.125166e-15 7.154897e-15 5.634003e-15 1.570263e-15 4.923059e-15
-4.713177e-16
E:\\R-3.1.2\\scripts\\mPDBS\\summary_pdb_93\\bc88c8a505f3a288.final.pdb
3.636225e-16 4.318190e-15 4.300001e-15 -3.366317e-16 5.479905e-15
-2.896323e-17
E:\\R-3.1.2\\scripts\\mPDBS\\summary_pdb_93\\bdac0e64f4ef427e.final.pdb
3.200531e-15 5.117809e-15 7.819281e-15 -5.570160e-15 1.023500e-15

```
-1.543801e-15
E:\\R-3.1.2\\scripts\\mPDBS\\summary_pdb_93\\ca2a1f55fc9d0681.final.pdb
7.138046e-16  6.283977e-15 -3.122349e-16 -3.228283e-15  8.732611e-15
-3.455032e-15
E:\\R-3.1.2\\scripts\\mPDBS\\summary_pdb_93\\ce34248de3e81a3c.final.pdb
7.421354e-15 -5.995918e-15  2.995256e-15 -8.192465e-15 -1.103249e-15
6.358247e-15
E:\\R-3.1.2\\scripts\\mPDBS\\summary_pdb_93\\d02e697772494a61.final.pdb
-1.544573e-15  1.296622e-14  6.436747e-15  1.003268e-14  1.824227e-14
-3.372643e-15
E:\\R-3.1.2\\scripts\\mPDBS\\summary_pdb_93\\d04221245d015047.final.pdb
-1.570072e-15  3.759297e-15  1.584721e-15  5.241218e-16  5.351401e-15
-1.137863e-15
E:\\R-3.1.2\\scripts\\mPDBS\\summary_pdb_93\\d678141e4018054a.final.pdb
-2.700157e-15  3.315197e-15  3.773738e-15  7.611338e-15  7.944920e-15
-1.260986e-14
E:\\R-3.1.2\\scripts\\mPDBS\\summary_pdb_93\\d71b40af0f0efe39.final.pdb
1.056445e-14  1.537163e-14 -4.294179e-16  2.100324e-16  1.004148e-14
7.679702e-15
E:\\R-3.1.2\\scripts\\mPDBS\\summary_pdb_93\\dc0809eff10197e2.final.pdb
4.529082e-15  7.914182e-15  6.969737e-15 -2.245022e-15 -7.205733e-16
-3.747082e-15
E:\\R-3.1.2\\scripts\\mPDBS\\summary_pdb_93\\e1163918d3707839.final.pdb
2.862695e-15  2.022379e-14  5.330819e-15  8.012996e-15  9.309345e-15
-1.482713e-14
E:\\R-3.1.2\\scripts\\mPDBS\\summary_pdb_93\\e4b48b06824ef848.final.pdb
-2.263026e-17  3.998751e-16  4.765847e-16  1.491020e-15  1.112383e-14
6.241926e-15
E:\\R-3.1.2\\scripts\\mPDBS\\summary_pdb_93\\e529e19ad281fea2.final.pdb
-3.028361e-15  1.624442e-15  4.997963e-15  4.561950e-16 -1.518366e-16
5.153908e-16
E:\\R-3.1.2\\scripts\\mPDBS\\summary_pdb_93\\e5602082718ec3a1.final.pdb
5.765319e-15  7.642266e-15 -1.415519e-15  3.370605e-15  4.038449e-15
-2.056845e-15
E:\\R-3.1.2\\scripts\\mPDBS\\summary_pdb_93\\e5d589d996d0a960.final.pdb
7.247313e-16 -5.716436e-15  9.167052e-15 -1.291956e-14 -1.320820e-15
-1.092971e-14
E:\\R-3.1.2\\scripts\\mPDBS\\summary_pdb_93\\e9df9e72307a2fbd.final.pdb
-1.137453e-15  3.804585e-15 -2.202250e-15 -8.408998e-15  3.818371e-15
4.364785e-17
E:\\R-3.1.2\\scripts\\mPDBS\\summary_pdb_93\\ea00f8694090cae7.final.pdb
5.765319e-15  7.642266e-15 -1.415519e-15  3.370605e-15  4.038449e-15
-2.056845e-15
E:\\R-3.1.2\\scripts\\mPDBS\\summary_pdb_93\\eba517be32db050e.final.pdb
1.125166e-15  7.154897e-15  5.634003e-15  1.570263e-15  4.923059e-15
-4.713177e-16
E:\\R-3.1.2\\scripts\\mPDBS\\summary_pdb_93\\ec04b9545899c29d.final.pdb
1.365629e-15  4.430002e-15  1.385624e-15  5.038118e-16  6.481745e-15
-1.945510e-15
E:\\R-3.1.2\\scripts\\mPDBS\\summary_pdb_93\\ee6862cbcad52078.final.pdb
-3.042461e-16  4.993431e-15  4.057140e-15  8.156584e-16  5.375821e-15
-1.626210e-15
E:\\R-3.1.2\\scripts\\mPDBS\\summary_pdb_93\\ef6a42fc83b81e5d.final.pdb
1.101904e-15  4.677703e-15  1.955003e-15  1.113284e-15  6.068841e-15
```

```
-9.775597e-16
E:\\R-3.1.2\\scripts\\mPDBS\\summary_pdb_93\\ef8e88d4ad93a0ad.final.pdb
-1.570072e-15  3.759297e-15  1.584721e-15  5.241218e-16  5.351401e-15
-1.137863e-15
E:\\R-3.1.2\\scripts\\mPDBS\\summary_pdb_93\\f14fcae8deec4fa3.final.pdb
1.178640e-15  8.077711e-15  3.342728e-15  4.845163e-15  7.966353e-15
-3.509495e-15
E:\\R-3.1.2\\scripts\\mPDBS\\summary_pdb_93\\f83584e22003fb92.final.pdb
-7.805624e-15 -3.318062e-15  3.184140e-15  5.654245e-16 -2.090326e-17
6.749008e-16
E:\\R-3.1.2\\scripts\\mPDBS\\summary_pdb_93\\f8e2bf87e52caa29.final.pdb
-3.028361e-15  1.624442e-15  4.997963e-15  4.561950e-16 -1.518366e-16
5.153908e-16

[,91]         [,92]         [,93]         [,94]         [,95]         [,96]
E:\\R-3.1.2\\scripts\\40.1ns_data\\NAT0.pdb
-3.069108e-15  3.458943e-15 -2.631622e-15  2.931621e-15  1.756424e-16
2.365704e-16
E:\\R-3.1.2\\scripts\\40.1ns_data\\LJP6.pdb
-4.847009e-15  3.073952e-15 -2.985604e-15  1.513908e-15 -5.968444e-16
-5.033627e-16
E:\\R-3.1.2\\scripts\\40.1ns_data\\3WY9.pdb
-5.130456e-15  2.164221e-15  5.908894e-16  4.022822e-15  8.567966e-16
-1.737340e-15
E:\\R-3.1.2\\scripts\\40.1ns_data\\ZSP9.pdb
-4.618802e-15  3.283268e-15 -4.678940e-15  1.309894e-15 -4.572341e-16
-5.948341e-16
E:\\R-3.1.2\\scripts\\mPDBS\\summary_pdb_93\\013d855ad26c877c.final.pdb
3.079050e-16 -1.946358e-15  2.238959e-15 -2.440883e-15 -1.935899e-15
-4.849148e-17
E:\\R-3.1.2\\scripts\\mPDBS\\summary_pdb_93\\0a2a705342ca0d54.final.pdb
-3.330593e-15  2.250116e-15 -3.591213e-15  3.549810e-16 -7.989843e-17
4.053567e-16
E:\\R-3.1.2\\scripts\\mPDBS\\summary_pdb_93\\0a59fceadb4194d9.final.pdb
-6.037265e-15  6.347589e-15 -5.533661e-16  1.202749e-16  3.802202e-16
-4.426609e-15
E:\\R-3.1.2\\scripts\\mPDBS\\summary_pdb_93\\0dba59f3306825e8.final.pdb
-4.497279e-15  2.199631e-15 -6.073836e-15  5.463108e-16 -1.346093e-15
-3.365283e-15
E:\\R-3.1.2\\scripts\\mPDBS\\summary_pdb_93\\126f26a78b57339c.final.pdb
-3.158073e-15  2.296342e-15 -5.644275e-15  5.540146e-15  2.220498e-15
6.391095e-16
E:\\R-3.1.2\\scripts\\mPDBS\\summary_pdb_93\\12f06f3e342df706.final.pdb
-6.268781e-15  2.706782e-15 -4.205089e-15  1.898885e-15 -1.563087e-15
-1.021345e-15
E:\\R-3.1.2\\scripts\\mPDBS\\summary_pdb_93\\18618a2a251b867b.final.pdb
9.825690e-16  1.296100e-15 -3.078516e-15  7.039532e-15  7.269907e-15
3.731576e-15
E:\\R-3.1.2\\scripts\\mPDBS\\summary_pdb_93\\1f305f598641eeb2.final.pdb
-5.298314e-15  1.645939e-15 -3.869340e-15  2.981852e-15 -3.843322e-16
-8.440275e-16
E:\\R-3.1.2\\scripts\\mPDBS\\summary_pdb_93\\1f8a00b551be0a39.final.pdb
9.825690e-16  1.296100e-15 -3.078516e-15  7.039532e-15  7.269907e-15
3.731576e-15
```

E:\\R-3.1.2\\scripts\\mPDBS\\summary_pdb_93\\219f45328d47579a.final.pdb
-9.145890e-15 -9.573316e-16 7.823710e-16 1.081745e-15 -1.621068e-14
-3.380571e-15
E:\\R-3.1.2\\scripts\\mPDBS\\summary_pdb_93\\238dd3918e54336d.final.pdb
-5.318656e-15 -6.295943e-15 -2.558923e-15 2.898868e-15 -4.445347e-15
7.767088e-15
E:\\R-3.1.2\\scripts\\mPDBS\\summary_pdb_93\\2b3027889e2fdc42.final.pdb
-4.322925e-15 2.156063e-15 -2.444865e-15 1.883188e-15 -1.075646e-15
-1.093555e-15
E:\\R-3.1.2\\scripts\\mPDBS\\summary_pdb_93\\2fcb3b46e33793b0.final.pdb
-3.744002e-15 1.704919e-15 3.031830e-17 8.636183e-15 -5.182577e-15
3.093795e-15
E:\\R-3.1.2\\scripts\\mPDBS\\summary_pdb_93\\34eb328251842006.final.pdb
-1.396104e-14 2.520000e-15 -4.604435e-15 -2.073052e-14 -6.784401e-15
-6.747281e-15
E:\\R-3.1.2\\scripts\\mPDBS\\summary_pdb_93\\354b32871797b7ab.final.pdb
-3.387075e-15 4.057746e-15 4.875096e-15 3.659252e-15 -3.027706e-15
1.599909e-14
E:\\R-3.1.2\\scripts\\mPDBS\\summary_pdb_93\\397368adbc32d5de.final.pdb
-3.158073e-15 2.296342e-15 -5.644275e-15 5.540146e-15 2.220498e-15
6.391095e-16
E:\\R-3.1.2\\scripts\\mPDBS\\summary_pdb_93\\411ca13cd3df6edd.final.pdb
-5.298314e-15 7.616634e-16 4.542413e-16 -1.778663e-15 -3.912711e-16
6.239933e-15
E:\\R-3.1.2\\scripts\\mPDBS\\summary_pdb_93\\42357eee6642bf12.final.pdb
-1.682263e-14 3.168238e-15 7.141715e-15 1.330165e-14 -5.098489e-15
4.252354e-15
E:\\R-3.1.2\\scripts\\mPDBS\\summary_pdb_93\\44342d1f75a7fcfc.final.pdb
-2.141824e-15 -4.789253e-15 -3.397200e-16 -2.116693e-15 3.185924e-15
-4.070847e-15
E:\\R-3.1.2\\scripts\\mPDBS\\summary_pdb_93\\448ef21dffe8cdfb.final.pdb
-1.339600e-14 1.130141e-14 1.584631e-15 9.678378e-16 4.753919e-15
1.508767e-14
E:\\R-3.1.2\\scripts\\mPDBS\\summary_pdb_93\\472a40b3e8f113c7.final.pdb
-4.497279e-15 2.199631e-15 -6.073836e-15 5.463108e-16 -1.346093e-15
-3.365283e-15
E:\\R-3.1.2\\scripts\\mPDBS\\summary_pdb_93\\48a26225951ea153.final.pdb
-6.716843e-15 -5.357024e-15 -3.799684e-15 1.480298e-15 -1.411315e-15
1.362814e-15
E:\\R-3.1.2\\scripts\\mPDBS\\summary_pdb_93\\4ab4a5c7c6682ce6.final.pdb
-4.146865e-15 3.396157e-15 5.048560e-15 1.879170e-14 -2.227333e-15
1.511364e-14
E:\\R-3.1.2\\scripts\\mPDBS\\summary_pdb_93\\4eaa45b62cb6a25f.final.pdb
1.284102e-15 -6.222254e-17 -5.774155e-15 7.147781e-15 4.996691e-15
-3.735451e-15
E:\\R-3.1.2\\scripts\\mPDBS\\summary_pdb_93\\58c371611d85ac96.final.pdb
-3.158073e-15 2.296342e-15 -5.644275e-15 5.540146e-15 2.220498e-15
6.391095e-16
E:\\R-3.1.2\\scripts\\mPDBS\\summary_pdb_93\\59a647f04822d30a.final.pdb
-8.874285e-15 1.011662e-15 -2.768333e-15 8.731834e-15 -4.474615e-15
-1.078449e-15
E:\\R-3.1.2\\scripts\\mPDBS\\summary_pdb_93\\59b091fe385e05fc.final.pdb
-6.037265e-15 6.347589e-15 -5.533661e-16 1.202749e-16 3.802202e-16
-4.426609e-15

E:\\R-3.1.2\\scripts\\mPDBS\\summary_pdb_93\\5c35ac30f930d2db.final.pdb
1.284102e-15 -6.222254e-17 -5.774155e-15 7.147781e-15 4.996691e-15
-3.735451e-15
E:\\R-3.1.2\\scripts\\mPDBS\\summary_pdb_93\\5f60752431cf2dcb.final.pdb
-3.545265e-15 3.460519e-15 -8.126431e-15 7.852022e-15 6.137784e-15
-3.916924e-15
E:\\R-3.1.2\\scripts\\mPDBS\\summary_pdb_93\\626456dd6ff2868d.final.pdb
-4.497279e-15 2.199631e-15 -6.073836e-15 5.463108e-16 -1.346093e-15
-3.365283e-15
E:\\R-3.1.2\\scripts\\mPDBS\\summary_pdb_93\\6583d53ff3c8f7ef.final.pdb
-8.874285e-15 1.011662e-15 -2.768333e-15 8.731834e-15 -4.474615e-15
-1.078449e-15
E:\\R-3.1.2\\scripts\\mPDBS\\summary_pdb_93\\69760178986bef01.final.pdb
-1.577201e-15 1.541302e-15 -4.598591e-15 5.385023e-15 9.209096e-16
-6.620116e-15
E:\\R-3.1.2\\scripts\\mPDBS\\summary_pdb_93\\6a9d87d47926b1c8.final.pdb
-5.014227e-15 3.066559e-15 -2.126256e-15 9.830275e-16 -2.262027e-15
-1.446028e-15
E:\\R-3.1.2\\scripts\\mPDBS\\summary_pdb_93\\6bb3547ea982ffc4.final.pdb
5.999092e-16 -1.029070e-14 -1.351992e-14 -4.259992e-14 6.549408e-17
3.998577e-15
E:\\R-3.1.2\\scripts\\mPDBS\\summary_pdb_93\\716d9624611d1c42.final.pdb
4.120733e-16 -7.002899e-15 -2.227721e-15 -2.646524e-15 -2.672486e-16
-3.431852e-15
E:\\R-3.1.2\\scripts\\mPDBS\\summary_pdb_93\\75f27e70ad0f1be4.final.pdb
-8.874285e-15 1.011662e-15 -2.768333e-15 8.731834e-15 -4.474615e-15
-1.078449e-15
E:\\R-3.1.2\\scripts\\mPDBS\\summary_pdb_93\\769f910fffb22872.final.pdb
-4.301399e-15 2.111854e-15 -2.776240e-15 3.179339e-15 -3.363948e-15
8.307926e-16
E:\\R-3.1.2\\scripts\\mPDBS\\summary_pdb_93\\791f8c80dd5c3135.final.pdb
-6.037265e-15 6.347589e-15 -5.533661e-16 1.202749e-16 3.802202e-16
-4.426609e-15
E:\\R-3.1.2\\scripts\\mPDBS\\summary_pdb_93\\7fad98e66a3f6099.final.pdb
-4.454935e-15 -1.873935e-15 -3.981770e-15 -1.144349e-14 4.208678e-15
1.302157e-15
E:\\R-3.1.2\\scripts\\mPDBS\\summary_pdb_93\\804b89c94a633196.final.pdb
-3.744002e-15 1.704919e-15 3.031830e-17 8.636183e-15 -5.182577e-15
3.093795e-15
E:\\R-3.1.2\\scripts\\mPDBS\\summary_pdb_93\\813cc65a2298feba.final.pdb
-3.744002e-15 1.704919e-15 3.031830e-17 8.636183e-15 -5.182577e-15
3.093795e-15
E:\\R-3.1.2\\scripts\\mPDBS\\summary_pdb_93\\83c0eefcfe12d3f9.final.pdb
-5.298314e-15 7.616634e-16 4.542413e-16 -1.778663e-15 -3.912711e-16
6.239933e-15
E:\\R-3.1.2\\scripts\\mPDBS\\summary_pdb_93\\86d50469ef569760.final.pdb
-4.497279e-15 2.199631e-15 -6.073836e-15 5.463108e-16 -1.346093e-15
-3.365283e-15
E:\\R-3.1.2\\scripts\\mPDBS\\summary_pdb_93\\89d0666e1402203d.final.pdb
-8.874285e-15 1.011662e-15 -2.768333e-15 8.731834e-15 -4.474615e-15
-1.078449e-15
E:\\R-3.1.2\\scripts\\mPDBS\\summary_pdb_93\\8b34852716dadc62.final.pdb
-8.726128e-15 1.854105e-15 -3.862401e-15 -4.706443e-15 3.640226e-15
-5.940645e-15

E:\\R-3.1.2\\scripts\\mPDBS\\summary_pdb_93\\8b6ef9ac3fe74395.final.pdb
-6.768603e-15 9.226284e-16 -1.135843e-14 -1.641625e-14 -5.152941e-15
-1.480414e-14
E:\\R-3.1.2\\scripts\\mPDBS\\summary_pdb_93\\98bbe0d5986c138f.final.pdb
-6.037265e-15 6.347589e-15 -5.533661e-16 1.202749e-16 3.802202e-16
-4.426609e-15
E:\\R-3.1.2\\scripts\\mPDBS\\summary_pdb_93\\9c22f4909d67e2fa.final.pdb
-4.042374e-15 3.080555e-15 -5.260155e-15 8.640086e-15 1.622743e-15
-8.739079e-15
E:\\R-3.1.2\\scripts\\mPDBS\\summary_pdb_93\\9dda8ed0d81fb3ef.final.pdb
1.009665e-14 -1.653307e-15 -6.590579e-15 5.330041e-15 1.339130e-14
-3.565392e-15
E:\\R-3.1.2\\scripts\\mPDBS\\summary_pdb_93\\9f752b3616e6033b.final.pdb
-8.393860e-15 4.511648e-15 -2.956773e-15 1.004701e-15 -5.500918e-15
-2.823002e-16
E:\\R-3.1.2\\scripts\\mPDBS\\summary_pdb_93\\a02c70083816d6ba.final.pdb
1.284102e-15 -6.222254e-17 -5.774155e-15 7.147781e-15 4.996691e-15
-3.735451e-15
E:\\R-3.1.2\\scripts\\mPDBS\\summary_pdb_93\\a0c997d891e4e082.final.pdb
3.360614e-15 1.048411e-14 -1.165689e-16 -2.324677e-15 -9.515598e-15
5.849927e-16
E:\\R-3.1.2\\scripts\\mPDBS\\summary_pdb_93\\ab8039a2d823c616.final.pdb
-4.886695e-15 1.506916e-15 -2.580093e-15 -2.026766e-16 -3.332610e-15
-1.534514e-15
E:\\R-3.1.2\\scripts\\mPDBS\\summary_pdb_93\\acf4d74b9c71d4dc.final.pdb
-3.744002e-15 1.704919e-15 3.031830e-17 8.636183e-15 -5.182577e-15
3.093795e-15
E:\\R-3.1.2\\scripts\\mPDBS\\summary_pdb_93\\ad8a823e4b4c668e.final.pdb
-5.312336e-15 2.059655e-15 -3.347137e-15 3.914713e-15 -1.148138e-15
-1.034749e-15
E:\\R-3.1.2\\scripts\\mPDBS\\summary_pdb_93\\b16ababd03b7b857.final.pdb
-4.042374e-15 3.080555e-15 -5.260155e-15 8.640086e-15 1.622743e-15
-8.739079e-15
E:\\R-3.1.2\\scripts\\mPDBS\\summary_pdb_93\\b226923e697d3c63.final.pdb
1.445419e-16 1.016189e-14 4.682274e-16 7.861870e-15 2.413972e-15
7.676221e-16
E:\\R-3.1.2\\scripts\\mPDBS\\summary_pdb_93\\b28e48f6fa27a165.final.pdb
-6.037265e-15 6.347589e-15 -5.533661e-16 1.202749e-16 3.802202e-16
-4.426609e-15
E:\\R-3.1.2\\scripts\\mPDBS\\summary_pdb_93\\b42c4924e3cd1aec.final.pdb
-1.054699e-14 4.554835e-15 3.769376e-16 -7.601889e-15 -5.935685e-15
-5.287213e-15
E:\\R-3.1.2\\scripts\\mPDBS\\summary_pdb_93\\b4dd239fcabff1df.final.pdb
-6.037265e-15 6.347589e-15 -5.533661e-16 1.202749e-16 3.802202e-16
-4.426609e-15
E:\\R-3.1.2\\scripts\\mPDBS\\summary_pdb_93\\b669942ef97459a2.final.pdb
-8.726128e-15 1.854105e-15 -3.862401e-15 -4.706443e-15 3.640226e-15
-5.940645e-15
E:\\R-3.1.2\\scripts\\mPDBS\\summary_pdb_93\\b99f4d9d87c1017b.final.pdb
-6.037265e-15 6.347589e-15 -5.533661e-16 1.202749e-16 3.802202e-16
-4.426609e-15
E:\\R-3.1.2\\scripts\\mPDBS\\summary_pdb_93\\b9d2cbf3975403e7.final.pdb
-6.037265e-15 6.347589e-15 -5.533661e-16 1.202749e-16 3.802202e-16
-4.426609e-15

E:\\R-3.1.2\\scripts\\mPDBS\\summary_pdb_93\\bc88c8a505f3a288.final.pdb
-4.871531e-15 2.444895e-15 -3.004314e-15 1.256952e-15 -1.715326e-15
-1.300963e-15
E:\\R-3.1.2\\scripts\\mPDBS\\summary_pdb_93\\bdac0e64f4ef427e.final.pdb
-6.040342e-15 3.396708e-15 -1.183779e-14 3.296270e-15 2.904752e-16
-1.624067e-14
E:\\R-3.1.2\\scripts\\mPDBS\\summary_pdb_93\\ca2a1f55fc9d0681.final.pdb
-8.726128e-15 1.854105e-15 -3.862401e-15 -4.706443e-15 3.640226e-15
-5.940645e-15
E:\\R-3.1.2\\scripts\\mPDBS\\summary_pdb_93\\ce34248de3e81a3c.final.pdb
1.256038e-15 1.441518e-15 1.007169e-15 1.259348e-14 1.775091e-15
-6.173419e-15
E:\\R-3.1.2\\scripts\\mPDBS\\summary_pdb_93\\d02e697772494a61.final.pdb
-1.488392e-14 3.043808e-15 -2.383716e-15 7.689307e-15 -1.008667e-14
5.511947e-15
E:\\R-3.1.2\\scripts\\mPDBS\\summary_pdb_93\\d04221245d015047.final.pdb
-6.296592e-15 -2.642370e-16 -3.683160e-15 1.743249e-15 -7.205499e-16
-8.860528e-16
E:\\R-3.1.2\\scripts\\mPDBS\\summary_pdb_93\\d678141e4018054a.final.pdb
-3.818984e-15 -8.320089e-15 -1.098886e-14 -3.252791e-15 -3.971001e-15
7.249883e-15
E:\\R-3.1.2\\scripts\\mPDBS\\summary_pdb_93\\d71b40af0f0efe39.final.pdb
-9.476516e-15 1.066600e-14 3.266543e-16 2.118798e-14 9.039609e-15
1.624528e-14
E:\\R-3.1.2\\scripts\\mPDBS\\summary_pdb_93\\dc0809eff10197e2.final.pdb
-1.058668e-14 1.413124e-14 -5.571614e-15 1.843588e-14 -1.889041e-15
-1.242197e-14
E:\\R-3.1.2\\scripts\\mPDBS\\summary_pdb_93\\e1163918d3707839.final.pdb
-1.082628e-14 9.732117e-15 -1.277227e-14 -2.083300e-15 -1.582881e-14
-1.819222e-16
E:\\R-3.1.2\\scripts\\mPDBS\\summary_pdb_93\\e4b48b06824ef848.final.pdb
9.825690e-16 1.296100e-15 -3.078516e-15 7.039532e-15 7.269907e-15
3.731576e-15
E:\\R-3.1.2\\scripts\\mPDBS\\summary_pdb_93\\e529e19ad281fea2.final.pdb
3.079050e-16 -1.946358e-15 2.238959e-15 -2.440883e-15 -1.935899e-15
-4.849148e-17
E:\\R-3.1.2\\scripts\\mPDBS\\summary_pdb_93\\e5602082718ec3a1.final.pdb
-3.744002e-15 1.704919e-15 3.031830e-17 8.636183e-15 -5.182577e-15
3.093795e-15
E:\\R-3.1.2\\scripts\\mPDBS\\summary_pdb_93\\e5d589d996d0a960.final.pdb
-4.698862e-15 -3.011270e-16 -9.172821e-15 -1.139722e-14 -1.165088e-14
-5.885770e-15
E:\\R-3.1.2\\scripts\\mPDBS\\summary_pdb_93\\e9df9e72307a2fbd.final.pdb
1.284102e-15 -6.222254e-17 -5.774155e-15 7.147781e-15 4.996691e-15
-3.735451e-15
E:\\R-3.1.2\\scripts\\mPDBS\\summary_pdb_93\\ea00f8694090cae7.final.pdb
-3.744002e-15 1.704919e-15 3.031830e-17 8.636183e-15 -5.182577e-15
3.093795e-15
E:\\R-3.1.2\\scripts\\mPDBS\\summary_pdb_93\\eba517be32db050e.final.pdb
-6.037265e-15 6.347589e-15 -5.533661e-16 1.202749e-16 3.802202e-16
-4.426609e-15
E:\\R-3.1.2\\scripts\\mPDBS\\summary_pdb_93\\ec04b9545899c29d.final.pdb
-4.841053e-15 2.280612e-15 -3.392833e-15 5.178686e-15 6.939940e-16
-2.559903e-15

E:\\R-3.1.2\\scripts\\mPDBS\\summary_pdb_93\\ee6862cbcad52078.final.pdb
-5.263578e-15 2.469615e-15 -2.281151e-15 1.481599e-15 -1.710122e-15
-1.099409e-15
E:\\R-3.1.2\\scripts\\mPDBS\\summary_pdb_93\\ef6a42fc83b81e5d.final.pdb
-4.817045e-15 1.092704e-15 -2.687553e-15 3.178038e-15 -8.342879e-16
-6.770074e-16
E:\\R-3.1.2\\scripts\\mPDBS\\summary_pdb_93\\ef8e88d4ad93a0ad.final.pdb
-6.296592e-15 -2.642370e-16 -3.683160e-15 1.743249e-15 -7.205499e-16
-8.860528e-16
E:\\R-3.1.2\\scripts\\mPDBS\\summary_pdb_93\\f14fcae8deec4fa3.final.pdb
-8.393860e-15 4.511648e-15 -2.956773e-15 1.004701e-15 -5.500918e-15
-2.823002e-16
E:\\R-3.1.2\\scripts\\mPDBS\\summary_pdb_93\\f83584e22003fb92.final.pdb
-6.716843e-15 -5.357024e-15 -3.799684e-15 1.480298e-15 -1.411315e-15
1.362814e-15
E:\\R-3.1.2\\scripts\\mPDBS\\summary_pdb_93\\f8e2bf87e52caa29.final.pdb
3.079050e-16 -1.946358e-15 2.238959e-15 -2.440883e-15 -1.935899e-15
-4.849148e-17

[,97]   [,98]   [,99]   [,100]   [,101]   [,102]
E:\\R-3.1.2\\scripts\\40.1ns_data\\NAT0.pdb
-5.461351e-16 6.921627e-15 6.715626e-16 -1.626108e-16 -2.161749e-15
2.375584e-15
E:\\R-3.1.2\\scripts\\40.1ns_data\\LJP6.pdb
-2.841306e-16 5.401011e-15 3.654806e-15 -7.173433e-16 -3.388505e-15
5.853360e-15
E:\\R-3.1.2\\scripts\\40.1ns_data\\3WY9.pdb
3.458788e-16 4.884878e-15 2.676235e-15 1.614151e-15 -2.388286e-15
5.457615e-15
E:\\R-3.1.2\\scripts\\40.1ns_data\\ZSP9.pdb
-1.275494e-15 3.158931e-15 1.843600e-15 -3.152105e-16 -3.184814e-15
3.302489e-15
E:\\R-3.1.2\\scripts\\mPDBS\\summary_pdb_93\\013d855ad26c877c.final.pdb
-4.145285e-15 3.517140e-15 2.113609e-15 1.156713e-15 3.379748e-15
-2.272541e-15
E:\\R-3.1.2\\scripts\\mPDBS\\summary_pdb_93\\0a2a705342ca0d54.final.pdb
2.386486e-15 5.505293e-15 1.530603e-15 -1.129040e-15 -4.936490e-15
3.614021e-15
E:\\R-3.1.2\\scripts\\mPDBS\\summary_pdb_93\\0a59fceadb4194d9.final.pdb
2.787754e-15 7.298413e-15 1.314912e-15 2.543175e-15 -3.282728e-15
1.067992e-14
E:\\R-3.1.2\\scripts\\mPDBS\\summary_pdb_93\\0dba59f3306825e8.final.pdb
1.167019e-14 3.921330e-15 1.587988e-15 5.819650e-15 -4.454264e-15
1.111259e-14
E:\\R-3.1.2\\scripts\\mPDBS\\summary_pdb_93\\126f26a78b57339c.final.pdb
-3.218075e-15 4.175901e-15 -1.590025e-15 -6.324282e-15 -1.157704e-14
-4.987858e-16
E:\\R-3.1.2\\scripts\\mPDBS\\summary_pdb_93\\12f06f3e342df706.final.pdb
7.155269e-16 4.599550e-15 2.168330e-15 -3.961193e-16 -4.448599e-15
4.409392e-15
E:\\R-3.1.2\\scripts\\mPDBS\\summary_pdb_93\\18618a2a251b867b.final.pdb
4.169171e-15 2.212146e-15 4.559937e-15 6.385902e-16 -1.537484e-15
4.265986e-15
E:\\R-3.1.2\\scripts\\mPDBS\\summary_pdb_93\\1f305f598641eeb2.final.pdb

8.750204e-16 4.980489e-15 9.898742e-16 -6.801687e-16 -4.588908e-15
5.504236e-15
E:\\R-3.1.2\\scripts\\mPDBS\\summary_pdb_93\\1f8a00b551be0a39.final.pdb
4.169171e-15 2.212146e-15 4.559937e-15 6.385902e-16 -1.537484e-15
4.265986e-15
E:\\R-3.1.2\\scripts\\mPDBS\\summary_pdb_93\\219f45328d47579a.final.pdb
-1.848343e-14 7.606326e-15 -2.630716e-15 -8.010883e-15 -6.800747e-15
9.163769e-15
E:\\R-3.1.2\\scripts\\mPDBS\\summary_pdb_93\\238dd3918e54336d.final.pdb
7.295046e-16 -4.050605e-16 8.926155e-16 2.642054e-15 -8.824735e-15
-7.316971e-15
E:\\R-3.1.2\\scripts\\mPDBS\\summary_pdb_93\\2b3027889e2fdc42.final.pdb
-4.418204e-17 5.118082e-15 1.466809e-15 -5.628473e-16 -3.535564e-15
5.376000e-15
E:\\R-3.1.2\\scripts\\mPDBS\\summary_pdb_93\\2fcb3b46e33793b0.final.pdb
5.922198e-15 5.058551e-15 4.761163e-15 5.432997e-15 -3.971346e-15
8.904294e-15
E:\\R-3.1.2\\scripts\\mPDBS\\summary_pdb_93\\34eb328251842006.final.pdb
1.816577e-14 -9.176344e-16 -2.397358e-15 -1.195731e-15 9.695183e-15
4.930516e-15
E:\\R-3.1.2\\scripts\\mPDBS\\summary_pdb_93\\354b32871797b7ab.final.pdb
1.344310e-14 1.180248e-14 1.475442e-14 3.058296e-15 3.672336e-15
-9.043054e-15
E:\\R-3.1.2\\scripts\\mPDBS\\summary_pdb_93\\397368adbc32d5de.final.pdb
-3.218075e-15 4.175901e-15 -1.590025e-15 -6.324282e-15 -1.157704e-14
-4.987858e-16
E:\\R-3.1.2\\scripts\\mPDBS\\summary_pdb_93\\411ca13cd3df6edd.final.pdb
1.048626e-14 1.195756e-15 4.528710e-15 3.407707e-15 -4.384210e-15
5.492960e-15
E:\\R-3.1.2\\scripts\\mPDBS\\summary_pdb_93\\42357eee6642bf12.final.pdb
2.166129e-15 7.971464e-15 1.889200e-15 1.169747e-15 -5.838665e-15
9.475956e-15
E:\\R-3.1.2\\scripts\\mPDBS\\summary_pdb_93\\44342d1f75a7fcfc.final.pdb
-1.816234e-15 8.127996e-16 4.456111e-15 -4.985075e-15 -7.591310e-15
1.174615e-14
E:\\R-3.1.2\\scripts\\mPDBS\\summary_pdb_93\\448ef21dffe8cdfb.final.pdb
-2.017411e-14 6.763785e-15 2.497349e-15 -1.365590e-14 4.771660e-15
-2.951636e-14
E:\\R-3.1.2\\scripts\\mPDBS\\summary_pdb_93\\472a40b3e8f113c7.final.pdb
1.167019e-14 3.921330e-15 1.587988e-15 5.819650e-15 -4.454264e-15
1.111259e-14
E:\\R-3.1.2\\scripts\\mPDBS\\summary_pdb_93\\48a26225951ea153.final.pdb
1.740848e-15 3.100563e-17 1.022196e-14 3.871106e-15 1.155129e-15
3.211065e-15
E:\\R-3.1.2\\scripts\\mPDBS\\summary_pdb_93\\4ab4a5c7c6682ce6.final.pdb
-9.871607e-15 5.270511e-15 6.276946e-15 -9.918629e-15 -5.207134e-15
1.584231e-14
E:\\R-3.1.2\\scripts\\mPDBS\\summary_pdb_93\\4eaa45b62cb6a25f.final.pdb
-4.960152e-15 2.052901e-15 3.951828e-17 -2.241284e-15 -8.150238e-15
7.704834e-15
E:\\R-3.1.2\\scripts\\mPDBS\\summary_pdb_93\\58c371611d85ac96.final.pdb
-3.218075e-15 4.175901e-15 -1.590025e-15 -6.324282e-15 -1.157704e-14
-4.987858e-16
E:\\R-3.1.2\\scripts\\mPDBS\\summary_pdb_93\\59a647f04822d30a.final.pdb

-6.902443e-15 9.764840e-15 3.627780e-15 -4.723131e-15 -8.351119e-15
3.490171e-16
E:\\R-3.1.2\\scripts\\mPDBS\\summary_pdb_93\\59b091fe385e05fc.final.pdb
2.787754e-15 7.298413e-15 1.314912e-15 2.543175e-15 -3.282728e-15
1.067992e-14
E:\\R-3.1.2\\scripts\\mPDBS\\summary_pdb_93\\5c35ac30f930d2db.final.pdb
-4.960152e-15 2.052901e-15 3.951828e-17 -2.241284e-15 -8.150238e-15
7.704834e-15
E:\\R-3.1.2\\scripts\\mPDBS\\summary_pdb_93\\5f60752431cf2dcb.final.pdb
-1.136304e-15 3.707282e-16 3.156147e-15 3.834871e-15 -3.621135e-15
9.009879e-15
E:\\R-3.1.2\\scripts\\mPDBS\\summary_pdb_93\\626456dd6ff2868d.final.pdb
1.167019e-14 3.921330e-15 1.587988e-15 5.819650e-15 -4.454264e-15
1.111259e-14
E:\\R-3.1.2\\scripts\\mPDBS\\summary_pdb_93\\6583d53ff3c8f7ef.final.pdb
-6.902443e-15 9.764840e-15 3.627780e-15 -4.723131e-15 -8.351119e-15
3.490171e-16
E:\\R-3.1.2\\scripts\\mPDBS\\summary_pdb_93\\69760178986bef01.final.pdb
-7.308414e-15 4.774265e-15 2.228819e-16 7.138032e-15 2.052774e-15
1.479971e-14
E:\\R-3.1.2\\scripts\\mPDBS\\summary_pdb_93\\6a9d87d47926b1c8.final.pdb
1.665171e-15 4.975608e-15 1.140429e-15 -5.164275e-16 -4.554878e-15
5.593563e-15
E:\\R-3.1.2\\scripts\\mPDBS\\summary_pdb_93\\6bb3547ea982ffc4.final.pdb
1.804477e-14 -1.651836e-14 1.946763e-15 -1.161144e-14 3.974464e-15
-1.694573e-14
E:\\R-3.1.2\\scripts\\mPDBS\\summary_pdb_93\\716d9624611d1c42.final.pdb
-7.238734e-16 1.100176e-14 1.668513e-15 2.291835e-15 8.864970e-15
1.983381e-15
E:\\R-3.1.2\\scripts\\mPDBS\\summary_pdb_93\\75f27e70ad0f1be4.final.pdb
-6.902443e-15 9.764840e-15 3.627780e-15 -4.723131e-15 -8.351119e-15
3.490171e-16
E:\\R-3.1.2\\scripts\\mPDBS\\summary_pdb_93\\769f910fffb22872.final.pdb
-2.069328e-15 4.087598e-15 2.241479e-15 -3.666820e-16 -3.995587e-15
4.243435e-15
E:\\R-3.1.2\\scripts\\mPDBS\\summary_pdb_93\\791f8c80dd5c3135.final.pdb
2.787754e-15 7.298413e-15 1.314912e-15 2.543175e-15 -3.282728e-15
1.067992e-14
E:\\R-3.1.2\\scripts\\mPDBS\\summary_pdb_93\\7fad98e66a3f6099.final.pdb
2.550572e-14 -2.101817e-15 -2.297903e-15 1.759550e-15 6.706224e-15
6.256732e-15
E:\\R-3.1.2\\scripts\\mPDBS\\summary_pdb_93\\804b89c94a633196.final.pdb
5.922198e-15 5.058551e-15 4.761163e-15 5.432997e-15 -3.971346e-15
8.904294e-15
E:\\R-3.1.2\\scripts\\mPDBS\\summary_pdb_93\\813cc65a2298feba.final.pdb
5.922198e-15 5.058551e-15 4.761163e-15 5.432997e-15 -3.971346e-15
8.904294e-15
E:\\R-3.1.2\\scripts\\mPDBS\\summary_pdb_93\\83c0eefcfe12d3f9.final.pdb
1.048626e-14 1.195756e-15 4.528710e-15 3.407707e-15 -4.384210e-15
5.492960e-15
E:\\R-3.1.2\\scripts\\mPDBS\\summary_pdb_93\\86d50469ef569760.final.pdb
1.167019e-14 3.921330e-15 1.587988e-15 5.819650e-15 -4.454264e-15
1.111259e-14
E:\\R-3.1.2\\scripts\\mPDBS\\summary_pdb_93\\89d0666e1402203d.final.pdb

-6.902443e-15 9.764840e-15 3.627780e-15 -4.723131e-15 -8.351119e-15
3.490171e-16
E:\\R-3.1.2\\scripts\\mPDBS\\summary_pdb_93\\8b34852716dadc62.final.pdb
-4.166086e-15 7.627677e-15 -1.896706e-15 -3.844304e-15 -8.953472e-15
1.922243e-14
E:\\R-3.1.2\\scripts\\mPDBS\\summary_pdb_93\\8b6ef9ac3fe74395.final.pdb
5.519443e-15 2.647249e-15 -4.321207e-16 -2.654867e-15 2.937327e-15
3.752378e-15
E:\\R-3.1.2\\scripts\\mPDBS\\summary_pdb_93\\98bbe0d5986c138f.final.pdb
2.787754e-15 7.298413e-15 1.314912e-15 2.543175e-15 -3.282728e-15
1.067992e-14
E:\\R-3.1.2\\scripts\\mPDBS\\summary_pdb_93\\9c22f4909d67e2fa.final.pdb
-5.772440e-15 8.791893e-15 -1.698947e-15 3.334849e-15 -7.570898e-15
7.597671e-16
E:\\R-3.1.2\\scripts\\mPDBS\\summary_pdb_93\\9dda8ed0d81fb3ef.final.pdb
7.163129e-15 -9.015409e-16 4.597599e-15 3.404916e-15 -8.662936e-15
1.986899e-14
E:\\R-3.1.2\\scripts\\mPDBS\\summary_pdb_93\\9f752b3616e6033b.final.pdb
1.081148e-15 5.171179e-15 1.054098e-15 -1.636721e-15 -6.809780e-15
3.524342e-15
E:\\R-3.1.2\\scripts\\mPDBS\\summary_pdb_93\\a02c70083816d6ba.final.pdb
-4.960152e-15 2.052901e-15 3.951828e-17 -2.241284e-15 -8.150238e-15
7.704834e-15
E:\\R-3.1.2\\scripts\\mPDBS\\summary_pdb_93\\a0c997d891e4e082.final.pdb
-2.967890e-15 3.369530e-16 -9.487210e-16 -8.023879e-15 -1.241016e-14
3.373115e-17
E:\\R-3.1.2\\scripts\\mPDBS\\summary_pdb_93\\ab8039a2d823c616.final.pdb
1.376986e-16 6.149578e-15 4.181832e-15 5.109604e-16 -4.733576e-15
5.327240e-15
E:\\R-3.1.2\\scripts\\mPDBS\\summary_pdb_93\\acf4d74b9c71d4dc.final.pdb
5.922198e-15 5.058551e-15 4.761163e-15 5.432997e-15 -3.971346e-15
8.904294e-15
E:\\R-3.1.2\\scripts\\mPDBS\\summary_pdb_93\\ad8a823e4b4c668e.final.pdb
3.527464e-15 4.714100e-15 1.745962e-15 -1.246457e-15 -3.669806e-15
5.696701e-15
E:\\R-3.1.2\\scripts\\mPDBS\\summary_pdb_93\\b16ababd03b7b857.final.pdb
-5.772440e-15 8.791893e-15 -1.698947e-15 3.334849e-15 -7.570898e-15
7.597671e-16
E:\\R-3.1.2\\scripts\\mPDBS\\summary_pdb_93\\b226923e697d3c63.final.pdb
-1.882587e-15 1.116888e-14 -5.728525e-16 9.103832e-16 3.172649e-15
6.231464e-15
E:\\R-3.1.2\\scripts\\mPDBS\\summary_pdb_93\\b28e48f6fa27a165.final.pdb
2.787754e-15 7.298413e-15 1.314912e-15 2.543175e-15 -3.282728e-15
1.067992e-14
E:\\R-3.1.2\\scripts\\mPDBS\\summary_pdb_93\\b42c4924e3cd1aec.final.pdb
-4.251352e-15 2.793420e-15 -4.532650e-16 4.543762e-15 6.447373e-15
1.813991e-15
E:\\R-3.1.2\\scripts\\mPDBS\\summary_pdb_93\\b4dd239fcabff1df.final.pdb
2.787754e-15 7.298413e-15 1.314912e-15 2.543175e-15 -3.282728e-15
1.067992e-14
E:\\R-3.1.2\\scripts\\mPDBS\\summary_pdb_93\\b669942ef97459a2.final.pdb
-4.166086e-15 7.627677e-15 -1.896706e-15 -3.844304e-15 -8.953472e-15
1.922243e-14
E:\\R-3.1.2\\scripts\\mPDBS\\summary_pdb_93\\b99f4d9d87c1017b.final.pdb

2.787754e-15 7.298413e-15 1.314912e-15 2.543175e-15 -3.282728e-15
1.067992e-14
E:\\R-3.1.2\\scripts\\mPDBS\\summary_pdb_93\\b9d2cbf3975403e7.final.pdb
2.787754e-15 7.298413e-15 1.314912e-15 2.543175e-15 -3.282728e-15
1.067992e-14
E:\\R-3.1.2\\scripts\\mPDBS\\summary_pdb_93\\bc88c8a505f3a288.final.pdb
1.295038e-15 5.117215e-15 2.312270e-15 -8.560155e-16 -4.277158e-15
5.234620e-15
E:\\R-3.1.2\\scripts\\mPDBS\\summary_pdb_93\\bdac0e64f4ef427e.final.pdb
-1.694926e-14 8.637936e-15 -4.058388e-15 -1.000171e-14 -3.953132e-15
5.203261e-15
E:\\R-3.1.2\\scripts\\mPDBS\\summary_pdb_93\\ca2a1f55fc9d0681.final.pdb
-4.166086e-15 7.627677e-15 -1.896706e-15 -3.844304e-15 -8.953472e-15
1.922243e-14
E:\\R-3.1.2\\scripts\\mPDBS\\summary_pdb_93\\ce34248de3e81a3c.final.pdb
-2.613611e-15 5.686525e-15 5.348988e-16 1.764027e-16 -7.327850e-15
1.110829e-14
E:\\R-3.1.2\\scripts\\mPDBS\\summary_pdb_93\\d02e697772494a61.final.pdb
-5.002455e-15 1.252730e-14 3.357693e-16 -8.283234e-15 -9.327805e-15
-7.011660e-15
E:\\R-3.1.2\\scripts\\mPDBS\\summary_pdb_93\\d04221245d015047.final.pdb
3.957387e-16 3.110776e-15 2.987367e-15 -6.148752e-16 -6.751493e-15
5.352393e-15
E:\\R-3.1.2\\scripts\\mPDBS\\summary_pdb_93\\d678141e4018054a.final.pdb
-9.575870e-16 2.118899e-15 1.162212e-15 3.569292e-15 1.275841e-14
-1.116651e-14
E:\\R-3.1.2\\scripts\\mPDBS\\summary_pdb_93\\d71b40af0f0efe39.final.pdb
3.133597e-15 1.921347e-14 8.439037e-15 4.189051e-15 -4.722073e-15
1.723932e-15
E:\\R-3.1.2\\scripts\\mPDBS\\summary_pdb_93\\dc0809eff10197e2.final.pdb
2.683359e-15 2.169417e-15 -4.769239e-15 -1.186053e-14 -1.295384e-14
9.586238e-15
E:\\R-3.1.2\\scripts\\mPDBS\\summary_pdb_93\\e1163918d3707839.final.pdb
2.099668e-14 7.859680e-15 -2.076966e-15 1.298493e-14 3.807124e-15
-1.844104e-16
E:\\R-3.1.2\\scripts\\mPDBS\\summary_pdb_93\\e4b48b06824ef848.final.pdb
4.169171e-15 2.212146e-15 4.559937e-15 6.385902e-16 -1.537484e-15
4.265986e-15
E:\\R-3.1.2\\scripts\\mPDBS\\summary_pdb_93\\e529e19ad281fea2.final.pdb
-4.145285e-15 3.517140e-15 2.113609e-15 1.156713e-15 3.379748e-15
-2.272541e-15
E:\\R-3.1.2\\scripts\\mPDBS\\summary_pdb_93\\e5602082718ec3a1.final.pdb
5.922198e-15 5.058551e-15 4.761163e-15 5.432997e-15 -3.971346e-15
8.904294e-15
E:\\R-3.1.2\\scripts\\mPDBS\\summary_pdb_93\\e5d589d996d0a960.final.pdb
-1.940199e-14 -5.510259e-15 -1.783184e-15 -4.128731e-15 -8.653897e-15
3.445544e-15
E:\\R-3.1.2\\scripts\\mPDBS\\summary_pdb_93\\e9df9e72307a2fbd.final.pdb
-4.960152e-15 2.052901e-15 3.951828e-17 -2.241284e-15 -8.150238e-15
7.704834e-15
E:\\R-3.1.2\\scripts\\mPDBS\\summary_pdb_93\\ea00f8694090cae7.final.pdb
5.922198e-15 5.058551e-15 4.761163e-15 5.432997e-15 -3.971346e-15
8.904294e-15
E:\\R-3.1.2\\scripts\\mPDBS\\summary_pdb_93\\eba517be32db050e.final.pdb

2.787754e-15 7.298413e-15 1.314912e-15 2.543175e-15 -3.282728e-15
1.067992e-14
E:\\R-3.1.2\\scripts\\mPDBS\\summary_pdb_93\\ec04b9545899c29d.final.pdb
6.097771e-16 4.288318e-15 1.781167e-15 -1.140927e-15 -2.976946e-15
5.393593e-15
E:\\R-3.1.2\\scripts\\mPDBS\\summary_pdb_93\\ee6862cbcad52078.final.pdb
1.449028e-16 3.805547e-15 2.549168e-15 -3.954464e-16 -4.198662e-15
4.996963e-15
E:\\R-3.1.2\\scripts\\mPDBS\\summary_pdb_93\\ef6a42fc83b81e5d.final.pdb
5.969420e-16 4.103645e-15 2.128614e-15 -6.390336e-16 -3.716730e-15
5.378811e-15
E:\\R-3.1.2\\scripts\\mPDBS\\summary_pdb_93\\ef8e88d4ad93a0ad.final.pdb
3.957387e-16 3.110776e-15 2.987367e-15 -6.148752e-16 -6.751493e-15
5.352393e-15
E:\\R-3.1.2\\scripts\\mPDBS\\summary_pdb_93\\f14fcae8deec4fa3.final.pdb
1.081148e-15 5.171179e-15 1.054098e-15 -1.636721e-15 -6.809780e-15
3.524342e-15
E:\\R-3.1.2\\scripts\\mPDBS\\summary_pdb_93\\f83584e22003fb92.final.pdb
1.740848e-15 3.100563e-17 1.022196e-14 3.871106e-15 1.155129e-15
3.211065e-15
E:\\R-3.1.2\\scripts\\mPDBS\\summary_pdb_93\\f8e2bf87e52caa29.final.pdb
-4.145285e-15 3.517140e-15 2.113609e-15 1.156713e-15 3.379748e-15
-2.272541e-15

[,103]   [,104]   [,105]   [,106]   [,107]   [,108]
E:\\R-3.1.2\\scripts\\40.1ns_data\\NAT0.pdb
2.623570e-15 -5.335196e-15 1.454719e-15 6.613645e-16 2.335475e-15
-3.196789e-16
E:\\R-3.1.2\\scripts\\40.1ns_data\\LJP6.pdb
3.421912e-15 -7.343211e-16 3.917184e-15 2.985442e-15 8.607160e-16
9.909457e-16
E:\\R-3.1.2\\scripts\\40.1ns_data\\3WY9.pdb
-2.435572e-15 -4.701071e-16 1.735494e-15 2.024626e-15 5.914866e-15
2.187279e-15
E:\\R-3.1.2\\scripts\\40.1ns_data\\ZSP9.pdb
-7.749256e-16 -2.389095e-15 2.629972e-15 9.394176e-16 4.856879e-15
-1.350604e-15
E:\\R-3.1.2\\scripts\\mPDBS\\summary_pdb_93\\013d855ad26c877c.final.pdb
5.742297e-15 7.194734e-15 6.065433e-15 2.787198e-15 3.336287e-15
7.062835e-16
E:\\R-3.1.2\\scripts\\mPDBS\\summary_pdb_93\\0a2a705342ca0d54.final.pdb
-8.314984e-16 2.367243e-15 1.482606e-15 5.149478e-16 4.326284e-15
1.003023e-16
E:\\R-3.1.2\\scripts\\mPDBS\\summary_pdb_93\\0a59fceadb4194d9.final.pdb
-4.582408e-15 -7.639936e-17 7.672088e-15 -1.119113e-15 8.221024e-15
-2.696610e-16
E:\\R-3.1.2\\scripts\\mPDBS\\summary_pdb_93\\0dba59f3306825e8.final.pdb
-6.534341e-15 -4.219746e-15 5.160529e-16 -1.036133e-15 6.398074e-15
3.732166e-16
E:\\R-3.1.2\\scripts\\mPDBS\\summary_pdb_93\\126f26a78b57339c.final.pdb
7.948648e-15 1.062775e-14 -6.718178e-15 8.502678e-15 -2.723969e-15
7.201613e-16
E:\\R-3.1.2\\scripts\\mPDBS\\summary_pdb_93\\12f06f3e342df706.final.pdb
2.422955e-16 1.090487e-15 2.115997e-15 1.701932e-15 6.368487e-15

```
7.126597e-16
E:\\R-3.1.2\\scripts\\mPDBS\\summary_pdb_93\\18618a2a251b867b.final.pdb
8.000295e-15  1.137126e-14  4.391068e-15  1.389098e-15 -9.850821e-15
-2.866858e-15
E:\\R-3.1.2\\scripts\\mPDBS\\summary_pdb_93\\1f305f598641eeb2.final.pdb
-1.908056e-16  1.700333e-15  2.039423e-15  2.093065e-15  3.741223e-15
1.007356e-15
E:\\R-3.1.2\\scripts\\mPDBS\\summary_pdb_93\\1f8a00b551be0a39.final.pdb
8.000295e-15  1.137126e-14  4.391068e-15  1.389098e-15 -9.850821e-15
-2.866858e-15
E:\\R-3.1.2\\scripts\\mPDBS\\summary_pdb_93\\219f45328d47579a.final.pdb
4.669740e-15  1.394364e-14  8.685167e-15  3.183002e-15 -1.541068e-14
4.637872e-15
E:\\R-3.1.2\\scripts\\mPDBS\\summary_pdb_93\\238dd3918e54336d.final.pdb
4.370500e-15  5.989061e-15 -5.604835e-15  1.115449e-14 -4.147338e-15
3.335094e-15
E:\\R-3.1.2\\scripts\\mPDBS\\summary_pdb_93\\2b3027889e2fdc42.final.pdb
-5.563313e-16  2.798037e-15  9.801754e-16  1.020669e-15  4.423280e-15
1.260365e-15
E:\\R-3.1.2\\scripts\\mPDBS\\summary_pdb_93\\2fcb3b46e33793b0.final.pdb
-4.909128e-16 -1.626866e-15  9.394651e-15  9.880189e-19  9.463208e-15
6.326556e-16
E:\\R-3.1.2\\scripts\\mPDBS\\summary_pdb_93\\34eb328251842006.final.pdb
-7.581936e-15 -1.379723e-14  1.552046e-15 -1.377130e-14  1.229622e-14
1.309440e-14
E:\\R-3.1.2\\scripts\\mPDBS\\summary_pdb_93\\354b32871797b7ab.final.pdb
-4.617472e-15 -4.026963e-15 -2.972612e-15 -1.429940e-14  8.106932e-15
9.353355e-15
E:\\R-3.1.2\\scripts\\mPDBS\\summary_pdb_93\\397368adbc32d5de.final.pdb
7.948648e-15  1.062775e-14 -6.718178e-15  8.502678e-15 -2.723969e-15
7.201613e-16
E:\\R-3.1.2\\scripts\\mPDBS\\summary_pdb_93\\411ca13cd3df6edd.final.pdb
2.989159e-15 -4.404158e-15  8.873800e-15 -2.199942e-15  6.694589e-15
7.738083e-15
E:\\R-3.1.2\\scripts\\mPDBS\\summary_pdb_93\\42357eee6642bf12.final.pdb
-6.154765e-16  6.951048e-17  1.598100e-14 -8.400126e-15 -6.615435e-15
-9.476884e-16
E:\\R-3.1.2\\scripts\\mPDBS\\summary_pdb_93\\44342d1f75a7fcfc.final.pdb
1.663566e-14  8.154483e-15  7.662882e-15  2.021484e-15  1.629662e-14
-6.222545e-15
E:\\R-3.1.2\\scripts\\mPDBS\\summary_pdb_93\\448ef21dffe8cdfb.final.pdb
2.928227e-15  1.131417e-14 -1.258776e-14  3.800032e-15 -5.107602e-15
3.824547e-15
E:\\R-3.1.2\\scripts\\mPDBS\\summary_pdb_93\\472a40b3e8f113c7.final.pdb
-6.534341e-15 -4.219746e-15  5.160529e-16 -1.036133e-15  6.398074e-15
3.732166e-16
E:\\R-3.1.2\\scripts\\mPDBS\\summary_pdb_93\\48a26225951ea153.final.pdb
6.414655e-15  4.414156e-16 -5.784737e-16  2.453550e-15  9.975046e-15
2.240484e-15
E:\\R-3.1.2\\scripts\\mPDBS\\summary_pdb_93\\4ab4a5c7c6682ce6.final.pdb
1.731399e-15  5.947468e-15 -3.976447e-15 -1.010545e-15 -2.201817e-15
-5.122387e-15
E:\\R-3.1.2\\scripts\\mPDBS\\summary_pdb_93\\4eaa45b62cb6a25f.final.pdb
4.640747e-15  7.583541e-15  5.015139e-15  1.147642e-14  1.106673e-15
```

-1.594485e-15
E:\\R-3.1.2\\scripts\\mPDBS\\summary_pdb_93\\58c371611d85ac96.final.pdb
7.948648e-15 1.062775e-14 -6.718178e-15 8.502678e-15 -2.723969e-15
7.201613e-16
E:\\R-3.1.2\\scripts\\mPDBS\\summary_pdb_93\\59a647f04822d30a.final.pdb
-7.801389e-15 -1.174860e-15 -6.379271e-15 5.226828e-16 -3.479232e-15
4.006296e-16
E:\\R-3.1.2\\scripts\\mPDBS\\summary_pdb_93\\59b091fe385e05fc.final.pdb
-4.582408e-15 -7.639936e-17 7.672088e-15 -1.119113e-15 8.221024e-15
-2.696610e-16
E:\\R-3.1.2\\scripts\\mPDBS\\summary_pdb_93\\5c35ac30f930d2db.final.pdb
4.640747e-15 7.583541e-15 5.015139e-15 1.147642e-14 1.106673e-15
-1.594485e-15
E:\\R-3.1.2\\scripts\\mPDBS\\summary_pdb_93\\5f60752431cf2dcb.final.pdb
-7.812242e-15 6.480273e-15 8.643761e-15 6.370507e-15 2.182816e-15
-1.344722e-15
E:\\R-3.1.2\\scripts\\mPDBS\\summary_pdb_93\\626456dd6ff2868d.final.pdb
-6.534341e-15 -4.219746e-15 5.160529e-16 -1.036133e-15 6.398074e-15
3.732166e-16
E:\\R-3.1.2\\scripts\\mPDBS\\summary_pdb_93\\6583d53ff3c8f7ef.final.pdb
-7.801389e-15 -1.174860e-15 -6.379271e-15 5.226828e-16 -3.479232e-15
4.006296e-16
E:\\R-3.1.2\\scripts\\mPDBS\\summary_pdb_93\\69760178986bef01.final.pdb
7.316914e-15 1.123646e-14 1.851555e-15 8.531844e-15 -1.144231e-14
-6.730314e-15
E:\\R-3.1.2\\scripts\\mPDBS\\summary_pdb_93\\6a9d87d47926b1c8.final.pdb
2.690274e-16 2.446796e-15 5.399053e-16 1.990526e-15 2.953781e-15
9.144503e-16
E:\\R-3.1.2\\scripts\\mPDBS\\summary_pdb_93\\6bb3547ea982ffc4.final.pdb
9.257268e-15 -4.070820e-15 -1.872319e-14 -1.615448e-15 1.521732e-14
2.297197e-14
E:\\R-3.1.2\\scripts\\mPDBS\\summary_pdb_93\\716d9624611d1c42.final.pdb
7.703341e-15 1.643864e-15 4.066477e-15 1.030161e-15 2.814473e-15
4.626196e-15
E:\\R-3.1.2\\scripts\\mPDBS\\summary_pdb_93\\75f27e70ad0f1be4.final.pdb
-7.801389e-15 -1.174860e-15 -6.379271e-15 5.226828e-16 -3.479232e-15
4.006296e-16
E:\\R-3.1.2\\scripts\\mPDBS\\summary_pdb_93\\769f910fffb22872.final.pdb
1.509177e-15 1.354968e-15 2.878823e-15 1.624587e-15 4.906038e-15
2.594052e-15
E:\\R-3.1.2\\scripts\\mPDBS\\summary_pdb_93\\791f8c80dd5c3135.final.pdb
-4.582408e-15 -7.639936e-17 7.672088e-15 -1.119113e-15 8.221024e-15
-2.696610e-16
E:\\R-3.1.2\\scripts\\mPDBS\\summary_pdb_93\\7fad98e66a3f6099.final.pdb
5.524568e-15 -9.044010e-15 2.048147e-15 4.433039e-17 1.471073e-14
3.906061e-15
E:\\R-3.1.2\\scripts\\mPDBS\\summary_pdb_93\\804b89c94a633196.final.pdb
-4.909128e-16 -1.626866e-15 9.394651e-15 9.880189e-19 9.463208e-15
6.326556e-16
E:\\R-3.1.2\\scripts\\mPDBS\\summary_pdb_93\\813cc65a2298feba.final.pdb
-4.909128e-16 -1.626866e-15 9.394651e-15 9.880189e-19 9.463208e-15
6.326556e-16
E:\\R-3.1.2\\scripts\\mPDBS\\summary_pdb_93\\83c0eefcfe12d3f9.final.pdb
2.989159e-15 -4.404158e-15 8.873800e-15 -2.199942e-15 6.694589e-15

7.738083e-15
E:\\R-3.1.2\\scripts\\mPDBS\\summary_pdb_93\\86d50469ef569760.final.pdb
-6.534341e-15 -4.219746e-15 5.160529e-16 -1.036133e-15 6.398074e-15
3.732166e-16
E:\\R-3.1.2\\scripts\\mPDBS\\summary_pdb_93\\89d0666e1402203d.final.pdb
-7.801389e-15 -1.174860e-15 -6.379271e-15 5.226828e-16 -3.479232e-15
4.006296e-16
E:\\R-3.1.2\\scripts\\mPDBS\\summary_pdb_93\\8b34852716dadc62.final.pdb
-1.644504e-15 1.218080e-15 4.426403e-15 2.370620e-15 -4.333895e-16
1.201645e-15
E:\\R-3.1.2\\scripts\\mPDBS\\summary_pdb_93\\8b6ef9ac3fe74395.final.pdb
-2.522387e-15 -4.591080e-15 -7.804498e-15 4.289953e-15 1.675012e-14
7.238422e-15
E:\\R-3.1.2\\scripts\\mPDBS\\summary_pdb_93\\98bbe0d5986c138f.final.pdb
-4.582408e-15 -7.639936e-17 7.672088e-15 -1.119113e-15 8.221024e-15
-2.696610e-16
E:\\R-3.1.2\\scripts\\mPDBS\\summary_pdb_93\\9c22f4909d67e2fa.final.pdb
-2.182051e-15 1.026206e-14 3.177104e-16 6.342703e-15 -4.423687e-15
-5.164790e-15
E:\\R-3.1.2\\scripts\\mPDBS\\summary_pdb_93\\9dda8ed0d81fb3ef.final.pdb
1.590399e-16 -6.674530e-16 1.521744e-15 2.827263e-15 2.324462e-14
-1.645047e-14
E:\\R-3.1.2\\scripts\\mPDBS\\summary_pdb_93\\9f752b3616e6033b.final.pdb
-9.051591e-16 -1.282989e-15 -2.802234e-15 -4.561309e-16 6.792433e-15
3.943505e-16
E:\\R-3.1.2\\scripts\\mPDBS\\summary_pdb_93\\a02c70083816d6ba.final.pdb
4.640747e-15 7.583541e-15 5.015139e-15 1.147642e-14 1.106673e-15
-1.594485e-15
E:\\R-3.1.2\\scripts\\mPDBS\\summary_pdb_93\\a0c997d891e4e082.final.pdb
7.917389e-15 -8.955055e-15 -9.491874e-15 -6.069323e-15 2.127900e-14
1.092787e-14
E:\\R-3.1.2\\scripts\\mPDBS\\summary_pdb_93\\ab8039a2d823c616.final.pdb
4.702213e-16 2.367486e-15 1.584986e-15 6.444909e-16 4.962838e-15
6.756521e-16
E:\\R-3.1.2\\scripts\\mPDBS\\summary_pdb_93\\acf4d74b9c71d4dc.final.pdb
-4.909128e-16 -1.626866e-15 9.394651e-15 9.880189e-19 9.463208e-15
6.326556e-16
E:\\R-3.1.2\\scripts\\mPDBS\\summary_pdb_93\\ad8a823e4b4c668e.final.pdb
-3.996872e-16 1.382526e-15 1.079856e-15 1.654955e-15 3.241488e-15
1.971137e-15
E:\\R-3.1.2\\scripts\\mPDBS\\summary_pdb_93\\b16ababd03b7b857.final.pdb
-2.182051e-15 1.026206e-14 3.177104e-16 6.342703e-15 -4.423687e-15
-5.164790e-15
E:\\R-3.1.2\\scripts\\mPDBS\\summary_pdb_93\\b226923e697d3c63.final.pdb
6.841195e-15 3.789052e-15 4.396831e-15 -4.916976e-15 -3.899469e-15
1.989636e-15
E:\\R-3.1.2\\scripts\\mPDBS\\summary_pdb_93\\b28e48f6fa27a165.final.pdb
-4.582408e-15 -7.639936e-17 7.672088e-15 -1.119113e-15 8.221024e-15
-2.696610e-16
E:\\R-3.1.2\\scripts\\mPDBS\\summary_pdb_93\\b42c4924e3cd1aec.final.pdb
-3.773361e-15 3.194041e-15 1.019449e-14 5.349985e-15 -4.731734e-16
9.105472e-15
E:\\R-3.1.2\\scripts\\mPDBS\\summary_pdb_93\\b4dd239fcabff1df.final.pdb
-4.582408e-15 -7.639936e-17 7.672088e-15 -1.119113e-15 8.221024e-15

-2.696610e-16
E:\\R-3.1.2\\scripts\\mPDBS\\summary_pdb_93\\b669942ef97459a2.final.pdb
-1.644504e-15 1.218080e-15 4.426403e-15 2.370620e-15 -4.333895e-16
1.201645e-15
E:\\R-3.1.2\\scripts\\mPDBS\\summary_pdb_93\\b99f4d9d87c1017b.final.pdb
-4.582408e-15 -7.639936e-17 7.672088e-15 -1.119113e-15 8.221024e-15
-2.696610e-16
E:\\R-3.1.2\\scripts\\mPDBS\\summary_pdb_93\\b9d2cbf3975403e7.final.pdb
-4.582408e-15 -7.639936e-17 7.672088e-15 -1.119113e-15 8.221024e-15
-2.696610e-16
E:\\R-3.1.2\\scripts\\mPDBS\\summary_pdb_93\\bc88c8a505f3a288.final.pdb
8.161330e-17 2.460200e-15 1.524879e-15 1.548458e-15 3.140886e-15
4.120854e-16
E:\\R-3.1.2\\scripts\\mPDBS\\summary_pdb_93\\bdac0e64f4ef427e.final.pdb
-6.518643e-15 1.443104e-14 -1.228766e-14 6.757736e-15 -3.102695e-15
-1.013998e-14
E:\\R-3.1.2\\scripts\\mPDBS\\summary_pdb_93\\ca2a1f55fc9d0681.final.pdb
-1.644504e-15 1.218080e-15 4.426403e-15 2.370620e-15 -4.333895e-16
1.201645e-15
E:\\R-3.1.2\\scripts\\mPDBS\\summary_pdb_93\\ce34248de3e81a3c.final.pdb
-3.588153e-16 4.869397e-15 9.017819e-15 7.802673e-15 7.221994e-15
2.666007e-15
E:\\R-3.1.2\\scripts\\mPDBS\\summary_pdb_93\\d02e697772494a61.final.pdb
4.798110e-15 3.787697e-15 -3.393063e-15 -3.101468e-15 -1.106238e-14
9.999902e-15
E:\\R-3.1.2\\scripts\\mPDBS\\summary_pdb_93\\d04221245d015047.final.pdb
-1.986147e-15 1.186139e-15 1.388311e-15 4.443102e-15 6.808367e-15
4.709547e-16
E:\\R-3.1.2\\scripts\\mPDBS\\summary_pdb_93\\d678141e4018054a.final.pdb
3.037924e-15 2.806842e-15 -3.178525e-15 -8.717980e-15 2.359430e-15
5.987606e-15
E:\\R-3.1.2\\scripts\\mPDBS\\summary_pdb_93\\d71b40af0f0efe39.final.pdb
-6.496250e-15 1.344772e-14 1.049437e-14 -4.985187e-18 -3.771628e-15
-1.455851e-14
E:\\R-3.1.2\\scripts\\mPDBS\\summary_pdb_93\\dc0809eff10197e2.final.pdb
-1.329434e-14 -6.053802e-15 -7.801980e-15 1.868159e-15 5.585121e-15
-9.998875e-15
E:\\R-3.1.2\\scripts\\mPDBS\\summary_pdb_93\\e1163918d3707839.final.pdb
3.375434e-15 -1.271137e-15 1.387358e-16 2.210135e-15 1.417049e-14
1.156878e-14
E:\\R-3.1.2\\scripts\\mPDBS\\summary_pdb_93\\e4b48b06824ef848.final.pdb
8.000295e-15 1.137126e-14 4.391068e-15 1.389098e-15 -9.850821e-15
-2.866858e-15
E:\\R-3.1.2\\scripts\\mPDBS\\summary_pdb_93\\e529e19ad281fea2.final.pdb
5.742297e-15 7.194734e-15 6.065433e-15 2.787198e-15 3.336287e-15
7.062835e-16
E:\\R-3.1.2\\scripts\\mPDBS\\summary_pdb_93\\e5602082718ec3a1.final.pdb
-4.909128e-16 -1.626866e-15 9.394651e-15 9.880189e-19 9.463208e-15
6.326556e-16
E:\\R-3.1.2\\scripts\\mPDBS\\summary_pdb_93\\e5d589d996d0a960.final.pdb
-2.849894e-15 -2.508877e-15 -1.013218e-14 7.913262e-15 2.745228e-14
7.875990e-15
E:\\R-3.1.2\\scripts\\mPDBS\\summary_pdb_93\\e9df9e72307a2fbd.final.pdb
4.640747e-15 7.583541e-15 5.015139e-15 1.147642e-14 1.106673e-15

```
-1.594485e-15
E:\\R-3.1.2\\scripts\\mPDBS\\summary_pdb_93\\ea00f8694090cae7.final.pdb
-4.909128e-16 -1.626866e-15  9.394651e-15  9.880189e-19  9.463208e-15
6.326556e-16
E:\\R-3.1.2\\scripts\\mPDBS\\summary_pdb_93\\eba517be32db050e.final.pdb
-4.582408e-15 -7.639936e-17  7.672088e-15 -1.119113e-15  8.221024e-15
-2.696610e-16
E:\\R-3.1.2\\scripts\\mPDBS\\summary_pdb_93\\ec04b9545899c29d.final.pdb
-4.383555e-16  2.164483e-15  1.409204e-15  2.250467e-15  3.613835e-15
9.878774e-17
E:\\R-3.1.2\\scripts\\mPDBS\\summary_pdb_93\\ee6862cbcad52078.final.pdb
-5.824878e-17  2.497930e-15  2.084977e-15  1.809101e-15  3.121370e-15
1.048729e-15
E:\\R-3.1.2\\scripts\\mPDBS\\summary_pdb_93\\ef6a42fc83b81e5d.final.pdb
-1.474842e-16  2.132125e-15  2.059817e-15  1.466293e-15  5.019229e-15
1.197600e-15
E:\\R-3.1.2\\scripts\\mPDBS\\summary_pdb_93\\ef8e88d4ad93a0ad.final.pdb
-1.986147e-15  1.186139e-15  1.388311e-15  4.443102e-15  6.808367e-15
4.709547e-16
E:\\R-3.1.2\\scripts\\mPDBS\\summary_pdb_93\\f14fcae8deec4fa3.final.pdb
-9.051591e-16 -1.282989e-15 -2.802234e-15 -4.561309e-16  6.792433e-15
3.943505e-16
E:\\R-3.1.2\\scripts\\mPDBS\\summary_pdb_93\\f83584e22003fb92.final.pdb
6.414655e-15  4.414156e-16 -5.784737e-16  2.453550e-15  9.975046e-15
2.240484e-15
E:\\R-3.1.2\\scripts\\mPDBS\\summary_pdb_93\\f8e2bf87e52caa29.final.pdb
5.742297e-15  7.194734e-15  6.065433e-15  2.787198e-15  3.336287e-15
7.062835e-16

[,109]        [,110]        [,111]        [,112]        [,113]        [,114]
E:\\R-3.1.2\\scripts\\40.1ns_data\\NAT0.pdb
-6.074698e-15 -9.307826e-16 -2.461554e-16 -4.225360e-16  3.338557e-15
4.008082e-15
E:\\R-3.1.2\\scripts\\40.1ns_data\\LJP6.pdb
-3.245655e-15 -1.978608e-15 -9.854429e-18 -1.477060e-15  2.539679e-15
3.629983e-15
E:\\R-3.1.2\\scripts\\40.1ns_data\\3WY9.pdb
-5.133618e-15 -2.228342e-15  4.017876e-15 -2.705175e-15  2.870743e-15
1.688058e-15
E:\\R-3.1.2\\scripts\\40.1ns_data\\ZSP9.pdb
-7.050858e-15 -3.161435e-15  2.130990e-15  1.895083e-16  2.721493e-15
4.694963e-15
E:\\R-3.1.2\\scripts\\mPDBS\\summary_pdb_93\\013d855ad26c877c.final.pdb
-3.773367e-15 -5.190773e-15 -7.164501e-16  1.388905e-15  7.778501e-15
1.523863e-15
E:\\R-3.1.2\\scripts\\mPDBS\\summary_pdb_93\\0a2a705342ca0d54.final.pdb
-4.614589e-15 -1.746599e-15 -2.368379e-15 -2.333451e-15  1.953301e-15
2.382634e-15
E:\\R-3.1.2\\scripts\\mPDBS\\summary_pdb_93\\0a59fceadb4194d9.final.pdb
-3.615441e-15 -4.060020e-15  8.430791e-16  4.568430e-16  1.943400e-15
3.538898e-15
E:\\R-3.1.2\\scripts\\mPDBS\\summary_pdb_93\\0dba59f3306825e8.final.pdb
-7.271437e-15 -4.698111e-15 -6.668286e-15 -2.358965e-15  1.252471e-15
5.923121e-15
```

E:\\R-3.1.2\\scripts\\mPDBS\\summary_pdb_93\\126f26a78b57339c.final.pdb
1.278365e-15 4.455157e-15 -9.859744e-15 1.979361e-15 6.420212e-15
1.628597e-15
E:\\R-3.1.2\\scripts\\mPDBS\\summary_pdb_93\\12f06f3e342df706.final.pdb
-4.327492e-15 -2.349416e-15 -3.420489e-15 9.263112e-16 -3.226560e-16
3.906589e-15
E:\\R-3.1.2\\scripts\\mPDBS\\summary_pdb_93\\18618a2a251b867b.final.pdb
-6.994495e-15 1.109490e-15 -1.765349e-14 -4.345055e-15 2.356482e-15
4.556887e-15
E:\\R-3.1.2\\scripts\\mPDBS\\summary_pdb_93\\1f305f598641eeb2.final.pdb
-3.992217e-15 -2.117894e-15 -2.195955e-15 1.035386e-15 2.100479e-15
2.834015e-15
E:\\R-3.1.2\\scripts\\mPDBS\\summary_pdb_93\\1f8a00b551be0a39.final.pdb
-6.994495e-15 1.109490e-15 -1.765349e-14 -4.345055e-15 2.356482e-15
4.556887e-15
E:\\R-3.1.2\\scripts\\mPDBS\\summary_pdb_93\\219f45328d47579a.final.pdb
1.302923e-14 4.294449e-16 -5.703767e-15 2.342054e-15 2.090852e-15
5.730751e-17
E:\\R-3.1.2\\scripts\\mPDBS\\summary_pdb_93\\238dd3918e54336d.final.pdb
-7.425327e-15 1.266358e-14 -8.319947e-15 -1.450883e-14 -1.110580e-15
6.713875e-15
E:\\R-3.1.2\\scripts\\mPDBS\\summary_pdb_93\\2b3027889e2fdc42.final.pdb
-3.053824e-15 -1.200328e-15 -9.634185e-16 -3.018817e-16 2.735302e-15
4.070590e-15
E:\\R-3.1.2\\scripts\\mPDBS\\summary_pdb_93\\2fcb3b46e33793b0.final.pdb
-7.799068e-15 -5.330602e-15 7.331253e-16 -3.922917e-16 8.819949e-17
2.570337e-15
E:\\R-3.1.2\\scripts\\mPDBS\\summary_pdb_93\\34eb328251842006.final.pdb
-7.949188e-15 -1.015744e-14 6.390228e-15 -4.552844e-15 4.553566e-15
2.276568e-16
E:\\R-3.1.2\\scripts\\mPDBS\\summary_pdb_93\\354b32871797b7ab.final.pdb
-4.519814e-15 -3.790991e-15 -6.916005e-15 -4.347541e-16 5.254041e-15
-5.070653e-15
E:\\R-3.1.2\\scripts\\mPDBS\\summary_pdb_93\\397368adbc32d5de.final.pdb
1.278365e-15 4.455157e-15 -9.859744e-15 1.979361e-15 6.420212e-15
1.628597e-15
E:\\R-3.1.2\\scripts\\mPDBS\\summary_pdb_93\\411ca13cd3df6edd.final.pdb
-8.073371e-15 -3.166534e-15 -3.030791e-15 1.255045e-15 5.033896e-15
-1.038538e-15
E:\\R-3.1.2\\scripts\\mPDBS\\summary_pdb_93\\42357eee6642bf12.final.pdb
4.339280e-15 -1.803465e-15 -1.728761e-15 -7.076850e-16 5.245209e-15
4.692605e-15
E:\\R-3.1.2\\scripts\\mPDBS\\summary_pdb_93\\44342d1f75a7fcfc.final.pdb
-1.461246e-15 1.198409e-15 9.297174e-15 3.805162e-15 6.579020e-15
-4.252540e-15
E:\\R-3.1.2\\scripts\\mPDBS\\summary_pdb_93\\448ef21dffe8cdfb.final.pdb
-6.444249e-15 3.176482e-15 1.798245e-15 -1.122610e-15 7.138116e-15
1.002147e-15
E:\\R-3.1.2\\scripts\\mPDBS\\summary_pdb_93\\472a40b3e8f113c7.final.pdb
-7.271437e-15 -4.698111e-15 -6.668286e-15 -2.358965e-15 1.252471e-15
5.923121e-15
E:\\R-3.1.2\\scripts\\mPDBS\\summary_pdb_93\\48a26225951ea153.final.pdb
-9.748555e-15 3.748011e-16 2.165589e-15 -7.704814e-15 3.127349e-15
-2.150128e-15

E:\\R-3.1.2\\scripts\\mPDBS\\summary_pdb_93\\4ab4a5c7c6682ce6.final.pdb
-1.693276e-14 3.913924e-15 7.178710e-15 1.261186e-15 -8.678821e-15
9.178113e-15
E:\\R-3.1.2\\scripts\\mPDBS\\summary_pdb_93\\4eaa45b62cb6a25f.final.pdb
-4.665748e-15 2.460085e-15 -1.887692e-15 1.449372e-15 -1.591639e-16
7.359942e-15
E:\\R-3.1.2\\scripts\\mPDBS\\summary_pdb_93\\58c371611d85ac96.final.pdb
1.278365e-15 4.455157e-15 -9.859744e-15 1.979361e-15 6.420212e-15
1.628597e-15
E:\\R-3.1.2\\scripts\\mPDBS\\summary_pdb_93\\59a647f04822d30a.final.pdb
-4.263692e-15 -4.252918e-16 1.772811e-15 -1.258062e-15 4.120781e-16
6.450704e-15
E:\\R-3.1.2\\scripts\\mPDBS\\summary_pdb_93\\59b091fe385e05fc.final.pdb
-3.615441e-15 -4.060020e-15 8.430791e-16 4.568430e-16 1.943400e-15
3.538898e-15
E:\\R-3.1.2\\scripts\\mPDBS\\summary_pdb_93\\5c35ac30f930d2db.final.pdb
-4.665748e-15 2.460085e-15 -1.887692e-15 1.449372e-15 -1.591639e-16
7.359942e-15
E:\\R-3.1.2\\scripts\\mPDBS\\summary_pdb_93\\5f60752431cf2dcb.final.pdb
-7.986241e-15 -4.486595e-15 1.924845e-15 1.840077e-15 -5.173810e-15
1.092793e-16
E:\\R-3.1.2\\scripts\\mPDBS\\summary_pdb_93\\626456dd6ff2868d.final.pdb
-7.271437e-15 -4.698111e-15 -6.668286e-15 -2.358965e-15 1.252471e-15
5.923121e-15
E:\\R-3.1.2\\scripts\\mPDBS\\summary_pdb_93\\6583d53ff3c8f7ef.final.pdb
-4.263692e-15 -4.252918e-16 1.772811e-15 -1.258062e-15 4.120781e-16
6.450704e-15
E:\\R-3.1.2\\scripts\\mPDBS\\summary_pdb_93\\69760178986bef01.final.pdb
-8.558235e-15 6.389366e-15 -5.745508e-15 1.691398e-16 -2.058239e-16
5.839352e-15
E:\\R-3.1.2\\scripts\\mPDBS\\summary_pdb_93\\6a9d87d47926b1c8.final.pdb
-3.354214e-15 -1.796786e-15 -2.178388e-15 6.297463e-16 2.999337e-15
2.856781e-15
E:\\R-3.1.2\\scripts\\mPDBS\\summary_pdb_93\\6bb3547ea982ffc4.final.pdb
-8.670289e-15 -2.046833e-14 -3.323496e-15 -5.008244e-15 5.801174e-15
-3.669842e-15
E:\\R-3.1.2\\scripts\\mPDBS\\summary_pdb_93\\716d9624611d1c42.final.pdb
-8.661265e-15 1.110076e-14 -3.985141e-15 -7.039322e-15 9.745679e-15
6.344743e-15
E:\\R-3.1.2\\scripts\\mPDBS\\summary_pdb_93\\75f27e70ad0f1be4.final.pdb
-4.263692e-15 -4.252918e-16 1.772811e-15 -1.258062e-15 4.120781e-16
6.450704e-15
E:\\R-3.1.2\\scripts\\mPDBS\\summary_pdb_93\\769f910fffb22872.final.pdb
-3.708712e-15 -3.608958e-15 -1.364866e-15 -1.682688e-15 2.630569e-15
3.449700e-15
E:\\R-3.1.2\\scripts\\mPDBS\\summary_pdb_93\\791f8c80dd5c3135.final.pdb
-3.615441e-15 -4.060020e-15 8.430791e-16 4.568430e-16 1.943400e-15
3.538898e-15
E:\\R-3.1.2\\scripts\\mPDBS\\summary_pdb_93\\7fad98e66a3f6099.final.pdb
-3.866383e-15 -1.299002e-14 1.317340e-14 1.820661e-15 2.012062e-15
6.155908e-15
E:\\R-3.1.2\\scripts\\mPDBS\\summary_pdb_93\\804b89c94a633196.final.pdb
-7.799068e-15 -5.330602e-15 7.331253e-16 -3.922917e-16 8.819949e-17
2.570337e-15

E:\\R-3.1.2\\scripts\\mPDBS\\summary_pdb_93\\813cc65a2298feba.final.pdb
-7.799068e-15 -5.330602e-15 7.331253e-16 -3.922917e-16 8.819949e-17
2.570337e-15
E:\\R-3.1.2\\scripts\\mPDBS\\summary_pdb_93\\83c0eefcfe12d3f9.final.pdb
-8.073371e-15 -3.166534e-15 -3.030791e-15 1.255045e-15 5.033896e-15
-1.038538e-15
E:\\R-3.1.2\\scripts\\mPDBS\\summary_pdb_93\\86d50469ef569760.final.pdb
-7.271437e-15 -4.698111e-15 -6.668286e-15 -2.358965e-15 1.252471e-15
5.923121e-15
E:\\R-3.1.2\\scripts\\mPDBS\\summary_pdb_93\\89d0666e1402203d.final.pdb
-4.263692e-15 -4.252918e-16 1.772811e-15 -1.258062e-15 4.120781e-16
6.450704e-15
E:\\R-3.1.2\\scripts\\mPDBS\\summary_pdb_93\\8b34852716dadc62.final.pdb
-1.717995e-15 3.669144e-15 3.160871e-15 4.196052e-15 -1.965713e-15
2.112370e-15
E:\\R-3.1.2\\scripts\\mPDBS\\summary_pdb_93\\8b6ef9ac3fe74395.final.pdb
-5.396006e-15 -1.866220e-14 7.126406e-15 -2.817136e-15 4.440358e-15
3.721856e-15
E:\\R-3.1.2\\scripts\\mPDBS\\summary_pdb_93\\98bbe0d5986c138f.final.pdb
-3.615441e-15 -4.060020e-15 8.430791e-16 4.568430e-16 1.943400e-15
3.538898e-15
E:\\R-3.1.2\\scripts\\mPDBS\\summary_pdb_93\\9c22f4909d67e2fa.final.pdb
5.566977e-15 4.793244e-15 -1.058031e-14 1.461911e-15 4.212505e-15
3.295235e-15
E:\\R-3.1.2\\scripts\\mPDBS\\summary_pdb_93\\9dda8ed0d81fb3ef.final.pdb
-2.054169e-14 -1.139135e-14 -7.758627e-15 -1.137670e-14 1.252552e-15
-1.469393e-15
E:\\R-3.1.2\\scripts\\mPDBS\\summary_pdb_93\\9f752b3616e6033b.final.pdb
-1.812645e-15 -6.482444e-15 -2.651784e-15 3.265618e-16 3.330789e-15
3.039468e-15
E:\\R-3.1.2\\scripts\\mPDBS\\summary_pdb_93\\a02c70083816d6ba.final.pdb
-4.665748e-15 2.460085e-15 -1.887692e-15 1.449372e-15 -1.591639e-16
7.359942e-15
E:\\R-3.1.2\\scripts\\mPDBS\\summary_pdb_93\\a0c997d891e4e082.final.pdb
-8.117249e-15 -9.097218e-15 2.524609e-15 8.165855e-15 -1.570639e-15
4.658381e-16
E:\\R-3.1.2\\scripts\\mPDBS\\summary_pdb_93\\ab8039a2d823c616.final.pdb
-3.215354e-15 -4.643662e-16 -1.552208e-15 7.488818e-17 2.381928e-15
5.779730e-15
E:\\R-3.1.2\\scripts\\mPDBS\\summary_pdb_93\\acf4d74b9c71d4dc.final.pdb
-7.799068e-15 -5.330602e-15 7.331253e-16 -3.922917e-16 8.819949e-17
2.570337e-15
E:\\R-3.1.2\\scripts\\mPDBS\\summary_pdb_93\\ad8a823e4b4c668e.final.pdb
-5.118713e-15 -1.546135e-15 -1.096260e-15 7.649345e-17 1.875706e-15
3.759366e-15
E:\\R-3.1.2\\scripts\\mPDBS\\summary_pdb_93\\b16ababd03b7b857.final.pdb
5.566977e-15 4.793244e-15 -1.058031e-14 1.461911e-15 4.212505e-15
3.295235e-15
E:\\R-3.1.2\\scripts\\mPDBS\\summary_pdb_93\\b226923e697d3c63.final.pdb
-6.677343e-15 -3.089827e-15 -6.377787e-15 4.849899e-15 2.259559e-15
5.647960e-15
E:\\R-3.1.2\\scripts\\mPDBS\\summary_pdb_93\\b28e48f6fa27a165.final.pdb
-3.615441e-15 -4.060020e-15 8.430791e-16 4.568430e-16 1.943400e-15
3.538898e-15

E:\\R-3.1.2\\scripts\\mPDBS\\summary_pdb_93\\b42c4924e3cd1aec.final.pdb
5.046135e-15 3.995692e-16 -2.765876e-15 8.570230e-15 4.826082e-15
6.233430e-15
E:\\R-3.1.2\\scripts\\mPDBS\\summary_pdb_93\\b4dd239fcabff1df.final.pdb
-3.615441e-15 -4.060020e-15 8.430791e-16 4.568430e-16 1.943400e-15
3.538898e-15
E:\\R-3.1.2\\scripts\\mPDBS\\summary_pdb_93\\b669942ef97459a2.final.pdb
-1.717995e-15 3.669144e-15 3.160871e-15 4.196052e-15 -1.965713e-15
2.112370e-15
E:\\R-3.1.2\\scripts\\mPDBS\\summary_pdb_93\\b99f4d9d87c1017b.final.pdb
-3.615441e-15 -4.060020e-15 8.430791e-16 4.568430e-16 1.943400e-15
3.538898e-15
E:\\R-3.1.2\\scripts\\mPDBS\\summary_pdb_93\\b9d2cbf3975403e7.final.pdb
-3.615441e-15 -4.060020e-15 8.430791e-16 4.568430e-16 1.943400e-15
3.538898e-15
E:\\R-3.1.2\\scripts\\mPDBS\\summary_pdb_93\\bc88c8a505f3a288.final.pdb
-4.760358e-15 -1.419770e-15 -2.200493e-15 -9.894827e-16 2.484634e-15
3.309697e-15
E:\\R-3.1.2\\scripts\\mPDBS\\summary_pdb_93\\bdac0e64f4ef427e.final.pdb
3.051194e-15 2.176414e-15 -1.225811e-15 -4.117827e-15 -5.578256e-17
1.406375e-14
E:\\R-3.1.2\\scripts\\mPDBS\\summary_pdb_93\\ca2a1f55fc9d0681.final.pdb
-1.717995e-15 3.669144e-15 3.160871e-15 4.196052e-15 -1.965713e-15
2.112370e-15
E:\\R-3.1.2\\scripts\\mPDBS\\summary_pdb_93\\ce34248de3e81a3c.final.pdb
-8.016591e-15 1.681570e-15 3.949278e-15 4.628731e-15 -1.640217e-15
5.643936e-15
E:\\R-3.1.2\\scripts\\mPDBS\\summary_pdb_93\\d02e697772494a61.final.pdb
3.182507e-15 5.870405e-15 4.075519e-15 4.070923e-15 -9.613941e-16
7.239496e-15
E:\\R-3.1.2\\scripts\\mPDBS\\summary_pdb_93\\d04221245d015047.final.pdb
-3.582681e-15 -2.713435e-15 -4.643191e-16 -1.718599e-15 1.857174e-15
2.256073e-15
E:\\R-3.1.2\\scripts\\mPDBS\\summary_pdb_93\\d678141e4018054a.final.pdb
-9.751193e-15 -3.204457e-16 -9.410040e-15 -1.001482e-14 1.419281e-14
-5.552901e-15
E:\\R-3.1.2\\scripts\\mPDBS\\summary_pdb_93\\d71b40af0f0efe39.final.pdb
5.184980e-15 1.167523e-14 -1.438456e-14 -1.608002e-15 -3.687168e-16
6.606730e-15
E:\\R-3.1.2\\scripts\\mPDBS\\summary_pdb_93\\dc0809eff10197e2.final.pdb
4.638817e-15 -6.745912e-15 -1.636765e-15 1.999142e-15 6.725560e-15
1.203204e-14
E:\\R-3.1.2\\scripts\\mPDBS\\summary_pdb_93\\e1163918d3707839.final.pdb
-3.545300e-15 -1.016490e-14 -1.435643e-14 2.211601e-15 7.117652e-15
1.090677e-14
E:\\R-3.1.2\\scripts\\mPDBS\\summary_pdb_93\\e4b48b06824ef848.final.pdb
-6.994495e-15 1.109490e-15 -1.765349e-14 -4.345055e-15 2.356482e-15
4.556887e-15
E:\\R-3.1.2\\scripts\\mPDBS\\summary_pdb_93\\e529e19ad281fea2.final.pdb
-3.773367e-15 -5.190773e-15 -7.164501e-16 1.388905e-15 7.778501e-15
1.523863e-15
E:\\R-3.1.2\\scripts\\mPDBS\\summary_pdb_93\\e5602082718ec3a1.final.pdb
-7.799068e-15 -5.330602e-15 7.331253e-16 -3.922917e-16 8.819949e-17
2.570337e-15

E:\\R-3.1.2\\scripts\\mPDBS\\summary_pdb_93\\e5d589d996d0a960.final.pdb
-4.231761e-15 -6.512552e-15 5.592157e-15 1.119534e-15 4.960096e-15
-7.367200e-15
E:\\R-3.1.2\\scripts\\mPDBS\\summary_pdb_93\\e9df9e72307a2fbd.final.pdb
-4.665748e-15 2.460085e-15 -1.887692e-15 1.449372e-15 -1.591639e-16
7.359942e-15
E:\\R-3.1.2\\scripts\\mPDBS\\summary_pdb_93\\ea00f8694090cae7.final.pdb
-7.799068e-15 -5.330602e-15 7.331253e-16 -3.922917e-16 8.819949e-17
2.570337e-15
E:\\R-3.1.2\\scripts\\mPDBS\\summary_pdb_93\\eba517be32db050e.final.pdb
-3.615441e-15 -4.060020e-15 8.430791e-16 4.568430e-16 1.943400e-15
3.538898e-15
E:\\R-3.1.2\\scripts\\mPDBS\\summary_pdb_93\\ec04b9545899c29d.final.pdb
-5.105900e-15 -1.549393e-15 -2.684995e-15 -1.534773e-16 2.014963e-15
3.614431e-15
E:\\R-3.1.2\\scripts\\mPDBS\\summary_pdb_93\\ee6862cbcad52078.final.pdb
-2.764559e-15 -1.506507e-15 -2.295036e-15 -1.175965e-15 3.155972e-15
3.377568e-15
E:\\R-3.1.2\\scripts\\mPDBS\\summary_pdb_93\\ef6a42fc83b81e5d.final.pdb
-5.548386e-15 -1.954899e-15 -1.640470e-15 -4.265317e-16 1.314781e-15
3.195996e-15
E:\\R-3.1.2\\scripts\\mPDBS\\summary_pdb_93\\ef8e88d4ad93a0ad.final.pdb
-3.582681e-15 -2.713435e-15 -4.643191e-16 -1.718599e-15 1.857174e-15
2.256073e-15
E:\\R-3.1.2\\scripts\\mPDBS\\summary_pdb_93\\f14fcae8deec4fa3.final.pdb
-1.812645e-15 -6.482444e-15 -2.651784e-15 3.265618e-16 3.330789e-15
3.039468e-15
E:\\R-3.1.2\\scripts\\mPDBS\\summary_pdb_93\\f83584e22003fb92.final.pdb
-9.748555e-15 3.748011e-16 2.165589e-15 -7.704814e-15 3.127349e-15
-2.150128e-15
E:\\R-3.1.2\\scripts\\mPDBS\\summary_pdb_93\\f8e2bf87e52caa29.final.pdb
-3.773367e-15 -5.190773e-15 -7.164501e-16 1.388905e-15 7.778501e-15
1.523863e-15

[,115]   [,116]   [,117]   [,118]   [,119]   [,120]
E:\\R-3.1.2\\scripts\\40.1ns_data\\NAT0.pdb
-3.597986e-15 -9.556393e-16 -1.228577e-15 3.532331e-15 4.849061e-16
4.026758e-15
E:\\R-3.1.2\\scripts\\40.1ns_data\\LJP6.pdb
-4.350206e-15 9.158436e-16 -3.579347e-15 -2.801097e-15 -4.421689e-15
2.321945e-15
E:\\R-3.1.2\\scripts\\40.1ns_data\\3WY9.pdb
-2.047815e-15 -5.720133e-16 -5.128771e-15 3.638372e-15 9.573840e-16
-4.434567e-17
E:\\R-3.1.2\\scripts\\40.1ns_data\\ZSP9.pdb
-5.694403e-16 -9.625673e-16 -1.299226e-15 3.002602e-15 4.625883e-16
2.940310e-15
E:\\R-3.1.2\\scripts\\mPDBS\\summary_pdb_93\\013d855ad26c877c.final.pdb
-1.808252e-16 1.781058e-15 -8.104150e-15 -2.758432e-14 -5.648382e-15
-6.184684e-15
E:\\R-3.1.2\\scripts\\mPDBS\\summary_pdb_93\\0a2a705342ca0d54.final.pdb
-1.154504e-15 -1.128802e-15 -4.356433e-15 4.880888e-15 -2.949580e-15
2.038258e-15
E:\\R-3.1.2\\scripts\\mPDBS\\summary_pdb_93\\0a59fceadb4194d9.final.pdb

-4.091504e-15 -5.684307e-16 -6.250152e-15 -6.548067e-15 1.431167e-15
3.862926e-15
E:\\R-3.1.2\\scripts\\mPDBS\\summary_pdb_93\\0dba59f3306825e8.final.pdb
-2.453313e-15 -2.290338e-15 -4.089566e-15 1.794523e-14 -1.316778e-15
4.862035e-15
E:\\R-3.1.2\\scripts\\mPDBS\\summary_pdb_93\\126f26a78b57339c.final.pdb
-7.119502e-15 9.801552e-15 -4.661591e-15 2.013967e-16 -1.471602e-15
-4.016714e-15
E:\\R-3.1.2\\scripts\\mPDBS\\summary_pdb_93\\12f06f3e342df706.final.pdb
-3.614775e-15 1.663861e-16 -4.127449e-15 -1.442613e-15 -1.746333e-15
1.274763e-15
E:\\R-3.1.2\\scripts\\mPDBS\\summary_pdb_93\\18618a2a251b867b.final.pdb
-1.029951e-14 1.496227e-14 -7.060756e-15 9.179547e-15 -4.802270e-15
-8.278633e-16
E:\\R-3.1.2\\scripts\\mPDBS\\summary_pdb_93\\1f305f598641eeb2.final.pdb
-3.595296e-15 8.227936e-16 -3.878389e-15 3.126175e-15 -3.982840e-15
1.089089e-15
E:\\R-3.1.2\\scripts\\mPDBS\\summary_pdb_93\\1f8a00b551be0a39.final.pdb
-1.029951e-14 1.496227e-14 -7.060756e-15 9.179547e-15 -4.802270e-15
-8.278633e-16
E:\\R-3.1.2\\scripts\\mPDBS\\summary_pdb_93\\219f45328d47579a.final.pdb
2.198603e-15 2.875863e-15 -1.156361e-14 -4.097279e-14 -6.152177e-15
-1.424065e-14
E:\\R-3.1.2\\scripts\\mPDBS\\summary_pdb_93\\238dd3918e54336d.final.pdb
4.923962e-15 -3.307736e-16 -6.222613e-15 4.187175e-14 -4.034297e-15
3.261628e-15
E:\\R-3.1.2\\scripts\\mPDBS\\summary_pdb_93\\2b3027889e2fdc42.final.pdb
-2.838925e-15 1.211396e-15 -5.003536e-15 -9.468616e-16 -1.419418e-15
8.093794e-16
E:\\R-3.1.2\\scripts\\mPDBS\\summary_pdb_93\\2fcb3b46e33793b0.final.pdb
-1.787714e-15 -4.046575e-15 -4.449980e-15 6.038775e-15 -2.893433e-15
1.474198e-15
E:\\R-3.1.2\\scripts\\mPDBS\\summary_pdb_93\\34eb328251842006.final.pdb
-2.061476e-15 -3.665721e-15 -3.997028e-15 -3.060312e-14 8.860984e-15
6.764896e-15
E:\\R-3.1.2\\scripts\\mPDBS\\summary_pdb_93\\354b32871797b7ab.final.pdb
6.693927e-15 -3.757885e-15 -6.473586e-15 3.195433e-15 1.673974e-15
1.266493e-14
E:\\R-3.1.2\\scripts\\mPDBS\\summary_pdb_93\\397368adbc32d5de.final.pdb
-7.119502e-15 9.801552e-15 -4.661591e-15 2.013967e-16 -1.471602e-15
-4.016714e-15
E:\\R-3.1.2\\scripts\\mPDBS\\summary_pdb_93\\411ca13cd3df6edd.final.pdb
-2.731621e-15 2.346748e-15 -3.546189e-15 -1.173412e-14 -1.271033e-15
3.610076e-15
E:\\R-3.1.2\\scripts\\mPDBS\\summary_pdb_93\\42357eee6642bf12.final.pdb
-4.286718e-16 9.474595e-16 -1.737390e-14 -1.161532e-15 -4.623699e-15
-7.821973e-15
E:\\R-3.1.2\\scripts\\mPDBS\\summary_pdb_93\\44342d1f75a7fcfc.final.pdb
-1.430751e-14 3.479965e-15 3.074995e-15 -3.368783e-14 -5.326652e-15
-8.503857e-15
E:\\R-3.1.2\\scripts\\mPDBS\\summary_pdb_93\\448ef21dffe8cdfb.final.pdb
1.764999e-15 8.596089e-15 -6.555501e-15 1.630660e-14 1.283781e-15
2.016758e-14
E:\\R-3.1.2\\scripts\\mPDBS\\summary_pdb_93\\472a40b3e8f113c7.final.pdb

-2.453313e-15 -2.290338e-15 -4.089566e-15 1.794523e-14 -1.316778e-15
4.862035e-15
E:\\R-3.1.2\\scripts\\mPDBS\\summary_pdb_93\\48a26225951ea153.final.pdb
-9.356281e-15 4.043765e-15 -1.688479e-15 4.856656e-15 -5.614846e-15
1.081731e-15
E:\\R-3.1.2\\scripts\\mPDBS\\summary_pdb_93\\4ab4a5c7c6682ce6.final.pdb
-1.276082e-14 1.034626e-14 8.379159e-16 5.604844e-16 -1.087207e-14
7.451023e-16
E:\\R-3.1.2\\scripts\\mPDBS\\summary_pdb_93\\4eaa45b62cb6a25f.final.pdb
-8.630728e-15 5.407526e-15 -1.836887e-15 3.241186e-15 -3.499622e-15
-4.306521e-15
E:\\R-3.1.2\\scripts\\mPDBS\\summary_pdb_93\\58c371611d85ac96.final.pdb
-7.119502e-15 9.801552e-15 -4.661591e-15 2.013967e-16 -1.471602e-15
-4.016714e-15
E:\\R-3.1.2\\scripts\\mPDBS\\summary_pdb_93\\59a647f04822d30a.final.pdb
1.017033e-15 1.523319e-16 -2.240198e-15 3.345959e-17 -2.591860e-15
4.200390e-15
E:\\R-3.1.2\\scripts\\mPDBS\\summary_pdb_93\\59b091fe385e05fc.final.pdb
-4.091504e-15 -5.684307e-16 -6.250152e-15 -6.548067e-15 1.431167e-15
3.862926e-15
E:\\R-3.1.2\\scripts\\mPDBS\\summary_pdb_93\\5c35ac30f930d2db.final.pdb
-8.630728e-15 5.407526e-15 -1.836887e-15 3.241186e-15 -3.499622e-15
-4.306521e-15
E:\\R-3.1.2\\scripts\\mPDBS\\summary_pdb_93\\5f60752431cf2dcb.final.pdb
-1.817030e-14 5.149596e-15 -2.168296e-15 -4.232458e-16 4.128525e-15
3.612138e-16
E:\\R-3.1.2\\scripts\\mPDBS\\summary_pdb_93\\626456dd6ff2868d.final.pdb
-2.453313e-15 -2.290338e-15 -4.089566e-15 1.794523e-14 -1.316778e-15
4.862035e-15
E:\\R-3.1.2\\scripts\\mPDBS\\summary_pdb_93\\6583d53ff3c8f7ef.final.pdb
1.017033e-15 1.523319e-16 -2.240198e-15 3.345959e-17 -2.591860e-15
4.200390e-15
E:\\R-3.1.2\\scripts\\mPDBS\\summary_pdb_93\\69760178986bef01.final.pdb
-7.941439e-15 1.518510e-14 -1.578002e-15 -2.909548e-14 -1.134224e-14
8.032261e-15
E:\\R-3.1.2\\scripts\\mPDBS\\summary_pdb_93\\6a9d87d47926b1c8.final.pdb
-3.445358e-15 1.205997e-15 -5.451541e-15 3.748690e-16 -2.862850e-15
8.994927e-16
E:\\R-3.1.2\\scripts\\mPDBS\\summary_pdb_93\\6bb3547ea982ffc4.final.pdb
-5.221961e-15 -9.736322e-15 5.997400e-15 6.070268e-15 -2.418316e-15
3.370972e-15
E:\\R-3.1.2\\scripts\\mPDBS\\summary_pdb_93\\716d9624611d1c42.final.pdb
3.806805e-15 5.291843e-15 -4.733301e-15 -3.187096e-14 -6.763370e-15
4.946522e-15
E:\\R-3.1.2\\scripts\\mPDBS\\summary_pdb_93\\75f27e70ad0f1be4.final.pdb
1.017033e-15 1.523319e-16 -2.240198e-15 3.345959e-17 -2.591860e-15
4.200390e-15
E:\\R-3.1.2\\scripts\\mPDBS\\summary_pdb_93\\769f910fffb22872.final.pdb
-3.178473e-15 2.101028e-15 -2.935584e-15 3.850911e-15 -1.677817e-15
2.692975e-15
E:\\R-3.1.2\\scripts\\mPDBS\\summary_pdb_93\\791f8c80dd5c3135.final.pdb
-4.091504e-15 -5.684307e-16 -6.250152e-15 -6.548067e-15 1.431167e-15
3.862926e-15
E:\\R-3.1.2\\scripts\\mPDBS\\summary_pdb_93\\7fad98e66a3f6099.final.pdb

7.507345e-15 -9.033170e-15 3.644289e-15 -5.838810e-14 -2.372866e-15
6.217508e-15
E:\\R-3.1.2\\scripts\\mPDBS\\summary_pdb_93\\804b89c94a633196.final.pdb
-1.787714e-15 -4.046575e-15 -4.449980e-15 6.038775e-15 -2.893433e-15
1.474198e-15
E:\\R-3.1.2\\scripts\\mPDBS\\summary_pdb_93\\813cc65a2298feba.final.pdb
-1.787714e-15 -4.046575e-15 -4.449980e-15 6.038775e-15 -2.893433e-15
1.474198e-15
E:\\R-3.1.2\\scripts\\mPDBS\\summary_pdb_93\\83c0eefcfe12d3f9.final.pdb
-2.731621e-15 2.346748e-15 -3.546189e-15 -1.173412e-14 -1.271033e-15
3.610076e-15
E:\\R-3.1.2\\scripts\\mPDBS\\summary_pdb_93\\86d50469ef569760.final.pdb
-2.453313e-15 -2.290338e-15 -4.089566e-15 1.794523e-14 -1.316778e-15
4.862035e-15
E:\\R-3.1.2\\scripts\\mPDBS\\summary_pdb_93\\89d0666e1402203d.final.pdb
1.017033e-15 1.523319e-16 -2.240198e-15 3.345959e-17 -2.591860e-15
4.200390e-15
E:\\R-3.1.2\\scripts\\mPDBS\\summary_pdb_93\\8b34852716dadc62.final.pdb
-1.056195e-14 5.157868e-15 9.372035e-16 -4.142317e-15 -6.952686e-15
9.364338e-16
E:\\R-3.1.2\\scripts\\mPDBS\\summary_pdb_93\\8b6ef9ac3fe74395.final.pdb
-1.898576e-15 -1.113054e-14 9.255486e-15 -2.161899e-14 6.540050e-15
7.128481e-15
E:\\R-3.1.2\\scripts\\mPDBS\\summary_pdb_93\\98bbe0d5986c138f.final.pdb
-4.091504e-15 -5.684307e-16 -6.250152e-15 -6.548067e-15 1.431167e-15
3.862926e-15
E:\\R-3.1.2\\scripts\\mPDBS\\summary_pdb_93\\9c22f4909d67e2fa.final.pdb
-1.111281e-15 1.171039e-15 -1.265978e-14 1.838437e-14 -3.766433e-15
-2.354120e-15
E:\\R-3.1.2\\scripts\\mPDBS\\summary_pdb_93\\9dda8ed0d81fb3ef.final.pdb
-2.052292e-14 2.201908e-15 8.625716e-16 1.565009e-14 -3.082353e-15
9.881176e-15
E:\\R-3.1.2\\scripts\\mPDBS\\summary_pdb_93\\9f752b3616e6033b.final.pdb
1.936069e-15 -4.590864e-15 -3.173172e-15 1.021981e-14 -2.152070e-15
2.049833e-15
E:\\R-3.1.2\\scripts\\mPDBS\\summary_pdb_93\\a02c70083816d6ba.final.pdb
-8.630728e-15 5.407526e-15 -1.836887e-15 3.241186e-15 -3.499622e-15
-4.306521e-15
E:\\R-3.1.2\\scripts\\mPDBS\\summary_pdb_93\\a0c997d891e4e082.final.pdb
1.636165e-15 -8.119298e-15 4.957136e-15 2.544535e-14 -3.142212e-15
-1.489240e-15
E:\\R-3.1.2\\scripts\\mPDBS\\summary_pdb_93\\ab8039a2d823c616.final.pdb
-4.875879e-15 1.593852e-15 -2.995053e-15 9.894307e-16 -3.940402e-15
1.672944e-15
E:\\R-3.1.2\\scripts\\mPDBS\\summary_pdb_93\\acf4d74b9c71d4dc.final.pdb
-1.787714e-15 -4.046575e-15 -4.449980e-15 6.038775e-15 -2.893433e-15
1.474198e-15
E:\\R-3.1.2\\scripts\\mPDBS\\summary_pdb_93\\ad8a823e4b4c668e.final.pdb
-2.458829e-15 -3.558594e-16 -3.240832e-15 -2.919811e-16 -2.888265e-15
1.001626e-15
E:\\R-3.1.2\\scripts\\mPDBS\\summary_pdb_93\\b16ababd03b7b857.final.pdb
-1.111281e-15 1.171039e-15 -1.265978e-14 1.838437e-14 -3.766433e-15
-2.354120e-15
E:\\R-3.1.2\\scripts\\mPDBS\\summary_pdb_93\\b226923e697d3c63.final.pdb

-4.934803e-15 7.587790e-15 -3.925482e-15 -1.807516e-15 -2.011812e-15
-1.880032e-15
E:\\R-3.1.2\\scripts\\mPDBS\\summary_pdb_93\\b28e48f6fa27a165.final.pdb
-4.091504e-15 -5.684307e-16 -6.250152e-15 -6.548067e-15 1.431167e-15
3.862926e-15
E:\\R-3.1.2\\scripts\\mPDBS\\summary_pdb_93\\b42c4924e3cd1aec.final.pdb
6.977435e-15 2.943068e-15 -2.405214e-15 -2.253551e-14 8.934163e-16
-3.924838e-15
E:\\R-3.1.2\\scripts\\mPDBS\\summary_pdb_93\\b4dd239fcabff1df.final.pdb
-4.091504e-15 -5.684307e-16 -6.250152e-15 -6.548067e-15 1.431167e-15
3.862926e-15
E:\\R-3.1.2\\scripts\\mPDBS\\summary_pdb_93\\b669942ef97459a2.final.pdb
-1.056195e-14 5.157868e-15 9.372035e-16 -4.142317e-15 -6.952686e-15
9.364338e-16
E:\\R-3.1.2\\scripts\\mPDBS\\summary_pdb_93\\b99f4d9d87c1017b.final.pdb
-4.091504e-15 -5.684307e-16 -6.250152e-15 -6.548067e-15 1.431167e-15
3.862926e-15
E:\\R-3.1.2\\scripts\\mPDBS\\summary_pdb_93\\b9d2cbf3975403e7.final.pdb
-4.091504e-15 -5.684307e-16 -6.250152e-15 -6.548067e-15 1.431167e-15
3.862926e-15
E:\\R-3.1.2\\scripts\\mPDBS\\summary_pdb_93\\bc88c8a505f3a288.final.pdb
-4.225186e-15 1.082592e-15 -5.386476e-15 5.089759e-15 -9.581982e-16
7.857437e-16
E:\\R-3.1.2\\scripts\\mPDBS\\summary_pdb_93\\bdac0e64f4ef427e.final.pdb
-8.304723e-16 2.124270e-15 -2.165349e-15 -1.682770e-14 -5.096532e-15
-1.734607e-15
E:\\R-3.1.2\\scripts\\mPDBS\\summary_pdb_93\\ca2a1f55fc9d0681.final.pdb
-1.056195e-14 5.157868e-15 9.372035e-16 -4.142317e-15 -6.952686e-15
9.364338e-16
E:\\R-3.1.2\\scripts\\mPDBS\\summary_pdb_93\\ce34248de3e81a3c.final.pdb
-8.725636e-15 4.608239e-16 -4.663148e-15 -2.183693e-14 -1.018179e-15
-9.763327e-15
E:\\R-3.1.2\\scripts\\mPDBS\\summary_pdb_93\\d02e697772494a61.final.pdb
4.962126e-15 -3.320746e-16 -9.515552e-15 -3.444430e-15 -7.220116e-15
-4.654783e-15
E:\\R-3.1.2\\scripts\\mPDBS\\summary_pdb_93\\d04221245d015047.final.pdb
-2.185431e-15 -1.675668e-15 -3.667909e-15 2.422963e-15 -2.262707e-15
1.445912e-15
E:\\R-3.1.2\\scripts\\mPDBS\\summary_pdb_93\\d678141e4018054a.final.pdb
-5.427709e-15 6.358604e-15 -8.157251e-15 -1.456391e-14 -3.814470e-15
-1.500778e-15
E:\\R-3.1.2\\scripts\\mPDBS\\summary_pdb_93\\d71b40af0f0efe39.final.pdb
3.815956e-15 4.515180e-15 -1.129808e-14 3.802657e-14 -1.604790e-15
7.877964e-15
E:\\R-3.1.2\\scripts\\mPDBS\\summary_pdb_93\\dc0809eff10197e2.final.pdb
1.858342e-15 -3.507825e-15 2.737623e-15 7.690762e-16 7.911125e-15
5.663273e-15
E:\\R-3.1.2\\scripts\\mPDBS\\summary_pdb_93\\e1163918d3707839.final.pdb
1.289284e-14 -6.071422e-15 -1.171461e-14 2.291858e-14 7.908638e-15
-1.171831e-15
E:\\R-3.1.2\\scripts\\mPDBS\\summary_pdb_93\\e4b48b06824ef848.final.pdb
-1.029951e-14 1.496227e-14 -7.060756e-15 9.179547e-15 -4.802270e-15
-8.278633e-16
E:\\R-3.1.2\\scripts\\mPDBS\\summary_pdb_93\\e529e19ad281fea2.final.pdb

-1.808252e-16 1.781058e-15 -8.104150e-15 -2.758432e-14 -5.648382e-15
-6.184684e-15
E:\\R-3.1.2\\scripts\\mPDBS\\summary_pdb_93\\e5602082718ec3a1.final.pdb
-1.787714e-15 -4.046575e-15 -4.449980e-15 6.038775e-15 -2.893433e-15
1.474198e-15
E:\\R-3.1.2\\scripts\\mPDBS\\summary_pdb_93\\e5d589d996d0a960.final.pdb
-8.090813e-15 -9.606722e-15 1.057570e-14 1.156166e-15 -5.510458e-15
-7.863347e-15
E:\\R-3.1.2\\scripts\\mPDBS\\summary_pdb_93\\e9df9e72307a2fbd.final.pdb
-8.630728e-15 5.407526e-15 -1.836887e-15 3.241186e-15 -3.499622e-15
-4.306521e-15
E:\\R-3.1.2\\scripts\\mPDBS\\summary_pdb_93\\ea00f8694090cae7.final.pdb
-1.787714e-15 -4.046575e-15 -4.449980e-15 6.038775e-15 -2.893433e-15
1.474198e-15
E:\\R-3.1.2\\scripts\\mPDBS\\summary_pdb_93\\eba517be32db050e.final.pdb
-4.091504e-15 -5.684307e-16 -6.250152e-15 -6.548067e-15 1.431167e-15
3.862926e-15
E:\\R-3.1.2\\scripts\\mPDBS\\summary_pdb_93\\ec04b9545899c29d.final.pdb
-3.458553e-15 1.383552e-15 -3.363432e-15 2.177578e-15 -1.436534e-15
5.982370e-16
E:\\R-3.1.2\\scripts\\mPDBS\\summary_pdb_93\\ee6862cbcad52078.final.pdb
-3.080160e-15 1.497625e-15 -4.630787e-15 4.194500e-16 -1.146632e-15
1.505437e-15
E:\\R-3.1.2\\scripts\\mPDBS\\summary_pdb_93\\ef6a42fc83b81e5d.final.pdb
-2.890617e-15 5.154903e-16 -2.872050e-15 -1.837464e-15 -3.048899e-15
7.858632e-16
E:\\R-3.1.2\\scripts\\mPDBS\\summary_pdb_93\\ef8e88d4ad93a0ad.final.pdb
-2.185431e-15 -1.675668e-15 -3.667909e-15 2.422963e-15 -2.262707e-15
1.445912e-15
E:\\R-3.1.2\\scripts\\mPDBS\\summary_pdb_93\\f14fcae8deec4fa3.final.pdb
1.936069e-15 -4.590864e-15 -3.173172e-15 1.021981e-14 -2.152070e-15
2.049833e-15
E:\\R-3.1.2\\scripts\\mPDBS\\summary_pdb_93\\f83584e22003fb92.final.pdb
-9.356281e-15 4.043765e-15 -1.688479e-15 4.856656e-15 -5.614846e-15
1.081731e-15
E:\\R-3.1.2\\scripts\\mPDBS\\summary_pdb_93\\f8e2bf87e52caa29.final.pdb
-1.808252e-16 1.781058e-15 -8.104150e-15 -2.758432e-14 -5.648382e-15
-6.184684e-15

[,121]   [,122]   [,123]   [,124]   [,125]   [,126]
E:\\R-3.1.2\\scripts\\40.1ns_data\\NAT0.pdb
-1.757053e-15 -6.399413e-15 6.449325e-16 3.840800e-15 -4.224505e-16
3.552208e-15
E:\\R-3.1.2\\scripts\\40.1ns_data\\LJP6.pdb
-1.453982e-15 -5.589621e-15 2.030586e-15 4.797310e-15 3.094064e-16
4.782699e-16
E:\\R-3.1.2\\scripts\\40.1ns_data\\3WY9.pdb
-2.735631e-15 -4.133733e-15 -6.352389e-16 3.476615e-15 -2.178295e-15
-2.214716e-15
E:\\R-3.1.2\\scripts\\40.1ns_data\\ZSP9.pdb
-9.687162e-16 -7.371682e-15 5.695813e-16 3.182785e-15 -1.133933e-15
1.328971e-15
E:\\R-3.1.2\\scripts\\mPDBS\\summary_pdb_93\\013d855ad26c877c.final.pdb
-1.538119e-15 -5.329706e-15 -2.551533e-15 1.674050e-14 -9.165652e-16

-2.041918e-16
E:\\R-3.1.2\\scripts\\mPDBS\\summary_pdb_93\\0a2a705342ca0d54.final.pdb
-2.506089e-15 -4.952795e-15 -1.198980e-16 5.779979e-15 -6.793869e-16
-1.017435e-15
E:\\R-3.1.2\\scripts\\mPDBS\\summary_pdb_93\\0a59fceadb4194d9.final.pdb
-2.262792e-15 -5.461392e-15 -1.227610e-15 2.397127e-15 -6.713368e-16
-3.154410e-15
E:\\R-3.1.2\\scripts\\mPDBS\\summary_pdb_93\\0dba59f3306825e8.final.pdb
1.194070e-15 -7.258719e-15 -1.992477e-16 -6.929797e-15 -2.956600e-15
7.568450e-16
E:\\R-3.1.2\\scripts\\mPDBS\\summary_pdb_93\\126f26a78b57339c.final.pdb
-4.616332e-15 -5.286338e-15 2.621846e-15 8.162622e-15 5.685792e-15
-4.206199e-15
E:\\R-3.1.2\\scripts\\mPDBS\\summary_pdb_93\\12f06f3e342df706.final.pdb
-3.078358e-15 -5.640396e-15 3.597530e-16 4.266812e-15 -2.087115e-15
-1.602904e-15
E:\\R-3.1.2\\scripts\\mPDBS\\summary_pdb_93\\18618a2a251b867b.final.pdb
-1.793208e-15 -1.321993e-15 -2.393033e-15 1.484662e-15 -7.356986e-15
-2.476580e-15
E:\\R-3.1.2\\scripts\\mPDBS\\summary_pdb_93\\1f305f598641eeb2.final.pdb
-2.998196e-15 -5.464370e-15 7.779243e-16 4.231853e-15 5.400835e-16
-4.437636e-16
E:\\R-3.1.2\\scripts\\mPDBS\\summary_pdb_93\\1f8a00b551be0a39.final.pdb
-1.793208e-15 -1.321993e-15 -2.393033e-15 1.484662e-15 -7.356986e-15
-2.476580e-15
E:\\R-3.1.2\\scripts\\mPDBS\\summary_pdb_93\\219f45328d47579a.final.pdb
7.564946e-16 -1.541697e-14 7.862341e-15 2.945079e-14 1.856155e-15
-4.894337e-15
E:\\R-3.1.2\\scripts\\mPDBS\\summary_pdb_93\\238dd3918e54336d.final.pdb
1.478870e-14 -1.256942e-14 1.256127e-14 6.631533e-15 5.002944e-15
-8.810546e-16
E:\\R-3.1.2\\scripts\\mPDBS\\summary_pdb_93\\2b3027889e2fdc42.final.pdb
-2.575421e-15 -6.673963e-15 1.402197e-16 5.215945e-15 1.445026e-15
-1.870714e-15
E:\\R-3.1.2\\scripts\\mPDBS\\summary_pdb_93\\2fcb3b46e33793b0.final.pdb
-2.059710e-15 -6.907660e-15 -2.604787e-15 5.217610e-15 -6.489016e-15
3.060378e-15
E:\\R-3.1.2\\scripts\\mPDBS\\summary_pdb_93\\34eb328251842006.final.pdb
-5.592438e-15 -8.349849e-15 7.980499e-15 9.155105e-15 5.957782e-15
-5.526648e-15
E:\\R-3.1.2\\scripts\\mPDBS\\summary_pdb_93\\354b32871797b7ab.final.pdb
-1.374649e-14 -1.084870e-14 -1.493607e-14 3.296754e-15 -5.435632e-15
8.529531e-15
E:\\R-3.1.2\\scripts\\mPDBS\\summary_pdb_93\\397368adbc32d5de.final.pdb
-4.616332e-15 -5.286338e-15 2.621846e-15 8.162622e-15 5.685792e-15
-4.206199e-15
E:\\R-3.1.2\\scripts\\mPDBS\\summary_pdb_93\\411ca13cd3df6edd.final.pdb
-8.810441e-15 -1.246745e-14 1.146987e-15 1.877508e-15 -4.528778e-15
2.420265e-15
E:\\R-3.1.2\\scripts\\mPDBS\\summary_pdb_93\\42357eee6642bf12.final.pdb
-4.051942e-15 -2.720076e-15 4.570417e-15 1.218277e-14 -1.765182e-15
-7.367865e-15
E:\\R-3.1.2\\scripts\\mPDBS\\summary_pdb_93\\44342d1f75a7fcfc.final.pdb
-6.262635e-15 1.957565e-15 8.520355e-16 2.370632e-14 1.211844e-14

-1.054074e-14
E:\\R-3.1.2\\scripts\\mPDBS\\summary_pdb_93\\448ef21dffe8cdfb.final.pdb
-1.489666e-14 -7.379505e-15 -2.316822e-15 9.992002e-15 5.182204e-15
9.652327e-15
E:\\R-3.1.2\\scripts\\mPDBS\\summary_pdb_93\\472a40b3e8f113c7.final.pdb
1.194070e-15 -7.258719e-15 -1.992477e-16 -6.929797e-15 -2.956600e-15
7.568450e-16
E:\\R-3.1.2\\scripts\\mPDBS\\summary_pdb_93\\48a26225951ea153.final.pdb
-1.679235e-14 2.527873e-16 -5.603450e-15 1.358187e-14 1.269819e-15
6.862681e-16
E:\\R-3.1.2\\scripts\\mPDBS\\summary_pdb_93\\4ab4a5c7c6682ce6.final.pdb
-7.062292e-15 1.472543e-14 -8.341606e-15 1.314345e-14 -1.080796e-14
1.405003e-14
E:\\R-3.1.2\\scripts\\mPDBS\\summary_pdb_93\\4eaa45b62cb6a25f.final.pdb
-6.020854e-16 -1.204507e-15 2.527829e-15 3.123770e-15 -2.458847e-15
-1.319362e-15
E:\\R-3.1.2\\scripts\\mPDBS\\summary_pdb_93\\58c371611d85ac96.final.pdb
-4.616332e-15 -5.286338e-15 2.621846e-15 8.162622e-15 5.685792e-15
-4.206199e-15
E:\\R-3.1.2\\scripts\\mPDBS\\summary_pdb_93\\59a647f04822d30a.final.pdb
-3.129725e-15 -4.663875e-15 -1.891430e-15 6.888397e-15 -5.995538e-15
4.695463e-16
E:\\R-3.1.2\\scripts\\mPDBS\\summary_pdb_93\\59b091fe385e05fc.final.pdb
-2.262792e-15 -5.461392e-15 -1.227610e-15 2.397127e-15 -6.713368e-16
-3.154410e-15
E:\\R-3.1.2\\scripts\\mPDBS\\summary_pdb_93\\5c35ac30f930d2db.final.pdb
-6.020854e-16 -1.204507e-15 2.527829e-15 3.123770e-15 -2.458847e-15
-1.319362e-15
E:\\R-3.1.2\\scripts\\mPDBS\\summary_pdb_93\\5f60752431cf2dcb.final.pdb
-4.885642e-15 -1.019513e-14 5.444169e-17 -5.983723e-15 1.112582e-15
-2.583890e-15
E:\\R-3.1.2\\scripts\\mPDBS\\summary_pdb_93\\626456dd6ff2868d.final.pdb
1.194070e-15 -7.258719e-15 -1.992477e-16 -6.929797e-15 -2.956600e-15
7.568450e-16
E:\\R-3.1.2\\scripts\\mPDBS\\summary_pdb_93\\6583d53ff3c8f7ef.final.pdb
-3.129725e-15 -4.663875e-15 -1.891430e-15 6.888397e-15 -5.995538e-15
4.695463e-16
E:\\R-3.1.2\\scripts\\mPDBS\\summary_pdb_93\\69760178986bef01.final.pdb
4.276947e-15 -7.699684e-15 6.889619e-16 -2.040930e-15 6.322293e-15
3.524610e-15
E:\\R-3.1.2\\scripts\\mPDBS\\summary_pdb_93\\6a9d87d47926b1c8.final.pdb
-3.046407e-15 -5.119805e-15 9.435425e-17 5.996820e-15 2.318218e-16
-9.310409e-16
E:\\R-3.1.2\\scripts\\mPDBS\\summary_pdb_93\\6bb3547ea982ffc4.final.pdb
-4.938191e-15 -2.538784e-14 9.685022e-15 1.403124e-15 1.187307e-14
9.157139e-16
E:\\R-3.1.2\\scripts\\mPDBS\\summary_pdb_93\\716d9624611d1c42.final.pdb
-1.447474e-15 -4.477184e-16 1.861156e-15 1.649645e-14 -6.204481e-15
3.312435e-15
E:\\R-3.1.2\\scripts\\mPDBS\\summary_pdb_93\\75f27e70ad0f1be4.final.pdb
-3.129725e-15 -4.663875e-15 -1.891430e-15 6.888397e-15 -5.995538e-15
4.695463e-16
E:\\R-3.1.2\\scripts\\mPDBS\\summary_pdb_93\\769f910fffb22872.final.pdb
-6.604602e-16 -4.207705e-15 6.436003e-16 4.224712e-15 -1.117186e-15

3.102536e-16
E:\\R-3.1.2\\scripts\\mPDBS\\summary_pdb_93\\791f8c80dd5c3135.final.pdb
-2.262792e-15 -5.461392e-15 -1.227610e-15 2.397127e-15 -6.713368e-16
-3.154410e-15
E:\\R-3.1.2\\scripts\\mPDBS\\summary_pdb_93\\7fad98e66a3f6099.final.pdb
4.555142e-16 -7.826586e-16 1.284416e-14 1.460685e-15 9.538776e-15
2.860840e-15
E:\\R-3.1.2\\scripts\\mPDBS\\summary_pdb_93\\804b89c94a633196.final.pdb
-2.059710e-15 -6.907660e-15 -2.604787e-15 5.217610e-15 -6.489016e-15
3.060378e-15
E:\\R-3.1.2\\scripts\\mPDBS\\summary_pdb_93\\813cc65a2298feba.final.pdb
-2.059710e-15 -6.907660e-15 -2.604787e-15 5.217610e-15 -6.489016e-15
3.060378e-15
E:\\R-3.1.2\\scripts\\mPDBS\\summary_pdb_93\\83c0eefcfe12d3f9.final.pdb
-8.810441e-15 -1.246745e-14 1.146987e-15 1.877508e-15 -4.528778e-15
2.420265e-15
E:\\R-3.1.2\\scripts\\mPDBS\\summary_pdb_93\\86d50469ef569760.final.pdb
1.194070e-15 -7.258719e-15 -1.992477e-16 -6.929797e-15 -2.956600e-15
7.568450e-16
E:\\R-3.1.2\\scripts\\mPDBS\\summary_pdb_93\\89d0666e1402203d.final.pdb
-3.129725e-15 -4.663875e-15 -1.891430e-15 6.888397e-15 -5.995538e-15
4.695463e-16
E:\\R-3.1.2\\scripts\\mPDBS\\summary_pdb_93\\8b34852716dadc62.final.pdb
-6.087738e-15 -1.897778e-15 5.815561e-15 6.570260e-15 1.775207e-15
-4.412811e-15
E:\\R-3.1.2\\scripts\\mPDBS\\summary_pdb_93\\8b6ef9ac3fe74395.final.pdb
1.844150e-15 -1.550789e-14 8.112364e-15 1.100121e-15 5.680619e-15
-6.548403e-15
E:\\R-3.1.2\\scripts\\mPDBS\\summary_pdb_93\\98bbe0d5986c138f.final.pdb
-2.262792e-15 -5.461392e-15 -1.227610e-15 2.397127e-15 -6.713368e-16
-3.154410e-15
E:\\R-3.1.2\\scripts\\mPDBS\\summary_pdb_93\\9c22f4909d67e2fa.final.pdb
2.615776e-15 -3.043563e-15 4.110302e-16 6.573350e-15 8.531955e-15
-5.114507e-15
E:\\R-3.1.2\\scripts\\mPDBS\\summary_pdb_93\\9dda8ed0d81fb3ef.final.pdb
-8.424512e-16 1.412491e-14 -1.173165e-14 -1.036337e-14 -1.023821e-15
-1.279055e-15
E:\\R-3.1.2\\scripts\\mPDBS\\summary_pdb_93\\9f752b3616e6033b.final.pdb
8.874386e-16 -8.282750e-15 7.066740e-16 4.732343e-15 1.823681e-15
-4.257779e-16
E:\\R-3.1.2\\scripts\\mPDBS\\summary_pdb_93\\a02c70083816d6ba.final.pdb
-6.020854e-16 -1.204507e-15 2.527829e-15 3.123770e-15 -2.458847e-15
-1.319362e-15
E:\\R-3.1.2\\scripts\\mPDBS\\summary_pdb_93\\a0c997d891e4e082.final.pdb
-1.696866e-15 -1.595261e-14 -3.617271e-15 3.867576e-15 3.407271e-15
7.315095e-15
E:\\R-3.1.2\\scripts\\mPDBS\\summary_pdb_93\\ab8039a2d823c616.final.pdb
-3.317505e-15 -5.590239e-15 -7.779622e-16 6.734803e-15 -7.959636e-16
3.059449e-16
E:\\R-3.1.2\\scripts\\mPDBS\\summary_pdb_93\\acf4d74b9c71d4dc.final.pdb
-2.059710e-15 -6.907660e-15 -2.604787e-15 5.217610e-15 -6.489016e-15
3.060378e-15
E:\\R-3.1.2\\scripts\\mPDBS\\summary_pdb_93\\ad8a823e4b4c668e.final.pdb
-1.462237e-15 -5.905113e-15 -5.805972e-18 5.278549e-15 -2.777182e-17

-4.342507e-16
E:\\R-3.1.2\\scripts\\mPDBS\\summary_pdb_93\\b16ababd03b7b857.final.pdb
2.615776e-15 -3.043563e-15 4.110302e-16 6.573350e-15 8.531955e-15
-5.114507e-15
E:\\R-3.1.2\\scripts\\mPDBS\\summary_pdb_93\\b226923e697d3c63.final.pdb
1.863270e-15 -1.048474e-14 3.059905e-15 -1.646280e-15 -5.723187e-15
-3.914967e-15
E:\\R-3.1.2\\scripts\\mPDBS\\summary_pdb_93\\b28e48f6fa27a165.final.pdb
-2.262792e-15 -5.461392e-15 -1.227610e-15 2.397127e-15 -6.713368e-16
-3.154410e-15
E:\\R-3.1.2\\scripts\\mPDBS\\summary_pdb_93\\b42c4924e3cd1aec.final.pdb
-1.221556e-15 -1.427685e-14 4.638503e-15 1.268980e-14 1.025709e-14
9.353195e-16
E:\\R-3.1.2\\scripts\\mPDBS\\summary_pdb_93\\b4dd239fcabff1df.final.pdb
-2.262792e-15 -5.461392e-15 -1.227610e-15 2.397127e-15 -6.713368e-16
-3.154410e-15
E:\\R-3.1.2\\scripts\\mPDBS\\summary_pdb_93\\b669942ef97459a2.final.pdb
-6.087738e-15 -1.897778e-15 5.815561e-15 6.570260e-15 1.775207e-15
-4.412811e-15
E:\\R-3.1.2\\scripts\\mPDBS\\summary_pdb_93\\b99f4d9d87c1017b.final.pdb
-2.262792e-15 -5.461392e-15 -1.227610e-15 2.397127e-15 -6.713368e-16
-3.154410e-15
E:\\R-3.1.2\\scripts\\mPDBS\\summary_pdb_93\\b9d2cbf3975403e7.final.pdb
-2.262792e-15 -5.461392e-15 -1.227610e-15 2.397127e-15 -6.713368e-16
-3.154410e-15
E:\\R-3.1.2\\scripts\\mPDBS\\summary_pdb_93\\bc88c8a505f3a288.final.pdb
-2.735531e-15 -5.210724e-15 7.885726e-16 4.258377e-15 8.899143e-16
-1.217591e-15
E:\\R-3.1.2\\scripts\\mPDBS\\summary_pdb_93\\bdac0e64f4ef427e.final.pdb
1.443526e-14 9.111644e-15 -1.310683e-15 9.003860e-15 8.216235e-15
-2.528034e-15
E:\\R-3.1.2\\scripts\\mPDBS\\summary_pdb_93\\ca2a1f55fc9d0681.final.pdb
-6.087738e-15 -1.897778e-15 5.815561e-15 6.570260e-15 1.775207e-15
-4.412811e-15
E:\\R-3.1.2\\scripts\\mPDBS\\summary_pdb_93\\ce34248de3e81a3c.final.pdb
-4.965882e-15 3.146307e-15 -3.192290e-15 1.222073e-14 -4.802181e-15
-3.325563e-15
E:\\R-3.1.2\\scripts\\mPDBS\\summary_pdb_93\\d02e697772494a61.final.pdb
-4.259865e-15 -1.777533e-14 8.874986e-15 1.247321e-14 -1.023834e-14
-1.080548e-15
E:\\R-3.1.2\\scripts\\mPDBS\\summary_pdb_93\\d04221245d015047.final.pdb
-2.845253e-15 -3.726278e-15 -2.345601e-16 6.997017e-15 1.377645e-15
1.068356e-15
E:\\R-3.1.2\\scripts\\mPDBS\\summary_pdb_93\\d678141e4018054a.final.pdb
-3.034369e-15 -1.513819e-14 6.113702e-15 1.008751e-14 3.184825e-15
1.678826e-15
E:\\R-3.1.2\\scripts\\mPDBS\\summary_pdb_93\\d71b40af0f0efe39.final.pdb
-3.092528e-15 6.534191e-15 -8.169142e-15 2.672461e-15 -1.148093e-14
1.172827e-15
E:\\R-3.1.2\\scripts\\mPDBS\\summary_pdb_93\\dc0809eff10197e2.final.pdb
3.006267e-15 4.475882e-15 -4.849830e-15 2.369872e-15 1.373511e-15
-5.527208e-16
E:\\R-3.1.2\\scripts\\mPDBS\\summary_pdb_93\\e1163918d3707839.final.pdb
-9.396634e-16 -1.927285e-14 6.166795e-15 -4.893791e-15 -1.341275e-15

2.465346e-15
E:\\R-3.1.2\\scripts\\mPDBS\\summary_pdb_93\\e4b48b06824ef848.final.pdb
-1.793208e-15 -1.321993e-15 -2.393033e-15 1.484662e-15 -7.356986e-15
-2.476580e-15
E:\\R-3.1.2\\scripts\\mPDBS\\summary_pdb_93\\e529e19ad281fea2.final.pdb
-1.538119e-15 -5.329706e-15 -2.551533e-15 1.674050e-14 -9.165652e-16
-2.041918e-16
E:\\R-3.1.2\\scripts\\mPDBS\\summary_pdb_93\\e5602082718ec3a1.final.pdb
-2.059710e-15 -6.907660e-15 -2.604787e-15 5.217610e-15 -6.489016e-15
3.060378e-15
E:\\R-3.1.2\\scripts\\mPDBS\\summary_pdb_93\\e5d589d996d0a960.final.pdb
-9.488401e-15 -1.034426e-14 -5.832023e-15 5.360601e-15 1.093890e-14
6.919464e-15
E:\\R-3.1.2\\scripts\\mPDBS\\summary_pdb_93\\e9df9e72307a2fbd.final.pdb
-6.020854e-16 -1.204507e-15 2.527829e-15 3.123770e-15 -2.458847e-15
-1.319362e-15
E:\\R-3.1.2\\scripts\\mPDBS\\summary_pdb_93\\ea00f8694090cae7.final.pdb
-2.059710e-15 -6.907660e-15 -2.604787e-15 5.217610e-15 -6.489016e-15
3.060378e-15
E:\\R-3.1.2\\scripts\\mPDBS\\summary_pdb_93\\eba517be32db050e.final.pdb
-2.262792e-15 -5.461392e-15 -1.227610e-15 2.397127e-15 -6.713368e-16
-3.154410e-15
E:\\R-3.1.2\\scripts\\mPDBS\\summary_pdb_93\\ec04b9545899c29d.final.pdb
-2.834388e-15 -5.971856e-15 -1.350221e-16 4.888694e-15 4.715108e-16
-6.545545e-16
E:\\R-3.1.2\\scripts\\mPDBS\\summary_pdb_93\\ee6862cbcad52078.final.pdb
-2.558996e-15 -5.352104e-15 -3.498733e-17 5.984102e-15 1.439832e-16
-8.888608e-16
E:\\R-3.1.2\\scripts\\mPDBS\\summary_pdb_93\\ef6a42fc83b81e5d.final.pdb
-2.113752e-15 -5.554718e-15 5.915586e-16 4.935136e-15 -2.741104e-16
-1.659280e-16
E:\\R-3.1.2\\scripts\\mPDBS\\summary_pdb_93\\ef8e88d4ad93a0ad.final.pdb
-2.845253e-15 -3.726278e-15 -2.345601e-16 6.997017e-15 1.377645e-15
1.068356e-15
E:\\R-3.1.2\\scripts\\mPDBS\\summary_pdb_93\\f14fcae8deec4fa3.final.pdb
8.874386e-16 -8.282750e-15 7.066740e-16 4.732343e-15 1.823681e-15
-4.257779e-16
E:\\R-3.1.2\\scripts\\mPDBS\\summary_pdb_93\\f83584e22003fb92.final.pdb
-1.679235e-14 2.527873e-16 -5.603450e-15 1.358187e-14 1.269819e-15
6.862681e-16
E:\\R-3.1.2\\scripts\\mPDBS\\summary_pdb_93\\f8e2bf87e52caa29.final.pdb
-1.538119e-15 -5.329706e-15 -2.551533e-15 1.674050e-14 -9.165652e-16
-2.041918e-16

[,127]   [,128]   [,129]   [,130]   [,131]   [,132]
E:\\R-3.1.2\\scripts\\40.1ns_data\\NAT0.pdb
-8.939742e-15 -1.450618e-15 7.011569e-15 1.869591e-15 8.781945e-15
5.133857e-16
E:\\R-3.1.2\\scripts\\40.1ns_data\\LJP6.pdb
-9.196208e-15 -1.089236e-16 5.730704e-15 9.585842e-16 4.171892e-15
-4.516574e-16
E:\\R-3.1.2\\scripts\\40.1ns_data\\3WY9.pdb
-5.499706e-15 -1.323283e-15 5.946544e-15 1.704267e-15 4.520106e-15
-5.157450e-16

E:\\R-3.1.2\\scripts\\40.1ns_data\\ZSP9.pdb
-7.578054e-15 1.262613e-15 4.933958e-15 4.172151e-15 6.894975e-15
-4.826474e-16
E:\\R-3.1.2\\scripts\\mPDBS\\summary_pdb_93\\013d855ad26c877c.final.pdb
-1.136101e-14 1.449385e-15 4.974558e-16 -5.127062e-15 3.129546e-15
-1.256974e-16
E:\\R-3.1.2\\scripts\\mPDBS\\summary_pdb_93\\0a2a705342ca0d54.final.pdb
-8.215876e-15 -2.052308e-15 3.683535e-15 1.214531e-15 4.856472e-15
-1.073259e-15
E:\\R-3.1.2\\scripts\\mPDBS\\summary_pdb_93\\0a59fceadb4194d9.final.pdb
-9.378224e-15 -1.984583e-15 6.362861e-15 2.555830e-15 1.236234e-15
-4.477137e-15
E:\\R-3.1.2\\scripts\\mPDBS\\summary_pdb_93\\0dba59f3306825e8.final.pdb
-7.620951e-15 3.534523e-15 3.424802e-15 7.730709e-15 1.179717e-15
-4.087806e-15
E:\\R-3.1.2\\scripts\\mPDBS\\summary_pdb_93\\126f26a78b57339c.final.pdb
-5.095411e-15 -5.418332e-16 6.993778e-15 5.180509e-15 6.202609e-15
-2.929011e-15
E:\\R-3.1.2\\scripts\\mPDBS\\summary_pdb_93\\12f06f3e342df706.final.pdb
-6.912122e-15 -1.203730e-15 4.272582e-15 2.088950e-15 5.423293e-15
-7.046303e-16
E:\\R-3.1.2\\scripts\\mPDBS\\summary_pdb_93\\18618a2a251b867b.final.pdb
-1.156275e-14 -4.135107e-17 2.358542e-15 6.183898e-15 9.531152e-15
4.493651e-15
E:\\R-3.1.2\\scripts\\mPDBS\\summary_pdb_93\\1f305f598641eeb2.final.pdb
-6.989829e-15 -1.487392e-16 3.703108e-15 2.519168e-15 5.245201e-15
-1.507046e-15
E:\\R-3.1.2\\scripts\\mPDBS\\summary_pdb_93\\1f8a00b551be0a39.final.pdb
-1.156275e-14 -4.135107e-17 2.358542e-15 6.183898e-15 9.531152e-15
4.493651e-15
E:\\R-3.1.2\\scripts\\mPDBS\\summary_pdb_93\\219f45328d47579a.final.pdb
-4.723961e-15 4.850228e-15 -1.078185e-14 -6.017174e-15 1.298684e-15
6.278148e-15
E:\\R-3.1.2\\scripts\\mPDBS\\summary_pdb_93\\238dd3918e54336d.final.pdb
-8.583829e-15 4.787886e-15 -2.191711e-15 1.411942e-14 1.376267e-14
3.301363e-15
E:\\R-3.1.2\\scripts\\mPDBS\\summary_pdb_93\\2b3027889e2fdc42.final.pdb
-8.165869e-15 9.906848e-16 3.539707e-15 2.828022e-15 3.937198e-15
-2.108372e-15
E:\\R-3.1.2\\scripts\\mPDBS\\summary_pdb_93\\2fcb3b46e33793b0.final.pdb
-7.940458e-15 -3.101239e-15 -2.525417e-15 1.853035e-15 4.572128e-15
1.199122e-15
E:\\R-3.1.2\\scripts\\mPDBS\\summary_pdb_93\\34eb328251842006.final.pdb
-6.884183e-15 6.990107e-16 1.286881e-14 -4.966296e-16 6.172776e-15
-9.354036e-16
E:\\R-3.1.2\\scripts\\mPDBS\\summary_pdb_93\\354b32871797b7ab.final.pdb
-4.058805e-15 1.771552e-15 9.888933e-15 4.832472e-15 -4.890106e-15
-4.944685e-15
E:\\R-3.1.2\\scripts\\mPDBS\\summary_pdb_93\\397368adbc32d5de.final.pdb
-5.095411e-15 -5.418332e-16 6.993778e-15 5.180509e-15 6.202609e-15
-2.929011e-15
E:\\R-3.1.2\\scripts\\mPDBS\\summary_pdb_93\\411ca13cd3df6edd.final.pdb
-6.809201e-15 -1.429209e-15 1.512369e-15 6.797152e-15 4.600751e-15
4.397952e-15

E:\\R-3.1.2\\scripts\\mPDBS\\summary_pdb_93\\42357eee6642bf12.final.pdb
-1.386531e-14 -4.144852e-15 9.558776e-15 -4.294208e-15 7.354863e-15
-8.725539e-16
E:\\R-3.1.2\\scripts\\mPDBS\\summary_pdb_93\\44342d1f75a7fcfc.final.pdb
-6.683213e-15 -2.095667e-14 6.043104e-15 -5.614128e-15 7.757900e-15
-2.179893e-16
E:\\R-3.1.2\\scripts\\mPDBS\\summary_pdb_93\\448ef21dffe8cdfb.final.pdb
-7.773924e-15 1.076614e-14 1.525550e-14 6.620055e-15 1.645845e-14
2.038728e-15
E:\\R-3.1.2\\scripts\\mPDBS\\summary_pdb_93\\472a40b3e8f113c7.final.pdb
-7.620951e-15 3.534523e-15 3.424802e-15 7.730709e-15 1.179717e-15
-4.087806e-15
E:\\R-3.1.2\\scripts\\mPDBS\\summary_pdb_93\\48a26225951ea153.final.pdb
-5.256755e-17 -5.884024e-15 7.814963e-16 2.127194e-15 1.007812e-14
1.453015e-15
E:\\R-3.1.2\\scripts\\mPDBS\\summary_pdb_93\\4ab4a5c7c6682ce6.final.pdb
-1.063913e-15 -1.440921e-14 -4.246362e-15 -1.369807e-15 8.922656e-15
1.234326e-15
E:\\R-3.1.2\\scripts\\mPDBS\\summary_pdb_93\\4eaa45b62cb6a25f.final.pdb
-6.934868e-15 -3.293949e-15 2.077690e-15 1.481068e-15 6.517435e-15
-1.586261e-15
E:\\R-3.1.2\\scripts\\mPDBS\\summary_pdb_93\\58c371611d85ac96.final.pdb
-5.095411e-15 -5.418332e-16 6.993778e-15 5.180509e-15 6.202609e-15
-2.929011e-15
E:\\R-3.1.2\\scripts\\mPDBS\\summary_pdb_93\\59a647f04822d30a.final.pdb
-6.169735e-15 2.814244e-15 1.980382e-15 1.158232e-15 7.246156e-15
-5.145589e-17
E:\\R-3.1.2\\scripts\\mPDBS\\summary_pdb_93\\59b091fe385e05fc.final.pdb
-9.378224e-15 -1.984583e-15 6.362861e-15 2.555830e-15 1.236234e-15
-4.477137e-15
E:\\R-3.1.2\\scripts\\mPDBS\\summary_pdb_93\\5c35ac30f930d2db.final.pdb
-6.934868e-15 -3.293949e-15 2.077690e-15 1.481068e-15 6.517435e-15
-1.586261e-15
E:\\R-3.1.2\\scripts\\mPDBS\\summary_pdb_93\\5f60752431cf2dcb.final.pdb
-7.621082e-15 -1.880353e-15 4.161126e-15 2.669804e-15 6.808036e-15
3.778372e-16
E:\\R-3.1.2\\scripts\\mPDBS\\summary_pdb_93\\626456dd6ff2868d.final.pdb
-7.620951e-15 3.534523e-15 3.424802e-15 7.730709e-15 1.179717e-15
-4.087806e-15
E:\\R-3.1.2\\scripts\\mPDBS\\summary_pdb_93\\6583d53ff3c8f7ef.final.pdb
-6.169735e-15 2.814244e-15 1.980382e-15 1.158232e-15 7.246156e-15
-5.145589e-17
E:\\R-3.1.2\\scripts\\mPDBS\\summary_pdb_93\\69760178986bef01.final.pdb
-8.111713e-15 7.709332e-15 -2.536614e-15 1.614476e-14 3.869753e-15
2.727082e-15
E:\\R-3.1.2\\scripts\\mPDBS\\summary_pdb_93\\6a9d87d47926b1c8.final.pdb
-7.891785e-15 -4.308109e-16 2.993506e-15 2.169029e-15 4.218085e-15
-2.221243e-15
E:\\R-3.1.2\\scripts\\mPDBS\\summary_pdb_93\\6bb3547ea982ffc4.final.pdb
-8.122104e-15 4.557601e-15 1.880647e-14 1.696036e-15 -1.555720e-15
6.388989e-15
E:\\R-3.1.2\\scripts\\mPDBS\\summary_pdb_93\\716d9624611d1c42.final.pdb
-8.319959e-15 1.281831e-15 5.052665e-15 2.248426e-15 4.596263e-15
-2.939821e-15

E:\\R-3.1.2\\scripts\\mPDBS\\summary_pdb_93\\75f27e70ad0f1be4.final.pdb
-6.169735e-15 2.814244e-15 1.980382e-15 1.158232e-15 7.246156e-15
-5.145589e-17
E:\\R-3.1.2\\scripts\\mPDBS\\summary_pdb_93\\769f910fffb22872.final.pdb
-6.525007e-15 -2.176904e-15 4.669736e-15 2.922696e-15 3.981771e-15
-9.195533e-16
E:\\R-3.1.2\\scripts\\mPDBS\\summary_pdb_93\\791f8c80dd5c3135.final.pdb
-9.378224e-15 -1.984583e-15 6.362861e-15 2.555830e-15 1.236234e-15
-4.477137e-15
E:\\R-3.1.2\\scripts\\mPDBS\\summary_pdb_93\\7fad98e66a3f6099.final.pdb
-9.415274e-16 -1.211394e-14 8.453305e-15 3.921927e-15 -1.171047e-14
-4.399114e-15
E:\\R-3.1.2\\scripts\\mPDBS\\summary_pdb_93\\804b89c94a633196.final.pdb
-7.940458e-15 -3.101239e-15 -2.525417e-15 1.853035e-15 4.572128e-15
1.199122e-15
E:\\R-3.1.2\\scripts\\mPDBS\\summary_pdb_93\\813cc65a2298feba.final.pdb
-7.940458e-15 -3.101239e-15 -2.525417e-15 1.853035e-15 4.572128e-15
1.199122e-15
E:\\R-3.1.2\\scripts\\mPDBS\\summary_pdb_93\\83c0eefcfe12d3f9.final.pdb
-6.809201e-15 -1.429209e-15 1.512369e-15 6.797152e-15 4.600751e-15
4.397952e-15
E:\\R-3.1.2\\scripts\\mPDBS\\summary_pdb_93\\86d50469ef569760.final.pdb
-7.620951e-15 3.534523e-15 3.424802e-15 7.730709e-15 1.179717e-15
-4.087806e-15
E:\\R-3.1.2\\scripts\\mPDBS\\summary_pdb_93\\89d0666e1402203d.final.pdb
-6.169735e-15 2.814244e-15 1.980382e-15 1.158232e-15 7.246156e-15
-5.145589e-17
E:\\R-3.1.2\\scripts\\mPDBS\\summary_pdb_93\\8b34852716dadc62.final.pdb
-4.047738e-15 -9.328896e-15 5.189766e-15 1.811401e-15 3.871300e-15
-2.020525e-15
E:\\R-3.1.2\\scripts\\mPDBS\\summary_pdb_93\\8b6ef9ac3fe74395.final.pdb
-2.049252e-14 -7.062694e-15 1.727017e-14 -2.023040e-15 -3.384869e-15
1.204812e-15
E:\\R-3.1.2\\scripts\\mPDBS\\summary_pdb_93\\98bbe0d5986c138f.final.pdb
-9.378224e-15 -1.984583e-15 6.362861e-15 2.555830e-15 1.236234e-15
-4.477137e-15
E:\\R-3.1.2\\scripts\\mPDBS\\summary_pdb_93\\9c22f4909d67e2fa.final.pdb
-4.002527e-15 9.307103e-15 4.403719e-15 1.303209e-15 6.137716e-15
-1.214437e-14
E:\\R-3.1.2\\scripts\\mPDBS\\summary_pdb_93\\9dda8ed0d81fb3ef.final.pdb
-5.590554e-15 -9.359828e-15 1.513978e-14 7.980740e-15 8.790060e-15
-1.685497e-16
E:\\R-3.1.2\\scripts\\mPDBS\\summary_pdb_93\\9f752b3616e6033b.final.pdb
-7.524106e-15 1.962190e-15 3.812068e-15 2.589672e-15 6.108987e-15
9.517900e-16
E:\\R-3.1.2\\scripts\\mPDBS\\summary_pdb_93\\a02c70083816d6ba.final.pdb
-6.934868e-15 -3.293949e-15 2.077690e-15 1.481068e-15 6.517435e-15
-1.586261e-15
E:\\R-3.1.2\\scripts\\mPDBS\\summary_pdb_93\\a0c997d891e4e082.final.pdb
-2.379010e-15 -2.152144e-15 -1.712500e-15 -6.562952e-15 3.855748e-15
2.024315e-15
E:\\R-3.1.2\\scripts\\mPDBS\\summary_pdb_93\\ab8039a2d823c616.final.pdb
-7.428898e-15 -2.675922e-15 5.005433e-15 2.637940e-15 5.387509e-15
-2.118205e-15

E:\\R-3.1.2\\scripts\\mPDBS\\summary_pdb_93\\acf4d74b9c71d4dc.final.pdb
-7.940458e-15 -3.101239e-15 -2.525417e-15 1.853035e-15 4.572128e-15
1.199122e-15
E:\\R-3.1.2\\scripts\\mPDBS\\summary_pdb_93\\ad8a823e4b4c668e.final.pdb
-7.382886e-15 -2.049879e-16 5.476811e-15 3.127681e-15 5.986846e-15
-1.030078e-15
E:\\R-3.1.2\\scripts\\mPDBS\\summary_pdb_93\\b16ababd03b7b857.final.pdb
-4.002527e-15 9.307103e-15 4.403719e-15 1.303209e-15 6.137716e-15
-1.214437e-14
E:\\R-3.1.2\\scripts\\mPDBS\\summary_pdb_93\\b226923e697d3c63.final.pdb
-2.142674e-14 -7.401434e-15 1.822847e-15 -3.442333e-15 1.722797e-15
4.331323e-15
E:\\R-3.1.2\\scripts\\mPDBS\\summary_pdb_93\\b28e48f6fa27a165.final.pdb
-9.378224e-15 -1.984583e-15 6.362861e-15 2.555830e-15 1.236234e-15
-4.477137e-15
E:\\R-3.1.2\\scripts\\mPDBS\\summary_pdb_93\\b42c4924e3cd1aec.final.pdb
-1.838022e-15 2.888729e-15 8.551969e-16 1.192140e-16 -2.547879e-16
-8.905485e-15
E:\\R-3.1.2\\scripts\\mPDBS\\summary_pdb_93\\b4dd239fcabff1df.final.pdb
-9.378224e-15 -1.984583e-15 6.362861e-15 2.555830e-15 1.236234e-15
-4.477137e-15
E:\\R-3.1.2\\scripts\\mPDBS\\summary_pdb_93\\b669942ef97459a2.final.pdb
-4.047738e-15 -9.328896e-15 5.189766e-15 1.811401e-15 3.871300e-15
-2.020525e-15
E:\\R-3.1.2\\scripts\\mPDBS\\summary_pdb_93\\b99f4d9d87c1017b.final.pdb
-9.378224e-15 -1.984583e-15 6.362861e-15 2.555830e-15 1.236234e-15
-4.477137e-15
E:\\R-3.1.2\\scripts\\mPDBS\\summary_pdb_93\\b9d2cbf3975403e7.final.pdb
-9.378224e-15 -1.984583e-15 6.362861e-15 2.555830e-15 1.236234e-15
-4.477137e-15
E:\\R-3.1.2\\scripts\\mPDBS\\summary_pdb_93\\bc88c8a505f3a288.final.pdb
-8.031970e-15 -4.529304e-16 3.619938e-15 2.998262e-15 3.913346e-15
-7.266650e-16
E:\\R-3.1.2\\scripts\\mPDBS\\summary_pdb_93\\bdac0e64f4ef427e.final.pdb
-3.946799e-15 4.918036e-15 1.170192e-14 -5.666755e-15 4.909532e-15
-1.301260e-14
E:\\R-3.1.2\\scripts\\mPDBS\\summary_pdb_93\\ca2a1f55fc9d0681.final.pdb
-4.047738e-15 -9.328896e-15 5.189766e-15 1.811401e-15 3.871300e-15
-2.020525e-15
E:\\R-3.1.2\\scripts\\mPDBS\\summary_pdb_93\\ce34248de3e81a3c.final.pdb
-8.202804e-15 -1.258571e-14 5.606522e-15 -6.989974e-15 2.209879e-15
-8.527650e-15
E:\\R-3.1.2\\scripts\\mPDBS\\summary_pdb_93\\d02e697772494a61.final.pdb
-1.296401e-14 4.602271e-15 -3.598572e-15 -1.730753e-15 5.028339e-15
4.463628e-15
E:\\R-3.1.2\\scripts\\mPDBS\\summary_pdb_93\\d04221245d015047.final.pdb
-3.496172e-15 5.314729e-16 4.287908e-15 3.512322e-15 5.351080e-15
-3.249245e-15
E:\\R-3.1.2\\scripts\\mPDBS\\summary_pdb_93\\d678141e4018054a.final.pdb
-1.298869e-14 1.939244e-15 1.082565e-14 1.353287e-15 4.603239e-15
2.544409e-15
E:\\R-3.1.2\\scripts\\mPDBS\\summary_pdb_93\\d71b40af0f0efe39.final.pdb
1.188254e-15 1.945956e-15 -1.433067e-14 1.120201e-14 6.441575e-15
-1.626067e-15

E:\\R-3.1.2\\scripts\\mPDBS\\summary_pdb_93\\dc0809eff10197e2.final.pdb
-4.203512e-15 7.163701e-15 9.687419e-17 5.201121e-15 5.437558e-15
-5.364561e-15
E:\\R-3.1.2\\scripts\\mPDBS\\summary_pdb_93\\e1163918d3707839.final.pdb
-3.737544e-15 1.036539e-14 1.171587e-14 9.224778e-15 2.303929e-15
-1.065756e-14
E:\\R-3.1.2\\scripts\\mPDBS\\summary_pdb_93\\e4b48b06824ef848.final.pdb
-1.156275e-14 -4.135107e-17 2.358542e-15 6.183898e-15 9.531152e-15
4.493651e-15
E:\\R-3.1.2\\scripts\\mPDBS\\summary_pdb_93\\e529e19ad281fea2.final.pdb
-1.136101e-14 1.449385e-15 4.974558e-16 -5.127062e-15 3.129546e-15
-1.256974e-16
E:\\R-3.1.2\\scripts\\mPDBS\\summary_pdb_93\\e5602082718ec3a1.final.pdb
-7.940458e-15 -3.101239e-15 -2.525417e-15 1.853035e-15 4.572128e-15
1.199122e-15
E:\\R-3.1.2\\scripts\\mPDBS\\summary_pdb_93\\e5d589d996d0a960.final.pdb
-3.598586e-15 4.547446e-15 1.709065e-15 -9.059504e-15 8.400077e-15
1.282659e-15
E:\\R-3.1.2\\scripts\\mPDBS\\summary_pdb_93\\e9df9e72307a2fbd.final.pdb
-6.934868e-15 -3.293949e-15 2.077690e-15 1.481068e-15 6.517435e-15
-1.586261e-15
E:\\R-3.1.2\\scripts\\mPDBS\\summary_pdb_93\\ea00f8694090cae7.final.pdb
-7.940458e-15 -3.101239e-15 -2.525417e-15 1.853035e-15 4.572128e-15
1.199122e-15
E:\\R-3.1.2\\scripts\\mPDBS\\summary_pdb_93\\eba517be32db050e.final.pdb
-9.378224e-15 -1.984583e-15 6.362861e-15 2.555830e-15 1.236234e-15
-4.477137e-15
E:\\R-3.1.2\\scripts\\mPDBS\\summary_pdb_93\\ec04b9545899c29d.final.pdb
-7.958235e-15 -3.932593e-16 4.026488e-15 1.963144e-15 6.399611e-15
-1.780975e-15
E:\\R-3.1.2\\scripts\\mPDBS\\summary_pdb_93\\ee6862cbcad52078.final.pdb
-7.723298e-15 -7.638699e-17 2.805810e-15 3.025940e-15 3.545151e-15
-1.840357e-15
E:\\R-3.1.2\\scripts\\mPDBS\\summary_pdb_93\\ef6a42fc83b81e5d.final.pdb
-7.730531e-15 -1.264548e-15 5.045521e-15 1.394486e-15 4.698212e-15
-8.518991e-16
E:\\R-3.1.2\\scripts\\mPDBS\\summary_pdb_93\\ef8e88d4ad93a0ad.final.pdb
-3.496172e-15 5.314729e-16 4.287908e-15 3.512322e-15 5.351080e-15
-3.249245e-15
E:\\R-3.1.2\\scripts\\mPDBS\\summary_pdb_93\\f14fcae8deec4fa3.final.pdb
-7.524106e-15 1.962190e-15 3.812068e-15 2.589672e-15 6.108987e-15
9.517900e-16
E:\\R-3.1.2\\scripts\\mPDBS\\summary_pdb_93\\f83584e22003fb92.final.pdb
-5.256755e-17 -5.884024e-15 7.814963e-16 2.127194e-15 1.007812e-14
1.453015e-15
E:\\R-3.1.2\\scripts\\mPDBS\\summary_pdb_93\\f8e2bf87e52caa29.final.pdb
-1.136101e-14 1.449385e-15 4.974558e-16 -5.127062e-15 3.129546e-15
-1.256974e-16

[,133]   [,134]   [,135]   [,136]   [,137]   [,138]
E:\\R-3.1.2\\scripts\\40.1ns_data\\NAT0.pdb
-5.383499e-15 5.346826e-15 2.698860e-15 6.052310e-17 3.719675e-15
-2.898946e-15
E:\\R-3.1.2\\scripts\\40.1ns_data\\LJP6.pdb

-4.072226e-15 5.722436e-15 2.144525e-15 -6.868074e-16 3.917434e-15
-2.988341e-15
E:\\R-3.1.2\\scripts\\40.1ns_data\\3WY9.pdb
-4.656236e-15 3.996145e-15 9.566815e-16 -1.384081e-15 5.019825e-15
-3.148144e-15
E:\\R-3.1.2\\scripts\\40.1ns_data\\ZSP9.pdb
-3.093207e-15 4.084113e-15 7.119749e-16 -1.964838e-15 4.059527e-15
-3.932775e-15
E:\\R-3.1.2\\scripts\\mPDBS\\summary_pdb_93\\013d855ad26c877c.final.pdb
-8.728513e-15 1.154755e-14 -3.695679e-15 -4.164576e-16 2.875614e-15
-3.920446e-15
E:\\R-3.1.2\\scripts\\mPDBS\\summary_pdb_93\\0a2a705342ca0d54.final.pdb
-4.483282e-15 5.717531e-15 9.827075e-16 -1.604424e-15 3.285622e-15
-4.791710e-15
E:\\R-3.1.2\\scripts\\mPDBS\\summary_pdb_93\\0a59fceadb4194d9.final.pdb
-3.816824e-15 4.591773e-15 7.589867e-16 3.472701e-16 2.348624e-15
-7.019075e-15
E:\\R-3.1.2\\scripts\\mPDBS\\summary_pdb_93\\0dba59f3306825e8.final.pdb
2.241878e-15 2.186981e-15 8.284794e-16 1.809193e-15 4.727865e-15
-3.408703e-15
E:\\R-3.1.2\\scripts\\mPDBS\\summary_pdb_93\\126f26a78b57339c.final.pdb
-5.874558e-16 7.313089e-15 1.806863e-15 -5.412027e-15 8.537318e-15
2.077617e-16
E:\\R-3.1.2\\scripts\\mPDBS\\summary_pdb_93\\12f06f3e342df706.final.pdb
-4.149348e-15 5.845684e-15 1.569044e-15 -2.545945e-15 3.483597e-15
-2.683154e-15
E:\\R-3.1.2\\scripts\\mPDBS\\summary_pdb_93\\18618a2a251b867b.final.pdb
-3.180478e-15 6.457424e-15 3.641107e-15 -7.340819e-15 3.452856e-15
-1.296419e-15
E:\\R-3.1.2\\scripts\\mPDBS\\summary_pdb_93\\1f305f598641eeb2.final.pdb
-3.706819e-15 5.630144e-15 8.588406e-16 -1.885269e-15 4.892576e-15
-3.555373e-15
E:\\R-3.1.2\\scripts\\mPDBS\\summary_pdb_93\\1f8a00b551be0a39.final.pdb
-3.180478e-15 6.457424e-15 3.641107e-15 -7.340819e-15 3.452856e-15
-1.296419e-15
E:\\R-3.1.2\\scripts\\mPDBS\\summary_pdb_93\\219f45328d47579a.final.pdb
-1.795829e-14 2.467605e-15 9.691078e-15 -1.041235e-14 5.875967e-15
2.192306e-15
E:\\R-3.1.2\\scripts\\mPDBS\\summary_pdb_93\\238dd3918e54336d.final.pdb
4.116934e-15 1.128520e-14 1.203076e-15 -7.260577e-15 -1.580308e-15
5.008630e-15
E:\\R-3.1.2\\scripts\\mPDBS\\summary_pdb_93\\2b3027889e2fdc42.final.pdb
-2.666702e-15 6.180780e-15 8.292430e-16 -1.200103e-15 3.386206e-15
-4.081755e-15
E:\\R-3.1.2\\scripts\\mPDBS\\summary_pdb_93\\2fcb3b46e33793b0.final.pdb
-7.585660e-15 3.055814e-15 3.433170e-15 3.010121e-15 2.666492e-15
-6.979717e-15
E:\\R-3.1.2\\scripts\\mPDBS\\summary_pdb_93\\34eb328251842006.final.pdb
-8.829188e-15 3.186827e-15 -2.999320e-15 5.564551e-15 -1.712522e-15
-1.105195e-14
E:\\R-3.1.2\\scripts\\mPDBS\\summary_pdb_93\\354b32871797b7ab.final.pdb
-1.300375e-14 -7.050117e-15 -1.314623e-16 4.251685e-15 -1.942759e-16
-9.415245e-15
E:\\R-3.1.2\\scripts\\mPDBS\\summary_pdb_93\\397368adbc32d5de.final.pdb

-5.874558e-16 7.313089e-15 1.806863e-15 -5.412027e-15 8.537318e-15
2.077617e-16
E:\\R-3.1.2\\scripts\\mPDBS\\summary_pdb_93\\411ca13cd3df6edd.final.pdb
-1.147318e-14 1.145883e-15 5.674433e-15 6.362908e-15 3.116219e-15
-8.962072e-15
E:\\R-3.1.2\\scripts\\mPDBS\\summary_pdb_93\\42357eee6642bf12.final.pdb
-1.657896e-14 -6.569895e-15 3.610011e-15 -1.235437e-15 -2.667238e-16
-9.617481e-15
E:\\R-3.1.2\\scripts\\mPDBS\\summary_pdb_93\\44342d1f75a7fcfc.final.pdb
-1.080662e-14 1.877031e-14 1.850498e-15 1.110733e-14 4.220631e-15
-1.054137e-14
E:\\R-3.1.2\\scripts\\mPDBS\\summary_pdb_93\\448ef21dffe8cdfb.final.pdb
-5.247253e-15 1.190290e-14 -2.811834e-15 -2.293961e-14 -1.188931e-15
-1.315845e-15
E:\\R-3.1.2\\scripts\\mPDBS\\summary_pdb_93\\472a40b3e8f113c7.final.pdb
2.241878e-15 2.186981e-15 8.284794e-16 1.809193e-15 4.727865e-15
-3.408703e-15
E:\\R-3.1.2\\scripts\\mPDBS\\summary_pdb_93\\48a26225951ea153.final.pdb
-1.667501e-15 1.360800e-14 -6.408023e-15 3.742991e-15 2.356430e-15
-5.471268e-15
E:\\R-3.1.2\\scripts\\mPDBS\\summary_pdb_93\\4ab4a5c7c6682ce6.final.pdb
-7.387572e-15 4.926109e-15 -3.147507e-15 -5.472743e-15 3.703078e-15
-7.239406e-15
E:\\R-3.1.2\\scripts\\mPDBS\\summary_pdb_93\\4eaa45b62cb6a25f.final.pdb
1.098685e-15 1.111070e-14 -2.338110e-16 -6.378917e-15 6.625789e-15
-7.806590e-16
E:\\R-3.1.2\\scripts\\mPDBS\\summary_pdb_93\\58c371611d85ac96.final.pdb
-5.874558e-16 7.313089e-15 1.806863e-15 -5.412027e-15 8.537318e-15
2.077617e-16
E:\\R-3.1.2\\scripts\\mPDBS\\summary_pdb_93\\59a647f04822d30a.final.pdb
-2.315153e-15 -1.626570e-15 -1.605072e-15 -3.129117e-15 -4.264767e-16
2.659599e-16
E:\\R-3.1.2\\scripts\\mPDBS\\summary_pdb_93\\59b091fe385e05fc.final.pdb
-3.816824e-15 4.591773e-15 7.589867e-16 3.472701e-16 2.348624e-15
-7.019075e-15
E:\\R-3.1.2\\scripts\\mPDBS\\summary_pdb_93\\5c35ac30f930d2db.final.pdb
1.098685e-15 1.111070e-14 -2.338110e-16 -6.378917e-15 6.625789e-15
-7.806590e-16
E:\\R-3.1.2\\scripts\\mPDBS\\summary_pdb_93\\5f60752431cf2dcb.final.pdb
9.469099e-15 6.193713e-15 -2.555261e-15 -1.175947e-15 5.872745e-15
3.584836e-15
E:\\R-3.1.2\\scripts\\mPDBS\\summary_pdb_93\\626456dd6ff2868d.final.pdb
2.241878e-15 2.186981e-15 8.284794e-16 1.809193e-15 4.727865e-15
-3.408703e-15
E:\\R-3.1.2\\scripts\\mPDBS\\summary_pdb_93\\6583d53ff3c8f7ef.final.pdb
-2.315153e-15 -1.626570e-15 -1.605072e-15 -3.129117e-15 -4.264767e-16
2.659599e-16
E:\\R-3.1.2\\scripts\\mPDBS\\summary_pdb_93\\69760178986bef01.final.pdb
2.174154e-15 1.691657e-14 5.023092e-15 -1.960309e-15 9.414417e-15
5.369989e-15
E:\\R-3.1.2\\scripts\\mPDBS\\summary_pdb_93\\6a9d87d47926b1c8.final.pdb
-2.870352e-15 5.807349e-15 1.203180e-15 -1.543594e-15 4.710085e-15
-3.684958e-15
E:\\R-3.1.2\\scripts\\mPDBS\\summary_pdb_93\\6bb3547ea982ffc4.final.pdb

2.311112e-15 1.476985e-14 -1.125064e-15 1.695444e-15 8.130896e-15
-4.729341e-15
E:\\R-3.1.2\\scripts\\mPDBS\\summary_pdb_93\\716d9624611d1c42.final.pdb
-1.585786e-14 1.199463e-14 1.286284e-15 -2.339947e-15 5.452075e-15
-3.807255e-15
E:\\R-3.1.2\\scripts\\mPDBS\\summary_pdb_93\\75f27e70ad0f1be4.final.pdb
-2.315153e-15 -1.626570e-15 -1.605072e-15 -3.129117e-15 -4.264767e-16
2.659599e-16
E:\\R-3.1.2\\scripts\\mPDBS\\summary_pdb_93\\769f910fffb22872.final.pdb
-3.562984e-15 4.965562e-15 1.480508e-15 -1.809436e-15 3.440062e-15
-3.234538e-15
E:\\R-3.1.2\\scripts\\mPDBS\\summary_pdb_93\\791f8c80dd5c3135.final.pdb
-3.816824e-15 4.591773e-15 7.589867e-16 3.472701e-16 2.348624e-15
-7.019075e-15
E:\\R-3.1.2\\scripts\\mPDBS\\summary_pdb_93\\7fad98e66a3f6099.final.pdb
-2.970219e-15 1.329259e-14 -6.415374e-15 8.914451e-15 6.724239e-15
-8.741256e-15
E:\\R-3.1.2\\scripts\\mPDBS\\summary_pdb_93\\804b89c94a633196.final.pdb
-7.585660e-15 3.055814e-15 3.433170e-15 3.010121e-15 2.666492e-15
-6.979717e-15
E:\\R-3.1.2\\scripts\\mPDBS\\summary_pdb_93\\813cc65a2298feba.final.pdb
-7.585660e-15 3.055814e-15 3.433170e-15 3.010121e-15 2.666492e-15
-6.979717e-15
E:\\R-3.1.2\\scripts\\mPDBS\\summary_pdb_93\\83c0eefcfe12d3f9.final.pdb
-1.147318e-14 1.145883e-15 5.674433e-15 6.362908e-15 3.116219e-15
-8.962072e-15
E:\\R-3.1.2\\scripts\\mPDBS\\summary_pdb_93\\86d50469ef569760.final.pdb
2.241878e-15 2.186981e-15 8.284794e-16 1.809193e-15 4.727865e-15
-3.408703e-15
E:\\R-3.1.2\\scripts\\mPDBS\\summary_pdb_93\\89d0666e1402203d.final.pdb
-2.315153e-15 -1.626570e-15 -1.605072e-15 -3.129117e-15 -4.264767e-16
2.659599e-16
E:\\R-3.1.2\\scripts\\mPDBS\\summary_pdb_93\\8b34852716dadc62.final.pdb
-7.300330e-16 5.127074e-15 4.022976e-15 -3.328559e-15 6.788629e-15
-4.353346e-15
E:\\R-3.1.2\\scripts\\mPDBS\\summary_pdb_93\\8b6ef9ac3fe74395.final.pdb
9.851702e-15 1.554889e-14 2.454558e-15 7.156794e-16 5.165100e-15
-2.396362e-15
E:\\R-3.1.2\\scripts\\mPDBS\\summary_pdb_93\\98bbe0d5986c138f.final.pdb
-3.816824e-15 4.591773e-15 7.589867e-16 3.472701e-16 2.348624e-15
-7.019075e-15
E:\\R-3.1.2\\scripts\\mPDBS\\summary_pdb_93\\9c22f4909d67e2fa.final.pdb
-9.946104e-17 6.798480e-15 -1.635692e-15 -9.914870e-15 6.944754e-15
3.638525e-15
E:\\R-3.1.2\\scripts\\mPDBS\\summary_pdb_93\\9dda8ed0d81fb3ef.final.pdb
-3.731121e-15 1.658629e-14 -4.173330e-15 7.118328e-15 -1.218378e-15
-1.109605e-14
E:\\R-3.1.2\\scripts\\mPDBS\\summary_pdb_93\\9f752b3616e6033b.final.pdb
-4.199011e-15 3.993937e-15 3.163306e-15 -1.182976e-15 2.482233e-15
-3.893481e-15
E:\\R-3.1.2\\scripts\\mPDBS\\summary_pdb_93\\a02c70083816d6ba.final.pdb
1.098685e-15 1.111070e-14 -2.338110e-16 -6.378917e-15 6.625789e-15
-7.806590e-16
E:\\R-3.1.2\\scripts\\mPDBS\\summary_pdb_93\\a0c997d891e4e082.final.pdb

-6.523432e-15 9.069649e-15 -8.004510e-16 -4.012154e-15 4.148993e-16
-8.550792e-15
E:\\R-3.1.2\\scripts\\mPDBS\\summary_pdb_93\\ab8039a2d823c616.final.pdb
-5.459179e-15 6.114547e-15 6.775334e-16 -1.578554e-15 1.988077e-15
-3.085545e-15
E:\\R-3.1.2\\scripts\\mPDBS\\summary_pdb_93\\acf4d74b9c71d4dc.final.pdb
-7.585660e-15 3.055814e-15 3.433170e-15 3.010121e-15 2.666492e-15
-6.979717e-15
E:\\R-3.1.2\\scripts\\mPDBS\\summary_pdb_93\\ad8a823e4b4c668e.final.pdb
-3.934784e-15 5.432546e-15 2.037787e-15 -3.024961e-15 4.717521e-15
-2.865158e-15
E:\\R-3.1.2\\scripts\\mPDBS\\summary_pdb_93\\b16ababd03b7b857.final.pdb
-9.946104e-17 6.798480e-15 -1.635692e-15 -9.914870e-15 6.944754e-15
3.638525e-15
E:\\R-3.1.2\\scripts\\mPDBS\\summary_pdb_93\\b226923e697d3c63.final.pdb
-7.211410e-15 7.908338e-15 5.745819e-15 -7.563287e-15 2.774088e-15
-6.493859e-17
E:\\R-3.1.2\\scripts\\mPDBS\\summary_pdb_93\\b28e48f6fa27a165.final.pdb
-3.816824e-15 4.591773e-15 7.589867e-16 3.472701e-16 2.348624e-15
-7.019075e-15
E:\\R-3.1.2\\scripts\\mPDBS\\summary_pdb_93\\b42c4924e3cd1aec.final.pdb
-1.851115e-15 1.213412e-14 -1.653644e-15 -4.413463e-15 7.668827e-15
-6.152796e-16
E:\\R-3.1.2\\scripts\\mPDBS\\summary_pdb_93\\b4dd239fcabff1df.final.pdb
-3.816824e-15 4.591773e-15 7.589867e-16 3.472701e-16 2.348624e-15
-7.019075e-15
E:\\R-3.1.2\\scripts\\mPDBS\\summary_pdb_93\\b669942ef97459a2.final.pdb
-7.300330e-16 5.127074e-15 4.022976e-15 -3.328559e-15 6.788629e-15
-4.353346e-15
E:\\R-3.1.2\\scripts\\mPDBS\\summary_pdb_93\\b99f4d9d87c1017b.final.pdb
-3.816824e-15 4.591773e-15 7.589867e-16 3.472701e-16 2.348624e-15
-7.019075e-15
E:\\R-3.1.2\\scripts\\mPDBS\\summary_pdb_93\\b9d2cbf3975403e7.final.pdb
-3.816824e-15 4.591773e-15 7.589867e-16 3.472701e-16 2.348624e-15
-7.019075e-15
E:\\R-3.1.2\\scripts\\mPDBS\\summary_pdb_93\\bc88c8a505f3a288.final.pdb
-3.415235e-15 5.878938e-15 5.603609e-16 -1.506065e-15 3.304457e-15
-2.767702e-15
E:\\R-3.1.2\\scripts\\mPDBS\\summary_pdb_93\\bdac0e64f4ef427e.final.pdb
1.467908e-14 1.473484e-14 -5.341061e-15 -1.725318e-14 8.636109e-15
9.495386e-15
E:\\R-3.1.2\\scripts\\mPDBS\\summary_pdb_93\\ca2a1f55fc9d0681.final.pdb
-7.300330e-16 5.127074e-15 4.022976e-15 -3.328559e-15 6.788629e-15
-4.353346e-15
E:\\R-3.1.2\\scripts\\mPDBS\\summary_pdb_93\\ce34248de3e81a3c.final.pdb
-7.908928e-18 6.143649e-15 -6.824434e-15 1.336683e-15 4.900632e-15
-6.724372e-15
E:\\R-3.1.2\\scripts\\mPDBS\\summary_pdb_93\\d02e697772494a61.final.pdb
-4.548010e-15 -1.056537e-14 8.436873e-15 -7.170371e-15 4.237087e-15
3.383194e-15
E:\\R-3.1.2\\scripts\\mPDBS\\summary_pdb_93\\d04221245d015047.final.pdb
-3.695833e-15 4.767535e-15 -1.982546e-17 7.687437e-16 4.671911e-15
-4.349291e-15
E:\\R-3.1.2\\scripts\\mPDBS\\summary_pdb_93\\d678141e4018054a.final.pdb

-1.614897e-14 3.789083e-15 3.727245e-16 4.650152e-15 7.587453e-15
5.028800e-15
E:\\R-3.1.2\\scripts\\mPDBS\\summary_pdb_93\\d71b40af0f0efe39.final.pdb
-1.062804e-14 -3.564282e-15 7.579671e-15 -1.367724e-14 1.087019e-15
-1.509224e-15
E:\\R-3.1.2\\scripts\\mPDBS\\summary_pdb_93\\dc0809eff10197e2.final.pdb
3.455833e-15 4.114761e-15 2.736331e-15 -6.193354e-15 -5.215076e-16
-4.210714e-15
E:\\R-3.1.2\\scripts\\mPDBS\\summary_pdb_93\\e1163918d3707839.final.pdb
-9.381549e-15 6.994138e-15 6.745887e-15 -2.466569e-16 1.608722e-14
-5.946085e-15
E:\\R-3.1.2\\scripts\\mPDBS\\summary_pdb_93\\e4b48b06824ef848.final.pdb
-3.180478e-15 6.457424e-15 3.641107e-15 -7.340819e-15 3.452856e-15
-1.296419e-15
E:\\R-3.1.2\\scripts\\mPDBS\\summary_pdb_93\\e529e19ad281fea2.final.pdb
-8.728513e-15 1.154755e-14 -3.695679e-15 -4.164576e-16 2.875614e-15
-3.920446e-15
E:\\R-3.1.2\\scripts\\mPDBS\\summary_pdb_93\\e5602082718ec3a1.final.pdb
-7.585660e-15 3.055814e-15 3.433170e-15 3.010121e-15 2.666492e-15
-6.979717e-15
E:\\R-3.1.2\\scripts\\mPDBS\\summary_pdb_93\\e5d589d996d0a960.final.pdb
8.571689e-15 1.538818e-14 -1.382403e-15 4.685859e-15 5.512945e-15
-1.718319e-15
E:\\R-3.1.2\\scripts\\mPDBS\\summary_pdb_93\\e9df9e72307a2fbd.final.pdb
1.098685e-15 1.111070e-14 -2.338110e-16 -6.378917e-15 6.625789e-15
-7.806590e-16
E:\\R-3.1.2\\scripts\\mPDBS\\summary_pdb_93\\ea00f8694090cae7.final.pdb
-7.585660e-15 3.055814e-15 3.433170e-15 3.010121e-15 2.666492e-15
-6.979717e-15
E:\\R-3.1.2\\scripts\\mPDBS\\summary_pdb_93\\eba517be32db050e.final.pdb
-3.816824e-15 4.591773e-15 7.589867e-16 3.472701e-16 2.348624e-15
-7.019075e-15
E:\\R-3.1.2\\scripts\\mPDBS\\summary_pdb_93\\ec04b9545899c29d.final.pdb
-4.853054e-15 6.132848e-15 1.467124e-15 -1.291611e-15 4.344663e-15
-2.794960e-15
E:\\R-3.1.2\\scripts\\mPDBS\\summary_pdb_93\\ee6862cbcad52078.final.pdb
-2.841909e-15 6.297007e-15 2.351002e-16 -1.137219e-15 3.882770e-15
-3.751724e-15
E:\\R-3.1.2\\scripts\\mPDBS\\summary_pdb_93\\ef6a42fc83b81e5d.final.pdb
-4.726116e-15 5.836393e-15 4.604910e-16 -2.414638e-15 3.696151e-15
-3.185099e-15
E:\\R-3.1.2\\scripts\\mPDBS\\summary_pdb_93\\ef8e88d4ad93a0ad.final.pdb
-3.695833e-15 4.767535e-15 -1.982546e-17 7.687437e-16 4.671911e-15
-4.349291e-15
E:\\R-3.1.2\\scripts\\mPDBS\\summary_pdb_93\\f14fcae8deec4fa3.final.pdb
-4.199011e-15 3.993937e-15 3.163306e-15 -1.182976e-15 2.482233e-15
-3.893481e-15
E:\\R-3.1.2\\scripts\\mPDBS\\summary_pdb_93\\f83584e22003fb92.final.pdb
-1.667501e-15 1.360800e-14 -6.408023e-15 3.742991e-15 2.356430e-15
-5.471268e-15
E:\\R-3.1.2\\scripts\\mPDBS\\summary_pdb_93\\f8e2bf87e52caa29.final.pdb
-8.728513e-15 1.154755e-14 -3.695679e-15 -4.164576e-16 2.875614e-15
-3.920446e-15

```
[,139]        [,140]        [,141]        [,142]        [,143]        [,144]
E:\\R-3.1.2\\scripts\\40.1ns_data\\NAT0.pdb
-3.288542e-15 -2.032610e-15 -1.527557e-15  2.969145e-15  3.068799e-15
-6.069221e-15
E:\\R-3.1.2\\scripts\\40.1ns_data\\LJP6.pdb
-3.713563e-15 -1.650947e-15 -1.428573e-15  1.244485e-15  3.328927e-15
-6.414186e-15
E:\\R-3.1.2\\scripts\\40.1ns_data\\3WY9.pdb
-4.239893e-15  3.909437e-16 -3.958424e-15  5.774024e-16  2.653798e-15
-5.281706e-15
E:\\R-3.1.2\\scripts\\40.1ns_data\\ZSP9.pdb
-3.457080e-15 -2.984113e-15 -5.159466e-17  1.565271e-15  1.153094e-15
-4.637888e-15
E:\\R-3.1.2\\scripts\\mPDBS\\summary_pdb_93\\013d855ad26c877c.final.pdb
6.429217e-15 -2.197369e-15 -2.976903e-15  2.031772e-15  2.838080e-15
-5.663505e-15
E:\\R-3.1.2\\scripts\\mPDBS\\summary_pdb_93\\0a2a705342ca0d54.final.pdb
-1.425700e-15 -8.120655e-16 -8.042208e-16  1.451919e-15  3.011361e-15
-6.038223e-15
E:\\R-3.1.2\\scripts\\mPDBS\\summary_pdb_93\\0a59fceadb4194d9.final.pdb
-3.671885e-15  7.408616e-16 -4.052371e-15  2.691037e-15 -5.707766e-17
-8.440647e-15
E:\\R-3.1.2\\scripts\\mPDBS\\summary_pdb_93\\0dba59f3306825e8.final.pdb
-3.028930e-15  3.188080e-15 -8.540595e-15  3.679759e-15 -3.117226e-15
-4.518587e-15
E:\\R-3.1.2\\scripts\\mPDBS\\summary_pdb_93\\126f26a78b57339c.final.pdb
3.180730e-15 -1.932390e-15  1.031672e-14 -1.543277e-15  4.640240e-15
-3.039736e-15
E:\\R-3.1.2\\scripts\\mPDBS\\summary_pdb_93\\12f06f3e342df706.final.pdb
-1.919554e-15 -1.952213e-15 -9.039674e-16  7.874114e-16  2.743780e-15
-5.816178e-15
E:\\R-3.1.2\\scripts\\mPDBS\\summary_pdb_93\\18618a2a251b867b.final.pdb
6.799875e-15  1.203911e-15  3.500680e-17 -3.792446e-16 -2.773478e-15
-1.081993e-15
E:\\R-3.1.2\\scripts\\mPDBS\\summary_pdb_93\\1f305f598641eeb2.final.pdb
-3.352761e-15 -1.524367e-15 -7.628655e-16  2.272702e-15  2.506832e-15
-4.961715e-15
E:\\R-3.1.2\\scripts\\mPDBS\\summary_pdb_93\\1f8a00b551be0a39.final.pdb
6.799875e-15  1.203911e-15  3.500680e-17 -3.792446e-16 -2.773478e-15
-1.081993e-15
E:\\R-3.1.2\\scripts\\mPDBS\\summary_pdb_93\\219f45328d47579a.final.pdb
-5.477515e-15 -1.190744e-14  2.036733e-14 -8.886941e-15  6.532916e-16
-2.266766e-15
E:\\R-3.1.2\\scripts\\mPDBS\\summary_pdb_93\\238dd3918e54336d.final.pdb
-1.790903e-15 -4.045240e-15  8.494342e-15 -1.982199e-15 -1.135273e-14
4.205921e-15
E:\\R-3.1.2\\scripts\\mPDBS\\summary_pdb_93\\2b3027889e2fdc42.final.pdb
4.641294e-16 -2.206089e-16 -6.673535e-17  2.686918e-15 -1.050424e-15
-5.915757e-15
E:\\R-3.1.2\\scripts\\mPDBS\\summary_pdb_93\\2fcb3b46e33793b0.final.pdb
-4.391860e-15  2.147175e-15 -6.234183e-15  5.046525e-15  4.130203e-16
-7.376450e-15
E:\\R-3.1.2\\scripts\\mPDBS\\summary_pdb_93\\34eb328251842006.final.pdb
-9.064155e-15  5.379717e-16 -6.924201e-15  6.351238e-15  3.163212e-15
```

-5.829799e-15
E:\\R-3.1.2\\scripts\\mPDBS\\summary_pdb_93\\354b32871797b7ab.final.pdb
-1.358924e-14 1.164191e-16 -9.541513e-15 3.548205e-15 1.303096e-14
-6.993608e-15
E:\\R-3.1.2\\scripts\\mPDBS\\summary_pdb_93\\397368adbc32d5de.final.pdb
3.180730e-15 -1.932390e-15 1.031672e-14 -1.543277e-15 4.640240e-15
-3.039736e-15
E:\\R-3.1.2\\scripts\\mPDBS\\summary_pdb_93\\411ca13cd3df6edd.final.pdb
2.600174e-16 6.562480e-15 -2.241284e-15 7.211243e-15 -4.176954e-16
-9.014897e-15
E:\\R-3.1.2\\scripts\\mPDBS\\summary_pdb_93\\42357eee6642bf12.final.pdb
-1.452882e-14 2.253792e-15 4.332094e-15 -4.317721e-16 2.020706e-14
-3.718150e-15
E:\\R-3.1.2\\scripts\\mPDBS\\summary_pdb_93\\44342d1f75a7fcfc.final.pdb
1.274067e-14 -2.502846e-15 -4.544237e-15 1.194005e-14 8.406184e-15
-1.101736e-14
E:\\R-3.1.2\\scripts\\mPDBS\\summary_pdb_93\\448ef21dffe8cdfb.final.pdb
1.896078e-15 1.229506e-15 1.991287e-14 -2.161251e-15 1.878862e-15
-8.438101e-15
E:\\R-3.1.2\\scripts\\mPDBS\\summary_pdb_93\\472a40b3e8f113c7.final.pdb
-3.028930e-15 3.188080e-15 -8.540595e-15 3.679759e-15 -3.117226e-15
-4.518587e-15
E:\\R-3.1.2\\scripts\\mPDBS\\summary_pdb_93\\48a26225951ea153.final.pdb
1.525980e-14 1.493732e-15 -2.948515e-16 8.529033e-15 -2.596062e-15
-1.047981e-14
E:\\R-3.1.2\\scripts\\mPDBS\\summary_pdb_93\\4ab4a5c7c6682ce6.final.pdb
-5.733147e-15 -1.438861e-15 6.155549e-15 9.836509e-15 9.533895e-15
-1.182438e-14
E:\\R-3.1.2\\scripts\\mPDBS\\summary_pdb_93\\4eaa45b62cb6a25f.final.pdb
4.168153e-15 -1.255313e-15 1.873943e-15 7.820803e-16 -3.380387e-15
-5.153500e-15
E:\\R-3.1.2\\scripts\\mPDBS\\summary_pdb_93\\58c371611d85ac96.final.pdb
3.180730e-15 -1.932390e-15 1.031672e-14 -1.543277e-15 4.640240e-15
-3.039736e-15
E:\\R-3.1.2\\scripts\\mPDBS\\summary_pdb_93\\59a647f04822d30a.final.pdb
-1.418138e-15 -4.418414e-15 1.717681e-15 7.805707e-16 1.268665e-15
-6.442895e-15
E:\\R-3.1.2\\scripts\\mPDBS\\summary_pdb_93\\59b091fe385e05fc.final.pdb
-3.671885e-15 7.408616e-16 -4.052371e-15 2.691037e-15 -5.707766e-17
-8.440647e-15
E:\\R-3.1.2\\scripts\\mPDBS\\summary_pdb_93\\5c35ac30f930d2db.final.pdb
4.168153e-15 -1.255313e-15 1.873943e-15 7.820803e-16 -3.380387e-15
-5.153500e-15
E:\\R-3.1.2\\scripts\\mPDBS\\summary_pdb_93\\5f60752431cf2dcb.final.pdb
7.121283e-15 4.905149e-15 -5.048981e-15 2.061024e-15 -1.072700e-14
-1.011569e-14
E:\\R-3.1.2\\scripts\\mPDBS\\summary_pdb_93\\626456dd6ff2868d.final.pdb
-3.028930e-15 3.188080e-15 -8.540595e-15 3.679759e-15 -3.117226e-15
-4.518587e-15
E:\\R-3.1.2\\scripts\\mPDBS\\summary_pdb_93\\6583d53ff3c8f7ef.final.pdb
-1.418138e-15 -4.418414e-15 1.717681e-15 7.805707e-16 1.268665e-15
-6.442895e-15
E:\\R-3.1.2\\scripts\\mPDBS\\summary_pdb_93\\69760178986bef01.final.pdb
3.640315e-15 1.465743e-15 4.587005e-15 2.836208e-15 -1.984388e-14

-8.316174e-15
E:\\R-3.1.2\\scripts\\mPDBS\\summary_pdb_93\\6a9d87d47926b1c8.final.pdb
-6.577794e-16 -1.138754e-15 -2.852385e-17 2.359851e-15 2.414129e-17
-6.217749e-15
E:\\R-3.1.2\\scripts\\mPDBS\\summary_pdb_93\\6bb3547ea982ffc4.final.pdb
1.461490e-14 -6.597858e-15 -2.388830e-15 5.616443e-15 -8.952409e-15
-7.706697e-15
E:\\R-3.1.2\\scripts\\mPDBS\\summary_pdb_93\\716d9624611d1c42.final.pdb
-4.601762e-15 -6.051364e-15 -4.405290e-15 3.814612e-15 5.732074e-16
-2.980231e-16
E:\\R-3.1.2\\scripts\\mPDBS\\summary_pdb_93\\75f27e70ad0f1be4.final.pdb
-1.418138e-15 -4.418414e-15 1.717681e-15 7.805707e-16 1.268665e-15
-6.442895e-15
E:\\R-3.1.2\\scripts\\mPDBS\\summary_pdb_93\\769f910fffb22872.final.pdb
-3.300010e-15 3.210523e-17 -1.436039e-15 1.829817e-15 1.355381e-15
-5.323826e-15
E:\\R-3.1.2\\scripts\\mPDBS\\summary_pdb_93\\791f8c80dd5c3135.final.pdb
-3.671885e-15 7.408616e-16 -4.052371e-15 2.691037e-15 -5.707766e-17
-8.440647e-15
E:\\R-3.1.2\\scripts\\mPDBS\\summary_pdb_93\\7fad98e66a3f6099.final.pdb
-1.485629e-14 1.376323e-16 -1.366480e-15 8.819066e-15 1.060986e-14
-1.472892e-14
E:\\R-3.1.2\\scripts\\mPDBS\\summary_pdb_93\\804b89c94a633196.final.pdb
-4.391860e-15 2.147175e-15 -6.234183e-15 5.046525e-15 4.130203e-16
-7.376450e-15
E:\\R-3.1.2\\scripts\\mPDBS\\summary_pdb_93\\813cc65a2298feba.final.pdb
-4.391860e-15 2.147175e-15 -6.234183e-15 5.046525e-15 4.130203e-16
-7.376450e-15
E:\\R-3.1.2\\scripts\\mPDBS\\summary_pdb_93\\83c0eefcfe12d3f9.final.pdb
2.600174e-16 6.562480e-15 -2.241284e-15 7.211243e-15 -4.176954e-16
-9.014897e-15
E:\\R-3.1.2\\scripts\\mPDBS\\summary_pdb_93\\86d50469ef569760.final.pdb
-3.028930e-15 3.188080e-15 -8.540595e-15 3.679759e-15 -3.117226e-15
-4.518587e-15
E:\\R-3.1.2\\scripts\\mPDBS\\summary_pdb_93\\89d0666e1402203d.final.pdb
-1.418138e-15 -4.418414e-15 1.717681e-15 7.805707e-16 1.268665e-15
-6.442895e-15
E:\\R-3.1.2\\scripts\\mPDBS\\summary_pdb_93\\8b34852716dadc62.final.pdb
-5.875049e-15 -3.966858e-15 5.899340e-15 2.626586e-15 1.063542e-15
-7.431962e-15
E:\\R-3.1.2\\scripts\\mPDBS\\summary_pdb_93\\8b6ef9ac3fe74395.final.pdb
3.671361e-15 -1.317460e-14 -6.351811e-15 3.441816e-15 -8.901614e-15
-9.871778e-15
E:\\R-3.1.2\\scripts\\mPDBS\\summary_pdb_93\\98bbe0d5986c138f.final.pdb
-3.671885e-15 7.408616e-16 -4.052371e-15 2.691037e-15 -5.707766e-17
-8.440647e-15
E:\\R-3.1.2\\scripts\\mPDBS\\summary_pdb_93\\9c22f4909d67e2fa.final.pdb
-6.157917e-15 -3.182763e-15 -3.946705e-16 -7.264916e-15 2.770726e-15
3.750066e-15
E:\\R-3.1.2\\scripts\\mPDBS\\summary_pdb_93\\9dda8ed0d81fb3ef.final.pdb
1.994541e-14 -2.392615e-17 -1.357944e-14 1.572689e-15 -1.574949e-16
-1.441872e-14
E:\\R-3.1.2\\scripts\\mPDBS\\summary_pdb_93\\9f752b3616e6033b.final.pdb
-4.000254e-15 -2.226245e-15 1.380138e-15 1.322492e-15 3.410081e-15

-6.439218e-15
E:\\R-3.1.2\\scripts\\mPDBS\\summary_pdb_93\\a02c70083816d6ba.final.pdb
4.168153e-15 -1.255313e-15 1.873943e-15 7.820803e-16 -3.380387e-15
-5.153500e-15
E:\\R-3.1.2\\scripts\\mPDBS\\summary_pdb_93\\a0c997d891e4e082.final.pdb
3.798223e-15 -1.077263e-14 -9.057719e-16 4.725693e-15 -4.484129e-16
-4.214029e-15
E:\\R-3.1.2\\scripts\\mPDBS\\summary_pdb_93\\ab8039a2d823c616.final.pdb
-6.382237e-16 -2.942951e-15 -1.642860e-16 2.007490e-15 7.494137e-16
-6.333428e-15
E:\\R-3.1.2\\scripts\\mPDBS\\summary_pdb_93\\acf4d74b9c71d4dc.final.pdb
-4.391860e-15 2.147175e-15 -6.234183e-15 5.046525e-15 4.130203e-16
-7.376450e-15
E:\\R-3.1.2\\scripts\\mPDBS\\summary_pdb_93\\ad8a823e4b4c668e.final.pdb
-2.667580e-15 -3.312224e-15 -1.732176e-15 1.537566e-15 2.491441e-15
-6.086826e-15
E:\\R-3.1.2\\scripts\\mPDBS\\summary_pdb_93\\b16ababd03b7b857.final.pdb
-6.157917e-15 -3.182763e-15 -3.946705e-16 -7.264916e-15 2.770726e-15
3.750066e-15
E:\\R-3.1.2\\scripts\\mPDBS\\summary_pdb_93\\b226923e697d3c63.final.pdb
-2.247661e-15 -3.633886e-15 -3.618545e-15 -1.026903e-15 1.512858e-16
1.772435e-16
E:\\R-3.1.2\\scripts\\mPDBS\\summary_pdb_93\\b28e48f6fa27a165.final.pdb
-3.671885e-15 7.408616e-16 -4.052371e-15 2.691037e-15 -5.707766e-17
-8.440647e-15
E:\\R-3.1.2\\scripts\\mPDBS\\summary_pdb_93\\b42c4924e3cd1aec.final.pdb
-1.381978e-14 3.312585e-16 2.776188e-15 7.978095e-16 2.881957e-15
-5.261869e-16
E:\\R-3.1.2\\scripts\\mPDBS\\summary_pdb_93\\b4dd239fcabff1df.final.pdb
-3.671885e-15 7.408616e-16 -4.052371e-15 2.691037e-15 -5.707766e-17
-8.440647e-15
E:\\R-3.1.2\\scripts\\mPDBS\\summary_pdb_93\\b669942ef97459a2.final.pdb
-5.875049e-15 -3.966858e-15 5.899340e-15 2.626586e-15 1.063542e-15
-7.431962e-15
E:\\R-3.1.2\\scripts\\mPDBS\\summary_pdb_93\\b99f4d9d87c1017b.final.pdb
-3.671885e-15 7.408616e-16 -4.052371e-15 2.691037e-15 -5.707766e-17
-8.440647e-15
E:\\R-3.1.2\\scripts\\mPDBS\\summary_pdb_93\\b9d2cbf3975403e7.final.pdb
-3.671885e-15 7.408616e-16 -4.052371e-15 2.691037e-15 -5.707766e-17
-8.440647e-15
E:\\R-3.1.2\\scripts\\mPDBS\\summary_pdb_93\\bc88c8a505f3a288.final.pdb
-3.803556e-16 -7.358217e-16 1.705965e-16 2.382582e-15 -8.940817e-16
-6.225405e-15
E:\\R-3.1.2\\scripts\\mPDBS\\summary_pdb_93\\bdac0e64f4ef427e.final.pdb
-8.936294e-15 -1.873499e-14 -1.463477e-15 -7.100334e-15 -3.310997e-15
1.081194e-15
E:\\R-3.1.2\\scripts\\mPDBS\\summary_pdb_93\\ca2a1f55fc9d0681.final.pdb
-5.875049e-15 -3.966858e-15 5.899340e-15 2.626586e-15 1.063542e-15
-7.431962e-15
E:\\R-3.1.2\\scripts\\mPDBS\\summary_pdb_93\\ce34248de3e81a3c.final.pdb
-2.407124e-15 -4.755145e-15 -3.812272e-15 3.341465e-15 7.413353e-15
-6.805053e-15
E:\\R-3.1.2\\scripts\\mPDBS\\summary_pdb_93\\d02e697772494a61.final.pdb
-1.111461e-14 -4.427747e-15 9.023649e-15 3.058907e-15 -1.713739e-15

-1.839590e-15
E:\\R-3.1.2\\scripts\\mPDBS\\summary_pdb_93\\d04221245d015047.final.pdb
-2.039121e-15 -1.526481e-16 -2.973652e-17 2.581575e-15 1.175288e-15
-7.527351e-15
E:\\R-3.1.2\\scripts\\mPDBS\\summary_pdb_93\\d678141e4018054a.final.pdb
-2.340599e-15 -1.297573e-16 -7.481954e-15 1.613813e-15 7.037332e-15
2.131552e-15
E:\\R-3.1.2\\scripts\\mPDBS\\summary_pdb_93\\d71b40af0f0efe39.final.pdb
-1.144045e-14 1.638767e-14 -1.141256e-16 -5.014320e-15 5.094907e-15
-3.216124e-17
E:\\R-3.1.2\\scripts\\mPDBS\\summary_pdb_93\\dc0809eff10197e2.final.pdb
3.301804e-15 2.967952e-16 5.317039e-16 1.259017e-15 6.929777e-15
-4.447953e-15
E:\\R-3.1.2\\scripts\\mPDBS\\summary_pdb_93\\e1163918d3707839.final.pdb
-2.373004e-14 9.049545e-15 -5.262737e-15 3.565561e-15 4.241981e-15
-1.225280e-15
E:\\R-3.1.2\\scripts\\mPDBS\\summary_pdb_93\\e4b48b06824ef848.final.pdb
6.799875e-15 1.203911e-15 3.500680e-17 -3.792446e-16 -2.773478e-15
-1.081993e-15
E:\\R-3.1.2\\scripts\\mPDBS\\summary_pdb_93\\e529e19ad281fea2.final.pdb
6.429217e-15 -2.197369e-15 -2.976903e-15 2.031772e-15 2.838080e-15
-5.663505e-15
E:\\R-3.1.2\\scripts\\mPDBS\\summary_pdb_93\\e5602082718ec3a1.final.pdb
-4.391860e-15 2.147175e-15 -6.234183e-15 5.046525e-15 4.130203e-16
-7.376450e-15
E:\\R-3.1.2\\scripts\\mPDBS\\summary_pdb_93\\e5d589d996d0a960.final.pdb
9.476452e-15 -9.390467e-15 -2.622775e-17 3.928571e-15 -6.279616e-15
-9.920381e-16
E:\\R-3.1.2\\scripts\\mPDBS\\summary_pdb_93\\e9df9e72307a2fbd.final.pdb
4.168153e-15 -1.255313e-15 1.873943e-15 7.820803e-16 -3.380387e-15
-5.153500e-15
E:\\R-3.1.2\\scripts\\mPDBS\\summary_pdb_93\\ea00f8694090cae7.final.pdb
-4.391860e-15 2.147175e-15 -6.234183e-15 5.046525e-15 4.130203e-16
-7.376450e-15
E:\\R-3.1.2\\scripts\\mPDBS\\summary_pdb_93\\eba517be32db050e.final.pdb
-3.671885e-15 7.408616e-16 -4.052371e-15 2.691037e-15 -5.707766e-17
-8.440647e-15
E:\\R-3.1.2\\scripts\\mPDBS\\summary_pdb_93\\ec04b9545899c29d.final.pdb
-2.455069e-15 -3.144694e-15 -1.052238e-15 1.956713e-15 6.831954e-16
-5.362162e-15
E:\\R-3.1.2\\scripts\\mPDBS\\summary_pdb_93\\ee6862cbcad52078.final.pdb
-1.148345e-16 -4.540179e-17 3.463755e-17 2.241744e-15 -3.146841e-16
-6.027647e-15
E:\\R-3.1.2\\scripts\\mPDBS\\summary_pdb_93\\ef6a42fc83b81e5d.final.pdb
-2.475257e-15 -9.245948e-16 -7.912637e-16 1.426386e-15 1.983351e-15
-5.043234e-15
E:\\R-3.1.2\\scripts\\mPDBS\\summary_pdb_93\\ef8e88d4ad93a0ad.final.pdb
-2.039121e-15 -1.526481e-16 -2.973652e-17 2.581575e-15 1.175288e-15
-7.527351e-15
E:\\R-3.1.2\\scripts\\mPDBS\\summary_pdb_93\\f14fcae8deec4fa3.final.pdb
-4.000254e-15 -2.226245e-15 1.380138e-15 1.322492e-15 3.410081e-15
-6.439218e-15
E:\\R-3.1.2\\scripts\\mPDBS\\summary_pdb_93\\f83584e22003fb92.final.pdb
1.525980e-14 1.493732e-15 -2.948515e-16 8.529033e-15 -2.596062e-15

-1.047981e-14
E:\\R-3.1.2\\scripts\\mPDBS\\summary_pdb_93\\f8e2bf87e52caa29.final.pdb
6.429217e-15 -2.197369e-15 -2.976903e-15 2.031772e-15 2.838080e-15
-5.663505e-15

[,145]   [,146]   [,147]   [,148]   [,149]   [,150]
E:\\R-3.1.2\\scripts\\40.1ns_data\\NAT0.pdb
-5.405877e-15 8.245838e-16 1.918716e-15 2.724410e-15 -1.013779e-15
6.573935e-15
E:\\R-3.1.2\\scripts\\40.1ns_data\\LJP6.pdb
-4.515866e-15 3.133767e-16 -3.990364e-15 3.860591e-15 -1.038785e-15
3.141096e-15
E:\\R-3.1.2\\scripts\\40.1ns_data\\3WY9.pdb
-2.913846e-15 8.853759e-16 4.272124e-16 2.276395e-15 -2.459240e-15
4.895015e-15
E:\\R-3.1.2\\scripts\\40.1ns_data\\ZSP9.pdb
-4.227719e-15 3.710725e-15 -3.581465e-16 2.856150e-15 -1.978506e-15
3.838970e-15
E:\\R-3.1.2\\scripts\\mPDBS\\summary_pdb_93\\013d855ad26c877c.final.pdb
-4.850544e-15 -4.659222e-15 -3.583279e-15 3.495473e-15 -6.142366e-15
7.886011e-15
E:\\R-3.1.2\\scripts\\mPDBS\\summary_pdb_93\\0a2a705342ca0d54.final.pdb
-3.274826e-15 -1.067360e-16 -2.580501e-15 3.322850e-15 -1.821251e-15
2.889187e-15
E:\\R-3.1.2\\scripts\\mPDBS\\summary_pdb_93\\0a59fceadb4194d9.final.pdb
-3.256977e-15 -1.895173e-16 -1.291048e-15 1.006076e-15 -1.110022e-15
2.513393e-16
E:\\R-3.1.2\\scripts\\mPDBS\\summary_pdb_93\\0dba59f3306825e8.final.pdb
-2.063711e-15 -3.722472e-15 -4.579010e-15 6.166948e-15 -1.068733e-15
-8.032185e-16
E:\\R-3.1.2\\scripts\\mPDBS\\summary_pdb_93\\126f26a78b57339c.final.pdb
-8.906978e-15 8.962911e-15 -3.961882e-15 -1.714498e-15 -3.293074e-16
3.792498e-15
E:\\R-3.1.2\\scripts\\mPDBS\\summary_pdb_93\\12f06f3e342df706.final.pdb
-4.615334e-15 -1.284201e-16 -2.801028e-15 2.208940e-15 -3.147664e-15
3.173248e-15
E:\\R-3.1.2\\scripts\\mPDBS\\summary_pdb_93\\18618a2a251b867b.final.pdb
-3.744301e-15 4.685202e-15 -3.951946e-15 3.116182e-15 1.235536e-15
1.253831e-15
E:\\R-3.1.2\\scripts\\mPDBS\\summary_pdb_93\\1f305f598641eeb2.final.pdb
-3.694698e-15 9.476937e-16 -3.040532e-15 2.882660e-15 -2.069225e-15
3.650994e-15
E:\\R-3.1.2\\scripts\\mPDBS\\summary_pdb_93\\1f8a00b551be0a39.final.pdb
-3.744301e-15 4.685202e-15 -3.951946e-15 3.116182e-15 1.235536e-15
1.253831e-15
E:\\R-3.1.2\\scripts\\mPDBS\\summary_pdb_93\\219f45328d47579a.final.pdb
-5.781867e-15 -7.090247e-15 -3.959307e-16 4.834611e-15 -5.925072e-15
1.976611e-14
E:\\R-3.1.2\\scripts\\mPDBS\\summary_pdb_93\\238dd3918e54336d.final.pdb
-7.797919e-16 -6.920678e-15 -6.056550e-15 7.524896e-15 3.033962e-15
3.177384e-15
E:\\R-3.1.2\\scripts\\mPDBS\\summary_pdb_93\\2b3027889e2fdc42.final.pdb
-4.102004e-15 -1.227533e-15 -4.217310e-15 2.238001e-15 -1.148511e-15
3.215548e-15

E:\\R-3.1.2\\scripts\\mPDBS\\summary_pdb_93\\2fcb3b46e33793b0.final.pdb
-2.097018e-15 -4.755210e-15 -2.769915e-15 3.864514e-15 -2.910132e-15
6.082895e-16
E:\\R-3.1.2\\scripts\\mPDBS\\summary_pdb_93\\34eb328251842006.final.pdb
1.752401e-15 -6.164326e-16 1.478060e-15 9.069136e-15 -7.934745e-15
7.590569e-15
E:\\R-3.1.2\\scripts\\mPDBS\\summary_pdb_93\\354b32871797b7ab.final.pdb
2.488384e-15 1.149019e-14 -1.640241e-14 5.460012e-15 -7.579143e-16
1.321396e-14
E:\\R-3.1.2\\scripts\\mPDBS\\summary_pdb_93\\397368adbc32d5de.final.pdb
-8.906978e-15 8.962911e-15 -3.961882e-15 -1.714498e-15 -3.293074e-16
3.792498e-15
E:\\R-3.1.2\\scripts\\mPDBS\\summary_pdb_93\\411ca13cd3df6edd.final.pdb
-1.506561e-15 9.353338e-16 -3.279490e-15 1.611704e-15 -7.412390e-15
-2.335970e-15
E:\\R-3.1.2\\scripts\\mPDBS\\summary_pdb_93\\42357eee6642bf12.final.pdb
4.445911e-15 5.726388e-15 3.579328e-15 1.008468e-14 -2.039245e-15
8.739035e-15
E:\\R-3.1.2\\scripts\\mPDBS\\summary_pdb_93\\44342d1f75a7fcfc.final.pdb
-1.556558e-14 3.420459e-15 2.269527e-15 -2.312286e-15 -8.083549e-15
-8.470822e-15
E:\\R-3.1.2\\scripts\\mPDBS\\summary_pdb_93\\448ef21dffe8cdfb.final.pdb
-5.438111e-17 9.780906e-15 -1.440644e-14 -2.037016e-15 6.525462e-15
1.898768e-14
E:\\R-3.1.2\\scripts\\mPDBS\\summary_pdb_93\\472a40b3e8f113c7.final.pdb
-2.063711e-15 -3.722472e-15 -4.579010e-15 6.166948e-15 -1.068733e-15
-8.032185e-16
E:\\R-3.1.2\\scripts\\mPDBS\\summary_pdb_93\\48a26225951ea153.final.pdb
-6.791693e-15 9.421729e-16 -8.883716e-15 1.303852e-15 -2.561009e-15
-8.662563e-16
E:\\R-3.1.2\\scripts\\mPDBS\\summary_pdb_93\\4ab4a5c7c6682ce6.final.pdb
3.536194e-15 2.977247e-15 -7.112140e-15 -2.328590e-15 -2.638875e-15
2.224367e-16
E:\\R-3.1.2\\scripts\\mPDBS\\summary_pdb_93\\4eaa45b62cb6a25f.final.pdb
-8.084322e-15 2.786712e-16 -3.013548e-16 -6.173203e-16 -2.543566e-16
-1.352081e-15
E:\\R-3.1.2\\scripts\\mPDBS\\summary_pdb_93\\58c371611d85ac96.final.pdb
-8.906978e-15 8.962911e-15 -3.961882e-15 -1.714498e-15 -3.293074e-16
3.792498e-15
E:\\R-3.1.2\\scripts\\mPDBS\\summary_pdb_93\\59a647f04822d30a.final.pdb
-1.075437e-15 -9.805348e-16 -6.333834e-15 4.369001e-15 6.658762e-18
1.076379e-14
E:\\R-3.1.2\\scripts\\mPDBS\\summary_pdb_93\\59b091fe385e05fc.final.pdb
-3.256977e-15 -1.895173e-16 -1.291048e-15 1.006076e-15 -1.110022e-15
2.513393e-16
E:\\R-3.1.2\\scripts\\mPDBS\\summary_pdb_93\\5c35ac30f930d2db.final.pdb
-8.084322e-15 2.786712e-16 -3.013548e-16 -6.173203e-16 -2.543566e-16
-1.352081e-15
E:\\R-3.1.2\\scripts\\mPDBS\\summary_pdb_93\\5f60752431cf2dcb.final.pdb
-9.117158e-15 2.779193e-15 -3.080752e-15 -3.732000e-15 1.073677e-15
-8.945530e-15
E:\\R-3.1.2\\scripts\\mPDBS\\summary_pdb_93\\626456dd6ff2868d.final.pdb
-2.063711e-15 -3.722472e-15 -4.579010e-15 6.166948e-15 -1.068733e-15
-8.032185e-16

E:\\R-3.1.2\\scripts\\mPDBS\\summary_pdb_93\\6583d53ff3c8f7ef.final.pdb
-1.075437e-15 -9.805348e-16 -6.333834e-15 4.369001e-15 6.658762e-18
1.076379e-14
E:\\R-3.1.2\\scripts\\mPDBS\\summary_pdb_93\\69760178986bef01.final.pdb
-8.641314e-15 -2.605395e-15 -5.932960e-15 3.454534e-15 2.807431e-15
-4.031528e-15
E:\\R-3.1.2\\scripts\\mPDBS\\summary_pdb_93\\6a9d87d47926b1c8.final.pdb
-3.925719e-15 -2.146439e-16 -4.305357e-15 2.230264e-15 -6.998877e-16
3.546167e-15
E:\\R-3.1.2\\scripts\\mPDBS\\summary_pdb_93\\6bb3547ea982ffc4.final.pdb
-1.513645e-14 -6.391809e-15 -1.585009e-14 1.075882e-14 -3.275594e-15
2.993568e-15
E:\\R-3.1.2\\scripts\\mPDBS\\summary_pdb_93\\716d9624611d1c42.final.pdb
-1.171474e-15 4.613859e-16 -7.373578e-16 7.511339e-15 -2.405528e-15
1.051937e-14
E:\\R-3.1.2\\scripts\\mPDBS\\summary_pdb_93\\75f27e70ad0f1be4.final.pdb
-1.075437e-15 -9.805348e-16 -6.333834e-15 4.369001e-15 6.658762e-18
1.076379e-14
E:\\R-3.1.2\\scripts\\mPDBS\\summary_pdb_93\\769f910fffb22872.final.pdb
-3.771840e-15 6.374420e-16 -2.760625e-15 2.640326e-15 -1.878673e-15
3.397950e-15
E:\\R-3.1.2\\scripts\\mPDBS\\summary_pdb_93\\791f8c80dd5c3135.final.pdb
-3.256977e-15 -1.895173e-16 -1.291048e-15 1.006076e-15 -1.110022e-15
2.513393e-16
E:\\R-3.1.2\\scripts\\mPDBS\\summary_pdb_93\\7fad98e66a3f6099.final.pdb
-2.143973e-15 -1.006307e-15 -3.263878e-15 5.837735e-15 -1.035380e-14
3.700339e-15
E:\\R-3.1.2\\scripts\\mPDBS\\summary_pdb_93\\804b89c94a633196.final.pdb
-2.097018e-15 -4.755210e-15 -2.769915e-15 3.864514e-15 -2.910132e-15
6.082895e-16
E:\\R-3.1.2\\scripts\\mPDBS\\summary_pdb_93\\813cc65a2298feba.final.pdb
-2.097018e-15 -4.755210e-15 -2.769915e-15 3.864514e-15 -2.910132e-15
6.082895e-16
E:\\R-3.1.2\\scripts\\mPDBS\\summary_pdb_93\\83c0eefcfe12d3f9.final.pdb
-1.506561e-15 9.353338e-16 -3.279490e-15 1.611704e-15 -7.412390e-15
-2.335970e-15
E:\\R-3.1.2\\scripts\\mPDBS\\summary_pdb_93\\86d50469ef569760.final.pdb
-2.063711e-15 -3.722472e-15 -4.579010e-15 6.166948e-15 -1.068733e-15
-8.032185e-16
E:\\R-3.1.2\\scripts\\mPDBS\\summary_pdb_93\\89d0666e1402203d.final.pdb
-1.075437e-15 -9.805348e-16 -6.333834e-15 4.369001e-15 6.658762e-18
1.076379e-14
E:\\R-3.1.2\\scripts\\mPDBS\\summary_pdb_93\\8b34852716dadc62.final.pdb
-5.247276e-15 6.120639e-15 -1.472342e-15 -6.214814e-16 9.561325e-16
-8.488782e-16
E:\\R-3.1.2\\scripts\\mPDBS\\summary_pdb_93\\8b6ef9ac3fe74395.final.pdb
-1.085102e-14 -1.063922e-14 -1.074973e-14 5.933282e-15 -9.302099e-16
5.231369e-15
E:\\R-3.1.2\\scripts\\mPDBS\\summary_pdb_93\\98bbe0d5986c138f.final.pdb
-3.256977e-15 -1.895173e-16 -1.291048e-15 1.006076e-15 -1.110022e-15
2.513393e-16
E:\\R-3.1.2\\scripts\\mPDBS\\summary_pdb_93\\9c22f4909d67e2fa.final.pdb
-8.144264e-15 3.772257e-15 1.881096e-15 3.666159e-15 6.235438e-15
8.213751e-15

E:\\R-3.1.2\\scripts\\mPDBS\\summary_pdb_93\\9dda8ed0d81fb3ef.final.pdb
-1.282580e-14 -3.339108e-15 1.938411e-15 -6.814490e-15 -6.346658e-15
-3.078457e-14
E:\\R-3.1.2\\scripts\\mPDBS\\summary_pdb_93\\9f752b3616e6033b.final.pdb
-4.412479e-15 -2.633498e-15 -4.717255e-15 3.966904e-15 -4.125744e-15
5.657181e-15
E:\\R-3.1.2\\scripts\\mPDBS\\summary_pdb_93\\a02c70083816d6ba.final.pdb
-8.084322e-15 2.786712e-16 -3.013548e-16 -6.173203e-16 -2.543566e-16
-1.352081e-15
E:\\R-3.1.2\\scripts\\mPDBS\\summary_pdb_93\\a0c997d891e4e082.final.pdb
-1.650893e-14 -2.407964e-15 -6.244783e-15 -7.542186e-15 -2.453755e-15
1.115565e-15
E:\\R-3.1.2\\scripts\\mPDBS\\summary_pdb_93\\ab8039a2d823c616.final.pdb
-4.835972e-15 9.179350e-16 -2.721777e-15 2.586594e-15 -1.785181e-15
3.512519e-15
E:\\R-3.1.2\\scripts\\mPDBS\\summary_pdb_93\\acf4d74b9c71d4dc.final.pdb
-2.097018e-15 -4.755210e-15 -2.769915e-15 3.864514e-15 -2.910132e-15
6.082895e-16
E:\\R-3.1.2\\scripts\\mPDBS\\summary_pdb_93\\ad8a823e4b4c668e.final.pdb
-3.964489e-15 4.823833e-16 -2.587990e-15 2.543284e-15 -3.111382e-15
3.251774e-15
E:\\R-3.1.2\\scripts\\mPDBS\\summary_pdb_93\\b16ababd03b7b857.final.pdb
-8.144264e-15 3.772257e-15 1.881096e-15 3.666159e-15 6.235438e-15
8.213751e-15
E:\\R-3.1.2\\scripts\\mPDBS\\summary_pdb_93\\b226923e697d3c63.final.pdb
-8.332436e-15 1.872868e-15 -2.300169e-15 -7.478909e-16 8.033030e-17
4.719148e-15
E:\\R-3.1.2\\scripts\\mPDBS\\summary_pdb_93\\b28e48f6fa27a165.final.pdb
-3.256977e-15 -1.895173e-16 -1.291048e-15 1.006076e-15 -1.110022e-15
2.513393e-16
E:\\R-3.1.2\\scripts\\mPDBS\\summary_pdb_93\\b42c4924e3cd1aec.final.pdb
-4.056560e-15 1.156211e-15 -6.413415e-15 2.555619e-15 -1.000980e-14
2.014388e-14
E:\\R-3.1.2\\scripts\\mPDBS\\summary_pdb_93\\b4dd239fcabff1df.final.pdb
-3.256977e-15 -1.895173e-16 -1.291048e-15 1.006076e-15 -1.110022e-15
2.513393e-16
E:\\R-3.1.2\\scripts\\mPDBS\\summary_pdb_93\\b669942ef97459a2.final.pdb
-5.247276e-15 6.120639e-15 -1.472342e-15 -6.214814e-16 9.561325e-16
-8.488782e-16
E:\\R-3.1.2\\scripts\\mPDBS\\summary_pdb_93\\b99f4d9d87c1017b.final.pdb
-3.256977e-15 -1.895173e-16 -1.291048e-15 1.006076e-15 -1.110022e-15
2.513393e-16
E:\\R-3.1.2\\scripts\\mPDBS\\summary_pdb_93\\b9d2cbf3975403e7.final.pdb
-3.256977e-15 -1.895173e-16 -1.291048e-15 1.006076e-15 -1.110022e-15
2.513393e-16
E:\\R-3.1.2\\scripts\\mPDBS\\summary_pdb_93\\bc88c8a505f3a288.final.pdb
-4.273091e-15 2.164079e-16 -3.871232e-15 2.241524e-15 -2.693282e-15
3.442363e-15
E:\\R-3.1.2\\scripts\\mPDBS\\summary_pdb_93\\bdac0e64f4ef427e.final.pdb
-8.624999e-15 -2.265665e-15 -2.961819e-15 4.973002e-15 1.352551e-14
1.221749e-14
E:\\R-3.1.2\\scripts\\mPDBS\\summary_pdb_93\\ca2a1f55fc9d0681.final.pdb
-5.247276e-15 6.120639e-15 -1.472342e-15 -6.214814e-16 9.561325e-16
-8.488782e-16

E:\\R-3.1.2\\scripts\\mPDBS\\summary_pdb_93\\ce34248de3e81a3c.final.pdb
-3.612207e-15 9.124232e-15 2.792593e-15 -4.164473e-15 -3.290659e-15
-1.275245e-15
E:\\R-3.1.2\\scripts\\mPDBS\\summary_pdb_93\\d02e697772494a61.final.pdb
3.515383e-15 3.885782e-15 -5.759913e-15 1.101429e-14 7.912221e-15
2.150474e-14
E:\\R-3.1.2\\scripts\\mPDBS\\summary_pdb_93\\d04221245d015047.final.pdb
-3.089973e-15 3.398384e-16 -3.153506e-15 3.577392e-15 -2.526613e-15
1.349753e-15
E:\\R-3.1.2\\scripts\\mPDBS\\summary_pdb_93\\d678141e4018054a.final.pdb
4.452526e-15 2.776203e-15 -2.678728e-15 1.050662e-14 -1.368550e-14
2.843717e-15
E:\\R-3.1.2\\scripts\\mPDBS\\summary_pdb_93\\d71b40af0f0efe39.final.pdb
6.678768e-15 4.073364e-15 -1.459775e-15 -1.715807e-15 3.750111e-15
6.463137e-15
E:\\R-3.1.2\\scripts\\mPDBS\\summary_pdb_93\\dc0809eff10197e2.final.pdb
-1.054661e-14 -7.086264e-15 -7.059824e-15 -4.433356e-17 -2.251842e-16
1.348337e-14
E:\\R-3.1.2\\scripts\\mPDBS\\summary_pdb_93\\e1163918d3707839.final.pdb
3.874162e-15 -2.285914e-15 5.812916e-15 1.271242e-14 -1.283620e-14
1.169664e-14
E:\\R-3.1.2\\scripts\\mPDBS\\summary_pdb_93\\e4b48b06824ef848.final.pdb
-3.744301e-15 4.685202e-15 -3.951946e-15 3.116182e-15 1.235536e-15
1.253831e-15
E:\\R-3.1.2\\scripts\\mPDBS\\summary_pdb_93\\e529e19ad281fea2.final.pdb
-4.850544e-15 -4.659222e-15 -3.583279e-15 3.495473e-15 -6.142366e-15
7.886011e-15
E:\\R-3.1.2\\scripts\\mPDBS\\summary_pdb_93\\e5602082718ec3a1.final.pdb
-2.097018e-15 -4.755210e-15 -2.769915e-15 3.864514e-15 -2.910132e-15
6.082895e-16
E:\\R-3.1.2\\scripts\\mPDBS\\summary_pdb_93\\e5d589d996d0a960.final.pdb
-1.840040e-14 -1.674896e-15 -1.010601e-14 -3.963276e-15 -1.785368e-15
-7.343756e-15
E:\\R-3.1.2\\scripts\\mPDBS\\summary_pdb_93\\e9df9e72307a2fbd.final.pdb
-8.084322e-15 2.786712e-16 -3.013548e-16 -6.173203e-16 -2.543566e-16
-1.352081e-15
E:\\R-3.1.2\\scripts\\mPDBS\\summary_pdb_93\\ea00f8694090cae7.final.pdb
-2.097018e-15 -4.755210e-15 -2.769915e-15 3.864514e-15 -2.910132e-15
6.082895e-16
E:\\R-3.1.2\\scripts\\mPDBS\\summary_pdb_93\\eba517be32db050e.final.pdb
-3.256977e-15 -1.895173e-16 -1.291048e-15 1.006076e-15 -1.110022e-15
2.513393e-16
E:\\R-3.1.2\\scripts\\mPDBS\\summary_pdb_93\\ec04b9545899c29d.final.pdb
-5.255254e-15 4.033408e-16 -2.780253e-15 2.445193e-15 -2.882198e-15
3.019119e-15
E:\\R-3.1.2\\scripts\\mPDBS\\summary_pdb_93\\ee6862cbcad52078.final.pdb
-4.545009e-15 -6.683010e-16 -3.879255e-15 2.416406e-15 -1.008215e-15
3.117103e-15
E:\\R-3.1.2\\scripts\\mPDBS\\summary_pdb_93\\ef6a42fc83b81e5d.final.pdb
-3.955287e-15 3.893765e-16 -2.927809e-15 2.652740e-15 -2.306336e-15
3.102613e-15
E:\\R-3.1.2\\scripts\\mPDBS\\summary_pdb_93\\ef8e88d4ad93a0ad.final.pdb
-3.089973e-15 3.398384e-16 -3.153506e-15 3.577392e-15 -2.526613e-15
1.349753e-15

E:\\R-3.1.2\\scripts\\mPDBS\\summary_pdb_93\\f14fcae8deec4fa3.final.pdb
-4.412479e-15 -2.633498e-15 -4.717255e-15 3.966904e-15 -4.125744e-15
5.657181e-15
E:\\R-3.1.2\\scripts\\mPDBS\\summary_pdb_93\\f83584e22003fb92.final.pdb
-6.791693e-15 9.421729e-16 -8.883716e-15 1.303852e-15 -2.561009e-15
-8.662563e-16
E:\\R-3.1.2\\scripts\\mPDBS\\summary_pdb_93\\f8e2bf87e52caa29.final.pdb
-4.850544e-15 -4.659222e-15 -3.583279e-15 3.495473e-15 -6.142366e-15
7.886011e-15

[,151]   [,152]   [,153]   [,154]   [,155]   [,156]
E:\\R-3.1.2\\scripts\\40.1ns_data\\NAT0.pdb
-3.730918e-15 -1.388556e-15 2.815549e-15 -3.114208e-15 -7.452288e-16
-2.648681e-15
E:\\R-3.1.2\\scripts\\40.1ns_data\\LJP6.pdb
-4.683882e-15 -3.091374e-15 -9.383258e-16 -2.907476e-15 -2.041168e-15
-8.451626e-16
E:\\R-3.1.2\\scripts\\40.1ns_data\\3WY9.pdb
-2.313717e-15 -5.765007e-15 3.507595e-15 -2.120251e-15 -8.757185e-16
-1.143746e-15
E:\\R-3.1.2\\scripts\\40.1ns_data\\ZSP9.pdb
-4.378965e-15 -4.125126e-15 1.597779e-15 -1.166707e-15 -3.053995e-17
-1.432078e-15
E:\\R-3.1.2\\scripts\\mPDBS\\summary_pdb_93\\013d855ad26c877c.final.pdb
-3.322380e-15 -6.418342e-15 4.469830e-15 -6.005348e-15 -3.101220e-16
-4.166517e-15
E:\\R-3.1.2\\scripts\\mPDBS\\summary_pdb_93\\0a2a705342ca0d54.final.pdb
-2.645282e-15 -4.351670e-15 5.629607e-16 -3.375691e-15 2.086013e-16
-2.343797e-15
E:\\R-3.1.2\\scripts\\mPDBS\\summary_pdb_93\\0a59fceadb4194d9.final.pdb
-3.106258e-15 9.600951e-16 -1.444855e-15 -3.251757e-15 -4.144616e-16
-2.211058e-16
E:\\R-3.1.2\\scripts\\mPDBS\\summary_pdb_93\\0dba59f3306825e8.final.pdb
-3.019237e-15 4.218982e-15 -3.509898e-15 8.285949e-16 3.478514e-15
-3.287012e-15
E:\\R-3.1.2\\scripts\\mPDBS\\summary_pdb_93\\126f26a78b57339c.final.pdb
-7.050952e-15 -1.507458e-14 1.497046e-15 -3.661303e-15 -4.047963e-15
-6.311507e-16
E:\\R-3.1.2\\scripts\\mPDBS\\summary_pdb_93\\12f06f3e342df706.final.pdb
-2.480266e-15 -4.106857e-15 -5.142009e-16 -2.800413e-15 -3.536660e-16
-2.097033e-15
E:\\R-3.1.2\\scripts\\mPDBS\\summary_pdb_93\\18618a2a251b867b.final.pdb
-3.856945e-15 -9.412754e-15 5.336238e-15 -1.435976e-15 6.076701e-16
-3.036829e-15
E:\\R-3.1.2\\scripts\\mPDBS\\summary_pdb_93\\1f305f598641eeb2.final.pdb
-2.223610e-15 -3.267546e-15 7.834379e-16 -3.530682e-15 2.048506e-16
-1.886482e-15
E:\\R-3.1.2\\scripts\\mPDBS\\summary_pdb_93\\1f8a00b551be0a39.final.pdb
-3.856945e-15 -9.412754e-15 5.336238e-15 -1.435976e-15 6.076701e-16
-3.036829e-15
E:\\R-3.1.2\\scripts\\mPDBS\\summary_pdb_93\\219f45328d47579a.final.pdb
-9.558562e-15 -1.148828e-14 -1.092706e-15 -1.576540e-14 1.233526e-15
6.099793e-15
E:\\R-3.1.2\\scripts\\mPDBS\\summary_pdb_93\\238dd3918e54336d.final.pdb

-7.496193e-15 -1.365667e-14 -8.853595e-15 -5.895475e-15 2.754228e-15
-1.650518e-15
E:\\R-3.1.2\\scripts\\mPDBS\\summary_pdb_93\\2b3027889e2fdc42.final.pdb
-3.873440e-15 -4.003347e-15 -1.831792e-16 -2.883562e-15 -6.759169e-16
-1.959299e-15
E:\\R-3.1.2\\scripts\\mPDBS\\summary_pdb_93\\2fcb3b46e33793b0.final.pdb
-4.106219e-15 2.946234e-15 -3.882103e-17 -1.661517e-15 3.386333e-15
-3.652431e-15
E:\\R-3.1.2\\scripts\\mPDBS\\summary_pdb_93\\34eb328251842006.final.pdb
-1.754172e-15 -2.735437e-15 6.930695e-15 1.597019e-15 1.737876e-15
-3.629824e-16
E:\\R-3.1.2\\scripts\\mPDBS\\summary_pdb_93\\354b32871797b7ab.final.pdb
-5.044700e-15 -6.592983e-15 1.169872e-15 -1.241095e-15 -6.454597e-15
-5.522687e-15
E:\\R-3.1.2\\scripts\\mPDBS\\summary_pdb_93\\397368adbc32d5de.final.pdb
-7.050952e-15 -1.507458e-14 1.497046e-15 -3.661303e-15 -4.047963e-15
-6.311507e-16
E:\\R-3.1.2\\scripts\\mPDBS\\summary_pdb_93\\411ca13cd3df6edd.final.pdb
-4.468993e-15 -5.945996e-15 -2.128253e-16 4.544890e-15 1.413465e-15
-5.747977e-15
E:\\R-3.1.2\\scripts\\mPDBS\\summary_pdb_93\\42357eee6642bf12.final.pdb
-1.356352e-15 1.134019e-15 5.681590e-15 -2.214971e-15 2.475071e-16
4.998703e-15
E:\\R-3.1.2\\scripts\\mPDBS\\summary_pdb_93\\44342d1f75a7fcfc.final.pdb
-1.055770e-15 -8.507081e-15 6.595885e-15 2.340793e-15 -2.263102e-16
-5.185277e-15
E:\\R-3.1.2\\scripts\\mPDBS\\summary_pdb_93\\448ef21dffe8cdfb.final.pdb
-5.910905e-17 -2.301390e-14 3.576343e-15 -7.913460e-15 -1.399039e-14
4.417503e-15
E:\\R-3.1.2\\scripts\\mPDBS\\summary_pdb_93\\472a40b3e8f113c7.final.pdb
-3.019237e-15 4.218982e-15 -3.509898e-15 8.285949e-16 3.478514e-15
-3.287012e-15
E:\\R-3.1.2\\scripts\\mPDBS\\summary_pdb_93\\48a26225951ea153.final.pdb
4.991477e-16 -1.110877e-14 1.187662e-14 6.895291e-15 -5.418651e-15
-1.033931e-14
E:\\R-3.1.2\\scripts\\mPDBS\\summary_pdb_93\\4ab4a5c7c6682ce6.final.pdb
-9.731967e-15 4.765457e-15 1.351868e-14 -4.002176e-15 4.258887e-16
-7.545758e-15
E:\\R-3.1.2\\scripts\\mPDBS\\summary_pdb_93\\4eaa45b62cb6a25f.final.pdb
-2.100440e-15 -3.842596e-15 -2.285791e-16 -5.218166e-15 8.657188e-16
-4.509609e-15
E:\\R-3.1.2\\scripts\\mPDBS\\summary_pdb_93\\58c371611d85ac96.final.pdb
-7.050952e-15 -1.507458e-14 1.497046e-15 -3.661303e-15 -4.047963e-15
-6.311507e-16
E:\\R-3.1.2\\scripts\\mPDBS\\summary_pdb_93\\59a647f04822d30a.final.pdb
-2.567148e-15 -1.299738e-15 4.836876e-15 -6.312225e-15 -1.797970e-15
7.062779e-16
E:\\R-3.1.2\\scripts\\mPDBS\\summary_pdb_93\\59b091fe385e05fc.final.pdb
-3.106258e-15 9.600951e-16 -1.444855e-15 -3.251757e-15 -4.144616e-16
-2.211058e-16
E:\\R-3.1.2\\scripts\\mPDBS\\summary_pdb_93\\5c35ac30f930d2db.final.pdb
-2.100440e-15 -3.842596e-15 -2.285791e-16 -5.218166e-15 8.657188e-16
-4.509609e-15
E:\\R-3.1.2\\scripts\\mPDBS\\summary_pdb_93\\5f60752431cf2dcb.final.pdb

3.669979e-15 3.691845e-15 -1.781854e-15 -1.298360e-15 -2.248147e-15
-3.427566e-15
E:\\R-3.1.2\\scripts\\mPDBS\\summary_pdb_93\\626456dd6ff2868d.final.pdb
-3.019237e-15 4.218982e-15 -3.509898e-15 8.285949e-16 3.478514e-15
-3.287012e-15
E:\\R-3.1.2\\scripts\\mPDBS\\summary_pdb_93\\6583d53ff3c8f7ef.final.pdb
-2.567148e-15 -1.299738e-15 4.836876e-15 -6.312225e-15 -1.797970e-15
7.062779e-16
E:\\R-3.1.2\\scripts\\mPDBS\\summary_pdb_93\\69760178986bef01.final.pdb
1.869469e-16 -8.014389e-15 -7.816835e-15 -7.777480e-15 1.372749e-15
-1.671388e-15
E:\\R-3.1.2\\scripts\\mPDBS\\summary_pdb_93\\6a9d87d47926b1c8.final.pdb
-3.675613e-15 -4.157960e-15 2.619229e-16 -2.663837e-15 -3.391244e-16
-1.231365e-15
E:\\R-3.1.2\\scripts\\mPDBS\\summary_pdb_93\\6bb3547ea982ffc4.final.pdb
4.023185e-15 -1.661730e-14 -4.785455e-15 5.097127e-15 4.107934e-15
-7.648861e-15
E:\\R-3.1.2\\scripts\\mPDBS\\summary_pdb_93\\716d9624611d1c42.final.pdb
-6.127305e-15 -1.460909e-14 6.931131e-15 -5.365202e-15 4.051014e-15
-7.227478e-15
E:\\R-3.1.2\\scripts\\mPDBS\\summary_pdb_93\\75f27e70ad0f1be4.final.pdb
-2.567148e-15 -1.299738e-15 4.836876e-15 -6.312225e-15 -1.797970e-15
7.062779e-16
E:\\R-3.1.2\\scripts\\mPDBS\\summary_pdb_93\\769f910fffb22872.final.pdb
-3.756331e-15 -3.363358e-15 -7.928081e-16 -2.387689e-15 4.131100e-16
-1.394046e-15
E:\\R-3.1.2\\scripts\\mPDBS\\summary_pdb_93\\791f8c80dd5c3135.final.pdb
-3.106258e-15 9.600951e-16 -1.444855e-15 -3.251757e-15 -4.144616e-16
-2.211058e-16
E:\\R-3.1.2\\scripts\\mPDBS\\summary_pdb_93\\7fad98e66a3f6099.final.pdb
6.949414e-15 -2.858398e-15 -3.691004e-15 -8.902006e-16 -3.663661e-15
-3.718564e-15
E:\\R-3.1.2\\scripts\\mPDBS\\summary_pdb_93\\804b89c94a633196.final.pdb
-4.106219e-15 2.946234e-15 -3.882103e-17 -1.661517e-15 3.386333e-15
-3.652431e-15
E:\\R-3.1.2\\scripts\\mPDBS\\summary_pdb_93\\813cc65a2298feba.final.pdb
-4.106219e-15 2.946234e-15 -3.882103e-17 -1.661517e-15 3.386333e-15
-3.652431e-15
E:\\R-3.1.2\\scripts\\mPDBS\\summary_pdb_93\\83c0eefcfe12d3f9.final.pdb
-4.468993e-15 -5.945996e-15 -2.128253e-16 4.544890e-15 1.413465e-15
-5.747977e-15
E:\\R-3.1.2\\scripts\\mPDBS\\summary_pdb_93\\86d50469ef569760.final.pdb
-3.019237e-15 4.218982e-15 -3.509898e-15 8.285949e-16 3.478514e-15
-3.287012e-15
E:\\R-3.1.2\\scripts\\mPDBS\\summary_pdb_93\\89d0666e1402203d.final.pdb
-2.567148e-15 -1.299738e-15 4.836876e-15 -6.312225e-15 -1.797970e-15
7.062779e-16
E:\\R-3.1.2\\scripts\\mPDBS\\summary_pdb_93\\8b34852716dadc62.final.pdb
-1.446454e-15 -2.865090e-15 4.503710e-16 -2.408315e-15 -1.425789e-15
-5.461424e-17
E:\\R-3.1.2\\scripts\\mPDBS\\summary_pdb_93\\8b6ef9ac3fe74395.final.pdb
5.178630e-15 -1.895572e-15 -3.395225e-15 -1.008803e-14 4.943051e-15
7.022268e-15
E:\\R-3.1.2\\scripts\\mPDBS\\summary_pdb_93\\98bbe0d5986c138f.final.pdb

-3.106258e-15 9.600951e-16 -1.444855e-15 -3.251757e-15 -4.144616e-16
-2.211058e-16
E:\\R-3.1.2\\scripts\\mPDBS\\summary_pdb_93\\9c22f4909d67e2fa.final.pdb
-2.973228e-15 -4.749887e-15 -4.377632e-15 -7.930591e-15 -3.968298e-15
7.398891e-16
E:\\R-3.1.2\\scripts\\mPDBS\\summary_pdb_93\\9dda8ed0d81fb3ef.final.pdb
-5.783325e-15 -7.433685e-15 1.112752e-14 6.789274e-15 2.158609e-15
-1.277599e-14
E:\\R-3.1.2\\scripts\\mPDBS\\summary_pdb_93\\9f752b3616e6033b.final.pdb
-3.850631e-15 -1.632768e-15 -2.412035e-15 -3.050276e-15 3.489295e-16
1.922218e-15
E:\\R-3.1.2\\scripts\\mPDBS\\summary_pdb_93\\a02c70083816d6ba.final.pdb
-2.100440e-15 -3.842596e-15 -2.285791e-16 -5.218166e-15 8.657188e-16
-4.509609e-15
E:\\R-3.1.2\\scripts\\mPDBS\\summary_pdb_93\\a0c997d891e4e082.final.pdb
-8.168866e-15 -3.235373e-15 -6.351116e-15 -5.902966e-15 3.926086e-15
-2.440512e-15
E:\\R-3.1.2\\scripts\\mPDBS\\summary_pdb_93\\ab8039a2d823c616.final.pdb
-4.825187e-15 -3.666001e-15 -7.615732e-16 -3.574533e-15 -4.972425e-16
-1.389270e-15
E:\\R-3.1.2\\scripts\\mPDBS\\summary_pdb_93\\acf4d74b9c71d4dc.final.pdb
-4.106219e-15 2.946234e-15 -3.882103e-17 -1.661517e-15 3.386333e-15
-3.652431e-15
E:\\R-3.1.2\\scripts\\mPDBS\\summary_pdb_93\\ad8a823e4b4c668e.final.pdb
-4.000141e-15 -4.429719e-15 -9.868781e-16 -3.157109e-15 2.297576e-17
-2.495387e-15
E:\\R-3.1.2\\scripts\\mPDBS\\summary_pdb_93\\b16ababd03b7b857.final.pdb
-2.973228e-15 -4.749887e-15 -4.377632e-15 -7.930591e-15 -3.968298e-15
7.398891e-16
E:\\R-3.1.2\\scripts\\mPDBS\\summary_pdb_93\\b226923e697d3c63.final.pdb
-2.563895e-15 -2.733035e-15 -1.100815e-15 -1.195658e-14 6.770101e-15
4.659279e-15
E:\\R-3.1.2\\scripts\\mPDBS\\summary_pdb_93\\b28e48f6fa27a165.final.pdb
-3.106258e-15 9.600951e-16 -1.444855e-15 -3.251757e-15 -4.144616e-16
-2.211058e-16
E:\\R-3.1.2\\scripts\\mPDBS\\summary_pdb_93\\b42c4924e3cd1aec.final.pdb
-3.417379e-15 1.483608e-15 -9.478327e-15 -9.271663e-15 -8.302108e-16
7.106147e-16
E:\\R-3.1.2\\scripts\\mPDBS\\summary_pdb_93\\b4dd239fcabff1df.final.pdb
-3.106258e-15 9.600951e-16 -1.444855e-15 -3.251757e-15 -4.144616e-16
-2.211058e-16
E:\\R-3.1.2\\scripts\\mPDBS\\summary_pdb_93\\b669942ef97459a2.final.pdb
-1.446454e-15 -2.865090e-15 4.503710e-16 -2.408315e-15 -1.425789e-15
-5.461424e-17
E:\\R-3.1.2\\scripts\\mPDBS\\summary_pdb_93\\b99f4d9d87c1017b.final.pdb
-3.106258e-15 9.600951e-16 -1.444855e-15 -3.251757e-15 -4.144616e-16
-2.211058e-16
E:\\R-3.1.2\\scripts\\mPDBS\\summary_pdb_93\\b9d2cbf3975403e7.final.pdb
-3.106258e-15 9.600951e-16 -1.444855e-15 -3.251757e-15 -4.144616e-16
-2.211058e-16
E:\\R-3.1.2\\scripts\\mPDBS\\summary_pdb_93\\bc88c8a505f3a288.final.pdb
-3.570080e-15 -3.131703e-15 1.200331e-15 -2.699681e-15 -4.069264e-16
-1.372962e-15
E:\\R-3.1.2\\scripts\\mPDBS\\summary_pdb_93\\bdac0e64f4ef427e.final.pdb

-6.267973e-16 8.723177e-16 -9.815755e-16 -1.897514e-14 -3.724190e-15
6.765451e-15
E:\\R-3.1.2\\scripts\\mPDBS\\summary_pdb_93\\ca2a1f55fc9d0681.final.pdb
-1.446454e-15 -2.865090e-15 4.503710e-16 -2.408315e-15 -1.425789e-15
-5.461424e-17
E:\\R-3.1.2\\scripts\\mPDBS\\summary_pdb_93\\ce34248de3e81a3c.final.pdb
-7.107139e-15 -2.721079e-15 9.989559e-15 -6.607799e-15 -1.384634e-15
-5.649936e-15
E:\\R-3.1.2\\scripts\\mPDBS\\summary_pdb_93\\d02e697772494a61.final.pdb
-4.111484e-16 -3.976911e-15 -9.207043e-16 -6.939779e-15 -1.776003e-15
8.240875e-15
E:\\R-3.1.2\\scripts\\mPDBS\\summary_pdb_93\\d04221245d015047.final.pdb
-2.111212e-15 -3.197209e-15 9.381916e-16 -1.444672e-17 -1.631968e-15
-3.094503e-15
E:\\R-3.1.2\\scripts\\mPDBS\\summary_pdb_93\\d678141e4018054a.final.pdb
-4.287613e-15 -1.791798e-14 1.044552e-14 7.434855e-15 -9.254849e-18
-6.529970e-15
E:\\R-3.1.2\\scripts\\mPDBS\\summary_pdb_93\\d71b40af0f0efe39.final.pdb
-8.432800e-15 9.682449e-16 4.080998e-16 5.058554e-16 -6.772153e-15
1.528937e-15
E:\\R-3.1.2\\scripts\\mPDBS\\summary_pdb_93\\dc0809eff10197e2.final.pdb
-9.393342e-15 1.144550e-14 -3.061735e-16 -7.958264e-15 3.526817e-15
5.978547e-15
E:\\R-3.1.2\\scripts\\mPDBS\\summary_pdb_93\\e1163918d3707839.final.pdb
-4.214918e-15 9.098486e-16 -9.042911e-15 2.831720e-15 4.299366e-15
-2.851166e-16
E:\\R-3.1.2\\scripts\\mPDBS\\summary_pdb_93\\e4b48b06824ef848.final.pdb
-3.856945e-15 -9.412754e-15 5.336238e-15 -1.435976e-15 6.076701e-16
-3.036829e-15
E:\\R-3.1.2\\scripts\\mPDBS\\summary_pdb_93\\e529e19ad281fea2.final.pdb
-3.322380e-15 -6.418342e-15 4.469830e-15 -6.005348e-15 -3.101220e-16
-4.166517e-15
E:\\R-3.1.2\\scripts\\mPDBS\\summary_pdb_93\\e5602082718ec3a1.final.pdb
-4.106219e-15 2.946234e-15 -3.882103e-17 -1.661517e-15 3.386333e-15
-3.652431e-15
E:\\R-3.1.2\\scripts\\mPDBS\\summary_pdb_93\\e5d589d996d0a960.final.pdb
-1.200914e-14 -8.275001e-15 6.918575e-15 -1.182735e-15 2.264620e-15
-1.459942e-15
E:\\R-3.1.2\\scripts\\mPDBS\\summary_pdb_93\\e9df9e72307a2fbd.final.pdb
-2.100440e-15 -3.842596e-15 -2.285791e-16 -5.218166e-15 8.657188e-16
-4.509609e-15
E:\\R-3.1.2\\scripts\\mPDBS\\summary_pdb_93\\ea00f8694090cae7.final.pdb
-4.106219e-15 2.946234e-15 -3.882103e-17 -1.661517e-15 3.386333e-15
-3.652431e-15
E:\\R-3.1.2\\scripts\\mPDBS\\summary_pdb_93\\eba517be32db050e.final.pdb
-3.106258e-15 9.600951e-16 -1.444855e-15 -3.251757e-15 -4.144616e-16
-2.211058e-16
E:\\R-3.1.2\\scripts\\mPDBS\\summary_pdb_93\\ec04b9545899c29d.final.pdb
-2.656486e-15 -3.443423e-15 1.278958e-15 -2.615746e-15 -2.530906e-18
-1.122554e-15
E:\\R-3.1.2\\scripts\\mPDBS\\summary_pdb_93\\ee6862cbcad52078.final.pdb
-3.767622e-15 -3.079209e-15 3.800740e-16 -2.844314e-15 -1.837434e-16
-1.168699e-15
E:\\R-3.1.2\\scripts\\mPDBS\\summary_pdb_93\\ef6a42fc83b81e5d.final.pdb

-3.287089e-15 -3.072809e-15 6.797079e-16 -3.248545e-15 -9.277870e-17
-1.036693e-15
E:\\R-3.1.2\\scripts\\mPDBS\\summary_pdb_93\\ef8e88d4ad93a0ad.final.pdb
-2.111212e-15 -3.197209e-15 9.381916e-16 -1.444672e-17 -1.631968e-15
-3.094503e-15
E:\\R-3.1.2\\scripts\\mPDBS\\summary_pdb_93\\f14fcae8deec4fa3.final.pdb
-3.850631e-15 -1.632768e-15 -2.412035e-15 -3.050276e-15 3.489295e-16
1.922218e-15
E:\\R-3.1.2\\scripts\\mPDBS\\summary_pdb_93\\f83584e22003fb92.final.pdb
4.991477e-16 -1.110877e-14 1.187662e-14 6.895291e-15 -5.418651e-15
-1.033931e-14
E:\\R-3.1.2\\scripts\\mPDBS\\summary_pdb_93\\f8e2bf87e52caa29.final.pdb
-3.322380e-15 -6.418342e-15 4.469830e-15 -6.005348e-15 -3.101220e-16
-4.166517e-15

[,157]   [,158]   [,159]   [,160]   [,161]   [,162]
E:\\R-3.1.2\\scripts\\40.1ns_data\\NAT0.pdb
4.690205e-15 -6.534357e-15 1.338617e-15 -7.762143e-15 -1.044754e-15
-1.198958e-15
E:\\R-3.1.2\\scripts\\40.1ns_data\\LJP6.pdb
3.380345e-15 -1.595905e-15 -6.201563e-16 -9.514872e-15 -2.644529e-15
-2.243057e-15
E:\\R-3.1.2\\scripts\\40.1ns_data\\3WY9.pdb
3.716387e-15 1.595317e-15 1.738744e-15 -7.880882e-15 -1.193133e-15
-9.040189e-16
E:\\R-3.1.2\\scripts\\40.1ns_data\\ZSP9.pdb
4.988838e-15 -1.619947e-15 3.444585e-16 -8.107343e-15 -1.799229e-15
-2.690997e-15
E:\\R-3.1.2\\scripts\\mPDBS\\summary_pdb_93\\013d855ad26c877c.final.pdb
3.995852e-15 -1.495796e-15 -1.230523e-15 -1.156829e-14 -4.046036e-15
-7.488434e-15
E:\\R-3.1.2\\scripts\\mPDBS\\summary_pdb_93\\0a2a705342ca0d54.final.pdb
3.154184e-15 2.007571e-15 5.351961e-16 -8.358849e-15 -3.163431e-15
-3.493401e-15
E:\\R-3.1.2\\scripts\\mPDBS\\summary_pdb_93\\0a59fceadb4194d9.final.pdb
2.603008e-15 5.263913e-15 4.211303e-16 -7.683813e-15 -2.557961e-15
-4.533316e-15
E:\\R-3.1.2\\scripts\\mPDBS\\summary_pdb_93\\0dba59f3306825e8.final.pdb
2.349715e-16 5.304320e-15 2.998733e-15 -1.348169e-14 -1.889775e-15
4.808858e-16
E:\\R-3.1.2\\scripts\\mPDBS\\summary_pdb_93\\126f26a78b57339c.final.pdb
4.261265e-15 -2.024236e-15 8.979828e-16 -1.028113e-14 -3.674155e-15
-4.160801e-15
E:\\R-3.1.2\\scripts\\mPDBS\\summary_pdb_93\\12f06f3e342df706.final.pdb
2.853210e-15 2.025894e-15 -4.540300e-16 -8.842836e-15 -2.051907e-15
-3.607459e-15
E:\\R-3.1.2\\scripts\\mPDBS\\summary_pdb_93\\18618a2a251b867b.final.pdb
5.231536e-15 -5.108184e-16 -4.902471e-15 -1.291166e-14 -5.566067e-16
-9.190250e-16
E:\\R-3.1.2\\scripts\\mPDBS\\summary_pdb_93\\1f305f598641eeb2.final.pdb
2.922224e-15 1.264464e-15 -2.137074e-16 -8.963127e-15 -3.226364e-15
-3.262944e-15
E:\\R-3.1.2\\scripts\\mPDBS\\summary_pdb_93\\1f8a00b551be0a39.final.pdb
5.231536e-15 -5.108184e-16 -4.902471e-15 -1.291166e-14 -5.566067e-16

-9.190250e-16
E:\\R-3.1.2\\scripts\\mPDBS\\summary_pdb_93\\219f45328d47579a.final.pdb
3.636035e-15 6.365384e-16 4.563650e-15 -1.124780e-14 -1.151260e-14
-1.360505e-14
E:\\R-3.1.2\\scripts\\mPDBS\\summary_pdb_93\\238dd3918e54336d.final.pdb
-5.001153e-15 6.879266e-15 5.423205e-15 -1.470597e-14 -6.029576e-15
1.412666e-15
E:\\R-3.1.2\\scripts\\mPDBS\\summary_pdb_93\\2b3027889e2fdc42.final.pdb
2.351473e-15 3.482894e-15 7.411868e-16 -9.963240e-15 -3.029806e-15
-4.210007e-15
E:\\R-3.1.2\\scripts\\mPDBS\\summary_pdb_93\\2fcb3b46e33793b0.final.pdb
3.027911e-16 1.160381e-15 -1.651352e-16 -1.058336e-14 -3.708617e-15
3.869143e-16
E:\\R-3.1.2\\scripts\\mPDBS\\summary_pdb_93\\34eb328251842006.final.pdb
3.660655e-15 7.839056e-15 -9.057591e-15 -4.914226e-15 -8.296457e-15
-8.097575e-15
E:\\R-3.1.2\\scripts\\mPDBS\\summary_pdb_93\\354b32871797b7ab.final.pdb
4.834766e-15 7.405828e-15 -9.081917e-15 -1.559929e-16 1.448172e-15
2.102305e-16
E:\\R-3.1.2\\scripts\\mPDBS\\summary_pdb_93\\397368adbc32d5de.final.pdb
4.261265e-15 -2.024236e-15 8.979828e-16 -1.028113e-14 -3.674155e-15
-4.160801e-15
E:\\R-3.1.2\\scripts\\mPDBS\\summary_pdb_93\\411ca13cd3df6edd.final.pdb
-1.794490e-15 -4.743534e-15 -5.547982e-15 -1.156174e-14 -7.577701e-15
4.261853e-15
E:\\R-3.1.2\\scripts\\mPDBS\\summary_pdb_93\\42357eee6642bf12.final.pdb
2.462571e-16 4.677215e-15 -4.057618e-15 -1.861792e-15 -1.460350e-15
-4.180222e-15
E:\\R-3.1.2\\scripts\\mPDBS\\summary_pdb_93\\44342d1f75a7fcfc.final.pdb
-1.465057e-15 -5.290629e-15 -3.264279e-15 -1.734117e-14 -1.028296e-14
-2.433375e-15
E:\\R-3.1.2\\scripts\\mPDBS\\summary_pdb_93\\448ef21dffe8cdfb.final.pdb
1.387874e-14 4.844934e-15 -9.719992e-15 3.284021e-15 4.507033e-15
-9.662339e-15
E:\\R-3.1.2\\scripts\\mPDBS\\summary_pdb_93\\472a40b3e8f113c7.final.pdb
2.349715e-16 5.304320e-15 2.998733e-15 -1.348169e-14 -1.889775e-15
4.808858e-16
E:\\R-3.1.2\\scripts\\mPDBS\\summary_pdb_93\\48a26225951ea153.final.pdb
1.960293e-15 1.785467e-15 -7.496148e-15 -1.525328e-14 4.585904e-17
3.761048e-15
E:\\R-3.1.2\\scripts\\mPDBS\\summary_pdb_93\\4ab4a5c7c6682ce6.final.pdb
1.916034e-16 1.893469e-14 -7.321137e-15 -5.826358e-15 3.309842e-15
1.156127e-15
E:\\R-3.1.2\\scripts\\mPDBS\\summary_pdb_93\\4eaa45b62cb6a25f.final.pdb
3.802605e-15 -1.012243e-15 2.419300e-15 -1.299555e-14 -2.673965e-15
-2.778048e-15
E:\\R-3.1.2\\scripts\\mPDBS\\summary_pdb_93\\58c371611d85ac96.final.pdb
4.261265e-15 -2.024236e-15 8.979828e-16 -1.028113e-14 -3.674155e-15
-4.160801e-15
E:\\R-3.1.2\\scripts\\mPDBS\\summary_pdb_93\\59a647f04822d30a.final.pdb
4.137233e-15 5.944308e-15 1.478455e-15 -2.389593e-15 4.484738e-15
-7.490094e-15
E:\\R-3.1.2\\scripts\\mPDBS\\summary_pdb_93\\59b091fe385e05fc.final.pdb
2.603008e-15 5.263913e-15 4.211303e-16 -7.683813e-15 -2.557961e-15

-4.533316e-15
E:\\R-3.1.2\\scripts\\mPDBS\\summary_pdb_93\\5c35ac30f930d2db.final.pdb
3.802605e-15 -1.012243e-15 2.419300e-15 -1.299555e-14 -2.673965e-15
-2.778048e-15
E:\\R-3.1.2\\scripts\\mPDBS\\summary_pdb_93\\5f60752431cf2dcb.final.pdb
7.195001e-16 3.997841e-15 3.998877e-16 -1.090629e-14 2.175180e-15
-6.920394e-16
E:\\R-3.1.2\\scripts\\mPDBS\\summary_pdb_93\\626456dd6ff2868d.final.pdb
2.349715e-16 5.304320e-15 2.998733e-15 -1.348169e-14 -1.889775e-15
4.808858e-16
E:\\R-3.1.2\\scripts\\mPDBS\\summary_pdb_93\\6583d53ff3c8f7ef.final.pdb
4.137233e-15 5.944308e-15 1.478455e-15 -2.389593e-15 4.484738e-15
-7.490094e-15
E:\\R-3.1.2\\scripts\\mPDBS\\summary_pdb_93\\69760178986bef01.final.pdb
5.017891e-15 1.484232e-15 9.044565e-15 -1.509077e-14 -1.304390e-15
1.551065e-15
E:\\R-3.1.2\\scripts\\mPDBS\\summary_pdb_93\\6a9d87d47926b1c8.final.pdb
2.158780e-15 3.339529e-15 4.781797e-16 -1.036092e-14 -3.731401e-15
-3.588342e-15
E:\\R-3.1.2\\scripts\\mPDBS\\summary_pdb_93\\6bb3547ea982ffc4.final.pdb
9.614468e-16 -3.472310e-16 3.513757e-15 -2.676530e-14 -1.365352e-15
-1.715488e-15
E:\\R-3.1.2\\scripts\\mPDBS\\summary_pdb_93\\716d9624611d1c42.final.pdb
9.748736e-15 -3.733496e-15 -6.153030e-15 -1.123936e-14 -8.916642e-15
-3.143854e-15
E:\\R-3.1.2\\scripts\\mPDBS\\summary_pdb_93\\75f27e70ad0f1be4.final.pdb
4.137233e-15 5.944308e-15 1.478455e-15 -2.389593e-15 4.484738e-15
-7.490094e-15
E:\\R-3.1.2\\scripts\\mPDBS\\summary_pdb_93\\769f910fffb22872.final.pdb
2.265158e-15 2.162174e-15 -8.432179e-16 -1.035988e-14 -2.781823e-15
-3.730578e-15
E:\\R-3.1.2\\scripts\\mPDBS\\summary_pdb_93\\791f8c80dd5c3135.final.pdb
2.603008e-15 5.263913e-15 4.211303e-16 -7.683813e-15 -2.557961e-15
-4.533316e-15
E:\\R-3.1.2\\scripts\\mPDBS\\summary_pdb_93\\7fad98e66a3f6099.final.pdb
-1.693986e-15 -1.493051e-15 -2.760754e-16 -4.407415e-15 -1.048463e-14
1.023193e-15
E:\\R-3.1.2\\scripts\\mPDBS\\summary_pdb_93\\804b89c94a633196.final.pdb
3.027911e-16 1.160381e-15 -1.651352e-16 -1.058336e-14 -3.708617e-15
3.869143e-16
E:\\R-3.1.2\\scripts\\mPDBS\\summary_pdb_93\\813cc65a2298feba.final.pdb
3.027911e-16 1.160381e-15 -1.651352e-16 -1.058336e-14 -3.708617e-15
3.869143e-16
E:\\R-3.1.2\\scripts\\mPDBS\\summary_pdb_93\\83c0eefcfe12d3f9.final.pdb
-1.794490e-15 -4.743534e-15 -5.547982e-15 -1.156174e-14 -7.577701e-15
4.261853e-15
E:\\R-3.1.2\\scripts\\mPDBS\\summary_pdb_93\\86d50469ef569760.final.pdb
2.349715e-16 5.304320e-15 2.998733e-15 -1.348169e-14 -1.889775e-15
4.808858e-16
E:\\R-3.1.2\\scripts\\mPDBS\\summary_pdb_93\\89d0666e1402203d.final.pdb
4.137233e-15 5.944308e-15 1.478455e-15 -2.389593e-15 4.484738e-15
-7.490094e-15
E:\\R-3.1.2\\scripts\\mPDBS\\summary_pdb_93\\8b34852716dadc62.final.pdb
8.336165e-16 4.678400e-15 8.618211e-16 -9.226805e-15 -4.232503e-15

-2.017412e-15
E:\\R-3.1.2\\scripts\\mPDBS\\summary_pdb_93\\8b6ef9ac3fe74395.final.pdb
3.246927e-15 -2.976058e-16 1.159752e-14 -1.283055e-14 6.449697e-15
-1.144959e-14
E:\\R-3.1.2\\scripts\\mPDBS\\summary_pdb_93\\98bbe0d5986c138f.final.pdb
2.603008e-15 5.263913e-15 4.211303e-16 -7.683813e-15 -2.557961e-15
-4.533316e-15
E:\\R-3.1.2\\scripts\\mPDBS\\summary_pdb_93\\9c22f4909d67e2fa.final.pdb
9.369323e-15 1.795832e-15 3.386711e-15 -8.015968e-15 -5.460904e-15
-8.809072e-15
E:\\R-3.1.2\\scripts\\mPDBS\\summary_pdb_93\\9dda8ed0d81fb3ef.final.pdb
-1.155409e-15 -4.303029e-15 1.509680e-15 -1.521354e-14 2.822873e-15
1.056382e-14
E:\\R-3.1.2\\scripts\\mPDBS\\summary_pdb_93\\9f752b3616e6033b.final.pdb
7.103554e-16 2.311395e-15 3.513226e-15 -8.629186e-15 -2.655763e-15
-4.472175e-15
E:\\R-3.1.2\\scripts\\mPDBS\\summary_pdb_93\\a02c70083816d6ba.final.pdb
3.802605e-15 -1.012243e-15 2.419300e-15 -1.299555e-14 -2.673965e-15
-2.778048e-15
E:\\R-3.1.2\\scripts\\mPDBS\\summary_pdb_93\\a0c997d891e4e082.final.pdb
3.321584e-15 -3.798750e-16 4.877265e-15 -1.280361e-14 -1.364872e-15
-4.886386e-15
E:\\R-3.1.2\\scripts\\mPDBS\\summary_pdb_93\\ab8039a2d823c616.final.pdb
2.460294e-15 1.324816e-15 5.404562e-16 -8.595170e-15 -3.249216e-15
-3.697884e-15
E:\\R-3.1.2\\scripts\\mPDBS\\summary_pdb_93\\acf4d74b9c71d4dc.final.pdb
3.027911e-16 1.160381e-15 -1.651352e-16 -1.058336e-14 -3.708617e-15
3.869143e-16
E:\\R-3.1.2\\scripts\\mPDBS\\summary_pdb_93\\ad8a823e4b4c668e.final.pdb
2.527236e-15 1.415926e-15 -6.962852e-16 -8.164467e-15 -3.641178e-15
-3.078091e-15
E:\\R-3.1.2\\scripts\\mPDBS\\summary_pdb_93\\b16ababd03b7b857.final.pdb
9.369323e-15 1.795832e-15 3.386711e-15 -8.015968e-15 -5.460904e-15
-8.809072e-15
E:\\R-3.1.2\\scripts\\mPDBS\\summary_pdb_93\\b226923e697d3c63.final.pdb
6.714164e-15 9.970931e-16 1.929483e-15 -1.001891e-14 2.055691e-15
-6.176474e-15
E:\\R-3.1.2\\scripts\\mPDBS\\summary_pdb_93\\b28e48f6fa27a165.final.pdb
2.603008e-15 5.263913e-15 4.211303e-16 -7.683813e-15 -2.557961e-15
-4.533316e-15
E:\\R-3.1.2\\scripts\\mPDBS\\summary_pdb_93\\b42c4924e3cd1aec.final.pdb
4.112946e-15 4.976007e-15 -1.765478e-15 -1.410765e-14 -1.187934e-14
-8.124352e-15
E:\\R-3.1.2\\scripts\\mPDBS\\summary_pdb_93\\b4dd239fcabff1df.final.pdb
2.603008e-15 5.263913e-15 4.211303e-16 -7.683813e-15 -2.557961e-15
-4.533316e-15
E:\\R-3.1.2\\scripts\\mPDBS\\summary_pdb_93\\b669942ef97459a2.final.pdb
8.336165e-16 4.678400e-15 8.618211e-16 -9.226805e-15 -4.232503e-15
-2.017412e-15
E:\\R-3.1.2\\scripts\\mPDBS\\summary_pdb_93\\b99f4d9d87c1017b.final.pdb
2.603008e-15 5.263913e-15 4.211303e-16 -7.683813e-15 -2.557961e-15
-4.533316e-15
E:\\R-3.1.2\\scripts\\mPDBS\\summary_pdb_93\\b9d2cbf3975403e7.final.pdb
2.603008e-15 5.263913e-15 4.211303e-16 -7.683813e-15 -2.557961e-15

```
-4.533316e-15
E:\\R-3.1.2\\scripts\\mPDBS\\summary_pdb_93\\bc88c8a505f3a288.final.pdb
2.377494e-15  2.160168e-15  5.859291e-16 -1.051575e-14 -3.038696e-15
-3.152042e-15
E:\\R-3.1.2\\scripts\\mPDBS\\summary_pdb_93\\bdac0e64f4ef427e.final.pdb
1.220213e-14  1.332936e-14  1.007060e-14 -5.316738e-15  7.186097e-15
-1.814253e-14
E:\\R-3.1.2\\scripts\\mPDBS\\summary_pdb_93\\ca2a1f55fc9d0681.final.pdb
8.336165e-16  4.678400e-15  8.618211e-16 -9.226805e-15 -4.232503e-15
-2.017412e-15
E:\\R-3.1.2\\scripts\\mPDBS\\summary_pdb_93\\ce34248de3e81a3c.final.pdb
2.729684e-15  5.657468e-16 -2.754113e-15 -8.214679e-16  2.306214e-15
-4.502267e-15
E:\\R-3.1.2\\scripts\\mPDBS\\summary_pdb_93\\d02e697772494a61.final.pdb
6.313581e-15  5.152349e-15  5.477652e-16  1.189297e-15  9.205934e-16
-1.277130e-14
E:\\R-3.1.2\\scripts\\mPDBS\\summary_pdb_93\\d04221245d015047.final.pdb
1.760333e-15  2.846711e-15 -1.249778e-15 -9.021912e-15 -3.491643e-15
-2.641654e-15
E:\\R-3.1.2\\scripts\\mPDBS\\summary_pdb_93\\d678141e4018054a.final.pdb
5.353691e-15 -3.899520e-15 -1.272768e-14 -1.294232e-14 -9.369905e-15
8.311516e-17
E:\\R-3.1.2\\scripts\\mPDBS\\summary_pdb_93\\d71b40af0f0efe39.final.pdb
2.647079e-15  6.684685e-15 -8.441493e-15 -6.764425e-15 -1.552958e-14
9.091336e-16
E:\\R-3.1.2\\scripts\\mPDBS\\summary_pdb_93\\dc0809eff10197e2.final.pdb
4.201772e-15  3.598564e-15  6.483228e-15 -9.334379e-15 -1.497873e-15
-1.045596e-14
E:\\R-3.1.2\\scripts\\mPDBS\\summary_pdb_93\\e1163918d3707839.final.pdb
1.034407e-14 -1.847528e-15 -6.535205e-16 -1.476164e-14 -1.219159e-14
-1.835112e-15
E:\\R-3.1.2\\scripts\\mPDBS\\summary_pdb_93\\e4b48b06824ef848.final.pdb
5.231536e-15 -5.108184e-16 -4.902471e-15 -1.291166e-14 -5.566067e-16
-9.190250e-16
E:\\R-3.1.2\\scripts\\mPDBS\\summary_pdb_93\\e529e19ad281fea2.final.pdb
3.995852e-15 -1.495796e-15 -1.230523e-15 -1.156829e-14 -4.046036e-15
-7.488434e-15
E:\\R-3.1.2\\scripts\\mPDBS\\summary_pdb_93\\e5602082718ec3a1.final.pdb
3.027911e-16  1.160381e-15 -1.651352e-16 -1.058336e-14 -3.708617e-15
3.869143e-16
E:\\R-3.1.2\\scripts\\mPDBS\\summary_pdb_93\\e5d589d996d0a960.final.pdb
-8.686916e-15 -4.018040e-15  8.541652e-15 -1.486824e-14  7.355143e-15
-3.386056e-15
E:\\R-3.1.2\\scripts\\mPDBS\\summary_pdb_93\\e9df9e72307a2fbd.final.pdb
3.802605e-15 -1.012243e-15  2.419300e-15 -1.299555e-14 -2.673965e-15
-2.778048e-15
E:\\R-3.1.2\\scripts\\mPDBS\\summary_pdb_93\\ea00f8694090cae7.final.pdb
3.027911e-16  1.160381e-15 -1.651352e-16 -1.058336e-14 -3.708617e-15
3.869143e-16
E:\\R-3.1.2\\scripts\\mPDBS\\summary_pdb_93\\eba517be32db050e.final.pdb
2.603008e-15  5.263913e-15  4.211303e-16 -7.683813e-15 -2.557961e-15
-4.533316e-15
E:\\R-3.1.2\\scripts\\mPDBS\\summary_pdb_93\\ec04b9545899c29d.final.pdb
2.959355e-15  1.997812e-15 -5.615802e-16 -8.929500e-15 -2.851408e-15
```

```
-3.533190e-15
E:\\R-3.1.2\\scripts\\mPDBS\\summary_pdb_93\\ee6862cbcad52078.final.pdb
2.391371e-15  3.479750e-15 -4.811235e-17 -1.022518e-14 -2.467755e-15
-3.580085e-15
E:\\R-3.1.2\\scripts\\mPDBS\\summary_pdb_93\\ef6a42fc83b81e5d.final.pdb
3.065733e-15  1.426326e-15 -1.302466e-16 -8.782152e-15 -2.616591e-15
-3.564261e-15
E:\\R-3.1.2\\scripts\\mPDBS\\summary_pdb_93\\ef8e88d4ad93a0ad.final.pdb
1.760333e-15  2.846711e-15 -1.249778e-15 -9.021912e-15 -3.491643e-15
-2.641654e-15
E:\\R-3.1.2\\scripts\\mPDBS\\summary_pdb_93\\f14fcae8deec4fa3.final.pdb
7.103554e-16  2.311395e-15  3.513226e-15 -8.629186e-15 -2.655763e-15
-4.472175e-15
E:\\R-3.1.2\\scripts\\mPDBS\\summary_pdb_93\\f83584e22003fb92.final.pdb
1.960293e-15  1.785467e-15 -7.496148e-15 -1.525328e-14  4.585904e-17
3.761048e-15
E:\\R-3.1.2\\scripts\\mPDBS\\summary_pdb_93\\f8e2bf87e52caa29.final.pdb
3.995852e-15 -1.495796e-15 -1.230523e-15 -1.156829e-14 -4.046036e-15
-7.488434e-15

[,163]        [,164]        [,165]        [,166]        [,167]        [,168]
E:\\R-3.1.2\\scripts\\40.1ns_data\\NAT0.pdb
-5.058544e-16  9.829777e-16  1.354420e-15  3.450064e-16  8.483427e-16
-1.805788e-15
E:\\R-3.1.2\\scripts\\40.1ns_data\\LJP6.pdb
8.803022e-17  1.967174e-15  2.969696e-15  6.935043e-16  1.448505e-15
-8.416395e-16
E:\\R-3.1.2\\scripts\\40.1ns_data\\3WY9.pdb
-1.292204e-15  7.298064e-16 -1.454697e-15  1.218533e-15  2.185470e-15
-1.052062e-15
E:\\R-3.1.2\\scripts\\40.1ns_data\\ZSP9.pdb
1.108406e-15  2.402949e-15  1.560218e-15  2.068487e-15  5.099573e-16
-2.020024e-15
E:\\R-3.1.2\\scripts\\mPDBS\\summary_pdb_93\\013d855ad26c877c.final.pdb
5.007534e-15 -1.427416e-15  5.909791e-16 -5.620224e-15 -8.448860e-15
3.674704e-16
E:\\R-3.1.2\\scripts\\mPDBS\\summary_pdb_93\\0a2a705342ca0d54.final.pdb
-1.121854e-17  1.573120e-15  7.678223e-16  2.966506e-16  1.019104e-15
-1.942092e-16
E:\\R-3.1.2\\scripts\\mPDBS\\summary_pdb_93\\0a59fceadb4194d9.final.pdb
-9.188711e-16  9.195418e-16 -4.997710e-16  1.913250e-15  1.948321e-15
-6.389162e-16
E:\\R-3.1.2\\scripts\\mPDBS\\summary_pdb_93\\0dba59f3306825e8.final.pdb
-6.288985e-15  4.599447e-15  3.172248e-15  4.881792e-15  4.284010e-15
2.486418e-16
E:\\R-3.1.2\\scripts\\mPDBS\\summary_pdb_93\\126f26a78b57339c.final.pdb
-2.703041e-15  3.822725e-15  1.293108e-15 -6.181484e-16 -1.779662e-15
-6.369762e-15
E:\\R-3.1.2\\scripts\\mPDBS\\summary_pdb_93\\12f06f3e342df706.final.pdb
-1.544741e-15  9.199971e-16  7.149132e-16  9.740601e-16  6.356486e-16
-4.977858e-16
E:\\R-3.1.2\\scripts\\mPDBS\\summary_pdb_93\\18618a2a251b867b.final.pdb
-1.918364e-16  7.734822e-15  9.744110e-16  4.463531e-15  2.332048e-16
-4.336842e-15
```

E:\\R-3.1.2\\scripts\\mPDBS\\summary_pdb_93\\1f305f598641eeb2.final.pdb
-1.956212e-15 1.356626e-15 9.389632e-16 5.209701e-16 1.048899e-15
-7.793622e-16
E:\\R-3.1.2\\scripts\\mPDBS\\summary_pdb_93\\1f8a00b551be0a39.final.pdb
-1.918364e-16 7.734822e-15 9.744110e-16 4.463531e-15 2.332048e-16
-4.336842e-15
E:\\R-3.1.2\\scripts\\mPDBS\\summary_pdb_93\\219f45328d47579a.final.pdb
2.266643e-14 1.809833e-16 4.634143e-15 -2.499886e-15 -9.859942e-15
1.168066e-14
E:\\R-3.1.2\\scripts\\mPDBS\\summary_pdb_93\\238dd3918e54336d.final.pdb
9.330817e-16 5.842687e-15 -4.754849e-16 4.233876e-15 8.091437e-15
-1.906132e-15
E:\\R-3.1.2\\scripts\\mPDBS\\summary_pdb_93\\2b3027889e2fdc42.final.pdb
-1.234293e-15 1.276461e-15 4.966108e-16 9.548153e-16 5.978926e-16
-7.993781e-16
E:\\R-3.1.2\\scripts\\mPDBS\\summary_pdb_93\\2fcb3b46e33793b0.final.pdb
-3.295418e-15 1.669743e-15 -1.996189e-15 1.266901e-15 1.687277e-15
9.657697e-16
E:\\R-3.1.2\\scripts\\mPDBS\\summary_pdb_93\\34eb328251842006.final.pdb
6.703542e-15 4.228517e-15 3.866212e-16 -9.709608e-16 1.145908e-14
9.313408e-15
E:\\R-3.1.2\\scripts\\mPDBS\\summary_pdb_93\\354b32871797b7ab.final.pdb
-3.135338e-15 -4.549312e-15 -1.126012e-14 1.169734e-14 7.088742e-15
-4.774954e-15
E:\\R-3.1.2\\scripts\\mPDBS\\summary_pdb_93\\397368adbc32d5de.final.pdb
-2.703041e-15 3.822725e-15 1.293108e-15 -6.181484e-16 -1.779662e-15
-6.369762e-15
E:\\R-3.1.2\\scripts\\mPDBS\\summary_pdb_93\\411ca13cd3df6edd.final.pdb
-4.848267e-15 5.984868e-15 -4.689781e-15 1.271238e-15 2.123506e-15
-1.485828e-15
E:\\R-3.1.2\\scripts\\mPDBS\\summary_pdb_93\\42357eee6642bf12.final.pdb
9.212321e-15 9.462441e-15 -3.486759e-15 5.654522e-15 1.188819e-14
4.505958e-15
E:\\R-3.1.2\\scripts\\mPDBS\\summary_pdb_93\\44342d1f75a7fcfc.final.pdb
-1.336982e-15 5.962480e-15 -2.838668e-15 -1.045667e-14 -1.475828e-15
-1.256416e-14
E:\\R-3.1.2\\scripts\\mPDBS\\summary_pdb_93\\448ef21dffe8cdfb.final.pdb
-4.336253e-15 -1.412968e-14 -9.247333e-15 1.086490e-15 2.762806e-15
-4.044112e-15
E:\\R-3.1.2\\scripts\\mPDBS\\summary_pdb_93\\472a40b3e8f113c7.final.pdb
-6.288985e-15 4.599447e-15 3.172248e-15 4.881792e-15 4.284010e-15
2.486418e-16
E:\\R-3.1.2\\scripts\\mPDBS\\summary_pdb_93\\48a26225951ea153.final.pdb
-7.028269e-15 1.060488e-15 -1.976238e-15 -1.126853e-15 1.010071e-14
5.376671e-15
E:\\R-3.1.2\\scripts\\mPDBS\\summary_pdb_93\\4ab4a5c7c6682ce6.final.pdb
-5.652071e-15 -4.303587e-15 -1.381835e-15 9.169161e-15 7.129878e-15
-8.633576e-15
E:\\R-3.1.2\\scripts\\mPDBS\\summary_pdb_93\\4eaa45b62cb6a25f.final.pdb
-4.413097e-15 2.322795e-15 4.578774e-15 -1.919804e-15 -3.533431e-15
-2.153786e-15
E:\\R-3.1.2\\scripts\\mPDBS\\summary_pdb_93\\58c371611d85ac96.final.pdb
-2.703041e-15 3.822725e-15 1.293108e-15 -6.181484e-16 -1.779662e-15
-6.369762e-15

E:\\R-3.1.2\\scripts\\mPDBS\\summary_pdb_93\\59a647f04822d30a.final.pdb
3.821293e-16 -3.335204e-15 2.010751e-15 5.919122e-15 1.773639e-16
2.922146e-15
E:\\R-3.1.2\\scripts\\mPDBS\\summary_pdb_93\\59b091fe385e05fc.final.pdb
-9.188711e-16 9.195418e-16 -4.997710e-16 1.913250e-15 1.948321e-15
-6.389162e-16
E:\\R-3.1.2\\scripts\\mPDBS\\summary_pdb_93\\5c35ac30f930d2db.final.pdb
-4.413097e-15 2.322795e-15 4.578774e-15 -1.919804e-15 -3.533431e-15
-2.153786e-15
E:\\R-3.1.2\\scripts\\mPDBS\\summary_pdb_93\\5f60752431cf2dcb.final.pdb
-5.865612e-15 1.546666e-15 5.942286e-15 1.255085e-15 -3.781618e-16
-6.534772e-16
E:\\R-3.1.2\\scripts\\mPDBS\\summary_pdb_93\\626456dd6ff2868d.final.pdb
-6.288985e-15 4.599447e-15 3.172248e-15 4.881792e-15 4.284010e-15
2.486418e-16
E:\\R-3.1.2\\scripts\\mPDBS\\summary_pdb_93\\6583d53ff3c8f7ef.final.pdb
3.821293e-16 -3.335204e-15 2.010751e-15 5.919122e-15 1.773639e-16
2.922146e-15
E:\\R-3.1.2\\scripts\\mPDBS\\summary_pdb_93\\69760178986bef01.final.pdb
-1.000731e-14 -1.630105e-15 7.762298e-16 3.487517e-15 -8.040474e-15
-7.419869e-15
E:\\R-3.1.2\\scripts\\mPDBS\\summary_pdb_93\\6a9d87d47926b1c8.final.pdb
-1.481795e-15 1.832997e-15 1.057186e-15 1.425356e-15 2.036730e-15
-1.313043e-15
E:\\R-3.1.2\\scripts\\mPDBS\\summary_pdb_93\\6bb3547ea982ffc4.final.pdb
-1.480875e-15 1.178703e-14 5.149873e-15 -1.184198e-15 5.145271e-15
-1.023227e-15
E:\\R-3.1.2\\scripts\\mPDBS\\summary_pdb_93\\716d9624611d1c42.final.pdb
-6.790871e-16 3.434045e-15 1.091348e-15 -2.581256e-15 -2.571774e-15
-5.156819e-15
E:\\R-3.1.2\\scripts\\mPDBS\\summary_pdb_93\\75f27e70ad0f1be4.final.pdb
3.821293e-16 -3.335204e-15 2.010751e-15 5.919122e-15 1.773639e-16
2.922146e-15
E:\\R-3.1.2\\scripts\\mPDBS\\summary_pdb_93\\769f910fffb22872.final.pdb
-1.146964e-15 6.714619e-16 9.564132e-16 1.148908e-15 2.418496e-15
-9.116305e-16
E:\\R-3.1.2\\scripts\\mPDBS\\summary_pdb_93\\791f8c80dd5c3135.final.pdb
-9.188711e-16 9.195418e-16 -4.997710e-16 1.913250e-15 1.948321e-15
-6.389162e-16
E:\\R-3.1.2\\scripts\\mPDBS\\summary_pdb_93\\7fad98e66a3f6099.final.pdb
-1.044218e-14 -7.117574e-16 -1.666400e-15 -5.755671e-15 1.279055e-14
3.670399e-15
E:\\R-3.1.2\\scripts\\mPDBS\\summary_pdb_93\\804b89c94a633196.final.pdb
-3.295418e-15 1.669743e-15 -1.996189e-15 1.266901e-15 1.687277e-15
9.657697e-16
E:\\R-3.1.2\\scripts\\mPDBS\\summary_pdb_93\\813cc65a2298feba.final.pdb
-3.295418e-15 1.669743e-15 -1.996189e-15 1.266901e-15 1.687277e-15
9.657697e-16
E:\\R-3.1.2\\scripts\\mPDBS\\summary_pdb_93\\83c0eefcfe12d3f9.final.pdb
-4.848267e-15 5.984868e-15 -4.689781e-15 1.271238e-15 2.123506e-15
-1.485828e-15
E:\\R-3.1.2\\scripts\\mPDBS\\summary_pdb_93\\86d50469ef569760.final.pdb
-6.288985e-15 4.599447e-15 3.172248e-15 4.881792e-15 4.284010e-15
2.486418e-16

E:\\R-3.1.2\\scripts\\mPDBS\\summary_pdb_93\\89d0666e1402203d.final.pdb
3.821293e-16 -3.335204e-15 2.010751e-15 5.919122e-15 1.773639e-16
2.922146e-15
E:\\R-3.1.2\\scripts\\mPDBS\\summary_pdb_93\\8b34852716dadc62.final.pdb
-1.005583e-15 4.666478e-15 2.597359e-15 4.184706e-15 5.475914e-15
-1.598152e-15
E:\\R-3.1.2\\scripts\\mPDBS\\summary_pdb_93\\8b6ef9ac3fe74395.final.pdb
2.065361e-15 9.596638e-15 5.799769e-15 1.371055e-15 1.741216e-15
-1.119569e-15
E:\\R-3.1.2\\scripts\\mPDBS\\summary_pdb_93\\98bbe0d5986c138f.final.pdb
-9.188711e-16 9.195418e-16 -4.997710e-16 1.913250e-15 1.948321e-15
-6.389162e-16
E:\\R-3.1.2\\scripts\\mPDBS\\summary_pdb_93\\9c22f4909d67e2fa.final.pdb
1.246819e-15 -4.240026e-15 5.856254e-15 -3.086821e-15 -1.546735e-15
5.417070e-15
E:\\R-3.1.2\\scripts\\mPDBS\\summary_pdb_93\\9dda8ed0d81fb3ef.final.pdb
-1.304607e-14 7.116938e-15 -1.563521e-16 -1.026845e-14 7.406878e-15
-4.303411e-15
E:\\R-3.1.2\\scripts\\mPDBS\\summary_pdb_93\\9f752b3616e6033b.final.pdb
1.144889e-15 1.376775e-16 -1.169510e-15 1.439219e-15 1.453820e-15
1.508258e-15
E:\\R-3.1.2\\scripts\\mPDBS\\summary_pdb_93\\a02c70083816d6ba.final.pdb
-4.413097e-15 2.322795e-15 4.578774e-15 -1.919804e-15 -3.533431e-15
-2.153786e-15
E:\\R-3.1.2\\scripts\\mPDBS\\summary_pdb_93\\a0c997d891e4e082.final.pdb
-5.938161e-15 -1.361902e-14 -1.071197e-14 1.473251e-15 1.618492e-16
-2.739383e-15
E:\\R-3.1.2\\scripts\\mPDBS\\summary_pdb_93\\ab8039a2d823c616.final.pdb
2.627432e-16 1.799636e-15 3.928624e-16 1.993234e-15 -3.563724e-16
2.989856e-16
E:\\R-3.1.2\\scripts\\mPDBS\\summary_pdb_93\\acf4d74b9c71d4dc.final.pdb
-3.295418e-15 1.669743e-15 -1.996189e-15 1.266901e-15 1.687277e-15
9.657697e-16
E:\\R-3.1.2\\scripts\\mPDBS\\summary_pdb_93\\ad8a823e4b4c668e.final.pdb
-1.645174e-15 1.329310e-15 1.275395e-15 1.441761e-15 1.942452e-15
-8.695961e-17
E:\\R-3.1.2\\scripts\\mPDBS\\summary_pdb_93\\b16ababd03b7b857.final.pdb
1.246819e-15 -4.240026e-15 5.856254e-15 -3.086821e-15 -1.546735e-15
5.417070e-15
E:\\R-3.1.2\\scripts\\mPDBS\\summary_pdb_93\\b226923e697d3c63.final.pdb
1.333592e-16 3.370803e-15 -5.941438e-15 7.535450e-15 8.734488e-16
-8.419042e-15
E:\\R-3.1.2\\scripts\\mPDBS\\summary_pdb_93\\b28e48f6fa27a165.final.pdb
-9.188711e-16 9.195418e-16 -4.997710e-16 1.913250e-15 1.948321e-15
-6.389162e-16
E:\\R-3.1.2\\scripts\\mPDBS\\summary_pdb_93\\b42c4924e3cd1aec.final.pdb
1.114856e-14 -1.986457e-15 3.056789e-15 2.051122e-15 1.838768e-15
5.394994e-15
E:\\R-3.1.2\\scripts\\mPDBS\\summary_pdb_93\\b4dd239fcabff1df.final.pdb
-9.188711e-16 9.195418e-16 -4.997710e-16 1.913250e-15 1.948321e-15
-6.389162e-16
E:\\R-3.1.2\\scripts\\mPDBS\\summary_pdb_93\\b669942ef97459a2.final.pdb
-1.005583e-15 4.666478e-15 2.597359e-15 4.184706e-15 5.475914e-15
-1.598152e-15

E:\\R-3.1.2\\scripts\\mPDBS\\summary_pdb_93\\b99f4d9d87c1017b.final.pdb
-9.188711e-16 9.195418e-16 -4.997710e-16 1.913250e-15 1.948321e-15
-6.389162e-16
E:\\R-3.1.2\\scripts\\mPDBS\\summary_pdb_93\\b9d2cbf3975403e7.final.pdb
-9.188711e-16 9.195418e-16 -4.997710e-16 1.913250e-15 1.948321e-15
-6.389162e-16
E:\\R-3.1.2\\scripts\\mPDBS\\summary_pdb_93\\bc88c8a505f3a288.final.pdb
-1.176613e-15 1.180618e-15 9.805805e-17 5.380480e-16 1.380605e-15
-6.046554e-16
E:\\R-3.1.2\\scripts\\mPDBS\\summary_pdb_93\\bdac0e64f4ef427e.final.pdb
6.858352e-15 -7.019487e-15 1.450081e-14 5.018882e-16 -7.288670e-16
1.842767e-16
E:\\R-3.1.2\\scripts\\mPDBS\\summary_pdb_93\\ca2a1f55fc9d0681.final.pdb
-1.005583e-15 4.666478e-15 2.597359e-15 4.184706e-15 5.475914e-15
-1.598152e-15
E:\\R-3.1.2\\scripts\\mPDBS\\summary_pdb_93\\ce34248de3e81a3c.final.pdb
-4.967416e-16 1.374203e-15 1.471826e-15 -5.747597e-17 5.203632e-16
-2.410671e-15
E:\\R-3.1.2\\scripts\\mPDBS\\summary_pdb_93\\d02e697772494a61.final.pdb
-2.043704e-15 -2.222042e-15 -1.594382e-15 1.141910e-14 -5.201749e-15
-2.897526e-15
E:\\R-3.1.2\\scripts\\mPDBS\\summary_pdb_93\\d04221245d015047.final.pdb
-1.691558e-15 1.338677e-16 2.551726e-15 -1.454962e-15 2.191476e-15
2.541116e-15
E:\\R-3.1.2\\scripts\\mPDBS\\summary_pdb_93\\d678141e4018054a.final.pdb
6.693561e-15 1.111921e-14 4.652340e-15 -5.215583e-15 7.101057e-15
-3.821606e-15
E:\\R-3.1.2\\scripts\\mPDBS\\summary_pdb_93\\d71b40af0f0efe39.final.pdb
-4.334202e-15 -5.286666e-15 -4.675073e-15 -4.236769e-16 1.313105e-15
2.010753e-15
E:\\R-3.1.2\\scripts\\mPDBS\\summary_pdb_93\\dc0809eff10197e2.final.pdb
-1.073678e-15 -3.530154e-15 2.613481e-15 -1.117084e-15 8.269732e-16
-6.176798e-16
E:\\R-3.1.2\\scripts\\mPDBS\\summary_pdb_93\\e1163918d3707839.final.pdb
-4.731104e-15 4.298013e-15 4.874780e-15 -4.642272e-16 7.096715e-15
1.014987e-14
E:\\R-3.1.2\\scripts\\mPDBS\\summary_pdb_93\\e4b48b06824ef848.final.pdb
-1.918364e-16 7.734822e-15 9.744110e-16 4.463531e-15 2.332048e-16
-4.336842e-15
E:\\R-3.1.2\\scripts\\mPDBS\\summary_pdb_93\\e529e19ad281fea2.final.pdb
5.007534e-15 -1.427416e-15 5.909791e-16 -5.620224e-15 -8.448860e-15
3.674704e-16
E:\\R-3.1.2\\scripts\\mPDBS\\summary_pdb_93\\e5602082718ec3a1.final.pdb
-3.295418e-15 1.669743e-15 -1.996189e-15 1.266901e-15 1.687277e-15
9.657697e-16
E:\\R-3.1.2\\scripts\\mPDBS\\summary_pdb_93\\e5d589d996d0a960.final.pdb
-5.271475e-15 -1.951359e-15 -5.347474e-15 -4.094031e-15 -1.895263e-15
-2.841302e-15
E:\\R-3.1.2\\scripts\\mPDBS\\summary_pdb_93\\e9df9e72307a2fbd.final.pdb
-4.413097e-15 2.322795e-15 4.578774e-15 -1.919804e-15 -3.533431e-15
-2.153786e-15
E:\\R-3.1.2\\scripts\\mPDBS\\summary_pdb_93\\ea00f8694090cae7.final.pdb
-3.295418e-15 1.669743e-15 -1.996189e-15 1.266901e-15 1.687277e-15
9.657697e-16

E:\\R-3.1.2\\scripts\\mPDBS\\summary_pdb_93\\eba517be32db050e.final.pdb
-9.188711e-16 9.195418e-16 -4.997710e-16 1.913250e-15 1.948321e-15
-6.389162e-16
E:\\R-3.1.2\\scripts\\mPDBS\\summary_pdb_93\\ec04b9545899c29d.final.pdb
-7.575840e-16 1.100917e-15 3.939889e-16 1.212817e-15 4.011103e-15
4.497313e-16
E:\\R-3.1.2\\scripts\\mPDBS\\summary_pdb_93\\ee6862cbcad52078.final.pdb
-4.661083e-16 1.155031e-15 -2.772749e-18 1.155176e-15 1.499027e-15
-1.163236e-15
E:\\R-3.1.2\\scripts\\mPDBS\\summary_pdb_93\\ef6a42fc83b81e5d.final.pdb
-4.055056e-16 7.872547e-16 5.765088e-16 7.035181e-16 1.451794e-15
1.023154e-16
E:\\R-3.1.2\\scripts\\mPDBS\\summary_pdb_93\\ef8e88d4ad93a0ad.final.pdb
-1.691558e-15 1.338677e-16 2.551726e-15 -1.454962e-15 2.191476e-15
2.541116e-15
E:\\R-3.1.2\\scripts\\mPDBS\\summary_pdb_93\\f14fcae8deec4fa3.final.pdb
1.144889e-15 1.376775e-16 -1.169510e-15 1.439219e-15 1.453820e-15
1.508258e-15
E:\\R-3.1.2\\scripts\\mPDBS\\summary_pdb_93\\f83584e22003fb92.final.pdb
-7.028269e-15 1.060488e-15 -1.976238e-15 -1.126853e-15 1.010071e-14
5.376671e-15
E:\\R-3.1.2\\scripts\\mPDBS\\summary_pdb_93\\f8e2bf87e52caa29.final.pdb
5.007534e-15 -1.427416e-15 5.909791e-16 -5.620224e-15 -8.448860e-15
3.674704e-16

[,169]   [,170]   [,171]   [,172]   [,173]   [,174]
E:\\R-3.1.2\\scripts\\40.1ns_data\\NAT0.pdb
3.345670e-15 -3.797836e-15 -5.297242e-15 -6.494311e-16 -8.721304e-16
-1.253715e-15
E:\\R-3.1.2\\scripts\\40.1ns_data\\LJP6.pdb
4.237359e-15 -2.589776e-15 -3.709290e-15 -1.101963e-16 -6.443982e-16
-2.739941e-15
E:\\R-3.1.2\\scripts\\40.1ns_data\\3WY9.pdb
-2.418186e-15 -1.831066e-15 -4.070676e-15 8.294453e-16 -2.404010e-16
-2.894450e-16
E:\\R-3.1.2\\scripts\\40.1ns_data\\ZSP9.pdb
-5.362025e-16 -1.482970e-15 -5.916293e-15 -3.640705e-16 -1.577135e-15
-1.712355e-15
E:\\R-3.1.2\\scripts\\mPDBS\\summary_pdb_93\\013d855ad26c877c.final.pdb
-1.363300e-15 -2.904064e-16 -5.764877e-15 -5.236631e-15 -3.398148e-15
-2.781186e-15
E:\\R-3.1.2\\scripts\\mPDBS\\summary_pdb_93\\0a2a705342ca0d54.final.pdb
1.599278e-16 -2.152209e-15 -4.922176e-15 -6.373595e-16 -1.732777e-15
-1.096310e-16
E:\\R-3.1.2\\scripts\\mPDBS\\summary_pdb_93\\0a59fceadb4194d9.final.pdb
3.864137e-16 -2.630688e-15 -9.677398e-16 -7.140869e-15 -1.585073e-16
9.347810e-17
E:\\R-3.1.2\\scripts\\mPDBS\\summary_pdb_93\\0dba59f3306825e8.final.pdb
1.696752e-15 -5.310696e-15 -3.459430e-15 -1.352585e-15 2.551799e-16
-2.309341e-15
E:\\R-3.1.2\\scripts\\mPDBS\\summary_pdb_93\\126f26a78b57339c.final.pdb
1.948504e-15 3.355766e-15 -7.866386e-15 1.019460e-14 -6.707566e-15
1.771812e-15
E:\\R-3.1.2\\scripts\\mPDBS\\summary_pdb_93\\12f06f3e342df706.final.pdb

3.402560e-15 -1.413651e-15 -3.820735e-15 -6.683677e-16 -3.073068e-15
-8.416843e-16
E:\\R-3.1.2\\scripts\\mPDBS\\summary_pdb_93\\18618a2a251b867b.final.pdb
4.902625e-15 2.447257e-15 -9.382946e-15 6.755294e-15 -6.028483e-15
-1.700208e-15
E:\\R-3.1.2\\scripts\\mPDBS\\summary_pdb_93\\1f305f598641eeb2.final.pdb
8.091554e-16 -1.580009e-15 -3.877148e-15 -8.438859e-16 -1.030372e-15
-7.246392e-16
E:\\R-3.1.2\\scripts\\mPDBS\\summary_pdb_93\\1f8a00b551be0a39.final.pdb
4.902625e-15 2.447257e-15 -9.382946e-15 6.755294e-15 -6.028483e-15
-1.700208e-15
E:\\R-3.1.2\\scripts\\mPDBS\\summary_pdb_93\\219f45328d47579a.final.pdb
4.789142e-15 9.901137e-16 -1.308613e-15 9.955264e-15 -5.931667e-15
2.747567e-16
E:\\R-3.1.2\\scripts\\mPDBS\\summary_pdb_93\\238dd3918e54336d.final.pdb
8.398234e-15 4.972822e-15 -1.646001e-14 1.169107e-14 -1.325372e-14
-1.002896e-14
E:\\R-3.1.2\\scripts\\mPDBS\\summary_pdb_93\\2b3027889e2fdc42.final.pdb
6.043383e-16 -1.393722e-15 -3.889773e-15 -2.488128e-16 -2.589505e-15
-3.738130e-16
E:\\R-3.1.2\\scripts\\mPDBS\\summary_pdb_93\\2fcb3b46e33793b0.final.pdb
2.242037e-15 -5.648803e-15 -3.174585e-15 -9.276377e-15 4.064511e-15
5.174228e-16
E:\\R-3.1.2\\scripts\\mPDBS\\summary_pdb_93\\34eb328251842006.final.pdb
-1.221308e-14 -1.692313e-15 2.521337e-15 -1.189475e-14 -1.539461e-14
-2.806050e-15
E:\\R-3.1.2\\scripts\\mPDBS\\summary_pdb_93\\354b32871797b7ab.final.pdb
-3.768204e-15 -9.473407e-15 1.680157e-16 -8.428100e-15 -1.305454e-15
1.111827e-14
E:\\R-3.1.2\\scripts\\mPDBS\\summary_pdb_93\\397368adbc32d5de.final.pdb
1.948504e-15 3.355766e-15 -7.866386e-15 1.019460e-14 -6.707566e-15
1.771812e-15
E:\\R-3.1.2\\scripts\\mPDBS\\summary_pdb_93\\411ca13cd3df6edd.final.pdb
2.011102e-15 -3.955930e-15 -7.359714e-15 -8.993617e-15 -2.779356e-16
1.078389e-15
E:\\R-3.1.2\\scripts\\mPDBS\\summary_pdb_93\\42357eee6642bf12.final.pdb
5.420278e-15 1.944165e-15 -6.403929e-16 -6.432282e-15 -6.482498e-16
-3.309033e-15
E:\\R-3.1.2\\scripts\\mPDBS\\summary_pdb_93\\44342d1f75a7fcfc.final.pdb
-3.062659e-15 1.247148e-15 -8.688801e-15 -1.154716e-14 6.251001e-15
1.771960e-15
E:\\R-3.1.2\\scripts\\mPDBS\\summary_pdb_93\\448ef21dffe8cdfb.final.pdb
-8.586107e-15 5.527586e-15 -1.169208e-14 3.836398e-15 -1.696901e-14
2.928688e-15
E:\\R-3.1.2\\scripts\\mPDBS\\summary_pdb_93\\472a40b3e8f113c7.final.pdb
1.696752e-15 -5.310696e-15 -3.459430e-15 -1.352585e-15 2.551799e-16
-2.309341e-15
E:\\R-3.1.2\\scripts\\mPDBS\\summary_pdb_93\\48a26225951ea153.final.pdb
-2.263160e-15 -4.771371e-16 -8.337170e-15 1.279912e-15 -4.054479e-15
5.490420e-15
E:\\R-3.1.2\\scripts\\mPDBS\\summary_pdb_93\\4ab4a5c7c6682ce6.final.pdb
-4.123028e-15 -5.488722e-15 9.546827e-16 -5.947868e-15 4.866509e-15
1.121912e-14
E:\\R-3.1.2\\scripts\\mPDBS\\summary_pdb_93\\4eaa45b62cb6a25f.final.pdb

4.888066e-15 1.753724e-15 -6.103985e-15 4.922549e-15 -1.322151e-15
-1.818975e-15
E:\\R-3.1.2\\scripts\\mPDBS\\summary_pdb_93\\58c371611d85ac96.final.pdb
1.948504e-15 3.355766e-15 -7.866386e-15 1.019460e-14 -6.707566e-15
1.771812e-15
E:\\R-3.1.2\\scripts\\mPDBS\\summary_pdb_93\\59a647f04822d30a.final.pdb
1.763341e-15 -1.563538e-15 -2.162771e-15 7.302782e-15 -2.475526e-15
3.308925e-15
E:\\R-3.1.2\\scripts\\mPDBS\\summary_pdb_93\\59b091fe385e05fc.final.pdb
3.864137e-16 -2.630688e-15 -9.677398e-16 -7.140869e-15 -1.585073e-16
9.347810e-17
E:\\R-3.1.2\\scripts\\mPDBS\\summary_pdb_93\\5c35ac30f930d2db.final.pdb
4.888066e-15 1.753724e-15 -6.103985e-15 4.922549e-15 -1.322151e-15
-1.818975e-15
E:\\R-3.1.2\\scripts\\mPDBS\\summary_pdb_93\\5f60752431cf2dcb.final.pdb
1.800939e-16 2.028149e-15 -1.655149e-15 2.120417e-15 -3.004980e-15
-4.975313e-15
E:\\R-3.1.2\\scripts\\mPDBS\\summary_pdb_93\\626456dd6ff2868d.final.pdb
1.696752e-15 -5.310696e-15 -3.459430e-15 -1.352585e-15 2.551799e-16
-2.309341e-15
E:\\R-3.1.2\\scripts\\mPDBS\\summary_pdb_93\\6583d53ff3c8f7ef.final.pdb
1.763341e-15 -1.563538e-15 -2.162771e-15 7.302782e-15 -2.475526e-15
3.308925e-15
E:\\R-3.1.2\\scripts\\mPDBS\\summary_pdb_93\\69760178986bef01.final.pdb
7.543256e-16 -3.686334e-15 -1.242820e-14 2.792601e-15 1.530567e-15
-2.387613e-15
E:\\R-3.1.2\\scripts\\mPDBS\\summary_pdb_93\\6a9d87d47926b1c8.final.pdb
3.282722e-16 -1.893074e-15 -3.966728e-15 -6.266033e-16 -2.927604e-15
-7.504754e-16
E:\\R-3.1.2\\scripts\\mPDBS\\summary_pdb_93\\6bb3547ea982ffc4.final.pdb
-4.407647e-15 2.977639e-16 -9.447614e-15 1.472575e-14 -3.074548e-14
-2.902180e-15
E:\\R-3.1.2\\scripts\\mPDBS\\summary_pdb_93\\716d9624611d1c42.final.pdb
1.444491e-15 -2.569844e-15 -6.074032e-15 -2.706451e-15 -9.639647e-15
2.774347e-15
E:\\R-3.1.2\\scripts\\mPDBS\\summary_pdb_93\\75f27e70ad0f1be4.final.pdb
1.763341e-15 -1.563538e-15 -2.162771e-15 7.302782e-15 -2.475526e-15
3.308925e-15
E:\\R-3.1.2\\scripts\\mPDBS\\summary_pdb_93\\769f910fffb22872.final.pdb
1.713119e-15 -1.788829e-15 -4.018314e-15 -1.071128e-15 -1.816574e-15
1.779553e-16
E:\\R-3.1.2\\scripts\\mPDBS\\summary_pdb_93\\791f8c80dd5c3135.final.pdb
3.864137e-16 -2.630688e-15 -9.677398e-16 -7.140869e-15 -1.585073e-16
9.347810e-17
E:\\R-3.1.2\\scripts\\mPDBS\\summary_pdb_93\\7fad98e66a3f6099.final.pdb
-8.825274e-15 -8.240354e-15 -1.969447e-15 -1.334386e-14 -2.401947e-15
-7.093505e-15
E:\\R-3.1.2\\scripts\\mPDBS\\summary_pdb_93\\804b89c94a633196.final.pdb
2.242037e-15 -5.648803e-15 -3.174585e-15 -9.276377e-15 4.064511e-15
5.174228e-16
E:\\R-3.1.2\\scripts\\mPDBS\\summary_pdb_93\\813cc65a2298feba.final.pdb
2.242037e-15 -5.648803e-15 -3.174585e-15 -9.276377e-15 4.064511e-15
5.174228e-16
E:\\R-3.1.2\\scripts\\mPDBS\\summary_pdb_93\\83c0eefcfe12d3f9.final.pdb

2.011102e-15 -3.955930e-15 -7.359714e-15 -8.993617e-15 -2.779356e-16
1.078389e-15
E:\\R-3.1.2\\scripts\\mPDBS\\summary_pdb_93\\86d50469ef569760.final.pdb
1.696752e-15 -5.310696e-15 -3.459430e-15 -1.352585e-15 2.551799e-16
-2.309341e-15
E:\\R-3.1.2\\scripts\\mPDBS\\summary_pdb_93\\89d0666e1402203d.final.pdb
1.763341e-15 -1.563538e-15 -2.162771e-15 7.302782e-15 -2.475526e-15
3.308925e-15
E:\\R-3.1.2\\scripts\\mPDBS\\summary_pdb_93\\8b34852716dadc62.final.pdb
2.536940e-15 -1.170615e-15 -3.302955e-15 3.881501e-15 1.649776e-15
1.828874e-15
E:\\R-3.1.2\\scripts\\mPDBS\\summary_pdb_93\\8b6ef9ac3fe74395.final.pdb
-1.935768e-15 -5.645663e-15 2.821852e-15 1.039525e-14 -1.351035e-14
-4.680306e-15
E:\\R-3.1.2\\scripts\\mPDBS\\summary_pdb_93\\98bbe0d5986c138f.final.pdb
3.864137e-16 -2.630688e-15 -9.677398e-16 -7.140869e-15 -1.585073e-16
9.347810e-17
E:\\R-3.1.2\\scripts\\mPDBS\\summary_pdb_93\\9c22f4909d67e2fa.final.pdb
1.560941e-15 4.214401e-15 -5.183937e-15 3.799101e-15 1.615081e-15
-5.060255e-15
E:\\R-3.1.2\\scripts\\mPDBS\\summary_pdb_93\\9dda8ed0d81fb3ef.final.pdb
6.013848e-15 -1.158480e-15 -1.125489e-14 -5.930339e-15 4.785376e-15
1.762528e-15
E:\\R-3.1.2\\scripts\\mPDBS\\summary_pdb_93\\9f752b3616e6033b.final.pdb
1.318256e-15 -2.232934e-15 -5.179484e-15 -5.876140e-16 4.287448e-16
-7.288470e-16
E:\\R-3.1.2\\scripts\\mPDBS\\summary_pdb_93\\a02c70083816d6ba.final.pdb
4.888066e-15 1.753724e-15 -6.103985e-15 4.922549e-15 -1.322151e-15
-1.818975e-15
E:\\R-3.1.2\\scripts\\mPDBS\\summary_pdb_93\\a0c997d891e4e082.final.pdb
-1.148185e-15 2.744579e-15 -2.984312e-15 2.004336e-16 5.158338e-16
7.021779e-16
E:\\R-3.1.2\\scripts\\mPDBS\\summary_pdb_93\\ab8039a2d823c616.final.pdb
1.054046e-15 -1.466896e-15 -4.462622e-15 -1.176720e-15 -2.620329e-15
-9.504714e-16
E:\\R-3.1.2\\scripts\\mPDBS\\summary_pdb_93\\acf4d74b9c71d4dc.final.pdb
2.242037e-15 -5.648803e-15 -3.174585e-15 -9.276377e-15 4.064511e-15
5.174228e-16
E:\\R-3.1.2\\scripts\\mPDBS\\summary_pdb_93\\ad8a823e4b4c668e.final.pdb
6.471548e-16 -2.005246e-15 -4.303291e-15 -6.834825e-16 -1.783085e-15
-1.355233e-15
E:\\R-3.1.2\\scripts\\mPDBS\\summary_pdb_93\\b16ababd03b7b857.final.pdb
1.560941e-15 4.214401e-15 -5.183937e-15 3.799101e-15 1.615081e-15
-5.060255e-15
E:\\R-3.1.2\\scripts\\mPDBS\\summary_pdb_93\\b226923e697d3c63.final.pdb
3.160878e-15 1.917510e-15 -1.602455e-15 7.042357e-15 -7.390865e-15
-7.499594e-15
E:\\R-3.1.2\\scripts\\mPDBS\\summary_pdb_93\\b28e48f6fa27a165.final.pdb
3.864137e-16 -2.630688e-15 -9.677398e-16 -7.140869e-15 -1.585073e-16
9.347810e-17
E:\\R-3.1.2\\scripts\\mPDBS\\summary_pdb_93\\b42c4924e3cd1aec.final.pdb
-6.435875e-16 -9.357646e-17 1.562006e-15 -2.585792e-15 1.587847e-15
-5.366313e-16
E:\\R-3.1.2\\scripts\\mPDBS\\summary_pdb_93\\b4dd239fcabff1df.final.pdb

3.864137e-16 -2.630688e-15 -9.677398e-16 -7.140869e-15 -1.585073e-16
9.347810e-17
E:\\R-3.1.2\\scripts\\mPDBS\\summary_pdb_93\\b669942ef97459a2.final.pdb
2.536940e-15 -1.170615e-15 -3.302955e-15 3.881501e-15 1.649776e-15
1.828874e-15
E:\\R-3.1.2\\scripts\\mPDBS\\summary_pdb_93\\b99f4d9d87c1017b.final.pdb
3.864137e-16 -2.630688e-15 -9.677398e-16 -7.140869e-15 -1.585073e-16
9.347810e-17
E:\\R-3.1.2\\scripts\\mPDBS\\summary_pdb_93\\b9d2cbf3975403e7.final.pdb
3.864137e-16 -2.630688e-15 -9.677398e-16 -7.140869e-15 -1.585073e-16
9.347810e-17
E:\\R-3.1.2\\scripts\\mPDBS\\summary_pdb_93\\bc88c8a505f3a288.final.pdb
-4.698892e-16 -1.402178e-15 -4.387313e-15 -4.760616e-16 -3.326329e-15
6.442148e-17
E:\\R-3.1.2\\scripts\\mPDBS\\summary_pdb_93\\bdac0e64f4ef427e.final.pdb
-1.643310e-15 3.754265e-15 3.067275e-15 1.449671e-14 1.439441e-15
-2.559760e-15
E:\\R-3.1.2\\scripts\\mPDBS\\summary_pdb_93\\ca2a1f55fc9d0681.final.pdb
2.536940e-15 -1.170615e-15 -3.302955e-15 3.881501e-15 1.649776e-15
1.828874e-15
E:\\R-3.1.2\\scripts\\mPDBS\\summary_pdb_93\\ce34248de3e81a3c.final.pdb
1.824924e-15 1.533406e-15 2.848714e-15 -2.408824e-15 1.401416e-15
2.643633e-15
E:\\R-3.1.2\\scripts\\mPDBS\\summary_pdb_93\\d02e697772494a61.final.pdb
-2.563014e-16 -1.459533e-15 -2.203188e-15 6.422717e-15 -1.357204e-14
-2.566178e-15
E:\\R-3.1.2\\scripts\\mPDBS\\summary_pdb_93\\d04221245d015047.final.pdb
1.108690e-15 -2.771841e-15 -4.333927e-15 -3.288095e-15 -2.786696e-16
3.172424e-15
E:\\R-3.1.2\\scripts\\mPDBS\\summary_pdb_93\\d678141e4018054a.final.pdb
-4.085995e-15 1.260247e-15 -9.051918e-15 -1.584519e-15 -2.050982e-14
-6.674833e-15
E:\\R-3.1.2\\scripts\\mPDBS\\summary_pdb_93\\d71b40af0f0efe39.final.pdb
6.129119e-15 -6.880744e-15 -1.051523e-14 -1.076752e-14 8.315304e-15
-3.589222e-15
E:\\R-3.1.2\\scripts\\mPDBS\\summary_pdb_93\\dc0809eff10197e2.final.pdb
-5.102007e-15 -6.030129e-15 4.445243e-15 -2.406333e-15 7.032281e-15
1.468646e-15
E:\\R-3.1.2\\scripts\\mPDBS\\summary_pdb_93\\e1163918d3707839.final.pdb
1.621961e-15 -4.666741e-15 1.623588e-15 -8.914497e-15 -3.644079e-15
8.190551e-16
E:\\R-3.1.2\\scripts\\mPDBS\\summary_pdb_93\\e4b48b06824ef848.final.pdb
4.902625e-15 2.447257e-15 -9.382946e-15 6.755294e-15 -6.028483e-15
-1.700208e-15
E:\\R-3.1.2\\scripts\\mPDBS\\summary_pdb_93\\e529e19ad281fea2.final.pdb
-1.363300e-15 -2.904064e-16 -5.764877e-15 -5.236631e-15 -3.398148e-15
-2.781186e-15
E:\\R-3.1.2\\scripts\\mPDBS\\summary_pdb_93\\e5602082718ec3a1.final.pdb
2.242037e-15 -5.648803e-15 -3.174585e-15 -9.276377e-15 4.064511e-15
5.174228e-16
E:\\R-3.1.2\\scripts\\mPDBS\\summary_pdb_93\\e5d589d996d0a960.final.pdb
-1.536939e-15 -3.932023e-15 -7.382007e-15 3.369986e-15 -7.874081e-15
6.561185e-16
E:\\R-3.1.2\\scripts\\mPDBS\\summary_pdb_93\\e9df9e72307a2fbd.final.pdb

4.888066e-15 1.753724e-15 -6.103985e-15 4.922549e-15 -1.322151e-15
-1.818975e-15
E:\\R-3.1.2\\scripts\\mPDBS\\summary_pdb_93\\ea00f8694090cae7.final.pdb
2.242037e-15 -5.648803e-15 -3.174585e-15 -9.276377e-15 4.064511e-15
5.174228e-16
E:\\R-3.1.2\\scripts\\mPDBS\\summary_pdb_93\\eba517be32db050e.final.pdb
3.864137e-16 -2.630688e-15 -9.677398e-16 -7.140869e-15 -1.585073e-16
9.347810e-17
E:\\R-3.1.2\\scripts\\mPDBS\\summary_pdb_93\\ec04b9545899c29d.final.pdb
2.567340e-16 -1.755225e-15 -3.433456e-15 -7.499656e-17 -2.301186e-15
2.254544e-16
E:\\R-3.1.2\\scripts\\mPDBS\\summary_pdb_93\\ee6862cbcad52078.final.pdb
1.470218e-16 -1.144463e-15 -4.317491e-15 -6.794579e-16 -3.234172e-15
-4.035202e-16
E:\\R-3.1.2\\scripts\\mPDBS\\summary_pdb_93\\ef6a42fc83b81e5d.final.pdb
1.230866e-15 -1.376616e-15 -4.120337e-15 -1.088692e-15 -2.323330e-15
-5.719873e-16
E:\\R-3.1.2\\scripts\\mPDBS\\summary_pdb_93\\ef8e88d4ad93a0ad.final.pdb
1.108690e-15 -2.771841e-15 -4.333927e-15 -3.288095e-15 -2.786696e-16
3.172424e-15
E:\\R-3.1.2\\scripts\\mPDBS\\summary_pdb_93\\f14fcae8deec4fa3.final.pdb
1.318256e-15 -2.232934e-15 -5.179484e-15 -5.876140e-16 4.287448e-16
-7.288470e-16
E:\\R-3.1.2\\scripts\\mPDBS\\summary_pdb_93\\f83584e22003fb92.final.pdb
-2.263160e-15 -4.771371e-16 -8.337170e-15 1.279912e-15 -4.054479e-15
5.490420e-15
E:\\R-3.1.2\\scripts\\mPDBS\\summary_pdb_93\\f8e2bf87e52caa29.final.pdb
-1.363300e-15 -2.904064e-16 -5.764877e-15 -5.236631e-15 -3.398148e-15
-2.781186e-15

[,175]   [,176]   [,177]   [,178]   [,179]   [,180]
E:\\R-3.1.2\\scripts\\40.1ns_data\\NAT0.pdb
5.229238e-15 -1.177024e-15 1.574820e-15 3.233389e-15 1.597243e-15
3.987872e-15
E:\\R-3.1.2\\scripts\\40.1ns_data\\LJP6.pdb
5.747173e-15 -1.047029e-15 6.274861e-15 1.928472e-15 6.530793e-16
6.809031e-16
E:\\R-3.1.2\\scripts\\40.1ns_data\\3WY9.pdb
7.152002e-15 9.195877e-17 9.260012e-16 2.039279e-15 5.403761e-16
1.100012e-15
E:\\R-3.1.2\\scripts\\40.1ns_data\\ZSP9.pdb
5.876755e-15 2.150475e-15 -4.124108e-15 1.754921e-15 3.801974e-16
3.699172e-15
E:\\R-3.1.2\\scripts\\mPDBS\\summary_pdb_93\\013d855ad26c877c.final.pdb
8.673630e-15 -1.373866e-15 9.411105e-15 3.185277e-15 -2.717000e-15
2.541251e-15
E:\\R-3.1.2\\scripts\\mPDBS\\summary_pdb_93\\0a2a705342ca0d54.final.pdb
6.876050e-15 1.197021e-16 1.964286e-15 3.143031e-15 -1.200905e-15
3.628984e-15
E:\\R-3.1.2\\scripts\\mPDBS\\summary_pdb_93\\0a59fceadb4194d9.final.pdb
7.003696e-15 1.446641e-15 -5.975764e-16 9.801638e-16 1.722560e-16
2.097505e-15
E:\\R-3.1.2\\scripts\\mPDBS\\summary_pdb_93\\0dba59f3306825e8.final.pdb
4.600499e-15 3.481625e-15 3.270184e-15 1.929337e-15 8.409182e-16

5.408749e-15
E:\\R-3.1.2\\scripts\\mPDBS\\summary_pdb_93\\126f26a78b57339c.final.pdb
1.224239e-14 -2.098330e-15 -6.034534e-16 7.817856e-15 2.984169e-15
-1.636390e-18
E:\\R-3.1.2\\scripts\\mPDBS\\summary_pdb_93\\12f06f3e342df706.final.pdb
6.325275e-15 7.021355e-16 2.371946e-15 3.641330e-15 1.200868e-16
3.700542e-15
E:\\R-3.1.2\\scripts\\mPDBS\\summary_pdb_93\\18618a2a251b867b.final.pdb
3.630450e-15 -2.582270e-15 7.372796e-16 3.783838e-15 -2.413914e-15
3.391810e-15
E:\\R-3.1.2\\scripts\\mPDBS\\summary_pdb_93\\1f305f598641eeb2.final.pdb
8.433422e-15 -3.872874e-16 1.936891e-15 2.765586e-15 -4.333498e-16
8.341815e-16
E:\\R-3.1.2\\scripts\\mPDBS\\summary_pdb_93\\1f8a00b551be0a39.final.pdb
3.630450e-15 -2.582270e-15 7.372796e-16 3.783838e-15 -2.413914e-15
3.391810e-15
E:\\R-3.1.2\\scripts\\mPDBS\\summary_pdb_93\\219f45328d47579a.final.pdb
3.808335e-15 -1.656846e-14 4.513786e-15 6.161783e-15 -1.275143e-14
1.699791e-14
E:\\R-3.1.2\\scripts\\mPDBS\\summary_pdb_93\\238dd3918e54336d.final.pdb
3.550729e-15 -1.010161e-15 1.364537e-14 7.227337e-15 2.642502e-15
1.854377e-14
E:\\R-3.1.2\\scripts\\mPDBS\\summary_pdb_93\\2b3027889e2fdc42.final.pdb
6.823719e-15 1.349728e-16 3.407900e-15 3.779140e-15 5.812171e-16
3.449939e-15
E:\\R-3.1.2\\scripts\\mPDBS\\summary_pdb_93\\2fcb3b46e33793b0.final.pdb
5.803581e-15 2.214798e-15 3.213647e-15 -6.548303e-17 -4.308722e-15
6.867300e-16
E:\\R-3.1.2\\scripts\\mPDBS\\summary_pdb_93\\34eb328251842006.final.pdb
1.443633e-14 -6.122041e-15 2.703264e-15 -8.316974e-17 7.983719e-15
5.080644e-15
E:\\R-3.1.2\\scripts\\mPDBS\\summary_pdb_93\\354b32871797b7ab.final.pdb
4.850984e-15 -5.475079e-15 -1.208635e-15 -3.057750e-16 -1.042828e-14
-7.187349e-15
E:\\R-3.1.2\\scripts\\mPDBS\\summary_pdb_93\\397368adbc32d5de.final.pdb
1.224239e-14 -2.098330e-15 -6.034534e-16 7.817856e-15 2.984169e-15
-1.636390e-18
E:\\R-3.1.2\\scripts\\mPDBS\\summary_pdb_93\\411ca13cd3df6edd.final.pdb
1.054068e-14 2.535071e-15 1.487598e-15 5.765790e-15 -3.983461e-15
1.609386e-15
E:\\R-3.1.2\\scripts\\mPDBS\\summary_pdb_93\\42357eee6642bf12.final.pdb
8.339545e-16 -4.631146e-15 -8.976008e-15 -2.363711e-16 -7.294514e-15
7.221549e-15
E:\\R-3.1.2\\scripts\\mPDBS\\summary_pdb_93\\44342d1f75a7fcfc.final.pdb
1.216066e-14 6.389138e-15 -4.350483e-15 6.664376e-15 5.241485e-15
-1.570999e-14
E:\\R-3.1.2\\scripts\\mPDBS\\summary_pdb_93\\448ef21dffe8cdfb.final.pdb
1.344677e-14 -8.686204e-15 3.952640e-15 -1.016111e-15 3.140181e-15
-2.886800e-15
E:\\R-3.1.2\\scripts\\mPDBS\\summary_pdb_93\\472a40b3e8f113c7.final.pdb
4.600499e-15 3.481625e-15 3.270184e-15 1.929337e-15 8.409182e-16
5.408749e-15
E:\\R-3.1.2\\scripts\\mPDBS\\summary_pdb_93\\48a26225951ea153.final.pdb
9.654787e-15 7.854492e-15 9.952144e-15 3.347194e-15 5.836562e-15

-3.999831e-15
E:\\R-3.1.2\\scripts\\mPDBS\\summary_pdb_93\\4ab4a5c7c6682ce6.final.pdb
1.023705e-14 6.726209e-15 -1.221916e-14 5.729249e-15 -1.046688e-14
-1.560547e-14
E:\\R-3.1.2\\scripts\\mPDBS\\summary_pdb_93\\4eaa45b62cb6a25f.final.pdb
9.332642e-15 5.010891e-15 4.113496e-15 6.879016e-15 1.771164e-15
2.493926e-15
E:\\R-3.1.2\\scripts\\mPDBS\\summary_pdb_93\\58c371611d85ac96.final.pdb
1.224239e-14 -2.098330e-15 -6.034534e-16 7.817856e-15 2.984169e-15
-1.636390e-18
E:\\R-3.1.2\\scripts\\mPDBS\\summary_pdb_93\\59a647f04822d30a.final.pdb
8.384171e-15 -3.032145e-15 3.630979e-15 6.764990e-15 -1.137960e-15
2.246136e-15
E:\\R-3.1.2\\scripts\\mPDBS\\summary_pdb_93\\59b091fe385e05fc.final.pdb
7.003696e-15 1.446641e-15 -5.975764e-16 9.801638e-16 1.722560e-16
2.097505e-15
E:\\R-3.1.2\\scripts\\mPDBS\\summary_pdb_93\\5c35ac30f930d2db.final.pdb
9.332642e-15 5.010891e-15 4.113496e-15 6.879016e-15 1.771164e-15
2.493926e-15
E:\\R-3.1.2\\scripts\\mPDBS\\summary_pdb_93\\5f60752431cf2dcb.final.pdb
9.445609e-15 4.091135e-15 3.368545e-15 6.755592e-15 1.313355e-14
-3.740772e-15
E:\\R-3.1.2\\scripts\\mPDBS\\summary_pdb_93\\626456dd6ff2868d.final.pdb
4.600499e-15 3.481625e-15 3.270184e-15 1.929337e-15 8.409182e-16
5.408749e-15
E:\\R-3.1.2\\scripts\\mPDBS\\summary_pdb_93\\6583d53ff3c8f7ef.final.pdb
8.384171e-15 -3.032145e-15 3.630979e-15 6.764990e-15 -1.137960e-15
2.246136e-15
E:\\R-3.1.2\\scripts\\mPDBS\\summary_pdb_93\\69760178986bef01.final.pdb
3.223913e-15 -7.265854e-16 -1.449259e-15 2.232658e-15 -6.202473e-15
-1.919583e-15
E:\\R-3.1.2\\scripts\\mPDBS\\summary_pdb_93\\6a9d87d47926b1c8.final.pdb
7.471900e-15 1.637496e-16 2.963138e-15 3.071652e-15 5.312700e-16
1.762577e-15
E:\\R-3.1.2\\scripts\\mPDBS\\summary_pdb_93\\6bb3547ea982ffc4.final.pdb
1.246483e-14 -1.071685e-15 2.025274e-14 3.354534e-15 1.733433e-14
1.217176e-14
E:\\R-3.1.2\\scripts\\mPDBS\\summary_pdb_93\\716d9624611d1c42.final.pdb
1.179226e-14 -5.759276e-15 4.900306e-15 8.404447e-15 -1.032035e-14
5.549323e-15
E:\\R-3.1.2\\scripts\\mPDBS\\summary_pdb_93\\75f27e70ad0f1be4.final.pdb
8.384171e-15 -3.032145e-15 3.630979e-15 6.764990e-15 -1.137960e-15
2.246136e-15
E:\\R-3.1.2\\scripts\\mPDBS\\summary_pdb_93\\769f910fffb22872.final.pdb
7.683197e-15 1.046000e-16 3.126861e-15 4.007184e-15 6.110098e-16
1.803670e-15
E:\\R-3.1.2\\scripts\\mPDBS\\summary_pdb_93\\791f8c80dd5c3135.final.pdb
7.003696e-15 1.446641e-15 -5.975764e-16 9.801638e-16 1.722560e-16
2.097505e-15
E:\\R-3.1.2\\scripts\\mPDBS\\summary_pdb_93\\7fad98e66a3f6099.final.pdb
1.204104e-14 7.073964e-15 8.356093e-15 -1.007017e-15 -1.556001e-15
1.185318e-15
E:\\R-3.1.2\\scripts\\mPDBS\\summary_pdb_93\\804b89c94a633196.final.pdb
5.803581e-15 2.214798e-15 3.213647e-15 -6.548303e-17 -4.308722e-15

6.867300e-16
E:\\R-3.1.2\\scripts\\mPDBS\\summary_pdb_93\\813cc65a2298feba.final.pdb
5.803581e-15 2.214798e-15 3.213647e-15 -6.548303e-17 -4.308722e-15
6.867300e-16
E:\\R-3.1.2\\scripts\\mPDBS\\summary_pdb_93\\83c0eefcfe12d3f9.final.pdb
1.054068e-14 2.535071e-15 1.487598e-15 5.765790e-15 -3.983461e-15
1.609386e-15
E:\\R-3.1.2\\scripts\\mPDBS\\summary_pdb_93\\86d50469ef569760.final.pdb
4.600499e-15 3.481625e-15 3.270184e-15 1.929337e-15 8.409182e-16
5.408749e-15
E:\\R-3.1.2\\scripts\\mPDBS\\summary_pdb_93\\89d0666e1402203d.final.pdb
8.384171e-15 -3.032145e-15 3.630979e-15 6.764990e-15 -1.137960e-15
2.246136e-15
E:\\R-3.1.2\\scripts\\mPDBS\\summary_pdb_93\\8b34852716dadc62.final.pdb
9.075270e-15 -3.699401e-16 -8.436755e-15 7.164844e-15 4.543386e-17
-1.528512e-15
E:\\R-3.1.2\\scripts\\mPDBS\\summary_pdb_93\\8b6ef9ac3fe74395.final.pdb
5.640968e-15 -5.325267e-15 1.744926e-14 -5.534209e-15 1.039922e-14
1.095635e-14
E:\\R-3.1.2\\scripts\\mPDBS\\summary_pdb_93\\98bbe0d5986c138f.final.pdb
7.003696e-15 1.446641e-15 -5.975764e-16 9.801638e-16 1.722560e-16
2.097505e-15
E:\\R-3.1.2\\scripts\\mPDBS\\summary_pdb_93\\9c22f4909d67e2fa.final.pdb
6.856992e-15 -3.748748e-15 1.505812e-15 3.678918e-15 3.798508e-15
4.432649e-15
E:\\R-3.1.2\\scripts\\mPDBS\\summary_pdb_93\\9dda8ed0d81fb3ef.final.pdb
1.706343e-14 2.042283e-14 8.162077e-15 -1.203464e-14 1.549023e-14
-4.518418e-15
E:\\R-3.1.2\\scripts\\mPDBS\\summary_pdb_93\\9f752b3616e6033b.final.pdb
4.330330e-15 -5.318827e-16 2.236222e-15 1.058291e-15 -6.894108e-16
6.389713e-15
E:\\R-3.1.2\\scripts\\mPDBS\\summary_pdb_93\\a02c70083816d6ba.final.pdb
9.332642e-15 5.010891e-15 4.113496e-15 6.879016e-15 1.771164e-15
2.493926e-15
E:\\R-3.1.2\\scripts\\mPDBS\\summary_pdb_93\\a0c997d891e4e082.final.pdb
1.179018e-14 8.277475e-15 5.228222e-15 6.744448e-15 -8.776450e-16
4.067918e-16
E:\\R-3.1.2\\scripts\\mPDBS\\summary_pdb_93\\ab8039a2d823c616.final.pdb
6.257283e-15 5.315007e-16 2.182915e-15 3.766362e-15 -2.149807e-16
3.781913e-15
E:\\R-3.1.2\\scripts\\mPDBS\\summary_pdb_93\\acf4d74b9c71d4dc.final.pdb
5.803581e-15 2.214798e-15 3.213647e-15 -6.548303e-17 -4.308722e-15
6.867300e-16
E:\\R-3.1.2\\scripts\\mPDBS\\summary_pdb_93\\ad8a823e4b4c668e.final.pdb
6.729730e-15 2.432540e-16 2.461236e-15 3.683748e-15 -4.312563e-16
2.663727e-15
E:\\R-3.1.2\\scripts\\mPDBS\\summary_pdb_93\\b16ababd03b7b857.final.pdb
6.856992e-15 -3.748748e-15 1.505812e-15 3.678918e-15 3.798508e-15
4.432649e-15
E:\\R-3.1.2\\scripts\\mPDBS\\summary_pdb_93\\b226923e697d3c63.final.pdb
4.602040e-15 -6.896025e-15 7.938391e-16 3.826802e-15 -7.898611e-15
5.814462e-15
E:\\R-3.1.2\\scripts\\mPDBS\\summary_pdb_93\\b28e48f6fa27a165.final.pdb
7.003696e-15 1.446641e-15 -5.975764e-16 9.801638e-16 1.722560e-16

2.097505e-15
E:\\R-3.1.2\\scripts\\mPDBS\\summary_pdb_93\\b42c4924e3cd1aec.final.pdb
1.251855e-15 -3.238079e-16 5.106362e-15 8.891327e-15 -5.738447e-15
6.952224e-15
E:\\R-3.1.2\\scripts\\mPDBS\\summary_pdb_93\\b4dd239fcabff1df.final.pdb
7.003696e-15 1.446641e-15 -5.975764e-16 9.801638e-16 1.722560e-16
2.097505e-15
E:\\R-3.1.2\\scripts\\mPDBS\\summary_pdb_93\\b669942ef97459a2.final.pdb
9.075270e-15 -3.699401e-16 -8.436755e-15 7.164844e-15 4.543386e-17
-1.528512e-15
E:\\R-3.1.2\\scripts\\mPDBS\\summary_pdb_93\\b99f4d9d87c1017b.final.pdb
7.003696e-15 1.446641e-15 -5.975764e-16 9.801638e-16 1.722560e-16
2.097505e-15
E:\\R-3.1.2\\scripts\\mPDBS\\summary_pdb_93\\b9d2cbf3975403e7.final.pdb
7.003696e-15 1.446641e-15 -5.975764e-16 9.801638e-16 1.722560e-16
2.097505e-15
E:\\R-3.1.2\\scripts\\mPDBS\\summary_pdb_93\\bc88c8a505f3a288.final.pdb
7.242221e-15 5.565106e-16 2.970750e-15 3.640796e-15 -4.743622e-16
3.962333e-15
E:\\R-3.1.2\\scripts\\mPDBS\\summary_pdb_93\\bdac0e64f4ef427e.final.pdb
5.623604e-15 -6.863010e-15 4.789644e-15 -8.148836e-16 6.663404e-15
-6.067232e-16
E:\\R-3.1.2\\scripts\\mPDBS\\summary_pdb_93\\ca2a1f55fc9d0681.final.pdb
9.075270e-15 -3.699401e-16 -8.436755e-15 7.164844e-15 4.543386e-17
-1.528512e-15
E:\\R-3.1.2\\scripts\\mPDBS\\summary_pdb_93\\ce34248de3e81a3c.final.pdb
1.209645e-14 5.535249e-15 6.076902e-16 7.627253e-15 8.315941e-17
-3.332180e-15
E:\\R-3.1.2\\scripts\\mPDBS\\summary_pdb_93\\d02e697772494a61.final.pdb
3.980940e-15 -1.962721e-14 -6.853857e-15 1.512033e-14 -1.068538e-14
7.039949e-15
E:\\R-3.1.2\\scripts\\mPDBS\\summary_pdb_93\\d04221245d015047.final.pdb
8.809928e-15 2.636154e-15 2.334703e-15 3.915114e-15 2.113018e-15
1.506606e-15
E:\\R-3.1.2\\scripts\\mPDBS\\summary_pdb_93\\d678141e4018054a.final.pdb
1.735612e-14 -9.911165e-15 4.781964e-15 1.040079e-14 4.170414e-15
7.064444e-15
E:\\R-3.1.2\\scripts\\mPDBS\\summary_pdb_93\\d71b40af0f0efe39.final.pdb
3.897391e-15 -4.081948e-15 -3.966047e-15 8.913465e-15 -1.178824e-14
3.732315e-15
E:\\R-3.1.2\\scripts\\mPDBS\\summary_pdb_93\\dc0809eff10197e2.final.pdb
2.047198e-15 8.687651e-15 7.914943e-15 -6.591878e-16 2.013197e-15
1.717783e-15
E:\\R-3.1.2\\scripts\\mPDBS\\summary_pdb_93\\e1163918d3707839.final.pdb
4.339663e-15 -2.753288e-15 3.753277e-15 -2.939487e-15 -6.392438e-15
1.600401e-14
E:\\R-3.1.2\\scripts\\mPDBS\\summary_pdb_93\\e4b48b06824ef848.final.pdb
3.630450e-15 -2.582270e-15 7.372796e-16 3.783838e-15 -2.413914e-15
3.391810e-15
E:\\R-3.1.2\\scripts\\mPDBS\\summary_pdb_93\\e529e19ad281fea2.final.pdb
8.673630e-15 -1.373866e-15 9.411105e-15 3.185277e-15 -2.717000e-15
2.541251e-15
E:\\R-3.1.2\\scripts\\mPDBS\\summary_pdb_93\\e5602082718ec3a1.final.pdb
5.803581e-15 2.214798e-15 3.213647e-15 -6.548303e-17 -4.308722e-15

```
6.867300e-16
E:\\R-3.1.2\\scripts\\mPDBS\\summary_pdb_93\\e5d589d996d0a960.final.pdb
6.927313e-15  7.227140e-15  7.622009e-15  7.814990e-15  5.860779e-15
1.365209e-15
E:\\R-3.1.2\\scripts\\mPDBS\\summary_pdb_93\\e9df9e72307a2fbd.final.pdb
9.332642e-15  5.010891e-15  4.113496e-15  6.879016e-15  1.771164e-15
2.493926e-15
E:\\R-3.1.2\\scripts\\mPDBS\\summary_pdb_93\\ea00f8694090cae7.final.pdb
5.803581e-15  2.214798e-15  3.213647e-15 -6.548303e-17 -4.308722e-15
6.867300e-16
E:\\R-3.1.2\\scripts\\mPDBS\\summary_pdb_93\\eba517be32db050e.final.pdb
7.003696e-15  1.446641e-15 -5.975764e-16  9.801638e-16  1.722560e-16
2.097505e-15
E:\\R-3.1.2\\scripts\\mPDBS\\summary_pdb_93\\ec04b9545899c29d.final.pdb
7.119557e-15  5.656716e-16  2.132178e-15  3.668509e-15 -1.942707e-16
2.336342e-15
E:\\R-3.1.2\\scripts\\mPDBS\\summary_pdb_93\\ee6862cbcad52078.final.pdb
7.449520e-15  5.636664e-16  2.530130e-15  3.778273e-15  1.005935e-17
3.198187e-15
E:\\R-3.1.2\\scripts\\mPDBS\\summary_pdb_93\\ef6a42fc83b81e5d.final.pdb
7.793352e-15  3.470277e-16  2.370522e-15  3.644193e-15 -1.383908e-16
2.646529e-15
E:\\R-3.1.2\\scripts\\mPDBS\\summary_pdb_93\\ef8e88d4ad93a0ad.final.pdb
8.809928e-15  2.636154e-15  2.334703e-15  3.915114e-15  2.113018e-15
1.506606e-15
E:\\R-3.1.2\\scripts\\mPDBS\\summary_pdb_93\\f14fcae8deec4fa3.final.pdb
4.330330e-15 -5.318827e-16  2.236222e-15  1.058291e-15 -6.894108e-16
6.389713e-15
E:\\R-3.1.2\\scripts\\mPDBS\\summary_pdb_93\\f83584e22003fb92.final.pdb
9.654787e-15  7.854492e-15  9.952144e-15  3.347194e-15  5.836562e-15
-3.999831e-15
E:\\R-3.1.2\\scripts\\mPDBS\\summary_pdb_93\\f8e2bf87e52caa29.final.pdb
8.673630e-15 -1.373866e-15  9.411105e-15  3.185277e-15 -2.717000e-15
2.541251e-15

[,181]        [,182]        [,183]        [,184]        [,185]        [,186]
E:\\R-3.1.2\\scripts\\40.1ns_data\\NAT0.pdb
2.685693e-15 -3.259846e-15 -5.894756e-16  4.742069e-16 -2.988996e-15
-3.034482e-15
E:\\R-3.1.2\\scripts\\40.1ns_data\\LJP6.pdb
2.495006e-17 -4.565918e-16  6.080186e-16  6.449192e-16 -2.291067e-15
-1.541283e-15
E:\\R-3.1.2\\scripts\\40.1ns_data\\3WY9.pdb
4.338186e-16 -3.838825e-15  1.733153e-15  1.243522e-15 -2.194694e-15
-3.399078e-15
E:\\R-3.1.2\\scripts\\40.1ns_data\\ZSP9.pdb
1.804308e-15 -1.950416e-15 -1.664034e-16  1.513576e-15 -2.017381e-15
-2.023916e-15
E:\\R-3.1.2\\scripts\\mPDBS\\summary_pdb_93\\013d855ad26c877c.final.pdb
6.242387e-15  1.812233e-15 -2.899318e-15 -8.763821e-16 -6.892373e-15
-6.925049e-16
E:\\R-3.1.2\\scripts\\mPDBS\\summary_pdb_93\\0a2a705342ca0d54.final.pdb
2.058056e-15 -2.409723e-15  7.770014e-16  4.285920e-16 -2.565142e-15
-1.589180e-15
```

E:\\R-3.1.2\\scripts\\mPDBS\\summary_pdb_93\\0a59fceadb4194d9.final.pdb
2.066859e-15 -3.156211e-15 2.445986e-15 -4.341032e-16 -5.315917e-15
-2.374464e-15
E:\\R-3.1.2\\scripts\\mPDBS\\summary_pdb_93\\0dba59f3306825e8.final.pdb
3.848469e-15 -3.548062e-15 1.593615e-15 2.407798e-16 -3.440273e-15
-3.738679e-15
E:\\R-3.1.2\\scripts\\mPDBS\\summary_pdb_93\\126f26a78b57339c.final.pdb
-3.051394e-15 -4.596703e-15 2.830318e-16 4.454771e-16 9.558877e-15
-5.272609e-15
E:\\R-3.1.2\\scripts\\mPDBS\\summary_pdb_93\\12f06f3e342df706.final.pdb
1.954407e-15 -2.541562e-15 6.243458e-16 -2.470828e-16 -3.134132e-15
-1.588963e-15
E:\\R-3.1.2\\scripts\\mPDBS\\summary_pdb_93\\18618a2a251b867b.final.pdb
7.100783e-16 -1.714701e-15 -2.269356e-15 1.408526e-15 8.646766e-15
-5.364852e-15
E:\\R-3.1.2\\scripts\\mPDBS\\summary_pdb_93\\1f305f598641eeb2.final.pdb
2.832388e-15 -2.872598e-15 7.337267e-16 1.072134e-15 -2.363768e-15
-2.202901e-15
E:\\R-3.1.2\\scripts\\mPDBS\\summary_pdb_93\\1f8a00b551be0a39.final.pdb
7.100783e-16 -1.714701e-15 -2.269356e-15 1.408526e-15 8.646766e-15
-5.364852e-15
E:\\R-3.1.2\\scripts\\mPDBS\\summary_pdb_93\\219f45328d47579a.final.pdb
-1.759941e-15 -3.940306e-15 -4.776535e-15 6.036415e-15 -3.199137e-16
1.164372e-15
E:\\R-3.1.2\\scripts\\mPDBS\\summary_pdb_93\\238dd3918e54336d.final.pdb
8.928848e-16 -9.357413e-15 -6.115742e-15 4.417051e-15 5.265029e-15
-6.300360e-15
E:\\R-3.1.2\\scripts\\mPDBS\\summary_pdb_93\\2b3027889e2fdc42.final.pdb
2.111094e-15 -2.235073e-15 3.552103e-16 1.432277e-17 -2.680872e-15
-1.275083e-15
E:\\R-3.1.2\\scripts\\mPDBS\\summary_pdb_93\\2fcb3b46e33793b0.final.pdb
8.907389e-15 2.099120e-15 -1.118958e-15 -2.889974e-15 -1.141902e-14
7.877628e-16
E:\\R-3.1.2\\scripts\\mPDBS\\summary_pdb_93\\34eb328251842006.final.pdb
-6.406094e-15 -7.850516e-15 7.355501e-15 6.448222e-15 -2.935614e-15
-4.280041e-15
E:\\R-3.1.2\\scripts\\mPDBS\\summary_pdb_93\\354b32871797b7ab.final.pdb
3.797631e-15 2.235843e-16 1.778349e-15 3.727654e-15 -1.792431e-14
1.994100e-15
E:\\R-3.1.2\\scripts\\mPDBS\\summary_pdb_93\\397368adbc32d5de.final.pdb
-3.051394e-15 -4.596703e-15 2.830318e-16 4.454771e-16 9.558877e-15
-5.272609e-15
E:\\R-3.1.2\\scripts\\mPDBS\\summary_pdb_93\\411ca13cd3df6edd.final.pdb
5.173397e-15 3.201536e-15 -8.075751e-16 -5.261341e-15 -7.650338e-15
1.568822e-15
E:\\R-3.1.2\\scripts\\mPDBS\\summary_pdb_93\\42357eee6642bf12.final.pdb
-1.415152e-16 -7.102562e-15 4.628095e-15 3.982181e-15 -6.608733e-15
-2.397025e-17
E:\\R-3.1.2\\scripts\\mPDBS\\summary_pdb_93\\44342d1f75a7fcfc.final.pdb
1.474622e-14 -8.954815e-15 1.705911e-15 -8.434920e-15 -2.645653e-15
-7.148705e-15
E:\\R-3.1.2\\scripts\\mPDBS\\summary_pdb_93\\448ef21dffe8cdfb.final.pdb
-9.550069e-15 4.545080e-15 9.428161e-15 8.670223e-15 5.567388e-15
3.518218e-15

E:\\R-3.1.2\\scripts\\mPDBS\\summary_pdb_93\\472a40b3e8f113c7.final.pdb
3.848469e-15 -3.548062e-15 1.593615e-15 2.407798e-16 -3.440273e-15
-3.738679e-15
E:\\R-3.1.2\\scripts\\mPDBS\\summary_pdb_93\\48a26225951ea153.final.pdb
6.345554e-15 -5.971667e-15 -1.093284e-15 -6.919367e-15 -5.354081e-15
-8.332816e-17
E:\\R-3.1.2\\scripts\\mPDBS\\summary_pdb_93\\4ab4a5c7c6682ce6.final.pdb
8.095939e-15 2.128820e-15 2.014286e-15 5.751996e-15 -1.694076e-14
6.579022e-15
E:\\R-3.1.2\\scripts\\mPDBS\\summary_pdb_93\\4eaa45b62cb6a25f.final.pdb
2.256763e-15 -2.412762e-15 -2.214117e-15 -1.466108e-15 1.647703e-15
-2.453514e-15
E:\\R-3.1.2\\scripts\\mPDBS\\summary_pdb_93\\58c371611d85ac96.final.pdb
-3.051394e-15 -4.596703e-15 2.830318e-16 4.454771e-16 9.558877e-15
-5.272609e-15
E:\\R-3.1.2\\scripts\\mPDBS\\summary_pdb_93\\59a647f04822d30a.final.pdb
-1.634775e-15 -2.175515e-15 3.042516e-15 4.169581e-15 -3.318726e-15
1.599506e-15
E:\\R-3.1.2\\scripts\\mPDBS\\summary_pdb_93\\59b091fe385e05fc.final.pdb
2.066859e-15 -3.156211e-15 2.445986e-15 -4.341032e-16 -5.315917e-15
-2.374464e-15
E:\\R-3.1.2\\scripts\\mPDBS\\summary_pdb_93\\5c35ac30f930d2db.final.pdb
2.256763e-15 -2.412762e-15 -2.214117e-15 -1.466108e-15 1.647703e-15
-2.453514e-15
E:\\R-3.1.2\\scripts\\mPDBS\\summary_pdb_93\\5f60752431cf2dcb.final.pdb
2.853427e-15 1.679888e-15 4.658879e-15 1.610806e-15 6.850937e-15
-1.011751e-14
E:\\R-3.1.2\\scripts\\mPDBS\\summary_pdb_93\\626456dd6ff2868d.final.pdb
3.848469e-15 -3.548062e-15 1.593615e-15 2.407798e-16 -3.440273e-15
-3.738679e-15
E:\\R-3.1.2\\scripts\\mPDBS\\summary_pdb_93\\6583d53ff3c8f7ef.final.pdb
-1.634775e-15 -2.175515e-15 3.042516e-15 4.169581e-15 -3.318726e-15
1.599506e-15
E:\\R-3.1.2\\scripts\\mPDBS\\summary_pdb_93\\69760178986bef01.final.pdb
1.208412e-14 -4.178994e-15 1.934580e-15 3.976074e-15 -3.961031e-16
-5.105500e-15
E:\\R-3.1.2\\scripts\\mPDBS\\summary_pdb_93\\6a9d87d47926b1c8.final.pdb
1.802362e-15 -2.528912e-15 9.144711e-16 6.328273e-16 -2.063770e-15
-1.907245e-15
E:\\R-3.1.2\\scripts\\mPDBS\\summary_pdb_93\\6bb3547ea982ffc4.final.pdb
-4.339409e-15 -8.441113e-15 2.124225e-15 3.453100e-15 1.131496e-14
-5.102284e-16
E:\\R-3.1.2\\scripts\\mPDBS\\summary_pdb_93\\716d9624611d1c42.final.pdb
-1.606548e-16 -6.913065e-15 -5.622393e-15 1.454681e-15 -8.296669e-15
1.011171e-15
E:\\R-3.1.2\\scripts\\mPDBS\\summary_pdb_93\\75f27e70ad0f1be4.final.pdb
-1.634775e-15 -2.175515e-15 3.042516e-15 4.169581e-15 -3.318726e-15
1.599506e-15
E:\\R-3.1.2\\scripts\\mPDBS\\summary_pdb_93\\769f910fffb22872.final.pdb
1.837215e-15 -2.318819e-15 7.555680e-16 -8.188589e-16 -1.705196e-15
-1.112828e-15
E:\\R-3.1.2\\scripts\\mPDBS\\summary_pdb_93\\791f8c80dd5c3135.final.pdb
2.066859e-15 -3.156211e-15 2.445986e-15 -4.341032e-16 -5.315917e-15
-2.374464e-15

E:\\R-3.1.2\\scripts\\mPDBS\\summary_pdb_93\\7fad98e66a3f6099.final.pdb
5.565262e-15 -3.956277e-15 -8.161024e-16 -1.783056e-15 -1.882933e-14
4.563384e-15
E:\\R-3.1.2\\scripts\\mPDBS\\summary_pdb_93\\804b89c94a633196.final.pdb
8.907389e-15 2.099120e-15 -1.118958e-15 -2.889974e-15 -1.141902e-14
7.877628e-16
E:\\R-3.1.2\\scripts\\mPDBS\\summary_pdb_93\\813cc65a2298feba.final.pdb
8.907389e-15 2.099120e-15 -1.118958e-15 -2.889974e-15 -1.141902e-14
7.877628e-16
E:\\R-3.1.2\\scripts\\mPDBS\\summary_pdb_93\\83c0eefcfe12d3f9.final.pdb
5.173397e-15 3.201536e-15 -8.075751e-16 -5.261341e-15 -7.650338e-15
1.568822e-15
E:\\R-3.1.2\\scripts\\mPDBS\\summary_pdb_93\\86d50469ef569760.final.pdb
3.848469e-15 -3.548062e-15 1.593615e-15 2.407798e-16 -3.440273e-15
-3.738679e-15
E:\\R-3.1.2\\scripts\\mPDBS\\summary_pdb_93\\89d0666e1402203d.final.pdb
-1.634775e-15 -2.175515e-15 3.042516e-15 4.169581e-15 -3.318726e-15
1.599506e-15
E:\\R-3.1.2\\scripts\\mPDBS\\summary_pdb_93\\8b34852716dadc62.final.pdb
-6.509372e-16 -1.124784e-14 1.635783e-15 3.556258e-15 3.007673e-16
-2.660868e-15
E:\\R-3.1.2\\scripts\\mPDBS\\summary_pdb_93\\8b6ef9ac3fe74395.final.pdb
-8.610711e-15 -4.341888e-15 -6.362825e-16 2.913352e-15 6.356664e-15
1.775757e-15
E:\\R-3.1.2\\scripts\\mPDBS\\summary_pdb_93\\98bbe0d5986c138f.final.pdb
2.066859e-15 -3.156211e-15 2.445986e-15 -4.341032e-16 -5.315917e-15
-2.374464e-15
E:\\R-3.1.2\\scripts\\mPDBS\\summary_pdb_93\\9c22f4909d67e2fa.final.pdb
1.853570e-15 -1.057824e-14 2.294544e-15 3.963051e-15 6.803378e-15
-1.378717e-14
E:\\R-3.1.2\\scripts\\mPDBS\\summary_pdb_93\\9dda8ed0d81fb3ef.final.pdb
1.441228e-14 -8.423989e-15 1.346390e-15 -1.049577e-14 1.084876e-14
-7.464966e-15
E:\\R-3.1.2\\scripts\\mPDBS\\summary_pdb_93\\9f752b3616e6033b.final.pdb
3.277747e-15 -3.139622e-15 2.382143e-15 -4.865438e-16 -2.889927e-15
9.563240e-17
E:\\R-3.1.2\\scripts\\mPDBS\\summary_pdb_93\\a02c70083816d6ba.final.pdb
2.256763e-15 -2.412762e-15 -2.214117e-15 -1.466108e-15 1.647703e-15
-2.453514e-15
E:\\R-3.1.2\\scripts\\mPDBS\\summary_pdb_93\\a0c997d891e4e082.final.pdb
3.733534e-15 2.775748e-15 -7.068839e-16 5.024404e-15 -8.536468e-15
2.016951e-16
E:\\R-3.1.2\\scripts\\mPDBS\\summary_pdb_93\\ab8039a2d823c616.final.pdb
1.176985e-15 -3.168715e-15 5.611990e-16 -1.095100e-16 -2.408557e-15
-1.873251e-15
E:\\R-3.1.2\\scripts\\mPDBS\\summary_pdb_93\\acf4d74b9c71d4dc.final.pdb
8.907389e-15 2.099120e-15 -1.118958e-15 -2.889974e-15 -1.141902e-14
7.877628e-16
E:\\R-3.1.2\\scripts\\mPDBS\\summary_pdb_93\\ad8a823e4b4c668e.final.pdb
1.578903e-15 -1.703223e-15 8.545705e-16 -1.229312e-16 -2.342951e-15
-1.200658e-15
E:\\R-3.1.2\\scripts\\mPDBS\\summary_pdb_93\\b16ababd03b7b857.final.pdb
1.853570e-15 -1.057824e-14 2.294544e-15 3.963051e-15 6.803378e-15
-1.378717e-14

E:\\R-3.1.2\\scripts\\mPDBS\\summary_pdb_93\\b226923e697d3c63.final.pdb
-4.076832e-15 4.919938e-15 -2.052801e-15 8.248783e-15 1.549775e-15
-5.570323e-15
E:\\R-3.1.2\\scripts\\mPDBS\\summary_pdb_93\\b28e48f6fa27a165.final.pdb
2.066859e-15 -3.156211e-15 2.445986e-15 -4.341032e-16 -5.315917e-15
-2.374464e-15
E:\\R-3.1.2\\scripts\\mPDBS\\summary_pdb_93\\b42c4924e3cd1aec.final.pdb
-8.667266e-16 -3.237600e-15 -5.908628e-16 -3.202126e-15 -9.335615e-15
-3.828227e-15
E:\\R-3.1.2\\scripts\\mPDBS\\summary_pdb_93\\b4dd239fcabff1df.final.pdb
2.066859e-15 -3.156211e-15 2.445986e-15 -4.341032e-16 -5.315917e-15
-2.374464e-15
E:\\R-3.1.2\\scripts\\mPDBS\\summary_pdb_93\\b669942ef97459a2.final.pdb
-6.509372e-16 -1.124784e-14 1.635783e-15 3.556258e-15 3.007673e-16
-2.660868e-15
E:\\R-3.1.2\\scripts\\mPDBS\\summary_pdb_93\\b99f4d9d87c1017b.final.pdb
2.066859e-15 -3.156211e-15 2.445986e-15 -4.341032e-16 -5.315917e-15
-2.374464e-15
E:\\R-3.1.2\\scripts\\mPDBS\\summary_pdb_93\\b9d2cbf3975403e7.final.pdb
2.066859e-15 -3.156211e-15 2.445986e-15 -4.341032e-16 -5.315917e-15
-2.374464e-15
E:\\R-3.1.2\\scripts\\mPDBS\\summary_pdb_93\\bc88c8a505f3a288.final.pdb
1.835707e-15 -3.112409e-15 4.341402e-16 7.012733e-16 -2.697352e-15
-2.055058e-15
E:\\R-3.1.2\\scripts\\mPDBS\\summary_pdb_93\\bdac0e64f4ef427e.final.pdb
-4.309035e-15 -1.564450e-14 4.001078e-15 1.343638e-14 9.531231e-15
-9.807278e-15
E:\\R-3.1.2\\scripts\\mPDBS\\summary_pdb_93\\ca2a1f55fc9d0681.final.pdb
-6.509372e-16 -1.124784e-14 1.635783e-15 3.556258e-15 3.007673e-16
-2.660868e-15
E:\\R-3.1.2\\scripts\\mPDBS\\summary_pdb_93\\ce34248de3e81a3c.final.pdb
-2.175327e-15 -7.201503e-16 -6.369919e-15 -8.672223e-16 -8.461990e-15
1.568546e-15
E:\\R-3.1.2\\scripts\\mPDBS\\summary_pdb_93\\d02e697772494a61.final.pdb
-7.267254e-15 2.835958e-15 6.930477e-16 1.399619e-14 -8.260610e-15
5.757703e-15
E:\\R-3.1.2\\scripts\\mPDBS\\summary_pdb_93\\d04221245d015047.final.pdb
4.861104e-15 -5.110305e-15 1.275905e-15 -3.243288e-15 -3.863122e-15
-1.023020e-15
E:\\R-3.1.2\\scripts\\mPDBS\\summary_pdb_93\\d678141e4018054a.final.pdb
1.917894e-16 -3.190813e-15 3.539823e-16 6.332807e-17 2.197035e-15
-9.228392e-15
E:\\R-3.1.2\\scripts\\mPDBS\\summary_pdb_93\\d71b40af0f0efe39.final.pdb
9.768041e-15 5.001330e-15 -2.760575e-15 -7.687903e-15 -5.796940e-15
-7.054979e-15
E:\\R-3.1.2\\scripts\\mPDBS\\summary_pdb_93\\dc0809eff10197e2.final.pdb
1.939661e-15 8.346248e-16 4.761524e-15 -2.351779e-15 1.178804e-15
-4.053089e-16
E:\\R-3.1.2\\scripts\\mPDBS\\summary_pdb_93\\e1163918d3707839.final.pdb
-4.152293e-15 3.990126e-15 4.090289e-15 -1.249677e-14 -8.544581e-15
3.372610e-15
E:\\R-3.1.2\\scripts\\mPDBS\\summary_pdb_93\\e4b48b06824ef848.final.pdb
7.100783e-16 -1.714701e-15 -2.269356e-15 1.408526e-15 8.646766e-15
-5.364852e-15

E:\\R-3.1.2\\scripts\\mPDBS\\summary_pdb_93\\e529e19ad281fea2.final.pdb
6.242387e-15 1.812233e-15 -2.899318e-15 -8.763821e-16 -6.892373e-15
-6.925049e-16
E:\\R-3.1.2\\scripts\\mPDBS\\summary_pdb_93\\e5602082718ec3a1.final.pdb
8.907389e-15 2.099120e-15 -1.118958e-15 -2.889974e-15 -1.141902e-14
7.877628e-16
E:\\R-3.1.2\\scripts\\mPDBS\\summary_pdb_93\\e5d589d996d0a960.final.pdb
6.615585e-15 -3.406728e-15 -9.684522e-15 1.141631e-15 -1.393103e-15
3.394804e-15
E:\\R-3.1.2\\scripts\\mPDBS\\summary_pdb_93\\e9df9e72307a2fbd.final.pdb
2.256763e-15 -2.412762e-15 -2.214117e-15 -1.466108e-15 1.647703e-15
-2.453514e-15
E:\\R-3.1.2\\scripts\\mPDBS\\summary_pdb_93\\ea00f8694090cae7.final.pdb
8.907389e-15 2.099120e-15 -1.118958e-15 -2.889974e-15 -1.141902e-14
7.877628e-16
E:\\R-3.1.2\\scripts\\mPDBS\\summary_pdb_93\\eba517be32db050e.final.pdb
2.066859e-15 -3.156211e-15 2.445986e-15 -4.341032e-16 -5.315917e-15
-2.374464e-15
E:\\R-3.1.2\\scripts\\mPDBS\\summary_pdb_93\\ec04b9545899c29d.final.pdb
2.188555e-15 -3.002978e-15 2.530802e-16 -5.731529e-16 -2.453099e-15
-1.370341e-15
E:\\R-3.1.2\\scripts\\mPDBS\\summary_pdb_93\\ee6862cbcad52078.final.pdb
2.554316e-15 -3.293254e-15 3.920731e-16 9.985962e-18 -2.646178e-15
-1.876599e-15
E:\\R-3.1.2\\scripts\\mPDBS\\summary_pdb_93\\ef6a42fc83b81e5d.final.pdb
2.417914e-15 -3.186614e-15 7.113326e-16 -1.371126e-16 -2.677509e-15
-1.827317e-15
E:\\R-3.1.2\\scripts\\mPDBS\\summary_pdb_93\\ef8e88d4ad93a0ad.final.pdb
4.861104e-15 -5.110305e-15 1.275905e-15 -3.243288e-15 -3.863122e-15
-1.023020e-15
E:\\R-3.1.2\\scripts\\mPDBS\\summary_pdb_93\\f14fcae8deec4fa3.final.pdb
3.277747e-15 -3.139622e-15 2.382143e-15 -4.865438e-16 -2.889927e-15
9.563240e-17
E:\\R-3.1.2\\scripts\\mPDBS\\summary_pdb_93\\f83584e22003fb92.final.pdb
6.345554e-15 -5.971667e-15 -1.093284e-15 -6.919367e-15 -5.354081e-15
-8.332816e-17
E:\\R-3.1.2\\scripts\\mPDBS\\summary_pdb_93\\f8e2bf87e52caa29.final.pdb
6.242387e-15 1.812233e-15 -2.899318e-15 -8.763821e-16 -6.892373e-15
-6.925049e-16

[,187]   [,188]   [,189]   [,190]   [,191]   [,192]
E:\\R-3.1.2\\scripts\\40.1ns_data\\NAT0.pdb
-6.237227e-16 -2.707291e-17 -3.877353e-15 -2.814228e-15 5.990393e-16
-2.357426e-15
E:\\R-3.1.2\\scripts\\40.1ns_data\\LJP6.pdb
-1.169529e-15 1.021489e-15 -3.917838e-15 -1.582042e-15 2.836847e-15
-2.139999e-15
E:\\R-3.1.2\\scripts\\40.1ns_data\\3WY9.pdb
3.271837e-15 1.753722e-15 -4.895569e-15 -1.412040e-15 2.809031e-16
-2.318618e-15
E:\\R-3.1.2\\scripts\\40.1ns_data\\ZSP9.pdb
2.609231e-16 6.158182e-16 -6.098184e-15 -1.079871e-15 2.878452e-16
-1.573075e-15
E:\\R-3.1.2\\scripts\\mPDBS\\summary_pdb_93\\013d855ad26c877c.final.pdb

5.473436e-16 3.331073e-15 -3.310404e-15 -1.779031e-15 -1.428031e-15
3.514159e-15
E:\\R-3.1.2\\scripts\\mPDBS\\summary_pdb_93\\0a2a705342ca0d54.final.pdb
1.569019e-15 -1.283254e-16 -2.556293e-15 -1.382837e-15 -3.076241e-15
-3.032101e-15
E:\\R-3.1.2\\scripts\\mPDBS\\summary_pdb_93\\0a59fceadb4194d9.final.pdb
2.426487e-15 -7.190066e-16 -9.195785e-16 -2.403085e-15 -2.934562e-15
4.064930e-16
E:\\R-3.1.2\\scripts\\mPDBS\\summary_pdb_93\\0dba59f3306825e8.final.pdb
-1.563815e-15 -4.634777e-15 -2.831620e-15 1.499816e-16 -2.531315e-15
-2.515740e-15
E:\\R-3.1.2\\scripts\\mPDBS\\summary_pdb_93\\126f26a78b57339c.final.pdb
-1.079482e-16 5.327957e-15 1.534052e-16 -4.657805e-15 -3.944247e-15
-1.151549e-14
E:\\R-3.1.2\\scripts\\mPDBS\\summary_pdb_93\\12f06f3e342df706.final.pdb
-1.110695e-15 7.550466e-17 -2.032298e-15 -1.264008e-15 -2.354163e-15
-3.000659e-15
E:\\R-3.1.2\\scripts\\mPDBS\\summary_pdb_93\\18618a2a251b867b.final.pdb
3.417138e-15 4.289494e-15 -3.704240e-15 -1.554874e-15 -4.836901e-15
-2.083476e-15
E:\\R-3.1.2\\scripts\\mPDBS\\summary_pdb_93\\1f305f598641eeb2.final.pdb
5.378561e-16 7.974649e-16 -2.909209e-15 -1.973440e-15 -1.959619e-15
-3.121239e-15
E:\\R-3.1.2\\scripts\\mPDBS\\summary_pdb_93\\1f8a00b551be0a39.final.pdb
3.417138e-15 4.289494e-15 -3.704240e-15 -1.554874e-15 -4.836901e-15
-2.083476e-15
E:\\R-3.1.2\\scripts\\mPDBS\\summary_pdb_93\\219f45328d47579a.final.pdb
4.554559e-15 -1.664431e-15 1.961797e-15 -9.897958e-15 7.159794e-15
-9.013055e-15
E:\\R-3.1.2\\scripts\\mPDBS\\summary_pdb_93\\238dd3918e54336d.final.pdb
-4.430007e-15 4.146134e-15 -3.701424e-15 6.408876e-15 -3.756387e-15
-1.922884e-14
E:\\R-3.1.2\\scripts\\mPDBS\\summary_pdb_93\\2b3027889e2fdc42.final.pdb
1.524865e-15 -5.796850e-17 -1.915635e-15 -9.632648e-16 -3.810164e-15
-3.082036e-15
E:\\R-3.1.2\\scripts\\mPDBS\\summary_pdb_93\\2fcb3b46e33793b0.final.pdb
1.129397e-15 -3.827308e-15 -5.976200e-15 -2.290894e-15 -3.212090e-15
4.553178e-15
E:\\R-3.1.2\\scripts\\mPDBS\\summary_pdb_93\\34eb328251842006.final.pdb
-6.960910e-15 7.338996e-15 -3.942530e-15 -2.136832e-15 5.131972e-15
-4.577389e-15
E:\\R-3.1.2\\scripts\\mPDBS\\summary_pdb_93\\354b32871797b7ab.final.pdb
2.284406e-15 4.382180e-16 -4.483206e-15 1.960763e-15 -8.620880e-15
1.093520e-14
E:\\R-3.1.2\\scripts\\mPDBS\\summary_pdb_93\\397368adbc32d5de.final.pdb
-1.079482e-16 5.327957e-15 1.534052e-16 -4.657805e-15 -3.944247e-15
-1.151549e-14
E:\\R-3.1.2\\scripts\\mPDBS\\summary_pdb_93\\411ca13cd3df6edd.final.pdb
1.118555e-15 1.558792e-15 -7.122419e-15 -3.497394e-15 -5.662387e-15
1.847009e-15
E:\\R-3.1.2\\scripts\\mPDBS\\summary_pdb_93\\42357eee6642bf12.final.pdb
8.028018e-15 3.268902e-15 2.198923e-15 5.891183e-15 5.049133e-15
6.494813e-15
E:\\R-3.1.2\\scripts\\mPDBS\\summary_pdb_93\\44342d1f75a7fcfc.final.pdb

4.557493e-15 2.279457e-15 -3.423550e-15 -4.317283e-15 -9.090690e-16
-7.339740e-16
E:\\R-3.1.2\\scripts\\mPDBS\\summary_pdb_93\\448ef21dffe8cdfb.final.pdb
1.642875e-15 1.878308e-14 -2.957781e-15 1.378046e-15 -1.576455e-14
-1.030299e-14
E:\\R-3.1.2\\scripts\\mPDBS\\summary_pdb_93\\472a40b3e8f113c7.final.pdb
-1.563815e-15 -4.634777e-15 -2.831620e-15 1.499816e-16 -2.531315e-15
-2.515740e-15
E:\\R-3.1.2\\scripts\\mPDBS\\summary_pdb_93\\48a26225951ea153.final.pdb
-5.761407e-15 -2.609311e-16 -7.308890e-15 -8.025324e-15 -9.815776e-15
-5.208807e-15
E:\\R-3.1.2\\scripts\\mPDBS\\summary_pdb_93\\4ab4a5c7c6682ce6.final.pdb
1.100643e-14 -1.086664e-16 -4.572175e-16 2.232517e-15 -1.596935e-14
9.190174e-15
E:\\R-3.1.2\\scripts\\mPDBS\\summary_pdb_93\\4eaa45b62cb6a25f.final.pdb
8.701730e-16 -4.752223e-16 -3.829700e-16 -4.059161e-15 -3.156067e-15
-6.469283e-15
E:\\R-3.1.2\\scripts\\mPDBS\\summary_pdb_93\\58c371611d85ac96.final.pdb
-1.079482e-16 5.327957e-15 1.534052e-16 -4.657805e-15 -3.944247e-15
-1.151549e-14
E:\\R-3.1.2\\scripts\\mPDBS\\summary_pdb_93\\59a647f04822d30a.final.pdb
1.166006e-16 -3.733094e-15 7.538686e-17 -7.827841e-16 -2.610833e-15
-6.094270e-15
E:\\R-3.1.2\\scripts\\mPDBS\\summary_pdb_93\\59b091fe385e05fc.final.pdb
2.426487e-15 -7.190066e-16 -9.195785e-16 -2.403085e-15 -2.934562e-15
4.064930e-16
E:\\R-3.1.2\\scripts\\mPDBS\\summary_pdb_93\\5c35ac30f930d2db.final.pdb
8.701730e-16 -4.752223e-16 -3.829700e-16 -4.059161e-15 -3.156067e-15
-6.469283e-15
E:\\R-3.1.2\\scripts\\mPDBS\\summary_pdb_93\\5f60752431cf2dcb.final.pdb
2.740481e-16 -4.454617e-15 -6.621075e-15 -2.766279e-15 2.783221e-15
-4.248142e-15
E:\\R-3.1.2\\scripts\\mPDBS\\summary_pdb_93\\626456dd6ff2868d.final.pdb
-1.563815e-15 -4.634777e-15 -2.831620e-15 1.499816e-16 -2.531315e-15
-2.515740e-15
E:\\R-3.1.2\\scripts\\mPDBS\\summary_pdb_93\\6583d53ff3c8f7ef.final.pdb
1.166006e-16 -3.733094e-15 7.538686e-17 -7.827841e-16 -2.610833e-15
-6.094270e-15
E:\\R-3.1.2\\scripts\\mPDBS\\summary_pdb_93\\69760178986bef01.final.pdb
-4.800012e-15 -1.251507e-15 -3.758350e-15 4.389397e-15 -8.049144e-15
-1.407667e-14
E:\\R-3.1.2\\scripts\\mPDBS\\summary_pdb_93\\6a9d87d47926b1c8.final.pdb
4.714494e-16 1.317276e-15 -1.976835e-15 -2.831141e-15 -3.278113e-15
-3.313713e-15
E:\\R-3.1.2\\scripts\\mPDBS\\summary_pdb_93\\6bb3547ea982ffc4.final.pdb
-1.045123e-14 7.957247e-15 -1.334120e-14 2.520105e-15 1.902662e-15
-1.678193e-14
E:\\R-3.1.2\\scripts\\mPDBS\\summary_pdb_93\\716d9624611d1c42.final.pdb
-7.781574e-15 8.930401e-15 2.470205e-16 -9.881874e-16 -1.032565e-14
-4.537841e-15
E:\\R-3.1.2\\scripts\\mPDBS\\summary_pdb_93\\75f27e70ad0f1be4.final.pdb
1.166006e-16 -3.733094e-15 7.538686e-17 -7.827841e-16 -2.610833e-15
-6.094270e-15
E:\\R-3.1.2\\scripts\\mPDBS\\summary_pdb_93\\769f910fffb22872.final.pdb

-1.264687e-16 1.042959e-15 -2.572224e-15 -9.698385e-16 -3.388407e-15
-3.169412e-15
E:\\R-3.1.2\\scripts\\mPDBS\\summary_pdb_93\\791f8c80dd5c3135.final.pdb
2.426487e-15 -7.190066e-16 -9.195785e-16 -2.403085e-15 -2.934562e-15
4.064930e-16
E:\\R-3.1.2\\scripts\\mPDBS\\summary_pdb_93\\7fad98e66a3f6099.final.pdb
-1.345795e-15 1.244409e-15 -3.567292e-15 -3.612135e-15 2.683571e-15
-2.420708e-15
E:\\R-3.1.2\\scripts\\mPDBS\\summary_pdb_93\\804b89c94a633196.final.pdb
1.129397e-15 -3.827308e-15 -5.976200e-15 -2.290894e-15 -3.212090e-15
4.553178e-15
E:\\R-3.1.2\\scripts\\mPDBS\\summary_pdb_93\\813cc65a2298feba.final.pdb
1.129397e-15 -3.827308e-15 -5.976200e-15 -2.290894e-15 -3.212090e-15
4.553178e-15
E:\\R-3.1.2\\scripts\\mPDBS\\summary_pdb_93\\83c0eefcfe12d3f9.final.pdb
1.118555e-15 1.558792e-15 -7.122419e-15 -3.497394e-15 -5.662387e-15
1.847009e-15
E:\\R-3.1.2\\scripts\\mPDBS\\summary_pdb_93\\86d50469ef569760.final.pdb
-1.563815e-15 -4.634777e-15 -2.831620e-15 1.499816e-16 -2.531315e-15
-2.515740e-15
E:\\R-3.1.2\\scripts\\mPDBS\\summary_pdb_93\\89d0666e1402203d.final.pdb
1.166006e-16 -3.733094e-15 7.538686e-17 -7.827841e-16 -2.610833e-15
-6.094270e-15
E:\\R-3.1.2\\scripts\\mPDBS\\summary_pdb_93\\8b34852716dadc62.final.pdb
2.907488e-15 -2.560960e-15 4.214602e-16 -4.769814e-15 -1.071441e-15
-1.026136e-14
E:\\R-3.1.2\\scripts\\mPDBS\\summary_pdb_93\\8b6ef9ac3fe74395.final.pdb
-6.487643e-15 -3.054250e-15 -1.107632e-14 -2.474496e-15 5.038053e-15
-8.805768e-15
E:\\R-3.1.2\\scripts\\mPDBS\\summary_pdb_93\\98bbe0d5986c138f.final.pdb
2.426487e-15 -7.190066e-16 -9.195785e-16 -2.403085e-15 -2.934562e-15
4.064930e-16
E:\\R-3.1.2\\scripts\\mPDBS\\summary_pdb_93\\9c22f4909d67e2fa.final.pdb
-3.561295e-15 2.548668e-15 6.318869e-15 -3.288794e-15 2.003356e-15
-1.180180e-14
E:\\R-3.1.2\\scripts\\mPDBS\\summary_pdb_93\\9dda8ed0d81fb3ef.final.pdb
4.245676e-15 3.088113e-16 -7.961804e-15 -3.171498e-15 -1.470793e-14
-1.101735e-15
E:\\R-3.1.2\\scripts\\mPDBS\\summary_pdb_93\\9f752b3616e6033b.final.pdb
2.464445e-15 -2.016890e-15 -3.158286e-15 -1.927382e-15 -2.735659e-16
-4.043667e-15
E:\\R-3.1.2\\scripts\\mPDBS\\summary_pdb_93\\a02c70083816d6ba.final.pdb
8.701730e-16 -4.752223e-16 -3.829700e-16 -4.059161e-15 -3.156067e-15
-6.469283e-15
E:\\R-3.1.2\\scripts\\mPDBS\\summary_pdb_93\\a0c997d891e4e082.final.pdb
6.802817e-15 -5.251453e-15 -2.803527e-15 5.710690e-15 2.700209e-15
-6.231420e-15
E:\\R-3.1.2\\scripts\\mPDBS\\summary_pdb_93\\ab8039a2d823c616.final.pdb
-4.023569e-16 4.845190e-16 -2.487157e-15 -1.644104e-15 -1.768005e-15
-3.265043e-15
E:\\R-3.1.2\\scripts\\mPDBS\\summary_pdb_93\\acf4d74b9c71d4dc.final.pdb
1.129397e-15 -3.827308e-15 -5.976200e-15 -2.290894e-15 -3.212090e-15
4.553178e-15
E:\\R-3.1.2\\scripts\\mPDBS\\summary_pdb_93\\ad8a823e4b4c668e.final.pdb

6.098225e-16 6.703048e-16 -2.521618e-15 -1.123549e-15 -2.759359e-15
-2.934111e-15
E:\\R-3.1.2\\scripts\\mPDBS\\summary_pdb_93\\b16ababd03b7b857.final.pdb
-3.561295e-15 2.548668e-15 6.318869e-15 -3.288794e-15 2.003356e-15
-1.180180e-14
E:\\R-3.1.2\\scripts\\mPDBS\\summary_pdb_93\\b226923e697d3c63.final.pdb
9.528776e-15 -1.515250e-15 -4.493116e-15 8.211323e-15 1.936745e-15
7.231937e-16
E:\\R-3.1.2\\scripts\\mPDBS\\summary_pdb_93\\b28e48f6fa27a165.final.pdb
2.426487e-15 -7.190066e-16 -9.195785e-16 -2.403085e-15 -2.934562e-15
4.064930e-16
E:\\R-3.1.2\\scripts\\mPDBS\\summary_pdb_93\\b42c4924e3cd1aec.final.pdb
-5.290533e-15 -5.250219e-15 1.443433e-15 -4.649049e-15 2.842704e-15
-1.795625e-15
E:\\R-3.1.2\\scripts\\mPDBS\\summary_pdb_93\\b4dd239fcabff1df.final.pdb
2.426487e-15 -7.190066e-16 -9.195785e-16 -2.403085e-15 -2.934562e-15
4.064930e-16
E:\\R-3.1.2\\scripts\\mPDBS\\summary_pdb_93\\b669942ef97459a2.final.pdb
2.907488e-15 -2.560960e-15 4.214602e-16 -4.769814e-15 -1.071441e-15
-1.026136e-14
E:\\R-3.1.2\\scripts\\mPDBS\\summary_pdb_93\\b99f4d9d87c1017b.final.pdb
2.426487e-15 -7.190066e-16 -9.195785e-16 -2.403085e-15 -2.934562e-15
4.064930e-16
E:\\R-3.1.2\\scripts\\mPDBS\\summary_pdb_93\\b9d2cbf3975403e7.final.pdb
2.426487e-15 -7.190066e-16 -9.195785e-16 -2.403085e-15 -2.934562e-15
4.064930e-16
E:\\R-3.1.2\\scripts\\mPDBS\\summary_pdb_93\\bc88c8a505f3a288.final.pdb
1.592953e-15 -2.844583e-16 -1.768617e-15 -3.065316e-15 -2.088017e-15
-3.437654e-15
E:\\R-3.1.2\\scripts\\mPDBS\\summary_pdb_93\\bdac0e64f4ef427e.final.pdb
-1.859573e-16 5.101514e-16 9.051276e-15 1.716751e-15 2.335556e-15
-1.266222e-14
E:\\R-3.1.2\\scripts\\mPDBS\\summary_pdb_93\\ca2a1f55fc9d0681.final.pdb
2.907488e-15 -2.560960e-15 4.214602e-16 -4.769814e-15 -1.071441e-15
-1.026136e-14
E:\\R-3.1.2\\scripts\\mPDBS\\summary_pdb_93\\ce34248de3e81a3c.final.pdb
5.791451e-16 2.228224e-15 1.988093e-15 -4.176112e-15 -1.918758e-16
3.222211e-15
E:\\R-3.1.2\\scripts\\mPDBS\\summary_pdb_93\\d02e697772494a61.final.pdb
4.679845e-16 5.367379e-15 -9.553572e-16 1.015621e-15 5.864389e-15
-7.228025e-15
E:\\R-3.1.2\\scripts\\mPDBS\\summary_pdb_93\\d04221245d015047.final.pdb
-2.677447e-15 1.267638e-15 -2.397602e-15 -5.226742e-15 -4.863185e-15
-4.278122e-15
E:\\R-3.1.2\\scripts\\mPDBS\\summary_pdb_93\\d678141e4018054a.final.pdb
-7.252958e-15 1.415380e-14 -1.168501e-14 1.874966e-15 -2.786125e-15
-6.010932e-16
E:\\R-3.1.2\\scripts\\mPDBS\\summary_pdb_93\\d71b40af0f0efe39.final.pdb
1.273633e-14 -3.927808e-15 3.346773e-16 -1.088317e-14 -1.336470e-14
7.099707e-15
E:\\R-3.1.2\\scripts\\mPDBS\\summary_pdb_93\\dc0809eff10197e2.final.pdb
4.451188e-15 -9.591100e-15 6.980665e-15 -4.699343e-15 4.943691e-16
-2.462284e-15
E:\\R-3.1.2\\scripts\\mPDBS\\summary_pdb_93\\e1163918d3707839.final.pdb

```
-8.746102e-15  4.925669e-15 -3.033888e-15 -8.016147e-15 -5.828311e-15
8.064604e-16
E:\\R-3.1.2\\scripts\\mPDBS\\summary_pdb_93\\e4b48b06824ef848.final.pdb
3.417138e-15  4.289494e-15 -3.704240e-15 -1.554874e-15 -4.836901e-15
-2.083476e-15
E:\\R-3.1.2\\scripts\\mPDBS\\summary_pdb_93\\e529e19ad281fea2.final.pdb
5.473436e-16  3.331073e-15 -3.310404e-15 -1.779031e-15 -1.428031e-15
3.514159e-15
E:\\R-3.1.2\\scripts\\mPDBS\\summary_pdb_93\\e5602082718ec3a1.final.pdb
1.129397e-15 -3.827308e-15 -5.976200e-15 -2.290894e-15 -3.212090e-15
4.553178e-15
E:\\R-3.1.2\\scripts\\mPDBS\\summary_pdb_93\\e5d589d996d0a960.final.pdb
-7.353988e-15 -1.542878e-16 -1.336982e-14  7.239750e-15  5.737662e-16
-9.530381e-15
E:\\R-3.1.2\\scripts\\mPDBS\\summary_pdb_93\\e9df9e72307a2fbd.final.pdb
8.701730e-16 -4.752223e-16 -3.829700e-16 -4.059161e-15 -3.156067e-15
-6.469283e-15
E:\\R-3.1.2\\scripts\\mPDBS\\summary_pdb_93\\ea00f8694090cae7.final.pdb
1.129397e-15 -3.827308e-15 -5.976200e-15 -2.290894e-15 -3.212090e-15
4.553178e-15
E:\\R-3.1.2\\scripts\\mPDBS\\summary_pdb_93\\eba517be32db050e.final.pdb
2.426487e-15 -7.190066e-16 -9.195785e-16 -2.403085e-15 -2.934562e-15
4.064930e-16
E:\\R-3.1.2\\scripts\\mPDBS\\summary_pdb_93\\ec04b9545899c29d.final.pdb
-1.626899e-16  1.432912e-15 -3.458245e-15 -2.851442e-15 -2.111232e-15
-3.231976e-15
E:\\R-3.1.2\\scripts\\mPDBS\\summary_pdb_93\\ee6862cbcad52078.final.pdb
3.743095e-16  4.970346e-16 -1.982964e-15 -2.079559e-15 -3.331814e-15
-3.265917e-15
E:\\R-3.1.2\\scripts\\mPDBS\\summary_pdb_93\\ef6a42fc83b81e5d.final.pdb
5.019349e-16 -3.405913e-16 -2.576344e-15 -1.761306e-15 -2.794698e-15
-3.365436e-15
E:\\R-3.1.2\\scripts\\mPDBS\\summary_pdb_93\\ef8e88d4ad93a0ad.final.pdb
-2.677447e-15  1.267638e-15 -2.397602e-15 -5.226742e-15 -4.863185e-15
-4.278122e-15
E:\\R-3.1.2\\scripts\\mPDBS\\summary_pdb_93\\f14fcae8deec4fa3.final.pdb
2.464445e-15 -2.016890e-15 -3.158286e-15 -1.927382e-15 -2.735659e-16
-4.043667e-15
E:\\R-3.1.2\\scripts\\mPDBS\\summary_pdb_93\\f83584e22003fb92.final.pdb
-5.761407e-15 -2.609311e-16 -7.308890e-15 -8.025324e-15 -9.815776e-15
-5.208807e-15
E:\\R-3.1.2\\scripts\\mPDBS\\summary_pdb_93\\f8e2bf87e52caa29.final.pdb
5.473436e-16  3.331073e-15 -3.310404e-15 -1.779031e-15 -1.428031e-15
3.514159e-15

[,193]        [,194]        [,195]        [,196]        [,197]        [,198]
E:\\R-3.1.2\\scripts\\40.1ns_data\\NAT0.pdb
-4.080303e-17  2.022778e-15 -3.261915e-15 -1.525614e-15  2.832372e-15
-2.242904e-15
E:\\R-3.1.2\\scripts\\40.1ns_data\\LJP6.pdb
3.878913e-16  1.168315e-15 -8.471389e-16 -2.641525e-15  4.633591e-15
-3.904770e-16
E:\\R-3.1.2\\scripts\\40.1ns_data\\3WY9.pdb
-6.652198e-16  5.540525e-15 -2.482741e-15 -5.198723e-15  3.319309e-15
```

-7.199446e-16
E:\\R-3.1.2\\scripts\\40.1ns_data\\ZSP9.pdb
3.721739e-16 3.384158e-15 -6.252370e-16 -2.830383e-15 3.423215e-15
-1.707653e-17
E:\\R-3.1.2\\scripts\\mPDBS\\summary_pdb_93\\013d855ad26c877c.final.pdb
5.768168e-17 5.305246e-15 6.992885e-16 7.203684e-16 5.720762e-15
-3.521917e-15
E:\\R-3.1.2\\scripts\\mPDBS\\summary_pdb_93\\0a2a705342ca0d54.final.pdb
-1.019283e-16 2.216910e-15 -3.316360e-15 -3.213508e-15 3.341505e-15
-1.668032e-15
E:\\R-3.1.2\\scripts\\mPDBS\\summary_pdb_93\\0a59fceadb4194d9.final.pdb
3.522056e-16 3.715892e-15 -4.232725e-15 -2.191076e-15 5.741876e-15
-4.402782e-15
E:\\R-3.1.2\\scripts\\mPDBS\\summary_pdb_93\\0dba59f3306825e8.final.pdb
-8.686607e-16 8.157817e-16 -4.234265e-15 9.285352e-16 3.691135e-15
2.389635e-16
E:\\R-3.1.2\\scripts\\mPDBS\\summary_pdb_93\\126f26a78b57339c.final.pdb
7.719541e-16 -4.304255e-15 -4.858766e-15 -6.010359e-15 6.177211e-15
-4.306446e-15
E:\\R-3.1.2\\scripts\\mPDBS\\summary_pdb_93\\12f06f3e342df706.final.pdb
1.950234e-17 3.293306e-15 -2.438590e-15 -2.608090e-15 3.117942e-15
-4.645679e-16
E:\\R-3.1.2\\scripts\\mPDBS\\summary_pdb_93\\18618a2a251b867b.final.pdb
1.151682e-15 6.974669e-15 -1.034289e-15 -5.009355e-16 4.373507e-15
9.732017e-17
E:\\R-3.1.2\\scripts\\mPDBS\\summary_pdb_93\\1f305f598641eeb2.final.pdb
4.592282e-17 3.023439e-15 -2.749407e-15 -2.984778e-15 3.313541e-15
-1.332024e-15
E:\\R-3.1.2\\scripts\\mPDBS\\summary_pdb_93\\1f8a00b551be0a39.final.pdb
1.151682e-15 6.974669e-15 -1.034289e-15 -5.009355e-16 4.373507e-15
9.732017e-17
E:\\R-3.1.2\\scripts\\mPDBS\\summary_pdb_93\\219f45328d47579a.final.pdb
-6.868938e-16 -2.912486e-15 6.904199e-15 2.796254e-15 -7.966776e-15
-1.596479e-16
E:\\R-3.1.2\\scripts\\mPDBS\\summary_pdb_93\\238dd3918e54336d.final.pdb
2.589565e-15 -2.137065e-15 -8.667546e-15 -1.724869e-15 -1.052018e-14
-2.213561e-15
E:\\R-3.1.2\\scripts\\mPDBS\\summary_pdb_93\\2b3027889e2fdc42.final.pdb
-1.530327e-16 1.465956e-15 -3.102553e-15 -2.737947e-15 3.616189e-15
-2.027945e-15
E:\\R-3.1.2\\scripts\\mPDBS\\summary_pdb_93\\2fcb3b46e33793b0.final.pdb
-6.653138e-16 6.336761e-15 -2.722216e-16 -1.881494e-15 2.050662e-15
-2.426176e-16
E:\\R-3.1.2\\scripts\\mPDBS\\summary_pdb_93\\34eb328251842006.final.pdb
5.654468e-15 4.589797e-15 1.688262e-16 -3.843332e-15 -1.144478e-15
-3.109356e-15
E:\\R-3.1.2\\scripts\\mPDBS\\summary_pdb_93\\354b32871797b7ab.final.pdb
-5.985855e-15 1.467863e-14 -6.937229e-15 -9.922335e-15 1.459058e-14
1.278351e-15
E:\\R-3.1.2\\scripts\\mPDBS\\summary_pdb_93\\397368adbc32d5de.final.pdb
7.719541e-16 -4.304255e-15 -4.858766e-15 -6.010359e-15 6.177211e-15
-4.306446e-15
E:\\R-3.1.2\\scripts\\mPDBS\\summary_pdb_93\\411ca13cd3df6edd.final.pdb
8.755543e-16 4.420759e-15 6.818763e-16 -1.215360e-15 2.598618e-15

-4.953693e-15
E:\\R-3.1.2\\scripts\\mPDBS\\summary_pdb_93\\42357eee6642bf12.final.pdb
3.863074e-15 1.328436e-14 -4.101726e-15 -1.684782e-15 -2.399870e-15
-7.532200e-16
E:\\R-3.1.2\\scripts\\mPDBS\\summary_pdb_93\\44342d1f75a7fcfc.final.pdb
2.292814e-15 -5.246291e-16 -8.327956e-15 -8.224838e-15 8.077000e-15
-4.638469e-15
E:\\R-3.1.2\\scripts\\mPDBS\\summary_pdb_93\\448ef21dffe8cdfb.final.pdb
-3.322910e-15 1.711633e-14 5.292206e-16 -1.185962e-14 2.846901e-15
-2.816947e-15
E:\\R-3.1.2\\scripts\\mPDBS\\summary_pdb_93\\472a40b3e8f113c7.final.pdb
-8.686607e-16 8.157817e-16 -4.234265e-15 9.285352e-16 3.691135e-15
2.389635e-16
E:\\R-3.1.2\\scripts\\mPDBS\\summary_pdb_93\\48a26225951ea153.final.pdb
-2.262456e-15 2.000251e-15 -1.641916e-15 -1.026665e-14 4.637942e-15
2.491010e-15
E:\\R-3.1.2\\scripts\\mPDBS\\summary_pdb_93\\4ab4a5c7c6682ce6.final.pdb
-1.236856e-15 1.390687e-14 1.344606e-15 -9.694911e-15 6.680281e-15
1.466714e-15
E:\\R-3.1.2\\scripts\\mPDBS\\summary_pdb_93\\4eaa45b62cb6a25f.final.pdb
7.049851e-17 -1.757872e-15 -1.834540e-15 -2.634328e-15 3.653043e-15
-3.176419e-15
E:\\R-3.1.2\\scripts\\mPDBS\\summary_pdb_93\\58c371611d85ac96.final.pdb
7.719541e-16 -4.304255e-15 -4.858766e-15 -6.010359e-15 6.177211e-15
-4.306446e-15
E:\\R-3.1.2\\scripts\\mPDBS\\summary_pdb_93\\59a647f04822d30a.final.pdb
-1.023977e-15 5.404911e-16 -1.786313e-15 -1.006364e-14 2.872829e-15
2.976967e-15
E:\\R-3.1.2\\scripts\\mPDBS\\summary_pdb_93\\59b091fe385e05fc.final.pdb
3.522056e-16 3.715892e-15 -4.232725e-15 -2.191076e-15 5.741876e-15
-4.402782e-15
E:\\R-3.1.2\\scripts\\mPDBS\\summary_pdb_93\\5c35ac30f930d2db.final.pdb
7.049851e-17 -1.757872e-15 -1.834540e-15 -2.634328e-15 3.653043e-15
-3.176419e-15
E:\\R-3.1.2\\scripts\\mPDBS\\summary_pdb_93\\5f60752431cf2dcb.final.pdb
4.267287e-16 -2.191022e-15 1.512242e-15 -3.375271e-15 4.220359e-15
-3.565748e-17
E:\\R-3.1.2\\scripts\\mPDBS\\summary_pdb_93\\626456dd6ff2868d.final.pdb
-8.686607e-16 8.157817e-16 -4.234265e-15 9.285352e-16 3.691135e-15
2.389635e-16
E:\\R-3.1.2\\scripts\\mPDBS\\summary_pdb_93\\6583d53ff3c8f7ef.final.pdb
-1.023977e-15 5.404911e-16 -1.786313e-15 -1.006364e-14 2.872829e-15
2.976967e-15
E:\\R-3.1.2\\scripts\\mPDBS\\summary_pdb_93\\69760178986bef01.final.pdb
-8.682311e-15 -6.552534e-15 -5.452959e-15 -2.682279e-15 -1.868824e-15
-2.641366e-15
E:\\R-3.1.2\\scripts\\mPDBS\\summary_pdb_93\\6a9d87d47926b1c8.final.pdb
2.133731e-16 1.281555e-15 -2.811792e-15 -3.338885e-15 3.746863e-15
-1.974977e-15
E:\\R-3.1.2\\scripts\\mPDBS\\summary_pdb_93\\6bb3547ea982ffc4.final.pdb
-1.304171e-16 -5.972483e-15 -8.586097e-16 2.659446e-15 3.004297e-15
3.297958e-15
E:\\R-3.1.2\\scripts\\mPDBS\\summary_pdb_93\\716d9624611d1c42.final.pdb
-2.896568e-15 5.061857e-15 -2.927782e-15 -2.280227e-15 6.522120e-15

-4.656961e-15
E:\\R-3.1.2\\scripts\\mPDBS\\summary_pdb_93\\75f27e70ad0f1be4.final.pdb
-1.023977e-15 5.404911e-16 -1.786313e-15 -1.006364e-14 2.872829e-15
2.976967e-15
E:\\R-3.1.2\\scripts\\mPDBS\\summary_pdb_93\\769f910fffb22872.final.pdb
-1.111970e-16 2.299590e-15 -2.781156e-15 -4.563658e-15 2.729748e-15
-1.731078e-15
E:\\R-3.1.2\\scripts\\mPDBS\\summary_pdb_93\\791f8c80dd5c3135.final.pdb
3.522056e-16 3.715892e-15 -4.232725e-15 -2.191076e-15 5.741876e-15
-4.402782e-15
E:\\R-3.1.2\\scripts\\mPDBS\\summary_pdb_93\\7fad98e66a3f6099.final.pdb
3.804289e-16 2.236784e-15 -4.170015e-15 -1.145328e-14 2.484982e-16
-1.338718e-15
E:\\R-3.1.2\\scripts\\mPDBS\\summary_pdb_93\\804b89c94a633196.final.pdb
-6.653138e-16 6.336761e-15 -2.722216e-16 -1.881494e-15 2.050662e-15
-2.426176e-16
E:\\R-3.1.2\\scripts\\mPDBS\\summary_pdb_93\\813cc65a2298feba.final.pdb
-6.653138e-16 6.336761e-15 -2.722216e-16 -1.881494e-15 2.050662e-15
-2.426176e-16
E:\\R-3.1.2\\scripts\\mPDBS\\summary_pdb_93\\83c0eefcfe12d3f9.final.pdb
8.755543e-16 4.420759e-15 6.818763e-16 -1.215360e-15 2.598618e-15
-4.953693e-15
E:\\R-3.1.2\\scripts\\mPDBS\\summary_pdb_93\\86d50469ef569760.final.pdb
-8.686607e-16 8.157817e-16 -4.234265e-15 9.285352e-16 3.691135e-15
2.389635e-16
E:\\R-3.1.2\\scripts\\mPDBS\\summary_pdb_93\\89d0666e1402203d.final.pdb
-1.023977e-15 5.404911e-16 -1.786313e-15 -1.006364e-14 2.872829e-15
2.976967e-15
E:\\R-3.1.2\\scripts\\mPDBS\\summary_pdb_93\\8b34852716dadc62.final.pdb
1.627991e-15 -5.379561e-15 -5.066997e-15 -4.636235e-15 3.330888e-15
-1.873258e-15
E:\\R-3.1.2\\scripts\\mPDBS\\summary_pdb_93\\8b6ef9ac3fe74395.final.pdb
4.934647e-15 -1.868610e-15 -3.725654e-15 -5.123908e-15 8.765998e-15
6.713806e-15
E:\\R-3.1.2\\scripts\\mPDBS\\summary_pdb_93\\98bbe0d5986c138f.final.pdb
3.522056e-16 3.715892e-15 -4.232725e-15 -2.191076e-15 5.741876e-15
-4.402782e-15
E:\\R-3.1.2\\scripts\\mPDBS\\summary_pdb_93\\9c22f4909d67e2fa.final.pdb
-2.366644e-15 -3.285750e-15 -8.869511e-15 -5.318794e-16 1.215176e-15
-3.862986e-15
E:\\R-3.1.2\\scripts\\mPDBS\\summary_pdb_93\\9dda8ed0d81fb3ef.final.pdb
9.115404e-16 -5.940181e-16 -7.621056e-15 -9.787824e-15 1.300025e-14
-1.122044e-14
E:\\R-3.1.2\\scripts\\mPDBS\\summary_pdb_93\\9f752b3616e6033b.final.pdb
-1.980226e-16 2.544942e-15 -2.841246e-15 -3.093719e-15 6.814402e-16
1.357990e-15
E:\\R-3.1.2\\scripts\\mPDBS\\summary_pdb_93\\a02c70083816d6ba.final.pdb
7.049851e-17 -1.757872e-15 -1.834540e-15 -2.634328e-15 3.653043e-15
-3.176419e-15
E:\\R-3.1.2\\scripts\\mPDBS\\summary_pdb_93\\a0c997d891e4e082.final.pdb
-2.145649e-15 -9.433855e-15 -6.084019e-15 -9.135185e-15 5.697794e-16
-3.543990e-15
E:\\R-3.1.2\\scripts\\mPDBS\\summary_pdb_93\\ab8039a2d823c616.final.pdb
2.232130e-17 2.291141e-15 -2.525233e-15 -2.330298e-15 3.622134e-15

-1.667019e-15
E:\\R-3.1.2\\scripts\\mPDBS\\summary_pdb_93\\acf4d74b9c71d4dc.final.pdb
-6.653138e-16 6.336761e-15 -2.722216e-16 -1.881494e-15 2.050662e-15
-2.426176e-16
E:\\R-3.1.2\\scripts\\mPDBS\\summary_pdb_93\\ad8a823e4b4c668e.final.pdb
-6.258835e-17 2.338462e-15 -2.612368e-15 -4.131012e-15 3.359831e-15
-1.545890e-15
E:\\R-3.1.2\\scripts\\mPDBS\\summary_pdb_93\\b16ababd03b7b857.final.pdb
-2.366644e-15 -3.285750e-15 -8.869511e-15 -5.318794e-16 1.215176e-15
-3.862986e-15
E:\\R-3.1.2\\scripts\\mPDBS\\summary_pdb_93\\b226923e697d3c63.final.pdb
1.897297e-15 5.820989e-15 -2.370915e-15 -4.800494e-15 2.707272e-15
9.143806e-16
E:\\R-3.1.2\\scripts\\mPDBS\\summary_pdb_93\\b28e48f6fa27a165.final.pdb
3.522056e-16 3.715892e-15 -4.232725e-15 -2.191076e-15 5.741876e-15
-4.402782e-15
E:\\R-3.1.2\\scripts\\mPDBS\\summary_pdb_93\\b42c4924e3cd1aec.final.pdb
-3.400626e-16 2.825122e-15 8.270475e-16 6.555440e-15 -1.095459e-15
-3.104374e-17
E:\\R-3.1.2\\scripts\\mPDBS\\summary_pdb_93\\b4dd239fcabff1df.final.pdb
3.522056e-16 3.715892e-15 -4.232725e-15 -2.191076e-15 5.741876e-15
-4.402782e-15
E:\\R-3.1.2\\scripts\\mPDBS\\summary_pdb_93\\b669942ef97459a2.final.pdb
1.627991e-15 -5.379561e-15 -5.066997e-15 -4.636235e-15 3.330888e-15
-1.873258e-15
E:\\R-3.1.2\\scripts\\mPDBS\\summary_pdb_93\\b99f4d9d87c1017b.final.pdb
3.522056e-16 3.715892e-15 -4.232725e-15 -2.191076e-15 5.741876e-15
-4.402782e-15
E:\\R-3.1.2\\scripts\\mPDBS\\summary_pdb_93\\b9d2cbf3975403e7.final.pdb
3.522056e-16 3.715892e-15 -4.232725e-15 -2.191076e-15 5.741876e-15
-4.402782e-15
E:\\R-3.1.2\\scripts\\mPDBS\\summary_pdb_93\\bc88c8a505f3a288.final.pdb
-4.544409e-16 1.322841e-15 -3.034899e-15 -2.012399e-15 3.942860e-15
-1.812406e-15
E:\\R-3.1.2\\scripts\\mPDBS\\summary_pdb_93\\bdac0e64f4ef427e.final.pdb
-1.304993e-15 -1.170255e-15 -9.905141e-15 -1.252229e-14 6.018409e-15
4.738207e-15
E:\\R-3.1.2\\scripts\\mPDBS\\summary_pdb_93\\ca2a1f55fc9d0681.final.pdb
1.627991e-15 -5.379561e-15 -5.066997e-15 -4.636235e-15 3.330888e-15
-1.873258e-15
E:\\R-3.1.2\\scripts\\mPDBS\\summary_pdb_93\\ce34248de3e81a3c.final.pdb
4.394230e-15 1.342288e-16 -5.894109e-15 -1.150082e-14 1.231965e-14
-7.419963e-15
E:\\R-3.1.2\\scripts\\mPDBS\\summary_pdb_93\\d02e697772494a61.final.pdb
-6.335511e-16 4.741114e-15 -1.426810e-15 -7.824157e-15 -1.558599e-15
6.772307e-15
E:\\R-3.1.2\\scripts\\mPDBS\\summary_pdb_93\\d04221245d015047.final.pdb
-1.038896e-15 1.779398e-15 -3.738672e-15 -3.011611e-15 3.766875e-15
-2.156211e-15
E:\\R-3.1.2\\scripts\\mPDBS\\summary_pdb_93\\d678141e4018054a.final.pdb
5.352032e-15 9.278186e-15 5.269889e-15 7.808799e-15 4.342983e-15
-2.978859e-16
E:\\R-3.1.2\\scripts\\mPDBS\\summary_pdb_93\\d71b40af0f0efe39.final.pdb
-5.888016e-15 1.946488e-14 1.365500e-15 4.010058e-15 1.616052e-15

-2.404057e-16
E:\\R-3.1.2\\scripts\\mPDBS\\summary_pdb_93\\dc0809eff10197e2.final.pdb
-1.240651e-15 -1.293933e-15 -1.053140e-15 -4.847132e-15 5.242034e-15
1.608502e-15
E:\\R-3.1.2\\scripts\\mPDBS\\summary_pdb_93\\e1163918d3707839.final.pdb
-1.613350e-15 1.513472e-14 4.105675e-15 6.942283e-15 -6.221567e-15
4.009024e-16
E:\\R-3.1.2\\scripts\\mPDBS\\summary_pdb_93\\e4b48b06824ef848.final.pdb
1.151682e-15 6.974669e-15 -1.034289e-15 -5.009355e-16 4.373507e-15
9.732017e-17
E:\\R-3.1.2\\scripts\\mPDBS\\summary_pdb_93\\e529e19ad281fea2.final.pdb
5.768168e-17 5.305246e-15 6.992885e-16 7.203684e-16 5.720762e-15
-3.521917e-15
E:\\R-3.1.2\\scripts\\mPDBS\\summary_pdb_93\\e5602082718ec3a1.final.pdb
-6.653138e-16 6.336761e-15 -2.722216e-16 -1.881494e-15 2.050662e-15
-2.426176e-16
E:\\R-3.1.2\\scripts\\mPDBS\\summary_pdb_93\\e5d589d996d0a960.final.pdb
-3.716579e-15 -1.321308e-14 -1.121802e-14 -8.697310e-15 1.047107e-14
2.823314e-16
E:\\R-3.1.2\\scripts\\mPDBS\\summary_pdb_93\\e9df9e72307a2fbd.final.pdb
7.049851e-17 -1.757872e-15 -1.834540e-15 -2.634328e-15 3.653043e-15
-3.176419e-15
E:\\R-3.1.2\\scripts\\mPDBS\\summary_pdb_93\\ea00f8694090cae7.final.pdb
-6.653138e-16 6.336761e-15 -2.722216e-16 -1.881494e-15 2.050662e-15
-2.426176e-16
E:\\R-3.1.2\\scripts\\mPDBS\\summary_pdb_93\\eba517be32db050e.final.pdb
3.522056e-16 3.715892e-15 -4.232725e-15 -2.191076e-15 5.741876e-15
-4.402782e-15
E:\\R-3.1.2\\scripts\\mPDBS\\summary_pdb_93\\ec04b9545899c29d.final.pdb
4.644154e-16 3.725443e-15 -2.658879e-15 -4.484774e-15 3.002066e-15
-1.479538e-15
E:\\R-3.1.2\\scripts\\mPDBS\\summary_pdb_93\\ee6862cbcad52078.final.pdb
-4.336242e-16 1.956883e-15 -3.238729e-15 -2.215362e-15 3.540512e-15
-1.908249e-15
E:\\R-3.1.2\\scripts\\mPDBS\\summary_pdb_93\\ef6a42fc83b81e5d.final.pdb
-1.645397e-16 2.771434e-15 -3.102947e-15 -3.865865e-15 3.197147e-15
-1.658220e-15
E:\\R-3.1.2\\scripts\\mPDBS\\summary_pdb_93\\ef8e88d4ad93a0ad.final.pdb
-1.038896e-15 1.779398e-15 -3.738672e-15 -3.011611e-15 3.766875e-15
-2.156211e-15
E:\\R-3.1.2\\scripts\\mPDBS\\summary_pdb_93\\f14fcae8deec4fa3.final.pdb
-1.980226e-16 2.544942e-15 -2.841246e-15 -3.093719e-15 6.814402e-16
1.357990e-15
E:\\R-3.1.2\\scripts\\mPDBS\\summary_pdb_93\\f83584e22003fb92.final.pdb
-2.262456e-15 2.000251e-15 -1.641916e-15 -1.026665e-14 4.637942e-15
2.491010e-15
E:\\R-3.1.2\\scripts\\mPDBS\\summary_pdb_93\\f8e2bf87e52caa29.final.pdb
5.768168e-17 5.305246e-15 6.992885e-16 7.203684e-16 5.720762e-15
-3.521917e-15

[,199]   [,200]   [,201]   [,202]   [,203]   [,204]
E:\\R-3.1.2\\scripts\\40.1ns_data\\NAT0.pdb
1.397640e-15 2.793338e-15 -1.086582e-15 -2.284171e-17 -1.923126e-15
3.212395e-15

E:\\R-3.1.2\\scripts\\40.1ns_data\\LJP6.pdb
1.719704e-15 2.051376e-15 -3.172800e-16 2.369319e-16 -1.055997e-15
3.567740e-15
E:\\R-3.1.2\\scripts\\40.1ns_data\\3WY9.pdb
2.614003e-15 -2.188979e-16 9.722707e-16 1.791321e-15 -2.797266e-15
4.281680e-15
E:\\R-3.1.2\\scripts\\40.1ns_data\\ZSP9.pdb
3.827699e-15 2.466437e-15 4.568592e-16 -8.024922e-16 -1.609863e-15
2.951379e-15
E:\\R-3.1.2\\scripts\\mPDBS\\summary_pdb_93\\013d855ad26c877c.final.pdb
-1.595256e-15 1.420533e-15 4.482756e-15 6.463851e-16 -8.274452e-16
8.635576e-16
E:\\R-3.1.2\\scripts\\mPDBS\\summary_pdb_93\\0a2a705342ca0d54.final.pdb
7.174265e-16 2.340347e-15 6.789029e-16 2.637470e-16 -1.004943e-15
2.439330e-15
E:\\R-3.1.2\\scripts\\mPDBS\\summary_pdb_93\\0a59fceadb4194d9.final.pdb
8.604284e-16 -7.888016e-16 -6.959781e-16 1.964777e-15 -2.437199e-15
4.573013e-15
E:\\R-3.1.2\\scripts\\mPDBS\\summary_pdb_93\\0dba59f3306825e8.final.pdb
7.827762e-15 1.807376e-15 -1.364346e-15 -4.291434e-16 -9.176509e-16
7.195755e-16
E:\\R-3.1.2\\scripts\\mPDBS\\summary_pdb_93\\126f26a78b57339c.final.pdb
-4.372115e-15 8.347718e-15 4.140040e-15 -9.271175e-16 -9.892676e-15
-2.295374e-15
E:\\R-3.1.2\\scripts\\mPDBS\\summary_pdb_93\\12f06f3e342df706.final.pdb
-1.609271e-15 1.000274e-15 9.288115e-16 5.785698e-17 -7.421326e-16
2.676554e-15
E:\\R-3.1.2\\scripts\\mPDBS\\summary_pdb_93\\18618a2a251b867b.final.pdb
2.821303e-16 3.274328e-15 6.566568e-15 -5.699968e-15 -7.540865e-15
-5.183123e-16
E:\\R-3.1.2\\scripts\\mPDBS\\summary_pdb_93\\1f305f598641eeb2.final.pdb
9.581533e-16 2.726731e-15 9.642772e-16 1.686763e-16 -1.686479e-15
2.789668e-15
E:\\R-3.1.2\\scripts\\mPDBS\\summary_pdb_93\\1f8a00b551be0a39.final.pdb
2.821303e-16 3.274328e-15 6.566568e-15 -5.699968e-15 -7.540865e-15
-5.183123e-16
E:\\R-3.1.2\\scripts\\mPDBS\\summary_pdb_93\\219f45328d47579a.final.pdb
-5.790501e-15 5.638349e-15 -1.453293e-15 -5.265550e-15 3.519842e-15
7.423164e-15
E:\\R-3.1.2\\scripts\\mPDBS\\summary_pdb_93\\238dd3918e54336d.final.pdb
1.127484e-14 5.441458e-15 1.488147e-15 -7.318595e-15 -6.884596e-15
-3.097995e-16
E:\\R-3.1.2\\scripts\\mPDBS\\summary_pdb_93\\2b3027889e2fdc42.final.pdb
1.016554e-15 2.411763e-15 8.019556e-16 5.311206e-17 -2.700009e-15
2.498284e-15
E:\\R-3.1.2\\scripts\\mPDBS\\summary_pdb_93\\2fcb3b46e33793b0.final.pdb
6.724374e-15 1.380586e-15 -8.582667e-16 2.403000e-15 4.298832e-16
4.364797e-15
E:\\R-3.1.2\\scripts\\mPDBS\\summary_pdb_93\\34eb328251842006.final.pdb
4.867578e-15 -5.550509e-16 -1.637278e-15 -2.799082e-16 3.626738e-15
1.315847e-14
E:\\R-3.1.2\\scripts\\mPDBS\\summary_pdb_93\\354b32871797b7ab.final.pdb
5.167938e-15 7.264385e-15 -3.908743e-15 5.123635e-15 4.757459e-15
1.797732e-14

E:\\R-3.1.2\\scripts\\mPDBS\\summary_pdb_93\\397368adbc32d5de.final.pdb
-4.372115e-15 8.347718e-15 4.140040e-15 -9.271175e-16 -9.892676e-15
-2.295374e-15
E:\\R-3.1.2\\scripts\\mPDBS\\summary_pdb_93\\411ca13cd3df6edd.final.pdb
3.925831e-15 3.768866e-15 1.120728e-15 1.145434e-15 -2.189983e-15
3.448863e-15
E:\\R-3.1.2\\scripts\\mPDBS\\summary_pdb_93\\42357eee6642bf12.final.pdb
4.333517e-15 -5.489684e-15 -1.786512e-15 4.018165e-16 -9.780826e-16
1.645308e-14
E:\\R-3.1.2\\scripts\\mPDBS\\summary_pdb_93\\44342d1f75a7fcfc.final.pdb
-8.807884e-15 4.043013e-15 -9.589096e-15 9.006721e-15 -1.519843e-14
-2.193936e-15
E:\\R-3.1.2\\scripts\\mPDBS\\summary_pdb_93\\448ef21dffe8cdfb.final.pdb
-3.274572e-15 8.254741e-16 1.430929e-14 -1.493189e-15 -3.067319e-15
9.834380e-15
E:\\R-3.1.2\\scripts\\mPDBS\\summary_pdb_93\\472a40b3e8f113c7.final.pdb
7.827762e-15 1.807376e-15 -1.364346e-15 -4.291434e-16 -9.176509e-16
7.195755e-16
E:\\R-3.1.2\\scripts\\mPDBS\\summary_pdb_93\\48a26225951ea153.final.pdb
4.040177e-15 1.038629e-14 1.487822e-15 4.362165e-15 -4.191872e-15
-2.993912e-16
E:\\R-3.1.2\\scripts\\mPDBS\\summary_pdb_93\\4ab4a5c7c6682ce6.final.pdb
-1.520229e-15 -5.831912e-15 3.748209e-15 -8.507896e-16 -2.412549e-15
1.682128e-14
E:\\R-3.1.2\\scripts\\mPDBS\\summary_pdb_93\\4eaa45b62cb6a25f.final.pdb
-2.769289e-15 3.150489e-15 4.284899e-15 -6.417184e-16 -4.988651e-15
-4.754062e-15
E:\\R-3.1.2\\scripts\\mPDBS\\summary_pdb_93\\58c371611d85ac96.final.pdb
-4.372115e-15 8.347718e-15 4.140040e-15 -9.271175e-16 -9.892676e-15
-2.295374e-15
E:\\R-3.1.2\\scripts\\mPDBS\\summary_pdb_93\\59a647f04822d30a.final.pdb
-1.329491e-15 6.848061e-15 -1.756277e-15 3.438259e-15 4.998957e-15
7.562149e-15
E:\\R-3.1.2\\scripts\\mPDBS\\summary_pdb_93\\59b091fe385e05fc.final.pdb
8.604284e-16 -7.888016e-16 -6.959781e-16 1.964777e-15 -2.437199e-15
4.573013e-15
E:\\R-3.1.2\\scripts\\mPDBS\\summary_pdb_93\\5c35ac30f930d2db.final.pdb
-2.769289e-15 3.150489e-15 4.284899e-15 -6.417184e-16 -4.988651e-15
-4.754062e-15
E:\\R-3.1.2\\scripts\\mPDBS\\summary_pdb_93\\5f60752431cf2dcb.final.pdb
-1.790984e-15 7.020631e-15 6.435149e-15 -1.137910e-15 -8.537980e-15
-7.209635e-15
E:\\R-3.1.2\\scripts\\mPDBS\\summary_pdb_93\\626456dd6ff2868d.final.pdb
7.827762e-15 1.807376e-15 -1.364346e-15 -4.291434e-16 -9.176509e-16
7.195755e-16
E:\\R-3.1.2\\scripts\\mPDBS\\summary_pdb_93\\6583d53ff3c8f7ef.final.pdb
-1.329491e-15 6.848061e-15 -1.756277e-15 3.438259e-15 4.998957e-15
7.562149e-15
E:\\R-3.1.2\\scripts\\mPDBS\\summary_pdb_93\\69760178986bef01.final.pdb
-2.972429e-15 1.585881e-17 -3.073181e-15 -7.064065e-16 -6.856753e-15
-5.180274e-15
E:\\R-3.1.2\\scripts\\mPDBS\\summary_pdb_93\\6a9d87d47926b1c8.final.pdb
1.349003e-15 2.220674e-15 9.650624e-16 3.808640e-16 -3.196644e-15
2.405727e-15

E:\\R-3.1.2\\scripts\\mPDBS\\summary_pdb_93\\6bb3547ea982ffc4.final.pdb
8.698482e-15 4.819398e-15 4.269116e-15 -7.807039e-15 -4.675018e-15
-4.743732e-15
E:\\R-3.1.2\\scripts\\mPDBS\\summary_pdb_93\\716d9624611d1c42.final.pdb
1.305498e-15 2.619638e-15 1.651107e-15 -6.730037e-16 3.579931e-15
6.401439e-15
E:\\R-3.1.2\\scripts\\mPDBS\\summary_pdb_93\\75f27e70ad0f1be4.final.pdb
-1.329491e-15 6.848061e-15 -1.756277e-15 3.438259e-15 4.998957e-15
7.562149e-15
E:\\R-3.1.2\\scripts\\mPDBS\\summary_pdb_93\\769f910fffb22872.final.pdb
-4.143612e-16 1.675780e-15 1.612523e-15 6.812119e-16 -1.674464e-15
2.612864e-15
E:\\R-3.1.2\\scripts\\mPDBS\\summary_pdb_93\\791f8c80dd5c3135.final.pdb
8.604284e-16 -7.888016e-16 -6.959781e-16 1.964777e-15 -2.437199e-15
4.573013e-15
E:\\R-3.1.2\\scripts\\mPDBS\\summary_pdb_93\\7fad98e66a3f6099.final.pdb
-5.743918e-15 8.759647e-16 -3.799944e-15 5.528563e-15 -4.937691e-15
7.229170e-15
E:\\R-3.1.2\\scripts\\mPDBS\\summary_pdb_93\\804b89c94a633196.final.pdb
6.724374e-15 1.380586e-15 -8.582667e-16 2.403000e-15 4.298832e-16
4.364797e-15
E:\\R-3.1.2\\scripts\\mPDBS\\summary_pdb_93\\813cc65a2298feba.final.pdb
6.724374e-15 1.380586e-15 -8.582667e-16 2.403000e-15 4.298832e-16
4.364797e-15
E:\\R-3.1.2\\scripts\\mPDBS\\summary_pdb_93\\83c0eefcfe12d3f9.final.pdb
3.925831e-15 3.768866e-15 1.120728e-15 1.145434e-15 -2.189983e-15
3.448863e-15
E:\\R-3.1.2\\scripts\\mPDBS\\summary_pdb_93\\86d50469ef569760.final.pdb
7.827762e-15 1.807376e-15 -1.364346e-15 -4.291434e-16 -9.176509e-16
7.195755e-16
E:\\R-3.1.2\\scripts\\mPDBS\\summary_pdb_93\\89d0666e1402203d.final.pdb
-1.329491e-15 6.848061e-15 -1.756277e-15 3.438259e-15 4.998957e-15
7.562149e-15
E:\\R-3.1.2\\scripts\\mPDBS\\summary_pdb_93\\8b34852716dadc62.final.pdb
-1.872915e-15 7.352685e-16 -4.493163e-15 3.525570e-16 -4.378770e-15
3.917238e-15
E:\\R-3.1.2\\scripts\\mPDBS\\summary_pdb_93\\8b6ef9ac3fe74395.final.pdb
2.317152e-15 5.985075e-15 6.983461e-16 -3.418240e-15 -2.946070e-15
1.089054e-15
E:\\R-3.1.2\\scripts\\mPDBS\\summary_pdb_93\\98bbe0d5986c138f.final.pdb
8.604284e-16 -7.888016e-16 -6.959781e-16 1.964777e-15 -2.437199e-15
4.573013e-15
E:\\R-3.1.2\\scripts\\mPDBS\\summary_pdb_93\\9c22f4909d67e2fa.final.pdb
-6.651142e-16 3.719427e-15 -8.479682e-17 3.033681e-15 -2.052506e-15
-4.073766e-15
E:\\R-3.1.2\\scripts\\mPDBS\\summary_pdb_93\\9dda8ed0d81fb3ef.final.pdb
1.710179e-15 7.877186e-15 1.303450e-15 7.287502e-15 -4.506894e-15
-9.246454e-15
E:\\R-3.1.2\\scripts\\mPDBS\\summary_pdb_93\\9f752b3616e6033b.final.pdb
2.907173e-15 1.885376e-15 -3.172400e-15 1.282001e-15 -1.410897e-15
4.618853e-15
E:\\R-3.1.2\\scripts\\mPDBS\\summary_pdb_93\\a02c70083816d6ba.final.pdb
-2.769289e-15 3.150489e-15 4.284899e-15 -6.417184e-16 -4.988651e-15
-4.754062e-15

E:\\R-3.1.2\\scripts\\mPDBS\\summary_pdb_93\\a0c997d891e4e082.final.pdb
3.393317e-15 1.007139e-15 2.787715e-15 5.823664e-15 -1.027542e-15
-1.576737e-15
E:\\R-3.1.2\\scripts\\mPDBS\\summary_pdb_93\\ab8039a2d823c616.final.pdb
2.497332e-15 3.270086e-15 1.254436e-15 1.152921e-15 -3.330821e-15
2.959999e-15
E:\\R-3.1.2\\scripts\\mPDBS\\summary_pdb_93\\acf4d74b9c71d4dc.final.pdb
6.724374e-15 1.380586e-15 -8.582667e-16 2.403000e-15 4.298832e-16
4.364797e-15
E:\\R-3.1.2\\scripts\\mPDBS\\summary_pdb_93\\ad8a823e4b4c668e.final.pdb
1.144606e-15 2.265886e-15 9.044810e-16 3.527203e-16 -1.151054e-15
2.888691e-15
E:\\R-3.1.2\\scripts\\mPDBS\\summary_pdb_93\\b16ababd03b7b857.final.pdb
-6.651142e-16 3.719427e-15 -8.479682e-17 3.033681e-15 -2.052506e-15
-4.073766e-15
E:\\R-3.1.2\\scripts\\mPDBS\\summary_pdb_93\\b226923e697d3c63.final.pdb
-1.687711e-15 3.768059e-15 5.949906e-15 -4.001103e-15 -4.279645e-15
2.015371e-15
E:\\R-3.1.2\\scripts\\mPDBS\\summary_pdb_93\\b28e48f6fa27a165.final.pdb
8.604284e-16 -7.888016e-16 -6.959781e-16 1.964777e-15 -2.437199e-15
4.573013e-15
E:\\R-3.1.2\\scripts\\mPDBS\\summary_pdb_93\\b42c4924e3cd1aec.final.pdb
-1.542156e-16 3.568566e-15 -1.972142e-15 -5.402759e-15 -2.826814e-15
7.304976e-15
E:\\R-3.1.2\\scripts\\mPDBS\\summary_pdb_93\\b4dd239fcabff1df.final.pdb
8.604284e-16 -7.888016e-16 -6.959781e-16 1.964777e-15 -2.437199e-15
4.573013e-15
E:\\R-3.1.2\\scripts\\mPDBS\\summary_pdb_93\\b669942ef97459a2.final.pdb
-1.872915e-15 7.352685e-16 -4.493163e-15 3.525570e-16 -4.378770e-15
3.917238e-15
E:\\R-3.1.2\\scripts\\mPDBS\\summary_pdb_93\\b99f4d9d87c1017b.final.pdb
8.604284e-16 -7.888016e-16 -6.959781e-16 1.964777e-15 -2.437199e-15
4.573013e-15
E:\\R-3.1.2\\scripts\\mPDBS\\summary_pdb_93\\b9d2cbf3975403e7.final.pdb
8.604284e-16 -7.888016e-16 -6.959781e-16 1.964777e-15 -2.437199e-15
4.573013e-15
E:\\R-3.1.2\\scripts\\mPDBS\\summary_pdb_93\\bc88c8a505f3a288.final.pdb
-4.986773e-17 2.198826e-15 -3.634949e-17 3.558213e-16 -3.032209e-15
2.463590e-15
E:\\R-3.1.2\\scripts\\mPDBS\\summary_pdb_93\\bdac0e64f4ef427e.final.pdb
-9.076788e-15 -5.063598e-16 1.903955e-15 -1.381191e-15 -1.605815e-15
2.245832e-15
E:\\R-3.1.2\\scripts\\mPDBS\\summary_pdb_93\\ca2a1f55fc9d0681.final.pdb
-1.872915e-15 7.352685e-16 -4.493163e-15 3.525570e-16 -4.378770e-15
3.917238e-15
E:\\R-3.1.2\\scripts\\mPDBS\\summary_pdb_93\\ce34248de3e81a3c.final.pdb
-2.091094e-15 5.376911e-16 9.239061e-15 2.233895e-15 -2.537891e-15
2.454889e-15
E:\\R-3.1.2\\scripts\\mPDBS\\summary_pdb_93\\d02e697772494a61.final.pdb
2.593467e-16 -4.101256e-15 2.883299e-15 -4.820501e-16 5.504288e-16
9.840068e-15
E:\\R-3.1.2\\scripts\\mPDBS\\summary_pdb_93\\d04221245d015047.final.pdb
1.250976e-16 2.436552e-15 -9.315325e-16 2.028089e-15 -1.277342e-15
2.893646e-15

E:\\R-3.1.2\\scripts\\mPDBS\\summary_pdb_93\\d678141e4018054a.final.pdb
5.730626e-16 6.443873e-15 1.064094e-14 -7.782105e-15 8.182784e-16
2.784043e-15
E:\\R-3.1.2\\scripts\\mPDBS\\summary_pdb_93\\d71b40af0f0efe39.final.pdb
-6.299316e-15 7.392940e-15 -2.309242e-15 8.147890e-16 -3.391009e-15
2.426403e-15
E:\\R-3.1.2\\scripts\\mPDBS\\summary_pdb_93\\dc0809eff10197e2.final.pdb
-4.565281e-15 1.383677e-15 -7.143244e-15 3.820647e-15 -2.878992e-15
2.125934e-15
E:\\R-3.1.2\\scripts\\mPDBS\\summary_pdb_93\\e1163918d3707839.final.pdb
1.130710e-14 5.922050e-17 9.366336e-15 -5.433984e-15 1.941940e-15
8.364025e-15
E:\\R-3.1.2\\scripts\\mPDBS\\summary_pdb_93\\e4b48b06824ef848.final.pdb
2.821303e-16 3.274328e-15 6.566568e-15 -5.699968e-15 -7.540865e-15
-5.183123e-16
E:\\R-3.1.2\\scripts\\mPDBS\\summary_pdb_93\\e529e19ad281fea2.final.pdb
-1.595256e-15 1.420533e-15 4.482756e-15 6.463851e-16 -8.274452e-16
8.635576e-16
E:\\R-3.1.2\\scripts\\mPDBS\\summary_pdb_93\\e5602082718ec3a1.final.pdb
6.724374e-15 1.380586e-15 -8.582667e-16 2.403000e-15 4.298832e-16
4.364797e-15
E:\\R-3.1.2\\scripts\\mPDBS\\summary_pdb_93\\e5d589d996d0a960.final.pdb
4.721081e-15 -4.847278e-16 8.432284e-15 4.822055e-16 -3.195942e-15
-1.337646e-14
E:\\R-3.1.2\\scripts\\mPDBS\\summary_pdb_93\\e9df9e72307a2fbd.final.pdb
-2.769289e-15 3.150489e-15 4.284899e-15 -6.417184e-16 -4.988651e-15
-4.754062e-15
E:\\R-3.1.2\\scripts\\mPDBS\\summary_pdb_93\\ea00f8694090cae7.final.pdb
6.724374e-15 1.380586e-15 -8.582667e-16 2.403000e-15 4.298832e-16
4.364797e-15
E:\\R-3.1.2\\scripts\\mPDBS\\summary_pdb_93\\eba517be32db050e.final.pdb
8.604284e-16 -7.888016e-16 -6.959781e-16 1.964777e-15 -2.437199e-15
4.573013e-15
E:\\R-3.1.2\\scripts\\mPDBS\\summary_pdb_93\\ec04b9545899c29d.final.pdb
7.643205e-16 2.911973e-15 1.363731e-15 6.175985e-16 -1.773404e-15
2.321116e-15
E:\\R-3.1.2\\scripts\\mPDBS\\summary_pdb_93\\ee6862cbcad52078.final.pdb
1.495337e-15 2.456866e-15 5.186536e-16 4.174040e-16 -2.893431e-15
2.480937e-15
E:\\R-3.1.2\\scripts\\mPDBS\\summary_pdb_93\\ef6a42fc83b81e5d.final.pdb
-5.331898e-16 2.047878e-15 9.628693e-16 1.321719e-16 -1.340096e-15
2.592914e-15
E:\\R-3.1.2\\scripts\\mPDBS\\summary_pdb_93\\ef8e88d4ad93a0ad.final.pdb
1.250976e-16 2.436552e-15 -9.315325e-16 2.028089e-15 -1.277342e-15
2.893646e-15
E:\\R-3.1.2\\scripts\\mPDBS\\summary_pdb_93\\f14fcae8deec4fa3.final.pdb
2.907173e-15 1.885376e-15 -3.172400e-15 1.282001e-15 -1.410897e-15
4.618853e-15
E:\\R-3.1.2\\scripts\\mPDBS\\summary_pdb_93\\f83584e22003fb92.final.pdb
4.040177e-15 1.038629e-14 1.487822e-15 4.362165e-15 -4.191872e-15
-2.993912e-16
E:\\R-3.1.2\\scripts\\mPDBS\\summary_pdb_93\\f8e2bf87e52caa29.final.pdb
-1.595256e-15 1.420533e-15 4.482756e-15 6.463851e-16 -8.274452e-16
8.635576e-16

```
[,205]        [,206]        [,207]        [,208]        [,209]        [,210]
E:\\R-3.1.2\\scripts\\40.1ns_data\\NAT0.pdb
-3.082224e-15 -1.700446e-15  1.350312e-15  2.491929e-15 -4.050201e-15
-1.208971e-15
E:\\R-3.1.2\\scripts\\40.1ns_data\\LJP6.pdb
-2.993554e-15 -4.962661e-16 -4.724211e-16  3.914347e-15 -7.607428e-16
-2.682001e-15
E:\\R-3.1.2\\scripts\\40.1ns_data\\3WY9.pdb
-4.388972e-15 -1.886893e-15  2.183733e-15  2.745943e-15 -3.387800e-15
-2.817730e-15
E:\\R-3.1.2\\scripts\\40.1ns_data\\ZSP9.pdb
-4.571149e-15 -2.091296e-15 -2.331323e-15  3.546617e-15  1.133203e-16
-1.407602e-15
E:\\R-3.1.2\\scripts\\mPDBS\\summary_pdb_93\\013d855ad26c877c.final.pdb
-3.864948e-15 -8.310774e-16  2.320694e-15  9.384456e-15 -4.341363e-15
1.703990e-15
E:\\R-3.1.2\\scripts\\mPDBS\\summary_pdb_93\\0a2a705342ca0d54.final.pdb
-3.877562e-15 -2.419269e-15  7.025253e-16  3.806045e-15 -2.257942e-15
-3.336047e-15
E:\\R-3.1.2\\scripts\\mPDBS\\summary_pdb_93\\0a59fceadb4194d9.final.pdb
-8.783506e-16  2.385302e-15  2.315716e-15 -9.591589e-17 -4.244270e-15
-3.334424e-15
E:\\R-3.1.2\\scripts\\mPDBS\\summary_pdb_93\\0dba59f3306825e8.final.pdb
-3.684537e-15 -4.668286e-15  2.483758e-15 -3.803025e-16 -1.642133e-15
-5.611339e-15
E:\\R-3.1.2\\scripts\\mPDBS\\summary_pdb_93\\126f26a78b57339c.final.pdb
-8.222574e-15 -1.014481e-14 -2.766274e-15  8.357066e-15 -4.575417e-17
-6.681663e-15
E:\\R-3.1.2\\scripts\\mPDBS\\summary_pdb_93\\12f06f3e342df706.final.pdb
-2.726139e-15 -2.846011e-15  1.600028e-15  3.247464e-15 -3.383669e-15
-3.159973e-15
E:\\R-3.1.2\\scripts\\mPDBS\\summary_pdb_93\\18618a2a251b867b.final.pdb
-7.666454e-15 -7.585078e-15 -5.821828e-15  6.823339e-15  5.265107e-15
-1.284014e-16
E:\\R-3.1.2\\scripts\\mPDBS\\summary_pdb_93\\1f305f598641eeb2.final.pdb
-3.208110e-15 -2.401736e-15  1.121782e-15  3.146925e-15 -2.799416e-15
-3.476218e-15
E:\\R-3.1.2\\scripts\\mPDBS\\summary_pdb_93\\1f8a00b551be0a39.final.pdb
-7.666454e-15 -7.585078e-15 -5.821828e-15  6.823339e-15  5.265107e-15
-1.284014e-16
E:\\R-3.1.2\\scripts\\mPDBS\\summary_pdb_93\\219f45328d47579a.final.pdb
-9.512299e-17 -6.747505e-16  7.470447e-15  8.107591e-15 -5.509750e-15
-1.315339e-14
E:\\R-3.1.2\\scripts\\mPDBS\\summary_pdb_93\\238dd3918e54336d.final.pdb
-1.391653e-14 -1.588220e-14  1.853900e-15  1.074480e-14  1.037313e-15
-2.033992e-14
E:\\R-3.1.2\\scripts\\mPDBS\\summary_pdb_93\\2b3027889e2fdc42.final.pdb
-3.162114e-15 -2.805858e-15  2.046942e-15  4.144616e-15 -3.368776e-15
-4.309338e-15
E:\\R-3.1.2\\scripts\\mPDBS\\summary_pdb_93\\2fcb3b46e33793b0.final.pdb
-2.798972e-16  1.713028e-15  4.504436e-16  7.126010e-17 -2.657168e-15
-1.458735e-15
E:\\R-3.1.2\\scripts\\mPDBS\\summary_pdb_93\\34eb328251842006.final.pdb
```

-1.474841e-15 -2.641464e-15 6.747055e-15 1.591552e-15 -6.159577e-15
4.218663e-16
E:\\R-3.1.2\\scripts\\mPDBS\\summary_pdb_93\\354b32871797b7ab.final.pdb
5.435465e-15 3.147326e-15 -6.883065e-15 -2.193741e-15 -3.967261e-15
4.954722e-15
E:\\R-3.1.2\\scripts\\mPDBS\\summary_pdb_93\\397368adbc32d5de.final.pdb
-8.222574e-15 -1.014481e-14 -2.766274e-15 8.357066e-15 -4.575417e-17
-6.681663e-15
E:\\R-3.1.2\\scripts\\mPDBS\\summary_pdb_93\\411ca13cd3df6edd.final.pdb
-4.207311e-15 -5.550259e-15 -3.372128e-15 2.260842e-16 2.286360e-15
-7.110688e-16
E:\\R-3.1.2\\scripts\\mPDBS\\summary_pdb_93\\42357eee6642bf12.final.pdb
6.171063e-15 4.032819e-15 9.634307e-16 2.634253e-15 -6.225272e-15
-1.164432e-14
E:\\R-3.1.2\\scripts\\mPDBS\\summary_pdb_93\\44342d1f75a7fcfc.final.pdb
-4.463814e-15 -3.373447e-15 4.852386e-15 1.055612e-14 -6.097150e-16
1.043070e-15
E:\\R-3.1.2\\scripts\\mPDBS\\summary_pdb_93\\448ef21dffe8cdfb.final.pdb
-7.049044e-16 -2.368007e-16 -2.250521e-15 4.579806e-15 -1.099387e-15
1.126026e-14
E:\\R-3.1.2\\scripts\\mPDBS\\summary_pdb_93\\472a40b3e8f113c7.final.pdb
-3.684537e-15 -4.668286e-15 2.483758e-15 -3.803025e-16 -1.642133e-15
-5.611339e-15
E:\\R-3.1.2\\scripts\\mPDBS\\summary_pdb_93\\48a26225951ea153.final.pdb
-5.443275e-15 -1.047507e-14 -6.397692e-15 9.519656e-15 -3.717456e-15
3.075813e-15
E:\\R-3.1.2\\scripts\\mPDBS\\summary_pdb_93\\4ab4a5c7c6682ce6.final.pdb
5.735011e-15 1.058167e-14 -8.758009e-15 5.359464e-15 -6.897912e-15
-2.466285e-15
E:\\R-3.1.2\\scripts\\mPDBS\\summary_pdb_93\\4eaa45b62cb6a25f.final.pdb
-7.279190e-15 -5.057048e-15 -1.926581e-15 7.521145e-15 -2.905827e-15
-4.874655e-15
E:\\R-3.1.2\\scripts\\mPDBS\\summary_pdb_93\\58c371611d85ac96.final.pdb
-8.222574e-15 -1.014481e-14 -2.766274e-15 8.357066e-15 -4.575417e-17
-6.681663e-15
E:\\R-3.1.2\\scripts\\mPDBS\\summary_pdb_93\\59a647f04822d30a.final.pdb
-2.265920e-15 -2.613530e-16 2.198259e-15 7.569794e-15 -7.703867e-15
-4.777795e-15
E:\\R-3.1.2\\scripts\\mPDBS\\summary_pdb_93\\59b091fe385e05fc.final.pdb
-8.783506e-16 2.385302e-15 2.315716e-15 -9.591589e-17 -4.244270e-15
-3.334424e-15
E:\\R-3.1.2\\scripts\\mPDBS\\summary_pdb_93\\5c35ac30f930d2db.final.pdb
-7.279190e-15 -5.057048e-15 -1.926581e-15 7.521145e-15 -2.905827e-15
-4.874655e-15
E:\\R-3.1.2\\scripts\\mPDBS\\summary_pdb_93\\5f60752431cf2dcb.final.pdb
-4.549767e-15 -5.880910e-15 -3.615093e-15 3.027154e-15 1.241321e-15
6.839407e-15
E:\\R-3.1.2\\scripts\\mPDBS\\summary_pdb_93\\626456dd6ff2868d.final.pdb
-3.684537e-15 -4.668286e-15 2.483758e-15 -3.803025e-16 -1.642133e-15
-5.611339e-15
E:\\R-3.1.2\\scripts\\mPDBS\\summary_pdb_93\\6583d53ff3c8f7ef.final.pdb
-2.265920e-15 -2.613530e-16 2.198259e-15 7.569794e-15 -7.703867e-15
-4.777795e-15
E:\\R-3.1.2\\scripts\\mPDBS\\summary_pdb_93\\69760178986bef01.final.pdb

-1.144137e-14 -5.308701e-15 9.530757e-15 -8.842541e-16 5.131009e-15
-4.063266e-15
E:\\R-3.1.2\\scripts\\mPDBS\\summary_pdb_93\\6a9d87d47926b1c8.final.pdb
-3.212692e-15 -2.366308e-15 1.648597e-15 4.256180e-15 -3.032948e-15
-4.388359e-15
E:\\R-3.1.2\\scripts\\mPDBS\\summary_pdb_93\\6bb3547ea982ffc4.final.pdb
-7.316239e-15 -2.207810e-14 7.815505e-15 1.672814e-14 -9.974742e-16
-5.262634e-15
E:\\R-3.1.2\\scripts\\mPDBS\\summary_pdb_93\\716d9624611d1c42.final.pdb
-8.663664e-15 -5.739530e-15 4.264780e-15 6.794973e-15 -3.634445e-15
-7.078279e-15
E:\\R-3.1.2\\scripts\\mPDBS\\summary_pdb_93\\75f27e70ad0f1be4.final.pdb
-2.265920e-15 -2.613530e-16 2.198259e-15 7.569794e-15 -7.703867e-15
-4.777795e-15
E:\\R-3.1.2\\scripts\\mPDBS\\summary_pdb_93\\769f910fffb22872.final.pdb
-2.240239e-15 -3.055715e-15 2.026440e-15 2.898093e-15 -2.584625e-15
-5.182518e-15
E:\\R-3.1.2\\scripts\\mPDBS\\summary_pdb_93\\791f8c80dd5c3135.final.pdb
-8.783506e-16 2.385302e-15 2.315716e-15 -9.591589e-17 -4.244270e-15
-3.334424e-15
E:\\R-3.1.2\\scripts\\mPDBS\\summary_pdb_93\\7fad98e66a3f6099.final.pdb
5.771585e-15 -2.379046e-15 4.242557e-15 -3.625188e-16 -1.086421e-14
-1.744847e-15
E:\\R-3.1.2\\scripts\\mPDBS\\summary_pdb_93\\804b89c94a633196.final.pdb
-2.798972e-16 1.713028e-15 4.504436e-16 7.126010e-17 -2.657168e-15
-1.458735e-15
E:\\R-3.1.2\\scripts\\mPDBS\\summary_pdb_93\\813cc65a2298feba.final.pdb
-2.798972e-16 1.713028e-15 4.504436e-16 7.126010e-17 -2.657168e-15
-1.458735e-15
E:\\R-3.1.2\\scripts\\mPDBS\\summary_pdb_93\\83c0eefcfe12d3f9.final.pdb
-4.207311e-15 -5.550259e-15 -3.372128e-15 2.260842e-16 2.286360e-15
-7.110688e-16
E:\\R-3.1.2\\scripts\\mPDBS\\summary_pdb_93\\86d50469ef569760.final.pdb
-3.684537e-15 -4.668286e-15 2.483758e-15 -3.803025e-16 -1.642133e-15
-5.611339e-15
E:\\R-3.1.2\\scripts\\mPDBS\\summary_pdb_93\\89d0666e1402203d.final.pdb
-2.265920e-15 -2.613530e-16 2.198259e-15 7.569794e-15 -7.703867e-15
-4.777795e-15
E:\\R-3.1.2\\scripts\\mPDBS\\summary_pdb_93\\8b34852716dadc62.final.pdb
-2.576671e-15 -1.263757e-15 6.534062e-16 4.083675e-15 -3.552286e-15
-1.252107e-14
E:\\R-3.1.2\\scripts\\mPDBS\\summary_pdb_93\\8b6ef9ac3fe74395.final.pdb
-1.932574e-15 -7.918721e-15 1.043480e-14 5.284635e-15 -1.074259e-14
-1.129080e-14
E:\\R-3.1.2\\scripts\\mPDBS\\summary_pdb_93\\98bbe0d5986c138f.final.pdb
-8.783506e-16 2.385302e-15 2.315716e-15 -9.591589e-17 -4.244270e-15
-3.334424e-15
E:\\R-3.1.2\\scripts\\mPDBS\\summary_pdb_93\\9c22f4909d67e2fa.final.pdb
-6.161477e-15 -3.336752e-15 6.072249e-15 4.550750e-15 -2.329522e-15
-3.412901e-15
E:\\R-3.1.2\\scripts\\mPDBS\\summary_pdb_93\\9dda8ed0d81fb3ef.final.pdb
-1.067239e-14 -9.562940e-15 -3.639790e-15 4.465506e-15 7.932389e-15
3.831638e-15
E:\\R-3.1.2\\scripts\\mPDBS\\summary_pdb_93\\9f752b3616e6033b.final.pdb

-2.907092e-16 -1.401111e-15 5.467196e-15 9.660744e-16 -2.257922e-15
-5.181570e-15
E:\\R-3.1.2\\scripts\\mPDBS\\summary_pdb_93\\a02c70083816d6ba.final.pdb
-7.279190e-15 -5.057048e-15 -1.926581e-15 7.521145e-15 -2.905827e-15
-4.874655e-15
E:\\R-3.1.2\\scripts\\mPDBS\\summary_pdb_93\\a0c997d891e4e082.final.pdb
-5.508047e-15 4.955216e-15 4.144426e-15 -1.696349e-15 2.001994e-15
9.662181e-16
E:\\R-3.1.2\\scripts\\mPDBS\\summary_pdb_93\\ab8039a2d823c616.final.pdb
-2.807082e-15 -2.633332e-15 9.304714e-16 2.153436e-15 -1.714434e-15
-4.572754e-15
E:\\R-3.1.2\\scripts\\mPDBS\\summary_pdb_93\\acf4d74b9c71d4dc.final.pdb
-2.798972e-16 1.713028e-15 4.504436e-16 7.126010e-17 -2.657168e-15
-1.458735e-15
E:\\R-3.1.2\\scripts\\mPDBS\\summary_pdb_93\\ad8a823e4b4c668e.final.pdb
-3.535219e-15 -2.852267e-15 1.610490e-15 3.985931e-15 -1.997177e-15
-3.296901e-15
E:\\R-3.1.2\\scripts\\mPDBS\\summary_pdb_93\\b16ababd03b7b857.final.pdb
-6.161477e-15 -3.336752e-15 6.072249e-15 4.550750e-15 -2.329522e-15
-3.412901e-15
E:\\R-3.1.2\\scripts\\mPDBS\\summary_pdb_93\\b226923e697d3c63.final.pdb
-2.895624e-15 2.633900e-15 5.787890e-15 1.525498e-16 1.031442e-15
-3.776686e-16
E:\\R-3.1.2\\scripts\\mPDBS\\summary_pdb_93\\b28e48f6fa27a165.final.pdb
-8.783506e-16 2.385302e-15 2.315716e-15 -9.591589e-17 -4.244270e-15
-3.334424e-15
E:\\R-3.1.2\\scripts\\mPDBS\\summary_pdb_93\\b42c4924e3cd1aec.final.pdb
-3.448134e-15 -2.664812e-15 3.026101e-15 -3.597055e-15 -6.724832e-15
-5.177648e-15
E:\\R-3.1.2\\scripts\\mPDBS\\summary_pdb_93\\b4dd239fcabff1df.final.pdb
-8.783506e-16 2.385302e-15 2.315716e-15 -9.591589e-17 -4.244270e-15
-3.334424e-15
E:\\R-3.1.2\\scripts\\mPDBS\\summary_pdb_93\\b669942ef97459a2.final.pdb
-2.576671e-15 -1.263757e-15 6.534062e-16 4.083675e-15 -3.552286e-15
-1.252107e-14
E:\\R-3.1.2\\scripts\\mPDBS\\summary_pdb_93\\b99f4d9d87c1017b.final.pdb
-8.783506e-16 2.385302e-15 2.315716e-15 -9.591589e-17 -4.244270e-15
-3.334424e-15
E:\\R-3.1.2\\scripts\\mPDBS\\summary_pdb_93\\b9d2cbf3975403e7.final.pdb
-8.783506e-16 2.385302e-15 2.315716e-15 -9.591589e-17 -4.244270e-15
-3.334424e-15
E:\\R-3.1.2\\scripts\\mPDBS\\summary_pdb_93\\bc88c8a505f3a288.final.pdb
-3.307831e-15 -2.484067e-15 5.466773e-16 3.851881e-15 -3.587785e-15
-4.001859e-15
E:\\R-3.1.2\\scripts\\mPDBS\\summary_pdb_93\\bdac0e64f4ef427e.final.pdb
-1.489255e-16 1.612414e-15 1.263547e-14 8.584416e-15 -9.709361e-15
-8.056755e-15
E:\\R-3.1.2\\scripts\\mPDBS\\summary_pdb_93\\ca2a1f55fc9d0681.final.pdb
-2.576671e-15 -1.263757e-15 6.534062e-16 4.083675e-15 -3.552286e-15
-1.252107e-14
E:\\R-3.1.2\\scripts\\mPDBS\\summary_pdb_93\\ce34248de3e81a3c.final.pdb
-6.347302e-15 2.603266e-15 -1.061602e-14 9.315626e-15 -9.985086e-15
-8.344113e-15
E:\\R-3.1.2\\scripts\\mPDBS\\summary_pdb_93\\d02e697772494a61.final.pdb

4.819349e-15 4.854681e-15 5.080485e-15 9.017020e-15 -8.659791e-15
-8.494359e-15
E:\\R-3.1.2\\scripts\\mPDBS\\summary_pdb_93\\d04221245d015047.final.pdb
-2.624936e-15 -4.759235e-15 -4.231008e-16 4.649269e-15 -3.948107e-15
-4.751430e-15
E:\\R-3.1.2\\scripts\\mPDBS\\summary_pdb_93\\d678141e4018054a.final.pdb
-6.258431e-15 -2.087719e-14 4.711227e-16 1.119309e-14 3.908785e-15
2.131747e-15
E:\\R-3.1.2\\scripts\\mPDBS\\summary_pdb_93\\d71b40af0f0efe39.final.pdb
7.439798e-15 2.763379e-15 -5.759886e-15 -4.431776e-15 2.208427e-15
9.273482e-15
E:\\R-3.1.2\\scripts\\mPDBS\\summary_pdb_93\\dc0809eff10197e2.final.pdb
-2.429652e-15 3.462529e-15 8.375913e-15 -7.199213e-15 -1.857408e-15
-3.541061e-15
E:\\R-3.1.2\\scripts\\mPDBS\\summary_pdb_93\\e1163918d3707839.final.pdb
1.373530e-15 -6.665085e-15 6.555396e-15 -1.099218e-14 -7.792554e-15
-1.285796e-15
E:\\R-3.1.2\\scripts\\mPDBS\\summary_pdb_93\\e4b48b06824ef848.final.pdb
-7.666454e-15 -7.585078e-15 -5.821828e-15 6.823339e-15 5.265107e-15
-1.284014e-16
E:\\R-3.1.2\\scripts\\mPDBS\\summary_pdb_93\\e529e19ad281fea2.final.pdb
-3.864948e-15 -8.310774e-16 2.320694e-15 9.384456e-15 -4.341363e-15
1.703990e-15
E:\\R-3.1.2\\scripts\\mPDBS\\summary_pdb_93\\e5602082718ec3a1.final.pdb
-2.798972e-16 1.713028e-15 4.504436e-16 7.126010e-17 -2.657168e-15
-1.458735e-15
E:\\R-3.1.2\\scripts\\mPDBS\\summary_pdb_93\\e5d589d996d0a960.final.pdb
-5.943419e-15 -7.349957e-15 3.532385e-15 3.850709e-15 1.481827e-15
-8.703708e-15
E:\\R-3.1.2\\scripts\\mPDBS\\summary_pdb_93\\e9df9e72307a2fbd.final.pdb
-7.279190e-15 -5.057048e-15 -1.926581e-15 7.521145e-15 -2.905827e-15
-4.874655e-15
E:\\R-3.1.2\\scripts\\mPDBS\\summary_pdb_93\\ea00f8694090cae7.final.pdb
-2.798972e-16 1.713028e-15 4.504436e-16 7.126010e-17 -2.657168e-15
-1.458735e-15
E:\\R-3.1.2\\scripts\\mPDBS\\summary_pdb_93\\eba517be32db050e.final.pdb
-8.783506e-16 2.385302e-15 2.315716e-15 -9.591589e-17 -4.244270e-15
-3.334424e-15
E:\\R-3.1.2\\scripts\\mPDBS\\summary_pdb_93\\ec04b9545899c29d.final.pdb
-3.605994e-15 -2.580678e-15 2.139278e-15 3.350946e-15 -2.994092e-15
-3.499303e-15
E:\\R-3.1.2\\scripts\\mPDBS\\summary_pdb_93\\ee6862cbcad52078.final.pdb
-3.428394e-15 -3.151936e-15 1.013806e-15 3.936449e-15 -3.119301e-15
-4.217398e-15
E:\\R-3.1.2\\scripts\\mPDBS\\summary_pdb_93\\ef6a42fc83b81e5d.final.pdb
-2.654838e-15 -2.789435e-15 9.122597e-16 3.423281e-15 -2.605659e-15
-3.368431e-15
E:\\R-3.1.2\\scripts\\mPDBS\\summary_pdb_93\\ef8e88d4ad93a0ad.final.pdb
-2.624936e-15 -4.759235e-15 -4.231008e-16 4.649269e-15 -3.948107e-15
-4.751430e-15
E:\\R-3.1.2\\scripts\\mPDBS\\summary_pdb_93\\f14fcae8deec4fa3.final.pdb
-2.907092e-16 -1.401111e-15 5.467196e-15 9.660744e-16 -2.257922e-15
-5.181570e-15
E:\\R-3.1.2\\scripts\\mPDBS\\summary_pdb_93\\f83584e22003fb92.final.pdb

-5.443275e-15 -1.047507e-14 -6.397692e-15 9.519656e-15 -3.717456e-15
3.075813e-15
E:\\R-3.1.2\\scripts\\mPDBS\\summary_pdb_93\\f8e2bf87e52caa29.final.pdb
-3.864948e-15 -8.310774e-16 2.320694e-15 9.384456e-15 -4.341363e-15
1.703990e-15

[,211]   [,212]   [,213]   [,214]   [,215]   [,216]
E:\\R-3.1.2\\scripts\\40.1ns_data\\NAT0.pdb
-7.055221e-16 6.020783e-15 4.628840e-16 -5.438607e-16 -1.599182e-15
-5.785822e-16
E:\\R-3.1.2\\scripts\\40.1ns_data\\LJP6.pdb
7.274193e-16 7.200297e-15 1.872892e-15 -1.112042e-15 -2.101026e-15
3.078226e-17
E:\\R-3.1.2\\scripts\\40.1ns_data\\3WY9.pdb
1.878978e-15 6.618537e-15 -5.244881e-16 1.481759e-15 -1.184666e-15
-6.956020e-16
E:\\R-3.1.2\\scripts\\40.1ns_data\\ZSP9.pdb
-8.161891e-16 6.163862e-15 1.540809e-16 -1.062240e-15 -1.378087e-15
-1.315528e-15
E:\\R-3.1.2\\scripts\\mPDBS\\summary_pdb_93\\013d855ad26c877c.final.pdb
-5.471620e-15 8.015015e-15 1.085506e-14 -4.137125e-16 -1.041469e-15
4.736208e-16
E:\\R-3.1.2\\scripts\\mPDBS\\summary_pdb_93\\0a2a705342ca0d54.final.pdb
-4.028647e-16 6.700037e-15 1.691567e-15 -6.802210e-16 -1.477748e-15
-1.618779e-15
E:\\R-3.1.2\\scripts\\mPDBS\\summary_pdb_93\\0a59fceadb4194d9.final.pdb
-1.407701e-15 6.545667e-15 -5.593764e-16 -3.451367e-15 -1.022104e-15
-4.084307e-15
E:\\R-3.1.2\\scripts\\mPDBS\\summary_pdb_93\\0dba59f3306825e8.final.pdb
4.695595e-15 6.092942e-15 -7.291020e-15 -3.647237e-15 -1.353502e-15
4.996416e-16
E:\\R-3.1.2\\scripts\\mPDBS\\summary_pdb_93\\126f26a78b57339c.final.pdb
9.863221e-16 5.975848e-15 2.842368e-15 4.619588e-15 -3.442109e-15
-5.579372e-15
E:\\R-3.1.2\\scripts\\mPDBS\\summary_pdb_93\\12f06f3e342df706.final.pdb
-1.052512e-16 6.819408e-15 1.794783e-15 -2.365655e-16 -1.838788e-15
-2.036956e-15
E:\\R-3.1.2\\scripts\\mPDBS\\summary_pdb_93\\18618a2a251b867b.final.pdb
-4.903112e-16 5.654377e-15 3.713126e-15 2.407398e-15 -4.368992e-15
-6.803263e-16
E:\\R-3.1.2\\scripts\\mPDBS\\summary_pdb_93\\1f305f598641eeb2.final.pdb
9.082160e-16 7.060643e-15 5.305189e-16 -4.769764e-16 -1.819135e-15
-1.808679e-15
E:\\R-3.1.2\\scripts\\mPDBS\\summary_pdb_93\\1f8a00b551be0a39.final.pdb
-4.903112e-16 5.654377e-15 3.713126e-15 2.407398e-15 -4.368992e-15
-6.803263e-16
E:\\R-3.1.2\\scripts\\mPDBS\\summary_pdb_93\\219f45328d47579a.final.pdb
-1.544682e-14 1.983344e-15 1.346846e-14 5.452968e-15 -7.160531e-15
1.605152e-15
E:\\R-3.1.2\\scripts\\mPDBS\\summary_pdb_93\\238dd3918e54336d.final.pdb
9.415889e-15 6.343572e-15 -6.456789e-16 8.869940e-15 -1.554230e-15
7.544628e-15
E:\\R-3.1.2\\scripts\\mPDBS\\summary_pdb_93\\2b3027889e2fdc42.final.pdb
3.491402e-16 6.290012e-15 1.779898e-15 -3.783046e-16 -1.849350e-15

-2.312830e-15
E:\\R-3.1.2\\scripts\\mPDBS\\summary_pdb_93\\2fcb3b46e33793b0.final.pdb
-1.865607e-15 7.988720e-15 -1.382881e-15 -3.002734e-15 -1.732615e-15
2.878544e-15
E:\\R-3.1.2\\scripts\\mPDBS\\summary_pdb_93\\34eb328251842006.final.pdb
1.986935e-15 2.751276e-15 2.974631e-15 -3.071737e-15 8.609920e-16
-7.873645e-15
E:\\R-3.1.2\\scripts\\mPDBS\\summary_pdb_93\\354b32871797b7ab.final.pdb
-2.886557e-15 5.230548e-15 1.713212e-14 -8.554207e-15 -4.326727e-15
-8.050776e-15
E:\\R-3.1.2\\scripts\\mPDBS\\summary_pdb_93\\397368adbc32d5de.final.pdb
9.863221e-16 5.975848e-15 2.842368e-15 4.619588e-15 -3.442109e-15
-5.579372e-15
E:\\R-3.1.2\\scripts\\mPDBS\\summary_pdb_93\\411ca13cd3df6edd.final.pdb
-2.106300e-15 3.882629e-15 -3.193932e-15 -4.657007e-16 -2.452309e-15
2.056827e-15
E:\\R-3.1.2\\scripts\\mPDBS\\summary_pdb_93\\42357eee6642bf12.final.pdb
-1.452415e-14 9.554600e-15 3.280247e-15 -2.923734e-15 -2.698526e-15
-8.742723e-15
E:\\R-3.1.2\\scripts\\mPDBS\\summary_pdb_93\\44342d1f75a7fcfc.final.pdb
-9.420395e-15 1.233244e-14 9.014005e-16 5.838452e-16 -2.046821e-15
1.281554e-15
E:\\R-3.1.2\\scripts\\mPDBS\\summary_pdb_93\\448ef21dffe8cdfb.final.pdb
7.435980e-15 1.056045e-14 1.684126e-14 1.598208e-14 6.168616e-15
-1.900152e-14
E:\\R-3.1.2\\scripts\\mPDBS\\summary_pdb_93\\472a40b3e8f113c7.final.pdb
4.695595e-15 6.092942e-15 -7.291020e-15 -3.647237e-15 -1.353502e-15
4.996416e-16
E:\\R-3.1.2\\scripts\\mPDBS\\summary_pdb_93\\48a26225951ea153.final.pdb
5.532494e-15 8.148985e-15 -1.491143e-16 -9.420897e-16 -1.060593e-15
-8.266052e-16
E:\\R-3.1.2\\scripts\\mPDBS\\summary_pdb_93\\4ab4a5c7c6682ce6.final.pdb
-4.193562e-15 -4.487006e-16 6.644014e-15 -1.333983e-15 -4.360428e-15
-4.118734e-15
E:\\R-3.1.2\\scripts\\mPDBS\\summary_pdb_93\\4eaa45b62cb6a25f.final.pdb
2.986123e-16 5.933978e-15 -8.704095e-16 1.967324e-15 -2.550262e-15
1.865987e-15
E:\\R-3.1.2\\scripts\\mPDBS\\summary_pdb_93\\58c371611d85ac96.final.pdb
9.863221e-16 5.975848e-15 2.842368e-15 4.619588e-15 -3.442109e-15
-5.579372e-15
E:\\R-3.1.2\\scripts\\mPDBS\\summary_pdb_93\\59a647f04822d30a.final.pdb
2.277713e-15 9.453665e-15 9.578487e-15 5.213952e-16 -4.088699e-15
-1.957608e-15
E:\\R-3.1.2\\scripts\\mPDBS\\summary_pdb_93\\59b091fe385e05fc.final.pdb
-1.407701e-15 6.545667e-15 -5.593764e-16 -3.451367e-15 -1.022104e-15
-4.084307e-15
E:\\R-3.1.2\\scripts\\mPDBS\\summary_pdb_93\\5c35ac30f930d2db.final.pdb
2.986123e-16 5.933978e-15 -8.704095e-16 1.967324e-15 -2.550262e-15
1.865987e-15
E:\\R-3.1.2\\scripts\\mPDBS\\summary_pdb_93\\5f60752431cf2dcb.final.pdb
7.834036e-15 4.376375e-15 -4.419864e-15 -4.546220e-18 -4.425694e-15
-2.693115e-15
E:\\R-3.1.2\\scripts\\mPDBS\\summary_pdb_93\\626456dd6ff2868d.final.pdb
4.695595e-15 6.092942e-15 -7.291020e-15 -3.647237e-15 -1.353502e-15

4.996416e-16
E:\\R-3.1.2\\scripts\\mPDBS\\summary_pdb_93\\6583d53ff3c8f7ef.final.pdb
2.277713e-15 9.453665e-15 9.578487e-15 5.213952e-16 -4.088699e-15
-1.957608e-15
E:\\R-3.1.2\\scripts\\mPDBS\\summary_pdb_93\\69760178986bef01.final.pdb
6.402138e-15 9.459839e-15 -6.865113e-17 7.897770e-15 2.091326e-15
5.492427e-15
E:\\R-3.1.2\\scripts\\mPDBS\\summary_pdb_93\\6a9d87d47926b1c8.final.pdb
1.047037e-15 6.512745e-15 1.642806e-15 -5.984606e-16 -1.982123e-15
-2.269519e-15
E:\\R-3.1.2\\scripts\\mPDBS\\summary_pdb_93\\6bb3547ea982ffc4.final.pdb
9.744571e-15 4.858386e-15 -2.693408e-18 7.031016e-15 1.615647e-15
5.005326e-15
E:\\R-3.1.2\\scripts\\mPDBS\\summary_pdb_93\\716d9624611d1c42.final.pdb
-5.565402e-15 4.493035e-15 1.386065e-14 1.637370e-15 -5.859920e-16
-3.415889e-16
E:\\R-3.1.2\\scripts\\mPDBS\\summary_pdb_93\\75f27e70ad0f1be4.final.pdb
2.277713e-15 9.453665e-15 9.578487e-15 5.213952e-16 -4.088699e-15
-1.957608e-15
E:\\R-3.1.2\\scripts\\mPDBS\\summary_pdb_93\\769f910fffb22872.final.pdb
4.483911e-16 6.703017e-15 2.419456e-15 3.591230e-16 -1.722997e-15
-1.641905e-15
E:\\R-3.1.2\\scripts\\mPDBS\\summary_pdb_93\\791f8c80dd5c3135.final.pdb
-1.407701e-15 6.545667e-15 -5.593764e-16 -3.451367e-15 -1.022104e-15
-4.084307e-15
E:\\R-3.1.2\\scripts\\mPDBS\\summary_pdb_93\\7fad98e66a3f6099.final.pdb
3.353718e-15 5.849250e-15 2.144288e-15 -1.258678e-15 3.043776e-15
-3.368168e-15
E:\\R-3.1.2\\scripts\\mPDBS\\summary_pdb_93\\804b89c94a633196.final.pdb
-1.865607e-15 7.988720e-15 -1.382881e-15 -3.002734e-15 -1.732615e-15
2.878544e-15
E:\\R-3.1.2\\scripts\\mPDBS\\summary_pdb_93\\813cc65a2298feba.final.pdb
-1.865607e-15 7.988720e-15 -1.382881e-15 -3.002734e-15 -1.732615e-15
2.878544e-15
E:\\R-3.1.2\\scripts\\mPDBS\\summary_pdb_93\\83c0eefcfe12d3f9.final.pdb
-2.106300e-15 3.882629e-15 -3.193932e-15 -4.657007e-16 -2.452309e-15
2.056827e-15
E:\\R-3.1.2\\scripts\\mPDBS\\summary_pdb_93\\86d50469ef569760.final.pdb
4.695595e-15 6.092942e-15 -7.291020e-15 -3.647237e-15 -1.353502e-15
4.996416e-16
E:\\R-3.1.2\\scripts\\mPDBS\\summary_pdb_93\\89d0666e1402203d.final.pdb
2.277713e-15 9.453665e-15 9.578487e-15 5.213952e-16 -4.088699e-15
-1.957608e-15
E:\\R-3.1.2\\scripts\\mPDBS\\summary_pdb_93\\8b34852716dadc62.final.pdb
-1.749380e-15 4.978975e-15 -3.233831e-15 -1.184744e-15 -4.847962e-15
-2.225012e-15
E:\\R-3.1.2\\scripts\\mPDBS\\summary_pdb_93\\8b6ef9ac3fe74395.final.pdb
1.394073e-14 1.611159e-14 5.701559e-16 -2.779602e-15 -7.762309e-17
1.341885e-15
E:\\R-3.1.2\\scripts\\mPDBS\\summary_pdb_93\\98bbe0d5986c138f.final.pdb
-1.407701e-15 6.545667e-15 -5.593764e-16 -3.451367e-15 -1.022104e-15
-4.084307e-15
E:\\R-3.1.2\\scripts\\mPDBS\\summary_pdb_93\\9c22f4909d67e2fa.final.pdb
-2.246162e-15 9.734719e-15 4.787097e-15 1.395224e-15 -3.054801e-16

-5.734033e-15
E:\\R-3.1.2\\scripts\\mPDBS\\summary_pdb_93\\9dda8ed0d81fb3ef.final.pdb
1.714993e-14 -1.597385e-16 -2.132561e-14 4.911113e-15 5.292794e-15
5.653383e-15
E:\\R-3.1.2\\scripts\\mPDBS\\summary_pdb_93\\9f752b3616e6033b.final.pdb
5.950288e-16 9.548159e-15 -3.045937e-15 6.767467e-16 -6.389945e-16
-1.727703e-15
E:\\R-3.1.2\\scripts\\mPDBS\\summary_pdb_93\\a02c70083816d6ba.final.pdb
2.986123e-16 5.933978e-15 -8.704095e-16 1.967324e-15 -2.550262e-15
1.865987e-15
E:\\R-3.1.2\\scripts\\mPDBS\\summary_pdb_93\\a0c997d891e4e082.final.pdb
-1.703068e-15 1.457802e-15 -4.971040e-15 1.420934e-15 -4.786541e-15
9.868239e-15
E:\\R-3.1.2\\scripts\\mPDBS\\summary_pdb_93\\ab8039a2d823c616.final.pdb
-1.856808e-15 7.413541e-15 2.107933e-15 -2.621810e-16 -1.784602e-15
-1.739447e-15
E:\\R-3.1.2\\scripts\\mPDBS\\summary_pdb_93\\acf4d74b9c71d4dc.final.pdb
-1.865607e-15 7.988720e-15 -1.382881e-15 -3.002734e-15 -1.732615e-15
2.878544e-15
E:\\R-3.1.2\\scripts\\mPDBS\\summary_pdb_93\\ad8a823e4b4c668e.final.pdb
1.367427e-15 6.437874e-15 2.048269e-15 -1.222242e-16 -2.275906e-15
-1.456457e-15
E:\\R-3.1.2\\scripts\\mPDBS\\summary_pdb_93\\b16ababd03b7b857.final.pdb
-2.246162e-15 9.734719e-15 4.787097e-15 1.395224e-15 -3.054801e-16
-5.734033e-15
E:\\R-3.1.2\\scripts\\mPDBS\\summary_pdb_93\\b226923e697d3c63.final.pdb
-3.296170e-15 5.867558e-15 8.157315e-15 3.326009e-15 -6.532274e-15
1.928281e-15
E:\\R-3.1.2\\scripts\\mPDBS\\summary_pdb_93\\b28e48f6fa27a165.final.pdb
-1.407701e-15 6.545667e-15 -5.593764e-16 -3.451367e-15 -1.022104e-15
-4.084307e-15
E:\\R-3.1.2\\scripts\\mPDBS\\summary_pdb_93\\b42c4924e3cd1aec.final.pdb
-9.107928e-15 6.827728e-15 4.584652e-15 -5.735319e-15 -6.434262e-15
-4.506134e-15
E:\\R-3.1.2\\scripts\\mPDBS\\summary_pdb_93\\b4dd239fcabff1df.final.pdb
-1.407701e-15 6.545667e-15 -5.593764e-16 -3.451367e-15 -1.022104e-15
-4.084307e-15
E:\\R-3.1.2\\scripts\\mPDBS\\summary_pdb_93\\b669942ef97459a2.final.pdb
-1.749380e-15 4.978975e-15 -3.233831e-15 -1.184744e-15 -4.847962e-15
-2.225012e-15
E:\\R-3.1.2\\scripts\\mPDBS\\summary_pdb_93\\b99f4d9d87c1017b.final.pdb
-1.407701e-15 6.545667e-15 -5.593764e-16 -3.451367e-15 -1.022104e-15
-4.084307e-15
E:\\R-3.1.2\\scripts\\mPDBS\\summary_pdb_93\\b9d2cbf3975403e7.final.pdb
-1.407701e-15 6.545667e-15 -5.593764e-16 -3.451367e-15 -1.022104e-15
-4.084307e-15
E:\\R-3.1.2\\scripts\\mPDBS\\summary_pdb_93\\bc88c8a505f3a288.final.pdb
-4.264980e-16 6.760773e-15 1.359661e-15 -3.665952e-16 -1.716860e-15
-1.965560e-15
E:\\R-3.1.2\\scripts\\mPDBS\\summary_pdb_93\\bdac0e64f4ef427e.final.pdb
5.792980e-15 1.214078e-14 1.076756e-14 -9.924033e-17 -9.121454e-16
-8.812733e-15
E:\\R-3.1.2\\scripts\\mPDBS\\summary_pdb_93\\ca2a1f55fc9d0681.final.pdb
-1.749380e-15 4.978975e-15 -3.233831e-15 -1.184744e-15 -4.847962e-15

```
-2.225012e-15
E:\\R-3.1.2\\scripts\\mPDBS\\summary_pdb_93\\ce34248de3e81a3c.final.pdb
-1.819864e-15  4.484905e-15  5.846732e-15 -1.041535e-14 -5.529991e-15
-1.108717e-15
E:\\R-3.1.2\\scripts\\mPDBS\\summary_pdb_93\\d02e697772494a61.final.pdb
-7.983387e-15  1.419363e-14  2.152322e-14  7.913327e-16 -3.777278e-15
-6.194382e-15
E:\\R-3.1.2\\scripts\\mPDBS\\summary_pdb_93\\d04221245d015047.final.pdb
2.259445e-16  7.847656e-15 -7.105590e-16 -1.333806e-15  1.470122e-16
-2.943562e-15
E:\\R-3.1.2\\scripts\\mPDBS\\summary_pdb_93\\d678141e4018054a.final.pdb
-4.568026e-16 -9.948115e-16  6.271442e-15  4.150982e-15 -1.063475e-15
-9.048297e-15
E:\\R-3.1.2\\scripts\\mPDBS\\summary_pdb_93\\d71b40af0f0efe39.final.pdb
-7.962798e-15  3.789199e-15  5.781647e-15  3.470844e-15 -2.295380e-15
-1.266567e-14
E:\\R-3.1.2\\scripts\\mPDBS\\summary_pdb_93\\dc0809eff10197e2.final.pdb
2.037109e-15  1.037498e-14 -4.296832e-15 -1.681212e-15 -4.351514e-15
-3.291369e-15
E:\\R-3.1.2\\scripts\\mPDBS\\summary_pdb_93\\e1163918d3707839.final.pdb
-2.303475e-15  9.298408e-15 -1.145373e-14 -4.040494e-15  6.610099e-15
-1.098402e-14
E:\\R-3.1.2\\scripts\\mPDBS\\summary_pdb_93\\e4b48b06824ef848.final.pdb
-4.903112e-16  5.654377e-15  3.713126e-15  2.407398e-15 -4.368992e-15
-6.803263e-16
E:\\R-3.1.2\\scripts\\mPDBS\\summary_pdb_93\\e529e19ad281fea2.final.pdb
-5.471620e-15  8.015015e-15  1.085506e-14 -4.137125e-16 -1.041469e-15
4.736208e-16
E:\\R-3.1.2\\scripts\\mPDBS\\summary_pdb_93\\e5602082718ec3a1.final.pdb
-1.865607e-15  7.988720e-15 -1.382881e-15 -3.002734e-15 -1.732615e-15
2.878544e-15
E:\\R-3.1.2\\scripts\\mPDBS\\summary_pdb_93\\e5d589d996d0a960.final.pdb
8.911722e-15  1.141495e-14 -6.829032e-15 -4.013336e-15  2.209152e-15
4.384398e-15
E:\\R-3.1.2\\scripts\\mPDBS\\summary_pdb_93\\e9df9e72307a2fbd.final.pdb
2.986123e-16  5.933978e-15 -8.704095e-16  1.967324e-15 -2.550262e-15
1.865987e-15
E:\\R-3.1.2\\scripts\\mPDBS\\summary_pdb_93\\ea00f8694090cae7.final.pdb
-1.865607e-15  7.988720e-15 -1.382881e-15 -3.002734e-15 -1.732615e-15
2.878544e-15
E:\\R-3.1.2\\scripts\\mPDBS\\summary_pdb_93\\eba517be32db050e.final.pdb
-1.407701e-15  6.545667e-15 -5.593764e-16 -3.451367e-15 -1.022104e-15
-4.084307e-15
E:\\R-3.1.2\\scripts\\mPDBS\\summary_pdb_93\\ec04b9545899c29d.final.pdb
3.114039e-16  7.312366e-15  1.489472e-15  2.873370e-17 -1.874216e-15
-1.711711e-15
E:\\R-3.1.2\\scripts\\mPDBS\\summary_pdb_93\\ee6862cbcad52078.final.pdb
-7.155463e-16  6.720441e-15  1.592548e-15 -6.168291e-16 -1.769661e-15
-2.002206e-15
E:\\R-3.1.2\\scripts\\mPDBS\\summary_pdb_93\\ef6a42fc83b81e5d.final.pdb
-3.059968e-16  6.949131e-15  1.211655e-15 -4.071911e-16 -1.695241e-15
-1.571866e-15
E:\\R-3.1.2\\scripts\\mPDBS\\summary_pdb_93\\ef8e88d4ad93a0ad.final.pdb
2.259445e-16  7.847656e-15 -7.105590e-16 -1.333806e-15  1.470122e-16
```

-2.943562e-15
E:\\R-3.1.2\\scripts\\mPDBS\\summary_pdb_93\\f14fcae8deec4fa3.final.pdb
5.950288e-16 9.548159e-15 -3.045937e-15 6.767467e-16 -6.389945e-16
-1.727703e-15
E:\\R-3.1.2\\scripts\\mPDBS\\summary_pdb_93\\f83584e22003fb92.final.pdb
5.532494e-15 8.148985e-15 -1.491143e-16 -9.420897e-16 -1.060593e-15
-8.266052e-16
E:\\R-3.1.2\\scripts\\mPDBS\\summary_pdb_93\\f8e2bf87e52caa29.final.pdb
-5.471620e-15 8.015015e-15 1.085506e-14 -4.137125e-16 -1.041469e-15
4.736208e-16

[,217]   [,218]   [,219]   [,220]   [,221]   [,222]
E:\\R-3.1.2\\scripts\\40.1ns_data\\NAT0.pdb
5.331560e-16 -7.942865e-17 -4.403767e-15 -4.628510e-15 -4.218822e-16
-5.157976e-16
E:\\R-3.1.2\\scripts\\40.1ns_data\\LJP6.pdb
2.875535e-15 -8.965068e-16 -6.958700e-15 -4.467057e-15 -3.640165e-15
-1.580122e-15
E:\\R-3.1.2\\scripts\\40.1ns_data\\3WY9.pdb
2.837331e-15 2.059161e-15 -7.022411e-15 -3.159445e-15 -1.112586e-15
-2.557268e-15
E:\\R-3.1.2\\scripts\\40.1ns_data\\ZSP9.pdb
9.150113e-16 -9.587049e-16 -5.259641e-15 -5.568626e-15 -9.596441e-16
-3.604911e-16
E:\\R-3.1.2\\scripts\\mPDBS\\summary_pdb_93\\013d855ad26c877c.final.pdb
-1.204714e-15 -6.980609e-15 -8.728824e-15 5.191045e-16 1.664050e-15
9.000060e-15
E:\\R-3.1.2\\scripts\\mPDBS\\summary_pdb_93\\0a2a705342ca0d54.final.pdb
1.091298e-15 -3.098188e-15 -5.210807e-15 -2.638430e-15 -1.127733e-15
-3.823419e-16
E:\\R-3.1.2\\scripts\\mPDBS\\summary_pdb_93\\0a59fceadb4194d9.final.pdb
-8.813847e-16 -2.764615e-15 -6.437572e-15 -3.405714e-16 -4.822105e-16
1.874222e-15
E:\\R-3.1.2\\scripts\\mPDBS\\summary_pdb_93\\0dba59f3306825e8.final.pdb
3.725371e-15 -5.205987e-15 -4.256707e-15 -5.850800e-15 -2.884829e-15
-6.652350e-15
E:\\R-3.1.2\\scripts\\mPDBS\\summary_pdb_93\\126f26a78b57339c.final.pdb
5.184707e-15 1.139762e-16 -9.934890e-15 -8.520540e-15 -2.481939e-15
-4.815277e-15
E:\\R-3.1.2\\scripts\\mPDBS\\summary_pdb_93\\12f06f3e342df706.final.pdb
1.625376e-15 -2.710911e-15 -5.460824e-15 -4.187538e-15 -1.624948e-15
-3.398412e-16
E:\\R-3.1.2\\scripts\\mPDBS\\summary_pdb_93\\18618a2a251b867b.final.pdb
2.595597e-15 -8.386331e-15 -4.290203e-15 -7.361846e-15 1.033666e-15
-7.626453e-15
E:\\R-3.1.2\\scripts\\mPDBS\\summary_pdb_93\\1f305f598641eeb2.final.pdb
1.304866e-15 -2.043519e-15 -5.316359e-15 -4.623581e-15 -1.080055e-15
-1.533537e-15
E:\\R-3.1.2\\scripts\\mPDBS\\summary_pdb_93\\1f8a00b551be0a39.final.pdb
2.595597e-15 -8.386331e-15 -4.290203e-15 -7.361846e-15 1.033666e-15
-7.626453e-15
E:\\R-3.1.2\\scripts\\mPDBS\\summary_pdb_93\\219f45328d47579a.final.pdb
1.006038e-14 -2.483983e-16 -3.733138e-15 -3.974817e-15 -4.210615e-16
6.058375e-15

E:\\R-3.1.2\\scripts\\mPDBS\\summary_pdb_93\\238dd3918e54336d.final.pdb
1.093902e-14 -1.314820e-15 8.290881e-15 -8.437393e-15 -6.464404e-15
-1.174856e-14
E:\\R-3.1.2\\scripts\\mPDBS\\summary_pdb_93\\2b3027889e2fdc42.final.pdb
1.767321e-15 -2.827065e-15 -6.619285e-15 -4.307450e-15 -1.829006e-15
-9.642378e-17
E:\\R-3.1.2\\scripts\\mPDBS\\summary_pdb_93\\2fcb3b46e33793b0.final.pdb
7.324074e-16 -5.523375e-15 -4.851453e-15 -1.861902e-15 -4.824425e-16
-7.043393e-16
E:\\R-3.1.2\\scripts\\mPDBS\\summary_pdb_93\\34eb328251842006.final.pdb
-6.977107e-16 -9.807439e-15 3.835668e-15 6.754099e-15 1.708356e-15
-7.874269e-16
E:\\R-3.1.2\\scripts\\mPDBS\\summary_pdb_93\\354b32871797b7ab.final.pdb
-9.910736e-15 -5.534395e-15 -7.312626e-15 -7.918161e-15 6.606675e-15
5.416435e-15
E:\\R-3.1.2\\scripts\\mPDBS\\summary_pdb_93\\397368adbc32d5de.final.pdb
5.184707e-15 1.139762e-16 -9.934890e-15 -8.520540e-15 -2.481939e-15
-4.815277e-15
E:\\R-3.1.2\\scripts\\mPDBS\\summary_pdb_93\\411ca13cd3df6edd.final.pdb
2.347001e-15 -8.419929e-15 -5.713177e-15 -3.438765e-15 9.487044e-16
-6.970161e-15
E:\\R-3.1.2\\scripts\\mPDBS\\summary_pdb_93\\42357eee6642bf12.final.pdb
2.995768e-15 -6.923921e-16 -9.683765e-16 6.449315e-15 4.073669e-15
-2.169428e-15
E:\\R-3.1.2\\scripts\\mPDBS\\summary_pdb_93\\44342d1f75a7fcfc.final.pdb
-3.258236e-15 1.596825e-15 -6.607137e-15 7.340795e-16 7.839492e-16
3.729711e-15
E:\\R-3.1.2\\scripts\\mPDBS\\summary_pdb_93\\448ef21dffe8cdfb.final.pdb
-1.383754e-14 4.541491e-15 -1.056216e-14 -1.134737e-14 5.484573e-15
5.649953e-15
E:\\R-3.1.2\\scripts\\mPDBS\\summary_pdb_93\\472a40b3e8f113c7.final.pdb
3.725371e-15 -5.205987e-15 -4.256707e-15 -5.850800e-15 -2.884829e-15
-6.652350e-15
E:\\R-3.1.2\\scripts\\mPDBS\\summary_pdb_93\\48a26225951ea153.final.pdb
1.210687e-16 -6.212378e-15 -1.066900e-14 -9.074470e-15 -1.069631e-15
-4.647471e-15
E:\\R-3.1.2\\scripts\\mPDBS\\summary_pdb_93\\4ab4a5c7c6682ce6.final.pdb
-4.273006e-15 -5.270605e-15 -6.756877e-15 -6.244582e-15 -4.492484e-15
-4.508231e-15
E:\\R-3.1.2\\scripts\\mPDBS\\summary_pdb_93\\4eaa45b62cb6a25f.final.pdb
4.324405e-15 -3.639643e-15 -8.512837e-15 -6.349104e-15 -3.830723e-15
-1.706339e-15
E:\\R-3.1.2\\scripts\\mPDBS\\summary_pdb_93\\58c371611d85ac96.final.pdb
5.184707e-15 1.139762e-16 -9.934890e-15 -8.520540e-15 -2.481939e-15
-4.815277e-15
E:\\R-3.1.2\\scripts\\mPDBS\\summary_pdb_93\\59a647f04822d30a.final.pdb
1.664042e-15 4.666522e-16 -2.102060e-15 -7.988110e-15 -4.185600e-15
9.211932e-16
E:\\R-3.1.2\\scripts\\mPDBS\\summary_pdb_93\\59b091fe385e05fc.final.pdb
-8.813847e-16 -2.764615e-15 -6.437572e-15 -3.405714e-16 -4.822105e-16
1.874222e-15
E:\\R-3.1.2\\scripts\\mPDBS\\summary_pdb_93\\5c35ac30f930d2db.final.pdb
4.324405e-15 -3.639643e-15 -8.512837e-15 -6.349104e-15 -3.830723e-15
-1.706339e-15

E:\\R-3.1.2\\scripts\\mPDBS\\summary_pdb_93\\5f60752431cf2dcb.final.pdb
3.907405e-15 -5.071436e-15 -2.309916e-15 -4.882728e-15 -2.883924e-15
-2.124872e-15
E:\\R-3.1.2\\scripts\\mPDBS\\summary_pdb_93\\626456dd6ff2868d.final.pdb
3.725371e-15 -5.205987e-15 -4.256707e-15 -5.850800e-15 -2.884829e-15
-6.652350e-15
E:\\R-3.1.2\\scripts\\mPDBS\\summary_pdb_93\\6583d53ff3c8f7ef.final.pdb
1.664042e-15 4.666522e-16 -2.102060e-15 -7.988110e-15 -4.185600e-15
9.211932e-16
E:\\R-3.1.2\\scripts\\mPDBS\\summary_pdb_93\\69760178986bef01.final.pdb
2.764778e-15 4.020239e-15 -3.905648e-15 -1.568554e-14 1.299138e-15
-5.960158e-16
E:\\R-3.1.2\\scripts\\mPDBS\\summary_pdb_93\\6a9d87d47926b1c8.final.pdb
2.169757e-15 -3.091793e-15 -6.531363e-15 -3.926992e-15 -1.581184e-15
-1.711857e-15
E:\\R-3.1.2\\scripts\\mPDBS\\summary_pdb_93\\6bb3547ea982ffc4.final.pdb
7.140896e-16 -5.181524e-15 -7.422474e-15 -8.377664e-15 5.023162e-15
-1.304135e-14
E:\\R-3.1.2\\scripts\\mPDBS\\summary_pdb_93\\716d9624611d1c42.final.pdb
-2.345189e-15 -7.768064e-15 -8.889288e-15 -9.384769e-15 -1.666798e-15
3.831301e-15
E:\\R-3.1.2\\scripts\\mPDBS\\summary_pdb_93\\75f27e70ad0f1be4.final.pdb
1.664042e-15 4.666522e-16 -2.102060e-15 -7.988110e-15 -4.185600e-15
9.211932e-16
E:\\R-3.1.2\\scripts\\mPDBS\\summary_pdb_93\\769f910fffb22872.final.pdb
2.501705e-15 -2.833915e-15 -5.789654e-15 -3.831250e-15 -1.253567e-15
-6.702120e-16
E:\\R-3.1.2\\scripts\\mPDBS\\summary_pdb_93\\791f8c80dd5c3135.final.pdb
-8.813847e-16 -2.764615e-15 -6.437572e-15 -3.405714e-16 -4.822105e-16
1.874222e-15
E:\\R-3.1.2\\scripts\\mPDBS\\summary_pdb_93\\7fad98e66a3f6099.final.pdb
3.107419e-15 -1.733588e-15 -7.050559e-15 3.182007e-15 1.995181e-15
4.702034e-15
E:\\R-3.1.2\\scripts\\mPDBS\\summary_pdb_93\\804b89c94a633196.final.pdb
7.324074e-16 -5.523375e-15 -4.851453e-15 -1.861902e-15 -4.824425e-16
-7.043393e-16
E:\\R-3.1.2\\scripts\\mPDBS\\summary_pdb_93\\813cc65a2298feba.final.pdb
7.324074e-16 -5.523375e-15 -4.851453e-15 -1.861902e-15 -4.824425e-16
-7.043393e-16
E:\\R-3.1.2\\scripts\\mPDBS\\summary_pdb_93\\83c0eefcfe12d3f9.final.pdb
2.347001e-15 -8.419929e-15 -5.713177e-15 -3.438765e-15 9.487044e-16
-6.970161e-15
E:\\R-3.1.2\\scripts\\mPDBS\\summary_pdb_93\\86d50469ef569760.final.pdb
3.725371e-15 -5.205987e-15 -4.256707e-15 -5.850800e-15 -2.884829e-15
-6.652350e-15
E:\\R-3.1.2\\scripts\\mPDBS\\summary_pdb_93\\89d0666e1402203d.final.pdb
1.664042e-15 4.666522e-16 -2.102060e-15 -7.988110e-15 -4.185600e-15
9.211932e-16
E:\\R-3.1.2\\scripts\\mPDBS\\summary_pdb_93\\8b34852716dadc62.final.pdb
4.968602e-15 2.189206e-15 -4.317158e-15 -5.761560e-15 -3.071517e-15
-7.723031e-15
E:\\R-3.1.2\\scripts\\mPDBS\\summary_pdb_93\\8b6ef9ac3fe74395.final.pdb
4.771412e-15 2.268779e-15 -2.444297e-15 3.463340e-15 2.658894e-15
-5.282088e-15

E:\\R-3.1.2\\scripts\\mPDBS\\summary_pdb_93\\98bbe0d5986c138f.final.pdb
-8.813847e-16 -2.764615e-15 -6.437572e-15 -3.405714e-16 -4.822105e-16
1.874222e-15
E:\\R-3.1.2\\scripts\\mPDBS\\summary_pdb_93\\9c22f4909d67e2fa.final.pdb
1.725320e-15 2.666689e-15 -6.378660e-15 -9.155546e-15 -2.991720e-15
7.942390e-15
E:\\R-3.1.2\\scripts\\mPDBS\\summary_pdb_93\\9dda8ed0d81fb3ef.final.pdb
1.051251e-15 -5.674703e-15 -1.350982e-14 -2.042779e-15 -5.799771e-16
-1.048288e-14
E:\\R-3.1.2\\scripts\\mPDBS\\summary_pdb_93\\9f752b3616e6033b.final.pdb
1.570243e-15 1.627102e-15 -5.132978e-15 -3.330020e-15 -1.679456e-15
-2.530945e-15
E:\\R-3.1.2\\scripts\\mPDBS\\summary_pdb_93\\a02c70083816d6ba.final.pdb
4.324405e-15 -3.639643e-15 -8.512837e-15 -6.349104e-15 -3.830723e-15
-1.706339e-15
E:\\R-3.1.2\\scripts\\mPDBS\\summary_pdb_93\\a0c997d891e4e082.final.pdb
2.320811e-15 2.021246e-15 -1.011655e-14 -3.538058e-15 -3.747934e-15
1.387552e-15
E:\\R-3.1.2\\scripts\\mPDBS\\summary_pdb_93\\ab8039a2d823c616.final.pdb
1.651642e-15 -2.387120e-15 -5.459900e-15 -5.473142e-15 -6.566565e-16
-4.052843e-16
E:\\R-3.1.2\\scripts\\mPDBS\\summary_pdb_93\\acf4d74b9c71d4dc.final.pdb
7.324074e-16 -5.523375e-15 -4.851453e-15 -1.861902e-15 -4.824425e-16
-7.043393e-16
E:\\R-3.1.2\\scripts\\mPDBS\\summary_pdb_93\\ad8a823e4b4c668e.final.pdb
7.060433e-16 -2.954735e-15 -5.339449e-15 -4.239237e-15 -1.739717e-15
-9.279119e-16
E:\\R-3.1.2\\scripts\\mPDBS\\summary_pdb_93\\b16ababd03b7b857.final.pdb
1.725320e-15 2.666689e-15 -6.378660e-15 -9.155546e-15 -2.991720e-15
7.942390e-15
E:\\R-3.1.2\\scripts\\mPDBS\\summary_pdb_93\\b226923e697d3c63.final.pdb
4.913830e-15 -4.496825e-15 -1.121291e-15 1.148245e-15 2.287738e-15
-2.361771e-15
E:\\R-3.1.2\\scripts\\mPDBS\\summary_pdb_93\\b28e48f6fa27a165.final.pdb
-8.813847e-16 -2.764615e-15 -6.437572e-15 -3.405714e-16 -4.822105e-16
1.874222e-15
E:\\R-3.1.2\\scripts\\mPDBS\\summary_pdb_93\\b42c4924e3cd1aec.final.pdb
9.202793e-16 3.222365e-16 -3.195587e-15 -4.571572e-15 1.839962e-15
8.290299e-15
E:\\R-3.1.2\\scripts\\mPDBS\\summary_pdb_93\\b4dd239fcabff1df.final.pdb
-8.813847e-16 -2.764615e-15 -6.437572e-15 -3.405714e-16 -4.822105e-16
1.874222e-15
E:\\R-3.1.2\\scripts\\mPDBS\\summary_pdb_93\\b669942ef97459a2.final.pdb
4.968602e-15 2.189206e-15 -4.317158e-15 -5.761560e-15 -3.071517e-15
-7.723031e-15
E:\\R-3.1.2\\scripts\\mPDBS\\summary_pdb_93\\b99f4d9d87c1017b.final.pdb
-8.813847e-16 -2.764615e-15 -6.437572e-15 -3.405714e-16 -4.822105e-16
1.874222e-15
E:\\R-3.1.2\\scripts\\mPDBS\\summary_pdb_93\\b9d2cbf3975403e7.final.pdb
-8.813847e-16 -2.764615e-15 -6.437572e-15 -3.405714e-16 -4.822105e-16
1.874222e-15
E:\\R-3.1.2\\scripts\\mPDBS\\summary_pdb_93\\bc88c8a505f3a288.final.pdb
2.011484e-15 -2.088940e-15 -6.381844e-15 -3.784865e-15 -1.704540e-15
-1.001082e-15

E:\\R-3.1.2\\scripts\\mPDBS\\summary_pdb_93\\bdac0e64f4ef427e.final.pdb
-1.330027e-16 1.185595e-14 -2.969495e-15 -8.477269e-15 -5.071653e-15
7.764581e-15
E:\\R-3.1.2\\scripts\\mPDBS\\summary_pdb_93\\ca2a1f55fc9d0681.final.pdb
4.968602e-15 2.189206e-15 -4.317158e-15 -5.761560e-15 -3.071517e-15
-7.723031e-15
E:\\R-3.1.2\\scripts\\mPDBS\\summary_pdb_93\\ce34248de3e81a3c.final.pdb
4.924932e-15 -6.674820e-15 -1.152629e-14 4.323355e-15 -3.868150e-15
3.160064e-15
E:\\R-3.1.2\\scripts\\mPDBS\\summary_pdb_93\\d02e697772494a61.final.pdb
3.716066e-15 -2.758977e-15 -4.989633e-16 -6.321031e-15 -6.902422e-15
8.299186e-16
E:\\R-3.1.2\\scripts\\mPDBS\\summary_pdb_93\\d04221245d015047.final.pdb
9.342615e-17 -2.135151e-15 -7.146702e-15 -6.022182e-15 -2.315052e-15
-1.494126e-16
E:\\R-3.1.2\\scripts\\mPDBS\\summary_pdb_93\\d678141e4018054a.final.pdb
1.421474e-15 -1.554835e-14 -1.166473e-15 -6.069389e-15 -4.858268e-16
-6.278067e-15
E:\\R-3.1.2\\scripts\\mPDBS\\summary_pdb_93\\d71b40af0f0efe39.final.pdb
-4.045055e-15 -9.493940e-15 -4.987065e-15 -1.053082e-14 -7.595359e-15
2.950476e-15
E:\\R-3.1.2\\scripts\\mPDBS\\summary_pdb_93\\dc0809eff10197e2.final.pdb
3.697552e-15 3.115695e-15 -9.351607e-15 -4.356580e-15 -5.756313e-15
5.236169e-15
E:\\R-3.1.2\\scripts\\mPDBS\\summary_pdb_93\\e1163918d3707839.final.pdb
-4.237487e-15 -2.547863e-15 -1.405496e-14 -4.974028e-15 9.765035e-16
-7.442776e-15
E:\\R-3.1.2\\scripts\\mPDBS\\summary_pdb_93\\e4b48b06824ef848.final.pdb
2.595597e-15 -8.386331e-15 -4.290203e-15 -7.361846e-15 1.033666e-15
-7.626453e-15
E:\\R-3.1.2\\scripts\\mPDBS\\summary_pdb_93\\e529e19ad281fea2.final.pdb
-1.204714e-15 -6.980609e-15 -8.728824e-15 5.191045e-16 1.664050e-15
9.000060e-15
E:\\R-3.1.2\\scripts\\mPDBS\\summary_pdb_93\\e5602082718ec3a1.final.pdb
7.324074e-16 -5.523375e-15 -4.851453e-15 -1.861902e-15 -4.824425e-16
-7.043393e-16
E:\\R-3.1.2\\scripts\\mPDBS\\summary_pdb_93\\e5d589d996d0a960.final.pdb
1.014425e-14 3.040212e-15 -1.402285e-14 5.386863e-16 -8.785185e-15
-1.607802e-15
E:\\R-3.1.2\\scripts\\mPDBS\\summary_pdb_93\\e9df9e72307a2fbd.final.pdb
4.324405e-15 -3.639643e-15 -8.512837e-15 -6.349104e-15 -3.830723e-15
-1.706339e-15
E:\\R-3.1.2\\scripts\\mPDBS\\summary_pdb_93\\ea00f8694090cae7.final.pdb
7.324074e-16 -5.523375e-15 -4.851453e-15 -1.861902e-15 -4.824425e-16
-7.043393e-16
E:\\R-3.1.2\\scripts\\mPDBS\\summary_pdb_93\\eba517be32db050e.final.pdb
-8.813847e-16 -2.764615e-15 -6.437572e-15 -3.405714e-16 -4.822105e-16
1.874222e-15
E:\\R-3.1.2\\scripts\\mPDBS\\summary_pdb_93\\ec04b9545899c29d.final.pdb
9.350245e-16 -2.720902e-15 -5.797889e-15 -4.442335e-15 -1.101695e-15
-1.221190e-15
E:\\R-3.1.2\\scripts\\mPDBS\\summary_pdb_93\\ee6862cbcad52078.final.pdb
1.861213e-15 -3.204367e-15 -6.470532e-15 -4.288802e-15 -2.351809e-15
-4.971449e-16

E:\\R-3.1.2\\scripts\\mPDBS\\summary_pdb_93\\ef6a42fc83b81e5d.final.pdb
8.923155e-16 -2.967055e-15 -5.467646e-15 -4.139163e-15 -1.503150e-15
-7.780390e-17
E:\\R-3.1.2\\scripts\\mPDBS\\summary_pdb_93\\ef8e88d4ad93a0ad.final.pdb
9.342615e-17 -2.135151e-15 -7.146702e-15 -6.022182e-15 -2.315052e-15
-1.494126e-16
E:\\R-3.1.2\\scripts\\mPDBS\\summary_pdb_93\\f14fcae8deec4fa3.final.pdb
1.570243e-15 1.627102e-15 -5.132978e-15 -3.330020e-15 -1.679456e-15
-2.530945e-15
E:\\R-3.1.2\\scripts\\mPDBS\\summary_pdb_93\\f83584e22003fb92.final.pdb
1.210687e-16 -6.212378e-15 -1.066900e-14 -9.074470e-15 -1.069631e-15
-4.647471e-15
E:\\R-3.1.2\\scripts\\mPDBS\\summary_pdb_93\\f8e2bf87e52caa29.final.pdb
-1.204714e-15 -6.980609e-15 -8.728824e-15 5.191045e-16 1.664050e-15
9.000060e-15

[,223]   [,224]   [,225]   [,226]   [,227]   [,228]
E:\\R-3.1.2\\scripts\\40.1ns_data\\NAT0.pdb
2.936322e-15 -5.482694e-16 -3.605151e-15 9.057935e-16 1.845067e-15
-2.058795e-15
E:\\R-3.1.2\\scripts\\40.1ns_data\\LJP6.pdb
2.894697e-15 3.204892e-16 -3.907434e-15 -1.639793e-15 1.763597e-15
-2.856312e-15
E:\\R-3.1.2\\scripts\\40.1ns_data\\3WY9.pdb
2.139327e-15 1.237876e-15 -2.839191e-15 -4.790808e-16 2.242899e-15
-2.024548e-15
E:\\R-3.1.2\\scripts\\40.1ns_data\\ZSP9.pdb
2.141026e-15 -1.975491e-16 -3.991877e-15 1.435218e-15 1.606004e-15
-1.834378e-15
E:\\R-3.1.2\\scripts\\mPDBS\\summary_pdb_93\\013d855ad26c877c.final.pdb
-1.317911e-15 1.214248e-15 -3.351407e-15 4.079697e-15 -5.725813e-16
-2.332850e-15
E:\\R-3.1.2\\scripts\\mPDBS\\summary_pdb_93\\0a2a705342ca0d54.final.pdb
3.294822e-15 1.584917e-15 -3.782031e-15 3.129152e-16 3.158248e-15
-3.242812e-15
E:\\R-3.1.2\\scripts\\mPDBS\\summary_pdb_93\\0a59fceadb4194d9.final.pdb
3.205303e-16 2.039753e-15 -3.093375e-15 -9.030038e-16 4.674488e-15
-1.038002e-15
E:\\R-3.1.2\\scripts\\mPDBS\\summary_pdb_93\\0dba59f3306825e8.final.pdb
9.944751e-16 -1.084261e-15 -4.076522e-15 -5.589652e-15 8.637066e-15
-5.217986e-16
E:\\R-3.1.2\\scripts\\mPDBS\\summary_pdb_93\\126f26a78b57339c.final.pdb
6.233557e-15 9.091531e-16 -5.824906e-15 4.827363e-15 1.974426e-15
-3.549960e-18
E:\\R-3.1.2\\scripts\\mPDBS\\summary_pdb_93\\12f06f3e342df706.final.pdb
2.002751e-15 8.466833e-16 -3.417956e-15 1.459480e-16 2.953334e-15
-2.353332e-15
E:\\R-3.1.2\\scripts\\mPDBS\\summary_pdb_93\\18618a2a251b867b.final.pdb
4.584086e-15 5.495175e-15 7.943032e-16 5.909860e-15 -4.449986e-15
2.069203e-15
E:\\R-3.1.2\\scripts\\mPDBS\\summary_pdb_93\\1f305f598641eeb2.final.pdb
2.085571e-15 4.964637e-16 -3.551497e-15 6.878139e-16 2.472320e-15
-1.656000e-15
E:\\R-3.1.2\\scripts\\mPDBS\\summary_pdb_93\\1f8a00b551be0a39.final.pdb

4.584086e-15 5.495175e-15 7.943032e-16 5.909860e-15 -4.449986e-15
2.069203e-15
E:\\R-3.1.2\\scripts\\mPDBS\\summary_pdb_93\\219f45328d47579a.final.pdb
-6.638530e-15 -1.356755e-14 -2.017846e-15 1.738980e-15 -3.418431e-15
-1.368901e-15
E:\\R-3.1.2\\scripts\\mPDBS\\summary_pdb_93\\238dd3918e54336d.final.pdb
9.391074e-15 5.575228e-15 -7.341495e-15 -1.081435e-14 1.635893e-14
6.883179e-15
E:\\R-3.1.2\\scripts\\mPDBS\\summary_pdb_93\\2b3027889e2fdc42.final.pdb
2.266050e-15 4.440788e-16 -3.124383e-15 8.510185e-16 3.838568e-15
-1.682452e-15
E:\\R-3.1.2\\scripts\\mPDBS\\summary_pdb_93\\2fcb3b46e33793b0.final.pdb
-1.253772e-15 1.707301e-15 -2.316374e-15 -1.768554e-15 5.092620e-15
3.927089e-16
E:\\R-3.1.2\\scripts\\mPDBS\\summary_pdb_93\\34eb328251842006.final.pdb
-8.802979e-16 -5.156989e-15 -7.642219e-15 -3.515609e-15 -1.771597e-15
-1.541524e-15
E:\\R-3.1.2\\scripts\\mPDBS\\summary_pdb_93\\354b32871797b7ab.final.pdb
-6.691489e-15 3.168773e-15 5.007960e-15 3.040319e-15 -6.639659e-15
-1.095694e-14
E:\\R-3.1.2\\scripts\\mPDBS\\summary_pdb_93\\397368adbc32d5de.final.pdb
6.233557e-15 9.091531e-16 -5.824906e-15 4.827363e-15 1.974426e-15
-3.549960e-18
E:\\R-3.1.2\\scripts\\mPDBS\\summary_pdb_93\\411ca13cd3df6edd.final.pdb
-8.541351e-16 -1.994816e-15 -3.291505e-15 -1.326200e-15 1.586310e-15
3.201660e-15
E:\\R-3.1.2\\scripts\\mPDBS\\summary_pdb_93\\42357eee6642bf12.final.pdb
-3.699828e-15 -2.877692e-15 -3.824467e-15 -1.058821e-14 -7.952419e-15
9.336566e-15
E:\\R-3.1.2\\scripts\\mPDBS\\summary_pdb_93\\44342d1f75a7fcfc.final.pdb
4.873138e-15 1.034057e-14 -8.943570e-15 5.596374e-15 -2.811702e-15
-5.415562e-15
E:\\R-3.1.2\\scripts\\mPDBS\\summary_pdb_93\\448ef21dffe8cdfb.final.pdb
7.056421e-15 8.904668e-15 3.273447e-16 1.301476e-14 -7.282030e-15
-8.541117e-15
E:\\R-3.1.2\\scripts\\mPDBS\\summary_pdb_93\\472a40b3e8f113c7.final.pdb
9.944751e-16 -1.084261e-15 -4.076522e-15 -5.589652e-15 8.637066e-15
-5.217986e-16
E:\\R-3.1.2\\scripts\\mPDBS\\summary_pdb_93\\48a26225951ea153.final.pdb
9.662260e-15 5.095740e-15 -9.698562e-16 -1.724683e-16 -4.992476e-15
-4.148795e-15
E:\\R-3.1.2\\scripts\\mPDBS\\summary_pdb_93\\4ab4a5c7c6682ce6.final.pdb
9.788005e-15 8.364995e-15 3.834034e-15 8.508446e-15 3.228631e-15
-1.253866e-14
E:\\R-3.1.2\\scripts\\mPDBS\\summary_pdb_93\\4eaa45b62cb6a25f.final.pdb
7.005779e-15 1.487718e-15 -3.669344e-15 2.945776e-15 6.751755e-15
1.782534e-16
E:\\R-3.1.2\\scripts\\mPDBS\\summary_pdb_93\\58c371611d85ac96.final.pdb
6.233557e-15 9.091531e-16 -5.824906e-15 4.827363e-15 1.974426e-15
-3.549960e-18
E:\\R-3.1.2\\scripts\\mPDBS\\summary_pdb_93\\59a647f04822d30a.final.pdb
6.831207e-15 -3.038488e-15 1.449158e-15 1.750492e-15 2.041621e-15
-6.000164e-15
E:\\R-3.1.2\\scripts\\mPDBS\\summary_pdb_93\\59b091fe385e05fc.final.pdb

3.205303e-16 2.039753e-15 -3.093375e-15 -9.030038e-16 4.674488e-15
-1.038002e-15
E:\\R-3.1.2\\scripts\\mPDBS\\summary_pdb_93\\5c35ac30f930d2db.final.pdb
7.005779e-15 1.487718e-15 -3.669344e-15 2.945776e-15 6.751755e-15
1.782534e-16
E:\\R-3.1.2\\scripts\\mPDBS\\summary_pdb_93\\5f60752431cf2dcb.final.pdb
1.220179e-14 2.554898e-15 -2.376688e-15 2.129387e-15 6.461378e-15
-1.181526e-15
E:\\R-3.1.2\\scripts\\mPDBS\\summary_pdb_93\\626456dd6ff2868d.final.pdb
9.944751e-16 -1.084261e-15 -4.076522e-15 -5.589652e-15 8.637066e-15
-5.217986e-16
E:\\R-3.1.2\\scripts\\mPDBS\\summary_pdb_93\\6583d53ff3c8f7ef.final.pdb
6.831207e-15 -3.038488e-15 1.449158e-15 1.750492e-15 2.041621e-15
-6.000164e-15
E:\\R-3.1.2\\scripts\\mPDBS\\summary_pdb_93\\69760178986bef01.final.pdb
6.365695e-15 5.639896e-15 -3.889361e-15 7.470661e-15 1.611584e-14
-4.086383e-15
E:\\R-3.1.2\\scripts\\mPDBS\\summary_pdb_93\\6a9d87d47926b1c8.final.pdb
2.145681e-15 2.013859e-16 -4.149163e-15 5.425957e-16 4.002318e-15
-1.743478e-15
E:\\R-3.1.2\\scripts\\mPDBS\\summary_pdb_93\\6bb3547ea982ffc4.final.pdb
1.142725e-15 -8.014705e-15 -1.147422e-14 1.581428e-15 -4.436986e-15
-7.974212e-15
E:\\R-3.1.2\\scripts\\mPDBS\\summary_pdb_93\\716d9624611d1c42.final.pdb
-4.792242e-15 6.065325e-16 -4.592146e-15 2.366828e-15 2.086622e-15
-4.809701e-15
E:\\R-3.1.2\\scripts\\mPDBS\\summary_pdb_93\\75f27e70ad0f1be4.final.pdb
6.831207e-15 -3.038488e-15 1.449158e-15 1.750492e-15 2.041621e-15
-6.000164e-15
E:\\R-3.1.2\\scripts\\mPDBS\\summary_pdb_93\\769f910fffb22872.final.pdb
1.298601e-15 3.732409e-16 -3.258011e-15 1.266655e-15 2.920854e-15
-2.275845e-15
E:\\R-3.1.2\\scripts\\mPDBS\\summary_pdb_93\\791f8c80dd5c3135.final.pdb
3.205303e-16 2.039753e-15 -3.093375e-15 -9.030038e-16 4.674488e-15
-1.038002e-15
E:\\R-3.1.2\\scripts\\mPDBS\\summary_pdb_93\\7fad98e66a3f6099.final.pdb
-2.009867e-15 3.689847e-15 -6.062530e-15 -3.838305e-15 2.218953e-15
-1.465668e-16
E:\\R-3.1.2\\scripts\\mPDBS\\summary_pdb_93\\804b89c94a633196.final.pdb
-1.253772e-15 1.707301e-15 -2.316374e-15 -1.768554e-15 5.092620e-15
3.927089e-16
E:\\R-3.1.2\\scripts\\mPDBS\\summary_pdb_93\\813cc65a2298feba.final.pdb
-1.253772e-15 1.707301e-15 -2.316374e-15 -1.768554e-15 5.092620e-15
3.927089e-16
E:\\R-3.1.2\\scripts\\mPDBS\\summary_pdb_93\\83c0eefcfe12d3f9.final.pdb
-8.541351e-16 -1.994816e-15 -3.291505e-15 -1.326200e-15 1.586310e-15
3.201660e-15
E:\\R-3.1.2\\scripts\\mPDBS\\summary_pdb_93\\86d50469ef569760.final.pdb
9.944751e-16 -1.084261e-15 -4.076522e-15 -5.589652e-15 8.637066e-15
-5.217986e-16
E:\\R-3.1.2\\scripts\\mPDBS\\summary_pdb_93\\89d0666e1402203d.final.pdb
6.831207e-15 -3.038488e-15 1.449158e-15 1.750492e-15 2.041621e-15
-6.000164e-15
E:\\R-3.1.2\\scripts\\mPDBS\\summary_pdb_93\\8b34852716dadc62.final.pdb

5.874207e-15 -1.977252e-15 -3.759664e-15 -4.002878e-15 1.889453e-15
-3.258884e-15
E:\\R-3.1.2\\scripts\\mPDBS\\summary_pdb_93\\8b6ef9ac3fe74395.final.pdb
2.074066e-15 -2.405464e-15 -5.521865e-15 -2.241811e-15 2.301504e-15
-3.602467e-15
E:\\R-3.1.2\\scripts\\mPDBS\\summary_pdb_93\\98bbe0d5986c138f.final.pdb
3.205303e-16 2.039753e-15 -3.093375e-15 -9.030038e-16 4.674488e-15
-1.038002e-15
E:\\R-3.1.2\\scripts\\mPDBS\\summary_pdb_93\\9c22f4909d67e2fa.final.pdb
1.167794e-15 1.127795e-15 -8.089750e-15 -1.023491e-15 3.718286e-15
-6.121297e-16
E:\\R-3.1.2\\scripts\\mPDBS\\summary_pdb_93\\9dda8ed0d81fb3ef.final.pdb
1.073003e-14 1.597348e-14 -5.604010e-15 1.847171e-14 5.840449e-15
-1.461260e-15
E:\\R-3.1.2\\scripts\\mPDBS\\summary_pdb_93\\9f752b3616e6033b.final.pdb
1.635529e-16 4.592906e-16 -4.209141e-15 -3.044060e-15 2.126872e-15
-2.365918e-15
E:\\R-3.1.2\\scripts\\mPDBS\\summary_pdb_93\\a02c70083816d6ba.final.pdb
7.005779e-15 1.487718e-15 -3.669344e-15 2.945776e-15 6.751755e-15
1.782534e-16
E:\\R-3.1.2\\scripts\\mPDBS\\summary_pdb_93\\a0c997d891e4e082.final.pdb
8.178037e-15 -3.619799e-15 -5.398178e-15 9.344138e-15 8.174656e-15
-9.120456e-15
E:\\R-3.1.2\\scripts\\mPDBS\\summary_pdb_93\\ab8039a2d823c616.final.pdb
2.363570e-15 1.990405e-15 -3.699667e-15 3.834234e-16 2.844935e-15
-2.519400e-15
E:\\R-3.1.2\\scripts\\mPDBS\\summary_pdb_93\\acf4d74b9c71d4dc.final.pdb
-1.253772e-15 1.707301e-15 -2.316374e-15 -1.768554e-15 5.092620e-15
3.927089e-16
E:\\R-3.1.2\\scripts\\mPDBS\\summary_pdb_93\\ad8a823e4b4c668e.final.pdb
2.280086e-15 4.989283e-16 -2.511374e-15 6.249179e-16 2.310728e-15
-2.297856e-15
E:\\R-3.1.2\\scripts\\mPDBS\\summary_pdb_93\\b16ababd03b7b857.final.pdb
1.167794e-15 1.127795e-15 -8.089750e-15 -1.023491e-15 3.718286e-15
-6.121297e-16
E:\\R-3.1.2\\scripts\\mPDBS\\summary_pdb_93\\b226923e697d3c63.final.pdb
4.208007e-15 -2.020910e-15 -1.858959e-15 1.019621e-14 1.429264e-15
-9.135213e-16
E:\\R-3.1.2\\scripts\\mPDBS\\summary_pdb_93\\b28e48f6fa27a165.final.pdb
3.205303e-16 2.039753e-15 -3.093375e-15 -9.030038e-16 4.674488e-15
-1.038002e-15
E:\\R-3.1.2\\scripts\\mPDBS\\summary_pdb_93\\b42c4924e3cd1aec.final.pdb
-3.078943e-15 -8.504710e-15 -1.213859e-15 -1.256358e-14 5.700803e-15
-1.858078e-15
E:\\R-3.1.2\\scripts\\mPDBS\\summary_pdb_93\\b4dd239fcabff1df.final.pdb
3.205303e-16 2.039753e-15 -3.093375e-15 -9.030038e-16 4.674488e-15
-1.038002e-15
E:\\R-3.1.2\\scripts\\mPDBS\\summary_pdb_93\\b669942ef97459a2.final.pdb
5.874207e-15 -1.977252e-15 -3.759664e-15 -4.002878e-15 1.889453e-15
-3.258884e-15
E:\\R-3.1.2\\scripts\\mPDBS\\summary_pdb_93\\b99f4d9d87c1017b.final.pdb
3.205303e-16 2.039753e-15 -3.093375e-15 -9.030038e-16 4.674488e-15
-1.038002e-15
E:\\R-3.1.2\\scripts\\mPDBS\\summary_pdb_93\\b9d2cbf3975403e7.final.pdb

3.205303e-16 2.039753e-15 -3.093375e-15 -9.030038e-16 4.674488e-15
-1.038002e-15
E:\\R-3.1.2\\scripts\\mPDBS\\summary_pdb_93\\bc88c8a505f3a288.final.pdb
2.451936e-15 3.621673e-16 -3.266847e-15 -1.048853e-17 3.453242e-15
-1.978223e-15
E:\\R-3.1.2\\scripts\\mPDBS\\summary_pdb_93\\bdac0e64f4ef427e.final.pdb
8.317212e-15 5.617042e-15 -2.788652e-15 4.248334e-15 5.034507e-15
-1.380253e-14
E:\\R-3.1.2\\scripts\\mPDBS\\summary_pdb_93\\ca2a1f55fc9d0681.final.pdb
5.874207e-15 -1.977252e-15 -3.759664e-15 -4.002878e-15 1.889453e-15
-3.258884e-15
E:\\R-3.1.2\\scripts\\mPDBS\\summary_pdb_93\\ce34248de3e81a3c.final.pdb
7.860537e-15 3.513707e-15 -6.799378e-16 2.470529e-15 4.700476e-15
2.290267e-15
E:\\R-3.1.2\\scripts\\mPDBS\\summary_pdb_93\\d02e697772494a61.final.pdb
-1.971365e-15 -5.612003e-15 -3.521634e-15 -4.244949e-15 -2.010920e-15
-1.762683e-15
E:\\R-3.1.2\\scripts\\mPDBS\\summary_pdb_93\\d04221245d015047.final.pdb
1.256491e-15 1.737894e-15 -3.698149e-15 -3.131994e-15 3.532536e-15
-2.087887e-15
E:\\R-3.1.2\\scripts\\mPDBS\\summary_pdb_93\\d678141e4018054a.final.pdb
-3.020097e-16 -2.808435e-15 -8.973009e-15 -5.856119e-15 -9.840983e-15
-5.418312e-15
E:\\R-3.1.2\\scripts\\mPDBS\\summary_pdb_93\\d71b40af0f0efe39.final.pdb
-6.776735e-15 8.154621e-15 1.697485e-15 -2.018017e-15 2.522445e-16
-4.471785e-15
E:\\R-3.1.2\\scripts\\mPDBS\\summary_pdb_93\\dc0809eff10197e2.final.pdb
4.939590e-15 -1.130471e-15 -3.122129e-15 5.563775e-15 1.743660e-14
-4.057787e-15
E:\\R-3.1.2\\scripts\\mPDBS\\summary_pdb_93\\e1163918d3707839.final.pdb
-1.276564e-14 -7.562755e-15 -8.274183e-15 -1.211429e-14 -8.447421e-16
6.218794e-15
E:\\R-3.1.2\\scripts\\mPDBS\\summary_pdb_93\\e4b48b06824ef848.final.pdb
4.584086e-15 5.495175e-15 7.943032e-16 5.909860e-15 -4.449986e-15
2.069203e-15
E:\\R-3.1.2\\scripts\\mPDBS\\summary_pdb_93\\e529e19ad281fea2.final.pdb
-1.317911e-15 1.214248e-15 -3.351407e-15 4.079697e-15 -5.725813e-16
-2.332850e-15
E:\\R-3.1.2\\scripts\\mPDBS\\summary_pdb_93\\e5602082718ec3a1.final.pdb
-1.253772e-15 1.707301e-15 -2.316374e-15 -1.768554e-15 5.092620e-15
3.927089e-16
E:\\R-3.1.2\\scripts\\mPDBS\\summary_pdb_93\\e5d589d996d0a960.final.pdb
6.879512e-15 -7.057171e-16 -1.462864e-14 -1.331301e-15 1.949767e-14
-1.019112e-14
E:\\R-3.1.2\\scripts\\mPDBS\\summary_pdb_93\\e9df9e72307a2fbd.final.pdb
7.005779e-15 1.487718e-15 -3.669344e-15 2.945776e-15 6.751755e-15
1.782534e-16
E:\\R-3.1.2\\scripts\\mPDBS\\summary_pdb_93\\ea00f8694090cae7.final.pdb
-1.253772e-15 1.707301e-15 -2.316374e-15 -1.768554e-15 5.092620e-15
3.927089e-16
E:\\R-3.1.2\\scripts\\mPDBS\\summary_pdb_93\\eba517be32db050e.final.pdb
3.205303e-16 2.039753e-15 -3.093375e-15 -9.030038e-16 4.674488e-15
-1.038002e-15
E:\\R-3.1.2\\scripts\\mPDBS\\summary_pdb_93\\ec04b9545899c29d.final.pdb

2.058333e-15 7.534042e-17 -3.774094e-15 -4.959747e-16 2.402877e-15
-2.237549e-15
E:\\R-3.1.2\\scripts\\mPDBS\\summary_pdb_93\\ee6862cbcad52078.final.pdb
2.253446e-15 7.499864e-16 -3.025720e-15 9.446224e-17 3.725160e-15
-1.875874e-15
E:\\R-3.1.2\\scripts\\mPDBS\\summary_pdb_93\\ef6a42fc83b81e5d.final.pdb
2.020788e-15 1.165901e-15 -3.325882e-15 3.914875e-16 2.744997e-15
-2.141404e-15
E:\\R-3.1.2\\scripts\\mPDBS\\summary_pdb_93\\ef8e88d4ad93a0ad.final.pdb
1.256491e-15 1.737894e-15 -3.698149e-15 -3.131994e-15 3.532536e-15
-2.087887e-15
E:\\R-3.1.2\\scripts\\mPDBS\\summary_pdb_93\\f14fcae8deec4fa3.final.pdb
1.635529e-16 4.592906e-16 -4.209141e-15 -3.044060e-15 2.126872e-15
-2.365918e-15
E:\\R-3.1.2\\scripts\\mPDBS\\summary_pdb_93\\f83584e22003fb92.final.pdb
9.662260e-15 5.095740e-15 -9.698562e-16 -1.724683e-16 -4.992476e-15
-4.148795e-15
E:\\R-3.1.2\\scripts\\mPDBS\\summary_pdb_93\\f8e2bf87e52caa29.final.pdb
-1.317911e-15 1.214248e-15 -3.351407e-15 4.079697e-15 -5.725813e-16
-2.332850e-15

[,229]   [,230]   [,231]   [,232]   [,233]   [,234]
E:\\R-3.1.2\\scripts\\40.1ns_data\\NAT0.pdb
-5.995569e-16 -7.184444e-17 -2.427700e-15 2.660570e-15 5.131006e-15
4.856505e-16
E:\\R-3.1.2\\scripts\\40.1ns_data\\LJP6.pdb
-5.781698e-16 -5.881805e-16 6.550597e-17 5.929997e-16 3.345668e-15
9.780251e-16
E:\\R-3.1.2\\scripts\\40.1ns_data\\3WY9.pdb
2.007567e-16 -3.933349e-16 -1.362569e-15 2.596302e-16 2.506672e-15
-4.182889e-16
E:\\R-3.1.2\\scripts\\40.1ns_data\\ZSP9.pdb
-5.422932e-16 3.048204e-16 1.692821e-15 1.203839e-15 1.274741e-15
6.842157e-16
E:\\R-3.1.2\\scripts\\mPDBS\\summary_pdb_93\\013d855ad26c877c.final.pdb
-9.636576e-17 -2.453431e-16 -1.439767e-15 3.149712e-15 8.158424e-15
1.148797e-15
E:\\R-3.1.2\\scripts\\mPDBS\\summary_pdb_93\\0a2a705342ca0d54.final.pdb
9.142207e-17 1.010547e-15 -8.446427e-17 7.460611e-16 1.837929e-15
9.864838e-16
E:\\R-3.1.2\\scripts\\mPDBS\\summary_pdb_93\\0a59fceadb4194d9.final.pdb
-2.693257e-15 2.928102e-15 -3.890328e-16 1.518499e-15 3.917119e-17
-2.180641e-16
E:\\R-3.1.2\\scripts\\mPDBS\\summary_pdb_93\\0dba59f3306825e8.final.pdb
-5.430571e-16 1.537953e-15 1.251440e-15 3.446637e-16 -3.286414e-15
-2.705759e-15
E:\\R-3.1.2\\scripts\\mPDBS\\summary_pdb_93\\126f26a78b57339c.final.pdb
3.056060e-15 2.439738e-15 -1.685772e-15 -1.295084e-15 7.059477e-15
5.990301e-15
E:\\R-3.1.2\\scripts\\mPDBS\\summary_pdb_93\\12f06f3e342df706.final.pdb
-5.512930e-16 1.124226e-15 2.430190e-16 5.166440e-16 2.822385e-15
1.192265e-15
E:\\R-3.1.2\\scripts\\mPDBS\\summary_pdb_93\\18618a2a251b867b.final.pdb
1.186505e-15 4.675259e-15 2.370665e-15 3.018739e-15 3.373711e-15

6.059261e-15
E:\\R-3.1.2\\scripts\\mPDBS\\summary_pdb_93\\1f305f598641eeb2.final.pdb
-2.274993e-16 2.053919e-15 4.027641e-16 -1.054095e-15 2.446498e-15
1.146598e-15
E:\\R-3.1.2\\scripts\\mPDBS\\summary_pdb_93\\1f8a00b551be0a39.final.pdb
1.186505e-15 4.675259e-15 2.370665e-15 3.018739e-15 3.373711e-15
6.059261e-15
E:\\R-3.1.2\\scripts\\mPDBS\\summary_pdb_93\\219f45328d47579a.final.pdb
5.503745e-15 4.769375e-16 2.101813e-15 -1.201765e-15 -3.217338e-15
1.245008e-14
E:\\R-3.1.2\\scripts\\mPDBS\\summary_pdb_93\\238dd3918e54336d.final.pdb
7.856897e-15 -2.371641e-15 7.440587e-15 7.818364e-15 8.976033e-15
8.127962e-15
E:\\R-3.1.2\\scripts\\mPDBS\\summary_pdb_93\\2b3027889e2fdc42.final.pdb
-6.458498e-16 1.822812e-15 2.843652e-16 2.626672e-16 2.430488e-15
1.438705e-15
E:\\R-3.1.2\\scripts\\mPDBS\\summary_pdb_93\\2fcb3b46e33793b0.final.pdb
7.664973e-16 1.162272e-15 9.860648e-16 1.391865e-15 1.457706e-15
-2.271675e-15
E:\\R-3.1.2\\scripts\\mPDBS\\summary_pdb_93\\34eb328251842006.final.pdb
-6.459655e-15 9.263803e-17 1.098379e-14 -9.143185e-15 3.204381e-15
3.387632e-15
E:\\R-3.1.2\\scripts\\mPDBS\\summary_pdb_93\\354b32871797b7ab.final.pdb
-7.345936e-15 2.394183e-15 6.716843e-15 -6.673375e-15 3.423994e-15
-1.597289e-15
E:\\R-3.1.2\\scripts\\mPDBS\\summary_pdb_93\\397368adbc32d5de.final.pdb
3.056060e-15 2.439738e-15 -1.685772e-15 -1.295084e-15 7.059477e-15
5.990301e-15
E:\\R-3.1.2\\scripts\\mPDBS\\summary_pdb_93\\411ca13cd3df6edd.final.pdb
2.357672e-15 -1.280917e-16 4.571847e-15 -2.732006e-15 5.438896e-15
-5.884507e-16
E:\\R-3.1.2\\scripts\\mPDBS\\summary_pdb_93\\42357eee6642bf12.final.pdb
-4.034987e-15 7.022572e-15 1.672132e-15 8.105610e-15 -1.094116e-14
1.018098e-14
E:\\R-3.1.2\\scripts\\mPDBS\\summary_pdb_93\\44342d1f75a7fcfc.final.pdb
5.274968e-17 -3.785232e-15 4.093594e-15 6.883543e-15 1.395006e-14
4.199376e-15
E:\\R-3.1.2\\scripts\\mPDBS\\summary_pdb_93\\448ef21dffe8cdfb.final.pdb
2.096511e-16 1.036671e-14 -1.843703e-15 -1.788785e-14 1.873902e-14
1.053102e-14
E:\\R-3.1.2\\scripts\\mPDBS\\summary_pdb_93\\472a40b3e8f113c7.final.pdb
-5.430571e-16 1.537953e-15 1.251440e-15 3.446637e-16 -3.286414e-15
-2.705759e-15
E:\\R-3.1.2\\scripts\\mPDBS\\summary_pdb_93\\48a26225951ea153.final.pdb
-4.542039e-15 4.669493e-15 2.896208e-15 -3.579528e-15 8.830750e-15
-1.666125e-15
E:\\R-3.1.2\\scripts\\mPDBS\\summary_pdb_93\\4ab4a5c7c6682ce6.final.pdb
-3.876762e-15 1.200500e-14 5.654276e-15 -5.232906e-15 -7.441766e-16
6.564605e-16
E:\\R-3.1.2\\scripts\\mPDBS\\summary_pdb_93\\4eaa45b62cb6a25f.final.pdb
1.997718e-15 3.369640e-16 -3.599720e-15 4.128314e-15 6.433739e-15
1.888799e-16
E:\\R-3.1.2\\scripts\\mPDBS\\summary_pdb_93\\58c371611d85ac96.final.pdb
3.056060e-15 2.439738e-15 -1.685772e-15 -1.295084e-15 7.059477e-15

5.990301e-15
E:\\R-3.1.2\\scripts\\mPDBS\\summary_pdb_93\\59a647f04822d30a.final.pdb
4.621444e-16 4.609095e-15 -2.442194e-15 -4.680991e-15 3.801064e-15
-2.716928e-17
E:\\R-3.1.2\\scripts\\mPDBS\\summary_pdb_93\\59b091fe385e05fc.final.pdb
-2.693257e-15 2.928102e-15 -3.890328e-16 1.518499e-15 3.917119e-17
-2.180641e-16
E:\\R-3.1.2\\scripts\\mPDBS\\summary_pdb_93\\5c35ac30f930d2db.final.pdb
1.997718e-15 3.369640e-16 -3.599720e-15 4.128314e-15 6.433739e-15
1.888799e-16
E:\\R-3.1.2\\scripts\\mPDBS\\summary_pdb_93\\5f60752431cf2dcb.final.pdb
8.373532e-16 3.007743e-16 -4.365437e-15 -1.562097e-15 5.064515e-15
7.660655e-16
E:\\R-3.1.2\\scripts\\mPDBS\\summary_pdb_93\\626456dd6ff2868d.final.pdb
-5.430571e-16 1.537953e-15 1.251440e-15 3.446637e-16 -3.286414e-15
-2.705759e-15
E:\\R-3.1.2\\scripts\\mPDBS\\summary_pdb_93\\6583d53ff3c8f7ef.final.pdb
4.621444e-16 4.609095e-15 -2.442194e-15 -4.680991e-15 3.801064e-15
-2.716928e-17
E:\\R-3.1.2\\scripts\\mPDBS\\summary_pdb_93\\69760178986bef01.final.pdb
5.010820e-15 -3.280925e-16 2.368735e-15 -1.538200e-15 4.455578e-15
2.370727e-15
E:\\R-3.1.2\\scripts\\mPDBS\\summary_pdb_93\\6a9d87d47926b1c8.final.pdb
8.968333e-17 1.974832e-15 2.519139e-16 -6.740526e-16 1.754693e-15
1.198995e-15
E:\\R-3.1.2\\scripts\\mPDBS\\summary_pdb_93\\6bb3547ea982ffc4.final.pdb
-1.175304e-15 -1.381061e-14 8.382560e-15 -1.142652e-14 1.095756e-14
1.209324e-14
E:\\R-3.1.2\\scripts\\mPDBS\\summary_pdb_93\\716d9624611d1c42.final.pdb
-4.140870e-15 -3.126768e-15 8.659193e-15 -1.163653e-15 9.740895e-15
-5.293393e-16
E:\\R-3.1.2\\scripts\\mPDBS\\summary_pdb_93\\75f27e70ad0f1be4.final.pdb
4.621444e-16 4.609095e-15 -2.442194e-15 -4.680991e-15 3.801064e-15
-2.716928e-17
E:\\R-3.1.2\\scripts\\mPDBS\\summary_pdb_93\\769f910fffb22872.final.pdb
-6.696491e-16 1.034183e-15 5.416703e-16 1.448442e-16 1.723122e-15
1.414701e-15
E:\\R-3.1.2\\scripts\\mPDBS\\summary_pdb_93\\791f8c80dd5c3135.final.pdb
-2.693257e-15 2.928102e-15 -3.890328e-16 1.518499e-15 3.917119e-17
-2.180641e-16
E:\\R-3.1.2\\scripts\\mPDBS\\summary_pdb_93\\7fad98e66a3f6099.final.pdb
-6.637820e-15 -8.409248e-15 4.192474e-15 -4.171982e-16 6.975350e-15
-5.694751e-16
E:\\R-3.1.2\\scripts\\mPDBS\\summary_pdb_93\\804b89c94a633196.final.pdb
7.664973e-16 1.162272e-15 9.860648e-16 1.391865e-15 1.457706e-15
-2.271675e-15
E:\\R-3.1.2\\scripts\\mPDBS\\summary_pdb_93\\813cc65a2298feba.final.pdb
7.664973e-16 1.162272e-15 9.860648e-16 1.391865e-15 1.457706e-15
-2.271675e-15
E:\\R-3.1.2\\scripts\\mPDBS\\summary_pdb_93\\83c0eefcfe12d3f9.final.pdb
2.357672e-15 -1.280917e-16 4.571847e-15 -2.732006e-15 5.438896e-15
-5.884507e-16
E:\\R-3.1.2\\scripts\\mPDBS\\summary_pdb_93\\86d50469ef569760.final.pdb
-5.430571e-16 1.537953e-15 1.251440e-15 3.446637e-16 -3.286414e-15

-2.705759e-15
E:\\R-3.1.2\\scripts\\mPDBS\\summary_pdb_93\\89d0666e1402203d.final.pdb
4.621444e-16 4.609095e-15 -2.442194e-15 -4.680991e-15 3.801064e-15
-2.716928e-17
E:\\R-3.1.2\\scripts\\mPDBS\\summary_pdb_93\\8b34852716dadc62.final.pdb
-3.142354e-16 3.295981e-15 2.413309e-15 -9.999673e-17 -3.073392e-15
3.042651e-15
E:\\R-3.1.2\\scripts\\mPDBS\\summary_pdb_93\\8b6ef9ac3fe74395.final.pdb
-7.683512e-15 -4.101766e-15 -3.838765e-15 -4.208298e-15 1.891594e-15
3.821921e-15
E:\\R-3.1.2\\scripts\\mPDBS\\summary_pdb_93\\98bbe0d5986c138f.final.pdb
-2.693257e-15 2.928102e-15 -3.890328e-16 1.518499e-15 3.917119e-17
-2.180641e-16
E:\\R-3.1.2\\scripts\\mPDBS\\summary_pdb_93\\9c22f4909d67e2fa.final.pdb
2.708520e-15 9.368659e-16 -3.464531e-15 5.961778e-15 1.691026e-15
2.328378e-15
E:\\R-3.1.2\\scripts\\mPDBS\\summary_pdb_93\\9dda8ed0d81fb3ef.final.pdb
-2.623061e-15 4.430147e-15 1.670510e-15 -7.185281e-15 1.105134e-14
-6.046226e-15
E:\\R-3.1.2\\scripts\\mPDBS\\summary_pdb_93\\9f752b3616e6033b.final.pdb
1.141776e-15 1.144005e-15 -1.849549e-16 -6.917868e-16 -1.657289e-15
3.100205e-15
E:\\R-3.1.2\\scripts\\mPDBS\\summary_pdb_93\\a02c70083816d6ba.final.pdb
1.997718e-15 3.369640e-16 -3.599720e-15 4.128314e-15 6.433739e-15
1.888799e-16
E:\\R-3.1.2\\scripts\\mPDBS\\summary_pdb_93\\a0c997d891e4e082.final.pdb
4.078388e-15 -1.028508e-15 4.270915e-15 -8.466679e-15 1.021784e-14
-7.124792e-15
E:\\R-3.1.2\\scripts\\mPDBS\\summary_pdb_93\\ab8039a2d823c616.final.pdb
-7.337354e-16 4.250274e-16 3.096198e-16 -3.410169e-16 1.751093e-15
8.758064e-16
E:\\R-3.1.2\\scripts\\mPDBS\\summary_pdb_93\\acf4d74b9c71d4dc.final.pdb
7.664973e-16 1.162272e-15 9.860648e-16 1.391865e-15 1.457706e-15
-2.271675e-15
E:\\R-3.1.2\\scripts\\mPDBS\\summary_pdb_93\\ad8a823e4b4c668e.final.pdb
-9.684876e-17 1.653704e-15 4.715113e-16 6.784016e-17 1.631274e-15
9.375865e-16
E:\\R-3.1.2\\scripts\\mPDBS\\summary_pdb_93\\b16ababd03b7b857.final.pdb
2.708520e-15 9.368659e-16 -3.464531e-15 5.961778e-15 1.691026e-15
2.328378e-15
E:\\R-3.1.2\\scripts\\mPDBS\\summary_pdb_93\\b226923e697d3c63.final.pdb
-4.295034e-16 4.362656e-15 2.770162e-15 -2.028274e-15 3.092429e-15
1.778949e-15
E:\\R-3.1.2\\scripts\\mPDBS\\summary_pdb_93\\b28e48f6fa27a165.final.pdb
-2.693257e-15 2.928102e-15 -3.890328e-16 1.518499e-15 3.917119e-17
-2.180641e-16
E:\\R-3.1.2\\scripts\\mPDBS\\summary_pdb_93\\b42c4924e3cd1aec.final.pdb
-3.548103e-15 -2.065958e-15 2.279778e-15 1.120192e-14 -2.613510e-15
-4.288120e-16
E:\\R-3.1.2\\scripts\\mPDBS\\summary_pdb_93\\b4dd239fcabff1df.final.pdb
-2.693257e-15 2.928102e-15 -3.890328e-16 1.518499e-15 3.917119e-17
-2.180641e-16
E:\\R-3.1.2\\scripts\\mPDBS\\summary_pdb_93\\b669942ef97459a2.final.pdb
-3.142354e-16 3.295981e-15 2.413309e-15 -9.999673e-17 -3.073392e-15

3.042651e-15
E:\\R-3.1.2\\scripts\\mPDBS\\summary_pdb_93\\b99f4d9d87c1017b.final.pdb
-2.693257e-15 2.928102e-15 -3.890328e-16 1.518499e-15 3.917119e-17
-2.180641e-16
E:\\R-3.1.2\\scripts\\mPDBS\\summary_pdb_93\\b9d2cbf3975403e7.final.pdb
-2.693257e-15 2.928102e-15 -3.890328e-16 1.518499e-15 3.917119e-17
-2.180641e-16
E:\\R-3.1.2\\scripts\\mPDBS\\summary_pdb_93\\bc88c8a505f3a288.final.pdb
-1.753061e-16 1.137976e-15 -2.858979e-17 9.906006e-16 2.880215e-15
1.630880e-15
E:\\R-3.1.2\\scripts\\mPDBS\\summary_pdb_93\\bdac0e64f4ef427e.final.pdb
-6.887536e-15 6.064980e-15 -6.880960e-15 3.575882e-15 -4.573060e-15
4.837764e-15
E:\\R-3.1.2\\scripts\\mPDBS\\summary_pdb_93\\ca2a1f55fc9d0681.final.pdb
-3.142354e-16 3.295981e-15 2.413309e-15 -9.999673e-17 -3.073392e-15
3.042651e-15
E:\\R-3.1.2\\scripts\\mPDBS\\summary_pdb_93\\ce34248de3e81a3c.final.pdb
-4.950279e-15 4.489981e-15 -6.331036e-15 7.974928e-15 4.775361e-15
-5.534508e-15
E:\\R-3.1.2\\scripts\\mPDBS\\summary_pdb_93\\d02e697772494a61.final.pdb
4.436023e-15 1.914861e-15 -2.920482e-15 -3.265652e-15 -1.634403e-15
1.034971e-14
E:\\R-3.1.2\\scripts\\mPDBS\\summary_pdb_93\\d04221245d015047.final.pdb
-4.509754e-16 2.413093e-16 -3.278368e-16 1.136751e-15 2.997925e-15
1.571376e-15
E:\\R-3.1.2\\scripts\\mPDBS\\summary_pdb_93\\d678141e4018054a.final.pdb
-5.108464e-15 -1.780385e-16 1.140990e-14 -6.971873e-15 2.235029e-15
7.887594e-15
E:\\R-3.1.2\\scripts\\mPDBS\\summary_pdb_93\\d71b40af0f0efe39.final.pdb
6.286005e-15 9.724796e-15 -8.509595e-16 4.850731e-15 1.835591e-15
-4.809029e-16
E:\\R-3.1.2\\scripts\\mPDBS\\summary_pdb_93\\dc0809eff10197e2.final.pdb
1.226814e-15 4.949323e-15 -6.274966e-15 -3.327147e-15 2.642894e-15
-8.480627e-15
E:\\R-3.1.2\\scripts\\mPDBS\\summary_pdb_93\\e1163918d3707839.final.pdb
-6.089472e-15 2.836370e-15 -1.028123e-15 2.201307e-15 -1.033737e-14
6.739301e-16
E:\\R-3.1.2\\scripts\\mPDBS\\summary_pdb_93\\e4b48b06824ef848.final.pdb
1.186505e-15 4.675259e-15 2.370665e-15 3.018739e-15 3.373711e-15
6.059261e-15
E:\\R-3.1.2\\scripts\\mPDBS\\summary_pdb_93\\e529e19ad281fea2.final.pdb
-9.636576e-17 -2.453431e-16 -1.439767e-15 3.149712e-15 8.158424e-15
1.148797e-15
E:\\R-3.1.2\\scripts\\mPDBS\\summary_pdb_93\\e5602082718ec3a1.final.pdb
7.664973e-16 1.162272e-15 9.860648e-16 1.391865e-15 1.457706e-15
-2.271675e-15
E:\\R-3.1.2\\scripts\\mPDBS\\summary_pdb_93\\e5d589d996d0a960.final.pdb
-1.460684e-15 -3.057603e-15 -6.227116e-15 -5.142709e-15 5.801108e-16
-3.572260e-15
E:\\R-3.1.2\\scripts\\mPDBS\\summary_pdb_93\\e9df9e72307a2fbd.final.pdb
1.997718e-15 3.369640e-16 -3.599720e-15 4.128314e-15 6.433739e-15
1.888799e-16
E:\\R-3.1.2\\scripts\\mPDBS\\summary_pdb_93\\ea00f8694090cae7.final.pdb
7.664973e-16 1.162272e-15 9.860648e-16 1.391865e-15 1.457706e-15

-2.271675e-15
E:\\R-3.1.2\\scripts\\mPDBS\\summary_pdb_93\\eba517be32db050e.final.pdb
-2.693257e-15 2.928102e-15 -3.890328e-16 1.518499e-15 3.917119e-17
-2.180641e-16
E:\\R-3.1.2\\scripts\\mPDBS\\summary_pdb_93\\ec04b9545899c29d.final.pdb
-1.215900e-16 1.939355e-15 6.618768e-16 -1.234388e-16 2.562905e-15
1.248611e-15
E:\\R-3.1.2\\scripts\\mPDBS\\summary_pdb_93\\ee6862cbcad52078.final.pdb
-4.389841e-16 1.601038e-15 1.350705e-16 2.530178e-16 2.659471e-15
1.771989e-15
E:\\R-3.1.2\\scripts\\mPDBS\\summary_pdb_93\\ef6a42fc83b81e5d.final.pdb
-4.692885e-16 1.251186e-15 3.322566e-16 6.678168e-17 2.554488e-15
1.438174e-15
E:\\R-3.1.2\\scripts\\mPDBS\\summary_pdb_93\\ef8e88d4ad93a0ad.final.pdb
-4.509754e-16 2.413093e-16 -3.278368e-16 1.136751e-15 2.997925e-15
1.571376e-15
E:\\R-3.1.2\\scripts\\mPDBS\\summary_pdb_93\\f14fcae8deec4fa3.final.pdb
1.141776e-15 1.144005e-15 -1.849549e-16 -6.917868e-16 -1.657289e-15
3.100205e-15
E:\\R-3.1.2\\scripts\\mPDBS\\summary_pdb_93\\f83584e22003fb92.final.pdb
-4.542039e-15 4.669493e-15 2.896208e-15 -3.579528e-15 8.830750e-15
-1.666125e-15
E:\\R-3.1.2\\scripts\\mPDBS\\summary_pdb_93\\f8e2bf87e52caa29.final.pdb
-9.636576e-17 -2.453431e-16 -1.439767e-15 3.149712e-15 8.158424e-15
1.148797e-15

[,235]   [,236]   [,237]   [,238]   [,239]   [,240]
E:\\R-3.1.2\\scripts\\40.1ns_data\\NAT0.pdb
1.197883e-15 3.359361e-15 -3.526243e-15 -1.836930e-15 2.226985e-16
1.622307e-15
E:\\R-3.1.2\\scripts\\40.1ns_data\\LJP6.pdb
6.734242e-16 2.625628e-15 -1.985496e-15 -2.087814e-15 -5.124494e-16
1.222607e-15
E:\\R-3.1.2\\scripts\\40.1ns_data\\3WY9.pdb
1.877720e-15 3.451091e-15 -2.499307e-15 -1.840119e-15 -3.754325e-16
1.266015e-15
E:\\R-3.1.2\\scripts\\40.1ns_data\\ZSP9.pdb
3.108431e-15 3.799901e-15 -4.316488e-15 2.731703e-16 -6.037163e-16
1.980129e-15
E:\\R-3.1.2\\scripts\\mPDBS\\summary_pdb_93\\013d855ad26c877c.final.pdb
3.558266e-15 4.269913e-15 -2.583671e-16 2.780369e-15 -5.180421e-15
-8.039302e-16
E:\\R-3.1.2\\scripts\\mPDBS\\summary_pdb_93\\0a2a705342ca0d54.final.pdb
1.340580e-15 2.165635e-15 -1.096953e-15 -1.658966e-15 6.080862e-16
3.155657e-15
E:\\R-3.1.2\\scripts\\mPDBS\\summary_pdb_93\\0a59fceadb4194d9.final.pdb
-1.507070e-15 2.353007e-15 3.805941e-16 -2.722855e-15 -2.127129e-15
5.346888e-15
E:\\R-3.1.2\\scripts\\mPDBS\\summary_pdb_93\\0dba59f3306825e8.final.pdb
4.874921e-15 3.499695e-15 7.123031e-17 -8.087737e-16 -6.389150e-16
2.006322e-15
E:\\R-3.1.2\\scripts\\mPDBS\\summary_pdb_93\\126f26a78b57339c.final.pdb
3.656495e-15 1.604185e-15 -5.232470e-15 1.231794e-16 1.216334e-14
6.576017e-15

E:\\R-3.1.2\\scripts\\mPDBS\\summary_pdb_93\\12f06f3e342df706.final.pdb
1.783802e-15 1.433582e-15 -1.971470e-15 -1.395207e-15 5.178806e-16
3.025878e-15
E:\\R-3.1.2\\scripts\\mPDBS\\summary_pdb_93\\18618a2a251b867b.final.pdb
8.133746e-15 3.148742e-15 -6.536093e-15 -7.101668e-16 1.302357e-14
1.450461e-15
E:\\R-3.1.2\\scripts\\mPDBS\\summary_pdb_93\\1f305f598641eeb2.final.pdb
1.851698e-15 3.070123e-15 -1.625913e-15 -1.202068e-15 -3.085752e-17
1.570244e-15
E:\\R-3.1.2\\scripts\\mPDBS\\summary_pdb_93\\1f8a00b551be0a39.final.pdb
8.133746e-15 3.148742e-15 -6.536093e-15 -7.101668e-16 1.302357e-14
1.450461e-15
E:\\R-3.1.2\\scripts\\mPDBS\\summary_pdb_93\\219f45328d47579a.final.pdb
6.872945e-15 1.131762e-15 2.389157e-17 1.127463e-14 4.564567e-15
-8.614166e-15
E:\\R-3.1.2\\scripts\\mPDBS\\summary_pdb_93\\238dd3918e54336d.final.pdb
8.140378e-15 8.559197e-15 -3.023150e-15 1.271423e-14 8.361009e-15
-7.875825e-15
E:\\R-3.1.2\\scripts\\mPDBS\\summary_pdb_93\\2b3027889e2fdc42.final.pdb
1.545827e-15 1.921495e-15 -1.331144e-15 -1.459235e-16 1.128327e-16
3.319430e-15
E:\\R-3.1.2\\scripts\\mPDBS\\summary_pdb_93\\2fcb3b46e33793b0.final.pdb
3.768568e-15 2.924406e-15 -3.411811e-15 -4.007116e-15 -6.855260e-15
3.836934e-16
E:\\R-3.1.2\\scripts\\mPDBS\\summary_pdb_93\\34eb328251842006.final.pdb
-8.027632e-15 -6.038083e-15 2.776206e-15 -1.956421e-15 -6.187940e-15
-1.959940e-15
E:\\R-3.1.2\\scripts\\mPDBS\\summary_pdb_93\\354b32871797b7ab.final.pdb
-3.998430e-15 3.206559e-15 -3.715246e-15 -1.678472e-14 -1.358210e-14
-1.481313e-15
E:\\R-3.1.2\\scripts\\mPDBS\\summary_pdb_93\\397368adbc32d5de.final.pdb
3.656495e-15 1.604185e-15 -5.232470e-15 1.231794e-16 1.216334e-14
6.576017e-15
E:\\R-3.1.2\\scripts\\mPDBS\\summary_pdb_93\\411ca13cd3df6edd.final.pdb
6.933354e-15 -1.699174e-15 -9.161769e-15 -7.372886e-15 1.596313e-15
-1.377485e-15
E:\\R-3.1.2\\scripts\\mPDBS\\summary_pdb_93\\42357eee6642bf12.final.pdb
4.289323e-15 -7.308252e-16 -5.852695e-15 1.105933e-15 6.132692e-15
-5.463636e-15
E:\\R-3.1.2\\scripts\\mPDBS\\summary_pdb_93\\44342d1f75a7fcfc.final.pdb
-1.563034e-15 -2.267765e-15 -7.868246e-15 -9.595260e-16 5.438856e-15
7.967235e-15
E:\\R-3.1.2\\scripts\\mPDBS\\summary_pdb_93\\448ef21dffe8cdfb.final.pdb
-4.887702e-15 4.978319e-15 -8.461156e-17 -2.027797e-15 -4.027660e-15
3.507336e-16
E:\\R-3.1.2\\scripts\\mPDBS\\summary_pdb_93\\472a40b3e8f113c7.final.pdb
4.874921e-15 3.499695e-15 7.123031e-17 -8.087737e-16 -6.389150e-16
2.006322e-15
E:\\R-3.1.2\\scripts\\mPDBS\\summary_pdb_93\\48a26225951ea153.final.pdb
-1.345958e-15 -1.440399e-15 -8.379543e-15 -4.122036e-16 3.198038e-15
2.450334e-15
E:\\R-3.1.2\\scripts\\mPDBS\\summary_pdb_93\\4ab4a5c7c6682ce6.final.pdb
-4.609463e-15 -4.844222e-16 -2.878450e-15 -1.178049e-14 -5.513488e-15
9.665560e-15

E:\\R-3.1.2\\scripts\\mPDBS\\summary_pdb_93\\4eaa45b62cb6a25f.final.pdb
5.431310e-15 2.480360e-15 -3.282649e-15 -2.395735e-16 6.439207e-15
7.718576e-15
E:\\R-3.1.2\\scripts\\mPDBS\\summary_pdb_93\\58c371611d85ac96.final.pdb
3.656495e-15 1.604185e-15 -5.232470e-15 1.231794e-16 1.216334e-14
6.576017e-15
E:\\R-3.1.2\\scripts\\mPDBS\\summary_pdb_93\\59a647f04822d30a.final.pdb
-1.552626e-15 -6.752884e-16 -2.179437e-15 -2.927570e-15 -1.474017e-15
6.543894e-15
E:\\R-3.1.2\\scripts\\mPDBS\\summary_pdb_93\\59b091fe385e05fc.final.pdb
-1.507070e-15 2.353007e-15 3.805941e-16 -2.722855e-15 -2.127129e-15
5.346888e-15
E:\\R-3.1.2\\scripts\\mPDBS\\summary_pdb_93\\5c35ac30f930d2db.final.pdb
5.431310e-15 2.480360e-15 -3.282649e-15 -2.395735e-16 6.439207e-15
7.718576e-15
E:\\R-3.1.2\\scripts\\mPDBS\\summary_pdb_93\\5f60752431cf2dcb.final.pdb
5.335432e-15 -3.411067e-15 -1.306204e-15 -2.077035e-15 5.336509e-15
6.011446e-15
E:\\R-3.1.2\\scripts\\mPDBS\\summary_pdb_93\\626456dd6ff2868d.final.pdb
4.874921e-15 3.499695e-15 7.123031e-17 -8.087737e-16 -6.389150e-16
2.006322e-15
E:\\R-3.1.2\\scripts\\mPDBS\\summary_pdb_93\\6583d53ff3c8f7ef.final.pdb
-1.552626e-15 -6.752884e-16 -2.179437e-15 -2.927570e-15 -1.474017e-15
6.543894e-15
E:\\R-3.1.2\\scripts\\mPDBS\\summary_pdb_93\\69760178986bef01.final.pdb
5.288819e-15 7.714003e-15 -4.373609e-15 1.440798e-15 -4.586990e-15
-6.998671e-15
E:\\R-3.1.2\\scripts\\mPDBS\\summary_pdb_93\\6a9d87d47926b1c8.final.pdb
1.588704e-15 2.998903e-15 -1.376180e-15 5.377783e-16 3.533468e-16
2.180228e-15
E:\\R-3.1.2\\scripts\\mPDBS\\summary_pdb_93\\6bb3547ea982ffc4.final.pdb
1.093827e-14 -4.211751e-15 -6.262698e-15 6.327385e-15 8.923588e-15
-8.837237e-15
E:\\R-3.1.2\\scripts\\mPDBS\\summary_pdb_93\\716d9624611d1c42.final.pdb
-3.156470e-16 6.752244e-15 -5.552807e-15 -1.502407e-15 -2.138848e-15
1.693285e-15
E:\\R-3.1.2\\scripts\\mPDBS\\summary_pdb_93\\75f27e70ad0f1be4.final.pdb
-1.552626e-15 -6.752884e-16 -2.179437e-15 -2.927570e-15 -1.474017e-15
6.543894e-15
E:\\R-3.1.2\\scripts\\mPDBS\\summary_pdb_93\\769f910fffb22872.final.pdb
3.155523e-15 2.323664e-15 -9.869291e-16 -1.390395e-15 6.267998e-16
2.485007e-15
E:\\R-3.1.2\\scripts\\mPDBS\\summary_pdb_93\\791f8c80dd5c3135.final.pdb
-1.507070e-15 2.353007e-15 3.805941e-16 -2.722855e-15 -2.127129e-15
5.346888e-15
E:\\R-3.1.2\\scripts\\mPDBS\\summary_pdb_93\\7fad98e66a3f6099.final.pdb
-7.814171e-15 1.159035e-15 -2.811955e-15 -3.368493e-15 -1.660564e-14
-1.123684e-14
E:\\R-3.1.2\\scripts\\mPDBS\\summary_pdb_93\\804b89c94a633196.final.pdb
3.768568e-15 2.924406e-15 -3.411811e-15 -4.007116e-15 -6.855260e-15
3.836934e-16
E:\\R-3.1.2\\scripts\\mPDBS\\summary_pdb_93\\813cc65a2298feba.final.pdb
3.768568e-15 2.924406e-15 -3.411811e-15 -4.007116e-15 -6.855260e-15
3.836934e-16

E:\\R-3.1.2\\scripts\\mPDBS\\summary_pdb_93\\83c0eefcfe12d3f9.final.pdb
6.933354e-15 -1.699174e-15 -9.161769e-15 -7.372886e-15 1.596313e-15
-1.377485e-15
E:\\R-3.1.2\\scripts\\mPDBS\\summary_pdb_93\\86d50469ef569760.final.pdb
4.874921e-15 3.499695e-15 7.123031e-17 -8.087737e-16 -6.389150e-16
2.006322e-15
E:\\R-3.1.2\\scripts\\mPDBS\\summary_pdb_93\\89d0666e1402203d.final.pdb
-1.552626e-15 -6.752884e-16 -2.179437e-15 -2.927570e-15 -1.474017e-15
6.543894e-15
E:\\R-3.1.2\\scripts\\mPDBS\\summary_pdb_93\\8b34852716dadc62.final.pdb
-9.238591e-16 1.009272e-15 -3.880187e-15 -2.548214e-15 8.063362e-15
5.796031e-15
E:\\R-3.1.2\\scripts\\mPDBS\\summary_pdb_93\\8b6ef9ac3fe74395.final.pdb
-2.620920e-15 5.667211e-15 -2.528024e-16 4.837631e-15 -1.495186e-15
1.512753e-15
E:\\R-3.1.2\\scripts\\mPDBS\\summary_pdb_93\\98bbe0d5986c138f.final.pdb
-1.507070e-15 2.353007e-15 3.805941e-16 -2.722855e-15 -2.127129e-15
5.346888e-15
E:\\R-3.1.2\\scripts\\mPDBS\\summary_pdb_93\\9c22f4909d67e2fa.final.pdb
2.574644e-15 8.165656e-15 6.497146e-15 7.294390e-15 3.629247e-16
3.747105e-15
E:\\R-3.1.2\\scripts\\mPDBS\\summary_pdb_93\\9dda8ed0d81fb3ef.final.pdb
6.876612e-15 -1.575176e-15 -8.762712e-15 3.730733e-15 1.066428e-14
1.121854e-14
E:\\R-3.1.2\\scripts\\mPDBS\\summary_pdb_93\\9f752b3616e6033b.final.pdb
1.196878e-15 2.489050e-15 -7.206889e-16 1.084293e-15 -1.304717e-15
-1.425058e-17
E:\\R-3.1.2\\scripts\\mPDBS\\summary_pdb_93\\a02c70083816d6ba.final.pdb
5.431310e-15 2.480360e-15 -3.282649e-15 -2.395735e-16 6.439207e-15
7.718576e-15
E:\\R-3.1.2\\scripts\\mPDBS\\summary_pdb_93\\a0c997d891e4e082.final.pdb
-3.171135e-15 5.629010e-15 -2.656050e-15 -5.865048e-15 -6.705081e-15
6.606876e-15
E:\\R-3.1.2\\scripts\\mPDBS\\summary_pdb_93\\ab8039a2d823c616.final.pdb
1.718834e-15 2.009726e-15 -1.589412e-15 -8.696949e-16 -1.981332e-16
3.234791e-15
E:\\R-3.1.2\\scripts\\mPDBS\\summary_pdb_93\\acf4d74b9c71d4dc.final.pdb
3.768568e-15 2.924406e-15 -3.411811e-15 -4.007116e-15 -6.855260e-15
3.836934e-16
E:\\R-3.1.2\\scripts\\mPDBS\\summary_pdb_93\\ad8a823e4b4c668e.final.pdb
1.899667e-15 2.319467e-15 -1.502703e-15 -8.109857e-16 1.015295e-15
2.662515e-15
E:\\R-3.1.2\\scripts\\mPDBS\\summary_pdb_93\\b16ababd03b7b857.final.pdb
2.574644e-15 8.165656e-15 6.497146e-15 7.294390e-15 3.629247e-16
3.747105e-15
E:\\R-3.1.2\\scripts\\mPDBS\\summary_pdb_93\\b226923e697d3c63.final.pdb
5.563644e-15 8.766161e-15 -5.473677e-15 1.500529e-15 6.717781e-15
9.808528e-16
E:\\R-3.1.2\\scripts\\mPDBS\\summary_pdb_93\\b28e48f6fa27a165.final.pdb
-1.507070e-15 2.353007e-15 3.805941e-16 -2.722855e-15 -2.127129e-15
5.346888e-15
E:\\R-3.1.2\\scripts\\mPDBS\\summary_pdb_93\\b42c4924e3cd1aec.final.pdb
-5.609060e-15 4.996173e-15 8.623337e-15 -6.878249e-16 -8.239438e-15
-3.583638e-15

E:\\R-3.1.2\\scripts\\mPDBS\\summary_pdb_93\\b4dd239fcabff1df.final.pdb
-1.507070e-15 2.353007e-15 3.805941e-16 -2.722855e-15 -2.127129e-15
5.346888e-15
E:\\R-3.1.2\\scripts\\mPDBS\\summary_pdb_93\\b669942ef97459a2.final.pdb
-9.238591e-16 1.009272e-15 -3.880187e-15 -2.548214e-15 8.063362e-15
5.796031e-15
E:\\R-3.1.2\\scripts\\mPDBS\\summary_pdb_93\\b99f4d9d87c1017b.final.pdb
-1.507070e-15 2.353007e-15 3.805941e-16 -2.722855e-15 -2.127129e-15
5.346888e-15
E:\\R-3.1.2\\scripts\\mPDBS\\summary_pdb_93\\b9d2cbf3975403e7.final.pdb
-1.507070e-15 2.353007e-15 3.805941e-16 -2.722855e-15 -2.127129e-15
5.346888e-15
E:\\R-3.1.2\\scripts\\mPDBS\\summary_pdb_93\\bc88c8a505f3a288.final.pdb
1.301231e-15 1.971910e-15 -1.847875e-15 -5.589775e-16 1.190096e-15
2.677799e-15
E:\\R-3.1.2\\scripts\\mPDBS\\summary_pdb_93\\bdac0e64f4ef427e.final.pdb
-5.426984e-15 1.261815e-14 1.566408e-14 1.114160e-14 -7.145826e-15
8.027969e-15
E:\\R-3.1.2\\scripts\\mPDBS\\summary_pdb_93\\ca2a1f55fc9d0681.final.pdb
-9.238591e-16 1.009272e-15 -3.880187e-15 -2.548214e-15 8.063362e-15
5.796031e-15
E:\\R-3.1.2\\scripts\\mPDBS\\summary_pdb_93\\ce34248de3e81a3c.final.pdb
-4.569234e-15 2.629963e-15 -4.855184e-15 -9.360400e-15 1.085934e-15
1.353673e-14
E:\\R-3.1.2\\scripts\\mPDBS\\summary_pdb_93\\d02e697772494a61.final.pdb
3.885101e-15 3.122574e-15 -5.970445e-15 -5.692919e-15 -1.700387e-15
4.100821e-16
E:\\R-3.1.2\\scripts\\mPDBS\\summary_pdb_93\\d04221245d015047.final.pdb
1.130980e-15 1.663654e-16 -8.541060e-16 -2.440704e-15 -1.622341e-15
3.213757e-15
E:\\R-3.1.2\\scripts\\mPDBS\\summary_pdb_93\\d678141e4018054a.final.pdb
9.988678e-15 2.650065e-15 -3.419605e-15 6.605777e-15 9.131486e-15
-4.737792e-15
E:\\R-3.1.2\\scripts\\mPDBS\\summary_pdb_93\\d71b40af0f0efe39.final.pdb
6.610182e-15 8.891375e-15 6.112218e-15 4.748755e-16 1.650332e-15
3.484015e-15
E:\\R-3.1.2\\scripts\\mPDBS\\summary_pdb_93\\dc0809eff10197e2.final.pdb
-9.463932e-15 4.636603e-16 8.216381e-15 1.271698e-16 -5.709859e-15
1.145523e-14
E:\\R-3.1.2\\scripts\\mPDBS\\summary_pdb_93\\e1163918d3707839.final.pdb
4.337845e-15 7.511522e-15 5.017280e-15 -4.502482e-15 -8.954144e-15
-2.398576e-17
E:\\R-3.1.2\\scripts\\mPDBS\\summary_pdb_93\\e4b48b06824ef848.final.pdb
8.133746e-15 3.148742e-15 -6.536093e-15 -7.101668e-16 1.302357e-14
1.450461e-15
E:\\R-3.1.2\\scripts\\mPDBS\\summary_pdb_93\\e529e19ad281fea2.final.pdb
3.558266e-15 4.269913e-15 -2.583671e-16 2.780369e-15 -5.180421e-15
-8.039302e-16
E:\\R-3.1.2\\scripts\\mPDBS\\summary_pdb_93\\e5602082718ec3a1.final.pdb
3.768568e-15 2.924406e-15 -3.411811e-15 -4.007116e-15 -6.855260e-15
3.836934e-16
E:\\R-3.1.2\\scripts\\mPDBS\\summary_pdb_93\\e5d589d996d0a960.final.pdb
4.105731e-15 6.763881e-15 -2.448524e-15 -2.437573e-15 -5.238682e-15
3.724774e-16

E:\\R-3.1.2\\scripts\\mPDBS\\summary_pdb_93\\e9df9e72307a2fbd.final.pdb
5.431310e-15 2.480360e-15 -3.282649e-15 -2.395735e-16 6.439207e-15
7.718576e-15
E:\\R-3.1.2\\scripts\\mPDBS\\summary_pdb_93\\ea00f8694090cae7.final.pdb
3.768568e-15 2.924406e-15 -3.411811e-15 -4.007116e-15 -6.855260e-15
3.836934e-16
E:\\R-3.1.2\\scripts\\mPDBS\\summary_pdb_93\\eba517be32db050e.final.pdb
-1.507070e-15 2.353007e-15 3.805941e-16 -2.722855e-15 -2.127129e-15
5.346888e-15
E:\\R-3.1.2\\scripts\\mPDBS\\summary_pdb_93\\ec04b9545899c29d.final.pdb
2.054298e-15 2.580788e-15 -2.886987e-15 -1.119988e-15 6.683667e-16
2.182799e-15
E:\\R-3.1.2\\scripts\\mPDBS\\summary_pdb_93\\ee6862cbcad52078.final.pdb
1.226854e-15 2.250659e-15 -1.900784e-15 -3.801112e-16 9.589441e-16
2.809855e-15
E:\\R-3.1.2\\scripts\\mPDBS\\summary_pdb_93\\ef6a42fc83b81e5d.final.pdb
2.082814e-15 2.109209e-15 -1.406515e-15 -1.705898e-15 -3.836767e-16
2.836071e-15
E:\\R-3.1.2\\scripts\\mPDBS\\summary_pdb_93\\ef8e88d4ad93a0ad.final.pdb
1.130980e-15 1.663654e-16 -8.541060e-16 -2.440704e-15 -1.622341e-15
3.213757e-15
E:\\R-3.1.2\\scripts\\mPDBS\\summary_pdb_93\\f14fcae8deec4fa3.final.pdb
1.196878e-15 2.489050e-15 -7.206889e-16 1.084293e-15 -1.304717e-15
-1.425058e-17
E:\\R-3.1.2\\scripts\\mPDBS\\summary_pdb_93\\f83584e22003fb92.final.pdb
-1.345958e-15 -1.440399e-15 -8.379543e-15 -4.122036e-16 3.198038e-15
2.450334e-15
E:\\R-3.1.2\\scripts\\mPDBS\\summary_pdb_93\\f8e2bf87e52caa29.final.pdb
3.558266e-15 4.269913e-15 -2.583671e-16 2.780369e-15 -5.180421e-15
-8.039302e-16

[,241]   [,242]   [,243]   [,244]   [,245]   [,246]
E:\\R-3.1.2\\scripts\\40.1ns_data\\NAT0.pdb
9.577049e-16 2.846266e-15 -3.289882e-15 -9.263143e-16 2.094474e-15
1.728885e-15
E:\\R-3.1.2\\scripts\\40.1ns_data\\LJP6.pdb
1.364021e-15 1.386572e-15 -3.209861e-15 -4.246749e-16 2.977114e-15
1.533098e-15
E:\\R-3.1.2\\scripts\\40.1ns_data\\3WY9.pdb
2.083837e-15 1.054919e-15 -5.842792e-15 -2.724825e-15 3.368529e-15
-1.762642e-15
E:\\R-3.1.2\\scripts\\40.1ns_data\\ZSP9.pdb
-2.053755e-16 -6.485787e-16 -1.876678e-15 -1.201227e-15 3.003719e-15
-9.361091e-16
E:\\R-3.1.2\\scripts\\mPDBS\\summary_pdb_93\\013d855ad26c877c.final.pdb
-7.070083e-16 -2.463310e-16 -9.378834e-15 1.812542e-15 9.161101e-15
-9.902243e-16
E:\\R-3.1.2\\scripts\\mPDBS\\summary_pdb_93\\0a2a705342ca0d54.final.pdb
1.624002e-15 1.411686e-15 -3.270914e-15 -2.366741e-15 2.246393e-15
-1.826893e-15
E:\\R-3.1.2\\scripts\\mPDBS\\summary_pdb_93\\0a59fceadb4194d9.final.pdb
2.556243e-16 1.247237e-15 -5.938303e-15 2.782492e-15 5.970575e-15
-6.986539e-16
E:\\R-3.1.2\\scripts\\mPDBS\\summary_pdb_93\\0dba59f3306825e8.final.pdb

8.108747e-16 -2.554381e-15 -5.473104e-15 -2.146097e-15 1.113719e-15
1.750205e-15
E:\\R-3.1.2\\scripts\\mPDBS\\summary_pdb_93\\126f26a78b57339c.final.pdb
8.155911e-15 4.624772e-15 4.210203e-15 -1.336027e-14 -3.174951e-15
-8.767532e-15
E:\\R-3.1.2\\scripts\\mPDBS\\summary_pdb_93\\12f06f3e342df706.final.pdb
1.590608e-15 1.178365e-15 -4.245395e-15 -1.541094e-15 2.535224e-15
-2.690243e-15
E:\\R-3.1.2\\scripts\\mPDBS\\summary_pdb_93\\18618a2a251b867b.final.pdb
3.554548e-15 2.462491e-15 -3.014769e-15 -1.392440e-14 -3.844191e-15
-8.665505e-15
E:\\R-3.1.2\\scripts\\mPDBS\\summary_pdb_93\\1f305f598641eeb2.final.pdb
2.019670e-15 5.500309e-16 -3.807218e-15 -2.412012e-15 2.645133e-15
-1.803075e-15
E:\\R-3.1.2\\scripts\\mPDBS\\summary_pdb_93\\1f8a00b551be0a39.final.pdb
3.554548e-15 2.462491e-15 -3.014769e-15 -1.392440e-14 -3.844191e-15
-8.665505e-15
E:\\R-3.1.2\\scripts\\mPDBS\\summary_pdb_93\\219f45328d47579a.final.pdb
5.431547e-16 2.597719e-15 1.199109e-15 -4.910140e-15 7.542234e-15
1.115818e-14
E:\\R-3.1.2\\scripts\\mPDBS\\summary_pdb_93\\238dd3918e54336d.final.pdb
2.078060e-15 -1.781590e-15 4.267455e-15 -2.012285e-14 -6.453298e-15
-1.240988e-14
E:\\R-3.1.2\\scripts\\mPDBS\\summary_pdb_93\\2b3027889e2fdc42.final.pdb
1.410787e-15 1.779797e-15 -3.115148e-15 -2.537987e-15 2.888405e-15
-2.937857e-15
E:\\R-3.1.2\\scripts\\mPDBS\\summary_pdb_93\\2fcb3b46e33793b0.final.pdb
-3.489812e-15 -2.948039e-15 -8.051652e-15 3.772277e-15 6.450249e-15
1.899693e-15
E:\\R-3.1.2\\scripts\\mPDBS\\summary_pdb_93\\34eb328251842006.final.pdb
-2.358462e-16 6.961578e-15 -5.996157e-15 6.828849e-15 1.398983e-14
-1.641391e-15
E:\\R-3.1.2\\scripts\\mPDBS\\summary_pdb_93\\354b32871797b7ab.final.pdb
4.271314e-15 4.060639e-15 -1.375516e-14 -4.408922e-15 -1.781762e-15
-1.093520e-14
E:\\R-3.1.2\\scripts\\mPDBS\\summary_pdb_93\\397368adbc32d5de.final.pdb
8.155911e-15 4.624772e-15 4.210203e-15 -1.336027e-14 -3.174951e-15
-8.767532e-15
E:\\R-3.1.2\\scripts\\mPDBS\\summary_pdb_93\\411ca13cd3df6edd.final.pdb
-4.208204e-15 6.844720e-16 -3.210693e-17 -1.406686e-15 5.623219e-15
-4.067214e-15
E:\\R-3.1.2\\scripts\\mPDBS\\summary_pdb_93\\42357eee6642bf12.final.pdb
6.837870e-16 4.893772e-15 -6.725669e-15 8.425602e-15 1.048211e-14
7.336685e-16
E:\\R-3.1.2\\scripts\\mPDBS\\summary_pdb_93\\44342d1f75a7fcfc.final.pdb
-1.325134e-15 2.838421e-16 -4.128835e-15 9.642521e-15 1.519254e-14
-1.079308e-15
E:\\R-3.1.2\\scripts\\mPDBS\\summary_pdb_93\\448ef21dffe8cdfb.final.pdb
3.339795e-15 1.713832e-14 3.314690e-15 -1.486212e-14 -4.798566e-15
-2.494038e-14
E:\\R-3.1.2\\scripts\\mPDBS\\summary_pdb_93\\472a40b3e8f113c7.final.pdb
8.108747e-16 -2.554381e-15 -5.473104e-15 -2.146097e-15 1.113719e-15
1.750205e-15
E:\\R-3.1.2\\scripts\\mPDBS\\summary_pdb_93\\48a26225951ea153.final.pdb

1.027847e-15 1.781532e-15 -2.961192e-15 -3.965664e-15 4.513407e-15
-5.280601e-15
E:\\R-3.1.2\\scripts\\mPDBS\\summary_pdb_93\\4ab4a5c7c6682ce6.final.pdb
-6.360255e-15 1.020017e-15 -6.090151e-15 9.250114e-15 9.814461e-16
-4.119774e-15
E:\\R-3.1.2\\scripts\\mPDBS\\summary_pdb_93\\4eaa45b62cb6a25f.final.pdb
2.945443e-15 -9.960803e-16 -1.070587e-15 -4.437924e-15 2.871123e-16
-1.533322e-15
E:\\R-3.1.2\\scripts\\mPDBS\\summary_pdb_93\\58c371611d85ac96.final.pdb
8.155911e-15 4.624772e-15 4.210203e-15 -1.336027e-14 -3.174951e-15
-8.767532e-15
E:\\R-3.1.2\\scripts\\mPDBS\\summary_pdb_93\\59a647f04822d30a.final.pdb
1.518077e-15 3.434967e-15 3.925811e-15 -1.421979e-15 -2.740166e-15
6.474221e-17
E:\\R-3.1.2\\scripts\\mPDBS\\summary_pdb_93\\59b091fe385e05fc.final.pdb
2.556243e-16 1.247237e-15 -5.938303e-15 2.782492e-15 5.970575e-15
-6.986539e-16
E:\\R-3.1.2\\scripts\\mPDBS\\summary_pdb_93\\5c35ac30f930d2db.final.pdb
2.945443e-15 -9.960803e-16 -1.070587e-15 -4.437924e-15 2.871123e-16
-1.533322e-15
E:\\R-3.1.2\\scripts\\mPDBS\\summary_pdb_93\\5f60752431cf2dcb.final.pdb
4.935426e-15 -3.698754e-16 -3.146195e-16 -1.466935e-15 1.703625e-16
-6.668614e-15
E:\\R-3.1.2\\scripts\\mPDBS\\summary_pdb_93\\626456dd6ff2868d.final.pdb
8.108747e-16 -2.554381e-15 -5.473104e-15 -2.146097e-15 1.113719e-15
1.750205e-15
E:\\R-3.1.2\\scripts\\mPDBS\\summary_pdb_93\\6583d53ff3c8f7ef.final.pdb
1.518077e-15 3.434967e-15 3.925811e-15 -1.421979e-15 -2.740166e-15
6.474221e-17
E:\\R-3.1.2\\scripts\\mPDBS\\summary_pdb_93\\69760178986bef01.final.pdb
-9.697137e-16 -2.548449e-15 5.990346e-15 -1.242682e-14 -2.687513e-15
-5.248048e-16
E:\\R-3.1.2\\scripts\\mPDBS\\summary_pdb_93\\6a9d87d47926b1c8.final.pdb
1.147628e-15 1.179612e-15 -3.413039e-15 -2.580700e-15 3.089135e-15
-2.100556e-15
E:\\R-3.1.2\\scripts\\mPDBS\\summary_pdb_93\\6bb3547ea982ffc4.final.pdb
5.228434e-15 6.973837e-15 7.511520e-16 -9.874425e-16 4.747351e-16
-4.602769e-15
E:\\R-3.1.2\\scripts\\mPDBS\\summary_pdb_93\\716d9624611d1c42.final.pdb
1.382441e-15 2.207056e-15 -9.330485e-16 -8.809070e-15 3.385673e-15
-4.725074e-16
E:\\R-3.1.2\\scripts\\mPDBS\\summary_pdb_93\\75f27e70ad0f1be4.final.pdb
1.518077e-15 3.434967e-15 3.925811e-15 -1.421979e-15 -2.740166e-15
6.474221e-17
E:\\R-3.1.2\\scripts\\mPDBS\\summary_pdb_93\\769f910fffb22872.final.pdb
1.856091e-15 1.129356e-15 -3.796858e-15 -1.633421e-15 2.817364e-15
-2.712802e-15
E:\\R-3.1.2\\scripts\\mPDBS\\summary_pdb_93\\791f8c80dd5c3135.final.pdb
2.556243e-16 1.247237e-15 -5.938303e-15 2.782492e-15 5.970575e-15
-6.986539e-16
E:\\R-3.1.2\\scripts\\mPDBS\\summary_pdb_93\\7fad98e66a3f6099.final.pdb
3.064210e-15 -9.326385e-16 -1.134946e-14 9.536199e-15 1.278048e-14
6.176309e-15
E:\\R-3.1.2\\scripts\\mPDBS\\summary_pdb_93\\804b89c94a633196.final.pdb

-3.489812e-15 -2.948039e-15 -8.051652e-15 3.772277e-15 6.450249e-15
1.899693e-15
E:\\R-3.1.2\\scripts\\mPDBS\\summary_pdb_93\\813cc65a2298feba.final.pdb
-3.489812e-15 -2.948039e-15 -8.051652e-15 3.772277e-15 6.450249e-15
1.899693e-15
E:\\R-3.1.2\\scripts\\mPDBS\\summary_pdb_93\\83c0eefcfe12d3f9.final.pdb
-4.208204e-15 6.844720e-16 -3.210693e-17 -1.406686e-15 5.623219e-15
-4.067214e-15
E:\\R-3.1.2\\scripts\\mPDBS\\summary_pdb_93\\86d50469ef569760.final.pdb
8.108747e-16 -2.554381e-15 -5.473104e-15 -2.146097e-15 1.113719e-15
1.750205e-15
E:\\R-3.1.2\\scripts\\mPDBS\\summary_pdb_93\\89d0666e1402203d.final.pdb
1.518077e-15 3.434967e-15 3.925811e-15 -1.421979e-15 -2.740166e-15
6.474221e-17
E:\\R-3.1.2\\scripts\\mPDBS\\summary_pdb_93\\8b34852716dadc62.final.pdb
2.980707e-15 1.334126e-15 1.806348e-15 -3.140596e-15 8.063257e-16
2.755779e-15
E:\\R-3.1.2\\scripts\\mPDBS\\summary_pdb_93\\8b6ef9ac3fe74395.final.pdb
5.561408e-15 1.056704e-15 -1.612044e-15 3.583736e-15 -1.270239e-16
1.221407e-14
E:\\R-3.1.2\\scripts\\mPDBS\\summary_pdb_93\\98bbe0d5986c138f.final.pdb
2.556243e-16 1.247237e-15 -5.938303e-15 2.782492e-15 5.970575e-15
-6.986539e-16
E:\\R-3.1.2\\scripts\\mPDBS\\summary_pdb_93\\9c22f4909d67e2fa.final.pdb
9.523975e-15 5.777865e-16 -9.197247e-15 -1.173398e-14 2.899486e-15
-3.997825e-15
E:\\R-3.1.2\\scripts\\mPDBS\\summary_pdb_93\\9dda8ed0d81fb3ef.final.pdb
9.875231e-16 -3.050965e-16 -1.533099e-16 3.533205e-15 5.705335e-15
-1.525590e-14
E:\\R-3.1.2\\scripts\\mPDBS\\summary_pdb_93\\9f752b3616e6033b.final.pdb
4.907088e-17 1.665390e-15 -3.954991e-15 3.452272e-17 3.164793e-15
3.051507e-16
E:\\R-3.1.2\\scripts\\mPDBS\\summary_pdb_93\\a02c70083816d6ba.final.pdb
2.945443e-15 -9.960803e-16 -1.070587e-15 -4.437924e-15 2.871123e-16
-1.533322e-15
E:\\R-3.1.2\\scripts\\mPDBS\\summary_pdb_93\\a0c997d891e4e082.final.pdb
-1.789565e-15 2.610686e-15 -5.089122e-15 -9.339137e-15 -3.086511e-15
-2.868326e-15
E:\\R-3.1.2\\scripts\\mPDBS\\summary_pdb_93\\ab8039a2d823c616.final.pdb
1.813130e-15 1.310511e-15 -2.541193e-15 -1.198568e-15 3.175623e-15
-2.004293e-16
E:\\R-3.1.2\\scripts\\mPDBS\\summary_pdb_93\\acf4d74b9c71d4dc.final.pdb
-3.489812e-15 -2.948039e-15 -8.051652e-15 3.772277e-15 6.450249e-15
1.899693e-15
E:\\R-3.1.2\\scripts\\mPDBS\\summary_pdb_93\\ad8a823e4b4c668e.final.pdb
8.374263e-16 1.688794e-15 -3.439280e-15 -2.339680e-15 2.682773e-15
-2.177410e-15
E:\\R-3.1.2\\scripts\\mPDBS\\summary_pdb_93\\b16ababd03b7b857.final.pdb
9.523975e-15 5.777865e-16 -9.197247e-15 -1.173398e-14 2.899486e-15
-3.997825e-15
E:\\R-3.1.2\\scripts\\mPDBS\\summary_pdb_93\\b226923e697d3c63.final.pdb
3.993690e-15 3.454049e-15 -2.082892e-15 -1.354295e-14 -4.794729e-15
-1.646562e-15
E:\\R-3.1.2\\scripts\\mPDBS\\summary_pdb_93\\b28e48f6fa27a165.final.pdb

2.556243e-16 1.247237e-15 -5.938303e-15 2.782492e-15 5.970575e-15
-6.986539e-16
E:\\R-3.1.2\\scripts\\mPDBS\\summary_pdb_93\\b42c4924e3cd1aec.final.pdb
2.477271e-15 1.400137e-15 -1.322556e-14 -2.159182e-15 7.960693e-15
8.606195e-15
E:\\R-3.1.2\\scripts\\mPDBS\\summary_pdb_93\\b4dd239fcabff1df.final.pdb
2.556243e-16 1.247237e-15 -5.938303e-15 2.782492e-15 5.970575e-15
-6.986539e-16
E:\\R-3.1.2\\scripts\\mPDBS\\summary_pdb_93\\b669942ef97459a2.final.pdb
2.980707e-15 1.334126e-15 1.806348e-15 -3.140596e-15 8.063257e-16
2.755779e-15
E:\\R-3.1.2\\scripts\\mPDBS\\summary_pdb_93\\b99f4d9d87c1017b.final.pdb
2.556243e-16 1.247237e-15 -5.938303e-15 2.782492e-15 5.970575e-15
-6.986539e-16
E:\\R-3.1.2\\scripts\\mPDBS\\summary_pdb_93\\b9d2cbf3975403e7.final.pdb
2.556243e-16 1.247237e-15 -5.938303e-15 2.782492e-15 5.970575e-15
-6.986539e-16
E:\\R-3.1.2\\scripts\\mPDBS\\summary_pdb_93\\bc88c8a505f3a288.final.pdb
1.625514e-15 1.346116e-15 -3.535765e-15 -2.127182e-15 2.858698e-15
-1.626297e-15
E:\\R-3.1.2\\scripts\\mPDBS\\summary_pdb_93\\bdac0e64f4ef427e.final.pdb
1.215880e-14 7.746776e-16 -1.178123e-14 -3.781034e-15 -1.916540e-15
2.902146e-15
E:\\R-3.1.2\\scripts\\mPDBS\\summary_pdb_93\\ca2a1f55fc9d0681.final.pdb
2.980707e-15 1.334126e-15 1.806348e-15 -3.140596e-15 8.063257e-16
2.755779e-15
E:\\R-3.1.2\\scripts\\mPDBS\\summary_pdb_93\\ce34248de3e81a3c.final.pdb
6.219490e-15 -2.124116e-15 -6.378239e-15 4.174266e-16 1.182945e-15
-2.510603e-15
E:\\R-3.1.2\\scripts\\mPDBS\\summary_pdb_93\\d02e697772494a61.final.pdb
1.935812e-15 2.432053e-15 2.065224e-15 -7.615061e-15 -4.897684e-15
6.556231e-16
E:\\R-3.1.2\\scripts\\mPDBS\\summary_pdb_93\\d04221245d015047.final.pdb
1.058851e-15 2.965649e-16 -4.856669e-15 1.794318e-15 5.410164e-15
-1.713000e-15
E:\\R-3.1.2\\scripts\\mPDBS\\summary_pdb_93\\d678141e4018054a.final.pdb
5.981837e-15 4.813218e-15 1.977956e-15 -8.520209e-15 4.675031e-15
-1.252165e-14
E:\\R-3.1.2\\scripts\\mPDBS\\summary_pdb_93\\d71b40af0f0efe39.final.pdb
1.281582e-15 -1.483196e-15 -1.327801e-14 -1.010287e-14 5.698430e-15
-1.007331e-14
E:\\R-3.1.2\\scripts\\mPDBS\\summary_pdb_93\\dc0809eff10197e2.final.pdb
-2.141385e-15 3.692325e-15 -6.668610e-15 3.447437e-15 5.077607e-15
1.101681e-14
E:\\R-3.1.2\\scripts\\mPDBS\\summary_pdb_93\\e1163918d3707839.final.pdb
2.179332e-15 3.428896e-15 -1.234812e-14 5.881864e-16 7.703520e-15
4.453267e-15
E:\\R-3.1.2\\scripts\\mPDBS\\summary_pdb_93\\e4b48b06824ef848.final.pdb
3.554548e-15 2.462491e-15 -3.014769e-15 -1.392440e-14 -3.844191e-15
-8.665505e-15
E:\\R-3.1.2\\scripts\\mPDBS\\summary_pdb_93\\e529e19ad281fea2.final.pdb
-7.070083e-16 -2.463310e-16 -9.378834e-15 1.812542e-15 9.161101e-15
-9.902243e-16
E:\\R-3.1.2\\scripts\\mPDBS\\summary_pdb_93\\e5602082718ec3a1.final.pdb

```
-3.489812e-15 -2.948039e-15 -8.051652e-15  3.772277e-15  6.450249e-15
1.899693e-15
E:\\R-3.1.2\\scripts\\mPDBS\\summary_pdb_93\\e5d589d996d0a960.final.pdb
8.382737e-15 -5.214724e-15 -4.991929e-15 -3.321891e-15  2.837335e-16
-9.620344e-17
E:\\R-3.1.2\\scripts\\mPDBS\\summary_pdb_93\\e9df9e72307a2fbd.final.pdb
2.945443e-15 -9.960803e-16 -1.070587e-15 -4.437924e-15  2.871123e-16
-1.533322e-15
E:\\R-3.1.2\\scripts\\mPDBS\\summary_pdb_93\\ea00f8694090cae7.final.pdb
-3.489812e-15 -2.948039e-15 -8.051652e-15  3.772277e-15  6.450249e-15
1.899693e-15
E:\\R-3.1.2\\scripts\\mPDBS\\summary_pdb_93\\eba517be32db050e.final.pdb
2.556243e-16  1.247237e-15 -5.938303e-15  2.782492e-15  5.970575e-15
-6.986539e-16
E:\\R-3.1.2\\scripts\\mPDBS\\summary_pdb_93\\ec04b9545899c29d.final.pdb
1.734918e-15  1.838154e-15 -2.995194e-15 -2.573835e-15  2.499566e-15
-8.589980e-16
E:\\R-3.1.2\\scripts\\mPDBS\\summary_pdb_93\\ee6862cbcad52078.final.pdb
1.567075e-15  1.296676e-15 -3.773693e-15 -2.002743e-15  3.158588e-15
-1.843571e-15
E:\\R-3.1.2\\scripts\\mPDBS\\summary_pdb_93\\ef6a42fc83b81e5d.final.pdb
1.823890e-15  1.474566e-15 -4.363516e-15 -1.976408e-15  2.362216e-15
-1.254658e-15
E:\\R-3.1.2\\scripts\\mPDBS\\summary_pdb_93\\ef8e88d4ad93a0ad.final.pdb
1.058851e-15  2.965649e-16 -4.856669e-15  1.794318e-15  5.410164e-15
-1.713000e-15
E:\\R-3.1.2\\scripts\\mPDBS\\summary_pdb_93\\f14fcae8deec4fa3.final.pdb
4.907088e-17  1.665390e-15 -3.954991e-15  3.452272e-17  3.164793e-15
3.051507e-16
E:\\R-3.1.2\\scripts\\mPDBS\\summary_pdb_93\\f83584e22003fb92.final.pdb
1.027847e-15  1.781532e-15 -2.961192e-15 -3.965664e-15  4.513407e-15
-5.280601e-15
E:\\R-3.1.2\\scripts\\mPDBS\\summary_pdb_93\\f8e2bf87e52caa29.final.pdb
-7.070083e-16 -2.463310e-16 -9.378834e-15  1.812542e-15  9.161101e-15
-9.902243e-16

[,247]        [,248]        [,249]        [,250]        [,251]        [,252]
E:\\R-3.1.2\\scripts\\40.1ns_data\\NAT0.pdb
-1.830343e-15  9.955871e-16 -6.698090e-15 -2.304634e-15 -1.830411e-15
-8.233149e-16
E:\\R-3.1.2\\scripts\\40.1ns_data\\LJP6.pdb
-6.330259e-16  8.000712e-16 -5.710651e-15 -7.229063e-16  8.952373e-16
4.901157e-16
E:\\R-3.1.2\\scripts\\40.1ns_data\\3WY9.pdb
-1.349177e-15  4.388453e-16 -6.486206e-15  1.607687e-16 -1.353248e-15
-1.160166e-15
E:\\R-3.1.2\\scripts\\40.1ns_data\\ZSP9.pdb
-1.641830e-15  9.540744e-16 -5.029367e-15 -3.805275e-16 -1.667816e-16
-1.417869e-15
E:\\R-3.1.2\\scripts\\mPDBS\\summary_pdb_93\\013d855ad26c877c.final.pdb
9.524728e-16 -1.291017e-15  4.589369e-15 -7.415281e-16 -2.350601e-15
-3.148164e-15
E:\\R-3.1.2\\scripts\\mPDBS\\summary_pdb_93\\0a2a705342ca0d54.final.pdb
-1.420647e-15  8.258900e-16 -5.361472e-15 -2.311871e-15 -7.240984e-16
```

-7.835917e-16
E:\\R-3.1.2\\scripts\\mPDBS\\summary_pdb_93\\0a59fceadb4194d9.final.pdb
-5.405752e-15 1.991396e-15 -6.285984e-15 -5.749792e-16 -7.263660e-15
4.181825e-15
E:\\R-3.1.2\\scripts\\mPDBS\\summary_pdb_93\\0dba59f3306825e8.final.pdb
-4.366189e-15 2.997220e-15 -7.460020e-15 -1.274360e-16 -5.848671e-15
-1.144559e-15
E:\\R-3.1.2\\scripts\\mPDBS\\summary_pdb_93\\126f26a78b57339c.final.pdb
1.965551e-15 3.053165e-15 -9.314439e-15 -1.790168e-15 8.121491e-15
-7.215657e-15
E:\\R-3.1.2\\scripts\\mPDBS\\summary_pdb_93\\12f06f3e342df706.final.pdb
-4.396608e-16 4.168205e-16 -5.305310e-15 -1.807066e-15 -8.557206e-16
-1.222477e-15
E:\\R-3.1.2\\scripts\\mPDBS\\summary_pdb_93\\18618a2a251b867b.final.pdb
-1.823740e-15 -3.591370e-15 2.494603e-15 -5.108058e-15 9.562717e-15
-7.433012e-15
E:\\R-3.1.2\\scripts\\mPDBS\\summary_pdb_93\\1f305f598641eeb2.final.pdb
-3.658126e-16 5.639333e-16 -5.284628e-15 -1.210362e-15 -8.158255e-16
-1.369298e-15
E:\\R-3.1.2\\scripts\\mPDBS\\summary_pdb_93\\1f8a00b551be0a39.final.pdb
-1.823740e-15 -3.591370e-15 2.494603e-15 -5.108058e-15 9.562717e-15
-7.433012e-15
E:\\R-3.1.2\\scripts\\mPDBS\\summary_pdb_93\\219f45328d47579a.final.pdb
9.637770e-15 -1.380196e-14 -1.038445e-15 -5.188476e-15 2.646066e-16
-1.386103e-14
E:\\R-3.1.2\\scripts\\mPDBS\\summary_pdb_93\\238dd3918e54336d.final.pdb
8.681070e-15 -6.109546e-15 -6.140050e-15 -6.655619e-15 1.408775e-14
-2.068977e-14
E:\\R-3.1.2\\scripts\\mPDBS\\summary_pdb_93\\2b3027889e2fdc42.final.pdb
-9.327672e-16 8.915816e-16 -5.116563e-15 -2.663153e-15 -1.760249e-15
-1.812079e-15
E:\\R-3.1.2\\scripts\\mPDBS\\summary_pdb_93\\2fcb3b46e33793b0.final.pdb
-2.260131e-15 -1.025073e-15 -2.620093e-15 -7.489254e-16 -7.378284e-15
3.219045e-15
E:\\R-3.1.2\\scripts\\mPDBS\\summary_pdb_93\\34eb328251842006.final.pdb
-1.767621e-15 -5.677975e-15 -2.807598e-15 -7.414252e-15 -1.007631e-15
-1.280678e-15
E:\\R-3.1.2\\scripts\\mPDBS\\summary_pdb_93\\354b32871797b7ab.final.pdb
-9.279573e-15 7.582375e-16 5.911904e-15 -1.320557e-14 -7.555897e-16
3.450053e-15
E:\\R-3.1.2\\scripts\\mPDBS\\summary_pdb_93\\397368adbc32d5de.final.pdb
1.965551e-15 3.053165e-15 -9.314439e-15 -1.790168e-15 8.121491e-15
-7.215657e-15
E:\\R-3.1.2\\scripts\\mPDBS\\summary_pdb_93\\411ca13cd3df6edd.final.pdb
-2.339928e-15 -2.906815e-15 -3.706030e-15 -1.962365e-15 -1.237363e-15
8.194890e-16
E:\\R-3.1.2\\scripts\\mPDBS\\summary_pdb_93\\42357eee6642bf12.final.pdb
-4.281948e-15 -1.064337e-14 8.388591e-16 -9.692866e-15 3.883949e-15
3.044768e-15
E:\\R-3.1.2\\scripts\\mPDBS\\summary_pdb_93\\44342d1f75a7fcfc.final.pdb
1.032935e-15 8.626860e-15 -4.351946e-15 7.677793e-16 -6.207264e-15
7.516404e-15
E:\\R-3.1.2\\scripts\\mPDBS\\summary_pdb_93\\448ef21dffe8cdfb.final.pdb
1.920553e-15 -4.663656e-15 1.244871e-15 -1.636144e-14 1.933602e-14

-9.288311e-15
E:\\R-3.1.2\\scripts\\mPDBS\\summary_pdb_93\\472a40b3e8f113c7.final.pdb
-4.366189e-15 2.997220e-15 -7.460020e-15 -1.274360e-16 -5.848671e-15
-1.144559e-15
E:\\R-3.1.2\\scripts\\mPDBS\\summary_pdb_93\\48a26225951ea153.final.pdb
4.375487e-15 9.523132e-15 -1.601146e-15 -8.228145e-15 7.857925e-15
5.467116e-16
E:\\R-3.1.2\\scripts\\mPDBS\\summary_pdb_93\\4ab4a5c7c6682ce6.final.pdb
-6.309080e-15 -6.179034e-15 -3.888224e-15 -1.241708e-14 -3.245718e-15
4.060045e-15
E:\\R-3.1.2\\scripts\\mPDBS\\summary_pdb_93\\4eaa45b62cb6a25f.final.pdb
1.351680e-15 2.991477e-15 -7.986917e-15 3.657244e-15 4.793165e-16
-3.027404e-15
E:\\R-3.1.2\\scripts\\mPDBS\\summary_pdb_93\\58c371611d85ac96.final.pdb
1.965551e-15 3.053165e-15 -9.314439e-15 -1.790168e-15 8.121491e-15
-7.215657e-15
E:\\R-3.1.2\\scripts\\mPDBS\\summary_pdb_93\\59a647f04822d30a.final.pdb
7.189502e-16 7.815728e-16 -1.003924e-14 -4.694865e-15 8.981631e-16
-2.418357e-15
E:\\R-3.1.2\\scripts\\mPDBS\\summary_pdb_93\\59b091fe385e05fc.final.pdb
-5.405752e-15 1.991396e-15 -6.285984e-15 -5.749792e-16 -7.263660e-15
4.181825e-15
E:\\R-3.1.2\\scripts\\mPDBS\\summary_pdb_93\\5c35ac30f930d2db.final.pdb
1.351680e-15 2.991477e-15 -7.986917e-15 3.657244e-15 4.793165e-16
-3.027404e-15
E:\\R-3.1.2\\scripts\\mPDBS\\summary_pdb_93\\5f60752431cf2dcb.final.pdb
-9.445083e-17 9.584075e-15 -7.942523e-15 -1.916049e-16 2.404692e-15
2.160234e-15
E:\\R-3.1.2\\scripts\\mPDBS\\summary_pdb_93\\626456dd6ff2868d.final.pdb
-4.366189e-15 2.997220e-15 -7.460020e-15 -1.274360e-16 -5.848671e-15
-1.144559e-15
E:\\R-3.1.2\\scripts\\mPDBS\\summary_pdb_93\\6583d53ff3c8f7ef.final.pdb
7.189502e-16 7.815728e-16 -1.003924e-14 -4.694865e-15 8.981631e-16
-2.418357e-15
E:\\R-3.1.2\\scripts\\mPDBS\\summary_pdb_93\\69760178986bef01.final.pdb
2.960621e-15 4.132785e-15 -8.657533e-15 1.159842e-15 2.865183e-15
-5.009645e-15
E:\\R-3.1.2\\scripts\\mPDBS\\summary_pdb_93\\6a9d87d47926b1c8.final.pdb
-6.989840e-16 6.427663e-16 -4.918650e-15 -1.908130e-15 -1.553062e-15
-1.596888e-15
E:\\R-3.1.2\\scripts\\mPDBS\\summary_pdb_93\\6bb3547ea982ffc4.final.pdb
2.586532e-15 7.813918e-16 2.774841e-16 -3.953179e-15 1.074347e-14
-8.655149e-15
E:\\R-3.1.2\\scripts\\mPDBS\\summary_pdb_93\\716d9624611d1c42.final.pdb
2.388806e-15 -6.351962e-15 -5.103174e-16 -3.703986e-15 1.290349e-15
-9.573436e-15
E:\\R-3.1.2\\scripts\\mPDBS\\summary_pdb_93\\75f27e70ad0f1be4.final.pdb
7.189502e-16 7.815728e-16 -1.003924e-14 -4.694865e-15 8.981631e-16
-2.418357e-15
E:\\R-3.1.2\\scripts\\mPDBS\\summary_pdb_93\\769f910fffb22872.final.pdb
-2.144664e-15 6.394038e-16 -5.406195e-15 -1.981219e-15 -1.368426e-15
-9.876470e-16
E:\\R-3.1.2\\scripts\\mPDBS\\summary_pdb_93\\791f8c80dd5c3135.final.pdb
-5.405752e-15 1.991396e-15 -6.285984e-15 -5.749792e-16 -7.263660e-15

4.181825e-15
E:\\R-3.1.2\\scripts\\mPDBS\\summary_pdb_93\\7fad98e66a3f6099.final.pdb
8.273250e-15 4.187114e-15 -4.632798e-15 -9.536241e-15 -6.193081e-15
4.915613e-15
E:\\R-3.1.2\\scripts\\mPDBS\\summary_pdb_93\\804b89c94a633196.final.pdb
-2.260131e-15 -1.025073e-15 -2.620093e-15 -7.489254e-16 -7.378284e-15
3.219045e-15
E:\\R-3.1.2\\scripts\\mPDBS\\summary_pdb_93\\813cc65a2298feba.final.pdb
-2.260131e-15 -1.025073e-15 -2.620093e-15 -7.489254e-16 -7.378284e-15
3.219045e-15
E:\\R-3.1.2\\scripts\\mPDBS\\summary_pdb_93\\83c0eefcfe12d3f9.final.pdb
-2.339928e-15 -2.906815e-15 -3.706030e-15 -1.962365e-15 -1.237363e-15
8.194890e-16
E:\\R-3.1.2\\scripts\\mPDBS\\summary_pdb_93\\86d50469ef569760.final.pdb
-4.366189e-15 2.997220e-15 -7.460020e-15 -1.274360e-16 -5.848671e-15
-1.144559e-15
E:\\R-3.1.2\\scripts\\mPDBS\\summary_pdb_93\\89d0666e1402203d.final.pdb
7.189502e-16 7.815728e-16 -1.003924e-14 -4.694865e-15 8.981631e-16
-2.418357e-15
E:\\R-3.1.2\\scripts\\mPDBS\\summary_pdb_93\\8b34852716dadc62.final.pdb
2.239934e-16 1.816404e-15 -1.415947e-14 -5.789225e-16 -4.792891e-16
1.482587e-15
E:\\R-3.1.2\\scripts\\mPDBS\\summary_pdb_93\\8b6ef9ac3fe74395.final.pdb
2.260030e-15 5.630995e-15 -9.713532e-15 1.234011e-15 -6.081722e-15
8.182845e-15
E:\\R-3.1.2\\scripts\\mPDBS\\summary_pdb_93\\98bbe0d5986c138f.final.pdb
-5.405752e-15 1.991396e-15 -6.285984e-15 -5.749792e-16 -7.263660e-15
4.181825e-15
E:\\R-3.1.2\\scripts\\mPDBS\\summary_pdb_93\\9c22f4909d67e2fa.final.pdb
2.329948e-15 6.287003e-15 -5.746932e-15 1.971121e-15 2.656874e-15
-1.109676e-14
E:\\R-3.1.2\\scripts\\mPDBS\\summary_pdb_93\\9dda8ed0d81fb3ef.final.pdb
-1.266271e-14 1.271170e-14 -1.306264e-14 5.957638e-15 -6.453071e-16
9.684158e-15
E:\\R-3.1.2\\scripts\\mPDBS\\summary_pdb_93\\9f752b3616e6033b.final.pdb
-2.228861e-15 2.349455e-16 -6.343311e-15 -2.542586e-15 -3.044675e-15
-5.679991e-16
E:\\R-3.1.2\\scripts\\mPDBS\\summary_pdb_93\\a02c70083816d6ba.final.pdb
1.351680e-15 2.991477e-15 -7.986917e-15 3.657244e-15 4.793165e-16
-3.027404e-15
E:\\R-3.1.2\\scripts\\mPDBS\\summary_pdb_93\\a0c997d891e4e082.final.pdb
-2.896614e-15 7.425435e-15 -1.331045e-14 -4.726050e-16 -7.896161e-15
-2.839583e-15
E:\\R-3.1.2\\scripts\\mPDBS\\summary_pdb_93\\ab8039a2d823c616.final.pdb
-6.614233e-16 9.356176e-16 -5.234232e-15 -1.843470e-15 -1.253471e-15
-1.811760e-15
E:\\R-3.1.2\\scripts\\mPDBS\\summary_pdb_93\\acf4d74b9c71d4dc.final.pdb
-2.260131e-15 -1.025073e-15 -2.620093e-15 -7.489254e-16 -7.378284e-15
3.219045e-15
E:\\R-3.1.2\\scripts\\mPDBS\\summary_pdb_93\\ad8a823e4b4c668e.final.pdb
-1.089536e-15 1.002707e-15 -4.888209e-15 -2.260629e-15 -6.695879e-16
-7.779398e-16
E:\\R-3.1.2\\scripts\\mPDBS\\summary_pdb_93\\b16ababd03b7b857.final.pdb
2.329948e-15 6.287003e-15 -5.746932e-15 1.971121e-15 2.656874e-15

-1.109676e-14
E:\\R-3.1.2\\scripts\\mPDBS\\summary_pdb_93\\b226923e697d3c63.final.pdb
2.750311e-15 -6.714750e-16 -5.636509e-15 -8.315233e-15 -3.588609e-16
-3.970067e-15
E:\\R-3.1.2\\scripts\\mPDBS\\summary_pdb_93\\b28e48f6fa27a165.final.pdb
-5.405752e-15 1.991396e-15 -6.285984e-15 -5.749792e-16 -7.263660e-15
4.181825e-15
E:\\R-3.1.2\\scripts\\mPDBS\\summary_pdb_93\\b42c4924e3cd1aec.final.pdb
-4.407125e-16 4.053912e-15 -3.302068e-16 8.792353e-16 -5.418832e-15
-6.985017e-15
E:\\R-3.1.2\\scripts\\mPDBS\\summary_pdb_93\\b4dd239fcabff1df.final.pdb
-5.405752e-15 1.991396e-15 -6.285984e-15 -5.749792e-16 -7.263660e-15
4.181825e-15
E:\\R-3.1.2\\scripts\\mPDBS\\summary_pdb_93\\b669942ef97459a2.final.pdb
2.239934e-16 1.816404e-15 -1.415947e-14 -5.789225e-16 -4.792891e-16
1.482587e-15
E:\\R-3.1.2\\scripts\\mPDBS\\summary_pdb_93\\b99f4d9d87c1017b.final.pdb
-5.405752e-15 1.991396e-15 -6.285984e-15 -5.749792e-16 -7.263660e-15
4.181825e-15
E:\\R-3.1.2\\scripts\\mPDBS\\summary_pdb_93\\b9d2cbf3975403e7.final.pdb
-5.405752e-15 1.991396e-15 -6.285984e-15 -5.749792e-16 -7.263660e-15
4.181825e-15
E:\\R-3.1.2\\scripts\\mPDBS\\summary_pdb_93\\bc88c8a505f3a288.final.pdb
-1.126189e-15 8.432262e-16 -4.731888e-15 -2.435036e-15 -1.938926e-15
-1.881251e-15
E:\\R-3.1.2\\scripts\\mPDBS\\summary_pdb_93\\bdac0e64f4ef427e.final.pdb
1.275236e-15 8.082008e-15 -8.084038e-15 -2.541762e-15 4.730741e-16
-6.017924e-15
E:\\R-3.1.2\\scripts\\mPDBS\\summary_pdb_93\\ca2a1f55fc9d0681.final.pdb
2.239934e-16 1.816404e-15 -1.415947e-14 -5.789225e-16 -4.792891e-16
1.482587e-15
E:\\R-3.1.2\\scripts\\mPDBS\\summary_pdb_93\\ce34248de3e81a3c.final.pdb
-3.262306e-15 3.588011e-15 -7.271747e-15 3.046470e-15 -7.108048e-16
7.875143e-15
E:\\R-3.1.2\\scripts\\mPDBS\\summary_pdb_93\\d02e697772494a61.final.pdb
1.013477e-14 -1.448696e-14 -1.818854e-15 -1.215296e-14 2.350178e-15
-7.833738e-15
E:\\R-3.1.2\\scripts\\mPDBS\\summary_pdb_93\\d04221245d015047.final.pdb
-2.632936e-15 1.274539e-15 -5.231847e-15 5.543513e-16 -1.819424e-15
-7.579151e-16
E:\\R-3.1.2\\scripts\\mPDBS\\summary_pdb_93\\d678141e4018054a.final.pdb
2.973204e-15 -4.913441e-15 4.819546e-15 -1.623959e-14 1.416064e-14
-1.419065e-14
E:\\R-3.1.2\\scripts\\mPDBS\\summary_pdb_93\\d71b40af0f0efe39.final.pdb
3.533784e-15 -4.620173e-15 5.390170e-16 -1.056884e-14 -8.229270e-15
-1.291722e-14
E:\\R-3.1.2\\scripts\\mPDBS\\summary_pdb_93\\dc0809eff10197e2.final.pdb
-5.434558e-15 6.789427e-15 -1.476090e-14 4.991231e-15 -1.890200e-14
-1.273044e-16
E:\\R-3.1.2\\scripts\\mPDBS\\summary_pdb_93\\e1163918d3707839.final.pdb
-5.252835e-15 -8.332379e-16 -1.430889e-15 -8.051812e-16 -2.852526e-15
-9.750671e-16
E:\\R-3.1.2\\scripts\\mPDBS\\summary_pdb_93\\e4b48b06824ef848.final.pdb
-1.823740e-15 -3.591370e-15 2.494603e-15 -5.108058e-15 9.562717e-15

-7.433012e-15
E:\\R-3.1.2\\scripts\\mPDBS\\summary_pdb_93\\e529e19ad281fea2.final.pdb
9.524728e-16 -1.291017e-15 4.589369e-15 -7.415281e-16 -2.350601e-15
-3.148164e-15
E:\\R-3.1.2\\scripts\\mPDBS\\summary_pdb_93\\e5602082718ec3a1.final.pdb
-2.260131e-15 -1.025073e-15 -2.620093e-15 -7.489254e-16 -7.378284e-15
3.219045e-15
E:\\R-3.1.2\\scripts\\mPDBS\\summary_pdb_93\\e5d589d996d0a960.final.pdb
1.901677e-15 9.320244e-15 -1.041187e-14 8.463452e-15 -5.751500e-15
3.182137e-15
E:\\R-3.1.2\\scripts\\mPDBS\\summary_pdb_93\\e9df9e72307a2fbd.final.pdb
1.351680e-15 2.991477e-15 -7.986917e-15 3.657244e-15 4.793165e-16
-3.027404e-15
E:\\R-3.1.2\\scripts\\mPDBS\\summary_pdb_93\\ea00f8694090cae7.final.pdb
-2.260131e-15 -1.025073e-15 -2.620093e-15 -7.489254e-16 -7.378284e-15
3.219045e-15
E:\\R-3.1.2\\scripts\\mPDBS\\summary_pdb_93\\eba517be32db050e.final.pdb
-5.405752e-15 1.991396e-15 -6.285984e-15 -5.749792e-16 -7.263660e-15
4.181825e-15
E:\\R-3.1.2\\scripts\\mPDBS\\summary_pdb_93\\ec04b9545899c29d.final.pdb
-9.446496e-16 7.794044e-16 -5.339004e-15 -1.507311e-15 -9.753382e-16
-1.129024e-15
E:\\R-3.1.2\\scripts\\mPDBS\\summary_pdb_93\\ee6862cbcad52078.final.pdb
-8.625109e-16 6.847158e-16 -5.254691e-15 -2.724302e-15 -1.695197e-15
-1.756134e-15
E:\\R-3.1.2\\scripts\\mPDBS\\summary_pdb_93\\ef6a42fc83b81e5d.final.pdb
-9.902058e-16 7.458724e-16 -5.354370e-15 -1.568788e-15 -1.010639e-15
-1.745287e-15
E:\\R-3.1.2\\scripts\\mPDBS\\summary_pdb_93\\ef8e88d4ad93a0ad.final.pdb
-2.632936e-15 1.274539e-15 -5.231847e-15 5.543513e-16 -1.819424e-15
-7.579151e-16
E:\\R-3.1.2\\scripts\\mPDBS\\summary_pdb_93\\f14fcae8deec4fa3.final.pdb
-2.228861e-15 2.349455e-16 -6.343311e-15 -2.542586e-15 -3.044675e-15
-5.679991e-16
E:\\R-3.1.2\\scripts\\mPDBS\\summary_pdb_93\\f83584e22003fb92.final.pdb
4.375487e-15 9.523132e-15 -1.601146e-15 -8.228145e-15 7.857925e-15
5.467116e-16
E:\\R-3.1.2\\scripts\\mPDBS\\summary_pdb_93\\f8e2bf87e52caa29.final.pdb
9.524728e-16 -1.291017e-15 4.589369e-15 -7.415281e-16 -2.350601e-15
-3.148164e-15

[,253] [,254] [,255] [,256] [,257] [,258]
E:\\R-3.1.2\\scripts\\40.1ns_data\\NAT0.pdb
5.274630e-15 -2.440283e-16 4.348793e-15 -3.798746e-15 1.540038e-15
-3.981575e-15
E:\\R-3.1.2\\scripts\\40.1ns_data\\LJP6.pdb
6.422916e-15 5.956632e-16 5.109090e-15 -2.954917e-15 2.246143e-15
-2.756152e-15
E:\\R-3.1.2\\scripts\\40.1ns_data\\3WY9.pdb
5.756084e-15 9.141924e-16 4.771762e-15 -1.760196e-15 2.673831e-15
-2.870898e-15
E:\\R-3.1.2\\scripts\\40.1ns_data\\ZSP9.pdb
2.833860e-15 -3.293564e-16 3.952578e-15 -1.979444e-15 1.616409e-15
-2.979867e-15

E:\\R-3.1.2\\scripts\\mPDBS\\summary_pdb_93\\013d855ad26c877c.final.pdb
-2.295226e-15 1.619429e-15 -1.402173e-15 4.937522e-15 8.456392e-15
-6.294969e-15
E:\\R-3.1.2\\scripts\\mPDBS\\summary_pdb_93\\0a2a705342ca0d54.final.pdb
2.774645e-15 -4.447790e-17 4.947623e-15 -1.441527e-15 2.031294e-15
-3.110523e-15
E:\\R-3.1.2\\scripts\\mPDBS\\summary_pdb_93\\0a59fceadb4194d9.final.pdb
3.345867e-15 1.793807e-15 6.528700e-15 2.166893e-15 1.119596e-15
1.392785e-15
E:\\R-3.1.2\\scripts\\mPDBS\\summary_pdb_93\\0dba59f3306825e8.final.pdb
-3.627438e-16 7.173727e-16 5.025411e-15 1.288688e-16 -7.706019e-16
-1.959896e-15
E:\\R-3.1.2\\scripts\\mPDBS\\summary_pdb_93\\126f26a78b57339c.final.pdb
2.296154e-15 -3.630712e-15 4.400911e-15 -4.775086e-15 9.155493e-16
-4.901119e-15
E:\\R-3.1.2\\scripts\\mPDBS\\summary_pdb_93\\12f06f3e342df706.final.pdb
2.612665e-15 -8.650021e-16 4.931360e-15 -1.133831e-15 1.885144e-15
-2.074893e-15
E:\\R-3.1.2\\scripts\\mPDBS\\summary_pdb_93\\18618a2a251b867b.final.pdb
-1.679261e-16 -5.546785e-15 -6.158830e-16 -1.607892e-15 2.623241e-15
-1.012531e-14
E:\\R-3.1.2\\scripts\\mPDBS\\summary_pdb_93\\1f305f598641eeb2.final.pdb
2.474992e-15 4.550736e-16 3.724286e-15 -1.736307e-15 2.241383e-15
-2.504573e-15
E:\\R-3.1.2\\scripts\\mPDBS\\summary_pdb_93\\1f8a00b551be0a39.final.pdb
-1.679261e-16 -5.546785e-15 -6.158830e-16 -1.607892e-15 2.623241e-15
-1.012531e-14
E:\\R-3.1.2\\scripts\\mPDBS\\summary_pdb_93\\219f45328d47579a.final.pdb
6.391174e-15 -6.408774e-16 2.364241e-16 -2.122759e-14 8.217651e-15
1.047797e-14
E:\\R-3.1.2\\scripts\\mPDBS\\summary_pdb_93\\238dd3918e54336d.final.pdb
-5.666021e-15 -1.230683e-15 5.852158e-15 -1.442997e-14 8.973990e-15
1.242683e-16
E:\\R-3.1.2\\scripts\\mPDBS\\summary_pdb_93\\2b3027889e2fdc42.final.pdb
1.366331e-15 -4.058224e-16 4.666040e-15 -1.255608e-15 2.863427e-15
-1.690686e-15
E:\\R-3.1.2\\scripts\\mPDBS\\summary_pdb_93\\2fcb3b46e33793b0.final.pdb
1.711714e-15 1.471622e-15 9.079622e-16 8.519002e-16 2.310772e-15
-2.599983e-15
E:\\R-3.1.2\\scripts\\mPDBS\\summary_pdb_93\\34eb328251842006.final.pdb
4.169610e-15 2.746698e-15 7.442381e-15 5.720383e-15 7.142644e-15
1.171873e-15
E:\\R-3.1.2\\scripts\\mPDBS\\summary_pdb_93\\354b32871797b7ab.final.pdb
6.144675e-15 -9.851947e-16 -7.143909e-15 3.902878e-16 7.630804e-15
-3.058065e-15
E:\\R-3.1.2\\scripts\\mPDBS\\summary_pdb_93\\397368adbc32d5de.final.pdb
2.296154e-15 -3.630712e-15 4.400911e-15 -4.775086e-15 9.155493e-16
-4.901119e-15
E:\\R-3.1.2\\scripts\\mPDBS\\summary_pdb_93\\411ca13cd3df6edd.final.pdb
1.040589e-16 -3.672701e-15 -1.877136e-15 3.771331e-15 2.451284e-15
-9.520662e-15
E:\\R-3.1.2\\scripts\\mPDBS\\summary_pdb_93\\42357eee6642bf12.final.pdb
8.555535e-15 -4.665828e-15 7.990624e-15 -3.362055e-15 6.361421e-15
9.113020e-15

E:\\R-3.1.2\\scripts\\mPDBS\\summary_pdb_93\\44342d1f75a7fcfc.final.pdb
-1.672982e-15 -2.127997e-15 -4.794456e-15 1.153717e-14 4.496172e-15
-1.826111e-14
E:\\R-3.1.2\\scripts\\mPDBS\\summary_pdb_93\\448ef21dffe8cdfb.final.pdb
1.115555e-14 2.399679e-16 7.992573e-15 -1.264772e-14 1.425781e-14
-6.779096e-16
E:\\R-3.1.2\\scripts\\mPDBS\\summary_pdb_93\\472a40b3e8f113c7.final.pdb
-3.627438e-16 7.173727e-16 5.025411e-15 1.288688e-16 -7.706019e-16
-1.959896e-15
E:\\R-3.1.2\\scripts\\mPDBS\\summary_pdb_93\\48a26225951ea153.final.pdb
-5.563022e-15 -1.819622e-15 -6.169693e-15 1.004265e-14 -2.018294e-16
-1.745846e-14
E:\\R-3.1.2\\scripts\\mPDBS\\summary_pdb_93\\4ab4a5c7c6682ce6.final.pdb
1.091773e-14 8.136498e-16 8.319217e-15 -4.206097e-15 2.691906e-15
4.851972e-16
E:\\R-3.1.2\\scripts\\mPDBS\\summary_pdb_93\\4eaa45b62cb6a25f.final.pdb
-2.920169e-16 -1.846485e-15 6.698967e-15 -5.457039e-16 -1.276134e-15
-5.466295e-15
E:\\R-3.1.2\\scripts\\mPDBS\\summary_pdb_93\\58c371611d85ac96.final.pdb
2.296154e-15 -3.630712e-15 4.400911e-15 -4.775086e-15 9.155493e-16
-4.901119e-15
E:\\R-3.1.2\\scripts\\mPDBS\\summary_pdb_93\\59a647f04822d30a.final.pdb
8.455201e-15 -9.214964e-16 1.075647e-14 -7.661010e-15 -1.137776e-15
3.910827e-15
E:\\R-3.1.2\\scripts\\mPDBS\\summary_pdb_93\\59b091fe385e05fc.final.pdb
3.345867e-15 1.793807e-15 6.528700e-15 2.166893e-15 1.119596e-15
1.392785e-15
E:\\R-3.1.2\\scripts\\mPDBS\\summary_pdb_93\\5c35ac30f930d2db.final.pdb
-2.920169e-16 -1.846485e-15 6.698967e-15 -5.457039e-16 -1.276134e-15
-5.466295e-15
E:\\R-3.1.2\\scripts\\mPDBS\\summary_pdb_93\\5f60752431cf2dcb.final.pdb
4.484865e-15 -3.095856e-15 1.232757e-14 8.422219e-15 -3.745335e-15
-8.927918e-15
E:\\R-3.1.2\\scripts\\mPDBS\\summary_pdb_93\\626456dd6ff2868d.final.pdb
-3.627438e-16 7.173727e-16 5.025411e-15 1.288688e-16 -7.706019e-16
-1.959896e-15
E:\\R-3.1.2\\scripts\\mPDBS\\summary_pdb_93\\6583d53ff3c8f7ef.final.pdb
8.455201e-15 -9.214964e-16 1.075647e-14 -7.661010e-15 -1.137776e-15
3.910827e-15
E:\\R-3.1.2\\scripts\\mPDBS\\summary_pdb_93\\69760178986bef01.final.pdb
1.116081e-15 -7.626600e-15 1.371844e-15 -1.036795e-14 -4.626980e-15
-1.081464e-14
E:\\R-3.1.2\\scripts\\mPDBS\\summary_pdb_93\\6a9d87d47926b1c8.final.pdb
1.411445e-15 7.060970e-16 4.372288e-15 -1.277884e-15 2.445575e-15
-1.698820e-15
E:\\R-3.1.2\\scripts\\mPDBS\\summary_pdb_93\\6bb3547ea982ffc4.final.pdb
-7.854304e-15 -2.823707e-15 -4.362035e-16 -7.658264e-16 1.086285e-14
-2.223509e-14
E:\\R-3.1.2\\scripts\\mPDBS\\summary_pdb_93\\716d9624611d1c42.final.pdb
6.678232e-16 1.122124e-15 -4.174420e-15 -3.347988e-15 5.965213e-15
-2.578397e-15
E:\\R-3.1.2\\scripts\\mPDBS\\summary_pdb_93\\75f27e70ad0f1be4.final.pdb
8.455201e-15 -9.214964e-16 1.075647e-14 -7.661010e-15 -1.137776e-15
3.910827e-15

E:\\R-3.1.2\\scripts\\mPDBS\\summary_pdb_93\\769f910fffb22872.final.pdb
1.637271e-15 -4.250719e-17 4.489625e-15 -2.938394e-16 2.200402e-15
-1.732715e-15
E:\\R-3.1.2\\scripts\\mPDBS\\summary_pdb_93\\791f8c80dd5c3135.final.pdb
3.345867e-15 1.793807e-15 6.528700e-15 2.166893e-15 1.119596e-15
1.392785e-15
E:\\R-3.1.2\\scripts\\mPDBS\\summary_pdb_93\\7fad98e66a3f6099.final.pdb
2.964794e-15 2.556334e-15 2.646377e-15 6.727031e-15 4.027634e-15
3.706172e-15
E:\\R-3.1.2\\scripts\\mPDBS\\summary_pdb_93\\804b89c94a633196.final.pdb
1.711714e-15 1.471622e-15 9.079622e-16 8.519002e-16 2.310772e-15
-2.599983e-15
E:\\R-3.1.2\\scripts\\mPDBS\\summary_pdb_93\\813cc65a2298feba.final.pdb
1.711714e-15 1.471622e-15 9.079622e-16 8.519002e-16 2.310772e-15
-2.599983e-15
E:\\R-3.1.2\\scripts\\mPDBS\\summary_pdb_93\\83c0eefcfe12d3f9.final.pdb
1.040589e-16 -3.672701e-15 -1.877136e-15 3.771331e-15 2.451284e-15
-9.520662e-15
E:\\R-3.1.2\\scripts\\mPDBS\\summary_pdb_93\\86d50469ef569760.final.pdb
-3.627438e-16 7.173727e-16 5.025411e-15 1.288688e-16 -7.706019e-16
-1.959896e-15
E:\\R-3.1.2\\scripts\\mPDBS\\summary_pdb_93\\89d0666e1402203d.final.pdb
8.455201e-15 -9.214964e-16 1.075647e-14 -7.661010e-15 -1.137776e-15
3.910827e-15
E:\\R-3.1.2\\scripts\\mPDBS\\summary_pdb_93\\8b34852716dadc62.final.pdb
4.102163e-15 -3.014373e-15 6.740970e-15 -5.962094e-15 -3.399938e-15
4.583343e-16
E:\\R-3.1.2\\scripts\\mPDBS\\summary_pdb_93\\8b6ef9ac3fe74395.final.pdb
3.590169e-15 8.815907e-15 9.381902e-15 -1.032557e-14 -5.985637e-15
3.465377e-15
E:\\R-3.1.2\\scripts\\mPDBS\\summary_pdb_93\\98bbe0d5986c138f.final.pdb
3.345867e-15 1.793807e-15 6.528700e-15 2.166893e-15 1.119596e-15
1.392785e-15
E:\\R-3.1.2\\scripts\\mPDBS\\summary_pdb_93\\9c22f4909d67e2fa.final.pdb
4.620625e-16 -3.151436e-16 3.885181e-15 -1.499517e-15 5.839199e-15
8.182894e-16
E:\\R-3.1.2\\scripts\\mPDBS\\summary_pdb_93\\9dda8ed0d81fb3ef.final.pdb
-6.058797e-15 1.018792e-15 -1.518648e-15 2.702305e-14 -7.288672e-15
-1.885135e-14
E:\\R-3.1.2\\scripts\\mPDBS\\summary_pdb_93\\9f752b3616e6033b.final.pdb
3.618418e-15 1.584708e-15 5.110612e-15 -5.749816e-15 3.403819e-15
1.199856e-15
E:\\R-3.1.2\\scripts\\mPDBS\\summary_pdb_93\\a02c70083816d6ba.final.pdb
-2.920169e-16 -1.846485e-15 6.698967e-15 -5.457039e-16 -1.276134e-15
-5.466295e-15
E:\\R-3.1.2\\scripts\\mPDBS\\summary_pdb_93\\a0c997d891e4e082.final.pdb
4.954692e-15 2.631016e-15 5.932057e-15 -1.487751e-15 7.343065e-15
-7.526251e-15
E:\\R-3.1.2\\scripts\\mPDBS\\summary_pdb_93\\ab8039a2d823c616.final.pdb
3.759467e-15 6.709500e-17 4.882766e-15 -1.040951e-15 2.430328e-15
-1.732275e-15
E:\\R-3.1.2\\scripts\\mPDBS\\summary_pdb_93\\acf4d74b9c71d4dc.final.pdb
1.711714e-15 1.471622e-15 9.079622e-16 8.519002e-16 2.310772e-15
-2.599983e-15

E:\\R-3.1.2\\scripts\\mPDBS\\summary_pdb_93\\ad8a823e4b4c668e.final.pdb
2.987861e-15 9.931633e-16 4.563423e-15 -1.126570e-15 2.017030e-15
-1.979405e-15
E:\\R-3.1.2\\scripts\\mPDBS\\summary_pdb_93\\b16ababd03b7b857.final.pdb
4.620625e-16 -3.151436e-16 3.885181e-15 -1.499517e-15 5.839199e-15
8.182894e-16
E:\\R-3.1.2\\scripts\\mPDBS\\summary_pdb_93\\b226923e697d3c63.final.pdb
1.283863e-14 4.810276e-16 7.570219e-15 -9.460786e-15 3.814426e-15
1.356013e-15
E:\\R-3.1.2\\scripts\\mPDBS\\summary_pdb_93\\b28e48f6fa27a165.final.pdb
3.345867e-15 1.793807e-15 6.528700e-15 2.166893e-15 1.119596e-15
1.392785e-15
E:\\R-3.1.2\\scripts\\mPDBS\\summary_pdb_93\\b42c4924e3cd1aec.final.pdb
-1.472932e-16 9.095043e-16 9.921478e-16 -6.617682e-15 3.333907e-15
2.448889e-15
E:\\R-3.1.2\\scripts\\mPDBS\\summary_pdb_93\\b4dd239fcabff1df.final.pdb
3.345867e-15 1.793807e-15 6.528700e-15 2.166893e-15 1.119596e-15
1.392785e-15
E:\\R-3.1.2\\scripts\\mPDBS\\summary_pdb_93\\b669942ef97459a2.final.pdb
4.102163e-15 -3.014373e-15 6.740970e-15 -5.962094e-15 -3.399938e-15
4.583343e-16
E:\\R-3.1.2\\scripts\\mPDBS\\summary_pdb_93\\b99f4d9d87c1017b.final.pdb
3.345867e-15 1.793807e-15 6.528700e-15 2.166893e-15 1.119596e-15
1.392785e-15
E:\\R-3.1.2\\scripts\\mPDBS\\summary_pdb_93\\b9d2cbf3975403e7.final.pdb
3.345867e-15 1.793807e-15 6.528700e-15 2.166893e-15 1.119596e-15
1.392785e-15
E:\\R-3.1.2\\scripts\\mPDBS\\summary_pdb_93\\bc88c8a505f3a288.final.pdb
2.345149e-15 -2.938736e-17 4.696398e-15 -1.719755e-15 2.490461e-15
-1.539332e-15
E:\\R-3.1.2\\scripts\\mPDBS\\summary_pdb_93\\bdac0e64f4ef427e.final.pdb
6.917402e-15 8.199747e-15 1.378351e-14 -1.084111e-14 7.113566e-16
1.235247e-14
E:\\R-3.1.2\\scripts\\mPDBS\\summary_pdb_93\\ca2a1f55fc9d0681.final.pdb
4.102163e-15 -3.014373e-15 6.740970e-15 -5.962094e-15 -3.399938e-15
4.583343e-16
E:\\R-3.1.2\\scripts\\mPDBS\\summary_pdb_93\\ce34248de3e81a3c.final.pdb
1.090418e-15 9.983308e-16 9.179178e-15 7.793535e-15 -4.774484e-15
4.327101e-16
E:\\R-3.1.2\\scripts\\mPDBS\\summary_pdb_93\\d02e697772494a61.final.pdb
1.325461e-14 -2.965407e-15 1.391732e-14 -2.358584e-14 1.101576e-14
1.112155e-14
E:\\R-3.1.2\\scripts\\mPDBS\\summary_pdb_93\\d04221245d015047.final.pdb
-1.200107e-15 -7.204695e-16 2.810422e-15 1.381482e-15 1.396433e-15
-3.029848e-15
E:\\R-3.1.2\\scripts\\mPDBS\\summary_pdb_93\\d678141e4018054a.final.pdb
1.620857e-15 -2.566952e-15 -1.242398e-15 1.087657e-14 1.518651e-14
-1.217679e-14
E:\\R-3.1.2\\scripts\\mPDBS\\summary_pdb_93\\d71b40af0f0efe39.final.pdb
7.729600e-15 2.160907e-15 -4.916771e-15 -2.589852e-15 1.256116e-14
7.897972e-15
E:\\R-3.1.2\\scripts\\mPDBS\\summary_pdb_93\\dc0809eff10197e2.final.pdb
7.224808e-15 4.170146e-15 1.150641e-14 -6.306082e-15 -7.023368e-15
1.243381e-14

E:\\R-3.1.2\\scripts\\mPDBS\\summary_pdb_93\\e1163918d3707839.final.pdb
2.544130e-15 6.336587e-15 4.122456e-15 -6.069510e-15 5.513588e-15
1.733654e-16
E:\\R-3.1.2\\scripts\\mPDBS\\summary_pdb_93\\e4b48b06824ef848.final.pdb
-1.679261e-16 -5.546785e-15 -6.158830e-16 -1.607892e-15 2.623241e-15
-1.012531e-14
E:\\R-3.1.2\\scripts\\mPDBS\\summary_pdb_93\\e529e19ad281fea2.final.pdb
-2.295226e-15 1.619429e-15 -1.402173e-15 4.937522e-15 8.456392e-15
-6.294969e-15
E:\\R-3.1.2\\scripts\\mPDBS\\summary_pdb_93\\e5602082718ec3a1.final.pdb
1.711714e-15 1.471622e-15 9.079622e-16 8.519002e-16 2.310772e-15
-2.599983e-15
E:\\R-3.1.2\\scripts\\mPDBS\\summary_pdb_93\\e5d589d996d0a960.final.pdb
-9.076852e-15 6.478074e-15 7.564828e-16 2.228331e-15 -2.604053e-15
-9.107178e-15
E:\\R-3.1.2\\scripts\\mPDBS\\summary_pdb_93\\e9df9e72307a2fbd.final.pdb
-2.920169e-16 -1.846485e-15 6.698967e-15 -5.457039e-16 -1.276134e-15
-5.466295e-15
E:\\R-3.1.2\\scripts\\mPDBS\\summary_pdb_93\\ea00f8694090cae7.final.pdb
1.711714e-15 1.471622e-15 9.079622e-16 8.519002e-16 2.310772e-15
-2.599983e-15
E:\\R-3.1.2\\scripts\\mPDBS\\summary_pdb_93\\eba517be32db050e.final.pdb
3.345867e-15 1.793807e-15 6.528700e-15 2.166893e-15 1.119596e-15
1.392785e-15
E:\\R-3.1.2\\scripts\\mPDBS\\summary_pdb_93\\ec04b9545899c29d.final.pdb
2.648214e-15 -5.060301e-16 3.766838e-15 -1.400393e-15 2.190725e-15
-1.630313e-15
E:\\R-3.1.2\\scripts\\mPDBS\\summary_pdb_93\\ee6862cbcad52078.final.pdb
2.227838e-15 -4.933668e-17 4.296111e-15 -1.267859e-15 2.297040e-15
-1.770917e-15
E:\\R-3.1.2\\scripts\\mPDBS\\summary_pdb_93\\ef6a42fc83b81e5d.final.pdb
2.589634e-15 -5.880777e-16 4.259991e-15 -1.320037e-15 1.822233e-15
-2.017643e-15
E:\\R-3.1.2\\scripts\\mPDBS\\summary_pdb_93\\ef8e88d4ad93a0ad.final.pdb
-1.200107e-15 -7.204695e-16 2.810422e-15 1.381482e-15 1.396433e-15
-3.029848e-15
E:\\R-3.1.2\\scripts\\mPDBS\\summary_pdb_93\\f14fcae8deec4fa3.final.pdb
3.618418e-15 1.584708e-15 5.110612e-15 -5.749816e-15 3.403819e-15
1.199856e-15
E:\\R-3.1.2\\scripts\\mPDBS\\summary_pdb_93\\f83584e22003fb92.final.pdb
-5.563022e-15 -1.819622e-15 -6.169693e-15 1.004265e-14 -2.018294e-16
-1.745846e-14
E:\\R-3.1.2\\scripts\\mPDBS\\summary_pdb_93\\f8e2bf87e52caa29.final.pdb
-2.295226e-15 1.619429e-15 -1.402173e-15 4.937522e-15 8.456392e-15
-6.294969e-15

[,259]   [,260]   [,261]   [,262]   [,263]   [,264]
E:\\R-3.1.2\\scripts\\40.1ns_data\\NAT0.pdb
5.437359e-15 2.419651e-15 5.322648e-16 -2.120845e-15 -1.006496e-15
1.298576e-15
E:\\R-3.1.2\\scripts\\40.1ns_data\\LJP6.pdb
1.964593e-15 2.465064e-15 1.137365e-17 -1.175831e-15 1.113160e-15
4.288511e-16
E:\\R-3.1.2\\scripts\\40.1ns_data\\3WY9.pdb

3.205525e-15 2.813994e-15 2.201469e-15 -2.778396e-15 8.808601e-16
1.474498e-16
E:\\R-3.1.2\\scripts\\40.1ns_data\\ZSP9.pdb
3.258728e-15 7.682056e-16 3.004728e-16 -1.797158e-15 -4.963422e-16
3.873400e-16
E:\\R-3.1.2\\scripts\\mPDBS\\summary_pdb_93\\013d855ad26c877c.final.pdb
1.358541e-15 4.759859e-15 3.357828e-15 -7.809132e-15 2.424808e-15
1.063359e-15
E:\\R-3.1.2\\scripts\\mPDBS\\summary_pdb_93\\0a2a705342ca0d54.final.pdb
2.170288e-15 1.206245e-15 3.390490e-15 -1.253412e-15 2.578361e-16
3.873153e-16
E:\\R-3.1.2\\scripts\\mPDBS\\summary_pdb_93\\0a59fceadb4194d9.final.pdb
-1.097630e-15 -2.911882e-16 7.860995e-15 -4.272911e-15 4.260584e-16
-3.396807e-15
E:\\R-3.1.2\\scripts\\mPDBS\\summary_pdb_93\\0dba59f3306825e8.final.pdb
2.052431e-15 2.891561e-15 3.533035e-15 2.713701e-15 1.977249e-15
2.031335e-15
E:\\R-3.1.2\\scripts\\mPDBS\\summary_pdb_93\\126f26a78b57339c.final.pdb
9.949326e-15 5.962889e-15 -6.121135e-15 -6.346326e-15 9.355477e-16
4.728288e-15
E:\\R-3.1.2\\scripts\\mPDBS\\summary_pdb_93\\12f06f3e342df706.final.pdb
2.400897e-15 2.117842e-15 2.579073e-15 -1.453339e-15 1.050605e-15
1.185153e-15
E:\\R-3.1.2\\scripts\\mPDBS\\summary_pdb_93\\18618a2a251b867b.final.pdb
9.840992e-15 8.653242e-15 -6.491788e-15 -3.827049e-15 3.956855e-15
7.251050e-15
E:\\R-3.1.2\\scripts\\mPDBS\\summary_pdb_93\\1f305f598641eeb2.final.pdb
2.780456e-15 2.264950e-15 1.211644e-15 -2.806360e-15 5.369818e-16
9.483940e-16
E:\\R-3.1.2\\scripts\\mPDBS\\summary_pdb_93\\1f8a00b551be0a39.final.pdb
9.840992e-15 8.653242e-15 -6.491788e-15 -3.827049e-15 3.956855e-15
7.251050e-15
E:\\R-3.1.2\\scripts\\mPDBS\\summary_pdb_93\\219f45328d47579a.final.pdb
1.079216e-14 -1.489342e-18 -8.071573e-15 -4.855778e-15 1.277555e-14
-7.426806e-16
E:\\R-3.1.2\\scripts\\mPDBS\\summary_pdb_93\\238dd3918e54336d.final.pdb
7.224055e-15 9.034252e-15 -1.280563e-14 -1.978739e-15 1.803429e-15
1.812997e-14
E:\\R-3.1.2\\scripts\\mPDBS\\summary_pdb_93\\2b3027889e2fdc42.final.pdb
2.384394e-15 2.166481e-15 2.233552e-15 -2.611479e-15 9.724963e-16
8.093548e-16
E:\\R-3.1.2\\scripts\\mPDBS\\summary_pdb_93\\2fcb3b46e33793b0.final.pdb
-4.069967e-15 1.659342e-16 3.026165e-15 6.353316e-16 -1.104067e-15
-6.093877e-16
E:\\R-3.1.2\\scripts\\mPDBS\\summary_pdb_93\\34eb328251842006.final.pdb
4.569741e-15 -6.845236e-15 3.765696e-15 -7.812450e-15 -1.939608e-16
3.322411e-15
E:\\R-3.1.2\\scripts\\mPDBS\\summary_pdb_93\\354b32871797b7ab.final.pdb
-2.543235e-15 7.172292e-15 1.490691e-15 -5.433831e-15 -1.293511e-14
7.942434e-16
E:\\R-3.1.2\\scripts\\mPDBS\\summary_pdb_93\\397368adbc32d5de.final.pdb
9.949326e-15 5.962889e-15 -6.121135e-15 -6.346326e-15 9.355477e-16
4.728288e-15
E:\\R-3.1.2\\scripts\\mPDBS\\summary_pdb_93\\411ca13cd3df6edd.final.pdb

-1.711176e-15 -2.917537e-15 -1.060410e-15 3.139741e-16 -4.513666e-15
2.432125e-15
E:\\R-3.1.2\\scripts\\mPDBS\\summary_pdb_93\\42357eee6642bf12.final.pdb
-1.509823e-15 -1.352399e-15 3.979405e-15 -6.776447e-15 4.445202e-15
-6.391531e-15
E:\\R-3.1.2\\scripts\\mPDBS\\summary_pdb_93\\44342d1f75a7fcfc.final.pdb
9.378754e-16 -2.404852e-15 1.883621e-15 -6.448467e-15 -6.476370e-17
1.964228e-16
E:\\R-3.1.2\\scripts\\mPDBS\\summary_pdb_93\\448ef21dffe8cdfb.final.pdb
6.532663e-15 4.542641e-15 3.324538e-15 -1.593128e-14 -9.697887e-15
5.149894e-15
E:\\R-3.1.2\\scripts\\mPDBS\\summary_pdb_93\\472a40b3e8f113c7.final.pdb
2.052431e-15 2.891561e-15 3.533035e-15 2.713701e-15 1.977249e-15
2.031335e-15
E:\\R-3.1.2\\scripts\\mPDBS\\summary_pdb_93\\48a26225951ea153.final.pdb
1.132082e-14 1.058427e-15 -5.589184e-15 8.484265e-16 -5.668025e-15
5.616274e-15
E:\\R-3.1.2\\scripts\\mPDBS\\summary_pdb_93\\4ab4a5c7c6682ce6.final.pdb
-9.696418e-15 4.228166e-15 1.164703e-15 -1.360160e-16 -1.173140e-14
-6.020059e-15
E:\\R-3.1.2\\scripts\\mPDBS\\summary_pdb_93\\4eaa45b62cb6a25f.final.pdb
3.512468e-15 2.351062e-15 1.874065e-15 1.823362e-16 4.123290e-15
3.113939e-16
E:\\R-3.1.2\\scripts\\mPDBS\\summary_pdb_93\\58c371611d85ac96.final.pdb
9.949326e-15 5.962889e-15 -6.121135e-15 -6.346326e-15 9.355477e-16
4.728288e-15
E:\\R-3.1.2\\scripts\\mPDBS\\summary_pdb_93\\59a647f04822d30a.final.pdb
3.059442e-15 3.091229e-16 2.724094e-15 -1.430779e-15 2.280582e-15
-1.654527e-15
E:\\R-3.1.2\\scripts\\mPDBS\\summary_pdb_93\\59b091fe385e05fc.final.pdb
-1.097630e-15 -2.911882e-16 7.860995e-15 -4.272911e-15 4.260584e-16
-3.396807e-15
E:\\R-3.1.2\\scripts\\mPDBS\\summary_pdb_93\\5c35ac30f930d2db.final.pdb
3.512468e-15 2.351062e-15 1.874065e-15 1.823362e-16 4.123290e-15
3.113939e-16
E:\\R-3.1.2\\scripts\\mPDBS\\summary_pdb_93\\5f60752431cf2dcb.final.pdb
1.295290e-14 -5.943984e-16 4.170249e-15 -3.372810e-15 1.830363e-15
3.780996e-16
E:\\R-3.1.2\\scripts\\mPDBS\\summary_pdb_93\\626456dd6ff2868d.final.pdb
2.052431e-15 2.891561e-15 3.533035e-15 2.713701e-15 1.977249e-15
2.031335e-15
E:\\R-3.1.2\\scripts\\mPDBS\\summary_pdb_93\\6583d53ff3c8f7ef.final.pdb
3.059442e-15 3.091229e-16 2.724094e-15 -1.430779e-15 2.280582e-15
-1.654527e-15
E:\\R-3.1.2\\scripts\\mPDBS\\summary_pdb_93\\69760178986bef01.final.pdb
5.364018e-15 -9.732773e-16 -5.457875e-15 3.776367e-15 6.059823e-16
4.431082e-15
E:\\R-3.1.2\\scripts\\mPDBS\\summary_pdb_93\\6a9d87d47926b1c8.final.pdb
2.058069e-15 2.293949e-15 5.297946e-16 -3.279335e-15 5.582453e-16
1.595377e-15
E:\\R-3.1.2\\scripts\\mPDBS\\summary_pdb_93\\6bb3547ea982ffc4.final.pdb
1.293609e-14 -6.134042e-16 2.757419e-15 -2.861377e-15 -4.008497e-17
1.603523e-14
E:\\R-3.1.2\\scripts\\mPDBS\\summary_pdb_93\\716d9624611d1c42.final.pdb

-4.518806e-15 1.140325e-15 -1.648881e-15 1.968168e-16 -8.020800e-16
4.618306e-15
E:\\R-3.1.2\\scripts\\mPDBS\\summary_pdb_93\\75f27e70ad0f1be4.final.pdb
3.059442e-15 3.091229e-16 2.724094e-15 -1.430779e-15 2.280582e-15
-1.654527e-15
E:\\R-3.1.2\\scripts\\mPDBS\\summary_pdb_93\\769f910fffb22872.final.pdb
2.206257e-15 1.496207e-15 2.218259e-15 -1.639996e-15 1.716886e-16
4.069697e-16
E:\\R-3.1.2\\scripts\\mPDBS\\summary_pdb_93\\791f8c80dd5c3135.final.pdb
-1.097630e-15 -2.911882e-16 7.860995e-15 -4.272911e-15 4.260584e-16
-3.396807e-15
E:\\R-3.1.2\\scripts\\mPDBS\\summary_pdb_93\\7fad98e66a3f6099.final.pdb
-2.825411e-15 -8.859054e-15 5.576495e-15 -1.227116e-15 -7.545839e-15
-5.404190e-15
E:\\R-3.1.2\\scripts\\mPDBS\\summary_pdb_93\\804b89c94a633196.final.pdb
-4.069967e-15 1.659342e-16 3.026165e-15 6.353316e-16 -1.104067e-15
-6.093877e-16
E:\\R-3.1.2\\scripts\\mPDBS\\summary_pdb_93\\813cc65a2298feba.final.pdb
-4.069967e-15 1.659342e-16 3.026165e-15 6.353316e-16 -1.104067e-15
-6.093877e-16
E:\\R-3.1.2\\scripts\\mPDBS\\summary_pdb_93\\83c0eefcfe12d3f9.final.pdb
-1.711176e-15 -2.917537e-15 -1.060410e-15 3.139741e-16 -4.513666e-15
2.432125e-15
E:\\R-3.1.2\\scripts\\mPDBS\\summary_pdb_93\\86d50469ef569760.final.pdb
2.052431e-15 2.891561e-15 3.533035e-15 2.713701e-15 1.977249e-15
2.031335e-15
E:\\R-3.1.2\\scripts\\mPDBS\\summary_pdb_93\\89d0666e1402203d.final.pdb
3.059442e-15 3.091229e-16 2.724094e-15 -1.430779e-15 2.280582e-15
-1.654527e-15
E:\\R-3.1.2\\scripts\\mPDBS\\summary_pdb_93\\8b34852716dadc62.final.pdb
4.633141e-15 -1.530625e-15 -1.820652e-15 -2.597857e-16 1.656479e-17
-1.809816e-15
E:\\R-3.1.2\\scripts\\mPDBS\\summary_pdb_93\\8b6ef9ac3fe74395.final.pdb
4.442566e-16 3.674342e-16 1.080895e-14 -5.579305e-15 5.038533e-15
-9.215140e-16
E:\\R-3.1.2\\scripts\\mPDBS\\summary_pdb_93\\98bbe0d5986c138f.final.pdb
-1.097630e-15 -2.911882e-16 7.860995e-15 -4.272911e-15 4.260584e-16
-3.396807e-15
E:\\R-3.1.2\\scripts\\mPDBS\\summary_pdb_93\\9c22f4909d67e2fa.final.pdb
1.493545e-14 8.587583e-15 -1.166228e-15 -5.475232e-15 1.040409e-14
3.990503e-15
E:\\R-3.1.2\\scripts\\mPDBS\\summary_pdb_93\\9dda8ed0d81fb3ef.final.pdb
6.492026e-15 2.032571e-15 8.670726e-15 5.650205e-16 -3.714622e-15
1.266192e-15
E:\\R-3.1.2\\scripts\\mPDBS\\summary_pdb_93\\9f752b3616e6033b.final.pdb
2.130124e-15 2.454046e-15 4.707171e-15 -1.342479e-15 2.136243e-15
7.868299e-16
E:\\R-3.1.2\\scripts\\mPDBS\\summary_pdb_93\\a02c70083816d6ba.final.pdb
3.512468e-15 2.351062e-15 1.874065e-15 1.823362e-16 4.123290e-15
3.113939e-16
E:\\R-3.1.2\\scripts\\mPDBS\\summary_pdb_93\\a0c997d891e4e082.final.pdb
-5.004066e-15 3.358150e-15 5.609941e-15 2.385419e-15 3.880477e-15
4.161080e-15
E:\\R-3.1.2\\scripts\\mPDBS\\summary_pdb_93\\ab8039a2d823c616.final.pdb

1.860452e-15 2.109582e-15 2.267996e-15 -2.483083e-15 2.670479e-16
4.719195e-16
E:\\R-3.1.2\\scripts\\mPDBS\\summary_pdb_93\\acf4d74b9c71d4dc.final.pdb
-4.069967e-15 1.659342e-16 3.026165e-15 6.353316e-16 -1.104067e-15
-6.093877e-16
E:\\R-3.1.2\\scripts\\mPDBS\\summary_pdb_93\\ad8a823e4b4c668e.final.pdb
1.353682e-15 2.450081e-15 2.613559e-15 -1.750746e-16 4.057261e-16
6.609874e-16
E:\\R-3.1.2\\scripts\\mPDBS\\summary_pdb_93\\b16ababd03b7b857.final.pdb
1.493545e-14 8.587583e-15 -1.166228e-15 -5.475232e-15 1.040409e-14
3.990503e-15
E:\\R-3.1.2\\scripts\\mPDBS\\summary_pdb_93\\b226923e697d3c63.final.pdb
-4.575726e-15 2.671383e-15 2.185462e-15 -1.868363e-15 1.057169e-14
-2.123756e-16
E:\\R-3.1.2\\scripts\\mPDBS\\summary_pdb_93\\b28e48f6fa27a165.final.pdb
-1.097630e-15 -2.911882e-16 7.860995e-15 -4.272911e-15 4.260584e-16
-3.396807e-15
E:\\R-3.1.2\\scripts\\mPDBS\\summary_pdb_93\\b42c4924e3cd1aec.final.pdb
3.452140e-15 8.223365e-15 1.437797e-15 -3.089175e-15 4.122859e-15
-2.742835e-16
E:\\R-3.1.2\\scripts\\mPDBS\\summary_pdb_93\\b4dd239fcabff1df.final.pdb
-1.097630e-15 -2.911882e-16 7.860995e-15 -4.272911e-15 4.260584e-16
-3.396807e-15
E:\\R-3.1.2\\scripts\\mPDBS\\summary_pdb_93\\b669942ef97459a2.final.pdb
4.633141e-15 -1.530625e-15 -1.820652e-15 -2.597857e-16 1.656479e-17
-1.809816e-15
E:\\R-3.1.2\\scripts\\mPDBS\\summary_pdb_93\\b99f4d9d87c1017b.final.pdb
-1.097630e-15 -2.911882e-16 7.860995e-15 -4.272911e-15 4.260584e-16
-3.396807e-15
E:\\R-3.1.2\\scripts\\mPDBS\\summary_pdb_93\\b9d2cbf3975403e7.final.pdb
-1.097630e-15 -2.911882e-16 7.860995e-15 -4.272911e-15 4.260584e-16
-3.396807e-15
E:\\R-3.1.2\\scripts\\mPDBS\\summary_pdb_93\\bc88c8a505f3a288.final.pdb
2.217210e-15 2.080179e-15 1.584765e-15 -2.575918e-15 7.660642e-16
1.426862e-15
E:\\R-3.1.2\\scripts\\mPDBS\\summary_pdb_93\\bdac0e64f4ef427e.final.pdb
1.029983e-14 1.495315e-14 9.594262e-15 -8.388700e-15 9.804742e-15
-4.323485e-15
E:\\R-3.1.2\\scripts\\mPDBS\\summary_pdb_93\\ca2a1f55fc9d0681.final.pdb
4.633141e-15 -1.530625e-15 -1.820652e-15 -2.597857e-16 1.656479e-17
-1.809816e-15
E:\\R-3.1.2\\scripts\\mPDBS\\summary_pdb_93\\ce34248de3e81a3c.final.pdb
-4.771679e-15 2.515398e-15 1.847486e-15 -5.462322e-15 -3.227879e-15
-6.224070e-15
E:\\R-3.1.2\\scripts\\mPDBS\\summary_pdb_93\\d02e697772494a61.final.pdb
-2.085338e-15 -3.715966e-15 -3.568231e-15 -5.317214e-15 2.885896e-15
2.510522e-15
E:\\R-3.1.2\\scripts\\mPDBS\\summary_pdb_93\\d04221245d015047.final.pdb
5.340958e-15 1.817715e-15 2.456214e-15 -2.256701e-15 -2.007175e-15
1.342154e-15
E:\\R-3.1.2\\scripts\\mPDBS\\summary_pdb_93\\d678141e4018054a.final.pdb
1.376453e-14 1.304721e-15 -5.670851e-15 -4.861251e-15 -5.759100e-15
1.401763e-14
E:\\R-3.1.2\\scripts\\mPDBS\\summary_pdb_93\\d71b40af0f0efe39.final.pdb

6.058883e-15 1.032105e-14 -1.857786e-15 4.600281e-17 6.064568e-16
7.746052e-16
E:\\R-3.1.2\\scripts\\mPDBS\\summary_pdb_93\\dc0809eff10197e2.final.pdb
-6.416594e-15 6.676666e-15 1.233050e-14 1.471032e-15 6.543680e-15
-6.036144e-15
E:\\R-3.1.2\\scripts\\mPDBS\\summary_pdb_93\\e1163918d3707839.final.pdb
6.748479e-16 1.706876e-15 6.066906e-15 4.677182e-15 5.405687e-15
3.102780e-17
E:\\R-3.1.2\\scripts\\mPDBS\\summary_pdb_93\\e4b48b06824ef848.final.pdb
9.840992e-15 8.653242e-15 -6.491788e-15 -3.827049e-15 3.956855e-15
7.251050e-15
E:\\R-3.1.2\\scripts\\mPDBS\\summary_pdb_93\\e529e19ad281fea2.final.pdb
1.358541e-15 4.759859e-15 3.357828e-15 -7.809132e-15 2.424808e-15
1.063359e-15
E:\\R-3.1.2\\scripts\\mPDBS\\summary_pdb_93\\e5602082718ec3a1.final.pdb
-4.069967e-15 1.659342e-16 3.026165e-15 6.353316e-16 -1.104067e-15
-6.093877e-16
E:\\R-3.1.2\\scripts\\mPDBS\\summary_pdb_93\\e5d589d996d0a960.final.pdb
-1.910501e-16 7.183914e-15 5.571780e-15 -1.588030e-16 -1.218491e-14
4.806500e-15
E:\\R-3.1.2\\scripts\\mPDBS\\summary_pdb_93\\e9df9e72307a2fbd.final.pdb
3.512468e-15 2.351062e-15 1.874065e-15 1.823362e-16 4.123290e-15
3.113939e-16
E:\\R-3.1.2\\scripts\\mPDBS\\summary_pdb_93\\ea00f8694090cae7.final.pdb
-4.069967e-15 1.659342e-16 3.026165e-15 6.353316e-16 -1.104067e-15
-6.093877e-16
E:\\R-3.1.2\\scripts\\mPDBS\\summary_pdb_93\\eba517be32db050e.final.pdb
-1.097630e-15 -2.911882e-16 7.860995e-15 -4.272911e-15 4.260584e-16
-3.396807e-15
E:\\R-3.1.2\\scripts\\mPDBS\\summary_pdb_93\\ec04b9545899c29d.final.pdb
2.264071e-15 2.112802e-15 2.783075e-15 -1.993697e-15 5.319812e-16
1.372565e-15
E:\\R-3.1.2\\scripts\\mPDBS\\summary_pdb_93\\ee6862cbcad52078.final.pdb
2.232172e-15 1.994310e-15 2.632538e-15 -2.841764e-15 1.008926e-15
9.400012e-16
E:\\R-3.1.2\\scripts\\mPDBS\\summary_pdb_93\\ef6a42fc83b81e5d.final.pdb
2.721253e-15 1.928153e-15 2.959853e-15 -2.135260e-15 7.536883e-16
9.666891e-16
E:\\R-3.1.2\\scripts\\mPDBS\\summary_pdb_93\\ef8e88d4ad93a0ad.final.pdb
5.340958e-15 1.817715e-15 2.456214e-15 -2.256701e-15 -2.007175e-15
1.342154e-15
E:\\R-3.1.2\\scripts\\mPDBS\\summary_pdb_93\\f14fcae8deec4fa3.final.pdb
2.130124e-15 2.454046e-15 4.707171e-15 -1.342479e-15 2.136243e-15
7.868299e-16
E:\\R-3.1.2\\scripts\\mPDBS\\summary_pdb_93\\f83584e22003fb92.final.pdb
1.132082e-14 1.058427e-15 -5.589184e-15 8.484265e-16 -5.668025e-15
5.616274e-15
E:\\R-3.1.2\\scripts\\mPDBS\\summary_pdb_93\\f8e2bf87e52caa29.final.pdb
1.358541e-15 4.759859e-15 3.357828e-15 -7.809132e-15 2.424808e-15
1.063359e-15

[,265]   [,266]   [,267]   [,268]   [,269]   [,270]
E:\\R-3.1.2\\scripts\\40.1ns_data\\NAT0.pdb
-2.585055e-17 1.181280e-15 1.091869e-15 4.512695e-16 9.154409e-16

4.699255e-15
E:\\R-3.1.2\\scripts\\40.1ns_data\\LJP6.pdb
-1.397377e-15 3.647895e-15 -3.160254e-15 3.345055e-16 -9.166203e-17
3.350811e-15
E:\\R-3.1.2\\scripts\\40.1ns_data\\3WY9.pdb
-5.820884e-15 2.551223e-15 -8.191451e-16 2.729830e-15 2.244991e-16
1.370245e-15
E:\\R-3.1.2\\scripts\\40.1ns_data\\ZSP9.pdb
-2.103860e-15 2.147074e-15 -2.220567e-15 2.607976e-15 1.900543e-16
3.788583e-15
E:\\R-3.1.2\\scripts\\mPDBS\\summary_pdb_93\\013d855ad26c877c.final.pdb
-4.275490e-15 3.361343e-15 1.171044e-14 -1.357106e-15 4.210196e-15
9.896963e-15
E:\\R-3.1.2\\scripts\\mPDBS\\summary_pdb_93\\0a2a705342ca0d54.final.pdb
-2.455890e-15 2.496660e-15 -1.383787e-15 9.865413e-16 1.192139e-15
3.562472e-15
E:\\R-3.1.2\\scripts\\mPDBS\\summary_pdb_93\\0a59fceadb4194d9.final.pdb
-1.864305e-15 5.344767e-15 1.995654e-15 -2.358439e-15 4.424901e-15
2.889771e-15
E:\\R-3.1.2\\scripts\\mPDBS\\summary_pdb_93\\0dba59f3306825e8.final.pdb
-4.331002e-15 3.533080e-15 -1.153397e-15 -2.802131e-15 -4.770273e-16
-1.191390e-15
E:\\R-3.1.2\\scripts\\mPDBS\\summary_pdb_93\\126f26a78b57339c.final.pdb
1.081986e-15 -3.915148e-16 -1.302172e-14 6.396674e-15 -4.950445e-15
-3.398392e-15
E:\\R-3.1.2\\scripts\\mPDBS\\summary_pdb_93\\12f06f3e342df706.final.pdb
-2.014945e-15 2.581228e-15 -3.704920e-16 1.302261e-15 1.120365e-15
3.171292e-15
E:\\R-3.1.2\\scripts\\mPDBS\\summary_pdb_93\\18618a2a251b867b.final.pdb
-2.224781e-15 1.922725e-15 -8.704582e-15 4.778200e-15 -1.595883e-15
9.786225e-16
E:\\R-3.1.2\\scripts\\mPDBS\\summary_pdb_93\\1f305f598641eeb2.final.pdb
-2.094459e-15 3.577506e-15 1.051256e-16 5.793154e-16 7.245716e-16
2.914498e-15
E:\\R-3.1.2\\scripts\\mPDBS\\summary_pdb_93\\1f8a00b551be0a39.final.pdb
-2.224781e-15 1.922725e-15 -8.704582e-15 4.778200e-15 -1.595883e-15
9.786225e-16
E:\\R-3.1.2\\scripts\\mPDBS\\summary_pdb_93\\219f45328d47579a.final.pdb
-9.942478e-15 1.890499e-15 1.545104e-14 -6.292358e-15 -1.121276e-14
-6.567942e-15
E:\\R-3.1.2\\scripts\\mPDBS\\summary_pdb_93\\238dd3918e54336d.final.pdb
-1.619453e-14 3.021539e-15 -9.731727e-15 2.454551e-15 -1.160026e-14
-1.344256e-15
E:\\R-3.1.2\\scripts\\mPDBS\\summary_pdb_93\\2b3027889e2fdc42.final.pdb
-2.008070e-15 3.758362e-15 -3.345130e-16 9.229145e-17 3.504691e-16
1.808170e-15
E:\\R-3.1.2\\scripts\\mPDBS\\summary_pdb_93\\2fcb3b46e33793b0.final.pdb
-2.686867e-15 5.307025e-15 8.477768e-15 -1.421688e-15 3.291962e-15
3.341240e-15
E:\\R-3.1.2\\scripts\\mPDBS\\summary_pdb_93\\34eb328251842006.final.pdb
-2.245542e-15 -3.851213e-16 1.535531e-14 -7.283497e-15 1.244242e-14
7.440181e-15
E:\\R-3.1.2\\scripts\\mPDBS\\summary_pdb_93\\354b32871797b7ab.final.pdb
5.836061e-15 3.319346e-15 1.622495e-14 1.202127e-14 9.631597e-15

1.517120e-14
E:\\R-3.1.2\\scripts\\mPDBS\\summary_pdb_93\\397368adbc32d5de.final.pdb
1.081986e-15 -3.915148e-16 -1.302172e-14 6.396674e-15 -4.950445e-15
-3.398392e-15
E:\\R-3.1.2\\scripts\\mPDBS\\summary_pdb_93\\411ca13cd3df6edd.final.pdb
-7.446270e-16 -1.827242e-15 3.638540e-15 2.866982e-15 1.964465e-15
-1.501674e-15
E:\\R-3.1.2\\scripts\\mPDBS\\summary_pdb_93\\42357eee6642bf12.final.pdb
-7.234307e-15 4.949759e-15 1.229761e-14 2.129307e-15 5.401721e-15
7.672166e-15
E:\\R-3.1.2\\scripts\\mPDBS\\summary_pdb_93\\44342d1f75a7fcfc.final.pdb
7.310792e-15 4.310545e-15 4.179160e-15 5.115227e-15 1.419559e-14
9.296963e-16
E:\\R-3.1.2\\scripts\\mPDBS\\summary_pdb_93\\448ef21dffe8cdfb.final.pdb
-1.549756e-15 7.076443e-15 -8.447495e-15 1.428190e-14 -4.827959e-16
2.152982e-14
E:\\R-3.1.2\\scripts\\mPDBS\\summary_pdb_93\\472a40b3e8f113c7.final.pdb
-4.331002e-15 3.533080e-15 -1.153397e-15 -2.802131e-15 -4.770273e-16
-1.191390e-15
E:\\R-3.1.2\\scripts\\mPDBS\\summary_pdb_93\\48a26225951ea153.final.pdb
7.113540e-15 4.361612e-15 8.448821e-16 5.331590e-15 1.121417e-14
4.666127e-15
E:\\R-3.1.2\\scripts\\mPDBS\\summary_pdb_93\\4ab4a5c7c6682ce6.final.pdb
3.844533e-15 1.282534e-14 -8.300241e-15 1.094425e-14 3.452555e-15
5.551914e-15
E:\\R-3.1.2\\scripts\\mPDBS\\summary_pdb_93\\4eaa45b62cb6a25f.final.pdb
-2.075037e-15 2.321880e-15 -1.106615e-14 -2.878931e-16 -4.198703e-15
-6.230261e-16
E:\\R-3.1.2\\scripts\\mPDBS\\summary_pdb_93\\58c371611d85ac96.final.pdb
1.081986e-15 -3.915148e-16 -1.302172e-14 6.396674e-15 -4.950445e-15
-3.398392e-15
E:\\R-3.1.2\\scripts\\mPDBS\\summary_pdb_93\\59a647f04822d30a.final.pdb
1.416202e-15 1.737083e-15 -5.718138e-16 3.322824e-15 -4.071914e-15
8.045532e-15
E:\\R-3.1.2\\scripts\\mPDBS\\summary_pdb_93\\59b091fe385e05fc.final.pdb
-1.864305e-15 5.344767e-15 1.995654e-15 -2.358439e-15 4.424901e-15
2.889771e-15
E:\\R-3.1.2\\scripts\\mPDBS\\summary_pdb_93\\5c35ac30f930d2db.final.pdb
-2.075037e-15 2.321880e-15 -1.106615e-14 -2.878931e-16 -4.198703e-15
-6.230261e-16
E:\\R-3.1.2\\scripts\\mPDBS\\summary_pdb_93\\5f60752431cf2dcb.final.pdb
8.095105e-16 -9.740882e-16 -1.580281e-14 -6.654482e-15 2.068608e-15
-2.985242e-15
E:\\R-3.1.2\\scripts\\mPDBS\\summary_pdb_93\\626456dd6ff2868d.final.pdb
-4.331002e-15 3.533080e-15 -1.153397e-15 -2.802131e-15 -4.770273e-16
-1.191390e-15
E:\\R-3.1.2\\scripts\\mPDBS\\summary_pdb_93\\6583d53ff3c8f7ef.final.pdb
1.416202e-15 1.737083e-15 -5.718138e-16 3.322824e-15 -4.071914e-15
8.045532e-15
E:\\R-3.1.2\\scripts\\mPDBS\\summary_pdb_93\\69760178986bef01.final.pdb
-8.812244e-15 4.806813e-15 -1.795949e-14 2.442858e-15 -1.263983e-14
-4.311771e-15
E:\\R-3.1.2\\scripts\\mPDBS\\summary_pdb_93\\6a9d87d47926b1c8.final.pdb
-2.025771e-15 4.318259e-15 6.890962e-16 -5.005862e-16 3.925029e-16

9.462232e-16
E:\\R-3.1.2\\scripts\\mPDBS\\summary_pdb_93\\6bb3547ea982ffc4.final.pdb
-4.897664e-16 2.455766e-15 6.162304e-15 1.804346e-15 7.867400e-16
-1.890534e-17
E:\\R-3.1.2\\scripts\\mPDBS\\summary_pdb_93\\716d9624611d1c42.final.pdb
-4.384902e-15 -1.440729e-15 1.952086e-15 9.112420e-15 -8.869273e-16
5.389136e-15
E:\\R-3.1.2\\scripts\\mPDBS\\summary_pdb_93\\75f27e70ad0f1be4.final.pdb
1.416202e-15 1.737083e-15 -5.718138e-16 3.322824e-15 -4.071914e-15
8.045532e-15
E:\\R-3.1.2\\scripts\\mPDBS\\summary_pdb_93\\769f910fffb22872.final.pdb
-1.856043e-15 3.559003e-15 -5.633084e-16 9.978043e-16 6.356218e-16
2.710310e-15
E:\\R-3.1.2\\scripts\\mPDBS\\summary_pdb_93\\791f8c80dd5c3135.final.pdb
-1.864305e-15 5.344767e-15 1.995654e-15 -2.358439e-15 4.424901e-15
2.889771e-15
E:\\R-3.1.2\\scripts\\mPDBS\\summary_pdb_93\\7fad98e66a3f6099.final.pdb
-1.059059e-15 8.750114e-16 5.705888e-15 -1.814093e-15 1.012262e-14
5.920844e-16
E:\\R-3.1.2\\scripts\\mPDBS\\summary_pdb_93\\804b89c94a633196.final.pdb
-2.686867e-15 5.307025e-15 8.477768e-15 -1.421688e-15 3.291962e-15
3.341240e-15
E:\\R-3.1.2\\scripts\\mPDBS\\summary_pdb_93\\813cc65a2298feba.final.pdb
-2.686867e-15 5.307025e-15 8.477768e-15 -1.421688e-15 3.291962e-15
3.341240e-15
E:\\R-3.1.2\\scripts\\mPDBS\\summary_pdb_93\\83c0eefcfe12d3f9.final.pdb
-7.446270e-16 -1.827242e-15 3.638540e-15 2.866982e-15 1.964465e-15
-1.501674e-15
E:\\R-3.1.2\\scripts\\mPDBS\\summary_pdb_93\\86d50469ef569760.final.pdb
-4.331002e-15 3.533080e-15 -1.153397e-15 -2.802131e-15 -4.770273e-16
-1.191390e-15
E:\\R-3.1.2\\scripts\\mPDBS\\summary_pdb_93\\89d0666e1402203d.final.pdb
1.416202e-15 1.737083e-15 -5.718138e-16 3.322824e-15 -4.071914e-15
8.045532e-15
E:\\R-3.1.2\\scripts\\mPDBS\\summary_pdb_93\\8b34852716dadc62.final.pdb
1.166821e-15 2.453405e-15 -8.991764e-15 1.887297e-15 -1.610366e-15
-5.658506e-15
E:\\R-3.1.2\\scripts\\mPDBS\\summary_pdb_93\\8b6ef9ac3fe74395.final.pdb
-6.775298e-16 5.936522e-15 2.683646e-16 -1.996633e-15 -1.013833e-15
-8.046774e-16
E:\\R-3.1.2\\scripts\\mPDBS\\summary_pdb_93\\98bbe0d5986c138f.final.pdb
-1.864305e-15 5.344767e-15 1.995654e-15 -2.358439e-15 4.424901e-15
2.889771e-15
E:\\R-3.1.2\\scripts\\mPDBS\\summary_pdb_93\\9c22f4909d67e2fa.final.pdb
-6.097333e-15 4.274214e-15 -3.389909e-15 -4.725035e-15 -5.099010e-16
6.169706e-15
E:\\R-3.1.2\\scripts\\mPDBS\\summary_pdb_93\\9dda8ed0d81fb3ef.final.pdb
5.816111e-15 1.840452e-14 -1.728587e-14 6.053279e-15 1.358887e-14
-4.865581e-15
E:\\R-3.1.2\\scripts\\mPDBS\\summary_pdb_93\\9f752b3616e6033b.final.pdb
-2.928149e-15 5.724572e-15 4.503820e-15 2.623483e-16 1.159145e-15
2.936183e-15
E:\\R-3.1.2\\scripts\\mPDBS\\summary_pdb_93\\a02c70083816d6ba.final.pdb
-2.075037e-15 2.321880e-15 -1.106615e-14 -2.878931e-16 -4.198703e-15

-6.230261e-16
E:\\R-3.1.2\\scripts\\mPDBS\\summary_pdb_93\\a0c997d891e4e082.final.pdb
4.350161e-15 1.011887e-14 6.473432e-16 -5.313729e-15 -3.518944e-15
-2.929992e-15
E:\\R-3.1.2\\scripts\\mPDBS\\summary_pdb_93\\ab8039a2d823c616.final.pdb
-2.194192e-15 2.559379e-15 -1.037507e-15 1.604348e-15 1.045330e-15
2.809774e-15
E:\\R-3.1.2\\scripts\\mPDBS\\summary_pdb_93\\acf4d74b9c71d4dc.final.pdb
-2.686867e-15 5.307025e-15 8.477768e-15 -1.421688e-15 3.291962e-15
3.341240e-15
E:\\R-3.1.2\\scripts\\mPDBS\\summary_pdb_93\\ad8a823e4b4c668e.final.pdb
-2.449117e-15 2.381812e-15 -5.964774e-16 1.323420e-15 8.819369e-16
1.388334e-15
E:\\R-3.1.2\\scripts\\mPDBS\\summary_pdb_93\\b16ababd03b7b857.final.pdb
-6.097333e-15 4.274214e-15 -3.389909e-15 -4.725035e-15 -5.099010e-16
6.169706e-15
E:\\R-3.1.2\\scripts\\mPDBS\\summary_pdb_93\\b226923e697d3c63.final.pdb
-3.537410e-15 5.620695e-15 -8.931229e-15 -5.972170e-16 -8.425853e-15
-5.184770e-15
E:\\R-3.1.2\\scripts\\mPDBS\\summary_pdb_93\\b28e48f6fa27a165.final.pdb
-1.864305e-15 5.344767e-15 1.995654e-15 -2.358439e-15 4.424901e-15
2.889771e-15
E:\\R-3.1.2\\scripts\\mPDBS\\summary_pdb_93\\b42c4924e3cd1aec.final.pdb
-1.325902e-14 -4.403404e-15 9.414772e-15 -3.343718e-15 -1.114428e-15
6.728443e-15
E:\\R-3.1.2\\scripts\\mPDBS\\summary_pdb_93\\b4dd239fcabff1df.final.pdb
-1.864305e-15 5.344767e-15 1.995654e-15 -2.358439e-15 4.424901e-15
2.889771e-15
E:\\R-3.1.2\\scripts\\mPDBS\\summary_pdb_93\\b669942ef97459a2.final.pdb
1.166821e-15 2.453405e-15 -8.991764e-15 1.887297e-15 -1.610366e-15
-5.658506e-15
E:\\R-3.1.2\\scripts\\mPDBS\\summary_pdb_93\\b99f4d9d87c1017b.final.pdb
-1.864305e-15 5.344767e-15 1.995654e-15 -2.358439e-15 4.424901e-15
2.889771e-15
E:\\R-3.1.2\\scripts\\mPDBS\\summary_pdb_93\\b9d2cbf3975403e7.final.pdb
-1.864305e-15 5.344767e-15 1.995654e-15 -2.358439e-15 4.424901e-15
2.889771e-15
E:\\R-3.1.2\\scripts\\mPDBS\\summary_pdb_93\\bc88c8a505f3a288.final.pdb
-2.451834e-15 2.719263e-15 -2.536315e-16 3.681125e-16 6.672730e-16
2.331623e-15
E:\\R-3.1.2\\scripts\\mPDBS\\summary_pdb_93\\bdac0e64f4ef427e.final.pdb
-4.125603e-15 1.532527e-14 -1.200238e-14 -2.446910e-15 -1.236967e-15
9.849922e-15
E:\\R-3.1.2\\scripts\\mPDBS\\summary_pdb_93\\ca2a1f55fc9d0681.final.pdb
1.166821e-15 2.453405e-15 -8.991764e-15 1.887297e-15 -1.610366e-15
-5.658506e-15
E:\\R-3.1.2\\scripts\\mPDBS\\summary_pdb_93\\ce34248de3e81a3c.final.pdb
4.713320e-15 -2.032921e-16 -6.838244e-15 1.552524e-15 3.057208e-15
3.213820e-15
E:\\R-3.1.2\\scripts\\mPDBS\\summary_pdb_93\\d02e697772494a61.final.pdb
1.068571e-15 -3.608575e-15 5.056791e-15 -5.361121e-16 -1.032871e-14
4.402883e-15
E:\\R-3.1.2\\scripts\\mPDBS\\summary_pdb_93\\d04221245d015047.final.pdb
-1.152382e-15 2.926706e-15 1.490419e-15 1.554041e-15 4.471192e-15

5.264842e-15
E:\\R-3.1.2\\scripts\\mPDBS\\summary_pdb_93\\d678141e4018054a.final.pdb
-2.666856e-15 -5.460956e-15 -3.908378e-15 6.863225e-15 9.718239e-15
1.527438e-15
E:\\R-3.1.2\\scripts\\mPDBS\\summary_pdb_93\\d71b40af0f0efe39.final.pdb
-6.003351e-15 8.163177e-16 3.238034e-15 1.516684e-15 3.551497e-15
3.534038e-15
E:\\R-3.1.2\\scripts\\mPDBS\\summary_pdb_93\\dc0809eff10197e2.final.pdb
-6.299572e-15 4.763714e-15 -1.404424e-15 -6.134897e-15 -6.576467e-15
1.605703e-15
E:\\R-3.1.2\\scripts\\mPDBS\\summary_pdb_93\\e1163918d3707839.final.pdb
-1.025274e-14 1.664658e-15 6.683017e-15 -2.083875e-15 5.070729e-15
5.675466e-15
E:\\R-3.1.2\\scripts\\mPDBS\\summary_pdb_93\\e4b48b06824ef848.final.pdb
-2.224781e-15 1.922725e-15 -8.704582e-15 4.778200e-15 -1.595883e-15
9.786225e-16
E:\\R-3.1.2\\scripts\\mPDBS\\summary_pdb_93\\e529e19ad281fea2.final.pdb
-4.275490e-15 3.361343e-15 1.171044e-14 -1.357106e-15 4.210196e-15
9.896963e-15
E:\\R-3.1.2\\scripts\\mPDBS\\summary_pdb_93\\e5602082718ec3a1.final.pdb
-2.686867e-15 5.307025e-15 8.477768e-15 -1.421688e-15 3.291962e-15
3.341240e-15
E:\\R-3.1.2\\scripts\\mPDBS\\summary_pdb_93\\e5d589d996d0a960.final.pdb
1.494546e-15 5.012406e-15 -1.083752e-14 -1.198886e-16 -5.494590e-16
-1.768803e-15
E:\\R-3.1.2\\scripts\\mPDBS\\summary_pdb_93\\e9df9e72307a2fbd.final.pdb
-2.075037e-15 2.321880e-15 -1.106615e-14 -2.878931e-16 -4.198703e-15
-6.230261e-16
E:\\R-3.1.2\\scripts\\mPDBS\\summary_pdb_93\\ea00f8694090cae7.final.pdb
-2.686867e-15 5.307025e-15 8.477768e-15 -1.421688e-15 3.291962e-15
3.341240e-15
E:\\R-3.1.2\\scripts\\mPDBS\\summary_pdb_93\\eba517be32db050e.final.pdb
-1.864305e-15 5.344767e-15 1.995654e-15 -2.358439e-15 4.424901e-15
2.889771e-15
E:\\R-3.1.2\\scripts\\mPDBS\\summary_pdb_93\\ec04b9545899c29d.final.pdb
-2.476518e-15 3.100576e-15 -2.357111e-16 1.088934e-15 1.131389e-15
2.510029e-15
E:\\R-3.1.2\\scripts\\mPDBS\\summary_pdb_93\\ee6862cbcad52078.final.pdb
-2.376048e-15 3.491215e-15 2.457520e-16 6.540324e-17 7.065211e-16
2.081389e-15
E:\\R-3.1.2\\scripts\\mPDBS\\summary_pdb_93\\ef6a42fc83b81e5d.final.pdb
-1.866343e-15 3.280580e-15 -9.221793e-16 1.238497e-15 9.875539e-16
2.932788e-15
E:\\R-3.1.2\\scripts\\mPDBS\\summary_pdb_93\\ef8e88d4ad93a0ad.final.pdb
-1.152382e-15 2.926706e-15 1.490419e-15 1.554041e-15 4.471192e-15
5.264842e-15
E:\\R-3.1.2\\scripts\\mPDBS\\summary_pdb_93\\f14fcae8deec4fa3.final.pdb
-2.928149e-15 5.724572e-15 4.503820e-15 2.623483e-16 1.159145e-15
2.936183e-15
E:\\R-3.1.2\\scripts\\mPDBS\\summary_pdb_93\\f83584e22003fb92.final.pdb
7.113540e-15 4.361612e-15 8.448821e-16 5.331590e-15 1.121417e-14
4.666127e-15
E:\\R-3.1.2\\scripts\\mPDBS\\summary_pdb_93\\f8e2bf87e52caa29.final.pdb
-4.275490e-15 3.361343e-15 1.171044e-14 -1.357106e-15 4.210196e-15

9.896963e-15

```
[,271]        [,272]        [,273]        [,274]        [,275]        [,276]
E:\\R-3.1.2\\scripts\\40.1ns_data\\NAT0.pdb
-1.014296e-15 -1.008829e-15 -1.780005e-16  1.167532e-15  2.642532e-16
-4.161912e-15
E:\\R-3.1.2\\scripts\\40.1ns_data\\LJP6.pdb
3.104699e-16 -9.999192e-16 -2.044181e-15  7.668618e-16  3.916358e-15
-5.073136e-15
E:\\R-3.1.2\\scripts\\40.1ns_data\\3WY9.pdb
-3.039367e-16  1.079657e-15 -3.795633e-15  1.583719e-15 -6.638684e-16
-1.206250e-15
E:\\R-3.1.2\\scripts\\40.1ns_data\\ZSP9.pdb
1.830857e-16 -2.567689e-15 -4.223516e-16 -1.343173e-15  5.157419e-16
-4.660903e-15
E:\\R-3.1.2\\scripts\\mPDBS\\summary_pdb_93\\013d855ad26c877c.final.pdb
-7.009163e-15  1.645293e-15  4.197798e-15  4.278150e-15 -2.033380e-15
-5.263727e-15
E:\\R-3.1.2\\scripts\\mPDBS\\summary_pdb_93\\0a2a705342ca0d54.final.pdb
8.986295e-16 -1.985607e-15 -3.671149e-16 -4.167730e-17  1.340980e-15
-4.622689e-15
E:\\R-3.1.2\\scripts\\mPDBS\\summary_pdb_93\\0a59fceadb4194d9.final.pdb
6.413976e-15 -4.970416e-15  5.600297e-16 -2.657846e-15  3.838051e-15
-6.498951e-15
E:\\R-3.1.2\\scripts\\mPDBS\\summary_pdb_93\\0dba59f3306825e8.final.pdb
3.705356e-15 -1.046998e-15 -7.392249e-16 -3.966990e-15  3.000787e-15
1.397611e-15
E:\\R-3.1.2\\scripts\\mPDBS\\summary_pdb_93\\126f26a78b57339c.final.pdb
-2.843225e-15 -3.001597e-15 -2.174709e-15  7.148034e-15  7.239628e-16
-7.019918e-15
E:\\R-3.1.2\\scripts\\mPDBS\\summary_pdb_93\\12f06f3e342df706.final.pdb
8.160133e-16 -1.126485e-15  1.975376e-16  4.900154e-16  1.151028e-15
-3.557135e-15
E:\\R-3.1.2\\scripts\\mPDBS\\summary_pdb_93\\18618a2a251b867b.final.pdb
-8.759534e-15  7.168895e-15 -1.931846e-16  4.388353e-15  2.258743e-15
-4.588619e-15
E:\\R-3.1.2\\scripts\\mPDBS\\summary_pdb_93\\1f305f598641eeb2.final.pdb
6.306956e-16 -1.316312e-15 -3.704619e-16  1.341863e-15  9.631566e-16
-4.520134e-15
E:\\R-3.1.2\\scripts\\mPDBS\\summary_pdb_93\\1f8a00b551be0a39.final.pdb
-8.759534e-15  7.168895e-15 -1.931846e-16  4.388353e-15  2.258743e-15
-4.588619e-15
E:\\R-3.1.2\\scripts\\mPDBS\\summary_pdb_93\\219f45328d47579a.final.pdb
-1.546236e-15 -8.138888e-15 -1.019436e-15 -1.357746e-16 -1.171375e-14
5.665779e-16
E:\\R-3.1.2\\scripts\\mPDBS\\summary_pdb_93\\238dd3918e54336d.final.pdb
-7.008013e-15 -2.926478e-15 -7.100076e-15 -2.584012e-15 -1.105455e-14
3.312211e-15
E:\\R-3.1.2\\scripts\\mPDBS\\summary_pdb_93\\2b3027889e2fdc42.final.pdb
7.002307e-16 -2.554813e-15 -2.630688e-17  2.512856e-16  8.413158e-16
-3.444970e-15
E:\\R-3.1.2\\scripts\\mPDBS\\summary_pdb_93\\2fcb3b46e33793b0.final.pdb
8.458013e-16  2.000479e-15  2.193459e-15 -1.367775e-15  2.916455e-15
-9.257868e-16
```

E:\\R-3.1.2\\scripts\\mPDBS\\summary_pdb_93\\34eb328251842006.final.pdb
6.998553e-15 -6.273657e-15 1.246306e-14 4.396823e-15 -2.345379e-15
-2.931823e-15
E:\\R-3.1.2\\scripts\\mPDBS\\summary_pdb_93\\354b32871797b7ab.final.pdb
8.050629e-15 -7.500690e-15 2.535260e-15 2.379419e-16 1.950629e-15
-1.052887e-14
E:\\R-3.1.2\\scripts\\mPDBS\\summary_pdb_93\\397368adbc32d5de.final.pdb
-2.843225e-15 -3.001597e-15 -2.174709e-15 7.148034e-15 7.239628e-16
-7.019918e-15
E:\\R-3.1.2\\scripts\\mPDBS\\summary_pdb_93\\411ca13cd3df6edd.final.pdb
-1.623578e-15 6.300425e-15 6.456542e-15 7.509021e-15 6.463097e-15
-2.347164e-16
E:\\R-3.1.2\\scripts\\mPDBS\\summary_pdb_93\\42357eee6642bf12.final.pdb
7.995584e-15 -7.283679e-15 7.932598e-16 -1.420835e-15 -1.387067e-15
-1.672541e-16
E:\\R-3.1.2\\scripts\\mPDBS\\summary_pdb_93\\44342d1f75a7fcfc.final.pdb
-8.678456e-15 6.954544e-15 2.272668e-15 3.578660e-15 1.287960e-14
-7.300323e-15
E:\\R-3.1.2\\scripts\\mPDBS\\summary_pdb_93\\448ef21dffe8cdfb.final.pdb
5.743171e-16 -1.054504e-14 2.325298e-15 9.208834e-15 -1.506569e-14
-1.661463e-14
E:\\R-3.1.2\\scripts\\mPDBS\\summary_pdb_93\\472a40b3e8f113c7.final.pdb
3.705356e-15 -1.046998e-15 -7.392249e-16 -3.966990e-15 3.000787e-15
1.397611e-15
E:\\R-3.1.2\\scripts\\mPDBS\\summary_pdb_93\\48a26225951ea153.final.pdb
-1.070601e-14 1.247743e-14 4.621017e-15 7.354328e-15 9.171458e-15
-4.636725e-15
E:\\R-3.1.2\\scripts\\mPDBS\\summary_pdb_93\\4ab4a5c7c6682ce6.final.pdb
1.896907e-15 3.236468e-15 2.203734e-15 4.434088e-17 2.476033e-15
-6.940120e-15
E:\\R-3.1.2\\scripts\\mPDBS\\summary_pdb_93\\4eaa45b62cb6a25f.final.pdb
-4.449355e-15 3.793661e-15 -2.038772e-15 2.149065e-15 2.512680e-15
-4.366698e-15
E:\\R-3.1.2\\scripts\\mPDBS\\summary_pdb_93\\58c371611d85ac96.final.pdb
-2.843225e-15 -3.001597e-15 -2.174709e-15 7.148034e-15 7.239628e-16
-7.019918e-15
E:\\R-3.1.2\\scripts\\mPDBS\\summary_pdb_93\\59a647f04822d30a.final.pdb
2.338339e-15 -4.057481e-15 -3.932438e-15 -3.468025e-16 -4.261203e-15
-2.156868e-15
E:\\R-3.1.2\\scripts\\mPDBS\\summary_pdb_93\\59b091fe385e05fc.final.pdb
6.413976e-15 -4.970416e-15 5.600297e-16 -2.657846e-15 3.838051e-15
-6.498951e-15
E:\\R-3.1.2\\scripts\\mPDBS\\summary_pdb_93\\5c35ac30f930d2db.final.pdb
-4.449355e-15 3.793661e-15 -2.038772e-15 2.149065e-15 2.512680e-15
-4.366698e-15
E:\\R-3.1.2\\scripts\\mPDBS\\summary_pdb_93\\5f60752431cf2dcb.final.pdb
-6.417567e-15 -2.691639e-15 2.177724e-15 -1.509037e-15 6.197338e-15
-4.692945e-15
E:\\R-3.1.2\\scripts\\mPDBS\\summary_pdb_93\\626456dd6ff2868d.final.pdb
3.705356e-15 -1.046998e-15 -7.392249e-16 -3.966990e-15 3.000787e-15
1.397611e-15
E:\\R-3.1.2\\scripts\\mPDBS\\summary_pdb_93\\6583d53ff3c8f7ef.final.pdb
2.338339e-15 -4.057481e-15 -3.932438e-15 -3.468025e-16 -4.261203e-15
-2.156868e-15

E:\\R-3.1.2\\scripts\\mPDBS\\summary_pdb_93\\69760178986bef01.final.pdb
-1.866319e-15 5.909956e-15 -3.947983e-15 -3.872437e-15 3.599169e-15
-9.984472e-15
E:\\R-3.1.2\\scripts\\mPDBS\\summary_pdb_93\\6a9d87d47926b1c8.final.pdb
8.335217e-16 -1.967702e-15 2.261488e-16 6.315448e-16 4.915098e-16
-4.001499e-15
E:\\R-3.1.2\\scripts\\mPDBS\\summary_pdb_93\\6bb3547ea982ffc4.final.pdb
-1.048146e-14 -1.033844e-15 5.108580e-15 6.306028e-15 -7.839426e-15
3.401573e-15
E:\\R-3.1.2\\scripts\\mPDBS\\summary_pdb_93\\716d9624611d1c42.final.pdb
6.466609e-16 5.767740e-15 4.407712e-15 8.605269e-15 -1.547335e-15
-6.851808e-15
E:\\R-3.1.2\\scripts\\mPDBS\\summary_pdb_93\\75f27e70ad0f1be4.final.pdb
2.338339e-15 -4.057481e-15 -3.932438e-15 -3.468025e-16 -4.261203e-15
-2.156868e-15
E:\\R-3.1.2\\scripts\\mPDBS\\summary_pdb_93\\769f910fffb22872.final.pdb
8.490997e-16 -1.202446e-15 6.893560e-16 7.680654e-17 6.220316e-16
-4.574307e-15
E:\\R-3.1.2\\scripts\\mPDBS\\summary_pdb_93\\791f8c80dd5c3135.final.pdb
6.413976e-15 -4.970416e-15 5.600297e-16 -2.657846e-15 3.838051e-15
-6.498951e-15
E:\\R-3.1.2\\scripts\\mPDBS\\summary_pdb_93\\7fad98e66a3f6099.final.pdb
6.239255e-15 -5.091285e-15 7.547632e-15 -1.416685e-15 6.290394e-15
-4.762844e-15
E:\\R-3.1.2\\scripts\\mPDBS\\summary_pdb_93\\804b89c94a633196.final.pdb
8.458013e-16 2.000479e-15 2.193459e-15 -1.367775e-15 2.916455e-15
-9.257868e-16
E:\\R-3.1.2\\scripts\\mPDBS\\summary_pdb_93\\813cc65a2298feba.final.pdb
8.458013e-16 2.000479e-15 2.193459e-15 -1.367775e-15 2.916455e-15
-9.257868e-16
E:\\R-3.1.2\\scripts\\mPDBS\\summary_pdb_93\\83c0eefcfe12d3f9.final.pdb
-1.623578e-15 6.300425e-15 6.456542e-15 7.509021e-15 6.463097e-15
-2.347164e-16
E:\\R-3.1.2\\scripts\\mPDBS\\summary_pdb_93\\86d50469ef569760.final.pdb
3.705356e-15 -1.046998e-15 -7.392249e-16 -3.966990e-15 3.000787e-15
1.397611e-15
E:\\R-3.1.2\\scripts\\mPDBS\\summary_pdb_93\\89d0666e1402203d.final.pdb
2.338339e-15 -4.057481e-15 -3.932438e-15 -3.468025e-16 -4.261203e-15
-2.156868e-15
E:\\R-3.1.2\\scripts\\mPDBS\\summary_pdb_93\\8b34852716dadc62.final.pdb
5.392512e-15 -3.321653e-15 -1.425174e-15 -8.091945e-16 5.667727e-15
-5.304229e-15
E:\\R-3.1.2\\scripts\\mPDBS\\summary_pdb_93\\8b6ef9ac3fe74395.final.pdb
9.476806e-15 -1.614017e-14 -3.894202e-15 -6.778963e-15 -2.158062e-15
-4.828423e-15
E:\\R-3.1.2\\scripts\\mPDBS\\summary_pdb_93\\98bbe0d5986c138f.final.pdb
6.413976e-15 -4.970416e-15 5.600297e-16 -2.657846e-15 3.838051e-15
-6.498951e-15
E:\\R-3.1.2\\scripts\\mPDBS\\summary_pdb_93\\9c22f4909d67e2fa.final.pdb
-2.277568e-15 -3.709363e-15 -4.774925e-15 -1.131745e-15 -3.694576e-15
-6.266784e-15
E:\\R-3.1.2\\scripts\\mPDBS\\summary_pdb_93\\9dda8ed0d81fb3ef.final.pdb
-9.425688e-15 2.236322e-14 3.937569e-15 -3.461520e-16 1.323175e-14
-6.869136e-15

E:\\R-3.1.2\\scripts\\mPDBS\\summary_pdb_93\\9f752b3616e6033b.final.pdb
4.170571e-15 -4.902384e-15 -1.944815e-15 -3.414968e-15 -1.684277e-15
-1.920950e-15
E:\\R-3.1.2\\scripts\\mPDBS\\summary_pdb_93\\a02c70083816d6ba.final.pdb
-4.449355e-15 3.793661e-15 -2.038772e-15 2.149065e-15 2.512680e-15
-4.366698e-15
E:\\R-3.1.2\\scripts\\mPDBS\\summary_pdb_93\\a0c997d891e4e082.final.pdb
-1.566343e-15 -6.494511e-15 1.474859e-15 4.913053e-15 1.199205e-15
-7.083484e-15
E:\\R-3.1.2\\scripts\\mPDBS\\summary_pdb_93\\ab8039a2d823c616.final.pdb
6.389242e-16 -1.377511e-15 -5.890283e-16 -3.109579e-16 1.317166e-15
-4.193868e-15
E:\\R-3.1.2\\scripts\\mPDBS\\summary_pdb_93\\acf4d74b9c71d4dc.final.pdb
8.458013e-16 2.000479e-15 2.193459e-15 -1.367775e-15 2.916455e-15
-9.257868e-16
E:\\R-3.1.2\\scripts\\mPDBS\\summary_pdb_93\\ad8a823e4b4c668e.final.pdb
6.427694e-16 -9.617691e-16 1.076466e-16 5.024623e-16 1.246404e-15
-4.410937e-15
E:\\R-3.1.2\\scripts\\mPDBS\\summary_pdb_93\\b16ababd03b7b857.final.pdb
-2.277568e-15 -3.709363e-15 -4.774925e-15 -1.131745e-15 -3.694576e-15
-6.266784e-15
E:\\R-3.1.2\\scripts\\mPDBS\\summary_pdb_93\\b226923e697d3c63.final.pdb
1.861724e-15 -1.354338e-14 -1.796559e-15 2.423226e-15 1.302490e-16
-6.252984e-15
E:\\R-3.1.2\\scripts\\mPDBS\\summary_pdb_93\\b28e48f6fa27a165.final.pdb
6.413976e-15 -4.970416e-15 5.600297e-16 -2.657846e-15 3.838051e-15
-6.498951e-15
E:\\R-3.1.2\\scripts\\mPDBS\\summary_pdb_93\\b42c4924e3cd1aec.final.pdb
1.046595e-14 -1.592516e-14 1.923011e-16 -5.006812e-15 9.967434e-16
-9.558500e-15
E:\\R-3.1.2\\scripts\\mPDBS\\summary_pdb_93\\b4dd239fcabff1df.final.pdb
6.413976e-15 -4.970416e-15 5.600297e-16 -2.657846e-15 3.838051e-15
-6.498951e-15
E:\\R-3.1.2\\scripts\\mPDBS\\summary_pdb_93\\b669942ef97459a2.final.pdb
5.392512e-15 -3.321653e-15 -1.425174e-15 -8.091945e-16 5.667727e-15
-5.304229e-15
E:\\R-3.1.2\\scripts\\mPDBS\\summary_pdb_93\\b99f4d9d87c1017b.final.pdb
6.413976e-15 -4.970416e-15 5.600297e-16 -2.657846e-15 3.838051e-15
-6.498951e-15
E:\\R-3.1.2\\scripts\\mPDBS\\summary_pdb_93\\b9d2cbf3975403e7.final.pdb
6.413976e-15 -4.970416e-15 5.600297e-16 -2.657846e-15 3.838051e-15
-6.498951e-15
E:\\R-3.1.2\\scripts\\mPDBS\\summary_pdb_93\\bc88c8a505f3a288.final.pdb
5.905095e-16 -1.737325e-15 -2.977633e-17 1.069783e-16 1.186526e-15
-4.113272e-15
E:\\R-3.1.2\\scripts\\mPDBS\\summary_pdb_93\\bdac0e64f4ef427e.final.pdb
4.994393e-15 -1.655239e-14 -1.295762e-14 -1.045393e-14 -9.479879e-15
-1.107457e-14
E:\\R-3.1.2\\scripts\\mPDBS\\summary_pdb_93\\ca2a1f55fc9d0681.final.pdb
5.392512e-15 -3.321653e-15 -1.425174e-15 -8.091945e-16 5.667727e-15
-5.304229e-15
E:\\R-3.1.2\\scripts\\mPDBS\\summary_pdb_93\\ce34248de3e81a3c.final.pdb
1.317668e-16 2.435329e-15 1.396912e-15 7.465273e-15 8.688896e-15
-1.419531e-14

E:\\R-3.1.2\\scripts\\mPDBS\\summary_pdb_93\\d02e697772494a61.final.pdb
1.236260e-15 -1.011517e-14 -2.733777e-15 6.471245e-15 -1.106843e-14
3.473541e-15
E:\\R-3.1.2\\scripts\\mPDBS\\summary_pdb_93\\d04221245d015047.final.pdb
6.950118e-17 3.209532e-15 5.970889e-16 -2.269323e-16 1.518394e-15
-4.817935e-15
E:\\R-3.1.2\\scripts\\mPDBS\\summary_pdb_93\\d678141e4018054a.final.pdb
-1.280266e-14 2.428609e-15 9.946614e-15 1.373653e-14 -1.777353e-15
2.918418e-15
E:\\R-3.1.2\\scripts\\mPDBS\\summary_pdb_93\\d71b40af0f0efe39.final.pdb
1.043386e-16 -3.129527e-15 -3.048883e-15 -3.998578e-15 -2.017663e-16
6.207217e-15
E:\\R-3.1.2\\scripts\\mPDBS\\summary_pdb_93\\dc0809eff10197e2.final.pdb
1.553156e-14 -1.020225e-14 -3.089849e-15 -1.318076e-14 -3.012572e-15
-5.246763e-15
E:\\R-3.1.2\\scripts\\mPDBS\\summary_pdb_93\\e1163918d3707839.final.pdb
1.381917e-14 -5.626972e-15 -1.139967e-15 6.296646e-15 2.176355e-15
-3.939556e-16
E:\\R-3.1.2\\scripts\\mPDBS\\summary_pdb_93\\e4b48b06824ef848.final.pdb
-8.759534e-15 7.168895e-15 -1.931846e-16 4.388353e-15 2.258743e-15
-4.588619e-15
E:\\R-3.1.2\\scripts\\mPDBS\\summary_pdb_93\\e529e19ad281fea2.final.pdb
-7.009163e-15 1.645293e-15 4.197798e-15 4.278150e-15 -2.033380e-15
-5.263727e-15
E:\\R-3.1.2\\scripts\\mPDBS\\summary_pdb_93\\e5602082718ec3a1.final.pdb
8.458013e-16 2.000479e-15 2.193459e-15 -1.367775e-15 2.916455e-15
-9.257868e-16
E:\\R-3.1.2\\scripts\\mPDBS\\summary_pdb_93\\e5d589d996d0a960.final.pdb
-2.250491e-15 1.334559e-15 4.893559e-15 5.067624e-15 -8.795713e-17
-1.002030e-14
E:\\R-3.1.2\\scripts\\mPDBS\\summary_pdb_93\\e9df9e72307a2fbd.final.pdb
-4.449355e-15 3.793661e-15 -2.038772e-15 2.149065e-15 2.512680e-15
-4.366698e-15
E:\\R-3.1.2\\scripts\\mPDBS\\summary_pdb_93\\ea00f8694090cae7.final.pdb
8.458013e-16 2.000479e-15 2.193459e-15 -1.367775e-15 2.916455e-15
-9.257868e-16
E:\\R-3.1.2\\scripts\\mPDBS\\summary_pdb_93\\eba517be32db050e.final.pdb
6.413976e-15 -4.970416e-15 5.600297e-16 -2.657846e-15 3.838051e-15
-6.498951e-15
E:\\R-3.1.2\\scripts\\mPDBS\\summary_pdb_93\\ec04b9545899c29d.final.pdb
6.916526e-16 -8.236225e-17 -7.153929e-16 -8.314556e-18 1.138991e-15
-4.831812e-15
E:\\R-3.1.2\\scripts\\mPDBS\\summary_pdb_93\\ee6862cbcad52078.final.pdb
8.060489e-16 -2.093377e-15 -2.899848e-16 2.059659e-16 6.721802e-16
-4.153605e-15
E:\\R-3.1.2\\scripts\\mPDBS\\summary_pdb_93\\ef6a42fc83b81e5d.final.pdb
8.113694e-16 -1.519467e-15 4.200401e-16 3.503507e-16 1.149388e-15
-4.757754e-15
E:\\R-3.1.2\\scripts\\mPDBS\\summary_pdb_93\\ef8e88d4ad93a0ad.final.pdb
6.950118e-17 3.209532e-15 5.970889e-16 -2.269323e-16 1.518394e-15
-4.817935e-15
E:\\R-3.1.2\\scripts\\mPDBS\\summary_pdb_93\\f14fcae8deec4fa3.final.pdb
4.170571e-15 -4.902384e-15 -1.944815e-15 -3.414968e-15 -1.684277e-15
-1.920950e-15

E:\\R-3.1.2\\scripts\\mPDBS\\summary_pdb_93\\f83584e22003fb92.final.pdb
-1.070601e-14 1.247743e-14 4.621017e-15 7.354328e-15 9.171458e-15
-4.636725e-15
E:\\R-3.1.2\\scripts\\mPDBS\\summary_pdb_93\\f8e2bf87e52caa29.final.pdb
-7.009163e-15 1.645293e-15 4.197798e-15 4.278150e-15 -2.033380e-15
-5.263727e-15

[,277]   [,278]   [,279]   [,280]   [,281]   [,282]
E:\\R-3.1.2\\scripts\\40.1ns_data\\NAT0.pdb
2.287094e-15 -2.447151e-15 -1.171340e-15 2.027297e-15 2.577746e-15
5.479544e-15
E:\\R-3.1.2\\scripts\\40.1ns_data\\LJP6.pdb
1.540708e-15 -1.131489e-15 4.040004e-16 -8.699543e-17 3.419153e-15
3.772206e-15
E:\\R-3.1.2\\scripts\\40.1ns_data\\3WY9.pdb
1.027455e-15 -3.071166e-15 1.748731e-15 2.420050e-15 1.511836e-15
6.932832e-16
E:\\R-3.1.2\\scripts\\40.1ns_data\\ZSP9.pdb
5.140376e-15 -2.217553e-15 -1.047328e-15 1.688386e-15 2.950198e-15
3.688596e-15
E:\\R-3.1.2\\scripts\\mPDBS\\summary_pdb_93\\013d855ad26c877c.final.pdb
-2.196190e-16 -6.837929e-15 -5.680076e-16 -5.577207e-16 -2.528984e-15
3.144614e-15
E:\\R-3.1.2\\scripts\\mPDBS\\summary_pdb_93\\0a2a705342ca0d54.final.pdb
6.960550e-17 -2.636582e-15 3.722544e-17 1.459748e-15 2.219976e-15
3.519921e-15
E:\\R-3.1.2\\scripts\\mPDBS\\summary_pdb_93\\0a59fceadb4194d9.final.pdb
-3.377303e-16 -6.198967e-15 7.231648e-16 2.342636e-15 -3.241131e-16
3.148487e-15
E:\\R-3.1.2\\scripts\\mPDBS\\summary_pdb_93\\0dba59f3306825e8.final.pdb
2.694716e-15 -2.726634e-15 -3.504894e-15 5.212773e-16 2.883354e-15
3.234819e-15
E:\\R-3.1.2\\scripts\\mPDBS\\summary_pdb_93\\126f26a78b57339c.final.pdb
-3.023436e-16 8.265224e-15 1.410011e-15 1.912526e-15 4.905824e-15
3.727914e-15
E:\\R-3.1.2\\scripts\\mPDBS\\summary_pdb_93\\12f06f3e342df706.final.pdb
2.722215e-15 -2.695346e-15 -2.286209e-16 1.827509e-15 3.052860e-15
2.959605e-15
E:\\R-3.1.2\\scripts\\mPDBS\\summary_pdb_93\\18618a2a251b867b.final.pdb
3.782903e-15 5.486344e-15 5.094421e-15 8.590611e-16 5.026697e-15
2.288878e-15
E:\\R-3.1.2\\scripts\\mPDBS\\summary_pdb_93\\1f305f598641eeb2.final.pdb
2.340057e-15 -2.373709e-15 1.758700e-16 1.171464e-15 2.382776e-15
2.928455e-15
E:\\R-3.1.2\\scripts\\mPDBS\\summary_pdb_93\\1f8a00b551be0a39.final.pdb
3.782903e-15 5.486344e-15 5.094421e-15 8.590611e-16 5.026697e-15
2.288878e-15
E:\\R-3.1.2\\scripts\\mPDBS\\summary_pdb_93\\219f45328d47579a.final.pdb
-7.283563e-15 1.521619e-14 -1.248719e-14 1.262955e-14 -9.172938e-15
2.770317e-15
E:\\R-3.1.2\\scripts\\mPDBS\\summary_pdb_93\\238dd3918e54336d.final.pdb
-1.891826e-15 8.057424e-15 -2.950546e-15 -1.703823e-15 1.564304e-14
1.170870e-14
E:\\R-3.1.2\\scripts\\mPDBS\\summary_pdb_93\\2b3027889e2fdc42.final.pdb

1.335031e-15 -1.646402e-15 -8.896944e-16 1.886187e-15 1.866680e-15
3.084736e-15
E:\\R-3.1.2\\scripts\\mPDBS\\summary_pdb_93\\2fcb3b46e33793b0.final.pdb
4.543156e-15 -8.476466e-15 -1.314257e-15 7.273744e-16 1.962973e-15
2.137421e-15
E:\\R-3.1.2\\scripts\\mPDBS\\summary_pdb_93\\34eb328251842006.final.pdb
1.298035e-14 -1.328379e-14 1.206953e-14 2.611309e-15 -4.226612e-15
-4.645942e-15
E:\\R-3.1.2\\scripts\\mPDBS\\summary_pdb_93\\354b32871797b7ab.final.pdb
2.468458e-14 -8.862995e-15 3.890650e-15 -6.567083e-15 2.050598e-15
1.006698e-15
E:\\R-3.1.2\\scripts\\mPDBS\\summary_pdb_93\\397368adbc32d5de.final.pdb
-3.023436e-16 8.265224e-15 1.410011e-15 1.912526e-15 4.905824e-15
3.727914e-15
E:\\R-3.1.2\\scripts\\mPDBS\\summary_pdb_93\\411ca13cd3df6edd.final.pdb
8.511661e-15 -8.209969e-15 5.280728e-15 -3.102895e-15 2.056865e-15
4.196108e-16
E:\\R-3.1.2\\scripts\\mPDBS\\summary_pdb_93\\42357eee6642bf12.final.pdb
-7.116429e-15 -1.167227e-15 4.364389e-15 6.091286e-15 -3.305903e-15
-8.630764e-16
E:\\R-3.1.2\\scripts\\mPDBS\\summary_pdb_93\\44342d1f75a7fcfc.final.pdb
-6.048488e-15 -1.171901e-14 8.310368e-15 3.520416e-15 -2.439230e-15
3.477676e-15
E:\\R-3.1.2\\scripts\\mPDBS\\summary_pdb_93\\448ef21dffe8cdfb.final.pdb
1.193655e-14 -2.259217e-15 1.871139e-14 -7.490011e-15 1.685037e-14
7.129955e-15
E:\\R-3.1.2\\scripts\\mPDBS\\summary_pdb_93\\472a40b3e8f113c7.final.pdb
2.694716e-15 -2.726634e-15 -3.504894e-15 5.212773e-16 2.883354e-15
3.234819e-15
E:\\R-3.1.2\\scripts\\mPDBS\\summary_pdb_93\\48a26225951ea153.final.pdb
5.559083e-15 -1.199056e-14 5.602075e-15 2.225871e-16 2.872820e-15
-3.819464e-15
E:\\R-3.1.2\\scripts\\mPDBS\\summary_pdb_93\\4ab4a5c7c6682ce6.final.pdb
2.098965e-14 -7.971788e-15 3.935768e-15 1.614153e-15 9.652028e-15
-3.209303e-16
E:\\R-3.1.2\\scripts\\mPDBS\\summary_pdb_93\\4eaa45b62cb6a25f.final.pdb
-3.112841e-15 1.716650e-15 -2.869567e-15 3.081666e-15 4.317123e-15
5.032774e-15
E:\\R-3.1.2\\scripts\\mPDBS\\summary_pdb_93\\58c371611d85ac96.final.pdb
-3.023436e-16 8.265224e-15 1.410011e-15 1.912526e-15 4.905824e-15
3.727914e-15
E:\\R-3.1.2\\scripts\\mPDBS\\summary_pdb_93\\59a647f04822d30a.final.pdb
5.917635e-15 -1.300261e-15 -4.290663e-15 5.520552e-15 7.203231e-15
5.454393e-15
E:\\R-3.1.2\\scripts\\mPDBS\\summary_pdb_93\\59b091fe385e05fc.final.pdb
-3.377303e-16 -6.198967e-15 7.231648e-16 2.342636e-15 -3.241131e-16
3.148487e-15
E:\\R-3.1.2\\scripts\\mPDBS\\summary_pdb_93\\5c35ac30f930d2db.final.pdb
-3.112841e-15 1.716650e-15 -2.869567e-15 3.081666e-15 4.317123e-15
5.032774e-15
E:\\R-3.1.2\\scripts\\mPDBS\\summary_pdb_93\\5f60752431cf2dcb.final.pdb
3.464947e-15 7.932004e-15 1.542098e-15 7.155495e-15 4.141020e-15
4.240264e-15
E:\\R-3.1.2\\scripts\\mPDBS\\summary_pdb_93\\626456dd6ff2868d.final.pdb

2.694716e-15 -2.726634e-15 -3.504894e-15 5.212773e-16 2.883354e-15
3.234819e-15
E:\\R-3.1.2\\scripts\\mPDBS\\summary_pdb_93\\6583d53ff3c8f7ef.final.pdb
5.917635e-15 -1.300261e-15 -4.290663e-15 5.520552e-15 7.203231e-15
5.454393e-15
E:\\R-3.1.2\\scripts\\mPDBS\\summary_pdb_93\\69760178986bef01.final.pdb
1.307242e-14 1.533858e-14 -3.806725e-15 -1.486159e-15 5.605243e-15
8.007314e-15
E:\\R-3.1.2\\scripts\\mPDBS\\summary_pdb_93\\6a9d87d47926b1c8.final.pdb
1.127068e-15 -2.250670e-15 -5.380836e-16 1.010469e-15 2.244759e-15
3.502400e-15
E:\\R-3.1.2\\scripts\\mPDBS\\summary_pdb_93\\6bb3547ea982ffc4.final.pdb
-1.097468e-14 -2.155603e-15 8.413606e-15 -5.139961e-15 8.204277e-15
4.832179e-15
E:\\R-3.1.2\\scripts\\mPDBS\\summary_pdb_93\\716d9624611d1c42.final.pdb
1.274523e-14 -6.912675e-15 3.007506e-15 -4.794798e-15 -9.892624e-16
-3.142285e-15
E:\\R-3.1.2\\scripts\\mPDBS\\summary_pdb_93\\75f27e70ad0f1be4.final.pdb
5.917635e-15 -1.300261e-15 -4.290663e-15 5.520552e-15 7.203231e-15
5.454393e-15
E:\\R-3.1.2\\scripts\\mPDBS\\summary_pdb_93\\769f910fffb22872.final.pdb
3.248338e-15 -2.200432e-15 9.146049e-16 1.513052e-15 1.497457e-15
3.480417e-15
E:\\R-3.1.2\\scripts\\mPDBS\\summary_pdb_93\\791f8c80dd5c3135.final.pdb
-3.377303e-16 -6.198967e-15 7.231648e-16 2.342636e-15 -3.241131e-16
3.148487e-15
E:\\R-3.1.2\\scripts\\mPDBS\\summary_pdb_93\\7fad98e66a3f6099.final.pdb
1.433652e-14 -1.306193e-14 -1.765399e-15 2.966199e-15 -7.070532e-15
1.094272e-15
E:\\R-3.1.2\\scripts\\mPDBS\\summary_pdb_93\\804b89c94a633196.final.pdb
4.543156e-15 -8.476466e-15 -1.314257e-15 7.273744e-16 1.962973e-15
2.137421e-15
E:\\R-3.1.2\\scripts\\mPDBS\\summary_pdb_93\\813cc65a2298feba.final.pdb
4.543156e-15 -8.476466e-15 -1.314257e-15 7.273744e-16 1.962973e-15
2.137421e-15
E:\\R-3.1.2\\scripts\\mPDBS\\summary_pdb_93\\83c0eefcfe12d3f9.final.pdb
8.511661e-15 -8.209969e-15 5.280728e-15 -3.102895e-15 2.056865e-15
4.196108e-16
E:\\R-3.1.2\\scripts\\mPDBS\\summary_pdb_93\\86d50469ef569760.final.pdb
2.694716e-15 -2.726634e-15 -3.504894e-15 5.212773e-16 2.883354e-15
3.234819e-15
E:\\R-3.1.2\\scripts\\mPDBS\\summary_pdb_93\\89d0666e1402203d.final.pdb
5.917635e-15 -1.300261e-15 -4.290663e-15 5.520552e-15 7.203231e-15
5.454393e-15
E:\\R-3.1.2\\scripts\\mPDBS\\summary_pdb_93\\8b34852716dadc62.final.pdb
-5.806205e-15 1.095738e-15 -1.229256e-15 6.368695e-15 3.843750e-16
2.487835e-15
E:\\R-3.1.2\\scripts\\mPDBS\\summary_pdb_93\\8b6ef9ac3fe74395.final.pdb
-6.773869e-15 -1.286890e-14 -4.175940e-15 3.237182e-15 3.774352e-15
6.478587e-15
E:\\R-3.1.2\\scripts\\mPDBS\\summary_pdb_93\\98bbe0d5986c138f.final.pdb
-3.377303e-16 -6.198967e-15 7.231648e-16 2.342636e-15 -3.241131e-16
3.148487e-15
E:\\R-3.1.2\\scripts\\mPDBS\\summary_pdb_93\\9c22f4909d67e2fa.final.pdb

-1.030456e-14 1.090538e-14 -5.704843e-15 5.039897e-15 -2.207193e-15
6.321357e-15
E:\\R-3.1.2\\scripts\\mPDBS\\summary_pdb_93\\9dda8ed0d81fb3ef.final.pdb
-1.078201e-14 -1.189031e-14 1.493224e-14 -1.553000e-15 4.737159e-15
4.386020e-15
E:\\R-3.1.2\\scripts\\mPDBS\\summary_pdb_93\\9f752b3616e6033b.final.pdb
-3.193089e-15 -3.853780e-15 -1.382323e-15 3.519696e-15 2.228588e-15
5.177419e-15
E:\\R-3.1.2\\scripts\\mPDBS\\summary_pdb_93\\a02c70083816d6ba.final.pdb
-3.112841e-15 1.716650e-15 -2.869567e-15 3.081666e-15 4.317123e-15
5.032774e-15
E:\\R-3.1.2\\scripts\\mPDBS\\summary_pdb_93\\a0c997d891e4e082.final.pdb
-5.150034e-15 -1.054386e-14 5.537141e-17 -1.649800e-15 1.012705e-14
6.518143e-15
E:\\R-3.1.2\\scripts\\mPDBS\\summary_pdb_93\\ab8039a2d823c616.final.pdb
2.397964e-15 -9.191777e-16 5.094353e-16 1.767388e-15 1.602510e-15
3.206773e-15
E:\\R-3.1.2\\scripts\\mPDBS\\summary_pdb_93\\acf4d74b9c71d4dc.final.pdb
4.543156e-15 -8.476466e-15 -1.314257e-15 7.273744e-16 1.962973e-15
2.137421e-15
E:\\R-3.1.2\\scripts\\mPDBS\\summary_pdb_93\\ad8a823e4b4c668e.final.pdb
1.574758e-15 -2.280550e-15 -9.550067e-18 1.525956e-15 2.386015e-15
3.690267e-15
E:\\R-3.1.2\\scripts\\mPDBS\\summary_pdb_93\\b16ababd03b7b857.final.pdb
-1.030456e-14 1.090538e-14 -5.704843e-15 5.039897e-15 -2.207193e-15
6.321357e-15
E:\\R-3.1.2\\scripts\\mPDBS\\summary_pdb_93\\b226923e697d3c63.final.pdb
-4.502640e-16 3.113092e-15 1.609131e-15 2.666932e-15 7.724461e-15
4.808425e-15
E:\\R-3.1.2\\scripts\\mPDBS\\summary_pdb_93\\b28e48f6fa27a165.final.pdb
-3.377303e-16 -6.198967e-15 7.231648e-16 2.342636e-15 -3.241131e-16
3.148487e-15
E:\\R-3.1.2\\scripts\\mPDBS\\summary_pdb_93\\b42c4924e3cd1aec.final.pdb
3.258087e-15 -3.561040e-15 -7.394517e-15 2.218723e-15 -1.273297e-14
1.149460e-15
E:\\R-3.1.2\\scripts\\mPDBS\\summary_pdb_93\\b4dd239fcabff1df.final.pdb
-3.377303e-16 -6.198967e-15 7.231648e-16 2.342636e-15 -3.241131e-16
3.148487e-15
E:\\R-3.1.2\\scripts\\mPDBS\\summary_pdb_93\\b669942ef97459a2.final.pdb
-5.806205e-15 1.095738e-15 -1.229256e-15 6.368695e-15 3.843750e-16
2.487835e-15
E:\\R-3.1.2\\scripts\\mPDBS\\summary_pdb_93\\b99f4d9d87c1017b.final.pdb
-3.377303e-16 -6.198967e-15 7.231648e-16 2.342636e-15 -3.241131e-16
3.148487e-15
E:\\R-3.1.2\\scripts\\mPDBS\\summary_pdb_93\\b9d2cbf3975403e7.final.pdb
-3.377303e-16 -6.198967e-15 7.231648e-16 2.342636e-15 -3.241131e-16
3.148487e-15
E:\\R-3.1.2\\scripts\\mPDBS\\summary_pdb_93\\bc88c8a505f3a288.final.pdb
1.542439e-15 -1.286447e-15 -6.091029e-16 2.133602e-15 2.599004e-15
3.750219e-15
E:\\R-3.1.2\\scripts\\mPDBS\\summary_pdb_93\\bdac0e64f4ef427e.final.pdb
-6.988310e-15 9.607159e-15 -1.079712e-14 1.092278e-14 2.084729e-15
9.190373e-15
E:\\R-3.1.2\\scripts\\mPDBS\\summary_pdb_93\\ca2a1f55fc9d0681.final.pdb

-5.806205e-15 1.095738e-15 -1.229256e-15 6.368695e-15 3.843750e-16
2.487835e-15
E:\\R-3.1.2\\scripts\\mPDBS\\summary_pdb_93\\ce34248de3e81a3c.final.pdb
7.492816e-15 -8.525428e-15 -3.558976e-15 2.094588e-15 1.860492e-15
-7.867690e-16
E:\\R-3.1.2\\scripts\\mPDBS\\summary_pdb_93\\d02e697772494a61.final.pdb
1.477366e-14 8.978563e-15 -7.718433e-15 5.960185e-15 1.451547e-14
3.990044e-15
E:\\R-3.1.2\\scripts\\mPDBS\\summary_pdb_93\\d04221245d015047.final.pdb
9.475187e-16 -4.794984e-15 -3.254004e-16 1.462294e-15 6.346428e-16
3.170994e-15
E:\\R-3.1.2\\scripts\\mPDBS\\summary_pdb_93\\d678141e4018054a.final.pdb
1.354889e-14 9.997566e-16 1.410173e-14 -4.483674e-15 -2.174000e-15
-8.154953e-15
E:\\R-3.1.2\\scripts\\mPDBS\\summary_pdb_93\\d71b40af0f0efe39.final.pdb
9.142867e-15 6.387035e-15 -2.148096e-15 7.186206e-15 -4.257419e-16
5.603289e-15
E:\\R-3.1.2\\scripts\\mPDBS\\summary_pdb_93\\dc0809eff10197e2.final.pdb
2.024889e-15 -1.031610e-14 -6.378753e-15 7.435881e-15 -3.841519e-16
8.960611e-15
E:\\R-3.1.2\\scripts\\mPDBS\\summary_pdb_93\\e1163918d3707839.final.pdb
6.984273e-15 -6.780687e-15 -1.571051e-15 -4.613485e-15 -4.827835e-15
-8.810455e-15
E:\\R-3.1.2\\scripts\\mPDBS\\summary_pdb_93\\e4b48b06824ef848.final.pdb
3.782903e-15 5.486344e-15 5.094421e-15 8.590611e-16 5.026697e-15
2.288878e-15
E:\\R-3.1.2\\scripts\\mPDBS\\summary_pdb_93\\e529e19ad281fea2.final.pdb
-2.196190e-16 -6.837929e-15 -5.680076e-16 -5.577207e-16 -2.528984e-15
3.144614e-15
E:\\R-3.1.2\\scripts\\mPDBS\\summary_pdb_93\\e5602082718ec3a1.final.pdb
4.543156e-15 -8.476466e-15 -1.314257e-15 7.273744e-16 1.962973e-15
2.137421e-15
E:\\R-3.1.2\\scripts\\mPDBS\\summary_pdb_93\\e5d589d996d0a960.final.pdb
2.103766e-15 -6.441426e-15 -1.384920e-15 -2.501065e-15 9.337199e-15
3.812249e-15
E:\\R-3.1.2\\scripts\\mPDBS\\summary_pdb_93\\e9df9e72307a2fbd.final.pdb
-3.112841e-15 1.716650e-15 -2.869567e-15 3.081666e-15 4.317123e-15
5.032774e-15
E:\\R-3.1.2\\scripts\\mPDBS\\summary_pdb_93\\ea00f8694090cae7.final.pdb
4.543156e-15 -8.476466e-15 -1.314257e-15 7.273744e-16 1.962973e-15
2.137421e-15
E:\\R-3.1.2\\scripts\\mPDBS\\summary_pdb_93\\eba517be32db050e.final.pdb
-3.377303e-16 -6.198967e-15 7.231648e-16 2.342636e-15 -3.241131e-16
3.148487e-15
E:\\R-3.1.2\\scripts\\mPDBS\\summary_pdb_93\\ec04b9545899c29d.final.pdb
6.926477e-16 -2.660285e-15 3.445176e-16 1.841204e-15 1.884569e-15
3.107312e-15
E:\\R-3.1.2\\scripts\\mPDBS\\summary_pdb_93\\ee6862cbcad52078.final.pdb
1.046579e-15 -1.817055e-15 1.637164e-16 2.059876e-15 2.252819e-15
3.839774e-15
E:\\R-3.1.2\\scripts\\mPDBS\\summary_pdb_93\\ef6a42fc83b81e5d.final.pdb
1.620950e-15 -2.606791e-15 3.856956e-17 1.663322e-15 2.059615e-15
2.911210e-15
E:\\R-3.1.2\\scripts\\mPDBS\\summary_pdb_93\\ef8e88d4ad93a0ad.final.pdb

9.475187e-16 -4.794984e-15 -3.254004e-16 1.462294e-15 6.346428e-16
3.170994e-15
E:\\R-3.1.2\\scripts\\mPDBS\\summary_pdb_93\\f14fcae8deec4fa3.final.pdb
-3.193089e-15 -3.853780e-15 -1.382323e-15 3.519696e-15 2.228588e-15
5.177419e-15
E:\\R-3.1.2\\scripts\\mPDBS\\summary_pdb_93\\f83584e22003fb92.final.pdb
5.559083e-15 -1.199056e-14 5.602075e-15 2.225871e-16 2.872820e-15
-3.819464e-15
E:\\R-3.1.2\\scripts\\mPDBS\\summary_pdb_93\\f8e2bf87e52caa29.final.pdb
-2.196190e-16 -6.837929e-15 -5.680076e-16 -5.577207e-16 -2.528984e-15
3.144614e-15

[,283]   [,284]   [,285]   [,286]   [,287]   [,288]
E:\\R-3.1.2\\scripts\\40.1ns_data\\NAT0.pdb
-4.357841e-15 -6.654154e-16 5.899414e-15 -2.518842e-16 -2.549867e-15
-2.208174e-15
E:\\R-3.1.2\\scripts\\40.1ns_data\\LJP6.pdb
-3.480887e-15 9.160306e-16 6.572227e-15 1.147196e-15 -2.369877e-15
-1.766598e-15
E:\\R-3.1.2\\scripts\\40.1ns_data\\3WY9.pdb
-2.195357e-15 -6.008495e-16 6.460744e-15 3.280540e-17 -5.863507e-15
-8.165617e-16
E:\\R-3.1.2\\scripts\\40.1ns_data\\ZSP9.pdb
-3.904554e-15 -1.042633e-15 4.310059e-15 6.934605e-16 -2.389335e-15
-2.766406e-15
E:\\R-3.1.2\\scripts\\mPDBS\\summary_pdb_93\\013d855ad26c877c.final.pdb
-4.187488e-15 -6.676418e-15 5.562716e-15 4.196882e-15 -3.784581e-15
9.243499e-16
E:\\R-3.1.2\\scripts\\mPDBS\\summary_pdb_93\\0a2a705342ca0d54.final.pdb
-3.410563e-15 -1.445801e-15 5.786363e-15 -1.047936e-18 -2.211300e-15
-2.505368e-15
E:\\R-3.1.2\\scripts\\mPDBS\\summary_pdb_93\\0a59fceadb4194d9.final.pdb
-4.173920e-15 -4.395092e-15 7.633631e-15 -8.522954e-16 -1.943324e-15
-4.821320e-15
E:\\R-3.1.2\\scripts\\mPDBS\\summary_pdb_93\\0dba59f3306825e8.final.pdb
-2.027757e-15 2.326796e-15 4.136774e-15 4.481446e-16 1.785616e-15
-1.066679e-15
E:\\R-3.1.2\\scripts\\mPDBS\\summary_pdb_93\\126f26a78b57339c.final.pdb
1.087372e-15 -8.464465e-16 5.265211e-15 -2.350398e-15 -3.742080e-15
-3.475993e-15
E:\\R-3.1.2\\scripts\\mPDBS\\summary_pdb_93\\12f06f3e342df706.final.pdb
-2.985122e-15 -7.108201e-16 5.365259e-15 -5.023830e-16 -2.016144e-15
-2.867546e-15
E:\\R-3.1.2\\scripts\\mPDBS\\summary_pdb_93\\18618a2a251b867b.final.pdb
2.496564e-15 -7.114940e-15 5.445912e-15 -1.613458e-15 -2.119024e-15
6.994574e-16
E:\\R-3.1.2\\scripts\\mPDBS\\summary_pdb_93\\1f305f598641eeb2.final.pdb
-3.250259e-15 -1.317874e-15 5.844472e-15 -1.511112e-16 -1.940743e-15
-2.454601e-15
E:\\R-3.1.2\\scripts\\mPDBS\\summary_pdb_93\\1f8a00b551be0a39.final.pdb
2.496564e-15 -7.114940e-15 5.445912e-15 -1.613458e-15 -2.119024e-15
6.994574e-16
E:\\R-3.1.2\\scripts\\mPDBS\\summary_pdb_93\\219f45328d47579a.final.pdb
-7.243513e-15 4.840576e-15 -1.985007e-20 1.130248e-14 -5.403230e-15

-1.000966e-14
E:\\R-3.1.2\\scripts\\mPDBS\\summary_pdb_93\\238dd3918e54336d.final.pdb
3.412893e-15 6.614005e-15 6.533382e-15 7.781278e-16 5.457874e-15
2.822631e-15
E:\\R-3.1.2\\scripts\\mPDBS\\summary_pdb_93\\2b3027889e2fdc42.final.pdb
-2.599008e-15 -4.301647e-16 5.041954e-15 -1.332525e-16 -1.151422e-15
-2.709131e-15
E:\\R-3.1.2\\scripts\\mPDBS\\summary_pdb_93\\2fcb3b46e33793b0.final.pdb
-5.394377e-15 4.133798e-16 4.869558e-15 4.716545e-16 -1.062973e-15
-1.541703e-15
E:\\R-3.1.2\\scripts\\mPDBS\\summary_pdb_93\\34eb328251842006.final.pdb
-1.366672e-14 -7.919046e-15 1.636184e-14 4.878233e-15 -1.144325e-15
-6.862665e-15
E:\\R-3.1.2\\scripts\\mPDBS\\summary_pdb_93\\354b32871797b7ab.final.pdb
-2.882325e-15 -1.204703e-14 8.882086e-15 -2.102615e-15 -6.735986e-15
-3.824386e-15
E:\\R-3.1.2\\scripts\\mPDBS\\summary_pdb_93\\397368adbc32d5de.final.pdb
1.087372e-15 -8.464465e-16 5.265211e-15 -2.350398e-15 -3.742080e-15
-3.475993e-15
E:\\R-3.1.2\\scripts\\mPDBS\\summary_pdb_93\\411ca13cd3df6edd.final.pdb
-7.343772e-15 -2.240313e-15 8.237523e-15 3.680048e-16 -5.711165e-15
-6.832315e-16
E:\\R-3.1.2\\scripts\\mPDBS\\summary_pdb_93\\42357eee6642bf12.final.pdb
-8.925437e-15 -1.211240e-14 1.084159e-14 9.361052e-15 -2.350447e-15
-9.717965e-15
E:\\R-3.1.2\\scripts\\mPDBS\\summary_pdb_93\\44342d1f75a7fcfc.final.pdb
4.917016e-15 -5.615400e-15 1.357520e-14 -9.455585e-15 -3.626685e-15
-4.134118e-15
E:\\R-3.1.2\\scripts\\mPDBS\\summary_pdb_93\\448ef21dffe8cdfb.final.pdb
-4.247725e-15 -1.688181e-14 8.182880e-15 -2.288291e-15 -1.168642e-14
-2.419473e-15
E:\\R-3.1.2\\scripts\\mPDBS\\summary_pdb_93\\472a40b3e8f113c7.final.pdb
-2.027757e-15 2.326796e-15 4.136774e-15 4.481446e-16 1.785616e-15
-1.066679e-15
E:\\R-3.1.2\\scripts\\mPDBS\\summary_pdb_93\\48a26225951ea153.final.pdb
1.940330e-15 2.841524e-15 7.471434e-15 -2.127317e-15 6.665679e-16
4.721773e-15
E:\\R-3.1.2\\scripts\\mPDBS\\summary_pdb_93\\4ab4a5c7c6682ce6.final.pdb
-7.273995e-15 1.070161e-14 2.634630e-17 -1.176301e-14 1.335455e-15
-1.551317e-15
E:\\R-3.1.2\\scripts\\mPDBS\\summary_pdb_93\\4eaa45b62cb6a25f.final.pdb
-1.252119e-15 3.270039e-15 1.640049e-15 -2.189568e-15 -9.506836e-16
3.752710e-16
E:\\R-3.1.2\\scripts\\mPDBS\\summary_pdb_93\\58c371611d85ac96.final.pdb
1.087372e-15 -8.464465e-16 5.265211e-15 -2.350398e-15 -3.742080e-15
-3.475993e-15
E:\\R-3.1.2\\scripts\\mPDBS\\summary_pdb_93\\59a647f04822d30a.final.pdb
-8.808550e-15 5.976022e-15 2.291691e-15 2.631968e-15 -7.756667e-16
-2.548798e-15
E:\\R-3.1.2\\scripts\\mPDBS\\summary_pdb_93\\59b091fe385e05fc.final.pdb
-4.173920e-15 -4.395092e-15 7.633631e-15 -8.522954e-16 -1.943324e-15
-4.821320e-15
E:\\R-3.1.2\\scripts\\mPDBS\\summary_pdb_93\\5c35ac30f930d2db.final.pdb
-1.252119e-15 3.270039e-15 1.640049e-15 -2.189568e-15 -9.506836e-16

3.752710e-16
E:\\R-3.1.2\\scripts\\mPDBS\\summary_pdb_93\\5f60752431cf2dcb.final.pdb
-2.316603e-15 1.898096e-15 7.860225e-15 -4.896438e-15 7.724241e-16
3.822421e-16
E:\\R-3.1.2\\scripts\\mPDBS\\summary_pdb_93\\626456dd6ff2868d.final.pdb
-2.027757e-15 2.326796e-15 4.136774e-15 4.481446e-16 1.785616e-15
-1.066679e-15
E:\\R-3.1.2\\scripts\\mPDBS\\summary_pdb_93\\6583d53ff3c8f7ef.final.pdb
-8.808550e-15 5.976022e-15 2.291691e-15 2.631968e-15 -7.756667e-16
-2.548798e-15
E:\\R-3.1.2\\scripts\\mPDBS\\summary_pdb_93\\69760178986bef01.final.pdb
1.081996e-14 -9.825146e-16 -2.385632e-15 -3.107408e-15 3.114400e-15
4.727797e-15
E:\\R-3.1.2\\scripts\\mPDBS\\summary_pdb_93\\6a9d87d47926b1c8.final.pdb
-2.853919e-15 -3.641933e-16 4.841939e-15 1.077051e-16 -9.457060e-16
-2.541411e-15
E:\\R-3.1.2\\scripts\\mPDBS\\summary_pdb_93\\6bb3547ea982ffc4.final.pdb
3.275632e-16 2.324865e-15 6.288877e-15 -2.186214e-15 -4.573141e-15
7.260219e-16
E:\\R-3.1.2\\scripts\\mPDBS\\summary_pdb_93\\716d9624611d1c42.final.pdb
-1.893114e-15 -2.080710e-15 2.845139e-15 -3.206707e-16 -3.261675e-15
-1.251380e-15
E:\\R-3.1.2\\scripts\\mPDBS\\summary_pdb_93\\75f27e70ad0f1be4.final.pdb
-8.808550e-15 5.976022e-15 2.291691e-15 2.631968e-15 -7.756667e-16
-2.548798e-15
E:\\R-3.1.2\\scripts\\mPDBS\\summary_pdb_93\\769f910fffb22872.final.pdb
-4.009733e-15 -1.123747e-15 5.407314e-15 -3.108971e-16 -2.106014e-15
-2.572719e-15
E:\\R-3.1.2\\scripts\\mPDBS\\summary_pdb_93\\791f8c80dd5c3135.final.pdb
-4.173920e-15 -4.395092e-15 7.633631e-15 -8.522954e-16 -1.943324e-15
-4.821320e-15
E:\\R-3.1.2\\scripts\\mPDBS\\summary_pdb_93\\7fad98e66a3f6099.final.pdb
-6.443572e-15 -2.953273e-15 1.284687e-14 6.357190e-15 1.094120e-15
-2.477881e-16
E:\\R-3.1.2\\scripts\\mPDBS\\summary_pdb_93\\804b89c94a633196.final.pdb
-5.394377e-15 4.133798e-16 4.869558e-15 4.716545e-16 -1.062973e-15
-1.541703e-15
E:\\R-3.1.2\\scripts\\mPDBS\\summary_pdb_93\\813cc65a2298feba.final.pdb
-5.394377e-15 4.133798e-16 4.869558e-15 4.716545e-16 -1.062973e-15
-1.541703e-15
E:\\R-3.1.2\\scripts\\mPDBS\\summary_pdb_93\\83c0eefcfe12d3f9.final.pdb
-7.343772e-15 -2.240313e-15 8.237523e-15 3.680048e-16 -5.711165e-15
-6.832315e-16
E:\\R-3.1.2\\scripts\\mPDBS\\summary_pdb_93\\86d50469ef569760.final.pdb
-2.027757e-15 2.326796e-15 4.136774e-15 4.481446e-16 1.785616e-15
-1.066679e-15
E:\\R-3.1.2\\scripts\\mPDBS\\summary_pdb_93\\89d0666e1402203d.final.pdb
-8.808550e-15 5.976022e-15 2.291691e-15 2.631968e-15 -7.756667e-16
-2.548798e-15
E:\\R-3.1.2\\scripts\\mPDBS\\summary_pdb_93\\8b34852716dadc62.final.pdb
-1.390635e-15 4.871619e-15 7.360621e-15 -3.197286e-15 -2.704022e-15
-6.059356e-15
E:\\R-3.1.2\\scripts\\mPDBS\\summary_pdb_93\\8b6ef9ac3fe74395.final.pdb
-4.064742e-15 3.628429e-15 9.403816e-15 1.791921e-15 -1.751484e-15

-7.984208e-15
E:\\R-3.1.2\\scripts\\mPDBS\\summary_pdb_93\\98bbe0d5986c138f.final.pdb
-4.173920e-15 -4.395092e-15 7.633631e-15 -8.522954e-16 -1.943324e-15
-4.821320e-15
E:\\R-3.1.2\\scripts\\mPDBS\\summary_pdb_93\\9c22f4909d67e2fa.final.pdb
3.976816e-15 -7.393526e-15 5.120659e-15 5.159311e-15 -6.171494e-16
-1.634619e-15
E:\\R-3.1.2\\scripts\\mPDBS\\summary_pdb_93\\9dda8ed0d81fb3ef.final.pdb
1.763289e-15 -3.661017e-15 1.614282e-15 -1.129743e-14 -1.675121e-15
4.948189e-15
E:\\R-3.1.2\\scripts\\mPDBS\\summary_pdb_93\\9f752b3616e6033b.final.pdb
-2.220634e-15 4.472508e-16 6.025416e-15 1.505682e-15 -2.679935e-15
-5.046771e-15
E:\\R-3.1.2\\scripts\\mPDBS\\summary_pdb_93\\a02c70083816d6ba.final.pdb
-1.252119e-15 3.270039e-15 1.640049e-15 -2.189568e-15 -9.506836e-16
3.752710e-16
E:\\R-3.1.2\\scripts\\mPDBS\\summary_pdb_93\\a0c997d891e4e082.final.pdb
-1.666175e-15 3.109037e-15 8.124215e-15 2.423012e-15 4.803933e-15
1.497784e-15
E:\\R-3.1.2\\scripts\\mPDBS\\summary_pdb_93\\ab8039a2d823c616.final.pdb
-2.847521e-15 5.300451e-16 5.148730e-15 -4.572792e-16 -2.986710e-15
-2.696885e-15
E:\\R-3.1.2\\scripts\\mPDBS\\summary_pdb_93\\acf4d74b9c71d4dc.final.pdb
-5.394377e-15 4.133798e-16 4.869558e-15 4.716545e-16 -1.062973e-15
-1.541703e-15
E:\\R-3.1.2\\scripts\\mPDBS\\summary_pdb_93\\ad8a823e4b4c668e.final.pdb
-3.175832e-15 -9.114367e-16 5.400044e-15 -1.160022e-16 -1.731012e-15
-3.134848e-15
E:\\R-3.1.2\\scripts\\mPDBS\\summary_pdb_93\\b16ababd03b7b857.final.pdb
3.976816e-15 -7.393526e-15 5.120659e-15 5.159311e-15 -6.171494e-16
-1.634619e-15
E:\\R-3.1.2\\scripts\\mPDBS\\summary_pdb_93\\b226923e697d3c63.final.pdb
-3.760721e-15 -1.225018e-14 1.077232e-14 5.824510e-15 3.895076e-15
-3.237212e-15
E:\\R-3.1.2\\scripts\\mPDBS\\summary_pdb_93\\b28e48f6fa27a165.final.pdb
-4.173920e-15 -4.395092e-15 7.633631e-15 -8.522954e-16 -1.943324e-15
-4.821320e-15
E:\\R-3.1.2\\scripts\\mPDBS\\summary_pdb_93\\b42c4924e3cd1aec.final.pdb
8.308747e-16 -2.866405e-15 1.371007e-14 4.234202e-15 -5.999786e-15
-4.179329e-15
E:\\R-3.1.2\\scripts\\mPDBS\\summary_pdb_93\\b4dd239fcabff1df.final.pdb
-4.173920e-15 -4.395092e-15 7.633631e-15 -8.522954e-16 -1.943324e-15
-4.821320e-15
E:\\R-3.1.2\\scripts\\mPDBS\\summary_pdb_93\\b669942ef97459a2.final.pdb
-1.390635e-15 4.871619e-15 7.360621e-15 -3.197286e-15 -2.704022e-15
-6.059356e-15
E:\\R-3.1.2\\scripts\\mPDBS\\summary_pdb_93\\b99f4d9d87c1017b.final.pdb
-4.173920e-15 -4.395092e-15 7.633631e-15 -8.522954e-16 -1.943324e-15
-4.821320e-15
E:\\R-3.1.2\\scripts\\mPDBS\\summary_pdb_93\\b9d2cbf3975403e7.final.pdb
-4.173920e-15 -4.395092e-15 7.633631e-15 -8.522954e-16 -1.943324e-15
-4.821320e-15
E:\\R-3.1.2\\scripts\\mPDBS\\summary_pdb_93\\bc88c8a505f3a288.final.pdb
-2.399081e-15 -4.005660e-16 5.867682e-15 -1.709828e-16 -1.416835e-15

-2.642399e-15
E:\\R-3.1.2\\scripts\\mPDBS\\summary_pdb_93\\bdac0e64f4ef427e.final.pdb
3.793152e-15 6.059341e-16 2.750159e-15 -8.788277e-16 4.820341e-15
-5.791613e-15
E:\\R-3.1.2\\scripts\\mPDBS\\summary_pdb_93\\ca2a1f55fc9d0681.final.pdb
-1.390635e-15 4.871619e-15 7.360621e-15 -3.197286e-15 -2.704022e-15
-6.059356e-15
E:\\R-3.1.2\\scripts\\mPDBS\\summary_pdb_93\\ce34248de3e81a3c.final.pdb
-7.907875e-15 1.491803e-15 5.288627e-15 -1.077641e-15 2.665510e-16
-4.554934e-16
E:\\R-3.1.2\\scripts\\mPDBS\\summary_pdb_93\\d02e697772494a61.final.pdb
-1.055553e-14 4.657237e-15 1.771566e-15 6.036942e-15 2.392618e-15
-9.139589e-15
E:\\R-3.1.2\\scripts\\mPDBS\\summary_pdb_93\\d04221245d015047.final.pdb
-2.047814e-15 2.376984e-15 4.595787e-15 -2.260741e-15 -3.682334e-15
-2.242279e-15
E:\\R-3.1.2\\scripts\\mPDBS\\summary_pdb_93\\d678141e4018054a.final.pdb
-2.325512e-15 -3.240434e-15 1.260362e-14 -2.440240e-15 -2.012510e-15
1.111800e-15
E:\\R-3.1.2\\scripts\\mPDBS\\summary_pdb_93\\d71b40af0f0efe39.final.pdb
-4.520563e-15 -6.771735e-15 7.020045e-15 -3.186702e-15 -7.246063e-15
-6.472170e-15
E:\\R-3.1.2\\scripts\\mPDBS\\summary_pdb_93\\dc0809eff10197e2.final.pdb
-5.632198e-15 5.330630e-15 3.131924e-16 1.626114e-15 -6.920544e-16
-7.948001e-15
E:\\R-3.1.2\\scripts\\mPDBS\\summary_pdb_93\\e1163918d3707839.final.pdb
-2.605179e-15 -5.898702e-15 7.720075e-15 5.742978e-15 -2.422787e-15
-6.242133e-15
E:\\R-3.1.2\\scripts\\mPDBS\\summary_pdb_93\\e4b48b06824ef848.final.pdb
2.496564e-15 -7.114940e-15 5.445912e-15 -1.613458e-15 -2.119024e-15
6.994574e-16
E:\\R-3.1.2\\scripts\\mPDBS\\summary_pdb_93\\e529e19ad281fea2.final.pdb
-4.187488e-15 -6.676418e-15 5.562716e-15 4.196882e-15 -3.784581e-15
9.243499e-16
E:\\R-3.1.2\\scripts\\mPDBS\\summary_pdb_93\\e5602082718ec3a1.final.pdb
-5.394377e-15 4.133798e-16 4.869558e-15 4.716545e-16 -1.062973e-15
-1.541703e-15
E:\\R-3.1.2\\scripts\\mPDBS\\summary_pdb_93\\e5d589d996d0a960.final.pdb
-2.140200e-16 1.348578e-14 -6.120836e-16 -6.827656e-15 -2.456534e-15
-2.676830e-15
E:\\R-3.1.2\\scripts\\mPDBS\\summary_pdb_93\\e9df9e72307a2fbd.final.pdb
-1.252119e-15 3.270039e-15 1.640049e-15 -2.189568e-15 -9.506836e-16
3.752710e-16
E:\\R-3.1.2\\scripts\\mPDBS\\summary_pdb_93\\ea00f8694090cae7.final.pdb
-5.394377e-15 4.133798e-16 4.869558e-15 4.716545e-16 -1.062973e-15
-1.541703e-15
E:\\R-3.1.2\\scripts\\mPDBS\\summary_pdb_93\\eba517be32db050e.final.pdb
-4.173920e-15 -4.395092e-15 7.633631e-15 -8.522954e-16 -1.943324e-15
-4.821320e-15
E:\\R-3.1.2\\scripts\\mPDBS\\summary_pdb_93\\ec04b9545899c29d.final.pdb
-2.762171e-15 -9.021563e-16 5.538226e-15 -1.114970e-16 -1.832981e-15
-2.693864e-15
E:\\R-3.1.2\\scripts\\mPDBS\\summary_pdb_93\\ee6862cbcad52078.final.pdb
-2.876781e-15 -9.794215e-16 5.533748e-15 -8.988446e-17 -1.274588e-15

-2.928953e-15
E:\\R-3.1.2\\scripts\\mPDBS\\summary_pdb_93\\ef6a42fc83b81e5d.final.pdb
-2.724303e-15 -7.929567e-16 5.416421e-15 -3.323643e-16 -2.010604e-15
-2.560901e-15
E:\\R-3.1.2\\scripts\\mPDBS\\summary_pdb_93\\ef8e88d4ad93a0ad.final.pdb
-2.047814e-15 2.376984e-15 4.595787e-15 -2.260741e-15 -3.682334e-15
-2.242279e-15
E:\\R-3.1.2\\scripts\\mPDBS\\summary_pdb_93\\f14fcae8deec4fa3.final.pdb
-2.220634e-15 4.472508e-16 6.025416e-15 1.505682e-15 -2.679935e-15
-5.046771e-15
E:\\R-3.1.2\\scripts\\mPDBS\\summary_pdb_93\\f83584e22003fb92.final.pdb
1.940330e-15 2.841524e-15 7.471434e-15 -2.127317e-15 6.665679e-16
4.721773e-15
E:\\R-3.1.2\\scripts\\mPDBS\\summary_pdb_93\\f8e2bf87e52caa29.final.pdb
-4.187488e-15 -6.676418e-15 5.562716e-15 4.196882e-15 -3.784581e-15
9.243499e-16

[,289]   [,290]   [,291]   [,292]   [,293]   [,294]
E:\\R-3.1.2\\scripts\\40.1ns_data\\NAT0.pdb
2.219551e-16 -2.348830e-15 -1.220880e-16 -3.537356e-15 -6.680600e-15
-1.737500e-15
E:\\R-3.1.2\\scripts\\40.1ns_data\\LJP6.pdb
3.521458e-16 -2.086584e-15 1.283206e-15 -4.000718e-15 -4.156933e-15
-1.930549e-15
E:\\R-3.1.2\\scripts\\40.1ns_data\\3WY9.pdb
-2.756548e-16 -3.958098e-15 1.067678e-15 -3.703432e-15 -5.360230e-15
-1.829011e-15
E:\\R-3.1.2\\scripts\\40.1ns_data\\ZSP9.pdb
1.207528e-15 -2.749073e-15 9.789569e-16 -2.613560e-15 -4.755868e-15
-1.466441e-15
E:\\R-3.1.2\\scripts\\mPDBS\\summary_pdb_93\\013d855ad26c877c.final.pdb
-3.622216e-15 2.054050e-15 2.101737e-15 3.992862e-15 -1.039002e-14
-5.630861e-16
E:\\R-3.1.2\\scripts\\mPDBS\\summary_pdb_93\\0a2a705342ca0d54.final.pdb
-7.230511e-16 -2.449923e-15 1.129655e-15 -3.828507e-15 -3.636085e-15
-1.976365e-15
E:\\R-3.1.2\\scripts\\mPDBS\\summary_pdb_93\\0a59fceadb4194d9.final.pdb
3.526917e-16 -2.933912e-15 3.854940e-15 8.214515e-16 -7.041740e-15
-2.475405e-15
E:\\R-3.1.2\\scripts\\mPDBS\\summary_pdb_93\\0dba59f3306825e8.final.pdb
-1.468991e-15 -6.674644e-15 1.708171e-15 -4.710246e-15 -3.459800e-15
-2.670775e-15
E:\\R-3.1.2\\scripts\\mPDBS\\summary_pdb_93\\126f26a78b57339c.final.pdb
2.442377e-15 -2.322548e-15 -9.687240e-16 -1.419398e-14 3.804355e-15
-8.399049e-15
E:\\R-3.1.2\\scripts\\mPDBS\\summary_pdb_93\\12f06f3e342df706.final.pdb
-7.869106e-16 -2.251731e-15 1.228100e-15 -3.930856e-15 -3.617870e-15
-1.728517e-15
E:\\R-3.1.2\\scripts\\mPDBS\\summary_pdb_93\\18618a2a251b867b.final.pdb
-3.302642e-15 -5.100140e-15 -1.492378e-15 -9.666895e-15 -2.379140e-15
-4.432710e-15
E:\\R-3.1.2\\scripts\\mPDBS\\summary_pdb_93\\1f305f598641eeb2.final.pdb
-1.183169e-16 -2.040197e-15 1.600170e-15 -3.975091e-15 -3.681925e-15
-1.350459e-15

E:\\R-3.1.2\\scripts\\mPDBS\\summary_pdb_93\\1f8a00b551be0a39.final.pdb
-3.302642e-15 -5.100140e-15 -1.492378e-15 -9.666895e-15 -2.379140e-15
-4.432710e-15
E:\\R-3.1.2\\scripts\\mPDBS\\summary_pdb_93\\219f45328d47579a.final.pdb
1.216584e-15 -1.472656e-14 5.618504e-15 3.424404e-15 -1.986756e-15
5.167353e-15
E:\\R-3.1.2\\scripts\\mPDBS\\summary_pdb_93\\238dd3918e54336d.final.pdb
-8.072210e-15 -5.999602e-15 -1.976116e-15 -1.470584e-14 4.480294e-15
-1.887876e-15
E:\\R-3.1.2\\scripts\\mPDBS\\summary_pdb_93\\2b3027889e2fdc42.final.pdb
-7.456051e-16 -2.752850e-15 1.166336e-15 -4.422184e-15 -3.261777e-15
-1.933954e-15
E:\\R-3.1.2\\scripts\\mPDBS\\summary_pdb_93\\2fcb3b46e33793b0.final.pdb
-2.525679e-15 -2.057761e-15 2.784553e-15 1.357449e-15 -8.376086e-15
2.354911e-15
E:\\R-3.1.2\\scripts\\mPDBS\\summary_pdb_93\\34eb328251842006.final.pdb
1.974333e-15 3.189393e-15 9.438045e-15 2.881227e-15 7.384845e-16
-1.977430e-15
E:\\R-3.1.2\\scripts\\mPDBS\\summary_pdb_93\\354b32871797b7ab.final.pdb
7.052782e-16 2.227667e-15 5.554323e-15 7.404242e-15 -4.356419e-15
1.607839e-15
E:\\R-3.1.2\\scripts\\mPDBS\\summary_pdb_93\\397368adbc32d5de.final.pdb
2.442377e-15 -2.322548e-15 -9.687240e-16 -1.419398e-14 3.804355e-15
-8.399049e-15
E:\\R-3.1.2\\scripts\\mPDBS\\summary_pdb_93\\411ca13cd3df6edd.final.pdb
2.607202e-16 1.967557e-15 5.903586e-15 -6.787945e-15 1.264639e-15
-3.584566e-15
E:\\R-3.1.2\\scripts\\mPDBS\\summary_pdb_93\\42357eee6642bf12.final.pdb
-3.112618e-17 -8.026942e-15 7.268984e-15 8.780438e-15 -9.586124e-15
5.120389e-16
E:\\R-3.1.2\\scripts\\mPDBS\\summary_pdb_93\\44342d1f75a7fcfc.final.pdb
-2.967168e-15 4.541817e-15 5.459882e-15 -8.068033e-15 -8.812774e-15
-7.990180e-15
E:\\R-3.1.2\\scripts\\mPDBS\\summary_pdb_93\\448ef21dffe8cdfb.final.pdb
2.599995e-15 1.513530e-14 1.213978e-15 -2.516188e-15 -1.357395e-15
1.253361e-15
E:\\R-3.1.2\\scripts\\mPDBS\\summary_pdb_93\\472a40b3e8f113c7.final.pdb
-1.468991e-15 -6.674644e-15 1.708171e-15 -4.710246e-15 -3.459800e-15
-2.670775e-15
E:\\R-3.1.2\\scripts\\mPDBS\\summary_pdb_93\\48a26225951ea153.final.pdb
-3.211731e-15 9.910631e-15 -4.404512e-15 -1.350455e-14 1.516519e-15
1.827360e-15
E:\\R-3.1.2\\scripts\\mPDBS\\summary_pdb_93\\4ab4a5c7c6682ce6.final.pdb
-3.486908e-15 3.189319e-15 -8.394425e-15 -7.268963e-15 -7.002973e-15
8.604711e-15
E:\\R-3.1.2\\scripts\\mPDBS\\summary_pdb_93\\4eaa45b62cb6a25f.final.pdb
-1.237829e-15 -3.117124e-15 -3.554710e-15 -1.039879e-14 -2.848944e-15
-3.398898e-15
E:\\R-3.1.2\\scripts\\mPDBS\\summary_pdb_93\\58c371611d85ac96.final.pdb
2.442377e-15 -2.322548e-15 -9.687240e-16 -1.419398e-14 3.804355e-15
-8.399049e-15
E:\\R-3.1.2\\scripts\\mPDBS\\summary_pdb_93\\59a647f04822d30a.final.pdb
2.325690e-15 -3.004470e-16 -1.376880e-15 -3.154429e-15 -2.108068e-15
1.484446e-15

E:\\R-3.1.2\\scripts\\mPDBS\\summary_pdb_93\\59b091fe385e05fc.final.pdb
3.526917e-16 -2.933912e-15 3.854940e-15 8.214515e-16 -7.041740e-15
-2.475405e-15
E:\\R-3.1.2\\scripts\\mPDBS\\summary_pdb_93\\5c35ac30f930d2db.final.pdb
-1.237829e-15 -3.117124e-15 -3.554710e-15 -1.039879e-14 -2.848944e-15
-3.398898e-15
E:\\R-3.1.2\\scripts\\mPDBS\\summary_pdb_93\\5f60752431cf2dcb.final.pdb
1.862337e-15 -1.414184e-15 -1.255373e-15 -7.626250e-15 -5.850459e-15
-6.131245e-15
E:\\R-3.1.2\\scripts\\mPDBS\\summary_pdb_93\\626456dd6ff2868d.final.pdb
-1.468991e-15 -6.674644e-15 1.708171e-15 -4.710246e-15 -3.459800e-15
-2.670775e-15
E:\\R-3.1.2\\scripts\\mPDBS\\summary_pdb_93\\6583d53ff3c8f7ef.final.pdb
2.325690e-15 -3.004470e-16 -1.376880e-15 -3.154429e-15 -2.108068e-15
1.484446e-15
E:\\R-3.1.2\\scripts\\mPDBS\\summary_pdb_93\\69760178986bef01.final.pdb
-2.571718e-15 -7.511614e-15 -2.471946e-15 -1.129078e-14 -2.458823e-15
9.137831e-16
E:\\R-3.1.2\\scripts\\mPDBS\\summary_pdb_93\\6a9d87d47926b1c8.final.pdb
-8.310461e-16 -2.434872e-15 1.187320e-15 -4.113935e-15 -3.405156e-15
-1.932000e-15
E:\\R-3.1.2\\scripts\\mPDBS\\summary_pdb_93\\6bb3547ea982ffc4.final.pdb
-3.914084e-16 3.011117e-15 7.829858e-15 -1.723104e-14 1.006984e-14
-4.443991e-15
E:\\R-3.1.2\\scripts\\mPDBS\\summary_pdb_93\\716d9624611d1c42.final.pdb
-3.713281e-15 -8.951768e-16 1.089539e-15 -5.908440e-15 1.068485e-15
-9.004032e-16
E:\\R-3.1.2\\scripts\\mPDBS\\summary_pdb_93\\75f27e70ad0f1be4.final.pdb
2.325690e-15 -3.004470e-16 -1.376880e-15 -3.154429e-15 -2.108068e-15
1.484446e-15
E:\\R-3.1.2\\scripts\\mPDBS\\summary_pdb_93\\769f910fffb22872.final.pdb
-1.565039e-16 -2.405138e-15 1.868865e-15 -4.472482e-15 -2.744299e-15
-1.913350e-15
E:\\R-3.1.2\\scripts\\mPDBS\\summary_pdb_93\\791f8c80dd5c3135.final.pdb
3.526917e-16 -2.933912e-15 3.854940e-15 8.214515e-16 -7.041740e-15
-2.475405e-15
E:\\R-3.1.2\\scripts\\mPDBS\\summary_pdb_93\\7fad98e66a3f6099.final.pdb
-5.630535e-15 2.955803e-15 2.928659e-15 -3.895660e-16 4.081301e-15
8.835252e-16
E:\\R-3.1.2\\scripts\\mPDBS\\summary_pdb_93\\804b89c94a633196.final.pdb
-2.525679e-15 -2.057761e-15 2.784553e-15 1.357449e-15 -8.376086e-15
2.354911e-15
E:\\R-3.1.2\\scripts\\mPDBS\\summary_pdb_93\\813cc65a2298feba.final.pdb
-2.525679e-15 -2.057761e-15 2.784553e-15 1.357449e-15 -8.376086e-15
2.354911e-15
E:\\R-3.1.2\\scripts\\mPDBS\\summary_pdb_93\\83c0eefcfe12d3f9.final.pdb
2.607202e-16 1.967557e-15 5.903586e-15 -6.787945e-15 1.264639e-15
-3.584566e-15
E:\\R-3.1.2\\scripts\\mPDBS\\summary_pdb_93\\86d50469ef569760.final.pdb
-1.468991e-15 -6.674644e-15 1.708171e-15 -4.710246e-15 -3.459800e-15
-2.670775e-15
E:\\R-3.1.2\\scripts\\mPDBS\\summary_pdb_93\\89d0666e1402203d.final.pdb
2.325690e-15 -3.004470e-16 -1.376880e-15 -3.154429e-15 -2.108068e-15
1.484446e-15

E:\\R-3.1.2\\scripts\\mPDBS\\summary_pdb_93\\8b34852716dadc62.final.pdb
1.935596e-15 -5.884344e-15 1.437106e-15 -1.245008e-14 2.316749e-15
-2.438130e-15
E:\\R-3.1.2\\scripts\\mPDBS\\summary_pdb_93\\8b6ef9ac3fe74395.final.pdb
5.585928e-17 -1.833585e-15 8.374291e-15 -4.194427e-15 5.760118e-16
5.030363e-16
E:\\R-3.1.2\\scripts\\mPDBS\\summary_pdb_93\\98bbe0d5986c138f.final.pdb
3.526917e-16 -2.933912e-15 3.854940e-15 8.214515e-16 -7.041740e-15
-2.475405e-15
E:\\R-3.1.2\\scripts\\mPDBS\\summary_pdb_93\\9c22f4909d67e2fa.final.pdb
-4.704657e-16 -8.842915e-15 -1.481566e-15 1.177038e-15 -8.291085e-15
-5.169344e-15
E:\\R-3.1.2\\scripts\\mPDBS\\summary_pdb_93\\9dda8ed0d81fb3ef.final.pdb
-5.431776e-15 8.383145e-15 -3.550055e-15 -1.124431e-14 -2.301490e-15
-9.579837e-15
E:\\R-3.1.2\\scripts\\mPDBS\\summary_pdb_93\\9f752b3616e6033b.final.pdb
2.360633e-16 -3.814811e-15 4.417563e-15 -1.474831e-15 -4.718928e-15
-8.301124e-16
E:\\R-3.1.2\\scripts\\mPDBS\\summary_pdb_93\\a02c70083816d6ba.final.pdb
-1.237829e-15 -3.117124e-15 -3.554710e-15 -1.039879e-14 -2.848944e-15
-3.398898e-15
E:\\R-3.1.2\\scripts\\mPDBS\\summary_pdb_93\\a0c997d891e4e082.final.pdb
4.487942e-15 6.392014e-15 -2.676113e-15 -9.718692e-15 5.144910e-15
-1.687609e-15
E:\\R-3.1.2\\scripts\\mPDBS\\summary_pdb_93\\ab8039a2d823c616.final.pdb
-8.359239e-16 -2.878998e-15 1.216389e-15 -4.351024e-15 -3.591157e-15
-1.923206e-15
E:\\R-3.1.2\\scripts\\mPDBS\\summary_pdb_93\\acf4d74b9c71d4dc.final.pdb
-2.525679e-15 -2.057761e-15 2.784553e-15 1.357449e-15 -8.376086e-15
2.354911e-15
E:\\R-3.1.2\\scripts\\mPDBS\\summary_pdb_93\\ad8a823e4b4c668e.final.pdb
-7.368159e-18 -2.254931e-15 1.544747e-15 -3.783823e-15 -3.339658e-15
-1.496164e-15
E:\\R-3.1.2\\scripts\\mPDBS\\summary_pdb_93\\b16ababd03b7b857.final.pdb
-4.704657e-16 -8.842915e-15 -1.481566e-15 1.177038e-15 -8.291085e-15
-5.169344e-15
E:\\R-3.1.2\\scripts\\mPDBS\\summary_pdb_93\\b226923e697d3c63.final.pdb
2.689589e-16 -5.963127e-15 1.704422e-15 -2.197512e-15 2.746556e-15
-3.883330e-15
E:\\R-3.1.2\\scripts\\mPDBS\\summary_pdb_93\\b28e48f6fa27a165.final.pdb
3.526917e-16 -2.933912e-15 3.854940e-15 8.214515e-16 -7.041740e-15
-2.475405e-15
E:\\R-3.1.2\\scripts\\mPDBS\\summary_pdb_93\\b42c4924e3cd1aec.final.pdb
2.439098e-15 -6.563666e-15 7.581015e-15 1.549057e-15 -8.870020e-15
1.726700e-16
E:\\R-3.1.2\\scripts\\mPDBS\\summary_pdb_93\\b4dd239fcabff1df.final.pdb
3.526917e-16 -2.933912e-15 3.854940e-15 8.214515e-16 -7.041740e-15
-2.475405e-15
E:\\R-3.1.2\\scripts\\mPDBS\\summary_pdb_93\\b669942ef97459a2.final.pdb
1.935596e-15 -5.884344e-15 1.437106e-15 -1.245008e-14 2.316749e-15
-2.438130e-15
E:\\R-3.1.2\\scripts\\mPDBS\\summary_pdb_93\\b99f4d9d87c1017b.final.pdb
3.526917e-16 -2.933912e-15 3.854940e-15 8.214515e-16 -7.041740e-15
-2.475405e-15

E:\\R-3.1.2\\scripts\\mPDBS\\summary_pdb_93\\b9d2cbf3975403e7.final.pdb
3.526917e-16 -2.933912e-15 3.854940e-15 8.214515e-16 -7.041740e-15
-2.475405e-15
E:\\R-3.1.2\\scripts\\mPDBS\\summary_pdb_93\\bc88c8a505f3a288.final.pdb
-6.547489e-16 -2.610603e-15 1.160915e-15 -3.628548e-15 -3.324227e-15
-2.094525e-15
E:\\R-3.1.2\\scripts\\mPDBS\\summary_pdb_93\\bdac0e64f4ef427e.final.pdb
-2.485781e-15 -1.026626e-14 -7.117466e-15 1.572989e-15 -1.036668e-14
4.113378e-15
E:\\R-3.1.2\\scripts\\mPDBS\\summary_pdb_93\\ca2a1f55fc9d0681.final.pdb
1.935596e-15 -5.884344e-15 1.437106e-15 -1.245008e-14 2.316749e-15
-2.438130e-15
E:\\R-3.1.2\\scripts\\mPDBS\\summary_pdb_93\\ce34248de3e81a3c.final.pdb
-4.492983e-16 3.787459e-15 -6.844130e-15 -6.965940e-15 -3.807231e-15
-1.629899e-15
E:\\R-3.1.2\\scripts\\mPDBS\\summary_pdb_93\\d02e697772494a61.final.pdb
1.924134e-15 -9.515019e-15 3.696641e-16 -2.037156e-15 8.547215e-16
3.377010e-15
E:\\R-3.1.2\\scripts\\mPDBS\\summary_pdb_93\\d04221245d015047.final.pdb
-3.788243e-16 -1.195666e-15 1.400487e-15 -4.158036e-15 -4.807198e-15
-1.016271e-15
E:\\R-3.1.2\\scripts\\mPDBS\\summary_pdb_93\\d678141e4018054a.final.pdb
-3.833828e-15 1.789111e-15 4.085434e-15 -9.149064e-15 4.898802e-15
-1.082137e-14
E:\\R-3.1.2\\scripts\\mPDBS\\summary_pdb_93\\d71b40af0f0efe39.final.pdb
-5.333807e-15 -1.288835e-14 3.601701e-15 8.206991e-15 -9.818654e-15
-5.364939e-15
E:\\R-3.1.2\\scripts\\mPDBS\\summary_pdb_93\\dc0809eff10197e2.final.pdb
4.213750e-15 -5.750210e-15 1.373716e-15 1.074490e-15 -9.075265e-15
-4.924036e-15
E:\\R-3.1.2\\scripts\\mPDBS\\summary_pdb_93\\e1163918d3707839.final.pdb
3.378884e-15 -6.816177e-15 6.496162e-15 2.913825e-16 -5.947011e-15
4.693359e-15
E:\\R-3.1.2\\scripts\\mPDBS\\summary_pdb_93\\e4b48b06824ef848.final.pdb
-3.302642e-15 -5.100140e-15 -1.492378e-15 -9.666895e-15 -2.379140e-15
-4.432710e-15
E:\\R-3.1.2\\scripts\\mPDBS\\summary_pdb_93\\e529e19ad281fea2.final.pdb
-3.622216e-15 2.054050e-15 2.101737e-15 3.992862e-15 -1.039002e-14
-5.630861e-16
E:\\R-3.1.2\\scripts\\mPDBS\\summary_pdb_93\\e5602082718ec3a1.final.pdb
-2.525679e-15 -2.057761e-15 2.784553e-15 1.357449e-15 -8.376086e-15
2.354911e-15
E:\\R-3.1.2\\scripts\\mPDBS\\summary_pdb_93\\e5d589d996d0a960.final.pdb
5.747230e-16 2.129660e-15 -4.430569e-15 -1.578850e-14 4.726452e-15
-1.579522e-16
E:\\R-3.1.2\\scripts\\mPDBS\\summary_pdb_93\\e9df9e72307a2fbd.final.pdb
-1.237829e-15 -3.117124e-15 -3.554710e-15 -1.039879e-14 -2.848944e-15
-3.398898e-15
E:\\R-3.1.2\\scripts\\mPDBS\\summary_pdb_93\\ea00f8694090cae7.final.pdb
-2.525679e-15 -2.057761e-15 2.784553e-15 1.357449e-15 -8.376086e-15
2.354911e-15
E:\\R-3.1.2\\scripts\\mPDBS\\summary_pdb_93\\eba517be32db050e.final.pdb
3.526917e-16 -2.933912e-15 3.854940e-15 8.214515e-16 -7.041740e-15
-2.475405e-15

E:\\R-3.1.2\\scripts\\mPDBS\\summary_pdb_93\\ec04b9545899c29d.final.pdb
-2.510245e-16 -1.815294e-15 1.103991e-15 -3.575967e-15 -3.101197e-15
-1.331444e-15
E:\\R-3.1.2\\scripts\\mPDBS\\summary_pdb_93\\ee6862cbcad52078.final.pdb
-6.987675e-16 -2.549888e-15 1.415269e-15 -3.709646e-15 -3.879339e-15
-2.057770e-15
E:\\R-3.1.2\\scripts\\mPDBS\\summary_pdb_93\\ef6a42fc83b81e5d.final.pdb
-4.377460e-16 -2.511607e-15 1.486576e-15 -3.748452e-15 -3.033130e-15
-1.503846e-15
E:\\R-3.1.2\\scripts\\mPDBS\\summary_pdb_93\\ef8e88d4ad93a0ad.final.pdb
-3.788243e-16 -1.195666e-15 1.400487e-15 -4.158036e-15 -4.807198e-15
-1.016271e-15
E:\\R-3.1.2\\scripts\\mPDBS\\summary_pdb_93\\f14fcae8deec4fa3.final.pdb
2.360633e-16 -3.814811e-15 4.417563e-15 -1.474831e-15 -4.718928e-15
-8.301124e-16
E:\\R-3.1.2\\scripts\\mPDBS\\summary_pdb_93\\f83584e22003fb92.final.pdb
-3.211731e-15 9.910631e-15 -4.404512e-15 -1.350455e-14 1.516519e-15
1.827360e-15
E:\\R-3.1.2\\scripts\\mPDBS\\summary_pdb_93\\f8e2bf87e52caa29.final.pdb
-3.622216e-15 2.054050e-15 2.101737e-15 3.992862e-15 -1.039002e-14
-5.630861e-16

[,295]   [,296]   [,297]   [,298]   [,299]   [,300]
E:\\R-3.1.2\\scripts\\40.1ns_data\\NAT0.pdb
-2.556051e-15 -1.201131e-15 4.166771e-15 7.121965e-15 2.366355e-15
5.481125e-15
E:\\R-3.1.2\\scripts\\40.1ns_data\\LJP6.pdb
-3.072605e-15 -5.378747e-16 2.326628e-15 6.419229e-15 4.135679e-15
6.024693e-15
E:\\R-3.1.2\\scripts\\40.1ns_data\\3WY9.pdb
-1.538763e-15 -3.441760e-16 1.905950e-15 6.213231e-15 4.276460e-15
4.067971e-15
E:\\R-3.1.2\\scripts\\40.1ns_data\\ZSP9.pdb
-8.974435e-16 1.850251e-16 2.580926e-15 5.306211e-15 2.734662e-15
4.634349e-15
E:\\R-3.1.2\\scripts\\mPDBS\\summary_pdb_93\\013d855ad26c877c.final.pdb
-3.390420e-15 -2.019331e-15 1.356135e-17 3.697899e-15 2.567197e-16
2.096838e-15
E:\\R-3.1.2\\scripts\\mPDBS\\summary_pdb_93\\0a2a705342ca0d54.final.pdb
-2.259110e-15 -1.357948e-16 2.268252e-15 6.751135e-15 3.005256e-15
5.610304e-15
E:\\R-3.1.2\\scripts\\mPDBS\\summary_pdb_93\\0a59fceadb4194d9.final.pdb
-6.935746e-15 -3.224066e-15 2.113072e-15 5.043687e-15 2.949472e-15
8.261183e-15
E:\\R-3.1.2\\scripts\\mPDBS\\summary_pdb_93\\0dba59f3306825e8.final.pdb
-2.502242e-15 -6.151442e-15 8.653106e-16 8.431959e-15 4.584855e-15
4.894079e-15
E:\\R-3.1.2\\scripts\\mPDBS\\summary_pdb_93\\126f26a78b57339c.final.pdb
1.286833e-16 6.684102e-15 8.371025e-15 5.940029e-15 1.825777e-15
3.131817e-15
E:\\R-3.1.2\\scripts\\mPDBS\\summary_pdb_93\\12f06f3e342df706.final.pdb
-2.750904e-15 -7.873461e-16 2.006742e-15 6.490927e-15 3.106303e-15
4.832172e-15
E:\\R-3.1.2\\scripts\\mPDBS\\summary_pdb_93\\18618a2a251b867b.final.pdb

2.576406e-15 3.640988e-15 3.628063e-15 9.381132e-15 2.066856e-17
5.273092e-15
E:\\R-3.1.2\\scripts\\mPDBS\\summary_pdb_93\\1f305f598641eeb2.final.pdb
-2.745022e-15 -4.413567e-16 2.750704e-15 6.460621e-15 3.184212e-15
5.178279e-15
E:\\R-3.1.2\\scripts\\mPDBS\\summary_pdb_93\\1f8a00b551be0a39.final.pdb
2.576406e-15 3.640988e-15 3.628063e-15 9.381132e-15 2.066856e-17
5.273092e-15
E:\\R-3.1.2\\scripts\\mPDBS\\summary_pdb_93\\219f45328d47579a.final.pdb
-7.834333e-15 -7.804603e-15 -1.289589e-15 1.610081e-14 1.430584e-14
-5.247543e-15
E:\\R-3.1.2\\scripts\\mPDBS\\summary_pdb_93\\238dd3918e54336d.final.pdb
-4.711180e-15 6.594632e-15 1.893521e-15 2.056990e-14 2.866639e-15
-4.384843e-15
E:\\R-3.1.2\\scripts\\mPDBS\\summary_pdb_93\\2b3027889e2fdc42.final.pdb
-2.351305e-15 -5.834915e-16 2.946281e-15 6.437971e-15 2.836715e-15
3.996582e-15
E:\\R-3.1.2\\scripts\\mPDBS\\summary_pdb_93\\2fcb3b46e33793b0.final.pdb
-1.627860e-15 -5.211846e-15 -2.555815e-15 5.447543e-15 4.260019e-16
3.312584e-15
E:\\R-3.1.2\\scripts\\mPDBS\\summary_pdb_93\\34eb328251842006.final.pdb
-1.277329e-14 2.796842e-15 3.571333e-15 6.252124e-15 4.885845e-15
1.216010e-14
E:\\R-3.1.2\\scripts\\mPDBS\\summary_pdb_93\\354b32871797b7ab.final.pdb
-3.489714e-15 4.656274e-15 -1.736080e-15 3.588071e-15 -1.518373e-15
5.725096e-15
E:\\R-3.1.2\\scripts\\mPDBS\\summary_pdb_93\\397368adbc32d5de.final.pdb
1.286833e-16 6.684102e-15 8.371025e-15 5.940029e-15 1.825777e-15
3.131817e-15
E:\\R-3.1.2\\scripts\\mPDBS\\summary_pdb_93\\411ca13cd3df6edd.final.pdb
2.256836e-15 7.604814e-16 -9.490271e-16 1.380483e-15 -4.917814e-15
3.250784e-15
E:\\R-3.1.2\\scripts\\mPDBS\\summary_pdb_93\\42357eee6642bf12.final.pdb
-9.648538e-15 2.784195e-15 -3.283960e-15 1.427969e-14 9.121426e-15
9.517326e-15
E:\\R-3.1.2\\scripts\\mPDBS\\summary_pdb_93\\44342d1f75a7fcfc.final.pdb
-7.507784e-16 4.112614e-15 1.117157e-14 -3.971149e-15 -1.062373e-14
8.912774e-15
E:\\R-3.1.2\\scripts\\mPDBS\\summary_pdb_93\\448ef21dffe8cdfb.final.pdb
3.705411e-16 2.270679e-14 5.266053e-15 -2.166159e-15 -2.941096e-15
-8.177929e-16
E:\\R-3.1.2\\scripts\\mPDBS\\summary_pdb_93\\472a40b3e8f113c7.final.pdb
-2.502242e-15 -6.151442e-15 8.653106e-16 8.431959e-15 4.584855e-15
4.894079e-15
E:\\R-3.1.2\\scripts\\mPDBS\\summary_pdb_93\\48a26225951ea153.final.pdb
1.597431e-14 4.006587e-15 7.375030e-15 -7.420734e-15 -2.262504e-15
5.569976e-15
E:\\R-3.1.2\\scripts\\mPDBS\\summary_pdb_93\\4ab4a5c7c6682ce6.final.pdb
-6.209996e-15 8.016549e-16 -6.759233e-15 5.050116e-15 1.759858e-15
2.349457e-15
E:\\R-3.1.2\\scripts\\mPDBS\\summary_pdb_93\\4eaa45b62cb6a25f.final.pdb
1.508204e-16 -2.537787e-15 4.072750e-15 6.126405e-15 2.863105e-15
4.418741e-15
E:\\R-3.1.2\\scripts\\mPDBS\\summary_pdb_93\\58c371611d85ac96.final.pdb

1.286833e-16 6.684102e-15 8.371025e-15 5.940029e-15 1.825777e-15
3.131817e-15
E:\\R-3.1.2\\scripts\\mPDBS\\summary_pdb_93\\59a647f04822d30a.final.pdb
8.122074e-16 6.214826e-16 6.085358e-15 4.764051e-15 8.854617e-15
6.781232e-15
E:\\R-3.1.2\\scripts\\mPDBS\\summary_pdb_93\\59b091fe385e05fc.final.pdb
-6.935746e-15 -3.224066e-15 2.113072e-15 5.043687e-15 2.949472e-15
8.261183e-15
E:\\R-3.1.2\\scripts\\mPDBS\\summary_pdb_93\\5c35ac30f930d2db.final.pdb
1.508204e-16 -2.537787e-15 4.072750e-15 6.126405e-15 2.863105e-15
4.418741e-15
E:\\R-3.1.2\\scripts\\mPDBS\\summary_pdb_93\\5f60752431cf2dcb.final.pdb
-1.135443e-15 2.416309e-15 8.093128e-15 4.332497e-15 -2.683770e-15
6.879471e-15
E:\\R-3.1.2\\scripts\\mPDBS\\summary_pdb_93\\626456dd6ff2868d.final.pdb
-2.502242e-15 -6.151442e-15 8.653106e-16 8.431959e-15 4.584855e-15
4.894079e-15
E:\\R-3.1.2\\scripts\\mPDBS\\summary_pdb_93\\6583d53ff3c8f7ef.final.pdb
8.122074e-16 6.214826e-16 6.085358e-15 4.764051e-15 8.854617e-15
6.781232e-15
E:\\R-3.1.2\\scripts\\mPDBS\\summary_pdb_93\\69760178986bef01.final.pdb
5.146660e-15 -1.590289e-15 6.359112e-16 5.772031e-15 -3.908447e-15
-3.359304e-15
E:\\R-3.1.2\\scripts\\mPDBS\\summary_pdb_93\\6a9d87d47926b1c8.final.pdb
-2.962594e-15 -7.821477e-16 2.596131e-15 5.615636e-15 2.868346e-15
4.263724e-15
E:\\R-3.1.2\\scripts\\mPDBS\\summary_pdb_93\\6bb3547ea982ffc4.final.pdb
-1.961057e-15 7.031093e-15 2.515549e-15 4.072357e-15 -8.932150e-16
-3.667964e-15
E:\\R-3.1.2\\scripts\\mPDBS\\summary_pdb_93\\716d9624611d1c42.final.pdb
-2.977285e-15 -1.917356e-15 -5.280844e-17 7.658396e-15 4.985443e-15
3.006917e-15
E:\\R-3.1.2\\scripts\\mPDBS\\summary_pdb_93\\75f27e70ad0f1be4.final.pdb
8.122074e-16 6.214826e-16 6.085358e-15 4.764051e-15 8.854617e-15
6.781232e-15
E:\\R-3.1.2\\scripts\\mPDBS\\summary_pdb_93\\769f910fffb22872.final.pdb
-2.878216e-15 -3.672532e-16 2.038406e-15 6.563074e-15 3.393405e-15
5.404823e-15
E:\\R-3.1.2\\scripts\\mPDBS\\summary_pdb_93\\791f8c80dd5c3135.final.pdb
-6.935746e-15 -3.224066e-15 2.113072e-15 5.043687e-15 2.949472e-15
8.261183e-15
E:\\R-3.1.2\\scripts\\mPDBS\\summary_pdb_93\\7fad98e66a3f6099.final.pdb
-5.327134e-15 -2.793784e-15 -4.127579e-15 2.817018e-15 2.410155e-16
3.269442e-15
E:\\R-3.1.2\\scripts\\mPDBS\\summary_pdb_93\\804b89c94a633196.final.pdb
-1.627860e-15 -5.211846e-15 -2.555815e-15 5.447543e-15 4.260019e-16
3.312584e-15
E:\\R-3.1.2\\scripts\\mPDBS\\summary_pdb_93\\813cc65a2298feba.final.pdb
-1.627860e-15 -5.211846e-15 -2.555815e-15 5.447543e-15 4.260019e-16
3.312584e-15
E:\\R-3.1.2\\scripts\\mPDBS\\summary_pdb_93\\83c0eefcfe12d3f9.final.pdb
2.256836e-15 7.604814e-16 -9.490271e-16 1.380483e-15 -4.917814e-15
3.250784e-15
E:\\R-3.1.2\\scripts\\mPDBS\\summary_pdb_93\\86d50469ef569760.final.pdb

-2.502242e-15 -6.151442e-15 8.653106e-16 8.431959e-15 4.584855e-15
4.894079e-15
E:\\R-3.1.2\\scripts\\mPDBS\\summary_pdb_93\\89d0666e1402203d.final.pdb
8.122074e-16 6.214826e-16 6.085358e-15 4.764051e-15 8.854617e-15
6.781232e-15
E:\\R-3.1.2\\scripts\\mPDBS\\summary_pdb_93\\8b34852716dadc62.final.pdb
-2.148277e-15 -1.572396e-15 4.828903e-15 6.224699e-15 5.817522e-15
8.319863e-15
E:\\R-3.1.2\\scripts\\mPDBS\\summary_pdb_93\\8b6ef9ac3fe74395.final.pdb
-9.183463e-15 -2.827166e-15 1.006285e-14 7.735708e-16 1.190860e-14
9.647767e-15
E:\\R-3.1.2\\scripts\\mPDBS\\summary_pdb_93\\98bbe0d5986c138f.final.pdb
-6.935746e-15 -3.224066e-15 2.113072e-15 5.043687e-15 2.949472e-15
8.261183e-15
E:\\R-3.1.2\\scripts\\mPDBS\\summary_pdb_93\\9c22f4909d67e2fa.final.pdb
-3.222505e-15 -2.085170e-15 7.536156e-15 1.403963e-14 7.472882e-15
6.402451e-15
E:\\R-3.1.2\\scripts\\mPDBS\\summary_pdb_93\\9dda8ed0d81fb3ef.final.pdb
1.208809e-15 -6.566019e-15 9.714833e-15 -9.137589e-15 -1.101404e-14
1.693912e-14
E:\\R-3.1.2\\scripts\\mPDBS\\summary_pdb_93\\9f752b3616e6033b.final.pdb
-2.636291e-15 -1.247119e-15 2.027665e-15 6.596829e-15 4.158222e-15
4.278455e-15
E:\\R-3.1.2\\scripts\\mPDBS\\summary_pdb_93\\a02c70083816d6ba.final.pdb
1.508204e-16 -2.537787e-15 4.072750e-15 6.126405e-15 2.863105e-15
4.418741e-15
E:\\R-3.1.2\\scripts\\mPDBS\\summary_pdb_93\\a0c997d891e4e082.final.pdb
-5.793787e-15 -4.599470e-15 -4.039326e-15 1.259507e-15 -3.287022e-15
6.460502e-15
E:\\R-3.1.2\\scripts\\mPDBS\\summary_pdb_93\\ab8039a2d823c616.final.pdb
-2.916965e-15 -2.971928e-16 1.966178e-15 6.693095e-15 3.624550e-15
5.090612e-15
E:\\R-3.1.2\\scripts\\mPDBS\\summary_pdb_93\\acf4d74b9c71d4dc.final.pdb
-1.627860e-15 -5.211846e-15 -2.555815e-15 5.447543e-15 4.260019e-16
3.312584e-15
E:\\R-3.1.2\\scripts\\mPDBS\\summary_pdb_93\\ad8a823e4b4c668e.final.pdb
-2.607660e-15 -3.728379e-16 2.379678e-15 6.978417e-15 2.982249e-15
6.061057e-15
E:\\R-3.1.2\\scripts\\mPDBS\\summary_pdb_93\\b16ababd03b7b857.final.pdb
-3.222505e-15 -2.085170e-15 7.536156e-15 1.403963e-14 7.472882e-15
6.402451e-15
E:\\R-3.1.2\\scripts\\mPDBS\\summary_pdb_93\\b226923e697d3c63.final.pdb
-1.222879e-14 4.646528e-16 8.559994e-17 1.090671e-14 5.479279e-15
7.969626e-15
E:\\R-3.1.2\\scripts\\mPDBS\\summary_pdb_93\\b28e48f6fa27a165.final.pdb
-6.935746e-15 -3.224066e-15 2.113072e-15 5.043687e-15 2.949472e-15
8.261183e-15
E:\\R-3.1.2\\scripts\\mPDBS\\summary_pdb_93\\b42c4924e3cd1aec.final.pdb
-7.568892e-15 -6.845797e-15 -2.162766e-17 1.042749e-14 9.803077e-15
2.479009e-15
E:\\R-3.1.2\\scripts\\mPDBS\\summary_pdb_93\\b4dd239fcabff1df.final.pdb
-6.935746e-15 -3.224066e-15 2.113072e-15 5.043687e-15 2.949472e-15
8.261183e-15
E:\\R-3.1.2\\scripts\\mPDBS\\summary_pdb_93\\b669942ef97459a2.final.pdb

-2.148277e-15 -1.572396e-15 4.828903e-15 6.224699e-15 5.817522e-15
8.319863e-15
E:\\R-3.1.2\\scripts\\mPDBS\\summary_pdb_93\\b99f4d9d87c1017b.final.pdb
-6.935746e-15 -3.224066e-15 2.113072e-15 5.043687e-15 2.949472e-15
8.261183e-15
E:\\R-3.1.2\\scripts\\mPDBS\\summary_pdb_93\\b9d2cbf3975403e7.final.pdb
-6.935746e-15 -3.224066e-15 2.113072e-15 5.043687e-15 2.949472e-15
8.261183e-15
E:\\R-3.1.2\\scripts\\mPDBS\\summary_pdb_93\\bc88c8a505f3a288.final.pdb
-3.131931e-15 -8.884233e-16 2.141044e-15 6.248887e-15 3.074806e-15
4.175367e-15
E:\\R-3.1.2\\scripts\\mPDBS\\summary_pdb_93\\bdac0e64f4ef427e.final.pdb
-1.393030e-14 -5.027586e-15 1.019581e-14 1.440219e-14 2.017583e-14
1.056394e-14
E:\\R-3.1.2\\scripts\\mPDBS\\summary_pdb_93\\ca2a1f55fc9d0681.final.pdb
-2.148277e-15 -1.572396e-15 4.828903e-15 6.224699e-15 5.817522e-15
8.319863e-15
E:\\R-3.1.2\\scripts\\mPDBS\\summary_pdb_93\\ce34248de3e81a3c.final.pdb
-4.303665e-15 -2.686926e-15 1.996868e-15 4.377753e-15 4.011617e-15
1.173468e-14
E:\\R-3.1.2\\scripts\\mPDBS\\summary_pdb_93\\d02e697772494a61.final.pdb
-1.406532e-15 1.071547e-14 -2.205631e-15 1.781516e-14 1.046126e-14
-5.212306e-15
E:\\R-3.1.2\\scripts\\mPDBS\\summary_pdb_93\\d04221245d015047.final.pdb
3.419301e-16 -1.542887e-15 3.534477e-15 4.538135e-15 2.388993e-15
4.251332e-15
E:\\R-3.1.2\\scripts\\mPDBS\\summary_pdb_93\\d678141e4018054a.final.pdb
-4.144339e-15 1.048321e-14 4.997888e-15 1.293740e-14 5.037239e-16
3.475020e-16
E:\\R-3.1.2\\scripts\\mPDBS\\summary_pdb_93\\d71b40af0f0efe39.final.pdb
2.282208e-16 -1.556986e-15 1.661780e-15 1.315407e-14 3.584785e-15
-3.296406e-15
E:\\R-3.1.2\\scripts\\mPDBS\\summary_pdb_93\\dc0809eff10197e2.final.pdb
-9.736487e-15 -9.674255e-15 7.590155e-15 2.346144e-16 8.689229e-15
1.165887e-14
E:\\R-3.1.2\\scripts\\mPDBS\\summary_pdb_93\\e1163918d3707839.final.pdb
1.503712e-15 -6.541353e-15 -5.167685e-15 1.256814e-14 1.338181e-14
-1.583279e-15
E:\\R-3.1.2\\scripts\\mPDBS\\summary_pdb_93\\e4b48b06824ef848.final.pdb
2.576406e-15 3.640988e-15 3.628063e-15 9.381132e-15 2.066856e-17
5.273092e-15
E:\\R-3.1.2\\scripts\\mPDBS\\summary_pdb_93\\e529e19ad281fea2.final.pdb
-3.390420e-15 -2.019331e-15 1.356135e-17 3.697899e-15 2.567197e-16
2.096838e-15
E:\\R-3.1.2\\scripts\\mPDBS\\summary_pdb_93\\e5602082718ec3a1.final.pdb
-1.627860e-15 -5.211846e-15 -2.555815e-15 5.447543e-15 4.260019e-16
3.312584e-15
E:\\R-3.1.2\\scripts\\mPDBS\\summary_pdb_93\\e5d589d996d0a960.final.pdb
-1.869627e-15 1.269069e-15 -4.757146e-16 -1.108661e-15 1.241914e-15
1.054893e-15
E:\\R-3.1.2\\scripts\\mPDBS\\summary_pdb_93\\e9df9e72307a2fbd.final.pdb
1.508204e-16 -2.537787e-15 4.072750e-15 6.126405e-15 2.863105e-15
4.418741e-15
E:\\R-3.1.2\\scripts\\mPDBS\\summary_pdb_93\\ea00f8694090cae7.final.pdb

-1.627860e-15 -5.211846e-15 -2.555815e-15 5.447543e-15 4.260019e-16
3.312584e-15
E:\\R-3.1.2\\scripts\\mPDBS\\summary_pdb_93\\eba517be32db050e.final.pdb
-6.935746e-15 -3.224066e-15 2.113072e-15 5.043687e-15 2.949472e-15
8.261183e-15
E:\\R-3.1.2\\scripts\\mPDBS\\summary_pdb_93\\ec04b9545899c29d.final.pdb
-2.605198e-15 -7.029788e-16 3.095562e-15 6.971487e-15 3.450086e-15
5.087708e-15
E:\\R-3.1.2\\scripts\\mPDBS\\summary_pdb_93\\ee6862cbcad52078.final.pdb
-3.010067e-15 -8.101981e-16 3.075843e-15 5.896304e-15 2.943401e-15
3.832000e-15
E:\\R-3.1.2\\scripts\\mPDBS\\summary_pdb_93\\ef6a42fc83b81e5d.final.pdb
-2.523248e-15 -8.427300e-16 2.065403e-15 6.416056e-15 3.163987e-15
5.026978e-15
E:\\R-3.1.2\\scripts\\mPDBS\\summary_pdb_93\\ef8e88d4ad93a0ad.final.pdb
3.419301e-16 -1.542887e-15 3.534477e-15 4.538135e-15 2.388993e-15
4.251332e-15
E:\\R-3.1.2\\scripts\\mPDBS\\summary_pdb_93\\f14fcae8deec4fa3.final.pdb
-2.636291e-15 -1.247119e-15 2.027665e-15 6.596829e-15 4.158222e-15
4.278455e-15
E:\\R-3.1.2\\scripts\\mPDBS\\summary_pdb_93\\f83584e22003fb92.final.pdb
1.597431e-14 4.006587e-15 7.375030e-15 -7.420734e-15 -2.262504e-15
5.569976e-15
E:\\R-3.1.2\\scripts\\mPDBS\\summary_pdb_93\\f8e2bf87e52caa29.final.pdb
-3.390420e-15 -2.019331e-15 1.356135e-17 3.697899e-15 2.567197e-16
2.096838e-15

[,301]   [,302]   [,303]   [,304]   [,305]   [,306]
E:\\R-3.1.2\\scripts\\40.1ns_data\\NAT0.pdb
9.077777e-16 -1.446101e-15 1.004205e-16 -1.469041e-15 -2.046460e-17
-3.034336e-16
E:\\R-3.1.2\\scripts\\40.1ns_data\\LJP6.pdb
1.876014e-15 -1.223794e-15 -5.144782e-16 -3.058261e-15 -8.815124e-16
7.312161e-16
E:\\R-3.1.2\\scripts\\40.1ns_data\\3WY9.pdb
2.285212e-16 -3.034429e-15 3.941874e-15 -3.246771e-16 9.329254e-16
-2.463170e-16
E:\\R-3.1.2\\scripts\\40.1ns_data\\ZSP9.pdb
2.120201e-15 -5.066522e-16 3.665681e-16 -2.493294e-15 -5.591783e-17
1.193458e-15
E:\\R-3.1.2\\scripts\\mPDBS\\summary_pdb_93\\013d855ad26c877c.final.pdb
1.993575e-15 -2.637406e-15 -2.820683e-15 -8.631575e-15 3.851054e-15
2.554400e-15
E:\\R-3.1.2\\scripts\\mPDBS\\summary_pdb_93\\0a2a705342ca0d54.final.pdb
3.813078e-16 -2.127216e-15 1.251424e-15 -2.334266e-15 -3.997453e-16
-7.120301e-16
E:\\R-3.1.2\\scripts\\mPDBS\\summary_pdb_93\\0a59fceadb4194d9.final.pdb
1.176859e-15 -4.533359e-16 -2.228465e-15 -5.578940e-15 2.982245e-15
5.244846e-16
E:\\R-3.1.2\\scripts\\mPDBS\\summary_pdb_93\\0dba59f3306825e8.final.pdb
2.642362e-15 2.632684e-15 -7.829697e-15 -2.889640e-15 1.084170e-15
-6.836525e-15
E:\\R-3.1.2\\scripts\\mPDBS\\summary_pdb_93\\126f26a78b57339c.final.pdb
3.994186e-16 -8.959606e-15 1.244618e-14 5.217373e-15 -6.751440e-15

-9.224191e-16
E:\\R-3.1.2\\scripts\\mPDBS\\summary_pdb_93\\12f06f3e342df706.final.pdb
4.616878e-16 -1.851395e-15 1.345099e-15 -2.078503e-15 2.588126e-16
-1.007800e-15
E:\\R-3.1.2\\scripts\\mPDBS\\summary_pdb_93\\18618a2a251b867b.final.pdb
2.311534e-15 -5.404588e-15 5.488636e-15 1.993311e-15 -6.532884e-15
-3.954635e-15
E:\\R-3.1.2\\scripts\\mPDBS\\summary_pdb_93\\1f305f598641eeb2.final.pdb
3.511265e-16 -1.855746e-15 7.879035e-16 -1.753539e-15 3.801567e-16
-2.694080e-16
E:\\R-3.1.2\\scripts\\mPDBS\\summary_pdb_93\\1f8a00b551be0a39.final.pdb
2.311534e-15 -5.404588e-15 5.488636e-15 1.993311e-15 -6.532884e-15
-3.954635e-15
E:\\R-3.1.2\\scripts\\mPDBS\\summary_pdb_93\\219f45328d47579a.final.pdb
-2.441774e-15 -2.438293e-15 1.328647e-14 -6.608499e-15 -3.840856e-15
9.752780e-15
E:\\R-3.1.2\\scripts\\mPDBS\\summary_pdb_93\\238dd3918e54336d.final.pdb
8.772373e-15 -3.400197e-15 7.711934e-15 7.208032e-15 -1.421704e-14
-1.301312e-15
E:\\R-3.1.2\\scripts\\mPDBS\\summary_pdb_93\\2b3027889e2fdc42.final.pdb
4.882280e-16 -2.266989e-15 -3.664171e-17 -2.207722e-15 -1.166894e-16
-8.893151e-16
E:\\R-3.1.2\\scripts\\mPDBS\\summary_pdb_93\\2fcb3b46e33793b0.final.pdb
1.069302e-15 3.356230e-15 -1.050081e-14 -7.467718e-15 5.932139e-15
-3.073588e-15
E:\\R-3.1.2\\scripts\\mPDBS\\summary_pdb_93\\34eb328251842006.final.pdb
-2.519959e-15 -1.697151e-15 4.765480e-15 -4.808995e-15 -2.729787e-15
4.375087e-15
E:\\R-3.1.2\\scripts\\mPDBS\\summary_pdb_93\\354b32871797b7ab.final.pdb
-6.769804e-15 -7.916256e-15 1.107094e-15 -1.134487e-14 -2.429454e-15
6.605542e-15
E:\\R-3.1.2\\scripts\\mPDBS\\summary_pdb_93\\397368adbc32d5de.final.pdb
3.994186e-16 -8.959606e-15 1.244618e-14 5.217373e-15 -6.751440e-15
-9.224191e-16
E:\\R-3.1.2\\scripts\\mPDBS\\summary_pdb_93\\411ca13cd3df6edd.final.pdb
2.165593e-15 -1.401047e-16 -5.311818e-15 -6.668661e-15 2.493782e-15
-6.192188e-15
E:\\R-3.1.2\\scripts\\mPDBS\\summary_pdb_93\\42357eee6642bf12.final.pdb
6.470923e-16 2.916724e-16 1.234410e-14 -5.424126e-15 -7.526969e-15
8.363566e-15
E:\\R-3.1.2\\scripts\\mPDBS\\summary_pdb_93\\44342d1f75a7fcfc.final.pdb
-6.185033e-15 -4.069731e-15 -2.556176e-15 -3.909316e-15 -5.497292e-15
-7.878414e-15
E:\\R-3.1.2\\scripts\\mPDBS\\summary_pdb_93\\448ef21dffe8cdfb.final.pdb
6.737851e-16 -1.695825e-14 2.109197e-14 5.778631e-15 -5.931635e-15
2.410779e-14
E:\\R-3.1.2\\scripts\\mPDBS\\summary_pdb_93\\472a40b3e8f113c7.final.pdb
2.642362e-15 2.632684e-15 -7.829697e-15 -2.889640e-15 1.084170e-15
-6.836525e-15
E:\\R-3.1.2\\scripts\\mPDBS\\summary_pdb_93\\48a26225951ea153.final.pdb
-7.530531e-15 -4.422817e-15 -6.248253e-15 -3.674431e-15 -3.304244e-15
-5.548349e-15
E:\\R-3.1.2\\scripts\\mPDBS\\summary_pdb_93\\4ab4a5c7c6682ce6.final.pdb
-8.013990e-15 1.971323e-16 -3.628197e-15 4.360420e-15 5.745508e-15

6.678272e-15
E:\\R-3.1.2\\scripts\\mPDBS\\summary_pdb_93\\4eaa45b62cb6a25f.final.pdb
2.785026e-15 -1.680792e-15 5.138190e-16 1.537733e-15 2.487118e-15
-5.306804e-15
E:\\R-3.1.2\\scripts\\mPDBS\\summary_pdb_93\\58c371611d85ac96.final.pdb
3.994186e-16 -8.959606e-15 1.244618e-14 5.217373e-15 -6.751440e-15
-9.224191e-16
E:\\R-3.1.2\\scripts\\mPDBS\\summary_pdb_93\\59a647f04822d30a.final.pdb
-2.817067e-15 -1.683185e-15 6.306093e-15 2.062469e-15 1.062999e-15
1.510343e-15
E:\\R-3.1.2\\scripts\\mPDBS\\summary_pdb_93\\59b091fe385e05fc.final.pdb
1.176859e-15 -4.533359e-16 -2.228465e-15 -5.578940e-15 2.982245e-15
5.244846e-16
E:\\R-3.1.2\\scripts\\mPDBS\\summary_pdb_93\\5c35ac30f930d2db.final.pdb
2.785026e-15 -1.680792e-15 5.138190e-16 1.537733e-15 2.487118e-15
-5.306804e-15
E:\\R-3.1.2\\scripts\\mPDBS\\summary_pdb_93\\5f60752431cf2dcb.final.pdb
-1.190297e-15 5.122194e-15 4.022645e-15 1.663187e-15 1.295229e-15
-6.483889e-15
E:\\R-3.1.2\\scripts\\mPDBS\\summary_pdb_93\\626456dd6ff2868d.final.pdb
2.642362e-15 2.632684e-15 -7.829697e-15 -2.889640e-15 1.084170e-15
-6.836525e-15
E:\\R-3.1.2\\scripts\\mPDBS\\summary_pdb_93\\6583d53ff3c8f7ef.final.pdb
-2.817067e-15 -1.683185e-15 6.306093e-15 2.062469e-15 1.062999e-15
1.510343e-15
E:\\R-3.1.2\\scripts\\mPDBS\\summary_pdb_93\\69760178986bef01.final.pdb
3.556919e-15 2.944439e-15 2.957340e-15 -3.468252e-15 -2.706566e-15
-5.071727e-15
E:\\R-3.1.2\\scripts\\mPDBS\\summary_pdb_93\\6a9d87d47926b1c8.final.pdb
4.427867e-16 -2.064080e-15 -7.750102e-16 -1.794379e-15 -6.668343e-17
-4.020020e-16
E:\\R-3.1.2\\scripts\\mPDBS\\summary_pdb_93\\6bb3547ea982ffc4.final.pdb
1.811593e-15 -1.267101e-14 4.930399e-16 4.494051e-15 -1.590128e-14
-2.674119e-15
E:\\R-3.1.2\\scripts\\mPDBS\\summary_pdb_93\\716d9624611d1c42.final.pdb
3.884582e-15 -9.025777e-15 7.453185e-16 -1.727980e-15 6.042259e-16
1.702271e-15
E:\\R-3.1.2\\scripts\\mPDBS\\summary_pdb_93\\75f27e70ad0f1be4.final.pdb
-2.817067e-15 -1.683185e-15 6.306093e-15 2.062469e-15 1.062999e-15
1.510343e-15
E:\\R-3.1.2\\scripts\\mPDBS\\summary_pdb_93\\769f910fffb22872.final.pdb
7.024388e-17 -2.470303e-15 1.637550e-15 -2.820004e-15 4.375870e-16
-7.714175e-16
E:\\R-3.1.2\\scripts\\mPDBS\\summary_pdb_93\\791f8c80dd5c3135.final.pdb
1.176859e-15 -4.533359e-16 -2.228465e-15 -5.578940e-15 2.982245e-15
5.244846e-16
E:\\R-3.1.2\\scripts\\mPDBS\\summary_pdb_93\\7fad98e66a3f6099.final.pdb
-8.321778e-15 -9.515238e-17 3.176840e-15 -1.083027e-14 4.232207e-15
5.016775e-15
E:\\R-3.1.2\\scripts\\mPDBS\\summary_pdb_93\\804b89c94a633196.final.pdb
1.069302e-15 3.356230e-15 -1.050081e-14 -7.467718e-15 5.932139e-15
-3.073588e-15
E:\\R-3.1.2\\scripts\\mPDBS\\summary_pdb_93\\813cc65a2298feba.final.pdb
1.069302e-15 3.356230e-15 -1.050081e-14 -7.467718e-15 5.932139e-15

-3.073588e-15
E:\\R-3.1.2\\scripts\\mPDBS\\summary_pdb_93\\83c0eefcfe12d3f9.final.pdb
2.165593e-15 -1.401047e-16 -5.311818e-15 -6.668661e-15 2.493782e-15
-6.192188e-15
E:\\R-3.1.2\\scripts\\mPDBS\\summary_pdb_93\\86d50469ef569760.final.pdb
2.642362e-15 2.632684e-15 -7.829697e-15 -2.889640e-15 1.084170e-15
-6.836525e-15
E:\\R-3.1.2\\scripts\\mPDBS\\summary_pdb_93\\89d0666e1402203d.final.pdb
-2.817067e-15 -1.683185e-15 6.306093e-15 2.062469e-15 1.062999e-15
1.510343e-15
E:\\R-3.1.2\\scripts\\mPDBS\\summary_pdb_93\\8b34852716dadc62.final.pdb
-3.571083e-15 -2.202691e-15 6.845558e-15 2.534698e-15 -4.013898e-15
-1.646778e-15
E:\\R-3.1.2\\scripts\\mPDBS\\summary_pdb_93\\8b6ef9ac3fe74395.final.pdb
-1.865451e-16 -3.089181e-15 -7.945472e-16 3.981917e-15 1.710657e-15
1.627599e-15
E:\\R-3.1.2\\scripts\\mPDBS\\summary_pdb_93\\98bbe0d5986c138f.final.pdb
1.176859e-15 -4.533359e-16 -2.228465e-15 -5.578940e-15 2.982245e-15
5.244846e-16
E:\\R-3.1.2\\scripts\\mPDBS\\summary_pdb_93\\9c22f4909d67e2fa.final.pdb
2.284449e-15 -3.325924e-15 9.456317e-15 -2.717394e-15 -5.524923e-15
1.792311e-15
E:\\R-3.1.2\\scripts\\mPDBS\\summary_pdb_93\\9dda8ed0d81fb3ef.final.pdb
7.822913e-15 -2.382712e-15 -1.329758e-14 -1.209656e-14 -1.780468e-16
-6.619872e-15
E:\\R-3.1.2\\scripts\\mPDBS\\summary_pdb_93\\9f752b3616e6033b.final.pdb
-5.299610e-17 -1.617280e-15 -5.311832e-16 -4.168217e-15 -1.192803e-15
1.009128e-15
E:\\R-3.1.2\\scripts\\mPDBS\\summary_pdb_93\\a02c70083816d6ba.final.pdb
2.785026e-15 -1.680792e-15 5.138190e-16 1.537733e-15 2.487118e-15
-5.306804e-15
E:\\R-3.1.2\\scripts\\mPDBS\\summary_pdb_93\\a0c997d891e4e082.final.pdb
5.430581e-15 -7.372915e-15 -7.914449e-15 -6.390006e-15 7.031905e-15
-5.697739e-15
E:\\R-3.1.2\\scripts\\mPDBS\\summary_pdb_93\\ab8039a2d823c616.final.pdb
-1.395892e-16 -1.921113e-15 8.966454e-16 -2.304407e-15 4.233815e-17
-6.627041e-16
E:\\R-3.1.2\\scripts\\mPDBS\\summary_pdb_93\\acf4d74b9c71d4dc.final.pdb
1.069302e-15 3.356230e-15 -1.050081e-14 -7.467718e-15 5.932139e-15
-3.073588e-15
E:\\R-3.1.2\\scripts\\mPDBS\\summary_pdb_93\\ad8a823e4b4c668e.final.pdb
8.456032e-17 -2.293909e-15 7.553788e-16 -2.522315e-15 3.471217e-16
-6.095664e-16
E:\\R-3.1.2\\scripts\\mPDBS\\summary_pdb_93\\b16ababd03b7b857.final.pdb
2.284449e-15 -3.325924e-15 9.456317e-15 -2.717394e-15 -5.524923e-15
1.792311e-15
E:\\R-3.1.2\\scripts\\mPDBS\\summary_pdb_93\\b226923e697d3c63.final.pdb
7.233948e-15 -5.266690e-15 7.198174e-15 -7.075587e-16 9.576956e-16
-8.805396e-16
E:\\R-3.1.2\\scripts\\mPDBS\\summary_pdb_93\\b28e48f6fa27a165.final.pdb
1.176859e-15 -4.533359e-16 -2.228465e-15 -5.578940e-15 2.982245e-15
5.244846e-16
E:\\R-3.1.2\\scripts\\mPDBS\\summary_pdb_93\\b42c4924e3cd1aec.final.pdb
-3.760784e-15 2.312317e-15 4.033171e-15 -7.464957e-15 2.503185e-15

-1.136845e-15
E:\\R-3.1.2\\scripts\\mPDBS\\summary_pdb_93\\b4dd239fcabff1df.final.pdb
1.176859e-15 -4.533359e-16 -2.228465e-15 -5.578940e-15 2.982245e-15
5.244846e-16
E:\\R-3.1.2\\scripts\\mPDBS\\summary_pdb_93\\b669942ef97459a2.final.pdb
-3.571083e-15 -2.202691e-15 6.845558e-15 2.534698e-15 -4.013898e-15
-1.646778e-15
E:\\R-3.1.2\\scripts\\mPDBS\\summary_pdb_93\\b99f4d9d87c1017b.final.pdb
1.176859e-15 -4.533359e-16 -2.228465e-15 -5.578940e-15 2.982245e-15
5.244846e-16
E:\\R-3.1.2\\scripts\\mPDBS\\summary_pdb_93\\b9d2cbf3975403e7.final.pdb
1.176859e-15 -4.533359e-16 -2.228465e-15 -5.578940e-15 2.982245e-15
5.244846e-16
E:\\R-3.1.2\\scripts\\mPDBS\\summary_pdb_93\\bc88c8a505f3a288.final.pdb
2.209857e-16 -1.930887e-15 3.085683e-16 -2.259005e-15 -1.967171e-16
-3.103511e-16
E:\\R-3.1.2\\scripts\\mPDBS\\summary_pdb_93\\bdac0e64f4ef427e.final.pdb
-5.167978e-15 -3.371895e-15 1.194001e-14 7.376311e-15 -3.595179e-15
1.248168e-14
E:\\R-3.1.2\\scripts\\mPDBS\\summary_pdb_93\\ca2a1f55fc9d0681.final.pdb
-3.571083e-15 -2.202691e-15 6.845558e-15 2.534698e-15 -4.013898e-15
-1.646778e-15
E:\\R-3.1.2\\scripts\\mPDBS\\summary_pdb_93\\ce34248de3e81a3c.final.pdb
2.932270e-16 -2.432091e-15 3.114935e-15 9.138711e-16 1.042614e-14
-1.243626e-15
E:\\R-3.1.2\\scripts\\mPDBS\\summary_pdb_93\\d02e697772494a61.final.pdb
-3.715034e-15 -5.882587e-15 1.151271e-14 1.369416e-14 -1.517126e-15
5.849218e-15
E:\\R-3.1.2\\scripts\\mPDBS\\summary_pdb_93\\d04221245d015047.final.pdb
-2.416441e-15 -9.891329e-16 -8.020826e-16 -2.495552e-15 1.086236e-16
-6.809187e-16
E:\\R-3.1.2\\scripts\\mPDBS\\summary_pdb_93\\d678141e4018054a.final.pdb
7.286640e-16 -6.990779e-15 9.883179e-15 4.085216e-15 -1.162881e-14
9.301956e-16
E:\\R-3.1.2\\scripts\\mPDBS\\summary_pdb_93\\d71b40af0f0efe39.final.pdb
-7.152160e-15 2.875102e-16 -5.667475e-15 -6.974430e-15 2.282569e-15
8.451962e-16
E:\\R-3.1.2\\scripts\\mPDBS\\summary_pdb_93\\dc0809eff10197e2.final.pdb
-4.486062e-16 3.564172e-15 -6.658028e-15 -6.042759e-15 1.450365e-14
-4.660154e-15
E:\\R-3.1.2\\scripts\\mPDBS\\summary_pdb_93\\e1163918d3707839.final.pdb
3.339439e-15 1.434790e-16 -1.025358e-14 -4.260056e-15 3.965557e-15
-2.607023e-15
E:\\R-3.1.2\\scripts\\mPDBS\\summary_pdb_93\\e4b48b06824ef848.final.pdb
2.311534e-15 -5.404588e-15 5.488636e-15 1.993311e-15 -6.532884e-15
-3.954635e-15
E:\\R-3.1.2\\scripts\\mPDBS\\summary_pdb_93\\e529e19ad281fea2.final.pdb
1.993575e-15 -2.637406e-15 -2.820683e-15 -8.631575e-15 3.851054e-15
2.554400e-15
E:\\R-3.1.2\\scripts\\mPDBS\\summary_pdb_93\\e5602082718ec3a1.final.pdb
1.069302e-15 3.356230e-15 -1.050081e-14 -7.467718e-15 5.932139e-15
-3.073588e-15
E:\\R-3.1.2\\scripts\\mPDBS\\summary_pdb_93\\e5d589d996d0a960.final.pdb
-1.039878e-15 -4.184979e-15 -1.195691e-14 -7.023791e-16 9.200840e-15

-3.591437e-15
E:\\R-3.1.2\\scripts\\mPDBS\\summary_pdb_93\\e9df9e72307a2fbd.final.pdb
2.785026e-15 -1.680792e-15 5.138190e-16 1.537733e-15 2.487118e-15
-5.306804e-15
E:\\R-3.1.2\\scripts\\mPDBS\\summary_pdb_93\\ea00f8694090cae7.final.pdb
1.069302e-15 3.356230e-15 -1.050081e-14 -7.467718e-15 5.932139e-15
-3.073588e-15
E:\\R-3.1.2\\scripts\\mPDBS\\summary_pdb_93\\eba517be32db050e.final.pdb
1.176859e-15 -4.533359e-16 -2.228465e-15 -5.578940e-15 2.982245e-15
5.244846e-16
E:\\R-3.1.2\\scripts\\mPDBS\\summary_pdb_93\\ec04b9545899c29d.final.pdb
7.124739e-17 -2.100819e-15 1.002609e-15 -2.025406e-15 6.093685e-16
-7.504354e-16
E:\\R-3.1.2\\scripts\\mPDBS\\summary_pdb_93\\ee6862cbcad52078.final.pdb
4.135807e-16 -2.349822e-15 -2.625894e-16 -2.125973e-15 1.169155e-16
-6.069888e-16
E:\\R-3.1.2\\scripts\\mPDBS\\summary_pdb_93\\ef6a42fc83b81e5d.final.pdb
-1.847876e-16 -1.864451e-15 1.309254e-15 -2.305117e-15 2.714195e-16
-7.878973e-16
E:\\R-3.1.2\\scripts\\mPDBS\\summary_pdb_93\\ef8e88d4ad93a0ad.final.pdb
-2.416441e-15 -9.891329e-16 -8.020826e-16 -2.495552e-15 1.086236e-16
-6.809187e-16
E:\\R-3.1.2\\scripts\\mPDBS\\summary_pdb_93\\f14fcae8deec4fa3.final.pdb
-5.299610e-17 -1.617280e-15 -5.311832e-16 -4.168217e-15 -1.192803e-15
1.009128e-15
E:\\R-3.1.2\\scripts\\mPDBS\\summary_pdb_93\\f83584e22003fb92.final.pdb
-7.530531e-15 -4.422817e-15 -6.248253e-15 -3.674431e-15 -3.304244e-15
-5.548349e-15
E:\\R-3.1.2\\scripts\\mPDBS\\summary_pdb_93\\f8e2bf87e52caa29.final.pdb
1.993575e-15 -2.637406e-15 -2.820683e-15 -8.631575e-15 3.851054e-15
2.554400e-15

[,307]   [,308]   [,309]   [,310]   [,311]   [,312]
E:\\R-3.1.2\\scripts\\40.1ns_data\\NAT0.pdb
1.677986e-15 -2.031532e-15 -1.640750e-15 -5.766136e-16 7.054827e-16
4.076707e-15
E:\\R-3.1.2\\scripts\\40.1ns_data\\LJP6.pdb
3.026929e-15 -1.875297e-15 -1.324503e-17 -2.915430e-15 -6.544817e-16
1.810309e-15
E:\\R-3.1.2\\scripts\\40.1ns_data\\3WY9.pdb
2.889347e-15 -1.159361e-15 -1.776836e-15 -2.432970e-15 -2.097165e-15
3.048159e-15
E:\\R-3.1.2\\scripts\\40.1ns_data\\ZSP9.pdb
2.665908e-15 -1.110514e-15 -1.534805e-15 -2.738140e-15 2.514053e-16
2.171465e-15
E:\\R-3.1.2\\scripts\\mPDBS\\summary_pdb_93\\013d855ad26c877c.final.pdb
6.891432e-15 -3.578437e-15 -2.999269e-15 1.720922e-15 7.956751e-15
3.739181e-15
E:\\R-3.1.2\\scripts\\mPDBS\\summary_pdb_93\\0a2a705342ca0d54.final.pdb
4.413548e-15 -1.871678e-15 -2.152924e-15 -2.065968e-15 8.303382e-16
2.346216e-15
E:\\R-3.1.2\\scripts\\mPDBS\\summary_pdb_93\\0a59fceadb4194d9.final.pdb
-4.428864e-16 -2.665069e-15 -4.755747e-15 -1.900480e-15 4.594944e-15
2.916125e-15

E:\\R-3.1.2\\scripts\\mPDBS\\summary_pdb_93\\0dba59f3306825e8.final.pdb
2.783606e-15 -3.083174e-15 -2.267216e-15 -2.168328e-15 -2.555674e-15
5.295228e-15
E:\\R-3.1.2\\scripts\\mPDBS\\summary_pdb_93\\126f26a78b57339c.final.pdb
5.733504e-15 1.284426e-15 6.336145e-15 -6.732819e-15 -4.020143e-16
2.080099e-17
E:\\R-3.1.2\\scripts\\mPDBS\\summary_pdb_93\\12f06f3e342df706.final.pdb
4.746615e-15 -1.510422e-15 -1.622966e-15 -1.792098e-15 8.893188e-16
2.086875e-15
E:\\R-3.1.2\\scripts\\mPDBS\\summary_pdb_93\\18618a2a251b867b.final.pdb
4.704953e-15 1.778926e-15 9.210642e-15 -3.365209e-15 1.164981e-15
5.438860e-15
E:\\R-3.1.2\\scripts\\mPDBS\\summary_pdb_93\\1f305f598641eeb2.final.pdb
3.562548e-15 -1.296614e-15 -1.197343e-15 -1.753178e-15 7.570614e-16
2.023134e-15
E:\\R-3.1.2\\scripts\\mPDBS\\summary_pdb_93\\1f8a00b551be0a39.final.pdb
4.704953e-15 1.778926e-15 9.210642e-15 -3.365209e-15 1.164981e-15
5.438860e-15
E:\\R-3.1.2\\scripts\\mPDBS\\summary_pdb_93\\219f45328d47579a.final.pdb
1.134716e-14 2.520887e-15 -1.333571e-15 1.962749e-15 -1.119971e-14
-4.539717e-15
E:\\R-3.1.2\\scripts\\mPDBS\\summary_pdb_93\\238dd3918e54336d.final.pdb
7.814831e-15 -1.325429e-15 8.442463e-15 1.662425e-15 -1.006737e-14
2.338462e-15
E:\\R-3.1.2\\scripts\\mPDBS\\summary_pdb_93\\2b3027889e2fdc42.final.pdb
3.885249e-15 -9.901933e-16 -1.281963e-15 -2.203623e-15 1.311979e-15
2.552700e-15
E:\\R-3.1.2\\scripts\\mPDBS\\summary_pdb_93\\2fcb3b46e33793b0.final.pdb
3.111520e-15 -2.226426e-15 -5.077052e-15 -2.880748e-15 1.551565e-15
1.512258e-15
E:\\R-3.1.2\\scripts\\mPDBS\\summary_pdb_93\\34eb328251842006.final.pdb
-1.029041e-16 -1.385618e-15 -9.652242e-15 3.588237e-15 7.700790e-16
7.450620e-15
E:\\R-3.1.2\\scripts\\mPDBS\\summary_pdb_93\\354b32871797b7ab.final.pdb
-5.116921e-15 3.719193e-15 -1.627454e-14 6.720895e-15 1.698546e-14
1.715426e-15
E:\\R-3.1.2\\scripts\\mPDBS\\summary_pdb_93\\397368adbc32d5de.final.pdb
5.733504e-15 1.284426e-15 6.336145e-15 -6.732819e-15 -4.020143e-16
2.080099e-17
E:\\R-3.1.2\\scripts\\mPDBS\\summary_pdb_93\\411ca13cd3df6edd.final.pdb
5.872333e-15 6.475768e-16 -4.208823e-15 -1.019044e-14 8.932372e-16
8.764814e-16
E:\\R-3.1.2\\scripts\\mPDBS\\summary_pdb_93\\42357eee6642bf12.final.pdb
-6.217403e-15 -3.179930e-15 -6.432308e-15 1.349446e-15 1.303662e-15
6.171729e-15
E:\\R-3.1.2\\scripts\\mPDBS\\summary_pdb_93\\44342d1f75a7fcfc.final.pdb
1.229458e-14 -1.400060e-15 1.788795e-15 -4.984286e-15 1.341995e-14
1.745658e-15
E:\\R-3.1.2\\scripts\\mPDBS\\summary_pdb_93\\448ef21dffe8cdfb.final.pdb
-4.757185e-15 1.081783e-14 5.849104e-15 -3.588515e-15 1.397912e-14
4.983116e-16
E:\\R-3.1.2\\scripts\\mPDBS\\summary_pdb_93\\472a40b3e8f113c7.final.pdb
2.783606e-15 -3.083174e-15 -2.267216e-15 -2.168328e-15 -2.555674e-15
5.295228e-15

E:\\R-3.1.2\\scripts\\mPDBS\\summary_pdb_93\\48a26225951ea153.final.pdb
1.166895e-14 -2.973416e-15 5.698131e-15 -3.064913e-15 1.023366e-14
4.467458e-16
E:\\R-3.1.2\\scripts\\mPDBS\\summary_pdb_93\\4ab4a5c7c6682ce6.final.pdb
-4.463201e-15 7.608361e-15 -7.802719e-15 -3.730013e-15 -5.894149e-15
-3.828550e-15
E:\\R-3.1.2\\scripts\\mPDBS\\summary_pdb_93\\4eaa45b62cb6a25f.final.pdb
6.558202e-15 -3.147710e-15 4.269787e-15 -5.656140e-15 -2.153309e-15
1.029343e-15
E:\\R-3.1.2\\scripts\\mPDBS\\summary_pdb_93\\58c371611d85ac96.final.pdb
5.733504e-15 1.284426e-15 6.336145e-15 -6.732819e-15 -4.020143e-16
2.080099e-17
E:\\R-3.1.2\\scripts\\mPDBS\\summary_pdb_93\\59a647f04822d30a.final.pdb
-2.228521e-15 -1.612564e-15 -1.770423e-15 -2.398171e-16 -4.175355e-15
2.824656e-15
E:\\R-3.1.2\\scripts\\mPDBS\\summary_pdb_93\\59b091fe385e05fc.final.pdb
-4.428864e-16 -2.665069e-15 -4.755747e-15 -1.900480e-15 4.594944e-15
2.916125e-15
E:\\R-3.1.2\\scripts\\mPDBS\\summary_pdb_93\\5c35ac30f930d2db.final.pdb
6.558202e-15 -3.147710e-15 4.269787e-15 -5.656140e-15 -2.153309e-15
1.029343e-15
E:\\R-3.1.2\\scripts\\mPDBS\\summary_pdb_93\\5f60752431cf2dcb.final.pdb
3.827211e-15 -3.092056e-15 2.706037e-15 -4.373584e-15 -8.329869e-15
5.272695e-15
E:\\R-3.1.2\\scripts\\mPDBS\\summary_pdb_93\\626456dd6ff2868d.final.pdb
2.783606e-15 -3.083174e-15 -2.267216e-15 -2.168328e-15 -2.555674e-15
5.295228e-15
E:\\R-3.1.2\\scripts\\mPDBS\\summary_pdb_93\\6583d53ff3c8f7ef.final.pdb
-2.228521e-15 -1.612564e-15 -1.770423e-15 -2.398171e-16 -4.175355e-15
2.824656e-15
E:\\R-3.1.2\\scripts\\mPDBS\\summary_pdb_93\\69760178986bef01.final.pdb
-1.364253e-15 3.831475e-15 5.788151e-15 -1.493220e-15 2.658930e-15
-2.344482e-15
E:\\R-3.1.2\\scripts\\mPDBS\\summary_pdb_93\\6a9d87d47926b1c8.final.pdb
2.923252e-15 -1.106890e-15 -7.935685e-16 -1.951921e-15 1.735165e-15
2.310636e-15
E:\\R-3.1.2\\scripts\\mPDBS\\summary_pdb_93\\6bb3547ea982ffc4.final.pdb
2.279234e-14 8.068385e-15 5.834935e-15 -4.829745e-15 -4.811890e-15
4.654974e-15
E:\\R-3.1.2\\scripts\\mPDBS\\summary_pdb_93\\716d9624611d1c42.final.pdb
5.290450e-15 1.456389e-15 -2.198460e-15 -8.317880e-17 1.026897e-14
-1.968041e-15
E:\\R-3.1.2\\scripts\\mPDBS\\summary_pdb_93\\75f27e70ad0f1be4.final.pdb
-2.228521e-15 -1.612564e-15 -1.770423e-15 -2.398171e-16 -4.175355e-15
2.824656e-15
E:\\R-3.1.2\\scripts\\mPDBS\\summary_pdb_93\\769f910fffb22872.final.pdb
4.381861e-15 -1.199160e-15 -1.195964e-15 -1.821955e-15 1.488073e-15
1.781195e-15
E:\\R-3.1.2\\scripts\\mPDBS\\summary_pdb_93\\791f8c80dd5c3135.final.pdb
-4.428864e-16 -2.665069e-15 -4.755747e-15 -1.900480e-15 4.594944e-15
2.916125e-15
E:\\R-3.1.2\\scripts\\mPDBS\\summary_pdb_93\\7fad98e66a3f6099.final.pdb
9.195179e-15 -2.114635e-15 -1.012960e-14 2.154276e-15 5.818142e-15
1.439607e-15

E:\\R-3.1.2\\scripts\\mPDBS\\summary_pdb_93\\804b89c94a633196.final.pdb
3.111520e-15 -2.226426e-15 -5.077052e-15 -2.880748e-15 1.551565e-15
1.512258e-15
E:\\R-3.1.2\\scripts\\mPDBS\\summary_pdb_93\\813cc65a2298feba.final.pdb
3.111520e-15 -2.226426e-15 -5.077052e-15 -2.880748e-15 1.551565e-15
1.512258e-15
E:\\R-3.1.2\\scripts\\mPDBS\\summary_pdb_93\\83c0eefcfe12d3f9.final.pdb
5.872333e-15 6.475768e-16 -4.208823e-15 -1.019044e-14 8.932372e-16
8.764814e-16
E:\\R-3.1.2\\scripts\\mPDBS\\summary_pdb_93\\86d50469ef569760.final.pdb
2.783606e-15 -3.083174e-15 -2.267216e-15 -2.168328e-15 -2.555674e-15
5.295228e-15
E:\\R-3.1.2\\scripts\\mPDBS\\summary_pdb_93\\89d0666e1402203d.final.pdb
-2.228521e-15 -1.612564e-15 -1.770423e-15 -2.398171e-16 -4.175355e-15
2.824656e-15
E:\\R-3.1.2\\scripts\\mPDBS\\summary_pdb_93\\8b34852716dadc62.final.pdb
2.167831e-15 -7.760544e-19 1.245166e-16 -4.025665e-15 -4.742012e-15
-4.818071e-16
E:\\R-3.1.2\\scripts\\mPDBS\\summary_pdb_93\\8b6ef9ac3fe74395.final.pdb
1.055743e-14 -1.592600e-15 5.932617e-15 -4.536675e-15 -3.254176e-15
4.336156e-15
E:\\R-3.1.2\\scripts\\mPDBS\\summary_pdb_93\\98bbe0d5986c138f.final.pdb
-4.428864e-16 -2.665069e-15 -4.755747e-15 -1.900480e-15 4.594944e-15
2.916125e-15
E:\\R-3.1.2\\scripts\\mPDBS\\summary_pdb_93\\9c22f4909d67e2fa.final.pdb
1.355980e-15 -7.380290e-15 3.066608e-15 7.730556e-15 7.614857e-15
4.851600e-15
E:\\R-3.1.2\\scripts\\mPDBS\\summary_pdb_93\\9dda8ed0d81fb3ef.final.pdb
1.341912e-14 -3.126110e-15 1.435773e-14 -8.829084e-15 9.432683e-15
3.240990e-15
E:\\R-3.1.2\\scripts\\mPDBS\\summary_pdb_93\\9f752b3616e6033b.final.pdb
4.517684e-15 -1.135592e-15 -1.641285e-15 -1.560545e-15 2.992011e-17
2.711535e-15
E:\\R-3.1.2\\scripts\\mPDBS\\summary_pdb_93\\a02c70083816d6ba.final.pdb
6.558202e-15 -3.147710e-15 4.269787e-15 -5.656140e-15 -2.153309e-15
1.029343e-15
E:\\R-3.1.2\\scripts\\mPDBS\\summary_pdb_93\\a0c997d891e4e082.final.pdb
8.976702e-15 6.732911e-16 -2.577931e-15 1.769280e-15 6.340919e-15
-3.173052e-15
E:\\R-3.1.2\\scripts\\mPDBS\\summary_pdb_93\\ab8039a2d823c616.final.pdb
3.554390e-15 -1.142964e-15 -7.013938e-16 -1.698650e-15 1.495402e-15
1.626928e-15
E:\\R-3.1.2\\scripts\\mPDBS\\summary_pdb_93\\acf4d74b9c71d4dc.final.pdb
3.111520e-15 -2.226426e-15 -5.077052e-15 -2.880748e-15 1.551565e-15
1.512258e-15
E:\\R-3.1.2\\scripts\\mPDBS\\summary_pdb_93\\ad8a823e4b4c668e.final.pdb
3.794038e-15 -1.821619e-15 -1.849608e-15 -1.525512e-15 1.437670e-15
1.982608e-15
E:\\R-3.1.2\\scripts\\mPDBS\\summary_pdb_93\\b16ababd03b7b857.final.pdb
1.355980e-15 -7.380290e-15 3.066608e-15 7.730556e-15 7.614857e-15
4.851600e-15
E:\\R-3.1.2\\scripts\\mPDBS\\summary_pdb_93\\b226923e697d3c63.final.pdb
4.415903e-15 3.755105e-15 3.000500e-15 1.291727e-15 1.621657e-15
1.926069e-15

E:\\R-3.1.2\\scripts\\mPDBS\\summary_pdb_93\\b28e48f6fa27a165.final.pdb
-4.428864e-16 -2.665069e-15 -4.755747e-15 -1.900480e-15 4.594944e-15
2.916125e-15
E:\\R-3.1.2\\scripts\\mPDBS\\summary_pdb_93\\b42c4924e3cd1aec.final.pdb
6.510582e-15 -8.177191e-15 -1.022156e-14 4.041697e-15 2.763791e-16
1.773414e-15
E:\\R-3.1.2\\scripts\\mPDBS\\summary_pdb_93\\b4dd239fcabff1df.final.pdb
-4.428864e-16 -2.665069e-15 -4.755747e-15 -1.900480e-15 4.594944e-15
2.916125e-15
E:\\R-3.1.2\\scripts\\mPDBS\\summary_pdb_93\\b669942ef97459a2.final.pdb
2.167831e-15 -7.760544e-19 1.245166e-16 -4.025665e-15 -4.742012e-15
-4.818071e-16
E:\\R-3.1.2\\scripts\\mPDBS\\summary_pdb_93\\b99f4d9d87c1017b.final.pdb
-4.428864e-16 -2.665069e-15 -4.755747e-15 -1.900480e-15 4.594944e-15
2.916125e-15
E:\\R-3.1.2\\scripts\\mPDBS\\summary_pdb_93\\b9d2cbf3975403e7.final.pdb
-4.428864e-16 -2.665069e-15 -4.755747e-15 -1.900480e-15 4.594944e-15
2.916125e-15
E:\\R-3.1.2\\scripts\\mPDBS\\summary_pdb_93\\bc88c8a505f3a288.final.pdb
2.883446e-15 -1.237825e-15 -1.260712e-15 -2.271277e-15 1.479814e-15
2.321982e-15
E:\\R-3.1.2\\scripts\\mPDBS\\summary_pdb_93\\bdac0e64f4ef427e.final.pdb
-3.726718e-16 -5.771334e-15 6.243754e-15 1.250191e-14 1.894606e-15
3.658110e-15
E:\\R-3.1.2\\scripts\\mPDBS\\summary_pdb_93\\ca2a1f55fc9d0681.final.pdb
2.167831e-15 -7.760544e-19 1.245166e-16 -4.025665e-15 -4.742012e-15
-4.818071e-16
E:\\R-3.1.2\\scripts\\mPDBS\\summary_pdb_93\\ce34248de3e81a3c.final.pdb
-2.467522e-15 -9.986646e-15 -6.238324e-15 -1.878868e-15 4.015019e-15
5.721724e-16
E:\\R-3.1.2\\scripts\\mPDBS\\summary_pdb_93\\d02e697772494a61.final.pdb
-9.136887e-15 7.408904e-15 -6.059825e-15 -1.357729e-15 -6.271798e-15
1.745186e-15
E:\\R-3.1.2\\scripts\\mPDBS\\summary_pdb_93\\d04221245d015047.final.pdb
3.075173e-15 -3.117080e-15 -2.000268e-15 -3.484331e-15 7.516397e-16
2.039792e-15
E:\\R-3.1.2\\scripts\\mPDBS\\summary_pdb_93\\d678141e4018054a.final.pdb
2.002902e-14 5.184879e-15 -1.428711e-16 -1.489906e-15 -4.397168e-15
5.291111e-15
E:\\R-3.1.2\\scripts\\mPDBS\\summary_pdb_93\\d71b40af0f0efe39.final.pdb
3.640268e-15 2.817470e-15 -1.638511e-15 -5.063971e-15 4.271180e-15
1.582493e-15
E:\\R-3.1.2\\scripts\\mPDBS\\summary_pdb_93\\dc0809eff10197e2.final.pdb
-3.508306e-15 -5.523964e-15 -1.367103e-15 -3.661236e-15 -1.020339e-15
9.263290e-15
E:\\R-3.1.2\\scripts\\mPDBS\\summary_pdb_93\\e1163918d3707839.final.pdb
1.035646e-14 -4.280135e-15 -6.667548e-15 -7.667686e-15 2.514172e-15
-2.300584e-15
E:\\R-3.1.2\\scripts\\mPDBS\\summary_pdb_93\\e4b48b06824ef848.final.pdb
4.704953e-15 1.778926e-15 9.210642e-15 -3.365209e-15 1.164981e-15
5.438860e-15
E:\\R-3.1.2\\scripts\\mPDBS\\summary_pdb_93\\e529e19ad281fea2.final.pdb
6.891432e-15 -3.578437e-15 -2.999269e-15 1.720922e-15 7.956751e-15
3.739181e-15

E:\\R-3.1.2\\scripts\\mPDBS\\summary_pdb_93\\e5602082718ec3a1.final.pdb
3.111520e-15 -2.226426e-15 -5.077052e-15 -2.880748e-15 1.551565e-15
1.512258e-15
E:\\R-3.1.2\\scripts\\mPDBS\\summary_pdb_93\\e5d589d996d0a960.final.pdb
1.030699e-14 -4.390303e-15 -1.937929e-15 -4.698946e-15 -1.057077e-15
-5.516051e-15
E:\\R-3.1.2\\scripts\\mPDBS\\summary_pdb_93\\e9df9e72307a2fbd.final.pdb
6.558202e-15 -3.147710e-15 4.269787e-15 -5.656140e-15 -2.153309e-15
1.029343e-15
E:\\R-3.1.2\\scripts\\mPDBS\\summary_pdb_93\\ea00f8694090cae7.final.pdb
3.111520e-15 -2.226426e-15 -5.077052e-15 -2.880748e-15 1.551565e-15
1.512258e-15
E:\\R-3.1.2\\scripts\\mPDBS\\summary_pdb_93\\eba517be32db050e.final.pdb
-4.428864e-16 -2.665069e-15 -4.755747e-15 -1.900480e-15 4.594944e-15
2.916125e-15
E:\\R-3.1.2\\scripts\\mPDBS\\summary_pdb_93\\ec04b9545899c29d.final.pdb
3.188153e-15 -1.282099e-15 -1.442994e-15 -1.823620e-15 1.080857e-15
2.070052e-15
E:\\R-3.1.2\\scripts\\mPDBS\\summary_pdb_93\\ee6862cbcad52078.final.pdb
3.016152e-15 -1.122032e-15 -1.036933e-15 -2.367988e-15 1.099476e-15
2.657651e-15
E:\\R-3.1.2\\scripts\\mPDBS\\summary_pdb_93\\ef6a42fc83b81e5d.final.pdb
4.022339e-15 -1.258575e-15 -9.648122e-16 -2.080212e-15 1.061765e-15
1.679714e-15
E:\\R-3.1.2\\scripts\\mPDBS\\summary_pdb_93\\ef8e88d4ad93a0ad.final.pdb
3.075173e-15 -3.117080e-15 -2.000268e-15 -3.484331e-15 7.516397e-16
2.039792e-15
E:\\R-3.1.2\\scripts\\mPDBS\\summary_pdb_93\\f14fcae8deec4fa3.final.pdb
4.517684e-15 -1.135592e-15 -1.641285e-15 -1.560545e-15 2.992011e-17
2.711535e-15
E:\\R-3.1.2\\scripts\\mPDBS\\summary_pdb_93\\f83584e22003fb92.final.pdb
1.166895e-14 -2.973416e-15 5.698131e-15 -3.064913e-15 1.023366e-14
4.467458e-16
E:\\R-3.1.2\\scripts\\mPDBS\\summary_pdb_93\\f8e2bf87e52caa29.final.pdb
6.891432e-15 -3.578437e-15 -2.999269e-15 1.720922e-15 7.956751e-15
3.739181e-15

[,313]   [,314]   [,315]   [,316]   [,317]   [,318]
E:\\R-3.1.2\\scripts\\40.1ns_data\\NAT0.pdb
-9.069796e-16 -3.647812e-15 1.222678e-15 4.766130e-15 -3.871517e-15
-3.104430e-15
E:\\R-3.1.2\\scripts\\40.1ns_data\\LJP6.pdb
-3.873900e-16 -3.172672e-15 2.512873e-15 3.829188e-15 -1.221721e-15
-3.221972e-15
E:\\R-3.1.2\\scripts\\40.1ns_data\\3WY9.pdb
-1.435864e-15 -2.593414e-15 1.936826e-15 5.284643e-15 -3.325983e-15
-2.292371e-15
E:\\R-3.1.2\\scripts\\40.1ns_data\\ZSP9.pdb
-2.393537e-15 -3.615599e-15 3.270428e-15 6.887379e-15 -1.679813e-15
-1.959025e-15
E:\\R-3.1.2\\scripts\\mPDBS\\summary_pdb_93\\013d855ad26c877c.final.pdb
-3.628344e-15 -6.080195e-15 2.698818e-15 8.869666e-15 1.737778e-15
-5.730117e-15
E:\\R-3.1.2\\scripts\\mPDBS\\summary_pdb_93\\0a2a705342ca0d54.final.pdb

-3.226258e-16 -2.372366e-15 2.491758e-15 4.793473e-15 -1.633525e-15
-2.612357e-15
E:\\R-3.1.2\\scripts\\mPDBS\\summary_pdb_93\\0a59fceadb4194d9.final.pdb
-5.609907e-16 -1.129007e-15 6.334313e-15 2.586441e-16 -6.944518e-16
-2.881062e-15
E:\\R-3.1.2\\scripts\\mPDBS\\summary_pdb_93\\0dba59f3306825e8.final.pdb
4.285464e-15 -9.141886e-16 1.904315e-15 8.276390e-15 -4.947846e-15
-4.175805e-15
E:\\R-3.1.2\\scripts\\mPDBS\\summary_pdb_93\\126f26a78b57339c.final.pdb
5.469847e-15 -1.611114e-15 3.062243e-15 7.966525e-15 -4.430696e-15
-1.807908e-15
E:\\R-3.1.2\\scripts\\mPDBS\\summary_pdb_93\\12f06f3e342df706.final.pdb
-3.516824e-16 -2.008508e-15 2.401119e-15 5.223576e-15 -2.968910e-15
-2.008240e-15
E:\\R-3.1.2\\scripts\\mPDBS\\summary_pdb_93\\18618a2a251b867b.final.pdb
4.108664e-15 -9.246423e-16 3.170128e-15 1.030809e-14 -5.081387e-15
-8.603183e-16
E:\\R-3.1.2\\scripts\\mPDBS\\summary_pdb_93\\1f305f598641eeb2.final.pdb
-4.653503e-16 -2.101557e-15 2.193526e-15 5.373729e-15 -2.838697e-15
-2.533832e-15
E:\\R-3.1.2\\scripts\\mPDBS\\summary_pdb_93\\1f8a00b551be0a39.final.pdb
4.108664e-15 -9.246423e-16 3.170128e-15 1.030809e-14 -5.081387e-15
-8.603183e-16
E:\\R-3.1.2\\scripts\\mPDBS\\summary_pdb_93\\219f45328d47579a.final.pdb
-5.394364e-15 -3.277462e-15 -5.471348e-15 2.896291e-15 -7.659367e-15
3.998852e-15
E:\\R-3.1.2\\scripts\\mPDBS\\summary_pdb_93\\238dd3918e54336d.final.pdb
4.974078e-15 2.187784e-15 -1.348344e-15 1.038975e-14 -9.732266e-15
9.561676e-15
E:\\R-3.1.2\\scripts\\mPDBS\\summary_pdb_93\\2b3027889e2fdc42.final.pdb
6.203560e-16 -2.244000e-15 1.884314e-15 6.668664e-15 -2.448067e-15
-2.484244e-15
E:\\R-3.1.2\\scripts\\mPDBS\\summary_pdb_93\\2fcb3b46e33793b0.final.pdb
-2.442068e-15 -2.857897e-15 7.224803e-16 4.629532e-15 -4.032186e-15
-5.137651e-15
E:\\R-3.1.2\\scripts\\mPDBS\\summary_pdb_93\\34eb328251842006.final.pdb
-4.014065e-15 -8.264395e-15 2.674005e-15 3.459257e-15 1.670060e-15
-1.564959e-15
E:\\R-3.1.2\\scripts\\mPDBS\\summary_pdb_93\\354b32871797b7ab.final.pdb
-4.334173e-15 2.738526e-15 3.307587e-15 -7.709669e-15 4.729284e-15
-1.983186e-15
E:\\R-3.1.2\\scripts\\mPDBS\\summary_pdb_93\\397368adbc32d5de.final.pdb
5.469847e-15 -1.611114e-15 3.062243e-15 7.966525e-15 -4.430696e-15
-1.807908e-15
E:\\R-3.1.2\\scripts\\mPDBS\\summary_pdb_93\\411ca13cd3df6edd.final.pdb
1.571649e-15 -7.052892e-15 -7.754534e-16 5.765126e-15 -3.730548e-15
-7.393659e-15
E:\\R-3.1.2\\scripts\\mPDBS\\summary_pdb_93\\42357eee6642bf12.final.pdb
-1.002193e-16 -2.659556e-15 2.584592e-15 -1.094187e-14 -4.086958e-16
4.568109e-15
E:\\R-3.1.2\\scripts\\mPDBS\\summary_pdb_93\\44342d1f75a7fcfc.final.pdb
-2.002762e-15 -1.084720e-14 6.038531e-15 1.020136e-14 -4.524310e-15
-8.374233e-15
E:\\R-3.1.2\\scripts\\mPDBS\\summary_pdb_93\\448ef21dffe8cdfb.final.pdb

-6.601067e-15 -1.313993e-15 3.411302e-15 -5.256657e-15 1.444956e-14
5.818862e-15
E:\\R-3.1.2\\scripts\\mPDBS\\summary_pdb_93\\472a40b3e8f113c7.final.pdb
4.285464e-15 -9.141886e-16 1.904315e-15 8.276390e-15 -4.947846e-15
-4.175805e-15
E:\\R-3.1.2\\scripts\\mPDBS\\summary_pdb_93\\48a26225951ea153.final.pdb
5.952679e-15 -1.530907e-14 -1.996263e-15 1.624737e-14 -3.204291e-16
-8.219239e-15
E:\\R-3.1.2\\scripts\\mPDBS\\summary_pdb_93\\4ab4a5c7c6682ce6.final.pdb
-5.274813e-15 2.409975e-15 -1.704777e-15 -1.858949e-15 -2.963120e-15
7.204518e-16
E:\\R-3.1.2\\scripts\\mPDBS\\summary_pdb_93\\4eaa45b62cb6a25f.final.pdb
3.629943e-15 -2.257478e-15 3.018321e-15 1.160268e-14 -4.999140e-15
-3.864060e-15
E:\\R-3.1.2\\scripts\\mPDBS\\summary_pdb_93\\58c371611d85ac96.final.pdb
5.469847e-15 -1.611114e-15 3.062243e-15 7.966525e-15 -4.430696e-15
-1.807908e-15
E:\\R-3.1.2\\scripts\\mPDBS\\summary_pdb_93\\59a647f04822d30a.final.pdb
1.517425e-15 -4.340249e-15 -5.246713e-15 4.567842e-15 1.051091e-15
-1.631363e-15
E:\\R-3.1.2\\scripts\\mPDBS\\summary_pdb_93\\59b091fe385e05fc.final.pdb
-5.609907e-16 -1.129007e-15 6.334313e-15 2.586441e-16 -6.944518e-16
-2.881062e-15
E:\\R-3.1.2\\scripts\\mPDBS\\summary_pdb_93\\5c35ac30f930d2db.final.pdb
3.629943e-15 -2.257478e-15 3.018321e-15 1.160268e-14 -4.999140e-15
-3.864060e-15
E:\\R-3.1.2\\scripts\\mPDBS\\summary_pdb_93\\5f60752431cf2dcb.final.pdb
7.224072e-15 -7.010583e-15 4.388245e-15 6.986814e-15 -2.856315e-15
-5.480289e-15
E:\\R-3.1.2\\scripts\\mPDBS\\summary_pdb_93\\626456dd6ff2868d.final.pdb
4.285464e-15 -9.141886e-16 1.904315e-15 8.276390e-15 -4.947846e-15
-4.175805e-15
E:\\R-3.1.2\\scripts\\mPDBS\\summary_pdb_93\\6583d53ff3c8f7ef.final.pdb
1.517425e-15 -4.340249e-15 -5.246713e-15 4.567842e-15 1.051091e-15
-1.631363e-15
E:\\R-3.1.2\\scripts\\mPDBS\\summary_pdb_93\\69760178986bef01.final.pdb
1.322169e-15 -2.034460e-16 7.358087e-16 1.426864e-14 -1.174534e-14
-1.424396e-15
E:\\R-3.1.2\\scripts\\mPDBS\\summary_pdb_93\\6a9d87d47926b1c8.final.pdb
9.673721e-16 -2.957259e-15 2.812443e-15 5.777304e-15 -2.800056e-15
-2.706494e-15
E:\\R-3.1.2\\scripts\\mPDBS\\summary_pdb_93\\6bb3547ea982ffc4.final.pdb
9.094870e-15 -1.154081e-14 -5.566970e-15 2.639768e-14 2.497298e-15
-9.817493e-16
E:\\R-3.1.2\\scripts\\mPDBS\\summary_pdb_93\\716d9624611d1c42.final.pdb
-3.756511e-15 8.524851e-16 1.942718e-15 8.952473e-15 -4.631127e-15
-1.056310e-15
E:\\R-3.1.2\\scripts\\mPDBS\\summary_pdb_93\\75f27e70ad0f1be4.final.pdb
1.517425e-15 -4.340249e-15 -5.246713e-15 4.567842e-15 1.051091e-15
-1.631363e-15
E:\\R-3.1.2\\scripts\\mPDBS\\summary_pdb_93\\769f910fffb22872.final.pdb
2.792624e-16 -1.570393e-15 2.327052e-15 5.801390e-15 -3.531506e-15
-2.212535e-15
E:\\R-3.1.2\\scripts\\mPDBS\\summary_pdb_93\\791f8c80dd5c3135.final.pdb

-5.609907e-16 -1.129007e-15 6.334313e-15 2.586441e-16 -6.944518e-16
-2.881062e-15
E:\\R-3.1.2\\scripts\\mPDBS\\summary_pdb_93\\7fad98e66a3f6099.final.pdb
1.140176e-15 -1.085251e-14 2.534991e-15 -4.005238e-16 -3.169541e-15
1.312864e-15
E:\\R-3.1.2\\scripts\\mPDBS\\summary_pdb_93\\804b89c94a633196.final.pdb
-2.442068e-15 -2.857897e-15 7.224803e-16 4.629532e-15 -4.032186e-15
-5.137651e-15
E:\\R-3.1.2\\scripts\\mPDBS\\summary_pdb_93\\813cc65a2298feba.final.pdb
-2.442068e-15 -2.857897e-15 7.224803e-16 4.629532e-15 -4.032186e-15
-5.137651e-15
E:\\R-3.1.2\\scripts\\mPDBS\\summary_pdb_93\\83c0eefcfe12d3f9.final.pdb
1.571649e-15 -7.052892e-15 -7.754534e-16 5.765126e-15 -3.730548e-15
-7.393659e-15
E:\\R-3.1.2\\scripts\\mPDBS\\summary_pdb_93\\86d50469ef569760.final.pdb
4.285464e-15 -9.141886e-16 1.904315e-15 8.276390e-15 -4.947846e-15
-4.175805e-15
E:\\R-3.1.2\\scripts\\mPDBS\\summary_pdb_93\\89d0666e1402203d.final.pdb
1.517425e-15 -4.340249e-15 -5.246713e-15 4.567842e-15 1.051091e-15
-1.631363e-15
E:\\R-3.1.2\\scripts\\mPDBS\\summary_pdb_93\\8b34852716dadc62.final.pdb
4.551470e-15 -2.094618e-15 1.874337e-15 3.045730e-15 -6.058343e-15
-1.503215e-16
E:\\R-3.1.2\\scripts\\mPDBS\\summary_pdb_93\\8b6ef9ac3fe74395.final.pdb
5.068836e-15 -6.234612e-15 5.802643e-16 1.285428e-14 1.384979e-16
-2.701741e-16
E:\\R-3.1.2\\scripts\\mPDBS\\summary_pdb_93\\98bbe0d5986c138f.final.pdb
-5.609907e-16 -1.129007e-15 6.334313e-15 2.586441e-16 -6.944518e-16
-2.881062e-15
E:\\R-3.1.2\\scripts\\mPDBS\\summary_pdb_93\\9c22f4909d67e2fa.final.pdb
-1.251276e-16 2.688014e-15 7.935894e-15 5.116773e-15 -1.411114e-15
-9.495953e-16
E:\\R-3.1.2\\scripts\\mPDBS\\summary_pdb_93\\9dda8ed0d81fb3ef.final.pdb
9.874889e-15 -1.196696e-14 3.499329e-15 1.446422e-14 -6.215462e-15
-4.952925e-15
E:\\R-3.1.2\\scripts\\mPDBS\\summary_pdb_93\\9f752b3616e6033b.final.pdb
-9.921951e-16 -2.715358e-15 1.883214e-16 4.098395e-15 -2.199868e-15
-1.180146e-15
E:\\R-3.1.2\\scripts\\mPDBS\\summary_pdb_93\\a02c70083816d6ba.final.pdb
3.629943e-15 -2.257478e-15 3.018321e-15 1.160268e-14 -4.999140e-15
-3.864060e-15
E:\\R-3.1.2\\scripts\\mPDBS\\summary_pdb_93\\a0c997d891e4e082.final.pdb
-3.835405e-15 -3.507477e-15 1.080156e-15 2.699181e-15 -3.511991e-15
-7.019970e-15
E:\\R-3.1.2\\scripts\\mPDBS\\summary_pdb_93\\ab8039a2d823c616.final.pdb
6.586132e-16 -1.687752e-15 3.090267e-15 4.526604e-15 -2.783312e-15
-2.681860e-15
E:\\R-3.1.2\\scripts\\mPDBS\\summary_pdb_93\\acf4d74b9c71d4dc.final.pdb
-2.442068e-15 -2.857897e-15 7.224803e-16 4.629532e-15 -4.032186e-15
-5.137651e-15
E:\\R-3.1.2\\scripts\\mPDBS\\summary_pdb_93\\ad8a823e4b4c668e.final.pdb
-2.146744e-16 -2.931586e-15 2.012735e-15 5.029099e-15 -2.753664e-15
-2.190191e-15
E:\\R-3.1.2\\scripts\\mPDBS\\summary_pdb_93\\b16ababd03b7b857.final.pdb

-1.251276e-16 2.688014e-15 7.935894e-15 5.116773e-15 -1.411114e-15
-9.495953e-16
E:\\R-3.1.2\\scripts\\mPDBS\\summary_pdb_93\\b226923e697d3c63.final.pdb
-1.441363e-15 -2.729241e-17 4.254368e-15 -1.142543e-15 -7.501001e-15
-1.653047e-15
E:\\R-3.1.2\\scripts\\mPDBS\\summary_pdb_93\\b28e48f6fa27a165.final.pdb
-5.609907e-16 -1.129007e-15 6.334313e-15 2.586441e-16 -6.944518e-16
-2.881062e-15
E:\\R-3.1.2\\scripts\\mPDBS\\summary_pdb_93\\b42c4924e3cd1aec.final.pdb
-3.345005e-15 6.781064e-15 3.200957e-15 5.213701e-15 -3.138361e-15
-9.902958e-15
E:\\R-3.1.2\\scripts\\mPDBS\\summary_pdb_93\\b4dd239fcabff1df.final.pdb
-5.609907e-16 -1.129007e-15 6.334313e-15 2.586441e-16 -6.944518e-16
-2.881062e-15
E:\\R-3.1.2\\scripts\\mPDBS\\summary_pdb_93\\b669942ef97459a2.final.pdb
4.551470e-15 -2.094618e-15 1.874337e-15 3.045730e-15 -6.058343e-15
-1.503215e-16
E:\\R-3.1.2\\scripts\\mPDBS\\summary_pdb_93\\b99f4d9d87c1017b.final.pdb
-5.609907e-16 -1.129007e-15 6.334313e-15 2.586441e-16 -6.944518e-16
-2.881062e-15
E:\\R-3.1.2\\scripts\\mPDBS\\summary_pdb_93\\b9d2cbf3975403e7.final.pdb
-5.609907e-16 -1.129007e-15 6.334313e-15 2.586441e-16 -6.944518e-16
-2.881062e-15
E:\\R-3.1.2\\scripts\\mPDBS\\summary_pdb_93\\bc88c8a505f3a288.final.pdb
1.011319e-15 -2.261348e-15 2.479324e-15 6.026274e-15 -2.602756e-15
-2.358476e-15
E:\\R-3.1.2\\scripts\\mPDBS\\summary_pdb_93\\bdac0e64f4ef427e.final.pdb
-3.568337e-15 9.334607e-15 9.054357e-15 4.514508e-16 2.450177e-15
7.298581e-15
E:\\R-3.1.2\\scripts\\mPDBS\\summary_pdb_93\\ca2a1f55fc9d0681.final.pdb
4.551470e-15 -2.094618e-15 1.874337e-15 3.045730e-15 -6.058343e-15
-1.503215e-16
E:\\R-3.1.2\\scripts\\mPDBS\\summary_pdb_93\\ce34248de3e81a3c.final.pdb
1.486854e-15 -2.100758e-15 7.685758e-15 2.179148e-15 -2.428896e-15
-4.848423e-15
E:\\R-3.1.2\\scripts\\mPDBS\\summary_pdb_93\\d02e697772494a61.final.pdb
-5.898735e-15 -1.821161e-15 4.974021e-16 -2.631947e-15 1.784563e-15
7.664756e-15
E:\\R-3.1.2\\scripts\\mPDBS\\summary_pdb_93\\d04221245d015047.final.pdb
3.591565e-16 -2.702565e-15 1.635268e-15 7.495691e-15 -9.584782e-16
-2.658875e-15
E:\\R-3.1.2\\scripts\\mPDBS\\summary_pdb_93\\d678141e4018054a.final.pdb
5.540964e-17 -6.391403e-15 -1.819050e-15 9.402293e-15 -2.938333e-15
-1.693766e-15
E:\\R-3.1.2\\scripts\\mPDBS\\summary_pdb_93\\d71b40af0f0efe39.final.pdb
-5.388272e-15 4.870491e-15 6.586841e-15 -7.184451e-15 -1.031215e-15
-2.029237e-15
E:\\R-3.1.2\\scripts\\mPDBS\\summary_pdb_93\\dc0809eff10197e2.final.pdb
-1.446224e-15 5.081117e-15 -6.746373e-16 1.081079e-14 -4.332616e-15
-8.854587e-15
E:\\R-3.1.2\\scripts\\mPDBS\\summary_pdb_93\\e1163918d3707839.final.pdb
-8.509060e-15 1.991492e-15 4.659859e-15 6.332164e-15 -8.901593e-16
-1.059338e-14
E:\\R-3.1.2\\scripts\\mPDBS\\summary_pdb_93\\e4b48b06824ef848.final.pdb

4.108664e-15 -9.246423e-16 3.170128e-15 1.030809e-14 -5.081387e-15
-8.603183e-16
E:\\R-3.1.2\\scripts\\mPDBS\\summary_pdb_93\\e529e19ad281fea2.final.pdb
-3.628344e-15 -6.080195e-15 2.698818e-15 8.869666e-15 1.737778e-15
-5.730117e-15
E:\\R-3.1.2\\scripts\\mPDBS\\summary_pdb_93\\e5602082718ec3a1.final.pdb
-2.442068e-15 -2.857897e-15 7.224803e-16 4.629532e-15 -4.032186e-15
-5.137651e-15
E:\\R-3.1.2\\scripts\\mPDBS\\summary_pdb_93\\e5d589d996d0a960.final.pdb
7.604629e-16 -3.571914e-15 9.143515e-15 1.190339e-14 -1.236799e-16
-3.749289e-15
E:\\R-3.1.2\\scripts\\mPDBS\\summary_pdb_93\\e9df9e72307a2fbd.final.pdb
3.629943e-15 -2.257478e-15 3.018321e-15 1.160268e-14 -4.999140e-15
-3.864060e-15
E:\\R-3.1.2\\scripts\\mPDBS\\summary_pdb_93\\ea00f8694090cae7.final.pdb
-2.442068e-15 -2.857897e-15 7.224803e-16 4.629532e-15 -4.032186e-15
-5.137651e-15
E:\\R-3.1.2\\scripts\\mPDBS\\summary_pdb_93\\eba517be32db050e.final.pdb
-5.609907e-16 -1.129007e-15 6.334313e-15 2.586441e-16 -6.944518e-16
-2.881062e-15
E:\\R-3.1.2\\scripts\\mPDBS\\summary_pdb_93\\ec04b9545899c29d.final.pdb
6.643749e-16 -2.760783e-15 1.982747e-15 5.406581e-15 -2.078350e-15
-2.867354e-15
E:\\R-3.1.2\\scripts\\mPDBS\\summary_pdb_93\\ee6862cbcad52078.final.pdb
5.891310e-16 -2.336374e-15 2.470216e-15 5.819300e-15 -2.713670e-15
-2.470799e-15
E:\\R-3.1.2\\scripts\\mPDBS\\summary_pdb_93\\ef6a42fc83b81e5d.final.pdb
1.237878e-16 -2.017084e-15 2.516571e-15 5.153579e-15 -2.522859e-15
-2.371696e-15
E:\\R-3.1.2\\scripts\\mPDBS\\summary_pdb_93\\ef8e88d4ad93a0ad.final.pdb
3.591565e-16 -2.702565e-15 1.635268e-15 7.495691e-15 -9.584782e-16
-2.658875e-15
E:\\R-3.1.2\\scripts\\mPDBS\\summary_pdb_93\\f14fcae8deec4fa3.final.pdb
-9.921951e-16 -2.715358e-15 1.883214e-16 4.098395e-15 -2.199868e-15
-1.180146e-15
E:\\R-3.1.2\\scripts\\mPDBS\\summary_pdb_93\\f83584e22003fb92.final.pdb
5.952679e-15 -1.530907e-14 -1.996263e-15 1.624737e-14 -3.204291e-16
-8.219239e-15
E:\\R-3.1.2\\scripts\\mPDBS\\summary_pdb_93\\f8e2bf87e52caa29.final.pdb
-3.628344e-15 -6.080195e-15 2.698818e-15 8.869666e-15 1.737778e-15
-5.730117e-15

[,319]   [,320]   [,321]   [,322]   [,323]   [,324]
E:\\R-3.1.2\\scripts\\40.1ns_data\\NAT0.pdb
3.064788e-15 1.092381e-16 1.305187e-15 3.866342e-15 -3.042695e-15
-1.605949e-15
E:\\R-3.1.2\\scripts\\40.1ns_data\\LJP6.pdb
2.514944e-15 6.590715e-16 1.183432e-15 2.776624e-15 -6.520026e-16
-1.935384e-15
E:\\R-3.1.2\\scripts\\40.1ns_data\\3WY9.pdb
2.754733e-15 1.321280e-15 1.743533e-15 1.825715e-15 -1.296138e-15
1.395320e-18
E:\\R-3.1.2\\scripts\\40.1ns_data\\ZSP9.pdb
4.380174e-15 1.380712e-15 9.795954e-16 6.935742e-16 -2.158030e-15

-1.791895e-15
E:\\R-3.1.2\\scripts\\mPDBS\\summary_pdb_93\\013d855ad26c877c.final.pdb
4.705107e-15 6.305881e-15 -1.532657e-16 4.776989e-16 -2.929314e-15
-3.151715e-15
E:\\R-3.1.2\\scripts\\mPDBS\\summary_pdb_93\\0a2a705342ca0d54.final.pdb
2.327738e-15 2.473800e-16 8.768544e-16 2.326925e-15 -1.903335e-15
-1.972174e-15
E:\\R-3.1.2\\scripts\\mPDBS\\summary_pdb_93\\0a59fceadb4194d9.final.pdb
4.853416e-15 -5.914584e-16 1.186637e-15 2.259672e-15 -2.451338e-15
-2.344014e-15
E:\\R-3.1.2\\scripts\\mPDBS\\summary_pdb_93\\0dba59f3306825e8.final.pdb
-1.387242e-15 -6.172852e-15 4.044479e-16 2.609674e-15 -8.771366e-16
-9.876470e-16
E:\\R-3.1.2\\scripts\\mPDBS\\summary_pdb_93\\126f26a78b57339c.final.pdb
5.435056e-16 1.307764e-15 3.890375e-15 2.837357e-15 -1.229285e-15
3.022166e-15
E:\\R-3.1.2\\scripts\\mPDBS\\summary_pdb_93\\12f06f3e342df706.final.pdb
3.188161e-15 -3.643269e-16 1.424593e-15 2.812864e-15 -1.846089e-15
-1.827758e-15
E:\\R-3.1.2\\scripts\\mPDBS\\summary_pdb_93\\18618a2a251b867b.final.pdb
-2.215564e-15 5.169506e-15 1.527123e-15 -1.274800e-15 -4.921987e-16
-2.309456e-15
E:\\R-3.1.2\\scripts\\mPDBS\\summary_pdb_93\\1f305f598641eeb2.final.pdb
2.397656e-15 9.687616e-17 1.305677e-15 2.965880e-15 -1.920071e-15
-1.592921e-15
E:\\R-3.1.2\\scripts\\mPDBS\\summary_pdb_93\\1f8a00b551be0a39.final.pdb
-2.215564e-15 5.169506e-15 1.527123e-15 -1.274800e-15 -4.921987e-16
-2.309456e-15
E:\\R-3.1.2\\scripts\\mPDBS\\summary_pdb_93\\219f45328d47579a.final.pdb
-2.031702e-15 1.282910e-15 7.763841e-15 9.781867e-15 5.096010e-15
-6.641791e-15
E:\\R-3.1.2\\scripts\\mPDBS\\summary_pdb_93\\238dd3918e54336d.final.pdb
-6.659076e-15 -1.490208e-15 3.350271e-15 3.252585e-15 1.227863e-14
-7.838751e-15
E:\\R-3.1.2\\scripts\\mPDBS\\summary_pdb_93\\2b3027889e2fdc42.final.pdb
2.442150e-15 -7.613713e-17 1.288118e-15 2.505569e-15 -1.896660e-15
-1.637982e-15
E:\\R-3.1.2\\scripts\\mPDBS\\summary_pdb_93\\2fcb3b46e33793b0.final.pdb
1.488660e-15 -5.241548e-16 6.260995e-16 4.026664e-15 -3.849517e-15
-1.428122e-15
E:\\R-3.1.2\\scripts\\mPDBS\\summary_pdb_93\\34eb328251842006.final.pdb
1.505230e-14 9.558106e-15 -3.830700e-15 -2.164086e-15 4.554552e-15
-8.783663e-15
E:\\R-3.1.2\\scripts\\mPDBS\\summary_pdb_93\\354b32871797b7ab.final.pdb
1.788383e-14 1.042074e-14 -5.263742e-15 7.304073e-15 -3.538818e-15
-7.433519e-15
E:\\R-3.1.2\\scripts\\mPDBS\\summary_pdb_93\\397368adbc32d5de.final.pdb
5.435056e-16 1.307764e-15 3.890375e-15 2.837357e-15 -1.229285e-15
3.022166e-15
E:\\R-3.1.2\\scripts\\mPDBS\\summary_pdb_93\\411ca13cd3df6edd.final.pdb
1.986526e-15 5.451480e-15 2.496645e-16 1.994869e-15 -1.856320e-15
2.700519e-15
E:\\R-3.1.2\\scripts\\mPDBS\\summary_pdb_93\\42357eee6642bf12.final.pdb
9.469644e-15 8.079799e-15 3.049850e-15 2.883167e-16 2.690041e-15

-1.301625e-14
E:\\R-3.1.2\\scripts\\mPDBS\\summary_pdb_93\\44342d1f75a7fcfc.final.pdb
9.845627e-15 4.404223e-15 1.779254e-15 -1.727847e-15 -4.391977e-15
-2.805650e-15
E:\\R-3.1.2\\scripts\\mPDBS\\summary_pdb_93\\448ef21dffe8cdfb.final.pdb
1.019697e-14 2.143484e-14 4.220880e-15 -1.450726e-15 2.375104e-15
-1.459347e-15
E:\\R-3.1.2\\scripts\\mPDBS\\summary_pdb_93\\472a40b3e8f113c7.final.pdb
-1.387242e-15 -6.172852e-15 4.044479e-16 2.609674e-15 -8.771366e-16
-9.876470e-16
E:\\R-3.1.2\\scripts\\mPDBS\\summary_pdb_93\\48a26225951ea153.final.pdb
6.022620e-15 5.846209e-15 -6.725437e-15 -6.273413e-16 -7.821174e-15
3.736192e-15
E:\\R-3.1.2\\scripts\\mPDBS\\summary_pdb_93\\4ab4a5c7c6682ce6.final.pdb
7.121577e-15 6.954063e-16 -4.858269e-15 5.190509e-15 -4.468448e-15
-2.860281e-15
E:\\R-3.1.2\\scripts\\mPDBS\\summary_pdb_93\\4eaa45b62cb6a25f.final.pdb
-4.137612e-15 -4.905811e-15 1.392392e-15 1.661668e-15 -4.640367e-15
3.336512e-15
E:\\R-3.1.2\\scripts\\mPDBS\\summary_pdb_93\\58c371611d85ac96.final.pdb
5.435056e-16 1.307764e-15 3.890375e-15 2.837357e-15 -1.229285e-15
3.022166e-15
E:\\R-3.1.2\\scripts\\mPDBS\\summary_pdb_93\\59a647f04822d30a.final.pdb
2.944897e-15 -4.690490e-16 -1.883614e-15 1.479657e-15 -8.322427e-15
-6.441848e-16
E:\\R-3.1.2\\scripts\\mPDBS\\summary_pdb_93\\59b091fe385e05fc.final.pdb
4.853416e-15 -5.914584e-16 1.186637e-15 2.259672e-15 -2.451338e-15
-2.344014e-15
E:\\R-3.1.2\\scripts\\mPDBS\\summary_pdb_93\\5c35ac30f930d2db.final.pdb
-4.137612e-15 -4.905811e-15 1.392392e-15 1.661668e-15 -4.640367e-15
3.336512e-15
E:\\R-3.1.2\\scripts\\mPDBS\\summary_pdb_93\\5f60752431cf2dcb.final.pdb
-4.142780e-15 -2.759655e-15 1.467094e-15 -2.998242e-15 -3.754719e-15
2.914542e-15
E:\\R-3.1.2\\scripts\\mPDBS\\summary_pdb_93\\626456dd6ff2868d.final.pdb
-1.387242e-15 -6.172852e-15 4.044479e-16 2.609674e-15 -8.771366e-16
-9.876470e-16
E:\\R-3.1.2\\scripts\\mPDBS\\summary_pdb_93\\6583d53ff3c8f7ef.final.pdb
2.944897e-15 -4.690490e-16 -1.883614e-15 1.479657e-15 -8.322427e-15
-6.441848e-16
E:\\R-3.1.2\\scripts\\mPDBS\\summary_pdb_93\\69760178986bef01.final.pdb
-8.167075e-15 -4.835960e-15 3.986019e-15 4.607290e-15 2.631460e-15
3.904529e-15
E:\\R-3.1.2\\scripts\\mPDBS\\summary_pdb_93\\6a9d87d47926b1c8.final.pdb
2.826402e-15 2.409087e-16 1.315178e-15 2.490412e-15 -1.558016e-15
-1.359745e-15
E:\\R-3.1.2\\scripts\\mPDBS\\summary_pdb_93\\6bb3547ea982ffc4.final.pdb
1.788146e-15 1.138952e-14 4.694240e-15 3.828600e-15 1.189792e-14
-8.231423e-15
E:\\R-3.1.2\\scripts\\mPDBS\\summary_pdb_93\\716d9624611d1c42.final.pdb
8.813869e-15 1.727099e-15 -2.646369e-15 3.601079e-15 3.287391e-15
-5.493663e-15
E:\\R-3.1.2\\scripts\\mPDBS\\summary_pdb_93\\75f27e70ad0f1be4.final.pdb
2.944897e-15 -4.690490e-16 -1.883614e-15 1.479657e-15 -8.322427e-15

-6.441848e-16
E:\\R-3.1.2\\scripts\\mPDBS\\summary_pdb_93\\769f910fffb22872.final.pdb
2.996412e-15 7.070928e-16 1.200127e-15 2.384816e-15 -2.311056e-15
-1.531433e-15
E:\\R-3.1.2\\scripts\\mPDBS\\summary_pdb_93\\791f8c80dd5c3135.final.pdb
4.853416e-15 -5.914584e-16 1.186637e-15 2.259672e-15 -2.451338e-15
-2.344014e-15
E:\\R-3.1.2\\scripts\\mPDBS\\summary_pdb_93\\7fad98e66a3f6099.final.pdb
1.242367e-14 -2.078649e-16 1.927084e-15 1.057683e-14 4.254099e-15
-5.004258e-15
E:\\R-3.1.2\\scripts\\mPDBS\\summary_pdb_93\\804b89c94a633196.final.pdb
1.488660e-15 -5.241548e-16 6.260995e-16 4.026664e-15 -3.849517e-15
-1.428122e-15
E:\\R-3.1.2\\scripts\\mPDBS\\summary_pdb_93\\813cc65a2298feba.final.pdb
1.488660e-15 -5.241548e-16 6.260995e-16 4.026664e-15 -3.849517e-15
-1.428122e-15
E:\\R-3.1.2\\scripts\\mPDBS\\summary_pdb_93\\83c0eefcfe12d3f9.final.pdb
1.986526e-15 5.451480e-15 2.496645e-16 1.994869e-15 -1.856320e-15
2.700519e-15
E:\\R-3.1.2\\scripts\\mPDBS\\summary_pdb_93\\86d50469ef569760.final.pdb
-1.387242e-15 -6.172852e-15 4.044479e-16 2.609674e-15 -8.771366e-16
-9.876470e-16
E:\\R-3.1.2\\scripts\\mPDBS\\summary_pdb_93\\89d0666e1402203d.final.pdb
2.944897e-15 -4.690490e-16 -1.883614e-15 1.479657e-15 -8.322427e-15
-6.441848e-16
E:\\R-3.1.2\\scripts\\mPDBS\\summary_pdb_93\\8b34852716dadc62.final.pdb
2.085405e-15 -4.791575e-15 3.033462e-15 3.944264e-15 -9.347484e-16
-3.577981e-16
E:\\R-3.1.2\\scripts\\mPDBS\\summary_pdb_93\\8b6ef9ac3fe74395.final.pdb
5.894158e-15 -1.709293e-16 1.133696e-14 9.342054e-15 -3.533464e-15
-1.014103e-14
E:\\R-3.1.2\\scripts\\mPDBS\\summary_pdb_93\\98bbe0d5986c138f.final.pdb
4.853416e-15 -5.914584e-16 1.186637e-15 2.259672e-15 -2.451338e-15
-2.344014e-15
E:\\R-3.1.2\\scripts\\mPDBS\\summary_pdb_93\\9c22f4909d67e2fa.final.pdb
-1.034495e-15 -5.230731e-15 9.093729e-17 -1.147800e-15 -7.215710e-17
-1.163577e-15
E:\\R-3.1.2\\scripts\\mPDBS\\summary_pdb_93\\9dda8ed0d81fb3ef.final.pdb
-2.521986e-16 7.249251e-15 -5.003515e-15 -2.290331e-15 -3.888474e-15
4.105271e-16
E:\\R-3.1.2\\scripts\\mPDBS\\summary_pdb_93\\9f752b3616e6033b.final.pdb
1.988389e-15 3.897840e-16 4.096422e-15 3.489443e-15 -1.233379e-15
-3.775813e-15
E:\\R-3.1.2\\scripts\\mPDBS\\summary_pdb_93\\a02c70083816d6ba.final.pdb
-4.137612e-15 -4.905811e-15 1.392392e-15 1.661668e-15 -4.640367e-15
3.336512e-15
E:\\R-3.1.2\\scripts\\mPDBS\\summary_pdb_93\\a0c997d891e4e082.final.pdb
-8.833143e-16 -1.738874e-15 -3.555063e-15 6.990788e-15 -2.001789e-15
2.557196e-15
E:\\R-3.1.2\\scripts\\mPDBS\\summary_pdb_93\\ab8039a2d823c616.final.pdb
2.004133e-15 -3.161300e-16 8.246797e-16 1.469813e-15 -1.980539e-15
-2.602739e-15
E:\\R-3.1.2\\scripts\\mPDBS\\summary_pdb_93\\acf4d74b9c71d4dc.final.pdb
1.488660e-15 -5.241548e-16 6.260995e-16 4.026664e-15 -3.849517e-15

-1.428122e-15
E:\\R-3.1.2\\scripts\\mPDBS\\summary_pdb_93\\ad8a823e4b4c668e.final.pdb
3.318818e-15 -2.379781e-16 1.230186e-15 2.268154e-15 -2.101859e-15
-1.809238e-15
E:\\R-3.1.2\\scripts\\mPDBS\\summary_pdb_93\\b16ababd03b7b857.final.pdb
-1.034495e-15 -5.230731e-15 9.093729e-17 -1.147800e-15 -7.215710e-17
-1.163577e-15
E:\\R-3.1.2\\scripts\\mPDBS\\summary_pdb_93\\b226923e697d3c63.final.pdb
2.985761e-16 2.436450e-15 6.489029e-15 5.177870e-15 4.311564e-15
-6.055655e-15
E:\\R-3.1.2\\scripts\\mPDBS\\summary_pdb_93\\b28e48f6fa27a165.final.pdb
4.853416e-15 -5.914584e-16 1.186637e-15 2.259672e-15 -2.451338e-15
-2.344014e-15
E:\\R-3.1.2\\scripts\\mPDBS\\summary_pdb_93\\b42c4924e3cd1aec.final.pdb
3.709042e-15 -8.110343e-15 -3.488177e-16 7.642479e-15 -1.433406e-15
6.698683e-16
E:\\R-3.1.2\\scripts\\mPDBS\\summary_pdb_93\\b4dd239fcabff1df.final.pdb
4.853416e-15 -5.914584e-16 1.186637e-15 2.259672e-15 -2.451338e-15
-2.344014e-15
E:\\R-3.1.2\\scripts\\mPDBS\\summary_pdb_93\\b669942ef97459a2.final.pdb
2.085405e-15 -4.791575e-15 3.033462e-15 3.944264e-15 -9.347484e-16
-3.577981e-16
E:\\R-3.1.2\\scripts\\mPDBS\\summary_pdb_93\\b99f4d9d87c1017b.final.pdb
4.853416e-15 -5.914584e-16 1.186637e-15 2.259672e-15 -2.451338e-15
-2.344014e-15
E:\\R-3.1.2\\scripts\\mPDBS\\summary_pdb_93\\b9d2cbf3975403e7.final.pdb
4.853416e-15 -5.914584e-16 1.186637e-15 2.259672e-15 -2.451338e-15
-2.344014e-15
E:\\R-3.1.2\\scripts\\mPDBS\\summary_pdb_93\\bc88c8a505f3a288.final.pdb
2.231381e-15 -2.638396e-16 1.209189e-15 2.395631e-15 -1.482061e-15
-1.731657e-15
E:\\R-3.1.2\\scripts\\mPDBS\\summary_pdb_93\\bdac0e64f4ef427e.final.pdb
5.916542e-15 -9.805684e-15 2.500902e-15 6.427304e-15 -1.744431e-15
-1.212529e-14
E:\\R-3.1.2\\scripts\\mPDBS\\summary_pdb_93\\ca2a1f55fc9d0681.final.pdb
2.085405e-15 -4.791575e-15 3.033462e-15 3.944264e-15 -9.347484e-16
-3.577981e-16
E:\\R-3.1.2\\scripts\\mPDBS\\summary_pdb_93\\ce34248de3e81a3c.final.pdb
7.242014e-15 -2.431205e-15 -5.346125e-15 2.524463e-15 -1.246696e-14
2.150747e-15
E:\\R-3.1.2\\scripts\\mPDBS\\summary_pdb_93\\d02e697772494a61.final.pdb
3.516811e-15 6.930859e-17 8.764343e-15 1.392961e-15 6.074135e-16
-6.287908e-15
E:\\R-3.1.2\\scripts\\mPDBS\\summary_pdb_93\\d04221245d015047.final.pdb
3.247062e-15 -8.186035e-16 -6.129724e-16 1.827817e-15 -4.081722e-15
-1.524418e-16
E:\\R-3.1.2\\scripts\\mPDBS\\summary_pdb_93\\d678141e4018054a.final.pdb
6.892611e-15 1.231366e-14 1.117992e-16 -6.099297e-15 1.479702e-14
-1.005308e-14
E:\\R-3.1.2\\scripts\\mPDBS\\summary_pdb_93\\d71b40af0f0efe39.final.pdb
-7.341839e-16 -4.578306e-15 5.067898e-15 -6.534467e-16 1.118037e-15
1.123836e-15
E:\\R-3.1.2\\scripts\\mPDBS\\summary_pdb_93\\dc0809eff10197e2.final.pdb
-1.162616e-15 -1.288155e-14 2.145546e-15 3.564907e-15 -1.401970e-14

1.823549e-15
E:\\R-3.1.2\\scripts\\mPDBS\\summary_pdb_93\\e1163918d3707839.final.pdb
2.286569e-15 -5.692572e-15 3.595510e-15 6.704861e-15 2.605464e-15
6.447365e-15
E:\\R-3.1.2\\scripts\\mPDBS\\summary_pdb_93\\e4b48b06824ef848.final.pdb
-2.215564e-15 5.169506e-15 1.527123e-15 -1.274800e-15 -4.921987e-16
-2.309456e-15
E:\\R-3.1.2\\scripts\\mPDBS\\summary_pdb_93\\e529e19ad281fea2.final.pdb
4.705107e-15 6.305881e-15 -1.532657e-16 4.776989e-16 -2.929314e-15
-3.151715e-15
E:\\R-3.1.2\\scripts\\mPDBS\\summary_pdb_93\\e5602082718ec3a1.final.pdb
1.488660e-15 -5.241548e-16 6.260995e-16 4.026664e-15 -3.849517e-15
-1.428122e-15
E:\\R-3.1.2\\scripts\\mPDBS\\summary_pdb_93\\e5d589d996d0a960.final.pdb
2.052409e-15 -3.666092e-15 5.116392e-15 8.241137e-15 -5.841712e-15
2.982287e-16
E:\\R-3.1.2\\scripts\\mPDBS\\summary_pdb_93\\e9df9e72307a2fbd.final.pdb
-4.137612e-15 -4.905811e-15 1.392392e-15 1.661668e-15 -4.640367e-15
3.336512e-15
E:\\R-3.1.2\\scripts\\mPDBS\\summary_pdb_93\\ea00f8694090cae7.final.pdb
1.488660e-15 -5.241548e-16 6.260995e-16 4.026664e-15 -3.849517e-15
-1.428122e-15
E:\\R-3.1.2\\scripts\\mPDBS\\summary_pdb_93\\eba517be32db050e.final.pdb
4.853416e-15 -5.914584e-16 1.186637e-15 2.259672e-15 -2.451338e-15
-2.344014e-15
E:\\R-3.1.2\\scripts\\mPDBS\\summary_pdb_93\\ec04b9545899c29d.final.pdb
1.839878e-15 -9.001196e-17 1.116590e-15 1.856525e-15 -2.165460e-15
-2.144721e-15
E:\\R-3.1.2\\scripts\\mPDBS\\summary_pdb_93\\ee6862cbcad52078.final.pdb
2.209697e-15 2.393473e-17 1.089493e-15 2.520748e-15 -1.718851e-15
-1.770688e-15
E:\\R-3.1.2\\scripts\\mPDBS\\summary_pdb_93\\ef6a42fc83b81e5d.final.pdb
2.801256e-15 -8.638061e-17 9.603016e-16 2.443580e-15 -1.755077e-15
-2.037105e-15
E:\\R-3.1.2\\scripts\\mPDBS\\summary_pdb_93\\ef8e88d4ad93a0ad.final.pdb
3.247062e-15 -8.186035e-16 -6.129724e-16 1.827817e-15 -4.081722e-15
-1.524418e-16
E:\\R-3.1.2\\scripts\\mPDBS\\summary_pdb_93\\f14fcae8deec4fa3.final.pdb
1.988389e-15 3.897840e-16 4.096422e-15 3.489443e-15 -1.233379e-15
-3.775813e-15
E:\\R-3.1.2\\scripts\\mPDBS\\summary_pdb_93\\f83584e22003fb92.final.pdb
6.022620e-15 5.846209e-15 -6.725437e-15 -6.273413e-16 -7.821174e-15
3.736192e-15
E:\\R-3.1.2\\scripts\\mPDBS\\summary_pdb_93\\f8e2bf87e52caa29.final.pdb
4.705107e-15 6.305881e-15 -1.532657e-16 4.776989e-16 -2.929314e-15
-3.151715e-15

[,325]   [,326]   [,327]   [,328]   [,329]   [,330]
E:\\R-3.1.2\\scripts\\40.1ns_data\\NAT0.pdb
-3.505140e-17 5.824961e-16 1.509201e-15 3.362865e-15 6.425219e-16
2.192034e-15
E:\\R-3.1.2\\scripts\\40.1ns_data\\LJP6.pdb
-1.332322e-15 -4.381804e-16 1.546128e-15 4.106159e-15 5.048053e-17
1.465252e-15

E:\\R-3.1.2\\scripts\\40.1ns_data\\3WY9.pdb
7.169514e-16 8.406279e-16 1.896975e-15 4.933572e-15 -2.380282e-15
1.573708e-15
E:\\R-3.1.2\\scripts\\40.1ns_data\\ZSP9.pdb
-1.080220e-15 7.733239e-16 1.616576e-15 3.983577e-15 -1.185557e-15
1.202467e-15
E:\\R-3.1.2\\scripts\\mPDBS\\summary_pdb_93\\013d855ad26c877c.final.pdb
1.219351e-15 -3.459694e-15 5.075000e-15 7.911795e-15 2.378156e-15
2.095660e-15
E:\\R-3.1.2\\scripts\\mPDBS\\summary_pdb_93\\0a2a705342ca0d54.final.pdb
-1.869964e-15 -4.623240e-16 -2.471361e-17 4.800724e-15 1.620171e-15
1.149312e-15
E:\\R-3.1.2\\scripts\\mPDBS\\summary_pdb_93\\0a59fceadb4194d9.final.pdb
3.731954e-16 6.079961e-16 -9.220951e-16 4.159586e-15 4.773881e-16
3.288006e-15
E:\\R-3.1.2\\scripts\\mPDBS\\summary_pdb_93\\0dba59f3306825e8.final.pdb
1.916710e-15 1.768112e-15 3.055782e-15 3.916727e-15 -2.477335e-15
4.388964e-15
E:\\R-3.1.2\\scripts\\mPDBS\\summary_pdb_93\\126f26a78b57339c.final.pdb
-4.416549e-15 -4.715309e-15 -3.735877e-15 2.732345e-15 -1.512395e-15
-3.057770e-15
E:\\R-3.1.2\\scripts\\mPDBS\\summary_pdb_93\\12f06f3e342df706.final.pdb
-5.614408e-16 -8.663791e-16 6.005458e-16 3.872430e-15 9.305104e-16
1.128495e-15
E:\\R-3.1.2\\scripts\\mPDBS\\summary_pdb_93\\18618a2a251b867b.final.pdb
-3.985631e-15 -8.475483e-16 1.805239e-15 -2.458180e-15 6.631134e-16
-4.044802e-15
E:\\R-3.1.2\\scripts\\mPDBS\\summary_pdb_93\\1f305f598641eeb2.final.pdb
-1.629882e-16 -1.069678e-16 8.191163e-16 4.526034e-15 5.755111e-16
1.050473e-15
E:\\R-3.1.2\\scripts\\mPDBS\\summary_pdb_93\\1f8a00b551be0a39.final.pdb
-3.985631e-15 -8.475483e-16 1.805239e-15 -2.458180e-15 6.631134e-16
-4.044802e-15
E:\\R-3.1.2\\scripts\\mPDBS\\summary_pdb_93\\219f45328d47579a.final.pdb
-4.376478e-15 3.463785e-15 3.909110e-15 1.733221e-14 -4.985690e-15
-8.228389e-15
E:\\R-3.1.2\\scripts\\mPDBS\\summary_pdb_93\\238dd3918e54336d.final.pdb
-1.009228e-14 8.934936e-16 1.334672e-15 2.466713e-15 1.913089e-15
-6.187921e-15
E:\\R-3.1.2\\scripts\\mPDBS\\summary_pdb_93\\2b3027889e2fdc42.final.pdb
-8.851297e-16 -6.480521e-16 5.678155e-16 4.747441e-15 -1.341418e-18
8.728374e-16
E:\\R-3.1.2\\scripts\\mPDBS\\summary_pdb_93\\2fcb3b46e33793b0.final.pdb
1.790310e-15 2.127790e-15 3.528754e-15 7.376184e-15 2.561769e-15
4.192057e-15
E:\\R-3.1.2\\scripts\\mPDBS\\summary_pdb_93\\34eb328251842006.final.pdb
5.344479e-15 8.915146e-15 -3.745080e-15 3.104869e-15 6.019968e-15
7.238233e-15
E:\\R-3.1.2\\scripts\\mPDBS\\summary_pdb_93\\354b32871797b7ab.final.pdb
-8.233077e-16 4.666906e-15 2.898341e-16 -3.493009e-15 2.772349e-15
1.621131e-15
E:\\R-3.1.2\\scripts\\mPDBS\\summary_pdb_93\\397368adbc32d5de.final.pdb
-4.416549e-15 -4.715309e-15 -3.735877e-15 2.732345e-15 -1.512395e-15
-3.057770e-15

E:\\R-3.1.2\\scripts\\mPDBS\\summary_pdb_93\\411ca13cd3df6edd.final.pdb
6.503802e-16 6.735107e-15 -8.859000e-16 5.533474e-15 -2.489163e-16
7.274227e-15
E:\\R-3.1.2\\scripts\\mPDBS\\summary_pdb_93\\42357eee6642bf12.final.pdb
-2.128936e-15 1.096915e-14 -4.297527e-15 -3.822850e-16 1.953853e-15
-5.090869e-15
E:\\R-3.1.2\\scripts\\mPDBS\\summary_pdb_93\\44342d1f75a7fcfc.final.pdb
2.472618e-16 -9.150231e-15 -8.018169e-15 6.902660e-17 4.780682e-15
1.335727e-14
E:\\R-3.1.2\\scripts\\mPDBS\\summary_pdb_93\\448ef21dffe8cdfb.final.pdb
2.221204e-16 -8.500800e-16 3.063848e-15 -3.535226e-15 6.626227e-15
-1.546409e-14
E:\\R-3.1.2\\scripts\\mPDBS\\summary_pdb_93\\472a40b3e8f113c7.final.pdb
1.916710e-15 1.768112e-15 3.055782e-15 3.916727e-15 -2.477335e-15
4.388964e-15
E:\\R-3.1.2\\scripts\\mPDBS\\summary_pdb_93\\48a26225951ea153.final.pdb
6.657131e-16 -5.292449e-15 7.881254e-16 -3.216241e-15 6.656527e-15
3.262419e-15
E:\\R-3.1.2\\scripts\\mPDBS\\summary_pdb_93\\4ab4a5c7c6682ce6.final.pdb
-4.305526e-15 8.097853e-16 -1.043586e-15 3.578890e-15 -3.778861e-16
-9.497064e-15
E:\\R-3.1.2\\scripts\\mPDBS\\summary_pdb_93\\4eaa45b62cb6a25f.final.pdb
-3.527850e-15 -4.706862e-15 1.125212e-15 7.704415e-15 6.254117e-16
2.840926e-16
E:\\R-3.1.2\\scripts\\mPDBS\\summary_pdb_93\\58c371611d85ac96.final.pdb
-4.416549e-15 -4.715309e-15 -3.735877e-15 2.732345e-15 -1.512395e-15
-3.057770e-15
E:\\R-3.1.2\\scripts\\mPDBS\\summary_pdb_93\\59a647f04822d30a.final.pdb
-1.559453e-15 -5.201986e-16 -2.160008e-15 1.929518e-15 -1.099163e-15
-2.303812e-15
E:\\R-3.1.2\\scripts\\mPDBS\\summary_pdb_93\\59b091fe385e05fc.final.pdb
3.731954e-16 6.079961e-16 -9.220951e-16 4.159586e-15 4.773881e-16
3.288006e-15
E:\\R-3.1.2\\scripts\\mPDBS\\summary_pdb_93\\5c35ac30f930d2db.final.pdb
-3.527850e-15 -4.706862e-15 1.125212e-15 7.704415e-15 6.254117e-16
2.840926e-16
E:\\R-3.1.2\\scripts\\mPDBS\\summary_pdb_93\\5f60752431cf2dcb.final.pdb
-2.343327e-15 -6.071192e-16 3.883402e-15 1.822420e-15 3.373976e-16
7.421674e-17
E:\\R-3.1.2\\scripts\\mPDBS\\summary_pdb_93\\626456dd6ff2868d.final.pdb
1.916710e-15 1.768112e-15 3.055782e-15 3.916727e-15 -2.477335e-15
4.388964e-15
E:\\R-3.1.2\\scripts\\mPDBS\\summary_pdb_93\\6583d53ff3c8f7ef.final.pdb
-1.559453e-15 -5.201986e-16 -2.160008e-15 1.929518e-15 -1.099163e-15
-2.303812e-15
E:\\R-3.1.2\\scripts\\mPDBS\\summary_pdb_93\\69760178986bef01.final.pdb
-6.892185e-15 -2.663128e-16 1.112312e-14 5.493671e-15 -7.948378e-15
-1.333540e-15
E:\\R-3.1.2\\scripts\\mPDBS\\summary_pdb_93\\6a9d87d47926b1c8.final.pdb
2.914909e-16 -5.370107e-16 7.917512e-16 4.852177e-15 -6.693196e-16
8.661745e-16
E:\\R-3.1.2\\scripts\\mPDBS\\summary_pdb_93\\6bb3547ea982ffc4.final.pdb
1.191745e-14 -4.283021e-15 2.195825e-15 -5.875305e-16 6.326106e-16
1.909539e-15

E:\\R-3.1.2\\scripts\\mPDBS\\summary_pdb_93\\716d9624611d1c42.final.pdb
-5.811256e-15 -3.434955e-16 -1.972808e-15 7.459187e-15 9.709511e-16
4.090102e-15
E:\\R-3.1.2\\scripts\\mPDBS\\summary_pdb_93\\75f27e70ad0f1be4.final.pdb
-1.559453e-15 -5.201986e-16 -2.160008e-15 1.929518e-15 -1.099163e-15
-2.303812e-15
E:\\R-3.1.2\\scripts\\mPDBS\\summary_pdb_93\\769f910fffb22872.final.pdb
-1.055792e-15 3.269950e-16 1.138347e-15 4.669365e-15 1.018027e-15
8.349753e-16
E:\\R-3.1.2\\scripts\\mPDBS\\summary_pdb_93\\791f8c80dd5c3135.final.pdb
3.731954e-16 6.079961e-16 -9.220951e-16 4.159586e-15 4.773881e-16
3.288006e-15
E:\\R-3.1.2\\scripts\\mPDBS\\summary_pdb_93\\7fad98e66a3f6099.final.pdb
1.031123e-15 7.435629e-15 1.698403e-15 1.239584e-14 5.494940e-15
8.732381e-15
E:\\R-3.1.2\\scripts\\mPDBS\\summary_pdb_93\\804b89c94a633196.final.pdb
1.790310e-15 2.127790e-15 3.528754e-15 7.376184e-15 2.561769e-15
4.192057e-15
E:\\R-3.1.2\\scripts\\mPDBS\\summary_pdb_93\\813cc65a2298feba.final.pdb
1.790310e-15 2.127790e-15 3.528754e-15 7.376184e-15 2.561769e-15
4.192057e-15
E:\\R-3.1.2\\scripts\\mPDBS\\summary_pdb_93\\83c0eefcfe12d3f9.final.pdb
6.503802e-16 6.735107e-15 -8.859000e-16 5.533474e-15 -2.489163e-16
7.274227e-15
E:\\R-3.1.2\\scripts\\mPDBS\\summary_pdb_93\\86d50469ef569760.final.pdb
1.916710e-15 1.768112e-15 3.055782e-15 3.916727e-15 -2.477335e-15
4.388964e-15
E:\\R-3.1.2\\scripts\\mPDBS\\summary_pdb_93\\89d0666e1402203d.final.pdb
-1.559453e-15 -5.201986e-16 -2.160008e-15 1.929518e-15 -1.099163e-15
-2.303812e-15
E:\\R-3.1.2\\scripts\\mPDBS\\summary_pdb_93\\8b34852716dadc62.final.pdb
-4.128566e-15 1.424793e-15 -6.740809e-15 2.298649e-15 -3.171492e-15
2.618663e-15
E:\\R-3.1.2\\scripts\\mPDBS\\summary_pdb_93\\8b6ef9ac3fe74395.final.pdb
8.542109e-15 -7.239900e-15 -8.584568e-15 -6.515549e-16 2.533611e-15
-9.745480e-16
E:\\R-3.1.2\\scripts\\mPDBS\\summary_pdb_93\\98bbe0d5986c138f.final.pdb
3.731954e-16 6.079961e-16 -9.220951e-16 4.159586e-15 4.773881e-16
3.288006e-15
E:\\R-3.1.2\\scripts\\mPDBS\\summary_pdb_93\\9c22f4909d67e2fa.final.pdb
-4.367091e-15 -4.956984e-15 8.733033e-15 6.187032e-15 3.506475e-16
1.034860e-15
E:\\R-3.1.2\\scripts\\mPDBS\\summary_pdb_93\\9dda8ed0d81fb3ef.final.pdb
6.908817e-15 -1.098099e-14 -2.823757e-15 -8.693712e-15 1.675147e-15
5.364967e-15
E:\\R-3.1.2\\scripts\\mPDBS\\summary_pdb_93\\9f752b3616e6033b.final.pdb
2.165896e-15 -2.444317e-16 1.616959e-15 2.961739e-15 1.048096e-15
1.069207e-15
E:\\R-3.1.2\\scripts\\mPDBS\\summary_pdb_93\\a02c70083816d6ba.final.pdb
-3.527850e-15 -4.706862e-15 1.125212e-15 7.704415e-15 6.254117e-16
2.840926e-16
E:\\R-3.1.2\\scripts\\mPDBS\\summary_pdb_93\\a0c997d891e4e082.final.pdb
4.392288e-15 -6.986800e-15 1.311020e-14 1.164678e-14 -6.452913e-15
2.788501e-15

E:\\R-3.1.2\\scripts\\mPDBS\\summary_pdb_93\\ab8039a2d823c616.final.pdb
-1.256731e-15 -4.709347e-16 1.036420e-15 4.045009e-15 7.984476e-16
9.566331e-16
E:\\R-3.1.2\\scripts\\mPDBS\\summary_pdb_93\\acf4d74b9c71d4dc.final.pdb
1.790310e-15 2.127790e-15 3.528754e-15 7.376184e-15 2.561769e-15
4.192057e-15
E:\\R-3.1.2\\scripts\\mPDBS\\summary_pdb_93\\ad8a823e4b4c668e.final.pdb
-1.388887e-15 -3.899055e-17 1.309495e-15 4.660803e-15 1.069587e-15
1.376709e-15
E:\\R-3.1.2\\scripts\\mPDBS\\summary_pdb_93\\b16ababd03b7b857.final.pdb
-4.367091e-15 -4.956984e-15 8.733033e-15 6.187032e-15 3.506475e-16
1.034860e-15
E:\\R-3.1.2\\scripts\\mPDBS\\summary_pdb_93\\b226923e697d3c63.final.pdb
-1.844706e-15 1.499589e-15 7.403414e-15 6.092854e-15 -8.358710e-15
-6.323166e-15
E:\\R-3.1.2\\scripts\\mPDBS\\summary_pdb_93\\b28e48f6fa27a165.final.pdb
3.731954e-16 6.079961e-16 -9.220951e-16 4.159586e-15 4.773881e-16
3.288006e-15
E:\\R-3.1.2\\scripts\\mPDBS\\summary_pdb_93\\b42c4924e3cd1aec.final.pdb
-3.106067e-15 2.776508e-15 1.514245e-15 6.585298e-15 1.250303e-15
9.461926e-16
E:\\R-3.1.2\\scripts\\mPDBS\\summary_pdb_93\\b4dd239fcabff1df.final.pdb
3.731954e-16 6.079961e-16 -9.220951e-16 4.159586e-15 4.773881e-16
3.288006e-15
E:\\R-3.1.2\\scripts\\mPDBS\\summary_pdb_93\\b669942ef97459a2.final.pdb
-4.128566e-15 1.424793e-15 -6.740809e-15 2.298649e-15 -3.171492e-15
2.618663e-15
E:\\R-3.1.2\\scripts\\mPDBS\\summary_pdb_93\\b99f4d9d87c1017b.final.pdb
3.731954e-16 6.079961e-16 -9.220951e-16 4.159586e-15 4.773881e-16
3.288006e-15
E:\\R-3.1.2\\scripts\\mPDBS\\summary_pdb_93\\b9d2cbf3975403e7.final.pdb
3.731954e-16 6.079961e-16 -9.220951e-16 4.159586e-15 4.773881e-16
3.288006e-15
E:\\R-3.1.2\\scripts\\mPDBS\\summary_pdb_93\\bc88c8a505f3a288.final.pdb
-4.214164e-16 -8.175129e-16 8.500333e-16 3.924314e-15 4.416094e-16
7.631162e-16
E:\\R-3.1.2\\scripts\\mPDBS\\summary_pdb_93\\bdac0e64f4ef427e.final.pdb
-2.905152e-15 -1.296479e-14 4.483178e-15 2.288674e-15 1.817790e-15
-1.140181e-14
E:\\R-3.1.2\\scripts\\mPDBS\\summary_pdb_93\\ca2a1f55fc9d0681.final.pdb
-4.128566e-15 1.424793e-15 -6.740809e-15 2.298649e-15 -3.171492e-15
2.618663e-15
E:\\R-3.1.2\\scripts\\mPDBS\\summary_pdb_93\\ce34248de3e81a3c.final.pdb
-7.814287e-15 -4.481154e-15 -5.334929e-15 6.674112e-15 4.928970e-15
9.112724e-16
E:\\R-3.1.2\\scripts\\mPDBS\\summary_pdb_93\\d02e697772494a61.final.pdb
-6.420741e-15 6.770303e-15 4.875242e-15 1.402431e-14 -5.289684e-16
-2.149485e-15
E:\\R-3.1.2\\scripts\\mPDBS\\summary_pdb_93\\d04221245d015047.final.pdb
-6.921868e-16 -1.215375e-15 -1.241163e-15 3.632361e-15 3.672668e-15
3.485860e-15
E:\\R-3.1.2\\scripts\\mPDBS\\summary_pdb_93\\d678141e4018054a.final.pdb
-4.172335e-15 6.621729e-15 -1.703526e-15 4.138860e-15 1.198280e-15
8.269512e-16

E:\\R-3.1.2\\scripts\\mPDBS\\summary_pdb_93\\d71b40af0f0efe39.final.pdb
-6.070713e-15 6.556988e-15 8.613949e-15 9.316562e-15 4.015662e-15
5.131621e-15
E:\\R-3.1.2\\scripts\\mPDBS\\summary_pdb_93\\dc0809eff10197e2.final.pdb
7.451332e-15 -5.250804e-15 -2.601904e-15 2.622520e-15 -1.573620e-16
1.582468e-15
E:\\R-3.1.2\\scripts\\mPDBS\\summary_pdb_93\\e1163918d3707839.final.pdb
8.640613e-15 4.965566e-15 1.115671e-14 1.698627e-14 4.112163e-15
1.845647e-15
E:\\R-3.1.2\\scripts\\mPDBS\\summary_pdb_93\\e4b48b06824ef848.final.pdb
-3.985631e-15 -8.475483e-16 1.805239e-15 -2.458180e-15 6.631134e-16
-4.044802e-15
E:\\R-3.1.2\\scripts\\mPDBS\\summary_pdb_93\\e529e19ad281fea2.final.pdb
1.219351e-15 -3.459694e-15 5.075000e-15 7.911795e-15 2.378156e-15
2.095660e-15
E:\\R-3.1.2\\scripts\\mPDBS\\summary_pdb_93\\e5602082718ec3a1.final.pdb
1.790310e-15 2.127790e-15 3.528754e-15 7.376184e-15 2.561769e-15
4.192057e-15
E:\\R-3.1.2\\scripts\\mPDBS\\summary_pdb_93\\e5d589d996d0a960.final.pdb
-7.386500e-16 -1.049228e-14 4.073243e-15 1.493040e-15 6.178340e-15
2.268500e-15
E:\\R-3.1.2\\scripts\\mPDBS\\summary_pdb_93\\e9df9e72307a2fbd.final.pdb
-3.527850e-15 -4.706862e-15 1.125212e-15 7.704415e-15 6.254117e-16
2.840926e-16
E:\\R-3.1.2\\scripts\\mPDBS\\summary_pdb_93\\ea00f8694090cae7.final.pdb
1.790310e-15 2.127790e-15 3.528754e-15 7.376184e-15 2.561769e-15
4.192057e-15
E:\\R-3.1.2\\scripts\\mPDBS\\summary_pdb_93\\eba517be32db050e.final.pdb
3.731954e-16 6.079961e-16 -9.220951e-16 4.159586e-15 4.773881e-16
3.288006e-15
E:\\R-3.1.2\\scripts\\mPDBS\\summary_pdb_93\\ec04b9545899c29d.final.pdb
-9.089314e-16 -2.259462e-16 1.142038e-15 4.620941e-15 1.021102e-15
1.121833e-15
E:\\R-3.1.2\\scripts\\mPDBS\\summary_pdb_93\\ee6862cbcad52078.final.pdb
-1.333773e-15 -6.884929e-16 8.993645e-16 4.512603e-15 5.957829e-16
8.663322e-16
E:\\R-3.1.2\\scripts\\mPDBS\\summary_pdb_93\\ef6a42fc83b81e5d.final.pdb
-8.624784e-16 -3.525558e-16 8.210006e-16 4.177787e-15 1.198221e-15
1.270391e-15
E:\\R-3.1.2\\scripts\\mPDBS\\summary_pdb_93\\ef8e88d4ad93a0ad.final.pdb
-6.921868e-16 -1.215375e-15 -1.241163e-15 3.632361e-15 3.672668e-15
3.485860e-15
E:\\R-3.1.2\\scripts\\mPDBS\\summary_pdb_93\\f14fcae8deec4fa3.final.pdb
2.165896e-15 -2.444317e-16 1.616959e-15 2.961739e-15 1.048096e-15
1.069207e-15
E:\\R-3.1.2\\scripts\\mPDBS\\summary_pdb_93\\f83584e22003fb92.final.pdb
6.657131e-16 -5.292449e-15 7.881254e-16 -3.216241e-15 6.656527e-15
3.262419e-15
E:\\R-3.1.2\\scripts\\mPDBS\\summary_pdb_93\\f8e2bf87e52caa29.final.pdb
1.219351e-15 -3.459694e-15 5.075000e-15 7.911795e-15 2.378156e-15
2.095660e-15

[,331]   [,332]   [,333]   [,334]   [,335]   [,336]
E:\\R-3.1.2\\scripts\\40.1ns_data\\NAT0.pdb

-3.686830e-17 7.013935e-15 -3.383055e-15 3.108844e-15 3.989305e-15
2.423560e-15
E:\\R-3.1.2\\scripts\\40.1ns_data\\LJP6.pdb
-1.277018e-17 4.936807e-15 -3.896135e-15 3.561037e-15 2.308313e-15
3.568902e-15
E:\\R-3.1.2\\scripts\\40.1ns_data\\3WY9.pdb
-1.030829e-15 5.695105e-15 -3.233199e-15 2.175627e-15 -5.677459e-16
4.876241e-15
E:\\R-3.1.2\\scripts\\40.1ns_data\\ZSP9.pdb
-2.935994e-16 8.166766e-15 -3.149844e-15 1.959412e-15 2.247519e-15
3.599482e-15
E:\\R-3.1.2\\scripts\\mPDBS\\summary_pdb_93\\013d855ad26c877c.final.pdb
-3.402730e-15 5.001753e-15 -5.104434e-15 1.788910e-15 3.385671e-15
5.827016e-15
E:\\R-3.1.2\\scripts\\mPDBS\\summary_pdb_93\\0a2a705342ca0d54.final.pdb
-4.908357e-16 6.194051e-15 -3.633518e-15 1.887102e-15 2.979180e-15
5.287684e-15
E:\\R-3.1.2\\scripts\\mPDBS\\summary_pdb_93\\0a59fceadb4194d9.final.pdb
2.642743e-15 8.102821e-15 -4.181548e-15 2.514179e-15 1.459889e-15
5.866279e-15
E:\\R-3.1.2\\scripts\\mPDBS\\summary_pdb_93\\0dba59f3306825e8.final.pdb
1.874146e-17 2.557528e-15 -9.394405e-15 9.506045e-16 4.912851e-16
7.065175e-15
E:\\R-3.1.2\\scripts\\mPDBS\\summary_pdb_93\\126f26a78b57339c.final.pdb
-2.656175e-15 3.313434e-15 1.394816e-15 6.615913e-15 7.928826e-17
1.008822e-15
E:\\R-3.1.2\\scripts\\mPDBS\\summary_pdb_93\\12f06f3e342df706.final.pdb
-4.158089e-16 5.944468e-15 -3.928312e-15 2.923518e-15 3.021898e-15
5.274674e-15
E:\\R-3.1.2\\scripts\\mPDBS\\summary_pdb_93\\18618a2a251b867b.final.pdb
-8.125957e-15 -5.176974e-16 -5.057522e-15 3.578656e-15 2.812667e-15
5.370753e-16
E:\\R-3.1.2\\scripts\\mPDBS\\summary_pdb_93\\1f305f598641eeb2.final.pdb
-1.606766e-16 5.349098e-15 -3.908047e-15 2.814366e-15 3.675179e-15
4.740894e-15
E:\\R-3.1.2\\scripts\\mPDBS\\summary_pdb_93\\1f8a00b551be0a39.final.pdb
-8.125957e-15 -5.176974e-16 -5.057522e-15 3.578656e-15 2.812667e-15
5.370753e-16
E:\\R-3.1.2\\scripts\\mPDBS\\summary_pdb_93\\219f45328d47579a.final.pdb
3.267788e-16 4.955166e-15 -8.504479e-15 4.557249e-15 7.967921e-15
9.159218e-15
E:\\R-3.1.2\\scripts\\mPDBS\\summary_pdb_93\\238dd3918e54336d.final.pdb
-2.594468e-15 3.796804e-15 -1.017973e-14 6.657403e-15 6.076530e-15
4.222628e-15
E:\\R-3.1.2\\scripts\\mPDBS\\summary_pdb_93\\2b3027889e2fdc42.final.pdb
-1.478578e-16 6.022237e-15 -4.282054e-15 3.596998e-15 7.081031e-16
5.169354e-15
E:\\R-3.1.2\\scripts\\mPDBS\\summary_pdb_93\\2fcb3b46e33793b0.final.pdb
-6.434718e-16 2.852831e-15 -6.822383e-15 1.897564e-15 3.961408e-15
6.515083e-15
E:\\R-3.1.2\\scripts\\mPDBS\\summary_pdb_93\\34eb328251842006.final.pdb
6.274878e-15 1.097067e-14 -2.095890e-15 1.882327e-16 3.066878e-15
7.497555e-15
E:\\R-3.1.2\\scripts\\mPDBS\\summary_pdb_93\\354b32871797b7ab.final.pdb

2.487561e-15 8.477914e-15 -2.718894e-15 -1.807997e-15 2.105116e-15
4.634855e-15
E:\\R-3.1.2\\scripts\\mPDBS\\summary_pdb_93\\397368adbc32d5de.final.pdb
-2.656175e-15 3.313434e-15 1.394816e-15 6.615913e-15 7.928826e-17
1.008822e-15
E:\\R-3.1.2\\scripts\\mPDBS\\summary_pdb_93\\411ca13cd3df6edd.final.pdb
-2.608127e-15 5.790425e-16 -1.345102e-15 6.122348e-15 2.930115e-15
2.682211e-15
E:\\R-3.1.2\\scripts\\mPDBS\\summary_pdb_93\\42357eee6642bf12.final.pdb
8.100658e-15 4.891311e-15 -9.828999e-15 8.152150e-15 6.169328e-15
1.105480e-16
E:\\R-3.1.2\\scripts\\mPDBS\\summary_pdb_93\\44342d1f75a7fcfc.final.pdb
-2.811431e-15 -4.067822e-15 1.824969e-16 1.370240e-14 -7.888208e-16
1.057605e-14
E:\\R-3.1.2\\scripts\\mPDBS\\summary_pdb_93\\448ef21dffe8cdfb.final.pdb
-5.712098e-16 2.631844e-14 1.011546e-14 4.017991e-16 7.789943e-15
-8.768699e-15
E:\\R-3.1.2\\scripts\\mPDBS\\summary_pdb_93\\472a40b3e8f113c7.final.pdb
1.874146e-17 2.557528e-15 -9.394405e-15 9.506045e-16 4.912851e-16
7.065175e-15
E:\\R-3.1.2\\scripts\\mPDBS\\summary_pdb_93\\48a26225951ea153.final.pdb
-9.540328e-15 7.295961e-16 -9.002106e-16 1.531376e-15 -5.903145e-15
-3.418628e-16
E:\\R-3.1.2\\scripts\\mPDBS\\summary_pdb_93\\4ab4a5c7c6682ce6.final.pdb
-2.988320e-15 7.372686e-15 3.721081e-15 -2.060740e-15 -4.913246e-15
3.519834e-15
E:\\R-3.1.2\\scripts\\mPDBS\\summary_pdb_93\\4eaa45b62cb6a25f.final.pdb
-4.120813e-15 2.567277e-15 -2.649126e-15 3.447077e-15 2.330739e-15
3.779636e-15
E:\\R-3.1.2\\scripts\\mPDBS\\summary_pdb_93\\58c371611d85ac96.final.pdb
-2.656175e-15 3.313434e-15 1.394816e-15 6.615913e-15 7.928826e-17
1.008822e-15
E:\\R-3.1.2\\scripts\\mPDBS\\summary_pdb_93\\59a647f04822d30a.final.pdb
1.831827e-15 1.013870e-14 -6.768540e-15 -2.662986e-15 -9.466806e-16
5.250017e-15
E:\\R-3.1.2\\scripts\\mPDBS\\summary_pdb_93\\59b091fe385e05fc.final.pdb
2.642743e-15 8.102821e-15 -4.181548e-15 2.514179e-15 1.459889e-15
5.866279e-15
E:\\R-3.1.2\\scripts\\mPDBS\\summary_pdb_93\\5c35ac30f930d2db.final.pdb
-4.120813e-15 2.567277e-15 -2.649126e-15 3.447077e-15 2.330739e-15
3.779636e-15
E:\\R-3.1.2\\scripts\\mPDBS\\summary_pdb_93\\5f60752431cf2dcb.final.pdb
-5.062510e-15 8.834517e-15 -1.922972e-15 3.992541e-15 -6.385858e-16
-1.899709e-15
E:\\R-3.1.2\\scripts\\mPDBS\\summary_pdb_93\\626456dd6ff2868d.final.pdb
1.874146e-17 2.557528e-15 -9.394405e-15 9.506045e-16 4.912851e-16
7.065175e-15
E:\\R-3.1.2\\scripts\\mPDBS\\summary_pdb_93\\6583d53ff3c8f7ef.final.pdb
1.831827e-15 1.013870e-14 -6.768540e-15 -2.662986e-15 -9.466806e-16
5.250017e-15
E:\\R-3.1.2\\scripts\\mPDBS\\summary_pdb_93\\69760178986bef01.final.pdb
-5.553260e-15 6.946526e-16 -4.278869e-15 5.378382e-15 5.196633e-15
4.074338e-15
E:\\R-3.1.2\\scripts\\mPDBS\\summary_pdb_93\\6a9d87d47926b1c8.final.pdb

-5.658875e-16 5.752455e-15 -4.371404e-15 3.447007e-15 8.815979e-16
5.218562e-15
E:\\R-3.1.2\\scripts\\mPDBS\\summary_pdb_93\\6bb3547ea982ffc4.final.pdb
-6.896917e-15 3.854494e-15 -3.862572e-15 1.382667e-14 1.457880e-16
8.771837e-15
E:\\R-3.1.2\\scripts\\mPDBS\\summary_pdb_93\\716d9624611d1c42.final.pdb
-2.943782e-16 3.059406e-15 -3.155601e-15 -6.733498e-16 5.005988e-15
8.310006e-15
E:\\R-3.1.2\\scripts\\mPDBS\\summary_pdb_93\\75f27e70ad0f1be4.final.pdb
1.831827e-15 1.013870e-14 -6.768540e-15 -2.662986e-15 -9.466806e-16
5.250017e-15
E:\\R-3.1.2\\scripts\\mPDBS\\summary_pdb_93\\769f910fffb22872.final.pdb
-9.792792e-17 5.757014e-15 -4.071820e-15 2.667844e-15 2.998607e-15
5.254222e-15
E:\\R-3.1.2\\scripts\\mPDBS\\summary_pdb_93\\791f8c80dd5c3135.final.pdb
2.642743e-15 8.102821e-15 -4.181548e-15 2.514179e-15 1.459889e-15
5.866279e-15
E:\\R-3.1.2\\scripts\\mPDBS\\summary_pdb_93\\7fad98e66a3f6099.final.pdb
4.206450e-15 1.187882e-14 -2.843538e-15 8.466924e-15 4.459433e-15
1.117900e-14
E:\\R-3.1.2\\scripts\\mPDBS\\summary_pdb_93\\804b89c94a633196.final.pdb
-6.434718e-16 2.852831e-15 -6.822383e-15 1.897564e-15 3.961408e-15
6.515083e-15
E:\\R-3.1.2\\scripts\\mPDBS\\summary_pdb_93\\813cc65a2298feba.final.pdb
-6.434718e-16 2.852831e-15 -6.822383e-15 1.897564e-15 3.961408e-15
6.515083e-15
E:\\R-3.1.2\\scripts\\mPDBS\\summary_pdb_93\\83c0eefcfe12d3f9.final.pdb
-2.608127e-15 5.790425e-16 -1.345102e-15 6.122348e-15 2.930115e-15
2.682211e-15
E:\\R-3.1.2\\scripts\\mPDBS\\summary_pdb_93\\86d50469ef569760.final.pdb
1.874146e-17 2.557528e-15 -9.394405e-15 9.506045e-16 4.912851e-16
7.065175e-15
E:\\R-3.1.2\\scripts\\mPDBS\\summary_pdb_93\\89d0666e1402203d.final.pdb
1.831827e-15 1.013870e-14 -6.768540e-15 -2.662986e-15 -9.466806e-16
5.250017e-15
E:\\R-3.1.2\\scripts\\mPDBS\\summary_pdb_93\\8b34852716dadc62.final.pdb
1.518536e-15 3.191102e-15 -1.253920e-15 7.171991e-15 5.700239e-16
5.750503e-15
E:\\R-3.1.2\\scripts\\mPDBS\\summary_pdb_93\\8b6ef9ac3fe74395.final.pdb
3.344512e-15 1.503289e-14 -5.265276e-15 8.902784e-15 5.783104e-16
1.304388e-14
E:\\R-3.1.2\\scripts\\mPDBS\\summary_pdb_93\\98bbe0d5986c138f.final.pdb
2.642743e-15 8.102821e-15 -4.181548e-15 2.514179e-15 1.459889e-15
5.866279e-15
E:\\R-3.1.2\\scripts\\mPDBS\\summary_pdb_93\\9c22f4909d67e2fa.final.pdb
1.228864e-15 3.104800e-15 -9.787513e-15 -4.272632e-16 7.761754e-15
2.359119e-15
E:\\R-3.1.2\\scripts\\mPDBS\\summary_pdb_93\\9dda8ed0d81fb3ef.final.pdb
-1.138270e-14 -1.024033e-15 -4.151601e-15 4.658577e-15 -4.405936e-15
3.099579e-16
E:\\R-3.1.2\\scripts\\mPDBS\\summary_pdb_93\\9f752b3616e6033b.final.pdb
1.820633e-15 6.879914e-15 -6.389571e-15 4.761708e-15 4.209823e-15
6.951723e-15
E:\\R-3.1.2\\scripts\\mPDBS\\summary_pdb_93\\a02c70083816d6ba.final.pdb

-4.120813e-15 2.567277e-15 -2.649126e-15 3.447077e-15 2.330739e-15
3.779636e-15
E:\\R-3.1.2\\scripts\\mPDBS\\summary_pdb_93\\a0c997d891e4e082.final.pdb
-6.003888e-15 -1.769000e-15 2.193766e-15 7.308444e-15 7.502729e-15
9.863199e-15
E:\\R-3.1.2\\scripts\\mPDBS\\summary_pdb_93\\ab8039a2d823c616.final.pdb
-2.343606e-16 5.536355e-15 -4.044679e-15 3.110467e-15 2.930106e-15
4.036697e-15
E:\\R-3.1.2\\scripts\\mPDBS\\summary_pdb_93\\acf4d74b9c71d4dc.final.pdb
-6.434718e-16 2.852831e-15 -6.822383e-15 1.897564e-15 3.961408e-15
6.515083e-15
E:\\R-3.1.2\\scripts\\mPDBS\\summary_pdb_93\\ad8a823e4b4c668e.final.pdb
-7.752929e-16 5.858482e-15 -3.911048e-15 2.760443e-15 3.637195e-15
4.592321e-15
E:\\R-3.1.2\\scripts\\mPDBS\\summary_pdb_93\\b16ababd03b7b857.final.pdb
1.228864e-15 3.104800e-15 -9.787513e-15 -4.272632e-16 7.761754e-15
2.359119e-15
E:\\R-3.1.2\\scripts\\mPDBS\\summary_pdb_93\\b226923e697d3c63.final.pdb
-3.144769e-15 1.687773e-15 -3.581365e-15 7.686456e-15 6.155819e-15
6.448277e-15
E:\\R-3.1.2\\scripts\\mPDBS\\summary_pdb_93\\b28e48f6fa27a165.final.pdb
2.642743e-15 8.102821e-15 -4.181548e-15 2.514179e-15 1.459889e-15
5.866279e-15
E:\\R-3.1.2\\scripts\\mPDBS\\summary_pdb_93\\b42c4924e3cd1aec.final.pdb
4.747437e-15 3.014620e-15 9.210013e-16 -3.285359e-16 1.207448e-14
9.139267e-15
E:\\R-3.1.2\\scripts\\mPDBS\\summary_pdb_93\\b4dd239fcabff1df.final.pdb
2.642743e-15 8.102821e-15 -4.181548e-15 2.514179e-15 1.459889e-15
5.866279e-15
E:\\R-3.1.2\\scripts\\mPDBS\\summary_pdb_93\\b669942ef97459a2.final.pdb
1.518536e-15 3.191102e-15 -1.253920e-15 7.171991e-15 5.700239e-16
5.750503e-15
E:\\R-3.1.2\\scripts\\mPDBS\\summary_pdb_93\\b99f4d9d87c1017b.final.pdb
2.642743e-15 8.102821e-15 -4.181548e-15 2.514179e-15 1.459889e-15
5.866279e-15
E:\\R-3.1.2\\scripts\\mPDBS\\summary_pdb_93\\b9d2cbf3975403e7.final.pdb
2.642743e-15 8.102821e-15 -4.181548e-15 2.514179e-15 1.459889e-15
5.866279e-15
E:\\R-3.1.2\\scripts\\mPDBS\\summary_pdb_93\\bc88c8a505f3a288.final.pdb
-7.809234e-16 6.147137e-15 -4.449184e-15 2.949513e-15 1.024148e-15
4.867078e-15
E:\\R-3.1.2\\scripts\\mPDBS\\summary_pdb_93\\bdac0e64f4ef427e.final.pdb
4.896259e-15 1.569239e-14 -8.784084e-15 -5.127632e-15 3.699682e-15
8.209474e-15
E:\\R-3.1.2\\scripts\\mPDBS\\summary_pdb_93\\ca2a1f55fc9d0681.final.pdb
1.518536e-15 3.191102e-15 -1.253920e-15 7.171991e-15 5.700239e-16
5.750503e-15
E:\\R-3.1.2\\scripts\\mPDBS\\summary_pdb_93\\ce34248de3e81a3c.final.pdb
-1.014898e-15 6.548804e-15 1.907649e-15 1.833972e-15 -1.392364e-15
1.880736e-15
E:\\R-3.1.2\\scripts\\mPDBS\\summary_pdb_93\\d02e697772494a61.final.pdb
6.259560e-15 1.522863e-14 -9.843198e-15 5.281117e-15 9.226667e-16
5.743981e-15
E:\\R-3.1.2\\scripts\\mPDBS\\summary_pdb_93\\d04221245d015047.final.pdb

7.511249e-16 6.252362e-15 -4.514409e-15 1.722634e-15 1.772504e-15
4.695458e-15
E:\\R-3.1.2\\scripts\\mPDBS\\summary_pdb_93\\d678141e4018054a.final.pdb
-3.226573e-15 3.534885e-15 -3.654411e-15 1.960202e-16 -2.596443e-15
7.099985e-17
E:\\R-3.1.2\\scripts\\mPDBS\\summary_pdb_93\\d71b40af0f0efe39.final.pdb
4.294426e-16 8.129253e-15 -1.041136e-14 -4.216813e-15 1.406037e-15
3.891676e-15
E:\\R-3.1.2\\scripts\\mPDBS\\summary_pdb_93\\dc0809eff10197e2.final.pdb
5.674204e-15 1.050057e-14 -3.135032e-15 -2.503121e-15 3.630521e-15
1.385736e-14
E:\\R-3.1.2\\scripts\\mPDBS\\summary_pdb_93\\e1163918d3707839.final.pdb
6.419873e-15 8.089189e-16 -2.474720e-15 -3.698453e-15 1.203805e-14
4.381354e-15
E:\\R-3.1.2\\scripts\\mPDBS\\summary_pdb_93\\e4b48b06824ef848.final.pdb
-8.125957e-15 -5.176974e-16 -5.057522e-15 3.578656e-15 2.812667e-15
5.370753e-16
E:\\R-3.1.2\\scripts\\mPDBS\\summary_pdb_93\\e529e19ad281fea2.final.pdb
-3.402730e-15 5.001753e-15 -5.104434e-15 1.788910e-15 3.385671e-15
5.827016e-15
E:\\R-3.1.2\\scripts\\mPDBS\\summary_pdb_93\\e5602082718ec3a1.final.pdb
-6.434718e-16 2.852831e-15 -6.822383e-15 1.897564e-15 3.961408e-15
6.515083e-15
E:\\R-3.1.2\\scripts\\mPDBS\\summary_pdb_93\\e5d589d996d0a960.final.pdb
-2.935037e-15 7.728749e-15 2.683597e-15 1.113079e-14 2.455904e-15
2.635911e-16
E:\\R-3.1.2\\scripts\\mPDBS\\summary_pdb_93\\e9df9e72307a2fbd.final.pdb
-4.120813e-15 2.567277e-15 -2.649126e-15 3.447077e-15 2.330739e-15
3.779636e-15
E:\\R-3.1.2\\scripts\\mPDBS\\summary_pdb_93\\ea00f8694090cae7.final.pdb
-6.434718e-16 2.852831e-15 -6.822383e-15 1.897564e-15 3.961408e-15
6.515083e-15
E:\\R-3.1.2\\scripts\\mPDBS\\summary_pdb_93\\eba517be32db050e.final.pdb
2.642743e-15 8.102821e-15 -4.181548e-15 2.514179e-15 1.459889e-15
5.866279e-15
E:\\R-3.1.2\\scripts\\mPDBS\\summary_pdb_93\\ec04b9545899c29d.final.pdb
-4.726984e-16 5.113202e-15 -4.301763e-15 3.057265e-15 3.358469e-15
5.633824e-15
E:\\R-3.1.2\\scripts\\mPDBS\\summary_pdb_93\\ee6862cbcad52078.final.pdb
-6.097008e-16 5.872834e-15 -4.509411e-15 3.083602e-15 1.283381e-15
4.747816e-15
E:\\R-3.1.2\\scripts\\mPDBS\\summary_pdb_93\\ef6a42fc83b81e5d.final.pdb
-2.939246e-16 5.145958e-15 -4.067103e-15 2.249071e-15 3.239842e-15
5.486241e-15
E:\\R-3.1.2\\scripts\\mPDBS\\summary_pdb_93\\ef8e88d4ad93a0ad.final.pdb
7.511249e-16 6.252362e-15 -4.514409e-15 1.722634e-15 1.772504e-15
4.695458e-15
E:\\R-3.1.2\\scripts\\mPDBS\\summary_pdb_93\\f14fcae8deec4fa3.final.pdb
1.820633e-15 6.879914e-15 -6.389571e-15 4.761708e-15 4.209823e-15
6.951723e-15
E:\\R-3.1.2\\scripts\\mPDBS\\summary_pdb_93\\f83584e22003fb92.final.pdb
-9.540328e-15 7.295961e-16 -9.002106e-16 1.531376e-15 -5.903145e-15
-3.418628e-16
E:\\R-3.1.2\\scripts\\mPDBS\\summary_pdb_93\\f8e2bf87e52caa29.final.pdb

```
-3.402730e-15  5.001753e-15 -5.104434e-15  1.788910e-15  3.385671e-15
5.827016e-15

[,337]        [,338]        [,339]        [,340]        [,341]        [,342]
E:\\R-3.1.2\\scripts\\40.1ns_data\\NAT0.pdb
-4.127416e-17  4.691285e-15  6.832256e-17  1.882362e-15 -1.191352e-15
2.599943e-15
E:\\R-3.1.2\\scripts\\40.1ns_data\\LJP6.pdb
-1.164624e-15  4.156895e-15  6.814344e-16  2.914916e-15 -2.948139e-15
2.990301e-15
E:\\R-3.1.2\\scripts\\40.1ns_data\\3WY9.pdb
-6.331658e-16  4.997560e-15  1.846777e-15  1.766267e-15 -1.527606e-15
1.781405e-15
E:\\R-3.1.2\\scripts\\40.1ns_data\\ZSP9.pdb
-2.461753e-16  5.551140e-15  8.871103e-17  3.712447e-15 -1.614374e-15
3.440180e-15
E:\\R-3.1.2\\scripts\\mPDBS\\summary_pdb_93\\013d855ad26c877c.final.pdb
-3.125066e-16  3.176888e-15  1.923547e-15  4.477032e-15  1.731310e-15
-1.756216e-15
E:\\R-3.1.2\\scripts\\mPDBS\\summary_pdb_93\\0a2a705342ca0d54.final.pdb
-7.642945e-16  4.546171e-15  1.720305e-15  2.600302e-15 -1.999872e-15
2.840887e-15
E:\\R-3.1.2\\scripts\\mPDBS\\summary_pdb_93\\0a59fceadb4194d9.final.pdb
-2.489238e-16  1.140046e-15  2.863581e-16  3.432587e-15 -2.251432e-15
-5.924479e-16
E:\\R-3.1.2\\scripts\\mPDBS\\summary_pdb_93\\0dba59f3306825e8.final.pdb
-3.010435e-15  7.802095e-15  2.785704e-15  2.300822e-15 -1.833981e-15
1.563167e-16
E:\\R-3.1.2\\scripts\\mPDBS\\summary_pdb_93\\126f26a78b57339c.final.pdb
-5.721591e-15  1.103519e-14  5.510087e-15  3.629620e-15  2.918511e-15
9.731557e-15
E:\\R-3.1.2\\scripts\\mPDBS\\summary_pdb_93\\12f06f3e342df706.final.pdb
-4.704758e-16  5.643167e-15  2.016076e-15  2.303882e-15 -1.735977e-15
2.896615e-15
E:\\R-3.1.2\\scripts\\mPDBS\\summary_pdb_93\\18618a2a251b867b.final.pdb
-5.693387e-15  1.841724e-14  5.642967e-15  5.220386e-15  6.353469e-15
2.656690e-15
E:\\R-3.1.2\\scripts\\mPDBS\\summary_pdb_93\\1f305f598641eeb2.final.pdb
-1.346477e-15  5.472753e-15  2.231330e-15  2.964551e-15 -1.702795e-15
2.311329e-15
E:\\R-3.1.2\\scripts\\mPDBS\\summary_pdb_93\\1f8a00b551be0a39.final.pdb
-5.693387e-15  1.841724e-14  5.642967e-15  5.220386e-15  6.353469e-15
2.656690e-15
E:\\R-3.1.2\\scripts\\mPDBS\\summary_pdb_93\\219f45328d47579a.final.pdb
-3.954510e-15  1.434791e-14  4.545972e-15 -1.605050e-15 -9.534451e-15
1.747275e-14
E:\\R-3.1.2\\scripts\\mPDBS\\summary_pdb_93\\238dd3918e54336d.final.pdb
-1.964782e-15  8.588190e-15  2.652521e-15 -2.306312e-15  5.910768e-15
1.450875e-14
E:\\R-3.1.2\\scripts\\mPDBS\\summary_pdb_93\\2b3027889e2fdc42.final.pdb
-1.037789e-15  5.028591e-15  1.755669e-15  3.214445e-15 -1.259170e-15
2.981082e-15
E:\\R-3.1.2\\scripts\\mPDBS\\summary_pdb_93\\2fcb3b46e33793b0.final.pdb
2.397490e-15  2.109124e-15  2.703175e-15  5.154639e-15 -3.896787e-15
```

-2.608346e-15
E:\\R-3.1.2\\scripts\\mPDBS\\summary_pdb_93\\34eb328251842006.final.pdb
-5.520692e-15 -1.742225e-15 -7.182497e-15 7.218847e-16 -9.021246e-16
5.250960e-16
E:\\R-3.1.2\\scripts\\mPDBS\\summary_pdb_93\\354b32871797b7ab.final.pdb
9.197251e-15 -3.055290e-15 5.838438e-15 7.153272e-15 -4.984666e-15
2.025637e-15
E:\\R-3.1.2\\scripts\\mPDBS\\summary_pdb_93\\397368adbc32d5de.final.pdb
-5.721591e-15 1.103519e-14 5.510087e-15 3.629620e-15 2.918511e-15
9.731557e-15
E:\\R-3.1.2\\scripts\\mPDBS\\summary_pdb_93\\411ca13cd3df6edd.final.pdb
-3.335554e-15 4.760215e-15 1.064728e-15 7.663917e-15 -9.085088e-16
-1.060539e-15
E:\\R-3.1.2\\scripts\\mPDBS\\summary_pdb_93\\42357eee6642bf12.final.pdb
-3.453257e-16 -9.842416e-16 -1.366145e-15 5.224739e-15 -1.989828e-15
2.437295e-15
E:\\R-3.1.2\\scripts\\mPDBS\\summary_pdb_93\\44342d1f75a7fcfc.final.pdb
6.972057e-15 4.569553e-15 -3.343571e-15 5.101223e-15 3.416987e-15
-2.087324e-15
E:\\R-3.1.2\\scripts\\mPDBS\\summary_pdb_93\\448ef21dffe8cdfb.final.pdb
4.213745e-15 -9.868708e-15 3.993809e-15 4.792082e-15 5.627712e-15
1.161986e-14
E:\\R-3.1.2\\scripts\\mPDBS\\summary_pdb_93\\472a40b3e8f113c7.final.pdb
-3.010435e-15 7.802095e-15 2.785704e-15 2.300822e-15 -1.833981e-15
1.563167e-16
E:\\R-3.1.2\\scripts\\mPDBS\\summary_pdb_93\\48a26225951ea153.final.pdb
-1.276314e-15 1.153521e-14 -2.575936e-15 9.251717e-15 1.656467e-15
1.031687e-15
E:\\R-3.1.2\\scripts\\mPDBS\\summary_pdb_93\\4ab4a5c7c6682ce6.final.pdb
1.764210e-14 6.663099e-16 2.541976e-15 8.112145e-15 -9.063224e-15
-2.045481e-15
E:\\R-3.1.2\\scripts\\mPDBS\\summary_pdb_93\\4eaa45b62cb6a25f.final.pdb
-4.460444e-15 1.143464e-14 3.218139e-15 2.535872e-15 3.735700e-16
2.104133e-15
E:\\R-3.1.2\\scripts\\mPDBS\\summary_pdb_93\\58c371611d85ac96.final.pdb
-5.721591e-15 1.103519e-14 5.510087e-15 3.629620e-15 2.918511e-15
9.731557e-15
E:\\R-3.1.2\\scripts\\mPDBS\\summary_pdb_93\\59a647f04822d30a.final.pdb
-2.539649e-15 7.783887e-15 2.108665e-15 -1.280824e-15 -6.191646e-15
7.826838e-15
E:\\R-3.1.2\\scripts\\mPDBS\\summary_pdb_93\\59b091fe385e05fc.final.pdb
-2.489238e-16 1.140046e-15 2.863581e-16 3.432587e-15 -2.251432e-15
-5.924479e-16
E:\\R-3.1.2\\scripts\\mPDBS\\summary_pdb_93\\5c35ac30f930d2db.final.pdb
-4.460444e-15 1.143464e-14 3.218139e-15 2.535872e-15 3.735700e-16
2.104133e-15
E:\\R-3.1.2\\scripts\\mPDBS\\summary_pdb_93\\5f60752431cf2dcb.final.pdb
-8.360649e-15 1.268761e-14 8.702909e-16 2.866583e-15 -5.348980e-16
-3.027249e-15
E:\\R-3.1.2\\scripts\\mPDBS\\summary_pdb_93\\626456dd6ff2868d.final.pdb
-3.010435e-15 7.802095e-15 2.785704e-15 2.300822e-15 -1.833981e-15
1.563167e-16
E:\\R-3.1.2\\scripts\\mPDBS\\summary_pdb_93\\6583d53ff3c8f7ef.final.pdb
-2.539649e-15 7.783887e-15 2.108665e-15 -1.280824e-15 -6.191646e-15

7.826838e-15
E:\\R-3.1.2\\scripts\\mPDBS\\summary_pdb_93\\69760178986bef01.final.pdb
-4.729133e-16 1.702226e-14 3.083810e-15 6.810762e-15 -3.909643e-15
5.329948e-15
E:\\R-3.1.2\\scripts\\mPDBS\\summary_pdb_93\\6a9d87d47926b1c8.final.pdb
-6.967478e-16 5.381288e-15 1.985129e-15 3.474362e-15 -1.213600e-15
2.938379e-15
E:\\R-3.1.2\\scripts\\mPDBS\\summary_pdb_93\\6bb3547ea982ffc4.final.pdb
2.942801e-15 1.043234e-14 -8.263699e-15 2.925174e-15 3.141919e-15
4.322528e-15
E:\\R-3.1.2\\scripts\\mPDBS\\summary_pdb_93\\716d9624611d1c42.final.pdb
3.071140e-16 6.071535e-15 4.349279e-15 5.719335e-15 7.550858e-16
7.294572e-15
E:\\R-3.1.2\\scripts\\mPDBS\\summary_pdb_93\\75f27e70ad0f1be4.final.pdb
-2.539649e-15 7.783887e-15 2.108665e-15 -1.280824e-15 -6.191646e-15
7.826838e-15
E:\\R-3.1.2\\scripts\\mPDBS\\summary_pdb_93\\769f910fffb22872.final.pdb
-8.673861e-16 4.252623e-15 1.948874e-15 2.739731e-15 -1.449209e-15
2.111770e-15
E:\\R-3.1.2\\scripts\\mPDBS\\summary_pdb_93\\791f8c80dd5c3135.final.pdb
-2.489238e-16 1.140046e-15 2.863581e-16 3.432587e-15 -2.251432e-15
-5.924479e-16
E:\\R-3.1.2\\scripts\\mPDBS\\summary_pdb_93\\7fad98e66a3f6099.final.pdb
7.998788e-15 -1.070057e-14 -4.559068e-15 5.272212e-15 -1.207900e-14
8.426140e-16
E:\\R-3.1.2\\scripts\\mPDBS\\summary_pdb_93\\804b89c94a633196.final.pdb
2.397490e-15 2.109124e-15 2.703175e-15 5.154639e-15 -3.896787e-15
-2.608346e-15
E:\\R-3.1.2\\scripts\\mPDBS\\summary_pdb_93\\813cc65a2298feba.final.pdb
2.397490e-15 2.109124e-15 2.703175e-15 5.154639e-15 -3.896787e-15
-2.608346e-15
E:\\R-3.1.2\\scripts\\mPDBS\\summary_pdb_93\\83c0eefcfe12d3f9.final.pdb
-3.335554e-15 4.760215e-15 1.064728e-15 7.663917e-15 -9.085088e-16
-1.060539e-15
E:\\R-3.1.2\\scripts\\mPDBS\\summary_pdb_93\\86d50469ef569760.final.pdb
-3.010435e-15 7.802095e-15 2.785704e-15 2.300822e-15 -1.833981e-15
1.563167e-16
E:\\R-3.1.2\\scripts\\mPDBS\\summary_pdb_93\\89d0666e1402203d.final.pdb
-2.539649e-15 7.783887e-15 2.108665e-15 -1.280824e-15 -6.191646e-15
7.826838e-15
E:\\R-3.1.2\\scripts\\mPDBS\\summary_pdb_93\\8b34852716dadc62.final.pdb
-1.159127e-15 1.068733e-14 -1.911190e-15 9.089034e-16 -6.435121e-15
7.730605e-15
E:\\R-3.1.2\\scripts\\mPDBS\\summary_pdb_93\\8b6ef9ac3fe74395.final.pdb
3.279489e-15 9.465486e-16 -7.328288e-15 -4.869779e-17 -8.553789e-15
3.098799e-15
E:\\R-3.1.2\\scripts\\mPDBS\\summary_pdb_93\\98bbe0d5986c138f.final.pdb
-2.489238e-16 1.140046e-15 2.863581e-16 3.432587e-15 -2.251432e-15
-5.924479e-16
E:\\R-3.1.2\\scripts\\mPDBS\\summary_pdb_93\\9c22f4909d67e2fa.final.pdb
-1.124221e-14 9.205444e-15 7.357438e-15 -2.567916e-15 4.736390e-15
7.811270e-15
E:\\R-3.1.2\\scripts\\mPDBS\\summary_pdb_93\\9dda8ed0d81fb3ef.final.pdb
-5.441624e-15 1.311063e-14 -1.201188e-15 8.927156e-15 3.450272e-15

-9.133988e-15
E:\\R-3.1.2\\scripts\\mPDBS\\summary_pdb_93\\9f752b3616e6033b.final.pdb
2.324968e-15 2.991896e-15 1.492301e-15 1.061818e-15 -2.682038e-15
4.190968e-15
E:\\R-3.1.2\\scripts\\mPDBS\\summary_pdb_93\\a02c70083816d6ba.final.pdb
-4.460444e-15 1.143464e-14 3.218139e-15 2.535872e-15 3.735700e-16
2.104133e-15
E:\\R-3.1.2\\scripts\\mPDBS\\summary_pdb_93\\a0c997d891e4e082.final.pdb
3.539928e-15 3.484576e-15 2.579492e-15 1.365048e-15 -3.115807e-15
-1.830190e-15
E:\\R-3.1.2\\scripts\\mPDBS\\summary_pdb_93\\ab8039a2d823c616.final.pdb
-1.369773e-15 5.652438e-15 2.005298e-15 2.599304e-15 -1.786033e-15
2.522448e-15
E:\\R-3.1.2\\scripts\\mPDBS\\summary_pdb_93\\acf4d74b9c71d4dc.final.pdb
2.397490e-15 2.109124e-15 2.703175e-15 5.154639e-15 -3.896787e-15
-2.608346e-15
E:\\R-3.1.2\\scripts\\mPDBS\\summary_pdb_93\\ad8a823e4b4c668e.final.pdb
-7.117778e-16 6.140608e-15 1.978336e-15 2.668492e-15 -1.789104e-15
2.765899e-15
E:\\R-3.1.2\\scripts\\mPDBS\\summary_pdb_93\\b16ababd03b7b857.final.pdb
-1.124221e-14 9.205444e-15 7.357438e-15 -2.567916e-15 4.736390e-15
7.811270e-15
E:\\R-3.1.2\\scripts\\mPDBS\\summary_pdb_93\\b226923e697d3c63.final.pdb
-2.218075e-15 7.093521e-15 4.361203e-15 3.267187e-15 -4.465674e-16
2.231921e-15
E:\\R-3.1.2\\scripts\\mPDBS\\summary_pdb_93\\b28e48f6fa27a165.final.pdb
-2.489238e-16 1.140046e-15 2.863581e-16 3.432587e-15 -2.251432e-15
-5.924479e-16
E:\\R-3.1.2\\scripts\\mPDBS\\summary_pdb_93\\b42c4924e3cd1aec.final.pdb
2.382939e-16 2.277116e-15 2.835514e-15 5.636203e-16 -4.541255e-17
2.790068e-15
E:\\R-3.1.2\\scripts\\mPDBS\\summary_pdb_93\\b4dd239fcabff1df.final.pdb
-2.489238e-16 1.140046e-15 2.863581e-16 3.432587e-15 -2.251432e-15
-5.924479e-16
E:\\R-3.1.2\\scripts\\mPDBS\\summary_pdb_93\\b669942ef97459a2.final.pdb
-1.159127e-15 1.068733e-14 -1.911190e-15 9.089034e-16 -6.435121e-15
7.730605e-15
E:\\R-3.1.2\\scripts\\mPDBS\\summary_pdb_93\\b99f4d9d87c1017b.final.pdb
-2.489238e-16 1.140046e-15 2.863581e-16 3.432587e-15 -2.251432e-15
-5.924479e-16
E:\\R-3.1.2\\scripts\\mPDBS\\summary_pdb_93\\b9d2cbf3975403e7.final.pdb
-2.489238e-16 1.140046e-15 2.863581e-16 3.432587e-15 -2.251432e-15
-5.924479e-16
E:\\R-3.1.2\\scripts\\mPDBS\\summary_pdb_93\\bc88c8a505f3a288.final.pdb
-6.075779e-16 5.880070e-15 1.629034e-15 3.124023e-15 -1.442617e-15
3.430593e-15
E:\\R-3.1.2\\scripts\\mPDBS\\summary_pdb_93\\bdac0e64f4ef427e.final.pdb
3.109811e-15 4.650115e-15 3.626048e-15 -6.214305e-15 -3.709437e-15
8.097933e-15
E:\\R-3.1.2\\scripts\\mPDBS\\summary_pdb_93\\ca2a1f55fc9d0681.final.pdb
-1.159127e-15 1.068733e-14 -1.911190e-15 9.089034e-16 -6.435121e-15
7.730605e-15
E:\\R-3.1.2\\scripts\\mPDBS\\summary_pdb_93\\ce34248de3e81a3c.final.pdb
-2.775789e-15 3.396384e-15 7.525403e-16 6.740531e-15 -4.696866e-15

-3.804715e-15
E:\\R-3.1.2\\scripts\\mPDBS\\summary_pdb_93\\d02e697772494a61.final.pdb
2.295699e-15 5.685631e-16 5.200830e-15 -7.173055e-16 -8.116640e-15
1.410456e-14
E:\\R-3.1.2\\scripts\\mPDBS\\summary_pdb_93\\d04221245d015047.final.pdb
-8.564428e-17 3.589526e-15 1.538658e-15 3.222937e-15 -2.352963e-15
3.018362e-15
E:\\R-3.1.2\\scripts\\mPDBS\\summary_pdb_93\\d678141e4018054a.final.pdb
-5.860117e-15 4.633544e-15 2.091861e-15 5.745764e-15 1.006605e-14
1.314980e-15
E:\\R-3.1.2\\scripts\\mPDBS\\summary_pdb_93\\d71b40af0f0efe39.final.pdb
-4.447093e-15 -1.087080e-16 1.810663e-14 -8.105656e-16 3.533729e-15
1.250384e-14
E:\\R-3.1.2\\scripts\\mPDBS\\summary_pdb_93\\dc0809eff10197e2.final.pdb
1.197080e-15 1.324335e-15 8.847663e-15 -5.939934e-15 -5.725330e-15
4.103758e-15
E:\\R-3.1.2\\scripts\\mPDBS\\summary_pdb_93\\e1163918d3707839.final.pdb
-2.622482e-15 -5.091447e-15 4.634422e-15 1.205573e-14 4.354930e-15
-4.802166e-15
E:\\R-3.1.2\\scripts\\mPDBS\\summary_pdb_93\\e4b48b06824ef848.final.pdb
-5.693387e-15 1.841724e-14 5.642967e-15 5.220386e-15 6.353469e-15
2.656690e-15
E:\\R-3.1.2\\scripts\\mPDBS\\summary_pdb_93\\e529e19ad281fea2.final.pdb
-3.125066e-16 3.176888e-15 1.923547e-15 4.477032e-15 1.731310e-15
-1.756216e-15
E:\\R-3.1.2\\scripts\\mPDBS\\summary_pdb_93\\e5602082718ec3a1.final.pdb
2.397490e-15 2.109124e-15 2.703175e-15 5.154639e-15 -3.896787e-15
-2.608346e-15
E:\\R-3.1.2\\scripts\\mPDBS\\summary_pdb_93\\e5d589d996d0a960.final.pdb
7.274549e-15 -3.443211e-15 3.647987e-16 5.516680e-15 -1.091232e-14
1.220331e-15
E:\\R-3.1.2\\scripts\\mPDBS\\summary_pdb_93\\e9df9e72307a2fbd.final.pdb
-4.460444e-15 1.143464e-14 3.218139e-15 2.535872e-15 3.735700e-16
2.104133e-15
E:\\R-3.1.2\\scripts\\mPDBS\\summary_pdb_93\\ea00f8694090cae7.final.pdb
2.397490e-15 2.109124e-15 2.703175e-15 5.154639e-15 -3.896787e-15
-2.608346e-15
E:\\R-3.1.2\\scripts\\mPDBS\\summary_pdb_93\\eba517be32db050e.final.pdb
-2.489238e-16 1.140046e-15 2.863581e-16 3.432587e-15 -2.251432e-15
-5.924479e-16
E:\\R-3.1.2\\scripts\\mPDBS\\summary_pdb_93\\ec04b9545899c29d.final.pdb
-1.089203e-15 6.169402e-15 2.283004e-15 3.170476e-15 -1.893869e-15
2.760361e-15
E:\\R-3.1.2\\scripts\\mPDBS\\summary_pdb_93\\ee6862cbcad52078.final.pdb
-4.278171e-16 5.254919e-15 2.144247e-15 3.116867e-15 -1.209947e-15
3.255819e-15
E:\\R-3.1.2\\scripts\\mPDBS\\summary_pdb_93\\ef6a42fc83b81e5d.final.pdb
-1.226691e-15 5.065883e-15 2.512659e-15 3.104891e-15 -2.017006e-15
2.538512e-15
E:\\R-3.1.2\\scripts\\mPDBS\\summary_pdb_93\\ef8e88d4ad93a0ad.final.pdb
-8.564428e-17 3.589526e-15 1.538658e-15 3.222937e-15 -2.352963e-15
3.018362e-15
E:\\R-3.1.2\\scripts\\mPDBS\\summary_pdb_93\\f14fcae8deec4fa3.final.pdb
2.324968e-15 2.991896e-15 1.492301e-15 1.061818e-15 -2.682038e-15

4.190968e-15
E:\\R-3.1.2\\scripts\\mPDBS\\summary_pdb_93\\f83584e22003fb92.final.pdb
-1.276314e-15 1.153521e-14 -2.575936e-15 9.251717e-15 1.656467e-15
1.031687e-15
E:\\R-3.1.2\\scripts\\mPDBS\\summary_pdb_93\\f8e2bf87e52caa29.final.pdb
-3.125066e-16 3.176888e-15 1.923547e-15 4.477032e-15 1.731310e-15
-1.756216e-15

[,343] [,344] [,345] [,346] [,347] [,348]
E:\\R-3.1.2\\scripts\\40.1ns_data\\NAT0.pdb
1.361823e-15 -3.728493e-15 -1.760666e-16 -4.112965e-15 -6.609370e-15
-1.864093e-15
E:\\R-3.1.2\\scripts\\40.1ns_data\\LJP6.pdb
1.335926e-15 -2.612523e-15 -1.094274e-15 -4.887879e-15 -7.023779e-15
-1.199711e-15
E:\\R-3.1.2\\scripts\\40.1ns_data\\3WY9.pdb
1.982186e-15 -1.699691e-15 1.599448e-15 -5.477150e-15 -6.091806e-15
-8.008548e-16
E:\\R-3.1.2\\scripts\\40.1ns_data\\ZSP9.pdb
2.133139e-15 -2.596159e-15 1.729914e-15 -3.727302e-15 -6.240803e-15
-9.284051e-16
E:\\R-3.1.2\\scripts\\mPDBS\\summary_pdb_93\\013d855ad26c877c.final.pdb
1.366827e-15 2.016202e-15 -3.580534e-16 -1.419161e-16 -4.742652e-15
-8.581627e-15
E:\\R-3.1.2\\scripts\\mPDBS\\summary_pdb_93\\0a2a705342ca0d54.final.pdb
1.369811e-15 -2.710749e-15 1.142185e-15 -4.912892e-15 -6.774269e-15
-1.138857e-15
E:\\R-3.1.2\\scripts\\mPDBS\\summary_pdb_93\\0a59fceadb4194d9.final.pdb
2.921327e-16 -4.427475e-16 3.775726e-16 -6.132561e-15 -5.662254e-15
-8.331360e-16
E:\\R-3.1.2\\scripts\\mPDBS\\summary_pdb_93\\0dba59f3306825e8.final.pdb
1.437084e-15 -3.738743e-15 -3.671375e-15 -4.513419e-15 -5.126460e-15
1.417319e-15
E:\\R-3.1.2\\scripts\\mPDBS\\summary_pdb_93\\126f26a78b57339c.final.pdb
2.442790e-15 -7.605442e-15 7.157404e-16 -7.414744e-15 -1.243789e-14
2.793404e-16
E:\\R-3.1.2\\scripts\\mPDBS\\summary_pdb_93\\12f06f3e342df706.final.pdb
1.816502e-15 -2.397631e-15 -2.082968e-16 -4.915494e-15 -6.316736e-15
-1.257685e-15
E:\\R-3.1.2\\scripts\\mPDBS\\summary_pdb_93\\18618a2a251b867b.final.pdb
1.605850e-15 -7.406274e-15 -2.213435e-15 -3.705299e-15 -9.126225e-15
-1.388052e-15
E:\\R-3.1.2\\scripts\\mPDBS\\summary_pdb_93\\1f305f598641eeb2.final.pdb
1.750564e-15 -2.334174e-15 3.649408e-16 -4.941873e-15 -6.352681e-15
-1.295023e-15
E:\\R-3.1.2\\scripts\\mPDBS\\summary_pdb_93\\1f8a00b551be0a39.final.pdb
1.605850e-15 -7.406274e-15 -2.213435e-15 -3.705299e-15 -9.126225e-15
-1.388052e-15
E:\\R-3.1.2\\scripts\\mPDBS\\summary_pdb_93\\219f45328d47579a.final.pdb
6.882347e-15 -4.870195e-15 1.200976e-14 -4.340592e-15 -1.274271e-15
-1.435952e-15
E:\\R-3.1.2\\scripts\\mPDBS\\summary_pdb_93\\238dd3918e54336d.final.pdb
7.652785e-16 1.759484e-15 -1.827261e-15 6.497527e-15 -1.052859e-14
-2.902661e-15

E:\\R-3.1.2\\scripts\\mPDBS\\summary_pdb_93\\2b3027889e2fdc42.final.pdb
1.525521e-15 -2.627198e-15 -3.553481e-16 -4.908714e-15 -7.149780e-15
-1.095079e-15
E:\\R-3.1.2\\scripts\\mPDBS\\summary_pdb_93\\2fcb3b46e33793b0.final.pdb
5.137060e-16 3.055611e-15 -3.289486e-16 -3.283478e-15 -3.941849e-15
-8.648118e-16
E:\\R-3.1.2\\scripts\\mPDBS\\summary_pdb_93\\34eb328251842006.final.pdb
1.906481e-15 -4.479765e-15 2.959330e-15 -6.144047e-15 -5.944141e-15
-5.784471e-15
E:\\R-3.1.2\\scripts\\mPDBS\\summary_pdb_93\\354b32871797b7ab.final.pdb
5.105976e-16 -5.670976e-15 -1.212382e-16 -1.768256e-14 -1.788066e-14
-4.848867e-15
E:\\R-3.1.2\\scripts\\mPDBS\\summary_pdb_93\\397368adbc32d5de.final.pdb
2.442790e-15 -7.605442e-15 7.157404e-16 -7.414744e-15 -1.243789e-14
2.793404e-16
E:\\R-3.1.2\\scripts\\mPDBS\\summary_pdb_93\\411ca13cd3df6edd.final.pdb
-1.554636e-16 2.211669e-15 -1.763565e-15 -5.908982e-15 -7.008840e-15
-5.733783e-16
E:\\R-3.1.2\\scripts\\mPDBS\\summary_pdb_93\\42357eee6642bf12.final.pdb
1.789512e-15 1.032386e-15 5.310573e-15 -8.457314e-15 -5.450269e-15
-2.458304e-15
E:\\R-3.1.2\\scripts\\mPDBS\\summary_pdb_93\\44342d1f75a7fcfc.final.pdb
6.379213e-15 -4.089091e-15 8.086356e-16 -3.178281e-15 -8.506495e-15
-2.896408e-15
E:\\R-3.1.2\\scripts\\mPDBS\\summary_pdb_93\\448ef21dffe8cdfb.final.pdb
-6.282941e-15 2.095442e-15 3.307032e-15 -8.269073e-15 -1.725672e-14
-8.501065e-15
E:\\R-3.1.2\\scripts\\mPDBS\\summary_pdb_93\\472a40b3e8f113c7.final.pdb
1.437084e-15 -3.738743e-15 -3.671375e-15 -4.513419e-15 -5.126460e-15
1.417319e-15
E:\\R-3.1.2\\scripts\\mPDBS\\summary_pdb_93\\48a26225951ea153.final.pdb
1.095310e-15 -8.069459e-15 -6.653262e-15 -8.855210e-15 -8.929172e-15
5.587943e-15
E:\\R-3.1.2\\scripts\\mPDBS\\summary_pdb_93\\4ab4a5c7c6682ce6.final.pdb
1.149553e-15 6.988787e-15 -2.417170e-15 -6.356563e-15 -3.670160e-15
1.157110e-15
E:\\R-3.1.2\\scripts\\mPDBS\\summary_pdb_93\\4eaa45b62cb6a25f.final.pdb
1.978607e-15 -2.025889e-15 -1.404066e-15 -6.297948e-16 -4.159865e-15
1.616466e-17
E:\\R-3.1.2\\scripts\\mPDBS\\summary_pdb_93\\58c371611d85ac96.final.pdb
2.442790e-15 -7.605442e-15 7.157404e-16 -7.414744e-15 -1.243789e-14
2.793404e-16
E:\\R-3.1.2\\scripts\\mPDBS\\summary_pdb_93\\59a647f04822d30a.final.pdb
2.601852e-15 -6.849470e-15 -8.436885e-16 -8.595823e-15 -7.123921e-15
1.714505e-16
E:\\R-3.1.2\\scripts\\mPDBS\\summary_pdb_93\\59b091fe385e05fc.final.pdb
2.921327e-16 -4.427475e-16 3.775726e-16 -6.132561e-15 -5.662254e-15
-8.331360e-16
E:\\R-3.1.2\\scripts\\mPDBS\\summary_pdb_93\\5c35ac30f930d2db.final.pdb
1.978607e-15 -2.025889e-15 -1.404066e-15 -6.297948e-16 -4.159865e-15
1.616466e-17
E:\\R-3.1.2\\scripts\\mPDBS\\summary_pdb_93\\5f60752431cf2dcb.final.pdb
2.381155e-15 -3.216854e-15 -1.000198e-15 -2.558005e-15 -9.823046e-15
-5.950209e-16

E:\\R-3.1.2\\scripts\\mPDBS\\summary_pdb_93\\626456dd6ff2868d.final.pdb
1.437084e-15 -3.738743e-15 -3.671375e-15 -4.513419e-15 -5.126460e-15
1.417319e-15
E:\\R-3.1.2\\scripts\\mPDBS\\summary_pdb_93\\6583d53ff3c8f7ef.final.pdb
2.601852e-15 -6.849470e-15 -8.436885e-16 -8.595823e-15 -7.123921e-15
1.714505e-16
E:\\R-3.1.2\\scripts\\mPDBS\\summary_pdb_93\\69760178986bef01.final.pdb
1.639981e-15 -6.025357e-16 4.153749e-15 -3.314994e-15 -1.099796e-14
3.143263e-15
E:\\R-3.1.2\\scripts\\mPDBS\\summary_pdb_93\\6a9d87d47926b1c8.final.pdb
1.367261e-15 -2.592091e-15 -7.657134e-16 -5.038173e-15 -6.562811e-15
-9.592522e-16
E:\\R-3.1.2\\scripts\\mPDBS\\summary_pdb_93\\6bb3547ea982ffc4.final.pdb
4.647464e-15 -9.611891e-15 -5.378855e-15 -5.588222e-15 -6.420765e-15
-8.325818e-15
E:\\R-3.1.2\\scripts\\mPDBS\\summary_pdb_93\\716d9624611d1c42.final.pdb
1.682929e-15 -3.573340e-15 1.984958e-16 -2.891939e-15 -4.565966e-15
-4.280441e-15
E:\\R-3.1.2\\scripts\\mPDBS\\summary_pdb_93\\75f27e70ad0f1be4.final.pdb
2.601852e-15 -6.849470e-15 -8.436885e-16 -8.595823e-15 -7.123921e-15
1.714505e-16
E:\\R-3.1.2\\scripts\\mPDBS\\summary_pdb_93\\769f910fffb22872.final.pdb
1.714704e-15 -2.329172e-15 3.886500e-16 -4.240895e-15 -6.402709e-15
-1.404532e-15
E:\\R-3.1.2\\scripts\\mPDBS\\summary_pdb_93\\791f8c80dd5c3135.final.pdb
2.921327e-16 -4.427475e-16 3.775726e-16 -6.132561e-15 -5.662254e-15
-8.331360e-16
E:\\R-3.1.2\\scripts\\mPDBS\\summary_pdb_93\\7fad98e66a3f6099.final.pdb
1.568080e-15 3.274698e-15 5.942730e-15 -5.710848e-15 -1.186965e-14
-1.883575e-15
E:\\R-3.1.2\\scripts\\mPDBS\\summary_pdb_93\\804b89c94a633196.final.pdb
5.137060e-16 3.055611e-15 -3.289486e-16 -3.283478e-15 -3.941849e-15
-8.648118e-16
E:\\R-3.1.2\\scripts\\mPDBS\\summary_pdb_93\\813cc65a2298feba.final.pdb
5.137060e-16 3.055611e-15 -3.289486e-16 -3.283478e-15 -3.941849e-15
-8.648118e-16
E:\\R-3.1.2\\scripts\\mPDBS\\summary_pdb_93\\83c0eefcfe12d3f9.final.pdb
-1.554636e-16 2.211669e-15 -1.763565e-15 -5.908982e-15 -7.008840e-15
-5.733783e-16
E:\\R-3.1.2\\scripts\\mPDBS\\summary_pdb_93\\86d50469ef569760.final.pdb
1.437084e-15 -3.738743e-15 -3.671375e-15 -4.513419e-15 -5.126460e-15
1.417319e-15
E:\\R-3.1.2\\scripts\\mPDBS\\summary_pdb_93\\89d0666e1402203d.final.pdb
2.601852e-15 -6.849470e-15 -8.436885e-16 -8.595823e-15 -7.123921e-15
1.714505e-16
E:\\R-3.1.2\\scripts\\mPDBS\\summary_pdb_93\\8b34852716dadc62.final.pdb
4.406425e-15 -7.500181e-15 2.335587e-15 -9.826855e-15 -6.161861e-15
4.488545e-15
E:\\R-3.1.2\\scripts\\mPDBS\\summary_pdb_93\\8b6ef9ac3fe74395.final.pdb
3.167980e-15 -7.822695e-15 5.037961e-15 -1.191564e-14 -2.130417e-15
-2.152253e-15
E:\\R-3.1.2\\scripts\\mPDBS\\summary_pdb_93\\98bbe0d5986c138f.final.pdb
2.921327e-16 -4.427475e-16 3.775726e-16 -6.132561e-15 -5.662254e-15
-8.331360e-16

E:\\R-3.1.2\\scripts\\mPDBS\\summary_pdb_93\\9c22f4909d67e2fa.final.pdb
3.741159e-15 -8.733569e-15 1.335519e-15 1.279279e-15 -9.985192e-15
-3.291690e-15
E:\\R-3.1.2\\scripts\\mPDBS\\summary_pdb_93\\9dda8ed0d81fb3ef.final.pdb
-5.488031e-15 -3.216092e-15 -1.317020e-14 -4.334475e-15 -1.290818e-16
1.131358e-14
E:\\R-3.1.2\\scripts\\mPDBS\\summary_pdb_93\\9f752b3616e6033b.final.pdb
1.810553e-15 -3.085193e-15 1.674397e-15 -5.881705e-15 -5.935805e-15
2.784463e-16
E:\\R-3.1.2\\scripts\\mPDBS\\summary_pdb_93\\a02c70083816d6ba.final.pdb
1.978607e-15 -2.025889e-15 -1.404066e-15 -6.297948e-16 -4.159865e-15
1.616466e-17
E:\\R-3.1.2\\scripts\\mPDBS\\summary_pdb_93\\a0c997d891e4e082.final.pdb
-3.089117e-15 -3.018263e-15 -3.719349e-15 -2.732989e-16 -8.632012e-15
-4.511079e-15
E:\\R-3.1.2\\scripts\\mPDBS\\summary_pdb_93\\ab8039a2d823c616.final.pdb
1.275881e-15 -2.103196e-15 4.344461e-16 -4.530744e-15 -6.749837e-15
-1.312248e-15
E:\\R-3.1.2\\scripts\\mPDBS\\summary_pdb_93\\acf4d74b9c71d4dc.final.pdb
5.137060e-16 3.055611e-15 -3.289486e-16 -3.283478e-15 -3.941849e-15
-8.648118e-16
E:\\R-3.1.2\\scripts\\mPDBS\\summary_pdb_93\\ad8a823e4b4c668e.final.pdb
1.779123e-15 -2.797008e-15 -8.239708e-16 -4.524816e-15 -6.167307e-15
-1.020656e-15
E:\\R-3.1.2\\scripts\\mPDBS\\summary_pdb_93\\b16ababd03b7b857.final.pdb
3.741159e-15 -8.733569e-15 1.335519e-15 1.279279e-15 -9.985192e-15
-3.291690e-15
E:\\R-3.1.2\\scripts\\mPDBS\\summary_pdb_93\\b226923e697d3c63.final.pdb
-5.765947e-16 -5.765702e-15 4.892175e-15 -6.113243e-16 -1.069528e-14
-7.571488e-15
E:\\R-3.1.2\\scripts\\mPDBS\\summary_pdb_93\\b28e48f6fa27a165.final.pdb
2.921327e-16 -4.427475e-16 3.775726e-16 -6.132561e-15 -5.662254e-15
-8.331360e-16
E:\\R-3.1.2\\scripts\\mPDBS\\summary_pdb_93\\b42c4924e3cd1aec.final.pdb
4.266753e-15 -8.880928e-16 1.104177e-14 -4.062557e-15 -1.409621e-14
-4.479343e-15
E:\\R-3.1.2\\scripts\\mPDBS\\summary_pdb_93\\b4dd239fcabff1df.final.pdb
2.921327e-16 -4.427475e-16 3.775726e-16 -6.132561e-15 -5.662254e-15
-8.331360e-16
E:\\R-3.1.2\\scripts\\mPDBS\\summary_pdb_93\\b669942ef97459a2.final.pdb
4.406425e-15 -7.500181e-15 2.335587e-15 -9.826855e-15 -6.161861e-15
4.488545e-15
E:\\R-3.1.2\\scripts\\mPDBS\\summary_pdb_93\\b99f4d9d87c1017b.final.pdb
2.921327e-16 -4.427475e-16 3.775726e-16 -6.132561e-15 -5.662254e-15
-8.331360e-16
E:\\R-3.1.2\\scripts\\mPDBS\\summary_pdb_93\\b9d2cbf3975403e7.final.pdb
2.921327e-16 -4.427475e-16 3.775726e-16 -6.132561e-15 -5.662254e-15
-8.331360e-16
E:\\R-3.1.2\\scripts\\mPDBS\\summary_pdb_93\\bc88c8a505f3a288.final.pdb
1.086202e-15 -2.565615e-15 -6.528531e-16 -4.763431e-15 -6.397777e-15
-1.033497e-15
E:\\R-3.1.2\\scripts\\mPDBS\\summary_pdb_93\\bdac0e64f4ef427e.final.pdb
6.111659e-15 -1.069077e-14 4.171791e-15 -1.825009e-15 -5.180441e-15
-3.419626e-15

E:\\R-3.1.2\\scripts\\mPDBS\\summary_pdb_93\\ca2a1f55fc9d0681.final.pdb
4.406425e-15 -7.500181e-15 2.335587e-15 -9.826855e-15 -6.161861e-15
4.488545e-15
E:\\R-3.1.2\\scripts\\mPDBS\\summary_pdb_93\\ce34248de3e81a3c.final.pdb
5.545696e-16 1.695360e-15 -1.932462e-15 -6.234800e-15 -4.106408e-15
-1.602004e-15
E:\\R-3.1.2\\scripts\\mPDBS\\summary_pdb_93\\d02e697772494a61.final.pdb
6.407467e-15 -3.117691e-15 2.786236e-15 -4.667154e-15 -5.725138e-15
-9.662011e-15
E:\\R-3.1.2\\scripts\\mPDBS\\summary_pdb_93\\d04221245d015047.final.pdb
2.557407e-15 -1.457011e-15 -1.089508e-15 -5.465326e-15 -5.582369e-15
7.139086e-16
E:\\R-3.1.2\\scripts\\mPDBS\\summary_pdb_93\\d678141e4018054a.final.pdb
4.711333e-15 -3.468144e-15 -3.349135e-15 1.829522e-15 -9.580942e-15
-1.095342e-14
E:\\R-3.1.2\\scripts\\mPDBS\\summary_pdb_93\\d71b40af0f0efe39.final.pdb
1.735090e-15 -1.922065e-15 3.723733e-16 4.180821e-15 -1.110362e-14
-2.603957e-16
E:\\R-3.1.2\\scripts\\mPDBS\\summary_pdb_93\\dc0809eff10197e2.final.pdb
-1.389938e-15 -7.085670e-15 4.118985e-15 -7.350915e-15 -7.055215e-15
4.582203e-15
E:\\R-3.1.2\\scripts\\mPDBS\\summary_pdb_93\\e1163918d3707839.final.pdb
1.146853e-15 3.416190e-15 3.703889e-15 -1.621801e-15 -2.993978e-15
-5.146326e-16
E:\\R-3.1.2\\scripts\\mPDBS\\summary_pdb_93\\e4b48b06824ef848.final.pdb
1.605850e-15 -7.406274e-15 -2.213435e-15 -3.705299e-15 -9.126225e-15
-1.388052e-15
E:\\R-3.1.2\\scripts\\mPDBS\\summary_pdb_93\\e529e19ad281fea2.final.pdb
1.366827e-15 2.016202e-15 -3.580534e-16 -1.419161e-16 -4.742652e-15
-8.581627e-15
E:\\R-3.1.2\\scripts\\mPDBS\\summary_pdb_93\\e5602082718ec3a1.final.pdb
5.137060e-16 3.055611e-15 -3.289486e-16 -3.283478e-15 -3.941849e-15
-8.648118e-16
E:\\R-3.1.2\\scripts\\mPDBS\\summary_pdb_93\\e5d589d996d0a960.final.pdb
1.779242e-16 1.084852e-15 5.702311e-16 -4.891547e-15 -1.709023e-15
-4.660874e-16
E:\\R-3.1.2\\scripts\\mPDBS\\summary_pdb_93\\e9df9e72307a2fbd.final.pdb
1.978607e-15 -2.025889e-15 -1.404066e-15 -6.297948e-16 -4.159865e-15
1.616466e-17
E:\\R-3.1.2\\scripts\\mPDBS\\summary_pdb_93\\ea00f8694090cae7.final.pdb
5.137060e-16 3.055611e-15 -3.289486e-16 -3.283478e-15 -3.941849e-15
-8.648118e-16
E:\\R-3.1.2\\scripts\\mPDBS\\summary_pdb_93\\eba517be32db050e.final.pdb
2.921327e-16 -4.427475e-16 3.775726e-16 -6.132561e-15 -5.662254e-15
-8.331360e-16
E:\\R-3.1.2\\scripts\\mPDBS\\summary_pdb_93\\ec04b9545899c29d.final.pdb
1.583570e-15 -2.347429e-15 6.326020e-18 -4.600755e-15 -6.746618e-15
-9.014759e-16
E:\\R-3.1.2\\scripts\\mPDBS\\summary_pdb_93\\ee6862cbcad52078.final.pdb
1.141280e-15 -2.558676e-15 -5.123405e-16 -4.711823e-15 -6.732579e-15
-7.065012e-16
E:\\R-3.1.2\\scripts\\mPDBS\\summary_pdb_93\\ef6a42fc83b81e5d.final.pdb
1.886875e-15 -2.515221e-15 7.390641e-16 -4.491996e-15 -6.846798e-15
-1.500809e-15

E:\\R-3.1.2\\scripts\\mPDBS\\summary_pdb_93\\ef8e88d4ad93a0ad.final.pdb
2.557407e-15 -1.457011e-15 -1.089508e-15 -5.465326e-15 -5.582369e-15
7.139086e-16
E:\\R-3.1.2\\scripts\\mPDBS\\summary_pdb_93\\f14fcae8deec4fa3.final.pdb
1.810553e-15 -3.085193e-15 1.674397e-15 -5.881705e-15 -5.935805e-15
2.784463e-16
E:\\R-3.1.2\\scripts\\mPDBS\\summary_pdb_93\\f83584e22003fb92.final.pdb
1.095310e-15 -8.069459e-15 -6.653262e-15 -8.855210e-15 -8.929172e-15
5.587943e-15
E:\\R-3.1.2\\scripts\\mPDBS\\summary_pdb_93\\f8e2bf87e52caa29.final.pdb
1.366827e-15 2.016202e-15 -3.580534e-16 -1.419161e-16 -4.742652e-15
-8.581627e-15

[,349]   [,350]   [,351]   [,352]   [,353]   [,354]
E:\\R-3.1.2\\scripts\\40.1ns_data\\NAT0.pdb
8.704329e-16 5.413606e-15 -2.051666e-15 -5.883462e-15 -5.442054e-15
-2.966244e-15
E:\\R-3.1.2\\scripts\\40.1ns_data\\LJP6.pdb
-2.247161e-16 6.166899e-15 2.516945e-17 -5.512208e-15 -6.741806e-15
-1.665839e-15
E:\\R-3.1.2\\scripts\\40.1ns_data\\3WY9.pdb
2.856430e-15 5.101587e-15 -6.553178e-16 -5.905213e-15 -5.379643e-15
-3.525953e-15
E:\\R-3.1.2\\scripts\\40.1ns_data\\ZSP9.pdb
1.459179e-17 4.655806e-15 -1.677958e-15 -6.986109e-15 -4.104027e-15
-4.276501e-15
E:\\R-3.1.2\\scripts\\mPDBS\\summary_pdb_93\\013d855ad26c877c.final.pdb
-2.853884e-15 6.604071e-15 -2.890721e-15 -2.310004e-15 -7.948927e-15
-3.397691e-15
E:\\R-3.1.2\\scripts\\mPDBS\\summary_pdb_93\\0a2a705342ca0d54.final.pdb
-1.037528e-15 4.981328e-15 -1.260685e-15 -4.789475e-15 -7.049393e-15
-2.163543e-15
E:\\R-3.1.2\\scripts\\mPDBS\\summary_pdb_93\\0a59fceadb4194d9.final.pdb
-3.345888e-15 3.808341e-15 -5.941472e-15 -5.702466e-15 -3.731616e-15
-2.383149e-15
E:\\R-3.1.2\\scripts\\mPDBS\\summary_pdb_93\\0dba59f3306825e8.final.pdb
4.418077e-15 5.091392e-15 -1.239264e-15 -2.295258e-15 -4.528052e-15
-4.232093e-15
E:\\R-3.1.2\\scripts\\mPDBS\\summary_pdb_93\\126f26a78b57339c.final.pdb
3.017946e-16 7.510464e-15 1.282157e-15 -5.879197e-15 -3.691698e-15
-1.222347e-15
E:\\R-3.1.2\\scripts\\mPDBS\\summary_pdb_93\\12f06f3e342df706.final.pdb
-6.777895e-16 4.911939e-15 -9.957062e-16 -4.332375e-15 -6.467827e-15
-1.863436e-15
E:\\R-3.1.2\\scripts\\mPDBS\\summary_pdb_93\\18618a2a251b867b.final.pdb
4.233040e-15 1.106754e-14 4.970025e-15 -7.471646e-15 -7.301070e-15
2.083006e-15
E:\\R-3.1.2\\scripts\\mPDBS\\summary_pdb_93\\1f305f598641eeb2.final.pdb
4.654821e-16 4.892884e-15 -8.904925e-16 -4.880142e-15 -5.530244e-15
-2.026893e-15
E:\\R-3.1.2\\scripts\\mPDBS\\summary_pdb_93\\1f8a00b551be0a39.final.pdb
4.233040e-15 1.106754e-14 4.970025e-15 -7.471646e-15 -7.301070e-15
2.083006e-15
E:\\R-3.1.2\\scripts\\mPDBS\\summary_pdb_93\\219f45328d47579a.final.pdb

3.323256e-15 -1.198721e-15 1.084158e-15 -1.020537e-14 -1.494660e-14
-2.541009e-15
E:\\R-3.1.2\\scripts\\mPDBS\\summary_pdb_93\\238dd3918e54336d.final.pdb
-1.574662e-16 1.098272e-14 4.901851e-15 -4.109122e-15 -1.298615e-14
-2.500243e-15
E:\\R-3.1.2\\scripts\\mPDBS\\summary_pdb_93\\2b3027889e2fdc42.final.pdb
-1.951821e-15 5.721741e-15 -1.650633e-15 -4.303411e-15 -5.068871e-15
-1.677117e-15
E:\\R-3.1.2\\scripts\\mPDBS\\summary_pdb_93\\2fcb3b46e33793b0.final.pdb
-8.112744e-16 3.318623e-15 -1.098659e-15 -2.356987e-15 -5.620450e-15
-2.949766e-15
E:\\R-3.1.2\\scripts\\mPDBS\\summary_pdb_93\\34eb328251842006.final.pdb
-2.824349e-15 5.705028e-16 1.430575e-14 -1.296683e-14 -3.079822e-15
7.943670e-15
E:\\R-3.1.2\\scripts\\mPDBS\\summary_pdb_93\\354b32871797b7ab.final.pdb
-2.363866e-15 5.241534e-16 7.490627e-15 -8.038461e-15 1.056207e-15
-1.423710e-15
E:\\R-3.1.2\\scripts\\mPDBS\\summary_pdb_93\\397368adbc32d5de.final.pdb
3.017946e-16 7.510464e-15 1.282157e-15 -5.879197e-15 -3.691698e-15
-1.222347e-15
E:\\R-3.1.2\\scripts\\mPDBS\\summary_pdb_93\\411ca13cd3df6edd.final.pdb
-4.771865e-15 2.252202e-15 7.981751e-15 -4.682384e-15 -1.523250e-15
4.110559e-15
E:\\R-3.1.2\\scripts\\mPDBS\\summary_pdb_93\\42357eee6642bf12.final.pdb
-1.822330e-15 -4.213748e-15 -1.226261e-15 -1.389125e-14 -6.527333e-15
2.825249e-17
E:\\R-3.1.2\\scripts\\mPDBS\\summary_pdb_93\\44342d1f75a7fcfc.final.pdb
-3.620090e-15 1.035345e-14 -1.398234e-15 3.651156e-15 -5.960270e-15
-2.642467e-15
E:\\R-3.1.2\\scripts\\mPDBS\\summary_pdb_93\\448ef21dffe8cdfb.final.pdb
-1.707431e-14 9.277398e-15 6.374529e-15 -1.453475e-14 1.946414e-15
6.301515e-15
E:\\R-3.1.2\\scripts\\mPDBS\\summary_pdb_93\\472a40b3e8f113c7.final.pdb
4.418077e-15 5.091392e-15 -1.239264e-15 -2.295258e-15 -4.528052e-15
-4.232093e-15
E:\\R-3.1.2\\scripts\\mPDBS\\summary_pdb_93\\48a26225951ea153.final.pdb
1.375379e-15 1.061388e-14 1.203980e-14 7.736909e-16 2.388705e-15
7.954068e-15
E:\\R-3.1.2\\scripts\\mPDBS\\summary_pdb_93\\4ab4a5c7c6682ce6.final.pdb
-2.555295e-15 7.235944e-15 -5.497114e-16 -8.962668e-15 3.753735e-15
1.023203e-14
E:\\R-3.1.2\\scripts\\mPDBS\\summary_pdb_93\\4eaa45b62cb6a25f.final.pdb
1.334565e-15 9.246994e-15 -6.940104e-15 -1.817309e-15 -8.472846e-15
-1.873871e-15
E:\\R-3.1.2\\scripts\\mPDBS\\summary_pdb_93\\58c371611d85ac96.final.pdb
3.017946e-16 7.510464e-15 1.282157e-15 -5.879197e-15 -3.691698e-15
-1.222347e-15
E:\\R-3.1.2\\scripts\\mPDBS\\summary_pdb_93\\59a647f04822d30a.final.pdb
3.787179e-15 4.520230e-15 2.011811e-16 -4.509417e-15 -6.380073e-15
3.831521e-15
E:\\R-3.1.2\\scripts\\mPDBS\\summary_pdb_93\\59b091fe385e05fc.final.pdb
-3.345888e-15 3.808341e-15 -5.941472e-15 -5.702466e-15 -3.731616e-15
-2.383149e-15
E:\\R-3.1.2\\scripts\\mPDBS\\summary_pdb_93\\5c35ac30f930d2db.final.pdb

1.334565e-15 9.246994e-15 -6.940104e-15 -1.817309e-15 -8.472846e-15
-1.873871e-15
E:\\R-3.1.2\\scripts\\mPDBS\\summary_pdb_93\\5f60752431cf2dcb.final.pdb
1.752608e-16 7.504050e-15 -1.084611e-15 -1.627941e-15 -3.100945e-15
-5.467810e-16
E:\\R-3.1.2\\scripts\\mPDBS\\summary_pdb_93\\626456dd6ff2868d.final.pdb
4.418077e-15 5.091392e-15 -1.239264e-15 -2.295258e-15 -4.528052e-15
-4.232093e-15
E:\\R-3.1.2\\scripts\\mPDBS\\summary_pdb_93\\6583d53ff3c8f7ef.final.pdb
3.787179e-15 4.520230e-15 2.011811e-16 -4.509417e-15 -6.380073e-15
3.831521e-15
E:\\R-3.1.2\\scripts\\mPDBS\\summary_pdb_93\\69760178986bef01.final.pdb
-6.123891e-15 1.130999e-14 -6.030851e-15 1.915324e-15 -1.002781e-14
-5.237917e-15
E:\\R-3.1.2\\scripts\\mPDBS\\summary_pdb_93\\6a9d87d47926b1c8.final.pdb
3.815919e-16 5.437469e-15 -1.084006e-15 -4.593767e-15 -4.465385e-15
-1.550210e-15
E:\\R-3.1.2\\scripts\\mPDBS\\summary_pdb_93\\6bb3547ea982ffc4.final.pdb
-4.771054e-15 1.081040e-14 1.781956e-14 -2.006137e-15 -1.243600e-14
-5.143761e-15
E:\\R-3.1.2\\scripts\\mPDBS\\summary_pdb_93\\716d9624611d1c42.final.pdb
-4.872134e-15 8.710333e-15 5.919994e-16 -7.378983e-15 -8.837892e-15
8.028338e-16
E:\\R-3.1.2\\scripts\\mPDBS\\summary_pdb_93\\75f27e70ad0f1be4.final.pdb
3.787179e-15 4.520230e-15 2.011811e-16 -4.509417e-15 -6.380073e-15
3.831521e-15
E:\\R-3.1.2\\scripts\\mPDBS\\summary_pdb_93\\769f910fffb22872.final.pdb
2.568368e-16 5.641106e-15 -3.707553e-16 -4.087634e-15 -4.846437e-15
-1.503535e-15
E:\\R-3.1.2\\scripts\\mPDBS\\summary_pdb_93\\791f8c80dd5c3135.final.pdb
-3.345888e-15 3.808341e-15 -5.941472e-15 -5.702466e-15 -3.731616e-15
-2.383149e-15
E:\\R-3.1.2\\scripts\\mPDBS\\summary_pdb_93\\7fad98e66a3f6099.final.pdb
-1.429177e-14 -2.583241e-15 -1.370034e-15 -5.258389e-15 -8.098374e-16
-4.400598e-15
E:\\R-3.1.2\\scripts\\mPDBS\\summary_pdb_93\\804b89c94a633196.final.pdb
-8.112744e-16 3.318623e-15 -1.098659e-15 -2.356987e-15 -5.620450e-15
-2.949766e-15
E:\\R-3.1.2\\scripts\\mPDBS\\summary_pdb_93\\813cc65a2298feba.final.pdb
-8.112744e-16 3.318623e-15 -1.098659e-15 -2.356987e-15 -5.620450e-15
-2.949766e-15
E:\\R-3.1.2\\scripts\\mPDBS\\summary_pdb_93\\83c0eefcfe12d3f9.final.pdb
-4.771865e-15 2.252202e-15 7.981751e-15 -4.682384e-15 -1.523250e-15
4.110559e-15
E:\\R-3.1.2\\scripts\\mPDBS\\summary_pdb_93\\86d50469ef569760.final.pdb
4.418077e-15 5.091392e-15 -1.239264e-15 -2.295258e-15 -4.528052e-15
-4.232093e-15
E:\\R-3.1.2\\scripts\\mPDBS\\summary_pdb_93\\89d0666e1402203d.final.pdb
3.787179e-15 4.520230e-15 2.011811e-16 -4.509417e-15 -6.380073e-15
3.831521e-15
E:\\R-3.1.2\\scripts\\mPDBS\\summary_pdb_93\\8b34852716dadc62.final.pdb
3.407573e-15 2.547538e-15 -2.000716e-15 -7.253244e-15 -4.662882e-15
1.546638e-15
E:\\R-3.1.2\\scripts\\mPDBS\\summary_pdb_93\\8b6ef9ac3fe74395.final.pdb

-3.719631e-15 4.465312e-15 -2.554451e-15 -6.474557e-15 -1.087555e-14
-1.567234e-14
E:\\R-3.1.2\\scripts\\mPDBS\\summary_pdb_93\\98bbe0d5986c138f.final.pdb
-3.345888e-15 3.808341e-15 -5.941472e-15 -5.702466e-15 -3.731616e-15
-2.383149e-15
E:\\R-3.1.2\\scripts\\mPDBS\\summary_pdb_93\\9c22f4909d67e2fa.final.pdb
9.918858e-15 7.358577e-15 -7.653746e-15 -1.156992e-15 -9.650212e-15
-6.336813e-15
E:\\R-3.1.2\\scripts\\mPDBS\\summary_pdb_93\\9dda8ed0d81fb3ef.final.pdb
-5.082245e-15 2.049031e-14 9.982865e-16 4.154226e-15 2.675401e-15
7.751996e-15
E:\\R-3.1.2\\scripts\\mPDBS\\summary_pdb_93\\9f752b3616e6033b.final.pdb
-1.093541e-15 3.189405e-15 2.005022e-16 -4.372105e-15 -6.483438e-15
-5.177149e-15
E:\\R-3.1.2\\scripts\\mPDBS\\summary_pdb_93\\a02c70083816d6ba.final.pdb
1.334565e-15 9.246994e-15 -6.940104e-15 -1.817309e-15 -8.472846e-15
-1.873871e-15
E:\\R-3.1.2\\scripts\\mPDBS\\summary_pdb_93\\a0c997d891e4e082.final.pdb
-8.840653e-16 4.727169e-15 -4.746173e-16 -3.295323e-15 -4.780559e-15
2.820710e-15
E:\\R-3.1.2\\scripts\\mPDBS\\summary_pdb_93\\ab8039a2d823c616.final.pdb
-2.456870e-16 6.111045e-15 -6.363802e-16 -4.282381e-15 -5.656377e-15
-9.340993e-16
E:\\R-3.1.2\\scripts\\mPDBS\\summary_pdb_93\\acf4d74b9c71d4dc.final.pdb
-8.112744e-16 3.318623e-15 -1.098659e-15 -2.356987e-15 -5.620450e-15
-2.949766e-15
E:\\R-3.1.2\\scripts\\mPDBS\\summary_pdb_93\\ad8a823e4b4c668e.final.pdb
-2.454492e-16 5.532996e-15 -6.926187e-16 -4.736210e-15 -5.818206e-15
-1.593392e-15
E:\\R-3.1.2\\scripts\\mPDBS\\summary_pdb_93\\b16ababd03b7b857.final.pdb
9.918858e-15 7.358577e-15 -7.653746e-15 -1.156992e-15 -9.650212e-15
-6.336813e-15
E:\\R-3.1.2\\scripts\\mPDBS\\summary_pdb_93\\b226923e697d3c63.final.pdb
-1.192012e-16 3.407405e-15 -2.392664e-15 -1.349702e-14 -7.851118e-15
-4.620919e-15
E:\\R-3.1.2\\scripts\\mPDBS\\summary_pdb_93\\b28e48f6fa27a165.final.pdb
-3.345888e-15 3.808341e-15 -5.941472e-15 -5.702466e-15 -3.731616e-15
-2.383149e-15
E:\\R-3.1.2\\scripts\\mPDBS\\summary_pdb_93\\b42c4924e3cd1aec.final.pdb
5.125518e-15 -2.593871e-15 -2.402205e-15 -6.823538e-15 4.475820e-16
-1.025704e-14
E:\\R-3.1.2\\scripts\\mPDBS\\summary_pdb_93\\b4dd239fcabff1df.final.pdb
-3.345888e-15 3.808341e-15 -5.941472e-15 -5.702466e-15 -3.731616e-15
-2.383149e-15
E:\\R-3.1.2\\scripts\\mPDBS\\summary_pdb_93\\b669942ef97459a2.final.pdb
3.407573e-15 2.547538e-15 -2.000716e-15 -7.253244e-15 -4.662882e-15
1.546638e-15
E:\\R-3.1.2\\scripts\\mPDBS\\summary_pdb_93\\b99f4d9d87c1017b.final.pdb
-3.345888e-15 3.808341e-15 -5.941472e-15 -5.702466e-15 -3.731616e-15
-2.383149e-15
E:\\R-3.1.2\\scripts\\mPDBS\\summary_pdb_93\\b9d2cbf3975403e7.final.pdb
-3.345888e-15 3.808341e-15 -5.941472e-15 -5.702466e-15 -3.731616e-15
-2.383149e-15
E:\\R-3.1.2\\scripts\\mPDBS\\summary_pdb_93\\bc88c8a505f3a288.final.pdb

-3.233492e-16 5.779854e-15 -1.894145e-15 -4.624335e-15 -5.483904e-15
-1.501042e-15
E:\\R-3.1.2\\scripts\\mPDBS\\summary_pdb_93\\bdac0e64f4ef427e.final.pdb
1.338462e-14 1.007299e-14 -1.655201e-14 -4.614296e-15 -1.087514e-14
-1.277784e-14
E:\\R-3.1.2\\scripts\\mPDBS\\summary_pdb_93\\ca2a1f55fc9d0681.final.pdb
3.407573e-15 2.547538e-15 -2.000716e-15 -7.253244e-15 -4.662882e-15
1.546638e-15
E:\\R-3.1.2\\scripts\\mPDBS\\summary_pdb_93\\ce34248de3e81a3c.final.pdb
-6.009860e-16 2.982100e-15 -1.032244e-14 -7.015310e-15 -4.590838e-15
1.801643e-15
E:\\R-3.1.2\\scripts\\mPDBS\\summary_pdb_93\\d02e697772494a61.final.pdb
-2.101224e-15 -1.809127e-15 -1.676221e-15 -1.058745e-14 -1.904033e-14
-2.044011e-15
E:\\R-3.1.2\\scripts\\mPDBS\\summary_pdb_93\\d04221245d015047.final.pdb
-1.293460e-15 5.724635e-15 -7.910030e-17 -1.251819e-15 -4.499533e-15
-7.666985e-16
E:\\R-3.1.2\\scripts\\mPDBS\\summary_pdb_93\\d678141e4018054a.final.pdb
-6.507926e-15 6.723314e-15 2.501985e-14 -1.004170e-14 -2.666161e-15
5.410042e-15
E:\\R-3.1.2\\scripts\\mPDBS\\summary_pdb_93\\d71b40af0f0efe39.final.pdb
8.997280e-15 6.072324e-15 -1.678816e-15 -4.070562e-15 3.960006e-15
-3.207072e-15
E:\\R-3.1.2\\scripts\\mPDBS\\summary_pdb_93\\dc0809eff10197e2.final.pdb
6.653471e-15 5.563456e-15 -1.417162e-14 -2.196164e-16 8.282238e-16
-8.088859e-15
E:\\R-3.1.2\\scripts\\mPDBS\\summary_pdb_93\\e1163918d3707839.final.pdb
-1.509799e-15 -1.425752e-15 4.522811e-15 -1.036888e-14 -1.384286e-15
-1.148349e-14
E:\\R-3.1.2\\scripts\\mPDBS\\summary_pdb_93\\e4b48b06824ef848.final.pdb
4.233040e-15 1.106754e-14 4.970025e-15 -7.471646e-15 -7.301070e-15
2.083006e-15
E:\\R-3.1.2\\scripts\\mPDBS\\summary_pdb_93\\e529e19ad281fea2.final.pdb
-2.853884e-15 6.604071e-15 -2.890721e-15 -2.310004e-15 -7.948927e-15
-3.397691e-15
E:\\R-3.1.2\\scripts\\mPDBS\\summary_pdb_93\\e5602082718ec3a1.final.pdb
-8.112744e-16 3.318623e-15 -1.098659e-15 -2.356987e-15 -5.620450e-15
-2.949766e-15
E:\\R-3.1.2\\scripts\\mPDBS\\summary_pdb_93\\e5d589d996d0a960.final.pdb
-5.170636e-15 6.233519e-15 -2.986831e-15 5.784438e-15 -6.064487e-15
-1.681593e-14
E:\\R-3.1.2\\scripts\\mPDBS\\summary_pdb_93\\e9df9e72307a2fbd.final.pdb
1.334565e-15 9.246994e-15 -6.940104e-15 -1.817309e-15 -8.472846e-15
-1.873871e-15
E:\\R-3.1.2\\scripts\\mPDBS\\summary_pdb_93\\ea00f8694090cae7.final.pdb
-8.112744e-16 3.318623e-15 -1.098659e-15 -2.356987e-15 -5.620450e-15
-2.949766e-15
E:\\R-3.1.2\\scripts\\mPDBS\\summary_pdb_93\\eba517be32db050e.final.pdb
-3.345888e-15 3.808341e-15 -5.941472e-15 -5.702466e-15 -3.731616e-15
-2.383149e-15
E:\\R-3.1.2\\scripts\\mPDBS\\summary_pdb_93\\ec04b9545899c29d.final.pdb
-7.751435e-16 5.151453e-15 -1.172720e-15 -4.512453e-15 -5.446792e-15
-2.024906e-15
E:\\R-3.1.2\\scripts\\mPDBS\\summary_pdb_93\\ee6862cbcad52078.final.pdb

-7.706910e-16 5.690299e-15 -1.597724e-15 -4.255272e-15 -5.207866e-15
-1.618136e-15
E:\\R-3.1.2\\scripts\\mPDBS\\summary_pdb_93\\ef6a42fc83b81e5d.final.pdb
-2.974074e-16 5.406918e-15 -7.051233e-16 -4.434579e-15 -5.835446e-15
-1.983186e-15
E:\\R-3.1.2\\scripts\\mPDBS\\summary_pdb_93\\ef8e88d4ad93a0ad.final.pdb
-1.293460e-15 5.724635e-15 -7.910030e-17 -1.251819e-15 -4.499533e-15
-7.666985e-16
E:\\R-3.1.2\\scripts\\mPDBS\\summary_pdb_93\\f14fcae8deec4fa3.final.pdb
-1.093541e-15 3.189405e-15 2.005022e-16 -4.372105e-15 -6.483438e-15
-5.177149e-15
E:\\R-3.1.2\\scripts\\mPDBS\\summary_pdb_93\\f83584e22003fb92.final.pdb
1.375379e-15 1.061388e-14 1.203980e-14 7.736909e-16 2.388705e-15
7.954068e-15
E:\\R-3.1.2\\scripts\\mPDBS\\summary_pdb_93\\f8e2bf87e52caa29.final.pdb
-2.853884e-15 6.604071e-15 -2.890721e-15 -2.310004e-15 -7.948927e-15
-3.397691e-15

[,355]   [,356]   [,357]   [,358]   [,359]   [,360]
E:\\R-3.1.2\\scripts\\40.1ns_data\\NAT0.pdb
7.669987e-16 4.304123e-15 -2.074902e-15 -3.117567e-15 1.507909e-15
-2.840503e-15
E:\\R-3.1.2\\scripts\\40.1ns_data\\LJP6.pdb
4.132903e-15 4.847800e-15 -1.882754e-15 -3.453894e-15 1.737353e-15
-1.875871e-15
E:\\R-3.1.2\\scripts\\40.1ns_data\\3WY9.pdb
-8.841720e-16 4.620940e-15 -2.433879e-15 -3.789690e-15 1.458896e-15
-2.209024e-15
E:\\R-3.1.2\\scripts\\40.1ns_data\\ZSP9.pdb
1.942059e-15 4.809396e-15 -6.807039e-16 -3.331274e-15 2.851184e-15
-1.956797e-15
E:\\R-3.1.2\\scripts\\mPDBS\\summary_pdb_93\\013d855ad26c877c.final.pdb
6.984648e-15 8.573614e-15 -2.851833e-15 -1.429247e-15 3.251683e-15
1.888461e-15
E:\\R-3.1.2\\scripts\\mPDBS\\summary_pdb_93\\0a2a705342ca0d54.final.pdb
1.951441e-15 4.660499e-15 -7.457946e-16 -3.318896e-15 2.287753e-15
-1.906389e-15
E:\\R-3.1.2\\scripts\\mPDBS\\summary_pdb_93\\0a59fceadb4194d9.final.pdb
7.699776e-15 5.339085e-15 2.081076e-15 -5.111001e-15 -1.414262e-15
-3.114716e-15
E:\\R-3.1.2\\scripts\\mPDBS\\summary_pdb_93\\0dba59f3306825e8.final.pdb
-1.505089e-15 1.306424e-15 -1.176090e-15 -3.239431e-15 6.565365e-16
-4.656650e-15
E:\\R-3.1.2\\scripts\\mPDBS\\summary_pdb_93\\126f26a78b57339c.final.pdb
-6.511501e-15 -5.727154e-16 -2.366111e-15 -4.006829e-15 4.198842e-15
-2.404762e-15
E:\\R-3.1.2\\scripts\\mPDBS\\summary_pdb_93\\12f06f3e342df706.final.pdb
3.063398e-15 4.589809e-15 -1.103581e-15 -2.795010e-15 2.720567e-15
-1.912135e-15
E:\\R-3.1.2\\scripts\\mPDBS\\summary_pdb_93\\18618a2a251b867b.final.pdb
-8.563658e-15 6.642496e-16 -5.950060e-16 5.571765e-15 3.709262e-15
-3.470357e-15
E:\\R-3.1.2\\scripts\\mPDBS\\summary_pdb_93\\1f305f598641eeb2.final.pdb
2.457322e-15 4.424519e-15 -1.234660e-15 -3.573824e-15 2.690799e-15

-2.527373e-15
E:\\R-3.1.2\\scripts\\mPDBS\\summary_pdb_93\\1f8a00b551be0a39.final.pdb
-8.563658e-15 6.642496e-16 -5.950060e-16 5.571765e-15 3.709262e-15
-3.470357e-15
E:\\R-3.1.2\\scripts\\mPDBS\\summary_pdb_93\\219f45328d47579a.final.pdb
1.118397e-14 1.536449e-14 -1.950756e-14 -1.351617e-14 4.427419e-15
-1.173561e-15
E:\\R-3.1.2\\scripts\\mPDBS\\summary_pdb_93\\238dd3918e54336d.final.pdb
-1.155999e-14 -3.713135e-15 -7.499802e-15 -6.122345e-15 1.151394e-15
1.230007e-15
E:\\R-3.1.2\\scripts\\mPDBS\\summary_pdb_93\\2b3027889e2fdc42.final.pdb
2.887653e-15 4.226694e-15 -1.018875e-15 -3.860699e-15 1.448032e-15
-1.226578e-15
E:\\R-3.1.2\\scripts\\mPDBS\\summary_pdb_93\\2fcb3b46e33793b0.final.pdb
9.800407e-15 5.122746e-15 -9.321208e-17 -3.455033e-16 2.482633e-15
-2.522169e-15
E:\\R-3.1.2\\scripts\\mPDBS\\summary_pdb_93\\34eb328251842006.final.pdb
9.042365e-15 1.097251e-14 -7.068030e-15 -4.906436e-15 1.452317e-15
2.907630e-15
E:\\R-3.1.2\\scripts\\mPDBS\\summary_pdb_93\\354b32871797b7ab.final.pdb
1.430637e-14 8.093179e-15 -1.486346e-14 3.178292e-15 6.967584e-15
6.960997e-15
E:\\R-3.1.2\\scripts\\mPDBS\\summary_pdb_93\\397368adbc32d5de.final.pdb
-6.511501e-15 -5.727154e-16 -2.366111e-15 -4.006829e-15 4.198842e-15
-2.404762e-15
E:\\R-3.1.2\\scripts\\mPDBS\\summary_pdb_93\\411ca13cd3df6edd.final.pdb
2.986413e-15 6.619043e-16 -4.478593e-15 4.764775e-15 4.019598e-15
-1.873599e-15
E:\\R-3.1.2\\scripts\\mPDBS\\summary_pdb_93\\42357eee6642bf12.final.pdb
1.364215e-14 8.582655e-15 -1.892578e-15 -3.595810e-15 -1.319917e-15
-1.315034e-15
E:\\R-3.1.2\\scripts\\mPDBS\\summary_pdb_93\\44342d1f75a7fcfc.final.pdb
3.669164e-15 -6.060550e-15 1.944296e-14 -4.041371e-15 5.016397e-15
2.842222e-15
E:\\R-3.1.2\\scripts\\mPDBS\\summary_pdb_93\\448ef21dffe8cdfb.final.pdb
6.899949e-15 1.065781e-14 -6.039844e-15 -1.623127e-15 3.044683e-15
6.789827e-15
E:\\R-3.1.2\\scripts\\mPDBS\\summary_pdb_93\\472a40b3e8f113c7.final.pdb
-1.505089e-15 1.306424e-15 -1.176090e-15 -3.239431e-15 6.565365e-16
-4.656650e-15
E:\\R-3.1.2\\scripts\\mPDBS\\summary_pdb_93\\48a26225951ea153.final.pdb
-6.884797e-16 -3.482054e-16 4.832347e-15 5.958269e-15 1.120932e-14
1.691661e-15
E:\\R-3.1.2\\scripts\\mPDBS\\summary_pdb_93\\4ab4a5c7c6682ce6.final.pdb
1.885883e-14 8.255292e-15 3.941344e-15 8.651418e-16 1.562062e-15
-7.560361e-15
E:\\R-3.1.2\\scripts\\mPDBS\\summary_pdb_93\\4eaa45b62cb6a25f.final.pdb
-4.071290e-15 1.792663e-15 3.040621e-15 -3.069532e-15 1.198761e-15
-6.696589e-15
E:\\R-3.1.2\\scripts\\mPDBS\\summary_pdb_93\\58c371611d85ac96.final.pdb
-6.511501e-15 -5.727154e-16 -2.366111e-15 -4.006829e-15 4.198842e-15
-2.404762e-15
E:\\R-3.1.2\\scripts\\mPDBS\\summary_pdb_93\\59a647f04822d30a.final.pdb
2.759302e-15 6.714387e-15 -6.239620e-15 -6.176815e-15 8.441609e-15

-5.309476e-16
E:\\R-3.1.2\\scripts\\mPDBS\\summary_pdb_93\\59b091fe385e05fc.final.pdb
7.699776e-15 5.339085e-15 2.081076e-15 -5.111001e-15 -1.414262e-15
-3.114716e-15
E:\\R-3.1.2\\scripts\\mPDBS\\summary_pdb_93\\5c35ac30f930d2db.final.pdb
-4.071290e-15 1.792663e-15 3.040621e-15 -3.069532e-15 1.198761e-15
-6.696589e-15
E:\\R-3.1.2\\scripts\\mPDBS\\summary_pdb_93\\5f60752431cf2dcb.final.pdb
-6.422070e-15 -5.076760e-16 8.682427e-15 -2.838161e-15 -2.857091e-16
-2.408266e-15
E:\\R-3.1.2\\scripts\\mPDBS\\summary_pdb_93\\626456dd6ff2868d.final.pdb
-1.505089e-15 1.306424e-15 -1.176090e-15 -3.239431e-15 6.565365e-16
-4.656650e-15
E:\\R-3.1.2\\scripts\\mPDBS\\summary_pdb_93\\6583d53ff3c8f7ef.final.pdb
2.759302e-15 6.714387e-15 -6.239620e-15 -6.176815e-15 8.441609e-15
-5.309476e-16
E:\\R-3.1.2\\scripts\\mPDBS\\summary_pdb_93\\69760178986bef01.final.pdb
-4.973239e-15 -4.137520e-15 -2.908615e-16 -6.165173e-15 -2.106860e-15
-3.897423e-15
E:\\R-3.1.2\\scripts\\mPDBS\\summary_pdb_93\\6a9d87d47926b1c8.final.pdb
2.507327e-15 3.937999e-15 -1.519566e-15 -4.177699e-15 1.999212e-15
-1.461073e-15
E:\\R-3.1.2\\scripts\\mPDBS\\summary_pdb_93\\6bb3547ea982ffc4.final.pdb
-1.646424e-14 -6.217932e-15 -3.925805e-15 -1.427872e-15 4.707054e-15
1.418968e-14
E:\\R-3.1.2\\scripts\\mPDBS\\summary_pdb_93\\716d9624611d1c42.final.pdb
2.717321e-15 6.472851e-15 -1.441889e-14 1.013576e-15 5.714699e-15
7.078396e-16
E:\\R-3.1.2\\scripts\\mPDBS\\summary_pdb_93\\75f27e70ad0f1be4.final.pdb
2.759302e-15 6.714387e-15 -6.239620e-15 -6.176815e-15 8.441609e-15
-5.309476e-16
E:\\R-3.1.2\\scripts\\mPDBS\\summary_pdb_93\\769f910fffb22872.final.pdb
2.144790e-15 3.456287e-15 -1.248997e-15 -3.395671e-15 2.239084e-15
-2.783623e-15
E:\\R-3.1.2\\scripts\\mPDBS\\summary_pdb_93\\791f8c80dd5c3135.final.pdb
7.699776e-15 5.339085e-15 2.081076e-15 -5.111001e-15 -1.414262e-15
-3.114716e-15
E:\\R-3.1.2\\scripts\\mPDBS\\summary_pdb_93\\7fad98e66a3f6099.final.pdb
1.979045e-14 1.083638e-14 -3.436084e-15 -5.436936e-15 -5.613746e-15
5.667371e-15
E:\\R-3.1.2\\scripts\\mPDBS\\summary_pdb_93\\804b89c94a633196.final.pdb
9.800407e-15 5.122746e-15 -9.321208e-17 -3.455033e-16 2.482633e-15
-2.522169e-15
E:\\R-3.1.2\\scripts\\mPDBS\\summary_pdb_93\\813cc65a2298feba.final.pdb
9.800407e-15 5.122746e-15 -9.321208e-17 -3.455033e-16 2.482633e-15
-2.522169e-15
E:\\R-3.1.2\\scripts\\mPDBS\\summary_pdb_93\\83c0eefcfe12d3f9.final.pdb
2.986413e-15 6.619043e-16 -4.478593e-15 4.764775e-15 4.019598e-15
-1.873599e-15
E:\\R-3.1.2\\scripts\\mPDBS\\summary_pdb_93\\86d50469ef569760.final.pdb
-1.505089e-15 1.306424e-15 -1.176090e-15 -3.239431e-15 6.565365e-16
-4.656650e-15
E:\\R-3.1.2\\scripts\\mPDBS\\summary_pdb_93\\89d0666e1402203d.final.pdb
2.759302e-15 6.714387e-15 -6.239620e-15 -6.176815e-15 8.441609e-15

-5.309476e-16
E:\\R-3.1.2\\scripts\\mPDBS\\summary_pdb_93\\8b34852716dadc62.final.pdb
7.295376e-16 2.023662e-15 2.449893e-15 -7.849917e-15 2.503449e-15
-7.440110e-15
E:\\R-3.1.2\\scripts\\mPDBS\\summary_pdb_93\\8b6ef9ac3fe74395.final.pdb
-1.778224e-15 3.117924e-15 1.638164e-15 -6.303554e-15 1.755257e-15
4.883279e-15
E:\\R-3.1.2\\scripts\\mPDBS\\summary_pdb_93\\98bbe0d5986c138f.final.pdb
7.699776e-15 5.339085e-15 2.081076e-15 -5.111001e-15 -1.414262e-15
-3.114716e-15
E:\\R-3.1.2\\scripts\\mPDBS\\summary_pdb_93\\9c22f4909d67e2fa.final.pdb
-3.890031e-15 6.408176e-15 -1.696097e-15 -1.298492e-14 1.818233e-15
-5.698447e-15
E:\\R-3.1.2\\scripts\\mPDBS\\summary_pdb_93\\9dda8ed0d81fb3ef.final.pdb
3.233164e-16 -6.800844e-15 1.964245e-14 5.734013e-15 -5.711469e-16
-2.718109e-15
E:\\R-3.1.2\\scripts\\mPDBS\\summary_pdb_93\\9f752b3616e6033b.final.pdb
5.114305e-15 4.522256e-15 2.623663e-16 -5.832873e-15 2.353342e-15
-9.888772e-16
E:\\R-3.1.2\\scripts\\mPDBS\\summary_pdb_93\\a02c70083816d6ba.final.pdb
-4.071290e-15 1.792663e-15 3.040621e-15 -3.069532e-15 1.198761e-15
-6.696589e-15
E:\\R-3.1.2\\scripts\\mPDBS\\summary_pdb_93\\a0c997d891e4e082.final.pdb
1.067478e-15 9.769088e-15 7.455251e-15 -5.951049e-15 6.092437e-16
-3.804468e-15
E:\\R-3.1.2\\scripts\\mPDBS\\summary_pdb_93\\ab8039a2d823c616.final.pdb
1.312942e-15 4.104782e-15 -1.299899e-15 -4.275740e-15 2.417693e-15
-2.025401e-15
E:\\R-3.1.2\\scripts\\mPDBS\\summary_pdb_93\\acf4d74b9c71d4dc.final.pdb
9.800407e-15 5.122746e-15 -9.321208e-17 -3.455033e-16 2.482633e-15
-2.522169e-15
E:\\R-3.1.2\\scripts\\mPDBS\\summary_pdb_93\\ad8a823e4b4c668e.final.pdb
1.992501e-15 4.081001e-15 -1.372986e-15 -2.760996e-15 1.789930e-15
-2.355429e-15
E:\\R-3.1.2\\scripts\\mPDBS\\summary_pdb_93\\b16ababd03b7b857.final.pdb
-3.890031e-15 6.408176e-15 -1.696097e-15 -1.298492e-14 1.818233e-15
-5.698447e-15
E:\\R-3.1.2\\scripts\\mPDBS\\summary_pdb_93\\b226923e697d3c63.final.pdb
-2.201140e-15 8.986007e-15 2.709819e-15 -2.462555e-15 -5.548069e-15
-3.458077e-15
E:\\R-3.1.2\\scripts\\mPDBS\\summary_pdb_93\\b28e48f6fa27a165.final.pdb
7.699776e-15 5.339085e-15 2.081076e-15 -5.111001e-15 -1.414262e-15
-3.114716e-15
E:\\R-3.1.2\\scripts\\mPDBS\\summary_pdb_93\\b42c4924e3cd1aec.final.pdb
-1.668580e-15 6.381754e-15 -9.624065e-15 -5.246136e-15 3.086518e-15
-1.104608e-14
E:\\R-3.1.2\\scripts\\mPDBS\\summary_pdb_93\\b4dd239fcabff1df.final.pdb
7.699776e-15 5.339085e-15 2.081076e-15 -5.111001e-15 -1.414262e-15
-3.114716e-15
E:\\R-3.1.2\\scripts\\mPDBS\\summary_pdb_93\\b669942ef97459a2.final.pdb
7.295376e-16 2.023662e-15 2.449893e-15 -7.849917e-15 2.503449e-15
-7.440110e-15
E:\\R-3.1.2\\scripts\\mPDBS\\summary_pdb_93\\b99f4d9d87c1017b.final.pdb
7.699776e-15 5.339085e-15 2.081076e-15 -5.111001e-15 -1.414262e-15

-3.114716e-15
E:\\R-3.1.2\\scripts\\mPDBS\\summary_pdb_93\\b9d2cbf3975403e7.final.pdb
7.699776e-15 5.339085e-15 2.081076e-15 -5.111001e-15 -1.414262e-15
-3.114716e-15
E:\\R-3.1.2\\scripts\\mPDBS\\summary_pdb_93\\bc88c8a505f3a288.final.pdb
2.522493e-15 4.120876e-15 -1.095203e-15 -3.718018e-15 2.045644e-15
-1.587401e-15
E:\\R-3.1.2\\scripts\\mPDBS\\summary_pdb_93\\bdac0e64f4ef427e.final.pdb
-2.974187e-16 1.136818e-14 4.621333e-15 -1.946953e-14 -2.437911e-15
-5.228771e-15
E:\\R-3.1.2\\scripts\\mPDBS\\summary_pdb_93\\ca2a1f55fc9d0681.final.pdb
7.295376e-16 2.023662e-15 2.449893e-15 -7.849917e-15 2.503449e-15
-7.440110e-15
E:\\R-3.1.2\\scripts\\mPDBS\\summary_pdb_93\\ce34248de3e81a3c.final.pdb
5.688097e-15 9.110274e-15 1.913116e-15 1.427334e-16 1.565543e-15
-6.621858e-15
E:\\R-3.1.2\\scripts\\mPDBS\\summary_pdb_93\\d02e697772494a61.final.pdb
8.805662e-15 1.309547e-14 -1.332847e-14 -1.161535e-14 4.041443e-15
7.443787e-15
E:\\R-3.1.2\\scripts\\mPDBS\\summary_pdb_93\\d04221245d015047.final.pdb
4.245492e-15 2.521413e-15 -2.465633e-15 -3.593560e-15 5.116055e-15
-1.941375e-15
E:\\R-3.1.2\\scripts\\mPDBS\\summary_pdb_93\\d678141e4018054a.final.pdb
-7.632826e-15 1.225299e-15 -1.033173e-14 3.024907e-15 7.084698e-15
8.616239e-15
E:\\R-3.1.2\\scripts\\mPDBS\\summary_pdb_93\\d71b40af0f0efe39.final.pdb
1.765924e-14 9.338046e-15 -6.312514e-15 -1.680161e-15 1.267598e-15
-7.298816e-15
E:\\R-3.1.2\\scripts\\mPDBS\\summary_pdb_93\\dc0809eff10197e2.final.pdb
4.300415e-15 7.910516e-15 3.989284e-16 -7.704271e-15 1.856143e-15
-7.649926e-15
E:\\R-3.1.2\\scripts\\mPDBS\\summary_pdb_93\\e1163918d3707839.final.pdb
7.123282e-16 5.443702e-15 -5.700121e-15 -2.859111e-16 -2.095101e-15
-1.147803e-14
E:\\R-3.1.2\\scripts\\mPDBS\\summary_pdb_93\\e4b48b06824ef848.final.pdb
-8.563658e-15 6.642496e-16 -5.950060e-16 5.571765e-15 3.709262e-15
-3.470357e-15
E:\\R-3.1.2\\scripts\\mPDBS\\summary_pdb_93\\e529e19ad281fea2.final.pdb
6.984648e-15 8.573614e-15 -2.851833e-15 -1.429247e-15 3.251683e-15
1.888461e-15
E:\\R-3.1.2\\scripts\\mPDBS\\summary_pdb_93\\e5602082718ec3a1.final.pdb
9.800407e-15 5.122746e-15 -9.321208e-17 -3.455033e-16 2.482633e-15
-2.522169e-15
E:\\R-3.1.2\\scripts\\mPDBS\\summary_pdb_93\\e5d589d996d0a960.final.pdb
-2.241955e-15 3.229311e-15 5.739848e-15 -4.995339e-15 3.354600e-15
-8.772554e-16
E:\\R-3.1.2\\scripts\\mPDBS\\summary_pdb_93\\e9df9e72307a2fbd.final.pdb
-4.071290e-15 1.792663e-15 3.040621e-15 -3.069532e-15 1.198761e-15
-6.696589e-15
E:\\R-3.1.2\\scripts\\mPDBS\\summary_pdb_93\\ea00f8694090cae7.final.pdb
9.800407e-15 5.122746e-15 -9.321208e-17 -3.455033e-16 2.482633e-15
-2.522169e-15
E:\\R-3.1.2\\scripts\\mPDBS\\summary_pdb_93\\eba517be32db050e.final.pdb
7.699776e-15 5.339085e-15 2.081076e-15 -5.111001e-15 -1.414262e-15

-3.114716e-15
E:\\R-3.1.2\\scripts\\mPDBS\\summary_pdb_93\\ec04b9545899c29d.final.pdb
1.740152e-15 4.437996e-15 -9.244269e-16 -3.742998e-15 2.507987e-15
-2.043193e-15
E:\\R-3.1.2\\scripts\\mPDBS\\summary_pdb_93\\ee6862cbcad52078.final.pdb
2.364634e-15 4.024599e-15 -1.008467e-15 -3.879781e-15 2.238198e-15
-1.923828e-15
E:\\R-3.1.2\\scripts\\mPDBS\\summary_pdb_93\\ef6a42fc83b81e5d.final.pdb
2.418009e-15 4.143238e-15 -8.656228e-16 -3.589526e-15 2.274646e-15
-2.366964e-15
E:\\R-3.1.2\\scripts\\mPDBS\\summary_pdb_93\\ef8e88d4ad93a0ad.final.pdb
4.245492e-15 2.521413e-15 -2.465633e-15 -3.593560e-15 5.116055e-15
-1.941375e-15
E:\\R-3.1.2\\scripts\\mPDBS\\summary_pdb_93\\f14fcae8deec4fa3.final.pdb
5.114305e-15 4.522256e-15 2.623663e-16 -5.832873e-15 2.353342e-15
-9.888772e-16
E:\\R-3.1.2\\scripts\\mPDBS\\summary_pdb_93\\f83584e22003fb92.final.pdb
-6.884797e-16 -3.482054e-16 4.832347e-15 5.958269e-15 1.120932e-14
1.691661e-15
E:\\R-3.1.2\\scripts\\mPDBS\\summary_pdb_93\\f8e2bf87e52caa29.final.pdb
6.984648e-15 8.573614e-15 -2.851833e-15 -1.429247e-15 3.251683e-15
1.888461e-15

[,361]   [,362]   [,363]   [,364]   [,365]   [,366]
E:\\R-3.1.2\\scripts\\40.1ns_data\\NAT0.pdb
7.407243e-16 1.269333e-15 -2.837951e-15 1.810862e-15 2.262039e-15
3.406482e-15
E:\\R-3.1.2\\scripts\\40.1ns_data\\LJP6.pdb
-9.889118e-16 1.174489e-15 -4.866572e-16 1.685343e-15 1.987814e-15
2.860452e-15
E:\\R-3.1.2\\scripts\\40.1ns_data\\3WY9.pdb
-1.162884e-15 -2.264837e-15 -1.179220e-15 -1.818242e-15 4.090100e-15
3.906640e-15
E:\\R-3.1.2\\scripts\\40.1ns_data\\ZSP9.pdb
-1.561821e-16 -1.450426e-15 -1.446970e-15 2.543309e-15 2.587299e-15
1.190772e-15
E:\\R-3.1.2\\scripts\\mPDBS\\summary_pdb_93\\013d855ad26c877c.final.pdb
-7.477714e-16 5.443569e-15 -8.505122e-15 1.118734e-14 1.851008e-15
5.073652e-15
E:\\R-3.1.2\\scripts\\mPDBS\\summary_pdb_93\\0a2a705342ca0d54.final.pdb
-5.704270e-17 -4.406298e-16 -1.360004e-15 3.009080e-15 2.762770e-15
4.195141e-15
E:\\R-3.1.2\\scripts\\mPDBS\\summary_pdb_93\\0a59fceadb4194d9.final.pdb
-2.229849e-15 2.161873e-16 2.139798e-15 5.487342e-15 4.052888e-15
3.941449e-15
E:\\R-3.1.2\\scripts\\mPDBS\\summary_pdb_93\\0dba59f3306825e8.final.pdb
-2.083508e-15 1.980194e-15 -8.220314e-16 2.936993e-15 7.416868e-15
3.956490e-15
E:\\R-3.1.2\\scripts\\mPDBS\\summary_pdb_93\\126f26a78b57339c.final.pdb
3.559113e-15 -8.665750e-15 -2.570633e-15 -4.851482e-15 2.561811e-15
5.370290e-15
E:\\R-3.1.2\\scripts\\mPDBS\\summary_pdb_93\\12f06f3e342df706.final.pdb
-5.509114e-17 5.941328e-16 -1.938535e-15 2.520321e-15 3.166093e-15
3.936667e-15

E:\\R-3.1.2\\scripts\\mPDBS\\summary_pdb_93\\18618a2a251b867b.final.pdb
2.470840e-15 -9.733991e-15 -8.062502e-15 5.392861e-15 5.623832e-15
4.376648e-15
E:\\R-3.1.2\\scripts\\mPDBS\\summary_pdb_93\\1f305f598641eeb2.final.pdb
-1.668964e-16 6.292732e-17 -1.642430e-15 1.513502e-15 2.783403e-15
4.178557e-15
E:\\R-3.1.2\\scripts\\mPDBS\\summary_pdb_93\\1f8a00b551be0a39.final.pdb
2.470840e-15 -9.733991e-15 -8.062502e-15 5.392861e-15 5.623832e-15
4.376648e-15
E:\\R-3.1.2\\scripts\\mPDBS\\summary_pdb_93\\219f45328d47579a.final.pdb
9.019833e-15 7.377776e-15 -8.650182e-15 -7.717373e-15 -8.717713e-15
1.000257e-14
E:\\R-3.1.2\\scripts\\mPDBS\\summary_pdb_93\\238dd3918e54336d.final.pdb
5.568384e-15 -3.109278e-15 -7.797566e-15 3.816803e-15 2.761820e-15
5.681810e-15
E:\\R-3.1.2\\scripts\\mPDBS\\summary_pdb_93\\2b3027889e2fdc42.final.pdb
-8.546467e-16 4.419724e-16 -2.276808e-15 4.228367e-15 3.125244e-15
4.079793e-15
E:\\R-3.1.2\\scripts\\mPDBS\\summary_pdb_93\\2fcb3b46e33793b0.final.pdb
-4.059616e-15 5.921088e-15 -2.772025e-16 8.951996e-15 6.295351e-15
2.679756e-15
E:\\R-3.1.2\\scripts\\mPDBS\\summary_pdb_93\\34eb328251842006.final.pdb
1.083728e-14 7.364521e-16 -1.557670e-15 6.558913e-15 -7.674272e-15
3.504692e-15
E:\\R-3.1.2\\scripts\\mPDBS\\summary_pdb_93\\354b32871797b7ab.final.pdb
6.723683e-15 -1.550812e-15 -9.403009e-15 1.627149e-14 -1.372680e-15
2.053316e-14
E:\\R-3.1.2\\scripts\\mPDBS\\summary_pdb_93\\397368adbc32d5de.final.pdb
3.559113e-15 -8.665750e-15 -2.570633e-15 -4.851482e-15 2.561811e-15
5.370290e-15
E:\\R-3.1.2\\scripts\\mPDBS\\summary_pdb_93\\411ca13cd3df6edd.final.pdb
1.382645e-15 -7.411712e-16 2.137243e-16 7.678275e-15 8.823276e-15
1.307590e-15
E:\\R-3.1.2\\scripts\\mPDBS\\summary_pdb_93\\42357eee6642bf12.final.pdb
5.020112e-15 -5.512815e-15 -2.089136e-15 9.032576e-15 -6.954636e-15
8.056684e-15
E:\\R-3.1.2\\scripts\\mPDBS\\summary_pdb_93\\44342d1f75a7fcfc.final.pdb
-6.721900e-15 -7.898557e-15 2.074473e-15 1.299010e-14 3.942383e-15
-1.129500e-14
E:\\R-3.1.2\\scripts\\mPDBS\\summary_pdb_93\\448ef21dffe8cdfb.final.pdb
7.127616e-15 -5.978247e-15 -7.160585e-15 -2.316768e-15 -6.718545e-15
1.519752e-14
E:\\R-3.1.2\\scripts\\mPDBS\\summary_pdb_93\\472a40b3e8f113c7.final.pdb
-2.083508e-15 1.980194e-15 -8.220314e-16 2.936993e-15 7.416868e-15
3.956490e-15
E:\\R-3.1.2\\scripts\\mPDBS\\summary_pdb_93\\48a26225951ea153.final.pdb
-1.885129e-16 -1.018278e-14 -1.014725e-14 1.348832e-14 1.170211e-15
1.923532e-15
E:\\R-3.1.2\\scripts\\mPDBS\\summary_pdb_93\\4ab4a5c7c6682ce6.final.pdb
-9.654275e-15 5.126169e-15 5.296338e-15 1.120512e-14 4.458731e-15
3.115149e-15
E:\\R-3.1.2\\scripts\\mPDBS\\summary_pdb_93\\4eaa45b62cb6a25f.final.pdb
-4.811144e-15 -3.921773e-17 7.884915e-16 3.935650e-16 7.820487e-15
-4.834525e-16

E:\\R-3.1.2\\scripts\\mPDBS\\summary_pdb_93\\58c371611d85ac96.final.pdb
3.559113e-15 -8.665750e-15 -2.570633e-15 -4.851482e-15 2.561811e-15
5.370290e-15
E:\\R-3.1.2\\scripts\\mPDBS\\summary_pdb_93\\59a647f04822d30a.final.pdb
-2.354733e-15 6.559496e-15 -3.769811e-15 4.341737e-16 -4.618911e-15
1.058681e-14
E:\\R-3.1.2\\scripts\\mPDBS\\summary_pdb_93\\59b091fe385e05fc.final.pdb
-2.229849e-15 2.161873e-16 2.139798e-15 5.487342e-15 4.052888e-15
3.941449e-15
E:\\R-3.1.2\\scripts\\mPDBS\\summary_pdb_93\\5c35ac30f930d2db.final.pdb
-4.811144e-15 -3.921773e-17 7.884915e-16 3.935650e-16 7.820487e-15
-4.834525e-16
E:\\R-3.1.2\\scripts\\mPDBS\\summary_pdb_93\\5f60752431cf2dcb.final.pdb
-5.898290e-15 -2.551517e-15 -2.836084e-16 -1.240559e-15 5.507103e-15
-2.940211e-15
E:\\R-3.1.2\\scripts\\mPDBS\\summary_pdb_93\\626456dd6ff2868d.final.pdb
-2.083508e-15 1.980194e-15 -8.220314e-16 2.936993e-15 7.416868e-15
3.956490e-15
E:\\R-3.1.2\\scripts\\mPDBS\\summary_pdb_93\\6583d53ff3c8f7ef.final.pdb
-2.354733e-15 6.559496e-15 -3.769811e-15 4.341737e-16 -4.618911e-15
1.058681e-14
E:\\R-3.1.2\\scripts\\mPDBS\\summary_pdb_93\\69760178986bef01.final.pdb
-9.253347e-15 2.386162e-15 2.454212e-15 -9.703763e-15 1.098512e-14
-9.163124e-15
E:\\R-3.1.2\\scripts\\mPDBS\\summary_pdb_93\\6a9d87d47926b1c8.final.pdb
-5.851411e-16 -7.389312e-16 -2.577572e-15 3.253146e-15 3.161158e-15
4.898445e-15
E:\\R-3.1.2\\scripts\\mPDBS\\summary_pdb_93\\6bb3547ea982ffc4.final.pdb
5.918040e-15 -8.874380e-15 -1.406368e-14 -1.964217e-15 3.754713e-15
-2.060290e-15
E:\\R-3.1.2\\scripts\\mPDBS\\summary_pdb_93\\716d9624611d1c42.final.pdb
6.633786e-15 3.519093e-15 -4.378423e-15 6.377913e-15 5.305441e-15
8.955222e-15
E:\\R-3.1.2\\scripts\\mPDBS\\summary_pdb_93\\75f27e70ad0f1be4.final.pdb
-2.354733e-15 6.559496e-15 -3.769811e-15 4.341737e-16 -4.618911e-15
1.058681e-14
E:\\R-3.1.2\\scripts\\mPDBS\\summary_pdb_93\\769f910fffb22872.final.pdb
-4.424109e-16 -7.126157e-16 -2.141891e-15 2.448601e-15 2.785824e-15
3.544848e-15
E:\\R-3.1.2\\scripts\\mPDBS\\summary_pdb_93\\791f8c80dd5c3135.final.pdb
-2.229849e-15 2.161873e-16 2.139798e-15 5.487342e-15 4.052888e-15
3.941449e-15
E:\\R-3.1.2\\scripts\\mPDBS\\summary_pdb_93\\7fad98e66a3f6099.final.pdb
4.500268e-15 8.367938e-15 -2.241800e-16 -8.971423e-16 -1.701232e-15
-8.229351e-16
E:\\R-3.1.2\\scripts\\mPDBS\\summary_pdb_93\\804b89c94a633196.final.pdb
-4.059616e-15 5.921088e-15 -2.772025e-16 8.951996e-15 6.295351e-15
2.679756e-15
E:\\R-3.1.2\\scripts\\mPDBS\\summary_pdb_93\\813cc65a2298feba.final.pdb
-4.059616e-15 5.921088e-15 -2.772025e-16 8.951996e-15 6.295351e-15
2.679756e-15
E:\\R-3.1.2\\scripts\\mPDBS\\summary_pdb_93\\83c0eefcfe12d3f9.final.pdb
1.382645e-15 -7.411712e-16 2.137243e-16 7.678275e-15 8.823276e-15
1.307590e-15

E:\\R-3.1.2\\scripts\\mPDBS\\summary_pdb_93\\86d50469ef569760.final.pdb
-2.083508e-15 1.980194e-15 -8.220314e-16 2.936993e-15 7.416868e-15
3.956490e-15
E:\\R-3.1.2\\scripts\\mPDBS\\summary_pdb_93\\89d0666e1402203d.final.pdb
-2.354733e-15 6.559496e-15 -3.769811e-15 4.341737e-16 -4.618911e-15
1.058681e-14
E:\\R-3.1.2\\scripts\\mPDBS\\summary_pdb_93\\8b34852716dadc62.final.pdb
-4.026159e-17 -5.706763e-15 5.060542e-15 -7.090727e-15 1.851856e-15
4.109169e-15
E:\\R-3.1.2\\scripts\\mPDBS\\summary_pdb_93\\8b6ef9ac3fe74395.final.pdb
-1.742145e-15 6.496416e-16 -3.683326e-15 -8.124064e-15 -6.895687e-16
3.817460e-15
E:\\R-3.1.2\\scripts\\mPDBS\\summary_pdb_93\\98bbe0d5986c138f.final.pdb
-2.229849e-15 2.161873e-16 2.139798e-15 5.487342e-15 4.052888e-15
3.941449e-15
E:\\R-3.1.2\\scripts\\mPDBS\\summary_pdb_93\\9c22f4909d67e2fa.final.pdb
3.543677e-15 -1.251180e-15 -5.638365e-15 -2.925655e-15 -2.311914e-15
6.764163e-15
E:\\R-3.1.2\\scripts\\mPDBS\\summary_pdb_93\\9dda8ed0d81fb3ef.final.pdb
-6.083088e-15 -1.283899e-14 -4.378698e-15 1.361851e-14 1.130108e-14
-1.644886e-14
E:\\R-3.1.2\\scripts\\mPDBS\\summary_pdb_93\\9f752b3616e6033b.final.pdb
-1.236424e-16 3.853575e-16 -2.136222e-15 9.422097e-16 1.041154e-15
3.993393e-15
E:\\R-3.1.2\\scripts\\mPDBS\\summary_pdb_93\\a02c70083816d6ba.final.pdb
-4.811144e-15 -3.921773e-17 7.884915e-16 3.935650e-16 7.820487e-15
-4.834525e-16
E:\\R-3.1.2\\scripts\\mPDBS\\summary_pdb_93\\a0c997d891e4e082.final.pdb
-1.257576e-15 7.130894e-15 -2.372285e-15 1.854128e-15 1.094004e-14
6.230443e-16
E:\\R-3.1.2\\scripts\\mPDBS\\summary_pdb_93\\ab8039a2d823c616.final.pdb
-8.501326e-16 9.049710e-16 -2.142434e-15 3.840387e-15 2.831074e-15
3.633223e-15
E:\\R-3.1.2\\scripts\\mPDBS\\summary_pdb_93\\acf4d74b9c71d4dc.final.pdb
-4.059616e-15 5.921088e-15 -2.772025e-16 8.951996e-15 6.295351e-15
2.679756e-15
E:\\R-3.1.2\\scripts\\mPDBS\\summary_pdb_93\\ad8a823e4b4c668e.final.pdb
-1.988387e-16 -2.898181e-16 -2.255276e-15 3.663299e-15 2.828775e-15
3.444798e-15
E:\\R-3.1.2\\scripts\\mPDBS\\summary_pdb_93\\b16ababd03b7b857.final.pdb
3.543677e-15 -1.251180e-15 -5.638365e-15 -2.925655e-15 -2.311914e-15
6.764163e-15
E:\\R-3.1.2\\scripts\\mPDBS\\summary_pdb_93\\b226923e697d3c63.final.pdb
4.200570e-15 2.635606e-15 -4.860952e-15 -2.876980e-15 7.187183e-15
4.871759e-15
E:\\R-3.1.2\\scripts\\mPDBS\\summary_pdb_93\\b28e48f6fa27a165.final.pdb
-2.229849e-15 2.161873e-16 2.139798e-15 5.487342e-15 4.052888e-15
3.941449e-15
E:\\R-3.1.2\\scripts\\mPDBS\\summary_pdb_93\\b42c4924e3cd1aec.final.pdb
3.590381e-15 3.627813e-15 -1.416658e-15 2.344104e-15 2.783587e-15
9.776256e-15
E:\\R-3.1.2\\scripts\\mPDBS\\summary_pdb_93\\b4dd239fcabff1df.final.pdb
-2.229849e-15 2.161873e-16 2.139798e-15 5.487342e-15 4.052888e-15
3.941449e-15

E:\\R-3.1.2\\scripts\\mPDBS\\summary_pdb_93\\b669942ef97459a2.final.pdb
-4.026159e-17 -5.706763e-15 5.060542e-15 -7.090727e-15 1.851856e-15
4.109169e-15
E:\\R-3.1.2\\scripts\\mPDBS\\summary_pdb_93\\b99f4d9d87c1017b.final.pdb
-2.229849e-15 2.161873e-16 2.139798e-15 5.487342e-15 4.052888e-15
3.941449e-15
E:\\R-3.1.2\\scripts\\mPDBS\\summary_pdb_93\\b9d2cbf3975403e7.final.pdb
-2.229849e-15 2.161873e-16 2.139798e-15 5.487342e-15 4.052888e-15
3.941449e-15
E:\\R-3.1.2\\scripts\\mPDBS\\summary_pdb_93\\bc88c8a505f3a288.final.pdb
-6.909322e-16 3.009177e-16 -2.098132e-15 3.618611e-15 3.232363e-15
4.237219e-15
E:\\R-3.1.2\\scripts\\mPDBS\\summary_pdb_93\\bdac0e64f4ef427e.final.pdb
-3.372232e-15 -1.236639e-16 -5.693009e-15 -1.167256e-14 -8.961976e-15
6.481403e-15
E:\\R-3.1.2\\scripts\\mPDBS\\summary_pdb_93\\ca2a1f55fc9d0681.final.pdb
-4.026159e-17 -5.706763e-15 5.060542e-15 -7.090727e-15 1.851856e-15
4.109169e-15
E:\\R-3.1.2\\scripts\\mPDBS\\summary_pdb_93\\ce34248de3e81a3c.final.pdb
-4.082322e-15 -1.091744e-15 1.619244e-15 9.405679e-15 5.084330e-15
7.026896e-15
E:\\R-3.1.2\\scripts\\mPDBS\\summary_pdb_93\\d02e697772494a61.final.pdb
1.508263e-15 1.067657e-14 -5.221353e-16 -7.143180e-15 -4.722645e-15
1.840427e-14
E:\\R-3.1.2\\scripts\\mPDBS\\summary_pdb_93\\d04221245d015047.final.pdb
-1.948863e-15 -4.533175e-16 -6.583778e-16 4.830914e-15 1.464980e-15
2.274501e-15
E:\\R-3.1.2\\scripts\\mPDBS\\summary_pdb_93\\d678141e4018054a.final.pdb
1.765959e-14 -6.313746e-15 -1.413439e-14 8.593402e-15 -2.842849e-16
9.091810e-15
E:\\R-3.1.2\\scripts\\mPDBS\\summary_pdb_93\\d71b40af0f0efe39.final.pdb
6.653606e-15 4.465994e-15 -4.488165e-15 4.987506e-15 3.543660e-15
1.644873e-14
E:\\R-3.1.2\\scripts\\mPDBS\\summary_pdb_93\\dc0809eff10197e2.final.pdb
-8.312157e-15 1.268578e-14 3.850664e-15 -2.166997e-15 2.226991e-15
4.182759e-16
E:\\R-3.1.2\\scripts\\mPDBS\\summary_pdb_93\\e1163918d3707839.final.pdb
5.616538e-15 -3.941429e-16 3.703378e-15 -2.662308e-15 1.205829e-14
1.799998e-15
E:\\R-3.1.2\\scripts\\mPDBS\\summary_pdb_93\\e4b48b06824ef848.final.pdb
2.470840e-15 -9.733991e-15 -8.062502e-15 5.392861e-15 5.623832e-15
4.376648e-15
E:\\R-3.1.2\\scripts\\mPDBS\\summary_pdb_93\\e529e19ad281fea2.final.pdb
-7.477714e-16 5.443569e-15 -8.505122e-15 1.118734e-14 1.851008e-15
5.073652e-15
E:\\R-3.1.2\\scripts\\mPDBS\\summary_pdb_93\\e5602082718ec3a1.final.pdb
-4.059616e-15 5.921088e-15 -2.772025e-16 8.951996e-15 6.295351e-15
2.679756e-15
E:\\R-3.1.2\\scripts\\mPDBS\\summary_pdb_93\\e5d589d996d0a960.final.pdb
-5.453571e-15 -5.202803e-15 -3.860271e-15 -3.199389e-15 9.425027e-15
-3.321511e-15
E:\\R-3.1.2\\scripts\\mPDBS\\summary_pdb_93\\e9df9e72307a2fbd.final.pdb
-4.811144e-15 -3.921773e-17 7.884915e-16 3.935650e-16 7.820487e-15
-4.834525e-16

E:\\R-3.1.2\\scripts\\mPDBS\\summary_pdb_93\\ea00f8694090cae7.final.pdb
-4.059616e-15 5.921088e-15 -2.772025e-16 8.951996e-15 6.295351e-15
2.679756e-15
E:\\R-3.1.2\\scripts\\mPDBS\\summary_pdb_93\\eba517be32db050e.final.pdb
-2.229849e-15 2.161873e-16 2.139798e-15 5.487342e-15 4.052888e-15
3.941449e-15
E:\\R-3.1.2\\scripts\\mPDBS\\summary_pdb_93\\ec04b9545899c29d.final.pdb
-2.331665e-16 -5.795415e-16 -1.865084e-15 2.429106e-15 2.816547e-15
4.320985e-15
E:\\R-3.1.2\\scripts\\mPDBS\\summary_pdb_93\\ee6862cbcad52078.final.pdb
-4.439510e-16 6.092957e-17 -2.136295e-15 3.912213e-15 3.104427e-15
4.125330e-15
E:\\R-3.1.2\\scripts\\mPDBS\\summary_pdb_93\\ef6a42fc83b81e5d.final.pdb
-2.609154e-16 9.349380e-16 -1.971021e-15 2.745239e-15 2.778452e-15
3.669314e-15
E:\\R-3.1.2\\scripts\\mPDBS\\summary_pdb_93\\ef8e88d4ad93a0ad.final.pdb
-1.948863e-15 -4.533175e-16 -6.583778e-16 4.830914e-15 1.464980e-15
2.274501e-15
E:\\R-3.1.2\\scripts\\mPDBS\\summary_pdb_93\\f14fcae8deec4fa3.final.pdb
-1.236424e-16 3.853575e-16 -2.136222e-15 9.422097e-16 1.041154e-15
3.993393e-15
E:\\R-3.1.2\\scripts\\mPDBS\\summary_pdb_93\\f83584e22003fb92.final.pdb
-1.885129e-16 -1.018278e-14 -1.014725e-14 1.348832e-14 1.170211e-15
1.923532e-15
E:\\R-3.1.2\\scripts\\mPDBS\\summary_pdb_93\\f8e2bf87e52caa29.final.pdb
-7.477714e-16 5.443569e-15 -8.505122e-15 1.118734e-14 1.851008e-15
5.073652e-15

[,367]   [,368]   [,369]   [,370]   [,371]   [,372]
E:\\R-3.1.2\\scripts\\40.1ns_data\\NAT0.pdb
-3.125680e-15 -2.371614e-15 -1.035778e-15 -3.298870e-15 6.049830e-15
8.484831e-16
E:\\R-3.1.2\\scripts\\40.1ns_data\\LJP6.pdb
-4.814704e-15 -2.204909e-15 1.925716e-15 -3.920659e-15 3.841835e-15
6.694435e-16
E:\\R-3.1.2\\scripts\\40.1ns_data\\3WY9.pdb
-1.216774e-15 -2.666463e-15 5.819473e-18 -4.069877e-15 7.113058e-15
-8.402211e-16
E:\\R-3.1.2\\scripts\\40.1ns_data\\ZSP9.pdb
-1.020211e-15 -2.427527e-15 1.282094e-15 -3.493025e-15 3.700964e-15
1.382878e-15
E:\\R-3.1.2\\scripts\\mPDBS\\summary_pdb_93\\013d855ad26c877c.final.pdb
-5.042902e-15 6.882978e-15 2.658705e-15 -2.855707e-16 8.370611e-15
-1.293885e-15
E:\\R-3.1.2\\scripts\\mPDBS\\summary_pdb_93\\0a2a705342ca0d54.final.pdb
-8.260472e-16 -2.939592e-15 5.281154e-16 -3.494723e-15 5.731293e-15
4.475521e-16
E:\\R-3.1.2\\scripts\\mPDBS\\summary_pdb_93\\0a59fceadb4194d9.final.pdb
-5.480934e-15 -8.302190e-15 -5.701056e-15 -3.148997e-15 7.640028e-15
2.653387e-15
E:\\R-3.1.2\\scripts\\mPDBS\\summary_pdb_93\\0dba59f3306825e8.final.pdb
9.835271e-16 -2.589479e-15 -3.165629e-15 -5.618978e-15 3.624407e-15
2.149541e-15
E:\\R-3.1.2\\scripts\\mPDBS\\summary_pdb_93\\126f26a78b57339c.final.pdb

-7.147671e-16 -4.441297e-15 4.562781e-15 -8.073612e-15 7.987237e-15
-4.260895e-16
E:\\R-3.1.2\\scripts\\mPDBS\\summary_pdb_93\\12f06f3e342df706.final.pdb
-2.834423e-15 -2.684154e-15 -1.226228e-16 -2.958368e-15 4.288870e-15
2.788502e-16
E:\\R-3.1.2\\scripts\\mPDBS\\summary_pdb_93\\18618a2a251b867b.final.pdb
9.527282e-16 7.479583e-16 7.979461e-15 -8.550531e-15 6.915254e-15
-7.264023e-15
E:\\R-3.1.2\\scripts\\mPDBS\\summary_pdb_93\\1f305f598641eeb2.final.pdb
-2.502807e-15 -3.091506e-15 2.486537e-16 -3.606035e-15 5.914954e-15
3.523423e-16
E:\\R-3.1.2\\scripts\\mPDBS\\summary_pdb_93\\1f8a00b551be0a39.final.pdb
9.527282e-16 7.479583e-16 7.979461e-15 -8.550531e-15 6.915254e-15
-7.264023e-15
E:\\R-3.1.2\\scripts\\mPDBS\\summary_pdb_93\\219f45328d47579a.final.pdb
-1.784951e-14 -5.687928e-16 -1.035951e-15 -3.730346e-15 2.171312e-15
-1.152147e-14
E:\\R-3.1.2\\scripts\\mPDBS\\summary_pdb_93\\238dd3918e54336d.final.pdb
8.746401e-15 2.308680e-15 1.536543e-14 -1.811959e-15 -3.865526e-15
-5.372311e-15
E:\\R-3.1.2\\scripts\\mPDBS\\summary_pdb_93\\2b3027889e2fdc42.final.pdb
-2.882318e-15 -2.665206e-15 -6.233037e-16 -3.778702e-15 4.815030e-15
-9.875153e-17
E:\\R-3.1.2\\scripts\\mPDBS\\summary_pdb_93\\2fcb3b46e33793b0.final.pdb
-7.731263e-15 4.247781e-16 -3.320540e-15 -1.308393e-15 5.828217e-15
-2.171680e-15
E:\\R-3.1.2\\scripts\\mPDBS\\summary_pdb_93\\34eb328251842006.final.pdb
-2.390256e-16 -1.155670e-14 -4.199282e-15 4.524962e-15 -2.021015e-15
1.538223e-15
E:\\R-3.1.2\\scripts\\mPDBS\\summary_pdb_93\\354b32871797b7ab.final.pdb
-1.103216e-14 1.186070e-15 3.380959e-15 -5.954039e-15 1.728678e-14
-9.265342e-15
E:\\R-3.1.2\\scripts\\mPDBS\\summary_pdb_93\\397368adbc32d5de.final.pdb
-7.147671e-16 -4.441297e-15 4.562781e-15 -8.073612e-15 7.987237e-15
-4.260895e-16
E:\\R-3.1.2\\scripts\\mPDBS\\summary_pdb_93\\411ca13cd3df6edd.final.pdb
-9.040979e-15 1.765719e-15 -1.814149e-15 -2.424057e-16 6.024241e-15
-7.539348e-15
E:\\R-3.1.2\\scripts\\mPDBS\\summary_pdb_93\\42357eee6642bf12.final.pdb
-2.170491e-15 -1.222106e-14 1.050081e-15 8.998892e-16 4.973580e-15
-7.692641e-15
E:\\R-3.1.2\\scripts\\mPDBS\\summary_pdb_93\\44342d1f75a7fcfc.final.pdb
-3.331410e-15 -1.258894e-15 -8.452372e-15 -6.392571e-16 8.402175e-15
-1.138799e-15
E:\\R-3.1.2\\scripts\\mPDBS\\summary_pdb_93\\448ef21dffe8cdfb.final.pdb
9.907405e-15 -9.846848e-16 1.954832e-14 4.810844e-15 3.756958e-15
1.658589e-15
E:\\R-3.1.2\\scripts\\mPDBS\\summary_pdb_93\\472a40b3e8f113c7.final.pdb
9.835271e-16 -2.589479e-15 -3.165629e-15 -5.618978e-15 3.624407e-15
2.149541e-15
E:\\R-3.1.2\\scripts\\mPDBS\\summary_pdb_93\\48a26225951ea153.final.pdb
2.035623e-15 6.111194e-15 2.429376e-15 -3.958029e-15 -1.039661e-15
2.006769e-15
E:\\R-3.1.2\\scripts\\mPDBS\\summary_pdb_93\\4ab4a5c7c6682ce6.final.pdb

-7.913870e-15 -1.700850e-14 1.234714e-15 1.146444e-15 2.116933e-15
4.769407e-15
E:\\R-3.1.2\\scripts\\mPDBS\\summary_pdb_93\\4eaa45b62cb6a25f.final.pdb
-1.976038e-15 -2.433430e-15 5.637683e-16 -5.576086e-15 4.344012e-15
4.099835e-15
E:\\R-3.1.2\\scripts\\mPDBS\\summary_pdb_93\\58c371611d85ac96.final.pdb
-7.147671e-16 -4.441297e-15 4.562781e-15 -8.073612e-15 7.987237e-15
-4.260895e-16
E:\\R-3.1.2\\scripts\\mPDBS\\summary_pdb_93\\59a647f04822d30a.final.pdb
1.709697e-15 -3.830621e-15 5.544244e-15 -6.949314e-15 -1.145743e-15
2.005821e-15
E:\\R-3.1.2\\scripts\\mPDBS\\summary_pdb_93\\59b091fe385e05fc.final.pdb
-5.480934e-15 -8.302190e-15 -5.701056e-15 -3.148997e-15 7.640028e-15
2.653387e-15
E:\\R-3.1.2\\scripts\\mPDBS\\summary_pdb_93\\5c35ac30f930d2db.final.pdb
-1.976038e-15 -2.433430e-15 5.637683e-16 -5.576086e-15 4.344012e-15
4.099835e-15
E:\\R-3.1.2\\scripts\\mPDBS\\summary_pdb_93\\5f60752431cf2dcb.final.pdb
1.345827e-15 -7.488037e-15 3.175633e-16 -5.860235e-15 2.233223e-15
8.159699e-15
E:\\R-3.1.2\\scripts\\mPDBS\\summary_pdb_93\\626456dd6ff2868d.final.pdb
9.835271e-16 -2.589479e-15 -3.165629e-15 -5.618978e-15 3.624407e-15
2.149541e-15
E:\\R-3.1.2\\scripts\\mPDBS\\summary_pdb_93\\6583d53ff3c8f7ef.final.pdb
1.709697e-15 -3.830621e-15 5.544244e-15 -6.949314e-15 -1.145743e-15
2.005821e-15
E:\\R-3.1.2\\scripts\\mPDBS\\summary_pdb_93\\69760178986bef01.final.pdb
3.558743e-16 2.653425e-15 -3.354118e-15 -8.435683e-15 1.155462e-14
-3.493856e-15
E:\\R-3.1.2\\scripts\\mPDBS\\summary_pdb_93\\6a9d87d47926b1c8.final.pdb
-2.064382e-15 -3.064794e-15 2.129618e-16 -3.390726e-15 4.857796e-15
2.556568e-16
E:\\R-3.1.2\\scripts\\mPDBS\\summary_pdb_93\\6bb3547ea982ffc4.final.pdb
1.182765e-14 1.256600e-14 7.357259e-15 -6.317741e-16 -1.390526e-14
-4.202666e-15
E:\\R-3.1.2\\scripts\\mPDBS\\summary_pdb_93\\716d9624611d1c42.final.pdb
-1.066366e-14 3.256840e-15 4.244657e-16 3.387989e-16 9.057625e-15
-5.404971e-15
E:\\R-3.1.2\\scripts\\mPDBS\\summary_pdb_93\\75f27e70ad0f1be4.final.pdb
1.709697e-15 -3.830621e-15 5.544244e-15 -6.949314e-15 -1.145743e-15
2.005821e-15
E:\\R-3.1.2\\scripts\\mPDBS\\summary_pdb_93\\769f910fffb22872.final.pdb
-1.284198e-15 -2.610033e-15 -1.782932e-16 -2.941737e-15 5.131091e-15
-1.737743e-16
E:\\R-3.1.2\\scripts\\mPDBS\\summary_pdb_93\\791f8c80dd5c3135.final.pdb
-5.480934e-15 -8.302190e-15 -5.701056e-15 -3.148997e-15 7.640028e-15
2.653387e-15
E:\\R-3.1.2\\scripts\\mPDBS\\summary_pdb_93\\7fad98e66a3f6099.final.pdb
-1.146181e-14 -3.393930e-15 -9.135131e-15 4.503515e-15 9.752891e-15
2.617068e-15
E:\\R-3.1.2\\scripts\\mPDBS\\summary_pdb_93\\804b89c94a633196.final.pdb
-7.731263e-15 4.247781e-16 -3.320540e-15 -1.308393e-15 5.828217e-15
-2.171680e-15
E:\\R-3.1.2\\scripts\\mPDBS\\summary_pdb_93\\813cc65a2298feba.final.pdb

-7.731263e-15 4.247781e-16 -3.320540e-15 -1.308393e-15 5.828217e-15
-2.171680e-15
E:\\R-3.1.2\\scripts\\mPDBS\\summary_pdb_93\\83c0eefcfe12d3f9.final.pdb
-9.040979e-15 1.765719e-15 -1.814149e-15 -2.424057e-16 6.024241e-15
-7.539348e-15
E:\\R-3.1.2\\scripts\\mPDBS\\summary_pdb_93\\86d50469ef569760.final.pdb
9.835271e-16 -2.589479e-15 -3.165629e-15 -5.618978e-15 3.624407e-15
2.149541e-15
E:\\R-3.1.2\\scripts\\mPDBS\\summary_pdb_93\\89d0666e1402203d.final.pdb
1.709697e-15 -3.830621e-15 5.544244e-15 -6.949314e-15 -1.145743e-15
2.005821e-15
E:\\R-3.1.2\\scripts\\mPDBS\\summary_pdb_93\\8b34852716dadc62.final.pdb
-5.365100e-15 -1.307311e-14 -5.420423e-15 -6.617514e-15 2.958985e-15
8.068398e-16
E:\\R-3.1.2\\scripts\\mPDBS\\summary_pdb_93\\8b6ef9ac3fe74395.final.pdb
1.680012e-15 -5.285649e-15 1.126677e-16 -9.412431e-15 -4.143143e-15
1.396271e-16
E:\\R-3.1.2\\scripts\\mPDBS\\summary_pdb_93\\98bbe0d5986c138f.final.pdb
-5.480934e-15 -8.302190e-15 -5.701056e-15 -3.148997e-15 7.640028e-15
2.653387e-15
E:\\R-3.1.2\\scripts\\mPDBS\\summary_pdb_93\\9c22f4909d67e2fa.final.pdb
4.034499e-15 -4.950591e-16 -6.567484e-18 -6.736777e-15 1.113213e-14
5.509242e-15
E:\\R-3.1.2\\scripts\\mPDBS\\summary_pdb_93\\9dda8ed0d81fb3ef.final.pdb
7.366196e-15 -3.819912e-16 -1.224209e-14 -9.446557e-15 2.509320e-15
9.216633e-15
E:\\R-3.1.2\\scripts\\mPDBS\\summary_pdb_93\\9f752b3616e6033b.final.pdb
2.481953e-16 -2.088845e-15 -1.161810e-15 -2.936579e-15 2.999991e-15
-1.246983e-15
E:\\R-3.1.2\\scripts\\mPDBS\\summary_pdb_93\\a02c70083816d6ba.final.pdb
-1.976038e-15 -2.433430e-15 5.637683e-16 -5.576086e-15 4.344012e-15
4.099835e-15
E:\\R-3.1.2\\scripts\\mPDBS\\summary_pdb_93\\a0c997d891e4e082.final.pdb
-5.170481e-15 -6.209591e-16 -4.313681e-15 -5.026877e-15 1.661291e-14
6.368425e-17
E:\\R-3.1.2\\scripts\\mPDBS\\summary_pdb_93\\ab8039a2d823c616.final.pdb
-1.943028e-15 -2.952427e-15 -8.520401e-16 -3.555398e-15 5.546246e-15
-6.728737e-17
E:\\R-3.1.2\\scripts\\mPDBS\\summary_pdb_93\\acf4d74b9c71d4dc.final.pdb
-7.731263e-15 4.247781e-16 -3.320540e-15 -1.308393e-15 5.828217e-15
-2.171680e-15
E:\\R-3.1.2\\scripts\\mPDBS\\summary_pdb_93\\ad8a823e4b4c668e.final.pdb
-1.745777e-15 -2.967807e-15 -8.433725e-17 -2.798669e-15 4.690501e-15
-3.970045e-16
E:\\R-3.1.2\\scripts\\mPDBS\\summary_pdb_93\\b16ababd03b7b857.final.pdb
4.034499e-15 -4.950591e-16 -6.567484e-18 -6.736777e-15 1.113213e-14
5.509242e-15
E:\\R-3.1.2\\scripts\\mPDBS\\summary_pdb_93\\b226923e697d3c63.final.pdb
-2.818799e-15 -5.341132e-15 2.438872e-15 -7.653178e-15 1.877495e-14
-9.494095e-15
E:\\R-3.1.2\\scripts\\mPDBS\\summary_pdb_93\\b28e48f6fa27a165.final.pdb
-5.480934e-15 -8.302190e-15 -5.701056e-15 -3.148997e-15 7.640028e-15
2.653387e-15
E:\\R-3.1.2\\scripts\\mPDBS\\summary_pdb_93\\b42c4924e3cd1aec.final.pdb

-9.025640e-15 -9.053440e-15 4.810198e-16 -5.234106e-15 1.025746e-14
-1.691742e-15
E:\\R-3.1.2\\scripts\\mPDBS\\summary_pdb_93\\b4dd239fcabff1df.final.pdb
-5.480934e-15 -8.302190e-15 -5.701056e-15 -3.148997e-15 7.640028e-15
2.653387e-15
E:\\R-3.1.2\\scripts\\mPDBS\\summary_pdb_93\\b669942ef97459a2.final.pdb
-5.365100e-15 -1.307311e-14 -5.420423e-15 -6.617514e-15 2.958985e-15
8.068398e-16
E:\\R-3.1.2\\scripts\\mPDBS\\summary_pdb_93\\b99f4d9d87c1017b.final.pdb
-5.480934e-15 -8.302190e-15 -5.701056e-15 -3.148997e-15 7.640028e-15
2.653387e-15
E:\\R-3.1.2\\scripts\\mPDBS\\summary_pdb_93\\b9d2cbf3975403e7.final.pdb
-5.480934e-15 -8.302190e-15 -5.701056e-15 -3.148997e-15 7.640028e-15
2.653387e-15
E:\\R-3.1.2\\scripts\\mPDBS\\summary_pdb_93\\bc88c8a505f3a288.final.pdb
-2.485934e-15 -2.881179e-15 -6.482403e-16 -3.529444e-15 4.600792e-15
5.487340e-16
E:\\R-3.1.2\\scripts\\mPDBS\\summary_pdb_93\\bdac0e64f4ef427e.final.pdb
1.141228e-14 -1.224131e-14 2.329021e-15 -9.875542e-15 5.749287e-15
1.415467e-14
E:\\R-3.1.2\\scripts\\mPDBS\\summary_pdb_93\\ca2a1f55fc9d0681.final.pdb
-5.365100e-15 -1.307311e-14 -5.420423e-15 -6.617514e-15 2.958985e-15
8.068398e-16
E:\\R-3.1.2\\scripts\\mPDBS\\summary_pdb_93\\ce34248de3e81a3c.final.pdb
-1.318350e-14 -1.089193e-14 8.833793e-16 -6.425534e-15 1.477373e-14
6.474619e-15
E:\\R-3.1.2\\scripts\\mPDBS\\summary_pdb_93\\d02e697772494a61.final.pdb
-6.434164e-15 1.048403e-15 1.038287e-14 4.916166e-15 2.384683e-15
-7.028104e-15
E:\\R-3.1.2\\scripts\\mPDBS\\summary_pdb_93\\d04221245d015047.final.pdb
-2.675949e-15 -2.321855e-15 -1.473938e-15 -1.782944e-15 3.620251e-16
3.271188e-15
E:\\R-3.1.2\\scripts\\mPDBS\\summary_pdb_93\\d678141e4018054a.final.pdb
3.909389e-15 7.821075e-15 6.315482e-15 8.446300e-15 3.690569e-16
-3.656102e-15
E:\\R-3.1.2\\scripts\\mPDBS\\summary_pdb_93\\d71b40af0f0efe39.final.pdb
-6.182421e-15 4.201383e-15 -3.419756e-15 3.531369e-15 9.617552e-15
-7.124447e-16
E:\\R-3.1.2\\scripts\\mPDBS\\summary_pdb_93\\dc0809eff10197e2.final.pdb
2.591077e-15 -1.233120e-14 -5.567283e-15 -1.058745e-14 6.186714e-15
5.359809e-15
E:\\R-3.1.2\\scripts\\mPDBS\\summary_pdb_93\\e1163918d3707839.final.pdb
-3.844021e-15 -3.831055e-15 -4.632511e-15 5.074055e-15 8.227836e-15
4.558467e-15
E:\\R-3.1.2\\scripts\\mPDBS\\summary_pdb_93\\e4b48b06824ef848.final.pdb
9.527282e-16 7.479583e-16 7.979461e-15 -8.550531e-15 6.915254e-15
-7.264023e-15
E:\\R-3.1.2\\scripts\\mPDBS\\summary_pdb_93\\e529e19ad281fea2.final.pdb
-5.042902e-15 6.882978e-15 2.658705e-15 -2.855707e-16 8.370611e-15
-1.293885e-15
E:\\R-3.1.2\\scripts\\mPDBS\\summary_pdb_93\\e5602082718ec3a1.final.pdb
-7.731263e-15 4.247781e-16 -3.320540e-15 -1.308393e-15 5.828217e-15
-2.171680e-15
E:\\R-3.1.2\\scripts\\mPDBS\\summary_pdb_93\\e5d589d996d0a960.final.pdb

3.512445e-15 6.279676e-15 1.103850e-15 -8.007382e-16 5.879230e-15
4.234793e-15
E:\\R-3.1.2\\scripts\\mPDBS\\summary_pdb_93\\e9df9e72307a2fbd.final.pdb
-1.976038e-15 -2.433430e-15 5.637683e-16 -5.576086e-15 4.344012e-15
4.099835e-15
E:\\R-3.1.2\\scripts\\mPDBS\\summary_pdb_93\\ea00f8694090cae7.final.pdb
-7.731263e-15 4.247781e-16 -3.320540e-15 -1.308393e-15 5.828217e-15
-2.171680e-15
E:\\R-3.1.2\\scripts\\mPDBS\\summary_pdb_93\\eba517be32db050e.final.pdb
-5.480934e-15 -8.302190e-15 -5.701056e-15 -3.148997e-15 7.640028e-15
2.653387e-15
E:\\R-3.1.2\\scripts\\mPDBS\\summary_pdb_93\\ec04b9545899c29d.final.pdb
-2.923750e-15 -3.025710e-15 -6.541812e-17 -3.193637e-15 4.897167e-15
-1.578129e-16
E:\\R-3.1.2\\scripts\\mPDBS\\summary_pdb_93\\ee6862cbcad52078.final.pdb
-3.022831e-15 -3.116234e-15 -4.465787e-16 -3.405302e-15 5.452541e-15
2.980665e-16
E:\\R-3.1.2\\scripts\\mPDBS\\summary_pdb_93\\ef6a42fc83b81e5d.final.pdb
-2.471616e-15 -2.776132e-15 -4.198534e-16 -3.145784e-15 5.003589e-15
2.577382e-16
E:\\R-3.1.2\\scripts\\mPDBS\\summary_pdb_93\\ef8e88d4ad93a0ad.final.pdb
-2.675949e-15 -2.321855e-15 -1.473938e-15 -1.782944e-15 3.620251e-16
3.271188e-15
E:\\R-3.1.2\\scripts\\mPDBS\\summary_pdb_93\\f14fcae8deec4fa3.final.pdb
2.481953e-16 -2.088845e-15 -1.161810e-15 -2.936579e-15 2.999991e-15
-1.246983e-15
E:\\R-3.1.2\\scripts\\mPDBS\\summary_pdb_93\\f83584e22003fb92.final.pdb
2.035623e-15 6.111194e-15 2.429376e-15 -3.958029e-15 -1.039661e-15
2.006769e-15
E:\\R-3.1.2\\scripts\\mPDBS\\summary_pdb_93\\f8e2bf87e52caa29.final.pdb
-5.042902e-15 6.882978e-15 2.658705e-15 -2.855707e-16 8.370611e-15
-1.293885e-15

[,373]   [,374]   [,375]   [,376]   [,377]   [,378]
E:\\R-3.1.2\\scripts\\40.1ns_data\\NAT0.pdb
2.936254e-15 4.103013e-15 4.538489e-15 2.987438e-15 1.164889e-15
5.321202e-15
E:\\R-3.1.2\\scripts\\40.1ns_data\\LJP6.pdb
2.093810e-15 1.580440e-15 5.070715e-15 3.542618e-15 -8.329069e-16
4.600105e-15
E:\\R-3.1.2\\scripts\\40.1ns_data\\3WY9.pdb
2.437362e-15 4.412126e-15 5.509850e-15 2.457727e-15 6.258335e-16
5.039683e-15
E:\\R-3.1.2\\scripts\\40.1ns_data\\ZSP9.pdb
3.921120e-15 2.048079e-15 2.980872e-15 1.966760e-15 5.029583e-16
5.234649e-15
E:\\R-3.1.2\\scripts\\mPDBS\\summary_pdb_93\\013d855ad26c877c.final.pdb
3.083988e-15 8.236863e-15 6.478100e-15 1.681830e-15 -2.574941e-15
2.744457e-15
E:\\R-3.1.2\\scripts\\mPDBS\\summary_pdb_93\\0a2a705342ca0d54.final.pdb
1.734100e-15 3.673439e-15 3.858024e-15 2.168849e-15 9.022116e-16
3.829136e-15
E:\\R-3.1.2\\scripts\\mPDBS\\summary_pdb_93\\0a59fceadb4194d9.final.pdb
1.483973e-15 4.571282e-15 2.853922e-15 4.559063e-15 6.556881e-17

8.840183e-16
E:\\R-3.1.2\\scripts\\mPDBS\\summary_pdb_93\\0dba59f3306825e8.final.pdb
4.385343e-16 1.107149e-15 2.327774e-15 -3.142436e-15 1.902082e-16
2.520678e-15
E:\\R-3.1.2\\scripts\\mPDBS\\summary_pdb_93\\126f26a78b57339c.final.pdb
5.545126e-15 4.765772e-16 6.027831e-15 5.290923e-15 -2.548486e-15
9.922743e-15
E:\\R-3.1.2\\scripts\\mPDBS\\summary_pdb_93\\12f06f3e342df706.final.pdb
2.278587e-15 2.977382e-15 4.372424e-15 1.912543e-15 7.794799e-16
3.960541e-15
E:\\R-3.1.2\\scripts\\mPDBS\\summary_pdb_93\\18618a2a251b867b.final.pdb
6.559046e-15 -2.213443e-15 8.991660e-15 -1.431342e-15 -1.537741e-15
6.332897e-15
E:\\R-3.1.2\\scripts\\mPDBS\\summary_pdb_93\\1f305f598641eeb2.final.pdb
1.959345e-15 3.012016e-15 3.945267e-15 1.878877e-15 2.449193e-16
3.932121e-15
E:\\R-3.1.2\\scripts\\mPDBS\\summary_pdb_93\\1f8a00b551be0a39.final.pdb
6.559046e-15 -2.213443e-15 8.991660e-15 -1.431342e-15 -1.537741e-15
6.332897e-15
E:\\R-3.1.2\\scripts\\mPDBS\\summary_pdb_93\\219f45328d47579a.final.pdb
-4.392403e-15 5.799466e-15 -2.815588e-15 -7.245947e-15 4.012498e-15
-1.878963e-15
E:\\R-3.1.2\\scripts\\mPDBS\\summary_pdb_93\\238dd3918e54336d.final.pdb
1.276456e-15 4.133698e-15 1.393231e-15 4.624548e-15 9.945253e-15
1.540756e-14
E:\\R-3.1.2\\scripts\\mPDBS\\summary_pdb_93\\2b3027889e2fdc42.final.pdb
1.786248e-15 3.648409e-15 4.164343e-15 2.032438e-15 3.824691e-17
3.410643e-15
E:\\R-3.1.2\\scripts\\mPDBS\\summary_pdb_93\\2fcb3b46e33793b0.final.pdb
-1.605512e-15 4.038973e-15 2.659837e-15 -4.285897e-15 1.359479e-15
-5.547409e-16
E:\\R-3.1.2\\scripts\\mPDBS\\summary_pdb_93\\34eb328251842006.final.pdb
7.012517e-16 7.494120e-15 3.974519e-15 5.639465e-15 -2.674080e-15
-3.379190e-15
E:\\R-3.1.2\\scripts\\mPDBS\\summary_pdb_93\\354b32871797b7ab.final.pdb
-5.778387e-16 9.326072e-15 8.164107e-15 2.953539e-15 -5.985577e-15
-1.893623e-15
E:\\R-3.1.2\\scripts\\mPDBS\\summary_pdb_93\\397368adbc32d5de.final.pdb
5.545126e-15 4.765772e-16 6.027831e-15 5.290923e-15 -2.548486e-15
9.922743e-15
E:\\R-3.1.2\\scripts\\mPDBS\\summary_pdb_93\\411ca13cd3df6edd.final.pdb
-1.164459e-15 3.767488e-15 7.258697e-15 -5.129840e-15 -4.663914e-15
4.774196e-16
E:\\R-3.1.2\\scripts\\mPDBS\\summary_pdb_93\\42357eee6642bf12.final.pdb
1.700347e-15 5.314504e-15 -2.660216e-15 6.210322e-15 4.748444e-15
-4.492612e-15
E:\\R-3.1.2\\scripts\\mPDBS\\summary_pdb_93\\44342d1f75a7fcfc.final.pdb
7.178003e-15 8.512994e-15 6.831944e-15 2.305581e-15 -3.527699e-15
3.882978e-15
E:\\R-3.1.2\\scripts\\mPDBS\\summary_pdb_93\\448ef21dffe8cdfb.final.pdb
3.874479e-15 9.954380e-15 1.584547e-14 3.079498e-14 1.724638e-15
1.466225e-14
E:\\R-3.1.2\\scripts\\mPDBS\\summary_pdb_93\\472a40b3e8f113c7.final.pdb
4.385343e-16 1.107149e-15 2.327774e-15 -3.142436e-15 1.902082e-16

2.520678e-15
E:\\R-3.1.2\\scripts\\mPDBS\\summary_pdb_93\\48a26225951ea153.final.pdb
4.774309e-15 4.009232e-15 9.839058e-15 -7.045587e-15 -2.982774e-15
6.646336e-15
E:\\R-3.1.2\\scripts\\mPDBS\\summary_pdb_93\\4ab4a5c7c6682ce6.final.pdb
-2.800749e-16 -7.758932e-16 2.574864e-15 4.005926e-15 6.926573e-15
-2.135320e-15
E:\\R-3.1.2\\scripts\\mPDBS\\summary_pdb_93\\4eaa45b62cb6a25f.final.pdb
4.545884e-15 -9.569347e-16 4.876427e-15 6.032060e-16 2.342737e-16
7.857257e-15
E:\\R-3.1.2\\scripts\\mPDBS\\summary_pdb_93\\58c371611d85ac96.final.pdb
5.545126e-15 4.765772e-16 6.027831e-15 5.290923e-15 -2.548486e-15
9.922743e-15
E:\\R-3.1.2\\scripts\\mPDBS\\summary_pdb_93\\59a647f04822d30a.final.pdb
1.940873e-15 6.248312e-15 -2.562017e-15 3.347499e-15 3.021316e-15
1.772254e-15
E:\\R-3.1.2\\scripts\\mPDBS\\summary_pdb_93\\59b091fe385e05fc.final.pdb
1.483973e-15 4.571282e-15 2.853922e-15 4.559063e-15 6.556881e-17
8.840183e-16
E:\\R-3.1.2\\scripts\\mPDBS\\summary_pdb_93\\5c35ac30f930d2db.final.pdb
4.545884e-15 -9.569347e-16 4.876427e-15 6.032060e-16 2.342737e-16
7.857257e-15
E:\\R-3.1.2\\scripts\\mPDBS\\summary_pdb_93\\5f60752431cf2dcb.final.pdb
9.876459e-15 4.459864e-16 6.677054e-16 -3.213131e-15 2.201519e-15
2.491664e-15
E:\\R-3.1.2\\scripts\\mPDBS\\summary_pdb_93\\626456dd6ff2868d.final.pdb
4.385343e-16 1.107149e-15 2.327774e-15 -3.142436e-15 1.902082e-16
2.520678e-15
E:\\R-3.1.2\\scripts\\mPDBS\\summary_pdb_93\\6583d53ff3c8f7ef.final.pdb
1.940873e-15 6.248312e-15 -2.562017e-15 3.347499e-15 3.021316e-15
1.772254e-15
E:\\R-3.1.2\\scripts\\mPDBS\\summary_pdb_93\\69760178986bef01.final.pdb
-1.417752e-15 1.058482e-15 6.404039e-16 -6.936492e-15 4.351274e-15
1.044589e-14
E:\\R-3.1.2\\scripts\\mPDBS\\summary_pdb_93\\6a9d87d47926b1c8.final.pdb
1.766899e-15 3.286829e-15 3.770088e-15 2.349699e-15 6.538131e-16
3.336865e-15
E:\\R-3.1.2\\scripts\\mPDBS\\summary_pdb_93\\6bb3547ea982ffc4.final.pdb
3.022453e-15 8.894267e-17 1.252528e-14 6.305934e-15 6.045057e-16
1.626919e-14
E:\\R-3.1.2\\scripts\\mPDBS\\summary_pdb_93\\716d9624611d1c42.final.pdb
-2.715465e-15 3.282259e-15 1.109746e-14 3.186914e-15 -9.545594e-15
7.289476e-15
E:\\R-3.1.2\\scripts\\mPDBS\\summary_pdb_93\\75f27e70ad0f1be4.final.pdb
1.940873e-15 6.248312e-15 -2.562017e-15 3.347499e-15 3.021316e-15
1.772254e-15
E:\\R-3.1.2\\scripts\\mPDBS\\summary_pdb_93\\769f910fffb22872.final.pdb
2.043778e-15 3.127336e-15 4.819758e-15 2.370522e-15 1.197918e-15
3.254196e-15
E:\\R-3.1.2\\scripts\\mPDBS\\summary_pdb_93\\791f8c80dd5c3135.final.pdb
1.483973e-15 4.571282e-15 2.853922e-15 4.559063e-15 6.556881e-17
8.840183e-16
E:\\R-3.1.2\\scripts\\mPDBS\\summary_pdb_93\\7fad98e66a3f6099.final.pdb
-1.559351e-15 5.903962e-15 7.748486e-15 2.076273e-15 2.162397e-15

-4.225641e-15
E:\\R-3.1.2\\scripts\\mPDBS\\summary_pdb_93\\804b89c94a633196.final.pdb
-1.605512e-15 4.038973e-15 2.659837e-15 -4.285897e-15 1.359479e-15
-5.547409e-16
E:\\R-3.1.2\\scripts\\mPDBS\\summary_pdb_93\\813cc65a2298feba.final.pdb
-1.605512e-15 4.038973e-15 2.659837e-15 -4.285897e-15 1.359479e-15
-5.547409e-16
E:\\R-3.1.2\\scripts\\mPDBS\\summary_pdb_93\\83c0eefcfe12d3f9.final.pdb
-1.164459e-15 3.767488e-15 7.258697e-15 -5.129840e-15 -4.663914e-15
4.774196e-16
E:\\R-3.1.2\\scripts\\mPDBS\\summary_pdb_93\\86d50469ef569760.final.pdb
4.385343e-16 1.107149e-15 2.327774e-15 -3.142436e-15 1.902082e-16
2.520678e-15
E:\\R-3.1.2\\scripts\\mPDBS\\summary_pdb_93\\89d0666e1402203d.final.pdb
1.940873e-15 6.248312e-15 -2.562017e-15 3.347499e-15 3.021316e-15
1.772254e-15
E:\\R-3.1.2\\scripts\\mPDBS\\summary_pdb_93\\8b34852716dadc62.final.pdb
2.257717e-15 -3.741638e-16 2.676533e-16 7.200815e-16 3.457627e-15
2.818429e-15
E:\\R-3.1.2\\scripts\\mPDBS\\summary_pdb_93\\8b6ef9ac3fe74395.final.pdb
2.306520e-15 -5.721491e-16 1.119537e-15 1.340996e-14 7.358371e-15
2.011561e-15
E:\\R-3.1.2\\scripts\\mPDBS\\summary_pdb_93\\98bbe0d5986c138f.final.pdb
1.483973e-15 4.571282e-15 2.853922e-15 4.559063e-15 6.556881e-17
8.840183e-16
E:\\R-3.1.2\\scripts\\mPDBS\\summary_pdb_93\\9c22f4909d67e2fa.final.pdb
6.145666e-15 5.882550e-15 2.996861e-15 1.853506e-15 -8.089252e-16
1.084933e-14
E:\\R-3.1.2\\scripts\\mPDBS\\summary_pdb_93\\9dda8ed0d81fb3ef.final.pdb
5.906885e-15 1.091847e-15 8.105069e-15 -7.011185e-15 -1.112964e-15
1.596359e-14
E:\\R-3.1.2\\scripts\\mPDBS\\summary_pdb_93\\9f752b3616e6033b.final.pdb
5.545287e-17 4.548238e-15 3.063657e-15 4.033663e-15 3.634037e-15
2.726906e-15
E:\\R-3.1.2\\scripts\\mPDBS\\summary_pdb_93\\a02c70083816d6ba.final.pdb
4.545884e-15 -9.569347e-16 4.876427e-15 6.032060e-16 2.342737e-16
7.857257e-15
E:\\R-3.1.2\\scripts\\mPDBS\\summary_pdb_93\\a0c997d891e4e082.final.pdb
-8.443955e-16 1.089539e-15 1.358841e-14 -4.105565e-16 6.077225e-15
1.556333e-14
E:\\R-3.1.2\\scripts\\mPDBS\\summary_pdb_93\\ab8039a2d823c616.final.pdb
1.776555e-15 3.217611e-15 4.183119e-15 1.931754e-15 1.427359e-16
3.405090e-15
E:\\R-3.1.2\\scripts\\mPDBS\\summary_pdb_93\\acf4d74b9c71d4dc.final.pdb
-1.605512e-15 4.038973e-15 2.659837e-15 -4.285897e-15 1.359479e-15
-5.547409e-16
E:\\R-3.1.2\\scripts\\mPDBS\\summary_pdb_93\\ad8a823e4b4c668e.final.pdb
2.225094e-15 2.853367e-15 4.904430e-15 2.698684e-15 4.776305e-16
3.414689e-15
E:\\R-3.1.2\\scripts\\mPDBS\\summary_pdb_93\\b16ababd03b7b857.final.pdb
6.145666e-15 5.882550e-15 2.996861e-15 1.853506e-15 -8.089252e-16
1.084933e-14
E:\\R-3.1.2\\scripts\\mPDBS\\summary_pdb_93\\b226923e697d3c63.final.pdb
2.832304e-15 -1.369292e-15 9.169810e-15 3.364846e-15 5.039233e-15

7.288241e-15
E:\\R-3.1.2\\scripts\\mPDBS\\summary_pdb_93\\b28e48f6fa27a165.final.pdb
1.483973e-15 4.571282e-15 2.853922e-15 4.559063e-15 6.556881e-17
8.840183e-16
E:\\R-3.1.2\\scripts\\mPDBS\\summary_pdb_93\\b42c4924e3cd1aec.final.pdb
2.983067e-16 5.993742e-15 5.478481e-15 4.021873e-15 -4.736801e-15
-3.705569e-15
E:\\R-3.1.2\\scripts\\mPDBS\\summary_pdb_93\\b4dd239fcabff1df.final.pdb
1.483973e-15 4.571282e-15 2.853922e-15 4.559063e-15 6.556881e-17
8.840183e-16
E:\\R-3.1.2\\scripts\\mPDBS\\summary_pdb_93\\b669942ef97459a2.final.pdb
2.257717e-15 -3.741638e-16 2.676533e-16 7.200815e-16 3.457627e-15
2.818429e-15
E:\\R-3.1.2\\scripts\\mPDBS\\summary_pdb_93\\b99f4d9d87c1017b.final.pdb
1.483973e-15 4.571282e-15 2.853922e-15 4.559063e-15 6.556881e-17
8.840183e-16
E:\\R-3.1.2\\scripts\\mPDBS\\summary_pdb_93\\b9d2cbf3975403e7.final.pdb
1.483973e-15 4.571282e-15 2.853922e-15 4.559063e-15 6.556881e-17
8.840183e-16
E:\\R-3.1.2\\scripts\\mPDBS\\summary_pdb_93\\bc88c8a505f3a288.final.pdb
1.982055e-15 3.321847e-15 3.454245e-15 2.105947e-15 5.352452e-16
3.536844e-15
E:\\R-3.1.2\\scripts\\mPDBS\\summary_pdb_93\\bdac0e64f4ef427e.final.pdb
9.041353e-15 1.721816e-15 -9.859013e-16 1.661340e-14 1.139789e-14
9.960285e-15
E:\\R-3.1.2\\scripts\\mPDBS\\summary_pdb_93\\ca2a1f55fc9d0681.final.pdb
2.257717e-15 -3.741638e-16 2.676533e-16 7.200815e-16 3.457627e-15
2.818429e-15
E:\\R-3.1.2\\scripts\\mPDBS\\summary_pdb_93\\ce34248de3e81a3c.final.pdb
6.801805e-15 -4.306549e-16 3.140397e-15 3.070856e-15 -5.247441e-16
8.504507e-16
E:\\R-3.1.2\\scripts\\mPDBS\\summary_pdb_93\\d02e697772494a61.final.pdb
5.764954e-16 2.048560e-15 -8.731852e-16 8.537651e-15 5.777580e-15
-1.920596e-15
E:\\R-3.1.2\\scripts\\mPDBS\\summary_pdb_93\\d04221245d015047.final.pdb
1.660545e-15 4.683886e-15 2.529548e-15 1.608662e-15 -1.021264e-15
2.497396e-15
E:\\R-3.1.2\\scripts\\mPDBS\\summary_pdb_93\\d678141e4018054a.final.pdb
7.057596e-15 3.010402e-15 1.327429e-14 1.265804e-15 -8.801805e-15
4.651769e-15
E:\\R-3.1.2\\scripts\\mPDBS\\summary_pdb_93\\d71b40af0f0efe39.final.pdb
2.608097e-16 9.459511e-15 8.510358e-15 -4.606045e-15 -8.143188e-15
-5.009165e-15
E:\\R-3.1.2\\scripts\\mPDBS\\summary_pdb_93\\dc0809eff10197e2.final.pdb
-3.270523e-15 5.427267e-15 -9.262290e-16 6.573450e-15 2.804439e-16
-3.059209e-15
E:\\R-3.1.2\\scripts\\mPDBS\\summary_pdb_93\\e1163918d3707839.final.pdb
-4.197229e-15 -4.409395e-15 1.700745e-14 7.260385e-15 -8.691103e-15
3.023857e-15
E:\\R-3.1.2\\scripts\\mPDBS\\summary_pdb_93\\e4b48b06824ef848.final.pdb
6.559046e-15 -2.213443e-15 8.991660e-15 -1.431342e-15 -1.537741e-15
6.332897e-15
E:\\R-3.1.2\\scripts\\mPDBS\\summary_pdb_93\\e529e19ad281fea2.final.pdb
3.083988e-15 8.236863e-15 6.478100e-15 1.681830e-15 -2.574941e-15

```
2.744457e-15
E:\\R-3.1.2\\scripts\\mPDBS\\summary_pdb_93\\e5602082718ec3a1.final.pdb
-1.605512e-15  4.038973e-15  2.659837e-15 -4.285897e-15  1.359479e-15
-5.547409e-16
E:\\R-3.1.2\\scripts\\mPDBS\\summary_pdb_93\\e5d589d996d0a960.final.pdb
7.748355e-16  1.911863e-16  2.522774e-15 -3.474802e-15  8.739161e-15
1.136021e-14
E:\\R-3.1.2\\scripts\\mPDBS\\summary_pdb_93\\e9df9e72307a2fbd.final.pdb
4.545884e-15 -9.569347e-16  4.876427e-15  6.032060e-16  2.342737e-16
7.857257e-15
E:\\R-3.1.2\\scripts\\mPDBS\\summary_pdb_93\\ea00f8694090cae7.final.pdb
-1.605512e-15  4.038973e-15  2.659837e-15 -4.285897e-15  1.359479e-15
-5.547409e-16
E:\\R-3.1.2\\scripts\\mPDBS\\summary_pdb_93\\eba517be32db050e.final.pdb
1.483973e-15  4.571282e-15  2.853922e-15  4.559063e-15  6.556881e-17
8.840183e-16
E:\\R-3.1.2\\scripts\\mPDBS\\summary_pdb_93\\ec04b9545899c29d.final.pdb
2.117815e-15  2.998308e-15  4.146159e-15  1.951697e-15  5.037984e-16
3.567259e-15
E:\\R-3.1.2\\scripts\\mPDBS\\summary_pdb_93\\ee6862cbcad52078.final.pdb
1.951481e-15  3.633664e-15  3.692228e-15  1.878698e-15  5.205000e-16
3.317402e-15
E:\\R-3.1.2\\scripts\\mPDBS\\summary_pdb_93\\ef6a42fc83b81e5d.final.pdb
2.095169e-15  3.092208e-15  4.483276e-15  1.752960e-15  5.512999e-16
3.932473e-15
E:\\R-3.1.2\\scripts\\mPDBS\\summary_pdb_93\\ef8e88d4ad93a0ad.final.pdb
1.660545e-15  4.683886e-15  2.529548e-15  1.608662e-15 -1.021264e-15
2.497396e-15
E:\\R-3.1.2\\scripts\\mPDBS\\summary_pdb_93\\f14fcae8deec4fa3.final.pdb
5.545287e-17  4.548238e-15  3.063657e-15  4.033663e-15  3.634037e-15
2.726906e-15
E:\\R-3.1.2\\scripts\\mPDBS\\summary_pdb_93\\f83584e22003fb92.final.pdb
4.774309e-15  4.009232e-15  9.839058e-15 -7.045587e-15 -2.982774e-15
6.646336e-15
E:\\R-3.1.2\\scripts\\mPDBS\\summary_pdb_93\\f8e2bf87e52caa29.final.pdb
3.083988e-15  8.236863e-15  6.478100e-15  1.681830e-15 -2.574941e-15
2.744457e-15

[,379]        [,380]        [,381]        [,382]        [,383]        [,384]
E:\\R-3.1.2\\scripts\\40.1ns_data\\NAT0.pdb
3.441226e-15 -2.775875e-15 -3.154402e-15 -3.409216e-15  1.794918e-15
2.701986e-15
E:\\R-3.1.2\\scripts\\40.1ns_data\\LJP6.pdb
3.052546e-15 -6.215171e-15 -3.132960e-15 -3.215251e-15 -6.321638e-16
1.652117e-15
E:\\R-3.1.2\\scripts\\40.1ns_data\\3WY9.pdb
3.012395e-15 -5.158294e-15 -1.562470e-15 -3.269999e-15  1.794162e-16
5.566061e-16
E:\\R-3.1.2\\scripts\\40.1ns_data\\ZSP9.pdb
2.480146e-15 -4.104481e-15 -4.005864e-15 -2.284672e-15 -6.672996e-16
7.760043e-16
E:\\R-3.1.2\\scripts\\mPDBS\\summary_pdb_93\\013d855ad26c877c.final.pdb
5.364017e-15 -7.535191e-15 -1.513216e-15 -3.188974e-16  4.101895e-15
7.279758e-16
```

E:\\R-3.1.2\\scripts\\mPDBS\\summary_pdb_93\\0a2a705342ca0d54.final.pdb
2.607285e-15 -4.047227e-15 -3.758721e-15 -2.495484e-15 5.686483e-17
1.047416e-15
E:\\R-3.1.2\\scripts\\mPDBS\\summary_pdb_93\\0a59fceadb4194d9.final.pdb
7.224002e-16 -4.047281e-15 -4.217903e-15 -3.057120e-15 3.014346e-16
2.434758e-15
E:\\R-3.1.2\\scripts\\mPDBS\\summary_pdb_93\\0dba59f3306825e8.final.pdb
-5.722066e-16 -2.783784e-15 -7.525398e-16 -6.656710e-15 3.005550e-15
1.519010e-15
E:\\R-3.1.2\\scripts\\mPDBS\\summary_pdb_93\\126f26a78b57339c.final.pdb
1.502306e-15 -2.499289e-15 -4.628779e-15 -1.800351e-15 -2.437579e-15
3.116690e-15
E:\\R-3.1.2\\scripts\\mPDBS\\summary_pdb_93\\12f06f3e342df706.final.pdb
3.200560e-15 -3.952685e-15 -3.433244e-15 -3.042790e-15 7.179300e-16
1.416912e-15
E:\\R-3.1.2\\scripts\\mPDBS\\summary_pdb_93\\18618a2a251b867b.final.pdb
9.919055e-15 -7.988560e-15 -3.134017e-15 -3.010615e-15 2.352245e-15
4.140054e-15
E:\\R-3.1.2\\scripts\\mPDBS\\summary_pdb_93\\1f305f598641eeb2.final.pdb
3.104161e-15 -4.057050e-15 -3.219863e-15 -2.371968e-15 3.846454e-16
1.659974e-15
E:\\R-3.1.2\\scripts\\mPDBS\\summary_pdb_93\\1f8a00b551be0a39.final.pdb
9.919055e-15 -7.988560e-15 -3.134017e-15 -3.010615e-15 2.352245e-15
4.140054e-15
E:\\R-3.1.2\\scripts\\mPDBS\\summary_pdb_93\\219f45328d47579a.final.pdb
2.540390e-15 -1.493768e-15 -3.993256e-15 2.095876e-15 -2.053886e-15
6.549522e-15
E:\\R-3.1.2\\scripts\\mPDBS\\summary_pdb_93\\238dd3918e54336d.final.pdb
2.182821e-15 -2.656033e-15 -1.172596e-15 -8.071839e-16 1.442252e-16
9.055994e-16
E:\\R-3.1.2\\scripts\\mPDBS\\summary_pdb_93\\2b3027889e2fdc42.final.pdb
2.181086e-15 -3.846053e-15 -3.842335e-15 -2.680685e-15 7.598352e-16
1.028765e-15
E:\\R-3.1.2\\scripts\\mPDBS\\summary_pdb_93\\2fcb3b46e33793b0.final.pdb
4.183159e-15 -4.823798e-15 -1.519834e-15 -4.459707e-15 3.685824e-15
-6.263918e-16
E:\\R-3.1.2\\scripts\\mPDBS\\summary_pdb_93\\34eb328251842006.final.pdb
6.238885e-15 -7.744074e-15 -5.436628e-15 2.183759e-16 -4.699375e-15
-1.536839e-15
E:\\R-3.1.2\\scripts\\mPDBS\\summary_pdb_93\\354b32871797b7ab.final.pdb
4.723818e-15 -2.320345e-14 2.659852e-17 -9.851479e-16 -2.520072e-15
5.403018e-15
E:\\R-3.1.2\\scripts\\mPDBS\\summary_pdb_93\\397368adbc32d5de.final.pdb
1.502306e-15 -2.499289e-15 -4.628779e-15 -1.800351e-15 -2.437579e-15
3.116690e-15
E:\\R-3.1.2\\scripts\\mPDBS\\summary_pdb_93\\411ca13cd3df6edd.final.pdb
6.965872e-15 -7.217716e-15 -3.263444e-16 -4.293174e-15 4.072234e-15
-2.881207e-16
E:\\R-3.1.2\\scripts\\mPDBS\\summary_pdb_93\\42357eee6642bf12.final.pdb
1.089609e-14 -1.666540e-14 -5.068390e-15 2.108674e-16 -4.531158e-15
3.608787e-15
E:\\R-3.1.2\\scripts\\mPDBS\\summary_pdb_93\\44342d1f75a7fcfc.final.pdb
8.582515e-15 -8.361074e-15 -3.408298e-15 -5.194766e-15 -5.179454e-15
-6.185877e-15

E:\\R-3.1.2\\scripts\\mPDBS\\summary_pdb_93\\448ef21dffe8cdfb.final.pdb
8.717726e-15 -9.798985e-15 -1.153092e-14 1.069657e-14 -2.678876e-15
7.107005e-15
E:\\R-3.1.2\\scripts\\mPDBS\\summary_pdb_93\\472a40b3e8f113c7.final.pdb
-5.722066e-16 -2.783784e-15 -7.525398e-16 -6.656710e-15 3.005550e-15
1.519010e-15
E:\\R-3.1.2\\scripts\\mPDBS\\summary_pdb_93\\48a26225951ea153.final.pdb
7.649802e-15 -9.073642e-15 -2.438727e-15 -5.649664e-15 -9.120045e-16
-1.039179e-14
E:\\R-3.1.2\\scripts\\mPDBS\\summary_pdb_93\\4ab4a5c7c6682ce6.final.pdb
1.354476e-14 -9.766046e-15 -1.574489e-14 -1.191463e-15 -9.570224e-17
-7.465123e-15
E:\\R-3.1.2\\scripts\\mPDBS\\summary_pdb_93\\4eaa45b62cb6a25f.final.pdb
2.542621e-15 2.440099e-15 -4.546783e-15 -3.621928e-15 2.843910e-15
2.212455e-19
E:\\R-3.1.2\\scripts\\mPDBS\\summary_pdb_93\\58c371611d85ac96.final.pdb
1.502306e-15 -2.499289e-15 -4.628779e-15 -1.800351e-15 -2.437579e-15
3.116690e-15
E:\\R-3.1.2\\scripts\\mPDBS\\summary_pdb_93\\59a647f04822d30a.final.pdb
5.038475e-15 -5.478797e-16 -7.713881e-15 7.319166e-16 1.370428e-15
4.423692e-15
E:\\R-3.1.2\\scripts\\mPDBS\\summary_pdb_93\\59b091fe385e05fc.final.pdb
7.224002e-16 -4.047281e-15 -4.217903e-15 -3.057120e-15 3.014346e-16
2.434758e-15
E:\\R-3.1.2\\scripts\\mPDBS\\summary_pdb_93\\5c35ac30f930d2db.final.pdb
2.542621e-15 2.440099e-15 -4.546783e-15 -3.621928e-15 2.843910e-15
2.212455e-19
E:\\R-3.1.2\\scripts\\mPDBS\\summary_pdb_93\\5f60752431cf2dcb.final.pdb
2.138692e-15 3.131058e-15 -6.774538e-15 -1.107803e-14 1.649775e-15
1.841919e-15
E:\\R-3.1.2\\scripts\\mPDBS\\summary_pdb_93\\626456dd6ff2868d.final.pdb
-5.722066e-16 -2.783784e-15 -7.525398e-16 -6.656710e-15 3.005550e-15
1.519010e-15
E:\\R-3.1.2\\scripts\\mPDBS\\summary_pdb_93\\6583d53ff3c8f7ef.final.pdb
5.038475e-15 -5.478797e-16 -7.713881e-15 7.319166e-16 1.370428e-15
4.423692e-15
E:\\R-3.1.2\\scripts\\mPDBS\\summary_pdb_93\\69760178986bef01.final.pdb
3.275327e-15 -2.103638e-15 2.107606e-16 -6.838249e-15 2.216316e-15
1.966984e-15
E:\\R-3.1.2\\scripts\\mPDBS\\summary_pdb_93\\6a9d87d47926b1c8.final.pdb
2.307218e-15 -3.935640e-15 -3.761418e-15 -2.574905e-15 1.974660e-16
9.933885e-16
E:\\R-3.1.2\\scripts\\mPDBS\\summary_pdb_93\\6bb3547ea982ffc4.final.pdb
7.004082e-16 -4.199481e-15 2.908100e-15 -2.810109e-15 4.770301e-15
5.855178e-15
E:\\R-3.1.2\\scripts\\mPDBS\\summary_pdb_93\\716d9624611d1c42.final.pdb
7.129709e-15 -9.698957e-15 -5.451462e-16 5.198881e-15 -7.753615e-18
6.571031e-16
E:\\R-3.1.2\\scripts\\mPDBS\\summary_pdb_93\\75f27e70ad0f1be4.final.pdb
5.038475e-15 -5.478797e-16 -7.713881e-15 7.319166e-16 1.370428e-15
4.423692e-15
E:\\R-3.1.2\\scripts\\mPDBS\\summary_pdb_93\\769f910fffb22872.final.pdb
2.916844e-15 -4.335233e-15 -3.242438e-15 -2.297644e-15 2.165835e-16
1.527561e-15

E:\\R-3.1.2\\scripts\\mPDBS\\summary_pdb_93\\791f8c80dd5c3135.final.pdb
7.224002e-16 -4.047281e-15 -4.217903e-15 -3.057120e-15 3.014346e-16
2.434758e-15
E:\\R-3.1.2\\scripts\\mPDBS\\summary_pdb_93\\7fad98e66a3f6099.final.pdb
-3.613462e-15 -8.362305e-15 -1.111083e-15 1.001402e-15 -2.922280e-15
-3.185082e-15
E:\\R-3.1.2\\scripts\\mPDBS\\summary_pdb_93\\804b89c94a633196.final.pdb
4.183159e-15 -4.823798e-15 -1.519834e-15 -4.459707e-15 3.685824e-15
-6.263918e-16
E:\\R-3.1.2\\scripts\\mPDBS\\summary_pdb_93\\813cc65a2298feba.final.pdb
4.183159e-15 -4.823798e-15 -1.519834e-15 -4.459707e-15 3.685824e-15
-6.263918e-16
E:\\R-3.1.2\\scripts\\mPDBS\\summary_pdb_93\\83c0eefcfe12d3f9.final.pdb
6.965872e-15 -7.217716e-15 -3.263444e-16 -4.293174e-15 4.072234e-15
-2.881207e-16
E:\\R-3.1.2\\scripts\\mPDBS\\summary_pdb_93\\86d50469ef569760.final.pdb
-5.722066e-16 -2.783784e-15 -7.525398e-16 -6.656710e-15 3.005550e-15
1.519010e-15
E:\\R-3.1.2\\scripts\\mPDBS\\summary_pdb_93\\89d0666e1402203d.final.pdb
5.038475e-15 -5.478797e-16 -7.713881e-15 7.319166e-16 1.370428e-15
4.423692e-15
E:\\R-3.1.2\\scripts\\mPDBS\\summary_pdb_93\\8b34852716dadc62.final.pdb
2.215982e-15 -1.652723e-15 -5.939910e-15 -2.451765e-15 -3.556646e-15
1.195068e-15
E:\\R-3.1.2\\scripts\\mPDBS\\summary_pdb_93\\8b6ef9ac3fe74395.final.pdb
-1.029078e-14 5.035807e-15 2.686210e-15 9.737314e-16 5.866382e-15
1.344644e-14
E:\\R-3.1.2\\scripts\\mPDBS\\summary_pdb_93\\98bbe0d5986c138f.final.pdb
7.224002e-16 -4.047281e-15 -4.217903e-15 -3.057120e-15 3.014346e-16
2.434758e-15
E:\\R-3.1.2\\scripts\\mPDBS\\summary_pdb_93\\9c22f4909d67e2fa.final.pdb
-4.067592e-17 -1.904258e-15 -2.978737e-15 -3.414536e-15 -5.983741e-15
1.574105e-15
E:\\R-3.1.2\\scripts\\mPDBS\\summary_pdb_93\\9dda8ed0d81fb3ef.final.pdb
2.284385e-15 9.432852e-15 -2.220662e-15 -1.766569e-14 5.752773e-15
-7.377632e-15
E:\\R-3.1.2\\scripts\\mPDBS\\summary_pdb_93\\9f752b3616e6033b.final.pdb
6.879343e-16 -4.312170e-15 -2.733816e-15 -2.615509e-15 1.127580e-15
2.152420e-15
E:\\R-3.1.2\\scripts\\mPDBS\\summary_pdb_93\\a02c70083816d6ba.final.pdb
2.542621e-15 2.440099e-15 -4.546783e-15 -3.621928e-15 2.843910e-15
2.212455e-19
E:\\R-3.1.2\\scripts\\mPDBS\\summary_pdb_93\\a0c997d891e4e082.final.pdb
-3.679612e-15 -1.980784e-15 -9.918138e-15 -6.687436e-15 7.243844e-15
-1.713826e-15
E:\\R-3.1.2\\scripts\\mPDBS\\summary_pdb_93\\ab8039a2d823c616.final.pdb
3.662389e-15 -3.542035e-15 -3.204975e-15 -2.383587e-15 2.539922e-16
1.910011e-15
E:\\R-3.1.2\\scripts\\mPDBS\\summary_pdb_93\\acf4d74b9c71d4dc.final.pdb
4.183159e-15 -4.823798e-15 -1.519834e-15 -4.459707e-15 3.685824e-15
-6.263918e-16
E:\\R-3.1.2\\scripts\\mPDBS\\summary_pdb_93\\ad8a823e4b4c668e.final.pdb
2.595875e-15 -4.111677e-15 -3.794046e-15 -3.072656e-15 -7.473522e-16
3.046707e-16

E:\\R-3.1.2\\scripts\\mPDBS\\summary_pdb_93\\b16ababd03b7b857.final.pdb
-4.067592e-17 -1.904258e-15 -2.978737e-15 -3.414536e-15 -5.983741e-15
1.574105e-15
E:\\R-3.1.2\\scripts\\mPDBS\\summary_pdb_93\\b226923e697d3c63.final.pdb
-4.900885e-16 -7.564836e-15 -8.277666e-15 -4.853026e-15 7.516336e-15
1.288828e-14
E:\\R-3.1.2\\scripts\\mPDBS\\summary_pdb_93\\b28e48f6fa27a165.final.pdb
7.224002e-16 -4.047281e-15 -4.217903e-15 -3.057120e-15 3.014346e-16
2.434758e-15
E:\\R-3.1.2\\scripts\\mPDBS\\summary_pdb_93\\b42c4924e3cd1aec.final.pdb
-2.514294e-15 -1.469628e-14 5.778798e-15 1.569788e-15 -2.795294e-15
4.129656e-15
E:\\R-3.1.2\\scripts\\mPDBS\\summary_pdb_93\\b4dd239fcabff1df.final.pdb
7.224002e-16 -4.047281e-15 -4.217903e-15 -3.057120e-15 3.014346e-16
2.434758e-15
E:\\R-3.1.2\\scripts\\mPDBS\\summary_pdb_93\\b669942ef97459a2.final.pdb
2.215982e-15 -1.652723e-15 -5.939910e-15 -2.451765e-15 -3.556646e-15
1.195068e-15
E:\\R-3.1.2\\scripts\\mPDBS\\summary_pdb_93\\b99f4d9d87c1017b.final.pdb
7.224002e-16 -4.047281e-15 -4.217903e-15 -3.057120e-15 3.014346e-16
2.434758e-15
E:\\R-3.1.2\\scripts\\mPDBS\\summary_pdb_93\\b9d2cbf3975403e7.final.pdb
7.224002e-16 -4.047281e-15 -4.217903e-15 -3.057120e-15 3.014346e-16
2.434758e-15
E:\\R-3.1.2\\scripts\\mPDBS\\summary_pdb_93\\bc88c8a505f3a288.final.pdb
2.135549e-15 -4.248509e-15 -3.325171e-15 -2.527162e-15 9.144425e-16
1.385684e-15
E:\\R-3.1.2\\scripts\\mPDBS\\summary_pdb_93\\bdac0e64f4ef427e.final.pdb
-4.856051e-15 2.289436e-15 -9.719872e-15 5.380310e-16 -8.407584e-15
5.648537e-15
E:\\R-3.1.2\\scripts\\mPDBS\\summary_pdb_93\\ca2a1f55fc9d0681.final.pdb
2.215982e-15 -1.652723e-15 -5.939910e-15 -2.451765e-15 -3.556646e-15
1.195068e-15
E:\\R-3.1.2\\scripts\\mPDBS\\summary_pdb_93\\ce34248de3e81a3c.final.pdb
3.063402e-15 -3.340178e-15 -6.278226e-15 -1.210083e-15 2.917822e-15
-2.934684e-15
E:\\R-3.1.2\\scripts\\mPDBS\\summary_pdb_93\\d02e697772494a61.final.pdb
8.497214e-15 -5.948538e-15 -1.379618e-14 7.831739e-15 -5.873741e-17
8.354502e-15
E:\\R-3.1.2\\scripts\\mPDBS\\summary_pdb_93\\d04221245d015047.final.pdb
3.765822e-15 -2.634775e-15 -1.745856e-15 -2.073071e-15 -1.857383e-15
-2.602005e-15
E:\\R-3.1.2\\scripts\\mPDBS\\summary_pdb_93\\d678141e4018054a.final.pdb
1.190884e-14 -1.299991e-14 -2.848744e-15 -3.768455e-15 -5.323853e-18
2.484153e-15
E:\\R-3.1.2\\scripts\\mPDBS\\summary_pdb_93\\d71b40af0f0efe39.final.pdb
2.196827e-15 -7.608344e-15 -6.734690e-15 -3.687223e-15 -3.194503e-15
3.729219e-16
E:\\R-3.1.2\\scripts\\mPDBS\\summary_pdb_93\\dc0809eff10197e2.final.pdb
-8.031087e-15 5.511784e-15 -2.243510e-15 -9.341066e-16 2.606299e-15
1.736271e-15
E:\\R-3.1.2\\scripts\\mPDBS\\summary_pdb_93\\e1163918d3707839.final.pdb
-6.026287e-16 -1.221650e-14 4.766587e-15 -2.373238e-15 5.375440e-16
6.090352e-16

E:\\R-3.1.2\\scripts\\mPDBS\\summary_pdb_93\\e4b48b06824ef848.final.pdb
9.919055e-15 -7.988560e-15 -3.134017e-15 -3.010615e-15 2.352245e-15
4.140054e-15
E:\\R-3.1.2\\scripts\\mPDBS\\summary_pdb_93\\e529e19ad281fea2.final.pdb
5.364017e-15 -7.535191e-15 -1.513216e-15 -3.188974e-16 4.101895e-15
7.279758e-16
E:\\R-3.1.2\\scripts\\mPDBS\\summary_pdb_93\\e5602082718ec3a1.final.pdb
4.183159e-15 -4.823798e-15 -1.519834e-15 -4.459707e-15 3.685824e-15
-6.263918e-16
E:\\R-3.1.2\\scripts\\mPDBS\\summary_pdb_93\\e5d589d996d0a960.final.pdb
-9.835267e-15 1.301056e-15 1.401323e-15 -8.509683e-15 5.855098e-15
-9.283493e-15
E:\\R-3.1.2\\scripts\\mPDBS\\summary_pdb_93\\e9df9e72307a2fbd.final.pdb
2.542621e-15 2.440099e-15 -4.546783e-15 -3.621928e-15 2.843910e-15
2.212455e-19
E:\\R-3.1.2\\scripts\\mPDBS\\summary_pdb_93\\ea00f8694090cae7.final.pdb
4.183159e-15 -4.823798e-15 -1.519834e-15 -4.459707e-15 3.685824e-15
-6.263918e-16
E:\\R-3.1.2\\scripts\\mPDBS\\summary_pdb_93\\eba517be32db050e.final.pdb
7.224002e-16 -4.047281e-15 -4.217903e-15 -3.057120e-15 3.014346e-16
2.434758e-15
E:\\R-3.1.2\\scripts\\mPDBS\\summary_pdb_93\\ec04b9545899c29d.final.pdb
3.168443e-15 -3.940131e-15 -3.562688e-15 -2.334207e-15 6.804418e-16
9.473062e-16
E:\\R-3.1.2\\scripts\\mPDBS\\summary_pdb_93\\ee6862cbcad52078.final.pdb
2.318346e-15 -4.009117e-15 -3.580392e-15 -2.981660e-15 7.364165e-16
1.064327e-15
E:\\R-3.1.2\\scripts\\mPDBS\\summary_pdb_93\\ef6a42fc83b81e5d.final.pdb
2.898846e-15 -3.602312e-15 -3.176518e-15 -2.221316e-15 7.831876e-16
1.515418e-15
E:\\R-3.1.2\\scripts\\mPDBS\\summary_pdb_93\\ef8e88d4ad93a0ad.final.pdb
3.765822e-15 -2.634775e-15 -1.745856e-15 -2.073071e-15 -1.857383e-15
-2.602005e-15
E:\\R-3.1.2\\scripts\\mPDBS\\summary_pdb_93\\f14fcae8deec4fa3.final.pdb
6.879343e-16 -4.312170e-15 -2.733816e-15 -2.615509e-15 1.127580e-15
2.152420e-15
E:\\R-3.1.2\\scripts\\mPDBS\\summary_pdb_93\\f83584e22003fb92.final.pdb
7.649802e-15 -9.073642e-15 -2.438727e-15 -5.649664e-15 -9.120045e-16
-1.039179e-14
E:\\R-3.1.2\\scripts\\mPDBS\\summary_pdb_93\\f8e2bf87e52caa29.final.pdb
5.364017e-15 -7.535191e-15 -1.513216e-15 -3.188974e-16 4.101895e-15
7.279758e-16

[,385]   [,386]   [,387]   [,388]   [,389]   [,390]
E:\\R-3.1.2\\scripts\\40.1ns_data\\NAT0.pdb
-1.952134e-15 -2.630558e-15 -1.922942e-15 1.003815e-17 -5.782407e-15
-3.503299e-15
E:\\R-3.1.2\\scripts\\40.1ns_data\\LJP6.pdb
-2.218173e-15 -2.920699e-15 -1.380874e-15 -1.065125e-15 -5.302634e-15
-1.832041e-15
E:\\R-3.1.2\\scripts\\40.1ns_data\\3WY9.pdb
-2.989188e-15 -1.450634e-15 -2.454794e-15 1.921481e-16 -6.647053e-15
-2.072250e-15
E:\\R-3.1.2\\scripts\\40.1ns_data\\ZSP9.pdb

-2.216006e-15 -2.733997e-15 -2.813873e-15 4.009687e-16 -6.139132e-15
-2.362856e-15
E:\\R-3.1.2\\scripts\\mPDBS\\summary_pdb_93\\013d855ad26c877c.final.pdb
-4.388509e-15 -7.724801e-15 -4.860078e-15 -6.220849e-15 -7.604817e-15
-4.646147e-15
E:\\R-3.1.2\\scripts\\mPDBS\\summary_pdb_93\\0a2a705342ca0d54.final.pdb
-2.233577e-15 -3.411097e-15 -1.956825e-15 -9.510450e-16 -6.620652e-15
-2.009794e-15
E:\\R-3.1.2\\scripts\\mPDBS\\summary_pdb_93\\0a59fceadb4194d9.final.pdb
-2.403735e-15 -3.235488e-15 4.054143e-16 -3.326907e-15 -6.151999e-15
-2.687002e-15
E:\\R-3.1.2\\scripts\\mPDBS\\summary_pdb_93\\0dba59f3306825e8.final.pdb
-1.383968e-15 -4.709852e-15 1.669421e-15 3.799781e-15 -4.356981e-15
-4.238298e-16
E:\\R-3.1.2\\scripts\\mPDBS\\summary_pdb_93\\126f26a78b57339c.final.pdb
1.719724e-15 1.841386e-15 -2.562328e-15 2.861079e-15 -7.765279e-15
-1.730945e-16
E:\\R-3.1.2\\scripts\\mPDBS\\summary_pdb_93\\12f06f3e342df706.final.pdb
-2.115291e-15 -2.699508e-15 -1.750447e-15 -1.021247e-15 -6.768537e-15
-2.520941e-15
E:\\R-3.1.2\\scripts\\mPDBS\\summary_pdb_93\\18618a2a251b867b.final.pdb
-4.595798e-15 -4.572269e-17 -2.920014e-15 -9.620180e-16 -4.949573e-15
-3.973437e-16
E:\\R-3.1.2\\scripts\\mPDBS\\summary_pdb_93\\1f305f598641eeb2.final.pdb
-1.851916e-15 -2.834723e-15 -1.837340e-15 -7.893024e-16 -6.770831e-15
-1.876052e-15
E:\\R-3.1.2\\scripts\\mPDBS\\summary_pdb_93\\1f8a00b551be0a39.final.pdb
-4.595798e-15 -4.572269e-17 -2.920014e-15 -9.620180e-16 -4.949573e-15
-3.973437e-16
E:\\R-3.1.2\\scripts\\mPDBS\\summary_pdb_93\\219f45328d47579a.final.pdb
-4.179225e-15 -8.507313e-15 3.252096e-15 1.285026e-14 -4.955040e-15
2.676763e-15
E:\\R-3.1.2\\scripts\\mPDBS\\summary_pdb_93\\238dd3918e54336d.final.pdb
8.102726e-15 -9.525135e-15 -1.458905e-14 1.281762e-14 -9.936732e-15
-1.775798e-15
E:\\R-3.1.2\\scripts\\mPDBS\\summary_pdb_93\\2b3027889e2fdc42.final.pdb
-1.896057e-15 -2.815631e-15 -1.127105e-15 3.545687e-18 -6.805557e-15
-2.087587e-15
E:\\R-3.1.2\\scripts\\mPDBS\\summary_pdb_93\\2fcb3b46e33793b0.final.pdb
-5.439324e-15 -6.102074e-15 -2.136393e-16 -1.679216e-15 -5.950740e-15
-3.395670e-15
E:\\R-3.1.2\\scripts\\mPDBS\\summary_pdb_93\\34eb328251842006.final.pdb
-2.505756e-15 1.640852e-15 1.001015e-15 -1.022892e-14 -1.326224e-14
-5.146704e-15
E:\\R-3.1.2\\scripts\\mPDBS\\summary_pdb_93\\354b32871797b7ab.final.pdb
-1.118485e-14 4.442365e-16 1.882734e-15 -1.596030e-14 -1.510448e-14
-1.443738e-14
E:\\R-3.1.2\\scripts\\mPDBS\\summary_pdb_93\\397368adbc32d5de.final.pdb
1.719724e-15 1.841386e-15 -2.562328e-15 2.861079e-15 -7.765279e-15
-1.730945e-16
E:\\R-3.1.2\\scripts\\mPDBS\\summary_pdb_93\\411ca13cd3df6edd.final.pdb
-6.707678e-15 -1.545877e-15 2.571351e-15 -4.635835e-15 -1.078281e-14
-2.182299e-15
E:\\R-3.1.2\\scripts\\mPDBS\\summary_pdb_93\\42357eee6642bf12.final.pdb

-8.126249e-15 1.221247e-15 -5.140086e-15 -4.603064e-15 -7.777900e-15
-5.338179e-15
E:\\R-3.1.2\\scripts\\mPDBS\\summary_pdb_93\\44342d1f75a7fcfc.final.pdb
-2.080740e-15 -1.722066e-15 -3.303738e-15 -8.038589e-16 -9.502220e-15
-4.831927e-15
E:\\R-3.1.2\\scripts\\mPDBS\\summary_pdb_93\\448ef21dffe8cdfb.final.pdb
-1.754771e-15 4.667956e-15 -1.327264e-14 -1.937339e-14 -1.672511e-14
-1.105968e-14
E:\\R-3.1.2\\scripts\\mPDBS\\summary_pdb_93\\472a40b3e8f113c7.final.pdb
-1.383968e-15 -4.709852e-15 1.669421e-15 3.799781e-15 -4.356981e-15
-4.238298e-16
E:\\R-3.1.2\\scripts\\mPDBS\\summary_pdb_93\\48a26225951ea153.final.pdb
-3.592617e-15 4.519647e-15 1.010074e-15 -8.515248e-15 -8.363772e-15
-3.048624e-15
E:\\R-3.1.2\\scripts\\mPDBS\\summary_pdb_93\\4ab4a5c7c6682ce6.final.pdb
-5.207027e-15 6.494782e-15 -3.193768e-15 -1.164728e-14 -5.179240e-15
-4.367656e-15
E:\\R-3.1.2\\scripts\\mPDBS\\summary_pdb_93\\4eaa45b62cb6a25f.final.pdb
1.263417e-15 -3.500985e-15 -3.136308e-15 3.340337e-15 -2.784910e-15
1.655180e-15
E:\\R-3.1.2\\scripts\\mPDBS\\summary_pdb_93\\58c371611d85ac96.final.pdb
1.719724e-15 1.841386e-15 -2.562328e-15 2.861079e-15 -7.765279e-15
-1.730945e-16
E:\\R-3.1.2\\scripts\\mPDBS\\summary_pdb_93\\59a647f04822d30a.final.pdb
-1.012150e-15 -1.520404e-15 -5.886741e-15 3.683437e-15 -1.028198e-14
6.866651e-17
E:\\R-3.1.2\\scripts\\mPDBS\\summary_pdb_93\\59b091fe385e05fc.final.pdb
-2.403735e-15 -3.235488e-15 4.054143e-16 -3.326907e-15 -6.151999e-15
-2.687002e-15
E:\\R-3.1.2\\scripts\\mPDBS\\summary_pdb_93\\5c35ac30f930d2db.final.pdb
1.263417e-15 -3.500985e-15 -3.136308e-15 3.340337e-15 -2.784910e-15
1.655180e-15
E:\\R-3.1.2\\scripts\\mPDBS\\summary_pdb_93\\5f60752431cf2dcb.final.pdb
3.059335e-15 -1.512822e-15 -3.728737e-15 -2.978720e-15 -3.817339e-15
1.195176e-15
E:\\R-3.1.2\\scripts\\mPDBS\\summary_pdb_93\\626456dd6ff2868d.final.pdb
-1.383968e-15 -4.709852e-15 1.669421e-15 3.799781e-15 -4.356981e-15
-4.238298e-16
E:\\R-3.1.2\\scripts\\mPDBS\\summary_pdb_93\\6583d53ff3c8f7ef.final.pdb
-1.012150e-15 -1.520404e-15 -5.886741e-15 3.683437e-15 -1.028198e-14
6.866651e-17
E:\\R-3.1.2\\scripts\\mPDBS\\summary_pdb_93\\69760178986bef01.final.pdb
1.503057e-15 -1.061777e-14 -3.215410e-15 1.409153e-14 -4.227456e-15
2.313919e-15
E:\\R-3.1.2\\scripts\\mPDBS\\summary_pdb_93\\6a9d87d47926b1c8.final.pdb
-1.862480e-15 -2.508433e-15 -1.493739e-15 7.424541e-17 -6.628602e-15
-2.091699e-15
E:\\R-3.1.2\\scripts\\mPDBS\\summary_pdb_93\\6bb3547ea982ffc4.final.pdb
3.514589e-15 -2.266539e-15 -1.687806e-15 2.280389e-15 -9.273241e-15
-8.415207e-16
E:\\R-3.1.2\\scripts\\mPDBS\\summary_pdb_93\\716d9624611d1c42.final.pdb
-4.388104e-15 -2.492995e-15 -4.476156e-16 -2.831052e-15 -8.803801e-15
-7.477642e-15
E:\\R-3.1.2\\scripts\\mPDBS\\summary_pdb_93\\75f27e70ad0f1be4.final.pdb

-1.012150e-15 -1.520404e-15 -5.886741e-15 3.683437e-15 -1.028198e-14
6.866651e-17
E:\\R-3.1.2\\scripts\\mPDBS\\summary_pdb_93\\769f910fffb22872.final.pdb
-2.281840e-15 -3.112741e-15 -2.301206e-15 -7.060379e-16 -6.869478e-15
-1.613846e-15
E:\\R-3.1.2\\scripts\\mPDBS\\summary_pdb_93\\791f8c80dd5c3135.final.pdb
-2.403735e-15 -3.235488e-15 4.054143e-16 -3.326907e-15 -6.151999e-15
-2.687002e-15
E:\\R-3.1.2\\scripts\\mPDBS\\summary_pdb_93\\7fad98e66a3f6099.final.pdb
-4.041312e-15 -2.951500e-16 -8.250328e-16 -6.211174e-15 -1.001504e-14
-9.719235e-15
E:\\R-3.1.2\\scripts\\mPDBS\\summary_pdb_93\\804b89c94a633196.final.pdb
-5.439324e-15 -6.102074e-15 -2.136393e-16 -1.679216e-15 -5.950740e-15
-3.395670e-15
E:\\R-3.1.2\\scripts\\mPDBS\\summary_pdb_93\\813cc65a2298feba.final.pdb
-5.439324e-15 -6.102074e-15 -2.136393e-16 -1.679216e-15 -5.950740e-15
-3.395670e-15
E:\\R-3.1.2\\scripts\\mPDBS\\summary_pdb_93\\83c0eefcfe12d3f9.final.pdb
-6.707678e-15 -1.545877e-15 2.571351e-15 -4.635835e-15 -1.078281e-14
-2.182299e-15
E:\\R-3.1.2\\scripts\\mPDBS\\summary_pdb_93\\86d50469ef569760.final.pdb
-1.383968e-15 -4.709852e-15 1.669421e-15 3.799781e-15 -4.356981e-15
-4.238298e-16
E:\\R-3.1.2\\scripts\\mPDBS\\summary_pdb_93\\89d0666e1402203d.final.pdb
-1.012150e-15 -1.520404e-15 -5.886741e-15 3.683437e-15 -1.028198e-14
6.866651e-17
E:\\R-3.1.2\\scripts\\mPDBS\\summary_pdb_93\\8b34852716dadc62.final.pdb
-5.820979e-16 1.717192e-15 2.457835e-15 4.581402e-15 -6.264292e-15
1.850134e-15
E:\\R-3.1.2\\scripts\\mPDBS\\summary_pdb_93\\8b6ef9ac3fe74395.final.pdb
9.966674e-16 -5.830959e-15 -6.677777e-15 3.921638e-15 -8.158883e-15
1.102269e-16
E:\\R-3.1.2\\scripts\\mPDBS\\summary_pdb_93\\98bbe0d5986c138f.final.pdb
-2.403735e-15 -3.235488e-15 4.054143e-16 -3.326907e-15 -6.151999e-15
-2.687002e-15
E:\\R-3.1.2\\scripts\\mPDBS\\summary_pdb_93\\9c22f4909d67e2fa.final.pdb
3.176072e-15 -7.792156e-15 -2.729476e-15 6.733434e-15 -2.369837e-15
-1.052520e-15
E:\\R-3.1.2\\scripts\\mPDBS\\summary_pdb_93\\9dda8ed0d81fb3ef.final.pdb
5.057051e-15 -7.583728e-16 7.273847e-15 -4.477786e-15 -1.055851e-15
1.376524e-14
E:\\R-3.1.2\\scripts\\mPDBS\\summary_pdb_93\\9f752b3616e6033b.final.pdb
-2.962556e-15 -4.738778e-15 -1.387289e-15 2.340861e-15 -6.513991e-15
-1.159799e-15
E:\\R-3.1.2\\scripts\\mPDBS\\summary_pdb_93\\a02c70083816d6ba.final.pdb
1.263417e-15 -3.500985e-15 -3.136308e-15 3.340337e-15 -2.784910e-15
1.655180e-15
E:\\R-3.1.2\\scripts\\mPDBS\\summary_pdb_93\\a0c997d891e4e082.final.pdb
-3.943995e-15 -1.028505e-14 -2.505150e-15 -2.090206e-15 -1.194020e-14
3.237702e-15
E:\\R-3.1.2\\scripts\\mPDBS\\summary_pdb_93\\ab8039a2d823c616.final.pdb
-1.606346e-15 -2.658309e-15 -1.793426e-15 -5.008744e-16 -6.521791e-15
-1.884462e-15
E:\\R-3.1.2\\scripts\\mPDBS\\summary_pdb_93\\acf4d74b9c71d4dc.final.pdb

-5.439324e-15 -6.102074e-15 -2.136393e-16 -1.679216e-15 -5.950740e-15
-3.395670e-15
E:\\R-3.1.2\\scripts\\mPDBS\\summary_pdb_93\\ad8a823e4b4c668e.final.pdb
-2.243087e-15 -3.236697e-15 -2.285339e-15 -4.410905e-16 -6.823612e-15
-1.107810e-15
E:\\R-3.1.2\\scripts\\mPDBS\\summary_pdb_93\\b16ababd03b7b857.final.pdb
3.176072e-15 -7.792156e-15 -2.729476e-15 6.733434e-15 -2.369837e-15
-1.052520e-15
E:\\R-3.1.2\\scripts\\mPDBS\\summary_pdb_93\\b226923e697d3c63.final.pdb
-8.631760e-15 -8.182149e-15 -8.165680e-15 -5.034265e-16 -1.110987e-14
-2.890311e-15
E:\\R-3.1.2\\scripts\\mPDBS\\summary_pdb_93\\b28e48f6fa27a165.final.pdb
-2.403735e-15 -3.235488e-15 4.054143e-16 -3.326907e-15 -6.151999e-15
-2.687002e-15
E:\\R-3.1.2\\scripts\\mPDBS\\summary_pdb_93\\b42c4924e3cd1aec.final.pdb
-4.080117e-15 -9.534854e-15 -1.843589e-15 -3.976126e-15 -7.384556e-15
-9.597687e-15
E:\\R-3.1.2\\scripts\\mPDBS\\summary_pdb_93\\b4dd239fcabff1df.final.pdb
-2.403735e-15 -3.235488e-15 4.054143e-16 -3.326907e-15 -6.151999e-15
-2.687002e-15
E:\\R-3.1.2\\scripts\\mPDBS\\summary_pdb_93\\b669942ef97459a2.final.pdb
-5.820979e-16 1.717192e-15 2.457835e-15 4.581402e-15 -6.264292e-15
1.850134e-15
E:\\R-3.1.2\\scripts\\mPDBS\\summary_pdb_93\\b99f4d9d87c1017b.final.pdb
-2.403735e-15 -3.235488e-15 4.054143e-16 -3.326907e-15 -6.151999e-15
-2.687002e-15
E:\\R-3.1.2\\scripts\\mPDBS\\summary_pdb_93\\b9d2cbf3975403e7.final.pdb
-2.403735e-15 -3.235488e-15 4.054143e-16 -3.326907e-15 -6.151999e-15
-2.687002e-15
E:\\R-3.1.2\\scripts\\mPDBS\\summary_pdb_93\\bc88c8a505f3a288.final.pdb
-1.712556e-15 -2.851274e-15 -1.681946e-15 -1.310038e-16 -6.444734e-15
-1.851326e-15
E:\\R-3.1.2\\scripts\\mPDBS\\summary_pdb_93\\bdac0e64f4ef427e.final.pdb
8.374767e-15 -5.713144e-15 -1.043956e-14 7.520348e-15 1.265709e-15
-1.756846e-16
E:\\R-3.1.2\\scripts\\mPDBS\\summary_pdb_93\\ca2a1f55fc9d0681.final.pdb
-5.820979e-16 1.717192e-15 2.457835e-15 4.581402e-15 -6.264292e-15
1.850134e-15
E:\\R-3.1.2\\scripts\\mPDBS\\summary_pdb_93\\ce34248de3e81a3c.final.pdb
-1.720827e-15 1.806456e-15 -4.659342e-15 -1.114959e-14 -5.254702e-15
-2.034471e-15
E:\\R-3.1.2\\scripts\\mPDBS\\summary_pdb_93\\d02e697772494a61.final.pdb
-6.512319e-15 1.956566e-15 -1.110919e-14 7.572686e-15 -1.036955e-14
-1.010941e-14
E:\\R-3.1.2\\scripts\\mPDBS\\summary_pdb_93\\d04221245d015047.final.pdb
4.535295e-16 -1.160402e-15 4.716350e-16 -5.393636e-16 -4.892019e-15
-1.466868e-15
E:\\R-3.1.2\\scripts\\mPDBS\\summary_pdb_93\\d678141e4018054a.final.pdb
-2.850160e-15 5.021156e-15 -2.151214e-15 -1.343900e-14 -9.737262e-15
-9.005188e-15
E:\\R-3.1.2\\scripts\\mPDBS\\summary_pdb_93\\d71b40af0f0efe39.final.pdb
-8.017215e-15 -1.852026e-15 4.832210e-15 -3.907988e-15 -3.737138e-15
-1.304926e-14
E:\\R-3.1.2\\scripts\\mPDBS\\summary_pdb_93\\dc0809eff10197e2.final.pdb

-2.092589e-15 -7.931061e-15 -2.143507e-15 1.047474e-14 -7.598917e-15
3.539288e-15
E:\\R-3.1.2\\scripts\\mPDBS\\summary_pdb_93\\e1163918d3707839.final.pdb
-7.423796e-15 -2.754254e-15 7.719942e-15 -8.489009e-15 3.930611e-15
-1.007127e-14
E:\\R-3.1.2\\scripts\\mPDBS\\summary_pdb_93\\e4b48b06824ef848.final.pdb
-4.595798e-15 -4.572269e-17 -2.920014e-15 -9.620180e-16 -4.949573e-15
-3.973437e-16
E:\\R-3.1.2\\scripts\\mPDBS\\summary_pdb_93\\e529e19ad281fea2.final.pdb
-4.388509e-15 -7.724801e-15 -4.860078e-15 -6.220849e-15 -7.604817e-15
-4.646147e-15
E:\\R-3.1.2\\scripts\\mPDBS\\summary_pdb_93\\e5602082718ec3a1.final.pdb
-5.439324e-15 -6.102074e-15 -2.136393e-16 -1.679216e-15 -5.950740e-15
-3.395670e-15
E:\\R-3.1.2\\scripts\\mPDBS\\summary_pdb_93\\e5d589d996d0a960.final.pdb
2.623913e-15 -5.420812e-15 -1.977396e-15 -3.240316e-15 -6.954428e-15
2.285853e-15
E:\\R-3.1.2\\scripts\\mPDBS\\summary_pdb_93\\e9df9e72307a2fbd.final.pdb
1.263417e-15 -3.500985e-15 -3.136308e-15 3.340337e-15 -2.784910e-15
1.655180e-15
E:\\R-3.1.2\\scripts\\mPDBS\\summary_pdb_93\\ea00f8694090cae7.final.pdb
-5.439324e-15 -6.102074e-15 -2.136393e-16 -1.679216e-15 -5.950740e-15
-3.395670e-15
E:\\R-3.1.2\\scripts\\mPDBS\\summary_pdb_93\\eba517be32db050e.final.pdb
-2.403735e-15 -3.235488e-15 4.054143e-16 -3.326907e-15 -6.151999e-15
-2.687002e-15
E:\\R-3.1.2\\scripts\\mPDBS\\summary_pdb_93\\ec04b9545899c29d.final.pdb
-2.148394e-15 -3.227372e-15 -1.773712e-15 -5.514566e-16 -6.372779e-15
-2.200352e-15
E:\\R-3.1.2\\scripts\\mPDBS\\summary_pdb_93\\ee6862cbcad52078.final.pdb
-1.800051e-15 -2.804843e-15 -1.461148e-15 -5.348335e-17 -6.556190e-15
-2.334853e-15
E:\\R-3.1.2\\scripts\\mPDBS\\summary_pdb_93\\ef6a42fc83b81e5d.final.pdb
-1.955820e-15 -3.114069e-15 -1.462087e-15 -7.406240e-16 -6.584549e-15
-2.098308e-15
E:\\R-3.1.2\\scripts\\mPDBS\\summary_pdb_93\\ef8e88d4ad93a0ad.final.pdb
4.535295e-16 -1.160402e-15 4.716350e-16 -5.393636e-16 -4.892019e-15
-1.466868e-15
E:\\R-3.1.2\\scripts\\mPDBS\\summary_pdb_93\\f14fcae8deec4fa3.final.pdb
-2.962556e-15 -4.738778e-15 -1.387289e-15 2.340861e-15 -6.513991e-15
-1.159799e-15
E:\\R-3.1.2\\scripts\\mPDBS\\summary_pdb_93\\f83584e22003fb92.final.pdb
-3.592617e-15 4.519647e-15 1.010074e-15 -8.515248e-15 -8.363772e-15
-3.048624e-15
E:\\R-3.1.2\\scripts\\mPDBS\\summary_pdb_93\\f8e2bf87e52caa29.final.pdb
-4.388509e-15 -7.724801e-15 -4.860078e-15 -6.220849e-15 -7.604817e-15
-4.646147e-15

[,391]   [,392]   [,393]   [,394]   [,395]   [,396]
E:\\R-3.1.2\\scripts\\40.1ns_data\\NAT0.pdb
-1.428484e-15 -3.832286e-15 1.433047e-15 3.665639e-15 -2.874962e-15
-4.619117e-15
E:\\R-3.1.2\\scripts\\40.1ns_data\\LJP6.pdb
-1.268876e-15 -4.471831e-15 2.040733e-15 2.897955e-15 -3.890779e-15

-5.082462e-15
E:\\R-3.1.2\\scripts\\40.1ns_data\\3WY9.pdb
-3.723011e-15 -4.443236e-15 1.544809e-15 2.496855e-15 -3.949910e-15
-4.126439e-15
E:\\R-3.1.2\\scripts\\40.1ns_data\\ZSP9.pdb
-1.898790e-15 -3.543229e-15 7.557361e-16 2.417883e-15 -4.238950e-15
-4.853838e-15
E:\\R-3.1.2\\scripts\\mPDBS\\summary_pdb_93\\013d855ad26c877c.final.pdb
-1.004812e-15 -3.419541e-15 -2.896498e-15 5.075082e-17 -7.077362e-15
5.638113e-15
E:\\R-3.1.2\\scripts\\mPDBS\\summary_pdb_93\\0a2a705342ca0d54.final.pdb
-1.341478e-15 -3.575904e-15 6.493495e-16 2.197272e-15 -4.710887e-15
-4.071619e-15
E:\\R-3.1.2\\scripts\\mPDBS\\summary_pdb_93\\0a59fceadb4194d9.final.pdb
-5.900370e-16 -4.376841e-15 4.591781e-16 3.239322e-15 -3.580103e-15
-4.335099e-15
E:\\R-3.1.2\\scripts\\mPDBS\\summary_pdb_93\\0dba59f3306825e8.final.pdb
1.009202e-15 -5.038038e-15 -2.370877e-15 1.584030e-15 -6.641079e-15
-1.179239e-14
E:\\R-3.1.2\\scripts\\mPDBS\\summary_pdb_93\\126f26a78b57339c.final.pdb
-2.484725e-16 -2.162084e-15 2.198710e-15 1.919048e-15 -3.085764e-15
-3.146960e-15
E:\\R-3.1.2\\scripts\\mPDBS\\summary_pdb_93\\12f06f3e342df706.final.pdb
-7.811624e-16 -3.804237e-15 7.989694e-16 2.214836e-15 -4.733005e-15
-4.491856e-15
E:\\R-3.1.2\\scripts\\mPDBS\\summary_pdb_93\\18618a2a251b867b.final.pdb
2.044645e-16 -8.055898e-16 -6.385670e-16 -4.182297e-15 -4.032156e-15
-3.945023e-15
E:\\R-3.1.2\\scripts\\mPDBS\\summary_pdb_93\\1f305f598641eeb2.final.pdb
-8.499183e-16 -4.053291e-15 5.579850e-16 2.453764e-15 -4.488247e-15
-4.111300e-15
E:\\R-3.1.2\\scripts\\mPDBS\\summary_pdb_93\\1f8a00b551be0a39.final.pdb
2.044645e-16 -8.055898e-16 -6.385670e-16 -4.182297e-15 -4.032156e-15
-3.945023e-15
E:\\R-3.1.2\\scripts\\mPDBS\\summary_pdb_93\\219f45328d47579a.final.pdb
-1.473150e-14 -9.571471e-15 -6.818363e-16 6.168619e-15 -7.806323e-15
8.267667e-15
E:\\R-3.1.2\\scripts\\mPDBS\\summary_pdb_93\\238dd3918e54336d.final.pdb
-9.776265e-15 -8.575089e-15 5.686550e-15 -3.779018e-16 -8.326740e-15
-1.511857e-14
E:\\R-3.1.2\\scripts\\mPDBS\\summary_pdb_93\\2b3027889e2fdc42.final.pdb
-2.210269e-15 -3.859243e-15 6.669112e-16 2.021438e-15 -4.940994e-15
-3.776084e-15
E:\\R-3.1.2\\scripts\\mPDBS\\summary_pdb_93\\2fcb3b46e33793b0.final.pdb
-1.373805e-15 -3.857267e-15 -1.376232e-15 1.053817e-16 -6.546496e-15
-1.380845e-15
E:\\R-3.1.2\\scripts\\mPDBS\\summary_pdb_93\\34eb328251842006.final.pdb
-6.189083e-15 -1.145693e-14 3.886029e-15 -4.715835e-15 -3.769534e-15
7.012861e-16
E:\\R-3.1.2\\scripts\\mPDBS\\summary_pdb_93\\354b32871797b7ab.final.pdb
-2.444403e-15 -2.878627e-15 -4.918684e-15 -1.792468e-15 -8.413471e-15
1.211831e-14
E:\\R-3.1.2\\scripts\\mPDBS\\summary_pdb_93\\397368adbc32d5de.final.pdb
-2.484725e-16 -2.162084e-15 2.198710e-15 1.919048e-15 -3.085764e-15

-3.146960e-15
E:\\R-3.1.2\\scripts\\mPDBS\\summary_pdb_93\\411ca13cd3df6edd.final.pdb
-3.713947e-15 -9.548577e-16 -2.363615e-15 -8.261190e-15 -6.702621e-15
5.697260e-15
E:\\R-3.1.2\\scripts\\mPDBS\\summary_pdb_93\\42357eee6642bf12.final.pdb
-6.067816e-15 -6.851870e-15 5.322954e-15 7.993161e-16 9.467226e-16
-3.777686e-15
E:\\R-3.1.2\\scripts\\mPDBS\\summary_pdb_93\\44342d1f75a7fcfc.final.pdb
1.028725e-14 3.125762e-15 -4.030341e-16 4.207578e-15 -2.445967e-15
7.025148e-15
E:\\R-3.1.2\\scripts\\mPDBS\\summary_pdb_93\\448ef21dffe8cdfb.final.pdb
-1.056437e-14 3.563764e-16 1.000355e-14 -4.721703e-15 4.687254e-16
5.100082e-15
E:\\R-3.1.2\\scripts\\mPDBS\\summary_pdb_93\\472a40b3e8f113c7.final.pdb
1.009202e-15 -5.038038e-15 -2.370877e-15 1.584030e-15 -6.641079e-15
-1.179239e-14
E:\\R-3.1.2\\scripts\\mPDBS\\summary_pdb_93\\48a26225951ea153.final.pdb
4.187391e-15 2.413517e-15 8.455877e-16 -2.812044e-15 -6.425917e-15
5.156874e-15
E:\\R-3.1.2\\scripts\\mPDBS\\summary_pdb_93\\4ab4a5c7c6682ce6.final.pdb
-5.591819e-17 -3.709457e-15 7.128794e-15 4.364574e-15 -5.581163e-15
-7.466422e-15
E:\\R-3.1.2\\scripts\\mPDBS\\summary_pdb_93\\4eaa45b62cb6a25f.final.pdb
3.007973e-16 -2.807286e-15 2.093286e-15 3.817519e-15 -4.240160e-15
-9.344988e-15
E:\\R-3.1.2\\scripts\\mPDBS\\summary_pdb_93\\58c371611d85ac96.final.pdb
-2.484725e-16 -2.162084e-15 2.198710e-15 1.919048e-15 -3.085764e-15
-3.146960e-15
E:\\R-3.1.2\\scripts\\mPDBS\\summary_pdb_93\\59a647f04822d30a.final.pdb
-6.948960e-15 -6.995482e-15 6.017611e-15 4.182208e-15 -3.408305e-15
-6.304468e-15
E:\\R-3.1.2\\scripts\\mPDBS\\summary_pdb_93\\59b091fe385e05fc.final.pdb
-5.900370e-16 -4.376841e-15 4.591781e-16 3.239322e-15 -3.580103e-15
-4.335099e-15
E:\\R-3.1.2\\scripts\\mPDBS\\summary_pdb_93\\5c35ac30f930d2db.final.pdb
3.007973e-16 -2.807286e-15 2.093286e-15 3.817519e-15 -4.240160e-15
-9.344988e-15
E:\\R-3.1.2\\scripts\\mPDBS\\summary_pdb_93\\5f60752431cf2dcb.final.pdb
1.380303e-15 -4.004597e-15 6.732591e-15 -1.023212e-16 -5.212222e-15
-8.957034e-15
E:\\R-3.1.2\\scripts\\mPDBS\\summary_pdb_93\\626456dd6ff2868d.final.pdb
1.009202e-15 -5.038038e-15 -2.370877e-15 1.584030e-15 -6.641079e-15
-1.179239e-14
E:\\R-3.1.2\\scripts\\mPDBS\\summary_pdb_93\\6583d53ff3c8f7ef.final.pdb
-6.948960e-15 -6.995482e-15 6.017611e-15 4.182208e-15 -3.408305e-15
-6.304468e-15
E:\\R-3.1.2\\scripts\\mPDBS\\summary_pdb_93\\69760178986bef01.final.pdb
-1.592022e-15 1.698571e-15 2.131320e-15 5.958656e-15 -9.998503e-15
-7.775167e-15
E:\\R-3.1.2\\scripts\\mPDBS\\summary_pdb_93\\6a9d87d47926b1c8.final.pdb
-2.866364e-16 -3.928032e-15 5.698048e-16 1.913844e-15 -5.045134e-15
-3.875544e-15
E:\\R-3.1.2\\scripts\\mPDBS\\summary_pdb_93\\6bb3547ea982ffc4.final.pdb
5.407751e-15 -1.629378e-15 4.177588e-16 -1.167451e-14 -1.404548e-14

4.275839e-15
E:\\R-3.1.2\\scripts\\mPDBS\\summary_pdb_93\\716d9624611d1c42.final.pdb
-8.858035e-15 -3.568965e-15 -4.912608e-15 -5.407725e-16 -6.385329e-15
1.911442e-15
E:\\R-3.1.2\\scripts\\mPDBS\\summary_pdb_93\\75f27e70ad0f1be4.final.pdb
-6.948960e-15 -6.995482e-15 6.017611e-15 4.182208e-15 -3.408305e-15
-6.304468e-15
E:\\R-3.1.2\\scripts\\mPDBS\\summary_pdb_93\\769f910fffb22872.final.pdb
-9.118301e-16 -4.406008e-15 6.045792e-16 2.057829e-15 -4.723444e-15
-3.802775e-15
E:\\R-3.1.2\\scripts\\mPDBS\\summary_pdb_93\\791f8c80dd5c3135.final.pdb
-5.900370e-16 -4.376841e-15 4.591781e-16 3.239322e-15 -3.580103e-15
-4.335099e-15
E:\\R-3.1.2\\scripts\\mPDBS\\summary_pdb_93\\7fad98e66a3f6099.final.pdb
-4.126217e-16 -1.962022e-15 1.691773e-15 5.413312e-15 -3.328624e-15
7.104103e-16
E:\\R-3.1.2\\scripts\\mPDBS\\summary_pdb_93\\804b89c94a633196.final.pdb
-1.373805e-15 -3.857267e-15 -1.376232e-15 1.053817e-16 -6.546496e-15
-1.380845e-15
E:\\R-3.1.2\\scripts\\mPDBS\\summary_pdb_93\\813cc65a2298feba.final.pdb
-1.373805e-15 -3.857267e-15 -1.376232e-15 1.053817e-16 -6.546496e-15
-1.380845e-15
E:\\R-3.1.2\\scripts\\mPDBS\\summary_pdb_93\\83c0eefcfe12d3f9.final.pdb
-3.713947e-15 -9.548577e-16 -2.363615e-15 -8.261190e-15 -6.702621e-15
5.697260e-15
E:\\R-3.1.2\\scripts\\mPDBS\\summary_pdb_93\\86d50469ef569760.final.pdb
1.009202e-15 -5.038038e-15 -2.370877e-15 1.584030e-15 -6.641079e-15
-1.179239e-14
E:\\R-3.1.2\\scripts\\mPDBS\\summary_pdb_93\\89d0666e1402203d.final.pdb
-6.948960e-15 -6.995482e-15 6.017611e-15 4.182208e-15 -3.408305e-15
-6.304468e-15
E:\\R-3.1.2\\scripts\\mPDBS\\summary_pdb_93\\8b34852716dadc62.final.pdb
-5.307292e-16 -3.338585e-15 3.735998e-15 5.451366e-15 -1.823712e-15
-7.618911e-15
E:\\R-3.1.2\\scripts\\mPDBS\\summary_pdb_93\\8b6ef9ac3fe74395.final.pdb
1.133757e-15 -6.219645e-15 4.923029e-15 1.348624e-16 -2.791189e-15
-7.352354e-15
E:\\R-3.1.2\\scripts\\mPDBS\\summary_pdb_93\\98bbe0d5986c138f.final.pdb
-5.900370e-16 -4.376841e-15 4.591781e-16 3.239322e-15 -3.580103e-15
-4.335099e-15
E:\\R-3.1.2\\scripts\\mPDBS\\summary_pdb_93\\9c22f4909d67e2fa.final.pdb
-1.222018e-16 -6.391698e-15 -1.102362e-15 1.236435e-14 -3.502057e-15
-1.117336e-14
E:\\R-3.1.2\\scripts\\mPDBS\\summary_pdb_93\\9dda8ed0d81fb3ef.final.pdb
6.614842e-15 1.108966e-14 -7.667189e-15 2.498875e-15 -2.932124e-15
-4.403644e-15
E:\\R-3.1.2\\scripts\\mPDBS\\summary_pdb_93\\9f752b3616e6033b.final.pdb
-5.739433e-16 -4.340015e-15 -4.159590e-17 3.691563e-15 -3.525434e-15
-4.240224e-15
E:\\R-3.1.2\\scripts\\mPDBS\\summary_pdb_93\\a02c70083816d6ba.final.pdb
3.007973e-16 -2.807286e-15 2.093286e-15 3.817519e-15 -4.240160e-15
-9.344988e-15
E:\\R-3.1.2\\scripts\\mPDBS\\summary_pdb_93\\a0c997d891e4e082.final.pdb
-5.172435e-16 -1.512129e-15 2.462269e-15 5.160288e-15 -7.406717e-15

-2.241115e-15
E:\\R-3.1.2\\scripts\\mPDBS\\summary_pdb_93\\ab8039a2d823c616.final.pdb
-2.174772e-15 -3.677281e-15 3.847206e-16 2.627158e-15 -4.632894e-15
-4.911288e-15
E:\\R-3.1.2\\scripts\\mPDBS\\summary_pdb_93\\acf4d74b9c71d4dc.final.pdb
-1.373805e-15 -3.857267e-15 -1.376232e-15 1.053817e-16 -6.546496e-15
-1.380845e-15
E:\\R-3.1.2\\scripts\\mPDBS\\summary_pdb_93\\ad8a823e4b4c668e.final.pdb
-2.308490e-15 -3.727770e-15 9.379336e-16 2.249264e-15 -4.318670e-15
-4.106602e-15
E:\\R-3.1.2\\scripts\\mPDBS\\summary_pdb_93\\b16ababd03b7b857.final.pdb
-1.222018e-16 -6.391698e-15 -1.102362e-15 1.236435e-14 -3.502057e-15
-1.117336e-14
E:\\R-3.1.2\\scripts\\mPDBS\\summary_pdb_93\\b226923e697d3c63.final.pdb
-4.136106e-15 -2.552856e-15 3.749243e-15 -1.263415e-15 -1.226457e-15
-6.273243e-15
E:\\R-3.1.2\\scripts\\mPDBS\\summary_pdb_93\\b28e48f6fa27a165.final.pdb
-5.900370e-16 -4.376841e-15 4.591781e-16 3.239322e-15 -3.580103e-15
-4.335099e-15
E:\\R-3.1.2\\scripts\\mPDBS\\summary_pdb_93\\b42c4924e3cd1aec.final.pdb
-7.338391e-17 -1.171111e-14 1.095550e-15 4.307867e-15 -7.795855e-15
-3.346330e-15
E:\\R-3.1.2\\scripts\\mPDBS\\summary_pdb_93\\b4dd239fcabff1df.final.pdb
-5.900370e-16 -4.376841e-15 4.591781e-16 3.239322e-15 -3.580103e-15
-4.335099e-15
E:\\R-3.1.2\\scripts\\mPDBS\\summary_pdb_93\\b669942ef97459a2.final.pdb
-5.307292e-16 -3.338585e-15 3.735998e-15 5.451366e-15 -1.823712e-15
-7.618911e-15
E:\\R-3.1.2\\scripts\\mPDBS\\summary_pdb_93\\b99f4d9d87c1017b.final.pdb
-5.900370e-16 -4.376841e-15 4.591781e-16 3.239322e-15 -3.580103e-15
-4.335099e-15
E:\\R-3.1.2\\scripts\\mPDBS\\summary_pdb_93\\b9d2cbf3975403e7.final.pdb
-5.900370e-16 -4.376841e-15 4.591781e-16 3.239322e-15 -3.580103e-15
-4.335099e-15
E:\\R-3.1.2\\scripts\\mPDBS\\summary_pdb_93\\bc88c8a505f3a288.final.pdb
-1.130403e-15 -4.024692e-15 6.341683e-16 2.492523e-15 -4.526395e-15
-4.043665e-15
E:\\R-3.1.2\\scripts\\mPDBS\\summary_pdb_93\\bdac0e64f4ef427e.final.pdb
5.104520e-15 -1.059298e-14 6.778161e-15 2.276976e-14 -3.925648e-16
-2.458928e-14
E:\\R-3.1.2\\scripts\\mPDBS\\summary_pdb_93\\ca2a1f55fc9d0681.final.pdb
-5.307292e-16 -3.338585e-15 3.735998e-15 5.451366e-15 -1.823712e-15
-7.618911e-15
E:\\R-3.1.2\\scripts\\mPDBS\\summary_pdb_93\\ce34248de3e81a3c.final.pdb
-1.466852e-15 -5.799518e-15 5.148563e-15 5.443904e-15 -1.271762e-15
-5.532583e-15
E:\\R-3.1.2\\scripts\\mPDBS\\summary_pdb_93\\d02e697772494a61.final.pdb
-1.290571e-14 -1.157790e-14 9.441359e-15 1.494541e-16 -2.616031e-15
-5.456164e-15
E:\\R-3.1.2\\scripts\\mPDBS\\summary_pdb_93\\d04221245d015047.final.pdb
-9.040868e-16 -4.402179e-15 2.487617e-16 4.121188e-15 -6.244452e-15
-3.363481e-15
E:\\R-3.1.2\\scripts\\mPDBS\\summary_pdb_93\\d678141e4018054a.final.pdb
-1.186716e-15 -6.409564e-15 -4.166690e-15 -1.384873e-14 -8.052404e-15

4.194242e-15
E:\\R-3.1.2\\scripts\\mPDBS\\summary_pdb_93\\d71b40af0f0efe39.final.pdb
-6.629704e-15 -1.831720e-15 -1.125056e-14 3.495558e-15 1.884272e-16
-3.722546e-17
E:\\R-3.1.2\\scripts\\mPDBS\\summary_pdb_93\\dc0809eff10197e2.final.pdb
-3.758296e-15 -5.342942e-15 5.987288e-16 1.528403e-14 2.164492e-16
-1.735285e-14
E:\\R-3.1.2\\scripts\\mPDBS\\summary_pdb_93\\e1163918d3707839.final.pdb
6.478667e-15 -1.018629e-14 -4.376635e-15 -5.211103e-15 -5.829112e-15
-9.447353e-15
E:\\R-3.1.2\\scripts\\mPDBS\\summary_pdb_93\\e4b48b06824ef848.final.pdb
2.044645e-16 -8.055898e-16 -6.385670e-16 -4.182297e-15 -4.032156e-15
-3.945023e-15
E:\\R-3.1.2\\scripts\\mPDBS\\summary_pdb_93\\e529e19ad281fea2.final.pdb
-1.004812e-15 -3.419541e-15 -2.896498e-15 5.075082e-17 -7.077362e-15
5.638113e-15
E:\\R-3.1.2\\scripts\\mPDBS\\summary_pdb_93\\e5602082718ec3a1.final.pdb
-1.373805e-15 -3.857267e-15 -1.376232e-15 1.053817e-16 -6.546496e-15
-1.380845e-15
E:\\R-3.1.2\\scripts\\mPDBS\\summary_pdb_93\\e5d589d996d0a960.final.pdb
5.230897e-15 6.834479e-16 -8.909004e-16 7.430652e-15 -8.415921e-15
-2.373938e-15
E:\\R-3.1.2\\scripts\\mPDBS\\summary_pdb_93\\e9df9e72307a2fbd.final.pdb
3.007973e-16 -2.807286e-15 2.093286e-15 3.817519e-15 -4.240160e-15
-9.344988e-15
E:\\R-3.1.2\\scripts\\mPDBS\\summary_pdb_93\\ea00f8694090cae7.final.pdb
-1.373805e-15 -3.857267e-15 -1.376232e-15 1.053817e-16 -6.546496e-15
-1.380845e-15
E:\\R-3.1.2\\scripts\\mPDBS\\summary_pdb_93\\eba517be32db050e.final.pdb
-5.900370e-16 -4.376841e-15 4.591781e-16 3.239322e-15 -3.580103e-15
-4.335099e-15
E:\\R-3.1.2\\scripts\\mPDBS\\summary_pdb_93\\ec04b9545899c29d.final.pdb
-1.175801e-15 -3.855633e-15 1.369420e-15 2.019658e-15 -4.822539e-15
-4.064072e-15
E:\\R-3.1.2\\scripts\\mPDBS\\summary_pdb_93\\ee6862cbcad52078.final.pdb
-1.618728e-15 -3.873771e-15 7.987502e-16 2.225593e-15 -4.895457e-15
-3.677639e-15
E:\\R-3.1.2\\scripts\\mPDBS\\summary_pdb_93\\ef6a42fc83b81e5d.final.pdb
-1.327296e-15 -3.838537e-15 8.459227e-16 2.142938e-15 -4.444587e-15
-4.092908e-15
E:\\R-3.1.2\\scripts\\mPDBS\\summary_pdb_93\\ef8e88d4ad93a0ad.final.pdb
-9.040868e-16 -4.402179e-15 2.487617e-16 4.121188e-15 -6.244452e-15
-3.363481e-15
E:\\R-3.1.2\\scripts\\mPDBS\\summary_pdb_93\\f14fcae8deec4fa3.final.pdb
-5.739433e-16 -4.340015e-15 -4.159590e-17 3.691563e-15 -3.525434e-15
-4.240224e-15
E:\\R-3.1.2\\scripts\\mPDBS\\summary_pdb_93\\f83584e22003fb92.final.pdb
4.187391e-15 2.413517e-15 8.455877e-16 -2.812044e-15 -6.425917e-15
5.156874e-15
E:\\R-3.1.2\\scripts\\mPDBS\\summary_pdb_93\\f8e2bf87e52caa29.final.pdb
-1.004812e-15 -3.419541e-15 -2.896498e-15 5.075082e-17 -7.077362e-15
5.638113e-15

[,397] [,398] [,399] [,400] [,401] [,402]

E:\\R-3.1.2\\scripts\\40.1ns_data\\NAT0.pdb
4.408834e-15 5.435902e-15 4.520983e-16 -1.745167e-15 -2.413804e-16
3.266649e-15
E:\\R-3.1.2\\scripts\\40.1ns_data\\LJP6.pdb
3.507346e-15 6.241411e-15 -2.153966e-15 -1.105425e-15 3.973839e-16
3.570710e-15
E:\\R-3.1.2\\scripts\\40.1ns_data\\3WY9.pdb
2.413479e-15 5.008036e-15 -1.859027e-15 -6.544472e-16 -4.026726e-16
2.866285e-15
E:\\R-3.1.2\\scripts\\40.1ns_data\\ZSP9.pdb
3.567915e-15 5.696612e-15 -1.933332e-15 -2.174113e-15 -2.465619e-16
2.667703e-15
E:\\R-3.1.2\\scripts\\mPDBS\\summary_pdb_93\\013d855ad26c877c.final.pdb
2.077878e-15 6.071029e-15 3.606101e-15 1.176715e-15 -2.656580e-15
2.316098e-15
E:\\R-3.1.2\\scripts\\mPDBS\\summary_pdb_93\\0a2a705342ca0d54.final.pdb
2.467272e-15 5.419113e-15 -1.370795e-15 -5.348466e-16 3.369941e-17
4.018319e-15
E:\\R-3.1.2\\scripts\\mPDBS\\summary_pdb_93\\0a59fceadb4194d9.final.pdb
3.321663e-17 6.396621e-15 -2.655675e-15 4.574960e-16 -2.802873e-16
3.779362e-15
E:\\R-3.1.2\\scripts\\mPDBS\\summary_pdb_93\\0dba59f3306825e8.final.pdb
-9.891129e-16 9.257716e-15 -3.535755e-15 -7.922508e-17 -1.406712e-15
4.881754e-15
E:\\R-3.1.2\\scripts\\mPDBS\\summary_pdb_93\\126f26a78b57339c.final.pdb
6.162325e-15 6.872688e-15 3.188033e-15 -4.094731e-16 5.922061e-15
3.330043e-15
E:\\R-3.1.2\\scripts\\mPDBS\\summary_pdb_93\\12f06f3e342df706.final.pdb
2.827112e-15 5.546832e-15 -1.123597e-15 -9.997525e-16 1.418377e-17
3.680048e-15
E:\\R-3.1.2\\scripts\\mPDBS\\summary_pdb_93\\18618a2a251b867b.final.pdb
3.379291e-15 7.438908e-15 2.045520e-15 -5.407875e-15 7.893076e-15
2.502549e-15
E:\\R-3.1.2\\scripts\\mPDBS\\summary_pdb_93\\1f305f598641eeb2.final.pdb
2.964297e-15 5.956502e-15 -7.802713e-16 -8.058879e-16 7.455170e-17
3.674803e-15
E:\\R-3.1.2\\scripts\\mPDBS\\summary_pdb_93\\1f8a00b551be0a39.final.pdb
3.379291e-15 7.438908e-15 2.045520e-15 -5.407875e-15 7.893076e-15
2.502549e-15
E:\\R-3.1.2\\scripts\\mPDBS\\summary_pdb_93\\219f45328d47579a.final.pdb
1.335242e-14 -7.058328e-15 2.433137e-15 -3.626043e-15 -7.032156e-15
6.389687e-15
E:\\R-3.1.2\\scripts\\mPDBS\\summary_pdb_93\\238dd3918e54336d.final.pdb
1.014884e-14 9.638168e-15 2.676865e-15 4.298391e-15 -2.528272e-15
9.931559e-15
E:\\R-3.1.2\\scripts\\mPDBS\\summary_pdb_93\\2b3027889e2fdc42.final.pdb
2.426302e-15 5.832836e-15 -1.030290e-16 -7.539915e-16 1.769208e-16
3.965411e-15
E:\\R-3.1.2\\scripts\\mPDBS\\summary_pdb_93\\2fcb3b46e33793b0.final.pdb
-1.304498e-16 6.386713e-15 -4.183799e-15 -3.028069e-15 -3.794749e-15
2.913259e-15
E:\\R-3.1.2\\scripts\\mPDBS\\summary_pdb_93\\34eb328251842006.final.pdb
5.609435e-15 -7.746269e-16 -1.327974e-14 -1.577365e-15 -5.079525e-15
9.401481e-15

E:\\R-3.1.2\\scripts\\mPDBS\\summary_pdb_93\\354b32871797b7ab.final.pdb
-3.090682e-15 -4.781027e-15 -1.725122e-14 -9.745609e-15 -6.068898e-15
7.719740e-15
E:\\R-3.1.2\\scripts\\mPDBS\\summary_pdb_93\\397368adbc32d5de.final.pdb
6.162325e-15 6.872688e-15 3.188033e-15 -4.094731e-16 5.922061e-15
3.330043e-15
E:\\R-3.1.2\\scripts\\mPDBS\\summary_pdb_93\\411ca13cd3df6edd.final.pdb
2.498483e-15 5.727302e-15 -4.999119e-15 -9.199564e-15 -7.741618e-16
2.423200e-15
E:\\R-3.1.2\\scripts\\mPDBS\\summary_pdb_93\\42357eee6642bf12.final.pdb
3.867673e-15 2.497903e-15 -8.483336e-15 -5.065995e-15 2.751286e-15
7.701092e-15
E:\\R-3.1.2\\scripts\\mPDBS\\summary_pdb_93\\44342d1f75a7fcfc.final.pdb
-6.973710e-19 1.417199e-14 -1.622009e-15 -4.580297e-15 3.891870e-15
-1.972129e-15
E:\\R-3.1.2\\scripts\\mPDBS\\summary_pdb_93\\448ef21dffe8cdfb.final.pdb
1.174894e-14 -6.717388e-15 9.376535e-15 -2.264790e-15 6.695991e-15
9.444493e-15
E:\\R-3.1.2\\scripts\\mPDBS\\summary_pdb_93\\472a40b3e8f113c7.final.pdb
-9.891129e-16 9.257716e-15 -3.535755e-15 -7.922508e-17 -1.406712e-15
4.881754e-15
E:\\R-3.1.2\\scripts\\mPDBS\\summary_pdb_93\\48a26225951ea153.final.pdb
2.901616e-15 1.112548e-14 -1.915381e-15 -4.506198e-15 3.335419e-15
5.935424e-16
E:\\R-3.1.2\\scripts\\mPDBS\\summary_pdb_93\\4ab4a5c7c6682ce6.final.pdb
-1.491275e-15 3.474365e-15 -6.040728e-16 -1.740006e-15 -6.939573e-16
3.059940e-17
E:\\R-3.1.2\\scripts\\mPDBS\\summary_pdb_93\\4eaa45b62cb6a25f.final.pdb
3.606638e-15 1.103640e-14 5.852257e-15 2.935870e-15 2.633776e-15
-2.682277e-16
E:\\R-3.1.2\\scripts\\mPDBS\\summary_pdb_93\\58c371611d85ac96.final.pdb
6.162325e-15 6.872688e-15 3.188033e-15 -4.094731e-16 5.922061e-15
3.330043e-15
E:\\R-3.1.2\\scripts\\mPDBS\\summary_pdb_93\\59a647f04822d30a.final.pdb
4.214681e-15 5.643221e-15 6.105802e-16 1.272226e-15 5.472148e-16
3.928099e-15
E:\\R-3.1.2\\scripts\\mPDBS\\summary_pdb_93\\59b091fe385e05fc.final.pdb
3.321663e-17 6.396621e-15 -2.655675e-15 4.574960e-16 -2.802873e-16
3.779362e-15
E:\\R-3.1.2\\scripts\\mPDBS\\summary_pdb_93\\5c35ac30f930d2db.final.pdb
3.606638e-15 1.103640e-14 5.852257e-15 2.935870e-15 2.633776e-15
-2.682277e-16
E:\\R-3.1.2\\scripts\\mPDBS\\summary_pdb_93\\5f60752431cf2dcb.final.pdb
1.845407e-15 1.355236e-14 5.865604e-15 7.758649e-15 8.782669e-15
9.283429e-16
E:\\R-3.1.2\\scripts\\mPDBS\\summary_pdb_93\\626456dd6ff2868d.final.pdb
-9.891129e-16 9.257716e-15 -3.535755e-15 -7.922508e-17 -1.406712e-15
4.881754e-15
E:\\R-3.1.2\\scripts\\mPDBS\\summary_pdb_93\\6583d53ff3c8f7ef.final.pdb
4.214681e-15 5.643221e-15 6.105802e-16 1.272226e-15 5.472148e-16
3.928099e-15
E:\\R-3.1.2\\scripts\\mPDBS\\summary_pdb_93\\69760178986bef01.final.pdb
3.402528e-15 1.083239e-14 1.211362e-14 1.061039e-15 -9.769635e-16
2.420396e-15

E:\\R-3.1.2\\scripts\\mPDBS\\summary_pdb_93\\6a9d87d47926b1c8.final.pdb
2.366750e-15 6.147574e-15 -5.190713e-16 -5.933537e-16 -5.254332e-17
3.578109e-15
E:\\R-3.1.2\\scripts\\mPDBS\\summary_pdb_93\\6bb3547ea982ffc4.final.pdb
1.879344e-15 3.892186e-15 -5.428591e-16 -5.842428e-15 -6.396132e-16
-2.470826e-16
E:\\R-3.1.2\\scripts\\mPDBS\\summary_pdb_93\\716d9624611d1c42.final.pdb
3.863724e-15 -8.471423e-16 -4.233956e-15 -8.888841e-15 -8.859686e-15
3.825331e-15
E:\\R-3.1.2\\scripts\\mPDBS\\summary_pdb_93\\75f27e70ad0f1be4.final.pdb
4.214681e-15 5.643221e-15 6.105802e-16 1.272226e-15 5.472148e-16
3.928099e-15
E:\\R-3.1.2\\scripts\\mPDBS\\summary_pdb_93\\769f910fffb22872.final.pdb
2.775153e-15 5.701366e-15 -2.889840e-16 -1.160404e-15 1.566425e-16
4.099362e-15
E:\\R-3.1.2\\scripts\\mPDBS\\summary_pdb_93\\791f8c80dd5c3135.final.pdb
3.321663e-17 6.396621e-15 -2.655675e-15 4.574960e-16 -2.802873e-16
3.779362e-15
E:\\R-3.1.2\\scripts\\mPDBS\\summary_pdb_93\\7fad98e66a3f6099.final.pdb
-4.395850e-16 2.381256e-16 -1.164504e-14 -2.842992e-15 -5.865427e-15
1.025976e-14
E:\\R-3.1.2\\scripts\\mPDBS\\summary_pdb_93\\804b89c94a633196.final.pdb
-1.304498e-16 6.386713e-15 -4.183799e-15 -3.028069e-15 -3.794749e-15
2.913259e-15
E:\\R-3.1.2\\scripts\\mPDBS\\summary_pdb_93\\813cc65a2298feba.final.pdb
-1.304498e-16 6.386713e-15 -4.183799e-15 -3.028069e-15 -3.794749e-15
2.913259e-15
E:\\R-3.1.2\\scripts\\mPDBS\\summary_pdb_93\\83c0eefcfe12d3f9.final.pdb
2.498483e-15 5.727302e-15 -4.999119e-15 -9.199564e-15 -7.741618e-16
2.423200e-15
E:\\R-3.1.2\\scripts\\mPDBS\\summary_pdb_93\\86d50469ef569760.final.pdb
-9.891129e-16 9.257716e-15 -3.535755e-15 -7.922508e-17 -1.406712e-15
4.881754e-15
E:\\R-3.1.2\\scripts\\mPDBS\\summary_pdb_93\\89d0666e1402203d.final.pdb
4.214681e-15 5.643221e-15 6.105802e-16 1.272226e-15 5.472148e-16
3.928099e-15
E:\\R-3.1.2\\scripts\\mPDBS\\summary_pdb_93\\8b34852716dadc62.final.pdb
4.359015e-15 5.942624e-15 -2.570506e-15 -3.359401e-15 3.488487e-15
2.422332e-15
E:\\R-3.1.2\\scripts\\mPDBS\\summary_pdb_93\\8b6ef9ac3fe74395.final.pdb
-4.612686e-15 2.937656e-15 -3.354841e-15 -1.239135e-15 8.040273e-15
1.219856e-15
E:\\R-3.1.2\\scripts\\mPDBS\\summary_pdb_93\\98bbe0d5986c138f.final.pdb
3.321663e-17 6.396621e-15 -2.655675e-15 4.574960e-16 -2.802873e-16
3.779362e-15
E:\\R-3.1.2\\scripts\\mPDBS\\summary_pdb_93\\9c22f4909d67e2fa.final.pdb
7.845361e-15 9.004411e-15 5.130799e-15 8.499602e-15 -3.386438e-15
7.682447e-15
E:\\R-3.1.2\\scripts\\mPDBS\\summary_pdb_93\\9dda8ed0d81fb3ef.final.pdb
-4.109356e-15 2.820525e-14 -3.385355e-15 5.992626e-15 9.792694e-16
-1.123859e-14
E:\\R-3.1.2\\scripts\\mPDBS\\summary_pdb_93\\9f752b3616e6033b.final.pdb
2.724322e-15 4.223442e-15 -1.818242e-15 -2.426358e-15 1.318988e-16
5.371290e-15

E:\\R-3.1.2\\scripts\\mPDBS\\summary_pdb_93\\a02c70083816d6ba.final.pdb
3.606638e-15 1.103640e-14 5.852257e-15 2.935870e-15 2.633776e-15
-2.682277e-16
E:\\R-3.1.2\\scripts\\mPDBS\\summary_pdb_93\\a0c997d891e4e082.final.pdb
-1.147640e-15 8.579920e-15 -2.591613e-15 -1.334288e-15 -8.645614e-15
-4.798198e-17
E:\\R-3.1.2\\scripts\\mPDBS\\summary_pdb_93\\ab8039a2d823c616.final.pdb
2.691750e-15 6.107289e-15 -1.120134e-15 -1.782931e-15 -1.141354e-16
4.083262e-15
E:\\R-3.1.2\\scripts\\mPDBS\\summary_pdb_93\\acf4d74b9c71d4dc.final.pdb
-1.304498e-16 6.386713e-15 -4.183799e-15 -3.028069e-15 -3.794749e-15
2.913259e-15
E:\\R-3.1.2\\scripts\\mPDBS\\summary_pdb_93\\ad8a823e4b4c668e.final.pdb
3.120872e-15 5.206546e-15 -6.218148e-16 -9.602952e-16 7.344653e-17
4.018469e-15
E:\\R-3.1.2\\scripts\\mPDBS\\summary_pdb_93\\b16ababd03b7b857.final.pdb
7.845361e-15 9.004411e-15 5.130799e-15 8.499602e-15 -3.386438e-15
7.682447e-15
E:\\R-3.1.2\\scripts\\mPDBS\\summary_pdb_93\\b226923e697d3c63.final.pdb
-3.511324e-15 4.764801e-15 -3.062697e-15 -5.623733e-15 5.791609e-15
1.639565e-15
E:\\R-3.1.2\\scripts\\mPDBS\\summary_pdb_93\\b28e48f6fa27a165.final.pdb
3.321663e-17 6.396621e-15 -2.655675e-15 4.574960e-16 -2.802873e-16
3.779362e-15
E:\\R-3.1.2\\scripts\\mPDBS\\summary_pdb_93\\b42c4924e3cd1aec.final.pdb
7.727048e-15 -3.562306e-15 -4.184195e-15 4.519189e-16 -1.833693e-15
1.002652e-14
E:\\R-3.1.2\\scripts\\mPDBS\\summary_pdb_93\\b4dd239fcabff1df.final.pdb
3.321663e-17 6.396621e-15 -2.655675e-15 4.574960e-16 -2.802873e-16
3.779362e-15
E:\\R-3.1.2\\scripts\\mPDBS\\summary_pdb_93\\b669942ef97459a2.final.pdb
4.359015e-15 5.942624e-15 -2.570506e-15 -3.359401e-15 3.488487e-15
2.422332e-15
E:\\R-3.1.2\\scripts\\mPDBS\\summary_pdb_93\\b99f4d9d87c1017b.final.pdb
3.321663e-17 6.396621e-15 -2.655675e-15 4.574960e-16 -2.802873e-16
3.779362e-15
E:\\R-3.1.2\\scripts\\mPDBS\\summary_pdb_93\\b9d2cbf3975403e7.final.pdb
3.321663e-17 6.396621e-15 -2.655675e-15 4.574960e-16 -2.802873e-16
3.779362e-15
E:\\R-3.1.2\\scripts\\mPDBS\\summary_pdb_93\\bc88c8a505f3a288.final.pdb
2.348618e-15 6.447361e-15 -1.163812e-15 -6.045884e-16 6.199535e-17
4.115031e-15
E:\\R-3.1.2\\scripts\\mPDBS\\summary_pdb_93\\bdac0e64f4ef427e.final.pdb
1.842310e-15 6.361994e-15 9.978917e-15 1.527261e-14 5.364218e-16
5.768614e-15
E:\\R-3.1.2\\scripts\\mPDBS\\summary_pdb_93\\ca2a1f55fc9d0681.final.pdb
4.359015e-15 5.942624e-15 -2.570506e-15 -3.359401e-15 3.488487e-15
2.422332e-15
E:\\R-3.1.2\\scripts\\mPDBS\\summary_pdb_93\\ce34248de3e81a3c.final.pdb
-4.024878e-16 1.116809e-14 -6.077648e-16 5.809910e-15 2.872159e-15
-2.428106e-15
E:\\R-3.1.2\\scripts\\mPDBS\\summary_pdb_93\\d02e697772494a61.final.pdb
4.408636e-15 -6.298736e-15 5.213031e-15 -9.555545e-15 5.434895e-16
1.201756e-14

E:\\R-3.1.2\\scripts\\mPDBS\\summary_pdb_93\\d04221245d015047.final.pdb
4.455517e-15 6.375087e-15 5.586449e-16 1.000650e-15 -2.279179e-15
4.661792e-15
E:\\R-3.1.2\\scripts\\mPDBS\\summary_pdb_93\\d678141e4018054a.final.pdb
3.718864e-15 1.957889e-15 -6.656310e-16 -4.405046e-15 -8.033478e-16
8.910354e-15
E:\\R-3.1.2\\scripts\\mPDBS\\summary_pdb_93\\d71b40af0f0efe39.final.pdb
5.730581e-15 -1.235204e-15 -2.231754e-15 -7.081718e-15 1.254852e-15
1.319521e-14
E:\\R-3.1.2\\scripts\\mPDBS\\summary_pdb_93\\dc0809eff10197e2.final.pdb
2.381082e-15 5.999806e-15 -6.691456e-15 3.723975e-15 4.386761e-16
6.820859e-15
E:\\R-3.1.2\\scripts\\mPDBS\\summary_pdb_93\\e1163918d3707839.final.pdb
3.195951e-15 -5.717699e-15 1.916445e-16 -6.352100e-15 -5.014200e-15
8.940149e-15
E:\\R-3.1.2\\scripts\\mPDBS\\summary_pdb_93\\e4b48b06824ef848.final.pdb
3.379291e-15 7.438908e-15 2.045520e-15 -5.407875e-15 7.893076e-15
2.502549e-15
E:\\R-3.1.2\\scripts\\mPDBS\\summary_pdb_93\\e529e19ad281fea2.final.pdb
2.077878e-15 6.071029e-15 3.606101e-15 1.176715e-15 -2.656580e-15
2.316098e-15
E:\\R-3.1.2\\scripts\\mPDBS\\summary_pdb_93\\e5602082718ec3a1.final.pdb
-1.304498e-16 6.386713e-15 -4.183799e-15 -3.028069e-15 -3.794749e-15
2.913259e-15
E:\\R-3.1.2\\scripts\\mPDBS\\summary_pdb_93\\e5d589d996d0a960.final.pdb
-7.549016e-16 1.049207e-14 5.133702e-15 8.439348e-16 1.455942e-15
-3.332753e-15
E:\\R-3.1.2\\scripts\\mPDBS\\summary_pdb_93\\e9df9e72307a2fbd.final.pdb
3.606638e-15 1.103640e-14 5.852257e-15 2.935870e-15 2.633776e-15
-2.682277e-16
E:\\R-3.1.2\\scripts\\mPDBS\\summary_pdb_93\\ea00f8694090cae7.final.pdb
-1.304498e-16 6.386713e-15 -4.183799e-15 -3.028069e-15 -3.794749e-15
2.913259e-15
E:\\R-3.1.2\\scripts\\mPDBS\\summary_pdb_93\\eba517be32db050e.final.pdb
3.321663e-17 6.396621e-15 -2.655675e-15 4.574960e-16 -2.802873e-16
3.779362e-15
E:\\R-3.1.2\\scripts\\mPDBS\\summary_pdb_93\\ec04b9545899c29d.final.pdb
3.192929e-15 5.781566e-15 -9.298542e-16 -9.824806e-16 1.693154e-17
3.768939e-15
E:\\R-3.1.2\\scripts\\mPDBS\\summary_pdb_93\\ee6862cbcad52078.final.pdb
2.092333e-15 6.172841e-15 -6.494669e-16 -7.884691e-16 2.599984e-17
4.092913e-15
E:\\R-3.1.2\\scripts\\mPDBS\\summary_pdb_93\\ef6a42fc83b81e5d.final.pdb
2.778175e-15 5.490380e-15 -1.190606e-15 -9.264333e-16 1.791939e-16
3.697774e-15
E:\\R-3.1.2\\scripts\\mPDBS\\summary_pdb_93\\ef8e88d4ad93a0ad.final.pdb
4.455517e-15 6.375087e-15 5.586449e-16 1.000650e-15 -2.279179e-15
4.661792e-15
E:\\R-3.1.2\\scripts\\mPDBS\\summary_pdb_93\\f14fcae8deec4fa3.final.pdb
2.724322e-15 4.223442e-15 -1.818242e-15 -2.426358e-15 1.318988e-16
5.371290e-15
E:\\R-3.1.2\\scripts\\mPDBS\\summary_pdb_93\\f83584e22003fb92.final.pdb
2.901616e-15 1.112548e-14 -1.915381e-15 -4.506198e-15 3.335419e-15
5.935424e-16

E:\\R-3.1.2\\scripts\\mPDBS\\summary_pdb_93\\f8e2bf87e52caa29.final.pdb
2.077878e-15 6.071029e-15 3.606101e-15 1.176715e-15 -2.656580e-15
2.316098e-15

[,403]   [,404]   [,405]   [,406]   [,407]   [,408]
E:\\R-3.1.2\\scripts\\40.1ns_data\\NAT0.pdb
5.787658e-15 -2.763987e-15 4.035083e-15 6.813531e-15 7.633636e-16
3.517014e-15
E:\\R-3.1.2\\scripts\\40.1ns_data\\LJP6.pdb
6.013541e-15 -1.596291e-15 2.378808e-15 4.320496e-15 -1.290876e-15
1.334159e-15
E:\\R-3.1.2\\scripts\\40.1ns_data\\3WY9.pdb
7.351378e-15 -1.201662e-15 1.556168e-15 5.229446e-15 -1.065797e-15
-1.444708e-15
E:\\R-3.1.2\\scripts\\40.1ns_data\\ZSP9.pdb
5.025083e-15 -2.405142e-15 1.659952e-15 6.259588e-15 4.682453e-16
4.120558e-15
E:\\R-3.1.2\\scripts\\mPDBS\\summary_pdb_93\\013d855ad26c877c.final.pdb
9.254462e-15 3.786660e-15 8.548036e-15 3.115077e-15 -2.176502e-15
1.083103e-15
E:\\R-3.1.2\\scripts\\mPDBS\\summary_pdb_93\\0a2a705342ca0d54.final.pdb
5.164042e-15 -2.331514e-15 3.142868e-15 4.828427e-15 -3.498538e-16
2.178704e-15
E:\\R-3.1.2\\scripts\\mPDBS\\summary_pdb_93\\0a59fceadb4194d9.final.pdb
5.544622e-15 -3.760150e-15 1.517804e-15 6.014290e-15 -8.897879e-16
-2.294997e-15
E:\\R-3.1.2\\scripts\\mPDBS\\summary_pdb_93\\0dba59f3306825e8.final.pdb
3.647836e-15 -6.031007e-15 -2.227264e-16 4.690206e-15 -2.343035e-15
-2.736758e-15
E:\\R-3.1.2\\scripts\\mPDBS\\summary_pdb_93\\126f26a78b57339c.final.pdb
-5.439068e-16 -2.825374e-15 -2.666365e-16 5.452834e-15 1.439096e-15
5.444717e-16
E:\\R-3.1.2\\scripts\\mPDBS\\summary_pdb_93\\12f06f3e342df706.final.pdb
4.813411e-15 -2.393639e-15 2.472723e-15 5.208223e-15 -1.867898e-16
1.708594e-15
E:\\R-3.1.2\\scripts\\mPDBS\\summary_pdb_93\\18618a2a251b867b.final.pdb
4.066064e-15 -9.926226e-16 -3.843069e-15 1.112365e-14 -2.312143e-15
1.066807e-16
E:\\R-3.1.2\\scripts\\mPDBS\\summary_pdb_93\\1f305f598641eeb2.final.pdb
4.444586e-15 -2.146612e-15 2.639143e-15 4.834341e-15 -1.442814e-16
1.835829e-15
E:\\R-3.1.2\\scripts\\mPDBS\\summary_pdb_93\\1f8a00b551be0a39.final.pdb
4.066064e-15 -9.926226e-16 -3.843069e-15 1.112365e-14 -2.312143e-15
1.066807e-16
E:\\R-3.1.2\\scripts\\mPDBS\\summary_pdb_93\\219f45328d47579a.final.pdb
-3.657219e-15 1.158391e-14 1.406332e-14 -4.136445e-15 5.784994e-15
4.526803e-15
E:\\R-3.1.2\\scripts\\mPDBS\\summary_pdb_93\\238dd3918e54336d.final.pdb
7.583029e-16 -1.512016e-15 1.372223e-14 -7.331263e-15 8.475550e-15
-1.679170e-15
E:\\R-3.1.2\\scripts\\mPDBS\\summary_pdb_93\\2b3027889e2fdc42.final.pdb
3.505671e-15 -2.764547e-15 3.094234e-15 4.225139e-15 -3.737077e-16
-1.326641e-16
E:\\R-3.1.2\\scripts\\mPDBS\\summary_pdb_93\\2fcb3b46e33793b0.final.pdb

5.887876e-15 -1.828290e-15 2.642613e-15 5.542108e-15 -2.588073e-15
4.411117e-16
E:\\R-3.1.2\\scripts\\mPDBS\\summary_pdb_93\\34eb328251842006.final.pdb
1.422954e-14 1.715146e-15 7.323105e-15 6.909365e-16 1.149490e-16
6.261878e-15
E:\\R-3.1.2\\scripts\\mPDBS\\summary_pdb_93\\354b32871797b7ab.final.pdb
1.517536e-15 1.369605e-15 2.417717e-15 -5.504403e-15 -7.398244e-15
4.368848e-15
E:\\R-3.1.2\\scripts\\mPDBS\\summary_pdb_93\\397368adbc32d5de.final.pdb
-5.439068e-16 -2.825374e-15 -2.666365e-16 5.452834e-15 1.439096e-15
5.444717e-16
E:\\R-3.1.2\\scripts\\mPDBS\\summary_pdb_93\\411ca13cd3df6edd.final.pdb
7.616311e-15 1.719355e-15 3.272991e-16 7.027682e-15 -4.937322e-15
-1.025597e-15
E:\\R-3.1.2\\scripts\\mPDBS\\summary_pdb_93\\42357eee6642bf12.final.pdb
6.548118e-15 2.166854e-15 4.553746e-15 5.621788e-16 5.293363e-15
4.504882e-15
E:\\R-3.1.2\\scripts\\mPDBS\\summary_pdb_93\\44342d1f75a7fcfc.final.pdb
9.722340e-15 -9.161633e-15 -1.098868e-14 1.483505e-14 -5.924462e-15
1.887039e-15
E:\\R-3.1.2\\scripts\\mPDBS\\summary_pdb_93\\448ef21dffe8cdfb.final.pdb
6.057879e-15 7.988510e-15 2.317480e-14 -2.153125e-15 1.104122e-14
3.650350e-15
E:\\R-3.1.2\\scripts\\mPDBS\\summary_pdb_93\\472a40b3e8f113c7.final.pdb
3.647836e-15 -6.031007e-15 -2.227264e-16 4.690206e-15 -2.343035e-15
-2.736758e-15
E:\\R-3.1.2\\scripts\\mPDBS\\summary_pdb_93\\48a26225951ea153.final.pdb
8.266620e-15 -4.543506e-15 -8.295960e-15 1.031402e-14 -8.465109e-15
3.238771e-15
E:\\R-3.1.2\\scripts\\mPDBS\\summary_pdb_93\\4ab4a5c7c6682ce6.final.pdb
-4.464382e-15 -1.156144e-14 4.376612e-16 6.483259e-15 6.727818e-16
4.507966e-16
E:\\R-3.1.2\\scripts\\mPDBS\\summary_pdb_93\\4eaa45b62cb6a25f.final.pdb
2.986196e-15 -5.028421e-15 -3.515517e-16 9.582813e-15 -6.816972e-17
-3.301287e-17
E:\\R-3.1.2\\scripts\\mPDBS\\summary_pdb_93\\58c371611d85ac96.final.pdb
-5.439068e-16 -2.825374e-15 -2.666365e-16 5.452834e-15 1.439096e-15
5.444717e-16
E:\\R-3.1.2\\scripts\\mPDBS\\summary_pdb_93\\59a647f04822d30a.final.pdb
2.317673e-15 -4.880152e-16 8.716044e-15 3.284293e-16 2.741391e-16
2.586132e-15
E:\\R-3.1.2\\scripts\\mPDBS\\summary_pdb_93\\59b091fe385e05fc.final.pdb
5.544622e-15 -3.760150e-15 1.517804e-15 6.014290e-15 -8.897879e-16
-2.294997e-15
E:\\R-3.1.2\\scripts\\mPDBS\\summary_pdb_93\\5c35ac30f930d2db.final.pdb
2.986196e-15 -5.028421e-15 -3.515517e-16 9.582813e-15 -6.816972e-17
-3.301287e-17
E:\\R-3.1.2\\scripts\\mPDBS\\summary_pdb_93\\5f60752431cf2dcb.final.pdb
6.461832e-15 -9.952283e-15 -3.203184e-15 1.239551e-14 -1.949486e-15
-9.941055e-16
E:\\R-3.1.2\\scripts\\mPDBS\\summary_pdb_93\\626456dd6ff2868d.final.pdb
3.647836e-15 -6.031007e-15 -2.227264e-16 4.690206e-15 -2.343035e-15
-2.736758e-15
E:\\R-3.1.2\\scripts\\mPDBS\\summary_pdb_93\\6583d53ff3c8f7ef.final.pdb

2.317673e-15 -4.880152e-16 8.716044e-15 3.284293e-16 2.741391e-16
2.586132e-15
E:\\R-3.1.2\\scripts\\mPDBS\\summary_pdb_93\\69760178986bef01.final.pdb
6.018099e-15 -3.633952e-15 -3.796054e-15 9.194626e-15 1.691382e-15
-3.234678e-15
E:\\R-3.1.2\\scripts\\mPDBS\\summary_pdb_93\\6a9d87d47926b1c8.final.pdb
3.012276e-15 -2.217353e-15 3.237505e-15 3.759744e-15 -4.257319e-16
-4.304429e-16
E:\\R-3.1.2\\scripts\\mPDBS\\summary_pdb_93\\6bb3547ea982ffc4.final.pdb
4.147648e-15 2.114664e-15 7.521950e-15 3.299517e-15 1.447716e-15
-5.177657e-16
E:\\R-3.1.2\\scripts\\mPDBS\\summary_pdb_93\\716d9624611d1c42.final.pdb
2.753644e-15 2.340963e-15 3.841962e-15 -2.439046e-16 -9.626449e-16
3.805007e-15
E:\\R-3.1.2\\scripts\\mPDBS\\summary_pdb_93\\75f27e70ad0f1be4.final.pdb
2.317673e-15 -4.880152e-16 8.716044e-15 3.284293e-16 2.741391e-16
2.586132e-15
E:\\R-3.1.2\\scripts\\mPDBS\\summary_pdb_93\\769f910fffb22872.final.pdb
3.585112e-15 -2.436285e-15 2.420366e-15 4.877239e-15 -1.181511e-16
1.844006e-15
E:\\R-3.1.2\\scripts\\mPDBS\\summary_pdb_93\\791f8c80dd5c3135.final.pdb
5.544622e-15 -3.760150e-15 1.517804e-15 6.014290e-15 -8.897879e-16
-2.294997e-15
E:\\R-3.1.2\\scripts\\mPDBS\\summary_pdb_93\\7fad98e66a3f6099.final.pdb
8.129084e-15 -8.137434e-15 6.192974e-15 -1.057128e-15 -9.553152e-16
6.778534e-15
E:\\R-3.1.2\\scripts\\mPDBS\\summary_pdb_93\\804b89c94a633196.final.pdb
5.887876e-15 -1.828290e-15 2.642613e-15 5.542108e-15 -2.588073e-15
4.411117e-16
E:\\R-3.1.2\\scripts\\mPDBS\\summary_pdb_93\\813cc65a2298feba.final.pdb
5.887876e-15 -1.828290e-15 2.642613e-15 5.542108e-15 -2.588073e-15
4.411117e-16
E:\\R-3.1.2\\scripts\\mPDBS\\summary_pdb_93\\83c0eefcfe12d3f9.final.pdb
7.616311e-15 1.719355e-15 3.272991e-16 7.027682e-15 -4.937322e-15
-1.025597e-15
E:\\R-3.1.2\\scripts\\mPDBS\\summary_pdb_93\\86d50469ef569760.final.pdb
3.647836e-15 -6.031007e-15 -2.227264e-16 4.690206e-15 -2.343035e-15
-2.736758e-15
E:\\R-3.1.2\\scripts\\mPDBS\\summary_pdb_93\\89d0666e1402203d.final.pdb
2.317673e-15 -4.880152e-16 8.716044e-15 3.284293e-16 2.741391e-16
2.586132e-15
E:\\R-3.1.2\\scripts\\mPDBS\\summary_pdb_93\\8b34852716dadc62.final.pdb
-5.181718e-17 -6.590974e-15 -4.771595e-15 7.235198e-15 -7.167402e-16
-5.580890e-16
E:\\R-3.1.2\\scripts\\mPDBS\\summary_pdb_93\\8b6ef9ac3fe74395.final.pdb
3.933250e-15 -2.395828e-15 1.283915e-14 4.922926e-15 3.240131e-15
-5.104521e-15
E:\\R-3.1.2\\scripts\\mPDBS\\summary_pdb_93\\98bbe0d5986c138f.final.pdb
5.544622e-15 -3.760150e-15 1.517804e-15 6.014290e-15 -8.897879e-16
-2.294997e-15
E:\\R-3.1.2\\scripts\\mPDBS\\summary_pdb_93\\9c22f4909d67e2fa.final.pdb
3.971440e-15 -2.040293e-15 7.252013e-16 5.516338e-16 1.664601e-15
5.320021e-15
E:\\R-3.1.2\\scripts\\mPDBS\\summary_pdb_93\\9dda8ed0d81fb3ef.final.pdb

1.320762e-14 -1.329474e-14 -2.316514e-14 2.509009e-14 -8.468867e-15
-4.558326e-15
E:\\R-3.1.2\\scripts\\mPDBS\\summary_pdb_93\\9f752b3616e6033b.final.pdb
3.954110e-15 -1.328137e-15 5.458568e-15 3.858355e-15 1.661079e-15
6.660103e-16
E:\\R-3.1.2\\scripts\\mPDBS\\summary_pdb_93\\a02c70083816d6ba.final.pdb
2.986196e-15 -5.028421e-15 -3.515517e-16 9.582813e-15 -6.816972e-17
-3.301287e-17
E:\\R-3.1.2\\scripts\\mPDBS\\summary_pdb_93\\a0c997d891e4e082.final.pdb
3.445436e-15 -3.165713e-15 -1.197131e-16 8.116393e-15 -3.619302e-15
8.440424e-16
E:\\R-3.1.2\\scripts\\mPDBS\\summary_pdb_93\\ab8039a2d823c616.final.pdb
4.617893e-15 -1.226360e-15 2.469145e-15 4.671775e-15 2.480471e-16
8.180215e-16
E:\\R-3.1.2\\scripts\\mPDBS\\summary_pdb_93\\acf4d74b9c71d4dc.final.pdb
5.887876e-15 -1.828290e-15 2.642613e-15 5.542108e-15 -2.588073e-15
4.411117e-16
E:\\R-3.1.2\\scripts\\mPDBS\\summary_pdb_93\\ad8a823e4b4c668e.final.pdb
4.151902e-15 -3.019448e-15 3.116935e-15 4.722376e-15 -5.325093e-16
9.928321e-16
E:\\R-3.1.2\\scripts\\mPDBS\\summary_pdb_93\\b16ababd03b7b857.final.pdb
3.971440e-15 -2.040293e-15 7.252013e-16 5.516338e-16 1.664601e-15
5.320021e-15
E:\\R-3.1.2\\scripts\\mPDBS\\summary_pdb_93\\b226923e697d3c63.final.pdb
8.118034e-17 -1.842462e-15 4.350395e-15 6.311165e-15 3.723678e-16
1.367489e-15
E:\\R-3.1.2\\scripts\\mPDBS\\summary_pdb_93\\b28e48f6fa27a165.final.pdb
5.544622e-15 -3.760150e-15 1.517804e-15 6.014290e-15 -8.897879e-16
-2.294997e-15
E:\\R-3.1.2\\scripts\\mPDBS\\summary_pdb_93\\b42c4924e3cd1aec.final.pdb
-1.367531e-15 -2.069222e-16 6.897403e-15 -5.368724e-16 3.354574e-15
-6.959646e-16
E:\\R-3.1.2\\scripts\\mPDBS\\summary_pdb_93\\b4dd239fcabff1df.final.pdb
5.544622e-15 -3.760150e-15 1.517804e-15 6.014290e-15 -8.897879e-16
-2.294997e-15
E:\\R-3.1.2\\scripts\\mPDBS\\summary_pdb_93\\b669942ef97459a2.final.pdb
-5.181718e-17 -6.590974e-15 -4.771595e-15 7.235198e-15 -7.167402e-16
-5.580890e-16
E:\\R-3.1.2\\scripts\\mPDBS\\summary_pdb_93\\b99f4d9d87c1017b.final.pdb
5.544622e-15 -3.760150e-15 1.517804e-15 6.014290e-15 -8.897879e-16
-2.294997e-15
E:\\R-3.1.2\\scripts\\mPDBS\\summary_pdb_93\\b9d2cbf3975403e7.final.pdb
5.544622e-15 -3.760150e-15 1.517804e-15 6.014290e-15 -8.897879e-16
-2.294997e-15
E:\\R-3.1.2\\scripts\\mPDBS\\summary_pdb_93\\bc88c8a505f3a288.final.pdb
3.627102e-15 -2.230923e-15 2.415740e-15 4.669337e-15 -3.218828e-16
1.045057e-17
E:\\R-3.1.2\\scripts\\mPDBS\\summary_pdb_93\\bdac0e64f4ef427e.final.pdb
-2.908039e-15 -1.045877e-14 7.714836e-15 -1.346662e-16 1.146145e-14
2.642476e-15
E:\\R-3.1.2\\scripts\\mPDBS\\summary_pdb_93\\ca2a1f55fc9d0681.final.pdb
-5.181718e-17 -6.590974e-15 -4.771595e-15 7.235198e-15 -7.167402e-16
-5.580890e-16
E:\\R-3.1.2\\scripts\\mPDBS\\summary_pdb_93\\ce34248de3e81a3c.final.pdb

5.676041e-15 -5.867295e-15 -2.942683e-15 7.844932e-15 -4.666825e-15
1.387084e-15
E:\\R-3.1.2\\scripts\\mPDBS\\summary_pdb_93\\d02e697772494a61.final.pdb
-6.268738e-16 5.143251e-15 2.370904e-14 -1.027162e-14 3.533323e-15
6.470561e-15
E:\\R-3.1.2\\scripts\\mPDBS\\summary_pdb_93\\d04221245d015047.final.pdb
4.354431e-15 -3.181325e-15 1.868280e-15 4.275716e-15 -7.518042e-16
1.190987e-15
E:\\R-3.1.2\\scripts\\mPDBS\\summary_pdb_93\\d678141e4018054a.final.pdb
5.498097e-15 -6.962099e-16 6.296815e-15 -1.313102e-15 -2.431895e-15
8.112842e-15
E:\\R-3.1.2\\scripts\\mPDBS\\summary_pdb_93\\d71b40af0f0efe39.final.pdb
-5.877614e-15 -3.285276e-15 7.469563e-15 -2.144107e-15 -3.819418e-15
6.244523e-15
E:\\R-3.1.2\\scripts\\mPDBS\\summary_pdb_93\\dc0809eff10197e2.final.pdb
1.796076e-15 -7.828518e-15 5.708825e-15 8.341527e-15 3.354516e-16
-4.068858e-15
E:\\R-3.1.2\\scripts\\mPDBS\\summary_pdb_93\\e1163918d3707839.final.pdb
1.898954e-15 2.095056e-15 4.886859e-15 5.489557e-15 1.065407e-14
7.304580e-15
E:\\R-3.1.2\\scripts\\mPDBS\\summary_pdb_93\\e4b48b06824ef848.final.pdb
4.066064e-15 -9.926226e-16 -3.843069e-15 1.112365e-14 -2.312143e-15
1.066807e-16
E:\\R-3.1.2\\scripts\\mPDBS\\summary_pdb_93\\e529e19ad281fea2.final.pdb
9.254462e-15 3.786660e-15 8.548036e-15 3.115077e-15 -2.176502e-15
1.083103e-15
E:\\R-3.1.2\\scripts\\mPDBS\\summary_pdb_93\\e5602082718ec3a1.final.pdb
5.887876e-15 -1.828290e-15 2.642613e-15 5.542108e-15 -2.588073e-15
4.411117e-16
E:\\R-3.1.2\\scripts\\mPDBS\\summary_pdb_93\\e5d589d996d0a960.final.pdb
5.009843e-15 -5.120601e-15 4.364258e-15 5.855019e-15 -4.001235e-15
-7.261061e-16
E:\\R-3.1.2\\scripts\\mPDBS\\summary_pdb_93\\e9df9e72307a2fbd.final.pdb
2.986196e-15 -5.028421e-15 -3.515517e-16 9.582813e-15 -6.816972e-17
-3.301287e-17
E:\\R-3.1.2\\scripts\\mPDBS\\summary_pdb_93\\ea00f8694090cae7.final.pdb
5.887876e-15 -1.828290e-15 2.642613e-15 5.542108e-15 -2.588073e-15
4.411117e-16
E:\\R-3.1.2\\scripts\\mPDBS\\summary_pdb_93\\eba517be32db050e.final.pdb
5.544622e-15 -3.760150e-15 1.517804e-15 6.014290e-15 -8.897879e-16
-2.294997e-15
E:\\R-3.1.2\\scripts\\mPDBS\\summary_pdb_93\\ec04b9545899c29d.final.pdb
4.179031e-15 -2.604812e-15 2.732983e-15 4.557685e-15 -6.734536e-16
1.803772e-15
E:\\R-3.1.2\\scripts\\mPDBS\\summary_pdb_93\\ee6862cbcad52078.final.pdb
3.917451e-15 -2.534134e-15 2.742519e-15 4.393190e-15 -4.600102e-16
-2.532274e-16
E:\\R-3.1.2\\scripts\\mPDBS\\summary_pdb_93\\ef6a42fc83b81e5d.final.pdb
3.833394e-15 -2.689921e-15 2.582780e-15 4.699756e-15 -6.149326e-16
1.662728e-15
E:\\R-3.1.2\\scripts\\mPDBS\\summary_pdb_93\\ef8e88d4ad93a0ad.final.pdb
4.354431e-15 -3.181325e-15 1.868280e-15 4.275716e-15 -7.518042e-16
1.190987e-15
E:\\R-3.1.2\\scripts\\mPDBS\\summary_pdb_93\\f14fcae8deec4fa3.final.pdb

3.954110e-15 -1.328137e-15 5.458568e-15 3.858355e-15 1.661079e-15
6.660103e-16
E:\\R-3.1.2\\scripts\\mPDBS\\summary_pdb_93\\f83584e22003fb92.final.pdb
8.266620e-15 -4.543506e-15 -8.295960e-15 1.031402e-14 -8.465109e-15
3.238771e-15
E:\\R-3.1.2\\scripts\\mPDBS\\summary_pdb_93\\f8e2bf87e52caa29.final.pdb
9.254462e-15 3.786660e-15 8.548036e-15 3.115077e-15 -2.176502e-15
1.083103e-15

[,409]   [,410]   [,411]   [,412]   [,413]   [,414]
E:\\R-3.1.2\\scripts\\40.1ns_data\\NAT0.pdb
2.938602e-15 4.186516e-15 -9.487381e-16 1.460247e-15 -1.823364e-15
-4.717062e-15
E:\\R-3.1.2\\scripts\\40.1ns_data\\LJP6.pdb
2.829167e-15 5.134185e-15 -1.156597e-15 2.491220e-15 -1.119059e-15
-2.718557e-15
E:\\R-3.1.2\\scripts\\40.1ns_data\\3WY9.pdb
2.399002e-15 7.698710e-15 -2.232831e-15 1.106732e-15 -1.502182e-15
-2.389896e-15
E:\\R-3.1.2\\scripts\\40.1ns_data\\ZSP9.pdb
2.376563e-15 5.902574e-15 -4.052324e-15 1.914708e-15 -1.790823e-15
-2.941987e-15
E:\\R-3.1.2\\scripts\\mPDBS\\summary_pdb_93\\013d855ad26c877c.final.pdb
6.285408e-16 4.606030e-15 4.358225e-15 -1.716597e-15 -7.290207e-15
-9.261984e-15
E:\\R-3.1.2\\scripts\\mPDBS\\summary_pdb_93\\0a2a705342ca0d54.final.pdb
2.584241e-15 5.061542e-15 -1.957375e-15 1.507545e-15 -1.790680e-15
-3.858728e-15
E:\\R-3.1.2\\scripts\\mPDBS\\summary_pdb_93\\0a59fceadb4194d9.final.pdb
2.085716e-15 7.716473e-15 -1.636316e-15 4.436454e-15 -3.984704e-15
-9.300323e-16
E:\\R-3.1.2\\scripts\\mPDBS\\summary_pdb_93\\0dba59f3306825e8.final.pdb
4.201204e-15 4.538376e-15 -3.187823e-15 2.845726e-15 -2.457268e-15
1.441477e-15
E:\\R-3.1.2\\scripts\\mPDBS\\summary_pdb_93\\126f26a78b57339c.final.pdb
4.888154e-15 3.617671e-15 -6.548253e-15 9.982454e-16 -2.367062e-15
-7.076666e-15
E:\\R-3.1.2\\scripts\\mPDBS\\summary_pdb_93\\12f06f3e342df706.final.pdb
3.037437e-15 4.864651e-15 -1.713863e-15 1.564357e-15 -2.200942e-15
-4.648027e-15
E:\\R-3.1.2\\scripts\\mPDBS\\summary_pdb_93\\18618a2a251b867b.final.pdb
6.303159e-15 -4.716757e-16 -9.220209e-15 -2.777165e-15 -5.514704e-17
-8.370489e-15
E:\\R-3.1.2\\scripts\\mPDBS\\summary_pdb_93\\1f305f598641eeb2.final.pdb
2.412714e-15 5.113111e-15 -2.428466e-15 1.635421e-15 -1.738355e-15
-3.533209e-15
E:\\R-3.1.2\\scripts\\mPDBS\\summary_pdb_93\\1f8a00b551be0a39.final.pdb
6.303159e-15 -4.716757e-16 -9.220209e-15 -2.777165e-15 -5.514704e-17
-8.370489e-15
E:\\R-3.1.2\\scripts\\mPDBS\\summary_pdb_93\\219f45328d47579a.final.pdb
8.620853e-15 3.845004e-15 1.401185e-15 -8.430858e-15 4.469471e-15
-1.932677e-14
E:\\R-3.1.2\\scripts\\mPDBS\\summary_pdb_93\\238dd3918e54336d.final.pdb
9.401913e-15 -5.758859e-15 -3.812689e-15 4.220481e-15 9.111195e-16

-1.230960e-14
E:\\R-3.1.2\\scripts\\mPDBS\\summary_pdb_93\\2b3027889e2fdc42.final.pdb
2.598761e-15 5.244492e-15 -1.888176e-15 1.238925e-15 -2.477229e-15
-4.279566e-15
E:\\R-3.1.2\\scripts\\mPDBS\\summary_pdb_93\\2fcb3b46e33793b0.final.pdb
-3.498328e-16 6.105373e-15 6.003615e-16 3.950939e-16 -1.846775e-15
-3.321790e-15
E:\\R-3.1.2\\scripts\\mPDBS\\summary_pdb_93\\34eb328251842006.final.pdb
4.730285e-15 5.934039e-15 7.210572e-15 1.228430e-15 2.837263e-15
-1.247571e-14
E:\\R-3.1.2\\scripts\\mPDBS\\summary_pdb_93\\354b32871797b7ab.final.pdb
5.309706e-16 5.367667e-15 -5.901036e-15 -3.357532e-15 4.273692e-15
-1.822263e-15
E:\\R-3.1.2\\scripts\\mPDBS\\summary_pdb_93\\397368adbc32d5de.final.pdb
4.888154e-15 3.617671e-15 -6.548253e-15 9.982454e-16 -2.367062e-15
-7.076666e-15
E:\\R-3.1.2\\scripts\\mPDBS\\summary_pdb_93\\411ca13cd3df6edd.final.pdb
1.633823e-15 4.583587e-15 -1.866376e-17 -1.795862e-15 2.747917e-16
-1.210426e-14
E:\\R-3.1.2\\scripts\\mPDBS\\summary_pdb_93\\42357eee6642bf12.final.pdb
1.959392e-15 8.015954e-15 -2.127799e-15 2.398374e-15 4.909570e-15
-6.306686e-15
E:\\R-3.1.2\\scripts\\mPDBS\\summary_pdb_93\\44342d1f75a7fcfc.final.pdb
-3.884614e-15 -1.322560e-15 2.830265e-15 6.653158e-15 -6.190567e-15
-5.088785e-15
E:\\R-3.1.2\\scripts\\mPDBS\\summary_pdb_93\\448ef21dffe8cdfb.final.pdb
4.654844e-15 1.008136e-14 -3.753794e-15 1.930324e-15 -1.344573e-15
-7.832071e-15
E:\\R-3.1.2\\scripts\\mPDBS\\summary_pdb_93\\472a40b3e8f113c7.final.pdb
4.201204e-15 4.538376e-15 -3.187823e-15 2.845726e-15 -2.457268e-15
1.441477e-15
E:\\R-3.1.2\\scripts\\mPDBS\\summary_pdb_93\\48a26225951ea153.final.pdb
-9.769380e-16 3.844570e-15 -1.706030e-15 -4.182520e-15 3.203557e-15
-7.599827e-15
E:\\R-3.1.2\\scripts\\mPDBS\\summary_pdb_93\\4ab4a5c7c6682ce6.final.pdb
-5.841978e-15 5.182392e-15 -6.808462e-15 1.147432e-15 1.130169e-14
2.404248e-15
E:\\R-3.1.2\\scripts\\mPDBS\\summary_pdb_93\\4eaa45b62cb6a25f.final.pdb
2.590521e-15 4.708039e-15 -2.486070e-15 1.704391e-15 -5.531833e-15
-3.446832e-15
E:\\R-3.1.2\\scripts\\mPDBS\\summary_pdb_93\\58c371611d85ac96.final.pdb
4.888154e-15 3.617671e-15 -6.548253e-15 9.982454e-16 -2.367062e-15
-7.076666e-15
E:\\R-3.1.2\\scripts\\mPDBS\\summary_pdb_93\\59a647f04822d30a.final.pdb
3.355688e-15 1.236788e-14 -2.469315e-15 -4.894663e-17 -2.349889e-15
6.878444e-16
E:\\R-3.1.2\\scripts\\mPDBS\\summary_pdb_93\\59b091fe385e05fc.final.pdb
2.085716e-15 7.716473e-15 -1.636316e-15 4.436454e-15 -3.984704e-15
-9.300323e-16
E:\\R-3.1.2\\scripts\\mPDBS\\summary_pdb_93\\5c35ac30f930d2db.final.pdb
2.590521e-15 4.708039e-15 -2.486070e-15 1.704391e-15 -5.531833e-15
-3.446832e-15
E:\\R-3.1.2\\scripts\\mPDBS\\summary_pdb_93\\5f60752431cf2dcb.final.pdb
5.458013e-17 6.775449e-15 -8.171479e-15 1.735423e-16 -3.901839e-15

-9.973016e-16
E:\\R-3.1.2\\scripts\\mPDBS\\summary_pdb_93\\626456dd6ff2868d.final.pdb
4.201204e-15 4.538376e-15 -3.187823e-15 2.845726e-15 -2.457268e-15
1.441477e-15
E:\\R-3.1.2\\scripts\\mPDBS\\summary_pdb_93\\6583d53ff3c8f7ef.final.pdb
3.355688e-15 1.236788e-14 -2.469315e-15 -4.894663e-17 -2.349889e-15
6.878444e-16
E:\\R-3.1.2\\scripts\\mPDBS\\summary_pdb_93\\69760178986bef01.final.pdb
7.341078e-15 4.391273e-15 -4.401566e-15 7.193485e-15 -4.807280e-15
4.631025e-15
E:\\R-3.1.2\\scripts\\mPDBS\\summary_pdb_93\\6a9d87d47926b1c8.final.pdb
2.800415e-15 5.588317e-15 -2.142055e-15 1.636624e-15 -2.009709e-15
-3.568621e-15
E:\\R-3.1.2\\scripts\\mPDBS\\summary_pdb_93\\6bb3547ea982ffc4.final.pdb
7.630718e-15 -1.242312e-14 1.322052e-15 -5.078487e-15 2.682605e-15
-1.512529e-14
E:\\R-3.1.2\\scripts\\mPDBS\\summary_pdb_93\\716d9624611d1c42.final.pdb
8.443703e-15 -6.938365e-16 4.492327e-15 1.310416e-16 -7.923469e-16
-9.027337e-15
E:\\R-3.1.2\\scripts\\mPDBS\\summary_pdb_93\\75f27e70ad0f1be4.final.pdb
3.355688e-15 1.236788e-14 -2.469315e-15 -4.894663e-17 -2.349889e-15
6.878444e-16
E:\\R-3.1.2\\scripts\\mPDBS\\summary_pdb_93\\769f910fffb22872.final.pdb
2.682791e-15 5.058189e-15 -2.040893e-15 1.524516e-15 -1.823698e-15
-4.212140e-15
E:\\R-3.1.2\\scripts\\mPDBS\\summary_pdb_93\\791f8c80dd5c3135.final.pdb
2.085716e-15 7.716473e-15 -1.636316e-15 4.436454e-15 -3.984704e-15
-9.300323e-16
E:\\R-3.1.2\\scripts\\mPDBS\\summary_pdb_93\\7fad98e66a3f6099.final.pdb
-2.943928e-15 7.734623e-15 6.293835e-15 3.270623e-15 3.084465e-15
-6.037907e-15
E:\\R-3.1.2\\scripts\\mPDBS\\summary_pdb_93\\804b89c94a633196.final.pdb
-3.498328e-16 6.105373e-15 6.003615e-16 3.950939e-16 -1.846775e-15
-3.321790e-15
E:\\R-3.1.2\\scripts\\mPDBS\\summary_pdb_93\\813cc65a2298feba.final.pdb
-3.498328e-16 6.105373e-15 6.003615e-16 3.950939e-16 -1.846775e-15
-3.321790e-15
E:\\R-3.1.2\\scripts\\mPDBS\\summary_pdb_93\\83c0eefcfe12d3f9.final.pdb
1.633823e-15 4.583587e-15 -1.866376e-17 -1.795862e-15 2.747917e-16
-1.210426e-14
E:\\R-3.1.2\\scripts\\mPDBS\\summary_pdb_93\\86d50469ef569760.final.pdb
4.201204e-15 4.538376e-15 -3.187823e-15 2.845726e-15 -2.457268e-15
1.441477e-15
E:\\R-3.1.2\\scripts\\mPDBS\\summary_pdb_93\\89d0666e1402203d.final.pdb
3.355688e-15 1.236788e-14 -2.469315e-15 -4.894663e-17 -2.349889e-15
6.878444e-16
E:\\R-3.1.2\\scripts\\mPDBS\\summary_pdb_93\\8b34852716dadc62.final.pdb
3.396302e-15 5.555466e-15 -7.469572e-15 4.549757e-15 3.543878e-15
-1.751648e-15
E:\\R-3.1.2\\scripts\\mPDBS\\summary_pdb_93\\8b6ef9ac3fe74395.final.pdb
5.825409e-15 2.408075e-15 -3.617188e-15 1.072701e-15 -3.067163e-15
1.658285e-15
E:\\R-3.1.2\\scripts\\mPDBS\\summary_pdb_93\\98bbe0d5986c138f.final.pdb
2.085716e-15 7.716473e-15 -1.636316e-15 4.436454e-15 -3.984704e-15

-9.300323e-16
E:\\R-3.1.2\\scripts\\mPDBS\\summary_pdb_93\\9c22f4909d67e2fa.final.pdb
6.095099e-15 7.045593e-15 -1.331145e-15 5.293953e-15 -1.056159e-14
-1.856266e-16
E:\\R-3.1.2\\scripts\\mPDBS\\summary_pdb_93\\9dda8ed0d81fb3ef.final.pdb
2.488327e-15 2.734525e-15 -7.340689e-15 6.911631e-15 -7.111271e-15
5.991567e-16
E:\\R-3.1.2\\scripts\\mPDBS\\summary_pdb_93\\9f752b3616e6033b.final.pdb
2.206292e-15 5.610313e-15 -2.121310e-15 1.817509e-15 -1.638045e-15
-3.405977e-15
E:\\R-3.1.2\\scripts\\mPDBS\\summary_pdb_93\\a02c70083816d6ba.final.pdb
2.590521e-15 4.708039e-15 -2.486070e-15 1.704391e-15 -5.531833e-15
-3.446832e-15
E:\\R-3.1.2\\scripts\\mPDBS\\summary_pdb_93\\a0c997d891e4e082.final.pdb
-1.345704e-16 8.493585e-15 7.810994e-16 1.216127e-15 -7.776419e-15
-1.193673e-14
E:\\R-3.1.2\\scripts\\mPDBS\\summary_pdb_93\\ab8039a2d823c616.final.pdb
2.491801e-15 5.383226e-15 -1.959059e-15 1.167988e-15 -1.619018e-15
-3.764668e-15
E:\\R-3.1.2\\scripts\\mPDBS\\summary_pdb_93\\acf4d74b9c71d4dc.final.pdb
-3.498328e-16 6.105373e-15 6.003615e-16 3.950939e-16 -1.846775e-15
-3.321790e-15
E:\\R-3.1.2\\scripts\\mPDBS\\summary_pdb_93\\ad8a823e4b4c668e.final.pdb
2.413611e-15 5.074796e-15 -2.953058e-15 1.368547e-15 -2.122258e-15
-3.771867e-15
E:\\R-3.1.2\\scripts\\mPDBS\\summary_pdb_93\\b16ababd03b7b857.final.pdb
6.095099e-15 7.045593e-15 -1.331145e-15 5.293953e-15 -1.056159e-14
-1.856266e-16
E:\\R-3.1.2\\scripts\\mPDBS\\summary_pdb_93\\b226923e697d3c63.final.pdb
7.432722e-15 4.984461e-15 -7.011851e-15 5.174406e-16 -4.929857e-15
-8.431814e-15
E:\\R-3.1.2\\scripts\\mPDBS\\summary_pdb_93\\b28e48f6fa27a165.final.pdb
2.085716e-15 7.716473e-15 -1.636316e-15 4.436454e-15 -3.984704e-15
-9.300323e-16
E:\\R-3.1.2\\scripts\\mPDBS\\summary_pdb_93\\b42c4924e3cd1aec.final.pdb
4.083405e-15 3.146955e-15 1.093103e-15 4.810114e-16 -1.819497e-15
-7.272151e-15
E:\\R-3.1.2\\scripts\\mPDBS\\summary_pdb_93\\b4dd239fcabff1df.final.pdb
2.085716e-15 7.716473e-15 -1.636316e-15 4.436454e-15 -3.984704e-15
-9.300323e-16
E:\\R-3.1.2\\scripts\\mPDBS\\summary_pdb_93\\b669942ef97459a2.final.pdb
3.396302e-15 5.555466e-15 -7.469572e-15 4.549757e-15 3.543878e-15
-1.751648e-15
E:\\R-3.1.2\\scripts\\mPDBS\\summary_pdb_93\\b99f4d9d87c1017b.final.pdb
2.085716e-15 7.716473e-15 -1.636316e-15 4.436454e-15 -3.984704e-15
-9.300323e-16
E:\\R-3.1.2\\scripts\\mPDBS\\summary_pdb_93\\b9d2cbf3975403e7.final.pdb
2.085716e-15 7.716473e-15 -1.636316e-15 4.436454e-15 -3.984704e-15
-9.300323e-16
E:\\R-3.1.2\\scripts\\mPDBS\\summary_pdb_93\\bc88c8a505f3a288.final.pdb
2.516361e-15 4.966069e-15 -1.777533e-15 1.063284e-15 -1.916046e-15
-3.296195e-15
E:\\R-3.1.2\\scripts\\mPDBS\\summary_pdb_93\\bdac0e64f4ef427e.final.pdb
3.344261e-15 5.927998e-15 -6.644277e-15 7.890834e-15 -5.699162e-15

1.446129e-14
E:\\R-3.1.2\\scripts\\mPDBS\\summary_pdb_93\\ca2a1f55fc9d0681.final.pdb
3.396302e-15 5.555466e-15 -7.469572e-15 4.549757e-15 3.543878e-15
-1.751648e-15
E:\\R-3.1.2\\scripts\\mPDBS\\summary_pdb_93\\ce34248de3e81a3c.final.pdb
-4.075321e-15 1.066464e-14 -4.376689e-15 6.069191e-16 -4.080041e-15
-4.370064e-15
E:\\R-3.1.2\\scripts\\mPDBS\\summary_pdb_93\\d02e697772494a61.final.pdb
1.675454e-15 9.668037e-15 -6.705986e-18 -2.985561e-15 2.705691e-15
-4.825137e-15
E:\\R-3.1.2\\scripts\\mPDBS\\summary_pdb_93\\d04221245d015047.final.pdb
8.854470e-16 4.829849e-15 -1.296305e-16 1.663686e-15 -8.774241e-16
-2.621268e-15
E:\\R-3.1.2\\scripts\\mPDBS\\summary_pdb_93\\d678141e4018054a.final.pdb
7.528369e-15 -7.094945e-15 -6.223265e-16 -7.636502e-15 5.700519e-15
-2.139357e-14
E:\\R-3.1.2\\scripts\\mPDBS\\summary_pdb_93\\d71b40af0f0efe39.final.pdb
3.001434e-15 3.713963e-15 -3.806417e-15 -2.033533e-16 -7.754117e-16
-5.801895e-15
E:\\R-3.1.2\\scripts\\mPDBS\\summary_pdb_93\\dc0809eff10197e2.final.pdb
3.741768e-15 1.534712e-14 1.881220e-15 7.754715e-15 -9.243652e-15
9.770131e-15
E:\\R-3.1.2\\scripts\\mPDBS\\summary_pdb_93\\e1163918d3707839.final.pdb
3.801946e-15 2.838175e-15 5.369196e-15 -1.313994e-15 -2.024195e-15
-8.249017e-15
E:\\R-3.1.2\\scripts\\mPDBS\\summary_pdb_93\\e4b48b06824ef848.final.pdb
6.303159e-15 -4.716757e-16 -9.220209e-15 -2.777165e-15 -5.514704e-17
-8.370489e-15
E:\\R-3.1.2\\scripts\\mPDBS\\summary_pdb_93\\e529e19ad281fea2.final.pdb
6.285408e-16 4.606030e-15 4.358225e-15 -1.716597e-15 -7.290207e-15
-9.261984e-15
E:\\R-3.1.2\\scripts\\mPDBS\\summary_pdb_93\\e5602082718ec3a1.final.pdb
-3.498328e-16 6.105373e-15 6.003615e-16 3.950939e-16 -1.846775e-15
-3.321790e-15
E:\\R-3.1.2\\scripts\\mPDBS\\summary_pdb_93\\e5d589d996d0a960.final.pdb
-8.386492e-15 -2.893382e-16 -5.455064e-15 9.204506e-16 1.595294e-15
-2.852170e-15
E:\\R-3.1.2\\scripts\\mPDBS\\summary_pdb_93\\e9df9e72307a2fbd.final.pdb
2.590521e-15 4.708039e-15 -2.486070e-15 1.704391e-15 -5.531833e-15
-3.446832e-15
E:\\R-3.1.2\\scripts\\mPDBS\\summary_pdb_93\\ea00f8694090cae7.final.pdb
-3.498328e-16 6.105373e-15 6.003615e-16 3.950939e-16 -1.846775e-15
-3.321790e-15
E:\\R-3.1.2\\scripts\\mPDBS\\summary_pdb_93\\eba517be32db050e.final.pdb
2.085716e-15 7.716473e-15 -1.636316e-15 4.436454e-15 -3.984704e-15
-9.300323e-16
E:\\R-3.1.2\\scripts\\mPDBS\\summary_pdb_93\\ec04b9545899c29d.final.pdb
2.402024e-15 5.189159e-15 -1.908999e-15 1.431640e-15 -2.108763e-15
-4.607399e-15
E:\\R-3.1.2\\scripts\\mPDBS\\summary_pdb_93\\ee6862cbcad52078.final.pdb
2.789580e-15 5.143878e-15 -1.530498e-15 1.573293e-15 -1.879617e-15
-4.042994e-15
E:\\R-3.1.2\\scripts\\mPDBS\\summary_pdb_93\\ef6a42fc83b81e5d.final.pdb
2.544881e-15 5.312326e-15 -2.310480e-15 1.005400e-15 -2.376207e-15

-4.215826e-15
E:\\R-3.1.2\\scripts\\mPDBS\\summary_pdb_93\\ef8e88d4ad93a0ad.final.pdb
8.854470e-16 4.829849e-15 -1.296305e-16 1.663686e-15 -8.774241e-16
-2.621268e-15
E:\\R-3.1.2\\scripts\\mPDBS\\summary_pdb_93\\f14fcae8deec4fa3.final.pdb
2.206292e-15 5.610313e-15 -2.121310e-15 1.817509e-15 -1.638045e-15
-3.405977e-15
E:\\R-3.1.2\\scripts\\mPDBS\\summary_pdb_93\\f83584e22003fb92.final.pdb
-9.769380e-16 3.844570e-15 -1.706030e-15 -4.182520e-15 3.203557e-15
-7.599827e-15
E:\\R-3.1.2\\scripts\\mPDBS\\summary_pdb_93\\f8e2bf87e52caa29.final.pdb
6.285408e-16 4.606030e-15 4.358225e-15 -1.716597e-15 -7.290207e-15
-9.261984e-15

[,415]   [,416]   [,417]   [,418]   [,419]   [,420]
E:\\R-3.1.2\\scripts\\40.1ns_data\\NAT0.pdb
-3.477605e-18 1.233621e-16 -2.157887e-15 2.948752e-15 -3.338856e-16
1.875616e-15
E:\\R-3.1.2\\scripts\\40.1ns_data\\LJP6.pdb
-6.383400e-16 2.166692e-16 -5.068050e-16 2.872994e-15 1.555856e-15
-9.575476e-17
E:\\R-3.1.2\\scripts\\40.1ns_data\\3WY9.pdb
-8.234601e-16 1.329297e-15 3.287087e-16 1.143384e-15 4.151776e-16
2.016728e-16
E:\\R-3.1.2\\scripts\\40.1ns_data\\ZSP9.pdb
-2.516325e-15 9.571833e-16 -7.672047e-16 -6.412372e-16 1.233718e-15
7.428607e-16
E:\\R-3.1.2\\scripts\\mPDBS\\summary_pdb_93\\013d855ad26c877c.final.pdb
1.089519e-15 2.116945e-15 -5.755528e-15 -1.717084e-15 1.000193e-14
-4.704746e-15
E:\\R-3.1.2\\scripts\\mPDBS\\summary_pdb_93\\0a2a705342ca0d54.final.pdb
-1.273044e-15 8.130249e-16 -1.137198e-15 1.750761e-15 2.804070e-15
3.102060e-16
E:\\R-3.1.2\\scripts\\mPDBS\\summary_pdb_93\\0a59fceadb4194d9.final.pdb
5.387904e-16 2.930544e-15 -2.196617e-15 -6.484778e-16 3.617676e-15
-3.831048e-15
E:\\R-3.1.2\\scripts\\mPDBS\\summary_pdb_93\\0dba59f3306825e8.final.pdb
-2.115235e-16 -1.197678e-15 1.754921e-16 1.120924e-15 3.874891e-15
-1.708879e-15
E:\\R-3.1.2\\scripts\\mPDBS\\summary_pdb_93\\126f26a78b57339c.final.pdb
7.578617e-16 1.219659e-15 -6.274681e-16 8.404160e-15 7.224209e-15
1.432452e-14
E:\\R-3.1.2\\scripts\\mPDBS\\summary_pdb_93\\12f06f3e342df706.final.pdb
-1.175899e-15 1.084292e-15 -5.803518e-16 3.445586e-15 3.178770e-15
8.076379e-16
E:\\R-3.1.2\\scripts\\mPDBS\\summary_pdb_93\\18618a2a251b867b.final.pdb
4.298759e-15 -1.053995e-16 -3.760402e-15 -3.499380e-15 3.068512e-15
1.288880e-14
E:\\R-3.1.2\\scripts\\mPDBS\\summary_pdb_93\\1f305f598641eeb2.final.pdb
-1.168835e-15 6.436278e-16 -6.231454e-16 5.324553e-16 2.998147e-15
9.903440e-16
E:\\R-3.1.2\\scripts\\mPDBS\\summary_pdb_93\\1f8a00b551be0a39.final.pdb
4.298759e-15 -1.053995e-16 -3.760402e-15 -3.499380e-15 3.068512e-15
1.288880e-14

E:\\R-3.1.2\\scripts\\mPDBS\\summary_pdb_93\\219f45328d47579a.final.pdb
-1.064961e-15 -1.769690e-15 3.213337e-16 8.932400e-15 8.284515e-15
1.504094e-15
E:\\R-3.1.2\\scripts\\mPDBS\\summary_pdb_93\\238dd3918e54336d.final.pdb
-4.356816e-15 -2.087578e-15 -2.695565e-16 7.942307e-15 3.496245e-15
1.117431e-14
E:\\R-3.1.2\\scripts\\mPDBS\\summary_pdb_93\\2b3027889e2fdc42.final.pdb
-7.547712e-16 5.721872e-16 -1.235364e-15 2.220321e-15 5.540616e-15
6.551636e-16
E:\\R-3.1.2\\scripts\\mPDBS\\summary_pdb_93\\2fcb3b46e33793b0.final.pdb
1.018651e-15 -6.153728e-17 -2.229499e-15 -1.965547e-15 2.367575e-15
-8.812578e-15
E:\\R-3.1.2\\scripts\\mPDBS\\summary_pdb_93\\34eb328251842006.final.pdb
-3.191110e-15 4.401356e-15 -2.577614e-15 -4.841921e-15 -4.791228e-15
-3.477884e-15
E:\\R-3.1.2\\scripts\\mPDBS\\summary_pdb_93\\354b32871797b7ab.final.pdb
2.004911e-15 3.034911e-15 -5.725150e-15 5.667196e-15 9.606845e-15
-2.740482e-15
E:\\R-3.1.2\\scripts\\mPDBS\\summary_pdb_93\\397368adbc32d5de.final.pdb
7.578617e-16 1.219659e-15 -6.274681e-16 8.404160e-15 7.224209e-15
1.432452e-14
E:\\R-3.1.2\\scripts\\mPDBS\\summary_pdb_93\\411ca13cd3df6edd.final.pdb
4.510975e-15 2.482001e-15 -1.856751e-15 -2.941328e-15 3.840789e-15
-2.747335e-15
E:\\R-3.1.2\\scripts\\mPDBS\\summary_pdb_93\\42357eee6642bf12.final.pdb
1.366357e-15 7.432574e-15 -8.469024e-15 -9.900025e-15 4.692074e-16
2.721667e-15
E:\\R-3.1.2\\scripts\\mPDBS\\summary_pdb_93\\44342d1f75a7fcfc.final.pdb
2.390569e-16 -9.184698e-16 -9.092294e-15 -1.086134e-15 5.672840e-15
4.562006e-17
E:\\R-3.1.2\\scripts\\mPDBS\\summary_pdb_93\\448ef21dffe8cdfb.final.pdb
-7.823234e-15 7.677498e-15 -1.310096e-15 1.663495e-15 2.132520e-15
7.965667e-15
E:\\R-3.1.2\\scripts\\mPDBS\\summary_pdb_93\\472a40b3e8f113c7.final.pdb
-2.115235e-16 -1.197678e-15 1.754921e-16 1.120924e-15 3.874891e-15
-1.708879e-15
E:\\R-3.1.2\\scripts\\mPDBS\\summary_pdb_93\\48a26225951ea153.final.pdb
-1.060950e-15 -3.107812e-15 -2.283137e-15 4.055659e-15 7.551161e-15
3.046914e-15
E:\\R-3.1.2\\scripts\\mPDBS\\summary_pdb_93\\4ab4a5c7c6682ce6.final.pdb
-5.200913e-15 -7.235817e-15 2.867312e-16 1.041994e-15 -8.942971e-16
-2.648015e-15
E:\\R-3.1.2\\scripts\\mPDBS\\summary_pdb_93\\4eaa45b62cb6a25f.final.pdb
2.721393e-16 -1.276094e-15 3.353230e-16 1.751491e-15 4.396170e-15
4.590460e-15
E:\\R-3.1.2\\scripts\\mPDBS\\summary_pdb_93\\58c371611d85ac96.final.pdb
7.578617e-16 1.219659e-15 -6.274681e-16 8.404160e-15 7.224209e-15
1.432452e-14
E:\\R-3.1.2\\scripts\\mPDBS\\summary_pdb_93\\59a647f04822d30a.final.pdb
-1.435819e-15 6.831136e-16 3.153987e-15 7.589218e-15 8.312720e-17
2.764799e-15
E:\\R-3.1.2\\scripts\\mPDBS\\summary_pdb_93\\59b091fe385e05fc.final.pdb
5.387904e-16 2.930544e-15 -2.196617e-15 -6.484778e-16 3.617676e-15
-3.831048e-15

E:\\R-3.1.2\\scripts\\mPDBS\\summary_pdb_93\\5c35ac30f930d2db.final.pdb
2.721393e-16 -1.276094e-15 3.353230e-16 1.751491e-15 4.396170e-15
4.590460e-15
E:\\R-3.1.2\\scripts\\mPDBS\\summary_pdb_93\\5f60752431cf2dcb.final.pdb
-2.735741e-15 -8.379982e-16 -1.222850e-15 9.159255e-16 -5.712687e-16
3.469788e-15
E:\\R-3.1.2\\scripts\\mPDBS\\summary_pdb_93\\626456dd6ff2868d.final.pdb
-2.115235e-16 -1.197678e-15 1.754921e-16 1.120924e-15 3.874891e-15
-1.708879e-15
E:\\R-3.1.2\\scripts\\mPDBS\\summary_pdb_93\\6583d53ff3c8f7ef.final.pdb
-1.435819e-15 6.831136e-16 3.153987e-15 7.589218e-15 8.312720e-17
2.764799e-15
E:\\R-3.1.2\\scripts\\mPDBS\\summary_pdb_93\\69760178986bef01.final.pdb
-9.259252e-15 -4.875075e-15 -5.568588e-17 7.104648e-15 9.256859e-15
1.672943e-15
E:\\R-3.1.2\\scripts\\mPDBS\\summary_pdb_93\\6a9d87d47926b1c8.final.pdb
6.086754e-16 5.912558e-16 -1.009975e-15 3.206929e-15 4.789958e-15
7.693907e-16
E:\\R-3.1.2\\scripts\\mPDBS\\summary_pdb_93\\6bb3547ea982ffc4.final.pdb
-1.298601e-14 1.782268e-16 -1.257496e-14 1.240177e-14 4.806901e-15
6.530796e-15
E:\\R-3.1.2\\scripts\\mPDBS\\summary_pdb_93\\716d9624611d1c42.final.pdb
8.251041e-16 -5.730192e-16 -8.548718e-16 2.834963e-15 1.045550e-14
4.248462e-15
E:\\R-3.1.2\\scripts\\mPDBS\\summary_pdb_93\\75f27e70ad0f1be4.final.pdb
-1.435819e-15 6.831136e-16 3.153987e-15 7.589218e-15 8.312720e-17
2.764799e-15
E:\\R-3.1.2\\scripts\\mPDBS\\summary_pdb_93\\769f910fffb22872.final.pdb
-8.974964e-16 8.521679e-16 -1.561207e-15 3.244357e-15 3.310072e-15
6.609524e-16
E:\\R-3.1.2\\scripts\\mPDBS\\summary_pdb_93\\791f8c80dd5c3135.final.pdb
5.387904e-16 2.930544e-15 -2.196617e-15 -6.484778e-16 3.617676e-15
-3.831048e-15
E:\\R-3.1.2\\scripts\\mPDBS\\summary_pdb_93\\7fad98e66a3f6099.final.pdb
-6.419519e-15 -1.692739e-15 -3.076255e-15 1.473251e-14 7.962213e-15
-1.199771e-14
E:\\R-3.1.2\\scripts\\mPDBS\\summary_pdb_93\\804b89c94a633196.final.pdb
1.018651e-15 -6.153728e-17 -2.229499e-15 -1.965547e-15 2.367575e-15
-8.812578e-15
E:\\R-3.1.2\\scripts\\mPDBS\\summary_pdb_93\\813cc65a2298feba.final.pdb
1.018651e-15 -6.153728e-17 -2.229499e-15 -1.965547e-15 2.367575e-15
-8.812578e-15
E:\\R-3.1.2\\scripts\\mPDBS\\summary_pdb_93\\83c0eefcfe12d3f9.final.pdb
4.510975e-15 2.482001e-15 -1.856751e-15 -2.941328e-15 3.840789e-15
-2.747335e-15
E:\\R-3.1.2\\scripts\\mPDBS\\summary_pdb_93\\86d50469ef569760.final.pdb
-2.115235e-16 -1.197678e-15 1.754921e-16 1.120924e-15 3.874891e-15
-1.708879e-15
E:\\R-3.1.2\\scripts\\mPDBS\\summary_pdb_93\\89d0666e1402203d.final.pdb
-1.435819e-15 6.831136e-16 3.153987e-15 7.589218e-15 8.312720e-17
2.764799e-15
E:\\R-3.1.2\\scripts\\mPDBS\\summary_pdb_93\\8b34852716dadc62.final.pdb
-2.671105e-15 2.342331e-16 2.624257e-15 4.210069e-15 2.554058e-15
7.603110e-15

E:\\R-3.1.2\\scripts\\mPDBS\\summary_pdb_93\\8b6ef9ac3fe74395.final.pdb
-1.114413e-14 9.173644e-15 -4.723159e-15 1.613129e-14 -3.315898e-15
-4.172318e-16
E:\\R-3.1.2\\scripts\\mPDBS\\summary_pdb_93\\98bbe0d5986c138f.final.pdb
5.387904e-16 2.930544e-15 -2.196617e-15 -6.484778e-16 3.617676e-15
-3.831048e-15
E:\\R-3.1.2\\scripts\\mPDBS\\summary_pdb_93\\9c22f4909d67e2fa.final.pdb
1.103002e-15 -1.660085e-15 1.331940e-16 -7.430001e-16 8.126424e-15
7.131699e-15
E:\\R-3.1.2\\scripts\\mPDBS\\summary_pdb_93\\9dda8ed0d81fb3ef.final.pdb
-4.326193e-15 -8.031487e-15 -1.028470e-14 -2.493429e-15 7.726798e-16
-1.832869e-15
E:\\R-3.1.2\\scripts\\mPDBS\\summary_pdb_93\\9f752b3616e6033b.final.pdb
-3.119764e-15 1.772207e-15 -1.759394e-15 2.217452e-15 1.635661e-15
-2.801403e-15
E:\\R-3.1.2\\scripts\\mPDBS\\summary_pdb_93\\a02c70083816d6ba.final.pdb
2.721393e-16 -1.276094e-15 3.353230e-16 1.751491e-15 4.396170e-15
4.590460e-15
E:\\R-3.1.2\\scripts\\mPDBS\\summary_pdb_93\\a0c997d891e4e082.final.pdb
-2.487651e-15 3.758059e-16 3.009126e-15 7.356159e-15 5.106680e-15
-9.537634e-15
E:\\R-3.1.2\\scripts\\mPDBS\\summary_pdb_93\\ab8039a2d823c616.final.pdb
-5.406405e-16 2.277039e-16 -1.710406e-15 4.291336e-15 1.680167e-15
7.115463e-16
E:\\R-3.1.2\\scripts\\mPDBS\\summary_pdb_93\\acf4d74b9c71d4dc.final.pdb
1.018651e-15 -6.153728e-17 -2.229499e-15 -1.965547e-15 2.367575e-15
-8.812578e-15
E:\\R-3.1.2\\scripts\\mPDBS\\summary_pdb_93\\ad8a823e4b4c668e.final.pdb
-1.115560e-15 8.859251e-16 -1.076606e-15 1.548758e-15 3.776887e-15
9.449409e-16
E:\\R-3.1.2\\scripts\\mPDBS\\summary_pdb_93\\b16ababd03b7b857.final.pdb
1.103002e-15 -1.660085e-15 1.331940e-16 -7.430001e-16 8.126424e-15
7.131699e-15
E:\\R-3.1.2\\scripts\\mPDBS\\summary_pdb_93\\b226923e697d3c63.final.pdb
-5.035132e-16 5.088010e-15 -4.607432e-16 6.042712e-15 1.916296e-15
3.146221e-15
E:\\R-3.1.2\\scripts\\mPDBS\\summary_pdb_93\\b28e48f6fa27a165.final.pdb
5.387904e-16 2.930544e-15 -2.196617e-15 -6.484778e-16 3.617676e-15
-3.831048e-15
E:\\R-3.1.2\\scripts\\mPDBS\\summary_pdb_93\\b42c4924e3cd1aec.final.pdb
2.582018e-15 9.135120e-15 1.021795e-15 5.978961e-15 5.566588e-15
-1.621449e-15
E:\\R-3.1.2\\scripts\\mPDBS\\summary_pdb_93\\b4dd239fcabff1df.final.pdb
5.387904e-16 2.930544e-15 -2.196617e-15 -6.484778e-16 3.617676e-15
-3.831048e-15
E:\\R-3.1.2\\scripts\\mPDBS\\summary_pdb_93\\b669942ef97459a2.final.pdb
-2.671105e-15 2.342331e-16 2.624257e-15 4.210069e-15 2.554058e-15
7.603110e-15
E:\\R-3.1.2\\scripts\\mPDBS\\summary_pdb_93\\b99f4d9d87c1017b.final.pdb
5.387904e-16 2.930544e-15 -2.196617e-15 -6.484778e-16 3.617676e-15
-3.831048e-15
E:\\R-3.1.2\\scripts\\mPDBS\\summary_pdb_93\\b9d2cbf3975403e7.final.pdb
5.387904e-16 2.930544e-15 -2.196617e-15 -6.484778e-16 3.617676e-15
-3.831048e-15

E:\\R-3.1.2\\scripts\\mPDBS\\summary_pdb_93\\bc88c8a505f3a288.final.pdb
-1.157552e-15 8.083264e-16 -1.205657e-15 3.191766e-15 4.455113e-15
6.508268e-16
E:\\R-3.1.2\\scripts\\mPDBS\\summary_pdb_93\\bdac0e64f4ef427e.final.pdb
-5.514642e-15 -3.777315e-15 1.450500e-15 1.086837e-14 1.253883e-15
9.874730e-15
E:\\R-3.1.2\\scripts\\mPDBS\\summary_pdb_93\\ca2a1f55fc9d0681.final.pdb
-2.671105e-15 2.342331e-16 2.624257e-15 4.210069e-15 2.554058e-15
7.603110e-15
E:\\R-3.1.2\\scripts\\mPDBS\\summary_pdb_93\\ce34248de3e81a3c.final.pdb
5.451304e-15 5.046212e-15 -3.569654e-16 -2.335818e-15 5.357121e-15
4.613996e-15
E:\\R-3.1.2\\scripts\\mPDBS\\summary_pdb_93\\d02e697772494a61.final.pdb
-2.559298e-16 8.180842e-16 4.993161e-15 4.854499e-15 3.431193e-15
5.672634e-15
E:\\R-3.1.2\\scripts\\mPDBS\\summary_pdb_93\\d04221245d015047.final.pdb
-8.917052e-16 -6.789060e-16 -1.879325e-15 5.282873e-16 3.580123e-15
-2.360464e-16
E:\\R-3.1.2\\scripts\\mPDBS\\summary_pdb_93\\d678141e4018054a.final.pdb
-3.541791e-15 -3.027971e-15 -5.136776e-15 5.525550e-15 4.579803e-15
9.979128e-15
E:\\R-3.1.2\\scripts\\mPDBS\\summary_pdb_93\\d71b40af0f0efe39.final.pdb
1.143268e-14 -7.609075e-15 8.792440e-15 -1.630818e-15 6.291515e-15
-2.869901e-15
E:\\R-3.1.2\\scripts\\mPDBS\\summary_pdb_93\\dc0809eff10197e2.final.pdb
9.425697e-16 4.251966e-15 8.372216e-15 5.633594e-15 -1.807924e-15
-6.025188e-15
E:\\R-3.1.2\\scripts\\mPDBS\\summary_pdb_93\\e1163918d3707839.final.pdb
7.137206e-16 5.306152e-15 -1.979710e-15 -8.454412e-16 6.356366e-16
-9.100709e-15
E:\\R-3.1.2\\scripts\\mPDBS\\summary_pdb_93\\e4b48b06824ef848.final.pdb
4.298759e-15 -1.053995e-16 -3.760402e-15 -3.499380e-15 3.068512e-15
1.288880e-14
E:\\R-3.1.2\\scripts\\mPDBS\\summary_pdb_93\\e529e19ad281fea2.final.pdb
1.089519e-15 2.116945e-15 -5.755528e-15 -1.717084e-15 1.000193e-14
-4.704746e-15
E:\\R-3.1.2\\scripts\\mPDBS\\summary_pdb_93\\e5602082718ec3a1.final.pdb
1.018651e-15 -6.153728e-17 -2.229499e-15 -1.965547e-15 2.367575e-15
-8.812578e-15
E:\\R-3.1.2\\scripts\\mPDBS\\summary_pdb_93\\e5d589d996d0a960.final.pdb
-8.609355e-15 2.352647e-15 1.209439e-15 -8.479863e-15 5.138475e-15
1.162997e-15
E:\\R-3.1.2\\scripts\\mPDBS\\summary_pdb_93\\e9df9e72307a2fbd.final.pdb
2.721393e-16 -1.276094e-15 3.353230e-16 1.751491e-15 4.396170e-15
4.590460e-15
E:\\R-3.1.2\\scripts\\mPDBS\\summary_pdb_93\\ea00f8694090cae7.final.pdb
1.018651e-15 -6.153728e-17 -2.229499e-15 -1.965547e-15 2.367575e-15
-8.812578e-15
E:\\R-3.1.2\\scripts\\mPDBS\\summary_pdb_93\\eba517be32db050e.final.pdb
5.387904e-16 2.930544e-15 -2.196617e-15 -6.484778e-16 3.617676e-15
-3.831048e-15
E:\\R-3.1.2\\scripts\\mPDBS\\summary_pdb_93\\ec04b9545899c29d.final.pdb
-1.056782e-15 8.769690e-16 -1.102157e-15 3.432780e-16 3.055142e-15
6.965750e-16

E:\\R-3.1.2\\scripts\\mPDBS\\summary_pdb_93\\ee6862cbcad52078.final.pdb
-5.670958e-16 7.565015e-16 -1.415992e-15 2.701273e-15 5.351098e-15
6.480079e-16
E:\\R-3.1.2\\scripts\\mPDBS\\summary_pdb_93\\ef6a42fc83b81e5d.final.pdb
-1.234683e-15 6.648178e-16 -1.117768e-15 4.596923e-16 2.772742e-15
8.634814e-16
E:\\R-3.1.2\\scripts\\mPDBS\\summary_pdb_93\\ef8e88d4ad93a0ad.final.pdb
-8.917052e-16 -6.789060e-16 -1.879325e-15 5.282873e-16 3.580123e-15
-2.360464e-16
E:\\R-3.1.2\\scripts\\mPDBS\\summary_pdb_93\\f14fcae8deec4fa3.final.pdb
-3.119764e-15 1.772207e-15 -1.759394e-15 2.217452e-15 1.635661e-15
-2.801403e-15
E:\\R-3.1.2\\scripts\\mPDBS\\summary_pdb_93\\f83584e22003fb92.final.pdb
-1.060950e-15 -3.107812e-15 -2.283137e-15 4.055659e-15 7.551161e-15
3.046914e-15
E:\\R-3.1.2\\scripts\\mPDBS\\summary_pdb_93\\f8e2bf87e52caa29.final.pdb
1.089519e-15 2.116945e-15 -5.755528e-15 -1.717084e-15 1.000193e-14
-4.704746e-15

[,421]   [,422]   [,423]   [,424]   [,425]   [,426]
E:\\R-3.1.2\\scripts\\40.1ns_data\\NAT0.pdb
2.101406e-15 1.645458e-15 2.096550e-15 -3.773798e-15 -2.759502e-15
3.243479e-15
E:\\R-3.1.2\\scripts\\40.1ns_data\\LJP6.pdb
1.532089e-15 1.525934e-15 2.110792e-15 -5.006908e-15 -2.485776e-15
2.626323e-15
E:\\R-3.1.2\\scripts\\40.1ns_data\\3WY9.pdb
2.218948e-15 1.978121e-15 4.290272e-15 -3.629972e-15 -3.198957e-15
3.260054e-15
E:\\R-3.1.2\\scripts\\40.1ns_data\\ZSP9.pdb
1.437461e-15 4.774178e-16 1.490519e-16 -4.131756e-15 -2.931128e-15
3.153911e-15
E:\\R-3.1.2\\scripts\\mPDBS\\summary_pdb_93\\013d855ad26c877c.final.pdb
-6.461622e-16 5.486557e-15 5.061185e-15 -6.691956e-15 -9.702341e-15
-4.792859e-15
E:\\R-3.1.2\\scripts\\mPDBS\\summary_pdb_93\\0a2a705342ca0d54.final.pdb
1.708958e-15 4.192585e-16 5.851745e-16 -4.193180e-15 -1.754916e-15
2.792383e-15
E:\\R-3.1.2\\scripts\\mPDBS\\summary_pdb_93\\0a59fceadb4194d9.final.pdb
5.038215e-15 1.183497e-15 -8.767795e-17 -4.005818e-15 -2.436181e-15
2.721150e-15
E:\\R-3.1.2\\scripts\\mPDBS\\summary_pdb_93\\0dba59f3306825e8.final.pdb
4.015040e-15 -9.163069e-16 3.954431e-15 -4.628069e-15 -1.163165e-15
1.795620e-15
E:\\R-3.1.2\\scripts\\mPDBS\\summary_pdb_93\\126f26a78b57339c.final.pdb
1.161853e-15 -9.833106e-16 -2.462311e-15 2.169879e-15 2.229087e-15
6.446414e-15
E:\\R-3.1.2\\scripts\\mPDBS\\summary_pdb_93\\12f06f3e342df706.final.pdb
1.216297e-15 -3.372121e-17 1.114265e-15 -3.484545e-15 -2.023581e-15
2.807345e-15
E:\\R-3.1.2\\scripts\\mPDBS\\summary_pdb_93\\18618a2a251b867b.final.pdb
-8.654128e-16 -2.603823e-15 5.681753e-15 -2.310479e-15 2.844820e-15
-1.175023e-15
E:\\R-3.1.2\\scripts\\mPDBS\\summary_pdb_93\\1f305f598641eeb2.final.pdb

1.627323e-15 7.172054e-16 7.592662e-16 -4.021944e-15 -2.224416e-15
2.803895e-15
E:\\R-3.1.2\\scripts\\mPDBS\\summary_pdb_93\\1f8a00b551be0a39.final.pdb
-8.654128e-16 -2.603823e-15 5.681753e-15 -2.310479e-15 2.844820e-15
-1.175023e-15
E:\\R-3.1.2\\scripts\\mPDBS\\summary_pdb_93\\219f45328d47579a.final.pdb
1.102997e-16 4.638577e-17 -3.478865e-16 -5.680693e-15 -1.337885e-15
7.722358e-15
E:\\R-3.1.2\\scripts\\mPDBS\\summary_pdb_93\\238dd3918e54336d.final.pdb
-4.196583e-16 8.015922e-15 2.092002e-15 2.705394e-15 -2.833650e-15
2.628342e-15
E:\\R-3.1.2\\scripts\\mPDBS\\summary_pdb_93\\2b3027889e2fdc42.final.pdb
2.296485e-15 9.783660e-16 -1.535974e-16 -3.315398e-15 -1.845508e-15
2.624873e-15
E:\\R-3.1.2\\scripts\\mPDBS\\summary_pdb_93\\2fcb3b46e33793b0.final.pdb
2.208889e-15 5.472025e-16 4.971175e-15 -8.191352e-15 -3.875873e-15
4.359976e-16
E:\\R-3.1.2\\scripts\\mPDBS\\summary_pdb_93\\34eb328251842006.final.pdb
5.871609e-15 2.790244e-15 -1.031077e-14 -4.385645e-15 -2.500664e-15
-1.691854e-16
E:\\R-3.1.2\\scripts\\mPDBS\\summary_pdb_93\\354b32871797b7ab.final.pdb
3.281322e-15 6.199909e-15 -4.921917e-15 -8.912046e-15 -1.596667e-16
1.355061e-15
E:\\R-3.1.2\\scripts\\mPDBS\\summary_pdb_93\\397368adbc32d5de.final.pdb
1.161853e-15 -9.833106e-16 -2.462311e-15 2.169879e-15 2.229087e-15
6.446414e-15
E:\\R-3.1.2\\scripts\\mPDBS\\summary_pdb_93\\411ca13cd3df6edd.final.pdb
3.674297e-15 -1.198580e-15 5.125565e-15 -8.920803e-15 1.781060e-15
1.053559e-15
E:\\R-3.1.2\\scripts\\mPDBS\\summary_pdb_93\\42357eee6642bf12.final.pdb
2.537798e-15 2.312667e-15 -1.130422e-14 -2.692987e-15 1.612803e-15
-4.203402e-16
E:\\R-3.1.2\\scripts\\mPDBS\\summary_pdb_93\\44342d1f75a7fcfc.final.pdb
-1.572573e-15 -3.703866e-15 6.610991e-16 1.365904e-15 -2.937164e-15
-2.072017e-15
E:\\R-3.1.2\\scripts\\mPDBS\\summary_pdb_93\\448ef21dffe8cdfb.final.pdb
-1.345993e-15 1.509806e-14 -5.312266e-15 -4.430905e-15 -2.342377e-15
1.084087e-14
E:\\R-3.1.2\\scripts\\mPDBS\\summary_pdb_93\\472a40b3e8f113c7.final.pdb
4.015040e-15 -9.163069e-16 3.954431e-15 -4.628069e-15 -1.163165e-15
1.795620e-15
E:\\R-3.1.2\\scripts\\mPDBS\\summary_pdb_93\\48a26225951ea153.final.pdb
-1.414956e-15 -8.240117e-16 3.104213e-15 -3.028735e-15 -5.076025e-16
1.215410e-15
E:\\R-3.1.2\\scripts\\mPDBS\\summary_pdb_93\\4ab4a5c7c6682ce6.final.pdb
1.984546e-15 -3.171768e-16 5.968445e-15 -9.764585e-15 4.449239e-16
-5.359491e-17
E:\\R-3.1.2\\scripts\\mPDBS\\summary_pdb_93\\4eaa45b62cb6a25f.final.pdb
1.779018e-16 -1.854629e-15 3.346642e-15 -2.335709e-15 -3.557556e-15
2.462681e-15
E:\\R-3.1.2\\scripts\\mPDBS\\summary_pdb_93\\58c371611d85ac96.final.pdb
1.161853e-15 -9.833106e-16 -2.462311e-15 2.169879e-15 2.229087e-15
6.446414e-15
E:\\R-3.1.2\\scripts\\mPDBS\\summary_pdb_93\\59a647f04822d30a.final.pdb

-6.161350e-15 3.102839e-16 -2.407627e-15 -6.595609e-15 -3.427223e-15
6.670122e-15
E:\\R-3.1.2\\scripts\\mPDBS\\summary_pdb_93\\59b091fe385e05fc.final.pdb
5.038215e-15 1.183497e-15 -8.767795e-17 -4.005818e-15 -2.436181e-15
2.721150e-15
E:\\R-3.1.2\\scripts\\mPDBS\\summary_pdb_93\\5c35ac30f930d2db.final.pdb
1.779018e-16 -1.854629e-15 3.346642e-15 -2.335709e-15 -3.557556e-15
2.462681e-15
E:\\R-3.1.2\\scripts\\mPDBS\\summary_pdb_93\\5f60752431cf2dcb.final.pdb
4.172699e-15 -3.670786e-15 4.172583e-15 -6.334696e-15 -2.429723e-15
4.441238e-15
E:\\R-3.1.2\\scripts\\mPDBS\\summary_pdb_93\\626456dd6ff2868d.final.pdb
4.015040e-15 -9.163069e-16 3.954431e-15 -4.628069e-15 -1.163165e-15
1.795620e-15
E:\\R-3.1.2\\scripts\\mPDBS\\summary_pdb_93\\6583d53ff3c8f7ef.final.pdb
-6.161350e-15 3.102839e-16 -2.407627e-15 -6.595609e-15 -3.427223e-15
6.670122e-15
E:\\R-3.1.2\\scripts\\mPDBS\\summary_pdb_93\\69760178986bef01.final.pdb
-5.016126e-15 -4.824965e-15 1.221447e-14 -4.167517e-15 -7.874083e-16
6.921847e-15
E:\\R-3.1.2\\scripts\\mPDBS\\summary_pdb_93\\6a9d87d47926b1c8.final.pdb
2.012735e-15 1.205910e-15 6.116189e-16 -3.712568e-15 -2.084303e-15
2.728902e-15
E:\\R-3.1.2\\scripts\\mPDBS\\summary_pdb_93\\6bb3547ea982ffc4.final.pdb
-2.829612e-15 6.553992e-15 1.903121e-15 -3.755927e-15 -5.678126e-16
6.414584e-15
E:\\R-3.1.2\\scripts\\mPDBS\\summary_pdb_93\\716d9624611d1c42.final.pdb
4.343703e-16 1.889437e-15 1.514986e-15 -1.749822e-15 -2.896147e-15
-2.828121e-15
E:\\R-3.1.2\\scripts\\mPDBS\\summary_pdb_93\\75f27e70ad0f1be4.final.pdb
-6.161350e-15 3.102839e-16 -2.407627e-15 -6.595609e-15 -3.427223e-15
6.670122e-15
E:\\R-3.1.2\\scripts\\mPDBS\\summary_pdb_93\\769f910fffb22872.final.pdb
1.263960e-15 1.266995e-15 7.369236e-16 -3.252000e-15 -2.430041e-15
3.923329e-15
E:\\R-3.1.2\\scripts\\mPDBS\\summary_pdb_93\\791f8c80dd5c3135.final.pdb
5.038215e-15 1.183497e-15 -8.767795e-17 -4.005818e-15 -2.436181e-15
2.721150e-15
E:\\R-3.1.2\\scripts\\mPDBS\\summary_pdb_93\\7fad98e66a3f6099.final.pdb
4.780554e-15 6.320368e-15 -8.236197e-15 -5.838836e-15 -4.925498e-15
5.197895e-15
E:\\R-3.1.2\\scripts\\mPDBS\\summary_pdb_93\\804b89c94a633196.final.pdb
2.208889e-15 5.472025e-16 4.971175e-15 -8.191352e-15 -3.875873e-15
4.359976e-16
E:\\R-3.1.2\\scripts\\mPDBS\\summary_pdb_93\\813cc65a2298feba.final.pdb
2.208889e-15 5.472025e-16 4.971175e-15 -8.191352e-15 -3.875873e-15
4.359976e-16
E:\\R-3.1.2\\scripts\\mPDBS\\summary_pdb_93\\83c0eefcfe12d3f9.final.pdb
3.674297e-15 -1.198580e-15 5.125565e-15 -8.920803e-15 1.781060e-15
1.053559e-15
E:\\R-3.1.2\\scripts\\mPDBS\\summary_pdb_93\\86d50469ef569760.final.pdb
4.015040e-15 -9.163069e-16 3.954431e-15 -4.628069e-15 -1.163165e-15
1.795620e-15
E:\\R-3.1.2\\scripts\\mPDBS\\summary_pdb_93\\89d0666e1402203d.final.pdb

-6.161350e-15 3.102839e-16 -2.407627e-15 -6.595609e-15 -3.427223e-15
6.670122e-15
E:\\R-3.1.2\\scripts\\mPDBS\\summary_pdb_93\\8b34852716dadc62.final.pdb
2.782649e-15 -2.439991e-15 -2.057925e-15 -7.537248e-16 4.168040e-15
8.958694e-15
E:\\R-3.1.2\\scripts\\mPDBS\\summary_pdb_93\\8b6ef9ac3fe74395.final.pdb
-5.740453e-15 4.651617e-15 -3.127246e-15 -5.858711e-15 -4.611308e-15
1.220220e-14
E:\\R-3.1.2\\scripts\\mPDBS\\summary_pdb_93\\98bbe0d5986c138f.final.pdb
5.038215e-15 1.183497e-15 -8.767795e-17 -4.005818e-15 -2.436181e-15
2.721150e-15
E:\\R-3.1.2\\scripts\\mPDBS\\summary_pdb_93\\9c22f4909d67e2fa.final.pdb
3.221100e-15 2.167217e-15 -4.287912e-15 5.375921e-15 -6.799749e-15
2.092658e-15
E:\\R-3.1.2\\scripts\\mPDBS\\summary_pdb_93\\9dda8ed0d81fb3ef.final.pdb
2.628494e-15 -1.754208e-14 5.427252e-15 -1.363250e-15 -3.949267e-16
-2.004669e-16
E:\\R-3.1.2\\scripts\\mPDBS\\summary_pdb_93\\9f752b3616e6033b.final.pdb
8.155054e-16 1.957094e-15 -1.113211e-15 -3.155734e-15 -1.348273e-15
5.128855e-15
E:\\R-3.1.2\\scripts\\mPDBS\\summary_pdb_93\\a02c70083816d6ba.final.pdb
1.779018e-16 -1.854629e-15 3.346642e-15 -2.335709e-15 -3.557556e-15
2.462681e-15
E:\\R-3.1.2\\scripts\\mPDBS\\summary_pdb_93\\a0c997d891e4e082.final.pdb
4.870452e-15 5.459182e-15 3.251065e-15 2.619825e-15 -4.334526e-15
1.464388e-14
E:\\R-3.1.2\\scripts\\mPDBS\\summary_pdb_93\\ab8039a2d823c616.final.pdb
1.921422e-15 7.929929e-16 6.351098e-17 -2.730062e-15 -2.358026e-15
2.963118e-15
E:\\R-3.1.2\\scripts\\mPDBS\\summary_pdb_93\\acf4d74b9c71d4dc.final.pdb
2.208889e-15 5.472025e-16 4.971175e-15 -8.191352e-15 -3.875873e-15
4.359976e-16
E:\\R-3.1.2\\scripts\\mPDBS\\summary_pdb_93\\ad8a823e4b4c668e.final.pdb
1.541486e-15 7.441994e-16 -1.888610e-16 -3.937179e-15 -2.056080e-15
2.643079e-15
E:\\R-3.1.2\\scripts\\mPDBS\\summary_pdb_93\\b16ababd03b7b857.final.pdb
3.221100e-15 2.167217e-15 -4.287912e-15 5.375921e-15 -6.799749e-15
2.092658e-15
E:\\R-3.1.2\\scripts\\mPDBS\\summary_pdb_93\\b226923e697d3c63.final.pdb
3.806371e-15 2.942944e-15 3.591914e-15 7.834708e-16 -2.734961e-16
9.857677e-15
E:\\R-3.1.2\\scripts\\mPDBS\\summary_pdb_93\\b28e48f6fa27a165.final.pdb
5.038215e-15 1.183497e-15 -8.767795e-17 -4.005818e-15 -2.436181e-15
2.721150e-15
E:\\R-3.1.2\\scripts\\mPDBS\\summary_pdb_93\\b42c4924e3cd1aec.final.pdb
7.218138e-15 1.082076e-14 -2.585436e-15 -3.479612e-15 -6.951965e-15
-2.462330e-15
E:\\R-3.1.2\\scripts\\mPDBS\\summary_pdb_93\\b4dd239fcabff1df.final.pdb
5.038215e-15 1.183497e-15 -8.767795e-17 -4.005818e-15 -2.436181e-15
2.721150e-15
E:\\R-3.1.2\\scripts\\mPDBS\\summary_pdb_93\\b669942ef97459a2.final.pdb
2.782649e-15 -2.439991e-15 -2.057925e-15 -7.537248e-16 4.168040e-15
8.958694e-15
E:\\R-3.1.2\\scripts\\mPDBS\\summary_pdb_93\\b99f4d9d87c1017b.final.pdb

5.038215e-15 1.183497e-15 -8.767795e-17 -4.005818e-15 -2.436181e-15
2.721150e-15
E:\\R-3.1.2\\scripts\\mPDBS\\summary_pdb_93\\b9d2cbf3975403e7.final.pdb
5.038215e-15 1.183497e-15 -8.767795e-17 -4.005818e-15 -2.436181e-15
2.721150e-15
E:\\R-3.1.2\\scripts\\mPDBS\\summary_pdb_93\\bc88c8a505f3a288.final.pdb
1.468588e-15 1.088629e-15 1.347806e-15 -3.661475e-15 -1.745978e-15
2.466362e-15
E:\\R-3.1.2\\scripts\\mPDBS\\summary_pdb_93\\bdac0e64f4ef427e.final.pdb
-2.474864e-15 6.028061e-15 -6.880456e-15 4.734073e-15 -1.184237e-14
7.524511e-15
E:\\R-3.1.2\\scripts\\mPDBS\\summary_pdb_93\\ca2a1f55fc9d0681.final.pdb
2.782649e-15 -2.439991e-15 -2.057925e-15 -7.537248e-16 4.168040e-15
8.958694e-15
E:\\R-3.1.2\\scripts\\mPDBS\\summary_pdb_93\\ce34248de3e81a3c.final.pdb
2.177429e-15 1.238447e-15 -3.607779e-15 -2.779261e-15 -5.864117e-15
-1.367366e-15
E:\\R-3.1.2\\scripts\\mPDBS\\summary_pdb_93\\d02e697772494a61.final.pdb
-4.900449e-15 1.051306e-14 -2.876246e-15 -5.283008e-15 1.088126e-15
1.091685e-14
E:\\R-3.1.2\\scripts\\mPDBS\\summary_pdb_93\\d04221245d015047.final.pdb
5.561943e-16 -2.862220e-16 -8.047181e-16 -5.023366e-15 -2.477505e-15
1.272630e-15
E:\\R-3.1.2\\scripts\\mPDBS\\summary_pdb_93\\d678141e4018054a.final.pdb
8.659572e-15 1.121053e-15 7.782062e-15 -2.856671e-15 3.198075e-15
-4.617037e-15
E:\\R-3.1.2\\scripts\\mPDBS\\summary_pdb_93\\d71b40af0f0efe39.final.pdb
1.506605e-14 1.857822e-15 6.282188e-15 -4.766633e-15 1.809857e-15
-2.628112e-15
E:\\R-3.1.2\\scripts\\mPDBS\\summary_pdb_93\\dc0809eff10197e2.final.pdb
-2.364421e-15 -5.240038e-15 -6.989366e-15 -5.248653e-15 -4.829024e-15
3.794512e-15
E:\\R-3.1.2\\scripts\\mPDBS\\summary_pdb_93\\e1163918d3707839.final.pdb
1.315263e-14 5.343367e-15 2.889351e-15 -8.996248e-17 -1.773381e-15
1.476360e-15
E:\\R-3.1.2\\scripts\\mPDBS\\summary_pdb_93\\e4b48b06824ef848.final.pdb
-8.654128e-16 -2.603823e-15 5.681753e-15 -2.310479e-15 2.844820e-15
-1.175023e-15
E:\\R-3.1.2\\scripts\\mPDBS\\summary_pdb_93\\e529e19ad281fea2.final.pdb
-6.461622e-16 5.486557e-15 5.061185e-15 -6.691956e-15 -9.702341e-15
-4.792859e-15
E:\\R-3.1.2\\scripts\\mPDBS\\summary_pdb_93\\e5602082718ec3a1.final.pdb
2.208889e-15 5.472025e-16 4.971175e-15 -8.191352e-15 -3.875873e-15
4.359976e-16
E:\\R-3.1.2\\scripts\\mPDBS\\summary_pdb_93\\e5d589d996d0a960.final.pdb
2.103575e-15 3.602283e-15 -3.601434e-16 -2.582216e-15 -4.267652e-15
8.185068e-15
E:\\R-3.1.2\\scripts\\mPDBS\\summary_pdb_93\\e9df9e72307a2fbd.final.pdb
1.779018e-16 -1.854629e-15 3.346642e-15 -2.335709e-15 -3.557556e-15
2.462681e-15
E:\\R-3.1.2\\scripts\\mPDBS\\summary_pdb_93\\ea00f8694090cae7.final.pdb
2.208889e-15 5.472025e-16 4.971175e-15 -8.191352e-15 -3.875873e-15
4.359976e-16
E:\\R-3.1.2\\scripts\\mPDBS\\summary_pdb_93\\eba517be32db050e.final.pdb

5.038215e-15 1.183497e-15 -8.767795e-17 -4.005818e-15 -2.436181e-15
2.721150e-15
E:\\R-3.1.2\\scripts\\mPDBS\\summary_pdb_93\\ec04b9545899c29d.final.pdb
1.423295e-15 6.034521e-16 2.204793e-16 -3.338666e-15 -1.645879e-15
3.540681e-15
E:\\R-3.1.2\\scripts\\mPDBS\\summary_pdb_93\\ee6862cbcad52078.final.pdb
1.862370e-15 1.327479e-15 8.091741e-16 -3.423818e-15 -1.764843e-15
2.942327e-15
E:\\R-3.1.2\\scripts\\mPDBS\\summary_pdb_93\\ef6a42fc83b81e5d.final.pdb
1.123448e-15 3.942125e-16 2.572725e-16 -3.653155e-15 -2.265893e-15
2.947547e-15
E:\\R-3.1.2\\scripts\\mPDBS\\summary_pdb_93\\ef8e88d4ad93a0ad.final.pdb
5.561943e-16 -2.862220e-16 -8.047181e-16 -5.023366e-15 -2.477505e-15
1.272630e-15
E:\\R-3.1.2\\scripts\\mPDBS\\summary_pdb_93\\f14fcae8deec4fa3.final.pdb
8.155054e-16 1.957094e-15 -1.113211e-15 -3.155734e-15 -1.348273e-15
5.128855e-15
E:\\R-3.1.2\\scripts\\mPDBS\\summary_pdb_93\\f83584e22003fb92.final.pdb
-1.414956e-15 -8.240117e-16 3.104213e-15 -3.028735e-15 -5.076025e-16
1.215410e-15
E:\\R-3.1.2\\scripts\\mPDBS\\summary_pdb_93\\f8e2bf87e52caa29.final.pdb
-6.461622e-16 5.486557e-15 5.061185e-15 -6.691956e-15 -9.702341e-15
-4.792859e-15

[,427]   [,428]   [,429]   [,430]   [,431]   [,432]
E:\\R-3.1.2\\scripts\\40.1ns_data\\NAT0.pdb
-5.056797e-16 -3.143311e-15 -1.617422e-15 -2.266858e-15 7.179719e-15
1.416024e-15
E:\\R-3.1.2\\scripts\\40.1ns_data\\LJP6.pdb
-4.338807e-16 -4.091202e-15 -9.337065e-16 -2.078876e-15 -3.230116e-16
1.806350e-15
E:\\R-3.1.2\\scripts\\40.1ns_data\\3WY9.pdb
4.724122e-16 -3.864070e-15 -1.491372e-15 -3.666856e-15 6.186004e-15
1.543782e-15
E:\\R-3.1.2\\scripts\\40.1ns_data\\ZSP9.pdb
3.700775e-16 -4.276098e-15 -4.241145e-16 -2.714436e-16 5.671208e-15
1.827721e-15
E:\\R-3.1.2\\scripts\\mPDBS\\summary_pdb_93\\013d855ad26c877c.final.pdb
-3.816285e-16 -4.997813e-15 -2.692240e-15 -1.469688e-15 -7.492981e-16
5.563390e-15
E:\\R-3.1.2\\scripts\\mPDBS\\summary_pdb_93\\0a2a705342ca0d54.final.pdb
9.038696e-16 -3.643667e-15 -6.904654e-17 -3.221984e-17 5.080453e-15
2.182972e-15
E:\\R-3.1.2\\scripts\\mPDBS\\summary_pdb_93\\0a59fceadb4194d9.final.pdb
1.697222e-15 -4.364413e-15 -3.014531e-15 -2.770007e-15 4.180453e-15
2.553193e-15
E:\\R-3.1.2\\scripts\\mPDBS\\summary_pdb_93\\0dba59f3306825e8.final.pdb
1.162275e-15 -2.591317e-15 1.639365e-15 -7.549819e-16 2.950055e-16
-2.935849e-17
E:\\R-3.1.2\\scripts\\mPDBS\\summary_pdb_93\\126f26a78b57339c.final.pdb
-5.313014e-15 5.847265e-17 2.844564e-15 -1.228128e-15 6.867873e-15
5.407265e-15
E:\\R-3.1.2\\scripts\\mPDBS\\summary_pdb_93\\12f06f3e342df706.final.pdb
5.066180e-16 -3.536114e-15 -3.279540e-16 6.248067e-16 3.418588e-15

2.363817e-15
E:\\R-3.1.2\\scripts\\mPDBS\\summary_pdb_93\\18618a2a251b867b.final.pdb
3.283895e-15 7.234054e-16 5.644216e-15 1.763571e-15 2.345642e-15
6.503585e-15
E:\\R-3.1.2\\scripts\\mPDBS\\summary_pdb_93\\1f305f598641eeb2.final.pdb
2.367380e-16 -3.342583e-15 -4.814963e-16 -8.911268e-16 3.940713e-15
2.462639e-15
E:\\R-3.1.2\\scripts\\mPDBS\\summary_pdb_93\\1f8a00b551be0a39.final.pdb
3.283895e-15 7.234054e-16 5.644216e-15 1.763571e-15 2.345642e-15
6.503585e-15
E:\\R-3.1.2\\scripts\\mPDBS\\summary_pdb_93\\219f45328d47579a.final.pdb
8.254477e-15 -8.948420e-15 5.031550e-15 -4.600141e-15 1.272917e-14
-1.411517e-15
E:\\R-3.1.2\\scripts\\mPDBS\\summary_pdb_93\\238dd3918e54336d.final.pdb
-1.230991e-15 -3.652743e-15 1.360607e-14 -2.512401e-15 -1.812149e-15
-9.254636e-15
E:\\R-3.1.2\\scripts\\mPDBS\\summary_pdb_93\\2b3027889e2fdc42.final.pdb
-1.290078e-16 -3.534348e-15 -4.011703e-16 -1.355774e-15 2.841247e-15
2.584852e-15
E:\\R-3.1.2\\scripts\\mPDBS\\summary_pdb_93\\2fcb3b46e33793b0.final.pdb
3.758227e-15 -6.329343e-15 -3.316034e-15 -7.905129e-16 9.257631e-16
-1.083136e-15
E:\\R-3.1.2\\scripts\\mPDBS\\summary_pdb_93\\34eb328251842006.final.pdb
4.217575e-15 -7.419509e-15 -9.415678e-15 -4.660926e-15 1.039542e-14
3.120635e-15
E:\\R-3.1.2\\scripts\\mPDBS\\summary_pdb_93\\354b32871797b7ab.final.pdb
7.271620e-15 -1.251681e-14 -1.346961e-14 -9.624315e-16 1.193022e-15
-1.658377e-15
E:\\R-3.1.2\\scripts\\mPDBS\\summary_pdb_93\\397368adbc32d5de.final.pdb
-5.313014e-15 5.847265e-17 2.844564e-15 -1.228128e-15 6.867873e-15
5.407265e-15
E:\\R-3.1.2\\scripts\\mPDBS\\summary_pdb_93\\411ca13cd3df6edd.final.pdb
5.884531e-16 -7.845058e-15 -6.896070e-15 -5.431332e-15 2.273643e-15
1.914466e-15
E:\\R-3.1.2\\scripts\\mPDBS\\summary_pdb_93\\42357eee6642bf12.final.pdb
1.013291e-14 -8.765054e-15 -1.656216e-15 3.232485e-15 1.082569e-14
2.113375e-15
E:\\R-3.1.2\\scripts\\mPDBS\\summary_pdb_93\\44342d1f75a7fcfc.final.pdb
-1.191658e-16 6.204391e-15 1.132282e-15 8.916267e-15 9.009288e-15
4.669798e-16
E:\\R-3.1.2\\scripts\\mPDBS\\summary_pdb_93\\448ef21dffe8cdfb.final.pdb
-6.761137e-15 -1.253705e-14 -9.710225e-15 -1.651752e-14 3.944182e-15
1.145198e-14
E:\\R-3.1.2\\scripts\\mPDBS\\summary_pdb_93\\472a40b3e8f113c7.final.pdb
1.162275e-15 -2.591317e-15 1.639365e-15 -7.549819e-16 2.950055e-16
-2.935849e-17
E:\\R-3.1.2\\scripts\\mPDBS\\summary_pdb_93\\48a26225951ea153.final.pdb
1.450785e-16 1.345429e-15 -5.453119e-15 9.477574e-15 -1.743628e-15
5.692609e-15
E:\\R-3.1.2\\scripts\\mPDBS\\summary_pdb_93\\4ab4a5c7c6682ce6.final.pdb
3.537546e-15 -1.168300e-14 -6.052833e-15 3.559709e-15 -2.128403e-15
3.852953e-15
E:\\R-3.1.2\\scripts\\mPDBS\\summary_pdb_93\\4eaa45b62cb6a25f.final.pdb
-4.773432e-15 7.572912e-16 4.185325e-15 1.251943e-15 1.481854e-15

5.740653e-15
E:\\R-3.1.2\\scripts\\mPDBS\\summary_pdb_93\\58c371611d85ac96.final.pdb
-5.313014e-15 5.847265e-17 2.844564e-15 -1.228128e-15 6.867873e-15
5.407265e-15
E:\\R-3.1.2\\scripts\\mPDBS\\summary_pdb_93\\59a647f04822d30a.final.pdb
-1.886849e-15 -5.785150e-15 -2.093870e-15 -2.569511e-16 2.531831e-15
3.527032e-15
E:\\R-3.1.2\\scripts\\mPDBS\\summary_pdb_93\\59b091fe385e05fc.final.pdb
1.697222e-15 -4.364413e-15 -3.014531e-15 -2.770007e-15 4.180453e-15
2.553193e-15
E:\\R-3.1.2\\scripts\\mPDBS\\summary_pdb_93\\5c35ac30f930d2db.final.pdb
-4.773432e-15 7.572912e-16 4.185325e-15 1.251943e-15 1.481854e-15
5.740653e-15
E:\\R-3.1.2\\scripts\\mPDBS\\summary_pdb_93\\5f60752431cf2dcb.final.pdb
-2.952854e-15 -1.757589e-15 5.034076e-15 3.040409e-15 9.786758e-15
6.757004e-15
E:\\R-3.1.2\\scripts\\mPDBS\\summary_pdb_93\\626456dd6ff2868d.final.pdb
1.162275e-15 -2.591317e-15 1.639365e-15 -7.549819e-16 2.950055e-16
-2.935849e-17
E:\\R-3.1.2\\scripts\\mPDBS\\summary_pdb_93\\6583d53ff3c8f7ef.final.pdb
-1.886849e-15 -5.785150e-15 -2.093870e-15 -2.569511e-16 2.531831e-15
3.527032e-15
E:\\R-3.1.2\\scripts\\mPDBS\\summary_pdb_93\\69760178986bef01.final.pdb
-5.564208e-15 -6.727447e-16 9.632322e-15 -2.468643e-15 4.364700e-15
-4.040146e-15
E:\\R-3.1.2\\scripts\\mPDBS\\summary_pdb_93\\6a9d87d47926b1c8.final.pdb
-5.680150e-17 -3.655137e-15 -4.861053e-16 -1.304456e-15 3.311690e-15
2.191088e-15
E:\\R-3.1.2\\scripts\\mPDBS\\summary_pdb_93\\6bb3547ea982ffc4.final.pdb
-5.334976e-16 1.341522e-16 4.891141e-15 -7.352490e-15 5.582978e-15
3.141493e-15
E:\\R-3.1.2\\scripts\\mPDBS\\summary_pdb_93\\716d9624611d1c42.final.pdb
-4.188570e-16 -3.439837e-15 -6.003102e-15 -3.848611e-15 -2.156813e-15
2.079756e-15
E:\\R-3.1.2\\scripts\\mPDBS\\summary_pdb_93\\75f27e70ad0f1be4.final.pdb
-1.886849e-15 -5.785150e-15 -2.093870e-15 -2.569511e-16 2.531831e-15
3.527032e-15
E:\\R-3.1.2\\scripts\\mPDBS\\summary_pdb_93\\769f910fffb22872.final.pdb
7.611738e-16 -3.124645e-15 -7.059562e-16 -3.957263e-16 2.899019e-15
2.348922e-15
E:\\R-3.1.2\\scripts\\mPDBS\\summary_pdb_93\\791f8c80dd5c3135.final.pdb
1.697222e-15 -4.364413e-15 -3.014531e-15 -2.770007e-15 4.180453e-15
2.553193e-15
E:\\R-3.1.2\\scripts\\mPDBS\\summary_pdb_93\\7fad98e66a3f6099.final.pdb
1.348035e-15 -1.068628e-14 -8.784682e-15 4.074747e-15 1.198968e-14
4.895663e-16
E:\\R-3.1.2\\scripts\\mPDBS\\summary_pdb_93\\804b89c94a633196.final.pdb
3.758227e-15 -6.329343e-15 -3.316034e-15 -7.905129e-16 9.257631e-16
-1.083136e-15
E:\\R-3.1.2\\scripts\\mPDBS\\summary_pdb_93\\813cc65a2298feba.final.pdb
3.758227e-15 -6.329343e-15 -3.316034e-15 -7.905129e-16 9.257631e-16
-1.083136e-15
E:\\R-3.1.2\\scripts\\mPDBS\\summary_pdb_93\\83c0eefcfe12d3f9.final.pdb
5.884531e-16 -7.845058e-15 -6.896070e-15 -5.431332e-15 2.273643e-15

1.914466e-15
E:\\R-3.1.2\\scripts\\mPDBS\\summary_pdb_93\\86d50469ef569760.final.pdb
1.162275e-15 -2.591317e-15 1.639365e-15 -7.549819e-16 2.950055e-16
-2.935849e-17
E:\\R-3.1.2\\scripts\\mPDBS\\summary_pdb_93\\89d0666e1402203d.final.pdb
-1.886849e-15 -5.785150e-15 -2.093870e-15 -2.569511e-16 2.531831e-15
3.527032e-15
E:\\R-3.1.2\\scripts\\mPDBS\\summary_pdb_93\\8b34852716dadc62.final.pdb
1.287980e-15 -1.250506e-15 1.766705e-15 1.568711e-15 1.002959e-14
1.862425e-15
E:\\R-3.1.2\\scripts\\mPDBS\\summary_pdb_93\\8b6ef9ac3fe74395.final.pdb
4.760691e-15 1.025468e-15 3.116478e-15 -9.294004e-15 1.053089e-14
3.891426e-15
E:\\R-3.1.2\\scripts\\mPDBS\\summary_pdb_93\\98bbe0d5986c138f.final.pdb
1.697222e-15 -4.364413e-15 -3.014531e-15 -2.770007e-15 4.180453e-15
2.553193e-15
E:\\R-3.1.2\\scripts\\mPDBS\\summary_pdb_93\\9c22f4909d67e2fa.final.pdb
-3.877159e-15 3.487891e-15 7.431662e-15 4.053269e-15 3.740352e-15
1.626502e-15
E:\\R-3.1.2\\scripts\\mPDBS\\summary_pdb_93\\9dda8ed0d81fb3ef.final.pdb
3.869831e-15 1.007933e-14 -9.450495e-16 4.420731e-15 1.621985e-16
8.408978e-15
E:\\R-3.1.2\\scripts\\mPDBS\\summary_pdb_93\\9f752b3616e6033b.final.pdb
2.959637e-15 -4.177094e-15 2.426520e-16 -2.700239e-15 5.591638e-15
-4.214195e-16
E:\\R-3.1.2\\scripts\\mPDBS\\summary_pdb_93\\a02c70083816d6ba.final.pdb
-4.773432e-15 7.572912e-16 4.185325e-15 1.251943e-15 1.481854e-15
5.740653e-15
E:\\R-3.1.2\\scripts\\mPDBS\\summary_pdb_93\\a0c997d891e4e082.final.pdb
-5.044026e-15 -2.801342e-15 -3.857039e-15 -7.683199e-15 4.525669e-15
-9.003944e-16
E:\\R-3.1.2\\scripts\\mPDBS\\summary_pdb_93\\ab8039a2d823c616.final.pdb
1.116844e-15 -2.862491e-15 1.113581e-16 -4.537394e-16 4.063365e-15
2.612854e-15
E:\\R-3.1.2\\scripts\\mPDBS\\summary_pdb_93\\acf4d74b9c71d4dc.final.pdb
3.758227e-15 -6.329343e-15 -3.316034e-15 -7.905129e-16 9.257631e-16
-1.083136e-15
E:\\R-3.1.2\\scripts\\mPDBS\\summary_pdb_93\\ad8a823e4b4c668e.final.pdb
1.114547e-16 -3.464335e-15 -5.945278e-16 -1.381087e-15 3.837065e-15
2.579816e-15
E:\\R-3.1.2\\scripts\\mPDBS\\summary_pdb_93\\b16ababd03b7b857.final.pdb
-3.877159e-15 3.487891e-15 7.431662e-15 4.053269e-15 3.740352e-15
1.626502e-15
E:\\R-3.1.2\\scripts\\mPDBS\\summary_pdb_93\\b226923e697d3c63.final.pdb
4.980488e-15 -3.366946e-15 3.538994e-15 -6.667188e-15 1.184990e-14
1.134848e-15
E:\\R-3.1.2\\scripts\\mPDBS\\summary_pdb_93\\b28e48f6fa27a165.final.pdb
1.697222e-15 -4.364413e-15 -3.014531e-15 -2.770007e-15 4.180453e-15
2.553193e-15
E:\\R-3.1.2\\scripts\\mPDBS\\summary_pdb_93\\b42c4924e3cd1aec.final.pdb
1.875766e-15 -6.021506e-15 4.507524e-16 1.811707e-15 7.355246e-16
-5.379472e-15
E:\\R-3.1.2\\scripts\\mPDBS\\summary_pdb_93\\b4dd239fcabff1df.final.pdb
1.697222e-15 -4.364413e-15 -3.014531e-15 -2.770007e-15 4.180453e-15

2.553193e-15
E:\\R-3.1.2\\scripts\\mPDBS\\summary_pdb_93\\b669942ef97459a2.final.pdb
1.287980e-15 -1.250506e-15 1.766705e-15 1.568711e-15 1.002959e-14
1.862425e-15
E:\\R-3.1.2\\scripts\\mPDBS\\summary_pdb_93\\b99f4d9d87c1017b.final.pdb
1.697222e-15 -4.364413e-15 -3.014531e-15 -2.770007e-15 4.180453e-15
2.553193e-15
E:\\R-3.1.2\\scripts\\mPDBS\\summary_pdb_93\\b9d2cbf3975403e7.final.pdb
1.697222e-15 -4.364413e-15 -3.014531e-15 -2.770007e-15 4.180453e-15
2.553193e-15
E:\\R-3.1.2\\scripts\\mPDBS\\summary_pdb_93\\bc88c8a505f3a288.final.pdb
6.528122e-17 -3.731673e-15 -6.169266e-16 -1.449449e-15 3.525161e-15
2.151605e-15
E:\\R-3.1.2\\scripts\\mPDBS\\summary_pdb_93\\bdac0e64f4ef427e.final.pdb
-1.607273e-15 5.204833e-15 1.337005e-14 5.987052e-15 4.088598e-15
3.891184e-15
E:\\R-3.1.2\\scripts\\mPDBS\\summary_pdb_93\\ca2a1f55fc9d0681.final.pdb
1.287980e-15 -1.250506e-15 1.766705e-15 1.568711e-15 1.002959e-14
1.862425e-15
E:\\R-3.1.2\\scripts\\mPDBS\\summary_pdb_93\\ce34248de3e81a3c.final.pdb
-4.536481e-15 -3.157834e-15 -6.226232e-15 5.315406e-15 1.078625e-15
1.072890e-14
E:\\R-3.1.2\\scripts\\mPDBS\\summary_pdb_93\\d02e697772494a61.final.pdb
-4.902533e-15 -1.417211e-14 -6.871829e-16 -9.661196e-15 9.512126e-15
4.321310e-15
E:\\R-3.1.2\\scripts\\mPDBS\\summary_pdb_93\\d04221245d015047.final.pdb
-7.367077e-16 -3.285330e-15 -1.834351e-15 8.464359e-16 5.184576e-16
2.888242e-15
E:\\R-3.1.2\\scripts\\mPDBS\\summary_pdb_93\\d678141e4018054a.final.pdb
5.603567e-15 -6.435204e-15 -1.294362e-15 -2.224445e-15 6.036393e-15
7.066644e-15
E:\\R-3.1.2\\scripts\\mPDBS\\summary_pdb_93\\d71b40af0f0efe39.final.pdb
7.548782e-15 -9.218954e-15 -2.216316e-15 3.755568e-17 -2.119288e-16
-1.859023e-15
E:\\R-3.1.2\\scripts\\mPDBS\\summary_pdb_93\\dc0809eff10197e2.final.pdb
-3.856950e-15 -1.028081e-15 -1.755731e-15 -8.338542e-15 6.930675e-16
5.391549e-16
E:\\R-3.1.2\\scripts\\mPDBS\\summary_pdb_93\\e1163918d3707839.final.pdb
2.508940e-15 -4.367014e-15 -4.731734e-15 -4.049924e-15 -7.598071e-16
-4.042851e-16
E:\\R-3.1.2\\scripts\\mPDBS\\summary_pdb_93\\e4b48b06824ef848.final.pdb
3.283895e-15 7.234054e-16 5.644216e-15 1.763571e-15 2.345642e-15
6.503585e-15
E:\\R-3.1.2\\scripts\\mPDBS\\summary_pdb_93\\e529e19ad281fea2.final.pdb
-3.816285e-16 -4.997813e-15 -2.692240e-15 -1.469688e-15 -7.492981e-16
5.563390e-15
E:\\R-3.1.2\\scripts\\mPDBS\\summary_pdb_93\\e5602082718ec3a1.final.pdb
3.758227e-15 -6.329343e-15 -3.316034e-15 -7.905129e-16 9.257631e-16
-1.083136e-15
E:\\R-3.1.2\\scripts\\mPDBS\\summary_pdb_93\\e5d589d996d0a960.final.pdb
-6.061601e-15 -5.155676e-16 -1.853709e-15 -4.243314e-15 -2.814967e-15
4.527480e-15
E:\\R-3.1.2\\scripts\\mPDBS\\summary_pdb_93\\e9df9e72307a2fbd.final.pdb
-4.773432e-15 7.572912e-16 4.185325e-15 1.251943e-15 1.481854e-15

5.740653e-15
E:\\R-3.1.2\\scripts\\mPDBS\\summary_pdb_93\\ea00f8694090cae7.final.pdb
3.758227e-15 -6.329343e-15 -3.316034e-15 -7.905129e-16 9.257631e-16
-1.083136e-15
E:\\R-3.1.2\\scripts\\mPDBS\\summary_pdb_93\\eba517be32db050e.final.pdb
1.697222e-15 -4.364413e-15 -3.014531e-15 -2.770007e-15 4.180453e-15
2.553193e-15
E:\\R-3.1.2\\scripts\\mPDBS\\summary_pdb_93\\ec04b9545899c29d.final.pdb
7.247772e-16 -3.128421e-15 -6.024416e-16 -1.415746e-15 3.551848e-15
2.356094e-15
E:\\R-3.1.2\\scripts\\mPDBS\\summary_pdb_93\\ee6862cbcad52078.final.pdb
1.654615e-16 -3.605472e-15 -6.331896e-16 -1.283349e-15 3.349521e-15
2.549724e-15
E:\\R-3.1.2\\scripts\\mPDBS\\summary_pdb_93\\ef6a42fc83b81e5d.final.pdb
6.501515e-16 -3.394394e-15 -2.963446e-16 -4.659826e-16 3.707400e-15
2.495073e-15
E:\\R-3.1.2\\scripts\\mPDBS\\summary_pdb_93\\ef8e88d4ad93a0ad.final.pdb
-7.367077e-16 -3.285330e-15 -1.834351e-15 8.464359e-16 5.184576e-16
2.888242e-15
E:\\R-3.1.2\\scripts\\mPDBS\\summary_pdb_93\\f14fcae8deec4fa3.final.pdb
2.959637e-15 -4.177094e-15 2.426520e-16 -2.700239e-15 5.591638e-15
-4.214195e-16
E:\\R-3.1.2\\scripts\\mPDBS\\summary_pdb_93\\f83584e22003fb92.final.pdb
1.450785e-16 1.345429e-15 -5.453119e-15 9.477574e-15 -1.743628e-15
5.692609e-15
E:\\R-3.1.2\\scripts\\mPDBS\\summary_pdb_93\\f8e2bf87e52caa29.final.pdb
-3.816285e-16 -4.997813e-15 -2.692240e-15 -1.469688e-15 -7.492981e-16
5.563390e-15

[,433]   [,434]   [,435]   [,436]   [,437]   [,438]
E:\\R-3.1.2\\scripts\\40.1ns_data\\NAT0.pdb
-5.348085e-16 2.938489e-15 2.742343e-15 1.587535e-15 5.344041e-15
-8.220074e-16
E:\\R-3.1.2\\scripts\\40.1ns_data\\LJP6.pdb
-1.410484e-15 3.281009e-15 1.682708e-15 1.028292e-15 4.686187e-15
-3.132263e-15
E:\\R-3.1.2\\scripts\\40.1ns_data\\3WY9.pdb
-1.770144e-15 2.908571e-15 2.637917e-15 1.252819e-15 9.078986e-15
-2.516133e-15
E:\\R-3.1.2\\scripts\\40.1ns_data\\ZSP9.pdb
-1.859573e-15 1.974734e-15 2.148383e-15 9.017062e-16 2.707260e-15
-1.344984e-15
E:\\R-3.1.2\\scripts\\mPDBS\\summary_pdb_93\\013d855ad26c877c.final.pdb
-4.170885e-15 8.508651e-15 5.182988e-15 -7.512994e-15 1.655473e-15
4.038134e-16
E:\\R-3.1.2\\scripts\\mPDBS\\summary_pdb_93\\0a2a705342ca0d54.final.pdb
-2.018879e-15 2.549947e-15 2.456057e-15 -1.610141e-16 3.689539e-15
-1.464175e-15
E:\\R-3.1.2\\scripts\\mPDBS\\summary_pdb_93\\0a59fceadb4194d9.final.pdb
-6.659073e-16 5.084697e-15 5.645689e-16 1.842238e-15 1.632583e-15
-1.875456e-15
E:\\R-3.1.2\\scripts\\mPDBS\\summary_pdb_93\\0dba59f3306825e8.final.pdb
5.597057e-16 3.549944e-15 2.650469e-16 -1.236765e-15 7.358810e-15
3.502897e-15

E:\\R-3.1.2\\scripts\\mPDBS\\summary_pdb_93\\126f26a78b57339c.final.pdb
-3.607859e-15 -1.712340e-15 6.128412e-15 -1.025345e-15 3.463055e-15
-3.700542e-15
E:\\R-3.1.2\\scripts\\mPDBS\\summary_pdb_93\\12f06f3e342df706.final.pdb
-1.961878e-15 2.436106e-15 2.485331e-15 7.317356e-17 2.962581e-15
-1.370934e-15
E:\\R-3.1.2\\scripts\\mPDBS\\summary_pdb_93\\18618a2a251b867b.final.pdb
-6.597043e-15 -1.427554e-15 2.448715e-15 -7.004296e-15 2.210000e-15
4.087408e-16
E:\\R-3.1.2\\scripts\\mPDBS\\summary_pdb_93\\1f305f598641eeb2.final.pdb
-1.703033e-15 2.488569e-15 2.495576e-15 -2.577492e-16 3.378777e-15
-1.562498e-15
E:\\R-3.1.2\\scripts\\mPDBS\\summary_pdb_93\\1f8a00b551be0a39.final.pdb
-6.597043e-15 -1.427554e-15 2.448715e-15 -7.004296e-15 2.210000e-15
4.087408e-16
E:\\R-3.1.2\\scripts\\mPDBS\\summary_pdb_93\\219f45328d47579a.final.pdb
-3.783639e-15 -4.167621e-16 1.447879e-14 -2.990702e-15 1.297399e-14
-7.791513e-15
E:\\R-3.1.2\\scripts\\mPDBS\\summary_pdb_93\\238dd3918e54336d.final.pdb
-2.738875e-15 -6.769707e-16 6.272677e-15 -8.961837e-15 1.260102e-14
-4.052774e-16
E:\\R-3.1.2\\scripts\\mPDBS\\summary_pdb_93\\2b3027889e2fdc42.final.pdb
-1.357981e-15 2.446399e-15 2.847465e-15 -1.152112e-15 3.470306e-15
-1.635630e-15
E:\\R-3.1.2\\scripts\\mPDBS\\summary_pdb_93\\2fcb3b46e33793b0.final.pdb
-9.241418e-16 4.999581e-15 1.222289e-15 -1.642059e-15 4.202337e-15
1.087726e-15
E:\\R-3.1.2\\scripts\\mPDBS\\summary_pdb_93\\34eb328251842006.final.pdb
3.486439e-15 9.212135e-15 1.620101e-16 3.556500e-15 -7.968688e-15
-6.899868e-15
E:\\R-3.1.2\\scripts\\mPDBS\\summary_pdb_93\\354b32871797b7ab.final.pdb
-5.575211e-16 3.742915e-15 -1.165020e-15 -3.653028e-15 1.236550e-14
-1.201651e-14
E:\\R-3.1.2\\scripts\\mPDBS\\summary_pdb_93\\397368adbc32d5de.final.pdb
-3.607859e-15 -1.712340e-15 6.128412e-15 -1.025345e-15 3.463055e-15
-3.700542e-15
E:\\R-3.1.2\\scripts\\mPDBS\\summary_pdb_93\\411ca13cd3df6edd.final.pdb
-2.392694e-15 3.691166e-15 4.181727e-15 -2.509203e-15 -1.058212e-15
-4.121649e-15
E:\\R-3.1.2\\scripts\\mPDBS\\summary_pdb_93\\42357eee6642bf12.final.pdb
-1.233413e-17 1.942792e-15 1.045162e-15 -5.514814e-16 5.230043e-15
-6.280600e-15
E:\\R-3.1.2\\scripts\\mPDBS\\summary_pdb_93\\44342d1f75a7fcfc.final.pdb
-4.900947e-15 6.861489e-15 6.863871e-18 -6.135434e-15 -3.110651e-15
4.132605e-15
E:\\R-3.1.2\\scripts\\mPDBS\\summary_pdb_93\\448ef21dffe8cdfb.final.pdb
-7.901200e-15 1.589982e-15 8.825582e-15 5.040096e-15 -6.555551e-15
-2.660497e-14
E:\\R-3.1.2\\scripts\\mPDBS\\summary_pdb_93\\472a40b3e8f113c7.final.pdb
5.597057e-16 3.549944e-15 2.650469e-16 -1.236765e-15 7.358810e-15
3.502897e-15
E:\\R-3.1.2\\scripts\\mPDBS\\summary_pdb_93\\48a26225951ea153.final.pdb
-3.041096e-15 5.116355e-15 1.225499e-15 -8.550707e-15 -5.572591e-15
2.395217e-15

E:\\R-3.1.2\\scripts\\mPDBS\\summary_pdb_93\\4ab4a5c7c6682ce6.final.pdb
5.678116e-15 -1.577531e-14 -5.948733e-15 1.889610e-16 -2.636233e-15
-8.206053e-15
E:\\R-3.1.2\\scripts\\mPDBS\\summary_pdb_93\\4eaa45b62cb6a25f.final.pdb
-2.386380e-15 -1.018684e-15 3.301025e-15 -1.166953e-15 1.395329e-15
3.887439e-15
E:\\R-3.1.2\\scripts\\mPDBS\\summary_pdb_93\\58c371611d85ac96.final.pdb
-3.607859e-15 -1.712340e-15 6.128412e-15 -1.025345e-15 3.463055e-15
-3.700542e-15
E:\\R-3.1.2\\scripts\\mPDBS\\summary_pdb_93\\59a647f04822d30a.final.pdb
1.726130e-15 -2.549819e-15 2.700889e-15 6.973872e-15 7.518217e-15
-1.580490e-15
E:\\R-3.1.2\\scripts\\mPDBS\\summary_pdb_93\\59b091fe385e05fc.final.pdb
-6.659073e-16 5.084697e-15 5.645689e-16 1.842238e-15 1.632583e-15
-1.875456e-15
E:\\R-3.1.2\\scripts\\mPDBS\\summary_pdb_93\\5c35ac30f930d2db.final.pdb
-2.386380e-15 -1.018684e-15 3.301025e-15 -1.166953e-15 1.395329e-15
3.887439e-15
E:\\R-3.1.2\\scripts\\mPDBS\\summary_pdb_93\\5f60752431cf2dcb.final.pdb
-3.963423e-15 3.829523e-15 7.717474e-15 2.522386e-15 2.868147e-15
1.224863e-15
E:\\R-3.1.2\\scripts\\mPDBS\\summary_pdb_93\\626456dd6ff2868d.final.pdb
5.597057e-16 3.549944e-15 2.650469e-16 -1.236765e-15 7.358810e-15
3.502897e-15
E:\\R-3.1.2\\scripts\\mPDBS\\summary_pdb_93\\6583d53ff3c8f7ef.final.pdb
1.726130e-15 -2.549819e-15 2.700889e-15 6.973872e-15 7.518217e-15
-1.580490e-15
E:\\R-3.1.2\\scripts\\mPDBS\\summary_pdb_93\\69760178986bef01.final.pdb
-3.493661e-15 8.724253e-16 5.104375e-15 -4.256905e-15 9.278411e-15
4.043820e-16
E:\\R-3.1.2\\scripts\\mPDBS\\summary_pdb_93\\6a9d87d47926b1c8.final.pdb
-1.408230e-15 2.784931e-15 2.573964e-15 -1.445149e-15 4.094928e-15
-1.663110e-15
E:\\R-3.1.2\\scripts\\mPDBS\\summary_pdb_93\\6bb3547ea982ffc4.final.pdb
-8.967099e-15 3.156033e-15 6.827828e-15 -9.430179e-15 -3.850130e-15
-4.434103e-15
E:\\R-3.1.2\\scripts\\mPDBS\\summary_pdb_93\\716d9624611d1c42.final.pdb
1.150894e-15 -1.862105e-15 -1.330844e-15 -5.861315e-15 -2.439022e-15
-2.521706e-15
E:\\R-3.1.2\\scripts\\mPDBS\\summary_pdb_93\\75f27e70ad0f1be4.final.pdb
1.726130e-15 -2.549819e-15 2.700889e-15 6.973872e-15 7.518217e-15
-1.580490e-15
E:\\R-3.1.2\\scripts\\mPDBS\\summary_pdb_93\\769f910fffb22872.final.pdb
-2.131485e-15 2.378863e-15 2.448715e-15 2.306902e-17 3.624884e-15
-2.225654e-15
E:\\R-3.1.2\\scripts\\mPDBS\\summary_pdb_93\\791f8c80dd5c3135.final.pdb
-6.659073e-16 5.084697e-15 5.645689e-16 1.842238e-15 1.632583e-15
-1.875456e-15
E:\\R-3.1.2\\scripts\\mPDBS\\summary_pdb_93\\7fad98e66a3f6099.final.pdb
3.544841e-15 7.868102e-15 5.246838e-15 -5.705070e-15 -6.573303e-15
-6.807301e-15
E:\\R-3.1.2\\scripts\\mPDBS\\summary_pdb_93\\804b89c94a633196.final.pdb
-9.241418e-16 4.999581e-15 1.222289e-15 -1.642059e-15 4.202337e-15
1.087726e-15

E:\\R-3.1.2\\scripts\\mPDBS\\summary_pdb_93\\813cc65a2298feba.final.pdb
-9.241418e-16 4.999581e-15 1.222289e-15 -1.642059e-15 4.202337e-15
1.087726e-15
E:\\R-3.1.2\\scripts\\mPDBS\\summary_pdb_93\\83c0eefcfe12d3f9.final.pdb
-2.392694e-15 3.691166e-15 4.181727e-15 -2.509203e-15 -1.058212e-15
-4.121649e-15
E:\\R-3.1.2\\scripts\\mPDBS\\summary_pdb_93\\86d50469ef569760.final.pdb
5.597057e-16 3.549944e-15 2.650469e-16 -1.236765e-15 7.358810e-15
3.502897e-15
E:\\R-3.1.2\\scripts\\mPDBS\\summary_pdb_93\\89d0666e1402203d.final.pdb
1.726130e-15 -2.549819e-15 2.700889e-15 6.973872e-15 7.518217e-15
-1.580490e-15
E:\\R-3.1.2\\scripts\\mPDBS\\summary_pdb_93\\8b34852716dadc62.final.pdb
1.912854e-16 -4.110319e-15 1.361067e-15 4.224776e-15 1.642319e-15
-3.269466e-15
E:\\R-3.1.2\\scripts\\mPDBS\\summary_pdb_93\\8b6ef9ac3fe74395.final.pdb
-7.203146e-15 3.933661e-15 2.796218e-15 5.397825e-15 -5.881647e-15
-1.846930e-15
E:\\R-3.1.2\\scripts\\mPDBS\\summary_pdb_93\\98bbe0d5986c138f.final.pdb
-6.659073e-16 5.084697e-15 5.645689e-16 1.842238e-15 1.632583e-15
-1.875456e-15
E:\\R-3.1.2\\scripts\\mPDBS\\summary_pdb_93\\9c22f4909d67e2fa.final.pdb
-7.164629e-16 1.001228e-14 5.818439e-15 -1.832878e-15 1.477418e-14
4.529634e-15
E:\\R-3.1.2\\scripts\\mPDBS\\summary_pdb_93\\9dda8ed0d81fb3ef.final.pdb
-7.839488e-15 7.607204e-15 -3.148598e-15 -6.766873e-15 -1.592234e-14
5.082366e-15
E:\\R-3.1.2\\scripts\\mPDBS\\summary_pdb_93\\9f752b3616e6033b.final.pdb
-2.870598e-15 4.303535e-15 2.844968e-15 1.499349e-15 5.268640e-15
-2.549832e-15
E:\\R-3.1.2\\scripts\\mPDBS\\summary_pdb_93\\a02c70083816d6ba.final.pdb
-2.386380e-15 -1.018684e-15 3.301025e-15 -1.166953e-15 1.395329e-15
3.887439e-15
E:\\R-3.1.2\\scripts\\mPDBS\\summary_pdb_93\\a0c997d891e4e082.final.pdb
-4.437249e-15 -3.065009e-15 -1.353464e-15 8.439553e-16 1.740488e-15
-6.495989e-15
E:\\R-3.1.2\\scripts\\mPDBS\\summary_pdb_93\\ab8039a2d823c616.final.pdb
-1.117829e-15 1.971977e-15 2.468999e-15 -8.441475e-16 3.361370e-15
-1.367713e-15
E:\\R-3.1.2\\scripts\\mPDBS\\summary_pdb_93\\acf4d74b9c71d4dc.final.pdb
-9.241418e-16 4.999581e-15 1.222289e-15 -1.642059e-15 4.202337e-15
1.087726e-15
E:\\R-3.1.2\\scripts\\mPDBS\\summary_pdb_93\\ad8a823e4b4c668e.final.pdb
-2.137199e-15 2.759731e-15 2.674512e-15 3.518020e-18 2.360811e-15
-1.586541e-15
E:\\R-3.1.2\\scripts\\mPDBS\\summary_pdb_93\\b16ababd03b7b857.final.pdb
-7.164629e-16 1.001228e-14 5.818439e-15 -1.832878e-15 1.477418e-14
4.529634e-15
E:\\R-3.1.2\\scripts\\mPDBS\\summary_pdb_93\\b226923e697d3c63.final.pdb
-9.587682e-15 -7.575530e-15 2.337465e-15 -1.588724e-15 5.697408e-15
-1.021139e-14
E:\\R-3.1.2\\scripts\\mPDBS\\summary_pdb_93\\b28e48f6fa27a165.final.pdb
-6.659073e-16 5.084697e-15 5.645689e-16 1.842238e-15 1.632583e-15
-1.875456e-15

E:\\R-3.1.2\\scripts\\mPDBS\\summary_pdb_93\\b42c4924e3cd1aec.final.pdb
-2.523493e-16 6.231134e-15 5.919669e-15 -1.659630e-15 1.294552e-14
4.174782e-16
E:\\R-3.1.2\\scripts\\mPDBS\\summary_pdb_93\\b4dd239fcabff1df.final.pdb
-6.659073e-16 5.084697e-15 5.645689e-16 1.842238e-15 1.632583e-15
-1.875456e-15
E:\\R-3.1.2\\scripts\\mPDBS\\summary_pdb_93\\b669942ef97459a2.final.pdb
1.912854e-16 -4.110319e-15 1.361067e-15 4.224776e-15 1.642319e-15
-3.269466e-15
E:\\R-3.1.2\\scripts\\mPDBS\\summary_pdb_93\\b99f4d9d87c1017b.final.pdb
-6.659073e-16 5.084697e-15 5.645689e-16 1.842238e-15 1.632583e-15
-1.875456e-15
E:\\R-3.1.2\\scripts\\mPDBS\\summary_pdb_93\\b9d2cbf3975403e7.final.pdb
-6.659073e-16 5.084697e-15 5.645689e-16 1.842238e-15 1.632583e-15
-1.875456e-15
E:\\R-3.1.2\\scripts\\mPDBS\\summary_pdb_93\\bc88c8a505f3a288.final.pdb
-1.311279e-15 2.274661e-15 2.438938e-15 -1.261183e-15 3.029903e-15
-1.091144e-15
E:\\R-3.1.2\\scripts\\mPDBS\\summary_pdb_93\\bdac0e64f4ef427e.final.pdb
-2.281924e-16 3.841326e-16 -1.611528e-16 3.527634e-15 1.107369e-14
2.472252e-15
E:\\R-3.1.2\\scripts\\mPDBS\\summary_pdb_93\\ca2a1f55fc9d0681.final.pdb
1.912854e-16 -4.110319e-15 1.361067e-15 4.224776e-15 1.642319e-15
-3.269466e-15
E:\\R-3.1.2\\scripts\\mPDBS\\summary_pdb_93\\ce34248de3e81a3c.final.pdb
2.654282e-15 -2.206411e-15 -2.375483e-15 7.920331e-16 -6.278610e-15
3.762718e-15
E:\\R-3.1.2\\scripts\\mPDBS\\summary_pdb_93\\d02e697772494a61.final.pdb
3.703888e-15 -7.507444e-15 4.825917e-15 6.643735e-15 1.234342e-14
-9.591506e-15
E:\\R-3.1.2\\scripts\\mPDBS\\summary_pdb_93\\d04221245d015047.final.pdb
1.112131e-15 5.887899e-15 2.610379e-15 -2.732066e-16 2.388407e-15
1.336287e-15
E:\\R-3.1.2\\scripts\\mPDBS\\summary_pdb_93\\d678141e4018054a.final.pdb
-3.564998e-15 4.084366e-15 9.404994e-15 -1.009570e-14 -2.270629e-15
-6.283207e-15
E:\\R-3.1.2\\scripts\\mPDBS\\summary_pdb_93\\d71b40af0f0efe39.final.pdb
-3.869754e-15 5.336446e-15 9.813586e-15 -5.628413e-15 1.686438e-14
-9.011758e-15
E:\\R-3.1.2\\scripts\\mPDBS\\summary_pdb_93\\dc0809eff10197e2.final.pdb
2.320932e-15 3.107759e-15 -2.865428e-15 1.116022e-14 4.277117e-15
2.400831e-15
E:\\R-3.1.2\\scripts\\mPDBS\\summary_pdb_93\\e1163918d3707839.final.pdb
-1.287503e-16 8.687720e-15 1.589799e-15 2.274722e-15 3.440533e-15
3.137958e-15
E:\\R-3.1.2\\scripts\\mPDBS\\summary_pdb_93\\e4b48b06824ef848.final.pdb
-6.597043e-15 -1.427554e-15 2.448715e-15 -7.004296e-15 2.210000e-15
4.087408e-16
E:\\R-3.1.2\\scripts\\mPDBS\\summary_pdb_93\\e529e19ad281fea2.final.pdb
-4.170885e-15 8.508651e-15 5.182988e-15 -7.512994e-15 1.655473e-15
4.038134e-16
E:\\R-3.1.2\\scripts\\mPDBS\\summary_pdb_93\\e5602082718ec3a1.final.pdb
-9.241418e-16 4.999581e-15 1.222289e-15 -1.642059e-15 4.202337e-15
1.087726e-15

E:\\R-3.1.2\\scripts\\mPDBS\\summary_pdb_93\\e5d589d996d0a960.final.pdb
-4.048224e-15 2.483751e-15 -2.360181e-16 -3.782781e-15 -7.245945e-15
-3.375377e-15
E:\\R-3.1.2\\scripts\\mPDBS\\summary_pdb_93\\e9df9e72307a2fbd.final.pdb
-2.386380e-15 -1.018684e-15 3.301025e-15 -1.166953e-15 1.395329e-15
3.887439e-15
E:\\R-3.1.2\\scripts\\mPDBS\\summary_pdb_93\\ea00f8694090cae7.final.pdb
-9.241418e-16 4.999581e-15 1.222289e-15 -1.642059e-15 4.202337e-15
1.087726e-15
E:\\R-3.1.2\\scripts\\mPDBS\\summary_pdb_93\\eba517be32db050e.final.pdb
-6.659073e-16 5.084697e-15 5.645689e-16 1.842238e-15 1.632583e-15
-1.875456e-15
E:\\R-3.1.2\\scripts\\mPDBS\\summary_pdb_93\\ec04b9545899c29d.final.pdb
-1.967530e-15 2.390673e-15 2.785891e-15 -6.086048e-16 3.098358e-15
-1.087611e-15
E:\\R-3.1.2\\scripts\\mPDBS\\summary_pdb_93\\ee6862cbcad52078.final.pdb
-1.416609e-15 2.614884e-15 2.779811e-15 -1.186589e-15 3.073922e-15
-1.770722e-15
E:\\R-3.1.2\\scripts\\mPDBS\\summary_pdb_93\\ef6a42fc83b81e5d.final.pdb
-1.663137e-15 1.995706e-15 2.490348e-15 -4.433548e-16 3.332149e-15
-1.439173e-15
E:\\R-3.1.2\\scripts\\mPDBS\\summary_pdb_93\\ef8e88d4ad93a0ad.final.pdb
1.112131e-15 5.887899e-15 2.610379e-15 -2.732066e-16 2.388407e-15
1.336287e-15
E:\\R-3.1.2\\scripts\\mPDBS\\summary_pdb_93\\f14fcae8deec4fa3.final.pdb
-2.870598e-15 4.303535e-15 2.844968e-15 1.499349e-15 5.268640e-15
-2.549832e-15
E:\\R-3.1.2\\scripts\\mPDBS\\summary_pdb_93\\f83584e22003fb92.final.pdb
-3.041096e-15 5.116355e-15 1.225499e-15 -8.550707e-15 -5.572591e-15
2.395217e-15
E:\\R-3.1.2\\scripts\\mPDBS\\summary_pdb_93\\f8e2bf87e52caa29.final.pdb
-4.170885e-15 8.508651e-15 5.182988e-15 -7.512994e-15 1.655473e-15
4.038134e-16

[,439]   [,440]   [,441]   [,442]   [,443]   [,444]
E:\\R-3.1.2\\scripts\\40.1ns_data\\NAT0.pdb
9.204451e-16 -2.076272e-15 1.491398e-15 -1.791224e-16 -7.181547e-16
-7.192636e-15
E:\\R-3.1.2\\scripts\\40.1ns_data\\LJP6.pdb
2.552330e-15 -1.555134e-15 1.070253e-15 6.646319e-16 -3.311059e-16
-5.071333e-15
E:\\R-3.1.2\\scripts\\40.1ns_data\\3WY9.pdb
1.853429e-15 -9.838743e-16 1.914723e-15 -6.034488e-16 -8.773586e-16
-3.665242e-15
E:\\R-3.1.2\\scripts\\40.1ns_data\\ZSP9.pdb
2.273104e-15 -1.927712e-15 2.877240e-15 -1.172460e-15 -1.419098e-15
-3.683706e-15
E:\\R-3.1.2\\scripts\\mPDBS\\summary_pdb_93\\013d855ad26c877c.final.pdb
-3.323806e-15 -4.021633e-15 2.817857e-15 6.097112e-15 -5.015774e-15
-1.360952e-15
E:\\R-3.1.2\\scripts\\mPDBS\\summary_pdb_93\\0a2a705342ca0d54.final.pdb
2.501221e-15 -2.299067e-15 1.681325e-15 4.115323e-16 -1.425779e-15
-4.350739e-15
E:\\R-3.1.2\\scripts\\mPDBS\\summary_pdb_93\\0a59fceadb4194d9.final.pdb

-1.021265e-15 -2.934483e-15 4.984304e-15 3.527760e-15 3.171570e-17
-3.170825e-15
E:\\R-3.1.2\\scripts\\mPDBS\\summary_pdb_93\\0dba59f3306825e8.final.pdb
3.718304e-15 2.773279e-15 1.648653e-15 -8.140266e-16 -3.584627e-15
-4.336003e-15
E:\\R-3.1.2\\scripts\\mPDBS\\summary_pdb_93\\126f26a78b57339c.final.pdb
7.284896e-15 -8.185511e-15 -2.087074e-15 -2.153342e-15 9.472142e-17
-9.504828e-15
E:\\R-3.1.2\\scripts\\mPDBS\\summary_pdb_93\\12f06f3e342df706.final.pdb
2.158180e-15 -2.556159e-15 1.428489e-15 2.833254e-16 -4.100988e-16
-4.017428e-15
E:\\R-3.1.2\\scripts\\mPDBS\\summary_pdb_93\\18618a2a251b867b.final.pdb
3.231163e-15 -5.065828e-15 4.949755e-16 -4.067036e-15 -1.452419e-15
-1.114712e-14
E:\\R-3.1.2\\scripts\\mPDBS\\summary_pdb_93\\1f305f598641eeb2.final.pdb
2.198628e-15 -2.617670e-15 1.092099e-15 1.808533e-16 -1.593516e-15
-4.035318e-15
E:\\R-3.1.2\\scripts\\mPDBS\\summary_pdb_93\\1f8a00b551be0a39.final.pdb
3.231163e-15 -5.065828e-15 4.949755e-16 -4.067036e-15 -1.452419e-15
-1.114712e-14
E:\\R-3.1.2\\scripts\\mPDBS\\summary_pdb_93\\219f45328d47579a.final.pdb
1.442948e-14 -6.103870e-16 9.111738e-16 -1.024154e-15 2.658954e-15
-3.330420e-15
E:\\R-3.1.2\\scripts\\mPDBS\\summary_pdb_93\\238dd3918e54336d.final.pdb
9.842441e-15 3.412714e-16 -4.747928e-15 -1.174138e-14 3.581827e-15
-4.201820e-15
E:\\R-3.1.2\\scripts\\mPDBS\\summary_pdb_93\\2b3027889e2fdc42.final.pdb
2.639001e-15 -2.498322e-15 1.871343e-15 2.897361e-16 -1.814030e-15
-3.942154e-15
E:\\R-3.1.2\\scripts\\mPDBS\\summary_pdb_93\\2fcb3b46e33793b0.final.pdb
-4.077942e-16 1.297601e-15 2.986417e-15 2.817633e-15 -3.347321e-15
-1.544255e-15
E:\\R-3.1.2\\scripts\\mPDBS\\summary_pdb_93\\34eb328251842006.final.pdb
-4.202398e-16 -5.653186e-15 4.081705e-15 5.108395e-16 4.226183e-15
-2.033697e-15
E:\\R-3.1.2\\scripts\\mPDBS\\summary_pdb_93\\354b32871797b7ab.final.pdb
-9.630178e-16 -9.623215e-16 4.073505e-15 5.873555e-15 -7.520005e-15
1.828448e-15
E:\\R-3.1.2\\scripts\\mPDBS\\summary_pdb_93\\397368adbc32d5de.final.pdb
7.284896e-15 -8.185511e-15 -2.087074e-15 -2.153342e-15 9.472142e-17
-9.504828e-15
E:\\R-3.1.2\\scripts\\mPDBS\\summary_pdb_93\\411ca13cd3df6edd.final.pdb
4.094681e-15 -1.834767e-15 8.310231e-16 3.480406e-15 -4.384686e-15
-5.444564e-15
E:\\R-3.1.2\\scripts\\mPDBS\\summary_pdb_93\\42357eee6642bf12.final.pdb
-1.106746e-15 -2.464688e-15 7.875613e-15 1.619999e-16 6.485547e-15
-5.203067e-15
E:\\R-3.1.2\\scripts\\mPDBS\\summary_pdb_93\\44342d1f75a7fcfc.final.pdb
-2.993162e-15 -7.610190e-15 1.264217e-15 -6.381008e-15 -7.203765e-15
-9.152469e-15
E:\\R-3.1.2\\scripts\\mPDBS\\summary_pdb_93\\448ef21dffe8cdfb.final.pdb
-8.397403e-16 -1.642954e-14 -7.005590e-15 6.231569e-15 4.660162e-15
-8.503659e-16
E:\\R-3.1.2\\scripts\\mPDBS\\summary_pdb_93\\472a40b3e8f113c7.final.pdb

3.718304e-15 2.773279e-15 1.648653e-15 -8.140266e-16 -3.584627e-15
-4.336003e-15
E:\\R-3.1.2\\scripts\\mPDBS\\summary_pdb_93\\48a26225951ea153.final.pdb
5.094069e-15 -3.709593e-15 -1.837496e-15 -6.558085e-15 -8.622820e-15
-8.114164e-15
E:\\R-3.1.2\\scripts\\mPDBS\\summary_pdb_93\\4ab4a5c7c6682ce6.final.pdb
-2.176286e-15 -7.474274e-15 5.337543e-15 7.089522e-16 -6.146814e-15
4.395511e-15
E:\\R-3.1.2\\scripts\\mPDBS\\summary_pdb_93\\4eaa45b62cb6a25f.final.pdb
2.153413e-15 -3.889830e-15 3.746408e-16 -3.652698e-16 -2.441602e-15
-6.190609e-15
E:\\R-3.1.2\\scripts\\mPDBS\\summary_pdb_93\\58c371611d85ac96.final.pdb
7.284896e-15 -8.185511e-15 -2.087074e-15 -2.153342e-15 9.472142e-17
-9.504828e-15
E:\\R-3.1.2\\scripts\\mPDBS\\summary_pdb_93\\59a647f04822d30a.final.pdb
2.674613e-15 -3.082996e-15 2.841081e-15 2.329906e-15 -1.246850e-17
1.700828e-15
E:\\R-3.1.2\\scripts\\mPDBS\\summary_pdb_93\\59b091fe385e05fc.final.pdb
-1.021265e-15 -2.934483e-15 4.984304e-15 3.527760e-15 3.171570e-17
-3.170825e-15
E:\\R-3.1.2\\scripts\\mPDBS\\summary_pdb_93\\5c35ac30f930d2db.final.pdb
2.153413e-15 -3.889830e-15 3.746408e-16 -3.652698e-16 -2.441602e-15
-6.190609e-15
E:\\R-3.1.2\\scripts\\mPDBS\\summary_pdb_93\\5f60752431cf2dcb.final.pdb
-2.947111e-15 -5.273373e-15 1.330043e-15 -5.843616e-16 2.562350e-15
-1.096982e-14
E:\\R-3.1.2\\scripts\\mPDBS\\summary_pdb_93\\626456dd6ff2868d.final.pdb
3.718304e-15 2.773279e-15 1.648653e-15 -8.140266e-16 -3.584627e-15
-4.336003e-15
E:\\R-3.1.2\\scripts\\mPDBS\\summary_pdb_93\\6583d53ff3c8f7ef.final.pdb
2.674613e-15 -3.082996e-15 2.841081e-15 2.329906e-15 -1.246850e-17
1.700828e-15
E:\\R-3.1.2\\scripts\\mPDBS\\summary_pdb_93\\69760178986bef01.final.pdb
6.352989e-15 2.517865e-15 -8.133873e-16 -4.453716e-15 -8.699702e-15
-4.303744e-15
E:\\R-3.1.2\\scripts\\mPDBS\\summary_pdb_93\\6a9d87d47926b1c8.final.pdb
2.628031e-15 -2.597967e-15 1.930546e-15 9.136495e-16 -1.548062e-15
-3.532175e-15
E:\\R-3.1.2\\scripts\\mPDBS\\summary_pdb_93\\6bb3547ea982ffc4.final.pdb
9.095197e-15 -1.629938e-15 -1.247496e-14 -7.877076e-15 -1.444058e-14
-7.314415e-15
E:\\R-3.1.2\\scripts\\mPDBS\\summary_pdb_93\\716d9624611d1c42.final.pdb
8.018943e-15 -1.412190e-15 -9.623936e-16 5.936782e-16 -8.773201e-15
5.966925e-16
E:\\R-3.1.2\\scripts\\mPDBS\\summary_pdb_93\\75f27e70ad0f1be4.final.pdb
2.674613e-15 -3.082996e-15 2.841081e-15 2.329906e-15 -1.246850e-17
1.700828e-15
E:\\R-3.1.2\\scripts\\mPDBS\\summary_pdb_93\\769f910fffb22872.final.pdb
1.623942e-15 -2.180698e-15 1.599561e-15 -2.046200e-16 -1.832323e-15
-4.417582e-15
E:\\R-3.1.2\\scripts\\mPDBS\\summary_pdb_93\\791f8c80dd5c3135.final.pdb
-1.021265e-15 -2.934483e-15 4.984304e-15 3.527760e-15 3.171570e-17
-3.170825e-15
E:\\R-3.1.2\\scripts\\mPDBS\\summary_pdb_93\\7fad98e66a3f6099.final.pdb

3.440625e-16 -6.416686e-16 2.639396e-15 -6.676164e-16 1.166542e-15
1.781590e-15
E:\\R-3.1.2\\scripts\\mPDBS\\summary_pdb_93\\804b89c94a633196.final.pdb
-4.077942e-16 1.297601e-15 2.986417e-15 2.817633e-15 -3.347321e-15
-1.544255e-15
E:\\R-3.1.2\\scripts\\mPDBS\\summary_pdb_93\\813cc65a2298feba.final.pdb
-4.077942e-16 1.297601e-15 2.986417e-15 2.817633e-15 -3.347321e-15
-1.544255e-15
E:\\R-3.1.2\\scripts\\mPDBS\\summary_pdb_93\\83c0eefcfe12d3f9.final.pdb
4.094681e-15 -1.834767e-15 8.310231e-16 3.480406e-15 -4.384686e-15
-5.444564e-15
E:\\R-3.1.2\\scripts\\mPDBS\\summary_pdb_93\\86d50469ef569760.final.pdb
3.718304e-15 2.773279e-15 1.648653e-15 -8.140266e-16 -3.584627e-15
-4.336003e-15
E:\\R-3.1.2\\scripts\\mPDBS\\summary_pdb_93\\89d0666e1402203d.final.pdb
2.674613e-15 -3.082996e-15 2.841081e-15 2.329906e-15 -1.246850e-17
1.700828e-15
E:\\R-3.1.2\\scripts\\mPDBS\\summary_pdb_93\\8b34852716dadc62.final.pdb
8.027299e-15 -3.134617e-15 1.029649e-15 -4.645147e-15 2.591686e-16
-5.801267e-15
E:\\R-3.1.2\\scripts\\mPDBS\\summary_pdb_93\\8b6ef9ac3fe74395.final.pdb
-3.688773e-16 2.095143e-15 2.084372e-16 4.847438e-15 3.161003e-16
1.078810e-15
E:\\R-3.1.2\\scripts\\mPDBS\\summary_pdb_93\\98bbe0d5986c138f.final.pdb
-1.021265e-15 -2.934483e-15 4.984304e-15 3.527760e-15 3.171570e-17
-3.170825e-15
E:\\R-3.1.2\\scripts\\mPDBS\\summary_pdb_93\\9c22f4909d67e2fa.final.pdb
4.243867e-15 -3.032865e-15 7.395164e-16 -4.796014e-15 2.956664e-15
-1.008462e-14
E:\\R-3.1.2\\scripts\\mPDBS\\summary_pdb_93\\9dda8ed0d81fb3ef.final.pdb
-2.147485e-15 -7.072128e-15 -3.264279e-15 -1.275622e-14 -1.133674e-14
-1.645763e-14
E:\\R-3.1.2\\scripts\\mPDBS\\summary_pdb_93\\9f752b3616e6033b.final.pdb
3.102897e-15 -6.970479e-16 6.434583e-16 -1.121673e-15 3.202696e-16
-3.615260e-15
E:\\R-3.1.2\\scripts\\mPDBS\\summary_pdb_93\\a02c70083816d6ba.final.pdb
2.153413e-15 -3.889830e-15 3.746408e-16 -3.652698e-16 -2.441602e-15
-6.190609e-15
E:\\R-3.1.2\\scripts\\mPDBS\\summary_pdb_93\\a0c997d891e4e082.final.pdb
9.116511e-15 -1.012926e-14 -4.327194e-15 1.448735e-17 -3.583880e-15
4.336143e-15
E:\\R-3.1.2\\scripts\\mPDBS\\summary_pdb_93\\ab8039a2d823c616.final.pdb
2.036237e-15 -2.514524e-15 1.184456e-15 -2.031554e-17 -1.266290e-15
-4.427382e-15
E:\\R-3.1.2\\scripts\\mPDBS\\summary_pdb_93\\acf4d74b9c71d4dc.final.pdb
-4.077942e-16 1.297601e-15 2.986417e-15 2.817633e-15 -3.347321e-15
-1.544255e-15
E:\\R-3.1.2\\scripts\\mPDBS\\summary_pdb_93\\ad8a823e4b4c668e.final.pdb
2.297110e-15 -2.362789e-15 2.091906e-15 -2.418653e-16 -5.659313e-16
-4.327897e-15
E:\\R-3.1.2\\scripts\\mPDBS\\summary_pdb_93\\b16ababd03b7b857.final.pdb
4.243867e-15 -3.032865e-15 7.395164e-16 -4.796014e-15 2.956664e-15
-1.008462e-14
E:\\R-3.1.2\\scripts\\mPDBS\\summary_pdb_93\\b226923e697d3c63.final.pdb

5.820100e-15 -6.367979e-15 2.628478e-16 3.341196e-15 1.804654e-15
-1.400532e-15
E:\\R-3.1.2\\scripts\\mPDBS\\summary_pdb_93\\b28e48f6fa27a165.final.pdb
-1.021265e-15 -2.934483e-15 4.984304e-15 3.527760e-15 3.171570e-17
-3.170825e-15
E:\\R-3.1.2\\scripts\\mPDBS\\summary_pdb_93\\b42c4924e3cd1aec.final.pdb
2.168074e-15 -1.384652e-16 3.961278e-15 6.952185e-15 1.044845e-15
-1.383808e-15
E:\\R-3.1.2\\scripts\\mPDBS\\summary_pdb_93\\b4dd239fcabff1df.final.pdb
-1.021265e-15 -2.934483e-15 4.984304e-15 3.527760e-15 3.171570e-17
-3.170825e-15
E:\\R-3.1.2\\scripts\\mPDBS\\summary_pdb_93\\b669942ef97459a2.final.pdb
8.027299e-15 -3.134617e-15 1.029649e-15 -4.645147e-15 2.591686e-16
-5.801267e-15
E:\\R-3.1.2\\scripts\\mPDBS\\summary_pdb_93\\b99f4d9d87c1017b.final.pdb
-1.021265e-15 -2.934483e-15 4.984304e-15 3.527760e-15 3.171570e-17
-3.170825e-15
E:\\R-3.1.2\\scripts\\mPDBS\\summary_pdb_93\\b9d2cbf3975403e7.final.pdb
-1.021265e-15 -2.934483e-15 4.984304e-15 3.527760e-15 3.171570e-17
-3.170825e-15
E:\\R-3.1.2\\scripts\\mPDBS\\summary_pdb_93\\bc88c8a505f3a288.final.pdb
2.346267e-15 -2.728200e-15 9.415314e-16 8.433297e-16 -1.405503e-15
-4.185937e-15
E:\\R-3.1.2\\scripts\\mPDBS\\summary_pdb_93\\bdac0e64f4ef427e.final.pdb
-2.183284e-15 -3.315055e-15 4.694252e-15 -5.906075e-15 2.653954e-15
-1.926816e-15
E:\\R-3.1.2\\scripts\\mPDBS\\summary_pdb_93\\ca2a1f55fc9d0681.final.pdb
8.027299e-15 -3.134617e-15 1.029649e-15 -4.645147e-15 2.591686e-16
-5.801267e-15
E:\\R-3.1.2\\scripts\\mPDBS\\summary_pdb_93\\ce34248de3e81a3c.final.pdb
-5.410227e-15 -7.210215e-15 9.910511e-15 8.618779e-15 2.368730e-15
-5.068257e-16
E:\\R-3.1.2\\scripts\\mPDBS\\summary_pdb_93\\d02e697772494a61.final.pdb
6.397280e-15 -2.446314e-16 3.605633e-15 6.681379e-15 6.451060e-15
5.708655e-15
E:\\R-3.1.2\\scripts\\mPDBS\\summary_pdb_93\\d04221245d015047.final.pdb
6.805505e-16 -1.426147e-15 2.171080e-15 -6.915451e-16 -2.171815e-15
-5.230857e-15
E:\\R-3.1.2\\scripts\\mPDBS\\summary_pdb_93\\d678141e4018054a.final.pdb
6.361999e-15 -5.160269e-15 -4.610878e-15 -5.803879e-15 -6.281308e-15
-1.183443e-14
E:\\R-3.1.2\\scripts\\mPDBS\\summary_pdb_93\\d71b40af0f0efe39.final.pdb
5.878933e-15 6.144080e-16 1.193371e-17 2.420523e-16 3.319455e-15
-1.256053e-14
E:\\R-3.1.2\\scripts\\mPDBS\\summary_pdb_93\\dc0809eff10197e2.final.pdb
-1.180135e-15 -3.349600e-15 6.329744e-15 6.145745e-15 5.939580e-15
3.688146e-16
E:\\R-3.1.2\\scripts\\mPDBS\\summary_pdb_93\\e1163918d3707839.final.pdb
6.026099e-15 5.172391e-15 3.261652e-16 6.522841e-15 -4.394380e-15
-7.410238e-15
E:\\R-3.1.2\\scripts\\mPDBS\\summary_pdb_93\\e4b48b06824ef848.final.pdb
3.231163e-15 -5.065828e-15 4.949755e-16 -4.067036e-15 -1.452419e-15
-1.114712e-14
E:\\R-3.1.2\\scripts\\mPDBS\\summary_pdb_93\\e529e19ad281fea2.final.pdb

-3.323806e-15 -4.021633e-15 2.817857e-15 6.097112e-15 -5.015774e-15
-1.360952e-15
E:\\R-3.1.2\\scripts\\mPDBS\\summary_pdb_93\\e5602082718ec3a1.final.pdb
-4.077942e-16 1.297601e-15 2.986417e-15 2.817633e-15 -3.347321e-15
-1.544255e-15
E:\\R-3.1.2\\scripts\\mPDBS\\summary_pdb_93\\e5d589d996d0a960.final.pdb
2.148423e-15 2.516953e-15 -1.488341e-15 1.305416e-15 -4.122774e-15
-5.213240e-15
E:\\R-3.1.2\\scripts\\mPDBS\\summary_pdb_93\\e9df9e72307a2fbd.final.pdb
2.153413e-15 -3.889830e-15 3.746408e-16 -3.652698e-16 -2.441602e-15
-6.190609e-15
E:\\R-3.1.2\\scripts\\mPDBS\\summary_pdb_93\\ea00f8694090cae7.final.pdb
-4.077942e-16 1.297601e-15 2.986417e-15 2.817633e-15 -3.347321e-15
-1.544255e-15
E:\\R-3.1.2\\scripts\\mPDBS\\summary_pdb_93\\eba517be32db050e.final.pdb
-1.021265e-15 -2.934483e-15 4.984304e-15 3.527760e-15 3.171570e-17
-3.170825e-15
E:\\R-3.1.2\\scripts\\mPDBS\\summary_pdb_93\\ec04b9545899c29d.final.pdb
2.150293e-15 -2.326223e-15 1.729123e-15 2.768609e-16 -8.217170e-16
-4.216313e-15
E:\\R-3.1.2\\scripts\\mPDBS\\summary_pdb_93\\ee6862cbcad52078.final.pdb
2.602139e-15 -2.714607e-15 1.654503e-15 5.735260e-16 -1.148764e-15
-3.797928e-15
E:\\R-3.1.2\\scripts\\mPDBS\\summary_pdb_93\\ef6a42fc83b81e5d.final.pdb
2.273596e-15 -2.484438e-15 1.351929e-15 8.692196e-17 -1.572982e-15
-4.431758e-15
E:\\R-3.1.2\\scripts\\mPDBS\\summary_pdb_93\\ef8e88d4ad93a0ad.final.pdb
6.805505e-16 -1.426147e-15 2.171080e-15 -6.915451e-16 -2.171815e-15
-5.230857e-15
E:\\R-3.1.2\\scripts\\mPDBS\\summary_pdb_93\\f14fcae8deec4fa3.final.pdb
3.102897e-15 -6.970479e-16 6.434583e-16 -1.121673e-15 3.202696e-16
-3.615260e-15
E:\\R-3.1.2\\scripts\\mPDBS\\summary_pdb_93\\f83584e22003fb92.final.pdb
5.094069e-15 -3.709593e-15 -1.837496e-15 -6.558085e-15 -8.622820e-15
-8.114164e-15
E:\\R-3.1.2\\scripts\\mPDBS\\summary_pdb_93\\f8e2bf87e52caa29.final.pdb
-3.323806e-15 -4.021633e-15 2.817857e-15 6.097112e-15 -5.015774e-15
-1.360952e-15

[,445]   [,446]   [,447]   [,448]   [,449]   [,450]
E:\\R-3.1.2\\scripts\\40.1ns_data\\NAT0.pdb
1.065248e-16 2.347266e-15 -2.016216e-15 4.450307e-15 1.597092e-15
-9.529326e-16
E:\\R-3.1.2\\scripts\\40.1ns_data\\LJP6.pdb
6.448918e-17 -1.979120e-17 -2.538396e-15 4.305096e-15 2.493031e-15
-3.132573e-16
E:\\R-3.1.2\\scripts\\40.1ns_data\\3WY9.pdb
9.921073e-16 8.256363e-16 -3.881529e-16 4.364010e-15 2.305326e-15
-1.608440e-15
E:\\R-3.1.2\\scripts\\40.1ns_data\\ZSP9.pdb
2.495167e-16 -4.475503e-16 -1.579832e-15 4.611265e-15 2.322659e-15
-1.831311e-15
E:\\R-3.1.2\\scripts\\mPDBS\\summary_pdb_93\\013d855ad26c877c.final.pdb
3.731374e-17 -3.332121e-15 3.525666e-15 1.098645e-14 5.949322e-16

-2.496650e-15
E:\\R-3.1.2\\scripts\\mPDBS\\summary_pdb_93\\0a2a705342ca0d54.final.pdb
5.414671e-16 2.368087e-18 -3.098641e-16 5.043833e-15 8.808299e-16
-1.171025e-15
E:\\R-3.1.2\\scripts\\mPDBS\\summary_pdb_93\\0a59fceadb4194d9.final.pdb
4.006402e-16 -5.942305e-15 2.840036e-15 5.472564e-15 1.874676e-15
-4.065572e-15
E:\\R-3.1.2\\scripts\\mPDBS\\summary_pdb_93\\0dba59f3306825e8.final.pdb
4.499023e-15 -2.988646e-15 -1.227476e-15 -1.113248e-15 4.919164e-15
-1.795822e-15
E:\\R-3.1.2\\scripts\\mPDBS\\summary_pdb_93\\126f26a78b57339c.final.pdb
-5.518572e-15 7.560641e-15 -3.047635e-15 -1.117314e-15 -2.871696e-15
-1.690302e-15
E:\\R-3.1.2\\scripts\\mPDBS\\summary_pdb_93\\12f06f3e342df706.final.pdb
1.121231e-16 -1.047511e-16 -1.006356e-15 4.697159e-15 1.978693e-15
-1.622676e-15
E:\\R-3.1.2\\scripts\\mPDBS\\summary_pdb_93\\18618a2a251b867b.final.pdb
-7.729016e-15 3.995096e-15 -5.070242e-15 9.661993e-15 -1.038674e-15
-9.397402e-16
E:\\R-3.1.2\\scripts\\mPDBS\\summary_pdb_93\\1f305f598641eeb2.final.pdb
-2.329606e-17 5.237044e-16 -1.999919e-16 4.470507e-15 1.472986e-15
-1.400766e-15
E:\\R-3.1.2\\scripts\\mPDBS\\summary_pdb_93\\1f8a00b551be0a39.final.pdb
-7.729016e-15 3.995096e-15 -5.070242e-15 9.661993e-15 -1.038674e-15
-9.397402e-16
E:\\R-3.1.2\\scripts\\mPDBS\\summary_pdb_93\\219f45328d47579a.final.pdb
-2.584819e-15 2.023545e-15 -5.072038e-15 8.241183e-15 9.763064e-16
1.389228e-14
E:\\R-3.1.2\\scripts\\mPDBS\\summary_pdb_93\\238dd3918e54336d.final.pdb
-3.587923e-15 6.656991e-15 2.977946e-15 -6.158161e-15 4.216553e-15
5.104881e-15
E:\\R-3.1.2\\scripts\\mPDBS\\summary_pdb_93\\2b3027889e2fdc42.final.pdb
3.316849e-16 -4.063689e-16 -2.512416e-16 4.772007e-15 1.424516e-15
-1.870605e-15
E:\\R-3.1.2\\scripts\\mPDBS\\summary_pdb_93\\2fcb3b46e33793b0.final.pdb
2.891039e-15 -4.826183e-15 -1.171437e-15 9.055381e-15 5.749947e-15
-1.759853e-15
E:\\R-3.1.2\\scripts\\mPDBS\\summary_pdb_93\\34eb328251842006.final.pdb
9.612712e-15 -2.300126e-14 3.426654e-15 1.219121e-14 -3.736914e-15
4.183232e-15
E:\\R-3.1.2\\scripts\\mPDBS\\summary_pdb_93\\354b32871797b7ab.final.pdb
-7.687680e-15 -1.020256e-14 3.928348e-15 2.125317e-14 -1.115206e-15
-1.127313e-14
E:\\R-3.1.2\\scripts\\mPDBS\\summary_pdb_93\\397368adbc32d5de.final.pdb
-5.518572e-15 7.560641e-15 -3.047635e-15 -1.117314e-15 -2.871696e-15
-1.690302e-15
E:\\R-3.1.2\\scripts\\mPDBS\\summary_pdb_93\\411ca13cd3df6edd.final.pdb
4.075422e-15 -6.545727e-15 -2.983789e-15 1.071264e-14 2.745189e-15
7.852841e-16
E:\\R-3.1.2\\scripts\\mPDBS\\summary_pdb_93\\42357eee6642bf12.final.pdb
-6.985510e-15 -2.087749e-14 5.946627e-15 1.774486e-14 -3.159227e-17
7.585433e-16
E:\\R-3.1.2\\scripts\\mPDBS\\summary_pdb_93\\44342d1f75a7fcfc.final.pdb
-2.171067e-15 9.305690e-16 -9.774844e-15 5.130388e-15 4.378544e-15

-1.400828e-14
E:\\R-3.1.2\\scripts\\mPDBS\\summary_pdb_93\\448ef21dffe8cdfb.final.pdb
-1.039000e-14 2.272306e-15 1.506557e-14 1.895545e-14 -1.333158e-14
5.239756e-15
E:\\R-3.1.2\\scripts\\mPDBS\\summary_pdb_93\\472a40b3e8f113c7.final.pdb
4.499023e-15 -2.988646e-15 -1.227476e-15 -1.113248e-15 4.919164e-15
-1.795822e-15
E:\\R-3.1.2\\scripts\\mPDBS\\summary_pdb_93\\48a26225951ea153.final.pdb
5.280851e-15 -1.740371e-15 -1.122684e-14 9.335630e-15 1.212446e-15
-3.356396e-15
E:\\R-3.1.2\\scripts\\mPDBS\\summary_pdb_93\\4ab4a5c7c6682ce6.final.pdb
-3.811604e-15 8.469636e-15 3.603295e-15 2.413332e-14 -9.244687e-16
-1.038300e-14
E:\\R-3.1.2\\scripts\\mPDBS\\summary_pdb_93\\4eaa45b62cb6a25f.final.pdb
1.398257e-16 1.126204e-14 -2.747735e-15 -9.158867e-16 1.390386e-15
-2.531698e-15
E:\\R-3.1.2\\scripts\\mPDBS\\summary_pdb_93\\58c371611d85ac96.final.pdb
-5.518572e-15 7.560641e-15 -3.047635e-15 -1.117314e-15 -2.871696e-15
-1.690302e-15
E:\\R-3.1.2\\scripts\\mPDBS\\summary_pdb_93\\59a647f04822d30a.final.pdb
8.407347e-17 2.570750e-15 -1.475927e-15 5.603100e-15 2.104105e-15
8.348779e-16
E:\\R-3.1.2\\scripts\\mPDBS\\summary_pdb_93\\59b091fe385e05fc.final.pdb
4.006402e-16 -5.942305e-15 2.840036e-15 5.472564e-15 1.874676e-15
-4.065572e-15
E:\\R-3.1.2\\scripts\\mPDBS\\summary_pdb_93\\5c35ac30f930d2db.final.pdb
1.398257e-16 1.126204e-14 -2.747735e-15 -9.158867e-16 1.390386e-15
-2.531698e-15
E:\\R-3.1.2\\scripts\\mPDBS\\summary_pdb_93\\5f60752431cf2dcb.final.pdb
-3.463576e-15 4.088076e-15 -5.026577e-15 4.496887e-16 2.581076e-15
-5.597497e-15
E:\\R-3.1.2\\scripts\\mPDBS\\summary_pdb_93\\626456dd6ff2868d.final.pdb
4.499023e-15 -2.988646e-15 -1.227476e-15 -1.113248e-15 4.919164e-15
-1.795822e-15
E:\\R-3.1.2\\scripts\\mPDBS\\summary_pdb_93\\6583d53ff3c8f7ef.final.pdb
8.407347e-17 2.570750e-15 -1.475927e-15 5.603100e-15 2.104105e-15
8.348779e-16
E:\\R-3.1.2\\scripts\\mPDBS\\summary_pdb_93\\69760178986bef01.final.pdb
-4.980534e-15 2.400011e-14 -9.910207e-15 -5.890039e-15 -2.058515e-15
-7.782390e-16
E:\\R-3.1.2\\scripts\\mPDBS\\summary_pdb_93\\6a9d87d47926b1c8.final.pdb
-2.311347e-16 -7.417453e-16 4.773234e-16 4.895237e-15 1.342381e-15
-1.301724e-15
E:\\R-3.1.2\\scripts\\mPDBS\\summary_pdb_93\\6bb3547ea982ffc4.final.pdb
8.287874e-15 1.898565e-15 -5.861339e-15 1.388443e-15 -3.309325e-15
8.375052e-16
E:\\R-3.1.2\\scripts\\mPDBS\\summary_pdb_93\\716d9624611d1c42.final.pdb
3.306616e-15 4.271523e-15 4.125824e-15 1.031045e-14 -3.433644e-15
-9.272962e-16
E:\\R-3.1.2\\scripts\\mPDBS\\summary_pdb_93\\75f27e70ad0f1be4.final.pdb
8.407347e-17 2.570750e-15 -1.475927e-15 5.603100e-15 2.104105e-15
8.348779e-16
E:\\R-3.1.2\\scripts\\mPDBS\\summary_pdb_93\\769f910fffb22872.final.pdb
1.332312e-15 4.705721e-16 -3.197015e-16 4.950376e-15 1.387987e-15

-1.589232e-15
E:\\R-3.1.2\\scripts\\mPDBS\\summary_pdb_93\\791f8c80dd5c3135.final.pdb
4.006402e-16 -5.942305e-15 2.840036e-15 5.472564e-15 1.874676e-15
-4.065572e-15
E:\\R-3.1.2\\scripts\\mPDBS\\summary_pdb_93\\7fad98e66a3f6099.final.pdb
6.965577e-15 -3.597167e-15 1.738784e-15 8.675808e-15 4.553761e-15
9.019563e-16
E:\\R-3.1.2\\scripts\\mPDBS\\summary_pdb_93\\804b89c94a633196.final.pdb
2.891039e-15 -4.826183e-15 -1.171437e-15 9.055381e-15 5.749947e-15
-1.759853e-15
E:\\R-3.1.2\\scripts\\mPDBS\\summary_pdb_93\\813cc65a2298feba.final.pdb
2.891039e-15 -4.826183e-15 -1.171437e-15 9.055381e-15 5.749947e-15
-1.759853e-15
E:\\R-3.1.2\\scripts\\mPDBS\\summary_pdb_93\\83c0eefcfe12d3f9.final.pdb
4.075422e-15 -6.545727e-15 -2.983789e-15 1.071264e-14 2.745189e-15
7.852841e-16
E:\\R-3.1.2\\scripts\\mPDBS\\summary_pdb_93\\86d50469ef569760.final.pdb
4.499023e-15 -2.988646e-15 -1.227476e-15 -1.113248e-15 4.919164e-15
-1.795822e-15
E:\\R-3.1.2\\scripts\\mPDBS\\summary_pdb_93\\89d0666e1402203d.final.pdb
8.407347e-17 2.570750e-15 -1.475927e-15 5.603100e-15 2.104105e-15
8.348779e-16
E:\\R-3.1.2\\scripts\\mPDBS\\summary_pdb_93\\8b34852716dadc62.final.pdb
-5.714687e-16 7.282187e-15 -4.377206e-15 -1.051118e-15 2.323001e-15
-7.543695e-17
E:\\R-3.1.2\\scripts\\mPDBS\\summary_pdb_93\\8b6ef9ac3fe74395.final.pdb
1.503423e-15 -1.030798e-15 -7.531599e-15 1.738956e-15 4.694032e-15
2.900096e-15
E:\\R-3.1.2\\scripts\\mPDBS\\summary_pdb_93\\98bbe0d5986c138f.final.pdb
4.006402e-16 -5.942305e-15 2.840036e-15 5.472564e-15 1.874676e-15
-4.065572e-15
E:\\R-3.1.2\\scripts\\mPDBS\\summary_pdb_93\\9c22f4909d67e2fa.final.pdb
-3.330980e-15 2.803998e-15 3.543108e-15 -1.005059e-14 -1.268744e-15
-9.611810e-17
E:\\R-3.1.2\\scripts\\mPDBS\\summary_pdb_93\\9dda8ed0d81fb3ef.final.pdb
7.529507e-15 9.179923e-16 -1.272257e-14 1.176641e-14 8.131402e-15
-1.586821e-14
E:\\R-3.1.2\\scripts\\mPDBS\\summary_pdb_93\\9f752b3616e6033b.final.pdb
9.853273e-16 -2.760222e-15 -1.313309e-15 3.868167e-15 4.119912e-15
1.304878e-15
E:\\R-3.1.2\\scripts\\mPDBS\\summary_pdb_93\\a02c70083816d6ba.final.pdb
1.398257e-16 1.126204e-14 -2.747735e-15 -9.158867e-16 1.390386e-15
-2.531698e-15
E:\\R-3.1.2\\scripts\\mPDBS\\summary_pdb_93\\a0c997d891e4e082.final.pdb
7.606917e-15 8.602982e-15 -2.216808e-15 -4.098827e-15 2.628788e-16
-4.322041e-15
E:\\R-3.1.2\\scripts\\mPDBS\\summary_pdb_93\\ab8039a2d823c616.final.pdb
-3.524276e-16 7.247355e-16 -7.292822e-16 4.192446e-15 7.375425e-16
-1.220518e-15
E:\\R-3.1.2\\scripts\\mPDBS\\summary_pdb_93\\acf4d74b9c71d4dc.final.pdb
2.891039e-15 -4.826183e-15 -1.171437e-15 9.055381e-15 5.749947e-15
-1.759853e-15
E:\\R-3.1.2\\scripts\\mPDBS\\summary_pdb_93\\ad8a823e4b4c668e.final.pdb
-2.941938e-16 9.541515e-19 -5.081967e-16 5.968375e-15 1.704399e-15

-1.946775e-15
E:\\R-3.1.2\\scripts\\mPDBS\\summary_pdb_93\\b16ababd03b7b857.final.pdb
-3.330980e-15 2.803998e-15 3.543108e-15 -1.005059e-14 -1.268744e-15
-9.611810e-17
E:\\R-3.1.2\\scripts\\mPDBS\\summary_pdb_93\\b226923e697d3c63.final.pdb
-8.900927e-15 4.494125e-15 -1.410875e-15 8.776452e-15 -1.155224e-15
-3.130863e-15
E:\\R-3.1.2\\scripts\\mPDBS\\summary_pdb_93\\b28e48f6fa27a165.final.pdb
4.006402e-16 -5.942305e-15 2.840036e-15 5.472564e-15 1.874676e-15
-4.065572e-15
E:\\R-3.1.2\\scripts\\mPDBS\\summary_pdb_93\\b42c4924e3cd1aec.final.pdb
1.330794e-16 -1.151099e-15 5.201891e-15 -4.448484e-15 -1.990276e-15
-1.607450e-15
E:\\R-3.1.2\\scripts\\mPDBS\\summary_pdb_93\\b4dd239fcabff1df.final.pdb
4.006402e-16 -5.942305e-15 2.840036e-15 5.472564e-15 1.874676e-15
-4.065572e-15
E:\\R-3.1.2\\scripts\\mPDBS\\summary_pdb_93\\b669942ef97459a2.final.pdb
-5.714687e-16 7.282187e-15 -4.377206e-15 -1.051118e-15 2.323001e-15
-7.543695e-17
E:\\R-3.1.2\\scripts\\mPDBS\\summary_pdb_93\\b99f4d9d87c1017b.final.pdb
4.006402e-16 -5.942305e-15 2.840036e-15 5.472564e-15 1.874676e-15
-4.065572e-15
E:\\R-3.1.2\\scripts\\mPDBS\\summary_pdb_93\\b9d2cbf3975403e7.final.pdb
4.006402e-16 -5.942305e-15 2.840036e-15 5.472564e-15 1.874676e-15
-4.065572e-15
E:\\R-3.1.2\\scripts\\mPDBS\\summary_pdb_93\\bc88c8a505f3a288.final.pdb
3.928339e-16 -8.187994e-16 -4.411939e-16 4.385651e-15 1.445549e-15
-1.292481e-15
E:\\R-3.1.2\\scripts\\mPDBS\\summary_pdb_93\\bdac0e64f4ef427e.final.pdb
-9.411186e-15 1.472632e-14 4.280365e-15 -5.824210e-15 -1.389958e-15
-5.081415e-15
E:\\R-3.1.2\\scripts\\mPDBS\\summary_pdb_93\\ca2a1f55fc9d0681.final.pdb
-5.714687e-16 7.282187e-15 -4.377206e-15 -1.051118e-15 2.323001e-15
-7.543695e-17
E:\\R-3.1.2\\scripts\\mPDBS\\summary_pdb_93\\ce34248de3e81a3c.final.pdb
-2.395837e-15 2.757721e-15 2.949242e-15 8.428239e-15 -3.369712e-17
-8.992745e-15
E:\\R-3.1.2\\scripts\\mPDBS\\summary_pdb_93\\d02e697772494a61.final.pdb
-7.754729e-15 7.355129e-16 6.568824e-15 7.005788e-15 -1.197953e-15
1.298660e-14
E:\\R-3.1.2\\scripts\\mPDBS\\summary_pdb_93\\d04221245d015047.final.pdb
3.403682e-15 -2.304959e-15 -9.747456e-16 3.049101e-15 2.793840e-15
-9.514729e-16
E:\\R-3.1.2\\scripts\\mPDBS\\summary_pdb_93\\d678141e4018054a.final.pdb
5.609343e-15 -1.345442e-14 7.522060e-15 1.220703e-14 -3.193859e-15
-4.954026e-16
E:\\R-3.1.2\\scripts\\mPDBS\\summary_pdb_93\\d71b40af0f0efe39.final.pdb
-5.964467e-15 -4.917019e-15 6.792487e-15 1.158823e-14 8.907684e-15
3.000734e-15
E:\\R-3.1.2\\scripts\\mPDBS\\summary_pdb_93\\dc0809eff10197e2.final.pdb
6.386755e-15 2.487258e-15 -4.426308e-15 -8.032104e-16 3.587234e-15
-4.643308e-16
E:\\R-3.1.2\\scripts\\mPDBS\\summary_pdb_93\\e1163918d3707839.final.pdb
1.138623e-14 -1.614171e-14 7.925407e-15 -2.576717e-15 -6.110678e-15

4.626279e-15
E:\\R-3.1.2\\scripts\\mPDBS\\summary_pdb_93\\e4b48b06824ef848.final.pdb
-7.729016e-15 3.995096e-15 -5.070242e-15 9.661993e-15 -1.038674e-15
-9.397402e-16
E:\\R-3.1.2\\scripts\\mPDBS\\summary_pdb_93\\e529e19ad281fea2.final.pdb
3.731374e-17 -3.332121e-15 3.525666e-15 1.098645e-14 5.949322e-16
-2.496650e-15
E:\\R-3.1.2\\scripts\\mPDBS\\summary_pdb_93\\e5602082718ec3a1.final.pdb
2.891039e-15 -4.826183e-15 -1.171437e-15 9.055381e-15 5.749947e-15
-1.759853e-15
E:\\R-3.1.2\\scripts\\mPDBS\\summary_pdb_93\\e5d589d996d0a960.final.pdb
2.266054e-15 1.278203e-14 -8.769850e-15 -2.161904e-15 7.547701e-15
-1.133644e-15
E:\\R-3.1.2\\scripts\\mPDBS\\summary_pdb_93\\e9df9e72307a2fbd.final.pdb
1.398257e-16 1.126204e-14 -2.747735e-15 -9.158867e-16 1.390386e-15
-2.531698e-15
E:\\R-3.1.2\\scripts\\mPDBS\\summary_pdb_93\\ea00f8694090cae7.final.pdb
2.891039e-15 -4.826183e-15 -1.171437e-15 9.055381e-15 5.749947e-15
-1.759853e-15
E:\\R-3.1.2\\scripts\\mPDBS\\summary_pdb_93\\eba517be32db050e.final.pdb
4.006402e-16 -5.942305e-15 2.840036e-15 5.472564e-15 1.874676e-15
-4.065572e-15
E:\\R-3.1.2\\scripts\\mPDBS\\summary_pdb_93\\ec04b9545899c29d.final.pdb
-4.849158e-16 6.521459e-16 -3.049883e-16 4.989876e-15 1.469413e-15
-1.695216e-15
E:\\R-3.1.2\\scripts\\mPDBS\\summary_pdb_93\\ee6862cbcad52078.final.pdb
-1.856964e-16 -7.043076e-16 -2.225814e-17 4.649438e-15 1.186208e-15
-1.704614e-15
E:\\R-3.1.2\\scripts\\mPDBS\\summary_pdb_93\\ef6a42fc83b81e5d.final.pdb
-1.140135e-16 2.988344e-16 -6.623093e-16 4.546890e-15 1.334428e-15
-1.376565e-15
E:\\R-3.1.2\\scripts\\mPDBS\\summary_pdb_93\\ef8e88d4ad93a0ad.final.pdb
3.403682e-15 -2.304959e-15 -9.747456e-16 3.049101e-15 2.793840e-15
-9.514729e-16
E:\\R-3.1.2\\scripts\\mPDBS\\summary_pdb_93\\f14fcae8deec4fa3.final.pdb
9.853273e-16 -2.760222e-15 -1.313309e-15 3.868167e-15 4.119912e-15
1.304878e-15
E:\\R-3.1.2\\scripts\\mPDBS\\summary_pdb_93\\f83584e22003fb92.final.pdb
5.280851e-15 -1.740371e-15 -1.122684e-14 9.335630e-15 1.212446e-15
-3.356396e-15
E:\\R-3.1.2\\scripts\\mPDBS\\summary_pdb_93\\f8e2bf87e52caa29.final.pdb
3.731374e-17 -3.332121e-15 3.525666e-15 1.098645e-14 5.949322e-16
-2.496650e-15

[,451]   [,452]   [,453]   [,454]   [,455]   [,456]
E:\\R-3.1.2\\scripts\\40.1ns_data\\NAT0.pdb
-1.196792e-15 5.479134e-15 -8.750414e-16 -2.034391e-15 1.866013e-15
4.325719e-15
E:\\R-3.1.2\\scripts\\40.1ns_data\\LJP6.pdb
1.162559e-16 6.276449e-15 -1.826631e-15 -1.362652e-15 2.252939e-15
4.777097e-15
E:\\R-3.1.2\\scripts\\40.1ns_data\\3WY9.pdb
-1.696162e-17 3.726412e-15 -9.296267e-16 -8.774900e-16 1.890139e-15
4.583443e-15

E:\\R-3.1.2\\scripts\\40.1ns_data\\ZSP9.pdb
-3.046569e-16 5.712765e-15 -1.285741e-15 -3.018118e-15 2.506568e-15
4.330281e-15
E:\\R-3.1.2\\scripts\\mPDBS\\summary_pdb_93\\013d855ad26c877c.final.pdb
-8.010021e-15 1.048691e-14 1.731394e-15 -7.118517e-15 3.251762e-16
-2.903806e-16
E:\\R-3.1.2\\scripts\\mPDBS\\summary_pdb_93\\0a2a705342ca0d54.final.pdb
-1.266627e-15 5.764920e-15 -8.045184e-16 -6.737978e-16 2.057571e-15
3.764553e-15
E:\\R-3.1.2\\scripts\\mPDBS\\summary_pdb_93\\0a59fceadb4194d9.final.pdb
-1.619344e-15 4.791082e-15 -1.311831e-15 -2.730852e-16 1.356033e-16
1.695450e-15
E:\\R-3.1.2\\scripts\\mPDBS\\summary_pdb_93\\0dba59f3306825e8.final.pdb
1.342741e-15 4.810893e-15 -2.778887e-15 -9.402990e-16 1.817038e-15
1.277809e-15
E:\\R-3.1.2\\scripts\\mPDBS\\summary_pdb_93\\126f26a78b57339c.final.pdb
8.304895e-18 -2.996028e-16 -6.003998e-16 3.923215e-15 6.181820e-15
8.408390e-15
E:\\R-3.1.2\\scripts\\mPDBS\\summary_pdb_93\\12f06f3e342df706.final.pdb
-6.423438e-16 5.182921e-15 -6.975461e-17 -1.004913e-15 1.778497e-15
4.131013e-15
E:\\R-3.1.2\\scripts\\mPDBS\\summary_pdb_93\\18618a2a251b867b.final.pdb
-3.405450e-15 6.329177e-15 2.514573e-16 2.118843e-15 4.539348e-15
3.049541e-15
E:\\R-3.1.2\\scripts\\mPDBS\\summary_pdb_93\\1f305f598641eeb2.final.pdb
-6.246818e-16 4.789434e-15 -1.950715e-16 -1.272359e-15 2.434181e-15
3.666553e-15
E:\\R-3.1.2\\scripts\\mPDBS\\summary_pdb_93\\1f8a00b551be0a39.final.pdb
-3.405450e-15 6.329177e-15 2.514573e-16 2.118843e-15 4.539348e-15
3.049541e-15
E:\\R-3.1.2\\scripts\\mPDBS\\summary_pdb_93\\219f45328d47579a.final.pdb
2.015769e-15 1.417767e-14 7.737354e-15 -7.364418e-15 4.285062e-15
1.357223e-14
E:\\R-3.1.2\\scripts\\mPDBS\\summary_pdb_93\\238dd3918e54336d.final.pdb
2.087327e-15 7.170255e-15 4.829795e-15 1.522570e-15 8.301055e-15
1.206693e-14
E:\\R-3.1.2\\scripts\\mPDBS\\summary_pdb_93\\2b3027889e2fdc42.final.pdb
-1.361520e-15 6.538816e-15 -3.510650e-16 -1.216775e-15 1.940041e-15
3.592804e-15
E:\\R-3.1.2\\scripts\\mPDBS\\summary_pdb_93\\2fcb3b46e33793b0.final.pdb
-1.608270e-15 8.362964e-15 1.001888e-15 -4.051820e-15 -1.444661e-15
-6.945420e-16
E:\\R-3.1.2\\scripts\\mPDBS\\summary_pdb_93\\34eb328251842006.final.pdb
-5.873728e-15 1.456573e-14 7.222219e-15 -5.512570e-15 -1.571092e-15
-3.243299e-15
E:\\R-3.1.2\\scripts\\mPDBS\\summary_pdb_93\\354b32871797b7ab.final.pdb
-1.077015e-14 5.599701e-15 1.403926e-15 2.429128e-15 -7.008293e-15
2.618805e-15
E:\\R-3.1.2\\scripts\\mPDBS\\summary_pdb_93\\397368adbc32d5de.final.pdb
8.304895e-18 -2.996028e-16 -6.003998e-16 3.923215e-15 6.181820e-15
8.408390e-15
E:\\R-3.1.2\\scripts\\mPDBS\\summary_pdb_93\\411ca13cd3df6edd.final.pdb
-3.745883e-15 6.015883e-15 4.469925e-15 2.778328e-16 -3.365216e-15
-3.927199e-15

E:\\R-3.1.2\\scripts\\mPDBS\\summary_pdb_93\\42357eee6642bf12.final.pdb
-1.116756e-16 1.280738e-14 7.665636e-15 1.282823e-15 7.294243e-16
6.347690e-15
E:\\R-3.1.2\\scripts\\mPDBS\\summary_pdb_93\\44342d1f75a7fcfc.final.pdb
-7.059997e-15 1.570834e-15 6.069292e-15 -5.614132e-15 -2.418676e-15
-3.126648e-15
E:\\R-3.1.2\\scripts\\mPDBS\\summary_pdb_93\\448ef21dffe8cdfb.final.pdb
-1.080838e-14 6.558852e-15 2.474668e-15 1.222639e-14 1.258039e-15
1.164975e-14
E:\\R-3.1.2\\scripts\\mPDBS\\summary_pdb_93\\472a40b3e8f113c7.final.pdb
1.342741e-15 4.810893e-15 -2.778887e-15 -9.402990e-16 1.817038e-15
1.277809e-15
E:\\R-3.1.2\\scripts\\mPDBS\\summary_pdb_93\\48a26225951ea153.final.pdb
-6.242165e-15 8.171190e-15 7.418108e-16 -2.309867e-15 3.618201e-17
1.829024e-15
E:\\R-3.1.2\\scripts\\mPDBS\\summary_pdb_93\\4ab4a5c7c6682ce6.final.pdb
2.281660e-15 9.588321e-15 3.401607e-15 -2.355616e-15 -9.773843e-16
2.126957e-15
E:\\R-3.1.2\\scripts\\mPDBS\\summary_pdb_93\\4eaa45b62cb6a25f.final.pdb
2.304029e-15 2.652406e-15 -3.349921e-15 -2.277394e-15 5.758296e-15
4.287175e-15
E:\\R-3.1.2\\scripts\\mPDBS\\summary_pdb_93\\58c371611d85ac96.final.pdb
8.304895e-18 -2.996028e-16 -6.003998e-16 3.923215e-15 6.181820e-15
8.408390e-15
E:\\R-3.1.2\\scripts\\mPDBS\\summary_pdb_93\\59a647f04822d30a.final.pdb
4.413464e-15 9.652911e-15 -1.044181e-14 -1.549025e-15 2.645849e-15
5.020481e-15
E:\\R-3.1.2\\scripts\\mPDBS\\summary_pdb_93\\59b091fe385e05fc.final.pdb
-1.619344e-15 4.791082e-15 -1.311831e-15 -2.730852e-16 1.356033e-16
1.695450e-15
E:\\R-3.1.2\\scripts\\mPDBS\\summary_pdb_93\\5c35ac30f930d2db.final.pdb
2.304029e-15 2.652406e-15 -3.349921e-15 -2.277394e-15 5.758296e-15
4.287175e-15
E:\\R-3.1.2\\scripts\\mPDBS\\summary_pdb_93\\5f60752431cf2dcb.final.pdb
5.657738e-15 3.606924e-15 -8.932604e-16 -7.113308e-15 6.835324e-15
1.293439e-15
E:\\R-3.1.2\\scripts\\mPDBS\\summary_pdb_93\\626456dd6ff2868d.final.pdb
1.342741e-15 4.810893e-15 -2.778887e-15 -9.402990e-16 1.817038e-15
1.277809e-15
E:\\R-3.1.2\\scripts\\mPDBS\\summary_pdb_93\\6583d53ff3c8f7ef.final.pdb
4.413464e-15 9.652911e-15 -1.044181e-14 -1.549025e-15 2.645849e-15
5.020481e-15
E:\\R-3.1.2\\scripts\\mPDBS\\summary_pdb_93\\69760178986bef01.final.pdb
4.388720e-16 -3.098107e-15 -2.712887e-15 2.335789e-15 3.886250e-15
4.026555e-15
E:\\R-3.1.2\\scripts\\mPDBS\\summary_pdb_93\\6a9d87d47926b1c8.final.pdb
-1.395954e-15 5.709479e-15 -2.447163e-17 -1.244960e-15 1.949094e-15
3.643105e-15
E:\\R-3.1.2\\scripts\\mPDBS\\summary_pdb_93\\6bb3547ea982ffc4.final.pdb
-5.067386e-15 9.357867e-15 1.063935e-14 -3.061559e-15 1.493331e-15
7.275878e-16
E:\\R-3.1.2\\scripts\\mPDBS\\summary_pdb_93\\716d9624611d1c42.final.pdb
-1.033056e-14 1.551277e-15 9.232662e-16 -5.855438e-16 -9.435397e-17
4.228591e-15

E:\\R-3.1.2\\scripts\\mPDBS\\summary_pdb_93\\75f27e70ad0f1be4.final.pdb
4.413464e-15 9.652911e-15 -1.044181e-14 -1.549025e-15 2.645849e-15
5.020481e-15
E:\\R-3.1.2\\scripts\\mPDBS\\summary_pdb_93\\769f910fffb22872.final.pdb
-9.306504e-16 4.185059e-15 -5.359987e-16 -7.391142e-16 2.905022e-15
4.030961e-15
E:\\R-3.1.2\\scripts\\mPDBS\\summary_pdb_93\\791f8c80dd5c3135.final.pdb
-1.619344e-15 4.791082e-15 -1.311831e-15 -2.730852e-16 1.356033e-16
1.695450e-15
E:\\R-3.1.2\\scripts\\mPDBS\\summary_pdb_93\\7fad98e66a3f6099.final.pdb
-6.715953e-15 8.467394e-15 1.531655e-14 -3.556166e-15 3.269935e-15
6.026943e-15
E:\\R-3.1.2\\scripts\\mPDBS\\summary_pdb_93\\804b89c94a633196.final.pdb
-1.608270e-15 8.362964e-15 1.001888e-15 -4.051820e-15 -1.444661e-15
-6.945420e-16
E:\\R-3.1.2\\scripts\\mPDBS\\summary_pdb_93\\813cc65a2298feba.final.pdb
-1.608270e-15 8.362964e-15 1.001888e-15 -4.051820e-15 -1.444661e-15
-6.945420e-16
E:\\R-3.1.2\\scripts\\mPDBS\\summary_pdb_93\\83c0eefcfe12d3f9.final.pdb
-3.745883e-15 6.015883e-15 4.469925e-15 2.778328e-16 -3.365216e-15
-3.927199e-15
E:\\R-3.1.2\\scripts\\mPDBS\\summary_pdb_93\\86d50469ef569760.final.pdb
1.342741e-15 4.810893e-15 -2.778887e-15 -9.402990e-16 1.817038e-15
1.277809e-15
E:\\R-3.1.2\\scripts\\mPDBS\\summary_pdb_93\\89d0666e1402203d.final.pdb
4.413464e-15 9.652911e-15 -1.044181e-14 -1.549025e-15 2.645849e-15
5.020481e-15
E:\\R-3.1.2\\scripts\\mPDBS\\summary_pdb_93\\8b34852716dadc62.final.pdb
5.495423e-15 3.381332e-16 2.697312e-17 2.485052e-15 4.009310e-15
7.973871e-15
E:\\R-3.1.2\\scripts\\mPDBS\\summary_pdb_93\\8b6ef9ac3fe74395.final.pdb
1.259780e-15 5.599966e-15 -1.738585e-15 -2.717823e-16 5.353046e-15
5.100612e-15
E:\\R-3.1.2\\scripts\\mPDBS\\summary_pdb_93\\98bbe0d5986c138f.final.pdb
-1.619344e-15 4.791082e-15 -1.311831e-15 -2.730852e-16 1.356033e-16
1.695450e-15
E:\\R-3.1.2\\scripts\\mPDBS\\summary_pdb_93\\9c22f4909d67e2fa.final.pdb
-9.547129e-16 1.749331e-15 -5.453669e-15 -2.324469e-15 7.449917e-15
1.091119e-14
E:\\R-3.1.2\\scripts\\mPDBS\\summary_pdb_93\\9dda8ed0d81fb3ef.final.pdb
3.925916e-16 6.106644e-16 -1.095897e-14 8.689630e-16 8.299190e-16
-6.073076e-15
E:\\R-3.1.2\\scripts\\mPDBS\\summary_pdb_93\\9f752b3616e6033b.final.pdb
5.642607e-18 7.590072e-15 4.570361e-16 1.800952e-15 6.508804e-16
5.117719e-15
E:\\R-3.1.2\\scripts\\mPDBS\\summary_pdb_93\\a02c70083816d6ba.final.pdb
2.304029e-15 2.652406e-15 -3.349921e-15 -2.277394e-15 5.758296e-15
4.287175e-15
E:\\R-3.1.2\\scripts\\mPDBS\\summary_pdb_93\\a0c997d891e4e082.final.pdb
2.041915e-15 7.034029e-15 3.321550e-15 1.638889e-15 -4.897857e-17
3.689832e-15
E:\\R-3.1.2\\scripts\\mPDBS\\summary_pdb_93\\ab8039a2d823c616.final.pdb
-1.342964e-15 5.522217e-15 6.791319e-17 -1.478192e-15 2.055349e-15
3.693736e-15

E:\\R-3.1.2\\scripts\\mPDBS\\summary_pdb_93\\acf4d74b9c71d4dc.final.pdb
-1.608270e-15 8.362964e-15 1.001888e-15 -4.051820e-15 -1.444661e-15
-6.945420e-16
E:\\R-3.1.2\\scripts\\mPDBS\\summary_pdb_93\\ad8a823e4b4c668e.final.pdb
-1.057906e-15 5.675612e-15 -3.483468e-16 -9.739709e-16 2.339648e-15
3.760617e-15
E:\\R-3.1.2\\scripts\\mPDBS\\summary_pdb_93\\b16ababd03b7b857.final.pdb
-9.547129e-16 1.749331e-15 -5.453669e-15 -2.324469e-15 7.449917e-15
1.091119e-14
E:\\R-3.1.2\\scripts\\mPDBS\\summary_pdb_93\\b226923e697d3c63.final.pdb
-1.378127e-15 6.716891e-15 7.829061e-15 2.488544e-15 4.384259e-15
6.932580e-15
E:\\R-3.1.2\\scripts\\mPDBS\\summary_pdb_93\\b28e48f6fa27a165.final.pdb
-1.619344e-15 4.791082e-15 -1.311831e-15 -2.730852e-16 1.356033e-16
1.695450e-15
E:\\R-3.1.2\\scripts\\mPDBS\\summary_pdb_93\\b42c4924e3cd1aec.final.pdb
-2.937861e-15 3.605664e-15 9.343746e-15 -3.510346e-15 -2.299956e-15
7.845044e-15
E:\\R-3.1.2\\scripts\\mPDBS\\summary_pdb_93\\b4dd239fcabff1df.final.pdb
-1.619344e-15 4.791082e-15 -1.311831e-15 -2.730852e-16 1.356033e-16
1.695450e-15
E:\\R-3.1.2\\scripts\\mPDBS\\summary_pdb_93\\b669942ef97459a2.final.pdb
5.495423e-15 3.381332e-16 2.697312e-17 2.485052e-15 4.009310e-15
7.973871e-15
E:\\R-3.1.2\\scripts\\mPDBS\\summary_pdb_93\\b99f4d9d87c1017b.final.pdb
-1.619344e-15 4.791082e-15 -1.311831e-15 -2.730852e-16 1.356033e-16
1.695450e-15
E:\\R-3.1.2\\scripts\\mPDBS\\summary_pdb_93\\b9d2cbf3975403e7.final.pdb
-1.619344e-15 4.791082e-15 -1.311831e-15 -2.730852e-16 1.356033e-16
1.695450e-15
E:\\R-3.1.2\\scripts\\mPDBS\\summary_pdb_93\\bc88c8a505f3a288.final.pdb
-1.450859e-15 6.004521e-15 7.952592e-17 -1.159529e-15 2.232776e-15
4.076792e-15
E:\\R-3.1.2\\scripts\\mPDBS\\summary_pdb_93\\bdac0e64f4ef427e.final.pdb
5.143274e-15 2.583733e-15 -8.950655e-15 -2.771377e-15 1.452737e-14
1.820310e-14
E:\\R-3.1.2\\scripts\\mPDBS\\summary_pdb_93\\ca2a1f55fc9d0681.final.pdb
5.495423e-15 3.381332e-16 2.697312e-17 2.485052e-15 4.009310e-15
7.973871e-15
E:\\R-3.1.2\\scripts\\mPDBS\\summary_pdb_93\\ce34248de3e81a3c.final.pdb
2.797327e-16 3.637749e-15 -6.105789e-15 -3.665914e-15 7.449003e-15
3.003819e-15
E:\\R-3.1.2\\scripts\\mPDBS\\summary_pdb_93\\d02e697772494a61.final.pdb
-4.401654e-16 1.213503e-14 1.604944e-15 -5.170307e-16 8.102429e-15
1.031919e-14
E:\\R-3.1.2\\scripts\\mPDBS\\summary_pdb_93\\d04221245d015047.final.pdb
-1.136098e-15 4.293166e-15 -2.928659e-15 -2.380465e-15 1.266809e-15
2.934360e-15
E:\\R-3.1.2\\scripts\\mPDBS\\summary_pdb_93\\d678141e4018054a.final.pdb
-1.141684e-14 6.466247e-15 1.473684e-14 -4.564809e-15 3.531941e-15
1.349453e-15
E:\\R-3.1.2\\scripts\\mPDBS\\summary_pdb_93\\d71b40af0f0efe39.final.pdb
-8.652744e-15 4.943994e-15 4.391259e-15 7.757029e-16 3.944922e-16
9.078616e-15

E:\\R-3.1.2\\scripts\\mPDBS\\summary_pdb_93\\dc0809eff10197e2.final.pdb
5.624608e-15 3.350448e-15 -1.200495e-14 -1.660485e-15 1.991839e-15
3.186952e-15
E:\\R-3.1.2\\scripts\\mPDBS\\summary_pdb_93\\e1163918d3707839.final.pdb
-8.916585e-15 6.083717e-15 9.476452e-15 4.400426e-15 -5.264598e-15
2.566281e-15
E:\\R-3.1.2\\scripts\\mPDBS\\summary_pdb_93\\e4b48b06824ef848.final.pdb
-3.405450e-15 6.329177e-15 2.514573e-16 2.118843e-15 4.539348e-15
3.049541e-15
E:\\R-3.1.2\\scripts\\mPDBS\\summary_pdb_93\\e529e19ad281fea2.final.pdb
-8.010021e-15 1.048691e-14 1.731394e-15 -7.118517e-15 3.251762e-16
-2.903806e-16
E:\\R-3.1.2\\scripts\\mPDBS\\summary_pdb_93\\e5602082718ec3a1.final.pdb
-1.608270e-15 8.362964e-15 1.001888e-15 -4.051820e-15 -1.444661e-15
-6.945420e-16
E:\\R-3.1.2\\scripts\\mPDBS\\summary_pdb_93\\e5d589d996d0a960.final.pdb
4.266957e-16 -4.906832e-15 4.089008e-16 -6.950580e-17 1.374931e-14
7.753786e-15
E:\\R-3.1.2\\scripts\\mPDBS\\summary_pdb_93\\e9df9e72307a2fbd.final.pdb
2.304029e-15 2.652406e-15 -3.349921e-15 -2.277394e-15 5.758296e-15
4.287175e-15
E:\\R-3.1.2\\scripts\\mPDBS\\summary_pdb_93\\ea00f8694090cae7.final.pdb
-1.608270e-15 8.362964e-15 1.001888e-15 -4.051820e-15 -1.444661e-15
-6.945420e-16
E:\\R-3.1.2\\scripts\\mPDBS\\summary_pdb_93\\eba517be32db050e.final.pdb
-1.619344e-15 4.791082e-15 -1.311831e-15 -2.730852e-16 1.356033e-16
1.695450e-15
E:\\R-3.1.2\\scripts\\mPDBS\\summary_pdb_93\\ec04b9545899c29d.final.pdb
-1.343216e-15 6.125350e-15 -4.558699e-16 -1.121198e-15 1.647516e-15
3.700790e-15
E:\\R-3.1.2\\scripts\\mPDBS\\summary_pdb_93\\ee6862cbcad52078.final.pdb
-1.458014e-15 6.008857e-15 -6.890136e-17 -8.973689e-16 2.229415e-15
3.662627e-15
E:\\R-3.1.2\\scripts\\mPDBS\\summary_pdb_93\\ef6a42fc83b81e5d.final.pdb
-1.082872e-15 5.702075e-15 -1.906261e-16 -8.345240e-16 2.341995e-15
3.703098e-15
E:\\R-3.1.2\\scripts\\mPDBS\\summary_pdb_93\\ef8e88d4ad93a0ad.final.pdb
-1.136098e-15 4.293166e-15 -2.928659e-15 -2.380465e-15 1.266809e-15
2.934360e-15
E:\\R-3.1.2\\scripts\\mPDBS\\summary_pdb_93\\f14fcae8deec4fa3.final.pdb
5.642607e-18 7.590072e-15 4.570361e-16 1.800952e-15 6.508804e-16
5.117719e-15
E:\\R-3.1.2\\scripts\\mPDBS\\summary_pdb_93\\f83584e22003fb92.final.pdb
-6.242165e-15 8.171190e-15 7.418108e-16 -2.309867e-15 3.618201e-17
1.829024e-15
E:\\R-3.1.2\\scripts\\mPDBS\\summary_pdb_93\\f8e2bf87e52caa29.final.pdb
-8.010021e-15 1.048691e-14 1.731394e-15 -7.118517e-15 3.251762e-16
-2.903806e-16

[,457]   [,458]   [,459]   [,460]   [,461]   [,462]
E:\\R-3.1.2\\scripts\\40.1ns_data\\NAT0.pdb
9.344500e-16 2.245656e-15 3.336993e-15 -3.549203e-15 -4.629968e-15
-5.263659e-17
E:\\R-3.1.2\\scripts\\40.1ns_data\\LJP6.pdb

-8.815142e-16 2.348266e-15 3.357352e-15 -3.882974e-15 -4.068253e-15
-8.131627e-16
E:\\R-3.1.2\\scripts\\40.1ns_data\\3WY9.pdb
1.439680e-15 3.349631e-15 4.708953e-15 -3.674572e-15 -4.624067e-15
5.519804e-16
E:\\R-3.1.2\\scripts\\40.1ns_data\\ZSP9.pdb
1.030574e-15 3.683724e-15 4.509764e-15 -3.003130e-15 -3.944233e-15
6.167109e-16
E:\\R-3.1.2\\scripts\\mPDBS\\summary_pdb_93\\013d855ad26c877c.final.pdb
4.771051e-15 7.083161e-15 1.674003e-15 -5.030047e-15 2.836272e-15
8.989695e-16
E:\\R-3.1.2\\scripts\\mPDBS\\summary_pdb_93\\0a2a705342ca0d54.final.pdb
2.993922e-18 3.507957e-15 3.940731e-15 -3.911600e-15 -3.532313e-15
9.919553e-16
E:\\R-3.1.2\\scripts\\mPDBS\\summary_pdb_93\\0a59fceadb4194d9.final.pdb
-1.736948e-15 4.262518e-15 3.776169e-15 -5.628440e-15 -3.761068e-15
3.299587e-15
E:\\R-3.1.2\\scripts\\mPDBS\\summary_pdb_93\\0dba59f3306825e8.final.pdb
-2.717750e-15 8.728507e-16 1.798903e-15 -6.603441e-15 -8.146262e-15
2.115878e-15
E:\\R-3.1.2\\scripts\\mPDBS\\summary_pdb_93\\126f26a78b57339c.final.pdb
1.627585e-16 2.795753e-16 5.862818e-15 5.133481e-15 -3.562472e-15
-7.624619e-16
E:\\R-3.1.2\\scripts\\mPDBS\\summary_pdb_93\\12f06f3e342df706.final.pdb
-4.471668e-16 3.216957e-15 3.943333e-15 -3.638056e-15 -3.444499e-15
8.505753e-16
E:\\R-3.1.2\\scripts\\mPDBS\\summary_pdb_93\\18618a2a251b867b.final.pdb
3.631453e-15 2.903880e-15 7.495157e-15 -2.987903e-15 -2.035924e-15
3.044108e-15
E:\\R-3.1.2\\scripts\\mPDBS\\summary_pdb_93\\1f305f598641eeb2.final.pdb
1.300596e-16 2.570049e-15 3.659743e-15 -3.911952e-15 -3.369440e-15
7.934928e-16
E:\\R-3.1.2\\scripts\\mPDBS\\summary_pdb_93\\1f8a00b551be0a39.final.pdb
3.631453e-15 2.903880e-15 7.495157e-15 -2.987903e-15 -2.035924e-15
3.044108e-15
E:\\R-3.1.2\\scripts\\mPDBS\\summary_pdb_93\\219f45328d47579a.final.pdb
-3.295597e-15 4.273794e-15 1.021676e-14 -5.943292e-15 -1.345899e-15
-6.134273e-15
E:\\R-3.1.2\\scripts\\mPDBS\\summary_pdb_93\\238dd3918e54336d.final.pdb
-7.278806e-16 -2.583568e-15 8.445069e-15 5.754707e-15 -8.168675e-15
-3.801937e-15
E:\\R-3.1.2\\scripts\\mPDBS\\summary_pdb_93\\2b3027889e2fdc42.final.pdb
-3.831043e-16 2.421109e-15 4.177107e-15 -3.655409e-15 -3.834143e-15
1.123810e-15
E:\\R-3.1.2\\scripts\\mPDBS\\summary_pdb_93\\2fcb3b46e33793b0.final.pdb
-7.303632e-16 3.123426e-15 2.405104e-15 -8.600910e-15 -1.603491e-15
8.325240e-16
E:\\R-3.1.2\\scripts\\mPDBS\\summary_pdb_93\\34eb328251842006.final.pdb
5.630657e-16 1.573939e-14 1.209069e-15 -9.726782e-15 -2.812554e-15
2.228798e-15
E:\\R-3.1.2\\scripts\\mPDBS\\summary_pdb_93\\354b32871797b7ab.final.pdb
-4.171470e-15 1.188190e-14 5.778525e-15 -1.333111e-14 2.117018e-15
5.203532e-15
E:\\R-3.1.2\\scripts\\mPDBS\\summary_pdb_93\\397368adbc32d5de.final.pdb

1.627585e-16 2.795753e-16 5.862818e-15 5.133481e-15 -3.562472e-15
-7.624619e-16
E:\\R-3.1.2\\scripts\\mPDBS\\summary_pdb_93\\411ca13cd3df6edd.final.pdb
-1.053889e-15 5.029887e-15 2.060436e-15 -4.758281e-15 -1.328321e-15
-1.790812e-15
E:\\R-3.1.2\\scripts\\mPDBS\\summary_pdb_93\\42357eee6642bf12.final.pdb
-2.160986e-15 1.120118e-14 1.104390e-14 -9.627062e-15 4.107636e-17
3.959900e-15
E:\\R-3.1.2\\scripts\\mPDBS\\summary_pdb_93\\44342d1f75a7fcfc.final.pdb
4.792668e-15 4.321489e-15 1.563502e-15 -3.636260e-15 -3.430824e-15
8.673582e-15
E:\\R-3.1.2\\scripts\\mPDBS\\summary_pdb_93\\448ef21dffe8cdfb.final.pdb
8.682246e-15 8.076061e-15 9.280247e-15 4.572580e-15 1.011110e-14
-5.400337e-15
E:\\R-3.1.2\\scripts\\mPDBS\\summary_pdb_93\\472a40b3e8f113c7.final.pdb
-2.717750e-15 8.728507e-16 1.798903e-15 -6.603441e-15 -8.146262e-15
2.115878e-15
E:\\R-3.1.2\\scripts\\mPDBS\\summary_pdb_93\\48a26225951ea153.final.pdb
5.974657e-15 4.162771e-15 2.599051e-15 -3.599897e-15 -3.031834e-15
4.433229e-15
E:\\R-3.1.2\\scripts\\mPDBS\\summary_pdb_93\\4ab4a5c7c6682ce6.final.pdb
6.863995e-15 -5.502258e-15 1.124827e-14 -4.426580e-15 -6.317213e-15
8.603719e-16
E:\\R-3.1.2\\scripts\\mPDBS\\summary_pdb_93\\4eaa45b62cb6a25f.final.pdb
2.296989e-15 -2.830290e-15 2.270589e-15 1.990075e-15 -3.772693e-15
-6.242719e-16
E:\\R-3.1.2\\scripts\\mPDBS\\summary_pdb_93\\58c371611d85ac96.final.pdb
1.627585e-16 2.795753e-16 5.862818e-15 5.133481e-15 -3.562472e-15
-7.624619e-16
E:\\R-3.1.2\\scripts\\mPDBS\\summary_pdb_93\\59a647f04822d30a.final.pdb
-2.594651e-15 -1.707542e-15 7.631436e-16 -2.659610e-15 -4.822939e-15
1.605018e-15
E:\\R-3.1.2\\scripts\\mPDBS\\summary_pdb_93\\59b091fe385e05fc.final.pdb
-1.736948e-15 4.262518e-15 3.776169e-15 -5.628440e-15 -3.761068e-15
3.299587e-15
E:\\R-3.1.2\\scripts\\mPDBS\\summary_pdb_93\\5c35ac30f930d2db.final.pdb
2.296989e-15 -2.830290e-15 2.270589e-15 1.990075e-15 -3.772693e-15
-6.242719e-16
E:\\R-3.1.2\\scripts\\mPDBS\\summary_pdb_93\\5f60752431cf2dcb.final.pdb
5.773443e-15 -8.415665e-15 -1.168355e-15 -2.520569e-15 -5.521824e-15
3.357684e-15
E:\\R-3.1.2\\scripts\\mPDBS\\summary_pdb_93\\626456dd6ff2868d.final.pdb
-2.717750e-15 8.728507e-16 1.798903e-15 -6.603441e-15 -8.146262e-15
2.115878e-15
E:\\R-3.1.2\\scripts\\mPDBS\\summary_pdb_93\\6583d53ff3c8f7ef.final.pdb
-2.594651e-15 -1.707542e-15 7.631436e-16 -2.659610e-15 -4.822939e-15
1.605018e-15
E:\\R-3.1.2\\scripts\\mPDBS\\summary_pdb_93\\69760178986bef01.final.pdb
2.722239e-15 -1.200627e-14 4.996822e-15 -4.767768e-19 -4.858059e-15
-1.516165e-15
E:\\R-3.1.2\\scripts\\mPDBS\\summary_pdb_93\\6a9d87d47926b1c8.final.pdb
-1.737778e-16 2.248486e-15 3.821049e-15 -3.829835e-15 -3.822681e-15
8.534330e-16
E:\\R-3.1.2\\scripts\\mPDBS\\summary_pdb_93\\6bb3547ea982ffc4.final.pdb

8.472731e-15 4.249716e-15 4.984101e-15 -3.287286e-15 -8.237400e-16
-2.331332e-15
E:\\R-3.1.2\\scripts\\mPDBS\\summary_pdb_93\\716d9624611d1c42.final.pdb
-9.216137e-16 1.293531e-14 3.559525e-15 -8.515482e-16 -7.316017e-16
-4.337549e-15
E:\\R-3.1.2\\scripts\\mPDBS\\summary_pdb_93\\75f27e70ad0f1be4.final.pdb
-2.594651e-15 -1.707542e-15 7.631436e-16 -2.659610e-15 -4.822939e-15
1.605018e-15
E:\\R-3.1.2\\scripts\\mPDBS\\summary_pdb_93\\769f910fffb22872.final.pdb
8.437745e-17 2.598569e-15 4.056176e-15 -3.832605e-15 -2.972133e-15
7.824624e-16
E:\\R-3.1.2\\scripts\\mPDBS\\summary_pdb_93\\791f8c80dd5c3135.final.pdb
-1.736948e-15 4.262518e-15 3.776169e-15 -5.628440e-15 -3.761068e-15
3.299587e-15
E:\\R-3.1.2\\scripts\\mPDBS\\summary_pdb_93\\7fad98e66a3f6099.final.pdb
-4.272300e-17 1.042133e-14 3.161306e-15 -8.984738e-15 -2.704959e-15
-4.996291e-15
E:\\R-3.1.2\\scripts\\mPDBS\\summary_pdb_93\\804b89c94a633196.final.pdb
-7.303632e-16 3.123426e-15 2.405104e-15 -8.600910e-15 -1.603491e-15
8.325240e-16
E:\\R-3.1.2\\scripts\\mPDBS\\summary_pdb_93\\813cc65a2298feba.final.pdb
-7.303632e-16 3.123426e-15 2.405104e-15 -8.600910e-15 -1.603491e-15
8.325240e-16
E:\\R-3.1.2\\scripts\\mPDBS\\summary_pdb_93\\83c0eefcfe12d3f9.final.pdb
-1.053889e-15 5.029887e-15 2.060436e-15 -4.758281e-15 -1.328321e-15
-1.790812e-15
E:\\R-3.1.2\\scripts\\mPDBS\\summary_pdb_93\\86d50469ef569760.final.pdb
-2.717750e-15 8.728507e-16 1.798903e-15 -6.603441e-15 -8.146262e-15
2.115878e-15
E:\\R-3.1.2\\scripts\\mPDBS\\summary_pdb_93\\89d0666e1402203d.final.pdb
-2.594651e-15 -1.707542e-15 7.631436e-16 -2.659610e-15 -4.822939e-15
1.605018e-15
E:\\R-3.1.2\\scripts\\mPDBS\\summary_pdb_93\\8b34852716dadc62.final.pdb
-4.074910e-15 -1.627982e-15 7.363377e-15 -9.542489e-16 -1.058242e-14
2.092801e-15
E:\\R-3.1.2\\scripts\\mPDBS\\summary_pdb_93\\8b6ef9ac3fe74395.final.pdb
-4.204040e-15 -2.117454e-16 5.051526e-15 -5.777861e-15 -3.638938e-15
1.994914e-15
E:\\R-3.1.2\\scripts\\mPDBS\\summary_pdb_93\\98bbe0d5986c138f.final.pdb
-1.736948e-15 4.262518e-15 3.776169e-15 -5.628440e-15 -3.761068e-15
3.299587e-15
E:\\R-3.1.2\\scripts\\mPDBS\\summary_pdb_93\\9c22f4909d67e2fa.final.pdb
4.314678e-16 6.027353e-15 8.441780e-16 -2.204876e-15 -4.608358e-15
3.660557e-15
E:\\R-3.1.2\\scripts\\mPDBS\\summary_pdb_93\\9dda8ed0d81fb3ef.final.pdb
1.296842e-14 1.517309e-15 9.316286e-16 1.788871e-15 -1.003948e-14
5.945730e-15
E:\\R-3.1.2\\scripts\\mPDBS\\summary_pdb_93\\9f752b3616e6033b.final.pdb
-2.312439e-15 3.306251e-15 5.951470e-15 -6.165142e-15 -4.013451e-15
1.307997e-15
E:\\R-3.1.2\\scripts\\mPDBS\\summary_pdb_93\\a02c70083816d6ba.final.pdb
2.296989e-15 -2.830290e-15 2.270589e-15 1.990075e-15 -3.772693e-15
-6.242719e-16
E:\\R-3.1.2\\scripts\\mPDBS\\summary_pdb_93\\a0c997d891e4e082.final.pdb

-3.225297e-15 3.745105e-16 9.610230e-15 -4.166575e-17 -2.851001e-17
-2.409360e-15
E:\\R-3.1.2\\scripts\\mPDBS\\summary_pdb_93\\ab8039a2d823c616.final.pdb
9.615621e-17 3.080679e-15 3.171153e-15 -3.525966e-15 -3.372732e-15
4.412240e-16
E:\\R-3.1.2\\scripts\\mPDBS\\summary_pdb_93\\acf4d74b9c71d4dc.final.pdb
-7.303632e-16 3.123426e-15 2.405104e-15 -8.600910e-15 -1.603491e-15
8.325240e-16
E:\\R-3.1.2\\scripts\\mPDBS\\summary_pdb_93\\ad8a823e4b4c668e.final.pdb
-3.844292e-16 2.795199e-15 3.776813e-15 -4.001798e-15 -3.752795e-15
1.014939e-15
E:\\R-3.1.2\\scripts\\mPDBS\\summary_pdb_93\\b16ababd03b7b857.final.pdb
4.314678e-16 6.027353e-15 8.441780e-16 -2.204876e-15 -4.608358e-15
3.660557e-15
E:\\R-3.1.2\\scripts\\mPDBS\\summary_pdb_93\\b226923e697d3c63.final.pdb
-3.458110e-15 3.109785e-15 1.234069e-14 -4.539292e-15 9.848066e-16
-2.211374e-15
E:\\R-3.1.2\\scripts\\mPDBS\\summary_pdb_93\\b28e48f6fa27a165.final.pdb
-1.736948e-15 4.262518e-15 3.776169e-15 -5.628440e-15 -3.761068e-15
3.299587e-15
E:\\R-3.1.2\\scripts\\mPDBS\\summary_pdb_93\\b42c4924e3cd1aec.final.pdb
-7.341180e-15 4.046536e-15 4.413978e-15 -6.020529e-15 4.958314e-16
3.037466e-15
E:\\R-3.1.2\\scripts\\mPDBS\\summary_pdb_93\\b4dd239fcabff1df.final.pdb
-1.736948e-15 4.262518e-15 3.776169e-15 -5.628440e-15 -3.761068e-15
3.299587e-15
E:\\R-3.1.2\\scripts\\mPDBS\\summary_pdb_93\\b669942ef97459a2.final.pdb
-4.074910e-15 -1.627982e-15 7.363377e-15 -9.542489e-16 -1.058242e-14
2.092801e-15
E:\\R-3.1.2\\scripts\\mPDBS\\summary_pdb_93\\b99f4d9d87c1017b.final.pdb
-1.736948e-15 4.262518e-15 3.776169e-15 -5.628440e-15 -3.761068e-15
3.299587e-15
E:\\R-3.1.2\\scripts\\mPDBS\\summary_pdb_93\\b9d2cbf3975403e7.final.pdb
-1.736948e-15 4.262518e-15 3.776169e-15 -5.628440e-15 -3.761068e-15
3.299587e-15
E:\\R-3.1.2\\scripts\\mPDBS\\summary_pdb_93\\bc88c8a505f3a288.final.pdb
-2.550600e-16 2.455803e-15 4.012308e-15 -3.500693e-15 -4.186394e-15
1.276032e-15
E:\\R-3.1.2\\scripts\\mPDBS\\summary_pdb_93\\bdac0e64f4ef427e.final.pdb
2.602474e-15 -1.553389e-15 6.401907e-15 -1.992915e-15 -9.275414e-15
7.564118e-15
E:\\R-3.1.2\\scripts\\mPDBS\\summary_pdb_93\\ca2a1f55fc9d0681.final.pdb
-4.074910e-15 -1.627982e-15 7.363377e-15 -9.542489e-16 -1.058242e-14
2.092801e-15
E:\\R-3.1.2\\scripts\\mPDBS\\summary_pdb_93\\ce34248de3e81a3c.final.pdb
-3.912444e-16 3.318770e-16 3.400633e-15 2.192639e-15 -2.949004e-15
2.818621e-15
E:\\R-3.1.2\\scripts\\mPDBS\\summary_pdb_93\\d02e697772494a61.final.pdb
-5.644954e-15 2.232891e-15 6.216383e-15 -6.804691e-15 -1.392412e-15
-5.653754e-15
E:\\R-3.1.2\\scripts\\mPDBS\\summary_pdb_93\\d04221245d015047.final.pdb
1.236412e-15 2.788378e-15 1.011206e-15 -3.714943e-15 -5.208394e-15
2.508581e-15
E:\\R-3.1.2\\scripts\\mPDBS\\summary_pdb_93\\d678141e4018054a.final.pdb

8.840701e-15 1.681203e-14 4.634548e-15 -5.816678e-15 -5.184835e-15
-3.354889e-15
E:\\R-3.1.2\\scripts\\mPDBS\\summary_pdb_93\\d71b40af0f0efe39.final.pdb
-2.564505e-15 1.371107e-14 9.976702e-16 -1.118892e-14 -7.662438e-15
-3.102076e-16
E:\\R-3.1.2\\scripts\\mPDBS\\summary_pdb_93\\dc0809eff10197e2.final.pdb
-8.584631e-15 -2.457088e-15 -3.615074e-15 -1.666271e-15 -6.519894e-15
1.931446e-15
E:\\R-3.1.2\\scripts\\mPDBS\\summary_pdb_93\\e1163918d3707839.final.pdb
-3.103251e-15 1.510800e-14 7.464164e-15 -1.371750e-14 5.379078e-15
-3.849820e-15
E:\\R-3.1.2\\scripts\\mPDBS\\summary_pdb_93\\e4b48b06824ef848.final.pdb
3.631453e-15 2.903880e-15 7.495157e-15 -2.987903e-15 -2.035924e-15
3.044108e-15
E:\\R-3.1.2\\scripts\\mPDBS\\summary_pdb_93\\e529e19ad281fea2.final.pdb
4.771051e-15 7.083161e-15 1.674003e-15 -5.030047e-15 2.836272e-15
8.989695e-16
E:\\R-3.1.2\\scripts\\mPDBS\\summary_pdb_93\\e5602082718ec3a1.final.pdb
-7.303632e-16 3.123426e-15 2.405104e-15 -8.600910e-15 -1.603491e-15
8.325240e-16
E:\\R-3.1.2\\scripts\\mPDBS\\summary_pdb_93\\e5d589d996d0a960.final.pdb
3.629781e-15 -6.299814e-15 3.772888e-15 5.071252e-15 -9.696679e-15
-1.551894e-15
E:\\R-3.1.2\\scripts\\mPDBS\\summary_pdb_93\\e9df9e72307a2fbd.final.pdb
2.296989e-15 -2.830290e-15 2.270589e-15 1.990075e-15 -3.772693e-15
-6.242719e-16
E:\\R-3.1.2\\scripts\\mPDBS\\summary_pdb_93\\ea00f8694090cae7.final.pdb
-7.303632e-16 3.123426e-15 2.405104e-15 -8.600910e-15 -1.603491e-15
8.325240e-16
E:\\R-3.1.2\\scripts\\mPDBS\\summary_pdb_93\\eba517be32db050e.final.pdb
-1.736948e-15 4.262518e-15 3.776169e-15 -5.628440e-15 -3.761068e-15
3.299587e-15
E:\\R-3.1.2\\scripts\\mPDBS\\summary_pdb_93\\ec04b9545899c29d.final.pdb
-1.408851e-16 2.810545e-15 3.764866e-15 -4.252954e-15 -3.719221e-15
1.075493e-15
E:\\R-3.1.2\\scripts\\mPDBS\\summary_pdb_93\\ee6862cbcad52078.final.pdb
-1.730944e-16 2.611928e-15 4.094274e-15 -3.446158e-15 -4.081992e-15
8.445198e-16
E:\\R-3.1.2\\scripts\\mPDBS\\summary_pdb_93\\ef6a42fc83b81e5d.final.pdb
-3.998054e-17 3.197048e-15 3.740890e-15 -3.975612e-15 -3.606933e-15
1.211373e-15
E:\\R-3.1.2\\scripts\\mPDBS\\summary_pdb_93\\ef8e88d4ad93a0ad.final.pdb
1.236412e-15 2.788378e-15 1.011206e-15 -3.714943e-15 -5.208394e-15
2.508581e-15
E:\\R-3.1.2\\scripts\\mPDBS\\summary_pdb_93\\f14fcae8deec4fa3.final.pdb
-2.312439e-15 3.306251e-15 5.951470e-15 -6.165142e-15 -4.013451e-15
1.307997e-15
E:\\R-3.1.2\\scripts\\mPDBS\\summary_pdb_93\\f83584e22003fb92.final.pdb
5.974657e-15 4.162771e-15 2.599051e-15 -3.599897e-15 -3.031834e-15
4.433229e-15
E:\\R-3.1.2\\scripts\\mPDBS\\summary_pdb_93\\f8e2bf87e52caa29.final.pdb
4.771051e-15 7.083161e-15 1.674003e-15 -5.030047e-15 2.836272e-15
8.989695e-16

[,463]   [,464]   [,465]   [,466]   [,467]   [,468]
E:\\R-3.1.2\\scripts\\40.1ns_data\\NAT0.pdb
8.204892e-16 1.661357e-15 8.178827e-16 -3.119249e-15 2.375306e-15
4.352905e-15
E:\\R-3.1.2\\scripts\\40.1ns_data\\LJP6.pdb
1.522864e-15 1.308703e-15 -2.442542e-15 -4.787092e-15 -9.842770e-16
3.587162e-15
E:\\R-3.1.2\\scripts\\40.1ns_data\\3WY9.pdb
6.324499e-16 2.383103e-15 8.954546e-16 -4.041555e-15 -1.172003e-15
4.738142e-15
E:\\R-3.1.2\\scripts\\40.1ns_data\\ZSP9.pdb
1.778841e-15 9.770576e-16 -2.756143e-15 -3.881096e-15 1.160053e-15
4.612059e-15
E:\\R-3.1.2\\scripts\\mPDBS\\summary_pdb_93\\013d855ad26c877c.final.pdb
1.667210e-15 3.947890e-16 -1.903573e-16 -7.102138e-15 2.666266e-15
-2.682058e-15
E:\\R-3.1.2\\scripts\\mPDBS\\summary_pdb_93\\0a2a705342ca0d54.final.pdb
8.259068e-16 1.646371e-15 3.456590e-17 -4.241864e-15 -1.333186e-16
3.649442e-15
E:\\R-3.1.2\\scripts\\mPDBS\\summary_pdb_93\\0a59fceadb4194d9.final.pdb
2.418970e-15 3.729164e-15 5.223790e-16 -4.268239e-15 -2.696265e-15
7.246149e-15
E:\\R-3.1.2\\scripts\\mPDBS\\summary_pdb_93\\0dba59f3306825e8.final.pdb
2.440897e-15 2.178085e-15 1.086399e-15 -2.601832e-15 -3.642959e-16
4.659292e-15
E:\\R-3.1.2\\scripts\\mPDBS\\summary_pdb_93\\126f26a78b57339c.final.pdb
-8.537771e-16 1.787664e-15 -4.128613e-15 -3.277995e-15 -1.422477e-15
6.724480e-15
E:\\R-3.1.2\\scripts\\mPDBS\\summary_pdb_93\\12f06f3e342df706.final.pdb
9.022346e-16 1.994637e-15 -1.251371e-16 -4.086552e-15 -5.774078e-16
4.083774e-15
E:\\R-3.1.2\\scripts\\mPDBS\\summary_pdb_93\\18618a2a251b867b.final.pdb
2.133946e-15 4.717639e-16 1.283298e-15 1.227748e-15 -1.275744e-15
9.168127e-16
E:\\R-3.1.2\\scripts\\mPDBS\\summary_pdb_93\\1f305f598641eeb2.final.pdb
1.556722e-15 2.215749e-15 -3.037626e-16 -4.281639e-15 -8.972253e-16
4.041065e-15
E:\\R-3.1.2\\scripts\\mPDBS\\summary_pdb_93\\1f8a00b551be0a39.final.pdb
2.133946e-15 4.717639e-16 1.283298e-15 1.227748e-15 -1.275744e-15
9.168127e-16
E:\\R-3.1.2\\scripts\\mPDBS\\summary_pdb_93\\219f45328d47579a.final.pdb
2.426001e-14 -6.466673e-15 -2.770451e-15 -2.190322e-14 1.632888e-14
-1.162786e-14
E:\\R-3.1.2\\scripts\\mPDBS\\summary_pdb_93\\238dd3918e54336d.final.pdb
1.266897e-15 2.270645e-15 -1.652334e-14 -5.344229e-15 4.579599e-15
1.994057e-15
E:\\R-3.1.2\\scripts\\mPDBS\\summary_pdb_93\\2b3027889e2fdc42.final.pdb
2.100215e-15 1.667340e-15 -1.363916e-15 -4.337059e-15 1.365388e-16
3.977280e-15
E:\\R-3.1.2\\scripts\\mPDBS\\summary_pdb_93\\2fcb3b46e33793b0.final.pdb
3.511755e-15 1.384816e-15 2.911547e-15 -5.844625e-15 -1.892957e-15
2.603847e-15
E:\\R-3.1.2\\scripts\\mPDBS\\summary_pdb_93\\34eb328251842006.final.pdb
3.816342e-15 8.078268e-15 2.205245e-16 -9.936599e-15 -5.422362e-16

1.110598e-14
E:\\R-3.1.2\\scripts\\mPDBS\\summary_pdb_93\\354b32871797b7ab.final.pdb
1.976750e-15 -4.904277e-15 -4.690427e-17 -1.616856e-14 -7.715955e-15
5.025185e-15
E:\\R-3.1.2\\scripts\\mPDBS\\summary_pdb_93\\397368adbc32d5de.final.pdb
-8.537771e-16 1.787664e-15 -4.128613e-15 -3.277995e-15 -1.422477e-15
6.724480e-15
E:\\R-3.1.2\\scripts\\mPDBS\\summary_pdb_93\\411ca13cd3df6edd.final.pdb
2.620541e-15 1.291249e-15 3.972764e-15 -5.208795e-15 -7.355601e-15
5.668236e-15
E:\\R-3.1.2\\scripts\\mPDBS\\summary_pdb_93\\42357eee6642bf12.final.pdb
8.104910e-15 6.705397e-15 6.224387e-16 -9.347361e-15 -4.077062e-16
6.753106e-15
E:\\R-3.1.2\\scripts\\mPDBS\\summary_pdb_93\\44342d1f75a7fcfc.final.pdb
-1.019047e-14 7.936013e-15 7.331832e-15 9.454268e-15 -1.110501e-14
1.328148e-14
E:\\R-3.1.2\\scripts\\mPDBS\\summary_pdb_93\\448ef21dffe8cdfb.final.pdb
-5.756872e-15 1.797680e-15 -1.308000e-14 -1.036011e-14 5.373910e-16
-2.159464e-16
E:\\R-3.1.2\\scripts\\mPDBS\\summary_pdb_93\\472a40b3e8f113c7.final.pdb
2.440897e-15 2.178085e-15 1.086399e-15 -2.601832e-15 -3.642959e-16
4.659292e-15
E:\\R-3.1.2\\scripts\\mPDBS\\summary_pdb_93\\48a26225951ea153.final.pdb
-9.227530e-15 4.592952e-15 2.435454e-15 4.631028e-15 -4.474790e-15
6.483088e-15
E:\\R-3.1.2\\scripts\\mPDBS\\summary_pdb_93\\4ab4a5c7c6682ce6.final.pdb
-9.144924e-16 4.157296e-15 5.735024e-15 -1.636946e-15 -5.778368e-15
8.585124e-16
E:\\R-3.1.2\\scripts\\mPDBS\\summary_pdb_93\\4eaa45b62cb6a25f.final.pdb
-8.107333e-16 2.154798e-15 -1.149429e-15 3.215317e-16 -6.599677e-16
3.920600e-15
E:\\R-3.1.2\\scripts\\mPDBS\\summary_pdb_93\\58c371611d85ac96.final.pdb
-8.537771e-16 1.787664e-15 -4.128613e-15 -3.277995e-15 -1.422477e-15
6.724480e-15
E:\\R-3.1.2\\scripts\\mPDBS\\summary_pdb_93\\59a647f04822d30a.final.pdb
1.384473e-15 1.189153e-16 -2.388800e-15 -7.385560e-15 6.227050e-15
4.103278e-15
E:\\R-3.1.2\\scripts\\mPDBS\\summary_pdb_93\\59b091fe385e05fc.final.pdb
2.418970e-15 3.729164e-15 5.223790e-16 -4.268239e-15 -2.696265e-15
7.246149e-15
E:\\R-3.1.2\\scripts\\mPDBS\\summary_pdb_93\\5c35ac30f930d2db.final.pdb
-8.107333e-16 2.154798e-15 -1.149429e-15 3.215317e-16 -6.599677e-16
3.920600e-15
E:\\R-3.1.2\\scripts\\mPDBS\\summary_pdb_93\\5f60752431cf2dcb.final.pdb
-3.883434e-15 6.340548e-16 7.342016e-16 4.218084e-15 -2.069270e-15
4.710226e-15
E:\\R-3.1.2\\scripts\\mPDBS\\summary_pdb_93\\626456dd6ff2868d.final.pdb
2.440897e-15 2.178085e-15 1.086399e-15 -2.601832e-15 -3.642959e-16
4.659292e-15
E:\\R-3.1.2\\scripts\\mPDBS\\summary_pdb_93\\6583d53ff3c8f7ef.final.pdb
1.384473e-15 1.189153e-16 -2.388800e-15 -7.385560e-15 6.227050e-15
4.103278e-15
E:\\R-3.1.2\\scripts\\mPDBS\\summary_pdb_93\\69760178986bef01.final.pdb
6.022356e-15 -3.724472e-15 -6.930169e-15 2.232298e-15 6.683051e-15

-1.870135e-15
E:\\R-3.1.2\\scripts\\mPDBS\\summary_pdb_93\\6a9d87d47926b1c8.final.pdb
1.848922e-15 1.810215e-15 -7.016670e-16 -4.801082e-15 3.295935e-16
3.952392e-15
E:\\R-3.1.2\\scripts\\mPDBS\\summary_pdb_93\\6bb3547ea982ffc4.final.pdb
1.463454e-15 -8.159145e-16 -8.033390e-15 -7.647447e-16 3.385624e-15
-1.528939e-15
E:\\R-3.1.2\\scripts\\mPDBS\\summary_pdb_93\\716d9624611d1c42.final.pdb
5.161848e-15 2.744885e-15 -2.562640e-15 -9.201655e-15 -1.352396e-15
3.925046e-15
E:\\R-3.1.2\\scripts\\mPDBS\\summary_pdb_93\\75f27e70ad0f1be4.final.pdb
1.384473e-15 1.189153e-16 -2.388800e-15 -7.385560e-15 6.227050e-15
4.103278e-15
E:\\R-3.1.2\\scripts\\mPDBS\\summary_pdb_93\\769f910fffb22872.final.pdb
1.422927e-15 2.213047e-15 -1.338303e-15 -3.842361e-15 -7.106575e-16
3.462736e-15
E:\\R-3.1.2\\scripts\\mPDBS\\summary_pdb_93\\791f8c80dd5c3135.final.pdb
2.418970e-15 3.729164e-15 5.223790e-16 -4.268239e-15 -2.696265e-15
7.246149e-15
E:\\R-3.1.2\\scripts\\mPDBS\\summary_pdb_93\\7fad98e66a3f6099.final.pdb
1.333141e-16 5.537771e-15 -6.310301e-15 -9.889844e-15 -4.648367e-15
9.405805e-15
E:\\R-3.1.2\\scripts\\mPDBS\\summary_pdb_93\\804b89c94a633196.final.pdb
3.511755e-15 1.384816e-15 2.911547e-15 -5.844625e-15 -1.892957e-15
2.603847e-15
E:\\R-3.1.2\\scripts\\mPDBS\\summary_pdb_93\\813cc65a2298feba.final.pdb
3.511755e-15 1.384816e-15 2.911547e-15 -5.844625e-15 -1.892957e-15
2.603847e-15
E:\\R-3.1.2\\scripts\\mPDBS\\summary_pdb_93\\83c0eefcfe12d3f9.final.pdb
2.620541e-15 1.291249e-15 3.972764e-15 -5.208795e-15 -7.355601e-15
5.668236e-15
E:\\R-3.1.2\\scripts\\mPDBS\\summary_pdb_93\\86d50469ef569760.final.pdb
2.440897e-15 2.178085e-15 1.086399e-15 -2.601832e-15 -3.642959e-16
4.659292e-15
E:\\R-3.1.2\\scripts\\mPDBS\\summary_pdb_93\\89d0666e1402203d.final.pdb
1.384473e-15 1.189153e-16 -2.388800e-15 -7.385560e-15 6.227050e-15
4.103278e-15
E:\\R-3.1.2\\scripts\\mPDBS\\summary_pdb_93\\8b34852716dadc62.final.pdb
2.410206e-15 3.447402e-15 2.915884e-15 -1.876919e-16 -3.207877e-15
9.373605e-15
E:\\R-3.1.2\\scripts\\mPDBS\\summary_pdb_93\\8b6ef9ac3fe74395.final.pdb
2.883040e-15 7.875709e-16 -6.076626e-15 4.871920e-16 1.117167e-14
4.261766e-15
E:\\R-3.1.2\\scripts\\mPDBS\\summary_pdb_93\\98bbe0d5986c138f.final.pdb
2.418970e-15 3.729164e-15 5.223790e-16 -4.268239e-15 -2.696265e-15
7.246149e-15
E:\\R-3.1.2\\scripts\\mPDBS\\summary_pdb_93\\9c22f4909d67e2fa.final.pdb
6.115143e-16 2.979081e-15 -4.988817e-15 -7.443552e-15 3.111721e-15
3.678508e-15
E:\\R-3.1.2\\scripts\\mPDBS\\summary_pdb_93\\9dda8ed0d81fb3ef.final.pdb
-6.762222e-15 7.702151e-15 2.278582e-15 1.923976e-14 -1.308259e-14
1.298268e-14
E:\\R-3.1.2\\scripts\\mPDBS\\summary_pdb_93\\9f752b3616e6033b.final.pdb
3.081070e-15 1.272567e-15 -4.055492e-17 -5.174212e-15 1.748447e-15

2.925603e-15
E:\\R-3.1.2\\scripts\\mPDBS\\summary_pdb_93\\a02c70083816d6ba.final.pdb
-8.107333e-16 2.154798e-15 -1.149429e-15 3.215317e-16 -6.599677e-16
3.920600e-15
E:\\R-3.1.2\\scripts\\mPDBS\\summary_pdb_93\\a0c997d891e4e082.final.pdb
1.607898e-15 -4.660806e-15 -4.979547e-15 -8.214350e-15 -5.207466e-15
9.731351e-15
E:\\R-3.1.2\\scripts\\mPDBS\\summary_pdb_93\\ab8039a2d823c616.final.pdb
1.545231e-15 2.070304e-15 -1.942372e-15 -4.098312e-15 -1.329058e-15
4.170695e-15
E:\\R-3.1.2\\scripts\\mPDBS\\summary_pdb_93\\acf4d74b9c71d4dc.final.pdb
3.511755e-15 1.384816e-15 2.911547e-15 -5.844625e-15 -1.892957e-15
2.603847e-15
E:\\R-3.1.2\\scripts\\mPDBS\\summary_pdb_93\\ad8a823e4b4c668e.final.pdb
1.400742e-15 1.875593e-15 -3.759412e-16 -3.872853e-15 -5.863387e-17
4.079649e-15
E:\\R-3.1.2\\scripts\\mPDBS\\summary_pdb_93\\b16ababd03b7b857.final.pdb
6.115143e-16 2.979081e-15 -4.988817e-15 -7.443552e-15 3.111721e-15
3.678508e-15
E:\\R-3.1.2\\scripts\\mPDBS\\summary_pdb_93\\b226923e697d3c63.final.pdb
1.036058e-14 -5.649528e-15 -5.894893e-15 -5.929747e-15 1.843838e-15
4.916647e-15
E:\\R-3.1.2\\scripts\\mPDBS\\summary_pdb_93\\b28e48f6fa27a165.final.pdb
2.418970e-15 3.729164e-15 5.223790e-16 -4.268239e-15 -2.696265e-15
7.246149e-15
E:\\R-3.1.2\\scripts\\mPDBS\\summary_pdb_93\\b42c4924e3cd1aec.final.pdb
2.258123e-15 -1.074957e-15 -1.287576e-15 -1.329183e-14 4.346176e-15
-4.386675e-15
E:\\R-3.1.2\\scripts\\mPDBS\\summary_pdb_93\\b4dd239fcabff1df.final.pdb
2.418970e-15 3.729164e-15 5.223790e-16 -4.268239e-15 -2.696265e-15
7.246149e-15
E:\\R-3.1.2\\scripts\\mPDBS\\summary_pdb_93\\b669942ef97459a2.final.pdb
2.410206e-15 3.447402e-15 2.915884e-15 -1.876919e-16 -3.207877e-15
9.373605e-15
E:\\R-3.1.2\\scripts\\mPDBS\\summary_pdb_93\\b99f4d9d87c1017b.final.pdb
2.418970e-15 3.729164e-15 5.223790e-16 -4.268239e-15 -2.696265e-15
7.246149e-15
E:\\R-3.1.2\\scripts\\mPDBS\\summary_pdb_93\\b9d2cbf3975403e7.final.pdb
2.418970e-15 3.729164e-15 5.223790e-16 -4.268239e-15 -2.696265e-15
7.246149e-15
E:\\R-3.1.2\\scripts\\mPDBS\\summary_pdb_93\\bc88c8a505f3a288.final.pdb
1.898770e-15 2.373966e-15 -4.247800e-16 -4.680426e-15 -6.375815e-16
3.756970e-15
E:\\R-3.1.2\\scripts\\mPDBS\\summary_pdb_93\\bdac0e64f4ef427e.final.pdb
-1.058591e-15 5.289834e-15 -1.131774e-14 -2.319992e-15 1.325812e-14
3.677880e-16
E:\\R-3.1.2\\scripts\\mPDBS\\summary_pdb_93\\ca2a1f55fc9d0681.final.pdb
2.410206e-15 3.447402e-15 2.915884e-15 -1.876919e-16 -3.207877e-15
9.373605e-15
E:\\R-3.1.2\\scripts\\mPDBS\\summary_pdb_93\\ce34248de3e81a3c.final.pdb
-4.333437e-15 3.802411e-15 2.646797e-16 -3.022938e-15 -5.814744e-15
1.245508e-14
E:\\R-3.1.2\\scripts\\mPDBS\\summary_pdb_93\\d02e697772494a61.final.pdb
1.001337e-14 -3.505224e-16 -3.659497e-15 -1.961927e-14 6.238862e-15

4.376700e-15
E:\\R-3.1.2\\scripts\\mPDBS\\summary_pdb_93\\d04221245d015047.final.pdb
-1.022810e-15 4.606087e-15 2.350315e-16 -3.709273e-15 -9.816796e-16
3.573082e-15
E:\\R-3.1.2\\scripts\\mPDBS\\summary_pdb_93\\d678141e4018054a.final.pdb
8.710658e-16 7.801104e-15 5.382478e-15 -4.655622e-15 -5.438020e-15
-2.599685e-15
E:\\R-3.1.2\\scripts\\mPDBS\\summary_pdb_93\\d71b40af0f0efe39.final.pdb
4.424477e-15 1.841532e-15 5.266131e-15 -1.170674e-14 -8.821883e-15
-2.631482e-15
E:\\R-3.1.2\\scripts\\mPDBS\\summary_pdb_93\\dc0809eff10197e2.final.pdb
1.070744e-15 -3.914609e-16 9.349682e-16 -4.244039e-15 7.347420e-15
6.645533e-15
E:\\R-3.1.2\\scripts\\mPDBS\\summary_pdb_93\\e1163918d3707839.final.pdb
5.718896e-15 8.646867e-15 2.279883e-15 -1.302295e-14 2.077592e-15
-2.517077e-15
E:\\R-3.1.2\\scripts\\mPDBS\\summary_pdb_93\\e4b48b06824ef848.final.pdb
2.133946e-15 4.717639e-16 1.283298e-15 1.227748e-15 -1.275744e-15
9.168127e-16
E:\\R-3.1.2\\scripts\\mPDBS\\summary_pdb_93\\e529e19ad281fea2.final.pdb
1.667210e-15 3.947890e-16 -1.903573e-16 -7.102138e-15 2.666266e-15
-2.682058e-15
E:\\R-3.1.2\\scripts\\mPDBS\\summary_pdb_93\\e5602082718ec3a1.final.pdb
3.511755e-15 1.384816e-15 2.911547e-15 -5.844625e-15 -1.892957e-15
2.603847e-15
E:\\R-3.1.2\\scripts\\mPDBS\\summary_pdb_93\\e5d589d996d0a960.final.pdb
-6.675613e-15 -3.044420e-15 -9.186542e-15 6.526161e-15 -6.495744e-15
4.378932e-15
E:\\R-3.1.2\\scripts\\mPDBS\\summary_pdb_93\\e9df9e72307a2fbd.final.pdb
-8.107333e-16 2.154798e-15 -1.149429e-15 3.215317e-16 -6.599677e-16
3.920600e-15
E:\\R-3.1.2\\scripts\\mPDBS\\summary_pdb_93\\ea00f8694090cae7.final.pdb
3.511755e-15 1.384816e-15 2.911547e-15 -5.844625e-15 -1.892957e-15
2.603847e-15
E:\\R-3.1.2\\scripts\\mPDBS\\summary_pdb_93\\eba517be32db050e.final.pdb
2.418970e-15 3.729164e-15 5.223790e-16 -4.268239e-15 -2.696265e-15
7.246149e-15
E:\\R-3.1.2\\scripts\\mPDBS\\summary_pdb_93\\ec04b9545899c29d.final.pdb
2.106985e-15 1.820679e-15 -1.066832e-15 -4.691751e-15 -7.747273e-16
4.130817e-15
E:\\R-3.1.2\\scripts\\mPDBS\\summary_pdb_93\\ee6862cbcad52078.final.pdb
2.163966e-15 2.033472e-15 -6.354405e-16 -4.605643e-15 1.942184e-16
4.152921e-15
E:\\R-3.1.2\\scripts\\mPDBS\\summary_pdb_93\\ef6a42fc83b81e5d.final.pdb
1.311254e-15 1.965466e-15 -1.225763e-15 -4.065246e-15 -9.647944e-16
3.478349e-15
E:\\R-3.1.2\\scripts\\mPDBS\\summary_pdb_93\\ef8e88d4ad93a0ad.final.pdb
-1.022810e-15 4.606087e-15 2.350315e-16 -3.709273e-15 -9.816796e-16
3.573082e-15
E:\\R-3.1.2\\scripts\\mPDBS\\summary_pdb_93\\f14fcae8deec4fa3.final.pdb
3.081070e-15 1.272567e-15 -4.055492e-17 -5.174212e-15 1.748447e-15
2.925603e-15
E:\\R-3.1.2\\scripts\\mPDBS\\summary_pdb_93\\f83584e22003fb92.final.pdb
-9.227530e-15 4.592952e-15 2.435454e-15 4.631028e-15 -4.474790e-15

6.483088e-15
E:\\R-3.1.2\\scripts\\mPDBS\\summary_pdb_93\\f8e2bf87e52caa29.final.pdb
1.667210e-15 3.947890e-16 -1.903573e-16 -7.102138e-15 2.666266e-15
-2.682058e-15

[,469]   [,470]   [,471]   [,472]   [,473]   [,474]
E:\\R-3.1.2\\scripts\\40.1ns_data\\NAT0.pdb
2.393472e-15 2.294814e-15 -4.093609e-15 -1.155675e-16 1.206354e-15
-7.921290e-15
E:\\R-3.1.2\\scripts\\40.1ns_data\\LJP6.pdb
3.020530e-15 2.776404e-15 -3.391082e-15 2.736938e-16 3.189873e-15
-6.817945e-15
E:\\R-3.1.2\\scripts\\40.1ns_data\\3WY9.pdb
3.015410e-15 2.283214e-15 -3.373793e-15 -1.065427e-15 4.051908e-16
-7.430639e-15
E:\\R-3.1.2\\scripts\\40.1ns_data\\ZSP9.pdb
3.499762e-15 2.842796e-15 -2.814616e-15 1.010001e-16 3.686835e-16
-1.069612e-14
E:\\R-3.1.2\\scripts\\mPDBS\\summary_pdb_93\\013d855ad26c877c.final.pdb
-1.585050e-15 8.195050e-15 -7.436719e-15 -4.536761e-15 2.662255e-15
-5.284425e-16
E:\\R-3.1.2\\scripts\\mPDBS\\summary_pdb_93\\0a2a705342ca0d54.final.pdb
2.403965e-15 3.132782e-15 -3.089866e-15 5.871050e-16 1.398496e-15
-6.601502e-15
E:\\R-3.1.2\\scripts\\mPDBS\\summary_pdb_93\\0a59fceadb4194d9.final.pdb
5.089143e-15 6.110459e-16 -4.702213e-15 2.295486e-15 -2.042641e-15
-3.922824e-15
E:\\R-3.1.2\\scripts\\mPDBS\\summary_pdb_93\\0dba59f3306825e8.final.pdb
6.024748e-15 -1.230570e-15 -3.196621e-15 4.555359e-15 -2.784777e-15
-6.596505e-15
E:\\R-3.1.2\\scripts\\mPDBS\\summary_pdb_93\\126f26a78b57339c.final.pdb
-2.320979e-15 8.035017e-16 2.426853e-16 6.941037e-15 3.909088e-15
-5.120689e-15
E:\\R-3.1.2\\scripts\\mPDBS\\summary_pdb_93\\12f06f3e342df706.final.pdb
2.205149e-15 2.939360e-15 -3.227018e-15 9.045594e-16 1.219711e-15
-6.248486e-15
E:\\R-3.1.2\\scripts\\mPDBS\\summary_pdb_93\\18618a2a251b867b.final.pdb
5.735302e-16 8.097750e-15 -3.121951e-15 2.164546e-15 5.407079e-15
-5.866279e-15
E:\\R-3.1.2\\scripts\\mPDBS\\summary_pdb_93\\1f305f598641eeb2.final.pdb
2.096860e-15 2.855159e-15 -3.022061e-15 5.133596e-16 1.184013e-15
-5.697002e-15
E:\\R-3.1.2\\scripts\\mPDBS\\summary_pdb_93\\1f8a00b551be0a39.final.pdb
5.735302e-16 8.097750e-15 -3.121951e-15 2.164546e-15 5.407079e-15
-5.866279e-15
E:\\R-3.1.2\\scripts\\mPDBS\\summary_pdb_93\\219f45328d47579a.final.pdb
-2.843694e-15 -1.535783e-15 2.031875e-15 -4.819482e-15 1.035716e-14
-6.223934e-15
E:\\R-3.1.2\\scripts\\mPDBS\\summary_pdb_93\\238dd3918e54336d.final.pdb
-1.201506e-16 2.305329e-15 -1.115889e-15 6.634463e-15 5.969830e-15
-1.313984e-14
E:\\R-3.1.2\\scripts\\mPDBS\\summary_pdb_93\\2b3027889e2fdc42.final.pdb
1.748743e-15 2.213822e-15 -3.115375e-15 1.488840e-15 1.709023e-15
-4.760261e-15

E:\\R-3.1.2\\scripts\\mPDBS\\summary_pdb_93\\2fcb3b46e33793b0.final.pdb
2.501267e-15 3.719919e-15 -3.514289e-15 -2.009362e-15 -3.069528e-15
-4.897294e-15
E:\\R-3.1.2\\scripts\\mPDBS\\summary_pdb_93\\34eb328251842006.final.pdb
8.139133e-15 7.679052e-15 -9.881906e-15 -7.854197e-15 1.264621e-14
-1.527411e-14
E:\\R-3.1.2\\scripts\\mPDBS\\summary_pdb_93\\354b32871797b7ab.final.pdb
3.600328e-15 6.439516e-15 -5.682773e-15 -4.952797e-15 5.960597e-15
-9.045801e-15
E:\\R-3.1.2\\scripts\\mPDBS\\summary_pdb_93\\397368adbc32d5de.final.pdb
-2.320979e-15 8.035017e-16 2.426853e-16 6.941037e-15 3.909088e-15
-5.120689e-15
E:\\R-3.1.2\\scripts\\mPDBS\\summary_pdb_93\\411ca13cd3df6edd.final.pdb
-2.249734e-15 4.467910e-15 -9.822428e-16 9.786992e-16 -7.914029e-16
-1.283432e-15
E:\\R-3.1.2\\scripts\\mPDBS\\summary_pdb_93\\42357eee6642bf12.final.pdb
9.674757e-15 4.802441e-15 -8.536232e-15 -2.137095e-15 6.187443e-15
-2.690953e-16
E:\\R-3.1.2\\scripts\\mPDBS\\summary_pdb_93\\44342d1f75a7fcfc.final.pdb
-5.212327e-16 1.255337e-14 2.391978e-15 1.943999e-15 -2.682562e-15
5.098364e-15
E:\\R-3.1.2\\scripts\\mPDBS\\summary_pdb_93\\448ef21dffe8cdfb.final.pdb
-1.111263e-14 1.007118e-14 -1.196586e-14 -1.241076e-14 1.854513e-14
-8.687665e-15
E:\\R-3.1.2\\scripts\\mPDBS\\summary_pdb_93\\472a40b3e8f113c7.final.pdb
6.024748e-15 -1.230570e-15 -3.196621e-15 4.555359e-15 -2.784777e-15
-6.596505e-15
E:\\R-3.1.2\\scripts\\mPDBS\\summary_pdb_93\\48a26225951ea153.final.pdb
-4.683449e-15 1.592453e-14 -2.866984e-15 5.885184e-16 5.497659e-15
-6.109009e-15
E:\\R-3.1.2\\scripts\\mPDBS\\summary_pdb_93\\4ab4a5c7c6682ce6.final.pdb
5.072195e-15 4.485235e-15 -1.721455e-15 -7.716075e-15 9.803678e-15
1.965656e-15
E:\\R-3.1.2\\scripts\\mPDBS\\summary_pdb_93\\4eaa45b62cb6a25f.final.pdb
1.361748e-15 1.582073e-15 -1.434977e-15 4.607697e-15 -1.683245e-15
-2.130760e-15
E:\\R-3.1.2\\scripts\\mPDBS\\summary_pdb_93\\58c371611d85ac96.final.pdb
-2.320979e-15 8.035017e-16 2.426853e-16 6.941037e-15 3.909088e-15
-5.120689e-15
E:\\R-3.1.2\\scripts\\mPDBS\\summary_pdb_93\\59a647f04822d30a.final.pdb
5.004566e-15 9.225864e-16 -3.249668e-15 -4.423303e-15 8.894115e-15
-1.216213e-14
E:\\R-3.1.2\\scripts\\mPDBS\\summary_pdb_93\\59b091fe385e05fc.final.pdb
5.089143e-15 6.110459e-16 -4.702213e-15 2.295486e-15 -2.042641e-15
-3.922824e-15
E:\\R-3.1.2\\scripts\\mPDBS\\summary_pdb_93\\5c35ac30f930d2db.final.pdb
1.361748e-15 1.582073e-15 -1.434977e-15 4.607697e-15 -1.683245e-15
-2.130760e-15
E:\\R-3.1.2\\scripts\\mPDBS\\summary_pdb_93\\5f60752431cf2dcb.final.pdb
3.214000e-15 5.332224e-17 -4.925854e-15 4.872739e-15 1.272687e-16
-7.780680e-15
E:\\R-3.1.2\\scripts\\mPDBS\\summary_pdb_93\\626456dd6ff2868d.final.pdb
6.024748e-15 -1.230570e-15 -3.196621e-15 4.555359e-15 -2.784777e-15
-6.596505e-15

E:\\R-3.1.2\\scripts\\mPDBS\\summary_pdb_93\\6583d53ff3c8f7ef.final.pdb
5.004566e-15 9.225864e-16 -3.249668e-15 -4.423303e-15 8.894115e-15
-1.216213e-14
E:\\R-3.1.2\\scripts\\mPDBS\\summary_pdb_93\\69760178986bef01.final.pdb
-6.654910e-15 -1.168918e-15 5.226178e-16 3.286146e-15 -3.311341e-15
-1.255833e-14
E:\\R-3.1.2\\scripts\\mPDBS\\summary_pdb_93\\6a9d87d47926b1c8.final.pdb
1.529240e-15 2.701072e-15 -2.922318e-15 1.128846e-15 1.657417e-15
-6.008868e-15
E:\\R-3.1.2\\scripts\\mPDBS\\summary_pdb_93\\6bb3547ea982ffc4.final.pdb
-6.382603e-16 6.708307e-15 1.906377e-16 -3.945524e-15 8.516805e-15
-1.000138e-14
E:\\R-3.1.2\\scripts\\mPDBS\\summary_pdb_93\\716d9624611d1c42.final.pdb
4.158093e-16 5.569092e-15 -3.613861e-15 -3.073162e-15 6.575778e-15
-9.004961e-15
E:\\R-3.1.2\\scripts\\mPDBS\\summary_pdb_93\\75f27e70ad0f1be4.final.pdb
5.004566e-15 9.225864e-16 -3.249668e-15 -4.423303e-15 8.894115e-15
-1.216213e-14
E:\\R-3.1.2\\scripts\\mPDBS\\summary_pdb_93\\769f910fffb22872.final.pdb
2.307820e-15 2.756319e-15 -2.931674e-15 5.855135e-16 1.197231e-15
-3.338787e-15
E:\\R-3.1.2\\scripts\\mPDBS\\summary_pdb_93\\791f8c80dd5c3135.final.pdb
5.089143e-15 6.110459e-16 -4.702213e-15 2.295486e-15 -2.042641e-15
-3.922824e-15
E:\\R-3.1.2\\scripts\\mPDBS\\summary_pdb_93\\7fad98e66a3f6099.final.pdb
-2.208393e-15 6.533512e-15 -3.137721e-15 2.303539e-15 -2.675569e-16
-1.582067e-14
E:\\R-3.1.2\\scripts\\mPDBS\\summary_pdb_93\\804b89c94a633196.final.pdb
2.501267e-15 3.719919e-15 -3.514289e-15 -2.009362e-15 -3.069528e-15
-4.897294e-15
E:\\R-3.1.2\\scripts\\mPDBS\\summary_pdb_93\\813cc65a2298feba.final.pdb
2.501267e-15 3.719919e-15 -3.514289e-15 -2.009362e-15 -3.069528e-15
-4.897294e-15
E:\\R-3.1.2\\scripts\\mPDBS\\summary_pdb_93\\83c0eefcfe12d3f9.final.pdb
-2.249734e-15 4.467910e-15 -9.822428e-16 9.786992e-16 -7.914029e-16
-1.283432e-15
E:\\R-3.1.2\\scripts\\mPDBS\\summary_pdb_93\\86d50469ef569760.final.pdb
6.024748e-15 -1.230570e-15 -3.196621e-15 4.555359e-15 -2.784777e-15
-6.596505e-15
E:\\R-3.1.2\\scripts\\mPDBS\\summary_pdb_93\\89d0666e1402203d.final.pdb
5.004566e-15 9.225864e-16 -3.249668e-15 -4.423303e-15 8.894115e-15
-1.216213e-14
E:\\R-3.1.2\\scripts\\mPDBS\\summary_pdb_93\\8b34852716dadc62.final.pdb
2.988508e-15 -1.235319e-15 4.527456e-15 5.696713e-15 1.697492e-15
-3.785337e-15
E:\\R-3.1.2\\scripts\\mPDBS\\summary_pdb_93\\8b6ef9ac3fe74395.final.pdb
5.482500e-15 1.302156e-15 3.229167e-15 3.948150e-16 2.126820e-15
-2.207476e-14
E:\\R-3.1.2\\scripts\\mPDBS\\summary_pdb_93\\98bbe0d5986c138f.final.pdb
5.089143e-15 6.110459e-16 -4.702213e-15 2.295486e-15 -2.042641e-15
-3.922824e-15
E:\\R-3.1.2\\scripts\\mPDBS\\summary_pdb_93\\9c22f4909d67e2fa.final.pdb
2.779365e-15 3.870815e-16 -8.591282e-15 4.862311e-15 -3.848270e-16
-4.309223e-15

E:\\R-3.1.2\\scripts\\mPDBS\\summary_pdb_93\\9dda8ed0d81fb3ef.final.pdb
4.433924e-15 8.726023e-15 -4.346555e-15 1.367680e-14 -1.645854e-14
-4.292236e-17
E:\\R-3.1.2\\scripts\\mPDBS\\summary_pdb_93\\9f752b3616e6033b.final.pdb
1.892637e-15 2.497473e-15 -1.798978e-15 -3.244561e-16 -3.169551e-16
-6.597061e-15
E:\\R-3.1.2\\scripts\\mPDBS\\summary_pdb_93\\a02c70083816d6ba.final.pdb
1.361748e-15 1.582073e-15 -1.434977e-15 4.607697e-15 -1.683245e-15
-2.130760e-15
E:\\R-3.1.2\\scripts\\mPDBS\\summary_pdb_93\\a0c997d891e4e082.final.pdb
-3.289956e-15 3.044195e-15 5.519625e-15 -4.592934e-15 -8.181996e-15
4.897230e-15
E:\\R-3.1.2\\scripts\\mPDBS\\summary_pdb_93\\ab8039a2d823c616.final.pdb
2.431568e-15 2.740726e-15 -3.046006e-15 7.166211e-16 1.470656e-15
-3.686616e-15
E:\\R-3.1.2\\scripts\\mPDBS\\summary_pdb_93\\acf4d74b9c71d4dc.final.pdb
2.501267e-15 3.719919e-15 -3.514289e-15 -2.009362e-15 -3.069528e-15
-4.897294e-15
E:\\R-3.1.2\\scripts\\mPDBS\\summary_pdb_93\\ad8a823e4b4c668e.final.pdb
2.394794e-15 2.637826e-15 -3.276977e-15 3.966722e-16 1.469398e-15
-6.282463e-15
E:\\R-3.1.2\\scripts\\mPDBS\\summary_pdb_93\\b16ababd03b7b857.final.pdb
2.779365e-15 3.870815e-16 -8.591282e-15 4.862311e-15 -3.848270e-16
-4.309223e-15
E:\\R-3.1.2\\scripts\\mPDBS\\summary_pdb_93\\b226923e697d3c63.final.pdb
2.372178e-15 4.676552e-16 1.479839e-15 1.090955e-16 7.518654e-16
-8.017880e-15
E:\\R-3.1.2\\scripts\\mPDBS\\summary_pdb_93\\b28e48f6fa27a165.final.pdb
5.089143e-15 6.110459e-16 -4.702213e-15 2.295486e-15 -2.042641e-15
-3.922824e-15
E:\\R-3.1.2\\scripts\\mPDBS\\summary_pdb_93\\b42c4924e3cd1aec.final.pdb
1.947794e-15 1.726956e-15 -2.134241e-15 4.658408e-15 3.357962e-15
4.233171e-15
E:\\R-3.1.2\\scripts\\mPDBS\\summary_pdb_93\\b4dd239fcabff1df.final.pdb
5.089143e-15 6.110459e-16 -4.702213e-15 2.295486e-15 -2.042641e-15
-3.922824e-15
E:\\R-3.1.2\\scripts\\mPDBS\\summary_pdb_93\\b669942ef97459a2.final.pdb
2.988508e-15 -1.235319e-15 4.527456e-15 5.696713e-15 1.697492e-15
-3.785337e-15
E:\\R-3.1.2\\scripts\\mPDBS\\summary_pdb_93\\b99f4d9d87c1017b.final.pdb
5.089143e-15 6.110459e-16 -4.702213e-15 2.295486e-15 -2.042641e-15
-3.922824e-15
E:\\R-3.1.2\\scripts\\mPDBS\\summary_pdb_93\\b9d2cbf3975403e7.final.pdb
5.089143e-15 6.110459e-16 -4.702213e-15 2.295486e-15 -2.042641e-15
-3.922824e-15
E:\\R-3.1.2\\scripts\\mPDBS\\summary_pdb_93\\bc88c8a505f3a288.final.pdb
2.332938e-15 2.389137e-15 -3.021483e-15 1.268530e-15 1.409675e-15
-6.338860e-15
E:\\R-3.1.2\\scripts\\mPDBS\\summary_pdb_93\\bdac0e64f4ef427e.final.pdb
9.665323e-15 -6.495242e-16 -7.022441e-15 -3.351197e-17 7.028080e-15
-7.106898e-15
E:\\R-3.1.2\\scripts\\mPDBS\\summary_pdb_93\\ca2a1f55fc9d0681.final.pdb
2.988508e-15 -1.235319e-15 4.527456e-15 5.696713e-15 1.697492e-15
-3.785337e-15

E:\\R-3.1.2\\scripts\\mPDBS\\summary_pdb_93\\ce34248de3e81a3c.final.pdb
5.331042e-15 3.362447e-15 -3.976978e-15 5.635877e-15 -1.734054e-15
-1.076715e-15
E:\\R-3.1.2\\scripts\\mPDBS\\summary_pdb_93\\d02e697772494a61.final.pdb
-3.565067e-16 -2.962159e-15 -2.905256e-15 -1.532540e-14 1.738951e-14
-1.895203e-14
E:\\R-3.1.2\\scripts\\mPDBS\\summary_pdb_93\\d04221245d015047.final.pdb
2.098095e-15 3.228075e-15 -4.544373e-15 1.407475e-15 1.438780e-15
-4.517115e-15
E:\\R-3.1.2\\scripts\\mPDBS\\summary_pdb_93\\d678141e4018054a.final.pdb
-1.050061e-16 5.028419e-15 -1.081915e-14 -5.128739e-16 1.474566e-14
-1.315121e-14
E:\\R-3.1.2\\scripts\\mPDBS\\summary_pdb_93\\d71b40af0f0efe39.final.pdb
-2.276371e-15 1.655547e-15 -5.736615e-15 5.580810e-15 1.196471e-15
3.363966e-15
E:\\R-3.1.2\\scripts\\mPDBS\\summary_pdb_93\\dc0809eff10197e2.final.pdb
7.609775e-15 -7.367587e-16 1.468153e-15 1.909303e-15 -2.716556e-15
-4.099872e-15
E:\\R-3.1.2\\scripts\\mPDBS\\summary_pdb_93\\e1163918d3707839.final.pdb
2.534293e-15 -7.776594e-16 -1.310778e-14 1.038914e-16 -4.875903e-15
-9.777323e-15
E:\\R-3.1.2\\scripts\\mPDBS\\summary_pdb_93\\e4b48b06824ef848.final.pdb
5.735302e-16 8.097750e-15 -3.121951e-15 2.164546e-15 5.407079e-15
-5.866279e-15
E:\\R-3.1.2\\scripts\\mPDBS\\summary_pdb_93\\e529e19ad281fea2.final.pdb
-1.585050e-15 8.195050e-15 -7.436719e-15 -4.536761e-15 2.662255e-15
-5.284425e-16
E:\\R-3.1.2\\scripts\\mPDBS\\summary_pdb_93\\e5602082718ec3a1.final.pdb
2.501267e-15 3.719919e-15 -3.514289e-15 -2.009362e-15 -3.069528e-15
-4.897294e-15
E:\\R-3.1.2\\scripts\\mPDBS\\summary_pdb_93\\e5d589d996d0a960.final.pdb
-8.100329e-15 1.270927e-15 6.802938e-15 2.414934e-15 -9.379786e-15
1.191489e-14
E:\\R-3.1.2\\scripts\\mPDBS\\summary_pdb_93\\e9df9e72307a2fbd.final.pdb
1.361748e-15 1.582073e-15 -1.434977e-15 4.607697e-15 -1.683245e-15
-2.130760e-15
E:\\R-3.1.2\\scripts\\mPDBS\\summary_pdb_93\\ea00f8694090cae7.final.pdb
2.501267e-15 3.719919e-15 -3.514289e-15 -2.009362e-15 -3.069528e-15
-4.897294e-15
E:\\R-3.1.2\\scripts\\mPDBS\\summary_pdb_93\\eba517be32db050e.final.pdb
5.089143e-15 6.110459e-16 -4.702213e-15 2.295486e-15 -2.042641e-15
-3.922824e-15
E:\\R-3.1.2\\scripts\\mPDBS\\summary_pdb_93\\ec04b9545899c29d.final.pdb
2.122594e-15 2.516255e-15 -3.289241e-15 1.105261e-15 1.449549e-15
-7.236387e-15
E:\\R-3.1.2\\scripts\\mPDBS\\summary_pdb_93\\ee6862cbcad52078.final.pdb
2.221807e-15 2.255889e-15 -3.043492e-15 1.396032e-15 1.442418e-15
-5.466728e-15
E:\\R-3.1.2\\scripts\\mPDBS\\summary_pdb_93\\ef6a42fc83b81e5d.final.pdb
2.117244e-15 2.880569e-15 -3.406988e-15 5.772735e-16 1.233009e-15
-5.907479e-15
E:\\R-3.1.2\\scripts\\mPDBS\\summary_pdb_93\\ef8e88d4ad93a0ad.final.pdb
2.098095e-15 3.228075e-15 -4.544373e-15 1.407475e-15 1.438780e-15
-4.517115e-15

E:\\R-3.1.2\\scripts\\mPDBS\\summary_pdb_93\\f14fcae8deec4fa3.final.pdb
1.892637e-15 2.497473e-15 -1.798978e-15 -3.244561e-16 -3.169551e-16
-6.597061e-15
E:\\R-3.1.2\\scripts\\mPDBS\\summary_pdb_93\\f83584e22003fb92.final.pdb
-4.683449e-15 1.592453e-14 -2.866984e-15 5.885184e-16 5.497659e-15
-6.109009e-15
E:\\R-3.1.2\\scripts\\mPDBS\\summary_pdb_93\\f8e2bf87e52caa29.final.pdb
-1.585050e-15 8.195050e-15 -7.436719e-15 -4.536761e-15 2.662255e-15
-5.284425e-16

[,475]   [,476]   [,477]   [,478]   [,479]   [,480]
E:\\R-3.1.2\\scripts\\40.1ns_data\\NAT0.pdb
7.563350e-16 -1.305494e-15 1.172107e-16 -4.841819e-16 1.408977e-15
-4.720162e-16
E:\\R-3.1.2\\scripts\\40.1ns_data\\LJP6.pdb
2.149159e-15 -1.531032e-15 -1.681435e-15 -9.519452e-16 -1.036541e-16
2.190780e-16
E:\\R-3.1.2\\scripts\\40.1ns_data\\3WY9.pdb
1.145222e-15 1.833781e-15 7.761031e-16 -1.802171e-15 9.355485e-16
2.997332e-17
E:\\R-3.1.2\\scripts\\40.1ns_data\\ZSP9.pdb
8.223793e-16 -1.345574e-15 1.668070e-15 -6.066841e-16 1.237653e-16
3.524749e-15
E:\\R-3.1.2\\scripts\\mPDBS\\summary_pdb_93\\013d855ad26c877c.final.pdb
2.940416e-16 4.231719e-15 -1.067797e-15 -4.275020e-15 -3.201903e-15
-2.696876e-16
E:\\R-3.1.2\\scripts\\mPDBS\\summary_pdb_93\\0a2a705342ca0d54.final.pdb
1.740692e-15 -5.933394e-16 -4.407903e-16 -1.559065e-15 -1.949636e-16
8.136850e-16
E:\\R-3.1.2\\scripts\\mPDBS\\summary_pdb_93\\0a59fceadb4194d9.final.pdb
-8.046581e-16 1.151611e-15 -2.197065e-15 -5.504564e-15 -2.711239e-15
-9.372551e-16
E:\\R-3.1.2\\scripts\\mPDBS\\summary_pdb_93\\0dba59f3306825e8.final.pdb
-1.332262e-15 -2.520692e-15 3.662360e-15 1.498139e-15 -1.661469e-15
2.559701e-15
E:\\R-3.1.2\\scripts\\mPDBS\\summary_pdb_93\\126f26a78b57339c.final.pdb
9.081609e-15 -2.079205e-15 9.547409e-15 4.850608e-16 8.646258e-15
-3.036897e-15
E:\\R-3.1.2\\scripts\\mPDBS\\summary_pdb_93\\12f06f3e342df706.final.pdb
1.377756e-15 -5.469355e-16 -7.122745e-16 -1.327480e-15 -5.228261e-17
7.691785e-16
E:\\R-3.1.2\\scripts\\mPDBS\\summary_pdb_93\\18618a2a251b867b.final.pdb
7.915135e-15 2.154982e-15 4.625843e-15 4.702753e-15 5.125065e-15
1.956251e-15
E:\\R-3.1.2\\scripts\\mPDBS\\summary_pdb_93\\1f305f598641eeb2.final.pdb
1.595345e-15 -2.721637e-16 3.372259e-16 -1.423803e-15 5.505008e-17
3.524437e-16
E:\\R-3.1.2\\scripts\\mPDBS\\summary_pdb_93\\1f8a00b551be0a39.final.pdb
7.915135e-15 2.154982e-15 4.625843e-15 4.702753e-15 5.125065e-15
1.956251e-15
E:\\R-3.1.2\\scripts\\mPDBS\\summary_pdb_93\\219f45328d47579a.final.pdb
6.344788e-15 2.635667e-15 -7.624147e-15 -1.160816e-15 5.830973e-15
-4.248761e-15
E:\\R-3.1.2\\scripts\\mPDBS\\summary_pdb_93\\238dd3918e54336d.final.pdb

1.573821e-14 -9.157454e-16 5.682050e-15 1.006978e-14 4.845217e-15
4.982950e-15
E:\\R-3.1.2\\scripts\\mPDBS\\summary_pdb_93\\2b3027889e2fdc42.final.pdb
2.089132e-15 2.595297e-16 -7.199475e-16 -1.292655e-15 -4.235720e-16
-1.790454e-16
E:\\R-3.1.2\\scripts\\mPDBS\\summary_pdb_93\\2fcb3b46e33793b0.final.pdb
-2.265282e-15 2.307370e-15 -4.740743e-15 -1.458498e-15 -4.991261e-15
2.560259e-15
E:\\R-3.1.2\\scripts\\mPDBS\\summary_pdb_93\\34eb328251842006.final.pdb
-1.671635e-15 7.144640e-15 -1.324990e-14 -1.853326e-15 8.788463e-16
-7.775615e-15
E:\\R-3.1.2\\scripts\\mPDBS\\summary_pdb_93\\354b32871797b7ab.final.pdb
2.049849e-15 5.083761e-15 -1.368383e-14 -1.113832e-14 -1.697167e-15
-3.980510e-15
E:\\R-3.1.2\\scripts\\mPDBS\\summary_pdb_93\\397368adbc32d5de.final.pdb
9.081609e-15 -2.079205e-15 9.547409e-15 4.850608e-16 8.646258e-15
-3.036897e-15
E:\\R-3.1.2\\scripts\\mPDBS\\summary_pdb_93\\411ca13cd3df6edd.final.pdb
2.373043e-15 4.762871e-15 -3.254519e-15 1.414204e-15 2.421213e-15
-3.117600e-15
E:\\R-3.1.2\\scripts\\mPDBS\\summary_pdb_93\\42357eee6642bf12.final.pdb
9.027933e-15 6.684228e-15 -1.176034e-14 -8.151931e-15 5.703825e-15
-8.371995e-15
E:\\R-3.1.2\\scripts\\mPDBS\\summary_pdb_93\\44342d1f75a7fcfc.final.pdb
-6.371279e-15 3.865119e-15 -1.786282e-15 -1.988629e-15 -8.901692e-15
-9.192868e-16
E:\\R-3.1.2\\scripts\\mPDBS\\summary_pdb_93\\448ef21dffe8cdfb.final.pdb
2.033123e-14 7.438682e-15 -1.090855e-14 -8.133714e-15 6.471792e-15
-7.130340e-15
E:\\R-3.1.2\\scripts\\mPDBS\\summary_pdb_93\\472a40b3e8f113c7.final.pdb
-1.332262e-15 -2.520692e-15 3.662360e-15 1.498139e-15 -1.661469e-15
2.559701e-15
E:\\R-3.1.2\\scripts\\mPDBS\\summary_pdb_93\\48a26225951ea153.final.pdb
-3.633938e-15 1.850705e-15 -5.690364e-15 5.084189e-15 -2.822695e-15
2.706559e-15
E:\\R-3.1.2\\scripts\\mPDBS\\summary_pdb_93\\4ab4a5c7c6682ce6.final.pdb
-5.866504e-15 3.606351e-15 -1.330150e-14 -4.052976e-15 -1.222247e-14
4.569811e-15
E:\\R-3.1.2\\scripts\\mPDBS\\summary_pdb_93\\4eaa45b62cb6a25f.final.pdb
8.764172e-16 -4.327548e-15 8.302926e-15 2.850488e-15 3.668879e-17
3.599472e-15
E:\\R-3.1.2\\scripts\\mPDBS\\summary_pdb_93\\58c371611d85ac96.final.pdb
9.081609e-15 -2.079205e-15 9.547409e-15 4.850608e-16 8.646258e-15
-3.036897e-15
E:\\R-3.1.2\\scripts\\mPDBS\\summary_pdb_93\\59a647f04822d30a.final.pdb
2.237058e-16 -1.687731e-15 -1.787150e-15 8.216225e-16 1.937294e-15
1.143760e-15
E:\\R-3.1.2\\scripts\\mPDBS\\summary_pdb_93\\59b091fe385e05fc.final.pdb
-8.046581e-16 1.151611e-15 -2.197065e-15 -5.504564e-15 -2.711239e-15
-9.372551e-16
E:\\R-3.1.2\\scripts\\mPDBS\\summary_pdb_93\\5c35ac30f930d2db.final.pdb
8.764172e-16 -4.327548e-15 8.302926e-15 2.850488e-15 3.668879e-17
3.599472e-15
E:\\R-3.1.2\\scripts\\mPDBS\\summary_pdb_93\\5f60752431cf2dcb.final.pdb

-2.276873e-15 -4.600551e-15 4.665802e-15 2.864480e-15 -6.472928e-16
2.717327e-15
E:\\R-3.1.2\\scripts\\mPDBS\\summary_pdb_93\\626456dd6ff2868d.final.pdb
-1.332262e-15 -2.520692e-15 3.662360e-15 1.498139e-15 -1.661469e-15
2.559701e-15
E:\\R-3.1.2\\scripts\\mPDBS\\summary_pdb_93\\6583d53ff3c8f7ef.final.pdb
2.237058e-16 -1.687731e-15 -1.787150e-15 8.216225e-16 1.937294e-15
1.143760e-15
E:\\R-3.1.2\\scripts\\mPDBS\\summary_pdb_93\\69760178986bef01.final.pdb
2.293232e-15 -3.763722e-15 3.708224e-15 3.374976e-15 -5.960321e-15
4.806288e-15
E:\\R-3.1.2\\scripts\\mPDBS\\summary_pdb_93\\6a9d87d47926b1c8.final.pdb
1.464438e-15 3.337951e-16 3.464362e-16 -1.020679e-15 -3.743040e-16
-2.183507e-16
E:\\R-3.1.2\\scripts\\mPDBS\\summary_pdb_93\\6bb3547ea982ffc4.final.pdb
8.853680e-15 -5.823493e-16 3.948655e-15 7.237700e-15 6.431215e-15
-3.732408e-15
E:\\R-3.1.2\\scripts\\mPDBS\\summary_pdb_93\\716d9624611d1c42.final.pdb
1.509324e-15 1.353020e-15 -1.167639e-15 7.862810e-16 -8.988277e-16
-2.246286e-15
E:\\R-3.1.2\\scripts\\mPDBS\\summary_pdb_93\\75f27e70ad0f1be4.final.pdb
2.237058e-16 -1.687731e-15 -1.787150e-15 8.216225e-16 1.937294e-15
1.143760e-15
E:\\R-3.1.2\\scripts\\mPDBS\\summary_pdb_93\\769f910fffb22872.final.pdb
1.779255e-15 -5.282016e-16 -5.167451e-16 -6.263179e-16 3.849333e-16
3.313299e-16
E:\\R-3.1.2\\scripts\\mPDBS\\summary_pdb_93\\791f8c80dd5c3135.final.pdb
-8.046581e-16 1.151611e-15 -2.197065e-15 -5.504564e-15 -2.711239e-15
-9.372551e-16
E:\\R-3.1.2\\scripts\\mPDBS\\summary_pdb_93\\7fad98e66a3f6099.final.pdb
3.216609e-16 -1.231757e-15 -1.397678e-14 -9.824870e-15 -9.282714e-15
-4.440225e-15
E:\\R-3.1.2\\scripts\\mPDBS\\summary_pdb_93\\804b89c94a633196.final.pdb
-2.265282e-15 2.307370e-15 -4.740743e-15 -1.458498e-15 -4.991261e-15
2.560259e-15
E:\\R-3.1.2\\scripts\\mPDBS\\summary_pdb_93\\813cc65a2298feba.final.pdb
-2.265282e-15 2.307370e-15 -4.740743e-15 -1.458498e-15 -4.991261e-15
2.560259e-15
E:\\R-3.1.2\\scripts\\mPDBS\\summary_pdb_93\\83c0eefcfe12d3f9.final.pdb
2.373043e-15 4.762871e-15 -3.254519e-15 1.414204e-15 2.421213e-15
-3.117600e-15
E:\\R-3.1.2\\scripts\\mPDBS\\summary_pdb_93\\86d50469ef569760.final.pdb
-1.332262e-15 -2.520692e-15 3.662360e-15 1.498139e-15 -1.661469e-15
2.559701e-15
E:\\R-3.1.2\\scripts\\mPDBS\\summary_pdb_93\\89d0666e1402203d.final.pdb
2.237058e-16 -1.687731e-15 -1.787150e-15 8.216225e-16 1.937294e-15
1.143760e-15
E:\\R-3.1.2\\scripts\\mPDBS\\summary_pdb_93\\8b34852716dadc62.final.pdb
5.892054e-16 -4.293253e-15 1.013768e-15 -2.580691e-16 2.018757e-15
-6.051236e-16
E:\\R-3.1.2\\scripts\\mPDBS\\summary_pdb_93\\8b6ef9ac3fe74395.final.pdb
4.459969e-15 -1.318126e-15 4.542103e-15 -7.447743e-15 3.860983e-15
2.085723e-15
E:\\R-3.1.2\\scripts\\mPDBS\\summary_pdb_93\\98bbe0d5986c138f.final.pdb

-8.046581e-16 1.151611e-15 -2.197065e-15 -5.504564e-15 -2.711239e-15
-9.372551e-16
E:\\R-3.1.2\\scripts\\mPDBS\\summary_pdb_93\\9c22f4909d67e2fa.final.pdb
2.577090e-15 -5.371817e-15 9.434983e-15 -2.094968e-16 1.098486e-15
1.573957e-16
E:\\R-3.1.2\\scripts\\mPDBS\\summary_pdb_93\\9dda8ed0d81fb3ef.final.pdb
-9.475296e-15 -4.066905e-15 1.150300e-14 1.206731e-15 -4.432177e-15
3.810599e-15
E:\\R-3.1.2\\scripts\\mPDBS\\summary_pdb_93\\9f752b3616e6033b.final.pdb
4.088243e-15 2.189692e-16 -4.131893e-15 -3.100674e-15 3.904254e-16
4.941397e-16
E:\\R-3.1.2\\scripts\\mPDBS\\summary_pdb_93\\a02c70083816d6ba.final.pdb
8.764172e-16 -4.327548e-15 8.302926e-15 2.850488e-15 3.668879e-17
3.599472e-15
E:\\R-3.1.2\\scripts\\mPDBS\\summary_pdb_93\\a0c997d891e4e082.final.pdb
-3.839411e-15 -6.111157e-15 5.806799e-15 1.511092e-15 5.939950e-16
4.826094e-15
E:\\R-3.1.2\\scripts\\mPDBS\\summary_pdb_93\\ab8039a2d823c616.final.pdb
2.128580e-15 -4.179288e-17 1.065411e-17 -1.840393e-15 -7.890133e-16
6.633536e-16
E:\\R-3.1.2\\scripts\\mPDBS\\summary_pdb_93\\acf4d74b9c71d4dc.final.pdb
-2.265282e-15 2.307370e-15 -4.740743e-15 -1.458498e-15 -4.991261e-15
2.560259e-15
E:\\R-3.1.2\\scripts\\mPDBS\\summary_pdb_93\\ad8a823e4b4c668e.final.pdb
1.789065e-15 -9.405491e-16 -7.005505e-16 -3.816693e-16 -3.057296e-16
4.602966e-16
E:\\R-3.1.2\\scripts\\mPDBS\\summary_pdb_93\\b16ababd03b7b857.final.pdb
2.577090e-15 -5.371817e-15 9.434983e-15 -2.094968e-16 1.098486e-15
1.573957e-16
E:\\R-3.1.2\\scripts\\mPDBS\\summary_pdb_93\\b226923e697d3c63.final.pdb
6.313182e-15 -1.344690e-15 8.231312e-15 -2.710274e-15 7.037814e-15
1.255103e-18
E:\\R-3.1.2\\scripts\\mPDBS\\summary_pdb_93\\b28e48f6fa27a165.final.pdb
-8.046581e-16 1.151611e-15 -2.197065e-15 -5.504564e-15 -2.711239e-15
-9.372551e-16
E:\\R-3.1.2\\scripts\\mPDBS\\summary_pdb_93\\b42c4924e3cd1aec.final.pdb
9.005732e-16 5.027817e-15 -4.074383e-15 -3.577636e-15 1.693565e-15
3.925308e-15
E:\\R-3.1.2\\scripts\\mPDBS\\summary_pdb_93\\b4dd239fcabff1df.final.pdb
-8.046581e-16 1.151611e-15 -2.197065e-15 -5.504564e-15 -2.711239e-15
-9.372551e-16
E:\\R-3.1.2\\scripts\\mPDBS\\summary_pdb_93\\b669942ef97459a2.final.pdb
5.892054e-16 -4.293253e-15 1.013768e-15 -2.580691e-16 2.018757e-15
-6.051236e-16
E:\\R-3.1.2\\scripts\\mPDBS\\summary_pdb_93\\b99f4d9d87c1017b.final.pdb
-8.046581e-16 1.151611e-15 -2.197065e-15 -5.504564e-15 -2.711239e-15
-9.372551e-16
E:\\R-3.1.2\\scripts\\mPDBS\\summary_pdb_93\\b9d2cbf3975403e7.final.pdb
-8.046581e-16 1.151611e-15 -2.197065e-15 -5.504564e-15 -2.711239e-15
-9.372551e-16
E:\\R-3.1.2\\scripts\\mPDBS\\summary_pdb_93\\bc88c8a505f3a288.final.pdb
1.898529e-15 6.472404e-16 -4.532337e-16 -1.525108e-15 -4.383172e-16
-2.690884e-16
E:\\R-3.1.2\\scripts\\mPDBS\\summary_pdb_93\\bdac0e64f4ef427e.final.pdb

-1.601317e-15 -8.524677e-15 7.505103e-15 -7.237729e-15 -5.072793e-15
7.189911e-15
E:\\R-3.1.2\\scripts\\mPDBS\\summary_pdb_93\\ca2a1f55fc9d0681.final.pdb
5.892054e-16 -4.293253e-15 1.013768e-15 -2.580691e-16 2.018757e-15
-6.051236e-16
E:\\R-3.1.2\\scripts\\mPDBS\\summary_pdb_93\\ce34248de3e81a3c.final.pdb
-2.755118e-15 -3.004579e-15 5.793681e-15 -6.618829e-15 8.771730e-16
2.626044e-15
E:\\R-3.1.2\\scripts\\mPDBS\\summary_pdb_93\\d02e697772494a61.final.pdb
1.342842e-14 -1.134321e-15 -9.370146e-15 -1.427963e-15 1.570059e-15
-6.990546e-15
E:\\R-3.1.2\\scripts\\mPDBS\\summary_pdb_93\\d04221245d015047.final.pdb
6.284916e-17 7.956449e-16 -3.234580e-15 -1.101401e-15 -2.573610e-15
7.078601e-16
E:\\R-3.1.2\\scripts\\mPDBS\\summary_pdb_93\\d678141e4018054a.final.pdb
7.504727e-15 5.450528e-15 -1.219495e-15 1.267292e-15 6.199341e-15
-8.368801e-15
E:\\R-3.1.2\\scripts\\mPDBS\\summary_pdb_93\\d71b40af0f0efe39.final.pdb
3.659485e-15 3.974249e-15 -8.364134e-15 -1.745114e-15 -8.240037e-15
-1.157040e-15
E:\\R-3.1.2\\scripts\\mPDBS\\summary_pdb_93\\dc0809eff10197e2.final.pdb
-8.649041e-15 7.852830e-17 5.782853e-15 -3.749136e-15 -2.142917e-15
5.978510e-15
E:\\R-3.1.2\\scripts\\mPDBS\\summary_pdb_93\\e1163918d3707839.final.pdb
8.069752e-15 5.423334e-15 -7.364719e-15 -2.384146e-15 2.697536e-15
-2.560444e-15
E:\\R-3.1.2\\scripts\\mPDBS\\summary_pdb_93\\e4b48b06824ef848.final.pdb
7.915135e-15 2.154982e-15 4.625843e-15 4.702753e-15 5.125065e-15
1.956251e-15
E:\\R-3.1.2\\scripts\\mPDBS\\summary_pdb_93\\e529e19ad281fea2.final.pdb
2.940416e-16 4.231719e-15 -1.067797e-15 -4.275020e-15 -3.201903e-15
-2.696876e-16
E:\\R-3.1.2\\scripts\\mPDBS\\summary_pdb_93\\e5602082718ec3a1.final.pdb
-2.265282e-15 2.307370e-15 -4.740743e-15 -1.458498e-15 -4.991261e-15
2.560259e-15
E:\\R-3.1.2\\scripts\\mPDBS\\summary_pdb_93\\e5d589d996d0a960.final.pdb
-2.704368e-15 -8.554390e-15 7.753033e-15 -6.361820e-15 -1.742849e-15
7.203609e-15
E:\\R-3.1.2\\scripts\\mPDBS\\summary_pdb_93\\e9df9e72307a2fbd.final.pdb
8.764172e-16 -4.327548e-15 8.302926e-15 2.850488e-15 3.668879e-17
3.599472e-15
E:\\R-3.1.2\\scripts\\mPDBS\\summary_pdb_93\\ea00f8694090cae7.final.pdb
-2.265282e-15 2.307370e-15 -4.740743e-15 -1.458498e-15 -4.991261e-15
2.560259e-15
E:\\R-3.1.2\\scripts\\mPDBS\\summary_pdb_93\\eba517be32db050e.final.pdb
-8.046581e-16 1.151611e-15 -2.197065e-15 -5.504564e-15 -2.711239e-15
-9.372551e-16
E:\\R-3.1.2\\scripts\\mPDBS\\summary_pdb_93\\ec04b9545899c29d.final.pdb
1.502631e-15 -1.694146e-16 -9.544825e-16 -1.273923e-15 -5.316515e-16
8.331092e-16
E:\\R-3.1.2\\scripts\\mPDBS\\summary_pdb_93\\ee6862cbcad52078.final.pdb
1.302597e-15 5.132330e-16 -7.693871e-16 -1.712458e-15 -6.542902e-16
-7.122149e-17
E:\\R-3.1.2\\scripts\\mPDBS\\summary_pdb_93\\ef6a42fc83b81e5d.final.pdb

1.490098e-15 -4.006995e-16 -3.055425e-16 -1.049590e-15 -4.277846e-16
6.312744e-16
E:\\R-3.1.2\\scripts\\mPDBS\\summary_pdb_93\\ef8e88d4ad93a0ad.final.pdb
6.284916e-17 7.956449e-16 -3.234580e-15 -1.101401e-15 -2.573610e-15
7.078601e-16
E:\\R-3.1.2\\scripts\\mPDBS\\summary_pdb_93\\f14fcae8deec4fa3.final.pdb
4.088243e-15 2.189692e-16 -4.131893e-15 -3.100674e-15 3.904254e-16
4.941397e-16
E:\\R-3.1.2\\scripts\\mPDBS\\summary_pdb_93\\f83584e22003fb92.final.pdb
-3.633938e-15 1.850705e-15 -5.690364e-15 5.084189e-15 -2.822695e-15
2.706559e-15
E:\\R-3.1.2\\scripts\\mPDBS\\summary_pdb_93\\f8e2bf87e52caa29.final.pdb
2.940416e-16 4.231719e-15 -1.067797e-15 -4.275020e-15 -3.201903e-15
-2.696876e-16

[,481]   [,482]   [,483]   [,484]   [,485]   [,486]
E:\\R-3.1.2\\scripts\\40.1ns_data\\NAT0.pdb
2.986533e-15 -9.343543e-16 1.083120e-15 3.572086e-15 -5.711582e-16
2.291289e-15
E:\\R-3.1.2\\scripts\\40.1ns_data\\LJP6.pdb
2.638387e-15 -3.037850e-15 1.255229e-15 2.297521e-15 -3.405289e-16
2.857688e-15
E:\\R-3.1.2\\scripts\\40.1ns_data\\3WY9.pdb
4.016679e-15 -1.155050e-15 1.335277e-15 2.826555e-15 1.020205e-15
3.524409e-15
E:\\R-3.1.2\\scripts\\40.1ns_data\\ZSP9.pdb
4.747305e-15 -1.241150e-15 -7.870123e-16 3.544662e-15 1.878494e-16
2.496475e-15
E:\\R-3.1.2\\scripts\\mPDBS\\summary_pdb_93\\013d855ad26c877c.final.pdb
4.960464e-15 3.955908e-15 4.034733e-15 4.179101e-15 -1.086541e-16
2.970826e-15
E:\\R-3.1.2\\scripts\\mPDBS\\summary_pdb_93\\0a2a705342ca0d54.final.pdb
3.514814e-15 -8.717159e-16 1.005899e-15 2.456438e-15 -1.031455e-15
2.438579e-15
E:\\R-3.1.2\\scripts\\mPDBS\\summary_pdb_93\\0a59fceadb4194d9.final.pdb
4.438836e-15 -5.090693e-15 -8.951511e-16 9.156208e-16 -1.352391e-15
5.094685e-16
E:\\R-3.1.2\\scripts\\mPDBS\\summary_pdb_93\\0dba59f3306825e8.final.pdb
2.227407e-15 -4.053311e-15 -3.662604e-16 2.316442e-15 -3.232623e-17
-1.020200e-16
E:\\R-3.1.2\\scripts\\mPDBS\\summary_pdb_93\\126f26a78b57339c.final.pdb
3.844928e-15 -8.744298e-16 3.566575e-15 -1.230201e-15 -1.676844e-15
9.266710e-16
E:\\R-3.1.2\\scripts\\mPDBS\\summary_pdb_93\\12f06f3e342df706.final.pdb
3.352021e-15 -1.128455e-15 1.094153e-15 2.754810e-15 -8.202520e-16
2.499945e-15
E:\\R-3.1.2\\scripts\\mPDBS\\summary_pdb_93\\18618a2a251b867b.final.pdb
2.006270e-15 5.322634e-15 4.363756e-15 -1.842096e-16 -6.534611e-16
3.095631e-15
E:\\R-3.1.2\\scripts\\mPDBS\\summary_pdb_93\\1f305f598641eeb2.final.pdb
3.702028e-15 -1.265827e-15 1.356811e-15 2.815699e-15 -4.413380e-16
3.034805e-15
E:\\R-3.1.2\\scripts\\mPDBS\\summary_pdb_93\\1f8a00b551be0a39.final.pdb
2.006270e-15 5.322634e-15 4.363756e-15 -1.842096e-16 -6.534611e-16

3.095631e-15
E:\\R-3.1.2\\scripts\\mPDBS\\summary_pdb_93\\219f45328d47579a.final.pdb
3.862040e-15 1.580186e-15 7.633834e-15 2.557970e-15 -6.504737e-15
7.436545e-15
E:\\R-3.1.2\\scripts\\mPDBS\\summary_pdb_93\\238dd3918e54336d.final.pdb
1.745190e-15 9.158326e-15 5.722494e-15 2.956957e-15 -1.433273e-15
1.136667e-14
E:\\R-3.1.2\\scripts\\mPDBS\\summary_pdb_93\\2b3027889e2fdc42.final.pdb
3.257842e-15 -1.816836e-15 1.053811e-15 2.787821e-15 -3.956914e-16
2.037651e-15
E:\\R-3.1.2\\scripts\\mPDBS\\summary_pdb_93\\2fcb3b46e33793b0.final.pdb
-7.099590e-17 -1.390728e-15 -1.293847e-15 6.012360e-15 1.527573e-15
2.086562e-15
E:\\R-3.1.2\\scripts\\mPDBS\\summary_pdb_93\\34eb328251842006.final.pdb
1.079429e-14 -6.629437e-15 -9.753411e-16 1.885203e-15 -4.024736e-15
1.197150e-14
E:\\R-3.1.2\\scripts\\mPDBS\\summary_pdb_93\\354b32871797b7ab.final.pdb
-3.562448e-15 -4.929804e-16 -9.202398e-15 6.918112e-15 1.668490e-15
9.187321e-15
E:\\R-3.1.2\\scripts\\mPDBS\\summary_pdb_93\\397368adbc32d5de.final.pdb
3.844928e-15 -8.744298e-16 3.566575e-15 -1.230201e-15 -1.676844e-15
9.266710e-16
E:\\R-3.1.2\\scripts\\mPDBS\\summary_pdb_93\\411ca13cd3df6edd.final.pdb
-3.375536e-15 -5.747486e-15 -3.070421e-15 4.520932e-15 -8.307834e-16
2.958043e-15
E:\\R-3.1.2\\scripts\\mPDBS\\summary_pdb_93\\42357eee6642bf12.final.pdb
6.243587e-15 -1.370772e-15 -3.603711e-16 6.262903e-15 2.199224e-15
9.896814e-15
E:\\R-3.1.2\\scripts\\mPDBS\\summary_pdb_93\\44342d1f75a7fcfc.final.pdb
1.699069e-15 6.168316e-17 -4.388104e-15 4.841910e-15 -2.034650e-15
-2.389548e-15
E:\\R-3.1.2\\scripts\\mPDBS\\summary_pdb_93\\448ef21dffe8cdfb.final.pdb
6.598149e-15 9.021083e-15 2.434941e-15 5.056528e-15 -7.414452e-16
1.373436e-14
E:\\R-3.1.2\\scripts\\mPDBS\\summary_pdb_93\\472a40b3e8f113c7.final.pdb
2.227407e-15 -4.053311e-15 -3.662604e-16 2.316442e-15 -3.232623e-17
-1.020200e-16
E:\\R-3.1.2\\scripts\\mPDBS\\summary_pdb_93\\48a26225951ea153.final.pdb
1.541333e-15 2.316143e-15 -2.091026e-15 1.412164e-14 3.583331e-15
4.674756e-15
E:\\R-3.1.2\\scripts\\mPDBS\\summary_pdb_93\\4ab4a5c7c6682ce6.final.pdb
-8.602580e-15 -2.732319e-15 1.208218e-15 8.804741e-15 6.814627e-15
1.003462e-14
E:\\R-3.1.2\\scripts\\mPDBS\\summary_pdb_93\\4eaa45b62cb6a25f.final.pdb
4.676242e-15 -4.608880e-16 6.100791e-15 7.047664e-16 -2.915309e-16
-1.081699e-15
E:\\R-3.1.2\\scripts\\mPDBS\\summary_pdb_93\\58c371611d85ac96.final.pdb
3.844928e-15 -8.744298e-16 3.566575e-15 -1.230201e-15 -1.676844e-15
9.266710e-16
E:\\R-3.1.2\\scripts\\mPDBS\\summary_pdb_93\\59a647f04822d30a.final.pdb
6.041516e-15 4.355161e-16 4.640264e-15 9.075069e-15 4.414835e-15
3.406705e-15
E:\\R-3.1.2\\scripts\\mPDBS\\summary_pdb_93\\59b091fe385e05fc.final.pdb
4.438836e-15 -5.090693e-15 -8.951511e-16 9.156208e-16 -1.352391e-15

5.094685e-16
E:\\R-3.1.2\\scripts\\mPDBS\\summary_pdb_93\\5c35ac30f930d2db.final.pdb
4.676242e-15 -4.608880e-16 6.100791e-15 7.047664e-16 -2.915309e-16
-1.081699e-15
E:\\R-3.1.2\\scripts\\mPDBS\\summary_pdb_93\\5f60752431cf2dcb.final.pdb
2.146035e-15 -4.855942e-15 6.731354e-15 -2.640613e-15 1.528564e-15
-2.231188e-15
E:\\R-3.1.2\\scripts\\mPDBS\\summary_pdb_93\\626456dd6ff2868d.final.pdb
2.227407e-15 -4.053311e-15 -3.662604e-16 2.316442e-15 -3.232623e-17
-1.020200e-16
E:\\R-3.1.2\\scripts\\mPDBS\\summary_pdb_93\\6583d53ff3c8f7ef.final.pdb
6.041516e-15 4.355161e-16 4.640264e-15 9.075069e-15 4.414835e-15
3.406705e-15
E:\\R-3.1.2\\scripts\\mPDBS\\summary_pdb_93\\69760178986bef01.final.pdb
-5.486528e-15 5.316918e-15 5.564539e-15 6.695207e-15 -8.218985e-16
-1.438907e-15
E:\\R-3.1.2\\scripts\\mPDBS\\summary_pdb_93\\6a9d87d47926b1c8.final.pdb
3.086854e-15 -1.228313e-15 1.046889e-15 2.769204e-15 -2.162069e-16
2.291248e-15
E:\\R-3.1.2\\scripts\\mPDBS\\summary_pdb_93\\6bb3547ea982ffc4.final.pdb
2.930937e-15 1.581588e-15 2.017454e-15 1.494747e-15 -5.474276e-15
2.402605e-15
E:\\R-3.1.2\\scripts\\mPDBS\\summary_pdb_93\\716d9624611d1c42.final.pdb
4.242096e-15 1.073777e-15 3.967946e-16 5.858664e-15 -3.191402e-15
8.496992e-15
E:\\R-3.1.2\\scripts\\mPDBS\\summary_pdb_93\\75f27e70ad0f1be4.final.pdb
6.041516e-15 4.355161e-16 4.640264e-15 9.075069e-15 4.414835e-15
3.406705e-15
E:\\R-3.1.2\\scripts\\mPDBS\\summary_pdb_93\\769f910fffb22872.final.pdb
3.749694e-15 -9.947953e-16 1.415051e-15 2.876276e-15 -4.871445e-16
3.070152e-15
E:\\R-3.1.2\\scripts\\mPDBS\\summary_pdb_93\\791f8c80dd5c3135.final.pdb
4.438836e-15 -5.090693e-15 -8.951511e-16 9.156208e-16 -1.352391e-15
5.094685e-16
E:\\R-3.1.2\\scripts\\mPDBS\\summary_pdb_93\\7fad98e66a3f6099.final.pdb
-3.102632e-15 -7.603255e-15 -6.738787e-15 9.344012e-15 1.779687e-15
8.520819e-15
E:\\R-3.1.2\\scripts\\mPDBS\\summary_pdb_93\\804b89c94a633196.final.pdb
-7.099590e-17 -1.390728e-15 -1.293847e-15 6.012360e-15 1.527573e-15
2.086562e-15
E:\\R-3.1.2\\scripts\\mPDBS\\summary_pdb_93\\813cc65a2298feba.final.pdb
-7.099590e-17 -1.390728e-15 -1.293847e-15 6.012360e-15 1.527573e-15
2.086562e-15
E:\\R-3.1.2\\scripts\\mPDBS\\summary_pdb_93\\83c0eefcfe12d3f9.final.pdb
-3.375536e-15 -5.747486e-15 -3.070421e-15 4.520932e-15 -8.307834e-16
2.958043e-15
E:\\R-3.1.2\\scripts\\mPDBS\\summary_pdb_93\\86d50469ef569760.final.pdb
2.227407e-15 -4.053311e-15 -3.662604e-16 2.316442e-15 -3.232623e-17
-1.020200e-16
E:\\R-3.1.2\\scripts\\mPDBS\\summary_pdb_93\\89d0666e1402203d.final.pdb
6.041516e-15 4.355161e-16 4.640264e-15 9.075069e-15 4.414835e-15
3.406705e-15
E:\\R-3.1.2\\scripts\\mPDBS\\summary_pdb_93\\8b34852716dadc62.final.pdb
1.030554e-15 -6.789187e-15 -1.137722e-15 1.778334e-15 -1.414518e-15

1.827437e-15
E:\\R-3.1.2\\scripts\\mPDBS\\summary_pdb_93\\8b6ef9ac3fe74395.final.pdb
7.502078e-15 -1.602239e-15 -1.146827e-15 1.601048e-15 4.307784e-15
-6.862657e-15
E:\\R-3.1.2\\scripts\\mPDBS\\summary_pdb_93\\98bbe0d5986c138f.final.pdb
4.438836e-15 -5.090693e-15 -8.951511e-16 9.156208e-16 -1.352391e-15
5.094685e-16
E:\\R-3.1.2\\scripts\\mPDBS\\summary_pdb_93\\9c22f4909d67e2fa.final.pdb
1.406315e-14 5.128797e-15 7.257798e-15 -8.337259e-16 -5.588696e-15
3.783555e-15
E:\\R-3.1.2\\scripts\\mPDBS\\summary_pdb_93\\9dda8ed0d81fb3ef.final.pdb
4.449392e-15 1.250836e-15 7.545926e-15 -2.907967e-15 9.523272e-16
-3.956112e-15
E:\\R-3.1.2\\scripts\\mPDBS\\summary_pdb_93\\9f752b3616e6033b.final.pdb
3.448285e-15 -5.203056e-16 -2.156810e-15 3.985484e-15 -1.365297e-15
1.762355e-15
E:\\R-3.1.2\\scripts\\mPDBS\\summary_pdb_93\\a02c70083816d6ba.final.pdb
4.676242e-15 -4.608880e-16 6.100791e-15 7.047664e-16 -2.915309e-16
-1.081699e-15
E:\\R-3.1.2\\scripts\\mPDBS\\summary_pdb_93\\a0c997d891e4e082.final.pdb
-5.052992e-15 4.060559e-15 3.278696e-15 2.983963e-15 -1.072891e-14
1.719520e-15
E:\\R-3.1.2\\scripts\\mPDBS\\summary_pdb_93\\ab8039a2d823c616.final.pdb
3.221151e-15 -5.520705e-16 1.399882e-15 2.956207e-15 -3.932139e-16
2.075355e-15
E:\\R-3.1.2\\scripts\\mPDBS\\summary_pdb_93\\acf4d74b9c71d4dc.final.pdb
-7.099590e-17 -1.390728e-15 -1.293847e-15 6.012360e-15 1.527573e-15
2.086562e-15
E:\\R-3.1.2\\scripts\\mPDBS\\summary_pdb_93\\ad8a823e4b4c668e.final.pdb
3.606476e-15 -1.527590e-15 1.262372e-15 3.035313e-15 -5.800459e-16
2.389878e-15
E:\\R-3.1.2\\scripts\\mPDBS\\summary_pdb_93\\b16ababd03b7b857.final.pdb
1.406315e-14 5.128797e-15 7.257798e-15 -8.337259e-16 -5.588696e-15
3.783555e-15
E:\\R-3.1.2\\scripts\\mPDBS\\summary_pdb_93\\b226923e697d3c63.final.pdb
-4.370322e-15 3.997337e-15 5.946186e-15 -2.049713e-15 -4.728242e-15
2.501505e-15
E:\\R-3.1.2\\scripts\\mPDBS\\summary_pdb_93\\b28e48f6fa27a165.final.pdb
4.438836e-15 -5.090693e-15 -8.951511e-16 9.156208e-16 -1.352391e-15
5.094685e-16
E:\\R-3.1.2\\scripts\\mPDBS\\summary_pdb_93\\b42c4924e3cd1aec.final.pdb
2.732151e-15 -6.503378e-15 -8.395858e-15 -1.003241e-15 -6.460363e-15
2.258101e-15
E:\\R-3.1.2\\scripts\\mPDBS\\summary_pdb_93\\b4dd239fcabff1df.final.pdb
4.438836e-15 -5.090693e-15 -8.951511e-16 9.156208e-16 -1.352391e-15
5.094685e-16
E:\\R-3.1.2\\scripts\\mPDBS\\summary_pdb_93\\b669942ef97459a2.final.pdb
1.030554e-15 -6.789187e-15 -1.137722e-15 1.778334e-15 -1.414518e-15
1.827437e-15
E:\\R-3.1.2\\scripts\\mPDBS\\summary_pdb_93\\b99f4d9d87c1017b.final.pdb
4.438836e-15 -5.090693e-15 -8.951511e-16 9.156208e-16 -1.352391e-15
5.094685e-16
E:\\R-3.1.2\\scripts\\mPDBS\\summary_pdb_93\\b9d2cbf3975403e7.final.pdb
4.438836e-15 -5.090693e-15 -8.951511e-16 9.156208e-16 -1.352391e-15

5.094685e-16
E:\\R-3.1.2\\scripts\\mPDBS\\summary_pdb_93\\bc88c8a505f3a288.final.pdb
3.103587e-15 -1.125549e-15 1.281385e-15 2.954788e-15 -4.472994e-16
2.133170e-15
E:\\R-3.1.2\\scripts\\mPDBS\\summary_pdb_93\\bdac0e64f4ef427e.final.pdb
1.579278e-14 6.420299e-15 1.059432e-14 -7.114279e-16 -3.177390e-16
3.188545e-15
E:\\R-3.1.2\\scripts\\mPDBS\\summary_pdb_93\\ca2a1f55fc9d0681.final.pdb
1.030554e-15 -6.789187e-15 -1.137722e-15 1.778334e-15 -1.414518e-15
1.827437e-15
E:\\R-3.1.2\\scripts\\mPDBS\\summary_pdb_93\\ce34248de3e81a3c.final.pdb
6.219737e-15 -5.994511e-15 4.891112e-15 2.109398e-15 4.847092e-15
1.246338e-15
E:\\R-3.1.2\\scripts\\mPDBS\\summary_pdb_93\\d02e697772494a61.final.pdb
3.553639e-15 1.294824e-15 3.583580e-15 1.232273e-14 3.838985e-15
8.765017e-15
E:\\R-3.1.2\\scripts\\mPDBS\\summary_pdb_93\\d04221245d015047.final.pdb
5.679742e-15 -2.829762e-15 -3.052427e-16 4.432457e-15 8.468966e-16
2.987603e-15
E:\\R-3.1.2\\scripts\\mPDBS\\summary_pdb_93\\d678141e4018054a.final.pdb
1.311742e-15 -5.647191e-16 2.377474e-15 -1.819758e-15 -2.519689e-15
1.850236e-14
E:\\R-3.1.2\\scripts\\mPDBS\\summary_pdb_93\\d71b40af0f0efe39.final.pdb
-1.503000e-15 -1.893183e-15 -4.736025e-15 -2.432105e-15 -3.152309e-15
9.159803e-15
E:\\R-3.1.2\\scripts\\mPDBS\\summary_pdb_93\\dc0809eff10197e2.final.pdb
8.682830e-15 -1.664163e-15 -1.416027e-15 -7.764728e-16 -1.528328e-15
-9.455166e-15
E:\\R-3.1.2\\scripts\\mPDBS\\summary_pdb_93\\e1163918d3707839.final.pdb
7.760247e-15 -1.315691e-14 -9.192963e-15 2.882106e-15 -6.557508e-15
4.499689e-15
E:\\R-3.1.2\\scripts\\mPDBS\\summary_pdb_93\\e4b48b06824ef848.final.pdb
2.006270e-15 5.322634e-15 4.363756e-15 -1.842096e-16 -6.534611e-16
3.095631e-15
E:\\R-3.1.2\\scripts\\mPDBS\\summary_pdb_93\\e529e19ad281fea2.final.pdb
4.960464e-15 3.955908e-15 4.034733e-15 4.179101e-15 -1.086541e-16
2.970826e-15
E:\\R-3.1.2\\scripts\\mPDBS\\summary_pdb_93\\e5602082718ec3a1.final.pdb
-7.099590e-17 -1.390728e-15 -1.293847e-15 6.012360e-15 1.527573e-15
2.086562e-15
E:\\R-3.1.2\\scripts\\mPDBS\\summary_pdb_93\\e5d589d996d0a960.final.pdb
-1.271199e-15 -5.456316e-16 7.207072e-16 3.164145e-17 3.691865e-15
-5.412838e-15
E:\\R-3.1.2\\scripts\\mPDBS\\summary_pdb_93\\e9df9e72307a2fbd.final.pdb
4.676242e-15 -4.608880e-16 6.100791e-15 7.047664e-16 -2.915309e-16
-1.081699e-15
E:\\R-3.1.2\\scripts\\mPDBS\\summary_pdb_93\\ea00f8694090cae7.final.pdb
-7.099590e-17 -1.390728e-15 -1.293847e-15 6.012360e-15 1.527573e-15
2.086562e-15
E:\\R-3.1.2\\scripts\\mPDBS\\summary_pdb_93\\eba517be32db050e.final.pdb
4.438836e-15 -5.090693e-15 -8.951511e-16 9.156208e-16 -1.352391e-15
5.094685e-16
E:\\R-3.1.2\\scripts\\mPDBS\\summary_pdb_93\\ec04b9545899c29d.final.pdb
3.579076e-15 -9.167009e-16 9.147285e-16 2.820957e-15 -5.308614e-16

2.815924e-15
E:\\R-3.1.2\\scripts\\mPDBS\\summary_pdb_93\\ee6862cbcad52078.final.pdb
3.266597e-15 -1.301189e-15 1.291468e-15 3.018539e-15 -6.712956e-16
2.260130e-15
E:\\R-3.1.2\\scripts\\mPDBS\\summary_pdb_93\\ef6a42fc83b81e5d.final.pdb
3.421018e-15 -1.164798e-15 9.665168e-16 2.634716e-15 -6.313433e-16
2.559601e-15
E:\\R-3.1.2\\scripts\\mPDBS\\summary_pdb_93\\ef8e88d4ad93a0ad.final.pdb
5.679742e-15 -2.829762e-15 -3.052427e-16 4.432457e-15 8.468966e-16
2.987603e-15
E:\\R-3.1.2\\scripts\\mPDBS\\summary_pdb_93\\f14fcae8deec4fa3.final.pdb
3.448285e-15 -5.203056e-16 -2.156810e-15 3.985484e-15 -1.365297e-15
1.762355e-15
E:\\R-3.1.2\\scripts\\mPDBS\\summary_pdb_93\\f83584e22003fb92.final.pdb
1.541333e-15 2.316143e-15 -2.091026e-15 1.412164e-14 3.583331e-15
4.674756e-15
E:\\R-3.1.2\\scripts\\mPDBS\\summary_pdb_93\\f8e2bf87e52caa29.final.pdb
4.960464e-15 3.955908e-15 4.034733e-15 4.179101e-15 -1.086541e-16
2.970826e-15

[,487]   [,488]   [,489]   [,490]   [,491]   [,492]
E:\\R-3.1.2\\scripts\\40.1ns_data\\NAT0.pdb
2.365853e-15 -5.159901e-16 -7.606496e-16 2.141377e-15 -2.541388e-15
-6.515941e-16
E:\\R-3.1.2\\scripts\\40.1ns_data\\LJP6.pdb
3.344055e-15 -1.395397e-15 -1.975390e-15 2.950234e-15 -4.848534e-15
-2.141291e-16
E:\\R-3.1.2\\scripts\\40.1ns_data\\3WY9.pdb
6.033938e-16 -1.054681e-15 -1.114549e-15 1.443740e-15 -4.015174e-15
1.562720e-16
E:\\R-3.1.2\\scripts\\40.1ns_data\\ZSP9.pdb
1.565495e-15 -2.028086e-15 3.485852e-16 4.528133e-15 -4.792460e-15
4.660499e-16
E:\\R-3.1.2\\scripts\\mPDBS\\summary_pdb_93\\013d855ad26c877c.final.pdb
-1.375418e-15 -9.803430e-16 3.988293e-15 -2.051355e-15 -2.662658e-15
-3.758579e-15
E:\\R-3.1.2\\scripts\\mPDBS\\summary_pdb_93\\0a2a705342ca0d54.final.pdb
8.763043e-16 -2.763935e-15 -2.027631e-15 2.659697e-15 -3.652322e-15
1.654314e-15
E:\\R-3.1.2\\scripts\\mPDBS\\summary_pdb_93\\0a59fceadb4194d9.final.pdb
-1.220526e-15 -2.460596e-15 1.613698e-15 5.603104e-15 -1.532368e-15
1.658939e-15
E:\\R-3.1.2\\scripts\\mPDBS\\summary_pdb_93\\0dba59f3306825e8.final.pdb
-4.318376e-15 -8.093577e-15 -3.908168e-15 5.532856e-15 -4.145846e-15
6.763386e-15
E:\\R-3.1.2\\scripts\\mPDBS\\summary_pdb_93\\126f26a78b57339c.final.pdb
4.749891e-15 8.998137e-17 -1.125646e-14 5.249283e-15 -8.518217e-15
-2.422625e-15
E:\\R-3.1.2\\scripts\\mPDBS\\summary_pdb_93\\12f06f3e342df706.final.pdb
5.865513e-16 -1.580420e-15 -1.825536e-15 2.360240e-15 -4.067354e-15
1.898476e-15
E:\\R-3.1.2\\scripts\\mPDBS\\summary_pdb_93\\18618a2a251b867b.final.pdb
4.851797e-15 -5.298517e-15 -7.040155e-15 5.123931e-15 -5.057824e-15
-3.514732e-16

E:\\R-3.1.2\\scripts\\mPDBS\\summary_pdb_93\\1f305f598641eeb2.final.pdb
1.376839e-15 -9.421241e-16 -2.109787e-15 2.730848e-15 -4.355337e-15
1.552063e-15
E:\\R-3.1.2\\scripts\\mPDBS\\summary_pdb_93\\1f8a00b551be0a39.final.pdb
4.851797e-15 -5.298517e-15 -7.040155e-15 5.123931e-15 -5.057824e-15
-3.514732e-16
E:\\R-3.1.2\\scripts\\mPDBS\\summary_pdb_93\\219f45328d47579a.final.pdb
-9.406252e-15 7.829784e-15 -8.554384e-15 1.055921e-14 -1.221913e-14
2.606098e-15
E:\\R-3.1.2\\scripts\\mPDBS\\summary_pdb_93\\238dd3918e54336d.final.pdb
3.871701e-15 6.111657e-16 -6.524758e-15 6.666951e-15 -1.211288e-14
6.603769e-15
E:\\R-3.1.2\\scripts\\mPDBS\\summary_pdb_93\\2b3027889e2fdc42.final.pdb
-6.928447e-16 -1.478309e-15 -1.983685e-15 3.364484e-15 -3.496942e-15
1.495225e-15
E:\\R-3.1.2\\scripts\\mPDBS\\summary_pdb_93\\2fcb3b46e33793b0.final.pdb
-3.233188e-15 -2.132144e-15 3.097853e-15 9.544917e-16 -1.611005e-15
2.702184e-15
E:\\R-3.1.2\\scripts\\mPDBS\\summary_pdb_93\\34eb328251842006.final.pdb
1.055505e-15 1.075362e-14 2.306442e-15 5.676839e-15 2.578550e-15
1.228157e-14
E:\\R-3.1.2\\scripts\\mPDBS\\summary_pdb_93\\354b32871797b7ab.final.pdb
-5.437017e-16 4.586160e-15 1.590137e-14 -3.866426e-15 6.214844e-15
-1.239588e-15
E:\\R-3.1.2\\scripts\\mPDBS\\summary_pdb_93\\397368adbc32d5de.final.pdb
4.749891e-15 8.998137e-17 -1.125646e-14 5.249283e-15 -8.518217e-15
-2.422625e-15
E:\\R-3.1.2\\scripts\\mPDBS\\summary_pdb_93\\411ca13cd3df6edd.final.pdb
5.346311e-16 4.598583e-15 -6.300679e-16 2.847888e-15 -9.925756e-16
6.406470e-15
E:\\R-3.1.2\\scripts\\mPDBS\\summary_pdb_93\\42357eee6642bf12.final.pdb
-2.637436e-15 6.525859e-15 -3.448818e-15 1.486797e-14 4.539321e-16
3.694152e-15
E:\\R-3.1.2\\scripts\\mPDBS\\summary_pdb_93\\44342d1f75a7fcfc.final.pdb
5.738608e-15 -5.361982e-15 -2.251665e-15 -1.185198e-15 -1.697822e-15
-3.123018e-15
E:\\R-3.1.2\\scripts\\mPDBS\\summary_pdb_93\\448ef21dffe8cdfb.final.pdb
1.852718e-14 2.032298e-14 1.331884e-14 -4.624379e-15 2.668558e-15
-9.034087e-15
E:\\R-3.1.2\\scripts\\mPDBS\\summary_pdb_93\\472a40b3e8f113c7.final.pdb
-4.318376e-15 -8.093577e-15 -3.908168e-15 5.532856e-15 -4.145846e-15
6.763386e-15
E:\\R-3.1.2\\scripts\\mPDBS\\summary_pdb_93\\48a26225951ea153.final.pdb
7.072211e-15 -7.380155e-16 -1.466131e-16 -5.973532e-15 -8.757751e-16
4.218647e-16
E:\\R-3.1.2\\scripts\\mPDBS\\summary_pdb_93\\4ab4a5c7c6682ce6.final.pdb
4.720400e-15 1.270576e-14 7.688458e-15 -8.461971e-15 3.619643e-15
-7.026581e-15
E:\\R-3.1.2\\scripts\\mPDBS\\summary_pdb_93\\4eaa45b62cb6a25f.final.pdb
2.250558e-15 -5.871186e-15 -8.254655e-15 1.276569e-15 -8.505586e-15
5.948818e-16
E:\\R-3.1.2\\scripts\\mPDBS\\summary_pdb_93\\58c371611d85ac96.final.pdb
4.749891e-15 8.998137e-17 -1.125646e-14 5.249283e-15 -8.518217e-15
-2.422625e-15

E:\\R-3.1.2\\scripts\\mPDBS\\summary_pdb_93\\59a647f04822d30a.final.pdb
-4.207862e-15 -2.623721e-15 -3.084332e-15 -4.063075e-16 -2.211300e-15
4.709692e-15
E:\\R-3.1.2\\scripts\\mPDBS\\summary_pdb_93\\59b091fe385e05fc.final.pdb
-1.220526e-15 -2.460596e-15 1.613698e-15 5.603104e-15 -1.532368e-15
1.658939e-15
E:\\R-3.1.2\\scripts\\mPDBS\\summary_pdb_93\\5c35ac30f930d2db.final.pdb
2.250558e-15 -5.871186e-15 -8.254655e-15 1.276569e-15 -8.505586e-15
5.948818e-16
E:\\R-3.1.2\\scripts\\mPDBS\\summary_pdb_93\\5f60752431cf2dcb.final.pdb
4.583734e-15 -1.160842e-14 -5.461950e-15 5.430958e-15 -3.965439e-15
-1.222942e-15
E:\\R-3.1.2\\scripts\\mPDBS\\summary_pdb_93\\626456dd6ff2868d.final.pdb
-4.318376e-15 -8.093577e-15 -3.908168e-15 5.532856e-15 -4.145846e-15
6.763386e-15
E:\\R-3.1.2\\scripts\\mPDBS\\summary_pdb_93\\6583d53ff3c8f7ef.final.pdb
-4.207862e-15 -2.623721e-15 -3.084332e-15 -4.063075e-16 -2.211300e-15
4.709692e-15
E:\\R-3.1.2\\scripts\\mPDBS\\summary_pdb_93\\69760178986bef01.final.pdb
2.836995e-15 -1.779938e-14 -4.185240e-15 7.316507e-15 -8.537610e-15
3.746017e-16
E:\\R-3.1.2\\scripts\\mPDBS\\summary_pdb_93\\6a9d87d47926b1c8.final.pdb
-1.005055e-15 -9.135562e-16 -2.294876e-15 3.334148e-15 -4.058243e-15
1.174324e-15
E:\\R-3.1.2\\scripts\\mPDBS\\summary_pdb_93\\6bb3547ea982ffc4.final.pdb
-1.611989e-15 6.019906e-15 -8.283713e-15 7.450958e-15 -4.061702e-15
5.227311e-15
E:\\R-3.1.2\\scripts\\mPDBS\\summary_pdb_93\\716d9624611d1c42.final.pdb
1.658231e-15 7.553333e-15 1.659267e-16 -4.601856e-15 -6.828600e-15
8.535962e-15
E:\\R-3.1.2\\scripts\\mPDBS\\summary_pdb_93\\75f27e70ad0f1be4.final.pdb
-4.207862e-15 -2.623721e-15 -3.084332e-15 -4.063075e-16 -2.211300e-15
4.709692e-15
E:\\R-3.1.2\\scripts\\mPDBS\\summary_pdb_93\\769f910fffb22872.final.pdb
-6.771459e-16 -1.205437e-15 -1.995146e-15 2.200475e-15 -3.362566e-15
2.257334e-15
E:\\R-3.1.2\\scripts\\mPDBS\\summary_pdb_93\\791f8c80dd5c3135.final.pdb
-1.220526e-15 -2.460596e-15 1.613698e-15 5.603104e-15 -1.532368e-15
1.658939e-15
E:\\R-3.1.2\\scripts\\mPDBS\\summary_pdb_93\\7fad98e66a3f6099.final.pdb
3.189599e-15 9.452678e-15 6.905845e-15 -2.796438e-15 2.806583e-15
1.067773e-15
E:\\R-3.1.2\\scripts\\mPDBS\\summary_pdb_93\\804b89c94a633196.final.pdb
-3.233188e-15 -2.132144e-15 3.097853e-15 9.544917e-16 -1.611005e-15
2.702184e-15
E:\\R-3.1.2\\scripts\\mPDBS\\summary_pdb_93\\813cc65a2298feba.final.pdb
-3.233188e-15 -2.132144e-15 3.097853e-15 9.544917e-16 -1.611005e-15
2.702184e-15
E:\\R-3.1.2\\scripts\\mPDBS\\summary_pdb_93\\83c0eefcfe12d3f9.final.pdb
5.346311e-16 4.598583e-15 -6.300679e-16 2.847888e-15 -9.925756e-16
6.406470e-15
E:\\R-3.1.2\\scripts\\mPDBS\\summary_pdb_93\\86d50469ef569760.final.pdb
-4.318376e-15 -8.093577e-15 -3.908168e-15 5.532856e-15 -4.145846e-15
6.763386e-15

E:\\R-3.1.2\\scripts\\mPDBS\\summary_pdb_93\\89d0666e1402203d.final.pdb
-4.207862e-15 -2.623721e-15 -3.084332e-15 -4.063075e-16 -2.211300e-15
4.709692e-15
E:\\R-3.1.2\\scripts\\mPDBS\\summary_pdb_93\\8b34852716dadc62.final.pdb
4.096886e-15 7.509660e-16 -1.051279e-14 8.018286e-15 -7.040689e-15
4.820282e-15
E:\\R-3.1.2\\scripts\\mPDBS\\summary_pdb_93\\8b6ef9ac3fe74395.final.pdb
-7.599752e-15 -2.686211e-15 -7.476288e-15 1.142841e-14 -2.780477e-15
2.982192e-15
E:\\R-3.1.2\\scripts\\mPDBS\\summary_pdb_93\\98bbe0d5986c138f.final.pdb
-1.220526e-15 -2.460596e-15 1.613698e-15 5.603104e-15 -1.532368e-15
1.658939e-15
E:\\R-3.1.2\\scripts\\mPDBS\\summary_pdb_93\\9c22f4909d67e2fa.final.pdb
3.071231e-15 -1.251663e-14 -5.545407e-15 4.426947e-15 -1.203626e-14
6.196489e-16
E:\\R-3.1.2\\scripts\\mPDBS\\summary_pdb_93\\9dda8ed0d81fb3ef.final.pdb
4.002042e-15 -6.391541e-15 4.280493e-16 1.908722e-15 6.877696e-17
9.204054e-16
E:\\R-3.1.2\\scripts\\mPDBS\\summary_pdb_93\\9f752b3616e6033b.final.pdb
-1.409635e-15 -1.559101e-15 -1.430893e-15 5.369201e-15 -3.023634e-15
1.932463e-15
E:\\R-3.1.2\\scripts\\mPDBS\\summary_pdb_93\\a02c70083816d6ba.final.pdb
2.250558e-15 -5.871186e-15 -8.254655e-15 1.276569e-15 -8.505586e-15
5.948818e-16
E:\\R-3.1.2\\scripts\\mPDBS\\summary_pdb_93\\a0c997d891e4e082.final.pdb
-6.155298e-16 -7.960225e-16 1.790741e-15 9.271754e-16 -7.462016e-15
-4.316199e-15
E:\\R-3.1.2\\scripts\\mPDBS\\summary_pdb_93\\ab8039a2d823c616.final.pdb
4.187448e-16 -2.231506e-15 -1.588500e-15 3.534926e-15 -4.043309e-15
2.100110e-15
E:\\R-3.1.2\\scripts\\mPDBS\\summary_pdb_93\\acf4d74b9c71d4dc.final.pdb
-3.233188e-15 -2.132144e-15 3.097853e-15 9.544917e-16 -1.611005e-15
2.702184e-15
E:\\R-3.1.2\\scripts\\mPDBS\\summary_pdb_93\\ad8a823e4b4c668e.final.pdb
1.570364e-15 -1.465646e-15 -2.055344e-15 2.420087e-15 -3.656393e-15
2.169856e-15
E:\\R-3.1.2\\scripts\\mPDBS\\summary_pdb_93\\b16ababd03b7b857.final.pdb
3.071231e-15 -1.251663e-14 -5.545407e-15 4.426947e-15 -1.203626e-14
6.196489e-16
E:\\R-3.1.2\\scripts\\mPDBS\\summary_pdb_93\\b226923e697d3c63.final.pdb
-7.614546e-16 -1.578117e-15 -6.020785e-15 1.146138e-14 -8.758580e-15
-2.312685e-15
E:\\R-3.1.2\\scripts\\mPDBS\\summary_pdb_93\\b28e48f6fa27a165.final.pdb
-1.220526e-15 -2.460596e-15 1.613698e-15 5.603104e-15 -1.532368e-15
1.658939e-15
E:\\R-3.1.2\\scripts\\mPDBS\\summary_pdb_93\\b42c4924e3cd1aec.final.pdb
3.236324e-15 -2.040421e-15 1.114566e-15 2.993290e-15 -7.987496e-15
3.659444e-16
E:\\R-3.1.2\\scripts\\mPDBS\\summary_pdb_93\\b4dd239fcabff1df.final.pdb
-1.220526e-15 -2.460596e-15 1.613698e-15 5.603104e-15 -1.532368e-15
1.658939e-15
E:\\R-3.1.2\\scripts\\mPDBS\\summary_pdb_93\\b669942ef97459a2.final.pdb
4.096886e-15 7.509660e-16 -1.051279e-14 8.018286e-15 -7.040689e-15
4.820282e-15

E:\\R-3.1.2\\scripts\\mPDBS\\summary_pdb_93\\b99f4d9d87c1017b.final.pdb
-1.220526e-15 -2.460596e-15 1.613698e-15 5.603104e-15 -1.532368e-15
1.658939e-15
E:\\R-3.1.2\\scripts\\mPDBS\\summary_pdb_93\\b9d2cbf3975403e7.final.pdb
-1.220526e-15 -2.460596e-15 1.613698e-15 5.603104e-15 -1.532368e-15
1.658939e-15
E:\\R-3.1.2\\scripts\\mPDBS\\summary_pdb_93\\bc88c8a505f3a288.final.pdb
-2.980324e-16 -2.232046e-15 -1.907791e-15 4.020576e-15 -3.909156e-15
1.762372e-15
E:\\R-3.1.2\\scripts\\mPDBS\\summary_pdb_93\\bdac0e64f4ef427e.final.pdb
1.717486e-16 -1.067696e-14 -1.879936e-15 2.444999e-15 -1.022954e-14
-5.424345e-15
E:\\R-3.1.2\\scripts\\mPDBS\\summary_pdb_93\\ca2a1f55fc9d0681.final.pdb
4.096886e-15 7.509660e-16 -1.051279e-14 8.018286e-15 -7.040689e-15
4.820282e-15
E:\\R-3.1.2\\scripts\\mPDBS\\summary_pdb_93\\ce34248de3e81a3c.final.pdb
3.828470e-15 -9.673578e-16 -3.267138e-15 -1.891470e-15 -4.110966e-15
-5.836367e-15
E:\\R-3.1.2\\scripts\\mPDBS\\summary_pdb_93\\d02e697772494a61.final.pdb
-6.132016e-15 5.045119e-15 -7.579470e-15 3.381384e-15 -4.119274e-15
3.456980e-15
E:\\R-3.1.2\\scripts\\mPDBS\\summary_pdb_93\\d04221245d015047.final.pdb
1.001232e-15 -1.456952e-15 -6.534844e-16 -5.317270e-16 -2.299479e-15
3.165663e-15
E:\\R-3.1.2\\scripts\\mPDBS\\summary_pdb_93\\d678141e4018054a.final.pdb
8.606083e-15 1.173311e-14 6.496512e-16 -2.144641e-15 -1.734515e-15
5.094061e-15
E:\\R-3.1.2\\scripts\\mPDBS\\summary_pdb_93\\d71b40af0f0efe39.final.pdb
6.764313e-15 3.142731e-15 5.942176e-15 -6.477936e-15 -4.250783e-15
3.158876e-15
E:\\R-3.1.2\\scripts\\mPDBS\\summary_pdb_93\\dc0809eff10197e2.final.pdb
-1.309863e-14 -1.351265e-14 -1.829623e-15 1.870545e-15 -2.230312e-15
5.479704e-15
E:\\R-3.1.2\\scripts\\mPDBS\\summary_pdb_93\\e1163918d3707839.final.pdb
3.180813e-15 5.029879e-15 -1.247260e-15 6.504370e-15 -6.440990e-15
3.478472e-15
E:\\R-3.1.2\\scripts\\mPDBS\\summary_pdb_93\\e4b48b06824ef848.final.pdb
4.851797e-15 -5.298517e-15 -7.040155e-15 5.123931e-15 -5.057824e-15
-3.514732e-16
E:\\R-3.1.2\\scripts\\mPDBS\\summary_pdb_93\\e529e19ad281fea2.final.pdb
-1.375418e-15 -9.803430e-16 3.988293e-15 -2.051355e-15 -2.662658e-15
-3.758579e-15
E:\\R-3.1.2\\scripts\\mPDBS\\summary_pdb_93\\e5602082718ec3a1.final.pdb
-3.233188e-15 -2.132144e-15 3.097853e-15 9.544917e-16 -1.611005e-15
2.702184e-15
E:\\R-3.1.2\\scripts\\mPDBS\\summary_pdb_93\\e5d589d996d0a960.final.pdb
4.027319e-15 -3.166143e-15 -4.617809e-15 3.045355e-15 -1.071353e-14
-4.990943e-15
E:\\R-3.1.2\\scripts\\mPDBS\\summary_pdb_93\\e9df9e72307a2fbd.final.pdb
2.250558e-15 -5.871186e-15 -8.254655e-15 1.276569e-15 -8.505586e-15
5.948818e-16
E:\\R-3.1.2\\scripts\\mPDBS\\summary_pdb_93\\ea00f8694090cae7.final.pdb
-3.233188e-15 -2.132144e-15 3.097853e-15 9.544917e-16 -1.611005e-15
2.702184e-15

E:\\R-3.1.2\\scripts\\mPDBS\\summary_pdb_93\\eba517be32db050e.final.pdb
-1.220526e-15 -2.460596e-15 1.613698e-15 5.603104e-15 -1.532368e-15
1.658939e-15
E:\\R-3.1.2\\scripts\\mPDBS\\summary_pdb_93\\ec04b9545899c29d.final.pdb
8.935258e-16 -1.045990e-15 -1.651450e-15 2.128124e-15 -3.897451e-15
1.820076e-15
E:\\R-3.1.2\\scripts\\mPDBS\\summary_pdb_93\\ee6862cbcad52078.final.pdb
-1.133356e-15 -1.584561e-15 -2.084733e-15 3.262963e-15 -3.656537e-15
1.371843e-15
E:\\R-3.1.2\\scripts\\mPDBS\\summary_pdb_93\\ef6a42fc83b81e5d.final.pdb
1.079749e-15 -1.940093e-15 -2.197241e-15 2.954619e-15 -3.851324e-15
1.512270e-15
E:\\R-3.1.2\\scripts\\mPDBS\\summary_pdb_93\\ef8e88d4ad93a0ad.final.pdb
1.001232e-15 -1.456952e-15 -6.534844e-16 -5.317270e-16 -2.299479e-15
3.165663e-15
E:\\R-3.1.2\\scripts\\mPDBS\\summary_pdb_93\\f14fcae8deec4fa3.final.pdb
-1.409635e-15 -1.559101e-15 -1.430893e-15 5.369201e-15 -3.023634e-15
1.932463e-15
E:\\R-3.1.2\\scripts\\mPDBS\\summary_pdb_93\\f83584e22003fb92.final.pdb
7.072211e-15 -7.380155e-16 -1.466131e-16 -5.973532e-15 -8.757751e-16
4.218647e-16
E:\\R-3.1.2\\scripts\\mPDBS\\summary_pdb_93\\f8e2bf87e52caa29.final.pdb
-1.375418e-15 -9.803430e-16 3.988293e-15 -2.051355e-15 -2.662658e-15
-3.758579e-15

[,493]   [,494]   [,495]   [,496]   [,497]   [,498]
E:\\R-3.1.2\\scripts\\40.1ns_data\\NAT0.pdb
-1.402340e-15 -1.504247e-15 -5.009432e-15 -2.794393e-15 1.120564e-15
9.712524e-16
E:\\R-3.1.2\\scripts\\40.1ns_data\\LJP6.pdb
-1.182561e-15 2.453247e-15 -4.054685e-15 -4.313124e-15 1.563333e-15
-1.742479e-16
E:\\R-3.1.2\\scripts\\40.1ns_data\\3WY9.pdb
5.338721e-16 3.970834e-15 -4.634139e-15 -4.327117e-15 1.110200e-15
-8.443095e-16
E:\\R-3.1.2\\scripts\\40.1ns_data\\ZSP9.pdb
5.124247e-16 1.421756e-15 -5.149753e-15 -3.473999e-15 -4.611973e-16
8.622637e-16
E:\\R-3.1.2\\scripts\\mPDBS\\summary_pdb_93\\013d855ad26c877c.final.pdb
-2.636683e-15 2.639399e-15 1.025359e-16 -1.064934e-14 6.213533e-15
-3.854003e-15
E:\\R-3.1.2\\scripts\\mPDBS\\summary_pdb_93\\0a2a705342ca0d54.final.pdb
-2.737433e-16 3.079137e-16 -5.463342e-15 -4.181650e-15 6.940293e-17
-9.810037e-17
E:\\R-3.1.2\\scripts\\mPDBS\\summary_pdb_93\\0a59fceadb4194d9.final.pdb
-1.718058e-15 3.572392e-15 -7.236052e-15 -9.214396e-15 8.612185e-16
1.842892e-15
E:\\R-3.1.2\\scripts\\mPDBS\\summary_pdb_93\\0dba59f3306825e8.final.pdb
3.370665e-15 1.681832e-15 -9.271910e-15 -3.595952e-15 -1.575375e-15
2.283448e-15
E:\\R-3.1.2\\scripts\\mPDBS\\summary_pdb_93\\126f26a78b57339c.final.pdb
1.902818e-15 -5.801548e-15 -8.019873e-15 2.531742e-15 -1.058861e-15
4.723969e-16
E:\\R-3.1.2\\scripts\\mPDBS\\summary_pdb_93\\12f06f3e342df706.final.pdb

-1.136067e-16 -7.892967e-17 -5.351452e-15 -4.123536e-15 3.929289e-16
1.518662e-15
E:\\R-3.1.2\\scripts\\mPDBS\\summary_pdb_93\\18618a2a251b867b.final.pdb
8.505614e-15 -6.950257e-15 -9.083500e-15 -4.438870e-15 1.371403e-15
-2.052059e-15
E:\\R-3.1.2\\scripts\\mPDBS\\summary_pdb_93\\1f305f598641eeb2.final.pdb
-2.503055e-16 -5.701845e-17 -4.550678e-15 -4.261630e-15 8.125632e-16
3.093346e-16
E:\\R-3.1.2\\scripts\\mPDBS\\summary_pdb_93\\1f8a00b551be0a39.final.pdb
8.505614e-15 -6.950257e-15 -9.083500e-15 -4.438870e-15 1.371403e-15
-2.052059e-15
E:\\R-3.1.2\\scripts\\mPDBS\\summary_pdb_93\\219f45328d47579a.final.pdb
-1.564745e-14 -6.825540e-15 8.141404e-15 -1.434571e-14 1.364288e-15
-5.135901e-15
E:\\R-3.1.2\\scripts\\mPDBS\\summary_pdb_93\\238dd3918e54336d.final.pdb
-4.124076e-16 -7.076858e-15 -5.731958e-16 -2.263142e-15 1.060343e-14
8.049949e-15
E:\\R-3.1.2\\scripts\\mPDBS\\summary_pdb_93\\2b3027889e2fdc42.final.pdb
-2.523901e-17 -1.166655e-15 -5.209461e-15 -5.104402e-15 1.246327e-15
4.466564e-16
E:\\R-3.1.2\\scripts\\mPDBS\\summary_pdb_93\\2fcb3b46e33793b0.final.pdb
-1.568695e-15 3.072423e-15 -2.198393e-15 -8.563744e-15 1.825642e-15
-1.243463e-16
E:\\R-3.1.2\\scripts\\mPDBS\\summary_pdb_93\\34eb328251842006.final.pdb
-2.185759e-16 7.723286e-15 -1.971779e-15 -8.593789e-15 9.400069e-16
-2.472206e-15
E:\\R-3.1.2\\scripts\\mPDBS\\summary_pdb_93\\354b32871797b7ab.final.pdb
-6.152790e-15 7.725840e-15 6.177509e-15 -7.270754e-15 2.199589e-16
4.324157e-15
E:\\R-3.1.2\\scripts\\mPDBS\\summary_pdb_93\\397368adbc32d5de.final.pdb
1.902818e-15 -5.801548e-15 -8.019873e-15 2.531742e-15 -1.058861e-15
4.723969e-16
E:\\R-3.1.2\\scripts\\mPDBS\\summary_pdb_93\\411ca13cd3df6edd.final.pdb
8.688622e-16 1.951574e-15 -2.975116e-15 -3.773088e-15 -4.811068e-16
-3.187868e-15
E:\\R-3.1.2\\scripts\\mPDBS\\summary_pdb_93\\42357eee6642bf12.final.pdb
-3.551774e-15 -2.700573e-15 -1.130428e-15 -2.262227e-14 7.726631e-15
-6.170718e-16
E:\\R-3.1.2\\scripts\\mPDBS\\summary_pdb_93\\44342d1f75a7fcfc.final.pdb
4.595036e-15 5.549490e-15 -1.172714e-14 3.962945e-15 1.703012e-15
8.624201e-15
E:\\R-3.1.2\\scripts\\mPDBS\\summary_pdb_93\\448ef21dffe8cdfb.final.pdb
-3.279133e-15 -8.598797e-15 7.915045e-15 -4.799394e-15 6.249187e-15
-7.344265e-15
E:\\R-3.1.2\\scripts\\mPDBS\\summary_pdb_93\\472a40b3e8f113c7.final.pdb
3.370665e-15 1.681832e-15 -9.271910e-15 -3.595952e-15 -1.575375e-15
2.283448e-15
E:\\R-3.1.2\\scripts\\mPDBS\\summary_pdb_93\\48a26225951ea153.final.pdb
7.215930e-15 1.005219e-15 -5.938045e-15 5.217202e-15 5.559071e-16
-6.615403e-15
E:\\R-3.1.2\\scripts\\mPDBS\\summary_pdb_93\\4ab4a5c7c6682ce6.final.pdb
-4.220756e-15 -5.454604e-15 1.718865e-15 -1.044746e-14 3.198150e-15
3.032849e-15
E:\\R-3.1.2\\scripts\\mPDBS\\summary_pdb_93\\4eaa45b62cb6a25f.final.pdb

2.470683e-15 -3.648959e-15 -9.373203e-15 -2.089352e-15 5.634341e-16
-1.017274e-15
E:\\R-3.1.2\\scripts\\mPDBS\\summary_pdb_93\\58c371611d85ac96.final.pdb
1.902818e-15 -5.801548e-15 -8.019873e-15 2.531742e-15 -1.058861e-15
4.723969e-16
E:\\R-3.1.2\\scripts\\mPDBS\\summary_pdb_93\\59a647f04822d30a.final.pdb
-1.166414e-15 -6.704597e-15 -1.064665e-15 -3.829433e-15 3.619881e-15
-5.845127e-15
E:\\R-3.1.2\\scripts\\mPDBS\\summary_pdb_93\\59b091fe385e05fc.final.pdb
-1.718058e-15 3.572392e-15 -7.236052e-15 -9.214396e-15 8.612185e-16
1.842892e-15
E:\\R-3.1.2\\scripts\\mPDBS\\summary_pdb_93\\5c35ac30f930d2db.final.pdb
2.470683e-15 -3.648959e-15 -9.373203e-15 -2.089352e-15 5.634341e-16
-1.017274e-15
E:\\R-3.1.2\\scripts\\mPDBS\\summary_pdb_93\\5f60752431cf2dcb.final.pdb
4.734132e-15 -4.347867e-15 -8.756280e-15 -7.026163e-15 -9.245936e-16
-8.958563e-16
E:\\R-3.1.2\\scripts\\mPDBS\\summary_pdb_93\\626456dd6ff2868d.final.pdb
3.370665e-15 1.681832e-15 -9.271910e-15 -3.595952e-15 -1.575375e-15
2.283448e-15
E:\\R-3.1.2\\scripts\\mPDBS\\summary_pdb_93\\6583d53ff3c8f7ef.final.pdb
-1.166414e-15 -6.704597e-15 -1.064665e-15 -3.829433e-15 3.619881e-15
-5.845127e-15
E:\\R-3.1.2\\scripts\\mPDBS\\summary_pdb_93\\69760178986bef01.final.pdb
1.313224e-16 -7.044581e-15 -8.635802e-16 -8.881293e-16 3.027166e-15
7.575676e-15
E:\\R-3.1.2\\scripts\\mPDBS\\summary_pdb_93\\6a9d87d47926b1c8.final.pdb
-5.865106e-16 -4.100278e-16 -5.029210e-15 -4.691646e-15 1.161585e-15
1.705550e-16
E:\\R-3.1.2\\scripts\\mPDBS\\summary_pdb_93\\6bb3547ea982ffc4.final.pdb
1.830292e-15 -2.256364e-15 -1.324045e-15 6.946114e-15 -3.498665e-15
-4.726267e-17
E:\\R-3.1.2\\scripts\\mPDBS\\summary_pdb_93\\716d9624611d1c42.final.pdb
-1.354910e-15 3.926547e-15 -3.608922e-15 4.157166e-15 -1.422333e-16
1.308760e-15
E:\\R-3.1.2\\scripts\\mPDBS\\summary_pdb_93\\75f27e70ad0f1be4.final.pdb
-1.166414e-15 -6.704597e-15 -1.064665e-15 -3.829433e-15 3.619881e-15
-5.845127e-15
E:\\R-3.1.2\\scripts\\mPDBS\\summary_pdb_93\\769f910fffb22872.final.pdb
3.677007e-16 -1.133729e-15 -5.040510e-15 -3.752136e-15 1.220482e-15
4.736987e-16
E:\\R-3.1.2\\scripts\\mPDBS\\summary_pdb_93\\791f8c80dd5c3135.final.pdb
-1.718058e-15 3.572392e-15 -7.236052e-15 -9.214396e-15 8.612185e-16
1.842892e-15
E:\\R-3.1.2\\scripts\\mPDBS\\summary_pdb_93\\7fad98e66a3f6099.final.pdb
-6.440263e-15 9.164475e-15 2.994273e-15 -5.808919e-15 -4.558933e-15
2.442795e-15
E:\\R-3.1.2\\scripts\\mPDBS\\summary_pdb_93\\804b89c94a633196.final.pdb
-1.568695e-15 3.072423e-15 -2.198393e-15 -8.563744e-15 1.825642e-15
-1.243463e-16
E:\\R-3.1.2\\scripts\\mPDBS\\summary_pdb_93\\813cc65a2298feba.final.pdb
-1.568695e-15 3.072423e-15 -2.198393e-15 -8.563744e-15 1.825642e-15
-1.243463e-16
E:\\R-3.1.2\\scripts\\mPDBS\\summary_pdb_93\\83c0eefcfe12d3f9.final.pdb

8.688622e-16 1.951574e-15 -2.975116e-15 -3.773088e-15 -4.811068e-16
-3.187868e-15
E:\\R-3.1.2\\scripts\\mPDBS\\summary_pdb_93\\86d50469ef569760.final.pdb
3.370665e-15 1.681832e-15 -9.271910e-15 -3.595952e-15 -1.575375e-15
2.283448e-15
E:\\R-3.1.2\\scripts\\mPDBS\\summary_pdb_93\\89d0666e1402203d.final.pdb
-1.166414e-15 -6.704597e-15 -1.064665e-15 -3.829433e-15 3.619881e-15
-5.845127e-15
E:\\R-3.1.2\\scripts\\mPDBS\\summary_pdb_93\\8b34852716dadc62.final.pdb
-1.353590e-15 -2.971354e-15 -9.133818e-15 -7.436106e-16 -1.511966e-15
5.076355e-15
E:\\R-3.1.2\\scripts\\mPDBS\\summary_pdb_93\\8b6ef9ac3fe74395.final.pdb
-1.836817e-15 -4.541211e-15 -1.012704e-14 -1.974132e-15 3.038422e-15
-8.791823e-16
E:\\R-3.1.2\\scripts\\mPDBS\\summary_pdb_93\\98bbe0d5986c138f.final.pdb
-1.718058e-15 3.572392e-15 -7.236052e-15 -9.214396e-15 8.612185e-16
1.842892e-15
E:\\R-3.1.2\\scripts\\mPDBS\\summary_pdb_93\\9c22f4909d67e2fa.final.pdb
3.977222e-16 1.863320e-15 -7.763386e-15 -2.872333e-15 1.576275e-15
1.886198e-15
E:\\R-3.1.2\\scripts\\mPDBS\\summary_pdb_93\\9dda8ed0d81fb3ef.final.pdb
6.077954e-15 1.267920e-14 -1.997619e-14 8.478149e-16 -7.517044e-15
-6.035947e-15
E:\\R-3.1.2\\scripts\\mPDBS\\summary_pdb_93\\9f752b3616e6033b.final.pdb
-7.751721e-16 -3.263374e-16 -3.967988e-15 -5.393127e-15 1.302072e-15
1.305867e-15
E:\\R-3.1.2\\scripts\\mPDBS\\summary_pdb_93\\a02c70083816d6ba.final.pdb
2.470683e-15 -3.648959e-15 -9.373203e-15 -2.089352e-15 5.634341e-16
-1.017274e-15
E:\\R-3.1.2\\scripts\\mPDBS\\summary_pdb_93\\a0c997d891e4e082.final.pdb
-6.422163e-15 6.052684e-15 1.331575e-15 2.128840e-15 5.260701e-15
-8.486770e-16
E:\\R-3.1.2\\scripts\\mPDBS\\summary_pdb_93\\ab8039a2d823c616.final.pdb
-3.130629e-16 -7.697665e-16 -5.095792e-15 -3.893903e-15 5.491916e-16
-4.181374e-17
E:\\R-3.1.2\\scripts\\mPDBS\\summary_pdb_93\\acf4d74b9c71d4dc.final.pdb
-1.568695e-15 3.072423e-15 -2.198393e-15 -8.563744e-15 1.825642e-15
-1.243463e-16
E:\\R-3.1.2\\scripts\\mPDBS\\summary_pdb_93\\ad8a823e4b4c668e.final.pdb
2.144400e-16 -2.477043e-16 -4.930739e-15 -3.330225e-15 7.965111e-16
6.982607e-16
E:\\R-3.1.2\\scripts\\mPDBS\\summary_pdb_93\\b16ababd03b7b857.final.pdb
3.977222e-16 1.863320e-15 -7.763386e-15 -2.872333e-15 1.576275e-15
1.886198e-15
E:\\R-3.1.2\\scripts\\mPDBS\\summary_pdb_93\\b226923e697d3c63.final.pdb
-2.265904e-15 -5.075692e-15 -4.407694e-15 -9.092367e-15 6.947081e-15
-9.740239e-16
E:\\R-3.1.2\\scripts\\mPDBS\\summary_pdb_93\\b28e48f6fa27a165.final.pdb
-1.718058e-15 3.572392e-15 -7.236052e-15 -9.214396e-15 8.612185e-16
1.842892e-15
E:\\R-3.1.2\\scripts\\mPDBS\\summary_pdb_93\\b42c4924e3cd1aec.final.pdb
-2.930032e-15 3.108517e-15 -3.132239e-15 -9.184741e-15 6.896774e-15
9.160014e-15
E:\\R-3.1.2\\scripts\\mPDBS\\summary_pdb_93\\b4dd239fcabff1df.final.pdb

-1.718058e-15 3.572392e-15 -7.236052e-15 -9.214396e-15 8.612185e-16
1.842892e-15
E:\\R-3.1.2\\scripts\\mPDBS\\summary_pdb_93\\b669942ef97459a2.final.pdb
-1.353590e-15 -2.971354e-15 -9.133818e-15 -7.436106e-16 -1.511966e-15
5.076355e-15
E:\\R-3.1.2\\scripts\\mPDBS\\summary_pdb_93\\b99f4d9d87c1017b.final.pdb
-1.718058e-15 3.572392e-15 -7.236052e-15 -9.214396e-15 8.612185e-16
1.842892e-15
E:\\R-3.1.2\\scripts\\mPDBS\\summary_pdb_93\\b9d2cbf3975403e7.final.pdb
-1.718058e-15 3.572392e-15 -7.236052e-15 -9.214396e-15 8.612185e-16
1.842892e-15
E:\\R-3.1.2\\scripts\\mPDBS\\summary_pdb_93\\bc88c8a505f3a288.final.pdb
-2.030482e-16 3.267249e-16 -5.368622e-15 -5.015064e-15 9.744092e-16
4.065409e-16
E:\\R-3.1.2\\scripts\\mPDBS\\summary_pdb_93\\bdac0e64f4ef427e.final.pdb
-3.310791e-15 -5.821721e-15 -8.436025e-15 -4.510563e-15 3.855702e-15
5.817082e-15
E:\\R-3.1.2\\scripts\\mPDBS\\summary_pdb_93\\ca2a1f55fc9d0681.final.pdb
-1.353590e-15 -2.971354e-15 -9.133818e-15 -7.436106e-16 -1.511966e-15
5.076355e-15
E:\\R-3.1.2\\scripts\\mPDBS\\summary_pdb_93\\ce34248de3e81a3c.final.pdb
-2.670121e-15 7.477356e-16 -7.705574e-15 -8.517638e-15 5.420995e-15
-2.144168e-15
E:\\R-3.1.2\\scripts\\mPDBS\\summary_pdb_93\\d02e697772494a61.final.pdb
-3.442102e-15 -1.753030e-14 8.546245e-15 -5.649322e-15 3.548305e-15
-1.891751e-15
E:\\R-3.1.2\\scripts\\mPDBS\\summary_pdb_93\\d04221245d015047.final.pdb
7.491842e-16 1.171172e-15 -5.491960e-15 -3.207169e-15 -9.062414e-16
7.646158e-16
E:\\R-3.1.2\\scripts\\mPDBS\\summary_pdb_93\\d678141e4018054a.final.pdb
5.254734e-15 3.402077e-15 -3.919390e-15 2.269964e-15 -8.614677e-15
5.330684e-16
E:\\R-3.1.2\\scripts\\mPDBS\\summary_pdb_93\\d71b40af0f0efe39.final.pdb
3.538739e-15 3.501152e-15 -1.043269e-14 -5.937332e-15 -1.013899e-14
1.131008e-15
E:\\R-3.1.2\\scripts\\mPDBS\\summary_pdb_93\\dc0809eff10197e2.final.pdb
4.594099e-15 8.695538e-16 -1.625227e-14 -2.581828e-15 -2.902238e-16
-2.542563e-15
E:\\R-3.1.2\\scripts\\mPDBS\\summary_pdb_93\\e1163918d3707839.final.pdb
3.494083e-15 3.767712e-15 -6.777760e-15 -2.480035e-15 -4.894142e-15
4.898658e-16
E:\\R-3.1.2\\scripts\\mPDBS\\summary_pdb_93\\e4b48b06824ef848.final.pdb
8.505614e-15 -6.950257e-15 -9.083500e-15 -4.438870e-15 1.371403e-15
-2.052059e-15
E:\\R-3.1.2\\scripts\\mPDBS\\summary_pdb_93\\e529e19ad281fea2.final.pdb
-2.636683e-15 2.639399e-15 1.025359e-16 -1.064934e-14 6.213533e-15
-3.854003e-15
E:\\R-3.1.2\\scripts\\mPDBS\\summary_pdb_93\\e5602082718ec3a1.final.pdb
-1.568695e-15 3.072423e-15 -2.198393e-15 -8.563744e-15 1.825642e-15
-1.243463e-16
E:\\R-3.1.2\\scripts\\mPDBS\\summary_pdb_93\\e5d589d996d0a960.final.pdb
-8.920934e-16 -2.083449e-15 -7.519838e-15 5.612794e-15 1.923678e-15
4.501460e-15
E:\\R-3.1.2\\scripts\\mPDBS\\summary_pdb_93\\e9df9e72307a2fbd.final.pdb

2.470683e-15 -3.648959e-15 -9.373203e-15 -2.089352e-15 5.634341e-16
-1.017274e-15
E:\\R-3.1.2\\scripts\\mPDBS\\summary_pdb_93\\ea00f8694090cae7.final.pdb
-1.568695e-15 3.072423e-15 -2.198393e-15 -8.563744e-15 1.825642e-15
-1.243463e-16
E:\\R-3.1.2\\scripts\\mPDBS\\summary_pdb_93\\eba517be32db050e.final.pdb
-1.718058e-15 3.572392e-15 -7.236052e-15 -9.214396e-15 8.612185e-16
1.842892e-15
E:\\R-3.1.2\\scripts\\mPDBS\\summary_pdb_93\\ec04b9545899c29d.final.pdb
4.919338e-17 -1.404033e-16 -5.204583e-15 -4.263114e-15 4.037037e-16
2.038533e-16
E:\\R-3.1.2\\scripts\\mPDBS\\summary_pdb_93\\ee6862cbcad52078.final.pdb
-1.182093e-16 -5.666577e-16 -5.424133e-15 -5.172490e-15 1.159591e-15
1.654144e-16
E:\\R-3.1.2\\scripts\\mPDBS\\summary_pdb_93\\ef6a42fc83b81e5d.final.pdb
2.906682e-16 -1.468882e-16 -4.979578e-15 -4.529509e-15 4.572036e-16
6.764446e-16
E:\\R-3.1.2\\scripts\\mPDBS\\summary_pdb_93\\ef8e88d4ad93a0ad.final.pdb
7.491842e-16 1.171172e-15 -5.491960e-15 -3.207169e-15 -9.062414e-16
7.646158e-16
E:\\R-3.1.2\\scripts\\mPDBS\\summary_pdb_93\\f14fcae8deec4fa3.final.pdb
-7.751721e-16 -3.263374e-16 -3.967988e-15 -5.393127e-15 1.302072e-15
1.305867e-15
E:\\R-3.1.2\\scripts\\mPDBS\\summary_pdb_93\\f83584e22003fb92.final.pdb
7.215930e-15 1.005219e-15 -5.938045e-15 5.217202e-15 5.559071e-16
-6.615403e-15
E:\\R-3.1.2\\scripts\\mPDBS\\summary_pdb_93\\f8e2bf87e52caa29.final.pdb
-2.636683e-15 2.639399e-15 1.025359e-16 -1.064934e-14 6.213533e-15
-3.854003e-15

[,499]   [,500]   [,501]   [,502]   [,503]   [,504]
E:\\R-3.1.2\\scripts\\40.1ns_data\\NAT0.pdb
5.861136e-15 7.542632e-15 -2.608869e-15 2.379704e-15 1.186207e-15
-2.604352e-15
E:\\R-3.1.2\\scripts\\40.1ns_data\\LJP6.pdb
4.538503e-15 7.607097e-15 -1.661762e-15 1.929369e-15 2.849050e-16
-3.710914e-15
E:\\R-3.1.2\\scripts\\40.1ns_data\\3WY9.pdb
5.601894e-15 6.820576e-15 -3.981965e-15 4.275861e-15 1.039958e-15
-3.463453e-15
E:\\R-3.1.2\\scripts\\40.1ns_data\\ZSP9.pdb
5.085702e-15 8.306675e-15 -1.766347e-15 1.762462e-15 1.312178e-15
-3.727110e-15
E:\\R-3.1.2\\scripts\\mPDBS\\summary_pdb_93\\013d855ad26c877c.final.pdb
5.805107e-15 4.650785e-15 4.073056e-15 -3.505749e-15 -9.562700e-16
-6.662243e-15
E:\\R-3.1.2\\scripts\\mPDBS\\summary_pdb_93\\0a2a705342ca0d54.final.pdb
4.292124e-15 7.499897e-15 -2.532794e-15 3.037825e-15 6.921875e-16
-4.402275e-15
E:\\R-3.1.2\\scripts\\mPDBS\\summary_pdb_93\\0a59fceadb4194d9.final.pdb
5.619594e-15 5.307889e-15 -2.388776e-15 6.835437e-15 8.611761e-16
-4.758571e-15
E:\\R-3.1.2\\scripts\\mPDBS\\summary_pdb_93\\0dba59f3306825e8.final.pdb
4.163625e-15 1.082293e-14 -8.561610e-16 5.895585e-15 6.607124e-15

-5.771029e-15
E:\\R-3.1.2\\scripts\\mPDBS\\summary_pdb_93\\126f26a78b57339c.final.pdb
9.041898e-15 9.221412e-15 -9.379099e-16 3.257070e-15 -3.421149e-15
5.173775e-15
E:\\R-3.1.2\\scripts\\mPDBS\\summary_pdb_93\\12f06f3e342df706.final.pdb
4.392033e-15 7.050295e-15 -2.268791e-15 3.772264e-15 1.092339e-15
-2.945974e-15
E:\\R-3.1.2\\scripts\\mPDBS\\summary_pdb_93\\18618a2a251b867b.final.pdb
5.553188e-15 1.005751e-14 5.550949e-16 -1.291881e-15 -1.455426e-15
-4.760738e-15
E:\\R-3.1.2\\scripts\\mPDBS\\summary_pdb_93\\1f305f598641eeb2.final.pdb
4.652853e-15 6.880245e-15 -2.414433e-15 3.078219e-15 6.898977e-16
-4.195348e-15
E:\\R-3.1.2\\scripts\\mPDBS\\summary_pdb_93\\1f8a00b551be0a39.final.pdb
5.553188e-15 1.005751e-14 5.550949e-16 -1.291881e-15 -1.455426e-15
-4.760738e-15
E:\\R-3.1.2\\scripts\\mPDBS\\summary_pdb_93\\219f45328d47579a.final.pdb
-4.358915e-16 4.017255e-15 -4.292852e-15 8.511180e-15 -1.413914e-15
1.501612e-15
E:\\R-3.1.2\\scripts\\mPDBS\\summary_pdb_93\\238dd3918e54336d.final.pdb
1.280494e-14 1.448699e-14 -5.047782e-15 8.866407e-17 6.414541e-15
-9.506075e-15
E:\\R-3.1.2\\scripts\\mPDBS\\summary_pdb_93\\2b3027889e2fdc42.final.pdb
4.751162e-15 7.706642e-15 -2.579379e-15 3.578928e-15 7.975877e-16
-3.113186e-15
E:\\R-3.1.2\\scripts\\mPDBS\\summary_pdb_93\\2fcb3b46e33793b0.final.pdb
2.005231e-15 7.091882e-15 -1.290333e-15 8.170067e-16 5.022370e-15
-9.154055e-15
E:\\R-3.1.2\\scripts\\mPDBS\\summary_pdb_93\\34eb328251842006.final.pdb
4.751108e-15 -4.397671e-15 -1.480247e-15 1.697145e-15 -5.186235e-15
-4.735318e-15
E:\\R-3.1.2\\scripts\\mPDBS\\summary_pdb_93\\354b32871797b7ab.final.pdb
3.818457e-15 3.814569e-15 9.767690e-15 8.351457e-15 -7.020905e-15
-1.967098e-15
E:\\R-3.1.2\\scripts\\mPDBS\\summary_pdb_93\\397368adbc32d5de.final.pdb
9.041898e-15 9.221412e-15 -9.379099e-16 3.257070e-15 -3.421149e-15
5.173775e-15
E:\\R-3.1.2\\scripts\\mPDBS\\summary_pdb_93\\411ca13cd3df6edd.final.pdb
4.049372e-15 8.936262e-15 4.879428e-15 -3.786082e-15 2.609207e-15
-4.289890e-15
E:\\R-3.1.2\\scripts\\mPDBS\\summary_pdb_93\\42357eee6642bf12.final.pdb
1.864015e-16 -2.962594e-16 -2.101787e-15 1.102259e-14 -5.628522e-15
-9.063460e-15
E:\\R-3.1.2\\scripts\\mPDBS\\summary_pdb_93\\44342d1f75a7fcfc.final.pdb
9.941148e-15 7.566853e-15 -2.079420e-15 -1.146620e-14 1.901073e-15
-6.630414e-16
E:\\R-3.1.2\\scripts\\mPDBS\\summary_pdb_93\\448ef21dffe8cdfb.final.pdb
2.984916e-15 -2.282564e-15 -7.969885e-15 -1.393898e-15 -1.554191e-14
-9.588169e-15
E:\\R-3.1.2\\scripts\\mPDBS\\summary_pdb_93\\472a40b3e8f113c7.final.pdb
4.163625e-15 1.082293e-14 -8.561610e-16 5.895585e-15 6.607124e-15
-5.771029e-15
E:\\R-3.1.2\\scripts\\mPDBS\\summary_pdb_93\\48a26225951ea153.final.pdb
1.989808e-15 1.163579e-14 -6.880842e-15 -6.774336e-15 -3.524910e-15

-3.979246e-15
E:\\R-3.1.2\\scripts\\mPDBS\\summary_pdb_93\\4ab4a5c7c6682ce6.final.pdb
-7.532517e-15 4.398881e-15 -1.200284e-14 7.257343e-15 -3.167012e-15
-4.480829e-15
E:\\R-3.1.2\\scripts\\mPDBS\\summary_pdb_93\\4eaa45b62cb6a25f.final.pdb
5.809255e-15 1.117432e-14 -3.997449e-15 1.770349e-15 3.407738e-15
-2.367385e-15
E:\\R-3.1.2\\scripts\\mPDBS\\summary_pdb_93\\58c371611d85ac96.final.pdb
9.041898e-15 9.221412e-15 -9.379099e-16 3.257070e-15 -3.421149e-15
5.173775e-15
E:\\R-3.1.2\\scripts\\mPDBS\\summary_pdb_93\\59a647f04822d30a.final.pdb
-3.594227e-16 7.674406e-15 -3.347937e-15 5.271713e-15 -1.199311e-15
-4.353232e-15
E:\\R-3.1.2\\scripts\\mPDBS\\summary_pdb_93\\59b091fe385e05fc.final.pdb
5.619594e-15 5.307889e-15 -2.388776e-15 6.835437e-15 8.611761e-16
-4.758571e-15
E:\\R-3.1.2\\scripts\\mPDBS\\summary_pdb_93\\5c35ac30f930d2db.final.pdb
5.809255e-15 1.117432e-14 -3.997449e-15 1.770349e-15 3.407738e-15
-2.367385e-15
E:\\R-3.1.2\\scripts\\mPDBS\\summary_pdb_93\\5f60752431cf2dcb.final.pdb
2.990661e-15 8.875163e-15 1.522556e-15 3.767710e-15 -7.856343e-16
4.306471e-15
E:\\R-3.1.2\\scripts\\mPDBS\\summary_pdb_93\\626456dd6ff2868d.final.pdb
4.163625e-15 1.082293e-14 -8.561610e-16 5.895585e-15 6.607124e-15
-5.771029e-15
E:\\R-3.1.2\\scripts\\mPDBS\\summary_pdb_93\\6583d53ff3c8f7ef.final.pdb
-3.594227e-16 7.674406e-15 -3.347937e-15 5.271713e-15 -1.199311e-15
-4.353232e-15
E:\\R-3.1.2\\scripts\\mPDBS\\summary_pdb_93\\69760178986bef01.final.pdb
8.847055e-16 1.449608e-14 -3.983361e-15 -7.245175e-15 2.899120e-15
-4.213178e-16
E:\\R-3.1.2\\scripts\\mPDBS\\summary_pdb_93\\6a9d87d47926b1c8.final.pdb
4.288322e-15 7.351380e-15 -2.228544e-15 3.066251e-15 1.793983e-17
-3.766990e-15
E:\\R-3.1.2\\scripts\\mPDBS\\summary_pdb_93\\6bb3547ea982ffc4.final.pdb
-1.506244e-16 1.164902e-14 -8.841960e-16 -2.672514e-15 6.340895e-15
7.471216e-15
E:\\R-3.1.2\\scripts\\mPDBS\\summary_pdb_93\\716d9624611d1c42.final.pdb
8.378482e-15 5.033120e-15 8.989122e-17 -2.467320e-15 4.905259e-16
-2.951612e-15
E:\\R-3.1.2\\scripts\\mPDBS\\summary_pdb_93\\75f27e70ad0f1be4.final.pdb
-3.594227e-16 7.674406e-15 -3.347937e-15 5.271713e-15 -1.199311e-15
-4.353232e-15
E:\\R-3.1.2\\scripts\\mPDBS\\summary_pdb_93\\769f910fffb22872.final.pdb
3.697142e-15 6.981156e-15 -2.774598e-15 3.736215e-15 1.647045e-15
-4.124094e-15
E:\\R-3.1.2\\scripts\\mPDBS\\summary_pdb_93\\791f8c80dd5c3135.final.pdb
5.619594e-15 5.307889e-15 -2.388776e-15 6.835437e-15 8.611761e-16
-4.758571e-15
E:\\R-3.1.2\\scripts\\mPDBS\\summary_pdb_93\\7fad98e66a3f6099.final.pdb
9.039026e-16 2.735160e-15 -6.661085e-15 9.939824e-15 -1.594253e-15
-1.890454e-15
E:\\R-3.1.2\\scripts\\mPDBS\\summary_pdb_93\\804b89c94a633196.final.pdb
2.005231e-15 7.091882e-15 -1.290333e-15 8.170067e-16 5.022370e-15

-9.154055e-15
E:\\R-3.1.2\\scripts\\mPDBS\\summary_pdb_93\\813cc65a2298feba.final.pdb
2.005231e-15 7.091882e-15 -1.290333e-15 8.170067e-16 5.022370e-15
-9.154055e-15
E:\\R-3.1.2\\scripts\\mPDBS\\summary_pdb_93\\83c0eefcfe12d3f9.final.pdb
4.049372e-15 8.936262e-15 4.879428e-15 -3.786082e-15 2.609207e-15
-4.289890e-15
E:\\R-3.1.2\\scripts\\mPDBS\\summary_pdb_93\\86d50469ef569760.final.pdb
4.163625e-15 1.082293e-14 -8.561610e-16 5.895585e-15 6.607124e-15
-5.771029e-15
E:\\R-3.1.2\\scripts\\mPDBS\\summary_pdb_93\\89d0666e1402203d.final.pdb
-3.594227e-16 7.674406e-15 -3.347937e-15 5.271713e-15 -1.199311e-15
-4.353232e-15
E:\\R-3.1.2\\scripts\\mPDBS\\summary_pdb_93\\8b34852716dadc62.final.pdb
2.418529e-15 9.607231e-15 -7.795546e-15 1.044732e-14 -1.219529e-16
1.404340e-15
E:\\R-3.1.2\\scripts\\mPDBS\\summary_pdb_93\\8b6ef9ac3fe74395.final.pdb
3.203163e-15 4.964221e-15 -1.004241e-14 1.371441e-14 7.950247e-15
2.447218e-15
E:\\R-3.1.2\\scripts\\mPDBS\\summary_pdb_93\\98bbe0d5986c138f.final.pdb
5.619594e-15 5.307889e-15 -2.388776e-15 6.835437e-15 8.611761e-16
-4.758571e-15
E:\\R-3.1.2\\scripts\\mPDBS\\summary_pdb_93\\9c22f4909d67e2fa.final.pdb
1.273309e-14 6.357412e-15 -8.798536e-16 2.396472e-15 -2.796571e-15
-6.086196e-15
E:\\R-3.1.2\\scripts\\mPDBS\\summary_pdb_93\\9dda8ed0d81fb3ef.final.pdb
8.775970e-15 1.864443e-14 -9.395400e-15 -7.485010e-15 4.819041e-15
-3.570004e-15
E:\\R-3.1.2\\scripts\\mPDBS\\summary_pdb_93\\9f752b3616e6033b.final.pdb
2.091505e-15 7.154202e-15 -5.640338e-15 4.341117e-15 2.910102e-15
-6.016420e-15
E:\\R-3.1.2\\scripts\\mPDBS\\summary_pdb_93\\a02c70083816d6ba.final.pdb
5.809255e-15 1.117432e-14 -3.997449e-15 1.770349e-15 3.407738e-15
-2.367385e-15
E:\\R-3.1.2\\scripts\\mPDBS\\summary_pdb_93\\a0c997d891e4e082.final.pdb
-2.968891e-15 1.353016e-14 -1.109852e-14 6.138880e-15 1.738941e-15
-4.285746e-15
E:\\R-3.1.2\\scripts\\mPDBS\\summary_pdb_93\\ab8039a2d823c616.final.pdb
4.548981e-15 6.867767e-15 -2.664834e-15 2.160725e-15 1.174370e-15
-3.719793e-15
E:\\R-3.1.2\\scripts\\mPDBS\\summary_pdb_93\\acf4d74b9c71d4dc.final.pdb
2.005231e-15 7.091882e-15 -1.290333e-15 8.170067e-16 5.022370e-15
-9.154055e-15
E:\\R-3.1.2\\scripts\\mPDBS\\summary_pdb_93\\ad8a823e4b4c668e.final.pdb
4.058319e-15 7.825258e-15 -2.443786e-15 3.624448e-15 1.169113e-15
-3.713413e-15
E:\\R-3.1.2\\scripts\\mPDBS\\summary_pdb_93\\b16ababd03b7b857.final.pdb
1.273309e-14 6.357412e-15 -8.798536e-16 2.396472e-15 -2.796571e-15
-6.086196e-15
E:\\R-3.1.2\\scripts\\mPDBS\\summary_pdb_93\\b226923e697d3c63.final.pdb
-2.053576e-16 7.472127e-15 -5.608390e-15 1.310095e-14 -4.216932e-17
-1.820536e-15
E:\\R-3.1.2\\scripts\\mPDBS\\summary_pdb_93\\b28e48f6fa27a165.final.pdb
5.619594e-15 5.307889e-15 -2.388776e-15 6.835437e-15 8.611761e-16

-4.758571e-15
E:\\R-3.1.2\\scripts\\mPDBS\\summary_pdb_93\\b42c4924e3cd1aec.final.pdb
1.024721e-14 1.411373e-15 2.595334e-15 1.132633e-14 4.774128e-15
4.020789e-16
E:\\R-3.1.2\\scripts\\mPDBS\\summary_pdb_93\\b4dd239fcabff1df.final.pdb
5.619594e-15 5.307889e-15 -2.388776e-15 6.835437e-15 8.611761e-16
-4.758571e-15
E:\\R-3.1.2\\scripts\\mPDBS\\summary_pdb_93\\b669942ef97459a2.final.pdb
2.418529e-15 9.607231e-15 -7.795546e-15 1.044732e-14 -1.219529e-16
1.404340e-15
E:\\R-3.1.2\\scripts\\mPDBS\\summary_pdb_93\\b99f4d9d87c1017b.final.pdb
5.619594e-15 5.307889e-15 -2.388776e-15 6.835437e-15 8.611761e-16
-4.758571e-15
E:\\R-3.1.2\\scripts\\mPDBS\\summary_pdb_93\\b9d2cbf3975403e7.final.pdb
5.619594e-15 5.307889e-15 -2.388776e-15 6.835437e-15 8.611761e-16
-4.758571e-15
E:\\R-3.1.2\\scripts\\mPDBS\\summary_pdb_93\\bc88c8a505f3a288.final.pdb
4.492024e-15 7.057544e-15 -2.099728e-15 3.496095e-15 6.268834e-17
-3.362552e-15
E:\\R-3.1.2\\scripts\\mPDBS\\summary_pdb_93\\bdac0e64f4ef427e.final.pdb
6.663492e-15 1.353105e-15 -1.584878e-14 1.385345e-14 -1.847840e-15
-2.125823e-15
E:\\R-3.1.2\\scripts\\mPDBS\\summary_pdb_93\\ca2a1f55fc9d0681.final.pdb
2.418529e-15 9.607231e-15 -7.795546e-15 1.044732e-14 -1.219529e-16
1.404340e-15
E:\\R-3.1.2\\scripts\\mPDBS\\summary_pdb_93\\ce34248de3e81a3c.final.pdb
7.893775e-15 5.638443e-15 -2.374431e-17 8.033678e-15 -5.545111e-15
-1.158816e-15
E:\\R-3.1.2\\scripts\\mPDBS\\summary_pdb_93\\d02e697772494a61.final.pdb
-5.769394e-15 1.398503e-15 -9.416912e-16 5.316253e-15 -6.730326e-15
-7.027155e-15
E:\\R-3.1.2\\scripts\\mPDBS\\summary_pdb_93\\d04221245d015047.final.pdb
6.273716e-15 7.215765e-15 -1.839431e-15 2.571406e-17 1.312199e-15
-4.340390e-15
E:\\R-3.1.2\\scripts\\mPDBS\\summary_pdb_93\\d678141e4018054a.final.pdb
1.153310e-14 2.877355e-15 9.702131e-15 -2.093125e-15 -2.379343e-15
7.662550e-15
E:\\R-3.1.2\\scripts\\mPDBS\\summary_pdb_93\\d71b40af0f0efe39.final.pdb
9.902222e-15 4.267252e-15 2.198321e-15 4.070904e-15 2.252952e-15
-1.461239e-14
E:\\R-3.1.2\\scripts\\mPDBS\\summary_pdb_93\\dc0809eff10197e2.final.pdb
3.439692e-15 5.859120e-15 -8.078922e-15 7.548057e-15 7.203537e-15
-7.611674e-15
E:\\R-3.1.2\\scripts\\mPDBS\\summary_pdb_93\\e1163918d3707839.final.pdb
8.118916e-15 -3.603713e-15 -8.005127e-15 4.812878e-15 9.247980e-15
-6.947077e-15
E:\\R-3.1.2\\scripts\\mPDBS\\summary_pdb_93\\e4b48b06824ef848.final.pdb
5.553188e-15 1.005751e-14 5.550949e-16 -1.291881e-15 -1.455426e-15
-4.760738e-15
E:\\R-3.1.2\\scripts\\mPDBS\\summary_pdb_93\\e529e19ad281fea2.final.pdb
5.805107e-15 4.650785e-15 4.073056e-15 -3.505749e-15 -9.562700e-16
-6.662243e-15
E:\\R-3.1.2\\scripts\\mPDBS\\summary_pdb_93\\e5602082718ec3a1.final.pdb
2.005231e-15 7.091882e-15 -1.290333e-15 8.170067e-16 5.022370e-15

-9.154055e-15
E:\\R-3.1.2\\scripts\\mPDBS\\summary_pdb_93\\e5d589d996d0a960.final.pdb
8.007503e-16 1.419551e-14 -6.811036e-15 -4.974483e-15 4.373477e-15
2.108592e-15
E:\\R-3.1.2\\scripts\\mPDBS\\summary_pdb_93\\e9df9e72307a2fbd.final.pdb
5.809255e-15 1.117432e-14 -3.997449e-15 1.770349e-15 3.407738e-15
-2.367385e-15
E:\\R-3.1.2\\scripts\\mPDBS\\summary_pdb_93\\ea00f8694090cae7.final.pdb
2.005231e-15 7.091882e-15 -1.290333e-15 8.170067e-16 5.022370e-15
-9.154055e-15
E:\\R-3.1.2\\scripts\\mPDBS\\summary_pdb_93\\eba517be32db050e.final.pdb
5.619594e-15 5.307889e-15 -2.388776e-15 6.835437e-15 8.611761e-16
-4.758571e-15
E:\\R-3.1.2\\scripts\\mPDBS\\summary_pdb_93\\ec04b9545899c29d.final.pdb
4.556350e-15 7.270215e-15 -3.097269e-15 1.594966e-15 9.322303e-16
-4.252184e-15
E:\\R-3.1.2\\scripts\\mPDBS\\summary_pdb_93\\ee6862cbcad52078.final.pdb
5.035210e-15 7.457212e-15 -2.369260e-15 3.395914e-15 7.887514e-16
-3.810111e-15
E:\\R-3.1.2\\scripts\\mPDBS\\summary_pdb_93\\ef6a42fc83b81e5d.final.pdb
4.417364e-15 6.971801e-15 -2.802462e-15 3.179802e-15 1.398031e-15
-3.908121e-15
E:\\R-3.1.2\\scripts\\mPDBS\\summary_pdb_93\\ef8e88d4ad93a0ad.final.pdb
6.273716e-15 7.215765e-15 -1.839431e-15 2.571406e-17 1.312199e-15
-4.340390e-15
E:\\R-3.1.2\\scripts\\mPDBS\\summary_pdb_93\\f14fcae8deec4fa3.final.pdb
2.091505e-15 7.154202e-15 -5.640338e-15 4.341117e-15 2.910102e-15
-6.016420e-15
E:\\R-3.1.2\\scripts\\mPDBS\\summary_pdb_93\\f83584e22003fb92.final.pdb
1.989808e-15 1.163579e-14 -6.880842e-15 -6.774336e-15 -3.524910e-15
-3.979246e-15
E:\\R-3.1.2\\scripts\\mPDBS\\summary_pdb_93\\f8e2bf87e52caa29.final.pdb
5.805107e-15 4.650785e-15 4.073056e-15 -3.505749e-15 -9.562700e-16
-6.662243e-15

[,505]   [,506]   [,507]   [,508]   [,509]   [,510]
E:\\R-3.1.2\\scripts\\40.1ns_data\\NAT0.pdb
1.763418e-15 1.118261e-15 2.773329e-15 -2.329621e-15 1.504885e-15
1.682464e-15
E:\\R-3.1.2\\scripts\\40.1ns_data\\LJP6.pdb
3.010891e-15 1.123026e-15 1.354674e-15 -1.428173e-15 1.568915e-15
2.558257e-15
E:\\R-3.1.2\\scripts\\40.1ns_data\\3WY9.pdb
2.982655e-15 1.916873e-15 2.700480e-15 -3.625510e-15 -5.603173e-16
3.483720e-15
E:\\R-3.1.2\\scripts\\40.1ns_data\\ZSP9.pdb
3.084436e-15 2.634679e-16 3.541619e-15 -4.153680e-15 4.303434e-16
-2.323908e-15
E:\\R-3.1.2\\scripts\\mPDBS\\summary_pdb_93\\013d855ad26c877c.final.pdb
-2.915828e-16 5.851886e-16 1.507922e-14 -6.229188e-15 -9.491457e-16
6.346774e-16
E:\\R-3.1.2\\scripts\\mPDBS\\summary_pdb_93\\0a2a705342ca0d54.final.pdb
1.496975e-15 3.993151e-16 3.173080e-15 -3.775402e-15 4.517577e-17
1.424031e-15

E:\\R-3.1.2\\scripts\\mPDBS\\summary_pdb_93\\0a59fceadb4194d9.final.pdb
4.605032e-15 -1.407280e-15 5.352627e-15 1.230533e-15 1.115365e-15
2.208860e-15
E:\\R-3.1.2\\scripts\\mPDBS\\summary_pdb_93\\0dba59f3306825e8.final.pdb
1.187230e-14 -1.040247e-15 1.982062e-15 -1.977381e-15 2.837756e-15
4.108981e-16
E:\\R-3.1.2\\scripts\\mPDBS\\summary_pdb_93\\126f26a78b57339c.final.pdb
-1.932198e-15 -3.545979e-16 -5.176709e-15 -6.618363e-15 1.795621e-15
-2.369864e-15
E:\\R-3.1.2\\scripts\\mPDBS\\summary_pdb_93\\12f06f3e342df706.final.pdb
2.060543e-15 9.745928e-16 3.006113e-15 -3.335162e-15 3.809532e-16
1.219767e-15
E:\\R-3.1.2\\scripts\\mPDBS\\summary_pdb_93\\18618a2a251b867b.final.pdb
-2.366915e-15 2.099847e-15 -2.722136e-15 -1.078421e-14 5.065422e-15
-5.856505e-15
E:\\R-3.1.2\\scripts\\mPDBS\\summary_pdb_93\\1f305f598641eeb2.final.pdb
1.975334e-15 4.631920e-16 2.609605e-15 -2.657995e-15 -3.485805e-16
2.236134e-15
E:\\R-3.1.2\\scripts\\mPDBS\\summary_pdb_93\\1f8a00b551be0a39.final.pdb
-2.366915e-15 2.099847e-15 -2.722136e-15 -1.078421e-14 5.065422e-15
-5.856505e-15
E:\\R-3.1.2\\scripts\\mPDBS\\summary_pdb_93\\219f45328d47579a.final.pdb
2.566732e-15 8.751478e-15 9.759192e-16 -2.332156e-15 -6.926813e-15
1.124937e-14
E:\\R-3.1.2\\scripts\\mPDBS\\summary_pdb_93\\238dd3918e54336d.final.pdb
-2.857531e-15 4.793056e-15 -4.962690e-15 -1.632818e-14 -2.964488e-15
7.791200e-15
E:\\R-3.1.2\\scripts\\mPDBS\\summary_pdb_93\\2b3027889e2fdc42.final.pdb
1.890623e-15 7.413923e-16 3.390221e-15 -3.617315e-15 1.187661e-16
1.449918e-15
E:\\R-3.1.2\\scripts\\mPDBS\\summary_pdb_93\\2fcb3b46e33793b0.final.pdb
4.882730e-15 1.214515e-16 7.452953e-15 1.361360e-15 -1.167370e-15
8.205993e-16
E:\\R-3.1.2\\scripts\\mPDBS\\summary_pdb_93\\34eb328251842006.final.pdb
-3.031519e-15 6.113151e-15 6.588870e-15 6.285113e-16 -3.790712e-15
1.429796e-14
E:\\R-3.1.2\\scripts\\mPDBS\\summary_pdb_93\\354b32871797b7ab.final.pdb
-7.080242e-15 2.715785e-15 5.644800e-15 4.197839e-15 -1.906652e-15
8.150323e-15
E:\\R-3.1.2\\scripts\\mPDBS\\summary_pdb_93\\397368adbc32d5de.final.pdb
-1.932198e-15 -3.545979e-16 -5.176709e-15 -6.618363e-15 1.795621e-15
-2.369864e-15
E:\\R-3.1.2\\scripts\\mPDBS\\summary_pdb_93\\411ca13cd3df6edd.final.pdb
4.122488e-15 1.834057e-15 1.257822e-15 -7.439983e-16 -3.504476e-15
-3.512740e-15
E:\\R-3.1.2\\scripts\\mPDBS\\summary_pdb_93\\42357eee6642bf12.final.pdb
-4.059351e-15 6.992629e-15 2.492287e-15 -4.143500e-16 -1.189098e-15
1.087292e-14
E:\\R-3.1.2\\scripts\\mPDBS\\summary_pdb_93\\44342d1f75a7fcfc.final.pdb
-5.237977e-16 -4.066206e-15 9.374287e-15 -1.198529e-15 -4.079288e-16
-3.855903e-15
E:\\R-3.1.2\\scripts\\mPDBS\\summary_pdb_93\\448ef21dffe8cdfb.final.pdb
-3.013093e-14 1.127919e-14 -1.990881e-15 -1.163345e-14 -7.323904e-15
3.627382e-15

E:\\R-3.1.2\\scripts\\mPDBS\\summary_pdb_93\\472a40b3e8f113c7.final.pdb
1.187230e-14 -1.040247e-15 1.982062e-15 -1.977381e-15 2.837756e-15
4.108981e-16
E:\\R-3.1.2\\scripts\\mPDBS\\summary_pdb_93\\48a26225951ea153.final.pdb
-4.640610e-15 2.405860e-15 5.601993e-15 -1.069889e-14 -1.633738e-15
-1.500846e-15
E:\\R-3.1.2\\scripts\\mPDBS\\summary_pdb_93\\4ab4a5c7c6682ce6.final.pdb
-9.689881e-15 5.193048e-15 3.032003e-15 9.226602e-15 -1.139532e-15
5.349656e-15
E:\\R-3.1.2\\scripts\\mPDBS\\summary_pdb_93\\4eaa45b62cb6a25f.final.pdb
4.247285e-15 -2.724091e-15 3.472709e-15 -5.880200e-15 2.030954e-15
-3.055123e-15
E:\\R-3.1.2\\scripts\\mPDBS\\summary_pdb_93\\58c371611d85ac96.final.pdb
-1.932198e-15 -3.545979e-16 -5.176709e-15 -6.618363e-15 1.795621e-15
-2.369864e-15
E:\\R-3.1.2\\scripts\\mPDBS\\summary_pdb_93\\59a647f04822d30a.final.pdb
4.459128e-16 1.216244e-15 3.665312e-15 -4.471519e-15 -1.169472e-15
7.870828e-15
E:\\R-3.1.2\\scripts\\mPDBS\\summary_pdb_93\\59b091fe385e05fc.final.pdb
4.605032e-15 -1.407280e-15 5.352627e-15 1.230533e-15 1.115365e-15
2.208860e-15
E:\\R-3.1.2\\scripts\\mPDBS\\summary_pdb_93\\5c35ac30f930d2db.final.pdb
4.247285e-15 -2.724091e-15 3.472709e-15 -5.880200e-15 2.030954e-15
-3.055123e-15
E:\\R-3.1.2\\scripts\\mPDBS\\summary_pdb_93\\5f60752431cf2dcb.final.pdb
6.611373e-15 -7.498447e-15 1.215011e-15 -7.883824e-15 2.577872e-15
-6.516666e-15
E:\\R-3.1.2\\scripts\\mPDBS\\summary_pdb_93\\626456dd6ff2868d.final.pdb
1.187230e-14 -1.040247e-15 1.982062e-15 -1.977381e-15 2.837756e-15
4.108981e-16
E:\\R-3.1.2\\scripts\\mPDBS\\summary_pdb_93\\6583d53ff3c8f7ef.final.pdb
4.459128e-16 1.216244e-15 3.665312e-15 -4.471519e-15 -1.169472e-15
7.870828e-15
E:\\R-3.1.2\\scripts\\mPDBS\\summary_pdb_93\\69760178986bef01.final.pdb
3.803672e-15 -4.829592e-15 1.103182e-15 -9.441155e-15 -1.228822e-15
-9.026860e-15
E:\\R-3.1.2\\scripts\\mPDBS\\summary_pdb_93\\6a9d87d47926b1c8.final.pdb
1.953583e-15 8.688924e-17 2.955675e-15 -3.135038e-15 2.621250e-16
1.810820e-15
E:\\R-3.1.2\\scripts\\mPDBS\\summary_pdb_93\\6bb3547ea982ffc4.final.pdb
-1.517060e-15 3.748035e-15 -2.847039e-15 -1.249803e-14 5.838065e-16
-1.265237e-15
E:\\R-3.1.2\\scripts\\mPDBS\\summary_pdb_93\\716d9624611d1c42.final.pdb
-2.234415e-15 9.731477e-15 7.539379e-15 -2.310862e-15 -2.539996e-15
7.328162e-15
E:\\R-3.1.2\\scripts\\mPDBS\\summary_pdb_93\\75f27e70ad0f1be4.final.pdb
4.459128e-16 1.216244e-15 3.665312e-15 -4.471519e-15 -1.169472e-15
7.870828e-15
E:\\R-3.1.2\\scripts\\mPDBS\\summary_pdb_93\\769f910fffb22872.final.pdb
1.721612e-15 7.016596e-16 3.009173e-15 -2.989774e-15 -3.427951e-16
1.092798e-15
E:\\R-3.1.2\\scripts\\mPDBS\\summary_pdb_93\\791f8c80dd5c3135.final.pdb
4.605032e-15 -1.407280e-15 5.352627e-15 1.230533e-15 1.115365e-15
2.208860e-15

E:\\R-3.1.2\\scripts\\mPDBS\\summary_pdb_93\\7fad98e66a3f6099.final.pdb
-2.715704e-15 2.510087e-15 7.637937e-15 -8.001145e-16 -1.243510e-14
1.007712e-14
E:\\R-3.1.2\\scripts\\mPDBS\\summary_pdb_93\\804b89c94a633196.final.pdb
4.882730e-15 1.214515e-16 7.452953e-15 1.361360e-15 -1.167370e-15
8.205993e-16
E:\\R-3.1.2\\scripts\\mPDBS\\summary_pdb_93\\813cc65a2298feba.final.pdb
4.882730e-15 1.214515e-16 7.452953e-15 1.361360e-15 -1.167370e-15
8.205993e-16
E:\\R-3.1.2\\scripts\\mPDBS\\summary_pdb_93\\83c0eefcfe12d3f9.final.pdb
4.122488e-15 1.834057e-15 1.257822e-15 -7.439983e-16 -3.504476e-15
-3.512740e-15
E:\\R-3.1.2\\scripts\\mPDBS\\summary_pdb_93\\86d50469ef569760.final.pdb
1.187230e-14 -1.040247e-15 1.982062e-15 -1.977381e-15 2.837756e-15
4.108981e-16
E:\\R-3.1.2\\scripts\\mPDBS\\summary_pdb_93\\89d0666e1402203d.final.pdb
4.459128e-16 1.216244e-15 3.665312e-15 -4.471519e-15 -1.169472e-15
7.870828e-15
E:\\R-3.1.2\\scripts\\mPDBS\\summary_pdb_93\\8b34852716dadc62.final.pdb
4.702319e-15 -5.498865e-16 -7.625263e-15 2.719648e-15 -4.075611e-16
4.744544e-15
E:\\R-3.1.2\\scripts\\mPDBS\\summary_pdb_93\\8b6ef9ac3fe74395.final.pdb
1.746357e-15 -2.264693e-15 1.628924e-16 -5.431527e-17 1.350008e-15
5.692283e-15
E:\\R-3.1.2\\scripts\\mPDBS\\summary_pdb_93\\98bbe0d5986c138f.final.pdb
4.605032e-15 -1.407280e-15 5.352627e-15 1.230533e-15 1.115365e-15
2.208860e-15
E:\\R-3.1.2\\scripts\\mPDBS\\summary_pdb_93\\9c22f4909d67e2fa.final.pdb
5.758549e-15 -1.865891e-15 5.129074e-15 -1.092970e-14 3.598566e-15
4.404538e-15
E:\\R-3.1.2\\scripts\\mPDBS\\summary_pdb_93\\9dda8ed0d81fb3ef.final.pdb
1.203083e-14 -3.906178e-15 -4.529955e-15 -8.055187e-15 1.278031e-14
-9.044460e-15
E:\\R-3.1.2\\scripts\\mPDBS\\summary_pdb_93\\9f752b3616e6033b.final.pdb
3.191591e-15 1.830044e-15 8.239198e-16 -2.521013e-15 -8.750250e-16
3.209558e-15
E:\\R-3.1.2\\scripts\\mPDBS\\summary_pdb_93\\a02c70083816d6ba.final.pdb
4.247285e-15 -2.724091e-15 3.472709e-15 -5.880200e-15 2.030954e-15
-3.055123e-15
E:\\R-3.1.2\\scripts\\mPDBS\\summary_pdb_93\\a0c997d891e4e082.final.pdb
1.099172e-14 -7.154349e-15 -1.552685e-15 -4.459463e-15 -8.904784e-15
-2.755295e-15
E:\\R-3.1.2\\scripts\\mPDBS\\summary_pdb_93\\ab8039a2d823c616.final.pdb
1.586271e-15 9.361849e-16 3.002048e-15 -2.101811e-15 -5.544752e-16
2.088053e-15
E:\\R-3.1.2\\scripts\\mPDBS\\summary_pdb_93\\acf4d74b9c71d4dc.final.pdb
4.882730e-15 1.214515e-16 7.452953e-15 1.361360e-15 -1.167370e-15
8.205993e-16
E:\\R-3.1.2\\scripts\\mPDBS\\summary_pdb_93\\ad8a823e4b4c668e.final.pdb
1.860304e-15 5.665879e-16 3.243369e-15 -3.927791e-15 1.260594e-16
1.388029e-15
E:\\R-3.1.2\\scripts\\mPDBS\\summary_pdb_93\\b16ababd03b7b857.final.pdb
5.758549e-15 -1.865891e-15 5.129074e-15 -1.092970e-14 3.598566e-15
4.404538e-15

E:\\R-3.1.2\\scripts\\mPDBS\\summary_pdb_93\\b226923e697d3c63.final.pdb
4.907838e-15 3.253677e-15 -7.232871e-15 -6.083836e-15 -5.839999e-15
-2.927825e-15
E:\\R-3.1.2\\scripts\\mPDBS\\summary_pdb_93\\b28e48f6fa27a165.final.pdb
4.605032e-15 -1.407280e-15 5.352627e-15 1.230533e-15 1.115365e-15
2.208860e-15
E:\\R-3.1.2\\scripts\\mPDBS\\summary_pdb_93\\b42c4924e3cd1aec.final.pdb
6.041914e-15 -1.603983e-15 1.001332e-14 2.580837e-15 -4.443112e-15
3.738717e-15
E:\\R-3.1.2\\scripts\\mPDBS\\summary_pdb_93\\b4dd239fcabff1df.final.pdb
4.605032e-15 -1.407280e-15 5.352627e-15 1.230533e-15 1.115365e-15
2.208860e-15
E:\\R-3.1.2\\scripts\\mPDBS\\summary_pdb_93\\b669942ef97459a2.final.pdb
4.702319e-15 -5.498865e-16 -7.625263e-15 2.719648e-15 -4.075611e-16
4.744544e-15
E:\\R-3.1.2\\scripts\\mPDBS\\summary_pdb_93\\b99f4d9d87c1017b.final.pdb
4.605032e-15 -1.407280e-15 5.352627e-15 1.230533e-15 1.115365e-15
2.208860e-15
E:\\R-3.1.2\\scripts\\mPDBS\\summary_pdb_93\\b9d2cbf3975403e7.final.pdb
4.605032e-15 -1.407280e-15 5.352627e-15 1.230533e-15 1.115365e-15
2.208860e-15
E:\\R-3.1.2\\scripts\\mPDBS\\summary_pdb_93\\bc88c8a505f3a288.final.pdb
2.326906e-15 6.105291e-16 3.752995e-15 -2.608952e-15 3.158741e-16
1.781250e-15
E:\\R-3.1.2\\scripts\\mPDBS\\summary_pdb_93\\bdac0e64f4ef427e.final.pdb
-2.592844e-15 -2.354866e-15 4.051377e-15 -6.207239e-15 5.979908e-15
1.403225e-14
E:\\R-3.1.2\\scripts\\mPDBS\\summary_pdb_93\\ca2a1f55fc9d0681.final.pdb
4.702319e-15 -5.498865e-16 -7.625263e-15 2.719648e-15 -4.075611e-16
4.744544e-15
E:\\R-3.1.2\\scripts\\mPDBS\\summary_pdb_93\\ce34248de3e81a3c.final.pdb
-1.964164e-15 -7.954284e-15 1.102016e-14 1.017898e-15 3.732090e-15
3.539286e-15
E:\\R-3.1.2\\scripts\\mPDBS\\summary_pdb_93\\d02e697772494a61.final.pdb
-1.218340e-14 7.489250e-15 4.028382e-15 -3.046265e-15 -8.373356e-15
1.188515e-14
E:\\R-3.1.2\\scripts\\mPDBS\\summary_pdb_93\\d04221245d015047.final.pdb
1.329315e-15 1.385782e-18 6.413165e-15 -2.075953e-15 1.848767e-15
3.201746e-15
E:\\R-3.1.2\\scripts\\mPDBS\\summary_pdb_93\\d678141e4018054a.final.pdb
-3.676100e-16 1.566145e-14 1.660961e-15 -1.269083e-14 -2.215845e-15
7.612737e-16
E:\\R-3.1.2\\scripts\\mPDBS\\summary_pdb_93\\d71b40af0f0efe39.final.pdb
6.460087e-15 1.716075e-14 -2.635083e-15 4.096537e-16 -2.989832e-15
2.869301e-15
E:\\R-3.1.2\\scripts\\mPDBS\\summary_pdb_93\\dc0809eff10197e2.final.pdb
1.247500e-14 -5.225810e-15 4.821803e-15 4.360385e-15 2.858014e-15
2.008948e-15
E:\\R-3.1.2\\scripts\\mPDBS\\summary_pdb_93\\e1163918d3707839.final.pdb
3.120640e-15 9.472227e-15 8.890082e-15 5.867721e-16 -4.606210e-16
-5.838691e-15
E:\\R-3.1.2\\scripts\\mPDBS\\summary_pdb_93\\e4b48b06824ef848.final.pdb
-2.366915e-15 2.099847e-15 -2.722136e-15 -1.078421e-14 5.065422e-15
-5.856505e-15

E:\\R-3.1.2\\scripts\\mPDBS\\summary_pdb_93\\e529e19ad281fea2.final.pdb
-2.915828e-16 5.851886e-16 1.507922e-14 -6.229188e-15 -9.491457e-16
6.346774e-16
E:\\R-3.1.2\\scripts\\mPDBS\\summary_pdb_93\\e5602082718ec3a1.final.pdb
4.882730e-15 1.214515e-16 7.452953e-15 1.361360e-15 -1.167370e-15
8.205993e-16
E:\\R-3.1.2\\scripts\\mPDBS\\summary_pdb_93\\e5d589d996d0a960.final.pdb
9.011330e-16 -7.459775e-15 -2.103145e-15 3.049093e-15 1.116167e-15
1.626057e-15
E:\\R-3.1.2\\scripts\\mPDBS\\summary_pdb_93\\e9df9e72307a2fbd.final.pdb
4.247285e-15 -2.724091e-15 3.472709e-15 -5.880200e-15 2.030954e-15
-3.055123e-15
E:\\R-3.1.2\\scripts\\mPDBS\\summary_pdb_93\\ea00f8694090cae7.final.pdb
4.882730e-15 1.214515e-16 7.452953e-15 1.361360e-15 -1.167370e-15
8.205993e-16
E:\\R-3.1.2\\scripts\\mPDBS\\summary_pdb_93\\eba517be32db050e.final.pdb
4.605032e-15 -1.407280e-15 5.352627e-15 1.230533e-15 1.115365e-15
2.208860e-15
E:\\R-3.1.2\\scripts\\mPDBS\\summary_pdb_93\\ec04b9545899c29d.final.pdb
1.729608e-15 7.337741e-16 2.976627e-15 -3.189992e-15 -3.055801e-16
2.072515e-15
E:\\R-3.1.2\\scripts\\mPDBS\\summary_pdb_93\\ee6862cbcad52078.final.pdb
2.157337e-15 5.418991e-16 3.401063e-15 -3.074617e-15 -8.332915e-17
1.841098e-15
E:\\R-3.1.2\\scripts\\mPDBS\\summary_pdb_93\\ef6a42fc83b81e5d.final.pdb
1.877520e-15 7.096827e-16 3.248348e-15 -2.827740e-15 -2.772596e-17
1.802299e-15
E:\\R-3.1.2\\scripts\\mPDBS\\summary_pdb_93\\ef8e88d4ad93a0ad.final.pdb
1.329315e-15 1.385782e-18 6.413165e-15 -2.075953e-15 1.848767e-15
3.201746e-15
E:\\R-3.1.2\\scripts\\mPDBS\\summary_pdb_93\\f14fcae8deec4fa3.final.pdb
3.191591e-15 1.830044e-15 8.239198e-16 -2.521013e-15 -8.750250e-16
3.209558e-15
E:\\R-3.1.2\\scripts\\mPDBS\\summary_pdb_93\\f83584e22003fb92.final.pdb
-4.640610e-15 2.405860e-15 5.601993e-15 -1.069889e-14 -1.633738e-15
-1.500846e-15
E:\\R-3.1.2\\scripts\\mPDBS\\summary_pdb_93\\f8e2bf87e52caa29.final.pdb
-2.915828e-16 5.851886e-16 1.507922e-14 -6.229188e-15 -9.491457e-16
6.346774e-16

[,511]   [,512]   [,513]   [,514]   [,515]   [,516]
E:\\R-3.1.2\\scripts\\40.1ns_data\\NAT0.pdb
-1.055551e-15 8.503152e-16 1.231933e-15 -1.014081e-15 3.262304e-15
2.546579e-15
E:\\R-3.1.2\\scripts\\40.1ns_data\\LJP6.pdb
-6.226039e-16 6.213757e-16 1.496337e-15 -2.220376e-16 1.368819e-15
2.522866e-15
E:\\R-3.1.2\\scripts\\40.1ns_data\\3WY9.pdb
-1.582246e-16 1.671490e-15 2.129163e-15 1.913570e-16 3.675992e-15
2.784099e-15
E:\\R-3.1.2\\scripts\\40.1ns_data\\ZSP9.pdb
-3.221908e-16 1.359770e-15 1.682494e-15 -1.498366e-17 2.053644e-15
3.917484e-15
E:\\R-3.1.2\\scripts\\mPDBS\\summary_pdb_93\\013d855ad26c877c.final.pdb

9.100262e-16 -4.279379e-15 -6.415634e-17 1.187021e-15 6.123840e-15
-1.205487e-15
E:\\R-3.1.2\\scripts\\mPDBS\\summary_pdb_93\\0a2a705342ca0d54.final.pdb
-6.560537e-16 1.024281e-15 1.119858e-15 2.018962e-16 2.433290e-15
4.352822e-15
E:\\R-3.1.2\\scripts\\mPDBS\\summary_pdb_93\\0a59fceadb4194d9.final.pdb
-2.101202e-15 -4.099705e-16 -3.968801e-15 9.252245e-16 1.185382e-15
6.075950e-15
E:\\R-3.1.2\\scripts\\mPDBS\\summary_pdb_93\\0dba59f3306825e8.final.pdb
-1.221949e-15 -1.030242e-15 -3.674983e-15 -4.572261e-15 -1.108222e-15
8.615650e-15
E:\\R-3.1.2\\scripts\\mPDBS\\summary_pdb_93\\126f26a78b57339c.final.pdb
-4.490249e-15 2.830385e-15 1.270655e-14 -1.978416e-15 1.622666e-15
1.369494e-15
E:\\R-3.1.2\\scripts\\mPDBS\\summary_pdb_93\\12f06f3e342df706.final.pdb
-7.798154e-16 5.801917e-16 1.623525e-15 -2.340100e-17 2.495306e-15
4.817294e-15
E:\\R-3.1.2\\scripts\\mPDBS\\summary_pdb_93\\18618a2a251b867b.final.pdb
-2.938251e-15 5.341338e-15 1.342185e-14 -1.401911e-15 7.100926e-16
-1.608698e-15
E:\\R-3.1.2\\scripts\\mPDBS\\summary_pdb_93\\1f305f598641eeb2.final.pdb
-4.888453e-16 9.737241e-16 1.192416e-15 -7.981337e-17 2.306034e-15
3.273600e-15
E:\\R-3.1.2\\scripts\\mPDBS\\summary_pdb_93\\1f8a00b551be0a39.final.pdb
-2.938251e-15 5.341338e-15 1.342185e-14 -1.401911e-15 7.100926e-16
-1.608698e-15
E:\\R-3.1.2\\scripts\\mPDBS\\summary_pdb_93\\219f45328d47579a.final.pdb
1.708397e-14 5.360587e-15 2.948626e-15 1.453882e-15 1.344034e-14
-1.096727e-14
E:\\R-3.1.2\\scripts\\mPDBS\\summary_pdb_93\\238dd3918e54336d.final.pdb
5.150769e-15 -8.497229e-16 8.540019e-15 1.781961e-15 5.652295e-15
1.151518e-14
E:\\R-3.1.2\\scripts\\mPDBS\\summary_pdb_93\\2b3027889e2fdc42.final.pdb
-4.819188e-16 3.112408e-16 1.452102e-15 -4.265587e-16 2.732321e-15
4.312170e-15
E:\\R-3.1.2\\scripts\\mPDBS\\summary_pdb_93\\2fcb3b46e33793b0.final.pdb
2.726983e-16 1.081277e-15 -6.093423e-15 -3.261441e-16 1.258261e-15
4.779990e-15
E:\\R-3.1.2\\scripts\\mPDBS\\summary_pdb_93\\34eb328251842006.final.pdb
7.290995e-15 -9.233089e-15 -3.156879e-15 7.029628e-15 6.083092e-15
7.263757e-15
E:\\R-3.1.2\\scripts\\mPDBS\\summary_pdb_93\\354b32871797b7ab.final.pdb
1.193867e-15 7.175086e-15 -7.863765e-15 2.809953e-15 6.012604e-15
6.540349e-15
E:\\R-3.1.2\\scripts\\mPDBS\\summary_pdb_93\\397368adbc32d5de.final.pdb
-4.490249e-15 2.830385e-15 1.270655e-14 -1.978416e-15 1.622666e-15
1.369494e-15
E:\\R-3.1.2\\scripts\\mPDBS\\summary_pdb_93\\411ca13cd3df6edd.final.pdb
-1.001859e-16 2.364105e-15 9.863089e-16 -1.761194e-15 1.370584e-15
4.805360e-15
E:\\R-3.1.2\\scripts\\mPDBS\\summary_pdb_93\\42357eee6642bf12.final.pdb
5.635862e-15 5.577134e-15 1.676416e-15 8.438786e-15 7.463529e-15
4.659530e-15
E:\\R-3.1.2\\scripts\\mPDBS\\summary_pdb_93\\44342d1f75a7fcfc.final.pdb

-1.098956e-14 3.349282e-15 3.285854e-15 7.464929e-16 -1.139507e-15
4.895036e-15
E:\\R-3.1.2\\scripts\\mPDBS\\summary_pdb_93\\448ef21dffe8cdfb.final.pdb
7.877726e-15 5.528241e-15 1.832108e-14 1.258540e-14 1.670944e-14
1.265657e-15
E:\\R-3.1.2\\scripts\\mPDBS\\summary_pdb_93\\472a40b3e8f113c7.final.pdb
-1.221949e-15 -1.030242e-15 -3.674983e-15 -4.572261e-15 -1.108222e-15
8.615650e-15
E:\\R-3.1.2\\scripts\\mPDBS\\summary_pdb_93\\48a26225951ea153.final.pdb
-5.859562e-15 -9.503368e-16 1.023316e-14 -2.577182e-15 6.341734e-16
5.928065e-15
E:\\R-3.1.2\\scripts\\mPDBS\\summary_pdb_93\\4ab4a5c7c6682ce6.final.pdb
1.336687e-15 7.929605e-15 -1.145214e-15 4.913641e-15 7.723689e-15
-3.780033e-15
E:\\R-3.1.2\\scripts\\mPDBS\\summary_pdb_93\\4eaa45b62cb6a25f.final.pdb
-5.300897e-15 -6.619606e-16 5.508297e-15 -3.118682e-15 -2.402070e-16
3.320268e-15
E:\\R-3.1.2\\scripts\\mPDBS\\summary_pdb_93\\58c371611d85ac96.final.pdb
-4.490249e-15 2.830385e-15 1.270655e-14 -1.978416e-15 1.622666e-15
1.369494e-15
E:\\R-3.1.2\\scripts\\mPDBS\\summary_pdb_93\\59a647f04822d30a.final.pdb
-4.865792e-17 -1.344013e-15 3.151087e-15 -1.790107e-15 5.094222e-15
5.714693e-15
E:\\R-3.1.2\\scripts\\mPDBS\\summary_pdb_93\\59b091fe385e05fc.final.pdb
-2.101202e-15 -4.099705e-16 -3.968801e-15 9.252245e-16 1.185382e-15
6.075950e-15
E:\\R-3.1.2\\scripts\\mPDBS\\summary_pdb_93\\5c35ac30f930d2db.final.pdb
-5.300897e-15 -6.619606e-16 5.508297e-15 -3.118682e-15 -2.402070e-16
3.320268e-15
E:\\R-3.1.2\\scripts\\mPDBS\\summary_pdb_93\\5f60752431cf2dcb.final.pdb
-8.686170e-15 -5.321997e-16 1.087549e-15 -1.856787e-15 -1.770355e-16
7.052919e-15
E:\\R-3.1.2\\scripts\\mPDBS\\summary_pdb_93\\626456dd6ff2868d.final.pdb
-1.221949e-15 -1.030242e-15 -3.674983e-15 -4.572261e-15 -1.108222e-15
8.615650e-15
E:\\R-3.1.2\\scripts\\mPDBS\\summary_pdb_93\\6583d53ff3c8f7ef.final.pdb
-4.865792e-17 -1.344013e-15 3.151087e-15 -1.790107e-15 5.094222e-15
5.714693e-15
E:\\R-3.1.2\\scripts\\mPDBS\\summary_pdb_93\\69760178986bef01.final.pdb
-1.087387e-15 8.545878e-15 2.305174e-15 -9.720498e-15 2.484533e-15
1.956027e-15
E:\\R-3.1.2\\scripts\\mPDBS\\summary_pdb_93\\6a9d87d47926b1c8.final.pdb
-1.430323e-16 8.779537e-16 1.279495e-15 -6.097189e-16 2.628806e-15
4.585888e-15
E:\\R-3.1.2\\scripts\\mPDBS\\summary_pdb_93\\6bb3547ea982ffc4.final.pdb
6.077266e-15 -3.685221e-15 1.689248e-14 2.215031e-15 8.474202e-15
1.070704e-14
E:\\R-3.1.2\\scripts\\mPDBS\\summary_pdb_93\\716d9624611d1c42.final.pdb
2.676350e-15 -1.192696e-15 5.359089e-15 1.348982e-15 5.846358e-15
2.449831e-15
E:\\R-3.1.2\\scripts\\mPDBS\\summary_pdb_93\\75f27e70ad0f1be4.final.pdb
-4.865792e-17 -1.344013e-15 3.151087e-15 -1.790107e-15 5.094222e-15
5.714693e-15
E:\\R-3.1.2\\scripts\\mPDBS\\summary_pdb_93\\769f910fffb22872.final.pdb

1.825568e-16 7.837862e-16 1.257916e-15 -1.695075e-17 2.623493e-15
4.422502e-15
E:\\R-3.1.2\\scripts\\mPDBS\\summary_pdb_93\\791f8c80dd5c3135.final.pdb
-2.101202e-15 -4.099705e-16 -3.968801e-15 9.252245e-16 1.185382e-15
6.075950e-15
E:\\R-3.1.2\\scripts\\mPDBS\\summary_pdb_93\\7fad98e66a3f6099.final.pdb
5.665690e-15 2.240318e-15 -9.556633e-15 3.828174e-15 8.007982e-15
4.439662e-15
E:\\R-3.1.2\\scripts\\mPDBS\\summary_pdb_93\\804b89c94a633196.final.pdb
2.726983e-16 1.081277e-15 -6.093423e-15 -3.261441e-16 1.258261e-15
4.779990e-15
E:\\R-3.1.2\\scripts\\mPDBS\\summary_pdb_93\\813cc65a2298feba.final.pdb
2.726983e-16 1.081277e-15 -6.093423e-15 -3.261441e-16 1.258261e-15
4.779990e-15
E:\\R-3.1.2\\scripts\\mPDBS\\summary_pdb_93\\83c0eefcfe12d3f9.final.pdb
-1.001859e-16 2.364105e-15 9.863089e-16 -1.761194e-15 1.370584e-15
4.805360e-15
E:\\R-3.1.2\\scripts\\mPDBS\\summary_pdb_93\\86d50469ef569760.final.pdb
-1.221949e-15 -1.030242e-15 -3.674983e-15 -4.572261e-15 -1.108222e-15
8.615650e-15
E:\\R-3.1.2\\scripts\\mPDBS\\summary_pdb_93\\89d0666e1402203d.final.pdb
-4.865792e-17 -1.344013e-15 3.151087e-15 -1.790107e-15 5.094222e-15
5.714693e-15
E:\\R-3.1.2\\scripts\\mPDBS\\summary_pdb_93\\8b34852716dadc62.final.pdb
-2.641637e-15 7.107706e-15 4.222978e-15 -2.418221e-15 7.621301e-16
2.106131e-15
E:\\R-3.1.2\\scripts\\mPDBS\\summary_pdb_93\\8b6ef9ac3fe74395.final.pdb
-1.966827e-15 -1.217541e-15 6.956176e-15 3.261792e-15 1.518964e-15
1.054581e-14
E:\\R-3.1.2\\scripts\\mPDBS\\summary_pdb_93\\98bbe0d5986c138f.final.pdb
-2.101202e-15 -4.099705e-16 -3.968801e-15 9.252245e-16 1.185382e-15
6.075950e-15
E:\\R-3.1.2\\scripts\\mPDBS\\summary_pdb_93\\9c22f4909d67e2fa.final.pdb
-3.630508e-16 -2.500927e-15 -4.193592e-16 -2.961406e-15 5.374834e-16
2.464568e-15
E:\\R-3.1.2\\scripts\\mPDBS\\summary_pdb_93\\9dda8ed0d81fb3ef.final.pdb
-1.306177e-14 -1.078178e-14 8.906358e-15 -3.949837e-15 -9.881212e-15
1.007210e-14
E:\\R-3.1.2\\scripts\\mPDBS\\summary_pdb_93\\9f752b3616e6033b.final.pdb
2.412875e-15 2.390143e-15 3.484100e-16 7.645706e-16 3.840092e-15
4.447434e-15
E:\\R-3.1.2\\scripts\\mPDBS\\summary_pdb_93\\a02c70083816d6ba.final.pdb
-5.300897e-15 -6.619606e-16 5.508297e-15 -3.118682e-15 -2.402070e-16
3.320268e-15
E:\\R-3.1.2\\scripts\\mPDBS\\summary_pdb_93\\a0c997d891e4e082.final.pdb
-8.032993e-17 5.070861e-16 -2.550566e-15 -3.388028e-15 -8.215658e-18
-2.011894e-15
E:\\R-3.1.2\\scripts\\mPDBS\\summary_pdb_93\\ab8039a2d823c616.final.pdb
-3.752736e-16 1.602130e-15 1.156376e-15 3.089545e-17 3.177604e-15
4.443880e-15
E:\\R-3.1.2\\scripts\\mPDBS\\summary_pdb_93\\acf4d74b9c71d4dc.final.pdb
2.726983e-16 1.081277e-15 -6.093423e-15 -3.261441e-16 1.258261e-15
4.779990e-15
E:\\R-3.1.2\\scripts\\mPDBS\\summary_pdb_93\\ad8a823e4b4c668e.final.pdb

-3.116412e-16 9.207042e-16 1.412577e-15 -8.147117e-17 2.548615e-15
2.392579e-15
E:\\R-3.1.2\\scripts\\mPDBS\\summary_pdb_93\\b16ababd03b7b857.final.pdb
-3.630508e-16 -2.500927e-15 -4.193592e-16 -2.961406e-15 5.374834e-16
2.464568e-15
E:\\R-3.1.2\\scripts\\mPDBS\\summary_pdb_93\\b226923e697d3c63.final.pdb
1.294845e-15 9.006649e-15 5.444988e-15 3.206548e-15 4.532605e-15
-1.878626e-15
E:\\R-3.1.2\\scripts\\mPDBS\\summary_pdb_93\\b28e48f6fa27a165.final.pdb
-2.101202e-15 -4.099705e-16 -3.968801e-15 9.252245e-16 1.185382e-15
6.075950e-15
E:\\R-3.1.2\\scripts\\mPDBS\\summary_pdb_93\\b42c4924e3cd1aec.final.pdb
8.785924e-16 3.664134e-15 -1.080731e-14 2.564266e-15 6.024034e-15
8.117811e-17
E:\\R-3.1.2\\scripts\\mPDBS\\summary_pdb_93\\b4dd239fcabff1df.final.pdb
-2.101202e-15 -4.099705e-16 -3.968801e-15 9.252245e-16 1.185382e-15
6.075950e-15
E:\\R-3.1.2\\scripts\\mPDBS\\summary_pdb_93\\b669942ef97459a2.final.pdb
-2.641637e-15 7.107706e-15 4.222978e-15 -2.418221e-15 7.621301e-16
2.106131e-15
E:\\R-3.1.2\\scripts\\mPDBS\\summary_pdb_93\\b99f4d9d87c1017b.final.pdb
-2.101202e-15 -4.099705e-16 -3.968801e-15 9.252245e-16 1.185382e-15
6.075950e-15
E:\\R-3.1.2\\scripts\\mPDBS\\summary_pdb_93\\b9d2cbf3975403e7.final.pdb
-2.101202e-15 -4.099705e-16 -3.968801e-15 9.252245e-16 1.185382e-15
6.075950e-15
E:\\R-3.1.2\\scripts\\mPDBS\\summary_pdb_93\\bc88c8a505f3a288.final.pdb
-8.296089e-16 7.761467e-16 1.411336e-15 -5.280401e-16 2.850283e-15
4.366814e-15
E:\\R-3.1.2\\scripts\\mPDBS\\summary_pdb_93\\bdac0e64f4ef427e.final.pdb
4.297992e-16 -1.482211e-15 1.243698e-15 2.297842e-15 3.770574e-15
2.892611e-15
E:\\R-3.1.2\\scripts\\mPDBS\\summary_pdb_93\\ca2a1f55fc9d0681.final.pdb
-2.641637e-15 7.107706e-15 4.222978e-15 -2.418221e-15 7.621301e-16
2.106131e-15
E:\\R-3.1.2\\scripts\\mPDBS\\summary_pdb_93\\ce34248de3e81a3c.final.pdb
-1.209493e-14 -5.924943e-15 1.089308e-15 -7.932246e-16 -3.561752e-15
2.621798e-15
E:\\R-3.1.2\\scripts\\mPDBS\\summary_pdb_93\\d02e697772494a61.final.pdb
1.081638e-14 9.318359e-15 6.090156e-15 8.165875e-16 1.217139e-14
2.885794e-15
E:\\R-3.1.2\\scripts\\mPDBS\\summary_pdb_93\\d04221245d015047.final.pdb
-1.062183e-15 -1.798361e-15 1.009589e-16 -8.336474e-16 1.931205e-15
6.267074e-15
E:\\R-3.1.2\\scripts\\mPDBS\\summary_pdb_93\\d678141e4018054a.final.pdb
6.789798e-15 -2.383372e-15 1.071192e-14 7.878200e-15 1.150932e-14
4.833296e-15
E:\\R-3.1.2\\scripts\\mPDBS\\summary_pdb_93\\d71b40af0f0efe39.final.pdb
6.644058e-15 1.274814e-14 -8.879116e-15 1.243749e-15 6.859571e-15
-1.268816e-15
E:\\R-3.1.2\\scripts\\mPDBS\\summary_pdb_93\\dc0809eff10197e2.final.pdb
-3.946090e-15 -2.819991e-15 -6.948406e-15 -7.515996e-15 -2.029731e-15
1.907897e-15
E:\\R-3.1.2\\scripts\\mPDBS\\summary_pdb_93\\e1163918d3707839.final.pdb

6.880736e-15 9.362813e-16 -4.431877e-15 7.407180e-15 2.408870e-15
6.791522e-15
E:\\R-3.1.2\\scripts\\mPDBS\\summary_pdb_93\\e4b48b06824ef848.final.pdb
-2.938251e-15 5.341338e-15 1.342185e-14 -1.401911e-15 7.100926e-16
-1.608698e-15
E:\\R-3.1.2\\scripts\\mPDBS\\summary_pdb_93\\e529e19ad281fea2.final.pdb
9.100262e-16 -4.279379e-15 -6.415634e-17 1.187021e-15 6.123840e-15
-1.205487e-15
E:\\R-3.1.2\\scripts\\mPDBS\\summary_pdb_93\\e5602082718ec3a1.final.pdb
2.726983e-16 1.081277e-15 -6.093423e-15 -3.261441e-16 1.258261e-15
4.779990e-15
E:\\R-3.1.2\\scripts\\mPDBS\\summary_pdb_93\\e5d589d996d0a960.final.pdb
-6.130046e-15 -4.472055e-15 4.197808e-15 -5.153854e-15 -1.774619e-15
7.095006e-15
E:\\R-3.1.2\\scripts\\mPDBS\\summary_pdb_93\\e9df9e72307a2fbd.final.pdb
-5.300897e-15 -6.619606e-16 5.508297e-15 -3.118682e-15 -2.402070e-16
3.320268e-15
E:\\R-3.1.2\\scripts\\mPDBS\\summary_pdb_93\\ea00f8694090cae7.final.pdb
2.726983e-16 1.081277e-15 -6.093423e-15 -3.261441e-16 1.258261e-15
4.779990e-15
E:\\R-3.1.2\\scripts\\mPDBS\\summary_pdb_93\\eba517be32db050e.final.pdb
-2.101202e-15 -4.099705e-16 -3.968801e-15 9.252245e-16 1.185382e-15
6.075950e-15
E:\\R-3.1.2\\scripts\\mPDBS\\summary_pdb_93\\ec04b9545899c29d.final.pdb
-5.343805e-16 1.314451e-15 1.436205e-15 1.705647e-16 2.839082e-15
4.121349e-15
E:\\R-3.1.2\\scripts\\mPDBS\\summary_pdb_93\\ee6862cbcad52078.final.pdb
-6.579119e-16 5.163719e-16 1.590446e-15 -4.959477e-16 3.005974e-15
4.025941e-15
E:\\R-3.1.2\\scripts\\mPDBS\\summary_pdb_93\\ef6a42fc83b81e5d.final.pdb
-6.995094e-16 7.265403e-16 1.199315e-15 2.235253e-16 2.664692e-15
4.487121e-15
E:\\R-3.1.2\\scripts\\mPDBS\\summary_pdb_93\\ef8e88d4ad93a0ad.final.pdb
-1.062183e-15 -1.798361e-15 1.009589e-16 -8.336474e-16 1.931205e-15
6.267074e-15
E:\\R-3.1.2\\scripts\\mPDBS\\summary_pdb_93\\f14fcae8deec4fa3.final.pdb
2.412875e-15 2.390143e-15 3.484100e-16 7.645706e-16 3.840092e-15
4.447434e-15
E:\\R-3.1.2\\scripts\\mPDBS\\summary_pdb_93\\f83584e22003fb92.final.pdb
-5.859562e-15 -9.503368e-16 1.023316e-14 -2.577182e-15 6.341734e-16
5.928065e-15
E:\\R-3.1.2\\scripts\\mPDBS\\summary_pdb_93\\f8e2bf87e52caa29.final.pdb
9.100262e-16 -4.279379e-15 -6.415634e-17 1.187021e-15 6.123840e-15
-1.205487e-15

[,517]   [,518]   [,519]   [,520]   [,521]   [,522]
E:\\R-3.1.2\\scripts\\40.1ns_data\\NAT0.pdb
3.146662e-15 -2.404586e-15 5.672977e-15 2.983568e-15 1.182117e-15
1.745364e-17
E:\\R-3.1.2\\scripts\\40.1ns_data\\LJP6.pdb
3.891983e-15 -2.328053e-15 7.651098e-15 2.774656e-15 2.260631e-16
3.393686e-15
E:\\R-3.1.2\\scripts\\40.1ns_data\\3WY9.pdb
3.531602e-15 -2.265087e-15 6.395002e-15 4.745126e-15 2.419965e-15

5.014870e-15
E:\\R-3.1.2\\scripts\\40.1ns_data\\ZSP9.pdb
4.346994e-15 -3.135135e-15 6.093342e-15 3.633207e-15 2.478621e-18
4.357262e-15
E:\\R-3.1.2\\scripts\\mPDBS\\summary_pdb_93\\013d855ad26c877c.final.pdb
1.267226e-14 -8.384895e-15 -1.056913e-15 2.937513e-15 6.265515e-15
1.074192e-16
E:\\R-3.1.2\\scripts\\mPDBS\\summary_pdb_93\\0a2a705342ca0d54.final.pdb
4.260138e-15 -3.477061e-15 6.578290e-15 2.509911e-15 8.418197e-16
4.430892e-15
E:\\R-3.1.2\\scripts\\mPDBS\\summary_pdb_93\\0a59fceadb4194d9.final.pdb
3.866248e-15 -3.419953e-15 8.456022e-15 1.395441e-15 -8.017257e-16
5.682836e-15
E:\\R-3.1.2\\scripts\\mPDBS\\summary_pdb_93\\0dba59f3306825e8.final.pdb
6.345943e-15 -9.173442e-16 1.207494e-14 3.375531e-15 -4.954677e-15
4.347083e-15
E:\\R-3.1.2\\scripts\\mPDBS\\summary_pdb_93\\126f26a78b57339c.final.pdb
-3.034140e-15 -5.944139e-15 9.721357e-15 1.511028e-15 2.232283e-15
4.700100e-15
E:\\R-3.1.2\\scripts\\mPDBS\\summary_pdb_93\\12f06f3e342df706.final.pdb
4.152910e-15 -3.073088e-15 6.790794e-15 2.395365e-15 9.337600e-16
4.184995e-15
E:\\R-3.1.2\\scripts\\mPDBS\\summary_pdb_93\\18618a2a251b867b.final.pdb
4.826764e-15 -5.440171e-15 6.633408e-15 2.943242e-15 3.103613e-15
-1.783502e-15
E:\\R-3.1.2\\scripts\\mPDBS\\summary_pdb_93\\1f305f598641eeb2.final.pdb
4.115920e-15 -3.316154e-15 6.514493e-15 2.451893e-15 1.334843e-15
3.715240e-15
E:\\R-3.1.2\\scripts\\mPDBS\\summary_pdb_93\\1f8a00b551be0a39.final.pdb
4.826764e-15 -5.440171e-15 6.633408e-15 2.943242e-15 3.103613e-15
-1.783502e-15
E:\\R-3.1.2\\scripts\\mPDBS\\summary_pdb_93\\219f45328d47579a.final.pdb
-2.906546e-15 -1.863255e-15 1.962759e-17 7.709834e-15 -2.379406e-16
3.564738e-15
E:\\R-3.1.2\\scripts\\mPDBS\\summary_pdb_93\\238dd3918e54336d.final.pdb
-1.681767e-16 -1.789963e-15 -1.008413e-15 6.584161e-15 1.964291e-15
1.031758e-14
E:\\R-3.1.2\\scripts\\mPDBS\\summary_pdb_93\\2b3027889e2fdc42.final.pdb
4.242358e-15 -3.757140e-15 6.809335e-15 1.946107e-15 7.933525e-16
4.044389e-15
E:\\R-3.1.2\\scripts\\mPDBS\\summary_pdb_93\\2fcb3b46e33793b0.final.pdb
9.501803e-15 -3.041634e-15 5.167480e-15 4.535296e-15 9.011617e-16
4.766483e-15
E:\\R-3.1.2\\scripts\\mPDBS\\summary_pdb_93\\34eb328251842006.final.pdb
2.448811e-15 -3.004835e-15 -1.543777e-15 -9.810792e-16 2.082118e-15
2.540954e-15
E:\\R-3.1.2\\scripts\\mPDBS\\summary_pdb_93\\354b32871797b7ab.final.pdb
9.483796e-15 -6.947888e-15 2.843817e-15 -3.538121e-15 2.432958e-15
-1.699329e-15
E:\\R-3.1.2\\scripts\\mPDBS\\summary_pdb_93\\397368adbc32d5de.final.pdb
-3.034140e-15 -5.944139e-15 9.721357e-15 1.511028e-15 2.232283e-15
4.700100e-15
E:\\R-3.1.2\\scripts\\mPDBS\\summary_pdb_93\\411ca13cd3df6edd.final.pdb
5.815949e-15 -7.921411e-15 6.093389e-15 4.626911e-15 4.028868e-15

4.115094e-15
E:\\R-3.1.2\\scripts\\mPDBS\\summary_pdb_93\\42357eee6642bf12.final.pdb
-4.252102e-15 -1.081574e-15 2.434700e-15 4.486292e-15 3.708654e-15
3.611119e-15
E:\\R-3.1.2\\scripts\\mPDBS\\summary_pdb_93\\44342d1f75a7fcfc.final.pdb
7.823345e-15 -6.953269e-15 7.036228e-15 -7.607477e-16 9.826649e-15
2.982142e-15
E:\\R-3.1.2\\scripts\\mPDBS\\summary_pdb_93\\448ef21dffe8cdfb.final.pdb
-2.012641e-15 -1.144810e-14 -8.997404e-15 -3.826072e-15 2.060415e-14
-4.134017e-16
E:\\R-3.1.2\\scripts\\mPDBS\\summary_pdb_93\\472a40b3e8f113c7.final.pdb
6.345943e-15 -9.173442e-16 1.207494e-14 3.375531e-15 -4.954677e-15
4.347083e-15
E:\\R-3.1.2\\scripts\\mPDBS\\summary_pdb_93\\48a26225951ea153.final.pdb
1.275660e-14 -5.446110e-15 7.767770e-15 4.570744e-15 6.871824e-15
-4.745454e-15
E:\\R-3.1.2\\scripts\\mPDBS\\summary_pdb_93\\4ab4a5c7c6682ce6.final.pdb
4.042664e-15 -5.094992e-15 -5.173846e-15 -1.025137e-15 2.277386e-15
2.663534e-15
E:\\R-3.1.2\\scripts\\mPDBS\\summary_pdb_93\\4eaa45b62cb6a25f.final.pdb
4.442147e-15 -3.451336e-15 8.064250e-15 4.224171e-15 4.041986e-16
5.213978e-15
E:\\R-3.1.2\\scripts\\mPDBS\\summary_pdb_93\\58c371611d85ac96.final.pdb
-3.034140e-15 -5.944139e-15 9.721357e-15 1.511028e-15 2.232283e-15
4.700100e-15
E:\\R-3.1.2\\scripts\\mPDBS\\summary_pdb_93\\59a647f04822d30a.final.pdb
5.177892e-15 -9.006016e-16 5.818452e-15 4.058538e-16 4.801155e-15
2.432235e-15
E:\\R-3.1.2\\scripts\\mPDBS\\summary_pdb_93\\59b091fe385e05fc.final.pdb
3.866248e-15 -3.419953e-15 8.456022e-15 1.395441e-15 -8.017257e-16
5.682836e-15
E:\\R-3.1.2\\scripts\\mPDBS\\summary_pdb_93\\5c35ac30f930d2db.final.pdb
4.442147e-15 -3.451336e-15 8.064250e-15 4.224171e-15 4.041986e-16
5.213978e-15
E:\\R-3.1.2\\scripts\\mPDBS\\summary_pdb_93\\5f60752431cf2dcb.final.pdb
5.959842e-15 2.473899e-16 6.364485e-15 7.021384e-15 4.711120e-15
7.132723e-15
E:\\R-3.1.2\\scripts\\mPDBS\\summary_pdb_93\\626456dd6ff2868d.final.pdb
6.345943e-15 -9.173442e-16 1.207494e-14 3.375531e-15 -4.954677e-15
4.347083e-15
E:\\R-3.1.2\\scripts\\mPDBS\\summary_pdb_93\\6583d53ff3c8f7ef.final.pdb
5.177892e-15 -9.006016e-16 5.818452e-15 4.058538e-16 4.801155e-15
2.432235e-15
E:\\R-3.1.2\\scripts\\mPDBS\\summary_pdb_93\\69760178986bef01.final.pdb
8.236384e-15 -6.709055e-15 1.032006e-14 5.762150e-15 4.430225e-15
6.028649e-15
E:\\R-3.1.2\\scripts\\mPDBS\\summary_pdb_93\\6a9d87d47926b1c8.final.pdb
4.144362e-15 -3.655605e-15 7.027332e-15 2.021037e-15 1.122060e-15
3.579468e-15
E:\\R-3.1.2\\scripts\\mPDBS\\summary_pdb_93\\6bb3547ea982ffc4.final.pdb
5.682375e-15 -3.350445e-15 5.473302e-15 4.953380e-15 4.825603e-15
9.357338e-16
E:\\R-3.1.2\\scripts\\mPDBS\\summary_pdb_93\\716d9624611d1c42.final.pdb
5.434546e-15 -8.494099e-15 4.709559e-15 -2.960540e-15 -2.827120e-15

1.299716e-15
E:\\R-3.1.2\\scripts\\mPDBS\\summary_pdb_93\\75f27e70ad0f1be4.final.pdb
5.177892e-15 -9.006016e-16 5.818452e-15 4.058538e-16 4.801155e-15
2.432235e-15
E:\\R-3.1.2\\scripts\\mPDBS\\summary_pdb_93\\769f910fffb22872.final.pdb
4.535114e-15 -2.919184e-15 6.709085e-15 2.333758e-15 1.941348e-15
4.262443e-15
E:\\R-3.1.2\\scripts\\mPDBS\\summary_pdb_93\\791f8c80dd5c3135.final.pdb
3.866248e-15 -3.419953e-15 8.456022e-15 1.395441e-15 -8.017257e-16
5.682836e-15
E:\\R-3.1.2\\scripts\\mPDBS\\summary_pdb_93\\7fad98e66a3f6099.final.pdb
1.658007e-15 -2.341140e-15 3.116448e-15 3.377025e-15 2.307387e-15
8.677504e-15
E:\\R-3.1.2\\scripts\\mPDBS\\summary_pdb_93\\804b89c94a633196.final.pdb
9.501803e-15 -3.041634e-15 5.167480e-15 4.535296e-15 9.011617e-16
4.766483e-15
E:\\R-3.1.2\\scripts\\mPDBS\\summary_pdb_93\\813cc65a2298feba.final.pdb
9.501803e-15 -3.041634e-15 5.167480e-15 4.535296e-15 9.011617e-16
4.766483e-15
E:\\R-3.1.2\\scripts\\mPDBS\\summary_pdb_93\\83c0eefcfe12d3f9.final.pdb
5.815949e-15 -7.921411e-15 6.093389e-15 4.626911e-15 4.028868e-15
4.115094e-15
E:\\R-3.1.2\\scripts\\mPDBS\\summary_pdb_93\\86d50469ef569760.final.pdb
6.345943e-15 -9.173442e-16 1.207494e-14 3.375531e-15 -4.954677e-15
4.347083e-15
E:\\R-3.1.2\\scripts\\mPDBS\\summary_pdb_93\\89d0666e1402203d.final.pdb
5.177892e-15 -9.006016e-16 5.818452e-15 4.058538e-16 4.801155e-15
2.432235e-15
E:\\R-3.1.2\\scripts\\mPDBS\\summary_pdb_93\\8b34852716dadc62.final.pdb
-5.966292e-15 -1.444387e-15 1.254266e-14 2.226379e-15 -1.822354e-15
5.696294e-15
E:\\R-3.1.2\\scripts\\mPDBS\\summary_pdb_93\\8b6ef9ac3fe74395.final.pdb
1.069612e-15 3.365333e-15 1.779727e-14 4.181017e-15 -3.464063e-15
7.855434e-15
E:\\R-3.1.2\\scripts\\mPDBS\\summary_pdb_93\\98bbe0d5986c138f.final.pdb
3.866248e-15 -3.419953e-15 8.456022e-15 1.395441e-15 -8.017257e-16
5.682836e-15
E:\\R-3.1.2\\scripts\\mPDBS\\summary_pdb_93\\9c22f4909d67e2fa.final.pdb
4.535181e-15 -1.321222e-15 7.472494e-15 1.076094e-15 1.213412e-15
6.365396e-16
E:\\R-3.1.2\\scripts\\mPDBS\\summary_pdb_93\\9dda8ed0d81fb3ef.final.pdb
1.509411e-14 -5.750021e-15 2.158340e-14 5.698387e-15 6.295619e-15
9.989557e-15
E:\\R-3.1.2\\scripts\\mPDBS\\summary_pdb_93\\9f752b3616e6033b.final.pdb
3.018664e-15 -1.682643e-15 8.525691e-15 3.046918e-15 8.788437e-16
4.071162e-15
E:\\R-3.1.2\\scripts\\mPDBS\\summary_pdb_93\\a02c70083816d6ba.final.pdb
4.442147e-15 -3.451336e-15 8.064250e-15 4.224171e-15 4.041986e-16
5.213978e-15
E:\\R-3.1.2\\scripts\\mPDBS\\summary_pdb_93\\a0c997d891e4e082.final.pdb
7.499935e-15 -6.642728e-15 5.146728e-15 6.080432e-15 -1.567108e-16
4.281422e-15
E:\\R-3.1.2\\scripts\\mPDBS\\summary_pdb_93\\ab8039a2d823c616.final.pdb
4.540606e-15 -3.577255e-15 6.679573e-15 2.503595e-15 6.282516e-16

2.834229e-15
E:\\R-3.1.2\\scripts\\mPDBS\\summary_pdb_93\\acf4d74b9c71d4dc.final.pdb
9.501803e-15 -3.041634e-15 5.167480e-15 4.535296e-15 9.011617e-16
4.766483e-15
E:\\R-3.1.2\\scripts\\mPDBS\\summary_pdb_93\\ad8a823e4b4c668e.final.pdb
4.696093e-15 -2.885936e-15 7.056164e-15 2.427771e-15 1.404301e-15
4.347755e-15
E:\\R-3.1.2\\scripts\\mPDBS\\summary_pdb_93\\b16ababd03b7b857.final.pdb
4.535181e-15 -1.321222e-15 7.472494e-15 1.076094e-15 1.213412e-15
6.365396e-16
E:\\R-3.1.2\\scripts\\mPDBS\\summary_pdb_93\\b226923e697d3c63.final.pdb
-4.922684e-16 -3.285629e-15 7.463186e-15 6.660562e-15 -1.468136e-15
6.282887e-15
E:\\R-3.1.2\\scripts\\mPDBS\\summary_pdb_93\\b28e48f6fa27a165.final.pdb
3.866248e-15 -3.419953e-15 8.456022e-15 1.395441e-15 -8.017257e-16
5.682836e-15
E:\\R-3.1.2\\scripts\\mPDBS\\summary_pdb_93\\b42c4924e3cd1aec.final.pdb
3.660551e-15 -3.448693e-15 -9.389468e-16 6.517989e-15 -6.860524e-15
1.360609e-15
E:\\R-3.1.2\\scripts\\mPDBS\\summary_pdb_93\\b4dd239fcabff1df.final.pdb
3.866248e-15 -3.419953e-15 8.456022e-15 1.395441e-15 -8.017257e-16
5.682836e-15
E:\\R-3.1.2\\scripts\\mPDBS\\summary_pdb_93\\b669942ef97459a2.final.pdb
-5.966292e-15 -1.444387e-15 1.254266e-14 2.226379e-15 -1.822354e-15
5.696294e-15
E:\\R-3.1.2\\scripts\\mPDBS\\summary_pdb_93\\b99f4d9d87c1017b.final.pdb
3.866248e-15 -3.419953e-15 8.456022e-15 1.395441e-15 -8.017257e-16
5.682836e-15
E:\\R-3.1.2\\scripts\\mPDBS\\summary_pdb_93\\b9d2cbf3975403e7.final.pdb
3.866248e-15 -3.419953e-15 8.456022e-15 1.395441e-15 -8.017257e-16
5.682836e-15
E:\\R-3.1.2\\scripts\\mPDBS\\summary_pdb_93\\bc88c8a505f3a288.final.pdb
4.304808e-15 -4.317239e-15 6.684002e-15 2.231848e-15 6.302885e-16
3.324046e-15
E:\\R-3.1.2\\scripts\\mPDBS\\summary_pdb_93\\bdac0e64f4ef427e.final.pdb
2.122832e-15 6.277301e-15 7.678059e-15 -1.961732e-15 -1.945520e-15
1.327827e-15
E:\\R-3.1.2\\scripts\\mPDBS\\summary_pdb_93\\ca2a1f55fc9d0681.final.pdb
-5.966292e-15 -1.444387e-15 1.254266e-14 2.226379e-15 -1.822354e-15
5.696294e-15
E:\\R-3.1.2\\scripts\\mPDBS\\summary_pdb_93\\ce34248de3e81a3c.final.pdb
4.391379e-15 -6.682817e-15 6.823872e-15 1.868408e-15 -2.536279e-15
5.923506e-15
E:\\R-3.1.2\\scripts\\mPDBS\\summary_pdb_93\\d02e697772494a61.final.pdb
-6.013111e-15 -1.151151e-15 -8.587930e-16 -4.962158e-15 4.025142e-15
-6.840331e-16
E:\\R-3.1.2\\scripts\\mPDBS\\summary_pdb_93\\d04221245d015047.final.pdb
5.652455e-15 -2.950369e-15 6.331544e-15 7.513612e-16 2.382924e-15
2.772212e-15
E:\\R-3.1.2\\scripts\\mPDBS\\summary_pdb_93\\d678141e4018054a.final.pdb
4.380659e-15 -6.074104e-15 -1.974061e-15 1.534091e-15 4.325883e-15
1.125583e-15
E:\\R-3.1.2\\scripts\\mPDBS\\summary_pdb_93\\d71b40af0f0efe39.final.pdb
2.048060e-15 -3.132487e-15 1.974355e-15 -2.574768e-15 -2.442311e-16

-1.318146e-15
E:\\R-3.1.2\\scripts\\mPDBS\\summary_pdb_93\\dc0809eff10197e2.final.pdb
6.724784e-15 -1.846956e-15 1.373598e-14 -1.462791e-15 -3.956085e-15
5.603098e-15
E:\\R-3.1.2\\scripts\\mPDBS\\summary_pdb_93\\e1163918d3707839.final.pdb
4.117868e-15 1.867367e-15 9.026172e-15 6.931287e-15 -1.366237e-14
1.390099e-15
E:\\R-3.1.2\\scripts\\mPDBS\\summary_pdb_93\\e4b48b06824ef848.final.pdb
4.826764e-15 -5.440171e-15 6.633408e-15 2.943242e-15 3.103613e-15
-1.783502e-15
E:\\R-3.1.2\\scripts\\mPDBS\\summary_pdb_93\\e529e19ad281fea2.final.pdb
1.267226e-14 -8.384895e-15 -1.056913e-15 2.937513e-15 6.265515e-15
1.074192e-16
E:\\R-3.1.2\\scripts\\mPDBS\\summary_pdb_93\\e5602082718ec3a1.final.pdb
9.501803e-15 -3.041634e-15 5.167480e-15 4.535296e-15 9.011617e-16
4.766483e-15
E:\\R-3.1.2\\scripts\\mPDBS\\summary_pdb_93\\e5d589d996d0a960.final.pdb
5.289076e-15 -7.408101e-15 1.503943e-14 4.262634e-15 -4.677354e-16
9.384803e-16
E:\\R-3.1.2\\scripts\\mPDBS\\summary_pdb_93\\e9df9e72307a2fbd.final.pdb
4.442147e-15 -3.451336e-15 8.064250e-15 4.224171e-15 4.041986e-16
5.213978e-15
E:\\R-3.1.2\\scripts\\mPDBS\\summary_pdb_93\\ea00f8694090cae7.final.pdb
9.501803e-15 -3.041634e-15 5.167480e-15 4.535296e-15 9.011617e-16
4.766483e-15
E:\\R-3.1.2\\scripts\\mPDBS\\summary_pdb_93\\eba517be32db050e.final.pdb
3.866248e-15 -3.419953e-15 8.456022e-15 1.395441e-15 -8.017257e-16
5.682836e-15
E:\\R-3.1.2\\scripts\\mPDBS\\summary_pdb_93\\ec04b9545899c29d.final.pdb
4.238539e-15 -2.915266e-15 6.298103e-15 2.353948e-15 1.009049e-15
3.710726e-15
E:\\R-3.1.2\\scripts\\mPDBS\\summary_pdb_93\\ee6862cbcad52078.final.pdb
4.107591e-15 -3.894617e-15 6.615046e-15 1.992185e-15 7.855463e-16
3.117180e-15
E:\\R-3.1.2\\scripts\\mPDBS\\summary_pdb_93\\ef6a42fc83b81e5d.final.pdb
4.362617e-15 -2.902921e-15 6.750935e-15 2.475409e-15 1.270877e-15
3.402454e-15
E:\\R-3.1.2\\scripts\\mPDBS\\summary_pdb_93\\ef8e88d4ad93a0ad.final.pdb
5.652455e-15 -2.950369e-15 6.331544e-15 7.513612e-16 2.382924e-15
2.772212e-15
E:\\R-3.1.2\\scripts\\mPDBS\\summary_pdb_93\\f14fcae8deec4fa3.final.pdb
3.018664e-15 -1.682643e-15 8.525691e-15 3.046918e-15 8.788437e-16
4.071162e-15
E:\\R-3.1.2\\scripts\\mPDBS\\summary_pdb_93\\f83584e22003fb92.final.pdb
1.275660e-14 -5.446110e-15 7.767770e-15 4.570744e-15 6.871824e-15
-4.745454e-15
E:\\R-3.1.2\\scripts\\mPDBS\\summary_pdb_93\\f8e2bf87e52caa29.final.pdb
1.267226e-14 -8.384895e-15 -1.056913e-15 2.937513e-15 6.265515e-15
1.074192e-16

[,523]   [,524]   [,525]   [,526]   [,527]   [,528]
E:\\R-3.1.2\\scripts\\40.1ns_data\\NAT0.pdb
2.397957e-15 -3.116221e-15 -5.942950e-16 3.471684e-15 -3.700205e-15
-2.078429e-16

E:\\R-3.1.2\\scripts\\40.1ns_data\\LJP6.pdb
1.657687e-15 -2.950368e-15 -4.658134e-16 3.324519e-15 -4.054742e-15
5.741875e-16
E:\\R-3.1.2\\scripts\\40.1ns_data\\3WY9.pdb
2.556508e-15 -3.943590e-15 2.398522e-15 3.488375e-15 -4.291511e-15
-3.982282e-16
E:\\R-3.1.2\\scripts\\40.1ns_data\\ZSP9.pdb
1.267951e-15 -4.416293e-15 -2.100635e-15 3.016202e-15 -4.243077e-15
-5.379293e-16
E:\\R-3.1.2\\scripts\\mPDBS\\summary_pdb_93\\013d855ad26c877c.final.pdb
9.131821e-16 -1.929138e-15 -2.305312e-15 -1.092548e-15 -6.253215e-15
6.235171e-15
E:\\R-3.1.2\\scripts\\mPDBS\\summary_pdb_93\\0a2a705342ca0d54.final.pdb
1.756455e-15 -3.258296e-15 -6.927995e-17 1.882969e-15 -3.060216e-15
-7.158091e-17
E:\\R-3.1.2\\scripts\\mPDBS\\summary_pdb_93\\0a59fceadb4194d9.final.pdb
2.484178e-15 -1.374528e-16 -3.042087e-15 -2.230720e-15 2.016745e-15
4.504225e-16
E:\\R-3.1.2\\scripts\\mPDBS\\summary_pdb_93\\0dba59f3306825e8.final.pdb
1.271403e-15 -6.000534e-15 -4.416470e-15 7.556919e-16 3.763079e-15
-2.270179e-15
E:\\R-3.1.2\\scripts\\mPDBS\\summary_pdb_93\\126f26a78b57339c.final.pdb
3.292789e-15 -2.412258e-15 7.247810e-15 9.092339e-15 -1.004619e-14
-9.134079e-16
E:\\R-3.1.2\\scripts\\mPDBS\\summary_pdb_93\\12f06f3e342df706.final.pdb
2.150237e-15 -2.141134e-15 -3.806628e-16 1.956912e-15 -3.276189e-15
-6.290730e-17
E:\\R-3.1.2\\scripts\\mPDBS\\summary_pdb_93\\18618a2a251b867b.final.pdb
-1.762495e-16 -9.877366e-15 3.250942e-15 1.184130e-14 -7.190708e-15
1.777415e-15
E:\\R-3.1.2\\scripts\\mPDBS\\summary_pdb_93\\1f305f598641eeb2.final.pdb
2.016526e-15 -2.478426e-15 3.098620e-16 2.591522e-15 -3.427880e-15
-4.557445e-16
E:\\R-3.1.2\\scripts\\mPDBS\\summary_pdb_93\\1f8a00b551be0a39.final.pdb
-1.762495e-16 -9.877366e-15 3.250942e-15 1.184130e-14 -7.190708e-15
1.777415e-15
E:\\R-3.1.2\\scripts\\mPDBS\\summary_pdb_93\\219f45328d47579a.final.pdb
3.478391e-15 2.099907e-15 1.482939e-15 1.079315e-14 -8.550324e-15
-5.847491e-15
E:\\R-3.1.2\\scripts\\mPDBS\\summary_pdb_93\\238dd3918e54336d.final.pdb
-3.509725e-15 -2.912577e-15 1.395734e-14 3.568569e-15 -1.153361e-14
-5.555003e-16
E:\\R-3.1.2\\scripts\\mPDBS\\summary_pdb_93\\2b3027889e2fdc42.final.pdb
1.690759e-15 -1.437194e-15 -4.027052e-16 1.255207e-15 -2.600222e-15
1.574711e-16
E:\\R-3.1.2\\scripts\\mPDBS\\summary_pdb_93\\2fcb3b46e33793b0.final.pdb
7.415042e-16 -2.832309e-15 -4.628028e-15 1.278228e-15 1.752004e-15
-7.627906e-16
E:\\R-3.1.2\\scripts\\mPDBS\\summary_pdb_93\\34eb328251842006.final.pdb
1.030501e-15 7.560451e-15 9.270694e-15 -6.672712e-15 2.155835e-15
-1.604324e-15
E:\\R-3.1.2\\scripts\\mPDBS\\summary_pdb_93\\354b32871797b7ab.final.pdb
-2.445100e-17 1.131986e-14 -9.012914e-15 -2.332793e-15 -3.296308e-15
8.912072e-16

E:\\R-3.1.2\\scripts\\mPDBS\\summary_pdb_93\\397368adbc32d5de.final.pdb
3.292789e-15 -2.412258e-15 7.247810e-15 9.092339e-15 -1.004619e-14
-9.134079e-16
E:\\R-3.1.2\\scripts\\mPDBS\\summary_pdb_93\\411ca13cd3df6edd.final.pdb
-1.787096e-17 1.043930e-15 -5.462240e-16 9.361280e-15 -3.843082e-16
-2.343340e-15
E:\\R-3.1.2\\scripts\\mPDBS\\summary_pdb_93\\42357eee6642bf12.final.pdb
1.161179e-15 3.980589e-15 1.615460e-15 6.354241e-16 3.616933e-15
-2.406737e-15
E:\\R-3.1.2\\scripts\\mPDBS\\summary_pdb_93\\44342d1f75a7fcfc.final.pdb
4.917394e-16 2.022329e-15 -8.256049e-15 2.204559e-15 1.898938e-15
2.656404e-15
E:\\R-3.1.2\\scripts\\mPDBS\\summary_pdb_93\\448ef21dffe8cdfb.final.pdb
1.712949e-15 7.478887e-15 2.403914e-14 2.265069e-15 -3.071508e-14
1.055975e-14
E:\\R-3.1.2\\scripts\\mPDBS\\summary_pdb_93\\472a40b3e8f113c7.final.pdb
1.271403e-15 -6.000534e-15 -4.416470e-15 7.556919e-16 3.763079e-15
-2.270179e-15
E:\\R-3.1.2\\scripts\\mPDBS\\summary_pdb_93\\48a26225951ea153.final.pdb
9.719327e-16 -4.660311e-15 -1.529050e-16 5.880955e-15 -4.510153e-15
1.533974e-15
E:\\R-3.1.2\\scripts\\mPDBS\\summary_pdb_93\\4ab4a5c7c6682ce6.final.pdb
1.437502e-15 2.574810e-16 6.926019e-15 -2.381339e-15 -2.219115e-15
-5.680428e-15
E:\\R-3.1.2\\scripts\\mPDBS\\summary_pdb_93\\4eaa45b62cb6a25f.final.pdb
2.629893e-15 -8.642469e-15 2.109265e-15 5.219976e-15 -5.417442e-15
-3.621938e-17
E:\\R-3.1.2\\scripts\\mPDBS\\summary_pdb_93\\58c371611d85ac96.final.pdb
3.292789e-15 -2.412258e-15 7.247810e-15 9.092339e-15 -1.004619e-14
-9.134079e-16
E:\\R-3.1.2\\scripts\\mPDBS\\summary_pdb_93\\59a647f04822d30a.final.pdb
4.932596e-18 6.649080e-16 3.051746e-15 -1.018124e-15 -3.591462e-15
1.223523e-15
E:\\R-3.1.2\\scripts\\mPDBS\\summary_pdb_93\\59b091fe385e05fc.final.pdb
2.484178e-15 -1.374528e-16 -3.042087e-15 -2.230720e-15 2.016745e-15
4.504225e-16
E:\\R-3.1.2\\scripts\\mPDBS\\summary_pdb_93\\5c35ac30f930d2db.final.pdb
2.629893e-15 -8.642469e-15 2.109265e-15 5.219976e-15 -5.417442e-15
-3.621938e-17
E:\\R-3.1.2\\scripts\\mPDBS\\summary_pdb_93\\5f60752431cf2dcb.final.pdb
2.691254e-15 -7.978045e-15 4.520045e-16 1.025193e-14 7.344916e-16
-1.251405e-16
E:\\R-3.1.2\\scripts\\mPDBS\\summary_pdb_93\\626456dd6ff2868d.final.pdb
1.271403e-15 -6.000534e-15 -4.416470e-15 7.556919e-16 3.763079e-15
-2.270179e-15
E:\\R-3.1.2\\scripts\\mPDBS\\summary_pdb_93\\6583d53ff3c8f7ef.final.pdb
4.932596e-18 6.649080e-16 3.051746e-15 -1.018124e-15 -3.591462e-15
1.223523e-15
E:\\R-3.1.2\\scripts\\mPDBS\\summary_pdb_93\\69760178986bef01.final.pdb
1.220629e-15 -7.028583e-15 -4.080892e-15 1.130858e-14 -1.230040e-14
-3.499727e-16
E:\\R-3.1.2\\scripts\\mPDBS\\summary_pdb_93\\6a9d87d47926b1c8.final.pdb
1.476534e-15 -2.224908e-15 1.037500e-15 1.815500e-15 -2.992802e-15
-6.323827e-16

E:\\R-3.1.2\\scripts\\mPDBS\\summary_pdb_93\\6bb3547ea982ffc4.final.pdb
-3.270396e-15 5.542160e-16 3.923942e-15 8.280113e-15 -1.753970e-14
-5.624007e-15
E:\\R-3.1.2\\scripts\\mPDBS\\summary_pdb_93\\716d9624611d1c42.final.pdb
4.367431e-15 2.857905e-15 1.777333e-15 -1.271842e-15 -1.290130e-14
-9.614941e-16
E:\\R-3.1.2\\scripts\\mPDBS\\summary_pdb_93\\75f27e70ad0f1be4.final.pdb
4.932596e-18 6.649080e-16 3.051746e-15 -1.018124e-15 -3.591462e-15
1.223523e-15
E:\\R-3.1.2\\scripts\\mPDBS\\summary_pdb_93\\769f910fffb22872.final.pdb
1.820417e-15 -2.687371e-15 3.001777e-16 2.089873e-15 -3.871748e-15
-7.171178e-16
E:\\R-3.1.2\\scripts\\mPDBS\\summary_pdb_93\\791f8c80dd5c3135.final.pdb
2.484178e-15 -1.374528e-16 -3.042087e-15 -2.230720e-15 2.016745e-15
4.504225e-16
E:\\R-3.1.2\\scripts\\mPDBS\\summary_pdb_93\\7fad98e66a3f6099.final.pdb
5.874154e-15 1.296866e-14 -1.376635e-15 -5.993178e-15 7.432644e-16
-4.187832e-15
E:\\R-3.1.2\\scripts\\mPDBS\\summary_pdb_93\\804b89c94a633196.final.pdb
7.415042e-16 -2.832309e-15 -4.628028e-15 1.278228e-15 1.752004e-15
-7.627906e-16
E:\\R-3.1.2\\scripts\\mPDBS\\summary_pdb_93\\813cc65a2298feba.final.pdb
7.415042e-16 -2.832309e-15 -4.628028e-15 1.278228e-15 1.752004e-15
-7.627906e-16
E:\\R-3.1.2\\scripts\\mPDBS\\summary_pdb_93\\83c0eefcfe12d3f9.final.pdb
-1.787096e-17 1.043930e-15 -5.462240e-16 9.361280e-15 -3.843082e-16
-2.343340e-15
E:\\R-3.1.2\\scripts\\mPDBS\\summary_pdb_93\\86d50469ef569760.final.pdb
1.271403e-15 -6.000534e-15 -4.416470e-15 7.556919e-16 3.763079e-15
-2.270179e-15
E:\\R-3.1.2\\scripts\\mPDBS\\summary_pdb_93\\89d0666e1402203d.final.pdb
4.932596e-18 6.649080e-16 3.051746e-15 -1.018124e-15 -3.591462e-15
1.223523e-15
E:\\R-3.1.2\\scripts\\mPDBS\\summary_pdb_93\\8b34852716dadc62.final.pdb
3.694871e-15 -9.838514e-17 2.599697e-15 5.894436e-15 -6.251695e-17
-9.098137e-15
E:\\R-3.1.2\\scripts\\mPDBS\\summary_pdb_93\\8b6ef9ac3fe74395.final.pdb
1.897044e-15 -1.938791e-15 -1.856343e-15 1.824192e-15 -9.053977e-15
-5.025727e-15
E:\\R-3.1.2\\scripts\\mPDBS\\summary_pdb_93\\98bbe0d5986c138f.final.pdb
2.484178e-15 -1.374528e-16 -3.042087e-15 -2.230720e-15 2.016745e-15
4.504225e-16
E:\\R-3.1.2\\scripts\\mPDBS\\summary_pdb_93\\9c22f4909d67e2fa.final.pdb
4.234804e-15 -6.849495e-15 3.211377e-16 -2.331515e-15 -4.053682e-15
4.877663e-15
E:\\R-3.1.2\\scripts\\mPDBS\\summary_pdb_93\\9dda8ed0d81fb3ef.final.pdb
-6.780005e-15 -1.408111e-14 -7.607263e-15 5.629337e-15 8.580009e-15
6.333151e-15
E:\\R-3.1.2\\scripts\\mPDBS\\summary_pdb_93\\9f752b3616e6033b.final.pdb
8.625904e-16 -2.238549e-15 -2.631151e-15 1.609830e-15 -1.571499e-15
-6.148651e-16
E:\\R-3.1.2\\scripts\\mPDBS\\summary_pdb_93\\a02c70083816d6ba.final.pdb
2.629893e-15 -8.642469e-15 2.109265e-15 5.219976e-15 -5.417442e-15
-3.621938e-17

E:\\R-3.1.2\\scripts\\mPDBS\\summary_pdb_93\\a0c997d891e4e082.final.pdb
-2.186870e-15 9.694880e-16 3.880954e-15 -7.035268e-15 -9.449145e-16
-3.139191e-15
E:\\R-3.1.2\\scripts\\mPDBS\\summary_pdb_93\\ab8039a2d823c616.final.pdb
1.849831e-15 -3.045096e-15 8.017455e-17 1.613196e-15 -2.830993e-15
-8.768952e-16
E:\\R-3.1.2\\scripts\\mPDBS\\summary_pdb_93\\acf4d74b9c71d4dc.final.pdb
7.415042e-16 -2.832309e-15 -4.628028e-15 1.278228e-15 1.752004e-15
-7.627906e-16
E:\\R-3.1.2\\scripts\\mPDBS\\summary_pdb_93\\ad8a823e4b4c668e.final.pdb
1.857359e-15 -2.600634e-15 -3.077224e-16 3.254066e-15 -3.007120e-15
-5.295561e-16
E:\\R-3.1.2\\scripts\\mPDBS\\summary_pdb_93\\b16ababd03b7b857.final.pdb
4.234804e-15 -6.849495e-15 3.211377e-16 -2.331515e-15 -4.053682e-15
4.877663e-15
E:\\R-3.1.2\\scripts\\mPDBS\\summary_pdb_93\\b226923e697d3c63.final.pdb
6.188079e-16 1.084277e-15 4.321563e-15 6.102591e-15 -2.839893e-15
-2.238985e-15
E:\\R-3.1.2\\scripts\\mPDBS\\summary_pdb_93\\b28e48f6fa27a165.final.pdb
2.484178e-15 -1.374528e-16 -3.042087e-15 -2.230720e-15 2.016745e-15
4.504225e-16
E:\\R-3.1.2\\scripts\\mPDBS\\summary_pdb_93\\b42c4924e3cd1aec.final.pdb
7.550112e-15 -1.506532e-15 -6.717350e-15 1.215441e-15 -6.401280e-15
-3.093222e-16
E:\\R-3.1.2\\scripts\\mPDBS\\summary_pdb_93\\b4dd239fcabff1df.final.pdb
2.484178e-15 -1.374528e-16 -3.042087e-15 -2.230720e-15 2.016745e-15
4.504225e-16
E:\\R-3.1.2\\scripts\\mPDBS\\summary_pdb_93\\b669942ef97459a2.final.pdb
3.694871e-15 -9.838514e-17 2.599697e-15 5.894436e-15 -6.251695e-17
-9.098137e-15
E:\\R-3.1.2\\scripts\\mPDBS\\summary_pdb_93\\b99f4d9d87c1017b.final.pdb
2.484178e-15 -1.374528e-16 -3.042087e-15 -2.230720e-15 2.016745e-15
4.504225e-16
E:\\R-3.1.2\\scripts\\mPDBS\\summary_pdb_93\\b9d2cbf3975403e7.final.pdb
2.484178e-15 -1.374528e-16 -3.042087e-15 -2.230720e-15 2.016745e-15
4.504225e-16
E:\\R-3.1.2\\scripts\\mPDBS\\summary_pdb_93\\bc88c8a505f3a288.final.pdb
2.046377e-15 -2.024832e-15 -2.639273e-16 1.402116e-15 -2.300549e-15
1.919487e-16
E:\\R-3.1.2\\scripts\\mPDBS\\summary_pdb_93\\bdac0e64f4ef427e.final.pdb
4.920453e-15 -8.323143e-15 1.105233e-15 -1.170954e-14 -9.406171e-15
3.614567e-15
E:\\R-3.1.2\\scripts\\mPDBS\\summary_pdb_93\\ca2a1f55fc9d0681.final.pdb
3.694871e-15 -9.838514e-17 2.599697e-15 5.894436e-15 -6.251695e-17
-9.098137e-15
E:\\R-3.1.2\\scripts\\mPDBS\\summary_pdb_93\\ce34248de3e81a3c.final.pdb
4.054309e-15 -3.949387e-15 2.849771e-15 -2.582170e-15 -1.280074e-15
-1.917308e-15
E:\\R-3.1.2\\scripts\\mPDBS\\summary_pdb_93\\d02e697772494a61.final.pdb
5.266241e-15 8.563486e-15 1.320273e-14 -8.148605e-16 -1.199245e-14
-6.240189e-15
E:\\R-3.1.2\\scripts\\mPDBS\\summary_pdb_93\\d04221245d015047.final.pdb
2.163816e-15 -3.023845e-15 -1.413422e-15 6.044539e-16 -3.448988e-15
3.882197e-17

E:\\R-3.1.2\\scripts\\mPDBS\\summary_pdb_93\\d678141e4018054a.final.pdb
3.655286e-15 2.647980e-15 4.958696e-15 8.340734e-15 -8.201132e-15
-1.914905e-15
E:\\R-3.1.2\\scripts\\mPDBS\\summary_pdb_93\\d71b40af0f0efe39.final.pdb
6.441242e-15 -2.086743e-16 -1.450401e-15 6.819189e-15 7.449135e-15
5.408135e-15
E:\\R-3.1.2\\scripts\\mPDBS\\summary_pdb_93\\dc0809eff10197e2.final.pdb
2.523456e-15 -8.291545e-15 -3.319302e-15 -7.281936e-15 6.086008e-15
8.051450e-15
E:\\R-3.1.2\\scripts\\mPDBS\\summary_pdb_93\\e1163918d3707839.final.pdb
1.098595e-14 -1.065026e-14 -4.072764e-15 3.246803e-15 -9.494292e-15
-3.335547e-15
E:\\R-3.1.2\\scripts\\mPDBS\\summary_pdb_93\\e4b48b06824ef848.final.pdb
-1.762495e-16 -9.877366e-15 3.250942e-15 1.184130e-14 -7.190708e-15
1.777415e-15
E:\\R-3.1.2\\scripts\\mPDBS\\summary_pdb_93\\e529e19ad281fea2.final.pdb
9.131821e-16 -1.929138e-15 -2.305312e-15 -1.092548e-15 -6.253215e-15
6.235171e-15
E:\\R-3.1.2\\scripts\\mPDBS\\summary_pdb_93\\e5602082718ec3a1.final.pdb
7.415042e-16 -2.832309e-15 -4.628028e-15 1.278228e-15 1.752004e-15
-7.627906e-16
E:\\R-3.1.2\\scripts\\mPDBS\\summary_pdb_93\\e5d589d996d0a960.final.pdb
3.085912e-15 -1.523109e-14 7.911061e-16 3.850757e-15 -1.141141e-14
-6.463190e-15
E:\\R-3.1.2\\scripts\\mPDBS\\summary_pdb_93\\e9df9e72307a2fbd.final.pdb
2.629893e-15 -8.642469e-15 2.109265e-15 5.219976e-15 -5.417442e-15
-3.621938e-17
E:\\R-3.1.2\\scripts\\mPDBS\\summary_pdb_93\\ea00f8694090cae7.final.pdb
7.415042e-16 -2.832309e-15 -4.628028e-15 1.278228e-15 1.752004e-15
-7.627906e-16
E:\\R-3.1.2\\scripts\\mPDBS\\summary_pdb_93\\eba517be32db050e.final.pdb
2.484178e-15 -1.374528e-16 -3.042087e-15 -2.230720e-15 2.016745e-15
4.504225e-16
E:\\R-3.1.2\\scripts\\mPDBS\\summary_pdb_93\\ec04b9545899c29d.final.pdb
1.825742e-15 -2.700889e-15 2.111358e-16 2.430940e-15 -3.717229e-15
-5.606402e-16
E:\\R-3.1.2\\scripts\\mPDBS\\summary_pdb_93\\ee6862cbcad52078.final.pdb
2.070663e-15 -1.811461e-15 2.269994e-16 9.334155e-16 -2.699101e-15
6.551199e-16
E:\\R-3.1.2\\scripts\\mPDBS\\summary_pdb_93\\ef6a42fc83b81e5d.final.pdb
2.022512e-15 -2.577650e-15 1.024192e-16 2.208593e-15 -3.789782e-15
-4.597282e-16
E:\\R-3.1.2\\scripts\\mPDBS\\summary_pdb_93\\ef8e88d4ad93a0ad.final.pdb
2.163816e-15 -3.023845e-15 -1.413422e-15 6.044539e-16 -3.448988e-15
3.882197e-17
E:\\R-3.1.2\\scripts\\mPDBS\\summary_pdb_93\\f14fcae8deec4fa3.final.pdb
8.625904e-16 -2.238549e-15 -2.631151e-15 1.609830e-15 -1.571499e-15
-6.148651e-16
E:\\R-3.1.2\\scripts\\mPDBS\\summary_pdb_93\\f83584e22003fb92.final.pdb
9.719327e-16 -4.660311e-15 -1.529050e-16 5.880955e-15 -4.510153e-15
1.533974e-15
E:\\R-3.1.2\\scripts\\mPDBS\\summary_pdb_93\\f8e2bf87e52caa29.final.pdb
9.131821e-16 -1.929138e-15 -2.305312e-15 -1.092548e-15 -6.253215e-15
6.235171e-15

```
[,529]        [,530]       [,531]        [,532]        [,533]        [,534]
E:\\R-3.1.2\\scripts\\40.1ns_data\\NAT0.pdb
4.703212e-16  2.531191e-15 6.799759e-15  2.251188e-15 -7.018234e-15
-4.330471e-15
E:\\R-3.1.2\\scripts\\40.1ns_data\\LJP6.pdb
1.266440e-15  6.498693e-15 6.665861e-15  1.826047e-15 -8.294560e-15
-5.726055e-15
E:\\R-3.1.2\\scripts\\40.1ns_data\\3WY9.pdb
9.516732e-16  3.493727e-15 6.396429e-15  3.159667e-16 -7.246210e-15
-1.704945e-15
E:\\R-3.1.2\\scripts\\40.1ns_data\\ZSP9.pdb
9.658125e-16  2.505216e-15 7.363350e-15  1.059072e-15 -5.875841e-15
-3.829383e-15
E:\\R-3.1.2\\scripts\\mPDBS\\summary_pdb_93\\013d855ad26c877c.final.pdb
2.306067e-15  7.588664e-15 6.113568e-15  2.960493e-15 -6.733000e-15
-5.416049e-15
E:\\R-3.1.2\\scripts\\mPDBS\\summary_pdb_93\\0a2a705342ca0d54.final.pdb
1.116414e-15  5.000557e-15 7.109770e-15  8.416845e-16 -7.196333e-15
-4.367595e-15
E:\\R-3.1.2\\scripts\\mPDBS\\summary_pdb_93\\0a59fceadb4194d9.final.pdb
-6.598962e-16  4.487759e-15 6.445482e-15  4.339948e-15 -6.202377e-15
-4.795255e-15
E:\\R-3.1.2\\scripts\\mPDBS\\summary_pdb_93\\0dba59f3306825e8.final.pdb
-6.663816e-16  3.487778e-15 6.407604e-15  4.504397e-15 -6.521364e-15
-2.961415e-15
E:\\R-3.1.2\\scripts\\mPDBS\\summary_pdb_93\\126f26a78b57339c.final.pdb
3.960993e-15  5.297962e-15 4.934390e-15 -6.437371e-15 -1.098394e-14
-5.571307e-15
E:\\R-3.1.2\\scripts\\mPDBS\\summary_pdb_93\\12f06f3e342df706.final.pdb
1.261263e-15  5.122855e-15 6.928274e-15  6.543344e-16 -7.172915e-15
-4.482954e-15
E:\\R-3.1.2\\scripts\\mPDBS\\summary_pdb_93\\18618a2a251b867b.final.pdb
7.533646e-15  2.161462e-15 7.763653e-15 -3.632529e-15 -8.438628e-15
-9.535260e-15
E:\\R-3.1.2\\scripts\\mPDBS\\summary_pdb_93\\1f305f598641eeb2.final.pdb
1.430196e-15  4.945306e-15 6.947269e-15  9.964399e-16 -7.796484e-15
-5.164026e-15
E:\\R-3.1.2\\scripts\\mPDBS\\summary_pdb_93\\1f8a00b551be0a39.final.pdb
7.533646e-15  2.161462e-15 7.763653e-15 -3.632529e-15 -8.438628e-15
-9.535260e-15
E:\\R-3.1.2\\scripts\\mPDBS\\summary_pdb_93\\219f45328d47579a.final.pdb
-4.543942e-15  7.204336e-15 5.717332e-15 -8.273226e-15 -1.032148e-14
9.005338e-15
E:\\R-3.1.2\\scripts\\mPDBS\\summary_pdb_93\\238dd3918e54336d.final.pdb
9.887656e-15  1.110516e-16 2.156595e-15 -1.770182e-15 -1.959965e-14
-7.628902e-16
E:\\R-3.1.2\\scripts\\mPDBS\\summary_pdb_93\\2b3027889e2fdc42.final.pdb
1.349781e-15  4.966543e-15 6.945082e-15  1.339744e-15 -8.251085e-15
-3.686767e-15
E:\\R-3.1.2\\scripts\\mPDBS\\summary_pdb_93\\2fcb3b46e33793b0.final.pdb
-1.645469e-15  5.902873e-15 7.502381e-15  5.905707e-15 -1.691993e-15
-5.635871e-15
E:\\R-3.1.2\\scripts\\mPDBS\\summary_pdb_93\\34eb328251842006.final.pdb
```

1.436388e-15 7.834557e-15 1.065693e-14 5.624689e-15 -1.754253e-14
-9.257582e-15
E:\\R-3.1.2\\scripts\\mPDBS\\summary_pdb_93\\354b32871797b7ab.final.pdb
3.872726e-15 1.487750e-14 8.757903e-15 -8.963656e-16 3.543938e-15
-2.023656e-14
E:\\R-3.1.2\\scripts\\mPDBS\\summary_pdb_93\\397368adbc32d5de.final.pdb
3.960993e-15 5.297962e-15 4.934390e-15 -6.437371e-15 -1.098394e-14
-5.571307e-15
E:\\R-3.1.2\\scripts\\mPDBS\\summary_pdb_93\\411ca13cd3df6edd.final.pdb
-2.788651e-15 6.375585e-15 7.248677e-15 2.067632e-15 -3.790141e-15
-1.472149e-14
E:\\R-3.1.2\\scripts\\mPDBS\\summary_pdb_93\\42357eee6642bf12.final.pdb
3.603499e-15 5.350582e-15 1.129808e-14 4.309969e-15 -1.775193e-14
-2.760278e-15
E:\\R-3.1.2\\scripts\\mPDBS\\summary_pdb_93\\44342d1f75a7fcfc.final.pdb
-4.752971e-15 7.339422e-15 9.220169e-15 4.322834e-15 -5.856500e-15
-3.411635e-15
E:\\R-3.1.2\\scripts\\mPDBS\\summary_pdb_93\\448ef21dffe8cdfb.final.pdb
1.671398e-14 2.096890e-15 4.423247e-15 -8.338107e-15 -2.074620e-14
-1.894120e-14
E:\\R-3.1.2\\scripts\\mPDBS\\summary_pdb_93\\472a40b3e8f113c7.final.pdb
-6.663816e-16 3.487778e-15 6.407604e-15 4.504397e-15 -6.521364e-15
-2.961415e-15
E:\\R-3.1.2\\scripts\\mPDBS\\summary_pdb_93\\48a26225951ea153.final.pdb
4.431951e-15 8.987632e-15 1.298019e-14 1.999806e-15 -7.263594e-15
-7.978472e-15
E:\\R-3.1.2\\scripts\\mPDBS\\summary_pdb_93\\4ab4a5c7c6682ce6.final.pdb
8.648840e-17 1.303830e-14 1.473037e-14 4.527816e-15 -1.594315e-15
-2.332578e-15
E:\\R-3.1.2\\scripts\\mPDBS\\summary_pdb_93\\4eaa45b62cb6a25f.final.pdb
1.770241e-15 2.819440e-15 5.159436e-15 6.575130e-16 -5.875610e-15
-7.677538e-16
E:\\R-3.1.2\\scripts\\mPDBS\\summary_pdb_93\\58c371611d85ac96.final.pdb
3.960993e-15 5.297962e-15 4.934390e-15 -6.437371e-15 -1.098394e-14
-5.571307e-15
E:\\R-3.1.2\\scripts\\mPDBS\\summary_pdb_93\\59a647f04822d30a.final.pdb
4.593719e-15 7.813001e-15 8.090431e-15 1.739831e-15 -1.371740e-14
-5.580153e-16
E:\\R-3.1.2\\scripts\\mPDBS\\summary_pdb_93\\59b091fe385e05fc.final.pdb
-6.598962e-16 4.487759e-15 6.445482e-15 4.339948e-15 -6.202377e-15
-4.795255e-15
E:\\R-3.1.2\\scripts\\mPDBS\\summary_pdb_93\\5c35ac30f930d2db.final.pdb
1.770241e-15 2.819440e-15 5.159436e-15 6.575130e-16 -5.875610e-15
-7.677538e-16
E:\\R-3.1.2\\scripts\\mPDBS\\summary_pdb_93\\5f60752431cf2dcb.final.pdb
4.242593e-16 6.131597e-15 4.597890e-15 4.646800e-15 -4.536569e-15
-6.959272e-15
E:\\R-3.1.2\\scripts\\mPDBS\\summary_pdb_93\\626456dd6ff2868d.final.pdb
-6.663816e-16 3.487778e-15 6.407604e-15 4.504397e-15 -6.521364e-15
-2.961415e-15
E:\\R-3.1.2\\scripts\\mPDBS\\summary_pdb_93\\6583d53ff3c8f7ef.final.pdb
4.593719e-15 7.813001e-15 8.090431e-15 1.739831e-15 -1.371740e-14
-5.580153e-16
E:\\R-3.1.2\\scripts\\mPDBS\\summary_pdb_93\\69760178986bef01.final.pdb

-4.026247e-15 2.440183e-16 4.388727e-15 5.410632e-15 3.621894e-15
2.740872e-16
E:\\R-3.1.2\\scripts\\mPDBS\\summary_pdb_93\\6a9d87d47926b1c8.final.pdb
9.555849e-16 5.696949e-15 6.928455e-15 1.224902e-15 -8.685431e-15
-3.590253e-15
E:\\R-3.1.2\\scripts\\mPDBS\\summary_pdb_93\\6bb3547ea982ffc4.final.pdb
-2.981317e-15 7.395078e-15 2.686931e-15 -2.848719e-15 -1.395604e-14
-4.783264e-15
E:\\R-3.1.2\\scripts\\mPDBS\\summary_pdb_93\\716d9624611d1c42.final.pdb
1.454685e-15 4.344832e-15 5.680791e-15 -3.753598e-15 -2.296840e-16
-9.888353e-15
E:\\R-3.1.2\\scripts\\mPDBS\\summary_pdb_93\\75f27e70ad0f1be4.final.pdb
4.593719e-15 7.813001e-15 8.090431e-15 1.739831e-15 -1.371740e-14
-5.580153e-16
E:\\R-3.1.2\\scripts\\mPDBS\\summary_pdb_93\\769f910fffb22872.final.pdb
1.551886e-15 5.126104e-15 7.138936e-15 1.257223e-15 -8.410005e-15
-4.098637e-15
E:\\R-3.1.2\\scripts\\mPDBS\\summary_pdb_93\\791f8c80dd5c3135.final.pdb
-6.598962e-16 4.487759e-15 6.445482e-15 4.339948e-15 -6.202377e-15
-4.795255e-15
E:\\R-3.1.2\\scripts\\mPDBS\\summary_pdb_93\\7fad98e66a3f6099.final.pdb
-3.396706e-15 7.450438e-15 7.914388e-15 4.443581e-15 2.661875e-15
-6.140411e-15
E:\\R-3.1.2\\scripts\\mPDBS\\summary_pdb_93\\804b89c94a633196.final.pdb
-1.645469e-15 5.902873e-15 7.502381e-15 5.905707e-15 -1.691993e-15
-5.635871e-15
E:\\R-3.1.2\\scripts\\mPDBS\\summary_pdb_93\\813cc65a2298feba.final.pdb
-1.645469e-15 5.902873e-15 7.502381e-15 5.905707e-15 -1.691993e-15
-5.635871e-15
E:\\R-3.1.2\\scripts\\mPDBS\\summary_pdb_93\\83c0eefcfe12d3f9.final.pdb
-2.788651e-15 6.375585e-15 7.248677e-15 2.067632e-15 -3.790141e-15
-1.472149e-14
E:\\R-3.1.2\\scripts\\mPDBS\\summary_pdb_93\\86d50469ef569760.final.pdb
-6.663816e-16 3.487778e-15 6.407604e-15 4.504397e-15 -6.521364e-15
-2.961415e-15
E:\\R-3.1.2\\scripts\\mPDBS\\summary_pdb_93\\89d0666e1402203d.final.pdb
4.593719e-15 7.813001e-15 8.090431e-15 1.739831e-15 -1.371740e-14
-5.580153e-16
E:\\R-3.1.2\\scripts\\mPDBS\\summary_pdb_93\\8b34852716dadc62.final.pdb
-2.741814e-15 3.571405e-15 7.871877e-15 -2.146879e-15 -7.831179e-15
1.067101e-15
E:\\R-3.1.2\\scripts\\mPDBS\\summary_pdb_93\\8b6ef9ac3fe74395.final.pdb
-3.827341e-15 -2.030693e-15 1.741382e-15 8.947157e-16 -1.107388e-14
-1.846360e-15
E:\\R-3.1.2\\scripts\\mPDBS\\summary_pdb_93\\98bbe0d5986c138f.final.pdb
-6.598962e-16 4.487759e-15 6.445482e-15 4.339948e-15 -6.202377e-15
-4.795255e-15
E:\\R-3.1.2\\scripts\\mPDBS\\summary_pdb_93\\9c22f4909d67e2fa.final.pdb
8.638406e-15 3.125581e-15 4.621656e-15 -7.950958e-16 -1.366202e-14
4.316238e-15
E:\\R-3.1.2\\scripts\\mPDBS\\summary_pdb_93\\9dda8ed0d81fb3ef.final.pdb
3.414541e-15 7.937034e-15 8.147243e-15 8.753318e-15 -4.758211e-16
-8.432792e-15
E:\\R-3.1.2\\scripts\\mPDBS\\summary_pdb_93\\9f752b3616e6033b.final.pdb

8.033138e-16 3.641354e-15 7.326332e-15 2.288597e-16 -1.015953e-14
-1.479421e-15
E:\\R-3.1.2\\scripts\\mPDBS\\summary_pdb_93\\a02c70083816d6ba.final.pdb
1.770241e-15 2.819440e-15 5.159436e-15 6.575130e-16 -5.875610e-15
-7.677538e-16
E:\\R-3.1.2\\scripts\\mPDBS\\summary_pdb_93\\a0c997d891e4e082.final.pdb
-3.582584e-16 8.124685e-15 4.849659e-15 -7.456225e-16 -5.317469e-16
-1.569241e-16
E:\\R-3.1.2\\scripts\\mPDBS\\summary_pdb_93\\ab8039a2d823c616.final.pdb
1.215136e-15 4.353396e-15 6.038161e-15 1.421481e-15 -7.319286e-15
-4.118165e-15
E:\\R-3.1.2\\scripts\\mPDBS\\summary_pdb_93\\acf4d74b9c71d4dc.final.pdb
-1.645469e-15 5.902873e-15 7.502381e-15 5.905707e-15 -1.691993e-15
-5.635871e-15
E:\\R-3.1.2\\scripts\\mPDBS\\summary_pdb_93\\ad8a823e4b4c668e.final.pdb
1.471525e-15 5.416231e-15 7.156675e-15 9.412257e-16 -8.124422e-15
-4.536780e-15
E:\\R-3.1.2\\scripts\\mPDBS\\summary_pdb_93\\b16ababd03b7b857.final.pdb
8.638406e-15 3.125581e-15 4.621656e-15 -7.950958e-16 -1.366202e-14
4.316238e-15
E:\\R-3.1.2\\scripts\\mPDBS\\summary_pdb_93\\b226923e697d3c63.final.pdb
2.668609e-15 -1.372363e-16 2.170036e-15 -5.085005e-15 -1.926050e-15
-7.835180e-15
E:\\R-3.1.2\\scripts\\mPDBS\\summary_pdb_93\\b28e48f6fa27a165.final.pdb
-6.598962e-16 4.487759e-15 6.445482e-15 4.339948e-15 -6.202377e-15
-4.795255e-15
E:\\R-3.1.2\\scripts\\mPDBS\\summary_pdb_93\\b42c4924e3cd1aec.final.pdb
-2.908526e-15 1.349422e-15 6.037602e-15 -1.718774e-15 -6.627584e-15
-3.606792e-15
E:\\R-3.1.2\\scripts\\mPDBS\\summary_pdb_93\\b4dd239fcabff1df.final.pdb
-6.598962e-16 4.487759e-15 6.445482e-15 4.339948e-15 -6.202377e-15
-4.795255e-15
E:\\R-3.1.2\\scripts\\mPDBS\\summary_pdb_93\\b669942ef97459a2.final.pdb
-2.741814e-15 3.571405e-15 7.871877e-15 -2.146879e-15 -7.831179e-15
1.067101e-15
E:\\R-3.1.2\\scripts\\mPDBS\\summary_pdb_93\\b99f4d9d87c1017b.final.pdb
-6.598962e-16 4.487759e-15 6.445482e-15 4.339948e-15 -6.202377e-15
-4.795255e-15
E:\\R-3.1.2\\scripts\\mPDBS\\summary_pdb_93\\b9d2cbf3975403e7.final.pdb
-6.598962e-16 4.487759e-15 6.445482e-15 4.339948e-15 -6.202377e-15
-4.795255e-15
E:\\R-3.1.2\\scripts\\mPDBS\\summary_pdb_93\\bc88c8a505f3a288.final.pdb
8.332671e-16 4.920572e-15 6.384983e-15 1.069561e-15 -7.377218e-15
-4.350298e-15
E:\\R-3.1.2\\scripts\\mPDBS\\summary_pdb_93\\bdac0e64f4ef427e.final.pdb
9.513574e-15 2.075206e-15 7.802813e-15 5.313172e-16 -1.790559e-14
1.766407e-14
E:\\R-3.1.2\\scripts\\mPDBS\\summary_pdb_93\\ca2a1f55fc9d0681.final.pdb
-2.741814e-15 3.571405e-15 7.871877e-15 -2.146879e-15 -7.831179e-15
1.067101e-15
E:\\R-3.1.2\\scripts\\mPDBS\\summary_pdb_93\\ce34248de3e81a3c.final.pdb
4.028044e-15 1.025289e-14 8.590526e-15 3.510897e-15 -2.879046e-15
-7.520194e-15
E:\\R-3.1.2\\scripts\\mPDBS\\summary_pdb_93\\d02e697772494a61.final.pdb

3.597549e-15 5.337773e-15 5.814693e-15 -3.709602e-15 -1.601224e-14
-5.963567e-16
E:\\R-3.1.2\\scripts\\mPDBS\\summary_pdb_93\\d04221245d015047.final.pdb
7.953327e-16 6.868745e-15 8.155175e-15 3.324039e-15 -9.386643e-15
-3.522288e-15
E:\\R-3.1.2\\scripts\\mPDBS\\summary_pdb_93\\d678141e4018054a.final.pdb
2.928561e-15 1.211909e-14 5.398690e-15 -7.427154e-15 -9.260088e-15
-1.858638e-14
E:\\R-3.1.2\\scripts\\mPDBS\\summary_pdb_93\\d71b40af0f0efe39.final.pdb
8.640855e-15 2.394558e-16 6.233016e-15 -1.040103e-14 -1.909015e-15
-1.061184e-14
E:\\R-3.1.2\\scripts\\mPDBS\\summary_pdb_93\\dc0809eff10197e2.final.pdb
1.277860e-15 -1.315102e-15 8.358704e-15 3.760468e-15 -1.158852e-14
5.102376e-15
E:\\R-3.1.2\\scripts\\mPDBS\\summary_pdb_93\\e1163918d3707839.final.pdb
-3.907727e-15 -1.542362e-15 8.544278e-15 1.179949e-15 -6.212552e-15
-9.693936e-15
E:\\R-3.1.2\\scripts\\mPDBS\\summary_pdb_93\\e4b48b06824ef848.final.pdb
7.533646e-15 2.161462e-15 7.763653e-15 -3.632529e-15 -8.438628e-15
-9.535260e-15
E:\\R-3.1.2\\scripts\\mPDBS\\summary_pdb_93\\e529e19ad281fea2.final.pdb
2.306067e-15 7.588664e-15 6.113568e-15 2.960493e-15 -6.733000e-15
-5.416049e-15
E:\\R-3.1.2\\scripts\\mPDBS\\summary_pdb_93\\e5602082718ec3a1.final.pdb
-1.645469e-15 5.902873e-15 7.502381e-15 5.905707e-15 -1.691993e-15
-5.635871e-15
E:\\R-3.1.2\\scripts\\mPDBS\\summary_pdb_93\\e5d589d996d0a960.final.pdb
-2.617812e-15 5.198443e-15 5.797788e-15 -5.833240e-16 -2.997715e-15
-1.422470e-15
E:\\R-3.1.2\\scripts\\mPDBS\\summary_pdb_93\\e9df9e72307a2fbd.final.pdb
1.770241e-15 2.819440e-15 5.159436e-15 6.575130e-16 -5.875610e-15
-7.677538e-16
E:\\R-3.1.2\\scripts\\mPDBS\\summary_pdb_93\\ea00f8694090cae7.final.pdb
-1.645469e-15 5.902873e-15 7.502381e-15 5.905707e-15 -1.691993e-15
-5.635871e-15
E:\\R-3.1.2\\scripts\\mPDBS\\summary_pdb_93\\eba517be32db050e.final.pdb
-6.598962e-16 4.487759e-15 6.445482e-15 4.339948e-15 -6.202377e-15
-4.795255e-15
E:\\R-3.1.2\\scripts\\mPDBS\\summary_pdb_93\\ec04b9545899c29d.final.pdb
1.557085e-15 5.462451e-15 6.654513e-15 7.128748e-16 -7.805461e-15
-4.212210e-15
E:\\R-3.1.2\\scripts\\mPDBS\\summary_pdb_93\\ee6862cbcad52078.final.pdb
1.119063e-15 5.292671e-15 6.822784e-15 1.080403e-15 -8.051592e-15
-3.770033e-15
E:\\R-3.1.2\\scripts\\mPDBS\\summary_pdb_93\\ef6a42fc83b81e5d.final.pdb
1.677219e-15 5.153860e-15 6.912337e-15 1.041683e-15 -7.144091e-15
-4.113382e-15
E:\\R-3.1.2\\scripts\\mPDBS\\summary_pdb_93\\ef8e88d4ad93a0ad.final.pdb
7.953327e-16 6.868745e-15 8.155175e-15 3.324039e-15 -9.386643e-15
-3.522288e-15
E:\\R-3.1.2\\scripts\\mPDBS\\summary_pdb_93\\f14fcae8deec4fa3.final.pdb
8.033138e-16 3.641354e-15 7.326332e-15 2.288597e-16 -1.015953e-14
-1.479421e-15
E:\\R-3.1.2\\scripts\\mPDBS\\summary_pdb_93\\f83584e22003fb92.final.pdb

4.431951e-15 8.987632e-15 1.298019e-14 1.999806e-15 -7.263594e-15
-7.978472e-15
E:\\R-3.1.2\\scripts\\mPDBS\\summary_pdb_93\\f8e2bf87e52caa29.final.pdb
2.306067e-15 7.588664e-15 6.113568e-15 2.960493e-15 -6.733000e-15
-5.416049e-15

[,535]   [,536]   [,537]   [,538]   [,539]   [,540]
E:\\R-3.1.2\\scripts\\40.1ns_data\\NAT0.pdb
-1.773908e-16 -6.625266e-15 3.333977e-15 3.753675e-15 -4.747456e-16
1.498772e-15
E:\\R-3.1.2\\scripts\\40.1ns_data\\LJP6.pdb
-2.123176e-16 -4.943372e-15 2.693114e-15 1.758077e-15 1.516482e-16
1.720196e-15
E:\\R-3.1.2\\scripts\\40.1ns_data\\3WY9.pdb
8.121193e-16 -4.067136e-15 2.630061e-15 2.181850e-15 1.344700e-15
1.938640e-15
E:\\R-3.1.2\\scripts\\40.1ns_data\\ZSP9.pdb
5.216320e-16 -4.561179e-15 1.987361e-15 1.327529e-15 1.830152e-15
3.410698e-15
E:\\R-3.1.2\\scripts\\mPDBS\\summary_pdb_93\\013d855ad26c877c.final.pdb
-3.348172e-16 -1.417374e-14 5.247672e-15 8.827213e-15 3.026292e-15
6.557264e-15
E:\\R-3.1.2\\scripts\\mPDBS\\summary_pdb_93\\0a2a705342ca0d54.final.pdb
6.186822e-16 -4.938380e-15 3.407489e-15 2.188936e-15 1.433900e-15
3.299400e-15
E:\\R-3.1.2\\scripts\\mPDBS\\summary_pdb_93\\0a59fceadb4194d9.final.pdb
1.390547e-15 -3.715823e-15 3.555509e-17 4.973182e-16 6.095338e-15
2.321950e-15
E:\\R-3.1.2\\scripts\\mPDBS\\summary_pdb_93\\0dba59f3306825e8.final.pdb
5.177994e-16 -2.956690e-15 4.358627e-15 -2.248129e-15 4.931018e-15
3.283841e-15
E:\\R-3.1.2\\scripts\\mPDBS\\summary_pdb_93\\126f26a78b57339c.final.pdb
-3.217711e-15 -2.193195e-15 5.073766e-15 -1.968575e-15 -8.887355e-15
4.677691e-15
E:\\R-3.1.2\\scripts\\mPDBS\\summary_pdb_93\\12f06f3e342df706.final.pdb
5.618700e-16 -4.682292e-15 3.320970e-15 1.888090e-15 1.055731e-15
3.264705e-15
E:\\R-3.1.2\\scripts\\mPDBS\\summary_pdb_93\\18618a2a251b867b.final.pdb
-2.968143e-15 -8.311831e-15 3.044699e-15 2.003881e-16 -1.076938e-14
8.695197e-15
E:\\R-3.1.2\\scripts\\mPDBS\\summary_pdb_93\\1f305f598641eeb2.final.pdb
1.833065e-16 -4.674491e-15 2.552085e-15 1.877728e-15 5.754898e-16
2.988222e-15
E:\\R-3.1.2\\scripts\\mPDBS\\summary_pdb_93\\1f8a00b551be0a39.final.pdb
-2.968143e-15 -8.311831e-15 3.044699e-15 2.003881e-16 -1.076938e-14
8.695197e-15
E:\\R-3.1.2\\scripts\\mPDBS\\summary_pdb_93\\219f45328d47579a.final.pdb
1.102000e-14 -3.603608e-15 6.520168e-15 1.524030e-14 -4.124771e-16
-4.915317e-15
E:\\R-3.1.2\\scripts\\mPDBS\\summary_pdb_93\\238dd3918e54336d.final.pdb
6.133913e-16 2.367419e-15 8.808702e-15 5.547661e-15 -2.114674e-15
7.259840e-15
E:\\R-3.1.2\\scripts\\mPDBS\\summary_pdb_93\\2b3027889e2fdc42.final.pdb
9.490729e-18 -5.532512e-15 3.360586e-15 2.161182e-15 1.585491e-15

4.225809e-15
E:\\R-3.1.2\\scripts\\mPDBS\\summary_pdb_93\\2fcb3b46e33793b0.final.pdb
7.601020e-16 -6.388615e-15 3.171381e-15 4.274249e-15 3.481152e-15
-1.915263e-16
E:\\R-3.1.2\\scripts\\mPDBS\\summary_pdb_93\\34eb328251842006.final.pdb
1.378528e-16 -4.853457e-15 -9.678990e-15 -4.002516e-15 1.243051e-14
-7.872919e-15
E:\\R-3.1.2\\scripts\\mPDBS\\summary_pdb_93\\354b32871797b7ab.final.pdb
1.686766e-15 -1.465769e-15 -5.230062e-15 8.645641e-15 2.836208e-16
-4.159958e-15
E:\\R-3.1.2\\scripts\\mPDBS\\summary_pdb_93\\397368adbc32d5de.final.pdb
-3.217711e-15 -2.193195e-15 5.073766e-15 -1.968575e-15 -8.887355e-15
4.677691e-15
E:\\R-3.1.2\\scripts\\mPDBS\\summary_pdb_93\\411ca13cd3df6edd.final.pdb
-5.087868e-15 -3.555486e-15 1.429285e-15 -2.585979e-15 -5.251880e-15
-7.728032e-15
E:\\R-3.1.2\\scripts\\mPDBS\\summary_pdb_93\\42357eee6642bf12.final.pdb
5.861025e-15 -2.086264e-15 -1.593998e-14 -1.173672e-15 5.901070e-15
-3.785690e-15
E:\\R-3.1.2\\scripts\\mPDBS\\summary_pdb_93\\44342d1f75a7fcfc.final.pdb
-6.543941e-15 -1.359863e-14 4.745583e-15 -8.653809e-16 1.019031e-15
1.025708e-14
E:\\R-3.1.2\\scripts\\mPDBS\\summary_pdb_93\\448ef21dffe8cdfb.final.pdb
-2.169846e-15 -7.354964e-15 -2.342438e-15 1.361920e-14 -1.274719e-14
7.566157e-15
E:\\R-3.1.2\\scripts\\mPDBS\\summary_pdb_93\\472a40b3e8f113c7.final.pdb
5.177994e-16 -2.956690e-15 4.358627e-15 -2.248129e-15 4.931018e-15
3.283841e-15
E:\\R-3.1.2\\scripts\\mPDBS\\summary_pdb_93\\48a26225951ea153.final.pdb
-7.592068e-15 -1.685505e-14 4.178725e-15 5.661041e-16 -8.308505e-15
4.873728e-15
E:\\R-3.1.2\\scripts\\mPDBS\\summary_pdb_93\\4ab4a5c7c6682ce6.final.pdb
3.182551e-15 -6.888983e-15 6.406901e-15 1.872970e-15 -1.374268e-15
-7.381154e-16
E:\\R-3.1.2\\scripts\\mPDBS\\summary_pdb_93\\4eaa45b62cb6a25f.final.pdb
-2.229806e-15 -6.409839e-15 9.436158e-15 -5.120929e-16 -1.666642e-15
7.071509e-15
E:\\R-3.1.2\\scripts\\mPDBS\\summary_pdb_93\\58c371611d85ac96.final.pdb
-3.217711e-15 -2.193195e-15 5.073766e-15 -1.968575e-15 -8.887355e-15
4.677691e-15
E:\\R-3.1.2\\scripts\\mPDBS\\summary_pdb_93\\59a647f04822d30a.final.pdb
2.716881e-15 -2.878580e-15 -2.642604e-15 2.530340e-15 6.965663e-15
4.619224e-15
E:\\R-3.1.2\\scripts\\mPDBS\\summary_pdb_93\\59b091fe385e05fc.final.pdb
1.390547e-15 -3.715823e-15 3.555509e-17 4.973182e-16 6.095338e-15
2.321950e-15
E:\\R-3.1.2\\scripts\\mPDBS\\summary_pdb_93\\5c35ac30f930d2db.final.pdb
-2.229806e-15 -6.409839e-15 9.436158e-15 -5.120929e-16 -1.666642e-15
7.071509e-15
E:\\R-3.1.2\\scripts\\mPDBS\\summary_pdb_93\\5f60752431cf2dcb.final.pdb
-3.710103e-15 -4.786484e-15 5.040948e-15 -4.405351e-15 3.860345e-15
5.847709e-15
E:\\R-3.1.2\\scripts\\mPDBS\\summary_pdb_93\\626456dd6ff2868d.final.pdb
5.177994e-16 -2.956690e-15 4.358627e-15 -2.248129e-15 4.931018e-15

3.283841e-15
E:\\R-3.1.2\\scripts\\mPDBS\\summary_pdb_93\\6583d53ff3c8f7ef.final.pdb
2.716881e-15 -2.878580e-15 -2.642604e-15 2.530340e-15 6.965663e-15
4.619224e-15
E:\\R-3.1.2\\scripts\\mPDBS\\summary_pdb_93\\69760178986bef01.final.pdb
4.190176e-15 -5.924711e-15 1.775941e-14 1.140305e-14 -8.687721e-15
8.910004e-15
E:\\R-3.1.2\\scripts\\mPDBS\\summary_pdb_93\\6a9d87d47926b1c8.final.pdb
1.251013e-16 -5.462606e-15 3.203734e-15 1.589343e-15 1.163632e-15
3.714920e-15
E:\\R-3.1.2\\scripts\\mPDBS\\summary_pdb_93\\6bb3547ea982ffc4.final.pdb
1.281789e-15 -3.437029e-15 1.318609e-14 -2.906599e-15 -4.303332e-15
1.771668e-15
E:\\R-3.1.2\\scripts\\mPDBS\\summary_pdb_93\\716d9624611d1c42.final.pdb
-7.239938e-16 -7.878628e-15 6.528907e-15 4.870384e-15 -5.391369e-15
-2.326308e-15
E:\\R-3.1.2\\scripts\\mPDBS\\summary_pdb_93\\75f27e70ad0f1be4.final.pdb
2.716881e-15 -2.878580e-15 -2.642604e-15 2.530340e-15 6.965663e-15
4.619224e-15
E:\\R-3.1.2\\scripts\\mPDBS\\summary_pdb_93\\769f910fffb22872.final.pdb
3.010957e-17 -4.604727e-15 3.283874e-15 1.710329e-15 1.479506e-15
3.581232e-15
E:\\R-3.1.2\\scripts\\mPDBS\\summary_pdb_93\\791f8c80dd5c3135.final.pdb
1.390547e-15 -3.715823e-15 3.555509e-17 4.973182e-16 6.095338e-15
2.321950e-15
E:\\R-3.1.2\\scripts\\mPDBS\\summary_pdb_93\\7fad98e66a3f6099.final.pdb
5.497039e-16 -1.120011e-14 2.599982e-15 4.621798e-15 9.977246e-15
-5.162925e-15
E:\\R-3.1.2\\scripts\\mPDBS\\summary_pdb_93\\804b89c94a633196.final.pdb
7.601020e-16 -6.388615e-15 3.171381e-15 4.274249e-15 3.481152e-15
-1.915263e-16
E:\\R-3.1.2\\scripts\\mPDBS\\summary_pdb_93\\813cc65a2298feba.final.pdb
7.601020e-16 -6.388615e-15 3.171381e-15 4.274249e-15 3.481152e-15
-1.915263e-16
E:\\R-3.1.2\\scripts\\mPDBS\\summary_pdb_93\\83c0eefcfe12d3f9.final.pdb
-5.087868e-15 -3.555486e-15 1.429285e-15 -2.585979e-15 -5.251880e-15
-7.728032e-15
E:\\R-3.1.2\\scripts\\mPDBS\\summary_pdb_93\\86d50469ef569760.final.pdb
5.177994e-16 -2.956690e-15 4.358627e-15 -2.248129e-15 4.931018e-15
3.283841e-15
E:\\R-3.1.2\\scripts\\mPDBS\\summary_pdb_93\\89d0666e1402203d.final.pdb
2.716881e-15 -2.878580e-15 -2.642604e-15 2.530340e-15 6.965663e-15
4.619224e-15
E:\\R-3.1.2\\scripts\\mPDBS\\summary_pdb_93\\8b34852716dadc62.final.pdb
5.302512e-16 2.299097e-15 9.700167e-16 -4.025536e-15 -1.346584e-15
6.081813e-16
E:\\R-3.1.2\\scripts\\mPDBS\\summary_pdb_93\\8b6ef9ac3fe74395.final.pdb
7.358103e-15 3.549978e-16 2.206872e-15 1.039797e-15 1.256972e-14
8.286167e-15
E:\\R-3.1.2\\scripts\\mPDBS\\summary_pdb_93\\98bbe0d5986c138f.final.pdb
1.390547e-15 -3.715823e-15 3.555509e-17 4.973182e-16 6.095338e-15
2.321950e-15
E:\\R-3.1.2\\scripts\\mPDBS\\summary_pdb_93\\9c22f4909d67e2fa.final.pdb
-5.912476e-16 -8.949717e-15 1.999792e-15 6.056982e-15 2.442486e-15

1.281196e-14
E:\\R-3.1.2\\scripts\\mPDBS\\summary_pdb_93\\9dda8ed0d81fb3ef.final.pdb
-9.861034e-16 -1.503639e-14 9.361032e-15 -9.114594e-15 -8.223142e-15
6.210532e-17
E:\\R-3.1.2\\scripts\\mPDBS\\summary_pdb_93\\9f752b3616e6033b.final.pdb
2.628121e-15 -3.260179e-15 2.031086e-15 4.645676e-15 3.728516e-15
5.270628e-15
E:\\R-3.1.2\\scripts\\mPDBS\\summary_pdb_93\\a02c70083816d6ba.final.pdb
-2.229806e-15 -6.409839e-15 9.436158e-15 -5.120929e-16 -1.666642e-15
7.071509e-15
E:\\R-3.1.2\\scripts\\mPDBS\\summary_pdb_93\\a0c997d891e4e082.final.pdb
-3.555690e-15 -1.475271e-15 1.629356e-14 8.960708e-15 -1.522991e-15
3.041985e-15
E:\\R-3.1.2\\scripts\\mPDBS\\summary_pdb_93\\ab8039a2d823c616.final.pdb
-1.241261e-18 -5.023322e-15 3.106412e-15 1.755663e-15 1.218306e-15
3.078415e-15
E:\\R-3.1.2\\scripts\\mPDBS\\summary_pdb_93\\acf4d74b9c71d4dc.final.pdb
7.601020e-16 -6.388615e-15 3.171381e-15 4.274249e-15 3.481152e-15
-1.915263e-16
E:\\R-3.1.2\\scripts\\mPDBS\\summary_pdb_93\\ad8a823e4b4c668e.final.pdb
1.581549e-16 -4.929388e-15 2.899365e-15 2.144760e-15 1.289339e-15
3.122963e-15
E:\\R-3.1.2\\scripts\\mPDBS\\summary_pdb_93\\b16ababd03b7b857.final.pdb
-5.912476e-16 -8.949717e-15 1.999792e-15 6.056982e-15 2.442486e-15
1.281196e-14
E:\\R-3.1.2\\scripts\\mPDBS\\summary_pdb_93\\b226923e697d3c63.final.pdb
1.592129e-15 -5.350773e-16 7.272859e-15 5.103938e-15 4.491747e-16
4.635704e-15
E:\\R-3.1.2\\scripts\\mPDBS\\summary_pdb_93\\b28e48f6fa27a165.final.pdb
1.390547e-15 -3.715823e-15 3.555509e-17 4.973182e-16 6.095338e-15
2.321950e-15
E:\\R-3.1.2\\scripts\\mPDBS\\summary_pdb_93\\b42c4924e3cd1aec.final.pdb
1.158232e-15 2.093788e-15 8.374408e-15 9.410125e-15 5.856741e-15
2.590465e-15
E:\\R-3.1.2\\scripts\\mPDBS\\summary_pdb_93\\b4dd239fcabff1df.final.pdb
1.390547e-15 -3.715823e-15 3.555509e-17 4.973182e-16 6.095338e-15
2.321950e-15
E:\\R-3.1.2\\scripts\\mPDBS\\summary_pdb_93\\b669942ef97459a2.final.pdb
5.302512e-16 2.299097e-15 9.700167e-16 -4.025536e-15 -1.346584e-15
6.081813e-16
E:\\R-3.1.2\\scripts\\mPDBS\\summary_pdb_93\\b99f4d9d87c1017b.final.pdb
1.390547e-15 -3.715823e-15 3.555509e-17 4.973182e-16 6.095338e-15
2.321950e-15
E:\\R-3.1.2\\scripts\\mPDBS\\summary_pdb_93\\b9d2cbf3975403e7.final.pdb
1.390547e-15 -3.715823e-15 3.555509e-17 4.973182e-16 6.095338e-15
2.321950e-15
E:\\R-3.1.2\\scripts\\mPDBS\\summary_pdb_93\\bc88c8a505f3a288.final.pdb
4.464242e-16 -5.747292e-15 3.971209e-15 1.927896e-15 1.490948e-15
3.189312e-15
E:\\R-3.1.2\\scripts\\mPDBS\\summary_pdb_93\\bdac0e64f4ef427e.final.pdb
1.045092e-14 -6.231297e-15 3.621325e-15 9.294579e-15 1.289550e-14
2.545463e-14
E:\\R-3.1.2\\scripts\\mPDBS\\summary_pdb_93\\ca2a1f55fc9d0681.final.pdb
5.302512e-16 2.299097e-15 9.700167e-16 -4.025536e-15 -1.346584e-15

6.081813e-16
E:\\R-3.1.2\\scripts\\mPDBS\\summary_pdb_93\\ce34248de3e81a3c.final.pdb
-2.879717e-15 -5.773270e-15 -1.623215e-15 -4.048911e-15 2.881261e-15
1.937695e-15
E:\\R-3.1.2\\scripts\\mPDBS\\summary_pdb_93\\d02e697772494a61.final.pdb
-2.487662e-16 -2.136791e-15 -8.362024e-15 5.803099e-15 6.281387e-15
7.148818e-15
E:\\R-3.1.2\\scripts\\mPDBS\\summary_pdb_93\\d04221245d015047.final.pdb
-1.209372e-16 -6.558010e-15 1.678518e-15 7.728136e-16 1.993294e-15
2.275238e-15
E:\\R-3.1.2\\scripts\\mPDBS\\summary_pdb_93\\d678141e4018054a.final.pdb
-4.656280e-15 -6.957805e-15 3.600587e-15 -8.295848e-15 -6.406288e-15
-7.583939e-15
E:\\R-3.1.2\\scripts\\mPDBS\\summary_pdb_93\\d71b40af0f0efe39.final.pdb
-7.319565e-15 -1.336982e-14 3.224770e-15 7.725555e-15 1.316431e-15
4.725012e-15
E:\\R-3.1.2\\scripts\\mPDBS\\summary_pdb_93\\dc0809eff10197e2.final.pdb
2.944480e-15 -3.279893e-15 5.995983e-15 6.281771e-15 1.410003e-14
1.093161e-14
E:\\R-3.1.2\\scripts\\mPDBS\\summary_pdb_93\\e1163918d3707839.final.pdb
1.215044e-15 -5.755402e-15 5.679081e-15 -5.931580e-16 -2.081353e-15
-4.567872e-15
E:\\R-3.1.2\\scripts\\mPDBS\\summary_pdb_93\\e4b48b06824ef848.final.pdb
-2.968143e-15 -8.311831e-15 3.044699e-15 2.003881e-16 -1.076938e-14
8.695197e-15
E:\\R-3.1.2\\scripts\\mPDBS\\summary_pdb_93\\e529e19ad281fea2.final.pdb
-3.348172e-16 -1.417374e-14 5.247672e-15 8.827213e-15 3.026292e-15
6.557264e-15
E:\\R-3.1.2\\scripts\\mPDBS\\summary_pdb_93\\e5602082718ec3a1.final.pdb
7.601020e-16 -6.388615e-15 3.171381e-15 4.274249e-15 3.481152e-15
-1.915263e-16
E:\\R-3.1.2\\scripts\\mPDBS\\summary_pdb_93\\e5d589d996d0a960.final.pdb
-4.439550e-15 -2.389871e-15 1.613761e-14 5.814603e-15 -3.456552e-15
8.486048e-15
E:\\R-3.1.2\\scripts\\mPDBS\\summary_pdb_93\\e9df9e72307a2fbd.final.pdb
-2.229806e-15 -6.409839e-15 9.436158e-15 -5.120929e-16 -1.666642e-15
7.071509e-15
E:\\R-3.1.2\\scripts\\mPDBS\\summary_pdb_93\\ea00f8694090cae7.final.pdb
7.601020e-16 -6.388615e-15 3.171381e-15 4.274249e-15 3.481152e-15
-1.915263e-16
E:\\R-3.1.2\\scripts\\mPDBS\\summary_pdb_93\\eba517be32db050e.final.pdb
1.390547e-15 -3.715823e-15 3.555509e-17 4.973182e-16 6.095338e-15
2.321950e-15
E:\\R-3.1.2\\scripts\\mPDBS\\summary_pdb_93\\ec04b9545899c29d.final.pdb
8.790728e-17 -4.918940e-15 2.459652e-15 1.868977e-15 9.930105e-16
3.509001e-15
E:\\R-3.1.2\\scripts\\mPDBS\\summary_pdb_93\\ee6862cbcad52078.final.pdb
1.970577e-16 -5.280218e-15 3.894881e-15 1.873388e-15 1.707355e-15
3.747892e-15
E:\\R-3.1.2\\scripts\\mPDBS\\summary_pdb_93\\ef6a42fc83b81e5d.final.pdb
2.738382e-16 -4.843034e-15 3.146831e-15 1.916199e-15 1.105239e-15
3.164898e-15
E:\\R-3.1.2\\scripts\\mPDBS\\summary_pdb_93\\ef8e88d4ad93a0ad.final.pdb
-1.209372e-16 -6.558010e-15 1.678518e-15 7.728136e-16 1.993294e-15

2.275238e-15
E:\\R-3.1.2\\scripts\\mPDBS\\summary_pdb_93\\f14fcae8deec4fa3.final.pdb
2.628121e-15 -3.260179e-15 2.031086e-15 4.645676e-15 3.728516e-15
5.270628e-15
E:\\R-3.1.2\\scripts\\mPDBS\\summary_pdb_93\\f83584e22003fb92.final.pdb
-7.592068e-15 -1.685505e-14 4.178725e-15 5.661041e-16 -8.308505e-15
4.873728e-15
E:\\R-3.1.2\\scripts\\mPDBS\\summary_pdb_93\\f8e2bf87e52caa29.final.pdb
-3.348172e-16 -1.417374e-14 5.247672e-15 8.827213e-15 3.026292e-15
6.557264e-15

[,541]   [,542]   [,543]   [,544]   [,545]   [,546]
E:\\R-3.1.2\\scripts\\40.1ns_data\\NAT0.pdb
-4.570933e-15 -6.667402e-15 -4.230781e-16 3.647044e-15 -2.657388e-15
-8.666313e-15
E:\\R-3.1.2\\scripts\\40.1ns_data\\LJP6.pdb
-5.907700e-15 -6.325428e-15 -1.207127e-15 4.530549e-15 -3.150227e-16
-8.960374e-15
E:\\R-3.1.2\\scripts\\40.1ns_data\\3WY9.pdb
-4.822237e-15 -5.859367e-15 -2.633890e-15 3.432264e-15 -1.078116e-15
-8.415375e-15
E:\\R-3.1.2\\scripts\\40.1ns_data\\ZSP9.pdb
-5.599561e-15 -6.538302e-15 -1.364978e-15 3.665074e-15 -1.471380e-15
-9.741091e-15
E:\\R-3.1.2\\scripts\\mPDBS\\summary_pdb_93\\013d855ad26c877c.final.pdb
-2.623542e-15 -7.235117e-15 -3.232859e-15 2.452273e-15 3.154861e-15
-6.078888e-15
E:\\R-3.1.2\\scripts\\mPDBS\\summary_pdb_93\\0a2a705342ca0d54.final.pdb
-5.451378e-15 -6.570444e-15 -2.252243e-15 4.818681e-15 -1.718201e-15
-8.748800e-15
E:\\R-3.1.2\\scripts\\mPDBS\\summary_pdb_93\\0a59fceadb4194d9.final.pdb
-6.571215e-15 -6.490903e-15 -6.327923e-16 4.510190e-15 -2.573530e-15
-6.543436e-15
E:\\R-3.1.2\\scripts\\mPDBS\\summary_pdb_93\\0dba59f3306825e8.final.pdb
-5.917782e-15 -8.537894e-15 -2.910200e-15 2.405436e-15 -5.167041e-15
-7.267174e-15
E:\\R-3.1.2\\scripts\\mPDBS\\summary_pdb_93\\126f26a78b57339c.final.pdb
-6.146766e-15 -8.506841e-16 -3.289888e-15 4.508788e-15 4.400934e-15
-1.291890e-14
E:\\R-3.1.2\\scripts\\mPDBS\\summary_pdb_93\\12f06f3e342df706.final.pdb
-5.185098e-15 -6.651108e-15 -2.060339e-15 4.611165e-15 -1.299509e-15
-8.972579e-15
E:\\R-3.1.2\\scripts\\mPDBS\\summary_pdb_93\\18618a2a251b867b.final.pdb
-4.012329e-15 -1.210875e-14 -5.610340e-15 1.460159e-15 7.677116e-15
-6.998940e-15
E:\\R-3.1.2\\scripts\\mPDBS\\summary_pdb_93\\1f305f598641eeb2.final.pdb
-5.141069e-15 -6.443617e-15 -2.215594e-15 4.139958e-15 -7.668703e-16
-8.403727e-15
E:\\R-3.1.2\\scripts\\mPDBS\\summary_pdb_93\\1f8a00b551be0a39.final.pdb
-4.012329e-15 -1.210875e-14 -5.610340e-15 1.460159e-15 7.677116e-15
-6.998940e-15
E:\\R-3.1.2\\scripts\\mPDBS\\summary_pdb_93\\219f45328d47579a.final.pdb
1.299225e-15 -1.422908e-15 -4.853591e-15 6.267682e-15 8.592714e-16
-2.157395e-14

E:\\R-3.1.2\\scripts\\mPDBS\\summary_pdb_93\\238dd3918e54336d.final.pdb
-4.808303e-15 -7.402066e-15 -1.450498e-15 -3.473875e-15 -1.834917e-15
-1.568890e-14
E:\\R-3.1.2\\scripts\\mPDBS\\summary_pdb_93\\2b3027889e2fdc42.final.pdb
-4.756695e-15 -5.884834e-15 -2.398090e-15 5.099996e-15 -7.495762e-16
-9.062255e-15
E:\\R-3.1.2\\scripts\\mPDBS\\summary_pdb_93\\2fcb3b46e33793b0.final.pdb
-3.603670e-15 -8.200892e-15 -2.008294e-15 4.786142e-15 2.397030e-16
-4.920402e-15
E:\\R-3.1.2\\scripts\\mPDBS\\summary_pdb_93\\34eb328251842006.final.pdb
-7.608594e-15 -1.017055e-14 -1.132898e-15 1.737332e-15 -9.750084e-15
-1.160021e-14
E:\\R-3.1.2\\scripts\\mPDBS\\summary_pdb_93\\354b32871797b7ab.final.pdb
-1.020534e-14 -8.493133e-16 -3.370358e-15 7.817349e-15 -6.425175e-15
-8.741219e-15
E:\\R-3.1.2\\scripts\\mPDBS\\summary_pdb_93\\397368adbc32d5de.final.pdb
-6.146766e-15 -8.506841e-16 -3.289888e-15 4.508788e-15 4.400934e-15
-1.291890e-14
E:\\R-3.1.2\\scripts\\mPDBS\\summary_pdb_93\\411ca13cd3df6edd.final.pdb
-5.584724e-15 -5.439862e-15 -3.515986e-15 5.796619e-15 3.798813e-15
-7.944025e-15
E:\\R-3.1.2\\scripts\\mPDBS\\summary_pdb_93\\42357eee6642bf12.final.pdb
-4.240107e-15 -8.169609e-15 5.914234e-16 3.139590e-15 -6.958832e-15
-1.297090e-14
E:\\R-3.1.2\\scripts\\mPDBS\\summary_pdb_93\\44342d1f75a7fcfc.final.pdb
-1.947102e-15 -7.013257e-15 4.544864e-15 6.983603e-15 1.697725e-14
-3.739645e-15
E:\\R-3.1.2\\scripts\\mPDBS\\summary_pdb_93\\448ef21dffe8cdfb.final.pdb
-2.839091e-15 -3.872757e-15 -2.451516e-15 1.105977e-14 2.103663e-15
-9.767220e-15
E:\\R-3.1.2\\scripts\\mPDBS\\summary_pdb_93\\472a40b3e8f113c7.final.pdb
-5.917782e-15 -8.537894e-15 -2.910200e-15 2.405436e-15 -5.167041e-15
-7.267174e-15
E:\\R-3.1.2\\scripts\\mPDBS\\summary_pdb_93\\48a26225951ea153.final.pdb
-6.119320e-15 -6.063510e-15 -9.131636e-15 7.306347e-15 9.885146e-15
-9.578335e-15
E:\\R-3.1.2\\scripts\\mPDBS\\summary_pdb_93\\4ab4a5c7c6682ce6.final.pdb
-5.742141e-15 -6.377513e-15 3.042720e-15 1.686913e-14 -1.702257e-15
-3.464660e-15
E:\\R-3.1.2\\scripts\\mPDBS\\summary_pdb_93\\4eaa45b62cb6a25f.final.pdb
-4.603916e-15 -7.472921e-15 -3.017559e-15 3.265434e-15 2.422077e-15
-6.981342e-15
E:\\R-3.1.2\\scripts\\mPDBS\\summary_pdb_93\\58c371611d85ac96.final.pdb
-6.146766e-15 -8.506841e-16 -3.289888e-15 4.508788e-15 4.400934e-15
-1.291890e-14
E:\\R-3.1.2\\scripts\\mPDBS\\summary_pdb_93\\59a647f04822d30a.final.pdb
-4.370511e-15 -5.124143e-15 -3.659728e-15 7.832533e-15 -8.682781e-15
-1.135768e-14
E:\\R-3.1.2\\scripts\\mPDBS\\summary_pdb_93\\59b091fe385e05fc.final.pdb
-6.571215e-15 -6.490903e-15 -6.327923e-16 4.510190e-15 -2.573530e-15
-6.543436e-15
E:\\R-3.1.2\\scripts\\mPDBS\\summary_pdb_93\\5c35ac30f930d2db.final.pdb
-4.603916e-15 -7.472921e-15 -3.017559e-15 3.265434e-15 2.422077e-15
-6.981342e-15

E:\\R-3.1.2\\scripts\\mPDBS\\summary_pdb_93\\5f60752431cf2dcb.final.pdb
-6.700813e-15 -1.498060e-14 -8.338087e-15 1.955303e-15 2.619367e-15
-4.158721e-15
E:\\R-3.1.2\\scripts\\mPDBS\\summary_pdb_93\\626456dd6ff2868d.final.pdb
-5.917782e-15 -8.537894e-15 -2.910200e-15 2.405436e-15 -5.167041e-15
-7.267174e-15
E:\\R-3.1.2\\scripts\\mPDBS\\summary_pdb_93\\6583d53ff3c8f7ef.final.pdb
-4.370511e-15 -5.124143e-15 -3.659728e-15 7.832533e-15 -8.682781e-15
-1.135768e-14
E:\\R-3.1.2\\scripts\\mPDBS\\summary_pdb_93\\69760178986bef01.final.pdb
3.867181e-15 -9.917300e-15 -4.222982e-15 6.811332e-15 8.338216e-15
-4.505379e-15
E:\\R-3.1.2\\scripts\\mPDBS\\summary_pdb_93\\6a9d87d47926b1c8.final.pdb
-5.145830e-15 -5.095552e-15 -2.452884e-15 4.402970e-15 -4.805776e-16
-8.793730e-15
E:\\R-3.1.2\\scripts\\mPDBS\\summary_pdb_93\\6bb3547ea982ffc4.final.pdb
-2.645799e-15 -1.376647e-14 -5.461291e-15 2.453503e-15 5.719497e-15
-1.262934e-14
E:\\R-3.1.2\\scripts\\mPDBS\\summary_pdb_93\\716d9624611d1c42.final.pdb
-5.059331e-15 -8.467236e-16 2.041848e-15 5.092984e-15 -1.495723e-15
-9.816522e-15
E:\\R-3.1.2\\scripts\\mPDBS\\summary_pdb_93\\75f27e70ad0f1be4.final.pdb
-4.370511e-15 -5.124143e-15 -3.659728e-15 7.832533e-15 -8.682781e-15
-1.135768e-14
E:\\R-3.1.2\\scripts\\mPDBS\\summary_pdb_93\\769f910fffb22872.final.pdb
-4.748719e-15 -6.175341e-15 -2.348626e-15 4.320935e-15 -1.610865e-15
-8.727592e-15
E:\\R-3.1.2\\scripts\\mPDBS\\summary_pdb_93\\791f8c80dd5c3135.final.pdb
-6.571215e-15 -6.490903e-15 -6.327923e-16 4.510190e-15 -2.573530e-15
-6.543436e-15
E:\\R-3.1.2\\scripts\\mPDBS\\summary_pdb_93\\7fad98e66a3f6099.final.pdb
-7.080608e-15 -3.321208e-15 -3.180920e-15 3.990990e-15 -9.204988e-15
-6.836152e-15
E:\\R-3.1.2\\scripts\\mPDBS\\summary_pdb_93\\804b89c94a633196.final.pdb
-3.603670e-15 -8.200892e-15 -2.008294e-15 4.786142e-15 2.397030e-16
-4.920402e-15
E:\\R-3.1.2\\scripts\\mPDBS\\summary_pdb_93\\813cc65a2298feba.final.pdb
-3.603670e-15 -8.200892e-15 -2.008294e-15 4.786142e-15 2.397030e-16
-4.920402e-15
E:\\R-3.1.2\\scripts\\mPDBS\\summary_pdb_93\\83c0eefcfe12d3f9.final.pdb
-5.584724e-15 -5.439862e-15 -3.515986e-15 5.796619e-15 3.798813e-15
-7.944025e-15
E:\\R-3.1.2\\scripts\\mPDBS\\summary_pdb_93\\86d50469ef569760.final.pdb
-5.917782e-15 -8.537894e-15 -2.910200e-15 2.405436e-15 -5.167041e-15
-7.267174e-15
E:\\R-3.1.2\\scripts\\mPDBS\\summary_pdb_93\\89d0666e1402203d.final.pdb
-4.370511e-15 -5.124143e-15 -3.659728e-15 7.832533e-15 -8.682781e-15
-1.135768e-14
E:\\R-3.1.2\\scripts\\mPDBS\\summary_pdb_93\\8b34852716dadc62.final.pdb
-7.505497e-15 -3.109478e-15 -5.953622e-16 8.107271e-15 2.739638e-16
-1.194256e-14
E:\\R-3.1.2\\scripts\\mPDBS\\summary_pdb_93\\8b6ef9ac3fe74395.final.pdb
-6.707820e-15 -1.227230e-14 -3.757561e-15 6.361826e-15 4.650746e-16
-9.609951e-15

E:\\R-3.1.2\\scripts\\mPDBS\\summary_pdb_93\\98bbe0d5986c138f.final.pdb
-6.571215e-15 -6.490903e-15 -6.327923e-16 4.510190e-15 -2.573530e-15
-6.543436e-15
E:\\R-3.1.2\\scripts\\mPDBS\\summary_pdb_93\\9c22f4909d67e2fa.final.pdb
-3.580685e-15 -3.612982e-15 -3.245586e-15 -4.846777e-15 -5.535896e-15
-1.148199e-14
E:\\R-3.1.2\\scripts\\mPDBS\\summary_pdb_93\\9dda8ed0d81fb3ef.final.pdb
-1.247340e-14 -1.020363e-14 -4.788166e-15 -2.660159e-15 7.621994e-15
7.850910e-15
E:\\R-3.1.2\\scripts\\mPDBS\\summary_pdb_93\\9f752b3616e6033b.final.pdb
-3.025818e-15 -5.881946e-15 -1.109731e-15 6.400040e-15 -2.801335e-15
-1.052917e-14
E:\\R-3.1.2\\scripts\\mPDBS\\summary_pdb_93\\a02c70083816d6ba.final.pdb
-4.603916e-15 -7.472921e-15 -3.017559e-15 3.265434e-15 2.422077e-15
-6.981342e-15
E:\\R-3.1.2\\scripts\\mPDBS\\summary_pdb_93\\a0c997d891e4e082.final.pdb
-5.459880e-15 -2.037688e-15 -3.609046e-15 4.476562e-15 2.302547e-15
-1.043343e-14
E:\\R-3.1.2\\scripts\\mPDBS\\summary_pdb_93\\ab8039a2d823c616.final.pdb
-4.675351e-15 -6.269979e-15 -2.371538e-15 3.984768e-15 -1.694419e-15
-8.823602e-15
E:\\R-3.1.2\\scripts\\mPDBS\\summary_pdb_93\\acf4d74b9c71d4dc.final.pdb
-3.603670e-15 -8.200892e-15 -2.008294e-15 4.786142e-15 2.397030e-16
-4.920402e-15
E:\\R-3.1.2\\scripts\\mPDBS\\summary_pdb_93\\ad8a823e4b4c668e.final.pdb
-5.000151e-15 -6.107884e-15 -2.119351e-15 4.681650e-15 -1.794255e-15
-8.430663e-15
E:\\R-3.1.2\\scripts\\mPDBS\\summary_pdb_93\\b16ababd03b7b857.final.pdb
-3.580685e-15 -3.612982e-15 -3.245586e-15 -4.846777e-15 -5.535896e-15
-1.148199e-14
E:\\R-3.1.2\\scripts\\mPDBS\\summary_pdb_93\\b226923e697d3c63.final.pdb
-3.840553e-15 -1.080341e-14 -2.184562e-15 3.401399e-15 4.496835e-15
-9.223973e-15
E:\\R-3.1.2\\scripts\\mPDBS\\summary_pdb_93\\b28e48f6fa27a165.final.pdb
-6.571215e-15 -6.490903e-15 -6.327923e-16 4.510190e-15 -2.573530e-15
-6.543436e-15
E:\\R-3.1.2\\scripts\\mPDBS\\summary_pdb_93\\b42c4924e3cd1aec.final.pdb
-4.296351e-15 -2.669076e-15 -2.962152e-15 8.728951e-15 -4.060013e-15
-1.971732e-14
E:\\R-3.1.2\\scripts\\mPDBS\\summary_pdb_93\\b4dd239fcabff1df.final.pdb
-6.571215e-15 -6.490903e-15 -6.327923e-16 4.510190e-15 -2.573530e-15
-6.543436e-15
E:\\R-3.1.2\\scripts\\mPDBS\\summary_pdb_93\\b669942ef97459a2.final.pdb
-7.505497e-15 -3.109478e-15 -5.953622e-16 8.107271e-15 2.739638e-16
-1.194256e-14
E:\\R-3.1.2\\scripts\\mPDBS\\summary_pdb_93\\b99f4d9d87c1017b.final.pdb
-6.571215e-15 -6.490903e-15 -6.327923e-16 4.510190e-15 -2.573530e-15
-6.543436e-15
E:\\R-3.1.2\\scripts\\mPDBS\\summary_pdb_93\\b9d2cbf3975403e7.final.pdb
-6.571215e-15 -6.490903e-15 -6.327923e-16 4.510190e-15 -2.573530e-15
-6.543436e-15
E:\\R-3.1.2\\scripts\\mPDBS\\summary_pdb_93\\bc88c8a505f3a288.final.pdb
-4.538553e-15 -5.948151e-15 -2.120318e-15 4.757388e-15 -7.076718e-16
-8.785566e-15

E:\\R-3.1.2\\scripts\\mPDBS\\summary_pdb_93\\bdac0e64f4ef427e.final.pdb
-4.305799e-15 -8.462184e-15 -8.808807e-18 5.594887e-16 -1.169167e-14
-8.915470e-15
E:\\R-3.1.2\\scripts\\mPDBS\\summary_pdb_93\\ca2a1f55fc9d0681.final.pdb
-7.505497e-15 -3.109478e-15 -5.953622e-16 8.107271e-15 2.739638e-16
-1.194256e-14
E:\\R-3.1.2\\scripts\\mPDBS\\summary_pdb_93\\ce34248de3e81a3c.final.pdb
-1.368379e-14 -2.664025e-15 -3.658948e-15 2.646741e-15 -3.915203e-15
-6.968627e-15
E:\\R-3.1.2\\scripts\\mPDBS\\summary_pdb_93\\d02e697772494a61.final.pdb
2.174827e-15 -5.592750e-15 7.586727e-16 7.682132e-15 -7.155964e-15
-1.441561e-14
E:\\R-3.1.2\\scripts\\mPDBS\\summary_pdb_93\\d04221245d015047.final.pdb
-5.477227e-15 -4.780285e-15 -1.829220e-15 4.906774e-15 -3.397755e-15
-8.664008e-15
E:\\R-3.1.2\\scripts\\mPDBS\\summary_pdb_93\\d678141e4018054a.final.pdb
-8.888532e-15 -9.933490e-15 -3.030988e-15 -8.958963e-16 1.665269e-16
-1.109071e-14
E:\\R-3.1.2\\scripts\\mPDBS\\summary_pdb_93\\d71b40af0f0efe39.final.pdb
-4.008794e-15 -3.126407e-16 2.329294e-15 5.164991e-15 -2.993719e-15
-5.531366e-15
E:\\R-3.1.2\\scripts\\mPDBS\\summary_pdb_93\\dc0809eff10197e2.final.pdb
-3.136687e-15 -2.276280e-15 3.011226e-17 1.148656e-14 -8.467809e-15
-7.815385e-15
E:\\R-3.1.2\\scripts\\mPDBS\\summary_pdb_93\\e1163918d3707839.final.pdb
9.368756e-16 -8.655607e-15 -2.650909e-16 5.024449e-15 -9.371303e-15
-1.622792e-14
E:\\R-3.1.2\\scripts\\mPDBS\\summary_pdb_93\\e4b48b06824ef848.final.pdb
-4.012329e-15 -1.210875e-14 -5.610340e-15 1.460159e-15 7.677116e-15
-6.998940e-15
E:\\R-3.1.2\\scripts\\mPDBS\\summary_pdb_93\\e529e19ad281fea2.final.pdb
-2.623542e-15 -7.235117e-15 -3.232859e-15 2.452273e-15 3.154861e-15
-6.078888e-15
E:\\R-3.1.2\\scripts\\mPDBS\\summary_pdb_93\\e5602082718ec3a1.final.pdb
-3.603670e-15 -8.200892e-15 -2.008294e-15 4.786142e-15 2.397030e-16
-4.920402e-15
E:\\R-3.1.2\\scripts\\mPDBS\\summary_pdb_93\\e5d589d996d0a960.final.pdb
-1.009647e-14 2.678477e-15 -3.193891e-15 7.163733e-15 3.781095e-15
-7.514618e-15
E:\\R-3.1.2\\scripts\\mPDBS\\summary_pdb_93\\e9df9e72307a2fbd.final.pdb
-4.603916e-15 -7.472921e-15 -3.017559e-15 3.265434e-15 2.422077e-15
-6.981342e-15
E:\\R-3.1.2\\scripts\\mPDBS\\summary_pdb_93\\ea00f8694090cae7.final.pdb
-3.603670e-15 -8.200892e-15 -2.008294e-15 4.786142e-15 2.397030e-16
-4.920402e-15
E:\\R-3.1.2\\scripts\\mPDBS\\summary_pdb_93\\eba517be32db050e.final.pdb
-6.571215e-15 -6.490903e-15 -6.327923e-16 4.510190e-15 -2.573530e-15
-6.543436e-15
E:\\R-3.1.2\\scripts\\mPDBS\\summary_pdb_93\\ec04b9545899c29d.final.pdb
-4.807661e-15 -6.378348e-15 -2.347193e-15 4.598358e-15 -1.364850e-15
-8.973305e-15
E:\\R-3.1.2\\scripts\\mPDBS\\summary_pdb_93\\ee6862cbcad52078.final.pdb
-4.786185e-15 -5.322567e-15 -2.447530e-15 5.095876e-15 -2.242802e-16
-9.177614e-15

E:\\R-3.1.2\\scripts\\mPDBS\\summary_pdb_93\\ef6a42fc83b81e5d.final.pdb
-5.129925e-15 -6.474147e-15 -2.129400e-15 4.251329e-15 -1.385405e-15
-8.535037e-15
E:\\R-3.1.2\\scripts\\mPDBS\\summary_pdb_93\\ef8e88d4ad93a0ad.final.pdb
-5.477227e-15 -4.780285e-15 -1.829220e-15 4.906774e-15 -3.397755e-15
-8.664008e-15
E:\\R-3.1.2\\scripts\\mPDBS\\summary_pdb_93\\f14fcae8deec4fa3.final.pdb
-3.025818e-15 -5.881946e-15 -1.109731e-15 6.400040e-15 -2.801335e-15
-1.052917e-14
E:\\R-3.1.2\\scripts\\mPDBS\\summary_pdb_93\\f83584e22003fb92.final.pdb
-6.119320e-15 -6.063510e-15 -9.131636e-15 7.306347e-15 9.885146e-15
-9.578335e-15
E:\\R-3.1.2\\scripts\\mPDBS\\summary_pdb_93\\f8e2bf87e52caa29.final.pdb
-2.623542e-15 -7.235117e-15 -3.232859e-15 2.452273e-15 3.154861e-15
-6.078888e-15

[,547]   [,548]   [,549]   [,550]   [,551]   [,552]
E:\\R-3.1.2\\scripts\\40.1ns_data\\NAT0.pdb
-1.303975e-15 2.664924e-15 -1.238744e-15 7.472808e-16 3.008532e-15
-9.429505e-16
E:\\R-3.1.2\\scripts\\40.1ns_data\\LJP6.pdb
-6.329294e-16 2.203602e-15 3.717277e-16 4.641153e-16 4.524591e-15
-4.913043e-17
E:\\R-3.1.2\\scripts\\40.1ns_data\\3WY9.pdb
-3.938604e-16 2.684626e-15 1.085207e-15 1.087635e-15 3.069812e-15
-6.005848e-16
E:\\R-3.1.2\\scripts\\40.1ns_data\\ZSP9.pdb
-5.096440e-16 3.006542e-15 -1.309293e-15 3.565232e-16 3.670334e-15
-1.210599e-15
E:\\R-3.1.2\\scripts\\mPDBS\\summary_pdb_93\\013d855ad26c877c.final.pdb
-4.698110e-15 2.173804e-15 9.691367e-16 4.733391e-15 4.457776e-15
7.371180e-16
E:\\R-3.1.2\\scripts\\mPDBS\\summary_pdb_93\\0a2a705342ca0d54.final.pdb
-3.306001e-17 2.554614e-15 -6.094873e-16 -5.593619e-17 4.036401e-15
-5.503058e-16
E:\\R-3.1.2\\scripts\\mPDBS\\summary_pdb_93\\0a59fceadb4194d9.final.pdb
5.971158e-17 3.883651e-15 -3.727336e-15 -9.981011e-16 4.013922e-15
1.468859e-15
E:\\R-3.1.2\\scripts\\mPDBS\\summary_pdb_93\\0dba59f3306825e8.final.pdb
-3.070909e-16 -9.747193e-16 -3.561094e-15 3.578932e-15 6.766693e-15
-2.853760e-15
E:\\R-3.1.2\\scripts\\mPDBS\\summary_pdb_93\\126f26a78b57339c.final.pdb
3.849144e-15 1.714721e-16 4.295252e-15 7.790885e-16 5.571035e-15
-5.352412e-15
E:\\R-3.1.2\\scripts\\mPDBS\\summary_pdb_93\\12f06f3e342df706.final.pdb
-1.077851e-15 2.816935e-15 4.233236e-16 -1.747648e-16 3.227152e-15
-4.091698e-16
E:\\R-3.1.2\\scripts\\mPDBS\\summary_pdb_93\\18618a2a251b867b.final.pdb
-4.998953e-16 -2.016455e-15 1.417671e-15 4.593674e-15 6.431419e-15
-4.428449e-15
E:\\R-3.1.2\\scripts\\mPDBS\\summary_pdb_93\\1f305f598641eeb2.final.pdb
-1.478164e-16 2.955436e-15 -3.794773e-16 8.659548e-16 3.919692e-15
-7.805414e-16
E:\\R-3.1.2\\scripts\\mPDBS\\summary_pdb_93\\1f8a00b551be0a39.final.pdb

-4.998953e-16 -2.016455e-15 1.417671e-15 4.593674e-15 6.431419e-15
-4.428449e-15
E:\\R-3.1.2\\scripts\\mPDBS\\summary_pdb_93\\219f45328d47579a.final.pdb
-3.772184e-15 1.024011e-14 1.686166e-14 -4.206039e-15 -8.291990e-15
3.169756e-15
E:\\R-3.1.2\\scripts\\mPDBS\\summary_pdb_93\\238dd3918e54336d.final.pdb
-3.087402e-15 3.572037e-15 7.176919e-15 1.237090e-15 4.332894e-15
-6.112180e-15
E:\\R-3.1.2\\scripts\\mPDBS\\summary_pdb_93\\2b3027889e2fdc42.final.pdb
-7.739857e-16 2.323240e-15 -3.554674e-16 6.481514e-16 4.273046e-15
-5.943501e-16
E:\\R-3.1.2\\scripts\\mPDBS\\summary_pdb_93\\2fcb3b46e33793b0.final.pdb
-2.801943e-15 3.926881e-15 -2.640689e-15 2.171334e-15 3.300830e-15
2.768703e-15
E:\\R-3.1.2\\scripts\\mPDBS\\summary_pdb_93\\34eb328251842006.final.pdb
-1.273284e-15 8.768417e-15 -4.234519e-15 -7.051793e-15 -4.762333e-15
8.496493e-15
E:\\R-3.1.2\\scripts\\mPDBS\\summary_pdb_93\\354b32871797b7ab.final.pdb
5.159930e-15 9.638211e-15 4.680295e-15 -1.060042e-14 -4.273863e-15
3.891698e-15
E:\\R-3.1.2\\scripts\\mPDBS\\summary_pdb_93\\397368adbc32d5de.final.pdb
3.849144e-15 1.714721e-16 4.295252e-15 7.790885e-16 5.571035e-15
-5.352412e-15
E:\\R-3.1.2\\scripts\\mPDBS\\summary_pdb_93\\411ca13cd3df6edd.final.pdb
8.125157e-16 2.494460e-15 -6.932454e-16 1.077591e-15 2.768703e-15
2.261079e-15
E:\\R-3.1.2\\scripts\\mPDBS\\summary_pdb_93\\42357eee6642bf12.final.pdb
-1.592596e-15 1.760580e-14 2.608675e-15 -2.890273e-15 -9.239405e-16
1.104001e-14
E:\\R-3.1.2\\scripts\\mPDBS\\summary_pdb_93\\44342d1f75a7fcfc.final.pdb
-8.313266e-15 -4.161883e-15 -7.494933e-15 2.382168e-15 4.664927e-15
4.424301e-15
E:\\R-3.1.2\\scripts\\mPDBS\\summary_pdb_93\\448ef21dffe8cdfb.final.pdb
6.142725e-15 1.114680e-14 6.896820e-15 -1.099348e-14 4.049797e-15
1.681031e-15
E:\\R-3.1.2\\scripts\\mPDBS\\summary_pdb_93\\472a40b3e8f113c7.final.pdb
-3.070909e-16 -9.747193e-16 -3.561094e-15 3.578932e-15 6.766693e-15
-2.853760e-15
E:\\R-3.1.2\\scripts\\mPDBS\\summary_pdb_93\\48a26225951ea153.final.pdb
-4.153986e-15 -1.812341e-15 -4.995419e-15 5.785969e-15 7.690885e-15
2.949690e-15
E:\\R-3.1.2\\scripts\\mPDBS\\summary_pdb_93\\4ab4a5c7c6682ce6.final.pdb
4.265044e-15 8.040178e-15 -3.356613e-15 -5.936893e-15 -1.731284e-15
6.568608e-15
E:\\R-3.1.2\\scripts\\mPDBS\\summary_pdb_93\\4eaa45b62cb6a25f.final.pdb
-5.390707e-16 -1.672823e-15 -7.561114e-16 5.898100e-15 8.716180e-15
-4.782489e-15
E:\\R-3.1.2\\scripts\\mPDBS\\summary_pdb_93\\58c371611d85ac96.final.pdb
3.849144e-15 1.714721e-16 4.295252e-15 7.790885e-16 5.571035e-15
-5.352412e-15
E:\\R-3.1.2\\scripts\\mPDBS\\summary_pdb_93\\59a647f04822d30a.final.pdb
3.223946e-15 7.479847e-15 4.567033e-15 1.968501e-16 4.490371e-15
-5.968564e-16
E:\\R-3.1.2\\scripts\\mPDBS\\summary_pdb_93\\59b091fe385e05fc.final.pdb

5.971158e-17 3.883651e-15 -3.727336e-15 -9.981011e-16 4.013922e-15
1.468859e-15
E:\\R-3.1.2\\scripts\\mPDBS\\summary_pdb_93\\5c35ac30f930d2db.final.pdb
-5.390707e-16 -1.672823e-15 -7.561114e-16 5.898100e-15 8.716180e-15
-4.782489e-15
E:\\R-3.1.2\\scripts\\mPDBS\\summary_pdb_93\\5f60752431cf2dcb.final.pdb
3.855702e-15 -1.184610e-15 -3.493465e-15 5.494312e-15 1.410907e-14
-5.474597e-16
E:\\R-3.1.2\\scripts\\mPDBS\\summary_pdb_93\\626456dd6ff2868d.final.pdb
-3.070909e-16 -9.747193e-16 -3.561094e-15 3.578932e-15 6.766693e-15
-2.853760e-15
E:\\R-3.1.2\\scripts\\mPDBS\\summary_pdb_93\\6583d53ff3c8f7ef.final.pdb
3.223946e-15 7.479847e-15 4.567033e-15 1.968501e-16 4.490371e-15
-5.968564e-16
E:\\R-3.1.2\\scripts\\mPDBS\\summary_pdb_93\\69760178986bef01.final.pdb
-8.425913e-16 -8.993207e-16 6.962048e-15 2.101847e-15 7.130780e-15
-5.893981e-15
E:\\R-3.1.2\\scripts\\mPDBS\\summary_pdb_93\\6a9d87d47926b1c8.final.pdb
-6.073607e-16 2.372836e-15 -4.266652e-16 8.025073e-16 4.446500e-15
-1.916096e-16
E:\\R-3.1.2\\scripts\\mPDBS\\summary_pdb_93\\6bb3547ea982ffc4.final.pdb
1.671322e-15 -7.982612e-15 6.125100e-15 2.720744e-15 7.032724e-15
-3.602912e-15
E:\\R-3.1.2\\scripts\\mPDBS\\summary_pdb_93\\716d9624611d1c42.final.pdb
-2.785307e-15 3.060719e-15 3.273257e-15 -3.163260e-15 -4.731976e-15
-4.663335e-15
E:\\R-3.1.2\\scripts\\mPDBS\\summary_pdb_93\\75f27e70ad0f1be4.final.pdb
3.223946e-15 7.479847e-15 4.567033e-15 1.968501e-16 4.490371e-15
-5.968564e-16
E:\\R-3.1.2\\scripts\\mPDBS\\summary_pdb_93\\769f910fffb22872.final.pdb
-1.033702e-15 2.865721e-15 -5.319416e-16 4.802107e-16 4.495684e-15
-6.117323e-16
E:\\R-3.1.2\\scripts\\mPDBS\\summary_pdb_93\\791f8c80dd5c3135.final.pdb
5.971158e-17 3.883651e-15 -3.727336e-15 -9.981011e-16 4.013922e-15
1.468859e-15
E:\\R-3.1.2\\scripts\\mPDBS\\summary_pdb_93\\7fad98e66a3f6099.final.pdb
-5.972235e-15 1.345125e-14 -2.965119e-15 -6.622261e-15 3.968671e-15
1.066032e-14
E:\\R-3.1.2\\scripts\\mPDBS\\summary_pdb_93\\804b89c94a633196.final.pdb
-2.801943e-15 3.926881e-15 -2.640689e-15 2.171334e-15 3.300830e-15
2.768703e-15
E:\\R-3.1.2\\scripts\\mPDBS\\summary_pdb_93\\813cc65a2298feba.final.pdb
-2.801943e-15 3.926881e-15 -2.640689e-15 2.171334e-15 3.300830e-15
2.768703e-15
E:\\R-3.1.2\\scripts\\mPDBS\\summary_pdb_93\\83c0eefcfe12d3f9.final.pdb
8.125157e-16 2.494460e-15 -6.932454e-16 1.077591e-15 2.768703e-15
2.261079e-15
E:\\R-3.1.2\\scripts\\mPDBS\\summary_pdb_93\\86d50469ef569760.final.pdb
-3.070909e-16 -9.747193e-16 -3.561094e-15 3.578932e-15 6.766693e-15
-2.853760e-15
E:\\R-3.1.2\\scripts\\mPDBS\\summary_pdb_93\\89d0666e1402203d.final.pdb
3.223946e-15 7.479847e-15 4.567033e-15 1.968501e-16 4.490371e-15
-5.968564e-16
E:\\R-3.1.2\\scripts\\mPDBS\\summary_pdb_93\\8b34852716dadc62.final.pdb

3.279997e-15 3.992801e-15 -4.777023e-18 -1.916542e-15 2.157213e-15
-7.128872e-16
E:\\R-3.1.2\\scripts\\mPDBS\\summary_pdb_93\\8b6ef9ac3fe74395.final.pdb
9.096591e-16 6.407298e-15 -2.135098e-15 3.431490e-15 1.373807e-14
-4.519531e-15
E:\\R-3.1.2\\scripts\\mPDBS\\summary_pdb_93\\98bbe0d5986c138f.final.pdb
5.971158e-17 3.883651e-15 -3.727336e-15 -9.981011e-16 4.013922e-15
1.468859e-15
E:\\R-3.1.2\\scripts\\mPDBS\\summary_pdb_93\\9c22f4909d67e2fa.final.pdb
-3.909456e-15 -1.298743e-15 2.072988e-15 2.914663e-15 3.149909e-15
-5.008605e-15
E:\\R-3.1.2\\scripts\\mPDBS\\summary_pdb_93\\9dda8ed0d81fb3ef.final.pdb
-3.161365e-16 -1.341492e-14 -9.628643e-15 1.278530e-14 1.574547e-14
2.196292e-15
E:\\R-3.1.2\\scripts\\mPDBS\\summary_pdb_93\\9f752b3616e6033b.final.pdb
-2.803848e-15 3.158996e-15 -5.345329e-16 -6.892490e-16 3.641636e-15
9.371196e-16
E:\\R-3.1.2\\scripts\\mPDBS\\summary_pdb_93\\a02c70083816d6ba.final.pdb
-5.390707e-16 -1.672823e-15 -7.561114e-16 5.898100e-15 8.716180e-15
-4.782489e-15
E:\\R-3.1.2\\scripts\\mPDBS\\summary_pdb_93\\a0c997d891e4e082.final.pdb
-4.237668e-15 -3.422597e-15 -1.649092e-15 -5.706765e-16 3.694910e-16
-2.147362e-15
E:\\R-3.1.2\\scripts\\mPDBS\\summary_pdb_93\\ab8039a2d823c616.final.pdb
-7.869782e-16 2.685134e-15 -8.162077e-16 1.337519e-15 4.247032e-15
-8.448833e-16
E:\\R-3.1.2\\scripts\\mPDBS\\summary_pdb_93\\acf4d74b9c71d4dc.final.pdb
-2.801943e-15 3.926881e-15 -2.640689e-15 2.171334e-15 3.300830e-15
2.768703e-15
E:\\R-3.1.2\\scripts\\mPDBS\\summary_pdb_93\\ad8a823e4b4c668e.final.pdb
-9.376125e-16 2.519173e-15 -7.128877e-16 -2.996043e-16 4.638331e-15
-6.027102e-16
E:\\R-3.1.2\\scripts\\mPDBS\\summary_pdb_93\\b16ababd03b7b857.final.pdb
-3.909456e-15 -1.298743e-15 2.072988e-15 2.914663e-15 3.149909e-15
-5.008605e-15
E:\\R-3.1.2\\scripts\\mPDBS\\summary_pdb_93\\b226923e697d3c63.final.pdb
-2.911174e-15 6.987822e-15 3.614887e-15 4.162927e-16 3.885603e-15
-2.666707e-15
E:\\R-3.1.2\\scripts\\mPDBS\\summary_pdb_93\\b28e48f6fa27a165.final.pdb
5.971158e-17 3.883651e-15 -3.727336e-15 -9.981011e-16 4.013922e-15
1.468859e-15
E:\\R-3.1.2\\scripts\\mPDBS\\summary_pdb_93\\b42c4924e3cd1aec.final.pdb
-3.937751e-15 6.028981e-15 -2.642694e-15 -2.219620e-15 1.167725e-15
-4.260294e-15
E:\\R-3.1.2\\scripts\\mPDBS\\summary_pdb_93\\b4dd239fcabff1df.final.pdb
5.971158e-17 3.883651e-15 -3.727336e-15 -9.981011e-16 4.013922e-15
1.468859e-15
E:\\R-3.1.2\\scripts\\mPDBS\\summary_pdb_93\\b669942ef97459a2.final.pdb
3.279997e-15 3.992801e-15 -4.777023e-18 -1.916542e-15 2.157213e-15
-7.128872e-16
E:\\R-3.1.2\\scripts\\mPDBS\\summary_pdb_93\\b99f4d9d87c1017b.final.pdb
5.971158e-17 3.883651e-15 -3.727336e-15 -9.981011e-16 4.013922e-15
1.468859e-15
E:\\R-3.1.2\\scripts\\mPDBS\\summary_pdb_93\\b9d2cbf3975403e7.final.pdb

5.971158e-17 3.883651e-15 -3.727336e-15 -9.981011e-16 4.013922e-15
1.468859e-15
E:\\R-3.1.2\\scripts\\mPDBS\\summary_pdb_93\\bc88c8a505f3a288.final.pdb
-4.397261e-16 2.227220e-15 -4.762476e-16 6.438146e-16 4.541061e-15
-5.882244e-16
E:\\R-3.1.2\\scripts\\mPDBS\\summary_pdb_93\\bdac0e64f4ef427e.final.pdb
-2.187146e-15 3.624863e-15 -1.186991e-15 -2.130432e-16 6.433307e-15
-6.322929e-15
E:\\R-3.1.2\\scripts\\mPDBS\\summary_pdb_93\\ca2a1f55fc9d0681.final.pdb
3.279997e-15 3.992801e-15 -4.777023e-18 -1.916542e-15 2.157213e-15
-7.128872e-16
E:\\R-3.1.2\\scripts\\mPDBS\\summary_pdb_93\\ce34248de3e81a3c.final.pdb
3.375920e-15 6.619307e-15 -3.333395e-15 4.927559e-15 7.736250e-15
-1.996203e-15
E:\\R-3.1.2\\scripts\\mPDBS\\summary_pdb_93\\d02e697772494a61.final.pdb
1.224904e-15 1.505591e-14 1.143003e-14 -8.146029e-15 -4.524170e-15
4.511460e-16
E:\\R-3.1.2\\scripts\\mPDBS\\summary_pdb_93\\d04221245d015047.final.pdb
-1.315780e-16 1.556927e-15 -1.479089e-15 7.164883e-16 4.515480e-15
1.893100e-16
E:\\R-3.1.2\\scripts\\mPDBS\\summary_pdb_93\\d678141e4018054a.final.pdb
4.588716e-16 -2.435164e-16 1.483540e-15 -2.739872e-15 -3.091802e-15
1.723560e-15
E:\\R-3.1.2\\scripts\\mPDBS\\summary_pdb_93\\d71b40af0f0efe39.final.pdb
-8.494462e-15 5.866158e-16 -4.770573e-15 -3.115180e-15 -3.234432e-15
2.340640e-15
E:\\R-3.1.2\\scripts\\mPDBS\\summary_pdb_93\\dc0809eff10197e2.final.pdb
-4.083768e-15 4.927232e-16 -7.846248e-15 3.107214e-15 1.017488e-14
-5.907037e-15
E:\\R-3.1.2\\scripts\\mPDBS\\summary_pdb_93\\e1163918d3707839.final.pdb
-4.147653e-15 1.517399e-15 -9.779780e-15 -3.783039e-15 -1.076574e-15
-1.770966e-15
E:\\R-3.1.2\\scripts\\mPDBS\\summary_pdb_93\\e4b48b06824ef848.final.pdb
-4.998953e-16 -2.016455e-15 1.417671e-15 4.593674e-15 6.431419e-15
-4.428449e-15
E:\\R-3.1.2\\scripts\\mPDBS\\summary_pdb_93\\e529e19ad281fea2.final.pdb
-4.698110e-15 2.173804e-15 9.691367e-16 4.733391e-15 4.457776e-15
7.371180e-16
E:\\R-3.1.2\\scripts\\mPDBS\\summary_pdb_93\\e5602082718ec3a1.final.pdb
-2.801943e-15 3.926881e-15 -2.640689e-15 2.171334e-15 3.300830e-15
2.768703e-15
E:\\R-3.1.2\\scripts\\mPDBS\\summary_pdb_93\\e5d589d996d0a960.final.pdb
-3.438989e-15 -3.656362e-15 -4.047673e-15 6.980335e-15 1.311101e-14
-8.251665e-15
E:\\R-3.1.2\\scripts\\mPDBS\\summary_pdb_93\\e9df9e72307a2fbd.final.pdb
-5.390707e-16 -1.672823e-15 -7.561114e-16 5.898100e-15 8.716180e-15
-4.782489e-15
E:\\R-3.1.2\\scripts\\mPDBS\\summary_pdb_93\\ea00f8694090cae7.final.pdb
-2.801943e-15 3.926881e-15 -2.640689e-15 2.171334e-15 3.300830e-15
2.768703e-15
E:\\R-3.1.2\\scripts\\mPDBS\\summary_pdb_93\\eba517be32db050e.final.pdb
5.971158e-17 3.883651e-15 -3.727336e-15 -9.981011e-16 4.013922e-15
1.468859e-15
E:\\R-3.1.2\\scripts\\mPDBS\\summary_pdb_93\\ec04b9545899c29d.final.pdb

-7.902039e-16 2.843798e-15 -3.331269e-16 3.213165e-16 4.020911e-15
-2.603200e-16
E:\\R-3.1.2\\scripts\\mPDBS\\summary_pdb_93\\ee6862cbcad52078.final.pdb
-5.146987e-16 2.352757e-15 -3.077625e-16 9.096610e-16 4.140773e-15
-3.924988e-16
E:\\R-3.1.2\\scripts\\mPDBS\\summary_pdb_93\\ef6a42fc83b81e5d.final.pdb
-7.782643e-16 2.684673e-15 -1.873821e-16 7.022554e-16 3.922358e-15
-4.830646e-16
E:\\R-3.1.2\\scripts\\mPDBS\\summary_pdb_93\\ef8e88d4ad93a0ad.final.pdb
-1.315780e-16 1.556927e-15 -1.479089e-15 7.164883e-16 4.515480e-15
1.893100e-16
E:\\R-3.1.2\\scripts\\mPDBS\\summary_pdb_93\\f14fcae8deec4fa3.final.pdb
-2.803848e-15 3.158996e-15 -5.345329e-16 -6.892490e-16 3.641636e-15
9.371196e-16
E:\\R-3.1.2\\scripts\\mPDBS\\summary_pdb_93\\f83584e22003fb92.final.pdb
-4.153986e-15 -1.812341e-15 -4.995419e-15 5.785969e-15 7.690885e-15
2.949690e-15
E:\\R-3.1.2\\scripts\\mPDBS\\summary_pdb_93\\f8e2bf87e52caa29.final.pdb
-4.698110e-15 2.173804e-15 9.691367e-16 4.733391e-15 4.457776e-15
7.371180e-16

[,553]   [,554]   [,555]   [,556]   [,557]   [,558]
E:\\R-3.1.2\\scripts\\40.1ns_data\\NAT0.pdb
1.783181e-15 2.324833e-15 4.198824e-15 3.456071e-15 3.759209e-15
9.999428e-16
E:\\R-3.1.2\\scripts\\40.1ns_data\\LJP6.pdb
2.177262e-16 1.814972e-15 2.529672e-15 2.426461e-15 4.358801e-15
1.852256e-15
E:\\R-3.1.2\\scripts\\40.1ns_data\\3WY9.pdb
1.053028e-15 1.375240e-15 4.328539e-15 3.790954e-15 5.978728e-15
-2.323927e-15
E:\\R-3.1.2\\scripts\\40.1ns_data\\ZSP9.pdb
3.458719e-17 1.970729e-15 4.000716e-15 2.231746e-15 5.471897e-15
9.561468e-16
E:\\R-3.1.2\\scripts\\mPDBS\\summary_pdb_93\\013d855ad26c877c.final.pdb
-3.810586e-15 2.915038e-15 -1.344613e-15 5.060719e-15 3.711611e-15
7.543661e-15
E:\\R-3.1.2\\scripts\\mPDBS\\summary_pdb_93\\0a2a705342ca0d54.final.pdb
-1.306744e-15 1.273247e-15 3.607514e-15 2.906355e-15 2.544261e-15
3.348546e-15
E:\\R-3.1.2\\scripts\\mPDBS\\summary_pdb_93\\0a59fceadb4194d9.final.pdb
6.262265e-16 4.914433e-15 4.524121e-15 3.580135e-15 6.643957e-15
-7.322475e-16
E:\\R-3.1.2\\scripts\\mPDBS\\summary_pdb_93\\0dba59f3306825e8.final.pdb
9.774641e-17 3.631045e-15 2.411714e-15 2.833767e-15 1.359326e-15
-1.443291e-15
E:\\R-3.1.2\\scripts\\mPDBS\\summary_pdb_93\\126f26a78b57339c.final.pdb
3.875107e-15 -2.765964e-15 4.116723e-16 7.399560e-16 1.237028e-15
2.343176e-15
E:\\R-3.1.2\\scripts\\mPDBS\\summary_pdb_93\\12f06f3e342df706.final.pdb
-7.065298e-16 1.669848e-15 4.164578e-15 2.638340e-15 3.004397e-15
1.454011e-15
E:\\R-3.1.2\\scripts\\mPDBS\\summary_pdb_93\\18618a2a251b867b.final.pdb
3.102145e-16 -5.373005e-15 -1.869094e-15 4.715538e-15 -2.488922e-16

2.328009e-15
E:\\R-3.1.2\\scripts\\mPDBS\\summary_pdb_93\\1f305f598641eeb2.final.pdb
-5.095610e-16 1.877948e-15 2.748729e-15 2.322617e-15 3.079005e-15
2.763519e-15
E:\\R-3.1.2\\scripts\\mPDBS\\summary_pdb_93\\1f8a00b551be0a39.final.pdb
3.102145e-16 -5.373005e-15 -1.869094e-15 4.715538e-15 -2.488922e-16
2.328009e-15
E:\\R-3.1.2\\scripts\\mPDBS\\summary_pdb_93\\219f45328d47579a.final.pdb
-7.553862e-15 -1.510874e-15 1.435035e-14 -1.422374e-15 -3.150292e-15
2.506915e-15
E:\\R-3.1.2\\scripts\\mPDBS\\summary_pdb_93\\238dd3918e54336d.final.pdb
3.136371e-15 -5.440998e-15 1.001088e-14 1.369663e-15 -3.615631e-15
-1.140492e-14
E:\\R-3.1.2\\scripts\\mPDBS\\summary_pdb_93\\2b3027889e2fdc42.final.pdb
-1.164008e-15 1.486945e-15 3.354362e-15 2.738360e-15 2.334036e-15
3.419800e-16
E:\\R-3.1.2\\scripts\\mPDBS\\summary_pdb_93\\2fcb3b46e33793b0.final.pdb
-4.685040e-15 3.634789e-15 1.112668e-15 3.330925e-15 2.680019e-15
5.172685e-16
E:\\R-3.1.2\\scripts\\mPDBS\\summary_pdb_93\\34eb328251842006.final.pdb
-4.627742e-15 4.970778e-15 1.301337e-14 1.029058e-14 1.179707e-14
1.848161e-15
E:\\R-3.1.2\\scripts\\mPDBS\\summary_pdb_93\\354b32871797b7ab.final.pdb
-1.110662e-14 8.757445e-15 -1.434273e-15 -1.251012e-15 1.993519e-15
1.185759e-15
E:\\R-3.1.2\\scripts\\mPDBS\\summary_pdb_93\\397368adbc32d5de.final.pdb
3.875107e-15 -2.765964e-15 4.116723e-16 7.399560e-16 1.237028e-15
2.343176e-15
E:\\R-3.1.2\\scripts\\mPDBS\\summary_pdb_93\\411ca13cd3df6edd.final.pdb
-6.486551e-15 7.627379e-16 -3.161135e-15 5.245626e-15 3.475389e-15
2.902296e-15
E:\\R-3.1.2\\scripts\\mPDBS\\summary_pdb_93\\42357eee6642bf12.final.pdb
-1.606538e-15 5.890052e-15 9.403377e-15 3.331766e-15 1.876280e-14
-1.903448e-16
E:\\R-3.1.2\\scripts\\mPDBS\\summary_pdb_93\\44342d1f75a7fcfc.final.pdb
3.364659e-15 5.718980e-16 6.860170e-16 7.021395e-15 4.427624e-15
2.576057e-15
E:\\R-3.1.2\\scripts\\mPDBS\\summary_pdb_93\\448ef21dffe8cdfb.final.pdb
-5.993022e-15 -4.987654e-15 4.609004e-15 1.685540e-15 5.816399e-15
6.804774e-15
E:\\R-3.1.2\\scripts\\mPDBS\\summary_pdb_93\\472a40b3e8f113c7.final.pdb
9.774641e-17 3.631045e-15 2.411714e-15 2.833767e-15 1.359326e-15
-1.443291e-15
E:\\R-3.1.2\\scripts\\mPDBS\\summary_pdb_93\\48a26225951ea153.final.pdb
-6.126185e-15 -2.717266e-15 -3.928658e-15 4.016418e-15 -3.286468e-15
6.309212e-15
E:\\R-3.1.2\\scripts\\mPDBS\\summary_pdb_93\\4ab4a5c7c6682ce6.final.pdb
-1.267589e-14 -5.013190e-15 3.379784e-15 2.747898e-15 -3.425909e-15
1.011929e-14
E:\\R-3.1.2\\scripts\\mPDBS\\summary_pdb_93\\4eaa45b62cb6a25f.final.pdb
3.304593e-15 -1.254945e-15 -8.804711e-18 2.456092e-15 5.905613e-16
4.259039e-15
E:\\R-3.1.2\\scripts\\mPDBS\\summary_pdb_93\\58c371611d85ac96.final.pdb
3.875107e-15 -2.765964e-15 4.116723e-16 7.399560e-16 1.237028e-15

2.343176e-15
E:\\R-3.1.2\\scripts\\mPDBS\\summary_pdb_93\\59a647f04822d30a.final.pdb
-5.450338e-15 6.394438e-16 5.990693e-15 2.841622e-16 2.920431e-16
-1.039540e-15
E:\\R-3.1.2\\scripts\\mPDBS\\summary_pdb_93\\59b091fe385e05fc.final.pdb
6.262265e-16 4.914433e-15 4.524121e-15 3.580135e-15 6.643957e-15
-7.322475e-16
E:\\R-3.1.2\\scripts\\mPDBS\\summary_pdb_93\\5c35ac30f930d2db.final.pdb
3.304593e-15 -1.254945e-15 -8.804711e-18 2.456092e-15 5.905613e-16
4.259039e-15
E:\\R-3.1.2\\scripts\\mPDBS\\summary_pdb_93\\5f60752431cf2dcb.final.pdb
3.274661e-15 -2.778416e-15 3.072881e-15 1.046238e-14 2.078488e-15
2.637092e-15
E:\\R-3.1.2\\scripts\\mPDBS\\summary_pdb_93\\626456dd6ff2868d.final.pdb
9.774641e-17 3.631045e-15 2.411714e-15 2.833767e-15 1.359326e-15
-1.443291e-15
E:\\R-3.1.2\\scripts\\mPDBS\\summary_pdb_93\\6583d53ff3c8f7ef.final.pdb
-5.450338e-15 6.394438e-16 5.990693e-15 2.841622e-16 2.920431e-16
-1.039540e-15
E:\\R-3.1.2\\scripts\\mPDBS\\summary_pdb_93\\69760178986bef01.final.pdb
-6.540647e-16 -2.856771e-15 2.889905e-15 -5.600493e-15 -1.462847e-14
-3.035600e-15
E:\\R-3.1.2\\scripts\\mPDBS\\summary_pdb_93\\6a9d87d47926b1c8.final.pdb
-1.028956e-15 1.733800e-15 2.900784e-15 2.628203e-15 3.454004e-15
1.663599e-15
E:\\R-3.1.2\\scripts\\mPDBS\\summary_pdb_93\\6bb3547ea982ffc4.final.pdb
-3.707972e-15 -8.677462e-15 -1.166392e-15 1.063104e-14 -2.972978e-15
5.214616e-15
E:\\R-3.1.2\\scripts\\mPDBS\\summary_pdb_93\\716d9624611d1c42.final.pdb
-5.241964e-15 2.063956e-15 5.629663e-16 -4.978397e-15 6.273917e-16
2.802541e-15
E:\\R-3.1.2\\scripts\\mPDBS\\summary_pdb_93\\75f27e70ad0f1be4.final.pdb
-5.450338e-15 6.394438e-16 5.990693e-15 2.841622e-16 2.920431e-16
-1.039540e-15
E:\\R-3.1.2\\scripts\\mPDBS\\summary_pdb_93\\769f910fffb22872.final.pdb
-8.095495e-16 1.861877e-15 3.443009e-15 2.096539e-15 4.029370e-15
9.528069e-16
E:\\R-3.1.2\\scripts\\mPDBS\\summary_pdb_93\\791f8c80dd5c3135.final.pdb
6.262265e-16 4.914433e-15 4.524121e-15 3.580135e-15 6.643957e-15
-7.322475e-16
E:\\R-3.1.2\\scripts\\mPDBS\\summary_pdb_93\\7fad98e66a3f6099.final.pdb
-5.317590e-15 9.067895e-15 8.329161e-15 -1.266840e-16 8.143620e-15
7.239109e-15
E:\\R-3.1.2\\scripts\\mPDBS\\summary_pdb_93\\804b89c94a633196.final.pdb
-4.685040e-15 3.634789e-15 1.112668e-15 3.330925e-15 2.680019e-15
5.172685e-16
E:\\R-3.1.2\\scripts\\mPDBS\\summary_pdb_93\\813cc65a2298feba.final.pdb
-4.685040e-15 3.634789e-15 1.112668e-15 3.330925e-15 2.680019e-15
5.172685e-16
E:\\R-3.1.2\\scripts\\mPDBS\\summary_pdb_93\\83c0eefcfe12d3f9.final.pdb
-6.486551e-15 7.627379e-16 -3.161135e-15 5.245626e-15 3.475389e-15
2.902296e-15
E:\\R-3.1.2\\scripts\\mPDBS\\summary_pdb_93\\86d50469ef569760.final.pdb
9.774641e-17 3.631045e-15 2.411714e-15 2.833767e-15 1.359326e-15

-1.443291e-15
E:\\R-3.1.2\\scripts\\mPDBS\\summary_pdb_93\\89d0666e1402203d.final.pdb
-5.450338e-15 6.394438e-16 5.990693e-15 2.841622e-16 2.920431e-16
-1.039540e-15
E:\\R-3.1.2\\scripts\\mPDBS\\summary_pdb_93\\8b34852716dadc62.final.pdb
2.161913e-15 7.868071e-16 6.870432e-15 3.589103e-16 2.260216e-15
1.200533e-15
E:\\R-3.1.2\\scripts\\mPDBS\\summary_pdb_93\\8b6ef9ac3fe74395.final.pdb
1.011896e-15 -4.111709e-16 3.616768e-15 6.811871e-15 8.531089e-16
-4.851588e-15
E:\\R-3.1.2\\scripts\\mPDBS\\summary_pdb_93\\98bbe0d5986c138f.final.pdb
6.262265e-16 4.914433e-15 4.524121e-15 3.580135e-15 6.643957e-15
-7.322475e-16
E:\\R-3.1.2\\scripts\\mPDBS\\summary_pdb_93\\9c22f4909d67e2fa.final.pdb
9.777349e-15 7.203658e-15 8.783019e-15 -2.899768e-15 6.123879e-15
1.802985e-16
E:\\R-3.1.2\\scripts\\mPDBS\\summary_pdb_93\\9dda8ed0d81fb3ef.final.pdb
5.865263e-15 -5.044920e-15 -5.486360e-15 2.312488e-15 6.010559e-15
-6.199538e-16
E:\\R-3.1.2\\scripts\\mPDBS\\summary_pdb_93\\9f752b3616e6033b.final.pdb
-8.622562e-16 2.052399e-15 6.304469e-15 1.955022e-15 3.808571e-15
-1.254577e-15
E:\\R-3.1.2\\scripts\\mPDBS\\summary_pdb_93\\a02c70083816d6ba.final.pdb
3.304593e-15 -1.254945e-15 -8.804711e-18 2.456092e-15 5.905613e-16
4.259039e-15
E:\\R-3.1.2\\scripts\\mPDBS\\summary_pdb_93\\a0c997d891e4e082.final.pdb
3.365878e-15 2.881208e-15 7.766630e-16 4.524436e-15 -2.556369e-15
6.997483e-15
E:\\R-3.1.2\\scripts\\mPDBS\\summary_pdb_93\\ab8039a2d823c616.final.pdb
-1.092361e-15 8.897450e-16 3.489703e-15 2.750248e-15 4.142700e-15
2.146762e-15
E:\\R-3.1.2\\scripts\\mPDBS\\summary_pdb_93\\acf4d74b9c71d4dc.final.pdb
-4.685040e-15 3.634789e-15 1.112668e-15 3.330925e-15 2.680019e-15
5.172685e-16
E:\\R-3.1.2\\scripts\\mPDBS\\summary_pdb_93\\ad8a823e4b4c668e.final.pdb
-8.363566e-16 1.887257e-15 3.218966e-15 2.332824e-15 3.243979e-15
1.802662e-15
E:\\R-3.1.2\\scripts\\mPDBS\\summary_pdb_93\\b16ababd03b7b857.final.pdb
9.777349e-15 7.203658e-15 8.783019e-15 -2.899768e-15 6.123879e-15
1.802985e-16
E:\\R-3.1.2\\scripts\\mPDBS\\summary_pdb_93\\b226923e697d3c63.final.pdb
3.138277e-15 -2.244485e-16 2.900120e-15 5.392055e-15 3.861670e-15
3.317652e-15
E:\\R-3.1.2\\scripts\\mPDBS\\summary_pdb_93\\b28e48f6fa27a165.final.pdb
6.262265e-16 4.914433e-15 4.524121e-15 3.580135e-15 6.643957e-15
-7.322475e-16
E:\\R-3.1.2\\scripts\\mPDBS\\summary_pdb_93\\b42c4924e3cd1aec.final.pdb
3.171237e-15 7.166991e-15 7.528485e-15 5.243303e-15 -1.787457e-15
5.236039e-15
E:\\R-3.1.2\\scripts\\mPDBS\\summary_pdb_93\\b4dd239fcabff1df.final.pdb
6.262265e-16 4.914433e-15 4.524121e-15 3.580135e-15 6.643957e-15
-7.322475e-16
E:\\R-3.1.2\\scripts\\mPDBS\\summary_pdb_93\\b669942ef97459a2.final.pdb
2.161913e-15 7.868071e-16 6.870432e-15 3.589103e-16 2.260216e-15

1.200533e-15
E:\\R-3.1.2\\scripts\\mPDBS\\summary_pdb_93\\b99f4d9d87c1017b.final.pdb
6.262265e-16 4.914433e-15 4.524121e-15 3.580135e-15 6.643957e-15
-7.322475e-16
E:\\R-3.1.2\\scripts\\mPDBS\\summary_pdb_93\\b9d2cbf3975403e7.final.pdb
6.262265e-16 4.914433e-15 4.524121e-15 3.580135e-15 6.643957e-15
-7.322475e-16
E:\\R-3.1.2\\scripts\\mPDBS\\summary_pdb_93\\bc88c8a505f3a288.final.pdb
-7.051738e-16 1.321387e-15 3.510677e-15 2.847214e-15 3.034431e-15
1.146675e-15
E:\\R-3.1.2\\scripts\\mPDBS\\summary_pdb_93\\bdac0e64f4ef427e.final.pdb
1.112696e-14 4.269590e-15 1.577821e-14 -2.736704e-15 1.180784e-15
-1.943654e-15
E:\\R-3.1.2\\scripts\\mPDBS\\summary_pdb_93\\ca2a1f55fc9d0681.final.pdb
2.161913e-15 7.868071e-16 6.870432e-15 3.589103e-16 2.260216e-15
1.200533e-15
E:\\R-3.1.2\\scripts\\mPDBS\\summary_pdb_93\\ce34248de3e81a3c.final.pdb
2.030146e-15 5.354788e-15 -5.085975e-15 3.089163e-15 7.265170e-15
7.766660e-15
E:\\R-3.1.2\\scripts\\mPDBS\\summary_pdb_93\\d02e697772494a61.final.pdb
-1.102938e-14 2.888710e-15 8.658224e-15 -3.104162e-16 4.497670e-15
-3.074341e-15
E:\\R-3.1.2\\scripts\\mPDBS\\summary_pdb_93\\d04221245d015047.final.pdb
-2.254626e-15 2.075429e-15 3.772530e-15 1.447037e-15 1.912279e-15
7.507438e-16
E:\\R-3.1.2\\scripts\\mPDBS\\summary_pdb_93\\d678141e4018054a.final.pdb
-6.482482e-15 -6.049658e-15 2.501292e-15 9.875471e-15 1.014756e-14
1.196311e-14
E:\\R-3.1.2\\scripts\\mPDBS\\summary_pdb_93\\d71b40af0f0efe39.final.pdb
-4.211917e-15 4.601894e-15 1.122371e-14 3.394286e-16 8.998317e-15
-1.277487e-15
E:\\R-3.1.2\\scripts\\mPDBS\\summary_pdb_93\\dc0809eff10197e2.final.pdb
2.552069e-15 6.986533e-15 9.801291e-15 1.139302e-15 1.845791e-15
-6.897024e-15
E:\\R-3.1.2\\scripts\\mPDBS\\summary_pdb_93\\e1163918d3707839.final.pdb
1.536260e-15 6.006461e-15 1.192516e-15 3.756211e-15 8.057099e-15
9.805951e-15
E:\\R-3.1.2\\scripts\\mPDBS\\summary_pdb_93\\e4b48b06824ef848.final.pdb
3.102145e-16 -5.373005e-15 -1.869094e-15 4.715538e-15 -2.488922e-16
2.328009e-15
E:\\R-3.1.2\\scripts\\mPDBS\\summary_pdb_93\\e529e19ad281fea2.final.pdb
-3.810586e-15 2.915038e-15 -1.344613e-15 5.060719e-15 3.711611e-15
7.543661e-15
E:\\R-3.1.2\\scripts\\mPDBS\\summary_pdb_93\\e5602082718ec3a1.final.pdb
-4.685040e-15 3.634789e-15 1.112668e-15 3.330925e-15 2.680019e-15
5.172685e-16
E:\\R-3.1.2\\scripts\\mPDBS\\summary_pdb_93\\e5d589d996d0a960.final.pdb
-1.314885e-15 1.809912e-15 -5.244597e-15 4.192104e-16 -3.966285e-15
-1.989214e-15
E:\\R-3.1.2\\scripts\\mPDBS\\summary_pdb_93\\e9df9e72307a2fbd.final.pdb
3.304593e-15 -1.254945e-15 -8.804711e-18 2.456092e-15 5.905613e-16
4.259039e-15
E:\\R-3.1.2\\scripts\\mPDBS\\summary_pdb_93\\ea00f8694090cae7.final.pdb
-4.685040e-15 3.634789e-15 1.112668e-15 3.330925e-15 2.680019e-15

5.172685e-16
E:\\R-3.1.2\\scripts\\mPDBS\\summary_pdb_93\\eba517be32db050e.final.pdb
6.262265e-16 4.914433e-15 4.524121e-15 3.580135e-15 6.643957e-15
-7.322475e-16
E:\\R-3.1.2\\scripts\\mPDBS\\summary_pdb_93\\ec04b9545899c29d.final.pdb
-1.050212e-15 1.150428e-15 3.321279e-15 2.479021e-15 3.251481e-15
1.173533e-15
E:\\R-3.1.2\\scripts\\mPDBS\\summary_pdb_93\\ee6862cbcad52078.final.pdb
-1.142324e-15 1.445095e-15 3.536304e-15 2.815555e-15 2.246433e-15
6.446893e-16
E:\\R-3.1.2\\scripts\\mPDBS\\summary_pdb_93\\ef6a42fc83b81e5d.final.pdb
-8.390398e-16 1.332677e-15 3.594431e-15 2.430907e-15 2.938662e-15
4.542908e-16
E:\\R-3.1.2\\scripts\\mPDBS\\summary_pdb_93\\ef8e88d4ad93a0ad.final.pdb
-2.254626e-15 2.075429e-15 3.772530e-15 1.447037e-15 1.912279e-15
7.507438e-16
E:\\R-3.1.2\\scripts\\mPDBS\\summary_pdb_93\\f14fcae8deec4fa3.final.pdb
-8.622562e-16 2.052399e-15 6.304469e-15 1.955022e-15 3.808571e-15
-1.254577e-15
E:\\R-3.1.2\\scripts\\mPDBS\\summary_pdb_93\\f83584e22003fb92.final.pdb
-6.126185e-15 -2.717266e-15 -3.928658e-15 4.016418e-15 -3.286468e-15
6.309212e-15
E:\\R-3.1.2\\scripts\\mPDBS\\summary_pdb_93\\f8e2bf87e52caa29.final.pdb
-3.810586e-15 2.915038e-15 -1.344613e-15 5.060719e-15 3.711611e-15
7.543661e-15

[,559]   [,560]   [,561]   [,562]   [,563]   [,564]
E:\\R-3.1.2\\scripts\\40.1ns_data\\NAT0.pdb
-1.035561e-15 3.123533e-15 1.944256e-16 1.035845e-16 5.596453e-15
-2.817761e-15
E:\\R-3.1.2\\scripts\\40.1ns_data\\LJP6.pdb
-3.943712e-16 2.120548e-15 2.426646e-15 -1.246164e-15 5.987679e-15
-2.927114e-15
E:\\R-3.1.2\\scripts\\40.1ns_data\\3WY9.pdb
3.173699e-16 1.478752e-15 2.553134e-15 -1.110047e-15 4.371671e-15
-3.669137e-15
E:\\R-3.1.2\\scripts\\40.1ns_data\\ZSP9.pdb
-3.411479e-16 1.924482e-15 3.866022e-15 3.870465e-16 4.948230e-15
-2.357563e-15
E:\\R-3.1.2\\scripts\\mPDBS\\summary_pdb_93\\013d855ad26c877c.final.pdb
-2.434581e-15 2.298554e-15 2.757103e-15 3.903053e-15 4.149069e-15
-9.373358e-16
E:\\R-3.1.2\\scripts\\mPDBS\\summary_pdb_93\\0a2a705342ca0d54.final.pdb
-6.097528e-16 1.270055e-15 1.278808e-15 2.784577e-17 5.311294e-15
-2.503262e-15
E:\\R-3.1.2\\scripts\\mPDBS\\summary_pdb_93\\0a59fceadb4194d9.final.pdb
-1.651661e-15 -2.492474e-15 5.866262e-15 -3.858347e-15 2.581759e-15
-3.466347e-16
E:\\R-3.1.2\\scripts\\mPDBS\\summary_pdb_93\\0dba59f3306825e8.final.pdb
-1.946256e-15 1.775101e-15 2.275717e-15 -2.990738e-15 -1.817512e-15
-4.722502e-15
E:\\R-3.1.2\\scripts\\mPDBS\\summary_pdb_93\\126f26a78b57339c.final.pdb
-3.471837e-15 5.709020e-15 -3.515216e-16 5.633331e-15 1.264878e-14
-4.013000e-15

E:\\R-3.1.2\\scripts\\mPDBS\\summary_pdb_93\\12f06f3e342df706.final.pdb
-5.945740e-16 1.619819e-15 2.590259e-15 6.177060e-16 4.644293e-15
-2.553569e-15
E:\\R-3.1.2\\scripts\\mPDBS\\summary_pdb_93\\18618a2a251b867b.final.pdb
-5.078335e-15 4.970338e-15 -1.480273e-15 4.687718e-15 9.718433e-15
-5.112561e-15
E:\\R-3.1.2\\scripts\\mPDBS\\summary_pdb_93\\1f305f598641eeb2.final.pdb
-5.015120e-16 1.663772e-15 2.246740e-15 -1.515674e-16 4.634853e-15
-2.381174e-15
E:\\R-3.1.2\\scripts\\mPDBS\\summary_pdb_93\\1f8a00b551be0a39.final.pdb
-5.078335e-15 4.970338e-15 -1.480273e-15 4.687718e-15 9.718433e-15
-5.112561e-15
E:\\R-3.1.2\\scripts\\mPDBS\\summary_pdb_93\\219f45328d47579a.final.pdb
1.117814e-14 4.092846e-16 -6.488872e-15 -7.086667e-15 3.090033e-15
-4.698000e-16
E:\\R-3.1.2\\scripts\\mPDBS\\summary_pdb_93\\238dd3918e54336d.final.pdb
3.928781e-15 6.719558e-15 -7.153271e-15 6.703029e-15 8.092108e-15
-7.879455e-15
E:\\R-3.1.2\\scripts\\mPDBS\\summary_pdb_93\\2b3027889e2fdc42.final.pdb
-1.823074e-15 1.506931e-15 2.111887e-15 -3.941583e-16 4.288293e-15
-2.212980e-15
E:\\R-3.1.2\\scripts\\mPDBS\\summary_pdb_93\\2fcb3b46e33793b0.final.pdb
-1.103461e-15 8.007472e-16 2.370772e-15 -4.519601e-15 -6.959522e-16
-2.183416e-15
E:\\R-3.1.2\\scripts\\mPDBS\\summary_pdb_93\\34eb328251842006.final.pdb
7.688404e-15 -8.108957e-15 7.533221e-15 -6.246181e-15 3.400531e-15
-4.739522e-15
E:\\R-3.1.2\\scripts\\mPDBS\\summary_pdb_93\\354b32871797b7ab.final.pdb
7.992653e-16 -7.198235e-15 -2.246249e-15 -1.200308e-14 -2.078960e-16
-5.987638e-15
E:\\R-3.1.2\\scripts\\mPDBS\\summary_pdb_93\\397368adbc32d5de.final.pdb
-3.471837e-15 5.709020e-15 -3.515216e-16 5.633331e-15 1.264878e-14
-4.013000e-15
E:\\R-3.1.2\\scripts\\mPDBS\\summary_pdb_93\\411ca13cd3df6edd.final.pdb
-2.197313e-15 2.892824e-15 1.664740e-15 -2.579205e-15 4.290077e-15
-5.418675e-15
E:\\R-3.1.2\\scripts\\mPDBS\\summary_pdb_93\\42357eee6642bf12.final.pdb
7.931495e-15 -1.142525e-14 -3.939654e-15 -1.019323e-14 3.526370e-15
-1.334623e-15
E:\\R-3.1.2\\scripts\\mPDBS\\summary_pdb_93\\44342d1f75a7fcfc.final.pdb
-9.835942e-15 1.576854e-15 4.387810e-15 4.615366e-15 8.481095e-15
-7.695378e-15
E:\\R-3.1.2\\scripts\\mPDBS\\summary_pdb_93\\448ef21dffe8cdfb.final.pdb
-3.046351e-15 1.115166e-15 6.943503e-15 7.269580e-15 2.789142e-14
6.854493e-15
E:\\R-3.1.2\\scripts\\mPDBS\\summary_pdb_93\\472a40b3e8f113c7.final.pdb
-1.946256e-15 1.775101e-15 2.275717e-15 -2.990738e-15 -1.817512e-15
-4.722502e-15
E:\\R-3.1.2\\scripts\\mPDBS\\summary_pdb_93\\48a26225951ea153.final.pdb
-5.616697e-15 7.720710e-15 3.541299e-15 7.641081e-15 2.723573e-15
-4.306792e-15
E:\\R-3.1.2\\scripts\\mPDBS\\summary_pdb_93\\4ab4a5c7c6682ce6.final.pdb
-6.992025e-15 -1.736413e-15 9.764092e-16 -4.282348e-15 9.398776e-15
3.723805e-16

E:\\R-3.1.2\\scripts\\mPDBS\\summary_pdb_93\\4eaa45b62cb6a25f.final.pdb
-4.010093e-15 6.965566e-15 2.911084e-15 7.153096e-15 7.253150e-15
-2.031818e-15
E:\\R-3.1.2\\scripts\\mPDBS\\summary_pdb_93\\58c371611d85ac96.final.pdb
-3.471837e-15 5.709020e-15 -3.515216e-16 5.633331e-15 1.264878e-14
-4.013000e-15
E:\\R-3.1.2\\scripts\\mPDBS\\summary_pdb_93\\59a647f04822d30a.final.pdb
5.430709e-15 -2.346312e-16 -1.734462e-15 1.006274e-15 3.542338e-15
-4.859106e-15
E:\\R-3.1.2\\scripts\\mPDBS\\summary_pdb_93\\59b091fe385e05fc.final.pdb
-1.651661e-15 -2.492474e-15 5.866262e-15 -3.858347e-15 2.581759e-15
-3.466347e-16
E:\\R-3.1.2\\scripts\\mPDBS\\summary_pdb_93\\5c35ac30f930d2db.final.pdb
-4.010093e-15 6.965566e-15 2.911084e-15 7.153096e-15 7.253150e-15
-2.031818e-15
E:\\R-3.1.2\\scripts\\mPDBS\\summary_pdb_93\\5f60752431cf2dcb.final.pdb
-7.252334e-15 3.680887e-15 8.576790e-15 9.324499e-16 2.671479e-15
2.872863e-15
E:\\R-3.1.2\\scripts\\mPDBS\\summary_pdb_93\\626456dd6ff2868d.final.pdb
-1.946256e-15 1.775101e-15 2.275717e-15 -2.990738e-15 -1.817512e-15
-4.722502e-15
E:\\R-3.1.2\\scripts\\mPDBS\\summary_pdb_93\\6583d53ff3c8f7ef.final.pdb
5.430709e-15 -2.346312e-16 -1.734462e-15 1.006274e-15 3.542338e-15
-4.859106e-15
E:\\R-3.1.2\\scripts\\mPDBS\\summary_pdb_93\\69760178986bef01.final.pdb
-8.769357e-15 1.010237e-14 2.161682e-15 1.219113e-15 2.803592e-15
-9.251971e-16
E:\\R-3.1.2\\scripts\\mPDBS\\summary_pdb_93\\6a9d87d47926b1c8.final.pdb
-1.983878e-15 1.395630e-15 2.460465e-15 -9.031070e-16 4.604000e-15
-2.385830e-15
E:\\R-3.1.2\\scripts\\mPDBS\\summary_pdb_93\\6bb3547ea982ffc4.final.pdb
-6.155975e-15 5.825988e-15 1.830347e-15 5.576688e-15 6.362839e-15
-1.276308e-14
E:\\R-3.1.2\\scripts\\mPDBS\\summary_pdb_93\\716d9624611d1c42.final.pdb
2.339494e-15 4.791977e-15 -1.128554e-15 5.454061e-15 1.009479e-14
-8.793369e-15
E:\\R-3.1.2\\scripts\\mPDBS\\summary_pdb_93\\75f27e70ad0f1be4.final.pdb
5.430709e-15 -2.346312e-16 -1.734462e-15 1.006274e-15 3.542338e-15
-4.859106e-15
E:\\R-3.1.2\\scripts\\mPDBS\\summary_pdb_93\\769f910fffb22872.final.pdb
-8.280995e-16 1.618202e-15 1.921954e-15 -9.514347e-16 4.136527e-15
-3.109822e-15
E:\\R-3.1.2\\scripts\\mPDBS\\summary_pdb_93\\791f8c80dd5c3135.final.pdb
-1.651661e-15 -2.492474e-15 5.866262e-15 -3.858347e-15 2.581759e-15
-3.466347e-16
E:\\R-3.1.2\\scripts\\mPDBS\\summary_pdb_93\\7fad98e66a3f6099.final.pdb
1.884044e-15 -4.135181e-15 2.207999e-15 -8.056776e-15 1.418393e-15
-4.309183e-15
E:\\R-3.1.2\\scripts\\mPDBS\\summary_pdb_93\\804b89c94a633196.final.pdb
-1.103461e-15 8.007472e-16 2.370772e-15 -4.519601e-15 -6.959522e-16
-2.183416e-15
E:\\R-3.1.2\\scripts\\mPDBS\\summary_pdb_93\\813cc65a2298feba.final.pdb
-1.103461e-15 8.007472e-16 2.370772e-15 -4.519601e-15 -6.959522e-16
-2.183416e-15

E:\\R-3.1.2\\scripts\\mPDBS\\summary_pdb_93\\83c0eefcfe12d3f9.final.pdb
-2.197313e-15 2.892824e-15 1.664740e-15 -2.579205e-15 4.290077e-15
-5.418675e-15
E:\\R-3.1.2\\scripts\\mPDBS\\summary_pdb_93\\86d50469ef569760.final.pdb
-1.946256e-15 1.775101e-15 2.275717e-15 -2.990738e-15 -1.817512e-15
-4.722502e-15
E:\\R-3.1.2\\scripts\\mPDBS\\summary_pdb_93\\89d0666e1402203d.final.pdb
5.430709e-15 -2.346312e-16 -1.734462e-15 1.006274e-15 3.542338e-15
-4.859106e-15
E:\\R-3.1.2\\scripts\\mPDBS\\summary_pdb_93\\8b34852716dadc62.final.pdb
2.253371e-16 8.519215e-16 1.330806e-15 -3.468359e-15 5.446704e-15
-3.963242e-15
E:\\R-3.1.2\\scripts\\mPDBS\\summary_pdb_93\\8b6ef9ac3fe74395.final.pdb
2.308636e-15 -4.031725e-15 1.675692e-15 -4.382523e-15 1.923648e-15
-6.895637e-15
E:\\R-3.1.2\\scripts\\mPDBS\\summary_pdb_93\\98bbe0d5986c138f.final.pdb
-1.651661e-15 -2.492474e-15 5.866262e-15 -3.858347e-15 2.581759e-15
-3.466347e-16
E:\\R-3.1.2\\scripts\\mPDBS\\summary_pdb_93\\9c22f4909d67e2fa.final.pdb
1.498136e-15 4.372543e-15 3.674417e-15 5.703666e-15 3.311259e-15
-2.802199e-17
E:\\R-3.1.2\\scripts\\mPDBS\\summary_pdb_93\\9dda8ed0d81fb3ef.final.pdb
-8.394007e-15 5.317351e-15 1.559846e-14 1.195233e-14 5.866433e-15
-6.109624e-15
E:\\R-3.1.2\\scripts\\mPDBS\\summary_pdb_93\\9f752b3616e6033b.final.pdb
4.091830e-16 -9.855948e-16 1.000760e-15 -3.853289e-15 3.536583e-15
-3.024081e-15
E:\\R-3.1.2\\scripts\\mPDBS\\summary_pdb_93\\a02c70083816d6ba.final.pdb
-4.010093e-15 6.965566e-15 2.911084e-15 7.153096e-15 7.253150e-15
-2.031818e-15
E:\\R-3.1.2\\scripts\\mPDBS\\summary_pdb_93\\a0c997d891e4e082.final.pdb
-1.069119e-14 6.010374e-15 3.561940e-15 -8.840769e-15 3.209037e-15
-1.191350e-15
E:\\R-3.1.2\\scripts\\mPDBS\\summary_pdb_93\\ab8039a2d823c616.final.pdb
-8.992711e-16 1.582709e-15 1.973801e-15 -2.153445e-16 4.094614e-15
-3.984905e-15
E:\\R-3.1.2\\scripts\\mPDBS\\summary_pdb_93\\acf4d74b9c71d4dc.final.pdb
-1.103461e-15 8.007472e-16 2.370772e-15 -4.519601e-15 -6.959522e-16
-2.183416e-15
E:\\R-3.1.2\\scripts\\mPDBS\\summary_pdb_93\\ad8a823e4b4c668e.final.pdb
-1.376775e-15 1.581492e-15 2.658990e-15 -7.708862e-16 5.224196e-15
-2.150637e-15
E:\\R-3.1.2\\scripts\\mPDBS\\summary_pdb_93\\b16ababd03b7b857.final.pdb
1.498136e-15 4.372543e-15 3.674417e-15 5.703666e-15 3.311259e-15
-2.802199e-17
E:\\R-3.1.2\\scripts\\mPDBS\\summary_pdb_93\\b226923e697d3c63.final.pdb
-4.269593e-15 -4.941892e-16 -3.282269e-15 -1.198897e-14 9.150265e-15
-1.848493e-15
E:\\R-3.1.2\\scripts\\mPDBS\\summary_pdb_93\\b28e48f6fa27a165.final.pdb
-1.651661e-15 -2.492474e-15 5.866262e-15 -3.858347e-15 2.581759e-15
-3.466347e-16
E:\\R-3.1.2\\scripts\\mPDBS\\summary_pdb_93\\b42c4924e3cd1aec.final.pdb
4.392206e-15 -1.646479e-15 -1.830306e-15 -4.468025e-15 -3.733093e-15
1.610329e-15

E:\\R-3.1.2\\scripts\\mPDBS\\summary_pdb_93\\b4dd239fcabff1df.final.pdb
-1.651661e-15 -2.492474e-15 5.866262e-15 -3.858347e-15 2.581759e-15
-3.466347e-16
E:\\R-3.1.2\\scripts\\mPDBS\\summary_pdb_93\\b669942ef97459a2.final.pdb
2.253371e-16 8.519215e-16 1.330806e-15 -3.468359e-15 5.446704e-15
-3.963242e-15
E:\\R-3.1.2\\scripts\\mPDBS\\summary_pdb_93\\b99f4d9d87c1017b.final.pdb
-1.651661e-15 -2.492474e-15 5.866262e-15 -3.858347e-15 2.581759e-15
-3.466347e-16
E:\\R-3.1.2\\scripts\\mPDBS\\summary_pdb_93\\b9d2cbf3975403e7.final.pdb
-1.651661e-15 -2.492474e-15 5.866262e-15 -3.858347e-15 2.581759e-15
-3.466347e-16
E:\\R-3.1.2\\scripts\\mPDBS\\summary_pdb_93\\bc88c8a505f3a288.final.pdb
-1.499494e-15 1.494137e-15 1.860352e-15 3.098141e-16 4.678606e-15
-2.148145e-15
E:\\R-3.1.2\\scripts\\mPDBS\\summary_pdb_93\\bdac0e64f4ef427e.final.pdb
2.262174e-15 -1.370693e-15 6.140326e-15 4.302877e-15 4.865138e-15
1.027991e-15
E:\\R-3.1.2\\scripts\\mPDBS\\summary_pdb_93\\ca2a1f55fc9d0681.final.pdb
2.253371e-16 8.519215e-16 1.330806e-15 -3.468359e-15 5.446704e-15
-3.963242e-15
E:\\R-3.1.2\\scripts\\mPDBS\\summary_pdb_93\\ce34248de3e81a3c.final.pdb
-1.781288e-15 1.809632e-15 3.045222e-15 3.684230e-15 4.789620e-15
1.835976e-15
E:\\R-3.1.2\\scripts\\mPDBS\\summary_pdb_93\\d02e697772494a61.final.pdb
9.021227e-16 7.711913e-16 -6.476729e-15 -8.181766e-15 1.151385e-14
-3.044780e-15
E:\\R-3.1.2\\scripts\\mPDBS\\summary_pdb_93\\d04221245d015047.final.pdb
3.832859e-16 2.043061e-15 4.085827e-15 4.081186e-15 3.174343e-15
-3.522818e-15
E:\\R-3.1.2\\scripts\\mPDBS\\summary_pdb_93\\d678141e4018054a.final.pdb
-3.308278e-15 3.679929e-15 -2.462115e-15 9.898124e-15 1.226730e-14
-7.913659e-15
E:\\R-3.1.2\\scripts\\mPDBS\\summary_pdb_93\\d71b40af0f0efe39.final.pdb
3.329560e-15 3.216609e-15 1.358301e-15 -2.070543e-15 1.033839e-14
-1.216602e-15
E:\\R-3.1.2\\scripts\\mPDBS\\summary_pdb_93\\dc0809eff10197e2.final.pdb
2.659744e-15 -1.119248e-15 3.518364e-15 -4.136239e-15 1.266832e-15
-4.389246e-15
E:\\R-3.1.2\\scripts\\mPDBS\\summary_pdb_93\\e1163918d3707839.final.pdb
-2.037113e-15 1.469951e-15 5.913500e-15 -8.901580e-16 2.740895e-15
3.731896e-15
E:\\R-3.1.2\\scripts\\mPDBS\\summary_pdb_93\\e4b48b06824ef848.final.pdb
-5.078335e-15 4.970338e-15 -1.480273e-15 4.687718e-15 9.718433e-15
-5.112561e-15
E:\\R-3.1.2\\scripts\\mPDBS\\summary_pdb_93\\e529e19ad281fea2.final.pdb
-2.434581e-15 2.298554e-15 2.757103e-15 3.903053e-15 4.149069e-15
-9.373358e-16
E:\\R-3.1.2\\scripts\\mPDBS\\summary_pdb_93\\e5602082718ec3a1.final.pdb
-1.103461e-15 8.007472e-16 2.370772e-15 -4.519601e-15 -6.959522e-16
-2.183416e-15
E:\\R-3.1.2\\scripts\\mPDBS\\summary_pdb_93\\e5d589d996d0a960.final.pdb
-7.450761e-15 1.324639e-14 9.315339e-15 -2.875956e-15 3.345181e-15
-3.505827e-17

E:\\R-3.1.2\\scripts\\mPDBS\\summary_pdb_93\\e9df9e72307a2fbd.final.pdb
-4.010093e-15 6.965566e-15 2.911084e-15 7.153096e-15 7.253150e-15
-2.031818e-15
E:\\R-3.1.2\\scripts\\mPDBS\\summary_pdb_93\\ea00f8694090cae7.final.pdb
-1.103461e-15 8.007472e-16 2.370772e-15 -4.519601e-15 -6.959522e-16
-2.183416e-15
E:\\R-3.1.2\\scripts\\mPDBS\\summary_pdb_93\\eba517be32db050e.final.pdb
-1.651661e-15 -2.492474e-15 5.866262e-15 -3.858347e-15 2.581759e-15
-3.466347e-16
E:\\R-3.1.2\\scripts\\mPDBS\\summary_pdb_93\\ec04b9545899c29d.final.pdb
-9.320396e-16 1.445015e-15 1.903686e-15 7.299303e-17 4.858993e-15
-2.433962e-15
E:\\R-3.1.2\\scripts\\mPDBS\\summary_pdb_93\\ee6862cbcad52078.final.pdb
-1.660118e-15 1.472670e-15 2.071988e-15 -1.243546e-16 4.316483e-15
-2.506365e-15
E:\\R-3.1.2\\scripts\\mPDBS\\summary_pdb_93\\ef6a42fc83b81e5d.final.pdb
-5.146024e-16 1.407216e-15 2.300991e-15 -1.113407e-16 4.638729e-15
-2.961720e-15
E:\\R-3.1.2\\scripts\\mPDBS\\summary_pdb_93\\ef8e88d4ad93a0ad.final.pdb
3.832859e-16 2.043061e-15 4.085827e-15 4.081186e-15 3.174343e-15
-3.522818e-15
E:\\R-3.1.2\\scripts\\mPDBS\\summary_pdb_93\\f14fcae8deec4fa3.final.pdb
4.091830e-16 -9.855948e-16 1.000760e-15 -3.853289e-15 3.536583e-15
-3.024081e-15
E:\\R-3.1.2\\scripts\\mPDBS\\summary_pdb_93\\f83584e22003fb92.final.pdb
-5.616697e-15 7.720710e-15 3.541299e-15 7.641081e-15 2.723573e-15
-4.306792e-15
E:\\R-3.1.2\\scripts\\mPDBS\\summary_pdb_93\\f8e2bf87e52caa29.final.pdb
-2.434581e-15 2.298554e-15 2.757103e-15 3.903053e-15 4.149069e-15
-9.373358e-16

[,565] [,566] [,567] [,568] [,569] [,570]
E:\\R-3.1.2\\scripts\\40.1ns_data\\NAT0.pdb
4.788902e-15 -2.458703e-16 2.500896e-15 -5.827519e-15 -3.302107e-15
3.622487e-16
E:\\R-3.1.2\\scripts\\40.1ns_data\\LJP6.pdb
4.357789e-15 -1.783548e-15 2.990305e-15 -6.900520e-15 -4.476542e-15
2.225596e-15
E:\\R-3.1.2\\scripts\\40.1ns_data\\3WY9.pdb
8.496883e-15 -2.256474e-15 2.851270e-15 -5.845383e-15 -2.750534e-15
5.911098e-16
E:\\R-3.1.2\\scripts\\40.1ns_data\\ZSP9.pdb
7.134679e-15 -2.183100e-15 2.585739e-15 -6.067317e-15 -5.119390e-15
6.018939e-16
E:\\R-3.1.2\\scripts\\mPDBS\\summary_pdb_93\\013d855ad26c877c.final.pdb
1.212259e-15 4.425100e-15 1.259505e-15 -1.075138e-14 -2.773844e-16
5.161886e-15
E:\\R-3.1.2\\scripts\\mPDBS\\summary_pdb_93\\0a2a705342ca0d54.final.pdb
6.893053e-15 -1.254984e-15 2.617859e-15 -6.101840e-15 -4.061983e-15
1.152780e-15
E:\\R-3.1.2\\scripts\\mPDBS\\summary_pdb_93\\0a59fceadb4194d9.final.pdb
4.851114e-15 -3.704523e-15 3.156911e-15 -5.890108e-15 -6.084350e-15
3.069157e-15
E:\\R-3.1.2\\scripts\\mPDBS\\summary_pdb_93\\0dba59f3306825e8.final.pdb

8.264778e-15 -5.249331e-16 1.554408e-15 -4.687650e-15 -6.073096e-15
7.721678e-16
E:\\R-3.1.2\\scripts\\mPDBS\\summary_pdb_93\\126f26a78b57339c.final.pdb
5.753765e-15 -3.376005e-15 1.517212e-16 -2.613734e-15 -7.356032e-15
-4.233377e-15
E:\\R-3.1.2\\scripts\\mPDBS\\summary_pdb_93\\12f06f3e342df706.final.pdb
7.172344e-15 -1.441033e-15 1.631831e-15 -6.100159e-15 -3.662672e-15
7.058714e-16
E:\\R-3.1.2\\scripts\\mPDBS\\summary_pdb_93\\18618a2a251b867b.final.pdb
8.752093e-15 6.671901e-16 -3.678464e-15 -5.312343e-15 -3.793505e-15
-1.013259e-15
E:\\R-3.1.2\\scripts\\mPDBS\\summary_pdb_93\\1f305f598641eeb2.final.pdb
5.351745e-15 -1.479337e-15 2.043211e-15 -6.060509e-15 -3.611582e-15
1.145338e-15
E:\\R-3.1.2\\scripts\\mPDBS\\summary_pdb_93\\1f8a00b551be0a39.final.pdb
8.752093e-15 6.671901e-16 -3.678464e-15 -5.312343e-15 -3.793505e-15
-1.013259e-15
E:\\R-3.1.2\\scripts\\mPDBS\\summary_pdb_93\\219f45328d47579a.final.pdb
8.651460e-15 3.624250e-15 2.589223e-15 4.845879e-15 5.080331e-15
-7.032164e-16
E:\\R-3.1.2\\scripts\\mPDBS\\summary_pdb_93\\238dd3918e54336d.final.pdb
4.909661e-15 9.445548e-15 2.472509e-15 1.285467e-15 -6.760892e-15
2.020733e-15
E:\\R-3.1.2\\scripts\\mPDBS\\summary_pdb_93\\2b3027889e2fdc42.final.pdb
5.262244e-15 -1.514055e-15 1.582541e-15 -6.052250e-15 -3.873553e-15
1.623915e-15
E:\\R-3.1.2\\scripts\\mPDBS\\summary_pdb_93\\2fcb3b46e33793b0.final.pdb
5.511773e-15 -1.951182e-15 2.434391e-15 -8.740874e-15 -1.005811e-15
2.714396e-15
E:\\R-3.1.2\\scripts\\mPDBS\\summary_pdb_93\\34eb328251842006.final.pdb
1.643381e-15 4.314099e-15 6.644046e-15 -3.392212e-15 -1.586921e-15
1.310993e-14
E:\\R-3.1.2\\scripts\\mPDBS\\summary_pdb_93\\354b32871797b7ab.final.pdb
8.118055e-15 -6.693216e-15 -3.087269e-15 -8.958067e-15 -5.783999e-15
7.020450e-15
E:\\R-3.1.2\\scripts\\mPDBS\\summary_pdb_93\\397368adbc32d5de.final.pdb
5.753765e-15 -3.376005e-15 1.517212e-16 -2.613734e-15 -7.356032e-15
-4.233377e-15
E:\\R-3.1.2\\scripts\\mPDBS\\summary_pdb_93\\411ca13cd3df6edd.final.pdb
1.804669e-15 -4.888882e-15 3.586410e-15 -6.353298e-15 -1.225578e-15
3.366652e-15
E:\\R-3.1.2\\scripts\\mPDBS\\summary_pdb_93\\42357eee6642bf12.final.pdb
4.750366e-15 -3.927062e-15 1.952887e-15 -2.086592e-15 -3.913598e-15
4.972542e-15
E:\\R-3.1.2\\scripts\\mPDBS\\summary_pdb_93\\44342d1f75a7fcfc.final.pdb
4.724842e-16 -1.864988e-15 -5.884067e-15 -7.937981e-15 -1.111815e-14
-8.945366e-16
E:\\R-3.1.2\\scripts\\mPDBS\\summary_pdb_93\\448ef21dffe8cdfb.final.pdb
-3.428558e-15 -3.584424e-15 5.913379e-15 -1.724709e-14 4.934533e-15
8.044334e-15
E:\\R-3.1.2\\scripts\\mPDBS\\summary_pdb_93\\472a40b3e8f113c7.final.pdb
8.264778e-15 -5.249331e-16 1.554408e-15 -4.687650e-15 -6.073096e-15
7.721678e-16
E:\\R-3.1.2\\scripts\\mPDBS\\summary_pdb_93\\48a26225951ea153.final.pdb

3.534893e-15 -1.848236e-16 2.219618e-15 -1.240015e-14 -2.319458e-15
-1.628908e-15
E:\\R-3.1.2\\scripts\\mPDBS\\summary_pdb_93\\4ab4a5c7c6682ce6.final.pdb
6.586433e-15 -8.584513e-15 5.047978e-15 -1.572477e-14 1.002481e-14
-6.949746e-16
E:\\R-3.1.2\\scripts\\mPDBS\\summary_pdb_93\\4eaa45b62cb6a25f.final.pdb
6.523410e-15 -8.125032e-16 2.231447e-15 -6.221324e-15 -2.910754e-15
-3.717488e-15
E:\\R-3.1.2\\scripts\\mPDBS\\summary_pdb_93\\58c371611d85ac96.final.pdb
5.753765e-15 -3.376005e-15 1.517212e-16 -2.613734e-15 -7.356032e-15
-4.233377e-15
E:\\R-3.1.2\\scripts\\mPDBS\\summary_pdb_93\\59a647f04822d30a.final.pdb
4.337882e-15 -1.307274e-15 -2.097860e-16 -3.520291e-15 -1.940592e-15
-2.966447e-15
E:\\R-3.1.2\\scripts\\mPDBS\\summary_pdb_93\\59b091fe385e05fc.final.pdb
4.851114e-15 -3.704523e-15 3.156911e-15 -5.890108e-15 -6.084350e-15
3.069157e-15
E:\\R-3.1.2\\scripts\\mPDBS\\summary_pdb_93\\5c35ac30f930d2db.final.pdb
6.523410e-15 -8.125032e-16 2.231447e-15 -6.221324e-15 -2.910754e-15
-3.717488e-15
E:\\R-3.1.2\\scripts\\mPDBS\\summary_pdb_93\\5f60752431cf2dcb.final.pdb
2.420502e-15 2.632043e-15 3.025844e-15 -3.536780e-15 -4.717275e-15
-1.649598e-16
E:\\R-3.1.2\\scripts\\mPDBS\\summary_pdb_93\\626456dd6ff2868d.final.pdb
8.264778e-15 -5.249331e-16 1.554408e-15 -4.687650e-15 -6.073096e-15
7.721678e-16
E:\\R-3.1.2\\scripts\\mPDBS\\summary_pdb_93\\6583d53ff3c8f7ef.final.pdb
4.337882e-15 -1.307274e-15 -2.097860e-16 -3.520291e-15 -1.940592e-15
-2.966447e-15
E:\\R-3.1.2\\scripts\\mPDBS\\summary_pdb_93\\69760178986bef01.final.pdb
8.950720e-15 -2.440024e-16 -3.371748e-15 -6.159526e-15 -3.082978e-15
7.890809e-16
E:\\R-3.1.2\\scripts\\mPDBS\\summary_pdb_93\\6a9d87d47926b1c8.final.pdb
5.052070e-15 -1.094844e-15 1.450162e-15 -6.210250e-15 -3.981127e-15
9.590843e-16
E:\\R-3.1.2\\scripts\\mPDBS\\summary_pdb_93\\6bb3547ea982ffc4.final.pdb
2.148094e-15 1.222314e-14 -1.682328e-17 -6.836235e-15 3.512985e-15
-1.373501e-15
E:\\R-3.1.2\\scripts\\mPDBS\\summary_pdb_93\\716d9624611d1c42.final.pdb
6.688789e-15 -2.553859e-15 3.896064e-16 -8.631284e-15 -9.055089e-17
2.702104e-15
E:\\R-3.1.2\\scripts\\mPDBS\\summary_pdb_93\\75f27e70ad0f1be4.final.pdb
4.337882e-15 -1.307274e-15 -2.097860e-16 -3.520291e-15 -1.940592e-15
-2.966447e-15
E:\\R-3.1.2\\scripts\\mPDBS\\summary_pdb_93\\769f910fffb22872.final.pdb
5.818676e-15 -1.398378e-15 1.374406e-15 -5.826200e-15 -3.363186e-15
8.600257e-16
E:\\R-3.1.2\\scripts\\mPDBS\\summary_pdb_93\\791f8c80dd5c3135.final.pdb
4.851114e-15 -3.704523e-15 3.156911e-15 -5.890108e-15 -6.084350e-15
3.069157e-15
E:\\R-3.1.2\\scripts\\mPDBS\\summary_pdb_93\\7fad98e66a3f6099.final.pdb
-5.086641e-16 -7.895822e-15 1.119276e-14 -1.086616e-14 -3.308963e-15
6.773259e-15
E:\\R-3.1.2\\scripts\\mPDBS\\summary_pdb_93\\804b89c94a633196.final.pdb

5.511773e-15 -1.951182e-15 2.434391e-15 -8.740874e-15 -1.005811e-15
2.714396e-15
E:\\R-3.1.2\\scripts\\mPDBS\\summary_pdb_93\\813cc65a2298feba.final.pdb
5.511773e-15 -1.951182e-15 2.434391e-15 -8.740874e-15 -1.005811e-15
2.714396e-15
E:\\R-3.1.2\\scripts\\mPDBS\\summary_pdb_93\\83c0eefcfe12d3f9.final.pdb
1.804669e-15 -4.888882e-15 3.586410e-15 -6.353298e-15 -1.225578e-15
3.366652e-15
E:\\R-3.1.2\\scripts\\mPDBS\\summary_pdb_93\\86d50469ef569760.final.pdb
8.264778e-15 -5.249331e-16 1.554408e-15 -4.687650e-15 -6.073096e-15
7.721678e-16
E:\\R-3.1.2\\scripts\\mPDBS\\summary_pdb_93\\89d0666e1402203d.final.pdb
4.337882e-15 -1.307274e-15 -2.097860e-16 -3.520291e-15 -1.940592e-15
-2.966447e-15
E:\\R-3.1.2\\scripts\\mPDBS\\summary_pdb_93\\8b34852716dadc62.final.pdb
6.451560e-15 -7.103310e-15 2.044079e-15 3.718455e-16 -6.964802e-15
-6.178664e-15
E:\\R-3.1.2\\scripts\\mPDBS\\summary_pdb_93\\8b6ef9ac3fe74395.final.pdb
-2.305017e-15 -8.037238e-16 2.174114e-15 -3.188112e-15 -4.924875e-15
-9.593947e-15
E:\\R-3.1.2\\scripts\\mPDBS\\summary_pdb_93\\98bbe0d5986c138f.final.pdb
4.851114e-15 -3.704523e-15 3.156911e-15 -5.890108e-15 -6.084350e-15
3.069157e-15
E:\\R-3.1.2\\scripts\\mPDBS\\summary_pdb_93\\9c22f4909d67e2fa.final.pdb
1.009274e-14 5.794413e-15 -1.741413e-15 -2.659692e-15 -1.180783e-14
4.904050e-15
E:\\R-3.1.2\\scripts\\mPDBS\\summary_pdb_93\\9dda8ed0d81fb3ef.final.pdb
4.362602e-15 -7.865884e-15 6.935432e-15 -1.354748e-14 -7.989030e-15
-4.900230e-15
E:\\R-3.1.2\\scripts\\mPDBS\\summary_pdb_93\\9f752b3616e6033b.final.pdb
6.009244e-15 -2.337394e-15 1.665342e-15 -5.042451e-15 -4.507360e-15
8.742762e-16
E:\\R-3.1.2\\scripts\\mPDBS\\summary_pdb_93\\a02c70083816d6ba.final.pdb
6.523410e-15 -8.125032e-16 2.231447e-15 -6.221324e-15 -2.910754e-15
-3.717488e-15
E:\\R-3.1.2\\scripts\\mPDBS\\summary_pdb_93\\a0c997d891e4e082.final.pdb
3.120813e-15 -5.265646e-15 1.172667e-14 -1.071802e-14 -1.047046e-15
-2.321776e-16
E:\\R-3.1.2\\scripts\\mPDBS\\summary_pdb_93\\ab8039a2d823c616.final.pdb
5.483072e-15 -1.466168e-15 2.205150e-15 -6.535064e-15 -3.020945e-15
1.150614e-15
E:\\R-3.1.2\\scripts\\mPDBS\\summary_pdb_93\\acf4d74b9c71d4dc.final.pdb
5.511773e-15 -1.951182e-15 2.434391e-15 -8.740874e-15 -1.005811e-15
2.714396e-15
E:\\R-3.1.2\\scripts\\mPDBS\\summary_pdb_93\\ad8a823e4b4c668e.final.pdb
5.512771e-15 -2.118421e-15 1.899096e-15 -6.026870e-15 -3.208478e-15
1.008323e-15
E:\\R-3.1.2\\scripts\\mPDBS\\summary_pdb_93\\b16ababd03b7b857.final.pdb
1.009274e-14 5.794413e-15 -1.741413e-15 -2.659692e-15 -1.180783e-14
4.904050e-15
E:\\R-3.1.2\\scripts\\mPDBS\\summary_pdb_93\\b226923e697d3c63.final.pdb
6.750131e-16 -7.077886e-15 7.043472e-15 -3.647848e-15 -1.359025e-15
-3.868070e-15
E:\\R-3.1.2\\scripts\\mPDBS\\summary_pdb_93\\b28e48f6fa27a165.final.pdb

4.851114e-15 -3.704523e-15 3.156911e-15 -5.890108e-15 -6.084350e-15
3.069157e-15
E:\\R-3.1.2\\scripts\\mPDBS\\summary_pdb_93\\b42c4924e3cd1aec.final.pdb
8.423589e-15 5.617742e-15 1.494634e-15 5.117482e-16 -7.484117e-15
2.747515e-15
E:\\R-3.1.2\\scripts\\mPDBS\\summary_pdb_93\\b4dd239fcabff1df.final.pdb
4.851114e-15 -3.704523e-15 3.156911e-15 -5.890108e-15 -6.084350e-15
3.069157e-15
E:\\R-3.1.2\\scripts\\mPDBS\\summary_pdb_93\\b669942ef97459a2.final.pdb
6.451560e-15 -7.103310e-15 2.044079e-15 3.718455e-16 -6.964802e-15
-6.178664e-15
E:\\R-3.1.2\\scripts\\mPDBS\\summary_pdb_93\\b99f4d9d87c1017b.final.pdb
4.851114e-15 -3.704523e-15 3.156911e-15 -5.890108e-15 -6.084350e-15
3.069157e-15
E:\\R-3.1.2\\scripts\\mPDBS\\summary_pdb_93\\b9d2cbf3975403e7.final.pdb
4.851114e-15 -3.704523e-15 3.156911e-15 -5.890108e-15 -6.084350e-15
3.069157e-15
E:\\R-3.1.2\\scripts\\mPDBS\\summary_pdb_93\\bc88c8a505f3a288.final.pdb
5.442221e-15 -1.079833e-15 2.299145e-15 -5.924857e-15 -4.090610e-15
8.944640e-16
E:\\R-3.1.2\\scripts\\mPDBS\\summary_pdb_93\\bdac0e64f4ef427e.final.pdb
8.645118e-15 4.811746e-15 -2.656372e-15 -4.864580e-15 -9.272310e-15
-3.158077e-15
E:\\R-3.1.2\\scripts\\mPDBS\\summary_pdb_93\\ca2a1f55fc9d0681.final.pdb
6.451560e-15 -7.103310e-15 2.044079e-15 3.718455e-16 -6.964802e-15
-6.178664e-15
E:\\R-3.1.2\\scripts\\mPDBS\\summary_pdb_93\\ce34248de3e81a3c.final.pdb
4.065695e-15 -6.885688e-15 6.435413e-15 -8.033986e-15 -5.867444e-15
-2.666274e-15
E:\\R-3.1.2\\scripts\\mPDBS\\summary_pdb_93\\d02e697772494a61.final.pdb
4.317253e-15 -2.447716e-15 -2.799316e-15 -4.897087e-15 4.623014e-15
4.295389e-15
E:\\R-3.1.2\\scripts\\mPDBS\\summary_pdb_93\\d04221245d015047.final.pdb
7.178331e-15 6.237404e-16 2.341242e-16 -6.705609e-15 -4.274499e-15
2.938897e-15
E:\\R-3.1.2\\scripts\\mPDBS\\summary_pdb_93\\d678141e4018054a.final.pdb
-1.262468e-15 6.403784e-15 3.397182e-15 -5.964509e-15 2.351349e-15
1.028511e-14
E:\\R-3.1.2\\scripts\\mPDBS\\summary_pdb_93\\d71b40af0f0efe39.final.pdb
1.017714e-14 -6.401313e-15 2.087992e-15 -6.099883e-15 -4.270123e-15
5.859782e-15
E:\\R-3.1.2\\scripts\\mPDBS\\summary_pdb_93\\dc0809eff10197e2.final.pdb
1.013142e-14 -3.306096e-15 4.318322e-16 -7.119806e-15 -1.096366e-14
-4.167392e-15
E:\\R-3.1.2\\scripts\\mPDBS\\summary_pdb_93\\e1163918d3707839.final.pdb
1.235274e-14 -1.652484e-15 7.465552e-15 -1.037635e-14 4.347239e-15
1.127650e-14
E:\\R-3.1.2\\scripts\\mPDBS\\summary_pdb_93\\e4b48b06824ef848.final.pdb
8.752093e-15 6.671901e-16 -3.678464e-15 -5.312343e-15 -3.793505e-15
-1.013259e-15
E:\\R-3.1.2\\scripts\\mPDBS\\summary_pdb_93\\e529e19ad281fea2.final.pdb
1.212259e-15 4.425100e-15 1.259505e-15 -1.075138e-14 -2.773844e-16
5.161886e-15
E:\\R-3.1.2\\scripts\\mPDBS\\summary_pdb_93\\e5602082718ec3a1.final.pdb

5.511773e-15 -1.951182e-15 2.434391e-15 -8.740874e-15 -1.005811e-15
2.714396e-15
E:\\R-3.1.2\\scripts\\mPDBS\\summary_pdb_93\\e5d589d996d0a960.final.pdb
5.458784e-15 -5.200688e-15 1.034546e-14 -1.476947e-14 -2.330406e-15
-6.852339e-15
E:\\R-3.1.2\\scripts\\mPDBS\\summary_pdb_93\\e9df9e72307a2fbd.final.pdb
6.523410e-15 -8.125032e-16 2.231447e-15 -6.221324e-15 -2.910754e-15
-3.717488e-15
E:\\R-3.1.2\\scripts\\mPDBS\\summary_pdb_93\\ea00f8694090cae7.final.pdb
5.511773e-15 -1.951182e-15 2.434391e-15 -8.740874e-15 -1.005811e-15
2.714396e-15
E:\\R-3.1.2\\scripts\\mPDBS\\summary_pdb_93\\eba517be32db050e.final.pdb
4.851114e-15 -3.704523e-15 3.156911e-15 -5.890108e-15 -6.084350e-15
3.069157e-15
E:\\R-3.1.2\\scripts\\mPDBS\\summary_pdb_93\\ec04b9545899c29d.final.pdb
6.110335e-15 -1.471639e-15 1.939970e-15 -5.981972e-15 -3.411203e-15
1.352364e-15
E:\\R-3.1.2\\scripts\\mPDBS\\summary_pdb_93\\ee6862cbcad52078.final.pdb
5.574060e-15 -1.017437e-15 1.671988e-15 -5.684760e-15 -4.089851e-15
8.116309e-16
E:\\R-3.1.2\\scripts\\mPDBS\\summary_pdb_93\\ef6a42fc83b81e5d.final.pdb
6.025975e-15 -1.564477e-15 1.645511e-15 -6.074916e-15 -3.522455e-15
1.009429e-15
E:\\R-3.1.2\\scripts\\mPDBS\\summary_pdb_93\\ef8e88d4ad93a0ad.final.pdb
7.178331e-15 6.237404e-16 2.341242e-16 -6.705609e-15 -4.274499e-15
2.938897e-15
E:\\R-3.1.2\\scripts\\mPDBS\\summary_pdb_93\\f14fcae8deec4fa3.final.pdb
6.009244e-15 -2.337394e-15 1.665342e-15 -5.042451e-15 -4.507360e-15
8.742762e-16
E:\\R-3.1.2\\scripts\\mPDBS\\summary_pdb_93\\f83584e22003fb92.final.pdb
3.534893e-15 -1.848236e-16 2.219618e-15 -1.240015e-14 -2.319458e-15
-1.628908e-15
E:\\R-3.1.2\\scripts\\mPDBS\\summary_pdb_93\\f8e2bf87e52caa29.final.pdb
1.212259e-15 4.425100e-15 1.259505e-15 -1.075138e-14 -2.773844e-16
5.161886e-15

[,571]   [,572]   [,573]   [,574]   [,575]   [,576]
E:\\R-3.1.2\\scripts\\40.1ns_data\\NAT0.pdb
-3.585539e-15 -4.815011e-17 2.731931e-15 -2.262463e-15 2.779551e-15
3.323068e-15
E:\\R-3.1.2\\scripts\\40.1ns_data\\LJP6.pdb
-2.154182e-15 -1.322486e-15 3.015764e-15 -1.208080e-15 3.172815e-15
2.263677e-15
E:\\R-3.1.2\\scripts\\40.1ns_data\\3WY9.pdb
-2.668654e-15 -8.870173e-16 3.303270e-15 -1.676020e-15 3.250670e-15
2.064196e-15
E:\\R-3.1.2\\scripts\\40.1ns_data\\ZSP9.pdb
-6.717122e-16 -4.283124e-16 2.461818e-15 -2.906676e-16 3.429547e-15
1.178070e-15
E:\\R-3.1.2\\scripts\\mPDBS\\summary_pdb_93\\013d855ad26c877c.final.pdb
-7.284603e-16 -1.008733e-15 6.718917e-15 4.103759e-16 -4.518199e-16
2.190875e-15
E:\\R-3.1.2\\scripts\\mPDBS\\summary_pdb_93\\0a2a705342ca0d54.final.pdb
-1.819354e-15 -9.288135e-16 5.801688e-16 -1.232909e-15 2.697734e-15

1.938672e-15
E:\\R-3.1.2\\scripts\\mPDBS\\summary_pdb_93\\0a59fceadb4194d9.final.pdb
-6.632422e-15 -1.068861e-15 1.776834e-15 -4.300048e-15 1.648772e-15
6.488444e-15
E:\\R-3.1.2\\scripts\\mPDBS\\summary_pdb_93\\0dba59f3306825e8.final.pdb
-7.440728e-16 4.269814e-15 3.208704e-15 2.539315e-15 1.912608e-15
4.343667e-15
E:\\R-3.1.2\\scripts\\mPDBS\\summary_pdb_93\\126f26a78b57339c.final.pdb
-3.387438e-16 -6.091635e-15 -7.268592e-15 -3.896726e-15 5.290982e-15
-2.482470e-15
E:\\R-3.1.2\\scripts\\mPDBS\\summary_pdb_93\\12f06f3e342df706.final.pdb
-1.075266e-15 -1.015333e-15 1.051580e-15 -1.225319e-15 2.487832e-15
1.472465e-15
E:\\R-3.1.2\\scripts\\mPDBS\\summary_pdb_93\\18618a2a251b867b.final.pdb
4.863556e-15 -1.878859e-15 3.089071e-15 -2.674446e-15 6.423983e-15
-2.728038e-15
E:\\R-3.1.2\\scripts\\mPDBS\\summary_pdb_93\\1f305f598641eeb2.final.pdb
-2.110532e-15 -3.599138e-16 1.622379e-15 -1.419099e-15 2.568717e-15
1.783755e-15
E:\\R-3.1.2\\scripts\\mPDBS\\summary_pdb_93\\1f8a00b551be0a39.final.pdb
4.863556e-15 -1.878859e-15 3.089071e-15 -2.674446e-15 6.423983e-15
-2.728038e-15
E:\\R-3.1.2\\scripts\\mPDBS\\summary_pdb_93\\219f45328d47579a.final.pdb
2.782789e-15 -5.322892e-16 -5.803474e-15 -2.164170e-15 -4.924962e-15
-5.800337e-15
E:\\R-3.1.2\\scripts\\mPDBS\\summary_pdb_93\\238dd3918e54336d.final.pdb
1.583718e-14 -1.477171e-15 -2.058206e-15 -1.655514e-16 6.456125e-15
-4.132509e-15
E:\\R-3.1.2\\scripts\\mPDBS\\summary_pdb_93\\2b3027889e2fdc42.final.pdb
-1.723263e-15 -1.431255e-15 6.904636e-16 -1.126588e-15 2.234241e-15
1.906931e-15
E:\\R-3.1.2\\scripts\\mPDBS\\summary_pdb_93\\2fcb3b46e33793b0.final.pdb
-9.552062e-16 2.816582e-15 8.492317e-15 2.485330e-15 -9.146082e-16
1.103744e-15
E:\\R-3.1.2\\scripts\\mPDBS\\summary_pdb_93\\34eb328251842006.final.pdb
-2.007190e-15 9.528829e-16 -2.700805e-15 -1.362971e-14 3.287319e-15
9.253681e-15
E:\\R-3.1.2\\scripts\\mPDBS\\summary_pdb_93\\354b32871797b7ab.final.pdb
-1.525477e-14 -9.303939e-15 1.061232e-14 -3.166785e-15 8.035549e-16
-2.107830e-15
E:\\R-3.1.2\\scripts\\mPDBS\\summary_pdb_93\\397368adbc32d5de.final.pdb
-3.387438e-16 -6.091635e-15 -7.268592e-15 -3.896726e-15 5.290982e-15
-2.482470e-15
E:\\R-3.1.2\\scripts\\mPDBS\\summary_pdb_93\\411ca13cd3df6edd.final.pdb
4.266040e-15 5.342820e-16 4.920955e-15 -2.631454e-15 5.943908e-17
-4.759622e-15
E:\\R-3.1.2\\scripts\\mPDBS\\summary_pdb_93\\42357eee6642bf12.final.pdb
-2.373222e-15 -5.022417e-15 4.033493e-15 -1.610404e-14 1.078147e-15
8.236542e-15
E:\\R-3.1.2\\scripts\\mPDBS\\summary_pdb_93\\44342d1f75a7fcfc.final.pdb
-8.092115e-16 -2.813709e-15 -3.328099e-15 -2.435935e-15 9.196061e-15
8.355537e-15
E:\\R-3.1.2\\scripts\\mPDBS\\summary_pdb_93\\448ef21dffe8cdfb.final.pdb
-3.127080e-15 -2.182083e-14 -4.391040e-15 -1.565381e-14 1.033681e-14

-7.392932e-15
E:\\R-3.1.2\\scripts\\mPDBS\\summary_pdb_93\\472a40b3e8f113c7.final.pdb
-7.440728e-16 4.269814e-15 3.208704e-15 2.539315e-15 1.912608e-15
4.343667e-15
E:\\R-3.1.2\\scripts\\mPDBS\\summary_pdb_93\\48a26225951ea153.final.pdb
-8.094977e-16 -6.203154e-15 1.213916e-15 8.580924e-15 5.852875e-15
-3.822861e-15
E:\\R-3.1.2\\scripts\\mPDBS\\summary_pdb_93\\4ab4a5c7c6682ce6.final.pdb
-7.776288e-16 -1.934587e-15 9.049085e-15 3.290607e-16 2.092152e-15
-4.845163e-15
E:\\R-3.1.2\\scripts\\mPDBS\\summary_pdb_93\\4eaa45b62cb6a25f.final.pdb
3.012137e-15 2.447945e-15 7.890193e-16 2.429650e-15 2.994077e-15
2.159405e-16
E:\\R-3.1.2\\scripts\\mPDBS\\summary_pdb_93\\58c371611d85ac96.final.pdb
-3.387438e-16 -6.091635e-15 -7.268592e-15 -3.896726e-15 5.290982e-15
-2.482470e-15
E:\\R-3.1.2\\scripts\\mPDBS\\summary_pdb_93\\59a647f04822d30a.final.pdb
4.298248e-15 1.690397e-16 2.932085e-15 -2.701328e-16 1.109648e-15
-3.744587e-16
E:\\R-3.1.2\\scripts\\mPDBS\\summary_pdb_93\\59b091fe385e05fc.final.pdb
-6.632422e-15 -1.068861e-15 1.776834e-15 -4.300048e-15 1.648772e-15
6.488444e-15
E:\\R-3.1.2\\scripts\\mPDBS\\summary_pdb_93\\5c35ac30f930d2db.final.pdb
3.012137e-15 2.447945e-15 7.890193e-16 2.429650e-15 2.994077e-15
2.159405e-16
E:\\R-3.1.2\\scripts\\mPDBS\\summary_pdb_93\\5f60752431cf2dcb.final.pdb
-2.050181e-15 5.941396e-15 2.040806e-15 -1.295576e-15 5.085125e-16
4.629120e-15
E:\\R-3.1.2\\scripts\\mPDBS\\summary_pdb_93\\626456dd6ff2868d.final.pdb
-7.440728e-16 4.269814e-15 3.208704e-15 2.539315e-15 1.912608e-15
4.343667e-15
E:\\R-3.1.2\\scripts\\mPDBS\\summary_pdb_93\\6583d53ff3c8f7ef.final.pdb
4.298248e-15 1.690397e-16 2.932085e-15 -2.701328e-16 1.109648e-15
-3.744587e-16
E:\\R-3.1.2\\scripts\\mPDBS\\summary_pdb_93\\69760178986bef01.final.pdb
2.725804e-15 2.985871e-15 -4.321699e-15 6.554465e-15 8.108045e-15
-2.429355e-15
E:\\R-3.1.2\\scripts\\mPDBS\\summary_pdb_93\\6a9d87d47926b1c8.final.pdb
-1.659736e-15 -1.164587e-15 8.538172e-16 -1.274257e-15 2.390525e-15
1.925462e-15
E:\\R-3.1.2\\scripts\\mPDBS\\summary_pdb_93\\6bb3547ea982ffc4.final.pdb
9.238754e-15 -2.606816e-16 -9.784486e-15 1.919544e-16 2.385852e-15
6.152410e-16
E:\\R-3.1.2\\scripts\\mPDBS\\summary_pdb_93\\716d9624611d1c42.final.pdb
3.950206e-16 -4.027355e-15 2.739466e-15 -4.969524e-16 6.511523e-15
-5.054865e-15
E:\\R-3.1.2\\scripts\\mPDBS\\summary_pdb_93\\75f27e70ad0f1be4.final.pdb
4.298248e-15 1.690397e-16 2.932085e-15 -2.701328e-16 1.109648e-15
-3.744587e-16
E:\\R-3.1.2\\scripts\\mPDBS\\summary_pdb_93\\769f910fffb22872.final.pdb
-1.179949e-15 -5.914235e-16 1.729047e-15 -1.207954e-15 2.371235e-15
1.998216e-15
E:\\R-3.1.2\\scripts\\mPDBS\\summary_pdb_93\\791f8c80dd5c3135.final.pdb
-6.632422e-15 -1.068861e-15 1.776834e-15 -4.300048e-15 1.648772e-15

6.488444e-15
E:\\R-3.1.2\\scripts\\mPDBS\\summary_pdb_93\\7fad98e66a3f6099.final.pdb
-1.113760e-14 3.022982e-16 -3.611364e-15 -1.947234e-15 -1.955398e-15
3.720816e-15
E:\\R-3.1.2\\scripts\\mPDBS\\summary_pdb_93\\804b89c94a633196.final.pdb
-9.552062e-16 2.816582e-15 8.492317e-15 2.485330e-15 -9.146082e-16
1.103744e-15
E:\\R-3.1.2\\scripts\\mPDBS\\summary_pdb_93\\813cc65a2298feba.final.pdb
-9.552062e-16 2.816582e-15 8.492317e-15 2.485330e-15 -9.146082e-16
1.103744e-15
E:\\R-3.1.2\\scripts\\mPDBS\\summary_pdb_93\\83c0eefcfe12d3f9.final.pdb
4.266040e-15 5.342820e-16 4.920955e-15 -2.631454e-15 5.943908e-17
-4.759622e-15
E:\\R-3.1.2\\scripts\\mPDBS\\summary_pdb_93\\86d50469ef569760.final.pdb
-7.440728e-16 4.269814e-15 3.208704e-15 2.539315e-15 1.912608e-15
4.343667e-15
E:\\R-3.1.2\\scripts\\mPDBS\\summary_pdb_93\\89d0666e1402203d.final.pdb
4.298248e-15 1.690397e-16 2.932085e-15 -2.701328e-16 1.109648e-15
-3.744587e-16
E:\\R-3.1.2\\scripts\\mPDBS\\summary_pdb_93\\8b34852716dadc62.final.pdb
-2.499110e-15 -9.792100e-16 -6.315716e-15 -3.913631e-15 3.942618e-15
9.875171e-16
E:\\R-3.1.2\\scripts\\mPDBS\\summary_pdb_93\\8b6ef9ac3fe74395.final.pdb
-5.249452e-15 1.630611e-16 -4.785925e-15 -3.287511e-16 -6.464922e-15
6.449032e-15
E:\\R-3.1.2\\scripts\\mPDBS\\summary_pdb_93\\98bbe0d5986c138f.final.pdb
-6.632422e-15 -1.068861e-15 1.776834e-15 -4.300048e-15 1.648772e-15
6.488444e-15
E:\\R-3.1.2\\scripts\\mPDBS\\summary_pdb_93\\9c22f4909d67e2fa.final.pdb
-6.187241e-15 -1.848632e-15 -3.335895e-15 -2.111904e-15 9.420441e-15
9.191892e-15
E:\\R-3.1.2\\scripts\\mPDBS\\summary_pdb_93\\9dda8ed0d81fb3ef.final.pdb
-1.533187e-15 -5.006454e-15 2.958973e-15 7.202405e-15 7.106587e-15
1.515523e-15
E:\\R-3.1.2\\scripts\\mPDBS\\summary_pdb_93\\9f752b3616e6033b.final.pdb
-1.329558e-15 -2.010921e-15 -8.387152e-16 -1.168468e-15 2.601266e-15
2.668720e-15
E:\\R-3.1.2\\scripts\\mPDBS\\summary_pdb_93\\a02c70083816d6ba.final.pdb
3.012137e-15 2.447945e-15 7.890193e-16 2.429650e-15 2.994077e-15
2.159405e-16
E:\\R-3.1.2\\scripts\\mPDBS\\summary_pdb_93\\a0c997d891e4e082.final.pdb
1.583721e-16 8.789986e-16 6.335115e-17 1.474256e-15 1.494423e-15
-5.346719e-15
E:\\R-3.1.2\\scripts\\mPDBS\\summary_pdb_93\\ab8039a2d823c616.final.pdb
-1.671599e-15 -3.190448e-16 1.836672e-15 -1.759919e-15 2.398647e-15
1.438543e-15
E:\\R-3.1.2\\scripts\\mPDBS\\summary_pdb_93\\acf4d74b9c71d4dc.final.pdb
-9.552062e-16 2.816582e-15 8.492317e-15 2.485330e-15 -9.146082e-16
1.103744e-15
E:\\R-3.1.2\\scripts\\mPDBS\\summary_pdb_93\\ad8a823e4b4c668e.final.pdb
-1.499935e-15 -8.880652e-16 1.475073e-15 -1.168715e-15 2.401347e-15
1.509990e-15
E:\\R-3.1.2\\scripts\\mPDBS\\summary_pdb_93\\b16ababd03b7b857.final.pdb
-6.187241e-15 -1.848632e-15 -3.335895e-15 -2.111904e-15 9.420441e-15

9.191892e-15
E:\\R-3.1.2\\scripts\\mPDBS\\summary_pdb_93\\b226923e697d3c63.final.pdb
1.608288e-15 -1.727609e-15 4.108661e-15 -1.066199e-14 -1.153733e-15
-2.212398e-15
E:\\R-3.1.2\\scripts\\mPDBS\\summary_pdb_93\\b28e48f6fa27a165.final.pdb
-6.632422e-15 -1.068861e-15 1.776834e-15 -4.300048e-15 1.648772e-15
6.488444e-15
E:\\R-3.1.2\\scripts\\mPDBS\\summary_pdb_93\\b42c4924e3cd1aec.final.pdb
-1.235396e-14 5.108335e-18 4.139019e-15 -1.375802e-15 -3.426221e-15
6.103107e-15
E:\\R-3.1.2\\scripts\\mPDBS\\summary_pdb_93\\b4dd239fcabff1df.final.pdb
-6.632422e-15 -1.068861e-15 1.776834e-15 -4.300048e-15 1.648772e-15
6.488444e-15
E:\\R-3.1.2\\scripts\\mPDBS\\summary_pdb_93\\b669942ef97459a2.final.pdb
-2.499110e-15 -9.792100e-16 -6.315716e-15 -3.913631e-15 3.942618e-15
9.875171e-16
E:\\R-3.1.2\\scripts\\mPDBS\\summary_pdb_93\\b99f4d9d87c1017b.final.pdb
-6.632422e-15 -1.068861e-15 1.776834e-15 -4.300048e-15 1.648772e-15
6.488444e-15
E:\\R-3.1.2\\scripts\\mPDBS\\summary_pdb_93\\b9d2cbf3975403e7.final.pdb
-6.632422e-15 -1.068861e-15 1.776834e-15 -4.300048e-15 1.648772e-15
6.488444e-15
E:\\R-3.1.2\\scripts\\mPDBS\\summary_pdb_93\\bc88c8a505f3a288.final.pdb
-1.639129e-15 -1.314270e-15 2.442060e-16 -1.107723e-15 2.444577e-15
2.055358e-15
E:\\R-3.1.2\\scripts\\mPDBS\\summary_pdb_93\\bdac0e64f4ef427e.final.pdb
-1.194164e-14 -1.214211e-15 -5.577591e-15 1.060255e-15 7.736892e-15
1.422432e-14
E:\\R-3.1.2\\scripts\\mPDBS\\summary_pdb_93\\ca2a1f55fc9d0681.final.pdb
-2.499110e-15 -9.792100e-16 -6.315716e-15 -3.913631e-15 3.942618e-15
9.875171e-16
E:\\R-3.1.2\\scripts\\mPDBS\\summary_pdb_93\\ce34248de3e81a3c.final.pdb
-8.133982e-15 2.807225e-16 6.217506e-15 -1.350142e-17 -3.187986e-15
1.530929e-15
E:\\R-3.1.2\\scripts\\mPDBS\\summary_pdb_93\\d02e697772494a61.final.pdb
9.316354e-15 2.611572e-15 -3.750429e-15 -9.924238e-15 3.717864e-15
-2.376102e-15
E:\\R-3.1.2\\scripts\\mPDBS\\summary_pdb_93\\d04221245d015047.final.pdb
-2.294527e-15 -5.929143e-16 2.663138e-16 1.633850e-15 3.697521e-15
3.328633e-15
E:\\R-3.1.2\\scripts\\mPDBS\\summary_pdb_93\\d678141e4018054a.final.pdb
4.244459e-15 -1.168939e-15 8.421571e-16 -9.292091e-15 6.380786e-15
-5.730508e-16
E:\\R-3.1.2\\scripts\\mPDBS\\summary_pdb_93\\d71b40af0f0efe39.final.pdb
-2.761912e-15 -6.877168e-15 7.314647e-15 -4.096377e-15 6.375163e-15
-3.351288e-15
E:\\R-3.1.2\\scripts\\mPDBS\\summary_pdb_93\\dc0809eff10197e2.final.pdb
-6.790549e-15 1.219686e-16 2.616146e-15 3.145794e-15 -8.476417e-17
7.033304e-15
E:\\R-3.1.2\\scripts\\mPDBS\\summary_pdb_93\\e1163918d3707839.final.pdb
-1.108241e-14 1.890970e-15 -2.220761e-16 -9.121974e-16 3.966304e-15
8.684376e-15
E:\\R-3.1.2\\scripts\\mPDBS\\summary_pdb_93\\e4b48b06824ef848.final.pdb
4.863556e-15 -1.878859e-15 3.089071e-15 -2.674446e-15 6.423983e-15

-2.728038e-15
E:\\R-3.1.2\\scripts\\mPDBS\\summary_pdb_93\\e529e19ad281fea2.final.pdb
-7.284603e-16 -1.008733e-15 6.718917e-15 4.103759e-16 -4.518199e-16
2.190875e-15
E:\\R-3.1.2\\scripts\\mPDBS\\summary_pdb_93\\e5602082718ec3a1.final.pdb
-9.552062e-16 2.816582e-15 8.492317e-15 2.485330e-15 -9.146082e-16
1.103744e-15
E:\\R-3.1.2\\scripts\\mPDBS\\summary_pdb_93\\e5d589d996d0a960.final.pdb
-1.053330e-14 -4.111632e-15 -1.482227e-15 1.157390e-14 -3.219652e-15
-7.685647e-15
E:\\R-3.1.2\\scripts\\mPDBS\\summary_pdb_93\\e9df9e72307a2fbd.final.pdb
3.012137e-15 2.447945e-15 7.890193e-16 2.429650e-15 2.994077e-15
2.159405e-16
E:\\R-3.1.2\\scripts\\mPDBS\\summary_pdb_93\\ea00f8694090cae7.final.pdb
-9.552062e-16 2.816582e-15 8.492317e-15 2.485330e-15 -9.146082e-16
1.103744e-15
E:\\R-3.1.2\\scripts\\mPDBS\\summary_pdb_93\\eba517be32db050e.final.pdb
-6.632422e-15 -1.068861e-15 1.776834e-15 -4.300048e-15 1.648772e-15
6.488444e-15
E:\\R-3.1.2\\scripts\\mPDBS\\summary_pdb_93\\ec04b9545899c29d.final.pdb
-2.203062e-15 -7.133924e-16 1.699130e-15 -1.610207e-15 2.400282e-15
1.574503e-15
E:\\R-3.1.2\\scripts\\mPDBS\\summary_pdb_93\\ee6862cbcad52078.final.pdb
-1.982062e-15 -1.559679e-15 8.110269e-16 -1.422575e-15 2.396438e-15
1.992041e-15
E:\\R-3.1.2\\scripts\\mPDBS\\summary_pdb_93\\ef6a42fc83b81e5d.final.pdb
-1.263812e-15 -3.518148e-16 1.511773e-15 -1.345431e-15 2.299678e-15
1.655717e-15
E:\\R-3.1.2\\scripts\\mPDBS\\summary_pdb_93\\ef8e88d4ad93a0ad.final.pdb
-2.294527e-15 -5.929143e-16 2.663138e-16 1.633850e-15 3.697521e-15
3.328633e-15
E:\\R-3.1.2\\scripts\\mPDBS\\summary_pdb_93\\f14fcae8deec4fa3.final.pdb
-1.329558e-15 -2.010921e-15 -8.387152e-16 -1.168468e-15 2.601266e-15
2.668720e-15
E:\\R-3.1.2\\scripts\\mPDBS\\summary_pdb_93\\f83584e22003fb92.final.pdb
-8.094977e-16 -6.203154e-15 1.213916e-15 8.580924e-15 5.852875e-15
-3.822861e-15
E:\\R-3.1.2\\scripts\\mPDBS\\summary_pdb_93\\f8e2bf87e52caa29.final.pdb
-7.284603e-16 -1.008733e-15 6.718917e-15 4.103759e-16 -4.518199e-16
2.190875e-15

[,577]   [,578]   [,579]   [,580]   [,581]   [,582]
E:\\R-3.1.2\\scripts\\40.1ns_data\\NAT0.pdb
3.924499e-15 -6.675456e-15 -4.030380e-15 2.869976e-15 -6.248980e-16
-1.480543e-15
E:\\R-3.1.2\\scripts\\40.1ns_data\\LJP6.pdb
4.826478e-15 -7.216106e-15 -3.484421e-15 5.668369e-15 -1.240912e-15
-1.278354e-15
E:\\R-3.1.2\\scripts\\40.1ns_data\\3WY9.pdb
4.741611e-15 -7.531237e-15 -5.237744e-15 6.358379e-15 -1.333826e-15
-1.936680e-15
E:\\R-3.1.2\\scripts\\40.1ns_data\\ZSP9.pdb
4.933986e-15 -6.291904e-15 -4.415212e-15 2.470607e-15 -5.646285e-16
-1.927305e-15

E:\\R-3.1.2\\scripts\\mPDBS\\summary_pdb_93\\013d855ad26c877c.final.pdb
3.958470e-15 -7.817401e-15 -1.748404e-15 -3.486982e-15 -9.762256e-16
-2.756198e-15
E:\\R-3.1.2\\scripts\\mPDBS\\summary_pdb_93\\0a2a705342ca0d54.final.pdb
4.580960e-15 -6.279748e-15 -4.029561e-15 2.613515e-15 -1.147463e-15
-1.624362e-15
E:\\R-3.1.2\\scripts\\mPDBS\\summary_pdb_93\\0a59fceadb4194d9.final.pdb
6.125504e-15 -6.373302e-15 -5.577047e-15 6.590797e-15 -2.788962e-15
-3.121687e-15
E:\\R-3.1.2\\scripts\\mPDBS\\summary_pdb_93\\0dba59f3306825e8.final.pdb
6.580071e-15 -3.814526e-15 -5.614235e-15 1.296167e-14 -4.052107e-15
-2.594001e-15
E:\\R-3.1.2\\scripts\\mPDBS\\summary_pdb_93\\126f26a78b57339c.final.pdb
3.592118e-15 -1.126083e-14 -2.240849e-15 -1.187606e-15 1.932255e-15
-2.024578e-15
E:\\R-3.1.2\\scripts\\mPDBS\\summary_pdb_93\\12f06f3e342df706.final.pdb
4.501271e-15 -6.919427e-15 -3.725767e-15 1.496895e-15 -7.584515e-16
-1.790028e-15
E:\\R-3.1.2\\scripts\\mPDBS\\summary_pdb_93\\18618a2a251b867b.final.pdb
-4.756869e-16 -9.370055e-15 -4.703157e-15 -2.298152e-16 -2.114460e-15
-1.048752e-15
E:\\R-3.1.2\\scripts\\mPDBS\\summary_pdb_93\\1f305f598641eeb2.final.pdb
4.426244e-15 -6.404483e-15 -4.084939e-15 2.216710e-15 -1.036363e-15
-1.811765e-15
E:\\R-3.1.2\\scripts\\mPDBS\\summary_pdb_93\\1f8a00b551be0a39.final.pdb
-4.756869e-16 -9.370055e-15 -4.703157e-15 -2.298152e-16 -2.114460e-15
-1.048752e-15
E:\\R-3.1.2\\scripts\\mPDBS\\summary_pdb_93\\219f45328d47579a.final.pdb
9.300304e-17 -3.100746e-15 -4.926526e-15 -5.665024e-15 -1.196563e-16
9.497993e-15
E:\\R-3.1.2\\scripts\\mPDBS\\summary_pdb_93\\238dd3918e54336d.final.pdb
4.194215e-15 -9.838846e-15 -1.092303e-14 5.514835e-15 1.050631e-15
-1.684035e-15
E:\\R-3.1.2\\scripts\\mPDBS\\summary_pdb_93\\2b3027889e2fdc42.final.pdb
4.695116e-15 -6.810453e-15 -3.568888e-15 3.802121e-15 -1.537793e-15
-2.091261e-15
E:\\R-3.1.2\\scripts\\mPDBS\\summary_pdb_93\\2fcb3b46e33793b0.final.pdb
4.675177e-15 -5.136400e-15 -3.158596e-15 4.548179e-15 -4.270972e-15
-2.065902e-15
E:\\R-3.1.2\\scripts\\mPDBS\\summary_pdb_93\\34eb328251842006.final.pdb
1.198885e-14 -1.155040e-15 -7.508499e-15 1.195616e-14 -7.027863e-15
1.604146e-15
E:\\R-3.1.2\\scripts\\mPDBS\\summary_pdb_93\\354b32871797b7ab.final.pdb
1.157256e-17 -1.632900e-14 -5.005231e-16 -1.182816e-14 -1.703931e-15
-6.021995e-15
E:\\R-3.1.2\\scripts\\mPDBS\\summary_pdb_93\\397368adbc32d5de.final.pdb
3.592118e-15 -1.126083e-14 -2.240849e-15 -1.187606e-15 1.932255e-15
-2.024578e-15
E:\\R-3.1.2\\scripts\\mPDBS\\summary_pdb_93\\411ca13cd3df6edd.final.pdb
6.616657e-15 -1.073956e-14 -4.618583e-15 6.908270e-15 -6.947867e-15
-1.935364e-15
E:\\R-3.1.2\\scripts\\mPDBS\\summary_pdb_93\\42357eee6642bf12.final.pdb
3.402228e-15 -5.573937e-16 -1.151672e-14 3.612869e-16 -1.341115e-15
4.061590e-15

E:\\R-3.1.2\\scripts\\mPDBS\\summary_pdb_93\\44342d1f75a7fcfc.final.pdb
7.965842e-15 -1.333571e-14 1.333867e-15 -1.229409e-14 -2.457773e-15
-5.885455e-15
E:\\R-3.1.2\\scripts\\mPDBS\\summary_pdb_93\\448ef21dffe8cdfb.final.pdb
7.369207e-16 -1.568439e-14 -6.359161e-15 -4.755804e-16 6.937510e-15
6.167096e-15
E:\\R-3.1.2\\scripts\\mPDBS\\summary_pdb_93\\472a40b3e8f113c7.final.pdb
6.580071e-15 -3.814526e-15 -5.614235e-15 1.296167e-14 -4.052107e-15
-2.594001e-15
E:\\R-3.1.2\\scripts\\mPDBS\\summary_pdb_93\\48a26225951ea153.final.pdb
4.992296e-15 -8.291906e-15 4.436861e-15 -1.098473e-15 -2.820621e-15
-5.153915e-15
E:\\R-3.1.2\\scripts\\mPDBS\\summary_pdb_93\\4ab4a5c7c6682ce6.final.pdb
-3.280423e-15 -5.655068e-15 6.360778e-15 -6.558309e-15 -2.442284e-15
-2.433106e-15
E:\\R-3.1.2\\scripts\\mPDBS\\summary_pdb_93\\4eaa45b62cb6a25f.final.pdb
4.139239e-15 -5.701652e-15 -1.623323e-15 1.373519e-15 8.877462e-16
-4.514520e-15
E:\\R-3.1.2\\scripts\\mPDBS\\summary_pdb_93\\58c371611d85ac96.final.pdb
3.592118e-15 -1.126083e-14 -2.240849e-15 -1.187606e-15 1.932255e-15
-2.024578e-15
E:\\R-3.1.2\\scripts\\mPDBS\\summary_pdb_93\\59a647f04822d30a.final.pdb
4.924222e-15 -3.555341e-15 -3.453453e-16 -2.325554e-16 1.454462e-15
-2.393635e-16
E:\\R-3.1.2\\scripts\\mPDBS\\summary_pdb_93\\59b091fe385e05fc.final.pdb
6.125504e-15 -6.373302e-15 -5.577047e-15 6.590797e-15 -2.788962e-15
-3.121687e-15
E:\\R-3.1.2\\scripts\\mPDBS\\summary_pdb_93\\5c35ac30f930d2db.final.pdb
4.139239e-15 -5.701652e-15 -1.623323e-15 1.373519e-15 8.877462e-16
-4.514520e-15
E:\\R-3.1.2\\scripts\\mPDBS\\summary_pdb_93\\5f60752431cf2dcb.final.pdb
5.039360e-15 -4.230172e-15 6.760937e-16 8.479374e-15 -6.798542e-15
1.966949e-15
E:\\R-3.1.2\\scripts\\mPDBS\\summary_pdb_93\\626456dd6ff2868d.final.pdb
6.580071e-15 -3.814526e-15 -5.614235e-15 1.296167e-14 -4.052107e-15
-2.594001e-15
E:\\R-3.1.2\\scripts\\mPDBS\\summary_pdb_93\\6583d53ff3c8f7ef.final.pdb
4.924222e-15 -3.555341e-15 -3.453453e-16 -2.325554e-16 1.454462e-15
-2.393635e-16
E:\\R-3.1.2\\scripts\\mPDBS\\summary_pdb_93\\69760178986bef01.final.pdb
-7.900819e-16 -1.341191e-14 -5.412585e-16 9.626142e-15 4.319144e-15
3.515953e-16
E:\\R-3.1.2\\scripts\\mPDBS\\summary_pdb_93\\6a9d87d47926b1c8.final.pdb
4.638590e-15 -6.709780e-15 -3.606076e-15 3.565537e-15 -1.525049e-15
-1.801666e-15
E:\\R-3.1.2\\scripts\\mPDBS\\summary_pdb_93\\6bb3547ea982ffc4.final.pdb
2.480132e-15 -1.047948e-14 -4.516682e-15 -6.421335e-15 -7.852911e-15
7.242465e-15
E:\\R-3.1.2\\scripts\\mPDBS\\summary_pdb_93\\716d9624611d1c42.final.pdb
3.829824e-15 -1.229490e-14 -3.849149e-15 -3.676592e-15 5.331295e-15
-1.003734e-14
E:\\R-3.1.2\\scripts\\mPDBS\\summary_pdb_93\\75f27e70ad0f1be4.final.pdb
4.924222e-15 -3.555341e-15 -3.453453e-16 -2.325554e-16 1.454462e-15
-2.393635e-16

E:\\R-3.1.2\\scripts\\mPDBS\\summary_pdb_93\\769f910fffb22872.final.pdb
5.233613e-15 -6.040253e-15 -4.148696e-15 2.239238e-15 -1.174023e-15
-2.042315e-15
E:\\R-3.1.2\\scripts\\mPDBS\\summary_pdb_93\\791f8c80dd5c3135.final.pdb
6.125504e-15 -6.373302e-15 -5.577047e-15 6.590797e-15 -2.788962e-15
-3.121687e-15
E:\\R-3.1.2\\scripts\\mPDBS\\summary_pdb_93\\7fad98e66a3f6099.final.pdb
9.108707e-15 -7.322723e-15 -6.449153e-16 8.264004e-16 3.373461e-19
-2.844809e-15
E:\\R-3.1.2\\scripts\\mPDBS\\summary_pdb_93\\804b89c94a633196.final.pdb
4.675177e-15 -5.136400e-15 -3.158596e-15 4.548179e-15 -4.270972e-15
-2.065902e-15
E:\\R-3.1.2\\scripts\\mPDBS\\summary_pdb_93\\813cc65a2298feba.final.pdb
4.675177e-15 -5.136400e-15 -3.158596e-15 4.548179e-15 -4.270972e-15
-2.065902e-15
E:\\R-3.1.2\\scripts\\mPDBS\\summary_pdb_93\\83c0eefcfe12d3f9.final.pdb
6.616657e-15 -1.073956e-14 -4.618583e-15 6.908270e-15 -6.947867e-15
-1.935364e-15
E:\\R-3.1.2\\scripts\\mPDBS\\summary_pdb_93\\86d50469ef569760.final.pdb
6.580071e-15 -3.814526e-15 -5.614235e-15 1.296167e-14 -4.052107e-15
-2.594001e-15
E:\\R-3.1.2\\scripts\\mPDBS\\summary_pdb_93\\89d0666e1402203d.final.pdb
4.924222e-15 -3.555341e-15 -3.453453e-16 -2.325554e-16 1.454462e-15
-2.393635e-16
E:\\R-3.1.2\\scripts\\mPDBS\\summary_pdb_93\\8b34852716dadc62.final.pdb
4.323895e-15 -6.119989e-15 -3.852486e-15 3.139583e-15 -1.626169e-15
-1.492576e-15
E:\\R-3.1.2\\scripts\\mPDBS\\summary_pdb_93\\8b6ef9ac3fe74395.final.pdb
-3.512173e-16 -1.626515e-15 -7.470702e-15 1.346389e-16 -2.529855e-15
4.175298e-15
E:\\R-3.1.2\\scripts\\mPDBS\\summary_pdb_93\\98bbe0d5986c138f.final.pdb
6.125504e-15 -6.373302e-15 -5.577047e-15 6.590797e-15 -2.788962e-15
-3.121687e-15
E:\\R-3.1.2\\scripts\\mPDBS\\summary_pdb_93\\9c22f4909d67e2fa.final.pdb
9.382782e-15 -4.180134e-15 -6.950485e-15 5.851390e-15 5.475138e-15
-3.984506e-15
E:\\R-3.1.2\\scripts\\mPDBS\\summary_pdb_93\\9dda8ed0d81fb3ef.final.pdb
1.012471e-14 -1.514850e-14 -3.928334e-16 -5.752717e-16 -7.786844e-15
-8.742978e-15
E:\\R-3.1.2\\scripts\\mPDBS\\summary_pdb_93\\9f752b3616e6033b.final.pdb
5.082294e-15 -5.221371e-15 -6.903309e-15 7.234986e-15 -1.678124e-15
1.796473e-15
E:\\R-3.1.2\\scripts\\mPDBS\\summary_pdb_93\\a02c70083816d6ba.final.pdb
4.139239e-15 -5.701652e-15 -1.623323e-15 1.373519e-15 8.877462e-16
-4.514520e-15
E:\\R-3.1.2\\scripts\\mPDBS\\summary_pdb_93\\a0c997d891e4e082.final.pdb
9.495872e-15 -1.714252e-14 -2.768856e-15 4.203451e-15 -2.482457e-15
-1.027752e-14
E:\\R-3.1.2\\scripts\\mPDBS\\summary_pdb_93\\ab8039a2d823c616.final.pdb
4.694483e-15 -6.110978e-15 -4.324469e-15 -2.101194e-16 -4.430729e-16
-1.783447e-15
E:\\R-3.1.2\\scripts\\mPDBS\\summary_pdb_93\\acf4d74b9c71d4dc.final.pdb
4.675177e-15 -5.136400e-15 -3.158596e-15 4.548179e-15 -4.270972e-15
-2.065902e-15

E:\\R-3.1.2\\scripts\\mPDBS\\summary_pdb_93\\ad8a823e4b4c668e.final.pdb
4.936875e-15 -6.577254e-15 -4.168841e-15 4.522693e-15 -8.937528e-16
-1.930400e-15
E:\\R-3.1.2\\scripts\\mPDBS\\summary_pdb_93\\b16ababd03b7b857.final.pdb
9.382782e-15 -4.180134e-15 -6.950485e-15 5.851390e-15 5.475138e-15
-3.984506e-15
E:\\R-3.1.2\\scripts\\mPDBS\\summary_pdb_93\\b226923e697d3c63.final.pdb
1.072197e-16 -1.347579e-14 -1.028466e-14 5.595573e-15 -1.607325e-15
-3.099055e-15
E:\\R-3.1.2\\scripts\\mPDBS\\summary_pdb_93\\b28e48f6fa27a165.final.pdb
6.125504e-15 -6.373302e-15 -5.577047e-15 6.590797e-15 -2.788962e-15
-3.121687e-15
E:\\R-3.1.2\\scripts\\mPDBS\\summary_pdb_93\\b42c4924e3cd1aec.final.pdb
2.404644e-15 -2.128965e-15 -1.201179e-14 1.735055e-15 7.018087e-16
-5.151667e-15
E:\\R-3.1.2\\scripts\\mPDBS\\summary_pdb_93\\b4dd239fcabff1df.final.pdb
6.125504e-15 -6.373302e-15 -5.577047e-15 6.590797e-15 -2.788962e-15
-3.121687e-15
E:\\R-3.1.2\\scripts\\mPDBS\\summary_pdb_93\\b669942ef97459a2.final.pdb
4.323895e-15 -6.119989e-15 -3.852486e-15 3.139583e-15 -1.626169e-15
-1.492576e-15
E:\\R-3.1.2\\scripts\\mPDBS\\summary_pdb_93\\b99f4d9d87c1017b.final.pdb
6.125504e-15 -6.373302e-15 -5.577047e-15 6.590797e-15 -2.788962e-15
-3.121687e-15
E:\\R-3.1.2\\scripts\\mPDBS\\summary_pdb_93\\b9d2cbf3975403e7.final.pdb
6.125504e-15 -6.373302e-15 -5.577047e-15 6.590797e-15 -2.788962e-15
-3.121687e-15
E:\\R-3.1.2\\scripts\\mPDBS\\summary_pdb_93\\bc88c8a505f3a288.final.pdb
4.575745e-15 -6.814356e-15 -3.252951e-15 2.792186e-15 -1.375813e-15
-1.852737e-15
E:\\R-3.1.2\\scripts\\mPDBS\\summary_pdb_93\\bdac0e64f4ef427e.final.pdb
1.747180e-15 1.396135e-15 -4.685803e-15 4.399199e-16 9.860953e-15
-1.554592e-15
E:\\R-3.1.2\\scripts\\mPDBS\\summary_pdb_93\\ca2a1f55fc9d0681.final.pdb
4.323895e-15 -6.119989e-15 -3.852486e-15 3.139583e-15 -1.626169e-15
-1.492576e-15
E:\\R-3.1.2\\scripts\\mPDBS\\summary_pdb_93\\ce34248de3e81a3c.final.pdb
2.444809e-15 -6.000639e-15 2.701734e-15 -4.532223e-15 1.917034e-15
-1.347150e-14
E:\\R-3.1.2\\scripts\\mPDBS\\summary_pdb_93\\d02e697772494a61.final.pdb
1.454219e-15 -2.279789e-15 4.515506e-16 4.369917e-15 3.609349e-15
8.213430e-15
E:\\R-3.1.2\\scripts\\mPDBS\\summary_pdb_93\\d04221245d015047.final.pdb
6.606690e-15 -4.152909e-15 -2.161703e-15 1.781777e-15 -1.091209e-15
-1.095629e-15
E:\\R-3.1.2\\scripts\\mPDBS\\summary_pdb_93\\d678141e4018054a.final.pdb
8.175079e-15 -1.037141e-14 -7.640267e-15 3.193952e-15 -7.595925e-15
4.251026e-15
E:\\R-3.1.2\\scripts\\mPDBS\\summary_pdb_93\\d71b40af0f0efe39.final.pdb
9.049509e-15 -6.098871e-15 -9.248123e-15 1.143032e-14 -3.472905e-15
4.279293e-16
E:\\R-3.1.2\\scripts\\mPDBS\\summary_pdb_93\\dc0809eff10197e2.final.pdb
9.321179e-15 9.737226e-16 -3.823934e-15 1.367960e-14 1.851909e-15
-4.802763e-15

E:\\R-3.1.2\\scripts\\mPDBS\\summary_pdb_93\\e1163918d3707839.final.pdb
5.475972e-15 3.636389e-15 -1.770516e-14 2.278419e-14 1.493059e-15
2.008226e-16
E:\\R-3.1.2\\scripts\\mPDBS\\summary_pdb_93\\e4b48b06824ef848.final.pdb
-4.756869e-16 -9.370055e-15 -4.703157e-15 -2.298152e-16 -2.114460e-15
-1.048752e-15
E:\\R-3.1.2\\scripts\\mPDBS\\summary_pdb_93\\e529e19ad281fea2.final.pdb
3.958470e-15 -7.817401e-15 -1.748404e-15 -3.486982e-15 -9.762256e-16
-2.756198e-15
E:\\R-3.1.2\\scripts\\mPDBS\\summary_pdb_93\\e5602082718ec3a1.final.pdb
4.675177e-15 -5.136400e-15 -3.158596e-15 4.548179e-15 -4.270972e-15
-2.065902e-15
E:\\R-3.1.2\\scripts\\mPDBS\\summary_pdb_93\\e5d589d996d0a960.final.pdb
5.073192e-16 -5.459770e-15 3.104821e-15 6.478948e-15 -3.574865e-15
-3.678180e-15
E:\\R-3.1.2\\scripts\\mPDBS\\summary_pdb_93\\e9df9e72307a2fbd.final.pdb
4.139239e-15 -5.701652e-15 -1.623323e-15 1.373519e-15 8.877462e-16
-4.514520e-15
E:\\R-3.1.2\\scripts\\mPDBS\\summary_pdb_93\\ea00f8694090cae7.final.pdb
4.675177e-15 -5.136400e-15 -3.158596e-15 4.548179e-15 -4.270972e-15
-2.065902e-15
E:\\R-3.1.2\\scripts\\mPDBS\\summary_pdb_93\\eba517be32db050e.final.pdb
6.125504e-15 -6.373302e-15 -5.577047e-15 6.590797e-15 -2.788962e-15
-3.121687e-15
E:\\R-3.1.2\\scripts\\mPDBS\\summary_pdb_93\\ec04b9545899c29d.final.pdb
4.557436e-15 -5.963137e-15 -4.139186e-15 4.841511e-15 -9.962687e-16
-1.751609e-15
E:\\R-3.1.2\\scripts\\mPDBS\\summary_pdb_93\\ee6862cbcad52078.final.pdb
4.692839e-15 -6.558050e-15 -3.410378e-15 3.322578e-15 -1.379933e-15
-1.776842e-15
E:\\R-3.1.2\\scripts\\mPDBS\\summary_pdb_93\\ef6a42fc83b81e5d.final.pdb
4.773912e-15 -6.113111e-15 -4.246274e-15 1.163706e-16 -1.069506e-15
-1.870143e-15
E:\\R-3.1.2\\scripts\\mPDBS\\summary_pdb_93\\ef8e88d4ad93a0ad.final.pdb
6.606690e-15 -4.152909e-15 -2.161703e-15 1.781777e-15 -1.091209e-15
-1.095629e-15
E:\\R-3.1.2\\scripts\\mPDBS\\summary_pdb_93\\f14fcae8deec4fa3.final.pdb
5.082294e-15 -5.221371e-15 -6.903309e-15 7.234986e-15 -1.678124e-15
1.796473e-15
E:\\R-3.1.2\\scripts\\mPDBS\\summary_pdb_93\\f83584e22003fb92.final.pdb
4.992296e-15 -8.291906e-15 4.436861e-15 -1.098473e-15 -2.820621e-15
-5.153915e-15
E:\\R-3.1.2\\scripts\\mPDBS\\summary_pdb_93\\f8e2bf87e52caa29.final.pdb
3.958470e-15 -7.817401e-15 -1.748404e-15 -3.486982e-15 -9.762256e-16
-2.756198e-15

[,583] [,584] [,585] [,586] [,587] [,588]
E:\\R-3.1.2\\scripts\\40.1ns_data\\NAT0.pdb
-2.771174e-15 1.731720e-15 7.630072e-16 6.153774e-15 5.016277e-16
1.977820e-15
E:\\R-3.1.2\\scripts\\40.1ns_data\\LJP6.pdb
-1.069997e-15 2.979317e-15 -7.796672e-17 6.549207e-15 -1.344255e-15
1.312452e-15
E:\\R-3.1.2\\scripts\\40.1ns_data\\3WY9.pdb

-2.657123e-15 1.893182e-15 9.097618e-16 5.845698e-15 4.485075e-16
1.960059e-15
E:\\R-3.1.2\\scripts\\40.1ns_data\\ZSP9.pdb
-1.811382e-16 2.833567e-15 1.166199e-15 4.615598e-15 2.800748e-16
1.941222e-15
E:\\R-3.1.2\\scripts\\mPDBS\\summary_pdb_93\\013d855ad26c877c.final.pdb
-5.806404e-15 2.641172e-15 -4.562002e-15 1.075088e-14 -6.555953e-15
2.527223e-15
E:\\R-3.1.2\\scripts\\mPDBS\\summary_pdb_93\\0a2a705342ca0d54.final.pdb
-1.744621e-15 3.721404e-15 9.457525e-16 6.035520e-15 -2.373356e-16
1.982110e-15
E:\\R-3.1.2\\scripts\\mPDBS\\summary_pdb_93\\0a59fceadb4194d9.final.pdb
-3.880478e-15 6.036260e-16 2.432381e-15 9.005341e-15 2.009651e-15
-3.699581e-16
E:\\R-3.1.2\\scripts\\mPDBS\\summary_pdb_93\\0dba59f3306825e8.final.pdb
4.195185e-16 1.341105e-15 7.160026e-16 7.482661e-15 -1.183514e-15
2.926209e-15
E:\\R-3.1.2\\scripts\\mPDBS\\summary_pdb_93\\126f26a78b57339c.final.pdb
6.762575e-16 4.650280e-15 -2.674907e-16 9.123962e-16 -3.573312e-15
2.001818e-15
E:\\R-3.1.2\\scripts\\mPDBS\\summary_pdb_93\\12f06f3e342df706.final.pdb
-2.142740e-15 3.355134e-15 8.298513e-16 6.119220e-15 1.289079e-16
1.984874e-15
E:\\R-3.1.2\\scripts\\mPDBS\\summary_pdb_93\\18618a2a251b867b.final.pdb
2.089108e-15 9.465749e-15 -2.222278e-15 -2.260900e-15 -5.077357e-15
6.339524e-15
E:\\R-3.1.2\\scripts\\mPDBS\\summary_pdb_93\\1f305f598641eeb2.final.pdb
-1.468783e-15 3.070457e-15 9.924555e-16 6.226512e-15 -5.449195e-16
1.499317e-15
E:\\R-3.1.2\\scripts\\mPDBS\\summary_pdb_93\\1f8a00b551be0a39.final.pdb
2.089108e-15 9.465749e-15 -2.222278e-15 -2.260900e-15 -5.077357e-15
6.339524e-15
E:\\R-3.1.2\\scripts\\mPDBS\\summary_pdb_93\\219f45328d47579a.final.pdb
-2.115831e-15 1.626300e-15 8.757427e-15 7.156126e-15 -6.879940e-15
-9.756113e-15
E:\\R-3.1.2\\scripts\\mPDBS\\summary_pdb_93\\238dd3918e54336d.final.pdb
-4.344517e-15 1.293502e-14 -5.999093e-15 4.556209e-15 -5.599497e-15
-3.581257e-15
E:\\R-3.1.2\\scripts\\mPDBS\\summary_pdb_93\\2b3027889e2fdc42.final.pdb
-2.841379e-15 3.111331e-15 1.153578e-15 6.492594e-15 -7.611365e-16
1.933538e-15
E:\\R-3.1.2\\scripts\\mPDBS\\summary_pdb_93\\2fcb3b46e33793b0.final.pdb
-3.659739e-15 3.743530e-15 1.233690e-15 1.036209e-14 -8.693127e-16
2.399639e-15
E:\\R-3.1.2\\scripts\\mPDBS\\summary_pdb_93\\34eb328251842006.final.pdb
-8.806238e-15 9.286190e-15 8.424510e-15 7.701267e-15 1.810526e-15
1.313458e-15
E:\\R-3.1.2\\scripts\\mPDBS\\summary_pdb_93\\354b32871797b7ab.final.pdb
-1.068897e-14 7.014199e-15 5.114749e-15 8.358533e-15 1.120939e-14
5.499752e-15
E:\\R-3.1.2\\scripts\\mPDBS\\summary_pdb_93\\397368adbc32d5de.final.pdb
6.762575e-16 4.650280e-15 -2.674907e-16 9.123962e-16 -3.573312e-15
2.001818e-15
E:\\R-3.1.2\\scripts\\mPDBS\\summary_pdb_93\\411ca13cd3df6edd.final.pdb

-7.176481e-16 9.161749e-15 2.716784e-15 9.660397e-15 -2.717444e-15
2.905094e-15
E:\\R-3.1.2\\scripts\\mPDBS\\summary_pdb_93\\42357eee6642bf12.final.pdb
-8.587397e-15 5.575857e-15 1.051603e-14 4.366394e-15 -5.347114e-16
-7.116605e-15
E:\\R-3.1.2\\scripts\\mPDBS\\summary_pdb_93\\44342d1f75a7fcfc.final.pdb
-1.399363e-15 1.002412e-15 -4.674682e-15 5.304138e-15 2.465029e-15
1.479665e-14
E:\\R-3.1.2\\scripts\\mPDBS\\summary_pdb_93\\448ef21dffe8cdfb.final.pdb
-1.526677e-14 2.240308e-14 3.344849e-15 -2.013424e-15 9.632703e-15
-5.573150e-15
E:\\R-3.1.2\\scripts\\mPDBS\\summary_pdb_93\\472a40b3e8f113c7.final.pdb
4.195185e-16 1.341105e-15 7.160026e-16 7.482661e-15 -1.183514e-15
2.926209e-15
E:\\R-3.1.2\\scripts\\mPDBS\\summary_pdb_93\\48a26225951ea153.final.pdb
-2.927681e-15 1.063060e-14 2.533110e-16 1.217300e-15 3.014489e-15
1.266259e-14
E:\\R-3.1.2\\scripts\\mPDBS\\summary_pdb_93\\4ab4a5c7c6682ce6.final.pdb
-9.194319e-15 7.335307e-15 1.127016e-14 -3.053182e-15 7.110849e-15
3.816339e-15
E:\\R-3.1.2\\scripts\\mPDBS\\summary_pdb_93\\4eaa45b62cb6a25f.final.pdb
2.661027e-15 3.848993e-16 -2.755325e-15 4.650996e-15 -7.083481e-15
2.515269e-15
E:\\R-3.1.2\\scripts\\mPDBS\\summary_pdb_93\\58c371611d85ac96.final.pdb
6.762575e-16 4.650280e-15 -2.674907e-16 9.123962e-16 -3.573312e-15
2.001818e-15
E:\\R-3.1.2\\scripts\\mPDBS\\summary_pdb_93\\59a647f04822d30a.final.pdb
-9.224266e-15 -1.407986e-15 5.818553e-16 6.776309e-16 5.419725e-17
-1.143292e-15
E:\\R-3.1.2\\scripts\\mPDBS\\summary_pdb_93\\59b091fe385e05fc.final.pdb
-3.880478e-15 6.036260e-16 2.432381e-15 9.005341e-15 2.009651e-15
-3.699581e-16
E:\\R-3.1.2\\scripts\\mPDBS\\summary_pdb_93\\5c35ac30f930d2db.final.pdb
2.661027e-15 3.848993e-16 -2.755325e-15 4.650996e-15 -7.083481e-15
2.515269e-15
E:\\R-3.1.2\\scripts\\mPDBS\\summary_pdb_93\\5f60752431cf2dcb.final.pdb
-1.378336e-16 3.620085e-15 1.670393e-15 9.675251e-16 -6.731707e-15
1.426781e-15
E:\\R-3.1.2\\scripts\\mPDBS\\summary_pdb_93\\626456dd6ff2868d.final.pdb
4.195185e-16 1.341105e-15 7.160026e-16 7.482661e-15 -1.183514e-15
2.926209e-15
E:\\R-3.1.2\\scripts\\mPDBS\\summary_pdb_93\\6583d53ff3c8f7ef.final.pdb
-9.224266e-15 -1.407986e-15 5.818553e-16 6.776309e-16 5.419725e-17
-1.143292e-15
E:\\R-3.1.2\\scripts\\mPDBS\\summary_pdb_93\\69760178986bef01.final.pdb
6.941566e-16 1.954207e-15 -5.619882e-15 -1.083339e-16 2.290114e-15
5.767178e-15
E:\\R-3.1.2\\scripts\\mPDBS\\summary_pdb_93\\6a9d87d47926b1c8.final.pdb
-2.876456e-15 3.289390e-15 1.153898e-15 6.390653e-15 -1.113475e-15
1.446707e-15
E:\\R-3.1.2\\scripts\\mPDBS\\summary_pdb_93\\6bb3547ea982ffc4.final.pdb
1.934368e-15 1.216902e-14 6.366655e-16 1.347886e-16 -3.060776e-15
7.588030e-15
E:\\R-3.1.2\\scripts\\mPDBS\\summary_pdb_93\\716d9624611d1c42.final.pdb

-2.299733e-15 6.486499e-15 -2.231854e-15 8.408621e-15 1.735912e-15
5.405586e-15
E:\\R-3.1.2\\scripts\\mPDBS\\summary_pdb_93\\75f27e70ad0f1be4.final.pdb
-9.224266e-15 -1.407986e-15 5.818553e-16 6.776309e-16 5.419725e-17
-1.143292e-15
E:\\R-3.1.2\\scripts\\mPDBS\\summary_pdb_93\\769f910fffb22872.final.pdb
-1.904659e-15 3.017565e-15 8.096932e-16 6.299526e-15 -9.857950e-16
1.284865e-15
E:\\R-3.1.2\\scripts\\mPDBS\\summary_pdb_93\\791f8c80dd5c3135.final.pdb
-3.880478e-15 6.036260e-16 2.432381e-15 9.005341e-15 2.009651e-15
-3.699581e-16
E:\\R-3.1.2\\scripts\\mPDBS\\summary_pdb_93\\7fad98e66a3f6099.final.pdb
-5.329967e-15 5.351605e-15 8.204381e-15 1.720125e-14 4.246014e-15
8.361224e-16
E:\\R-3.1.2\\scripts\\mPDBS\\summary_pdb_93\\804b89c94a633196.final.pdb
-3.659739e-15 3.743530e-15 1.233690e-15 1.036209e-14 -8.693127e-16
2.399639e-15
E:\\R-3.1.2\\scripts\\mPDBS\\summary_pdb_93\\813cc65a2298feba.final.pdb
-3.659739e-15 3.743530e-15 1.233690e-15 1.036209e-14 -8.693127e-16
2.399639e-15
E:\\R-3.1.2\\scripts\\mPDBS\\summary_pdb_93\\83c0eefcfe12d3f9.final.pdb
-7.176481e-16 9.161749e-15 2.716784e-15 9.660397e-15 -2.717444e-15
2.905094e-15
E:\\R-3.1.2\\scripts\\mPDBS\\summary_pdb_93\\86d50469ef569760.final.pdb
4.195185e-16 1.341105e-15 7.160026e-16 7.482661e-15 -1.183514e-15
2.926209e-15
E:\\R-3.1.2\\scripts\\mPDBS\\summary_pdb_93\\89d0666e1402203d.final.pdb
-9.224266e-15 -1.407986e-15 5.818553e-16 6.776309e-16 5.419725e-17
-1.143292e-15
E:\\R-3.1.2\\scripts\\mPDBS\\summary_pdb_93\\8b34852716dadc62.final.pdb
2.139441e-15 -1.051246e-15 7.005874e-15 1.452553e-15 5.550899e-15
1.439418e-17
E:\\R-3.1.2\\scripts\\mPDBS\\summary_pdb_93\\8b6ef9ac3fe74395.final.pdb
-3.981714e-15 -4.810296e-15 9.136537e-16 5.269380e-15 3.905466e-15
-2.813327e-15
E:\\R-3.1.2\\scripts\\mPDBS\\summary_pdb_93\\98bbe0d5986c138f.final.pdb
-3.880478e-15 6.036260e-16 2.432381e-15 9.005341e-15 2.009651e-15
-3.699581e-16
E:\\R-3.1.2\\scripts\\mPDBS\\summary_pdb_93\\9c22f4909d67e2fa.final.pdb
1.234783e-15 -6.515817e-16 -6.302978e-15 8.631706e-15 -3.617332e-15
2.234298e-15
E:\\R-3.1.2\\scripts\\mPDBS\\summary_pdb_93\\9dda8ed0d81fb3ef.final.pdb
6.533962e-15 8.715210e-15 -5.915064e-15 4.023045e-15 5.369824e-15
8.065488e-15
E:\\R-3.1.2\\scripts\\mPDBS\\summary_pdb_93\\9f752b3616e6033b.final.pdb
-3.039525e-15 3.222619e-15 1.883103e-15 6.710711e-15 3.686507e-15
1.760031e-16
E:\\R-3.1.2\\scripts\\mPDBS\\summary_pdb_93\\a02c70083816d6ba.final.pdb
2.661027e-15 3.848993e-16 -2.755325e-15 4.650996e-15 -7.083481e-15
2.515269e-15
E:\\R-3.1.2\\scripts\\mPDBS\\summary_pdb_93\\a0c997d891e4e082.final.pdb
6.426453e-16 -7.101721e-16 -3.350456e-15 1.041697e-14 3.539622e-15
2.401113e-15
E:\\R-3.1.2\\scripts\\mPDBS\\summary_pdb_93\\ab8039a2d823c616.final.pdb

-2.068728e-15 2.833109e-15 7.476523e-16 6.196823e-15 -3.494116e-16
1.720126e-15
E:\\R-3.1.2\\scripts\\mPDBS\\summary_pdb_93\\acf4d74b9c71d4dc.final.pdb
-3.659739e-15 3.743530e-15 1.233690e-15 1.036209e-14 -8.693127e-16
2.399639e-15
E:\\R-3.1.2\\scripts\\mPDBS\\summary_pdb_93\\ad8a823e4b4c668e.final.pdb
-1.372982e-15 3.552705e-15 1.064594e-15 6.748453e-15 4.806479e-17
1.966315e-15
E:\\R-3.1.2\\scripts\\mPDBS\\summary_pdb_93\\b16ababd03b7b857.final.pdb
1.234783e-15 -6.515817e-16 -6.302978e-15 8.631706e-15 -3.617332e-15
2.234298e-15
E:\\R-3.1.2\\scripts\\mPDBS\\summary_pdb_93\\b226923e697d3c63.final.pdb
-1.184691e-15 1.963557e-15 1.051694e-15 4.747726e-15 -2.576294e-15
-3.042706e-15
E:\\R-3.1.2\\scripts\\mPDBS\\summary_pdb_93\\b28e48f6fa27a165.final.pdb
-3.880478e-15 6.036260e-16 2.432381e-15 9.005341e-15 2.009651e-15
-3.699581e-16
E:\\R-3.1.2\\scripts\\mPDBS\\summary_pdb_93\\b42c4924e3cd1aec.final.pdb
7.139643e-16 -4.680650e-15 1.435841e-15 1.606766e-14 -4.460536e-16
-1.498585e-15
E:\\R-3.1.2\\scripts\\mPDBS\\summary_pdb_93\\b4dd239fcabff1df.final.pdb
-3.880478e-15 6.036260e-16 2.432381e-15 9.005341e-15 2.009651e-15
-3.699581e-16
E:\\R-3.1.2\\scripts\\mPDBS\\summary_pdb_93\\b669942ef97459a2.final.pdb
2.139441e-15 -1.051246e-15 7.005874e-15 1.452553e-15 5.550899e-15
1.439418e-17
E:\\R-3.1.2\\scripts\\mPDBS\\summary_pdb_93\\b99f4d9d87c1017b.final.pdb
-3.880478e-15 6.036260e-16 2.432381e-15 9.005341e-15 2.009651e-15
-3.699581e-16
E:\\R-3.1.2\\scripts\\mPDBS\\summary_pdb_93\\b9d2cbf3975403e7.final.pdb
-3.880478e-15 6.036260e-16 2.432381e-15 9.005341e-15 2.009651e-15
-3.699581e-16
E:\\R-3.1.2\\scripts\\mPDBS\\summary_pdb_93\\bc88c8a505f3a288.final.pdb
-2.955437e-15 3.863985e-15 9.227652e-16 6.158226e-15 -1.133018e-15
1.508856e-15
E:\\R-3.1.2\\scripts\\mPDBS\\summary_pdb_93\\bdac0e64f4ef427e.final.pdb
-4.314597e-15 -9.731341e-15 -2.614292e-15 1.605209e-15 4.967164e-15
-2.925435e-16
E:\\R-3.1.2\\scripts\\mPDBS\\summary_pdb_93\\ca2a1f55fc9d0681.final.pdb
2.139441e-15 -1.051246e-15 7.005874e-15 1.452553e-15 5.550899e-15
1.439418e-17
E:\\R-3.1.2\\scripts\\mPDBS\\summary_pdb_93\\ce34248de3e81a3c.final.pdb
-3.016340e-15 -5.153255e-15 -1.532021e-15 7.599542e-15 -5.246058e-15
1.897485e-16
E:\\R-3.1.2\\scripts\\mPDBS\\summary_pdb_93\\d02e697772494a61.final.pdb
-1.424675e-14 5.442746e-15 6.912887e-15 -1.510119e-15 -5.663248e-15
-1.623621e-15
E:\\R-3.1.2\\scripts\\mPDBS\\summary_pdb_93\\d04221245d015047.final.pdb
-2.398325e-15 3.685617e-15 8.579834e-16 7.030791e-15 8.525349e-16
3.358681e-15
E:\\R-3.1.2\\scripts\\mPDBS\\summary_pdb_93\\d678141e4018054a.final.pdb
-2.314681e-15 2.150145e-14 5.082104e-15 3.153396e-15 -6.150907e-15
1.005995e-14
E:\\R-3.1.2\\scripts\\mPDBS\\summary_pdb_93\\d71b40af0f0efe39.final.pdb

-2.210692e-15 1.588587e-14 7.313110e-15 1.660678e-14 -1.457913e-15
1.353418e-15
E:\\R-3.1.2\\scripts\\mPDBS\\summary_pdb_93\\dc0809eff10197e2.final.pdb
-3.188640e-15 -8.476652e-15 -3.090070e-15 1.279905e-14 -3.117324e-15
-1.013177e-15
E:\\R-3.1.2\\scripts\\mPDBS\\summary_pdb_93\\e1163918d3707839.final.pdb
6.525288e-15 7.882217e-15 7.089792e-15 1.475968e-14 -2.899285e-16
6.040090e-15
E:\\R-3.1.2\\scripts\\mPDBS\\summary_pdb_93\\e4b48b06824ef848.final.pdb
2.089108e-15 9.465749e-15 -2.222278e-15 -2.260900e-15 -5.077357e-15
6.339524e-15
E:\\R-3.1.2\\scripts\\mPDBS\\summary_pdb_93\\e529e19ad281fea2.final.pdb
-5.806404e-15 2.641172e-15 -4.562002e-15 1.075088e-14 -6.555953e-15
2.527223e-15
E:\\R-3.1.2\\scripts\\mPDBS\\summary_pdb_93\\e5602082718ec3a1.final.pdb
-3.659739e-15 3.743530e-15 1.233690e-15 1.036209e-14 -8.693127e-16
2.399639e-15
E:\\R-3.1.2\\scripts\\mPDBS\\summary_pdb_93\\e5d589d996d0a960.final.pdb
3.565968e-15 2.683655e-16 -2.911656e-15 1.148562e-14 6.139175e-15
2.537136e-15
E:\\R-3.1.2\\scripts\\mPDBS\\summary_pdb_93\\e9df9e72307a2fbd.final.pdb
2.661027e-15 3.848993e-16 -2.755325e-15 4.650996e-15 -7.083481e-15
2.515269e-15
E:\\R-3.1.2\\scripts\\mPDBS\\summary_pdb_93\\ea00f8694090cae7.final.pdb
-3.659739e-15 3.743530e-15 1.233690e-15 1.036209e-14 -8.693127e-16
2.399639e-15
E:\\R-3.1.2\\scripts\\mPDBS\\summary_pdb_93\\eba517be32db050e.final.pdb
-3.880478e-15 6.036260e-16 2.432381e-15 9.005341e-15 2.009651e-15
-3.699581e-16
E:\\R-3.1.2\\scripts\\mPDBS\\summary_pdb_93\\ec04b9545899c29d.final.pdb
-1.517324e-15 3.095302e-15 5.666494e-16 6.254767e-15 2.710377e-16
1.835662e-15
E:\\R-3.1.2\\scripts\\mPDBS\\summary_pdb_93\\ee6862cbcad52078.final.pdb
-3.023959e-15 3.576942e-15 9.657809e-16 6.066720e-15 -7.147327e-16
1.187390e-15
E:\\R-3.1.2\\scripts\\mPDBS\\summary_pdb_93\\ef6a42fc83b81e5d.final.pdb
-1.855219e-15 3.322117e-15 8.374081e-16 6.155111e-15 -4.018437e-16
1.669432e-15
E:\\R-3.1.2\\scripts\\mPDBS\\summary_pdb_93\\ef8e88d4ad93a0ad.final.pdb
-2.398325e-15 3.685617e-15 8.579834e-16 7.030791e-15 8.525349e-16
3.358681e-15
E:\\R-3.1.2\\scripts\\mPDBS\\summary_pdb_93\\f14fcae8deec4fa3.final.pdb
-3.039525e-15 3.222619e-15 1.883103e-15 6.710711e-15 3.686507e-15
1.760031e-16
E:\\R-3.1.2\\scripts\\mPDBS\\summary_pdb_93\\f83584e22003fb92.final.pdb
-2.927681e-15 1.063060e-14 2.533110e-16 1.217300e-15 3.014489e-15
1.266259e-14
E:\\R-3.1.2\\scripts\\mPDBS\\summary_pdb_93\\f8e2bf87e52caa29.final.pdb
-5.806404e-15 2.641172e-15 -4.562002e-15 1.075088e-14 -6.555953e-15
2.527223e-15

[,589]   [,590]   [,591]   [,592]   [,593]   [,594]
E:\\R-3.1.2\\scripts\\40.1ns_data\\NAT0.pdb
-1.536767e-15 -3.137635e-15 9.744995e-16 -2.473478e-15 1.014024e-15

-4.252926e-15
E:\\R-3.1.2\\scripts\\40.1ns_data\\LJP6.pdb
-2.826937e-15 -2.942851e-15 1.765914e-15 -4.224957e-15 3.121494e-16
-4.645546e-15
E:\\R-3.1.2\\scripts\\40.1ns_data\\3WY9.pdb
-2.474076e-15 -3.511157e-15 -1.575482e-15 -3.782453e-15 -1.440360e-15
-6.878457e-15
E:\\R-3.1.2\\scripts\\40.1ns_data\\ZSP9.pdb
1.178947e-16 -4.837187e-15 -2.406787e-16 -3.698656e-15 1.084301e-15
-4.063157e-15
E:\\R-3.1.2\\scripts\\mPDBS\\summary_pdb_93\\013d855ad26c877c.final.pdb
-1.803285e-15 -3.116293e-15 7.820347e-16 -2.613403e-15 6.232523e-15
-1.176686e-14
E:\\R-3.1.2\\scripts\\mPDBS\\summary_pdb_93\\0a2a705342ca0d54.final.pdb
-3.037718e-15 -3.691858e-15 1.150731e-15 -3.598690e-15 -3.514956e-16
-3.750752e-15
E:\\R-3.1.2\\scripts\\mPDBS\\summary_pdb_93\\0a59fceadb4194d9.final.pdb
-6.770266e-15 -2.645935e-15 -3.308072e-15 -2.963108e-15 3.230069e-15
-7.642385e-15
E:\\R-3.1.2\\scripts\\mPDBS\\summary_pdb_93\\0dba59f3306825e8.final.pdb
-3.801253e-15 -5.305514e-15 3.666880e-15 -4.226696e-15 -4.586947e-15
-2.676913e-15
E:\\R-3.1.2\\scripts\\mPDBS\\summary_pdb_93\\126f26a78b57339c.final.pdb
-1.733462e-15 -8.439726e-15 2.096467e-15 -4.926657e-15 -3.913468e-15
1.460403e-15
E:\\R-3.1.2\\scripts\\mPDBS\\summary_pdb_93\\12f06f3e342df706.final.pdb
-2.506893e-15 -3.537034e-15 1.442544e-15 -3.816398e-15 -3.222222e-16
-4.396069e-15
E:\\R-3.1.2\\scripts\\mPDBS\\summary_pdb_93\\18618a2a251b867b.final.pdb
1.115152e-15 1.577637e-15 6.478444e-15 -1.813725e-15 -6.695899e-15
9.555663e-16
E:\\R-3.1.2\\scripts\\mPDBS\\summary_pdb_93\\1f305f598641eeb2.final.pdb
-1.884853e-15 -4.300833e-15 9.268699e-16 -4.299889e-15 -8.402498e-16
-4.734319e-15
E:\\R-3.1.2\\scripts\\mPDBS\\summary_pdb_93\\1f8a00b551be0a39.final.pdb
1.115152e-15 1.577637e-15 6.478444e-15 -1.813725e-15 -6.695899e-15
9.555663e-16
E:\\R-3.1.2\\scripts\\mPDBS\\summary_pdb_93\\219f45328d47579a.final.pdb
3.352930e-15 -1.328673e-14 4.622217e-15 -4.926815e-15 -2.883097e-15
-6.079399e-15
E:\\R-3.1.2\\scripts\\mPDBS\\summary_pdb_93\\238dd3918e54336d.final.pdb
4.407425e-15 -1.121807e-14 4.989300e-15 -4.945030e-15 -9.292683e-15
7.961772e-16
E:\\R-3.1.2\\scripts\\mPDBS\\summary_pdb_93\\2b3027889e2fdc42.final.pdb
-3.466702e-15 -5.239130e-15 -4.618174e-17 -3.606691e-15 -5.433886e-16
-4.713305e-15
E:\\R-3.1.2\\scripts\\mPDBS\\summary_pdb_93\\2fcb3b46e33793b0.final.pdb
-3.059043e-15 9.842596e-17 8.739608e-16 -2.280671e-15 -1.140357e-15
-8.418871e-15
E:\\R-3.1.2\\scripts\\mPDBS\\summary_pdb_93\\34eb328251842006.final.pdb
-8.780107e-15 -3.564927e-16 -2.124596e-16 -6.832803e-15 8.078386e-15
-7.187898e-15
E:\\R-3.1.2\\scripts\\mPDBS\\summary_pdb_93\\354b32871797b7ab.final.pdb
-1.004690e-14 -8.932337e-15 1.844297e-15 2.122581e-15 5.969868e-15

-5.519848e-15
E:\\R-3.1.2\\scripts\\mPDBS\\summary_pdb_93\\397368adbc32d5de.final.pdb
-1.733462e-15 -8.439726e-15 2.096467e-15 -4.926657e-15 -3.913468e-15
1.460403e-15
E:\\R-3.1.2\\scripts\\mPDBS\\summary_pdb_93\\411ca13cd3df6edd.final.pdb
-2.572020e-15 3.891833e-15 4.525174e-15 -7.445734e-16 -1.720161e-15
-5.218307e-15
E:\\R-3.1.2\\scripts\\mPDBS\\summary_pdb_93\\42357eee6642bf12.final.pdb
-4.638778e-15 -6.051339e-15 -8.191953e-15 -4.440129e-15 8.086999e-16
-1.249842e-14
E:\\R-3.1.2\\scripts\\mPDBS\\summary_pdb_93\\44342d1f75a7fcfc.final.pdb
-9.817000e-15 1.545737e-14 -9.232612e-15 -6.607406e-15 -2.828192e-15
1.428254e-15
E:\\R-3.1.2\\scripts\\mPDBS\\summary_pdb_93\\448ef21dffe8cdfb.final.pdb
3.449856e-15 -1.588185e-14 -1.075476e-14 -3.890494e-15 1.531696e-14
-8.470913e-15
E:\\R-3.1.2\\scripts\\mPDBS\\summary_pdb_93\\472a40b3e8f113c7.final.pdb
-3.801253e-15 -5.305514e-15 3.666880e-15 -4.226696e-15 -4.586947e-15
-2.676913e-15
E:\\R-3.1.2\\scripts\\mPDBS\\summary_pdb_93\\48a26225951ea153.final.pdb
-8.522336e-15 6.382650e-15 2.296996e-15 -1.449562e-15 -3.921763e-15
-1.777285e-15
E:\\R-3.1.2\\scripts\\mPDBS\\summary_pdb_93\\4ab4a5c7c6682ce6.final.pdb
-6.733152e-15 -1.061507e-14 -4.909214e-15 -2.350969e-16 -3.420807e-15
-1.033225e-14
E:\\R-3.1.2\\scripts\\mPDBS\\summary_pdb_93\\4eaa45b62cb6a25f.final.pdb
2.933520e-16 -2.116608e-15 3.184832e-15 -4.128495e-15 -2.795040e-15
-4.243959e-15
E:\\R-3.1.2\\scripts\\mPDBS\\summary_pdb_93\\58c371611d85ac96.final.pdb
-1.733462e-15 -8.439726e-15 2.096467e-15 -4.926657e-15 -3.913468e-15
1.460403e-15
E:\\R-3.1.2\\scripts\\mPDBS\\summary_pdb_93\\59a647f04822d30a.final.pdb
-2.421657e-15 -1.237627e-14 -1.549955e-15 -9.115816e-15 -2.355480e-15
-4.697054e-15
E:\\R-3.1.2\\scripts\\mPDBS\\summary_pdb_93\\59b091fe385e05fc.final.pdb
-6.770266e-15 -2.645935e-15 -3.308072e-15 -2.963108e-15 3.230069e-15
-7.642385e-15
E:\\R-3.1.2\\scripts\\mPDBS\\summary_pdb_93\\5c35ac30f930d2db.final.pdb
2.933520e-16 -2.116608e-15 3.184832e-15 -4.128495e-15 -2.795040e-15
-4.243959e-15
E:\\R-3.1.2\\scripts\\mPDBS\\summary_pdb_93\\5f60752431cf2dcb.final.pdb
-2.938405e-15 1.202014e-15 1.741364e-15 -1.162271e-15 -9.278575e-16
-2.718591e-15
E:\\R-3.1.2\\scripts\\mPDBS\\summary_pdb_93\\626456dd6ff2868d.final.pdb
-3.801253e-15 -5.305514e-15 3.666880e-15 -4.226696e-15 -4.586947e-15
-2.676913e-15
E:\\R-3.1.2\\scripts\\mPDBS\\summary_pdb_93\\6583d53ff3c8f7ef.final.pdb
-2.421657e-15 -1.237627e-14 -1.549955e-15 -9.115816e-15 -2.355480e-15
-4.697054e-15
E:\\R-3.1.2\\scripts\\mPDBS\\summary_pdb_93\\69760178986bef01.final.pdb
2.964154e-15 -3.033753e-15 2.841342e-15 -4.946200e-15 -1.024929e-14
7.452647e-16
E:\\R-3.1.2\\scripts\\mPDBS\\summary_pdb_93\\6a9d87d47926b1c8.final.pdb
-3.038625e-15 -5.837640e-15 -1.543365e-16 -4.226696e-15 -9.991326e-16

-4.781132e-15
E:\\R-3.1.2\\scripts\\mPDBS\\summary_pdb_93\\6bb3547ea982ffc4.final.pdb
4.377882e-15 -5.266459e-15 1.044528e-14 -2.359283e-15 -1.492951e-15
6.669276e-15
E:\\R-3.1.2\\scripts\\mPDBS\\summary_pdb_93\\716d9624611d1c42.final.pdb
-9.276441e-16 -3.910433e-15 7.970796e-15 -6.242409e-15 1.668590e-15
-5.348432e-15
E:\\R-3.1.2\\scripts\\mPDBS\\summary_pdb_93\\75f27e70ad0f1be4.final.pdb
-2.421657e-15 -1.237627e-14 -1.549955e-15 -9.115816e-15 -2.355480e-15
-4.697054e-15
E:\\R-3.1.2\\scripts\\mPDBS\\summary_pdb_93\\769f910fffb22872.final.pdb
-2.731598e-15 -3.459350e-15 3.485954e-16 -4.239736e-15 1.248167e-16
-4.990198e-15
E:\\R-3.1.2\\scripts\\mPDBS\\summary_pdb_93\\791f8c80dd5c3135.final.pdb
-6.770266e-15 -2.645935e-15 -3.308072e-15 -2.963108e-15 3.230069e-15
-7.642385e-15
E:\\R-3.1.2\\scripts\\mPDBS\\summary_pdb_93\\7fad98e66a3f6099.final.pdb
-7.699989e-15 4.535869e-15 4.773581e-16 -4.318866e-15 6.715066e-15
-9.948327e-15
E:\\R-3.1.2\\scripts\\mPDBS\\summary_pdb_93\\804b89c94a633196.final.pdb
-3.059043e-15 9.842596e-17 8.739608e-16 -2.280671e-15 -1.140357e-15
-8.418871e-15
E:\\R-3.1.2\\scripts\\mPDBS\\summary_pdb_93\\813cc65a2298feba.final.pdb
-3.059043e-15 9.842596e-17 8.739608e-16 -2.280671e-15 -1.140357e-15
-8.418871e-15
E:\\R-3.1.2\\scripts\\mPDBS\\summary_pdb_93\\83c0eefcfe12d3f9.final.pdb
-2.572020e-15 3.891833e-15 4.525174e-15 -7.445734e-16 -1.720161e-15
-5.218307e-15
E:\\R-3.1.2\\scripts\\mPDBS\\summary_pdb_93\\86d50469ef569760.final.pdb
-3.801253e-15 -5.305514e-15 3.666880e-15 -4.226696e-15 -4.586947e-15
-2.676913e-15
E:\\R-3.1.2\\scripts\\mPDBS\\summary_pdb_93\\89d0666e1402203d.final.pdb
-2.421657e-15 -1.237627e-14 -1.549955e-15 -9.115816e-15 -2.355480e-15
-4.697054e-15
E:\\R-3.1.2\\scripts\\mPDBS\\summary_pdb_93\\8b34852716dadc62.final.pdb
-4.587552e-15 -4.682472e-15 2.954306e-16 -4.848062e-15 -5.975031e-15
2.825016e-16
E:\\R-3.1.2\\scripts\\mPDBS\\summary_pdb_93\\8b6ef9ac3fe74395.final.pdb
-2.488300e-15 -2.987844e-15 1.750034e-15 -1.156825e-14 -3.694591e-16
1.089699e-15
E:\\R-3.1.2\\scripts\\mPDBS\\summary_pdb_93\\98bbe0d5986c138f.final.pdb
-6.770266e-15 -2.645935e-15 -3.308072e-15 -2.963108e-15 3.230069e-15
-7.642385e-15
E:\\R-3.1.2\\scripts\\mPDBS\\summary_pdb_93\\9c22f4909d67e2fa.final.pdb
-8.310080e-16 -9.939551e-15 -1.468539e-15 -6.776207e-15 1.001810e-15
-4.728247e-15
E:\\R-3.1.2\\scripts\\mPDBS\\summary_pdb_93\\9dda8ed0d81fb3ef.final.pdb
-6.832842e-15 1.794453e-14 -4.821034e-15 2.080089e-15 3.189103e-15
-4.327558e-15
E:\\R-3.1.2\\scripts\\mPDBS\\summary_pdb_93\\9f752b3616e6033b.final.pdb
-2.466430e-15 -6.256993e-15 -2.764787e-15 -4.733159e-15 -1.652295e-15
-3.640751e-15
E:\\R-3.1.2\\scripts\\mPDBS\\summary_pdb_93\\a02c70083816d6ba.final.pdb
2.933520e-16 -2.116608e-15 3.184832e-15 -4.128495e-15 -2.795040e-15

-4.243959e-15
E:\\R-3.1.2\\scripts\\mPDBS\\summary_pdb_93\\a0c997d891e4e082.final.pdb
-7.824294e-15 -9.781250e-15 5.503255e-15 8.000534e-15 2.367489e-15
-9.556892e-15
E:\\R-3.1.2\\scripts\\mPDBS\\summary_pdb_93\\ab8039a2d823c616.final.pdb
-3.432842e-15 -2.452044e-15 1.217377e-15 -4.173755e-15 -4.963229e-16
-3.902561e-15
E:\\R-3.1.2\\scripts\\mPDBS\\summary_pdb_93\\acf4d74b9c71d4dc.final.pdb
-3.059043e-15 9.842596e-17 8.739608e-16 -2.280671e-15 -1.140357e-15
-8.418871e-15
E:\\R-3.1.2\\scripts\\mPDBS\\summary_pdb_93\\ad8a823e4b4c668e.final.pdb
-2.343239e-15 -4.037528e-15 4.911381e-16 -4.115371e-15 1.888416e-16
-4.175360e-15
E:\\R-3.1.2\\scripts\\mPDBS\\summary_pdb_93\\b16ababd03b7b857.final.pdb
-8.310080e-16 -9.939551e-15 -1.468539e-15 -6.776207e-15 1.001810e-15
-4.728247e-15
E:\\R-3.1.2\\scripts\\mPDBS\\summary_pdb_93\\b226923e697d3c63.final.pdb
-5.108743e-15 -4.256680e-15 5.068218e-15 1.204054e-15 -1.798417e-15
-5.299871e-15
E:\\R-3.1.2\\scripts\\mPDBS\\summary_pdb_93\\b28e48f6fa27a165.final.pdb
-6.770266e-15 -2.645935e-15 -3.308072e-15 -2.963108e-15 3.230069e-15
-7.642385e-15
E:\\R-3.1.2\\scripts\\mPDBS\\summary_pdb_93\\b42c4924e3cd1aec.final.pdb
-5.057135e-15 -1.019312e-14 7.145156e-15 -4.830182e-15 4.641310e-15
-4.246893e-15
E:\\R-3.1.2\\scripts\\mPDBS\\summary_pdb_93\\b4dd239fcabff1df.final.pdb
-6.770266e-15 -2.645935e-15 -3.308072e-15 -2.963108e-15 3.230069e-15
-7.642385e-15
E:\\R-3.1.2\\scripts\\mPDBS\\summary_pdb_93\\b669942ef97459a2.final.pdb
-4.587552e-15 -4.682472e-15 2.954306e-16 -4.848062e-15 -5.975031e-15
2.825016e-16
E:\\R-3.1.2\\scripts\\mPDBS\\summary_pdb_93\\b99f4d9d87c1017b.final.pdb
-6.770266e-15 -2.645935e-15 -3.308072e-15 -2.963108e-15 3.230069e-15
-7.642385e-15
E:\\R-3.1.2\\scripts\\mPDBS\\summary_pdb_93\\b9d2cbf3975403e7.final.pdb
-6.770266e-15 -2.645935e-15 -3.308072e-15 -2.963108e-15 3.230069e-15
-7.642385e-15
E:\\R-3.1.2\\scripts\\mPDBS\\summary_pdb_93\\bc88c8a505f3a288.final.pdb
-4.297851e-15 -5.239563e-15 3.227316e-16 -3.582404e-15 -1.120808e-15
-4.737591e-15
E:\\R-3.1.2\\scripts\\mPDBS\\summary_pdb_93\\bdac0e64f4ef427e.final.pdb
-2.752214e-15 -1.708531e-14 -8.857133e-15 -1.183813e-14 1.894786e-15
-3.782823e-15
E:\\R-3.1.2\\scripts\\mPDBS\\summary_pdb_93\\ca2a1f55fc9d0681.final.pdb
-4.587552e-15 -4.682472e-15 2.954306e-16 -4.848062e-15 -5.975031e-15
2.825016e-16
E:\\R-3.1.2\\scripts\\mPDBS\\summary_pdb_93\\ce34248de3e81a3c.final.pdb
-7.600110e-15 -2.961191e-15 3.520475e-15 4.958958e-16 4.507915e-15
-1.411649e-14
E:\\R-3.1.2\\scripts\\mPDBS\\summary_pdb_93\\d02e697772494a61.final.pdb
3.237354e-15 -2.038262e-14 -1.257439e-15 -8.407538e-15 -9.669118e-15
-6.905995e-15
E:\\R-3.1.2\\scripts\\mPDBS\\summary_pdb_93\\d04221245d015047.final.pdb
-2.300935e-15 -4.230135e-15 -1.072214e-15 -5.800926e-15 -5.641940e-16

-4.578236e-15
E:\\R-3.1.2\\scripts\\mPDBS\\summary_pdb_93\\d678141e4018054a.final.pdb
1.671733e-15 -2.188636e-16 1.076461e-14 -9.745358e-16 -9.692483e-16
-1.175651e-15
E:\\R-3.1.2\\scripts\\mPDBS\\summary_pdb_93\\d71b40af0f0efe39.final.pdb
-6.594701e-15 -1.660964e-16 -5.165870e-15 -1.901207e-15 -2.863702e-15
-2.246502e-15
E:\\R-3.1.2\\scripts\\mPDBS\\summary_pdb_93\\dc0809eff10197e2.final.pdb
-6.204452e-15 -1.145341e-14 -6.057700e-15 -1.530355e-14 -1.140640e-16
-2.219359e-15
E:\\R-3.1.2\\scripts\\mPDBS\\summary_pdb_93\\e1163918d3707839.final.pdb
1.244837e-16 -6.990821e-15 5.843030e-15 -3.073774e-15 1.112883e-15
-6.026720e-15
E:\\R-3.1.2\\scripts\\mPDBS\\summary_pdb_93\\e4b48b06824ef848.final.pdb
1.115152e-15 1.577637e-15 6.478444e-15 -1.813725e-15 -6.695899e-15
9.555663e-16
E:\\R-3.1.2\\scripts\\mPDBS\\summary_pdb_93\\e529e19ad281fea2.final.pdb
-1.803285e-15 -3.116293e-15 7.820347e-16 -2.613403e-15 6.232523e-15
-1.176686e-14
E:\\R-3.1.2\\scripts\\mPDBS\\summary_pdb_93\\e5602082718ec3a1.final.pdb
-3.059043e-15 9.842596e-17 8.739608e-16 -2.280671e-15 -1.140357e-15
-8.418871e-15
E:\\R-3.1.2\\scripts\\mPDBS\\summary_pdb_93\\e5d589d996d0a960.final.pdb
-5.545720e-15 -9.958806e-15 3.707147e-15 1.194687e-15 -1.981659e-16
-8.354147e-15
E:\\R-3.1.2\\scripts\\mPDBS\\summary_pdb_93\\e9df9e72307a2fbd.final.pdb
2.933520e-16 -2.116608e-15 3.184832e-15 -4.128495e-15 -2.795040e-15
-4.243959e-15
E:\\R-3.1.2\\scripts\\mPDBS\\summary_pdb_93\\ea00f8694090cae7.final.pdb
-3.059043e-15 9.842596e-17 8.739608e-16 -2.280671e-15 -1.140357e-15
-8.418871e-15
E:\\R-3.1.2\\scripts\\mPDBS\\summary_pdb_93\\eba517be32db050e.final.pdb
-6.770266e-15 -2.645935e-15 -3.308072e-15 -2.963108e-15 3.230069e-15
-7.642385e-15
E:\\R-3.1.2\\scripts\\mPDBS\\summary_pdb_93\\ec04b9545899c29d.final.pdb
-2.573662e-15 -3.560405e-15 3.855625e-16 -4.192671e-15 -4.498861e-16
-4.781188e-15
E:\\R-3.1.2\\scripts\\mPDBS\\summary_pdb_93\\ee6862cbcad52078.final.pdb
-3.715201e-15 -4.595764e-15 -1.530434e-16 -3.896389e-15 -7.979592e-16
-4.800908e-15
E:\\R-3.1.2\\scripts\\mPDBS\\summary_pdb_93\\ef6a42fc83b81e5d.final.pdb
-2.568751e-15 -3.493177e-15 7.690761e-16 -4.073203e-15 -4.426547e-16
-4.514884e-15
E:\\R-3.1.2\\scripts\\mPDBS\\summary_pdb_93\\ef8e88d4ad93a0ad.final.pdb
-2.300935e-15 -4.230135e-15 -1.072214e-15 -5.800926e-15 -5.641940e-16
-4.578236e-15
E:\\R-3.1.2\\scripts\\mPDBS\\summary_pdb_93\\f14fcae8deec4fa3.final.pdb
-2.466430e-15 -6.256993e-15 -2.764787e-15 -4.733159e-15 -1.652295e-15
-3.640751e-15
E:\\R-3.1.2\\scripts\\mPDBS\\summary_pdb_93\\f83584e22003fb92.final.pdb
-8.522336e-15 6.382650e-15 2.296996e-15 -1.449562e-15 -3.921763e-15
-1.777285e-15
E:\\R-3.1.2\\scripts\\mPDBS\\summary_pdb_93\\f8e2bf87e52caa29.final.pdb
-1.803285e-15 -3.116293e-15 7.820347e-16 -2.613403e-15 6.232523e-15

-1.176686e-14

[,595] [,596] [,597] [,598] [,599] [,600]
E:\\R-3.1.2\\scripts\\40.1ns_data\\NAT0.pdb
1.908812e-15 -1.712480e-15 3.283681e-15 1.495863e-15 6.629299e-16
7.902959e-16
E:\\R-3.1.2\\scripts\\40.1ns_data\\LJP6.pdb
7.593686e-16 -1.522591e-15 3.079099e-15 1.038022e-15 4.900375e-16
5.403986e-16
E:\\R-3.1.2\\scripts\\40.1ns_data\\3WY9.pdb
1.021517e-15 -3.512149e-15 3.889682e-15 8.102555e-16 6.405374e-16
1.951216e-15
E:\\R-3.1.2\\scripts\\40.1ns_data\\ZSP9.pdb
9.097391e-16 -2.952804e-15 3.856814e-15 8.207561e-16 1.073902e-15
6.564357e-16
E:\\R-3.1.2\\scripts\\mPDBS\\summary_pdb_93\\013d855ad26c877c.final.pdb
1.577290e-15 7.398482e-16 6.199192e-15 3.621939e-16 3.933362e-15
3.303541e-15
E:\\R-3.1.2\\scripts\\mPDBS\\summary_pdb_93\\0a2a705342ca0d54.final.pdb
5.749030e-16 -2.313435e-15 3.261967e-15 1.429965e-15 5.890679e-16
7.498758e-16
E:\\R-3.1.2\\scripts\\mPDBS\\summary_pdb_93\\0a59fceadb4194d9.final.pdb
1.503743e-15 -4.415744e-15 7.299234e-15 3.941409e-15 9.843088e-16
2.635823e-16
E:\\R-3.1.2\\scripts\\mPDBS\\summary_pdb_93\\0dba59f3306825e8.final.pdb
1.657521e-15 -8.037853e-15 1.107778e-15 4.607496e-15 -9.880489e-16
1.751838e-16
E:\\R-3.1.2\\scripts\\mPDBS\\summary_pdb_93\\126f26a78b57339c.final.pdb
-4.470513e-16 -5.892000e-15 -6.730136e-15 -2.342257e-16 -2.372358e-15
3.079328e-15
E:\\R-3.1.2\\scripts\\mPDBS\\summary_pdb_93\\12f06f3e342df706.final.pdb
7.233845e-16 -2.816938e-15 3.311840e-15 1.395379e-15 6.979218e-16
5.304333e-16
E:\\R-3.1.2\\scripts\\mPDBS\\summary_pdb_93\\18618a2a251b867b.final.pdb
1.104577e-15 5.777882e-15 -6.090570e-15 -3.655476e-15 -2.257563e-15
2.715494e-15
E:\\R-3.1.2\\scripts\\mPDBS\\summary_pdb_93\\1f305f598641eeb2.final.pdb
1.225009e-15 -3.455397e-15 2.640402e-15 1.127466e-15 5.631410e-16
6.471789e-16
E:\\R-3.1.2\\scripts\\mPDBS\\summary_pdb_93\\1f8a00b551be0a39.final.pdb
1.104577e-15 5.777882e-15 -6.090570e-15 -3.655476e-15 -2.257563e-15
2.715494e-15
E:\\R-3.1.2\\scripts\\mPDBS\\summary_pdb_93\\219f45328d47579a.final.pdb
-5.558642e-15 -3.284704e-15 5.764438e-15 2.015216e-15 3.641471e-15
2.515904e-15
E:\\R-3.1.2\\scripts\\mPDBS\\summary_pdb_93\\238dd3918e54336d.final.pdb
5.670034e-15 -2.628111e-15 -1.472395e-15 -4.434052e-15 3.881514e-15
1.267987e-15
E:\\R-3.1.2\\scripts\\mPDBS\\summary_pdb_93\\2b3027889e2fdc42.final.pdb
8.284748e-16 -2.925616e-15 3.183169e-15 1.422916e-15 3.765050e-16
7.718563e-16
E:\\R-3.1.2\\scripts\\mPDBS\\summary_pdb_93\\2fcb3b46e33793b0.final.pdb
5.220125e-16 -6.659621e-16 6.682308e-15 -7.556517e-17 1.272643e-15
-8.468517e-16

E:\\R-3.1.2\\scripts\\mPDBS\\summary_pdb_93\\34eb328251842006.final.pdb
5.934414e-16 2.372903e-15 2.189801e-14 7.702960e-15 -3.987372e-15
3.445007e-15
E:\\R-3.1.2\\scripts\\mPDBS\\summary_pdb_93\\354b32871797b7ab.final.pdb
-7.155217e-15 2.153326e-15 5.272979e-15 -2.377233e-15 3.477532e-15
-4.162571e-15
E:\\R-3.1.2\\scripts\\mPDBS\\summary_pdb_93\\397368adbc32d5de.final.pdb
-4.470513e-16 -5.892000e-15 -6.730136e-15 -2.342257e-16 -2.372358e-15
3.079328e-15
E:\\R-3.1.2\\scripts\\mPDBS\\summary_pdb_93\\411ca13cd3df6edd.final.pdb
-1.881962e-15 1.407032e-15 5.456292e-15 -3.470744e-15 -4.132755e-15
1.700156e-15
E:\\R-3.1.2\\scripts\\mPDBS\\summary_pdb_93\\42357eee6642bf12.final.pdb
-1.597607e-15 1.012078e-15 1.937249e-14 2.949993e-16 1.784674e-15
-4.603289e-16
E:\\R-3.1.2\\scripts\\mPDBS\\summary_pdb_93\\44342d1f75a7fcfc.final.pdb
4.952429e-15 5.409688e-15 -4.167010e-15 5.548959e-15 -7.711334e-15
2.002694e-15
E:\\R-3.1.2\\scripts\\mPDBS\\summary_pdb_93\\448ef21dffe8cdfb.final.pdb
5.766562e-16 1.205997e-14 1.069993e-14 -1.267226e-14 9.580234e-15
-4.112193e-16
E:\\R-3.1.2\\scripts\\mPDBS\\summary_pdb_93\\472a40b3e8f113c7.final.pdb
1.657521e-15 -8.037853e-15 1.107778e-15 4.607496e-15 -9.880489e-16
1.751838e-16
E:\\R-3.1.2\\scripts\\mPDBS\\summary_pdb_93\\48a26225951ea153.final.pdb
1.898474e-15 5.258809e-15 -4.931435e-16 -5.614857e-15 -6.059319e-15
3.100072e-15
E:\\R-3.1.2\\scripts\\mPDBS\\summary_pdb_93\\4ab4a5c7c6682ce6.final.pdb
-2.006134e-15 1.303633e-15 8.540748e-16 -1.275546e-14 1.228928e-15
-1.252299e-14
E:\\R-3.1.2\\scripts\\mPDBS\\summary_pdb_93\\4eaa45b62cb6a25f.final.pdb
2.682481e-15 -5.848086e-15 -2.651671e-15 3.170092e-16 -5.442077e-16
1.844126e-15
E:\\R-3.1.2\\scripts\\mPDBS\\summary_pdb_93\\58c371611d85ac96.final.pdb
-4.470513e-16 -5.892000e-15 -6.730136e-15 -2.342257e-16 -2.372358e-15
3.079328e-15
E:\\R-3.1.2\\scripts\\mPDBS\\summary_pdb_93\\59a647f04822d30a.final.pdb
-6.579917e-16 -2.972496e-15 6.892719e-15 3.169785e-15 2.184830e-15
-4.693555e-15
E:\\R-3.1.2\\scripts\\mPDBS\\summary_pdb_93\\59b091fe385e05fc.final.pdb
1.503743e-15 -4.415744e-15 7.299234e-15 3.941409e-15 9.843088e-16
2.635823e-16
E:\\R-3.1.2\\scripts\\mPDBS\\summary_pdb_93\\5c35ac30f930d2db.final.pdb
2.682481e-15 -5.848086e-15 -2.651671e-15 3.170092e-16 -5.442077e-16
1.844126e-15
E:\\R-3.1.2\\scripts\\mPDBS\\summary_pdb_93\\5f60752431cf2dcb.final.pdb
4.277318e-15 -4.684802e-15 -4.031678e-15 1.194151e-15 -2.488548e-15
5.764035e-16
E:\\R-3.1.2\\scripts\\mPDBS\\summary_pdb_93\\626456dd6ff2868d.final.pdb
1.657521e-15 -8.037853e-15 1.107778e-15 4.607496e-15 -9.880489e-16
1.751838e-16
E:\\R-3.1.2\\scripts\\mPDBS\\summary_pdb_93\\6583d53ff3c8f7ef.final.pdb
-6.579917e-16 -2.972496e-15 6.892719e-15 3.169785e-15 2.184830e-15
-4.693555e-15

E:\\R-3.1.2\\scripts\\mPDBS\\summary_pdb_93\\69760178986bef01.final.pdb
-1.359374e-16 -1.001353e-14 -1.136778e-14 1.732026e-15 1.176114e-15
-4.942893e-15
E:\\R-3.1.2\\scripts\\mPDBS\\summary_pdb_93\\6a9d87d47926b1c8.final.pdb
1.140437e-15 -3.775637e-15 2.330325e-15 1.399884e-15 2.349311e-16
8.057557e-16
E:\\R-3.1.2\\scripts\\mPDBS\\summary_pdb_93\\6bb3547ea982ffc4.final.pdb
-2.115308e-16 1.026725e-15 -4.419259e-15 2.140784e-15 -7.789220e-15
3.634933e-15
E:\\R-3.1.2\\scripts\\mPDBS\\summary_pdb_93\\716d9624611d1c42.final.pdb
-2.443841e-15 -9.256561e-16 7.442216e-15 -9.420528e-16 7.829233e-16
4.021781e-15
E:\\R-3.1.2\\scripts\\mPDBS\\summary_pdb_93\\75f27e70ad0f1be4.final.pdb
-6.579917e-16 -2.972496e-15 6.892719e-15 3.169785e-15 2.184830e-15
-4.693555e-15
E:\\R-3.1.2\\scripts\\mPDBS\\summary_pdb_93\\769f910fffb22872.final.pdb
1.188873e-15 -1.991077e-15 2.744810e-15 1.699723e-15 2.356681e-16
5.828687e-16
E:\\R-3.1.2\\scripts\\mPDBS\\summary_pdb_93\\791f8c80dd5c3135.final.pdb
1.503743e-15 -4.415744e-15 7.299234e-15 3.941409e-15 9.843088e-16
2.635823e-16
E:\\R-3.1.2\\scripts\\mPDBS\\summary_pdb_93\\7fad98e66a3f6099.final.pdb
1.932529e-15 -9.593431e-15 1.024025e-14 -1.727084e-15 2.959532e-15
8.023763e-15
E:\\R-3.1.2\\scripts\\mPDBS\\summary_pdb_93\\804b89c94a633196.final.pdb
5.220125e-16 -6.659621e-16 6.682308e-15 -7.556517e-17 1.272643e-15
-8.468517e-16
E:\\R-3.1.2\\scripts\\mPDBS\\summary_pdb_93\\813cc65a2298feba.final.pdb
5.220125e-16 -6.659621e-16 6.682308e-15 -7.556517e-17 1.272643e-15
-8.468517e-16
E:\\R-3.1.2\\scripts\\mPDBS\\summary_pdb_93\\83c0eefcfe12d3f9.final.pdb
-1.881962e-15 1.407032e-15 5.456292e-15 -3.470744e-15 -4.132755e-15
1.700156e-15
E:\\R-3.1.2\\scripts\\mPDBS\\summary_pdb_93\\86d50469ef569760.final.pdb
1.657521e-15 -8.037853e-15 1.107778e-15 4.607496e-15 -9.880489e-16
1.751838e-16
E:\\R-3.1.2\\scripts\\mPDBS\\summary_pdb_93\\89d0666e1402203d.final.pdb
-6.579917e-16 -2.972496e-15 6.892719e-15 3.169785e-15 2.184830e-15
-4.693555e-15
E:\\R-3.1.2\\scripts\\mPDBS\\summary_pdb_93\\8b34852716dadc62.final.pdb
5.311198e-16 -7.479956e-15 -3.344394e-15 2.052073e-15 -4.484904e-15
-1.094483e-15
E:\\R-3.1.2\\scripts\\mPDBS\\summary_pdb_93\\8b6ef9ac3fe74395.final.pdb
4.968355e-15 -3.441870e-15 7.954120e-16 7.716258e-15 2.533619e-15
6.927529e-15
E:\\R-3.1.2\\scripts\\mPDBS\\summary_pdb_93\\98bbe0d5986c138f.final.pdb
1.503743e-15 -4.415744e-15 7.299234e-15 3.941409e-15 9.843088e-16
2.635823e-16
E:\\R-3.1.2\\scripts\\mPDBS\\summary_pdb_93\\9c22f4909d67e2fa.final.pdb
4.092697e-15 -9.829639e-15 9.937156e-16 1.003332e-14 2.992621e-15
3.320821e-15
E:\\R-3.1.2\\scripts\\mPDBS\\summary_pdb_93\\9dda8ed0d81fb3ef.final.pdb
5.262294e-15 -7.988015e-15 -5.386087e-15 -4.145544e-15 -4.274846e-15
3.790977e-15

E:\\R-3.1.2\\scripts\\mPDBS\\summary_pdb_93\\9f752b3616e6033b.final.pdb
1.043377e-15 -2.153744e-15 4.570756e-15 3.086949e-15 1.869297e-15
-2.414434e-17
E:\\R-3.1.2\\scripts\\mPDBS\\summary_pdb_93\\a02c70083816d6ba.final.pdb
2.682481e-15 -5.848086e-15 -2.651671e-15 3.170092e-16 -5.442077e-16
1.844126e-15
E:\\R-3.1.2\\scripts\\mPDBS\\summary_pdb_93\\a0c997d891e4e082.final.pdb
-8.795848e-15 -5.415307e-15 -8.172365e-15 1.815303e-17 -3.536146e-15
-3.072251e-15
E:\\R-3.1.2\\scripts\\mPDBS\\summary_pdb_93\\ab8039a2d823c616.final.pdb
6.158132e-16 -3.388283e-15 3.437271e-15 1.744420e-15 -4.338095e-16
8.169993e-16
E:\\R-3.1.2\\scripts\\mPDBS\\summary_pdb_93\\acf4d74b9c71d4dc.final.pdb
5.220125e-16 -6.659621e-16 6.682308e-15 -7.556517e-17 1.272643e-15
-8.468517e-16
E:\\R-3.1.2\\scripts\\mPDBS\\summary_pdb_93\\ad8a823e4b4c668e.final.pdb
1.248435e-15 -2.374348e-15 3.444468e-15 1.067413e-15 7.573326e-16
6.442505e-16
E:\\R-3.1.2\\scripts\\mPDBS\\summary_pdb_93\\b16ababd03b7b857.final.pdb
4.092697e-15 -9.829639e-15 9.937156e-16 1.003332e-14 2.992621e-15
3.320821e-15
E:\\R-3.1.2\\scripts\\mPDBS\\summary_pdb_93\\b226923e697d3c63.final.pdb
-9.623530e-15 7.094544e-16 -6.076507e-15 -2.432829e-15 8.608024e-16
3.402833e-15
E:\\R-3.1.2\\scripts\\mPDBS\\summary_pdb_93\\b28e48f6fa27a165.final.pdb
1.503743e-15 -4.415744e-15 7.299234e-15 3.941409e-15 9.843088e-16
2.635823e-16
E:\\R-3.1.2\\scripts\\mPDBS\\summary_pdb_93\\b42c4924e3cd1aec.final.pdb
2.340125e-15 -3.747050e-15 4.377370e-15 5.824563e-15 3.865560e-15
5.152735e-15
E:\\R-3.1.2\\scripts\\mPDBS\\summary_pdb_93\\b4dd239fcabff1df.final.pdb
1.503743e-15 -4.415744e-15 7.299234e-15 3.941409e-15 9.843088e-16
2.635823e-16
E:\\R-3.1.2\\scripts\\mPDBS\\summary_pdb_93\\b669942ef97459a2.final.pdb
5.311198e-16 -7.479956e-15 -3.344394e-15 2.052073e-15 -4.484904e-15
-1.094483e-15
E:\\R-3.1.2\\scripts\\mPDBS\\summary_pdb_93\\b99f4d9d87c1017b.final.pdb
1.503743e-15 -4.415744e-15 7.299234e-15 3.941409e-15 9.843088e-16
2.635823e-16
E:\\R-3.1.2\\scripts\\mPDBS\\summary_pdb_93\\b9d2cbf3975403e7.final.pdb
1.503743e-15 -4.415744e-15 7.299234e-15 3.941409e-15 9.843088e-16
2.635823e-16
E:\\R-3.1.2\\scripts\\mPDBS\\summary_pdb_93\\bc88c8a505f3a288.final.pdb
8.915483e-16 -3.688027e-15 2.859210e-15 1.451539e-15 3.038635e-16
7.599300e-16
E:\\R-3.1.2\\scripts\\mPDBS\\summary_pdb_93\\bdac0e64f4ef427e.final.pdb
8.555598e-15 -1.300245e-14 -3.500519e-15 1.145894e-14 9.756525e-15
-1.049814e-15
E:\\R-3.1.2\\scripts\\mPDBS\\summary_pdb_93\\ca2a1f55fc9d0681.final.pdb
5.311198e-16 -7.479956e-15 -3.344394e-15 2.052073e-15 -4.484904e-15
-1.094483e-15
E:\\R-3.1.2\\scripts\\mPDBS\\summary_pdb_93\\ce34248de3e81a3c.final.pdb
1.885346e-15 -7.969839e-15 3.860145e-15 -2.097713e-15 1.551309e-15
1.826555e-15

E:\\R-3.1.2\\scripts\\mPDBS\\summary_pdb_93\\d02e697772494a61.final.pdb
-4.907443e-15 2.527487e-15 1.014852e-14 2.664002e-15 1.903929e-15
-6.443292e-15
E:\\R-3.1.2\\scripts\\mPDBS\\summary_pdb_93\\d04221245d015047.final.pdb
4.290473e-15 -3.285067e-15 5.830322e-15 3.017490e-15 2.065203e-16
-1.648542e-16
E:\\R-3.1.2\\scripts\\mPDBS\\summary_pdb_93\\d678141e4018054a.final.pdb
-2.392713e-15 6.182258e-15 6.930152e-15 -3.791003e-15 -2.833420e-15
6.600266e-15
E:\\R-3.1.2\\scripts\\mPDBS\\summary_pdb_93\\d71b40af0f0efe39.final.pdb
-1.639468e-15 6.913044e-15 3.711203e-15 -6.107472e-15 5.661034e-15
5.217757e-15
E:\\R-3.1.2\\scripts\\mPDBS\\summary_pdb_93\\dc0809eff10197e2.final.pdb
2.973454e-15 -9.977578e-15 2.686538e-15 1.147543e-14 4.559961e-15
2.061283e-15
E:\\R-3.1.2\\scripts\\mPDBS\\summary_pdb_93\\e1163918d3707839.final.pdb
-1.587437e-16 1.729970e-17 2.116602e-14 5.320409e-15 7.066289e-16
6.823057e-15
E:\\R-3.1.2\\scripts\\mPDBS\\summary_pdb_93\\e4b48b06824ef848.final.pdb
1.104577e-15 5.777882e-15 -6.090570e-15 -3.655476e-15 -2.257563e-15
2.715494e-15
E:\\R-3.1.2\\scripts\\mPDBS\\summary_pdb_93\\e529e19ad281fea2.final.pdb
1.577290e-15 7.398482e-16 6.199192e-15 3.621939e-16 3.933362e-15
3.303541e-15
E:\\R-3.1.2\\scripts\\mPDBS\\summary_pdb_93\\e5602082718ec3a1.final.pdb
5.220125e-16 -6.659621e-16 6.682308e-15 -7.556517e-17 1.272643e-15
-8.468517e-16
E:\\R-3.1.2\\scripts\\mPDBS\\summary_pdb_93\\e5d589d996d0a960.final.pdb
2.492481e-15 -1.403889e-14 -1.436237e-14 -3.769805e-15 2.127229e-15
1.372700e-15
E:\\R-3.1.2\\scripts\\mPDBS\\summary_pdb_93\\e9df9e72307a2fbd.final.pdb
2.682481e-15 -5.848086e-15 -2.651671e-15 3.170092e-16 -5.442077e-16
1.844126e-15
E:\\R-3.1.2\\scripts\\mPDBS\\summary_pdb_93\\ea00f8694090cae7.final.pdb
5.220125e-16 -6.659621e-16 6.682308e-15 -7.556517e-17 1.272643e-15
-8.468517e-16
E:\\R-3.1.2\\scripts\\mPDBS\\summary_pdb_93\\eba517be32db050e.final.pdb
1.503743e-15 -4.415744e-15 7.299234e-15 3.941409e-15 9.843088e-16
2.635823e-16
E:\\R-3.1.2\\scripts\\mPDBS\\summary_pdb_93\\ec04b9545899c29d.final.pdb
1.159240e-15 -2.767799e-15 3.121430e-15 1.865056e-15 8.599351e-16
5.489960e-16
E:\\R-3.1.2\\scripts\\mPDBS\\summary_pdb_93\\ee6862cbcad52078.final.pdb
8.953972e-16 -3.555321e-15 2.710891e-15 1.295956e-15 3.021288e-16
6.345963e-16
E:\\R-3.1.2\\scripts\\mPDBS\\summary_pdb_93\\ef6a42fc83b81e5d.final.pdb
1.059718e-15 -2.730937e-15 3.329846e-15 1.417831e-15 6.799742e-16
7.003962e-16
E:\\R-3.1.2\\scripts\\mPDBS\\summary_pdb_93\\ef8e88d4ad93a0ad.final.pdb
4.290473e-15 -3.285067e-15 5.830322e-15 3.017490e-15 2.065203e-16
-1.648542e-16
E:\\R-3.1.2\\scripts\\mPDBS\\summary_pdb_93\\f14fcae8deec4fa3.final.pdb
1.043377e-15 -2.153744e-15 4.570756e-15 3.086949e-15 1.869297e-15
-2.414434e-17

E:\\R-3.1.2\\scripts\\mPDBS\\summary_pdb_93\\f83584e22003fb92.final.pdb
1.898474e-15 5.258809e-15 -4.931435e-16 -5.614857e-15 -6.059319e-15
3.100072e-15
E:\\R-3.1.2\\scripts\\mPDBS\\summary_pdb_93\\f8e2bf87e52caa29.final.pdb
1.577290e-15 7.398482e-16 6.199192e-15 3.621939e-16 3.933362e-15
3.303541e-15

[,601]   [,602]   [,603]   [,604]   [,605]   [,606]
E:\\R-3.1.2\\scripts\\40.1ns_data\\NAT0.pdb
1.916802e-15 -1.570946e-15 -2.431516e-15 -5.852141e-15 -2.663106e-15
1.604593e-15
E:\\R-3.1.2\\scripts\\40.1ns_data\\LJP6.pdb
6.771323e-16 -1.414104e-15 -2.437934e-15 -4.966656e-15 -2.986260e-15
2.469853e-15
E:\\R-3.1.2\\scripts\\40.1ns_data\\3WY9.pdb
1.897177e-15 1.680235e-15 -2.790354e-15 -5.675878e-15 -5.720361e-16
1.499355e-15
E:\\R-3.1.2\\scripts\\40.1ns_data\\ZSP9.pdb
2.030655e-15 -1.345068e-15 -1.691141e-15 -6.953534e-15 -3.128674e-15
1.665921e-15
E:\\R-3.1.2\\scripts\\mPDBS\\summary_pdb_93\\013d855ad26c877c.final.pdb
6.722003e-15 6.032666e-15 -3.093325e-15 -1.551044e-14 -7.410007e-15
2.355569e-15
E:\\R-3.1.2\\scripts\\mPDBS\\summary_pdb_93\\0a2a705342ca0d54.final.pdb
2.230537e-15 -2.212986e-15 -2.246582e-15 -4.926356e-15 -2.394120e-15
1.940944e-15
E:\\R-3.1.2\\scripts\\mPDBS\\summary_pdb_93\\0a59fceadb4194d9.final.pdb
3.507144e-15 -4.981087e-16 -7.516114e-16 -4.607619e-15 -6.193334e-15
-2.143250e-15
E:\\R-3.1.2\\scripts\\mPDBS\\summary_pdb_93\\0dba59f3306825e8.final.pdb
-2.267334e-15 -5.380324e-16 -1.759323e-15 3.327924e-16 -1.108190e-15
7.422758e-16
E:\\R-3.1.2\\scripts\\mPDBS\\summary_pdb_93\\126f26a78b57339c.final.pdb
1.753972e-15 -4.201118e-15 -4.341134e-15 3.521756e-15 2.986705e-16
1.107949e-14
E:\\R-3.1.2\\scripts\\mPDBS\\summary_pdb_93\\12f06f3e342df706.final.pdb
2.113443e-15 -7.868269e-16 -1.916876e-15 -4.601258e-15 -1.879775e-15
2.748892e-15
E:\\R-3.1.2\\scripts\\mPDBS\\summary_pdb_93\\18618a2a251b867b.final.pdb
4.253725e-15 -5.023049e-15 -2.174070e-15 1.100805e-15 4.878593e-15
9.157251e-15
E:\\R-3.1.2\\scripts\\mPDBS\\summary_pdb_93\\1f305f598641eeb2.final.pdb
2.503681e-15 -1.744923e-16 -2.396362e-15 -3.675762e-15 -2.353561e-15
1.678857e-15
E:\\R-3.1.2\\scripts\\mPDBS\\summary_pdb_93\\1f8a00b551be0a39.final.pdb
4.253725e-15 -5.023049e-15 -2.174070e-15 1.100805e-15 4.878593e-15
9.157251e-15
E:\\R-3.1.2\\scripts\\mPDBS\\summary_pdb_93\\219f45328d47579a.final.pdb
1.891249e-15 3.599289e-16 -1.677954e-15 -1.292649e-14 -1.917349e-15
7.783705e-15
E:\\R-3.1.2\\scripts\\mPDBS\\summary_pdb_93\\238dd3918e54336d.final.pdb
9.217854e-15 3.284022e-15 -7.538934e-15 -5.909691e-15 2.169334e-15
2.269495e-14
E:\\R-3.1.2\\scripts\\mPDBS\\summary_pdb_93\\2b3027889e2fdc42.final.pdb

2.627856e-15 -1.336847e-15 -2.279578e-15 -4.428780e-15 -2.546729e-15
2.424711e-15
E:\\R-3.1.2\\scripts\\mPDBS\\summary_pdb_93\\2fcb3b46e33793b0.final.pdb
2.519294e-15 1.594492e-15 -2.852037e-16 -8.043796e-15 -3.273182e-15
-3.018774e-15
E:\\R-3.1.2\\scripts\\mPDBS\\summary_pdb_93\\34eb328251842006.final.pdb
1.073044e-14 -1.799882e-15 -4.462946e-15 -1.601442e-14 -2.294672e-15
-8.448945e-16
E:\\R-3.1.2\\scripts\\mPDBS\\summary_pdb_93\\354b32871797b7ab.final.pdb
5.688492e-16 1.411082e-15 -3.312365e-15 -1.817552e-14 -1.472913e-14
-1.212079e-14
E:\\R-3.1.2\\scripts\\mPDBS\\summary_pdb_93\\397368adbc32d5de.final.pdb
1.753972e-15 -4.201118e-15 -4.341134e-15 3.521756e-15 2.986705e-16
1.107949e-14
E:\\R-3.1.2\\scripts\\mPDBS\\summary_pdb_93\\411ca13cd3df6edd.final.pdb
3.903386e-15 -5.734064e-15 2.496332e-16 -4.213201e-15 -2.764469e-16
1.566100e-15
E:\\R-3.1.2\\scripts\\mPDBS\\summary_pdb_93\\42357eee6642bf12.final.pdb
1.209024e-14 -2.506577e-15 1.609669e-15 -8.703893e-15 -3.975140e-15
-1.911999e-15
E:\\R-3.1.2\\scripts\\mPDBS\\summary_pdb_93\\44342d1f75a7fcfc.final.pdb
7.084059e-15 -4.642175e-15 -4.347390e-15 -5.538493e-17 -3.987570e-15
-8.294608e-16
E:\\R-3.1.2\\scripts\\mPDBS\\summary_pdb_93\\448ef21dffe8cdfb.final.pdb
1.669025e-14 4.307599e-15 1.090432e-15 -2.118693e-14 -5.037612e-15
3.812567e-15
E:\\R-3.1.2\\scripts\\mPDBS\\summary_pdb_93\\472a40b3e8f113c7.final.pdb
-2.267334e-15 -5.380324e-16 -1.759323e-15 3.327924e-16 -1.108190e-15
7.422758e-16
E:\\R-3.1.2\\scripts\\mPDBS\\summary_pdb_93\\48a26225951ea153.final.pdb
1.667687e-15 -3.584182e-15 -6.356178e-15 -5.713776e-15 4.121006e-15
7.125811e-15
E:\\R-3.1.2\\scripts\\mPDBS\\summary_pdb_93\\4ab4a5c7c6682ce6.final.pdb
7.867138e-15 -3.397941e-15 3.621247e-15 -1.049481e-14 4.322362e-15
-8.970441e-15
E:\\R-3.1.2\\scripts\\mPDBS\\summary_pdb_93\\4eaa45b62cb6a25f.final.pdb
1.681674e-15 -2.508525e-15 -3.010306e-15 2.828673e-15 1.370836e-15
8.217539e-15
E:\\R-3.1.2\\scripts\\mPDBS\\summary_pdb_93\\58c371611d85ac96.final.pdb
1.753972e-15 -4.201118e-15 -4.341134e-15 3.521756e-15 2.986705e-16
1.107949e-14
E:\\R-3.1.2\\scripts\\mPDBS\\summary_pdb_93\\59a647f04822d30a.final.pdb
1.785347e-15 -8.038821e-16 3.693234e-16 -7.295253e-15 4.536470e-16
2.561924e-15
E:\\R-3.1.2\\scripts\\mPDBS\\summary_pdb_93\\59b091fe385e05fc.final.pdb
3.507144e-15 -4.981087e-16 -7.516114e-16 -4.607619e-15 -6.193334e-15
-2.143250e-15
E:\\R-3.1.2\\scripts\\mPDBS\\summary_pdb_93\\5c35ac30f930d2db.final.pdb
1.681674e-15 -2.508525e-15 -3.010306e-15 2.828673e-15 1.370836e-15
8.217539e-15
E:\\R-3.1.2\\scripts\\mPDBS\\summary_pdb_93\\5f60752431cf2dcb.final.pdb
-2.147688e-15 -1.081288e-14 -2.054569e-15 3.264303e-15 1.100589e-15
3.313544e-15
E:\\R-3.1.2\\scripts\\mPDBS\\summary_pdb_93\\626456dd6ff2868d.final.pdb

-2.267334e-15 -5.380324e-16 -1.759323e-15 3.327924e-16 -1.108190e-15
7.422758e-16
E:\\R-3.1.2\\scripts\\mPDBS\\summary_pdb_93\\6583d53ff3c8f7ef.final.pdb
1.785347e-15 -8.038821e-16 3.693234e-16 -7.295253e-15 4.536470e-16
2.561924e-15
E:\\R-3.1.2\\scripts\\mPDBS\\summary_pdb_93\\69760178986bef01.final.pdb
-4.985835e-15 -4.411279e-15 -2.012214e-15 6.360825e-15 7.187112e-16
6.026760e-16
E:\\R-3.1.2\\scripts\\mPDBS\\summary_pdb_93\\6a9d87d47926b1c8.final.pdb
2.642150e-15 -2.520474e-17 -2.367018e-15 -4.073730e-15 -2.608943e-15
2.287914e-15
E:\\R-3.1.2\\scripts\\mPDBS\\summary_pdb_93\\6bb3547ea982ffc4.final.pdb
5.689516e-15 -6.287874e-15 -4.074474e-15 -8.928691e-15 5.730644e-15
1.095861e-14
E:\\R-3.1.2\\scripts\\mPDBS\\summary_pdb_93\\716d9624611d1c42.final.pdb
5.622789e-15 3.111097e-15 -7.525345e-15 -1.083699e-14 -7.811527e-16
6.419133e-15
E:\\R-3.1.2\\scripts\\mPDBS\\summary_pdb_93\\75f27e70ad0f1be4.final.pdb
1.785347e-15 -8.038821e-16 3.693234e-16 -7.295253e-15 4.536470e-16
2.561924e-15
E:\\R-3.1.2\\scripts\\mPDBS\\summary_pdb_93\\769f910fffb22872.final.pdb
2.134543e-15 -1.419161e-15 -2.034966e-15 -3.847385e-15 -2.198103e-15
2.052691e-15
E:\\R-3.1.2\\scripts\\mPDBS\\summary_pdb_93\\791f8c80dd5c3135.final.pdb
3.507144e-15 -4.981087e-16 -7.516114e-16 -4.607619e-15 -6.193334e-15
-2.143250e-15
E:\\R-3.1.2\\scripts\\mPDBS\\summary_pdb_93\\7fad98e66a3f6099.final.pdb
5.008043e-16 1.726679e-15 -7.987325e-15 -1.664056e-14 -6.878251e-15
-6.790882e-15
E:\\R-3.1.2\\scripts\\mPDBS\\summary_pdb_93\\804b89c94a633196.final.pdb
2.519294e-15 1.594492e-15 -2.852037e-16 -8.043796e-15 -3.273182e-15
-3.018774e-15
E:\\R-3.1.2\\scripts\\mPDBS\\summary_pdb_93\\813cc65a2298feba.final.pdb
2.519294e-15 1.594492e-15 -2.852037e-16 -8.043796e-15 -3.273182e-15
-3.018774e-15
E:\\R-3.1.2\\scripts\\mPDBS\\summary_pdb_93\\83c0eefcfe12d3f9.final.pdb
3.903386e-15 -5.734064e-15 2.496332e-16 -4.213201e-15 -2.764469e-16
1.566100e-15
E:\\R-3.1.2\\scripts\\mPDBS\\summary_pdb_93\\86d50469ef569760.final.pdb
-2.267334e-15 -5.380324e-16 -1.759323e-15 3.327924e-16 -1.108190e-15
7.422758e-16
E:\\R-3.1.2\\scripts\\mPDBS\\summary_pdb_93\\89d0666e1402203d.final.pdb
1.785347e-15 -8.038821e-16 3.693234e-16 -7.295253e-15 4.536470e-16
2.561924e-15
E:\\R-3.1.2\\scripts\\mPDBS\\summary_pdb_93\\8b34852716dadc62.final.pdb
-1.400372e-16 -6.908689e-15 -2.774532e-15 6.569515e-15 1.496657e-15
2.449074e-15
E:\\R-3.1.2\\scripts\\mPDBS\\summary_pdb_93\\8b6ef9ac3fe74395.final.pdb
-3.403601e-15 7.237874e-16 2.960207e-15 -1.161795e-14 2.373155e-15
4.832177e-15
E:\\R-3.1.2\\scripts\\mPDBS\\summary_pdb_93\\98bbe0d5986c138f.final.pdb
3.507144e-15 -4.981087e-16 -7.516114e-16 -4.607619e-15 -6.193334e-15
-2.143250e-15
E:\\R-3.1.2\\scripts\\mPDBS\\summary_pdb_93\\9c22f4909d67e2fa.final.pdb

1.801986e-15 8.658503e-15 -1.058838e-14 1.566681e-15 -1.003828e-14
7.685337e-15
E:\\R-3.1.2\\scripts\\mPDBS\\summary_pdb_93\\9dda8ed0d81fb3ef.final.pdb
2.775223e-15 -1.430068e-14 1.936971e-15 6.250498e-15 -2.790828e-15
2.447432e-15
E:\\R-3.1.2\\scripts\\mPDBS\\summary_pdb_93\\9f752b3616e6033b.final.pdb
1.261177e-15 1.176642e-15 8.921415e-16 -5.827255e-15 -3.093112e-15
3.386451e-18
E:\\R-3.1.2\\scripts\\mPDBS\\summary_pdb_93\\a02c70083816d6ba.final.pdb
1.681674e-15 -2.508525e-15 -3.010306e-15 2.828673e-15 1.370836e-15
8.217539e-15
E:\\R-3.1.2\\scripts\\mPDBS\\summary_pdb_93\\a0c997d891e4e082.final.pdb
3.705795e-15 7.162605e-15 -5.517249e-15 -4.877874e-15 -5.060482e-15
-1.750302e-15
E:\\R-3.1.2\\scripts\\mPDBS\\summary_pdb_93\\ab8039a2d823c616.final.pdb
2.637205e-15 1.016294e-16 -2.190266e-15 -4.741427e-15 -2.287309e-15
3.355423e-15
E:\\R-3.1.2\\scripts\\mPDBS\\summary_pdb_93\\acf4d74b9c71d4dc.final.pdb
2.519294e-15 1.594492e-15 -2.852037e-16 -8.043796e-15 -3.273182e-15
-3.018774e-15
E:\\R-3.1.2\\scripts\\mPDBS\\summary_pdb_93\\ad8a823e4b4c668e.final.pdb
1.414334e-15 -1.436379e-15 -2.197753e-15 -4.189216e-15 -2.140513e-15
2.294486e-15
E:\\R-3.1.2\\scripts\\mPDBS\\summary_pdb_93\\b16ababd03b7b857.final.pdb
1.801986e-15 8.658503e-15 -1.058838e-14 1.566681e-15 -1.003828e-14
7.685337e-15
E:\\R-3.1.2\\scripts\\mPDBS\\summary_pdb_93\\b226923e697d3c63.final.pdb
2.900991e-15 -2.317862e-15 4.357850e-16 -4.436537e-15 1.864302e-15
1.417873e-15
E:\\R-3.1.2\\scripts\\mPDBS\\summary_pdb_93\\b28e48f6fa27a165.final.pdb
3.507144e-15 -4.981087e-16 -7.516114e-16 -4.607619e-15 -6.193334e-15
-2.143250e-15
E:\\R-3.1.2\\scripts\\mPDBS\\summary_pdb_93\\b42c4924e3cd1aec.final.pdb
-7.352066e-16 6.869667e-15 -2.776381e-15 -8.793024e-15 -1.189455e-14
3.470485e-15
E:\\R-3.1.2\\scripts\\mPDBS\\summary_pdb_93\\b4dd239fcabff1df.final.pdb
3.507144e-15 -4.981087e-16 -7.516114e-16 -4.607619e-15 -6.193334e-15
-2.143250e-15
E:\\R-3.1.2\\scripts\\mPDBS\\summary_pdb_93\\b669942ef97459a2.final.pdb
-1.400372e-16 -6.908689e-15 -2.774532e-15 6.569515e-15 1.496657e-15
2.449074e-15
E:\\R-3.1.2\\scripts\\mPDBS\\summary_pdb_93\\b99f4d9d87c1017b.final.pdb
3.507144e-15 -4.981087e-16 -7.516114e-16 -4.607619e-15 -6.193334e-15
-2.143250e-15
E:\\R-3.1.2\\scripts\\mPDBS\\summary_pdb_93\\b9d2cbf3975403e7.final.pdb
3.507144e-15 -4.981087e-16 -7.516114e-16 -4.607619e-15 -6.193334e-15
-2.143250e-15
E:\\R-3.1.2\\scripts\\mPDBS\\summary_pdb_93\\bc88c8a505f3a288.final.pdb
2.762731e-15 7.543617e-16 -2.021212e-15 -4.757943e-15 -2.175606e-15
2.663669e-15
E:\\R-3.1.2\\scripts\\mPDBS\\summary_pdb_93\\bdac0e64f4ef427e.final.pdb
-4.273507e-16 1.069789e-14 -5.886734e-15 -6.166391e-15 -7.555238e-15
4.908045e-15
E:\\R-3.1.2\\scripts\\mPDBS\\summary_pdb_93\\ca2a1f55fc9d0681.final.pdb

-1.400372e-16 -6.908689e-15 -2.774532e-15 6.569515e-15 1.496657e-15
2.449074e-15
E:\\R-3.1.2\\scripts\\mPDBS\\summary_pdb_93\\ce34248de3e81a3c.final.pdb
3.368410e-15 -5.583383e-16 -6.550379e-15 -3.495683e-15 -3.684788e-15
6.291261e-15
E:\\R-3.1.2\\scripts\\mPDBS\\summary_pdb_93\\d02e697772494a61.final.pdb
5.280032e-15 5.562581e-15 -6.122532e-15 -1.065243e-14 7.577118e-15
1.710004e-15
E:\\R-3.1.2\\scripts\\mPDBS\\summary_pdb_93\\d04221245d015047.final.pdb
2.002297e-15 -4.615052e-16 -3.007104e-15 -4.483224e-15 -4.010974e-15
2.572629e-15
E:\\R-3.1.2\\scripts\\mPDBS\\summary_pdb_93\\d678141e4018054a.final.pdb
5.345660e-15 -5.218264e-15 -8.117990e-15 -1.460842e-14 3.336615e-15
1.070168e-14
E:\\R-3.1.2\\scripts\\mPDBS\\summary_pdb_93\\d71b40af0f0efe39.final.pdb
-1.047857e-15 1.106065e-15 -2.796272e-15 -3.646654e-15 -7.243506e-15
-2.138353e-15
E:\\R-3.1.2\\scripts\\mPDBS\\summary_pdb_93\\dc0809eff10197e2.final.pdb
-4.861739e-15 4.824778e-15 5.133127e-15 2.556099e-15 -5.762060e-15
-5.025626e-15
E:\\R-3.1.2\\scripts\\mPDBS\\summary_pdb_93\\e1163918d3707839.final.pdb
-8.555267e-17 5.471263e-15 6.245446e-16 -4.958852e-15 -6.026485e-17
2.637818e-15
E:\\R-3.1.2\\scripts\\mPDBS\\summary_pdb_93\\e4b48b06824ef848.final.pdb
4.253725e-15 -5.023049e-15 -2.174070e-15 1.100805e-15 4.878593e-15
9.157251e-15
E:\\R-3.1.2\\scripts\\mPDBS\\summary_pdb_93\\e529e19ad281fea2.final.pdb
6.722003e-15 6.032666e-15 -3.093325e-15 -1.551044e-14 -7.410007e-15
2.355569e-15
E:\\R-3.1.2\\scripts\\mPDBS\\summary_pdb_93\\e5602082718ec3a1.final.pdb
2.519294e-15 1.594492e-15 -2.852037e-16 -8.043796e-15 -3.273182e-15
-3.018774e-15
E:\\R-3.1.2\\scripts\\mPDBS\\summary_pdb_93\\e5d589d996d0a960.final.pdb
-1.083532e-14 -1.926005e-15 -4.850478e-15 -4.117386e-15 -3.293327e-15
5.385035e-15
E:\\R-3.1.2\\scripts\\mPDBS\\summary_pdb_93\\e9df9e72307a2fbd.final.pdb
1.681674e-15 -2.508525e-15 -3.010306e-15 2.828673e-15 1.370836e-15
8.217539e-15
E:\\R-3.1.2\\scripts\\mPDBS\\summary_pdb_93\\ea00f8694090cae7.final.pdb
2.519294e-15 1.594492e-15 -2.852037e-16 -8.043796e-15 -3.273182e-15
-3.018774e-15
E:\\R-3.1.2\\scripts\\mPDBS\\summary_pdb_93\\eba517be32db050e.final.pdb
3.507144e-15 -4.981087e-16 -7.516114e-16 -4.607619e-15 -6.193334e-15
-2.143250e-15
E:\\R-3.1.2\\scripts\\mPDBS\\summary_pdb_93\\ec04b9545899c29d.final.pdb
2.480103e-15 -1.617684e-15 -2.636952e-15 -3.532204e-15 -2.678288e-15
2.783101e-15
E:\\R-3.1.2\\scripts\\mPDBS\\summary_pdb_93\\ee6862cbcad52078.final.pdb
2.942492e-15 -5.182748e-16 -2.484600e-15 -4.558613e-15 -2.255620e-15
2.611627e-15
E:\\R-3.1.2\\scripts\\mPDBS\\summary_pdb_93\\ef6a42fc83b81e5d.final.pdb
2.202198e-15 -1.052917e-15 -2.103271e-15 -4.848158e-15 -2.343711e-15
2.418284e-15
E:\\R-3.1.2\\scripts\\mPDBS\\summary_pdb_93\\ef8e88d4ad93a0ad.final.pdb

2.002297e-15 -4.615052e-16 -3.007104e-15 -4.483224e-15 -4.010974e-15
2.572629e-15
E:\\R-3.1.2\\scripts\\mPDBS\\summary_pdb_93\\f14fcae8deec4fa3.final.pdb
1.261177e-15 1.176642e-15 8.921415e-16 -5.827255e-15 -3.093112e-15
3.386451e-18
E:\\R-3.1.2\\scripts\\mPDBS\\summary_pdb_93\\f83584e22003fb92.final.pdb
1.667687e-15 -3.584182e-15 -6.356178e-15 -5.713776e-15 4.121006e-15
7.125811e-15
E:\\R-3.1.2\\scripts\\mPDBS\\summary_pdb_93\\f8e2bf87e52caa29.final.pdb
6.722003e-15 6.032666e-15 -3.093325e-15 -1.551044e-14 -7.410007e-15
2.355569e-15

[,607]   [,608]   [,609]   [,610]   [,611]   [,612]
E:\\R-3.1.2\\scripts\\40.1ns_data\\NAT0.pdb
1.919439e-15 -3.240347e-15 4.100538e-15 3.004290e-15 2.169054e-15
-2.440541e-15
E:\\R-3.1.2\\scripts\\40.1ns_data\\LJP6.pdb
1.971670e-15 -1.573123e-15 3.146051e-15 2.197786e-15 3.407613e-16
-2.384431e-15
E:\\R-3.1.2\\scripts\\40.1ns_data\\3WY9.pdb
1.597567e-15 -2.818345e-15 4.333263e-15 2.566413e-15 2.727424e-15
-2.945268e-15
E:\\R-3.1.2\\scripts\\40.1ns_data\\ZSP9.pdb
9.626442e-16 -3.039765e-15 3.421941e-15 2.526394e-15 -6.373030e-16
-2.067004e-15
E:\\R-3.1.2\\scripts\\mPDBS\\summary_pdb_93\\013d855ad26c877c.final.pdb
-8.679516e-16 -5.965731e-15 8.117990e-16 3.099670e-15 -3.780482e-15
-8.045588e-17
E:\\R-3.1.2\\scripts\\mPDBS\\summary_pdb_93\\0a2a705342ca0d54.final.pdb
6.979695e-16 -1.871652e-15 3.601582e-15 2.616553e-15 7.566486e-16
-2.295452e-15
E:\\R-3.1.2\\scripts\\mPDBS\\summary_pdb_93\\0a59fceadb4194d9.final.pdb
-3.520195e-15 -2.072331e-15 2.979093e-15 4.979755e-15 -2.997242e-15
-3.430543e-15
E:\\R-3.1.2\\scripts\\mPDBS\\summary_pdb_93\\0dba59f3306825e8.final.pdb
-1.953888e-15 -5.514703e-15 -3.105671e-16 2.669459e-15 2.961185e-16
-1.607013e-15
E:\\R-3.1.2\\scripts\\mPDBS\\summary_pdb_93\\126f26a78b57339c.final.pdb
4.462528e-15 1.693453e-15 4.194943e-15 -1.140211e-15 1.409811e-15
-4.367229e-15
E:\\R-3.1.2\\scripts\\mPDBS\\summary_pdb_93\\12f06f3e342df706.final.pdb
1.161032e-15 -1.857286e-15 3.867428e-15 3.000686e-15 6.668767e-16
-2.206439e-15
E:\\R-3.1.2\\scripts\\mPDBS\\summary_pdb_93\\18618a2a251b867b.final.pdb
1.096380e-14 2.694859e-15 6.155271e-15 -8.219910e-16 3.663825e-15
-1.761748e-15
E:\\R-3.1.2\\scripts\\mPDBS\\summary_pdb_93\\1f305f598641eeb2.final.pdb
9.339241e-16 -2.310201e-15 3.356487e-15 2.646738e-15 9.882219e-16
-2.502680e-15
E:\\R-3.1.2\\scripts\\mPDBS\\summary_pdb_93\\1f8a00b551be0a39.final.pdb
1.096380e-14 2.694859e-15 6.155271e-15 -8.219910e-16 3.663825e-15
-1.761748e-15
E:\\R-3.1.2\\scripts\\mPDBS\\summary_pdb_93\\219f45328d47579a.final.pdb
4.433512e-15 -5.555981e-15 7.170619e-15 5.040904e-15 -9.195409e-15

1.297663e-15
E:\\R-3.1.2\\scripts\\mPDBS\\summary_pdb_93\\238dd3918e54336d.final.pdb
4.096759e-15 -3.833509e-15 5.568602e-15 2.816447e-15 8.778928e-15
1.067893e-15
E:\\R-3.1.2\\scripts\\mPDBS\\summary_pdb_93\\2b3027889e2fdc42.final.pdb
1.995398e-16 -1.762250e-15 3.559792e-15 2.710086e-15 -3.470183e-16
-2.373391e-15
E:\\R-3.1.2\\scripts\\mPDBS\\summary_pdb_93\\2fcb3b46e33793b0.final.pdb
-1.813878e-15 -3.147747e-15 2.182079e-15 4.508964e-15 -1.058752e-15
-2.282587e-15
E:\\R-3.1.2\\scripts\\mPDBS\\summary_pdb_93\\34eb328251842006.final.pdb
9.343930e-17 -4.861218e-15 7.001399e-15 1.032898e-14 4.887260e-15
-2.000358e-15
E:\\R-3.1.2\\scripts\\mPDBS\\summary_pdb_93\\354b32871797b7ab.final.pdb
-7.059534e-16 1.623266e-15 7.728765e-15 5.458642e-15 5.806875e-15
-1.332431e-14
E:\\R-3.1.2\\scripts\\mPDBS\\summary_pdb_93\\397368adbc32d5de.final.pdb
4.462528e-15 1.693453e-15 4.194943e-15 -1.140211e-15 1.409811e-15
-4.367229e-15
E:\\R-3.1.2\\scripts\\mPDBS\\summary_pdb_93\\411ca13cd3df6edd.final.pdb
2.094779e-15 1.211667e-15 3.614527e-15 3.835241e-15 8.008718e-16
-5.537742e-15
E:\\R-3.1.2\\scripts\\mPDBS\\summary_pdb_93\\42357eee6642bf12.final.pdb
3.640153e-15 -3.116714e-15 1.521957e-14 1.096362e-14 3.357698e-15
-3.392120e-15
E:\\R-3.1.2\\scripts\\mPDBS\\summary_pdb_93\\44342d1f75a7fcfc.final.pdb
5.310643e-15 1.492648e-16 -3.475668e-15 -5.351718e-16 -7.850627e-15
-5.575438e-15
E:\\R-3.1.2\\scripts\\mPDBS\\summary_pdb_93\\448ef21dffe8cdfb.final.pdb
4.587252e-15 1.010283e-14 2.222854e-14 1.497484e-15 1.964344e-14
-8.550143e-15
E:\\R-3.1.2\\scripts\\mPDBS\\summary_pdb_93\\472a40b3e8f113c7.final.pdb
-1.953888e-15 -5.514703e-15 -3.105671e-16 2.669459e-15 2.961185e-16
-1.607013e-15
E:\\R-3.1.2\\scripts\\mPDBS\\summary_pdb_93\\48a26225951ea153.final.pdb
2.682796e-15 -2.620391e-16 1.116868e-15 -4.792026e-16 7.314388e-15
-4.086756e-15
E:\\R-3.1.2\\scripts\\mPDBS\\summary_pdb_93\\4ab4a5c7c6682ce6.final.pdb
-1.275499e-16 7.748180e-15 1.269335e-14 6.136087e-15 3.144534e-15
-6.349711e-16
E:\\R-3.1.2\\scripts\\mPDBS\\summary_pdb_93\\4eaa45b62cb6a25f.final.pdb
1.603180e-15 -1.505416e-15 2.364940e-16 4.571094e-16 -3.402772e-15
1.343343e-15
E:\\R-3.1.2\\scripts\\mPDBS\\summary_pdb_93\\58c371611d85ac96.final.pdb
4.462528e-15 1.693453e-15 4.194943e-15 -1.140211e-15 1.409811e-15
-4.367229e-15
E:\\R-3.1.2\\scripts\\mPDBS\\summary_pdb_93\\59a647f04822d30a.final.pdb
-5.631312e-16 -2.309814e-15 8.617957e-15 3.116855e-15 4.505808e-15
1.051747e-15
E:\\R-3.1.2\\scripts\\mPDBS\\summary_pdb_93\\59b091fe385e05fc.final.pdb
-3.520195e-15 -2.072331e-15 2.979093e-15 4.979755e-15 -2.997242e-15
-3.430543e-15
E:\\R-3.1.2\\scripts\\mPDBS\\summary_pdb_93\\5c35ac30f930d2db.final.pdb
1.603180e-15 -1.505416e-15 2.364940e-16 4.571094e-16 -3.402772e-15

1.343343e-15
E:\\R-3.1.2\\scripts\\mPDBS\\summary_pdb_93\\5f60752431cf2dcb.final.pdb
-1.188054e-15 3.804580e-15 1.906757e-15 -4.508606e-15 -3.933610e-15
-4.271682e-15
E:\\R-3.1.2\\scripts\\mPDBS\\summary_pdb_93\\626456dd6ff2868d.final.pdb
-1.953888e-15 -5.514703e-15 -3.105671e-16 2.669459e-15 2.961185e-16
-1.607013e-15
E:\\R-3.1.2\\scripts\\mPDBS\\summary_pdb_93\\6583d53ff3c8f7ef.final.pdb
-5.631312e-16 -2.309814e-15 8.617957e-15 3.116855e-15 4.505808e-15
1.051747e-15
E:\\R-3.1.2\\scripts\\mPDBS\\summary_pdb_93\\69760178986bef01.final.pdb
1.848767e-15 1.227874e-15 -3.168200e-17 -5.013983e-15 -6.208106e-15
-4.350705e-15
E:\\R-3.1.2\\scripts\\mPDBS\\summary_pdb_93\\6a9d87d47926b1c8.final.pdb
-2.420417e-16 -1.777295e-15 3.294622e-15 2.560822e-15 -3.318514e-16
-2.439734e-15
E:\\R-3.1.2\\scripts\\mPDBS\\summary_pdb_93\\6bb3547ea982ffc4.final.pdb
3.346317e-15 -3.264388e-15 7.404742e-15 -2.332237e-15 7.197754e-15
-8.601858e-15
E:\\R-3.1.2\\scripts\\mPDBS\\summary_pdb_93\\716d9624611d1c42.final.pdb
7.420890e-15 -4.119311e-15 1.825659e-15 8.621299e-15 3.743842e-15
8.743214e-16
E:\\R-3.1.2\\scripts\\mPDBS\\summary_pdb_93\\75f27e70ad0f1be4.final.pdb
-5.631312e-16 -2.309814e-15 8.617957e-15 3.116855e-15 4.505808e-15
1.051747e-15
E:\\R-3.1.2\\scripts\\mPDBS\\summary_pdb_93\\769f910fffb22872.final.pdb
1.592379e-16 -2.243953e-15 4.668613e-15 2.530037e-15 7.473214e-16
-2.598508e-15
E:\\R-3.1.2\\scripts\\mPDBS\\summary_pdb_93\\791f8c80dd5c3135.final.pdb
-3.520195e-15 -2.072331e-15 2.979093e-15 4.979755e-15 -2.997242e-15
-3.430543e-15
E:\\R-3.1.2\\scripts\\mPDBS\\summary_pdb_93\\7fad98e66a3f6099.final.pdb
-3.709324e-15 -8.583066e-15 5.926243e-15 6.542093e-15 -2.612243e-15
-5.336144e-15
E:\\R-3.1.2\\scripts\\mPDBS\\summary_pdb_93\\804b89c94a633196.final.pdb
-1.813878e-15 -3.147747e-15 2.182079e-15 4.508964e-15 -1.058752e-15
-2.282587e-15
E:\\R-3.1.2\\scripts\\mPDBS\\summary_pdb_93\\813cc65a2298feba.final.pdb
-1.813878e-15 -3.147747e-15 2.182079e-15 4.508964e-15 -1.058752e-15
-2.282587e-15
E:\\R-3.1.2\\scripts\\mPDBS\\summary_pdb_93\\83c0eefcfe12d3f9.final.pdb
2.094779e-15 1.211667e-15 3.614527e-15 3.835241e-15 8.008718e-16
-5.537742e-15
E:\\R-3.1.2\\scripts\\mPDBS\\summary_pdb_93\\86d50469ef569760.final.pdb
-1.953888e-15 -5.514703e-15 -3.105671e-16 2.669459e-15 2.961185e-16
-1.607013e-15
E:\\R-3.1.2\\scripts\\mPDBS\\summary_pdb_93\\89d0666e1402203d.final.pdb
-5.631312e-16 -2.309814e-15 8.617957e-15 3.116855e-15 4.505808e-15
1.051747e-15
E:\\R-3.1.2\\scripts\\mPDBS\\summary_pdb_93\\8b34852716dadc62.final.pdb
2.148231e-15 2.433120e-16 3.363426e-15 2.343162e-15 -3.119603e-15
-3.649333e-15
E:\\R-3.1.2\\scripts\\mPDBS\\summary_pdb_93\\8b6ef9ac3fe74395.final.pdb
-5.117857e-15 -7.844740e-15 8.228059e-15 2.046078e-15 6.134404e-15

-6.142949e-15
E:\\R-3.1.2\\scripts\\mPDBS\\summary_pdb_93\\98bbe0d5986c138f.final.pdb
-3.520195e-15 -2.072331e-15 2.979093e-15 4.979755e-15 -2.997242e-15
-3.430543e-15
E:\\R-3.1.2\\scripts\\mPDBS\\summary_pdb_93\\9c22f4909d67e2fa.final.pdb
1.892576e-15 -8.711819e-15 -3.156098e-15 -3.381789e-16 -3.959209e-15
1.802930e-15
E:\\R-3.1.2\\scripts\\mPDBS\\summary_pdb_93\\9dda8ed0d81fb3ef.final.pdb
1.228459e-15 -2.906830e-15 -7.240451e-15 5.950742e-15 2.596312e-15
-2.899898e-15
E:\\R-3.1.2\\scripts\\mPDBS\\summary_pdb_93\\9f752b3616e6033b.final.pdb
-6.504922e-16 -3.453896e-15 5.311507e-15 2.716747e-15 2.389119e-15
-3.577996e-15
E:\\R-3.1.2\\scripts\\mPDBS\\summary_pdb_93\\a02c70083816d6ba.final.pdb
1.603180e-15 -1.505416e-15 2.364940e-16 4.571094e-16 -3.402772e-15
1.343343e-15
E:\\R-3.1.2\\scripts\\mPDBS\\summary_pdb_93\\a0c997d891e4e082.final.pdb
-3.365364e-15 -1.313389e-15 1.359840e-15 1.244793e-15 -1.080282e-14
5.710470e-17
E:\\R-3.1.2\\scripts\\mPDBS\\summary_pdb_93\\ab8039a2d823c616.final.pdb
1.486440e-15 -1.956321e-15 3.922460e-15 3.228775e-15 6.220657e-16
-1.951190e-15
E:\\R-3.1.2\\scripts\\mPDBS\\summary_pdb_93\\acf4d74b9c71d4dc.final.pdb
-1.813878e-15 -3.147747e-15 2.182079e-15 4.508964e-15 -1.058752e-15
-2.282587e-15
E:\\R-3.1.2\\scripts\\mPDBS\\summary_pdb_93\\ad8a823e4b4c668e.final.pdb
8.059848e-16 -1.604881e-15 3.696147e-15 2.418319e-15 1.111116e-16
-2.304037e-15
E:\\R-3.1.2\\scripts\\mPDBS\\summary_pdb_93\\b16ababd03b7b857.final.pdb
1.892576e-15 -8.711819e-15 -3.156098e-15 -3.381789e-16 -3.959209e-15
1.802930e-15
E:\\R-3.1.2\\scripts\\mPDBS\\summary_pdb_93\\b226923e697d3c63.final.pdb
7.541334e-15 -1.566051e-15 1.092254e-14 2.255456e-15 -5.659237e-15
-1.379603e-15
E:\\R-3.1.2\\scripts\\mPDBS\\summary_pdb_93\\b28e48f6fa27a165.final.pdb
-3.520195e-15 -2.072331e-15 2.979093e-15 4.979755e-15 -2.997242e-15
-3.430543e-15
E:\\R-3.1.2\\scripts\\mPDBS\\summary_pdb_93\\b42c4924e3cd1aec.final.pdb
-4.850283e-15 -5.041353e-15 -3.144769e-15 2.014980e-15 -1.360159e-15
-6.477034e-15
E:\\R-3.1.2\\scripts\\mPDBS\\summary_pdb_93\\b4dd239fcabff1df.final.pdb
-3.520195e-15 -2.072331e-15 2.979093e-15 4.979755e-15 -2.997242e-15
-3.430543e-15
E:\\R-3.1.2\\scripts\\mPDBS\\summary_pdb_93\\b669942ef97459a2.final.pdb
2.148231e-15 2.433120e-16 3.363426e-15 2.343162e-15 -3.119603e-15
-3.649333e-15
E:\\R-3.1.2\\scripts\\mPDBS\\summary_pdb_93\\b99f4d9d87c1017b.final.pdb
-3.520195e-15 -2.072331e-15 2.979093e-15 4.979755e-15 -2.997242e-15
-3.430543e-15
E:\\R-3.1.2\\scripts\\mPDBS\\summary_pdb_93\\b9d2cbf3975403e7.final.pdb
-3.520195e-15 -2.072331e-15 2.979093e-15 4.979755e-15 -2.997242e-15
-3.430543e-15
E:\\R-3.1.2\\scripts\\mPDBS\\summary_pdb_93\\bc88c8a505f3a288.final.pdb
5.088627e-16 -1.597939e-15 3.411473e-15 2.949261e-15 -3.435489e-16

-2.253939e-15
E:\\R-3.1.2\\scripts\\mPDBS\\summary_pdb_93\\bdac0e64f4ef427e.final.pdb
-1.637261e-15 -9.025045e-15 5.085139e-15 5.493490e-16 7.879313e-17
4.326384e-15
E:\\R-3.1.2\\scripts\\mPDBS\\summary_pdb_93\\ca2a1f55fc9d0681.final.pdb
2.148231e-15 2.433120e-16 3.363426e-15 2.343162e-15 -3.119603e-15
-3.649333e-15
E:\\R-3.1.2\\scripts\\mPDBS\\summary_pdb_93\\ce34248de3e81a3c.final.pdb
-6.149312e-15 -6.326579e-16 3.095559e-15 5.718896e-15 -5.460943e-15
8.293721e-16
E:\\R-3.1.2\\scripts\\mPDBS\\summary_pdb_93\\d02e697772494a61.final.pdb
5.823676e-15 2.377668e-15 2.205194e-14 4.398755e-16 1.833734e-16
2.940961e-15
E:\\R-3.1.2\\scripts\\mPDBS\\summary_pdb_93\\d04221245d015047.final.pdb
-1.900390e-15 -2.303049e-15 2.121383e-15 2.653823e-15 2.714142e-15
-2.734933e-15
E:\\R-3.1.2\\scripts\\mPDBS\\summary_pdb_93\\d678141e4018054a.final.pdb
1.153339e-14 -1.535584e-15 7.890465e-15 3.814012e-15 1.331882e-14
-3.004776e-15
E:\\R-3.1.2\\scripts\\mPDBS\\summary_pdb_93\\d71b40af0f0efe39.final.pdb
1.211649e-14 -1.541682e-15 2.149792e-15 2.265611e-15 1.353635e-15
5.862419e-17
E:\\R-3.1.2\\scripts\\mPDBS\\summary_pdb_93\\dc0809eff10197e2.final.pdb
-3.255182e-15 -5.766310e-15 -5.329073e-15 4.013308e-15 8.518001e-16
2.628778e-15
E:\\R-3.1.2\\scripts\\mPDBS\\summary_pdb_93\\e1163918d3707839.final.pdb
-4.779160e-15 -7.499348e-15 6.716266e-15 4.909366e-15 9.862635e-15
-5.837463e-15
E:\\R-3.1.2\\scripts\\mPDBS\\summary_pdb_93\\e4b48b06824ef848.final.pdb
1.096380e-14 2.694859e-15 6.155271e-15 -8.219910e-16 3.663825e-15
-1.761748e-15
E:\\R-3.1.2\\scripts\\mPDBS\\summary_pdb_93\\e529e19ad281fea2.final.pdb
-8.679516e-16 -5.965731e-15 8.117990e-16 3.099670e-15 -3.780482e-15
-8.045588e-17
E:\\R-3.1.2\\scripts\\mPDBS\\summary_pdb_93\\e5602082718ec3a1.final.pdb
-1.813878e-15 -3.147747e-15 2.182079e-15 4.508964e-15 -1.058752e-15
-2.282587e-15
E:\\R-3.1.2\\scripts\\mPDBS\\summary_pdb_93\\e5d589d996d0a960.final.pdb
-8.738071e-15 -2.692320e-15 -8.718372e-16 -2.465421e-15 -1.133660e-15
-5.141375e-15
E:\\R-3.1.2\\scripts\\mPDBS\\summary_pdb_93\\e9df9e72307a2fbd.final.pdb
1.603180e-15 -1.505416e-15 2.364940e-16 4.571094e-16 -3.402772e-15
1.343343e-15
E:\\R-3.1.2\\scripts\\mPDBS\\summary_pdb_93\\ea00f8694090cae7.final.pdb
-1.813878e-15 -3.147747e-15 2.182079e-15 4.508964e-15 -1.058752e-15
-2.282587e-15
E:\\R-3.1.2\\scripts\\mPDBS\\summary_pdb_93\\eba517be32db050e.final.pdb
-3.520195e-15 -2.072331e-15 2.979093e-15 4.979755e-15 -2.997242e-15
-3.430543e-15
E:\\R-3.1.2\\scripts\\mPDBS\\summary_pdb_93\\ec04b9545899c29d.final.pdb
2.261680e-16 -2.073350e-15 4.108543e-15 2.761236e-15 3.424721e-16
-2.172670e-15
E:\\R-3.1.2\\scripts\\mPDBS\\summary_pdb_93\\ee6862cbcad52078.final.pdb
1.452755e-16 -2.094774e-15 3.753647e-15 2.661297e-15 -2.999762e-17

-2.125705e-15
E:\\R-3.1.2\\scripts\\mPDBS\\summary_pdb_93\\ef6a42fc83b81e5d.final.pdb
7.189031e-16 -1.959295e-15 4.375444e-15 3.047527e-15 6.345643e-16
-2.412323e-15
E:\\R-3.1.2\\scripts\\mPDBS\\summary_pdb_93\\ef8e88d4ad93a0ad.final.pdb
-1.900390e-15 -2.303049e-15 2.121383e-15 2.653823e-15 2.714142e-15
-2.734933e-15
E:\\R-3.1.2\\scripts\\mPDBS\\summary_pdb_93\\f14fcae8deec4fa3.final.pdb
-6.504922e-16 -3.453896e-15 5.311507e-15 2.716747e-15 2.389119e-15
-3.577996e-15
E:\\R-3.1.2\\scripts\\mPDBS\\summary_pdb_93\\f83584e22003fb92.final.pdb
2.682796e-15 -2.620391e-16 1.116868e-15 -4.792026e-16 7.314388e-15
-4.086756e-15
E:\\R-3.1.2\\scripts\\mPDBS\\summary_pdb_93\\f8e2bf87e52caa29.final.pdb
-8.679516e-16 -5.965731e-15 8.117990e-16 3.099670e-15 -3.780482e-15
-8.045588e-17

[,613] [,614] [,615] [,616] [,617] [,618]
E:\\R-3.1.2\\scripts\\40.1ns_data\\NAT0.pdb
4.067712e-15 -3.084030e-15 -8.791014e-16 -4.838658e-15 -2.218035e-15
1.727360e-15
E:\\R-3.1.2\\scripts\\40.1ns_data\\LJP6.pdb
3.559076e-15 -1.439465e-15 -1.680219e-15 -3.582185e-15 -2.558288e-15
1.065558e-15
E:\\R-3.1.2\\scripts\\40.1ns_data\\3WY9.pdb
2.826139e-15 -2.480630e-15 -9.286779e-16 -4.100880e-15 -3.183385e-15
9.209628e-16
E:\\R-3.1.2\\scripts\\40.1ns_data\\ZSP9.pdb
2.612926e-15 -3.346903e-15 -1.002026e-15 -3.508040e-15 -1.259578e-15
6.435843e-17
E:\\R-3.1.2\\scripts\\mPDBS\\summary_pdb_93\\013d855ad26c877c.final.pdb
8.227777e-15 -2.946128e-15 4.171061e-15 -1.949405e-15 -8.296477e-16
1.293497e-15
E:\\R-3.1.2\\scripts\\mPDBS\\summary_pdb_93\\0a2a705342ca0d54.final.pdb
2.068460e-15 -2.198018e-15 -2.583325e-15 -3.664302e-15 -3.005465e-15
8.702104e-16
E:\\R-3.1.2\\scripts\\mPDBS\\summary_pdb_93\\0a59fceadb4194d9.final.pdb
4.401268e-15 -7.174095e-16 -2.289258e-15 -3.523123e-15 -6.385574e-15
2.094900e-16
E:\\R-3.1.2\\scripts\\mPDBS\\summary_pdb_93\\0dba59f3306825e8.final.pdb
-4.389017e-16 8.286307e-16 -2.701914e-15 -7.476451e-15 -2.119415e-15
-3.458166e-16
E:\\R-3.1.2\\scripts\\mPDBS\\summary_pdb_93\\126f26a78b57339c.final.pdb
-3.073513e-15 -1.841976e-15 -1.527506e-15 -4.796629e-15 -1.629789e-15
4.996373e-15
E:\\R-3.1.2\\scripts\\mPDBS\\summary_pdb_93\\12f06f3e342df706.final.pdb
1.952667e-15 -1.913957e-15 -2.367894e-15 -3.436619e-15 -2.509334e-15
8.441896e-16
E:\\R-3.1.2\\scripts\\mPDBS\\summary_pdb_93\\18618a2a251b867b.final.pdb
3.293764e-15 5.606486e-16 -1.440364e-15 -4.758764e-15 2.817576e-16
4.093499e-15
E:\\R-3.1.2\\scripts\\mPDBS\\summary_pdb_93\\1f305f598641eeb2.final.pdb
2.395951e-15 -1.122755e-15 -1.650273e-15 -3.973636e-15 -2.695018e-15
1.331107e-15

E:\\R-3.1.2\\scripts\\mPDBS\\summary_pdb_93\\1f8a00b551be0a39.final.pdb
3.293764e-15 5.606486e-16 -1.440364e-15 -4.758764e-15 2.817576e-16
4.093499e-15
E:\\R-3.1.2\\scripts\\mPDBS\\summary_pdb_93\\219f45328d47579a.final.pdb
4.582980e-15 -1.700998e-15 -2.002174e-16 -5.190157e-16 -2.944316e-15
1.169423e-14
E:\\R-3.1.2\\scripts\\mPDBS\\summary_pdb_93\\238dd3918e54336d.final.pdb
-3.020313e-15 -2.170674e-15 -4.608150e-15 -3.042279e-15 8.664453e-15
-4.132089e-15
E:\\R-3.1.2\\scripts\\mPDBS\\summary_pdb_93\\2b3027889e2fdc42.final.pdb
1.922348e-15 -1.156295e-15 -1.701621e-15 -3.197862e-15 -3.267192e-15
1.036953e-15
E:\\R-3.1.2\\scripts\\mPDBS\\summary_pdb_93\\2fcb3b46e33793b0.final.pdb
5.977287e-15 -1.459291e-15 -2.455185e-15 -3.614114e-15 -3.922552e-15
-1.246693e-15
E:\\R-3.1.2\\scripts\\mPDBS\\summary_pdb_93\\34eb328251842006.final.pdb
4.766540e-15 -3.537054e-15 -2.003861e-15 -4.381942e-15 -7.519653e-15
-7.718668e-17
E:\\R-3.1.2\\scripts\\mPDBS\\summary_pdb_93\\354b32871797b7ab.final.pdb
5.316591e-15 -2.633457e-15 -2.565503e-15 -1.265066e-15 -7.637947e-15
2.375526e-15
E:\\R-3.1.2\\scripts\\mPDBS\\summary_pdb_93\\397368adbc32d5de.final.pdb
-3.073513e-15 -1.841976e-15 -1.527506e-15 -4.796629e-15 -1.629789e-15
4.996373e-15
E:\\R-3.1.2\\scripts\\mPDBS\\summary_pdb_93\\411ca13cd3df6edd.final.pdb
3.745999e-15 -3.833260e-15 -3.388466e-15 -6.392599e-15 -4.099060e-15
2.110756e-15
E:\\R-3.1.2\\scripts\\mPDBS\\summary_pdb_93\\42357eee6642bf12.final.pdb
5.716903e-15 -1.752538e-15 -1.073452e-15 -6.149525e-16 -9.482133e-15
4.466225e-15
E:\\R-3.1.2\\scripts\\mPDBS\\summary_pdb_93\\44342d1f75a7fcfc.final.pdb
1.936481e-15 -6.731045e-15 -3.561422e-16 -6.341767e-15 -9.281583e-15
-6.457897e-15
E:\\R-3.1.2\\scripts\\mPDBS\\summary_pdb_93\\448ef21dffe8cdfb.final.pdb
6.986029e-15 -6.306109e-15 4.638099e-15 1.187675e-14 -2.348074e-15
5.544750e-15
E:\\R-3.1.2\\scripts\\mPDBS\\summary_pdb_93\\472a40b3e8f113c7.final.pdb
-4.389017e-16 8.286307e-16 -2.701914e-15 -7.476451e-15 -2.119415e-15
-3.458166e-16
E:\\R-3.1.2\\scripts\\mPDBS\\summary_pdb_93\\48a26225951ea153.final.pdb
-9.971916e-16 -6.958945e-15 -5.151986e-15 -8.912223e-16 -2.755015e-15
-1.461283e-16
E:\\R-3.1.2\\scripts\\mPDBS\\summary_pdb_93\\4ab4a5c7c6682ce6.final.pdb
1.323983e-14 5.122939e-15 -6.330955e-15 3.105254e-15 -8.114185e-15
-5.007778e-15
E:\\R-3.1.2\\scripts\\mPDBS\\summary_pdb_93\\4eaa45b62cb6a25f.final.pdb
1.730917e-15 -4.680879e-16 -2.994847e-15 -5.416801e-15 1.010083e-15
1.028947e-15
E:\\R-3.1.2\\scripts\\mPDBS\\summary_pdb_93\\58c371611d85ac96.final.pdb
-3.073513e-15 -1.841976e-15 -1.527506e-15 -4.796629e-15 -1.629789e-15
4.996373e-15
E:\\R-3.1.2\\scripts\\mPDBS\\summary_pdb_93\\59a647f04822d30a.final.pdb
-9.470627e-16 -3.844466e-15 -4.642210e-15 -1.565206e-15 -2.564300e-15
-1.179133e-15

E:\\R-3.1.2\\scripts\\mPDBS\\summary_pdb_93\\59b091fe385e05fc.final.pdb
4.401268e-15 -7.174095e-16 -2.289258e-15 -3.523123e-15 -6.385574e-15
2.094900e-16
E:\\R-3.1.2\\scripts\\mPDBS\\summary_pdb_93\\5c35ac30f930d2db.final.pdb
1.730917e-15 -4.680879e-16 -2.994847e-15 -5.416801e-15 1.010083e-15
1.028947e-15
E:\\R-3.1.2\\scripts\\mPDBS\\summary_pdb_93\\5f60752431cf2dcb.final.pdb
1.315156e-15 -3.511204e-15 -2.936341e-15 -4.806942e-15 1.731992e-16
1.274401e-15
E:\\R-3.1.2\\scripts\\mPDBS\\summary_pdb_93\\626456dd6ff2868d.final.pdb
-4.389017e-16 8.286307e-16 -2.701914e-15 -7.476451e-15 -2.119415e-15
-3.458166e-16
E:\\R-3.1.2\\scripts\\mPDBS\\summary_pdb_93\\6583d53ff3c8f7ef.final.pdb
-9.470627e-16 -3.844466e-15 -4.642210e-15 -1.565206e-15 -2.564300e-15
-1.179133e-15
E:\\R-3.1.2\\scripts\\mPDBS\\summary_pdb_93\\69760178986bef01.final.pdb
-7.365012e-16 1.350769e-15 -3.132792e-15 -2.717043e-16 2.619935e-18
3.382722e-15
E:\\R-3.1.2\\scripts\\mPDBS\\summary_pdb_93\\6a9d87d47926b1c8.final.pdb
2.320610e-15 8.530173e-17 -1.201378e-15 -3.582322e-15 -3.352152e-15
9.874268e-16
E:\\R-3.1.2\\scripts\\mPDBS\\summary_pdb_93\\6bb3547ea982ffc4.final.pdb
-5.476607e-15 -7.631885e-15 1.521025e-15 -5.763648e-15 3.064897e-16
5.362371e-15
E:\\R-3.1.2\\scripts\\mPDBS\\summary_pdb_93\\716d9624611d1c42.final.pdb
2.407164e-15 -1.605610e-15 -2.229008e-15 -5.573148e-15 -2.551401e-15
1.398434e-15
E:\\R-3.1.2\\scripts\\mPDBS\\summary_pdb_93\\75f27e70ad0f1be4.final.pdb
-9.470627e-16 -3.844466e-15 -4.642210e-15 -1.565206e-15 -2.564300e-15
-1.179133e-15
E:\\R-3.1.2\\scripts\\mPDBS\\summary_pdb_93\\769f910fffb22872.final.pdb
1.079389e-15 -2.397489e-15 -1.433859e-15 -3.970874e-15 -2.822313e-15
1.120508e-15
E:\\R-3.1.2\\scripts\\mPDBS\\summary_pdb_93\\791f8c80dd5c3135.final.pdb
4.401268e-15 -7.174095e-16 -2.289258e-15 -3.523123e-15 -6.385574e-15
2.094900e-16
E:\\R-3.1.2\\scripts\\mPDBS\\summary_pdb_93\\7fad98e66a3f6099.final.pdb
2.525839e-15 -5.252493e-15 -3.885302e-15 -6.027472e-16 -6.002882e-15
-1.076527e-15
E:\\R-3.1.2\\scripts\\mPDBS\\summary_pdb_93\\804b89c94a633196.final.pdb
5.977287e-15 -1.459291e-15 -2.455185e-15 -3.614114e-15 -3.922552e-15
-1.246693e-15
E:\\R-3.1.2\\scripts\\mPDBS\\summary_pdb_93\\813cc65a2298feba.final.pdb
5.977287e-15 -1.459291e-15 -2.455185e-15 -3.614114e-15 -3.922552e-15
-1.246693e-15
E:\\R-3.1.2\\scripts\\mPDBS\\summary_pdb_93\\83c0eefcfe12d3f9.final.pdb
3.745999e-15 -3.833260e-15 -3.388466e-15 -6.392599e-15 -4.099060e-15
2.110756e-15
E:\\R-3.1.2\\scripts\\mPDBS\\summary_pdb_93\\86d50469ef569760.final.pdb
-4.389017e-16 8.286307e-16 -2.701914e-15 -7.476451e-15 -2.119415e-15
-3.458166e-16
E:\\R-3.1.2\\scripts\\mPDBS\\summary_pdb_93\\89d0666e1402203d.final.pdb
-9.470627e-16 -3.844466e-15 -4.642210e-15 -1.565206e-15 -2.564300e-15
-1.179133e-15

E:\\R-3.1.2\\scripts\\mPDBS\\summary_pdb_93\\8b34852716dadc62.final.pdb
-1.687588e-15 8.284855e-18 -6.167493e-15 -5.971170e-15 -6.659729e-15
2.625210e-15
E:\\R-3.1.2\\scripts\\mPDBS\\summary_pdb_93\\8b6ef9ac3fe74395.final.pdb
-2.757162e-15 -6.868129e-15 -2.811171e-15 -4.265579e-15 -2.947967e-15
-5.038082e-15
E:\\R-3.1.2\\scripts\\mPDBS\\summary_pdb_93\\98bbe0d5986c138f.final.pdb
4.401268e-15 -7.174095e-16 -2.289258e-15 -3.523123e-15 -6.385574e-15
2.094900e-16
E:\\R-3.1.2\\scripts\\mPDBS\\summary_pdb_93\\9c22f4909d67e2fa.final.pdb
-2.097851e-15 9.237852e-16 3.750788e-15 -4.821590e-15 3.372783e-16
6.082839e-15
E:\\R-3.1.2\\scripts\\mPDBS\\summary_pdb_93\\9dda8ed0d81fb3ef.final.pdb
7.382211e-16 -4.076918e-15 -1.055534e-14 -2.495126e-15 -9.446381e-15
-2.570193e-16
E:\\R-3.1.2\\scripts\\mPDBS\\summary_pdb_93\\9f752b3616e6033b.final.pdb
9.398866e-16 -2.318760e-15 -1.043636e-15 -2.226662e-15 -4.026116e-15
5.716186e-16
E:\\R-3.1.2\\scripts\\mPDBS\\summary_pdb_93\\a02c70083816d6ba.final.pdb
1.730917e-15 -4.680879e-16 -2.994847e-15 -5.416801e-15 1.010083e-15
1.028947e-15
E:\\R-3.1.2\\scripts\\mPDBS\\summary_pdb_93\\a0c997d891e4e082.final.pdb
7.806513e-16 4.242314e-15 -7.451202e-15 -3.566716e-15 -1.925818e-15
3.008337e-15
E:\\R-3.1.2\\scripts\\mPDBS\\summary_pdb_93\\ab8039a2d823c616.final.pdb
2.080827e-15 -1.841795e-15 -2.594844e-15 -4.072821e-15 -2.756317e-15
6.348016e-16
E:\\R-3.1.2\\scripts\\mPDBS\\summary_pdb_93\\acf4d74b9c71d4dc.final.pdb
5.977287e-15 -1.459291e-15 -2.455185e-15 -3.614114e-15 -3.922552e-15
-1.246693e-15
E:\\R-3.1.2\\scripts\\mPDBS\\summary_pdb_93\\ad8a823e4b4c668e.final.pdb
1.680067e-15 -1.476857e-15 -1.937122e-15 -3.740879e-15 -2.270219e-15
1.064091e-15
E:\\R-3.1.2\\scripts\\mPDBS\\summary_pdb_93\\b16ababd03b7b857.final.pdb
-2.097851e-15 9.237852e-16 3.750788e-15 -4.821590e-15 3.372783e-16
6.082839e-15
E:\\R-3.1.2\\scripts\\mPDBS\\summary_pdb_93\\b226923e697d3c63.final.pdb
2.317687e-15 1.544019e-15 -3.266141e-15 -5.384416e-15 -3.120929e-15
2.968157e-15
E:\\R-3.1.2\\scripts\\mPDBS\\summary_pdb_93\\b28e48f6fa27a165.final.pdb
4.401268e-15 -7.174095e-16 -2.289258e-15 -3.523123e-15 -6.385574e-15
2.094900e-16
E:\\R-3.1.2\\scripts\\mPDBS\\summary_pdb_93\\b42c4924e3cd1aec.final.pdb
5.555115e-15 -1.300927e-15 2.701308e-15 -7.267350e-15 3.477309e-15
-1.457123e-15
E:\\R-3.1.2\\scripts\\mPDBS\\summary_pdb_93\\b4dd239fcabff1df.final.pdb
4.401268e-15 -7.174095e-16 -2.289258e-15 -3.523123e-15 -6.385574e-15
2.094900e-16
E:\\R-3.1.2\\scripts\\mPDBS\\summary_pdb_93\\b669942ef97459a2.final.pdb
-1.687588e-15 8.284855e-18 -6.167493e-15 -5.971170e-15 -6.659729e-15
2.625210e-15
E:\\R-3.1.2\\scripts\\mPDBS\\summary_pdb_93\\b99f4d9d87c1017b.final.pdb
4.401268e-15 -7.174095e-16 -2.289258e-15 -3.523123e-15 -6.385574e-15
2.094900e-16

E:\\R-3.1.2\\scripts\\mPDBS\\summary_pdb_93\\b9d2cbf3975403e7.final.pdb
4.401268e-15 -7.174095e-16 -2.289258e-15 -3.523123e-15 -6.385574e-15
2.094900e-16
E:\\R-3.1.2\\scripts\\mPDBS\\summary_pdb_93\\bc88c8a505f3a288.final.pdb
2.460546e-15 -1.929548e-15 -1.617486e-15 -3.629375e-15 -2.999177e-15
9.341708e-16
E:\\R-3.1.2\\scripts\\mPDBS\\summary_pdb_93\\bdac0e64f4ef427e.final.pdb
-1.909199e-15 3.894499e-15 2.375153e-15 3.182871e-16 -6.545498e-16
-7.719204e-16
E:\\R-3.1.2\\scripts\\mPDBS\\summary_pdb_93\\ca2a1f55fc9d0681.final.pdb
-1.687588e-15 8.284855e-18 -6.167493e-15 -5.971170e-15 -6.659729e-15
2.625210e-15
E:\\R-3.1.2\\scripts\\mPDBS\\summary_pdb_93\\ce34248de3e81a3c.final.pdb
6.071608e-15 -4.199657e-16 -6.011277e-15 -4.109029e-15 -1.735263e-15
-5.350310e-16
E:\\R-3.1.2\\scripts\\mPDBS\\summary_pdb_93\\d02e697772494a61.final.pdb
2.669881e-16 1.265813e-15 2.160741e-15 -1.266573e-15 -2.945184e-15
4.768777e-15
E:\\R-3.1.2\\scripts\\mPDBS\\summary_pdb_93\\d04221245d015047.final.pdb
1.953324e-15 -2.531781e-15 -1.157626e-15 -2.833361e-15 -3.132318e-15
3.837003e-16
E:\\R-3.1.2\\scripts\\mPDBS\\summary_pdb_93\\d678141e4018054a.final.pdb
-1.675397e-15 -6.575163e-15 4.835826e-15 -8.427040e-15 -3.268463e-15
5.322475e-15
E:\\R-3.1.2\\scripts\\mPDBS\\summary_pdb_93\\d71b40af0f0efe39.final.pdb
6.094829e-15 -5.165791e-16 4.954250e-15 -7.023425e-15 -4.159948e-15
3.622898e-16
E:\\R-3.1.2\\scripts\\mPDBS\\summary_pdb_93\\dc0809eff10197e2.final.pdb
8.980078e-16 3.498063e-15 -3.048563e-15 -3.781440e-15 -2.726159e-15
-9.047020e-15
E:\\R-3.1.2\\scripts\\mPDBS\\summary_pdb_93\\e1163918d3707839.final.pdb
2.073525e-15 -3.615745e-16 6.424892e-15 -7.212164e-15 -2.773767e-15
7.602471e-15
E:\\R-3.1.2\\scripts\\mPDBS\\summary_pdb_93\\e4b48b06824ef848.final.pdb
3.293764e-15 5.606486e-16 -1.440364e-15 -4.758764e-15 2.817576e-16
4.093499e-15
E:\\R-3.1.2\\scripts\\mPDBS\\summary_pdb_93\\e529e19ad281fea2.final.pdb
8.227777e-15 -2.946128e-15 4.171061e-15 -1.949405e-15 -8.296477e-16
1.293497e-15
E:\\R-3.1.2\\scripts\\mPDBS\\summary_pdb_93\\e5602082718ec3a1.final.pdb
5.977287e-15 -1.459291e-15 -2.455185e-15 -3.614114e-15 -3.922552e-15
-1.246693e-15
E:\\R-3.1.2\\scripts\\mPDBS\\summary_pdb_93\\e5d589d996d0a960.final.pdb
-3.206562e-15 -2.615219e-15 -1.841775e-16 -4.406704e-15 1.890101e-16
-2.673613e-16
E:\\R-3.1.2\\scripts\\mPDBS\\summary_pdb_93\\e9df9e72307a2fbd.final.pdb
1.730917e-15 -4.680879e-16 -2.994847e-15 -5.416801e-15 1.010083e-15
1.028947e-15
E:\\R-3.1.2\\scripts\\mPDBS\\summary_pdb_93\\ea00f8694090cae7.final.pdb
5.977287e-15 -1.459291e-15 -2.455185e-15 -3.614114e-15 -3.922552e-15
-1.246693e-15
E:\\R-3.1.2\\scripts\\mPDBS\\summary_pdb_93\\eba517be32db050e.final.pdb
4.401268e-15 -7.174095e-16 -2.289258e-15 -3.523123e-15 -6.385574e-15
2.094900e-16

E:\\R-3.1.2\\scripts\\mPDBS\\summary_pdb_93\\ec04b9545899c29d.final.pdb
1.967706e-15 -1.089725e-15 -1.685808e-15 -3.641236e-15 -3.079079e-15
1.067334e-15
E:\\R-3.1.2\\scripts\\mPDBS\\summary_pdb_93\\ee6862cbcad52078.final.pdb
2.381616e-15 -1.548342e-15 -1.885501e-15 -3.453734e-15 -3.127330e-15
9.059815e-16
E:\\R-3.1.2\\scripts\\mPDBS\\summary_pdb_93\\ef6a42fc83b81e5d.final.pdb
1.844402e-15 -1.736125e-15 -2.021063e-15 -3.961333e-15 -3.123721e-15
1.045589e-15
E:\\R-3.1.2\\scripts\\mPDBS\\summary_pdb_93\\ef8e88d4ad93a0ad.final.pdb
1.953324e-15 -2.531781e-15 -1.157626e-15 -2.833361e-15 -3.132318e-15
3.837003e-16
E:\\R-3.1.2\\scripts\\mPDBS\\summary_pdb_93\\f14fcae8deec4fa3.final.pdb
9.398866e-16 -2.318760e-15 -1.043636e-15 -2.226662e-15 -4.026116e-15
5.716186e-16
E:\\R-3.1.2\\scripts\\mPDBS\\summary_pdb_93\\f83584e22003fb92.final.pdb
-9.971916e-16 -6.958945e-15 -5.151986e-15 -8.912223e-16 -2.755015e-15
-1.461283e-16
E:\\R-3.1.2\\scripts\\mPDBS\\summary_pdb_93\\f8e2bf87e52caa29.final.pdb
8.227777e-15 -2.946128e-15 4.171061e-15 -1.949405e-15 -8.296477e-16
1.293497e-15

[,619]   [,620]   [,621]   [,622]   [,623]   [,624]
E:\\R-3.1.2\\scripts\\40.1ns_data\\NAT0.pdb
-6.039712e-17 -1.142138e-15 5.265571e-16 -1.130014e-15 5.291943e-15
2.137328e-15
E:\\R-3.1.2\\scripts\\40.1ns_data\\LJP6.pdb
-7.755982e-16 -4.502869e-15 2.255628e-16 -1.176838e-15 5.675056e-15
2.235108e-15
E:\\R-3.1.2\\scripts\\40.1ns_data\\3WY9.pdb
-3.159146e-16 -1.713630e-15 1.195736e-15 -9.785558e-16 4.705609e-15
-2.194794e-16
E:\\R-3.1.2\\scripts\\40.1ns_data\\ZSP9.pdb
-2.464118e-16 -2.321647e-15 1.212624e-15 -1.406331e-15 4.047187e-15
1.526082e-15
E:\\R-3.1.2\\scripts\\mPDBS\\summary_pdb_93\\013d855ad26c877c.final.pdb
-5.695170e-15 1.331915e-15 -4.011031e-15 -3.569372e-15 -1.587974e-15
3.698859e-15
E:\\R-3.1.2\\scripts\\mPDBS\\summary_pdb_93\\0a2a705342ca0d54.final.pdb
-9.023032e-16 -2.986365e-15 9.379125e-16 -5.037281e-16 4.203418e-15
9.264674e-16
E:\\R-3.1.2\\scripts\\mPDBS\\summary_pdb_93\\0a59fceadb4194d9.final.pdb
-1.225384e-15 9.016669e-16 -2.288276e-15 -4.748564e-15 6.422113e-15
-1.091288e-15
E:\\R-3.1.2\\scripts\\mPDBS\\summary_pdb_93\\0dba59f3306825e8.final.pdb
-3.198903e-15 -1.549461e-15 -1.635327e-15 -3.226764e-15 2.201530e-15
-2.354459e-15
E:\\R-3.1.2\\scripts\\mPDBS\\summary_pdb_93\\126f26a78b57339c.final.pdb
4.543168e-15 -5.756816e-15 8.629899e-15 4.110140e-15 5.093964e-15
3.997231e-15
E:\\R-3.1.2\\scripts\\mPDBS\\summary_pdb_93\\12f06f3e342df706.final.pdb
-6.609598e-16 -3.190520e-15 6.948344e-16 6.063730e-18 4.594273e-15
1.266256e-15
E:\\R-3.1.2\\scripts\\mPDBS\\summary_pdb_93\\18618a2a251b867b.final.pdb

-4.410820e-15 -5.954251e-15 4.824035e-15 2.286123e-15 3.501882e-15
-2.198154e-15
E:\\R-3.1.2\\scripts\\mPDBS\\summary_pdb_93\\1f305f598641eeb2.final.pdb
-2.979942e-16 -2.189803e-15 6.249064e-16 -1.103173e-15 4.684642e-15
9.584484e-16
E:\\R-3.1.2\\scripts\\mPDBS\\summary_pdb_93\\1f8a00b551be0a39.final.pdb
-4.410820e-15 -5.954251e-15 4.824035e-15 2.286123e-15 3.501882e-15
-2.198154e-15
E:\\R-3.1.2\\scripts\\mPDBS\\summary_pdb_93\\219f45328d47579a.final.pdb
3.664801e-15 -4.210563e-15 6.714939e-15 1.263488e-14 9.029836e-15
7.744526e-15
E:\\R-3.1.2\\scripts\\mPDBS\\summary_pdb_93\\238dd3918e54336d.final.pdb
8.622471e-16 -1.126774e-14 1.664341e-14 1.081429e-14 -3.158874e-15
-3.972230e-15
E:\\R-3.1.2\\scripts\\mPDBS\\summary_pdb_93\\2b3027889e2fdc42.final.pdb
-5.194601e-16 -2.357662e-15 8.303232e-16 4.090942e-16 4.678986e-15
1.553840e-15
E:\\R-3.1.2\\scripts\\mPDBS\\summary_pdb_93\\2fcb3b46e33793b0.final.pdb
-2.986816e-15 1.895470e-15 -4.695491e-15 -5.437379e-15 3.398345e-15
-1.026075e-15
E:\\R-3.1.2\\scripts\\mPDBS\\summary_pdb_93\\34eb328251842006.final.pdb
-6.019658e-15 -1.517812e-15 -7.903063e-16 -9.020831e-16 7.757336e-15
-1.149326e-15
E:\\R-3.1.2\\scripts\\mPDBS\\summary_pdb_93\\354b32871797b7ab.final.pdb
-4.331689e-15 3.881750e-15 -1.417368e-14 -1.672699e-15 6.604472e-15
7.242310e-15
E:\\R-3.1.2\\scripts\\mPDBS\\summary_pdb_93\\397368adbc32d5de.final.pdb
4.543168e-15 -5.756816e-15 8.629899e-15 4.110140e-15 5.093964e-15
3.997231e-15
E:\\R-3.1.2\\scripts\\mPDBS\\summary_pdb_93\\411ca13cd3df6edd.final.pdb
-6.223875e-16 2.012347e-15 -4.359388e-15 -5.699214e-15 2.075835e-15
-3.816255e-16
E:\\R-3.1.2\\scripts\\mPDBS\\summary_pdb_93\\42357eee6642bf12.final.pdb
-5.995803e-15 2.343113e-15 3.022608e-15 3.001136e-15 1.145927e-14
-1.037825e-14
E:\\R-3.1.2\\scripts\\mPDBS\\summary_pdb_93\\44342d1f75a7fcfc.final.pdb
-1.089836e-14 7.601900e-15 -1.416416e-15 -1.823547e-15 -6.355847e-16
2.903960e-15
E:\\R-3.1.2\\scripts\\mPDBS\\summary_pdb_93\\448ef21dffe8cdfb.final.pdb
1.187283e-14 -1.684128e-14 9.322810e-15 2.143362e-15 8.125466e-15
6.811405e-15
E:\\R-3.1.2\\scripts\\mPDBS\\summary_pdb_93\\472a40b3e8f113c7.final.pdb
-3.198903e-15 -1.549461e-15 -1.635327e-15 -3.226764e-15 2.201530e-15
-2.354459e-15
E:\\R-3.1.2\\scripts\\mPDBS\\summary_pdb_93\\48a26225951ea153.final.pdb
-2.248979e-15 -6.619877e-15 -9.976417e-16 7.665009e-15 2.115065e-15
7.003040e-15
E:\\R-3.1.2\\scripts\\mPDBS\\summary_pdb_93\\4ab4a5c7c6682ce6.final.pdb
5.563185e-15 -8.544516e-15 -3.112991e-16 4.760661e-15 1.606956e-14
5.358122e-15
E:\\R-3.1.2\\scripts\\mPDBS\\summary_pdb_93\\4eaa45b62cb6a25f.final.pdb
2.321951e-15 -4.704806e-15 4.456163e-15 -2.521777e-16 2.693213e-15
1.552851e-15
E:\\R-3.1.2\\scripts\\mPDBS\\summary_pdb_93\\58c371611d85ac96.final.pdb

4.543168e-15 -5.756816e-15 8.629899e-15 4.110140e-15 5.093964e-15
3.997231e-15
E:\\R-3.1.2\\scripts\\mPDBS\\summary_pdb_93\\59a647f04822d30a.final.pdb
4.441798e-15 -7.605553e-15 5.180304e-15 5.183651e-15 9.192491e-15
5.914572e-15
E:\\R-3.1.2\\scripts\\mPDBS\\summary_pdb_93\\59b091fe385e05fc.final.pdb
-1.225384e-15 9.016669e-16 -2.288276e-15 -4.748564e-15 6.422113e-15
-1.091288e-15
E:\\R-3.1.2\\scripts\\mPDBS\\summary_pdb_93\\5c35ac30f930d2db.final.pdb
2.321951e-15 -4.704806e-15 4.456163e-15 -2.521777e-16 2.693213e-15
1.552851e-15
E:\\R-3.1.2\\scripts\\mPDBS\\summary_pdb_93\\5f60752431cf2dcb.final.pdb
2.177590e-15 -3.596771e-15 8.094514e-15 2.381914e-16 8.036308e-16
-2.391950e-15
E:\\R-3.1.2\\scripts\\mPDBS\\summary_pdb_93\\626456dd6ff2868d.final.pdb
-3.198903e-15 -1.549461e-15 -1.635327e-15 -3.226764e-15 2.201530e-15
-2.354459e-15
E:\\R-3.1.2\\scripts\\mPDBS\\summary_pdb_93\\6583d53ff3c8f7ef.final.pdb
4.441798e-15 -7.605553e-15 5.180304e-15 5.183651e-15 9.192491e-15
5.914572e-15
E:\\R-3.1.2\\scripts\\mPDBS\\summary_pdb_93\\69760178986bef01.final.pdb
-3.399404e-16 -9.188811e-15 7.329242e-15 7.683645e-16 -1.114803e-15
2.166707e-16
E:\\R-3.1.2\\scripts\\mPDBS\\summary_pdb_93\\6a9d87d47926b1c8.final.pdb
-5.430414e-16 -2.217980e-15 1.071709e-15 -2.830472e-16 4.218796e-15
1.919032e-15
E:\\R-3.1.2\\scripts\\mPDBS\\summary_pdb_93\\6bb3547ea982ffc4.final.pdb
-6.818809e-15 -1.205083e-14 4.681508e-15 7.202730e-15 -1.142588e-14
1.057758e-14
E:\\R-3.1.2\\scripts\\mPDBS\\summary_pdb_93\\716d9624611d1c42.final.pdb
-3.393366e-15 -4.827774e-15 -7.711419e-15 3.000630e-15 4.125355e-15
7.239993e-15
E:\\R-3.1.2\\scripts\\mPDBS\\summary_pdb_93\\75f27e70ad0f1be4.final.pdb
4.441798e-15 -7.605553e-15 5.180304e-15 5.183651e-15 9.192491e-15
5.914572e-15
E:\\R-3.1.2\\scripts\\mPDBS\\summary_pdb_93\\769f910fffb22872.final.pdb
-7.757072e-16 -1.552932e-15 7.745396e-16 -1.700597e-15 4.628206e-15
7.529363e-16
E:\\R-3.1.2\\scripts\\mPDBS\\summary_pdb_93\\791f8c80dd5c3135.final.pdb
-1.225384e-15 9.016669e-16 -2.288276e-15 -4.748564e-15 6.422113e-15
-1.091288e-15
E:\\R-3.1.2\\scripts\\mPDBS\\summary_pdb_93\\7fad98e66a3f6099.final.pdb
2.995123e-16 4.255745e-15 -4.502451e-15 -2.059136e-15 7.401694e-15
3.906009e-16
E:\\R-3.1.2\\scripts\\mPDBS\\summary_pdb_93\\804b89c94a633196.final.pdb
-2.986816e-15 1.895470e-15 -4.695491e-15 -5.437379e-15 3.398345e-15
-1.026075e-15
E:\\R-3.1.2\\scripts\\mPDBS\\summary_pdb_93\\813cc65a2298feba.final.pdb
-2.986816e-15 1.895470e-15 -4.695491e-15 -5.437379e-15 3.398345e-15
-1.026075e-15
E:\\R-3.1.2\\scripts\\mPDBS\\summary_pdb_93\\83c0eefcfe12d3f9.final.pdb
-6.223875e-16 2.012347e-15 -4.359388e-15 -5.699214e-15 2.075835e-15
-3.816255e-16
E:\\R-3.1.2\\scripts\\mPDBS\\summary_pdb_93\\86d50469ef569760.final.pdb

-3.198903e-15 -1.549461e-15 -1.635327e-15 -3.226764e-15 2.201530e-15
-2.354459e-15
E:\\R-3.1.2\\scripts\\mPDBS\\summary_pdb_93\\89d0666e1402203d.final.pdb
4.441798e-15 -7.605553e-15 5.180304e-15 5.183651e-15 9.192491e-15
5.914572e-15
E:\\R-3.1.2\\scripts\\mPDBS\\summary_pdb_93\\8b34852716dadc62.final.pdb
3.780341e-15 -3.400640e-15 4.850693e-15 5.311835e-15 1.044045e-14
2.238674e-15
E:\\R-3.1.2\\scripts\\mPDBS\\summary_pdb_93\\8b6ef9ac3fe74395.final.pdb
3.034500e-16 -7.309612e-15 2.443496e-15 -4.411519e-15 9.984512e-15
2.953151e-15
E:\\R-3.1.2\\scripts\\mPDBS\\summary_pdb_93\\98bbe0d5986c138f.final.pdb
-1.225384e-15 9.016669e-16 -2.288276e-15 -4.748564e-15 6.422113e-15
-1.091288e-15
E:\\R-3.1.2\\scripts\\mPDBS\\summary_pdb_93\\9c22f4909d67e2fa.final.pdb
-3.912291e-15 -1.374917e-15 6.256903e-15 2.460383e-15 -1.403261e-15
-5.399190e-16
E:\\R-3.1.2\\scripts\\mPDBS\\summary_pdb_93\\9dda8ed0d81fb3ef.final.pdb
3.978519e-15 -2.759386e-15 -2.951429e-15 -1.209647e-14 -1.584207e-15
5.462785e-15
E:\\R-3.1.2\\scripts\\mPDBS\\summary_pdb_93\\9f752b3616e6033b.final.pdb
-9.916678e-16 -2.515127e-15 1.781984e-15 -9.392202e-16 5.079502e-15
2.217267e-16
E:\\R-3.1.2\\scripts\\mPDBS\\summary_pdb_93\\a02c70083816d6ba.final.pdb
2.321951e-15 -4.704806e-15 4.456163e-15 -2.521777e-16 2.693213e-15
1.552851e-15
E:\\R-3.1.2\\scripts\\mPDBS\\summary_pdb_93\\a0c997d891e4e082.final.pdb
7.327759e-15 2.694469e-15 1.735116e-15 -5.542899e-15 -3.047376e-15
5.958250e-15
E:\\R-3.1.2\\scripts\\mPDBS\\summary_pdb_93\\ab8039a2d823c616.final.pdb
-7.743751e-16 -2.178695e-15 1.430456e-15 -5.473290e-16 4.554074e-15
1.141357e-15
E:\\R-3.1.2\\scripts\\mPDBS\\summary_pdb_93\\acf4d74b9c71d4dc.final.pdb
-2.986816e-15 1.895470e-15 -4.695491e-15 -5.437379e-15 3.398345e-15
-1.026075e-15
E:\\R-3.1.2\\scripts\\mPDBS\\summary_pdb_93\\ad8a823e4b4c668e.final.pdb
-3.259870e-16 -2.556468e-15 1.578777e-16 -3.942975e-17 4.428965e-15
1.256004e-15
E:\\R-3.1.2\\scripts\\mPDBS\\summary_pdb_93\\b16ababd03b7b857.final.pdb
-3.912291e-15 -1.374917e-15 6.256903e-15 2.460383e-15 -1.403261e-15
-5.399190e-16
E:\\R-3.1.2\\scripts\\mPDBS\\summary_pdb_93\\b226923e697d3c63.final.pdb
3.555355e-15 2.385262e-15 3.730726e-15 -4.802176e-15 4.806189e-15
-6.335503e-15
E:\\R-3.1.2\\scripts\\mPDBS\\summary_pdb_93\\b28e48f6fa27a165.final.pdb
-1.225384e-15 9.016669e-16 -2.288276e-15 -4.748564e-15 6.422113e-15
-1.091288e-15
E:\\R-3.1.2\\scripts\\mPDBS\\summary_pdb_93\\b42c4924e3cd1aec.final.pdb
-3.674864e-15 2.472648e-15 -4.229487e-15 -3.706590e-15 5.946498e-15
-5.225388e-15
E:\\R-3.1.2\\scripts\\mPDBS\\summary_pdb_93\\b4dd239fcabff1df.final.pdb
-1.225384e-15 9.016669e-16 -2.288276e-15 -4.748564e-15 6.422113e-15
-1.091288e-15
E:\\R-3.1.2\\scripts\\mPDBS\\summary_pdb_93\\b669942ef97459a2.final.pdb

3.780341e-15 -3.400640e-15 4.850693e-15 5.311835e-15 1.044045e-14
2.238674e-15
E:\\R-3.1.2\\scripts\\mPDBS\\summary_pdb_93\\b99f4d9d87c1017b.final.pdb
-1.225384e-15 9.016669e-16 -2.288276e-15 -4.748564e-15 6.422113e-15
-1.091288e-15
E:\\R-3.1.2\\scripts\\mPDBS\\summary_pdb_93\\b9d2cbf3975403e7.final.pdb
-1.225384e-15 9.016669e-16 -2.288276e-15 -4.748564e-15 6.422113e-15
-1.091288e-15
E:\\R-3.1.2\\scripts\\mPDBS\\summary_pdb_93\\bc88c8a505f3a288.final.pdb
-5.001613e-16 -2.240459e-15 1.015505e-15 6.968415e-16 4.305804e-15
1.521531e-15
E:\\R-3.1.2\\scripts\\mPDBS\\summary_pdb_93\\bdac0e64f4ef427e.final.pdb
-1.035902e-15 -1.063444e-14 1.032244e-14 7.895380e-15 1.021256e-14
3.091725e-15
E:\\R-3.1.2\\scripts\\mPDBS\\summary_pdb_93\\ca2a1f55fc9d0681.final.pdb
3.780341e-15 -3.400640e-15 4.850693e-15 5.311835e-15 1.044045e-14
2.238674e-15
E:\\R-3.1.2\\scripts\\mPDBS\\summary_pdb_93\\ce34248de3e81a3c.final.pdb
4.088186e-15 7.827312e-16 -1.312179e-15 -2.261097e-15 1.025143e-14
2.577308e-15
E:\\R-3.1.2\\scripts\\mPDBS\\summary_pdb_93\\d02e697772494a61.final.pdb
-1.880351e-15 -6.239648e-15 9.675895e-15 1.543776e-14 9.143785e-15
3.869587e-15
E:\\R-3.1.2\\scripts\\mPDBS\\summary_pdb_93\\d04221245d015047.final.pdb
-1.956481e-15 -4.303406e-15 -2.841018e-16 1.028234e-15 4.627908e-15
3.857500e-15
E:\\R-3.1.2\\scripts\\mPDBS\\summary_pdb_93\\d678141e4018054a.final.pdb
-9.608099e-15 -4.270436e-15 -1.764741e-15 9.225971e-15 -3.672716e-15
-1.502508e-15
E:\\R-3.1.2\\scripts\\mPDBS\\summary_pdb_93\\d71b40af0f0efe39.final.pdb
6.446584e-16 5.810744e-15 -6.750492e-15 -3.579754e-16 6.125873e-15
-1.945117e-15
E:\\R-3.1.2\\scripts\\mPDBS\\summary_pdb_93\\dc0809eff10197e2.final.pdb
7.559175e-15 -4.814827e-16 9.789305e-16 -1.415300e-14 1.407097e-14
2.099962e-15
E:\\R-3.1.2\\scripts\\mPDBS\\summary_pdb_93\\e1163918d3707839.final.pdb
-9.674406e-15 -5.253810e-15 -9.091049e-15 -6.209145e-15 4.377875e-15
-1.397533e-14
E:\\R-3.1.2\\scripts\\mPDBS\\summary_pdb_93\\e4b48b06824ef848.final.pdb
-4.410820e-15 -5.954251e-15 4.824035e-15 2.286123e-15 3.501882e-15
-2.198154e-15
E:\\R-3.1.2\\scripts\\mPDBS\\summary_pdb_93\\e529e19ad281fea2.final.pdb
-5.695170e-15 1.331915e-15 -4.011031e-15 -3.569372e-15 -1.587974e-15
3.698859e-15
E:\\R-3.1.2\\scripts\\mPDBS\\summary_pdb_93\\e5602082718ec3a1.final.pdb
-2.986816e-15 1.895470e-15 -4.695491e-15 -5.437379e-15 3.398345e-15
-1.026075e-15
E:\\R-3.1.2\\scripts\\mPDBS\\summary_pdb_93\\e5d589d996d0a960.final.pdb
7.333410e-15 -1.906932e-15 -2.553208e-15 -5.991514e-15 -1.761625e-15
1.254970e-14
E:\\R-3.1.2\\scripts\\mPDBS\\summary_pdb_93\\e9df9e72307a2fbd.final.pdb
2.321951e-15 -4.704806e-15 4.456163e-15 -2.521777e-16 2.693213e-15
1.552851e-15
E:\\R-3.1.2\\scripts\\mPDBS\\summary_pdb_93\\ea00f8694090cae7.final.pdb

-2.986816e-15 1.895470e-15 -4.695491e-15 -5.437379e-15 3.398345e-15
-1.026075e-15
E:\\R-3.1.2\\scripts\\mPDBS\\summary_pdb_93\\eba517be32db050e.final.pdb
-1.225384e-15 9.016669e-16 -2.288276e-15 -4.748564e-15 6.422113e-15
-1.091288e-15
E:\\R-3.1.2\\scripts\\mPDBS\\summary_pdb_93\\ec04b9545899c29d.final.pdb
-1.326675e-16 -1.900048e-15 4.332332e-16 -2.596942e-16 4.434145e-15
1.590340e-15
E:\\R-3.1.2\\scripts\\mPDBS\\summary_pdb_93\\ee6862cbcad52078.final.pdb
-5.974143e-16 -2.280683e-15 8.572114e-16 2.220694e-16 4.362562e-15
1.784883e-15
E:\\R-3.1.2\\scripts\\mPDBS\\summary_pdb_93\\ef6a42fc83b81e5d.final.pdb
-4.780937e-16 -2.182312e-15 7.162095e-16 -9.514129e-16 4.431857e-15
5.161465e-16
E:\\R-3.1.2\\scripts\\mPDBS\\summary_pdb_93\\ef8e88d4ad93a0ad.final.pdb
-1.956481e-15 -4.303406e-15 -2.841018e-16 1.028234e-15 4.627908e-15
3.857500e-15
E:\\R-3.1.2\\scripts\\mPDBS\\summary_pdb_93\\f14fcae8deec4fa3.final.pdb
-9.916678e-16 -2.515127e-15 1.781984e-15 -9.392202e-16 5.079502e-15
2.217267e-16
E:\\R-3.1.2\\scripts\\mPDBS\\summary_pdb_93\\f83584e22003fb92.final.pdb
-2.248979e-15 -6.619877e-15 -9.976417e-16 7.665009e-15 2.115065e-15
7.003040e-15
E:\\R-3.1.2\\scripts\\mPDBS\\summary_pdb_93\\f8e2bf87e52caa29.final.pdb
-5.695170e-15 1.331915e-15 -4.011031e-15 -3.569372e-15 -1.587974e-15
3.698859e-15

[,625]   [,626]   [,627]   [,628]   [,629]   [,630]
E:\\R-3.1.2\\scripts\\40.1ns_data\\NAT0.pdb
-3.457228e-15 1.242097e-16 -1.263643e-15 1.959270e-15 2.461906e-15
3.540972e-15
E:\\R-3.1.2\\scripts\\40.1ns_data\\LJP6.pdb
-2.532754e-15 1.879292e-15 9.605515e-16 2.391257e-15 2.373495e-15
1.930169e-15
E:\\R-3.1.2\\scripts\\40.1ns_data\\3WY9.pdb
-3.424466e-15 -3.752059e-16 7.513571e-16 4.388059e-15 1.596723e-15
2.338530e-15
E:\\R-3.1.2\\scripts\\40.1ns_data\\ZSP9.pdb
-2.572089e-15 1.429306e-16 4.126551e-16 1.557038e-15 1.916967e-15
2.024411e-15
E:\\R-3.1.2\\scripts\\mPDBS\\summary_pdb_93\\013d855ad26c877c.final.pdb
-8.106838e-15 -3.630384e-15 3.517978e-15 1.059407e-14 3.907437e-15
1.021247e-14
E:\\R-3.1.2\\scripts\\mPDBS\\summary_pdb_93\\0a2a705342ca0d54.final.pdb
-2.825253e-15 1.642854e-15 6.125610e-17 2.766174e-15 1.515350e-15
1.740662e-15
E:\\R-3.1.2\\scripts\\mPDBS\\summary_pdb_93\\0a59fceadb4194d9.final.pdb
-1.619357e-15 3.103048e-15 -3.112446e-15 -6.183282e-16 3.411718e-15
2.885973e-15
E:\\R-3.1.2\\scripts\\mPDBS\\summary_pdb_93\\0dba59f3306825e8.final.pdb
-1.669712e-15 2.808910e-15 -5.326017e-16 2.714089e-15 1.458008e-15
3.158738e-16
E:\\R-3.1.2\\scripts\\mPDBS\\summary_pdb_93\\126f26a78b57339c.final.pdb
-6.360341e-16 -4.839486e-15 2.963083e-15 5.421992e-15 -3.422015e-16

-2.323074e-15
E:\\R-3.1.2\\scripts\\mPDBS\\summary_pdb_93\\12f06f3e342df706.final.pdb
-3.225432e-15 6.475566e-16 5.363535e-16 3.256233e-15 1.664970e-15
1.674742e-15
E:\\R-3.1.2\\scripts\\mPDBS\\summary_pdb_93\\18618a2a251b867b.final.pdb
-2.406447e-15 -4.503416e-15 3.470426e-15 9.605013e-15 5.446634e-16
-1.020063e-15
E:\\R-3.1.2\\scripts\\mPDBS\\summary_pdb_93\\1f305f598641eeb2.final.pdb
-2.762188e-15 1.798076e-16 2.249412e-16 3.121370e-15 1.827825e-15
1.430641e-15
E:\\R-3.1.2\\scripts\\mPDBS\\summary_pdb_93\\1f8a00b551be0a39.final.pdb
-2.406447e-15 -4.503416e-15 3.470426e-15 9.605013e-15 5.446634e-16
-1.020063e-15
E:\\R-3.1.2\\scripts\\mPDBS\\summary_pdb_93\\219f45328d47579a.final.pdb
-4.422453e-15 1.001939e-14 -3.867918e-15 -5.955205e-15 -5.569813e-15
3.948058e-15
E:\\R-3.1.2\\scripts\\mPDBS\\summary_pdb_93\\238dd3918e54336d.final.pdb
-1.144212e-14 -1.987498e-15 -2.588342e-15 2.120366e-15 4.352155e-15
1.957896e-15
E:\\R-3.1.2\\scripts\\mPDBS\\summary_pdb_93\\2b3027889e2fdc42.final.pdb
-2.887548e-15 5.582090e-16 1.193355e-15 3.048877e-15 1.408113e-15
1.661692e-15
E:\\R-3.1.2\\scripts\\mPDBS\\summary_pdb_93\\2fcb3b46e33793b0.final.pdb
-1.923883e-15 2.287497e-15 2.506103e-15 4.140520e-15 3.264217e-16
5.466475e-15
E:\\R-3.1.2\\scripts\\mPDBS\\summary_pdb_93\\34eb328251842006.final.pdb
-5.690659e-15 7.683960e-15 -7.449688e-15 -8.357026e-15 6.482185e-16
5.689662e-15
E:\\R-3.1.2\\scripts\\mPDBS\\summary_pdb_93\\354b32871797b7ab.final.pdb
5.546263e-15 7.567434e-15 2.561487e-15 5.655520e-16 -9.942783e-17
-4.041897e-15
E:\\R-3.1.2\\scripts\\mPDBS\\summary_pdb_93\\397368adbc32d5de.final.pdb
-6.360341e-16 -4.839486e-15 2.963083e-15 5.421992e-15 -3.422015e-16
-2.323074e-15
E:\\R-3.1.2\\scripts\\mPDBS\\summary_pdb_93\\411ca13cd3df6edd.final.pdb
1.740070e-15 -1.568794e-15 5.207718e-15 6.214382e-15 -3.143459e-15
7.191224e-15
E:\\R-3.1.2\\scripts\\mPDBS\\summary_pdb_93\\42357eee6642bf12.final.pdb
-2.615419e-15 9.131771e-15 -7.189561e-15 -1.058650e-14 -1.519802e-15
6.007584e-15
E:\\R-3.1.2\\scripts\\mPDBS\\summary_pdb_93\\44342d1f75a7fcfc.final.pdb
2.045177e-15 -1.859532e-14 4.825829e-15 8.904813e-15 -1.199538e-16
-1.615446e-15
E:\\R-3.1.2\\scripts\\mPDBS\\summary_pdb_93\\448ef21dffe8cdfb.final.pdb
-9.637331e-15 -2.165539e-15 3.150552e-15 5.720853e-15 8.220281e-15
-3.144906e-16
E:\\R-3.1.2\\scripts\\mPDBS\\summary_pdb_93\\472a40b3e8f113c7.final.pdb
-1.669712e-15 2.808910e-15 -5.326017e-16 2.714089e-15 1.458008e-15
3.158738e-16
E:\\R-3.1.2\\scripts\\mPDBS\\summary_pdb_93\\48a26225951ea153.final.pdb
-1.074112e-15 -1.189450e-14 1.542503e-14 1.378768e-14 -2.618614e-15
-5.205902e-16
E:\\R-3.1.2\\scripts\\mPDBS\\summary_pdb_93\\4ab4a5c7c6682ce6.final.pdb
-3.335264e-15 7.804914e-15 1.004596e-14 1.371413e-15 2.586879e-15

-1.495611e-15
E:\\R-3.1.2\\scripts\\mPDBS\\summary_pdb_93\\4eaa45b62cb6a25f.final.pdb
-5.790481e-15 -3.582502e-15 2.831020e-15 9.718387e-15 2.371252e-15
4.243576e-15
E:\\R-3.1.2\\scripts\\mPDBS\\summary_pdb_93\\58c371611d85ac96.final.pdb
-6.360341e-16 -4.839486e-15 2.963083e-15 5.421992e-15 -3.422015e-16
-2.323074e-15
E:\\R-3.1.2\\scripts\\mPDBS\\summary_pdb_93\\59a647f04822d30a.final.pdb
-1.061288e-14 8.475044e-15 6.750504e-15 -1.773762e-17 -1.033286e-15
1.517898e-15
E:\\R-3.1.2\\scripts\\mPDBS\\summary_pdb_93\\59b091fe385e05fc.final.pdb
-1.619357e-15 3.103048e-15 -3.112446e-15 -6.183282e-16 3.411718e-15
2.885973e-15
E:\\R-3.1.2\\scripts\\mPDBS\\summary_pdb_93\\5c35ac30f930d2db.final.pdb
-5.790481e-15 -3.582502e-15 2.831020e-15 9.718387e-15 2.371252e-15
4.243576e-15
E:\\R-3.1.2\\scripts\\mPDBS\\summary_pdb_93\\5f60752431cf2dcb.final.pdb
-9.523996e-15 -2.537829e-15 4.196186e-15 5.277186e-15 -1.581349e-15
2.250670e-15
E:\\R-3.1.2\\scripts\\mPDBS\\summary_pdb_93\\626456dd6ff2868d.final.pdb
-1.669712e-15 2.808910e-15 -5.326017e-16 2.714089e-15 1.458008e-15
3.158738e-16
E:\\R-3.1.2\\scripts\\mPDBS\\summary_pdb_93\\6583d53ff3c8f7ef.final.pdb
-1.061288e-14 8.475044e-15 6.750504e-15 -1.773762e-17 -1.033286e-15
1.517898e-15
E:\\R-3.1.2\\scripts\\mPDBS\\summary_pdb_93\\69760178986bef01.final.pdb
1.186332e-15 -1.852707e-15 4.199609e-15 1.296791e-14 -1.558384e-16
-2.690686e-15
E:\\R-3.1.2\\scripts\\mPDBS\\summary_pdb_93\\6a9d87d47926b1c8.final.pdb
-2.840434e-15 3.855658e-17 1.227492e-15 2.819907e-15 1.875642e-15
1.725770e-15
E:\\R-3.1.2\\scripts\\mPDBS\\summary_pdb_93\\6bb3547ea982ffc4.final.pdb
-3.119831e-15 -1.328795e-14 3.373062e-15 9.465939e-15 -1.236634e-15
2.979783e-16
E:\\R-3.1.2\\scripts\\mPDBS\\summary_pdb_93\\716d9624611d1c42.final.pdb
7.090292e-16 1.350553e-15 -1.900716e-15 5.278920e-15 4.342082e-15
2.461006e-15
E:\\R-3.1.2\\scripts\\mPDBS\\summary_pdb_93\\75f27e70ad0f1be4.final.pdb
-1.061288e-14 8.475044e-15 6.750504e-15 -1.773762e-17 -1.033286e-15
1.517898e-15
E:\\R-3.1.2\\scripts\\mPDBS\\summary_pdb_93\\769f910fffb22872.final.pdb
-3.343632e-15 1.560514e-16 2.759329e-16 3.013498e-15 1.794315e-15
2.447216e-15
E:\\R-3.1.2\\scripts\\mPDBS\\summary_pdb_93\\791f8c80dd5c3135.final.pdb
-1.619357e-15 3.103048e-15 -3.112446e-15 -6.183282e-16 3.411718e-15
2.885973e-15
E:\\R-3.1.2\\scripts\\mPDBS\\summary_pdb_93\\7fad98e66a3f6099.final.pdb
1.793247e-15 3.708268e-16 -1.395013e-15 5.674322e-16 3.174784e-15
2.803639e-15
E:\\R-3.1.2\\scripts\\mPDBS\\summary_pdb_93\\804b89c94a633196.final.pdb
-1.923883e-15 2.287497e-15 2.506103e-15 4.140520e-15 3.264217e-16
5.466475e-15
E:\\R-3.1.2\\scripts\\mPDBS\\summary_pdb_93\\813cc65a2298feba.final.pdb
-1.923883e-15 2.287497e-15 2.506103e-15 4.140520e-15 3.264217e-16

5.466475e-15
E:\\R-3.1.2\\scripts\\mPDBS\\summary_pdb_93\\83c0eefcfe12d3f9.final.pdb
1.740070e-15 -1.568794e-15 5.207718e-15 6.214382e-15 -3.143459e-15
7.191224e-15
E:\\R-3.1.2\\scripts\\mPDBS\\summary_pdb_93\\86d50469ef569760.final.pdb
-1.669712e-15 2.808910e-15 -5.326017e-16 2.714089e-15 1.458008e-15
3.158738e-16
E:\\R-3.1.2\\scripts\\mPDBS\\summary_pdb_93\\89d0666e1402203d.final.pdb
-1.061288e-14 8.475044e-15 6.750504e-15 -1.773762e-17 -1.033286e-15
1.517898e-15
E:\\R-3.1.2\\scripts\\mPDBS\\summary_pdb_93\\8b34852716dadc62.final.pdb
2.474942e-15 -1.879538e-16 -1.391821e-15 -4.591210e-15 -1.603458e-15
-5.771931e-15
E:\\R-3.1.2\\scripts\\mPDBS\\summary_pdb_93\\8b6ef9ac3fe74395.final.pdb
-6.164526e-15 -2.040914e-15 -6.631715e-15 -3.734926e-15 8.138423e-15
-6.621403e-15
E:\\R-3.1.2\\scripts\\mPDBS\\summary_pdb_93\\98bbe0d5986c138f.final.pdb
-1.619357e-15 3.103048e-15 -3.112446e-15 -6.183282e-16 3.411718e-15
2.885973e-15
E:\\R-3.1.2\\scripts\\mPDBS\\summary_pdb_93\\9c22f4909d67e2fa.final.pdb
-5.917217e-15 -6.346451e-16 -5.170049e-15 5.048648e-15 3.109785e-15
1.843505e-15
E:\\R-3.1.2\\scripts\\mPDBS\\summary_pdb_93\\9dda8ed0d81fb3ef.final.pdb
-1.891019e-15 -1.802719e-14 1.466366e-14 1.890723e-14 4.306627e-15
4.438305e-15
E:\\R-3.1.2\\scripts\\mPDBS\\summary_pdb_93\\9f752b3616e6033b.final.pdb
-1.903227e-15 2.480457e-15 -1.117745e-15 -3.119201e-16 1.862642e-15
-6.768267e-16
E:\\R-3.1.2\\scripts\\mPDBS\\summary_pdb_93\\a02c70083816d6ba.final.pdb
-5.790481e-15 -3.582502e-15 2.831020e-15 9.718387e-15 2.371252e-15
4.243576e-15
E:\\R-3.1.2\\scripts\\mPDBS\\summary_pdb_93\\a0c997d891e4e082.final.pdb
1.882078e-15 -6.559241e-15 -2.605769e-16 7.710521e-15 -1.589189e-15
1.803567e-15
E:\\R-3.1.2\\scripts\\mPDBS\\summary_pdb_93\\ab8039a2d823c616.final.pdb
-3.010601e-15 1.120379e-15 4.847037e-16 2.644695e-15 1.043905e-15
1.933237e-15
E:\\R-3.1.2\\scripts\\mPDBS\\summary_pdb_93\\acf4d74b9c71d4dc.final.pdb
-1.923883e-15 2.287497e-15 2.506103e-15 4.140520e-15 3.264217e-16
5.466475e-15
E:\\R-3.1.2\\scripts\\mPDBS\\summary_pdb_93\\ad8a823e4b4c668e.final.pdb
-3.064889e-15 -3.061171e-16 2.159102e-16 2.965051e-15 2.523199e-15
2.057433e-15
E:\\R-3.1.2\\scripts\\mPDBS\\summary_pdb_93\\b16ababd03b7b857.final.pdb
-5.917217e-15 -6.346451e-16 -5.170049e-15 5.048648e-15 3.109785e-15
1.843505e-15
E:\\R-3.1.2\\scripts\\mPDBS\\summary_pdb_93\\b226923e697d3c63.final.pdb
-2.196222e-15 2.698025e-16 -9.602734e-15 1.049941e-16 1.623877e-15
-2.712225e-15
E:\\R-3.1.2\\scripts\\mPDBS\\summary_pdb_93\\b28e48f6fa27a165.final.pdb
-1.619357e-15 3.103048e-15 -3.112446e-15 -6.183282e-16 3.411718e-15
2.885973e-15
E:\\R-3.1.2\\scripts\\mPDBS\\summary_pdb_93\\b42c4924e3cd1aec.final.pdb
-1.998355e-15 3.253527e-15 -1.458508e-14 -4.475543e-15 6.615761e-15

-3.668791e-16
E:\\R-3.1.2\\scripts\\mPDBS\\summary_pdb_93\\b4dd239fcabff1df.final.pdb
-1.619357e-15 3.103048e-15 -3.112446e-15 -6.183282e-16 3.411718e-15
2.885973e-15
E:\\R-3.1.2\\scripts\\mPDBS\\summary_pdb_93\\b669942ef97459a2.final.pdb
2.474942e-15 -1.879538e-16 -1.391821e-15 -4.591210e-15 -1.603458e-15
-5.771931e-15
E:\\R-3.1.2\\scripts\\mPDBS\\summary_pdb_93\\b99f4d9d87c1017b.final.pdb
-1.619357e-15 3.103048e-15 -3.112446e-15 -6.183282e-16 3.411718e-15
2.885973e-15
E:\\R-3.1.2\\scripts\\mPDBS\\summary_pdb_93\\b9d2cbf3975403e7.final.pdb
-1.619357e-15 3.103048e-15 -3.112446e-15 -6.183282e-16 3.411718e-15
2.885973e-15
E:\\R-3.1.2\\scripts\\mPDBS\\summary_pdb_93\\bc88c8a505f3a288.final.pdb
-3.119134e-15 1.687636e-16 8.034757e-16 2.920074e-15 1.390115e-15
1.653018e-15
E:\\R-3.1.2\\scripts\\mPDBS\\summary_pdb_93\\bdac0e64f4ef427e.final.pdb
-1.237668e-14 5.325865e-15 -1.055680e-14 -1.593608e-15 1.265684e-14
-8.471160e-15
E:\\R-3.1.2\\scripts\\mPDBS\\summary_pdb_93\\ca2a1f55fc9d0681.final.pdb
2.474942e-15 -1.879538e-16 -1.391821e-15 -4.591210e-15 -1.603458e-15
-5.771931e-15
E:\\R-3.1.2\\scripts\\mPDBS\\summary_pdb_93\\ce34248de3e81a3c.final.pdb
-4.491498e-15 -1.554221e-15 3.684955e-15 6.830700e-15 3.996842e-15
8.241936e-15
E:\\R-3.1.2\\scripts\\mPDBS\\summary_pdb_93\\d02e697772494a61.final.pdb
-5.410053e-15 1.650726e-14 2.131840e-15 -5.304250e-15 -6.248740e-15
1.558910e-15
E:\\R-3.1.2\\scripts\\mPDBS\\summary_pdb_93\\d04221245d015047.final.pdb
-3.587939e-15 1.097828e-15 3.842044e-15 4.457915e-15 1.520303e-15
3.423798e-15
E:\\R-3.1.2\\scripts\\mPDBS\\summary_pdb_93\\d678141e4018054a.final.pdb
-4.827668e-15 2.860899e-16 5.532028e-16 2.888240e-15 -1.873097e-15
5.220799e-15
E:\\R-3.1.2\\scripts\\mPDBS\\summary_pdb_93\\d71b40af0f0efe39.final.pdb
-1.314231e-15 6.333878e-15 1.270684e-15 2.049975e-15 1.640411e-15
-5.073808e-15
E:\\R-3.1.2\\scripts\\mPDBS\\summary_pdb_93\\dc0809eff10197e2.final.pdb
-7.105926e-15 4.726968e-15 -2.160970e-15 1.013241e-15 7.009998e-15
-7.202925e-15
E:\\R-3.1.2\\scripts\\mPDBS\\summary_pdb_93\\e1163918d3707839.final.pdb
7.921445e-15 1.092187e-14 -1.603010e-14 -6.660905e-16 5.078145e-15
9.385736e-15
E:\\R-3.1.2\\scripts\\mPDBS\\summary_pdb_93\\e4b48b06824ef848.final.pdb
-2.406447e-15 -4.503416e-15 3.470426e-15 9.605013e-15 5.446634e-16
-1.020063e-15
E:\\R-3.1.2\\scripts\\mPDBS\\summary_pdb_93\\e529e19ad281fea2.final.pdb
-8.106838e-15 -3.630384e-15 3.517978e-15 1.059407e-14 3.907437e-15
1.021247e-14
E:\\R-3.1.2\\scripts\\mPDBS\\summary_pdb_93\\e5602082718ec3a1.final.pdb
-1.923883e-15 2.287497e-15 2.506103e-15 4.140520e-15 3.264217e-16
5.466475e-15
E:\\R-3.1.2\\scripts\\mPDBS\\summary_pdb_93\\e5d589d996d0a960.final.pdb
5.013637e-16 -1.189781e-14 7.099122e-15 1.321901e-14 5.116216e-15

-2.230365e-15
E:\\R-3.1.2\\scripts\\mPDBS\\summary_pdb_93\\e9df9e72307a2fbd.final.pdb
-5.790481e-15 -3.582502e-15 2.831020e-15 9.718387e-15 2.371252e-15
4.243576e-15
E:\\R-3.1.2\\scripts\\mPDBS\\summary_pdb_93\\ea00f8694090cae7.final.pdb
-1.923883e-15 2.287497e-15 2.506103e-15 4.140520e-15 3.264217e-16
5.466475e-15
E:\\R-3.1.2\\scripts\\mPDBS\\summary_pdb_93\\eba517be32db050e.final.pdb
-1.619357e-15 3.103048e-15 -3.112446e-15 -6.183282e-16 3.411718e-15
2.885973e-15
E:\\R-3.1.2\\scripts\\mPDBS\\summary_pdb_93\\ec04b9545899c29d.final.pdb
-2.851513e-15 -1.621437e-16 2.886595e-16 3.195838e-15 1.702373e-15
2.365744e-15
E:\\R-3.1.2\\scripts\\mPDBS\\summary_pdb_93\\ee6862cbcad52078.final.pdb
-3.038578e-15 6.406084e-16 1.054360e-15 3.313423e-15 1.459287e-15
1.458729e-15
E:\\R-3.1.2\\scripts\\mPDBS\\summary_pdb_93\\ef6a42fc83b81e5d.final.pdb
-3.086567e-15 9.609631e-16 3.537786e-16 3.118882e-15 1.695869e-15
1.913788e-15
E:\\R-3.1.2\\scripts\\mPDBS\\summary_pdb_93\\ef8e88d4ad93a0ad.final.pdb
-3.587939e-15 1.097828e-15 3.842044e-15 4.457915e-15 1.520303e-15
3.423798e-15
E:\\R-3.1.2\\scripts\\mPDBS\\summary_pdb_93\\f14fcae8deec4fa3.final.pdb
-1.903227e-15 2.480457e-15 -1.117745e-15 -3.119201e-16 1.862642e-15
-6.768267e-16
E:\\R-3.1.2\\scripts\\mPDBS\\summary_pdb_93\\f83584e22003fb92.final.pdb
-1.074112e-15 -1.189450e-14 1.542503e-14 1.378768e-14 -2.618614e-15
-5.205902e-16
E:\\R-3.1.2\\scripts\\mPDBS\\summary_pdb_93\\f8e2bf87e52caa29.final.pdb
-8.106838e-15 -3.630384e-15 3.517978e-15 1.059407e-14 3.907437e-15
1.021247e-14

[,631]   [,632]   [,633]   [,634]   [,635]   [,636]
E:\\R-3.1.2\\scripts\\40.1ns_data\\NAT0.pdb
3.845779e-15 1.801813e-15 3.074905e-15 -1.032279e-15 1.859780e-15
-2.664911e-15
E:\\R-3.1.2\\scripts\\40.1ns_data\\LJP6.pdb
2.138492e-15 8.814888e-16 1.908498e-15 -1.549908e-15 -3.397431e-16
-7.590069e-16
E:\\R-3.1.2\\scripts\\40.1ns_data\\3WY9.pdb
1.869905e-15 -5.505726e-17 1.895689e-15 -2.137747e-15 2.062978e-15
-1.694605e-15
E:\\R-3.1.2\\scripts\\40.1ns_data\\ZSP9.pdb
3.503141e-15 1.204684e-15 2.930986e-15 -9.294519e-16 1.146189e-15
-1.957803e-15
E:\\R-3.1.2\\scripts\\mPDBS\\summary_pdb_93\\013d855ad26c877c.final.pdb
-1.750093e-15 -3.928847e-15 -6.538384e-15 -1.056556e-15 -6.352853e-15
2.902716e-15
E:\\R-3.1.2\\scripts\\mPDBS\\summary_pdb_93\\0a2a705342ca0d54.final.pdb
2.299450e-15 1.462433e-15 1.741317e-15 -9.704817e-16 1.606868e-15
-5.963963e-16
E:\\R-3.1.2\\scripts\\mPDBS\\summary_pdb_93\\0a59fceadb4194d9.final.pdb
1.633243e-15 1.790269e-15 7.667822e-16 3.672184e-16 -6.464781e-16
-7.297213e-15

E:\\R-3.1.2\\scripts\\mPDBS\\summary_pdb_93\\0dba59f3306825e8.final.pdb
5.724831e-15 4.847987e-15 3.006932e-15 -3.089652e-15 3.044144e-15
-4.210517e-15
E:\\R-3.1.2\\scripts\\mPDBS\\summary_pdb_93\\126f26a78b57339c.final.pdb
5.131122e-15 2.244848e-15 6.073490e-15 -1.592699e-16 7.127331e-15
4.915212e-15
E:\\R-3.1.2\\scripts\\mPDBS\\summary_pdb_93\\12f06f3e342df706.final.pdb
2.172816e-15 1.599706e-15 2.752661e-15 -6.944439e-16 1.371799e-15
-1.114645e-15
E:\\R-3.1.2\\scripts\\mPDBS\\summary_pdb_93\\18618a2a251b867b.final.pdb
5.728815e-15 -9.998046e-17 2.016322e-15 8.045010e-16 3.611222e-15
6.777100e-15
E:\\R-3.1.2\\scripts\\mPDBS\\summary_pdb_93\\1f305f598641eeb2.final.pdb
2.106980e-15 9.490559e-16 2.001503e-15 -5.638592e-17 1.573094e-15
-1.092820e-15
E:\\R-3.1.2\\scripts\\mPDBS\\summary_pdb_93\\1f8a00b551be0a39.final.pdb
5.728815e-15 -9.998046e-17 2.016322e-15 8.045010e-16 3.611222e-15
6.777100e-15
E:\\R-3.1.2\\scripts\\mPDBS\\summary_pdb_93\\219f45328d47579a.final.pdb
-1.722103e-14 -2.118063e-15 -7.747241e-15 -1.177628e-14 6.776837e-15
-4.261904e-16
E:\\R-3.1.2\\scripts\\mPDBS\\summary_pdb_93\\238dd3918e54336d.final.pdb
5.539407e-15 2.741671e-15 -8.720978e-16 -6.534162e-15 8.669244e-15
1.387726e-14
E:\\R-3.1.2\\scripts\\mPDBS\\summary_pdb_93\\2b3027889e2fdc42.final.pdb
2.398256e-15 7.618250e-16 9.814543e-16 -7.683749e-16 1.286953e-15
-1.001685e-15
E:\\R-3.1.2\\scripts\\mPDBS\\summary_pdb_93\\2fcb3b46e33793b0.final.pdb
-7.240887e-16 -5.245917e-16 -5.398672e-16 2.177004e-16 -2.839175e-15
-3.656741e-15
E:\\R-3.1.2\\scripts\\mPDBS\\summary_pdb_93\\34eb328251842006.final.pdb
-5.728952e-15 -7.508129e-15 2.080498e-15 3.336384e-15 3.606948e-15
-9.671600e-15
E:\\R-3.1.2\\scripts\\mPDBS\\summary_pdb_93\\354b32871797b7ab.final.pdb
-3.480252e-16 -4.112107e-15 -3.796360e-15 6.604111e-15 2.720955e-15
1.011823e-14
E:\\R-3.1.2\\scripts\\mPDBS\\summary_pdb_93\\397368adbc32d5de.final.pdb
5.131122e-15 2.244848e-15 6.073490e-15 -1.592699e-16 7.127331e-15
4.915212e-15
E:\\R-3.1.2\\scripts\\mPDBS\\summary_pdb_93\\411ca13cd3df6edd.final.pdb
-3.565132e-15 -3.336392e-15 1.030491e-15 4.021515e-15 -6.066299e-17
-1.041428e-15
E:\\R-3.1.2\\scripts\\mPDBS\\summary_pdb_93\\42357eee6642bf12.final.pdb
-5.264455e-15 -3.046659e-15 -1.125789e-15 5.714515e-15 4.161050e-15
-7.624580e-15
E:\\R-3.1.2\\scripts\\mPDBS\\summary_pdb_93\\44342d1f75a7fcfc.final.pdb
8.423526e-15 -3.893648e-16 6.080063e-15 8.419297e-15 -4.917099e-15
-4.187379e-15
E:\\R-3.1.2\\scripts\\mPDBS\\summary_pdb_93\\448ef21dffe8cdfb.final.pdb
-2.200338e-15 -1.243087e-14 -2.219080e-15 4.073991e-15 7.404269e-16
1.592829e-14
E:\\R-3.1.2\\scripts\\mPDBS\\summary_pdb_93\\472a40b3e8f113c7.final.pdb
5.724831e-15 4.847987e-15 3.006932e-15 -3.089652e-15 3.044144e-15
-4.210517e-15

E:\\R-3.1.2\\scripts\\mPDBS\\summary_pdb_93\\48a26225951ea153.final.pdb
8.792881e-15 -2.796526e-15 4.627843e-15 4.385489e-15 -2.699618e-16
7.586714e-15
E:\\R-3.1.2\\scripts\\mPDBS\\summary_pdb_93\\4ab4a5c7c6682ce6.final.pdb
-1.311208e-15 -1.486542e-15 -8.801493e-16 2.938946e-15 1.583199e-17
-2.461266e-15
E:\\R-3.1.2\\scripts\\mPDBS\\summary_pdb_93\\4eaa45b62cb6a25f.final.pdb
5.274647e-15 4.949314e-15 4.105739e-15 -2.473046e-15 7.704346e-16
-1.173740e-15
E:\\R-3.1.2\\scripts\\mPDBS\\summary_pdb_93\\58c371611d85ac96.final.pdb
5.131122e-15 2.244848e-15 6.073490e-15 -1.592699e-16 7.127331e-15
4.915212e-15
E:\\R-3.1.2\\scripts\\mPDBS\\summary_pdb_93\\59a647f04822d30a.final.pdb
3.048919e-15 1.958150e-15 -8.041874e-16 -1.253521e-15 4.943503e-15
8.060223e-16
E:\\R-3.1.2\\scripts\\mPDBS\\summary_pdb_93\\59b091fe385e05fc.final.pdb
1.633243e-15 1.790269e-15 7.667822e-16 3.672184e-16 -6.464781e-16
-7.297213e-15
E:\\R-3.1.2\\scripts\\mPDBS\\summary_pdb_93\\5c35ac30f930d2db.final.pdb
5.274647e-15 4.949314e-15 4.105739e-15 -2.473046e-15 7.704346e-16
-1.173740e-15
E:\\R-3.1.2\\scripts\\mPDBS\\summary_pdb_93\\5f60752431cf2dcb.final.pdb
4.206345e-15 8.632293e-15 4.193783e-15 3.458484e-15 3.849513e-15
-5.839598e-15
E:\\R-3.1.2\\scripts\\mPDBS\\summary_pdb_93\\626456dd6ff2868d.final.pdb
5.724831e-15 4.847987e-15 3.006932e-15 -3.089652e-15 3.044144e-15
-4.210517e-15
E:\\R-3.1.2\\scripts\\mPDBS\\summary_pdb_93\\6583d53ff3c8f7ef.final.pdb
3.048919e-15 1.958150e-15 -8.041874e-16 -1.253521e-15 4.943503e-15
8.060223e-16
E:\\R-3.1.2\\scripts\\mPDBS\\summary_pdb_93\\69760178986bef01.final.pdb
5.280609e-15 8.178425e-15 -2.379613e-17 -6.473069e-15 4.543365e-15
7.186937e-16
E:\\R-3.1.2\\scripts\\mPDBS\\summary_pdb_93\\6a9d87d47926b1c8.final.pdb
2.393728e-15 5.795134e-16 3.957398e-16 -9.919372e-16 1.509429e-15
-8.310592e-16
E:\\R-3.1.2\\scripts\\mPDBS\\summary_pdb_93\\6bb3547ea982ffc4.final.pdb
5.311617e-15 4.150641e-15 6.417260e-15 -3.337170e-16 4.152860e-15
7.142277e-15
E:\\R-3.1.2\\scripts\\mPDBS\\summary_pdb_93\\716d9624611d1c42.final.pdb
1.185324e-15 -4.864103e-15 7.820152e-16 -3.461978e-15 1.007087e-15
5.807986e-15
E:\\R-3.1.2\\scripts\\mPDBS\\summary_pdb_93\\75f27e70ad0f1be4.final.pdb
3.048919e-15 1.958150e-15 -8.041874e-16 -1.253521e-15 4.943503e-15
8.060223e-16
E:\\R-3.1.2\\scripts\\mPDBS\\summary_pdb_93\\769f910fffb22872.final.pdb
1.781885e-15 1.258835e-15 1.459475e-15 -1.283466e-16 1.521490e-15
-6.238809e-16
E:\\R-3.1.2\\scripts\\mPDBS\\summary_pdb_93\\791f8c80dd5c3135.final.pdb
1.633243e-15 1.790269e-15 7.667822e-16 3.672184e-16 -6.464781e-16
-7.297213e-15
E:\\R-3.1.2\\scripts\\mPDBS\\summary_pdb_93\\7fad98e66a3f6099.final.pdb
-1.172207e-15 -5.341419e-15 2.168456e-15 2.882894e-15 -1.235544e-15
-8.523080e-15

E:\\R-3.1.2\\scripts\\mPDBS\\summary_pdb_93\\804b89c94a633196.final.pdb
-7.240887e-16 -5.245917e-16 -5.398672e-16 2.177004e-16 -2.839175e-15
-3.656741e-15
E:\\R-3.1.2\\scripts\\mPDBS\\summary_pdb_93\\813cc65a2298feba.final.pdb
-7.240887e-16 -5.245917e-16 -5.398672e-16 2.177004e-16 -2.839175e-15
-3.656741e-15
E:\\R-3.1.2\\scripts\\mPDBS\\summary_pdb_93\\83c0eefcfe12d3f9.final.pdb
-3.565132e-15 -3.336392e-15 1.030491e-15 4.021515e-15 -6.066299e-17
-1.041428e-15
E:\\R-3.1.2\\scripts\\mPDBS\\summary_pdb_93\\86d50469ef569760.final.pdb
5.724831e-15 4.847987e-15 3.006932e-15 -3.089652e-15 3.044144e-15
-4.210517e-15
E:\\R-3.1.2\\scripts\\mPDBS\\summary_pdb_93\\89d0666e1402203d.final.pdb
3.048919e-15 1.958150e-15 -8.041874e-16 -1.253521e-15 4.943503e-15
8.060223e-16
E:\\R-3.1.2\\scripts\\mPDBS\\summary_pdb_93\\8b34852716dadc62.final.pdb
3.279653e-15 5.632809e-15 6.216881e-15 5.577062e-16 8.199738e-15
-6.147804e-15
E:\\R-3.1.2\\scripts\\mPDBS\\summary_pdb_93\\8b6ef9ac3fe74395.final.pdb
9.349962e-15 5.405798e-15 -6.823957e-16 -4.496412e-16 -2.231136e-15
-8.875111e-15
E:\\R-3.1.2\\scripts\\mPDBS\\summary_pdb_93\\98bbe0d5986c138f.final.pdb
1.633243e-15 1.790269e-15 7.667822e-16 3.672184e-16 -6.464781e-16
-7.297213e-15
E:\\R-3.1.2\\scripts\\mPDBS\\summary_pdb_93\\9c22f4909d67e2fa.final.pdb
6.190953e-15 3.943715e-15 3.269586e-15 -6.316569e-15 6.204968e-15
4.103761e-15
E:\\R-3.1.2\\scripts\\mPDBS\\summary_pdb_93\\9dda8ed0d81fb3ef.final.pdb
1.801741e-14 9.069896e-15 1.426189e-14 3.514584e-15 -7.651375e-15
-8.327947e-15
E:\\R-3.1.2\\scripts\\mPDBS\\summary_pdb_93\\9f752b3616e6033b.final.pdb
1.368386e-15 1.160203e-15 1.897470e-15 -1.689242e-15 6.252240e-16
-2.036748e-15
E:\\R-3.1.2\\scripts\\mPDBS\\summary_pdb_93\\a02c70083816d6ba.final.pdb
5.274647e-15 4.949314e-15 4.105739e-15 -2.473046e-15 7.704346e-16
-1.173740e-15
E:\\R-3.1.2\\scripts\\mPDBS\\summary_pdb_93\\a0c997d891e4e082.final.pdb
5.011960e-15 2.393241e-15 4.043094e-15 -5.894544e-15 -4.947076e-15
2.414775e-15
E:\\R-3.1.2\\scripts\\mPDBS\\summary_pdb_93\\ab8039a2d823c616.final.pdb
1.640091e-15 8.622540e-16 1.319145e-15 -4.267070e-17 8.965978e-16
-7.458917e-16
E:\\R-3.1.2\\scripts\\mPDBS\\summary_pdb_93\\acf4d74b9c71d4dc.final.pdb
-7.240887e-16 -5.245917e-16 -5.398672e-16 2.177004e-16 -2.839175e-15
-3.656741e-15
E:\\R-3.1.2\\scripts\\mPDBS\\summary_pdb_93\\ad8a823e4b4c668e.final.pdb
1.631781e-15 1.703930e-15 2.477376e-15 -3.675782e-16 1.321449e-15
-8.754230e-16
E:\\R-3.1.2\\scripts\\mPDBS\\summary_pdb_93\\b16ababd03b7b857.final.pdb
6.190953e-15 3.943715e-15 3.269586e-15 -6.316569e-15 6.204968e-15
4.103761e-15
E:\\R-3.1.2\\scripts\\mPDBS\\summary_pdb_93\\b226923e697d3c63.final.pdb
4.271899e-15 2.814143e-15 -2.479930e-15 -1.810801e-15 6.269012e-16
-1.575102e-15

E:\\R-3.1.2\\scripts\\mPDBS\\summary_pdb_93\\b28e48f6fa27a165.final.pdb
1.633243e-15 1.790269e-15 7.667822e-16 3.672184e-16 -6.464781e-16
-7.297213e-15
E:\\R-3.1.2\\scripts\\mPDBS\\summary_pdb_93\\b42c4924e3cd1aec.final.pdb
-8.527324e-15 1.211444e-15 -4.117076e-15 -4.219247e-15 1.176294e-15
-3.371604e-16
E:\\R-3.1.2\\scripts\\mPDBS\\summary_pdb_93\\b4dd239fcabff1df.final.pdb
1.633243e-15 1.790269e-15 7.667822e-16 3.672184e-16 -6.464781e-16
-7.297213e-15
E:\\R-3.1.2\\scripts\\mPDBS\\summary_pdb_93\\b669942ef97459a2.final.pdb
3.279653e-15 5.632809e-15 6.216881e-15 5.577062e-16 8.199738e-15
-6.147804e-15
E:\\R-3.1.2\\scripts\\mPDBS\\summary_pdb_93\\b99f4d9d87c1017b.final.pdb
1.633243e-15 1.790269e-15 7.667822e-16 3.672184e-16 -6.464781e-16
-7.297213e-15
E:\\R-3.1.2\\scripts\\mPDBS\\summary_pdb_93\\b9d2cbf3975403e7.final.pdb
1.633243e-15 1.790269e-15 7.667822e-16 3.672184e-16 -6.464781e-16
-7.297213e-15
E:\\R-3.1.2\\scripts\\mPDBS\\summary_pdb_93\\bc88c8a505f3a288.final.pdb
2.294607e-15 9.806797e-16 1.370776e-16 -1.230679e-15 1.466158e-15
-9.557147e-16
E:\\R-3.1.2\\scripts\\mPDBS\\summary_pdb_93\\bdac0e64f4ef427e.final.pdb
1.315283e-14 1.045952e-14 -1.169138e-15 -9.363177e-15 4.088091e-15
-5.095188e-16
E:\\R-3.1.2\\scripts\\mPDBS\\summary_pdb_93\\ca2a1f55fc9d0681.final.pdb
3.279653e-15 5.632809e-15 6.216881e-15 5.577062e-16 8.199738e-15
-6.147804e-15
E:\\R-3.1.2\\scripts\\mPDBS\\summary_pdb_93\\ce34248de3e81a3c.final.pdb
5.260567e-15 2.521685e-15 -5.614170e-17 4.404324e-15 -3.122706e-15
-2.289978e-15
E:\\R-3.1.2\\scripts\\mPDBS\\summary_pdb_93\\d02e697772494a61.final.pdb
-3.615596e-15 -7.071659e-15 -4.580069e-15 -2.978312e-16 1.019320e-14
4.982027e-15
E:\\R-3.1.2\\scripts\\mPDBS\\summary_pdb_93\\d04221245d015047.final.pdb
1.821803e-15 7.261714e-16 2.745521e-15 2.429082e-16 2.110647e-15
-6.105832e-16
E:\\R-3.1.2\\scripts\\mPDBS\\summary_pdb_93\\d678141e4018054a.final.pdb
-9.351710e-16 -5.837422e-15 2.741716e-15 4.651282e-15 8.493113e-15
8.521216e-15
E:\\R-3.1.2\\scripts\\mPDBS\\summary_pdb_93\\d71b40af0f0efe39.final.pdb
-5.557717e-15 -6.892234e-15 -3.197997e-15 -2.702165e-15 4.401802e-15
5.495997e-15
E:\\R-3.1.2\\scripts\\mPDBS\\summary_pdb_93\\dc0809eff10197e2.final.pdb
4.136540e-15 1.122409e-15 4.163986e-15 -8.712811e-15 -4.022449e-15
-1.167276e-14
E:\\R-3.1.2\\scripts\\mPDBS\\summary_pdb_93\\e1163918d3707839.final.pdb
-4.691851e-15 -3.991425e-15 8.618397e-15 -4.600019e-15 7.048824e-16
-6.458566e-15
E:\\R-3.1.2\\scripts\\mPDBS\\summary_pdb_93\\e4b48b06824ef848.final.pdb
5.728815e-15 -9.998046e-17 2.016322e-15 8.045010e-16 3.611222e-15
6.777100e-15
E:\\R-3.1.2\\scripts\\mPDBS\\summary_pdb_93\\e529e19ad281fea2.final.pdb
-1.750093e-15 -3.928847e-15 -6.538384e-15 -1.056556e-15 -6.352853e-15
2.902716e-15

E:\\R-3.1.2\\scripts\\mPDBS\\summary_pdb_93\\e5602082718ec3a1.final.pdb
-7.240887e-16 -5.245917e-16 -5.398672e-16 2.177004e-16 -2.839175e-15
-3.656741e-15
E:\\R-3.1.2\\scripts\\mPDBS\\summary_pdb_93\\e5d589d996d0a960.final.pdb
8.905907e-15 7.160777e-15 2.075922e-15 1.580170e-15 -5.562167e-15
5.956272e-15
E:\\R-3.1.2\\scripts\\mPDBS\\summary_pdb_93\\e9df9e72307a2fbd.final.pdb
5.274647e-15 4.949314e-15 4.105739e-15 -2.473046e-15 7.704346e-16
-1.173740e-15
E:\\R-3.1.2\\scripts\\mPDBS\\summary_pdb_93\\ea00f8694090cae7.final.pdb
-7.240887e-16 -5.245917e-16 -5.398672e-16 2.177004e-16 -2.839175e-15
-3.656741e-15
E:\\R-3.1.2\\scripts\\mPDBS\\summary_pdb_93\\eba517be32db050e.final.pdb
1.633243e-15 1.790269e-15 7.667822e-16 3.672184e-16 -6.464781e-16
-7.297213e-15
E:\\R-3.1.2\\scripts\\mPDBS\\summary_pdb_93\\ec04b9545899c29d.final.pdb
2.194134e-15 1.262526e-15 2.228977e-15 -2.074824e-16 1.731663e-15
-1.121689e-15
E:\\R-3.1.2\\scripts\\mPDBS\\summary_pdb_93\\ee6862cbcad52078.final.pdb
2.247769e-15 1.041302e-15 1.074696e-15 -9.971416e-16 1.450369e-15
-1.093408e-15
E:\\R-3.1.2\\scripts\\mPDBS\\summary_pdb_93\\ef6a42fc83b81e5d.final.pdb
2.327889e-15 1.068108e-15 1.577870e-15 -1.498138e-16 1.535110e-15
-1.110254e-15
E:\\R-3.1.2\\scripts\\mPDBS\\summary_pdb_93\\ef8e88d4ad93a0ad.final.pdb
1.821803e-15 7.261714e-16 2.745521e-15 2.429082e-16 2.110647e-15
-6.105832e-16
E:\\R-3.1.2\\scripts\\mPDBS\\summary_pdb_93\\f14fcae8deec4fa3.final.pdb
1.368386e-15 1.160203e-15 1.897470e-15 -1.689242e-15 6.252240e-16
-2.036748e-15
E:\\R-3.1.2\\scripts\\mPDBS\\summary_pdb_93\\f83584e22003fb92.final.pdb
8.792881e-15 -2.796526e-15 4.627843e-15 4.385489e-15 -2.699618e-16
7.586714e-15
E:\\R-3.1.2\\scripts\\mPDBS\\summary_pdb_93\\f8e2bf87e52caa29.final.pdb
-1.750093e-15 -3.928847e-15 -6.538384e-15 -1.056556e-15 -6.352853e-15
2.902716e-15

[,637] [,638] [,639] [,640] [,641] [,642]
E:\\R-3.1.2\\scripts\\40.1ns_data\\NAT0.pdb
-6.248499e-15 7.199058e-16 -2.984236e-15 4.810724e-15 5.822602e-15
4.652893e-15
E:\\R-3.1.2\\scripts\\40.1ns_data\\LJP6.pdb
-4.376650e-15 2.142370e-15 -2.726622e-15 5.004567e-15 7.330959e-15
4.621695e-15
E:\\R-3.1.2\\scripts\\40.1ns_data\\3WY9.pdb
-4.940608e-15 1.681431e-15 -3.623625e-15 6.183807e-15 3.348738e-15
4.229261e-15
E:\\R-3.1.2\\scripts\\40.1ns_data\\ZSP9.pdb
-5.507524e-15 8.339847e-16 -3.253285e-16 5.698276e-15 6.578813e-15
4.220213e-15
E:\\R-3.1.2\\scripts\\mPDBS\\summary_pdb_93\\013d855ad26c877c.final.pdb
-5.130455e-15 1.034526e-14 -2.081339e-15 -9.482246e-16 6.293881e-15
2.999577e-15
E:\\R-3.1.2\\scripts\\mPDBS\\summary_pdb_93\\0a2a705342ca0d54.final.pdb

-4.750487e-15 1.483998e-15 -2.490598e-15 5.595815e-15 5.926157e-15
3.397810e-15
E:\\R-3.1.2\\scripts\\mPDBS\\summary_pdb_93\\0a59fceadb4194d9.final.pdb
-2.683117e-15 2.085051e-15 1.339952e-15 8.000157e-15 6.087684e-15
-8.910590e-16
E:\\R-3.1.2\\scripts\\mPDBS\\summary_pdb_93\\0dba59f3306825e8.final.pdb
-2.590820e-15 -1.662939e-16 -1.640719e-15 6.524964e-15 6.711515e-15
1.425315e-15
E:\\R-3.1.2\\scripts\\mPDBS\\summary_pdb_93\\126f26a78b57339c.final.pdb
-8.437705e-15 -2.067117e-15 -5.443016e-15 1.039253e-14 4.839965e-15
1.070825e-14
E:\\R-3.1.2\\scripts\\mPDBS\\summary_pdb_93\\12f06f3e342df706.final.pdb
-5.096565e-15 1.755699e-15 -2.052580e-15 5.965311e-15 5.543109e-15
3.260984e-15
E:\\R-3.1.2\\scripts\\mPDBS\\summary_pdb_93\\18618a2a251b867b.final.pdb
-1.151961e-14 7.437272e-15 -8.526448e-15 5.281628e-15 5.496133e-15
9.214410e-15
E:\\R-3.1.2\\scripts\\mPDBS\\summary_pdb_93\\1f305f598641eeb2.final.pdb
-4.882933e-15 1.698879e-15 -2.954207e-15 5.698059e-15 5.157021e-15
3.136681e-15
E:\\R-3.1.2\\scripts\\mPDBS\\summary_pdb_93\\1f8a00b551be0a39.final.pdb
-1.151961e-14 7.437272e-15 -8.526448e-15 5.281628e-15 5.496133e-15
9.214410e-15
E:\\R-3.1.2\\scripts\\mPDBS\\summary_pdb_93\\219f45328d47579a.final.pdb
-6.609230e-15 -1.061833e-14 -1.711057e-14 5.293988e-15 6.921435e-15
1.609796e-14
E:\\R-3.1.2\\scripts\\mPDBS\\summary_pdb_93\\238dd3918e54336d.final.pdb
-1.041782e-14 -1.370518e-15 -1.091218e-14 1.208196e-14 1.260992e-14
1.301415e-15
E:\\R-3.1.2\\scripts\\mPDBS\\summary_pdb_93\\2b3027889e2fdc42.final.pdb
-4.355824e-15 1.873198e-15 -1.805536e-15 5.966193e-15 6.316234e-15
3.357713e-15
E:\\R-3.1.2\\scripts\\mPDBS\\summary_pdb_93\\2fcb3b46e33793b0.final.pdb
-2.549730e-15 2.997320e-15 -1.955006e-15 2.596373e-15 5.683509e-15
1.040268e-15
E:\\R-3.1.2\\scripts\\mPDBS\\summary_pdb_93\\34eb328251842006.final.pdb
-7.108202e-15 3.512017e-15 4.506145e-15 2.647673e-15 1.540616e-14
5.294474e-15
E:\\R-3.1.2\\scripts\\mPDBS\\summary_pdb_93\\354b32871797b7ab.final.pdb
2.878609e-15 9.159736e-15 7.467533e-15 -1.360855e-15 1.879224e-15
1.054786e-14
E:\\R-3.1.2\\scripts\\mPDBS\\summary_pdb_93\\397368adbc32d5de.final.pdb
-8.437705e-15 -2.067117e-15 -5.443016e-15 1.039253e-14 4.839965e-15
1.070825e-14
E:\\R-3.1.2\\scripts\\mPDBS\\summary_pdb_93\\411ca13cd3df6edd.final.pdb
-5.950655e-15 -2.163049e-15 -6.680580e-16 4.083898e-15 7.957515e-15
1.103618e-14
E:\\R-3.1.2\\scripts\\mPDBS\\summary_pdb_93\\42357eee6642bf12.final.pdb
-4.269703e-15 -2.200099e-15 -6.187461e-15 1.160725e-14 5.305526e-15
2.738774e-15
E:\\R-3.1.2\\scripts\\mPDBS\\summary_pdb_93\\44342d1f75a7fcfc.final.pdb
-1.127543e-14 2.104216e-14 -2.527968e-15 3.916174e-16 3.298100e-15
6.676187e-16
E:\\R-3.1.2\\scripts\\mPDBS\\summary_pdb_93\\448ef21dffe8cdfb.final.pdb

-9.760975e-15 4.737247e-15 6.375136e-15 2.095038e-15 3.602709e-15
5.802951e-15
E:\\R-3.1.2\\scripts\\mPDBS\\summary_pdb_93\\472a40b3e8f113c7.final.pdb
-2.590820e-15 -1.662939e-16 -1.640719e-15 6.524964e-15 6.711515e-15
1.425315e-15
E:\\R-3.1.2\\scripts\\mPDBS\\summary_pdb_93\\48a26225951ea153.final.pdb
-6.552851e-15 8.707574e-15 3.704813e-15 -2.330989e-15 9.389730e-15
3.195516e-15
E:\\R-3.1.2\\scripts\\mPDBS\\summary_pdb_93\\4ab4a5c7c6682ce6.final.pdb
-1.715652e-15 -3.022082e-15 -3.885234e-15 2.988730e-15 6.865689e-15
-7.544506e-15
E:\\R-3.1.2\\scripts\\mPDBS\\summary_pdb_93\\4eaa45b62cb6a25f.final.pdb
-6.840179e-15 -4.172390e-16 -6.089548e-15 7.683993e-15 4.864358e-15
1.576524e-15
E:\\R-3.1.2\\scripts\\mPDBS\\summary_pdb_93\\58c371611d85ac96.final.pdb
-8.437705e-15 -2.067117e-15 -5.443016e-15 1.039253e-14 4.839965e-15
1.070825e-14
E:\\R-3.1.2\\scripts\\mPDBS\\summary_pdb_93\\59a647f04822d30a.final.pdb
1.034171e-15 -4.082712e-15 -4.371111e-15 5.122208e-15 4.121226e-15
3.921960e-15
E:\\R-3.1.2\\scripts\\mPDBS\\summary_pdb_93\\59b091fe385e05fc.final.pdb
-2.683117e-15 2.085051e-15 1.339952e-15 8.000157e-15 6.087684e-15
-8.910590e-16
E:\\R-3.1.2\\scripts\\mPDBS\\summary_pdb_93\\5c35ac30f930d2db.final.pdb
-6.840179e-15 -4.172390e-16 -6.089548e-15 7.683993e-15 4.864358e-15
1.576524e-15
E:\\R-3.1.2\\scripts\\mPDBS\\summary_pdb_93\\5f60752431cf2dcb.final.pdb
-9.476308e-15 5.276754e-15 -2.595982e-15 3.612159e-15 7.626620e-15
-1.877484e-15
E:\\R-3.1.2\\scripts\\mPDBS\\summary_pdb_93\\626456dd6ff2868d.final.pdb
-2.590820e-15 -1.662939e-16 -1.640719e-15 6.524964e-15 6.711515e-15
1.425315e-15
E:\\R-3.1.2\\scripts\\mPDBS\\summary_pdb_93\\6583d53ff3c8f7ef.final.pdb
1.034171e-15 -4.082712e-15 -4.371111e-15 5.122208e-15 4.121226e-15
3.921960e-15
E:\\R-3.1.2\\scripts\\mPDBS\\summary_pdb_93\\69760178986bef01.final.pdb
-9.858874e-15 3.300182e-15 -1.438511e-14 -1.912718e-15 -2.553167e-15
7.855193e-16
E:\\R-3.1.2\\scripts\\mPDBS\\summary_pdb_93\\6a9d87d47926b1c8.final.pdb
-4.518965e-15 1.482127e-15 -2.379494e-15 5.658470e-15 6.154018e-15
3.348690e-15
E:\\R-3.1.2\\scripts\\mPDBS\\summary_pdb_93\\6bb3547ea982ffc4.final.pdb
-1.152472e-14 4.593171e-15 -2.322956e-15 -1.769774e-16 7.625292e-15
1.152587e-14
E:\\R-3.1.2\\scripts\\mPDBS\\summary_pdb_93\\716d9624611d1c42.final.pdb
-5.727570e-15 1.545580e-15 2.822076e-17 3.763947e-15 5.401620e-15
1.024693e-14
E:\\R-3.1.2\\scripts\\mPDBS\\summary_pdb_93\\75f27e70ad0f1be4.final.pdb
1.034171e-15 -4.082712e-15 -4.371111e-15 5.122208e-15 4.121226e-15
3.921960e-15
E:\\R-3.1.2\\scripts\\mPDBS\\summary_pdb_93\\769f910fffb22872.final.pdb
-4.902078e-15 1.699457e-15 -2.245231e-15 6.289503e-15 6.287926e-15
3.101461e-15
E:\\R-3.1.2\\scripts\\mPDBS\\summary_pdb_93\\791f8c80dd5c3135.final.pdb

-2.683117e-15 2.085051e-15 1.339952e-15 8.000157e-15 6.087684e-15
-8.910590e-16
E:\\R-3.1.2\\scripts\\mPDBS\\summary_pdb_93\\7fad98e66a3f6099.final.pdb
3.299266e-16 1.212582e-15 5.842610e-15 3.024135e-15 4.753925e-15
-1.062525e-15
E:\\R-3.1.2\\scripts\\mPDBS\\summary_pdb_93\\804b89c94a633196.final.pdb
-2.549730e-15 2.997320e-15 -1.955006e-15 2.596373e-15 5.683509e-15
1.040268e-15
E:\\R-3.1.2\\scripts\\mPDBS\\summary_pdb_93\\813cc65a2298feba.final.pdb
-2.549730e-15 2.997320e-15 -1.955006e-15 2.596373e-15 5.683509e-15
1.040268e-15
E:\\R-3.1.2\\scripts\\mPDBS\\summary_pdb_93\\83c0eefcfe12d3f9.final.pdb
-5.950655e-15 -2.163049e-15 -6.680580e-16 4.083898e-15 7.957515e-15
1.103618e-14
E:\\R-3.1.2\\scripts\\mPDBS\\summary_pdb_93\\86d50469ef569760.final.pdb
-2.590820e-15 -1.662939e-16 -1.640719e-15 6.524964e-15 6.711515e-15
1.425315e-15
E:\\R-3.1.2\\scripts\\mPDBS\\summary_pdb_93\\89d0666e1402203d.final.pdb
1.034171e-15 -4.082712e-15 -4.371111e-15 5.122208e-15 4.121226e-15
3.921960e-15
E:\\R-3.1.2\\scripts\\mPDBS\\summary_pdb_93\\8b34852716dadc62.final.pdb
-5.649680e-15 -7.021576e-15 -6.173853e-15 1.259185e-14 4.535990e-15
4.057819e-15
E:\\R-3.1.2\\scripts\\mPDBS\\summary_pdb_93\\8b6ef9ac3fe74395.final.pdb
2.160403e-15 3.660956e-15 -8.259197e-16 1.243188e-14 2.475901e-15
-3.249272e-15
E:\\R-3.1.2\\scripts\\mPDBS\\summary_pdb_93\\98bbe0d5986c138f.final.pdb
-2.683117e-15 2.085051e-15 1.339952e-15 8.000157e-15 6.087684e-15
-8.910590e-16
E:\\R-3.1.2\\scripts\\mPDBS\\summary_pdb_93\\9c22f4909d67e2fa.final.pdb
-8.330262e-15 7.722923e-15 -5.729981e-15 5.387543e-15 4.143834e-15
4.384598e-15
E:\\R-3.1.2\\scripts\\mPDBS\\summary_pdb_93\\9dda8ed0d81fb3ef.final.pdb
-4.536026e-15 1.103280e-14 5.626591e-16 9.976832e-15 4.724259e-15
-6.789090e-15
E:\\R-3.1.2\\scripts\\mPDBS\\summary_pdb_93\\9f752b3616e6033b.final.pdb
-3.817097e-15 3.220239e-16 -3.006906e-15 6.717309e-15 5.634984e-15
2.132235e-15
E:\\R-3.1.2\\scripts\\mPDBS\\summary_pdb_93\\a02c70083816d6ba.final.pdb
-6.840179e-15 -4.172390e-16 -6.089548e-15 7.683993e-15 4.864358e-15
1.576524e-15
E:\\R-3.1.2\\scripts\\mPDBS\\summary_pdb_93\\a0c997d891e4e082.final.pdb
-1.022102e-15 -4.987023e-15 -2.031958e-16 2.401666e-15 4.050379e-15
4.922694e-15
E:\\R-3.1.2\\scripts\\mPDBS\\summary_pdb_93\\ab8039a2d823c616.final.pdb
-4.652444e-15 1.378979e-15 -1.162281e-15 5.475596e-15 6.834284e-15
3.494366e-15
E:\\R-3.1.2\\scripts\\mPDBS\\summary_pdb_93\\acf4d74b9c71d4dc.final.pdb
-2.549730e-15 2.997320e-15 -1.955006e-15 2.596373e-15 5.683509e-15
1.040268e-15
E:\\R-3.1.2\\scripts\\mPDBS\\summary_pdb_93\\ad8a823e4b4c668e.final.pdb
-4.845494e-15 1.767487e-15 -1.336998e-15 5.525978e-15 6.310348e-15
3.539804e-15
E:\\R-3.1.2\\scripts\\mPDBS\\summary_pdb_93\\b16ababd03b7b857.final.pdb

-8.330262e-15 7.722923e-15 -5.729981e-15 5.387543e-15 4.143834e-15
4.384598e-15
E:\\R-3.1.2\\scripts\\mPDBS\\summary_pdb_93\\b226923e697d3c63.final.pdb
-2.852044e-15 7.873076e-16 -6.704965e-15 9.844993e-15 -1.927212e-15
8.083778e-15
E:\\R-3.1.2\\scripts\\mPDBS\\summary_pdb_93\\b28e48f6fa27a165.final.pdb
-2.683117e-15 2.085051e-15 1.339952e-15 8.000157e-15 6.087684e-15
-8.910590e-16
E:\\R-3.1.2\\scripts\\mPDBS\\summary_pdb_93\\b42c4924e3cd1aec.final.pdb
-8.628673e-15 2.284591e-15 2.275265e-15 6.211615e-15 1.294956e-14
2.091176e-15
E:\\R-3.1.2\\scripts\\mPDBS\\summary_pdb_93\\b4dd239fcabff1df.final.pdb
-2.683117e-15 2.085051e-15 1.339952e-15 8.000157e-15 6.087684e-15
-8.910590e-16
E:\\R-3.1.2\\scripts\\mPDBS\\summary_pdb_93\\b669942ef97459a2.final.pdb
-5.649680e-15 -7.021576e-15 -6.173853e-15 1.259185e-14 4.535990e-15
4.057819e-15
E:\\R-3.1.2\\scripts\\mPDBS\\summary_pdb_93\\b99f4d9d87c1017b.final.pdb
-2.683117e-15 2.085051e-15 1.339952e-15 8.000157e-15 6.087684e-15
-8.910590e-16
E:\\R-3.1.2\\scripts\\mPDBS\\summary_pdb_93\\b9d2cbf3975403e7.final.pdb
-2.683117e-15 2.085051e-15 1.339952e-15 8.000157e-15 6.087684e-15
-8.910590e-16
E:\\R-3.1.2\\scripts\\mPDBS\\summary_pdb_93\\bc88c8a505f3a288.final.pdb
-4.831572e-15 7.846587e-16 -2.379512e-15 5.583687e-15 6.646373e-15
3.335812e-15
E:\\R-3.1.2\\scripts\\mPDBS\\summary_pdb_93\\bdac0e64f4ef427e.final.pdb
-2.087859e-15 1.092479e-14 -5.737137e-15 1.000277e-14 5.574110e-17
-1.315411e-14
E:\\R-3.1.2\\scripts\\mPDBS\\summary_pdb_93\\ca2a1f55fc9d0681.final.pdb
-5.649680e-15 -7.021576e-15 -6.173853e-15 1.259185e-14 4.535990e-15
4.057819e-15
E:\\R-3.1.2\\scripts\\mPDBS\\summary_pdb_93\\ce34248de3e81a3c.final.pdb
8.641901e-16 -1.553425e-15 7.922352e-16 1.167652e-14 6.678929e-15
-2.238217e-15
E:\\R-3.1.2\\scripts\\mPDBS\\summary_pdb_93\\d02e697772494a61.final.pdb
-1.344344e-15 -8.108184e-15 -8.642298e-15 1.045650e-15 -3.708251e-17
1.493287e-14
E:\\R-3.1.2\\scripts\\mPDBS\\summary_pdb_93\\d04221245d015047.final.pdb
-5.339828e-15 1.918911e-15 -9.421880e-16 4.861504e-15 8.038942e-15
1.657944e-15
E:\\R-3.1.2\\scripts\\mPDBS\\summary_pdb_93\\d678141e4018054a.final.pdb
-1.624026e-14 7.607060e-15 2.096666e-15 1.339837e-15 1.307470e-14
1.648903e-14
E:\\R-3.1.2\\scripts\\mPDBS\\summary_pdb_93\\d71b40af0f0efe39.final.pdb
-7.576206e-15 5.152782e-15 3.833092e-15 5.091367e-15 7.546365e-15
1.184428e-14
E:\\R-3.1.2\\scripts\\mPDBS\\summary_pdb_93\\dc0809eff10197e2.final.pdb
2.944636e-15 1.144642e-15 7.543644e-16 8.035582e-15 7.874713e-15
-1.777039e-15
E:\\R-3.1.2\\scripts\\mPDBS\\summary_pdb_93\\e1163918d3707839.final.pdb
-9.556750e-15 -6.174788e-15 6.829781e-15 6.955811e-15 8.878597e-15
5.280899e-15
E:\\R-3.1.2\\scripts\\mPDBS\\summary_pdb_93\\e4b48b06824ef848.final.pdb

-1.151961e-14 7.437272e-15 -8.526448e-15 5.281628e-15 5.496133e-15
9.214410e-15
E:\\R-3.1.2\\scripts\\mPDBS\\summary_pdb_93\\e529e19ad281fea2.final.pdb
-5.130455e-15 1.034526e-14 -2.081339e-15 -9.482246e-16 6.293881e-15
2.999577e-15
E:\\R-3.1.2\\scripts\\mPDBS\\summary_pdb_93\\e5602082718ec3a1.final.pdb
-2.549730e-15 2.997320e-15 -1.955006e-15 2.596373e-15 5.683509e-15
1.040268e-15
E:\\R-3.1.2\\scripts\\mPDBS\\summary_pdb_93\\e5d589d996d0a960.final.pdb
-3.544667e-16 2.160243e-16 3.149950e-15 6.386638e-15 6.541245e-15
-4.020593e-15
E:\\R-3.1.2\\scripts\\mPDBS\\summary_pdb_93\\e9df9e72307a2fbd.final.pdb
-6.840179e-15 -4.172390e-16 -6.089548e-15 7.683993e-15 4.864358e-15
1.576524e-15
E:\\R-3.1.2\\scripts\\mPDBS\\summary_pdb_93\\ea00f8694090cae7.final.pdb
-2.549730e-15 2.997320e-15 -1.955006e-15 2.596373e-15 5.683509e-15
1.040268e-15
E:\\R-3.1.2\\scripts\\mPDBS\\summary_pdb_93\\eba517be32db050e.final.pdb
-2.683117e-15 2.085051e-15 1.339952e-15 8.000157e-15 6.087684e-15
-8.910590e-16
E:\\R-3.1.2\\scripts\\mPDBS\\summary_pdb_93\\ec04b9545899c29d.final.pdb
-4.731616e-15 1.686979e-15 -2.370108e-15 5.947937e-15 5.679225e-15
2.782247e-15
E:\\R-3.1.2\\scripts\\mPDBS\\summary_pdb_93\\ee6862cbcad52078.final.pdb
-4.604323e-15 1.413713e-15 -2.354142e-15 5.890733e-15 6.265493e-15
3.250593e-15
E:\\R-3.1.2\\scripts\\mPDBS\\summary_pdb_93\\ef6a42fc83b81e5d.final.pdb
-5.067310e-15 1.393712e-15 -2.193407e-15 5.948630e-15 5.860642e-15
3.226145e-15
E:\\R-3.1.2\\scripts\\mPDBS\\summary_pdb_93\\ef8e88d4ad93a0ad.final.pdb
-5.339828e-15 1.918911e-15 -9.421880e-16 4.861504e-15 8.038942e-15
1.657944e-15
E:\\R-3.1.2\\scripts\\mPDBS\\summary_pdb_93\\f14fcae8deec4fa3.final.pdb
-3.817097e-15 3.220239e-16 -3.006906e-15 6.717309e-15 5.634984e-15
2.132235e-15
E:\\R-3.1.2\\scripts\\mPDBS\\summary_pdb_93\\f83584e22003fb92.final.pdb
-6.552851e-15 8.707574e-15 3.704813e-15 -2.330989e-15 9.389730e-15
3.195516e-15
E:\\R-3.1.2\\scripts\\mPDBS\\summary_pdb_93\\f8e2bf87e52caa29.final.pdb
-5.130455e-15 1.034526e-14 -2.081339e-15 -9.482246e-16 6.293881e-15
2.999577e-15

[,643]   [,644]   [,645]   [,646]   [,647]   [,648]
E:\\R-3.1.2\\scripts\\40.1ns_data\\NAT0.pdb
-1.197020e-15 -7.212276e-15 -1.943966e-15 6.360827e-15 -6.279134e-16
2.134891e-15
E:\\R-3.1.2\\scripts\\40.1ns_data\\LJP6.pdb
-1.084678e-15 -6.329721e-15 -1.742054e-15 5.916898e-15 -4.861027e-15
2.864463e-15
E:\\R-3.1.2\\scripts\\40.1ns_data\\3WY9.pdb
-1.086493e-15 -7.014767e-15 -2.499228e-15 6.995659e-15 3.280922e-16
3.814168e-15
E:\\R-3.1.2\\scripts\\40.1ns_data\\ZSP9.pdb
-2.553219e-15 -6.347765e-15 -1.186554e-15 7.921310e-15 -1.445413e-15

3.741213e-15
E:\\R-3.1.2\\scripts\\mPDBS\\summary_pdb_93\\013d855ad26c877c.final.pdb
-1.349576e-15 -4.086620e-15 3.481620e-15 4.718781e-15 -4.271195e-15
3.801732e-15
E:\\R-3.1.2\\scripts\\mPDBS\\summary_pdb_93\\0a2a705342ca0d54.final.pdb
-7.192107e-16 -6.539107e-15 -1.782836e-15 6.425689e-15 -1.566428e-15
2.048987e-15
E:\\R-3.1.2\\scripts\\mPDBS\\summary_pdb_93\\0a59fceadb4194d9.final.pdb
-4.031140e-15 -9.201640e-15 -7.251496e-16 3.705589e-15 -1.160436e-15
-2.556605e-15
E:\\R-3.1.2\\scripts\\mPDBS\\summary_pdb_93\\0dba59f3306825e8.final.pdb
-1.677439e-15 -6.284949e-15 -1.417239e-15 4.322831e-15 -6.664397e-16
4.068879e-15
E:\\R-3.1.2\\scripts\\mPDBS\\summary_pdb_93\\126f26a78b57339c.final.pdb
5.679524e-15 -4.525072e-15 -1.245702e-14 1.220975e-14 -3.021477e-16
7.548734e-15
E:\\R-3.1.2\\scripts\\mPDBS\\summary_pdb_93\\12f06f3e342df706.final.pdb
-8.430194e-17 -5.977924e-15 -1.948502e-15 7.064610e-15 -1.817963e-15
2.749815e-15
E:\\R-3.1.2\\scripts\\mPDBS\\summary_pdb_93\\18618a2a251b867b.final.pdb
6.815671e-15 -1.364427e-15 -7.610611e-15 8.152123e-15 -1.893280e-15
6.382638e-15
E:\\R-3.1.2\\scripts\\mPDBS\\summary_pdb_93\\1f305f598641eeb2.final.pdb
-8.509710e-16 -6.711245e-15 -2.261557e-15 6.944340e-15 -1.713305e-15
3.001846e-15
E:\\R-3.1.2\\scripts\\mPDBS\\summary_pdb_93\\1f8a00b551be0a39.final.pdb
6.815671e-15 -1.364427e-15 -7.610611e-15 8.152123e-15 -1.893280e-15
6.382638e-15
E:\\R-3.1.2\\scripts\\mPDBS\\summary_pdb_93\\219f45328d47579a.final.pdb
5.686781e-15 1.034963e-15 -3.256978e-15 4.239450e-15 -5.398365e-15
5.074444e-15
E:\\R-3.1.2\\scripts\\mPDBS\\summary_pdb_93\\238dd3918e54336d.final.pdb
6.577995e-15 7.686831e-16 -1.428375e-14 1.384472e-14 -1.420644e-15
6.712023e-15
E:\\R-3.1.2\\scripts\\mPDBS\\summary_pdb_93\\2b3027889e2fdc42.final.pdb
-7.477420e-16 -6.668944e-15 -2.010580e-15 6.293688e-15 -2.870873e-15
2.096791e-15
E:\\R-3.1.2\\scripts\\mPDBS\\summary_pdb_93\\2fcb3b46e33793b0.final.pdb
-4.292662e-15 -5.648293e-15 6.405121e-15 4.103079e-15 -3.600684e-15
1.287940e-15
E:\\R-3.1.2\\scripts\\mPDBS\\summary_pdb_93\\34eb328251842006.final.pdb
-5.594677e-15 -1.517880e-14 8.352525e-15 -3.616201e-15 4.734797e-16
-5.444398e-15
E:\\R-3.1.2\\scripts\\mPDBS\\summary_pdb_93\\354b32871797b7ab.final.pdb
-1.862027e-15 -1.462126e-14 9.945420e-15 5.670528e-15 -1.119804e-14
-3.649545e-15
E:\\R-3.1.2\\scripts\\mPDBS\\summary_pdb_93\\397368adbc32d5de.final.pdb
5.679524e-15 -4.525072e-15 -1.245702e-14 1.220975e-14 -3.021477e-16
7.548734e-15
E:\\R-3.1.2\\scripts\\mPDBS\\summary_pdb_93\\411ca13cd3df6edd.final.pdb
-1.140670e-15 -5.487397e-15 9.170271e-15 3.049669e-15 -4.138448e-15
4.080844e-15
E:\\R-3.1.2\\scripts\\mPDBS\\summary_pdb_93\\42357eee6642bf12.final.pdb
8.062856e-15 -5.990976e-15 -3.532450e-15 -4.821113e-15 -8.523005e-15

-5.903152e-15
E:\\R-3.1.2\\scripts\\mPDBS\\summary_pdb_93\\44342d1f75a7fcfc.final.pdb
-4.739369e-15 -1.058516e-14 -4.675065e-15 1.107940e-14 1.084724e-14
7.092575e-15
E:\\R-3.1.2\\scripts\\mPDBS\\summary_pdb_93\\448ef21dffe8cdfb.final.pdb
3.926457e-15 -1.183865e-14 -3.604233e-15 1.274916e-14 -1.446005e-14
-6.115860e-15
E:\\R-3.1.2\\scripts\\mPDBS\\summary_pdb_93\\472a40b3e8f113c7.final.pdb
-1.677439e-15 -6.284949e-15 -1.417239e-15 4.322831e-15 -6.664397e-16
4.068879e-15
E:\\R-3.1.2\\scripts\\mPDBS\\summary_pdb_93\\48a26225951ea153.final.pdb
-4.839087e-15 -9.872978e-15 6.520790e-15 6.799793e-15 7.980672e-16
6.909781e-15
E:\\R-3.1.2\\scripts\\mPDBS\\summary_pdb_93\\4ab4a5c7c6682ce6.final.pdb
-5.348114e-15 -1.218127e-14 1.288035e-14 7.160838e-15 -1.239317e-14
-1.342407e-14
E:\\R-3.1.2\\scripts\\mPDBS\\summary_pdb_93\\4eaa45b62cb6a25f.final.pdb
-8.224944e-16 -2.396660e-15 -6.118119e-15 9.675896e-15 1.944220e-15
6.939207e-15
E:\\R-3.1.2\\scripts\\mPDBS\\summary_pdb_93\\58c371611d85ac96.final.pdb
5.679524e-15 -4.525072e-15 -1.245702e-14 1.220975e-14 -3.021477e-16
7.548734e-15
E:\\R-3.1.2\\scripts\\mPDBS\\summary_pdb_93\\59a647f04822d30a.final.pdb
1.161216e-16 -8.827017e-15 -1.424194e-15 7.100380e-15 -8.370031e-15
3.394145e-15
E:\\R-3.1.2\\scripts\\mPDBS\\summary_pdb_93\\59b091fe385e05fc.final.pdb
-4.031140e-15 -9.201640e-15 -7.251496e-16 3.705589e-15 -1.160436e-15
-2.556605e-15
E:\\R-3.1.2\\scripts\\mPDBS\\summary_pdb_93\\5c35ac30f930d2db.final.pdb
-8.224944e-16 -2.396660e-15 -6.118119e-15 9.675896e-15 1.944220e-15
6.939207e-15
E:\\R-3.1.2\\scripts\\mPDBS\\summary_pdb_93\\5f60752431cf2dcb.final.pdb
-1.969513e-15 -8.558325e-15 -4.541914e-15 5.355257e-15 3.592639e-15
9.574218e-15
E:\\R-3.1.2\\scripts\\mPDBS\\summary_pdb_93\\626456dd6ff2868d.final.pdb
-1.677439e-15 -6.284949e-15 -1.417239e-15 4.322831e-15 -6.664397e-16
4.068879e-15
E:\\R-3.1.2\\scripts\\mPDBS\\summary_pdb_93\\6583d53ff3c8f7ef.final.pdb
1.161216e-16 -8.827017e-15 -1.424194e-15 7.100380e-15 -8.370031e-15
3.394145e-15
E:\\R-3.1.2\\scripts\\mPDBS\\summary_pdb_93\\69760178986bef01.final.pdb
8.500137e-16 -1.918127e-16 -4.442659e-15 1.187241e-14 1.864405e-15
7.061994e-15
E:\\R-3.1.2\\scripts\\mPDBS\\summary_pdb_93\\6a9d87d47926b1c8.final.pdb
-2.254751e-16 -6.825749e-15 -2.447665e-15 6.949419e-15 -2.064627e-15
1.938639e-15
E:\\R-3.1.2\\scripts\\mPDBS\\summary_pdb_93\\6bb3547ea982ffc4.final.pdb
2.439024e-15 -5.196769e-15 -2.215259e-15 8.912017e-15 2.086362e-15
1.272108e-14
E:\\R-3.1.2\\scripts\\mPDBS\\summary_pdb_93\\716d9624611d1c42.final.pdb
7.355789e-17 -7.884988e-16 2.795967e-15 9.639265e-15 -2.105927e-15
4.711620e-17
E:\\R-3.1.2\\scripts\\mPDBS\\summary_pdb_93\\75f27e70ad0f1be4.final.pdb
1.161216e-16 -8.827017e-15 -1.424194e-15 7.100380e-15 -8.370031e-15

3.394145e-15
E:\\R-3.1.2\\scripts\\mPDBS\\summary_pdb_93\\769f910fffb22872.final.pdb
1.815750e-17 -6.961512e-15 -2.063832e-15 6.692462e-15 -2.330431e-15
2.325120e-15
E:\\R-3.1.2\\scripts\\mPDBS\\summary_pdb_93\\791f8c80dd5c3135.final.pdb
-4.031140e-15 -9.201640e-15 -7.251496e-16 3.705589e-15 -1.160436e-15
-2.556605e-15
E:\\R-3.1.2\\scripts\\mPDBS\\summary_pdb_93\\7fad98e66a3f6099.final.pdb
-1.009851e-14 -9.045976e-15 8.888299e-15 1.309040e-15 3.196042e-15
4.535292e-15
E:\\R-3.1.2\\scripts\\mPDBS\\summary_pdb_93\\804b89c94a633196.final.pdb
-4.292662e-15 -5.648293e-15 6.405121e-15 4.103079e-15 -3.600684e-15
1.287940e-15
E:\\R-3.1.2\\scripts\\mPDBS\\summary_pdb_93\\813cc65a2298feba.final.pdb
-4.292662e-15 -5.648293e-15 6.405121e-15 4.103079e-15 -3.600684e-15
1.287940e-15
E:\\R-3.1.2\\scripts\\mPDBS\\summary_pdb_93\\83c0eefcfe12d3f9.final.pdb
-1.140670e-15 -5.487397e-15 9.170271e-15 3.049669e-15 -4.138448e-15
4.080844e-15
E:\\R-3.1.2\\scripts\\mPDBS\\summary_pdb_93\\86d50469ef569760.final.pdb
-1.677439e-15 -6.284949e-15 -1.417239e-15 4.322831e-15 -6.664397e-16
4.068879e-15
E:\\R-3.1.2\\scripts\\mPDBS\\summary_pdb_93\\89d0666e1402203d.final.pdb
1.161216e-16 -8.827017e-15 -1.424194e-15 7.100380e-15 -8.370031e-15
3.394145e-15
E:\\R-3.1.2\\scripts\\mPDBS\\summary_pdb_93\\8b34852716dadc62.final.pdb
-8.162766e-16 -8.782505e-15 -7.802264e-15 8.904577e-15 3.636583e-15
1.825704e-15
E:\\R-3.1.2\\scripts\\mPDBS\\summary_pdb_93\\8b6ef9ac3fe74395.final.pdb
-4.887651e-15 -6.921375e-15 -1.285235e-14 1.212708e-14 -8.797860e-16
2.465157e-15
E:\\R-3.1.2\\scripts\\mPDBS\\summary_pdb_93\\98bbe0d5986c138f.final.pdb
-4.031140e-15 -9.201640e-15 -7.251496e-16 3.705589e-15 -1.160436e-15
-2.556605e-15
E:\\R-3.1.2\\scripts\\mPDBS\\summary_pdb_93\\9c22f4909d67e2fa.final.pdb
3.365274e-15 -5.056319e-15 -1.563107e-14 7.975850e-15 5.678352e-15
9.271137e-15
E:\\R-3.1.2\\scripts\\mPDBS\\summary_pdb_93\\9dda8ed0d81fb3ef.final.pdb
-1.071202e-14 -1.324492e-14 4.021971e-15 3.104336e-15 6.293586e-15
-1.251445e-15
E:\\R-3.1.2\\scripts\\mPDBS\\summary_pdb_93\\9f752b3616e6033b.final.pdb
-7.418022e-16 -7.953445e-15 -3.877918e-15 6.534009e-15 -2.969779e-15
1.738095e-15
E:\\R-3.1.2\\scripts\\mPDBS\\summary_pdb_93\\a02c70083816d6ba.final.pdb
-8.224944e-16 -2.396660e-15 -6.118119e-15 9.675896e-15 1.944220e-15
6.939207e-15
E:\\R-3.1.2\\scripts\\mPDBS\\summary_pdb_93\\a0c997d891e4e082.final.pdb
-1.096986e-14 -7.351470e-15 9.779018e-15 7.825845e-15 2.885760e-16
7.427745e-15
E:\\R-3.1.2\\scripts\\mPDBS\\summary_pdb_93\\ab8039a2d823c616.final.pdb
-1.132675e-15 -6.004457e-15 -2.422160e-15 6.266437e-15 -1.541557e-15
2.861953e-15
E:\\R-3.1.2\\scripts\\mPDBS\\summary_pdb_93\\acf4d74b9c71d4dc.final.pdb
-4.292662e-15 -5.648293e-15 6.405121e-15 4.103079e-15 -3.600684e-15

1.287940e-15
E:\\R-3.1.2\\scripts\\mPDBS\\summary_pdb_93\\ad8a823e4b4c668e.final.pdb
4.481940e-16 -6.604311e-15 -1.991375e-15 6.737231e-15 -2.358199e-15
2.189137e-15
E:\\R-3.1.2\\scripts\\mPDBS\\summary_pdb_93\\b16ababd03b7b857.final.pdb
3.365274e-15 -5.056319e-15 -1.563107e-14 7.975850e-15 5.678352e-15
9.271137e-15
E:\\R-3.1.2\\scripts\\mPDBS\\summary_pdb_93\\b226923e697d3c63.final.pdb
3.463271e-15 7.471622e-17 -5.356812e-15 3.548750e-15 -4.742724e-15
4.990090e-15
E:\\R-3.1.2\\scripts\\mPDBS\\summary_pdb_93\\b28e48f6fa27a165.final.pdb
-4.031140e-15 -9.201640e-15 -7.251496e-16 3.705589e-15 -1.160436e-15
-2.556605e-15
E:\\R-3.1.2\\scripts\\mPDBS\\summary_pdb_93\\b42c4924e3cd1aec.final.pdb
-3.474322e-15 -7.906746e-15 -3.870154e-15 9.886671e-15 3.509355e-15
2.374127e-15
E:\\R-3.1.2\\scripts\\mPDBS\\summary_pdb_93\\b4dd239fcabff1df.final.pdb
-4.031140e-15 -9.201640e-15 -7.251496e-16 3.705589e-15 -1.160436e-15
-2.556605e-15
E:\\R-3.1.2\\scripts\\mPDBS\\summary_pdb_93\\b669942ef97459a2.final.pdb
-8.162766e-16 -8.782505e-15 -7.802264e-15 8.904577e-15 3.636583e-15
1.825704e-15
E:\\R-3.1.2\\scripts\\mPDBS\\summary_pdb_93\\b99f4d9d87c1017b.final.pdb
-4.031140e-15 -9.201640e-15 -7.251496e-16 3.705589e-15 -1.160436e-15
-2.556605e-15
E:\\R-3.1.2\\scripts\\mPDBS\\summary_pdb_93\\b9d2cbf3975403e7.final.pdb
-4.031140e-15 -9.201640e-15 -7.251496e-16 3.705589e-15 -1.160436e-15
-2.556605e-15
E:\\R-3.1.2\\scripts\\mPDBS\\summary_pdb_93\\bc88c8a505f3a288.final.pdb
-8.748105e-16 -6.629479e-15 -2.489363e-15 6.276340e-15 -2.130146e-15
2.128883e-15
E:\\R-3.1.2\\scripts\\mPDBS\\summary_pdb_93\\bdac0e64f4ef427e.final.pdb
-3.036658e-16 -9.991173e-15 -2.476612e-14 1.397387e-14 2.783099e-15
9.444983e-17
E:\\R-3.1.2\\scripts\\mPDBS\\summary_pdb_93\\ca2a1f55fc9d0681.final.pdb
-8.162766e-16 -8.782505e-15 -7.802264e-15 8.904577e-15 3.636583e-15
1.825704e-15
E:\\R-3.1.2\\scripts\\mPDBS\\summary_pdb_93\\ce34248de3e81a3c.final.pdb
-4.326195e-15 -6.203627e-15 -5.309907e-16 7.433123e-15 -1.709105e-15
-1.722104e-15
E:\\R-3.1.2\\scripts\\mPDBS\\summary_pdb_93\\d02e697772494a61.final.pdb
1.165380e-14 8.588888e-16 -6.435859e-15 7.199864e-15 -1.568701e-14
7.388565e-15
E:\\R-3.1.2\\scripts\\mPDBS\\summary_pdb_93\\d04221245d015047.final.pdb
-2.523489e-15 -8.758331e-15 -4.836065e-16 7.817171e-15 -1.290022e-15
1.800302e-15
E:\\R-3.1.2\\scripts\\mPDBS\\summary_pdb_93\\d678141e4018054a.final.pdb
9.414647e-15 -3.578650e-15 5.065847e-15 -1.712088e-15 -4.085305e-15
7.600261e-15
E:\\R-3.1.2\\scripts\\mPDBS\\summary_pdb_93\\d71b40af0f0efe39.final.pdb
4.174261e-15 -8.207197e-15 -4.559472e-15 3.048115e-15 -3.524667e-15
4.727314e-15
E:\\R-3.1.2\\scripts\\mPDBS\\summary_pdb_93\\dc0809eff10197e2.final.pdb
-7.415883e-15 -1.378863e-14 -7.258213e-15 1.325446e-14 -2.687345e-16

5.939020e-16
E:\\R-3.1.2\\scripts\\mPDBS\\summary_pdb_93\\e1163918d3707839.final.pdb
-2.373283e-16 -3.442195e-16 2.185766e-15 -3.479807e-15 -1.705224e-15
-2.836982e-15
E:\\R-3.1.2\\scripts\\mPDBS\\summary_pdb_93\\e4b48b06824ef848.final.pdb
6.815671e-15 -1.364427e-15 -7.610611e-15 8.152123e-15 -1.893280e-15
6.382638e-15
E:\\R-3.1.2\\scripts\\mPDBS\\summary_pdb_93\\e529e19ad281fea2.final.pdb
-1.349576e-15 -4.086620e-15 3.481620e-15 4.718781e-15 -4.271195e-15
3.801732e-15
E:\\R-3.1.2\\scripts\\mPDBS\\summary_pdb_93\\e5602082718ec3a1.final.pdb
-4.292662e-15 -5.648293e-15 6.405121e-15 4.103079e-15 -3.600684e-15
1.287940e-15
E:\\R-3.1.2\\scripts\\mPDBS\\summary_pdb_93\\e5d589d996d0a960.final.pdb
-6.997662e-15 -9.492541e-15 -7.163748e-15 2.173743e-14 -1.686870e-15
2.980398e-15
E:\\R-3.1.2\\scripts\\mPDBS\\summary_pdb_93\\e9df9e72307a2fbd.final.pdb
-8.224944e-16 -2.396660e-15 -6.118119e-15 9.675896e-15 1.944220e-15
6.939207e-15
E:\\R-3.1.2\\scripts\\mPDBS\\summary_pdb_93\\ea00f8694090cae7.final.pdb
-4.292662e-15 -5.648293e-15 6.405121e-15 4.103079e-15 -3.600684e-15
1.287940e-15
E:\\R-3.1.2\\scripts\\mPDBS\\summary_pdb_93\\eba517be32db050e.final.pdb
-4.031140e-15 -9.201640e-15 -7.251496e-16 3.705589e-15 -1.160436e-15
-2.556605e-15
E:\\R-3.1.2\\scripts\\mPDBS\\summary_pdb_93\\ec04b9545899c29d.final.pdb
-9.914994e-16 -6.482539e-15 -2.301964e-15 6.923004e-15 -9.159245e-16
2.355913e-15
E:\\R-3.1.2\\scripts\\mPDBS\\summary_pdb_93\\ee6862cbcad52078.final.pdb
-7.559819e-16 -6.562258e-15 -2.341912e-15 6.588265e-15 -2.041675e-15
2.236436e-15
E:\\R-3.1.2\\scripts\\mPDBS\\summary_pdb_93\\ef6a42fc83b81e5d.final.pdb
-1.132398e-15 -6.793244e-15 -1.820971e-15 6.497739e-15 -1.114389e-15
2.273945e-15
E:\\R-3.1.2\\scripts\\mPDBS\\summary_pdb_93\\ef8e88d4ad93a0ad.final.pdb
-2.523489e-15 -8.758331e-15 -4.836065e-16 7.817171e-15 -1.290022e-15
1.800302e-15
E:\\R-3.1.2\\scripts\\mPDBS\\summary_pdb_93\\f14fcae8deec4fa3.final.pdb
-7.418022e-16 -7.953445e-15 -3.877918e-15 6.534009e-15 -2.969779e-15
1.738095e-15
E:\\R-3.1.2\\scripts\\mPDBS\\summary_pdb_93\\f83584e22003fb92.final.pdb
-4.839087e-15 -9.872978e-15 6.520790e-15 6.799793e-15 7.980672e-16
6.909781e-15
E:\\R-3.1.2\\scripts\\mPDBS\\summary_pdb_93\\f8e2bf87e52caa29.final.pdb
-1.349576e-15 -4.086620e-15 3.481620e-15 4.718781e-15 -4.271195e-15
3.801732e-15

[,649]   [,650]   [,651]   [,652]   [,653]   [,654]
E:\\R-3.1.2\\scripts\\40.1ns_data\\NAT0.pdb
-2.410009e-15 2.401845e-15 1.964122e-15 6.314300e-16 -1.801145e-16
-6.306628e-15
E:\\R-3.1.2\\scripts\\40.1ns_data\\LJP6.pdb
-1.658865e-15 1.797041e-15 2.942861e-15 -1.297293e-15 1.680064e-15
-5.920796e-15

E:\\R-3.1.2\\scripts\\40.1ns_data\\3WY9.pdb
-2.667900e-15 -3.993669e-15 1.792218e-15 -3.123820e-16 1.896199e-15
-7.030319e-15
E:\\R-3.1.2\\scripts\\40.1ns_data\\ZSP9.pdb
-3.283970e-15 7.637539e-16 3.097736e-15 -9.005245e-17 2.408397e-15
-6.558546e-15
E:\\R-3.1.2\\scripts\\mPDBS\\summary_pdb_93\\013d855ad26c877c.final.pdb
5.265724e-16 4.350829e-15 6.620662e-15 2.217374e-15 2.566329e-15
-1.083837e-14
E:\\R-3.1.2\\scripts\\mPDBS\\summary_pdb_93\\0a2a705342ca0d54.final.pdb
-2.641920e-15 5.850986e-16 3.655586e-15 -1.496289e-16 2.557741e-15
-5.582722e-15
E:\\R-3.1.2\\scripts\\mPDBS\\summary_pdb_93\\0a59fceadb4194d9.final.pdb
-3.073586e-15 7.546107e-16 7.906893e-15 -1.850723e-15 7.061375e-16
-9.447714e-15
E:\\R-3.1.2\\scripts\\mPDBS\\summary_pdb_93\\0dba59f3306825e8.final.pdb
-6.107010e-15 3.725461e-15 4.537203e-16 -1.949432e-15 -2.014208e-15
-8.695552e-15
E:\\R-3.1.2\\scripts\\mPDBS\\summary_pdb_93\\126f26a78b57339c.final.pdb
-7.837397e-15 1.705790e-16 -1.490905e-15 -4.844355e-16 3.852844e-15
-1.393993e-15
E:\\R-3.1.2\\scripts\\mPDBS\\summary_pdb_93\\12f06f3e342df706.final.pdb
-2.628476e-15 9.840850e-16 3.605930e-15 4.061638e-17 2.246358e-15
-5.720632e-15
E:\\R-3.1.2\\scripts\\mPDBS\\summary_pdb_93\\18618a2a251b867b.final.pdb
-7.820913e-15 5.144355e-15 -2.732303e-15 -1.459373e-15 7.040616e-15
-6.822339e-16
E:\\R-3.1.2\\scripts\\mPDBS\\summary_pdb_93\\1f305f598641eeb2.final.pdb
-2.845422e-15 1.163298e-15 3.503717e-15 -3.883146e-16 2.053587e-15
-5.859073e-15
E:\\R-3.1.2\\scripts\\mPDBS\\summary_pdb_93\\1f8a00b551be0a39.final.pdb
-7.820913e-15 5.144355e-15 -2.732303e-15 -1.459373e-15 7.040616e-15
-6.822339e-16
E:\\R-3.1.2\\scripts\\mPDBS\\summary_pdb_93\\219f45328d47579a.final.pdb
-4.432309e-15 6.722114e-16 1.219470e-14 7.718445e-15 8.240909e-15
3.334596e-15
E:\\R-3.1.2\\scripts\\mPDBS\\summary_pdb_93\\238dd3918e54336d.final.pdb
-9.460405e-15 5.509271e-15 -4.008489e-15 4.245540e-15 4.047865e-15
6.554351e-15
E:\\R-3.1.2\\scripts\\mPDBS\\summary_pdb_93\\2b3027889e2fdc42.final.pdb
-3.443950e-15 8.816792e-16 3.667663e-15 7.874474e-17 1.508447e-15
-6.386036e-15
E:\\R-3.1.2\\scripts\\mPDBS\\summary_pdb_93\\2fcb3b46e33793b0.final.pdb
-1.618049e-16 2.566689e-15 5.431862e-15 -4.039271e-16 2.120373e-15
-1.037803e-14
E:\\R-3.1.2\\scripts\\mPDBS\\summary_pdb_93\\34eb328251842006.final.pdb
-1.606893e-15 2.226821e-16 1.365206e-14 -6.164508e-16 9.553515e-15
-3.566824e-15
E:\\R-3.1.2\\scripts\\mPDBS\\summary_pdb_93\\354b32871797b7ab.final.pdb
3.696237e-15 -8.605963e-15 1.335070e-14 3.573374e-15 3.222992e-15
-3.854906e-15
E:\\R-3.1.2\\scripts\\mPDBS\\summary_pdb_93\\397368adbc32d5de.final.pdb
-7.837397e-15 1.705790e-16 -1.490905e-15 -4.844355e-16 3.852844e-15
-1.393993e-15

E:\\R-3.1.2\\scripts\\mPDBS\\summary_pdb_93\\411ca13cd3df6edd.final.pdb
-2.684960e-15 4.059852e-15 5.365943e-15 -1.565347e-15 1.023259e-14
-8.880636e-15
E:\\R-3.1.2\\scripts\\mPDBS\\summary_pdb_93\\42357eee6642bf12.final.pdb
-2.256162e-15 3.137857e-15 1.525540e-14 1.435982e-15 9.972093e-15
-4.331257e-16
E:\\R-3.1.2\\scripts\\mPDBS\\summary_pdb_93\\44342d1f75a7fcfc.final.pdb
-7.382962e-15 5.741351e-15 2.332602e-15 -5.614977e-15 1.090209e-15
-8.235662e-15
E:\\R-3.1.2\\scripts\\mPDBS\\summary_pdb_93\\448ef21dffe8cdfb.final.pdb
1.230218e-14 -1.561061e-14 1.400833e-14 1.016748e-14 1.176370e-14
3.533154e-15
E:\\R-3.1.2\\scripts\\mPDBS\\summary_pdb_93\\472a40b3e8f113c7.final.pdb
-6.107010e-15 3.725461e-15 4.537203e-16 -1.949432e-15 -2.014208e-15
-8.695552e-15
E:\\R-3.1.2\\scripts\\mPDBS\\summary_pdb_93\\48a26225951ea153.final.pdb
-2.099539e-15 3.426680e-17 -2.118074e-15 -3.842598e-15 5.052487e-15
-2.174669e-15
E:\\R-3.1.2\\scripts\\mPDBS\\summary_pdb_93\\4ab4a5c7c6682ce6.final.pdb
1.628046e-14 -1.233504e-14 8.492372e-16 -5.220231e-15 -1.824906e-15
-3.607500e-15
E:\\R-3.1.2\\scripts\\mPDBS\\summary_pdb_93\\4eaa45b62cb6a25f.final.pdb
-4.284358e-15 4.629760e-15 -3.743916e-15 -3.054581e-15 -3.195298e-16
-7.331291e-15
E:\\R-3.1.2\\scripts\\mPDBS\\summary_pdb_93\\58c371611d85ac96.final.pdb
-7.837397e-15 1.705790e-16 -1.490905e-15 -4.844355e-16 3.852844e-15
-1.393993e-15
E:\\R-3.1.2\\scripts\\mPDBS\\summary_pdb_93\\59a647f04822d30a.final.pdb
-1.129284e-15 -6.003594e-15 -1.856321e-15 2.387736e-15 -4.805249e-15
-1.061657e-14
E:\\R-3.1.2\\scripts\\mPDBS\\summary_pdb_93\\59b091fe385e05fc.final.pdb
-3.073586e-15 7.546107e-16 7.906893e-15 -1.850723e-15 7.061375e-16
-9.447714e-15
E:\\R-3.1.2\\scripts\\mPDBS\\summary_pdb_93\\5c35ac30f930d2db.final.pdb
-4.284358e-15 4.629760e-15 -3.743916e-15 -3.054581e-15 -3.195298e-16
-7.331291e-15
E:\\R-3.1.2\\scripts\\mPDBS\\summary_pdb_93\\5f60752431cf2dcb.final.pdb
-5.739269e-15 4.078832e-15 -1.393977e-15 -3.653748e-15 -3.297818e-15
-1.190178e-14
E:\\R-3.1.2\\scripts\\mPDBS\\summary_pdb_93\\626456dd6ff2868d.final.pdb
-6.107010e-15 3.725461e-15 4.537203e-16 -1.949432e-15 -2.014208e-15
-8.695552e-15
E:\\R-3.1.2\\scripts\\mPDBS\\summary_pdb_93\\6583d53ff3c8f7ef.final.pdb
-1.129284e-15 -6.003594e-15 -1.856321e-15 2.387736e-15 -4.805249e-15
-1.061657e-14
E:\\R-3.1.2\\scripts\\mPDBS\\summary_pdb_93\\69760178986bef01.final.pdb
-6.192024e-15 4.866952e-15 -4.024689e-15 1.665807e-15 -1.943888e-15
-3.802228e-15
E:\\R-3.1.2\\scripts\\mPDBS\\summary_pdb_93\\6a9d87d47926b1c8.final.pdb
-3.542221e-15 7.126801e-16 3.449588e-15 -9.121283e-18 1.052133e-15
-6.396068e-15
E:\\R-3.1.2\\scripts\\mPDBS\\summary_pdb_93\\6bb3547ea982ffc4.final.pdb
-2.417063e-15 2.134954e-15 2.831628e-15 6.482668e-16 7.532550e-15
-8.952859e-16

E:\\R-3.1.2\\scripts\\mPDBS\\summary_pdb_93\\716d9624611d1c42.final.pdb
-2.250306e-15 7.772192e-16 3.449154e-15 1.101459e-16 7.600149e-15
-1.376418e-16
E:\\R-3.1.2\\scripts\\mPDBS\\summary_pdb_93\\75f27e70ad0f1be4.final.pdb
-1.129284e-15 -6.003594e-15 -1.856321e-15 2.387736e-15 -4.805249e-15
-1.061657e-14
E:\\R-3.1.2\\scripts\\mPDBS\\summary_pdb_93\\769f910fffb22872.final.pdb
-3.612908e-15 7.626607e-16 3.444180e-15 -4.254892e-16 2.213531e-15
-6.618891e-15
E:\\R-3.1.2\\scripts\\mPDBS\\summary_pdb_93\\791f8c80dd5c3135.final.pdb
-3.073586e-15 7.546107e-16 7.906893e-15 -1.850723e-15 7.061375e-16
-9.447714e-15
E:\\R-3.1.2\\scripts\\mPDBS\\summary_pdb_93\\7fad98e66a3f6099.final.pdb
3.024298e-15 -2.964695e-16 1.389958e-14 2.089782e-15 8.809560e-15
-3.146303e-15
E:\\R-3.1.2\\scripts\\mPDBS\\summary_pdb_93\\804b89c94a633196.final.pdb
-1.618049e-16 2.566689e-15 5.431862e-15 -4.039271e-16 2.120373e-15
-1.037803e-14
E:\\R-3.1.2\\scripts\\mPDBS\\summary_pdb_93\\813cc65a2298feba.final.pdb
-1.618049e-16 2.566689e-15 5.431862e-15 -4.039271e-16 2.120373e-15
-1.037803e-14
E:\\R-3.1.2\\scripts\\mPDBS\\summary_pdb_93\\83c0eefcfe12d3f9.final.pdb
-2.684960e-15 4.059852e-15 5.365943e-15 -1.565347e-15 1.023259e-14
-8.880636e-15
E:\\R-3.1.2\\scripts\\mPDBS\\summary_pdb_93\\86d50469ef569760.final.pdb
-6.107010e-15 3.725461e-15 4.537203e-16 -1.949432e-15 -2.014208e-15
-8.695552e-15
E:\\R-3.1.2\\scripts\\mPDBS\\summary_pdb_93\\89d0666e1402203d.final.pdb
-1.129284e-15 -6.003594e-15 -1.856321e-15 2.387736e-15 -4.805249e-15
-1.061657e-14
E:\\R-3.1.2\\scripts\\mPDBS\\summary_pdb_93\\8b34852716dadc62.final.pdb
-6.871715e-15 -6.789788e-16 1.057757e-15 -4.478793e-15 1.045712e-15
-1.772064e-15
E:\\R-3.1.2\\scripts\\mPDBS\\summary_pdb_93\\8b6ef9ac3fe74395.final.pdb
-7.848726e-15 -3.782535e-15 6.608198e-15 1.562525e-15 -1.756629e-15
-1.372687e-14
E:\\R-3.1.2\\scripts\\mPDBS\\summary_pdb_93\\98bbe0d5986c138f.final.pdb
-3.073586e-15 7.546107e-16 7.906893e-15 -1.850723e-15 7.061375e-16
-9.447714e-15
E:\\R-3.1.2\\scripts\\mPDBS\\summary_pdb_93\\9c22f4909d67e2fa.final.pdb
-1.034593e-14 8.662290e-15 9.870668e-16 2.350368e-15 -2.815565e-15
-8.334704e-16
E:\\R-3.1.2\\scripts\\mPDBS\\summary_pdb_93\\9dda8ed0d81fb3ef.final.pdb
-4.857638e-15 -1.219227e-15 -2.468678e-15 -1.524007e-14 8.677307e-16
-1.179434e-14
E:\\R-3.1.2\\scripts\\mPDBS\\summary_pdb_93\\9f752b3616e6033b.final.pdb
-2.769403e-15 -6.859699e-16 6.303326e-15 1.575369e-15 2.079372e-15
-5.694067e-15
E:\\R-3.1.2\\scripts\\mPDBS\\summary_pdb_93\\a02c70083816d6ba.final.pdb
-4.284358e-15 4.629760e-15 -3.743916e-15 -3.054581e-15 -3.195298e-16
-7.331291e-15
E:\\R-3.1.2\\scripts\\mPDBS\\summary_pdb_93\\a0c997d891e4e082.final.pdb
5.055702e-16 -2.267487e-15 -1.323749e-15 -1.100327e-15 -2.851643e-15
-6.144955e-15

E:\\R-3.1.2\\scripts\\mPDBS\\summary_pdb_93\\ab8039a2d823c616.final.pdb
-2.903595e-15 1.469324e-15 3.124949e-15 -3.131851e-16 2.108582e-15
-6.178565e-15
E:\\R-3.1.2\\scripts\\mPDBS\\summary_pdb_93\\acf4d74b9c71d4dc.final.pdb
-1.618049e-16 2.566689e-15 5.431862e-15 -4.039271e-16 2.120373e-15
-1.037803e-14
E:\\R-3.1.2\\scripts\\mPDBS\\summary_pdb_93\\ad8a823e4b4c668e.final.pdb
-3.026480e-15 1.193534e-15 3.454415e-15 -1.776389e-16 2.034509e-15
-6.246190e-15
E:\\R-3.1.2\\scripts\\mPDBS\\summary_pdb_93\\b16ababd03b7b857.final.pdb
-1.034593e-14 8.662290e-15 9.870668e-16 2.350368e-15 -2.815565e-15
-8.334704e-16
E:\\R-3.1.2\\scripts\\mPDBS\\summary_pdb_93\\b226923e697d3c63.final.pdb
-7.395105e-15 8.392392e-16 3.367582e-15 2.409431e-15 3.289621e-15
-4.684249e-15
E:\\R-3.1.2\\scripts\\mPDBS\\summary_pdb_93\\b28e48f6fa27a165.final.pdb
-3.073586e-15 7.546107e-16 7.906893e-15 -1.850723e-15 7.061375e-16
-9.447714e-15
E:\\R-3.1.2\\scripts\\mPDBS\\summary_pdb_93\\b42c4924e3cd1aec.final.pdb
-5.162950e-15 6.639287e-15 1.035180e-14 2.006725e-16 1.284715e-15
-1.761132e-15
E:\\R-3.1.2\\scripts\\mPDBS\\summary_pdb_93\\b4dd239fcabff1df.final.pdb
-3.073586e-15 7.546107e-16 7.906893e-15 -1.850723e-15 7.061375e-16
-9.447714e-15
E:\\R-3.1.2\\scripts\\mPDBS\\summary_pdb_93\\b669942ef97459a2.final.pdb
-6.871715e-15 -6.789788e-16 1.057757e-15 -4.478793e-15 1.045712e-15
-1.772064e-15
E:\\R-3.1.2\\scripts\\mPDBS\\summary_pdb_93\\b99f4d9d87c1017b.final.pdb
-3.073586e-15 7.546107e-16 7.906893e-15 -1.850723e-15 7.061375e-16
-9.447714e-15
E:\\R-3.1.2\\scripts\\mPDBS\\summary_pdb_93\\b9d2cbf3975403e7.final.pdb
-3.073586e-15 7.546107e-16 7.906893e-15 -1.850723e-15 7.061375e-16
-9.447714e-15
E:\\R-3.1.2\\scripts\\mPDBS\\summary_pdb_93\\bc88c8a505f3a288.final.pdb
-2.789525e-15 7.938588e-16 3.464917e-15 -1.385624e-16 1.694496e-15
-6.403166e-15
E:\\R-3.1.2\\scripts\\mPDBS\\summary_pdb_93\\bdac0e64f4ef427e.final.pdb
-4.816935e-15 -3.110844e-15 -9.245985e-16 1.969574e-15 -1.440200e-14
-1.672209e-15
E:\\R-3.1.2\\scripts\\mPDBS\\summary_pdb_93\\ca2a1f55fc9d0681.final.pdb
-6.871715e-15 -6.789788e-16 1.057757e-15 -4.478793e-15 1.045712e-15
-1.772064e-15
E:\\R-3.1.2\\scripts\\mPDBS\\summary_pdb_93\\ce34248de3e81a3c.final.pdb
-5.700304e-16 1.631560e-15 -2.123279e-15 -6.521611e-15 -3.326482e-16
-1.202082e-14
E:\\R-3.1.2\\scripts\\mPDBS\\summary_pdb_93\\d02e697772494a61.final.pdb
4.648159e-16 -1.533706e-15 2.687110e-15 1.435724e-14 4.137637e-15
-4.850372e-15
E:\\R-3.1.2\\scripts\\mPDBS\\summary_pdb_93\\d04221245d015047.final.pdb
-1.334971e-15 1.822991e-15 3.463220e-15 -9.771615e-16 1.559759e-15
-6.661902e-15
E:\\R-3.1.2\\scripts\\mPDBS\\summary_pdb_93\\d678141e4018054a.final.pdb
5.054242e-16 4.283732e-15 6.782753e-15 1.722300e-15 1.626995e-14
-1.144298e-17

E:\\R-3.1.2\\scripts\\mPDBS\\summary_pdb_93\\d71b40af0f0efe39.final.pdb
-3.549243e-15 4.245665e-16 1.056582e-14 5.314972e-15 7.261494e-15
-1.146634e-15
E:\\R-3.1.2\\scripts\\mPDBS\\summary_pdb_93\\dc0809eff10197e2.final.pdb
-7.114105e-15 -6.943670e-15 5.461728e-16 7.354499e-16 -1.416612e-14
-1.640348e-14
E:\\R-3.1.2\\scripts\\mPDBS\\summary_pdb_93\\e1163918d3707839.final.pdb
2.552233e-15 1.119857e-14 1.402486e-14 -6.202854e-16 1.813669e-14
-7.029502e-16
E:\\R-3.1.2\\scripts\\mPDBS\\summary_pdb_93\\e4b48b06824ef848.final.pdb
-7.820913e-15 5.144355e-15 -2.732303e-15 -1.459373e-15 7.040616e-15
-6.822339e-16
E:\\R-3.1.2\\scripts\\mPDBS\\summary_pdb_93\\e529e19ad281fea2.final.pdb
5.265724e-16 4.350829e-15 6.620662e-15 2.217374e-15 2.566329e-15
-1.083837e-14
E:\\R-3.1.2\\scripts\\mPDBS\\summary_pdb_93\\e5602082718ec3a1.final.pdb
-1.618049e-16 2.566689e-15 5.431862e-15 -4.039271e-16 2.120373e-15
-1.037803e-14
E:\\R-3.1.2\\scripts\\mPDBS\\summary_pdb_93\\e5d589d996d0a960.final.pdb
-3.124187e-15 -1.158581e-15 -9.894654e-16 3.711708e-16 -4.089833e-15
-1.089170e-14
E:\\R-3.1.2\\scripts\\mPDBS\\summary_pdb_93\\e9df9e72307a2fbd.final.pdb
-4.284358e-15 4.629760e-15 -3.743916e-15 -3.054581e-15 -3.195298e-16
-7.331291e-15
E:\\R-3.1.2\\scripts\\mPDBS\\summary_pdb_93\\ea00f8694090cae7.final.pdb
-1.618049e-16 2.566689e-15 5.431862e-15 -4.039271e-16 2.120373e-15
-1.037803e-14
E:\\R-3.1.2\\scripts\\mPDBS\\summary_pdb_93\\eba517be32db050e.final.pdb
-3.073586e-15 7.546107e-16 7.906893e-15 -1.850723e-15 7.061375e-16
-9.447714e-15
E:\\R-3.1.2\\scripts\\mPDBS\\summary_pdb_93\\ec04b9545899c29d.final.pdb
-2.994545e-15 1.569341e-15 3.546475e-15 -4.528735e-16 1.727720e-15
-6.171774e-15
E:\\R-3.1.2\\scripts\\mPDBS\\summary_pdb_93\\ee6862cbcad52078.final.pdb
-3.532421e-15 9.289504e-16 3.142909e-15 8.032737e-18 1.369669e-15
-6.145234e-15
E:\\R-3.1.2\\scripts\\mPDBS\\summary_pdb_93\\ef6a42fc83b81e5d.final.pdb
-2.836619e-15 1.072092e-15 3.172479e-15 -3.821321e-16 2.328673e-15
-5.811486e-15
E:\\R-3.1.2\\scripts\\mPDBS\\summary_pdb_93\\ef8e88d4ad93a0ad.final.pdb
-1.334971e-15 1.822991e-15 3.463220e-15 -9.771615e-16 1.559759e-15
-6.661902e-15
E:\\R-3.1.2\\scripts\\mPDBS\\summary_pdb_93\\f14fcae8deec4fa3.final.pdb
-2.769403e-15 -6.859699e-16 6.303326e-15 1.575369e-15 2.079372e-15
-5.694067e-15
E:\\R-3.1.2\\scripts\\mPDBS\\summary_pdb_93\\f83584e22003fb92.final.pdb
-2.099539e-15 3.426680e-17 -2.118074e-15 -3.842598e-15 5.052487e-15
-2.174669e-15
E:\\R-3.1.2\\scripts\\mPDBS\\summary_pdb_93\\f8e2bf87e52caa29.final.pdb
5.265724e-16 4.350829e-15 6.620662e-15 2.217374e-15 2.566329e-15
-1.083837e-14

[,655]   [,656]   [,657]   [,658]   [,659]   [,660]
E:\\R-3.1.2\\scripts\\40.1ns_data\\NAT0.pdb

1.240934e-15 1.458725e-15 -1.108922e-15 3.780482e-15 -4.308912e-16
-4.069836e-16
E:\\R-3.1.2\\scripts\\40.1ns_data\\LJP6.pdb
2.365477e-15 1.263930e-15 -9.610689e-16 2.381608e-15 -3.845163e-16
-9.407860e-16
E:\\R-3.1.2\\scripts\\40.1ns_data\\3WY9.pdb
2.184944e-15 1.974286e-15 2.090575e-16 3.624406e-15 6.245562e-16
-1.592985e-15
E:\\R-3.1.2\\scripts\\40.1ns_data\\ZSP9.pdb
1.644612e-15 1.455023e-15 7.385519e-16 3.288887e-15 7.818715e-16
-1.677695e-15
E:\\R-3.1.2\\scripts\\mPDBS\\summary_pdb_93\\013d855ad26c877c.final.pdb
1.610739e-15 -3.556760e-15 3.733539e-15 2.955544e-15 4.155434e-15
1.107162e-15
E:\\R-3.1.2\\scripts\\mPDBS\\summary_pdb_93\\0a2a705342ca0d54.final.pdb
1.307336e-15 1.317747e-15 -4.349559e-16 2.989891e-15 -5.871747e-16
-1.273719e-15
E:\\R-3.1.2\\scripts\\mPDBS\\summary_pdb_93\\0a59fceadb4194d9.final.pdb
4.533856e-15 1.524829e-15 3.867471e-16 1.129367e-15 1.059235e-15
-9.806513e-17
E:\\R-3.1.2\\scripts\\mPDBS\\summary_pdb_93\\0dba59f3306825e8.final.pdb
1.762527e-15 1.735014e-15 3.129855e-15 4.138626e-15 -1.899185e-15
-2.289427e-15
E:\\R-3.1.2\\scripts\\mPDBS\\summary_pdb_93\\126f26a78b57339c.final.pdb
-2.650609e-15 4.827050e-15 -4.360681e-15 2.215251e-15 -2.321590e-15
-1.415343e-15
E:\\R-3.1.2\\scripts\\mPDBS\\summary_pdb_93\\12f06f3e342df706.final.pdb
1.287820e-15 1.498728e-15 -2.033703e-16 2.561848e-15 2.250554e-16
-1.650371e-15
E:\\R-3.1.2\\scripts\\mPDBS\\summary_pdb_93\\18618a2a251b867b.final.pdb
-2.449984e-15 -3.215433e-15 5.539617e-16 3.556076e-15 -2.552302e-15
-8.247183e-16
E:\\R-3.1.2\\scripts\\mPDBS\\summary_pdb_93\\1f305f598641eeb2.final.pdb
1.425889e-15 1.490373e-15 3.418335e-16 2.701941e-15 -2.483941e-16
-1.288401e-15
E:\\R-3.1.2\\scripts\\mPDBS\\summary_pdb_93\\1f8a00b551be0a39.final.pdb
-2.449984e-15 -3.215433e-15 5.539617e-16 3.556076e-15 -2.552302e-15
-8.247183e-16
E:\\R-3.1.2\\scripts\\mPDBS\\summary_pdb_93\\219f45328d47579a.final.pdb
9.266615e-15 5.909776e-15 1.878609e-15 9.293410e-15 7.673676e-16
-3.375825e-15
E:\\R-3.1.2\\scripts\\mPDBS\\summary_pdb_93\\238dd3918e54336d.final.pdb
-8.457055e-15 3.388301e-15 -3.652123e-15 9.361931e-15 -2.541401e-15
-5.062193e-15
E:\\R-3.1.2\\scripts\\mPDBS\\summary_pdb_93\\2b3027889e2fdc42.final.pdb
1.747456e-15 1.383313e-15 5.671594e-17 2.536662e-15 -7.679217e-17
-1.467629e-15
E:\\R-3.1.2\\scripts\\mPDBS\\summary_pdb_93\\2fcb3b46e33793b0.final.pdb
3.583885e-15 -1.652946e-15 4.789665e-15 2.622577e-15 4.190408e-16
-7.367588e-16
E:\\R-3.1.2\\scripts\\mPDBS\\summary_pdb_93\\34eb328251842006.final.pdb
7.097973e-15 6.626170e-15 9.579175e-17 5.702425e-15 1.232967e-15
6.822278e-16
E:\\R-3.1.2\\scripts\\mPDBS\\summary_pdb_93\\354b32871797b7ab.final.pdb

-8.334214e-15 -1.041953e-15 -2.047603e-15 6.123386e-15 8.699094e-15
2.456438e-15
E:\\R-3.1.2\\scripts\\mPDBS\\summary_pdb_93\\397368adbc32d5de.final.pdb
-2.650609e-15 4.827050e-15 -4.360681e-15 2.215251e-15 -2.321590e-15
-1.415343e-15
E:\\R-3.1.2\\scripts\\mPDBS\\summary_pdb_93\\411ca13cd3df6edd.final.pdb
2.281975e-15 1.118383e-15 1.431240e-15 1.527533e-15 -1.744593e-15
2.990879e-16
E:\\R-3.1.2\\scripts\\mPDBS\\summary_pdb_93\\42357eee6642bf12.final.pdb
5.831835e-15 2.144014e-15 -3.075511e-17 4.721587e-15 -1.841709e-15
-7.287567e-16
E:\\R-3.1.2\\scripts\\mPDBS\\summary_pdb_93\\44342d1f75a7fcfc.final.pdb
4.911824e-15 2.563616e-15 7.560880e-15 -2.993845e-15 4.732564e-16
-2.312748e-15
E:\\R-3.1.2\\scripts\\mPDBS\\summary_pdb_93\\448ef21dffe8cdfb.final.pdb
-7.765544e-15 -3.139604e-15 -1.951728e-14 -2.155285e-15 9.067505e-15
2.378805e-15
E:\\R-3.1.2\\scripts\\mPDBS\\summary_pdb_93\\472a40b3e8f113c7.final.pdb
1.762527e-15 1.735014e-15 3.129855e-15 4.138626e-15 -1.899185e-15
-2.289427e-15
E:\\R-3.1.2\\scripts\\mPDBS\\summary_pdb_93\\48a26225951ea153.final.pdb
-1.091082e-14 8.516205e-16 -7.586041e-16 2.114257e-15 -1.215421e-15
-2.106441e-15
E:\\R-3.1.2\\scripts\\mPDBS\\summary_pdb_93\\4ab4a5c7c6682ce6.final.pdb
8.200086e-15 -5.169294e-15 -6.404185e-15 -3.654620e-15 6.035873e-16
-4.179625e-15
E:\\R-3.1.2\\scripts\\mPDBS\\summary_pdb_93\\4eaa45b62cb6a25f.final.pdb
2.244564e-15 1.125638e-15 -8.467410e-16 8.741125e-16 -2.543648e-15
-1.950193e-15
E:\\R-3.1.2\\scripts\\mPDBS\\summary_pdb_93\\58c371611d85ac96.final.pdb
-2.650609e-15 4.827050e-15 -4.360681e-15 2.215251e-15 -2.321590e-15
-1.415343e-15
E:\\R-3.1.2\\scripts\\mPDBS\\summary_pdb_93\\59a647f04822d30a.final.pdb
3.010134e-15 4.003871e-15 -4.629021e-15 6.080060e-15 -7.651194e-16
-4.632073e-15
E:\\R-3.1.2\\scripts\\mPDBS\\summary_pdb_93\\59b091fe385e05fc.final.pdb
4.533856e-15 1.524829e-15 3.867471e-16 1.129367e-15 1.059235e-15
-9.806513e-17
E:\\R-3.1.2\\scripts\\mPDBS\\summary_pdb_93\\5c35ac30f930d2db.final.pdb
2.244564e-15 1.125638e-15 -8.467410e-16 8.741125e-16 -2.543648e-15
-1.950193e-15
E:\\R-3.1.2\\scripts\\mPDBS\\summary_pdb_93\\5f60752431cf2dcb.final.pdb
3.590666e-15 5.868225e-15 1.710463e-15 1.740890e-15 1.196338e-15
1.798693e-15
E:\\R-3.1.2\\scripts\\mPDBS\\summary_pdb_93\\626456dd6ff2868d.final.pdb
1.762527e-15 1.735014e-15 3.129855e-15 4.138626e-15 -1.899185e-15
-2.289427e-15
E:\\R-3.1.2\\scripts\\mPDBS\\summary_pdb_93\\6583d53ff3c8f7ef.final.pdb
3.010134e-15 4.003871e-15 -4.629021e-15 6.080060e-15 -7.651194e-16
-4.632073e-15
E:\\R-3.1.2\\scripts\\mPDBS\\summary_pdb_93\\69760178986bef01.final.pdb
-7.212012e-15 -2.723250e-15 2.318596e-15 2.707716e-15 1.335421e-15
-5.541534e-15
E:\\R-3.1.2\\scripts\\mPDBS\\summary_pdb_93\\6a9d87d47926b1c8.final.pdb

1.608137e-15 1.076578e-15 5.936892e-16 2.731331e-15 3.199589e-17
-1.467385e-15
E:\\R-3.1.2\\scripts\\mPDBS\\summary_pdb_93\\6bb3547ea982ffc4.final.pdb
-9.191728e-16 -8.813193e-16 5.601436e-15 1.164893e-16 1.066317e-15
-3.111513e-15
E:\\R-3.1.2\\scripts\\mPDBS\\summary_pdb_93\\716d9624611d1c42.final.pdb
-3.617110e-15 -2.345555e-15 -1.814061e-15 1.867958e-15 -6.908244e-16
-3.956469e-15
E:\\R-3.1.2\\scripts\\mPDBS\\summary_pdb_93\\75f27e70ad0f1be4.final.pdb
3.010134e-15 4.003871e-15 -4.629021e-15 6.080060e-15 -7.651194e-16
-4.632073e-15
E:\\R-3.1.2\\scripts\\mPDBS\\summary_pdb_93\\769f910fffb22872.final.pdb
2.120145e-15 1.841368e-15 -2.891139e-16 2.731649e-15 -2.002341e-16
-1.605344e-15
E:\\R-3.1.2\\scripts\\mPDBS\\summary_pdb_93\\791f8c80dd5c3135.final.pdb
4.533856e-15 1.524829e-15 3.867471e-16 1.129367e-15 1.059235e-15
-9.806513e-17
E:\\R-3.1.2\\scripts\\mPDBS\\summary_pdb_93\\7fad98e66a3f6099.final.pdb
-6.282205e-16 5.256157e-15 2.521079e-15 2.328877e-15 3.823197e-15
-1.808938e-15
E:\\R-3.1.2\\scripts\\mPDBS\\summary_pdb_93\\804b89c94a633196.final.pdb
3.583885e-15 -1.652946e-15 4.789665e-15 2.622577e-15 4.190408e-16
-7.367588e-16
E:\\R-3.1.2\\scripts\\mPDBS\\summary_pdb_93\\813cc65a2298feba.final.pdb
3.583885e-15 -1.652946e-15 4.789665e-15 2.622577e-15 4.190408e-16
-7.367588e-16
E:\\R-3.1.2\\scripts\\mPDBS\\summary_pdb_93\\83c0eefcfe12d3f9.final.pdb
2.281975e-15 1.118383e-15 1.431240e-15 1.527533e-15 -1.744593e-15
2.990879e-16
E:\\R-3.1.2\\scripts\\mPDBS\\summary_pdb_93\\86d50469ef569760.final.pdb
1.762527e-15 1.735014e-15 3.129855e-15 4.138626e-15 -1.899185e-15
-2.289427e-15
E:\\R-3.1.2\\scripts\\mPDBS\\summary_pdb_93\\89d0666e1402203d.final.pdb
3.010134e-15 4.003871e-15 -4.629021e-15 6.080060e-15 -7.651194e-16
-4.632073e-15
E:\\R-3.1.2\\scripts\\mPDBS\\summary_pdb_93\\8b34852716dadc62.final.pdb
4.367980e-15 6.331986e-15 -1.642690e-15 1.029668e-15 -4.873167e-15
-4.015386e-15
E:\\R-3.1.2\\scripts\\mPDBS\\summary_pdb_93\\8b6ef9ac3fe74395.final.pdb
1.026324e-14 6.293208e-16 3.300944e-15 -2.262486e-15 -2.430603e-15
-3.337944e-15
E:\\R-3.1.2\\scripts\\mPDBS\\summary_pdb_93\\98bbe0d5986c138f.final.pdb
4.533856e-15 1.524829e-15 3.867471e-16 1.129367e-15 1.059235e-15
-9.806513e-17
E:\\R-3.1.2\\scripts\\mPDBS\\summary_pdb_93\\9c22f4909d67e2fa.final.pdb
-6.581595e-15 5.453403e-15 -3.633618e-17 9.664687e-15 2.413322e-15
-6.057872e-16
E:\\R-3.1.2\\scripts\\mPDBS\\summary_pdb_93\\9dda8ed0d81fb3ef.final.pdb
-4.055829e-15 -4.445296e-16 3.499459e-15 2.002149e-16 2.444770e-15
-3.542667e-15
E:\\R-3.1.2\\scripts\\mPDBS\\summary_pdb_93\\9f752b3616e6033b.final.pdb
3.694802e-15 1.670652e-15 8.464159e-16 3.558853e-15 1.463547e-16
-2.519151e-15
E:\\R-3.1.2\\scripts\\mPDBS\\summary_pdb_93\\a02c70083816d6ba.final.pdb

2.244564e-15 1.125638e-15 -8.467410e-16 8.741125e-16 -2.543648e-15
-1.950193e-15
E:\\R-3.1.2\\scripts\\mPDBS\\summary_pdb_93\\a0c997d891e4e082.final.pdb
3.734010e-15 -4.535925e-15 5.110623e-16 2.271187e-15 2.552319e-15
-3.751392e-16
E:\\R-3.1.2\\scripts\\mPDBS\\summary_pdb_93\\ab8039a2d823c616.final.pdb
2.271638e-15 1.433638e-15 -7.678626e-16 2.942525e-15 6.906237e-17
-1.568846e-15
E:\\R-3.1.2\\scripts\\mPDBS\\summary_pdb_93\\acf4d74b9c71d4dc.final.pdb
3.583885e-15 -1.652946e-15 4.789665e-15 2.622577e-15 4.190408e-16
-7.367588e-16
E:\\R-3.1.2\\scripts\\mPDBS\\summary_pdb_93\\ad8a823e4b4c668e.final.pdb
1.483743e-15 1.709504e-15 -4.440677e-16 2.766925e-15 7.101394e-17
-1.378161e-15
E:\\R-3.1.2\\scripts\\mPDBS\\summary_pdb_93\\b16ababd03b7b857.final.pdb
-6.581595e-15 5.453403e-15 -3.633618e-17 9.664687e-15 2.413322e-15
-6.057872e-16
E:\\R-3.1.2\\scripts\\mPDBS\\summary_pdb_93\\b226923e697d3c63.final.pdb
9.533561e-15 -3.295060e-15 -6.721171e-16 1.782716e-15 2.310456e-16
-1.290562e-15
E:\\R-3.1.2\\scripts\\mPDBS\\summary_pdb_93\\b28e48f6fa27a165.final.pdb
4.533856e-15 1.524829e-15 3.867471e-16 1.129367e-15 1.059235e-15
-9.806513e-17
E:\\R-3.1.2\\scripts\\mPDBS\\summary_pdb_93\\b42c4924e3cd1aec.final.pdb
3.402615e-15 6.738716e-15 -4.984206e-15 5.236551e-15 3.700403e-16
4.096838e-15
E:\\R-3.1.2\\scripts\\mPDBS\\summary_pdb_93\\b4dd239fcabff1df.final.pdb
4.533856e-15 1.524829e-15 3.867471e-16 1.129367e-15 1.059235e-15
-9.806513e-17
E:\\R-3.1.2\\scripts\\mPDBS\\summary_pdb_93\\b669942ef97459a2.final.pdb
4.367980e-15 6.331986e-15 -1.642690e-15 1.029668e-15 -4.873167e-15
-4.015386e-15
E:\\R-3.1.2\\scripts\\mPDBS\\summary_pdb_93\\b99f4d9d87c1017b.final.pdb
4.533856e-15 1.524829e-15 3.867471e-16 1.129367e-15 1.059235e-15
-9.806513e-17
E:\\R-3.1.2\\scripts\\mPDBS\\summary_pdb_93\\b9d2cbf3975403e7.final.pdb
4.533856e-15 1.524829e-15 3.867471e-16 1.129367e-15 1.059235e-15
-9.806513e-17
E:\\R-3.1.2\\scripts\\mPDBS\\summary_pdb_93\\bc88c8a505f3a288.final.pdb
1.179768e-15 1.334063e-15 -6.558217e-16 2.741359e-15 1.487219e-16
-1.287218e-15
E:\\R-3.1.2\\scripts\\mPDBS\\summary_pdb_93\\bdac0e64f4ef427e.final.pdb
2.983184e-16 2.822803e-15 -3.706144e-15 5.555127e-15 3.538290e-15
-5.463447e-15
E:\\R-3.1.2\\scripts\\mPDBS\\summary_pdb_93\\ca2a1f55fc9d0681.final.pdb
4.367980e-15 6.331986e-15 -1.642690e-15 1.029668e-15 -4.873167e-15
-4.015386e-15
E:\\R-3.1.2\\scripts\\mPDBS\\summary_pdb_93\\ce34248de3e81a3c.final.pdb
-5.954719e-16 -7.242701e-17 -1.531349e-15 7.212237e-17 -3.185754e-15
1.945621e-15
E:\\R-3.1.2\\scripts\\mPDBS\\summary_pdb_93\\d02e697772494a61.final.pdb
5.668798e-15 -1.564768e-15 1.253675e-15 2.473344e-15 -2.576962e-15
-5.348856e-15
E:\\R-3.1.2\\scripts\\mPDBS\\summary_pdb_93\\d04221245d015047.final.pdb

-9.636212e-16 3.255586e-15 -4.777301e-16 2.978954e-15 -1.772684e-16
-1.705672e-15
E:\\R-3.1.2\\scripts\\mPDBS\\summary_pdb_93\\d678141e4018054a.final.pdb
-5.347460e-15 4.881659e-15 7.923378e-16 3.219877e-15 9.591570e-16
-1.077872e-15
E:\\R-3.1.2\\scripts\\mPDBS\\summary_pdb_93\\d71b40af0f0efe39.final.pdb
2.441158e-15 6.864802e-15 -5.139671e-15 6.373499e-15 3.971685e-15
-1.048950e-15
E:\\R-3.1.2\\scripts\\mPDBS\\summary_pdb_93\\dc0809eff10197e2.final.pdb
1.565169e-14 4.589802e-15 -5.133861e-15 3.830175e-15 -1.177180e-15
-1.729218e-15
E:\\R-3.1.2\\scripts\\mPDBS\\summary_pdb_93\\e1163918d3707839.final.pdb
9.744358e-16 -1.372938e-16 -1.825275e-15 -4.013004e-16 -4.201823e-15
2.641622e-15
E:\\R-3.1.2\\scripts\\mPDBS\\summary_pdb_93\\e4b48b06824ef848.final.pdb
-2.449984e-15 -3.215433e-15 5.539617e-16 3.556076e-15 -2.552302e-15
-8.247183e-16
E:\\R-3.1.2\\scripts\\mPDBS\\summary_pdb_93\\e529e19ad281fea2.final.pdb
1.610739e-15 -3.556760e-15 3.733539e-15 2.955544e-15 4.155434e-15
1.107162e-15
E:\\R-3.1.2\\scripts\\mPDBS\\summary_pdb_93\\e5602082718ec3a1.final.pdb
3.583885e-15 -1.652946e-15 4.789665e-15 2.622577e-15 4.190408e-16
-7.367588e-16
E:\\R-3.1.2\\scripts\\mPDBS\\summary_pdb_93\\e5d589d996d0a960.final.pdb
-3.322386e-15 -3.700564e-15 3.957586e-15 -4.282564e-15 -5.089795e-16
-2.071336e-15
E:\\R-3.1.2\\scripts\\mPDBS\\summary_pdb_93\\e9df9e72307a2fbd.final.pdb
2.244564e-15 1.125638e-15 -8.467410e-16 8.741125e-16 -2.543648e-15
-1.950193e-15
E:\\R-3.1.2\\scripts\\mPDBS\\summary_pdb_93\\ea00f8694090cae7.final.pdb
3.583885e-15 -1.652946e-15 4.789665e-15 2.622577e-15 4.190408e-16
-7.367588e-16
E:\\R-3.1.2\\scripts\\mPDBS\\summary_pdb_93\\eba517be32db050e.final.pdb
4.533856e-15 1.524829e-15 3.867471e-16 1.129367e-15 1.059235e-15
-9.806513e-17
E:\\R-3.1.2\\scripts\\mPDBS\\summary_pdb_93\\ec04b9545899c29d.final.pdb
1.641871e-15 1.696255e-15 6.340562e-17 2.366451e-15 -1.234885e-16
-1.687380e-15
E:\\R-3.1.2\\scripts\\mPDBS\\summary_pdb_93\\ee6862cbcad52078.final.pdb
1.744854e-15 1.529599e-15 -1.631603e-16 2.496763e-15 8.605500e-17
-1.484977e-15
E:\\R-3.1.2\\scripts\\mPDBS\\summary_pdb_93\\ef6a42fc83b81e5d.final.pdb
1.566768e-15 1.531774e-15 -2.956191e-16 2.679607e-15 1.210648e-18
-1.378312e-15
E:\\R-3.1.2\\scripts\\mPDBS\\summary_pdb_93\\ef8e88d4ad93a0ad.final.pdb
-9.636212e-16 3.255586e-15 -4.777301e-16 2.978954e-15 -1.772684e-16
-1.705672e-15
E:\\R-3.1.2\\scripts\\mPDBS\\summary_pdb_93\\f14fcae8deec4fa3.final.pdb
3.694802e-15 1.670652e-15 8.464159e-16 3.558853e-15 1.463547e-16
-2.519151e-15
E:\\R-3.1.2\\scripts\\mPDBS\\summary_pdb_93\\f83584e22003fb92.final.pdb
-1.091082e-14 8.516205e-16 -7.586041e-16 2.114257e-15 -1.215421e-15
-2.106441e-15
E:\\R-3.1.2\\scripts\\mPDBS\\summary_pdb_93\\f8e2bf87e52caa29.final.pdb

1.610739e-15 -3.556760e-15 3.733539e-15 2.955544e-15 4.155434e-15
1.107162e-15

[,661]   [,662]   [,663]   [,664]   [,665]   [,666]
E:\\R-3.1.2\\scripts\\40.1ns_data\\NAT0.pdb
-4.111380e-15 2.530384e-15 5.259921e-15 -1.844700e-16 -4.420322e-15
3.979172e-15
E:\\R-3.1.2\\scripts\\40.1ns_data\\LJP6.pdb
-3.123000e-15 2.286184e-15 6.738585e-15 5.123526e-16 -2.957255e-15
3.563307e-15
E:\\R-3.1.2\\scripts\\40.1ns_data\\3WY9.pdb
-2.911488e-15 3.094214e-15 5.597576e-15 7.813523e-16 -2.508734e-15
3.282996e-15
E:\\R-3.1.2\\scripts\\40.1ns_data\\ZSP9.pdb
-3.509087e-15 3.353390e-15 6.083073e-15 9.202229e-17 -1.814432e-15
3.355749e-15
E:\\R-3.1.2\\scripts\\mPDBS\\summary_pdb_93\\013d855ad26c877c.final.pdb
-1.756493e-15 1.434813e-15 4.423891e-15 1.894173e-15 -4.275879e-15
-2.168730e-15
E:\\R-3.1.2\\scripts\\mPDBS\\summary_pdb_93\\0a2a705342ca0d54.final.pdb
-3.378934e-15 3.067735e-15 5.051000e-15 -4.116125e-16 -3.596183e-15
3.524058e-15
E:\\R-3.1.2\\scripts\\mPDBS\\summary_pdb_93\\0a59fceadb4194d9.final.pdb
-2.919177e-15 5.252697e-15 3.297629e-15 -4.643337e-16 -5.276462e-15
8.779955e-16
E:\\R-3.1.2\\scripts\\mPDBS\\summary_pdb_93\\0dba59f3306825e8.final.pdb
-3.714996e-15 4.328332e-15 3.903474e-15 -1.426522e-15 -2.546360e-15
4.911544e-15
E:\\R-3.1.2\\scripts\\mPDBS\\summary_pdb_93\\126f26a78b57339c.final.pdb
-5.757741e-15 8.250577e-16 1.374413e-14 -1.670955e-15 -1.177663e-15
9.303105e-15
E:\\R-3.1.2\\scripts\\mPDBS\\summary_pdb_93\\12f06f3e342df706.final.pdb
-3.238747e-15 2.956713e-15 5.539758e-15 -2.081185e-17 -3.414905e-15
3.086528e-15
E:\\R-3.1.2\\scripts\\mPDBS\\summary_pdb_93\\18618a2a251b867b.final.pdb
-3.358312e-15 1.889030e-15 1.333295e-14 -2.450213e-15 -9.619995e-15
7.354608e-15
E:\\R-3.1.2\\scripts\\mPDBS\\summary_pdb_93\\1f305f598641eeb2.final.pdb
-3.209509e-15 2.804196e-15 5.683360e-15 -7.736910e-17 -3.204301e-15
3.575846e-15
E:\\R-3.1.2\\scripts\\mPDBS\\summary_pdb_93\\1f8a00b551be0a39.final.pdb
-3.358312e-15 1.889030e-15 1.333295e-14 -2.450213e-15 -9.619995e-15
7.354608e-15
E:\\R-3.1.2\\scripts\\mPDBS\\summary_pdb_93\\219f45328d47579a.final.pdb
-6.587684e-15 4.367988e-15 -8.763470e-15 1.563124e-15 1.030049e-14
-7.555146e-15
E:\\R-3.1.2\\scripts\\mPDBS\\summary_pdb_93\\238dd3918e54336d.final.pdb
1.612680e-15 -5.261462e-15 1.201071e-14 -1.849836e-15 4.306150e-15
-2.554534e-15
E:\\R-3.1.2\\scripts\\mPDBS\\summary_pdb_93\\2b3027889e2fdc42.final.pdb
-3.209960e-15 3.285087e-15 5.605245e-15 -1.230811e-16 -2.883411e-15
2.833191e-15
E:\\R-3.1.2\\scripts\\mPDBS\\summary_pdb_93\\2fcb3b46e33793b0.final.pdb
-7.253851e-16 5.094031e-15 1.117568e-15 1.188112e-15 -3.919007e-15

2.503353e-15
E:\\R-3.1.2\\scripts\\mPDBS\\summary_pdb_93\\34eb328251842006.final.pdb
1.992781e-16 6.201583e-15 5.083663e-15 -2.242247e-15 -6.305703e-15
-8.649105e-15
E:\\R-3.1.2\\scripts\\mPDBS\\summary_pdb_93\\354b32871797b7ab.final.pdb
3.891632e-15 3.273524e-15 3.068977e-15 -5.813287e-16 -3.409460e-15
9.217504e-15
E:\\R-3.1.2\\scripts\\mPDBS\\summary_pdb_93\\397368adbc32d5de.final.pdb
-5.757741e-15 8.250577e-16 1.374413e-14 -1.670955e-15 -1.177663e-15
9.303105e-15
E:\\R-3.1.2\\scripts\\mPDBS\\summary_pdb_93\\411ca13cd3df6edd.final.pdb
1.541139e-15 5.089694e-15 8.518332e-15 -7.852989e-16 -4.130643e-15
5.152167e-15
E:\\R-3.1.2\\scripts\\mPDBS\\summary_pdb_93\\42357eee6642bf12.final.pdb
-1.528587e-15 7.581784e-15 1.012580e-15 -1.213513e-15 -9.523229e-15
-1.487550e-14
E:\\R-3.1.2\\scripts\\mPDBS\\summary_pdb_93\\44342d1f75a7fcfc.final.pdb
-3.820467e-16 3.921478e-15 1.534157e-14 -2.316468e-15 -1.468081e-14
1.677590e-14
E:\\R-3.1.2\\scripts\\mPDBS\\summary_pdb_93\\448ef21dffe8cdfb.final.pdb
1.045768e-15 -3.777345e-15 1.381574e-14 4.245562e-15 -4.253809e-15
-7.888074e-15
E:\\R-3.1.2\\scripts\\mPDBS\\summary_pdb_93\\472a40b3e8f113c7.final.pdb
-3.714996e-15 4.328332e-15 3.903474e-15 -1.426522e-15 -2.546360e-15
4.911544e-15
E:\\R-3.1.2\\scripts\\mPDBS\\summary_pdb_93\\48a26225951ea153.final.pdb
-6.747495e-15 1.377758e-15 1.632627e-14 2.606073e-15 -1.413396e-14
1.143243e-14
E:\\R-3.1.2\\scripts\\mPDBS\\summary_pdb_93\\4ab4a5c7c6682ce6.final.pdb
6.246545e-15 3.153057e-15 -1.163139e-16 3.017135e-15 -1.456366e-14
4.247254e-15
E:\\R-3.1.2\\scripts\\mPDBS\\summary_pdb_93\\4eaa45b62cb6a25f.final.pdb
-6.133660e-15 1.165835e-15 7.974341e-15 5.576256e-16 -2.509139e-15
5.706587e-15
E:\\R-3.1.2\\scripts\\mPDBS\\summary_pdb_93\\58c371611d85ac96.final.pdb
-5.757741e-15 8.250577e-16 1.374413e-14 -1.670955e-15 -1.177663e-15
9.303105e-15
E:\\R-3.1.2\\scripts\\mPDBS\\summary_pdb_93\\59a647f04822d30a.final.pdb
-6.122680e-15 4.800982e-15 3.628284e-15 1.037186e-15 -1.594928e-15
7.229838e-16
E:\\R-3.1.2\\scripts\\mPDBS\\summary_pdb_93\\59b091fe385e05fc.final.pdb
-2.919177e-15 5.252697e-15 3.297629e-15 -4.643337e-16 -5.276462e-15
8.779955e-16
E:\\R-3.1.2\\scripts\\mPDBS\\summary_pdb_93\\5c35ac30f930d2db.final.pdb
-6.133660e-15 1.165835e-15 7.974341e-15 5.576256e-16 -2.509139e-15
5.706587e-15
E:\\R-3.1.2\\scripts\\mPDBS\\summary_pdb_93\\5f60752431cf2dcb.final.pdb
-6.428795e-15 5.295554e-15 9.216939e-15 -1.268403e-15 -5.606728e-15
5.387314e-15
E:\\R-3.1.2\\scripts\\mPDBS\\summary_pdb_93\\626456dd6ff2868d.final.pdb
-3.714996e-15 4.328332e-15 3.903474e-15 -1.426522e-15 -2.546360e-15
4.911544e-15
E:\\R-3.1.2\\scripts\\mPDBS\\summary_pdb_93\\6583d53ff3c8f7ef.final.pdb
-6.122680e-15 4.800982e-15 3.628284e-15 1.037186e-15 -1.594928e-15

7.229838e-16
E:\\R-3.1.2\\scripts\\mPDBS\\summary_pdb_93\\69760178986bef01.final.pdb
-6.348902e-15 3.397905e-15 3.460761e-16 2.369118e-15 -2.100737e-15
1.506848e-14
E:\\R-3.1.2\\scripts\\mPDBS\\summary_pdb_93\\6a9d87d47926b1c8.final.pdb
-3.107084e-15 3.239793e-15 5.487710e-15 -3.048602e-16 -3.288822e-15
3.202083e-15
E:\\R-3.1.2\\scripts\\mPDBS\\summary_pdb_93\\6bb3547ea982ffc4.final.pdb
-1.502893e-15 1.771929e-15 1.493768e-14 -3.400010e-15 3.569863e-15
1.411635e-14
E:\\R-3.1.2\\scripts\\mPDBS\\summary_pdb_93\\716d9624611d1c42.final.pdb
1.181220e-15 -2.322050e-15 8.550371e-15 1.571176e-15 8.212901e-16
5.079454e-15
E:\\R-3.1.2\\scripts\\mPDBS\\summary_pdb_93\\75f27e70ad0f1be4.final.pdb
-6.122680e-15 4.800982e-15 3.628284e-15 1.037186e-15 -1.594928e-15
7.229838e-16
E:\\R-3.1.2\\scripts\\mPDBS\\summary_pdb_93\\769f910fffb22872.final.pdb
-3.035979e-15 2.644936e-15 5.256513e-15 -4.880778e-16 -3.358511e-15
2.836195e-15
E:\\R-3.1.2\\scripts\\mPDBS\\summary_pdb_93\\791f8c80dd5c3135.final.pdb
-2.919177e-15 5.252697e-15 3.297629e-15 -4.643337e-16 -5.276462e-15
8.779955e-16
E:\\R-3.1.2\\scripts\\mPDBS\\summary_pdb_93\\7fad98e66a3f6099.final.pdb
-3.350514e-15 2.061901e-15 -1.793754e-17 4.060593e-15 -1.273290e-15
-6.294214e-15
E:\\R-3.1.2\\scripts\\mPDBS\\summary_pdb_93\\804b89c94a633196.final.pdb
-7.253851e-16 5.094031e-15 1.117568e-15 1.188112e-15 -3.919007e-15
2.503353e-15
E:\\R-3.1.2\\scripts\\mPDBS\\summary_pdb_93\\813cc65a2298feba.final.pdb
-7.253851e-16 5.094031e-15 1.117568e-15 1.188112e-15 -3.919007e-15
2.503353e-15
E:\\R-3.1.2\\scripts\\mPDBS\\summary_pdb_93\\83c0eefcfe12d3f9.final.pdb
1.541139e-15 5.089694e-15 8.518332e-15 -7.852989e-16 -4.130643e-15
5.152167e-15
E:\\R-3.1.2\\scripts\\mPDBS\\summary_pdb_93\\86d50469ef569760.final.pdb
-3.714996e-15 4.328332e-15 3.903474e-15 -1.426522e-15 -2.546360e-15
4.911544e-15
E:\\R-3.1.2\\scripts\\mPDBS\\summary_pdb_93\\89d0666e1402203d.final.pdb
-6.122680e-15 4.800982e-15 3.628284e-15 1.037186e-15 -1.594928e-15
7.229838e-16
E:\\R-3.1.2\\scripts\\mPDBS\\summary_pdb_93\\8b34852716dadc62.final.pdb
-5.934760e-15 4.861578e-15 5.589685e-15 -2.109598e-15 -3.596349e-15
9.543294e-15
E:\\R-3.1.2\\scripts\\mPDBS\\summary_pdb_93\\8b6ef9ac3fe74395.final.pdb
-7.196003e-15 8.616585e-15 8.727063e-15 1.097423e-16 -1.072979e-15
3.334627e-15
E:\\R-3.1.2\\scripts\\mPDBS\\summary_pdb_93\\98bbe0d5986c138f.final.pdb
-2.919177e-15 5.252697e-15 3.297629e-15 -4.643337e-16 -5.276462e-15
8.779955e-16
E:\\R-3.1.2\\scripts\\mPDBS\\summary_pdb_93\\9c22f4909d67e2fa.final.pdb
-1.089726e-14 -1.803663e-15 4.107797e-15 -1.078413e-15 8.818400e-16
9.614006e-16
E:\\R-3.1.2\\scripts\\mPDBS\\summary_pdb_93\\9dda8ed0d81fb3ef.final.pdb
-8.500661e-15 2.591812e-15 1.401017e-14 3.056296e-15 -2.167956e-14

1.957677e-14
E:\\R-3.1.2\\scripts\\mPDBS\\summary_pdb_93\\9f752b3616e6033b.final.pdb
-3.348745e-15 4.598317e-15 3.168486e-15 4.756276e-19 -2.486707e-15
1.515712e-15
E:\\R-3.1.2\\scripts\\mPDBS\\summary_pdb_93\\a02c70083816d6ba.final.pdb
-6.133660e-15 1.165835e-15 7.974341e-15 5.576256e-16 -2.509139e-15
5.706587e-15
E:\\R-3.1.2\\scripts\\mPDBS\\summary_pdb_93\\a0c997d891e4e082.final.pdb
2.850855e-15 4.666188e-15 -2.319238e-15 -1.495569e-15 3.804271e-16
1.371419e-14
E:\\R-3.1.2\\scripts\\mPDBS\\summary_pdb_93\\ab8039a2d823c616.final.pdb
-3.899930e-15 2.548091e-15 5.601076e-15 -5.014644e-16 -3.148000e-15
2.693550e-15
E:\\R-3.1.2\\scripts\\mPDBS\\summary_pdb_93\\acf4d74b9c71d4dc.final.pdb
-7.253851e-16 5.094031e-15 1.117568e-15 1.188112e-15 -3.919007e-15
2.503353e-15
E:\\R-3.1.2\\scripts\\mPDBS\\summary_pdb_93\\ad8a823e4b4c668e.final.pdb
-2.806726e-15 3.352112e-15 6.130924e-15 1.262389e-16 -3.205756e-15
2.970636e-15
E:\\R-3.1.2\\scripts\\mPDBS\\summary_pdb_93\\b16ababd03b7b857.final.pdb
-1.089726e-14 -1.803663e-15 4.107797e-15 -1.078413e-15 8.818400e-16
9.614006e-16
E:\\R-3.1.2\\scripts\\mPDBS\\summary_pdb_93\\b226923e697d3c63.final.pdb
3.108542e-15 7.034005e-15 3.296519e-15 -4.110985e-15 -2.328716e-15
1.675889e-15
E:\\R-3.1.2\\scripts\\mPDBS\\summary_pdb_93\\b28e48f6fa27a165.final.pdb
-2.919177e-15 5.252697e-15 3.297629e-15 -4.643337e-16 -5.276462e-15
8.779955e-16
E:\\R-3.1.2\\scripts\\mPDBS\\summary_pdb_93\\b42c4924e3cd1aec.final.pdb
1.407863e-15 -3.025740e-16 5.536047e-15 -8.390259e-16 4.561605e-15
-8.894377e-15
E:\\R-3.1.2\\scripts\\mPDBS\\summary_pdb_93\\b4dd239fcabff1df.final.pdb
-2.919177e-15 5.252697e-15 3.297629e-15 -4.643337e-16 -5.276462e-15
8.779955e-16
E:\\R-3.1.2\\scripts\\mPDBS\\summary_pdb_93\\b669942ef97459a2.final.pdb
-5.934760e-15 4.861578e-15 5.589685e-15 -2.109598e-15 -3.596349e-15
9.543294e-15
E:\\R-3.1.2\\scripts\\mPDBS\\summary_pdb_93\\b99f4d9d87c1017b.final.pdb
-2.919177e-15 5.252697e-15 3.297629e-15 -4.643337e-16 -5.276462e-15
8.779955e-16
E:\\R-3.1.2\\scripts\\mPDBS\\summary_pdb_93\\b9d2cbf3975403e7.final.pdb
-2.919177e-15 5.252697e-15 3.297629e-15 -4.643337e-16 -5.276462e-15
8.779955e-16
E:\\R-3.1.2\\scripts\\mPDBS\\summary_pdb_93\\bc88c8a505f3a288.final.pdb
-3.137752e-15 3.064777e-15 5.667695e-15 3.239354e-17 -3.030862e-15
2.975168e-15
E:\\R-3.1.2\\scripts\\mPDBS\\summary_pdb_93\\bdac0e64f4ef427e.final.pdb
-1.207937e-14 -1.141432e-15 3.681672e-16 6.422701e-16 -3.928548e-15
3.548979e-16
E:\\R-3.1.2\\scripts\\mPDBS\\summary_pdb_93\\ca2a1f55fc9d0681.final.pdb
-5.934760e-15 4.861578e-15 5.589685e-15 -2.109598e-15 -3.596349e-15
9.543294e-15
E:\\R-3.1.2\\scripts\\mPDBS\\summary_pdb_93\\ce34248de3e81a3c.final.pdb
-5.040815e-15 2.610184e-15 8.083695e-15 2.006933e-15 -6.704309e-15

2.854917e-15
E:\\R-3.1.2\\scripts\\mPDBS\\summary_pdb_93\\d02e697772494a61.final.pdb
-2.200243e-15 6.475677e-15 -2.120346e-15 -2.117417e-15 8.205809e-15
-2.394072e-15
E:\\R-3.1.2\\scripts\\mPDBS\\summary_pdb_93\\d04221245d015047.final.pdb
-5.245100e-15 1.633891e-15 6.616323e-15 1.489022e-15 -3.758623e-15
3.772494e-15
E:\\R-3.1.2\\scripts\\mPDBS\\summary_pdb_93\\d678141e4018054a.final.pdb
4.983553e-15 -1.034558e-15 1.886672e-14 -4.301164e-15 -3.652436e-15
-1.893767e-16
E:\\R-3.1.2\\scripts\\mPDBS\\summary_pdb_93\\d71b40af0f0efe39.final.pdb
3.642445e-16 -3.966585e-15 6.329577e-15 -1.285476e-15 5.787794e-16
-5.523109e-15
E:\\R-3.1.2\\scripts\\mPDBS\\summary_pdb_93\\dc0809eff10197e2.final.pdb
-4.927970e-15 4.040684e-15 -1.078210e-16 2.899384e-15 -1.290217e-15
-1.159138e-15
E:\\R-3.1.2\\scripts\\mPDBS\\summary_pdb_93\\e1163918d3707839.final.pdb
-1.469610e-15 3.664739e-15 6.304529e-15 -1.115262e-16 1.905744e-15
-9.234600e-15
E:\\R-3.1.2\\scripts\\mPDBS\\summary_pdb_93\\e4b48b06824ef848.final.pdb
-3.358312e-15 1.889030e-15 1.333295e-14 -2.450213e-15 -9.619995e-15
7.354608e-15
E:\\R-3.1.2\\scripts\\mPDBS\\summary_pdb_93\\e529e19ad281fea2.final.pdb
-1.756493e-15 1.434813e-15 4.423891e-15 1.894173e-15 -4.275879e-15
-2.168730e-15
E:\\R-3.1.2\\scripts\\mPDBS\\summary_pdb_93\\e5602082718ec3a1.final.pdb
-7.253851e-16 5.094031e-15 1.117568e-15 1.188112e-15 -3.919007e-15
2.503353e-15
E:\\R-3.1.2\\scripts\\mPDBS\\summary_pdb_93\\e5d589d996d0a960.final.pdb
-6.301220e-15 -5.617603e-16 5.181886e-15 2.444701e-15 1.219041e-15
1.867184e-14
E:\\R-3.1.2\\scripts\\mPDBS\\summary_pdb_93\\e9df9e72307a2fbd.final.pdb
-6.133660e-15 1.165835e-15 7.974341e-15 5.576256e-16 -2.509139e-15
5.706587e-15
E:\\R-3.1.2\\scripts\\mPDBS\\summary_pdb_93\\ea00f8694090cae7.final.pdb
-7.253851e-16 5.094031e-15 1.117568e-15 1.188112e-15 -3.919007e-15
2.503353e-15
E:\\R-3.1.2\\scripts\\mPDBS\\summary_pdb_93\\eba517be32db050e.final.pdb
-2.919177e-15 5.252697e-15 3.297629e-15 -4.643337e-16 -5.276462e-15
8.779955e-16
E:\\R-3.1.2\\scripts\\mPDBS\\summary_pdb_93\\ec04b9545899c29d.final.pdb
-3.592152e-15 2.628241e-15 6.152741e-15 -1.537017e-16 -2.570710e-15
3.031684e-15
E:\\R-3.1.2\\scripts\\mPDBS\\summary_pdb_93\\ee6862cbcad52078.final.pdb
-3.354159e-15 2.966331e-15 5.997292e-15 -5.277054e-17 -3.124538e-15
2.704442e-15
E:\\R-3.1.2\\scripts\\mPDBS\\summary_pdb_93\\ef6a42fc83b81e5d.final.pdb
-3.410571e-15 2.977135e-15 5.819431e-15 -2.611542e-16 -2.917891e-15
3.149692e-15
E:\\R-3.1.2\\scripts\\mPDBS\\summary_pdb_93\\ef8e88d4ad93a0ad.final.pdb
-5.245100e-15 1.633891e-15 6.616323e-15 1.489022e-15 -3.758623e-15
3.772494e-15
E:\\R-3.1.2\\scripts\\mPDBS\\summary_pdb_93\\f14fcae8deec4fa3.final.pdb
-3.348745e-15 4.598317e-15 3.168486e-15 4.756276e-19 -2.486707e-15

1.515712e-15
E:\\R-3.1.2\\scripts\\mPDBS\\summary_pdb_93\\f83584e22003fb92.final.pdb
-6.747495e-15 1.377758e-15 1.632627e-14 2.606073e-15 -1.413396e-14
1.143243e-14
E:\\R-3.1.2\\scripts\\mPDBS\\summary_pdb_93\\f8e2bf87e52caa29.final.pdb
-1.756493e-15 1.434813e-15 4.423891e-15 1.894173e-15 -4.275879e-15
-2.168730e-15

[,667]   [,668]   [,669]   [,670]   [,671]   [,672]
E:\\R-3.1.2\\scripts\\40.1ns_data\\NAT0.pdb
4.034550e-15 2.913687e-15 -2.439992e-15 2.299598e-15 -2.835000e-15
5.987426e-15
E:\\R-3.1.2\\scripts\\40.1ns_data\\LJP6.pdb
3.393920e-15 3.369108e-15 -1.629848e-15 9.817092e-16 -1.173311e-15
4.027790e-15
E:\\R-3.1.2\\scripts\\40.1ns_data\\3WY9.pdb
3.944051e-15 1.915584e-15 -1.672406e-15 1.765958e-17 -1.408611e-15
4.658105e-15
E:\\R-3.1.2\\scripts\\40.1ns_data\\ZSP9.pdb
6.300410e-15 4.336384e-15 -7.966633e-16 1.157595e-15 -1.027349e-15
3.203723e-15
E:\\R-3.1.2\\scripts\\mPDBS\\summary_pdb_93\\013d855ad26c877c.final.pdb
2.279809e-15 8.487080e-15 -3.847260e-15 -3.947896e-15 -4.967468e-15
1.026330e-14
E:\\R-3.1.2\\scripts\\mPDBS\\summary_pdb_93\\0a2a705342ca0d54.final.pdb
4.344412e-15 1.989211e-15 -2.039134e-15 4.813303e-16 -2.323774e-15
4.465301e-15
E:\\R-3.1.2\\scripts\\mPDBS\\summary_pdb_93\\0a59fceadb4194d9.final.pdb
-5.167919e-16 1.295647e-15 -2.672146e-15 1.396144e-15 -2.224178e-15
1.824592e-15
E:\\R-3.1.2\\scripts\\mPDBS\\summary_pdb_93\\0dba59f3306825e8.final.pdb
-2.983342e-15 -2.188408e-15 -2.546480e-16 2.193025e-15 -6.844361e-16
5.255151e-15
E:\\R-3.1.2\\scripts\\mPDBS\\summary_pdb_93\\126f26a78b57339c.final.pdb
7.081524e-15 -1.193978e-15 3.769910e-15 -1.187951e-15 1.276316e-15
6.261073e-15
E:\\R-3.1.2\\scripts\\mPDBS\\summary_pdb_93\\12f06f3e342df706.final.pdb
4.363494e-15 2.063371e-15 -1.415718e-15 3.451545e-16 -2.017609e-15
5.017810e-15
E:\\R-3.1.2\\scripts\\mPDBS\\summary_pdb_93\\18618a2a251b867b.final.pdb
7.610184e-15 -4.185142e-15 -3.731956e-16 -3.784502e-15 1.026442e-15
6.806091e-15
E:\\R-3.1.2\\scripts\\mPDBS\\summary_pdb_93\\1f305f598641eeb2.final.pdb
3.976065e-15 1.346727e-15 -1.573006e-15 4.611497e-16 -1.330273e-15
4.948938e-15
E:\\R-3.1.2\\scripts\\mPDBS\\summary_pdb_93\\1f8a00b551be0a39.final.pdb
7.610184e-15 -4.185142e-15 -3.731956e-16 -3.784502e-15 1.026442e-15
6.806091e-15
E:\\R-3.1.2\\scripts\\mPDBS\\summary_pdb_93\\219f45328d47579a.final.pdb
2.095702e-15 1.586212e-14 5.096813e-15 -2.163075e-15 -6.010646e-15
-5.946969e-15
E:\\R-3.1.2\\scripts\\mPDBS\\summary_pdb_93\\238dd3918e54336d.final.pdb
1.331416e-14 -7.931998e-15 3.757607e-15 1.018841e-15 -6.015159e-15
5.369283e-15

E:\\R-3.1.2\\scripts\\mPDBS\\summary_pdb_93\\2b3027889e2fdc42.final.pdb
2.704590e-15 2.118785e-16 -6.676728e-16 6.387618e-17 -2.321242e-15
4.933867e-15
E:\\R-3.1.2\\scripts\\mPDBS\\summary_pdb_93\\2fcb3b46e33793b0.final.pdb
3.192684e-16 4.073712e-15 -5.526441e-15 -6.499407e-16 -3.280970e-15
5.432926e-15
E:\\R-3.1.2\\scripts\\mPDBS\\summary_pdb_93\\34eb328251842006.final.pdb
2.713231e-15 -2.349146e-15 -1.929845e-16 3.772567e-15 -2.049187e-15
1.255205e-15
E:\\R-3.1.2\\scripts\\mPDBS\\summary_pdb_93\\354b32871797b7ab.final.pdb
-4.792075e-15 -7.023663e-15 -1.054789e-14 1.401796e-16 -9.580686e-15
8.518998e-15
E:\\R-3.1.2\\scripts\\mPDBS\\summary_pdb_93\\397368adbc32d5de.final.pdb
7.081524e-15 -1.193978e-15 3.769910e-15 -1.187951e-15 1.276316e-15
6.261073e-15
E:\\R-3.1.2\\scripts\\mPDBS\\summary_pdb_93\\411ca13cd3df6edd.final.pdb
-4.578878e-16 1.642931e-15 -4.983473e-15 -4.981545e-15 -1.004660e-15
7.983186e-15
E:\\R-3.1.2\\scripts\\mPDBS\\summary_pdb_93\\42357eee6642bf12.final.pdb
5.443563e-15 1.583646e-15 -5.859187e-15 3.659436e-15 -8.851296e-16
-5.981918e-15
E:\\R-3.1.2\\scripts\\mPDBS\\summary_pdb_93\\44342d1f75a7fcfc.final.pdb
1.138118e-14 9.772978e-16 -3.664892e-16 1.988997e-15 5.337848e-15
5.785011e-15
E:\\R-3.1.2\\scripts\\mPDBS\\summary_pdb_93\\448ef21dffe8cdfb.final.pdb
2.022349e-14 -5.694516e-15 -3.375384e-15 -1.165242e-14 -4.596758e-15
1.065097e-14
E:\\R-3.1.2\\scripts\\mPDBS\\summary_pdb_93\\472a40b3e8f113c7.final.pdb
-2.983342e-15 -2.188408e-15 -2.546480e-16 2.193025e-15 -6.844361e-16
5.255151e-15
E:\\R-3.1.2\\scripts\\mPDBS\\summary_pdb_93\\48a26225951ea153.final.pdb
8.153356e-15 -8.850317e-15 3.124459e-16 -2.145728e-15 3.433704e-15
1.111208e-14
E:\\R-3.1.2\\scripts\\mPDBS\\summary_pdb_93\\4ab4a5c7c6682ce6.final.pdb
2.723898e-15 -5.286190e-15 -7.875288e-15 -4.397189e-15 -5.225427e-15
-2.211316e-15
E:\\R-3.1.2\\scripts\\mPDBS\\summary_pdb_93\\4eaa45b62cb6a25f.final.pdb
6.325768e-15 4.570145e-15 1.289867e-15 -9.470583e-16 -5.655858e-16
6.763675e-15
E:\\R-3.1.2\\scripts\\mPDBS\\summary_pdb_93\\58c371611d85ac96.final.pdb
7.081524e-15 -1.193978e-15 3.769910e-15 -1.187951e-15 1.276316e-15
6.261073e-15
E:\\R-3.1.2\\scripts\\mPDBS\\summary_pdb_93\\59a647f04822d30a.final.pdb
3.630434e-15 1.574043e-15 -1.342271e-15 1.498504e-15 -2.337436e-15
7.890098e-15
E:\\R-3.1.2\\scripts\\mPDBS\\summary_pdb_93\\59b091fe385e05fc.final.pdb
-5.167919e-16 1.295647e-15 -2.672146e-15 1.396144e-15 -2.224178e-15
1.824592e-15
E:\\R-3.1.2\\scripts\\mPDBS\\summary_pdb_93\\5c35ac30f930d2db.final.pdb
6.325768e-15 4.570145e-15 1.289867e-15 -9.470583e-16 -5.655858e-16
6.763675e-15
E:\\R-3.1.2\\scripts\\mPDBS\\summary_pdb_93\\5f60752431cf2dcb.final.pdb
8.649996e-15 7.094190e-16 1.013979e-15 -3.516340e-15 1.770688e-15
5.447805e-15

E:\\R-3.1.2\\scripts\\mPDBS\\summary_pdb_93\\626456dd6ff2868d.final.pdb
-2.983342e-15 -2.188408e-15 -2.546480e-16 2.193025e-15 -6.844361e-16
5.255151e-15
E:\\R-3.1.2\\scripts\\mPDBS\\summary_pdb_93\\6583d53ff3c8f7ef.final.pdb
3.630434e-15 1.574043e-15 -1.342271e-15 1.498504e-15 -2.337436e-15
7.890098e-15
E:\\R-3.1.2\\scripts\\mPDBS\\summary_pdb_93\\69760178986bef01.final.pdb
5.187237e-15 9.362702e-16 5.016873e-15 -5.200225e-15 -1.984179e-15
3.963432e-15
E:\\R-3.1.2\\scripts\\mPDBS\\summary_pdb_93\\6a9d87d47926b1c8.final.pdb
2.567773e-15 -1.340615e-16 -2.511786e-16 -2.182404e-16 -1.326636e-15
4.982582e-15
E:\\R-3.1.2\\scripts\\mPDBS\\summary_pdb_93\\6bb3547ea982ffc4.final.pdb
1.246361e-14 -7.885293e-15 1.032523e-14 -7.777073e-15 8.097748e-15
6.339132e-15
E:\\R-3.1.2\\scripts\\mPDBS\\summary_pdb_93\\716d9624611d1c42.final.pdb
-1.849511e-16 1.947578e-15 -2.355721e-15 3.655375e-16 -5.846997e-15
8.673740e-15
E:\\R-3.1.2\\scripts\\mPDBS\\summary_pdb_93\\75f27e70ad0f1be4.final.pdb
3.630434e-15 1.574043e-15 -1.342271e-15 1.498504e-15 -2.337436e-15
7.890098e-15
E:\\R-3.1.2\\scripts\\mPDBS\\summary_pdb_93\\769f910fffb22872.final.pdb
4.590031e-15 1.073456e-15 -9.100925e-16 4.706887e-17 -2.414206e-15
4.766923e-15
E:\\R-3.1.2\\scripts\\mPDBS\\summary_pdb_93\\791f8c80dd5c3135.final.pdb
-5.167919e-16 1.295647e-15 -2.672146e-15 1.396144e-15 -2.224178e-15
1.824592e-15
E:\\R-3.1.2\\scripts\\mPDBS\\summary_pdb_93\\7fad98e66a3f6099.final.pdb
1.845787e-15 1.500790e-15 -4.193041e-15 2.812501e-15 -3.180018e-15
-1.457403e-15
E:\\R-3.1.2\\scripts\\mPDBS\\summary_pdb_93\\804b89c94a633196.final.pdb
3.192684e-16 4.073712e-15 -5.526441e-15 -6.499407e-16 -3.280970e-15
5.432926e-15
E:\\R-3.1.2\\scripts\\mPDBS\\summary_pdb_93\\813cc65a2298feba.final.pdb
3.192684e-16 4.073712e-15 -5.526441e-15 -6.499407e-16 -3.280970e-15
5.432926e-15
E:\\R-3.1.2\\scripts\\mPDBS\\summary_pdb_93\\83c0eefcfe12d3f9.final.pdb
-4.578878e-16 1.642931e-15 -4.983473e-15 -4.981545e-15 -1.004660e-15
7.983186e-15
E:\\R-3.1.2\\scripts\\mPDBS\\summary_pdb_93\\86d50469ef569760.final.pdb
-2.983342e-15 -2.188408e-15 -2.546480e-16 2.193025e-15 -6.844361e-16
5.255151e-15
E:\\R-3.1.2\\scripts\\mPDBS\\summary_pdb_93\\89d0666e1402203d.final.pdb
3.630434e-15 1.574043e-15 -1.342271e-15 1.498504e-15 -2.337436e-15
7.890098e-15
E:\\R-3.1.2\\scripts\\mPDBS\\summary_pdb_93\\8b34852716dadc62.final.pdb
3.941371e-15 -1.685570e-15 2.298896e-15 3.736308e-15 2.439281e-15
-2.555476e-15
E:\\R-3.1.2\\scripts\\mPDBS\\summary_pdb_93\\8b6ef9ac3fe74395.final.pdb
7.161551e-15 -4.981694e-15 2.581483e-16 5.540440e-15 7.667009e-15
-2.616397e-15
E:\\R-3.1.2\\scripts\\mPDBS\\summary_pdb_93\\98bbe0d5986c138f.final.pdb
-5.167919e-16 1.295647e-15 -2.672146e-15 1.396144e-15 -2.224178e-15
1.824592e-15

E:\\R-3.1.2\\scripts\\mPDBS\\summary_pdb_93\\9c22f4909d67e2fa.final.pdb
5.374253e-15 7.011249e-15 5.204558e-15 4.893802e-15 -4.624324e-15
1.005683e-14
E:\\R-3.1.2\\scripts\\mPDBS\\summary_pdb_93\\9dda8ed0d81fb3ef.final.pdb
5.481384e-15 -2.509280e-15 -3.069405e-15 -3.007874e-15 1.146108e-14
2.913827e-15
E:\\R-3.1.2\\scripts\\mPDBS\\summary_pdb_93\\9f752b3616e6033b.final.pdb
5.266456e-15 1.504286e-15 -1.139491e-15 1.489158e-15 -1.940709e-15
1.626617e-15
E:\\R-3.1.2\\scripts\\mPDBS\\summary_pdb_93\\a02c70083816d6ba.final.pdb
6.325768e-15 4.570145e-15 1.289867e-15 -9.470583e-16 -5.655858e-16
6.763675e-15
E:\\R-3.1.2\\scripts\\mPDBS\\summary_pdb_93\\a0c997d891e4e082.final.pdb
7.736870e-15 7.245309e-15 -5.406689e-15 3.246005e-15 -4.649101e-15
6.749716e-15
E:\\R-3.1.2\\scripts\\mPDBS\\summary_pdb_93\\ab8039a2d823c616.final.pdb
2.419942e-15 2.177531e-15 -2.126327e-15 2.397292e-16 -2.101402e-15
5.259372e-15
E:\\R-3.1.2\\scripts\\mPDBS\\summary_pdb_93\\acf4d74b9c71d4dc.final.pdb
3.192684e-16 4.073712e-15 -5.526441e-15 -6.499407e-16 -3.280970e-15
5.432926e-15
E:\\R-3.1.2\\scripts\\mPDBS\\summary_pdb_93\\ad8a823e4b4c668e.final.pdb
5.325295e-15 8.225654e-16 -8.554773e-16 2.190534e-16 -2.513244e-15
4.443107e-15
E:\\R-3.1.2\\scripts\\mPDBS\\summary_pdb_93\\b16ababd03b7b857.final.pdb
5.374253e-15 7.011249e-15 5.204558e-15 4.893802e-15 -4.624324e-15
1.005683e-14
E:\\R-3.1.2\\scripts\\mPDBS\\summary_pdb_93\\b226923e697d3c63.final.pdb
8.902259e-15 4.076598e-15 -1.047646e-14 2.411836e-15 6.151080e-17
-1.489552e-15
E:\\R-3.1.2\\scripts\\mPDBS\\summary_pdb_93\\b28e48f6fa27a165.final.pdb
-5.167919e-16 1.295647e-15 -2.672146e-15 1.396144e-15 -2.224178e-15
1.824592e-15
E:\\R-3.1.2\\scripts\\mPDBS\\summary_pdb_93\\b42c4924e3cd1aec.final.pdb
1.876629e-15 1.199993e-15 -2.016905e-16 5.543012e-15 -1.213194e-14
1.862151e-15
E:\\R-3.1.2\\scripts\\mPDBS\\summary_pdb_93\\b4dd239fcabff1df.final.pdb
-5.167919e-16 1.295647e-15 -2.672146e-15 1.396144e-15 -2.224178e-15
1.824592e-15
E:\\R-3.1.2\\scripts\\mPDBS\\summary_pdb_93\\b669942ef97459a2.final.pdb
3.941371e-15 -1.685570e-15 2.298896e-15 3.736308e-15 2.439281e-15
-2.555476e-15
E:\\R-3.1.2\\scripts\\mPDBS\\summary_pdb_93\\b99f4d9d87c1017b.final.pdb
-5.167919e-16 1.295647e-15 -2.672146e-15 1.396144e-15 -2.224178e-15
1.824592e-15
E:\\R-3.1.2\\scripts\\mPDBS\\summary_pdb_93\\b9d2cbf3975403e7.final.pdb
-5.167919e-16 1.295647e-15 -2.672146e-15 1.396144e-15 -2.224178e-15
1.824592e-15
E:\\R-3.1.2\\scripts\\mPDBS\\summary_pdb_93\\bc88c8a505f3a288.final.pdb
2.695916e-15 7.466070e-16 -8.218464e-16 -1.486274e-16 -2.243531e-15
4.842144e-15
E:\\R-3.1.2\\scripts\\mPDBS\\summary_pdb_93\\bdac0e64f4ef427e.final.pdb
1.084557e-14 -3.840937e-16 6.559811e-15 1.024889e-14 -1.594304e-15
2.807796e-18

E:\\R-3.1.2\\scripts\\mPDBS\\summary_pdb_93\\ca2a1f55fc9d0681.final.pdb
3.941371e-15 -1.685570e-15 2.298896e-15 3.736308e-15 2.439281e-15
-2.555476e-15
E:\\R-3.1.2\\scripts\\mPDBS\\summary_pdb_93\\ce34248de3e81a3c.final.pdb
6.606556e-16 2.162029e-15 -5.643544e-15 3.753183e-15 -4.916625e-15
6.579004e-15
E:\\R-3.1.2\\scripts\\mPDBS\\summary_pdb_93\\d02e697772494a61.final.pdb
4.146145e-15 4.198924e-15 9.664367e-16 -1.606663e-15 -5.617040e-15
8.897710e-15
E:\\R-3.1.2\\scripts\\mPDBS\\summary_pdb_93\\d04221245d015047.final.pdb
2.664538e-15 -2.278796e-16 2.194869e-15 -3.778324e-16 -2.925653e-15
6.407976e-15
E:\\R-3.1.2\\scripts\\mPDBS\\summary_pdb_93\\d678141e4018054a.final.pdb
7.168914e-15 -2.097279e-15 -1.417861e-15 -6.794132e-15 7.690929e-16
7.307740e-15
E:\\R-3.1.2\\scripts\\mPDBS\\summary_pdb_93\\d71b40af0f0efe39.final.pdb
-1.269514e-15 7.953390e-15 -9.227476e-15 -3.965449e-15 -8.929091e-15
8.910867e-15
E:\\R-3.1.2\\scripts\\mPDBS\\summary_pdb_93\\dc0809eff10197e2.final.pdb
3.189098e-15 8.461170e-16 -1.793863e-16 1.144076e-14 -4.589088e-15
7.240900e-15
E:\\R-3.1.2\\scripts\\mPDBS\\summary_pdb_93\\e1163918d3707839.final.pdb
-2.753348e-15 1.154890e-15 -8.019048e-16 -1.834333e-15 -3.975706e-15
8.534088e-16
E:\\R-3.1.2\\scripts\\mPDBS\\summary_pdb_93\\e4b48b06824ef848.final.pdb
7.610184e-15 -4.185142e-15 -3.731956e-16 -3.784502e-15 1.026442e-15
6.806091e-15
E:\\R-3.1.2\\scripts\\mPDBS\\summary_pdb_93\\e529e19ad281fea2.final.pdb
2.279809e-15 8.487080e-15 -3.847260e-15 -3.947896e-15 -4.967468e-15
1.026330e-14
E:\\R-3.1.2\\scripts\\mPDBS\\summary_pdb_93\\e5602082718ec3a1.final.pdb
3.192684e-16 4.073712e-15 -5.526441e-15 -6.499407e-16 -3.280970e-15
5.432926e-15
E:\\R-3.1.2\\scripts\\mPDBS\\summary_pdb_93\\e5d589d996d0a960.final.pdb
8.751023e-15 8.347215e-16 -3.921389e-16 2.757769e-15 -2.326616e-15
9.629339e-15
E:\\R-3.1.2\\scripts\\mPDBS\\summary_pdb_93\\e9df9e72307a2fbd.final.pdb
6.325768e-15 4.570145e-15 1.289867e-15 -9.470583e-16 -5.655858e-16
6.763675e-15
E:\\R-3.1.2\\scripts\\mPDBS\\summary_pdb_93\\ea00f8694090cae7.final.pdb
3.192684e-16 4.073712e-15 -5.526441e-15 -6.499407e-16 -3.280970e-15
5.432926e-15
E:\\R-3.1.2\\scripts\\mPDBS\\summary_pdb_93\\eba517be32db050e.final.pdb
-5.167919e-16 1.295647e-15 -2.672146e-15 1.396144e-15 -2.224178e-15
1.824592e-15
E:\\R-3.1.2\\scripts\\mPDBS\\summary_pdb_93\\ec04b9545899c29d.final.pdb
4.520347e-15 9.027047e-16 -1.140176e-15 4.251440e-16 -1.784059e-15
4.763259e-15
E:\\R-3.1.2\\scripts\\mPDBS\\summary_pdb_93\\ee6862cbcad52078.final.pdb
2.151213e-15 1.958323e-16 -6.000186e-16 -1.768167e-16 -1.692919e-15
4.615762e-15
E:\\R-3.1.2\\scripts\\mPDBS\\summary_pdb_93\\ef6a42fc83b81e5d.final.pdb
4.720135e-15 1.634422e-15 -1.178975e-15 3.168184e-16 -2.028921e-15
4.558106e-15

E:\\R-3.1.2\\scripts\\mPDBS\\summary_pdb_93\\ef8e88d4ad93a0ad.final.pdb
2.664538e-15 -2.278796e-16 2.194869e-15 -3.778324e-16 -2.925653e-15
6.407976e-15
E:\\R-3.1.2\\scripts\\mPDBS\\summary_pdb_93\\f14fcae8deec4fa3.final.pdb
5.266456e-15 1.504286e-15 -1.139491e-15 1.489158e-15 -1.940709e-15
1.626617e-15
E:\\R-3.1.2\\scripts\\mPDBS\\summary_pdb_93\\f83584e22003fb92.final.pdb
8.153356e-15 -8.850317e-15 3.124459e-16 -2.145728e-15 3.433704e-15
1.111208e-14
E:\\R-3.1.2\\scripts\\mPDBS\\summary_pdb_93\\f8e2bf87e52caa29.final.pdb
2.279809e-15 8.487080e-15 -3.847260e-15 -3.947896e-15 -4.967468e-15
1.026330e-14

[,673]   [,674]   [,675]   [,676]   [,677]   [,678]
E:\\R-3.1.2\\scripts\\40.1ns_data\\NAT0.pdb
2.047101e-15 -3.025189e-15 -2.971982e-15 2.188976e-15 2.403509e-15
-2.549375e-15
E:\\R-3.1.2\\scripts\\40.1ns_data\\LJP6.pdb
2.794338e-15 -4.212332e-15 -2.682709e-15 2.273582e-15 2.726817e-15
-3.290315e-17
E:\\R-3.1.2\\scripts\\40.1ns_data\\3WY9.pdb
6.260105e-16 -5.874945e-15 -3.162588e-15 1.554005e-15 1.766018e-15
-2.539624e-16
E:\\R-3.1.2\\scripts\\40.1ns_data\\ZSP9.pdb
2.218422e-15 -4.801896e-15 -2.214754e-15 2.681558e-15 1.942813e-15
-2.319183e-15
E:\\R-3.1.2\\scripts\\mPDBS\\summary_pdb_93\\013d855ad26c877c.final.pdb
1.909052e-15 -6.730263e-15 -1.049538e-15 2.878203e-15 1.950224e-15
-4.352856e-15
E:\\R-3.1.2\\scripts\\mPDBS\\summary_pdb_93\\0a2a705342ca0d54.final.pdb
8.414151e-16 -4.526182e-15 -2.171055e-15 2.111782e-15 1.322216e-15
-7.025414e-16
E:\\R-3.1.2\\scripts\\mPDBS\\summary_pdb_93\\0a59fceadb4194d9.final.pdb
5.675357e-15 -7.613441e-15 -5.246065e-15 5.903519e-15 -1.734701e-15
-1.218127e-15
E:\\R-3.1.2\\scripts\\mPDBS\\summary_pdb_93\\0dba59f3306825e8.final.pdb
2.691846e-15 -5.203706e-15 -4.314721e-15 3.951997e-15 8.885736e-16
-3.048789e-16
E:\\R-3.1.2\\scripts\\mPDBS\\summary_pdb_93\\126f26a78b57339c.final.pdb
-7.919674e-15 1.398651e-15 5.702603e-16 -2.049279e-15 -1.304551e-15
2.205700e-15
E:\\R-3.1.2\\scripts\\mPDBS\\summary_pdb_93\\12f06f3e342df706.final.pdb
9.790003e-16 -4.393476e-15 -2.249768e-15 2.071667e-15 1.491785e-15
-6.747859e-16
E:\\R-3.1.2\\scripts\\mPDBS\\summary_pdb_93\\18618a2a251b867b.final.pdb
-8.621746e-15 2.831771e-15 -4.332357e-16 -2.571718e-16 -4.275065e-16
-6.196173e-18
E:\\R-3.1.2\\scripts\\mPDBS\\summary_pdb_93\\1f305f598641eeb2.final.pdb
9.957216e-16 -4.030774e-15 -2.496246e-15 2.191799e-15 1.702144e-15
-8.519023e-16
E:\\R-3.1.2\\scripts\\mPDBS\\summary_pdb_93\\1f8a00b551be0a39.final.pdb
-8.621746e-15 2.831771e-15 -4.332357e-16 -2.571718e-16 -4.275065e-16
-6.196173e-18
E:\\R-3.1.2\\scripts\\mPDBS\\summary_pdb_93\\219f45328d47579a.final.pdb

-6.273961e-15 -1.047227e-14 5.239904e-15 2.726373e-15 8.574760e-15
1.737409e-15
E:\\R-3.1.2\\scripts\\mPDBS\\summary_pdb_93\\238dd3918e54336d.final.pdb
8.970681e-15 5.682347e-15 4.190397e-15 7.905824e-15 9.628171e-15
7.680354e-15
E:\\R-3.1.2\\scripts\\mPDBS\\summary_pdb_93\\2b3027889e2fdc42.final.pdb
1.721813e-15 -4.967121e-15 -2.070220e-15 2.222436e-15 1.088128e-15
-6.693035e-16
E:\\R-3.1.2\\scripts\\mPDBS\\summary_pdb_93\\2fcb3b46e33793b0.final.pdb
6.997431e-15 -9.314742e-15 -3.386593e-15 3.563965e-15 2.880021e-15
-2.827752e-15
E:\\R-3.1.2\\scripts\\mPDBS\\summary_pdb_93\\34eb328251842006.final.pdb
-3.382424e-15 -1.022187e-14 -3.387724e-15 4.212233e-15 -4.425853e-15
-1.141628e-14
E:\\R-3.1.2\\scripts\\mPDBS\\summary_pdb_93\\354b32871797b7ab.final.pdb
-8.744325e-15 -6.879262e-15 -3.950821e-15 -8.647060e-16 -4.550998e-15
-1.427277e-14
E:\\R-3.1.2\\scripts\\mPDBS\\summary_pdb_93\\397368adbc32d5de.final.pdb
-7.919674e-15 1.398651e-15 5.702603e-16 -2.049279e-15 -1.304551e-15
2.205700e-15
E:\\R-3.1.2\\scripts\\mPDBS\\summary_pdb_93\\411ca13cd3df6edd.final.pdb
-2.028986e-15 -8.672894e-15 -6.409589e-16 -1.321883e-15 -3.525879e-15
-4.107545e-15
E:\\R-3.1.2\\scripts\\mPDBS\\summary_pdb_93\\42357eee6642bf12.final.pdb
7.425141e-15 -5.255451e-15 -5.232239e-15 1.364795e-14 4.105619e-16
-3.964697e-15
E:\\R-3.1.2\\scripts\\mPDBS\\summary_pdb_93\\44342d1f75a7fcfc.final.pdb
9.202597e-15 -1.046229e-14 -4.401551e-16 -2.432000e-15 -8.000348e-15
-3.126909e-15
E:\\R-3.1.2\\scripts\\mPDBS\\summary_pdb_93\\448ef21dffe8cdfb.final.pdb
-7.418988e-15 9.919872e-15 -1.112370e-15 -5.484352e-15 9.962897e-15
-2.789588e-15
E:\\R-3.1.2\\scripts\\mPDBS\\summary_pdb_93\\472a40b3e8f113c7.final.pdb
2.691846e-15 -5.203706e-15 -4.314721e-15 3.951997e-15 8.885736e-16
-3.048789e-16
E:\\R-3.1.2\\scripts\\mPDBS\\summary_pdb_93\\48a26225951ea153.final.pdb
-7.190437e-15 2.526621e-16 2.747974e-15 -1.147494e-14 -2.733034e-15
-1.004895e-14
E:\\R-3.1.2\\scripts\\mPDBS\\summary_pdb_93\\4ab4a5c7c6682ce6.final.pdb
5.068634e-15 -6.858633e-15 -6.333505e-15 5.129080e-16 6.519052e-15
-3.836342e-15
E:\\R-3.1.2\\scripts\\mPDBS\\summary_pdb_93\\4eaa45b62cb6a25f.final.pdb
1.582918e-15 -2.184232e-15 -1.510715e-15 1.934704e-15 2.059166e-15
4.517534e-15
E:\\R-3.1.2\\scripts\\mPDBS\\summary_pdb_93\\58c371611d85ac96.final.pdb
-7.919674e-15 1.398651e-15 5.702603e-16 -2.049279e-15 -1.304551e-15
2.205700e-15
E:\\R-3.1.2\\scripts\\mPDBS\\summary_pdb_93\\59a647f04822d30a.final.pdb
-3.504360e-15 6.087196e-16 -3.190559e-15 4.475235e-15 7.242667e-15
-8.609265e-16
E:\\R-3.1.2\\scripts\\mPDBS\\summary_pdb_93\\59b091fe385e05fc.final.pdb
5.675357e-15 -7.613441e-15 -5.246065e-15 5.903519e-15 -1.734701e-15
-1.218127e-15
E:\\R-3.1.2\\scripts\\mPDBS\\summary_pdb_93\\5c35ac30f930d2db.final.pdb

1.582918e-15 -2.184232e-15 -1.510715e-15 1.934704e-15 2.059166e-15
4.517534e-15
E:\\R-3.1.2\\scripts\\mPDBS\\summary_pdb_93\\5f60752431cf2dcb.final.pdb
4.208947e-15 -8.442319e-16 -3.749870e-15 -1.071436e-15 2.729510e-15
4.118906e-15
E:\\R-3.1.2\\scripts\\mPDBS\\summary_pdb_93\\626456dd6ff2868d.final.pdb
2.691846e-15 -5.203706e-15 -4.314721e-15 3.951997e-15 8.885736e-16
-3.048789e-16
E:\\R-3.1.2\\scripts\\mPDBS\\summary_pdb_93\\6583d53ff3c8f7ef.final.pdb
-3.504360e-15 6.087196e-16 -3.190559e-15 4.475235e-15 7.242667e-15
-8.609265e-16
E:\\R-3.1.2\\scripts\\mPDBS\\summary_pdb_93\\69760178986bef01.final.pdb
6.141005e-15 4.680980e-15 -1.565942e-15 -5.188549e-15 7.472106e-15
4.429421e-15
E:\\R-3.1.2\\scripts\\mPDBS\\summary_pdb_93\\6a9d87d47926b1c8.final.pdb
8.458837e-16 -4.720586e-15 -2.475047e-15 2.159161e-15 1.407690e-15
-1.017417e-15
E:\\R-3.1.2\\scripts\\mPDBS\\summary_pdb_93\\6bb3547ea982ffc4.final.pdb
-1.273884e-14 -7.346266e-15 3.659467e-15 -5.378453e-15 4.621748e-15
-4.303492e-15
E:\\R-3.1.2\\scripts\\mPDBS\\summary_pdb_93\\716d9624611d1c42.final.pdb
-1.086808e-14 -6.182843e-15 1.868676e-16 1.410954e-15 -4.522068e-15
-6.307867e-15
E:\\R-3.1.2\\scripts\\mPDBS\\summary_pdb_93\\75f27e70ad0f1be4.final.pdb
-3.504360e-15 6.087196e-16 -3.190559e-15 4.475235e-15 7.242667e-15
-8.609265e-16
E:\\R-3.1.2\\scripts\\mPDBS\\summary_pdb_93\\769f910fffb22872.final.pdb
8.476760e-16 -4.928515e-15 -2.176850e-15 2.132410e-15 9.980025e-16
-2.828846e-16
E:\\R-3.1.2\\scripts\\mPDBS\\summary_pdb_93\\791f8c80dd5c3135.final.pdb
5.675357e-15 -7.613441e-15 -5.246065e-15 5.903519e-15 -1.734701e-15
-1.218127e-15
E:\\R-3.1.2\\scripts\\mPDBS\\summary_pdb_93\\7fad98e66a3f6099.final.pdb
1.096328e-14 -1.435156e-14 -1.972255e-15 -1.774571e-15 -2.763777e-15
-4.632134e-15
E:\\R-3.1.2\\scripts\\mPDBS\\summary_pdb_93\\804b89c94a633196.final.pdb
6.997431e-15 -9.314742e-15 -3.386593e-15 3.563965e-15 2.880021e-15
-2.827752e-15
E:\\R-3.1.2\\scripts\\mPDBS\\summary_pdb_93\\813cc65a2298feba.final.pdb
6.997431e-15 -9.314742e-15 -3.386593e-15 3.563965e-15 2.880021e-15
-2.827752e-15
E:\\R-3.1.2\\scripts\\mPDBS\\summary_pdb_93\\83c0eefcfe12d3f9.final.pdb
-2.028986e-15 -8.672894e-15 -6.409589e-16 -1.321883e-15 -3.525879e-15
-4.107545e-15
E:\\R-3.1.2\\scripts\\mPDBS\\summary_pdb_93\\86d50469ef569760.final.pdb
2.691846e-15 -5.203706e-15 -4.314721e-15 3.951997e-15 8.885736e-16
-3.048789e-16
E:\\R-3.1.2\\scripts\\mPDBS\\summary_pdb_93\\89d0666e1402203d.final.pdb
-3.504360e-15 6.087196e-16 -3.190559e-15 4.475235e-15 7.242667e-15
-8.609265e-16
E:\\R-3.1.2\\scripts\\mPDBS\\summary_pdb_93\\8b34852716dadc62.final.pdb
-8.682387e-16 -4.502619e-15 -2.081647e-15 1.985367e-15 -1.534850e-15
2.627953e-15
E:\\R-3.1.2\\scripts\\mPDBS\\summary_pdb_93\\8b6ef9ac3fe74395.final.pdb

7.797115e-16 -1.054195e-14 -2.139731e-15 8.252122e-15 9.788272e-15
2.220348e-15
E:\\R-3.1.2\\scripts\\mPDBS\\summary_pdb_93\\98bbe0d5986c138f.final.pdb
5.675357e-15 -7.613441e-15 -5.246065e-15 5.903519e-15 -1.734701e-15
-1.218127e-15
E:\\R-3.1.2\\scripts\\mPDBS\\summary_pdb_93\\9c22f4909d67e2fa.final.pdb
1.707067e-15 4.941216e-15 -2.093573e-15 3.011022e-15 -7.351428e-16
3.719094e-15
E:\\R-3.1.2\\scripts\\mPDBS\\summary_pdb_93\\9dda8ed0d81fb3ef.final.pdb
3.350163e-16 -7.402233e-15 -1.019385e-14 -4.778647e-15 -5.916078e-15
-5.531019e-15
E:\\R-3.1.2\\scripts\\mPDBS\\summary_pdb_93\\9f752b3616e6033b.final.pdb
4.666800e-15 -5.638218e-15 -2.486968e-15 2.977007e-15 4.296613e-15
8.388528e-16
E:\\R-3.1.2\\scripts\\mPDBS\\summary_pdb_93\\a02c70083816d6ba.final.pdb
1.582918e-15 -2.184232e-15 -1.510715e-15 1.934704e-15 2.059166e-15
4.517534e-15
E:\\R-3.1.2\\scripts\\mPDBS\\summary_pdb_93\\a0c997d891e4e082.final.pdb
6.313698e-15 -8.233983e-15 -1.031990e-14 -2.293144e-15 2.498109e-15
-2.196959e-15
E:\\R-3.1.2\\scripts\\mPDBS\\summary_pdb_93\\ab8039a2d823c616.final.pdb
7.274551e-16 -4.276212e-15 -2.202260e-15 2.232316e-15 7.928754e-16
-3.796198e-16
E:\\R-3.1.2\\scripts\\mPDBS\\summary_pdb_93\\acf4d74b9c71d4dc.final.pdb
6.997431e-15 -9.314742e-15 -3.386593e-15 3.563965e-15 2.880021e-15
-2.827752e-15
E:\\R-3.1.2\\scripts\\mPDBS\\summary_pdb_93\\ad8a823e4b4c668e.final.pdb
8.851465e-16 -4.307078e-15 -2.118825e-15 1.816737e-15 1.416196e-15
1.085681e-16
E:\\R-3.1.2\\scripts\\mPDBS\\summary_pdb_93\\b16ababd03b7b857.final.pdb
1.707067e-15 4.941216e-15 -2.093573e-15 3.011022e-15 -7.351428e-16
3.719094e-15
E:\\R-3.1.2\\scripts\\mPDBS\\summary_pdb_93\\b226923e697d3c63.final.pdb
1.789582e-15 -5.055153e-15 -1.011926e-14 8.033587e-15 5.018752e-15
9.670384e-16
E:\\R-3.1.2\\scripts\\mPDBS\\summary_pdb_93\\b28e48f6fa27a165.final.pdb
5.675357e-15 -7.613441e-15 -5.246065e-15 5.903519e-15 -1.734701e-15
-1.218127e-15
E:\\R-3.1.2\\scripts\\mPDBS\\summary_pdb_93\\b42c4924e3cd1aec.final.pdb
5.154512e-15 -9.250581e-15 4.452199e-15 5.275810e-15 -1.646490e-15
2.351565e-15
E:\\R-3.1.2\\scripts\\mPDBS\\summary_pdb_93\\b4dd239fcabff1df.final.pdb
5.675357e-15 -7.613441e-15 -5.246065e-15 5.903519e-15 -1.734701e-15
-1.218127e-15
E:\\R-3.1.2\\scripts\\mPDBS\\summary_pdb_93\\b669942ef97459a2.final.pdb
-8.682387e-16 -4.502619e-15 -2.081647e-15 1.985367e-15 -1.534850e-15
2.627953e-15
E:\\R-3.1.2\\scripts\\mPDBS\\summary_pdb_93\\b99f4d9d87c1017b.final.pdb
5.675357e-15 -7.613441e-15 -5.246065e-15 5.903519e-15 -1.734701e-15
-1.218127e-15
E:\\R-3.1.2\\scripts\\mPDBS\\summary_pdb_93\\b9d2cbf3975403e7.final.pdb
5.675357e-15 -7.613441e-15 -5.246065e-15 5.903519e-15 -1.734701e-15
-1.218127e-15
E:\\R-3.1.2\\scripts\\mPDBS\\summary_pdb_93\\bc88c8a505f3a288.final.pdb

8.226303e-16 -4.492674e-15 -1.939249e-15 2.258431e-15 1.366551e-15
-8.963354e-16
E:\\R-3.1.2\\scripts\\mPDBS\\summary_pdb_93\\bdac0e64f4ef427e.final.pdb
6.378460e-15 5.458164e-15 -6.116180e-15 8.014832e-15 1.097511e-14
6.456921e-15
E:\\R-3.1.2\\scripts\\mPDBS\\summary_pdb_93\\ca2a1f55fc9d0681.final.pdb
-8.682387e-16 -4.502619e-15 -2.081647e-15 1.985367e-15 -1.534850e-15
2.627953e-15
E:\\R-3.1.2\\scripts\\mPDBS\\summary_pdb_93\\ce34248de3e81a3c.final.pdb
1.630594e-15 -4.058200e-15 -4.626958e-15 6.186358e-15 -4.075100e-15
-1.636297e-15
E:\\R-3.1.2\\scripts\\mPDBS\\summary_pdb_93\\d02e697772494a61.final.pdb
-2.409539e-15 3.061179e-15 -6.646606e-16 5.924336e-15 1.081993e-14
2.690422e-15
E:\\R-3.1.2\\scripts\\mPDBS\\summary_pdb_93\\d04221245d015047.final.pdb
4.178070e-16 -4.282283e-15 4.327848e-16 7.475330e-17 2.672541e-16
-3.104477e-16
E:\\R-3.1.2\\scripts\\mPDBS\\summary_pdb_93\\d678141e4018054a.final.pdb
-1.721572e-14 -3.343944e-15 3.926774e-15 -4.646217e-15 -3.588309e-15
-7.416570e-15
E:\\R-3.1.2\\scripts\\mPDBS\\summary_pdb_93\\d71b40af0f0efe39.final.pdb
4.600677e-16 -4.538071e-15 1.546488e-15 -4.243853e-16 -4.071544e-15
2.987602e-15
E:\\R-3.1.2\\scripts\\mPDBS\\summary_pdb_93\\dc0809eff10197e2.final.pdb
6.432032e-15 -1.094315e-14 -7.764416e-15 5.058568e-15 7.146047e-15
4.895724e-15
E:\\R-3.1.2\\scripts\\mPDBS\\summary_pdb_93\\e1163918d3707839.final.pdb
2.504288e-15 -7.163709e-15 1.075776e-15 9.962471e-16 -4.191329e-15
-2.903780e-15
E:\\R-3.1.2\\scripts\\mPDBS\\summary_pdb_93\\e4b48b06824ef848.final.pdb
-8.621746e-15 2.831771e-15 -4.332357e-16 -2.571718e-16 -4.275065e-16
-6.196173e-18
E:\\R-3.1.2\\scripts\\mPDBS\\summary_pdb_93\\e529e19ad281fea2.final.pdb
1.909052e-15 -6.730263e-15 -1.049538e-15 2.878203e-15 1.950224e-15
-4.352856e-15
E:\\R-3.1.2\\scripts\\mPDBS\\summary_pdb_93\\e5602082718ec3a1.final.pdb
6.997431e-15 -9.314742e-15 -3.386593e-15 3.563965e-15 2.880021e-15
-2.827752e-15
E:\\R-3.1.2\\scripts\\mPDBS\\summary_pdb_93\\e5d589d996d0a960.final.pdb
4.075578e-15 -6.631003e-15 -2.765297e-15 -8.937574e-15 9.544709e-15
3.546437e-15
E:\\R-3.1.2\\scripts\\mPDBS\\summary_pdb_93\\e9df9e72307a2fbd.final.pdb
1.582918e-15 -2.184232e-15 -1.510715e-15 1.934704e-15 2.059166e-15
4.517534e-15
E:\\R-3.1.2\\scripts\\mPDBS\\summary_pdb_93\\ea00f8694090cae7.final.pdb
6.997431e-15 -9.314742e-15 -3.386593e-15 3.563965e-15 2.880021e-15
-2.827752e-15
E:\\R-3.1.2\\scripts\\mPDBS\\summary_pdb_93\\eba517be32db050e.final.pdb
5.675357e-15 -7.613441e-15 -5.246065e-15 5.903519e-15 -1.734701e-15
-1.218127e-15
E:\\R-3.1.2\\scripts\\mPDBS\\summary_pdb_93\\ec04b9545899c29d.final.pdb
7.232691e-16 -4.297078e-15 -2.023958e-15 1.696208e-15 1.783492e-15
-2.130073e-16
E:\\R-3.1.2\\scripts\\mPDBS\\summary_pdb_93\\ee6862cbcad52078.final.pdb

9.221058e-16 -4.692167e-15 -2.175171e-15 2.216364e-15 1.450251e-15
-8.022267e-16
E:\\R-3.1.2\\scripts\\mPDBS\\summary_pdb_93\\ef6a42fc83b81e5d.final.pdb
8.349909e-16 -4.068092e-15 -2.302183e-15 1.949396e-15 1.360560e-15
-4.257824e-16
E:\\R-3.1.2\\scripts\\mPDBS\\summary_pdb_93\\ef8e88d4ad93a0ad.final.pdb
4.178070e-16 -4.282283e-15 4.327848e-16 7.475330e-17 2.672541e-16
-3.104477e-16
E:\\R-3.1.2\\scripts\\mPDBS\\summary_pdb_93\\f14fcae8deec4fa3.final.pdb
4.666800e-15 -5.638218e-15 -2.486968e-15 2.977007e-15 4.296613e-15
8.388528e-16
E:\\R-3.1.2\\scripts\\mPDBS\\summary_pdb_93\\f83584e22003fb92.final.pdb
-7.190437e-15 2.526621e-16 2.747974e-15 -1.147494e-14 -2.733034e-15
-1.004895e-14
E:\\R-3.1.2\\scripts\\mPDBS\\summary_pdb_93\\f8e2bf87e52caa29.final.pdb
1.909052e-15 -6.730263e-15 -1.049538e-15 2.878203e-15 1.950224e-15
-4.352856e-15

[,679]   [,680]   [,681]   [,682]   [,683]   [,684]
E:\\R-3.1.2\\scripts\\40.1ns_data\\NAT0.pdb
-2.506860e-16 -3.196428e-15 1.463345e-15 -2.041479e-17 1.437240e-15
1.732772e-15
E:\\R-3.1.2\\scripts\\40.1ns_data\\LJP6.pdb
1.305232e-15 -5.219200e-15 4.364762e-16 -4.779181e-16 1.586128e-15
1.475173e-15
E:\\R-3.1.2\\scripts\\40.1ns_data\\3WY9.pdb
3.020227e-16 -2.794374e-15 5.844993e-16 -1.956919e-15 2.184398e-15
1.220630e-15
E:\\R-3.1.2\\scripts\\40.1ns_data\\ZSP9.pdb
2.699468e-15 -3.884780e-15 2.142465e-15 -1.307385e-16 2.082299e-15
1.078123e-15
E:\\R-3.1.2\\scripts\\mPDBS\\summary_pdb_93\\013d855ad26c877c.final.pdb
-4.703369e-15 2.023941e-15 9.053991e-16 -3.442803e-15 2.038630e-16
2.379866e-15
E:\\R-3.1.2\\scripts\\mPDBS\\summary_pdb_93\\0a2a705342ca0d54.final.pdb
1.557768e-15 -3.364927e-15 1.140049e-15 -4.471463e-16 2.990851e-15
7.671501e-16
E:\\R-3.1.2\\scripts\\mPDBS\\summary_pdb_93\\0a59fceadb4194d9.final.pdb
-4.974669e-17 -2.024460e-15 -7.515835e-16 -4.434795e-15 2.390653e-15
9.481173e-16
E:\\R-3.1.2\\scripts\\mPDBS\\summary_pdb_93\\0dba59f3306825e8.final.pdb
3.324932e-15 -2.835453e-15 -8.917744e-16 -2.251048e-15 2.715092e-15
-1.886687e-15
E:\\R-3.1.2\\scripts\\mPDBS\\summary_pdb_93\\126f26a78b57339c.final.pdb
8.502864e-15 -7.026599e-15 7.311299e-15 3.985717e-15 5.902430e-15
4.333029e-15
E:\\R-3.1.2\\scripts\\mPDBS\\summary_pdb_93\\12f06f3e342df706.final.pdb
1.266985e-15 -2.862724e-15 1.313088e-15 -1.765294e-16 3.309172e-15
6.988996e-16
E:\\R-3.1.2\\scripts\\mPDBS\\summary_pdb_93\\18618a2a251b867b.final.pdb
1.187108e-14 -4.724660e-15 4.734758e-15 3.141149e-15 6.822132e-15
5.446495e-15
E:\\R-3.1.2\\scripts\\mPDBS\\summary_pdb_93\\1f305f598641eeb2.final.pdb
5.944663e-16 -3.415164e-15 1.512884e-15 -5.404960e-16 2.952650e-15

1.589998e-15
E:\\R-3.1.2\\scripts\\mPDBS\\summary_pdb_93\\1f8a00b551be0a39.final.pdb
1.187108e-14 -4.724660e-15 4.734758e-15 3.141149e-15 6.822132e-15
5.446495e-15
E:\\R-3.1.2\\scripts\\mPDBS\\summary_pdb_93\\219f45328d47579a.final.pdb
3.561786e-16 -5.049504e-16 5.982613e-15 1.166194e-15 6.316549e-15
6.133156e-16
E:\\R-3.1.2\\scripts\\mPDBS\\summary_pdb_93\\238dd3918e54336d.final.pdb
3.141277e-15 -1.416736e-14 1.283564e-14 5.108786e-15 -2.606869e-15
4.656007e-15
E:\\R-3.1.2\\scripts\\mPDBS\\summary_pdb_93\\2b3027889e2fdc42.final.pdb
1.946703e-15 -3.544945e-15 1.480572e-15 -6.656743e-16 2.956823e-15
9.922172e-16
E:\\R-3.1.2\\scripts\\mPDBS\\summary_pdb_93\\2fcb3b46e33793b0.final.pdb
-2.188030e-15 -4.236335e-16 -4.304511e-15 -2.825127e-15 1.140514e-15
-9.661170e-16
E:\\R-3.1.2\\scripts\\mPDBS\\summary_pdb_93\\34eb328251842006.final.pdb
-1.207006e-14 -4.642150e-15 9.448641e-16 -5.438136e-15 2.776431e-15
1.080273e-14
E:\\R-3.1.2\\scripts\\mPDBS\\summary_pdb_93\\354b32871797b7ab.final.pdb
-6.049111e-16 9.555985e-15 3.155750e-15 -2.930916e-15 7.534644e-15
8.931901e-15
E:\\R-3.1.2\\scripts\\mPDBS\\summary_pdb_93\\397368adbc32d5de.final.pdb
8.502864e-15 -7.026599e-15 7.311299e-15 3.985717e-15 5.902430e-15
4.333029e-15
E:\\R-3.1.2\\scripts\\mPDBS\\summary_pdb_93\\411ca13cd3df6edd.final.pdb
8.774431e-16 -2.452338e-15 -3.840906e-15 1.495636e-15 3.064322e-15
5.316320e-15
E:\\R-3.1.2\\scripts\\mPDBS\\summary_pdb_93\\42357eee6642bf12.final.pdb
-2.395707e-15 -8.231150e-15 1.524436e-15 -8.643354e-15 5.201901e-15
9.008912e-15
E:\\R-3.1.2\\scripts\\mPDBS\\summary_pdb_93\\44342d1f75a7fcfc.final.pdb
2.772924e-15 -1.712955e-14 2.470778e-15 -1.022712e-15 2.816591e-16
2.899297e-15
E:\\R-3.1.2\\scripts\\mPDBS\\summary_pdb_93\\448ef21dffe8cdfb.final.pdb
-5.789316e-15 1.445097e-15 1.292910e-14 9.168751e-15 1.408529e-15
1.509482e-14
E:\\R-3.1.2\\scripts\\mPDBS\\summary_pdb_93\\472a40b3e8f113c7.final.pdb
3.324932e-15 -2.835453e-15 -8.917744e-16 -2.251048e-15 2.715092e-15
-1.886687e-15
E:\\R-3.1.2\\scripts\\mPDBS\\summary_pdb_93\\48a26225951ea153.final.pdb
6.942328e-16 -5.132108e-15 1.794557e-15 6.133141e-15 5.096171e-15
-2.558762e-15
E:\\R-3.1.2\\scripts\\mPDBS\\summary_pdb_93\\4ab4a5c7c6682ce6.final.pdb
2.587457e-15 -1.580435e-15 -7.638548e-15 2.169461e-15 4.002691e-15
2.312076e-15
E:\\R-3.1.2\\scripts\\mPDBS\\summary_pdb_93\\4eaa45b62cb6a25f.final.pdb
4.103996e-15 -4.699484e-15 -2.052765e-16 7.479988e-16 2.297080e-15
-2.074838e-15
E:\\R-3.1.2\\scripts\\mPDBS\\summary_pdb_93\\58c371611d85ac96.final.pdb
8.502864e-15 -7.026599e-15 7.311299e-15 3.985717e-15 5.902430e-15
4.333029e-15
E:\\R-3.1.2\\scripts\\mPDBS\\summary_pdb_93\\59a647f04822d30a.final.pdb
-5.642219e-15 -1.625892e-15 1.651989e-15 -3.306475e-15 1.071025e-16

-2.042930e-15
E:\\R-3.1.2\\scripts\\mPDBS\\summary_pdb_93\\59b091fe385e05fc.final.pdb
-4.974669e-17 -2.024460e-15 -7.515835e-16 -4.434795e-15 2.390653e-15
9.481173e-16
E:\\R-3.1.2\\scripts\\mPDBS\\summary_pdb_93\\5c35ac30f930d2db.final.pdb
4.103996e-15 -4.699484e-15 -2.052765e-16 7.479988e-16 2.297080e-15
-2.074838e-15
E:\\R-3.1.2\\scripts\\mPDBS\\summary_pdb_93\\5f60752431cf2dcb.final.pdb
2.525527e-15 -7.576374e-16 1.382477e-15 2.146507e-16 3.798798e-15
-1.888180e-16
E:\\R-3.1.2\\scripts\\mPDBS\\summary_pdb_93\\626456dd6ff2868d.final.pdb
3.324932e-15 -2.835453e-15 -8.917744e-16 -2.251048e-15 2.715092e-15
-1.886687e-15
E:\\R-3.1.2\\scripts\\mPDBS\\summary_pdb_93\\6583d53ff3c8f7ef.final.pdb
-5.642219e-15 -1.625892e-15 1.651989e-15 -3.306475e-15 1.071025e-16
-2.042930e-15
E:\\R-3.1.2\\scripts\\mPDBS\\summary_pdb_93\\69760178986bef01.final.pdb
1.118924e-14 -3.140886e-15 1.903500e-15 4.227757e-15 2.128314e-15
-3.299030e-15
E:\\R-3.1.2\\scripts\\mPDBS\\summary_pdb_93\\6a9d87d47926b1c8.final.pdb
7.833450e-16 -3.578620e-15 1.466148e-15 -9.322242e-16 2.704467e-15
1.586094e-15
E:\\R-3.1.2\\scripts\\mPDBS\\summary_pdb_93\\6bb3547ea982ffc4.final.pdb
-3.647829e-15 -7.504973e-15 8.507370e-15 5.498143e-15 7.943525e-15
7.962932e-15
E:\\R-3.1.2\\scripts\\mPDBS\\summary_pdb_93\\716d9624611d1c42.final.pdb
5.042828e-15 -1.966631e-15 6.265712e-16 -2.083800e-17 4.112132e-15
5.395392e-15
E:\\R-3.1.2\\scripts\\mPDBS\\summary_pdb_93\\75f27e70ad0f1be4.final.pdb
-5.642219e-15 -1.625892e-15 1.651989e-15 -3.306475e-15 1.071025e-16
-2.042930e-15
E:\\R-3.1.2\\scripts\\mPDBS\\summary_pdb_93\\769f910fffb22872.final.pdb
9.512294e-17 -3.961869e-15 1.530290e-15 -3.512829e-16 3.415785e-15
1.086321e-15
E:\\R-3.1.2\\scripts\\mPDBS\\summary_pdb_93\\791f8c80dd5c3135.final.pdb
-4.974669e-17 -2.024460e-15 -7.515835e-16 -4.434795e-15 2.390653e-15
9.481173e-16
E:\\R-3.1.2\\scripts\\mPDBS\\summary_pdb_93\\7fad98e66a3f6099.final.pdb
-1.266498e-14 -8.384855e-16 -1.057557e-15 -5.286862e-16 2.801678e-15
3.299489e-15
E:\\R-3.1.2\\scripts\\mPDBS\\summary_pdb_93\\804b89c94a633196.final.pdb
-2.188030e-15 -4.236335e-16 -4.304511e-15 -2.825127e-15 1.140514e-15
-9.661170e-16
E:\\R-3.1.2\\scripts\\mPDBS\\summary_pdb_93\\813cc65a2298feba.final.pdb
-2.188030e-15 -4.236335e-16 -4.304511e-15 -2.825127e-15 1.140514e-15
-9.661170e-16
E:\\R-3.1.2\\scripts\\mPDBS\\summary_pdb_93\\83c0eefcfe12d3f9.final.pdb
8.774431e-16 -2.452338e-15 -3.840906e-15 1.495636e-15 3.064322e-15
5.316320e-15
E:\\R-3.1.2\\scripts\\mPDBS\\summary_pdb_93\\86d50469ef569760.final.pdb
3.324932e-15 -2.835453e-15 -8.917744e-16 -2.251048e-15 2.715092e-15
-1.886687e-15
E:\\R-3.1.2\\scripts\\mPDBS\\summary_pdb_93\\89d0666e1402203d.final.pdb
-5.642219e-15 -1.625892e-15 1.651989e-15 -3.306475e-15 1.071025e-16

-2.042930e-15
E:\\R-3.1.2\\scripts\\mPDBS\\summary_pdb_93\\8b34852716dadc62.final.pdb
5.930476e-15 -1.135673e-14 2.189427e-15 1.110961e-15 5.721268e-15
1.831373e-16
E:\\R-3.1.2\\scripts\\mPDBS\\summary_pdb_93\\8b6ef9ac3fe74395.final.pdb
-1.220755e-14 -6.695245e-15 6.583948e-15 -1.542793e-15 2.203002e-15
-1.587232e-15
E:\\R-3.1.2\\scripts\\mPDBS\\summary_pdb_93\\98bbe0d5986c138f.final.pdb
-4.974669e-17 -2.024460e-15 -7.515835e-16 -4.434795e-15 2.390653e-15
9.481173e-16
E:\\R-3.1.2\\scripts\\mPDBS\\summary_pdb_93\\9c22f4909d67e2fa.final.pdb
6.821256e-15 -3.835997e-15 8.474330e-15 -3.972213e-15 1.726851e-15
-1.012467e-15
E:\\R-3.1.2\\scripts\\mPDBS\\summary_pdb_93\\9dda8ed0d81fb3ef.final.pdb
-4.920419e-16 1.668185e-15 -2.579869e-15 -2.995526e-15 2.100083e-15
-1.530156e-14
E:\\R-3.1.2\\scripts\\mPDBS\\summary_pdb_93\\9f752b3616e6033b.final.pdb
-4.229099e-16 -5.322002e-15 3.012409e-15 -2.440182e-16 1.969312e-15
-2.137345e-16
E:\\R-3.1.2\\scripts\\mPDBS\\summary_pdb_93\\a02c70083816d6ba.final.pdb
4.103996e-15 -4.699484e-15 -2.052765e-16 7.479988e-16 2.297080e-15
-2.074838e-15
E:\\R-3.1.2\\scripts\\mPDBS\\summary_pdb_93\\a0c997d891e4e082.final.pdb
-2.864692e-15 2.860072e-15 -2.393735e-15 -4.016535e-17 -3.189457e-16
-1.883904e-17
E:\\R-3.1.2\\scripts\\mPDBS\\summary_pdb_93\\ab8039a2d823c616.final.pdb
8.224632e-16 -3.224959e-15 1.372350e-15 -6.984929e-16 2.597992e-15
9.212998e-16
E:\\R-3.1.2\\scripts\\mPDBS\\summary_pdb_93\\acf4d74b9c71d4dc.final.pdb
-2.188030e-15 -4.236335e-16 -4.304511e-15 -2.825127e-15 1.140514e-15
-9.661170e-16
E:\\R-3.1.2\\scripts\\mPDBS\\summary_pdb_93\\ad8a823e4b4c668e.final.pdb
1.059801e-15 -3.603765e-15 1.128273e-15 -4.556398e-16 2.941119e-15
1.071026e-15
E:\\R-3.1.2\\scripts\\mPDBS\\summary_pdb_93\\b16ababd03b7b857.final.pdb
6.821256e-15 -3.835997e-15 8.474330e-15 -3.972213e-15 1.726851e-15
-1.012467e-15
E:\\R-3.1.2\\scripts\\mPDBS\\summary_pdb_93\\b226923e697d3c63.final.pdb
5.039124e-15 6.025231e-16 3.387146e-15 -3.407523e-15 4.370836e-15
8.497196e-15
E:\\R-3.1.2\\scripts\\mPDBS\\summary_pdb_93\\b28e48f6fa27a165.final.pdb
-4.974669e-17 -2.024460e-15 -7.515835e-16 -4.434795e-15 2.390653e-15
9.481173e-16
E:\\R-3.1.2\\scripts\\mPDBS\\summary_pdb_93\\b42c4924e3cd1aec.final.pdb
-1.838838e-15 -4.818813e-15 9.198036e-15 3.904770e-15 2.454834e-15
1.011843e-14
E:\\R-3.1.2\\scripts\\mPDBS\\summary_pdb_93\\b4dd239fcabff1df.final.pdb
-4.974669e-17 -2.024460e-15 -7.515835e-16 -4.434795e-15 2.390653e-15
9.481173e-16
E:\\R-3.1.2\\scripts\\mPDBS\\summary_pdb_93\\b669942ef97459a2.final.pdb
5.930476e-15 -1.135673e-14 2.189427e-15 1.110961e-15 5.721268e-15
1.831373e-16
E:\\R-3.1.2\\scripts\\mPDBS\\summary_pdb_93\\b99f4d9d87c1017b.final.pdb
-4.974669e-17 -2.024460e-15 -7.515835e-16 -4.434795e-15 2.390653e-15

9.481173e-16
E:\\R-3.1.2\\scripts\\mPDBS\\summary_pdb_93\\b9d2cbf3975403e7.final.pdb
-4.974669e-17 -2.024460e-15 -7.515835e-16 -4.434795e-15 2.390653e-15
9.481173e-16
E:\\R-3.1.2\\scripts\\mPDBS\\summary_pdb_93\\bc88c8a505f3a288.final.pdb
1.836765e-15 -3.107740e-15 1.893436e-15 -3.616640e-16 2.695314e-15
1.097791e-15
E:\\R-3.1.2\\scripts\\mPDBS\\summary_pdb_93\\bdac0e64f4ef427e.final.pdb
-6.846754e-16 -5.267524e-15 1.271920e-14 -4.879907e-15 8.770530e-16
-5.720371e-15
E:\\R-3.1.2\\scripts\\mPDBS\\summary_pdb_93\\ca2a1f55fc9d0681.final.pdb
5.930476e-15 -1.135673e-14 2.189427e-15 1.110961e-15 5.721268e-15
1.831373e-16
E:\\R-3.1.2\\scripts\\mPDBS\\summary_pdb_93\\ce34248de3e81a3c.final.pdb
2.044823e-16 8.174313e-16 -5.147891e-15 -5.755977e-15 3.897149e-15
-2.215668e-15
E:\\R-3.1.2\\scripts\\mPDBS\\summary_pdb_93\\d02e697772494a61.final.pdb
3.568019e-15 -6.852033e-15 -1.613741e-15 -2.566498e-15 4.494222e-15
9.293791e-15
E:\\R-3.1.2\\scripts\\mPDBS\\summary_pdb_93\\d04221245d015047.final.pdb
-3.701592e-16 -4.883734e-15 8.484630e-16 4.273426e-16 2.727229e-15
-1.072250e-15
E:\\R-3.1.2\\scripts\\mPDBS\\summary_pdb_93\\d678141e4018054a.final.pdb
9.827442e-15 -1.282147e-15 6.227260e-15 5.639846e-15 1.137247e-14
1.544501e-14
E:\\R-3.1.2\\scripts\\mPDBS\\summary_pdb_93\\d71b40af0f0efe39.final.pdb
1.548063e-14 -1.187458e-15 4.456929e-15 4.965307e-15 3.805379e-15
2.688385e-15
E:\\R-3.1.2\\scripts\\mPDBS\\summary_pdb_93\\dc0809eff10197e2.final.pdb
-8.295419e-15 -3.408064e-15 1.876814e-15 -2.089295e-15 -6.349012e-15
-5.888797e-15
E:\\R-3.1.2\\scripts\\mPDBS\\summary_pdb_93\\e1163918d3707839.final.pdb
9.053491e-15 -2.451241e-15 -4.114232e-15 3.718288e-15 1.010019e-14
7.764844e-15
E:\\R-3.1.2\\scripts\\mPDBS\\summary_pdb_93\\e4b48b06824ef848.final.pdb
1.187108e-14 -4.724660e-15 4.734758e-15 3.141149e-15 6.822132e-15
5.446495e-15
E:\\R-3.1.2\\scripts\\mPDBS\\summary_pdb_93\\e529e19ad281fea2.final.pdb
-4.703369e-15 2.023941e-15 9.053991e-16 -3.442803e-15 2.038630e-16
2.379866e-15
E:\\R-3.1.2\\scripts\\mPDBS\\summary_pdb_93\\e5602082718ec3a1.final.pdb
-2.188030e-15 -4.236335e-16 -4.304511e-15 -2.825127e-15 1.140514e-15
-9.661170e-16
E:\\R-3.1.2\\scripts\\mPDBS\\summary_pdb_93\\e5d589d996d0a960.final.pdb
-9.844414e-16 -3.643833e-15 1.197519e-15 5.803674e-15 2.834310e-15
-1.140537e-14
E:\\R-3.1.2\\scripts\\mPDBS\\summary_pdb_93\\e9df9e72307a2fbd.final.pdb
4.103996e-15 -4.699484e-15 -2.052765e-16 7.479988e-16 2.297080e-15
-2.074838e-15
E:\\R-3.1.2\\scripts\\mPDBS\\summary_pdb_93\\ea00f8694090cae7.final.pdb
-2.188030e-15 -4.236335e-16 -4.304511e-15 -2.825127e-15 1.140514e-15
-9.661170e-16
E:\\R-3.1.2\\scripts\\mPDBS\\summary_pdb_93\\eba517be32db050e.final.pdb
-4.974669e-17 -2.024460e-15 -7.515835e-16 -4.434795e-15 2.390653e-15

9.481173e-16
E:\\R-3.1.2\\scripts\\mPDBS\\summary_pdb_93\\ec04b9545899c29d.final.pdb
1.218612e-15 -3.262603e-15 1.284228e-15 -3.366285e-16 3.290537e-15
8.974263e-16
E:\\R-3.1.2\\scripts\\mPDBS\\summary_pdb_93\\ee6862cbcad52078.final.pdb
1.992023e-15 -3.126443e-15 1.377790e-15 -8.621318e-16 2.904348e-15
1.043961e-15
E:\\R-3.1.2\\scripts\\mPDBS\\summary_pdb_93\\ef6a42fc83b81e5d.final.pdb
9.824340e-16 -3.421015e-15 1.527688e-15 -5.728938e-16 3.354636e-15
1.274620e-15
E:\\R-3.1.2\\scripts\\mPDBS\\summary_pdb_93\\ef8e88d4ad93a0ad.final.pdb
-3.701592e-16 -4.883734e-15 8.484630e-16 4.273426e-16 2.727229e-15
-1.072250e-15
E:\\R-3.1.2\\scripts\\mPDBS\\summary_pdb_93\\f14fcae8deec4fa3.final.pdb
-4.229099e-16 -5.322002e-15 3.012409e-15 -2.440182e-16 1.969312e-15
-2.137345e-16
E:\\R-3.1.2\\scripts\\mPDBS\\summary_pdb_93\\f83584e22003fb92.final.pdb
6.942328e-16 -5.132108e-15 1.794557e-15 6.133141e-15 5.096171e-15
-2.558762e-15
E:\\R-3.1.2\\scripts\\mPDBS\\summary_pdb_93\\f8e2bf87e52caa29.final.pdb
-4.703369e-15 2.023941e-15 9.053991e-16 -3.442803e-15 2.038630e-16
2.379866e-15

[,685]   [,686]   [,687]   [,688]   [,689]   [,690]
E:\\R-3.1.2\\scripts\\40.1ns_data\\NAT0.pdb
-5.844826e-15 -3.047495e-15 -1.605519e-15 -3.477385e-15 1.249739e-15
3.253294e-15
E:\\R-3.1.2\\scripts\\40.1ns_data\\LJP6.pdb
-5.494620e-15 -1.745936e-15 4.303765e-16 -2.457789e-15 9.016984e-16
2.773428e-15
E:\\R-3.1.2\\scripts\\40.1ns_data\\3WY9.pdb
-6.049259e-15 -2.281434e-15 9.254519e-16 -1.663391e-15 2.369212e-15
3.047715e-15
E:\\R-3.1.2\\scripts\\40.1ns_data\\ZSP9.pdb
-4.814826e-15 -2.261875e-15 -5.763863e-16 -3.938861e-15 1.766549e-15
3.801646e-15
E:\\R-3.1.2\\scripts\\mPDBS\\summary_pdb_93\\013d855ad26c877c.final.pdb
-1.565152e-15 -8.604434e-16 -2.085346e-15 -4.599810e-15 -2.090418e-15
5.784065e-15
E:\\R-3.1.2\\scripts\\mPDBS\\summary_pdb_93\\0a2a705342ca0d54.final.pdb
-5.038932e-15 -2.479532e-15 5.846779e-17 -4.382824e-15 1.208496e-15
4.076587e-15
E:\\R-3.1.2\\scripts\\mPDBS\\summary_pdb_93\\0a59fceadb4194d9.final.pdb
-7.910279e-16 -7.432626e-16 1.164778e-15 -3.100922e-15 -1.012830e-15
2.470393e-15
E:\\R-3.1.2\\scripts\\mPDBS\\summary_pdb_93\\0dba59f3306825e8.final.pdb
-2.484965e-16 -3.726207e-15 3.576791e-15 -1.178935e-15 2.744690e-15
2.245229e-15
E:\\R-3.1.2\\scripts\\mPDBS\\summary_pdb_93\\126f26a78b57339c.final.pdb
-1.425704e-14 -6.719655e-15 -5.183020e-15 -3.676937e-15 5.220357e-15
5.238928e-15
E:\\R-3.1.2\\scripts\\mPDBS\\summary_pdb_93\\12f06f3e342df706.final.pdb
-5.426426e-15 -2.716481e-15 1.708996e-16 -3.604584e-15 1.995843e-15
3.599105e-15

E:\\R-3.1.2\\scripts\\mPDBS\\summary_pdb_93\\18618a2a251b867b.final.pdb
-1.140793e-14 -1.659294e-15 -4.969529e-15 -5.516097e-15 5.256794e-15
2.798240e-15
E:\\R-3.1.2\\scripts\\mPDBS\\summary_pdb_93\\1f305f598641eeb2.final.pdb
-5.455130e-15 -2.775652e-15 6.101877e-16 -3.872729e-15 1.239635e-15
4.189976e-15
E:\\R-3.1.2\\scripts\\mPDBS\\summary_pdb_93\\1f8a00b551be0a39.final.pdb
-1.140793e-14 -1.659294e-15 -4.969529e-15 -5.516097e-15 5.256794e-15
2.798240e-15
E:\\R-3.1.2\\scripts\\mPDBS\\summary_pdb_93\\219f45328d47579a.final.pdb
-1.471045e-14 -7.289242e-15 -4.395011e-15 -4.587580e-15 -1.495516e-15
7.239148e-15
E:\\R-3.1.2\\scripts\\mPDBS\\summary_pdb_93\\238dd3918e54336d.final.pdb
-1.081231e-14 -1.222332e-14 -8.199043e-15 -3.857912e-15 1.045131e-14
8.493787e-15
E:\\R-3.1.2\\scripts\\mPDBS\\summary_pdb_93\\2b3027889e2fdc42.final.pdb
-5.032860e-15 -3.079254e-15 -6.111456e-16 -3.990618e-15 1.224313e-15
4.638797e-15
E:\\R-3.1.2\\scripts\\mPDBS\\summary_pdb_93\\2fcb3b46e33793b0.final.pdb
1.106461e-15 -5.170419e-16 3.559218e-15 -2.859651e-15 -1.904552e-15
2.536351e-15
E:\\R-3.1.2\\scripts\\mPDBS\\summary_pdb_93\\34eb328251842006.final.pdb
-7.793818e-15 1.012554e-15 1.388517e-15 -6.530975e-15 -6.763149e-16
1.199206e-15
E:\\R-3.1.2\\scripts\\mPDBS\\summary_pdb_93\\354b32871797b7ab.final.pdb
-3.139132e-15 5.009184e-15 4.896394e-15 1.056132e-15 2.877184e-15
-1.788702e-15
E:\\R-3.1.2\\scripts\\mPDBS\\summary_pdb_93\\397368adbc32d5de.final.pdb
-1.425704e-14 -6.719655e-15 -5.183020e-15 -3.676937e-15 5.220357e-15
5.238928e-15
E:\\R-3.1.2\\scripts\\mPDBS\\summary_pdb_93\\411ca13cd3df6edd.final.pdb
-5.342590e-15 -2.545618e-15 8.575916e-17 4.085682e-16 1.786289e-15
1.553196e-15
E:\\R-3.1.2\\scripts\\mPDBS\\summary_pdb_93\\42357eee6642bf12.final.pdb
-5.596420e-15 3.544948e-15 -1.226754e-16 -3.266114e-15 -7.499677e-16
-3.728772e-16
E:\\R-3.1.2\\scripts\\mPDBS\\summary_pdb_93\\44342d1f75a7fcfc.final.pdb
-4.720593e-15 -5.072380e-16 -1.496597e-15 -5.989499e-15 2.250326e-15
7.086578e-15
E:\\R-3.1.2\\scripts\\mPDBS\\summary_pdb_93\\448ef21dffe8cdfb.final.pdb
-2.412683e-14 -4.199860e-15 -1.565155e-15 -5.874600e-15 2.479528e-15
1.072815e-14
E:\\R-3.1.2\\scripts\\mPDBS\\summary_pdb_93\\472a40b3e8f113c7.final.pdb
-2.484965e-16 -3.726207e-15 3.576791e-15 -1.178935e-15 2.744690e-15
2.245229e-15
E:\\R-3.1.2\\scripts\\mPDBS\\summary_pdb_93\\48a26225951ea153.final.pdb
-1.006638e-14 -5.513071e-15 -1.635870e-16 -6.841636e-15 6.376008e-15
7.564842e-15
E:\\R-3.1.2\\scripts\\mPDBS\\summary_pdb_93\\4ab4a5c7c6682ce6.final.pdb
-8.704189e-15 9.012552e-15 -1.515815e-15 -1.223953e-14 -7.532029e-15
3.717142e-15
E:\\R-3.1.2\\scripts\\mPDBS\\summary_pdb_93\\4eaa45b62cb6a25f.final.pdb
-5.241400e-15 -5.170515e-15 -4.332552e-15 -6.260281e-15 1.519112e-15
5.468080e-15

E:\\R-3.1.2\\scripts\\mPDBS\\summary_pdb_93\\58c371611d85ac96.final.pdb
-1.425704e-14 -6.719655e-15 -5.183020e-15 -3.676937e-15 5.220357e-15
5.238928e-15
E:\\R-3.1.2\\scripts\\mPDBS\\summary_pdb_93\\59a647f04822d30a.final.pdb
-7.320510e-15 1.389465e-16 -3.874902e-15 -1.131510e-14 1.301866e-15
1.398420e-15
E:\\R-3.1.2\\scripts\\mPDBS\\summary_pdb_93\\59b091fe385e05fc.final.pdb
-7.910279e-16 -7.432626e-16 1.164778e-15 -3.100922e-15 -1.012830e-15
2.470393e-15
E:\\R-3.1.2\\scripts\\mPDBS\\summary_pdb_93\\5c35ac30f930d2db.final.pdb
-5.241400e-15 -5.170515e-15 -4.332552e-15 -6.260281e-15 1.519112e-15
5.468080e-15
E:\\R-3.1.2\\scripts\\mPDBS\\summary_pdb_93\\5f60752431cf2dcb.final.pdb
1.589663e-15 -3.004472e-15 -2.792984e-15 -6.640350e-15 6.850033e-15
4.411823e-15
E:\\R-3.1.2\\scripts\\mPDBS\\summary_pdb_93\\626456dd6ff2868d.final.pdb
-2.484965e-16 -3.726207e-15 3.576791e-15 -1.178935e-15 2.744690e-15
2.245229e-15
E:\\R-3.1.2\\scripts\\mPDBS\\summary_pdb_93\\6583d53ff3c8f7ef.final.pdb
-7.320510e-15 1.389465e-16 -3.874902e-15 -1.131510e-14 1.301866e-15
1.398420e-15
E:\\R-3.1.2\\scripts\\mPDBS\\summary_pdb_93\\69760178986bef01.final.pdb
-6.419255e-15 -6.082392e-15 -1.972158e-15 -3.980752e-15 4.881022e-15
1.979475e-16
E:\\R-3.1.2\\scripts\\mPDBS\\summary_pdb_93\\6a9d87d47926b1c8.final.pdb
-5.113312e-15 -3.073266e-15 2.327867e-16 -3.989187e-15 1.056587e-15
3.980386e-15
E:\\R-3.1.2\\scripts\\mPDBS\\summary_pdb_93\\6bb3547ea982ffc4.final.pdb
-1.472652e-14 -7.971082e-15 1.117653e-16 4.057595e-15 1.098988e-14
9.289337e-15
E:\\R-3.1.2\\scripts\\mPDBS\\summary_pdb_93\\716d9624611d1c42.final.pdb
-1.529288e-14 -2.140394e-15 -4.179462e-15 -4.852067e-15 -1.557955e-15
4.168528e-15
E:\\R-3.1.2\\scripts\\mPDBS\\summary_pdb_93\\75f27e70ad0f1be4.final.pdb
-7.320510e-15 1.389465e-16 -3.874902e-15 -1.131510e-14 1.301866e-15
1.398420e-15
E:\\R-3.1.2\\scripts\\mPDBS\\summary_pdb_93\\769f910fffb22872.final.pdb
-4.923317e-15 -2.771591e-15 1.221482e-15 -4.169952e-15 1.984455e-15
4.058516e-15
E:\\R-3.1.2\\scripts\\mPDBS\\summary_pdb_93\\791f8c80dd5c3135.final.pdb
-7.910279e-16 -7.432626e-16 1.164778e-15 -3.100922e-15 -1.012830e-15
2.470393e-15
E:\\R-3.1.2\\scripts\\mPDBS\\summary_pdb_93\\7fad98e66a3f6099.final.pdb
2.262304e-15 -4.600026e-15 6.912678e-15 2.987267e-15 -1.463287e-15
7.987277e-15
E:\\R-3.1.2\\scripts\\mPDBS\\summary_pdb_93\\804b89c94a633196.final.pdb
1.106461e-15 -5.170419e-16 3.559218e-15 -2.859651e-15 -1.904552e-15
2.536351e-15
E:\\R-3.1.2\\scripts\\mPDBS\\summary_pdb_93\\813cc65a2298feba.final.pdb
1.106461e-15 -5.170419e-16 3.559218e-15 -2.859651e-15 -1.904552e-15
2.536351e-15
E:\\R-3.1.2\\scripts\\mPDBS\\summary_pdb_93\\83c0eefcfe12d3f9.final.pdb
-5.342590e-15 -2.545618e-15 8.575916e-17 4.085682e-16 1.786289e-15
1.553196e-15

E:\\R-3.1.2\\scripts\\mPDBS\\summary_pdb_93\\86d50469ef569760.final.pdb
-2.484965e-16 -3.726207e-15 3.576791e-15 -1.178935e-15 2.744690e-15
2.245229e-15
E:\\R-3.1.2\\scripts\\mPDBS\\summary_pdb_93\\89d0666e1402203d.final.pdb
-7.320510e-15 1.389465e-16 -3.874902e-15 -1.131510e-14 1.301866e-15
1.398420e-15
E:\\R-3.1.2\\scripts\\mPDBS\\summary_pdb_93\\8b34852716dadc62.final.pdb
-9.222950e-15 -4.881606e-15 -3.067426e-15 -4.413963e-15 2.596188e-15
2.614847e-15
E:\\R-3.1.2\\scripts\\mPDBS\\summary_pdb_93\\8b6ef9ac3fe74395.final.pdb
-2.160384e-15 -1.716135e-15 3.608521e-15 1.444698e-16 -6.698258e-16
5.312987e-15
E:\\R-3.1.2\\scripts\\mPDBS\\summary_pdb_93\\98bbe0d5986c138f.final.pdb
-7.910279e-16 -7.432626e-16 1.164778e-15 -3.100922e-15 -1.012830e-15
2.470393e-15
E:\\R-3.1.2\\scripts\\mPDBS\\summary_pdb_93\\9c22f4909d67e2fa.final.pdb
-6.091340e-15 -1.057097e-14 -4.204548e-16 -5.536329e-15 6.174489e-15
5.587729e-15
E:\\R-3.1.2\\scripts\\mPDBS\\summary_pdb_93\\9dda8ed0d81fb3ef.final.pdb
-2.836033e-15 -5.238015e-15 4.970344e-16 -5.352856e-15 2.246857e-15
1.310817e-15
E:\\R-3.1.2\\scripts\\mPDBS\\summary_pdb_93\\9f752b3616e6033b.final.pdb
-4.724164e-15 -3.244632e-15 3.310324e-15 -1.900426e-15 1.572279e-15
4.936978e-15
E:\\R-3.1.2\\scripts\\mPDBS\\summary_pdb_93\\a02c70083816d6ba.final.pdb
-5.241400e-15 -5.170515e-15 -4.332552e-15 -6.260281e-15 1.519112e-15
5.468080e-15
E:\\R-3.1.2\\scripts\\mPDBS\\summary_pdb_93\\a0c997d891e4e082.final.pdb
-4.191496e-15 -5.796301e-15 3.381879e-15 -1.792979e-15 -1.976810e-15
-1.863567e-15
E:\\R-3.1.2\\scripts\\mPDBS\\summary_pdb_93\\ab8039a2d823c616.final.pdb
-5.188962e-15 -2.783612e-15 3.636770e-16 -3.518812e-15 2.340267e-15
3.941685e-15
E:\\R-3.1.2\\scripts\\mPDBS\\summary_pdb_93\\acf4d74b9c71d4dc.final.pdb
1.106461e-15 -5.170419e-16 3.559218e-15 -2.859651e-15 -1.904552e-15
2.536351e-15
E:\\R-3.1.2\\scripts\\mPDBS\\summary_pdb_93\\ad8a823e4b4c668e.final.pdb
-5.390799e-15 -2.421893e-15 -3.881029e-16 -3.517349e-15 1.471181e-15
4.303638e-15
E:\\R-3.1.2\\scripts\\mPDBS\\summary_pdb_93\\b16ababd03b7b857.final.pdb
-6.091340e-15 -1.057097e-14 -4.204548e-16 -5.536329e-15 6.174489e-15
5.587729e-15
E:\\R-3.1.2\\scripts\\mPDBS\\summary_pdb_93\\b226923e697d3c63.final.pdb
-6.418887e-15 -4.262346e-16 -2.192004e-15 -1.395951e-15 -1.070368e-15
-1.668571e-15
E:\\R-3.1.2\\scripts\\mPDBS\\summary_pdb_93\\b28e48f6fa27a165.final.pdb
-7.910279e-16 -7.432626e-16 1.164778e-15 -3.100922e-15 -1.012830e-15
2.470393e-15
E:\\R-3.1.2\\scripts\\mPDBS\\summary_pdb_93\\b42c4924e3cd1aec.final.pdb
4.187342e-17 -4.480964e-15 6.304238e-15 -4.221209e-16 2.534170e-15
6.992470e-15
E:\\R-3.1.2\\scripts\\mPDBS\\summary_pdb_93\\b4dd239fcabff1df.final.pdb
-7.910279e-16 -7.432626e-16 1.164778e-15 -3.100922e-15 -1.012830e-15
2.470393e-15

E:\\R-3.1.2\\scripts\\mPDBS\\summary_pdb_93\\b669942ef97459a2.final.pdb
-9.222950e-15 -4.881606e-15 -3.067426e-15 -4.413963e-15 2.596188e-15
2.614847e-15
E:\\R-3.1.2\\scripts\\mPDBS\\summary_pdb_93\\b99f4d9d87c1017b.final.pdb
-7.910279e-16 -7.432626e-16 1.164778e-15 -3.100922e-15 -1.012830e-15
2.470393e-15
E:\\R-3.1.2\\scripts\\mPDBS\\summary_pdb_93\\b9d2cbf3975403e7.final.pdb
-7.910279e-16 -7.432626e-16 1.164778e-15 -3.100922e-15 -1.012830e-15
2.470393e-15
E:\\R-3.1.2\\scripts\\mPDBS\\summary_pdb_93\\bc88c8a505f3a288.final.pdb
-5.268783e-15 -2.817467e-15 -5.425156e-16 -3.947467e-15 1.192871e-15
4.273638e-15
E:\\R-3.1.2\\scripts\\mPDBS\\summary_pdb_93\\bdac0e64f4ef427e.final.pdb
-7.363109e-15 -4.264200e-15 2.659872e-15 -1.375545e-14 1.336129e-15
8.988221e-15
E:\\R-3.1.2\\scripts\\mPDBS\\summary_pdb_93\\ca2a1f55fc9d0681.final.pdb
-9.222950e-15 -4.881606e-15 -3.067426e-15 -4.413963e-15 2.596188e-15
2.614847e-15
E:\\R-3.1.2\\scripts\\mPDBS\\summary_pdb_93\\ce34248de3e81a3c.final.pdb
1.978869e-15 2.895006e-15 -9.501600e-15 -8.239119e-15 -4.357734e-15
-1.394012e-16
E:\\R-3.1.2\\scripts\\mPDBS\\summary_pdb_93\\d02e697772494a61.final.pdb
-1.222828e-14 6.899612e-16 -8.006489e-15 -4.905685e-15 1.964894e-16
4.533413e-15
E:\\R-3.1.2\\scripts\\mPDBS\\summary_pdb_93\\d04221245d015047.final.pdb
-5.136921e-15 -3.790961e-15 1.236026e-15 -4.689967e-15 2.362601e-15
5.987791e-15
E:\\R-3.1.2\\scripts\\mPDBS\\summary_pdb_93\\d678141e4018054a.final.pdb
-1.890608e-14 -1.878928e-15 -8.306608e-16 -1.036065e-15 1.002340e-14
8.113042e-15
E:\\R-3.1.2\\scripts\\mPDBS\\summary_pdb_93\\d71b40af0f0efe39.final.pdb
-4.402406e-15 -1.035947e-14 4.057848e-15 -1.576390e-15 3.433239e-15
1.786877e-14
E:\\R-3.1.2\\scripts\\mPDBS\\summary_pdb_93\\dc0809eff10197e2.final.pdb
1.221071e-15 -2.505535e-15 1.280872e-14 -1.244349e-14 -5.610963e-15
3.074653e-15
E:\\R-3.1.2\\scripts\\mPDBS\\summary_pdb_93\\e1163918d3707839.final.pdb
-9.991117e-15 -4.011722e-15 1.516521e-14 9.351311e-15 -2.215069e-15
9.045949e-15
E:\\R-3.1.2\\scripts\\mPDBS\\summary_pdb_93\\e4b48b06824ef848.final.pdb
-1.140793e-14 -1.659294e-15 -4.969529e-15 -5.516097e-15 5.256794e-15
2.798240e-15
E:\\R-3.1.2\\scripts\\mPDBS\\summary_pdb_93\\e529e19ad281fea2.final.pdb
-1.565152e-15 -8.604434e-16 -2.085346e-15 -4.599810e-15 -2.090418e-15
5.784065e-15
E:\\R-3.1.2\\scripts\\mPDBS\\summary_pdb_93\\e5602082718ec3a1.final.pdb
1.106461e-15 -5.170419e-16 3.559218e-15 -2.859651e-15 -1.904552e-15
2.536351e-15
E:\\R-3.1.2\\scripts\\mPDBS\\summary_pdb_93\\e5d589d996d0a960.final.pdb
2.327822e-15 -1.134234e-14 2.672196e-15 1.811166e-15 3.893460e-15
6.484054e-15
E:\\R-3.1.2\\scripts\\mPDBS\\summary_pdb_93\\e9df9e72307a2fbd.final.pdb
-5.241400e-15 -5.170515e-15 -4.332552e-15 -6.260281e-15 1.519112e-15
5.468080e-15

E:\\R-3.1.2\\scripts\\mPDBS\\summary_pdb_93\\ea00f8694090cae7.final.pdb
1.106461e-15 -5.170419e-16 3.559218e-15 -2.859651e-15 -1.904552e-15
2.536351e-15
E:\\R-3.1.2\\scripts\\mPDBS\\summary_pdb_93\\eba517be32db050e.final.pdb
-7.910279e-16 -7.432626e-16 1.164778e-15 -3.100922e-15 -1.012830e-15
2.470393e-15
E:\\R-3.1.2\\scripts\\mPDBS\\summary_pdb_93\\ec04b9545899c29d.final.pdb
-5.390196e-15 -3.041669e-15 -6.327042e-16 -3.422976e-15 1.490517e-15
4.180917e-15
E:\\R-3.1.2\\scripts\\mPDBS\\summary_pdb_93\\ee6862cbcad52078.final.pdb
-4.870664e-15 -2.974984e-15 -5.439251e-16 -3.850322e-15 1.280908e-15
4.186035e-15
E:\\R-3.1.2\\scripts\\mPDBS\\summary_pdb_93\\ef6a42fc83b81e5d.final.pdb
-5.101126e-15 -2.809229e-15 6.420456e-17 -3.620261e-15 1.536029e-15
3.692923e-15
E:\\R-3.1.2\\scripts\\mPDBS\\summary_pdb_93\\ef8e88d4ad93a0ad.final.pdb
-5.136921e-15 -3.790961e-15 1.236026e-15 -4.689967e-15 2.362601e-15
5.987791e-15
E:\\R-3.1.2\\scripts\\mPDBS\\summary_pdb_93\\f14fcae8deec4fa3.final.pdb
-4.724164e-15 -3.244632e-15 3.310324e-15 -1.900426e-15 1.572279e-15
4.936978e-15
E:\\R-3.1.2\\scripts\\mPDBS\\summary_pdb_93\\f83584e22003fb92.final.pdb
-1.006638e-14 -5.513071e-15 -1.635870e-16 -6.841636e-15 6.376008e-15
7.564842e-15
E:\\R-3.1.2\\scripts\\mPDBS\\summary_pdb_93\\f8e2bf87e52caa29.final.pdb
-1.565152e-15 -8.604434e-16 -2.085346e-15 -4.599810e-15 -2.090418e-15
5.784065e-15

[,691]   [,692]   [,693]   [,694]   [,695]   [,696]
E:\\R-3.1.2\\scripts\\40.1ns_data\\NAT0.pdb
-3.382935e-15 3.162660e-15 3.251027e-16 -3.112085e-15 2.582123e-15
1.651362e-15
E:\\R-3.1.2\\scripts\\40.1ns_data\\LJP6.pdb
-3.693158e-15 4.128276e-15 1.736202e-15 -2.369056e-15 3.875015e-16
-3.573194e-16
E:\\R-3.1.2\\scripts\\40.1ns_data\\3WY9.pdb
-2.861741e-15 4.120631e-15 3.338455e-16 -5.507494e-15 8.578511e-16
8.918744e-17
E:\\R-3.1.2\\scripts\\40.1ns_data\\ZSP9.pdb
-2.265078e-15 4.295390e-15 -3.362770e-16 -5.044208e-15 4.287164e-16
-1.999460e-16
E:\\R-3.1.2\\scripts\\mPDBS\\summary_pdb_93\\013d855ad26c877c.final.pdb
-4.156860e-15 9.194899e-15 3.799481e-15 -5.491993e-15 -2.814068e-15
-1.396738e-15
E:\\R-3.1.2\\scripts\\mPDBS\\summary_pdb_93\\0a2a705342ca0d54.final.pdb
-3.331959e-15 4.372603e-15 -4.954050e-17 -4.367520e-15 -3.026813e-16
3.462381e-16
E:\\R-3.1.2\\scripts\\mPDBS\\summary_pdb_93\\0a59fceadb4194d9.final.pdb
-4.657076e-15 5.993829e-15 4.218105e-15 2.977048e-16 1.770819e-15
-2.399680e-15
E:\\R-3.1.2\\scripts\\mPDBS\\summary_pdb_93\\0dba59f3306825e8.final.pdb
-4.416635e-15 1.453696e-15 1.871336e-15 1.705374e-15 8.924806e-15
-3.381261e-15
E:\\R-3.1.2\\scripts\\mPDBS\\summary_pdb_93\\126f26a78b57339c.final.pdb

-7.377408e-16 3.561819e-15 -2.921272e-15 -9.118975e-15 -9.120438e-15
6.132830e-15
E:\\R-3.1.2\\scripts\\mPDBS\\summary_pdb_93\\12f06f3e342df706.final.pdb
-3.122892e-15 4.699381e-15 1.439720e-15 -3.713908e-15 3.781976e-16
-3.930422e-17
E:\\R-3.1.2\\scripts\\mPDBS\\summary_pdb_93\\18618a2a251b867b.final.pdb
-3.134688e-16 1.745169e-15 -9.383338e-16 -7.561354e-15 -3.103755e-15
2.697921e-15
E:\\R-3.1.2\\scripts\\mPDBS\\summary_pdb_93\\1f305f598641eeb2.final.pdb
-3.276071e-15 4.533348e-15 4.869186e-16 -3.366473e-15 2.502163e-16
2.824543e-17
E:\\R-3.1.2\\scripts\\mPDBS\\summary_pdb_93\\1f8a00b551be0a39.final.pdb
-3.134688e-16 1.745169e-15 -9.383338e-16 -7.561354e-15 -3.103755e-15
2.697921e-15
E:\\R-3.1.2\\scripts\\mPDBS\\summary_pdb_93\\219f45328d47579a.final.pdb
-1.206435e-14 8.881037e-16 -9.762465e-15 -9.230698e-15 -9.949407e-15
1.100106e-14
E:\\R-3.1.2\\scripts\\mPDBS\\summary_pdb_93\\238dd3918e54336d.final.pdb
-3.164465e-15 -3.617624e-15 -8.440606e-15 -7.816356e-15 5.293391e-15
1.088180e-14
E:\\R-3.1.2\\scripts\\mPDBS\\summary_pdb_93\\2b3027889e2fdc42.final.pdb
-3.968411e-15 4.583282e-15 1.331959e-15 -3.650688e-15 3.607053e-16
2.826359e-16
E:\\R-3.1.2\\scripts\\mPDBS\\summary_pdb_93\\2fcb3b46e33793b0.final.pdb
-3.769600e-15 5.539488e-15 3.036962e-15 4.013460e-16 5.319078e-15
-3.765595e-15
E:\\R-3.1.2\\scripts\\mPDBS\\summary_pdb_93\\34eb328251842006.final.pdb
-9.533264e-15 8.973321e-15 3.897867e-15 4.344099e-15 -5.119774e-15
-4.012286e-16
E:\\R-3.1.2\\scripts\\mPDBS\\summary_pdb_93\\354b32871797b7ab.final.pdb
-3.265466e-15 5.910300e-15 5.787331e-15 1.062486e-14 -7.444610e-15
-1.183176e-14
E:\\R-3.1.2\\scripts\\mPDBS\\summary_pdb_93\\397368adbc32d5de.final.pdb
-7.377408e-16 3.561819e-15 -2.921272e-15 -9.118975e-15 -9.120438e-15
6.132830e-15
E:\\R-3.1.2\\scripts\\mPDBS\\summary_pdb_93\\411ca13cd3df6edd.final.pdb
-2.827625e-15 7.769909e-15 7.926636e-16 1.774922e-15 -2.809185e-15
-1.341319e-15
E:\\R-3.1.2\\scripts\\mPDBS\\summary_pdb_93\\42357eee6642bf12.final.pdb
-8.579717e-15 2.071159e-15 1.692173e-15 -2.053681e-17 -3.280567e-15
1.621280e-15
E:\\R-3.1.2\\scripts\\mPDBS\\summary_pdb_93\\44342d1f75a7fcfc.final.pdb
1.448923e-15 1.100267e-14 1.798823e-15 -5.549488e-15 -6.923973e-15
-1.385988e-15
E:\\R-3.1.2\\scripts\\mPDBS\\summary_pdb_93\\448ef21dffe8cdfb.final.pdb
-7.479740e-15 1.263277e-14 4.344076e-15 -1.158386e-14 -1.791581e-14
6.304475e-15
E:\\R-3.1.2\\scripts\\mPDBS\\summary_pdb_93\\472a40b3e8f113c7.final.pdb
-4.416635e-15 1.453696e-15 1.871336e-15 1.705374e-15 8.924806e-15
-3.381261e-15
E:\\R-3.1.2\\scripts\\mPDBS\\summary_pdb_93\\48a26225951ea153.final.pdb
1.566224e-15 5.642764e-15 3.947488e-15 -6.869902e-15 -7.707681e-16
-1.327621e-15
E:\\R-3.1.2\\scripts\\mPDBS\\summary_pdb_93\\4ab4a5c7c6682ce6.final.pdb

5.329888e-15 2.969410e-15 5.268791e-15 -6.886386e-15 5.385319e-15
4.240246e-15
E:\\R-3.1.2\\scripts\\mPDBS\\summary_pdb_93\\4eaa45b62cb6a25f.final.pdb
4.159526e-16 4.642434e-15 -9.767893e-16 -1.056360e-14 1.307720e-15
3.645005e-15
E:\\R-3.1.2\\scripts\\mPDBS\\summary_pdb_93\\58c371611d85ac96.final.pdb
-7.377408e-16 3.561819e-15 -2.921272e-15 -9.118975e-15 -9.120438e-15
6.132830e-15
E:\\R-3.1.2\\scripts\\mPDBS\\summary_pdb_93\\59a647f04822d30a.final.pdb
-9.427188e-16 5.714803e-15 -3.895841e-15 -4.831747e-15 4.301857e-15
-1.571604e-15
E:\\R-3.1.2\\scripts\\mPDBS\\summary_pdb_93\\59b091fe385e05fc.final.pdb
-4.657076e-15 5.993829e-15 4.218105e-15 2.977048e-16 1.770819e-15
-2.399680e-15
E:\\R-3.1.2\\scripts\\mPDBS\\summary_pdb_93\\5c35ac30f930d2db.final.pdb
4.159526e-16 4.642434e-15 -9.767893e-16 -1.056360e-14 1.307720e-15
3.645005e-15
E:\\R-3.1.2\\scripts\\mPDBS\\summary_pdb_93\\5f60752431cf2dcb.final.pdb
-6.768762e-16 7.264604e-15 3.275057e-15 -8.498059e-15 8.390669e-15
3.057611e-15
E:\\R-3.1.2\\scripts\\mPDBS\\summary_pdb_93\\626456dd6ff2868d.final.pdb
-4.416635e-15 1.453696e-15 1.871336e-15 1.705374e-15 8.924806e-15
-3.381261e-15
E:\\R-3.1.2\\scripts\\mPDBS\\summary_pdb_93\\6583d53ff3c8f7ef.final.pdb
-9.427188e-16 5.714803e-15 -3.895841e-15 -4.831747e-15 4.301857e-15
-1.571604e-15
E:\\R-3.1.2\\scripts\\mPDBS\\summary_pdb_93\\69760178986bef01.final.pdb
3.139378e-15 -1.363828e-15 -4.098894e-15 -7.099523e-15 -2.055885e-16
3.595464e-15
E:\\R-3.1.2\\scripts\\mPDBS\\summary_pdb_93\\6a9d87d47926b1c8.final.pdb
-3.822079e-15 4.240095e-15 8.474150e-16 -3.385714e-15 5.020727e-16
9.425730e-17
E:\\R-3.1.2\\scripts\\mPDBS\\summary_pdb_93\\6bb3547ea982ffc4.final.pdb
-6.940272e-15 -1.929186e-15 -1.039921e-15 -1.251092e-14 -3.891532e-15
2.721853e-15
E:\\R-3.1.2\\scripts\\mPDBS\\summary_pdb_93\\716d9624611d1c42.final.pdb
-1.619353e-15 3.496134e-15 1.465307e-15 -4.097479e-16 -1.243664e-14
-1.502110e-15
E:\\R-3.1.2\\scripts\\mPDBS\\summary_pdb_93\\75f27e70ad0f1be4.final.pdb
-9.427188e-16 5.714803e-15 -3.895841e-15 -4.831747e-15 4.301857e-15
-1.571604e-15
E:\\R-3.1.2\\scripts\\mPDBS\\summary_pdb_93\\769f910fffb22872.final.pdb
-3.592953e-15 4.473022e-15 6.697549e-16 -3.078991e-15 5.504396e-16
-5.330019e-16
E:\\R-3.1.2\\scripts\\mPDBS\\summary_pdb_93\\791f8c80dd5c3135.final.pdb
-4.657076e-15 5.993829e-15 4.218105e-15 2.977048e-16 1.770819e-15
-2.399680e-15
E:\\R-3.1.2\\scripts\\mPDBS\\summary_pdb_93\\7fad98e66a3f6099.final.pdb
-9.546695e-15 1.020596e-14 4.719050e-15 2.113804e-15 -2.546346e-15
-1.743687e-15
E:\\R-3.1.2\\scripts\\mPDBS\\summary_pdb_93\\804b89c94a633196.final.pdb
-3.769600e-15 5.539488e-15 3.036962e-15 4.013460e-16 5.319078e-15
-3.765595e-15
E:\\R-3.1.2\\scripts\\mPDBS\\summary_pdb_93\\813cc65a2298feba.final.pdb

-3.769600e-15 5.539488e-15 3.036962e-15 4.013460e-16 5.319078e-15
-3.765595e-15
E:\\R-3.1.2\\scripts\\mPDBS\\summary_pdb_93\\83c0eefcfe12d3f9.final.pdb
-2.827625e-15 7.769909e-15 7.926636e-16 1.774922e-15 -2.809185e-15
-1.341319e-15
E:\\R-3.1.2\\scripts\\mPDBS\\summary_pdb_93\\86d50469ef569760.final.pdb
-4.416635e-15 1.453696e-15 1.871336e-15 1.705374e-15 8.924806e-15
-3.381261e-15
E:\\R-3.1.2\\scripts\\mPDBS\\summary_pdb_93\\89d0666e1402203d.final.pdb
-9.427188e-16 5.714803e-15 -3.895841e-15 -4.831747e-15 4.301857e-15
-1.571604e-15
E:\\R-3.1.2\\scripts\\mPDBS\\summary_pdb_93\\8b34852716dadc62.final.pdb
-2.438200e-15 3.040924e-16 -3.617437e-15 -5.808964e-15 -8.044956e-16
3.244423e-15
E:\\R-3.1.2\\scripts\\mPDBS\\summary_pdb_93\\8b6ef9ac3fe74395.final.pdb
-1.151893e-14 -2.391162e-16 1.636082e-15 -1.367458e-14 1.147835e-14
-7.630733e-15
E:\\R-3.1.2\\scripts\\mPDBS\\summary_pdb_93\\98bbe0d5986c138f.final.pdb
-4.657076e-15 5.993829e-15 4.218105e-15 2.977048e-16 1.770819e-15
-2.399680e-15
E:\\R-3.1.2\\scripts\\mPDBS\\summary_pdb_93\\9c22f4909d67e2fa.final.pdb
-4.901714e-15 4.086657e-15 -2.188025e-15 -2.643202e-15 -4.571881e-15
2.960362e-15
E:\\R-3.1.2\\scripts\\mPDBS\\summary_pdb_93\\9dda8ed0d81fb3ef.final.pdb
1.403801e-14 4.059877e-15 6.749724e-15 -1.172456e-15 1.116464e-14
6.948125e-16
E:\\R-3.1.2\\scripts\\mPDBS\\summary_pdb_93\\9f752b3616e6033b.final.pdb
-6.610627e-15 3.321019e-15 -3.719135e-16 -2.756157e-15 2.920932e-15
-1.058428e-15
E:\\R-3.1.2\\scripts\\mPDBS\\summary_pdb_93\\a02c70083816d6ba.final.pdb
4.159526e-16 4.642434e-15 -9.767893e-16 -1.056360e-14 1.307720e-15
3.645005e-15
E:\\R-3.1.2\\scripts\\mPDBS\\summary_pdb_93\\a0c997d891e4e082.final.pdb
6.488645e-15 1.979507e-15 -3.830867e-15 -8.291412e-15 2.306037e-15
4.430519e-15
E:\\R-3.1.2\\scripts\\mPDBS\\summary_pdb_93\\ab8039a2d823c616.final.pdb
-3.827440e-15 4.909238e-15 7.561755e-16 -3.433644e-15 -4.202979e-16
3.452456e-16
E:\\R-3.1.2\\scripts\\mPDBS\\summary_pdb_93\\acf4d74b9c71d4dc.final.pdb
-3.769600e-15 5.539488e-15 3.036962e-15 4.013460e-16 5.319078e-15
-3.765595e-15
E:\\R-3.1.2\\scripts\\mPDBS\\summary_pdb_93\\ad8a823e4b4c668e.final.pdb
-3.438835e-15 4.354845e-15 1.897121e-16 -4.078748e-15 2.348641e-16
-7.815758e-17
E:\\R-3.1.2\\scripts\\mPDBS\\summary_pdb_93\\b16ababd03b7b857.final.pdb
-4.901714e-15 4.086657e-15 -2.188025e-15 -2.643202e-15 -4.571881e-15
2.960362e-15
E:\\R-3.1.2\\scripts\\mPDBS\\summary_pdb_93\\b226923e697d3c63.final.pdb
-9.582331e-16 -7.743640e-16 -3.046260e-15 -1.040048e-14 2.839919e-15
5.807476e-15
E:\\R-3.1.2\\scripts\\mPDBS\\summary_pdb_93\\b28e48f6fa27a165.final.pdb
-4.657076e-15 5.993829e-15 4.218105e-15 2.977048e-16 1.770819e-15
-2.399680e-15
E:\\R-3.1.2\\scripts\\mPDBS\\summary_pdb_93\\b42c4924e3cd1aec.final.pdb

-1.381447e-14 8.872216e-15 2.722662e-16 -3.092795e-15 1.747693e-15
-3.364007e-15
E:\\R-3.1.2\\scripts\\mPDBS\\summary_pdb_93\\b4dd239fcabff1df.final.pdb
-4.657076e-15 5.993829e-15 4.218105e-15 2.977048e-16 1.770819e-15
-2.399680e-15
E:\\R-3.1.2\\scripts\\mPDBS\\summary_pdb_93\\b669942ef97459a2.final.pdb
-2.438200e-15 3.040924e-16 -3.617437e-15 -5.808964e-15 -8.044956e-16
3.244423e-15
E:\\R-3.1.2\\scripts\\mPDBS\\summary_pdb_93\\b99f4d9d87c1017b.final.pdb
-4.657076e-15 5.993829e-15 4.218105e-15 2.977048e-16 1.770819e-15
-2.399680e-15
E:\\R-3.1.2\\scripts\\mPDBS\\summary_pdb_93\\b9d2cbf3975403e7.final.pdb
-4.657076e-15 5.993829e-15 4.218105e-15 2.977048e-16 1.770819e-15
-2.399680e-15
E:\\R-3.1.2\\scripts\\mPDBS\\summary_pdb_93\\bc88c8a505f3a288.final.pdb
-3.790571e-15 4.521048e-15 9.949889e-16 -3.793369e-15 7.106857e-16
-2.776798e-16
E:\\R-3.1.2\\scripts\\mPDBS\\summary_pdb_93\\bdac0e64f4ef427e.final.pdb
-4.222983e-15 -2.408581e-15 -1.584775e-15 -1.345779e-14 6.119653e-15
2.855411e-15
E:\\R-3.1.2\\scripts\\mPDBS\\summary_pdb_93\\ca2a1f55fc9d0681.final.pdb
-2.438200e-15 3.040924e-16 -3.617437e-15 -5.808964e-15 -8.044956e-16
3.244423e-15
E:\\R-3.1.2\\scripts\\mPDBS\\summary_pdb_93\\ce34248de3e81a3c.final.pdb
6.247466e-15 4.286314e-15 5.009591e-15 -7.600080e-15 2.035505e-15
-1.506406e-15
E:\\R-3.1.2\\scripts\\mPDBS\\summary_pdb_93\\d02e697772494a61.final.pdb
-1.135626e-14 1.047915e-15 -2.898164e-15 -6.268115e-15 -1.136212e-14
2.947171e-15
E:\\R-3.1.2\\scripts\\mPDBS\\summary_pdb_93\\d04221245d015047.final.pdb
-4.528584e-15 6.660658e-15 2.237196e-15 -2.136613e-15 -5.146235e-16
-1.946324e-15
E:\\R-3.1.2\\scripts\\mPDBS\\summary_pdb_93\\d678141e4018054a.final.pdb
-6.461802e-15 2.573564e-15 7.579474e-15 2.022058e-15 -9.582843e-15
6.827954e-15
E:\\R-3.1.2\\scripts\\mPDBS\\summary_pdb_93\\d71b40af0f0efe39.final.pdb
-1.623041e-14 1.414966e-14 2.969053e-15 8.494883e-15 -3.494816e-15
1.558517e-15
E:\\R-3.1.2\\scripts\\mPDBS\\summary_pdb_93\\dc0809eff10197e2.final.pdb
-6.514653e-15 1.221766e-14 -2.905772e-16 -5.348400e-15 1.387515e-14
-1.060646e-14
E:\\R-3.1.2\\scripts\\mPDBS\\summary_pdb_93\\e1163918d3707839.final.pdb
-1.676057e-14 1.789340e-15 9.258134e-15 5.433805e-15 -4.514225e-15
-1.196401e-16
E:\\R-3.1.2\\scripts\\mPDBS\\summary_pdb_93\\e4b48b06824ef848.final.pdb
-3.134688e-16 1.745169e-15 -9.383338e-16 -7.561354e-15 -3.103755e-15
2.697921e-15
E:\\R-3.1.2\\scripts\\mPDBS\\summary_pdb_93\\e529e19ad281fea2.final.pdb
-4.156860e-15 9.194899e-15 3.799481e-15 -5.491993e-15 -2.814068e-15
-1.396738e-15
E:\\R-3.1.2\\scripts\\mPDBS\\summary_pdb_93\\e5602082718ec3a1.final.pdb
-3.769600e-15 5.539488e-15 3.036962e-15 4.013460e-16 5.319078e-15
-3.765595e-15
E:\\R-3.1.2\\scripts\\mPDBS\\summary_pdb_93\\e5d589d996d0a960.final.pdb

-7.193449e-16 -4.297662e-15 4.114023e-15 -1.875294e-14 8.366747e-15
-2.601227e-15
E:\\R-3.1.2\\scripts\\mPDBS\\summary_pdb_93\\e9df9e72307a2fbd.final.pdb
4.159526e-16 4.642434e-15 -9.767893e-16 -1.056360e-14 1.307720e-15
3.645005e-15
E:\\R-3.1.2\\scripts\\mPDBS\\summary_pdb_93\\ea00f8694090cae7.final.pdb
-3.769600e-15 5.539488e-15 3.036962e-15 4.013460e-16 5.319078e-15
-3.765595e-15
E:\\R-3.1.2\\scripts\\mPDBS\\summary_pdb_93\\eba517be32db050e.final.pdb
-4.657076e-15 5.993829e-15 4.218105e-15 2.977048e-16 1.770819e-15
-2.399680e-15
E:\\R-3.1.2\\scripts\\mPDBS\\summary_pdb_93\\ec04b9545899c29d.final.pdb
-3.546647e-15 4.663127e-15 1.242844e-15 -3.859868e-15 1.857916e-16
-6.239426e-17
E:\\R-3.1.2\\scripts\\mPDBS\\summary_pdb_93\\ee6862cbcad52078.final.pdb
-3.820614e-15 4.377500e-15 1.437777e-15 -3.807952e-15 3.071457e-16
3.446523e-16
E:\\R-3.1.2\\scripts\\mPDBS\\summary_pdb_93\\ef6a42fc83b81e5d.final.pdb
-3.592076e-15 4.757733e-15 7.066177e-16 -3.523189e-15 -1.375951e-16
-2.892732e-16
E:\\R-3.1.2\\scripts\\mPDBS\\summary_pdb_93\\ef8e88d4ad93a0ad.final.pdb
-4.528584e-15 6.660658e-15 2.237196e-15 -2.136613e-15 -5.146235e-16
-1.946324e-15
E:\\R-3.1.2\\scripts\\mPDBS\\summary_pdb_93\\f14fcae8deec4fa3.final.pdb
-6.610627e-15 3.321019e-15 -3.719135e-16 -2.756157e-15 2.920932e-15
-1.058428e-15
E:\\R-3.1.2\\scripts\\mPDBS\\summary_pdb_93\\f83584e22003fb92.final.pdb
1.566224e-15 5.642764e-15 3.947488e-15 -6.869902e-15 -7.707681e-16
-1.327621e-15
E:\\R-3.1.2\\scripts\\mPDBS\\summary_pdb_93\\f8e2bf87e52caa29.final.pdb
-4.156860e-15 9.194899e-15 3.799481e-15 -5.491993e-15 -2.814068e-15
-1.396738e-15

[,697]   [,698]   [,699]   [,700]   [,701]   [,702]
E:\\R-3.1.2\\scripts\\40.1ns_data\\NAT0.pdb
-8.358865e-16 -5.711874e-16 -1.974734e-15 -9.327723e-16 4.062983e-15
-2.825867e-15
E:\\R-3.1.2\\scripts\\40.1ns_data\\LJP6.pdb
4.034041e-16 6.247668e-16 -1.422544e-15 -3.717330e-18 4.070565e-15
-2.812939e-15
E:\\R-3.1.2\\scripts\\40.1ns_data\\3WY9.pdb
2.027407e-16 1.348644e-15 -2.943347e-15 -4.139920e-16 1.218529e-15
-3.345999e-15
E:\\R-3.1.2\\scripts\\40.1ns_data\\ZSP9.pdb
-1.378398e-15 5.071584e-16 -1.485799e-15 1.343812e-16 2.692484e-15
-2.141775e-15
E:\\R-3.1.2\\scripts\\mPDBS\\summary_pdb_93\\013d855ad26c877c.final.pdb
2.182215e-15 -4.212361e-15 -3.699029e-15 2.069373e-15 -1.702460e-15
-2.157517e-15
E:\\R-3.1.2\\scripts\\mPDBS\\summary_pdb_93\\0a2a705342ca0d54.final.pdb
2.509091e-16 6.892202e-16 -2.283593e-15 -3.425841e-16 2.131451e-15
-3.290236e-15
E:\\R-3.1.2\\scripts\\mPDBS\\summary_pdb_93\\0a59fceadb4194d9.final.pdb
2.460495e-15 -2.142606e-15 2.113644e-15 1.992187e-16 2.566010e-15

-5.718835e-15
E:\\R-3.1.2\\scripts\\mPDBS\\summary_pdb_93\\0dba59f3306825e8.final.pdb
-6.245681e-16 9.224205e-16 -1.740713e-16 7.188908e-16 5.925792e-16
-5.188730e-15
E:\\R-3.1.2\\scripts\\mPDBS\\summary_pdb_93\\126f26a78b57339c.final.pdb
5.341580e-15 7.814043e-15 -1.223170e-14 -1.847633e-15 3.046346e-15
5.319545e-16
E:\\R-3.1.2\\scripts\\mPDBS\\summary_pdb_93\\12f06f3e342df706.final.pdb
-1.059024e-15 4.793186e-16 -2.087894e-15 1.817360e-16 1.153935e-15
-2.738593e-15
E:\\R-3.1.2\\scripts\\mPDBS\\summary_pdb_93\\18618a2a251b867b.final.pdb
-6.528748e-16 -3.930925e-16 -1.281907e-14 9.476056e-16 1.595239e-15
1.047047e-16
E:\\R-3.1.2\\scripts\\mPDBS\\summary_pdb_93\\1f305f598641eeb2.final.pdb
4.413278e-16 9.122041e-16 -2.132657e-15 2.844300e-16 2.031328e-15
-3.322627e-15
E:\\R-3.1.2\\scripts\\mPDBS\\summary_pdb_93\\1f8a00b551be0a39.final.pdb
-6.528748e-16 -3.930925e-16 -1.281907e-14 9.476056e-16 1.595239e-15
1.047047e-16
E:\\R-3.1.2\\scripts\\mPDBS\\summary_pdb_93\\219f45328d47579a.final.pdb
2.277482e-15 4.050356e-15 1.057198e-15 7.448194e-15 -5.463158e-15
5.058785e-15
E:\\R-3.1.2\\scripts\\mPDBS\\summary_pdb_93\\238dd3918e54336d.final.pdb
-4.560798e-15 3.210317e-15 -1.099631e-14 -1.283536e-15 -2.210118e-15
3.604220e-15
E:\\R-3.1.2\\scripts\\mPDBS\\summary_pdb_93\\2b3027889e2fdc42.final.pdb
1.849222e-15 1.507686e-15 -2.406611e-15 4.086866e-16 1.997021e-15
-2.831605e-15
E:\\R-3.1.2\\scripts\\mPDBS\\summary_pdb_93\\2fcb3b46e33793b0.final.pdb
5.218756e-15 -2.446221e-15 1.784348e-15 2.238162e-15 -2.263847e-15
-5.820845e-15
E:\\R-3.1.2\\scripts\\mPDBS\\summary_pdb_93\\34eb328251842006.final.pdb
-1.351294e-14 2.109713e-15 3.754042e-15 2.277392e-15 7.737539e-15
-3.443215e-15
E:\\R-3.1.2\\scripts\\mPDBS\\summary_pdb_93\\354b32871797b7ab.final.pdb
2.184669e-15 -8.382015e-15 6.006661e-16 -3.188292e-15 1.452299e-14
-9.863418e-15
E:\\R-3.1.2\\scripts\\mPDBS\\summary_pdb_93\\397368adbc32d5de.final.pdb
5.341580e-15 7.814043e-15 -1.223170e-14 -1.847633e-15 3.046346e-15
5.319545e-16
E:\\R-3.1.2\\scripts\\mPDBS\\summary_pdb_93\\411ca13cd3df6edd.final.pdb
5.650269e-15 1.518056e-15 -6.754394e-15 1.034264e-15 -2.837173e-15
-2.117861e-15
E:\\R-3.1.2\\scripts\\mPDBS\\summary_pdb_93\\42357eee6642bf12.final.pdb
5.437435e-15 -5.357367e-15 3.115562e-15 -1.804552e-15 1.195313e-15
-2.901966e-15
E:\\R-3.1.2\\scripts\\mPDBS\\summary_pdb_93\\44342d1f75a7fcfc.final.pdb
4.897428e-15 -5.596981e-15 -7.736736e-15 2.220059e-15 -5.363304e-15
-9.562465e-15
E:\\R-3.1.2\\scripts\\mPDBS\\summary_pdb_93\\448ef21dffe8cdfb.final.pdb
4.399967e-15 1.835077e-15 -1.005514e-14 -5.317859e-15 2.611103e-14
8.056075e-15
E:\\R-3.1.2\\scripts\\mPDBS\\summary_pdb_93\\472a40b3e8f113c7.final.pdb
-6.245681e-16 9.224205e-16 -1.740713e-16 7.188908e-16 5.925792e-16

-5.188730e-15
E:\\R-3.1.2\\scripts\\mPDBS\\summary_pdb_93\\48a26225951ea153.final.pdb
9.974730e-16 6.831552e-15 -1.383757e-14 6.364860e-15 2.358710e-15
-5.580708e-15
E:\\R-3.1.2\\scripts\\mPDBS\\summary_pdb_93\\4ab4a5c7c6682ce6.final.pdb
1.590605e-14 -5.801801e-15 6.383617e-15 -7.204892e-15 5.967620e-15
-1.044299e-14
E:\\R-3.1.2\\scripts\\mPDBS\\summary_pdb_93\\4eaa45b62cb6a25f.final.pdb
5.676668e-15 4.136183e-15 -5.469348e-15 2.295471e-16 -3.735405e-15
-1.096776e-15
E:\\R-3.1.2\\scripts\\mPDBS\\summary_pdb_93\\58c371611d85ac96.final.pdb
5.341580e-15 7.814043e-15 -1.223170e-14 -1.847633e-15 3.046346e-15
5.319545e-16
E:\\R-3.1.2\\scripts\\mPDBS\\summary_pdb_93\\59a647f04822d30a.final.pdb
4.841917e-15 7.400434e-15 4.900725e-15 -4.120789e-15 4.040632e-15
-9.328662e-16
E:\\R-3.1.2\\scripts\\mPDBS\\summary_pdb_93\\59b091fe385e05fc.final.pdb
2.460495e-15 -2.142606e-15 2.113644e-15 1.992187e-16 2.566010e-15
-5.718835e-15
E:\\R-3.1.2\\scripts\\mPDBS\\summary_pdb_93\\5c35ac30f930d2db.final.pdb
5.676668e-15 4.136183e-15 -5.469348e-15 2.295471e-16 -3.735405e-15
-1.096776e-15
E:\\R-3.1.2\\scripts\\mPDBS\\summary_pdb_93\\5f60752431cf2dcb.final.pdb
8.851674e-16 8.475093e-15 -8.019890e-15 -2.780304e-15 -3.368489e-15
-4.167789e-15
E:\\R-3.1.2\\scripts\\mPDBS\\summary_pdb_93\\626456dd6ff2868d.final.pdb
-6.245681e-16 9.224205e-16 -1.740713e-16 7.188908e-16 5.925792e-16
-5.188730e-15
E:\\R-3.1.2\\scripts\\mPDBS\\summary_pdb_93\\6583d53ff3c8f7ef.final.pdb
4.841917e-15 7.400434e-15 4.900725e-15 -4.120789e-15 4.040632e-15
-9.328662e-16
E:\\R-3.1.2\\scripts\\mPDBS\\summary_pdb_93\\69760178986bef01.final.pdb
-7.283374e-15 6.568631e-16 -9.064868e-15 -1.969015e-15 -3.372599e-15
-2.516096e-15
E:\\R-3.1.2\\scripts\\mPDBS\\summary_pdb_93\\6a9d87d47926b1c8.final.pdb
3.332770e-15 1.268931e-15 -2.574495e-15 2.565870e-16 2.231026e-15
-3.030083e-15
E:\\R-3.1.2\\scripts\\mPDBS\\summary_pdb_93\\6bb3547ea982ffc4.final.pdb
-5.352577e-15 8.836798e-15 -1.338140e-14 2.792054e-15 1.133157e-15
2.621720e-15
E:\\R-3.1.2\\scripts\\mPDBS\\summary_pdb_93\\716d9624611d1c42.final.pdb
1.679454e-15 -7.554186e-15 -2.175173e-15 2.060442e-15 3.195704e-15
-5.092570e-16
E:\\R-3.1.2\\scripts\\mPDBS\\summary_pdb_93\\75f27e70ad0f1be4.final.pdb
4.841917e-15 7.400434e-15 4.900725e-15 -4.120789e-15 4.040632e-15
-9.328662e-16
E:\\R-3.1.2\\scripts\\mPDBS\\summary_pdb_93\\769f910fffb22872.final.pdb
-7.684507e-16 1.060606e-15 -2.038850e-15 -6.113609e-17 2.012006e-15
-3.119713e-15
E:\\R-3.1.2\\scripts\\mPDBS\\summary_pdb_93\\791f8c80dd5c3135.final.pdb
2.460495e-15 -2.142606e-15 2.113644e-15 1.992187e-16 2.566010e-15
-5.718835e-15
E:\\R-3.1.2\\scripts\\mPDBS\\summary_pdb_93\\7fad98e66a3f6099.final.pdb
-1.009955e-14 2.937366e-16 8.174528e-15 4.674087e-15 9.269375e-17

-9.184249e-15
E:\\R-3.1.2\\scripts\\mPDBS\\summary_pdb_93\\804b89c94a633196.final.pdb
5.218756e-15 -2.446221e-15 1.784348e-15 2.238162e-15 -2.263847e-15
-5.820845e-15
E:\\R-3.1.2\\scripts\\mPDBS\\summary_pdb_93\\813cc65a2298feba.final.pdb
5.218756e-15 -2.446221e-15 1.784348e-15 2.238162e-15 -2.263847e-15
-5.820845e-15
E:\\R-3.1.2\\scripts\\mPDBS\\summary_pdb_93\\83c0eefcfe12d3f9.final.pdb
5.650269e-15 1.518056e-15 -6.754394e-15 1.034264e-15 -2.837173e-15
-2.117861e-15
E:\\R-3.1.2\\scripts\\mPDBS\\summary_pdb_93\\86d50469ef569760.final.pdb
-6.245681e-16 9.224205e-16 -1.740713e-16 7.188908e-16 5.925792e-16
-5.188730e-15
E:\\R-3.1.2\\scripts\\mPDBS\\summary_pdb_93\\89d0666e1402203d.final.pdb
4.841917e-15 7.400434e-15 4.900725e-15 -4.120789e-15 4.040632e-15
-9.328662e-16
E:\\R-3.1.2\\scripts\\mPDBS\\summary_pdb_93\\8b34852716dadc62.final.pdb
2.186460e-15 3.633985e-15 -1.563668e-15 -2.672120e-16 2.947262e-15
-4.387747e-15
E:\\R-3.1.2\\scripts\\mPDBS\\summary_pdb_93\\8b6ef9ac3fe74395.final.pdb
-1.713718e-15 3.935524e-15 4.427075e-15 4.218249e-15 1.402315e-15
-5.438640e-15
E:\\R-3.1.2\\scripts\\mPDBS\\summary_pdb_93\\98bbe0d5986c138f.final.pdb
2.460495e-15 -2.142606e-15 2.113644e-15 1.992187e-16 2.566010e-15
-5.718835e-15
E:\\R-3.1.2\\scripts\\mPDBS\\summary_pdb_93\\9c22f4909d67e2fa.final.pdb
-6.882241e-15 2.180287e-15 -5.601454e-15 1.771522e-15 4.614765e-15
6.000169e-16
E:\\R-3.1.2\\scripts\\mPDBS\\summary_pdb_93\\9dda8ed0d81fb3ef.final.pdb
5.137036e-15 7.913913e-15 -5.939887e-15 7.751225e-15 1.971975e-15
-1.035610e-14
E:\\R-3.1.2\\scripts\\mPDBS\\summary_pdb_93\\9f752b3616e6033b.final.pdb
-1.476621e-15 9.041775e-16 6.446171e-16 1.170821e-15 3.145194e-15
-2.809703e-15
E:\\R-3.1.2\\scripts\\mPDBS\\summary_pdb_93\\a02c70083816d6ba.final.pdb
5.676668e-15 4.136183e-15 -5.469348e-15 2.295471e-16 -3.735405e-15
-1.096776e-15
E:\\R-3.1.2\\scripts\\mPDBS\\summary_pdb_93\\a0c997d891e4e082.final.pdb
3.202667e-15 4.495894e-15 1.231132e-15 1.740064e-15 7.214223e-15
-5.155752e-15
E:\\R-3.1.2\\scripts\\mPDBS\\summary_pdb_93\\ab8039a2d823c616.final.pdb
2.698729e-15 1.449286e-15 -1.783600e-15 8.501510e-16 5.073482e-16
-2.964796e-15
E:\\R-3.1.2\\scripts\\mPDBS\\summary_pdb_93\\acf4d74b9c71d4dc.final.pdb
5.218756e-15 -2.446221e-15 1.784348e-15 2.238162e-15 -2.263847e-15
-5.820845e-15
E:\\R-3.1.2\\scripts\\mPDBS\\summary_pdb_93\\ad8a823e4b4c668e.final.pdb
-3.780571e-16 9.539556e-16 -2.102805e-15 -4.024381e-17 2.172816e-15
-2.384599e-15
E:\\R-3.1.2\\scripts\\mPDBS\\summary_pdb_93\\b16ababd03b7b857.final.pdb
-6.882241e-15 2.180287e-15 -5.601454e-15 1.771522e-15 4.614765e-15
6.000169e-16
E:\\R-3.1.2\\scripts\\mPDBS\\summary_pdb_93\\b226923e697d3c63.final.pdb
3.982578e-15 -7.272724e-15 -5.139976e-16 -1.336558e-15 4.074026e-15

-3.815977e-15
E:\\R-3.1.2\\scripts\\mPDBS\\summary_pdb_93\\b28e48f6fa27a165.final.pdb
2.460495e-15 -2.142606e-15 2.113644e-15 1.992187e-16 2.566010e-15
-5.718835e-15
E:\\R-3.1.2\\scripts\\mPDBS\\summary_pdb_93\\b42c4924e3cd1aec.final.pdb
-6.481491e-15 -6.287835e-15 -5.965800e-15 1.759490e-15 -3.230027e-15
-4.528297e-15
E:\\R-3.1.2\\scripts\\mPDBS\\summary_pdb_93\\b4dd239fcabff1df.final.pdb
2.460495e-15 -2.142606e-15 2.113644e-15 1.992187e-16 2.566010e-15
-5.718835e-15
E:\\R-3.1.2\\scripts\\mPDBS\\summary_pdb_93\\b669942ef97459a2.final.pdb
2.186460e-15 3.633985e-15 -1.563668e-15 -2.672120e-16 2.947262e-15
-4.387747e-15
E:\\R-3.1.2\\scripts\\mPDBS\\summary_pdb_93\\b99f4d9d87c1017b.final.pdb
2.460495e-15 -2.142606e-15 2.113644e-15 1.992187e-16 2.566010e-15
-5.718835e-15
E:\\R-3.1.2\\scripts\\mPDBS\\summary_pdb_93\\b9d2cbf3975403e7.final.pdb
2.460495e-15 -2.142606e-15 2.113644e-15 1.992187e-16 2.566010e-15
-5.718835e-15
E:\\R-3.1.2\\scripts\\mPDBS\\summary_pdb_93\\bc88c8a505f3a288.final.pdb
1.755981e-15 7.049422e-16 -2.055113e-15 4.737387e-16 1.572013e-15
-2.920075e-15
E:\\R-3.1.2\\scripts\\mPDBS\\summary_pdb_93\\bdac0e64f4ef427e.final.pdb
-9.236261e-15 3.644652e-16 7.359966e-15 -1.121130e-15 9.512757e-15
-6.427131e-15
E:\\R-3.1.2\\scripts\\mPDBS\\summary_pdb_93\\ca2a1f55fc9d0681.final.pdb
2.186460e-15 3.633985e-15 -1.563668e-15 -2.672120e-16 2.947262e-15
-4.387747e-15
E:\\R-3.1.2\\scripts\\mPDBS\\summary_pdb_93\\ce34248de3e81a3c.final.pdb
7.497664e-15 2.467742e-15 -9.891983e-16 -4.156247e-15 -3.590957e-15
-7.880069e-15
E:\\R-3.1.2\\scripts\\mPDBS\\summary_pdb_93\\d02e697772494a61.final.pdb
4.584664e-15 9.465025e-16 6.205207e-15 -7.579715e-15 5.439146e-15
5.970382e-15
E:\\R-3.1.2\\scripts\\mPDBS\\summary_pdb_93\\d04221245d015047.final.pdb
-1.000568e-15 3.553940e-15 -2.942612e-15 9.420914e-16 4.921694e-16
-2.404481e-15
E:\\R-3.1.2\\scripts\\mPDBS\\summary_pdb_93\\d678141e4018054a.final.pdb
-7.031990e-15 -3.777961e-15 -1.619103e-14 -7.890602e-16 2.133104e-15
2.007635e-15
E:\\R-3.1.2\\scripts\\mPDBS\\summary_pdb_93\\d71b40af0f0efe39.final.pdb
6.701220e-15 -1.064800e-14 -1.710481e-15 4.703053e-15 1.193639e-14
-1.593044e-15
E:\\R-3.1.2\\scripts\\mPDBS\\summary_pdb_93\\dc0809eff10197e2.final.pdb
6.883811e-15 6.352255e-15 1.032937e-14 1.147967e-15 9.084973e-15
-4.831558e-15
E:\\R-3.1.2\\scripts\\mPDBS\\summary_pdb_93\\e1163918d3707839.final.pdb
-7.404364e-15 -3.675341e-15 -6.105445e-15 4.754924e-15 -4.465151e-15
7.077485e-16
E:\\R-3.1.2\\scripts\\mPDBS\\summary_pdb_93\\e4b48b06824ef848.final.pdb
-6.528748e-16 -3.930925e-16 -1.281907e-14 9.476056e-16 1.595239e-15
1.047047e-16
E:\\R-3.1.2\\scripts\\mPDBS\\summary_pdb_93\\e529e19ad281fea2.final.pdb
2.182215e-15 -4.212361e-15 -3.699029e-15 2.069373e-15 -1.702460e-15

-2.157517e-15
E:\\R-3.1.2\\scripts\\mPDBS\\summary_pdb_93\\e5602082718ec3a1.final.pdb
5.218756e-15 -2.446221e-15 1.784348e-15 2.238162e-15 -2.263847e-15
-5.820845e-15
E:\\R-3.1.2\\scripts\\mPDBS\\summary_pdb_93\\e5d589d996d0a960.final.pdb
-5.898010e-15 1.009425e-14 -1.123661e-14 2.901069e-16 6.021432e-15
-7.882346e-15
E:\\R-3.1.2\\scripts\\mPDBS\\summary_pdb_93\\e9df9e72307a2fbd.final.pdb
5.676668e-15 4.136183e-15 -5.469348e-15 2.295471e-16 -3.735405e-15
-1.096776e-15
E:\\R-3.1.2\\scripts\\mPDBS\\summary_pdb_93\\ea00f8694090cae7.final.pdb
5.218756e-15 -2.446221e-15 1.784348e-15 2.238162e-15 -2.263847e-15
-5.820845e-15
E:\\R-3.1.2\\scripts\\mPDBS\\summary_pdb_93\\eba517be32db050e.final.pdb
2.460495e-15 -2.142606e-15 2.113644e-15 1.992187e-16 2.566010e-15
-5.718835e-15
E:\\R-3.1.2\\scripts\\mPDBS\\summary_pdb_93\\ec04b9545899c29d.final.pdb
1.033331e-15 8.319040e-16 -2.730324e-15 1.487992e-16 1.311045e-15
-2.562856e-15
E:\\R-3.1.2\\scripts\\mPDBS\\summary_pdb_93\\ee6862cbcad52078.final.pdb
2.090999e-15 3.796816e-16 -2.471230e-15 4.399116e-16 1.611912e-15
-3.038037e-15
E:\\R-3.1.2\\scripts\\mPDBS\\summary_pdb_93\\ef6a42fc83b81e5d.final.pdb
3.906303e-17 1.067978e-15 -2.072027e-15 4.133108e-16 1.992490e-15
-3.258057e-15
E:\\R-3.1.2\\scripts\\mPDBS\\summary_pdb_93\\ef8e88d4ad93a0ad.final.pdb
-1.000568e-15 3.553940e-15 -2.942612e-15 9.420914e-16 4.921694e-16
-2.404481e-15
E:\\R-3.1.2\\scripts\\mPDBS\\summary_pdb_93\\f14fcae8deec4fa3.final.pdb
-1.476621e-15 9.041775e-16 6.446171e-16 1.170821e-15 3.145194e-15
-2.809703e-15
E:\\R-3.1.2\\scripts\\mPDBS\\summary_pdb_93\\f83584e22003fb92.final.pdb
9.974730e-16 6.831552e-15 -1.383757e-14 6.364860e-15 2.358710e-15
-5.580708e-15
E:\\R-3.1.2\\scripts\\mPDBS\\summary_pdb_93\\f8e2bf87e52caa29.final.pdb
2.182215e-15 -4.212361e-15 -3.699029e-15 2.069373e-15 -1.702460e-15
-2.157517e-15

[,703]   [,704]   [,705]   [,706]   [,707]   [,708]
E:\\R-3.1.2\\scripts\\40.1ns_data\\NAT0.pdb
5.207988e-15 -4.463356e-16 3.276785e-15 3.132471e-15 -1.275777e-15
2.857617e-15
E:\\R-3.1.2\\scripts\\40.1ns_data\\LJP6.pdb
3.642992e-15 -4.466081e-16 3.842975e-15 3.804356e-15 -2.653304e-15
2.665648e-15
E:\\R-3.1.2\\scripts\\40.1ns_data\\3WY9.pdb
4.046487e-15 2.075212e-16 5.134759e-15 4.684645e-15 -6.587740e-16
3.491782e-15
E:\\R-3.1.2\\scripts\\40.1ns_data\\ZSP9.pdb
5.189503e-15 -6.891910e-16 3.386575e-15 1.959896e-15 -2.202375e-15
1.923894e-15
E:\\R-3.1.2\\scripts\\mPDBS\\summary_pdb_93\\013d855ad26c877c.final.pdb
1.103650e-14 -1.054479e-15 7.234083e-15 1.035641e-15 1.404506e-15
-3.708755e-15

E:\\R-3.1.2\\scripts\\mPDBS\\summary_pdb_93\\0a2a705342ca0d54.final.pdb
4.519944e-15 -4.568274e-16 3.563165e-15 3.444302e-15 -1.406536e-15
3.741902e-15
E:\\R-3.1.2\\scripts\\mPDBS\\summary_pdb_93\\0a59fceadb4194d9.final.pdb
5.943851e-15 6.087216e-16 -1.293218e-15 -3.691706e-16 -1.327763e-16
7.583226e-15
E:\\R-3.1.2\\scripts\\mPDBS\\summary_pdb_93\\0dba59f3306825e8.final.pdb
5.301283e-15 -1.479053e-15 -1.813367e-15 3.433028e-15 3.454573e-16
5.405482e-15
E:\\R-3.1.2\\scripts\\mPDBS\\summary_pdb_93\\126f26a78b57339c.final.pdb
7.469471e-15 9.677745e-16 7.228879e-15 9.634773e-15 -6.568364e-15
6.447502e-16
E:\\R-3.1.2\\scripts\\mPDBS\\summary_pdb_93\\12f06f3e342df706.final.pdb
4.479395e-15 -7.313474e-16 2.757386e-15 3.692205e-15 -1.412011e-15
2.963662e-15
E:\\R-3.1.2\\scripts\\mPDBS\\summary_pdb_93\\18618a2a251b867b.final.pdb
5.615754e-15 5.705461e-15 2.885518e-15 5.747583e-15 -1.501875e-15
-1.564107e-15
E:\\R-3.1.2\\scripts\\mPDBS\\summary_pdb_93\\1f305f598641eeb2.final.pdb
4.944694e-15 -9.047119e-16 2.275451e-15 3.407393e-15 -1.861766e-15
2.558415e-15
E:\\R-3.1.2\\scripts\\mPDBS\\summary_pdb_93\\1f8a00b551be0a39.final.pdb
5.615754e-15 5.705461e-15 2.885518e-15 5.747583e-15 -1.501875e-15
-1.564107e-15
E:\\R-3.1.2\\scripts\\mPDBS\\summary_pdb_93\\219f45328d47579a.final.pdb
-7.008570e-15 7.123714e-16 -1.196778e-16 4.790331e-15 -1.153533e-14
-6.644108e-15
E:\\R-3.1.2\\scripts\\mPDBS\\summary_pdb_93\\238dd3918e54336d.final.pdb
3.159620e-15 -1.527590e-15 -4.052296e-15 9.387836e-15 -3.155153e-15
-4.981701e-15
E:\\R-3.1.2\\scripts\\mPDBS\\summary_pdb_93\\2b3027889e2fdc42.final.pdb
4.544796e-15 -3.844076e-16 2.711391e-15 3.303781e-15 -7.016301e-16
3.109374e-15
E:\\R-3.1.2\\scripts\\mPDBS\\summary_pdb_93\\2fcb3b46e33793b0.final.pdb
3.065988e-15 -1.932536e-15 1.093237e-15 -1.189624e-15 1.861818e-15
5.696530e-15
E:\\R-3.1.2\\scripts\\mPDBS\\summary_pdb_93\\34eb328251842006.final.pdb
4.409759e-15 2.685018e-15 -1.166813e-14 5.505566e-15 -9.838599e-15
7.884204e-17
E:\\R-3.1.2\\scripts\\mPDBS\\summary_pdb_93\\354b32871797b7ab.final.pdb
2.629134e-15 -8.263433e-16 4.086335e-15 -1.543646e-15 -6.104740e-16
4.621379e-15
E:\\R-3.1.2\\scripts\\mPDBS\\summary_pdb_93\\397368adbc32d5de.final.pdb
7.469471e-15 9.677745e-16 7.228879e-15 9.634773e-15 -6.568364e-15
6.447502e-16
E:\\R-3.1.2\\scripts\\mPDBS\\summary_pdb_93\\411ca13cd3df6edd.final.pdb
4.826516e-15 2.033476e-15 8.189304e-17 2.173897e-15 -5.517614e-15
6.162086e-15
E:\\R-3.1.2\\scripts\\mPDBS\\summary_pdb_93\\42357eee6642bf12.final.pdb
7.596911e-15 4.841823e-16 -9.012372e-15 1.630880e-15 -5.680388e-15
1.527936e-15
E:\\R-3.1.2\\scripts\\mPDBS\\summary_pdb_93\\44342d1f75a7fcfc.final.pdb
1.266560e-14 -3.801297e-15 1.134408e-14 4.480287e-16 3.339044e-15
8.881073e-15

E:\\R-3.1.2\\scripts\\mPDBS\\summary_pdb_93\\448ef21dffe8cdfb.final.pdb
1.219375e-15 5.822979e-15 4.380538e-15 7.749406e-15 -4.228796e-15
-7.759720e-15
E:\\R-3.1.2\\scripts\\mPDBS\\summary_pdb_93\\472a40b3e8f113c7.final.pdb
5.301283e-15 -1.479053e-15 -1.813367e-15 3.433028e-15 3.454573e-16
5.405482e-15
E:\\R-3.1.2\\scripts\\mPDBS\\summary_pdb_93\\48a26225951ea153.final.pdb
4.667420e-15 -4.017569e-15 1.197048e-14 9.553122e-15 3.620433e-15
-3.979573e-15
E:\\R-3.1.2\\scripts\\mPDBS\\summary_pdb_93\\4ab4a5c7c6682ce6.final.pdb
-1.129005e-14 -5.781818e-15 1.155008e-14 -4.277709e-15 7.779533e-15
1.158565e-14
E:\\R-3.1.2\\scripts\\mPDBS\\summary_pdb_93\\4eaa45b62cb6a25f.final.pdb
7.338149e-15 -1.120596e-15 9.450968e-15 5.097255e-15 -6.891328e-16
3.190341e-15
E:\\R-3.1.2\\scripts\\mPDBS\\summary_pdb_93\\58c371611d85ac96.final.pdb
7.469471e-15 9.677745e-16 7.228879e-15 9.634773e-15 -6.568364e-15
6.447502e-16
E:\\R-3.1.2\\scripts\\mPDBS\\summary_pdb_93\\59a647f04822d30a.final.pdb
1.551770e-15 -7.012704e-15 8.628373e-15 4.980861e-15 -1.824360e-15
3.176108e-16
E:\\R-3.1.2\\scripts\\mPDBS\\summary_pdb_93\\59b091fe385e05fc.final.pdb
5.943851e-15 6.087216e-16 -1.293218e-15 -3.691706e-16 -1.327763e-16
7.583226e-15
E:\\R-3.1.2\\scripts\\mPDBS\\summary_pdb_93\\5c35ac30f930d2db.final.pdb
7.338149e-15 -1.120596e-15 9.450968e-15 5.097255e-15 -6.891328e-16
3.190341e-15
E:\\R-3.1.2\\scripts\\mPDBS\\summary_pdb_93\\5f60752431cf2dcb.final.pdb
6.106565e-15 2.810091e-15 3.438265e-15 4.311772e-15 5.655462e-16
2.787154e-15
E:\\R-3.1.2\\scripts\\mPDBS\\summary_pdb_93\\626456dd6ff2868d.final.pdb
5.301283e-15 -1.479053e-15 -1.813367e-15 3.433028e-15 3.454573e-16
5.405482e-15
E:\\R-3.1.2\\scripts\\mPDBS\\summary_pdb_93\\6583d53ff3c8f7ef.final.pdb
1.551770e-15 -7.012704e-15 8.628373e-15 4.980861e-15 -1.824360e-15
3.176108e-16
E:\\R-3.1.2\\scripts\\mPDBS\\summary_pdb_93\\69760178986bef01.final.pdb
-3.398926e-16 -3.468217e-15 6.400653e-15 2.261567e-15 1.197132e-14
4.884951e-15
E:\\R-3.1.2\\scripts\\mPDBS\\summary_pdb_93\\6a9d87d47926b1c8.final.pdb
4.905983e-15 -8.532515e-16 2.254559e-15 3.558037e-15 -1.326897e-15
2.651825e-15
E:\\R-3.1.2\\scripts\\mPDBS\\summary_pdb_93\\6bb3547ea982ffc4.final.pdb
3.388556e-15 4.136882e-15 8.664848e-15 1.325811e-14 -9.909508e-15
1.164046e-15
E:\\R-3.1.2\\scripts\\mPDBS\\summary_pdb_93\\716d9624611d1c42.final.pdb
5.599267e-15 -9.772444e-16 7.092460e-15 5.980468e-15 -6.196325e-15
2.021057e-15
E:\\R-3.1.2\\scripts\\mPDBS\\summary_pdb_93\\75f27e70ad0f1be4.final.pdb
1.551770e-15 -7.012704e-15 8.628373e-15 4.980861e-15 -1.824360e-15
3.176108e-16
E:\\R-3.1.2\\scripts\\mPDBS\\summary_pdb_93\\769f910fffb22872.final.pdb
4.496857e-15 2.163932e-17 3.183891e-15 3.393509e-15 -1.476261e-15
2.880146e-15

E:\\R-3.1.2\\scripts\\mPDBS\\summary_pdb_93\\791f8c80dd5c3135.final.pdb
5.943851e-15 6.087216e-16 -1.293218e-15 -3.691706e-16 -1.327763e-16
7.583226e-15
E:\\R-3.1.2\\scripts\\mPDBS\\summary_pdb_93\\7fad98e66a3f6099.final.pdb
2.602888e-15 -2.065760e-15 -3.485835e-15 9.723925e-16 6.879838e-16
3.653494e-15
E:\\R-3.1.2\\scripts\\mPDBS\\summary_pdb_93\\804b89c94a633196.final.pdb
3.065988e-15 -1.932536e-15 1.093237e-15 -1.189624e-15 1.861818e-15
5.696530e-15
E:\\R-3.1.2\\scripts\\mPDBS\\summary_pdb_93\\813cc65a2298feba.final.pdb
3.065988e-15 -1.932536e-15 1.093237e-15 -1.189624e-15 1.861818e-15
5.696530e-15
E:\\R-3.1.2\\scripts\\mPDBS\\summary_pdb_93\\83c0eefcfe12d3f9.final.pdb
4.826516e-15 2.033476e-15 8.189304e-17 2.173897e-15 -5.517614e-15
6.162086e-15
E:\\R-3.1.2\\scripts\\mPDBS\\summary_pdb_93\\86d50469ef569760.final.pdb
5.301283e-15 -1.479053e-15 -1.813367e-15 3.433028e-15 3.454573e-16
5.405482e-15
E:\\R-3.1.2\\scripts\\mPDBS\\summary_pdb_93\\89d0666e1402203d.final.pdb
1.551770e-15 -7.012704e-15 8.628373e-15 4.980861e-15 -1.824360e-15
3.176108e-16
E:\\R-3.1.2\\scripts\\mPDBS\\summary_pdb_93\\8b34852716dadc62.final.pdb
-3.028449e-16 -1.360944e-15 2.695254e-15 4.940889e-15 -4.505484e-15
8.699336e-15
E:\\R-3.1.2\\scripts\\mPDBS\\summary_pdb_93\\8b6ef9ac3fe74395.final.pdb
-9.679684e-17 3.955968e-15 1.986276e-15 2.870676e-15 -5.254623e-15
8.740437e-15
E:\\R-3.1.2\\scripts\\mPDBS\\summary_pdb_93\\98bbe0d5986c138f.final.pdb
5.943851e-15 6.087216e-16 -1.293218e-15 -3.691706e-16 -1.327763e-16
7.583226e-15
E:\\R-3.1.2\\scripts\\mPDBS\\summary_pdb_93\\9c22f4909d67e2fa.final.pdb
1.544075e-14 -3.582691e-15 8.503755e-16 8.531712e-15 -1.183416e-16
-8.831454e-15
E:\\R-3.1.2\\scripts\\mPDBS\\summary_pdb_93\\9dda8ed0d81fb3ef.final.pdb
7.631434e-15 -3.105456e-15 1.196772e-14 1.239551e-15 4.720670e-15
4.202632e-15
E:\\R-3.1.2\\scripts\\mPDBS\\summary_pdb_93\\9f752b3616e6033b.final.pdb
2.050655e-15 -5.685777e-16 -1.835574e-16 2.481810e-15 -5.669420e-16
3.608755e-15
E:\\R-3.1.2\\scripts\\mPDBS\\summary_pdb_93\\a02c70083816d6ba.final.pdb
7.338149e-15 -1.120596e-15 9.450968e-15 5.097255e-15 -6.891328e-16
3.190341e-15
E:\\R-3.1.2\\scripts\\mPDBS\\summary_pdb_93\\a0c997d891e4e082.final.pdb
-4.576316e-15 2.424735e-15 9.049102e-15 -2.136140e-15 5.720835e-15
1.279896e-14
E:\\R-3.1.2\\scripts\\mPDBS\\summary_pdb_93\\ab8039a2d823c616.final.pdb
5.286671e-15 -8.306901e-16 2.896905e-15 3.217271e-15 -1.635748e-15
3.297987e-15
E:\\R-3.1.2\\scripts\\mPDBS\\summary_pdb_93\\acf4d74b9c71d4dc.final.pdb
3.065988e-15 -1.932536e-15 1.093237e-15 -1.189624e-15 1.861818e-15
5.696530e-15
E:\\R-3.1.2\\scripts\\mPDBS\\summary_pdb_93\\ad8a823e4b4c668e.final.pdb
4.651538e-15 -3.396071e-16 2.018747e-15 3.314511e-15 -1.372602e-15
3.017603e-15

E:\\R-3.1.2\\scripts\\mPDBS\\summary_pdb_93\\b16ababd03b7b857.final.pdb
1.544075e-14 -3.582691e-15 8.503755e-16 8.531712e-15 -1.183416e-16
-8.831454e-15
E:\\R-3.1.2\\scripts\\mPDBS\\summary_pdb_93\\b226923e697d3c63.final.pdb
-7.519429e-16 1.087013e-14 -6.064277e-16 -1.225925e-15 -3.141910e-15
9.438462e-15
E:\\R-3.1.2\\scripts\\mPDBS\\summary_pdb_93\\b28e48f6fa27a165.final.pdb
5.943851e-15 6.087216e-16 -1.293218e-15 -3.691706e-16 -1.327763e-16
7.583226e-15
E:\\R-3.1.2\\scripts\\mPDBS\\summary_pdb_93\\b42c4924e3cd1aec.final.pdb
8.007326e-15 5.597001e-15 -3.390659e-15 -8.695480e-16 -4.825811e-15
1.886991e-16
E:\\R-3.1.2\\scripts\\mPDBS\\summary_pdb_93\\b4dd239fcabff1df.final.pdb
5.943851e-15 6.087216e-16 -1.293218e-15 -3.691706e-16 -1.327763e-16
7.583226e-15
E:\\R-3.1.2\\scripts\\mPDBS\\summary_pdb_93\\b669942ef97459a2.final.pdb
-3.028449e-16 -1.360944e-15 2.695254e-15 4.940889e-15 -4.505484e-15
8.699336e-15
E:\\R-3.1.2\\scripts\\mPDBS\\summary_pdb_93\\b99f4d9d87c1017b.final.pdb
5.943851e-15 6.087216e-16 -1.293218e-15 -3.691706e-16 -1.327763e-16
7.583226e-15
E:\\R-3.1.2\\scripts\\mPDBS\\summary_pdb_93\\b9d2cbf3975403e7.final.pdb
5.943851e-15 6.087216e-16 -1.293218e-15 -3.691706e-16 -1.327763e-16
7.583226e-15
E:\\R-3.1.2\\scripts\\mPDBS\\summary_pdb_93\\bc88c8a505f3a288.final.pdb
4.682707e-15 -7.413269e-16 2.683635e-15 3.539975e-15 -1.344643e-15
3.235250e-15
E:\\R-3.1.2\\scripts\\mPDBS\\summary_pdb_93\\bdac0e64f4ef427e.final.pdb
5.544800e-15 -7.062547e-15 8.499639e-15 4.640619e-15 6.065162e-15
-5.064719e-15
E:\\R-3.1.2\\scripts\\mPDBS\\summary_pdb_93\\ca2a1f55fc9d0681.final.pdb
-3.028449e-16 -1.360944e-15 2.695254e-15 4.940889e-15 -4.505484e-15
8.699336e-15
E:\\R-3.1.2\\scripts\\mPDBS\\summary_pdb_93\\ce34248de3e81a3c.final.pdb
1.169977e-14 -2.799313e-15 1.147246e-14 1.727079e-15 1.028047e-15
5.767064e-15
E:\\R-3.1.2\\scripts\\mPDBS\\summary_pdb_93\\d02e697772494a61.final.pdb
-8.762144e-16 -3.810053e-15 -2.111140e-15 9.316712e-15 -4.588901e-15
4.122995e-15
E:\\R-3.1.2\\scripts\\mPDBS\\summary_pdb_93\\d04221245d015047.final.pdb
6.089198e-15 -3.924353e-15 3.517068e-15 4.707344e-15 -9.509117e-16
8.241633e-16
E:\\R-3.1.2\\scripts\\mPDBS\\summary_pdb_93\\d678141e4018054a.final.pdb
7.853075e-15 5.517428e-15 -2.206662e-15 1.310695e-14 -1.241276e-14
-7.052344e-15
E:\\R-3.1.2\\scripts\\mPDBS\\summary_pdb_93\\d71b40af0f0efe39.final.pdb
4.789438e-15 3.110198e-15 -9.178626e-15 3.023775e-15 -6.477241e-15
-5.138365e-15
E:\\R-3.1.2\\scripts\\mPDBS\\summary_pdb_93\\dc0809eff10197e2.final.pdb
1.607957e-15 -9.401064e-16 4.293156e-15 -9.420552e-16 2.599539e-15
6.861795e-15
E:\\R-3.1.2\\scripts\\mPDBS\\summary_pdb_93\\e1163918d3707839.final.pdb
1.133789e-14 8.064645e-15 -1.470106e-14 5.214127e-15 -6.889699e-15
3.541269e-15

E:\\R-3.1.2\\scripts\\mPDBS\\summary_pdb_93\\e4b48b06824ef848.final.pdb
5.615754e-15 5.705461e-15 2.885518e-15 5.747583e-15 -1.501875e-15
-1.564107e-15
E:\\R-3.1.2\\scripts\\mPDBS\\summary_pdb_93\\e529e19ad281fea2.final.pdb
1.103650e-14 -1.054479e-15 7.234083e-15 1.035641e-15 1.404506e-15
-3.708755e-15
E:\\R-3.1.2\\scripts\\mPDBS\\summary_pdb_93\\e5602082718ec3a1.final.pdb
3.065988e-15 -1.932536e-15 1.093237e-15 -1.189624e-15 1.861818e-15
5.696530e-15
E:\\R-3.1.2\\scripts\\mPDBS\\summary_pdb_93\\e5d589d996d0a960.final.pdb
3.216857e-15 -3.801657e-15 1.582833e-14 6.339019e-15 4.892984e-15
5.801656e-15
E:\\R-3.1.2\\scripts\\mPDBS\\summary_pdb_93\\e9df9e72307a2fbd.final.pdb
7.338149e-15 -1.120596e-15 9.450968e-15 5.097255e-15 -6.891328e-16
3.190341e-15
E:\\R-3.1.2\\scripts\\mPDBS\\summary_pdb_93\\ea00f8694090cae7.final.pdb
3.065988e-15 -1.932536e-15 1.093237e-15 -1.189624e-15 1.861818e-15
5.696530e-15
E:\\R-3.1.2\\scripts\\mPDBS\\summary_pdb_93\\eba517be32db050e.final.pdb
5.943851e-15 6.087216e-16 -1.293218e-15 -3.691706e-16 -1.327763e-16
7.583226e-15
E:\\R-3.1.2\\scripts\\mPDBS\\summary_pdb_93\\ec04b9545899c29d.final.pdb
4.936974e-15 -3.795552e-16 2.802304e-15 3.292975e-15 -1.229133e-15
2.822985e-15
E:\\R-3.1.2\\scripts\\mPDBS\\summary_pdb_93\\ee6862cbcad52078.final.pdb
4.407211e-15 -3.592541e-16 2.262098e-15 3.700057e-15 -9.559026e-16
3.252489e-15
E:\\R-3.1.2\\scripts\\mPDBS\\summary_pdb_93\\ef6a42fc83b81e5d.final.pdb
4.534913e-15 -3.708419e-16 2.784037e-15 3.429884e-15 -1.420262e-15
3.168436e-15
E:\\R-3.1.2\\scripts\\mPDBS\\summary_pdb_93\\ef8e88d4ad93a0ad.final.pdb
6.089198e-15 -3.924353e-15 3.517068e-15 4.707344e-15 -9.509117e-16
8.241633e-16
E:\\R-3.1.2\\scripts\\mPDBS\\summary_pdb_93\\f14fcae8deec4fa3.final.pdb
2.050655e-15 -5.685777e-16 -1.835574e-16 2.481810e-15 -5.669420e-16
3.608755e-15
E:\\R-3.1.2\\scripts\\mPDBS\\summary_pdb_93\\f83584e22003fb92.final.pdb
4.667420e-15 -4.017569e-15 1.197048e-14 9.553122e-15 3.620433e-15
-3.979573e-15
E:\\R-3.1.2\\scripts\\mPDBS\\summary_pdb_93\\f8e2bf87e52caa29.final.pdb
1.103650e-14 -1.054479e-15 7.234083e-15 1.035641e-15 1.404506e-15
-3.708755e-15

[,709]   [,710]   [,711]   [,712]   [,713]   [,714]
E:\\R-3.1.2\\scripts\\40.1ns_data\\NAT0.pdb
-1.556992e-15 1.195780e-15 -4.090383e-15 2.391955e-15 -3.638363e-15
1.172652e-15
E:\\R-3.1.2\\scripts\\40.1ns_data\\LJP6.pdb
-2.603104e-15 9.875291e-16 -3.812094e-15 3.035703e-15 -3.688414e-15
1.062240e-15
E:\\R-3.1.2\\scripts\\40.1ns_data\\3WY9.pdb
-2.537363e-15 7.625650e-16 -1.901226e-15 3.029879e-15 -3.870749e-15
5.282179e-16
E:\\R-3.1.2\\scripts\\40.1ns_data\\ZSP9.pdb

-1.705454e-15 4.554415e-16 -3.433633e-15 7.579329e-16 -1.256099e-15
1.832346e-15
E:\\R-3.1.2\\scripts\\mPDBS\\summary_pdb_93\\013d855ad26c877c.final.pdb
4.717425e-15 7.457080e-16 -8.879882e-15 -2.289935e-15 -5.485118e-15
2.200083e-15
E:\\R-3.1.2\\scripts\\mPDBS\\summary_pdb_93\\0a2a705342ca0d54.final.pdb
-1.554188e-15 9.765021e-16 -3.695556e-15 1.646398e-15 -3.825645e-15
7.653746e-16
E:\\R-3.1.2\\scripts\\mPDBS\\summary_pdb_93\\0a59fceadb4194d9.final.pdb
-2.448880e-15 2.822946e-15 -1.187859e-15 4.944663e-15 -1.194582e-14
4.655144e-15
E:\\R-3.1.2\\scripts\\mPDBS\\summary_pdb_93\\0dba59f3306825e8.final.pdb
-2.573618e-15 3.583716e-15 1.923976e-15 6.598572e-15 -9.587739e-15
-9.900736e-16
E:\\R-3.1.2\\scripts\\mPDBS\\summary_pdb_93\\126f26a78b57339c.final.pdb
5.713273e-16 -9.041710e-15 -3.588108e-15 1.595033e-15 2.590887e-15
-1.548655e-15
E:\\R-3.1.2\\scripts\\mPDBS\\summary_pdb_93\\12f06f3e342df706.final.pdb
-1.459212e-15 1.029357e-15 -3.529022e-15 1.265626e-15 -4.426726e-15
1.928507e-15
E:\\R-3.1.2\\scripts\\mPDBS\\summary_pdb_93\\18618a2a251b867b.final.pdb
-9.571247e-16 -9.546669e-15 -6.442415e-16 -6.887746e-16 3.471615e-15
1.107636e-15
E:\\R-3.1.2\\scripts\\mPDBS\\summary_pdb_93\\1f305f598641eeb2.final.pdb
-1.349993e-15 5.401508e-16 -3.307261e-15 1.840498e-15 -4.632629e-15
6.333180e-16
E:\\R-3.1.2\\scripts\\mPDBS\\summary_pdb_93\\1f8a00b551be0a39.final.pdb
-9.571247e-16 -9.546669e-15 -6.442415e-16 -6.887746e-16 3.471615e-15
1.107636e-15
E:\\R-3.1.2\\scripts\\mPDBS\\summary_pdb_93\\219f45328d47579a.final.pdb
-5.143339e-15 8.263906e-15 -2.474714e-14 -1.193723e-14 -4.895035e-15
-1.025307e-14
E:\\R-3.1.2\\scripts\\mPDBS\\summary_pdb_93\\238dd3918e54336d.final.pdb
5.108334e-15 -3.510570e-15 -4.521997e-15 3.178389e-15 3.016605e-15
9.428359e-16
E:\\R-3.1.2\\scripts\\mPDBS\\summary_pdb_93\\2b3027889e2fdc42.final.pdb
-1.252463e-15 3.248631e-16 -3.661574e-15 1.628793e-15 -5.389431e-15
1.586418e-15
E:\\R-3.1.2\\scripts\\mPDBS\\summary_pdb_93\\2fcb3b46e33793b0.final.pdb
-3.218290e-15 5.444214e-15 -2.564800e-15 2.057446e-15 -8.310243e-15
1.427821e-15
E:\\R-3.1.2\\scripts\\mPDBS\\summary_pdb_93\\34eb328251842006.final.pdb
-5.874893e-15 1.628740e-15 -3.767046e-17 -5.182471e-15 -7.805749e-15
5.611451e-15
E:\\R-3.1.2\\scripts\\mPDBS\\summary_pdb_93\\354b32871797b7ab.final.pdb
-7.458331e-15 6.709393e-15 -3.627196e-15 -5.694641e-15 -4.989905e-15
-2.944915e-15
E:\\R-3.1.2\\scripts\\mPDBS\\summary_pdb_93\\397368adbc32d5de.final.pdb
5.713273e-16 -9.041710e-15 -3.588108e-15 1.595033e-15 2.590887e-15
-1.548655e-15
E:\\R-3.1.2\\scripts\\mPDBS\\summary_pdb_93\\411ca13cd3df6edd.final.pdb
-5.150664e-15 -4.972692e-15 3.256065e-15 1.413902e-16 -4.909766e-16
1.674152e-15
E:\\R-3.1.2\\scripts\\mPDBS\\summary_pdb_93\\42357eee6642bf12.final.pdb

-6.509658e-15 8.792853e-17 -1.055084e-14 3.168965e-15 -5.578491e-15
7.910310e-15
E:\\R-3.1.2\\scripts\\mPDBS\\summary_pdb_93\\44342d1f75a7fcfc.final.pdb
-4.557931e-15 -2.451364e-15 1.096179e-15 4.983737e-16 -4.903892e-15
-2.274981e-15
E:\\R-3.1.2\\scripts\\mPDBS\\summary_pdb_93\\448ef21dffe8cdfb.final.pdb
7.943788e-15 -1.152659e-14 -5.440971e-15 -1.286020e-14 9.914762e-15
1.500030e-14
E:\\R-3.1.2\\scripts\\mPDBS\\summary_pdb_93\\472a40b3e8f113c7.final.pdb
-2.573618e-15 3.583716e-15 1.923976e-15 6.598572e-15 -9.587739e-15
-9.900736e-16
E:\\R-3.1.2\\scripts\\mPDBS\\summary_pdb_93\\48a26225951ea153.final.pdb
5.855417e-17 -7.045110e-15 -1.028264e-15 -3.541334e-15 2.678768e-15
7.311996e-16
E:\\R-3.1.2\\scripts\\mPDBS\\summary_pdb_93\\4ab4a5c7c6682ce6.final.pdb
-9.135318e-15 8.757039e-16 -1.030149e-14 8.949634e-16 -8.039670e-16
6.609750e-15
E:\\R-3.1.2\\scripts\\mPDBS\\summary_pdb_93\\4eaa45b62cb6a25f.final.pdb
2.104874e-15 -4.018964e-15 -2.622791e-15 6.764631e-15 -2.476724e-15
2.267929e-15
E:\\R-3.1.2\\scripts\\mPDBS\\summary_pdb_93\\58c371611d85ac96.final.pdb
5.713273e-16 -9.041710e-15 -3.588108e-15 1.595033e-15 2.590887e-15
-1.548655e-15
E:\\R-3.1.2\\scripts\\mPDBS\\summary_pdb_93\\59a647f04822d30a.final.pdb
-2.701776e-15 1.732789e-15 -7.580908e-15 4.331137e-15 -1.268779e-15
1.591717e-15
E:\\R-3.1.2\\scripts\\mPDBS\\summary_pdb_93\\59b091fe385e05fc.final.pdb
-2.448880e-15 2.822946e-15 -1.187859e-15 4.944663e-15 -1.194582e-14
4.655144e-15
E:\\R-3.1.2\\scripts\\mPDBS\\summary_pdb_93\\5c35ac30f930d2db.final.pdb
2.104874e-15 -4.018964e-15 -2.622791e-15 6.764631e-15 -2.476724e-15
2.267929e-15
E:\\R-3.1.2\\scripts\\mPDBS\\summary_pdb_93\\5f60752431cf2dcb.final.pdb
-5.058438e-15 -1.042245e-14 4.362235e-15 9.738991e-15 -1.599127e-15
5.011978e-15
E:\\R-3.1.2\\scripts\\mPDBS\\summary_pdb_93\\626456dd6ff2868d.final.pdb
-2.573618e-15 3.583716e-15 1.923976e-15 6.598572e-15 -9.587739e-15
-9.900736e-16
E:\\R-3.1.2\\scripts\\mPDBS\\summary_pdb_93\\6583d53ff3c8f7ef.final.pdb
-2.701776e-15 1.732789e-15 -7.580908e-15 4.331137e-15 -1.268779e-15
1.591717e-15
E:\\R-3.1.2\\scripts\\mPDBS\\summary_pdb_93\\69760178986bef01.final.pdb
2.513997e-15 -4.459000e-15 -4.279145e-18 4.623357e-15 -1.231663e-15
1.113684e-16
E:\\R-3.1.2\\scripts\\mPDBS\\summary_pdb_93\\6a9d87d47926b1c8.final.pdb
-1.222377e-15 1.609978e-16 -3.806683e-15 1.784551e-15 -6.034158e-15
9.111834e-16
E:\\R-3.1.2\\scripts\\mPDBS\\summary_pdb_93\\6bb3547ea982ffc4.final.pdb
-5.761967e-16 -1.145740e-14 -3.552064e-15 -7.130493e-15 1.092235e-14
-7.794808e-15
E:\\R-3.1.2\\scripts\\mPDBS\\summary_pdb_93\\716d9624611d1c42.final.pdb
3.283522e-15 5.629898e-15 -5.432014e-15 -6.843556e-15 -2.639094e-15
-2.806637e-16
E:\\R-3.1.2\\scripts\\mPDBS\\summary_pdb_93\\75f27e70ad0f1be4.final.pdb

-2.701776e-15 1.732789e-15 -7.580908e-15 4.331137e-15 -1.268779e-15
1.591717e-15
E:\\R-3.1.2\\scripts\\mPDBS\\summary_pdb_93\\769f910fffb22872.final.pdb
-1.268074e-15 1.104574e-15 -2.670398e-15 1.686983e-15 -4.357626e-15
1.464989e-15
E:\\R-3.1.2\\scripts\\mPDBS\\summary_pdb_93\\791f8c80dd5c3135.final.pdb
-2.448880e-15 2.822946e-15 -1.187859e-15 4.944663e-15 -1.194582e-14
4.655144e-15
E:\\R-3.1.2\\scripts\\mPDBS\\summary_pdb_93\\7fad98e66a3f6099.final.pdb
-6.030379e-15 7.473389e-15 -3.994538e-15 -1.147522e-15 -4.727724e-15
5.019693e-15
E:\\R-3.1.2\\scripts\\mPDBS\\summary_pdb_93\\804b89c94a633196.final.pdb
-3.218290e-15 5.444214e-15 -2.564800e-15 2.057446e-15 -8.310243e-15
1.427821e-15
E:\\R-3.1.2\\scripts\\mPDBS\\summary_pdb_93\\813cc65a2298feba.final.pdb
-3.218290e-15 5.444214e-15 -2.564800e-15 2.057446e-15 -8.310243e-15
1.427821e-15
E:\\R-3.1.2\\scripts\\mPDBS\\summary_pdb_93\\83c0eefcfe12d3f9.final.pdb
-5.150664e-15 -4.972692e-15 3.256065e-15 1.413902e-16 -4.909766e-16
1.674152e-15
E:\\R-3.1.2\\scripts\\mPDBS\\summary_pdb_93\\86d50469ef569760.final.pdb
-2.573618e-15 3.583716e-15 1.923976e-15 6.598572e-15 -9.587739e-15
-9.900736e-16
E:\\R-3.1.2\\scripts\\mPDBS\\summary_pdb_93\\89d0666e1402203d.final.pdb
-2.701776e-15 1.732789e-15 -7.580908e-15 4.331137e-15 -1.268779e-15
1.591717e-15
E:\\R-3.1.2\\scripts\\mPDBS\\summary_pdb_93\\8b34852716dadc62.final.pdb
-8.337459e-15 -3.723138e-16 -3.161545e-15 5.009837e-15 -4.188540e-15
-7.960941e-16
E:\\R-3.1.2\\scripts\\mPDBS\\summary_pdb_93\\8b6ef9ac3fe74395.final.pdb
-4.324403e-15 3.110660e-15 -6.116951e-15 2.772753e-15 -4.617394e-15
2.762952e-15
E:\\R-3.1.2\\scripts\\mPDBS\\summary_pdb_93\\98bbe0d5986c138f.final.pdb
-2.448880e-15 2.822946e-15 -1.187859e-15 4.944663e-15 -1.194582e-14
4.655144e-15
E:\\R-3.1.2\\scripts\\mPDBS\\summary_pdb_93\\9c22f4909d67e2fa.final.pdb
7.605625e-15 4.724358e-15 -3.596960e-15 3.551206e-15 -1.132042e-14
-3.611550e-15
E:\\R-3.1.2\\scripts\\mPDBS\\summary_pdb_93\\9dda8ed0d81fb3ef.final.pdb
5.763674e-15 -1.191611e-14 1.261344e-14 1.787841e-14 -6.244833e-15
2.793881e-15
E:\\R-3.1.2\\scripts\\mPDBS\\summary_pdb_93\\9f752b3616e6033b.final.pdb
-2.412594e-15 4.717738e-15 -5.844188e-15 3.872148e-17 -6.722409e-15
1.512641e-16
E:\\R-3.1.2\\scripts\\mPDBS\\summary_pdb_93\\a02c70083816d6ba.final.pdb
2.104874e-15 -4.018964e-15 -2.622791e-15 6.764631e-15 -2.476724e-15
2.267929e-15
E:\\R-3.1.2\\scripts\\mPDBS\\summary_pdb_93\\a0c997d891e4e082.final.pdb
6.493087e-16 -1.881520e-15 -1.831921e-15 1.767224e-15 -9.923683e-15
2.586479e-15
E:\\R-3.1.2\\scripts\\mPDBS\\summary_pdb_93\\ab8039a2d823c616.final.pdb
-1.772040e-15 5.097556e-16 -2.371420e-15 1.883342e-15 -4.885612e-15
1.484929e-15
E:\\R-3.1.2\\scripts\\mPDBS\\summary_pdb_93\\acf4d74b9c71d4dc.final.pdb

-3.218290e-15 5.444214e-15 -2.564800e-15 2.057446e-15 -8.310243e-15
1.427821e-15
E:\\R-3.1.2\\scripts\\mPDBS\\summary_pdb_93\\ad8a823e4b4c668e.final.pdb
-1.376469e-15 5.092528e-16 -4.115137e-15 1.566469e-15 -4.464024e-15
1.726720e-15
E:\\R-3.1.2\\scripts\\mPDBS\\summary_pdb_93\\b16ababd03b7b857.final.pdb
7.605625e-15 4.724358e-15 -3.596960e-15 3.551206e-15 -1.132042e-14
-3.611550e-15
E:\\R-3.1.2\\scripts\\mPDBS\\summary_pdb_93\\b226923e697d3c63.final.pdb
-3.991977e-15 -5.150634e-15 -1.783462e-15 2.023304e-15 -2.907659e-15
9.123887e-15
E:\\R-3.1.2\\scripts\\mPDBS\\summary_pdb_93\\b28e48f6fa27a165.final.pdb
-2.448880e-15 2.822946e-15 -1.187859e-15 4.944663e-15 -1.194582e-14
4.655144e-15
E:\\R-3.1.2\\scripts\\mPDBS\\summary_pdb_93\\b42c4924e3cd1aec.final.pdb
-1.960507e-15 5.618610e-15 -5.901695e-15 -7.589558e-15 -1.369937e-14
1.170179e-15
E:\\R-3.1.2\\scripts\\mPDBS\\summary_pdb_93\\b4dd239fcabff1df.final.pdb
-2.448880e-15 2.822946e-15 -1.187859e-15 4.944663e-15 -1.194582e-14
4.655144e-15
E:\\R-3.1.2\\scripts\\mPDBS\\summary_pdb_93\\b669942ef97459a2.final.pdb
-8.337459e-15 -3.723138e-16 -3.161545e-15 5.009837e-15 -4.188540e-15
-7.960941e-16
E:\\R-3.1.2\\scripts\\mPDBS\\summary_pdb_93\\b99f4d9d87c1017b.final.pdb
-2.448880e-15 2.822946e-15 -1.187859e-15 4.944663e-15 -1.194582e-14
4.655144e-15
E:\\R-3.1.2\\scripts\\mPDBS\\summary_pdb_93\\b9d2cbf3975403e7.final.pdb
-2.448880e-15 2.822946e-15 -1.187859e-15 4.944663e-15 -1.194582e-14
4.655144e-15
E:\\R-3.1.2\\scripts\\mPDBS\\summary_pdb_93\\bc88c8a505f3a288.final.pdb
-1.223298e-15 4.845932e-16 -3.736601e-15 1.847260e-15 -5.785381e-15
1.057328e-15
E:\\R-3.1.2\\scripts\\mPDBS\\summary_pdb_93\\bdac0e64f4ef427e.final.pdb
6.022690e-15 1.130598e-14 -1.307289e-14 5.911406e-15 -1.262233e-14
-1.026812e-15
E:\\R-3.1.2\\scripts\\mPDBS\\summary_pdb_93\\ca2a1f55fc9d0681.final.pdb
-8.337459e-15 -3.723138e-16 -3.161545e-15 5.009837e-15 -4.188540e-15
-7.960941e-16
E:\\R-3.1.2\\scripts\\mPDBS\\summary_pdb_93\\ce34248de3e81a3c.final.pdb
1.695571e-15 -4.443946e-15 -6.739967e-15 1.269854e-14 -5.579026e-15
9.102013e-15
E:\\R-3.1.2\\scripts\\mPDBS\\summary_pdb_93\\d02e697772494a61.final.pdb
-7.499202e-15 8.401776e-15 -1.494248e-14 -4.629818e-15 5.399248e-15
4.605827e-16
E:\\R-3.1.2\\scripts\\mPDBS\\summary_pdb_93\\d04221245d015047.final.pdb
-8.599007e-16 3.024278e-15 -3.195414e-15 1.629747e-15 -4.745533e-15
-9.072545e-16
E:\\R-3.1.2\\scripts\\mPDBS\\summary_pdb_93\\d678141e4018054a.final.pdb
-1.878854e-15 -8.005322e-15 2.109800e-15 -1.108513e-14 1.224965e-14
-2.442312e-15
E:\\R-3.1.2\\scripts\\mPDBS\\summary_pdb_93\\d71b40af0f0efe39.final.pdb
-6.482021e-15 1.786465e-14 2.265966e-15 -9.357007e-15 -1.004191e-14
-3.576713e-15
E:\\R-3.1.2\\scripts\\mPDBS\\summary_pdb_93\\dc0809eff10197e2.final.pdb

-2.104495e-15 9.956716e-15 -1.606462e-15 6.097620e-15 -1.918183e-14
3.519616e-15
E:\\R-3.1.2\\scripts\\mPDBS\\summary_pdb_93\\e1163918d3707839.final.pdb
4.823129e-15 6.589188e-15 -5.778291e-16 -1.041418e-14 -1.255793e-14
2.332444e-15
E:\\R-3.1.2\\scripts\\mPDBS\\summary_pdb_93\\e4b48b06824ef848.final.pdb
-9.571247e-16 -9.546669e-15 -6.442415e-16 -6.887746e-16 3.471615e-15
1.107636e-15
E:\\R-3.1.2\\scripts\\mPDBS\\summary_pdb_93\\e529e19ad281fea2.final.pdb
4.717425e-15 7.457080e-16 -8.879882e-15 -2.289935e-15 -5.485118e-15
2.200083e-15
E:\\R-3.1.2\\scripts\\mPDBS\\summary_pdb_93\\e5602082718ec3a1.final.pdb
-3.218290e-15 5.444214e-15 -2.564800e-15 2.057446e-15 -8.310243e-15
1.427821e-15
E:\\R-3.1.2\\scripts\\mPDBS\\summary_pdb_93\\e5d589d996d0a960.final.pdb
3.895257e-15 4.463310e-15 -4.449442e-15 7.324867e-15 1.071426e-15
-2.340983e-15
E:\\R-3.1.2\\scripts\\mPDBS\\summary_pdb_93\\e9df9e72307a2fbd.final.pdb
2.104874e-15 -4.018964e-15 -2.622791e-15 6.764631e-15 -2.476724e-15
2.267929e-15
E:\\R-3.1.2\\scripts\\mPDBS\\summary_pdb_93\\ea00f8694090cae7.final.pdb
-3.218290e-15 5.444214e-15 -2.564800e-15 2.057446e-15 -8.310243e-15
1.427821e-15
E:\\R-3.1.2\\scripts\\mPDBS\\summary_pdb_93\\eba517be32db050e.final.pdb
-2.448880e-15 2.822946e-15 -1.187859e-15 4.944663e-15 -1.194582e-14
4.655144e-15
E:\\R-3.1.2\\scripts\\mPDBS\\summary_pdb_93\\ec04b9545899c29d.final.pdb
-1.229430e-15 9.035914e-16 -3.009911e-15 1.688394e-15 -4.776390e-15
1.605595e-15
E:\\R-3.1.2\\scripts\\mPDBS\\summary_pdb_93\\ee6862cbcad52078.final.pdb
-1.409347e-15 6.129085e-16 -3.484633e-15 1.946193e-15 -5.381625e-15
1.242943e-15
E:\\R-3.1.2\\scripts\\mPDBS\\summary_pdb_93\\ef6a42fc83b81e5d.final.pdb
-1.208551e-15 8.192523e-16 -2.791829e-15 1.501097e-15 -4.129943e-15
1.555195e-15
E:\\R-3.1.2\\scripts\\mPDBS\\summary_pdb_93\\ef8e88d4ad93a0ad.final.pdb
-8.599007e-16 3.024278e-15 -3.195414e-15 1.629747e-15 -4.745533e-15
-9.072545e-16
E:\\R-3.1.2\\scripts\\mPDBS\\summary_pdb_93\\f14fcae8deec4fa3.final.pdb
-2.412594e-15 4.717738e-15 -5.844188e-15 3.872148e-17 -6.722409e-15
1.512641e-16
E:\\R-3.1.2\\scripts\\mPDBS\\summary_pdb_93\\f83584e22003fb92.final.pdb
5.855417e-17 -7.045110e-15 -1.028264e-15 -3.541334e-15 2.678768e-15
7.311996e-16
E:\\R-3.1.2\\scripts\\mPDBS\\summary_pdb_93\\f8e2bf87e52caa29.final.pdb
4.717425e-15 7.457080e-16 -8.879882e-15 -2.289935e-15 -5.485118e-15
2.200083e-15

[,715]   [,716]   [,717]   [,718]   [,719]   [,720]
E:\\R-3.1.2\\scripts\\40.1ns_data\\NAT0.pdb
1.378013e-15 6.703139e-16 -6.560934e-16 -2.819488e-15 -4.108492e-15
-3.115866e-15
E:\\R-3.1.2\\scripts\\40.1ns_data\\LJP6.pdb
6.540940e-16 7.237277e-16 6.666247e-16 -2.218769e-15 -5.159401e-15

-1.394679e-15
E:\\R-3.1.2\\scripts\\40.1ns_data\\3WY9.pdb
1.544349e-15 3.350643e-16 -7.102270e-16 -2.504623e-15 -3.877686e-15
-1.709138e-15
E:\\R-3.1.2\\scripts\\40.1ns_data\\ZSP9.pdb
1.127511e-15 -4.441686e-16 -7.004290e-16 -8.283338e-16 -3.541696e-15
-3.655692e-15
E:\\R-3.1.2\\scripts\\mPDBS\\summary_pdb_93\\013d855ad26c877c.final.pdb
6.810739e-15 2.498196e-16 -2.446889e-15 -4.022234e-15 -3.469406e-15
-7.014087e-15
E:\\R-3.1.2\\scripts\\mPDBS\\summary_pdb_93\\0a2a705342ca0d54.final.pdb
2.111557e-15 6.325265e-16 -2.714696e-16 -1.015083e-15 -3.360428e-15
-2.372827e-15
E:\\R-3.1.2\\scripts\\mPDBS\\summary_pdb_93\\0a59fceadb4194d9.final.pdb
1.398546e-15 -5.895482e-16 -2.240973e-16 -3.618634e-15 -3.247524e-15
-2.952241e-16
E:\\R-3.1.2\\scripts\\mPDBS\\summary_pdb_93\\0dba59f3306825e8.final.pdb
1.131255e-15 -2.231702e-15 -1.966371e-15 -9.587126e-16 -3.731349e-15
-1.590582e-15
E:\\R-3.1.2\\scripts\\mPDBS\\summary_pdb_93\\126f26a78b57339c.final.pdb
-2.739347e-15 5.451171e-15 8.888757e-16 1.681103e-15 -1.633201e-15
2.374562e-15
E:\\R-3.1.2\\scripts\\mPDBS\\summary_pdb_93\\12f06f3e342df706.final.pdb
1.673973e-15 5.088191e-16 -4.877680e-16 -1.591445e-15 -3.516553e-15
-2.155987e-15
E:\\R-3.1.2\\scripts\\mPDBS\\summary_pdb_93\\18618a2a251b867b.final.pdb
-3.567983e-15 4.190029e-15 -3.594615e-16 2.325244e-15 1.821449e-15
1.346055e-15
E:\\R-3.1.2\\scripts\\mPDBS\\summary_pdb_93\\1f305f598641eeb2.final.pdb
1.865466e-15 6.577933e-16 -5.982785e-16 -1.813864e-15 -3.730035e-15
-2.033665e-15
E:\\R-3.1.2\\scripts\\mPDBS\\summary_pdb_93\\1f8a00b551be0a39.final.pdb
-3.567983e-15 4.190029e-15 -3.594615e-16 2.325244e-15 1.821449e-15
1.346055e-15
E:\\R-3.1.2\\scripts\\mPDBS\\summary_pdb_93\\219f45328d47579a.final.pdb
1.087187e-14 4.314081e-15 -2.666588e-15 3.308117e-15 -4.995258e-15
-1.208305e-14
E:\\R-3.1.2\\scripts\\mPDBS\\summary_pdb_93\\238dd3918e54336d.final.pdb
3.656723e-15 1.039331e-14 -4.378760e-15 6.724438e-15 -4.502597e-15
-5.834088e-15
E:\\R-3.1.2\\scripts\\mPDBS\\summary_pdb_93\\2b3027889e2fdc42.final.pdb
2.025215e-15 4.606100e-16 -7.365249e-18 -1.973466e-15 -3.721971e-15
-2.273785e-15
E:\\R-3.1.2\\scripts\\mPDBS\\summary_pdb_93\\2fcb3b46e33793b0.final.pdb
4.006115e-15 -2.837675e-15 -1.219310e-15 -3.529506e-15 -4.698011e-15
-4.947892e-15
E:\\R-3.1.2\\scripts\\mPDBS\\summary_pdb_93\\34eb328251842006.final.pdb
3.391375e-15 -1.483449e-15 -7.200040e-15 -6.263024e-15 -3.355383e-15
9.438472e-16
E:\\R-3.1.2\\scripts\\mPDBS\\summary_pdb_93\\354b32871797b7ab.final.pdb
-6.768959e-16 -8.890451e-16 4.265200e-15 -1.178376e-14 -2.672803e-15
-8.685149e-15
E:\\R-3.1.2\\scripts\\mPDBS\\summary_pdb_93\\397368adbc32d5de.final.pdb
-2.739347e-15 5.451171e-15 8.888757e-16 1.681103e-15 -1.633201e-15

2.374562e-15
E:\\R-3.1.2\\scripts\\mPDBS\\summary_pdb_93\\411ca13cd3df6edd.final.pdb
-1.580996e-15 -2.257193e-15 -2.952190e-15 -4.542151e-15 -7.142183e-16
-9.245257e-16
E:\\R-3.1.2\\scripts\\mPDBS\\summary_pdb_93\\42357eee6642bf12.final.pdb
-8.210552e-16 7.416392e-15 -3.575525e-15 -1.387298e-15 -2.855696e-15
-4.100452e-15
E:\\R-3.1.2\\scripts\\mPDBS\\summary_pdb_93\\44342d1f75a7fcfc.final.pdb
3.736519e-15 -4.011953e-16 6.582702e-15 -8.012368e-15 -2.403704e-15
9.318542e-16
E:\\R-3.1.2\\scripts\\mPDBS\\summary_pdb_93\\448ef21dffe8cdfb.final.pdb
-5.909026e-16 1.031413e-14 2.375038e-15 -9.311339e-15 -2.744659e-16
4.011283e-16
E:\\R-3.1.2\\scripts\\mPDBS\\summary_pdb_93\\472a40b3e8f113c7.final.pdb
1.131255e-15 -2.231702e-15 -1.966371e-15 -9.587126e-16 -3.731349e-15
-1.590582e-15
E:\\R-3.1.2\\scripts\\mPDBS\\summary_pdb_93\\48a26225951ea153.final.pdb
-4.950975e-15 -8.851018e-16 5.201142e-15 -7.494223e-15 -2.965632e-15
-6.718218e-16
E:\\R-3.1.2\\scripts\\mPDBS\\summary_pdb_93\\4ab4a5c7c6682ce6.final.pdb
2.603601e-15 -2.953564e-15 7.524193e-15 -6.338524e-15 -1.052713e-14
2.043338e-15
E:\\R-3.1.2\\scripts\\mPDBS\\summary_pdb_93\\4eaa45b62cb6a25f.final.pdb
1.220458e-15 4.381268e-16 -4.437071e-16 2.741301e-15 -3.449092e-15
7.021443e-16
E:\\R-3.1.2\\scripts\\mPDBS\\summary_pdb_93\\58c371611d85ac96.final.pdb
-2.739347e-15 5.451171e-15 8.888757e-16 1.681103e-15 -1.633201e-15
2.374562e-15
E:\\R-3.1.2\\scripts\\mPDBS\\summary_pdb_93\\59a647f04822d30a.final.pdb
5.924873e-15 9.271692e-16 -1.948668e-15 3.255800e-16 -4.274603e-15
-3.783504e-15
E:\\R-3.1.2\\scripts\\mPDBS\\summary_pdb_93\\59b091fe385e05fc.final.pdb
1.398546e-15 -5.895482e-16 -2.240973e-16 -3.618634e-15 -3.247524e-15
-2.952241e-16
E:\\R-3.1.2\\scripts\\mPDBS\\summary_pdb_93\\5c35ac30f930d2db.final.pdb
1.220458e-15 4.381268e-16 -4.437071e-16 2.741301e-15 -3.449092e-15
7.021443e-16
E:\\R-3.1.2\\scripts\\mPDBS\\summary_pdb_93\\5f60752431cf2dcb.final.pdb
1.943722e-15 -8.461111e-15 1.338245e-15 -3.561007e-15 4.387715e-15
2.355921e-15
E:\\R-3.1.2\\scripts\\mPDBS\\summary_pdb_93\\626456dd6ff2868d.final.pdb
1.131255e-15 -2.231702e-15 -1.966371e-15 -9.587126e-16 -3.731349e-15
-1.590582e-15
E:\\R-3.1.2\\scripts\\mPDBS\\summary_pdb_93\\6583d53ff3c8f7ef.final.pdb
5.924873e-15 9.271692e-16 -1.948668e-15 3.255800e-16 -4.274603e-15
-3.783504e-15
E:\\R-3.1.2\\scripts\\mPDBS\\summary_pdb_93\\69760178986bef01.final.pdb
-5.638494e-16 -1.225737e-16 4.068790e-16 8.597672e-15 5.167954e-15
-6.407579e-15
E:\\R-3.1.2\\scripts\\mPDBS\\summary_pdb_93\\6a9d87d47926b1c8.final.pdb
1.524603e-15 8.871137e-16 -6.869168e-17 -1.949673e-15 -4.377533e-15
-1.869548e-15
E:\\R-3.1.2\\scripts\\mPDBS\\summary_pdb_93\\6bb3547ea982ffc4.final.pdb
3.830053e-15 -5.520462e-15 -8.607201e-16 -8.558729e-15 5.759686e-15

1.943732e-15
E:\\R-3.1.2\\scripts\\mPDBS\\summary_pdb_93\\716d9624611d1c42.final.pdb
8.642905e-16 7.400116e-15 -2.751257e-15 7.369875e-16 -8.908941e-15
-2.415924e-15
E:\\R-3.1.2\\scripts\\mPDBS\\summary_pdb_93\\75f27e70ad0f1be4.final.pdb
5.924873e-15 9.271692e-16 -1.948668e-15 3.255800e-16 -4.274603e-15
-3.783504e-15
E:\\R-3.1.2\\scripts\\mPDBS\\summary_pdb_93\\769f910fffb22872.final.pdb
1.666545e-15 4.318585e-16 -4.226705e-16 -1.349768e-15 -3.361905e-15
-2.316217e-15
E:\\R-3.1.2\\scripts\\mPDBS\\summary_pdb_93\\791f8c80dd5c3135.final.pdb
1.398546e-15 -5.895482e-16 -2.240973e-16 -3.618634e-15 -3.247524e-15
-2.952241e-16
E:\\R-3.1.2\\scripts\\mPDBS\\summary_pdb_93\\7fad98e66a3f6099.final.pdb
1.652897e-15 -9.046934e-16 6.154708e-15 -4.749575e-15 -5.522482e-15
-8.866744e-15
E:\\R-3.1.2\\scripts\\mPDBS\\summary_pdb_93\\804b89c94a633196.final.pdb
4.006115e-15 -2.837675e-15 -1.219310e-15 -3.529506e-15 -4.698011e-15
-4.947892e-15
E:\\R-3.1.2\\scripts\\mPDBS\\summary_pdb_93\\813cc65a2298feba.final.pdb
4.006115e-15 -2.837675e-15 -1.219310e-15 -3.529506e-15 -4.698011e-15
-4.947892e-15
E:\\R-3.1.2\\scripts\\mPDBS\\summary_pdb_93\\83c0eefcfe12d3f9.final.pdb
-1.580996e-15 -2.257193e-15 -2.952190e-15 -4.542151e-15 -7.142183e-16
-9.245257e-16
E:\\R-3.1.2\\scripts\\mPDBS\\summary_pdb_93\\86d50469ef569760.final.pdb
1.131255e-15 -2.231702e-15 -1.966371e-15 -9.587126e-16 -3.731349e-15
-1.590582e-15
E:\\R-3.1.2\\scripts\\mPDBS\\summary_pdb_93\\89d0666e1402203d.final.pdb
5.924873e-15 9.271692e-16 -1.948668e-15 3.255800e-16 -4.274603e-15
-3.783504e-15
E:\\R-3.1.2\\scripts\\mPDBS\\summary_pdb_93\\8b34852716dadc62.final.pdb
-1.749698e-15 1.625769e-15 3.035967e-15 -1.669089e-17 -4.868014e-15
2.507842e-15
E:\\R-3.1.2\\scripts\\mPDBS\\summary_pdb_93\\8b6ef9ac3fe74395.final.pdb
8.142391e-15 2.703499e-15 4.300119e-15 -9.952230e-16 -3.136862e-15
1.508740e-15
E:\\R-3.1.2\\scripts\\mPDBS\\summary_pdb_93\\98bbe0d5986c138f.final.pdb
1.398546e-15 -5.895482e-16 -2.240973e-16 -3.618634e-15 -3.247524e-15
-2.952241e-16
E:\\R-3.1.2\\scripts\\mPDBS\\summary_pdb_93\\9c22f4909d67e2fa.final.pdb
1.781765e-15 4.745019e-15 -4.276651e-15 2.786189e-15 -3.101198e-15
-6.684567e-15
E:\\R-3.1.2\\scripts\\mPDBS\\summary_pdb_93\\9dda8ed0d81fb3ef.final.pdb
-1.110881e-14 -8.058482e-15 6.867088e-15 -1.606035e-15 1.116357e-14
1.338121e-14
E:\\R-3.1.2\\scripts\\mPDBS\\summary_pdb_93\\9f752b3616e6033b.final.pdb
3.607863e-15 1.120787e-15 -3.829966e-16 -2.846781e-15 -3.935064e-15
-4.297826e-15
E:\\R-3.1.2\\scripts\\mPDBS\\summary_pdb_93\\a02c70083816d6ba.final.pdb
1.220458e-15 4.381268e-16 -4.437071e-16 2.741301e-15 -3.449092e-15
7.021443e-16
E:\\R-3.1.2\\scripts\\mPDBS\\summary_pdb_93\\a0c997d891e4e082.final.pdb
4.438061e-15 -5.918649e-15 -2.417454e-15 4.047605e-15 8.613159e-16

1.357436e-15
E:\\R-3.1.2\\scripts\\mPDBS\\summary_pdb_93\\ab8039a2d823c616.final.pdb
1.970850e-15 9.858757e-16 -5.283401e-16 -5.461600e-16 -2.912575e-15
-1.916854e-15
E:\\R-3.1.2\\scripts\\mPDBS\\summary_pdb_93\\acf4d74b9c71d4dc.final.pdb
4.006115e-15 -2.837675e-15 -1.219310e-15 -3.529506e-15 -4.698011e-15
-4.947892e-15
E:\\R-3.1.2\\scripts\\mPDBS\\summary_pdb_93\\ad8a823e4b4c668e.final.pdb
1.883653e-15 5.136467e-16 -2.140457e-16 -8.184742e-16 -3.276503e-15
-2.669545e-15
E:\\R-3.1.2\\scripts\\mPDBS\\summary_pdb_93\\b16ababd03b7b857.final.pdb
1.781765e-15 4.745019e-15 -4.276651e-15 2.786189e-15 -3.101198e-15
-6.684567e-15
E:\\R-3.1.2\\scripts\\mPDBS\\summary_pdb_93\\b226923e697d3c63.final.pdb
4.292498e-15 3.669225e-15 5.448883e-16 8.487454e-15 2.332959e-15
3.083291e-15
E:\\R-3.1.2\\scripts\\mPDBS\\summary_pdb_93\\b28e48f6fa27a165.final.pdb
1.398546e-15 -5.895482e-16 -2.240973e-16 -3.618634e-15 -3.247524e-15
-2.952241e-16
E:\\R-3.1.2\\scripts\\mPDBS\\summary_pdb_93\\b42c4924e3cd1aec.final.pdb
8.640149e-15 2.439523e-15 -5.922052e-15 -6.659067e-15 -7.432667e-15
-6.916089e-15
E:\\R-3.1.2\\scripts\\mPDBS\\summary_pdb_93\\b4dd239fcabff1df.final.pdb
1.398546e-15 -5.895482e-16 -2.240973e-16 -3.618634e-15 -3.247524e-15
-2.952241e-16
E:\\R-3.1.2\\scripts\\mPDBS\\summary_pdb_93\\b669942ef97459a2.final.pdb
-1.749698e-15 1.625769e-15 3.035967e-15 -1.669089e-17 -4.868014e-15
2.507842e-15
E:\\R-3.1.2\\scripts\\mPDBS\\summary_pdb_93\\b99f4d9d87c1017b.final.pdb
1.398546e-15 -5.895482e-16 -2.240973e-16 -3.618634e-15 -3.247524e-15
-2.952241e-16
E:\\R-3.1.2\\scripts\\mPDBS\\summary_pdb_93\\b9d2cbf3975403e7.final.pdb
1.398546e-15 -5.895482e-16 -2.240973e-16 -3.618634e-15 -3.247524e-15
-2.952241e-16
E:\\R-3.1.2\\scripts\\mPDBS\\summary_pdb_93\\bc88c8a505f3a288.final.pdb
2.065114e-15 8.672942e-16 1.047413e-16 -2.113761e-15 -4.059375e-15
-1.636491e-15
E:\\R-3.1.2\\scripts\\mPDBS\\summary_pdb_93\\bdac0e64f4ef427e.final.pdb
8.542416e-15 6.953538e-15 3.609510e-15 5.414295e-15 -8.208224e-15
-5.408677e-15
E:\\R-3.1.2\\scripts\\mPDBS\\summary_pdb_93\\ca2a1f55fc9d0681.final.pdb
-1.749698e-15 1.625769e-15 3.035967e-15 -1.669089e-17 -4.868014e-15
2.507842e-15
E:\\R-3.1.2\\scripts\\mPDBS\\summary_pdb_93\\ce34248de3e81a3c.final.pdb
-2.994396e-15 5.278777e-16 4.389553e-16 2.669749e-15 -8.586535e-15
1.258662e-15
E:\\R-3.1.2\\scripts\\mPDBS\\summary_pdb_93\\d02e697772494a61.final.pdb
8.364762e-15 7.198818e-15 -2.552964e-15 2.333853e-15 -1.431766e-14
-1.269264e-14
E:\\R-3.1.2\\scripts\\mPDBS\\summary_pdb_93\\d04221245d015047.final.pdb
9.408583e-16 -6.927946e-16 -7.989570e-16 -5.115205e-15 -5.224094e-15
-3.873622e-15
E:\\R-3.1.2\\scripts\\mPDBS\\summary_pdb_93\\d678141e4018054a.final.pdb
-2.151670e-15 -2.577850e-17 -2.524381e-15 -1.126638e-14 -1.524943e-15

-8.498534e-16
E:\\R-3.1.2\\scripts\\mPDBS\\summary_pdb_93\\d71b40af0f0efe39.final.pdb
5.574536e-15 4.452976e-15 5.566093e-15 -7.209178e-15 -1.560681e-14
-7.941734e-15
E:\\R-3.1.2\\scripts\\mPDBS\\summary_pdb_93\\dc0809eff10197e2.final.pdb
1.089652e-14 8.171975e-16 -2.077172e-15 1.578625e-15 -8.132976e-15
5.762248e-16
E:\\R-3.1.2\\scripts\\mPDBS\\summary_pdb_93\\e1163918d3707839.final.pdb
-3.119542e-15 1.021820e-15 -9.970030e-15 -3.791569e-15 -1.039904e-14
-2.263073e-15
E:\\R-3.1.2\\scripts\\mPDBS\\summary_pdb_93\\e4b48b06824ef848.final.pdb
-3.567983e-15 4.190029e-15 -3.594615e-16 2.325244e-15 1.821449e-15
1.346055e-15
E:\\R-3.1.2\\scripts\\mPDBS\\summary_pdb_93\\e529e19ad281fea2.final.pdb
6.810739e-15 2.498196e-16 -2.446889e-15 -4.022234e-15 -3.469406e-15
-7.014087e-15
E:\\R-3.1.2\\scripts\\mPDBS\\summary_pdb_93\\e5602082718ec3a1.final.pdb
4.006115e-15 -2.837675e-15 -1.219310e-15 -3.529506e-15 -4.698011e-15
-4.947892e-15
E:\\R-3.1.2\\scripts\\mPDBS\\summary_pdb_93\\e5d589d996d0a960.final.pdb
3.418049e-15 -2.944986e-15 5.554869e-15 -2.925325e-16 -9.811258e-15
-5.115934e-15
E:\\R-3.1.2\\scripts\\mPDBS\\summary_pdb_93\\e9df9e72307a2fbd.final.pdb
1.220458e-15 4.381268e-16 -4.437071e-16 2.741301e-15 -3.449092e-15
7.021443e-16
E:\\R-3.1.2\\scripts\\mPDBS\\summary_pdb_93\\ea00f8694090cae7.final.pdb
4.006115e-15 -2.837675e-15 -1.219310e-15 -3.529506e-15 -4.698011e-15
-4.947892e-15
E:\\R-3.1.2\\scripts\\mPDBS\\summary_pdb_93\\eba517be32db050e.final.pdb
1.398546e-15 -5.895482e-16 -2.240973e-16 -3.618634e-15 -3.247524e-15
-2.952241e-16
E:\\R-3.1.2\\scripts\\mPDBS\\summary_pdb_93\\ec04b9545899c29d.final.pdb
1.792762e-15 2.154989e-16 -5.045110e-16 -2.121248e-15 -3.738573e-15
-2.161808e-15
E:\\R-3.1.2\\scripts\\mPDBS\\summary_pdb_93\\ee6862cbcad52078.final.pdb
1.977076e-15 7.466225e-16 -5.259391e-16 -2.069526e-15 -3.800034e-15
-2.073695e-15
E:\\R-3.1.2\\scripts\\mPDBS\\summary_pdb_93\\ef6a42fc83b81e5d.final.pdb
1.954076e-15 4.141860e-16 -3.862413e-16 -1.518686e-15 -3.201443e-15
-2.279354e-15
E:\\R-3.1.2\\scripts\\mPDBS\\summary_pdb_93\\ef8e88d4ad93a0ad.final.pdb
9.408583e-16 -6.927946e-16 -7.989570e-16 -5.115205e-15 -5.224094e-15
-3.873622e-15
E:\\R-3.1.2\\scripts\\mPDBS\\summary_pdb_93\\f14fcae8deec4fa3.final.pdb
3.607863e-15 1.120787e-15 -3.829966e-16 -2.846781e-15 -3.935064e-15
-4.297826e-15
E:\\R-3.1.2\\scripts\\mPDBS\\summary_pdb_93\\f83584e22003fb92.final.pdb
-4.950975e-15 -8.851018e-16 5.201142e-15 -7.494223e-15 -2.965632e-15
-6.718218e-16
E:\\R-3.1.2\\scripts\\mPDBS\\summary_pdb_93\\f8e2bf87e52caa29.final.pdb
6.810739e-15 2.498196e-16 -2.446889e-15 -4.022234e-15 -3.469406e-15
-7.014087e-15

[,721]   [,722]   [,723]   [,724]   [,725]   [,726]

E:\\R-3.1.2\\scripts\\40.1ns_data\\NAT0.pdb
-2.579616e-15 5.644753e-15 -7.123873e-16 -1.539164e-15 -1.434943e-16
3.735376e-15
E:\\R-3.1.2\\scripts\\40.1ns_data\\LJP6.pdb
-3.100126e-15 5.119800e-15 -1.006593e-15 -1.574128e-15 8.432697e-16
2.997254e-15
E:\\R-3.1.2\\scripts\\40.1ns_data\\3WY9.pdb
-2.887920e-15 4.596631e-15 -5.974554e-16 -1.243937e-15 -4.038097e-15
4.531923e-15
E:\\R-3.1.2\\scripts\\40.1ns_data\\ZSP9.pdb
-1.065558e-15 6.120990e-15 -8.754398e-16 -1.704931e-15 -3.840367e-16
4.456968e-15
E:\\R-3.1.2\\scripts\\mPDBS\\summary_pdb_93\\013d855ad26c877c.final.pdb
-2.645998e-15 9.348003e-15 -2.181667e-15 -2.273152e-15 1.243095e-15
5.944712e-15
E:\\R-3.1.2\\scripts\\mPDBS\\summary_pdb_93\\0a2a705342ca0d54.final.pdb
-3.535152e-15 4.514771e-15 -1.318108e-15 -1.321227e-15 -1.880861e-15
3.697270e-15
E:\\R-3.1.2\\scripts\\mPDBS\\summary_pdb_93\\0a59fceadb4194d9.final.pdb
-7.292862e-16 1.253574e-15 -4.396798e-16 -1.238526e-16 -4.957566e-15
5.884665e-15
E:\\R-3.1.2\\scripts\\mPDBS\\summary_pdb_93\\0dba59f3306825e8.final.pdb
-3.660811e-15 3.050089e-15 2.324277e-15 -1.624366e-15 -2.728554e-15
4.945728e-15
E:\\R-3.1.2\\scripts\\mPDBS\\summary_pdb_93\\126f26a78b57339c.final.pdb
-6.516166e-15 7.133845e-15 -5.499759e-15 -1.403099e-16 6.045244e-15
-4.172412e-15
E:\\R-3.1.2\\scripts\\mPDBS\\summary_pdb_93\\12f06f3e342df706.final.pdb
-3.780615e-15 5.480144e-15 -1.740513e-15 -1.328166e-15 -1.866550e-15
3.102476e-15
E:\\R-3.1.2\\scripts\\mPDBS\\summary_pdb_93\\18618a2a251b867b.final.pdb
-5.187773e-15 9.989343e-15 -7.603881e-15 8.624009e-16 1.958739e-15
1.471712e-15
E:\\R-3.1.2\\scripts\\mPDBS\\summary_pdb_93\\1f305f598641eeb2.final.pdb
-3.956544e-15 5.639032e-15 -1.893850e-15 -7.822468e-16 -1.237057e-15
4.227131e-15
E:\\R-3.1.2\\scripts\\mPDBS\\summary_pdb_93\\1f8a00b551be0a39.final.pdb
-5.187773e-15 9.989343e-15 -7.603881e-15 8.624009e-16 1.958739e-15
1.471712e-15
E:\\R-3.1.2\\scripts\\mPDBS\\summary_pdb_93\\219f45328d47579a.final.pdb
-1.970803e-14 1.573630e-15 2.006280e-15 -6.727945e-15 -1.111497e-14
5.305701e-15
E:\\R-3.1.2\\scripts\\mPDBS\\summary_pdb_93\\238dd3918e54336d.final.pdb
-8.709014e-15 1.028725e-14 -7.555082e-15 3.199878e-15 5.267624e-16
-3.442509e-15
E:\\R-3.1.2\\scripts\\mPDBS\\summary_pdb_93\\2b3027889e2fdc42.final.pdb
-3.584641e-15 5.247341e-15 -1.385971e-15 -8.073337e-16 -1.996393e-15
3.750305e-15
E:\\R-3.1.2\\scripts\\mPDBS\\summary_pdb_93\\2fcb3b46e33793b0.final.pdb
-3.004181e-15 7.669526e-15 5.373649e-16 -1.723376e-17 -4.267618e-15
9.245253e-15
E:\\R-3.1.2\\scripts\\mPDBS\\summary_pdb_93\\34eb328251842006.final.pdb
7.499167e-15 1.829051e-15 -9.216148e-15 9.038200e-16 -8.546267e-15
1.051685e-14

E:\\R-3.1.2\\scripts\\mPDBS\\summary_pdb_93\\354b32871797b7ab.final.pdb
9.033959e-16 1.307714e-14 -5.592045e-15 -4.845819e-15 -4.701528e-15
2.870996e-15
E:\\R-3.1.2\\scripts\\mPDBS\\summary_pdb_93\\397368adbc32d5de.final.pdb
-6.516166e-15 7.133845e-15 -5.499759e-15 -1.403099e-16 6.045244e-15
-4.172412e-15
E:\\R-3.1.2\\scripts\\mPDBS\\summary_pdb_93\\411ca13cd3df6edd.final.pdb
-1.066495e-15 1.349364e-14 -4.290804e-15 1.725296e-15 3.957139e-15
6.796907e-15
E:\\R-3.1.2\\scripts\\mPDBS\\summary_pdb_93\\42357eee6642bf12.final.pdb
1.800406e-15 4.109670e-16 -8.575249e-15 7.007868e-15 -1.611427e-14
7.446357e-15
E:\\R-3.1.2\\scripts\\mPDBS\\summary_pdb_93\\44342d1f75a7fcfc.final.pdb
8.868158e-15 1.664539e-14 -1.053654e-14 -2.023336e-15 5.972169e-15
2.503848e-16
E:\\R-3.1.2\\scripts\\mPDBS\\summary_pdb_93\\448ef21dffe8cdfb.final.pdb
-4.471757e-15 1.047544e-14 -1.791836e-14 1.140592e-14 1.060156e-14
4.971111e-15
E:\\R-3.1.2\\scripts\\mPDBS\\summary_pdb_93\\472a40b3e8f113c7.final.pdb
-3.660811e-15 3.050089e-15 2.324277e-15 -1.624366e-15 -2.728554e-15
4.945728e-15
E:\\R-3.1.2\\scripts\\mPDBS\\summary_pdb_93\\48a26225951ea153.final.pdb
5.620669e-15 1.522655e-14 -1.754166e-15 1.531074e-15 3.172216e-15
5.242601e-15
E:\\R-3.1.2\\scripts\\mPDBS\\summary_pdb_93\\4ab4a5c7c6682ce6.final.pdb
-2.050987e-15 9.663939e-15 -2.344297e-15 1.023507e-14 -1.300462e-14
1.407211e-14
E:\\R-3.1.2\\scripts\\mPDBS\\summary_pdb_93\\4eaa45b62cb6a25f.final.pdb
-7.429348e-15 4.547826e-15 4.898960e-16 -7.362589e-16 2.844086e-15
9.348278e-16
E:\\R-3.1.2\\scripts\\mPDBS\\summary_pdb_93\\58c371611d85ac96.final.pdb
-6.516166e-15 7.133845e-15 -5.499759e-15 -1.403099e-16 6.045244e-15
-4.172412e-15
E:\\R-3.1.2\\scripts\\mPDBS\\summary_pdb_93\\59a647f04822d30a.final.pdb
-4.221198e-15 3.742947e-15 -1.842106e-15 -6.320881e-16 -4.533317e-15
2.561036e-15
E:\\R-3.1.2\\scripts\\mPDBS\\summary_pdb_93\\59b091fe385e05fc.final.pdb
-7.292862e-16 1.253574e-15 -4.396798e-16 -1.238526e-16 -4.957566e-15
5.884665e-15
E:\\R-3.1.2\\scripts\\mPDBS\\summary_pdb_93\\5c35ac30f930d2db.final.pdb
-7.429348e-15 4.547826e-15 4.898960e-16 -7.362589e-16 2.844086e-15
9.348278e-16
E:\\R-3.1.2\\scripts\\mPDBS\\summary_pdb_93\\5f60752431cf2dcb.final.pdb
-1.963492e-15 4.583943e-15 -1.783014e-15 -8.927504e-16 -5.524965e-16
9.924021e-16
E:\\R-3.1.2\\scripts\\mPDBS\\summary_pdb_93\\626456dd6ff2868d.final.pdb
-3.660811e-15 3.050089e-15 2.324277e-15 -1.624366e-15 -2.728554e-15
4.945728e-15
E:\\R-3.1.2\\scripts\\mPDBS\\summary_pdb_93\\6583d53ff3c8f7ef.final.pdb
-4.221198e-15 3.742947e-15 -1.842106e-15 -6.320881e-16 -4.533317e-15
2.561036e-15
E:\\R-3.1.2\\scripts\\mPDBS\\summary_pdb_93\\69760178986bef01.final.pdb
-1.377731e-14 1.534294e-14 1.254762e-16 -2.880696e-15 5.378407e-15
1.130324e-15

E:\\R-3.1.2\\scripts\\mPDBS\\summary_pdb_93\\6a9d87d47926b1c8.final.pdb
-3.322540e-15 5.883109e-15 -1.729338e-15 1.234579e-17 -1.139114e-15
3.971247e-15
E:\\R-3.1.2\\scripts\\mPDBS\\summary_pdb_93\\6bb3547ea982ffc4.final.pdb
-7.917305e-15 1.952978e-14 -1.316301e-14 -3.102975e-15 1.073880e-14
-1.550146e-15
E:\\R-3.1.2\\scripts\\mPDBS\\summary_pdb_93\\716d9624611d1c42.final.pdb
-6.769544e-15 7.667631e-15 -6.044362e-15 -3.366466e-15 3.763929e-15
4.457079e-15
E:\\R-3.1.2\\scripts\\mPDBS\\summary_pdb_93\\75f27e70ad0f1be4.final.pdb
-4.221198e-15 3.742947e-15 -1.842106e-15 -6.320881e-16 -4.533317e-15
2.561036e-15
E:\\R-3.1.2\\scripts\\mPDBS\\summary_pdb_93\\769f910fffb22872.final.pdb
-3.638665e-15 5.588241e-15 -1.635998e-15 -1.344167e-15 -1.101313e-15
3.646621e-15
E:\\R-3.1.2\\scripts\\mPDBS\\summary_pdb_93\\791f8c80dd5c3135.final.pdb
-7.292862e-16 1.253574e-15 -4.396798e-16 -1.238526e-16 -4.957566e-15
5.884665e-15
E:\\R-3.1.2\\scripts\\mPDBS\\summary_pdb_93\\7fad98e66a3f6099.final.pdb
-1.826995e-16 4.745386e-15 -1.525069e-17 -1.976184e-15 -4.192431e-15
8.760032e-15
E:\\R-3.1.2\\scripts\\mPDBS\\summary_pdb_93\\804b89c94a633196.final.pdb
-3.004181e-15 7.669526e-15 5.373649e-16 -1.723376e-17 -4.267618e-15
9.245253e-15
E:\\R-3.1.2\\scripts\\mPDBS\\summary_pdb_93\\813cc65a2298feba.final.pdb
-3.004181e-15 7.669526e-15 5.373649e-16 -1.723376e-17 -4.267618e-15
9.245253e-15
E:\\R-3.1.2\\scripts\\mPDBS\\summary_pdb_93\\83c0eefcfe12d3f9.final.pdb
-1.066495e-15 1.349364e-14 -4.290804e-15 1.725296e-15 3.957139e-15
6.796907e-15
E:\\R-3.1.2\\scripts\\mPDBS\\summary_pdb_93\\86d50469ef569760.final.pdb
-3.660811e-15 3.050089e-15 2.324277e-15 -1.624366e-15 -2.728554e-15
4.945728e-15
E:\\R-3.1.2\\scripts\\mPDBS\\summary_pdb_93\\89d0666e1402203d.final.pdb
-4.221198e-15 3.742947e-15 -1.842106e-15 -6.320881e-16 -4.533317e-15
2.561036e-15
E:\\R-3.1.2\\scripts\\mPDBS\\summary_pdb_93\\8b34852716dadc62.final.pdb
-2.756116e-15 3.470628e-15 -3.187211e-16 -7.101019e-17 -4.408131e-15
2.159341e-15
E:\\R-3.1.2\\scripts\\mPDBS\\summary_pdb_93\\8b6ef9ac3fe74395.final.pdb
-3.415987e-15 3.193758e-15 -7.722887e-16 -3.194393e-16 -9.233210e-16
-5.860934e-17
E:\\R-3.1.2\\scripts\\mPDBS\\summary_pdb_93\\98bbe0d5986c138f.final.pdb
-7.292862e-16 1.253574e-15 -4.396798e-16 -1.238526e-16 -4.957566e-15
5.884665e-15
E:\\R-3.1.2\\scripts\\mPDBS\\summary_pdb_93\\9c22f4909d67e2fa.final.pdb
-7.166650e-15 -1.762708e-15 7.498685e-16 -7.374196e-15 3.905477e-16
-7.948930e-16
E:\\R-3.1.2\\scripts\\mPDBS\\summary_pdb_93\\9dda8ed0d81fb3ef.final.pdb
1.001481e-14 1.013909e-14 2.320796e-15 2.978739e-15 5.448716e-15
7.528417e-15
E:\\R-3.1.2\\scripts\\mPDBS\\summary_pdb_93\\9f752b3616e6033b.final.pdb
-4.101978e-15 4.184463e-15 -8.894456e-16 -1.465929e-15 -3.666033e-15
3.972235e-15

E:\\R-3.1.2\\scripts\\mPDBS\\summary_pdb_93\\a02c70083816d6ba.final.pdb
-7.429348e-15 4.547826e-15 4.898960e-16 -7.362589e-16 2.844086e-15
9.348278e-16
E:\\R-3.1.2\\scripts\\mPDBS\\summary_pdb_93\\a0c997d891e4e082.final.pdb
-9.218604e-15 1.529946e-14 1.185589e-15 -9.890105e-15 3.245435e-15
3.576892e-15
E:\\R-3.1.2\\scripts\\mPDBS\\summary_pdb_93\\ab8039a2d823c616.final.pdb
-3.631060e-15 6.249084e-15 -2.036660e-15 -1.433328e-15 -1.537198e-15
4.056976e-15
E:\\R-3.1.2\\scripts\\mPDBS\\summary_pdb_93\\acf4d74b9c71d4dc.final.pdb
-3.004181e-15 7.669526e-15 5.373649e-16 -1.723376e-17 -4.267618e-15
9.245253e-15
E:\\R-3.1.2\\scripts\\mPDBS\\summary_pdb_93\\ad8a823e4b4c668e.final.pdb
-3.628468e-15 5.655797e-15 -1.762946e-15 -1.573200e-15 -4.776690e-16
2.909634e-15
E:\\R-3.1.2\\scripts\\mPDBS\\summary_pdb_93\\b16ababd03b7b857.final.pdb
-7.166650e-15 -1.762708e-15 7.498685e-16 -7.374196e-15 3.905477e-16
-7.948930e-16
E:\\R-3.1.2\\scripts\\mPDBS\\summary_pdb_93\\b226923e697d3c63.final.pdb
-1.314288e-14 8.545638e-15 -7.945297e-15 -3.667854e-15 -3.099134e-15
1.277341e-15
E:\\R-3.1.2\\scripts\\mPDBS\\summary_pdb_93\\b28e48f6fa27a165.final.pdb
-7.292862e-16 1.253574e-15 -4.396798e-16 -1.238526e-16 -4.957566e-15
5.884665e-15
E:\\R-3.1.2\\scripts\\mPDBS\\summary_pdb_93\\b42c4924e3cd1aec.final.pdb
-3.884662e-15 1.587445e-15 5.443299e-15 -4.821445e-15 -2.050927e-15
-8.693792e-16
E:\\R-3.1.2\\scripts\\mPDBS\\summary_pdb_93\\b4dd239fcabff1df.final.pdb
-7.292862e-16 1.253574e-15 -4.396798e-16 -1.238526e-16 -4.957566e-15
5.884665e-15
E:\\R-3.1.2\\scripts\\mPDBS\\summary_pdb_93\\b669942ef97459a2.final.pdb
-2.756116e-15 3.470628e-15 -3.187211e-16 -7.101019e-17 -4.408131e-15
2.159341e-15
E:\\R-3.1.2\\scripts\\mPDBS\\summary_pdb_93\\b99f4d9d87c1017b.final.pdb
-7.292862e-16 1.253574e-15 -4.396798e-16 -1.238526e-16 -4.957566e-15
5.884665e-15
E:\\R-3.1.2\\scripts\\mPDBS\\summary_pdb_93\\b9d2cbf3975403e7.final.pdb
-7.292862e-16 1.253574e-15 -4.396798e-16 -1.238526e-16 -4.957566e-15
5.884665e-15
E:\\R-3.1.2\\scripts\\mPDBS\\summary_pdb_93\\bc88c8a505f3a288.final.pdb
-3.763318e-15 4.681279e-15 -1.845239e-15 -1.345098e-15 -1.998995e-15
3.706503e-15
E:\\R-3.1.2\\scripts\\mPDBS\\summary_pdb_93\\bdac0e64f4ef427e.final.pdb
-8.186884e-15 -7.601028e-15 3.997271e-15 -3.997557e-15 -9.105762e-15
-1.667676e-15
E:\\R-3.1.2\\scripts\\mPDBS\\summary_pdb_93\\ca2a1f55fc9d0681.final.pdb
-2.756116e-15 3.470628e-15 -3.187211e-16 -7.101019e-17 -4.408131e-15
2.159341e-15
E:\\R-3.1.2\\scripts\\mPDBS\\summary_pdb_93\\ce34248de3e81a3c.final.pdb
1.455412e-15 1.641861e-15 4.079154e-15 1.528900e-16 -4.092484e-15
1.654883e-15
E:\\R-3.1.2\\scripts\\mPDBS\\summary_pdb_93\\d02e697772494a61.final.pdb
-1.898031e-14 3.769031e-15 -1.345835e-14 -1.773575e-15 -7.070459e-15
4.415354e-15

E:\\R-3.1.2\\scripts\\mPDBS\\summary_pdb_93\\d04221245d015047.final.pdb
-1.429695e-15 3.966513e-15 5.402717e-16 -2.450422e-16 -5.640179e-16
4.680176e-15
E:\\R-3.1.2\\scripts\\mPDBS\\summary_pdb_93\\d678141e4018054a.final.pdb
-2.366517e-15 1.273522e-14 -1.564666e-14 -2.290081e-15 2.401798e-15
1.779796e-15
E:\\R-3.1.2\\scripts\\mPDBS\\summary_pdb_93\\d71b40af0f0efe39.final.pdb
-7.984522e-15 -5.331228e-15 -9.316474e-17 -2.334990e-15 -2.928341e-15
1.146484e-14
E:\\R-3.1.2\\scripts\\mPDBS\\summary_pdb_93\\dc0809eff10197e2.final.pdb
1.538083e-16 -7.988686e-15 7.826984e-15 -5.975549e-15 -5.954221e-16
4.324179e-15
E:\\R-3.1.2\\scripts\\mPDBS\\summary_pdb_93\\e1163918d3707839.final.pdb
-1.397121e-14 -4.036962e-15 2.183753e-15 3.781048e-15 -2.861477e-15
8.141425e-15
E:\\R-3.1.2\\scripts\\mPDBS\\summary_pdb_93\\e4b48b06824ef848.final.pdb
-5.187773e-15 9.989343e-15 -7.603881e-15 8.624009e-16 1.958739e-15
1.471712e-15
E:\\R-3.1.2\\scripts\\mPDBS\\summary_pdb_93\\e529e19ad281fea2.final.pdb
-2.645998e-15 9.348003e-15 -2.181667e-15 -2.273152e-15 1.243095e-15
5.944712e-15
E:\\R-3.1.2\\scripts\\mPDBS\\summary_pdb_93\\e5602082718ec3a1.final.pdb
-3.004181e-15 7.669526e-15 5.373649e-16 -1.723376e-17 -4.267618e-15
9.245253e-15
E:\\R-3.1.2\\scripts\\mPDBS\\summary_pdb_93\\e5d589d996d0a960.final.pdb
-5.158464e-15 1.359092e-14 9.828492e-15 -7.801667e-15 9.207925e-15
1.770830e-15
E:\\R-3.1.2\\scripts\\mPDBS\\summary_pdb_93\\e9df9e72307a2fbd.final.pdb
-7.429348e-15 4.547826e-15 4.898960e-16 -7.362589e-16 2.844086e-15
9.348278e-16
E:\\R-3.1.2\\scripts\\mPDBS\\summary_pdb_93\\ea00f8694090cae7.final.pdb
-3.004181e-15 7.669526e-15 5.373649e-16 -1.723376e-17 -4.267618e-15
9.245253e-15
E:\\R-3.1.2\\scripts\\mPDBS\\summary_pdb_93\\eba517be32db050e.final.pdb
-7.292862e-16 1.253574e-15 -4.396798e-16 -1.238526e-16 -4.957566e-15
5.884665e-15
E:\\R-3.1.2\\scripts\\mPDBS\\summary_pdb_93\\ec04b9545899c29d.final.pdb
-4.152677e-15 5.496536e-15 -2.132673e-15 -1.168118e-15 -1.409947e-15
3.734577e-15
E:\\R-3.1.2\\scripts\\mPDBS\\summary_pdb_93\\ee6862cbcad52078.final.pdb
-3.543875e-15 4.902998e-15 -1.744192e-15 -7.899864e-16 -1.947821e-15
3.839860e-15
E:\\R-3.1.2\\scripts\\mPDBS\\summary_pdb_93\\ef6a42fc83b81e5d.final.pdb
-4.258829e-15 5.536741e-15 -1.854140e-15 -1.250492e-15 -1.360220e-15
3.753307e-15
E:\\R-3.1.2\\scripts\\mPDBS\\summary_pdb_93\\ef8e88d4ad93a0ad.final.pdb
-1.429695e-15 3.966513e-15 5.402717e-16 -2.450422e-16 -5.640179e-16
4.680176e-15
E:\\R-3.1.2\\scripts\\mPDBS\\summary_pdb_93\\f14fcae8deec4fa3.final.pdb
-4.101978e-15 4.184463e-15 -8.894456e-16 -1.465929e-15 -3.666033e-15
3.972235e-15
E:\\R-3.1.2\\scripts\\mPDBS\\summary_pdb_93\\f83584e22003fb92.final.pdb
5.620669e-15 1.522655e-14 -1.754166e-15 1.531074e-15 3.172216e-15
5.242601e-15

E:\\R-3.1.2\\scripts\\mPDBS\\summary_pdb_93\\f8e2bf87e52caa29.final.pdb
-2.645998e-15 9.348003e-15 -2.181667e-15 -2.273152e-15 1.243095e-15
5.944712e-15

[,727]   [,728]   [,729]   [,730]   [,731]   [,732]
E:\\R-3.1.2\\scripts\\40.1ns_data\\NAT0.pdb
2.832754e-16 1.841655e-15 -4.611372e-15 -1.876526e-16 5.464485e-15
-2.681793e-15
E:\\R-3.1.2\\scripts\\40.1ns_data\\LJP6.pdb
1.908963e-16 2.677507e-15 -9.244638e-16 1.559969e-15 4.265522e-15
-3.003971e-15
E:\\R-3.1.2\\scripts\\40.1ns_data\\3WY9.pdb
8.446160e-16 1.907897e-15 -2.235410e-15 1.597948e-15 2.846759e-15
-1.874961e-15
E:\\R-3.1.2\\scripts\\40.1ns_data\\ZSP9.pdb
1.226312e-16 8.406694e-16 -7.801302e-16 -4.035229e-16 5.186335e-15
-2.158562e-15
E:\\R-3.1.2\\scripts\\mPDBS\\summary_pdb_93\\013d855ad26c877c.final.pdb
-7.174605e-16 3.517027e-15 -4.956937e-16 -8.382217e-16 5.378571e-15
-7.677485e-15
E:\\R-3.1.2\\scripts\\mPDBS\\summary_pdb_93\\0a2a705342ca0d54.final.pdb
4.456906e-16 1.047328e-15 -1.616266e-15 1.140426e-15 4.734172e-15
-2.188700e-15
E:\\R-3.1.2\\scripts\\mPDBS\\summary_pdb_93\\0a59fceadb4194d9.final.pdb
1.727360e-15 1.500238e-15 1.301249e-15 -8.919810e-16 4.691097e-15
-4.132905e-15
E:\\R-3.1.2\\scripts\\mPDBS\\summary_pdb_93\\0dba59f3306825e8.final.pdb
6.980738e-16 2.024298e-15 1.072496e-15 1.472430e-15 2.116424e-15
-1.795038e-15
E:\\R-3.1.2\\scripts\\mPDBS\\summary_pdb_93\\126f26a78b57339c.final.pdb
-4.025578e-15 2.063075e-16 -6.225453e-16 2.521504e-15 4.457867e-15
1.537149e-15
E:\\R-3.1.2\\scripts\\mPDBS\\summary_pdb_93\\12f06f3e342df706.final.pdb
-7.890061e-17 1.193912e-15 -1.357413e-15 5.536558e-16 4.859940e-15
-2.609371e-15
E:\\R-3.1.2\\scripts\\mPDBS\\summary_pdb_93\\18618a2a251b867b.final.pdb
-2.130652e-15 4.089389e-16 -7.026182e-16 -1.583134e-15 5.178604e-15
-1.817351e-16
E:\\R-3.1.2\\scripts\\mPDBS\\summary_pdb_93\\1f305f598641eeb2.final.pdb
1.408638e-16 8.804459e-16 -8.859049e-16 2.735273e-16 4.475693e-15
-2.459733e-15
E:\\R-3.1.2\\scripts\\mPDBS\\summary_pdb_93\\1f8a00b551be0a39.final.pdb
-2.130652e-15 4.089389e-16 -7.026182e-16 -1.583134e-15 5.178604e-15
-1.817351e-16
E:\\R-3.1.2\\scripts\\mPDBS\\summary_pdb_93\\219f45328d47579a.final.pdb
-4.293865e-15 -7.632214e-15 8.548923e-15 -3.849685e-15 5.489504e-15
8.910698e-15
E:\\R-3.1.2\\scripts\\mPDBS\\summary_pdb_93\\238dd3918e54336d.final.pdb
-2.549601e-15 -1.145728e-14 -3.367814e-15 8.214233e-15 8.803042e-15
1.232378e-15
E:\\R-3.1.2\\scripts\\mPDBS\\summary_pdb_93\\2b3027889e2fdc42.final.pdb
-9.965333e-18 9.876271e-16 -8.343585e-16 7.356233e-16 4.514589e-15
-2.029119e-15
E:\\R-3.1.2\\scripts\\mPDBS\\summary_pdb_93\\2fcb3b46e33793b0.final.pdb

3.272040e-15 3.782638e-15 4.394238e-16 -1.530585e-15 4.885955e-15
-5.404860e-15
E:\\R-3.1.2\\scripts\\mPDBS\\summary_pdb_93\\34eb328251842006.final.pdb
5.703046e-16 -5.432891e-15 3.436021e-15 -1.670615e-15 1.768964e-15
-3.176376e-15
E:\\R-3.1.2\\scripts\\mPDBS\\summary_pdb_93\\354b32871797b7ab.final.pdb
-7.542702e-15 4.416229e-15 -5.580204e-15 -4.103337e-15 1.284655e-14
-6.123804e-15
E:\\R-3.1.2\\scripts\\mPDBS\\summary_pdb_93\\397368adbc32d5de.final.pdb
-4.025578e-15 2.063075e-16 -6.225453e-16 2.521504e-15 4.457867e-15
1.537149e-15
E:\\R-3.1.2\\scripts\\mPDBS\\summary_pdb_93\\411ca13cd3df6edd.final.pdb
-8.465027e-17 6.607635e-15 4.793797e-15 -5.323992e-15 3.711114e-15
-4.512995e-15
E:\\R-3.1.2\\scripts\\mPDBS\\summary_pdb_93\\42357eee6642bf12.final.pdb
5.989487e-15 -7.350660e-15 1.742699e-15 -6.099557e-15 4.752038e-15
-1.124755e-15
E:\\R-3.1.2\\scripts\\mPDBS\\summary_pdb_93\\44342d1f75a7fcfc.final.pdb
5.318186e-15 3.505762e-15 -4.214117e-15 4.002290e-15 8.001517e-15
-1.394236e-14
E:\\R-3.1.2\\scripts\\mPDBS\\summary_pdb_93\\448ef21dffe8cdfb.final.pdb
-5.597601e-15 -3.993260e-15 -1.671179e-14 -5.902088e-15 1.062962e-14
-1.783191e-15
E:\\R-3.1.2\\scripts\\mPDBS\\summary_pdb_93\\472a40b3e8f113c7.final.pdb
6.980738e-16 2.024298e-15 1.072496e-15 1.472430e-15 2.116424e-15
-1.795038e-15
E:\\R-3.1.2\\scripts\\mPDBS\\summary_pdb_93\\48a26225951ea153.final.pdb
4.401954e-16 7.672384e-15 -2.656685e-15 1.617730e-15 3.517557e-15
-4.522351e-15
E:\\R-3.1.2\\scripts\\mPDBS\\summary_pdb_93\\4ab4a5c7c6682ce6.final.pdb
1.494709e-14 -1.691480e-15 -7.254393e-15 1.397403e-15 4.559348e-15
-3.396404e-15
E:\\R-3.1.2\\scripts\\mPDBS\\summary_pdb_93\\4eaa45b62cb6a25f.final.pdb
2.029502e-15 2.561810e-15 1.086438e-17 4.318095e-15 1.215012e-15
-1.837302e-15
E:\\R-3.1.2\\scripts\\mPDBS\\summary_pdb_93\\58c371611d85ac96.final.pdb
-4.025578e-15 2.063075e-16 -6.225453e-16 2.521504e-15 4.457867e-15
1.537149e-15
E:\\R-3.1.2\\scripts\\mPDBS\\summary_pdb_93\\59a647f04822d30a.final.pdb
1.013470e-15 4.358379e-15 -4.567133e-15 6.567946e-15 3.821775e-16
2.568525e-15
E:\\R-3.1.2\\scripts\\mPDBS\\summary_pdb_93\\59b091fe385e05fc.final.pdb
1.727360e-15 1.500238e-15 1.301249e-15 -8.919810e-16 4.691097e-15
-4.132905e-15
E:\\R-3.1.2\\scripts\\mPDBS\\summary_pdb_93\\5c35ac30f930d2db.final.pdb
2.029502e-15 2.561810e-15 1.086438e-17 4.318095e-15 1.215012e-15
-1.837302e-15
E:\\R-3.1.2\\scripts\\mPDBS\\summary_pdb_93\\5f60752431cf2dcb.final.pdb
1.833469e-15 4.045797e-15 -2.280828e-15 5.037916e-15 2.191209e-16
1.243667e-15
E:\\R-3.1.2\\scripts\\mPDBS\\summary_pdb_93\\626456dd6ff2868d.final.pdb
6.980738e-16 2.024298e-15 1.072496e-15 1.472430e-15 2.116424e-15
-1.795038e-15
E:\\R-3.1.2\\scripts\\mPDBS\\summary_pdb_93\\6583d53ff3c8f7ef.final.pdb

1.013470e-15 4.358379e-15 -4.567133e-15 6.567946e-15 3.821775e-16
2.568525e-15
E:\\R-3.1.2\\scripts\\mPDBS\\summary_pdb_93\\69760178986bef01.final.pdb
1.834242e-16 -9.827901e-16 -3.221369e-15 3.835681e-15 1.049067e-14
-1.427271e-15
E:\\R-3.1.2\\scripts\\mPDBS\\summary_pdb_93\\6a9d87d47926b1c8.final.pdb
2.626582e-16 1.290510e-15 -2.131506e-16 4.675914e-16 4.360722e-15
-2.175593e-15
E:\\R-3.1.2\\scripts\\mPDBS\\summary_pdb_93\\6bb3547ea982ffc4.final.pdb
-5.499897e-15 2.768358e-15 -1.100563e-14 8.959702e-15 1.318879e-14
1.999046e-15
E:\\R-3.1.2\\scripts\\mPDBS\\summary_pdb_93\\716d9624611d1c42.final.pdb
-3.273557e-15 -2.037878e-15 2.611255e-15 -1.078285e-15 7.061738e-15
-3.489526e-15
E:\\R-3.1.2\\scripts\\mPDBS\\summary_pdb_93\\75f27e70ad0f1be4.final.pdb
1.013470e-15 4.358379e-15 -4.567133e-15 6.567946e-15 3.821775e-16
2.568525e-15
E:\\R-3.1.2\\scripts\\mPDBS\\summary_pdb_93\\769f910fffb22872.final.pdb
-1.851593e-16 7.394038e-16 -1.416348e-15 8.500323e-16 4.748609e-15
-2.573485e-15
E:\\R-3.1.2\\scripts\\mPDBS\\summary_pdb_93\\791f8c80dd5c3135.final.pdb
1.727360e-15 1.500238e-15 1.301249e-15 -8.919810e-16 4.691097e-15
-4.132905e-15
E:\\R-3.1.2\\scripts\\mPDBS\\summary_pdb_93\\7fad98e66a3f6099.final.pdb
2.240171e-15 2.375591e-15 -1.735662e-15 8.637246e-16 5.122494e-15
-6.365367e-15
E:\\R-3.1.2\\scripts\\mPDBS\\summary_pdb_93\\804b89c94a633196.final.pdb
3.272040e-15 3.782638e-15 4.394238e-16 -1.530585e-15 4.885955e-15
-5.404860e-15
E:\\R-3.1.2\\scripts\\mPDBS\\summary_pdb_93\\813cc65a2298feba.final.pdb
3.272040e-15 3.782638e-15 4.394238e-16 -1.530585e-15 4.885955e-15
-5.404860e-15
E:\\R-3.1.2\\scripts\\mPDBS\\summary_pdb_93\\83c0eefcfe12d3f9.final.pdb
-8.465027e-17 6.607635e-15 4.793797e-15 -5.323992e-15 3.711114e-15
-4.512995e-15
E:\\R-3.1.2\\scripts\\mPDBS\\summary_pdb_93\\86d50469ef569760.final.pdb
6.980738e-16 2.024298e-15 1.072496e-15 1.472430e-15 2.116424e-15
-1.795038e-15
E:\\R-3.1.2\\scripts\\mPDBS\\summary_pdb_93\\89d0666e1402203d.final.pdb
1.013470e-15 4.358379e-15 -4.567133e-15 6.567946e-15 3.821775e-16
2.568525e-15
E:\\R-3.1.2\\scripts\\mPDBS\\summary_pdb_93\\8b34852716dadc62.final.pdb
2.508761e-15 -5.630475e-17 1.782100e-15 1.102725e-15 4.267526e-15
1.408700e-15
E:\\R-3.1.2\\scripts\\mPDBS\\summary_pdb_93\\8b6ef9ac3fe74395.final.pdb
1.337380e-15 1.009943e-14 -8.185104e-15 4.028114e-15 9.379495e-15
3.479123e-15
E:\\R-3.1.2\\scripts\\mPDBS\\summary_pdb_93\\98bbe0d5986c138f.final.pdb
1.727360e-15 1.500238e-15 1.301249e-15 -8.919810e-16 4.691097e-15
-4.132905e-15
E:\\R-3.1.2\\scripts\\mPDBS\\summary_pdb_93\\9c22f4909d67e2fa.final.pdb
-7.412122e-15 -7.063720e-15 9.865905e-17 4.117241e-15 2.209927e-15
-2.503969e-15
E:\\R-3.1.2\\scripts\\mPDBS\\summary_pdb_93\\9dda8ed0d81fb3ef.final.pdb

4.005813e-15 1.030588e-14 6.664117e-15 8.026415e-15 6.202825e-15
-3.970515e-15
E:\\R-3.1.2\\scripts\\mPDBS\\summary_pdb_93\\9f752b3616e6033b.final.pdb
-4.838439e-17 6.251989e-16 -3.447896e-15 -2.543071e-16 6.533137e-15
-1.720234e-15
E:\\R-3.1.2\\scripts\\mPDBS\\summary_pdb_93\\a02c70083816d6ba.final.pdb
2.029502e-15 2.561810e-15 1.086438e-17 4.318095e-15 1.215012e-15
-1.837302e-15
E:\\R-3.1.2\\scripts\\mPDBS\\summary_pdb_93\\a0c997d891e4e082.final.pdb
-6.286960e-15 6.742821e-15 1.382840e-15 2.747709e-15 5.374044e-15
-7.109461e-15
E:\\R-3.1.2\\scripts\\mPDBS\\summary_pdb_93\\ab8039a2d823c616.final.pdb
-1.113447e-16 1.546457e-15 -2.076253e-15 1.187108e-15 3.994722e-15
-2.083967e-15
E:\\R-3.1.2\\scripts\\mPDBS\\summary_pdb_93\\acf4d74b9c71d4dc.final.pdb
3.272040e-15 3.782638e-15 4.394238e-16 -1.530585e-15 4.885955e-15
-5.404860e-15
E:\\R-3.1.2\\scripts\\mPDBS\\summary_pdb_93\\ad8a823e4b4c668e.final.pdb
-6.451225e-18 7.369270e-16 -1.615791e-15 1.286642e-15 4.563176e-15
-2.251999e-15
E:\\R-3.1.2\\scripts\\mPDBS\\summary_pdb_93\\b16ababd03b7b857.final.pdb
-7.412122e-15 -7.063720e-15 9.865905e-17 4.117241e-15 2.209927e-15
-2.503969e-15
E:\\R-3.1.2\\scripts\\mPDBS\\summary_pdb_93\\b226923e697d3c63.final.pdb
-3.564466e-15 -1.485925e-17 4.164489e-15 -4.021890e-15 4.284221e-15
1.168603e-15
E:\\R-3.1.2\\scripts\\mPDBS\\summary_pdb_93\\b28e48f6fa27a165.final.pdb
1.727360e-15 1.500238e-15 1.301249e-15 -8.919810e-16 4.691097e-15
-4.132905e-15
E:\\R-3.1.2\\scripts\\mPDBS\\summary_pdb_93\\b42c4924e3cd1aec.final.pdb
-3.373646e-15 -4.321310e-15 3.173257e-15 -5.209742e-15 4.125060e-15
-6.486081e-15
E:\\R-3.1.2\\scripts\\mPDBS\\summary_pdb_93\\b4dd239fcabff1df.final.pdb
1.727360e-15 1.500238e-15 1.301249e-15 -8.919810e-16 4.691097e-15
-4.132905e-15
E:\\R-3.1.2\\scripts\\mPDBS\\summary_pdb_93\\b669942ef97459a2.final.pdb
2.508761e-15 -5.630475e-17 1.782100e-15 1.102725e-15 4.267526e-15
1.408700e-15
E:\\R-3.1.2\\scripts\\mPDBS\\summary_pdb_93\\b99f4d9d87c1017b.final.pdb
1.727360e-15 1.500238e-15 1.301249e-15 -8.919810e-16 4.691097e-15
-4.132905e-15
E:\\R-3.1.2\\scripts\\mPDBS\\summary_pdb_93\\b9d2cbf3975403e7.final.pdb
1.727360e-15 1.500238e-15 1.301249e-15 -8.919810e-16 4.691097e-15
-4.132905e-15
E:\\R-3.1.2\\scripts\\mPDBS\\summary_pdb_93\\bc88c8a505f3a288.final.pdb
4.393823e-16 1.371868e-15 -1.344096e-15 4.968820e-16 4.464065e-15
-2.091136e-15
E:\\R-3.1.2\\scripts\\mPDBS\\summary_pdb_93\\bdac0e64f4ef427e.final.pdb
9.891978e-16 -9.756878e-15 -9.882777e-15 1.225873e-14 6.460434e-15
1.243251e-15
E:\\R-3.1.2\\scripts\\mPDBS\\summary_pdb_93\\ca2a1f55fc9d0681.final.pdb
2.508761e-15 -5.630475e-17 1.782100e-15 1.102725e-15 4.267526e-15
1.408700e-15
E:\\R-3.1.2\\scripts\\mPDBS\\summary_pdb_93\\ce34248de3e81a3c.final.pdb

4.099808e-15 8.585977e-15 2.814162e-15 3.929889e-15 2.321761e-15
-5.482029e-15
E:\\R-3.1.2\\scripts\\mPDBS\\summary_pdb_93\\d02e697772494a61.final.pdb
-6.058428e-16 -1.833033e-15 -1.275874e-14 -8.375541e-16 3.580657e-15
3.942016e-15
E:\\R-3.1.2\\scripts\\mPDBS\\summary_pdb_93\\d04221245d015047.final.pdb
1.438631e-15 1.555998e-15 -2.787436e-15 2.673116e-15 4.694032e-15
-3.192456e-15
E:\\R-3.1.2\\scripts\\mPDBS\\summary_pdb_93\\d678141e4018054a.final.pdb
-6.968910e-15 -5.679233e-15 2.358266e-15 -1.048291e-15 4.260153e-15
-1.892101e-16
E:\\R-3.1.2\\scripts\\mPDBS\\summary_pdb_93\\d71b40af0f0efe39.final.pdb
-3.994909e-15 -6.802994e-15 2.369099e-15 -1.286773e-14 -4.458602e-15
-3.511444e-15
E:\\R-3.1.2\\scripts\\mPDBS\\summary_pdb_93\\dc0809eff10197e2.final.pdb
2.766807e-15 5.880749e-15 -4.096640e-15 3.426033e-15 -3.787283e-15
-3.514769e-15
E:\\R-3.1.2\\scripts\\mPDBS\\summary_pdb_93\\e1163918d3707839.final.pdb
-1.327540e-15 -6.178332e-15 4.813438e-15 -1.175138e-14 5.606371e-16
-4.029713e-15
E:\\R-3.1.2\\scripts\\mPDBS\\summary_pdb_93\\e4b48b06824ef848.final.pdb
-2.130652e-15 4.089389e-16 -7.026182e-16 -1.583134e-15 5.178604e-15
-1.817351e-16
E:\\R-3.1.2\\scripts\\mPDBS\\summary_pdb_93\\e529e19ad281fea2.final.pdb
-7.174605e-16 3.517027e-15 -4.956937e-16 -8.382217e-16 5.378571e-15
-7.677485e-15
E:\\R-3.1.2\\scripts\\mPDBS\\summary_pdb_93\\e5602082718ec3a1.final.pdb
3.272040e-15 3.782638e-15 4.394238e-16 -1.530585e-15 4.885955e-15
-5.404860e-15
E:\\R-3.1.2\\scripts\\mPDBS\\summary_pdb_93\\e5d589d996d0a960.final.pdb
-1.045875e-15 1.203934e-14 -1.275373e-14 5.879875e-16 1.466262e-14
-4.416532e-15
E:\\R-3.1.2\\scripts\\mPDBS\\summary_pdb_93\\e9df9e72307a2fbd.final.pdb
2.029502e-15 2.561810e-15 1.086438e-17 4.318095e-15 1.215012e-15
-1.837302e-15
E:\\R-3.1.2\\scripts\\mPDBS\\summary_pdb_93\\ea00f8694090cae7.final.pdb
3.272040e-15 3.782638e-15 4.394238e-16 -1.530585e-15 4.885955e-15
-5.404860e-15
E:\\R-3.1.2\\scripts\\mPDBS\\summary_pdb_93\\eba517be32db050e.final.pdb
1.727360e-15 1.500238e-15 1.301249e-15 -8.919810e-16 4.691097e-15
-4.132905e-15
E:\\R-3.1.2\\scripts\\mPDBS\\summary_pdb_93\\ec04b9545899c29d.final.pdb
-2.199189e-16 1.387665e-15 -1.044777e-15 1.619647e-15 4.433190e-15
-2.239912e-15
E:\\R-3.1.2\\scripts\\mPDBS\\summary_pdb_93\\ee6862cbcad52078.final.pdb
2.979481e-16 1.117731e-15 -4.248011e-16 6.935562e-16 4.422649e-15
-1.893160e-15
E:\\R-3.1.2\\scripts\\mPDBS\\summary_pdb_93\\ef6a42fc83b81e5d.final.pdb
2.996959e-16 9.640504e-16 -1.110983e-15 1.056681e-15 4.686810e-15
-2.705324e-15
E:\\R-3.1.2\\scripts\\mPDBS\\summary_pdb_93\\ef8e88d4ad93a0ad.final.pdb
1.438631e-15 1.555998e-15 -2.787436e-15 2.673116e-15 4.694032e-15
-3.192456e-15
E:\\R-3.1.2\\scripts\\mPDBS\\summary_pdb_93\\f14fcae8deec4fa3.final.pdb

-4.838439e-17 6.251989e-16 -3.447896e-15 -2.543071e-16 6.533137e-15
-1.720234e-15
E:\\R-3.1.2\\scripts\\mPDBS\\summary_pdb_93\\f83584e22003fb92.final.pdb
4.401954e-16 7.672384e-15 -2.656685e-15 1.617730e-15 3.517557e-15
-4.522351e-15
E:\\R-3.1.2\\scripts\\mPDBS\\summary_pdb_93\\f8e2bf87e52caa29.final.pdb
-7.174605e-16 3.517027e-15 -4.956937e-16 -8.382217e-16 5.378571e-15
-7.677485e-15

[,733]   [,734]   [,735]   [,736]   [,737]   [,738]
E:\\R-3.1.2\\scripts\\40.1ns_data\\NAT0.pdb
-3.096886e-15 3.002177e-15 4.034787e-16 1.985450e-15 1.687626e-15
3.971095e-15
E:\\R-3.1.2\\scripts\\40.1ns_data\\LJP6.pdb
-2.159088e-15 4.383300e-15 -2.146102e-15 1.472493e-15 1.030757e-15
3.312640e-15
E:\\R-3.1.2\\scripts\\40.1ns_data\\3WY9.pdb
-3.136789e-15 2.146842e-15 4.384870e-16 4.793012e-16 2.306492e-15
7.388504e-15
E:\\R-3.1.2\\scripts\\40.1ns_data\\ZSP9.pdb
-2.608067e-15 4.102107e-15 -8.470344e-16 3.154542e-16 8.304228e-16
3.501047e-15
E:\\R-3.1.2\\scripts\\mPDBS\\summary_pdb_93\\013d855ad26c877c.final.pdb
-8.236733e-15 1.413343e-16 -6.921566e-15 -5.515476e-16 -7.087165e-16
1.252974e-14
E:\\R-3.1.2\\scripts\\mPDBS\\summary_pdb_93\\0a2a705342ca0d54.final.pdb
-4.344043e-15 2.662265e-15 -1.654759e-17 8.089849e-16 1.334056e-15
3.330425e-15
E:\\R-3.1.2\\scripts\\mPDBS\\summary_pdb_93\\0a59fceadb4194d9.final.pdb
-1.605508e-15 6.113576e-15 6.102334e-16 2.952691e-15 2.501794e-15
3.334269e-15
E:\\R-3.1.2\\scripts\\mPDBS\\summary_pdb_93\\0dba59f3306825e8.final.pdb
-3.082869e-15 2.321014e-15 4.413117e-15 5.892516e-15 3.321046e-15
3.628871e-15
E:\\R-3.1.2\\scripts\\mPDBS\\summary_pdb_93\\126f26a78b57339c.final.pdb
-9.829209e-15 1.219357e-15 -4.875893e-15 -9.132374e-16 -1.302642e-15
-3.217866e-15
E:\\R-3.1.2\\scripts\\mPDBS\\summary_pdb_93\\12f06f3e342df706.final.pdb
-4.012006e-15 2.660747e-15 -5.768633e-16 7.179119e-16 1.040671e-15
3.609390e-15
E:\\R-3.1.2\\scripts\\mPDBS\\summary_pdb_93\\18618a2a251b867b.final.pdb
-1.331774e-14 2.452472e-15 -1.870788e-15 -4.378881e-15 -3.491687e-15
8.225960e-15
E:\\R-3.1.2\\scripts\\mPDBS\\summary_pdb_93\\1f305f598641eeb2.final.pdb
-3.771420e-15 3.248061e-15 -1.249030e-16 3.246099e-16 1.745776e-15
4.791116e-15
E:\\R-3.1.2\\scripts\\mPDBS\\summary_pdb_93\\1f8a00b551be0a39.final.pdb
-1.331774e-14 2.452472e-15 -1.870788e-15 -4.378881e-15 -3.491687e-15
8.225960e-15
E:\\R-3.1.2\\scripts\\mPDBS\\summary_pdb_93\\219f45328d47579a.final.pdb
-1.816277e-15 -4.935524e-16 -1.228136e-14 -8.927779e-15 1.618508e-14
-4.778599e-15
E:\\R-3.1.2\\scripts\\mPDBS\\summary_pdb_93\\238dd3918e54336d.final.pdb
-9.046658e-15 -5.852113e-15 9.814785e-15 -3.006084e-15 5.902068e-15

5.529670e-15
E:\\R-3.1.2\\scripts\\mPDBS\\summary_pdb_93\\2b3027889e2fdc42.final.pdb
-4.579746e-15 3.042139e-15 -2.923649e-16 9.453995e-16 1.643322e-15
3.363977e-15
E:\\R-3.1.2\\scripts\\mPDBS\\summary_pdb_93\\2fcb3b46e33793b0.final.pdb
-2.520685e-15 6.058313e-15 1.207365e-15 3.202730e-16 1.876314e-15
1.183930e-14
E:\\R-3.1.2\\scripts\\mPDBS\\summary_pdb_93\\34eb328251842006.final.pdb
3.146399e-15 2.803124e-15 1.528121e-16 8.252551e-16 5.250269e-15
8.851205e-15
E:\\R-3.1.2\\scripts\\mPDBS\\summary_pdb_93\\354b32871797b7ab.final.pdb
-2.323485e-15 1.451944e-14 -1.014807e-14 -6.103103e-15 -7.684208e-16
1.600862e-14
E:\\R-3.1.2\\scripts\\mPDBS\\summary_pdb_93\\397368adbc32d5de.final.pdb
-9.829209e-15 1.219357e-15 -4.875893e-15 -9.132374e-16 -1.302642e-15
-3.217866e-15
E:\\R-3.1.2\\scripts\\mPDBS\\summary_pdb_93\\411ca13cd3df6edd.final.pdb
-9.929689e-15 6.678586e-15 -3.044876e-15 -1.758793e-15 -1.602024e-15
1.545945e-14
E:\\R-3.1.2\\scripts\\mPDBS\\summary_pdb_93\\42357eee6642bf12.final.pdb
-8.303212e-16 1.414297e-14 1.727333e-15 -5.509324e-15 5.258905e-15
1.581271e-14
E:\\R-3.1.2\\scripts\\mPDBS\\summary_pdb_93\\44342d1f75a7fcfc.final.pdb
-1.083648e-14 -1.076136e-15 -1.192805e-14 2.333799e-15 -1.140648e-14
3.829364e-15
E:\\R-3.1.2\\scripts\\mPDBS\\summary_pdb_93\\448ef21dffe8cdfb.final.pdb
-3.839075e-15 5.902188e-15 2.824127e-15 -1.112413e-14 -3.028183e-15
1.336932e-14
E:\\R-3.1.2\\scripts\\mPDBS\\summary_pdb_93\\472a40b3e8f113c7.final.pdb
-3.082869e-15 2.321014e-15 4.413117e-15 5.892516e-15 3.321046e-15
3.628871e-15
E:\\R-3.1.2\\scripts\\mPDBS\\summary_pdb_93\\48a26225951ea153.final.pdb
-1.136945e-14 -2.494985e-16 -8.106427e-15 -2.890725e-15 -9.773977e-15
6.940976e-15
E:\\R-3.1.2\\scripts\\mPDBS\\summary_pdb_93\\4ab4a5c7c6682ce6.final.pdb
9.473059e-15 1.703830e-14 2.180962e-15 -4.397863e-15 1.900871e-16
8.571315e-15
E:\\R-3.1.2\\scripts\\mPDBS\\summary_pdb_93\\4eaa45b62cb6a25f.final.pdb
-8.047151e-15 6.559613e-16 3.148024e-16 2.411353e-15 -3.572913e-16
-5.320793e-16
E:\\R-3.1.2\\scripts\\mPDBS\\summary_pdb_93\\58c371611d85ac96.final.pdb
-9.829209e-15 1.219357e-15 -4.875893e-15 -9.132374e-16 -1.302642e-15
-3.217866e-15
E:\\R-3.1.2\\scripts\\mPDBS\\summary_pdb_93\\59a647f04822d30a.final.pdb
-1.004939e-15 3.633638e-15 5.345457e-15 -1.414722e-15 6.502593e-15
1.725672e-16
E:\\R-3.1.2\\scripts\\mPDBS\\summary_pdb_93\\59b091fe385e05fc.final.pdb
-1.605508e-15 6.113576e-15 6.102334e-16 2.952691e-15 2.501794e-15
3.334269e-15
E:\\R-3.1.2\\scripts\\mPDBS\\summary_pdb_93\\5c35ac30f930d2db.final.pdb
-8.047151e-15 6.559613e-16 3.148024e-16 2.411353e-15 -3.572913e-16
-5.320793e-16
E:\\R-3.1.2\\scripts\\mPDBS\\summary_pdb_93\\5f60752431cf2dcb.final.pdb
-5.336359e-15 1.536755e-15 1.334260e-15 3.542475e-15 1.384580e-15

9.445295e-16
E:\\R-3.1.2\\scripts\\mPDBS\\summary_pdb_93\\626456dd6ff2868d.final.pdb
-3.082869e-15 2.321014e-15 4.413117e-15 5.892516e-15 3.321046e-15
3.628871e-15
E:\\R-3.1.2\\scripts\\mPDBS\\summary_pdb_93\\6583d53ff3c8f7ef.final.pdb
-1.004939e-15 3.633638e-15 5.345457e-15 -1.414722e-15 6.502593e-15
1.725672e-16
E:\\R-3.1.2\\scripts\\mPDBS\\summary_pdb_93\\69760178986bef01.final.pdb
-1.819485e-14 -1.664827e-15 3.604340e-16 2.582970e-15 3.882220e-16
-1.061018e-14
E:\\R-3.1.2\\scripts\\mPDBS\\summary_pdb_93\\6a9d87d47926b1c8.final.pdb
-4.196562e-15 2.700810e-15 -1.191340e-15 4.264028e-16 1.987261e-15
3.035813e-15
E:\\R-3.1.2\\scripts\\mPDBS\\summary_pdb_93\\6bb3547ea982ffc4.final.pdb
-1.048199e-14 -1.063784e-14 -3.408709e-15 -2.599444e-15 -4.255493e-15
4.055866e-15
E:\\R-3.1.2\\scripts\\mPDBS\\summary_pdb_93\\716d9624611d1c42.final.pdb
-8.250640e-15 3.130207e-15 -6.062926e-15 2.501871e-16 -2.570156e-15
3.374660e-15
E:\\R-3.1.2\\scripts\\mPDBS\\summary_pdb_93\\75f27e70ad0f1be4.final.pdb
-1.004939e-15 3.633638e-15 5.345457e-15 -1.414722e-15 6.502593e-15
1.725672e-16
E:\\R-3.1.2\\scripts\\mPDBS\\summary_pdb_93\\769f910fffb22872.final.pdb
-4.775094e-15 3.579283e-15 1.785710e-16 2.014395e-16 1.332362e-15
4.159659e-15
E:\\R-3.1.2\\scripts\\mPDBS\\summary_pdb_93\\791f8c80dd5c3135.final.pdb
-1.605508e-15 6.113576e-15 6.102334e-16 2.952691e-15 2.501794e-15
3.334269e-15
E:\\R-3.1.2\\scripts\\mPDBS\\summary_pdb_93\\7fad98e66a3f6099.final.pdb
-2.765248e-15 6.151076e-15 -3.967772e-15 5.690817e-15 -6.714215e-16
5.389866e-15
E:\\R-3.1.2\\scripts\\mPDBS\\summary_pdb_93\\804b89c94a633196.final.pdb
-2.520685e-15 6.058313e-15 1.207365e-15 3.202730e-16 1.876314e-15
1.183930e-14
E:\\R-3.1.2\\scripts\\mPDBS\\summary_pdb_93\\813cc65a2298feba.final.pdb
-2.520685e-15 6.058313e-15 1.207365e-15 3.202730e-16 1.876314e-15
1.183930e-14
E:\\R-3.1.2\\scripts\\mPDBS\\summary_pdb_93\\83c0eefcfe12d3f9.final.pdb
-9.929689e-15 6.678586e-15 -3.044876e-15 -1.758793e-15 -1.602024e-15
1.545945e-14
E:\\R-3.1.2\\scripts\\mPDBS\\summary_pdb_93\\86d50469ef569760.final.pdb
-3.082869e-15 2.321014e-15 4.413117e-15 5.892516e-15 3.321046e-15
3.628871e-15
E:\\R-3.1.2\\scripts\\mPDBS\\summary_pdb_93\\89d0666e1402203d.final.pdb
-1.004939e-15 3.633638e-15 5.345457e-15 -1.414722e-15 6.502593e-15
1.725672e-16
E:\\R-3.1.2\\scripts\\mPDBS\\summary_pdb_93\\8b34852716dadc62.final.pdb
-7.992878e-17 5.034827e-15 -1.491865e-15 2.344042e-16 4.163980e-15
-8.427477e-15
E:\\R-3.1.2\\scripts\\mPDBS\\summary_pdb_93\\8b6ef9ac3fe74395.final.pdb
3.955006e-15 -4.274642e-15 1.069060e-14 9.051987e-16 1.991974e-15
-2.956408e-15
E:\\R-3.1.2\\scripts\\mPDBS\\summary_pdb_93\\98bbe0d5986c138f.final.pdb
-1.605508e-15 6.113576e-15 6.102334e-16 2.952691e-15 2.501794e-15

3.334269e-15
E:\\R-3.1.2\\scripts\\mPDBS\\summary_pdb_93\\9c22f4909d67e2fa.final.pdb
-8.581809e-15 -3.333913e-15 -5.573019e-15 4.700884e-15 5.646085e-15
-3.938121e-15
E:\\R-3.1.2\\scripts\\mPDBS\\summary_pdb_93\\9dda8ed0d81fb3ef.final.pdb
-1.788987e-14 3.093597e-15 9.074919e-16 3.893907e-16 -9.531890e-15
4.382091e-15
E:\\R-3.1.2\\scripts\\mPDBS\\summary_pdb_93\\9f752b3616e6033b.final.pdb
-1.977281e-15 2.774290e-15 1.666598e-15 5.039507e-16 3.758524e-15
5.163638e-15
E:\\R-3.1.2\\scripts\\mPDBS\\summary_pdb_93\\a02c70083816d6ba.final.pdb
-8.047151e-15 6.559613e-16 3.148024e-16 2.411353e-15 -3.572913e-16
-5.320793e-16
E:\\R-3.1.2\\scripts\\mPDBS\\summary_pdb_93\\a0c997d891e4e082.final.pdb
-1.048340e-14 5.503001e-16 -1.576081e-15 3.054148e-15 5.912991e-15
3.664054e-15
E:\\R-3.1.2\\scripts\\mPDBS\\summary_pdb_93\\ab8039a2d823c616.final.pdb
-4.209830e-15 3.795968e-15 -9.679523e-16 1.098247e-15 1.392085e-15
3.365790e-15
E:\\R-3.1.2\\scripts\\mPDBS\\summary_pdb_93\\acf4d74b9c71d4dc.final.pdb
-2.520685e-15 6.058313e-15 1.207365e-15 3.202730e-16 1.876314e-15
1.183930e-14
E:\\R-3.1.2\\scripts\\mPDBS\\summary_pdb_93\\ad8a823e4b4c668e.final.pdb
-4.660736e-15 3.410315e-15 -1.039709e-15 4.330666e-16 1.432056e-15
3.840464e-15
E:\\R-3.1.2\\scripts\\mPDBS\\summary_pdb_93\\b16ababd03b7b857.final.pdb
-8.581809e-15 -3.333913e-15 -5.573019e-15 4.700884e-15 5.646085e-15
-3.938121e-15
E:\\R-3.1.2\\scripts\\mPDBS\\summary_pdb_93\\b226923e697d3c63.final.pdb
-1.183829e-14 4.508264e-15 1.892706e-15 1.300121e-15 5.604874e-15
8.622514e-15
E:\\R-3.1.2\\scripts\\mPDBS\\summary_pdb_93\\b28e48f6fa27a165.final.pdb
-1.605508e-15 6.113576e-15 6.102334e-16 2.952691e-15 2.501794e-15
3.334269e-15
E:\\R-3.1.2\\scripts\\mPDBS\\summary_pdb_93\\b42c4924e3cd1aec.final.pdb
2.457623e-15 2.224609e-15 -2.888517e-15 1.513925e-15 6.678017e-15
3.224705e-15
E:\\R-3.1.2\\scripts\\mPDBS\\summary_pdb_93\\b4dd239fcabff1df.final.pdb
-1.605508e-15 6.113576e-15 6.102334e-16 2.952691e-15 2.501794e-15
3.334269e-15
E:\\R-3.1.2\\scripts\\mPDBS\\summary_pdb_93\\b669942ef97459a2.final.pdb
-7.992878e-17 5.034827e-15 -1.491865e-15 2.344042e-16 4.163980e-15
-8.427477e-15
E:\\R-3.1.2\\scripts\\mPDBS\\summary_pdb_93\\b99f4d9d87c1017b.final.pdb
-1.605508e-15 6.113576e-15 6.102334e-16 2.952691e-15 2.501794e-15
3.334269e-15
E:\\R-3.1.2\\scripts\\mPDBS\\summary_pdb_93\\b9d2cbf3975403e7.final.pdb
-1.605508e-15 6.113576e-15 6.102334e-16 2.952691e-15 2.501794e-15
3.334269e-15
E:\\R-3.1.2\\scripts\\mPDBS\\summary_pdb_93\\bc88c8a505f3a288.final.pdb
-4.356779e-15 2.929165e-15 -1.011083e-15 1.067264e-15 1.311990e-15
3.668529e-15
E:\\R-3.1.2\\scripts\\mPDBS\\summary_pdb_93\\bdac0e64f4ef427e.final.pdb
6.584879e-15 -2.654660e-15 5.433368e-15 2.503423e-15 7.534765e-15

-1.637284e-14
E:\\R-3.1.2\\scripts\\mPDBS\\summary_pdb_93\\ca2a1f55fc9d0681.final.pdb
-7.992878e-17 5.034827e-15 -1.491865e-15 2.344042e-16 4.163980e-15
-8.427477e-15
E:\\R-3.1.2\\scripts\\mPDBS\\summary_pdb_93\\ce34248de3e81a3c.final.pdb
-1.646299e-15 9.460600e-15 -3.029515e-15 5.593723e-16 -2.182639e-15
2.995296e-15
E:\\R-3.1.2\\scripts\\mPDBS\\summary_pdb_93\\d02e697772494a61.final.pdb
2.581193e-15 5.248382e-15 1.589593e-15 -2.418019e-16 7.079946e-15
4.081176e-15
E:\\R-3.1.2\\scripts\\mPDBS\\summary_pdb_93\\d04221245d015047.final.pdb
-1.894787e-15 2.753399e-15 -7.607613e-16 2.230793e-16 -1.429286e-16
3.147106e-15
E:\\R-3.1.2\\scripts\\mPDBS\\summary_pdb_93\\d678141e4018054a.final.pdb
-1.049574e-14 1.568445e-15 -1.003608e-14 -1.499881e-16 -5.694863e-15
1.596967e-14
E:\\R-3.1.2\\scripts\\mPDBS\\summary_pdb_93\\d71b40af0f0efe39.final.pdb
-5.840498e-18 7.903193e-15 -9.212656e-15 3.213855e-15 6.411067e-15
1.483837e-14
E:\\R-3.1.2\\scripts\\mPDBS\\summary_pdb_93\\dc0809eff10197e2.final.pdb
4.488616e-15 6.823214e-17 1.143828e-14 7.280369e-15 8.003860e-15
-3.115737e-15
E:\\R-3.1.2\\scripts\\mPDBS\\summary_pdb_93\\e1163918d3707839.final.pdb
-1.886976e-16 6.793870e-15 2.496065e-15 1.046813e-14 -1.860942e-15
1.347172e-14
E:\\R-3.1.2\\scripts\\mPDBS\\summary_pdb_93\\e4b48b06824ef848.final.pdb
-1.331774e-14 2.452472e-15 -1.870788e-15 -4.378881e-15 -3.491687e-15
8.225960e-15
E:\\R-3.1.2\\scripts\\mPDBS\\summary_pdb_93\\e529e19ad281fea2.final.pdb
-8.236733e-15 1.413343e-16 -6.921566e-15 -5.515476e-16 -7.087165e-16
1.252974e-14
E:\\R-3.1.2\\scripts\\mPDBS\\summary_pdb_93\\e5602082718ec3a1.final.pdb
-2.520685e-15 6.058313e-15 1.207365e-15 3.202730e-16 1.876314e-15
1.183930e-14
E:\\R-3.1.2\\scripts\\mPDBS\\summary_pdb_93\\e5d589d996d0a960.final.pdb
1.743541e-15 -3.399237e-15 -8.986423e-16 -7.469316e-16 -4.915244e-15
1.065731e-15
E:\\R-3.1.2\\scripts\\mPDBS\\summary_pdb_93\\e9df9e72307a2fbd.final.pdb
-8.047151e-15 6.559613e-16 3.148024e-16 2.411353e-15 -3.572913e-16
-5.320793e-16
E:\\R-3.1.2\\scripts\\mPDBS\\summary_pdb_93\\ea00f8694090cae7.final.pdb
-2.520685e-15 6.058313e-15 1.207365e-15 3.202730e-16 1.876314e-15
1.183930e-14
E:\\R-3.1.2\\scripts\\mPDBS\\summary_pdb_93\\eba517be32db050e.final.pdb
-1.605508e-15 6.113576e-15 6.102334e-16 2.952691e-15 2.501794e-15
3.334269e-15
E:\\R-3.1.2\\scripts\\mPDBS\\summary_pdb_93\\ec04b9545899c29d.final.pdb
-4.138717e-15 3.111486e-15 -6.679623e-16 4.767774e-16 1.682881e-15
3.685382e-15
E:\\R-3.1.2\\scripts\\mPDBS\\summary_pdb_93\\ee6862cbcad52078.final.pdb
-4.326422e-15 2.642394e-15 -7.714739e-16 1.502246e-15 1.746538e-15
3.543304e-15
E:\\R-3.1.2\\scripts\\mPDBS\\summary_pdb_93\\ef6a42fc83b81e5d.final.pdb
-4.238142e-15 3.036748e-15 -4.791884e-17 4.850668e-16 1.525567e-15

4.016436e-15
E:\\R-3.1.2\\scripts\\mPDBS\\summary_pdb_93\\ef8e88d4ad93a0ad.final.pdb
-1.894787e-15 2.753399e-15 -7.607613e-16 2.230793e-16 -1.429286e-16
3.147106e-15
E:\\R-3.1.2\\scripts\\mPDBS\\summary_pdb_93\\f14fcae8deec4fa3.final.pdb
-1.977281e-15 2.774290e-15 1.666598e-15 5.039507e-16 3.758524e-15
5.163638e-15
E:\\R-3.1.2\\scripts\\mPDBS\\summary_pdb_93\\f83584e22003fb92.final.pdb
-1.136945e-14 -2.494985e-16 -8.106427e-15 -2.890725e-15 -9.773977e-15
6.940976e-15
E:\\R-3.1.2\\scripts\\mPDBS\\summary_pdb_93\\f8e2bf87e52caa29.final.pdb
-8.236733e-15 1.413343e-16 -6.921566e-15 -5.515476e-16 -7.087165e-16
1.252974e-14

[,739]   [,740]   [,741]   [,742]   [,743]   [,744]
E:\\R-3.1.2\\scripts\\40.1ns_data\\NAT0.pdb
-1.460353e-15 -2.342355e-15 1.725937e-15 -1.926243e-15 -1.479574e-16
1.701267e-16
E:\\R-3.1.2\\scripts\\40.1ns_data\\LJP6.pdb
-1.681464e-15 -3.296285e-15 7.345286e-16 -2.055643e-15 1.011909e-15
-6.601845e-17
E:\\R-3.1.2\\scripts\\40.1ns_data\\3WY9.pdb
-2.930186e-15 -1.882008e-15 1.160358e-15 -1.513931e-15 5.817352e-16
-3.615768e-16
E:\\R-3.1.2\\scripts\\40.1ns_data\\ZSP9.pdb
-2.451341e-15 -2.807004e-15 7.906684e-16 -1.192556e-15 5.796875e-16
-4.398431e-16
E:\\R-3.1.2\\scripts\\mPDBS\\summary_pdb_93\\013d855ad26c877c.final.pdb
-9.494316e-16 -8.037384e-15 3.901084e-15 -3.854987e-15 -4.053336e-15
-2.507276e-15
E:\\R-3.1.2\\scripts\\mPDBS\\summary_pdb_93\\0a2a705342ca0d54.final.pdb
-1.394933e-15 -2.198712e-15 1.700383e-15 -8.315462e-16 5.139893e-16
-6.702486e-16
E:\\R-3.1.2\\scripts\\mPDBS\\summary_pdb_93\\0a59fceadb4194d9.final.pdb
1.305465e-15 -6.400169e-15 2.710021e-15 -1.949174e-15 -6.421426e-16
1.218452e-15
E:\\R-3.1.2\\scripts\\mPDBS\\summary_pdb_93\\0dba59f3306825e8.final.pdb
7.882572e-17 -3.475061e-15 2.463649e-15 7.585104e-16 1.172518e-15
1.050859e-15
E:\\R-3.1.2\\scripts\\mPDBS\\summary_pdb_93\\126f26a78b57339c.final.pdb
-3.821700e-15 3.093903e-15 -4.017278e-15 -2.454198e-15 -1.547745e-15
3.548319e-15
E:\\R-3.1.2\\scripts\\mPDBS\\summary_pdb_93\\12f06f3e342df706.final.pdb
-1.616978e-15 -2.203482e-15 1.384229e-15 -5.084540e-16 6.474546e-16
-4.620818e-16
E:\\R-3.1.2\\scripts\\mPDBS\\summary_pdb_93\\18618a2a251b867b.final.pdb
-3.026084e-15 3.201190e-15 1.790691e-15 -1.859534e-15 -1.184975e-15
1.321495e-15
E:\\R-3.1.2\\scripts\\mPDBS\\summary_pdb_93\\1f305f598641eeb2.final.pdb
-3.041936e-15 -2.005309e-15 1.112739e-15 -8.392666e-16 2.333833e-17
2.434236e-16
E:\\R-3.1.2\\scripts\\mPDBS\\summary_pdb_93\\1f8a00b551be0a39.final.pdb
-3.026084e-15 3.201190e-15 1.790691e-15 -1.859534e-15 -1.184975e-15
1.321495e-15

E:\\R-3.1.2\\scripts\\mPDBS\\summary_pdb_93\\219f45328d47579a.final.pdb
-2.562202e-16 -3.195268e-15 2.537199e-15 1.304057e-14 8.926540e-16
-8.689638e-15
E:\\R-3.1.2\\scripts\\mPDBS\\summary_pdb_93\\238dd3918e54336d.final.pdb
-2.443707e-15 4.625733e-15 8.071075e-15 1.279988e-14 2.587587e-15
-1.792866e-15
E:\\R-3.1.2\\scripts\\mPDBS\\summary_pdb_93\\2b3027889e2fdc42.final.pdb
-1.698209e-15 -2.339615e-15 1.552852e-15 -1.270463e-15 2.998123e-16
-3.429080e-16
E:\\R-3.1.2\\scripts\\mPDBS\\summary_pdb_93\\2fcb3b46e33793b0.final.pdb
-1.054810e-15 -5.939662e-15 3.218476e-15 1.304438e-16 2.256795e-15
-2.005645e-15
E:\\R-3.1.2\\scripts\\mPDBS\\summary_pdb_93\\34eb328251842006.final.pdb
-1.302907e-15 -3.621884e-16 8.547921e-15 -2.586477e-16 -2.143086e-15
-3.712330e-15
E:\\R-3.1.2\\scripts\\mPDBS\\summary_pdb_93\\354b32871797b7ab.final.pdb
-8.057901e-15 6.309255e-16 4.597034e-15 -8.206417e-15 -2.089575e-15
-1.878925e-17
E:\\R-3.1.2\\scripts\\mPDBS\\summary_pdb_93\\397368adbc32d5de.final.pdb
-3.821700e-15 3.093903e-15 -4.017278e-15 -2.454198e-15 -1.547745e-15
3.548319e-15
E:\\R-3.1.2\\scripts\\mPDBS\\summary_pdb_93\\411ca13cd3df6edd.final.pdb
-1.769950e-15 -3.890520e-15 -1.870119e-15 -1.107281e-15 2.905365e-15
1.011798e-15
E:\\R-3.1.2\\scripts\\mPDBS\\summary_pdb_93\\42357eee6642bf12.final.pdb
1.886383e-15 -1.820092e-15 9.318186e-15 8.519687e-15 1.171471e-15
3.171706e-15
E:\\R-3.1.2\\scripts\\mPDBS\\summary_pdb_93\\44342d1f75a7fcfc.final.pdb
4.535691e-15 -3.504778e-15 -1.130443e-15 -9.949523e-15 -6.284970e-15
-2.821901e-15
E:\\R-3.1.2\\scripts\\mPDBS\\summary_pdb_93\\448ef21dffe8cdfb.final.pdb
-2.165335e-14 6.539938e-15 -2.184449e-16 -8.358426e-15 1.279278e-15
-8.496690e-16
E:\\R-3.1.2\\scripts\\mPDBS\\summary_pdb_93\\472a40b3e8f113c7.final.pdb
7.882572e-17 -3.475061e-15 2.463649e-15 7.585104e-16 1.172518e-15
1.050859e-15
E:\\R-3.1.2\\scripts\\mPDBS\\summary_pdb_93\\48a26225951ea153.final.pdb
-8.859581e-15 4.829997e-15 -4.234186e-15 -9.357744e-15 -5.799094e-16
-4.989148e-15
E:\\R-3.1.2\\scripts\\mPDBS\\summary_pdb_93\\4ab4a5c7c6682ce6.final.pdb
-2.026298e-14 3.248293e-15 -2.327440e-15 -1.549539e-15 9.613466e-15
-2.127913e-15
E:\\R-3.1.2\\scripts\\mPDBS\\summary_pdb_93\\4eaa45b62cb6a25f.final.pdb
-8.449207e-16 -2.599130e-15 -2.726318e-15 -9.895337e-16 9.937879e-16
1.662212e-15
E:\\R-3.1.2\\scripts\\mPDBS\\summary_pdb_93\\58c371611d85ac96.final.pdb
-3.821700e-15 3.093903e-15 -4.017278e-15 -2.454198e-15 -1.547745e-15
3.548319e-15
E:\\R-3.1.2\\scripts\\mPDBS\\summary_pdb_93\\59a647f04822d30a.final.pdb
-1.029923e-14 3.480087e-15 6.448299e-16 -3.443587e-16 2.062086e-15
-3.774590e-16
E:\\R-3.1.2\\scripts\\mPDBS\\summary_pdb_93\\59b091fe385e05fc.final.pdb
1.305465e-15 -6.400169e-15 2.710021e-15 -1.949174e-15 -6.421426e-16
1.218452e-15

E:\\R-3.1.2\\scripts\\mPDBS\\summary_pdb_93\\5c35ac30f930d2db.final.pdb
-8.449207e-16 -2.599130e-15 -2.726318e-15 -9.895337e-16 9.937879e-16
1.662212e-15
E:\\R-3.1.2\\scripts\\mPDBS\\summary_pdb_93\\5f60752431cf2dcb.final.pdb
-8.021497e-18 2.603436e-15 -3.358721e-15 2.121387e-15 -1.058754e-15
1.642463e-15
E:\\R-3.1.2\\scripts\\mPDBS\\summary_pdb_93\\626456dd6ff2868d.final.pdb
7.882572e-17 -3.475061e-15 2.463649e-15 7.585104e-16 1.172518e-15
1.050859e-15
E:\\R-3.1.2\\scripts\\mPDBS\\summary_pdb_93\\6583d53ff3c8f7ef.final.pdb
-1.029923e-14 3.480087e-15 6.448299e-16 -3.443587e-16 2.062086e-15
-3.774590e-16
E:\\R-3.1.2\\scripts\\mPDBS\\summary_pdb_93\\69760178986bef01.final.pdb
-5.627612e-15 -5.038922e-17 -3.965289e-15 -4.630381e-16 3.416022e-15
-2.640953e-15
E:\\R-3.1.2\\scripts\\mPDBS\\summary_pdb_93\\6a9d87d47926b1c8.final.pdb
-3.233195e-15 -2.261189e-15 1.260618e-15 -1.407292e-15 -1.660375e-16
1.620485e-17
E:\\R-3.1.2\\scripts\\mPDBS\\summary_pdb_93\\6bb3547ea982ffc4.final.pdb
-2.341646e-15 1.003316e-15 4.071759e-16 5.982861e-15 -5.330049e-15
-3.097851e-15
E:\\R-3.1.2\\scripts\\mPDBS\\summary_pdb_93\\716d9624611d1c42.final.pdb
-2.828248e-15 -3.389599e-15 2.868936e-15 -6.308068e-15 8.153975e-16
-1.085932e-15
E:\\R-3.1.2\\scripts\\mPDBS\\summary_pdb_93\\75f27e70ad0f1be4.final.pdb
-1.029923e-14 3.480087e-15 6.448299e-16 -3.443587e-16 2.062086e-15
-3.774590e-16
E:\\R-3.1.2\\scripts\\mPDBS\\summary_pdb_93\\769f910fffb22872.final.pdb
-1.673433e-15 -1.676418e-15 1.135941e-15 -1.048550e-15 2.952866e-16
-3.956098e-16
E:\\R-3.1.2\\scripts\\mPDBS\\summary_pdb_93\\791f8c80dd5c3135.final.pdb
1.305465e-15 -6.400169e-15 2.710021e-15 -1.949174e-15 -6.421426e-16
1.218452e-15
E:\\R-3.1.2\\scripts\\mPDBS\\summary_pdb_93\\7fad98e66a3f6099.final.pdb
4.146824e-16 -3.642091e-15 7.057593e-16 5.783657e-16 3.353343e-15
-1.061061e-15
E:\\R-3.1.2\\scripts\\mPDBS\\summary_pdb_93\\804b89c94a633196.final.pdb
-1.054810e-15 -5.939662e-15 3.218476e-15 1.304438e-16 2.256795e-15
-2.005645e-15
E:\\R-3.1.2\\scripts\\mPDBS\\summary_pdb_93\\813cc65a2298feba.final.pdb
-1.054810e-15 -5.939662e-15 3.218476e-15 1.304438e-16 2.256795e-15
-2.005645e-15
E:\\R-3.1.2\\scripts\\mPDBS\\summary_pdb_93\\83c0eefcfe12d3f9.final.pdb
-1.769950e-15 -3.890520e-15 -1.870119e-15 -1.107281e-15 2.905365e-15
1.011798e-15
E:\\R-3.1.2\\scripts\\mPDBS\\summary_pdb_93\\86d50469ef569760.final.pdb
7.882572e-17 -3.475061e-15 2.463649e-15 7.585104e-16 1.172518e-15
1.050859e-15
E:\\R-3.1.2\\scripts\\mPDBS\\summary_pdb_93\\89d0666e1402203d.final.pdb
-1.029923e-14 3.480087e-15 6.448299e-16 -3.443587e-16 2.062086e-15
-3.774590e-16
E:\\R-3.1.2\\scripts\\mPDBS\\summary_pdb_93\\8b34852716dadc62.final.pdb
-3.867664e-15 1.478015e-15 -3.838162e-15 1.117501e-15 2.448482e-15
2.741425e-15

E:\\R-3.1.2\\scripts\\mPDBS\\summary_pdb_93\\8b6ef9ac3fe74395.final.pdb
-3.327316e-15 -4.082818e-15 -3.767942e-16 1.654595e-15 -4.953581e-15
4.852870e-15
E:\\R-3.1.2\\scripts\\mPDBS\\summary_pdb_93\\98bbe0d5986c138f.final.pdb
1.305465e-15 -6.400169e-15 2.710021e-15 -1.949174e-15 -6.421426e-16
1.218452e-15
E:\\R-3.1.2\\scripts\\mPDBS\\summary_pdb_93\\9c22f4909d67e2fa.final.pdb
4.284669e-15 -2.009646e-15 7.710109e-15 -2.802106e-15 -5.950399e-15
-1.065371e-15
E:\\R-3.1.2\\scripts\\mPDBS\\summary_pdb_93\\9dda8ed0d81fb3ef.final.pdb
1.657785e-15 -1.017609e-14 -3.718108e-15 -8.088598e-15 -9.000897e-15
7.681795e-15
E:\\R-3.1.2\\scripts\\mPDBS\\summary_pdb_93\\9f752b3616e6033b.final.pdb
-1.472225e-15 -4.171514e-15 2.693131e-15 2.064577e-16 8.502338e-16
-1.771978e-15
E:\\R-3.1.2\\scripts\\mPDBS\\summary_pdb_93\\a02c70083816d6ba.final.pdb
-8.449207e-16 -2.599130e-15 -2.726318e-15 -9.895337e-16 9.937879e-16
1.662212e-15
E:\\R-3.1.2\\scripts\\mPDBS\\summary_pdb_93\\a0c997d891e4e082.final.pdb
-7.316656e-15 -1.321400e-14 -8.530628e-17 1.093819e-15 9.282010e-16
-2.660944e-15
E:\\R-3.1.2\\scripts\\mPDBS\\summary_pdb_93\\ab8039a2d823c616.final.pdb
-1.410357e-15 -2.103962e-15 2.156499e-15 -2.083761e-15 2.213326e-17
-1.129067e-16
E:\\R-3.1.2\\scripts\\mPDBS\\summary_pdb_93\\acf4d74b9c71d4dc.final.pdb
-1.054810e-15 -5.939662e-15 3.218476e-15 1.304438e-16 2.256795e-15
-2.005645e-15
E:\\R-3.1.2\\scripts\\mPDBS\\summary_pdb_93\\ad8a823e4b4c668e.final.pdb
-2.413458e-15 -2.311157e-15 1.384613e-15 -1.304955e-15 4.403579e-16
-5.705973e-16
E:\\R-3.1.2\\scripts\\mPDBS\\summary_pdb_93\\b16ababd03b7b857.final.pdb
4.284669e-15 -2.009646e-15 7.710109e-15 -2.802106e-15 -5.950399e-15
-1.065371e-15
E:\\R-3.1.2\\scripts\\mPDBS\\summary_pdb_93\\b226923e697d3c63.final.pdb
-2.830812e-15 -6.125511e-15 2.316995e-15 9.283357e-15 -3.807303e-16
4.382351e-15
E:\\R-3.1.2\\scripts\\mPDBS\\summary_pdb_93\\b28e48f6fa27a165.final.pdb
1.305465e-15 -6.400169e-15 2.710021e-15 -1.949174e-15 -6.421426e-16
1.218452e-15
E:\\R-3.1.2\\scripts\\mPDBS\\summary_pdb_93\\b42c4924e3cd1aec.final.pdb
-5.340379e-16 -3.169976e-15 -4.344934e-16 1.389931e-15 -3.380128e-16
-6.447419e-15
E:\\R-3.1.2\\scripts\\mPDBS\\summary_pdb_93\\b4dd239fcabff1df.final.pdb
1.305465e-15 -6.400169e-15 2.710021e-15 -1.949174e-15 -6.421426e-16
1.218452e-15
E:\\R-3.1.2\\scripts\\mPDBS\\summary_pdb_93\\b669942ef97459a2.final.pdb
-3.867664e-15 1.478015e-15 -3.838162e-15 1.117501e-15 2.448482e-15
2.741425e-15
E:\\R-3.1.2\\scripts\\mPDBS\\summary_pdb_93\\b99f4d9d87c1017b.final.pdb
1.305465e-15 -6.400169e-15 2.710021e-15 -1.949174e-15 -6.421426e-16
1.218452e-15
E:\\R-3.1.2\\scripts\\mPDBS\\summary_pdb_93\\b9d2cbf3975403e7.final.pdb
1.305465e-15 -6.400169e-15 2.710021e-15 -1.949174e-15 -6.421426e-16
1.218452e-15

E:\\R-3.1.2\\scripts\\mPDBS\\summary_pdb_93\\bc88c8a505f3a288.final.pdb
-1.750251e-15 -2.669647e-15 1.283644e-15 -1.098725e-15 2.724383e-17
-2.818674e-16
E:\\R-3.1.2\\scripts\\mPDBS\\summary_pdb_93\\bdac0e64f4ef427e.final.pdb
-6.384748e-15 1.278088e-15 7.424747e-15 -1.347541e-15 -6.979307e-15
-4.703067e-16
E:\\R-3.1.2\\scripts\\mPDBS\\summary_pdb_93\\ca2a1f55fc9d0681.final.pdb
-3.867664e-15 1.478015e-15 -3.838162e-15 1.117501e-15 2.448482e-15
2.741425e-15
E:\\R-3.1.2\\scripts\\mPDBS\\summary_pdb_93\\ce34248de3e81a3c.final.pdb
-2.695281e-15 -4.684684e-15 -2.205421e-15 -4.948546e-15 3.282771e-16
6.732822e-15
E:\\R-3.1.2\\scripts\\mPDBS\\summary_pdb_93\\d02e697772494a61.final.pdb
-6.368518e-15 6.134075e-15 7.667752e-15 3.547731e-15 1.023035e-14
-1.457847e-15
E:\\R-3.1.2\\scripts\\mPDBS\\summary_pdb_93\\d04221245d015047.final.pdb
-1.125428e-15 -9.789938e-16 1.338902e-15 -4.391377e-15 4.546215e-16
-1.580212e-15
E:\\R-3.1.2\\scripts\\mPDBS\\summary_pdb_93\\d678141e4018054a.final.pdb
3.518722e-15 6.898084e-15 8.593513e-15 4.008506e-15 7.219448e-16
-1.681039e-15
E:\\R-3.1.2\\scripts\\mPDBS\\summary_pdb_93\\d71b40af0f0efe39.final.pdb
7.670288e-15 -1.167598e-15 4.312883e-15 -4.807023e-15 4.367471e-15
-5.934734e-15
E:\\R-3.1.2\\scripts\\mPDBS\\summary_pdb_93\\dc0809eff10197e2.final.pdb
-5.288667e-15 -7.913771e-15 -3.564760e-16 -7.262486e-15 -1.190844e-15
-7.348878e-17
E:\\R-3.1.2\\scripts\\mPDBS\\summary_pdb_93\\e1163918d3707839.final.pdb
5.191851e-15 -6.953408e-15 2.714789e-15 -1.403841e-15 7.746449e-15
-7.486735e-15
E:\\R-3.1.2\\scripts\\mPDBS\\summary_pdb_93\\e4b48b06824ef848.final.pdb
-3.026084e-15 3.201190e-15 1.790691e-15 -1.859534e-15 -1.184975e-15
1.321495e-15
E:\\R-3.1.2\\scripts\\mPDBS\\summary_pdb_93\\e529e19ad281fea2.final.pdb
-9.494316e-16 -8.037384e-15 3.901084e-15 -3.854987e-15 -4.053336e-15
-2.507276e-15
E:\\R-3.1.2\\scripts\\mPDBS\\summary_pdb_93\\e5602082718ec3a1.final.pdb
-1.054810e-15 -5.939662e-15 3.218476e-15 1.304438e-16 2.256795e-15
-2.005645e-15
E:\\R-3.1.2\\scripts\\mPDBS\\summary_pdb_93\\e5d589d996d0a960.final.pdb
2.595029e-16 -8.972533e-15 -5.647602e-15 -7.358697e-15 -2.260890e-15
1.644665e-15
E:\\R-3.1.2\\scripts\\mPDBS\\summary_pdb_93\\e9df9e72307a2fbd.final.pdb
-8.449207e-16 -2.599130e-15 -2.726318e-15 -9.895337e-16 9.937879e-16
1.662212e-15
E:\\R-3.1.2\\scripts\\mPDBS\\summary_pdb_93\\ea00f8694090cae7.final.pdb
-1.054810e-15 -5.939662e-15 3.218476e-15 1.304438e-16 2.256795e-15
-2.005645e-15
E:\\R-3.1.2\\scripts\\mPDBS\\summary_pdb_93\\eba517be32db050e.final.pdb
1.305465e-15 -6.400169e-15 2.710021e-15 -1.949174e-15 -6.421426e-16
1.218452e-15
E:\\R-3.1.2\\scripts\\mPDBS\\summary_pdb_93\\ec04b9545899c29d.final.pdb
-1.567066e-15 -1.813422e-15 1.563583e-15 -9.419710e-16 6.621637e-16
-4.260312e-16

E:\\R-3.1.2\\scripts\\mPDBS\\summary_pdb_93\\ee6862cbcad52078.final.pdb
-1.512160e-15 -2.411173e-15 1.456466e-15 -1.139058e-15 1.532281e-16
-6.405215e-16
E:\\R-3.1.2\\scripts\\mPDBS\\summary_pdb_93\\ef6a42fc83b81e5d.final.pdb
-1.366387e-15 -2.124844e-15 1.683572e-15 -7.011720e-16 6.453755e-16
-4.274311e-16
E:\\R-3.1.2\\scripts\\mPDBS\\summary_pdb_93\\ef8e88d4ad93a0ad.final.pdb
-1.125428e-15 -9.789938e-16 1.338902e-15 -4.391377e-15 4.546215e-16
-1.580212e-15
E:\\R-3.1.2\\scripts\\mPDBS\\summary_pdb_93\\f14fcae8deec4fa3.final.pdb
-1.472225e-15 -4.171514e-15 2.693131e-15 2.064577e-16 8.502338e-16
-1.771978e-15
E:\\R-3.1.2\\scripts\\mPDBS\\summary_pdb_93\\f83584e22003fb92.final.pdb
-8.859581e-15 4.829997e-15 -4.234186e-15 -9.357744e-15 -5.799094e-16
-4.989148e-15
E:\\R-3.1.2\\scripts\\mPDBS\\summary_pdb_93\\f8e2bf87e52caa29.final.pdb
-9.494316e-16 -8.037384e-15 3.901084e-15 -3.854987e-15 -4.053336e-15
-2.507276e-15

[,745]   [,746]   [,747]   [,748]   [,749]   [,750]
E:\\R-3.1.2\\scripts\\40.1ns_data\\NAT0.pdb
2.269540e-15 -5.886353e-16 -2.507816e-15 -3.775087e-15 -1.886931e-15
2.919503e-15
E:\\R-3.1.2\\scripts\\40.1ns_data\\LJP6.pdb
1.748523e-15 6.321305e-16 1.316411e-15 -2.896260e-15 -1.185189e-15
3.351630e-15
E:\\R-3.1.2\\scripts\\40.1ns_data\\3WY9.pdb
1.731865e-15 3.372779e-17 3.738850e-16 -1.390082e-15 -1.169893e-15
3.423881e-15
E:\\R-3.1.2\\scripts\\40.1ns_data\\ZSP9.pdb
2.473569e-15 4.424394e-16 -1.213470e-15 -2.875995e-15 -1.056613e-15
2.444950e-15
E:\\R-3.1.2\\scripts\\mPDBS\\summary_pdb_93\\013d855ad26c877c.final.pdb
-7.926781e-16 -2.657122e-16 7.477528e-15 -6.453139e-15 -6.087827e-15
-1.127292e-15
E:\\R-3.1.2\\scripts\\mPDBS\\summary_pdb_93\\0a2a705342ca0d54.final.pdb
1.453547e-15 1.288317e-16 8.614708e-16 -2.926530e-15 -9.876219e-16
3.001068e-15
E:\\R-3.1.2\\scripts\\mPDBS\\summary_pdb_93\\0a59fceadb4194d9.final.pdb
1.448193e-15 -1.919066e-15 5.933227e-15 -3.231895e-15 -4.755847e-15
1.944659e-15
E:\\R-3.1.2\\scripts\\mPDBS\\summary_pdb_93\\0dba59f3306825e8.final.pdb
4.788742e-16 -3.267218e-15 -1.485788e-15 -4.857194e-15 -2.606237e-15
3.220793e-15
E:\\R-3.1.2\\scripts\\mPDBS\\summary_pdb_93\\126f26a78b57339c.final.pdb
-2.636255e-15 8.684988e-16 -6.084351e-15 2.261569e-15 4.431536e-15
5.005823e-15
E:\\R-3.1.2\\scripts\\mPDBS\\summary_pdb_93\\12f06f3e342df706.final.pdb
1.039382e-15 2.682872e-16 1.172813e-15 -2.561587e-15 -1.281658e-15
3.371431e-15
E:\\R-3.1.2\\scripts\\mPDBS\\summary_pdb_93\\18618a2a251b867b.final.pdb
-3.341919e-15 9.087632e-15 -2.415775e-15 -3.121533e-15 5.888214e-15
3.066934e-15
E:\\R-3.1.2\\scripts\\mPDBS\\summary_pdb_93\\1f305f598641eeb2.final.pdb

5.583022e-16 -3.347053e-16 -3.639497e-16 -2.682140e-15 -1.526578e-15
2.315481e-15
E:\\R-3.1.2\\scripts\\mPDBS\\summary_pdb_93\\1f8a00b551be0a39.final.pdb
-3.341919e-15 9.087632e-15 -2.415775e-15 -3.121533e-15 5.888214e-15
3.066934e-15
E:\\R-3.1.2\\scripts\\mPDBS\\summary_pdb_93\\219f45328d47579a.final.pdb
6.321031e-15 -1.184844e-15 1.006707e-14 9.765521e-15 3.936852e-15
2.787951e-15
E:\\R-3.1.2\\scripts\\mPDBS\\summary_pdb_93\\238dd3918e54336d.final.pdb
4.121185e-15 7.542244e-16 -8.251079e-15 4.615669e-15 6.035000e-15
1.118987e-14
E:\\R-3.1.2\\scripts\\mPDBS\\summary_pdb_93\\2b3027889e2fdc42.final.pdb
8.891780e-16 -5.595305e-16 -1.671723e-16 -2.422429e-15 -1.674978e-15
2.746752e-15
E:\\R-3.1.2\\scripts\\mPDBS\\summary_pdb_93\\2fcb3b46e33793b0.final.pdb
1.605208e-15 -4.361866e-16 5.572274e-15 -7.660796e-15 -2.772110e-15
1.814579e-15
E:\\R-3.1.2\\scripts\\mPDBS\\summary_pdb_93\\34eb328251842006.final.pdb
2.635257e-15 6.035398e-15 -5.644463e-15 1.756657e-15 -3.980627e-15
-7.281429e-16
E:\\R-3.1.2\\scripts\\mPDBS\\summary_pdb_93\\354b32871797b7ab.final.pdb
4.261076e-15 3.739691e-15 3.595820e-16 -4.219275e-15 3.172238e-15
9.087659e-16
E:\\R-3.1.2\\scripts\\mPDBS\\summary_pdb_93\\397368adbc32d5de.final.pdb
-2.636255e-15 8.684988e-16 -6.084351e-15 2.261569e-15 4.431536e-15
5.005823e-15
E:\\R-3.1.2\\scripts\\mPDBS\\summary_pdb_93\\411ca13cd3df6edd.final.pdb
-5.393440e-16 4.632974e-15 -1.980902e-15 -7.578722e-15 3.391363e-15
3.342003e-15
E:\\R-3.1.2\\scripts\\mPDBS\\summary_pdb_93\\42357eee6642bf12.final.pdb
5.372827e-15 8.725478e-15 8.146440e-15 2.365584e-15 1.635989e-15
1.435392e-15
E:\\R-3.1.2\\scripts\\mPDBS\\summary_pdb_93\\44342d1f75a7fcfc.final.pdb
-5.937444e-15 7.986993e-15 4.179399e-15 -8.938811e-15 -3.278675e-15
7.289093e-16
E:\\R-3.1.2\\scripts\\mPDBS\\summary_pdb_93\\448ef21dffe8cdfb.final.pdb
2.348537e-15 1.198920e-14 -8.274289e-15 4.740742e-15 2.442469e-15
3.197587e-15
E:\\R-3.1.2\\scripts\\mPDBS\\summary_pdb_93\\472a40b3e8f113c7.final.pdb
4.788742e-16 -3.267218e-15 -1.485788e-15 -4.857194e-15 -2.606237e-15
3.220793e-15
E:\\R-3.1.2\\scripts\\mPDBS\\summary_pdb_93\\48a26225951ea153.final.pdb
-5.648127e-15 9.777904e-15 -9.081483e-15 -7.708999e-15 6.851132e-16
2.322178e-15
E:\\R-3.1.2\\scripts\\mPDBS\\summary_pdb_93\\4ab4a5c7c6682ce6.final.pdb
1.528816e-15 8.463811e-16 6.500201e-15 -1.015038e-14 4.516538e-15
-5.513634e-16
E:\\R-3.1.2\\scripts\\mPDBS\\summary_pdb_93\\4eaa45b62cb6a25f.final.pdb
-2.078254e-15 -3.459059e-15 1.867395e-15 -4.354259e-15 -2.554084e-15
3.467266e-15
E:\\R-3.1.2\\scripts\\mPDBS\\summary_pdb_93\\58c371611d85ac96.final.pdb
-2.636255e-15 8.684988e-16 -6.084351e-15 2.261569e-15 4.431536e-15
5.005823e-15
E:\\R-3.1.2\\scripts\\mPDBS\\summary_pdb_93\\59a647f04822d30a.final.pdb

9.398671e-16 -5.326576e-15 -2.068207e-15 -1.319906e-15 -1.009657e-15
-9.244990e-16
E:\\R-3.1.2\\scripts\\mPDBS\\summary_pdb_93\\59b091fe385e05fc.final.pdb
1.448193e-15 -1.919066e-15 5.933227e-15 -3.231895e-15 -4.755847e-15
1.944659e-15
E:\\R-3.1.2\\scripts\\mPDBS\\summary_pdb_93\\5c35ac30f930d2db.final.pdb
-2.078254e-15 -3.459059e-15 1.867395e-15 -4.354259e-15 -2.554084e-15
3.467266e-15
E:\\R-3.1.2\\scripts\\mPDBS\\summary_pdb_93\\5f60752431cf2dcb.final.pdb
-7.924865e-16 4.898168e-16 -4.718945e-15 -3.995770e-15 -2.955666e-15
1.314483e-15
E:\\R-3.1.2\\scripts\\mPDBS\\summary_pdb_93\\626456dd6ff2868d.final.pdb
4.788742e-16 -3.267218e-15 -1.485788e-15 -4.857194e-15 -2.606237e-15
3.220793e-15
E:\\R-3.1.2\\scripts\\mPDBS\\summary_pdb_93\\6583d53ff3c8f7ef.final.pdb
9.398671e-16 -5.326576e-15 -2.068207e-15 -1.319906e-15 -1.009657e-15
-9.244990e-16
E:\\R-3.1.2\\scripts\\mPDBS\\summary_pdb_93\\69760178986bef01.final.pdb
-1.185642e-16 3.772811e-15 5.293067e-16 -7.571428e-15 4.397393e-15
5.101750e-15
E:\\R-3.1.2\\scripts\\mPDBS\\summary_pdb_93\\6a9d87d47926b1c8.final.pdb
3.570099e-16 -3.731296e-16 5.717833e-16 -2.488103e-15 -1.941838e-15
2.219857e-15
E:\\R-3.1.2\\scripts\\mPDBS\\summary_pdb_93\\6bb3547ea982ffc4.final.pdb
1.509486e-15 1.311003e-14 -1.613359e-14 -7.323950e-16 -5.800827e-15
8.395971e-15
E:\\R-3.1.2\\scripts\\mPDBS\\summary_pdb_93\\716d9624611d1c42.final.pdb
3.541030e-17 2.055676e-15 6.816343e-15 -3.165922e-15 8.347051e-16
5.170556e-15
E:\\R-3.1.2\\scripts\\mPDBS\\summary_pdb_93\\75f27e70ad0f1be4.final.pdb
9.398671e-16 -5.326576e-15 -2.068207e-15 -1.319906e-15 -1.009657e-15
-9.244990e-16
E:\\R-3.1.2\\scripts\\mPDBS\\summary_pdb_93\\769f910fffb22872.final.pdb
5.098180e-16 2.703848e-16 9.412536e-16 -2.306755e-15 -1.158233e-15
2.947672e-15
E:\\R-3.1.2\\scripts\\mPDBS\\summary_pdb_93\\791f8c80dd5c3135.final.pdb
1.448193e-15 -1.919066e-15 5.933227e-15 -3.231895e-15 -4.755847e-15
1.944659e-15
E:\\R-3.1.2\\scripts\\mPDBS\\summary_pdb_93\\7fad98e66a3f6099.final.pdb
4.501205e-15 -9.541807e-16 -4.292556e-15 -3.798642e-15 -9.225213e-15
3.966463e-15
E:\\R-3.1.2\\scripts\\mPDBS\\summary_pdb_93\\804b89c94a633196.final.pdb
1.605208e-15 -4.361866e-16 5.572274e-15 -7.660796e-15 -2.772110e-15
1.814579e-15
E:\\R-3.1.2\\scripts\\mPDBS\\summary_pdb_93\\813cc65a2298feba.final.pdb
1.605208e-15 -4.361866e-16 5.572274e-15 -7.660796e-15 -2.772110e-15
1.814579e-15
E:\\R-3.1.2\\scripts\\mPDBS\\summary_pdb_93\\83c0eefcfe12d3f9.final.pdb
-5.393440e-16 4.632974e-15 -1.980902e-15 -7.578722e-15 3.391363e-15
3.342003e-15
E:\\R-3.1.2\\scripts\\mPDBS\\summary_pdb_93\\86d50469ef569760.final.pdb
4.788742e-16 -3.267218e-15 -1.485788e-15 -4.857194e-15 -2.606237e-15
3.220793e-15
E:\\R-3.1.2\\scripts\\mPDBS\\summary_pdb_93\\89d0666e1402203d.final.pdb

9.398671e-16 -5.326576e-15 -2.068207e-15 -1.319906e-15 -1.009657e-15
-9.244990e-16
E:\\R-3.1.2\\scripts\\mPDBS\\summary_pdb_93\\8b34852716dadc62.final.pdb
1.532349e-15 -1.313089e-15 2.944168e-15 1.203641e-15 2.161444e-15
4.346842e-15
E:\\R-3.1.2\\scripts\\mPDBS\\summary_pdb_93\\8b6ef9ac3fe74395.final.pdb
3.425068e-15 -7.477135e-17 -9.682651e-15 1.787662e-15 -1.256917e-14
3.886745e-15
E:\\R-3.1.2\\scripts\\mPDBS\\summary_pdb_93\\98bbe0d5986c138f.final.pdb
1.448193e-15 -1.919066e-15 5.933227e-15 -3.231895e-15 -4.755847e-15
1.944659e-15
E:\\R-3.1.2\\scripts\\mPDBS\\summary_pdb_93\\9c22f4909d67e2fa.final.pdb
-3.209857e-16 -5.621790e-15 3.523457e-15 4.062682e-15 -3.616053e-15
2.284256e-15
E:\\R-3.1.2\\scripts\\mPDBS\\summary_pdb_93\\9dda8ed0d81fb3ef.final.pdb
-1.121751e-14 4.145583e-15 -1.288922e-14 -1.693990e-14 -5.020338e-15
4.432544e-15
E:\\R-3.1.2\\scripts\\mPDBS\\summary_pdb_93\\9f752b3616e6033b.final.pdb
3.282663e-15 8.856766e-16 -1.189253e-16 -6.705985e-16 -1.263264e-15
3.405972e-15
E:\\R-3.1.2\\scripts\\mPDBS\\summary_pdb_93\\a02c70083816d6ba.final.pdb
-2.078254e-15 -3.459059e-15 1.867395e-15 -4.354259e-15 -2.554084e-15
3.467266e-15
E:\\R-3.1.2\\scripts\\mPDBS\\summary_pdb_93\\a0c997d891e4e082.final.pdb
5.286572e-15 -9.677140e-15 -5.914049e-16 -5.548875e-15 -5.149226e-15
-5.051146e-16
E:\\R-3.1.2\\scripts\\mPDBS\\summary_pdb_93\\ab8039a2d823c616.final.pdb
1.035415e-15 -4.370546e-16 -2.054549e-15 -2.693845e-15 -1.303344e-15
2.617605e-15
E:\\R-3.1.2\\scripts\\mPDBS\\summary_pdb_93\\acf4d74b9c71d4dc.final.pdb
1.605208e-15 -4.361866e-16 5.572274e-15 -7.660796e-15 -2.772110e-15
1.814579e-15
E:\\R-3.1.2\\scripts\\mPDBS\\summary_pdb_93\\ad8a823e4b4c668e.final.pdb
1.046343e-15 1.090309e-15 -5.283906e-16 -2.623989e-15 -1.270542e-15
3.061381e-15
E:\\R-3.1.2\\scripts\\mPDBS\\summary_pdb_93\\b16ababd03b7b857.final.pdb
-3.209857e-16 -5.621790e-15 3.523457e-15 4.062682e-15 -3.616053e-15
2.284256e-15
E:\\R-3.1.2\\scripts\\mPDBS\\summary_pdb_93\\b226923e697d3c63.final.pdb
5.214876e-15 7.216187e-16 5.485307e-15 -1.586511e-15 -1.131955e-15
-2.358138e-16
E:\\R-3.1.2\\scripts\\mPDBS\\summary_pdb_93\\b28e48f6fa27a165.final.pdb
1.448193e-15 -1.919066e-15 5.933227e-15 -3.231895e-15 -4.755847e-15
1.944659e-15
E:\\R-3.1.2\\scripts\\mPDBS\\summary_pdb_93\\b42c4924e3cd1aec.final.pdb
5.707104e-15 3.302759e-16 6.816951e-15 5.720795e-15 5.056240e-16
2.763957e-15
E:\\R-3.1.2\\scripts\\mPDBS\\summary_pdb_93\\b4dd239fcabff1df.final.pdb
1.448193e-15 -1.919066e-15 5.933227e-15 -3.231895e-15 -4.755847e-15
1.944659e-15
E:\\R-3.1.2\\scripts\\mPDBS\\summary_pdb_93\\b669942ef97459a2.final.pdb
1.532349e-15 -1.313089e-15 2.944168e-15 1.203641e-15 2.161444e-15
4.346842e-15
E:\\R-3.1.2\\scripts\\mPDBS\\summary_pdb_93\\b99f4d9d87c1017b.final.pdb

1.448193e-15 -1.919066e-15 5.933227e-15 -3.231895e-15 -4.755847e-15
1.944659e-15
E:\\R-3.1.2\\scripts\\mPDBS\\summary_pdb_93\\b9d2cbf3975403e7.final.pdb
1.448193e-15 -1.919066e-15 5.933227e-15 -3.231895e-15 -4.755847e-15
1.944659e-15
E:\\R-3.1.2\\scripts\\mPDBS\\summary_pdb_93\\bc88c8a505f3a288.final.pdb
1.006922e-15 2.807999e-17 -3.374598e-16 -2.497890e-15 -1.527527e-15
2.692325e-15
E:\\R-3.1.2\\scripts\\mPDBS\\summary_pdb_93\\bdac0e64f4ef427e.final.pdb
2.356994e-15 -8.947390e-15 3.572707e-15 7.487677e-15 -1.284652e-14
4.333056e-16
E:\\R-3.1.2\\scripts\\mPDBS\\summary_pdb_93\\ca2a1f55fc9d0681.final.pdb
1.532349e-15 -1.313089e-15 2.944168e-15 1.203641e-15 2.161444e-15
4.346842e-15
E:\\R-3.1.2\\scripts\\mPDBS\\summary_pdb_93\\ce34248de3e81a3c.final.pdb
-4.152590e-16 -9.384685e-15 2.190632e-15 -4.845335e-15 -4.485224e-15
-6.684199e-16
E:\\R-3.1.2\\scripts\\mPDBS\\summary_pdb_93\\d02e697772494a61.final.pdb
8.044262e-15 -5.259195e-15 9.539812e-15 3.811625e-15 5.480700e-16
1.004439e-15
E:\\R-3.1.2\\scripts\\mPDBS\\summary_pdb_93\\d04221245d015047.final.pdb
-5.516403e-16 6.853602e-17 -1.404990e-15 -2.800206e-15 -9.494608e-16
4.153485e-15
E:\\R-3.1.2\\scripts\\mPDBS\\summary_pdb_93\\d678141e4018054a.final.pdb
-3.984781e-16 1.088475e-14 -1.253305e-14 -2.097491e-15 3.815518e-15
5.113053e-15
E:\\R-3.1.2\\scripts\\mPDBS\\summary_pdb_93\\d71b40af0f0efe39.final.pdb
4.943197e-16 -3.446612e-15 1.227916e-14 -8.524549e-16 6.407696e-15
5.208456e-15
E:\\R-3.1.2\\scripts\\mPDBS\\summary_pdb_93\\dc0809eff10197e2.final.pdb
-1.238034e-15 -1.136250e-14 3.483490e-15 -1.461374e-15 -4.467508e-15
-1.529712e-15
E:\\R-3.1.2\\scripts\\mPDBS\\summary_pdb_93\\e1163918d3707839.final.pdb
5.828535e-15 8.258396e-15 4.977875e-16 7.612205e-16 -3.629089e-15
7.340157e-15
E:\\R-3.1.2\\scripts\\mPDBS\\summary_pdb_93\\e4b48b06824ef848.final.pdb
-3.341919e-15 9.087632e-15 -2.415775e-15 -3.121533e-15 5.888214e-15
3.066934e-15
E:\\R-3.1.2\\scripts\\mPDBS\\summary_pdb_93\\e529e19ad281fea2.final.pdb
-7.926781e-16 -2.657122e-16 7.477528e-15 -6.453139e-15 -6.087827e-15
-1.127292e-15
E:\\R-3.1.2\\scripts\\mPDBS\\summary_pdb_93\\e5602082718ec3a1.final.pdb
1.605208e-15 -4.361866e-16 5.572274e-15 -7.660796e-15 -2.772110e-15
1.814579e-15
E:\\R-3.1.2\\scripts\\mPDBS\\summary_pdb_93\\e5d589d996d0a960.final.pdb
3.294735e-15 -5.424755e-15 -8.058807e-15 -3.623690e-15 -7.912388e-16
7.903489e-15
E:\\R-3.1.2\\scripts\\mPDBS\\summary_pdb_93\\e9df9e72307a2fbd.final.pdb
-2.078254e-15 -3.459059e-15 1.867395e-15 -4.354259e-15 -2.554084e-15
3.467266e-15
E:\\R-3.1.2\\scripts\\mPDBS\\summary_pdb_93\\ea00f8694090cae7.final.pdb
1.605208e-15 -4.361866e-16 5.572274e-15 -7.660796e-15 -2.772110e-15
1.814579e-15
E:\\R-3.1.2\\scripts\\mPDBS\\summary_pdb_93\\eba517be32db050e.final.pdb

1.448193e-15 -1.919066e-15 5.933227e-15 -3.231895e-15 -4.755847e-15
1.944659e-15
E:\\R-3.1.2\\scripts\\mPDBS\\summary_pdb_93\\ec04b9545899c29d.final.pdb
6.562398e-16 4.391102e-17 -6.752244e-16 -2.486507e-15 -5.785782e-16
2.747260e-15
E:\\R-3.1.2\\scripts\\mPDBS\\summary_pdb_93\\ee6862cbcad52078.final.pdb
6.042496e-16 -6.687638e-16 1.978491e-15 -2.407034e-15 -1.208337e-15
2.455752e-15
E:\\R-3.1.2\\scripts\\mPDBS\\summary_pdb_93\\ef6a42fc83b81e5d.final.pdb
9.094549e-16 1.581970e-16 7.611812e-16 -2.558832e-15 -1.114865e-15
2.945720e-15
E:\\R-3.1.2\\scripts\\mPDBS\\summary_pdb_93\\ef8e88d4ad93a0ad.final.pdb
-5.516403e-16 6.853602e-17 -1.404990e-15 -2.800206e-15 -9.494608e-16
4.153485e-15
E:\\R-3.1.2\\scripts\\mPDBS\\summary_pdb_93\\f14fcae8deec4fa3.final.pdb
3.282663e-15 8.856766e-16 -1.189253e-16 -6.705985e-16 -1.263264e-15
3.405972e-15
E:\\R-3.1.2\\scripts\\mPDBS\\summary_pdb_93\\f83584e22003fb92.final.pdb
-5.648127e-15 9.777904e-15 -9.081483e-15 -7.708999e-15 6.851132e-16
2.322178e-15
E:\\R-3.1.2\\scripts\\mPDBS\\summary_pdb_93\\f8e2bf87e52caa29.final.pdb
-7.926781e-16 -2.657122e-16 7.477528e-15 -6.453139e-15 -6.087827e-15
-1.127292e-15

[,751]   [,752]   [,753]   [,754]   [,755]   [,756]
E:\\R-3.1.2\\scripts\\40.1ns_data\\NAT0.pdb
-4.231311e-15 -7.556943e-16 -2.979913e-15 3.262476e-15 2.740296e-16
-8.703776e-16
E:\\R-3.1.2\\scripts\\40.1ns_data\\LJP6.pdb
-4.557441e-15 -1.812304e-15 -1.505289e-15 3.593921e-15 3.536388e-16
1.943049e-15
E:\\R-3.1.2\\scripts\\40.1ns_data\\3WY9.pdb
-3.792481e-15 -1.961224e-15 -1.719650e-15 4.297806e-15 8.745152e-17
6.207814e-16
E:\\R-3.1.2\\scripts\\40.1ns_data\\ZSP9.pdb
-3.104221e-15 1.103233e-16 -1.782996e-15 2.701290e-15 4.097650e-16
1.241846e-15
E:\\R-3.1.2\\scripts\\mPDBS\\summary_pdb_93\\013d855ad26c877c.final.pdb
-3.809808e-15 5.838006e-15 -6.624265e-15 5.017601e-15 -1.160359e-15
3.375328e-15
E:\\R-3.1.2\\scripts\\mPDBS\\summary_pdb_93\\0a2a705342ca0d54.final.pdb
-4.515026e-15 -6.705391e-16 -1.834442e-15 3.511932e-15 6.178813e-16
6.889438e-16
E:\\R-3.1.2\\scripts\\mPDBS\\summary_pdb_93\\0a59fceadb4194d9.final.pdb
-4.753492e-15 -2.404577e-15 2.122541e-16 2.397077e-15 -2.247235e-15
5.051452e-17
E:\\R-3.1.2\\scripts\\mPDBS\\summary_pdb_93\\0dba59f3306825e8.final.pdb
-3.115919e-15 -4.152267e-15 3.010389e-15 4.795646e-16 4.922449e-15
-4.436286e-19
E:\\R-3.1.2\\scripts\\mPDBS\\summary_pdb_93\\126f26a78b57339c.final.pdb
-8.489634e-15 -2.407135e-15 -3.990521e-15 3.442798e-15 4.013454e-15
-3.289073e-15
E:\\R-3.1.2\\scripts\\mPDBS\\summary_pdb_93\\12f06f3e342df706.final.pdb
-4.433270e-15 -2.758895e-16 -1.673058e-15 3.653421e-15 1.181666e-15

6.145133e-16
E:\\R-3.1.2\\scripts\\mPDBS\\summary_pdb_93\\18618a2a251b867b.final.pdb
-4.280741e-15 2.285690e-16 -5.492047e-15 2.349380e-15 4.843890e-15
-7.868040e-17
E:\\R-3.1.2\\scripts\\mPDBS\\summary_pdb_93\\1f305f598641eeb2.final.pdb
-4.134654e-15 -1.232390e-15 -1.890293e-15 3.956247e-15 -9.172543e-17
8.019590e-16
E:\\R-3.1.2\\scripts\\mPDBS\\summary_pdb_93\\1f8a00b551be0a39.final.pdb
-4.280741e-15 2.285690e-16 -5.492047e-15 2.349380e-15 4.843890e-15
-7.868040e-17
E:\\R-3.1.2\\scripts\\mPDBS\\summary_pdb_93\\219f45328d47579a.final.pdb
2.862811e-15 -3.663119e-15 9.066856e-16 9.522887e-15 -6.680321e-15
1.424416e-15
E:\\R-3.1.2\\scripts\\mPDBS\\summary_pdb_93\\238dd3918e54336d.final.pdb
-3.342935e-15 1.390443e-14 -6.712762e-15 -9.031259e-16 1.001553e-14
-2.168861e-15
E:\\R-3.1.2\\scripts\\mPDBS\\summary_pdb_93\\2b3027889e2fdc42.final.pdb
-4.684513e-15 -1.391932e-15 -1.731829e-15 3.261728e-15 6.731722e-16
-2.483051e-16
E:\\R-3.1.2\\scripts\\mPDBS\\summary_pdb_93\\2fcb3b46e33793b0.final.pdb
-3.043850e-16 -3.522225e-15 -5.096702e-16 1.242272e-15 1.658611e-15
3.384962e-15
E:\\R-3.1.2\\scripts\\mPDBS\\summary_pdb_93\\34eb328251842006.final.pdb
-5.113725e-15 8.266097e-15 1.827509e-15 9.978082e-15 -6.086656e-15
9.311065e-16
E:\\R-3.1.2\\scripts\\mPDBS\\summary_pdb_93\\354b32871797b7ab.final.pdb
-4.813522e-15 -2.682824e-15 -8.864612e-15 5.948021e-15 -9.523773e-15
1.949923e-15
E:\\R-3.1.2\\scripts\\mPDBS\\summary_pdb_93\\397368adbc32d5de.final.pdb
-8.489634e-15 -2.407135e-15 -3.990521e-15 3.442798e-15 4.013454e-15
-3.289073e-15
E:\\R-3.1.2\\scripts\\mPDBS\\summary_pdb_93\\411ca13cd3df6edd.final.pdb
1.606823e-15 -6.063595e-15 -4.584959e-16 -1.615143e-15 9.127462e-15
2.012389e-15
E:\\R-3.1.2\\scripts\\mPDBS\\summary_pdb_93\\42357eee6642bf12.final.pdb
-3.790945e-15 7.307146e-15 4.333233e-16 7.884562e-15 -9.137103e-15
1.769947e-15
E:\\R-3.1.2\\scripts\\mPDBS\\summary_pdb_93\\44342d1f75a7fcfc.final.pdb
-1.299846e-14 -2.143473e-15 -1.102604e-14 3.361518e-15 1.272114e-14
9.970604e-16
E:\\R-3.1.2\\scripts\\mPDBS\\summary_pdb_93\\448ef21dffe8cdfb.final.pdb
-5.865909e-15 1.095925e-14 -1.723306e-14 1.290308e-14 -2.080346e-14
-5.068345e-15
E:\\R-3.1.2\\scripts\\mPDBS\\summary_pdb_93\\472a40b3e8f113c7.final.pdb
-3.115919e-15 -4.152267e-15 3.010389e-15 4.795646e-16 4.922449e-15
-4.436286e-19
E:\\R-3.1.2\\scripts\\mPDBS\\summary_pdb_93\\48a26225951ea153.final.pdb
-5.612590e-15 -6.416645e-16 -6.547855e-15 4.549761e-15 8.119473e-15
2.956596e-15
E:\\R-3.1.2\\scripts\\mPDBS\\summary_pdb_93\\4ab4a5c7c6682ce6.final.pdb
-3.778126e-15 -9.819608e-15 -3.642492e-15 4.687462e-15 -8.794877e-15
-3.670712e-15
E:\\R-3.1.2\\scripts\\mPDBS\\summary_pdb_93\\4eaa45b62cb6a25f.final.pdb
-5.402688e-15 -2.020160e-15 -7.379223e-17 1.219490e-15 5.293368e-15

-1.865926e-16
E:\\R-3.1.2\\scripts\\mPDBS\\summary_pdb_93\\58c371611d85ac96.final.pdb
-8.489634e-15 -2.407135e-15 -3.990521e-15 3.442798e-15 4.013454e-15
-3.289073e-15
E:\\R-3.1.2\\scripts\\mPDBS\\summary_pdb_93\\59a647f04822d30a.final.pdb
-6.151503e-15 2.343390e-15 3.174947e-15 6.336135e-15 1.376912e-15
5.685837e-16
E:\\R-3.1.2\\scripts\\mPDBS\\summary_pdb_93\\59b091fe385e05fc.final.pdb
-4.753492e-15 -2.404577e-15 2.122541e-16 2.397077e-15 -2.247235e-15
5.051452e-17
E:\\R-3.1.2\\scripts\\mPDBS\\summary_pdb_93\\5c35ac30f930d2db.final.pdb
-5.402688e-15 -2.020160e-15 -7.379223e-17 1.219490e-15 5.293368e-15
-1.865926e-16
E:\\R-3.1.2\\scripts\\mPDBS\\summary_pdb_93\\5f60752431cf2dcb.final.pdb
-6.458326e-15 -2.368400e-15 -6.388958e-15 2.333079e-15 3.264202e-15
-1.505929e-15
E:\\R-3.1.2\\scripts\\mPDBS\\summary_pdb_93\\626456dd6ff2868d.final.pdb
-3.115919e-15 -4.152267e-15 3.010389e-15 4.795646e-16 4.922449e-15
-4.436286e-19
E:\\R-3.1.2\\scripts\\mPDBS\\summary_pdb_93\\6583d53ff3c8f7ef.final.pdb
-6.151503e-15 2.343390e-15 3.174947e-15 6.336135e-15 1.376912e-15
5.685837e-16
E:\\R-3.1.2\\scripts\\mPDBS\\summary_pdb_93\\69760178986bef01.final.pdb
-2.699983e-15 -8.139442e-15 -8.042898e-15 -2.264990e-15 1.051093e-14
1.874480e-16
E:\\R-3.1.2\\scripts\\mPDBS\\summary_pdb_93\\6a9d87d47926b1c8.final.pdb
-4.933451e-15 -8.042502e-16 -1.643874e-15 3.532136e-15 5.275271e-16
-2.238164e-16
E:\\R-3.1.2\\scripts\\mPDBS\\summary_pdb_93\\6bb3547ea982ffc4.final.pdb
-3.566260e-15 7.812719e-15 -1.116759e-14 7.164750e-15 9.661658e-15
-4.023458e-15
E:\\R-3.1.2\\scripts\\mPDBS\\summary_pdb_93\\716d9624611d1c42.final.pdb
-4.378850e-15 -9.741157e-16 5.248908e-16 2.344897e-15 2.586792e-15
-5.812981e-18
E:\\R-3.1.2\\scripts\\mPDBS\\summary_pdb_93\\75f27e70ad0f1be4.final.pdb
-6.151503e-15 2.343390e-15 3.174947e-15 6.336135e-15 1.376912e-15
5.685837e-16
E:\\R-3.1.2\\scripts\\mPDBS\\summary_pdb_93\\769f910fffb22872.final.pdb
-4.615773e-15 -8.855572e-16 -1.659826e-15 3.063870e-15 3.176734e-17
8.882111e-16
E:\\R-3.1.2\\scripts\\mPDBS\\summary_pdb_93\\791f8c80dd5c3135.final.pdb
-4.753492e-15 -2.404577e-15 2.122541e-16 2.397077e-15 -2.247235e-15
5.051452e-17
E:\\R-3.1.2\\scripts\\mPDBS\\summary_pdb_93\\7fad98e66a3f6099.final.pdb
-1.517084e-15 1.548233e-15 -3.960354e-15 2.126176e-15 -4.113670e-15
-1.395260e-15
E:\\R-3.1.2\\scripts\\mPDBS\\summary_pdb_93\\804b89c94a633196.final.pdb
-3.043850e-16 -3.522225e-15 -5.096702e-16 1.242272e-15 1.658611e-15
3.384962e-15
E:\\R-3.1.2\\scripts\\mPDBS\\summary_pdb_93\\813cc65a2298feba.final.pdb
-3.043850e-16 -3.522225e-15 -5.096702e-16 1.242272e-15 1.658611e-15
3.384962e-15
E:\\R-3.1.2\\scripts\\mPDBS\\summary_pdb_93\\83c0eefcfe12d3f9.final.pdb
1.606823e-15 -6.063595e-15 -4.584959e-16 -1.615143e-15 9.127462e-15

2.012389e-15
E:\\R-3.1.2\\scripts\\mPDBS\\summary_pdb_93\\86d50469ef569760.final.pdb
-3.115919e-15 -4.152267e-15 3.010389e-15 4.795646e-16 4.922449e-15
-4.436286e-19
E:\\R-3.1.2\\scripts\\mPDBS\\summary_pdb_93\\89d0666e1402203d.final.pdb
-6.151503e-15 2.343390e-15 3.174947e-15 6.336135e-15 1.376912e-15
5.685837e-16
E:\\R-3.1.2\\scripts\\mPDBS\\summary_pdb_93\\8b34852716dadc62.final.pdb
-5.335083e-15 -8.237204e-15 3.254897e-15 2.807860e-15 3.117513e-15
-2.403810e-15
E:\\R-3.1.2\\scripts\\mPDBS\\summary_pdb_93\\8b6ef9ac3fe74395.final.pdb
-7.153035e-16 3.409993e-15 1.616791e-15 7.011431e-15 7.023619e-16
-2.492365e-15
E:\\R-3.1.2\\scripts\\mPDBS\\summary_pdb_93\\98bbe0d5986c138f.final.pdb
-4.753492e-15 -2.404577e-15 2.122541e-16 2.397077e-15 -2.247235e-15
5.051452e-17
E:\\R-3.1.2\\scripts\\mPDBS\\summary_pdb_93\\9c22f4909d67e2fa.final.pdb
-1.184573e-14 5.744667e-15 -3.499358e-15 6.121074e-15 -4.804969e-15
3.138442e-15
E:\\R-3.1.2\\scripts\\mPDBS\\summary_pdb_93\\9dda8ed0d81fb3ef.final.pdb
-9.492123e-15 -8.507739e-15 -3.575838e-15 -1.245060e-14 1.454780e-14
4.458385e-16
E:\\R-3.1.2\\scripts\\mPDBS\\summary_pdb_93\\9f752b3616e6033b.final.pdb
-3.495542e-15 -9.312689e-16 -1.528228e-15 4.542007e-15 -4.239455e-16
1.164461e-15
E:\\R-3.1.2\\scripts\\mPDBS\\summary_pdb_93\\a02c70083816d6ba.final.pdb
-5.402688e-15 -2.020160e-15 -7.379223e-17 1.219490e-15 5.293368e-15
-1.865926e-16
E:\\R-3.1.2\\scripts\\mPDBS\\summary_pdb_93\\a0c997d891e4e082.final.pdb
-2.564131e-15 -3.964445e-16 -9.201291e-16 2.246458e-15 1.702936e-15
1.885070e-15
E:\\R-3.1.2\\scripts\\mPDBS\\summary_pdb_93\\ab8039a2d823c616.final.pdb
-3.946478e-15 6.846135e-17 -1.631040e-15 3.696361e-15 1.568494e-15
8.479085e-16
E:\\R-3.1.2\\scripts\\mPDBS\\summary_pdb_93\\acf4d74b9c71d4dc.final.pdb
-3.043850e-16 -3.522225e-15 -5.096702e-16 1.242272e-15 1.658611e-15
3.384962e-15
E:\\R-3.1.2\\scripts\\mPDBS\\summary_pdb_93\\ad8a823e4b4c668e.final.pdb
-4.733552e-15 -9.465361e-16 -1.488159e-15 3.774389e-15 -9.684046e-17
5.880243e-16
E:\\R-3.1.2\\scripts\\mPDBS\\summary_pdb_93\\b16ababd03b7b857.final.pdb
-1.184573e-14 5.744667e-15 -3.499358e-15 6.121074e-15 -4.804969e-15
3.138442e-15
E:\\R-3.1.2\\scripts\\mPDBS\\summary_pdb_93\\b226923e697d3c63.final.pdb
-5.342379e-16 -6.589830e-16 -2.309598e-15 5.225261e-15 -2.823506e-16
-4.814046e-15
E:\\R-3.1.2\\scripts\\mPDBS\\summary_pdb_93\\b28e48f6fa27a165.final.pdb
-4.753492e-15 -2.404577e-15 2.122541e-16 2.397077e-15 -2.247235e-15
5.051452e-17
E:\\R-3.1.2\\scripts\\mPDBS\\summary_pdb_93\\b42c4924e3cd1aec.final.pdb
-4.149994e-16 -3.619116e-16 -1.883290e-15 1.153629e-14 -8.354886e-15
7.236382e-16
E:\\R-3.1.2\\scripts\\mPDBS\\summary_pdb_93\\b4dd239fcabff1df.final.pdb
-4.753492e-15 -2.404577e-15 2.122541e-16 2.397077e-15 -2.247235e-15

5.051452e-17
E:\\R-3.1.2\\scripts\\mPDBS\\summary_pdb_93\\b669942ef97459a2.final.pdb
-5.335083e-15 -8.237204e-15 3.254897e-15 2.807860e-15 3.117513e-15
-2.403810e-15
E:\\R-3.1.2\\scripts\\mPDBS\\summary_pdb_93\\b99f4d9d87c1017b.final.pdb
-4.753492e-15 -2.404577e-15 2.122541e-16 2.397077e-15 -2.247235e-15
5.051452e-17
E:\\R-3.1.2\\scripts\\mPDBS\\summary_pdb_93\\b9d2cbf3975403e7.final.pdb
-4.753492e-15 -2.404577e-15 2.122541e-16 2.397077e-15 -2.247235e-15
5.051452e-17
E:\\R-3.1.2\\scripts\\mPDBS\\summary_pdb_93\\bc88c8a505f3a288.final.pdb
-4.745913e-15 -6.819968e-16 -1.637828e-15 3.509360e-15 1.309816e-15
-1.730470e-18
E:\\R-3.1.2\\scripts\\mPDBS\\summary_pdb_93\\bdac0e64f4ef427e.final.pdb
-1.447949e-14 8.305119e-15 -4.315111e-15 1.310214e-14 -1.740296e-14
-9.351226e-16
E:\\R-3.1.2\\scripts\\mPDBS\\summary_pdb_93\\ca2a1f55fc9d0681.final.pdb
-5.335083e-15 -8.237204e-15 3.254897e-15 2.807860e-15 3.117513e-15
-2.403810e-15
E:\\R-3.1.2\\scripts\\mPDBS\\summary_pdb_93\\ce34248de3e81a3c.final.pdb
-6.380598e-15 2.369542e-15 2.861520e-15 -2.589263e-16 -3.695394e-16
3.754788e-15
E:\\R-3.1.2\\scripts\\mPDBS\\summary_pdb_93\\d02e697772494a61.final.pdb
-2.593535e-15 5.878727e-15 8.449945e-16 1.183105e-14 -3.746036e-15
8.454492e-17
E:\\R-3.1.2\\scripts\\mPDBS\\summary_pdb_93\\d04221245d015047.final.pdb
-5.622448e-15 -8.943102e-16 -1.417995e-15 2.395644e-15 3.481165e-16
2.906822e-15
E:\\R-3.1.2\\scripts\\mPDBS\\summary_pdb_93\\d678141e4018054a.final.pdb
-7.022973e-15 6.952181e-16 -1.043846e-14 3.524848e-15 5.492933e-15
-3.175543e-15
E:\\R-3.1.2\\scripts\\mPDBS\\summary_pdb_93\\d71b40af0f0efe39.final.pdb
-1.467685e-15 -1.374253e-14 -4.557808e-15 4.252479e-15 -6.741087e-15
-2.503962e-15
E:\\R-3.1.2\\scripts\\mPDBS\\summary_pdb_93\\dc0809eff10197e2.final.pdb
-5.766597e-15 -5.551643e-15 9.306369e-15 5.709910e-15 -2.722752e-15
-1.544048e-16
E:\\R-3.1.2\\scripts\\mPDBS\\summary_pdb_93\\e1163918d3707839.final.pdb
2.202698e-15 -6.868859e-15 3.151311e-15 1.063467e-14 -1.153550e-14
3.001330e-15
E:\\R-3.1.2\\scripts\\mPDBS\\summary_pdb_93\\e4b48b06824ef848.final.pdb
-4.280741e-15 2.285690e-16 -5.492047e-15 2.349380e-15 4.843890e-15
-7.868040e-17
E:\\R-3.1.2\\scripts\\mPDBS\\summary_pdb_93\\e529e19ad281fea2.final.pdb
-3.809808e-15 5.838006e-15 -6.624265e-15 5.017601e-15 -1.160359e-15
3.375328e-15
E:\\R-3.1.2\\scripts\\mPDBS\\summary_pdb_93\\e5602082718ec3a1.final.pdb
-3.043850e-16 -3.522225e-15 -5.096702e-16 1.242272e-15 1.658611e-15
3.384962e-15
E:\\R-3.1.2\\scripts\\mPDBS\\summary_pdb_93\\e5d589d996d0a960.final.pdb
3.831327e-16 -2.239027e-15 -5.907559e-15 -1.144941e-15 3.913785e-15
6.821717e-15
E:\\R-3.1.2\\scripts\\mPDBS\\summary_pdb_93\\e9df9e72307a2fbd.final.pdb
-5.402688e-15 -2.020160e-15 -7.379223e-17 1.219490e-15 5.293368e-15

```
-1.865926e-16
E:\\R-3.1.2\\scripts\\mPDBS\\summary_pdb_93\\ea00f8694090cae7.final.pdb
-3.043850e-16 -3.522225e-15 -5.096702e-16  1.242272e-15  1.658611e-15
3.384962e-15
E:\\R-3.1.2\\scripts\\mPDBS\\summary_pdb_93\\eba517be32db050e.final.pdb
-4.753492e-15 -2.404577e-15  2.122541e-16  2.397077e-15 -2.247235e-15
5.051452e-17
E:\\R-3.1.2\\scripts\\mPDBS\\summary_pdb_93\\ec04b9545899c29d.final.pdb
-4.641426e-15 -8.892676e-16 -1.529731e-15  3.245725e-15  5.676639e-16
2.823544e-16
E:\\R-3.1.2\\scripts\\mPDBS\\summary_pdb_93\\ee6862cbcad52078.final.pdb
-4.802065e-15 -1.247517e-15 -1.497641e-15  3.425768e-15  1.082567e-15
1.441896e-16
E:\\R-3.1.2\\scripts\\mPDBS\\summary_pdb_93\\ef6a42fc83b81e5d.final.pdb
-4.624674e-15 -9.081086e-16 -1.814325e-15  3.396394e-15  3.509565e-16
8.082105e-16
E:\\R-3.1.2\\scripts\\mPDBS\\summary_pdb_93\\ef8e88d4ad93a0ad.final.pdb
-5.622448e-15 -8.943102e-16 -1.417995e-15  2.395644e-15  3.481165e-16
2.906822e-15
E:\\R-3.1.2\\scripts\\mPDBS\\summary_pdb_93\\f14fcae8deec4fa3.final.pdb
-3.495542e-15 -9.312689e-16 -1.528228e-15  4.542007e-15 -4.239455e-16
1.164461e-15
E:\\R-3.1.2\\scripts\\mPDBS\\summary_pdb_93\\f83584e22003fb92.final.pdb
-5.612590e-15 -6.416645e-16 -6.547855e-15  4.549761e-15  8.119473e-15
2.956596e-15
E:\\R-3.1.2\\scripts\\mPDBS\\summary_pdb_93\\f8e2bf87e52caa29.final.pdb
-3.809808e-15  5.838006e-15 -6.624265e-15  5.017601e-15 -1.160359e-15
3.375328e-15

[,757]        [,758]        [,759]        [,760]        [,761]        [,762]
E:\\R-3.1.2\\scripts\\40.1ns_data\\NAT0.pdb
2.989794e-15 -2.851242e-16  1.617896e-16 -1.222998e-15 -2.438483e-15
2.537826e-15
E:\\R-3.1.2\\scripts\\40.1ns_data\\LJP6.pdb
4.912188e-15 -1.056987e-15 -1.278516e-15  3.355516e-16 -1.602441e-15
4.174227e-15
E:\\R-3.1.2\\scripts\\40.1ns_data\\3WY9.pdb
4.381881e-15 -1.442050e-15 -7.502764e-16  1.055850e-16 -6.865589e-16
5.849012e-15
E:\\R-3.1.2\\scripts\\40.1ns_data\\ZSP9.pdb
2.734508e-15 -1.253986e-15  3.174851e-16 -1.551718e-15 -9.380626e-16
3.339521e-15
E:\\R-3.1.2\\scripts\\mPDBS\\summary_pdb_93\\013d855ad26c877c.final.pdb
-1.668372e-15 -4.869415e-15 -1.101771e-15 -7.560594e-16 -6.169277e-15
3.717201e-15
E:\\R-3.1.2\\scripts\\mPDBS\\summary_pdb_93\\0a2a705342ca0d54.final.pdb
1.378879e-15 -1.101494e-15 -2.696722e-16 -1.471928e-15 -3.116697e-15
6.133325e-15
E:\\R-3.1.2\\scripts\\mPDBS\\summary_pdb_93\\0a59fceadb4194d9.final.pdb
1.555164e-15 -2.568204e-15 -1.440976e-15 -1.079654e-15 -1.733208e-15
1.561514e-15
E:\\R-3.1.2\\scripts\\mPDBS\\summary_pdb_93\\0dba59f3306825e8.final.pdb
9.527952e-16  1.763300e-15  2.730233e-15  3.643199e-15 -6.478058e-15
-2.761329e-16
```

E:\\R-3.1.2\\scripts\\mPDBS\\summary_pdb_93\\126f26a78b57339c.final.pdb
6.297912e-15 2.629795e-15 -1.093097e-15 -4.245456e-15 1.635244e-15
1.454459e-14
E:\\R-3.1.2\\scripts\\mPDBS\\summary_pdb_93\\12f06f3e342df706.final.pdb
1.362833e-15 -1.522056e-15 -5.012578e-16 -6.323218e-16 -1.960504e-15
6.049300e-15
E:\\R-3.1.2\\scripts\\mPDBS\\summary_pdb_93\\18618a2a251b867b.final.pdb
-1.101369e-15 5.906642e-15 5.207940e-15 -1.365195e-15 -2.130583e-16
1.290191e-14
E:\\R-3.1.2\\scripts\\mPDBS\\summary_pdb_93\\1f305f598641eeb2.final.pdb
1.714382e-15 -1.323796e-15 -7.308596e-16 -1.485519e-15 -2.217379e-15
5.164174e-15
E:\\R-3.1.2\\scripts\\mPDBS\\summary_pdb_93\\1f8a00b551be0a39.final.pdb
-1.101369e-15 5.906642e-15 5.207940e-15 -1.365195e-15 -2.130583e-16
1.290191e-14
E:\\R-3.1.2\\scripts\\mPDBS\\summary_pdb_93\\219f45328d47579a.final.pdb
2.367882e-15 9.698199e-17 -1.992012e-14 -1.360679e-15 -5.025713e-15
-1.328535e-15
E:\\R-3.1.2\\scripts\\mPDBS\\summary_pdb_93\\238dd3918e54336d.final.pdb
4.215363e-15 4.386845e-15 4.916445e-18 -4.882168e-15 -2.816543e-15
2.048355e-14
E:\\R-3.1.2\\scripts\\mPDBS\\summary_pdb_93\\2b3027889e2fdc42.final.pdb
1.148805e-15 -8.964725e-16 -5.606034e-16 -1.145574e-15 -3.103640e-15
5.117047e-15
E:\\R-3.1.2\\scripts\\mPDBS\\summary_pdb_93\\2fcb3b46e33793b0.final.pdb
-4.544501e-15 -2.252740e-15 1.312980e-16 9.986052e-16 -5.312126e-15
-3.478218e-15
E:\\R-3.1.2\\scripts\\mPDBS\\summary_pdb_93\\34eb328251842006.final.pdb
1.239792e-14 -6.223603e-15 7.014930e-16 -1.117763e-14 2.248691e-15
6.564092e-15
E:\\R-3.1.2\\scripts\\mPDBS\\summary_pdb_93\\354b32871797b7ab.final.pdb
-5.151616e-15 -5.923548e-15 4.181881e-15 9.108527e-18 7.411432e-16
2.309134e-15
E:\\R-3.1.2\\scripts\\mPDBS\\summary_pdb_93\\397368adbc32d5de.final.pdb
6.297912e-15 2.629795e-15 -1.093097e-15 -4.245456e-15 1.635244e-15
1.454459e-14
E:\\R-3.1.2\\scripts\\mPDBS\\summary_pdb_93\\411ca13cd3df6edd.final.pdb
-6.591537e-16 -1.002872e-15 3.324924e-15 -6.766884e-15 -1.240730e-15
-6.699294e-17
E:\\R-3.1.2\\scripts\\mPDBS\\summary_pdb_93\\42357eee6642bf12.final.pdb
3.592636e-15 -2.176778e-15 -5.162990e-15 -6.073166e-15 3.539288e-16
-9.196033e-16
E:\\R-3.1.2\\scripts\\mPDBS\\summary_pdb_93\\44342d1f75a7fcfc.final.pdb
-2.418063e-15 4.780023e-15 4.908242e-15 -5.480988e-16 8.365405e-15
3.373947e-15
E:\\R-3.1.2\\scripts\\mPDBS\\summary_pdb_93\\448ef21dffe8cdfb.final.pdb
1.300396e-14 -1.923395e-14 -1.022293e-15 -2.697902e-14 3.777675e-16
3.463366e-14
E:\\R-3.1.2\\scripts\\mPDBS\\summary_pdb_93\\472a40b3e8f113c7.final.pdb
9.527952e-16 1.763300e-15 2.730233e-15 3.643199e-15 -6.478058e-15
-2.761329e-16
E:\\R-3.1.2\\scripts\\mPDBS\\summary_pdb_93\\48a26225951ea153.final.pdb
3.430261e-16 -7.949911e-16 1.068561e-14 -4.434609e-15 1.340722e-15
9.845903e-15

E:\\R-3.1.2\\scripts\\mPDBS\\summary_pdb_93\\4ab4a5c7c6682ce6.final.pdb
-1.409550e-14 -2.690602e-15 3.032075e-15 -7.076524e-15 1.423608e-15
2.747502e-16
E:\\R-3.1.2\\scripts\\mPDBS\\summary_pdb_93\\4eaa45b62cb6a25f.final.pdb
5.499866e-16 1.900597e-15 -2.087044e-16 1.806282e-15 -2.940807e-15
7.216992e-15
E:\\R-3.1.2\\scripts\\mPDBS\\summary_pdb_93\\58c371611d85ac96.final.pdb
6.297912e-15 2.629795e-15 -1.093097e-15 -4.245456e-15 1.635244e-15
1.454459e-14
E:\\R-3.1.2\\scripts\\mPDBS\\summary_pdb_93\\59a647f04822d30a.final.pdb
1.393992e-15 -4.660342e-15 -3.405421e-15 -5.655125e-15 -6.296478e-15
5.216223e-15
E:\\R-3.1.2\\scripts\\mPDBS\\summary_pdb_93\\59b091fe385e05fc.final.pdb
1.555164e-15 -2.568204e-15 -1.440976e-15 -1.079654e-15 -1.733208e-15
1.561514e-15
E:\\R-3.1.2\\scripts\\mPDBS\\summary_pdb_93\\5c35ac30f930d2db.final.pdb
5.499866e-16 1.900597e-15 -2.087044e-16 1.806282e-15 -2.940807e-15
7.216992e-15
E:\\R-3.1.2\\scripts\\mPDBS\\summary_pdb_93\\5f60752431cf2dcb.final.pdb
4.184795e-15 -1.800263e-15 -8.265184e-16 -1.853567e-15 1.879508e-16
1.113215e-14
E:\\R-3.1.2\\scripts\\mPDBS\\summary_pdb_93\\626456dd6ff2868d.final.pdb
9.527952e-16 1.763300e-15 2.730233e-15 3.643199e-15 -6.478058e-15
-2.761329e-16
E:\\R-3.1.2\\scripts\\mPDBS\\summary_pdb_93\\6583d53ff3c8f7ef.final.pdb
1.393992e-15 -4.660342e-15 -3.405421e-15 -5.655125e-15 -6.296478e-15
5.216223e-15
E:\\R-3.1.2\\scripts\\mPDBS\\summary_pdb_93\\69760178986bef01.final.pdb
-4.860806e-15 1.019587e-15 1.088536e-15 4.275731e-15 -2.557945e-15
1.073196e-14
E:\\R-3.1.2\\scripts\\mPDBS\\summary_pdb_93\\6a9d87d47926b1c8.final.pdb
1.256806e-15 -9.159800e-16 -5.518635e-16 -1.594798e-15 -2.777893e-15
4.590742e-15
E:\\R-3.1.2\\scripts\\mPDBS\\summary_pdb_93\\6bb3547ea982ffc4.final.pdb
9.177552e-15 1.165380e-15 2.913997e-15 -1.109572e-14 -7.709583e-15
1.756842e-14
E:\\R-3.1.2\\scripts\\mPDBS\\summary_pdb_93\\716d9624611d1c42.final.pdb
-6.897783e-16 2.045511e-15 6.709988e-15 -9.619193e-16 -6.568595e-16
7.862845e-15
E:\\R-3.1.2\\scripts\\mPDBS\\summary_pdb_93\\75f27e70ad0f1be4.final.pdb
1.393992e-15 -4.660342e-15 -3.405421e-15 -5.655125e-15 -6.296478e-15
5.216223e-15
E:\\R-3.1.2\\scripts\\mPDBS\\summary_pdb_93\\769f910fffb22872.final.pdb
1.142496e-15 -9.233983e-16 -1.546206e-15 -6.678361e-16 -3.187242e-15
4.964144e-15
E:\\R-3.1.2\\scripts\\mPDBS\\summary_pdb_93\\791f8c80dd5c3135.final.pdb
1.555164e-15 -2.568204e-15 -1.440976e-15 -1.079654e-15 -1.733208e-15
1.561514e-15
E:\\R-3.1.2\\scripts\\mPDBS\\summary_pdb_93\\7fad98e66a3f6099.final.pdb
2.811024e-15 -1.017457e-14 -7.171862e-16 -3.664426e-15 -1.083346e-15
3.897742e-15
E:\\R-3.1.2\\scripts\\mPDBS\\summary_pdb_93\\804b89c94a633196.final.pdb
-4.544501e-15 -2.252740e-15 1.312980e-16 9.986052e-16 -5.312126e-15
-3.478218e-15

E:\\R-3.1.2\\scripts\\mPDBS\\summary_pdb_93\\813cc65a2298feba.final.pdb
-4.544501e-15 -2.252740e-15 1.312980e-16 9.986052e-16 -5.312126e-15
-3.478218e-15
E:\\R-3.1.2\\scripts\\mPDBS\\summary_pdb_93\\83c0eefcfe12d3f9.final.pdb
-6.591537e-16 -1.002872e-15 3.324924e-15 -6.766884e-15 -1.240730e-15
-6.699294e-17
E:\\R-3.1.2\\scripts\\mPDBS\\summary_pdb_93\\86d50469ef569760.final.pdb
9.527952e-16 1.763300e-15 2.730233e-15 3.643199e-15 -6.478058e-15
-2.761329e-16
E:\\R-3.1.2\\scripts\\mPDBS\\summary_pdb_93\\89d0666e1402203d.final.pdb
1.393992e-15 -4.660342e-15 -3.405421e-15 -5.655125e-15 -6.296478e-15
5.216223e-15
E:\\R-3.1.2\\scripts\\mPDBS\\summary_pdb_93\\8b34852716dadc62.final.pdb
3.317266e-15 4.275891e-15 -3.624378e-15 -2.623497e-15 3.930481e-15
3.242101e-15
E:\\R-3.1.2\\scripts\\mPDBS\\summary_pdb_93\\8b6ef9ac3fe74395.final.pdb
4.121952e-15 -1.025367e-15 -7.552153e-15 -8.360015e-15 -4.248867e-16
1.072614e-14
E:\\R-3.1.2\\scripts\\mPDBS\\summary_pdb_93\\98bbe0d5986c138f.final.pdb
1.555164e-15 -2.568204e-15 -1.440976e-15 -1.079654e-15 -1.733208e-15
1.561514e-15
E:\\R-3.1.2\\scripts\\mPDBS\\summary_pdb_93\\9c22f4909d67e2fa.final.pdb
1.062262e-14 -8.931509e-16 -3.846423e-15 9.536914e-15 -5.011151e-15
9.353857e-15
E:\\R-3.1.2\\scripts\\mPDBS\\summary_pdb_93\\9dda8ed0d81fb3ef.final.pdb
-2.615388e-15 -5.113176e-16 8.079316e-15 1.541376e-15 -4.313779e-16
2.041029e-15
E:\\R-3.1.2\\scripts\\mPDBS\\summary_pdb_93\\9f752b3616e6033b.final.pdb
3.068386e-15 -2.168424e-15 -3.914924e-15 -1.870444e-15 -3.963440e-15
3.250098e-15
E:\\R-3.1.2\\scripts\\mPDBS\\summary_pdb_93\\a02c70083816d6ba.final.pdb
5.499866e-16 1.900597e-15 -2.087044e-16 1.806282e-15 -2.940807e-15
7.216992e-15
E:\\R-3.1.2\\scripts\\mPDBS\\summary_pdb_93\\a0c997d891e4e082.final.pdb
1.758098e-15 -9.538475e-15 -7.271428e-15 3.106534e-16 -5.780752e-15
8.315724e-16
E:\\R-3.1.2\\scripts\\mPDBS\\summary_pdb_93\\ab8039a2d823c616.final.pdb
1.308805e-15 -1.304501e-15 -5.736606e-16 -7.961002e-16 -1.861144e-15
5.773474e-15
E:\\R-3.1.2\\scripts\\mPDBS\\summary_pdb_93\\acf4d74b9c71d4dc.final.pdb
-4.544501e-15 -2.252740e-15 1.312980e-16 9.986052e-16 -5.312126e-15
-3.478218e-15
E:\\R-3.1.2\\scripts\\mPDBS\\summary_pdb_93\\ad8a823e4b4c668e.final.pdb
1.332281e-15 -1.179589e-15 -6.395097e-16 -4.649255e-16 -1.700430e-15
5.646746e-15
E:\\R-3.1.2\\scripts\\mPDBS\\summary_pdb_93\\b16ababd03b7b857.final.pdb
1.062262e-14 -8.931509e-16 -3.846423e-15 9.536914e-15 -5.011151e-15
9.353857e-15
E:\\R-3.1.2\\scripts\\mPDBS\\summary_pdb_93\\b226923e697d3c63.final.pdb
-9.418132e-16 -1.507916e-15 -7.717076e-15 -4.395715e-15 -3.524480e-16
8.215235e-15
E:\\R-3.1.2\\scripts\\mPDBS\\summary_pdb_93\\b28e48f6fa27a165.final.pdb
1.555164e-15 -2.568204e-15 -1.440976e-15 -1.079654e-15 -1.733208e-15
1.561514e-15

E:\\R-3.1.2\\scripts\\mPDBS\\summary_pdb_93\\b42c4924e3cd1aec.final.pdb
4.176488e-15 4.140490e-16 -7.784730e-15 7.275504e-15 4.263651e-15
4.550415e-15
E:\\R-3.1.2\\scripts\\mPDBS\\summary_pdb_93\\b4dd239fcabff1df.final.pdb
1.555164e-15 -2.568204e-15 -1.440976e-15 -1.079654e-15 -1.733208e-15
1.561514e-15
E:\\R-3.1.2\\scripts\\mPDBS\\summary_pdb_93\\b669942ef97459a2.final.pdb
3.317266e-15 4.275891e-15 -3.624378e-15 -2.623497e-15 3.930481e-15
3.242101e-15
E:\\R-3.1.2\\scripts\\mPDBS\\summary_pdb_93\\b99f4d9d87c1017b.final.pdb
1.555164e-15 -2.568204e-15 -1.440976e-15 -1.079654e-15 -1.733208e-15
1.561514e-15
E:\\R-3.1.2\\scripts\\mPDBS\\summary_pdb_93\\b9d2cbf3975403e7.final.pdb
1.555164e-15 -2.568204e-15 -1.440976e-15 -1.079654e-15 -1.733208e-15
1.561514e-15
E:\\R-3.1.2\\scripts\\mPDBS\\summary_pdb_93\\bc88c8a505f3a288.final.pdb
9.280616e-16 -9.525257e-16 -4.183561e-16 -2.439677e-15 -2.646540e-15
5.128540e-15
E:\\R-3.1.2\\scripts\\mPDBS\\summary_pdb_93\\bdac0e64f4ef427e.final.pdb
6.528673e-15 -2.208071e-15 -9.217994e-15 9.973197e-15 -2.507945e-15
1.495777e-14
E:\\R-3.1.2\\scripts\\mPDBS\\summary_pdb_93\\ca2a1f55fc9d0681.final.pdb
3.317266e-15 4.275891e-15 -3.624378e-15 -2.623497e-15 3.930481e-15
3.242101e-15
E:\\R-3.1.2\\scripts\\mPDBS\\summary_pdb_93\\ce34248de3e81a3c.final.pdb
-3.231611e-15 -9.026143e-16 5.324219e-16 1.176789e-15 2.823896e-15
4.077196e-15
E:\\R-3.1.2\\scripts\\mPDBS\\summary_pdb_93\\d02e697772494a61.final.pdb
3.345399e-15 -3.023027e-15 -3.181771e-15 -1.157820e-14 -1.078933e-14
8.571207e-15
E:\\R-3.1.2\\scripts\\mPDBS\\summary_pdb_93\\d04221245d015047.final.pdb
2.648011e-15 -1.251701e-15 1.207900e-15 -7.301807e-16 -2.843865e-15
4.422450e-15
E:\\R-3.1.2\\scripts\\mPDBS\\summary_pdb_93\\d678141e4018054a.final.pdb
8.987668e-15 3.920256e-16 1.096325e-14 -1.655712e-14 -3.758213e-15
1.480860e-14
E:\\R-3.1.2\\scripts\\mPDBS\\summary_pdb_93\\d71b40af0f0efe39.final.pdb
-1.154043e-15 6.071096e-16 1.453792e-15 2.637078e-15 -4.274497e-15
1.880613e-15
E:\\R-3.1.2\\scripts\\mPDBS\\summary_pdb_93\\dc0809eff10197e2.final.pdb
7.804648e-16 -3.577356e-15 -8.257245e-15 6.978687e-15 -2.845523e-15
-2.919990e-15
E:\\R-3.1.2\\scripts\\mPDBS\\summary_pdb_93\\e1163918d3707839.final.pdb
9.341194e-15 -9.824034e-16 5.324575e-15 4.268158e-16 -1.147029e-14
5.103791e-15
E:\\R-3.1.2\\scripts\\mPDBS\\summary_pdb_93\\e4b48b06824ef848.final.pdb
-1.101369e-15 5.906642e-15 5.207940e-15 -1.365195e-15 -2.130583e-16
1.290191e-14
E:\\R-3.1.2\\scripts\\mPDBS\\summary_pdb_93\\e529e19ad281fea2.final.pdb
-1.668372e-15 -4.869415e-15 -1.101771e-15 -7.560594e-16 -6.169277e-15
3.717201e-15
E:\\R-3.1.2\\scripts\\mPDBS\\summary_pdb_93\\e5602082718ec3a1.final.pdb
-4.544501e-15 -2.252740e-15 1.312980e-16 9.986052e-16 -5.312126e-15
-3.478218e-15

E:\\R-3.1.2\\scripts\\mPDBS\\summary_pdb_93\\e5d589d996d0a960.final.pdb
2.545732e-15 -1.373401e-15 -5.657449e-15 4.900763e-15 1.675632e-15
8.636762e-15
E:\\R-3.1.2\\scripts\\mPDBS\\summary_pdb_93\\e9df9e72307a2fbd.final.pdb
5.499866e-16 1.900597e-15 -2.087044e-16 1.806282e-15 -2.940807e-15
7.216992e-15
E:\\R-3.1.2\\scripts\\mPDBS\\summary_pdb_93\\ea00f8694090cae7.final.pdb
-4.544501e-15 -2.252740e-15 1.312980e-16 9.986052e-16 -5.312126e-15
-3.478218e-15
E:\\R-3.1.2\\scripts\\mPDBS\\summary_pdb_93\\eba517be32db050e.final.pdb
1.555164e-15 -2.568204e-15 -1.440976e-15 -1.079654e-15 -1.733208e-15
1.561514e-15
E:\\R-3.1.2\\scripts\\mPDBS\\summary_pdb_93\\ec04b9545899c29d.final.pdb
2.444367e-15 -1.262670e-15 -8.510403e-16 -9.089212e-16 -2.617129e-15
5.596128e-15
E:\\R-3.1.2\\scripts\\mPDBS\\summary_pdb_93\\ee6862cbcad52078.final.pdb
1.442407e-15 -8.473581e-16 -3.645797e-16 -1.846402e-15 -3.004761e-15
5.587808e-15
E:\\R-3.1.2\\scripts\\mPDBS\\summary_pdb_93\\ef6a42fc83b81e5d.final.pdb
1.615208e-15 -1.155092e-15 -8.419085e-16 -1.163967e-15 -1.902679e-15
5.944588e-15
E:\\R-3.1.2\\scripts\\mPDBS\\summary_pdb_93\\ef8e88d4ad93a0ad.final.pdb
2.648011e-15 -1.251701e-15 1.207900e-15 -7.301807e-16 -2.843865e-15
4.422450e-15
E:\\R-3.1.2\\scripts\\mPDBS\\summary_pdb_93\\f14fcae8deec4fa3.final.pdb
3.068386e-15 -2.168424e-15 -3.914924e-15 -1.870444e-15 -3.963440e-15
3.250098e-15
E:\\R-3.1.2\\scripts\\mPDBS\\summary_pdb_93\\f83584e22003fb92.final.pdb
3.430261e-16 -7.949911e-16 1.068561e-14 -4.434609e-15 1.340722e-15
9.845903e-15
E:\\R-3.1.2\\scripts\\mPDBS\\summary_pdb_93\\f8e2bf87e52caa29.final.pdb
-1.668372e-15 -4.869415e-15 -1.101771e-15 -7.560594e-16 -6.169277e-15
3.717201e-15

[,763]   [,764]   [,765]   [,766]   [,767]   [,768]
E:\\R-3.1.2\\scripts\\40.1ns_data\\NAT0.pdb
7.647573e-16 -2.868502e-15 1.267309e-16 -2.555861e-15 -1.449246e-15
2.536390e-15
E:\\R-3.1.2\\scripts\\40.1ns_data\\LJP6.pdb
2.515575e-15 -3.361798e-15 2.475555e-16 -2.028857e-15 -4.141549e-16
1.192260e-15
E:\\R-3.1.2\\scripts\\40.1ns_data\\3WY9.pdb
1.042613e-15 -2.122558e-15 -7.607923e-16 -9.796925e-16 -2.274523e-15
4.364403e-15
E:\\R-3.1.2\\scripts\\40.1ns_data\\ZSP9.pdb
2.452433e-15 -1.804201e-18 4.228424e-17 -1.320003e-15 -4.905706e-16
2.963019e-15
E:\\R-3.1.2\\scripts\\mPDBS\\summary_pdb_93\\013d855ad26c877c.final.pdb
6.057480e-15 -2.613912e-15 -2.754749e-15 1.060266e-15 1.229226e-15
4.011337e-15
E:\\R-3.1.2\\scripts\\mPDBS\\summary_pdb_93\\0a2a705342ca0d54.final.pdb
9.423683e-16 -3.059844e-15 3.973774e-16 -1.766685e-15 -1.574148e-15
1.546645e-15
E:\\R-3.1.2\\scripts\\mPDBS\\summary_pdb_93\\0a59fceadb4194d9.final.pdb

2.533160e-15 -1.934182e-15 -7.543999e-17 -1.044311e-15 -1.523796e-15
2.560764e-16
E:\\R-3.1.2\\scripts\\mPDBS\\summary_pdb_93\\0dba59f3306825e8.final.pdb
2.701657e-15 -2.353703e-15 1.308840e-15 -2.350200e-15 -2.140639e-16
-6.533345e-16
E:\\R-3.1.2\\scripts\\mPDBS\\summary_pdb_93\\126f26a78b57339c.final.pdb
-8.831652e-15 -3.985211e-15 4.549304e-15 -5.247405e-15 -1.025047e-15
-1.415745e-15
E:\\R-3.1.2\\scripts\\mPDBS\\summary_pdb_93\\12f06f3e342df706.final.pdb
1.245511e-15 -2.661508e-15 3.583462e-16 -1.522523e-15 -1.347550e-15
2.129512e-15
E:\\R-3.1.2\\scripts\\mPDBS\\summary_pdb_93\\18618a2a251b867b.final.pdb
-9.454226e-15 -2.192096e-15 -1.893261e-15 4.833417e-16 1.989024e-16
3.393189e-15
E:\\R-3.1.2\\scripts\\mPDBS\\summary_pdb_93\\1f305f598641eeb2.final.pdb
9.481178e-16 -2.843308e-15 6.042977e-16 -1.362245e-15 -1.349773e-15
1.215116e-15
E:\\R-3.1.2\\scripts\\mPDBS\\summary_pdb_93\\1f8a00b551be0a39.final.pdb
-9.454226e-15 -2.192096e-15 -1.893261e-15 4.833417e-16 1.989024e-16
3.393189e-15
E:\\R-3.1.2\\scripts\\mPDBS\\summary_pdb_93\\219f45328d47579a.final.pdb
9.910939e-15 -1.694995e-14 2.791191e-15 -1.037105e-14 -1.490545e-14
1.016308e-14
E:\\R-3.1.2\\scripts\\mPDBS\\summary_pdb_93\\238dd3918e54336d.final.pdb
-5.877213e-15 -3.452499e-15 6.070035e-15 -1.319280e-14 3.030287e-15
9.301792e-15
E:\\R-3.1.2\\scripts\\mPDBS\\summary_pdb_93\\2b3027889e2fdc42.final.pdb
1.441586e-15 -4.014983e-15 4.334845e-16 -1.947451e-15 -2.027516e-15
7.921059e-16
E:\\R-3.1.2\\scripts\\mPDBS\\summary_pdb_93\\2fcb3b46e33793b0.final.pdb
6.474947e-15 -1.018379e-15 -1.073180e-15 -1.702251e-15 -1.328957e-15
3.546936e-16
E:\\R-3.1.2\\scripts\\mPDBS\\summary_pdb_93\\34eb328251842006.final.pdb
1.301177e-14 -4.553265e-15 -1.721601e-15 -9.484532e-16 -1.763326e-15
-1.047619e-14
E:\\R-3.1.2\\scripts\\mPDBS\\summary_pdb_93\\354b32871797b7ab.final.pdb
1.692048e-14 8.804152e-15 -1.198369e-14 9.507336e-15 1.260975e-15
-1.041736e-14
E:\\R-3.1.2\\scripts\\mPDBS\\summary_pdb_93\\397368adbc32d5de.final.pdb
-8.831652e-15 -3.985211e-15 4.549304e-15 -5.247405e-15 -1.025047e-15
-1.415745e-15
E:\\R-3.1.2\\scripts\\mPDBS\\summary_pdb_93\\411ca13cd3df6edd.final.pdb
1.489352e-15 -4.068885e-16 1.815135e-15 -5.147195e-15 -7.504264e-16
-1.597738e-15
E:\\R-3.1.2\\scripts\\mPDBS\\summary_pdb_93\\42357eee6642bf12.final.pdb
3.763261e-15 -6.430550e-15 -5.186276e-15 -2.332076e-15 -3.144861e-15
6.592556e-15
E:\\R-3.1.2\\scripts\\mPDBS\\summary_pdb_93\\44342d1f75a7fcfc.final.pdb
-1.153342e-14 3.859030e-15 1.300453e-15 -2.454276e-15 4.904718e-15
3.078994e-15
E:\\R-3.1.2\\scripts\\mPDBS\\summary_pdb_93\\448ef21dffe8cdfb.final.pdb
1.278583e-15 -4.633542e-15 -7.470840e-15 1.105144e-14 -2.262238e-15
4.210984e-15
E:\\R-3.1.2\\scripts\\mPDBS\\summary_pdb_93\\472a40b3e8f113c7.final.pdb

2.701657e-15 -2.353703e-15 1.308840e-15 -2.350200e-15 -2.140639e-16
-6.533345e-16
E:\\R-3.1.2\\scripts\\mPDBS\\summary_pdb_93\\48a26225951ea153.final.pdb
1.207832e-15 -1.364326e-15 -4.416835e-16 3.708614e-15 4.134222e-15
-6.441691e-15
E:\\R-3.1.2\\scripts\\mPDBS\\summary_pdb_93\\4ab4a5c7c6682ce6.final.pdb
6.338670e-16 -6.656685e-15 -4.128650e-15 5.469716e-15 -1.301546e-14
5.667130e-15
E:\\R-3.1.2\\scripts\\mPDBS\\summary_pdb_93\\4eaa45b62cb6a25f.final.pdb
-6.693013e-15 -4.603959e-15 4.821663e-15 -4.496003e-15 -1.770020e-15
5.726845e-15
E:\\R-3.1.2\\scripts\\mPDBS\\summary_pdb_93\\58c371611d85ac96.final.pdb
-8.831652e-15 -3.985211e-15 4.549304e-15 -5.247405e-15 -1.025047e-15
-1.415745e-15
E:\\R-3.1.2\\scripts\\mPDBS\\summary_pdb_93\\59a647f04822d30a.final.pdb
7.169930e-15 -6.599618e-15 -3.356412e-15 -1.295481e-15 -5.909549e-15
-2.584878e-15
E:\\R-3.1.2\\scripts\\mPDBS\\summary_pdb_93\\59b091fe385e05fc.final.pdb
2.533160e-15 -1.934182e-15 -7.543999e-17 -1.044311e-15 -1.523796e-15
2.560764e-16
E:\\R-3.1.2\\scripts\\mPDBS\\summary_pdb_93\\5c35ac30f930d2db.final.pdb
-6.693013e-15 -4.603959e-15 4.821663e-15 -4.496003e-15 -1.770020e-15
5.726845e-15
E:\\R-3.1.2\\scripts\\mPDBS\\summary_pdb_93\\5f60752431cf2dcb.final.pdb
-7.347008e-15 1.486866e-15 6.942320e-16 1.344325e-15 5.996770e-16
-7.882926e-16
E:\\R-3.1.2\\scripts\\mPDBS\\summary_pdb_93\\626456dd6ff2868d.final.pdb
2.701657e-15 -2.353703e-15 1.308840e-15 -2.350200e-15 -2.140639e-16
-6.533345e-16
E:\\R-3.1.2\\scripts\\mPDBS\\summary_pdb_93\\6583d53ff3c8f7ef.final.pdb
7.169930e-15 -6.599618e-15 -3.356412e-15 -1.295481e-15 -5.909549e-15
-2.584878e-15
E:\\R-3.1.2\\scripts\\mPDBS\\summary_pdb_93\\69760178986bef01.final.pdb
-2.314822e-15 -3.062014e-15 1.312927e-15 2.169522e-15 -8.610857e-16
9.081837e-15
E:\\R-3.1.2\\scripts\\mPDBS\\summary_pdb_93\\6a9d87d47926b1c8.final.pdb
1.561944e-15 -4.009497e-15 6.444414e-16 -1.913267e-15 -1.385870e-15
7.162297e-16
E:\\R-3.1.2\\scripts\\mPDBS\\summary_pdb_93\\6bb3547ea982ffc4.final.pdb
-6.102472e-15 -8.411162e-15 4.213347e-15 -3.174303e-17 8.534290e-15
-3.221616e-15
E:\\R-3.1.2\\scripts\\mPDBS\\summary_pdb_93\\716d9624611d1c42.final.pdb
5.826482e-15 -3.490923e-15 -1.161272e-15 -1.143610e-16 -5.169363e-15
1.355826e-15
E:\\R-3.1.2\\scripts\\mPDBS\\summary_pdb_93\\75f27e70ad0f1be4.final.pdb
7.169930e-15 -6.599618e-15 -3.356412e-15 -1.295481e-15 -5.909549e-15
-2.584878e-15
E:\\R-3.1.2\\scripts\\mPDBS\\summary_pdb_93\\769f910fffb22872.final.pdb
1.282845e-15 -3.505715e-15 -8.871485e-17 -1.085716e-15 -2.179187e-15
7.798279e-16
E:\\R-3.1.2\\scripts\\mPDBS\\summary_pdb_93\\791f8c80dd5c3135.final.pdb
2.533160e-15 -1.934182e-15 -7.543999e-17 -1.044311e-15 -1.523796e-15
2.560764e-16
E:\\R-3.1.2\\scripts\\mPDBS\\summary_pdb_93\\7fad98e66a3f6099.final.pdb

1.221918e-14 -5.744145e-15 1.681652e-15 4.393038e-15 -4.668946e-15
-3.955007e-15
E:\\R-3.1.2\\scripts\\mPDBS\\summary_pdb_93\\804b89c94a633196.final.pdb
6.474947e-15 -1.018379e-15 -1.073180e-15 -1.702251e-15 -1.328957e-15
3.546936e-16
E:\\R-3.1.2\\scripts\\mPDBS\\summary_pdb_93\\813cc65a2298feba.final.pdb
6.474947e-15 -1.018379e-15 -1.073180e-15 -1.702251e-15 -1.328957e-15
3.546936e-16
E:\\R-3.1.2\\scripts\\mPDBS\\summary_pdb_93\\83c0eefcfe12d3f9.final.pdb
1.489352e-15 -4.068885e-16 1.815135e-15 -5.147195e-15 -7.504264e-16
-1.597738e-15
E:\\R-3.1.2\\scripts\\mPDBS\\summary_pdb_93\\86d50469ef569760.final.pdb
2.701657e-15 -2.353703e-15 1.308840e-15 -2.350200e-15 -2.140639e-16
-6.533345e-16
E:\\R-3.1.2\\scripts\\mPDBS\\summary_pdb_93\\89d0666e1402203d.final.pdb
7.169930e-15 -6.599618e-15 -3.356412e-15 -1.295481e-15 -5.909549e-15
-2.584878e-15
E:\\R-3.1.2\\scripts\\mPDBS\\summary_pdb_93\\8b34852716dadc62.final.pdb
-5.394031e-15 -6.458471e-15 5.593362e-15 -5.598440e-15 -7.639881e-15
-1.796012e-16
E:\\R-3.1.2\\scripts\\mPDBS\\summary_pdb_93\\8b6ef9ac3fe74395.final.pdb
-4.467531e-15 -1.003336e-14 -9.395449e-17 7.412612e-15 2.148828e-15
-1.066303e-14
E:\\R-3.1.2\\scripts\\mPDBS\\summary_pdb_93\\98bbe0d5986c138f.final.pdb
2.533160e-15 -1.934182e-15 -7.543999e-17 -1.044311e-15 -1.523796e-15
2.560764e-16
E:\\R-3.1.2\\scripts\\mPDBS\\summary_pdb_93\\9c22f4909d67e2fa.final.pdb
3.949623e-15 -1.193581e-16 1.972995e-15 -5.775381e-15 1.454841e-15
4.465988e-15
E:\\R-3.1.2\\scripts\\mPDBS\\summary_pdb_93\\9dda8ed0d81fb3ef.final.pdb
-1.752733e-14 1.354957e-15 1.071523e-14 -1.205492e-15 4.947435e-15
-1.302050e-15
E:\\R-3.1.2\\scripts\\mPDBS\\summary_pdb_93\\9f752b3616e6033b.final.pdb
3.133804e-15 -5.306351e-15 7.944607e-16 -2.957813e-15 -2.344525e-15
2.366289e-15
E:\\R-3.1.2\\scripts\\mPDBS\\summary_pdb_93\\a02c70083816d6ba.final.pdb
-6.693013e-15 -4.603959e-15 4.821663e-15 -4.496003e-15 -1.770020e-15
5.726845e-15
E:\\R-3.1.2\\scripts\\mPDBS\\summary_pdb_93\\a0c997d891e4e082.final.pdb
3.940385e-15 -1.350936e-15 1.106822e-14 -4.233434e-15 -5.532925e-15
2.301702e-15
E:\\R-3.1.2\\scripts\\mPDBS\\summary_pdb_93\\ab8039a2d823c616.final.pdb
1.157455e-15 -2.997622e-15 -2.945768e-17 -1.774680e-15 -1.519208e-15
-4.608451e-16
E:\\R-3.1.2\\scripts\\mPDBS\\summary_pdb_93\\acf4d74b9c71d4dc.final.pdb
6.474947e-15 -1.018379e-15 -1.073180e-15 -1.702251e-15 -1.328957e-15
3.546936e-16
E:\\R-3.1.2\\scripts\\mPDBS\\summary_pdb_93\\ad8a823e4b4c668e.final.pdb
7.541192e-16 -3.123788e-15 6.564382e-16 -9.824416e-16 -2.005652e-15
1.993672e-15
E:\\R-3.1.2\\scripts\\mPDBS\\summary_pdb_93\\b16ababd03b7b857.final.pdb
3.949623e-15 -1.193581e-16 1.972995e-15 -5.775381e-15 1.454841e-15
4.465988e-15
E:\\R-3.1.2\\scripts\\mPDBS\\summary_pdb_93\\b226923e697d3c63.final.pdb

-7.070848e-15 -2.533860e-15 -1.182804e-15 7.279257e-15 -8.818897e-15
6.407061e-15
E:\\R-3.1.2\\scripts\\mPDBS\\summary_pdb_93\\b28e48f6fa27a165.final.pdb
2.533160e-15 -1.934182e-15 -7.543999e-17 -1.044311e-15 -1.523796e-15
2.560764e-16
E:\\R-3.1.2\\scripts\\mPDBS\\summary_pdb_93\\b42c4924e3cd1aec.final.pdb
8.313113e-15 1.519322e-15 -2.554320e-15 -3.751632e-15 1.593564e-15
3.329662e-15
E:\\R-3.1.2\\scripts\\mPDBS\\summary_pdb_93\\b4dd239fcabff1df.final.pdb
2.533160e-15 -1.934182e-15 -7.543999e-17 -1.044311e-15 -1.523796e-15
2.560764e-16
E:\\R-3.1.2\\scripts\\mPDBS\\summary_pdb_93\\b669942ef97459a2.final.pdb
-5.394031e-15 -6.458471e-15 5.593362e-15 -5.598440e-15 -7.639881e-15
-1.796012e-16
E:\\R-3.1.2\\scripts\\mPDBS\\summary_pdb_93\\b99f4d9d87c1017b.final.pdb
2.533160e-15 -1.934182e-15 -7.543999e-17 -1.044311e-15 -1.523796e-15
2.560764e-16
E:\\R-3.1.2\\scripts\\mPDBS\\summary_pdb_93\\b9d2cbf3975403e7.final.pdb
2.533160e-15 -1.934182e-15 -7.543999e-17 -1.044311e-15 -1.523796e-15
2.560764e-16
E:\\R-3.1.2\\scripts\\mPDBS\\summary_pdb_93\\bc88c8a505f3a288.final.pdb
1.762510e-15 -3.900708e-15 2.788773e-16 -2.425151e-15 -1.988268e-15
1.798246e-15
E:\\R-3.1.2\\scripts\\mPDBS\\summary_pdb_93\\bdac0e64f4ef427e.final.pdb
7.230374e-16 -8.149827e-15 -3.083941e-15 7.439959e-15 -9.711700e-16
4.030573e-15
E:\\R-3.1.2\\scripts\\mPDBS\\summary_pdb_93\\ca2a1f55fc9d0681.final.pdb
-5.394031e-15 -6.458471e-15 5.593362e-15 -5.598440e-15 -7.639881e-15
-1.796012e-16
E:\\R-3.1.2\\scripts\\mPDBS\\summary_pdb_93\\ce34248de3e81a3c.final.pdb
-1.784164e-17 -3.180752e-16 2.754978e-15 -1.263035e-15 -1.794185e-15
-3.848231e-15
E:\\R-3.1.2\\scripts\\mPDBS\\summary_pdb_93\\d02e697772494a61.final.pdb
1.384529e-14 -1.240541e-14 -7.238764e-15 -3.645163e-16 -1.274876e-14
1.294308e-15
E:\\R-3.1.2\\scripts\\mPDBS\\summary_pdb_93\\d04221245d015047.final.pdb
3.583032e-15 -3.116885e-15 8.645754e-16 -4.797219e-15 3.061546e-16
-3.836499e-16
E:\\R-3.1.2\\scripts\\mPDBS\\summary_pdb_93\\d678141e4018054a.final.pdb
-1.453958e-16 2.584733e-15 -4.152237e-15 3.128635e-15 -3.733957e-15
3.055837e-16
E:\\R-3.1.2\\scripts\\mPDBS\\summary_pdb_93\\d71b40af0f0efe39.final.pdb
8.866490e-15 2.368026e-15 -7.171401e-15 -2.647740e-15 -1.381785e-14
8.744177e-15
E:\\R-3.1.2\\scripts\\mPDBS\\summary_pdb_93\\dc0809eff10197e2.final.pdb
1.899218e-15 -7.346723e-15 1.342968e-15 -4.354096e-15 -5.706956e-15
-2.066464e-15
E:\\R-3.1.2\\scripts\\mPDBS\\summary_pdb_93\\e1163918d3707839.final.pdb
1.288411e-14 -3.745564e-15 -8.330403e-16 -1.681240e-15 4.451521e-15
-5.647924e-16
E:\\R-3.1.2\\scripts\\mPDBS\\summary_pdb_93\\e4b48b06824ef848.final.pdb
-9.454226e-15 -2.192096e-15 -1.893261e-15 4.833417e-16 1.989024e-16
3.393189e-15
E:\\R-3.1.2\\scripts\\mPDBS\\summary_pdb_93\\e529e19ad281fea2.final.pdb

6.057480e-15 -2.613912e-15 -2.754749e-15 1.060266e-15 1.229226e-15
4.011337e-15
E:\\R-3.1.2\\scripts\\mPDBS\\summary_pdb_93\\e5602082718ec3a1.final.pdb
6.474947e-15 -1.018379e-15 -1.073180e-15 -1.702251e-15 -1.328957e-15
3.546936e-16
E:\\R-3.1.2\\scripts\\mPDBS\\summary_pdb_93\\e5d589d996d0a960.final.pdb
-3.744270e-15 -3.070038e-15 9.175636e-15 2.815828e-15 4.190972e-15
-4.046432e-15
E:\\R-3.1.2\\scripts\\mPDBS\\summary_pdb_93\\e9df9e72307a2fbd.final.pdb
-6.693013e-15 -4.603959e-15 4.821663e-15 -4.496003e-15 -1.770020e-15
5.726845e-15
E:\\R-3.1.2\\scripts\\mPDBS\\summary_pdb_93\\ea00f8694090cae7.final.pdb
6.474947e-15 -1.018379e-15 -1.073180e-15 -1.702251e-15 -1.328957e-15
3.546936e-16
E:\\R-3.1.2\\scripts\\mPDBS\\summary_pdb_93\\eba517be32db050e.final.pdb
2.533160e-15 -1.934182e-15 -7.543999e-17 -1.044311e-15 -1.523796e-15
2.560764e-16
E:\\R-3.1.2\\scripts\\mPDBS\\summary_pdb_93\\ec04b9545899c29d.final.pdb
5.870883e-16 -2.537763e-15 4.539077e-16 -1.671916e-15 -2.095759e-15
9.053857e-16
E:\\R-3.1.2\\scripts\\mPDBS\\summary_pdb_93\\ee6862cbcad52078.final.pdb
1.351814e-15 -3.818092e-15 5.950306e-16 -1.835345e-15 -1.770126e-15
1.243134e-15
E:\\R-3.1.2\\scripts\\mPDBS\\summary_pdb_93\\ef6a42fc83b81e5d.final.pdb
1.246849e-15 -2.795780e-15 4.405927e-16 -1.836634e-15 -1.954974e-15
1.377440e-15
E:\\R-3.1.2\\scripts\\mPDBS\\summary_pdb_93\\ef8e88d4ad93a0ad.final.pdb
3.583032e-15 -3.116885e-15 8.645754e-16 -4.797219e-15 3.061546e-16
-3.836499e-16
E:\\R-3.1.2\\scripts\\mPDBS\\summary_pdb_93\\f14fcae8deec4fa3.final.pdb
3.133804e-15 -5.306351e-15 7.944607e-16 -2.957813e-15 -2.344525e-15
2.366289e-15
E:\\R-3.1.2\\scripts\\mPDBS\\summary_pdb_93\\f83584e22003fb92.final.pdb
1.207832e-15 -1.364326e-15 -4.416835e-16 3.708614e-15 4.134222e-15
-6.441691e-15
E:\\R-3.1.2\\scripts\\mPDBS\\summary_pdb_93\\f8e2bf87e52caa29.final.pdb
6.057480e-15 -2.613912e-15 -2.754749e-15 1.060266e-15 1.229226e-15
4.011337e-15

[,769]   [,770]   [,771]   [,772]   [,773]   [,774]
E:\\R-3.1.2\\scripts\\40.1ns_data\\NAT0.pdb
-2.288953e-15 -2.247030e-15 1.528971e-15 -5.346803e-15 3.119738e-15
3.769179e-16
E:\\R-3.1.2\\scripts\\40.1ns_data\\LJP6.pdb
-2.455315e-15 -2.184028e-15 1.121043e-15 -4.757293e-15 1.916610e-15
1.949063e-15
E:\\R-3.1.2\\scripts\\40.1ns_data\\3WY9.pdb
-2.396759e-15 -3.634253e-15 2.921620e-15 -3.280347e-15 2.548312e-15
1.029371e-15
E:\\R-3.1.2\\scripts\\40.1ns_data\\ZSP9.pdb
-1.982249e-15 -2.908153e-15 3.441023e-16 -3.819563e-15 5.324629e-16
8.399696e-16
E:\\R-3.1.2\\scripts\\mPDBS\\summary_pdb_93\\013d855ad26c877c.final.pdb
3.669386e-15 7.140844e-15 5.203129e-15 -2.899785e-15 -3.356977e-16

-1.066502e-15
E:\\R-3.1.2\\scripts\\mPDBS\\summary_pdb_93\\0a2a705342ca0d54.final.pdb
-5.836604e-16 -1.344782e-15 1.609559e-15 -3.531307e-15 2.567719e-15
4.820835e-16
E:\\R-3.1.2\\scripts\\mPDBS\\summary_pdb_93\\0a59fceadb4194d9.final.pdb
-1.317158e-15 -2.578827e-15 1.432212e-16 1.213103e-15 1.266555e-15
1.211776e-15
E:\\R-3.1.2\\scripts\\mPDBS\\summary_pdb_93\\0dba59f3306825e8.final.pdb
-3.642473e-15 -1.665262e-14 -3.866005e-15 -1.853747e-15 -7.988689e-16
7.601620e-16
E:\\R-3.1.2\\scripts\\mPDBS\\summary_pdb_93\\126f26a78b57339c.final.pdb
-2.891853e-15 -1.670684e-15 9.151794e-15 -3.997323e-15 1.054536e-14
3.275511e-15
E:\\R-3.1.2\\scripts\\mPDBS\\summary_pdb_93\\12f06f3e342df706.final.pdb
-8.103399e-16 -1.171743e-15 2.071429e-15 -3.613165e-15 2.498547e-15
9.309432e-16
E:\\R-3.1.2\\scripts\\mPDBS\\summary_pdb_93\\18618a2a251b867b.final.pdb
-1.524576e-15 -3.612359e-15 3.957020e-15 -9.318487e-15 5.152057e-15
-5.093640e-15
E:\\R-3.1.2\\scripts\\mPDBS\\summary_pdb_93\\1f305f598641eeb2.final.pdb
-1.218469e-15 -1.199738e-15 1.877953e-15 -3.252438e-15 2.344428e-15
1.662393e-15
E:\\R-3.1.2\\scripts\\mPDBS\\summary_pdb_93\\1f8a00b551be0a39.final.pdb
-1.524576e-15 -3.612359e-15 3.957020e-15 -9.318487e-15 5.152057e-15
-5.093640e-15
E:\\R-3.1.2\\scripts\\mPDBS\\summary_pdb_93\\219f45328d47579a.final.pdb
4.041403e-15 3.115634e-14 1.639420e-15 -3.253594e-15 3.238936e-15
9.387961e-15
E:\\R-3.1.2\\scripts\\mPDBS\\summary_pdb_93\\238dd3918e54336d.final.pdb
3.110453e-15 -9.479124e-15 -1.547176e-16 -8.847744e-16 1.428110e-14
3.891490e-15
E:\\R-3.1.2\\scripts\\mPDBS\\summary_pdb_93\\2b3027889e2fdc42.final.pdb
-4.039342e-16 -1.445186e-15 2.472521e-15 -2.382166e-15 3.071752e-15
4.996718e-16
E:\\R-3.1.2\\scripts\\mPDBS\\summary_pdb_93\\2fcb3b46e33793b0.final.pdb
-5.974375e-16 -3.892029e-15 -2.146605e-15 -4.165770e-15 -2.242181e-15
-2.182188e-15
E:\\R-3.1.2\\scripts\\mPDBS\\summary_pdb_93\\34eb328251842006.final.pdb
6.610745e-15 2.146798e-14 -5.809695e-15 1.838195e-15 3.109001e-15
5.333741e-15
E:\\R-3.1.2\\scripts\\mPDBS\\summary_pdb_93\\354b32871797b7ab.final.pdb
-8.338716e-16 -8.173850e-15 4.965627e-15 -1.010112e-14 -7.669156e-15
-5.749072e-15
E:\\R-3.1.2\\scripts\\mPDBS\\summary_pdb_93\\397368adbc32d5de.final.pdb
-2.891853e-15 -1.670684e-15 9.151794e-15 -3.997323e-15 1.054536e-14
3.275511e-15
E:\\R-3.1.2\\scripts\\mPDBS\\summary_pdb_93\\411ca13cd3df6edd.final.pdb
-8.085588e-16 -3.219824e-15 -1.345596e-15 -6.220225e-15 2.983674e-15
-2.628012e-15
E:\\R-3.1.2\\scripts\\mPDBS\\summary_pdb_93\\42357eee6642bf12.final.pdb
3.531200e-15 1.267629e-14 -1.245962e-15 -1.310818e-15 2.364297e-15
9.038662e-17
E:\\R-3.1.2\\scripts\\mPDBS\\summary_pdb_93\\44342d1f75a7fcfc.final.pdb
3.881659e-15 2.163375e-14 1.581475e-14 -7.490131e-15 8.261363e-15

-1.056633e-14
E:\\R-3.1.2\\scripts\\mPDBS\\summary_pdb_93\\448ef21dffe8cdfb.final.pdb
-1.251428e-15 -9.734578e-15 1.599513e-14 -8.194665e-15 1.152979e-14
1.011223e-14
E:\\R-3.1.2\\scripts\\mPDBS\\summary_pdb_93\\472a40b3e8f113c7.final.pdb
-3.642473e-15 -1.665262e-14 -3.866005e-15 -1.853747e-15 -7.988689e-16
7.601620e-16
E:\\R-3.1.2\\scripts\\mPDBS\\summary_pdb_93\\48a26225951ea153.final.pdb
5.078659e-15 3.068851e-16 9.679430e-15 -1.587116e-14 -3.890214e-16
-8.539539e-15
E:\\R-3.1.2\\scripts\\mPDBS\\summary_pdb_93\\4ab4a5c7c6682ce6.final.pdb
4.699297e-15 1.344223e-14 3.912929e-15 -5.755465e-15 6.460081e-15
-8.899212e-15
E:\\R-3.1.2\\scripts\\mPDBS\\summary_pdb_93\\4eaa45b62cb6a25f.final.pdb
-2.302478e-15 -4.418607e-15 1.741210e-15 -1.768426e-15 6.650335e-15
-3.167859e-16
E:\\R-3.1.2\\scripts\\mPDBS\\summary_pdb_93\\58c371611d85ac96.final.pdb
-2.891853e-15 -1.670684e-15 9.151794e-15 -3.997323e-15 1.054536e-14
3.275511e-15
E:\\R-3.1.2\\scripts\\mPDBS\\summary_pdb_93\\59a647f04822d30a.final.pdb
-6.871685e-16 -1.113680e-15 -2.710365e-15 1.512217e-15 1.965184e-15
5.995508e-15
E:\\R-3.1.2\\scripts\\mPDBS\\summary_pdb_93\\59b091fe385e05fc.final.pdb
-1.317158e-15 -2.578827e-15 1.432212e-16 1.213103e-15 1.266555e-15
1.211776e-15
E:\\R-3.1.2\\scripts\\mPDBS\\summary_pdb_93\\5c35ac30f930d2db.final.pdb
-2.302478e-15 -4.418607e-15 1.741210e-15 -1.768426e-15 6.650335e-15
-3.167859e-16
E:\\R-3.1.2\\scripts\\mPDBS\\summary_pdb_93\\5f60752431cf2dcb.final.pdb
-5.641789e-15 -6.228896e-15 -2.300941e-15 -5.137932e-15 9.091363e-15
-2.512917e-15
E:\\R-3.1.2\\scripts\\mPDBS\\summary_pdb_93\\626456dd6ff2868d.final.pdb
-3.642473e-15 -1.665262e-14 -3.866005e-15 -1.853747e-15 -7.988689e-16
7.601620e-16
E:\\R-3.1.2\\scripts\\mPDBS\\summary_pdb_93\\6583d53ff3c8f7ef.final.pdb
-6.871685e-16 -1.113680e-15 -2.710365e-15 1.512217e-15 1.965184e-15
5.995508e-15
E:\\R-3.1.2\\scripts\\mPDBS\\summary_pdb_93\\69760178986bef01.final.pdb
-8.683103e-15 -8.333689e-15 4.372421e-15 -1.165820e-14 8.883795e-15
-5.267702e-15
E:\\R-3.1.2\\scripts\\mPDBS\\summary_pdb_93\\6a9d87d47926b1c8.final.pdb
-3.053801e-16 -1.053122e-15 2.691683e-15 -2.507196e-15 2.831040e-15
1.288819e-15
E:\\R-3.1.2\\scripts\\mPDBS\\summary_pdb_93\\6bb3547ea982ffc4.final.pdb
1.445601e-15 -1.713459e-14 1.146031e-14 -1.071607e-14 1.117039e-14
5.081579e-15
E:\\R-3.1.2\\scripts\\mPDBS\\summary_pdb_93\\716d9624611d1c42.final.pdb
3.825139e-15 5.493498e-15 5.454574e-15 -6.330180e-16 5.129390e-16
-1.199297e-15
E:\\R-3.1.2\\scripts\\mPDBS\\summary_pdb_93\\75f27e70ad0f1be4.final.pdb
-6.871685e-16 -1.113680e-15 -2.710365e-15 1.512217e-15 1.965184e-15
5.995508e-15
E:\\R-3.1.2\\scripts\\mPDBS\\summary_pdb_93\\769f910fffb22872.final.pdb
-1.308766e-15 1.758436e-16 9.125807e-16 -2.824225e-15 2.827094e-15

6.149009e-16
E:\\R-3.1.2\\scripts\\mPDBS\\summary_pdb_93\\791f8c80dd5c3135.final.pdb
-1.317158e-15 -2.578827e-15 1.432212e-16 1.213103e-15 1.266555e-15
1.211776e-15
E:\\R-3.1.2\\scripts\\mPDBS\\summary_pdb_93\\7fad98e66a3f6099.final.pdb
5.774766e-15 1.098039e-14 5.109679e-15 -1.008917e-14 -9.715342e-16
-6.867185e-15
E:\\R-3.1.2\\scripts\\mPDBS\\summary_pdb_93\\804b89c94a633196.final.pdb
-5.974375e-16 -3.892029e-15 -2.146605e-15 -4.165770e-15 -2.242181e-15
-2.182188e-15
E:\\R-3.1.2\\scripts\\mPDBS\\summary_pdb_93\\813cc65a2298feba.final.pdb
-5.974375e-16 -3.892029e-15 -2.146605e-15 -4.165770e-15 -2.242181e-15
-2.182188e-15
E:\\R-3.1.2\\scripts\\mPDBS\\summary_pdb_93\\83c0eefcfe12d3f9.final.pdb
-8.085588e-16 -3.219824e-15 -1.345596e-15 -6.220225e-15 2.983674e-15
-2.628012e-15
E:\\R-3.1.2\\scripts\\mPDBS\\summary_pdb_93\\86d50469ef569760.final.pdb
-3.642473e-15 -1.665262e-14 -3.866005e-15 -1.853747e-15 -7.988689e-16
7.601620e-16
E:\\R-3.1.2\\scripts\\mPDBS\\summary_pdb_93\\89d0666e1402203d.final.pdb
-6.871685e-16 -1.113680e-15 -2.710365e-15 1.512217e-15 1.965184e-15
5.995508e-15
E:\\R-3.1.2\\scripts\\mPDBS\\summary_pdb_93\\8b34852716dadc62.final.pdb
-8.680544e-16 3.719937e-15 2.613476e-15 -2.660897e-15 6.996957e-15
2.779555e-15
E:\\R-3.1.2\\scripts\\mPDBS\\summary_pdb_93\\8b6ef9ac3fe74395.final.pdb
5.729574e-15 -6.841467e-15 2.221158e-15 4.368010e-15 -1.353468e-15
1.265874e-14
E:\\R-3.1.2\\scripts\\mPDBS\\summary_pdb_93\\98bbe0d5986c138f.final.pdb
-1.317158e-15 -2.578827e-15 1.432212e-16 1.213103e-15 1.266555e-15
1.211776e-15
E:\\R-3.1.2\\scripts\\mPDBS\\summary_pdb_93\\9c22f4909d67e2fa.final.pdb
-6.590663e-15 -3.312632e-15 3.342060e-15 -2.109093e-15 1.894701e-15
5.470111e-15
E:\\R-3.1.2\\scripts\\mPDBS\\summary_pdb_93\\9dda8ed0d81fb3ef.final.pdb
-1.117553e-16 -1.490560e-14 5.679191e-15 -5.253042e-15 -9.758227e-16
-2.593108e-15
E:\\R-3.1.2\\scripts\\mPDBS\\summary_pdb_93\\9f752b3616e6033b.final.pdb
-1.791522e-15 -1.034758e-15 2.354744e-15 -3.890316e-15 8.483677e-16
3.173155e-15
E:\\R-3.1.2\\scripts\\mPDBS\\summary_pdb_93\\a02c70083816d6ba.final.pdb
-2.302478e-15 -4.418607e-15 1.741210e-15 -1.768426e-15 6.650335e-15
-3.167859e-16
E:\\R-3.1.2\\scripts\\mPDBS\\summary_pdb_93\\a0c997d891e4e082.final.pdb
6.950007e-16 -1.134125e-14 -2.341675e-15 -3.239766e-15 8.447217e-15
3.807679e-15
E:\\R-3.1.2\\scripts\\mPDBS\\summary_pdb_93\\ab8039a2d823c616.final.pdb
-1.921477e-16 -1.654469e-17 8.280986e-16 -2.812536e-15 2.795271e-15
4.791783e-16
E:\\R-3.1.2\\scripts\\mPDBS\\summary_pdb_93\\acf4d74b9c71d4dc.final.pdb
-5.974375e-16 -3.892029e-15 -2.146605e-15 -4.165770e-15 -2.242181e-15
-2.182188e-15
E:\\R-3.1.2\\scripts\\mPDBS\\summary_pdb_93\\ad8a823e4b4c668e.final.pdb
-1.477689e-15 2.895213e-16 2.547356e-15 -3.125451e-15 2.606008e-15

5.218712e-16
E:\\R-3.1.2\\scripts\\mPDBS\\summary_pdb_93\\b16ababd03b7b857.final.pdb
-6.590663e-15 -3.312632e-15 3.342060e-15 -2.109093e-15 1.894701e-15
5.470111e-15
E:\\R-3.1.2\\scripts\\mPDBS\\summary_pdb_93\\b226923e697d3c63.final.pdb
2.052122e-15 -2.802867e-15 -2.628833e-15 3.069093e-16 8.616548e-15
2.921144e-15
E:\\R-3.1.2\\scripts\\mPDBS\\summary_pdb_93\\b28e48f6fa27a165.final.pdb
-1.317158e-15 -2.578827e-15 1.432212e-16 1.213103e-15 1.266555e-15
1.211776e-15
E:\\R-3.1.2\\scripts\\mPDBS\\summary_pdb_93\\b42c4924e3cd1aec.final.pdb
3.617385e-15 1.354256e-14 -2.395079e-15 -2.697794e-15 2.337800e-17
6.417174e-16
E:\\R-3.1.2\\scripts\\mPDBS\\summary_pdb_93\\b4dd239fcabff1df.final.pdb
-1.317158e-15 -2.578827e-15 1.432212e-16 1.213103e-15 1.266555e-15
1.211776e-15
E:\\R-3.1.2\\scripts\\mPDBS\\summary_pdb_93\\b669942ef97459a2.final.pdb
-8.680544e-16 3.719937e-15 2.613476e-15 -2.660897e-15 6.996957e-15
2.779555e-15
E:\\R-3.1.2\\scripts\\mPDBS\\summary_pdb_93\\b99f4d9d87c1017b.final.pdb
-1.317158e-15 -2.578827e-15 1.432212e-16 1.213103e-15 1.266555e-15
1.211776e-15
E:\\R-3.1.2\\scripts\\mPDBS\\summary_pdb_93\\b9d2cbf3975403e7.final.pdb
-1.317158e-15 -2.578827e-15 1.432212e-16 1.213103e-15 1.266555e-15
1.211776e-15
E:\\R-3.1.2\\scripts\\mPDBS\\summary_pdb_93\\bc88c8a505f3a288.final.pdb
-6.082792e-16 -1.614755e-15 2.025830e-15 -3.022116e-15 3.177787e-15
5.560503e-16
E:\\R-3.1.2\\scripts\\mPDBS\\summary_pdb_93\\bdac0e64f4ef427e.final.pdb
-6.764759e-16 8.947527e-15 7.719635e-15 3.393721e-15 2.595075e-16
9.460409e-15
E:\\R-3.1.2\\scripts\\mPDBS\\summary_pdb_93\\ca2a1f55fc9d0681.final.pdb
-8.680544e-16 3.719937e-15 2.613476e-15 -2.660897e-15 6.996957e-15
2.779555e-15
E:\\R-3.1.2\\scripts\\mPDBS\\summary_pdb_93\\ce34248de3e81a3c.final.pdb
3.334540e-15 1.195426e-14 1.039651e-15 3.210049e-15 1.899485e-15
-3.906306e-15
E:\\R-3.1.2\\scripts\\mPDBS\\summary_pdb_93\\d02e697772494a61.final.pdb
-6.903238e-15 1.028096e-15 1.739600e-15 -1.012927e-15 9.178412e-15
4.061493e-15
E:\\R-3.1.2\\scripts\\mPDBS\\summary_pdb_93\\d04221245d015047.final.pdb
-1.800399e-15 1.408964e-15 2.505142e-15 -4.140955e-15 1.020866e-15
-2.923400e-16
E:\\R-3.1.2\\scripts\\mPDBS\\summary_pdb_93\\d678141e4018054a.final.pdb
1.841756e-15 4.259117e-16 2.480947e-15 -9.510404e-15 9.931124e-15
3.390205e-16
E:\\R-3.1.2\\scripts\\mPDBS\\summary_pdb_93\\d71b40af0f0efe39.final.pdb
-7.559964e-15 -1.022652e-15 3.040028e-15 -5.760186e-15 -4.429258e-15
-3.749751e-15
E:\\R-3.1.2\\scripts\\mPDBS\\summary_pdb_93\\dc0809eff10197e2.final.pdb
7.088673e-16 -2.166384e-15 -4.808788e-15 6.323733e-15 -5.653453e-15
9.543265e-15
E:\\R-3.1.2\\scripts\\mPDBS\\summary_pdb_93\\e1163918d3707839.final.pdb
-9.533120e-15 -9.832834e-15 -1.808743e-15 -6.453904e-15 -2.816364e-15

3.887819e-15
E:\\R-3.1.2\\scripts\\mPDBS\\summary_pdb_93\\e4b48b06824ef848.final.pdb
-1.524576e-15 -3.612359e-15 3.957020e-15 -9.318487e-15 5.152057e-15
-5.093640e-15
E:\\R-3.1.2\\scripts\\mPDBS\\summary_pdb_93\\e529e19ad281fea2.final.pdb
3.669386e-15 7.140844e-15 5.203129e-15 -2.899785e-15 -3.356977e-16
-1.066502e-15
E:\\R-3.1.2\\scripts\\mPDBS\\summary_pdb_93\\e5602082718ec3a1.final.pdb
-5.974375e-16 -3.892029e-15 -2.146605e-15 -4.165770e-15 -2.242181e-15
-2.182188e-15
E:\\R-3.1.2\\scripts\\mPDBS\\summary_pdb_93\\e5d589d996d0a960.final.pdb
-1.552267e-15 2.619111e-15 7.125204e-15 -5.003759e-15 -1.587040e-16
-4.777806e-16
E:\\R-3.1.2\\scripts\\mPDBS\\summary_pdb_93\\e9df9e72307a2fbd.final.pdb
-2.302478e-15 -4.418607e-15 1.741210e-15 -1.768426e-15 6.650335e-15
-3.167859e-16
E:\\R-3.1.2\\scripts\\mPDBS\\summary_pdb_93\\ea00f8694090cae7.final.pdb
-5.974375e-16 -3.892029e-15 -2.146605e-15 -4.165770e-15 -2.242181e-15
-2.182188e-15
E:\\R-3.1.2\\scripts\\mPDBS\\summary_pdb_93\\eba517be32db050e.final.pdb
-1.317158e-15 -2.578827e-15 1.432212e-16 1.213103e-15 1.266555e-15
1.211776e-15
E:\\R-3.1.2\\scripts\\mPDBS\\summary_pdb_93\\ec04b9545899c29d.final.pdb
-1.352001e-15 8.257406e-16 1.091281e-15 -3.189534e-15 2.824740e-15
1.004275e-15
E:\\R-3.1.2\\scripts\\mPDBS\\summary_pdb_93\\ee6862cbcad52078.final.pdb
-8.253636e-16 -8.840026e-16 1.987232e-15 -2.416373e-15 3.362101e-15
5.825048e-16
E:\\R-3.1.2\\scripts\\mPDBS\\summary_pdb_93\\ef6a42fc83b81e5d.final.pdb
-1.236070e-15 -5.705212e-16 1.495014e-15 -3.156045e-15 2.778739e-15
4.553064e-16
E:\\R-3.1.2\\scripts\\mPDBS\\summary_pdb_93\\ef8e88d4ad93a0ad.final.pdb
-1.800399e-15 1.408964e-15 2.505142e-15 -4.140955e-15 1.020866e-15
-2.923400e-16
E:\\R-3.1.2\\scripts\\mPDBS\\summary_pdb_93\\f14fcae8deec4fa3.final.pdb
-1.791522e-15 -1.034758e-15 2.354744e-15 -3.890316e-15 8.483677e-16
3.173155e-15
E:\\R-3.1.2\\scripts\\mPDBS\\summary_pdb_93\\f83584e22003fb92.final.pdb
5.078659e-15 3.068851e-16 9.679430e-15 -1.587116e-14 -3.890214e-16
-8.539539e-15
E:\\R-3.1.2\\scripts\\mPDBS\\summary_pdb_93\\f8e2bf87e52caa29.final.pdb
3.669386e-15 7.140844e-15 5.203129e-15 -2.899785e-15 -3.356977e-16
-1.066502e-15

[,775]   [,776]   [,777]   [,778]   [,779]   [,780]
E:\\R-3.1.2\\scripts\\40.1ns_data\\NAT0.pdb
-5.425503e-15 -3.314613e-15 6.548647e-16 3.303885e-15 -1.159532e-15
1.363151e-15
E:\\R-3.1.2\\scripts\\40.1ns_data\\LJP6.pdb
-4.998829e-15 -4.275938e-15 7.183069e-16 2.371178e-15 -3.544020e-15
2.007843e-15
E:\\R-3.1.2\\scripts\\40.1ns_data\\3WY9.pdb
-5.750576e-15 -3.876246e-15 -1.194506e-15 9.844545e-16 -3.034293e-15
7.421947e-16

E:\\R-3.1.2\\scripts\\40.1ns_data\\ZSP9.pdb
-4.306104e-15 -4.202095e-15 -1.679366e-16 5.164702e-15 -3.953557e-15
1.653742e-15
E:\\R-3.1.2\\scripts\\mPDBS\\summary_pdb_93\\013d855ad26c877c.final.pdb
-7.079616e-15 -3.422422e-15 -3.398805e-15 5.417983e-16 7.766609e-16
-3.171893e-15
E:\\R-3.1.2\\scripts\\mPDBS\\summary_pdb_93\\0a2a705342ca0d54.final.pdb
-4.926265e-15 -3.623622e-15 -4.155644e-16 1.287464e-16 -2.850724e-15
1.998457e-15
E:\\R-3.1.2\\scripts\\mPDBS\\summary_pdb_93\\0a59fceadb4194d9.final.pdb
-4.477189e-15 -7.301586e-15 -1.412839e-15 2.896383e-15 -6.747475e-17
6.761747e-15
E:\\R-3.1.2\\scripts\\mPDBS\\summary_pdb_93\\0dba59f3306825e8.final.pdb
-3.070237e-15 -5.906112e-15 2.202617e-15 1.599980e-15 -4.956600e-15
1.888296e-15
E:\\R-3.1.2\\scripts\\mPDBS\\summary_pdb_93\\126f26a78b57339c.final.pdb
-9.147840e-15 -1.513792e-15 1.060518e-15 -6.620998e-16 -4.550241e-15
-5.045448e-15
E:\\R-3.1.2\\scripts\\mPDBS\\summary_pdb_93\\12f06f3e342df706.final.pdb
-4.860345e-15 -3.901828e-15 -4.689071e-16 6.970852e-16 -2.412489e-15
2.115442e-15
E:\\R-3.1.2\\scripts\\mPDBS\\summary_pdb_93\\18618a2a251b867b.final.pdb
-6.885432e-15 -3.135715e-15 -3.302731e-15 -2.138601e-15 -4.885770e-15
-1.014078e-14
E:\\R-3.1.2\\scripts\\mPDBS\\summary_pdb_93\\1f305f598641eeb2.final.pdb
-5.063652e-15 -3.906578e-15 -4.537807e-16 1.064140e-15 -2.611044e-15
1.503377e-15
E:\\R-3.1.2\\scripts\\mPDBS\\summary_pdb_93\\1f8a00b551be0a39.final.pdb
-6.885432e-15 -3.135715e-15 -3.302731e-15 -2.138601e-15 -4.885770e-15
-1.014078e-14
E:\\R-3.1.2\\scripts\\mPDBS\\summary_pdb_93\\219f45328d47579a.final.pdb
7.892473e-15 7.830841e-15 -7.604501e-15 -7.794955e-16 -5.601362e-16
4.464973e-15
E:\\R-3.1.2\\scripts\\mPDBS\\summary_pdb_93\\238dd3918e54336d.final.pdb
-4.721135e-15 3.216567e-16 -1.432382e-16 -5.478861e-15 -4.801427e-15
-6.691033e-15
E:\\R-3.1.2\\scripts\\mPDBS\\summary_pdb_93\\2b3027889e2fdc42.final.pdb
-5.005630e-15 -3.667123e-15 -8.458012e-16 4.599463e-18 -2.158033e-15
1.804721e-15
E:\\R-3.1.2\\scripts\\mPDBS\\summary_pdb_93\\2fcb3b46e33793b0.final.pdb
-1.751197e-15 -5.897173e-15 -3.591895e-15 3.529035e-16 -3.537386e-15
4.133218e-15
E:\\R-3.1.2\\scripts\\mPDBS\\summary_pdb_93\\34eb328251842006.final.pdb
-6.202778e-15 -1.826065e-15 3.489741e-15 6.402867e-15 -4.306476e-15
6.997294e-15
E:\\R-3.1.2\\scripts\\mPDBS\\summary_pdb_93\\354b32871797b7ab.final.pdb
6.443477e-15 -1.015253e-14 -1.480067e-15 8.190415e-15 -8.160589e-15
6.378546e-15
E:\\R-3.1.2\\scripts\\mPDBS\\summary_pdb_93\\397368adbc32d5de.final.pdb
-9.147840e-15 -1.513792e-15 1.060518e-15 -6.620998e-16 -4.550241e-15
-5.045448e-15
E:\\R-3.1.2\\scripts\\mPDBS\\summary_pdb_93\\411ca13cd3df6edd.final.pdb
-4.134274e-15 -2.142798e-15 -6.331241e-15 3.147395e-16 -1.022171e-14
-1.206207e-15

E:\\R-3.1.2\\scripts\\mPDBS\\summary_pdb_93\\42357eee6642bf12.final.pdb
-4.938090e-15 -4.243992e-15 -5.947024e-15 3.392828e-15 1.578707e-16
8.388851e-15
E:\\R-3.1.2\\scripts\\mPDBS\\summary_pdb_93\\44342d1f75a7fcfc.final.pdb
-1.585092e-14 -3.384741e-15 2.706728e-15 4.595146e-15 5.501776e-15
-3.923759e-15
E:\\R-3.1.2\\scripts\\mPDBS\\summary_pdb_93\\448ef21dffe8cdfb.final.pdb
-9.048745e-15 6.978116e-16 -4.774977e-15 -9.999926e-15 -2.387265e-15
1.659051e-16
E:\\R-3.1.2\\scripts\\mPDBS\\summary_pdb_93\\472a40b3e8f113c7.final.pdb
-3.070237e-15 -5.906112e-15 2.202617e-15 1.599980e-15 -4.956600e-15
1.888296e-15
E:\\R-3.1.2\\scripts\\mPDBS\\summary_pdb_93\\48a26225951ea153.final.pdb
-1.579821e-14 2.408312e-15 1.110818e-14 -6.736536e-15 -6.995743e-15
-1.056733e-14
E:\\R-3.1.2\\scripts\\mPDBS\\summary_pdb_93\\4ab4a5c7c6682ce6.final.pdb
8.311565e-16 -3.571608e-15 -1.026462e-14 -7.557749e-16 -7.651927e-15
1.557868e-14
E:\\R-3.1.2\\scripts\\mPDBS\\summary_pdb_93\\4eaa45b62cb6a25f.final.pdb
-8.969541e-15 -3.101314e-15 -1.537529e-15 -1.550057e-15 -2.535857e-15
-2.184050e-15
E:\\R-3.1.2\\scripts\\mPDBS\\summary_pdb_93\\58c371611d85ac96.final.pdb
-9.147840e-15 -1.513792e-15 1.060518e-15 -6.620998e-16 -4.550241e-15
-5.045448e-15
E:\\R-3.1.2\\scripts\\mPDBS\\summary_pdb_93\\59a647f04822d30a.final.pdb
-4.950132e-15 -3.118028e-15 2.052304e-15 -2.581405e-15 -1.649188e-15
4.565110e-15
E:\\R-3.1.2\\scripts\\mPDBS\\summary_pdb_93\\59b091fe385e05fc.final.pdb
-4.477189e-15 -7.301586e-15 -1.412839e-15 2.896383e-15 -6.747475e-17
6.761747e-15
E:\\R-3.1.2\\scripts\\mPDBS\\summary_pdb_93\\5c35ac30f930d2db.final.pdb
-8.969541e-15 -3.101314e-15 -1.537529e-15 -1.550057e-15 -2.535857e-15
-2.184050e-15
E:\\R-3.1.2\\scripts\\mPDBS\\summary_pdb_93\\5f60752431cf2dcb.final.pdb
-9.126898e-15 -4.822966e-15 3.611813e-15 -3.087431e-15 -4.248261e-15
-1.946359e-15
E:\\R-3.1.2\\scripts\\mPDBS\\summary_pdb_93\\626456dd6ff2868d.final.pdb
-3.070237e-15 -5.906112e-15 2.202617e-15 1.599980e-15 -4.956600e-15
1.888296e-15
E:\\R-3.1.2\\scripts\\mPDBS\\summary_pdb_93\\6583d53ff3c8f7ef.final.pdb
-4.950132e-15 -3.118028e-15 2.052304e-15 -2.581405e-15 -1.649188e-15
4.565110e-15
E:\\R-3.1.2\\scripts\\mPDBS\\summary_pdb_93\\69760178986bef01.final.pdb
3.856116e-15 1.969413e-15 -4.009003e-15 -1.378595e-15 9.029486e-16
-2.494905e-15
E:\\R-3.1.2\\scripts\\mPDBS\\summary_pdb_93\\6a9d87d47926b1c8.final.pdb
-5.119812e-15 -3.492678e-15 -2.266467e-16 5.973094e-16 -2.541431e-15
1.520480e-15
E:\\R-3.1.2\\scripts\\mPDBS\\summary_pdb_93\\6bb3547ea982ffc4.final.pdb
-7.976796e-15 5.347738e-15 3.047978e-15 -1.543309e-15 -1.176570e-14
-9.557107e-15
E:\\R-3.1.2\\scripts\\mPDBS\\summary_pdb_93\\716d9624611d1c42.final.pdb
-1.371599e-15 -2.516434e-15 -4.588875e-15 8.758996e-15 -3.793980e-15
-1.124913e-15

E:\\R-3.1.2\\scripts\\mPDBS\\summary_pdb_93\\75f27e70ad0f1be4.final.pdb
-4.950132e-15 -3.118028e-15 2.052304e-15 -2.581405e-15 -1.649188e-15
4.565110e-15
E:\\R-3.1.2\\scripts\\mPDBS\\summary_pdb_93\\769f910fffb22872.final.pdb
-4.574563e-15 -3.649627e-15 -1.594121e-16 -9.509683e-17 -1.842306e-15
1.707434e-15
E:\\R-3.1.2\\scripts\\mPDBS\\summary_pdb_93\\791f8c80dd5c3135.final.pdb
-4.477189e-15 -7.301586e-15 -1.412839e-15 2.896383e-15 -6.747475e-17
6.761747e-15
E:\\R-3.1.2\\scripts\\mPDBS\\summary_pdb_93\\7fad98e66a3f6099.final.pdb
-3.588743e-15 -3.079871e-16 1.041972e-15 3.390054e-15 -5.764318e-15
9.415064e-15
E:\\R-3.1.2\\scripts\\mPDBS\\summary_pdb_93\\804b89c94a633196.final.pdb
-1.751197e-15 -5.897173e-15 -3.591895e-15 3.529035e-16 -3.537386e-15
4.133218e-15
E:\\R-3.1.2\\scripts\\mPDBS\\summary_pdb_93\\813cc65a2298feba.final.pdb
-1.751197e-15 -5.897173e-15 -3.591895e-15 3.529035e-16 -3.537386e-15
4.133218e-15
E:\\R-3.1.2\\scripts\\mPDBS\\summary_pdb_93\\83c0eefcfe12d3f9.final.pdb
-4.134274e-15 -2.142798e-15 -6.331241e-15 3.147395e-16 -1.022171e-14
-1.206207e-15
E:\\R-3.1.2\\scripts\\mPDBS\\summary_pdb_93\\86d50469ef569760.final.pdb
-3.070237e-15 -5.906112e-15 2.202617e-15 1.599980e-15 -4.956600e-15
1.888296e-15
E:\\R-3.1.2\\scripts\\mPDBS\\summary_pdb_93\\89d0666e1402203d.final.pdb
-4.950132e-15 -3.118028e-15 2.052304e-15 -2.581405e-15 -1.649188e-15
4.565110e-15
E:\\R-3.1.2\\scripts\\mPDBS\\summary_pdb_93\\8b34852716dadc62.final.pdb
-5.250135e-15 -1.720827e-15 6.286868e-16 2.909886e-15 -4.505362e-15
6.117741e-15
E:\\R-3.1.2\\scripts\\mPDBS\\summary_pdb_93\\8b6ef9ac3fe74395.final.pdb
-8.839760e-15 -1.039029e-14 1.224445e-15 1.587365e-16 9.195270e-16
4.067304e-15
E:\\R-3.1.2\\scripts\\mPDBS\\summary_pdb_93\\98bbe0d5986c138f.final.pdb
-4.477189e-15 -7.301586e-15 -1.412839e-15 2.896383e-15 -6.747475e-17
6.761747e-15
E:\\R-3.1.2\\scripts\\mPDBS\\summary_pdb_93\\9c22f4909d67e2fa.final.pdb
-7.407697e-15 -2.308464e-15 9.858066e-15 1.818745e-15 5.010247e-15
-4.189118e-15
E:\\R-3.1.2\\scripts\\mPDBS\\summary_pdb_93\\9dda8ed0d81fb3ef.final.pdb
-1.288237e-14 -4.440971e-15 8.147038e-15 -6.583412e-15 -9.614783e-16
-9.728252e-15
E:\\R-3.1.2\\scripts\\mPDBS\\summary_pdb_93\\9f752b3616e6033b.final.pdb
-3.447048e-15 -3.638432e-15 4.059294e-16 -1.728669e-15 -7.080350e-16
4.464155e-15
E:\\R-3.1.2\\scripts\\mPDBS\\summary_pdb_93\\a02c70083816d6ba.final.pdb
-8.969541e-15 -3.101314e-15 -1.537529e-15 -1.550057e-15 -2.535857e-15
-2.184050e-15
E:\\R-3.1.2\\scripts\\mPDBS\\summary_pdb_93\\a0c997d891e4e082.final.pdb
-1.695165e-16 -2.821113e-15 -1.896878e-15 -2.543951e-15 -4.366995e-15
6.730275e-15
E:\\R-3.1.2\\scripts\\mPDBS\\summary_pdb_93\\ab8039a2d823c616.final.pdb
-5.015900e-15 -3.840263e-15 -4.911012e-16 1.104172e-15 -2.547162e-15
2.043052e-15

E:\\R-3.1.2\\scripts\\mPDBS\\summary_pdb_93\\acf4d74b9c71d4dc.final.pdb
-1.751197e-15 -5.897173e-15 -3.591895e-15 3.529035e-16 -3.537386e-15
4.133218e-15
E:\\R-3.1.2\\scripts\\mPDBS\\summary_pdb_93\\ad8a823e4b4c668e.final.pdb
-4.515194e-15 -3.793049e-15 -9.637222e-16 4.193882e-16 -1.829135e-15
2.041073e-15
E:\\R-3.1.2\\scripts\\mPDBS\\summary_pdb_93\\b16ababd03b7b857.final.pdb
-7.407697e-15 -2.308464e-15 9.858066e-15 1.818745e-15 5.010247e-15
-4.189118e-15
E:\\R-3.1.2\\scripts\\mPDBS\\summary_pdb_93\\b226923e697d3c63.final.pdb
6.476040e-16 -6.847466e-15 -9.314038e-15 2.355618e-15 -2.361183e-15
8.354008e-16
E:\\R-3.1.2\\scripts\\mPDBS\\summary_pdb_93\\b28e48f6fa27a165.final.pdb
-4.477189e-15 -7.301586e-15 -1.412839e-15 2.896383e-15 -6.747475e-17
6.761747e-15
E:\\R-3.1.2\\scripts\\mPDBS\\summary_pdb_93\\b42c4924e3cd1aec.final.pdb
-3.414251e-15 -4.590808e-15 -2.473373e-15 5.037501e-15 -3.116439e-15
4.906851e-15
E:\\R-3.1.2\\scripts\\mPDBS\\summary_pdb_93\\b4dd239fcabff1df.final.pdb
-4.477189e-15 -7.301586e-15 -1.412839e-15 2.896383e-15 -6.747475e-17
6.761747e-15
E:\\R-3.1.2\\scripts\\mPDBS\\summary_pdb_93\\b669942ef97459a2.final.pdb
-5.250135e-15 -1.720827e-15 6.286868e-16 2.909886e-15 -4.505362e-15
6.117741e-15
E:\\R-3.1.2\\scripts\\mPDBS\\summary_pdb_93\\b99f4d9d87c1017b.final.pdb
-4.477189e-15 -7.301586e-15 -1.412839e-15 2.896383e-15 -6.747475e-17
6.761747e-15
E:\\R-3.1.2\\scripts\\mPDBS\\summary_pdb_93\\b9d2cbf3975403e7.final.pdb
-4.477189e-15 -7.301586e-15 -1.412839e-15 2.896383e-15 -6.747475e-17
6.761747e-15
E:\\R-3.1.2\\scripts\\mPDBS\\summary_pdb_93\\bc88c8a505f3a288.final.pdb
-4.985247e-15 -4.135715e-15 -4.062656e-16 1.227580e-15 -3.249608e-15
1.771002e-15
E:\\R-3.1.2\\scripts\\mPDBS\\summary_pdb_93\\bdac0e64f4ef427e.final.pdb
-7.218612e-15 -6.260381e-15 1.011827e-14 3.407604e-16 1.442611e-14
6.727551e-15
E:\\R-3.1.2\\scripts\\mPDBS\\summary_pdb_93\\ca2a1f55fc9d0681.final.pdb
-5.250135e-15 -1.720827e-15 6.286868e-16 2.909886e-15 -4.505362e-15
6.117741e-15
E:\\R-3.1.2\\scripts\\mPDBS\\summary_pdb_93\\ce34248de3e81a3c.final.pdb
-1.191953e-14 -1.161305e-14 -2.582998e-15 6.661210e-15 -3.811765e-15
4.305223e-15
E:\\R-3.1.2\\scripts\\mPDBS\\summary_pdb_93\\d02e697772494a61.final.pdb
1.176925e-15 -2.238795e-15 -7.067387e-15 4.426410e-15 -4.321342e-15
1.004905e-14
E:\\R-3.1.2\\scripts\\mPDBS\\summary_pdb_93\\d04221245d015047.final.pdb
-6.679934e-15 -2.980708e-15 2.602995e-15 -1.045150e-15 -2.531658e-15
2.152340e-15
E:\\R-3.1.2\\scripts\\mPDBS\\summary_pdb_93\\d678141e4018054a.final.pdb
-7.484027e-15 3.388251e-15 7.842313e-16 3.351914e-15 -1.171639e-14
-1.320281e-14
E:\\R-3.1.2\\scripts\\mPDBS\\summary_pdb_93\\d71b40af0f0efe39.final.pdb
1.271306e-15 -3.122989e-15 9.103495e-16 -7.807059e-16 -5.138454e-15
5.963754e-17

E:\\R-3.1.2\\scripts\\mPDBS\\summary_pdb_93\\dc0809eff10197e2.final.pdb
-3.421751e-15 -1.204223e-14 2.065530e-15 -6.287579e-16 5.193955e-15
1.114621e-14
E:\\R-3.1.2\\scripts\\mPDBS\\summary_pdb_93\\e1163918d3707839.final.pdb
-5.197980e-15 -1.045467e-15 -1.595169e-15 1.802749e-16 -7.790651e-15
3.807904e-15
E:\\R-3.1.2\\scripts\\mPDBS\\summary_pdb_93\\e4b48b06824ef848.final.pdb
-6.885432e-15 -3.135715e-15 -3.302731e-15 -2.138601e-15 -4.885770e-15
-1.014078e-14
E:\\R-3.1.2\\scripts\\mPDBS\\summary_pdb_93\\e529e19ad281fea2.final.pdb
-7.079616e-15 -3.422422e-15 -3.398805e-15 5.417983e-16 7.766609e-16
-3.171893e-15
E:\\R-3.1.2\\scripts\\mPDBS\\summary_pdb_93\\e5602082718ec3a1.final.pdb
-1.751197e-15 -5.897173e-15 -3.591895e-15 3.529035e-16 -3.537386e-15
4.133218e-15
E:\\R-3.1.2\\scripts\\mPDBS\\summary_pdb_93\\e5d589d996d0a960.final.pdb
-6.516662e-15 -8.247967e-15 6.742360e-15 3.112332e-15 -5.781099e-15
2.230101e-15
E:\\R-3.1.2\\scripts\\mPDBS\\summary_pdb_93\\e9df9e72307a2fbd.final.pdb
-8.969541e-15 -3.101314e-15 -1.537529e-15 -1.550057e-15 -2.535857e-15
-2.184050e-15
E:\\R-3.1.2\\scripts\\mPDBS\\summary_pdb_93\\ea00f8694090cae7.final.pdb
-1.751197e-15 -5.897173e-15 -3.591895e-15 3.529035e-16 -3.537386e-15
4.133218e-15
E:\\R-3.1.2\\scripts\\mPDBS\\summary_pdb_93\\eba517be32db050e.final.pdb
-4.477189e-15 -7.301586e-15 -1.412839e-15 2.896383e-15 -6.747475e-17
6.761747e-15
E:\\R-3.1.2\\scripts\\mPDBS\\summary_pdb_93\\ec04b9545899c29d.final.pdb
-5.042723e-15 -4.228684e-15 -3.138592e-16 6.607933e-16 -2.839208e-15
2.022006e-15
E:\\R-3.1.2\\scripts\\mPDBS\\summary_pdb_93\\ee6862cbcad52078.final.pdb
-5.006063e-15 -3.893288e-15 -2.484058e-16 5.076693e-16 -2.468766e-15
1.793499e-15
E:\\R-3.1.2\\scripts\\mPDBS\\summary_pdb_93\\ef6a42fc83b81e5d.final.pdb
-4.764949e-15 -4.019340e-15 -5.057063e-16 2.778687e-16 -2.748374e-15
1.939887e-15
E:\\R-3.1.2\\scripts\\mPDBS\\summary_pdb_93\\ef8e88d4ad93a0ad.final.pdb
-6.679934e-15 -2.980708e-15 2.602995e-15 -1.045150e-15 -2.531658e-15
2.152340e-15
E:\\R-3.1.2\\scripts\\mPDBS\\summary_pdb_93\\f14fcae8deec4fa3.final.pdb
-3.447048e-15 -3.638432e-15 4.059294e-16 -1.728669e-15 -7.080350e-16
4.464155e-15
E:\\R-3.1.2\\scripts\\mPDBS\\summary_pdb_93\\f83584e22003fb92.final.pdb
-1.579821e-14 2.408312e-15 1.110818e-14 -6.736536e-15 -6.995743e-15
-1.056733e-14
E:\\R-3.1.2\\scripts\\mPDBS\\summary_pdb_93\\f8e2bf87e52caa29.final.pdb
-7.079616e-15 -3.422422e-15 -3.398805e-15 5.417983e-16 7.766609e-16
-3.171893e-15

[,781]   [,782]   [,783]   [,784]   [,785]   [,786]
E:\\R-3.1.2\\scripts\\40.1ns_data\\NAT0.pdb
-5.703916e-15 -9.660611e-16 3.608321e-15 -3.755028e-15 -8.343957e-16
-2.978764e-15
E:\\R-3.1.2\\scripts\\40.1ns_data\\LJP6.pdb

-4.620258e-15 3.235559e-16 1.052770e-15 -4.906675e-15 -1.731131e-15
-3.055892e-15
E:\\R-3.1.2\\scripts\\40.1ns_data\\3WY9.pdb
-6.121559e-15 -3.008526e-15 2.724080e-15 -3.007129e-15 -2.690170e-16
-2.907416e-15
E:\\R-3.1.2\\scripts\\40.1ns_data\\ZSP9.pdb
-5.296235e-15 -8.320598e-17 1.844665e-15 -3.463627e-15 -2.836134e-16
-2.443625e-15
E:\\R-3.1.2\\scripts\\mPDBS\\summary_pdb_93\\013d855ad26c877c.final.pdb
-2.847869e-15 -4.249285e-15 3.608123e-15 -2.256161e-15 -3.705893e-15
-2.805450e-15
E:\\R-3.1.2\\scripts\\mPDBS\\summary_pdb_93\\0a2a705342ca0d54.final.pdb
-6.173335e-15 -2.135706e-15 5.759818e-16 -4.828480e-15 -1.131884e-15
-2.804789e-15
E:\\R-3.1.2\\scripts\\mPDBS\\summary_pdb_93\\0a59fceadb4194d9.final.pdb
-8.388577e-15 2.585183e-15 5.494194e-15 -6.050429e-15 -1.954618e-15
-2.984374e-15
E:\\R-3.1.2\\scripts\\mPDBS\\summary_pdb_93\\0dba59f3306825e8.final.pdb
-6.486451e-15 -3.704582e-15 2.210804e-15 -4.126193e-15 -2.701489e-15
-2.004442e-15
E:\\R-3.1.2\\scripts\\mPDBS\\summary_pdb_93\\126f26a78b57339c.final.pdb
-5.154184e-15 -7.090762e-15 -1.801178e-15 -3.726990e-15 2.471023e-15
-5.160338e-15
E:\\R-3.1.2\\scripts\\mPDBS\\summary_pdb_93\\12f06f3e342df706.final.pdb
-6.756202e-15 -1.790496e-15 4.515154e-16 -4.999784e-15 -1.106730e-15
-3.060661e-15
E:\\R-3.1.2\\scripts\\mPDBS\\summary_pdb_93\\18618a2a251b867b.final.pdb
-2.897455e-15 -1.120828e-14 -8.068979e-15 -1.784543e-15 -4.076046e-15
-3.141984e-15
E:\\R-3.1.2\\scripts\\mPDBS\\summary_pdb_93\\1f305f598641eeb2.final.pdb
-6.201991e-15 -2.506216e-15 1.068152e-15 -4.941411e-15 -1.310201e-15
-3.444624e-15
E:\\R-3.1.2\\scripts\\mPDBS\\summary_pdb_93\\1f8a00b551be0a39.final.pdb
-2.897455e-15 -1.120828e-14 -8.068979e-15 -1.784543e-15 -4.076046e-15
-3.141984e-15
E:\\R-3.1.2\\scripts\\mPDBS\\summary_pdb_93\\219f45328d47579a.final.pdb
4.262327e-15 -1.609172e-14 -1.617738e-15 -5.640167e-15 2.902607e-15
-6.053534e-15
E:\\R-3.1.2\\scripts\\mPDBS\\summary_pdb_93\\238dd3918e54336d.final.pdb
3.317553e-15 -8.932947e-15 -4.983535e-15 -1.907909e-15 1.043851e-15
-2.053261e-15
E:\\R-3.1.2\\scripts\\mPDBS\\summary_pdb_93\\2b3027889e2fdc42.final.pdb
-6.414679e-15 -2.700953e-15 8.490385e-16 -4.581118e-15 -7.268678e-16
-3.402876e-15
E:\\R-3.1.2\\scripts\\mPDBS\\summary_pdb_93\\2fcb3b46e33793b0.final.pdb
-9.536996e-15 -5.737342e-16 2.600781e-15 -5.135700e-15 -3.364113e-15
-2.692187e-15
E:\\R-3.1.2\\scripts\\mPDBS\\summary_pdb_93\\34eb328251842006.final.pdb
-5.287184e-15 2.419618e-15 1.077005e-15 -7.579818e-15 -9.800842e-16
-3.915208e-15
E:\\R-3.1.2\\scripts\\mPDBS\\summary_pdb_93\\354b32871797b7ab.final.pdb
-1.689021e-14 4.672238e-16 1.385665e-15 -9.003570e-15 -3.435049e-15
5.027134e-16
E:\\R-3.1.2\\scripts\\mPDBS\\summary_pdb_93\\397368adbc32d5de.final.pdb

-5.154184e-15 -7.090762e-15 -1.801178e-15 -3.726990e-15 2.471023e-15
-5.160338e-15
E:\\R-3.1.2\\scripts\\mPDBS\\summary_pdb_93\\411ca13cd3df6edd.final.pdb
-1.178953e-14 -3.505417e-15 5.976087e-16 -4.972853e-15 -8.604736e-16
-4.974216e-15
E:\\R-3.1.2\\scripts\\mPDBS\\summary_pdb_93\\42357eee6642bf12.final.pdb
-9.305194e-15 4.617945e-15 2.687856e-15 -7.941024e-15 -1.466752e-15
-1.641006e-15
E:\\R-3.1.2\\scripts\\mPDBS\\summary_pdb_93\\44342d1f75a7fcfc.final.pdb
-2.392587e-15 5.895588e-15 9.236377e-16 -3.894112e-15 2.273877e-15
-3.801820e-15
E:\\R-3.1.2\\scripts\\mPDBS\\summary_pdb_93\\448ef21dffe8cdfb.final.pdb
-5.970405e-15 4.352880e-15 -6.587182e-15 -4.816511e-15 3.016200e-15
-9.442864e-15
E:\\R-3.1.2\\scripts\\mPDBS\\summary_pdb_93\\472a40b3e8f113c7.final.pdb
-6.486451e-15 -3.704582e-15 2.210804e-15 -4.126193e-15 -2.701489e-15
-2.004442e-15
E:\\R-3.1.2\\scripts\\mPDBS\\summary_pdb_93\\48a26225951ea153.final.pdb
-8.822475e-15 -7.075925e-15 -1.015084e-14 -1.886225e-15 1.462353e-15
-3.796870e-16
E:\\R-3.1.2\\scripts\\mPDBS\\summary_pdb_93\\4ab4a5c7c6682ce6.final.pdb
-2.290648e-14 8.265162e-16 -2.985127e-15 -1.197733e-14 1.420648e-15
-5.360698e-15
E:\\R-3.1.2\\scripts\\mPDBS\\summary_pdb_93\\4eaa45b62cb6a25f.final.pdb
-5.243930e-15 -5.021008e-15 1.026577e-15 -2.303201e-15 -1.306977e-15
-3.740854e-15
E:\\R-3.1.2\\scripts\\mPDBS\\summary_pdb_93\\58c371611d85ac96.final.pdb
-5.154184e-15 -7.090762e-15 -1.801178e-15 -3.726990e-15 2.471023e-15
-5.160338e-15
E:\\R-3.1.2\\scripts\\mPDBS\\summary_pdb_93\\59a647f04822d30a.final.pdb
-1.156103e-14 -4.814595e-15 -3.863765e-15 -8.129440e-15 -1.184294e-15
3.350615e-16
E:\\R-3.1.2\\scripts\\mPDBS\\summary_pdb_93\\59b091fe385e05fc.final.pdb
-8.388577e-15 2.585183e-15 5.494194e-15 -6.050429e-15 -1.954618e-15
-2.984374e-15
E:\\R-3.1.2\\scripts\\mPDBS\\summary_pdb_93\\5c35ac30f930d2db.final.pdb
-5.243930e-15 -5.021008e-15 1.026577e-15 -2.303201e-15 -1.306977e-15
-3.740854e-15
E:\\R-3.1.2\\scripts\\mPDBS\\summary_pdb_93\\5f60752431cf2dcb.final.pdb
-6.476695e-15 -3.773718e-15 -2.919052e-15 -5.748751e-15 -9.896362e-16
4.183274e-16
E:\\R-3.1.2\\scripts\\mPDBS\\summary_pdb_93\\626456dd6ff2868d.final.pdb
-6.486451e-15 -3.704582e-15 2.210804e-15 -4.126193e-15 -2.701489e-15
-2.004442e-15
E:\\R-3.1.2\\scripts\\mPDBS\\summary_pdb_93\\6583d53ff3c8f7ef.final.pdb
-1.156103e-14 -4.814595e-15 -3.863765e-15 -8.129440e-15 -1.184294e-15
3.350615e-16
E:\\R-3.1.2\\scripts\\mPDBS\\summary_pdb_93\\69760178986bef01.final.pdb
-4.678684e-15 -1.088262e-14 -3.036725e-16 -5.496090e-15 3.965780e-15
-6.679436e-15
E:\\R-3.1.2\\scripts\\mPDBS\\summary_pdb_93\\6a9d87d47926b1c8.final.pdb
-6.300836e-15 -3.798257e-15 1.092341e-15 -4.805988e-15 -8.093389e-16
-3.450334e-15
E:\\R-3.1.2\\scripts\\mPDBS\\summary_pdb_93\\6bb3547ea982ffc4.final.pdb

6.294123e-15 -8.440918e-15 -4.162229e-15 -8.081689e-16 1.337666e-15
-6.823309e-15
E:\\R-3.1.2\\scripts\\mPDBS\\summary_pdb_93\\716d9624611d1c42.final.pdb
-5.741822e-15 -5.803779e-15 2.950385e-15 -2.501348e-15 -1.975826e-15
-7.505456e-15
E:\\R-3.1.2\\scripts\\mPDBS\\summary_pdb_93\\75f27e70ad0f1be4.final.pdb
-1.156103e-14 -4.814595e-15 -3.863765e-15 -8.129440e-15 -1.184294e-15
3.350615e-16
E:\\R-3.1.2\\scripts\\mPDBS\\summary_pdb_93\\769f910fffb22872.final.pdb
-7.056455e-15 -2.402825e-15 7.486204e-16 -3.587788e-15 -1.485670e-15
-3.162367e-15
E:\\R-3.1.2\\scripts\\mPDBS\\summary_pdb_93\\791f8c80dd5c3135.final.pdb
-8.388577e-15 2.585183e-15 5.494194e-15 -6.050429e-15 -1.954618e-15
-2.984374e-15
E:\\R-3.1.2\\scripts\\mPDBS\\summary_pdb_93\\7fad98e66a3f6099.final.pdb
-6.966166e-15 6.235457e-15 4.339139e-15 -6.973297e-15 6.642666e-16
-1.363034e-15
E:\\R-3.1.2\\scripts\\mPDBS\\summary_pdb_93\\804b89c94a633196.final.pdb
-9.536996e-15 -5.737342e-16 2.600781e-15 -5.135700e-15 -3.364113e-15
-2.692187e-15
E:\\R-3.1.2\\scripts\\mPDBS\\summary_pdb_93\\813cc65a2298feba.final.pdb
-9.536996e-15 -5.737342e-16 2.600781e-15 -5.135700e-15 -3.364113e-15
-2.692187e-15
E:\\R-3.1.2\\scripts\\mPDBS\\summary_pdb_93\\83c0eefcfe12d3f9.final.pdb
-1.178953e-14 -3.505417e-15 5.976087e-16 -4.972853e-15 -8.604736e-16
-4.974216e-15
E:\\R-3.1.2\\scripts\\mPDBS\\summary_pdb_93\\86d50469ef569760.final.pdb
-6.486451e-15 -3.704582e-15 2.210804e-15 -4.126193e-15 -2.701489e-15
-2.004442e-15
E:\\R-3.1.2\\scripts\\mPDBS\\summary_pdb_93\\89d0666e1402203d.final.pdb
-1.156103e-14 -4.814595e-15 -3.863765e-15 -8.129440e-15 -1.184294e-15
3.350615e-16
E:\\R-3.1.2\\scripts\\mPDBS\\summary_pdb_93\\8b34852716dadc62.final.pdb
-7.572422e-15 -2.506216e-15 -8.370395e-17 -6.842668e-15 3.040486e-15
-3.812385e-15
E:\\R-3.1.2\\scripts\\mPDBS\\summary_pdb_93\\8b6ef9ac3fe74395.final.pdb
-1.014974e-15 -4.317397e-15 -4.280562e-15 -7.564664e-15 -2.826948e-15
-7.444067e-16
E:\\R-3.1.2\\scripts\\mPDBS\\summary_pdb_93\\98bbe0d5986c138f.final.pdb
-8.388577e-15 2.585183e-15 5.494194e-15 -6.050429e-15 -1.954618e-15
-2.984374e-15
E:\\R-3.1.2\\scripts\\mPDBS\\summary_pdb_93\\9c22f4909d67e2fa.final.pdb
5.237210e-15 -2.851426e-15 6.938023e-15 6.875499e-16 -1.633293e-15
-1.758039e-15
E:\\R-3.1.2\\scripts\\mPDBS\\summary_pdb_93\\9dda8ed0d81fb3ef.final.pdb
-5.851409e-15 2.738391e-15 3.480620e-15 -7.962906e-15 -1.026947e-14
3.173936e-15
E:\\R-3.1.2\\scripts\\mPDBS\\summary_pdb_93\\9f752b3616e6033b.final.pdb
-4.706643e-15 8.564272e-17 -4.671613e-16 -4.838628e-15 -1.179884e-16
-3.178691e-15
E:\\R-3.1.2\\scripts\\mPDBS\\summary_pdb_93\\a02c70083816d6ba.final.pdb
-5.243930e-15 -5.021008e-15 1.026577e-15 -2.303201e-15 -1.306977e-15
-3.740854e-15
E:\\R-3.1.2\\scripts\\mPDBS\\summary_pdb_93\\a0c997d891e4e082.final.pdb

-9.462347e-15 4.602223e-15 3.236083e-15 -7.413734e-15 4.255764e-15
-7.502502e-15
E:\\R-3.1.2\\scripts\\mPDBS\\summary_pdb_93\\ab8039a2d823c616.final.pdb
-6.176130e-15 -2.216541e-15 1.112348e-15 -5.116960e-15 -1.036010e-15
-2.933674e-15
E:\\R-3.1.2\\scripts\\mPDBS\\summary_pdb_93\\acf4d74b9c71d4dc.final.pdb
-9.536996e-15 -5.737342e-16 2.600781e-15 -5.135700e-15 -3.364113e-15
-2.692187e-15
E:\\R-3.1.2\\scripts\\mPDBS\\summary_pdb_93\\ad8a823e4b4c668e.final.pdb
-6.711048e-15 -2.586010e-15 1.014112e-15 -4.766524e-15 -1.637233e-15
-3.233313e-15
E:\\R-3.1.2\\scripts\\mPDBS\\summary_pdb_93\\b16ababd03b7b857.final.pdb
5.237210e-15 -2.851426e-15 6.938023e-15 6.875499e-16 -1.633293e-15
-1.758039e-15
E:\\R-3.1.2\\scripts\\mPDBS\\summary_pdb_93\\b226923e697d3c63.final.pdb
-3.701870e-15 -1.752808e-15 -3.445263e-15 -8.613428e-15 1.226104e-15
-5.181793e-15
E:\\R-3.1.2\\scripts\\mPDBS\\summary_pdb_93\\b28e48f6fa27a165.final.pdb
-8.388577e-15 2.585183e-15 5.494194e-15 -6.050429e-15 -1.954618e-15
-2.984374e-15
E:\\R-3.1.2\\scripts\\mPDBS\\summary_pdb_93\\b42c4924e3cd1aec.final.pdb
-4.418527e-15 -4.832376e-15 -1.716178e-15 5.064878e-17 4.117021e-15
-3.551153e-15
E:\\R-3.1.2\\scripts\\mPDBS\\summary_pdb_93\\b4dd239fcabff1df.final.pdb
-8.388577e-15 2.585183e-15 5.494194e-15 -6.050429e-15 -1.954618e-15
-2.984374e-15
E:\\R-3.1.2\\scripts\\mPDBS\\summary_pdb_93\\b669942ef97459a2.final.pdb
-7.572422e-15 -2.506216e-15 -8.370395e-17 -6.842668e-15 3.040486e-15
-3.812385e-15
E:\\R-3.1.2\\scripts\\mPDBS\\summary_pdb_93\\b99f4d9d87c1017b.final.pdb
-8.388577e-15 2.585183e-15 5.494194e-15 -6.050429e-15 -1.954618e-15
-2.984374e-15
E:\\R-3.1.2\\scripts\\mPDBS\\summary_pdb_93\\b9d2cbf3975403e7.final.pdb
-8.388577e-15 2.585183e-15 5.494194e-15 -6.050429e-15 -1.954618e-15
-2.984374e-15
E:\\R-3.1.2\\scripts\\mPDBS\\summary_pdb_93\\bc88c8a505f3a288.final.pdb
-6.385189e-15 -2.869654e-15 8.542427e-16 -4.654844e-15 -4.428069e-16
-2.988711e-15
E:\\R-3.1.2\\scripts\\mPDBS\\summary_pdb_93\\bdac0e64f4ef427e.final.pdb
5.212490e-15 -1.803653e-15 1.836635e-15 -5.755213e-15 -3.102604e-15
-2.288159e-17
E:\\R-3.1.2\\scripts\\mPDBS\\summary_pdb_93\\ca2a1f55fc9d0681.final.pdb
-7.572422e-15 -2.506216e-15 -8.370395e-17 -6.842668e-15 3.040486e-15
-3.812385e-15
E:\\R-3.1.2\\scripts\\mPDBS\\summary_pdb_93\\ce34248de3e81a3c.final.pdb
-1.899477e-14 -2.199753e-15 7.229096e-15 -7.807157e-15 -3.807887e-15
5.640537e-16
E:\\R-3.1.2\\scripts\\mPDBS\\summary_pdb_93\\d02e697772494a61.final.pdb
-8.629487e-15 -4.486850e-15 2.865544e-16 -5.320977e-15 3.407412e-15
-9.512572e-15
E:\\R-3.1.2\\scripts\\mPDBS\\summary_pdb_93\\d04221245d015047.final.pdb
-7.583642e-15 -2.260343e-15 2.162289e-15 -2.400383e-15 -1.998782e-15
-2.855177e-15
E:\\R-3.1.2\\scripts\\mPDBS\\summary_pdb_93\\d678141e4018054a.final.pdb

-2.489489e-16 -5.463797e-15 -5.456659e-15 2.259090e-16 -4.123133e-16
-5.170445e-15
E:\\R-3.1.2\\scripts\\mPDBS\\summary_pdb_93\\d71b40af0f0efe39.final.pdb
2.267156e-15 2.589390e-15 -2.421749e-15 3.704598e-15 -2.934401e-15
-2.978268e-15
E:\\R-3.1.2\\scripts\\mPDBS\\summary_pdb_93\\dc0809eff10197e2.final.pdb
-7.792407e-15 -2.266238e-15 -4.066734e-15 -6.705145e-15 -4.950312e-15
-7.273850e-16
E:\\R-3.1.2\\scripts\\mPDBS\\summary_pdb_93\\e1163918d3707839.final.pdb
-6.635504e-15 4.488293e-15 1.068232e-14 5.113439e-15 4.557207e-15
-1.333152e-14
E:\\R-3.1.2\\scripts\\mPDBS\\summary_pdb_93\\e4b48b06824ef848.final.pdb
-2.897455e-15 -1.120828e-14 -8.068979e-15 -1.784543e-15 -4.076046e-15
-3.141984e-15
E:\\R-3.1.2\\scripts\\mPDBS\\summary_pdb_93\\e529e19ad281fea2.final.pdb
-2.847869e-15 -4.249285e-15 3.608123e-15 -2.256161e-15 -3.705893e-15
-2.805450e-15
E:\\R-3.1.2\\scripts\\mPDBS\\summary_pdb_93\\e5602082718ec3a1.final.pdb
-9.536996e-15 -5.737342e-16 2.600781e-15 -5.135700e-15 -3.364113e-15
-2.692187e-15
E:\\R-3.1.2\\scripts\\mPDBS\\summary_pdb_93\\e5d589d996d0a960.final.pdb
-1.099589e-14 -6.216720e-15 3.249029e-15 -2.425768e-15 6.398100e-16
-4.898065e-15
E:\\R-3.1.2\\scripts\\mPDBS\\summary_pdb_93\\e9df9e72307a2fbd.final.pdb
-5.243930e-15 -5.021008e-15 1.026577e-15 -2.303201e-15 -1.306977e-15
-3.740854e-15
E:\\R-3.1.2\\scripts\\mPDBS\\summary_pdb_93\\ea00f8694090cae7.final.pdb
-9.536996e-15 -5.737342e-16 2.600781e-15 -5.135700e-15 -3.364113e-15
-2.692187e-15
E:\\R-3.1.2\\scripts\\mPDBS\\summary_pdb_93\\eba517be32db050e.final.pdb
-8.388577e-15 2.585183e-15 5.494194e-15 -6.050429e-15 -1.954618e-15
-2.984374e-15
E:\\R-3.1.2\\scripts\\mPDBS\\summary_pdb_93\\ec04b9545899c29d.final.pdb
-6.221122e-15 -2.426748e-15 1.086702e-15 -4.080595e-15 -1.218121e-15
-3.158238e-15
E:\\R-3.1.2\\scripts\\mPDBS\\summary_pdb_93\\ee6862cbcad52078.final.pdb
-6.473876e-15 -3.101240e-15 7.007197e-16 -4.300960e-15 -6.171466e-16
-3.312671e-15
E:\\R-3.1.2\\scripts\\mPDBS\\summary_pdb_93\\ef6a42fc83b81e5d.final.pdb
-6.900113e-15 -2.170806e-15 7.095892e-16 -4.064837e-15 -9.431352e-16
-3.045274e-15
E:\\R-3.1.2\\scripts\\mPDBS\\summary_pdb_93\\ef8e88d4ad93a0ad.final.pdb
-7.583642e-15 -2.260343e-15 2.162289e-15 -2.400383e-15 -1.998782e-15
-2.855177e-15
E:\\R-3.1.2\\scripts\\mPDBS\\summary_pdb_93\\f14fcae8deec4fa3.final.pdb
-4.706643e-15 8.564272e-17 -4.671613e-16 -4.838628e-15 -1.179884e-16
-3.178691e-15
E:\\R-3.1.2\\scripts\\mPDBS\\summary_pdb_93\\f83584e22003fb92.final.pdb
-8.822475e-15 -7.075925e-15 -1.015084e-14 -1.886225e-15 1.462353e-15
-3.796870e-16
E:\\R-3.1.2\\scripts\\mPDBS\\summary_pdb_93\\f8e2bf87e52caa29.final.pdb
-2.847869e-15 -4.249285e-15 3.608123e-15 -2.256161e-15 -3.705893e-15
-2.805450e-15

[,787] [,788] [,789] [,790] [,791] [,792]
E:\\R-3.1.2\\scripts\\40.1ns_data\\NAT0.pdb
2.609602e-15 4.946202e-15 6.372577e-15 3.893019e-15 -7.224477e-15
-1.730900e-15
E:\\R-3.1.2\\scripts\\40.1ns_data\\LJP6.pdb
1.702599e-15 2.689352e-15 5.015356e-15 3.587576e-15 -6.473057e-15
3.080785e-15
E:\\R-3.1.2\\scripts\\40.1ns_data\\3WY9.pdb
2.501703e-15 3.923107e-15 4.987632e-15 2.750377e-15 -6.280643e-15
2.983608e-15
E:\\R-3.1.2\\scripts\\40.1ns_data\\ZSP9.pdb
3.846526e-15 4.249584e-15 5.342010e-15 4.328670e-15 -5.489771e-15
1.458005e-15
E:\\R-3.1.2\\scripts\\mPDBS\\summary_pdb_93\\013d855ad26c877c.final.pdb
-1.099920e-15 3.367985e-15 8.706741e-15 5.494756e-15 -2.016872e-17
-3.561612e-15
E:\\R-3.1.2\\scripts\\mPDBS\\summary_pdb_93\\0a2a705342ca0d54.final.pdb
2.418453e-15 3.955389e-15 4.495561e-15 3.752448e-15 -6.416651e-15
4.029768e-15
E:\\R-3.1.2\\scripts\\mPDBS\\summary_pdb_93\\0a59fceadb4194d9.final.pdb
4.893281e-15 2.499442e-15 2.426018e-15 4.624824e-15 -7.434485e-15
5.209452e-15
E:\\R-3.1.2\\scripts\\mPDBS\\summary_pdb_93\\0dba59f3306825e8.final.pdb
4.416501e-15 3.358444e-15 6.527061e-15 3.888402e-15 -5.074286e-15
2.552421e-15
E:\\R-3.1.2\\scripts\\mPDBS\\summary_pdb_93\\126f26a78b57339c.final.pdb
1.687347e-15 4.861799e-15 7.234395e-15 4.672930e-15 -1.358121e-14
1.679258e-15
E:\\R-3.1.2\\scripts\\mPDBS\\summary_pdb_93\\12f06f3e342df706.final.pdb
1.913974e-15 3.720551e-15 4.892487e-15 4.355047e-15 -6.095023e-15
3.121153e-15
E:\\R-3.1.2\\scripts\\mPDBS\\summary_pdb_93\\18618a2a251b867b.final.pdb
-6.035652e-16 7.265178e-15 6.280061e-15 6.243407e-15 -8.286308e-15
-6.570798e-15
E:\\R-3.1.2\\scripts\\mPDBS\\summary_pdb_93\\1f305f598641eeb2.final.pdb
2.653669e-15 4.148634e-15 4.892520e-15 3.870648e-15 -6.113772e-15
2.664462e-15
E:\\R-3.1.2\\scripts\\mPDBS\\summary_pdb_93\\1f8a00b551be0a39.final.pdb
-6.035652e-16 7.265178e-15 6.280061e-15 6.243407e-15 -8.286308e-15
-6.570798e-15
E:\\R-3.1.2\\scripts\\mPDBS\\summary_pdb_93\\219f45328d47579a.final.pdb
1.794198e-15 5.955444e-15 1.098341e-14 3.320312e-15 1.499983e-14
4.766924e-16
E:\\R-3.1.2\\scripts\\mPDBS\\summary_pdb_93\\238dd3918e54336d.final.pdb
-3.134423e-15 1.066912e-14 1.539579e-14 -3.165602e-15 -2.086224e-15
-3.693420e-15
E:\\R-3.1.2\\scripts\\mPDBS\\summary_pdb_93\\2b3027889e2fdc42.final.pdb
2.222186e-15 3.656936e-15 5.156472e-15 3.938307e-15 -5.913783e-15
2.909534e-15
E:\\R-3.1.2\\scripts\\mPDBS\\summary_pdb_93\\2fcb3b46e33793b0.final.pdb
3.396130e-15 4.018530e-15 2.862027e-15 5.213324e-15 -7.448028e-16
3.397382e-15
E:\\R-3.1.2\\scripts\\mPDBS\\summary_pdb_93\\34eb328251842006.final.pdb
1.621716e-15 1.006675e-14 3.950725e-15 2.087317e-15 -4.758642e-15

4.716413e-15
E:\\R-3.1.2\\scripts\\mPDBS\\summary_pdb_93\\354b32871797b7ab.final.pdb
3.509071e-15 1.409399e-14 -2.104343e-15 -3.501102e-15 -8.945002e-15
9.002116e-15
E:\\R-3.1.2\\scripts\\mPDBS\\summary_pdb_93\\397368adbc32d5de.final.pdb
1.687347e-15 4.861799e-15 7.234395e-15 4.672930e-15 -1.358121e-14
1.679258e-15
E:\\R-3.1.2\\scripts\\mPDBS\\summary_pdb_93\\411ca13cd3df6edd.final.pdb
1.386020e-15 8.620534e-15 2.656462e-15 6.506994e-15 -5.608263e-15
3.954825e-15
E:\\R-3.1.2\\scripts\\mPDBS\\summary_pdb_93\\42357eee6642bf12.final.pdb
8.229331e-16 1.015965e-14 -4.580401e-16 5.989869e-15 -2.736401e-15
-3.251974e-15
E:\\R-3.1.2\\scripts\\mPDBS\\summary_pdb_93\\44342d1f75a7fcfc.final.pdb
2.907144e-15 -2.109156e-15 1.015883e-14 4.975012e-15 -1.904179e-14
5.263882e-15
E:\\R-3.1.2\\scripts\\mPDBS\\summary_pdb_93\\448ef21dffe8cdfb.final.pdb
-8.993265e-15 8.109875e-15 -2.453166e-15 -1.121886e-15 -8.954382e-15
2.103279e-15
E:\\R-3.1.2\\scripts\\mPDBS\\summary_pdb_93\\472a40b3e8f113c7.final.pdb
4.416501e-15 3.358444e-15 6.527061e-15 3.888402e-15 -5.074286e-15
2.552421e-15
E:\\R-3.1.2\\scripts\\mPDBS\\summary_pdb_93\\48a26225951ea153.final.pdb
-1.763341e-15 2.828605e-15 3.784740e-15 4.391070e-15 -1.102948e-14
-1.252935e-15
E:\\R-3.1.2\\scripts\\mPDBS\\summary_pdb_93\\4ab4a5c7c6682ce6.final.pdb
-1.459441e-15 6.923518e-15 -1.369201e-14 2.900043e-15 -6.703896e-15
1.263490e-14
E:\\R-3.1.2\\scripts\\mPDBS\\summary_pdb_93\\4eaa45b62cb6a25f.final.pdb
1.780690e-15 8.825480e-16 6.411956e-15 7.295600e-15 -7.828816e-15
1.981094e-15
E:\\R-3.1.2\\scripts\\mPDBS\\summary_pdb_93\\58c371611d85ac96.final.pdb
1.687347e-15 4.861799e-15 7.234395e-15 4.672930e-15 -1.358121e-14
1.679258e-15
E:\\R-3.1.2\\scripts\\mPDBS\\summary_pdb_93\\59a647f04822d30a.final.pdb
1.318137e-15 5.984630e-15 -1.390744e-15 -2.638532e-16 -4.140516e-15
3.987126e-15
E:\\R-3.1.2\\scripts\\mPDBS\\summary_pdb_93\\59b091fe385e05fc.final.pdb
4.893281e-15 2.499442e-15 2.426018e-15 4.624824e-15 -7.434485e-15
5.209452e-15
E:\\R-3.1.2\\scripts\\mPDBS\\summary_pdb_93\\5c35ac30f930d2db.final.pdb
1.780690e-15 8.825480e-16 6.411956e-15 7.295600e-15 -7.828816e-15
1.981094e-15
E:\\R-3.1.2\\scripts\\mPDBS\\summary_pdb_93\\5f60752431cf2dcb.final.pdb
3.764110e-15 -3.107321e-15 3.446487e-15 5.459849e-15 -1.255128e-14
7.696865e-15
E:\\R-3.1.2\\scripts\\mPDBS\\summary_pdb_93\\626456dd6ff2868d.final.pdb
4.416501e-15 3.358444e-15 6.527061e-15 3.888402e-15 -5.074286e-15
2.552421e-15
E:\\R-3.1.2\\scripts\\mPDBS\\summary_pdb_93\\6583d53ff3c8f7ef.final.pdb
1.318137e-15 5.984630e-15 -1.390744e-15 -2.638532e-16 -4.140516e-15
3.987126e-15
E:\\R-3.1.2\\scripts\\mPDBS\\summary_pdb_93\\69760178986bef01.final.pdb
6.286165e-15 7.118194e-17 6.466290e-15 5.131350e-15 -6.975044e-16

4.964555e-15
E:\\R-3.1.2\\scripts\\mPDBS\\summary_pdb_93\\6a9d87d47926b1c8.final.pdb
2.565117e-15 3.866501e-15 4.993132e-15 4.010266e-15 -5.562448e-15
2.224829e-15
E:\\R-3.1.2\\scripts\\mPDBS\\summary_pdb_93\\6bb3547ea982ffc4.final.pdb
4.963475e-15 4.120768e-15 1.998070e-14 -2.647213e-15 -6.720739e-15
4.792936e-15
E:\\R-3.1.2\\scripts\\mPDBS\\summary_pdb_93\\716d9624611d1c42.final.pdb
-1.923560e-15 1.230331e-14 7.566455e-15 2.295280e-15 -1.766725e-15
-1.495760e-15
E:\\R-3.1.2\\scripts\\mPDBS\\summary_pdb_93\\75f27e70ad0f1be4.final.pdb
1.318137e-15 5.984630e-15 -1.390744e-15 -2.638532e-16 -4.140516e-15
3.987126e-15
E:\\R-3.1.2\\scripts\\mPDBS\\summary_pdb_93\\769f910fffb22872.final.pdb
2.482725e-15 3.687310e-15 4.951805e-15 3.925600e-15 -5.747649e-15
2.855527e-15
E:\\R-3.1.2\\scripts\\mPDBS\\summary_pdb_93\\791f8c80dd5c3135.final.pdb
4.893281e-15 2.499442e-15 2.426018e-15 4.624824e-15 -7.434485e-15
5.209452e-15
E:\\R-3.1.2\\scripts\\mPDBS\\summary_pdb_93\\7fad98e66a3f6099.final.pdb
-6.619070e-16 3.435141e-15 5.039066e-15 6.907729e-15 -4.571677e-15
1.110052e-14
E:\\R-3.1.2\\scripts\\mPDBS\\summary_pdb_93\\804b89c94a633196.final.pdb
3.396130e-15 4.018530e-15 2.862027e-15 5.213324e-15 -7.448028e-16
3.397382e-15
E:\\R-3.1.2\\scripts\\mPDBS\\summary_pdb_93\\813cc65a2298feba.final.pdb
3.396130e-15 4.018530e-15 2.862027e-15 5.213324e-15 -7.448028e-16
3.397382e-15
E:\\R-3.1.2\\scripts\\mPDBS\\summary_pdb_93\\83c0eefcfe12d3f9.final.pdb
1.386020e-15 8.620534e-15 2.656462e-15 6.506994e-15 -5.608263e-15
3.954825e-15
E:\\R-3.1.2\\scripts\\mPDBS\\summary_pdb_93\\86d50469ef569760.final.pdb
4.416501e-15 3.358444e-15 6.527061e-15 3.888402e-15 -5.074286e-15
2.552421e-15
E:\\R-3.1.2\\scripts\\mPDBS\\summary_pdb_93\\89d0666e1402203d.final.pdb
1.318137e-15 5.984630e-15 -1.390744e-15 -2.638532e-16 -4.140516e-15
3.987126e-15
E:\\R-3.1.2\\scripts\\mPDBS\\summary_pdb_93\\8b34852716dadc62.final.pdb
6.109238e-15 4.148634e-15 1.823795e-15 3.065737e-15 -1.192336e-14
8.659666e-15
E:\\R-3.1.2\\scripts\\mPDBS\\summary_pdb_93\\8b6ef9ac3fe74395.final.pdb
1.005210e-14 -7.616099e-16 1.206073e-14 -3.029822e-15 -7.188882e-15
6.787238e-15
E:\\R-3.1.2\\scripts\\mPDBS\\summary_pdb_93\\98bbe0d5986c138f.final.pdb
4.893281e-15 2.499442e-15 2.426018e-15 4.624824e-15 -7.434485e-15
5.209452e-15
E:\\R-3.1.2\\scripts\\mPDBS\\summary_pdb_93\\9c22f4909d67e2fa.final.pdb
1.860250e-15 5.414932e-16 1.427998e-14 4.756875e-15 -5.966375e-15
-5.508363e-15
E:\\R-3.1.2\\scripts\\mPDBS\\summary_pdb_93\\9dda8ed0d81fb3ef.final.pdb
7.505408e-15 -6.268156e-15 2.828429e-15 4.838619e-15 -1.592167e-14
1.488874e-16
E:\\R-3.1.2\\scripts\\mPDBS\\summary_pdb_93\\9f752b3616e6033b.final.pdb
2.979956e-15 2.073488e-15 6.028500e-15 2.520329e-15 -4.235523e-15

2.673990e-15
E:\\R-3.1.2\\scripts\\mPDBS\\summary_pdb_93\\a02c70083816d6ba.final.pdb
1.780690e-15 8.825480e-16 6.411956e-15 7.295600e-15 -7.828816e-15
1.981094e-15
E:\\R-3.1.2\\scripts\\mPDBS\\summary_pdb_93\\a0c997d891e4e082.final.pdb
5.468705e-15 2.919515e-15 8.559483e-16 1.265879e-15 -7.674804e-15
1.492690e-14
E:\\R-3.1.2\\scripts\\mPDBS\\summary_pdb_93\\ab8039a2d823c616.final.pdb
2.327717e-15 3.880118e-15 5.541978e-15 2.728468e-15 -6.652944e-15
2.958775e-15
E:\\R-3.1.2\\scripts\\mPDBS\\summary_pdb_93\\acf4d74b9c71d4dc.final.pdb
3.396130e-15 4.018530e-15 2.862027e-15 5.213324e-15 -7.448028e-16
3.397382e-15
E:\\R-3.1.2\\scripts\\mPDBS\\summary_pdb_93\\ad8a823e4b4c668e.final.pdb
2.022469e-15 3.614647e-15 5.104333e-15 3.740753e-15 -5.841298e-15
2.849676e-15
E:\\R-3.1.2\\scripts\\mPDBS\\summary_pdb_93\\b16ababd03b7b857.final.pdb
1.860250e-15 5.414932e-16 1.427998e-14 4.756875e-15 -5.966375e-15
-5.508363e-15
E:\\R-3.1.2\\scripts\\mPDBS\\summary_pdb_93\\b226923e697d3c63.final.pdb
2.416868e-15 8.103811e-15 2.252717e-15 6.644407e-16 -2.961663e-15
4.807379e-15
E:\\R-3.1.2\\scripts\\mPDBS\\summary_pdb_93\\b28e48f6fa27a165.final.pdb
4.893281e-15 2.499442e-15 2.426018e-15 4.624824e-15 -7.434485e-15
5.209452e-15
E:\\R-3.1.2\\scripts\\mPDBS\\summary_pdb_93\\b42c4924e3cd1aec.final.pdb
2.073880e-15 6.477291e-15 1.304335e-14 5.056327e-15 -1.584509e-15
4.821799e-15
E:\\R-3.1.2\\scripts\\mPDBS\\summary_pdb_93\\b4dd239fcabff1df.final.pdb
4.893281e-15 2.499442e-15 2.426018e-15 4.624824e-15 -7.434485e-15
5.209452e-15
E:\\R-3.1.2\\scripts\\mPDBS\\summary_pdb_93\\b669942ef97459a2.final.pdb
6.109238e-15 4.148634e-15 1.823795e-15 3.065737e-15 -1.192336e-14
8.659666e-15
E:\\R-3.1.2\\scripts\\mPDBS\\summary_pdb_93\\b99f4d9d87c1017b.final.pdb
4.893281e-15 2.499442e-15 2.426018e-15 4.624824e-15 -7.434485e-15
5.209452e-15
E:\\R-3.1.2\\scripts\\mPDBS\\summary_pdb_93\\b9d2cbf3975403e7.final.pdb
4.893281e-15 2.499442e-15 2.426018e-15 4.624824e-15 -7.434485e-15
5.209452e-15
E:\\R-3.1.2\\scripts\\mPDBS\\summary_pdb_93\\bc88c8a505f3a288.final.pdb
2.123144e-15 3.958778e-15 5.404321e-15 4.041740e-15 -6.188194e-15
2.572130e-15
E:\\R-3.1.2\\scripts\\mPDBS\\summary_pdb_93\\bdac0e64f4ef427e.final.pdb
4.443795e-15 -4.365389e-15 1.114381e-14 -1.502441e-15 -6.495682e-15
5.853956e-16
E:\\R-3.1.2\\scripts\\mPDBS\\summary_pdb_93\\ca2a1f55fc9d0681.final.pdb
6.109238e-15 4.148634e-15 1.823795e-15 3.065737e-15 -1.192336e-14
8.659666e-15
E:\\R-3.1.2\\scripts\\mPDBS\\summary_pdb_93\\ce34248de3e81a3c.final.pdb
3.388208e-15 6.012017e-15 -3.236622e-15 9.467099e-15 -1.532655e-14
4.335619e-15
E:\\R-3.1.2\\scripts\\mPDBS\\summary_pdb_93\\d02e697772494a61.final.pdb
-3.023401e-15 1.070555e-14 -1.152438e-16 1.830402e-15 -8.233450e-16

3.126645e-15
E:\\R-3.1.2\\scripts\\mPDBS\\summary_pdb_93\\d04221245d015047.final.pdb
2.195282e-15 2.645429e-15 5.864962e-15 4.964309e-15 -7.178673e-15
2.702307e-15
E:\\R-3.1.2\\scripts\\mPDBS\\summary_pdb_93\\d678141e4018054a.final.pdb
-7.801985e-15 1.070835e-14 1.143290e-14 -1.920986e-15 -4.950875e-15
-6.690457e-15
E:\\R-3.1.2\\scripts\\mPDBS\\summary_pdb_93\\d71b40af0f0efe39.final.pdb
-1.088874e-14 3.894058e-15 5.598100e-15 5.479238e-15 -1.071050e-15
-1.879529e-15
E:\\R-3.1.2\\scripts\\mPDBS\\summary_pdb_93\\dc0809eff10197e2.final.pdb
2.875605e-15 -1.930998e-15 5.773706e-15 3.557781e-15 -4.448366e-15
6.915787e-15
E:\\R-3.1.2\\scripts\\mPDBS\\summary_pdb_93\\e1163918d3707839.final.pdb
3.273442e-15 3.793457e-15 9.486297e-15 1.472741e-14 2.280455e-15
-1.852116e-15
E:\\R-3.1.2\\scripts\\mPDBS\\summary_pdb_93\\e4b48b06824ef848.final.pdb
-6.035652e-16 7.265178e-15 6.280061e-15 6.243407e-15 -8.286308e-15
-6.570798e-15
E:\\R-3.1.2\\scripts\\mPDBS\\summary_pdb_93\\e529e19ad281fea2.final.pdb
-1.099920e-15 3.367985e-15 8.706741e-15 5.494756e-15 -2.016872e-17
-3.561612e-15
E:\\R-3.1.2\\scripts\\mPDBS\\summary_pdb_93\\e5602082718ec3a1.final.pdb
3.396130e-15 4.018530e-15 2.862027e-15 5.213324e-15 -7.448028e-16
3.397382e-15
E:\\R-3.1.2\\scripts\\mPDBS\\summary_pdb_93\\e5d589d996d0a960.final.pdb
5.133692e-15 -2.295505e-15 1.208069e-14 8.755567e-16 -1.299423e-14
9.127304e-15
E:\\R-3.1.2\\scripts\\mPDBS\\summary_pdb_93\\e9df9e72307a2fbd.final.pdb
1.780690e-15 8.825480e-16 6.411956e-15 7.295600e-15 -7.828816e-15
1.981094e-15
E:\\R-3.1.2\\scripts\\mPDBS\\summary_pdb_93\\ea00f8694090cae7.final.pdb
3.396130e-15 4.018530e-15 2.862027e-15 5.213324e-15 -7.448028e-16
3.397382e-15
E:\\R-3.1.2\\scripts\\mPDBS\\summary_pdb_93\\eba517be32db050e.final.pdb
4.893281e-15 2.499442e-15 2.426018e-15 4.624824e-15 -7.434485e-15
5.209452e-15
E:\\R-3.1.2\\scripts\\mPDBS\\summary_pdb_93\\ec04b9545899c29d.final.pdb
2.204581e-15 3.613696e-15 4.752128e-15 3.946321e-15 -6.308053e-15
2.820091e-15
E:\\R-3.1.2\\scripts\\mPDBS\\summary_pdb_93\\ee6862cbcad52078.final.pdb
1.931241e-15 3.717651e-15 5.313248e-15 3.851138e-15 -5.990327e-15
2.891861e-15
E:\\R-3.1.2\\scripts\\mPDBS\\summary_pdb_93\\ef6a42fc83b81e5d.final.pdb
2.110898e-15 3.906970e-15 4.946710e-15 3.871606e-15 -6.065211e-15
2.884855e-15
E:\\R-3.1.2\\scripts\\mPDBS\\summary_pdb_93\\ef8e88d4ad93a0ad.final.pdb
2.195282e-15 2.645429e-15 5.864962e-15 4.964309e-15 -7.178673e-15
2.702307e-15
E:\\R-3.1.2\\scripts\\mPDBS\\summary_pdb_93\\f14fcae8deec4fa3.final.pdb
2.979956e-15 2.073488e-15 6.028500e-15 2.520329e-15 -4.235523e-15
2.673990e-15
E:\\R-3.1.2\\scripts\\mPDBS\\summary_pdb_93\\f83584e22003fb92.final.pdb
-1.763341e-15 2.828605e-15 3.784740e-15 4.391070e-15 -1.102948e-14

-1.252935e-15
E:\\R-3.1.2\\scripts\\mPDBS\\summary_pdb_93\\f8e2bf87e52caa29.final.pdb
-1.099920e-15 3.367985e-15 8.706741e-15 5.494756e-15 -2.016872e-17
-3.561612e-15

[,793]   [,794]   [,795]   [,796]   [,797]   [,798]
E:\\R-3.1.2\\scripts\\40.1ns_data\\NAT0.pdb
8.938063e-16 3.639432e-15 -5.631291e-16 -6.222396e-15 5.665151e-15
-4.751665e-15
E:\\R-3.1.2\\scripts\\40.1ns_data\\LJP6.pdb
-9.180723e-16 3.485332e-15 -1.601077e-15 -6.487697e-15 4.475694e-15
-5.008160e-15
E:\\R-3.1.2\\scripts\\40.1ns_data\\3WY9.pdb
-2.216881e-16 2.399800e-15 -1.664885e-15 -7.715358e-15 2.822874e-15
-4.353370e-15
E:\\R-3.1.2\\scripts\\40.1ns_data\\ZSP9.pdb
-3.950518e-16 3.389899e-15 -8.644333e-16 -5.085955e-15 6.099590e-15
-4.859823e-15
E:\\R-3.1.2\\scripts\\mPDBS\\summary_pdb_93\\013d855ad26c877c.final.pdb
-1.103335e-15 1.248880e-16 3.920159e-15 -6.058927e-15 5.563275e-15
-2.877017e-15
E:\\R-3.1.2\\scripts\\mPDBS\\summary_pdb_93\\0a2a705342ca0d54.final.pdb
4.449822e-16 2.465759e-15 -7.636360e-16 -5.675348e-15 5.070818e-15
-3.690378e-15
E:\\R-3.1.2\\scripts\\mPDBS\\summary_pdb_93\\0a59fceadb4194d9.final.pdb
-1.921564e-15 1.007832e-15 7.446277e-16 -6.477917e-15 4.987828e-15
5.556692e-16
E:\\R-3.1.2\\scripts\\mPDBS\\summary_pdb_93\\0dba59f3306825e8.final.pdb
-3.994943e-17 3.646377e-15 -4.373554e-15 -5.883720e-15 1.767700e-15
-5.603135e-15
E:\\R-3.1.2\\scripts\\mPDBS\\summary_pdb_93\\126f26a78b57339c.final.pdb
4.239613e-15 8.565293e-15 -2.639944e-16 -1.235710e-16 5.489007e-15
-3.687024e-15
E:\\R-3.1.2\\scripts\\mPDBS\\summary_pdb_93\\12f06f3e342df706.final.pdb
6.117467e-17 2.759903e-15 -1.423915e-15 -5.997031e-15 5.178912e-15
-4.102483e-15
E:\\R-3.1.2\\scripts\\mPDBS\\summary_pdb_93\\18618a2a251b867b.final.pdb
-1.542626e-15 1.080737e-14 1.046943e-15 1.277517e-15 6.431940e-15
-6.540883e-15
E:\\R-3.1.2\\scripts\\mPDBS\\summary_pdb_93\\1f305f598641eeb2.final.pdb
4.220241e-16 2.780856e-15 -6.740203e-16 -6.362749e-15 5.072874e-15
-4.479997e-15
E:\\R-3.1.2\\scripts\\mPDBS\\summary_pdb_93\\1f8a00b551be0a39.final.pdb
-1.542626e-15 1.080737e-14 1.046943e-15 1.277517e-15 6.431940e-15
-6.540883e-15
E:\\R-3.1.2\\scripts\\mPDBS\\summary_pdb_93\\219f45328d47579a.final.pdb
1.117647e-14 -1.019068e-14 -3.361463e-15 -1.704108e-14 1.991093e-15
-8.081953e-15
E:\\R-3.1.2\\scripts\\mPDBS\\summary_pdb_93\\238dd3918e54336d.final.pdb
9.387098e-15 3.768809e-16 -7.014357e-15 1.084937e-14 1.001788e-14
-1.437239e-14
E:\\R-3.1.2\\scripts\\mPDBS\\summary_pdb_93\\2b3027889e2fdc42.final.pdb
4.602111e-16 1.759076e-15 -8.114192e-16 -4.883707e-15 5.367624e-15
-3.537302e-15

E:\\R-3.1.2\\scripts\\mPDBS\\summary_pdb_93\\2fcb3b46e33793b0.final.pdb
-3.838457e-15 3.210186e-16 -1.927358e-15 -9.741991e-15 4.488272e-15
-4.037643e-15
E:\\R-3.1.2\\scripts\\mPDBS\\summary_pdb_93\\34eb328251842006.final.pdb
1.102342e-15 -2.226385e-15 1.087196e-14 -1.002333e-14 5.726926e-15
-3.598071e-15
E:\\R-3.1.2\\scripts\\mPDBS\\summary_pdb_93\\354b32871797b7ab.final.pdb
4.143360e-15 -5.182480e-16 1.613206e-15 -9.324015e-15 1.786521e-14
5.161751e-15
E:\\R-3.1.2\\scripts\\mPDBS\\summary_pdb_93\\397368adbc32d5de.final.pdb
4.239613e-15 8.565293e-15 -2.639944e-16 -1.235710e-16 5.489007e-15
-3.687024e-15
E:\\R-3.1.2\\scripts\\mPDBS\\summary_pdb_93\\411ca13cd3df6edd.final.pdb
-3.753455e-15 5.914526e-15 2.400777e-15 -8.849475e-15 6.668495e-15
-4.362144e-15
E:\\R-3.1.2\\scripts\\mPDBS\\summary_pdb_93\\42357eee6642bf12.final.pdb
4.479930e-16 -8.926938e-15 4.100396e-15 -4.750068e-15 1.287289e-14
2.980985e-15
E:\\R-3.1.2\\scripts\\mPDBS\\summary_pdb_93\\44342d1f75a7fcfc.final.pdb
-3.457903e-15 1.053098e-14 1.151162e-14 7.031430e-16 7.074301e-15
-1.423429e-15
E:\\R-3.1.2\\scripts\\mPDBS\\summary_pdb_93\\448ef21dffe8cdfb.final.pdb
-3.117640e-16 -3.609865e-15 9.293701e-15 -2.749320e-15 2.623303e-14
-2.513688e-15
E:\\R-3.1.2\\scripts\\mPDBS\\summary_pdb_93\\472a40b3e8f113c7.final.pdb
-3.994943e-17 3.646377e-15 -4.373554e-15 -5.883720e-15 1.767700e-15
-5.603135e-15
E:\\R-3.1.2\\scripts\\mPDBS\\summary_pdb_93\\48a26225951ea153.final.pdb
-3.767310e-15 6.778498e-15 3.614728e-15 -3.475545e-15 3.718769e-15
-8.629150e-15
E:\\R-3.1.2\\scripts\\mPDBS\\summary_pdb_93\\4ab4a5c7c6682ce6.final.pdb
-1.494122e-14 -1.520389e-15 -8.285356e-15 -6.396331e-15 1.209744e-14
8.031900e-15
E:\\R-3.1.2\\scripts\\mPDBS\\summary_pdb_93\\4eaa45b62cb6a25f.final.pdb
-1.674903e-15 7.904214e-15 -4.190745e-15 -3.057760e-15 2.206875e-16
-5.037230e-15
E:\\R-3.1.2\\scripts\\mPDBS\\summary_pdb_93\\58c371611d85ac96.final.pdb
4.239613e-15 8.565293e-15 -2.639944e-16 -1.235710e-16 5.489007e-15
-3.687024e-15
E:\\R-3.1.2\\scripts\\mPDBS\\summary_pdb_93\\59a647f04822d30a.final.pdb
2.204235e-15 4.374956e-16 -5.484768e-15 -7.687281e-15 8.589582e-15
-7.043066e-15
E:\\R-3.1.2\\scripts\\mPDBS\\summary_pdb_93\\59b091fe385e05fc.final.pdb
-1.921564e-15 1.007832e-15 7.446277e-16 -6.477917e-15 4.987828e-15
5.556692e-16
E:\\R-3.1.2\\scripts\\mPDBS\\summary_pdb_93\\5c35ac30f930d2db.final.pdb
-1.674903e-15 7.904214e-15 -4.190745e-15 -3.057760e-15 2.206875e-16
-5.037230e-15
E:\\R-3.1.2\\scripts\\mPDBS\\summary_pdb_93\\5f60752431cf2dcb.final.pdb
-1.587246e-15 1.136673e-14 9.021322e-16 -1.632641e-16 -1.871221e-15
-2.662880e-15
E:\\R-3.1.2\\scripts\\mPDBS\\summary_pdb_93\\626456dd6ff2868d.final.pdb
-3.994943e-17 3.646377e-15 -4.373554e-15 -5.883720e-15 1.767700e-15
-5.603135e-15

E:\\R-3.1.2\\scripts\\mPDBS\\summary_pdb_93\\6583d53ff3c8f7ef.final.pdb
2.204235e-15 4.374956e-16 -5.484768e-15 -7.687281e-15 8.589582e-15
-7.043066e-15
E:\\R-3.1.2\\scripts\\mPDBS\\summary_pdb_93\\69760178986bef01.final.pdb
3.662256e-15 4.893554e-15 -4.172638e-15 -7.906013e-16 7.724068e-15
-9.575961e-15
E:\\R-3.1.2\\scripts\\mPDBS\\summary_pdb_93\\6a9d87d47926b1c8.final.pdb
4.665898e-16 1.463336e-15 -8.702802e-16 -5.362436e-15 5.426557e-15
-4.135450e-15
E:\\R-3.1.2\\scripts\\mPDBS\\summary_pdb_93\\6bb3547ea982ffc4.final.pdb
4.358366e-15 4.913557e-15 9.700534e-15 -1.216621e-14 5.742554e-15
-1.183374e-14
E:\\R-3.1.2\\scripts\\mPDBS\\summary_pdb_93\\716d9624611d1c42.final.pdb
3.413961e-15 1.418853e-15 -2.717368e-15 -6.918711e-15 8.703328e-15
-5.262471e-15
E:\\R-3.1.2\\scripts\\mPDBS\\summary_pdb_93\\75f27e70ad0f1be4.final.pdb
2.204235e-15 4.374956e-16 -5.484768e-15 -7.687281e-15 8.589582e-15
-7.043066e-15
E:\\R-3.1.2\\scripts\\mPDBS\\summary_pdb_93\\769f910fffb22872.final.pdb
3.889883e-16 1.921633e-15 -1.137942e-15 -5.307927e-15 4.685399e-15
-4.245843e-15
E:\\R-3.1.2\\scripts\\mPDBS\\summary_pdb_93\\791f8c80dd5c3135.final.pdb
-1.921564e-15 1.007832e-15 7.446277e-16 -6.477917e-15 4.987828e-15
5.556692e-16
E:\\R-3.1.2\\scripts\\mPDBS\\summary_pdb_93\\7fad98e66a3f6099.final.pdb
1.065481e-17 -5.895255e-15 -2.031395e-15 -9.028480e-15 6.502750e-15
1.530424e-15
E:\\R-3.1.2\\scripts\\mPDBS\\summary_pdb_93\\804b89c94a633196.final.pdb
-3.838457e-15 3.210186e-16 -1.927358e-15 -9.741991e-15 4.488272e-15
-4.037643e-15
E:\\R-3.1.2\\scripts\\mPDBS\\summary_pdb_93\\813cc65a2298feba.final.pdb
-3.838457e-15 3.210186e-16 -1.927358e-15 -9.741991e-15 4.488272e-15
-4.037643e-15
E:\\R-3.1.2\\scripts\\mPDBS\\summary_pdb_93\\83c0eefcfe12d3f9.final.pdb
-3.753455e-15 5.914526e-15 2.400777e-15 -8.849475e-15 6.668495e-15
-4.362144e-15
E:\\R-3.1.2\\scripts\\mPDBS\\summary_pdb_93\\86d50469ef569760.final.pdb
-3.994943e-17 3.646377e-15 -4.373554e-15 -5.883720e-15 1.767700e-15
-5.603135e-15
E:\\R-3.1.2\\scripts\\mPDBS\\summary_pdb_93\\89d0666e1402203d.final.pdb
2.204235e-15 4.374956e-16 -5.484768e-15 -7.687281e-15 8.589582e-15
-7.043066e-15
E:\\R-3.1.2\\scripts\\mPDBS\\summary_pdb_93\\8b34852716dadc62.final.pdb
1.056933e-15 4.796605e-15 -3.983873e-15 -6.236115e-15 3.650401e-15
-4.667347e-15
E:\\R-3.1.2\\scripts\\mPDBS\\summary_pdb_93\\8b6ef9ac3fe74395.final.pdb
1.511910e-15 -1.383463e-15 2.047319e-15 -1.595811e-14 5.563409e-15
-1.072861e-14
E:\\R-3.1.2\\scripts\\mPDBS\\summary_pdb_93\\98bbe0d5986c138f.final.pdb
-1.921564e-15 1.007832e-15 7.446277e-16 -6.477917e-15 4.987828e-15
5.556692e-16
E:\\R-3.1.2\\scripts\\mPDBS\\summary_pdb_93\\9c22f4909d67e2fa.final.pdb
1.102639e-14 2.720521e-15 -1.439467e-15 1.421389e-15 -3.760006e-16
-6.963362e-15

E:\\R-3.1.2\\scripts\\mPDBS\\summary_pdb_93\\9dda8ed0d81fb3ef.final.pdb
-1.947894e-14 2.261344e-14 1.242924e-15 -1.428941e-15 -3.126090e-15
-4.917741e-16
E:\\R-3.1.2\\scripts\\mPDBS\\summary_pdb_93\\9f752b3616e6033b.final.pdb
3.493418e-16 -9.626671e-16 -3.128558e-16 -8.099915e-15 6.624604e-15
-6.137565e-15
E:\\R-3.1.2\\scripts\\mPDBS\\summary_pdb_93\\a02c70083816d6ba.final.pdb
-1.674903e-15 7.904214e-15 -4.190745e-15 -3.057760e-15 2.206875e-16
-5.037230e-15
E:\\R-3.1.2\\scripts\\mPDBS\\summary_pdb_93\\a0c997d891e4e082.final.pdb
-5.548562e-15 5.741978e-15 -2.952412e-15 -1.404379e-14 4.498536e-15
-1.228156e-14
E:\\R-3.1.2\\scripts\\mPDBS\\summary_pdb_93\\ab8039a2d823c616.final.pdb
-3.808210e-16 2.240059e-15 -5.563254e-16 -5.414408e-15 4.665287e-15
-3.644696e-15
E:\\R-3.1.2\\scripts\\mPDBS\\summary_pdb_93\\acf4d74b9c71d4dc.final.pdb
-3.838457e-15 3.210186e-16 -1.927358e-15 -9.741991e-15 4.488272e-15
-4.037643e-15
E:\\R-3.1.2\\scripts\\mPDBS\\summary_pdb_93\\ad8a823e4b4c668e.final.pdb
3.638463e-16 2.432045e-15 -7.287962e-16 -5.335011e-15 5.056851e-15
-4.390857e-15
E:\\R-3.1.2\\scripts\\mPDBS\\summary_pdb_93\\b16ababd03b7b857.final.pdb
1.102639e-14 2.720521e-15 -1.439467e-15 1.421389e-15 -3.760006e-16
-6.963362e-15
E:\\R-3.1.2\\scripts\\mPDBS\\summary_pdb_93\\b226923e697d3c63.final.pdb
-1.329397e-15 1.881273e-15 -3.720988e-15 -7.775752e-15 1.038166e-14
-9.326829e-15
E:\\R-3.1.2\\scripts\\mPDBS\\summary_pdb_93\\b28e48f6fa27a165.final.pdb
-1.921564e-15 1.007832e-15 7.446277e-16 -6.477917e-15 4.987828e-15
5.556692e-16
E:\\R-3.1.2\\scripts\\mPDBS\\summary_pdb_93\\b42c4924e3cd1aec.final.pdb
1.222400e-14 -1.749611e-15 4.718009e-15 -5.989854e-15 3.100700e-15
-4.516549e-15
E:\\R-3.1.2\\scripts\\mPDBS\\summary_pdb_93\\b4dd239fcabff1df.final.pdb
-1.921564e-15 1.007832e-15 7.446277e-16 -6.477917e-15 4.987828e-15
5.556692e-16
E:\\R-3.1.2\\scripts\\mPDBS\\summary_pdb_93\\b669942ef97459a2.final.pdb
1.056933e-15 4.796605e-15 -3.983873e-15 -6.236115e-15 3.650401e-15
-4.667347e-15
E:\\R-3.1.2\\scripts\\mPDBS\\summary_pdb_93\\b99f4d9d87c1017b.final.pdb
-1.921564e-15 1.007832e-15 7.446277e-16 -6.477917e-15 4.987828e-15
5.556692e-16
E:\\R-3.1.2\\scripts\\mPDBS\\summary_pdb_93\\b9d2cbf3975403e7.final.pdb
-1.921564e-15 1.007832e-15 7.446277e-16 -6.477917e-15 4.987828e-15
5.556692e-16
E:\\R-3.1.2\\scripts\\mPDBS\\summary_pdb_93\\bc88c8a505f3a288.final.pdb
-2.232699e-16 2.272798e-15 -9.293804e-16 -5.468308e-15 5.312547e-15
-3.210849e-15
E:\\R-3.1.2\\scripts\\mPDBS\\summary_pdb_93\\bdac0e64f4ef427e.final.pdb
9.021048e-15 -4.564586e-15 -6.508329e-15 -2.429702e-16 2.199738e-15
-2.897279e-15
E:\\R-3.1.2\\scripts\\mPDBS\\summary_pdb_93\\ca2a1f55fc9d0681.final.pdb
1.056933e-15 4.796605e-15 -3.983873e-15 -6.236115e-15 3.650401e-15
-4.667347e-15

E:\\R-3.1.2\\scripts\\mPDBS\\summary_pdb_93\\ce34248de3e81a3c.final.pdb
-6.558226e-15 5.948097e-15 -5.748488e-15 -2.128895e-15 -3.727159e-16
4.664615e-15
E:\\R-3.1.2\\scripts\\mPDBS\\summary_pdb_93\\d02e697772494a61.final.pdb
7.532679e-15 -1.249824e-14 -9.140694e-15 -8.955970e-15 1.576415e-14
-6.642457e-15
E:\\R-3.1.2\\scripts\\mPDBS\\summary_pdb_93\\d04221245d015047.final.pdb
5.090921e-16 2.409574e-15 -1.490991e-16 -5.358029e-15 3.067389e-15
-1.824646e-15
E:\\R-3.1.2\\scripts\\mPDBS\\summary_pdb_93\\d678141e4018054a.final.pdb
3.777715e-15 5.010012e-15 6.763395e-15 -1.929936e-16 4.777524e-15
-2.971297e-15
E:\\R-3.1.2\\scripts\\mPDBS\\summary_pdb_93\\d71b40af0f0efe39.final.pdb
3.004707e-15 5.342899e-16 -1.067191e-14 -7.724264e-16 7.323872e-15
2.153907e-16
E:\\R-3.1.2\\scripts\\mPDBS\\summary_pdb_93\\dc0809eff10197e2.final.pdb
5.466563e-16 4.907943e-15 -3.429495e-15 -8.965175e-15 1.646748e-15
-7.374688e-15
E:\\R-3.1.2\\scripts\\mPDBS\\summary_pdb_93\\e1163918d3707839.final.pdb
1.811320e-15 -8.994684e-15 5.574095e-15 -1.746201e-14 -1.351034e-15
-1.508105e-15
E:\\R-3.1.2\\scripts\\mPDBS\\summary_pdb_93\\e4b48b06824ef848.final.pdb
-1.542626e-15 1.080737e-14 1.046943e-15 1.277517e-15 6.431940e-15
-6.540883e-15
E:\\R-3.1.2\\scripts\\mPDBS\\summary_pdb_93\\e529e19ad281fea2.final.pdb
-1.103335e-15 1.248880e-16 3.920159e-15 -6.058927e-15 5.563275e-15
-2.877017e-15
E:\\R-3.1.2\\scripts\\mPDBS\\summary_pdb_93\\e5602082718ec3a1.final.pdb
-3.838457e-15 3.210186e-16 -1.927358e-15 -9.741991e-15 4.488272e-15
-4.037643e-15
E:\\R-3.1.2\\scripts\\mPDBS\\summary_pdb_93\\e5d589d996d0a960.final.pdb
-4.265731e-15 4.939246e-15 -9.741772e-15 -7.580705e-15 -4.157150e-15
-8.974053e-15
E:\\R-3.1.2\\scripts\\mPDBS\\summary_pdb_93\\e9df9e72307a2fbd.final.pdb
-1.674903e-15 7.904214e-15 -4.190745e-15 -3.057760e-15 2.206875e-16
-5.037230e-15
E:\\R-3.1.2\\scripts\\mPDBS\\summary_pdb_93\\ea00f8694090cae7.final.pdb
-3.838457e-15 3.210186e-16 -1.927358e-15 -9.741991e-15 4.488272e-15
-4.037643e-15
E:\\R-3.1.2\\scripts\\mPDBS\\summary_pdb_93\\eba517be32db050e.final.pdb
-1.921564e-15 1.007832e-15 7.446277e-16 -6.477917e-15 4.987828e-15
5.556692e-16
E:\\R-3.1.2\\scripts\\mPDBS\\summary_pdb_93\\ec04b9545899c29d.final.pdb
3.142534e-16 2.083748e-15 -1.050381e-15 -5.598240e-15 4.433185e-15
-4.591034e-15
E:\\R-3.1.2\\scripts\\mPDBS\\summary_pdb_93\\ee6862cbcad52078.final.pdb
1.202053e-16 1.685242e-15 -7.554744e-16 -4.954397e-15 5.265384e-15
-3.153170e-15
E:\\R-3.1.2\\scripts\\mPDBS\\summary_pdb_93\\ef6a42fc83b81e5d.final.pdb
7.587071e-17 2.837079e-15 -1.239423e-15 -5.669617e-15 4.620564e-15
-4.089827e-15
E:\\R-3.1.2\\scripts\\mPDBS\\summary_pdb_93\\ef8e88d4ad93a0ad.final.pdb
5.090921e-16 2.409574e-15 -1.490991e-16 -5.358029e-15 3.067389e-15
-1.824646e-15

E:\\R-3.1.2\\scripts\\mPDBS\\summary_pdb_93\\f14fcae8deec4fa3.final.pdb
3.493418e-16 -9.626671e-16 -3.128558e-16 -8.099915e-15 6.624604e-15
-6.137565e-15
E:\\R-3.1.2\\scripts\\mPDBS\\summary_pdb_93\\f83584e22003fb92.final.pdb
-3.767310e-15 6.778498e-15 3.614728e-15 -3.475545e-15 3.718769e-15
-8.629150e-15
E:\\R-3.1.2\\scripts\\mPDBS\\summary_pdb_93\\f8e2bf87e52caa29.final.pdb
-1.103335e-15 1.248880e-16 3.920159e-15 -6.058927e-15 5.563275e-15
-2.877017e-15

[,799]   [,800]   [,801]   [,802]   [,803]   [,804]
E:\\R-3.1.2\\scripts\\40.1ns_data\\NAT0.pdb
-1.767887e-15 7.440187e-16 4.766214e-15 -6.968731e-15 -2.506496e-15
4.789891e-16
E:\\R-3.1.2\\scripts\\40.1ns_data\\LJP6.pdb
-1.591558e-15 1.011138e-15 4.033651e-15 -6.373600e-15 -8.519026e-16
-1.737762e-15
E:\\R-3.1.2\\scripts\\40.1ns_data\\3WY9.pdb
-6.630291e-17 1.887935e-15 5.046670e-15 -7.760469e-15 -2.262171e-15
-1.645058e-15
E:\\R-3.1.2\\scripts\\40.1ns_data\\ZSP9.pdb
-1.993676e-15 1.134875e-15 2.107430e-15 -6.761063e-15 -1.916213e-15
-2.377528e-15
E:\\R-3.1.2\\scripts\\mPDBS\\summary_pdb_93\\013d855ad26c877c.final.pdb
-3.345585e-15 2.143563e-15 2.824560e-15 -2.001935e-15 -7.110479e-16
-1.308135e-15
E:\\R-3.1.2\\scripts\\mPDBS\\summary_pdb_93\\0a2a705342ca0d54.final.pdb
-2.635065e-15 1.332221e-15 4.017231e-15 -3.876297e-15 -7.262536e-16
-1.761563e-15
E:\\R-3.1.2\\scripts\\mPDBS\\summary_pdb_93\\0a59fceadb4194d9.final.pdb
-6.220262e-15 -3.801143e-17 -4.288634e-16 -5.168945e-15 -4.469439e-16
-1.681225e-15
E:\\R-3.1.2\\scripts\\mPDBS\\summary_pdb_93\\0dba59f3306825e8.final.pdb
-2.057552e-15 1.904171e-15 -3.559223e-15 -4.664735e-15 -2.466588e-15
-1.875389e-15
E:\\R-3.1.2\\scripts\\mPDBS\\summary_pdb_93\\126f26a78b57339c.final.pdb
4.831428e-15 -4.482226e-16 1.043956e-14 3.883115e-15 -1.933594e-15
-2.313407e-15
E:\\R-3.1.2\\scripts\\mPDBS\\summary_pdb_93\\12f06f3e342df706.final.pdb
-2.020770e-15 8.476911e-16 1.934262e-15 -3.695236e-15 -8.884503e-16
-2.436804e-15
E:\\R-3.1.2\\scripts\\mPDBS\\summary_pdb_93\\18618a2a251b867b.final.pdb
3.651344e-15 1.049804e-15 -4.450668e-16 1.942732e-15 -8.280352e-15
-6.570827e-15
E:\\R-3.1.2\\scripts\\mPDBS\\summary_pdb_93\\1f305f598641eeb2.final.pdb
-1.838946e-15 1.525456e-15 4.282305e-15 -5.196161e-15 -1.375200e-15
-2.037633e-15
E:\\R-3.1.2\\scripts\\mPDBS\\summary_pdb_93\\1f8a00b551be0a39.final.pdb
3.651344e-15 1.049804e-15 -4.450668e-16 1.942732e-15 -8.280352e-15
-6.570827e-15
E:\\R-3.1.2\\scripts\\mPDBS\\summary_pdb_93\\219f45328d47579a.final.pdb
3.161339e-15 4.419361e-15 1.864442e-14 -7.569802e-15 3.832185e-15
-7.832555e-15
E:\\R-3.1.2\\scripts\\mPDBS\\summary_pdb_93\\238dd3918e54336d.final.pdb

4.082464e-15 1.084033e-14 4.613978e-15 1.869262e-15 1.793235e-15
-1.792248e-15
E:\\R-3.1.2\\scripts\\mPDBS\\summary_pdb_93\\2b3027889e2fdc42.final.pdb
-2.236944e-15 1.034807e-15 1.961152e-15 -2.065525e-15 -4.629901e-16
-2.929359e-15
E:\\R-3.1.2\\scripts\\mPDBS\\summary_pdb_93\\2fcb3b46e33793b0.final.pdb
-4.128781e-15 3.034665e-15 -1.317382e-15 -7.761817e-15 -3.142883e-15
-3.101018e-15
E:\\R-3.1.2\\scripts\\mPDBS\\summary_pdb_93\\34eb328251842006.final.pdb
-1.327225e-14 -2.151738e-16 5.003892e-15 -1.368814e-14 7.283717e-15
2.393165e-15
E:\\R-3.1.2\\scripts\\mPDBS\\summary_pdb_93\\354b32871797b7ab.final.pdb
-2.066098e-14 4.611737e-15 -1.405532e-14 7.564429e-15 -5.633279e-15
-7.441189e-15
E:\\R-3.1.2\\scripts\\mPDBS\\summary_pdb_93\\397368adbc32d5de.final.pdb
4.831428e-15 -4.482226e-16 1.043956e-14 3.883115e-15 -1.933594e-15
-2.313407e-15
E:\\R-3.1.2\\scripts\\mPDBS\\summary_pdb_93\\411ca13cd3df6edd.final.pdb
-4.059785e-15 1.256424e-16 2.860266e-15 -4.961106e-15 -6.251942e-15
-2.558917e-15
E:\\R-3.1.2\\scripts\\mPDBS\\summary_pdb_93\\42357eee6642bf12.final.pdb
-9.711010e-15 5.556848e-16 4.800756e-15 -2.979756e-15 7.086656e-16
-1.708749e-15
E:\\R-3.1.2\\scripts\\mPDBS\\summary_pdb_93\\44342d1f75a7fcfc.final.pdb
7.274574e-17 -6.271843e-15 2.216562e-14 -2.363176e-15 -7.220098e-15
-3.375159e-15
E:\\R-3.1.2\\scripts\\mPDBS\\summary_pdb_93\\448ef21dffe8cdfb.final.pdb
-1.047787e-14 8.748844e-15 6.058662e-15 5.527900e-15 8.200473e-15
-1.418336e-15
E:\\R-3.1.2\\scripts\\mPDBS\\summary_pdb_93\\472a40b3e8f113c7.final.pdb
-2.057552e-15 1.904171e-15 -3.559223e-15 -4.664735e-15 -2.466588e-15
-1.875389e-15
E:\\R-3.1.2\\scripts\\mPDBS\\summary_pdb_93\\48a26225951ea153.final.pdb
4.098049e-15 -4.934848e-16 8.901781e-15 2.548840e-15 1.591356e-15
-5.927828e-15
E:\\R-3.1.2\\scripts\\mPDBS\\summary_pdb_93\\4ab4a5c7c6682ce6.final.pdb
-8.332955e-15 2.157332e-15 -5.273563e-15 -2.169335e-15 -9.478742e-17
-7.222675e-15
E:\\R-3.1.2\\scripts\\mPDBS\\summary_pdb_93\\4eaa45b62cb6a25f.final.pdb
6.504366e-15 4.493767e-16 2.037705e-15 -4.517487e-15 -2.374818e-15
-1.234782e-15
E:\\R-3.1.2\\scripts\\mPDBS\\summary_pdb_93\\58c371611d85ac96.final.pdb
4.831428e-15 -4.482226e-16 1.043956e-14 3.883115e-15 -1.933594e-15
-2.313407e-15
E:\\R-3.1.2\\scripts\\mPDBS\\summary_pdb_93\\59a647f04822d30a.final.pdb
-8.898090e-16 1.631557e-15 5.524422e-15 2.112411e-15 -1.515338e-16
2.908009e-15
E:\\R-3.1.2\\scripts\\mPDBS\\summary_pdb_93\\59b091fe385e05fc.final.pdb
-6.220262e-15 -3.801143e-17 -4.288634e-16 -5.168945e-15 -4.469439e-16
-1.681225e-15
E:\\R-3.1.2\\scripts\\mPDBS\\summary_pdb_93\\5c35ac30f930d2db.final.pdb
6.504366e-15 4.493767e-16 2.037705e-15 -4.517487e-15 -2.374818e-15
-1.234782e-15
E:\\R-3.1.2\\scripts\\mPDBS\\summary_pdb_93\\5f60752431cf2dcb.final.pdb

6.663012e-15 -1.856309e-15 -3.577822e-15 -1.508400e-15 -4.793746e-15
-2.003991e-15
E:\\R-3.1.2\\scripts\\mPDBS\\summary_pdb_93\\626456dd6ff2868d.final.pdb
-2.057552e-15 1.904171e-15 -3.559223e-15 -4.664735e-15 -2.466588e-15
-1.875389e-15
E:\\R-3.1.2\\scripts\\mPDBS\\summary_pdb_93\\6583d53ff3c8f7ef.final.pdb
-8.898090e-16 1.631557e-15 5.524422e-15 2.112411e-15 -1.515338e-16
2.908009e-15
E:\\R-3.1.2\\scripts\\mPDBS\\summary_pdb_93\\69760178986bef01.final.pdb
5.133913e-15 4.594207e-15 -3.865663e-15 6.217690e-15 -9.647593e-15
-1.571658e-14
E:\\R-3.1.2\\scripts\\mPDBS\\summary_pdb_93\\6a9d87d47926b1c8.final.pdb
-1.941800e-15 1.139049e-15 2.688818e-15 -2.818122e-15 -2.019431e-17
-2.669893e-15
E:\\R-3.1.2\\scripts\\mPDBS\\summary_pdb_93\\6bb3547ea982ffc4.final.pdb
-1.769922e-15 2.425629e-15 2.971344e-14 2.048486e-15 -4.642463e-15
-4.526033e-15
E:\\R-3.1.2\\scripts\\mPDBS\\summary_pdb_93\\716d9624611d1c42.final.pdb
-6.246759e-15 4.349773e-15 8.739121e-16 -6.326594e-15 -5.627559e-16
-3.617717e-15
E:\\R-3.1.2\\scripts\\mPDBS\\summary_pdb_93\\75f27e70ad0f1be4.final.pdb
-8.898090e-16 1.631557e-15 5.524422e-15 2.112411e-15 -1.515338e-16
2.908009e-15
E:\\R-3.1.2\\scripts\\mPDBS\\summary_pdb_93\\769f910fffb22872.final.pdb
-2.098153e-15 1.257970e-15 -3.578969e-16 -3.445753e-15 -1.002970e-15
-1.939115e-15
E:\\R-3.1.2\\scripts\\mPDBS\\summary_pdb_93\\791f8c80dd5c3135.final.pdb
-6.220262e-15 -3.801143e-17 -4.288634e-16 -5.168945e-15 -4.469439e-16
-1.681225e-15
E:\\R-3.1.2\\scripts\\mPDBS\\summary_pdb_93\\7fad98e66a3f6099.final.pdb
-8.400024e-15 -9.173663e-16 3.899692e-15 -1.038073e-14 4.135349e-15
-3.920797e-15
E:\\R-3.1.2\\scripts\\mPDBS\\summary_pdb_93\\804b89c94a633196.final.pdb
-4.128781e-15 3.034665e-15 -1.317382e-15 -7.761817e-15 -3.142883e-15
-3.101018e-15
E:\\R-3.1.2\\scripts\\mPDBS\\summary_pdb_93\\813cc65a2298feba.final.pdb
-4.128781e-15 3.034665e-15 -1.317382e-15 -7.761817e-15 -3.142883e-15
-3.101018e-15
E:\\R-3.1.2\\scripts\\mPDBS\\summary_pdb_93\\83c0eefcfe12d3f9.final.pdb
-4.059785e-15 1.256424e-16 2.860266e-15 -4.961106e-15 -6.251942e-15
-2.558917e-15
E:\\R-3.1.2\\scripts\\mPDBS\\summary_pdb_93\\86d50469ef569760.final.pdb
-2.057552e-15 1.904171e-15 -3.559223e-15 -4.664735e-15 -2.466588e-15
-1.875389e-15
E:\\R-3.1.2\\scripts\\mPDBS\\summary_pdb_93\\89d0666e1402203d.final.pdb
-8.898090e-16 1.631557e-15 5.524422e-15 2.112411e-15 -1.515338e-16
2.908009e-15
E:\\R-3.1.2\\scripts\\mPDBS\\summary_pdb_93\\8b34852716dadc62.final.pdb
6.144324e-17 -2.506042e-15 1.555107e-14 -7.166807e-15 -2.419504e-15
-4.013483e-15
E:\\R-3.1.2\\scripts\\mPDBS\\summary_pdb_93\\8b6ef9ac3fe74395.final.pdb
-2.938967e-15 9.024384e-16 3.210453e-14 -7.797760e-15 -1.784420e-15
3.110843e-15
E:\\R-3.1.2\\scripts\\mPDBS\\summary_pdb_93\\98bbe0d5986c138f.final.pdb

-6.220262e-15 -3.801143e-17 -4.288634e-16 -5.168945e-15 -4.469439e-16
-1.681225e-15
E:\\R-3.1.2\\scripts\\mPDBS\\summary_pdb_93\\9c22f4909d67e2fa.final.pdb
3.796981e-15 2.990538e-15 -5.058747e-15 -3.268449e-15 4.201502e-15
-1.982121e-15
E:\\R-3.1.2\\scripts\\mPDBS\\summary_pdb_93\\9dda8ed0d81fb3ef.final.pdb
3.759860e-15 -8.777760e-15 8.610062e-15 -3.526312e-16 -1.709566e-14
-6.021914e-15
E:\\R-3.1.2\\scripts\\mPDBS\\summary_pdb_93\\9f752b3616e6033b.final.pdb
-3.856922e-15 1.361042e-15 9.590466e-15 -5.608876e-15 2.663138e-16
-2.156881e-15
E:\\R-3.1.2\\scripts\\mPDBS\\summary_pdb_93\\a02c70083816d6ba.final.pdb
6.504366e-15 4.493767e-16 2.037705e-15 -4.517487e-15 -2.374818e-15
-1.234782e-15
E:\\R-3.1.2\\scripts\\mPDBS\\summary_pdb_93\\a0c997d891e4e082.final.pdb
-3.846333e-16 1.902652e-15 1.092618e-14 -3.824903e-15 2.835735e-15
-3.855550e-16
E:\\R-3.1.2\\scripts\\mPDBS\\summary_pdb_93\\ab8039a2d823c616.final.pdb
-1.892338e-15 1.165286e-15 5.503433e-15 -4.075136e-15 -1.033783e-15
-2.341456e-15
E:\\R-3.1.2\\scripts\\mPDBS\\summary_pdb_93\\acf4d74b9c71d4dc.final.pdb
-4.128781e-15 3.034665e-15 -1.317382e-15 -7.761817e-15 -3.142883e-15
-3.101018e-15
E:\\R-3.1.2\\scripts\\mPDBS\\summary_pdb_93\\ad8a823e4b4c668e.final.pdb
-2.154571e-15 5.089130e-16 3.572125e-15 -4.396462e-15 -1.154936e-15
-2.788171e-15
E:\\R-3.1.2\\scripts\\mPDBS\\summary_pdb_93\\b16ababd03b7b857.final.pdb
3.796981e-15 2.990538e-15 -5.058747e-15 -3.268449e-15 4.201502e-15
-1.982121e-15
E:\\R-3.1.2\\scripts\\mPDBS\\summary_pdb_93\\b226923e697d3c63.final.pdb
5.688495e-16 4.143436e-15 3.902022e-15 -1.171425e-16 -4.553611e-15
-8.593765e-16
E:\\R-3.1.2\\scripts\\mPDBS\\summary_pdb_93\\b28e48f6fa27a165.final.pdb
-6.220262e-15 -3.801143e-17 -4.288634e-16 -5.168945e-15 -4.469439e-16
-1.681225e-15
E:\\R-3.1.2\\scripts\\mPDBS\\summary_pdb_93\\b42c4924e3cd1aec.final.pdb
-7.777949e-15 4.446254e-15 -3.903800e-15 -9.953025e-15 7.910810e-15
1.471658e-15
E:\\R-3.1.2\\scripts\\mPDBS\\summary_pdb_93\\b4dd239fcabff1df.final.pdb
-6.220262e-15 -3.801143e-17 -4.288634e-16 -5.168945e-15 -4.469439e-16
-1.681225e-15
E:\\R-3.1.2\\scripts\\mPDBS\\summary_pdb_93\\b669942ef97459a2.final.pdb
6.144324e-17 -2.506042e-15 1.555107e-14 -7.166807e-15 -2.419504e-15
-4.013483e-15
E:\\R-3.1.2\\scripts\\mPDBS\\summary_pdb_93\\b99f4d9d87c1017b.final.pdb
-6.220262e-15 -3.801143e-17 -4.288634e-16 -5.168945e-15 -4.469439e-16
-1.681225e-15
E:\\R-3.1.2\\scripts\\mPDBS\\summary_pdb_93\\b9d2cbf3975403e7.final.pdb
-6.220262e-15 -3.801143e-17 -4.288634e-16 -5.168945e-15 -4.469439e-16
-1.681225e-15
E:\\R-3.1.2\\scripts\\mPDBS\\summary_pdb_93\\bc88c8a505f3a288.final.pdb
-1.576597e-15 9.456852e-16 2.797722e-15 -2.114531e-15 -1.830491e-16
-2.351696e-15
E:\\R-3.1.2\\scripts\\mPDBS\\summary_pdb_93\\bdac0e64f4ef427e.final.pdb

1.799921e-15 3.269937e-15 6.154506e-15 -3.753305e-15 7.679189e-15
-1.733189e-15
E:\\R-3.1.2\\scripts\\mPDBS\\summary_pdb_93\\ca2a1f55fc9d0681.final.pdb
6.144324e-17 -2.506042e-15 1.555107e-14 -7.166807e-15 -2.419504e-15
-4.013483e-15
E:\\R-3.1.2\\scripts\\mPDBS\\summary_pdb_93\\ce34248de3e81a3c.final.pdb
-4.063435e-16 -4.014548e-15 -6.163071e-16 -4.176194e-15 -9.721926e-16
1.409586e-15
E:\\R-3.1.2\\scripts\\mPDBS\\summary_pdb_93\\d02e697772494a61.final.pdb
-1.089397e-15 6.058566e-15 5.887265e-15 -6.634344e-16 5.960908e-15
-2.097559e-15
E:\\R-3.1.2\\scripts\\mPDBS\\summary_pdb_93\\d04221245d015047.final.pdb
-2.839701e-15 1.332337e-16 3.048799e-15 -4.411239e-15 1.920162e-16
-2.474162e-15
E:\\R-3.1.2\\scripts\\mPDBS\\summary_pdb_93\\d678141e4018054a.final.pdb
-7.036440e-15 1.205983e-15 6.221197e-16 -2.015030e-15 5.298269e-16
2.375472e-16
E:\\R-3.1.2\\scripts\\mPDBS\\summary_pdb_93\\d71b40af0f0efe39.final.pdb
6.794754e-16 6.776387e-15 -2.243120e-14 -7.984269e-15 3.032967e-15
-2.508072e-15
E:\\R-3.1.2\\scripts\\mPDBS\\summary_pdb_93\\dc0809eff10197e2.final.pdb
-3.290003e-16 -1.516949e-15 -7.626511e-16 -7.784640e-15 3.829744e-15
4.050992e-15
E:\\R-3.1.2\\scripts\\mPDBS\\summary_pdb_93\\e1163918d3707839.final.pdb
-7.495189e-15 7.475081e-15 -1.064667e-14 -2.060076e-14 1.405130e-14
1.086983e-15
E:\\R-3.1.2\\scripts\\mPDBS\\summary_pdb_93\\e4b48b06824ef848.final.pdb
3.651344e-15 1.049804e-15 -4.450668e-16 1.942732e-15 -8.280352e-15
-6.570827e-15
E:\\R-3.1.2\\scripts\\mPDBS\\summary_pdb_93\\e529e19ad281fea2.final.pdb
-3.345585e-15 2.143563e-15 2.824560e-15 -2.001935e-15 -7.110479e-16
-1.308135e-15
E:\\R-3.1.2\\scripts\\mPDBS\\summary_pdb_93\\e5602082718ec3a1.final.pdb
-4.128781e-15 3.034665e-15 -1.317382e-15 -7.761817e-15 -3.142883e-15
-3.101018e-15
E:\\R-3.1.2\\scripts\\mPDBS\\summary_pdb_93\\e5d589d996d0a960.final.pdb
7.455443e-15 -2.996261e-16 3.133492e-14 -4.422258e-15 -1.092166e-15
-8.718184e-15
E:\\R-3.1.2\\scripts\\mPDBS\\summary_pdb_93\\e9df9e72307a2fbd.final.pdb
6.504366e-15 4.493767e-16 2.037705e-15 -4.517487e-15 -2.374818e-15
-1.234782e-15
E:\\R-3.1.2\\scripts\\mPDBS\\summary_pdb_93\\ea00f8694090cae7.final.pdb
-4.128781e-15 3.034665e-15 -1.317382e-15 -7.761817e-15 -3.142883e-15
-3.101018e-15
E:\\R-3.1.2\\scripts\\mPDBS\\summary_pdb_93\\eba517be32db050e.final.pdb
-6.220262e-15 -3.801143e-17 -4.288634e-16 -5.168945e-15 -4.469439e-16
-1.681225e-15
E:\\R-3.1.2\\scripts\\mPDBS\\summary_pdb_93\\ec04b9545899c29d.final.pdb
-2.093490e-15 1.576046e-15 4.958902e-15 -4.754492e-15 -4.989122e-16
-2.438409e-15
E:\\R-3.1.2\\scripts\\mPDBS\\summary_pdb_93\\ee6862cbcad52078.final.pdb
-1.498995e-15 1.485076e-15 3.916619e-15 -1.750672e-15 -4.839117e-17
-2.461851e-15
E:\\R-3.1.2\\scripts\\mPDBS\\summary_pdb_93\\ef6a42fc83b81e5d.final.pdb

-2.145845e-15 1.211241e-15 3.189613e-15 -3.373328e-15 -1.231303e-15
-1.745260e-15
E:\\R-3.1.2\\scripts\\mPDBS\\summary_pdb_93\\ef8e88d4ad93a0ad.final.pdb
-2.839701e-15 1.332337e-16 3.048799e-15 -4.411239e-15 1.920162e-16
-2.474162e-15
E:\\R-3.1.2\\scripts\\mPDBS\\summary_pdb_93\\f14fcae8deec4fa3.final.pdb
-3.856922e-15 1.361042e-15 9.590466e-15 -5.608876e-15 2.663138e-16
-2.156881e-15
E:\\R-3.1.2\\scripts\\mPDBS\\summary_pdb_93\\f83584e22003fb92.final.pdb
4.098049e-15 -4.934848e-16 8.901781e-15 2.548840e-15 1.591356e-15
-5.927828e-15
E:\\R-3.1.2\\scripts\\mPDBS\\summary_pdb_93\\f8e2bf87e52caa29.final.pdb
-3.345585e-15 2.143563e-15 2.824560e-15 -2.001935e-15 -7.110479e-16
-1.308135e-15

[,805]   [,806]   [,807]   [,808]   [,809]   [,810]
E:\\R-3.1.2\\scripts\\40.1ns_data\\NAT0.pdb
-1.830169e-15 3.474800e-15 4.514924e-15 -1.997220e-15 2.754455e-15
-1.388500e-15
E:\\R-3.1.2\\scripts\\40.1ns_data\\LJP6.pdb
-1.143950e-15 2.972295e-15 2.448423e-15 -1.242814e-15 4.304883e-15
-1.006685e-15
E:\\R-3.1.2\\scripts\\40.1ns_data\\3WY9.pdb
-1.196751e-15 2.215389e-15 2.939452e-15 -1.185723e-15 3.802871e-15
2.337373e-16
E:\\R-3.1.2\\scripts\\40.1ns_data\\ZSP9.pdb
-2.408432e-15 2.704431e-15 2.164627e-15 -8.521624e-16 1.833984e-15
-1.788727e-15
E:\\R-3.1.2\\scripts\\mPDBS\\summary_pdb_93\\013d855ad26c877c.final.pdb
8.042491e-15 2.413151e-15 -3.779130e-15 -4.692529e-16 -2.452661e-15
5.567735e-15
E:\\R-3.1.2\\scripts\\mPDBS\\summary_pdb_93\\0a2a705342ca0d54.final.pdb
-1.146173e-15 1.866477e-15 5.721257e-16 -1.099638e-15 2.857803e-15
-1.814960e-15
E:\\R-3.1.2\\scripts\\mPDBS\\summary_pdb_93\\0a59fceadb4194d9.final.pdb
-3.378996e-15 1.599718e-15 -3.117867e-15 -2.069792e-15 6.454652e-15
-3.144357e-15
E:\\R-3.1.2\\scripts\\mPDBS\\summary_pdb_93\\0dba59f3306825e8.final.pdb
4.045040e-16 6.268141e-15 -3.352388e-15 -1.712182e-15 1.821698e-15
-4.873566e-15
E:\\R-3.1.2\\scripts\\mPDBS\\summary_pdb_93\\126f26a78b57339c.final.pdb
-4.664575e-15 3.137073e-15 3.743065e-15 -3.602814e-15 6.312897e-15
-2.911811e-15
E:\\R-3.1.2\\scripts\\mPDBS\\summary_pdb_93\\12f06f3e342df706.final.pdb
-1.767420e-15 1.827988e-15 1.034430e-15 -1.167509e-15 3.496181e-15
-1.807805e-15
E:\\R-3.1.2\\scripts\\mPDBS\\summary_pdb_93\\18618a2a251b867b.final.pdb
-4.350491e-15 7.064351e-15 1.519809e-15 -5.914925e-15 2.950851e-15
-1.806251e-15
E:\\R-3.1.2\\scripts\\mPDBS\\summary_pdb_93\\1f305f598641eeb2.final.pdb
-7.386731e-16 1.625877e-15 2.847245e-16 -1.007658e-15 3.374050e-15
-1.865616e-15
E:\\R-3.1.2\\scripts\\mPDBS\\summary_pdb_93\\1f8a00b551be0a39.final.pdb
-4.350491e-15 7.064351e-15 1.519809e-15 -5.914925e-15 2.950851e-15

-1.806251e-15
E:\\R-3.1.2\\scripts\\mPDBS\\summary_pdb_93\\219f45328d47579a.final.pdb
-9.473051e-17 -3.747242e-15 1.114590e-14 1.254048e-14 -5.056137e-17
3.466240e-15
E:\\R-3.1.2\\scripts\\mPDBS\\summary_pdb_93\\238dd3918e54336d.final.pdb
5.092960e-15 5.600804e-15 6.603957e-15 8.776116e-16 -5.774498e-15
-3.553318e-15
E:\\R-3.1.2\\scripts\\mPDBS\\summary_pdb_93\\2b3027889e2fdc42.final.pdb
-7.891620e-16 2.823566e-15 1.460149e-16 -9.138157e-16 4.175659e-15
-1.903379e-15
E:\\R-3.1.2\\scripts\\mPDBS\\summary_pdb_93\\2fcb3b46e33793b0.final.pdb
1.815977e-16 1.439395e-15 -4.593318e-15 1.819074e-15 6.869629e-16
-1.921127e-15
E:\\R-3.1.2\\scripts\\mPDBS\\summary_pdb_93\\34eb328251842006.final.pdb
-4.632883e-16 -5.863947e-15 -1.422542e-15 -6.783490e-15 4.512380e-15
-3.280390e-15
E:\\R-3.1.2\\scripts\\mPDBS\\summary_pdb_93\\354b32871797b7ab.final.pdb
-1.535537e-14 -2.298180e-15 -4.323526e-15 -3.337833e-15 6.031844e-15
-5.440581e-15
E:\\R-3.1.2\\scripts\\mPDBS\\summary_pdb_93\\397368adbc32d5de.final.pdb
-4.664575e-15 3.137073e-15 3.743065e-15 -3.602814e-15 6.312897e-15
-2.911811e-15
E:\\R-3.1.2\\scripts\\mPDBS\\summary_pdb_93\\411ca13cd3df6edd.final.pdb
-4.885313e-15 -1.883180e-16 -7.164612e-15 -6.401134e-16 2.839755e-15
-3.291342e-15
E:\\R-3.1.2\\scripts\\mPDBS\\summary_pdb_93\\42357eee6642bf12.final.pdb
-9.017516e-15 -4.519653e-15 5.269539e-15 -2.164778e-15 1.421712e-14
-8.824661e-15
E:\\R-3.1.2\\scripts\\mPDBS\\summary_pdb_93\\44342d1f75a7fcfc.final.pdb
3.936743e-15 -5.350751e-17 -1.071043e-14 -1.847422e-14 2.116079e-15
-2.912765e-15
E:\\R-3.1.2\\scripts\\mPDBS\\summary_pdb_93\\448ef21dffe8cdfb.final.pdb
-1.116089e-14 -6.069790e-15 1.510273e-14 5.976339e-15 1.707143e-14
1.064001e-14
E:\\R-3.1.2\\scripts\\mPDBS\\summary_pdb_93\\472a40b3e8f113c7.final.pdb
4.045040e-16 6.268141e-15 -3.352388e-15 -1.712182e-15 1.821698e-15
-4.873566e-15
E:\\R-3.1.2\\scripts\\mPDBS\\summary_pdb_93\\48a26225951ea153.final.pdb
2.901029e-15 7.964202e-15 -3.352922e-15 -9.611286e-15 3.539883e-15
-2.047795e-15
E:\\R-3.1.2\\scripts\\mPDBS\\summary_pdb_93\\4ab4a5c7c6682ce6.final.pdb
-9.742110e-15 1.651283e-15 5.387583e-15 9.085821e-15 8.339054e-15
-3.640394e-15
E:\\R-3.1.2\\scripts\\mPDBS\\summary_pdb_93\\4eaa45b62cb6a25f.final.pdb
3.555281e-15 6.713437e-15 1.228022e-15 -1.811779e-16 7.637060e-16
-6.683567e-16
E:\\R-3.1.2\\scripts\\mPDBS\\summary_pdb_93\\58c371611d85ac96.final.pdb
-4.664575e-15 3.137073e-15 3.743065e-15 -3.602814e-15 6.312897e-15
-2.911811e-15
E:\\R-3.1.2\\scripts\\mPDBS\\summary_pdb_93\\59a647f04822d30a.final.pdb
2.402814e-15 -1.819781e-15 7.851111e-15 4.445815e-15 3.830854e-15
-4.791037e-15
E:\\R-3.1.2\\scripts\\mPDBS\\summary_pdb_93\\59b091fe385e05fc.final.pdb
-3.378996e-15 1.599718e-15 -3.117867e-15 -2.069792e-15 6.454652e-15

-3.144357e-15
E:\\R-3.1.2\\scripts\\mPDBS\\summary_pdb_93\\5c35ac30f930d2db.final.pdb
3.555281e-15 6.713437e-15 1.228022e-15 -1.811779e-16 7.637060e-16
-6.683567e-16
E:\\R-3.1.2\\scripts\\mPDBS\\summary_pdb_93\\5f60752431cf2dcb.final.pdb
2.887690e-16 3.924835e-15 -3.284165e-15 -8.648070e-15 2.729000e-15
3.031640e-15
E:\\R-3.1.2\\scripts\\mPDBS\\summary_pdb_93\\626456dd6ff2868d.final.pdb
4.045040e-16 6.268141e-15 -3.352388e-15 -1.712182e-15 1.821698e-15
-4.873566e-15
E:\\R-3.1.2\\scripts\\mPDBS\\summary_pdb_93\\6583d53ff3c8f7ef.final.pdb
2.402814e-15 -1.819781e-15 7.851111e-15 4.445815e-15 3.830854e-15
-4.791037e-15
E:\\R-3.1.2\\scripts\\mPDBS\\summary_pdb_93\\69760178986bef01.final.pdb
2.384392e-15 9.864058e-15 1.238707e-15 4.333288e-15 -3.939681e-15
9.209314e-16
E:\\R-3.1.2\\scripts\\mPDBS\\summary_pdb_93\\6a9d87d47926b1c8.final.pdb
-6.842518e-16 2.442750e-15 -6.180298e-16 -8.862370e-16 4.479294e-15
-1.527501e-15
E:\\R-3.1.2\\scripts\\mPDBS\\summary_pdb_93\\6bb3547ea982ffc4.final.pdb
2.228298e-15 6.461491e-15 -2.413426e-15 -7.853730e-15 -3.317761e-15
8.462338e-15
E:\\R-3.1.2\\scripts\\mPDBS\\summary_pdb_93\\716d9624611d1c42.final.pdb
1.284175e-15 4.733432e-15 3.175078e-15 2.152535e-15 7.397056e-16
-4.162475e-15
E:\\R-3.1.2\\scripts\\mPDBS\\summary_pdb_93\\75f27e70ad0f1be4.final.pdb
2.402814e-15 -1.819781e-15 7.851111e-15 4.445815e-15 3.830854e-15
-4.791037e-15
E:\\R-3.1.2\\scripts\\mPDBS\\summary_pdb_93\\769f910fffb22872.final.pdb
-8.943801e-16 2.604648e-15 2.044552e-16 -9.415809e-16 3.739499e-15
-1.291689e-15
E:\\R-3.1.2\\scripts\\mPDBS\\summary_pdb_93\\791f8c80dd5c3135.final.pdb
-3.378996e-15 1.599718e-15 -3.117867e-15 -2.069792e-15 6.454652e-15
-3.144357e-15
E:\\R-3.1.2\\scripts\\mPDBS\\summary_pdb_93\\7fad98e66a3f6099.final.pdb
-4.861547e-15 -2.643921e-15 -2.492546e-15 -3.686858e-15 6.977618e-15
3.222495e-15
E:\\R-3.1.2\\scripts\\mPDBS\\summary_pdb_93\\804b89c94a633196.final.pdb
1.815977e-16 1.439395e-15 -4.593318e-15 1.819074e-15 6.869629e-16
-1.921127e-15
E:\\R-3.1.2\\scripts\\mPDBS\\summary_pdb_93\\813cc65a2298feba.final.pdb
1.815977e-16 1.439395e-15 -4.593318e-15 1.819074e-15 6.869629e-16
-1.921127e-15
E:\\R-3.1.2\\scripts\\mPDBS\\summary_pdb_93\\83c0eefcfe12d3f9.final.pdb
-4.885313e-15 -1.883180e-16 -7.164612e-15 -6.401134e-16 2.839755e-15
-3.291342e-15
E:\\R-3.1.2\\scripts\\mPDBS\\summary_pdb_93\\86d50469ef569760.final.pdb
4.045040e-16 6.268141e-15 -3.352388e-15 -1.712182e-15 1.821698e-15
-4.873566e-15
E:\\R-3.1.2\\scripts\\mPDBS\\summary_pdb_93\\89d0666e1402203d.final.pdb
2.402814e-15 -1.819781e-15 7.851111e-15 4.445815e-15 3.830854e-15
-4.791037e-15
E:\\R-3.1.2\\scripts\\mPDBS\\summary_pdb_93\\8b34852716dadc62.final.pdb
-8.763504e-15 1.230361e-15 1.623931e-15 -2.084921e-15 8.352706e-15

-8.367360e-15
E:\\R-3.1.2\\scripts\\mPDBS\\summary_pdb_93\\8b6ef9ac3fe74395.final.pdb
-1.569696e-15 6.538086e-16 2.698524e-15 -6.679502e-16 8.998094e-15
4.721449e-15
E:\\R-3.1.2\\scripts\\mPDBS\\summary_pdb_93\\98bbe0d5986c138f.final.pdb
-3.378996e-15 1.599718e-15 -3.117867e-15 -2.069792e-15 6.454652e-15
-3.144357e-15
E:\\R-3.1.2\\scripts\\mPDBS\\summary_pdb_93\\9c22f4909d67e2fa.final.pdb
7.854822e-15 5.925769e-15 2.981786e-15 -6.465206e-15 -1.926398e-15
-2.617619e-15
E:\\R-3.1.2\\scripts\\mPDBS\\summary_pdb_93\\9dda8ed0d81fb3ef.final.pdb
-2.580396e-15 7.509345e-15 -9.382933e-15 -1.304437e-14 -2.316573e-15
-3.610341e-15
E:\\R-3.1.2\\scripts\\mPDBS\\summary_pdb_93\\9f752b3616e6033b.final.pdb
-2.841253e-15 1.948681e-16 2.311264e-15 4.472240e-16 4.854224e-15
-1.414562e-15
E:\\R-3.1.2\\scripts\\mPDBS\\summary_pdb_93\\a02c70083816d6ba.final.pdb
3.555281e-15 6.713437e-15 1.228022e-15 -1.811779e-16 7.637060e-16
-6.683567e-16
E:\\R-3.1.2\\scripts\\mPDBS\\summary_pdb_93\\a0c997d891e4e082.final.pdb
-1.757070e-15 -2.571944e-15 -3.924623e-15 1.299072e-15 -9.170928e-15
3.408787e-15
E:\\R-3.1.2\\scripts\\mPDBS\\summary_pdb_93\\ab8039a2d823c616.final.pdb
-1.293031e-15 2.975539e-15 7.769109e-16 -1.099942e-15 4.029234e-15
-1.932861e-15
E:\\R-3.1.2\\scripts\\mPDBS\\summary_pdb_93\\acf4d74b9c71d4dc.final.pdb
1.815977e-16 1.439395e-15 -4.593318e-15 1.819074e-15 6.869629e-16
-1.921127e-15
E:\\R-3.1.2\\scripts\\mPDBS\\summary_pdb_93\\ad8a823e4b4c668e.final.pdb
-1.391957e-15 2.428954e-15 1.102096e-15 -1.378754e-15 4.118233e-15
-1.877497e-15
E:\\R-3.1.2\\scripts\\mPDBS\\summary_pdb_93\\b16ababd03b7b857.final.pdb
7.854822e-15 5.925769e-15 2.981786e-15 -6.465206e-15 -1.926398e-15
-2.617619e-15
E:\\R-3.1.2\\scripts\\mPDBS\\summary_pdb_93\\b226923e697d3c63.final.pdb
-7.267510e-15 -4.580648e-15 2.463494e-15 -7.343946e-16 3.409966e-15
3.273910e-15
E:\\R-3.1.2\\scripts\\mPDBS\\summary_pdb_93\\b28e48f6fa27a165.final.pdb
-3.378996e-15 1.599718e-15 -3.117867e-15 -2.069792e-15 6.454652e-15
-3.144357e-15
E:\\R-3.1.2\\scripts\\mPDBS\\summary_pdb_93\\b42c4924e3cd1aec.final.pdb
-6.352907e-17 1.877619e-15 3.054411e-17 -1.801466e-15 2.500537e-15
1.357257e-15
E:\\R-3.1.2\\scripts\\mPDBS\\summary_pdb_93\\b4dd239fcabff1df.final.pdb
-3.378996e-15 1.599718e-15 -3.117867e-15 -2.069792e-15 6.454652e-15
-3.144357e-15
E:\\R-3.1.2\\scripts\\mPDBS\\summary_pdb_93\\b669942ef97459a2.final.pdb
-8.763504e-15 1.230361e-15 1.623931e-15 -2.084921e-15 8.352706e-15
-8.367360e-15
E:\\R-3.1.2\\scripts\\mPDBS\\summary_pdb_93\\b99f4d9d87c1017b.final.pdb
-3.378996e-15 1.599718e-15 -3.117867e-15 -2.069792e-15 6.454652e-15
-3.144357e-15
E:\\R-3.1.2\\scripts\\mPDBS\\summary_pdb_93\\b9d2cbf3975403e7.final.pdb
-3.378996e-15 1.599718e-15 -3.117867e-15 -2.069792e-15 6.454652e-15

-3.144357e-15
E:\\R-3.1.2\\scripts\\mPDBS\\summary_pdb_93\\bc88c8a505f3a288.final.pdb
-8.041240e-16 2.452335e-15 4.201012e-16 -7.124251e-16 3.783178e-15
-1.581588e-15
E:\\R-3.1.2\\scripts\\mPDBS\\summary_pdb_93\\bdac0e64f4ef427e.final.pdb
7.572279e-15 7.167723e-15 1.223437e-14 -1.298495e-15 3.973830e-15
1.934513e-15
E:\\R-3.1.2\\scripts\\mPDBS\\summary_pdb_93\\ca2a1f55fc9d0681.final.pdb
-8.763504e-15 1.230361e-15 1.623931e-15 -2.084921e-15 8.352706e-15
-8.367360e-15
E:\\R-3.1.2\\scripts\\mPDBS\\summary_pdb_93\\ce34248de3e81a3c.final.pdb
-1.695907e-15 4.597218e-15 -1.128833e-15 -8.486801e-16 3.204654e-15
-5.438973e-15
E:\\R-3.1.2\\scripts\\mPDBS\\summary_pdb_93\\d02e697772494a61.final.pdb
-8.423963e-16 -2.778778e-15 1.056433e-14 1.117363e-14 1.027169e-14
-3.252344e-15
E:\\R-3.1.2\\scripts\\mPDBS\\summary_pdb_93\\d04221245d015047.final.pdb
1.234461e-15 4.925530e-15 -5.228307e-16 -1.077499e-15 3.438777e-15
-3.266429e-15
E:\\R-3.1.2\\scripts\\mPDBS\\summary_pdb_93\\d678141e4018054a.final.pdb
2.034732e-15 7.420110e-16 -1.939454e-16 -1.148298e-14 3.053803e-15
1.984252e-15
E:\\R-3.1.2\\scripts\\mPDBS\\summary_pdb_93\\d71b40af0f0efe39.final.pdb
-5.301506e-15 1.655815e-15 -9.243907e-16 2.632353e-16 1.288199e-14
-2.075232e-15
E:\\R-3.1.2\\scripts\\mPDBS\\summary_pdb_93\\dc0809eff10197e2.final.pdb
3.035146e-15 -2.957005e-15 4.566988e-15 4.269760e-15 7.134973e-15
-7.951289e-16
E:\\R-3.1.2\\scripts\\mPDBS\\summary_pdb_93\\e1163918d3707839.final.pdb
-4.998120e-16 9.348640e-15 2.669059e-15 4.911913e-15 1.119063e-14
-1.374499e-15
E:\\R-3.1.2\\scripts\\mPDBS\\summary_pdb_93\\e4b48b06824ef848.final.pdb
-4.350491e-15 7.064351e-15 1.519809e-15 -5.914925e-15 2.950851e-15
-1.806251e-15
E:\\R-3.1.2\\scripts\\mPDBS\\summary_pdb_93\\e529e19ad281fea2.final.pdb
8.042491e-15 2.413151e-15 -3.779130e-15 -4.692529e-16 -2.452661e-15
5.567735e-15
E:\\R-3.1.2\\scripts\\mPDBS\\summary_pdb_93\\e5602082718ec3a1.final.pdb
1.815977e-16 1.439395e-15 -4.593318e-15 1.819074e-15 6.869629e-16
-1.921127e-15
E:\\R-3.1.2\\scripts\\mPDBS\\summary_pdb_93\\e5d589d996d0a960.final.pdb
-9.468136e-16 1.068037e-14 -2.468413e-15 4.837163e-15 -7.565355e-16
7.272620e-15
E:\\R-3.1.2\\scripts\\mPDBS\\summary_pdb_93\\e9df9e72307a2fbd.final.pdb
3.555281e-15 6.713437e-15 1.228022e-15 -1.811779e-16 7.637060e-16
-6.683567e-16
E:\\R-3.1.2\\scripts\\mPDBS\\summary_pdb_93\\ea00f8694090cae7.final.pdb
1.815977e-16 1.439395e-15 -4.593318e-15 1.819074e-15 6.869629e-16
-1.921127e-15
E:\\R-3.1.2\\scripts\\mPDBS\\summary_pdb_93\\eba517be32db050e.final.pdb
-3.378996e-15 1.599718e-15 -3.117867e-15 -2.069792e-15 6.454652e-15
-3.144357e-15
E:\\R-3.1.2\\scripts\\mPDBS\\summary_pdb_93\\ec04b9545899c29d.final.pdb
-1.118241e-15 2.293573e-15 8.168075e-16 -1.550690e-15 4.111011e-15

-2.044536e-15
E:\\R-3.1.2\\scripts\\mPDBS\\summary_pdb_93\\ee6862cbcad52078.final.pdb
-6.350969e-16 2.929709e-15 -2.920027e-16 -8.572203e-16 4.024304e-15
-1.512416e-15
E:\\R-3.1.2\\scripts\\mPDBS\\summary_pdb_93\\ef6a42fc83b81e5d.final.pdb
-1.660694e-15 2.254397e-15 2.968292e-16 -9.273778e-16 3.684205e-15
-1.932236e-15
E:\\R-3.1.2\\scripts\\mPDBS\\summary_pdb_93\\ef8e88d4ad93a0ad.final.pdb
1.234461e-15 4.925530e-15 -5.228307e-16 -1.077499e-15 3.438777e-15
-3.266429e-15
E:\\R-3.1.2\\scripts\\mPDBS\\summary_pdb_93\\f14fcae8deec4fa3.final.pdb
-2.841253e-15 1.948681e-16 2.311264e-15 4.472240e-16 4.854224e-15
-1.414562e-15
E:\\R-3.1.2\\scripts\\mPDBS\\summary_pdb_93\\f83584e22003fb92.final.pdb
2.901029e-15 7.964202e-15 -3.352922e-15 -9.611286e-15 3.539883e-15
-2.047795e-15
E:\\R-3.1.2\\scripts\\mPDBS\\summary_pdb_93\\f8e2bf87e52caa29.final.pdb
8.042491e-15 2.413151e-15 -3.779130e-15 -4.692529e-16 -2.452661e-15
5.567735e-15

[,811]   [,812]   [,813]   [,814]   [,815]   [,816]
E:\\R-3.1.2\\scripts\\40.1ns_data\\NAT0.pdb
1.386747e-15 -1.763868e-15 2.728116e-15 -1.689374e-15 -9.584328e-16
4.182086e-15
E:\\R-3.1.2\\scripts\\40.1ns_data\\LJP6.pdb
4.976743e-16 -6.492991e-16 5.275226e-15 -2.604683e-15 -1.438266e-15
1.629673e-15
E:\\R-3.1.2\\scripts\\40.1ns_data\\3WY9.pdb
1.187482e-15 2.195989e-16 1.834408e-15 -2.670499e-15 -1.510913e-15
1.983447e-15
E:\\R-3.1.2\\scripts\\40.1ns_data\\ZSP9.pdb
-1.308574e-16 2.087200e-17 3.931962e-15 -2.629172e-15 -1.691453e-15
2.839782e-15
E:\\R-3.1.2\\scripts\\mPDBS\\summary_pdb_93\\013d855ad26c877c.final.pdb
-4.565707e-15 -9.623237e-16 7.826802e-15 1.211395e-15 -1.414860e-15
1.227129e-15
E:\\R-3.1.2\\scripts\\mPDBS\\summary_pdb_93\\0a2a705342ca0d54.final.pdb
2.535969e-16 -1.618446e-15 4.980559e-15 -1.896674e-15 -1.553878e-15
1.905811e-15
E:\\R-3.1.2\\scripts\\mPDBS\\summary_pdb_93\\0a59fceadb4194d9.final.pdb
-7.481482e-16 -1.488682e-15 -1.587444e-15 7.286525e-16 -8.712242e-16
-4.926954e-16
E:\\R-3.1.2\\scripts\\mPDBS\\summary_pdb_93\\0dba59f3306825e8.final.pdb
-2.852771e-16 6.636735e-17 2.678143e-15 -5.168918e-15 -3.604081e-15
6.243124e-16
E:\\R-3.1.2\\scripts\\mPDBS\\summary_pdb_93\\126f26a78b57339c.final.pdb
-5.832158e-16 -2.388050e-15 8.748347e-15 -2.412442e-15 -4.332665e-15
2.242159e-15
E:\\R-3.1.2\\scripts\\mPDBS\\summary_pdb_93\\12f06f3e342df706.final.pdb
-3.409796e-16 -1.542335e-15 4.087962e-15 -1.735344e-15 -1.985933e-15
1.077698e-15
E:\\R-3.1.2\\scripts\\mPDBS\\summary_pdb_93\\18618a2a251b867b.final.pdb
-2.120777e-15 -3.327120e-16 2.601961e-15 -1.765095e-15 -3.132572e-15
3.636786e-15

E:\\R-3.1.2\\scripts\\mPDBS\\summary_pdb_93\\1f305f598641eeb2.final.pdb
-6.147726e-16 -8.988311e-16 5.171168e-15 -1.717452e-15 -1.609726e-15
1.716942e-15
E:\\R-3.1.2\\scripts\\mPDBS\\summary_pdb_93\\1f8a00b551be0a39.final.pdb
-2.120777e-15 -3.327120e-16 2.601961e-15 -1.765095e-15 -3.132572e-15
3.636786e-15
E:\\R-3.1.2\\scripts\\mPDBS\\summary_pdb_93\\219f45328d47579a.final.pdb
-5.822648e-15 -5.289461e-15 1.128767e-14 2.546643e-15 -8.997536e-15
1.534891e-16
E:\\R-3.1.2\\scripts\\mPDBS\\summary_pdb_93\\238dd3918e54336d.final.pdb
2.443743e-15 6.893716e-16 1.101464e-14 -6.946881e-16 -2.610718e-15
1.884960e-15
E:\\R-3.1.2\\scripts\\mPDBS\\summary_pdb_93\\2b3027889e2fdc42.final.pdb
-8.487622e-16 -1.368769e-15 3.657031e-15 -2.319257e-15 -1.932441e-15
9.293442e-16
E:\\R-3.1.2\\scripts\\mPDBS\\summary_pdb_93\\2fcb3b46e33793b0.final.pdb
-2.445773e-15 7.434875e-17 1.644475e-15 -2.591753e-15 -2.267186e-15
1.108054e-15
E:\\R-3.1.2\\scripts\\mPDBS\\summary_pdb_93\\34eb328251842006.final.pdb
-1.731537e-15 -1.457675e-14 4.902066e-16 6.521572e-15 7.997324e-15
-5.968975e-15
E:\\R-3.1.2\\scripts\\mPDBS\\summary_pdb_93\\354b32871797b7ab.final.pdb
-9.776036e-15 1.945787e-15 -3.451508e-15 1.702131e-16 -3.967686e-15
9.758827e-15
E:\\R-3.1.2\\scripts\\mPDBS\\summary_pdb_93\\397368adbc32d5de.final.pdb
-5.832158e-16 -2.388050e-15 8.748347e-15 -2.412442e-15 -4.332665e-15
2.242159e-15
E:\\R-3.1.2\\scripts\\mPDBS\\summary_pdb_93\\411ca13cd3df6edd.final.pdb
-3.121882e-15 -4.640847e-15 8.114014e-16 -1.337548e-15 -3.030464e-15
6.342577e-16
E:\\R-3.1.2\\scripts\\mPDBS\\summary_pdb_93\\42357eee6642bf12.final.pdb
-1.129249e-16 -8.580841e-15 -1.260880e-14 1.442044e-14 8.417533e-15
-2.232914e-15
E:\\R-3.1.2\\scripts\\mPDBS\\summary_pdb_93\\44342d1f75a7fcfc.final.pdb
-8.395300e-15 -2.602320e-15 3.144424e-15 -4.475550e-15 9.307450e-16
-3.764758e-15
E:\\R-3.1.2\\scripts\\mPDBS\\summary_pdb_93\\448ef21dffe8cdfb.final.pdb
1.099486e-14 -7.043222e-15 1.110392e-14 1.253504e-14 4.423643e-15
8.232563e-15
E:\\R-3.1.2\\scripts\\mPDBS\\summary_pdb_93\\472a40b3e8f113c7.final.pdb
-2.852771e-16 6.636735e-17 2.678143e-15 -5.168918e-15 -3.604081e-15
6.243124e-16
E:\\R-3.1.2\\scripts\\mPDBS\\summary_pdb_93\\48a26225951ea153.final.pdb
-2.655477e-15 -4.502547e-15 6.278198e-15 -9.409071e-15 4.523766e-15
1.864794e-15
E:\\R-3.1.2\\scripts\\mPDBS\\summary_pdb_93\\4ab4a5c7c6682ce6.final.pdb
6.086796e-15 8.558834e-16 4.328272e-15 -7.231504e-15 3.268893e-15
1.907357e-15
E:\\R-3.1.2\\scripts\\mPDBS\\summary_pdb_93\\4eaa45b62cb6a25f.final.pdb
1.390813e-15 1.301229e-15 9.406073e-15 -5.896982e-15 -3.264689e-15
6.031734e-16
E:\\R-3.1.2\\scripts\\mPDBS\\summary_pdb_93\\58c371611d85ac96.final.pdb
-5.832158e-16 -2.388050e-15 8.748347e-15 -2.412442e-15 -4.332665e-15
2.242159e-15

E:\\R-3.1.2\\scripts\\mPDBS\\summary_pdb_93\\59a647f04822d30a.final.pdb
1.490455e-15 1.190790e-17 6.756986e-15 -3.897887e-15 7.221445e-16
2.696219e-15
E:\\R-3.1.2\\scripts\\mPDBS\\summary_pdb_93\\59b091fe385e05fc.final.pdb
-7.481482e-16 -1.488682e-15 -1.587444e-15 7.286525e-16 -8.712242e-16
-4.926954e-16
E:\\R-3.1.2\\scripts\\mPDBS\\summary_pdb_93\\5c35ac30f930d2db.final.pdb
1.390813e-15 1.301229e-15 9.406073e-15 -5.896982e-15 -3.264689e-15
6.031734e-16
E:\\R-3.1.2\\scripts\\mPDBS\\summary_pdb_93\\5f60752431cf2dcb.final.pdb
3.630670e-15 -2.049308e-15 6.879620e-15 -6.138072e-15 -6.902464e-15
-7.947193e-16
E:\\R-3.1.2\\scripts\\mPDBS\\summary_pdb_93\\626456dd6ff2868d.final.pdb
-2.852771e-16 6.636735e-17 2.678143e-15 -5.168918e-15 -3.604081e-15
6.243124e-16
E:\\R-3.1.2\\scripts\\mPDBS\\summary_pdb_93\\6583d53ff3c8f7ef.final.pdb
1.490455e-15 1.190790e-17 6.756986e-15 -3.897887e-15 7.221445e-16
2.696219e-15
E:\\R-3.1.2\\scripts\\mPDBS\\summary_pdb_93\\69760178986bef01.final.pdb
-2.807486e-15 4.885393e-15 1.465444e-14 -8.427723e-15 -1.058408e-14
2.012349e-15
E:\\R-3.1.2\\scripts\\mPDBS\\summary_pdb_93\\6a9d87d47926b1c8.final.pdb
-1.116643e-15 -1.288235e-15 3.764486e-15 -1.697736e-15 -1.735784e-15
7.416231e-16
E:\\R-3.1.2\\scripts\\mPDBS\\summary_pdb_93\\6bb3547ea982ffc4.final.pdb
-1.266696e-15 -1.004490e-14 2.060356e-14 -3.335608e-15 -5.514835e-15
-3.627459e-15
E:\\R-3.1.2\\scripts\\mPDBS\\summary_pdb_93\\716d9624611d1c42.final.pdb
-7.773402e-15 -2.475400e-15 7.191464e-15 -1.659016e-15 1.673141e-15
1.458253e-15
E:\\R-3.1.2\\scripts\\mPDBS\\summary_pdb_93\\75f27e70ad0f1be4.final.pdb
1.490455e-15 1.190790e-17 6.756986e-15 -3.897887e-15 7.221445e-16
2.696219e-15
E:\\R-3.1.2\\scripts\\mPDBS\\summary_pdb_93\\769f910fffb22872.final.pdb
-2.346986e-16 -1.037118e-15 4.404122e-15 -1.795453e-15 -1.152819e-15
1.275611e-15
E:\\R-3.1.2\\scripts\\mPDBS\\summary_pdb_93\\791f8c80dd5c3135.final.pdb
-7.481482e-16 -1.488682e-15 -1.587444e-15 7.286525e-16 -8.712242e-16
-4.926954e-16
E:\\R-3.1.2\\scripts\\mPDBS\\summary_pdb_93\\7fad98e66a3f6099.final.pdb
-4.891404e-16 -5.791196e-15 9.487374e-15 -2.072943e-15 4.965031e-15
-5.058122e-16
E:\\R-3.1.2\\scripts\\mPDBS\\summary_pdb_93\\804b89c94a633196.final.pdb
-2.445773e-15 7.434875e-17 1.644475e-15 -2.591753e-15 -2.267186e-15
1.108054e-15
E:\\R-3.1.2\\scripts\\mPDBS\\summary_pdb_93\\813cc65a2298feba.final.pdb
-2.445773e-15 7.434875e-17 1.644475e-15 -2.591753e-15 -2.267186e-15
1.108054e-15
E:\\R-3.1.2\\scripts\\mPDBS\\summary_pdb_93\\83c0eefcfe12d3f9.final.pdb
-3.121882e-15 -4.640847e-15 8.114014e-16 -1.337548e-15 -3.030464e-15
6.342577e-16
E:\\R-3.1.2\\scripts\\mPDBS\\summary_pdb_93\\86d50469ef569760.final.pdb
-2.852771e-16 6.636735e-17 2.678143e-15 -5.168918e-15 -3.604081e-15
6.243124e-16

E:\\R-3.1.2\\scripts\\mPDBS\\summary_pdb_93\\89d0666e1402203d.final.pdb
1.490455e-15 1.190790e-17 6.756986e-15 -3.897887e-15 7.221445e-16
2.696219e-15
E:\\R-3.1.2\\scripts\\mPDBS\\summary_pdb_93\\8b34852716dadc62.final.pdb
7.903534e-16 -1.106998e-15 3.117255e-15 -5.554661e-15 -1.561153e-15
5.512078e-16
E:\\R-3.1.2\\scripts\\mPDBS\\summary_pdb_93\\8b6ef9ac3fe74395.final.pdb
2.235661e-15 -2.260208e-15 1.023647e-14 -3.277292e-15 -5.545273e-16
7.703768e-16
E:\\R-3.1.2\\scripts\\mPDBS\\summary_pdb_93\\98bbe0d5986c138f.final.pdb
-7.481482e-16 -1.488682e-15 -1.587444e-15 7.286525e-16 -8.712242e-16
-4.926954e-16
E:\\R-3.1.2\\scripts\\mPDBS\\summary_pdb_93\\9c22f4909d67e2fa.final.pdb
-2.740676e-15 1.294618e-15 7.739209e-15 1.490919e-15 -2.932452e-15
1.714340e-15
E:\\R-3.1.2\\scripts\\mPDBS\\summary_pdb_93\\9dda8ed0d81fb3ef.final.pdb
6.216709e-15 1.856190e-15 1.666846e-15 -1.008998e-14 3.384186e-15
-2.336431e-15
E:\\R-3.1.2\\scripts\\mPDBS\\summary_pdb_93\\9f752b3616e6033b.final.pdb
1.219580e-15 -1.080092e-15 3.325977e-15 -1.093151e-15 -2.014293e-15
1.495091e-15
E:\\R-3.1.2\\scripts\\mPDBS\\summary_pdb_93\\a02c70083816d6ba.final.pdb
1.390813e-15 1.301229e-15 9.406073e-15 -5.896982e-15 -3.264689e-15
6.031734e-16
E:\\R-3.1.2\\scripts\\mPDBS\\summary_pdb_93\\a0c997d891e4e082.final.pdb
-8.286522e-16 2.510442e-15 1.536382e-14 -1.233163e-14 -9.685109e-15
-3.078483e-15
E:\\R-3.1.2\\scripts\\mPDBS\\summary_pdb_93\\ab8039a2d823c616.final.pdb
-5.966329e-16 -1.242524e-15 3.958203e-15 -1.811396e-15 -1.754387e-15
5.017692e-16
E:\\R-3.1.2\\scripts\\mPDBS\\summary_pdb_93\\acf4d74b9c71d4dc.final.pdb
-2.445773e-15 7.434875e-17 1.644475e-15 -2.591753e-15 -2.267186e-15
1.108054e-15
E:\\R-3.1.2\\scripts\\mPDBS\\summary_pdb_93\\ad8a823e4b4c668e.final.pdb
3.663460e-17 -7.851744e-16 4.585143e-15 -1.657053e-15 -1.441423e-15
1.314074e-15
E:\\R-3.1.2\\scripts\\mPDBS\\summary_pdb_93\\b16ababd03b7b857.final.pdb
-2.740676e-15 1.294618e-15 7.739209e-15 1.490919e-15 -2.932452e-15
1.714340e-15
E:\\R-3.1.2\\scripts\\mPDBS\\summary_pdb_93\\b226923e697d3c63.final.pdb
-1.776463e-15 -6.593217e-16 4.336396e-15 -4.553281e-16 -5.521993e-15
-2.137371e-15
E:\\R-3.1.2\\scripts\\mPDBS\\summary_pdb_93\\b28e48f6fa27a165.final.pdb
-7.481482e-16 -1.488682e-15 -1.587444e-15 7.286525e-16 -8.712242e-16
-4.926954e-16
E:\\R-3.1.2\\scripts\\mPDBS\\summary_pdb_93\\b42c4924e3cd1aec.final.pdb
-4.792713e-15 -1.112301e-15 2.615144e-15 4.432587e-16 -1.027644e-14
6.087387e-15
E:\\R-3.1.2\\scripts\\mPDBS\\summary_pdb_93\\b4dd239fcabff1df.final.pdb
-7.481482e-16 -1.488682e-15 -1.587444e-15 7.286525e-16 -8.712242e-16
-4.926954e-16
E:\\R-3.1.2\\scripts\\mPDBS\\summary_pdb_93\\b669942ef97459a2.final.pdb
7.903534e-16 -1.106998e-15 3.117255e-15 -5.554661e-15 -1.561153e-15
5.512078e-16

E:\\R-3.1.2\\scripts\\mPDBS\\summary_pdb_93\\b99f4d9d87c1017b.final.pdb
-7.481482e-16 -1.488682e-15 -1.587444e-15 7.286525e-16 -8.712242e-16
-4.926954e-16
E:\\R-3.1.2\\scripts\\mPDBS\\summary_pdb_93\\b9d2cbf3975403e7.final.pdb
-7.481482e-16 -1.488682e-15 -1.587444e-15 7.286525e-16 -8.712242e-16
-4.926954e-16
E:\\R-3.1.2\\scripts\\mPDBS\\summary_pdb_93\\bc88c8a505f3a288.final.pdb
-6.995760e-16 -1.339659e-15 3.794345e-15 -1.921138e-15 -1.986651e-15
7.365731e-16
E:\\R-3.1.2\\scripts\\mPDBS\\summary_pdb_93\\bdac0e64f4ef427e.final.pdb
4.690879e-15 5.088242e-15 1.423363e-14 -2.937830e-15 2.831925e-15
2.671474e-15
E:\\R-3.1.2\\scripts\\mPDBS\\summary_pdb_93\\ca2a1f55fc9d0681.final.pdb
7.903534e-16 -1.106998e-15 3.117255e-15 -5.554661e-15 -1.561153e-15
5.512078e-16
E:\\R-3.1.2\\scripts\\mPDBS\\summary_pdb_93\\ce34248de3e81a3c.final.pdb
-1.354885e-15 3.758555e-15 2.754169e-16 -5.220446e-15 3.399173e-15
2.715708e-15
E:\\R-3.1.2\\scripts\\mPDBS\\summary_pdb_93\\d02e697772494a61.final.pdb
-4.999955e-15 -4.304030e-15 8.864454e-15 3.453036e-15 1.069064e-15
-8.706482e-16
E:\\R-3.1.2\\scripts\\mPDBS\\summary_pdb_93\\d04221245d015047.final.pdb
5.456465e-19 -1.777795e-15 4.527870e-15 -1.895530e-15 -3.102296e-16
2.521204e-15
E:\\R-3.1.2\\scripts\\mPDBS\\summary_pdb_93\\d678141e4018054a.final.pdb
-2.706783e-15 -1.526107e-14 4.519387e-15 3.933678e-15 2.069944e-15
-2.415813e-15
E:\\R-3.1.2\\scripts\\mPDBS\\summary_pdb_93\\d71b40af0f0efe39.final.pdb
-5.836828e-16 -8.234883e-16 -5.674060e-15 1.494741e-15 -4.070960e-15
6.878830e-15
E:\\R-3.1.2\\scripts\\mPDBS\\summary_pdb_93\\dc0809eff10197e2.final.pdb
3.741914e-15 4.856895e-15 2.990691e-15 -8.334634e-15 -5.232690e-15
4.378762e-15
E:\\R-3.1.2\\scripts\\mPDBS\\summary_pdb_93\\e1163918d3707839.final.pdb
1.847808e-15 -1.269158e-14 -1.621051e-15 8.162778e-15 4.836201e-16
-2.214133e-15
E:\\R-3.1.2\\scripts\\mPDBS\\summary_pdb_93\\e4b48b06824ef848.final.pdb
-2.120777e-15 -3.327120e-16 2.601961e-15 -1.765095e-15 -3.132572e-15
3.636786e-15
E:\\R-3.1.2\\scripts\\mPDBS\\summary_pdb_93\\e529e19ad281fea2.final.pdb
-4.565707e-15 -9.623237e-16 7.826802e-15 1.211395e-15 -1.414860e-15
1.227129e-15
E:\\R-3.1.2\\scripts\\mPDBS\\summary_pdb_93\\e5602082718ec3a1.final.pdb
-2.445773e-15 7.434875e-17 1.644475e-15 -2.591753e-15 -2.267186e-15
1.108054e-15
E:\\R-3.1.2\\scripts\\mPDBS\\summary_pdb_93\\e5d589d996d0a960.final.pdb
2.946795e-15 4.993798e-15 1.618196e-14 -1.616964e-14 -5.042158e-15
6.202822e-15
E:\\R-3.1.2\\scripts\\mPDBS\\summary_pdb_93\\e9df9e72307a2fbd.final.pdb
1.390813e-15 1.301229e-15 9.406073e-15 -5.896982e-15 -3.264689e-15
6.031734e-16
E:\\R-3.1.2\\scripts\\mPDBS\\summary_pdb_93\\ea00f8694090cae7.final.pdb
-2.445773e-15 7.434875e-17 1.644475e-15 -2.591753e-15 -2.267186e-15
1.108054e-15

E:\\R-3.1.2\\scripts\\mPDBS\\summary_pdb_93\\eba517be32db050e.final.pdb
-7.481482e-16 -1.488682e-15 -1.587444e-15 7.286525e-16 -8.712242e-16
-4.926954e-16
E:\\R-3.1.2\\scripts\\mPDBS\\summary_pdb_93\\ec04b9545899c29d.final.pdb
-3.565919e-16 -1.160765e-15 3.445399e-15 -2.045202e-15 -1.694325e-15
1.626762e-15
E:\\R-3.1.2\\scripts\\mPDBS\\summary_pdb_93\\ee6862cbcad52078.final.pdb
-8.370528e-16 -1.464396e-15 3.443254e-15 -1.786697e-15 -1.935043e-15
5.559450e-16
E:\\R-3.1.2\\scripts\\mPDBS\\summary_pdb_93\\ef6a42fc83b81e5d.final.pdb
-3.591650e-16 -1.249730e-15 4.126376e-15 -1.784177e-15 -2.021915e-15
1.192995e-15
E:\\R-3.1.2\\scripts\\mPDBS\\summary_pdb_93\\ef8e88d4ad93a0ad.final.pdb
5.456465e-19 -1.777795e-15 4.527870e-15 -1.895530e-15 -3.102296e-16
2.521204e-15
E:\\R-3.1.2\\scripts\\mPDBS\\summary_pdb_93\\f14fcae8deec4fa3.final.pdb
1.219580e-15 -1.080092e-15 3.325977e-15 -1.093151e-15 -2.014293e-15
1.495091e-15
E:\\R-3.1.2\\scripts\\mPDBS\\summary_pdb_93\\f83584e22003fb92.final.pdb
-2.655477e-15 -4.502547e-15 6.278198e-15 -9.409071e-15 4.523766e-15
1.864794e-15
E:\\R-3.1.2\\scripts\\mPDBS\\summary_pdb_93\\f8e2bf87e52caa29.final.pdb
-4.565707e-15 -9.623237e-16 7.826802e-15 1.211395e-15 -1.414860e-15
1.227129e-15

[,817]   [,818]   [,819]   [,820]   [,821]   [,822]
E:\\R-3.1.2\\scripts\\40.1ns_data\\NAT0.pdb
-1.032965e-15 -5.745692e-16 3.073974e-15 -4.004647e-15 2.427009e-15
-4.700713e-15
E:\\R-3.1.2\\scripts\\40.1ns_data\\LJP6.pdb
-9.726938e-17 -1.445900e-15 -4.235277e-16 -2.860531e-15 2.372330e-15
-3.659954e-15
E:\\R-3.1.2\\scripts\\40.1ns_data\\3WY9.pdb
-1.157743e-15 -8.802733e-16 3.497530e-15 -2.647300e-15 1.529430e-15
-5.031632e-15
E:\\R-3.1.2\\scripts\\40.1ns_data\\ZSP9.pdb
-1.295718e-15 -3.712390e-16 6.438738e-16 -3.064619e-15 2.280936e-15
-2.785136e-15
E:\\R-3.1.2\\scripts\\mPDBS\\summary_pdb_93\\013d855ad26c877c.final.pdb
2.336930e-15 -1.004061e-17 3.794588e-15 -2.876972e-15 3.285260e-15
1.043244e-15
E:\\R-3.1.2\\scripts\\mPDBS\\summary_pdb_93\\0a2a705342ca0d54.final.pdb
-3.780455e-16 -1.562752e-15 -3.138287e-16 -2.292599e-15 1.379363e-15
-4.009735e-15
E:\\R-3.1.2\\scripts\\mPDBS\\summary_pdb_93\\0a59fceadb4194d9.final.pdb
-1.839980e-15 -3.801862e-16 -4.068726e-15 -1.220578e-15 2.724983e-15
-3.582660e-15
E:\\R-3.1.2\\scripts\\mPDBS\\summary_pdb_93\\0dba59f3306825e8.final.pdb
1.130863e-15 -4.965278e-15 -6.362218e-15 9.914617e-16 7.876916e-16
-9.291372e-15
E:\\R-3.1.2\\scripts\\mPDBS\\summary_pdb_93\\126f26a78b57339c.final.pdb
4.339383e-16 -3.379034e-16 5.902711e-15 -2.862443e-15 1.616479e-15
-3.384204e-15
E:\\R-3.1.2\\scripts\\mPDBS\\summary_pdb_93\\12f06f3e342df706.final.pdb

-5.011024e-16 -8.029428e-16 1.455589e-15 -2.099611e-15 1.863392e-15
-3.679703e-15
E:\\R-3.1.2\\scripts\\mPDBS\\summary_pdb_93\\18618a2a251b867b.final.pdb
1.494795e-15 -2.650905e-16 2.280291e-15 -3.581195e-15 -1.331224e-15
-4.208494e-15
E:\\R-3.1.2\\scripts\\mPDBS\\summary_pdb_93\\1f305f598641eeb2.final.pdb
-3.727663e-16 -1.159985e-15 9.638347e-16 -1.815635e-15 1.722218e-15
-3.894519e-15
E:\\R-3.1.2\\scripts\\mPDBS\\summary_pdb_93\\1f8a00b551be0a39.final.pdb
1.494795e-15 -2.650905e-16 2.280291e-15 -3.581195e-15 -1.331224e-15
-4.208494e-15
E:\\R-3.1.2\\scripts\\mPDBS\\summary_pdb_93\\219f45328d47579a.final.pdb
2.787683e-16 6.777717e-15 8.125079e-15 4.492734e-15 1.114121e-14
4.625617e-15
E:\\R-3.1.2\\scripts\\mPDBS\\summary_pdb_93\\238dd3918e54336d.final.pdb
-5.417196e-15 3.212426e-15 5.887720e-15 2.614245e-15 7.109292e-15
1.703042e-15
E:\\R-3.1.2\\scripts\\mPDBS\\summary_pdb_93\\2b3027889e2fdc42.final.pdb
-3.997254e-16 -1.631355e-15 4.662753e-16 -1.068356e-15 2.287422e-15
-3.574637e-15
E:\\R-3.1.2\\scripts\\mPDBS\\summary_pdb_93\\2fcb3b46e33793b0.final.pdb
6.481770e-17 -5.107348e-15 -2.273159e-15 -8.112302e-16 1.864899e-15
-4.184217e-15
E:\\R-3.1.2\\scripts\\mPDBS\\summary_pdb_93\\34eb328251842006.final.pdb
-7.845850e-15 8.796396e-15 -4.213305e-15 1.948387e-15 5.073774e-15
2.900335e-15
E:\\R-3.1.2\\scripts\\mPDBS\\summary_pdb_93\\354b32871797b7ab.final.pdb
-4.110713e-15 5.915070e-16 2.713689e-16 5.386460e-15 -1.937271e-16
-7.431094e-15
E:\\R-3.1.2\\scripts\\mPDBS\\summary_pdb_93\\397368adbc32d5de.final.pdb
4.339383e-16 -3.379034e-16 5.902711e-15 -2.862443e-15 1.616479e-15
-3.384204e-15
E:\\R-3.1.2\\scripts\\mPDBS\\summary_pdb_93\\411ca13cd3df6edd.final.pdb
-6.897870e-16 -1.587594e-15 -9.222434e-16 -2.137209e-15 4.840793e-15
-1.369195e-15
E:\\R-3.1.2\\scripts\\mPDBS\\summary_pdb_93\\42357eee6642bf12.final.pdb
-5.903387e-15 7.597012e-15 -8.070518e-15 3.620907e-15 5.083209e-15
7.333020e-15
E:\\R-3.1.2\\scripts\\mPDBS\\summary_pdb_93\\44342d1f75a7fcfc.final.pdb
-4.691279e-16 -2.356916e-15 -1.217705e-15 -5.908702e-15 -8.611155e-16
2.241143e-15
E:\\R-3.1.2\\scripts\\mPDBS\\summary_pdb_93\\448ef21dffe8cdfb.final.pdb
-1.110854e-14 1.091109e-14 2.065121e-14 -3.546889e-15 5.289243e-15
8.356099e-15
E:\\R-3.1.2\\scripts\\mPDBS\\summary_pdb_93\\472a40b3e8f113c7.final.pdb
1.130863e-15 -4.965278e-15 -6.362218e-15 9.914617e-16 7.876916e-16
-9.291372e-15
E:\\R-3.1.2\\scripts\\mPDBS\\summary_pdb_93\\48a26225951ea153.final.pdb
2.840955e-15 -3.979737e-15 8.587817e-15 -4.582039e-15 -2.471575e-15
-2.390255e-15
E:\\R-3.1.2\\scripts\\mPDBS\\summary_pdb_93\\4ab4a5c7c6682ce6.final.pdb
-6.280743e-15 -1.065864e-14 4.343194e-15 -5.882597e-15 -1.905877e-15
-6.403491e-15
E:\\R-3.1.2\\scripts\\mPDBS\\summary_pdb_93\\4eaa45b62cb6a25f.final.pdb

3.759727e-15 -4.574933e-15 2.468396e-15 -6.580747e-15 4.604686e-16
-5.950507e-15
E:\\R-3.1.2\\scripts\\mPDBS\\summary_pdb_93\\58c371611d85ac96.final.pdb
4.339383e-16 -3.379034e-16 5.902711e-15 -2.862443e-15 1.616479e-15
-3.384204e-15
E:\\R-3.1.2\\scripts\\mPDBS\\summary_pdb_93\\59a647f04822d30a.final.pdb
7.649111e-16 -6.746201e-15 4.808291e-15 -9.638727e-16 1.421347e-15
-6.705694e-15
E:\\R-3.1.2\\scripts\\mPDBS\\summary_pdb_93\\59b091fe385e05fc.final.pdb
-1.839980e-15 -3.801862e-16 -4.068726e-15 -1.220578e-15 2.724983e-15
-3.582660e-15
E:\\R-3.1.2\\scripts\\mPDBS\\summary_pdb_93\\5c35ac30f930d2db.final.pdb
3.759727e-15 -4.574933e-15 2.468396e-15 -6.580747e-15 4.604686e-16
-5.950507e-15
E:\\R-3.1.2\\scripts\\mPDBS\\summary_pdb_93\\5f60752431cf2dcb.final.pdb
-1.691231e-15 -3.885113e-15 -2.652564e-16 -1.791698e-15 -2.927066e-15
-7.084315e-15
E:\\R-3.1.2\\scripts\\mPDBS\\summary_pdb_93\\626456dd6ff2868d.final.pdb
1.130863e-15 -4.965278e-15 -6.362218e-15 9.914617e-16 7.876916e-16
-9.291372e-15
E:\\R-3.1.2\\scripts\\mPDBS\\summary_pdb_93\\6583d53ff3c8f7ef.final.pdb
7.649111e-16 -6.746201e-15 4.808291e-15 -9.638727e-16 1.421347e-15
-6.705694e-15
E:\\R-3.1.2\\scripts\\mPDBS\\summary_pdb_93\\69760178986bef01.final.pdb
-5.280656e-16 -3.770810e-15 1.352832e-15 -1.646685e-15 4.295992e-17
-5.004777e-15
E:\\R-3.1.2\\scripts\\mPDBS\\summary_pdb_93\\6a9d87d47926b1c8.final.pdb
-4.789599e-16 -1.512311e-15 -1.271880e-16 -9.056753e-16 2.163785e-15
-3.658291e-15
E:\\R-3.1.2\\scripts\\mPDBS\\summary_pdb_93\\6bb3547ea982ffc4.final.pdb
3.278634e-15 2.324814e-15 6.158421e-15 9.441570e-16 -1.506854e-15
-3.878290e-15
E:\\R-3.1.2\\scripts\\mPDBS\\summary_pdb_93\\716d9624611d1c42.final.pdb
2.531303e-15 1.431815e-15 6.727848e-15 -4.668303e-15 5.725279e-15
-3.129796e-15
E:\\R-3.1.2\\scripts\\mPDBS\\summary_pdb_93\\75f27e70ad0f1be4.final.pdb
7.649111e-16 -6.746201e-15 4.808291e-15 -9.638727e-16 1.421347e-15
-6.705694e-15
E:\\R-3.1.2\\scripts\\mPDBS\\summary_pdb_93\\769f910fffb22872.final.pdb
-2.572754e-16 -6.658117e-16 8.612875e-16 -2.040978e-15 2.639547e-15
-4.071234e-15
E:\\R-3.1.2\\scripts\\mPDBS\\summary_pdb_93\\791f8c80dd5c3135.final.pdb
-1.839980e-15 -3.801862e-16 -4.068726e-15 -1.220578e-15 2.724983e-15
-3.582660e-15
E:\\R-3.1.2\\scripts\\mPDBS\\summary_pdb_93\\7fad98e66a3f6099.final.pdb
-5.883424e-15 5.093811e-15 4.464124e-16 -3.121504e-15 4.912443e-15
2.430888e-15
E:\\R-3.1.2\\scripts\\mPDBS\\summary_pdb_93\\804b89c94a633196.final.pdb
6.481770e-17 -5.107348e-15 -2.273159e-15 -8.112302e-16 1.864899e-15
-4.184217e-15
E:\\R-3.1.2\\scripts\\mPDBS\\summary_pdb_93\\813cc65a2298feba.final.pdb
6.481770e-17 -5.107348e-15 -2.273159e-15 -8.112302e-16 1.864899e-15
-4.184217e-15
E:\\R-3.1.2\\scripts\\mPDBS\\summary_pdb_93\\83c0eefcfe12d3f9.final.pdb

-6.897870e-16 -1.587594e-15 -9.222434e-16 -2.137209e-15 4.840793e-15
-1.369195e-15
E:\\R-3.1.2\\scripts\\mPDBS\\summary_pdb_93\\86d50469ef569760.final.pdb
1.130863e-15 -4.965278e-15 -6.362218e-15 9.914617e-16 7.876916e-16
-9.291372e-15
E:\\R-3.1.2\\scripts\\mPDBS\\summary_pdb_93\\89d0666e1402203d.final.pdb
7.649111e-16 -6.746201e-15 4.808291e-15 -9.638727e-16 1.421347e-15
-6.705694e-15
E:\\R-3.1.2\\scripts\\mPDBS\\summary_pdb_93\\8b34852716dadc62.final.pdb
-1.476050e-15 3.596330e-16 -1.652128e-15 -3.768934e-15 2.825502e-15
-4.505141e-15
E:\\R-3.1.2\\scripts\\mPDBS\\summary_pdb_93\\8b6ef9ac3fe74395.final.pdb
1.518827e-15 -2.214024e-15 2.978052e-15 -4.458615e-15 -4.171805e-15
-1.094729e-14
E:\\R-3.1.2\\scripts\\mPDBS\\summary_pdb_93\\98bbe0d5986c138f.final.pdb
-1.839980e-15 -3.801862e-16 -4.068726e-15 -1.220578e-15 2.724983e-15
-3.582660e-15
E:\\R-3.1.2\\scripts\\mPDBS\\summary_pdb_93\\9c22f4909d67e2fa.final.pdb
1.441754e-15 4.680829e-15 -8.840795e-16 2.154063e-15 3.848543e-15
-1.548522e-15
E:\\R-3.1.2\\scripts\\mPDBS\\summary_pdb_93\\9dda8ed0d81fb3ef.final.pdb
2.561759e-15 -9.381679e-15 6.800824e-16 -1.319584e-14 -2.367049e-15
-8.912696e-15
E:\\R-3.1.2\\scripts\\mPDBS\\summary_pdb_93\\9f752b3616e6033b.final.pdb
-1.728946e-15 -6.407682e-16 -1.378235e-15 3.339696e-16 2.763909e-15
-2.371623e-15
E:\\R-3.1.2\\scripts\\mPDBS\\summary_pdb_93\\a02c70083816d6ba.final.pdb
3.759727e-15 -4.574933e-15 2.468396e-15 -6.580747e-15 4.604686e-16
-5.950507e-15
E:\\R-3.1.2\\scripts\\mPDBS\\summary_pdb_93\\a0c997d891e4e082.final.pdb
-2.161158e-15 -8.204685e-16 -2.812643e-16 -9.312431e-15 2.106593e-15
-8.816970e-15
E:\\R-3.1.2\\scripts\\mPDBS\\summary_pdb_93\\ab8039a2d823c616.final.pdb
-1.616728e-16 -1.032972e-15 -1.737115e-16 -2.081887e-15 2.179030e-15
-3.735098e-15
E:\\R-3.1.2\\scripts\\mPDBS\\summary_pdb_93\\acf4d74b9c71d4dc.final.pdb
6.481770e-17 -5.107348e-15 -2.273159e-15 -8.112302e-16 1.864899e-15
-4.184217e-15
E:\\R-3.1.2\\scripts\\mPDBS\\summary_pdb_93\\ad8a823e4b4c668e.final.pdb
-8.688105e-16 -9.752521e-16 4.844115e-16 -1.801500e-15 2.043760e-15
-3.801044e-15
E:\\R-3.1.2\\scripts\\mPDBS\\summary_pdb_93\\b16ababd03b7b857.final.pdb
1.441754e-15 4.680829e-15 -8.840795e-16 2.154063e-15 3.848543e-15
-1.548522e-15
E:\\R-3.1.2\\scripts\\mPDBS\\summary_pdb_93\\b226923e697d3c63.final.pdb
-4.197482e-15 1.677183e-15 -5.728217e-16 -6.797531e-15 1.299927e-15
-9.057328e-15
E:\\R-3.1.2\\scripts\\mPDBS\\summary_pdb_93\\b28e48f6fa27a165.final.pdb
-1.839980e-15 -3.801862e-16 -4.068726e-15 -1.220578e-15 2.724983e-15
-3.582660e-15
E:\\R-3.1.2\\scripts\\mPDBS\\summary_pdb_93\\b42c4924e3cd1aec.final.pdb
-7.729379e-15 8.865443e-15 2.433657e-16 6.324351e-15 6.106173e-15
-2.241816e-15
E:\\R-3.1.2\\scripts\\mPDBS\\summary_pdb_93\\b4dd239fcabff1df.final.pdb

-1.839980e-15 -3.801862e-16 -4.068726e-15 -1.220578e-15 2.724983e-15
-3.582660e-15
E:\\R-3.1.2\\scripts\\mPDBS\\summary_pdb_93\\b669942ef97459a2.final.pdb
-1.476050e-15 3.596330e-16 -1.652128e-15 -3.768934e-15 2.825502e-15
-4.505141e-15
E:\\R-3.1.2\\scripts\\mPDBS\\summary_pdb_93\\b99f4d9d87c1017b.final.pdb
-1.839980e-15 -3.801862e-16 -4.068726e-15 -1.220578e-15 2.724983e-15
-3.582660e-15
E:\\R-3.1.2\\scripts\\mPDBS\\summary_pdb_93\\b9d2cbf3975403e7.final.pdb
-1.839980e-15 -3.801862e-16 -4.068726e-15 -1.220578e-15 2.724983e-15
-3.582660e-15
E:\\R-3.1.2\\scripts\\mPDBS\\summary_pdb_93\\bc88c8a505f3a288.final.pdb
-4.636934e-16 -1.276605e-15 7.043661e-16 -1.580967e-15 1.824216e-15
-3.705609e-15
E:\\R-3.1.2\\scripts\\mPDBS\\summary_pdb_93\\bdac0e64f4ef427e.final.pdb
-4.477931e-16 7.438742e-16 3.460969e-15 -2.745447e-15 -3.645486e-15
-6.773943e-15
E:\\R-3.1.2\\scripts\\mPDBS\\summary_pdb_93\\ca2a1f55fc9d0681.final.pdb
-1.476050e-15 3.596330e-16 -1.652128e-15 -3.768934e-15 2.825502e-15
-4.505141e-15
E:\\R-3.1.2\\scripts\\mPDBS\\summary_pdb_93\\ce34248de3e81a3c.final.pdb
4.002559e-15 -4.438103e-15 1.954972e-15 -1.138674e-14 -2.417263e-15
-7.451521e-15
E:\\R-3.1.2\\scripts\\mPDBS\\summary_pdb_93\\d02e697772494a61.final.pdb
3.049989e-15 -2.059399e-15 2.438222e-15 2.365312e-15 2.639777e-15
-1.466069e-16
E:\\R-3.1.2\\scripts\\mPDBS\\summary_pdb_93\\d04221245d015047.final.pdb
6.360831e-16 -2.128450e-15 1.037126e-15 1.244644e-16 2.021592e-15
-2.114866e-15
E:\\R-3.1.2\\scripts\\mPDBS\\summary_pdb_93\\d678141e4018054a.final.pdb
-3.621559e-15 2.304770e-15 8.420777e-15 3.727776e-15 5.557697e-15
2.471398e-15
E:\\R-3.1.2\\scripts\\mPDBS\\summary_pdb_93\\d71b40af0f0efe39.final.pdb
-3.212266e-15 1.165431e-15 -3.732644e-16 4.927443e-15 1.135893e-14
-1.624680e-15
E:\\R-3.1.2\\scripts\\mPDBS\\summary_pdb_93\\dc0809eff10197e2.final.pdb
-3.764561e-15 -6.766030e-15 -3.675801e-15 -3.100220e-15 -1.112106e-15
-1.279077e-14
E:\\R-3.1.2\\scripts\\mPDBS\\summary_pdb_93\\e1163918d3707839.final.pdb
1.107085e-15 1.181052e-15 -5.513314e-15 7.601108e-15 4.299241e-15
-2.067043e-15
E:\\R-3.1.2\\scripts\\mPDBS\\summary_pdb_93\\e4b48b06824ef848.final.pdb
1.494795e-15 -2.650905e-16 2.280291e-15 -3.581195e-15 -1.331224e-15
-4.208494e-15
E:\\R-3.1.2\\scripts\\mPDBS\\summary_pdb_93\\e529e19ad281fea2.final.pdb
2.336930e-15 -1.004061e-17 3.794588e-15 -2.876972e-15 3.285260e-15
1.043244e-15
E:\\R-3.1.2\\scripts\\mPDBS\\summary_pdb_93\\e5602082718ec3a1.final.pdb
6.481770e-17 -5.107348e-15 -2.273159e-15 -8.112302e-16 1.864899e-15
-4.184217e-15
E:\\R-3.1.2\\scripts\\mPDBS\\summary_pdb_93\\e5d589d996d0a960.final.pdb
5.552225e-15 9.358856e-16 6.144418e-16 -1.375832e-14 -5.769983e-15
-1.285251e-14
E:\\R-3.1.2\\scripts\\mPDBS\\summary_pdb_93\\e9df9e72307a2fbd.final.pdb

3.759727e-15 -4.574933e-15 2.468396e-15 -6.580747e-15 4.604686e-16
-5.950507e-15
E:\\R-3.1.2\\scripts\\mPDBS\\summary_pdb_93\\ea00f8694090cae7.final.pdb
6.481770e-17 -5.107348e-15 -2.273159e-15 -8.112302e-16 1.864899e-15
-4.184217e-15
E:\\R-3.1.2\\scripts\\mPDBS\\summary_pdb_93\\eba517be32db050e.final.pdb
-1.839980e-15 -3.801862e-16 -4.068726e-15 -1.220578e-15 2.724983e-15
-3.582660e-15
E:\\R-3.1.2\\scripts\\mPDBS\\summary_pdb_93\\ec04b9545899c29d.final.pdb
-2.115214e-16 -1.283123e-15 1.084273e-15 -1.215408e-15 1.871508e-15
-3.834726e-15
E:\\R-3.1.2\\scripts\\mPDBS\\summary_pdb_93\\ee6862cbcad52078.final.pdb
-2.251689e-16 -1.403239e-15 4.437239e-16 -1.125168e-15 2.021986e-15
-3.603477e-15
E:\\R-3.1.2\\scripts\\mPDBS\\summary_pdb_93\\ef6a42fc83b81e5d.final.pdb
-4.717306e-16 -8.904583e-16 7.775871e-16 -2.107114e-15 2.125190e-15
-3.770259e-15
E:\\R-3.1.2\\scripts\\mPDBS\\summary_pdb_93\\ef8e88d4ad93a0ad.final.pdb
6.360831e-16 -2.128450e-15 1.037126e-15 1.244644e-16 2.021592e-15
-2.114866e-15
E:\\R-3.1.2\\scripts\\mPDBS\\summary_pdb_93\\f14fcae8deec4fa3.final.pdb
-1.728946e-15 -6.407682e-16 -1.378235e-15 3.339696e-16 2.763909e-15
-2.371623e-15
E:\\R-3.1.2\\scripts\\mPDBS\\summary_pdb_93\\f83584e22003fb92.final.pdb
2.840955e-15 -3.979737e-15 8.587817e-15 -4.582039e-15 -2.471575e-15
-2.390255e-15
E:\\R-3.1.2\\scripts\\mPDBS\\summary_pdb_93\\f8e2bf87e52caa29.final.pdb
2.336930e-15 -1.004061e-17 3.794588e-15 -2.876972e-15 3.285260e-15
1.043244e-15

[,823]   [,824]   [,825]   [,826]   [,827]   [,828]
E:\\R-3.1.2\\scripts\\40.1ns_data\\NAT0.pdb
2.446896e-15 6.741100e-15 1.087528e-15 1.030971e-15 -2.621331e-15
-2.388885e-15
E:\\R-3.1.2\\scripts\\40.1ns_data\\LJP6.pdb
1.861324e-15 5.085527e-15 1.626140e-15 8.703131e-16 -1.168997e-15
1.347183e-15
E:\\R-3.1.2\\scripts\\40.1ns_data\\3WY9.pdb
1.060790e-15 5.434459e-15 1.330340e-15 -1.481904e-15 -2.665343e-15
6.284081e-16
E:\\R-3.1.2\\scripts\\40.1ns_data\\ZSP9.pdb
1.357857e-15 7.248158e-15 2.911533e-15 -3.884441e-16 -1.891503e-15
4.634334e-17
E:\\R-3.1.2\\scripts\\mPDBS\\summary_pdb_93\\013d855ad26c877c.final.pdb
7.608394e-15 6.248619e-15 9.723281e-15 -2.132417e-15 2.420487e-15
2.337725e-15
E:\\R-3.1.2\\scripts\\mPDBS\\summary_pdb_93\\0a2a705342ca0d54.final.pdb
1.105018e-15 4.560945e-15 6.979295e-16 3.987961e-16 -1.598328e-15
5.201213e-16
E:\\R-3.1.2\\scripts\\mPDBS\\summary_pdb_93\\0a59fceadb4194d9.final.pdb
1.420992e-15 3.244164e-15 -6.228492e-16 -2.541414e-15 -3.123020e-15
3.198002e-16
E:\\R-3.1.2\\scripts\\mPDBS\\summary_pdb_93\\0dba59f3306825e8.final.pdb
2.642748e-15 4.702981e-15 1.190176e-15 8.478376e-16 -1.255393e-15

4.530415e-15
E:\\R-3.1.2\\scripts\\mPDBS\\summary_pdb_93\\126f26a78b57339c.final.pdb
-3.860947e-15 8.176337e-16 -2.403574e-15 3.333480e-15 -1.929983e-15
-4.291684e-15
E:\\R-3.1.2\\scripts\\mPDBS\\summary_pdb_93\\12f06f3e342df706.final.pdb
1.775597e-15 4.533623e-15 1.435336e-15 -2.035642e-17 -2.583245e-15
2.852628e-16
E:\\R-3.1.2\\scripts\\mPDBS\\summary_pdb_93\\18618a2a251b867b.final.pdb
5.558143e-16 -2.688472e-15 3.503333e-15 2.700763e-15 -4.113059e-15
-3.971847e-16
E:\\R-3.1.2\\scripts\\mPDBS\\summary_pdb_93\\1f305f598641eeb2.final.pdb
1.349845e-15 4.729482e-15 2.208135e-15 3.083422e-16 -1.839417e-15
4.306917e-16
E:\\R-3.1.2\\scripts\\mPDBS\\summary_pdb_93\\1f8a00b551be0a39.final.pdb
5.558143e-16 -2.688472e-15 3.503333e-15 2.700763e-15 -4.113059e-15
-3.971847e-16
E:\\R-3.1.2\\scripts\\mPDBS\\summary_pdb_93\\219f45328d47579a.final.pdb
4.407102e-15 1.427874e-14 7.241086e-15 2.627669e-16 -1.348831e-15
4.355309e-15
E:\\R-3.1.2\\scripts\\mPDBS\\summary_pdb_93\\238dd3918e54336d.final.pdb
6.002614e-15 9.844354e-15 1.414448e-15 7.595483e-16 6.526054e-15
9.557119e-16
E:\\R-3.1.2\\scripts\\mPDBS\\summary_pdb_93\\2b3027889e2fdc42.final.pdb
1.327209e-15 3.869099e-15 8.383843e-16 -5.241471e-16 -2.021687e-15
3.559584e-16
E:\\R-3.1.2\\scripts\\mPDBS\\summary_pdb_93\\2fcb3b46e33793b0.final.pdb
7.348953e-15 7.584837e-15 5.141552e-15 -5.694278e-16 -2.944436e-15
4.004656e-15
E:\\R-3.1.2\\scripts\\mPDBS\\summary_pdb_93\\34eb328251842006.final.pdb
-2.548567e-15 6.899732e-15 3.415185e-15 -1.427356e-16 -1.281699e-15
-4.847700e-16
E:\\R-3.1.2\\scripts\\mPDBS\\summary_pdb_93\\354b32871797b7ab.final.pdb
5.366918e-15 1.044375e-14 -8.840239e-15 -7.793009e-16 5.759986e-16
-5.204383e-15
E:\\R-3.1.2\\scripts\\mPDBS\\summary_pdb_93\\397368adbc32d5de.final.pdb
-3.860947e-15 8.176337e-16 -2.403574e-15 3.333480e-15 -1.929983e-15
-4.291684e-15
E:\\R-3.1.2\\scripts\\mPDBS\\summary_pdb_93\\411ca13cd3df6edd.final.pdb
5.421458e-15 6.118562e-15 5.559892e-15 4.112808e-15 -5.904959e-15
-5.084984e-16
E:\\R-3.1.2\\scripts\\mPDBS\\summary_pdb_93\\42357eee6642bf12.final.pdb
8.200671e-16 -9.337201e-17 3.023679e-15 -3.011886e-15 -9.013217e-15
5.782044e-15
E:\\R-3.1.2\\scripts\\mPDBS\\summary_pdb_93\\44342d1f75a7fcfc.final.pdb
3.354868e-15 5.689717e-15 -1.757836e-15 4.710596e-15 -7.490887e-15
7.454058e-16
E:\\R-3.1.2\\scripts\\mPDBS\\summary_pdb_93\\448ef21dffe8cdfb.final.pdb
-8.188965e-15 -6.296421e-15 2.106009e-16 -1.134948e-15 6.448224e-15
-1.217966e-14
E:\\R-3.1.2\\scripts\\mPDBS\\summary_pdb_93\\472a40b3e8f113c7.final.pdb
2.642748e-15 4.702981e-15 1.190176e-15 8.478376e-16 -1.255393e-15
4.530415e-15
E:\\R-3.1.2\\scripts\\mPDBS\\summary_pdb_93\\48a26225951ea153.final.pdb
3.062366e-15 2.313919e-15 4.631791e-15 1.105626e-15 -2.523890e-15

1.856302e-16
E:\\R-3.1.2\\scripts\\mPDBS\\summary_pdb_93\\4ab4a5c7c6682ce6.final.pdb
-9.131471e-15 7.465101e-16 1.829151e-15 -6.193622e-15 -1.060013e-14
-4.404875e-15
E:\\R-3.1.2\\scripts\\mPDBS\\summary_pdb_93\\4eaa45b62cb6a25f.final.pdb
2.494838e-16 1.547652e-15 4.569309e-15 -2.682080e-16 -3.140755e-15
-1.943889e-15
E:\\R-3.1.2\\scripts\\mPDBS\\summary_pdb_93\\58c371611d85ac96.final.pdb
-3.860947e-15 8.176337e-16 -2.403574e-15 3.333480e-15 -1.929983e-15
-4.291684e-15
E:\\R-3.1.2\\scripts\\mPDBS\\summary_pdb_93\\59a647f04822d30a.final.pdb
-3.946800e-15 6.642229e-16 -1.180889e-16 9.419088e-16 -1.609307e-15
-2.174741e-16
E:\\R-3.1.2\\scripts\\mPDBS\\summary_pdb_93\\59b091fe385e05fc.final.pdb
1.420992e-15 3.244164e-15 -6.228492e-16 -2.541414e-15 -3.123020e-15
3.198002e-16
E:\\R-3.1.2\\scripts\\mPDBS\\summary_pdb_93\\5c35ac30f930d2db.final.pdb
2.494838e-16 1.547652e-15 4.569309e-15 -2.682080e-16 -3.140755e-15
-1.943889e-15
E:\\R-3.1.2\\scripts\\mPDBS\\summary_pdb_93\\5f60752431cf2dcb.final.pdb
-5.452562e-15 -1.976795e-15 -1.845880e-15 2.742191e-15 4.766177e-16
-3.771964e-15
E:\\R-3.1.2\\scripts\\mPDBS\\summary_pdb_93\\626456dd6ff2868d.final.pdb
2.642748e-15 4.702981e-15 1.190176e-15 8.478376e-16 -1.255393e-15
4.530415e-15
E:\\R-3.1.2\\scripts\\mPDBS\\summary_pdb_93\\6583d53ff3c8f7ef.final.pdb
-3.946800e-15 6.642229e-16 -1.180889e-16 9.419088e-16 -1.609307e-15
-2.174741e-16
E:\\R-3.1.2\\scripts\\mPDBS\\summary_pdb_93\\69760178986bef01.final.pdb
1.002555e-15 5.368018e-15 -2.985408e-15 6.157809e-15 -3.322262e-16
6.194311e-17
E:\\R-3.1.2\\scripts\\mPDBS\\summary_pdb_93\\6a9d87d47926b1c8.final.pdb
1.066969e-15 4.106669e-15 2.117982e-15 -2.539286e-16 -1.230022e-15
7.322922e-16
E:\\R-3.1.2\\scripts\\mPDBS\\summary_pdb_93\\6bb3547ea982ffc4.final.pdb
-1.672030e-15 1.437987e-14 5.687945e-15 5.762382e-15 4.809255e-15
-2.549136e-15
E:\\R-3.1.2\\scripts\\mPDBS\\summary_pdb_93\\716d9624611d1c42.final.pdb
8.577990e-15 9.701798e-15 9.728466e-15 -6.168845e-16 -3.033812e-15
-4.518708e-15
E:\\R-3.1.2\\scripts\\mPDBS\\summary_pdb_93\\75f27e70ad0f1be4.final.pdb
-3.946800e-15 6.642229e-16 -1.180889e-16 9.419088e-16 -1.609307e-15
-2.174741e-16
E:\\R-3.1.2\\scripts\\mPDBS\\summary_pdb_93\\769f910fffb22872.final.pdb
1.553389e-15 4.695378e-15 1.921834e-15 4.213366e-16 -2.218415e-15
-9.175868e-18
E:\\R-3.1.2\\scripts\\mPDBS\\summary_pdb_93\\791f8c80dd5c3135.final.pdb
1.420992e-15 3.244164e-15 -6.228492e-16 -2.541414e-15 -3.123020e-15
3.198002e-16
E:\\R-3.1.2\\scripts\\mPDBS\\summary_pdb_93\\7fad98e66a3f6099.final.pdb
2.930656e-15 7.835873e-15 6.740185e-16 -6.037905e-15 3.085590e-15
5.020671e-15
E:\\R-3.1.2\\scripts\\mPDBS\\summary_pdb_93\\804b89c94a633196.final.pdb
7.348953e-15 7.584837e-15 5.141552e-15 -5.694278e-16 -2.944436e-15

4.004656e-15
E:\\R-3.1.2\\scripts\\mPDBS\\summary_pdb_93\\813cc65a2298feba.final.pdb
7.348953e-15 7.584837e-15 5.141552e-15 -5.694278e-16 -2.944436e-15
4.004656e-15
E:\\R-3.1.2\\scripts\\mPDBS\\summary_pdb_93\\83c0eefcfe12d3f9.final.pdb
5.421458e-15 6.118562e-15 5.559892e-15 4.112808e-15 -5.904959e-15
-5.084984e-16
E:\\R-3.1.2\\scripts\\mPDBS\\summary_pdb_93\\86d50469ef569760.final.pdb
2.642748e-15 4.702981e-15 1.190176e-15 8.478376e-16 -1.255393e-15
4.530415e-15
E:\\R-3.1.2\\scripts\\mPDBS\\summary_pdb_93\\89d0666e1402203d.final.pdb
-3.946800e-15 6.642229e-16 -1.180889e-16 9.419088e-16 -1.609307e-15
-2.174741e-16
E:\\R-3.1.2\\scripts\\mPDBS\\summary_pdb_93\\8b34852716dadc62.final.pdb
-4.884751e-15 3.709464e-15 -3.082772e-15 8.703926e-16 -1.023895e-14
-9.796385e-16
E:\\R-3.1.2\\scripts\\mPDBS\\summary_pdb_93\\8b6ef9ac3fe74395.final.pdb
-4.465838e-15 3.439245e-15 -1.001020e-15 -7.994838e-15 3.183985e-15
3.218413e-15
E:\\R-3.1.2\\scripts\\mPDBS\\summary_pdb_93\\98bbe0d5986c138f.final.pdb
1.420992e-15 3.244164e-15 -6.228492e-16 -2.541414e-15 -3.123020e-15
3.198002e-16
E:\\R-3.1.2\\scripts\\mPDBS\\summary_pdb_93\\9c22f4909d67e2fa.final.pdb
6.373605e-15 5.138443e-15 -1.739385e-16 3.445806e-15 3.935369e-15
4.032818e-15
E:\\R-3.1.2\\scripts\\mPDBS\\summary_pdb_93\\9dda8ed0d81fb3ef.final.pdb
1.439517e-15 -1.856764e-15 -8.887387e-15 2.850991e-16 -7.842016e-16
2.333274e-15
E:\\R-3.1.2\\scripts\\mPDBS\\summary_pdb_93\\9f752b3616e6033b.final.pdb
2.103629e-15 5.833073e-15 -4.260398e-16 4.283518e-16 -1.394727e-15
5.051928e-15
E:\\R-3.1.2\\scripts\\mPDBS\\summary_pdb_93\\a02c70083816d6ba.final.pdb
2.494838e-16 1.547652e-15 4.569309e-15 -2.682080e-16 -3.140755e-15
-1.943889e-15
E:\\R-3.1.2\\scripts\\mPDBS\\summary_pdb_93\\a0c997d891e4e082.final.pdb
-1.664796e-15 1.513583e-14 -5.189383e-16 -2.618465e-16 -4.474563e-15
4.743054e-15
E:\\R-3.1.2\\scripts\\mPDBS\\summary_pdb_93\\ab8039a2d823c616.final.pdb
1.865800e-15 4.109496e-15 2.268627e-15 -1.840008e-16 -1.957804e-15
1.813007e-16
E:\\R-3.1.2\\scripts\\mPDBS\\summary_pdb_93\\acf4d74b9c71d4dc.final.pdb
7.348953e-15 7.584837e-15 5.141552e-15 -5.694278e-16 -2.944436e-15
4.004656e-15
E:\\R-3.1.2\\scripts\\mPDBS\\summary_pdb_93\\ad8a823e4b4c668e.final.pdb
1.261184e-15 4.476875e-15 2.047649e-15 3.727653e-16 -2.178758e-15
8.520178e-16
E:\\R-3.1.2\\scripts\\mPDBS\\summary_pdb_93\\b16ababd03b7b857.final.pdb
6.373605e-15 5.138443e-15 -1.739385e-16 3.445806e-15 3.935369e-15
4.032818e-15
E:\\R-3.1.2\\scripts\\mPDBS\\summary_pdb_93\\b226923e697d3c63.final.pdb
-3.262994e-15 1.034586e-15 -5.826966e-16 -1.722626e-15 -4.471492e-15
2.949399e-15
E:\\R-3.1.2\\scripts\\mPDBS\\summary_pdb_93\\b28e48f6fa27a165.final.pdb
1.420992e-15 3.244164e-15 -6.228492e-16 -2.541414e-15 -3.123020e-15

3.198002e-16
E:\\R-3.1.2\\scripts\\mPDBS\\summary_pdb_93\\b42c4924e3cd1aec.final.pdb
7.275343e-15 1.172265e-14 3.311161e-15 -6.935254e-15 2.742573e-15
-2.666822e-15
E:\\R-3.1.2\\scripts\\mPDBS\\summary_pdb_93\\b4dd239fcabff1df.final.pdb
1.420992e-15 3.244164e-15 -6.228492e-16 -2.541414e-15 -3.123020e-15
3.198002e-16
E:\\R-3.1.2\\scripts\\mPDBS\\summary_pdb_93\\b669942ef97459a2.final.pdb
-4.884751e-15 3.709464e-15 -3.082772e-15 8.703926e-16 -1.023895e-14
-9.796385e-16
E:\\R-3.1.2\\scripts\\mPDBS\\summary_pdb_93\\b99f4d9d87c1017b.final.pdb
1.420992e-15 3.244164e-15 -6.228492e-16 -2.541414e-15 -3.123020e-15
3.198002e-16
E:\\R-3.1.2\\scripts\\mPDBS\\summary_pdb_93\\b9d2cbf3975403e7.final.pdb
1.420992e-15 3.244164e-15 -6.228492e-16 -2.541414e-15 -3.123020e-15
3.198002e-16
E:\\R-3.1.2\\scripts\\mPDBS\\summary_pdb_93\\bc88c8a505f3a288.final.pdb
1.035992e-15 4.027826e-15 1.334922e-15 -5.558058e-16 -2.017676e-15
5.891407e-17
E:\\R-3.1.2\\scripts\\mPDBS\\summary_pdb_93\\bdac0e64f4ef427e.final.pdb
-5.718069e-15 1.755298e-15 -3.482666e-15 -7.963253e-15 7.161737e-15
2.563019e-15
E:\\R-3.1.2\\scripts\\mPDBS\\summary_pdb_93\\ca2a1f55fc9d0681.final.pdb
-4.884751e-15 3.709464e-15 -3.082772e-15 8.703926e-16 -1.023895e-14
-9.796385e-16
E:\\R-3.1.2\\scripts\\mPDBS\\summary_pdb_93\\ce34248de3e81a3c.final.pdb
-1.001571e-15 4.955200e-16 3.361633e-15 -9.283149e-15 -6.577965e-15
-6.262904e-15
E:\\R-3.1.2\\scripts\\mPDBS\\summary_pdb_93\\d02e697772494a61.final.pdb
-2.934682e-15 2.311317e-15 1.016743e-14 5.823206e-15 -8.701348e-15
5.489192e-16
E:\\R-3.1.2\\scripts\\mPDBS\\summary_pdb_93\\d04221245d015047.final.pdb
3.043243e-15 5.395359e-15 1.824673e-15 4.597984e-16 -1.466552e-15
-2.772355e-16
E:\\R-3.1.2\\scripts\\mPDBS\\summary_pdb_93\\d678141e4018054a.final.pdb
2.106119e-15 5.254702e-15 1.090550e-14 9.745956e-15 -1.094602e-15
-5.414253e-16
E:\\R-3.1.2\\scripts\\mPDBS\\summary_pdb_93\\d71b40af0f0efe39.final.pdb
1.457279e-14 -1.700940e-15 4.183285e-16 3.786057e-15 4.424326e-16
3.847311e-15
E:\\R-3.1.2\\scripts\\mPDBS\\summary_pdb_93\\dc0809eff10197e2.final.pdb
-4.849153e-15 -3.694651e-15 -4.263374e-15 -7.144788e-15 9.910405e-16
6.012073e-15
E:\\R-3.1.2\\scripts\\mPDBS\\summary_pdb_93\\e1163918d3707839.final.pdb
1.264258e-14 1.215113e-14 1.509687e-14 2.094849e-15 -1.852276e-15
1.260808e-15
E:\\R-3.1.2\\scripts\\mPDBS\\summary_pdb_93\\e4b48b06824ef848.final.pdb
5.558143e-16 -2.688472e-15 3.503333e-15 2.700763e-15 -4.113059e-15
-3.971847e-16
E:\\R-3.1.2\\scripts\\mPDBS\\summary_pdb_93\\e529e19ad281fea2.final.pdb
7.608394e-15 6.248619e-15 9.723281e-15 -2.132417e-15 2.420487e-15
2.337725e-15
E:\\R-3.1.2\\scripts\\mPDBS\\summary_pdb_93\\e5602082718ec3a1.final.pdb
7.348953e-15 7.584837e-15 5.141552e-15 -5.694278e-16 -2.944436e-15

4.004656e-15
E:\\R-3.1.2\\scripts\\mPDBS\\summary_pdb_93\\e5d589d996d0a960.final.pdb
8.456186e-16 1.302741e-14 2.053594e-15 -6.669890e-15 1.978456e-15
-5.142017e-15
E:\\R-3.1.2\\scripts\\mPDBS\\summary_pdb_93\\e9df9e72307a2fbd.final.pdb
2.494838e-16 1.547652e-15 4.569309e-15 -2.682080e-16 -3.140755e-15
-1.943889e-15
E:\\R-3.1.2\\scripts\\mPDBS\\summary_pdb_93\\ea00f8694090cae7.final.pdb
7.348953e-15 7.584837e-15 5.141552e-15 -5.694278e-16 -2.944436e-15
4.004656e-15
E:\\R-3.1.2\\scripts\\mPDBS\\summary_pdb_93\\eba517be32db050e.final.pdb
1.420992e-15 3.244164e-15 -6.228492e-16 -2.541414e-15 -3.123020e-15
3.198002e-16
E:\\R-3.1.2\\scripts\\mPDBS\\summary_pdb_93\\ec04b9545899c29d.final.pdb
2.131454e-15 4.297601e-15 1.661737e-15 7.541445e-17 -2.902977e-15
4.897510e-16
E:\\R-3.1.2\\scripts\\mPDBS\\summary_pdb_93\\ee6862cbcad52078.final.pdb
1.573865e-15 3.740295e-15 1.771503e-15 -6.071970e-16 -1.978970e-15
4.482782e-16
E:\\R-3.1.2\\scripts\\mPDBS\\summary_pdb_93\\ef6a42fc83b81e5d.final.pdb
1.944135e-15 4.415654e-15 1.688649e-15 -7.544484e-17 -1.945305e-15
-9.442126e-17
E:\\R-3.1.2\\scripts\\mPDBS\\summary_pdb_93\\ef8e88d4ad93a0ad.final.pdb
3.043243e-15 5.395359e-15 1.824673e-15 4.597984e-16 -1.466552e-15
-2.772355e-16
E:\\R-3.1.2\\scripts\\mPDBS\\summary_pdb_93\\f14fcae8deec4fa3.final.pdb
2.103629e-15 5.833073e-15 -4.260398e-16 4.283518e-16 -1.394727e-15
5.051928e-15
E:\\R-3.1.2\\scripts\\mPDBS\\summary_pdb_93\\f83584e22003fb92.final.pdb
3.062366e-15 2.313919e-15 4.631791e-15 1.105626e-15 -2.523890e-15
1.856302e-16
E:\\R-3.1.2\\scripts\\mPDBS\\summary_pdb_93\\f8e2bf87e52caa29.final.pdb
7.608394e-15 6.248619e-15 9.723281e-15 -2.132417e-15 2.420487e-15
2.337725e-15

[,829]   [,830]   [,831]   [,832]   [,833]   [,834]
E:\\R-3.1.2\\scripts\\40.1ns_data\\NAT0.pdb
-3.172565e-15 1.672364e-15 3.972757e-15 1.582404e-15 1.244574e-15
-2.748672e-15
E:\\R-3.1.2\\scripts\\40.1ns_data\\LJP6.pdb
-2.118199e-15 7.238491e-16 -4.908578e-16 1.522524e-15 1.233095e-15
-1.966263e-15
E:\\R-3.1.2\\scripts\\40.1ns_data\\3WY9.pdb
-1.609118e-15 5.550177e-16 2.988506e-15 -4.492531e-16 1.668723e-15
-3.693246e-15
E:\\R-3.1.2\\scripts\\40.1ns_data\\ZSP9.pdb
-2.127208e-15 -6.971233e-16 5.737556e-15 2.270473e-15 2.899206e-15
-4.132832e-15
E:\\R-3.1.2\\scripts\\mPDBS\\summary_pdb_93\\013d855ad26c877c.final.pdb
-4.192304e-15 2.908888e-17 8.495748e-15 -9.431959e-16 -1.700205e-15
-2.774982e-15
E:\\R-3.1.2\\scripts\\mPDBS\\summary_pdb_93\\0a2a705342ca0d54.final.pdb
-2.009454e-15 -2.171484e-16 5.916177e-15 8.127683e-17 1.650470e-15
-1.780112e-15

E:\\R-3.1.2\\scripts\\mPDBS\\summary_pdb_93\\0a59fceadb4194d9.final.pdb
-2.544875e-15 8.763751e-16 6.100835e-15 -5.028305e-15 -4.571263e-15
1.735807e-15
E:\\R-3.1.2\\scripts\\mPDBS\\summary_pdb_93\\0dba59f3306825e8.final.pdb
5.043364e-15 -1.293638e-15 6.594491e-15 -1.888837e-15 -2.291592e-16
1.398763e-15
E:\\R-3.1.2\\scripts\\mPDBS\\summary_pdb_93\\126f26a78b57339c.final.pdb
-1.156748e-14 4.165537e-15 2.939862e-15 6.724282e-15 8.209403e-15
-6.811683e-15
E:\\R-3.1.2\\scripts\\mPDBS\\summary_pdb_93\\12f06f3e342df706.final.pdb
-3.328712e-15 7.473578e-16 5.473822e-15 1.706151e-16 2.224121e-15
-2.933919e-15
E:\\R-3.1.2\\scripts\\mPDBS\\summary_pdb_93\\18618a2a251b867b.final.pdb
-1.074942e-15 -3.622298e-15 -8.084874e-16 3.147911e-15 5.348652e-15
-7.088455e-15
E:\\R-3.1.2\\scripts\\mPDBS\\summary_pdb_93\\1f305f598641eeb2.final.pdb
-3.071350e-15 4.333335e-16 5.612861e-15 6.950066e-16 1.873390e-15
-1.992875e-15
E:\\R-3.1.2\\scripts\\mPDBS\\summary_pdb_93\\1f8a00b551be0a39.final.pdb
-1.074942e-15 -3.622298e-15 -8.084874e-16 3.147911e-15 5.348652e-15
-7.088455e-15
E:\\R-3.1.2\\scripts\\mPDBS\\summary_pdb_93\\219f45328d47579a.final.pdb
-9.518463e-15 5.474260e-15 -6.101210e-15 8.711572e-15 1.847248e-14
-9.077592e-15
E:\\R-3.1.2\\scripts\\mPDBS\\summary_pdb_93\\238dd3918e54336d.final.pdb
2.950729e-15 1.419732e-14 -3.590198e-15 1.430454e-14 1.387112e-14
-6.117503e-15
E:\\R-3.1.2\\scripts\\mPDBS\\summary_pdb_93\\2b3027889e2fdc42.final.pdb
-2.478956e-15 1.980982e-16 5.656312e-15 -6.077957e-16 1.120148e-15
-1.743615e-15
E:\\R-3.1.2\\scripts\\mPDBS\\summary_pdb_93\\2fcb3b46e33793b0.final.pdb
3.354066e-15 -3.008358e-15 7.607793e-15 -5.334242e-15 -3.530707e-15
9.995229e-16
E:\\R-3.1.2\\scripts\\mPDBS\\summary_pdb_93\\34eb328251842006.final.pdb
2.390557e-15 -4.254002e-15 1.360388e-14 -3.647610e-15 -1.637266e-15
2.873514e-15
E:\\R-3.1.2\\scripts\\mPDBS\\summary_pdb_93\\354b32871797b7ab.final.pdb
1.177432e-14 -3.808505e-15 7.441659e-15 2.003703e-16 -6.401889e-15
-1.086706e-15
E:\\R-3.1.2\\scripts\\mPDBS\\summary_pdb_93\\397368adbc32d5de.final.pdb
-1.156748e-14 4.165537e-15 2.939862e-15 6.724282e-15 8.209403e-15
-6.811683e-15
E:\\R-3.1.2\\scripts\\mPDBS\\summary_pdb_93\\411ca13cd3df6edd.final.pdb
3.347798e-16 -8.678302e-15 9.215448e-15 -2.956803e-15 -1.033573e-15
-2.627784e-15
E:\\R-3.1.2\\scripts\\mPDBS\\summary_pdb_93\\42357eee6642bf12.final.pdb
1.896625e-15 5.536405e-15 8.518977e-15 4.424646e-16 -2.321445e-15
2.769113e-15
E:\\R-3.1.2\\scripts\\mPDBS\\summary_pdb_93\\44342d1f75a7fcfc.final.pdb
-1.353861e-14 6.495997e-17 1.049489e-14 -9.695157e-15 -5.014203e-15
-2.429396e-15
E:\\R-3.1.2\\scripts\\mPDBS\\summary_pdb_93\\448ef21dffe8cdfb.final.pdb
-3.862384e-15 3.556703e-15 1.268359e-14 8.733065e-15 4.385269e-15
-8.040121e-15

E:\\R-3.1.2\\scripts\\mPDBS\\summary_pdb_93\\472a40b3e8f113c7.final.pdb
5.043364e-15 -1.293638e-15 6.594491e-15 -1.888837e-15 -2.291592e-16
1.398763e-15
E:\\R-3.1.2\\scripts\\mPDBS\\summary_pdb_93\\48a26225951ea153.final.pdb
-3.616934e-15 -8.069594e-15 1.140172e-14 -3.856716e-15 -2.062094e-15
-3.742884e-15
E:\\R-3.1.2\\scripts\\mPDBS\\summary_pdb_93\\4ab4a5c7c6682ce6.final.pdb
3.816047e-15 -1.430681e-15 7.897702e-15 -2.609831e-15 -5.457182e-15
-3.206928e-15
E:\\R-3.1.2\\scripts\\mPDBS\\summary_pdb_93\\4eaa45b62cb6a25f.final.pdb
-6.454151e-15 2.143331e-15 2.845509e-15 2.365305e-15 3.474542e-15
-2.993817e-15
E:\\R-3.1.2\\scripts\\mPDBS\\summary_pdb_93\\58c371611d85ac96.final.pdb
-1.156748e-14 4.165537e-15 2.939862e-15 6.724282e-15 8.209403e-15
-6.811683e-15
E:\\R-3.1.2\\scripts\\mPDBS\\summary_pdb_93\\59a647f04822d30a.final.pdb
-1.079823e-15 4.834582e-15 3.595032e-15 1.145998e-15 7.204431e-15
-2.777208e-15
E:\\R-3.1.2\\scripts\\mPDBS\\summary_pdb_93\\59b091fe385e05fc.final.pdb
-2.544875e-15 8.763751e-16 6.100835e-15 -5.028305e-15 -4.571263e-15
1.735807e-15
E:\\R-3.1.2\\scripts\\mPDBS\\summary_pdb_93\\5c35ac30f930d2db.final.pdb
-6.454151e-15 2.143331e-15 2.845509e-15 2.365305e-15 3.474542e-15
-2.993817e-15
E:\\R-3.1.2\\scripts\\mPDBS\\summary_pdb_93\\5f60752431cf2dcb.final.pdb
-1.019041e-14 -1.889422e-15 8.268028e-15 -1.275277e-15 1.452494e-15
-2.778879e-15
E:\\R-3.1.2\\scripts\\mPDBS\\summary_pdb_93\\626456dd6ff2868d.final.pdb
5.043364e-15 -1.293638e-15 6.594491e-15 -1.888837e-15 -2.291592e-16
1.398763e-15
E:\\R-3.1.2\\scripts\\mPDBS\\summary_pdb_93\\6583d53ff3c8f7ef.final.pdb
-1.079823e-15 4.834582e-15 3.595032e-15 1.145998e-15 7.204431e-15
-2.777208e-15
E:\\R-3.1.2\\scripts\\mPDBS\\summary_pdb_93\\69760178986bef01.final.pdb
-3.033861e-15 -1.953782e-15 -3.923152e-16 1.113196e-15 7.090952e-15
-3.055256e-15
E:\\R-3.1.2\\scripts\\mPDBS\\summary_pdb_93\\6a9d87d47926b1c8.final.pdb
-2.193035e-15 1.024820e-15 5.727563e-15 -4.516186e-16 1.022444e-15
-1.458327e-15
E:\\R-3.1.2\\scripts\\mPDBS\\summary_pdb_93\\6bb3547ea982ffc4.final.pdb
-1.915949e-14 -7.109285e-15 1.475030e-14 8.657606e-15 1.035615e-14
-5.692648e-15
E:\\R-3.1.2\\scripts\\mPDBS\\summary_pdb_93\\716d9624611d1c42.final.pdb
-5.453479e-16 1.096771e-17 -1.810282e-15 5.998419e-15 3.924367e-15
-5.799466e-15
E:\\R-3.1.2\\scripts\\mPDBS\\summary_pdb_93\\75f27e70ad0f1be4.final.pdb
-1.079823e-15 4.834582e-15 3.595032e-15 1.145998e-15 7.204431e-15
-2.777208e-15
E:\\R-3.1.2\\scripts\\mPDBS\\summary_pdb_93\\769f910fffb22872.final.pdb
-1.529439e-15 1.530698e-15 5.234856e-15 1.889067e-16 1.648487e-15
-1.656819e-15
E:\\R-3.1.2\\scripts\\mPDBS\\summary_pdb_93\\791f8c80dd5c3135.final.pdb
-2.544875e-15 8.763751e-16 6.100835e-15 -5.028305e-15 -4.571263e-15
1.735807e-15

E:\\R-3.1.2\\scripts\\mPDBS\\summary_pdb_93\\7fad98e66a3f6099.final.pdb
3.890294e-15 -2.209982e-15 1.379610e-14 1.562120e-15 -4.494188e-15
6.086649e-16
E:\\R-3.1.2\\scripts\\mPDBS\\summary_pdb_93\\804b89c94a633196.final.pdb
3.354066e-15 -3.008358e-15 7.607793e-15 -5.334242e-15 -3.530707e-15
9.995229e-16
E:\\R-3.1.2\\scripts\\mPDBS\\summary_pdb_93\\813cc65a2298feba.final.pdb
3.354066e-15 -3.008358e-15 7.607793e-15 -5.334242e-15 -3.530707e-15
9.995229e-16
E:\\R-3.1.2\\scripts\\mPDBS\\summary_pdb_93\\83c0eefcfe12d3f9.final.pdb
3.347798e-16 -8.678302e-15 9.215448e-15 -2.956803e-15 -1.033573e-15
-2.627784e-15
E:\\R-3.1.2\\scripts\\mPDBS\\summary_pdb_93\\86d50469ef569760.final.pdb
5.043364e-15 -1.293638e-15 6.594491e-15 -1.888837e-15 -2.291592e-16
1.398763e-15
E:\\R-3.1.2\\scripts\\mPDBS\\summary_pdb_93\\89d0666e1402203d.final.pdb
-1.079823e-15 4.834582e-15 3.595032e-15 1.145998e-15 7.204431e-15
-2.777208e-15
E:\\R-3.1.2\\scripts\\mPDBS\\summary_pdb_93\\8b34852716dadc62.final.pdb
-8.331031e-15 3.646041e-15 4.433852e-16 2.025540e-15 5.145078e-15
-2.672887e-15
E:\\R-3.1.2\\scripts\\mPDBS\\summary_pdb_93\\8b6ef9ac3fe74395.final.pdb
-2.714589e-14 8.328635e-15 4.668076e-15 -2.462047e-15 5.661112e-15
-1.126547e-15
E:\\R-3.1.2\\scripts\\mPDBS\\summary_pdb_93\\98bbe0d5986c138f.final.pdb
-2.544875e-15 8.763751e-16 6.100835e-15 -5.028305e-15 -4.571263e-15
1.735807e-15
E:\\R-3.1.2\\scripts\\mPDBS\\summary_pdb_93\\9c22f4909d67e2fa.final.pdb
4.190203e-15 6.798901e-15 2.046703e-15 5.647642e-15 5.770663e-15
-9.988785e-16
E:\\R-3.1.2\\scripts\\mPDBS\\summary_pdb_93\\9dda8ed0d81fb3ef.final.pdb
-9.031796e-15 -4.411494e-15 1.613622e-14 -1.276280e-14 -1.326693e-14
1.211571e-15
E:\\R-3.1.2\\scripts\\mPDBS\\summary_pdb_93\\9f752b3616e6033b.final.pdb
-1.245787e-15 1.005563e-15 7.328530e-15 -1.739110e-15 2.983378e-15
-1.040915e-15
E:\\R-3.1.2\\scripts\\mPDBS\\summary_pdb_93\\a02c70083816d6ba.final.pdb
-6.454151e-15 2.143331e-15 2.845509e-15 2.365305e-15 3.474542e-15
-2.993817e-15
E:\\R-3.1.2\\scripts\\mPDBS\\summary_pdb_93\\a0c997d891e4e082.final.pdb
-2.987323e-15 -3.040903e-15 9.032159e-15 -2.811133e-15 -5.122243e-15
3.349398e-15
E:\\R-3.1.2\\scripts\\mPDBS\\summary_pdb_93\\ab8039a2d823c616.final.pdb
-3.115693e-15 5.959448e-16 3.940746e-15 1.760606e-16 1.969632e-15
-1.925246e-15
E:\\R-3.1.2\\scripts\\mPDBS\\summary_pdb_93\\acf4d74b9c71d4dc.final.pdb
3.354066e-15 -3.008358e-15 7.607793e-15 -5.334242e-15 -3.530707e-15
9.995229e-16
E:\\R-3.1.2\\scripts\\mPDBS\\summary_pdb_93\\ad8a823e4b4c668e.final.pdb
-2.029035e-15 5.282356e-16 5.149794e-15 2.158944e-16 3.175730e-15
-2.655669e-15
E:\\R-3.1.2\\scripts\\mPDBS\\summary_pdb_93\\b16ababd03b7b857.final.pdb
4.190203e-15 6.798901e-15 2.046703e-15 5.647642e-15 5.770663e-15
-9.988785e-16

E:\\R-3.1.2\\scripts\\mPDBS\\summary_pdb_93\\b226923e697d3c63.final.pdb
-7.671772e-15 6.834718e-15 -3.466593e-15 3.914833e-15 1.312984e-15
-2.523288e-15
E:\\R-3.1.2\\scripts\\mPDBS\\summary_pdb_93\\b28e48f6fa27a165.final.pdb
-2.544875e-15 8.763751e-16 6.100835e-15 -5.028305e-15 -4.571263e-15
1.735807e-15
E:\\R-3.1.2\\scripts\\mPDBS\\summary_pdb_93\\b42c4924e3cd1aec.final.pdb
-4.965604e-15 6.277872e-15 2.303531e-15 5.402359e-15 5.400860e-15
5.016359e-16
E:\\R-3.1.2\\scripts\\mPDBS\\summary_pdb_93\\b4dd239fcabff1df.final.pdb
-2.544875e-15 8.763751e-16 6.100835e-15 -5.028305e-15 -4.571263e-15
1.735807e-15
E:\\R-3.1.2\\scripts\\mPDBS\\summary_pdb_93\\b669942ef97459a2.final.pdb
-8.331031e-15 3.646041e-15 4.433852e-16 2.025540e-15 5.145078e-15
-2.672887e-15
E:\\R-3.1.2\\scripts\\mPDBS\\summary_pdb_93\\b99f4d9d87c1017b.final.pdb
-2.544875e-15 8.763751e-16 6.100835e-15 -5.028305e-15 -4.571263e-15
1.735807e-15
E:\\R-3.1.2\\scripts\\mPDBS\\summary_pdb_93\\b9d2cbf3975403e7.final.pdb
-2.544875e-15 8.763751e-16 6.100835e-15 -5.028305e-15 -4.571263e-15
1.735807e-15
E:\\R-3.1.2\\scripts\\mPDBS\\summary_pdb_93\\bc88c8a505f3a288.final.pdb
-1.980223e-15 1.195564e-15 5.505391e-15 -2.721267e-16 1.482597e-15
-2.185536e-15
E:\\R-3.1.2\\scripts\\mPDBS\\summary_pdb_93\\bdac0e64f4ef427e.final.pdb
-5.865122e-15 1.972780e-14 -7.066233e-15 8.320634e-15 7.205279e-15
-2.377550e-15
E:\\R-3.1.2\\scripts\\mPDBS\\summary_pdb_93\\ca2a1f55fc9d0681.final.pdb
-8.331031e-15 3.646041e-15 4.433852e-16 2.025540e-15 5.145078e-15
-2.672887e-15
E:\\R-3.1.2\\scripts\\mPDBS\\summary_pdb_93\\ce34248de3e81a3c.final.pdb
-9.529842e-15 2.222226e-15 8.694332e-16 -1.015417e-15 -6.118658e-15
-1.965879e-15
E:\\R-3.1.2\\scripts\\mPDBS\\summary_pdb_93\\d02e697772494a61.final.pdb
5.830370e-15 3.247330e-16 -3.030750e-15 7.750319e-15 1.361742e-14
-8.320602e-15
E:\\R-3.1.2\\scripts\\mPDBS\\summary_pdb_93\\d04221245d015047.final.pdb
-7.703464e-16 -1.819658e-15 8.078061e-15 -1.659277e-15 1.577259e-15
-1.375989e-15
E:\\R-3.1.2\\scripts\\mPDBS\\summary_pdb_93\\d678141e4018054a.final.pdb
-5.293808e-15 -7.787535e-15 8.296023e-15 1.303795e-14 9.250511e-15
-1.067958e-14
E:\\R-3.1.2\\scripts\\mPDBS\\summary_pdb_93\\d71b40af0f0efe39.final.pdb
3.017106e-14 1.100003e-15 1.802638e-16 2.412275e-15 1.982140e-15
-8.706282e-15
E:\\R-3.1.2\\scripts\\mPDBS\\summary_pdb_93\\dc0809eff10197e2.final.pdb
5.864328e-15 6.326774e-15 1.211588e-14 -1.071820e-14 2.591369e-15
3.551216e-15
E:\\R-3.1.2\\scripts\\mPDBS\\summary_pdb_93\\e1163918d3707839.final.pdb
2.552688e-15 -1.248229e-14 8.969205e-15 1.455429e-15 -2.360401e-15
4.485428e-15
E:\\R-3.1.2\\scripts\\mPDBS\\summary_pdb_93\\e4b48b06824ef848.final.pdb
-1.074942e-15 -3.622298e-15 -8.084874e-16 3.147911e-15 5.348652e-15
-7.088455e-15

E:\\R-3.1.2\\scripts\\mPDBS\\summary_pdb_93\\e529e19ad281fea2.final.pdb
-4.192304e-15 2.908888e-17 8.495748e-15 -9.431959e-16 -1.700205e-15
-2.774982e-15
E:\\R-3.1.2\\scripts\\mPDBS\\summary_pdb_93\\e5602082718ec3a1.final.pdb
3.354066e-15 -3.008358e-15 7.607793e-15 -5.334242e-15 -3.530707e-15
9.995229e-16
E:\\R-3.1.2\\scripts\\mPDBS\\summary_pdb_93\\e5d589d996d0a960.final.pdb
-1.664243e-14 -3.426885e-15 8.683461e-15 -3.468132e-15 -2.223871e-17
-5.955134e-15
E:\\R-3.1.2\\scripts\\mPDBS\\summary_pdb_93\\e9df9e72307a2fbd.final.pdb
-6.454151e-15 2.143331e-15 2.845509e-15 2.365305e-15 3.474542e-15
-2.993817e-15
E:\\R-3.1.2\\scripts\\mPDBS\\summary_pdb_93\\ea00f8694090cae7.final.pdb
3.354066e-15 -3.008358e-15 7.607793e-15 -5.334242e-15 -3.530707e-15
9.995229e-16
E:\\R-3.1.2\\scripts\\mPDBS\\summary_pdb_93\\eba517be32db050e.final.pdb
-2.544875e-15 8.763751e-16 6.100835e-15 -5.028305e-15 -4.571263e-15
1.735807e-15
E:\\R-3.1.2\\scripts\\mPDBS\\summary_pdb_93\\ec04b9545899c29d.final.pdb
-2.765974e-15 -8.128101e-16 4.548263e-15 -1.650195e-16 1.924691e-15
-1.778875e-15
E:\\R-3.1.2\\scripts\\mPDBS\\summary_pdb_93\\ee6862cbcad52078.final.pdb
-1.909966e-15 5.250936e-16 5.417354e-15 -5.739686e-16 1.175768e-15
-2.051962e-15
E:\\R-3.1.2\\scripts\\mPDBS\\summary_pdb_93\\ef6a42fc83b81e5d.final.pdb
-2.573743e-15 7.275206e-16 5.253938e-15 3.155416e-16 2.009635e-15
-1.620173e-15
E:\\R-3.1.2\\scripts\\mPDBS\\summary_pdb_93\\ef8e88d4ad93a0ad.final.pdb
-7.703464e-16 -1.819658e-15 8.078061e-15 -1.659277e-15 1.577259e-15
-1.375989e-15
E:\\R-3.1.2\\scripts\\mPDBS\\summary_pdb_93\\f14fcae8deec4fa3.final.pdb
-1.245787e-15 1.005563e-15 7.328530e-15 -1.739110e-15 2.983378e-15
-1.040915e-15
E:\\R-3.1.2\\scripts\\mPDBS\\summary_pdb_93\\f83584e22003fb92.final.pdb
-3.616934e-15 -8.069594e-15 1.140172e-14 -3.856716e-15 -2.062094e-15
-3.742884e-15
E:\\R-3.1.2\\scripts\\mPDBS\\summary_pdb_93\\f8e2bf87e52caa29.final.pdb
-4.192304e-15 2.908888e-17 8.495748e-15 -9.431959e-16 -1.700205e-15
-2.774982e-15

[,835]   [,836]   [,837]   [,838]   [,839]   [,840]
E:\\R-3.1.2\\scripts\\40.1ns_data\\NAT0.pdb
-2.605252e-15 -1.225258e-15 -3.125462e-15 2.544525e-15 6.436856e-16
2.935137e-15
E:\\R-3.1.2\\scripts\\40.1ns_data\\LJP6.pdb
-3.976582e-15 -1.059533e-15 -2.408976e-15 4.142297e-15 1.800257e-15
1.559023e-15
E:\\R-3.1.2\\scripts\\40.1ns_data\\3WY9.pdb
-2.029876e-15 -2.113253e-15 -2.774966e-15 3.695826e-15 2.495767e-16
4.170689e-15
E:\\R-3.1.2\\scripts\\40.1ns_data\\ZSP9.pdb
-2.847321e-15 -1.124466e-15 -3.437504e-15 4.655356e-15 1.163106e-15
1.089258e-15
E:\\R-3.1.2\\scripts\\mPDBS\\summary_pdb_93\\013d855ad26c877c.final.pdb

-7.179139e-15 -7.972457e-15 -8.038521e-15 -3.737154e-15 2.332645e-15
1.804075e-15
E:\\R-3.1.2\\scripts\\mPDBS\\summary_pdb_93\\0a2a705342ca0d54.final.pdb
-3.361738e-15 -2.298522e-15 -3.108578e-15 3.384849e-15 1.551489e-15
1.548400e-15
E:\\R-3.1.2\\scripts\\mPDBS\\summary_pdb_93\\0a59fceadb4194d9.final.pdb
-1.918800e-15 4.241256e-16 -5.939433e-15 1.623405e-15 1.721714e-15
-2.030959e-15
E:\\R-3.1.2\\scripts\\mPDBS\\summary_pdb_93\\0dba59f3306825e8.final.pdb
7.780371e-16 -3.106557e-15 -4.213498e-16 4.485436e-15 -1.666580e-16
3.855045e-16
E:\\R-3.1.2\\scripts\\mPDBS\\summary_pdb_93\\126f26a78b57339c.final.pdb
-7.188124e-16 -2.730990e-15 4.442815e-15 1.014627e-14 3.681826e-15
7.967981e-15
E:\\R-3.1.2\\scripts\\mPDBS\\summary_pdb_93\\12f06f3e342df706.final.pdb
-2.444069e-15 -1.783959e-15 -3.945582e-15 3.843683e-15 8.338556e-16
1.809042e-15
E:\\R-3.1.2\\scripts\\mPDBS\\summary_pdb_93\\18618a2a251b867b.final.pdb
-5.614998e-15 -6.020033e-15 4.511251e-15 6.674445e-15 6.481763e-15
7.518314e-15
E:\\R-3.1.2\\scripts\\mPDBS\\summary_pdb_93\\1f305f598641eeb2.final.pdb
-2.351969e-15 -2.359775e-15 -3.381892e-15 3.890963e-15 8.907489e-16
1.788961e-15
E:\\R-3.1.2\\scripts\\mPDBS\\summary_pdb_93\\1f8a00b551be0a39.final.pdb
-5.614998e-15 -6.020033e-15 4.511251e-15 6.674445e-15 6.481763e-15
7.518314e-15
E:\\R-3.1.2\\scripts\\mPDBS\\summary_pdb_93\\219f45328d47579a.final.pdb
-9.225455e-15 1.711290e-15 -6.243877e-15 -2.006938e-15 -3.020370e-15
1.147603e-14
E:\\R-3.1.2\\scripts\\mPDBS\\summary_pdb_93\\238dd3918e54336d.final.pdb
9.805383e-15 -2.434265e-15 8.832604e-15 -1.710300e-15 -6.655049e-15
2.285901e-15
E:\\R-3.1.2\\scripts\\mPDBS\\summary_pdb_93\\2b3027889e2fdc42.final.pdb
-2.216142e-15 -2.627687e-15 -3.320868e-15 3.579306e-15 1.017633e-15
1.782831e-15
E:\\R-3.1.2\\scripts\\mPDBS\\summary_pdb_93\\2fcb3b46e33793b0.final.pdb
-4.312207e-15 -6.429436e-15 -8.952089e-15 -6.236547e-16 5.000025e-16
-1.281933e-15
E:\\R-3.1.2\\scripts\\mPDBS\\summary_pdb_93\\34eb328251842006.final.pdb
-1.044472e-14 2.556741e-15 -1.040401e-14 7.555447e-15 1.375770e-15
-8.566061e-15
E:\\R-3.1.2\\scripts\\mPDBS\\summary_pdb_93\\354b32871797b7ab.final.pdb
-3.408337e-15 -4.478243e-15 6.320814e-16 1.282232e-15 7.626723e-15
7.073944e-16
E:\\R-3.1.2\\scripts\\mPDBS\\summary_pdb_93\\397368adbc32d5de.final.pdb
-7.188124e-16 -2.730990e-15 4.442815e-15 1.014627e-14 3.681826e-15
7.967981e-15
E:\\R-3.1.2\\scripts\\mPDBS\\summary_pdb_93\\411ca13cd3df6edd.final.pdb
-7.445334e-15 -1.072374e-14 -7.441145e-15 5.509026e-15 4.564893e-15
4.797959e-18
E:\\R-3.1.2\\scripts\\mPDBS\\summary_pdb_93\\42357eee6642bf12.final.pdb
-7.485702e-15 5.060309e-15 -1.159518e-14 -7.878114e-16 1.441138e-15
-3.926130e-15
E:\\R-3.1.2\\scripts\\mPDBS\\summary_pdb_93\\44342d1f75a7fcfc.final.pdb

-3.978669e-15 -1.005667e-14 -9.864099e-15 1.322353e-14 4.003909e-15
-7.393621e-15
E:\\R-3.1.2\\scripts\\mPDBS\\summary_pdb_93\\448ef21dffe8cdfb.final.pdb
-2.735343e-15 2.093506e-16 -2.328915e-15 -5.059343e-15 9.095124e-15
9.714479e-15
E:\\R-3.1.2\\scripts\\mPDBS\\summary_pdb_93\\472a40b3e8f113c7.final.pdb
7.780371e-16 -3.106557e-15 -4.213498e-16 4.485436e-15 -1.666580e-16
3.855045e-16
E:\\R-3.1.2\\scripts\\mPDBS\\summary_pdb_93\\48a26225951ea153.final.pdb
-5.950135e-15 -1.476121e-14 -3.118773e-15 1.621069e-14 5.684906e-15
2.772491e-15
E:\\R-3.1.2\\scripts\\mPDBS\\summary_pdb_93\\4ab4a5c7c6682ce6.final.pdb
1.263977e-15 -7.413008e-15 -7.094831e-15 -3.199823e-15 -2.562311e-15
-2.145891e-15
E:\\R-3.1.2\\scripts\\mPDBS\\summary_pdb_93\\4eaa45b62cb6a25f.final.pdb
-1.817650e-15 -3.479910e-15 -1.688979e-15 4.755148e-15 -1.366084e-15
4.700728e-15
E:\\R-3.1.2\\scripts\\mPDBS\\summary_pdb_93\\58c371611d85ac96.final.pdb
-7.188124e-16 -2.730990e-15 4.442815e-15 1.014627e-14 3.681826e-15
7.967981e-15
E:\\R-3.1.2\\scripts\\mPDBS\\summary_pdb_93\\59a647f04822d30a.final.pdb
-1.878786e-15 8.616810e-16 -3.783026e-15 5.373470e-15 -3.446945e-15
4.906003e-15
E:\\R-3.1.2\\scripts\\mPDBS\\summary_pdb_93\\59b091fe385e05fc.final.pdb
-1.918800e-15 4.241256e-16 -5.939433e-15 1.623405e-15 1.721714e-15
-2.030959e-15
E:\\R-3.1.2\\scripts\\mPDBS\\summary_pdb_93\\5c35ac30f930d2db.final.pdb
-1.817650e-15 -3.479910e-15 -1.688979e-15 4.755148e-15 -1.366084e-15
4.700728e-15
E:\\R-3.1.2\\scripts\\mPDBS\\summary_pdb_93\\5f60752431cf2dcb.final.pdb
3.351202e-16 -6.846967e-15 -1.955854e-15 3.689727e-15 1.867209e-15
3.865990e-15
E:\\R-3.1.2\\scripts\\mPDBS\\summary_pdb_93\\626456dd6ff2868d.final.pdb
7.780371e-16 -3.106557e-15 -4.213498e-16 4.485436e-15 -1.666580e-16
3.855045e-16
E:\\R-3.1.2\\scripts\\mPDBS\\summary_pdb_93\\6583d53ff3c8f7ef.final.pdb
-1.878786e-15 8.616810e-16 -3.783026e-15 5.373470e-15 -3.446945e-15
4.906003e-15
E:\\R-3.1.2\\scripts\\mPDBS\\summary_pdb_93\\69760178986bef01.final.pdb
4.659508e-15 -9.056048e-15 1.060368e-15 5.074969e-15 5.654250e-15
1.290876e-14
E:\\R-3.1.2\\scripts\\mPDBS\\summary_pdb_93\\6a9d87d47926b1c8.final.pdb
-2.042406e-15 -3.149058e-15 -2.653939e-15 3.466719e-15 1.150865e-15
1.725145e-15
E:\\R-3.1.2\\scripts\\mPDBS\\summary_pdb_93\\6bb3547ea982ffc4.final.pdb
1.852900e-15 -1.480786e-14 4.341611e-15 1.309244e-14 1.511488e-15
4.131845e-15
E:\\R-3.1.2\\scripts\\mPDBS\\summary_pdb_93\\716d9624611d1c42.final.pdb
-5.609072e-15 -4.050710e-16 -1.279312e-15 6.100992e-15 4.982950e-16
3.550704e-15
E:\\R-3.1.2\\scripts\\mPDBS\\summary_pdb_93\\75f27e70ad0f1be4.final.pdb
-1.878786e-15 8.616810e-16 -3.783026e-15 5.373470e-15 -3.446945e-15
4.906003e-15
E:\\R-3.1.2\\scripts\\mPDBS\\summary_pdb_93\\769f910fffb22872.final.pdb

-2.049383e-15 -1.138522e-15 -3.500569e-15 3.912766e-15 5.789323e-16
2.418227e-15
E:\\R-3.1.2\\scripts\\mPDBS\\summary_pdb_93\\791f8c80dd5c3135.final.pdb
-1.918800e-15 4.241256e-16 -5.939433e-15 1.623405e-15 1.721714e-15
-2.030959e-15
E:\\R-3.1.2\\scripts\\mPDBS\\summary_pdb_93\\7fad98e66a3f6099.final.pdb
9.512150e-16 -3.725124e-15 -1.427978e-14 -4.961150e-16 1.238679e-15
-3.886205e-15
E:\\R-3.1.2\\scripts\\mPDBS\\summary_pdb_93\\804b89c94a633196.final.pdb
-4.312207e-15 -6.429436e-15 -8.952089e-15 -6.236547e-16 5.000025e-16
-1.281933e-15
E:\\R-3.1.2\\scripts\\mPDBS\\summary_pdb_93\\813cc65a2298feba.final.pdb
-4.312207e-15 -6.429436e-15 -8.952089e-15 -6.236547e-16 5.000025e-16
-1.281933e-15
E:\\R-3.1.2\\scripts\\mPDBS\\summary_pdb_93\\83c0eefcfe12d3f9.final.pdb
-7.445334e-15 -1.072374e-14 -7.441145e-15 5.509026e-15 4.564893e-15
4.797959e-18
E:\\R-3.1.2\\scripts\\mPDBS\\summary_pdb_93\\86d50469ef569760.final.pdb
7.780371e-16 -3.106557e-15 -4.213498e-16 4.485436e-15 -1.666580e-16
3.855045e-16
E:\\R-3.1.2\\scripts\\mPDBS\\summary_pdb_93\\89d0666e1402203d.final.pdb
-1.878786e-15 8.616810e-16 -3.783026e-15 5.373470e-15 -3.446945e-15
4.906003e-15
E:\\R-3.1.2\\scripts\\mPDBS\\summary_pdb_93\\8b34852716dadc62.final.pdb
-3.153560e-18 1.879889e-15 -1.098996e-15 1.180477e-14 -1.628070e-15
1.841003e-15
E:\\R-3.1.2\\scripts\\mPDBS\\summary_pdb_93\\8b6ef9ac3fe74395.final.pdb
5.157289e-15 2.544177e-15 -5.025826e-15 3.730941e-15 -2.583572e-15
2.016068e-17
E:\\R-3.1.2\\scripts\\mPDBS\\summary_pdb_93\\98bbe0d5986c138f.final.pdb
-1.918800e-15 4.241256e-16 -5.939433e-15 1.623405e-15 1.721714e-15
-2.030959e-15
E:\\R-3.1.2\\scripts\\mPDBS\\summary_pdb_93\\9c22f4909d67e2fa.final.pdb
-1.804772e-15 6.098303e-15 3.055667e-15 5.319508e-15 1.185652e-15
7.680082e-15
E:\\R-3.1.2\\scripts\\mPDBS\\summary_pdb_93\\9dda8ed0d81fb3ef.final.pdb
7.422096e-15 -1.640619e-14 -7.311453e-15 1.492659e-14 5.433322e-15
2.684256e-15
E:\\R-3.1.2\\scripts\\mPDBS\\summary_pdb_93\\9f752b3616e6033b.final.pdb
-1.900062e-15 -5.250161e-16 -4.279538e-15 3.040376e-15 8.459844e-16
1.476407e-15
E:\\R-3.1.2\\scripts\\mPDBS\\summary_pdb_93\\a02c70083816d6ba.final.pdb
-1.817650e-15 -3.479910e-15 -1.688979e-15 4.755148e-15 -1.366084e-15
4.700728e-15
E:\\R-3.1.2\\scripts\\mPDBS\\summary_pdb_93\\a0c997d891e4e082.final.pdb
-3.758073e-15 -5.920776e-15 -3.016568e-15 1.411599e-16 3.711423e-15
3.290446e-16
E:\\R-3.1.2\\scripts\\mPDBS\\summary_pdb_93\\ab8039a2d823c616.final.pdb
-2.793512e-15 -1.700627e-15 -3.810204e-15 2.529874e-15 7.656455e-16
2.785847e-15
E:\\R-3.1.2\\scripts\\mPDBS\\summary_pdb_93\\acf4d74b9c71d4dc.final.pdb
-4.312207e-15 -6.429436e-15 -8.952089e-15 -6.236547e-16 5.000025e-16
-1.281933e-15
E:\\R-3.1.2\\scripts\\mPDBS\\summary_pdb_93\\ad8a823e4b4c668e.final.pdb

-2.949966e-15 -2.139390e-15 -3.847946e-15 3.422031e-15 1.198395e-15
1.939989e-15
E:\\R-3.1.2\\scripts\\mPDBS\\summary_pdb_93\\b16ababd03b7b857.final.pdb
-1.804772e-15 6.098303e-15 3.055667e-15 5.319508e-15 1.185652e-15
7.680082e-15
E:\\R-3.1.2\\scripts\\mPDBS\\summary_pdb_93\\b226923e697d3c63.final.pdb
-1.039072e-15 1.170895e-15 3.317384e-16 -7.381952e-15 6.576504e-15
3.187759e-15
E:\\R-3.1.2\\scripts\\mPDBS\\summary_pdb_93\\b28e48f6fa27a165.final.pdb
-1.918800e-15 4.241256e-16 -5.939433e-15 1.623405e-15 1.721714e-15
-2.030959e-15
E:\\R-3.1.2\\scripts\\mPDBS\\summary_pdb_93\\b42c4924e3cd1aec.final.pdb
-7.950373e-15 4.244825e-15 -2.260806e-15 -1.309119e-15 2.037167e-15
-3.136175e-15
E:\\R-3.1.2\\scripts\\mPDBS\\summary_pdb_93\\b4dd239fcabff1df.final.pdb
-1.918800e-15 4.241256e-16 -5.939433e-15 1.623405e-15 1.721714e-15
-2.030959e-15
E:\\R-3.1.2\\scripts\\mPDBS\\summary_pdb_93\\b669942ef97459a2.final.pdb
-3.153560e-18 1.879889e-15 -1.098996e-15 1.180477e-14 -1.628070e-15
1.841003e-15
E:\\R-3.1.2\\scripts\\mPDBS\\summary_pdb_93\\b99f4d9d87c1017b.final.pdb
-1.918800e-15 4.241256e-16 -5.939433e-15 1.623405e-15 1.721714e-15
-2.030959e-15
E:\\R-3.1.2\\scripts\\mPDBS\\summary_pdb_93\\b9d2cbf3975403e7.final.pdb
-1.918800e-15 4.241256e-16 -5.939433e-15 1.623405e-15 1.721714e-15
-2.030959e-15
E:\\R-3.1.2\\scripts\\mPDBS\\summary_pdb_93\\bc88c8a505f3a288.final.pdb
-2.337790e-15 -2.885727e-15 -2.414475e-15 3.572367e-15 1.567106e-15
1.627140e-15
E:\\R-3.1.2\\scripts\\mPDBS\\summary_pdb_93\\bdac0e64f4ef427e.final.pdb
1.066431e-14 1.544413e-14 2.349634e-15 -1.695908e-16 -6.881246e-15
7.508344e-15
E:\\R-3.1.2\\scripts\\mPDBS\\summary_pdb_93\\ca2a1f55fc9d0681.final.pdb
-3.153560e-18 1.879889e-15 -1.098996e-15 1.180477e-14 -1.628070e-15
1.841003e-15
E:\\R-3.1.2\\scripts\\mPDBS\\summary_pdb_93\\ce34248de3e81a3c.final.pdb
-5.664656e-15 -1.680272e-15 -3.771088e-15 2.822492e-15 -2.264822e-15
-1.033306e-15
E:\\R-3.1.2\\scripts\\mPDBS\\summary_pdb_93\\d02e697772494a61.final.pdb
-6.622448e-15 6.015573e-15 -1.671411e-16 -2.276687e-15 -3.989213e-15
6.398930e-15
E:\\R-3.1.2\\scripts\\mPDBS\\summary_pdb_93\\d04221245d015047.final.pdb
-2.602747e-15 -3.341242e-15 -4.713128e-15 7.708024e-15 -6.583456e-16
1.543351e-15
E:\\R-3.1.2\\scripts\\mPDBS\\summary_pdb_93\\d678141e4018054a.final.pdb
-2.973414e-15 -9.610311e-15 8.705273e-16 5.072165e-15 5.433998e-15
2.888761e-15
E:\\R-3.1.2\\scripts\\mPDBS\\summary_pdb_93\\d71b40af0f0efe39.final.pdb
-5.188402e-15 4.724165e-15 -1.138031e-15 -6.845356e-15 3.308897e-15
4.719824e-15
E:\\R-3.1.2\\scripts\\mPDBS\\summary_pdb_93\\dc0809eff10197e2.final.pdb
-4.220522e-15 8.188847e-15 -1.036758e-14 4.008399e-15 -2.377719e-15
7.173499e-16
E:\\R-3.1.2\\scripts\\mPDBS\\summary_pdb_93\\e1163918d3707839.final.pdb

-1.172773e-14 2.262470e-15 -4.352015e-15 2.447425e-15 9.099876e-15
-2.050672e-15
E:\\R-3.1.2\\scripts\\mPDBS\\summary_pdb_93\\e4b48b06824ef848.final.pdb
-5.614998e-15 -6.020033e-15 4.511251e-15 6.674445e-15 6.481763e-15
7.518314e-15
E:\\R-3.1.2\\scripts\\mPDBS\\summary_pdb_93\\e529e19ad281fea2.final.pdb
-7.179139e-15 -7.972457e-15 -8.038521e-15 -3.737154e-15 2.332645e-15
1.804075e-15
E:\\R-3.1.2\\scripts\\mPDBS\\summary_pdb_93\\e5602082718ec3a1.final.pdb
-4.312207e-15 -6.429436e-15 -8.952089e-15 -6.236547e-16 5.000025e-16
-1.281933e-15
E:\\R-3.1.2\\scripts\\mPDBS\\summary_pdb_93\\e5d589d996d0a960.final.pdb
1.768570e-15 -7.017566e-15 2.873055e-15 5.125661e-15 -4.200675e-15
5.496952e-15
E:\\R-3.1.2\\scripts\\mPDBS\\summary_pdb_93\\e9df9e72307a2fbd.final.pdb
-1.817650e-15 -3.479910e-15 -1.688979e-15 4.755148e-15 -1.366084e-15
4.700728e-15
E:\\R-3.1.2\\scripts\\mPDBS\\summary_pdb_93\\ea00f8694090cae7.final.pdb
-4.312207e-15 -6.429436e-15 -8.952089e-15 -6.236547e-16 5.000025e-16
-1.281933e-15
E:\\R-3.1.2\\scripts\\mPDBS\\summary_pdb_93\\eba517be32db050e.final.pdb
-1.918800e-15 4.241256e-16 -5.939433e-15 1.623405e-15 1.721714e-15
-2.030959e-15
E:\\R-3.1.2\\scripts\\mPDBS\\summary_pdb_93\\ec04b9545899c29d.final.pdb
-2.954315e-15 -2.118574e-15 -3.802975e-15 3.827398e-15 1.119281e-15
1.669442e-15
E:\\R-3.1.2\\scripts\\mPDBS\\summary_pdb_93\\ee6862cbcad52078.final.pdb
-1.982985e-15 -2.287247e-15 -2.937494e-15 3.570198e-15 1.174191e-15
1.899491e-15
E:\\R-3.1.2\\scripts\\mPDBS\\summary_pdb_93\\ef6a42fc83b81e5d.final.pdb
-2.809246e-15 -1.897463e-15 -3.924709e-15 3.875469e-15 9.805208e-16
1.871355e-15
E:\\R-3.1.2\\scripts\\mPDBS\\summary_pdb_93\\ef8e88d4ad93a0ad.final.pdb
-2.602747e-15 -3.341242e-15 -4.713128e-15 7.708024e-15 -6.583456e-16
1.543351e-15
E:\\R-3.1.2\\scripts\\mPDBS\\summary_pdb_93\\f14fcae8deec4fa3.final.pdb
-1.900062e-15 -5.250161e-16 -4.279538e-15 3.040376e-15 8.459844e-16
1.476407e-15
E:\\R-3.1.2\\scripts\\mPDBS\\summary_pdb_93\\f83584e22003fb92.final.pdb
-5.950135e-15 -1.476121e-14 -3.118773e-15 1.621069e-14 5.684906e-15
2.772491e-15
E:\\R-3.1.2\\scripts\\mPDBS\\summary_pdb_93\\f8e2bf87e52caa29.final.pdb
-7.179139e-15 -7.972457e-15 -8.038521e-15 -3.737154e-15 2.332645e-15
1.804075e-15

[,841]   [,842]   [,843]   [,844]   [,845]   [,846]
E:\\R-3.1.2\\scripts\\40.1ns_data\\NAT0.pdb
1.431399e-15 -2.087829e-15 2.310170e-15 3.306817e-16 -9.282537e-15
-2.030175e-15
E:\\R-3.1.2\\scripts\\40.1ns_data\\LJP6.pdb
1.019034e-15 -2.765100e-15 9.258004e-16 8.823633e-16 -7.201302e-15
-2.717779e-15
E:\\R-3.1.2\\scripts\\40.1ns_data\\3WY9.pdb
7.029134e-16 -3.063363e-15 1.344168e-15 3.077854e-15 -9.961488e-15

-4.056287e-15
E:\\R-3.1.2\\scripts\\40.1ns_data\\ZSP9.pdb
1.106821e-15 1.825262e-16 -1.169885e-15 3.499727e-16 -8.016109e-15
-2.462259e-15
E:\\R-3.1.2\\scripts\\mPDBS\\summary_pdb_93\\013d855ad26c877c.final.pdb
7.488056e-16 -9.787638e-15 2.013963e-15 4.061221e-15 -4.596015e-15
-4.364974e-15
E:\\R-3.1.2\\scripts\\mPDBS\\summary_pdb_93\\0a2a705342ca0d54.final.pdb
5.058340e-16 -3.664093e-15 -8.632646e-16 8.411427e-16 -7.111810e-15
-1.789721e-15
E:\\R-3.1.2\\scripts\\mPDBS\\summary_pdb_93\\0a59fceadb4194d9.final.pdb
-3.323121e-15 -3.778355e-15 -9.925518e-16 1.760900e-15 -6.165864e-15
-2.179938e-15
E:\\R-3.1.2\\scripts\\mPDBS\\summary_pdb_93\\0dba59f3306825e8.final.pdb
-8.905081e-16 -7.391984e-15 -2.787752e-15 2.052412e-15 -4.269887e-15
1.782886e-15
E:\\R-3.1.2\\scripts\\mPDBS\\summary_pdb_93\\126f26a78b57339c.final.pdb
2.810266e-16 1.801183e-15 1.276705e-15 7.034473e-16 -1.196729e-14
6.576794e-15
E:\\R-3.1.2\\scripts\\mPDBS\\summary_pdb_93\\12f06f3e342df706.final.pdb
8.566276e-16 -3.581694e-15 -1.152963e-15 1.041503e-15 -7.433602e-15
-2.458457e-15
E:\\R-3.1.2\\scripts\\mPDBS\\summary_pdb_93\\18618a2a251b867b.final.pdb
-2.961733e-16 -4.759686e-15 -1.286998e-15 -2.314103e-16 -1.227237e-14
-5.593527e-15
E:\\R-3.1.2\\scripts\\mPDBS\\summary_pdb_93\\1f305f598641eeb2.final.pdb
8.339364e-16 -3.825982e-15 1.726984e-16 1.414006e-15 -7.036426e-15
-2.423371e-15
E:\\R-3.1.2\\scripts\\mPDBS\\summary_pdb_93\\1f8a00b551be0a39.final.pdb
-2.961733e-16 -4.759686e-15 -1.286998e-15 -2.314103e-16 -1.227237e-14
-5.593527e-15
E:\\R-3.1.2\\scripts\\mPDBS\\summary_pdb_93\\219f45328d47579a.final.pdb
7.969061e-15 -9.299113e-15 -1.684966e-14 1.698450e-15 -8.486924e-15
-5.163379e-16
E:\\R-3.1.2\\scripts\\mPDBS\\summary_pdb_93\\238dd3918e54336d.final.pdb
8.089895e-15 2.261952e-15 -1.055002e-15 9.192781e-15 -1.400074e-14
1.153565e-14
E:\\R-3.1.2\\scripts\\mPDBS\\summary_pdb_93\\2b3027889e2fdc42.final.pdb
7.818307e-16 -4.860823e-15 -1.005562e-15 1.074600e-15 -6.421302e-15
-2.974798e-17
E:\\R-3.1.2\\scripts\\mPDBS\\summary_pdb_93\\2fcb3b46e33793b0.final.pdb
-6.995592e-16 -7.906052e-15 -1.967950e-15 -8.792984e-16 -3.337996e-15
-8.942462e-15
E:\\R-3.1.2\\scripts\\mPDBS\\summary_pdb_93\\34eb328251842006.final.pdb
2.264620e-15 -2.593861e-15 -1.673282e-15 1.063104e-14 -1.231372e-14
-1.558123e-14
E:\\R-3.1.2\\scripts\\mPDBS\\summary_pdb_93\\354b32871797b7ab.final.pdb
-5.781214e-16 1.636083e-15 1.958604e-15 -6.903846e-15 -8.306612e-15
-1.336286e-14
E:\\R-3.1.2\\scripts\\mPDBS\\summary_pdb_93\\397368adbc32d5de.final.pdb
2.810266e-16 1.801183e-15 1.276705e-15 7.034473e-16 -1.196729e-14
6.576794e-15
E:\\R-3.1.2\\scripts\\mPDBS\\summary_pdb_93\\411ca13cd3df6edd.final.pdb
-2.365490e-15 -2.654177e-15 -4.847618e-16 -5.209819e-15 -8.567753e-15

-1.087408e-14
E:\\R-3.1.2\\scripts\\mPDBS\\summary_pdb_93\\42357eee6642bf12.final.pdb
7.914812e-16 -4.964927e-15 -3.242320e-15 2.784653e-15 -8.436513e-15
-1.500386e-14
E:\\R-3.1.2\\scripts\\mPDBS\\summary_pdb_93\\44342d1f75a7fcfc.final.pdb
-7.926261e-15 -4.586040e-15 1.297937e-15 7.893715e-15 -5.087175e-15
-1.335472e-14
E:\\R-3.1.2\\scripts\\mPDBS\\summary_pdb_93\\448ef21dffe8cdfb.final.pdb
1.043216e-14 1.082549e-14 9.758780e-15 -6.381841e-15 -1.930439e-14
1.826701e-15
E:\\R-3.1.2\\scripts\\mPDBS\\summary_pdb_93\\472a40b3e8f113c7.final.pdb
-8.905081e-16 -7.391984e-15 -2.787752e-15 2.052412e-15 -4.269887e-15
1.782886e-15
E:\\R-3.1.2\\scripts\\mPDBS\\summary_pdb_93\\48a26225951ea153.final.pdb
4.551385e-15 -9.874174e-15 1.834180e-15 -1.619426e-15 -8.322559e-15
-4.697021e-15
E:\\R-3.1.2\\scripts\\mPDBS\\summary_pdb_93\\4ab4a5c7c6682ce6.final.pdb
1.379452e-14 -5.338939e-15 6.442712e-15 -4.171126e-15 -8.095475e-16
-9.798995e-15
E:\\R-3.1.2\\scripts\\mPDBS\\summary_pdb_93\\4eaa45b62cb6a25f.final.pdb
3.843791e-16 -4.399887e-15 2.451265e-15 7.875923e-16 -5.984684e-15
6.181399e-15
E:\\R-3.1.2\\scripts\\mPDBS\\summary_pdb_93\\58c371611d85ac96.final.pdb
2.810266e-16 1.801183e-15 1.276705e-15 7.034473e-16 -1.196729e-14
6.576794e-15
E:\\R-3.1.2\\scripts\\mPDBS\\summary_pdb_93\\59a647f04822d30a.final.pdb
7.956000e-15 -4.135012e-15 3.991096e-15 -3.061064e-15 -1.739159e-15
4.325587e-15
E:\\R-3.1.2\\scripts\\mPDBS\\summary_pdb_93\\59b091fe385e05fc.final.pdb
-3.323121e-15 -3.778355e-15 -9.925518e-16 1.760900e-15 -6.165864e-15
-2.179938e-15
E:\\R-3.1.2\\scripts\\mPDBS\\summary_pdb_93\\5c35ac30f930d2db.final.pdb
3.843791e-16 -4.399887e-15 2.451265e-15 7.875923e-16 -5.984684e-15
6.181399e-15
E:\\R-3.1.2\\scripts\\mPDBS\\summary_pdb_93\\5f60752431cf2dcb.final.pdb
-4.229365e-15 -5.197589e-15 -3.793213e-15 -7.420093e-16 -5.552728e-15
3.546288e-15
E:\\R-3.1.2\\scripts\\mPDBS\\summary_pdb_93\\626456dd6ff2868d.final.pdb
-8.905081e-16 -7.391984e-15 -2.787752e-15 2.052412e-15 -4.269887e-15
1.782886e-15
E:\\R-3.1.2\\scripts\\mPDBS\\summary_pdb_93\\6583d53ff3c8f7ef.final.pdb
7.956000e-15 -4.135012e-15 3.991096e-15 -3.061064e-15 -1.739159e-15
4.325587e-15
E:\\R-3.1.2\\scripts\\mPDBS\\summary_pdb_93\\69760178986bef01.final.pdb
2.190663e-15 -2.971362e-15 -1.684287e-15 -5.130647e-15 -6.731544e-15
9.973553e-15
E:\\R-3.1.2\\scripts\\mPDBS\\summary_pdb_93\\6a9d87d47926b1c8.final.pdb
8.361381e-16 -4.756072e-15 -4.267932e-16 1.976734e-15 -7.082308e-15
-5.494499e-16
E:\\R-3.1.2\\scripts\\mPDBS\\summary_pdb_93\\6bb3547ea982ffc4.final.pdb
1.389086e-15 -5.026340e-15 -6.823957e-15 9.466744e-15 -1.083960e-14
5.279498e-15
E:\\R-3.1.2\\scripts\\mPDBS\\summary_pdb_93\\716d9624611d1c42.final.pdb
6.073429e-15 -2.202589e-15 5.955933e-15 1.646054e-15 -1.042166e-14

-6.400616e-15
E:\\R-3.1.2\\scripts\\mPDBS\\summary_pdb_93\\75f27e70ad0f1be4.final.pdb
7.956000e-15 -4.135012e-15 3.991096e-15 -3.061064e-15 -1.739159e-15
4.325587e-15
E:\\R-3.1.2\\scripts\\mPDBS\\summary_pdb_93\\769f910fffb22872.final.pdb
3.366755e-16 -3.668545e-15 -4.803514e-16 6.963415e-16 -7.354863e-15
-2.201275e-15
E:\\R-3.1.2\\scripts\\mPDBS\\summary_pdb_93\\791f8c80dd5c3135.final.pdb
-3.323121e-15 -3.778355e-15 -9.925518e-16 1.760900e-15 -6.165864e-15
-2.179938e-15
E:\\R-3.1.2\\scripts\\mPDBS\\summary_pdb_93\\7fad98e66a3f6099.final.pdb
2.668129e-15 -3.055334e-15 -7.230993e-15 2.735861e-15 -1.321648e-14
-8.078334e-15
E:\\R-3.1.2\\scripts\\mPDBS\\summary_pdb_93\\804b89c94a633196.final.pdb
-6.995592e-16 -7.906052e-15 -1.967950e-15 -8.792984e-16 -3.337996e-15
-8.942462e-15
E:\\R-3.1.2\\scripts\\mPDBS\\summary_pdb_93\\813cc65a2298feba.final.pdb
-6.995592e-16 -7.906052e-15 -1.967950e-15 -8.792984e-16 -3.337996e-15
-8.942462e-15
E:\\R-3.1.2\\scripts\\mPDBS\\summary_pdb_93\\83c0eefcfe12d3f9.final.pdb
-2.365490e-15 -2.654177e-15 -4.847618e-16 -5.209819e-15 -8.567753e-15
-1.087408e-14
E:\\R-3.1.2\\scripts\\mPDBS\\summary_pdb_93\\86d50469ef569760.final.pdb
-8.905081e-16 -7.391984e-15 -2.787752e-15 2.052412e-15 -4.269887e-15
1.782886e-15
E:\\R-3.1.2\\scripts\\mPDBS\\summary_pdb_93\\89d0666e1402203d.final.pdb
7.956000e-15 -4.135012e-15 3.991096e-15 -3.061064e-15 -1.739159e-15
4.325587e-15
E:\\R-3.1.2\\scripts\\mPDBS\\summary_pdb_93\\8b34852716dadc62.final.pdb
1.271087e-15 -1.938603e-15 -1.707742e-15 -4.941897e-16 -7.119693e-15
1.781598e-15
E:\\R-3.1.2\\scripts\\mPDBS\\summary_pdb_93\\8b6ef9ac3fe74395.final.pdb
-3.189892e-15 -6.354460e-15 -6.323225e-15 7.178783e-15 -1.261015e-14
5.433112e-15
E:\\R-3.1.2\\scripts\\mPDBS\\summary_pdb_93\\98bbe0d5986c138f.final.pdb
-3.323121e-15 -3.778355e-15 -9.925518e-16 1.760900e-15 -6.165864e-15
-2.179938e-15
E:\\R-3.1.2\\scripts\\mPDBS\\summary_pdb_93\\9c22f4909d67e2fa.final.pdb
-5.133474e-16 -3.742716e-15 -2.427679e-16 8.605844e-15 -7.215536e-15
7.156639e-15
E:\\R-3.1.2\\scripts\\mPDBS\\summary_pdb_93\\9dda8ed0d81fb3ef.final.pdb
-1.581947e-14 1.096914e-15 5.061420e-15 1.056757e-14 -8.735732e-15
-1.061689e-14
E:\\R-3.1.2\\scripts\\mPDBS\\summary_pdb_93\\9f752b3616e6033b.final.pdb
8.046726e-16 -5.131899e-15 -5.156861e-15 9.052399e-16 -6.643683e-15
-1.533232e-15
E:\\R-3.1.2\\scripts\\mPDBS\\summary_pdb_93\\a02c70083816d6ba.final.pdb
3.843791e-16 -4.399887e-15 2.451265e-15 7.875923e-16 -5.984684e-15
6.181399e-15
E:\\R-3.1.2\\scripts\\mPDBS\\summary_pdb_93\\a0c997d891e4e082.final.pdb
4.157620e-15 3.515097e-15 -1.506698e-15 1.385412e-15 -4.108869e-15
5.780126e-15
E:\\R-3.1.2\\scripts\\mPDBS\\summary_pdb_93\\ab8039a2d823c616.final.pdb
8.766616e-16 -3.887835e-15 -7.867878e-16 5.669481e-16 -8.418789e-15

-6.600353e-16
E:\\R-3.1.2\\scripts\\mPDBS\\summary_pdb_93\\acf4d74b9c71d4dc.final.pdb
-6.995592e-16 -7.906052e-15 -1.967950e-15 -8.792984e-16 -3.337996e-15
-8.942462e-15
E:\\R-3.1.2\\scripts\\mPDBS\\summary_pdb_93\\ad8a823e4b4c668e.final.pdb
2.583070e-16 -3.694053e-15 -5.688877e-16 3.751767e-16 -6.918354e-15
-1.831652e-15
E:\\R-3.1.2\\scripts\\mPDBS\\summary_pdb_93\\b16ababd03b7b857.final.pdb
-5.133474e-16 -3.742716e-15 -2.427679e-16 8.605844e-15 -7.215536e-15
7.156639e-15
E:\\R-3.1.2\\scripts\\mPDBS\\summary_pdb_93\\b226923e697d3c63.final.pdb
-9.271941e-16 2.657298e-15 -5.691263e-15 3.427127e-15 -1.446986e-14
-3.120714e-15
E:\\R-3.1.2\\scripts\\mPDBS\\summary_pdb_93\\b28e48f6fa27a165.final.pdb
-3.323121e-15 -3.778355e-15 -9.925518e-16 1.760900e-15 -6.165864e-15
-2.179938e-15
E:\\R-3.1.2\\scripts\\mPDBS\\summary_pdb_93\\b42c4924e3cd1aec.final.pdb
6.706728e-15 -5.902695e-15 -6.720822e-15 4.984258e-15 -1.035987e-14
3.224904e-15
E:\\R-3.1.2\\scripts\\mPDBS\\summary_pdb_93\\b4dd239fcabff1df.final.pdb
-3.323121e-15 -3.778355e-15 -9.925518e-16 1.760900e-15 -6.165864e-15
-2.179938e-15
E:\\R-3.1.2\\scripts\\mPDBS\\summary_pdb_93\\b669942ef97459a2.final.pdb
1.271087e-15 -1.938603e-15 -1.707742e-15 -4.941897e-16 -7.119693e-15
1.781598e-15
E:\\R-3.1.2\\scripts\\mPDBS\\summary_pdb_93\\b99f4d9d87c1017b.final.pdb
-3.323121e-15 -3.778355e-15 -9.925518e-16 1.760900e-15 -6.165864e-15
-2.179938e-15
E:\\R-3.1.2\\scripts\\mPDBS\\summary_pdb_93\\b9d2cbf3975403e7.final.pdb
-3.323121e-15 -3.778355e-15 -9.925518e-16 1.760900e-15 -6.165864e-15
-2.179938e-15
E:\\R-3.1.2\\scripts\\mPDBS\\summary_pdb_93\\bc88c8a505f3a288.final.pdb
6.985097e-16 -4.623166e-15 -1.148677e-15 9.088254e-16 -6.747430e-15
5.135034e-17
E:\\R-3.1.2\\scripts\\mPDBS\\summary_pdb_93\\bdac0e64f4ef427e.final.pdb
8.012168e-15 -5.703821e-15 5.248473e-16 1.382671e-14 -5.166394e-15
1.513030e-14
E:\\R-3.1.2\\scripts\\mPDBS\\summary_pdb_93\\ca2a1f55fc9d0681.final.pdb
1.271087e-15 -1.938603e-15 -1.707742e-15 -4.941897e-16 -7.119693e-15
1.781598e-15
E:\\R-3.1.2\\scripts\\mPDBS\\summary_pdb_93\\ce34248de3e81a3c.final.pdb
-9.916391e-16 -4.270968e-15 1.258976e-14 -1.581713e-15 -4.439493e-15
1.017927e-15
E:\\R-3.1.2\\scripts\\mPDBS\\summary_pdb_93\\d02e697772494a61.final.pdb
1.150557e-14 -8.304249e-15 -1.342966e-15 -1.254330e-14 -2.950554e-15
4.065934e-15
E:\\R-3.1.2\\scripts\\mPDBS\\summary_pdb_93\\d04221245d015047.final.pdb
1.453186e-15 -6.428338e-15 4.292534e-16 3.237616e-16 -4.726430e-15
-2.095519e-15
E:\\R-3.1.2\\scripts\\mPDBS\\summary_pdb_93\\d678141e4018054a.final.pdb
4.827921e-15 -5.103120e-15 -7.006636e-15 5.534259e-15 -1.386502e-14
-1.407638e-14
E:\\R-3.1.2\\scripts\\mPDBS\\summary_pdb_93\\d71b40af0f0efe39.final.pdb
-2.763136e-15 -6.816541e-15 -1.225809e-14 -4.682488e-15 -9.543632e-15

-1.190487e-14
E:\\R-3.1.2\\scripts\\mPDBS\\summary_pdb_93\\dc0809eff10197e2.final.pdb
1.588386e-15 -3.724547e-15 -1.510351e-15 3.685104e-15 -4.115937e-15
3.499165e-15
E:\\R-3.1.2\\scripts\\mPDBS\\summary_pdb_93\\e1163918d3707839.final.pdb
3.057737e-15 -9.340050e-15 -7.243841e-15 1.794102e-15 -1.082130e-14
-5.176361e-15
E:\\R-3.1.2\\scripts\\mPDBS\\summary_pdb_93\\e4b48b06824ef848.final.pdb
-2.961733e-16 -4.759686e-15 -1.286998e-15 -2.314103e-16 -1.227237e-14
-5.593527e-15
E:\\R-3.1.2\\scripts\\mPDBS\\summary_pdb_93\\e529e19ad281fea2.final.pdb
7.488056e-16 -9.787638e-15 2.013963e-15 4.061221e-15 -4.596015e-15
-4.364974e-15
E:\\R-3.1.2\\scripts\\mPDBS\\summary_pdb_93\\e5602082718ec3a1.final.pdb
-6.995592e-16 -7.906052e-15 -1.967950e-15 -8.792984e-16 -3.337996e-15
-8.942462e-15
E:\\R-3.1.2\\scripts\\mPDBS\\summary_pdb_93\\e5d589d996d0a960.final.pdb
-2.106460e-15 -6.895779e-15 1.360987e-15 -3.147013e-15 -7.456876e-15
5.496426e-15
E:\\R-3.1.2\\scripts\\mPDBS\\summary_pdb_93\\e9df9e72307a2fbd.final.pdb
3.843791e-16 -4.399887e-15 2.451265e-15 7.875923e-16 -5.984684e-15
6.181399e-15
E:\\R-3.1.2\\scripts\\mPDBS\\summary_pdb_93\\ea00f8694090cae7.final.pdb
-6.995592e-16 -7.906052e-15 -1.967950e-15 -8.792984e-16 -3.337996e-15
-8.942462e-15
E:\\R-3.1.2\\scripts\\mPDBS\\summary_pdb_93\\eba517be32db050e.final.pdb
-3.323121e-15 -3.778355e-15 -9.925518e-16 1.760900e-15 -6.165864e-15
-2.179938e-15
E:\\R-3.1.2\\scripts\\mPDBS\\summary_pdb_93\\ec04b9545899c29d.final.pdb
5.600060e-16 -4.278994e-15 -5.772986e-16 9.756374e-16 -6.599854e-15
-2.315802e-15
E:\\R-3.1.2\\scripts\\mPDBS\\summary_pdb_93\\ee6862cbcad52078.final.pdb
6.207724e-16 -4.916334e-15 -1.208525e-15 8.813951e-16 -6.620362e-15
5.643948e-16
E:\\R-3.1.2\\scripts\\mPDBS\\summary_pdb_93\\ef6a42fc83b81e5d.final.pdb
9.791195e-16 -3.786506e-15 -8.246941e-16 6.613218e-16 -7.197871e-15
-1.875147e-15
E:\\R-3.1.2\\scripts\\mPDBS\\summary_pdb_93\\ef8e88d4ad93a0ad.final.pdb
1.453186e-15 -6.428338e-15 4.292534e-16 3.237616e-16 -4.726430e-15
-2.095519e-15
E:\\R-3.1.2\\scripts\\mPDBS\\summary_pdb_93\\f14fcae8deec4fa3.final.pdb
8.046726e-16 -5.131899e-15 -5.156861e-15 9.052399e-16 -6.643683e-15
-1.533232e-15
E:\\R-3.1.2\\scripts\\mPDBS\\summary_pdb_93\\f83584e22003fb92.final.pdb
4.551385e-15 -9.874174e-15 1.834180e-15 -1.619426e-15 -8.322559e-15
-4.697021e-15
E:\\R-3.1.2\\scripts\\mPDBS\\summary_pdb_93\\f8e2bf87e52caa29.final.pdb
7.488056e-16 -9.787638e-15 2.013963e-15 4.061221e-15 -4.596015e-15
-4.364974e-15

[,847]   [,848]   [,849]   [,850]   [,851]   [,852]
E:\\R-3.1.2\\scripts\\40.1ns_data\\NAT0.pdb
8.899940e-16 7.979942e-16 2.383313e-15 -1.149477e-15 -2.935809e-15
-3.976949e-15

E:\\R-3.1.2\\scripts\\40.1ns_data\\LJP6.pdb
6.361151e-17 3.469984e-17 2.587309e-15 -1.258487e-15 -4.596657e-15
-4.764745e-15
E:\\R-3.1.2\\scripts\\40.1ns_data\\3WY9.pdb
-1.305309e-16 2.191446e-16 2.735680e-15 -1.421121e-16 -8.167028e-15
-4.239329e-15
E:\\R-3.1.2\\scripts\\40.1ns_data\\ZSP9.pdb
3.745746e-16 -1.062086e-15 4.478754e-15 -1.429538e-15 -6.469917e-15
-5.122857e-15
E:\\R-3.1.2\\scripts\\mPDBS\\summary_pdb_93\\013d855ad26c877c.final.pdb
3.498961e-16 -2.838271e-15 2.887127e-15 2.251293e-15 -2.014056e-15
-2.175342e-15
E:\\R-3.1.2\\scripts\\mPDBS\\summary_pdb_93\\0a2a705342ca0d54.final.pdb
1.636391e-15 -1.156549e-15 2.394974e-15 -7.561468e-16 -5.701864e-15
-4.021029e-15
E:\\R-3.1.2\\scripts\\mPDBS\\summary_pdb_93\\0a59fceadb4194d9.final.pdb
6.015417e-15 1.478624e-15 -5.267374e-16 1.969938e-16 -7.454546e-15
-6.773389e-15
E:\\R-3.1.2\\scripts\\mPDBS\\summary_pdb_93\\0dba59f3306825e8.final.pdb
1.735940e-15 1.309886e-15 -1.027276e-15 2.775179e-15 -8.696210e-15
-7.025629e-15
E:\\R-3.1.2\\scripts\\mPDBS\\summary_pdb_93\\126f26a78b57339c.final.pdb
-3.734510e-15 -2.004303e-15 6.844899e-15 -9.845801e-15 -1.018764e-14
-2.504072e-15
E:\\R-3.1.2\\scripts\\mPDBS\\summary_pdb_93\\12f06f3e342df706.final.pdb
1.751751e-15 -8.282525e-16 2.372857e-15 -1.694199e-15 -6.351518e-15
-4.038376e-15
E:\\R-3.1.2\\scripts\\mPDBS\\summary_pdb_93\\18618a2a251b867b.final.pdb
-3.630372e-15 1.441514e-15 6.973772e-15 1.411649e-15 -5.739534e-15
-4.580627e-15
E:\\R-3.1.2\\scripts\\mPDBS\\summary_pdb_93\\1f305f598641eeb2.final.pdb
1.913419e-15 -6.508915e-16 2.837600e-15 -2.312537e-15 -5.599096e-15
-4.175912e-15
E:\\R-3.1.2\\scripts\\mPDBS\\summary_pdb_93\\1f8a00b551be0a39.final.pdb
-3.630372e-15 1.441514e-15 6.973772e-15 1.411649e-15 -5.739534e-15
-4.580627e-15
E:\\R-3.1.2\\scripts\\mPDBS\\summary_pdb_93\\219f45328d47579a.final.pdb
8.751943e-15 -6.188420e-15 -2.018600e-15 -1.019313e-14 -4.124364e-16
6.887558e-15
E:\\R-3.1.2\\scripts\\mPDBS\\summary_pdb_93\\238dd3918e54336d.final.pdb
-7.704511e-15 -5.258825e-15 7.132067e-15 -7.545944e-15 -8.898704e-15
-2.265710e-15
E:\\R-3.1.2\\scripts\\mPDBS\\summary_pdb_93\\2b3027889e2fdc42.final.pdb
2.309614e-15 -5.722530e-16 2.087925e-15 -3.236458e-15 -6.513459e-15
-4.194723e-15
E:\\R-3.1.2\\scripts\\mPDBS\\summary_pdb_93\\2fcb3b46e33793b0.final.pdb
3.519773e-15 -6.547946e-16 2.390042e-15 1.829983e-15 -2.375980e-15
-7.659236e-15
E:\\R-3.1.2\\scripts\\mPDBS\\summary_pdb_93\\34eb328251842006.final.pdb
5.759754e-15 6.605590e-15 -3.745206e-17 -2.887938e-15 -9.040035e-15
6.464234e-15
E:\\R-3.1.2\\scripts\\mPDBS\\summary_pdb_93\\354b32871797b7ab.final.pdb
6.911405e-15 3.064194e-15 2.572669e-15 2.718003e-15 -1.375275e-14
6.266539e-15

E:\\R-3.1.2\\scripts\\mPDBS\\summary_pdb_93\\397368adbc32d5de.final.pdb
-3.734510e-15 -2.004303e-15 6.844899e-15 -9.845801e-15 -1.018764e-14
-2.504072e-15
E:\\R-3.1.2\\scripts\\mPDBS\\summary_pdb_93\\411ca13cd3df6edd.final.pdb
-1.865676e-15 4.870871e-16 8.014882e-15 7.258311e-16 -5.205748e-15
-6.248039e-15
E:\\R-3.1.2\\scripts\\mPDBS\\summary_pdb_93\\42357eee6642bf12.final.pdb
1.329864e-14 2.405890e-15 -1.491051e-16 -8.434135e-15 -5.834323e-15
-2.451690e-15
E:\\R-3.1.2\\scripts\\mPDBS\\summary_pdb_93\\44342d1f75a7fcfc.final.pdb
-1.589419e-15 -1.321751e-15 6.328543e-15 4.897224e-16 -9.663232e-15
-1.127000e-14
E:\\R-3.1.2\\scripts\\mPDBS\\summary_pdb_93\\448ef21dffe8cdfb.final.pdb
3.417593e-16 -2.960242e-15 1.719677e-14 -1.494826e-14 -2.313530e-15
8.317567e-15
E:\\R-3.1.2\\scripts\\mPDBS\\summary_pdb_93\\472a40b3e8f113c7.final.pdb
1.735940e-15 1.309886e-15 -1.027276e-15 2.775179e-15 -8.696210e-15
-7.025629e-15
E:\\R-3.1.2\\scripts\\mPDBS\\summary_pdb_93\\48a26225951ea153.final.pdb
-1.043607e-15 -8.003691e-16 9.097942e-15 5.582499e-16 -5.247978e-15
-4.080231e-15
E:\\R-3.1.2\\scripts\\mPDBS\\summary_pdb_93\\4ab4a5c7c6682ce6.final.pdb
5.867184e-15 -6.828773e-16 7.571748e-15 -6.733707e-15 2.626763e-15
-1.118116e-14
E:\\R-3.1.2\\scripts\\mPDBS\\summary_pdb_93\\4eaa45b62cb6a25f.final.pdb
-3.463109e-15 -2.394980e-15 3.456944e-15 -3.322304e-15 -2.909860e-15
-9.395412e-15
E:\\R-3.1.2\\scripts\\mPDBS\\summary_pdb_93\\58c371611d85ac96.final.pdb
-3.734510e-15 -2.004303e-15 6.844899e-15 -9.845801e-15 -1.018764e-14
-2.504072e-15
E:\\R-3.1.2\\scripts\\mPDBS\\summary_pdb_93\\59a647f04822d30a.final.pdb
3.384033e-15 3.307792e-15 2.261484e-15 -1.058475e-14 -2.260299e-15
-1.996170e-15
E:\\R-3.1.2\\scripts\\mPDBS\\summary_pdb_93\\59b091fe385e05fc.final.pdb
6.015417e-15 1.478624e-15 -5.267374e-16 1.969938e-16 -7.454546e-15
-6.773389e-15
E:\\R-3.1.2\\scripts\\mPDBS\\summary_pdb_93\\5c35ac30f930d2db.final.pdb
-3.463109e-15 -2.394980e-15 3.456944e-15 -3.322304e-15 -2.909860e-15
-9.395412e-15
E:\\R-3.1.2\\scripts\\mPDBS\\summary_pdb_93\\5f60752431cf2dcb.final.pdb
1.491976e-15 1.582796e-15 7.371029e-15 -1.749710e-15 -9.225088e-15
-7.801859e-15
E:\\R-3.1.2\\scripts\\mPDBS\\summary_pdb_93\\626456dd6ff2868d.final.pdb
1.735940e-15 1.309886e-15 -1.027276e-15 2.775179e-15 -8.696210e-15
-7.025629e-15
E:\\R-3.1.2\\scripts\\mPDBS\\summary_pdb_93\\6583d53ff3c8f7ef.final.pdb
3.384033e-15 3.307792e-15 2.261484e-15 -1.058475e-14 -2.260299e-15
-1.996170e-15
E:\\R-3.1.2\\scripts\\mPDBS\\summary_pdb_93\\69760178986bef01.final.pdb
2.678982e-15 -2.446291e-15 1.644698e-15 -6.038795e-15 -1.046529e-14
-1.358919e-14
E:\\R-3.1.2\\scripts\\mPDBS\\summary_pdb_93\\6a9d87d47926b1c8.final.pdb
2.236408e-15 -7.596388e-16 2.209718e-15 -2.074240e-15 -7.006156e-15
-4.665538e-15

E:\\R-3.1.2\\scripts\\mPDBS\\summary_pdb_93\\6bb3547ea982ffc4.final.pdb
-9.114713e-15 -1.653961e-15 1.050638e-14 -1.155219e-14 -6.007260e-15
4.442318e-16
E:\\R-3.1.2\\scripts\\mPDBS\\summary_pdb_93\\716d9624611d1c42.final.pdb
-6.337265e-15 -3.534421e-15 9.499496e-16 -1.768358e-15 -4.850115e-15
-3.636910e-17
E:\\R-3.1.2\\scripts\\mPDBS\\summary_pdb_93\\75f27e70ad0f1be4.final.pdb
3.384033e-15 3.307792e-15 2.261484e-15 -1.058475e-14 -2.260299e-15
-1.996170e-15
E:\\R-3.1.2\\scripts\\mPDBS\\summary_pdb_93\\769f910fffb22872.final.pdb
1.852655e-15 -9.671498e-16 2.171190e-15 -1.801493e-15 -6.612967e-15
-4.651751e-15
E:\\R-3.1.2\\scripts\\mPDBS\\summary_pdb_93\\791f8c80dd5c3135.final.pdb
6.015417e-15 1.478624e-15 -5.267374e-16 1.969938e-16 -7.454546e-15
-6.773389e-15
E:\\R-3.1.2\\scripts\\mPDBS\\summary_pdb_93\\7fad98e66a3f6099.final.pdb
1.205744e-14 -6.694316e-15 3.065596e-15 -6.378985e-15 -1.750117e-14
-2.971041e-15
E:\\R-3.1.2\\scripts\\mPDBS\\summary_pdb_93\\804b89c94a633196.final.pdb
3.519773e-15 -6.547946e-16 2.390042e-15 1.829983e-15 -2.375980e-15
-7.659236e-15
E:\\R-3.1.2\\scripts\\mPDBS\\summary_pdb_93\\813cc65a2298feba.final.pdb
3.519773e-15 -6.547946e-16 2.390042e-15 1.829983e-15 -2.375980e-15
-7.659236e-15
E:\\R-3.1.2\\scripts\\mPDBS\\summary_pdb_93\\83c0eefcfe12d3f9.final.pdb
-1.865676e-15 4.870871e-16 8.014882e-15 7.258311e-16 -5.205748e-15
-6.248039e-15
E:\\R-3.1.2\\scripts\\mPDBS\\summary_pdb_93\\86d50469ef569760.final.pdb
1.735940e-15 1.309886e-15 -1.027276e-15 2.775179e-15 -8.696210e-15
-7.025629e-15
E:\\R-3.1.2\\scripts\\mPDBS\\summary_pdb_93\\89d0666e1402203d.final.pdb
3.384033e-15 3.307792e-15 2.261484e-15 -1.058475e-14 -2.260299e-15
-1.996170e-15
E:\\R-3.1.2\\scripts\\mPDBS\\summary_pdb_93\\8b34852716dadc62.final.pdb
4.123456e-15 -1.304744e-16 1.227777e-15 -6.142807e-15 -7.902809e-15
-7.957609e-15
E:\\R-3.1.2\\scripts\\mPDBS\\summary_pdb_93\\8b6ef9ac3fe74395.final.pdb
3.663760e-15 -3.258705e-15 2.741690e-15 -1.528742e-14 -4.337515e-15
-1.172956e-14
E:\\R-3.1.2\\scripts\\mPDBS\\summary_pdb_93\\98bbe0d5986c138f.final.pdb
6.015417e-15 1.478624e-15 -5.267374e-16 1.969938e-16 -7.454546e-15
-6.773389e-15
E:\\R-3.1.2\\scripts\\mPDBS\\summary_pdb_93\\9c22f4909d67e2fa.final.pdb
2.767770e-15 -7.176783e-16 -5.090953e-15 2.392033e-15 -1.199979e-14
4.250508e-15
E:\\R-3.1.2\\scripts\\mPDBS\\summary_pdb_93\\9dda8ed0d81fb3ef.final.pdb
3.795596e-15 6.084651e-15 7.104398e-15 8.371439e-15 -7.094106e-15
-2.471324e-14
E:\\R-3.1.2\\scripts\\mPDBS\\summary_pdb_93\\9f752b3616e6033b.final.pdb
4.434129e-15 -7.773067e-16 1.653925e-15 -2.286725e-15 -6.125357e-15
-3.367963e-15
E:\\R-3.1.2\\scripts\\mPDBS\\summary_pdb_93\\a02c70083816d6ba.final.pdb
-3.463109e-15 -2.394980e-15 3.456944e-15 -3.322304e-15 -2.909860e-15
-9.395412e-15

E:\\R-3.1.2\\scripts\\mPDBS\\summary_pdb_93\\a0c997d891e4e082.final.pdb
-2.408992e-15 -2.778692e-15 -6.043266e-16 -3.622745e-15 -9.204118e-15
-2.999818e-15
E:\\R-3.1.2\\scripts\\mPDBS\\summary_pdb_93\\ab8039a2d823c616.final.pdb
1.376175e-15 -1.116176e-15 2.486951e-15 -1.628680e-15 -5.470993e-15
-3.923558e-15
E:\\R-3.1.2\\scripts\\mPDBS\\summary_pdb_93\\acf4d74b9c71d4dc.final.pdb
3.519773e-15 -6.547946e-16 2.390042e-15 1.829983e-15 -2.375980e-15
-7.659236e-15
E:\\R-3.1.2\\scripts\\mPDBS\\summary_pdb_93\\ad8a823e4b4c668e.final.pdb
1.600170e-15 -8.679042e-16 2.476783e-15 -1.472052e-15 -6.308950e-15
-4.392301e-15
E:\\R-3.1.2\\scripts\\mPDBS\\summary_pdb_93\\b16ababd03b7b857.final.pdb
2.767770e-15 -7.176783e-16 -5.090953e-15 2.392033e-15 -1.199979e-14
4.250508e-15
E:\\R-3.1.2\\scripts\\mPDBS\\summary_pdb_93\\b226923e697d3c63.final.pdb
1.266369e-15 -2.492256e-15 5.467100e-16 -8.735103e-15 -1.352212e-14
-5.384085e-15
E:\\R-3.1.2\\scripts\\mPDBS\\summary_pdb_93\\b28e48f6fa27a165.final.pdb
6.015417e-15 1.478624e-15 -5.267374e-16 1.969938e-16 -7.454546e-15
-6.773389e-15
E:\\R-3.1.2\\scripts\\mPDBS\\summary_pdb_93\\b42c4924e3cd1aec.final.pdb
-1.646665e-15 -6.672289e-15 -5.520485e-15 3.961771e-15 -7.266711e-15
6.494434e-15
E:\\R-3.1.2\\scripts\\mPDBS\\summary_pdb_93\\b4dd239fcabff1df.final.pdb
6.015417e-15 1.478624e-15 -5.267374e-16 1.969938e-16 -7.454546e-15
-6.773389e-15
E:\\R-3.1.2\\scripts\\mPDBS\\summary_pdb_93\\b669942ef97459a2.final.pdb
4.123456e-15 -1.304744e-16 1.227777e-15 -6.142807e-15 -7.902809e-15
-7.957609e-15
E:\\R-3.1.2\\scripts\\mPDBS\\summary_pdb_93\\b99f4d9d87c1017b.final.pdb
6.015417e-15 1.478624e-15 -5.267374e-16 1.969938e-16 -7.454546e-15
-6.773389e-15
E:\\R-3.1.2\\scripts\\mPDBS\\summary_pdb_93\\b9d2cbf3975403e7.final.pdb
6.015417e-15 1.478624e-15 -5.267374e-16 1.969938e-16 -7.454546e-15
-6.773389e-15
E:\\R-3.1.2\\scripts\\mPDBS\\summary_pdb_93\\bc88c8a505f3a288.final.pdb
1.469574e-15 -4.473529e-16 1.887998e-15 -2.415066e-15 -6.917649e-15
-4.210336e-15
E:\\R-3.1.2\\scripts\\mPDBS\\summary_pdb_93\\bdac0e64f4ef427e.final.pdb
9.321555e-15 -3.671912e-15 -6.172987e-15 -9.437046e-15 -9.166989e-15
-6.075856e-16
E:\\R-3.1.2\\scripts\\mPDBS\\summary_pdb_93\\ca2a1f55fc9d0681.final.pdb
4.123456e-15 -1.304744e-16 1.227777e-15 -6.142807e-15 -7.902809e-15
-7.957609e-15
E:\\R-3.1.2\\scripts\\mPDBS\\summary_pdb_93\\ce34248de3e81a3c.final.pdb
8.339999e-16 -6.138223e-16 2.604387e-15 -3.438416e-15 -3.773331e-15
-1.129043e-14
E:\\R-3.1.2\\scripts\\mPDBS\\summary_pdb_93\\d02e697772494a61.final.pdb
2.563317e-15 -8.101269e-16 6.157152e-15 -1.990585e-14 -4.988637e-15
6.171984e-15
E:\\R-3.1.2\\scripts\\mPDBS\\summary_pdb_93\\d04221245d015047.final.pdb
1.608628e-15 5.411166e-17 3.378599e-15 1.311969e-16 -4.058061e-15
-3.686769e-15

E:\\R-3.1.2\\scripts\\mPDBS\\summary_pdb_93\\d678141e4018054a.final.pdb
-7.182852e-15 -7.800623e-16 1.272780e-14 -1.429243e-16 -1.190105e-14
1.012184e-14
E:\\R-3.1.2\\scripts\\mPDBS\\summary_pdb_93\\d71b40af0f0efe39.final.pdb
2.545229e-15 -4.316875e-15 4.594370e-15 1.823951e-14 -1.379863e-14
9.976681e-15
E:\\R-3.1.2\\scripts\\mPDBS\\summary_pdb_93\\dc0809eff10197e2.final.pdb
4.549794e-15 1.132600e-15 -8.889971e-15 -4.723019e-15 -9.361751e-15
-6.006068e-15
E:\\R-3.1.2\\scripts\\mPDBS\\summary_pdb_93\\e1163918d3707839.final.pdb
-1.768096e-15 -4.499765e-15 -1.503299e-15 1.734386e-15 1.981100e-15
2.832433e-15
E:\\R-3.1.2\\scripts\\mPDBS\\summary_pdb_93\\e4b48b06824ef848.final.pdb
-3.630372e-15 1.441514e-15 6.973772e-15 1.411649e-15 -5.739534e-15
-4.580627e-15
E:\\R-3.1.2\\scripts\\mPDBS\\summary_pdb_93\\e529e19ad281fea2.final.pdb
3.498961e-16 -2.838271e-15 2.887127e-15 2.251293e-15 -2.014056e-15
-2.175342e-15
E:\\R-3.1.2\\scripts\\mPDBS\\summary_pdb_93\\e5602082718ec3a1.final.pdb
3.519773e-15 -6.547946e-16 2.390042e-15 1.829983e-15 -2.375980e-15
-7.659236e-15
E:\\R-3.1.2\\scripts\\mPDBS\\summary_pdb_93\\e5d589d996d0a960.final.pdb
-2.349049e-15 -1.199583e-14 8.525794e-15 -1.331421e-15 -9.381896e-15
-9.782782e-15
E:\\R-3.1.2\\scripts\\mPDBS\\summary_pdb_93\\e9df9e72307a2fbd.final.pdb
-3.463109e-15 -2.394980e-15 3.456944e-15 -3.322304e-15 -2.909860e-15
-9.395412e-15
E:\\R-3.1.2\\scripts\\mPDBS\\summary_pdb_93\\ea00f8694090cae7.final.pdb
3.519773e-15 -6.547946e-16 2.390042e-15 1.829983e-15 -2.375980e-15
-7.659236e-15
E:\\R-3.1.2\\scripts\\mPDBS\\summary_pdb_93\\eba517be32db050e.final.pdb
6.015417e-15 1.478624e-15 -5.267374e-16 1.969938e-16 -7.454546e-15
-6.773389e-15
E:\\R-3.1.2\\scripts\\mPDBS\\summary_pdb_93\\ec04b9545899c29d.final.pdb
1.726505e-15 -5.870958e-16 2.465314e-15 -2.299713e-15 -5.740154e-15
-4.681522e-15
E:\\R-3.1.2\\scripts\\mPDBS\\summary_pdb_93\\ee6862cbcad52078.final.pdb
2.356885e-15 -7.118982e-16 2.280045e-15 -2.299273e-15 -6.580246e-15
-4.513045e-15
E:\\R-3.1.2\\scripts\\mPDBS\\summary_pdb_93\\ef6a42fc83b81e5d.final.pdb
1.781531e-15 -8.949419e-16 2.479104e-15 -1.444140e-15 -5.606827e-15
-3.930973e-15
E:\\R-3.1.2\\scripts\\mPDBS\\summary_pdb_93\\ef8e88d4ad93a0ad.final.pdb
1.608628e-15 5.411166e-17 3.378599e-15 1.311969e-16 -4.058061e-15
-3.686769e-15
E:\\R-3.1.2\\scripts\\mPDBS\\summary_pdb_93\\f14fcae8deec4fa3.final.pdb
4.434129e-15 -7.773067e-16 1.653925e-15 -2.286725e-15 -6.125357e-15
-3.367963e-15
E:\\R-3.1.2\\scripts\\mPDBS\\summary_pdb_93\\f83584e22003fb92.final.pdb
-1.043607e-15 -8.003691e-16 9.097942e-15 5.582499e-16 -5.247978e-15
-4.080231e-15
E:\\R-3.1.2\\scripts\\mPDBS\\summary_pdb_93\\f8e2bf87e52caa29.final.pdb
3.498961e-16 -2.838271e-15 2.887127e-15 2.251293e-15 -2.014056e-15
-2.175342e-15

[,853] [,854] [,855] [,856] [,857] [,858]
E:\\R-3.1.2\\scripts\\40.1ns_data\\NAT0.pdb
5.748219e-16 7.025258e-16 -2.326684e-15 -2.440972e-16 -4.750230e-15
-2.235209e-15
E:\\R-3.1.2\\scripts\\40.1ns_data\\LJP6.pdb
1.301334e-15 5.801608e-16 -1.056402e-15 -3.559041e-16 -2.934785e-15
-1.609355e-15
E:\\R-3.1.2\\scripts\\40.1ns_data\\3WY9.pdb
3.536878e-15 -8.245021e-16 -3.419996e-15 -1.412127e-15 -5.055368e-15
-3.128321e-15
E:\\R-3.1.2\\scripts\\40.1ns_data\\ZSP9.pdb
-4.260036e-16 1.820047e-15 7.252999e-16 -3.673584e-16 -3.304151e-15
-4.349954e-15
E:\\R-3.1.2\\scripts\\mPDBS\\summary_pdb_93\\013d855ad26c877c.final.pdb
-8.430003e-16 -5.570329e-15 -1.295509e-15 -1.229260e-15 -5.356623e-15
1.667343e-16
E:\\R-3.1.2\\scripts\\mPDBS\\summary_pdb_93\\0a2a705342ca0d54.final.pdb
7.811348e-16 3.375615e-16 -1.929513e-15 -1.383822e-15 -4.024393e-15
-2.465578e-15
E:\\R-3.1.2\\scripts\\mPDBS\\summary_pdb_93\\0a59fceadb4194d9.final.pdb
1.534402e-16 -9.095772e-16 -1.890353e-15 -8.639776e-16 -5.192844e-15
-1.021002e-15
E:\\R-3.1.2\\scripts\\mPDBS\\summary_pdb_93\\0dba59f3306825e8.final.pdb
4.125248e-15 -6.298369e-16 -2.690227e-15 7.691412e-16 -4.118031e-15
5.930426e-16
E:\\R-3.1.2\\scripts\\mPDBS\\summary_pdb_93\\126f26a78b57339c.final.pdb
5.537313e-15 3.614164e-15 -1.146106e-15 -1.030418e-15 -2.764079e-15
-1.224521e-14
E:\\R-3.1.2\\scripts\\mPDBS\\summary_pdb_93\\12f06f3e342df706.final.pdb
-5.441940e-16 -7.226697e-17 -1.327564e-15 -1.629069e-15 -3.480123e-15
-2.744435e-15
E:\\R-3.1.2\\scripts\\mPDBS\\summary_pdb_93\\18618a2a251b867b.final.pdb
3.322441e-15 2.404220e-15 -5.866842e-15 -1.978684e-15 -1.954838e-15
-1.151907e-14
E:\\R-3.1.2\\scripts\\mPDBS\\summary_pdb_93\\1f305f598641eeb2.final.pdb
5.465008e-16 -8.840450e-16 -1.892869e-15 -1.318665e-15 -3.862267e-15
-2.649825e-15
E:\\R-3.1.2\\scripts\\mPDBS\\summary_pdb_93\\1f8a00b551be0a39.final.pdb
3.322441e-15 2.404220e-15 -5.866842e-15 -1.978684e-15 -1.954838e-15
-1.151907e-14
E:\\R-3.1.2\\scripts\\mPDBS\\summary_pdb_93\\219f45328d47579a.final.pdb
-4.686438e-15 -4.019936e-15 -3.262519e-15 -4.277913e-15 -1.044906e-14
-2.784348e-15
E:\\R-3.1.2\\scripts\\mPDBS\\summary_pdb_93\\238dd3918e54336d.final.pdb
6.843874e-17 -4.168689e-15 5.402425e-15 -4.192379e-16 2.567902e-16
-4.838261e-15
E:\\R-3.1.2\\scripts\\mPDBS\\summary_pdb_93\\2b3027889e2fdc42.final.pdb
1.226367e-15 -6.866652e-16 -1.921361e-15 -1.546374e-15 -3.479804e-15
-1.662849e-15
E:\\R-3.1.2\\scripts\\mPDBS\\summary_pdb_93\\2fcb3b46e33793b0.final.pdb
-1.890220e-16 -4.107161e-15 -3.516570e-15 -3.247677e-15 -3.480627e-15
1.694357e-15
E:\\R-3.1.2\\scripts\\mPDBS\\summary_pdb_93\\34eb328251842006.final.pdb

-4.816486e-15 -3.102792e-15 4.276933e-15 -1.898682e-15 -6.191886e-15
3.784797e-15
E:\\R-3.1.2\\scripts\\mPDBS\\summary_pdb_93\\354b32871797b7ab.final.pdb
-1.671341e-14 8.337441e-16 2.730202e-15 -2.945958e-15 1.243902e-15
8.756400e-15
E:\\R-3.1.2\\scripts\\mPDBS\\summary_pdb_93\\397368adbc32d5de.final.pdb
5.537313e-15 3.614164e-15 -1.146106e-15 -1.030418e-15 -2.764079e-15
-1.224521e-14
E:\\R-3.1.2\\scripts\\mPDBS\\summary_pdb_93\\411ca13cd3df6edd.final.pdb
3.556812e-16 -1.460841e-15 -5.608180e-16 -5.798805e-15 -2.815361e-15
-5.350355e-15
E:\\R-3.1.2\\scripts\\mPDBS\\summary_pdb_93\\42357eee6642bf12.final.pdb
-8.577849e-15 -1.294364e-15 1.178567e-15 -1.275516e-15 -3.994896e-15
-5.349800e-15
E:\\R-3.1.2\\scripts\\mPDBS\\summary_pdb_93\\44342d1f75a7fcfc.final.pdb
1.261139e-14 -2.130148e-15 -1.383145e-14 -5.323156e-15 -1.368459e-14
-9.992095e-15
E:\\R-3.1.2\\scripts\\mPDBS\\summary_pdb_93\\448ef21dffe8cdfb.final.pdb
-2.221307e-14 -3.913836e-16 9.535489e-15 -8.819609e-15 1.190290e-14
-5.289640e-15
E:\\R-3.1.2\\scripts\\mPDBS\\summary_pdb_93\\472a40b3e8f113c7.final.pdb
4.125248e-15 -6.298369e-16 -2.690227e-15 7.691412e-16 -4.118031e-15
5.930426e-16
E:\\R-3.1.2\\scripts\\mPDBS\\summary_pdb_93\\48a26225951ea153.final.pdb
9.841003e-15 2.691674e-16 -1.971668e-15 9.845871e-16 -1.309231e-15
-7.384255e-17
E:\\R-3.1.2\\scripts\\mPDBS\\summary_pdb_93\\4ab4a5c7c6682ce6.final.pdb
-1.175441e-14 -3.900658e-15 -5.063111e-15 -1.164346e-14 1.180803e-14
8.186795e-15
E:\\R-3.1.2\\scripts\\mPDBS\\summary_pdb_93\\4eaa45b62cb6a25f.final.pdb
7.709759e-15 -8.030460e-18 -3.691744e-15 -7.296316e-16 -3.948559e-15
-7.420764e-15
E:\\R-3.1.2\\scripts\\mPDBS\\summary_pdb_93\\58c371611d85ac96.final.pdb
5.537313e-15 3.614164e-15 -1.146106e-15 -1.030418e-15 -2.764079e-15
-1.224521e-14
E:\\R-3.1.2\\scripts\\mPDBS\\summary_pdb_93\\59a647f04822d30a.final.pdb
-5.794030e-15 2.557074e-15 2.952000e-15 5.523529e-16 2.267923e-15
6.623086e-15
E:\\R-3.1.2\\scripts\\mPDBS\\summary_pdb_93\\59b091fe385e05fc.final.pdb
1.534402e-16 -9.095772e-16 -1.890353e-15 -8.639776e-16 -5.192844e-15
-1.021002e-15
E:\\R-3.1.2\\scripts\\mPDBS\\summary_pdb_93\\5c35ac30f930d2db.final.pdb
7.709759e-15 -8.030460e-18 -3.691744e-15 -7.296316e-16 -3.948559e-15
-7.420764e-15
E:\\R-3.1.2\\scripts\\mPDBS\\summary_pdb_93\\5f60752431cf2dcb.final.pdb
4.362471e-15 -4.098994e-15 -5.610380e-15 -4.836572e-15 -5.496306e-15
-9.473861e-15
E:\\R-3.1.2\\scripts\\mPDBS\\summary_pdb_93\\626456dd6ff2868d.final.pdb
4.125248e-15 -6.298369e-16 -2.690227e-15 7.691412e-16 -4.118031e-15
5.930426e-16
E:\\R-3.1.2\\scripts\\mPDBS\\summary_pdb_93\\6583d53ff3c8f7ef.final.pdb
-5.794030e-15 2.557074e-15 2.952000e-15 5.523529e-16 2.267923e-15
6.623086e-15
E:\\R-3.1.2\\scripts\\mPDBS\\summary_pdb_93\\69760178986bef01.final.pdb

-6.109927e-16 2.074506e-15 -1.476491e-14 -1.923426e-15 -4.684879e-15
-6.251273e-15
E:\\R-3.1.2\\scripts\\mPDBS\\summary_pdb_93\\6a9d87d47926b1c8.final.pdb
7.217202e-16 -1.395717e-15 -1.829587e-15 -1.307973e-15 -3.124901e-15
-1.574061e-15
E:\\R-3.1.2\\scripts\\mPDBS\\summary_pdb_93\\6bb3547ea982ffc4.final.pdb
-4.415124e-15 -7.058791e-15 -2.815726e-15 -8.102579e-15 -9.782316e-15
-2.579031e-15
E:\\R-3.1.2\\scripts\\mPDBS\\summary_pdb_93\\716d9624611d1c42.final.pdb
5.452124e-16 1.423498e-15 2.431581e-15 1.749089e-15 -2.227219e-15
2.073326e-15
E:\\R-3.1.2\\scripts\\mPDBS\\summary_pdb_93\\75f27e70ad0f1be4.final.pdb
-5.794030e-15 2.557074e-15 2.952000e-15 5.523529e-16 2.267923e-15
6.623086e-15
E:\\R-3.1.2\\scripts\\mPDBS\\summary_pdb_93\\769f910fffb22872.final.pdb
-3.230808e-16 -7.048377e-16 -2.238018e-15 -1.368495e-15 -3.235498e-15
-3.262221e-15
E:\\R-3.1.2\\scripts\\mPDBS\\summary_pdb_93\\791f8c80dd5c3135.final.pdb
[truncated: 577,620 more chars]
